# Global, regional, and national stillbirths at 20 weeks' gestation or longer in 204 countries and territories, 1990–2021: findings from the Global Burden of Disease Study 2021

**DOI:** 10.1016/S0140-6736(24)01925-1

**Published:** 2024-11-16

**Authors:** GBD 2021 Global Stillbirths Collaborators, Haley Comfort, Theresa A McHugh, Austin E Schumacher, Ashley Harris, Erin A May, Katherine R Paulson, William M Gardner, John E Fuller, Meghan E Frisch, Heather Jean Taylor, Andrew T Leever, Corey Teply, Nicholas Alexander Verghese, Tahiya Alam, Yohannes Habtegiorgis Abate, Hedayat Abbastabar, Samar Abd ElHafeez, Michael Abdelmasseh, Sherief Abd-Elsalam, Daba Abdissa, Meriem Abdoun, Rizwan Suliankatchi Abdulkader, Mesfin Abebe, Aidin Abedi, Hassan Abidi, Olumide Abiodun, Richard Gyan Aboagye, Hassan Abolhassani, Michael R M Abrigo, Eman Abu-Gharbieh, Niveen ME Abu-Rmeileh, Mesafint Molla Adane, Isaac Yeboah Addo, Bulcha Guye Adema, Miracle Ayomikun Adesina, Charles Oluwaseun Oluwaseun Adetunji, Daniel Adedayo Adeyinka, Qorinah Estiningtyas Sakilah Adnani, Saira Afzal, Suneth Buddhika Agampodi, Antonella Agodi, Williams Agyemang-Duah, Bright Opoku Ahinkorah, Aqeel Ahmad, Danish Ahmad, Ali Ahmadi, Ayman Ahmed, Haroon Ahmed, Luai A Ahmed, Marjan Ajami, Karolina Akinosoglou, Syed Mahfuz Al Hasan, Ziyad Al-Aly, Khurshid Alam, Fahad Mashhour Alanezi, Turki M Alanzi, Mohammed Albashtawy, Sharifullah Alemi, Abdelazeem M Algammal, Adel Ali Saeed Al-Gheethi, Abid Ali, Liaqat Ali, Mohammed Usman Ali, Sheikh Mohammad Alif, Syed Mohamed Aljunid, Joseph Uy Almazan, Hesham M Al-Mekhlafi, Louay Almidani, Sami Almustanyir, Khalid A Altirkawi, Hany Aly, Safwat Aly, Reza Amani, Edward Kwabena Ameyaw, Abebe Feyissa Amhare, Tarek Tawfik Amin, Sohrab Amiri, Catalina Liliana Andrei, Tudorel Andrei, Amir Anoushiravani, Adnan Ansar, Davood Anvari, Razique Anwer, Francis Appiah, Morteza Arab-Zozani, Aleksandr Y Aravkin, Demelash Areda, Brhane Berhe Aregawi, Anton A Artamonov, Umesh Raj Aryal, Zatollah Asemi, Mulu Tiruneh Asemu, Akeza Awealom Asgedom, Tahira Ashraf, Melash Belachew Asresie, Daniel Atlaw, Maha Moh'd Wahbi Atout, Alok Atreya, Madhu Sudhan Atteraya, Avinash Aujayeb, Beatriz Paulina Ayala Quintanilla, Haleh Ayatollahi, Seyed Mohammad Ayyoubzadeh, Sina Azadnajafabad, Rui M S Azevedo, Ahmed Y Azzam, Darshan B B, Mahsa Babaei, Muhammad Badar, Ashish D Badiye, Nayereh Baghcheghi, Soroush Baghdadi, Nasser Bagheri, Sara Bagherieh, Farshad Bahrami Asl, Ruhai Bai, Ravleen Kaur Bakshi, Kiran Bam, Maciej Banach, Aduragbemi Banke-Thomas, Hansi Bansal, Berihun Bantie Bantie, Martina Barchitta, Mainak Bardhan, Azadeh Bashiri, Afisu Basiru, Pritish Baskaran, Kavita Batra, Mojtaba Bayani, Nebiyou Simegnew Bayleyegn, Neeraj Bedi, Tahmina Begum, Amir Hossein Behnoush, Uzma Iqbal Belgaumi, Amiel Nazer C Bermudez, Kebede A Beyene, Bharti Bhandari Bhandari, Dinesh Bhandari, Nikha Bhardwaj, Pankaj Bhardwaj, Sonu Bhaskar, Suraj Bhattarai, Virginia Bodolica, Dejana Braithwaite, Hermann Brenner, Yasser Bustanji, Nadeem Shafique Butt, Zahid A Butt, Abdul Cadri, Ismael Campos-Nonato, Maria Sofia Cattaruzza, Francieli Cembranel, Ester Cerin, Pamela Roxana Chacón-Uscamaita, Jaykaran Charan, Vijay Kumar Chattu, Dhun Chauhan, Malizgani Paul Chavula, Simiao Chen, Gerald Chi, Abdulaal Chitheer, William C S Cho, Sonali Gajanan Choudhari, Dinh-Toi Chu, Natalia Cruz-Martins, Omid Dadras, Gizachew Worku Dagnew, Maxwell Ayindenaba Dalaba, Lalit Dandona, Aso Mohammad Darwesh, Jai K Das, Saswati Das, Nihar Ranjan Dash, Claudio Alberto Dávila-Cervantes, Kairat Davletov, Berhanu Gidisa Debela, Aklilu Tamire Debele, Msganaw Derese, Kebede Deribe, Emina Dervišević, Anteneh Mengist Dessie, Arkadeep Dhali, Vishal R Dhulipala, M Ashworth Dirac, Wanyue Dong, Bezabih Terefe Dora, Haneil Larson Dsouza, Andre Rodrigues Duraes, Sulagna Dutta, Arkadiusz Marian Dziedzic, Abdelaziz Ed-Dra, Kristina Edvardsson, Ebrahim Eini, Michael Ekholuenetale, Maysaa El Sayed Zaki, Islam Y Elgendy, Muhammed Elhadi, Mohammed Elshaer, Ibrahim Elsohaby, Theophilus I Emeto, Luchuo Engelbert Bain, Hawi Leul Esayas, Babak Eshrati, Francesco Esposito, Adeniyi Francis Fagbamigbe, Ildar Ravisovich Fakhradiyev, Ali Faramarzi, Andre Faro, Ali Fatehizadeh, Ginenus Fekadu, Florian Fischer, Artem Alekseevich Fomenkov, Takeshi Fukumoto, Peter Andras Gaal, Abhay Motiramji Gaidhane, Márió Gajdács, Yaseen Galali, Silvano Gallus, Balasankar Ganesan, Federica Gazzelloni, Mesfin Gebrehiwot, Amanuel Tesfay Gebremedhin, Teferi Gebru Gebremeskel, Yohannes Fikadu Geda, Kebede Embaye Gezae, Ramy Mohamed Ghazy, Gloria Gheno, Alessandro Gialluisi, Mika Gissler, James C Glasbey, Logan M Glasstetter, Mahaveer Golechha, Pouya Goleij, Davide Golinelli, Michal Grivna, Avirup Guha, Stefano Guicciardi, Hanbing Guo, Sapna Gupta, Veer Bala Gupta, Vivek Kumar Gupta, Sebastian Haller, Rabih Halwani, Samer Hamidi, Alexis J Handal, Josep Maria Haro, Nicholas Nathaniel Hartman, Taufiq Hasan, Ali Hasanpour- Dehkordi, Md Saquib Hasnain, Soheil Hassanipour, Wen-Qiang He, Mohammad Heidari, Brenda Yuliana Herrera-Serna, Claudiu Herteliu, Kamran Hessami, Kamal Hezam, Yuta Hiraike, Ramesh Holla, Md Mahbub Hossain, Hassan Hosseinzadeh, Mehdi Hosseinzadeh, Mihaela Hostiuc, Sorin Hostiuc, Chengxi Hu, Junjie Huang, M Mamun Huda, Md Nazmul Huda, Hong-Han Huynh, Bing-Fang Hwang, Pulwasha Maria Iftikhar, Olayinka Stephen Ilesanmi, Irena M Ilic, Milena D Ilic, Mustapha Immurana, Arad Iranmehr, Farideh Iravanpour, Masao Iwagami, Chidozie Declan Iwu, Assefa N Iyasu, Jalil Jaafari, Abdollah Jafarzadeh, Haitham Jahrami, Manthan Dilipkumar Janodia, Nilofer Javadi, Tahereh Javaheri, Sathish Kumar Jayapal, Alelign Tasew Jema, Mohammad Jokar, Nitin Joseph, Charity Ehimwenma Joshua, Mikk Jürisson, Ali Kabir, Zubair Kabir, Ibraheem M Karaye, Hanie Karimi, Hengameh Kasraei, Joonas H Kauppila, Evie Shoshannah Kendal, Mohammad Keykhaei, Nauman Khalid, Faham Khamesipour, M Nuruzzaman Khan, Maseer Khan, Yusra H Khan, Khaled Khatab, Haitham Khatatbeh, Moawiah Mohammad Khatatbeh, Sorour Khateri, Hamid Reza Khayat Kashani, Moein Khormali, Min Seo Kim, Thanh V Kim, Yun Jin Kim, Ruth W Kimokoti, Adnan Kisa, Sezer Kisa, Sonali Kochhar, Ali-Asghar Kolahi, Farzad Kompani, Hamid Reza Koohestani, Soewarta Kosen, Ai Koyanagi, Kewal Krishan, Vijay Krishnamoorthy, Barthelemy Kuate Defo, Raja Amir Hassan Kuchay, Mohammed Kuddus, G Anil Kumar, Om P Kurmi, Carlo La Vecchia, Ben Lacey, Chandrakant Lahariya, Tri Laksono, Dharmesh Kumar Lal, Savita Lasrado, Kamaluddin Latief, Kaveh Latifinaibin, Thao Thi Thu Le, Munjae Lee, Sang-woong Lee, Wei-Chen Lee, Yo Han Lee, Jacopo Lenzi, Ming-Chieh Li, Shanshan Li, Virendra S Ligade, Stephen S Lim, Gang Liu, Jue Liu, Xuefeng Liu, László Lorenzovici, Masoud Lotfizadeh, Ahmed M Afifi, Áurea M Madureira-Carvalho, Laura A Magee, Azeem Majeed, Elaheh Malakan Rad, Kashish Malhotra, Ahmad Azam Malik, Iram Malik, Tauqeer Hussain Mallhi, Joemer C Maravilla, Santi Martini, Francisco Rogerlândio Rogerlândio Martins-Melo, Miquel Martorell, Melvin Barrientos Marzan, Yasith Mathangasinghe, Rita Mattiello, Andrea Maugeri, Mahsa Mayeli, Maryam Mazaheri, Rishi P Mediratta, Kamran Mehrabani-Zeinabad, Gebrekiros Gebremichael Meles, Hadush Negash Meles, Max Alberto Mendez-Lopez, Walter Mendoza, Ritesh G Menezes, Atte Meretoja, Tuomo J Meretoja, Irmina Maria Michalek, Le Huu Nhat Minh, Reza Mirfakhraie, Mojgan Mirghafourvand, Andreea Mirica, Erkin M Mirrakhimov, Moonis Mirza, Eric Mishio Bawa, Sanjeev Misra, Biru Abdissa Mizana, Nouh Saad Mohamed, Sakineh Mohammad-Alizadeh-Charandabi, Ghada Mohammed, Salahuddin Mohammed, Shafiu Mohammed, Ali H Mokdad, Sabrina Molinaro, Sara Momtazmanesh, Lorenzo Monasta, Mohammad Ali Moni, AmirAli Moodi Ghalibaf, Paula Moraga, Negar Morovatdar, Abbas Mosapour, Simin Mouodi, Parsa Mousavi, Ulrich Otto Mueller, Faraz Mughal, Admir Mulita, Francesk Mulita, Moses K Muriithi, Tapas Sadasivan Nair, Hastyar Hama Rashid Najmuldeen, Gopal Nambi, Vinay Nangia, Gustavo G Nascimento, Javaid Nauman, Seyed Aria Nejadghaderi, Mohammad Hadi Nematollahi, Georges Nguefack-Tsague, Josephine W Ngunjiri, Dang H Nguyen, Hau Thi Hien Nguyen, Hien Quang Nguyen, Phat Tuan Nguyen, Robina Khan Niazi, Ali Nikoobar, Lawrence Achilles Nnyanzi, Efaq Ali Noman, Shuhei Nomura, Mamoona Noreen, Dieta Nurrika, Chimezie Igwegbe Nzoputam, Ogochukwu Janet Nzoputam, Bogdan Oancea, Kehinde O Obamiro, Ropo Ebenezer Ogunsakin, Sylvester Reuben Okeke, Akinkunmi Paul Okekunle, Osaretin Christabel Okonji, Patrick Godwin Okwute, Andrew T Olagunju, Babayemi Oluwaseun Olakunde, Matthew Idowu Olatubi, Isaac Iyinoluwa Olufadewa, Bolajoko Olubukunola Olusanya, Michal Ordak, Doris V Ortega-Altamirano, Wael M S Osman, Uchechukwu Levi Osuagwu, Adrian Otoiu, Nikita Otstavnov, Stanislav S Otstavnov, Amel Ouyahia, Mayowa O Owolabi, Alicia Padron-Monedero, Jagadish Rao Padubidri, Adrian Pana, Pragyan Paramita Parija, Romil R Parikh, Ava Pashaei, Sangram Kishor Patel, Shankargouda Patil, Shrikant Pawar, Paolo Pedersini, Veincent Christian Filipino Pepito, Prince Peprah, Gavin Pereira, Jeevan Pereira, Marcos Pereira, Maria Odete Pereira, Arokiasamy Perianayagam, Norberto Perico, Konrad Pesudovs, Ionela-Roxana Petcu, Fanny Emily Petermann-Rocha, Parmida Sadat Pezeshki, Tom Pham, My Kieu Phan, Anil K Philip, Manon Pigeolet, Zahra Zahid Piracha, Vivek Podder, Dimitri Poddighe, Pranil Man Singh Pradhan, Hadi Raeisi Shahraki, Pankaja Raghav, Mosiur Rahman, Vahid Rahmanian, Ivano Raimondo, Shakthi Kumaran Ramasamy, Chhabi Lal Ranabhat, Nemanja Rancic, Chythra R Rao, Sowmya J Rao, Davide Rasella, Ahmed Mustafa Rashid, Reza Rawassizadeh, Elrashdy Moustafa Mohamed Redwan, Giuseppe Remuzzi, Kannan RR Rengasamy, Andre M N Renzaho, Nazila Rezaei, Negar Rezaei, Mohsen Rezaeian, Hannah Elizabeth Robinson-Oden, Leonardo Roever, Peter Rohloff, Luca Ronfani, Godfrey M Rwegerera, Aly M A Saad, Zahra Saadatian, Siamak Sabour, Basema Ahmad Saddik, Malihe Sadeghi, Mohammad Reza Saeb, Umar Saeed, Amene Saghazadeh, Dominic Sagoe, Fatemeh Saheb Sharif-Askari, Narjes Saheb Sharif-Askari, Amirhossein Sahebkar, Harihar Sahoo, Soumya Swaroop Sahoo, Mohamed A Saleh, Sana Salehi, Marwa Rashad Salem, Abdallah M Samy, Rama Krishna Sanjeev, Yaser Sarikhani, Sachin C Sarode, Maheswar Satpathy, Monika Sawhney, Ganesh Kumar Saya, Mete Saylan, Markus P Schlaich, Ione Jayce Ceola Schneider, Art Schuermans, Pallav Sengupta, Subramanian Senthilkumaran, Sadaf G Sepanlou, Dragos Serban, SeyedAhmad SeyedAlinaghi, Allen Seylani, Mahan Shafie, Jaffer Shah, Pritik A Shah, Samiah Shahid, Masood Ali Shaikh, Sunder Sham, Mohd Shanawaz, Mohammed Shannawaz, Mequannent Melaku Sharew, Manoj Sharma, Adithi Shetty, B Suresh Kumar Shetty, Pavanchand H Shetty, Rahman Shiri, Reza Shirkoohi, Siddharudha Shivalli, Sina Shool, Seyed Afshin Shorofi, Kanwar Hamza Shuja, Kerem Shuval, Migbar Mekonnen Sibhat, Negussie Boti Sidamo, João Pedro Silva, Colin R Simpson, Jasvinder A Singh, Paramdeep Singh, Surjit Singh, Natia Skhvitaridze, Bogdan Socea, Abdullah Al Mamun Sohag, Hamidreza Soleimani, Yonatan Solomon, Suhang Song, Yi Song, Michael Spartalis, Chandrashekhar T Sreeramareddy, Andy Stergachis, Muhammad Suleman, Saima Sultana, Haitong Zhe Sun, Jing Sun, Mindy D Szeto, Rafael Tabarés-Seisdedos, Shima Tabatabai, Mohammad Tabish, Majid Taheri, Moslem Taheri Soodejani, Jacques Lukenze Tamuzi, Ker-Kan Tan, Ingan Ukur Tarigan, Razieh Tavakoli Oliaee, Birhan Tsegaw Taye, Yibekal Manaye Tefera, Mohamad-Hani Temsah, Masayuki Teramoto, Wegen Beyene Tesfamariam, Enoch Teye-Kwadjo, Samar Tharwat, Aravind Thavamani, Nihal Thomas, Mariya Vladimirovna Titova, Amir Tiyuri, Roman Topor-Madry, Marcos Roberto Tovani-Palone, Jaya Prasad Tripathy, Samuel Joseph Tromans, Chukwudi S Ubah, Muhammad Umair, Srikanth Umakanthan, Brigid Unim, Asokan Govindaraj Vaithinathan, Sahel Valadan Tahbaz, Mario Valenti, Rohollah Valizadeh, Jef Van den Eynde, Shoban Babu Varthya, Massimiliano Veroux, Georgios-Ioannis Verras, Leonardo Villani, Francesco S Violante, Vasily Vlassov, Mandaras Tariku Walde, Fang Wang, Shu Wang, Yanqing Wang, Yanzhong Wang, Emebet Gashaw Wassie, Kosala Gayan Weerakoon, Asrat Arja Wolde, Xiaoyue Xu, Vikas Yadav, Lin Yang, Yuichiro Yano, Sisay Shewasinad Yehualashet, Siyan Yi, Arzu Yiğit, Vahit Yiğit, Paul Yip, Naohiro Yonemoto, Nazar Zaki, Giulia Zamagni, Burhan Abdullah Zaman, Michael Zastrozhin, Haijun Zhang, Yunquan Zhang, Zhi-Jiang Zhang, Hanqing Zhao, Claire Chenwen Zhong, Magdalena Zielińska, Lilik Zuhriyah, Simon I Hay, Mohsen Naghavi, Christopher J L Murray, Rakhi Dandona, Nicholas J Kassebaum

## Abstract

**Background:**

Stillbirth is a devastating and often avoidable adverse pregnancy outcome. Monitoring stillbirth levels and trends—in a comprehensive manner that leaves no one uncounted—is imperative for continuing progress in pregnancy loss reduction. This analysis, completed as part of the Global Burden of Diseases, Injuries, and Risk Factors Study (GBD) 2021, methodically accounted for different stillbirth definitions with the aim of comprehensively estimating all stillbirths at 20 weeks or longer for 204 countries and territories from 1990 to 2021.

**Methods:**

We extracted data on stillbirths from 11 412 sources across 185 of 204 countries and territories, including 234 surveys, 231 published studies, 1633 vital statistics reports, and 10 585 unique location-year combinations from vital registration systems. Our final dataset comprised 11 different definitions, which were adjusted to match two gestational age thresholds: 20 weeks or longer (reference) and 28 weeks or longer (for comparisons). We modelled the ratio of stillbirth rate to neonatal mortality rate with spatiotemporal Gaussian process regression for each location and year, and then used final GBD 2021 assessments of fertility and all-cause neonatal mortality to calculate total stillbirths. Secondary analyses evaluated the number of stillbirths missed with the more restrictive gestational age definition, trends in stillbirths as a function of Socio-demographic Index, and progress in reducing stillbirths relative to neonatal deaths.

**Findings:**

In 2021, the global stillbirth rate was 23·0 (95% uncertainty interval [UI] 19·7–27·2) per 1000 births (stillbirths plus livebirths) at 20 weeks' gestation or longer, compared to 16·1 (13·9–19·0) per 1000 births at 28 weeks' gestation or longer. The global neonatal mortality rate in 2021 was 17·1 (14·8–19·9) per 1000 livebirths, corresponding to 2·19 million (1·90–2·55) neonatal deaths. The estimated number of stillbirths occurring at 20 weeks' gestation or longer decreased from 5·08 million (95% UI 4·07–6·35) in 1990 to 3·04 million (2·61–3·62) in 2021, corresponding to a 39·8% (31·8–48·0) reduction, which lagged behind a global improvement in neonatal deaths of 45·6% (36·3–53·1) for the same period (down from 4·03 million [3·86–4·22] neonatal deaths in 1990). Stillbirths in south Asia and sub-Saharan Africa comprised 77·4% (2·35 million of 3·04 million) of the global total, an increase from 60·3% (3·07 million of 5·08 million) in 1990. In 2021, 0·926 million (0·792–1·10) stillbirths, corresponding to 30·5% of the global total (3·04 million), occurred between 20 weeks' gestation and 28 weeks' gestation, with substantial variation at the country level.

**Interpretation:**

Despite the gradual global decline in stillbirths between 1990 and 2021, the overall number of stillbirths remains substantially high. Counting all stillbirths is paramount to progress, as nearly a third—close to 1 million in total—are left uncounted at the 28 weeks or longer threshold. Our findings draw attention to the differential progress in reducing stillbirths, with a high burden concentrated in countries with low development status. Scarce data availability and poor data quality constrain our capacity to precisely account for stillbirths in many locations. Addressing inequities in universal maternal health coverage, strengthening the quality of maternal health care, and improving the robustness of data systems are urgently needed to reduce the global burden of stillbirths.

**Funding:**

Bill & Melinda Gates Foundation.

## Introduction

Improvements in maternal, neonatal, and child health have long been the focus of large-scale global public health efforts.^1^, ^2^ Corresponding mortality reduction targets have been the cornerstones of the global health agenda put forward in recent decades through both the Millennium Development Goals (MDGs) and the UN Sustainable Development Goals (SDGs).^3^, ^4^ Stillbirth, meanwhile, has not historically received nearly as much attention despite being a potentially devastating and stigmatising experience for families, and being largely avoidable through improvements in antenatal, pregnancy, and delivery care.^5^, ^6^ This changed when, in 2014, UNICEF and WHO articulated in the Every Newborn Action Plan (ENAP) an absolute goal for every country to reduce their annual stillbirth rate (SBR) to 12 or fewer stillbirths per 1000 livebirths by 2030.^7^, ^8^, ^9^

**Research in context**

**Evidence before this study**

In 2014, UNICEF and WHO's Every Newborn Action Plan (ENAP) set a country target of 12 or fewer stillbirths per 1000 births by 2030, thereby emphasising stillbirth as a major global public health issue. One of the challenges of tracking progress towards the ENAP stillbirth targets, as is common in many topics of global health, is the need for clear definitions of who and what should be counted. In the case of stillbirths, gestational age at the time of fetal death is a paramount consideration. A stillbirth cutoff of 28 weeks' completed gestation or longer has been recommended by WHO for international comparison due to poor data availability in many countries, and this is the definition that was used by the *Lancet* Stillbirth Epidemiology Investigator Group (LSEIG), the UN Inter-agency Group for Child Mortality Estimation (UN IGME), and previous iterations of the Global Burden of Diseases, Injuries, and Risk Factors Study (GBD). Unfortunately, stillbirth assessments restricting to 28 weeks' gestation or longer leave many fetal losses uncounted. Although the International Classification of Diseases, version 11 (ICD-11), released in 2022, articulated a definition of stillbirth to include any fetal death at 22 weeks' gestation or longer, it will be quite some time before the world fully transitions to ICD-11-based data collection and reporting. ICD-10, which was released in 1994 and represents the bulk of recent and contemporary data, has defined stillbirth with a cutoff of 20 weeks' gestation or longer. To our knowledge, no previous estimates have been produced to evaluate the global, regional, and national magnitude of stillbirths at 20 weeks' gestation or longer, and to date, there have been no analyses of stillbirths that comprehensively account for heterogeneous case definitions, leverage assessments of reporting completeness, or are internally consistent with data on livebirths and neonatal deaths.

**Added value of this study**

Here, we provide global estimates of levels of and trends in stillbirths down to 20 weeks' gestation—a time period that is otherwise invisible within previous assessments of pregnancy loss and neonatal mortality. We accomplished this through three important advancements in stillbirth estimation. First, we compiled the largest global dataset on stillbirths, including a total of 11 412 sources from 185 of 204 countries and territories. Second, we developed and implemented Bayesian meta-regression techniques to evaluate and standardise each of 11 different definitions of stillbirth that appeared in the raw data before modelling, rather than lumping definitions based on gestational age and birthweight together and labelling them as equivalent. Third, our incorporation of stillbirth estimation into GBD 2021 allowed us to leverage comprehensive assessments of age-specific death registration completeness and use these assessments to adjust for likely under-reporting patterns of stillbirths. Additionally, we completed secondary analyses to quantify progress towards WHO ENAP goals, to evaluate the historical relationship between stillbirths and Socio-demographic Index (SDI), and to quantify comparative progress in stillbirths and neonatal mortality, thus providing a clear assessment of progress, opportunities, and challenges for the remaining decade of the UN Sustainable Development Goals (SDG) era.

**Implications of all the available evidence**

Of the approximately 3·0 million stillbirths that occurred globally after at least 20 weeks' gestation in 2021, nearly a third occurred before 28 weeks' gestation, highlighting the potential undercounting of stillbirths using a 28 weeks or longer threshold. Stillbirths decreased annually, but the total number of stillbirths affecting women and families remained high, and the ratio of stillbirths to neonatal deaths continued to increase. Only 129 (63%) of 204 countries and territories achieved the 2030 ENAP target for stillbirths occurring at 28 weeks or longer in 2021 (≤12 stillbirths per 1000 births). This means that 75 countries and territories are still working towards this target, of which 42 are in sub-Saharan Africa. Furthermore, based on the 20 weeks or longer threshold, 26 countries currently achieving the target would no longer be achieving it and should continue to focus on stillbirth prevention. As we consider strategies for continued decreases in stillbirths, addressing inequity in universal access to high-quality maternal care, especially antenatal care and delivery in facilities with skilled providers, must be a central goal of the global health community for all countries to reach the ENAP target by 2030. To accurately track progress and prevent misclassification of stillbirths, it is also necessary to ensure that stillbirth reporting is comprehensive and accurately reflects gestational age.

Stillbirth and miscarriage are defined as fetal loss before or during delivery, and their primary differentiation is gestational age at the end of pregnancy;^10^ both are differentiated from abortion by intentionality. Conceptually, threshold definitions used for stillbirth data collection should allow for all pregnancy losses to be counted in a comparable manner, but unfortunately this has not historically been the case. WHO has articulated that 28 weeks or longer of completed gestation (or, if information on gestational age is not available, ≥1000 g in birthweight) is the only threshold of stillbirth that should be tracked for international comparison.^10^ This threshold definition contrasts with that of the International Classification of Diseases, version 10 (ICD-10), which proposes a stillbirth threshold definition of 20 weeks' gestation or longer, and the more recent ICD-11, which defines stillbirths as fetal deaths at 22 weeks of completed gestation or longer (or, if information on gestational age is not available, ≥500 g birthweight).^11^ Although only reporting on fetal deaths at 28 weeks' completed gestation or longer and counting gestational age and birthweight as equivalent stillbirth criteria might be considered pragmatic, this functionally means all fetal deaths occurring between 20 weeks and less than 28 weeks are left uncounted, creates potential comparability issues in allowing for interchange between birthweight and gestational age, and actively ignores the large volume of data that are available to generate complete and comprehensive estimates of all pregnancy losses. Although the ICD-11 definition might more appropriately reflect the limits of extrauterine viability, the reality is that it will be quite some time before ICD-11 supplants ICD-10 as the primary method of data collection and reporting. In this setting, the most appropriate approach to ensure no unintentional pregnancy losses are missed is a gestational age threshold of 20 weeks or longer, as has been adopted by the US Centers for Disease Control and Prevention and more than 30 other countries and territories in reporting stillbirths.

Numerous efforts have previously estimated levels of and trends in stillbirths. These include work by the *Lancet* Stillbirth Epidemiology Investigator Group (LSEIG), which estimated levels and trends for 195 countries in the years 2000 and 2015, and the UN Inter-agency Group for Child Mortality Estimation (IGME), which published new estimates in January, 2023, for 2000 to 2021.^12^, ^13^, ^14^ The Global Burden of Diseases, Injuries, and Risk Factors Study (GBD) also used a definition of 28 weeks or longer for GBD 2015 and GBD 2016, introduced data definition adjustment, and modelled data in a manner that ensured internal consistency with corresponding estimates of neonatal mortality, but this analysis has not been updated since GBD 2016.^15^, ^16^

Comprehensive and timely accounting of levels of and trends in stillbirths is crucial for monitoring progress towards ENAP's SBR target of 12 or fewer stillbirths by 2030, complementing other assessments of maternal, neonatal, and child health, and identifying priorities and opportunities for further investment in population health and health system strengthening.^17^ We therefore undertook this analysis to update and improve upon previous stillbirth assessments, with an explicit aim of estimating stillbirths with the most inclusive gestation threshold of 20 weeks or longer. In addition to substantially increasing the size of our input dataset, we implemented new methods to account for different stillbirth definitions over time and geography and leveraged the collective advances between GBD 2016 and GBD 2021, including internally consistent estimates of fertility, population, and all-cause mortality, as well as updated completeness assessments for all data sources.^18^, ^19^ To help facilitate comparisons and highlight the importance of full enumeration of fetal loss, we also produced stillbirth estimates at 22 weeks or longer (the ICD-11 threshold) and 28 weeks or longer (the WHO benchmarking threshold). This manuscript was produced as part of the GBD Collaborator Network and in accordance with the GBD Protocol.^20^

## Methods

### Overview

A detailed description of all analytical procedures is given in appendix 1. Below is a summary of each of the main components of our stillbirth analysis. We chose to model the ratio of SBR over neonatal mortality rate (NMR)—where NMR is defined as the number of deaths in the first 28 days over the number of livebirths—to leverage the extensive GBD 2021 efforts to maximise data quality, estimate and correct for completeness, and generate internally consistent estimates of fertility, all-cause mortality, and population size. At the end of this process, NMR data and results are inclusive of all deaths following livebirths so building on this relationship ensures all of these insights are propagated into SBR estimates. Our final estimation spans from 1990 to 2021 and covers 204 countries and territories, including 22 with subnational locations.

This study complies with the GATHER recommendations.^21^ The GATHER checklist is included in appendix 1 (table S1). Input data sources are shown for each reporting location in appendix 2 (figure S4). Input data sources and results are available for download from the Global Health Data Exchange.

### Definitions and data seeking

To maximise both the comparability and comprehensiveness of our estimates, our analysis estimated stillbirths for three gestational age thresholds. A model estimating stillbirths at 20 weeks or longer was our primary model representing full enumeration of fetal loss, and we added modelled estimates at both 22 weeks or longer (the ICD-11 threshold) and 28 weeks or longer (the WHO benchmarking threshold) for additional comparisons. A full list of definitions is included in appendix 1 (table S2).

Our data seeking built on the approach used throughout GBD: namely, to identify, review, and extract all available data sources globally. For stillbirths, this included household surveys, national reports, vital registration, sample registration, and any additional sources listed on the Global Health Data Exchange, supplemented with published studies that were representative of the general population, identified through a systematic literature review through PubMed (appendix 1 figure S2).

Data were extracted for the following univariate categorisations of fetal death: completed gestation of 20 weeks or longer, 22 weeks or longer, 24 weeks or longer, 26 weeks or longer, or 28 weeks or longer; and birthweight of 500 g or greater and 1000 g or greater. We also included data reported with a combination of criteria (≥22 weeks or ≥500 g, ≥28 weeks or ≥1000 g, ≥22 weeks and ≥500 g, and ≥28 weeks and ≥1000 g). Whenever multiple definitions were reported, each was extracted as a separate observation. Uncommonly used thresholds outside those listed above (eg, ≥12 weeks, ≥32 weeks, and ≥2000 g) were excluded. Sources reporting on only lifetime incidence of stillbirth were excluded as well since the stillbirth counts could not be split into annual data. About 20% of reported observations do not have explicit documentation of their case definition, so we manually assigned them in comparison with their closest neighbours in time and space. Most (80%) observations with missing definitions were assigned to the 28 weeks or longer definition, with nearly all the rest (20%) assigned to the 22 weeks or longer definition, rather than the 20 weeks or longer definition, since 22 weeks or longer was more commonly seen among other datasets from the same locations. Of these, only data from eight locations had no other data with a known definition.

In total, we extracted stillbirth data from 11 412 sources across 185 of 204 countries and territories, including 234 surveys, 231 published studies, 1633 vital statistics reports, and for 10 585 unique location-year combinations of vital registration. The 19 countries with no data were Bhutan, Central African Republic, Chad, Djibouti, Dominica, Equatorial Guinea, Eritrea, Federated States of Micronesia, Laos, Libya, Nauru, Niue, North Korea, São Tomé and Príncipe, Somalia, South Sudan, Syria, Tokelau, and Tuvalu. appendix 1 (tables S3 and S4) shows the distribution of data by definition and location, and also maps available data by source type (figure S3).

### Data processing

We completed several data processing steps to standardise and deduplicate input data before modelling for each definition (appendix 1 figure S1). First, we adjusted surveys reporting only period incidence of stillbirths by applying the ratio of the number of women with a birth in the previous 5 years over the total number of births observed among this group to the reported SBR. Second, we adjusted all SBR data using source type-location-year-specific completeness estimates from the GBD 2021 demographics analysis. Third, we matched each observation of SBR with a GBD 2021 NMR (neonatal [<28 days] deaths per 1000 livebirths) estimate from the same location-year to calculate the SBR to NMR ratio. Next, we developed statistical crosswalk models to standardise all data to each of the three gestational age thresholds (≥20, >22, or ≥28 weeks). Crosswalks allowed us to impute the implied value of a data point if it were to meet our case definition, thereby rendering it comparable to the other data points in the model and allowing us to incorporate as much input data as possible. This started with excluding observations considered implausible by any of the following criteria: SBR less than 1 (per 1000 births), SBR greater than 200 (per 1000 births), SBR greater than 50 (per 1000 births) for the high-income GBD super-region, observation from a location-year with a major mortality shock where the with-shock death rate among all ages of the population was more than 500 per 100 000 people, and an SBR to NMR ratio less than 0·5 (appendix 1 table S5). Then, for each reference definition (≥20, ≥22, or ≥28 weeks), we made direct comparisons by pairing data points with the specified reference definition to data points with alternate definitions based on location-year and source. By calculating the mean ratio of each matched pair and standard error (SE) of the ratio using the delta method, we could adjust the data points with alternate definitions to the reference definition. This process was repeated for indirect comparisons (eg, ≥26 weeks to ≥24 weeks) to maximise the size of the dataset (appendix 1 tables S6 and S7).^22^

Logit-transformed means and SEs were then analysed with a flexible network meta-regression tool called meta-regression Bayesian, regularised, trimmed (MR-BRT) to calculate the pooled difference in the SBR to NMR ratio between the different definitions.^23^ We included ordinal priors to ensure relationships between effect sizes (eg, ≥22 weeks will have a higher SBR than ≥24 weeks) and also a fixed effect of summary exposure values (SEVs) for short gestation for birthweight from GBD 2019 to account for location-specific differences (appendix 1 table S8).^24^ appendix 1 (figure S4) illustrates MR-BRT outputs that were used to crosswalk all data to the corresponding reference definitions, including uncertainty, by location, year, and definition. This was followed by another round of systematic removal of outliers following the same criteria as above. Finally, we deduplicated for sources where multiple different definitions were extracted and for location-years covered by both vital registration (preferred) and tabulated vital statistics reports. appendix 1 (figure S5) maps the final distribution of the proportion of outliers within the dataset and the volume of included data by location.

### Modelling the SBR to NMR ratio

We implemented a comprehensive, three-stage modelling process that consisted of an ensemble linear mixed-effects model (stage 1), spatiotemporal smoothing (stage 2), and Gaussian process regression (GPR; stage 3; appendix 1 section 6, appendix 2 figure S1). To enhance the predictive accuracy of the stage 1 model, we use an approach that ranks models built from all possible combinations of candidate covariates and then combines the highest ranked models into a single ensemble. Every combination of ten candidate covariates—selected from among the most predictive and influential for GBD cause-specific mortality models of neonatal and maternal disorders—was tested in the ensemble model, and we retained only those with statistically significant beta coefficients in the pre-specified direction (appendix 1 table S9).^25^, ^26^ The full set of candidate covariates included the proportion of births with one or more visits of antenatal care; four or more visits of antenatal care; in-facility delivery; skilled birth attendance; maternal care and immunisation (a composite metric of vaccine and maternal care coverage); Healthcare Access and Quality (HAQ) Index; Socio-demographic Index (SDI; a composite of total fertility rate in those aged <25 years, mean years of education for those aged ≥15 years, and per-capita income); maternal education (years per capita); population with at least 12 years of education (among women of reproductive age); and education relative inequality (Gini coefficient). Retained models were ranked by out-of-sample predictive validity, and the top 50 were combined into a final ensemble model to produce initial estimates. The second stage, spatiotemporal smoothing, used time and space weight hyperparameters based on data density, and residuals were combined with stage 1 predictions to better reflect local trends (appendix 1 table S10). The final stage, GPR, incorporated data, data variance, a scale parameter, an amplitude parameter, and a prior to smooth residuals and generate final estimates of the SBR to NMR ratio. NMR estimates were used to transform to SBR, and stillbirth counts were aggregated to produce results for the regional, super-regional, and global locations. appendix 2 (figure S4) shows a complete set of results and data inputs with definitions for each GBD reporting location.

### Uncertainty

These estimates reflect uncertainty in input data, variable sample size, crosswalks from non-reference definitions, and spatiotemporal-GPR results. The model uncertainty was derived by generating 1000 draws of the SBR to NMR ratio for each location. The means of these draw-level estimates were used as the final estimates. The 95% uncertainty intervals (UIs) for our estimates were assigned on the basis of 0·025 and 0·975 quantiles of the draws, which were also used to test for statistical significance.

Count data (ie, total stillbirths) are presented to three significant figures and rates are presented to 1 decimal place.

### Role of the funding source

The funder of the study had no role in study design, data collection, data analysis, data interpretation, writing of the manuscript, or the decision to submit the manuscript for publication.

## Results

### Total number of stillbirths, SBR, and time trends

**Figure 1:**
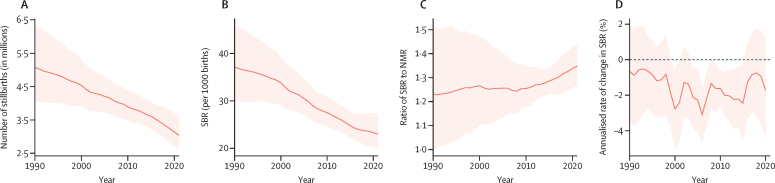
Global trends in the number of stillbirths, SBR, SBR to NMR ratio, and annualised rate of change in SBR, 1990–2021 (A) The solid line represents estimates for the total number of stillbirths (in millions) for the 20 weeks or longer definition, and the shaded area indicates the 95% UI, between 1990 and 2021. (B) The solid line represents the SBR estimates per 1000 births for the 20 weeks or longer definition, and the shaded area indicates the 95% UI, between 1990 and 2021. (C) The solid line represents the SBR to NMR ratio estimates (neonatal deaths per 1000 livebirths) for the 20 weeks or longer definition, and the shaded area indicates the 95% UI, between 1990 and 2021. (D) The solid line represents the annualised rate of change in SBR between adjacent years for the 20 weeks or longer definition, and the shaded area indicates the 95% UI, between 1990 and 2021. NMR=neonatal mortality rate. SBR=stillbirth rate (stillbirths at ≥20 weeks' gestation per 1000 births). UI=uncertainty interval.

Stillbirths and SBR have steadily declined globally over the past three decades (figure 1). The estimated number of stillbirths occurring at 20 weeks' gestation or longer decreased from 5·08 million (95% UI 4·07–6·35) in 1990 to 4·54 million (3·91–5·30) in 2000, 3·61 million (3·23–4·02) in 2015, and 3·04 million (2·61–3·62) in 2021; this corresponds to a decline of 39·8% (31·8–48·0) between 1990 and 2021. SBR similarly declined globally, dropping from 37·1 (30·0–46·0) per 1000 births in 1990 to 33·8 (29·2–39·2) per 1000 births in 2000, 24·8 (22·3–27·6) per 1000 births in 2015, and 23·0 (19·7–27·2) per 1000 births in 2021, corresponding to a total decline of 37·8% (29·9–46·0) between 1990 and 2021. In comparison, the global SBR in 2021 was 22·1 (19·1–26·2) per 1000 births at 22 weeks' gestation or longer and 16·1 (13·9–19·0) per 1000 births at 28 weeks' gestation or longer.

The annualised rate of change was always negative, indicating a decreasing global SBR annually for every year over the past three decades. Although the rate varied by year, SBR declined on average by about 1·5% annually. The first 10-year period, 1990 to 2000, saw an average decrease in the SBR of 0·9% (0·2–2·2) per year. In the early 2000s, there was wide variation in the annual decrease in SBR. This period, 2000 to 2010, had an average annual decrease of 2·1% (1·2–3·0) per year. Most recently, from 2010 to 2021, the SBR continued to decrease at varying levels but averaged at a 1·6% (0·6–2·7) decline per year (figure 1D).

The burden of stillbirths is unequally distributed around the world and concentrated in certain regions (table; appendix 2 figures S1 and S2). While south Asia contributed almost a third (30·3%) of the total number of stillbirths at 20 weeks' gestation or longer in 2021 (0·922 [95% UI 0·764–1·13] of 3·04 million), the region was ranked fifth highest in terms of SBR compared with other regions. Together, the regions of western and eastern sub-Saharan Africa contributed more than another third, (25·5% [0·775 of 3·04 million] for western sub-Saharan Africa and 15·0% [0·456 of 3·04 million] for eastern sub-Saharan Africa), and were ranked first and third, respectively, according to SBR. The global reduction in SBR between 1990 and 2021 was 39%, but only 36% in sub-Saharan Africa and 20% in central Europe, eastern Europe, and central Asia.

**Table:** Global, SDI quintile, super-regional, regional, and country-level stillbirth counts and rates for 20 weeks' gestation or longer in 1990, 2000, 2015, 2020, and 2021

			**Total stillbirths (in thousands)**					**Stillbirth rate (per 1000 births)**				
			1990	2000	2015	2020	2021	1990	2000	2015	2020	2021
**Global**			**5080 (4070–6350)**	**4540 (3910–5300)**	**3610 (3230–4020)**	**3140 (2710–3700)**	**3040 (2610–3620)**	**37·1 (30·0–46·0)**	**33·8 (29·2–39·2)**	**24·8 (22·3–27·6)**	**23·3 (20·3–27·4)**	**23·0 (19·7–27·2)**
	Low SDI		1420 (1110–1800)	1480 (1250–1750)	1460 (1320–1620)	1390 (1210–1620)	1370 (1180–1610)	60·5 (48·0–75·9)	50·7 (43·2–59·6)	40·7 (37·0–44·9)	37·2 (32·6–43·2)	36·3 (31·5–42·5)
	Low-middle SDI		2040 (1600–2580)	1900 (1640–2230)	1340 (1180–1520)	1120 (946–1330)	1070 (904–1300)	51·1 (40·6–63·9)	45·2 (39·1–52·7)	30·9 (27·4–34·9)	27·3 (23·2–32·4)	26·6 (22·5–32·0)
	Middle SDI		1150 (919–1450)	844 (714–1010)	616 (534–714)	490 (416–586)	465 (392–560)	27·1 (21·7–33·8)	22·7 (19·3–27·2)	15·8 (13·7–18·2)	14·6 (12·4–17·4)	14·3 (12·1–17·2)
	High-middle SDI		370 (302–456)	232 (187–294)	125 (108–148)	86·4 (73·8–103)	79·9 (67·3–95·7)	20·0 (16·4–24·5)	16·0 (12·9–20·2)	8·1 (7·0–9·5)	7·0 (6·0–8·4)	6·8 (5·8–8·2)
	High SDI		103 (94·0–114)	78·7 (74·4–83·4)	65·9 (62·4–69·8)	56·0 (50·0–62·7)	53·7 (45·9–62·6)	8·2 (7·5–9·1)	6·9 (6·5–7·3)	5·8 (5·4–6·1)	5·4 (4·8–6·1)	5·2 (4·5–6·1)
**Central Europe, eastern Europe, and central Asia**			**93·4 (85·2–103)**	**57·3 (54·0–60·6)**	**67·9 (64·7–71·2)**	**57·0 (51·7–62·9)**	**56·0 (48·8–64·1)**	**13·9 (12·7–15·2)**	**12·3 (11·6–13·0)**	**11·7 (11·2–12·3)**	**11·2 (10·2–12·3)**	**11·3 (9·8–12·9)**
	Central Asia		38·8 (33·7–45·5)	29·4 (26·5–32·5)	43·7 (41·5–45·9)	38·9 (34·4–43·6)	38·7 (32·8–45·4)	19·3 (16·8–22·6)	19·6 (17·8–21·7)	20·9 (19·9–21·9)	18·2 (16·2–20·4)	18·3 (15·6–21·4)
		Armenia	1·65 (1·51–1·81)	0·872 (0·744–1·02)	1·09 (0·936–1·28)	0·849 (0·741–0·963)	0·801 (0·668–0·958)	21·1 (19·3–23·1)	20·0 (17·1–23·3)	24·3 (20·9–28·5)	22·6 (19·8–25·6)	22·3 (18·7–26·6)
		Azerbaijan	5·36 (4·65–6·20)	3·75 (3·41–4·14)	4·23 (3·90–4·58)	2·99 (2·36–3·74)	2·94 (2·19–3·79)	27·6 (24·1–31·9)	27·3 (24·9–30·0)	24·1 (22·2–26·0)	20·4 (16·2–25·4)	20·7 (15·6–26·6)
		Georgia	1·67 (1·39–1·97)	1·47 (1·32–1·65)	0·549 (0·492–0·613)	0·478 (0·450–0·506)	0·429 (0·390–0·476)	19·3 (16·1–22·7)	28·9 (26·0–32·1)	9·8 (8·8–10·9)	10·1 (9·5–10·6)	9·4 (8·5–10·4)
		Kazakhstan	6·58 (5·84–7·40)	3·86 (3·51–4·24)	4·46 (4·06–4·89)	4·01 (3·39–4·73)	3·78 (2·96–4·81)	17·3 (15·4–19·4)	17·1 (15·6–18·8)	11·1 (10·1–12·2)	9·3 (7·9–11·0)	8·8 (6·9–11·2)
		Kyrgyzstan	3·55 (2·48–5·17)	1·74 (1·38–2·20)	2·08 (1·86–2·33)	1·77 (1·56–2·02)	1·70 (1·41–2·03)	25·9 (18·3–37·4)	15·9 (12·6–20·0)	11·8 (10·6–13·3)	10·8 (9·5–12·3)	10·6 (8·8–12·6)
		Mongolia	1·78 (1·25–2·64)	0·972 (0·707–1·30)	0·885 (0·822–0·953)	0·651 (0·582–0·728)	0·611 (0·496–0·749)	24·2 (17·2–35·6)	17·9 (13·1–23·8)	10·5 (9·8–11·3)	8·0 (7·1–8·9)	7·6 (6·2–9·3)
		Tajikistan	6·35 (5·18–7·70)	6·15 (5·29–7·16)	11·6 (9·86–13·4)	10·1 (7·50–13·1)	10·4 (7·38–14·3)	29·2 (23·9–35·1)	30·3 (26·2–35·1)	39·9 (34·0–45·8)	33·8 (25·4–43·6)	34·9 (25·1–47·5)
		Turkmenistan	2·98 (2·72–3·27)	2·32 (1·91–2·79)	2·23 (1·52–3·09)	2·02 (1·43–2·80)	2·01 (1·41–2·80)	23·4 (21·4–25·7)	20·6 (17·1–24·7)	19·3 (13·3–26·6)	17·8 (12·7–24·5)	17·8 (12·6–24·7)
		Uzbekistan	8·89 (6·33–12·7)	8·22 (6·23–10·7)	16·5 (15·6–17·5)	16·0 (14·3–17·9)	16·0 (13·0–19·9)	12·4 (8·9–17·7)	14·7 (11·2–19·0)	22·2 (20·9–23·4)	19·7 (17·6–22·0)	19·8 (16·2–24·4)
	Central Europe		16·5 (14·0–19·5)	9·63 (9·24–10·1)	6·11 (5·90–6·33)	5·76 (5·36–6·17)	5·34 (4·77–5·97)	9·6 (8·1–11·3)	7·9 (7·6–8·3)	5·3 (5·1–5·5)	5·4 (5·0–5·7)	5·1 (4·6–5·7)
		Albania	0·711 (0·507–0·966)	0·526 (0·381–0·727)	0·270 (0·239–0·301)	0·212 (0·174–0·258)	0·20 (0·160–0·247)	8·9 (6·4–12·1)	10·0 (7·3–13·8)	8·4 (7·4–9·3)	7·4 (6·1–9·0)	7·1 (5·7–8·8)
		Bosnia and Herzegovina	0·860 (0·803–0·920)	0·364 (0·327–0·404)	0·199 (0·184–0·215)	0·128 (0·114–0·145)	0·114 (0·0971–0·134)	12·8 (11·9–13·6)	9·1 (8·2–10·1)	6·5 (6·0–7·0)	4·7 (4·1–5·3)	4·3 (3·6–5·0)
		Bulgaria	0·926 (0·681–1·29)	0·839 (0·776–0·903)	0·688 (0·638–0·741)	0·500 (0·431–0·574)	0·502 (0·391–0·621)	9·1 (6·7–12·6)	11·8 (10·9–12·7)	10·3 (9·6–11·1)	8·3 (7·2–9·5)	8·6 (6·7–10·6)
		Croatia	0·348 (0·323–0·377)	0·274 (0·254–0·296)	0·182 (0·169–0·195)	0·149 (0·125–0·176)	0·144 (0·111–0·181)	6·5 (6·0–7·0)	6·4 (5·9–6·9)	4·8 (4·4–5·1)	4·2 (3·5–4·9)	4·1 (3·2–5·2)
		Czechia	0·873 (0·797–0·950)	0·367 (0·329–0·406)	0·370 (0·329–0·416)	0·456 (0·398–0·519)	0·428 (0·354–0·513)	6·7 (6·2–7·3)	4·0 (3·6–4·4)	3·3 (2·9–3·7)	4·1 (3·6–4·7)	4·0 (3·3–4·8)
		Hungary	1·04 (0·738–1·49)	0·700 (0·630–0·782)	0·574 (0·516–0·639)	0·545 (0·457–0·639)	0·513 (0·400–0·642)	8·3 (5·9–11·8)	7·2 (6·5–8·1)	6·1 (5·5–6·8)	6·0 (5·0–7·0)	5·8 (4·5–7·2)
		Montenegro	0·0830 (0·0600–0·114)	0·0597 (0·0499–0·0710)	0·0296 (0·0227–0·0381)	0·0224 (0·0160–0·0308)	0·0214 (0·0153–0·0298)	8·6 (6·2–11·8)	6·8 (5·7–8·1)	3·9 (3·0–5·0)	3·2 (2·3–4·3)	3·1 (2·2–4·3)
		North Macedonia	0·559 (0·422–0·718)	0·469 (0·362–0·600)	0·328 (0·292–0·370)	0·248 (0·218–0·282)	0·213 (0·171–0·261)	15·8 (12·0–20·2)	16·6 (12·9–21·2)	14·2 (12·6–16·0)	12·7 (11·2–14·4)	11·3 (9·1–13·8)
		Poland	5·43 (3·96–7·18)	2·54 (2·35–2·75)	1·24 (1·13–1·36)	1·36 (1·21–1·54)	1·22 (0·983–1·51)	9·8 (7·2–13·0)	6·8 (6·3–7·4)	3·2 (3·0–3·6)	3·8 (3·4–4·3)	3·6 (2·9–4·4)
		Romania	2·89 (2·69–3·09)	1·95 (1·83–2·07)	1·12 (1·05–1·20)	1·06 (0·946–1·18)	0·978 (0·788–1·20)	9·6 (9·0–10·3)	8·7 (8·2–9·3)	5·7 (5·3–6·1)	5·8 (5·2–6·5)	5·5 (4·4–6·7)
		Serbia	1·74 (1·24–2·46)	0·861 (0·748–0·991)	0·558 (0·519–0·597)	0·546 (0·494–0·600)	0·502 (0·432–0·580)	12·0 (8·6–16·9)	8·2 (7·1–9·4)	8·4 (7·8–9·0)	8·7 (7·9–9·5)	8·1 (7·0–9·3)
		Slovakia	0·632 (0·479–0·820)	0·429 (0·394–0·466)	0·344 (0·318–0·371)	0·340 (0·312–0·373)	0·329 (0·284–0·378)	7·9 (6·0–10·3)	8·0 (7·4–8·7)	6·0 (5·5–6·5)	5·9 (5·4–6·5)	5·8 (5·0–6·7)
		Slovenia	0·166 (0·139–0·195)	0·111 (0·101–0·122)	0·106 (0·0973–0·115)	0·107 (0·0964–0·117)	0·0972 (0·0848–0·112)	7·5 (6·3–8·8)	6·2 (5·6–6·8)	5·1 (4·7–5·5)	5·6 (5·0–6·1)	5·2 (4·5–5·9)
	Eastern Europe		38·1 (35·7–40·4)	18·3 (17·3–19·3)	18·1 (16·7–19·6)	12·4 (10·9–14·1)	12·0 (9·64–14·8)	12·7 (12·0–13·5)	9·4 (8·9–10·0)	7·1 (6·6–7·7)	6·6 (5·8–7·5)	6·6 (5·3–8·2)
		Belarus	1·46 (1·28–1·66)	0·830 (0·763–0·898)	0·541 (0·504–0·579)	0·397 (0·309–0·501)	0·362 (0·271–0·474)	10·4 (9·1–11·8)	8·9 (8·2–9·7)	4·6 (4·3–4·9)	4·6 (3·6–5·8)	4·4 (3·3–5·7)
		Estonia	0·239 (0·204–0·278)	0·0885 (0·0789–0·0987)	0·0493 (0·0444–0·0549)	0·0328 (0·0291–0·0366)	0·0329 (0·0278–0·0387)	11·2 (9·6–13·1)	6·9 (6·1–7·7)	3·5 (3·2–3·9)	2·4 (2·2–2·7)	2·5 (2·1–2·9)
		Latvia	0·301 (0·266–0·338)	0·157 (0·145–0·171)	0·125 (0·115–0·135)	0·0970 (0·0856–0·110)	0·0900 (0·0716–0·112)	8·2 (7·3–9·3)	7·8 (7·2–8·5)	5·6 (5·2–6·1)	5·5 (4·9–6·2)	5·3 (4·2–6·6)
		Lithuania	0·407 (0·371–0·446)	0·241 (0·222–0·261)	0·147 (0·135–0·158)	0·110 (0·0953–0·126)	0·106 (0·0840–0·132)	7·3 (6·6–7·9)	7·2 (6·6–7·8)	4·7 (4·4–5·1)	4·4 (3·8–5·1)	4·5 (3·5–5·6)
		Moldova	1·12 (1·05–1·20)	0·623 (0·556–0·691)	0·312 (0·292–0·334)	0·272 (0·254–0·291)	0·253 (0·225–0·282)	14·0 (13·1–14·9)	14·8 (13·3–16·4)	8·5 (8·0–9·1)	9·1 (8·5–9·8)	8·8 (7·9–9·9)
		Russia	25·7 (23·6–27·8)	13·5 (12·5–14·5)	13·5 (12·5–14·4)	9·42 (8·32–10·6)	9·24 (7·39–11·4)	12·9 (11·9–14·0)	10·0 (9·3–10·8)	7·1 (6·5–7·6)	6·6 (5·9–7·5)	6·8 (5·4–8·3)
		Ukraine	8·88 (8·21–9·58)	2·86 (2·66–3·07)	3·44 (2·52–4·64)	2·06 (1·44–2·81)	1·87 (1·33–2·57)	13·2 (12·3–14·3)	7·4 (6·9–8·0)	8·3 (6·1–11·1)	7·0 (4·9–9·5)	6·7 (4·8–9·1)
**High income**			**96·5 (88·6–107)**	**71·3 (69·5–73·3)**	**59·3 (57·2–61·8)**	**51·1 (46·2–56·1)**	**48·9 (42·4–56·3)**	**7·7 (7·1–8·5)**	**6·0 (5·8–6·2)**	**5·1 (4·9–5·3)**	**4·9 (4·4–5·4)**	**4·7 (4·1–5·4)**
	Australasia		3·15 (2·65–3·76)	1·83 (1·74–1·91)	2·69 (2·19–3·34)	2·70 (1·93–3·65)	2·42 (1·70–3·37)	9·9 (8·3–11·8)	6·0 (5·8–6·3)	7·3 (6·0–9·1)	7·6 (5·4–10·2)	6·7 (4·7–9·3)
		Australia	2·08 (1·59–2·71)	1·23 (1·16–1·31)	2·13 (1·64–2·79)	2·26 (1·60–3·09)	1·93 (1·35–2·69)	8·1 (6·2–10·5)	5·0 (4·7–5·4)	7·0 (5·4–9·1)	7·6 (5·4–10·3)	6·4 (4·5–8·9)
		New Zealand	1·07 (1·02–1·12)	0·595 (0·564–0·625)	0·560 (0·532–0·590)	0·437 (0·323–0·588)	0·483 (0·344–0·669)	17·5 (16·7–18·4)	10·4 (9·9–10·9)	9·3 (8·8–9·8)	7·5 (5·5–10·0)	8·2 (5·8–11·3)
	High-income Asia Pacific		10·3 (7·56–13·6)	7·10 (6·39–8·02)	3·88 (3·73–4·03)	2·67 (2·32–3·07)	2·42 (1·98–2·90)	5·2 (3·8–6·9)	3·9 (3·5–4·4)	2·6 (2·5–2·7)	2·2 (1·9–2·6)	2·1 (1·7–2·5)
		Brunei	0·0628 (0·0448–0·0891)	0·0595 (0·0521–0·0680)	0·0541 (0·0478–0·0613)	0·0549 (0·0414–0·0715)	0·0519 (0·0387–0·0689)	8·9 (6·4–12·6)	8·2 (7·1–9·3)	8·2 (7·3–9·3)	8·5 (6·4–11·1)	8·1 (6·0–10·7)
		Japan	5·69 (4·00–7·67)	4·23 (4·02–4·47)	2·48 (2·35–2·61)	1·74 (1·43–2·11)	1·65 (1·24–2·11)	4·6 (3·3–6·2)	3·6 (3·4–3·8)	2·5 (2·3–2·6)	2·1 (1·7–2·5)	2·0 (1·5–2·5)
		Singapore	0·461 (0·331–0·632)	0·307 (0·284–0·331)	0·207 (0·194–0·221)	0·170 (0·159–0·182)	0·171 (0·152–0·192)	8·5 (6·1–11·6)	5·2 (4·8–5·7)	3·4 (3·2–3·6)	3·0 (2·8–3·2)	3·1 (2·7–3·4)
		South Korea	4·09 (2·88–5·85)	2·50 (1·85–3·39)	1·14 (1·07–1·22)	0·703 (0·598–0·817)	0·549 (0·453–0·661)	6·0 (4·2–8·5)	4·3 (3·2–5·8)	2·8 (2·6–3·0)	2·5 (2·1–2·9)	2·0 (1·7–2·4)
	High-income North America		34·3 (32·7–35·9)	26·9 (25·9–28·0)	24·0 (22·9–25·0)	21·2 (18·7–23·7)	19·9 (16·3–24·1)	7·5 (7·2–7·9)	6·1 (5·9–6·4)	5·5 (5·2–5·7)	5·3 (4·6–5·9)	4·9 (4·0–6·0)
		Canada	2·50 (1·97–3·15)	1·62 (1·49–1·76)	2·13 (1·80–2·50)	2·28 (1·85–2·76)	2·11 (1·66–2·64)	6·2 (4·9–7·8)	4·9 (4·5–5·3)	5·6 (4·7–6·5)	6·3 (5·1–7·6)	5·8 (4·6–7·2)
		Greenland	0·0236 (0·0170–0·0323)	0·0139 (0·0102–0·0186)	0·00774 (0·00552–0·0109)	0·00725 (0·00513–0·0102)	0·00619 (0·00440–0·00885)	18·6 (13·4–25·2)	14·6 (10·8–19·4)	9·2 (6·6–12·9)	9·0 (6·4–12·6)	7·9 (5·6–11·2)
		USA	31·7 (30·3–33·2)	25·3 (24·3–26·4)	21·8 (20·9–22·7)	18·9 (16·6–21·3)	17·8 (14·4–21·7)	7·6 (7·3–8·0)	6·2 (6·0–6·5)	5·5 (5·2–5·7)	5·2 (4·5–5·8)	4·8 (3·9–5·9)
	Southern Latin America		15·2 (12·7–18·6)	10·7 (10·2–11·3)	7·43 (7·04–7·82)	5·42 (5·09–5·80)	5·19 (4·70–5·70)	14·5 (12·1–17·7)	10·7 (10·2–11·3)	7·2 (6·8–7·5)	6·9 (6·5–7·4)	6·7 (6·1–7·4)
		Argentina	8·59 (6·16–11·8)	7·45 (6·96–7·95)	5·87 (5·51–6·26)	4·43 (4·16–4·70)	4·29 (3·88–4·75)	12·5 (9·0–17·1)	10·7 (10·0–11·4)	7·8 (7·3–8·3)	8·1 (7·6–8·6)	7·9 (7·2–8·8)
		Chile	5·85 (5·31–6·45)	2·52 (2·30–2·77)	1·28 (1·17–1·41)	0·803 (0·629–1·01)	0·723 (0·541–0·939)	19·2 (17·5–21·2)	9·9 (9·0–10·9)	5·4 (4·9–5·9)	4·0 (3·2–5·1)	3·7 (2·7–4·7)
		Uruguay	0·784 (0·596–1·03)	0·772 (0·713–0·836)	0·280 (0·259–0·303)	0·196 (0·157–0·242)	0·176 (0·130–0·228)	14·0 (10·6–18·3)	14·4 (13·3–15·6)	5·9 (5·4–6·3)	5·4 (4·4–6·7)	4·9 (3·7–6·4)
	Western Europe		33·6 (30·6–37·5)	24·7 (23·9–25·6)	21·4 (19·8–23·3)	19·1 (17·2–21·3)	19·0 (16·6–22·0)	7·3 (6·6–8·1)	5·7 (5·5–5·9)	4·8 (4·5–5·3)	4·6 (4·2–5·2)	4·6 (4·0–5·4)
		Andorra	0·00469 (0·00335–0·00658)	0·00257 (0·00216–0·00307)	0·00361 (0·00297–0·00426)	0·00360 (0·00266–0·00473)	0·00213 (0·00151–0·00285)	7·6 (5·4–10·6)	3·6 (3·0–4·3)	5·7 (4·7–6·8)	6·5 (4·8–8·5)	3·9 (2·8–5·2)
		Austria	0·539 (0·503–0·578)	0·351 (0·331–0·373)	0·325 (0·306–0·345)	0·351 (0·315–0·388)	0·326 (0·264–0·399)	5·8 (5·4–6·2)	4·5 (4·2–4·8)	3·8 (3·6–4·0)	4·1 (3·7–4·6)	3·8 (3·1–4·6)
		Belgium	0·999 (0·710–1·37)	0·712 (0·625–0·812)	0·620 (0·538–0·711)	0·579 (0·440–0·745)	0·578 (0·420–0·762)	7·9 (5·7–10·8)	6·1 (5·3–6·9)	5·1 (4·4–5·8)	5·1 (3·9–6·5)	5·1 (3·7–6·7)
		Cyprus	0·181 (0·128–0·247)	0·0877 (0·0636–0·117)	0·0576 (0·0468–0·0696)	0·0518 (0·0402–0·0661)	0·0504 (0·0381–0·0648)	12·2 (8·7–16·6)	7·8 (5·7–10·4)	4·0 (3·2–4·8)	3·4 (2·7–4·4)	3·3 (2·5–4·3)
		Denmark	0·454 (0·396–0·520)	0·441 (0·392–0·498)	0·261 (0·229–0·297)	0·224 (0·167–0·294)	0·223 (0·166–0·298)	7·1 (6·2–8·1)	6·6 (5·9–7·5)	4·4 (3·8–5·0)	3·6 (2·7–4·7)	3·5 (2·6–4·7)
		Finland	0·315 (0·293–0·340)	0·241 (0·224–0·259)	0·165 (0·154–0·176)	0·134 (0·123–0·145)	0·138 (0·120–0·157)	4·8 (4·5–5·2)	4·3 (4·0–4·6)	3·0 (2·8–3·2)	2·8 (2·6–3·0)	2·8 (2·5–3·2)
		France	5·59 (5·23–5·96)	4·49 (4·22–4·78)	4·80 (3·33–6·69)	4·26 (2·94–5·82)	4·17 (2·95–5·76)	7·3 (6·8–7·8)	5·8 (5·4–6·1)	6·3 (4·4–8·8)	6·1 (4·2–8·3)	6·0 (4·2–8·2)
		Germany	5·51 (5·18–5·85)	3·73 (3·52–3·95)	3·26 (3·07–3·46)	4·06 (3·83–4·31)	4·04 (3·64–4·49)	6·4 (6·0–6·7)	4·9 (4·6–5·2)	4·3 (4·0–4·5)	5·1 (4·9–5·5)	5·1 (4·6–5·7)
		Greece	0·895 (0·751–1·05)	0·818 (0·703–0·943)	0·520 (0·449–0·602)	0·603 (0·497–0·734)	0·540 (0·417–0·697)	8·7 (7·3–10·2)	7·9 (6·8–9·1)	5·5 (4·8–6·4)	7·1 (5·9–8·6)	6·5 (5·0–8·4)
		Iceland	0·0243 (0·0212–0·0277)	0·0232 (0·0201–0·0262)	0·0158 (0·0138–0·0179)	0·0197 (0·0172–0·0226)	0·0226 (0·0186–0·0270)	5·2 (4·6–6·0)	5·6 (4·8–6·3)	3·8 (3·3–4·3)	4·3 (3·7–4·9)	4·8 (4·0–5·7)
		Ireland	0·406 (0·373–0·440)	0·388 (0·361–0·418)	0·289 (0·268–0·310)	0·219 (0·175–0·272)	0·219 (0·164–0·295)	7·5 (6·9–8·1)	6·7 (6·2–7·2)	4·4 (4·1–4·7)	3·8 (3·0–4·7)	3·8 (2·8–5·1)
		Israel	0·691 (0·631–0·751)	0·960 (0·899–1·02)	0·977 (0·911–1·05)	0·892 (0·672–1·17)	0·739 (0·547–0·982)	6·6 (6·1–7·2)	7·0 (6·6–7·5)	5·4 (5·1–5·8)	4·9 (3·7–6·4)	4·0 (3·0–5·3)
		Italy	4·36 (3·85–4·89)	2·50 (2·01–3·07)	1·81 (1·64–2·01)	1·34 (1·18–1·51)	1·30 (1·09–1·55)	7·7 (6·8–8·6)	4·6 (3·7–5·6)	3·7 (3·4–4·1)	3·3 (2·9–3·7)	3·3 (2·7–3·9)
		Luxembourg	0·0330 (0·0287–0·0378)	0·0324 (0·0284–0·0366)	0·0396 (0·0345–0·0451)	0·0519 (0·0434–0·0623)	0·0514 (0·0397–0·0649)	6·5 (5·7–7·5)	5·7 (5·0–6·5)	6·6 (5·8–7·5)	8·0 (6·7–9·6)	7·7 (6·0–9·7)
		Malta	0·0494 (0·0371–0·0648)	0·0266 (0·0238–0·0296)	0·0245 (0·0210–0·0282)	0·0254 (0·0204–0·0312)	0·0229 (0·0173–0·0296)	9·0 (6·8–11·8)	6·3 (5·7–7·1)	5·6 (4·8–6·4)	5·8 (4·6–7·1)	5·3 (4·0–6·8)
		Monaco	0·00164 (0·00115–0·00222)	0·00130 (0·000909–0·00184)	0·000903 (0·000662–0·00120)	0·000967 (0·000707–0·00130)	0·000975 (0·000713–0·00133)	5·3 (3·7–7·2)	4·3 (3·0–6·1)	3·1 (2·3–4·1)	3·3 (2·4–4·4)	3·3 (2·4–4·5)
		Netherlands	1·81 (1·30–2·47)	1·33 (1·19–1·50)	0·669 (0·620–0·725)	0·541 (0·478–0·608)	0·548 (0·430–0·672)	9·1 (6·5–12·4)	6·4 (5·8–7·3)	3·9 (3·6–4·2)	3·1 (2·7–3·5)	3·1 (2·4–3·8)
		Norway	0·448 (0·421–0·475)	0·355 (0·334–0·375)	0·227 (0·215–0·242)	0·168 (0·151–0·188)	0·177 (0·150–0·206)	7·6 (7·2–8·1)	6·1 (5·7–6·4)	3·9 (3·7–4·1)	3·1 (2·8–3·4)	3·2 (2·7–3·7)
		Portugal	1·25 (1·17–1·35)	0·758 (0·710–0·808)	0·302 (0·284–0·323)	0·230 (0·185–0·286)	0·212 (0·159–0·276)	10·7 (10·0–11·5)	6·5 (6·1–6·9)	3·5 (3·3–3·7)	2·8 (2·2–3·4)	2·6 (2·0–3·4)
		San Marino	0·00288 (0·00205–0·00409)	0·00219 (0·00169–0·00278)	0·00250 (0·00190–0·00316)	0·00327 (0·00242–0·00436)	0·00241 (0·00180–0·00322)	12·4 (8·9–17·6)	7·3 (5·7–9·3)	9·1 (6·9–11·4)	13·3 (9·9–17·6)	9·9 (7·4–13·2)
		Spain	2·63 (1·92–3·67)	1·73 (1·56–1·92)	1·57 (1·45–1·70)	1·10 (0·923–1·29)	1·07 (0·836–1·34)	6·6 (4·8–9·2)	4·3 (3·9–4·8)	3·7 (3·4–4·0)	3·2 (2·7–3·7)	3·2 (2·5–4·0)
		Sweden	0·598 (0·551–0·649)	0·465 (0·434–0·498)	0·446 (0·413–0·483)	0·378 (0·332–0·429)	0·361 (0·288–0·446)	4·8 (4·5–5·3)	5·1 (4·7–5·4)	3·9 (3·6–4·2)	3·3 (2·9–3·8)	3·2 (2·5–3·9)
		Switzerland	0·462 (0·400–0·534)	0·349 (0·307–0·396)	0·410 (0·359–0·464)	0·382 (0·317–0·465)	0·396 (0·315–0·493)	5·3 (4·6–6·1)	4·5 (4·0–5·1)	4·7 (4·1–5·3)	4·3 (3·6–5·3)	4·4 (3·5–5·5)
	UK		6·31 (4·70–8·63)	4·91 (4·71–5·11)	4·54 (4·36–4·71)	3·48 (3·14–3·85)	3·80 (3·15–4·62)	7·9 (5·9–10·8)	7·2 (6·9–7·5)	5·8 (5·6–6·1)	5·1 (4·6–5·6)	5·5 (4·6–6·7)
		England	5·24 (3·73–7·43)	4·17 (3·99–4·35)	3·94 (3·77–4·11)	3·01 (2·69–3·36)	3·34 (2·72–4·12)	7·8 (5·6–11·1)	7·3 (6·9–7·6)	5·9 (5·7–6·2)	5·1 (4·6–5·7)	5·7 (4·6–7·0)
		Northern Ireland	0·216 (0·195–0·237)	0·141 (0·128–0·155)	0·106 (0·0960–0·117)	0·0723 (0·0539–0·0957)	0·0641 (0·0470–0·0855)	8·2 (7·5–9·0)	6·4 (5·8–7·0)	4·4 (4·0–4·8)	3·4 (2·5–4·5)	3·1 (2·3–4·1)
		Scotland	0·574 (0·532–0·616)	0·394 (0·369–0·421)	0·275 (0·259–0·294)	0·230 (0·215–0·246)	0·226 (0·202–0·252)	8·6 (8·0–9·2)	7·3 (6·9–7·9)	5·0 (4·7–5·3)	4·8 (4·5–5·1)	4·7 (4·2–5·2)
		Wales	0·281 (0·200–0·398)	0·208 (0·192–0·226)	0·217 (0·200–0·235)	0·168 (0·149–0·189)	0·177 (0·143–0·219)	7·2 (5·1–10·2)	6·5 (6·0–7·0)	6·5 (6·0–7·0)	5·8 (5·2–6·6)	6·2 (5·0–7·6)
**Latin America and Caribbean**			**302 (246–369)**	**228 (214–243)**	**131 (119–144)**	**109 (95·0–125)**	**102 (87·5–118)**	**28·1 (23·0–34·2)**	**20·9 (19·7–22·3)**	**12·5 (11·4–13·7)**	**11·3 (9·9–12·9)**	**10·7 (9·2–12·5)**
	Andean Latin America		23·8 (21·4–26·8)	21·5 (19·9–23·2)	16·8 (14·5–19·4)	14·2 (11·6–17·1)	13·3 (11·0–16·2)	19·7 (17·7–22·1)	17·4 (16·1–18·8)	13·2 (11·4–15·2)	11·2 (9·2–13·5)	10·6 (8·8–12·8)
		Bolivia	6·78 (5·71–8·01)	6·89 (5·97–7·94)	5·50 (4·01–7·34)	4·59 (3·31–6·37)	4·43 (3·20–6·06)	29·0 (24·5–34·0)	27·9 (24·3–32·0)	20·9 (15·3–27·7)	18·3 (13·2–25·2)	17·8 (12·9–24·2)
		Ecuador	4·04 (2·85–5·81)	4·19 (3·84–4·58)	2·60 (2·44–2·76)	2·54 (2·39–2·70)	2·39 (2·15–2·65)	13·3 (9·4–19·0)	12·3 (11·3–13·5)	7·6 (7·1–8·1)	7·7 (7·2–8·2)	7·4 (6·6–8·2)
		Peru	13·0 (11·7–14·4)	10·5 (9·50–11·5)	8·72 (7·13–10·6)	7·10 (5·25–9·74)	6·48 (4·73–9·02)	19·3 (17·5–21·3)	16·1 (14·7–17·7)	13·0 (10·7–15·8)	10·4 (7·7–14·2)	9·5 (7·0–13·2)
	Caribbean		37·8 (29·7–48·0)	29·3 (24·1–35·8)	22·8 (18·0–28·8)	21·4 (16·5–28·7)	21·1 (16·1–28·0)	40·0 (31·7–50·3)	32·9 (27·2–40·0)	26·8 (21·3–33·7)	26·0 (20·2–34·5)	25·7 (19·8–34·0)
		Antigua and Barbuda	0·0236 (0·0165–0·0327)	0·0315 (0·0218–0·0445)	0·0215 (0·0151–0·0296)	0·0195 (0·0137–0·0274)	0·0184 (0·0130–0·0259)	19·6 (13·8–27·0)	21·7 (15·1–30·4)	19·0 (13·4–25·9)	18·1 (12·8–25·3)	17·4 (12·4–24·3)
		The Bahamas	0·0820 (0·0608–0·108)	0·0581 (0·0529–0·0643)	0·0602 (0·0473–0·0763)	0·0530 (0·0378–0·0754)	0·0525 (0·0373–0·0740)	15·0 (11·2–19·6)	13·0 (11·9–14·4)	14·2 (11·2–17·9)	13·4 (9·6–19·0)	13·4 (9·6–18·8)
		Barbados	0·0608 (0·0446–0·0810)	0·0413 (0·0330–0·0505)	0·0318 (0·0224–0·0437)	0·0298 (0·0210–0·0410)	0·0297 (0·0213–0·0410)	14·4 (10·6–19·1)	10·4 (8·4–12·8)	11·0 (7·7–15·0)	11·2 (7·9–15·3)	11·3 (8·1–15·5)
		Belize	0·198 (0·144–0·265)	0·161 (0·155–0·167)	0·0873 (0·0773–0·0991)	0·0818 (0·0635–0·105)	0·0794 (0·0607–0·106)	30·1 (22·1–40·1)	23·4 (22·6–24·3)	11·4 (10·1–13·0)	10·7 (8·4–13·7)	10·4 (8·0–13·8)
		Bermuda	0·0147 (0·0106–0·0201)	0·00790 (0·00563–0·0111)	0·00446 (0·00345–0·00557)	0·00376 (0·00277–0·00501)	0·00337 (0·00247–0·00451)	16·2 (11·8–22·0)	9·4 (6·7–13·1)	7·6 (5·9–9·5)	7·4 (5·4–9·8)	6·8 (5·0–9·1)
		Cuba	2·91 (2·75–3·08)	2·31 (2·18–2·43)	1·33 (1·23–1·43)	1·14 (1·01–1·30)	0·942 (0·804–1·11)	16·2 (15·3–17·1)	15·2 (14·4–16·0)	10·9 (10·1–11·7)	11·0 (9·7–12·4)	9·4 (8·0–11·0)
		Dominica	0·0390 (0·0275–0·0542)	0·0282 (0·0201–0·0393)	0·0332 (0·0229–0·0471)	0·0280 (0·0193–0·0407)	0·0275 (0·0192–0·0399)	20·0 (14·2–27·7)	23·0 (16·5–31·9)	39·6 (27·7–55·3)	40·9 (28·6–58·4)	41·2 (29·1–58·7)
		Dominican Republic	10·4 (7·27–14·8)	8·61 (6·83–10·9)	4·71 (4·24–5·21)	4·42 (3·47–5·59)	4·38 (3·37–5·66)	43·1 (30·7–60·6)	37·3 (29·8–46·7)	21·9 (19·8–24·2)	20·2 (16·0–25·5)	20·1 (15·5–25·8)
		Grenada	0·0706 (0·0514–0·0934)	0·0550 (0·0478–0·0632)	0·0383 (0·0271–0·0525)	0·0319 (0·0226–0·0451)	0·0301 (0·0212–0·0424)	28·2 (20·7–37·0)	23·4 (20·4–26·8)	23·1 (16·5–31·4)	22·3 (15·9–31·2)	21·6 (15·3–30·1)
		Guyana	1·12 (0·783–1·59)	0·621 (0·434–0·874)	0·428 (0·297–0·599)	0·403 (0·279–0·580)	0·388 (0·270–0·554)	39·6 (28·0–55·1)	29·8 (21·0–41·5)	26·2 (18·3–36·3)	25·0 (17·4–35·6)	24·6 (17·3–34·9)
		Haiti	17·3 (12·0–24·4)	12·9 (9·09–18·1)	13·4 (9·25–19·0)	13·0 (8·96–18·8)	12·9 (9·02–18·7)	63·3 (44·9–87·4)	44·5 (31·9–61·6)	38·8 (27·2–54·3)	36·4 (25·5–52·0)	36·1 (25·5–51·5)
		Jamaica	1·19 (0·901–1·54)	1·51 (1·25–1·82)	0·829 (0·765–0·899)	0·745 (0·602–0·914)	0·713 (0·555–0·901)	20·4 (15·5–26·3)	29·7 (24·6–35·4)	21·8 (20·2–23·6)	21·8 (17·7–26·6)	21·3 (16·6–26·7)
		Puerto Rico	1·69 (1·17–2·39)	0·903 (0·704–1·14)	0·267 (0·212–0·326)	0·170 (0·130–0·220)	0·154 (0·114–0·204)	25·2 (17·7–35·3)	15·5 (12·1–19·6)	8·6 (6·8–10·5)	8·5 (6·5–11·0)	8·0 (5·9–10·6)
		Saint Kitts and Nevis	0·0310 (0·0215–0·0431)	0·0235 (0·0162–0·0332)	0·0188 (0·0131–0·0260)	0·0160 (0·0112–0·0226)	0·0157 (0·0111–0·0222)	32·6 (22·8–44·7)	27·4 (19·0–38·3)	28·6 (20·2–39·2)	26·6 (18·8–37·2)	26·6 (19·0–37·3)
		Saint Lucia	0·0882 (0·0755–0·102)	0·0611 (0·0529–0·0703)	0·0411 (0·0306–0·0555)	0·0319 (0·0230–0·0436)	0·0307 (0·0220–0·0428)	24·3 (20·9–28·0)	21·6 (18·7–24·7)	20·9 (15·7–28·1)	18·2 (13·2–24·8)	18·0 (12·9–24·9)
		Saint Vincent and the Grenadines	0·0404 (0·0285–0·0562)	0·0298 (0·0269–0·0331)	0·0401 (0·0356–0·0450)	0·0136 (0·0121–0·0154)	0·0132 (0·0110–0·0154)	15·5 (11·0–21·4)	14·0 (12·6–15·5)	22·8 (20·3–25·6)	10·1 (8·9–11·3)	10·1 (8·4–11·7)
		Suriname	0·554 (0·385–0·764)	0·462 (0·396–0·541)	0·369 (0·286–0·470)	0·341 (0·245–0·471)	0·333 (0·238–0·465)	54·6 (38·7–73·9)	46·4 (40·1–54·0)	36·1 (28·3–45·6)	35·8 (26·0–48·9)	35·5 (25·7–49·0)
		Trinidad and Tobago	0·723 (0·652–0·802)	0·430 (0·390–0·473)	0·280 (0·200–0·389)	0·206 (0·144–0·283)	0·197 (0·140–0·278)	29·5 (26·7–32·6)	23·6 (21·5–25·9)	15·4 (11·0–21·2)	13·3 (9·4–18·3)	13·2 (9·4–18·5)
		Virgin Islands	0·0688 (0·0477–0·0949)	0·0402 (0·0292–0·0534)	0·0167 (0·0146–0·0194)	0·0112 (0·00832–0·0149)	0·00948 (0·00691–0·0125)	28·4 (19·9–38·8)	19·2 (14·0–25·3)	14·2 (12·4–16·5)	13·3 (9·9–17·6)	12·0 (8·8–15·7)
	Central Latin America		144 (119–173)	101 (93·5–111)	51·3 (45·3–58·6)	39·4 (32·2–47·9)	36·0 (29·1–44·5)	27·9 (23·2–33·4)	20·2 (18·7–22·1)	10·9 (9·7–12·5)	9·8 (8·0–11·9)	9·2 (7·4–11·3)
		Colombia	31·2 (21·5–44·4)	26·8 (20·5–34·1)	11·4 (9·90–13·2)	8·68 (6·97–10·9)	7·07 (5·51–8·99)	32·6 (22·8–45·8)	28·9 (22·3–36·5)	15·7 (13·6–18·0)	12·5 (10·0–15·6)	10·4 (8·1–13·1)
		Costa Rica	0·740 (0·685–0·802)	0·619 (0·576–0·665)	0·458 (0·427–0·491)	0·339 (0·301–0·378)	0·315 (0·253–0·387)	9·1 (8·4–9·9)	8·1 (7·5–8·7)	6·4 (6·0–6·9)	5·9 (5·2–6·6)	5·7 (4·6–7·0)
		El Salvador	3·39 (2·48–4·54)	2·42 (2·14–2·71)	2·47 (2·31–2·64)	1·73 (1·47–2·03)	1·64 (1·32–2·01)	18·8 (13·8–25·0)	15·4 (13·6–17·3)	18·4 (17·3–19·6)	14·4 (12·3–16·9)	14·0 (11·3–17·1)
		Guatemala	44·3 (31·4–60·1)	22·2 (19·1–26·0)	3·69 (3·06–4·38)	2·88 (2·27–3·66)	2·68 (2·01–3·48)	114·3 (84·3–149·6)	54·6 (47·3–63·4)	9·5 (7·9–11·3)	8·2 (6·4–10·4)	7·7 (5·8–10·0)
		Honduras	6·13 (4·53–8·28)	4·88 (3·61–6·50)	3·23 (2·37–4·46)	2·72 (1·94–3·80)	2·64 (1·88–3·75)	33·0 (24·6–44·1)	24·6 (18·3–32·5)	14·8 (10·9–20·3)	12·2 (8·7–17·0)	11·8 (8·5–16·7)
		Mexico	44·9 (43·0–47·0)	35·1 (33·5–36·9)	20·5 (17·0–24·4)	15·5 (11·7–20·4)	14·8 (11·2–19·6)	17·2 (16·5–18·0)	13·9 (13·3–14·6)	8·9 (7·4–10·5)	8·0 (6·1–10·5)	7·9 (6·0–10·4)
		Nicaragua	2·84 (2·05–3·87)	2·40 (1·81–3·10)	1·85 (1·28–2·54)	1·48 (1·05–2·04)	1·39 (0·987–1·93)	19·7 (14·3–26·6)	17·9 (13·5–23·0)	13·5 (9·4–18·4)	11·4 (8·1–15·6)	10·8 (7·7–15·0)
		Panama	0·578 (0·415–0·793)	0·763 (0·647–0·907)	0·661 (0·569–0·766)	0·634 (0·507–0·781)	0·604 (0·460–0·774)	9·8 (7·1–13·4)	11·9 (10·1–14·2)	8·7 (7·5–10·1)	8·9 (7·1–10·9)	8·6 (6·5–11·0)
		Venezuela	9·92 (7·68–12·4)	6·34 (5·85–6·85)	6·90 (4·92–9·40)	5·41 (3·89–7·50)	4·87 (3·44–6·72)	17·8 (13·8–22·2)	12·1 (11·1–13·0)	11·2 (8·0–15·1)	12·3 (8·9–17·0)	11·7 (8·3–16·0)
	Tropical Latin America		96·4 (70·4–130)	75·4 (72·7–78·3)	40·1 (39·0–41·3)	33·6 (31·1–36·3)	31·2 (26·7–36·6)	28·2 (20·8–37·8)	20·1 (19·4–20·8)	10·9 (10·6–11·2)	9·5 (8·8–10·2)	8·9 (7·7–10·5)
		Brazil	91·4 (65·7–125)	71·6 (69·0–74·3)	38·3 (37·3–39·5)	31·9 (29·5–34·5)	29·6 (25·1–35·0)	27·8 (20·2–37·8)	19·8 (19·1–20·5)	10·8 (10·5–11·1)	9·3 (8·6–10·1)	8·8 (7·5–10·4)
		Paraguay	5·03 (4·26–5·89)	3·80 (3·04–4·63)	1·78 (1·31–2·33)	1·72 (1·25–2·35)	1·63 (1·18–2·20)	37·7 (32·1–43·8)	28·1 (22·6–34·1)	13·1 (9·7–17·1)	13·1 (9·6–17·9)	12·5 (9·1–16·9)
**North Africa and Middle East**			**557 (454–684)**	**533 (470–614)**	**326 (280–380)**	**252 (212–303)**	**237 (199–286)**	**47·6 (39·2–58·0)**	**44·1 (39·1–50·5)**	**22·8 (19·7–26·5)**	**19·9 (16·8–23·8)**	**19·1 (16·1–23·0)**
	Afghanistan		38·7 (26·5–55·7)	56·0 (39·0–77·7)	56·4 (40·4–78·1)	50·8 (35·9–72·6)	50·2 (35·4–72·7)	79·2 (55·7–110·3)	66·7 (47·4–90·2)	50·4 (36·7–68·6)	41·1 (29·4–57·8)	39·7 (28·4–56·6)
	Algeria		30·9 (27·9–34·4)	27·6 (26·0–29·3)	22·3 (21·0–23·6)	17·0 (13·7–20·8)	15·6 (12·0–20·2)	38·5 (34·8–42·7)	40·5 (38·2–43·0)	21·6 (20·4–22·9)	17·8 (14·4–21·7)	16·9 (13·1–21·7)
	Bahrain		0·712 (0·499–0·999)	0·258 (0·197–0·330)	0·255 (0·198–0·321)	0·171 (0·125–0·235)	0·158 (0·113–0·222)	50·5 (35·9–69·5)	20·4 (15·6–25·8)	12·2 (9·5–15·3)	9·7 (7·1–13·3)	9·3 (6·6–13·0)
	Egypt		82·4 (59·9–113)	59·0 (44·1–76·9)	37·8 (27·4–51·5)	25·8 (18·2–36·2)	24·3 (17·3–34·6)	41·5 (30·5–56·1)	29·2 (22·0–37·7)	11·9 (8·7–16·2)	9·5 (6·8–13·3)	9·2 (6·6–13·1)
	Iran		41·2 (28·7–56·2)	23·4 (16·3–33·5)	18·4 (13·2–24·9)	6·35 (4·58–8·77)	4·25 (3·06–5·83)	26·1 (18·3–35·2)	20·6 (14·5–29·3)	12·1 (8·7–16·3)	5·7 (4·1–7·9)	4·1 (3·0–5·6)
	Iraq		23·7 (16·9–32·1)	26·1 (18·5–37·5)	18·4 (14·1–23·2)	14·0 (10·6–18·2)	13·1 (9·85–17·3)	30·5 (22·0–41·0)	27·2 (19·4–38·6)	17·3 (13·3–21·7)	14·5 (11·0–18·9)	13·9 (10·4–18·2)
	Jordan		4·75 (3·43–6·42)	3·67 (2·73–4·73)	3·08 (2·26–4·07)	2·57 (1·85–3·52)	2·53 (1·82–3·53)	34·3 (25·1–45·9)	23·9 (17·9–30·6)	14·4 (10·6–19·0)	12·0 (8·6–16·4)	11·8 (8·5–16·4)
	Kuwait		0·931 (0·666–1·29)	1·03 (0·926–1·14)	1·36 (1·23–1·50)	1·43 (1·30–1·58)	1·57 (1·35–1·81)	26·3 (19·0–36·1)	24·4 (22·0–27·0)	22·8 (20·6–25·0)	26·9 (24·5–29·6)	29·9 (25·9–34·4)
	Lebanon		1·04 (0·727–1·44)	0·905 (0·637–1·30)	0·658 (0·505–0·851)	0·502 (0·380–0·656)	0·474 (0·353–0·626)	11·7 (8·2–16·1)	9·8 (6·9–14·0)	6·3 (4·8–8·1)	5·9 (4·4–7·6)	5·8 (4·3–7·6)
	Libya		5·07 (3·55–7·09)	3·46 (2·46–4·84)	2·28 (1·59–3·22)	1·98 (1·38–2·86)	1·87 (1·31–2·69)	36·6 (26·0–50·5)	27·2 (19·5–37·6)	22·9 (16·0–32·1)	23·6 (16·6–33·8)	23·1 (16·3–32·9)
	Morocco		106 (76·1–144)	121 (110–134)	50·5 (47·7–53·8)	43·8 (40·0–48·3)	39·8 (33·9–46·9)	115·9 (86·5–152·0)	126·6 (116·3–138·7)	61·1 (57·9–64·8)	61·6 (56·5–67·5)	58·0 (49·8–67·6)
	Oman		1·20 (1·13–1·28)	0·750 (0·654–0·861)	0·757 (0·694–0·827)	1·07 (0·973–1·17)	0·985 (0·863–1·12)	16·1 (15·2–17·1)	13·2 (11·5–15·1)	9·4 (8·6–10·3)	12·7 (11·6–14·0)	11·9 (10·4–13·5)
	Palestine		2·33 (1·65–3·20)	2·39 (1·79–3·08)	1·59 (1·24–1·99)	0·981 (0·761–1·27)	0·954 (0·727–1·25)	25·8 (18·4–35·2)	20·0 (15·1–25·6)	12·1 (9·5–15·1)	8·0 (6·2–10·3)	7·9 (6·0–10·3)
	Qatar		0·374 (0·286–0·479)	0·365 (0·278–0·464)	0·467 (0·337–0·661)	0·592 (0·410–0·822)	0·523 (0·367–0·721)	31·2 (24·0–39·6)	26·9 (20·6–33·9)	13·8 (10·0–19·5)	15·5 (10·8–21·5)	13·5 (9·6–18·6)
	Saudi Arabia		11·0 (10·4–11·7)	10·2 (8·39–12·3)	6·92 (6·09–7·85)	4·65 (3·27–6·30)	4·43 (3·10–6·09)	21·0 (19·8–22·3)	20·2 (16·7–24·2)	13·1 (11·6–14·8)	9·7 (6·9–13·1)	9·5 (6·7–13·0)
	Sudan		51·0 (36·0–71·1)	65·2 (53·3–79·9)	30·0 (22·7–39·1)	24·5 (17·3–34·1)	23·1 (16·2–32·8)	52·8 (37·8–72·1)	54·0 (44·6–65·3)	23·7 (18·0–30·7)	20·2 (14·4–28·0)	19·4 (13·7–27·3)
	Syria		16·4 (11·5–23·0)	9·31 (6·63–13·0)	5·54 (3·85–7·79)	2·54 (1·77–3·65)	2·39 (1·68–3·41)	34·7 (24·7–48·0)	18·4 (13·2–25·5)	17·8 (12·4–24·9)	12·5 (8·8–17·9)	12·0 (8·5–17·1)
	Tunisia		6·41 (5·93–6·91)	4·25 (3·38–5·29)	4·99 (4·65–5·39)	4·55 (4·22–4·91)	4·35 (3·87–4·89)	28·3 (26·3–30·5)	22·2 (17·8–27·6)	24·3 (22·7–26·2)	25·7 (23·8–27·6)	25·5 (22·7–28·5)
	Türkiye		79·5 (56·5–114)	43·6 (32·6–57·7)	14·4 (13·0–16·0)	8·79 (7·59–10·2)	7·99 (6·55–9·68)	49·8 (36·0–69·7)	29·3 (22·1–38·5)	11·1 (10·0–12·3)	7·9 (6·9–9·2)	7·5 (6·2–9·1)
	United Arab Emirates		0·916 (0·647–1·31)	0·615 (0·454–0·833)	0·787 (0·726–0·849)	0·507 (0·433–0·597)	0·423 (0·344–0·517)	18·6 (13·2–26·3)	12·3 (9·1–16·6)	7·8 (7·2–8·4)	6·4 (5·5–7·5)	5·7 (4·6–6·9)
	Yemen		52·2 (43·0–63·1)	73·7 (62·4–86·1)	48·8 (35·3–67·4)	39·0 (27·9–54·2)	37·5 (26·9–52·5)	73·2 (61·1–87·2)	79·4 (68·1–91·5)	44·3 (32·5–60·3)	37·7 (27·3–51·7)	36·5 (26·4–50·4)
**South Asia**			**1760 (1380–2240)**	**1620 (1340–1960)**	**1170 (1000–1350)**	**963 (800–1170)**	**922 (764–1130)**	**51·1 (40·6–64·3)**	**44·4 (37·1–53·2)**	**32·9 (28·5–37·9)**	**28·8 (24·1–34·7)**	**28·0 (23·3–34·0)**
	Bangladesh		313 (225–419)	266 (220–318)	112 (90·0–138)	52·7 (41·1–66·5)	51·9 (39·2–67·9)	69·8 (51·3–91·6)	61·4 (51·3–72·4)	32·6 (26·4–40·0)	18·0 (14·1–22·6)	18·1 (13·8–23·6)
	Bhutan		2·23 (1·53–3·19)	1·38 (0·964–1·96)	0·523 (0·362–0·741)	0·387 (0·268–0·559)	0·365 (0·255–0·525)	90·6 (64·3–125·0)	71·8 (51·4–99·3)	35·4 (24·8–49·6)	29·2 (20·4–41·7)	28·1 (19·8–40·0)
	India		923 (721–1170)	874 (702–1090)	711 (599–834)	599 (495–738)	567 (466–700)	37·9 (29·8–47·4)	33·3 (26·9–41·1)	28·5 (24·1–33·2)	25·7 (21·3–31·5)	24·7 (20·4–30·3)
	Nepal		76·3 (54·8–108)	37·3 (33·1–42·0)	16·3 (13·7–19·3)	13·9 (11·2–17·1)	14·4 (11·5–17·8)	87·4 (64·6–119·3)	45·6 (40·6–51·0)	24·7 (20·8–29·2)	21·1 (17·1–25·9)	21·9 (17·6–26·9)
	Pakistan		444 (304–633)	438 (320–581)	327 (267–395)	297 (225–381)	289 (216–379)	95·1 (67·4–130·8)	87·0 (65·2–112·6)	51·4 (42·3–61·4)	45·7 (35·0–58·1)	44·5 (33·7–57·7)
**Southeast Asia, east Asia, and Oceania**			**966 (709–1300)**	**634 (489–830)**	**345 (268–441)**	**256 (198–338)**	**244 (190–318)**	**25·9 (19·2–34·6)**	**21·8 (16·9–28·4)**	**11·6 (9·0–14·7)**	**10·6 (8·2–13·9)**	**10·6 (8·3–13·8)**
	East Asia		634 (449–881)	387 (276–537)	163 (115–227)	91·8 (65·6–130)	80·9 (57·8–114)	26·0 (18·5–35·8)	23·3 (16·8–32·1)	9·4 (6·7–13·0)	7·4 (5·3–10·4)	7·2 (5·1–10·1)
		China	621 (438–863)	373 (266–519)	156 (110–219)	86·8 (61·3–124)	76·1 (54·1–108)	26·5 (18·9–36·5)	23·7 (17·0–32·7)	9·3 (6·6–13·0)	7·3 (5·2–10·4)	7·1 (5·0–10·0)
		North Korea	10·7 (7·56–14·9)	9·77 (6·96–13·6)	4·22 (2·94–5·93)	2·89 (2·02–4·15)	2·68 (1·89–3·83)	19·1 (13·5–26·3)	18·4 (13·2–25·4)	11·9 (8·3–16·6)	9·3 (6·5–13·3)	8·9 (6·3–12·6)
		Taiwan (province of China)	1·81 (1·26–2·50)	3·62 (2·57–5·08)	2·50 (2·24–2·76)	1·96 (1·71–2·24)	1·96 (1·66–2·30)	5·6 (3·9–7·7)	13·1 (9·4–18·4)	11·9 (10·7–13·1)	11·9 (10·4–13·6)	12·4 (10·5–14·4)
	Oceania		8·97 (6·38–12·5)	9·54 (6·84–13·2)	10·8 (7·56–15·1)	12·1 (8·53–17·4)	12·3 (8·71–17·7)	38·2 (27·5–52·5)	33·2 (24·1–45·5)	27·2 (19·2–37·7)	27·9 (19·8–39·5)	27·8 (19·8–39·4)
		American Samoa	0·0343 (0·0298–0·0394)	0·0314 (0·0260–0·0376)	0·00908 (0·00725–0·0113)	0·00969 (0·00728–0·0128)	0·00744 (0·00545–0·0100)	18·8 (16·4–21·5)	18·3 (15·2–21·8)	8·6 (6·9–10·7)	11·8 (8·9–15·6)	9·5 (7·0–12·7)
		Cook Islands	0·00602 (0·00476–0·00755)	0·00520 (0·00411–0·00632)	0·00215 (0·00188–0·00244)	0·00239 (0·00199–0·00284)	0·00245 (0·00200–0·00301)	13·7 (10·9–17·1)	14·8 (11·8–18·0)	9·0 (7·9–10·3)	10·8 (9·0–12·8)	11·2 (9·1–13·6)
		Federated States of Micronesia	0·0803 (0·0565–0·112)	0·0597 (0·0425–0·0833)	0·0309 (0·0215–0·0435)	0·0269 (0·0187–0·0386)	0·0260 (0·0183–0·0372)	24·9 (17·7–34·4)	21·2 (15·2–29·4)	15·0 (10·5–21·0)	14·1 (9·8–20·1)	13·8 (9·7–19·6)
		Fiji	0·190 (0·135–0·259)	0·191 (0·134–0·273)	0·198 (0·136–0·274)	0·186 (0·132–0·258)	0·182 (0·129–0·254)	10·0 (7·1–13·7)	10·6 (7·4–15·1)	10·3 (7·1–14·2)	10·8 (7·7–14·9)	10·8 (7·7–15·0)
		Guam	0·0879 (0·0615–0·125)	0·0676 (0·0498–0·0902)	0·0470 (0·0425–0·0520)	0·0438 (0·0375–0·0508)	0·0394 (0·0313–0·0496)	22·2 (15·6–31·2)	18·0 (13·3–23·9)	14·0 (12·7–15·4)	15·5 (13·3–18·0)	14·4 (11·4–18·0)
		Kiribati	0·0845 (0·0594–0·118)	0·0720 (0·0512–0·101)	0·0682 (0·0474–0·0961)	0·0683 (0·0475–0·0985)	0·0679 (0·0476–0·0975)	30·0 (21·3–41·4)	26·8 (19·2–37·1)	22·5 (15·7–31·4)	22·7 (15·9–32·5)	22·6 (16·0–32·2)
		Marshall Islands	0·0302 (0·0214–0·0424)	0·0348 (0·0256–0·0456)	0·0259 (0·0199–0·0327)	0·0226 (0·0162–0·0311)	0·0218 (0·0155–0·0307)	19·9 (14·2–27·7)	20·8 (15·4–27·1)	19·6 (15·2–24·7)	18·8 (13·5–25·7)	18·4 (13·1–25·7)
		Nauru	0·00768 (0·00541–0·0107)	0·00896 (0·00637–0·0125)	0·00652 (0·00453–0·00919)	0·00608 (0·00423–0·00876)	0·00600 (0·00421–0·00861)	21·0 (14·9–28·9)	24·1 (17·3–33·3)	20·7 (14·5–28·9)	20·3 (14·2–29·0)	20·1 (14·2–28·6)
		Niue	0·00109 (0·000765–0·00151)	0·000857 (0·000609–0·00120)	0·000599 (0·000416–0·000846)	0·000605 (0·000420–0·000873)	0·000604 (0·000423–0·000869)	23·5 (16·7–32·5)	27·6 (19·8–38·1)	24·7 (17·3–34·6)	25·9 (18·1–37·0)	26·0 (18·3–37·0)
		Northern Mariana Islands	0·0474 (0·0333–0·0682)	0·0541 (0·0383–0·0751)	0·00754 (0·00657–0·00874)	0·00807 (0·00612–0·0103)	0·00754 (0·00561–0·00992)	37·5 (26·7–53·2)	21·5 (15·3–29·5)	11·5 (10·0–13·3)	13·7 (10·4–17·4)	12·8 (9·6–16·8)
		Palau	0·00545 (0·00390–0·00765)	0·00555 (0·00411–0·00721)	0·00683 (0·00542–0·00861)	0·00467 (0·00337–0·00628)	0·00448 (0·00325–0·00606)	17·3 (12·5–24·2)	16·6 (12·4–21·5)	28·7 (22·9–36·0)	24·3 (17·7–32·5)	24·2 (17·7–32·5)
		Papua New Guinea	7·10 (4·96–9·96)	7·74 (5·48–10·9)	9·32 (6·46–13·2)	10·7 (7·40–15·5)	10·9 (7·62–15·7)	45·8 (32·5–63·3)	38·4 (27·5–53·1)	30·2 (21·1–42·2)	30·6 (21·4–43·8)	30·6 (21·6–43·5)
		Samoa	0·0623 (0·0442–0·0870)	0·0509 (0·0359–0·0706)	0·0447 (0·0317–0·0605)	0·0453 (0·0317–0·0648)	0·0449 (0·0318–0·0642)	11·2 (8·0–15·5)	9·0 (6·4–12·4)	7·5 (5·3–10·1)	7·3 (5·1–10·4)	7·1 (5·1–10·2)
		Solomon Islands	0·431 (0·303–0·601)	0·432 (0·308–0·604)	0·321 (0·224–0·452)	0·300 (0·209–0·431)	0·297 (0·209–0·425)	30·0 (21·3–41·4)	24·0 (17·2–33·3)	16·1 (11·3–22·5)	14·5 (10·2–20·8)	14·2 (10·1–20·3)
		Tokelau	0·000691 (0·000487–0·000961)	0·000491 (0·000350–0·000683)	0·000196 (0·000137–0·000275)	0·000185 (0·000129–0·000266)	0·000183 (0·000129–0·000262)	19·1 (13·5–26·3)	16·4 (11·8–22·7)	10·5 (7·4–14·7)	10·4 (7·3–14·8)	10·2 (7·2–14·6)
		Tonga	0·0706 (0·0487–0·0977)	0·0644 (0·0474–0·0847)	0·0524 (0·0436–0·0620)	0·0458 (0·0344–0·0602)	0·0445 (0·0333–0·0586)	20·9 (14·5–28·6)	19·7 (14·6–25·7)	16·1 (13·5–19·0)	15·0 (11·3–19·6)	14·7 (11·0–19·2)
		Tuvalu	0·0136 (0·00954–0·0190)	0·00737 (0·00524–0·0103)	0·00466 (0·00324–0·00656)	0·00445 (0·00310–0·00640)	0·00438 (0·00308–0·00628)	34·6 (24·6–47·8)	25·7 (18·4–35·5)	17·4 (12·2–24·4)	15·9 (11·1–22·8)	15·6 (11·0–22·1)
		Vanuatu	0·145 (0·102–0·201)	0·156 (0·111–0·218)	0·129 (0·0900–0·182)	0·133 (0·0928–0·192)	0·132 (0·0925–0·189)	22·8 (16·2–31·4)	21·0 (15·1–29·1)	15·1 (10·5–21·1)	15·1 (10·6–21·6)	14·8 (10·5–21·1)
	Southeast Asia		323 (257–404)	238 (201–285)	171 (143–205)	152 (124–188)	151 (123–189)	25·6 (20·5–31·8)	19·6 (16·7–23·4)	14·2 (11·9–17·0)	13·3 (10·9–16·4)	13·3 (10·9–16·6)
		Cambodia	18·9 (12·8–26·2)	11·3 (8·17–15·3)	6·08 (4·35–8·37)	5·85 (4·12–8·22)	5·78 (4·01–8·23)	41·9 (28·9–57·1)	30·1 (21·9–40·3)	16·4 (11·7–22·4)	15·4 (10·9–21·6)	15·3 (10·7–21·6)
		Indonesia	138 (108–171)	110 (87·1–138)	79·3 (64·5–97·3)	68·5 (53·8–88·1)	67·5 (52·7–87·9)	28·7 (22·8–35·5)	22·9 (18·2–28·5)	17·0 (13·8–20·7)	15·3 (12·0–19·5)	15·1 (11·9–19·6)
		Laos	11·4 (7·90–16·1)	8·67 (6·13–12·2)	4·23 (2·94–5·97)	3·80 (2·64–5·47)	3·72 (2·61–5·34)	61·6 (43·7–85·0)	42·9 (30·8–59·4)	23·3 (16·3–32·5)	20·9 (14·6–29·8)	20·5 (14·5–29·2)
		Malaysia	5·11 (4·56–5·70)	2·68 (2·49–2·87)	3·36 (3·05–3·69)	3·34 (2·97–3·71)	3·12 (2·51–3·84)	10·3 (9·2–11·5)	5·1 (4·7–5·4)	6·7 (6·1–7·4)	7·0 (6·2–7·7)	6·5 (5·3–8·0)
		Maldives	0·512 (0·371–0·712)	0·199 (0·173–0·227)	0·0994 (0·0907–0·109)	0·0665 (0·0534–0·0813)	0·0657 (0·0520–0·0825)	55·2 (40·7–75·2)	31·7 (27·6–36·0)	14·3 (13·1–15·7)	10·7 (8·6–13·0)	10·8 (8·6–13·6)
		Mauritius	0·615 (0·567–0·663)	0·405 (0·376–0·437)	0·191 (0·178–0·206)	0·196 (0·173–0·221)	0·176 (0·141–0·218)	26·1 (24·1–28·1)	19·7 (18·3–21·1)	14·5 (13·5–15·6)	14·9 (13·2–16·8)	13·7 (11·0–16·9)
		Myanmar	44·3 (31·4–61·1)	34·2 (24·3–49·2)	23·1 (17·1–30·3)	20·6 (14·8–27·8)	20·2 (14·7–27·4)	38·1 (27·3–51·9)	31·4 (22·5–44·5)	21·0 (15·6–27·4)	18·8 (13·5–25·2)	18·4 (13·5–24·9)
		Philippines	32·4 (25·5–41·3)	27·0 (25·6–28·4)	23·9 (22·7–25·2)	23·9 (19·9–28·4)	26·1 (21·2–31·5)	15·8 (12·4–20·0)	12·0 (11·4–12·6)	10·0 (9·5–10·5)	10·8 (9·0–12·8)	11·8 (9·6–14·2)
		Seychelles	0·0333 (0·0245–0·0454)	0·0161 (0·0125–0·0202)	0·0164 (0·0139–0·0192)	0·0145 (0·0119–0·0174)	0·0142 (0·0114–0·0173)	19·7 (14·5–26·6)	11·0 (8·5–13·7)	9·9 (8·4–11·5)	9·0 (7·4–10·8)	8·9 (7·1–10·8)
		Sri Lanka	3·43 (2·65–4·39)	5·07 (3·93–6·53)	1·67 (1·48–1·89)	1·53 (1·17–2·02)	1·40 (1·03–1·91)	9·5 (7·3–12·1)	14·4 (11·2–18·5)	4·9 (4·3–5·5)	5·0 (3·8–6·6)	4·7 (3·4–6·4)
		Thailand	18·6 (13·1–25·8)	9·10 (6·50–12·6)	3·42 (2·39–4·80)	2·61 (1·83–3·74)	2·52 (1·78–3·60)	17·7 (12·6–24·5)	10·4 (7·4–14·3)	5·1 (3·6–7·1)	4·4 (3·1–6·3)	4·4 (3·1–6·2)
		Timor-Leste	1·98 (1·38–2·78)	1·22 (0·862–1·70)	0·631 (0·439–0·888)	0·639 (0·445–0·919)	0·646 (0·454–0·925)	50·5 (35·8–69·7)	31·7 (22·8–43·9)	17·1 (11·9–23·9)	15·7 (11·0–22·5)	15·5 (10·9–22·1)
		Viet Nam	47·7 (33·4–65·8)	27·4 (21·9–33·9)	24·5 (18·2–32·2)	21·0 (15·0–28·9)	19·6 (14·1–27·4)	24·2 (17·1–33·1)	17·7 (14·1–21·7)	14·3 (10·7–18·7)	13·1 (9·4–18·0)	12·5 (9·1–17·4)
**Sub-Saharan Africa**			**1310 (1020–1690)**	**1390 (1190–1660)**	**1510 (1400–1650)**	**1450 (1280–1670)**	**1430 (1240–1660)**	**55·4 (43·8–70·5)**	**47·5 (40·6–56·2)**	**39·9 (37·0–43·2)**	**37·3 (33·0–42·8)**	**36·5 (31·9–42·2)**
	Central sub-Saharan Africa		126 (91·9–171)	137 (110–168)	152 (120–193)	146 (109–200)	142 (107–194)	46·2 (34·3–61·8)	39·7 (32·0–48·2)	33·1 (26·3–41·5)	31·7 (23·8–43·0)	30·8 (23·5–41·8)
		Angola	24·7 (17·5–34·4)	25·9 (18·3–36·5)	20·5 (19·7–21·4)	19·9 (15·6–25·0)	20·0 (15·4–25·8)	46·5 (33·5–63·7)	35·2 (25·1–48·8)	18·1 (17·4–18·8)	16·5 (13·0–20·6)	16·4 (12·6–21·0)
		Central African Republic	12·2 (8·37–17·4)	12·7 (8·91–18·1)	6·95 (4·81–9·85)	8·01 (5·53–11·6)	7·85 (5·47–11·4)	86·6 (61·4–119·5)	73·5 (52·7–101·7)	34·7 (24·3–48·5)	40·2 (28·1–57·4)	39·4 (27·8–56·1)
		Congo (Brazzaville)	3·90 (2·73–5·47)	4·81 (3·40–6·75)	2·82 (1·96–3·97)	3·12 (2·17–4·50)	3·05 (2·14–4·38)	40·6 (28·8–56·0)	38·6 (27·7–53·4)	19·1 (13·4–26·7)	23·4 (16·4–33·5)	23·1 (16·3–32·9)
		DR Congo	82·2 (57·9–114)	90·6 (71·7–113)	120 (90·4–156)	112 (79·0–161)	109 (77·6–155)	43·4 (31·0–59·3)	38·6 (30·8–47·6)	39·5 (30·2–51·1)	37·7 (26·8–53·1)	36·6 (26·5–51·5)
		Equatorial Guinea	1·09 (0·763–1·54)	1·33 (0·937–1·87)	0·886 (0·615–1·25)	1·18 (0·817–1·71)	1·17 (0·816–1·68)	50·7 (36·0–70·0)	45·0 (32·3–62·3)	22·3 (15·6–31·1)	30·5 (21·3–43·5)	30·2 (21·4–43·1)
		Gabon	1·62 (1·10–2·22)	1·78 (1·25–2·56)	1·50 (1·25–1·80)	1·25 (1·14–1·39)	1·19 (1·04–1·37)	42·9 (29·6–58·2)	40·2 (28·7–57·0)	30·7 (25·7–36·5)	27·8 (25·3–30·7)	26·7 (23·3–30·6)
	Eastern sub-Saharan Africa		566 (441–728)	552 (483–632)	484 (446–531)	461 (387–559)	456 (380–562)	58·9 (46·5–74·5)	46·7 (41·1–53·1)	34·7 (32·1–37·9)	32·6 (27·5–39·3)	32·0 (26·8–39·2)
		Burundi	19·4 (13·6–27·0)	17·7 (13·0–23·8)	24·7 (22·9–26·6)	22·5 (18·0–27·7)	22·0 (17·1–28·0)	69·5 (49·7–94·5)	61·6 (45·8–81·0)	53·2 (49·6–57·1)	46·4 (37·4–56·5)	44·8 (35·2–56·3)
		Comoros	1·82 (1·25–2·59)	1·51 (1·06–2·15)	0·896 (0·617–1·28)	0·774 (0·534–1·13)	0·758 (0·528–1·10)	81·9 (58·1–113·1)	69·3 (49·7–95·9)	46·6 (32·6–65·1)	42·6 (29·8–60·9)	41·9 (29·6–59·6)
		Djibouti	0·861 (0·599–1·22)	0·993 (0·700–1·40)	0·947 (0·655–1·34)	0·783 (0·543–1·13)	0·751 (0·525–1·08)	58·9 (41·8–81·3)	50·0 (35·8–69·2)	34·5 (24·2–48·3)	29·8 (20·9–42·6)	28·9 (20·4–41·2)
		Eritrea	8·61 (6·00–12·1)	6·50 (4·59–9·13)	5·57 (3·86–7·87)	5·19 (3·60–7·49)	5·14 (3·60–7·39)	55·0 (39·0–76·0)	41·7 (29·9–57·7)	28·4 (19·9–39·7)	26·0 (18·2–37·1)	25·6 (18·1–36·4)
		Ethiopia	175 (123–244)	171 (125–228)	90·6 (84·3–97·3)	86·7 (69·6–106)	87·0 (68·1–110)	67·2 (48·0–91·3)	52·9 (39·4–69·6)	27·0 (25·2–29·0)	24·5 (19·8–29·8)	24·2 (19·1–30·5)
		Kenya	45·5 (33·6–60·2)	53·3 (47·7–60·1)	42·6 (35·3–51·4)	35·9 (27·7–47·2)	34·8 (26·7–46·4)	43·8 (32·7–57·2)	42·1 (37·8–47·2)	31·4 (26·1–37·6)	29·0 (22·5–37·7)	28·5 (22·0–37·6)
		Madagascar	26·3 (19·0–37·0)	29·8 (25·6–34·7)	39·5 (32·0–48·5)	40·1 (37·2–43·3)	39·2 (36·2–42·1)	47·5 (34·8–65·7)	41·3 (35·7–47·7)	41·9 (34·2–50·9)	43·5 (40·4–46·8)	42·8 (39·6–45·8)
		Malawi	43·0 (33·0–55·4)	19·6 (18·3–20·9)	17·8 (16·8–18·9)	15·9 (12·4–20·3)	15·5 (11·9–20·4)	77·9 (60·9–98·2)	34·1 (32·0–36·3)	29·4 (27·8–31·2)	26·9 (21·2–34·2)	26·3 (20·3–34·4)
		Mozambique	40·0 (27·9–56·2)	40·4 (34·0–47·2)	57·5 (50·9–64·8)	52·3 (39·6–69·5)	50·7 (38·0–68·8)	60·2 (42·8–82·7)	46·7 (39·6–54·2)	52·1 (46·5–58·4)	45·7 (35·0–59·8)	43·8 (33·2–58·7)
		Rwanda	20·7 (14·6–29·9)	15·0 (13·8–16·3)	8·49 (8·08–8·96)	10·5 (9·09–12·1)	10·2 (8·35–12·3)	61·1 (43·9–85·9)	41·3 (38·3–44·8)	22·7 (21·6–23·9)	27·5 (24·0–31·7)	26·6 (21·9–31·9)
		Somalia	27·2 (18·8–38·4)	29·2 (20·5–41·2)	32·5 (22·4–46·1)	34·9 (24·2–50·7)	35·4 (24·7–51·2)	64·7 (45·9–89·4)	54·4 (39·0–75·3)	39·3 (27·5–55·0)	36·2 (25·3–51·7)	35·5 (25·1–50·6)
		South Sudan	24·5 (16·9–35·0)	28·4 (19·8–40·5)	32·5 (22·2–46·7)	27·5 (18·8–40·4)	28·2 (19·5–41·4)	86·8 (61·6–119·8)	79·5 (57·0–110·0)	69·8 (48·9–97·7)	68·7 (48·1–98·2)	68·3 (48·2–97·2)
		Tanzania	62·5 (44·3–86·5)	63·3 (58·9–68·0)	72·0 (66·7–77·4)	70·7 (55·1–91·1)	69·8 (53·5–92·0)	48·7 (35·0–66·3)	39·3 (36·6–42·1)	36·6 (34·0–39·2)	35·8 (28·2–45·7)	35·4 (27·3–46·2)
		Uganda	55·1 (40·5–73·2)	57·6 (53·5–61·9)	38·7 (37·2–40·3)	40·4 (32·0–50·7)	39·9 (31·1–51·1)	55·6 (41·5–72·6)	43·7 (40·7–46·8)	24·2 (23·3–25·2)	24·8 (19·8–31·0)	24·5 (19·2–31·1)
		Zambia	15·0 (10·5–20·7)	17·5 (13·4–22·5)	19·0 (17·2–20·9)	16·1 (13·7–18·8)	15·8 (13·0–18·9)	36·9 (26·2–50·3)	35·8 (27·7–45·5)	29·3 (26·6–32·1)	25·7 (22·0–29·9)	25·3 (20·9–30·3)
	Southern sub-Saharan Africa		76·8 (57·5–104)	65·6 (58·1–74·2)	59·4 (53·7–65·6)	58·1 (45·4–73·8)	57·2 (44·3–73·9)	44·9 (34·0–59·8)	38·3 (34·1–43·1)	32·5 (29·5–35·8)	33·8 (26·6–42·6)	33·7 (26·4–43·2)
		Botswana	1·84 (1·44–2·30)	1·39 (1·15–1·65)	1·32 (1·09–1·60)	1·25 (0·977–1·60)	1·25 (0·958–1·66)	37·8 (29·9–47·0)	28·0 (23·4–33·2)	26·2 (21·7–31·5)	25·0 (19·6–31·7)	25·1 (19·3–32·9)
		Eswatini	1·22 (0·865–1·71)	1·13 (0·827–1·50)	0·873 (0·622–1·23)	0·740 (0·523–1·05)	0·724 (0·511–1·04)	36·1 (25·9–49·9)	30·5 (22·4–40·0)	26·0 (18·6–36·2)	24·2 (17·2–33·9)	24·0 (17·1–34·2)
		Lesotho	2·08 (1·46–2·88)	1·93 (1·39–2·70)	1·96 (1·50–2·49)	1·91 (1·35–2·58)	1·90 (1·36–2·61)	36·8 (26·2–50·4)	36·0 (26·1–49·7)	40·8 (31·5–51·1)	42·5 (30·5–56·8)	42·8 (31·0–57·8)
		Namibia	1·39 (0·970–1·95)	1·27 (0·937–1·68)	1·05 (0·796–1·39)	0·965 (0·698–1·34)	0·956 (0·683–1·34)	26·4 (18·6–36·7)	21·8 (16·2–28·7)	17·1 (13·0–22·5)	16·3 (11·8–22·4)	16·2 (11·6–22·7)
		South Africa	55·4 (38·4–79·8)	41·7 (35·8–48·3)	36·2 (32·2–40·8)	36·5 (25·6–50·3)	35·6 (24·9–49·3)	50·0 (35·3–70·6)	38·9 (33·7–44·8)	31·9 (28·4–35·8)	34·9 (24·8–47·5)	34·8 (24·6–47·5)
		Zimbabwe	14·9 (11·2–19·4)	18·2 (14·6–22·6)	17·9 (14·7–21·9)	16·7 (12·5–22·3)	16·7 (12·3–22·8)	36·3 (27·5–46·7)	40·9 (33·0–50·3)	36·1 (29·7–43·7)	34·3 (26·0–45·3)	34·3 (25·5–46·4)
	Western sub-Saharan Africa		539 (415–707)	640 (514–805)	818 (764–880)	786 (711–872)	775 (696–871)	56·3 (44·0–72·6)	51·6 (41·9–64·1)	46·7 (43·7–50·0)	42·7 (38·8–47·1)	41·7 (37·5–46·5)
		Benin	9·35 (6·39–13·1)	11·3 (8·42–14·9)	15·3 (14·6–16·0)	15·0 (14·4–15·8)	15·0 (13·9–16·3)	38·0 (26·3–52·6)	35·4 (26·5–46·0)	31·7 (30·4–33·1)	28·5 (27·3–29·8)	27·9 (25·9–30·3)
		Burkina Faso	17·6 (13·7–21·8)	21·1 (18·7–23·9)	21·6 (16·6–27·4)	22·1 (16·0–30·5)	22·3 (16·1–31·5)	36·8 (29·0–45·2)	34·2 (30·4–38·6)	24·9 (19·3–31·4)	23·2 (16·8–31·7)	22·9 (16·6–32·0)
		Cabo Verde	0·615 (0·466–0·823)	0·455 (0·401–0·516)	0·196 (0·189–0·203)	0·142 (0·118–0·169)	0·132 (0·106–0·162)	46·6 (35·7–61·4)	34·6 (30·6–39·0)	18·9 (18·3–19·6)	16·0 (13·3–19·0)	15·3 (12·3–18·7)
		Cameroon	23·1 (16·2–32·5)	24·7 (17·2–34·5)	35·0 (29·6–41·4)	33·8 (29·1–39·3)	33·4 (27·8–39·8)	46·2 (32·8–63·7)	37·4 (26·5–51·7)	33·9 (28·9–39·9)	31·9 (27·6–36·9)	31·4 (26·2–37·2)
		Chad	17·8 (12·4–25·0)	21·1 (14·9–29·6)	30·1 (20·8–42·8)	32·8 (22·7–47·6)	33·5 (23·4–48·5)	52·2 (37·0–72·1)	45·8 (32·8–63·3)	41·0 (28·7–57·3)	37·9 (26·6–54·2)	37·5 (26·5–53·4)
		Côte d'Ivoire	22·3 (17·7–28·3)	26·7 (20·7–33·6)	28·9 (20·5–40·9)	25·7 (18·3–36·8)	25·3 (17·8–35·3)	38·8 (31·0–48·6)	37·3 (29·2–46·5)	30·6 (21·9–42·8)	26·5 (19·0–37·5)	25·9 (18·4–35·8)
		The Gambia	2·63 (1·83–3·76)	3·06 (2·31–4·02)	3·00 (2·88–3·12)	3·15 (2·83–3·52)	3·06 (2·63–3·53)	53·6 (38·0–75·0)	47·5 (36·3–61·4)	38·0 (36·6–39·5)	39·2 (35·3–43·7)	37·8 (32·7–43·4)
		Ghana	32·4 (23·1–46·0)	33·6 (31·3–36·0)	24·4 (23·3–25·5)	20·0 (15·8–25·1)	19·6 (15·0–25·4)	50·7 (36·7–70·5)	47·8 (44·6–50·9)	25·2 (24·1–26·3)	20·3 (16·1–25·4)	19·9 (15·2–25·6)
		Guinea	15·5 (10·8–22·0)	15·4 (12·3–19·0)	16·7 (14·4–19·2)	16·0 (13·5–18·7)	15·8 (13·0–19·0)	50·9 (36·0–71·0)	41·6 (33·4–50·9)	34·9 (30·3–40·0)	31·7 (27·0–36·8)	30·9 (25·5–37·0)
		Guinea-Bissau	2·87 (2·00–4·05)	3·21 (2·26–4·54)	2·85 (1·97–4·04)	2·40 (1·66–3·47)	2·33 (1·63–3·37)	58·9 (41·8–81·3)	55·9 (40·1–77·4)	38·9 (27·2–54·3)	32·3 (22·6–46·2)	31·4 (22·1–44·6)
		Liberia	6·58 (4·48–9·09)	5·03 (3·48–7·18)	4·12 (3·42–4·97)	3·40 (3·04–3·79)	3·31 (2·86–3·82)	51·7 (35·8–70·1)	36·8 (25·8–51·9)	23·8 (19·8–28·5)	20·4 (18·3–22·6)	19·8 (17·2–22·8)
		Mali	32·4 (27·6–37·8)	28·0 (22·7–34·2)	33·8 (29·3–38·9)	34·5 (29·8–40·6)	35·0 (28·8–42·8)	66·6 (57·5–77·0)	47·8 (39·1–58·0)	36·3 (31·7–41·7)	32·3 (28·0–37·8)	31·8 (26·4–38·7)
		Mauritania	3·04 (2·41–3·78)	3·31 (2·58–4·21)	5·48 (4·48–6·59)	4·55 (4·07–5·09)	4·34 (3·72–5·02)	33·5 (26·7–41·3)	30·7 (24·1–38·7)	38·4 (31·7–45·9)	32·4 (29·1–36·2)	31·1 (26·8–35·8)
		Niger	23·6 (16·8–33·3)	33·0 (28·0–38·3)	53·1 (50·9–55·4)	53·7 (42·7–66·1)	54·5 (42·2–68·4)	50·4 (36·5–69·7)	50·2 (43·0–57·8)	52·9 (50·8–55·0)	45·3 (36·4–55·2)	44·3 (34·7–55·0)
		Nigeria	246 (183–331)	321 (239–430)	490 (453–532)	481 (435–537)	472 (416–535)	55·9 (42·2–73·9)	53·3 (40·3–70·2)	57·4 (53·3–62·0)	54·9 (49·9–60·9)	53·6 (47·6–60·3)
		São Tomé and Príncipe	0·0943 (0·0665–0·131)	0·109 (0·0777–0·152)	0·0749 (0·0522–0·105)	0·0503 (0·0351–0·0721)	0·0479 (0·0337–0·0685)	19·8 (14·1–27·4)	18·8 (13·4–26·0)	13·3 (9·3–18·6)	10·0 (7·0–14·3)	9·6 (6·8–13·7)
		Senegal	12·7 (9·47–16·2)	13·9 (11·2–16·8)	24·2 (23·2–25·5)	22·3 (19·1–25·7)	21·1 (17·3–25·4)	35·1 (26·5–44·6)	32·5 (26·4–39·0)	47·3 (45·4–49·6)	44·3 (38·2–50·7)	42·2 (34·8–50·4)
		Sierra Leone	59·1 (38·0–90·7)	62·4 (41·6–95·2)	19·3 (17·0–21·9)	6·78 (6·08–7·54)	6·65 (5·73–7·66)	221·5 (156·2–306·7)	232·1 (169·3–318·1)	64·0 (56·8–72·0)	22·2 (19·9–24·6)	21·5 (18·6–24·7)
		Togo	11·3 (7·78–16·2)	12·5 (9·46–16·4)	9·90 (8·20–11·8)	8·22 (6·14–11·1)	7·91 (5·84–10·9)	66·1 (46·4–92·2)	59·3 (45·6–76·2)	37·4 (31·2–44·4)	32·4 (24·4–43·3)	31·4 (23·4–42·9)

Numbers in parentheses are 95% uncertainty intervals. Super-regions, regions, and countries are listed in alphabetical order. Total stillbirths are presented to three significant figures and stillbirth rates are presented to 1 decimal place. SDI=Socio-demographic Index.

**Figure 2:**
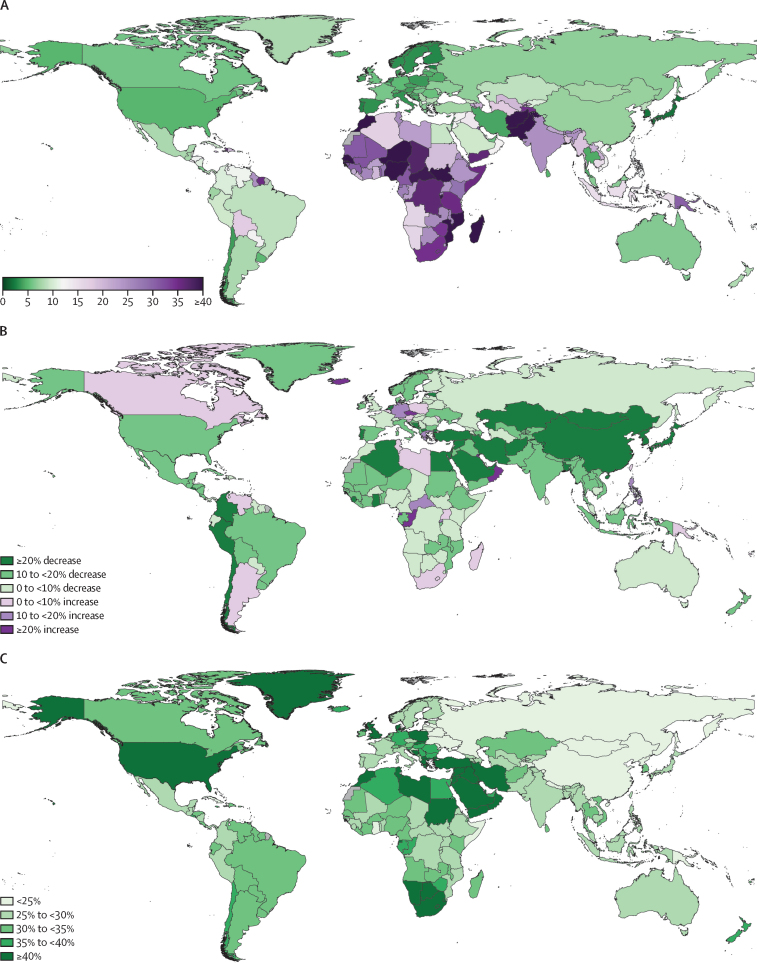
Maps of SBR, percentage change in SBR between 2015 and 2021, and percentage of stillbirths between 20 weeks' and less than 28 weeks' gestation in 2021 (A) The map shows SBR (per 1000 births) in 2021 for the 20 weeks or longer stillbirth definition. The colour scale diverges at an SBR of 12 to indicate whether countries met the ENAP target of 12 or fewer stillbirths per 1000 births in 2021. (B) The map shows the percentage change in SBR since the ENAP target was published, from 2015 to 2021. The colour scale diverges on the basis of whether SBR increased (purple) or decreased (green), with darker colours indicating a larger change in the respective direction. (C) The map shows the percentage of stillbirths that occurred between 20 weeks' and less than 28 weeks' gestation in 2021. Countries with a larger percentage of stillbirths between 20 weeks' and less than 28 weeks' gestation are darker in colour. ENAP=Every Newborn Action Plan. SBR=stillbirth rate (stillbirths at ≥20 weeks' gestation per 1000 births).

Quantifying stillbirths at any gestational age besides 20 weeks or longer misses a substantial number of fetal deaths; complete results for all thresholds are shown in appendix 2 (figure S2, tables S1 and S2). In 2021, there were approximately 1·4 times more stillbirths for the 20 weeks or longer threshold (3·04 million [95% UI 2·61–3·62]) than for the 28 weeks or longer threshold (2·11 million [1·82–2·51]) and 1·04 times more stillbirths than for the 22 weeks or longer threshold (2·93 million [2·51–3·48]). In 2021, 0·926 million (0·792–1·10) of 3·04 million stillbirths occurred globally between 20 weeks' gestation or longer and less than 28 weeks' gestation, representing 30·5% of all stillbirths; this is a slight increase from 29·3% (1·49 of 5·08 million stillbirths) in 1990. Moreover, in 2021, 0·109 million stillbirths occurred between 20 weeks' and 22 weeks' gestation (3·6% of the global total), down from 0·195 million in the same gestational range in 1990. By region, Oceania had the lowest percentage of stillbirths between 20 weeks' gestation or longer and less than 28 weeks' gestation, at 22·9% (2820 of 12 300), while southern sub-Saharan Africa had the highest percentage, at 46·2% (26 400 of 57 200). At the country level, we estimated that the average contribution of stillbirths occurring between 20 weeks' gestation or longer and less than 28 weeks' gestation ranged from 19·4% (520 of 2680) in North Korea and 19·5% (2520 of 12 900) in Haiti to 75·6% (395 of 523) in Qatar and 77·2% (1210 of 1570) in Kuwait (figure 2C).

Stillbirths were unequally distributed by country when evaluating SBR (per 1000 births) in 2021 (figure 2). The smallest estimated SBRs for the 20 weeks or longer threshold were 2·0 (95% UI 1·5–2·5) per 1000 births in Japan, 2·0 (1·7–2·4) per 1000 births in South Korea, 2·5 (2·1–2·9) per 1000 births in Estonia, 2·6 (2·0–3·4) per 1000 births in Portugal, and 2·8 (2·5–3·2) per 1000 births in Finland. Alternatively, the largest estimated SBRs for the 20 weeks or longer threshold were 68·3 (48·2–97·2) per 1000 births in South Sudan, 58·0 (49·8–67·6) per 1000 births in Morocco, 53·6 (47·6–60·3) per 1000 births in Nigeria, 44·8 (35·2–56·3) per 1000 births in Burundi, and 44·5 (33·7–57·7) per 1000 births in Pakistan (figure 2A; appendix 2 figure S2A).

The two countries or territories that showed the largest decreases in SBR per 1000 births between 2015 and 2021 for the 20 weeks or longer threshold were Iran with a 66·3% (95% UI 59·4–72·8) decrease and Sierra Leone with a 65·6% (56·1–73·6) decrease corresponding to declining annualised rates of 17·9% (13·7–22·2) and 18·2% (15·0–21·7), respectively. We estimated that the largest increases were seen in Kuwait [31·5% (10·0–56·9)] and Equatorial Guinea [37·0% (5·8–76·8)], where SBR increased annually, on average, by 4·5% (1·6–7·5) in Kuwait and by 5·1% (0·9–9·5) in Equatorial Guinea (figure 2B).

With just 9 years remaining from 2021 to 2030, only slightly more than half the countries (103 of 204) are estimated to have already met the ENAP target threshold of fewer than 12 stillbirths per 1000 births based on the more inclusive 20 weeks or longer threshold. Even with the threshold of 28 weeks or longer, this number increased to only 129 countries and territories with an SBR under the ENAP threshold in 2021, which corresponds to a total of 135 countries and territories estimated by GBD 2021 to have already met the SDG 3·2 target for neonatal mortality (<12 neonatal deaths per 1000 livebirths) and 138 countries and territories estimated by GBD 2021 to have met the SDG 3·2 target for under-5 mortality (<25 under-5 deaths per 1000 livebirths).^20^ Of the 101 countries not meeting the ENAP SBR target for 2030 based on the 20 weeks or longer definition, 45 are in sub-Saharan Africa; 21 are in southeast Asia, east Asia, and Oceania; and 15 are in Latin America and the Caribbean. At the regional level, (14 of 21 regions had at least one country still above the ENAP threshold in 2021, and four regions (central sub-Saharan Africa, eastern sub-Saharan Africa, southern sub-Saharan Africa, and south Asia) had all countries above the threshold. For the longer than 22 weeks threshold, the number was in the middle, with 106 countries and territories under the ENAP target level in 2021.

### SBR versus NMR and SDI

Between 1990 and 2021, the SBR to NMR ratio stayed relatively constant, from 1·23 (95% UI 1·00–1·53) in 1990 to 1·27 (1·10–1·47) in 2000, 1·26 (1·12–1·41) in 2010, and 1·34 (1·15–1·59) in 2021 (figure 1; appendix 2 tables S3A and S3B). Although there was a slight decrease in the SBR to NMR ratio in the 2000s, this rebounded in the 2010s.

**Figure 3:**
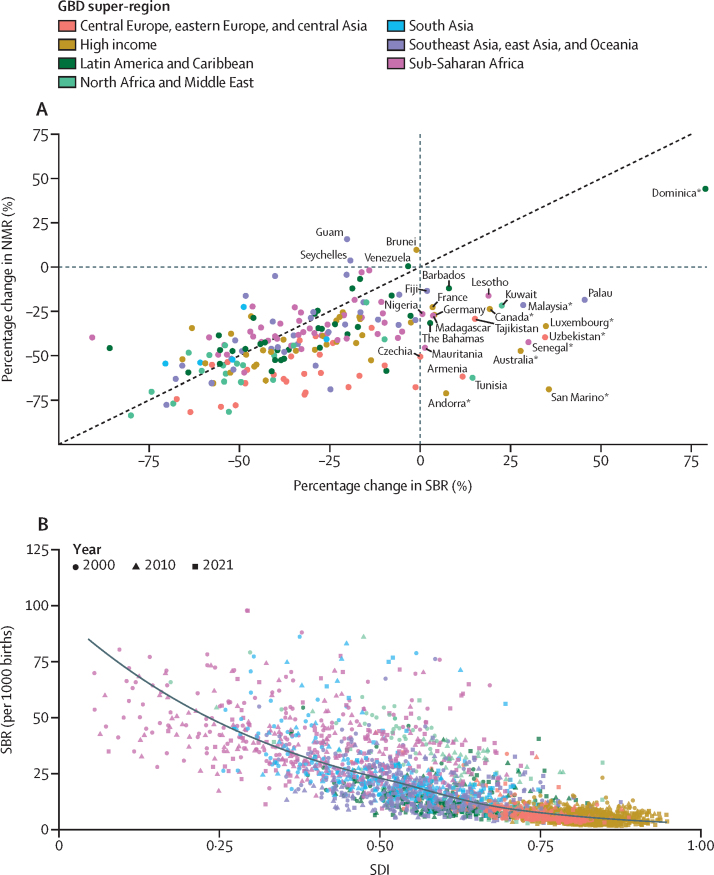
Comparative improvement in SBR and NMR and the historical association between SBR and SDI from 2000 to 2021 (A) Data points compare the percentage change in SBR and the percentage change in NMR for the 20 weeks or longer definition between 2000 and 2021 by GBD super-region, indicated by colour. Between 2000 and 2021, points in the bottom left quadrant had decreases in both SBR and NMR; points in the top left quadrant had a decrease in SBR but an increase in NMR; points in the top right quadrant had increases in both SBR and NMR; and points in the bottom right quadrant had an increase in SBR but a decrease in NMR. The diagonal line depicts where the change in SBR and NMR between 2000 and 2021 was equal. All data points not in the bottom left quadrant are labelled. (B) The scatter plot shows the spline fit between SBR and SDI, where each point represents a location. Point shape indicates year, and colour indicates GBD super-region. GBD=Global Burden of Diseases, Injuries, and Risk Factors Study. NMR=neonatal mortality rate (neonatal deaths per 1000 livebirths). SBR=stillbirth rate (stillbirths at ≥20 weeks' gestation per 1000 births). SDI=Socio-demographic Index. *Locations that had statistically significant increases in SBR.

**Figure 4:**
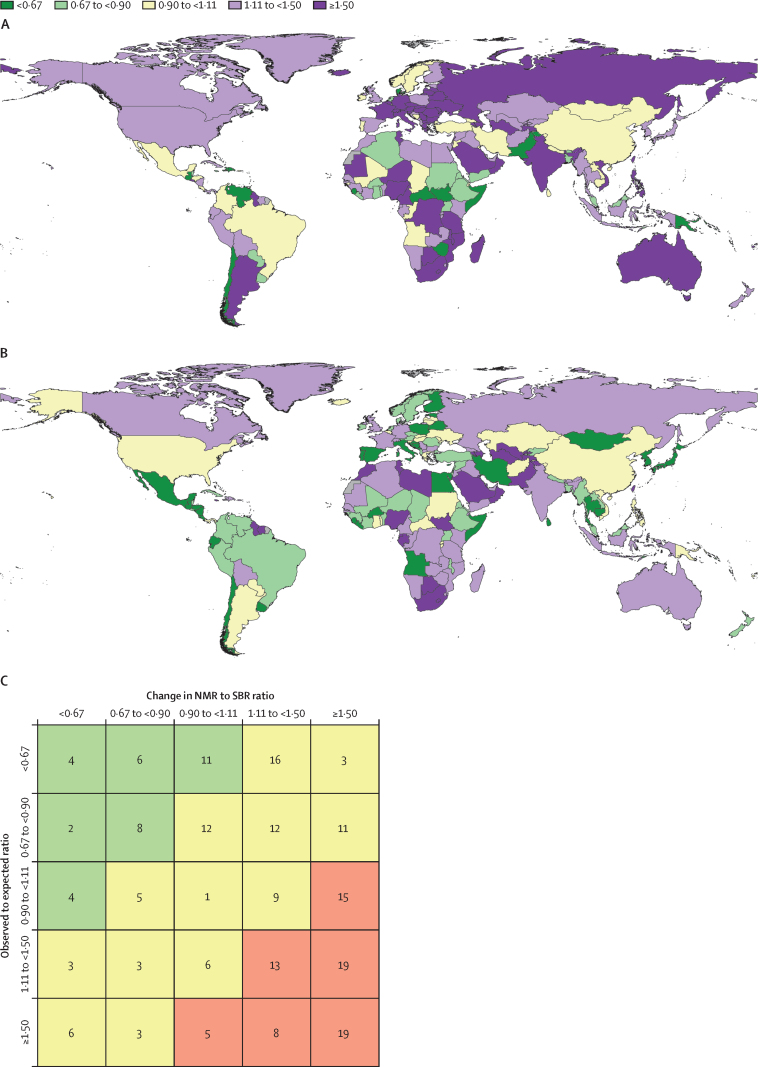
Country-specific relative changes in SBR and NMR between 2000 and 2021, ratio of observed to expected SBR based on SDI in 2021, and distribution of countries based on stillbirth performance (A) The map shows the ratio of absolute change in SBR over absolute change in NMR between 2000 and 2021. The colour scale diverges at a ratio of 1 based on whether the change in NMR was larger (purple) or change in SBR was larger (green), with darker colours indicating greater disparity in the change. (B) The map shows the ratio of the observed SBR for the 20 weeks or longer definition in 2021 compared to what SBR would be expected according to SDI, by country. The colour scale diverges at an observed to expected ratio of 1 according to whether SBR is smaller than expected (green) or larger than expected (purple). (C) The table depicts the number of countries in 25 scenarios based on their observed to expected ratio of SBR and the change in NMR to SBR ratio. The green boxes indicate countries where SBR improvements are greater than those in NMR and SDI, yellow boxes indicate where all three are similar, and red boxes indicate where SBR is not keeping up with NMR and SDI. NMR=neonatal mortality rate (neonatal deaths per 1000 livebirths). SBR=stillbirth rate (stillbirths ≥20 weeks' gestation per 1000 births). SDI=Socio-demographic Index.

Progress was unequal between stillbirths and neonatal deaths, as shown in figure 3A and figure 4A. While 180 countries had a decrease in SBR per 1000 births for the 20 weeks or longer threshold between 2000 and 2021, 24 countries had an increase. Of the countries with an increase, 22 had data available during the 2000–21 period. A larger decline in SBR than NMR was observed for 61 of the 204 countries and territories between 2000 and 2021. In 176 countries and territories both SBR and NMR decreased, in 23 SBR increased while NMR decreased, in four (Brunei, Guam, Seychelles, and Venezuela) SBR decreased while NMR increased, and in one (Dominica) both SBR and NMR increased. In 2021, the global NMR was 17·1 (95% UI 14·8–19·9) per 1000 livebirths, corresponding to 2·19 million (1·90–2·55) neonatal deaths. Globally, there was a 45·6% (36·3–53·1) reduction in neonatal deaths, with 4·03 million (3·86–4·22) neonatal deaths having occurred in 1990. The correlation between SBR (for the ≥20 weeks threshold) and NMR in 2021 was 0·85. Among the countries with declining SBRs, 142 had a relative decline in SBR greater than 20% and 45 had a relative decline greater than 50% over the 21-year period. Statistically significant increases in SBR were seen in Andorra, Australia, Canada, Dominica, Luxembourg, Malaysia, San Marino, Senegal, and Uzbekistan.

Locations with the lowest SDI status have had an increasing proportion of stillbirth burden over time. SDI has an established historical relationship with under-5 mortality, and we also estimated a correlation of –0·77 between SDI and SBR estimates at 20 weeks' gestation or longer over the entire time series, indicating that lower SDI is associated with a higher stillbirth burden (figure 3B). In addition to the overall negative correlation between SDI and stillbirth, we observed a rapidly increasing concentration of stillbirth burden in the lowest two SDI quintiles from 1990 to 2021. In 2021, 80·3% (2·44 of 3·04 million) of global stillbirths occurred in the low and low-middle SDI quintiles, up from 77·6% (2·80 of 3·61 million) in 2015, 74·5% (3·38 of 4·54 million) in 2000, and 67·9% (3·45 of 5·08 million) in 1990. This is comparable to trends in neonatal mortality, where 82·6% (1·81 of 2·19 million) of neonatal deaths occurred in the two lowest SDI quintiles in 2021, up from 67·7% (2·73 of 4·03 million) in 1990.^18^ Fertility also is correlated with SDI, but the trend in livebirths has been far less striking than that in stillbirths and neonatal deaths. In 2021, 58·4% (75·5 of 129 million) of livebirths occurred in low and low-middle SDI quintiles, up from 53·9% (76·5 of 142 million) in 2015, 52·3% (67·8 of 130 million) in 2000, and 45·3% (59·8 of 132 million) in 1990.^19^

Among the 204 countries and territories included in this study, 100 had an observed SBR lower than what would be expected based on the historical relationship with SDI. The country with the smallest observed to expected ratio based on the historical relationship with SDI was Portugal, with an observed to expected ratio of 0·34. The country with the largest observed to expected ratio based on the historical relationship with SDI was Kuwait, with an observed to expected ratio of 5·96 (figure 4B).

Differential progress was observed in relation to NMR and SDI between 2000 and 2021 (figure 4C). Chile (34·5% decrease in NMR; 63·1% decrease in SBR) and Guatemala (45·8% decrease in NMR; 85·9% decrease in SBR) were doing well with respect to SBR specifically, while Armenia (61·9% decrease in NMR; 11·7% increase in SBR) and Antigua and Barbuda (38·0% decrease in NMR; 20·1% decrease in SBR) were doing poorly. Dominica (44·1% increase in NMR; 78·9% increase in SBR) and Palau (18·6% decrease in NMR; 45·5% increase in SBR) were among the countries where SBR has a long way to go. Notably, China showed improvements in SBR and NMR (77·7% decrease in NMR; 70·2% decrease in SBR) in parallel. In Italy (46·7% decrease in NMR; 29·1% decrease in SBR; observed to expected ratio of 0·55) and Belarus (77·9% decrease in NMR; 51·1% decrease in SBR; observed to expected ratio of 0·66), stillbirths were comparatively low for the SDI level, but progress has not kept pace with NMR. appendix 2 (table S5) displays the full list of countries present in each group.

## Discussion

Stillbirth remains a major global public health concern. Although the worldwide number of stillbirths has declined gradually since 1990, the overall number of stillbirths is still substantially high. For 2021, we estimated 3·04 million stillbirths (corresponding to approximately 8328 per day; one every 10 s) according to the 20 weeks or longer threshold, nearly a third of which—926 000 in total—would have been missed by using the threshold of 28 weeks or longer and 110 000 would have been missed using the 22 weeks or longer threshold. The global SBR in 2021 was above the ENAP 2030 target threshold of 12 or fewer stillbirths per 1000 births for all three gestational time designations, with an estimated SBR of 23·0 per 1000 births (one in 44 births) for 20 weeks' gestation or longer, 22·1 per 1000 births (one in 45 births) for 22 weeks' gestation or longer, and 16·1 per 1000 births (one in 62 births) for 28 weeks' gestation or longer. Considerable variation in SBR was observed across countries and territories, ranging from 2·0 to 68·3 per 1000 births for 20 weeks' gestation or longer.

Stillbirth thresholds vary across countries, with gestational age cutoffs of 22 weeks or longer being particularly common in high-income nations where enhanced neonatal intensive care has made survival possible at earlier timepoints.^27^, ^28^ Although a stillbirth threshold of 28 weeks or longer does arguably have public health relevance by focusing on late gestation stillbirths and also allowing for international comparison in tracking progress, for example, towards the ENAP 2030 target, it is important to stress to individual countries that the insufficient data on the 20 weeks or longer (full enumeration) and 22 weeks or longer (ICD-11) thresholds need to be improved to address early gestation stillbirths as well. Smith and colleagues^29^ estimated that, in 2015, 32% of stillbirths (occurring at ≥22 weeks and <28 weeks) in developed countries were overlooked when using the 28 weeks or longer gestational age designation; this is even larger than our global estimate of 30·5% of all stillbirths occurring at 22 weeks' gestation or longer and less than 28 weeks' gestation in 2021. The adoption of a lower completed gestational age cutoff for stillbirths not only reflects current improvements in medical care that are possible in some high-income countries, but it indicates the trajectory that is needed to address stillbirths in many countries over time and provides a crucial piece of information to help understand the full burden of perinatal mortality and fetal losses.

Regardless of the gestational cutoff, countries in sub-Saharan Africa and south Asia collectively accounted for almost three-quarters of all stillbirths—a pattern also documented in a separate global assessment in 2019.^30^ The majority of stillbirths in these high-burden regions take place in rural areas with low HAQ Indices.^31^, ^32^ Structural inequalities such as reduced total health spending per capita and lowered health system inputs result in restricted access to and utilisation of medical services, including midwifery care, emergency obstetric care, and family planning services. A related contributor to disparities in SBRs is the poor focus on the quality of pre-conception, antenatal, and intrapartum care services. Correspondingly, our analyses indicate that the reduction in SBRs over the past 30 years has been slower in sub-Saharan Africa (36%), as well as central Europe, eastern Europe, and central Asia (20%), compared to globally (39%). Of notable concern is our observation that, over time, low SDI countries have been contributing an increasingly large proportion of stillbirths to the global total. As we consider strategies for continued decreases in stillbirths, universal access to high-quality medical care—especially antenatal care—must be a central goal.

Global reductions in stillbirths have not kept pace with declines in neonatal mortality and under-5 mortality, signifying insufficient attention and resource allotment towards improvements in the quality and coverage of antenatal care services and intrapartum care services—crucial pathways towards ending preventable stillbirths.^33^, ^34^, ^35^, ^36^ Stillbirth research has received minimal funding in both high-income countries and low-income and middle-income countries (LMICs). Direct investment in LMIC-led research is recommended to accelerate the slow global progress on stillbirth prevention.^37^ A recent review of policies from 155 countries highlighted that the current policy environment in many countries is not supportive for identifying stillbirths and recording causes of death, compared with that for neonatal and under-5 deaths, which is likely to contribute to continued slow progress in stillbirth reduction in these countries.^38^ Similarly, a recent analysis from India highlighted the invisibility of stillbirths in data collection in the sample registration system used to track perinatal mortality, compared with household surveys.^39^ Calls have been made to improve the counting of every stillbirth, along with neonatal deaths, through mortality audits to improve the quality of care for every pregnant woman and her baby, and to systematically capture and review the causes and avoidable factors linked to these deaths in order to effect change.^40^ Further insights can be gained from community-level assessments of the causes of stillbirths, allowing for focused efforts to promote maternal care and survival of newborns. In rural Ghana, a community-based verbal autopsy tool was used to identify infections (eg, syphilis, malaria, and HIV) as a major cause of death in the antepartum period, while labour and delivery (the intrapartum period) were documented as the riskiest timeframe for stillbirth occurrence.^41^ Additionally, population surveys in India have highlighted that the absence of timely care from a health-care provider and poor knowledge and performance on the part of the health-care provider are key risk factors associated with stillbirths, along with deferred and referred deliveries.^42^, ^43^ Studies such as these allow for evidence-based health interventions and policy strategies that are tailored to the unique circumstances of a particular setting.

There is mixed evidence of the impact of the COVID-19 pandemic on SBRs. The available literature captures SBRs in pregnant women with COVID-19, SBRs in pregnant women without COVID-19 during the same period, and population-level SBRs in pre-pandemic and pandemic periods, with a further breakdown of the pandemic between lockdown and post-lockdown periods. The reported data on SBRs during the pandemic have, however, been inconsistent, with some high-income countries and LMICs reporting a rise and others reporting no change.^44^, ^45^, ^46^, ^47^, ^48^, ^49^, ^50^ Additional population-level data are needed, particularly from LMICs, to better understand the observed increase in SBRs and the associated implications for reaching the ENAP target.

Two other sets of global estimates of stillbirths are available. The first is from the LSEIG, which used the thresholds of 28 weeks or longer or 1000 g or greater to model SBRs directly for 1995 and 2009,^12^, ^51^ and also compared data from 2000 and 2015 with a 28 weeks or longer stillbirth threshold.^12^ The total number of stillbirths for the 28 weeks or longer threshold in 2015 was 2·62 million, compared with 2·55 million presented in this study. A comparison of each set of estimates is shown in appendix 2 (figure S3; table S5). We calculated a correlation of 0·87 in country-specific estimates between the SBR estimates from the two analyses for the year 2015, with the greatest absolute differences in Djibouti and Angola (LSEIG estimates larger than GBD estimates) and Mozambique and South Sudan (LSEIG estimates smaller than GBD estimates).

The second set of stillbirth estimates is available from the UN IGME, from 2000 to 2021 for 195 countries.^13^, ^14^ The UN IGME estimated 1·9 million (90% UI 1·8–2·0) stillbirths for the 28 weeks or longer threshold in 2021, corresponding to an SBR of 13·9 (13·3–15·1) per 1000 births. At the country level, the calculated correlation was 0·84, with the largest percentage differences in Liberia and Sudan (UN IGME estimates larger than GBD estimates) and Morocco and South Sudan (UN IGME estimates smaller than GBD estimates). appendix 2 (figure S3, table S5) also illustrates a comparison between UN IGME and the GBD 2021 SBR estimates for 2021.

Although all analyses generally agree that stillbirths are a major problem globally, with the number of stillbirths of roughly equal magnitude to the total number of neonatal deaths, there are some important differences between the various analyses. First, the LSEIG and UN IGME estimates use only the 28 weeks or longer threshold, whereas GBD 2021 estimated for the 20 weeks or longer threshold in addition to the 28 weeks or longer threshold (and ≥22 weeks for comparison). Second, the LSEIG and UN IGME estimates are based on a statistical model to estimate SBR directly, whereas we modelled the SBR to NMR ratio, which allows us to directly leverage all the insights of the GBD demographics analysis to generate estimates that are internally consistent with other disease burden assessments. Relatedly, although the UN IGME estimates are reportedly adjusted for all data to a reference threshold of 28 weeks or longer, neither dataset adjusted data where gestational age and birthweight were considered equivalent and neither systematically accounted for known under-reporting or completeness of vital statistics. Fourth, the GBD 2021 dataset used for modelling included 11 412 source-location-years of data, which is much larger than the 2207 datapoints used in LSEIG estimates and 1531 datapoints used in UN IGME estimates.^12^, ^52^

We acknowledge several limitations to this analysis. First, as we worked within the framework of GBD 2021, our findings share the limitations of this broader research effort—most notably, those of GBD fertility and neonatal mortality estimates on which this analysis depends and makes an assumption that SBR completeness tracks with NMR, which might not always be the case. Second, the precision of our modelled estimates is hindered by a comparative sparsity of primary data on stillbirths, especially in sub-Saharan Africa and south Asia, where the estimated burden is the highest. Data also tend to be sparser in recent years, given the time required for countries to finalise and release data. Third, although we have undertaken extensive efforts to correct for biases and standardise data (especially with respect to stillbirth definitions), these adjustments are limited because not all data sources provide documentation on the definitions used, documentation in a foreign language could have been misinterpreted, we cannot fully control for potential misrepresentation of abortions as stillbirths in administrative locations where abortion is restricted, and our statistical approaches do not account for potential measurement error in gestational ages or weights used to inform adjustments. Household surveys remain an important source of stillbirth data in many countries, and classifying an adverse pregnancy outcome as stillbirth requires accurate reporting of vital status at birth, gestational age, or birthweight for every pregnancy by participating women. Addressing the issues identified in misclassification and misreporting of these parameters is a limitation, which is beyond the scope of the present analysis.^39^, ^42^, ^53^, ^54^ Fourth, in concentrating on maximising the comparability and comprehensiveness of stillbirth estimates for all gestational ages of 20 weeks or longer, we did not attempt to add estimates for other dimensions of stillbirth statistics including underlying cause, timing (ie, intrapartum *vs* antepartum), or preventable versus non-preventable stillbirths. Although we believe complete enumeration is an important prerequisite, addition of these other dimensions to stillbirth statistics in the future is likely to be very valuable in informing local policy, research, education, and clinical practice.

The burden of stillbirths is immense and unevenly distributed across the world. Including stillbirths from 20 weeks and beyond in our analysis allowed us to gauge more fully the magnitude of the problem; yet sparse data availability and poor data quality continue to constrain our capacity to make precise estimates for many locations. Expanded investment in recognising and counting each stillbirth is central to not only quantifying the burden of stillbirths but also to appropriately investing in stillbirth prevention. Detailed information on the timing, location, and possible cause of stillbirth, alongside demographic characteristics, will facilitate the prioritisation of regions, countries, and populations that are most in need of life-saving interventions. Further progress towards reaching the ENAP 2030 target rate of stillbirths will require enhanced access to and utilisation of high-quality health care during the antenatal period and the stages of labour and delivery.

### GBD 2021 Global Stillbirths Collaborators

### Affiliations

### Contributors

### Data sharing

To download the data used in these analyses and corresponding results, please visit the Global Health Data Exchange at http://ghdx.healthdata.org.

## Declaration of interests

S Afzal reports support for the present manuscript from King Edward Medical University, which provided study material, research articles, valid data sources, and authentic real-time information for this manuscript; reports payment or honoraria for lectures, presentations, speakers bureaus, manuscript writing, or educational events from King Edward Medical University and collaborative partners including University of Johns Hopkins, University of California, University of Massachusetts, King Edward Medical College Alumni Association of North America (KEMCAANA), and Kings Edward Medical College Alumni Association UK (KEMCA-UK), as well as participation in international scientific conferences, webinars, and meetings; support for attending meetings or travel, or both, from King Edward Medical University to discuss findings and gather the latest information from various sources; participation on a Data Safety Monitoring Board or Advisory Board with the National Bioethics Committee Pakistan, the King Edward Medical University Ethical Review Board, the Ethical Review Board Fatima Jinnah Medical University and Sir Ganga Ram Hospital, and the Technical Working Group on Infectious Diseases to formulate guidelines; leadership or fiduciary roles in board, society, committee, or advocacy groups paid or unpaid with the Pakistan Association of Medical Editors, the Society of Prevention, Advocacy, and Research at King Edward Medical University (SPARK), and the Pakistan Society of Infectious Diseases, as well as being a Fellow of the Faculty of Public Health Royal Colleges UK (FFPH); other support from serving as Dean of Public Health and Preventive Medicine and Chief Editor of the Annals of King Edward Medical University since 2014, Director of the Quality Enhancement Cell at King Edward Medical University, Advisory Board Member and Chair of the Scientific Session at KEMCA-UK, and Chairperson of the International Scientific Conference at KEMCAANA; other support from membership in the Research and Publications Committee of the Higher Education Commission (HEC) Pakistan, the Research and Journals Committee at the Pakistan Medical and Dental Council, and the National Bioethics Committee Pakistan; other support from serving on the Corona Experts Advisory Group, the Technical Working Group on Infectious Diseases, the Dengue Experts Advisory Group, and as Chair of the Punjab Residency Program Research Committee; all outside the submitted work. R Bai reports support for the present manuscript from the Social Science Fund of Jiangsu Province (grant number 21GLD008) and the Fundamental Research Funds for the Central Universities (grant number 30923011101). S Bhaskar reports grants or contracts from the Japan Society for the Promotion of Science (JSPS), Japanese Ministry of Education, Culture, Sports, Science and Technology (MEXT), including Grant-in-Aid for Scientific Research (KAKENHI) (P23712), and the JSPS and the Australian Academy of Science JSPS International Fellowship (P23712); leadership or fiduciary roles in board, society, committee, or advocacy groups paid or unpaid with Rotary District 9675, Sydney, Australia (District Chair, Diversity, Equity & Inclusion), Global Health & Migration Hub Community, Global Health Hub Germany, Berlin, Germany (Chair, Founding Member, and Manager), *PLOS One, BMC Neurology, Frontiers in Neurology, Frontiers in Stroke, Frontiers in Public Health, Journal of Aging Research*, and *BMC Medical Research Methodology* (Editorial Board Member), College of Reviewers, Canadian Institutes of Health Research (CIHR), Government of Canada (Member), World Headache Society, Bengaluru, India (Director of Research), Cariplo Foundation, Milan, Italy (Expert Adviser/Reviewer), National Cerebral and Cardiovascular Center, Department of Neurology, Suita, Osaka, Japan (Visiting Director), and Cardiff University Biobank, Cardiff, UK (Member, Scientific Review Committee); all outside the submitted work. A A Fomenkov reports support for the present manuscript from research carried out within the state assignment of the Ministry of Science and Higher Education of the Russian Federation (theme number 122042600086-7). A Guha reports grants or contracts from the American Heart Association and the US Department of Defense; consulting fees from Pfizer and Novartis; and leadership or fiduciary roles in board, society, committee, or advocacy groups paid or unpaid with ZERO Prostate Cancer Health Equity Task Force; all outside the submitted work. C Herteliu reports grants or contracts from the Romanian Ministry of Research, Innovation and Digitalization through UEFISCDI for the project “Analysis of the impact of Covid-19 on the main demographic indicators in Romania and the Republic of Moldova by using econometric modeling” (code PN-IV-P8-8.3-ROMD-2023-0208); a grant from the European Commission Horizon 4P-CAN (Personalised Cancer Primary Prevention Research through Citizen Participation and Digitally Enabled Social Innovation); the European Union – NextGenerationEU and Romanian Government under the National Recovery and Resilience Plan for Romania for the projects “Societal and Economic Resilience within multi-hazards environment in Romania” (contract number 760050/23.05.2023, cod PNRR-C9-I8-CF 267/29.11.2022) and “A better understanding of socio-economic systems using quantitative methods from Physics” (contract number 760034/23.05.2023, cod PNRR-C9-I8-CF 255/29.11.2022) within Component 9, Investment I8; all outside the submitted work. I M Ilic reports support for the present manuscript from the Ministry of Education, Science and Technological Development, Republic of Serbia, project number 175042, 2011–2023. M D Ilic reports support for the present manuscript from the Ministry of Science, Technological Development and Innovation of the Republic of Serbia (number 451-03-47/2023-01/200111). B Lacey reports support for the present manuscript from UK Biobank, funded largely by the UK Medical Research Council and Wellcome; and reports employment with the University of Oxford, with the post funded by a grant from UK Biobank; all outside the submitted work. M Lee reports support for the present manuscript from the Ministry of Education of the Republic of Korea and the National Research Foundation of Korea (NRF-2023S1A3A2A05095298). M-C Li reports support for the present manuscript from the National Science and Technology Council, Taiwan (NSTC 113-2314-B-003-002); and reports leadership or fiduciary roles in other board, society, committee, or advocacy groups, paid or unpaid with the *Journal of the American Heart Association* as Technical Editor; all outside the submitted work. L Monasta reports support for the present manuscript from the Italian Ministry of Health (Ricerca Corrente 34/2017), with payments made to the Institute for Maternal and Child Health IRCCS Burlo Garofolo. F Mughal reports support for the present manuscript from NIHR Doctoral Fellow, 300957, with payments made to Keele University. S Nomura reports support for the present manuscript from the Ministry of Education, Culture, Sports, Science and Technology of Japan (24H00663) and the Precursory Research for Embryonic Science and Technology from the Japan Science and Technology Agency (JPMJPR22R8). A P Okekunle reports support for the present manuscript from the National Research Foundation of Korea funded by the Ministry of Science and ICT (2020H1D3A1A04081265); and reports support for attending meetings or travel, or both, from the National Research Foundation of Korea funded by the Ministry of Science and ICT (2020H1D3A1A04081265); all outside the submitted work. M Pigeolet reports grants or contracts from the Belgian Kids' Fund for Pediatric Research. L Ronfani reports support for the present manuscript from the Italian Ministry of Health (Ricerca Corrente 34/2017), with payments made to the Institute for Maternal and Child Health IRCCS Burlo Garofolo. J P Silva reports support for the present manuscript from the Portuguese Foundation for Science and Technology, with payment of salary under the contract reference 2021.01789.CEECIND/CP1662/CT0014. C R Simpson reports grants or contracts from the Health Research Council (HRC) of New Zealand, the New Zealand Ministry of Health, the Ministry of Business, Innovation and Employment (MBIE) of New Zealand, the Chief Scientist Office of the UK, and the UK Medical Research Council; and leadership or fiduciary roles in board, society, committee, or advocacy groups, paid or unpaid with the New Zealand Government Data Ethics Advisory Group (Chair, remunerated under the NZ Cabinet Fees Framework); all outside the submitted work. J A Singh reports consulting fees from ROMTech, Atheneum, Clearview Healthcare Partners, American College of Rheumatology, Yale, Hulio, Horizon Pharmaceuticals, DINORA, ANI/Exeltis USA, Frictionless Solutions, Schipher, Crealta/Horizon, Medisys, Fidia, PK Med, Two Labs, Adept Field Solutions, Clinical Care Options, Putnam Associates, Focus Forward, Navigant Consulting, Spherix, MedIQ, Jupiter Life Science, UBM LLC, Trio Health, Medscape, WebMD, Practice Point Communications, and the US National Institutes of Health, with consultant fees paid directly for each entity; payment or honoraria for lectures, presentations, speakers bureaus, manuscript writing, or educational events from the speakers bureau of Simply Speaking; support for attending meetings or travel, or both, from OMERACT, as a past steering committee member and received support to attend meetings every 2 years; participation on a Data Safety Monitoring Board or Advisory Board with the FDA Arthritis Advisory Committee, as a member without financial support; leadership or fiduciary roles in board, society, committee, or advocacy groups paid or unpaid with OMERACT (an international organisation developing clinical trial measures funded at arm's length by pharmaceutical companies, with J A Singh previously receiving meeting support), as Chair of the Veterans Affairs Rheumatology Field Advisory Committee (without financial support), and as Editor and Director of the UAB Cochrane Musculoskeletal Group Satellite Center on Network Meta-analysis (without financial support); and stock or stock options in Atai Life Sciences, Kintara Therapeutics, Intelligent Biosolutions, Acumen Pharmaceutical, TPT Global Tech, Vaxart Pharmaceuticals, Atyu Biopharma, Adaptimmune Therapeutics, GeoVax Labs, Pieris Pharmaceuticals, Enzolytics, Seres Therapeutics, Tonix Pharmaceuticals Holding Corp., Aebona Pharmaceuticals, and Charlotte's Web Holdings, with previous stock ownership in Amarin, Viking, and Moderna Pharmaceuticals; all outside the submitted work. S J Tromans reports grants or contracts from NHS Digital via the Department of Health and Social Care for the 2023 Adult Psychiatric Morbidity Survey, with payments made to the University of Leicester; and reports leadership or fiduciary roles in board, society, committee, or advocacy groups paid or unpaid with the Neurodevelopmental Psychiatry Special Interest Group, the Faculty of Psychiatry of Intellectual Disability (both Royal College of Psychiatrists), and editorial board roles with *BMC Psychiatry, Advances in Mental Health and Intellectual Disabilities, Advances in Autism*, and *Progress in Neurology and Psychiatry*; all outside the submitted work. G Zamagni reports support for the present manuscript from the Italian Ministry of Health (Ricerca Corrente 34/2017), with payments made to the Institute for Maternal and Child Health IRCCS Burlo Garofolo. M Zielińska reports other financial or non-financial interests as an employee of AstraZeneca; all outside the submitted work. All other authors declare no competing interests.
