# Supplementary material for: Global, regional, and national stillbirths at 20 weeks' gestation or longer in 204 countries and territories, 1990–2021: findings from the Global Burden of Disease Study 2021
Source: Lancet. 2024 Nov 16;404(10466):1955–88. doi: 10.1016/S0140-6736(24)01925-1 (PMC11694012; doi:10.1016/S0140-6736(24)01925-1)
Supplement: Supplementary appendix 1 [file mmc1.pdf]

THE LANCET

Supplementary appendix 1

This appendix formed part of the original submission and has been peer reviewed. We post it as supplied by the authors.

Supplement to: GBD 2021 Global Stillbirths Collaborators. Global, regional, and national stillbirths at 20 weeks' gestation or longer in 204 countries and territories, 1990–2021: findings from the Global Burden of Disease Study 2021. *Lancet* 2024; published online Nov 4. https://doi.org/10.1016/S0140-6736(24)01925-1.

Appendix 1 to Stillbirths GBD 2021

Table of Contents

1 Overview 3
1.1 Appendix 1 Table S1: GATHER checklist of information that should be included in reports of global health estimates 4
1.2 Appendix 1 Figure S1: Analytical Flowchart for the GBD 2021 Stillbirth Estimation Process 6
2 Geographical units and time periods 6
3 Thresholds of stillbirth 7
3.1 Appendix 1 Table S2: Universal thresholds of stillbirth 7
4 Data seeking 7
4.1 Vital Registration 8
4.2 Household Surveys 9
4.3 Literature 9
4.3.1 Search string 9
4.3.2 Appendix 1 Figure S2: PRISMA diagram for systematic review 10
4.4 Data Coverage Visuals 11
4.4.1 Appendix 1 Table S3: Distribution of thresholds in stillbirth data 11
4.4.2 Appendix 1 Table S4: Distribution of data points assigned to each stillbirth threshold by location 11
4.4.3 Appendix 1 Figure S3A: Map of the number of years of vital registration data by location 37
4.4.3 Appendix 1 Figure S3B: Map of the number of years of survey data by location 38
4.4.4 Appendix 1 Figure S3C: Map of the number of years of scientific literature data by location 39
4.4.5 Appendix 1 Figure S3D: Map of the number of years of data by location 40
4.4.6 Appendix 1 Figure S3E: Dot plot of annual data coverage by GBD region 41
5 Data processing 41
5.1 Reassignment of thresholds in data (including unknowns) 41
5.2 Data adjustment for completeness 42
5.3 Standardising all data to be SBR/NMR 42
5.4 Outlier criteria (round 1) 43

5.4.1 Appendix 1 Table S5: Distribution of data points outliered by various SBR/NMR thresholds ... 45
5.5 Crosswalking all non-reference data to the reference threshold ... 76
5.5.1 Appendix 1 Table S6: Number of direct comparisons to each reference threshold for crosswalking ... 76
5.5.2 Appendix 1 Table S7: Number of indirect comparisons to each reference threshold for crosswalking ... 77
5.5.3 Appendix 1 Table S8: Beta coefficients from crosswalking comparing non-reference data to the reference threshold ... 78
5.5.4 Appendix 1 Figure S4: Dose response plots from crosswalking (≥20 weeks threshold) ... 78
5.6 Outlier criteria (round 2) ... 81
5.7 Data Coverage Visuals (Post-Outliering) ... 82
5.7.1 Appendix 1 Figure S5A: Map of the number of years of data included in the model by location ... 82
5.7.2 Appendix 1 Figure S5B: Map of the percentage of data outliered for each location 83
6 Modelling SBR/NMR using Spatiotemporal Gaussian Process Regression (ST-GPR) ... 84
6.1 Stage 1: Ensemble model ... 84
6.1.1 Appendix 1 Table S9: Covariates tested in the stage 1 ensemble model ... 85
6.1.2 Appendix 1 Figure S6: Standardised betas among covariates present in selected models (28 weeks threshold) ... 86
6.2 Stage 2: Spatiotemporal smoothing ... 86
6.2.1 Appendix 1 Table S10: Hyperparameter values by data density ... 87
6.3 Stage 3: Gaussian Process Regression ... 87
6.4 Raking and aggregating settings in ST-GPR ... 87
6.5 Calculation of stillbirth ratio from SBR/NMR ... 87
6.5.1 Uncertainty ... 88
6.6 Secondary analysis ... 88
6.6.1 Stillbirth ratio vs. SDI (Epi Transition) ... 88
7 References ... 89
8 Authorship appendix to "GBD 2021 Global Stillbirths" ... 91
8.1 GBD 2021 Global Stillbirths Collaborators ... 91
8.2 Author Affiliations ... 94
8.3 Authors' Contributions ... 113
8.3.1 Managing the overall research enterprise ... 113

8.3.2 Writing the first draft of the manuscript 113
8.3.3 Primary responsibility for applying analytical methods to produce estimates 113
8.3.4 Primary responsibility for seeking, cataloguing, extracting, or cleaning data; designing or coding figures and tables 113
8.3.5 Providing data or critical feedback on data sources 113
8.3.6 Developing methods or computational machinery 115
8.3.7 Providing critical feedback on methods or results 115
8.3.8 Drafting the work or revising it critically for important intellectual content 118
8.3.9 Managing the estimation or publications process 120

1 Overview

Below, we describe each step in our process of estimating stillbirth occurrence over time, emphasising changes we have made since last publishing stillbirth estimates for GBD 2016.[1] This analysis used Python version 3·6·2, Stata versions 13 and 15, and R versions 3·5·0, 3·6·3, 4·0·3, and 4·2·1. In accordance with the Guidelines for Accurate and Transparent Health Estimates Reporting (GATHER) recommendations, we have provided the GATHER checklist as Appendix 1 Table S1.[2] The GATHER recommendations can be found here: http://gather-statement.org/

1.1 Appendix 1 Table S1: GATHER checklist of information that should be included in reports of global health estimates

Item #	Checklist item	Reporting location
**Objectives and funding**		
1	Define the indicator(s), populations (including age, sex, and geographic entities), and time period(s) for which estimates were made.	Main text methods ("Overview" section, paragraph 2)<br>Appendix 1 section 2 ("Geographical units and time periods")
2	List the funding sources for the work.	Main text summary ("Funding" section)
**Data Inputs**		
For all data inputs from multiple sources that are synthesized as part of the study:		
3	Describe how the data were identified and how the data were accessed.	Main text methods ("Definitions and data seeking" section)<br>Appendix 1 section 4 ("Data seeking")
4	Specify the inclusion and exclusion criteria. Identify all ad-hoc exclusions.	Exclusion criteria summarized in Main text methods ("Data processing" section); Full inclusion and exclusion criteria provided in Appendix 1 sections 5.4 and 5.6 ("Data processing" → "Outlier criteria (round 1)" and "Outlier criteria (round 2)"); Reasons for exclusion and number of studies excluded also provided in Appendix 1figure 2 (PRISMA diagram)
5	Provide information on all included data sources and their main characteristics. For each data source used, report reference information or contact name/institution, population represented, data collection method, year(s) of data collection, sex and age range, diagnostic criteria or measurement method, and sample size, as relevant.	Citations are provided on the GHDx (URL will be provided at resubmission and will go live at publication)<br>Appendix 2 Figure 4 ("Model results, source list, and final estimates of # stillbirths, SBR, and SBR/NMR for both definitions, by location")
6	Identify and describe any categories of input data that have potentially important biases (e.g., based on characteristics listed in item 5).	Main text methods ("Data processing" section)<br>Appendix 1 section 5 ("Data processing")
For data inputs that contribute to the analysis but were not synthesized as part of the study:		
7	Describe and give sources for any other data inputs.	Appendix 1 section 4 ("Data seeking")<br>Citations are provided on the GHDx (URL will be provided at resubmission and will go live at publication)
For all data inputs:		
8	Provide all data inputs in a file format from which data can be efficiently extracted (e.g., a spreadsheet rather than a PDF), including all relevant meta-data listed in item 5. For any data inputs that cannot be shared because of ethical or legal reasons, such as third-party ownership, provide a contact name or the name of the institution that retains the right to the data.	Data inputs in excel format available on the GHDx (URL will be provided at resubmission and will go live at publication)
**Data analysis**		
9	Provide a conceptual overview of the data analysis method. A diagram may be helpful.	Main text methods ("Data processing" section)<br>Appendix 1 section 5 ("Data processing")<br>Appendix 1 figure 1 (Analytical flowchart)<br>Appendix 1 figure 2 (PRISMA diagram)
10	Provide a detailed description of all steps of the analysis, including mathematical formulae. This description should cover, as relevant, data cleaning, data pre-processing, data adjustments and weighting of data sources, and mathematical or statistical model(s).	Main text methods ("Data processing", "Modelling SBR/NMR", and "Calculating SBR and stillbirth counts, secondary analyses, and presentation of results" sections)<br>Appendix 1 sections 5 and 6 ("Data processing" and "Modelling SBR/NMR using ST-GPR")<br>Appendix 1 figure 1 (Analytical flowchart)
11	Describe how candidate models were evaluated and how the final model(s) were selected.	Appendix 1 section 6.1 ("Modelling SBR/NMR using ST-GPR" → "Stage 1: Ensemble model")
12	Provide the results of an evaluation of model performance, if done, as well as the results of any relevant sensitivity analysis.	Appendix 1 section 6.1 ("Modelling SBR/NMR using ST-GPR" → "Stage 1: Ensemble model")
13	Describe methods for calculating uncertainty of the estimates. State which sources of uncertainty were, and were not, accounted for in the uncertainty analysis.	Appendix 1 section 6.5.1 ("Modelling SBR/NMR using ST-GPR" → "Calculation of stillbirth ratio from SBR/NMR" → "Uncertainty")
14	State how analytic or statistical source code used to generate estimates can be accessed.	GitHub URL will be provided at resubmission
**Results and Discussion**		
15	Provide published estimates in a file format from which data can be efficiently extracted.	URL for access to estimates in excel files will be provided at resubmission and will go live at publication (https://vizhub.healthdata.org/gbd-results/)
16	Report a quantitative measure of the uncertainty of the estimates (e.g. uncertainty intervals).	95% UIs are given for all mean estimates in the Main text and Appendix 1<br>Online viz tools (see information above)
17	Interpret results in light of existing evidence. If updating a previous set of estimates, describe the reasons for changes in estimates.	Main text introduction (paragraphs 3-4)<br>Main text discussion ("Comparison to estimates from other groups" section)
18	Discuss limitations of the estimates. Include a discussion of any modelling assumptions or data limitations that affect interpretation of the estimates.	Main text discussion ("Limitations" section)

In GBD 2016, stillbirth estimation used data from vital registration, surveys, and scientific literature. Live birth counts came from the World Population Prospects 2015 or from interpolated census birth counts because GBD was not yet estimating this input.[3] A mixed effects generalised linear model was used to predict the ratio of the stillbirth rate over the neonatal mortality rate (SBR/NMR) in natural logarithmic space. The decision to include NMR in the model was based upon the fact that incorporating a relationally dependent variable led to more consistent results since the input stillbirth data were sparse. The model equation was:

$$log\left(\frac{SBR}{NMR}\right) = \left(\beta_1 * education_{cy}\right) + \alpha_{threshold} + \alpha_t + \gamma_{nmr} + \gamma_c + \gamma_{cs} + \epsilon_{cys}$$

Where β was a fixed covariate coefficient for the mean years of education among reproductive age women, α represented fixed effect dummy variables for stillbirth $threshold$ and the source type (t), γ represented the random effects for nmr, country (c), and country-source (cs), and ϵ was the residual for the country-year-source (cys). Data were assigned as either a reference source (generally complete vital registration) or not, and non-reference sources were adjusted based on the fixed and random effects. Additionally, data were multiplied by an adjustment factor based on the work from the *Lancet* Stillbirth Epidemiology Investigator Group to account for child mortality completeness.[4] These adjustment factors depended on stillbirth threshold, ranging from 0·46 to 1·15 for developed locations and 0·89 to 1 for other locations. Then, spatiotemporal smoothing and Gaussian Process Regression were run to generate second stage predictions and final estimates, respectively.

While both spatiotemporal smoothing and Gaussian Process Regression were again used in the estimation process, many updates and improvements were made to data processing. Appendix 1 Figure S1 illustrates the data processing and modeling step taken to produce estimates of stillbirth for GBD 2021. Each step will be discussed in detail below.

1.2 Appendix 1 Figure S1: Analytical Flowchart for the GBD 2021 Stillbirth Estimation Process

Caption: The boxes in this flowchart depict each step of data processing and modeling that was undertaken to generate stillbirth estimates.

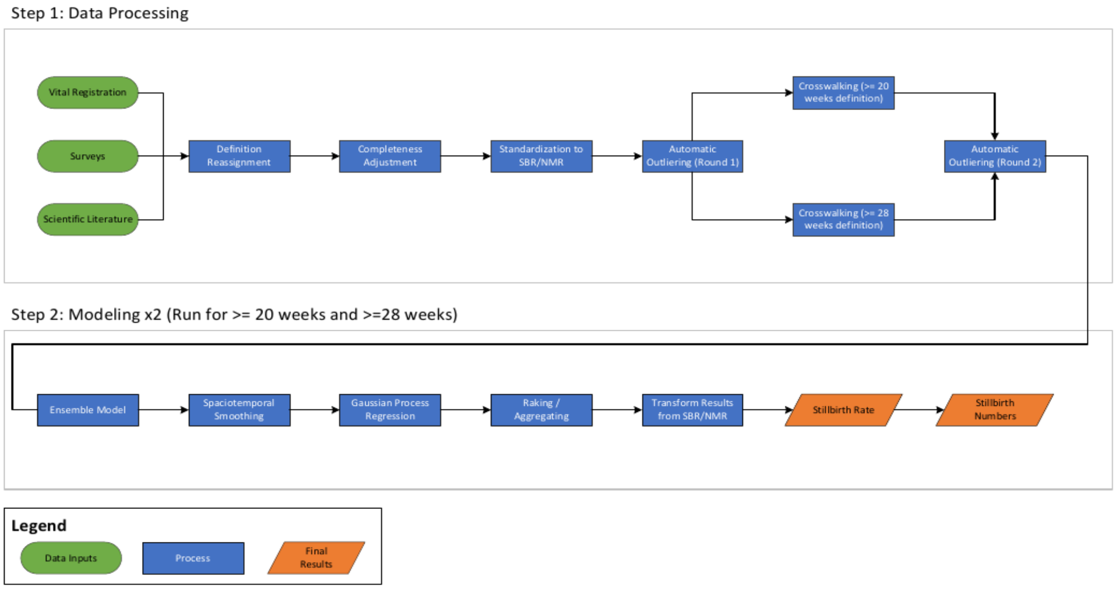

2 Geographical units and time periods

In this analysis, we present global, regional, national, territory, and for a subset of countries, subnational level results from 1990 to 2021. We estimated the number of stillbirths and the stillbirth rate (SBR) when fetal death occurs after at least 20 weeks of gestation and after at least 28 weeks of gestation.

Our analysis covers 204 countries and territories, grouped into seven super-regions and 21 regions. Since last publishing on stillbirth estimation in GBD 2016, we have added 9 new national locations (Cook Islands, Monaco, Nauru, Niue, Palau, Saint Kitts and Nevis, San Marino, Tokelau, Tuvalu). The GBD location hierarchy includes all WHO member states. Further, our subnational analysis has now expanded to include 11 additional countries (Ethiopia, Iran, Italy, New Zealand, Nigeria, Norway, Pakistan, Philippines, Poland, Russia, Ukraine), joining 11 countries (Brazil, China, India, Indonesia, Japan, Kenya, Mexico, South Africa, Sweden, UK, USA) which were previously estimated at the subnational level. These subnational analyses are generally performed at the first administrative level of organisation, but there were a few exceptions. Specifically, these countries were India (by state and urbanicity), Kenya (by district and province), New Zealand (by Maori ethnicity), the Philippines (by province), Sweden (by Stockholm and non-Stockholm), and the UK (by local government authorities). This appendix reports on data availability for all countries and subnational locations. Subnational estimates are presented for Brazil, Ethiopia, Indonesia, Iran, Japan, Kenya, Mexico, Norway, Pakistan, South Africa, Sweden, the UK, and the USA.

3 Thresholds of stillbirth

A stillbirth is defined as the death or loss of a fetus before or during delivery. The WHO uses a cutoff of at least 28 weeks completed gestation.[5] This threshold was pragmatically selected in 2011 for purposes of international comparison.[6] A substantial number of countries report stillbirths using a more inclusive threshold of 20 weeks of completed pregnancy, which is an inflection point used to differentiate whether a pregnancy loss is considered a miscarriage versus a stillbirth. Therefore, in this analysis, we chose to quantify stillbirths for both the ≥20 weeks and ≥28 weeks thresholds.

Stillbirths can be subcategorised in a number of different ways. The first example differentiates by gestational age into early, late, or term stillbirth. Fetal deaths between 20 and 27 weeks are early stillbirths, fetal deaths between 28 and 36 weeks are late stillbirths, and fetal deaths beyond 36 weeks are term stillbirths. Another heuristic differentiates by temporal relation to labor; antepartum stillbirths occur before onset of labor whereas intrapartum stillbirths are fetal deaths occurring after the start of labor.[7] A third categorisation differentiates according to when the stillbirth was detected. "Fresh" stillbirths, when the fetus is born with intact skin, meaning they most likely died in the 12 hours preceding delivery, are compared to "macerated" stillbirths, when degenerative changes of the fetus can be detected.[8] The various threshold(s) of stillbirth used by other organisations – Centers for Disease Control and Prevention (CDC), International Classification of Diseases (ICD-11), *Lancet* Stillbirth Epidemiology Investigator Group (LSEIG)[4], UN Inter-agency Group for Child Mortality Estimation (UN IGME), and the World Health Organization (WHO) – are shown in Appendix 1 Table S2.[5,9–11]

3.1 Appendix 1 Table S2: Universal thresholds of stillbirth

```
Caption: The rows in this table list the various stillbirth thresholds used
by different public and global health groups.
```

Group	Keyword	Description
Centers for Disease Control and Prevention (CDC)	stillbirth	A loss of a fetus at or after 20 weeks of pregnancy
Centers for Disease Control and Prevention (CDC)	early stillbirth	A fetal death occurring between 20 and 27 completed weeks of pregnancy
Centers for Disease Control and Prevention (CDC)	late stillbirth	A fetal death occurring between 28 and 36 completed weeks of pregnancy
Centers for Disease Control and Prevention (CDC)	term stillbirth	A fetal death occurring after 37 completed weeks of pregnancy
International Classification of Diseases (ICD–11)	stillbirth	A fetal death at 22 or more completed weeks of gestation
Lancet Stillbirth Epidemiology Investigator Group (LSEIG)	stillbirth	A birth with no signs of life after 28 weeks gestation or more
UN Inter–agency Group for Child Mortality Estimation (UN IGME)	stillbirth	A birth with no signs of life after 28 weeks of completed gestation
World Health Organization (WHO)	stillbirth	A birth with no signs of life at or after 28 weeks' gestation

4 Data seeking

Data seeking for this analysis utilised the Global Health Data Exchange (GHDx) as well as PubMed for recently published scientific literature articles.[12] During the GHDx cataloguing process, data sources are assigned relevant keywords. We began by compiling a filtered list of all data sources on the GHDx tagged with the "stillbirth" keyword. We also included all sources from the GBD 2016 dataset in our review.

Each data source was thoroughly reviewed, and extraction occurred if the source reported the necessary stillbirth variables. Whenever possible, we preferred to extract stillbirth counts along with corresponding live birth counts or total birth counts, so we could manually calculate stillbirth rate as `# stillbirths / # total births`. In cases where live births or total births were not reported, we calculated the missing variable using the equation `stillbirths + live births = total births`. Otherwise, we extracted stillbirth rates directly from the report.

Throughout the extraction process, many stillbirth thresholds were identified among data sources. Stillbirth thresholds included fetal death after or equal to 20 weeks of gestation, 22 weeks of gestation, 24 weeks of gestation, 26 weeks of gestation, 28 weeks of gestation, a birthweight of at least 500 grams, and a birthweight of at least 1000 grams. In several instances, data sources combined two different thresholds when reporting stillbirth counts; therefore, we also included 22 weeks of gestation OR a birthweight of at least 500 grams, 28 weeks of gestation OR a birthweight of at least 1000 grams, 22 weeks of gestation AND a birthweight of at least 500 grams, and 28 weeks of gestation AND a birthweight of at least 1000 grams. When available, we extracted live birth values with thresholds that matched the corresponding stillbirth threshold extracted. However, many sources did not report a threshold for live birth, so the value was extracted without confirmation of aligned thresholds. Lastly, we did not extract stillbirth data if it was reported in combination with abortion or miscarriage as this would bias the stillbirth rates upward (ie, descriptions such as "stillbirths + miscarriages" or "stillbirths, abortions, and miscarriages" were excluded).

The details of data seeking, usage considerations, and potential biases for vital registration data, household surveys, and published scientific literature are further described below. We extracted data from 1980 to 2021 from 234 surveys, 231 scientific literature articles, 1633 vital statistics reports, and for 10585 unique location and year combinations (location-years) of data from vital registration (VR) systems. These source counts include the sources identified in GBD 2016.

Our extractions from these sources amounted to a total of 14,459 unique location-years of data, of which, 3682 location-years were from national locations. This was an increase of 7159 location-years compared to what was included in GBD 2016. The dataset used in this analysis has no data for 19 national locations. These countries and territories are Bhutan, Central African Republic, Chad, Djibouti, Dominica, Equatorial Guinea, Eritrea, Federated States of Micronesia, Laos, Libya, Nauru, Niue, North Korea, Somalia, South Sudan, Syria, São Tomé and Príncipe, Tokelau, and Tuvalu.

4.1 Vital Registration

Vital registration data were extracted from multi-country sources, specifically the WHO Europe Health for All database (HFA-DB) and the United Nation Demographic Yearbooks (UNDYB). From the HFA-DB, we gathered tables on "Number of live births by sex" (HFA_19) and "Number of deadborn fetuses with a birth weight of 1000 g or more" (HFA_85). From the UNDYB website, we downloaded tables reporting "Vital statistics summary and life expectancy at birth" (Table 4) and "Late fetal deaths and late fetal death

ratios, by urban/rural residence" (Table 12) from each annual report released between 2000 and 2019. For the UNDYB data, we removed duplicate location-years by prioritising the most recently reported value for each location-year. In this analysis, we used 3657 more location-years of VR data than GBD 2016.

We also reviewed single-country vital statistics reports published by national ministries of health or statistical offices. In cases where we were missing one or more years of a series of annual reports, the reports for missing years were added to the list for review and the "stillbirth" keyword was added to the given source on the GHDx. We also visited statistical office or ministry of health websites of countries to add newly released reports to the source list for review and the GHDx. In total, 1615 more reports were included than what was used for GBD 2016, comprising 3769 additional location-years.

4.2 Household Surveys

Stillbirth data were extracted from a variety of survey series. For USAID Demographic Health Surveys (DHS) with individual level responses, also known as "microdata", stillbirth rates were calculated from the summary birth history information in the women's module using the DHS contraceptive calendar method (https://www.dhsprogram.com/data/calendar-tutorial/#example4), if the necessary variables were present. These variables were date of birth cmc (b3_01), age at death (b6_01), woman's individual sample weight (v005), date of interview cmc (v008), row of month of interview (v018), psu (v021), region (v024), and calendar (vcal_1).

In cases where survey microdata was unavailable for a DHS survey and for surveys from other series, we extracted stillbirth data from the published survey report, provided that the requisite data was available for extraction. We found that some survey series, like the UNICEF Multiple Indicator Cluster Surveys, often asked questions which grouped abortion, stillbirth, and miscarriage. These data sources were not included in this analysis as they did not match the thresholds about timing and the nature of pregnancy loss. Even so, we used 120 more surveys than GBD 2016, covering 1051 additional location-years.

4.3 Literature

We included scientific literature sources we had previously extracted for our stillbirth analysis for GBD 2016 as well as new sources. To identify new scientific literature sources with stillbirth information, we conducted a scientific literature search on PubMed on August 21, 2019 and set a time frame for the results from January 1, 2016 to August 21, 2019. Our search string is printed below. This search string is similar to the string used for GBD 2016, with only the term "f(o)etal loss" being added. In GBD 2016, the search string matched the one provided by Blencowe et al, with a modification to exclude the limitations on locations.

4.3.1 Search string

((stillbirth[MeSH Terms] OR stillbirth[All Fields] OR (still[All Fields] AND birth[All Fields]) OR still birth[All Fields]) OR (foetal loss[All Fields] OR fetal loss[All Fields]) OR

(foetal death[All Fields] OR fetal death[MeSH Terms] OR (fetal[All Fields] AND death[All Fields]) OR fetal death[All Fields]) OR (perinatal mortality[MeSH Terms] OR (perinatal[All Fields] AND mortality[All Fields]) OR perinatal mortality[All Fields]) AND (timing[All Fields] OR rate[All Fields] OR epidemiology[Subheading] OR epidemiology[All Fields] OR prevalence[All Fields] OR prevalence[MeSH Terms] OR incidence[All Fields] OR incidence[MeSH Terms]))

This query returned 6851 results. We first reviewed based on article title and abstract. Of the 441 sources selected and then retrieved for full text review, we extracted stillbirth data from 233. Literature sources were excluded if they only covered a limited study subpopulation (eg, high risks mothers or exclusively pregnancies of multiples), if the study had no information about stillbirth rate or was irrelevant (eg, non-human populations), or if the study contained no primary data (eg, meta-analysis). In total, we used 137 additional scientific literature articles compared to GBD 2016, including 472 additional location-years.

4.3.2 Appendix 1 Figure S2: PRISMA diagram for systematic review

```
Caption: The boxes in this diagram show the number of sources that made it
through each stage of our systematic review of scientific literature.
```

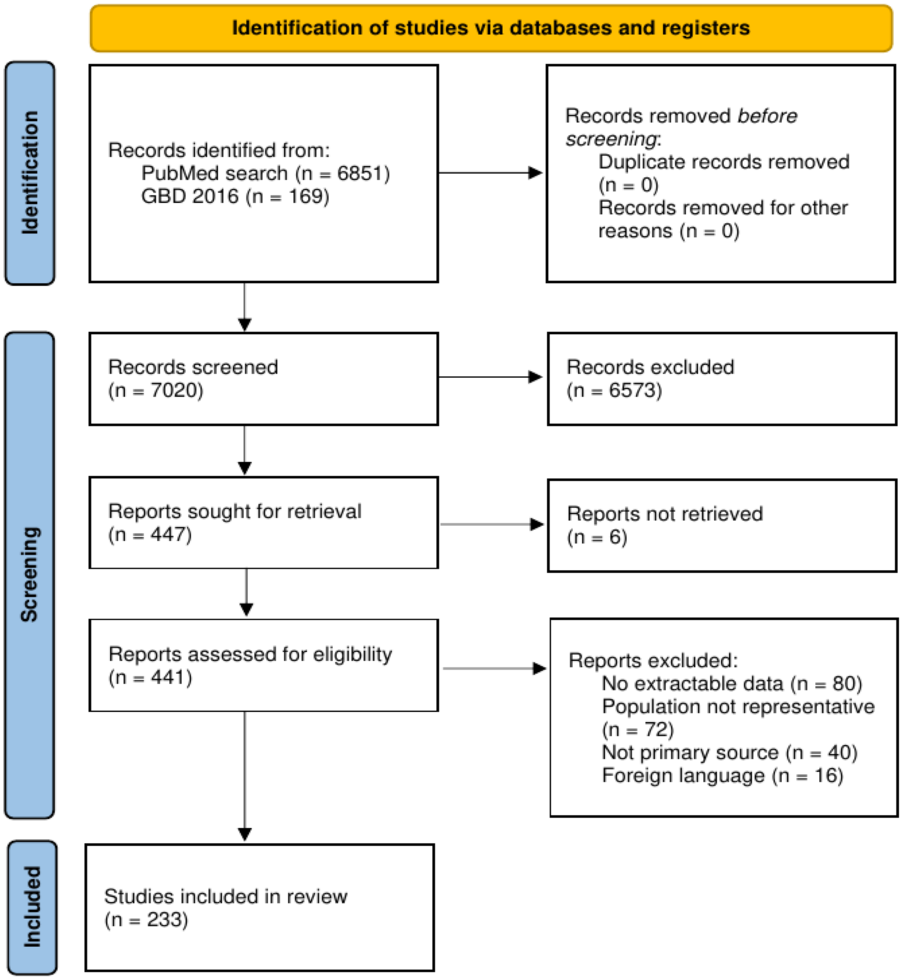

4.4 Data Coverage Visuals

4.4.1 Appendix 1 Table S3: Distribution of thresholds in stillbirth data

```
Caption: This table shows the distribution of data points by stillbirth
threshold, with the majority of stillbirth data assigned to 28 weeks'
gestation.
```

Threshold	# Data Points	Distribution
20 weeks	5796	23·7%
22 weeks	1804	7·4%
24 weeks	1358	5·5%
26 weeks	244	1%
28 weeks	10824	44·2%
500 grams	642	2·6%
1000 grams	1997	8·2%
22 weeks OR 500 grams	976	4%
22 weeks AND 500 grams	94	0·4%
28 weeks OR 1000 grams	97	0·4%
28 weeks AND 1000 grams	104	0·4%
Other	566	2·3%

4.4.2 Appendix 1 Table S4: Distribution of data points assigned to each stillbirth threshold by location

```
Caption: This table shows the distribution of data points by stillbirth
threshold for each location in the GBD location hierarchy.
```

	20 weeks	22 weeks	24 weeks	26 weeks	28 weeks	500 grams	1000 grams	22 weeks OR 500	22 weeks AND 500	28 weeks OR 1000	28 weeks AND 1000
**Global**	**5796**	**1804**	**1358**	**244**	**1082 4**	**642**	**1997**	**976**	**94**	**97**	**104**
**Central Europe, eastern Europe, and central Asia**	**20**	**243**	**26**	**15**	**752**	**34**	**860**	**386**	**30**	**12**	**16**
**Central Asia**	**20**	**58**	**0**	**0**	**131**	**0**	**247**	**47**	**0**	**1**	**0**
Armenia	0	0	0	0	4	0	40	47	0	0	0
Azerbaijan	0	0	0	0	28	0	27	0	0	0	0
Georgia	0	58	0	0	0	0	30	0	0	0	0
Kazakhstan	0	0	0	0	27	0	30	0	0	0	0
Kyrgyzstan	0	0	0	0	26	0	38	0	0	0	0
Mongolia	20	0	0	0	6	0	0	0	0	1	0
Tajikistan	0	0	0	0	22	0	28	0	0	0	0
Turkmenistan	0	0	0	0	1	0	29	0	0	0	0
Uzbekistan	0	0	0	0	17	0	25	0	0	0	0
**Central Europe**	**0**	**31**	**26**	**15**	**460**	**15**	**379**	**312**	**17**	**0**	**16**

Albania	0	0	0	0	6	0	0	0	0	0	0
Bosnia and Herzegovina	0	0	3	0	25	15	27	15	0	0	0
Bulgaria	0	0	0	0	38	0	41	0	0	0	0
Croatia	0	0	0	0	47	0	20	38	17	0	0
Czechia	0	11	9	15	25	0	102	0	0	0	16
Hungary	0	0	14	0	28	0	26	0	0	0	0
Montenegro	0	0	0	0	33	0	0	0	0	0	0
North Macedonia	0	0	0	0	49	0	0	0	0	0	0
Poland	0	0	0	0	32	0	24	259	0	0	0
Dolnośląskie	0	0	0	0	0	0	0	15	0	0	0
Kujawsko-Pomorskie	0	0	0	0	0	0	0	15	0	0	0
Łódzkie	0	0	0	0	0	0	0	15	0	0	0
Lubelskie	0	0	0	0	0	0	0	15	0	0	0
Lubuskie	0	0	0	0	0	0	0	15	0	0	0
Małopolskie	0	0	0	0	0	0	0	15	0	0	0
Mazowieckie	0	0	0	0	0	0	0	15	0	0	0
Opolskie	0	0	0	0	0	0	0	15	0	0	0
Podkarpackie	0	0	0	0	0	0	0	15	0	0	0
Podlaskie	0	0	0	0	0	0	0	15	0	0	0
Pomorskie	0	0	0	0	0	0	0	15	0	0	0
Śląskie	0	0	0	0	0	0	0	15	0	0	0
Świętokrzyskie	0	0	0	0	0	0	0	15	0	0	0
Warmińsko-Mazurskie	0	0	0	0	0	0	0	15	0	0	0
Wielkopolskie	0	0	0	0	0	0	0	15	0	0	0
Zachodniopomorskie	0	0	0	0	0	0	0	15	0	0	0
Romania	0	0	0	0	77	0	26	0	0	0	0
Serbia	0	0	0	0	50	0	21	0	0	0	0
Slovakia	0	0	0	0	26	0	35	0	0	0	0
Slovenia	0	20	0	0	24	0	57	0	0	0	0
**Eastern Europe**	**0**	**154**	**0**	**0**	**161**	**19**	**234**	**27**	**13**	**11**	**0**
Belarus	0	0	0	0	23	14	27	0	0	0	0
Estonia	0	30	0	0	24	5	71	0	13	0	0
Latvia	0	107	0	0	0	0	0	0	0	0	0
Lithuania	0	15	0	0	41	0	33	27	0	11	0
Moldova	0	0	0	0	2	0	42	0	0	0	0
Russia	0	2	0	0	43	0	30	0	0	0	0
Altay krai	0	0	0	0	0	0	0	0	0	0	0
Amur oblast	0	0	0	0	0	0	0	0	0	0	0
Arkhangelsk oblast without Nenets autonomous district	0	2	0	0	0	0	0	0	0	0	0
Astrakhan oblast	0	0	0	0	0	0	0	0	0	0	0
Belgorod oblast	0	0	0	0	0	0	0	0	0	0	0
Bryansk oblast	0	0	0	0	0	0	0	0	0	0	0

Chechen Republic	0	0	0	0	0	0	0	0	0	0	0
Chelyabinsk oblast	0	0	0	0	0	0	0	0	0	0	0
Chukotka Autonomous Okrug	0	0	0	0	0	0	0	0	0	0	0
Chuvash Republic	0	0	0	0	0	0	0	0	0	0	0
Irkutsk oblast	0	0	0	0	0	0	0	0	0	0	0
Ivanovo oblast	0	0	0	0	0	0	0	0	0	0	0
Jewish autonomous oblast	0	0	0	0	0	0	0	0	0	0	0
Kabardino-Balkarian Republic	0	0	0	0	0	0	0	0	0	0	0
Kaliningrad oblast	0	0	0	0	0	0	0	0	0	0	0
Kaluga oblast	0	0	0	0	0	0	0	0	0	0	0
Kamchatka krai	0	0	0	0	0	0	0	0	0	0	0
Karachayevo-Chircassian Republic	0	0	0	0	0	0	0	0	0	0	0
Kemerovo oblast	0	0	0	0	0	0	0	0	0	0	0
Khabarovsk krai	0	0	0	0	0	0	0	0	0	0	0
Khanty-Mansi autonomous area	0	0	0	0	0	0	0	0	0	0	0
Kirov oblast	0	0	0	0	0	0	0	0	0	0	0
Republic of Komi	0	0	0	0	0	0	0	0	0	0	0
Kostroma oblast	0	0	0	0	0	0	0	0	0	0	0
Krasnodar krai	0	0	0	0	0	0	0	0	0	0	0
Krasnoyarsk krai	0	0	0	0	0	0	0	0	0	0	0
Kurgan oblast	0	0	0	0	0	0	0	0	0	0	0
Kursk oblast	0	0	0	0	0	0	0	0	0	0	0
Leningrad oblast	0	0	0	0	0	0	0	0	0	0	0
Lipetzk oblast	0	0	0	0	0	0	0	0	0	0	0
Magadan oblast	0	0	0	0	0	0	0	0	0	0	0
Moscow, city of	0	0	0	0	0	0	0	0	0	0	0
Moscow oblast	0	0	0	0	0	0	0	0	0	0	0
Murmansk oblast	0	0	0	0	0	0	0	0	0	0	0
Nenets autonomous district	0	0	0	0	0	0	0	0	0	0	0
Nizhny Novgorod oblast	0	0	0	0	0	0	0	0	0	0	0
Novgorod oblast	0	0	0	0	0	0	0	0	0	0	0
Novosibirsk oblast	0	0	0	0	0	0	0	0	0	0	0
Omsk oblast	0	0	0	0	0	0	0	0	0	0	0
Orenburg oblast	0	0	0	0	0	0	0	0	0	0	0
Oryol oblast	0	0	0	0	0	0	0	0	0	0	0
Penza oblast	0	0	0	0	0	0	0	0	0	0	0
Perm krai	0	0	0	0	0	0	0	0	0	0	0
Primorye krai	0	0	0	0	0	0	0	0	0	0	0
Pskov oblast	0	0	0	0	0	0	0	0	0	0	0
Republic of Adygeya	0	0	0	0	0	0	0	0	0	0	0
Republic of Altay	0	0	0	0	0	0	0	0	0	0	0
Republic of Bashkortostan	0	0	0	0	0	0	0	0	0	0	0
Republic of Buryatia	0	0	0	0	0	0	0	0	0	0	0

Republic of Dagestan	0	0	0	0	0	0	0	0	0	0	0
Republic of Ingushetia	0	0	0	0	0	0	0	0	0	0	0
Republic of Kalmykia	0	0	0	0	0	0	0	0	0	0	0
Republic of Karelia	0	0	0	0	0	0	0	0	0	0	0
Republic of Khakasia	0	0	0	0	0	0	0	0	0	0	0
Republic of Mari El	0	0	0	0	0	0	0	0	0	0	0
Republic of Mordovia	0	0	0	0	0	0	0	0	0	0	0
Republic of North Ossetia - Alania	0	0	0	0	0	0	0	0	0	0	0
Republic of Sakha (Yakutia)	0	0	0	0	0	0	0	0	0	0	0
Republic of Tatarstan	0	0	0	0	0	0	0	0	0	0	0
Republic of Tuva	0	0	0	0	0	0	0	0	0	0	0
Rostov oblast	0	0	0	0	0	0	0	0	0	0	0
Ryazan oblast	0	0	0	0	0	0	0	0	0	0	0
St. Petersburg, city of	0	0	0	0	0	0	0	0	0	0	0
Sakhalin oblast	0	0	0	0	0	0	0	0	0	0	0
Samara oblast	0	0	0	0	0	0	0	0	0	0	0
Saratov oblast	0	0	0	0	0	0	0	0	0	0	0
Smolensk oblast	0	0	0	0	0	0	0	0	0	0	0
Stavropol krai	0	0	0	0	0	0	0	0	0	0	0
Sverdlovsk oblast	0	0	0	0	0	0	0	0	0	0	0
Tambov oblast	0	0	0	0	0	0	0	0	0	0	0
Tomsk oblast	0	0	0	0	0	0	0	0	0	0	0
Tula oblast	0	0	0	0	0	0	0	0	0	0	0
Tver oblast	0	0	0	0	0	0	0	0	0	0	0
Tyumen oblast without autonomous areas	0	0	0	0	0	0	0	0	0	0	0
Udmurtian Republic	0	0	0	0	0	0	0	0	0	0	0
Ulyanovsk oblast	0	0	0	0	0	0	0	0	0	0	0
Vladimir oblast	0	0	0	0	0	0	0	0	0	0	0
Volgograd oblast	0	0	0	0	0	0	0	0	0	0	0
Vologda oblast	0	0	0	0	0	0	0	0	0	0	0
Voronezh oblast	0	0	0	0	0	0	0	0	0	0	0
Yamal-Nenets Autonomous Okrug	0	0	0	0	0	0	0	0	0	0	0
Yaroslavl oblast	0	0	0	0	0	0	0	0	0	0	0
Trans-Baikal krai	0	0	0	0	0	0	0	0	0	0	0
Ukraine	0	0	0	0	28	0	31	0	0	0	0
Republic of Crimea	0	0	0	0	1	0	0	0	0	0	0
Sevastopol	0	0	0	0	1	0	0	0	0	0	0
Ukraine (without Republic of Crimea & Sevastopol)	0	0	0	0	8	0	0	0	0	0	0
**High income**	**2509**	**389**	**1294**	**184**	**3159**	**309**	**846**	**579**	**35**	**47**	**77**
**Australasia**	**196**	**103**	**104**	**102**	**163**	**89**	**106**	**0**	**0**	**0**	**0**
Australia	32	14	14	13	40	0	0	0	0	0	0
New Zealand	164	89	90	89	123	89	106	0	0	0	0

New Zealand Maori population	47	29	29	29	29	29	29	0	0	0	0
New Zealand non-Maori population	48	30	30	30	30	30	30	0	0	0	0
**High-income Asia Pacific**	**0**	**0**	**18**	**0**	**90**	**0**	**0**	**0**	**0**	**1**	**9**
Brunei	0	0	18	0	4	0	0	0	0	0	0
Japan	0	0	0	0	25	0	0	0	0	1	0
Aichi	0	0	0	0	0	0	0	0	0	0	0
Akita	0	0	0	0	0	0	0	0	0	0	0
Aomori	0	0	0	0	0	0	0	0	0	0	0
Chiba	0	0	0	0	0	0	0	0	0	0	0
Ehime	0	0	0	0	0	0	0	0	0	0	0
Fukui	0	0	0	0	0	0	0	0	0	0	0
Fukuoka	0	0	0	0	0	0	0	0	0	0	0
Fukushima	0	0	0	0	0	0	0	0	0	0	0
Gifu	0	0	0	0	0	0	0	0	0	0	0
Gunma	0	0	0	0	0	0	0	0	0	0	0
Hiroshima	0	0	0	0	0	0	0	0	0	0	0
Hokkaidō	0	0	0	0	0	0	0	0	0	0	0
Hyōgo	0	0	0	0	0	0	0	0	0	0	0
Ibaraki	0	0	0	0	0	0	0	0	0	0	0
Ishikawa	0	0	0	0	0	0	0	0	0	0	0
Iwate	0	0	0	0	0	0	0	0	0	0	0
Kagawa	0	0	0	0	0	0	0	0	0	0	0
Kagoshima	0	0	0	0	0	0	0	0	0	0	0
Kanagawa	0	0	0	0	0	0	0	0	0	0	0
Kōchi	0	0	0	0	0	0	0	0	0	0	0
Kumamoto	0	0	0	0	0	0	0	0	0	0	0
Kyōto	0	0	0	0	0	0	0	0	0	0	0
Mie	0	0	0	0	0	0	0	0	0	0	0
Miyagi	0	0	0	0	0	0	0	0	0	0	0
Miyazaki	0	0	0	0	0	0	0	0	0	0	0
Nagano	0	0	0	0	0	0	0	0	0	0	0
Nagasaki	0	0	0	0	0	0	0	0	0	0	0
Nara	0	0	0	0	0	0	0	0	0	0	0
Niigata	0	0	0	0	0	0	0	0	0	0	0
Ōita	0	0	0	0	0	0	0	0	0	0	0
Okayama	0	0	0	0	0	0	0	0	0	0	0
Okinawa	0	0	0	0	0	0	0	0	0	0	0
Ōsaka	0	0	0	0	0	0	0	0	0	0	0
Saga	0	0	0	0	0	0	0	0	0	0	0
Saitama	0	0	0	0	0	0	0	0	0	0	0
Shiga	0	0	0	0	0	0	0	0	0	0	0
Shimane	0	0	0	0	0	0	0	0	0	0	0
Shizuoka	0	0	0	0	0	0	0	0	0	0	0

Tochigi	0	0	0	0	0	0	0	0	0	0	0
Tokushima	0	0	0	0	0	0	0	0	0	0	0
Tōkyō	0	0	0	0	0	0	0	0	0	0	0
Tottori	0	0	0	0	0	0	0	0	0	0	0
Toyama	0	0	0	0	0	0	0	0	0	0	0
Wakayama	0	0	0	0	0	0	0	0	0	0	0
Yamagata	0	0	0	0	0	0	0	0	0	0	0
Yamaguchi	0	0	0	0	0	0	0	0	0	0	0
Yamanashi	0	0	0	0	0	0	0	0	0	0	0
South Korea	0	0	0	0	13	0	0	0	0	0	0
Singapore	0	0	0	0	48	0	0	0	0	0	9
**High-income North America**	**2246**	**1**	**0**	**0**	**2111**	**16**	**10**	**0**	**0**	**6**	**0**
Canada	47	0	0	0	55	16	10	0	0	6	0
Greenland	0	0	0	0	8	0	0	0	0	0	0
USA	2199	1	0	0	2048	0	0	0	0	0	0
Alabama	42	0	0	0	39	0	0	0	0	0	0
Alaska	42	0	0	0	39	0	0	0	0	0	0
Arizona	42	0	0	0	39	0	0	0	0	0	0
Arkansas	42	0	0	0	39	0	0	0	0	0	0
California	42	0	0	0	39	0	0	0	0	0	0
Colorado	42	0	0	0	39	0	0	0	0	0	0
Connecticut	42	0	0	0	39	0	0	0	0	0	0
Delaware	42	0	0	0	39	0	0	0	0	0	0
Washington, DC	42	0	0	0	39	0	0	0	0	0	0
Florida	42	0	0	0	39	0	0	0	0	0	0
Georgia	42	0	0	0	39	0	0	0	0	0	0
Hawaii	42	0	0	0	39	0	0	0	0	0	0
Idaho	42	0	0	0	39	0	0	0	0	0	0
Illinois	42	0	0	0	39	0	0	0	0	0	0
Indiana	42	0	0	0	39	0	0	0	0	0	0
Iowa	42	0	0	0	39	0	0	0	0	0	0
Kansas	42	0	0	0	39	0	0	0	0	0	0
Kentucky	42	0	0	0	39	0	0	0	0	0	0
Louisiana	42	0	0	0	39	0	0	0	0	0	0
Maine	42	0	0	0	39	0	0	0	0	0	0
Maryland	42	0	0	0	39	0	0	0	0	0	0
Massachusetts	42	0	0	0	39	0	0	0	0	0	0
Michigan	42	0	0	0	39	0	0	0	0	0	0
Minnesota	42	0	0	0	39	0	0	0	0	0	0
Mississippi	42	0	0	0	39	0	0	0	0	0	0
Missouri	42	0	0	0	39	0	0	0	0	0	0
Montana	42	0	0	0	39	0	0	0	0	0	0
Nebraska	42	0	0	0	39	0	0	0	0	0	0

Nevada	42	0	0	0	39	0	0	0	0	0	0
New Hampshire	42	0	0	0	39	0	0	0	0	0	0
New Jersey	42	0	0	0	39	0	0	0	0	0	0
New Mexico	42	0	0	0	39	0	0	0	0	0	0
New York	42	0	0	0	39	0	0	0	0	0	0
North Carolina	42	0	0	0	39	0	0	0	0	0	0
North Dakota	42	0	0	0	39	0	0	0	0	0	0
Ohio	42	0	0	0	39	0	0	0	0	0	0
Oklahoma	42	0	0	0	39	0	0	0	0	0	0
Oregon	42	0	0	0	39	0	0	0	0	0	0
Pennsylvania	42	0	0	0	39	0	0	0	0	0	0
Rhode Island	42	0	0	0	39	0	0	0	0	0	0
South Carolina	42	0	0	0	39	0	0	0	0	0	0
South Dakota	42	0	0	0	39	0	0	0	0	0	0
Tennessee	42	0	0	0	39	0	0	0	0	0	0
Texas	43	0	0	0	39	0	0	0	0	0	0
Utah	42	0	0	0	39	0	0	0	0	0	0
Vermont	42	0	0	0	39	0	0	0	0	0	0
Virginia	42	0	0	0	39	0	0	0	0	0	0
Washington	42	0	0	0	39	0	0	0	0	0	0
West Virginia	42	0	0	0	39	0	0	0	0	0	0
Wisconsin	42	0	0	0	39	0	0	0	0	0	0
Wyoming	42	0	0	0	39	0	0	0	0	0	0
**Southern Latin America**	**31**	**56**	**30**	**25**	**171**	**47**	**22**	**0**	**30**	**1**	**56**
Argentina	1	22	0	0	47	22	22	0	21	1	22
Chile	30	34	30	25	85	25	0	0	9	0	34
Uruguay	0	0	0	0	39	0	0	0	0	0	0
**Western Europe**	**36**	**229**	**1142**	**57**	**624**	**157**	**708**	**579**	**5**	**39**	**12**
Andorra	0	0	0	0	9	0	23	0	0	0	0
Austria	0	0	0	0	0	51	52	0	0	0	0
Belgium	0	0	0	0	19	0	22	0	0	0	0
Cyprus	0	6	0	0	6	0	11	0	0	0	0
Denmark	0	0	0	0	21	0	39	0	0	0	0
Finland	0	2	0	0	23	0	34	33	0	0	0
France	0	0	0	22	6	9	0	7	0	0	0
Germany	0	0	3	0	0	48	38	0	0	0	0
Greece	0	41	0	0	25	0	41	0	0	0	0
Iceland	0	0	0	0	68	0	37	0	0	0	0
Ireland	0	0	0	0	20	25	30	0	0	0	0
Israel	0	0	0	0	21	0	39	19	0	39	0
Italy	1	1	0	0	21	0	33	0	0	0	0
Abruzzo	0	0	0	0	0	0	0	0	0	0	0
Basilicata	0	0	0	0	0	0	0	0	0	0	0

Calabria	0	0	0	0	0	0	0	0	0	0	0
Campania	0	0	0	0	0	0	0	0	0	0	0
Emilia-Romagna	0	0	0	0	0	0	0	0	0	0	0
Friuli-Venezia Giulia	1	0	0	0	0	0	0	0	0	0	0
Lazio	0	0	0	0	0	0	0	0	0	0	0
Liguria	0	0	0	0	0	0	0	0	0	0	0
Lombardia	0	0	0	0	0	0	0	0	0	0	0
Marche	0	0	0	0	0	0	0	0	0	0	0
Molise	0	0	0	0	0	0	0	0	0	0	0
Piemonte	0	0	0	0	0	0	0	0	0	0	0
Provincia autonoma di Bolzano	0	0	0	0	0	0	0	0	0	0	0
Provincia autonoma di Trento	0	0	0	0	0	0	0	0	0	0	0
Puglia	0	0	0	0	0	0	0	0	0	0	0
Sardegna	0	0	0	0	0	0	0	0	0	0	0
Sicilia	0	0	0	0	0	0	0	0	0	0	0
Toscana	0	0	0	0	0	0	0	0	0	0	0
Umbria	0	0	0	0	0	0	0	0	0	0	0
Valle d'Aosta	0	0	0	0	0	0	0	0	0	0	0
Veneto	0	1	0	0	0	0	0	0	0	0	0
Luxembourg	0	0	0	0	64	0	31	0	0	0	0
Malta	0	34	0	0	12	16	48	15	0	0	0
Monaco	0	0	0	0	0	0	4	0	0	0	0
Netherlands	0	0	0	0	24	0	20	0	0	0	0
Norway	35	35	35	35	60	0	41	505	0	0	0
Agder	0	0	0	0	0	0	0	42	0	0	0
Innlandet	0	0	0	0	0	0	0	42	0	0	0
Møre og Romsdal	0	0	0	0	0	0	0	42	0	0	0
Nordland	0	0	0	0	0	0	0	42	0	0	0
Oslo	0	0	0	0	0	0	0	42	0	0	0
Rogaland	0	0	0	0	0	0	0	42	0	0	0
Troms og Finnmark	0	0	0	0	0	0	0	42	0	0	0
Trøndelag	0	0	0	0	0	0	0	42	0	0	0
Vestfold og Telemark	0	0	0	0	0	0	0	42	0	0	0
Vestland	0	0	0	0	0	0	0	42	0	0	0
Viken	0	0	0	0	0	0	0	42	0	0	0
Portugal	0	12	1	0	38	8	46	0	0	0	0
San Marino	0	0	0	0	7	0	11	0	0	0	0
Spain	0	1	0	0	33	0	25	0	0	0	0
Sweden	0	42	0	0	43	0	41	0	0	0	0
Stockholm	0	0	0	0	0	0	0	0	0	0	0
Sweden except Stockholm	0	0	0	0	0	0	0	0	0	0	0
Switzerland	0	42	0	0	25	0	40	0	0	0	0
UK	0	13	1103	0	79	0	2	0	5	0	12

England	0	9	973	0	0	0	0	0	0	0	0
East Midlands	0	1	67	0	0	0	0	0	0	0	0
Derby	0	0	5	0	0	0	0	0	0	0	0
Derbyshire	0	0	5	0	0	0	0	0	0	0	0
Leicester	0	0	5	0	0	0	0	0	0	0	0
Leicestershire	0	0	5	0	0	0	0	0	0	0	0
Lincolnshire	0	0	5	0	0	0	0	0	0	0	0
Northamptonshire	0	0	4	0	0	0	0	0	0	0	0
Nottingham	0	0	5	0	0	0	0	0	0	0	0
Nottinghamshire	0	0	5	0	0	0	0	0	0	0	0
Rutland	0	0	5	0	0	0	0	0	0	0	0
East of England	0	1	78	0	0	0	0	0	0	0	0
Bedford	0	0	5	0	0	0	0	0	0	0	0
Cambridgeshire	0	0	5	0	0	0	0	0	0	0	0
Central Bedfordshire	0	0	5	0	0	0	0	0	0	0	0
Essex	0	0	5	0	0	0	0	0	0	0	0
Hertfordshire	0	0	5	0	0	0	0	0	0	0	0
Luton	0	0	5	0	0	0	0	0	0	0	0
Norfolk	0	0	5	0	0	0	0	0	0	0	0
Peterborough	0	0	5	0	0	0	0	0	0	0	0
Southend-on-Sea	0	0	5	0	0	0	0	0	0	0	0
Suffolk	0	0	5	0	0	0	0	0	0	0	0
Thurrock	0	0	5	0	0	0	0	0	0	0	0
Greater London	0	1	187	0	0	0	0	0	0	0	0
Barking and Dagenham	0	0	5	0	0	0	0	0	0	0	0
Barnet	0	0	5	0	0	0	0	0	0	0	0
Bexley	0	0	5	0	0	0	0	0	0	0	0
Brent	0	0	5	0	0	0	0	0	0	0	0
Bromley	0	0	5	0	0	0	0	0	0	0	0
Camden	0	0	5	0	0	0	0	0	0	0	0
Croydon	0	0	5	0	0	0	0	0	0	0	0
Ealing	0	0	5	0	0	0	0	0	0	0	0
Enfield	0	0	5	0	0	0	0	0	0	0	0
Greenwich	0	0	5	0	0	0	0	0	0	0	0
Hackney	0	0	5	0	0	0	0	0	0	0	0
Hammersmith and Fulham	0	0	5	0	0	0	0	0	0	0	0
Haringey	0	0	5	0	0	0	0	0	0	0	0
Harrow	0	0	5	0	0	0	0	0	0	0	0
Havering	0	0	5	0	0	0	0	0	0	0	0
Hillingdon	0	0	5	0	0	0	0	0	0	0	0
Hounslow	0	0	5	0	0	0	0	0	0	0	0
Islington	0	0	5	0	0	0	0	0	0	0	0
Kensington and Chelsea	0	0	5	0	0	0	0	0	0	0	0

Kingston upon Thames	0	0	5	0	0	0	0	0	0	0	0
Lambeth	0	0	5	0	0	0	0	0	0	0	0
Lewisham	0	0	5	0	0	0	0	0	0	0	0
Merton	0	0	5	0	0	0	0	0	0	0	0
Newham	0	0	5	0	0	0	0	0	0	0	0
Redbridge	0	0	5	0	0	0	0	0	0	0	0
Richmond upon Thames	0	0	5	0	0	0	0	0	0	0	0
Southwark	0	0	5	0	0	0	0	0	0	0	0
Sutton	0	0	5	0	0	0	0	0	0	0	0
Tower Hamlets	0	0	5	0	0	0	0	0	0	0	0
Waltham Forest	0	0	5	0	0	0	0	0	0	0	0
Wandsworth	0	0	5	0	0	0	0	0	0	0	0
Westminster	0	0	5	0	0	0	0	0	0	0	0
North East England	0	1	83	0	0	0	0	0	0	0	0
County Durham	0	0	5	0	0	0	0	0	0	0	0
Darlington	0	0	5	0	0	0	0	0	0	0	0
Gateshead	0	0	5	0	0	0	0	0	0	0	0
Hartlepool	0	0	5	0	0	0	0	0	0	0	0
Middlesbrough	0	0	5	0	0	0	0	0	0	0	0
Newcastle upon Tyne	0	0	5	0	0	0	0	0	0	0	0
North Tyneside	0	0	5	0	0	0	0	0	0	0	0
Northumberland	0	0	5	0	0	0	0	0	0	0	0
Redcar and Cleveland	0	0	5	0	0	0	0	0	0	0	0
South Tyneside	0	0	5	0	0	0	0	0	0	0	0
Stockton-on-Tees	0	0	5	0	0	0	0	0	0	0	0
Sunderland	0	0	5	0	0	0	0	0	0	0	0
North West England	0	1	138	0	0	0	0	0	0	0	0
Blackburn with Darwen	0	0	5	0	0	0	0	0	0	0	0
Blackpool	0	0	5	0	0	0	0	0	0	0	0
Bolton	0	0	5	0	0	0	0	0	0	0	0
Bury	0	0	5	0	0	0	0	0	0	0	0
Cheshire East	0	0	5	0	0	0	0	0	0	0	0
Cheshire West and Chester	0	0	5	0	0	0	0	0	0	0	0
Cumbria	0	0	5	0	0	0	0	0	0	0	0
Halton	0	0	5	0	0	0	0	0	0	0	0
Knowsley	0	0	5	0	0	0	0	0	0	0	0
Lancashire	0	0	5	0	0	0	0	0	0	0	0
Liverpool	0	0	5	0	0	0	0	0	0	0	0
Manchester	0	0	5	0	0	0	0	0	0	0	0
Oldham	0	0	5	0	0	0	0	0	0	0	0
Rochdale	0	0	5	0	0	0	0	0	0	0	0
Salford	0	0	5	0	0	0	0	0	0	0	0
Sefton	0	0	5	0	0	0	0	0	0	0	0

St Helens	0	0	5	0	0	0	0	0	0	0	0
Stockport	0	0	5	0	0	0	0	0	0	0	0
Tameside	0	0	5	0	0	0	0	0	0	0	0
Trafford	0	0	5	0	0	0	0	0	0	0	0
Warrington	0	0	5	0	0	0	0	0	0	0	0
Wigan	0	0	5	0	0	0	0	0	0	0	0
Wirral	0	0	5	0	0	0	0	0	0	0	0
South East England	0	1	118	0	0	0	0	0	0	0	0
Bracknell Forest	0	0	5	0	0	0	0	0	0	0	0
Brighton and Hove	0	0	5	0	0	0	0	0	0	0	0
Buckinghamshire	0	0	5	0	0	0	0	0	0	0	0
East Sussex	0	0	5	0	0	0	0	0	0	0	0
Hampshire	0	0	5	0	0	0	0	0	0	0	0
Isle of Wight	0	0	5	0	0	0	0	0	0	0	0
Kent	0	0	5	0	0	0	0	0	0	0	0
Medway	0	0	5	0	0	0	0	0	0	0	0
Milton Keynes	0	0	5	0	0	0	0	0	0	0	0
Oxfordshire	0	0	5	0	0	0	0	0	0	0	0
Portsmouth	0	0	5	0	0	0	0	0	0	0	0
Reading	0	0	5	0	0	0	0	0	0	0	0
Slough	0	0	5	0	0	0	0	0	0	0	0
Southampton	0	0	5	0	0	0	0	0	0	0	0
Surrey	0	0	5	0	0	0	0	0	0	0	0
West Berkshire	0	0	5	0	0	0	0	0	0	0	0
West Sussex	0	0	5	0	0	0	0	0	0	0	0
Windsor and Maidenhead	0	0	5	0	0	0	0	0	0	0	0
Wokingham	0	0	5	0	0	0	0	0	0	0	0
South West England	0	1	87	0	0	0	0	0	0	0	0
Bath and North East Somerset	0	0	5	0	0	0	0	0	0	0	0
Bournemouth	0	0	2	0	0	0	0	0	0	0	0
Bristol, City of	0	0	5	0	0	0	0	0	0	0	0
Cornwall	0	0	0	0	0	0	0	0	0	0	0
Devon	0	0	5	0	0	0	0	0	0	0	0
Dorset	0	0	5	0	0	0	0	0	0	0	0
Gloucestershire	0	0	5	0	0	0	0	0	0	0	0
North Somerset	0	0	5	0	0	0	0	0	0	0	0
Plymouth	0	0	5	0	0	0	0	0	0	0	0
Poole	0	0	2	0	0	0	0	0	0	0	0
Somerset	0	0	5	0	0	0	0	0	0	0	0
South Gloucestershire	0	0	5	0	0	0	0	0	0	0	0
Swindon	0	0	5	0	0	0	0	0	0	0	0
Torbay	0	0	5	0	0	0	0	0	0	0	0
Wiltshire	0	0	5	0	0	0	0	0	0	0	0

West Midlands	0	1	93	0	0	0	0	0	0	0	0
Birmingham	0	0	5	0	0	0	0	0	0	0	0
Coventry	0	0	5	0	0	0	0	0	0	0	0
Dudley	0	0	5	0	0	0	0	0	0	0	0
Herefordshire, County of	0	0	5	0	0	0	0	0	0	0	0
Sandwell	0	0	5	0	0	0	0	0	0	0	0
Shropshire	0	0	5	0	0	0	0	0	0	0	0
Solihull	0	0	5	0	0	0	0	0	0	0	0
Staffordshire	0	0	5	0	0	0	0	0	0	0	0
Stoke-on-Trent	0	0	5	0	0	0	0	0	0	0	0
Telford and Wrekin	0	0	5	0	0	0	0	0	0	0	0
Walsall	0	0	5	0	0	0	0	0	0	0	0
Warwickshire	0	0	5	0	0	0	0	0	0	0	0
Wolverhampton	0	0	5	0	0	0	0	0	0	0	0
Worcestershire	0	0	5	0	0	0	0	0	0	0	0
Yorkshire and the Humber	0	1	98	0	0	0	0	0	0	0	0
Barnsley	0	0	5	0	0	0	0	0	0	0	0
Bradford	0	0	5	0	0	0	0	0	0	0	0
Calderdale	0	0	5	0	0	0	0	0	0	0	0
Doncaster	0	0	5	0	0	0	0	0	0	0	0
East Riding of Yorkshire	0	0	5	0	0	0	0	0	0	0	0
Kingston upon Hull, City of	0	0	5	0	0	0	0	0	0	0	0
Kirklees	0	0	5	0	0	0	0	0	0	0	0
Leeds	0	0	5	0	0	0	0	0	0	0	0
North East Lincolnshire	0	0	5	0	0	0	0	0	0	0	0
North Lincolnshire	0	0	5	0	0	0	0	0	0	0	0
North Yorkshire	0	0	5	0	0	0	0	0	0	0	0
Rotherham	0	0	5	0	0	0	0	0	0	0	0
Sheffield	0	0	5	0	0	0	0	0	0	0	0
Wakefield	0	0	5	0	0	0	0	0	0	0	0
York	0	0	5	0	0	0	0	0	0	0	0
Northern Ireland	0	1	29	0	36	0	0	0	0	0	12
Scotland	0	1	55	0	42	0	0	0	5	0	0
Wales	0	1	23	0	0	0	0	0	0	0	0
**Latin America and Caribbean**	**1027**	**998**	**25**	**24**	**1996**	**54**	**0**	**5**	**0**	**8**	**1**
**Andean Latin America**	**24**	**25**	**24**	**24**	**62**	**0**	**0**	**0**	**0**	**2**	**0**
Bolivia	0	0	0	0	3	0	0	0	0	0	0
Ecuador	24	25	24	24	52	0	0	0	0	1	0
Peru	0	0	0	0	7	0	0	0	0	1	0
**Caribbean**	**18**	**40**	**1**	**0**	**273**	**53**	**0**	**0**	**0**	**0**	**0**
Antigua and Barbuda	0	0	0	0	2	0	0	0	0	0	0
The Bahamas	0	30	0	0	2	0	0	0	0	0	0
Barbados	0	5	0	0	2	0	0	0	0	0	0

Belize	0	0	0	0	17	0	0	0	0	0	0
Bermuda	0	0	0	0	11	0	0	0	0	0	0
Cuba	0	0	0	0	6	53	0	0	0	0	0
Dominica	0	0	0	0	0	0	0	0	0	0	0
Dominican Republic	0	5	0	0	12	0	0	0	0	0	0
Grenada	0	0	0	0	5	0	0	0	0	0	0
Guyana	0	0	0	0	1	0	0	0	0	0	0
Haiti	0	0	0	0	17	0	0	0	0	0	0
Jamaica	0	0	0	0	20	0	0	0	0	0	0
Puerto Rico	14	0	0	0	44	0	0	0	0	0	0
Saint Kitts and Nevis	0	0	0	0	1	0	0	0	0	0	0
Saint Lucia	0	0	0	0	42	0	0	0	0	0	0
Saint Vincent and the Grenadines	0	0	0	0	37	0	0	0	0	0	0
Suriname	0	0	0	0	10	0	0	0	0	0	0
Trinidad and Tobago	0	0	1	0	36	0	0	0	0	0	0
Virgin Islands	4	0	0	0	8	0	0	0	0	0	0
**Central Latin America**	**983**	**65**	**0**	**0**	**1153**	**0**	**0**	**0**	**0**	**2**	**1**
Colombia	0	0	0	0	29	0	0	0	0	0	0
Costa Rica	0	41	0	0	22	0	0	0	0	0	0
El Salvador	0	2	0	0	32	0	0	0	0	0	0
Guatemala	23	0	0	0	34	0	0	0	0	0	1
Honduras	0	0	0	0	3	0	0	0	0	0	0
Mexico	960	0	0	0	985	0	0	0	0	1	0
Aguascalientes	30	0	0	0	30	0	0	0	0	0	0
Baja California	30	0	0	0	30	0	0	0	0	0	0
Baja California Sur	30	0	0	0	30	0	0	0	0	0	0
Campeche	30	0	0	0	30	0	0	0	0	0	0
Chiapas	30	0	0	0	30	0	0	0	0	0	0
Chihuahua	30	0	0	0	30	0	0	0	0	0	0
Coahuila	30	0	0	0	30	0	0	0	0	0	0
Colima	30	0	0	0	30	0	0	0	0	0	0
Durango	30	0	0	0	30	0	0	0	0	0	0
Guanajuato	30	0	0	0	30	0	0	0	0	0	0
Guerrero	30	0	0	0	30	0	0	0	0	0	0
Hidalgo	30	0	0	0	30	0	0	0	0	0	0
Jalisco	30	0	0	0	30	0	0	0	0	0	0
México	30	0	0	0	30	0	0	0	0	0	0
Mexico City	0	0	0	0	0	0	0	0	0	0	0
Michoacán de Ocampo	30	0	0	0	30	0	0	0	0	0	0
Morelos	30	0	0	0	30	0	0	0	0	0	0
Nayarit	30	0	0	0	30	0	0	0	0	0	0
Nuevo León	30	0	0	0	30	0	0	0	0	0	0
Oaxaca	30	0	0	0	30	0	0	0	0	0	0

Puebla	30	0	0	0	30	0	0	0	0	0	0
Querétaro	30	0	0	0	30	0	0	0	0	0	0
Quintana Roo	30	0	0	0	30	0	0	0	0	0	0
San Luis Potosí	30	0	0	0	30	0	0	0	0	0	0
Sinaloa	30	0	0	0	30	0	0	0	0	0	0
Sonora	30	0	0	0	30	0	0	0	0	0	0
Tabasco	30	0	0	0	30	0	0	0	0	0	0
Tamaulipas	30	0	0	0	30	0	0	0	0	0	0
Tlaxcala	30	0	0	0	30	0	0	0	0	0	0
Veracruz de Ignacio de la Llave	30	0	0	0	30	0	0	0	0	0	0
Yucatán	30	0	0	0	30	0	0	0	0	0	0
Zacatecas	30	0	0	0	30	0	0	0	0	0	0
Nicaragua	0	0	0	0	2	0	0	0	0	1	0
Panama	0	22	0	0	27	0	0	0	0	0	0
Venezuela	0	0	0	0	19	0	0	0	0	0	0
**Tropical Latin America**	**2**	**868**	**0**	**0**	**508**	**1**	**0**	**5**	**0**	**4**	**0**
Brazil	2	868	0	0	502	1	0	5	0	3	0
Acre	0	31	0	0	17	0	0	0	0	0	0
Alagoas	0	31	0	0	17	0	0	0	0	0	0
Amapá	0	31	0	0	17	0	0	0	0	0	0
Amazonas	0	31	0	0	17	0	0	0	0	0	0
Bahia	0	31	0	0	17	0	0	0	0	0	0
Ceará	0	31	0	0	17	0	0	0	0	0	0
Distrito Federal	0	31	0	0	17	0	0	0	0	0	0
Espírito Santo	0	31	0	0	17	0	0	0	0	0	0
Goiás	0	31	0	0	17	0	0	0	0	0	0
Maranhão	0	31	0	0	17	0	0	0	0	0	0
Mato Grosso	0	31	0	0	17	0	0	0	0	0	0
Mato Grosso do Sul	0	31	0	0	17	0	0	0	0	0	0
Minas Gerais	1	31	0	0	17	0	0	0	0	0	0
Pará	0	31	0	0	17	0	0	0	0	0	0
Paraíba	0	31	0	0	17	0	0	0	0	0	0
Paraná	0	31	0	0	17	0	0	0	0	0	0
Pernambuco	1	31	0	0	17	0	0	5	0	0	0
Piauí	0	31	0	0	17	0	0	0	0	0	0
Rio de Janeiro	0	31	0	0	17	0	0	0	0	0	0
Rio Grande do Norte	0	31	0	0	17	0	0	0	0	0	0
Rio Grande do Sul	0	31	0	0	19	1	0	0	0	2	0
Rondônia	0	31	0	0	17	0	0	0	0	0	0
Roraima	0	31	0	0	17	0	0	0	0	0	0
Santa Catarina	0	31	0	0	17	0	0	0	0	0	0
São Paulo	0	31	0	0	17	0	0	0	0	0	0
Sergipe	0	31	0	0	17	0	0	0	0	0	0

Tocantins	0	31	0	0	17	0	0	0	0	0	0
Paraguay	0	0	0	0	6	0	0	0	0	1	0
**North Africa and Middle East**	**15**	**79**	**2**	**3**	**408**	**2**	**17**	**0**	**1**	**5**	**0**
**North Africa and Middle East**	**15**	**79**	**2**	**3**	**408**	**2**	**17**	**0**	**1**	**5**	**0**
Afghanistan	0	2	0	0	2	0	0	0	0	1	0
Algeria	0	0	0	0	37	0	0	0	0	0	0
Bahrain	0	0	0	0	40	0	0	0	0	0	0
Egypt	0	0	0	0	24	0	0	0	0	0	0
Iran	11	0	0	0	1	0	3	0	0	0	0
Alborz	0	0	0	0	0	0	0	0	0	0	0
Ardebil	0	0	0	0	0	0	0	0	0	0	0
Bushehr	0	0	0	0	0	0	0	0	0	0	0
Chahar Mahaal and Bakhtiari	0	0	0	0	0	0	0	0	0	0	0
East Azarbayejan	0	0	0	0	0	0	0	0	0	0	0
Fars	0	0	0	0	0	0	0	0	0	0	0
Gilan	0	0	0	0	0	0	0	0	0	0	0
Golestan	0	0	0	0	0	0	0	0	0	0	0
Hamadan	0	0	0	0	0	0	0	0	0	0	0
Hormozgan	0	0	0	0	0	0	0	0	0	0	0
Ilam	0	0	0	0	0	0	0	0	0	0	0
Isfahan	0	0	0	0	0	0	0	0	0	0	0
Kerman	1	0	0	0	0	0	0	0	0	0	0
Kermanshah	0	0	0	0	0	0	0	0	0	0	0
Khorasan-e-Razavi	0	0	0	0	0	0	0	0	0	0	0
Khuzestan	0	0	0	0	0	0	0	0	0	0	0
Kohgiluyeh and Boyer-Ahmad	0	0	0	0	0	0	0	0	0	0	0
Kurdistan	0	0	0	0	0	0	0	0	0	0	0
Lorestan	0	0	0	0	0	0	0	0	0	0	0
Markazi	0	0	0	0	0	0	0	0	0	0	0
Mazandaran	10	0	0	0	0	0	0	0	0	0	0
North Khorasan	0	0	0	0	0	0	0	0	0	0	0
Qazvin	0	0	0	0	0	0	0	0	0	0	0
Qom	0	0	0	0	0	0	0	0	0	0	0
Semnan	0	0	0	0	0	0	0	0	0	0	0
Sistan and Baluchistan	0	0	0	0	0	0	0	0	0	0	0
South Khorasan	0	0	0	0	0	0	0	0	0	0	0
Tehran	0	0	0	0	0	0	0	0	0	0	0
West Azarbayejan	0	0	0	0	1	0	0	0	0	0	0
Yazd	0	0	0	0	0	0	0	0	0	0	0
Zanjan	0	0	0	0	0	0	0	0	0	0	0
Iraq	0	0	0	0	7	0	0	0	0	0	0
Jordan	1	1	0	0	7	0	0	0	0	1	0
Kuwait	0	0	0	0	45	0	0	0	0	0	0

Lebanon	0	0	0	0	10	0	0	0	0	1	0
Libya	0	0	0	0	0	0	0	0	0	0	0
Morocco	0	1	0	0	31	0	0	0	0	0	0
Oman	0	31	0	0	30	0	0	0	0	0	0
Palestine	0	0	0	0	19	0	0	0	0	1	0
Qatar	0	0	1	0	45	0	0	0	0	1	0
Saudi Arabia	0	40	1	0	12	1	0	0	0	0	0
Sudan	0	0	0	0	23	0	0	0	0	0	0
Syria	0	0	0	0	0	0	0	0	0	0	0
Tunisia	0	1	0	0	41	0	0	0	0	0	0
Türkiye	3	3	0	0	8	1	14	0	1	0	0
United Arab Emirates	0	0	0	0	24	0	0	0	0	0	0
Yemen	0	0	0	3	2	0	0	0	0	0	0
**South Asia**	**25**	**13**	**3**	**0**	**2710**	**1**	**8**	**2**	**25**	**4**	**5**
**South Asia**	**25**	**13**	**3**	**0**	**2710**	**1**	**8**	**2**	**25**	**4**	**5**
Bangladesh	0	6	1	0	33	0	1	0	0	0	0
Bhutan	0	0	0	0	0	0	0	0	0	0	0
India	22	6	0	0	2642	1	7	0	25	2	3
Andhra Pradesh	0	0	0	0	131	0	0	0	0	0	0
Andhra Pradesh, Rural	0	0	0	0	41	0	0	0	0	0	0
Andhra Pradesh, Urban	0	0	0	0	41	0	0	0	0	0	0
Arunachal Pradesh	0	0	0	0	8	0	0	0	0	0	0
Arunachal Pradesh, Rural	0	0	0	0	1	0	0	0	0	0	0
Arunachal Pradesh, Urban	0	0	0	0	1	0	0	0	0	0	0
Assam	0	0	0	0	147	0	0	0	0	0	0
Assam, Rural	0	0	0	0	46	0	0	0	0	0	0
Assam, Urban	0	0	0	0	46	0	0	0	0	0	0
Bihar	0	0	0	0	151	0	0	0	0	0	0
Bihar, Rural	0	0	0	0	48	0	0	0	0	0	0
Bihar, Urban	0	0	0	0	48	0	0	0	0	0	0
Chhattisgarh	0	0	0	0	75	0	0	0	0	0	0
Chhattisgarh, Rural	0	0	0	0	23	0	0	0	0	0	0
Chhattisgarh, Urban	0	0	0	0	23	0	0	0	0	0	0
Delhi	0	0	0	0	80	0	0	0	0	0	0
Delhi, Rural	0	0	0	0	28	0	0	0	0	0	0
Delhi, Urban	0	0	0	0	29	0	0	0	0	0	0
Goa	0	0	0	0	11	0	0	0	0	0	0
Goa, Rural	0	0	0	0	1	0	0	0	0	0	0
Goa, Urban	0	0	0	0	1	0	0	0	0	0	0
Gujarat	0	0	0	0	128	0	0	0	0	0	0
Gujarat, Rural	0	0	0	0	41	0	0	0	0	0	0
Gujarat, Urban	0	0	0	0	41	0	0	0	0	0	0
Haryana	0	0	0	0	129	0	0	0	0	0	0

Haryana, Rural	0	0	0	0	41	0	0	0	0	0	0
Haryana, Urban	0	0	0	0	41	0	0	0	0	0	0
Himachal Pradesh	0	0	0	0	100	0	0	0	0	0	0
Himachal Pradesh, Rural	0	0	0	0	31	0	0	0	0	0	0
Himachal Pradesh, Urban	0	0	0	0	31	0	0	0	0	0	0
Jammu & Kashmir and Ladakh	0	0	0	0	58	0	0	0	0	0	0
Jammu & Kashmir and Ladakh, Rural	0	0	0	0	17	0	0	0	0	0	0
Jammu & Kashmir and Ladakh, Urban	0	0	0	0	17	0	0	0	0	0	0
Jharkhand	0	0	0	0	68	0	0	0	0	0	0
Jharkhand, Rural	0	0	0	0	21	0	0	0	0	0	0
Jharkhand, Urban	0	0	0	0	21	0	0	0	0	0	0
Karnataka	6	6	0	0	132	0	0	0	0	0	1
Karnataka, Rural	0	0	0	0	41	0	0	0	0	0	0
Karnataka, Urban	0	0	0	0	41	0	0	0	0	0	0
Kerala	0	0	0	0	131	1	0	0	0	0	0
Kerala, Rural	0	0	0	0	41	0	0	0	0	0	0
Kerala, Urban	0	0	0	0	41	0	0	0	0	0	0
Madhya Pradesh	0	0	0	0	146	0	0	0	0	0	0
Madhya Pradesh, Rural	0	0	0	0	46	0	0	0	0	0	0
Madhya Pradesh, Urban	0	0	0	0	46	0	0	0	0	0	0
Maharashtra	6	0	0	0	139	0	0	0	0	0	1
Maharashtra, Rural	0	0	0	0	49	0	0	0	0	0	0
Maharashtra, Urban	0	0	0	0	41	0	0	0	0	0	0
Manipur	0	0	0	0	4	0	0	0	0	0	0
Manipur, Rural	0	0	0	0	1	0	0	0	0	0	0
Manipur, Urban	0	0	0	0	1	0	0	0	0	0	0
Meghalaya	0	0	0	0	10	0	0	0	0	0	0
Meghalaya, Rural	0	0	0	0	1	0	0	0	0	0	0
Meghalaya, Urban	0	0	0	0	1	0	0	0	0	0	0
Mizoram	0	0	0	0	11	0	0	0	0	0	0
Mizoram, Rural	0	0	0	0	1	0	0	0	0	0	0
Mizoram, Urban	0	0	0	0	1	0	0	0	0	0	0
Nagaland	0	0	0	0	11	0	0	0	0	0	0
Nagaland, Rural	0	0	0	0	1	0	0	0	0	0	0
Nagaland, Urban	0	0	0	0	1	0	0	0	0	0	0
Odisha	0	0	0	0	147	0	0	0	0	0	1
Odisha, Rural	0	0	0	0	47	0	0	0	0	0	0
Odisha, Urban	0	0	0	0	46	0	0	0	0	0	0
Other Union Territories	0	0	0	0	7	0	0	0	0	0	0
Other Union Territories, Rural	0	0	0	0	0	0	0	0	0	0	0
Other Union Territories, Urban	0	0	0	0	0	0	0	0	0	0	0
Punjab	10	0	0	0	131	0	1	0	0	0	0
Punjab, Rural	0	0	0	0	41	0	0	0	0	0	0

Punjab, Urban	0	0	0	0	41	0	0	0	0	0	0
Rajasthan	0	0	0	0	146	0	0	0	0	1	0
Rajasthan, Rural	0	0	0	0	46	0	0	0	0	1	0
Rajasthan, Urban	0	0	0	0	46	0	0	0	0	0	0
Sikkim	0	0	0	0	10	0	0	0	0	0	0
Sikkim, Rural	0	0	0	0	1	0	0	0	0	0	0
Sikkim, Urban	0	0	0	0	1	0	0	0	0	0	0
Tamil Nadu	0	0	0	0	130	0	0	0	25	0	0
Tamil Nadu, Rural	0	0	0	0	42	0	0	0	0	0	0
Tamil Nadu, Urban	0	0	0	0	41	0	0	0	0	0	0
Telangana	0	0	0	0	26	0	0	0	0	0	0
Telangana, Rural	0	0	0	0	7	0	0	0	0	0	0
Telangana, Urban	0	0	0	0	7	0	0	0	0	0	0
Tripura	0	0	0	0	11	0	0	0	0	0	0
Tripura, Rural	0	0	0	0	1	0	0	0	0	0	0
Tripura, Urban	0	0	0	0	1	0	0	0	0	0	0
Uttar Pradesh	0	0	0	0	146	0	4	0	0	0	0
Uttar Pradesh, Rural	0	0	0	0	49	0	0	0	0	0	0
Uttar Pradesh, Urban	0	0	0	0	46	0	0	0	0	0	0
Uttarakhand	0	0	0	0	42	0	0	0	0	0	0
Uttarakhand, Rural	0	0	0	0	12	0	0	0	0	0	0
Uttarakhand, Urban	0	0	0	0	12	0	0	0	0	0	0
West Bengal	0	0	0	0	128	0	0	0	0	0	0
West Bengal, Rural	0	0	0	0	40	0	0	0	0	0	0
West Bengal, Urban	0	0	0	0	40	0	0	0	0	0	0
Nepal	0	1	0	0	10	0	0	2	0	1	1
Pakistan	3	0	2	0	25	0	0	0	0	1	1
Azad Jammu & Kashmir	0	0	0	0	1	0	0	0	0	0	0
Balochistan	0	0	0	0	3	0	0	0	0	0	0
Gilgit-Baltistan	0	0	0	0	1	0	0	0	0	0	0
Islamabad Capital Territory	0	0	0	0	1	0	0	0	0	0	0
Khyber Pakhtunkhwa	0	0	0	0	4	0	0	0	0	0	0
Punjab	0	0	0	0	3	0	0	0	0	0	0
Sindh	2	0	2	0	9	0	0	0	0	0	0
**Southeast Asia, east Asia, and Oceania**	**2190**	**47**	**3**	**0**	**1207**	**0**	**0**	**3**	**1**	**7**	**0**
**East Asia**	**10**	**17**	**2**	**0**	**75**	**0**	**0**	**0**	**0**	**2**	**0**
China	3	17	2	0	74	0	0	0	0	2	0
Anhui	0	0	0	0	1	0	0	0	0	0	0
Beijing	0	0	0	0	0	0	0	0	0	0	0
Chongqing	0	0	0	0	0	0	0	0	0	0	0
Fujian	0	0	0	0	0	0	0	0	0	0	0
Gansu	0	0	0	0	0	0	0	0	0	0	0
Guangdong	1	0	0	0	0	0	0	0	0	0	0

Guangxi	0	0	0	0	0	0	0	0	0	0	0
Guizhou	0	0	0	0	0	0	0	0	0	0	0
Hainan	0	0	0	0	0	0	0	0	0	0	0
Hebei	0	0	0	0	1	0	0	0	0	0	0
Heilongjiang	0	0	0	0	0	0	0	0	0	0	0
Henan	0	0	0	0	0	0	0	0	0	0	0
Hong Kong Special Administrative Region of China	0	0	1	0	25	0	0	0	0	0	0
Hubei	1	0	0	0	0	0	0	0	0	0	0
Hunan	0	0	0	0	0	0	0	0	0	0	0
Inner Mongolia	0	0	0	0	0	0	0	0	0	0	0
Jiangsu	0	2	0	0	0	0	0	0	0	0	0
Jiangxi	0	0	0	0	0	0	0	0	0	0	0
Jilin	0	0	0	0	0	0	0	0	0	0	0
Liaoning	0	0	0	0	0	0	0	0	0	0	0
Macao Special Administrative Region of China	0	14	0	0	45	0	0	0	0	0	0
Ningxia	0	0	0	0	0	0	0	0	0	0	0
Qinghai	0	0	0	0	0	0	0	0	0	0	0
Shaanxi	0	0	0	0	0	0	0	0	0	0	0
Shandong	0	0	0	0	0	0	0	0	0	0	0
Shanghai	0	0	0	0	0	0	0	0	0	0	0
Shanxi	0	0	0	0	1	0	0	0	0	0	0
Sichuan	0	0	0	0	0	0	0	0	0	0	0
Tianjin	0	0	0	0	0	0	0	0	0	0	0
Tibet	0	0	0	0	0	0	0	0	0	0	0
Xinjiang	0	0	0	0	0	0	0	0	0	0	0
Yunnan	0	0	0	0	0	0	0	0	0	0	0
Zhejiang	0	0	0	0	0	0	0	0	0	0	0
North Korea	0	0	0	0	0	0	0	0	0	0	0
Taiwan (province of China)	7	0	0	0	1	0	0	0	0	0	0
**Oceania**	**33**	**1**	**0**	**0**	**187**	**0**	**0**	**0**	**0**	**0**	**0**
American Samoa	9	0	0	0	57	0	0	0	0	0	0
Cook Islands	0	0	0	0	36	0	0	0	0	0	0
Fiji	0	0	0	0	2	0	0	0	0	0	0
Guam	13	0	0	0	34	0	0	0	0	0	0
Kiribati	0	0	0	0	1	0	0	0	0	0	0
Marshall Islands	0	0	0	0	5	0	0	0	0	0	0
Federated States of Micronesia	0	0	0	0	0	0	0	0	0	0	0
Nauru	0	0	0	0	0	0	0	0	0	0	0
Niue	0	0	0	0	0	0	0	0	0	0	0
Northern Mariana Islands	11	0	0	0	15	0	0	0	0	0	0
Palau	0	0	0	0	15	0	0	0	0	0	0
Papua New Guinea	0	1	0	0	5	0	0	0	0	0	0

Samoa	0	0	0	0	2	0	0	0	0	0	0
Solomon Islands	0	0	0	0	4	0	0	0	0	0	0
Tokelau	0	0	0	0	0	0	0	0	0	0	0
Tonga	0	0	0	0	10	0	0	0	0	0	0
Tuvalu	0	0	0	0	0	0	0	0	0	0	0
Vanuatu	0	0	0	0	1	0	0	0	0	0	0
**Southeast Asia**	**2147**	**29**	**1**	**0**	**945**	**0**	**0**	**3**	**1**	**5**	**0**
Cambodia	0	0	0	0	8	0	0	0	0	1	0
Indonesia	0	0	0	0	401	0	0	0	0	0	0
Aceh	0	0	0	0	14	0	0	0	0	0	0
Bali	0	0	0	0	12	0	0	0	0	0	0
Bangka-Belitung Islands	0	0	0	0	14	0	0	0	0	0	0
Banten	0	0	0	0	12	0	0	0	0	0	0
Bengkulu	0	0	0	0	13	0	0	0	0	0	0
Gorontalo	0	0	0	0	12	0	0	0	0	0	0
Jakarta	0	0	0	0	14	0	0	0	0	0	0
Jambi	0	0	0	0	13	0	0	0	0	0	0
West Java	0	0	0	0	10	0	0	0	0	0	0
Central Java	0	0	0	0	15	0	0	0	0	0	0
East Java	0	0	0	0	15	0	0	0	0	0	0
West Kalimantan	0	0	0	0	12	0	0	0	0	0	0
South Kalimantan	0	0	0	0	13	0	0	0	0	0	0
Central Kalimantan	0	0	0	0	9	0	0	0	0	0	0
East Kalimantan	0	0	0	0	14	0	0	0	0	0	0
North Kalimantan	0	0	0	0	4	0	0	0	0	0	0
Riau Islands	0	0	0	0	13	0	0	0	0	0	0
Lampung	0	0	0	0	15	0	0	0	0	0	0
Maluku	0	0	0	0	9	0	0	0	0	0	0
North Maluku	0	0	0	0	7	0	0	0	0	0	0
West Nusa Tenggara	0	0	0	0	14	0	0	0	0	0	0
East Nusa Tenggara	0	0	0	0	11	0	0	0	0	0	0
Papua	0	0	0	0	6	0	0	0	0	0	0
West Papua	0	0	0	0	6	0	0	0	0	0	0
Riau	0	0	0	0	12	0	0	0	0	0	0
West Sulawesi	0	0	0	0	12	0	0	0	0	0	0
South Sulawesi	0	0	0	0	13	0	0	0	0	0	0
Central Sulawesi	0	0	0	0	15	0	0	0	0	0	0
Southeast Sulawesi	0	0	0	0	11	0	0	0	0	0	0
North Sulawesi	0	0	0	0	6	0	0	0	0	0	0
West Sumatra	0	0	0	0	9	0	0	0	0	0	0
South Sumatra	0	0	0	0	15	0	0	0	0	0	0
North Sumatra	0	0	0	0	13	0	0	0	0	0	0
Yogyakarta	0	0	0	0	12	0	0	0	0	0	0

Laos	0	0	0	0	0	0	0	0	0	0	0
Malaysia	0	0	0	0	57	0	0	0	0	0	0
Maldives	0	0	0	0	45	0	0	0	0	0	0
Mauritius	0	0	0	0	100	0	0	0	0	0	0
Myanmar	0	0	0	0	8	0	0	0	0	0	0
Philippines	2147	0	0	0	258	0	0	0	0	1	0
Abra	27	0	0	0	3	0	0	0	0	0	0
Agusan Del Norte	26	0	0	0	3	0	0	0	0	0	0
Agusan Del Sur	26	0	0	0	3	0	0	0	0	0	0
Aklan	27	0	0	0	3	0	0	0	0	0	0
Albay	26	0	0	0	3	0	0	0	0	0	0
Antique	27	0	0	0	3	0	0	0	0	0	0
Apayao	27	0	0	0	2	0	0	0	0	0	0
Aurora	25	0	0	0	3	0	0	0	0	0	0
Basilan	26	0	0	0	3	0	0	0	0	0	0
Bataan	26	0	0	0	3	0	0	0	0	0	0
Batanes	24	0	0	0	1	0	0	0	0	0	0
Batangas	26	0	0	0	3	0	0	0	0	0	0
Benguet	27	0	0	0	3	0	0	0	0	0	0
Biliran	27	0	0	0	2	0	0	0	0	0	0
Bohol	27	0	0	0	3	0	0	0	0	0	0
Bukidnon	26	0	0	0	3	0	0	0	0	0	0
Bulacan	27	0	0	0	3	0	0	0	0	0	0
Cagayan	26	0	0	0	3	0	0	0	0	0	0
Camarines Norte	25	0	0	0	3	0	0	0	0	0	0
Camarines Sur	25	0	0	0	3	0	0	0	0	0	0
Camiguin	26	0	0	0	3	0	0	0	0	0	0
Capiz	27	0	0	0	3	0	0	0	0	0	0
Catanduanes	25	0	0	0	3	0	0	0	0	0	0
Cavite	26	0	0	0	3	0	0	0	0	0	0
Cebu	27	0	0	0	3	0	0	0	0	0	0
Cotabato (North Cotabato)	27	0	0	0	3	0	0	0	0	0	0
Davao de Oro	22	0	0	0	1	0	0	0	0	0	0
Davao Del Norte	27	0	0	0	3	0	0	0	0	0	0
Davao Del Sur	27	0	0	0	3	0	0	0	0	0	0
Davao Occidental	3	0	0	0	0	0	0	0	0	0	0
Davao Oriental	27	0	0	0	3	0	0	0	0	0	0
Dinagat Islands	11	0	0	0	0	0	0	0	0	0	0
Eastern Samar	27	0	0	0	3	0	0	0	0	0	0
Guimaras	27	0	0	0	2	0	0	0	0	0	0
Ifugao	27	0	0	0	3	0	0	0	0	0	0
Ilocos Norte	26	0	0	0	3	0	0	0	0	0	0
Ilocos Sur	26	0	0	0	3	0	0	0	0	0	0

Iloilo	27	0	0	0	3	0	0	0	0	0	0
Isabela	26	0	0	0	3	0	0	0	0	0	0
Kalinga	25	0	0	0	3	0	0	0	0	0	0
La Union	26	0	0	0	3	0	0	0	0	0	0
Laguna	26	0	0	0	3	0	0	0	0	0	0
Lanao Del Norte	27	0	0	0	3	0	0	0	0	0	0
Lanao Del Sur	27	0	0	0	3	0	0	0	0	0	0
Leyte	27	0	0	0	3	0	0	0	0	0	0
Maguindanao	27	0	0	0	3	0	0	0	0	0	0
Marinduque	26	0	0	0	3	0	0	0	0	0	0
Masbate	26	0	0	0	3	0	0	0	0	0	0
Misamis Occidental	26	0	0	0	3	0	0	0	0	0	0
Misamis Oriental	26	0	0	0	3	0	0	0	0	0	0
Mountain Province	27	0	0	0	3	0	0	0	0	0	0
National Capital Region	27	0	0	0	3	0	0	0	0	0	0
Negros Occidental	27	0	0	0	3	0	0	0	0	0	0
Negros Oriental	27	0	0	0	3	0	0	0	0	0	0
Northern Samar	27	0	0	0	3	0	0	0	0	0	0
Nueva Ecija	24	0	0	0	3	0	0	0	0	0	0
Nueva Vizcaya	26	0	0	0	3	0	0	0	0	0	0
Occidental Mindoro	26	0	0	0	3	0	0	0	0	0	0
Oriental Mindoro	25	0	0	0	3	0	0	0	0	0	0
Palawan	24	0	0	0	3	0	0	0	0	0	0
Pampanga	27	0	0	0	3	0	0	0	0	0	0
Pangasinan	26	0	0	0	3	0	0	0	0	0	0
Quezon	27	0	0	0	3	0	0	0	0	0	0
Quirino	26	0	0	0	3	0	0	0	0	0	0
Rizal	26	0	0	0	3	0	0	0	0	0	0
Romblon	26	0	0	0	3	0	0	0	0	0	0
Samar (Western Samar)	27	0	0	0	3	0	0	0	0	0	0
Sarangani	26	0	0	0	2	0	0	0	0	0	0
Siquijor	27	0	0	0	3	0	0	0	0	0	0
Sorsogon	26	0	0	0	3	0	0	0	0	0	0
South Cotabato	26	0	0	0	3	0	0	0	0	0	0
Southern Leyte	27	0	0	0	3	0	0	0	0	0	0
Sultan Kudarat	27	0	0	0	3	0	0	0	0	0	0
Sulu	25	0	0	0	3	0	0	0	0	0	0
Surigao Del Norte	26	0	0	0	3	0	0	0	0	0	0
Surigao Del Sur	27	0	0	0	3	0	0	0	0	0	0
Tarlac	27	0	0	0	3	0	0	0	0	0	0
Tawi-Tawi	27	0	0	0	3	0	0	0	0	0	0
Zambales	27	0	0	0	3	0	0	0	0	0	0
Zamboanga Del Norte	27	0	0	0	3	0	0	0	0	0	0

Zamboanga Del Sur	27	0	0	0	3	0	0	0	0	0	0
Zamboanga Sibugay	19	0	0	0	1	0	0	0	0	0	0
Seychelles	0	7	0	0	5	0	0	0	0	0	0
Sri Lanka	0	22	0	0	49	0	0	0	0	1	0
Thailand	0	0	0	0	6	0	0	0	0	1	0
Timor-Leste	0	0	0	0	2	0	0	2	0	0	0
Viet Nam	0	0	1	0	6	0	0	1	1	1	0
**Sub-Saharan Africa**	**10**	**35**	**5**	**18**	**592**	**242**	**266**	**1**	**2**	**14**	**5**
**Central sub-Saharan Africa**	**1**	**0**	**1**	**0**	**5**	**0**	**0**	**0**	**0**	**2**	**1**
Angola	0	0	0	0	2	0	0	0	0	1	0
Central African Republic	0	0	0	0	0	0	0	0	0	0	0
Congo (Brazzaville)	0	0	0	0	1	0	0	0	0	0	0
DR Congo	1	0	1	0	1	0	0	0	0	1	1
Equatorial Guinea	0	0	0	0	0	0	0	0	0	0	0
Gabon	0	0	0	0	1	0	0	0	0	0	0
**Eastern sub-Saharan Africa**	**5**	**12**	**2**	**1**	**275**	**4**	**0**	**0**	**1**	**6**	**3**
Burundi	0	0	0	0	4	0	0	0	0	0	0
Comoros	0	0	0	0	1	0	0	0	0	0	0
Djibouti	0	0	0	0	0	0	0	0	0	0	0
Eritrea	0	0	0	0	0	0	0	0	0	0	0
Ethiopia	0	0	0	0	38	0	0	0	0	0	0
Addis Ababa	0	0	0	0	2	0	0	0	0	0	0
Afar	0	0	0	0	2	0	0	0	0	0	0
Amhara	0	0	0	0	7	0	0	0	0	0	0
Benishangul-Gumuz	0	0	0	0	2	0	0	0	0	0	0
Dire Dawa	0	0	0	0	2	0	0	0	0	0	0
Gambella	0	0	0	0	2	0	0	0	0	0	0
Harari	0	0	0	0	2	0	0	0	0	0	0
Oromia	0	0	0	0	4	0	0	0	0	0	0
Somali	0	0	0	0	2	0	0	0	0	0	0
Southern Nations, Nationalities, and Peoples	0	0	0	0	7	0	0	0	0	0	0
Tigray	0	0	0	0	3	0	0	0	0	0	0
Kenya	2	2	1	0	179	0	0	0	0	2	1
Baringo	0	0	0	0	4	0	0	0	0	0	0
Bomet	0	0	0	0	3	0	0	0	0	0	0
Bungoma	0	0	0	0	4	0	0	0	0	0	0
Busia	0	0	0	0	4	0	0	0	0	0	0
Elgeyo Marakwet	0	0	0	0	4	0	0	0	0	0	0
Embu	0	0	0	0	4	0	0	0	0	0	0
Garissa	0	0	0	0	3	0	0	0	0	0	0
Homa Bay	0	0	0	0	4	0	0	0	0	0	0
Isiolo	0	0	0	0	3	0	0	0	0	0	0
Kajiado	0	0	0	0	4	0	0	0	0	0	0

Kakamega	0	0	0	0	4	0	0	0	0	0	0
Kericho	0	0	0	0	4	0	0	0	0	0	0
Kiambu	0	0	0	0	5	0	0	0	0	0	0
Kilifi	0	1	0	0	4	0	0	0	0	0	0
Kirinyaga	0	0	0	0	4	0	0	0	0	0	0
Kisii	0	0	0	0	4	0	0	0	0	0	0
Kisumu	0	0	0	0	4	0	0	0	0	0	0
Kitui	0	0	0	0	4	0	0	0	0	0	0
Kwale	0	0	0	0	4	0	0	0	0	0	0
Laikipia	0	0	0	0	4	0	0	0	0	0	0
Lamu	0	0	0	0	3	0	0	0	0	0	0
Machakos	0	0	0	0	4	0	0	0	0	0	0
Makueni	0	0	0	0	3	0	0	0	0	0	0
Mandera	0	0	0	0	3	0	0	0	0	0	0
Marsabit	0	0	0	0	3	0	0	0	0	0	0
Meru	0	0	0	0	3	0	0	0	0	0	0
Migori	0	0	0	0	3	0	0	0	0	0	0
Mombasa	0	1	0	0	4	0	0	0	0	0	0
Murang'a	0	0	0	0	4	0	0	0	0	0	0
Nairobi	0	0	0	0	5	0	0	0	0	0	0
Nakuru	0	0	0	0	4	0	0	0	0	0	0
Nandi	0	0	0	0	4	0	0	0	0	0	0
Narok	0	0	0	0	4	0	0	0	0	0	0
Nyamira	0	0	0	0	2	0	0	0	0	0	0
Nyandarua	0	0	0	0	4	0	0	0	0	0	0
Nyeri	0	0	1	0	5	0	0	0	0	0	0
Samburu	0	0	0	0	3	0	0	0	0	0	0
Siaya	0	0	0	0	4	0	0	0	0	0	0
Taita Taveta	0	0	0	0	4	0	0	0	0	0	0
Tana River	0	0	0	0	3	0	0	0	0	0	0
Tharaka Nithi	0	0	0	0	3	0	0	0	0	0	0
Trans Nzoia	0	0	0	0	4	0	0	0	0	0	0
Turkana	0	0	0	0	3	0	0	0	0	0	0
Uasin Gishu	0	0	0	0	4	0	0	0	0	0	0
Vihiga	0	0	0	0	3	0	0	0	0	0	0
Wajir	0	0	0	0	3	0	0	0	0	0	0
West Pokot	0	0	0	0	4	0	0	0	0	0	0
Madagascar	0	0	0	0	3	0	0	0	0	0	0
Malawi	1	0	0	0	6	0	0	0	0	1	0
Mozambique	0	1	0	0	2	0	0	0	0	0	0
Rwanda	0	5	0	0	5	0	0	0	0	0	0
Somalia	0	0	0	0	0	0	0	0	0	0	0
South Sudan	0	0	0	0	0	0	0	0	0	0	0

Uganda	0	0	0	0	22	0	0	0	1	1	0
Tanzania	0	2	1	0	8	4	0	0	0	0	1
Zambia	2	2	0	1	7	0	0	0	0	2	1
**Southern sub-Saharan Africa**	**2**	**5**	**0**	**16**	**63**	**236**	**259**	**1**	**0**	**1**	**0**
Botswana	0	0	0	0	26	0	0	0	0	0	0
Eswatini	0	0	0	0	1	0	0	0	0	0	0
Lesotho	0	0	0	0	2	0	0	0	0	0	0
Namibia	0	0	0	0	2	0	0	0	0	0	0
South Africa	1	4	0	16	27	236	259	0	0	0	0
Eastern Cape	0	0	0	0	1	23	25	0	0	0	0
Free State	0	0	0	0	1	23	25	0	0	0	0
Gauteng	0	0	0	0	3	23	25	0	0	0	0
KwaZulu-Natal	0	0	0	0	1	23	25	0	0	0	0
Limpopo	1	0	0	0	1	23	26	0	0	0	0
Mpumalanga	0	0	0	0	1	23	26	0	0	0	0
North West	0	0	0	0	1	23	25	0	0	0	0
Northern Cape	0	0	0	0	1	23	25	0	0	0	0
Western Cape	0	1	0	0	2	23	27	0	0	0	0
Zimbabwe	1	1	0	0	5	0	0	1	0	1	0
**Western sub-Saharan Africa**	**2**	**18**	**2**	**1**	**249**	**2**	**7**	**0**	**1**	**5**	**1**
Benin	0	1	0	0	16	0	0	0	0	0	0
Burkina Faso	0	0	0	0	4	0	0	0	0	0	0
Cabo Verde	0	0	0	1	4	0	0	0	0	0	0
Cameroon	0	1	0	0	1	1	0	0	1	0	0
Chad	0	0	0	0	0	0	0	0	0	0	0
Côte d'Ivoire	0	1	0	0	1	0	0	0	0	0	0
The Gambia	0	5	0	0	3	0	0	0	0	0	0
Ghana	1	0	1	0	20	0	0	0	0	0	0
Guinea	1	0	0	0	2	0	0	0	0	0	0
Guinea-Bissau	0	0	0	0	0	0	7	0	0	0	0
Liberia	0	0	0	0	5	0	0	0	0	0	0
Mali	0	0	0	0	6	0	0	0	0	0	0
Mauritania	0	1	0	0	1	0	0	0	0	0	0
Niger	0	1	1	0	8	0	0	0	0	1	0
Nigeria	0	2	0	0	144	1	0	0	0	3	1
Abia	0	0	0	0	4	0	0	0	0	0	0
Adamawa	0	0	0	0	4	0	0	0	0	0	0
Akwa Ibom	0	0	0	0	4	0	0	0	0	0	0
Anambra	0	0	0	0	4	0	0	0	0	0	0
Bauchi	0	0	0	0	3	0	0	0	0	0	0
Bayelsa	0	0	0	0	4	0	0	0	0	0	0
Benue	0	0	0	0	4	0	0	0	0	0	0
Borno	0	0	0	0	4	0	0	0	0	0	0

Cross River	0	0	0	0	3	0	0	0	0	0	0
Delta	0	0	0	0	4	0	0	0	0	0	0
Ebonyi	0	0	0	0	4	0	0	0	0	0	0
Edo	0	0	0	0	4	1	0	0	0	0	0
Ekiti	0	0	0	0	3	0	0	0	0	1	0
Enugu	0	1	0	0	4	0	0	0	0	0	0
FCT (Abuja)	0	0	0	0	4	0	0	0	0	0	0
Gombe	0	0	0	0	3	0	0	0	0	0	0
Imo	0	0	0	0	4	0	0	0	0	0	0
Jigawa	0	1	0	0	4	0	0	0	0	0	0
Kaduna	0	0	0	0	4	0	0	0	0	0	0
Kano	0	0	0	0	4	0	0	0	0	0	0
Katsina	0	0	0	0	4	0	0	0	0	0	1
Kebbi	0	0	0	0	4	0	0	0	0	0	0
Kogi	0	0	0	0	4	0	0	0	0	0	0
Kwara	0	0	0	0	4	0	0	0	0	0	0
Lagos	0	0	0	0	3	0	0	0	0	0	0
Nasarawa	0	0	0	0	4	0	0	0	0	0	0
Niger	0	0	0	0	3	0	0	0	0	0	0
Ogun	0	0	0	0	3	0	0	0	0	0	0
Ondo	0	0	0	0	3	0	0	0	0	0	0
Osun	0	0	0	0	4	0	0	0	0	0	0
Oyo	0	0	0	0	4	0	0	0	0	0	0
Plateau	0	0	0	0	5	0	0	0	0	0	0
Rivers	0	0	0	0	4	0	0	0	0	1	0
Sokoto	0	0	0	0	4	0	0	0	0	0	0
Taraba	0	0	0	0	4	0	0	0	0	0	0
Yobe	0	0	0	0	4	0	0	0	0	0	0
Zamfara	0	0	0	0	4	0	0	0	0	0	0
São Tomé and Príncipe	0	0	0	0	0	0	0	0	0	0	0
Senegal	0	6	0	0	10	0	0	0	0	0	0
Sierra Leone	0	0	0	0	18	0	0	0	0	1	0
Togo	0	0	0	0	6	0	0	0	0	0	0

4.4.3 Appendix 1 Figure S3A: Map of the number of years of vital registration data by location

Caption: (A) The map shows the number of years of stillbirth data coming from vital registration for each location, indicated by colour. The number of years of data can range from 0 to 42, representing the number of years covered from 1980 to 2021. National locations and their corresponding subnational locations may be coloured differently due to disparity in data availability at the different administrative levels.

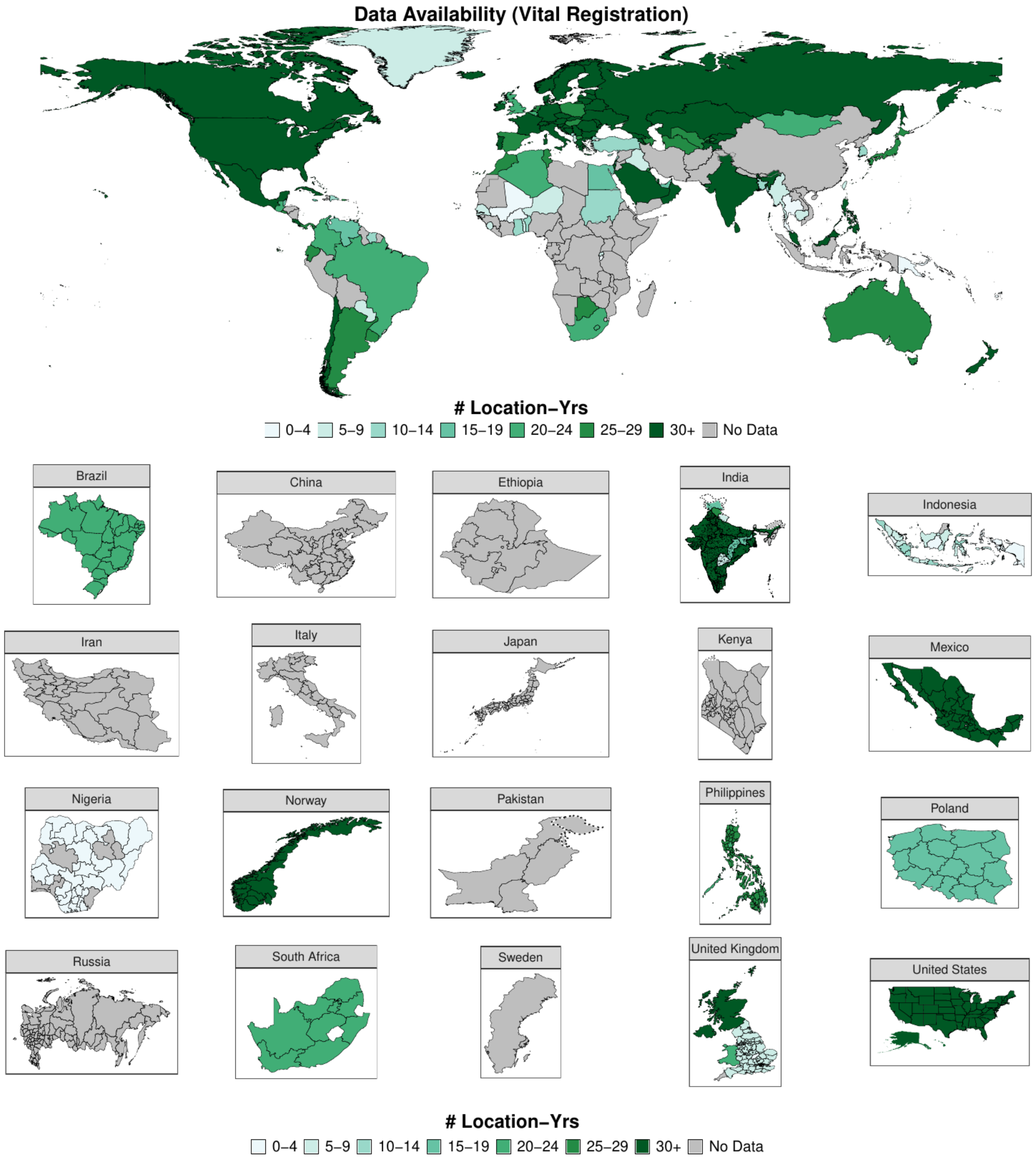

4.4.3 Appendix 1 Figure S3B: Map of the number of years of survey data by location

Caption: (B) The map shows the number of years of stillbirth data coming from surveys for each location, indicated by colour. The number of years of data can range from 0 to 42, representing the number of years covered from 1980 to 2021. National locations and their corresponding subnational locations may be coloured differently due to disparity in data availability at the different administrative levels.

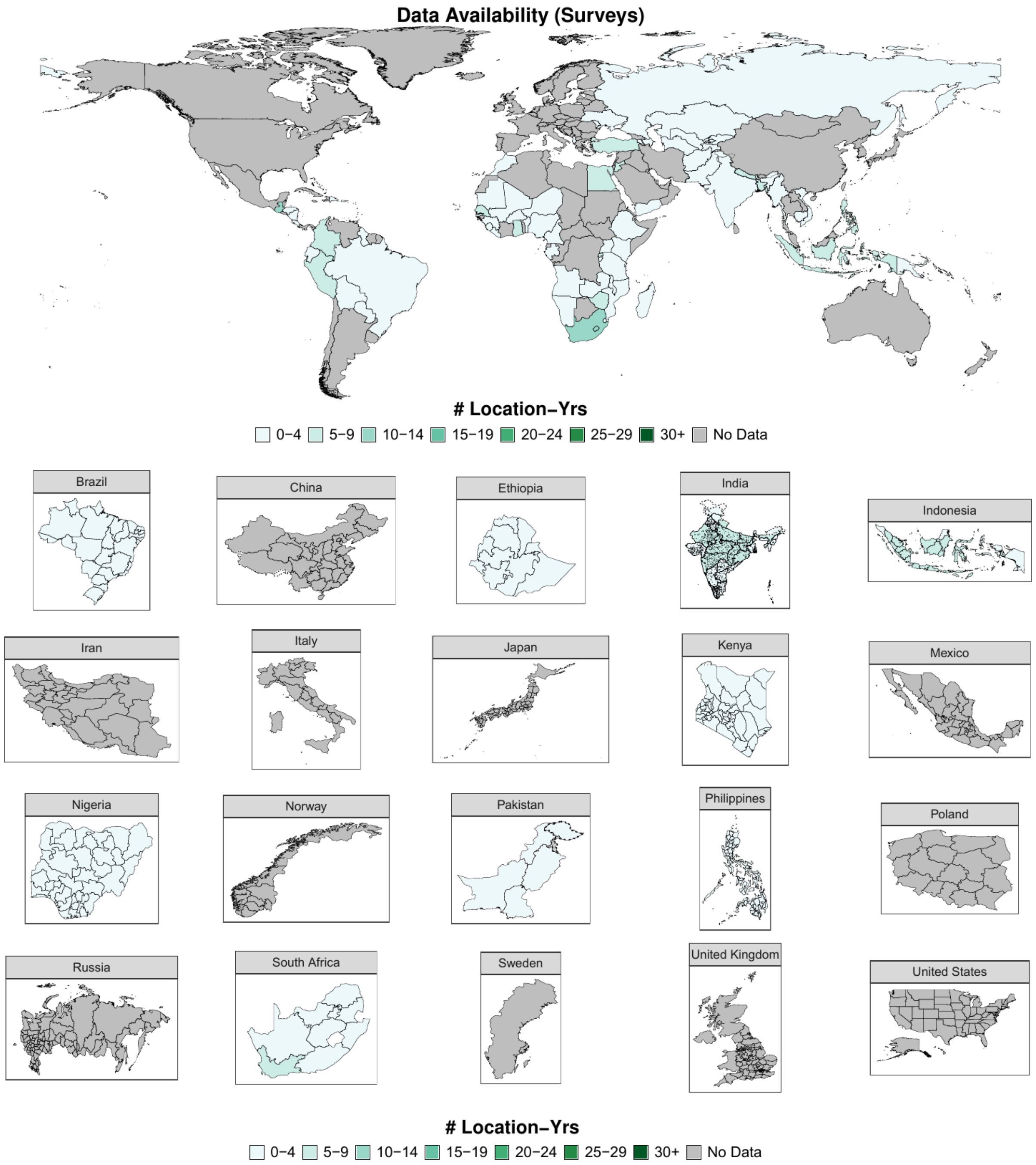

4.4.4 Appendix 1 Figure S3C: Map of the number of years of scientific literature data by location

```
Caption: (C) The map shows the number of years of stillbirth data coming from
scientific literature for each location, indicated by colour. The number of
years of data can range from 0 to 42, representing the number of years
covered from 1980 to 2021. National locations and their corresponding
subnational locations may be coloured differently due to disparity in data
availability at the different administrative levels.
```

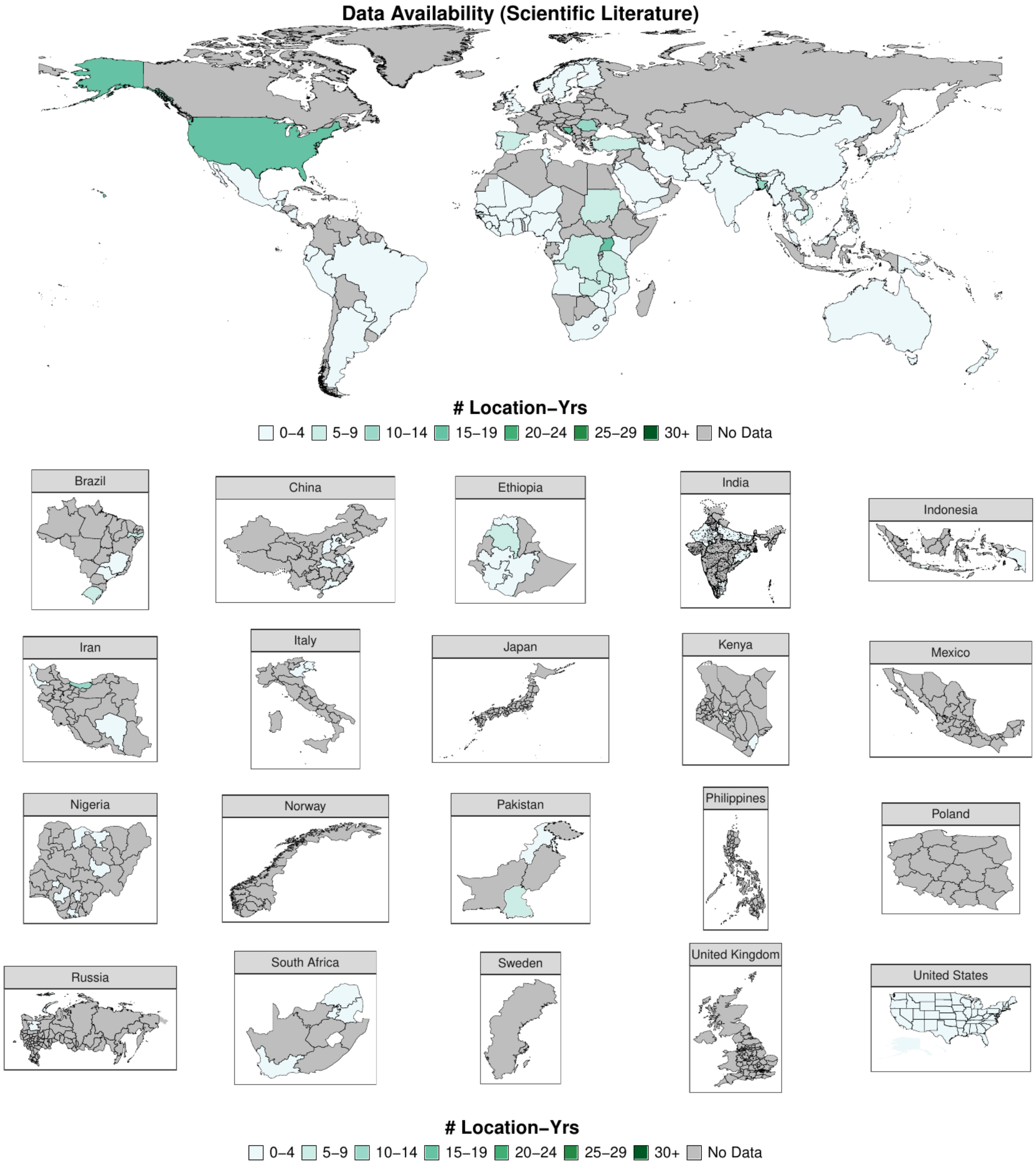

4.4.5 Appendix 1 Figure S3D: Map of the number of years of data by location

Caption: (D) The map shows the number of years of stillbirth data coming from all sources for each location, indicated by colour. The number of years of data can range from 0 to 42, representing the number of years covered from 19 80 to 2021. National locations and their corresponding subnational locations may be coloured differently due to disparity in data availability at the different administrative levels.

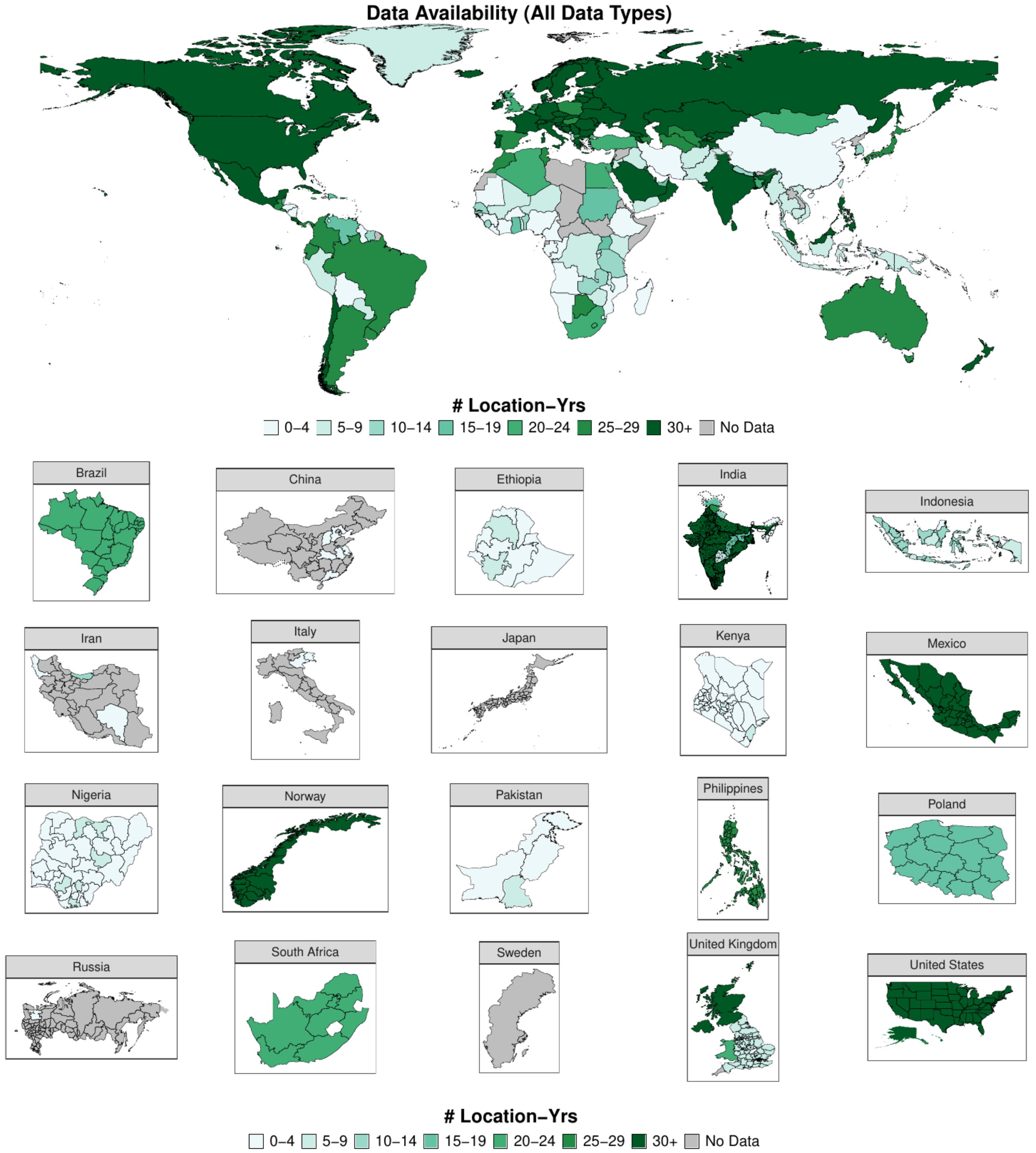

4.4.6 Appendix 1 Figure S3E: Dot plot of annual data coverage by GBD region

```
Caption: (E) This dot plot shows the number of location-years of data covered
for each GBD region, indicated by size and shade of the data point. Dots are
larger in size and darker in colour for regions with more data. The legend
provides examples of what the dot would look like if a region had 20, 40, or
60 data points available. GBD=Global Burden of Diseases, Injuries, and Risk
Factors Study.
```

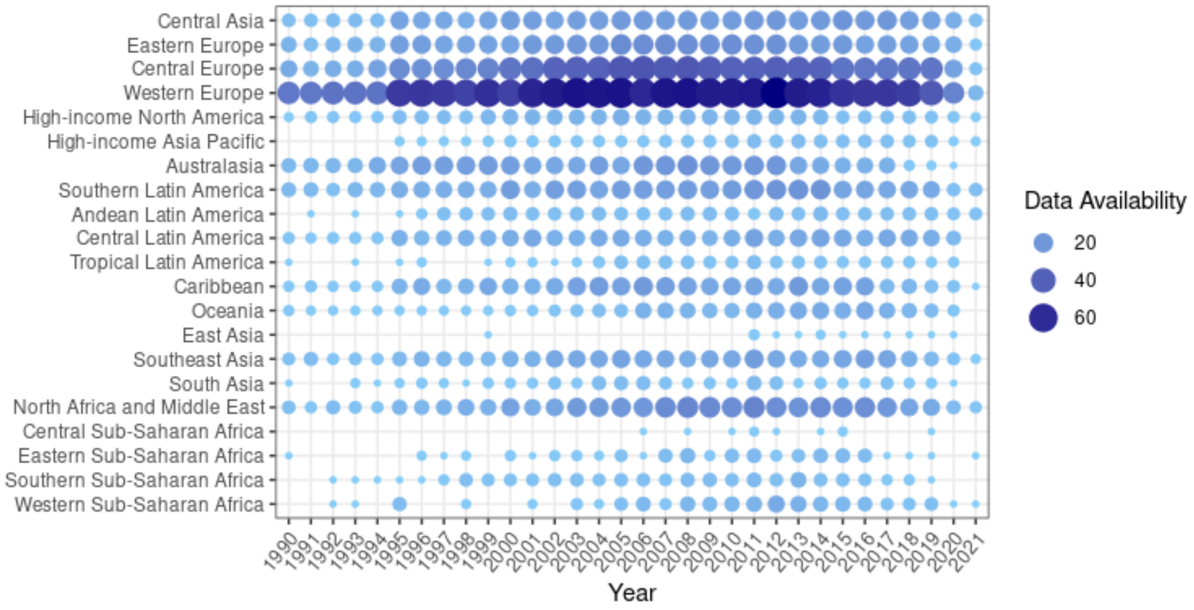

5 Data processing

Prior to modelling, we undertook a number of data processing steps to standardise and address potential biases in the input data. Steps included reassignment of stillbirth thresholds, data adjustment using birth and death completeness, standardisation of input data to the ratio of stillbirths-to-neonatal deaths (SBR/NMR) which is a dependent variable used in modelling, systematic outliering based on empirical plausibility bounds, and crosswalking of all non-reference data to the reference threshold. This multi-step process of data standardisation contrasts with the approach taken in GBD 2016. Data adjustment was limited to first applying global LSEIG-reported adjustment factors to the stillbirth data with non-standard thresholds to align the points with the reference threshold.[4] One set of adjustment factors was calculated for developed countries using meta-analyses and another for other countries. Then, a second stage of adjustment was performed during the first stage mixed effects regression of spatiotemporal Gaussian Process Regression (ST-GPR). Each step of the current data processing pipeline is described below in detail.

5.1 Reassignment of thresholds in data (including unknowns)

Differences in gestational age and birthweight-based thresholds are expected to lead to large differences in the reported rates of stillbirths. In order to facilitate standardisation of

input data, it was crucial to ensure that every datum had a precise threshold assigned. Acceptable thresholds were ≥20 weeks, ≥22 weeks, ≥24 weeks, ≥26 weeks, ≥28 weeks, ≥500 grams, or ≥1000 grams. We also accepted certain combinations of thresholds. These were ≥22 weeks OR ≥500 grams, ≥22 weeks AND ≥500 grams, ≥28 weeks OR ≥1000 grams, and ≥28 weeks AND ≥1000 grams.

We identified 4605 location-years of data where the source did not explicitly provide a threshold in its documentation. In these cases, each source was manually reviewed, the stillbirth values it reported were compared to other data points or estimates from the same or neighbouring locations, and that comparison was used to assign the most likely threshold. The majority of data points, approximately 80%, were reassigned to the ≥28 weeks threshold and 20% were reassigned to the ≥22 weeks threshold, rather than the ≥20 weeks threshold since ≥22 weeks was more commonly seen amongst other datasets. This review process ensured all data points were assigned to one of the thresholds listed above, unless we were confident the data point did not fit into any of the threshold categories. In these circumstances, the data point was assigned to the threshold called "other".

5.2 Data adjustment for completeness

Underreporting is a common problem in many data sources. GBD demographics analyses comprehensively account for this as part of the internally-consistent estimation process for population, fertility, and mortality. First, we adjusted household surveys reporting only period incidence of stillbirths by applying the ratio of the number of women with a birth in the past five years over the total number of births observed among this group to the reported SBR. Then, we adjusted all SBR data based on source type. We calculated completeness adjustments for vital registration data, surveys, and a combined value using all source types to adjust scientific literature data. These completeness estimates were calculated by taking the ratio of reported versus estimated probability of death (qx) from the final GBD 2021 with shock life table results.[13] We prioritised early neonatal mortality (enn), then infant mortality (inf), and lastly under-5 mortality (u5) data for a given location-year. If a location had greater than 6 data points, we fit a loess model to the data with constant tails based on the predicted value for the last year of observed data. For locations with 2 to 6 data points, we fit a linear model with constant tails based on the predicted value for the last year of observed data.

5.3 Standardising all data to be SBR/NMR

Neonatal mortality rate (NMR) is a probability of death in the neonatal period, defined as the number of deaths among infants less than 28 days divided by the number of live births. GBD has a specific two-step process for estimating probability of death for detailed age groups under 5 years. We first generate under-5 mortality rate for both sexes combined for all locations and then an age sex split model is set up to generate age and sex specific probabilities of death based on 5q0 and additional empirical data. Further information on how neonatal mortality rate was calculated can be found in the GBD 2019 Demographics Capstone appendix.[14]

The dependent variable in GBD 2021 models, like in GBD 2016, was the ratio of SBR/NMR. This model formulation was chosen instead of SBR directly or SBR + NMR combined (perinatal mortality) in order to facilitate SBR estimates are internally-consistent with other demographics assessments and because NMR data and results are not available by gestation age so they are assumed to be inclusive of all deaths following live birth in each location-year. A next step was therefore to transform all data into units of SBR/NMR. For our analysis, we used NMR estimates from the GBD life table estimates without fatal discontinuities. Stated differently, these NMR estimates excluded mortality shocks like natural disasters, humanitarian crises, and terrorism. This exclusion ensures that the effects of fatal discontinuities will not be spilled over to different years and countries in the modelling process and distort the relationship between the dependent variable and covariates.

5.4 Outlier criteria (round 1)

Even after application of rigorous inclusion criteria and completeness adjustments, there were some observations with implausible stillbirth rates in the dataset. Most of the time, implausibility was likely the result of severe under-reporting, but there was also the possibility of implausibly high reported stillbirth rates that could be the result of a) inadvertent sampling of a high risk subgroup (or inadequate documentation that the sample is from a high risk subgroup) or b) data derived from survey instruments that did not adequately differentiate between stillbirths and non-stillbirth adverse pregnancy outcomes (eg, elective and therapeutic abortions, miscarriages). We aimed to identify and exclude implausible data from the subset prior to crosswalking between thresholds to minimise the risk that these data would adversely influence subsequent statistical models.

Automatic outliering criteria were as follows: 1) SBR <1 per 1000 births, 2) SBR >200 per 1000 births, 3) SBR >50 per 1000 births for the High-income GBD super-region, 4) any observation from a unique, or rarely used, threshold (eg, >32 weeks, >2000 grams), 5) any data from a location-year with a major mortality shock, identified as anywhere the with shock death rate among all ages of the population was >500 per 100,000 persons, like the 1988 earthquake in Armenia with a ratio of 0·0073 versus the 1972 earthquake in Nicaragua which had a ratio of 0·0048, based on the premise that stillbirth data would be differentially affected by health service disruptions, 6) SBR/NMR <0.5, a criterion invoked as a lower limit observed in studies with comprehensive stillbirth assessments to reflect underreporting of stillbirths that was set at a more conservative 0·33 in the LSEIG analysis after identifying a median SBR/NMR of 0·9 in developed regions,[4] and 7) deduplication of repetitive data points (ex: from the same data collection system but reported in different publications).

Table S5 illustrates the distribution of source-location-years and unique observations (i.e. total # of data points) by location at several different SBR/NMR thresholds and the cumulative impact of outliering. Of the 24,502 data points (comprised of 18,656 unique location-years), 7,185 (29%) were outliered. Among these outliers, 66% were outliered based on criterion 6. Additionally, 8% of outliers did not meet any of the criteria but were instead manually outliered. For example, manually outliered data included statistical reports from Chile with reported values much lower that the values reported by vital

registration and Sierra Leone data that led to implausibly high estimates for the country. Four sources from two countries (India and Philippines) accounted for more than 70% of data outliered for having SBR/NMR <0.5. A majority of these data would still have been outliered at an SBR/NMR threshold of <0.33. In both of these countries, as was common across our dataset, there were other UN-outliered sources from the same locations in the same or adjacent years that guided estimates for those locations. While this cannot entirely make up for the data lost to outliering, it did help ensure that the impact of outliering implausibly low data was largely relegated to preventing downward bias in estimates. Exceptions to this pattern were largely in Africa, where in many cases there were not data source sthat overlapped, or were temporally adjacent, for the same locations. This highlights the importance and impact that targeted studies and improvements in routine data collection in comprehensively capturing stillbirth and other fetal losses can have on global and local assessments.

5.4.1 Appendix 1 Table S5: Distribution of data points outliered by various SBR/NMR thresholds

Caption: This table shows the distribution of data, outliered data, and outliered data for various SBR/NMR ratios for each location in the GBD location hierarchy. Each cell contains the number of unique source-location-years then the number of observations in parentheses.

Location	Unique source-location-years (total observations)	Unique source-location-years outliered (outliered observations) for reasons other than SBR/NMR	Unique source-location-years (# observations) where:							
			SBR/NMR < 0.5	Overlapping non-outlier (to SBR/NMR < 0.5)	SBR/NMR < 0.44	Overlapping non-outlier (to SBR/NMR < 0.44)	SBR/NMR < 0.33	Overlapping non-outlier (to SBR/NMR < 0.33)	SBR/NMR < 0.25	Overlapping non-outlier (to SBR/NMR < 0.25)
**Global**	**18656 (24502)**	**2358 (2468)**	**4497 (4717)**	**913 (1107)**	**3900 (4086)**	**705 (865)**	**2778 (2863)**	**445 (516)**	**1916 (1959)**	**311 (345)**
**Central Europe, eastern Europe, and central Asia**	**2284 (2395)**	**142 (157)**	**176 (179)**	**105 (108)**	**113 (114)**	**70 (71)**	**30 (30)**	**7 (7)**	**4 (4)**	**0 (0)**
**Central Asia**	**504 (504)**	**33 (33)**	**82 (82)**	**74 (74)**	**56 (56)**	**51 (51)**	**16 (16)**	**5 (5)**	**1 (1)**	**0 (0)**
Armenia	91 (91)	15 (15)	0 (0)	0 (0)	0 (0)	0 (0)	0 (0)	0 (0)	0 (0)	0 (0)
Azerbaijan	55 (55)	0 (0)	16 (16)	16 (16)	12 (12)	12 (12)	5 (5)	5 (5)	0 (0)	0 (0)
Georgia	88 (88)	15 (15)	0 (0)	0 (0)	0 (0)	0 (0)	0 (0)	0 (0)	0 (0)	0 (0)
Kazakhstan	57 (57)	1 (1)	0 (0)	0 (0)	0 (0)	0 (0)	0 (0)	0 (0)	0 (0)	0 (0)
Kyrgyzstan	64 (64)	0 (0)	26 (26)	22 (22)	25 (25)	21 (21)	4 (4)	0 (0)	1 (1)	0 (0)
Mongolia	27 (27)	0 (0)	1 (1)	1 (1)	0 (0)	0 (0)	0 (0)	0 (0)	0 (0)	0 (0)
Tajikistan	50 (50)	2 (2)	0 (0)	0 (0)	0 (0)	0 (0)	0 (0)	0 (0)	0 (0)	0 (0)
Turkmenistan	30 (30)	0 (0)	22 (22)	22 (22)	18 (18)	18 (18)	7 (7)	0 (0)	0 (0)	0 (0)
Uzbekistan	42 (42)	0 (0)	17 (17)	13 (13)	1 (1)	0 (0)	0 (0)	0 (0)	0 (0)	0 (0)
**Central Europe**	**1187 (1271)**	**72 (87)**	**88 (91)**	**31 (34)**	**57 (58)**	**19 (20)**	**14 (14)**	**2 (2)**	**3 (3)**	**0 (0)**
Albania	6 (6)	0 (0)	1 (1)	0 (0)	0 (0)	0 (0)	0 (0)	0 (0)	0 (0)	0 (0)
Bosnia and Herzegovina	70 (85)	15 (29)	16 (16)	13 (13)	15 (15)	12 (12)	7 (7)	0 (0)	3 (3)	0 (0)
Bulgaria	79 (79)	0 (0)	15 (15)	0 (0)	14 (14)	0 (0)	3 (3)	0 (0)	0 (0)	0 (0)
Croatia	122 (122)	0 (0)	17 (17)	0 (0)	11 (11)	0 (0)	0 (0)	0 (0)	0 (0)	0 (0)
Czechia	142 (178)	1 (1)	4 (4)	4 (4)	0 (0)	0 (0)	0 (0)	0 (0)	0 (0)	0 (0)
Hungary	68 (68)	0 (0)	5 (5)	2 (2)	3 (3)	1 (1)	0 (0)	0 (0)	0 (0)	0 (0)
Montenegro	20 (33)	1 (2)	6 (9)	5 (8)	2 (3)	2 (3)	1 (1)	1 (1)	0 (0)	0 (0)
North Macedonia	49 (49)	15 (15)	0 (0)	0 (0)	0 (0)	0 (0)	0 (0)	0 (0)	0 (0)	0 (0)
Poland	315 (315)	0 (0)	0 (0)	0 (0)	0 (0)	0 (0)	0 (0)	0 (0)	0 (0)	0 (0)

Dolnośląskie	15 (15)	0 (0)	0 (0)	0 (0)	0 (0)	0 (0)	0 (0)	0 (0)	0 (0)	0 (0)
Kujawsko-Pomorskie	15 (15)	0 (0)	0 (0)	0 (0)	0 (0)	0 (0)	0 (0)	0 (0)	0 (0)	0 (0)
Łódzkie	15 (15)	0 (0)	0 (0)	0 (0)	0 (0)	0 (0)	0 (0)	0 (0)	0 (0)	0 (0)
Lubelskie	15 (15)	0 (0)	0 (0)	0 (0)	0 (0)	0 (0)	0 (0)	0 (0)	0 (0)	0 (0)
Lubuskie	15 (15)	0 (0)	0 (0)	0 (0)	0 (0)	0 (0)	0 (0)	0 (0)	0 (0)	0 (0)
Małopolskie	15 (15)	0 (0)	0 (0)	0 (0)	0 (0)	0 (0)	0 (0)	0 (0)	0 (0)	0 (0)
Mazowieckie	15 (15)	0 (0)	0 (0)	0 (0)	0 (0)	0 (0)	0 (0)	0 (0)	0 (0)	0 (0)
Opolskie	15 (15)	0 (0)	0 (0)	0 (0)	0 (0)	0 (0)	0 (0)	0 (0)	0 (0)	0 (0)
Podkarpackie	15 (15)	0 (0)	0 (0)	0 (0)	0 (0)	0 (0)	0 (0)	0 (0)	0 (0)	0 (0)
Podlaskie	15 (15)	0 (0)	0 (0)	0 (0)	0 (0)	0 (0)	0 (0)	0 (0)	0 (0)	0 (0)
Pomorskie	15 (15)	0 (0)	0 (0)	0 (0)	0 (0)	0 (0)	0 (0)	0 (0)	0 (0)	0 (0)
Śląskie	15 (15)	0 (0)	0 (0)	0 (0)	0 (0)	0 (0)	0 (0)	0 (0)	0 (0)	0 (0)
Świętokrzyskie	15 (15)	0 (0)	0 (0)	0 (0)	0 (0)	0 (0)	0 (0)	0 (0)	0 (0)	0 (0)
Warmińsko-Mazurskie	15 (15)	0 (0)	0 (0)	0 (0)	0 (0)	0 (0)	0 (0)	0 (0)	0 (0)	0 (0)
Wielkopolskie	15 (15)	0 (0)	0 (0)	0 (0)	0 (0)	0 (0)	0 (0)	0 (0)	0 (0)	0 (0)
Zachodniopomorskie	15 (15)	0 (0)	0 (0)	0 (0)	0 (0)	0 (0)	0 (0)	0 (0)	0 (0)	0 (0)
Romania	103 (103)	26 (26)	1 (1)	1 (1)	0 (0)	0 (0)	0 (0)	0 (0)	0 (0)	0 (0)
Serbia	71 (71)	14 (14)	19 (19)	6 (6)	12 (12)	4 (4)	3 (3)	1 (1)	0 (0)	0 (0)
Slovakia	61 (61)	0 (0)	4 (4)	0 (0)	0 (0)	0 (0)	0 (0)	0 (0)	0 (0)	0 (0)
Slovenia	81 (101)	0 (0)	0 (0)	0 (0)	0 (0)	0 (0)	0 (0)	0 (0)	0 (0)	0 (0)
**Eastern Europe**	**593 (620)**	**37 (37)**	**6 (6)**	**0 (0)**	**0 (0)**	**0 (0)**	**0 (0)**	**0 (0)**	**0 (0)**	**0 (0)**
Belarus	64 (64)	0 (0)	0 (0)	0 (0)	0 (0)	0 (0)	0 (0)	0 (0)	0 (0)	0 (0)
Estonia	138 (143)	0 (0)	0 (0)	0 (0)	0 (0)	0 (0)	0 (0)	0 (0)	0 (0)	0 (0)
Latvia	101 (107)	24 (24)	2 (2)	0 (0)	0 (0)	0 (0)	0 (0)	0 (0)	0 (0)	0 (0)
Lithuania	112 (127)	0 (0)	0 (0)	0 (0)	0 (0)	0 (0)	0 (0)	0 (0)	0 (0)	0 (0)
Moldova	44 (44)	1 (1)	4 (4)	0 (0)	0 (0)	0 (0)	0 (0)	0 (0)	0 (0)	0 (0)
Russia	75 (76)	12 (12)	0 (0)	0 (0)	0 (0)	0 (0)	0 (0)	0 (0)	0 (0)	0 (0)
Altay krai	0 (0)	0 (0)	0 (0)	0 (0)	0 (0)	0 (0)	0 (0)	0 (0)	0 (0)	0 (0)
Amur oblast	0 (0)	0 (0)	0 (0)	0 (0)	0 (0)	0 (0)	0 (0)	0 (0)	0 (0)	0 (0)
Arkhangelsk oblast without Nenets autonomous district	1 (2)	0 (0)	0 (0)	0 (0)	0 (0)	0 (0)	0 (0)	0 (0)	0 (0)	0 (0)
Astrakhan oblast	0 (0)	0 (0)	0 (0)	0 (0)	0 (0)	0 (0)	0 (0)	0 (0)	0 (0)	0 (0)
Belgorod oblast	0 (0)	0 (0)	0 (0)	0 (0)	0 (0)	0 (0)	0 (0)	0 (0)	0 (0)	0 (0)
Bryansk oblast	0 (0)	0 (0)	0 (0)	0 (0)	0 (0)	0 (0)	0 (0)	0 (0)	0 (0)	0 (0)
Chechen Republic	0 (0)	0 (0)	0 (0)	0 (0)	0 (0)	0 (0)	0 (0)	0 (0)	0 (0)	0 (0)
Chelyabinsk oblast	0 (0)	0 (0)	0 (0)	0 (0)	0 (0)	0 (0)	0 (0)	0 (0)	0 (0)	0 (0)
Chukotka Autonomous Okrug	0 (0)	0 (0)	0 (0)	0 (0)	0 (0)	0 (0)	0 (0)	0 (0)	0 (0)	0 (0)

Chuvash Republic	0 (0)	0 (0)	0 (0)	0 (0)	0 (0)	0 (0)	0 (0)	0 (0)	0 (0)	0 (0)
Irkutsk oblast	0 (0)	0 (0)	0 (0)	0 (0)	0 (0)	0 (0)	0 (0)	0 (0)	0 (0)	0 (0)
Ivanovo oblast	0 (0)	0 (0)	0 (0)	0 (0)	0 (0)	0 (0)	0 (0)	0 (0)	0 (0)	0 (0)
Jewish autonomous oblast	0 (0)	0 (0)	0 (0)	0 (0)	0 (0)	0 (0)	0 (0)	0 (0)	0 (0)	0 (0)
Kabardino-Balkarian Republic	0 (0)	0 (0)	0 (0)	0 (0)	0 (0)	0 (0)	0 (0)	0 (0)	0 (0)	0 (0)
Kaliningrad oblast	0 (0)	0 (0)	0 (0)	0 (0)	0 (0)	0 (0)	0 (0)	0 (0)	0 (0)	0 (0)
Kaluga oblast	0 (0)	0 (0)	0 (0)	0 (0)	0 (0)	0 (0)	0 (0)	0 (0)	0 (0)	0 (0)
Kamchatka krai	0 (0)	0 (0)	0 (0)	0 (0)	0 (0)	0 (0)	0 (0)	0 (0)	0 (0)	0 (0)
Karachayevo-Chircassian Republic	0 (0)	0 (0)	0 (0)	0 (0)	0 (0)	0 (0)	0 (0)	0 (0)	0 (0)	0 (0)
Kemerovo oblast	0 (0)	0 (0)	0 (0)	0 (0)	0 (0)	0 (0)	0 (0)	0 (0)	0 (0)	0 (0)
Khabarovsk krai	0 (0)	0 (0)	0 (0)	0 (0)	0 (0)	0 (0)	0 (0)	0 (0)	0 (0)	0 (0)
Khanty-Mansi autonomous area	0 (0)	0 (0)	0 (0)	0 (0)	0 (0)	0 (0)	0 (0)	0 (0)	0 (0)	0 (0)
Kirov oblast	0 (0)	0 (0)	0 (0)	0 (0)	0 (0)	0 (0)	0 (0)	0 (0)	0 (0)	0 (0)
Republic of Komi	0 (0)	0 (0)	0 (0)	0 (0)	0 (0)	0 (0)	0 (0)	0 (0)	0 (0)	0 (0)
Kostroma oblast	0 (0)	0 (0)	0 (0)	0 (0)	0 (0)	0 (0)	0 (0)	0 (0)	0 (0)	0 (0)
Krasnodar krai	0 (0)	0 (0)	0 (0)	0 (0)	0 (0)	0 (0)	0 (0)	0 (0)	0 (0)	0 (0)
Krasnoyarsk krai	0 (0)	0 (0)	0 (0)	0 (0)	0 (0)	0 (0)	0 (0)	0 (0)	0 (0)	0 (0)
Kurgan oblast	0 (0)	0 (0)	0 (0)	0 (0)	0 (0)	0 (0)	0 (0)	0 (0)	0 (0)	0 (0)
Kursk oblast	0 (0)	0 (0)	0 (0)	0 (0)	0 (0)	0 (0)	0 (0)	0 (0)	0 (0)	0 (0)
Leningrad oblast	0 (0)	0 (0)	0 (0)	0 (0)	0 (0)	0 (0)	0 (0)	0 (0)	0 (0)	0 (0)
Lipetzk oblast	0 (0)	0 (0)	0 (0)	0 (0)	0 (0)	0 (0)	0 (0)	0 (0)	0 (0)	0 (0)
Magadan oblast	0 (0)	0 (0)	0 (0)	0 (0)	0 (0)	0 (0)	0 (0)	0 (0)	0 (0)	0 (0)
Moscow, city of	0 (0)	0 (0)	0 (0)	0 (0)	0 (0)	0 (0)	0 (0)	0 (0)	0 (0)	0 (0)
Moscow oblast	0 (0)	0 (0)	0 (0)	0 (0)	0 (0)	0 (0)	0 (0)	0 (0)	0 (0)	0 (0)
Murmansk oblast	0 (0)	0 (0)	0 (0)	0 (0)	0 (0)	0 (0)	0 (0)	0 (0)	0 (0)	0 (0)
Nenets autonomous district	0 (0)	0 (0)	0 (0)	0 (0)	0 (0)	0 (0)	0 (0)	0 (0)	0 (0)	0 (0)
Nizhny Novgorod oblast	0 (0)	0 (0)	0 (0)	0 (0)	0 (0)	0 (0)	0 (0)	0 (0)	0 (0)	0 (0)
Novgorod oblast	0 (0)	0 (0)	0 (0)	0 (0)	0 (0)	0 (0)	0 (0)	0 (0)	0 (0)	0 (0)
Novosibirsk oblast	0 (0)	0 (0)	0 (0)	0 (0)	0 (0)	0 (0)	0 (0)	0 (0)	0 (0)	0 (0)
Omsk oblast	0 (0)	0 (0)	0 (0)	0 (0)	0 (0)	0 (0)	0 (0)	0 (0)	0 (0)	0 (0)
Orenburg oblast	0 (0)	0 (0)	0 (0)	0 (0)	0 (0)	0 (0)	0 (0)	0 (0)	0 (0)	0 (0)
Oryol oblast	0 (0)	0 (0)	0 (0)	0 (0)	0 (0)	0 (0)	0 (0)	0 (0)	0 (0)	0 (0)
Penza oblast	0 (0)	0 (0)	0 (0)	0 (0)	0 (0)	0 (0)	0 (0)	0 (0)	0 (0)	0 (0)
Perm krai	0 (0)	0 (0)	0 (0)	0 (0)	0 (0)	0 (0)	0 (0)	0 (0)	0 (0)	0 (0)
Primorye krai	0 (0)	0 (0)	0 (0)	0 (0)	0 (0)	0 (0)	0 (0)	0 (0)	0 (0)	0 (0)
Pskov oblast	0 (0)	0 (0)	0 (0)	0 (0)	0 (0)	0 (0)	0 (0)	0 (0)	0 (0)	0 (0)

Republic of Adygeya	0 (0)	0 (0)	0 (0)	0 (0)	0 (0)	0 (0)	0 (0)	0 (0)	0 (0)	0 (0)
Republic of Altay	0 (0)	0 (0)	0 (0)	0 (0)	0 (0)	0 (0)	0 (0)	0 (0)	0 (0)	0 (0)
Republic of Bashkortostan	0 (0)	0 (0)	0 (0)	0 (0)	0 (0)	0 (0)	0 (0)	0 (0)	0 (0)	0 (0)
Republic of Buryatia	0 (0)	0 (0)	0 (0)	0 (0)	0 (0)	0 (0)	0 (0)	0 (0)	0 (0)	0 (0)
Republic of Dagestan	0 (0)	0 (0)	0 (0)	0 (0)	0 (0)	0 (0)	0 (0)	0 (0)	0 (0)	0 (0)
Republic of Ingushetia	0 (0)	0 (0)	0 (0)	0 (0)	0 (0)	0 (0)	0 (0)	0 (0)	0 (0)	0 (0)
Republic of Kalmykia	0 (0)	0 (0)	0 (0)	0 (0)	0 (0)	0 (0)	0 (0)	0 (0)	0 (0)	0 (0)
Republic of Karelia	0 (0)	0 (0)	0 (0)	0 (0)	0 (0)	0 (0)	0 (0)	0 (0)	0 (0)	0 (0)
Republic of Khakasia	0 (0)	0 (0)	0 (0)	0 (0)	0 (0)	0 (0)	0 (0)	0 (0)	0 (0)	0 (0)
Republic of Mari El	0 (0)	0 (0)	0 (0)	0 (0)	0 (0)	0 (0)	0 (0)	0 (0)	0 (0)	0 (0)
Republic of Mordovia	0 (0)	0 (0)	0 (0)	0 (0)	0 (0)	0 (0)	0 (0)	0 (0)	0 (0)	0 (0)
Republic of North Ossetia - Alania	0 (0)	0 (0)	0 (0)	0 (0)	0 (0)	0 (0)	0 (0)	0 (0)	0 (0)	0 (0)
Republic of Sakha (Yakutia)	0 (0)	0 (0)	0 (0)	0 (0)	0 (0)	0 (0)	0 (0)	0 (0)	0 (0)	0 (0)
Republic of Tatarstan	0 (0)	0 (0)	0 (0)	0 (0)	0 (0)	0 (0)	0 (0)	0 (0)	0 (0)	0 (0)
Republic of Tuva	0 (0)	0 (0)	0 (0)	0 (0)	0 (0)	0 (0)	0 (0)	0 (0)	0 (0)	0 (0)
Rostov oblast	0 (0)	0 (0)	0 (0)	0 (0)	0 (0)	0 (0)	0 (0)	0 (0)	0 (0)	0 (0)
Ryazan oblast	0 (0)	0 (0)	0 (0)	0 (0)	0 (0)	0 (0)	0 (0)	0 (0)	0 (0)	0 (0)
St. Petersburg, city of	0 (0)	0 (0)	0 (0)	0 (0)	0 (0)	0 (0)	0 (0)	0 (0)	0 (0)	0 (0)
Sakhalin oblast	0 (0)	0 (0)	0 (0)	0 (0)	0 (0)	0 (0)	0 (0)	0 (0)	0 (0)	0 (0)
Samara oblast	0 (0)	0 (0)	0 (0)	0 (0)	0 (0)	0 (0)	0 (0)	0 (0)	0 (0)	0 (0)
Saratov oblast	0 (0)	0 (0)	0 (0)	0 (0)	0 (0)	0 (0)	0 (0)	0 (0)	0 (0)	0 (0)
Smolensk oblast	0 (0)	0 (0)	0 (0)	0 (0)	0 (0)	0 (0)	0 (0)	0 (0)	0 (0)	0 (0)
Stavropol krai	0 (0)	0 (0)	0 (0)	0 (0)	0 (0)	0 (0)	0 (0)	0 (0)	0 (0)	0 (0)
Sverdlovsk oblast	0 (0)	0 (0)	0 (0)	0 (0)	0 (0)	0 (0)	0 (0)	0 (0)	0 (0)	0 (0)
Tambov oblast	0 (0)	0 (0)	0 (0)	0 (0)	0 (0)	0 (0)	0 (0)	0 (0)	0 (0)	0 (0)
Tomsk oblast	0 (0)	0 (0)	0 (0)	0 (0)	0 (0)	0 (0)	0 (0)	0 (0)	0 (0)	0 (0)
Tula oblast	0 (0)	0 (0)	0 (0)	0 (0)	0 (0)	0 (0)	0 (0)	0 (0)	0 (0)	0 (0)
Tver oblast	0 (0)	0 (0)	0 (0)	0 (0)	0 (0)	0 (0)	0 (0)	0 (0)	0 (0)	0 (0)
Tyumen oblast without autonomous areas	0 (0)	0 (0)	0 (0)	0 (0)	0 (0)	0 (0)	0 (0)	0 (0)	0 (0)	0 (0)
Udmurtian Republic	0 (0)	0 (0)	0 (0)	0 (0)	0 (0)	0 (0)	0 (0)	0 (0)	0 (0)	0 (0)
Ulyanovsk oblast	0 (0)	0 (0)	0 (0)	0 (0)	0 (0)	0 (0)	0 (0)	0 (0)	0 (0)	0 (0)
Vladimir oblast	0 (0)	0 (0)	0 (0)	0 (0)	0 (0)	0 (0)	0 (0)	0 (0)	0 (0)	0 (0)
Volgograd oblast	0 (0)	0 (0)	0 (0)	0 (0)	0 (0)	0 (0)	0 (0)	0 (0)	0 (0)	0 (0)
Vologda oblast	0 (0)	0 (0)	0 (0)	0 (0)	0 (0)	0 (0)	0 (0)	0 (0)	0 (0)	0 (0)
Voronezh oblast	0 (0)	0 (0)	0 (0)	0 (0)	0 (0)	0 (0)	0 (0)	0 (0)	0 (0)	0 (0)
Yamal-Nenets Autonomous Okrug	0 (0)	0 (0)	0 (0)	0 (0)	0 (0)	0 (0)	0 (0)	0 (0)	0 (0)	0 (0)

Yaroslavl oblast	0 (0)	0 (0)	0 (0)	0 (0)	0 (0)	0 (0)	0 (0)	0 (0)	0 (0)	0 (0)
Trans-Baikal krai	0 (0)	0 (0)	0 (0)	0 (0)	0 (0)	0 (0)	0 (0)	0 (0)	0 (0)	0 (0)
Ukraine	59 (59)	0 (0)	0 (0)	0 (0)	0 (0)	0 (0)	0 (0)	0 (0)	0 (0)	0 (0)
Republic of Crimea	1 (1)	0 (0)	0 (0)	0 (0)	0 (0)	0 (0)	0 (0)	0 (0)	0 (0)	0 (0)
Sevastopol	1 (1)	0 (0)	0 (0)	0 (0)	0 (0)	0 (0)	0 (0)	0 (0)	0 (0)	0 (0)
Ukraine (without Republic of Crimea & Sevastopol)	8 (8)	0 (0)	0 (0)	0 (0)	0 (0)	0 (0)	0 (0)	0 (0)	0 (0)	0 (0)
**High income**	**6130 (9535)**	**349 (388)**	**174 (176)**	**164 (166)**	**75 (75)**	**69 (69)**	**15 (15)**	**14 (14)**	**1 (1)**	**1 (1)**
**Australasia**	**231 (953)**	**101 (102)**	**2 (2)**	**2 (2)**	**0 (0)**	**0 (0)**	**0 (0)**	**0 (0)**	**0 (0)**	**0 (0)**
Australia	57 (113)	11 (12)	2 (2)	2 (2)	0 (0)	0 (0)	0 (0)	0 (0)	0 (0)	0 (0)
New Zealand	174 (840)	90 (90)	0 (0)	0 (0)	0 (0)	0 (0)	0 (0)	0 (0)	0 (0)	0 (0)
New Zealand Maori population	46 (250)	29 (29)	0 (0)	0 (0)	0 (0)	0 (0)	0 (0)	0 (0)	0 (0)	0 (0)
New Zealand non-Maori population	47 (258)	30 (30)	0 (0)	0 (0)	0 (0)	0 (0)	0 (0)	0 (0)	0 (0)	0 (0)
**High-income Asia Pacific**	**117 (118)**	**19 (19)**	**0 (0)**	**0 (0)**	**0 (0)**	**0 (0)**	**0 (0)**	**0 (0)**	**0 (0)**	**0 (0)**
Brunei	22 (22)	0 (0)	0 (0)	0 (0)	0 (0)	0 (0)	0 (0)	0 (0)	0 (0)	0 (0)
Japan	26 (26)	1 (1)	0 (0)	0 (0)	0 (0)	0 (0)	0 (0)	0 (0)	0 (0)	0 (0)
Aichi	0 (0)	0 (0)	0 (0)	0 (0)	0 (0)	0 (0)	0 (0)	0 (0)	0 (0)	0 (0)
Akita	0 (0)	0 (0)	0 (0)	0 (0)	0 (0)	0 (0)	0 (0)	0 (0)	0 (0)	0 (0)
Aomori	0 (0)	0 (0)	0 (0)	0 (0)	0 (0)	0 (0)	0 (0)	0 (0)	0 (0)	0 (0)
Chiba	0 (0)	0 (0)	0 (0)	0 (0)	0 (0)	0 (0)	0 (0)	0 (0)	0 (0)	0 (0)
Ehime	0 (0)	0 (0)	0 (0)	0 (0)	0 (0)	0 (0)	0 (0)	0 (0)	0 (0)	0 (0)
Fukui	0 (0)	0 (0)	0 (0)	0 (0)	0 (0)	0 (0)	0 (0)	0 (0)	0 (0)	0 (0)
Fukuoka	0 (0)	0 (0)	0 (0)	0 (0)	0 (0)	0 (0)	0 (0)	0 (0)	0 (0)	0 (0)
Fukushima	0 (0)	0 (0)	0 (0)	0 (0)	0 (0)	0 (0)	0 (0)	0 (0)	0 (0)	0 (0)
Gifu	0 (0)	0 (0)	0 (0)	0 (0)	0 (0)	0 (0)	0 (0)	0 (0)	0 (0)	0 (0)
Gunma	0 (0)	0 (0)	0 (0)	0 (0)	0 (0)	0 (0)	0 (0)	0 (0)	0 (0)	0 (0)
Hiroshima	0 (0)	0 (0)	0 (0)	0 (0)	0 (0)	0 (0)	0 (0)	0 (0)	0 (0)	0 (0)
Hokkaidō	0 (0)	0 (0)	0 (0)	0 (0)	0 (0)	0 (0)	0 (0)	0 (0)	0 (0)	0 (0)
Hyōgo	0 (0)	0 (0)	0 (0)	0 (0)	0 (0)	0 (0)	0 (0)	0 (0)	0 (0)	0 (0)
Ibaraki	0 (0)	0 (0)	0 (0)	0 (0)	0 (0)	0 (0)	0 (0)	0 (0)	0 (0)	0 (0)
Ishikawa	0 (0)	0 (0)	0 (0)	0 (0)	0 (0)	0 (0)	0 (0)	0 (0)	0 (0)	0 (0)
Iwate	0 (0)	0 (0)	0 (0)	0 (0)	0 (0)	0 (0)	0 (0)	0 (0)	0 (0)	0 (0)
Kagawa	0 (0)	0 (0)	0 (0)	0 (0)	0 (0)	0 (0)	0 (0)	0 (0)	0 (0)	0 (0)
Kagoshima	0 (0)	0 (0)	0 (0)	0 (0)	0 (0)	0 (0)	0 (0)	0 (0)	0 (0)	0 (0)
Kanagawa	0 (0)	0 (0)	0 (0)	0 (0)	0 (0)	0 (0)	0 (0)	0 (0)	0 (0)	0 (0)
Kōchi	0 (0)	0 (0)	0 (0)	0 (0)	0 (0)	0 (0)	0 (0)	0 (0)	0 (0)	0 (0)
Kumamoto	0 (0)	0 (0)	0 (0)	0 (0)	0 (0)	0 (0)	0 (0)	0 (0)	0 (0)	0 (0)

Kyōto	0 (0)	0 (0)	0 (0)	0 (0)	0 (0)	0 (0)	0 (0)	0 (0)	0 (0)	0 (0)
Mie	0 (0)	0 (0)	0 (0)	0 (0)	0 (0)	0 (0)	0 (0)	0 (0)	0 (0)	0 (0)
Miyagi	0 (0)	0 (0)	0 (0)	0 (0)	0 (0)	0 (0)	0 (0)	0 (0)	0 (0)	0 (0)
Miyazaki	0 (0)	0 (0)	0 (0)	0 (0)	0 (0)	0 (0)	0 (0)	0 (0)	0 (0)	0 (0)
Nagano	0 (0)	0 (0)	0 (0)	0 (0)	0 (0)	0 (0)	0 (0)	0 (0)	0 (0)	0 (0)
Nagasaki	0 (0)	0 (0)	0 (0)	0 (0)	0 (0)	0 (0)	0 (0)	0 (0)	0 (0)	0 (0)
Nara	0 (0)	0 (0)	0 (0)	0 (0)	0 (0)	0 (0)	0 (0)	0 (0)	0 (0)	0 (0)
Niigata	0 (0)	0 (0)	0 (0)	0 (0)	0 (0)	0 (0)	0 (0)	0 (0)	0 (0)	0 (0)
Ōita	0 (0)	0 (0)	0 (0)	0 (0)	0 (0)	0 (0)	0 (0)	0 (0)	0 (0)	0 (0)
Okayama	0 (0)	0 (0)	0 (0)	0 (0)	0 (0)	0 (0)	0 (0)	0 (0)	0 (0)	0 (0)
Okinawa	0 (0)	0 (0)	0 (0)	0 (0)	0 (0)	0 (0)	0 (0)	0 (0)	0 (0)	0 (0)
Ōsaka	0 (0)	0 (0)	0 (0)	0 (0)	0 (0)	0 (0)	0 (0)	0 (0)	0 (0)	0 (0)
Saga	0 (0)	0 (0)	0 (0)	0 (0)	0 (0)	0 (0)	0 (0)	0 (0)	0 (0)	0 (0)
Saitama	0 (0)	0 (0)	0 (0)	0 (0)	0 (0)	0 (0)	0 (0)	0 (0)	0 (0)	0 (0)
Shiga	0 (0)	0 (0)	0 (0)	0 (0)	0 (0)	0 (0)	0 (0)	0 (0)	0 (0)	0 (0)
Shimane	0 (0)	0 (0)	0 (0)	0 (0)	0 (0)	0 (0)	0 (0)	0 (0)	0 (0)	0 (0)
Shizuoka	0 (0)	0 (0)	0 (0)	0 (0)	0 (0)	0 (0)	0 (0)	0 (0)	0 (0)	0 (0)
Tochigi	0 (0)	0 (0)	0 (0)	0 (0)	0 (0)	0 (0)	0 (0)	0 (0)	0 (0)	0 (0)
Tokushima	0 (0)	0 (0)	0 (0)	0 (0)	0 (0)	0 (0)	0 (0)	0 (0)	0 (0)	0 (0)
Tōkyō	0 (0)	0 (0)	0 (0)	0 (0)	0 (0)	0 (0)	0 (0)	0 (0)	0 (0)	0 (0)
Tottori	0 (0)	0 (0)	0 (0)	0 (0)	0 (0)	0 (0)	0 (0)	0 (0)	0 (0)	0 (0)
Toyama	0 (0)	0 (0)	0 (0)	0 (0)	0 (0)	0 (0)	0 (0)	0 (0)	0 (0)	0 (0)
Wakayama	0 (0)	0 (0)	0 (0)	0 (0)	0 (0)	0 (0)	0 (0)	0 (0)	0 (0)	0 (0)
Yamagata	0 (0)	0 (0)	0 (0)	0 (0)	0 (0)	0 (0)	0 (0)	0 (0)	0 (0)	0 (0)
Yamaguchi	0 (0)	0 (0)	0 (0)	0 (0)	0 (0)	0 (0)	0 (0)	0 (0)	0 (0)	0 (0)
Yamanashi	0 (0)	0 (0)	0 (0)	0 (0)	0 (0)	0 (0)	0 (0)	0 (0)	0 (0)	0 (0)
South Korea	13 (13)	0 (0)	0 (0)	0 (0)	0 (0)	0 (0)	0 (0)	0 (0)	0 (0)	0 (0)
Singapore	56 (57)	18 (18)	0 (0)	0 (0)	0 (0)	0 (0)	0 (0)	0 (0)	0 (0)	0 (0)
**High-income North America**	**2299 (4390)**	**85 (85)**	**149 (149)**	**145 (145)**	**66 (66)**	**63 (63)**	**14 (14)**	**13 (13)**	**1 (1)**	**1 (1)**
Canada	71 (134)	15 (15)	0 (0)	0 (0)	0 (0)	0 (0)	0 (0)	0 (0)	0 (0)	0 (0)
Greenland	8 (8)	0 (0)	4 (4)	0 (0)	3 (3)	0 (0)	1 (1)	0 (0)	0 (0)	0 (0)
USA	2220 (4248)	70 (70)	145 (145)	145 (145)	63 (63)	63 (63)	13 (13)	13 (13)	1 (1)	1 (1)
Alabama	42 (81)	1 (1)	0 (0)	0 (0)	0 (0)	0 (0)	0 (0)	0 (0)	0 (0)	0 (0)
Alaska	42 (81)	1 (1)	7 (7)	7 (7)	3 (3)	3 (3)	1 (1)	1 (1)	0 (0)	0 (0)
Arizona	42 (81)	1 (1)	0 (0)	0 (0)	0 (0)	0 (0)	0 (0)	0 (0)	0 (0)	0 (0)
Arkansas	42 (81)	1 (1)	0 (0)	0 (0)	0 (0)	0 (0)	0 (0)	0 (0)	0 (0)	0 (0)

California	42 (81)	1 (1)	0 (0)	0 (0)	0 (0)	0 (0)	0 (0)	0 (0)	0 (0)	0 (0)
Colorado	42 (81)	1 (1)	0 (0)	0 (0)	0 (0)	0 (0)	0 (0)	0 (0)	0 (0)	0 (0)
Connecticut	42 (81)	1 (1)	13 (13)	13 (13)	9 (9)	9 (9)	2 (2)	2 (2)	0 (0)	0 (0)
Delaware	42 (81)	1 (1)	10 (10)	10 (10)	5 (5)	5 (5)	0 (0)	0 (0)	0 (0)	0 (0)
Washington, DC	42 (81)	1 (1)	5 (5)	5 (5)	3 (3)	3 (3)	0 (0)	0 (0)	0 (0)	0 (0)
Florida	42 (81)	1 (1)	3 (3)	3 (3)	0 (0)	0 (0)	0 (0)	0 (0)	0 (0)	0 (0)
Georgia	42 (81)	1 (1)	0 (0)	0 (0)	0 (0)	0 (0)	0 (0)	0 (0)	0 (0)	0 (0)
Hawaii	42 (81)	1 (1)	4 (4)	4 (4)	0 (0)	0 (0)	0 (0)	0 (0)	0 (0)	0 (0)
Idaho	42 (81)	1 (1)	0 (0)	0 (0)	0 (0)	0 (0)	0 (0)	0 (0)	0 (0)	0 (0)
Illinois	42 (81)	1 (1)	2 (2)	2 (2)	0 (0)	0 (0)	0 (0)	0 (0)	0 (0)	0 (0)
Indiana	42 (81)	1 (1)	1 (1)	1 (1)	0 (0)	0 (0)	0 (0)	0 (0)	0 (0)	0 (0)
Iowa	42 (81)	1 (1)	0 (0)	0 (0)	0 (0)	0 (0)	0 (0)	0 (0)	0 (0)	0 (0)
Kansas	42 (81)	1 (1)	0 (0)	0 (0)	0 (0)	0 (0)	0 (0)	0 (0)	0 (0)	0 (0)
Kentucky	42 (81)	1 (1)	0 (0)	0 (0)	0 (0)	0 (0)	0 (0)	0 (0)	0 (0)	0 (0)
Louisiana	42 (81)	1 (1)	7 (7)	7 (7)	1 (1)	1 (1)	0 (0)	0 (0)	0 (0)	0 (0)
Maine	42 (81)	1 (1)	2 (2)	2 (2)	1 (1)	1 (1)	0 (0)	0 (0)	0 (0)	0 (0)
Maryland	42 (81)	1 (1)	16 (16)	16 (16)	13 (13)	13 (13)	7 (7)	7 (7)	1 (1)	1 (1)
Massachusetts	42 (81)	1 (1)	0 (0)	0 (0)	0 (0)	0 (0)	0 (0)	0 (0)	0 (0)	0 (0)
Michigan	42 (81)	1 (1)	16 (16)	16 (16)	2 (2)	2 (2)	0 (0)	0 (0)	0 (0)	0 (0)
Minnesota	42 (81)	1 (1)	5 (5)	5 (5)	3 (3)	3 (3)	0 (0)	0 (0)	0 (0)	0 (0)
Mississippi	42 (81)	1 (1)	0 (0)	0 (0)	0 (0)	0 (0)	0 (0)	0 (0)	0 (0)	0 (0)
Missouri	42 (81)	1 (1)	0 (0)	0 (0)	0 (0)	0 (0)	0 (0)	0 (0)	0 (0)	0 (0)
Montana	42 (81)	1 (1)	1 (1)	1 (1)	0 (0)	0 (0)	0 (0)	0 (0)	0 (0)	0 (0)
Nebraska	42 (81)	1 (1)	0 (0)	0 (0)	0 (0)	0 (0)	0 (0)	0 (0)	0 (0)	0 (0)
Nevada	42 (81)	1 (1)	0 (0)	0 (0)	0 (0)	0 (0)	0 (0)	0 (0)	0 (0)	0 (0)
New Hampshire	42 (81)	1 (1)	0 (0)	0 (0)	0 (0)	0 (0)	0 (0)	0 (0)	0 (0)	0 (0)
New Jersey	42 (81)	1 (1)	8 (8)	8 (8)	5 (5)	5 (5)	0 (0)	0 (0)	0 (0)	0 (0)
New Mexico	42 (81)	1 (1)	9 (9)	9 (9)	3 (3)	3 (3)	0 (0)	0 (0)	0 (0)	0 (0)
New York	42 (81)	1 (1)	0 (0)	0 (0)	0 (0)	0 (0)	0 (0)	0 (0)	0 (0)	0 (0)
North Carolina	42 (81)	1 (1)	0 (0)	0 (0)	0 (0)	0 (0)	0 (0)	0 (0)	0 (0)	0 (0)
North Dakota	42 (81)	1 (1)	0 (0)	0 (0)	0 (0)	0 (0)	0 (0)	0 (0)	0 (0)	0 (0)
Ohio	42 (81)	1 (1)	1 (1)	1 (1)	0 (0)	0 (0)	0 (0)	0 (0)	0 (0)	0 (0)
Oklahoma	42 (81)	2 (2)	21 (21)	21 (21)	10 (10)	10 (10)	2 (2)	2 (2)	0 (0)	0 (0)
Oregon	42 (81)	1 (1)	0 (0)	0 (0)	0 (0)	0 (0)	0 (0)	0 (0)	0 (0)	0 (0)
Pennsylvania	42 (81)	1 (1)	0 (0)	0 (0)	0 (0)	0 (0)	0 (0)	0 (0)	0 (0)	0 (0)
Rhode Island	42 (81)	1 (1)	4 (4)	4 (4)	2 (2)	2 (2)	1 (1)	1 (1)	0 (0)	0 (0)

South Carolina	42 (81)	1 (1)	0 (0)	0 (0)	0 (0)	0 (0)	0 (0)	0 (0)	0 (0)	0 (0)
South Dakota	42 (81)	1 (1)	1 (1)	1 (1)	0 (0)	0 (0)	0 (0)	0 (0)	0 (0)	0 (0)
Tennessee	42 (81)	1 (1)	0 (0)	0 (0)	0 (0)	0 (0)	0 (0)	0 (0)	0 (0)	0 (0)
Texas	43 (82)	2 (2)	0 (0)	0 (0)	0 (0)	0 (0)	0 (0)	0 (0)	0 (0)	0 (0)
Utah	42 (81)	1 (1)	0 (0)	0 (0)	0 (0)	0 (0)	0 (0)	0 (0)	0 (0)	0 (0)
Vermont	42 (81)	1 (1)	2 (2)	2 (2)	2 (2)	2 (2)	0 (0)	0 (0)	0 (0)	0 (0)
Virginia	42 (81)	1 (1)	2 (2)	2 (2)	0 (0)	0 (0)	0 (0)	0 (0)	0 (0)	0 (0)
Washington	42 (81)	1 (1)	4 (4)	4 (4)	1 (1)	1 (1)	0 (0)	0 (0)	0 (0)	0 (0)
West Virginia	42 (81)	1 (1)	0 (0)	0 (0)	0 (0)	0 (0)	0 (0)	0 (0)	0 (0)	0 (0)
Wisconsin	42 (81)	1 (1)	0 (0)	0 (0)	0 (0)	0 (0)	0 (0)	0 (0)	0 (0)	0 (0)
Wyoming	42 (81)	1 (1)	1 (1)	1 (1)	0 (0)	0 (0)	0 (0)	0 (0)	0 (0)	0 (0)
**Southern Latin America**	**174 (472)**	**27 (59)**	**4 (4)**	**3 (3)**	**0 (0)**	**0 (0)**	**0 (0)**	**0 (0)**	**0 (0)**	**0 (0)**
Argentina	50 (158)	6 (6)	1 (1)	1 (1)	0 (0)	0 (0)	0 (0)	0 (0)	0 (0)	0 (0)
Chile	85 (275)	21 (53)	2 (2)	2 (2)	0 (0)	0 (0)	0 (0)	0 (0)	0 (0)	0 (0)
Uruguay	39 (39)	0 (0)	1 (1)	0 (0)	0 (0)	0 (0)	0 (0)	0 (0)	0 (0)	0 (0)
**Western Europe**	**3309 (3602)**	**117 (123)**	**19 (21)**	**14 (16)**	**9 (9)**	**6 (6)**	**1 (1)**	**1 (1)**	**0 (0)**	**0 (0)**
Andorra	32 (32)	10 (10)	0 (0)	0 (0)	0 (0)	0 (0)	0 (0)	0 (0)	0 (0)	0 (0)
Austria	103 (103)	8 (8)	0 (0)	0 (0)	0 (0)	0 (0)	0 (0)	0 (0)	0 (0)	0 (0)
Belgium	41 (41)	0 (0)	0 (0)	0 (0)	0 (0)	0 (0)	0 (0)	0 (0)	0 (0)	0 (0)
Cyprus	17 (23)	0 (0)	0 (0)	0 (0)	0 (0)	0 (0)	0 (0)	0 (0)	0 (0)	0 (0)
Denmark	60 (60)	0 (0)	0 (0)	0 (0)	0 (0)	0 (0)	0 (0)	0 (0)	0 (0)	0 (0)
Finland	92 (92)	2 (2)	0 (0)	0 (0)	0 (0)	0 (0)	0 (0)	0 (0)	0 (0)	0 (0)
France	57 (57)	29 (29)	0 (0)	0 (0)	0 (0)	0 (0)	0 (0)	0 (0)	0 (0)	0 (0)
Germany	89 (89)	17 (17)	0 (0)	0 (0)	0 (0)	0 (0)	0 (0)	0 (0)	0 (0)	0 (0)
Greece	107 (107)	0 (0)	0 (0)	0 (0)	0 (0)	0 (0)	0 (0)	0 (0)	0 (0)	0 (0)
Iceland	105 (105)	4 (4)	3 (3)	2 (2)	2 (2)	0 (0)	1 (1)	1 (1)	0 (0)	0 (0)
Ireland	75 (75)	0 (0)	0 (0)	0 (0)	0 (0)	0 (0)	0 (0)	0 (0)	0 (0)	0 (0)
Israel	111 (118)	0 (0)	1 (1)	1 (1)	0 (0)	0 (0)	0 (0)	0 (0)	0 (0)	0 (0)
Italy	56 (56)	2 (2)	0 (0)	0 (0)	0 (0)	0 (0)	0 (0)	0 (0)	0 (0)	0 (0)
Abruzzo	0 (0)	0 (0)	0 (0)	0 (0)	0 (0)	0 (0)	0 (0)	0 (0)	0 (0)	0 (0)
Basilicata	0 (0)	0 (0)	0 (0)	0 (0)	0 (0)	0 (0)	0 (0)	0 (0)	0 (0)	0 (0)
Calabria	0 (0)	0 (0)	0 (0)	0 (0)	0 (0)	0 (0)	0 (0)	0 (0)	0 (0)	0 (0)
Campania	0 (0)	0 (0)	0 (0)	0 (0)	0 (0)	0 (0)	0 (0)	0 (0)	0 (0)	0 (0)
Emilia-Romagna	0 (0)	0 (0)	0 (0)	0 (0)	0 (0)	0 (0)	0 (0)	0 (0)	0 (0)	0 (0)
Friuli-Venezia Giulia	0 (0)	0 (0)	0 (0)	0 (0)	0 (0)	0 (0)	0 (0)	0 (0)	0 (0)	0 (0)
Lazio	0 (0)	0 (0)	0 (0)	0 (0)	0 (0)	0 (0)	0 (0)	0 (0)	0 (0)	0 (0)

Liguria	0 (0)	0 (0)	0 (0)	0 (0)	0 (0)	0 (0)	0 (0)	0 (0)	0 (0)	0 (0)
Lombardia	0 (0)	0 (0)	0 (0)	0 (0)	0 (0)	0 (0)	0 (0)	0 (0)	0 (0)	0 (0)
Marche	0 (0)	0 (0)	0 (0)	0 (0)	0 (0)	0 (0)	0 (0)	0 (0)	0 (0)	0 (0)
Molise	0 (0)	0 (0)	0 (0)	0 (0)	0 (0)	0 (0)	0 (0)	0 (0)	0 (0)	0 (0)
Piemonte	0 (0)	0 (0)	0 (0)	0 (0)	0 (0)	0 (0)	0 (0)	0 (0)	0 (0)	0 (0)
Provincia autonoma di Bolzano	0 (0)	0 (0)	0 (0)	0 (0)	0 (0)	0 (0)	0 (0)	0 (0)	0 (0)	0 (0)
Provincia autonoma di Trento	0 (0)	0 (0)	0 (0)	0 (0)	0 (0)	0 (0)	0 (0)	0 (0)	0 (0)	0 (0)
Puglia	0 (0)	0 (0)	0 (0)	0 (0)	0 (0)	0 (0)	0 (0)	0 (0)	0 (0)	0 (0)
Sardegna	0 (0)	0 (0)	0 (0)	0 (0)	0 (0)	0 (0)	0 (0)	0 (0)	0 (0)	0 (0)
Sicilia	0 (0)	0 (0)	0 (0)	0 (0)	0 (0)	0 (0)	0 (0)	0 (0)	0 (0)	0 (0)
Toscana	0 (0)	0 (0)	0 (0)	0 (0)	0 (0)	0 (0)	0 (0)	0 (0)	0 (0)	0 (0)
Umbria	0 (0)	0 (0)	0 (0)	0 (0)	0 (0)	0 (0)	0 (0)	0 (0)	0 (0)	0 (0)
Valle d'Aosta	0 (0)	0 (0)	0 (0)	0 (0)	0 (0)	0 (0)	0 (0)	0 (0)	0 (0)	0 (0)
Veneto	0 (0)	0 (0)	0 (0)	0 (0)	0 (0)	0 (0)	0 (0)	0 (0)	0 (0)	0 (0)
Luxembourg	95 (95)	0 (0)	0 (0)	0 (0)	0 (0)	0 (0)	0 (0)	0 (0)	0 (0)	0 (0)
Malta	91 (125)	0 (0)	13 (15)	11 (13)	6 (6)	6 (6)	0 (0)	0 (0)	0 (0)	0 (0)
Monaco	4 (4)	2 (2)	0 (0)	0 (0)	0 (0)	0 (0)	0 (0)	0 (0)	0 (0)	0 (0)
Netherlands	44 (44)	0 (0)	0 (0)	0 (0)	0 (0)	0 (0)	0 (0)	0 (0)	0 (0)	0 (0)
Norway	606 (746)	1 (1)	0 (0)	0 (0)	0 (0)	0 (0)	0 (0)	0 (0)	0 (0)	0 (0)
Agder	42 (42)	0 (0)	0 (0)	0 (0)	0 (0)	0 (0)	0 (0)	0 (0)	0 (0)	0 (0)
Innlandet	42 (42)	0 (0)	0 (0)	0 (0)	0 (0)	0 (0)	0 (0)	0 (0)	0 (0)	0 (0)
Møre og Romsdal	42 (42)	0 (0)	0 (0)	0 (0)	0 (0)	0 (0)	0 (0)	0 (0)	0 (0)	0 (0)
Nordland	42 (42)	0 (0)	0 (0)	0 (0)	0 (0)	0 (0)	0 (0)	0 (0)	0 (0)	0 (0)
Oslo	42 (42)	0 (0)	0 (0)	0 (0)	0 (0)	0 (0)	0 (0)	0 (0)	0 (0)	0 (0)
Rogaland	42 (42)	0 (0)	0 (0)	0 (0)	0 (0)	0 (0)	0 (0)	0 (0)	0 (0)	0 (0)
Troms og Finnmark	42 (42)	1 (1)	0 (0)	0 (0)	0 (0)	0 (0)	0 (0)	0 (0)	0 (0)	0 (0)
Trøndelag	42 (42)	0 (0)	0 (0)	0 (0)	0 (0)	0 (0)	0 (0)	0 (0)	0 (0)	0 (0)
Vestfold og Telemark	42 (42)	0 (0)	0 (0)	0 (0)	0 (0)	0 (0)	0 (0)	0 (0)	0 (0)	0 (0)
Vestland	42 (42)	0 (0)	0 (0)	0 (0)	0 (0)	0 (0)	0 (0)	0 (0)	0 (0)	0 (0)
Viken	42 (42)	0 (0)	0 (0)	0 (0)	0 (0)	0 (0)	0 (0)	0 (0)	0 (0)	0 (0)
Portugal	77 (105)	2 (4)	0 (0)	0 (0)	0 (0)	0 (0)	0 (0)	0 (0)	0 (0)	0 (0)
San Marino	18 (18)	7 (7)	0 (0)	0 (0)	0 (0)	0 (0)	0 (0)	0 (0)	0 (0)	0 (0)
Spain	59 (59)	1 (1)	0 (0)	0 (0)	0 (0)	0 (0)	0 (0)	0 (0)	0 (0)	0 (0)
Sweden	115 (126)	1 (1)	0 (0)	0 (0)	0 (0)	0 (0)	0 (0)	0 (0)	0 (0)	0 (0)
Stockholm	0 (0)	0 (0)	0 (0)	0 (0)	0 (0)	0 (0)	0 (0)	0 (0)	0 (0)	0 (0)
Sweden except Stockholm	0 (0)	0 (0)	0 (0)	0 (0)	0 (0)	0 (0)	0 (0)	0 (0)	0 (0)	0 (0)

Switzerland	107 (107)	0 (0)	0 (0)	0 (0)	0 (0)	0 (0)	0 (0)	0 (0)	0 (0)	0 (0)
UK	1148 (1215)	31 (35)	2 (2)	0 (0)	1 (1)	0 (0)	0 (0)	0 (0)	0 (0)	0 (0)
England	983 (983)	11 (11)	2 (2)	0 (0)	1 (1)	0 (0)	0 (0)	0 (0)	0 (0)	0 (0)
East Midlands	68 (68)	0 (0)	0 (0)	0 (0)	0 (0)	0 (0)	0 (0)	0 (0)	0 (0)	0 (0)
Derby	5 (5)	0 (0)	0 (0)	0 (0)	0 (0)	0 (0)	0 (0)	0 (0)	0 (0)	0 (0)
Derbyshire	5 (5)	0 (0)	0 (0)	0 (0)	0 (0)	0 (0)	0 (0)	0 (0)	0 (0)	0 (0)
Leicester	5 (5)	0 (0)	0 (0)	0 (0)	0 (0)	0 (0)	0 (0)	0 (0)	0 (0)	0 (0)
Leicestershire	5 (5)	0 (0)	0 (0)	0 (0)	0 (0)	0 (0)	0 (0)	0 (0)	0 (0)	0 (0)
Lincolnshire	5 (5)	0 (0)	0 (0)	0 (0)	0 (0)	0 (0)	0 (0)	0 (0)	0 (0)	0 (0)
Northamptonshire	4 (4)	0 (0)	0 (0)	0 (0)	0 (0)	0 (0)	0 (0)	0 (0)	0 (0)	0 (0)
Nottingham	5 (5)	0 (0)	0 (0)	0 (0)	0 (0)	0 (0)	0 (0)	0 (0)	0 (0)	0 (0)
Nottinghamshire	5 (5)	0 (0)	0 (0)	0 (0)	0 (0)	0 (0)	0 (0)	0 (0)	0 (0)	0 (0)
Rutland	5 (5)	0 (0)	0 (0)	0 (0)	0 (0)	0 (0)	0 (0)	0 (0)	0 (0)	0 (0)
East of England	79 (79)	0 (0)	0 (0)	0 (0)	0 (0)	0 (0)	0 (0)	0 (0)	0 (0)	0 (0)
Bedford	5 (5)	0 (0)	0 (0)	0 (0)	0 (0)	0 (0)	0 (0)	0 (0)	0 (0)	0 (0)
Cambridgeshire	5 (5)	0 (0)	0 (0)	0 (0)	0 (0)	0 (0)	0 (0)	0 (0)	0 (0)	0 (0)
Central Bedfordshire	5 (5)	0 (0)	0 (0)	0 (0)	0 (0)	0 (0)	0 (0)	0 (0)	0 (0)	0 (0)
Essex	5 (5)	0 (0)	0 (0)	0 (0)	0 (0)	0 (0)	0 (0)	0 (0)	0 (0)	0 (0)
Hertfordshire	5 (5)	0 (0)	0 (0)	0 (0)	0 (0)	0 (0)	0 (0)	0 (0)	0 (0)	0 (0)
Luton	5 (5)	0 (0)	0 (0)	0 (0)	0 (0)	0 (0)	0 (0)	0 (0)	0 (0)	0 (0)
Norfolk	5 (5)	0 (0)	0 (0)	0 (0)	0 (0)	0 (0)	0 (0)	0 (0)	0 (0)	0 (0)
Peterborough	5 (5)	0 (0)	0 (0)	0 (0)	0 (0)	0 (0)	0 (0)	0 (0)	0 (0)	0 (0)
Southend-on-Sea	5 (5)	0 (0)	0 (0)	0 (0)	0 (0)	0 (0)	0 (0)	0 (0)	0 (0)	0 (0)
Suffolk	5 (5)	0 (0)	0 (0)	0 (0)	0 (0)	0 (0)	0 (0)	0 (0)	0 (0)	0 (0)
Thurrock	5 (5)	0 (0)	0 (0)	0 (0)	0 (0)	0 (0)	0 (0)	0 (0)	0 (0)	0 (0)
Greater London	188 (188)	4 (4)	0 (0)	0 (0)	0 (0)	0 (0)	0 (0)	0 (0)	0 (0)	0 (0)
Barking and Dagenham	5 (5)	0 (0)	0 (0)	0 (0)	0 (0)	0 (0)	0 (0)	0 (0)	0 (0)	0 (0)
Barnet	5 (5)	0 (0)	0 (0)	0 (0)	0 (0)	0 (0)	0 (0)	0 (0)	0 (0)	0 (0)
Bexley	5 (5)	0 (0)	0 (0)	0 (0)	0 (0)	0 (0)	0 (0)	0 (0)	0 (0)	0 (0)
Brent	5 (5)	0 (0)	0 (0)	0 (0)	0 (0)	0 (0)	0 (0)	0 (0)	0 (0)	0 (0)
Bromley	5 (5)	0 (0)	0 (0)	0 (0)	0 (0)	0 (0)	0 (0)	0 (0)	0 (0)	0 (0)
Camden	5 (5)	0 (0)	0 (0)	0 (0)	0 (0)	0 (0)	0 (0)	0 (0)	0 (0)	0 (0)
Croydon	5 (5)	0 (0)	0 (0)	0 (0)	0 (0)	0 (0)	0 (0)	0 (0)	0 (0)	0 (0)
Ealing	5 (5)	0 (0)	0 (0)	0 (0)	0 (0)	0 (0)	0 (0)	0 (0)	0 (0)	0 (0)
Enfield	5 (5)	0 (0)	0 (0)	0 (0)	0 (0)	0 (0)	0 (0)	0 (0)	0 (0)	0 (0)
Greenwich	5 (5)	0 (0)	0 (0)	0 (0)	0 (0)	0 (0)	0 (0)	0 (0)	0 (0)	0 (0)

Hackney	5 (5)	0 (0)	0 (0)	0 (0)	0 (0)	0 (0)	0 (0)	0 (0)	0 (0)	0 (0)
Hammersmith and Fulham	5 (5)	0 (0)	0 (0)	0 (0)	0 (0)	0 (0)	0 (0)	0 (0)	0 (0)	0 (0)
Haringey	5 (5)	0 (0)	0 (0)	0 (0)	0 (0)	0 (0)	0 (0)	0 (0)	0 (0)	0 (0)
Harrow	5 (5)	0 (0)	0 (0)	0 (0)	0 (0)	0 (0)	0 (0)	0 (0)	0 (0)	0 (0)
Havering	5 (5)	0 (0)	0 (0)	0 (0)	0 (0)	0 (0)	0 (0)	0 (0)	0 (0)	0 (0)
Hillingdon	5 (5)	0 (0)	0 (0)	0 (0)	0 (0)	0 (0)	0 (0)	0 (0)	0 (0)	0 (0)
Hounslow	5 (5)	0 (0)	0 (0)	0 (0)	0 (0)	0 (0)	0 (0)	0 (0)	0 (0)	0 (0)
Islington	5 (5)	0 (0)	0 (0)	0 (0)	0 (0)	0 (0)	0 (0)	0 (0)	0 (0)	0 (0)
Kensington and Chelsea	5 (5)	0 (0)	0 (0)	0 (0)	0 (0)	0 (0)	0 (0)	0 (0)	0 (0)	0 (0)
Kingston upon Thames	5 (5)	0 (0)	0 (0)	0 (0)	0 (0)	0 (0)	0 (0)	0 (0)	0 (0)	0 (0)
Lambeth	5 (5)	0 (0)	0 (0)	0 (0)	0 (0)	0 (0)	0 (0)	0 (0)	0 (0)	0 (0)
Lewisham	5 (5)	0 (0)	0 (0)	0 (0)	0 (0)	0 (0)	0 (0)	0 (0)	0 (0)	0 (0)
Merton	5 (5)	0 (0)	0 (0)	0 (0)	0 (0)	0 (0)	0 (0)	0 (0)	0 (0)	0 (0)
Newham	5 (5)	0 (0)	0 (0)	0 (0)	0 (0)	0 (0)	0 (0)	0 (0)	0 (0)	0 (0)
Redbridge	5 (5)	0 (0)	0 (0)	0 (0)	0 (0)	0 (0)	0 (0)	0 (0)	0 (0)	0 (0)
Richmond upon Thames	5 (5)	0 (0)	0 (0)	0 (0)	0 (0)	0 (0)	0 (0)	0 (0)	0 (0)	0 (0)
Southwark	5 (5)	0 (0)	0 (0)	0 (0)	0 (0)	0 (0)	0 (0)	0 (0)	0 (0)	0 (0)
Sutton	5 (5)	0 (0)	0 (0)	0 (0)	0 (0)	0 (0)	0 (0)	0 (0)	0 (0)	0 (0)
Tower Hamlets	5 (5)	0 (0)	0 (0)	0 (0)	0 (0)	0 (0)	0 (0)	0 (0)	0 (0)	0 (0)
Waltham Forest	5 (5)	0 (0)	0 (0)	0 (0)	0 (0)	0 (0)	0 (0)	0 (0)	0 (0)	0 (0)
Wandsworth	5 (5)	0 (0)	0 (0)	0 (0)	0 (0)	0 (0)	0 (0)	0 (0)	0 (0)	0 (0)
Westminster	5 (5)	0 (0)	0 (0)	0 (0)	0 (0)	0 (0)	0 (0)	0 (0)	0 (0)	0 (0)
North East England	84 (84)	1 (1)	1 (1)	0 (0)	1 (1)	0 (0)	0 (0)	0 (0)	0 (0)	0 (0)
County Durham	5 (5)	0 (0)	0 (0)	0 (0)	0 (0)	0 (0)	0 (0)	0 (0)	0 (0)	0 (0)
Darlington	5 (5)	0 (0)	0 (0)	0 (0)	0 (0)	0 (0)	0 (0)	0 (0)	0 (0)	0 (0)
Gateshead	5 (5)	0 (0)	0 (0)	0 (0)	0 (0)	0 (0)	0 (0)	0 (0)	0 (0)	0 (0)
Hartlepool	5 (5)	0 (0)	0 (0)	0 (0)	0 (0)	0 (0)	0 (0)	0 (0)	0 (0)	0 (0)
Middlesbrough	5 (5)	0 (0)	0 (0)	0 (0)	0 (0)	0 (0)	0 (0)	0 (0)	0 (0)	0 (0)
Newcastle upon Tyne	5 (5)	0 (0)	0 (0)	0 (0)	0 (0)	0 (0)	0 (0)	0 (0)	0 (0)	0 (0)
North Tyneside	5 (5)	0 (0)	0 (0)	0 (0)	0 (0)	0 (0)	0 (0)	0 (0)	0 (0)	0 (0)
Northumberland	5 (5)	0 (0)	0 (0)	0 (0)	0 (0)	0 (0)	0 (0)	0 (0)	0 (0)	0 (0)
Redcar and Cleveland	5 (5)	0 (0)	0 (0)	0 (0)	0 (0)	0 (0)	0 (0)	0 (0)	0 (0)	0 (0)
South Tyneside	5 (5)	0 (0)	0 (0)	0 (0)	0 (0)	0 (0)	0 (0)	0 (0)	0 (0)	0 (0)
Stockton-on-Tees	5 (5)	1 (1)	1 (1)	0 (0)	1 (1)	0 (0)	0 (0)	0 (0)	0 (0)	0 (0)
Sunderland	5 (5)	0 (0)	0 (0)	0 (0)	0 (0)	0 (0)	0 (0)	0 (0)	0 (0)	0 (0)
North West England	139 (139)	0 (0)	1 (1)	0 (0)	0 (0)	0 (0)	0 (0)	0 (0)	0 (0)	0 (0)

Blackburn with Darwen	5 (5)	0 (0)	0 (0)	0 (0)	0 (0)	0 (0)	0 (0)	0 (0)	0 (0)	0 (0)
Blackpool	5 (5)	0 (0)	0 (0)	0 (0)	0 (0)	0 (0)	0 (0)	0 (0)	0 (0)	0 (0)
Bolton	5 (5)	0 (0)	0 (0)	0 (0)	0 (0)	0 (0)	0 (0)	0 (0)	0 (0)	0 (0)
Bury	5 (5)	0 (0)	0 (0)	0 (0)	0 (0)	0 (0)	0 (0)	0 (0)	0 (0)	0 (0)
Cheshire East	5 (5)	0 (0)	0 (0)	0 (0)	0 (0)	0 (0)	0 (0)	0 (0)	0 (0)	0 (0)
Cheshire West and Chester	5 (5)	0 (0)	0 (0)	0 (0)	0 (0)	0 (0)	0 (0)	0 (0)	0 (0)	0 (0)
Cumbria	5 (5)	0 (0)	0 (0)	0 (0)	0 (0)	0 (0)	0 (0)	0 (0)	0 (0)	0 (0)
Halton	5 (5)	0 (0)	0 (0)	0 (0)	0 (0)	0 (0)	0 (0)	0 (0)	0 (0)	0 (0)
Knowsley	5 (5)	0 (0)	0 (0)	0 (0)	0 (0)	0 (0)	0 (0)	0 (0)	0 (0)	0 (0)
Lancashire	5 (5)	0 (0)	0 (0)	0 (0)	0 (0)	0 (0)	0 (0)	0 (0)	0 (0)	0 (0)
Liverpool	5 (5)	0 (0)	0 (0)	0 (0)	0 (0)	0 (0)	0 (0)	0 (0)	0 (0)	0 (0)
Manchester	5 (5)	0 (0)	0 (0)	0 (0)	0 (0)	0 (0)	0 (0)	0 (0)	0 (0)	0 (0)
Oldham	5 (5)	0 (0)	0 (0)	0 (0)	0 (0)	0 (0)	0 (0)	0 (0)	0 (0)	0 (0)
Rochdale	5 (5)	0 (0)	0 (0)	0 (0)	0 (0)	0 (0)	0 (0)	0 (0)	0 (0)	0 (0)
Salford	5 (5)	0 (0)	0 (0)	0 (0)	0 (0)	0 (0)	0 (0)	0 (0)	0 (0)	0 (0)
Sefton	5 (5)	0 (0)	0 (0)	0 (0)	0 (0)	0 (0)	0 (0)	0 (0)	0 (0)	0 (0)
St Helens	5 (5)	0 (0)	0 (0)	0 (0)	0 (0)	0 (0)	0 (0)	0 (0)	0 (0)	0 (0)
Stockport	5 (5)	0 (0)	0 (0)	0 (0)	0 (0)	0 (0)	0 (0)	0 (0)	0 (0)	0 (0)
Tameside	5 (5)	0 (0)	0 (0)	0 (0)	0 (0)	0 (0)	0 (0)	0 (0)	0 (0)	0 (0)
Trafford	5 (5)	0 (0)	0 (0)	0 (0)	0 (0)	0 (0)	0 (0)	0 (0)	0 (0)	0 (0)
Warrington	5 (5)	0 (0)	1 (1)	0 (0)	0 (0)	0 (0)	0 (0)	0 (0)	0 (0)	0 (0)
Wigan	5 (5)	0 (0)	0 (0)	0 (0)	0 (0)	0 (0)	0 (0)	0 (0)	0 (0)	0 (0)
Wirral	5 (5)	0 (0)	0 (0)	0 (0)	0 (0)	0 (0)	0 (0)	0 (0)	0 (0)	0 (0)
South East England	119 (119)	1 (1)	0 (0)	0 (0)	0 (0)	0 (0)	0 (0)	0 (0)	0 (0)	0 (0)
Bracknell Forest	5 (5)	0 (0)	0 (0)	0 (0)	0 (0)	0 (0)	0 (0)	0 (0)	0 (0)	0 (0)
Brighton and Hove	5 (5)	0 (0)	0 (0)	0 (0)	0 (0)	0 (0)	0 (0)	0 (0)	0 (0)	0 (0)
Buckinghamshire	5 (5)	0 (0)	0 (0)	0 (0)	0 (0)	0 (0)	0 (0)	0 (0)	0 (0)	0 (0)
East Sussex	5 (5)	0 (0)	0 (0)	0 (0)	0 (0)	0 (0)	0 (0)	0 (0)	0 (0)	0 (0)
Hampshire	5 (5)	0 (0)	0 (0)	0 (0)	0 (0)	0 (0)	0 (0)	0 (0)	0 (0)	0 (0)
Isle of Wight	5 (5)	1 (1)	0 (0)	0 (0)	0 (0)	0 (0)	0 (0)	0 (0)	0 (0)	0 (0)
Kent	5 (5)	0 (0)	0 (0)	0 (0)	0 (0)	0 (0)	0 (0)	0 (0)	0 (0)	0 (0)
Medway	5 (5)	0 (0)	0 (0)	0 (0)	0 (0)	0 (0)	0 (0)	0 (0)	0 (0)	0 (0)
Milton Keynes	5 (5)	0 (0)	0 (0)	0 (0)	0 (0)	0 (0)	0 (0)	0 (0)	0 (0)	0 (0)
Oxfordshire	5 (5)	0 (0)	0 (0)	0 (0)	0 (0)	0 (0)	0 (0)	0 (0)	0 (0)	0 (0)
Portsmouth	5 (5)	0 (0)	0 (0)	0 (0)	0 (0)	0 (0)	0 (0)	0 (0)	0 (0)	0 (0)
Reading	5 (5)	0 (0)	0 (0)	0 (0)	0 (0)	0 (0)	0 (0)	0 (0)	0 (0)	0 (0)

Slough	5 (5)	0 (0)	0 (0)	0 (0)	0 (0)	0 (0)	0 (0)	0 (0)	0 (0)	0 (0)
Southampton	5 (5)	0 (0)	0 (0)	0 (0)	0 (0)	0 (0)	0 (0)	0 (0)	0 (0)	0 (0)
Surrey	5 (5)	0 (0)	0 (0)	0 (0)	0 (0)	0 (0)	0 (0)	0 (0)	0 (0)	0 (0)
West Berkshire	5 (5)	0 (0)	0 (0)	0 (0)	0 (0)	0 (0)	0 (0)	0 (0)	0 (0)	0 (0)
West Sussex	5 (5)	0 (0)	0 (0)	0 (0)	0 (0)	0 (0)	0 (0)	0 (0)	0 (0)	0 (0)
Windsor and Maidenhead	5 (5)	0 (0)	0 (0)	0 (0)	0 (0)	0 (0)	0 (0)	0 (0)	0 (0)	0 (0)
Wokingham	5 (5)	0 (0)	0 (0)	0 (0)	0 (0)	0 (0)	0 (0)	0 (0)	0 (0)	0 (0)
South West England	88 (88)	1 (1)	0 (0)	0 (0)	0 (0)	0 (0)	0 (0)	0 (0)	0 (0)	0 (0)
Bath and North East Somerset	5 (5)	1 (1)	0 (0)	0 (0)	0 (0)	0 (0)	0 (0)	0 (0)	0 (0)	0 (0)
Bournemouth	2 (2)	0 (0)	0 (0)	0 (0)	0 (0)	0 (0)	0 (0)	0 (0)	0 (0)	0 (0)
Bristol, City of	5 (5)	0 (0)	0 (0)	0 (0)	0 (0)	0 (0)	0 (0)	0 (0)	0 (0)	0 (0)
Cornwall	0 (0)	0 (0)	0 (0)	0 (0)	0 (0)	0 (0)	0 (0)	0 (0)	0 (0)	0 (0)
Devon	5 (5)	0 (0)	0 (0)	0 (0)	0 (0)	0 (0)	0 (0)	0 (0)	0 (0)	0 (0)
Dorset	5 (5)	0 (0)	0 (0)	0 (0)	0 (0)	0 (0)	0 (0)	0 (0)	0 (0)	0 (0)
Gloucestershire	5 (5)	0 (0)	0 (0)	0 (0)	0 (0)	0 (0)	0 (0)	0 (0)	0 (0)	0 (0)
North Somerset	5 (5)	0 (0)	0 (0)	0 (0)	0 (0)	0 (0)	0 (0)	0 (0)	0 (0)	0 (0)
Plymouth	5 (5)	0 (0)	0 (0)	0 (0)	0 (0)	0 (0)	0 (0)	0 (0)	0 (0)	0 (0)
Poole	2 (2)	0 (0)	0 (0)	0 (0)	0 (0)	0 (0)	0 (0)	0 (0)	0 (0)	0 (0)
Somerset	5 (5)	0 (0)	0 (0)	0 (0)	0 (0)	0 (0)	0 (0)	0 (0)	0 (0)	0 (0)
South Gloucestershire	5 (5)	0 (0)	0 (0)	0 (0)	0 (0)	0 (0)	0 (0)	0 (0)	0 (0)	0 (0)
Swindon	5 (5)	0 (0)	0 (0)	0 (0)	0 (0)	0 (0)	0 (0)	0 (0)	0 (0)	0 (0)
Torbay	5 (5)	0 (0)	0 (0)	0 (0)	0 (0)	0 (0)	0 (0)	0 (0)	0 (0)	0 (0)
Wiltshire	5 (5)	0 (0)	0 (0)	0 (0)	0 (0)	0 (0)	0 (0)	0 (0)	0 (0)	0 (0)
West Midlands	94 (94)	1 (1)	0 (0)	0 (0)	0 (0)	0 (0)	0 (0)	0 (0)	0 (0)	0 (0)
Birmingham	5 (5)	0 (0)	0 (0)	0 (0)	0 (0)	0 (0)	0 (0)	0 (0)	0 (0)	0 (0)
Coventry	5 (5)	0 (0)	0 (0)	0 (0)	0 (0)	0 (0)	0 (0)	0 (0)	0 (0)	0 (0)
Dudley	5 (5)	0 (0)	0 (0)	0 (0)	0 (0)	0 (0)	0 (0)	0 (0)	0 (0)	0 (0)
Herefordshire, County of	5 (5)	0 (0)	0 (0)	0 (0)	0 (0)	0 (0)	0 (0)	0 (0)	0 (0)	0 (0)
Sandwell	5 (5)	0 (0)	0 (0)	0 (0)	0 (0)	0 (0)	0 (0)	0 (0)	0 (0)	0 (0)
Shropshire	5 (5)	1 (1)	0 (0)	0 (0)	0 (0)	0 (0)	0 (0)	0 (0)	0 (0)	0 (0)
Solihull	5 (5)	0 (0)	0 (0)	0 (0)	0 (0)	0 (0)	0 (0)	0 (0)	0 (0)	0 (0)
Staffordshire	5 (5)	0 (0)	0 (0)	0 (0)	0 (0)	0 (0)	0 (0)	0 (0)	0 (0)	0 (0)
Stoke-on-Trent	5 (5)	0 (0)	0 (0)	0 (0)	0 (0)	0 (0)	0 (0)	0 (0)	0 (0)	0 (0)
Telford and Wrekin	5 (5)	0 (0)	0 (0)	0 (0)	0 (0)	0 (0)	0 (0)	0 (0)	0 (0)	0 (0)
Walsall	5 (5)	0 (0)	0 (0)	0 (0)	0 (0)	0 (0)	0 (0)	0 (0)	0 (0)	0 (0)
Warwickshire	5 (5)	0 (0)	0 (0)	0 (0)	0 (0)	0 (0)	0 (0)	0 (0)	0 (0)	0 (0)

Wolverhampton	5 (5)	0 (0)	0 (0)	0 (0)	0 (0)	0 (0)	0 (0)	0 (0)	0 (0)	0 (0)
Worcestershire	5 (5)	0 (0)	0 (0)	0 (0)	0 (0)	0 (0)	0 (0)	0 (0)	0 (0)	0 (0)
Yorkshire and the Humber	99 (99)	1 (1)	0 (0)	0 (0)	0 (0)	0 (0)	0 (0)	0 (0)	0 (0)	0 (0)
Barnsley	5 (5)	0 (0)	0 (0)	0 (0)	0 (0)	0 (0)	0 (0)	0 (0)	0 (0)	0 (0)
Bradford	5 (5)	0 (0)	0 (0)	0 (0)	0 (0)	0 (0)	0 (0)	0 (0)	0 (0)	0 (0)
Calderdale	5 (5)	0 (0)	0 (0)	0 (0)	0 (0)	0 (0)	0 (0)	0 (0)	0 (0)	0 (0)
Doncaster	5 (5)	0 (0)	0 (0)	0 (0)	0 (0)	0 (0)	0 (0)	0 (0)	0 (0)	0 (0)
East Riding of Yorkshire	5 (5)	0 (0)	0 (0)	0 (0)	0 (0)	0 (0)	0 (0)	0 (0)	0 (0)	0 (0)
Kingston upon Hull, City of	5 (5)	0 (0)	0 (0)	0 (0)	0 (0)	0 (0)	0 (0)	0 (0)	0 (0)	0 (0)
Kirklees	5 (5)	0 (0)	0 (0)	0 (0)	0 (0)	0 (0)	0 (0)	0 (0)	0 (0)	0 (0)
Leeds	5 (5)	0 (0)	0 (0)	0 (0)	0 (0)	0 (0)	0 (0)	0 (0)	0 (0)	0 (0)
North East Lincolnshire	5 (5)	0 (0)	0 (0)	0 (0)	0 (0)	0 (0)	0 (0)	0 (0)	0 (0)	0 (0)
North Lincolnshire	5 (5)	0 (0)	0 (0)	0 (0)	0 (0)	0 (0)	0 (0)	0 (0)	0 (0)	0 (0)
North Yorkshire	5 (5)	0 (0)	0 (0)	0 (0)	0 (0)	0 (0)	0 (0)	0 (0)	0 (0)	0 (0)
Rotherham	5 (5)	0 (0)	0 (0)	0 (0)	0 (0)	0 (0)	0 (0)	0 (0)	0 (0)	0 (0)
Sheffield	5 (5)	0 (0)	0 (0)	0 (0)	0 (0)	0 (0)	0 (0)	0 (0)	0 (0)	0 (0)
Wakefield	5 (5)	0 (0)	0 (0)	0 (0)	0 (0)	0 (0)	0 (0)	0 (0)	0 (0)	0 (0)
York	5 (5)	1 (1)	0 (0)	0 (0)	0 (0)	0 (0)	0 (0)	0 (0)	0 (0)	0 (0)
Northern Ireland	50 (78)	0 (0)	0 (0)	0 (0)	0 (0)	0 (0)	0 (0)	0 (0)	0 (0)	0 (0)
Scotland	66 (103)	19 (21)	0 (0)	0 (0)	0 (0)	0 (0)	0 (0)	0 (0)	0 (0)	0 (0)
Wales	24 (24)	0 (0)	0 (0)	0 (0)	0 (0)	0 (0)	0 (0)	0 (0)	0 (0)	0 (0)
**Latin America and Caribbean**	**2586 (4595)**	**558 (573)**	**268 (397)**	**240 (357)**	**191 (294)**	**168 (257)**	**100 (145)**	**88 (127)**	**48 (69)**	**42 (61)**
**Andean Latin America**	**64 (161)**	**7 (7)**	**5 (9)**	**3 (3)**	**2 (6)**	**0 (0)**	**2 (2)**	**1 (1)**	**0 (0)**	**0 (0)**
Bolivia	3 (3)	0 (0)	0 (0)	0 (0)	0 (0)	0 (0)	0 (0)	0 (0)	0 (0)	0 (0)
Ecuador	53 (150)	6 (6)	5 (9)	3 (3)	2 (6)	0 (0)	2 (2)	1 (1)	0 (0)	0 (0)
Peru	8 (8)	1 (1)	0 (0)	0 (0)	0 (0)	0 (0)	0 (0)	0 (0)	0 (0)	0 (0)
**Caribbean**	**352 (385)**	**44 (56)**	**4 (4)**	**1 (1)**	**3 (3)**	**1 (1)**	**1 (1)**	**0 (0)**	**1 (1)**	**0 (0)**
Antigua and Barbuda	2 (2)	0 (0)	1 (1)	0 (0)	1 (1)	0 (0)	0 (0)	0 (0)	0 (0)	0 (0)
The Bahamas	32 (32)	3 (3)	0 (0)	0 (0)	0 (0)	0 (0)	0 (0)	0 (0)	0 (0)	0 (0)
Barbados	7 (7)	0 (0)	0 (0)	0 (0)	0 (0)	0 (0)	0 (0)	0 (0)	0 (0)	0 (0)
Belize	17 (17)	0 (0)	0 (0)	0 (0)	0 (0)	0 (0)	0 (0)	0 (0)	0 (0)	0 (0)
Bermuda	11 (11)	2 (2)	0 (0)	0 (0)	0 (0)	0 (0)	0 (0)	0 (0)	0 (0)	0 (0)
Cuba	59 (59)	11 (11)	0 (0)	0 (0)	0 (0)	0 (0)	0 (0)	0 (0)	0 (0)	0 (0)
Dominica	0 (0)	0 (0)	0 (0)	0 (0)	0 (0)	0 (0)	0 (0)	0 (0)	0 (0)	0 (0)
Dominican Republic	17 (17)	4 (4)	1 (1)	0 (0)	0 (0)	0 (0)	0 (0)	0 (0)	0 (0)	0 (0)
Grenada	5 (5)	0 (0)	0 (0)	0 (0)	0 (0)	0 (0)	0 (0)	0 (0)	0 (0)	0 (0)

Guyana	1 (1)	0 (0)	0 (0)	0 (0)	0 (0)	0 (0)	0 (0)	0 (0)	0 (0)	0 (0)
Haiti	0 (0)	0 (0)	0 (0)	0 (0)	0 (0)	0 (0)	0 (0)	0 (0)	0 (0)	0 (0)
Jamaica	20 (20)	0 (0)	0 (0)	0 (0)	0 (0)	0 (0)	0 (0)	0 (0)	0 (0)	0 (0)
Puerto Rico	43 (58)	2 (2)	0 (0)	0 (0)	0 (0)	0 (0)	0 (0)	0 (0)	0 (0)	0 (0)
Saint Kitts and Nevis	1 (1)	0 (0)	0 (0)	0 (0)	0 (0)	0 (0)	0 (0)	0 (0)	0 (0)	0 (0)
Saint Lucia	41 (42)	6 (6)	1 (1)	1 (1)	1 (1)	1 (1)	0 (0)	0 (0)	0 (0)	0 (0)
Saint Vincent and the Grenadines	37 (37)	6 (6)	1 (1)	0 (0)	1 (1)	0 (0)	1 (1)	0 (0)	1 (1)	0 (0)
Suriname	10 (10)	3 (3)	0 (0)	0 (0)	0 (0)	0 (0)	0 (0)	0 (0)	0 (0)	0 (0)
Trinidad and Tobago	37 (37)	2 (2)	0 (0)	0 (0)	0 (0)	0 (0)	0 (0)	0 (0)	0 (0)	0 (0)
Virgin Islands	7 (12)	0 (0)	0 (0)	0 (0)	0 (0)	0 (0)	0 (0)	0 (0)	0 (0)	0 (0)
**Central Latin America**	**1229 (2212)**	**30 (33)**	**152 (214)**	**139 (201)**	**106 (159)**	**95 (144)**	**53 (83)**	**49 (78)**	**33 (51)**	**31 (49)**
Colombia	29 (29)	11 (11)	1 (1)	0 (0)	0 (0)	0 (0)	0 (0)	0 (0)	0 (0)	0 (0)
Costa Rica	63 (63)	1 (1)	2 (2)	2 (2)	0 (0)	0 (0)	0 (0)	0 (0)	0 (0)	0 (0)
El Salvador	34 (34)	0 (0)	0 (0)	0 (0)	0 (0)	0 (0)	0 (0)	0 (0)	0 (0)	0 (0)
Guatemala	47 (66)	9 (9)	0 (0)	0 (0)	0 (0)	0 (0)	0 (0)	0 (0)	0 (0)	0 (0)
Honduras	3 (3)	0 (0)	0 (0)	0 (0)	0 (0)	0 (0)	0 (0)	0 (0)	0 (0)	0 (0)
Mexico	986 (1946)	9 (12)	137 (199)	137 (199)	99 (152)	95 (144)	50 (80)	49 (78)	31 (49)	31 (49)
Aguascalientes	30 (60)	0 (0)	0 (0)	0 (0)	0 (0)	0 (0)	0 (0)	0 (0)	0 (0)	0 (0)
Baja California	30 (60)	0 (0)	0 (0)	0 (0)	0 (0)	0 (0)	0 (0)	0 (0)	0 (0)	0 (0)
Baja California Sur	30 (60)	0 (0)	0 (0)	0 (0)	0 (0)	0 (0)	0 (0)	0 (0)	0 (0)	0 (0)
Campeche	30 (60)	0 (0)	3 (3)	3 (3)	1 (1)	1 (1)	0 (0)	0 (0)	0 (0)	0 (0)
Chiapas	30 (60)	0 (0)	0 (0)	0 (0)	0 (0)	0 (0)	0 (0)	0 (0)	0 (0)	0 (0)
Chihuahua	30 (60)	0 (0)	1 (1)	1 (1)	1 (1)	1 (1)	0 (0)	0 (0)	0 (0)	0 (0)
Coahuila	30 (60)	0 (0)	17 (27)	17 (27)	14 (22)	14 (22)	10 (15)	10 (15)	6 (9)	6 (9)
Colima	30 (60)	0 (0)	1 (1)	1 (1)	1 (1)	1 (1)	0 (0)	0 (0)	0 (0)	0 (0)
Durango	30 (60)	0 (0)	9 (11)	9 (11)	5 (6)	5 (6)	1 (1)	1 (1)	0 (0)	0 (0)
Guanajuato	30 (60)	0 (0)	2 (2)	2 (2)	1 (1)	1 (1)	0 (0)	0 (0)	0 (0)	0 (0)
Guerrero	30 (60)	0 (0)	22 (42)	22 (42)	20 (38)	20 (38)	17 (32)	17 (32)	15 (27)	15 (27)
Hidalgo	30 (60)	0 (0)	2 (2)	2 (2)	0 (0)	0 (0)	0 (0)	0 (0)	0 (0)	0 (0)
Jalisco	30 (60)	0 (0)	2 (2)	2 (2)	1 (1)	1 (1)	0 (0)	0 (0)	0 (0)	0 (0)
México	30 (60)	0 (0)	3 (4)	3 (4)	1 (2)	0 (0)	1 (1)	1 (1)	0 (0)	0 (0)
Mexico City	0 (0)	0 (0)	0 (0)	0 (0)	0 (0)	0 (0)	0 (0)	0 (0)	0 (0)	0 (0)
Michoacán de Ocampo	30 (60)	0 (0)	0 (0)	0 (0)	0 (0)	0 (0)	0 (0)	0 (0)	0 (0)	0 (0)
Morelos	30 (60)	0 (0)	5 (5)	5 (5)	5 (5)	5 (5)	0 (0)	0 (0)	0 (0)	0 (0)
Nayarit	30 (60)	0 (0)	10 (11)	10 (11)	5 (6)	5 (6)	1 (2)	0 (0)	0 (0)	0 (0)
Nuevo León	30 (60)	3 (6)	0 (0)	0 (0)	0 (0)	0 (0)	0 (0)	0 (0)	0 (0)	0 (0)

Oaxaca	30 (60)	0 (0)	4 (7)	4 (7)	4 (7)	4 (7)	0 (0)	0 (0)	0 (0)	0 (0)
Puebla	30 (60)	0 (0)	2 (2)	2 (2)	0 (0)	0 (0)	0 (0)	0 (0)	0 (0)	0 (0)
Querétaro	30 (60)	0 (0)	2 (2)	2 (2)	1 (1)	1 (1)	0 (0)	0 (0)	0 (0)	0 (0)
Quintana Roo	30 (60)	0 (0)	1 (1)	1 (1)	1 (1)	1 (1)	0 (0)	0 (0)	0 (0)	0 (0)
San Luis Potosí	30 (60)	0 (0)	0 (0)	0 (0)	0 (0)	0 (0)	0 (0)	0 (0)	0 (0)	0 (0)
Sinaloa	30 (60)	1 (1)	27 (41)	27 (41)	21 (34)	21 (34)	11 (18)	11 (18)	5 (7)	5 (7)
Sonora	30 (60)	0 (0)	2 (2)	2 (2)	1 (1)	1 (1)	0 (0)	0 (0)	0 (0)	0 (0)
Tabasco	30 (60)	0 (0)	2 (2)	2 (2)	0 (0)	0 (0)	0 (0)	0 (0)	0 (0)	0 (0)
Tamaulipas	30 (60)	0 (0)	10 (17)	10 (17)	9 (14)	9 (14)	6 (6)	6 (6)	2 (2)	2 (2)
Tlaxcala	30 (60)	0 (0)	5 (8)	5 (8)	3 (6)	0 (0)	3 (5)	3 (5)	3 (4)	3 (4)
Veracruz de Ignacio de la Llave	30 (60)	0 (0)	1 (1)	1 (1)	1 (1)	1 (1)	0 (0)	0 (0)	0 (0)	0 (0)
Yucatán	30 (60)	0 (0)	0 (0)	0 (0)	0 (0)	0 (0)	0 (0)	0 (0)	0 (0)	0 (0)
Zacatecas	30 (60)	0 (0)	1 (1)	1 (1)	0 (0)	0 (0)	0 (0)	0 (0)	0 (0)	0 (0)
Nicaragua	3 (3)	0 (0)	1 (1)	0 (0)	0 (0)	0 (0)	0 (0)	0 (0)	0 (0)	0 (0)
Panama	45 (49)	0 (0)	0 (0)	0 (0)	0 (0)	0 (0)	0 (0)	0 (0)	0 (0)	0 (0)
Venezuela	19 (19)	0 (0)	11 (11)	0 (0)	7 (7)	0 (0)	3 (3)	0 (0)	2 (2)	0 (0)
**Tropical Latin America**	**941 (1837)**	**477 (477)**	**107 (170)**	**97 (152)**	**80 (126)**	**72 (112)**	**44 (59)**	**38 (48)**	**14 (17)**	**11 (12)**
Brazil	934 (1830)	477 (477)	106 (169)	97 (152)	79 (125)	72 (112)	43 (58)	38 (48)	13 (16)	11 (12)
Acre	32 (64)	17 (17)	1 (1)	1 (1)	0 (0)	0 (0)	0 (0)	0 (0)	0 (0)	0 (0)
Alagoas	32 (64)	17 (17)	5 (9)	5 (9)	4 (7)	4 (7)	1 (1)	1 (1)	0 (0)	0 (0)
Amapá	32 (64)	17 (17)	15 (29)	9 (17)	15 (27)	9 (15)	12 (18)	7 (8)	5 (8)	3 (4)
Amazonas	32 (64)	16 (16)	3 (3)	3 (3)	2 (2)	2 (2)	0 (0)	0 (0)	0 (0)	0 (0)
Bahia	32 (64)	16 (16)	0 (0)	0 (0)	0 (0)	0 (0)	0 (0)	0 (0)	0 (0)	0 (0)
Ceará	32 (64)	17 (17)	3 (4)	3 (4)	1 (2)	1 (2)	1 (1)	1 (1)	0 (0)	0 (0)
Distrito Federal	32 (64)	16 (16)	0 (0)	0 (0)	0 (0)	0 (0)	0 (0)	0 (0)	0 (0)	0 (0)
Espírito Santo	32 (64)	17 (17)	0 (0)	0 (0)	0 (0)	0 (0)	0 (0)	0 (0)	0 (0)	0 (0)
Goiás	32 (64)	16 (16)	0 (0)	0 (0)	0 (0)	0 (0)	0 (0)	0 (0)	0 (0)	0 (0)
Maranhão	32 (64)	17 (17)	4 (6)	4 (6)	3 (3)	3 (3)	0 (0)	0 (0)	0 (0)	0 (0)
Mato Grosso	32 (64)	16 (16)	10 (15)	10 (15)	6 (10)	6 (10)	3 (3)	3 (3)	0 (0)	0 (0)
Mato Grosso do Sul	32 (64)	17 (17)	10 (19)	10 (19)	9 (16)	9 (16)	7 (11)	7 (11)	5 (5)	5 (5)
Minas Gerais	33 (65)	17 (17)	0 (0)	0 (0)	0 (0)	0 (0)	0 (0)	0 (0)	0 (0)	0 (0)
Pará	32 (64)	17 (17)	16 (27)	14 (23)	13 (21)	13 (21)	8 (9)	8 (9)	0 (0)	0 (0)
Paraíba	32 (64)	17 (17)	0 (0)	0 (0)	0 (0)	0 (0)	0 (0)	0 (0)	0 (0)	0 (0)
Paraná	32 (64)	16 (16)	0 (0)	0 (0)	0 (0)	0 (0)	0 (0)	0 (0)	0 (0)	0 (0)
Pernambuco	38 (70)	21 (21)	0 (0)	0 (0)	0 (0)	0 (0)	0 (0)	0 (0)	0 (0)	0 (0)
Piauí	32 (64)	16 (16)	10 (18)	10 (18)	9 (14)	9 (14)	4 (5)	4 (5)	0 (0)	0 (0)

Rio de Janeiro	32 (64)	16 (16)	0 (0)	0 (0)	0 (0)	0 (0)	0 (0)	0 (0)	0 (0)	0 (0)
Rio Grande do Norte	32 (64)	17 (17)	1 (1)	1 (1)	0 (0)	0 (0)	0 (0)	0 (0)	0 (0)	0 (0)
Rio Grande do Sul	37 (69)	21 (21)	0 (0)	0 (0)	0 (0)	0 (0)	0 (0)	0 (0)	0 (0)	0 (0)
Rondônia	32 (64)	16 (16)	1 (1)	1 (1)	0 (0)	0 (0)	0 (0)	0 (0)	0 (0)	0 (0)
Roraima	32 (64)	17 (17)	5 (5)	5 (5)	2 (2)	2 (2)	0 (0)	0 (0)	0 (0)	0 (0)
Santa Catarina	32 (64)	17 (17)	8 (15)	8 (15)	7 (12)	7 (12)	6 (9)	6 (9)	3 (3)	3 (3)
São Paulo	33 (65)	17 (17)	2 (2)	1 (1)	1 (1)	0 (0)	0 (0)	0 (0)	0 (0)	0 (0)
Sergipe	32 (64)	17 (17)	8 (10)	8 (10)	6 (7)	6 (7)	0 (0)	0 (0)	0 (0)	0 (0)
Tocantins	32 (64)	17 (17)	3 (3)	3 (3)	0 (0)	0 (0)	0 (0)	0 (0)	0 (0)	0 (0)
Paraguay	7 (7)	0 (0)	1 (1)	0 (0)	1 (1)	0 (0)	1 (1)	0 (0)	1 (1)	0 (0)
**North Africa and Middle East**	**528 (532)**	**53 (53)**	**40 (40)**	**16 (16)**	**32 (32)**	**10 (10)**	**24 (24)**	**10 (10)**	**15 (15)**	**6 (6)**
**North Africa and Middle East**	**528 (532)**	**53 (53)**	**40 (40)**	**16 (16)**	**32 (32)**	**10 (10)**	**24 (24)**	**10 (10)**	**15 (15)**	**6 (6)**
Afghanistan	5 (5)	1 (1)	0 (0)	0 (0)	0 (0)	0 (0)	0 (0)	0 (0)	0 (0)	0 (0)
Algeria	37 (37)	1 (1)	2 (2)	0 (0)	2 (2)	0 (0)	0 (0)	0 (0)	0 (0)	0 (0)
Bahrain	39 (40)	2 (2)	5 (5)	3 (3)	5 (5)	3 (3)	4 (4)	3 (3)	0 (0)	0 (0)
Egypt	24 (24)	1 (1)	16 (16)	6 (6)	16 (16)	6 (6)	16 (16)	6 (6)	15 (15)	6 (6)
Iran	15 (15)	1 (1)	2 (2)	0 (0)	1 (1)	0 (0)	0 (0)	0 (0)	0 (0)	0 (0)
Alborz	0 (0)	0 (0)	0 (0)	0 (0)	0 (0)	0 (0)	0 (0)	0 (0)	0 (0)	0 (0)
Ardebil	0 (0)	0 (0)	0 (0)	0 (0)	0 (0)	0 (0)	0 (0)	0 (0)	0 (0)	0 (0)
Bushehr	0 (0)	0 (0)	0 (0)	0 (0)	0 (0)	0 (0)	0 (0)	0 (0)	0 (0)	0 (0)
Chahar Mahaal and Bakhtiari	0 (0)	0 (0)	0 (0)	0 (0)	0 (0)	0 (0)	0 (0)	0 (0)	0 (0)	0 (0)
East Azarbayejan	0 (0)	0 (0)	0 (0)	0 (0)	0 (0)	0 (0)	0 (0)	0 (0)	0 (0)	0 (0)
Fars	0 (0)	0 (0)	0 (0)	0 (0)	0 (0)	0 (0)	0 (0)	0 (0)	0 (0)	0 (0)
Gilan	0 (0)	0 (0)	0 (0)	0 (0)	0 (0)	0 (0)	0 (0)	0 (0)	0 (0)	0 (0)
Golestan	0 (0)	0 (0)	0 (0)	0 (0)	0 (0)	0 (0)	0 (0)	0 (0)	0 (0)	0 (0)
Hamadan	0 (0)	0 (0)	0 (0)	0 (0)	0 (0)	0 (0)	0 (0)	0 (0)	0 (0)	0 (0)
Hormozgan	0 (0)	0 (0)	0 (0)	0 (0)	0 (0)	0 (0)	0 (0)	0 (0)	0 (0)	0 (0)
Ilam	0 (0)	0 (0)	0 (0)	0 (0)	0 (0)	0 (0)	0 (0)	0 (0)	0 (0)	0 (0)
Isfahan	0 (0)	0 (0)	0 (0)	0 (0)	0 (0)	0 (0)	0 (0)	0 (0)	0 (0)	0 (0)
Kerman	1 (1)	0 (0)	0 (0)	0 (0)	0 (0)	0 (0)	0 (0)	0 (0)	0 (0)	0 (0)
Kermanshah	0 (0)	0 (0)	0 (0)	0 (0)	0 (0)	0 (0)	0 (0)	0 (0)	0 (0)	0 (0)
Khorasan-e-Razavi	0 (0)	0 (0)	0 (0)	0 (0)	0 (0)	0 (0)	0 (0)	0 (0)	0 (0)	0 (0)
Khuzestan	0 (0)	0 (0)	0 (0)	0 (0)	0 (0)	0 (0)	0 (0)	0 (0)	0 (0)	0 (0)
Kohgiluyeh and Boyer-Ahmad	0 (0)	0 (0)	0 (0)	0 (0)	0 (0)	0 (0)	0 (0)	0 (0)	0 (0)	0 (0)
Kurdistan	0 (0)	0 (0)	0 (0)	0 (0)	0 (0)	0 (0)	0 (0)	0 (0)	0 (0)	0 (0)
Lorestan	0 (0)	0 (0)	0 (0)	0 (0)	0 (0)	0 (0)	0 (0)	0 (0)	0 (0)	0 (0)

Markazi	0 (0)	0 (0)	0 (0)	0 (0)	0 (0)	0 (0)	0 (0)	0 (0)	0 (0)	0 (0)
Mazandaran	10 (10)	0 (0)	0 (0)	0 (0)	0 (0)	0 (0)	0 (0)	0 (0)	0 (0)	0 (0)
North Khorasan	0 (0)	0 (0)	0 (0)	0 (0)	0 (0)	0 (0)	0 (0)	0 (0)	0 (0)	0 (0)
Qazvin	0 (0)	0 (0)	0 (0)	0 (0)	0 (0)	0 (0)	0 (0)	0 (0)	0 (0)	0 (0)
Qom	0 (0)	0 (0)	0 (0)	0 (0)	0 (0)	0 (0)	0 (0)	0 (0)	0 (0)	0 (0)
Semnan	0 (0)	0 (0)	0 (0)	0 (0)	0 (0)	0 (0)	0 (0)	0 (0)	0 (0)	0 (0)
Sistan and Baluchistan	0 (0)	0 (0)	0 (0)	0 (0)	0 (0)	0 (0)	0 (0)	0 (0)	0 (0)	0 (0)
South Khorasan	0 (0)	0 (0)	0 (0)	0 (0)	0 (0)	0 (0)	0 (0)	0 (0)	0 (0)	0 (0)
Tehran	0 (0)	0 (0)	0 (0)	0 (0)	0 (0)	0 (0)	0 (0)	0 (0)	0 (0)	0 (0)
West Azarbayejan	0 (0)	0 (0)	0 (0)	0 (0)	0 (0)	0 (0)	0 (0)	0 (0)	0 (0)	0 (0)
Yazd	0 (0)	0 (0)	0 (0)	0 (0)	0 (0)	0 (0)	0 (0)	0 (0)	0 (0)	0 (0)
Zanjan	0 (0)	0 (0)	0 (0)	0 (0)	0 (0)	0 (0)	0 (0)	0 (0)	0 (0)	0 (0)
Iraq	7 (7)	0 (0)	0 (0)	0 (0)	0 (0)	0 (0)	0 (0)	0 (0)	0 (0)	0 (0)
Jordan	10 (10)	1 (1)	0 (0)	0 (0)	0 (0)	0 (0)	0 (0)	0 (0)	0 (0)	0 (0)
Kuwait	45 (45)	10 (10)	0 (0)	0 (0)	0 (0)	0 (0)	0 (0)	0 (0)	0 (0)	0 (0)
Lebanon	11 (11)	1 (1)	5 (5)	0 (0)	5 (5)	0 (0)	2 (2)	0 (0)	0 (0)	0 (0)
Libya	0 (0)	0 (0)	0 (0)	0 (0)	0 (0)	0 (0)	0 (0)	0 (0)	0 (0)	0 (0)
Morocco	32 (32)	3 (3)	0 (0)	0 (0)	0 (0)	0 (0)	0 (0)	0 (0)	0 (0)	0 (0)
Oman	61 (61)	1 (1)	0 (0)	0 (0)	0 (0)	0 (0)	0 (0)	0 (0)	0 (0)	0 (0)
Palestine	20 (20)	1 (1)	2 (2)	0 (0)	1 (1)	0 (0)	1 (1)	0 (0)	0 (0)	0 (0)
Qatar	47 (47)	8 (8)	0 (0)	0 (0)	0 (0)	0 (0)	0 (0)	0 (0)	0 (0)	0 (0)
Saudi Arabia	53 (54)	2 (2)	0 (0)	0 (0)	0 (0)	0 (0)	0 (0)	0 (0)	0 (0)	0 (0)
Sudan	23 (23)	1 (1)	3 (3)	2 (2)	1 (1)	0 (0)	0 (0)	0 (0)	0 (0)	0 (0)
Syria	0 (0)	0 (0)	0 (0)	0 (0)	0 (0)	0 (0)	0 (0)	0 (0)	0 (0)	0 (0)
Tunisia	42 (42)	7 (7)	0 (0)	0 (0)	0 (0)	0 (0)	0 (0)	0 (0)	0 (0)	0 (0)
Türkiye	30 (30)	11 (11)	0 (0)	0 (0)	0 (0)	0 (0)	0 (0)	0 (0)	0 (0)	0 (0)
United Arab Emirates	22 (24)	0 (0)	5 (5)	5 (5)	1 (1)	1 (1)	1 (1)	1 (1)	0 (0)	0 (0)
Yemen	5 (5)	1 (1)	0 (0)	0 (0)	0 (0)	0 (0)	0 (0)	0 (0)	0 (0)	0 (0)
**South Asia**	**2775 (2796)**	**481 (488)**	**2035 (2040)**	**160 (160)**	**1893 (1898)**	**186 (186)**	**1389 (1393)**	**203 (203)**	**960 (963)**	**188 (189)**
**South Asia**	**2775 (2796)**	**481 (488)**	**2035 (2040)**	**160 (160)**	**1893 (1898)**	**186 (186)**	**1389 (1393)**	**203 (203)**	**960 (963)**	**188 (189)**
Bangladesh	41 (41)	8 (8)	2 (2)	0 (0)	0 (0)	0 (0)	0 (0)	0 (0)	0 (0)	0 (0)
Bhutan	0 (0)	0 (0)	0 (0)	0 (0)	0 (0)	0 (0)	0 (0)	0 (0)	0 (0)	0 (0)
India	2694 (2708)	461 (466)	2032 (2037)	160 (160)	1893 (1898)	186 (186)	1389 (1393)	203 (203)	960 (963)	188 (189)
Andhra Pradesh	131 (131)	3 (3)	125 (125)	4 (4)	117 (117)	4 (4)	98 (98)	4 (4)	74 (74)	3 (3)
Andhra Pradesh, Rural	41 (41)	0 (0)	40 (40)	1 (1)	40 (40)	1 (1)	37 (37)	1 (1)	30 (30)	1 (1)
Andhra Pradesh, Urban	41 (41)	2 (2)	38 (38)	1 (1)	30 (30)	1 (1)	17 (17)	1 (1)	12 (12)	0 (0)

Arunachal Pradesh	8 (8)	5 (5)	0 (0)	0 (0)	0 (0)	0 (0)	0 (0)	0 (0)	0 (0)	0 (0)
Arunachal Pradesh, Rural	1 (1)	0 (0)	0 (0)	0 (0)	0 (0)	0 (0)	0 (0)	0 (0)	0 (0)	0 (0)
Arunachal Pradesh, Urban	1 (1)	0 (0)	0 (0)	0 (0)	0 (0)	0 (0)	0 (0)	0 (0)	0 (0)	0 (0)
Assam	147 (147)	6 (6)	139 (139)	1 (1)	136 (136)	5 (5)	95 (95)	15 (15)	48 (48)	16 (16)
Assam, Rural	46 (46)	1 (1)	44 (44)	1 (1)	43 (43)	2 (2)	33 (33)	4 (4)	14 (14)	4 (4)
Assam, Urban	46 (46)	2 (2)	43 (43)	0 (0)	43 (43)	0 (0)	23 (23)	3 (3)	17 (17)	0 (0)
Bihar	150 (151)	14 (14)	117 (117)	12 (12)	113 (113)	13 (13)	104 (104)	16 (16)	99 (99)	15 (15)
Bihar, Rural	48 (48)	4 (4)	38 (38)	3 (3)	38 (38)	3 (3)	36 (36)	4 (4)	35 (35)	5 (5)
Bihar, Urban	48 (48)	7 (7)	35 (35)	4 (4)	32 (32)	4 (4)	27 (27)	5 (5)	25 (25)	4 (4)
Chhattisgarh	75 (75)	1 (1)	69 (69)	9 (9)	66 (66)	9 (9)	45 (45)	18 (18)	22 (22)	20 (20)
Chhattisgarh, Rural	23 (23)	0 (0)	22 (22)	1 (1)	22 (22)	1 (1)	16 (16)	6 (6)	7 (7)	6 (6)
Chhattisgarh, Urban	23 (23)	1 (1)	19 (19)	6 (6)	17 (17)	6 (6)	9 (9)	6 (6)	5 (5)	4 (4)
Delhi	72 (80)	23 (27)	42 (46)	3 (3)	38 (42)	3 (3)	11 (14)	2 (2)	5 (7)	1 (1)
Delhi, Rural	24 (28)	11 (11)	13 (17)	0 (0)	13 (17)	0 (0)	8 (11)	1 (1)	4 (6)	1 (1)
Delhi, Urban	25 (29)	9 (13)	14 (14)	0 (0)	12 (12)	0 (0)	2 (2)	0 (0)	1 (1)	0 (0)
Goa	11 (11)	1 (1)	1 (1)	1 (1)	1 (1)	1 (1)	0 (0)	0 (0)	0 (0)	0 (0)
Goa, Rural	1 (1)	0 (0)	0 (0)	0 (0)	0 (0)	0 (0)	0 (0)	0 (0)	0 (0)	0 (0)
Goa, Urban	0 (0)	0 (0)	0 (0)	0 (0)	0 (0)	0 (0)	0 (0)	0 (0)	0 (0)	0 (0)
Gujarat	128 (128)	1 (1)	126 (126)	1 (1)	124 (124)	2 (2)	114 (114)	5 (5)	94 (94)	6 (6)
Gujarat, Rural	41 (41)	0 (0)	41 (41)	0 (0)	41 (41)	0 (0)	40 (40)	1 (1)	38 (38)	1 (1)
Gujarat, Urban	41 (41)	1 (1)	40 (40)	0 (0)	38 (38)	1 (1)	32 (32)	1 (1)	23 (23)	1 (1)
Haryana	129 (129)	7 (7)	118 (118)	4 (4)	101 (101)	4 (4)	53 (53)	3 (3)	25 (25)	2 (2)
Haryana, Rural	41 (41)	2 (2)	38 (38)	1 (1)	31 (31)	1 (1)	15 (15)	1 (1)	10 (10)	0 (0)
Haryana, Urban	41 (41)	1 (1)	39 (39)	0 (0)	35 (35)	0 (0)	21 (21)	0 (0)	8 (8)	0 (0)
Himachal Pradesh	98 (100)	49 (50)	48 (49)	0 (0)	35 (36)	0 (0)	18 (19)	1 (1)	14 (15)	2 (3)
Himachal Pradesh, Rural	30 (31)	15 (16)	15 (15)	0 (0)	11 (11)	0 (0)	5 (5)	0 (0)	5 (5)	0 (0)
Himachal Pradesh, Urban	30 (31)	14 (14)	15 (16)	0 (0)	12 (13)	0 (0)	6 (7)	0 (0)	3 (4)	1 (2)
Jammu & Kashmir and Ladakh	58 (58)	1 (1)	48 (48)	8 (8)	45 (45)	8 (8)	36 (36)	7 (7)	22 (22)	7 (7)
Jammu & Kashmir and Ladakh, Rural	17 (17)	0 (0)	16 (16)	1 (1)	16 (16)	1 (1)	11 (11)	1 (1)	7 (7)	1 (1)
Jammu & Kashmir and Ladakh, Urban	17 (17)	1 (1)	15 (15)	1 (1)	13 (13)	0 (0)	13 (13)	0 (0)	7 (7)	0 (0)
Jharkhand	68 (68)	12 (12)	49 (49)	7 (7)	48 (48)	8 (8)	44 (44)	12 (12)	38 (38)	18 (18)
Jharkhand, Rural	21 (21)	4 (4)	16 (16)	0 (0)	15 (15)	1 (1)	14 (14)	2 (2)	12 (12)	4 (4)
Jharkhand, Urban	21 (21)	6 (6)	12 (12)	3 (3)	12 (12)	3 (3)	10 (10)	5 (5)	9 (9)	6 (6)
Karnataka	145 (145)	53 (53)	73 (73)	9 (9)	63 (63)	10 (10)	47 (47)	12 (12)	25 (25)	5 (5)
Karnataka, Rural	41 (41)	17 (17)	23 (23)	0 (0)	18 (18)	1 (1)	13 (13)	1 (1)	8 (8)	1 (1)
Karnataka, Urban	41 (41)	19 (19)	20 (20)	1 (1)	19 (19)	1 (1)	15 (15)	1 (1)	8 (8)	1 (1)

Kerala	132 (132)	94 (94)	24 (24)	2 (2)	19 (19)	1 (1)	11 (11)	1 (1)	5 (5)	0 (0)
Kerala, Rural	41 (41)	29 (29)	9 (9)	1 (1)	7 (7)	1 (1)	5 (5)	1 (1)	2 (2)	0 (0)
Kerala, Urban	41 (41)	32 (32)	8 (8)	0 (0)	6 (6)	0 (0)	4 (4)	0 (0)	2 (2)	0 (0)
Madhya Pradesh	146 (146)	4 (4)	141 (141)	5 (5)	140 (140)	6 (6)	131 (131)	8 (8)	111 (111)	16 (16)
Madhya Pradesh, Rural	46 (46)	0 (0)	46 (46)	0 (0)	46 (46)	0 (0)	45 (45)	1 (1)	45 (45)	1 (1)
Madhya Pradesh, Urban	46 (46)	4 (4)	41 (41)	5 (5)	40 (40)	6 (6)	33 (33)	5 (5)	19 (19)	4 (4)
Maharashtra	146 (146)	15 (15)	117 (117)	25 (25)	104 (104)	34 (34)	50 (50)	11 (11)	22 (22)	7 (7)
Maharashtra, Rural	49 (49)	0 (0)	48 (48)	1 (1)	42 (42)	9 (9)	21 (21)	6 (6)	11 (11)	6 (6)
Maharashtra, Urban	41 (41)	9 (9)	30 (30)	1 (1)	26 (26)	1 (1)	13 (13)	0 (0)	4 (4)	0 (0)
Manipur	4 (4)	1 (1)	0 (0)	0 (0)	0 (0)	0 (0)	0 (0)	0 (0)	0 (0)	0 (0)
Manipur, Rural	1 (1)	0 (0)	0 (0)	0 (0)	0 (0)	0 (0)	0 (0)	0 (0)	0 (0)	0 (0)
Manipur, Urban	1 (1)	0 (0)	0 (0)	0 (0)	0 (0)	0 (0)	0 (0)	0 (0)	0 (0)	0 (0)
Meghalaya	10 (10)	0 (0)	0 (0)	0 (0)	0 (0)	0 (0)	0 (0)	0 (0)	0 (0)	0 (0)
Meghalaya, Rural	1 (1)	0 (0)	0 (0)	0 (0)	0 (0)	0 (0)	0 (0)	0 (0)	0 (0)	0 (0)
Meghalaya, Urban	1 (1)	0 (0)	0 (0)	0 (0)	0 (0)	0 (0)	0 (0)	0 (0)	0 (0)	0 (0)
Mizoram	11 (11)	0 (0)	11 (11)	0 (0)	10 (10)	0 (0)	4 (4)	0 (0)	2 (2)	0 (0)
Mizoram, Rural	1 (1)	0 (0)	1 (1)	0 (0)	1 (1)	0 (0)	0 (0)	0 (0)	0 (0)	0 (0)
Mizoram, Urban	1 (1)	0 (0)	1 (1)	0 (0)	0 (0)	0 (0)	0 (0)	0 (0)	0 (0)	0 (0)
Nagaland	11 (11)	8 (8)	0 (0)	0 (0)	0 (0)	0 (0)	0 (0)	0 (0)	0 (0)	0 (0)
Nagaland, Rural	1 (1)	0 (0)	0 (0)	0 (0)	0 (0)	0 (0)	0 (0)	0 (0)	0 (0)	0 (0)
Nagaland, Urban	1 (1)	0 (0)	0 (0)	0 (0)	0 (0)	0 (0)	0 (0)	0 (0)	0 (0)	0 (0)
Odisha	148 (148)	14 (14)	120 (120)	15 (15)	109 (109)	14 (14)	58 (58)	15 (15)	27 (27)	6 (6)
Odisha, Rural	47 (47)	1 (1)	43 (43)	2 (2)	40 (40)	2 (2)	18 (18)	6 (6)	7 (7)	2 (2)
Odisha, Urban	46 (46)	11 (11)	31 (31)	0 (0)	29 (29)	0 (0)	21 (21)	1 (1)	13 (13)	1 (1)
Other Union Territories	7 (7)	0 (0)	2 (2)	0 (0)	2 (2)	0 (0)	0 (0)	0 (0)	0 (0)	0 (0)
Other Union Territories, Rural	0 (0)	0 (0)	0 (0)	0 (0)	0 (0)	0 (0)	0 (0)	0 (0)	0 (0)	0 (0)
Other Union Territories, Urban	0 (0)	0 (0)	0 (0)	0 (0)	0 (0)	0 (0)	0 (0)	0 (0)	0 (0)	0 (0)
Punjab	142 (142)	62 (62)	74 (74)	4 (4)	62 (62)	5 (5)	27 (27)	5 (5)	10 (10)	3 (3)
Punjab, Rural	41 (41)	16 (16)	24 (24)	1 (1)	20 (20)	1 (1)	11 (11)	1 (1)	3 (3)	1 (1)
Punjab, Urban	41 (41)	18 (18)	21 (21)	1 (1)	18 (18)	0 (0)	9 (9)	0 (0)	4 (4)	0 (0)
Rajasthan	147 (147)	2 (2)	141 (141)	3 (3)	138 (138)	6 (6)	126 (126)	10 (10)	101 (101)	19 (19)
Rajasthan, Rural	47 (47)	0 (0)	46 (46)	1 (1)	45 (45)	2 (2)	45 (45)	2 (2)	39 (39)	7 (7)
Rajasthan, Urban	46 (46)	1 (1)	44 (44)	1 (1)	44 (44)	1 (1)	32 (32)	5 (5)	24 (24)	3 (3)
Sikkim	10 (10)	0 (0)	4 (4)	0 (0)	4 (4)	0 (0)	0 (0)	0 (0)	0 (0)	0 (0)
Sikkim, Rural	1 (1)	0 (0)	1 (1)	0 (0)	1 (1)	0 (0)	0 (0)	0 (0)	0 (0)	0 (0)
Sikkim, Urban	1 (1)	0 (0)	0 (0)	0 (0)	0 (0)	0 (0)	0 (0)	0 (0)	0 (0)	0 (0)

Tamil Nadu	155 (155)	58 (58)	93 (93)	3 (3)	78 (78)	5 (5)	44 (44)	5 (5)	27 (27)	5 (5)
Tamil Nadu, Rural	42 (42)	14 (14)	28 (28)	0 (0)	24 (24)	1 (1)	17 (17)	1 (1)	12 (12)	1 (1)
Tamil Nadu, Urban	41 (41)	7 (7)	32 (32)	1 (1)	26 (26)	1 (1)	11 (11)	1 (1)	5 (5)	1 (1)
Telangana	26 (26)	5 (5)	18 (18)	3 (3)	18 (18)	3 (3)	16 (16)	4 (4)	15 (15)	5 (5)
Telangana, Rural	7 (7)	1 (1)	5 (5)	1 (1)	5 (5)	1 (1)	5 (5)	1 (1)	5 (5)	1 (1)
Telangana, Urban	7 (7)	3 (3)	3 (3)	0 (0)	3 (3)	0 (0)	2 (2)	0 (0)	2 (2)	0 (0)
Tripura	11 (11)	0 (0)	7 (7)	1 (1)	5 (5)	1 (1)	1 (1)	0 (0)	0 (0)	0 (0)
Tripura, Rural	1 (1)	0 (0)	0 (0)	0 (0)	0 (0)	0 (0)	0 (0)	0 (0)	0 (0)	0 (0)
Tripura, Urban	1 (1)	0 (0)	1 (1)	0 (0)	1 (1)	0 (0)	0 (0)	0 (0)	0 (0)	0 (0)
Uttar Pradesh	147 (150)	3 (3)	137 (137)	11 (11)	135 (135)	11 (11)	127 (127)	17 (17)	115 (115)	21 (21)
Uttar Pradesh, Rural	49 (49)	1 (1)	44 (44)	4 (4)	44 (44)	4 (4)	40 (40)	6 (6)	36 (36)	6 (6)
Uttar Pradesh, Urban	46 (46)	1 (1)	43 (43)	2 (2)	42 (42)	3 (3)	41 (41)	4 (4)	38 (38)	6 (6)
Uttarakhand	42 (42)	12 (12)	24 (24)	2 (2)	21 (21)	6 (6)	7 (7)	2 (2)	5 (5)	2 (2)
Uttarakhand, Rural	12 (12)	3 (3)	7 (7)	0 (0)	6 (6)	0 (0)	1 (1)	0 (0)	1 (1)	0 (0)
Uttarakhand, Urban	12 (12)	4 (4)	6 (6)	0 (0)	5 (5)	0 (0)	2 (2)	0 (0)	1 (1)	0 (0)
West Bengal	128 (128)	6 (6)	117 (117)	4 (4)	114 (114)	4 (4)	75 (75)	7 (7)	29 (29)	7 (7)
West Bengal, Rural	40 (40)	1 (1)	38 (38)	1 (1)	38 (38)	1 (1)	26 (26)	1 (1)	9 (9)	1 (1)
West Bengal, Urban	40 (40)	5 (5)	32 (32)	1 (1)	30 (30)	1 (1)	22 (22)	1 (1)	10 (10)	1 (1)
Nepal	14 (15)	3 (3)	1 (1)	0 (0)	0 (0)	0 (0)	0 (0)	0 (0)	0 (0)	0 (0)
Pakistan	26 (32)	9 (11)	0 (0)	0 (0)	0 (0)	0 (0)	0 (0)	0 (0)	0 (0)	0 (0)
Azad Jammu & Kashmir	0 (0)	0 (0)	0 (0)	0 (0)	0 (0)	0 (0)	0 (0)	0 (0)	0 (0)	0 (0)
Balochistan	2 (3)	1 (1)	0 (0)	0 (0)	0 (0)	0 (0)	0 (0)	0 (0)	0 (0)	0 (0)
Gilgit-Baltistan	1 (1)	0 (0)	0 (0)	0 (0)	0 (0)	0 (0)	0 (0)	0 (0)	0 (0)	0 (0)
Islamabad Capital Territory	1 (1)	0 (0)	0 (0)	0 (0)	0 (0)	0 (0)	0 (0)	0 (0)	0 (0)	0 (0)
Khyber Pakhtunkhwa	3 (4)	1 (1)	0 (0)	0 (0)	0 (0)	0 (0)	0 (0)	0 (0)	0 (0)	0 (0)
Punjab	2 (3)	0 (0)	0 (0)	0 (0)	0 (0)	0 (0)	0 (0)	0 (0)	0 (0)	0 (0)
Sindh	10 (13)	4 (6)	0 (0)	0 (0)	0 (0)	0 (0)	0 (0)	0 (0)	0 (0)	0 (0)
**Southeast Asia, east Asia, and Oceania**	**3404 (3458)**	**552 (552)**	**1580 (1581)**	**141 (141)**	**1404 (1405)**	**122 (122)**	**1101 (1101)**	**72 (72)**	**823 (823)**	**51 (51)**
**East Asia**	**91 (106)**	**20 (20)**	**1 (1)**	**1 (1)**	**1 (1)**	**1 (1)**	**0 (0)**	**0 (0)**	**0 (0)**	**0 (0)**
China	83 (98)	19 (19)	1 (1)	1 (1)	1 (1)	1 (1)	0 (0)	0 (0)	0 (0)	0 (0)
Anhui	0 (0)	0 (0)	0 (0)	0 (0)	0 (0)	0 (0)	0 (0)	0 (0)	0 (0)	0 (0)
Beijing	0 (0)	0 (0)	0 (0)	0 (0)	0 (0)	0 (0)	0 (0)	0 (0)	0 (0)	0 (0)
Chongqing	0 (0)	0 (0)	0 (0)	0 (0)	0 (0)	0 (0)	0 (0)	0 (0)	0 (0)	0 (0)
Fujian	0 (0)	0 (0)	0 (0)	0 (0)	0 (0)	0 (0)	0 (0)	0 (0)	0 (0)	0 (0)
Gansu	0 (0)	0 (0)	0 (0)	0 (0)	0 (0)	0 (0)	0 (0)	0 (0)	0 (0)	0 (0)
Guangdong	0 (0)	0 (0)	0 (0)	0 (0)	0 (0)	0 (0)	0 (0)	0 (0)	0 (0)	0 (0)

Guangxi	0 (0)	0 (0)	0 (0)	0 (0)	0 (0)	0 (0)	0 (0)	0 (0)	0 (0)	0 (0)
Guizhou	0 (0)	0 (0)	0 (0)	0 (0)	0 (0)	0 (0)	0 (0)	0 (0)	0 (0)	0 (0)
Hainan	0 (0)	0 (0)	0 (0)	0 (0)	0 (0)	0 (0)	0 (0)	0 (0)	0 (0)	0 (0)
Hebei	0 (0)	0 (0)	0 (0)	0 (0)	0 (0)	0 (0)	0 (0)	0 (0)	0 (0)	0 (0)
Heilongjiang	0 (0)	0 (0)	0 (0)	0 (0)	0 (0)	0 (0)	0 (0)	0 (0)	0 (0)	0 (0)
Henan	0 (0)	0 (0)	0 (0)	0 (0)	0 (0)	0 (0)	0 (0)	0 (0)	0 (0)	0 (0)
Hong Kong Special Administrative Region of China	26 (26)	0 (0)	0 (0)	0 (0)	0 (0)	0 (0)	0 (0)	0 (0)	0 (0)	0 (0)
Hubei	0 (0)	0 (0)	0 (0)	0 (0)	0 (0)	0 (0)	0 (0)	0 (0)	0 (0)	0 (0)
Hunan	0 (0)	0 (0)	0 (0)	0 (0)	0 (0)	0 (0)	0 (0)	0 (0)	0 (0)	0 (0)
Inner Mongolia	0 (0)	0 (0)	0 (0)	0 (0)	0 (0)	0 (0)	0 (0)	0 (0)	0 (0)	0 (0)
Jiangsu	2 (2)	1 (1)	0 (0)	0 (0)	0 (0)	0 (0)	0 (0)	0 (0)	0 (0)	0 (0)
Jiangxi	0 (0)	0 (0)	0 (0)	0 (0)	0 (0)	0 (0)	0 (0)	0 (0)	0 (0)	0 (0)
Jilin	0 (0)	0 (0)	0 (0)	0 (0)	0 (0)	0 (0)	0 (0)	0 (0)	0 (0)	0 (0)
Liaoning	0 (0)	0 (0)	0 (0)	0 (0)	0 (0)	0 (0)	0 (0)	0 (0)	0 (0)	0 (0)
Macao Special Administrative Region of China	44 (59)	11 (11)	0 (0)	0 (0)	0 (0)	0 (0)	0 (0)	0 (0)	0 (0)	0 (0)
Ningxia	0 (0)	0 (0)	0 (0)	0 (0)	0 (0)	0 (0)	0 (0)	0 (0)	0 (0)	0 (0)
Qinghai	0 (0)	0 (0)	0 (0)	0 (0)	0 (0)	0 (0)	0 (0)	0 (0)	0 (0)	0 (0)
Shaanxi	0 (0)	0 (0)	0 (0)	0 (0)	0 (0)	0 (0)	0 (0)	0 (0)	0 (0)	0 (0)
Shandong	0 (0)	0 (0)	0 (0)	0 (0)	0 (0)	0 (0)	0 (0)	0 (0)	0 (0)	0 (0)
Shanghai	0 (0)	0 (0)	0 (0)	0 (0)	0 (0)	0 (0)	0 (0)	0 (0)	0 (0)	0 (0)
Shanxi	0 (0)	0 (0)	0 (0)	0 (0)	0 (0)	0 (0)	0 (0)	0 (0)	0 (0)	0 (0)
Sichuan	0 (0)	0 (0)	0 (0)	0 (0)	0 (0)	0 (0)	0 (0)	0 (0)	0 (0)	0 (0)
Tianjin	0 (0)	0 (0)	0 (0)	0 (0)	0 (0)	0 (0)	0 (0)	0 (0)	0 (0)	0 (0)
Tibet	0 (0)	0 (0)	0 (0)	0 (0)	0 (0)	0 (0)	0 (0)	0 (0)	0 (0)	0 (0)
Xinjiang	0 (0)	0 (0)	0 (0)	0 (0)	0 (0)	0 (0)	0 (0)	0 (0)	0 (0)	0 (0)
Yunnan	0 (0)	0 (0)	0 (0)	0 (0)	0 (0)	0 (0)	0 (0)	0 (0)	0 (0)	0 (0)
Zhejiang	0 (0)	0 (0)	0 (0)	0 (0)	0 (0)	0 (0)	0 (0)	0 (0)	0 (0)	0 (0)
North Korea	0 (0)	0 (0)	0 (0)	0 (0)	0 (0)	0 (0)	0 (0)	0 (0)	0 (0)	0 (0)
Taiwan (province of China)	8 (8)	1 (1)	0 (0)	0 (0)	0 (0)	0 (0)	0 (0)	0 (0)	0 (0)	0 (0)
**Oceania**	**184 (221)**	**21 (21)**	**12 (12)**	**2 (2)**	**9 (9)**	**2 (2)**	**7 (7)**	**0 (0)**	**2 (2)**	**0 (0)**
American Samoa	56 (66)	8 (8)	2 (2)	2 (2)	2 (2)	2 (2)	0 (0)	0 (0)	0 (0)	0 (0)
Cook Islands	36 (36)	6 (6)	1 (1)	0 (0)	1 (1)	0 (0)	1 (1)	0 (0)	1 (1)	0 (0)
Fiji	2 (2)	0 (0)	1 (1)	0 (0)	0 (0)	0 (0)	0 (0)	0 (0)	0 (0)	0 (0)
Guam	32 (47)	1 (1)	0 (0)	0 (0)	0 (0)	0 (0)	0 (0)	0 (0)	0 (0)	0 (0)
Kiribati	1 (1)	0 (0)	1 (1)	0 (0)	1 (1)	0 (0)	1 (1)	0 (0)	1 (1)	0 (0)
Marshall Islands	5 (5)	0 (0)	0 (0)	0 (0)	0 (0)	0 (0)	0 (0)	0 (0)	0 (0)	0 (0)

Federated States of Micronesia	0 (0)	0 (0)	0 (0)	0 (0)	0 (0)	0 (0)	0 (0)	0 (0)	0 (0)	0 (0)
Nauru	0 (0)	0 (0)	0 (0)	0 (0)	0 (0)	0 (0)	0 (0)	0 (0)	0 (0)	0 (0)
Niue	0 (0)	0 (0)	0 (0)	0 (0)	0 (0)	0 (0)	0 (0)	0 (0)	0 (0)	0 (0)
Northern Mariana Islands	14 (26)	0 (0)	0 (0)	0 (0)	0 (0)	0 (0)	0 (0)	0 (0)	0 (0)	0 (0)
Palau	15 (15)	1 (1)	1 (1)	0 (0)	0 (0)	0 (0)	0 (0)	0 (0)	0 (0)	0 (0)
Papua New Guinea	6 (6)	1 (1)	5 (5)	0 (0)	5 (5)	0 (0)	5 (5)	0 (0)	0 (0)	0 (0)
Samoa	2 (2)	0 (0)	0 (0)	0 (0)	0 (0)	0 (0)	0 (0)	0 (0)	0 (0)	0 (0)
Solomon Islands	0 (0)	0 (0)	0 (0)	0 (0)	0 (0)	0 (0)	0 (0)	0 (0)	0 (0)	0 (0)
Tokelau	0 (0)	0 (0)	0 (0)	0 (0)	0 (0)	0 (0)	0 (0)	0 (0)	0 (0)	0 (0)
Tonga	10 (10)	0 (0)	0 (0)	0 (0)	0 (0)	0 (0)	0 (0)	0 (0)	0 (0)	0 (0)
Tuvalu	0 (0)	0 (0)	0 (0)	0 (0)	0 (0)	0 (0)	0 (0)	0 (0)	0 (0)	0 (0)
Vanuatu	1 (1)	0 (0)	1 (1)	0 (0)	0 (0)	0 (0)	0 (0)	0 (0)	0 (0)	0 (0)
**Southeast Asia**	**3129 (3131)**	**511 (511)**	**1567 (1568)**	**138 (138)**	**1394 (1395)**	**119 (119)**	**1094 (1094)**	**72 (72)**	**821 (821)**	**51 (51)**
Cambodia	9 (9)	1 (1)	6 (6)	0 (0)	5 (5)	0 (0)	4 (4)	0 (0)	0 (0)	0 (0)
Indonesia	399 (401)	21 (21)	173 (174)	13 (13)	123 (124)	14 (14)	83 (83)	9 (9)	53 (53)	3 (3)
Aceh	14 (14)	0 (0)	7 (7)	0 (0)	3 (3)	0 (0)	1 (1)	0 (0)	1 (1)	0 (0)
Bali	12 (12)	0 (0)	4 (4)	1 (1)	2 (2)	0 (0)	2 (2)	0 (0)	2 (2)	0 (0)
Bangka-Belitung Islands	14 (14)	1 (1)	6 (6)	0 (0)	4 (4)	1 (1)	2 (2)	0 (0)	2 (2)	0 (0)
Banten	12 (12)	1 (1)	10 (10)	0 (0)	9 (9)	0 (0)	8 (8)	0 (0)	7 (7)	0 (0)
Bengkulu	13 (13)	1 (1)	6 (6)	0 (0)	3 (3)	0 (0)	0 (0)	0 (0)	0 (0)	0 (0)
Gorontalo	12 (12)	2 (2)	5 (5)	1 (1)	2 (2)	1 (1)	1 (1)	1 (1)	0 (0)	0 (0)
Jakarta	14 (14)	2 (2)	11 (11)	0 (0)	9 (9)	1 (1)	8 (8)	1 (1)	8 (8)	1 (1)
Jambi	13 (13)	0 (0)	10 (10)	1 (1)	9 (9)	1 (1)	8 (8)	1 (1)	3 (3)	0 (0)
West Java	10 (10)	0 (0)	8 (8)	1 (1)	6 (6)	1 (1)	6 (6)	1 (1)	4 (4)	1 (1)
Central Java	15 (15)	1 (1)	11 (11)	0 (0)	3 (3)	0 (0)	1 (1)	0 (0)	0 (0)	0 (0)
East Java	15 (15)	0 (0)	11 (11)	1 (1)	9 (9)	2 (2)	1 (1)	1 (1)	0 (0)	0 (0)
West Kalimantan	12 (12)	0 (0)	0 (0)	0 (0)	0 (0)	0 (0)	0 (0)	0 (0)	0 (0)	0 (0)
South Kalimantan	12 (13)	0 (0)	10 (11)	1 (1)	8 (9)	1 (1)	4 (4)	1 (1)	1 (1)	0 (0)
Central Kalimantan	9 (9)	1 (1)	3 (3)	0 (0)	1 (1)	0 (0)	0 (0)	0 (0)	0 (0)	0 (0)
East Kalimantan	14 (14)	1 (1)	1 (1)	0 (0)	1 (1)	0 (0)	0 (0)	0 (0)	0 (0)	0 (0)
North Kalimantan	4 (4)	2 (2)	0 (0)	0 (0)	0 (0)	0 (0)	0 (0)	0 (0)	0 (0)	0 (0)
Riau Islands	13 (13)	1 (1)	1 (1)	0 (0)	0 (0)	0 (0)	0 (0)	0 (0)	0 (0)	0 (0)
Lampung	15 (15)	0 (0)	11 (11)	1 (1)	11 (11)	1 (1)	11 (11)	1 (1)	5 (5)	0 (0)
Maluku	9 (9)	0 (0)	1 (1)	0 (0)	0 (0)	0 (0)	0 (0)	0 (0)	0 (0)	0 (0)
North Maluku	7 (7)	2 (2)	0 (0)	0 (0)	0 (0)	0 (0)	0 (0)	0 (0)	0 (0)	0 (0)
West Nusa Tenggara	14 (14)	0 (0)	8 (8)	1 (1)	3 (3)	1 (1)	2 (2)	0 (0)	2 (2)	0 (0)

East Nusa Tenggara	11 (11)	0 (0)	2 (2)	0 (0)	2 (2)	0 (0)	1 (1)	0 (0)	1 (1)	0 (0)
Papua	6 (6)	1 (1)	2 (2)	1 (1)	1 (1)	0 (0)	0 (0)	0 (0)	0 (0)	0 (0)
West Papua	6 (6)	1 (1)	2 (2)	0 (0)	1 (1)	0 (0)	0 (0)	0 (0)	0 (0)	0 (0)
Riau	12 (12)	0 (0)	8 (8)	1 (1)	8 (8)	1 (1)	7 (7)	1 (1)	3 (3)	0 (0)
West Sulawesi	11 (12)	2 (2)	3 (3)	1 (1)	2 (2)	1 (1)	0 (0)	0 (0)	0 (0)	0 (0)
South Sulawesi	13 (13)	1 (1)	2 (2)	0 (0)	1 (1)	0 (0)	0 (0)	0 (0)	0 (0)	0 (0)
Central Sulawesi	15 (15)	0 (0)	0 (0)	0 (0)	0 (0)	0 (0)	0 (0)	0 (0)	0 (0)	0 (0)
Southeast Sulawesi	11 (11)	1 (1)	3 (3)	0 (0)	1 (1)	0 (0)	0 (0)	0 (0)	0 (0)	0 (0)
North Sulawesi	6 (6)	0 (0)	2 (2)	0 (0)	1 (1)	0 (0)	0 (0)	0 (0)	0 (0)	0 (0)
West Sumatra	9 (9)	0 (0)	0 (0)	0 (0)	0 (0)	0 (0)	0 (0)	0 (0)	0 (0)	0 (0)
South Sumatra	15 (15)	0 (0)	13 (13)	1 (1)	13 (13)	1 (1)	12 (12)	1 (1)	9 (9)	1 (1)
North Sumatra	13 (13)	0 (0)	11 (11)	1 (1)	9 (9)	1 (1)	8 (8)	0 (0)	5 (5)	0 (0)
Yogyakarta	12 (12)	0 (0)	0 (0)	0 (0)	0 (0)	0 (0)	0 (0)	0 (0)	0 (0)	0 (0)
Laos	0 (0)	0 (0)	0 (0)	0 (0)	0 (0)	0 (0)	0 (0)	0 (0)	0 (0)	0 (0)
Malaysia	57 (57)	15 (15)	0 (0)	0 (0)	0 (0)	0 (0)	0 (0)	0 (0)	0 (0)	0 (0)
Maldives	45 (45)	6 (6)	1 (1)	1 (1)	1 (1)	1 (1)	0 (0)	0 (0)	0 (0)	0 (0)
Mauritius	100 (100)	12 (12)	0 (0)	0 (0)	0 (0)	0 (0)	0 (0)	0 (0)	0 (0)	0 (0)
Myanmar	8 (8)	0 (0)	6 (6)	1 (1)	4 (4)	1 (1)	1 (1)	0 (0)	0 (0)	0 (0)
Philippines	2406 (2406)	445 (445)	1335 (1335)	83 (83)	1228 (1228)	76 (76)	992 (992)	55 (55)	763 (763)	48 (48)
Abra	30 (30)	19 (19)	10 (10)	1 (1)	10 (10)	1 (1)	10 (10)	1 (1)	9 (9)	1 (1)
Agusan Del Norte	29 (29)	5 (5)	22 (22)	1 (1)	21 (21)	1 (1)	21 (21)	1 (1)	20 (20)	1 (1)
Agusan Del Sur	29 (29)	4 (4)	22 (22)	0 (0)	16 (16)	0 (0)	11 (11)	0 (0)	6 (6)	0 (0)
Aklan	30 (30)	4 (4)	13 (13)	0 (0)	7 (7)	0 (0)	2 (2)	0 (0)	2 (2)	0 (0)
Albay	29 (29)	7 (7)	14 (14)	0 (0)	10 (10)	0 (0)	4 (4)	0 (0)	2 (2)	0 (0)
Antique	30 (30)	4 (4)	14 (14)	0 (0)	14 (14)	0 (0)	10 (10)	0 (0)	5 (5)	0 (0)
Apayao	29 (29)	5 (5)	17 (17)	0 (0)	15 (15)	0 (0)	8 (8)	0 (0)	5 (5)	0 (0)
Aurora	28 (28)	10 (10)	18 (18)	0 (0)	17 (17)	0 (0)	14 (14)	0 (0)	10 (10)	0 (0)
Basilan	29 (29)	15 (15)	12 (12)	0 (0)	12 (12)	0 (0)	9 (9)	0 (0)	7 (7)	0 (0)
Bataan	29 (29)	4 (4)	14 (14)	0 (0)	10 (10)	0 (0)	7 (7)	0 (0)	5 (5)	0 (0)
Batanes	25 (25)	4 (4)	1 (1)	0 (0)	0 (0)	0 (0)	0 (0)	0 (0)	0 (0)	0 (0)
Batangas	29 (29)	4 (4)	11 (11)	0 (0)	8 (8)	0 (0)	6 (6)	0 (0)	1 (1)	0 (0)
Benguet	30 (30)	1 (1)	8 (8)	1 (1)	8 (8)	1 (1)	3 (3)	1 (1)	1 (1)	1 (1)
Biliran	29 (29)	2 (2)	11 (11)	0 (0)	10 (10)	0 (0)	6 (6)	0 (0)	3 (3)	0 (0)
Bohol	30 (30)	3 (3)	25 (25)	0 (0)	23 (23)	0 (0)	10 (10)	0 (0)	4 (4)	0 (0)
Bukidnon	29 (29)	5 (5)	22 (22)	1 (1)	22 (22)	1 (1)	20 (20)	1 (1)	18 (18)	1 (1)
Bulacan	30 (30)	2 (2)	23 (23)	1 (1)	19 (19)	1 (1)	15 (15)	1 (1)	10 (10)	1 (1)

Cagayan	29 (29)	1 (1)	23 (23)	2 (2)	23 (23)	2 (2)	23 (23)	2 (2)	23 (23)	2 (2)
Camarines Norte	28 (28)	1 (1)	4 (4)	0 (0)	2 (2)	0 (0)	0 (0)	0 (0)	0 (0)	0 (0)
Camarines Sur	28 (28)	2 (2)	22 (22)	2 (2)	22 (22)	2 (2)	17 (17)	2 (2)	12 (12)	1 (1)
Camiguin	29 (29)	12 (12)	3 (3)	0 (0)	3 (3)	0 (0)	1 (1)	0 (0)	0 (0)	0 (0)
Capiz	30 (30)	3 (3)	25 (25)	1 (1)	24 (24)	1 (1)	23 (23)	1 (1)	19 (19)	1 (1)
Catanduanes	28 (28)	3 (3)	14 (14)	0 (0)	10 (10)	0 (0)	7 (7)	0 (0)	5 (5)	0 (0)
Cavite	29 (29)	4 (4)	17 (17)	0 (0)	16 (16)	0 (0)	8 (8)	0 (0)	7 (7)	0 (0)
Cebu	30 (30)	1 (1)	17 (17)	2 (2)	14 (14)	2 (2)	7 (7)	1 (1)	2 (2)	0 (0)
Cotabato (North Cotabato)	30 (30)	3 (3)	25 (25)	2 (2)	24 (24)	3 (3)	24 (24)	3 (3)	23 (23)	3 (3)
Davao de Oro	23 (23)	5 (5)	9 (9)	0 (0)	8 (8)	0 (0)	5 (5)	0 (0)	3 (3)	0 (0)
Davao Del Norte	30 (30)	6 (6)	15 (15)	0 (0)	14 (14)	0 (0)	13 (13)	1 (1)	12 (12)	1 (1)
Davao Del Sur	30 (30)	4 (4)	14 (14)	2 (2)	14 (14)	2 (2)	11 (11)	2 (2)	8 (8)	2 (2)
Davao Occidental	3 (3)	1 (1)	0 (0)	0 (0)	0 (0)	0 (0)	0 (0)	0 (0)	0 (0)	0 (0)
Davao Oriental	30 (30)	8 (8)	13 (13)	0 (0)	13 (13)	0 (0)	13 (13)	0 (0)	11 (11)	0 (0)
Dinagat Islands	11 (11)	4 (4)	6 (6)	0 (0)	6 (6)	0 (0)	6 (6)	0 (0)	5 (5)	0 (0)
Eastern Samar	30 (30)	3 (3)	25 (25)	1 (1)	25 (25)	1 (1)	23 (23)	1 (1)	18 (18)	1 (1)
Guimaras	29 (29)	5 (5)	8 (8)	0 (0)	7 (7)	0 (0)	3 (3)	0 (0)	1 (1)	0 (0)
Ifugao	30 (30)	4 (4)	11 (11)	1 (1)	8 (8)	1 (1)	4 (4)	0 (0)	1 (1)	0 (0)
Ilocos Norte	29 (29)	2 (2)	17 (17)	0 (0)	15 (15)	0 (0)	11 (11)	0 (0)	4 (4)	0 (0)
Ilocos Sur	29 (29)	7 (7)	16 (16)	0 (0)	16 (16)	0 (0)	14 (14)	0 (0)	5 (5)	0 (0)
Iloilo	30 (30)	3 (3)	19 (19)	1 (1)	18 (18)	1 (1)	15 (15)	1 (1)	11 (11)	1 (1)
Isabela	29 (29)	1 (1)	23 (23)	3 (3)	23 (23)	3 (3)	23 (23)	3 (3)	21 (21)	3 (3)
Kalinga	28 (28)	5 (5)	9 (9)	0 (0)	5 (5)	0 (0)	4 (4)	0 (0)	4 (4)	0 (0)
La Union	29 (29)	7 (7)	17 (17)	0 (0)	15 (15)	0 (0)	10 (10)	0 (0)	8 (8)	0 (0)
Laguna	29 (29)	7 (7)	7 (7)	1 (1)	5 (5)	1 (1)	1 (1)	1 (1)	1 (1)	1 (1)
Lanao Del Norte	30 (30)	2 (2)	26 (26)	2 (2)	26 (26)	2 (2)	22 (22)	2 (2)	19 (19)	1 (1)
Lanao Del Sur	30 (30)	11 (11)	18 (18)	0 (0)	18 (18)	0 (0)	18 (18)	0 (0)	18 (18)	0 (0)
Leyte	30 (30)	0 (0)	28 (28)	2 (2)	28 (28)	2 (2)	25 (25)	2 (2)	17 (17)	3 (3)
Maguindanao	30 (30)	17 (17)	11 (11)	0 (0)	10 (10)	1 (1)	10 (10)	1 (1)	10 (10)	1 (1)
Marinduque	29 (29)	3 (3)	0 (0)	0 (0)	0 (0)	0 (0)	0 (0)	0 (0)	0 (0)	0 (0)
Masbate	29 (29)	10 (10)	19 (19)	0 (0)	19 (19)	0 (0)	19 (19)	0 (0)	18 (18)	0 (0)
Misamis Occidental	29 (29)	12 (12)	14 (14)	0 (0)	14 (14)	0 (0)	13 (13)	0 (0)	11 (11)	0 (0)
Misamis Oriental	29 (29)	12 (12)	15 (15)	0 (0)	15 (15)	0 (0)	12 (12)	0 (0)	8 (8)	0 (0)
Mountain Province	30 (30)	3 (3)	0 (0)	0 (0)	0 (0)	0 (0)	0 (0)	0 (0)	0 (0)	0 (0)
National Capital Region	30 (30)	0 (0)	14 (14)	1 (1)	8 (8)	0 (0)	6 (6)	0 (0)	2 (2)	0 (0)
Negros Occidental	30 (30)	1 (1)	24 (24)	3 (3)	23 (23)	3 (3)	19 (19)	3 (3)	12 (12)	2 (2)

Negros Oriental	30 (30)	2 (2)	24 (24)	2 (2)	24 (24)	2 (2)	23 (23)	2 (2)	20 (20)	2 (2)
Northern Samar	30 (30)	7 (7)	18 (18)	1 (1)	18 (18)	1 (1)	16 (16)	1 (1)	15 (15)	1 (1)
Nueva Ecija	27 (27)	16 (16)	8 (8)	1 (1)	8 (8)	1 (1)	8 (8)	1 (1)	7 (7)	1 (1)
Nueva Vizcaya	29 (29)	11 (11)	9 (9)	0 (0)	8 (8)	0 (0)	6 (6)	0 (0)	6 (6)	0 (0)
Occidental Mindoro	29 (29)	5 (5)	16 (16)	0 (0)	16 (16)	0 (0)	16 (16)	0 (0)	15 (15)	0 (0)
Oriental Mindoro	28 (28)	4 (4)	20 (20)	0 (0)	20 (20)	0 (0)	19 (19)	0 (0)	16 (16)	0 (0)
Palawan	27 (27)	3 (3)	20 (20)	1 (1)	20 (20)	1 (1)	18 (18)	1 (1)	11 (11)	0 (0)
Pampanga	30 (30)	15 (15)	13 (13)	0 (0)	12 (12)	0 (0)	11 (11)	0 (0)	10 (10)	0 (0)
Pangasinan	29 (29)	3 (3)	20 (20)	2 (2)	19 (19)	2 (2)	16 (16)	2 (2)	13 (13)	2 (2)
Quezon	30 (30)	6 (6)	13 (13)	1 (1)	12 (12)	1 (1)	9 (9)	1 (1)	5 (5)	1 (1)
Quirino	29 (29)	6 (6)	21 (21)	0 (0)	19 (19)	0 (0)	18 (18)	0 (0)	18 (18)	0 (0)
Rizal	29 (29)	11 (11)	14 (14)	1 (1)	10 (10)	1 (1)	4 (4)	1 (1)	3 (3)	1 (1)
Romblon	29 (29)	4 (4)	21 (21)	0 (0)	18 (18)	0 (0)	9 (9)	0 (0)	2 (2)	0 (0)
Samar (Western Samar)	30 (30)	11 (11)	18 (18)	1 (1)	18 (18)	1 (1)	17 (17)	1 (1)	16 (16)	1 (1)
Sarangani	28 (28)	3 (3)	16 (16)	1 (1)	16 (16)	1 (1)	13 (13)	1 (1)	11 (11)	1 (1)
Siquijor	30 (30)	4 (4)	19 (19)	0 (0)	16 (16)	0 (0)	15 (15)	0 (0)	10 (10)	0 (0)
Sorsogon	29 (29)	1 (1)	22 (22)	1 (1)	22 (22)	1 (1)	21 (21)	1 (1)	15 (15)	1 (1)
South Cotabato	29 (29)	4 (4)	16 (16)	1 (1)	15 (15)	1 (1)	15 (15)	1 (1)	14 (14)	1 (1)
Southern Leyte	30 (30)	2 (2)	23 (23)	1 (1)	20 (20)	1 (1)	17 (17)	1 (1)	9 (9)	0 (0)
Sultan Kudarat	30 (30)	2 (2)	25 (25)	1 (1)	25 (25)	1 (1)	24 (24)	1 (1)	17 (17)	1 (1)
Sulu	28 (28)	7 (7)	18 (18)	1 (1)	18 (18)	1 (1)	18 (18)	1 (1)	18 (18)	1 (1)
Surigao Del Norte	29 (29)	3 (3)	23 (23)	1 (1)	22 (22)	1 (1)	18 (18)	1 (1)	16 (16)	0 (0)
Surigao Del Sur	30 (30)	1 (1)	25 (25)	2 (2)	24 (24)	2 (2)	16 (16)	2 (2)	12 (12)	2 (2)
Tarlac	30 (30)	3 (3)	23 (23)	0 (0)	22 (22)	0 (0)	17 (17)	0 (0)	11 (11)	0 (0)
Tawi-Tawi	30 (30)	13 (13)	9 (9)	0 (0)	6 (6)	0 (0)	2 (2)	0 (0)	2 (2)	0 (0)
Zambales	30 (30)	8 (8)	13 (13)	0 (0)	11 (11)	0 (0)	7 (7)	0 (0)	2 (2)	0 (0)
Zamboanga Del Norte	30 (30)	10 (10)	18 (18)	2 (2)	18 (18)	2 (2)	18 (18)	2 (2)	16 (16)	2 (2)
Zamboanga Del Sur	30 (30)	6 (6)	21 (21)	3 (3)	20 (20)	3 (3)	18 (18)	2 (2)	13 (13)	1 (1)
Zamboanga Sibugay	20 (20)	2 (2)	16 (16)	0 (0)	16 (16)	0 (0)	16 (16)	0 (0)	12 (12)	0 (0)
Seychelles	12 (12)	0 (0)	0 (0)	0 (0)	0 (0)	0 (0)	0 (0)	0 (0)	0 (0)	0 (0)
Sri Lanka	72 (72)	4 (4)	39 (39)	39 (39)	26 (26)	26 (26)	8 (8)	8 (8)	0 (0)	0 (0)
Thailand	7 (7)	4 (4)	3 (3)	0 (0)	3 (3)	0 (0)	3 (3)	0 (0)	3 (3)	0 (0)
Timor-Leste	4 (4)	2 (2)	2 (2)	0 (0)	2 (2)	0 (0)	2 (2)	0 (0)	1 (1)	0 (0)
Viet Nam	10 (10)	1 (1)	2 (2)	1 (1)	2 (2)	1 (1)	1 (1)	0 (0)	1 (1)	0 (0)
**Sub-Saharan Africa**	**949 (1191)**	**223 (257)**	**224 (304)**	**87 (159)**	**192 (268)**	**80 (150)**	**119 (155)**	**51 (83)**	**65 (84)**	**23 (37)**
**Central sub-Saharan Africa**	**10 (10)**	**5 (5)**	**1 (1)**	**0 (0)**	**1 (1)**	**0 (0)**	**0 (0)**	**0 (0)**	**0 (0)**	**0 (0)**

Angola	3 (3)	1 (1)	1 (1)	0 (0)	1 (1)	0 (0)	0 (0)	0 (0)	0 (0)	0 (0)
Central African Republic	0 (0)	0 (0)	0 (0)	0 (0)	0 (0)	0 (0)	0 (0)	0 (0)	0 (0)	0 (0)
Congo (Brazzaville)	0 (0)	0 (0)	0 (0)	0 (0)	0 (0)	0 (0)	0 (0)	0 (0)	0 (0)	0 (0)
DR Congo	5 (5)	3 (3)	0 (0)	0 (0)	0 (0)	0 (0)	0 (0)	0 (0)	0 (0)	0 (0)
Equatorial Guinea	0 (0)	0 (0)	0 (0)	0 (0)	0 (0)	0 (0)	0 (0)	0 (0)	0 (0)	0 (0)
Gabon	1 (1)	0 (0)	0 (0)	0 (0)	0 (0)	0 (0)	0 (0)	0 (0)	0 (0)	0 (0)
**Eastern sub-Saharan Africa**	**307 (310)**	**106 (109)**	**58 (58)**	**1 (1)**	**48 (48)**	**1 (1)**	**29 (29)**	**0 (0)**	**13 (13)**	**0 (0)**
Burundi	4 (4)	0 (0)	2 (2)	1 (1)	1 (1)	1 (1)	0 (0)	0 (0)	0 (0)	0 (0)
Comoros	1 (1)	0 (0)	1 (1)	0 (0)	1 (1)	0 (0)	1 (1)	0 (0)	0 (0)	0 (0)
Djibouti	0 (0)	0 (0)	0 (0)	0 (0)	0 (0)	0 (0)	0 (0)	0 (0)	0 (0)	0 (0)
Eritrea	0 (0)	0 (0)	0 (0)	0 (0)	0 (0)	0 (0)	0 (0)	0 (0)	0 (0)	0 (0)
Ethiopia	39 (39)	7 (7)	14 (14)	0 (0)	12 (12)	0 (0)	8 (8)	0 (0)	3 (3)	0 (0)
Addis Ababa	2 (2)	0 (0)	0 (0)	0 (0)	0 (0)	0 (0)	0 (0)	0 (0)	0 (0)	0 (0)
Afar	2 (2)	0 (0)	2 (2)	0 (0)	2 (2)	0 (0)	2 (2)	0 (0)	0 (0)	0 (0)
Amhara	7 (7)	1 (1)	3 (3)	0 (0)	2 (2)	0 (0)	1 (1)	0 (0)	1 (1)	0 (0)
Benishangul-Gumuz	2 (2)	0 (0)	0 (0)	0 (0)	0 (0)	0 (0)	0 (0)	0 (0)	0 (0)	0 (0)
Dire Dawa	2 (2)	0 (0)	1 (1)	0 (0)	1 (1)	0 (0)	1 (1)	0 (0)	0 (0)	0 (0)
Gambella	2 (2)	0 (0)	1 (1)	0 (0)	1 (1)	0 (0)	1 (1)	0 (0)	1 (1)	0 (0)
Harari	3 (3)	1 (1)	2 (2)	0 (0)	2 (2)	0 (0)	1 (1)	0 (0)	1 (1)	0 (0)
Oromia	4 (4)	0 (0)	1 (1)	0 (0)	1 (1)	0 (0)	1 (1)	0 (0)	0 (0)	0 (0)
Somali	2 (2)	0 (0)	1 (1)	0 (0)	1 (1)	0 (0)	1 (1)	0 (0)	0 (0)	0 (0)
Southern Nations, Nationalities, and Peoples	7 (7)	4 (4)	1 (1)	0 (0)	1 (1)	0 (0)	0 (0)	0 (0)	0 (0)	0 (0)
Tigray	3 (3)	1 (1)	1 (1)	0 (0)	1 (1)	0 (0)	0 (0)	0 (0)	0 (0)	0 (0)
Kenya	186 (187)	72 (73)	36 (36)	0 (0)	32 (32)	0 (0)	19 (19)	0 (0)	10 (10)	0 (0)
Baringo	4 (4)	3 (3)	0 (0)	0 (0)	0 (0)	0 (0)	0 (0)	0 (0)	0 (0)	0 (0)
Bomet	3 (3)	1 (1)	1 (1)	0 (0)	1 (1)	0 (0)	1 (1)	0 (0)	0 (0)	0 (0)
Bungoma	4 (4)	1 (1)	1 (1)	0 (0)	1 (1)	0 (0)	1 (1)	0 (0)	0 (0)	0 (0)
Busia	4 (4)	0 (0)	1 (1)	0 (0)	1 (1)	0 (0)	1 (1)	0 (0)	0 (0)	0 (0)
Elgeyo Marakwet	4 (4)	2 (2)	0 (0)	0 (0)	0 (0)	0 (0)	0 (0)	0 (0)	0 (0)	0 (0)
Embu	4 (4)	2 (2)	0 (0)	0 (0)	0 (0)	0 (0)	0 (0)	0 (0)	0 (0)	0 (0)
Garissa	3 (3)	0 (0)	2 (2)	0 (0)	2 (2)	0 (0)	1 (1)	0 (0)	1 (1)	0 (0)
Homa Bay	4 (4)	0 (0)	4 (4)	0 (0)	4 (4)	0 (0)	2 (2)	0 (0)	2 (2)	0 (0)
Isiolo	3 (3)	2 (2)	0 (0)	0 (0)	0 (0)	0 (0)	0 (0)	0 (0)	0 (0)	0 (0)
Kajiado	4 (4)	2 (2)	0 (0)	0 (0)	0 (0)	0 (0)	0 (0)	0 (0)	0 (0)	0 (0)
Kakamega	4 (4)	1 (1)	1 (1)	0 (0)	1 (1)	0 (0)	1 (1)	0 (0)	0 (0)	0 (0)
Kericho	4 (4)	2 (2)	0 (0)	0 (0)	0 (0)	0 (0)	0 (0)	0 (0)	0 (0)	0 (0)

Kiambu	5 (5)	3 (3)	1 (1)	0 (0)	1 (1)	0 (0)	1 (1)	0 (0)	1 (1)	0 (0)
Kilifi	5 (5)	2 (2)	0 (0)	0 (0)	0 (0)	0 (0)	0 (0)	0 (0)	0 (0)	0 (0)
Kirinyaga	4 (4)	3 (3)	1 (1)	0 (0)	1 (1)	0 (0)	0 (0)	0 (0)	0 (0)	0 (0)
Kisii	4 (4)	0 (0)	0 (0)	0 (0)	0 (0)	0 (0)	0 (0)	0 (0)	0 (0)	0 (0)
Kisumu	4 (4)	2 (2)	1 (1)	0 (0)	1 (1)	0 (0)	0 (0)	0 (0)	0 (0)	0 (0)
Kitui	4 (4)	3 (3)	0 (0)	0 (0)	0 (0)	0 (0)	0 (0)	0 (0)	0 (0)	0 (0)
Kwale	4 (4)	0 (0)	2 (2)	0 (0)	2 (2)	0 (0)	1 (1)	0 (0)	0 (0)	0 (0)
Laikipia	4 (4)	3 (3)	0 (0)	0 (0)	0 (0)	0 (0)	0 (0)	0 (0)	0 (0)	0 (0)
Lamu	3 (3)	2 (2)	1 (1)	0 (0)	0 (0)	0 (0)	0 (0)	0 (0)	0 (0)	0 (0)
Machakos	4 (4)	1 (1)	1 (1)	0 (0)	1 (1)	0 (0)	1 (1)	0 (0)	1 (1)	0 (0)
Makueni	3 (3)	0 (0)	0 (0)	0 (0)	0 (0)	0 (0)	0 (0)	0 (0)	0 (0)	0 (0)
Mandera	3 (3)	0 (0)	1 (1)	0 (0)	0 (0)	0 (0)	0 (0)	0 (0)	0 (0)	0 (0)
Marsabit	3 (3)	1 (1)	0 (0)	0 (0)	0 (0)	0 (0)	0 (0)	0 (0)	0 (0)	0 (0)
Meru	3 (3)	1 (1)	1 (1)	0 (0)	1 (1)	0 (0)	0 (0)	0 (0)	0 (0)	0 (0)
Migori	3 (3)	0 (0)	1 (1)	0 (0)	1 (1)	0 (0)	1 (1)	0 (0)	1 (1)	0 (0)
Mombasa	5 (5)	3 (3)	0 (0)	0 (0)	0 (0)	0 (0)	0 (0)	0 (0)	0 (0)	0 (0)
Murang'a	4 (4)	2 (2)	1 (1)	0 (0)	1 (1)	0 (0)	0 (0)	0 (0)	0 (0)	0 (0)
Nairobi	5 (5)	0 (0)	1 (1)	0 (0)	1 (1)	0 (0)	1 (1)	0 (0)	0 (0)	0 (0)
Nakuru	4 (4)	0 (0)	3 (3)	0 (0)	2 (2)	0 (0)	1 (1)	0 (0)	0 (0)	0 (0)
Nandi	4 (4)	2 (2)	1 (1)	0 (0)	0 (0)	0 (0)	0 (0)	0 (0)	0 (0)	0 (0)
Narok	4 (4)	3 (3)	0 (0)	0 (0)	0 (0)	0 (0)	0 (0)	0 (0)	0 (0)	0 (0)
Nyamira	0 (0)	0 (0)	0 (0)	0 (0)	0 (0)	0 (0)	0 (0)	0 (0)	0 (0)	0 (0)
Nyandarua	4 (4)	3 (3)	0 (0)	0 (0)	0 (0)	0 (0)	0 (0)	0 (0)	0 (0)	0 (0)
Nyeri	5 (6)	2 (3)	0 (0)	0 (0)	0 (0)	0 (0)	0 (0)	0 (0)	0 (0)	0 (0)
Samburu	3 (3)	2 (2)	0 (0)	0 (0)	0 (0)	0 (0)	0 (0)	0 (0)	0 (0)	0 (0)
Siaya	4 (4)	0 (0)	2 (2)	0 (0)	2 (2)	0 (0)	1 (1)	0 (0)	1 (1)	0 (0)
Taita Taveta	4 (4)	1 (1)	0 (0)	0 (0)	0 (0)	0 (0)	0 (0)	0 (0)	0 (0)	0 (0)
Tana River	3 (3)	1 (1)	1 (1)	0 (0)	1 (1)	0 (0)	1 (1)	0 (0)	0 (0)	0 (0)
Tharaka Nithi	3 (3)	2 (2)	1 (1)	0 (0)	1 (1)	0 (0)	1 (1)	0 (0)	0 (0)	0 (0)
Trans Nzoia	4 (4)	2 (2)	0 (0)	0 (0)	0 (0)	0 (0)	0 (0)	0 (0)	0 (0)	0 (0)
Turkana	3 (3)	1 (1)	1 (1)	0 (0)	1 (1)	0 (0)	1 (1)	0 (0)	1 (1)	0 (0)
Uasin Gishu	4 (4)	2 (2)	1 (1)	0 (0)	1 (1)	0 (0)	0 (0)	0 (0)	0 (0)	0 (0)
Vihiga	3 (3)	1 (1)	1 (1)	0 (0)	1 (1)	0 (0)	1 (1)	0 (0)	1 (1)	0 (0)
Wajir	3 (3)	2 (2)	0 (0)	0 (0)	0 (0)	0 (0)	0 (0)	0 (0)	0 (0)	0 (0)
West Pokot	4 (4)	2 (2)	1 (1)	0 (0)	1 (1)	0 (0)	1 (1)	0 (0)	1 (1)	0 (0)
Madagascar	3 (3)	0 (0)	0 (0)	0 (0)	0 (0)	0 (0)	0 (0)	0 (0)	0 (0)	0 (0)

Malawi	8 (8)	1 (1)	0 (0)	0 (0)	0 (0)	0 (0)	0 (0)	0 (0)	0 (0)	0 (0)
Mozambique	3 (3)	0 (0)	0 (0)	0 (0)	0 (0)	0 (0)	0 (0)	0 (0)	0 (0)	0 (0)
Rwanda	10 (10)	0 (0)	0 (0)	0 (0)	0 (0)	0 (0)	0 (0)	0 (0)	0 (0)	0 (0)
Somalia	0 (0)	0 (0)	0 (0)	0 (0)	0 (0)	0 (0)	0 (0)	0 (0)	0 (0)	0 (0)
South Sudan	0 (0)	0 (0)	0 (0)	0 (0)	0 (0)	0 (0)	0 (0)	0 (0)	0 (0)	0 (0)
Uganda	24 (24)	10 (10)	5 (5)	0 (0)	2 (2)	0 (0)	1 (1)	0 (0)	0 (0)	0 (0)
Tanzania	14 (16)	6 (8)	0 (0)	0 (0)	0 (0)	0 (0)	0 (0)	0 (0)	0 (0)	0 (0)
Zambia	15 (15)	10 (10)	0 (0)	0 (0)	0 (0)	0 (0)	0 (0)	0 (0)	0 (0)	0 (0)
**Southern sub-Saharan Africa**	**345 (583)**	**37 (68)**	**100 (180)**	**84 (156)**	**89 (165)**	**77 (147)**	**55 (91)**	**50 (82)**	**28 (47)**	**23 (37)**
Botswana	26 (26)	1 (1)	7 (7)	0 (0)	5 (5)	0 (0)	1 (1)	0 (0)	0 (0)	0 (0)
Eswatini	1 (1)	0 (0)	0 (0)	0 (0)	0 (0)	0 (0)	0 (0)	0 (0)	0 (0)	0 (0)
Lesotho	2 (2)	0 (0)	0 (0)	0 (0)	0 (0)	0 (0)	0 (0)	0 (0)	0 (0)	0 (0)
Namibia	2 (2)	0 (0)	0 (0)	0 (0)	0 (0)	0 (0)	0 (0)	0 (0)	0 (0)	0 (0)
South Africa	305 (543)	35 (66)	92 (172)	84 (156)	83 (159)	77 (147)	54 (90)	50 (82)	28 (47)	23 (37)
Eastern Cape	26 (49)	3 (6)	16 (27)	13 (21)	11 (21)	9 (17)	9 (17)	8 (15)	6 (8)	6 (8)
Free State	26 (49)	3 (6)	8 (16)	8 (16)	8 (15)	8 (15)	2 (4)	0 (0)	1 (2)	0 (0)
Gauteng	28 (51)	5 (8)	8 (16)	8 (16)	8 (16)	8 (16)	6 (10)	6 (10)	1 (2)	0 (0)
KwaZulu-Natal	26 (49)	3 (6)	9 (17)	9 (17)	9 (17)	9 (17)	9 (12)	9 (12)	4 (5)	4 (5)
Limpopo	28 (51)	4 (7)	14 (26)	10 (18)	12 (23)	9 (17)	9 (17)	9 (17)	8 (15)	8 (15)
Mpumalanga	27 (50)	3 (6)	8 (16)	8 (16)	8 (16)	8 (16)	2 (3)	2 (3)	1 (2)	0 (0)
North West	26 (49)	3 (6)	8 (16)	8 (16)	8 (16)	8 (16)	5 (9)	5 (9)	1 (2)	0 (0)
Northern Cape	26 (49)	3 (6)	8 (16)	8 (16)	8 (16)	8 (16)	7 (11)	7 (11)	4 (7)	4 (7)
Western Cape	29 (53)	4 (8)	1 (2)	0 (0)	1 (2)	0 (0)	1 (2)	0 (0)	1 (2)	0 (0)
Zimbabwe	9 (9)	1 (1)	1 (1)	0 (0)	1 (1)	0 (0)	0 (0)	0 (0)	0 (0)	0 (0)
**Western sub-Saharan Africa**	**287 (288)**	**75 (75)**	**65 (65)**	**2 (2)**	**54 (54)**	**2 (2)**	**35 (35)**	**1 (1)**	**24 (24)**	**0 (0)**
Benin	17 (17)	1 (1)	2 (2)	2 (2)	2 (2)	2 (2)	1 (1)	1 (1)	0 (0)	0 (0)
Burkina Faso	4 (4)	0 (0)	0 (0)	0 (0)	0 (0)	0 (0)	0 (0)	0 (0)	0 (0)	0 (0)
Cabo Verde	5 (5)	0 (0)	0 (0)	0 (0)	0 (0)	0 (0)	0 (0)	0 (0)	0 (0)	0 (0)
Cameroon	4 (4)	2 (2)	1 (1)	0 (0)	0 (0)	0 (0)	0 (0)	0 (0)	0 (0)	0 (0)
Chad	0 (0)	0 (0)	0 (0)	0 (0)	0 (0)	0 (0)	0 (0)	0 (0)	0 (0)	0 (0)
Côte d'Ivoire	2 (2)	1 (1)	0 (0)	0 (0)	0 (0)	0 (0)	0 (0)	0 (0)	0 (0)	0 (0)
The Gambia	8 (8)	2 (2)	0 (0)	0 (0)	0 (0)	0 (0)	0 (0)	0 (0)	0 (0)	0 (0)
Ghana	22 (22)	4 (4)	1 (1)	0 (0)	0 (0)	0 (0)	0 (0)	0 (0)	0 (0)	0 (0)
Guinea	3 (3)	1 (1)	0 (0)	0 (0)	0 (0)	0 (0)	0 (0)	0 (0)	0 (0)	0 (0)
Guinea-Bissau	0 (0)	0 (0)	0 (0)	0 (0)	0 (0)	0 (0)	0 (0)	0 (0)	0 (0)	0 (0)
Liberia	5 (5)	0 (0)	4 (4)	0 (0)	4 (4)	0 (0)	0 (0)	0 (0)	0 (0)	0 (0)

Mali	6 (6)	0 (0)	3 (3)	0 (0)	1 (1)	0 (0)	1 (1)	0 (0)	0 (0)	0 (0)
Mauritania	2 (2)	0 (0)	0 (0)	0 (0)	0 (0)	0 (0)	0 (0)	0 (0)	0 (0)	0 (0)
Niger	11 (11)	2 (2)	1 (1)	0 (0)	0 (0)	0 (0)	0 (0)	0 (0)	0 (0)	0 (0)
Nigeria	151 (151)	39 (39)	50 (50)	0 (0)	44 (44)	0 (0)	32 (32)	0 (0)	23 (23)	0 (0)
Abia	4 (4)	1 (1)	0 (0)	0 (0)	0 (0)	0 (0)	0 (0)	0 (0)	0 (0)	0 (0)
Adamawa	4 (4)	1 (1)	1 (1)	0 (0)	1 (1)	0 (0)	1 (1)	0 (0)	1 (1)	0 (0)
Akwa Ibom	4 (4)	1 (1)	2 (2)	0 (0)	2 (2)	0 (0)	1 (1)	0 (0)	0 (0)	0 (0)
Anambra	4 (4)	1 (1)	1 (1)	0 (0)	1 (1)	0 (0)	1 (1)	0 (0)	1 (1)	0 (0)
Bauchi	3 (3)	0 (0)	1 (1)	0 (0)	1 (1)	0 (0)	1 (1)	0 (0)	0 (0)	0 (0)
Bayelsa	4 (4)	2 (2)	1 (1)	0 (0)	1 (1)	0 (0)	1 (1)	0 (0)	1 (1)	0 (0)
Benue	4 (4)	1 (1)	0 (0)	0 (0)	0 (0)	0 (0)	0 (0)	0 (0)	0 (0)	0 (0)
Borno	4 (4)	0 (0)	2 (2)	0 (0)	1 (1)	0 (0)	1 (1)	0 (0)	1 (1)	0 (0)
Cross River	3 (3)	0 (0)	1 (1)	0 (0)	1 (1)	0 (0)	0 (0)	0 (0)	0 (0)	0 (0)
Delta	4 (4)	1 (1)	1 (1)	0 (0)	1 (1)	0 (0)	1 (1)	0 (0)	1 (1)	0 (0)
Ebonyi	4 (4)	1 (1)	1 (1)	0 (0)	1 (1)	0 (0)	1 (1)	0 (0)	0 (0)	0 (0)
Edo	5 (5)	3 (3)	0 (0)	0 (0)	0 (0)	0 (0)	0 (0)	0 (0)	0 (0)	0 (0)
Ekiti	4 (4)	3 (3)	1 (1)	0 (0)	1 (1)	0 (0)	1 (1)	0 (0)	1 (1)	0 (0)
Enugu	5 (5)	2 (2)	1 (1)	0 (0)	1 (1)	0 (0)	0 (0)	0 (0)	0 (0)	0 (0)
FCT (Abuja)	4 (4)	1 (1)	1 (1)	0 (0)	1 (1)	0 (0)	0 (0)	0 (0)	0 (0)	0 (0)
Gombe	3 (3)	0 (0)	1 (1)	0 (0)	1 (1)	0 (0)	1 (1)	0 (0)	1 (1)	0 (0)
Imo	4 (4)	1 (1)	1 (1)	0 (0)	1 (1)	0 (0)	0 (0)	0 (0)	0 (0)	0 (0)
Jigawa	5 (5)	1 (1)	3 (3)	0 (0)	2 (2)	0 (0)	1 (1)	0 (0)	1 (1)	0 (0)
Kaduna	4 (4)	1 (1)	1 (1)	0 (0)	0 (0)	0 (0)	0 (0)	0 (0)	0 (0)	0 (0)
Kano	4 (4)	1 (1)	2 (2)	0 (0)	2 (2)	0 (0)	1 (1)	0 (0)	0 (0)	0 (0)
Katsina	5 (5)	1 (1)	3 (3)	0 (0)	3 (3)	0 (0)	3 (3)	0 (0)	2 (2)	0 (0)
Kebbi	4 (4)	1 (1)	2 (2)	0 (0)	2 (2)	0 (0)	1 (1)	0 (0)	1 (1)	0 (0)
Kogi	4 (4)	2 (2)	1 (1)	0 (0)	1 (1)	0 (0)	1 (1)	0 (0)	1 (1)	0 (0)
Kwara	4 (4)	2 (2)	2 (2)	0 (0)	2 (2)	0 (0)	2 (2)	0 (0)	2 (2)	0 (0)
Lagos	3 (3)	0 (0)	1 (1)	0 (0)	1 (1)	0 (0)	1 (1)	0 (0)	0 (0)	0 (0)
Nasarawa	4 (4)	1 (1)	2 (2)	0 (0)	1 (1)	0 (0)	1 (1)	0 (0)	1 (1)	0 (0)
Niger	3 (3)	0 (0)	2 (2)	0 (0)	2 (2)	0 (0)	1 (1)	0 (0)	1 (1)	0 (0)
Ogun	3 (3)	0 (0)	2 (2)	0 (0)	2 (2)	0 (0)	2 (2)	0 (0)	1 (1)	0 (0)
Ondo	3 (3)	1 (1)	1 (1)	0 (0)	1 (1)	0 (0)	1 (1)	0 (0)	0 (0)	0 (0)
Osun	4 (4)	2 (2)	2 (2)	0 (0)	2 (2)	0 (0)	2 (2)	0 (0)	2 (2)	0 (0)
Oyo	4 (4)	1 (1)	2 (2)	0 (0)	1 (1)	0 (0)	0 (0)	0 (0)	0 (0)	0 (0)
Plateau	5 (5)	0 (0)	3 (3)	0 (0)	3 (3)	0 (0)	2 (2)	0 (0)	2 (2)	0 (0)

Rivers	5 (5)	2 (2)	2 (2)	0 (0)	1 (1)	0 (0)	1 (1)	0 (0)	1 (1)	0 (0)
Sokoto	4 (4)	0 (0)	1 (1)	0 (0)	1 (1)	0 (0)	1 (1)	0 (0)	1 (1)	0 (0)
Taraba	4 (4)	1 (1)	0 (0)	0 (0)	0 (0)	0 (0)	0 (0)	0 (0)	0 (0)	0 (0)
Yobe	4 (4)	1 (1)	0 (0)	0 (0)	0 (0)	0 (0)	0 (0)	0 (0)	0 (0)	0 (0)
Zamfara	4 (4)	1 (1)	1 (1)	0 (0)	1 (1)	0 (0)	1 (1)	0 (0)	0 (0)	0 (0)
São Tomé and Príncipe	0 (0)	0 (0)	0 (0)	0 (0)	0 (0)	0 (0)	0 (0)	0 (0)	0 (0)	0 (0)
Senegal	15 (16)	2 (2)	0 (0)	0 (0)	0 (0)	0 (0)	0 (0)	0 (0)	0 (0)	0 (0)
Sierra Leone	19 (19)	14 (14)	3 (3)	0 (0)	3 (3)	0 (0)	1 (1)	0 (0)	1 (1)	0 (0)
Togo	6 (6)	0 (0)	0 (0)	0 (0)	0 (0)	0 (0)	0 (0)	0 (0)	0 (0)	0 (0)

5.5 Crosswalking all non-reference data to the reference threshold

Since there were a variety of thresholds included in the dataset, it was important we standardised the data by adjusting it to a single reference threshold prior to using it in the model. This adjustment was done through a process called crosswalking where we utilised meta-regression—Bayesian, regularised, trimmed (MR-BRT), a flexible network meta-regression tool based on LimeTr.[15] It was first developed for GBD 2019 and has been extensively used in quantifying the statistical relationships and corresponding uncertainty between reference (i.e gold standard) data and other data that is non-standard on one or more dimensions such as threshold, diagnostic technology, or sampling strategy.

We ran two iterations of a MR-BRT network meta-analysis to standardise our input data to two reference thresholds: 1) ≥20 weeks' gestation and 2) ≥28 weeks' gestation. This was performed in six steps.

First, all non-outliered data were matched between reference and non-reference thresholds for the exact same location and exact same year both "within-study" (both points came from the same source) and "between-study" (points came from two unique sources). All of these matches are termed "direct comparisons". Second, to increase the empirical bases of the dataset, we also matched both within- and between-study to generate pairs of non-reference thresholds (eg, 500 grams to 1000 grams). All of these matches are termed "indirect comparisons". Deduplication was performed to ensure none of the indirect matches were inverted repeats of each other.

There were a total of 11,940 matches for each threshold (6932 within-study matches and 5008 between-study matches). As shown in Appendix 1 Table S5, there were 4751 direct comparisons (3831 within-study matches and 920 between-study matches) for the ≥20 weeks threshold and 7349 direct comparisons (4359 within-study matches and 2990 between-study matches) for the ≥28 weeks threshold. As shown in Appendix 1 Table S6, there were also 7189 (3101 within-study matches and 4088 between-study matches) and 4591 indirect comparisons (2573 within-study matches and 2018 between-study matches) for the ≥20 weeks and ≥28 weeks thresholds, respectively.

5.5.1 Appendix 1 Table S6: Number of direct comparisons to each reference threshold for crosswalking

```
Caption: This table depicts the number of direct matches that were made when
comparing data with the reference threshold (20 or 28 weeks gestation) to
data with other thresholds for crosswalking. The matches were either from the
same source (within-study) or across different sources (between-study).
```

Threshold	Within–Study (>=20 weeks)	Between–Study (>=20 weeks)	Within–Study (>=28 weeks)	Between–Study (>=28 weeks)
20 weeks	Reference	Reference	2994	386
22 weeks	194	85	621	631
24 weeks	196	80	242	161
26 weeks	189	74	182	148
28 weeks	2994	386	Reference	Reference
500 grams	109	96	128	169
1000 grams	133	140	120	1153
22 weeks OR 500 grams	0	36	0	186
28 weeks OR 1000 grams	6	6	0	31
22 weeks AND 500 grams	5	4	30	46
28 weeks AND 1000 grams	5	13	42	79
Total	3831	920	4359	2990

5.5.2 Appendix 1 Table S7: Number of indirect comparisons to each reference threshold for crosswalking

```
Caption: This table depicts the number of indirect matches that were made
when comparing data for crosswalking. The matches were either from the same
source (within-study) or across different sources (between-study).
```

Threshold	Within–Study (>=20 weeks)	Between–Study (>=20 weeks)	Within–Study (>=28 weeks)	Between–Study (>=28 weeks)
20 weeks	Reference	Reference	133	140
22 weeks	144	404	338	489
24 weeks	291	162	487	242
26 weeks	482	156	671	230
28 weeks	1195	2325	Reference	Reference
500 grams	762	418	743	345
1000 grams	0	0	0	0
22 weeks OR 500 grams	10	312	10	348
28 weeks OR 1000 grams	7	84	13	59
22 weeks AND 500 grams	51	61	56	65
28 weeks AND 1000 grams	159	166	122	100
Total	3101	4088	2573	2018

Third, for each match, we calculated the ratio of SBR/NMR for the non-reference to reference thresholds and then natural-log transformed that ratio, calculating the standard error of the ratio using the delta method.[16] All log-transformed ratios for within- and between-study matches for both direct and indirect comparisons were data inputs to the MR-BRT models.

Fourth, we specified a set of ordinal constraints to ensure that MR-BRT outputs were consistent with what is biologically known about the relationships between gestational ages and birthweights, including:

- Gestational age
  - 28 weeks ≤ 26 weeks ≤ 24 weeks ≤ 22 weeks ≤ 20 weeks
  - 22 weeks AND 500 grams ≤ 22 weeks ≤ 22 weeks OR 500 grams
  - 28 weeks AND 1000 grams ≤ 28 weeks ≤ 28 weeks OR 1000 grams
- Birthweight
  - 1000 grams ≤ 500 grams

- 22 weeks AND 500 grams ≤ 500 grams ≤ 22 weeks OR 500 grams
- 28 weeks AND 1000 grams ≤ 1000 grams ≤ 28 weeks OR 1000 grams

Fifth, we tested and found that the relationship between non-reference and reference thresholds in MR-BRT varied as a function of the summary exposure value (SEV) for short gestation for birthweight estimates from GBD 2019.[17] The addition of the SEV value as a fixed effect took gestational age into account, with higher SEV values corresponding to higher rates of preterm births. MR-BRT output betas (intercepts and SEV slopes, including gamma) are shown in Appendix 1 Table S8 and dose response plots showing the ratios of non-reference to reference as a function of short gestation for birthweight SEV are shown in Appendix 1 Figure S4.

Sixth, each non-reference observation was adjusted using the beta corresponding to that threshold, matched on the short gestation for birthweight SEV from the location-year of the source. Uncertainty was propagated again using Wilson's interval.

5.5.3 Appendix 1 Table S8: Beta coefficients from crosswalking comparing non-reference data to the reference threshold

```
Caption: This table shows the beta coefficients and 95% UIs that are output
when crosswalking for both the 20 and 28 week gestational age thresholds and
including a fixed effect for short gestation SEV. SEV=summary exposure value.
```

Threshold	Intercept (20 weeks)	SEV (20 weeks)	Intercept (28 weeks)	SEV (28 weeks)
20 weeks	0 (0 to 0)	0 (0 to 0)	0·16 (0·097 to 0·222)	1·494 (1·167 to 1·82)
22 weeks	0·008 (–0·079 to 0·095)	–0·341 (–0·832 to 0·15)	0·165 (0·096 to 0·235)	1·165 (0·763 to 1·566)
22 weeks AND 500 grams	0·064 (–0·201 to 0·328)	–1·804 (–3·589 to –0·019)	0·18 (–0·078 to 0·439)	0 (–1·762 to 1·762)
22 weeks OR 500 grams	0·008 (–0·123 to 0·138)	–0·341 (–1·278 to 0·596)	0·165 (0·045 to 0·286)	1·165 (0·269 to 2·061)
500 grams	0·037 (–0·131 to 0·206)	–0·927 (–1·827 to –0·027)	0·195 (0·036 to 0·354)	0·579 (–0·27 to 1·427)
24 weeks	–0·11 (–0·234 to 0·015)	–0·378 (–1·088 to 0·331)	0·047 (–0·069 to 0·164)	1·133 (0·466 to 1·8)
26 weeks	–0·104 (–0·234 to 0·026)	–1·067 (–1·829 to –0·306)	0·053 (–0·069 to 0·176)	0·441 (–0·287 to 1·168)
28 weeks	–0·16 (–0·223 to –0·098)	–1·49 (–1·817 to –1·163)	0 (0 to 0)	0 (0 to 0)
28 weeks AND 1000 grams	–0·27 (–0·622 to 0·082)	–2·266 (–4·536 to 0·005)	–0·222 (–0·57 to 0·126)	0 (–2·253 to 2·253)
28 weeks OR 1000 grams	–0·181 (–0·934 to 0·572)	–1·075 (–5·236 to 3·086)	–0·022 (–0·773 to 0·729)	0·431 (–3·72 to 4·583)
1000 grams	–0·344 (–0·431 to –0·257)	–0·8 (–1·284 to –0·316)	–0·188 (–0·254 to –0·122)	0·712 (0·33 to 1·094)

5.5.4 Appendix 1 Figure S4: Dose response plots from crosswalking (≥20 weeks threshold)

```
Caption: (A) This plot shows the adjustment of data from the ≥22 weeks
threshold to the ≥20 weeks threshold. (B) This plot shows the adjustment of
data from the ≥24 weeks threshold to the ≥20 weeks threshold. (C) This plot
shows the adjustment of data from the ≥26 weeks threshold to the ≥20 weeks
threshold. (D) This plot shows the adjustment of data from the ≥28 weeks
threshold to the ≥20 weeks threshold. (E) This plot shows the adjustment of
data from the ≥500 grams threshold to the ≥20 weeks threshold. (F) This plot
shows the adjustment of data from the ≥1000 grams threshold to the ≥20 weeks
threshold. (G) This plot shows the adjustment of data from the ≥22 weeks AND
≥500 grams threshold to the ≥20 weeks threshold. (H) This plot shows the
adjustment of data from the ≥28 weeks AND ≥1000 grams threshold to the ≥20
```

weeks threshold. (I) This plot shows the adjustment of data from the ≥22 weeks OR ≥500 grams threshold to the ≥20 weeks threshold. (J) This plot shows the adjustment of data from the ≥28 weeks OR ≥1000 grams threshold to the ≥20 weeks threshold.

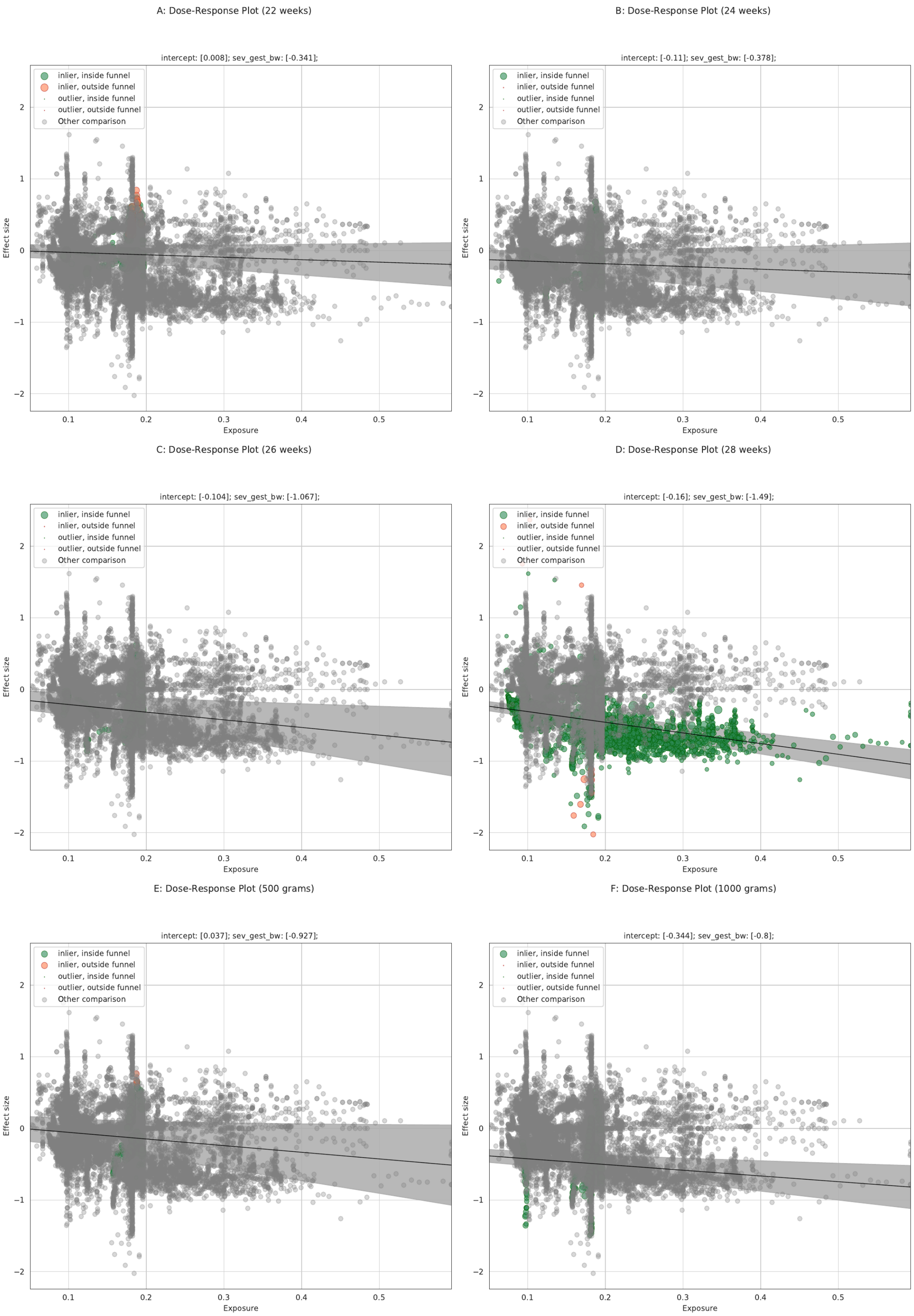
A: Dose-Response Plot (22 weeks)
intercept: [0.008]; sev_gest_bw: [-0.341];
B: Dose-Response Plot (24 weeks)
intercept: [-0.11]; sev_gest_bw: [-0.378];
C: Dose-Response Plot (26 weeks)
intercept: [-0.104]; sev_gest_bw: [-1.067];
D: Dose-Response Plot (28 weeks)
intercept: [-0.16]; sev_gest_bw: [-1.49];
E: Dose-Response Plot (500 grams)
intercept: [0.037]; sev_gest_bw: [-0.927];
F: Dose-Response Plot (1000 grams)
intercept: [-0.344]; sev_gest_bw: [-0.8];
inlier, inside funnel
inlier, outside funnel
outlier, inside funnel
outlier, outside funnel
Other comparison
Effect size
Exposure

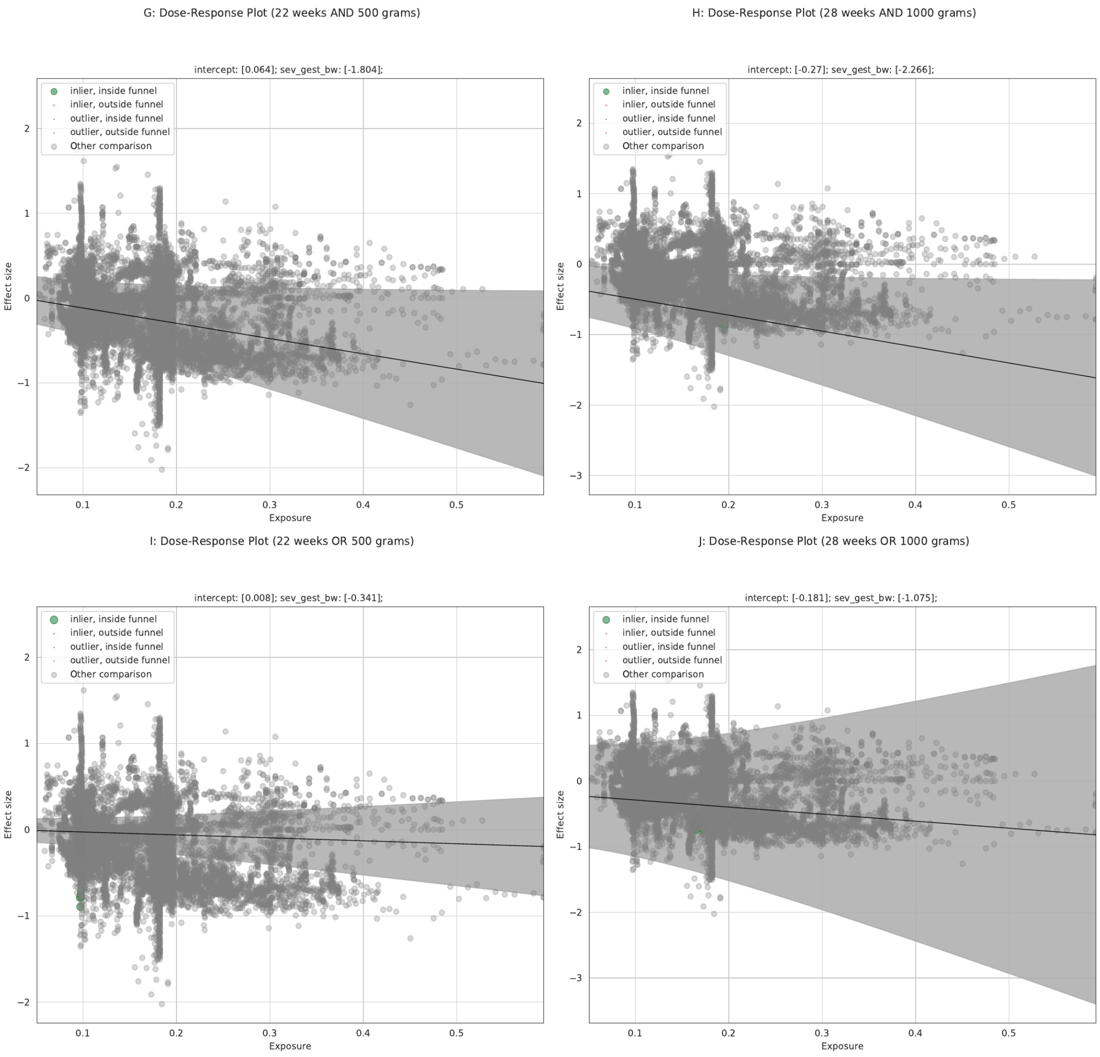

5.6 Outlier criteria (round 2)

After crosswalking, we again applied outlier criterion 1, 2, 3, and 6, described above, for consistency. Any location-year-source with an adjusted data point that met one of the outliering criteria was outliered in both datasets (≥20 weeks and ≥28 weeks), even if it only qualified as an outlier by a criterion for one of the two thresholds. This ensured the same data points were used in the model estimating stillbirth after 20 weeks' gestation and the model estimating stillbirth after 28 weeks' gestation.

We also performed prioritisation by location-year-threshold for data points from vital registration or vital statistics reports. The data we extracted directly from a data source released by a country-specific provider had the highest level of priority and then, in order of priority, we kept data from DYB or HFA-DB so only one vital registration data point for each location-year-threshold was included in our final dataset. In total, after this second round of outliering and prioritisation, 15,547 data points informed our model, covering 9519 location-years.

5.7 Data Coverage Visuals (Post-Outliering)

5.7.1 Appendix 1 Figure S5A: Map of the number of years of data included in the model by location

Caption: (A) The map shows the number of years of stillbirth data included in the stillbirth model, indicated by colour.

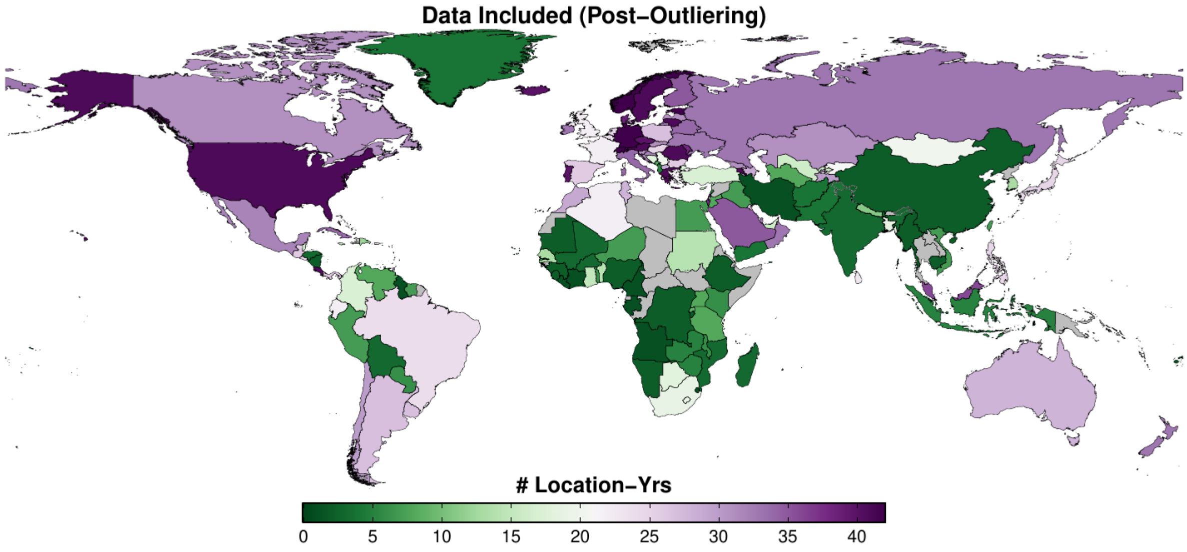

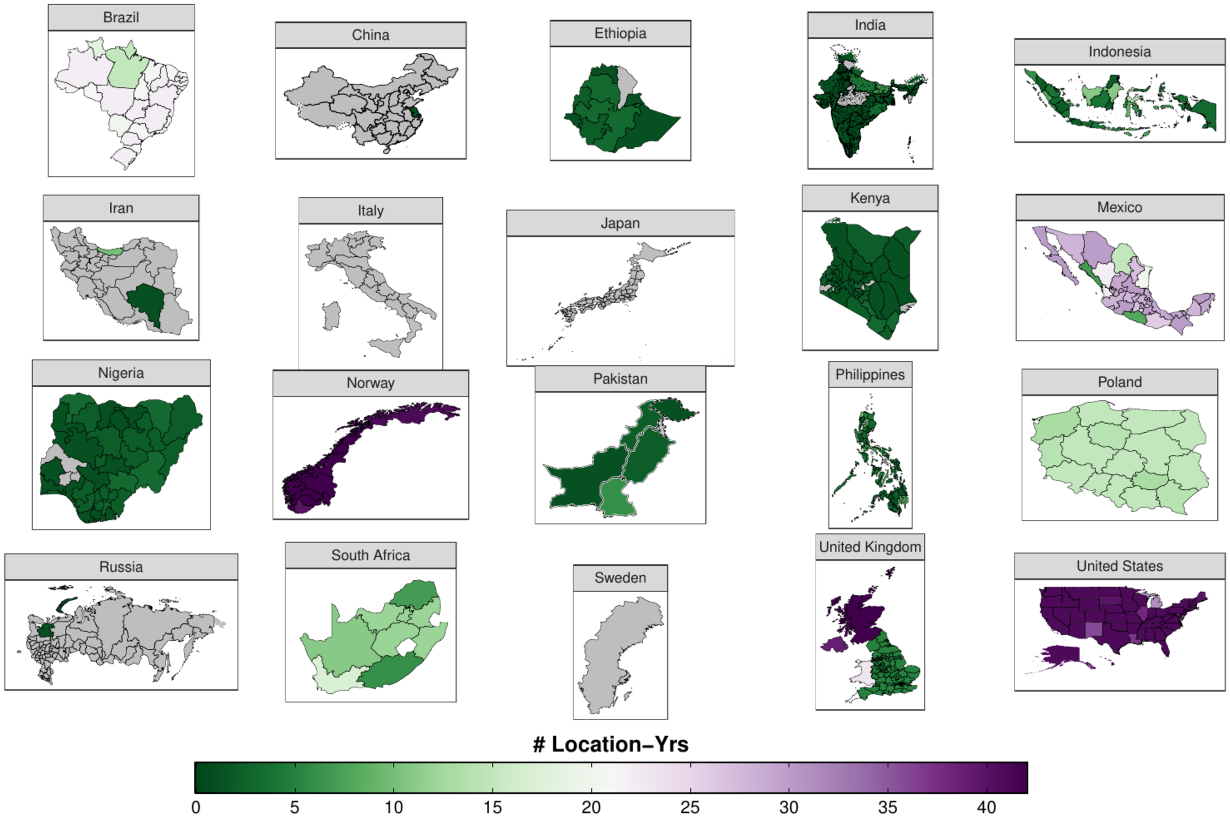

5.7.2 Appendix 1 Figure S5B: Map of the percentage of data outliered for each location

Caption: (B) The map shows the percentage of stillbirth data outliered prior to modelling, indicated by colour.

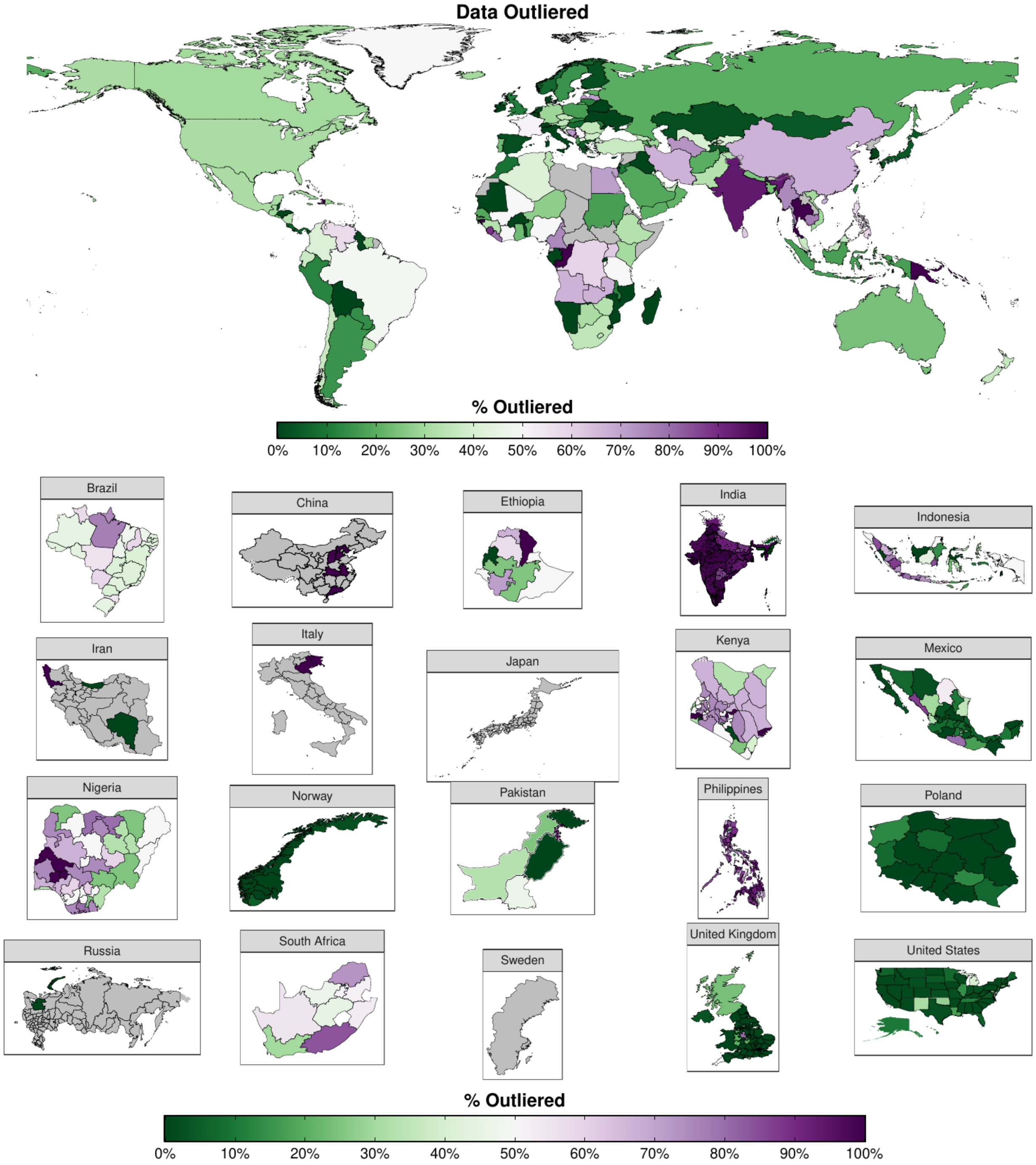

6 Modelling SBR/NMR using Spatiotemporal Gaussian Process Regression (ST-GPR)

To estimate stillbirth for GBD 2021, we implemented a three-stage model. The first stage utilised an ensemble model, the second stage was spatiotemporal (ST) smoothing, and the last stage was Gaussian Process Regression (GPR). The second and third stage are commonly referenced together as ST-GPR which is used across GBD processes to produce consistent time series estimates for locations with 95% uncertainty intervals (UIs).

For more detailed information on modelling with ST-GPR, please see Appendix 1 (sections 2·2·6 and 2·3·4) of the GBD 2019 Demographics Capstone.[14]

6.1 Stage 1: Ensemble model

In GBD 2016, for the stage 1 estimate, a mixed-effects generalised linear model was used to estimate the ratio of stillbirth rate to neonatal mortality rate in natural logarithmic space. The educational attainment among women of reproductive age covariate was included as a fixed effect. The model also included a random effect on smoothed neonatal mortality classified into 20 bins, random intercepts for each location, and data source-specific random effects nested within each location. Dummy variables were set on stillbirth threshold with a reference of ≥28 weeks gestation and data type with a reference of complete vital registration calculated according to child mortality completeness. If complete vital registration was not available, surveys were used as the reference for the data type dummy variable. The model was run using the lme4 package in R. The adjusted data and predictions from stage 1 without source type fixed effects or country source random effects were used as the input prior for stage 2 estimation, spatiotemporal smoothing (ST).

For GBD 2021, we substantially improved upon the approach used in GBD 2016 by implementing an ensemble linear regression model to create stage 1 predictions. We selected 10 potentially predictive covariates from the Global Burden of Disease covariates database. We tested all covariate combinations in linear regression models where our log-transformed SBR/NMR data points were regressed on the covariates with a nested geographical random effect at the super-region, region, and location levels.

For each covariate, a prior on the direction of the covariate's beta value was set. These were based on our hypotheses about how we would expect the covariate to affect the dependent variable of our model, SBR/NMR. If we expected a preferential decrease (ie, SBR improves faster than NMR), the direction was set as negative; if we expected a preferential increase (ie, NMR improves faster than SBR), the direction was set as positive. Therefore, for the maternal preventive care components, we expected a larger effect on stillbirths, and for the other health system components (education, SDI, HAQI), we expected a larger effect on neonatal mortality. Each covariate, its transformation (if applicable), and the direction of its prior are listed below in Appendix 1 Table S9.[18]

6.1.1 Appendix 1 Table S9: Covariates tested in the stage 1 ensemble model

```
Caption: This table lists each covariate tested for incorporation into the
stage 1 ensemble model, its description, any transformation performed on it,
and the directional prior that was specified.
```

Name	Description	Transformation	Direction
antenatal care coverage (1 visit)	proportion of women 15 to 49 years old who had at least one antenatal visit at a health facility during pregnancy	logit	−1
antenatal care coverage (4 visits)	proportion of women 15 to 49 years old who had four or more antenatal visits at a health facility during pregnancy	logit	−1
in−facility delivery	proportion of pregnant women 15 to 49 who delivered in a health facility	logit	−1
skilled birth attendance	proportion of pregnant women 15 to 49 who delivered with a skilled birth attendant (mainly nurses, doctors, midwives)	logit	−1
maternal care and immunisation	measure of health system access estimated using a principal component analysis of antenatal clinics, DTP3 immunisation, measules immunisation, in−facility delivery, and skilled birth attendance		−1
healthcare access and quality index	measure of personal health care access and quality based on risk−standardized mortality rates from causes that, in the presence of high−quality health care, should not result in death		+1
socio−demographic index	measure of development estimated using a principal component analysis of log−transformed lag−distributed income, total fertility rate (under age 25), and education years per capita over age 15		+1
maternal education	mean level of education attainment (among women of reproductive age)		+1
maternal education 12+	proportion of population with at least 12 years of education (among women of reproductive age)		+1
education relative inequality (gini)	average disproportionality of education		+1

Any candidate model containing a covariate combination where all betas were not statistically significant (p-value <0·05) and in the pre-specified direction was dropped from the ensemble candidates. In all, we tested 1024 models but dropped 913 of them after restricting the direction of the beta and checking for statistical significance (p-value <0·05).

The standardised distribution of beta values for the covariates present in the remaining 111 models are shown in Appendix 1 Figure 6. Standardisation was performed by multiplying each beta by the ratio of the standard deviation of the betas from the corresponding covariate and the standard deviation of the SBR/NMR values from the crosswalk dataset. One covariate, the proportion of skilled birth attendance, was not included in any of the final models and thus does not appear in Appendix 1 Figure 6.

6.1.2 Appendix 1 Figure S6: Standardised betas among covariates present in selected models (28 weeks threshold)

```
Caption: The plot shows the standardised betas for the various covariates
tested in the stage 1 ensemble model. The betas for each covariate are shown
and the direction of the prior set on the beta is indicated by colour.
```

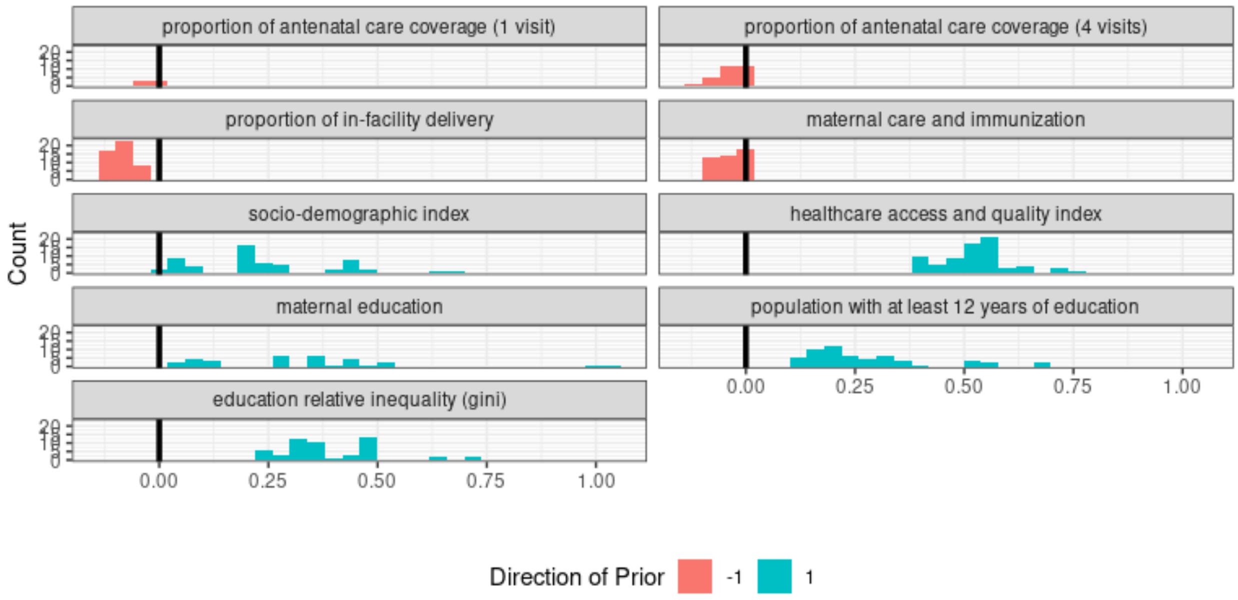

Remaining models were ranked in terms of their out-of-sample root mean square error (RMSE). The 50 top performers weighted by out-of-sample predictive validity were selected as the final set and combined into an ensemble model to produce initial estimates for every location and year combination in the analysis.

6.2 Stage 2: Spatiotemporal smoothing

For the stage 2 model, we used spatiotemporal smoothing (ST) which uses time and space weights to determine smoothness over time and weighting of regional data. By using regional information, we can estimate for locations without data. Hyperparameters were based on data density and selected during expert consultation which assisted with preliminary setting of hyperparameters for fertility estimation for GBD 2017.[19] The thresholds for each parameter are provided in Appendix 1 Table S10. The lambda parameters (λ) are the time weights, and the zeta parameters (ζ) are the space weights. The smoothed residuals from ST are added to the 1st stage prediction to get the 2nd stage prediction.

6.2.1 Appendix 1 Table S10: Hyperparameter values by data density

```
Caption: This table list the three hyperparameters (lambda, zeta, and scale)
which were set based on the data density category for each location.
```

Data Density Category	# Locations	Lambda	Zeta	Scale
0	311	1	0·6	15
1 to 4	299	0·8	0·7	15
5 to 9	193	0·6	0·8	15
10 to 19	63	0·4	0·9	10
20 plus	218	0·2	0·99	5

6.3 Stage 3: Gaussian Process Regression

The final step, Gaussian Process Regression (GPR), incorporates data, data variance, additional hyperparameters, and a prior to generate final draw-level estimates with 95% uncertainty intervals (UIs) for all locations and years in the time series. More specifically, the median absolute deviation (MAD) is used as the non-sampling variance input into GPR. The two additional hyperparameters are scale and amplitude where scale, also assigned based on data density, corresponds to correlation over time and amplitude was fixed at 1. We take 1000 realisations, called draws, from the Gaussian process posterior distribution. These draws are collapsed to estimate the mean and 95% uncertainty interval for each location and year.

6.4 Raking and aggregating settings in ST-GPR

After GPR, scaling or aggregating is performed at the draw level on all locations where subnational locations are also estimated to ensure consistency. In aggregation, the sum of the subnational estimates replaces the national estimate. In scaling, the proportion each subnational estimate contributes to the national estimate remains constant, but the subnational estimates are recalculated so their total is equal to the national estimate. The decision of whether to scale or aggregate was based on data availability at the national and subnational levels. We performed aggregation on 11 countries (Brazil, China, India, Indonesia, Kenya, Mexico, New Zealand, Nigeria, Norway, UK, USA) and scaling on 11 countries (Ethiopia, Iran, Italy, Japan, Pakistan, Philippines, Poland, Russia, South Africa, Sweden, Ukraine).

6.5 Calculation of stillbirth ratio from SBR/NMR

We merged the draw level estimates with live birth counts and neonatal mortality rates. The SBR/NMR estimates were multiplied by NMR to calculate SBR which was then used with live birth counts to produce stillbirth counts. These draw-level stillbirth counts were aggregated to get a final estimate for each location and year. This aggregation started with the most granular locations and aggregated up through each location level, eventually producing regional, super-regional, and global geographic aggregates. SBR was then recalculated for all locations and years from 1990 to 2021.

A complete set of results for each GBD reporting location for SBR/NMR and SBR along with a list of input data sources and model fit are shown in appendix 2.

6.5.1 Uncertainty

In stillbirth estimation, there is uncertainty in input data, variable sample size, crosswalks from non-reference thresholds, and ST-GPR results. The model uncertainty is derived by generating 1000 draws of the ratio of stillbirth rate over neonatal mortality rate for each location and year combination. The means of these draw-level estimates are used as the final estimates; the 95% uncertainty intervals for our estimates are assigned based on the 0·025 and 0·975 quantiles of the draws. Our use of a Bayesian framework where all estimates are generated at draw level and then we take the mean as the best estimate is different from traditional frequentist statistical models where predictions are made based on the standard deviation of coefficients to generate confidence intervals.[20]

Neonatal mortality rates (NMRs) were taken from the GBD life table estimates where uncertainty is included in each step of the mortality estimation process. The methods described in King and colleagues were used and uncertainty was incorporated in the entry parameters (5q0 and 45q15) as well as the coefficients.[21] Also, the model life table process was repeated 1000 times based on random pairs with different standard life tables and coefficients.

6.6 Secondary analysis

6.6.1 Stillbirth ratio vs. SDI (Epi Transition)

The Socio-demographic Index (SDI) is a composite indicator of development, over time and geography, calculated using lag-distributed income (LDI) per capita, mean years of schooling for those ages 15 and older, and total fertility rate (TFR) under the age of 25. The composite SDI is the geometric mean of the three indices for a given location-year, where an SDI of 0 indicates a theoretical minimum level and an SDI of 1 indicates a theoretical maximum level of development relevant to health. Quintiles of SDI were determined using country-level estimates of SDI in 2021 where only countries with populations greater than 1 million were included. For reporting, final SDI values were multiplied by 100. A full description of the methods used to calculate SDI can be found in the GBD 2019 Diseases and Injuries capstone.[22,23]

We employed spline models in MR-BRT to compare this historical relationship between stillbirth rate (SBR) and SDI. Using data from the years 1990 to 2021, we estimated models including an intercept and a cubic spline on SDI to predict the average relationship between development and the outcome of interest, SBR, over time. Models were fit in logit-space to constrain values between zero and one. To avoid bias from extremely small values, an offset of 1*10-7 was used. The splines contained 2 internal knots, placed at the tertiles of observed SDI values. We also included linear tails on the left and right side of each spline to control the behaviour at the extremities. For the outcome of SBR, we used the expected values predicted from the spline models to create aggregate expected values for a given aggregate location, region, super-region, and/or the globe. We calculated observed to

expected (O:E) ratios for each group and aggregate group and calculated the annualised rate of change in the O:E ratio between years y1 and y2 with the following formula:

$$\text{Annualised Rate of Change}_{OE,y1,y2} = \frac{log\left(\frac{OE_{y2}}{OE_{y1}}\right)}{y2 - y1}$$

7 References

1 Wang H, Abajobir AA, Abate KH, *et al.* Global, regional, and national under-5 mortality, adult mortality, age-specific mortality, and life expectancy, 1970: A systematic analysis for the Global Burden of Disease Study 2016. *Lancet* 2017; **390**: 1084–150.

2 Stevens GA, Alkema L, Black RE, *et al.* Guidelines for Accurate and Transparent Health Estimates Reporting: The GATHER statement. *Lancet* 2016; **388**: e19–23.

3 United Nations. World Population Prospects 2015: Data Booklet. United Nations, 2015 DOI:10.18356/9789210014380.

4 Blencowe H, Cousens S, Jassir FB, *et al.* National, regional, and worldwide estimates of stillbirth rates in 2015, with trends from 2000: A systematic analysis. *Lancet Glob Health* 2016; **4**: e98–108.

5 Stillbirth. https://www.who.int/health-topics/stillbirth (accessed Feb 1, 2023).

6 Cousens S, Blencowe H, Stanton C, *et al.* National, regional, and worldwide estimates of stillbirth rates in 2009 with trends since 1995: A systematic analysis. *Lancet* 2011; **377**: 1319–30.

7 Da Silva FT, Gonik B, McMillan M, *et al.* Stillbirth: Case definition and guidelines for data collection, analysis, and presentation of maternal immunization safety data. *Vaccine* 2016; **34**: 6057–68.

8 Atlas of Neonatal Pathology: Stillbirth. https://atlases.muni.cz/atlases/novo/atl_en/novormrtvoroz.html (accessed May 25, 2023).

9 CDC. What is Stillbirth? | CDC. Centers for Disease Control and Prevention. 2022; published online Sept. https://www.cdc.gov/ncbddd/stillbirth/facts.html (accessed Feb 6, 2023).

10 World Health Organization. International Classification of Diseases, Eleventh Revision (ICD-11). 2022. https://icd.who.int/browse11/l-m/en#/http%3a%2f%2fid.who.int%2ficd%2fentity%2f505744734 (accessed July 13, 2023).

11 Stillbirths and stillbirth rates. UNICEF DATA. https://data.unicef.org/topic/child-survival/stillbirths/ (accessed Feb 1, 2023).

12 Global Health Data Exchange | GHDx. https://ghdx.healthdata.org/ (accessed Feb 1, 2023).

13 Schumacher AE, Kyu HH, Aali A, *et al.* Global age-sex-specific mortality, life expectancy, and population estimates in 204 countries and territories and 811 subnational locations, 1950, and the impact of the COVID-19 pandemic: A comprehensive demographic analysis for the Global Burden of Disease Study 2021. *The Lancet* 2024; : S0140673624004768.

14 Wang H, Abbas KM, Abbasifard M, *et al.* Global age-sex-specific fertility, mortality, healthy life expectancy (HALE), and population estimates in 204 countries and territories, 1950: A comprehensive demographic analysis for the Global Burden of Disease Study 2019. *Lancet* 2020; **396**: 1160–203.

15 Zheng P, Barber R, Sorensen RJD, Murray CJL, Aravkin AY. Trimmed constrained mixed effects models: Formulations and algorithms. *Journal of Computational and Graphical Statistics* 2021; **30**: 544–56.

16 Oehlert GW. A note on the delta method. *The American Statistician* 1992; **46**: 27–9.

17 Murray CJL, Aravkin AY, Zheng P, *et al.* Global burden of 87 risk factors in 204 countries and territories, 1990: A systematic analysis for the Global Burden of Disease Study 2019. *Lancet* 2020; **396**: 1223–49.

18 Terms Defined. Institute for Health Metrics and Evaluation. https://www.healthdata.org/terms-defined (accessed May 24, 2023).

19 Murray CJL, Callender CSKH, Kulikoff XR, *et al.* Population and fertility by age and sex for 195 countries and territories, 1950: A systematic analysis for the Global Burden of Disease Study 2017. *Lancet* 2018; **392**: 1995–2051.

20 Gelman A, Carlin JB, Stern HS, Rubin DB. Bayesian Data Analysis, Second Edition. CRC Press, 2003.

21 King G, Tomz M, Wittenberg J. Making the Most of Statistical Analyses: Improving Interpretation and Presentation. *American Journal of Political Science* 2000; **44**: 341–55.

22 Vos T, Lim SS, Abbafati C, *et al.* Global burden of 369 diseases and injuries in 204 countries and territories, 1990: A systematic analysis for the Global Burden of Disease Study 2019. *The Lancet* 2020; **396**: 1204–22.

23 Disease Collaborative Network GB of. Global Burden of Disease Study 2019 (GBD 2019) Socio-Demographic Index (SDI) 1950–2019. 2020. DOI:10.6069/D8QB-JK35.

8 Authorship appendix to “GBD 2021 Global Stillbirths”

8.1 GBD 2021 Global Stillbirths Collaborators

Haley Comfort, Theresa A McHugh, Austin E Schumacher, Ashley Harris, Erin A May, Katherine R Paulson, William M Gardner, John E Fuller, Meghan E Frisch, Heather Jean Taylor, Andrew T Leever, Corey Teply, Nicholas Alexander Verghese, Tahiya Alam, Yohannes Habtegiorgis Abate, Hedayat Abbastabar, Samar Abd ElHafeez, Michael Abdelmasseh, Sherief Abd-Elsalam, Daba Abdissa, Meriem Abdoun, Rizwan Suliankatchi Abdulkader, Mesfin Abebe, Aidin Abedi, Hassan Abidi, Olumide Abiodun, Richard Gyan Aboagye, Hassan Abolhassani, Michael R M Abrigo, Eman Abu-Gharbieh, Niveen ME Abu-Rmeileh, Mesafint Molla Adane, Isaac Yeboah Addo, Bulcha Guye Adema, Miracle Ayomikun Adesina, Charles Oluwaseun Oluwaseun Adetunji, Daniel Adedayo Adeyinka, Qorinah Estiningtyas Sakilah Adnani, Saira Afzal, Suneth Buddhika Agampodi, Antonella Agodi, Williams Agyemang-Duah, Bright Opoku Ahinkorah, Aqeel Ahmad, Danish Ahmad, Ali Ahmadi, Ayman Ahmed, Haroon Ahmed, Luai A Ahmed, Marjan Ajami, Karolina Akinosoglou, Syed Mahfuz Al Hasan, Ziyad Al-Aly, Khurshid Alam, Fahad Mashhour Alanezi, Turki M Alanzi, Mohammed Albashtawy, Sharifullah Alemi, Abdelazeem M Algammal, Adel Ali Saeed Al-Gheethi, Abid Ali, Liaqat Ali, Mohammed Usman Ali, Sheikh Mohammad Alif, Syed Mohamed Aljunid, Joseph Uy Almazan, Hesham M Al-Mekhlafi, Louay Almidani, Sami Almustanyir, Khalid A Altirkawi, Hany Aly, Safwat Aly, Reza Amani, Edward Kwabena Ameyaw, Abebe Feyissa Amhare, Tarek Tawfik Amin, Sohrab Amiri, Catalina Liliana Andrei, Tudorel Andrei, Amir Anoushiravani, Adnan Ansar, Davood Anvari, Razique Anwer, Francis Appiah, Morteza Arab-Zozani, Aleksandr Y Aravkin, Demelash Areda, Brhane Berhe Aregawi, Anton A Artamonov, Umesh Raj Aryal, Zatollah Asemi, Mulu Tiruneh Asemu, Akeza Awealom Asgedom, Tahira Ashraf, Melash Belachew Asresie, Daniel Atlaw, Maha Moh'd Wahbi Atout, Alok Atreya, Madhu Sudhan Atteraya, Avinash Aujayeb, Beatriz Paulina Ayala Quintanilla, Haleh Ayatollahi, Seyed Mohammad Ayyoubzadeh, Sina Azadnajafabad, Rui M S Azevedo, Ahmed Y Azzam, Darshan B B, Mahsa Babaei, Muhammad Badar, Ashish D Badiye, Nayereh Baghcheghi, Soroush Baghdadi, Nasser Bagheri, Sara Bagherieh, Farshad Bahrami Asl, Ruhai Bai, Ravleen Kaur Bakshi, Kiran Bam, Maciej Banach, Aduragbemi Banke-Thomas, Hansi Bansal, Berihun Bantie Bantie, Martina Barchitta, Mainak Bardhan, Azadeh Bashiri, Afisu Basiru, Pritish Baskaran, Kavita Batra, Mojtaba Bayani, Nebiyou Simegnew Bayleyegn, Neeraj Bedi, Tahmina Begum, Amir Hossein Behnoush, Uzma Iqbal Belgaumi, Amiel Nazer C Bermudez, Kebede A Beyene, Bharti Bhandari Bhandari, Dinesh Bhandari, Nikha Bhardwaj, Pankaj Bhardwaj, Sonu Bhaskar, Suraj Bhattarai, Virginia Bodolica, Dejana Braithwaite, Hermann Brenner, Yasser Bustanji, Nadeem Shafique Butt, Zahid A Butt, Abdul Cadri, Ismael Campos-Nonato, Maria Sofia Cattaruzza, Francieli Cembranel, Ester Cerin, Pamela Roxana Chacón-Uscamaita, Jaykaran Charan, Vijay Kumar Chattu, Dhun Chauhan, Malizgani Paul Chavula, Simiao Chen, Gerald Chi, Abdulaal Chitheer, William C S Cho, Sonali Gajanan Choudhari, Dinh-Toi Chu, Natalia Cruz-Martins, Omid Dadras, Gizachew Worku Dagnew, Maxwell Ayindenaba Dalaba, Lalit Dandona, Aso Mohammad Darwesh, Jai K Das, Saswati Das, Nihar Ranjan Dash, Claudio Alberto Dávila-Cervantes, Kairat Davletov, Berhanu Gidisa Debela, Aklilu Tamire Debele, Msganaw Derese, Kebede Deribe, Emina Dervišević, Anteneh Mengist Dessie, Arkadeep Dhali, Vishal R Dhulipala, M Ashworth Dirac, Wanyue Dong, Bezabih Terefe Dora, Haneil Larson Dsouza,

Andre Rodrigues Duraes, Sulagna Dutta, Arkadiusz Marian Dziedzic, Abdelaziz Ed-Dra, Kristina Edvardsson, Ebrahim Eini, Michael Ekholuenetale, Maysaa El Sayed Zaki, Islam Y Elgendy, Muhammed Elhadi, Mohammed Elshaer, Ibrahim Elsohaby, Theophilus I Emeto, Luchuo Engelbert Bain, Hawi Leul Esayas, Babak Eshrati, Francesco Esposito, Adeniyi Francis Fagbamigbe, Ildar Ravisovich Fakhradiyev, Ali Faramarzi, Andre Faro, Ali Fatehizadeh, Ginenus Fekadu, Florian Fischer, Artem Alekseevich Fomenkov, Takeshi Fukumoto, Peter Andras Gaal, Abhay Motiramji Gaidhane, Márió Gajdács, Yaseen Galali, Silvano Gallus, Balasankar Ganesan, Federica Gazzelloni, Mesfin Gebrehiwot, Amanuel Tesfay Gebremedhin, Teferi Gebru Gebremeskel, Yohannes Fikadu Geda, Kebede Embaye Gezae, Ramy Mohamed Ghazy, Gloria Gheno, Alessandro Gialluisi, Mika Gissler, James C Glasbey, Logan M Glasstetter, Mahaveer Golechha, Pouya Goleij, Davide Golinelli, Michal Grivna, Avirup Guha, Stefano Guicciardi, Hanbing Guo, Sapna Gupta, Veer Bala Gupta, Vivek Kumar Gupta, Sebastian Haller, Rabih Halwani, Samer Hamidi, Alexis J Handal, Josep Maria Haro, Nicholas Nathaniel Hartman, Taufiq Hasan, Ali Hasanpour- Dehkordi, Md Saquib Hasnain, Soheil Hassanipour, Wen-Qiang He, Mohammad Heidari, Brenda Yuliana Herrera-Serna, Claudiu Herteliu, Kamran Hessami, Kamal Hezam, Yuta Hiraike, Ramesh Holla, Md Mahbub Hossain, Hassan Hosseinzadeh, Mehdi Hosseinzadeh, Mihaela Hostiuc, Sorin Hostiuc, Chengxi Hu, Junjie Huang, M Mamun Huda, Md Nazmul Huda, Hong-Han Huynh, Bing-Fang Hwang, Pulwasha Maria Iftikhar, Olayinka Stephen Ilesanmi, Irena M Ilic, Milena D Ilic, Mustapha Immurana, Arad Iranmehr, Farideh Iravanpour, Masao Iwagami, Chidozie Declan Iwu, Assefa N Iyasu, Jalil Jaafari, Abdollah Jafarzadeh, Haitham Jahrami, Manthan Dilipkumar Janodia, Nilofer Javadi, Tahereh Javaheri, Sathish Kumar Jayapal, Alelign Tasew Jema, Mohammad Jokar, Nitin Joseph, Charity Ehimwenma Joshua, Mikk Jürisson, Ali Kabir, Zubair Kabir, Ibraheem M Karaye, Hanie Karimi, Hengameh Kasraei, Joonas H Kauppila, Evie Shoshannah Kendal, Mohammad Keykhaei, Nauman Khalid, Faham Khamesipour, M Nuruzzaman Khan, Maseer Khan, Yusra H Khan, Khaled Khatab, Haitham Khatatbeh, Moawiah Mohammad Khatatbeh, Sorour Khateri, Hamid Reza Khayat Kashani, Moein Khormali, Min Seo Kim, Thanh V Kim, Yun Jin Kim, Ruth W Kimokoti, Adnan Kisa, Sezer Kisa, Sonali Kochhar, Ali-Asghar Kolahi, Farzad Kompani, Hamid Reza Koohestani, Soewarta Kosen, Ai Koyanagi, Kewal Krishan, Vijay Krishnamoorthy, Barthelemy Kuate Defo, Raja Amir Hassan Kuchay, Mohammed Kuddus, G Anil Kumar, Om P Kurmi, Carlo La Vecchia, Ben Lacey, Chandrakant Lahariya, Tri Laksono, Dharmesh Kumar Lal, Savita Lasrado, Kamaluddin Latief, Kaveh Latifinaibin, Thao Thi Thu Le, Munjae Lee, Sang-woong Lee, Wei-Chen Lee, Yo Han Lee, Jacopo Lenzi, Ming-Chieh Li, Shanshan Li, Virendra S Ligade, Stephen S Lim, Gang Liu, Jue Liu, Xuefeng Liu, László Lorenzovici, Masoud Lotfizadeh, Ahmed M. Afifi, Áurea M Madureira-Carvalho, Laura A Magee, Azeem Majeed, Elaheh Malakan Rad, Kashish Malhotra, Ahmad Azam Malik, Iram Malik, Tauqeer Hussain Mallhi, Joemer C Maravilla, Santi Martini, Francisco Rogerlândio Rogerlândio Martins-Melo, Miquel Martorell, Melvin Barrientos Marzan, Yasith Mathangasinghe, Rita Mattiello, Andrea Maugeri, Mahsa Mayeli, Maryam Mazaheri, Rishi P Mediratta, Kamran Mehrabani-Zeinabad, Gebrekiros Gebremichael Meles, Hadush Negash Meles, Max Alberto Mendez-Lopez, Walter Mendoza, Ritesh G Menezes, Atte Meretoja, Tuomo J Meretoja, Irmina Maria Michalek, Le Huu Nhat Minh, Reza Mirfakhraie, Mojgan Mirghafourvand, Andreea Mirica, Erkin M Mirrakhimov, Moonis Mirza, Eric Mishio Bawa, Sanjeev Misra, Biru Abdissa Mizana, Nouh Saad Mohamed, Sakineh Mohammad-Alizadeh-Charandabi, Ghada Mohammed, Salahuddin Mohammed, Shafiu Mohammed, Ali H Mokdad, Sabrina Molinaro, Sara Momtazmanesh,

Lorenzo Monasta, Mohammad Ali Moni, AmirAli Moodi Ghalibaf, Paula Moraga, Negar Morovatdar, Abbas Mosapour, Simin Mouodi, Parsa Mousavi, Ulrich Otto Mueller, Faraz Mughal, Admir Mulita, Francesk Mulita, Moses K Muriithi, Tapas Sadasivan Nair, Hastyar Hama Rashid Najmuldeen, Gopal Nambi, Vinay Nangia, Gustavo G Nascimento, Javaid Nauman, Seyed Aria Nejadghaderi, Mohammad Hadi Nematollahi, Georges Nguefack-Tsague, Josephine W Ngunjiri, Dang H Nguyen, Hau Thi Hien Nguyen, Hien Quang Nguyen, Phat Tuan Nguyen, Robina Khan Niazi, Ali Nikoobar, Lawrence Achilles Nnyanzi, Efaq Ali Noman, Shuhei Nomura, Mamoona Noreen, Dieta Nurrika, Chimezie Igwegbe Nzoputam, Ogochukwu Janet Nzoputam, Bogdan Oancea, Kehinde O Obamiro, Ropo Ebenezer Ogunsakin, Sylvester Reuben Okeke, Akinkunmi Paul Okekunle, Osaretin Christabel Okonji, Patrick Godwin Okwute, Andrew T Olagunju, Babayemi Oluwaseun Olakunde, Matthew Idowu Olatubi, Isaac Iyinoluwa Olufadewa, Bolajoko Olubukunola Olusanya, Michal Ordak, Doris V Ortega-Altamirano, Wael M S Osman, Uchechukwu Levi Osuagwu, Adrian Otoiu, Nikita Otstavnov, Stanislav S Otstavnov, Amel Ouyahia, Mayowa O Owolabi, Alicia Padron-Monedero, Jagadish Rao Padubidri, Adrian Pana, Pragyan Paramita Parija, Romil R Parikh, Ava Pashaei, Sangram Kishor Patel, Shankargouda Patil, Shrikant Pawar, Paolo Pedersini, Veincent Christian Filipino Pepito, Prince Peprah, Gavin Pereira, Jeevan Pereira, Marcos Pereira, Maria Odete Pereira, Arokiasamy Perianayagam, Norberto Perico, Konrad Pesudovs, Ionela-Roxana Petcu, Fanny Emily Petermann-Rocha, Parmida Sadat Pezeshki, Tom Pham, My Kieu Phan, Anil K Philip, Manon Pigeolet, Zahra Zahid Piracha, Vivek Podder, Dimitri Poddighe, Pranil Man Singh Pradhan, Hadi Raeisi Shahraki, Pankaja Raghav, Mosiur Rahman, Vahid Rahmanian, Ivano Raimondo, Shakthi Kumaran Ramasamy, Chhabi Lal Ranabhat, Nemanja Rancic, Chythra R Rao, Sowmya J Rao, Davide Rasella, Ahmed Mustafa Rashid, Reza Rawassizadeh, Elrashdy Moustafa Mohamed Redwan, Giuseppe Remuzzi, Kannan RR Rengasamy, Andre M N Renzaho, Nazila Rezaei, Negar Rezaei, Mohsen Rezaeian, Hannah Elizabeth Robinson-Oden, Leonardo Roever, Peter Rohloff, Luca Ronfani, Godfrey M Rwegerera, Aly M A Saad, Zahra Saadatian, Siamak Sabour, Basema Ahmad Saddik, Malihe Sadeghi, Mohammad Reza Saeb, Umar Saeed, Amene Saghazadeh, Dominic Sagoe, Fatemeh Saheb Sharif-Askari, Narjes Saheb Sharif-Askari, Amirhossein Sahebkar, Harihar Sahoo, Soumya Swaroop Sahoo, Mohamed A Saleh, Sana Salehi, Marwa Rashad Salem, Abdallah M Samy, Rama Krishna Sanjeev, Yaser Sarikhani, Sachin C Sarode, Maheswar Satpathy, Monika Sawhney, Ganesh Kumar Saya, Mete Saylan, Markus P Schlaich, Ione Jayce Ceola Schneider, Art Schuermans, Pallav Sengupta, Subramanian Senthilkumaran, Sadaf G Sepanlou, Dragos Serban, SeyedAhmad SeyedAlinaghi, Allen Seylani, Mahan Shafie, Jaffer Shah, Pritik A Shah, Samiah Shahid, Masood Ali Shaikh, Sunder Sham, Mohd Shanawaz, Mohammed Shannawaz, Mequannent Melaku Sharew, Manoj Sharma, Adithi Shetty, B Suresh Kumar Shetty, Pavanchand H Shetty, Rahman Shiri, Reza Shirkoohi, Siddharudha Shivalli, Sina Shool, Seyed Afshin Shorofi, Kanwar Hamza Shuja, Kerem Shuval, Migbar Mekonnen Sibhat, Negussie Boti Sidamo, João Pedro Silva, Colin R Simpson, Jasvinder A Singh, Paramdeep Singh, Surjit Singh, Natia Skhvitaridze, Bogdan Socea, Abdullah Al Mamun Sohag, Hamidreza Soleimani, Yonatan Solomon, Suhang Song, Yi Song, Michael Spartalis, Chandrashekhar T Sreeramareddy, Andy Stergachis, Muhammad Suleman, Saima Sultana, Haitong Zhe Sun, Jing Sun, Mindy D Szeto, Rafael Tabarés-Seisdedos, Shima Tabatabai, Mohammad Tabish, Majid Taheri, Moslem Taheri Soodejani, Jacques Lukenze Tamuzi, Ker-Kan Tan, Ingan Ukur Tarigan, Razieh Tavakoli Oliaee, Birhan Tsegaw Taye, Yibekal Manaye Tefera, Mohamad-

Hani Temsah, Masayuki Teramoto, Wegen Beyene Tesfamariam, Enoch Teye-Kwadjo, Samar Tharwat, Aravind Thavamani, Nihal Thomas, Mariya Vladimirovna Titova, Amir Tiyuri, Roman Topor-Madry, Marcos Roberto Tovani-Palone, Jaya Prasad Tripathy, Samuel Joseph Tromans, Chukwudi S Ubah, Muhammad Umair, Srikanth Umakanthan, Brigid Unim, Asokan Govindaraj Vaithinathan, Sahel Valadan Tahbaz, Mario Valenti, Rohollah Valizadeh, Jef Van den Eynde, Shoban Babu Varthya, Massimiliano Veroux, Georgios-Ioannis Verras, Leonardo Villani, Francesco S Violante, Vasily Vlassov, Mandaras Tariku Walde, Fang Wang, Shu Wang, Yanqing Wang, Yanzhong Wang, Emebet Gashaw Wassie, Kosala Gayan Weerakoon, Asrat Arja Wolde, Xiaoyue Xu, Vikas Yadav, Lin Yang, Yuichiro Yano, Sisay Shewasinad Yehualashet, Siyan Yi, Arzu Yiğit, Vahit Yiğit, Paul Yip, Naohiro Yonemoto, Nazar Zaki, Giulia Zamagni, Burhan Abdullah Zaman, Michael Zastrozhin, Haijun Zhang, Yunquan Zhang, Zhi-Jiang Zhang, Hanqing Zhao, Claire Chenwen Zhong, Magdalena Zielińska, Lilik Zuhriyah, Simon I Hay, Mohsen Naghavi, Christopher J L Murray, Rakhi Dandona, and Nicholas J Kassebaum.

8.2 Author Affiliations

Institute for Health Metrics and Evaluation (H Comfort MPH, T A McHugh PhD, A E Schumacher PhD, A Harris, E A May MS, K R Paulson MPH, W M Gardner MPH, J E Fuller MLIS, M E Frisch BA, H J Taylor BA, A T Leever BS, C Teply MS, N A Verghese BA, T Alam MPH, A Y Aravkin PhD, Prof L Dandona MD, M A Dirac MD, Prof S S Lim PhD, Prof A H Mokdad PhD, T Pham BS, H E Robinson-Oden MLIS, A A Wolde MPH, Prof S I Hay FMedSci, Prof M Naghavi PhD, Prof C J L Murray DPhil, Prof R Dandona PhD, N J Kassebaum MD), Department of Applied Mathematics (A Y Aravkin PhD), Department of Health Metrics Sciences, School of Medicine (M A Dirac MD, Prof S S Lim PhD, Prof A H Mokdad PhD, Prof A Stergachis PhD, Prof S I Hay FMedSci, Prof M Naghavi PhD, Prof C J L Murray DPhil), Department of Epidemiology (H Guo MPH), School of Health Systems and Public Health (C Iwu MPH), Department of Global Health (S Kochhar MD), Department of Anesthesiology & Pain Medicine (V Krishnamoorthy MD, N J Kassebaum MD), Department of Pharmacy (Prof A Stergachis PhD), University of Washington, Seattle, WA, United States of America; Department of Clinical Governance and Quality Improvement (Y H Abate MSc), Aleta Wondo General Hospital, Aleta Wondo, Ethiopia; Advanced Diagnostic and Interventional Radiology Research Center (H Abbastabar PhD), Research Center for Immunodeficiencies (H Abolhassani PhD, A Saghazadeh MD), Digestive Diseases Research Institute (A Anoushiravani MD, S G Sepanlou MD), Department of Health Information Management (S Ayyoubzadeh PhD), Neurosicence Institute (M Babaei MD), School of Medicine (A Behnoush BS, H Karimi MD, M Mayeli MD, S Momtazmanesh MD), Iranian Research Center for HIV/AIDS (IRCHA) (O Dadras PhD), Department of Neurosurgery (A Iranmehr MD), Non-communicable Diseases Research Center (M Keykhaei MD, S Momtazmanesh MD, P Mousavi MD, N Rezaei MD, N Rezaei PhD), Students' Scientific Research Center (SSRC) (M Keykhaei MD), Sina Trauma and Surgery Research Center (M Khormali MD, S Shool MD), Children's Medical Center (Prof F Kompani MD), Department of Pediatric Cardiology (Prof E Malakan Rad MD), Department of Internal Medicine (P Pezeshki BMedSc), Department of Master of Public Health (P Pezeshki BMedSc), Endocrinology and Metabolism Research Institute (N Rezaei PhD), Iranian Research Center for HIV/AIDS (Prof S SeyedAlinaghi PhD), Department of Neurology (M Shafie MD), Cancer Research Center (R Shirkoohi PhD),

Cancer Biology Research Center (R Shirkoohi PhD), Tehran University of Medical Sciences, Tehran, Iran; Department of Epidemiology (S Abd ElHafeez DrPH), Tropical Health Department (R M Ghazy PhD), Alexandria University, Alexandria, Egypt; Department of Surgery (M Abdelmasseh MD), Marshall University, Huntington, WV, United States of America; Department of Tropical Medicine and Infectious Diseases (S Abd-Elsalam PhD), Tanta University, Tanta, Egypt; Department of Public health (D Abdissa MSc), Department of Health, Jimma, Ethiopia; Department of Health (D Abdissa MSc), Diabetes Research Center, Jimma, Ethiopia; Department of Medicine (Prof M Abdoun PhD), University of Setif Algeria, Sétif, Algeria; Department of Health, Sétif, Algeria (Prof M Abdoun PhD); National Institute of Epidemiology (R Abdulkader PhD), Indian Council of Medical Research, Chennai, India; Department of Midwifery (M Abebe MSc), Department of Public Health (B G Debela MPH), Department of Pediatrics and Child Health Nursing (M M Sibhat MSc), Dilla University, Dilla, Ethiopia; Department of Neurosurgery (A Abedi MD), Keck School of Medicine (A Abedi MD), Mark and Mary Stevens Neuroimaging and Informatics Institute (S Salehi MD), University of Southern California, Los Angeles, CA, United States of America; Laboratory Technology Sciences Department (H Abidi PhD), Yasuj University of Medical Sciences, Yasuj, Iran; Department of Community Medicine (Prof O Abiodun MPH), Department of Medical Physiology (P G Okwute MSc), Babcock University, Ilishan-Remo, Nigeria; Department of Family and Community Health (R G Aboagye MPH), Institute of Health and Allied Sciences (M A Dalaba PhD), Institute of Health Research (M Immurana PhD), University of Health and Allied Sciences, Ho, Ghana; Department of Medical Biochemistry and Biophysics (H Abolhassani PhD), Department of Molecular Medicine and Surgery (Prof J H Kauppila MD), Karolinska Institute, Stockholm, Sweden; Philippine Institute for Development Studies, Quezon City, Philippines (M R M Abrigo PhD); Department of Clinical Sciences (Prof E Abu-Gharbieh PhD), Department of Basic Biomedical Sciences (Prof Y Bustanji PhD), Clinical Sciences Department (N R Dash MD, Prof R Halwani PhD, G Mohammed FRCOG, N Saheb Sharif-Askari PhD), College of Medicine (Prof R Halwani PhD, Prof B A Saddik PhD, Prof M A Saleh PhD), Sharjah Institute of Medical Sciences (F Saheb Sharif-Askari PhD), University of Sharjah, Sharjah, United Arab Emirates (K A Altirkawi MD); Department of Biopharmaceutics and Clinical Pharmacy (Prof E Abu-Gharbieh PhD), University of Jordan, Amman, Jordan; Institute of Community and Public Health (Prof N M Abu-Rmeileh PhD), Birzeit University, Ramallah, Palestine; College of Medicine and Health Sciences (M M Adane PhD), School of Public Health (M B Asresie MPH), Department of Reproductive Health (G W Dagnew MPH), Bahir Dar University, Bahir Dar, Ethiopia; School of Medicine (I Y Addo PhD), Faculty of Medicine and Health (W He PhD), Department of Public Health (M Khan PhD), School of Chemical & Biomolecular Engineering (E A Noman PhD), University of Sydney, Sydney, NSW, Australia (S R Okeke PhD); Centre for Social Research in Health (I Y Addo PhD, S R Okeke PhD), Ingham Institute for Applied Medical Research (M Huda PhD), School of Optometry and Vision Science (Prof K Pesudovs PhD), School of Population Health (Prof B A Saddik PhD, X Xu PhD), University of New South Wales, Sydney, NSW, Australia; Department of Nursing (B G Adema MSc), Wolaita Sodo University, Woliata Sodo, Ethiopia; Slum and Rural Health Initiative Research Academy (M A Adesina BPT, I I Olufadewa MHS), Slum and Rural Health Initiative, Ibadan, Nigeria; Department of Physiotherapy (M A Adesina BPT), Department of Epidemiology and Medical Statistics (A F Fagbamigbe PhD), College of Medicine (A P Okekunle PhD), Faculty of Public Health (I I Olufadewa MHS), Department of Medicine (Prof M O Owolabi

DrM), University of Ibadan, Ibadan, Nigeria; Department of Microbiology (Prof C O O Adetunji PhD), Edo State University Uzairue, Iyamho, Nigeria; Department of Community Health and Epidemiology (D A Adeyinka PhD), University of Saskatchewan, Saskatoon, SK, Canada; Department of Public Health (D A Adeyinka PhD), Federal Ministry of Health, Abuja, Nigeria; Department of Public Health (Q Adnani PhD), Universitas Padjadjaran (Padjadjaran University), Bandung, Indonesia; Department of Community Medicine (Prof S Afzal PhD), King Edward Memorial Hospital, Lahore, Pakistan; Department of Public Health (Prof S Afzal PhD), Public Health Institute, Lahore, Pakistan; Department of New Initiatives (Prof S B Agampodi MD), International Vaccine Institute, Seoul, South Korea; Department of Medical and Surgical Sciences and Advanced Technologies "GF Ingrassia" (Prof A Agodi PhD, M Barchitta PhD, A Maugeri PhD, Prof M Veroux PhD), University of Catania, Catania, Italy; Department of Public Health Sciences (W Agyemang-Duah PhD), Queen's University, Kingston, ON, Canada; School of Public Health (B O Ahinkorah MPhil), School of Life Sciences (G Liu PhD), University of Technology Sydney, Sydney, NSW, Australia; Department of Medical Biochemistry (A Ahmad PhD), Department of Pharmacology (M Tabish MPharm), Shaqra University, Shaqra, Saudi Arabia; School of Medicine and Psychology (D Ahmad PhD), Australian National University, Canberra, ACT, Australia; Public Health Foundation of India, Gandhinagar, India (D Ahmad PhD); Department of Epidemiology and Biostatistics (A Ahmadi PhD), Community-Oriented Nursing Midwifery Research Center (M Heidari PhD), Department of Community Health (M Lotfizadeh PhD), Social Determinants of Health Research Center (M Lotfizadeh PhD), Shahrekord University of Medical Sciences, Shahrekord, Iran; Department of Epidemiology (A Ahmadi PhD, Prof S Sabour PhD), National Nutrition and Food Technology Research Institute (M Ajami PhD), Department of Neurosurgery (H Khayat Kashani MD), Social Determinants of Health Research Center (A Kolahi MD, A Nikoobar BSc), Department of Genetics (R Mirfakhraie PhD), Department of Medical Education (S Tabatabai PhD), Medical Ethics and Law Research Center (M Taheri PhD), Shahid Beheshti University of Medical Sciences, Tehran, Iran; Institute of Endemic Diseases (A Ahmed MSc), University of Khartoum, Khartoum, Sudan; Swiss Tropical and Public Health Institute (A Ahmed MSc), University of Basel, Basel, Switzerland; Department of Biosciences (H Ahmed PhD), COMSATS Institute of Information Technology, Islamabad, Pakistan; Institute of Public Health (Prof L A Ahmed PhD, Prof M Grivna PhD), College of Medicine and Health Sciences (J Nauman PhD), Department of Computer Science and Software Engineering (Prof N Zaki PhD), United Arab Emirates University, Al Ain, United Arab Emirates; Department of Internal Medicine (K Akinosoglou PhD), University of Patras, Patras, Greece; Department of Internal Medicine and Infectious Diseases (K Akinosoglou PhD), University General Hospital of Patras, Patras, Greece; Division of Public Health Sciences (S Al Hasan PhD), Department of Research and Development (Z Al-Aly MD), Department of Surgery (S Azadnajafabad MD), Washington University in St. Louis, St. Louis, MO, United States of America; Clinical Epidemiology Center (Z Al-Aly MD), US Department of Veterans Affairs (VA), St. Louis, MO, United States of America; Murdoch Business School (K Alam PhD), Murdoch University, Perth, WA, Australia; Department of Health Information Management and Technology (T M Alanzi PhD), Division of Forensic Medicine (Prof R G Menezes MD), Imam Abdulrahman Bin Faisal University, Dammam, Saudi Arabia (F M Alanezi PhD); Department of Community and Mental Health (Prof M Albashtawy PhD), Al al-Bayt University, Mafraq, Jordan; Global Health Entrepreneurship (S Alemi PhD), Tokyo Medical and Dental University, Tokyo,

Japan; Department of Bacteriology, Immunology, and Mycology (Prof A M Algammal PhD), Suez Canal University, Ismailia, Egypt; Global Centre for Environmental Remediation (A A S Al-Gheethi PhD), University of Newcastle, Newcastle, NSW, Australia; Cooperative Research Centre for Contamination Assessment and Remediation of the Environment, Newcastle, NSW, Australia (A A S Al-Gheethi PhD); Department of Zoology (A Ali PhD), Abdul Wali Khan University Mardan, Mardan, Pakistan; Department of Biological Sciences (L Ali PhD), National University of Medical Sciences (NUMS), Rawalpindi, Pakistan; Department of Medical Rehabilitation (Physiotherapy) (M U Ali MSc), University of Maiduguri, Maiduguri, Nigeria; Department of Rehabilitation Sciences (M U Ali MSc), Hong Kong Polytechnic University, Hong Kong, China; Institute of Health and Wellbeing (S M Alif PhD), Federation University Australia, Melbourne, VIC, Australia; School of Public Health and Preventive Medicine (S M Alif PhD, S Li PhD), Monash University, Melbourne, VIC, Australia; Department of Public Health and Community Medicine (Prof S M Aljunid PhD, Prof C T Sreeramareddy MD), International Medical University, Kuala Lumpur, Malaysia; International Centre for Casemix and Clinical Coding (Prof S M Aljunid PhD), National University of Malaysia, Bandar Tun Razak, Malaysia; Department of Medicine (J U Almazan PhD), Nazarbayev University, Astana, Kazakhstan; Department of Parasitology (Prof H M Al-Mekhlafi PhD), University of Malaya, Kuala Lumpur, Malaysia; Department of Parasitology (Prof H M Al-Mekhlafi PhD), Sana'a University, Sana'a, Yemen; Wilmer Eye Institute (L Almidani MSc), Department of International Health (H Zhang MS), Johns Hopkins University, Baltimore, MD, United States of America; Doheny Image Reading and Research Lab (DIRRL) (L Almidani MSc), Department of Orthopedics (S Baghdadi MD), University of California Los Angeles, Los Angeles, CA, United States of America; College of Medicine (S Almustanyir MD), Alfaisal University, Riyadh, Saudi Arabia; Ministry of Health, Riyadh, Saudi Arabia (S Almustanyir MD); Department of Pediatrics (Prof H Aly MD), Lerner Research Institute (X Liu PhD), Cleveland Clinic, Cleveland, OH, United States of America; Department of Pediatric Cardiology (S Aly MD), Department of Pediatric Orthopedic Surgery (M Pigeolet MD), Boston Children's Hospital, Boston, MA, United States of America; Department of Pediatrics (S Aly MD), Division of Cardiovascular Medicine (G Chi MD), Division of Cardiology (I Y Elgendy MD), Maternal Fetal Care Center (K Hessami MD), Department of Global Health and Social Medicine (M Pigeolet MD), T.H. Chan School of Public Health (P M S Pradhan MD), Department of Global Health and Population (P Rohloff MD), Harvard University, Boston, MA, United States of America; Interdisciplinary Graduate Program in Human Toxicology (R Amani DVM), University of Iowa, Iowa City, IA, United States of America; Holden Comprehensive Cancer Center (R Amani DVM), University of Iowa Hospitals and Clinics, Iowa City, IA, United States of America; School of Graduate Studies (E K Ameyaw MPhil), Lingnan University, Hong Kong, China; Department of Public Health (A Amhare MSc), Salale University, Fitche, Ethiopia; School of Public Health (A Amhare MSc), Xi'an Jiaotong University, Xi'an, China; Public Health and Community Medicine Department (Prof T T Amin MD), Cairo University, Cairo, Egypt; Quran and Hadith Research Center (S Amiri PhD), Baqiyatallah University of Medical Sciences, Tehran, Iran; Department of Cardiology (Prof C Andrei PhD), Department of Internal Medicine (M Hostiuc PhD), Department of Legal Medicine and Bioethics (Prof S Hostiuc PhD), Department of General Surgery (D Serban PhD, B Socea PhD), Carol Davila University of Medicine and Pharmacy, Bucharest, Romania; Department of Statistics and Econometrics (Prof T Andrei PhD, Prof C Herteliu PhD, A Mirica PhD, A Otoiu PhD, I Petcu PhD), Bucharest

University of Economic Studies, Bucharest, Romania; School of Nursing and Midwifery (A Ansar PhD), The Judith Lumley Centre (B Ayala Quintanilla PhD), Centre for Alcohol Policy Research (CAPR) (M B Marzan MSc), La Trobe University, Melbourne, VIC, Australia; Special Interest Group International Health (A Ansar PhD), Public Health Association of Australia, Canberra, ACT, Australia; Department of Parasitology (D Anvari PhD), Iranshahr University of Medical Sciences, Iranshahr, Iran; Department of Pathology (R Anwer PhD), Imam Mohammad Ibn Saud Islamic University, Riyadh, Saudi Arabia; Department of Social Sciences (F Appiah MPhil), Berekum College of Education, Berekum, Ghana; School of Public Health (F Appiah MPhil), Kwame Nkrumah University of Science and Technology, Kumasi, Ghana; Social Determinants of Health Research Center (M Arab-Zozani PhD), Faculty of Medicine (A Moodi Ghalibaf MD), Department of Epidemiology and Biostatistics (A Tiyuri PhD), Birjand University of Medical Sciences, Birjand, Iran; Department of Health Metrics Sciences, School of Medicine (A Y Aravkin PhD, Prof R Dandona PhD, N J Kassebaum MD), Department of Family Medicine (M A Dirac MD), University of Washington, Seattle; WA (A Y Aravkin PhD, M A Dirac MD, Prof R Dandona PhD, N J Kassebaum MD), United States of America, ; College of Art and Science (D Areda PhD), Ottawa University, Surprise, AZ, United States of America; School of Life Sciences (D Areda PhD), Arizona State University, Tempe, AZ, United States of America; College of Medicine and Health Sciences (B B Aregawi PhD), Department of Medical Laboratory Sciences (H N Meles MSc), Adigrat University, Adigrat, Ethiopia; Institute for Biomedical Problems (A A Artamonov PhD), K.A. Timiryazev Institute of Plant Physiology (M V Titova PhD), Russian Academy of Sciences, Moscow, Russia; Department of Research (U R Aryal PhD), Nepal Health Research Council, Kathmandu, Nepal; Research Center for Biochemistry and Nutrition in Metabolic Diseases (Z Asemi PhD), Kashan University of Medical Sciences, Kashan, Iran; Department of Public Health (M T Asemu MSc, A M Dessie MPH), Department of Comprehensive Nursing (B B Bantie MSc), Debre Tabor University, Debre Tabor, Ethiopia; Department of Environmental Health (A A Asgedom PhD), Department of Biostatistics (K Gezae MSc), School of Public Health (G G Meles MPH), Department of Medical Biochemistry and Molecular Biology (W B Tesfamariam MSc), Mekelle University, Mekelle, Ethiopia; University Institute of Radiological Sciences and Medical Imaging Technology (T Ashraf MS), Institute of Molecular Biology and Biotechnology (S Shahid PhD), Research Centre for Health Sciences (RCHS) (S Shahid PhD), The University of Lahore, Lahore, Pakistan; Department of Biomedical Science (D Atlaw MSc), Madda Walabu University, Bale Robe, Ethiopia; Faculty of Nursing (M M W Atout PhD), Philadelphia University, Amman, Jordan; Department of Forensic Medicine (A Atreya MD), Lumbini Medical College, Palpa, Nepal; Department of Social Welfare (M S Atteraya PhD), Keimyung University, Daegu, South Korea; Northumbria HealthCare NHS Foundation Trust, Newcastle upon Tyne, United Kingdom (A Aujayeb MBBS); Universidad de San Martin de Porres, Lima, Peru (B Ayala Quintanilla PhD); Health Management and Economics Research Center (H Ayatollahi PhD), Department of Health Information Management (H Ayatollahi PhD), Preventive Medicine and Public Health Research Center (B Eshrati PhD), Minimally Invasive Surgery Research Center (A Kabir MD), Eye Research Center (H Kasraei MD), Research Center of Pediatric Infectious Diseases (F Khamesipour PhD), Department of Anesthesiology (K Latifinaibin MD), Center for Technology and Innovation in Cardiovascular Informatics (S Shool MD), Trauma and Injury Research Center (M Taheri PhD), Department of Epidemiology and Biostatistics (A Tiyuri PhD), Iran University of Medical Sciences, Tehran, Iran; Leeds Institute of Rheumatic and

Musculoskeletal Medicine (S Azadnajafabad MD), University of Leeds, Leeds, United Kingdom; Department of Sciences (Prof R M S Azevedo PhD), Cooperativa de Ensino Superior Politécnico e Universitário (Polytechnic and University Higher Education Cooperative), Gandra, Portugal; ASIDE Healthcare, Lewes, DE, United States of America (A Azzam MD); Faculty of Medicine (A Azzam MD), October 6 University, 6th of October City, Egypt; Kasturba Medical College, Mangalore (D B B MD, R Holla MD), Department of Pharmaceutical Regulatory Affairs and Management (V S Ligade PhD), Department of Community Medicine (C R Rao MD), Manipal Academy of Higher Education, Manipal, India (H L Dsouza MD); Department of Medicine (M Babaei MD), Division of Pediatric Hospital Medicine (R P Mediratta MD), Stanford University, Palo Alto, CA, United States of America; Gomal Center of Biochemistry and Biotechnology (M Badar PhD), Gomal University, Dera Ismail Khan, Pakistan; Department of Forensic Science (A D Badiye PhD), Government Institute of Forensic Science Nagpur, Nagpur, India; Rashtrasant Tukadoji Maharaj Nagpur University, Nagpur, India (A D Badiye PhD); Department of Nursing (N Baghcheghi PhD), Social Determinants of Health Research Center (H Koohestani PhD), Saveh University of Medical Sciences, Saveh, Iran; Orthopedic Institute for Children, Los Angeles, CA, United States of America (S Baghdadi MD); Health Research Institute (Prof N Bagheri PhD), University of Canberra, Canberra, ACT, Australia; School of Medicine (S Bagherieh BSc, N Javadi MD), Department of Pediatrics (N Javadi MD), Department of Epidemiology and Biostatistics (K Mehrabani-Zeinabad PhD), Isfahan University of Medical Sciences, Isfahan, Iran; Department of Environmental Health Engineering (F Bahrami Asl PhD), Urmia University of Medical Sciences, Urmia, Iran (R Valizadeh PhD); Department of Environmental Health Engineering (F Bahrami Asl PhD), Tehran University of Medical Sciences, Hamadan, Iran; School of Public Affairs (R Bai MD), Nanjing University of Information Science and Technology, Nanjing, China; Maternal and Child Health Unit (R K Bakshi MD), Indian Council of Medical Research, New Delhi, India (D K Lal MD); Department of Medicine (K Bam MPH), School of Nursing and Midwifery (D Bhandari PhD), Department of Anatomy and Developmental Biology (Y Mathangasinghe PhD), Monash University, Clayton, VIC, Australia; Department of Hypertension (Prof M Banach PhD), Medical University of Lodz, Lodz, Poland; Polish Mothers' Memorial Hospital Research Institute, Lodz, Poland (Prof M Banach PhD); Infectious Disease Epidemiology (A Banke-Thomas PhD), Department of Clinical Research (S Bhattarai MD), Department of Non-Communicable Disease Epidemiology (M Iwagami PhD), Medical Statistics Department (S Shivalli MD), London School of Hygiene & Tropical Medicine, London, United Kingdom; Department of Human Sciences (A Banke-Thomas PhD), University of Greenwich, London, United Kingdom; Department of Forensic Science (H Bansal MSc), Government Institute of Forensic Science, Nagpur, India; Miller School of Medicine (M Bardhan MD), University of Miami, Miami, FL, United States of America; Health Information Management (A Bashiri PhD), Student Research Committee (A Faramarzi MD), Department of Otolaryngology (A Faramarzi MD), Maternal Fetal Medicine Research Center (K Hessami MD), Basic Science Laboratory (F Iravanpour PhD), Health Policy Research Center (H Kasraei MD, Y Sarikhani PhD), Department of Epidemiology and Biostatistics (H Raeisi Shahraki PhD), Non-communicable Disease Research Center (S G Sepanlou MD), Basic Sciences in Infectious Diseases Research Center (R Tavakoli Oliaee PhD), Shiraz University of Medical Sciences, Shiraz, Iran; Department of Veterinary Physiology and Biochemistry (A Basiru PhD), University of Ilorin, Ilorin, Nigeria; Department of Community Medicine (P Baskaran MD),

Sri Manakula Vinayagar Medical College and Hospital, Puducherry, Puducherry, India; Department of Medical Education (K Batra PhD), Department of Social and Behavioral Health (Prof M Sharma PhD), University of Nevada Las Vegas, Las Vegas, NV, United States of America; School of Dentistry (M Bayani DMD), Arak University of Medical Sciences, Arak, Iran; Department of Surgery (N S Bayleyegn MD), Department of Midwifery (B A Mizana MSc), Jimma University, Jimma, Ethiopia; School of Public Health (Prof N Bedi MD), Dr. D.Y. Patil Vidyapeeth, Mumbai, India; Department of Epidemiology (M Khan MD), College of Nursing and Health Sciences (M Shanawaz MD), Jazan University, Jazan, Saudi Arabia (Prof N Bedi MD); Center of Research Excellence in Stillbirth (T Begum PhD), Institue for Social Science Research (M Huda PhD), School of Public Health (J C Maravilla PhD), The University of Queensland, Brisbane, QLD, Australia (M Moni PhD); Health System and Population Studies Division (T Begum PhD), International Centre for Diarrhoeal Disease Research, Dhaka, Bangladesh; Department of Epidemiology (S Nejadghaderi MD, H Soleimani MD), Non-Communicable Diseases Research Center (NCDRC), Tehran, Iran (A Behnoush BS); Department of Oral Pathology and Microbiology (U I Belgaumi MD), Krishna Vishwa Vidyapeeth (Deemed to be University), Karad, India; Department of Epidemiology and Biostatistics (A C Bermudez MD), University of the Philippines Manila, Manila, Philippines; Department of Epidemiology (A C Bermudez MD), Brown University, Providence, RI, United States of America; Department of Pharmaceutical and Administrative Sciences (K A Beyene PhD), University of Health Sciences and Pharmacy in St. Louis, St. Louis, MO, United States of America; School of Pharmacy (K A Beyene PhD), University of Auckland, Auckland, New Zealand; Department of Physiology (B B Bhandari MD), Government Institute of Medical Sciences, Greater Noida, India; School of Public Health (D Bhandari PhD), The University of Adelaide, Adelaide, SA, Australia; Department of Anatomy (N Bhardwaj MD), Department of Community Medicine and Family Medicine (Prof P Bhardwaj MD, Prof P Raghav MD), School of Public Health (Prof P Bhardwaj MD), Department of Pharmacology (J Charan MD, S Singh MD, S B Varthya MD), Department of Surgical Oncology (Prof S Misra MCh), All India Institute of Medical Sciences, Jodhpur, India; Global Health Neurology Lab (S Bhaskar MD), NSW Brain Clot Bank, Sydney, NSW, Australia; Division of Cerebrovascular Medicine and Neurology (S Bhaskar MD), National Cerebral and Cardiovascular Center, Suita, Japan; Global Health Division (S Bhattarai MD), Global Health Research and Medical Interventions for Development, Kathmandu, Nepal; School of Business Administration (Prof V Bodolica PhD), American University of Sharjah, Sharjah, United Arab Emirates; Department of Epidemiology (D Braithwaite PhD), University of Florida, Gainesville, FL, United States of America; Cancer Population Sciences Program (D Braithwaite PhD), University of Florida Health Cancer Center, Gainesville, FL, United States of America; Division of Clinical Epidemiology and Aging Research (Prof H Brenner MD), German Cancer Research Center, Heidelberg, Germany; School of Pharmacy (Prof Y Bustanji PhD), The University of Jordan, Amman, Jordan; Department of Family and Community Medicine (Prof N S Butt PhD), Rabigh Faculty of Medicine (Prof A Malik PhD), King Abdulaziz University, Jeddah, Saudi Arabia; School of Public Health Sciences (Z A Butt PhD), University of Waterloo, Waterloo, ON, Canada; Al Shifa School of Public Health (Z A Butt PhD), Al Shifa Trust Eye Hospital, Rawalpindi, Pakistan; Department of Family Medicine (A Cadri MPH), McGill University, Montreal, QC, Canada; Department of Public Health (A Cadri MPH), University of Ghana, Accra, Ghana; Center for Nutrition and Health Research (I Campos-Nonato PhD), Center for Health Systems Research (D V Ortega-Altamirano EdD), National Institute of Public Health,

Cuernavaca, Mexico; Department of Public Health and Infectious Diseases (M S Cattaruzza PhD), La Sapienza University, Rome, Italy; Department of Nutrition (Prof F Cembranel DSc), Federal University of Santa Catarina, Florianópolis, Brazil; Mary MacKillop Institute for Health Research (Prof E Cerin PhD), Australian Catholic University, Melbourne, VIC, Australia; School of Public Health (Prof E Cerin PhD), Centre for Suicide Research and Prevention (Prof P Yip PhD), Department of Social Work and Social Administration (Prof P Yip PhD), University of Hong Kong, Hong Kong, China; Carolina Health Informatics Program (P Chacón-Uscamaita DDS), University of North Carolina Chapel Hill, Chapel Hill, NC, United States of America; Temerty Faculty of Medicine (V Chattu MD), University of Toronto, Toronto, ON, Canada; Department of Community Medicine (V Chattu MD), Datta Meghe Institute of Medical Sciences, Sawangi, India; Department of Orthopaedic Surgery (D Chauhan BS), Oakland University William Beaumont School of Medicine, Auburn Hills, MI, United States of America; Department of Orthopaedic Surgery (D Chauhan BS), Henry Ford Health System, Detroit, MI, United States of America; School of Public Health (M P Chavula MPH), University of Zambia, Lusaka, Zambia; Department of Epidemiology and Global Health (M P Chavula MPH), Umeå University, Umea, Sweden; Heidelberg Institute of Global Health (HIGH) (S Chen DSc, Prof S Mohammed PhD), Heidelberg University, Heidelberg, Germany; Iraq Field Epidemiology Training Program (I-FETP) (A Chitheer MD), Ministry of Health, Baghdad, Iraq; Department of Clinical Oncology (W C S Cho PhD), Queen Elizabeth Hospital Birmingham, Hong Kong, China; Department of Community Medicine (Prof S G Choudhari MD), Jawaharlal Nehru Medical College, Wardha, India; The Interdisciplinary Research Group on Biomedicine and Health (D Chu PhD), Faculty of Applied Sciences (D Chu PhD), VNU International School (VNUIS), Hanoi, Vietnam; Department of Diagnostic and Therapeutic Technologies (Prof N Cruz-Martins PhD), Cooperativa de Ensino Superior Politécnico e Universitário (Polytechnic and University Higher Education Cooperative), Vila Nova de Famalicão, Portugal; Institute for Research and Innovation in Health (i3S) (Prof N Cruz-Martins PhD), Research Unit on Applied Molecular Biosciences (UCIBIO) (J Silva PhD), University of Porto, Porto, Portugal; Department of Global Public Health and Primary Care (O Dadras PhD), Department of Psychosocial Science (Prof D Sagoe PhD), University of Bergen, Bergen, Norway; Public Health Foundation of India, Gurugram, India (Prof L Dandona MD, G Kumar PhD, Prof R Dandona PhD); Indian Council of Medical Research, New Delhi (Prof L Dandona MD); India, (Prof L Dandona MD); Department of Information Technology (A M Darwesh PhD), University of Human Development, Sulaymaniyah, Iraq; Division of Women and Child Health (J K Das MD), Aga Khan University, Karachi, Pakistan; Department of Biochemistry (S Das MD), Ministry of Health and Welfare, New Delhi, India; Department of Population and Development (C A Dávila-Cervantes PhD), Latin American Faculty of Social Sciences Mexico, Mexico City, Mexico; Health Research Institute (Prof K Davletov PhD), Director of the Scientific and Technological Park (I R Fakhradiyev PhD), Kazakh National Medical University, Almaty, Kazakhstan; Department of Health Policy and Management (A T Debele MSc), Department of Psychiatry (M T Walde MSc), Haramaya University, Harar, Ethiopia; Department of Nursing (M Derese MSc), Mizan-Tepi University, Mizan-Aman, Ethiopia; Wellcome Trust Brighton and Sussex Centre for Global Health Research (K Deribe PhD), Brighton and Sussex Medical School, Brighton, United Kingdom; School of Public Health (K Deribe PhD), Addis Ababa University, Addis Ababa, Ethiopia; Department of Forensic Medicine (E Dervišević PhD), University of Sarajevo, Sarajevo, Bosnia and Herzegovina; Sheffield Teaching Hospitals NHS Foundation Trust, Sheffield,

United Kingdom (A Dhali MBBS); The Zena and Michael A. Wiener Cardiovascular Institute (V R Dhulipala MD), Icahn School of Medicine at Mount Sinai, New York, NY, United States of America; School of Elderly Care Services and Management (W Dong MD), Nanjing University of Chinese Medicine, Nanjing, China; Department of Midwifery (B T Dora MSc, H L Esayas MSc), School of Public Health (N Sidamo PhD), Arba Minch University, Arba Minch, Ethiopia; Department of Forensic Medicine and Toxicology (H L Dsouza MD), Kasturba Medical College Mangalore, Mangalore, India; School of Medicine (Prof A R Duraes PhD), Institute of Collective Health (Prof M Pereira PhD, Prof D Rasella PhD), Federal University of Bahia, Salvador, Brazil; Department of Internal Medicine (Prof A R Duraes PhD), Escola Bahiana de Medicina e Saúde Pública (Bahiana School of Medicine and Public Health), Salvador, Brazil; College of Medicine (S Dutta PhD), Ajman University, Ajman, United Arab Emirates; Department of Conservative Dentistry with Endodontics (A M Dziedzic DSc), Medical University of Silesia, Katowice, Poland; Higher School of Technology (Prof A Ed-Dra PhD), Sultan Moulay Slimane University, Beni Mellal, Morocco; School of Nursing and Midwifery (K Edvardsson PhD), La Trobe University, Bundoora, VIC, Australia; Private Orthodontist, Ahvaz, Iran (E Eini MSD); Faculty of Science and Health (M Ekholuenetale PhD), University of Portsmouth, Hampshire, United Kingdom; Department of Clinical Pathology (Prof M El Sayed Zaki PhD, Prof M Elshaer PhD), Faculty of Pharmacy (Prof M A Saleh PhD), Rheumatology and Immunology Unit (Prof S Tharwat MD), Mansoura University, Mansoura, Egypt; Division of Cardiovascular Medicine (I Y Elgendy MD), University of Kentucky, Lexington, KY, United States of America; Faculty of Medicine (M Elhadi MD), University of Tripoli, Tripoli, Libya; Houston Methodist Hospital, Houston, TX, United States of America (M Elhadi MD); Department of Infectious Diseases and Public Health (I Elsohaby PhD, G Fekadu PhD), City University of Hong Kong, Hong Kong, China; Department of Animal Medicine (I Elsohaby PhD), Cardiovascular Department (Prof A M A Saad MD), Zagazig University, Zagazig, Egypt; Department of Public Health and Tropical Medicine (T I Emeto PhD), James Cook University, Townsville, QLD, Australia (K O Obamiro PhD); Lincoln International Institute for Rural Health (L Engelbert Bain PhD), University of Lincoln, Lincoln, United Kingdom; Department of Biomedical and Neuromotor Sciences (DIBINEM) (F Esposito MD, J Lenzi PhD), Department of Biomedical and Neuromotor Sciences (S Guicciardi MD), Department of Medical and Surgical Sciences (Prof F S Violante MD), University of Bologna, Bologna, Italy; Research Centre for Healthcare and Community (A F Fagbamigbe PhD), Faculty of Health and Life Sciences (O P Kurmi PhD), Coventry University, Coventry, United Kingdom; Department of Psychology (A Faro PhD), Federal University of Sergipe, São Cristóvão, Brazil; School of Engineering (A Fatehizadeh PhD), Edith Cowan University, Joondalup, WA, Australia; Department of Pharmacy (G Fekadu PhD), Wollega University, Nekemte, Ethiopia; Institute of Public Health (F Fischer PhD), Charité Medical University Berlin, Berlin, Germany; Department of Cell Biology and Biotechnology (A A Fomenkov PhD), K.A. Timiryazev Institute of Plant Physiology, Moscow, Russia; Department of Dermatology (T Fukumoto PhD), Kobe University, Kobe, Japan; Health Services Management Training Centre (Prof P A Gaal PhD), Semmelweis University, Budapest, Hungary; Department of Applied Social Sciences (Prof P A Gaal PhD), Sapientia Hungarian University of Transylvania, Târgu-Mureș, Romania; Department of Community Medicine (Prof A M Gaidhane MD), Datta Meghe Institute of Medical Sciences, Wardha, India; Department of Oral Biology and Experimental Dental Research (M Gajdács PhD), University of Szeged, Szeged, Hungary; Department of Food Technology (Y Galali ResM),

Salahaddin University-Erbil, Erbil, Iraq; Department of Nutrition and Dietetics (Y Galali ResM), Cihan University-Erbil, Erbil, Iraq; Department of Medical Epidemiology (S Gallus PhD), Mario Negri Institute for Pharmacological Research, Milan, Italy; Institute of Health and Wellbeing (B Ganesan PhD), Federation University Australia, Churchill, VIC, Australia; Institute and Faculty of Actuaries, London, United Kingdom (F Gazzelloni BSc); Department of Environmental Health (M Gebrehiwot DSc), Wollo University, Dessie, Ethiopia; School of Nursing and Midwifery (A T Gebremedhin MPH), Edith Cowan University, Perth, WA, Australia; School of Population Health (A T Gebremedhin MPH), Curtin University, Perth, WA, Australia; Department of Reproductive and Family Health (T G Gebremeskel PhD), Axum College of Health Science, Axum, Ethiopia; College of Medicine and Public Health (T G Gebremeskel PhD), Department of Nursing and Health Sciences (S Shorofi PhD), Flinders University, Adelaide, SA, Australia; Department of Midwifery (Y F Geda MSc), Wolkite University, Wolkite, Ethiopia; Family and Community Medicine Department (R M Ghazy PhD), King Khalid University, Abha, Saudi Arabia; Department of Statistics (G Gheno PhD), Ronin Institute, Montclair, NJ, United States of America; Department of Epidemiology and Prevention (A Gialluisi PhD), IRCCS Neuromed, Pozzilli, Italy; Information Services Department (Prof M Gissler PhD), THL Finnish Institute for Health and Welfare, Helsinki, Finland; Department of Neurobiology, Care Sciences, and Society (Prof M Gissler PhD), Karolinska Institute, Sweden, Sweden; NIHR Global Health Research Unit on Global Surgery (J C Glasbey MSc), Institute of Applied Health Research (K Malhotra MBBS), University of Birmingham, Birmingham, United Kingdom; National Human Genome Research Institute (L M Glasstetter BS), National Institute of Health, Bethesda, MD, United States of America; Department of Health Systems and Policy Research (Prof M Golechha PhD), Indian Institute of Public Health, Gandhinagar, India; Department of Genetics (P Goleij MSc), Sana Institute of Higher Education, Sari, Iran; Universal Scientific Education and Research Network (USERN) (P Goleij MSc), Kermanshah University of Medical Sciences, Kermanshah, Iran; Department of Life Sciences, Health and Healthcare Professions (Prof D Golinelli MD), Link Campus University, Rome, Italy; Health Services Research, Evaluation and Policy Unit (Prof D Golinelli MD), AUSL della Romagna, Ravenna, Italy; Department of Public Health and Preventive Medicine (Prof M Grivna PhD), Charles University, Prague, Czech Republic; Harrington Heart and Vascular Institute (A Guha MD), Department of Quantitative Health Science (X Liu PhD), Department of Pediatrics (A Thavamani MD), Division of Pediatric Gastroenterology (A Thavamani MD), Case Western Reserve University, Cleveland, OH, United States of America; Division of Cardiovascular Medicine (A Guha MD), Ohio State University, Columbus, OH, United States of America; Department of the Health Directorate (S Guicciardi MD), Local Health Authority of Bologna, Bologna, Italy; Department of Toxicology (S Gupta MSc), Shriram Institute for Industrial Research, Delhi, India; School of Medicine (V Gupta PhD), Deakin University, Geelong, VIC, Australia; Faculty of Medicine Health and Human Sciences (Prof V K Gupta PhD), Australian Institute of Health Innovation (P Peprah MSc), Macquarie University, Sydney, NSW, Australia; Department of Infectious Disease Epidemiology (S Haller MD), Robert Koch Institute, Berlin, Germany; Department of Public Health (S Haller MD), Charité Insitute of Public Health, Berlin, Germany; School of Health and Environmental Studies (Prof S Hamidi DrPH), Hamdan Bin Mohammed Smart University, Dubai, United Arab Emirates; Department of Epidemiology (A J Handal PhD), University of Michigan School of Public Health, Ann Arbor, MI, United States of America; Research Unit (J M Haro MD), Parc Sanitari Sant Joan de Deu, Barcelona, Spain; Department

of Mental Health (J M Haro MD), Carlos III Health Institute (Prof R Tabarés-Seisdedos PhD), Biomedical Research Networking Center for Mental Health Network (CiberSAM), Madrid, Spain; University of Tulsa College of Law (N N Hartman BS), University of Tulsa, Tulsa, OK, United States of America; Department of Economics & Statistics (N N Hartman BS), Department of Anesthesiology (V Krishnamoorthy MD), Duke University, Durham, NC, United States of America; Biomedical Engineering Department (T Hasan PhD), Bangladesh University of Engineering and Technology, Dhaka, Bangladesh; Department of Medical Surgical (Prof A Hasanpour- Dehkordi PhD), Shahroud University of Medical Sciences, Shahrekord, Iran; Department of Pharmacy (Prof M S Hasnain PhD), Marwadi University, Rajkot, India; Gastrointestinal and Liver Diseases Research Center (S Hassanipour PhD), Caspian Digestive Disease Research Center (S Hassanipour PhD), Department of Environmental Health Engineering (J Jaafari PhD), Guilan University of Medical Sciences, Rasht, Iran; Departamento de Salud Oral (Department of Oral Health) (B Y Herrera-Serna PhD), Universidad Autónoma de Manizales (Autonomous University of Manizales), Manizales, Colombia; Babes-Bolyai University, Cluj-Napoca, Romania (Prof C Herteliu PhD); Department of Microbiology (K Hezam PhD), Faculty of Applied Sciences (E A Noman PhD), Taiz University, Taiz, Yemen; School of Medicine (K Hezam PhD), Nankai University, Tianjin, China; Graduate School of Medicine (Y Hiraike PhD), Department of Global Health Policy (Prof S Nomura PhD), University of Tokyo, Tokyo, Japan; Department of Decision and Information Sciences (M Hossain DrPH), University of Houston, Houston, TX, United States of America; Public Health Research Group (M Hossain DrPH), Nature Study Society of Bangladesh, Khulna, Bangladesh; School of Health and Society (H Hosseinzadeh PhD), University of Wollongong, Wollongong, NSW, Australia; School of Computer Science (Prof M Hosseinzadeh PhD), Faculty of Medicine (H T H Nguyen MD), Institute for Research and Training in Medicine, Biology and Pharmacy (H T H Nguyen MD), Duy Tan University, Da Nang, Vietnam; Jadara University Research Center (Prof M Hosseinzadeh PhD), Jadara University, Irbid, Jordan; Department of Clinical Legal Medicine (Prof S Hostiuc PhD), National Institute of Legal Medicine Mina Minovici, Bucharest, Romania; Department of Psychology (C Hu PhD), Tsinghua University, Beijing, China; Faculty of Medicine (J Huang MD), Jockey Club School of Public Health and Primary Care (C Zhong PhD), The Chinese University of Hong Kong, Hong Kong, China; Rural Health Research Institute (M Huda PhD, Prof J Sun PhD), Charles Sturt University, Orange, NSW, Australia; Research Division (M Huda PhD), ARCED Foundation, Dhaka, Bangladesh; International Master Program for Translational Science (H Huynh BS), Department of Global Health and Health Security (K Latief PhD), International Ph.D. Program in Medicine (L Minh MD), Research Center for Artificial Intelligence in Medicine (L Minh MD), Taipei Medical University, Taipei, Taiwan; Department of Occupational Safety and Health (Prof B Hwang PhD), China Medical University, Taichung, Taiwan; Department of Occupational Therapy (Prof B Hwang PhD), Asia University, Taichung, Taiwan; Health Policy and Management Department (P M Iftikhar MD), City University of New York, New York, NY, United States of America; West Africa RCC (O S Ilesanmi PhD), Africa Centre for Disease Control and Prevention, Abuja, Nigeria; Department of Community Medicine (O S Ilesanmi PhD), Department of Medicine (Prof M O Owolabi DrM), University College Hospital, Ibadan, Nigeria; Faculty of Medicine (I M Ilic PhD), University of Belgrade, Belgrade, Serbia; Faculty of Medical Sciences (Prof M D Ilic PhD), University of Kragujevac, Kragujevac, Serbia; Department of Health Services Research (M Iwagami PhD), University of Tsukuba, Tsukuba, Japan; Department of Nursing

(A N Iyasu MSc), Aksum University, Aksum, Ethiopia; Department of Immunology (Prof A Jafarzadeh PhD), HIV/STI Surveillance Research Center (S Nejadghaderi MD), Applied Cellular and Molecular Research Center (M Nematollahi PhD), Kerman University of Medical Sciences, Kerman, Iran; Department of Immunology (Prof A Jafarzadeh PhD), Department of Epidemiology and Biostatistics (Prof M Rezaeian PhD), Rafsanjan University of Medical Sciences, Rafsanjan, Iran; College of Medicine and Medical Sciences (H Jahrami PhD), Arabian Gulf University, Manama, Bahrain; Ministry of Health, Manama, Bahrain (H Jahrami PhD); School of Pharmaceutical Management (Prof M D Janodia PhD), IIHMR University, Jaipur, India; Health Informatic Lab (T Javaheri PhD), Department of Computer Science (R Rawassizadeh PhD), Boston University, Boston, MA, United States of America; Centre of Studies and Research (S Jayapal PhD), Ministry of Health, Muscat, Oman; Department of Public Health (A Jema MPH), Madda Walabu University, Goba, Ethiopia; Faculty of Veterinary Medicine (M Jokar DVM), Department of Oncology (L Yang PhD), University of Calgary, Calgary, AB, Canada; Young Researchers and Elite Club (M Jokar DVM), Islamic Azad University, Karaj, Iran; Department of Community Medicine (N Joseph MD), Department of Forensic Medicine and Toxicology (Prof J Padubidri MD, Prof B K Shetty MD, P H Shetty MD), Department of Obstetrics and Gynaecology (A Shetty MS), Manipal Academy of Higher Education, Mangalore, India; Department of Economics (C E Joshua BSc), National Open University, Benin City, Nigeria; Institute of Family Medicine and Public Health (M Jürisson PhD), University of Tartu, Tartu, Estonia; School of Public Health (Z Kabir PhD), University College Cork, Cork, Ireland; School of Health Professions and Human Services (I M Karaye MD), Hofstra University, Hempstead, NY, United States of America; Department of Anesthesiology (I M Karaye MD), Montefiore Medical Center, Bronx, NY, United States of America; Surgery Research Unit (Prof J H Kauppila MD), University of Oulu, Oulu, Finland; Department of Health Sciences and Biostatistics (E S Kendal PhD), Swinburne University of Technology, Hawthorn, VIC, Australia; College of Health Sciences (N Khalid PhD), Abu Dhabi University, Adu Dhabi, United Arab Emirates; Halal Research Center of the Islamic Republic of Iran (IRI) (F Khamesipour PhD), Iran Food and Drug Administration, Tehran, Iran; Population Science Department (M Khan PhD), Jatiya Kabi Kazi Nazrul Islam University, Mymensingh, Bangladesh; Department of Clinical Pharmacy (Y H Khan PhD, T Mallhi PhD), Jouf University, Sakaka, Saudi Arabia; College of Health, Wellbeing and Life Sciences (Prof K Khatab PhD), Sheffield Hallam University, Sheffield, United Kingdom; College of Arts and Sciences (Prof K Khatab PhD), Ohio University, Zanesville, OH, United States of America; Faculty of Nursing (H Khatatbeh PhD), Department of Basic Medical Sciences (Prof M M Khatatbeh PhD), Yarmouk University, Irbid, Jordan; School of Medicine (S Khateri MD), Kurdistan University of Medical Sciences, Sanandaj, Iran; Broad Institute of MIT and Harvard, Cambridge, MA, United States of America (M Kim MD); Division of Cardiology (D H Nguyen BS), Cardiovascular Research Center (A Schuermans BSc), Massachusetts General Hospital, Boston, MA, United States of America (M Kim MD); Department of Epidemiology (T V Kim MD), Pham Ngoc Thach University of Medicine, Ho Chi Minh City, Vietnam; Center of Excellence for Liver Disease in Viet Nam (T V Kim MD), Johns Hopkins University, Ho Chi Minh City, Vietnam; School of Traditional Chinese Medicine (Y Kim PhD), Xiamen University Malaysia, Sepang, Malaysia; Millennium Prevention, Inc., Westwood, MA, United States of America (R W Kimokoti MD); School of Health Sciences (Prof A Kisa PhD), Kristiania University College, Oslo, Norway; Department of International Health and Sustainable Development (Prof A Kisa PhD),

Tulane University, New Orleans, LA, United States of America; Department of Nursing and Health Promotion (S Kisa PhD), Oslo Metropolitan University, Oslo, Norway; Global Healthcare Consulting, New Delhi, India (S Kochhar MD); Independent Consultant, Jakarta, Indonesia (S Kosen MD); San Juan de Dios Sanitary Park, Barcelona, Spain (A Koyanagi MD); Department of Anthropology (Prof K Krishan PhD), Panjab University, Chandigarh, India; Department of Demography (Prof B Kuate Defo PhD), Department of Social and Preventive Medicine (Prof B Kuate Defo PhD), University of Montreal, Montreal, QC, Canada; Department of Biotechnology (R H Kuchay PhD), Baba Ghulam Shah Badshah University, Jammu and Kashmir, India; Department of Biochemistry (Prof M Kuddus PhD), University of Hail, Hail, Saudi Arabia; Department of Medicine (O P Kurmi PhD), Department of Psychiatry and Behavioural Neurosciences (A T Olagunju MD), McMaster University, Hamilton, ON, Canada; Department of Clinical Sciences and Community Health (Prof C La Vecchia MD), University of Milan, Milan, Italy; Nuffield Department of Population Health (B Lacey DPhil), University of Oxford, Oxford, United Kingdom; National Institute for Health Research (NIHR) Oxford Biomedical Research Centre, Oxford, United Kingdom (B Lacey DPhil); Integrated Department of Epidemiology, Health Policy, Preventive Medicine and Pediatrics (Prof C Lahariya MD), Foundation for People-centric Health Systems, New Delhi, India; Centre for Health: The Specialty Practice, New Delhi, India (Prof C Lahariya MD); Department of Physiotherapy (T Laksono MS), Universitas Aisyiyah Yogyakarta, Yogyakarta, Indonesia; Institute of Allied Health Sciences (T Laksono MS), National Cheng Kung University, Tainan, Taiwan; Department of Otorhinolaryngology (S Lasrado MS), Father Muller Medical College, Mangalore, India; Centre for Family Welfare (K Latief PhD), University of Indonesia, Depok, Indonesia; University of Medicine and Pharmacy at Ho Chi Minh City, Ho Chi Minh City, Vietnam (T T Le MD); Department of Medical Science (M Lee PhD), Ajou University School of Medicine, Suwon, South Korea; Pattern Recognition and Machine Learning Lab (Prof S Lee PhD), Gachon University, Seongnam, South Korea; Department of Family Medicine (W Lee PhD), University of Texas Medical Branch, Galveston, TX, United States of America; Department of Preventive Medicine (Prof Y Lee PhD), Korea University, Seoul, South Korea; Department of Health Promotion and Health Education (M Li PhD), National Taiwan Normal University, Taipei, Taiwan; Department of Epidemiology and Biostatistics (Prof J Liu PhD), Institute of Child and Adolescent Health (Y Song PhD), School of Public Health (H Zhang MS), Peking University, Beijing, China; Department of Health Economics (L Lorenzovici MSc), Syreon Research Romania, Targu Mures, Romania; Department of Doctoral Studies (L Lorenzovici MSc), George Emil Palade University of Medicine, Pharmacy, Science, and Technology of Targu Mures, Targu Mures, Romania; Department of Surgery (A M Afifi MD), Baylor College of Medicine, Toledo, OH, United States of America; Toxicology Research Unit (TOXRUN) (Á M Madureira-Carvalho PhD), Cooperativa de Ensino Superior Politécnico e Universitário (CESPU) (University Polytechnic Higher Education Cooperative), Gandra, Portugal; Laboratório de Farmacognosia (LAQV) (Associated Laboratory for Green Chemistry (Á M Madureira-Carvalho PhD), Universidade do Porto (University of Porto), Porto, Portugal; Department of Women and Children's Health (Prof L A Magee MD), School of Life Course and Population Sciences (Prof Y Wang PhD), King's College London, London, United Kingdom; Department of Obstetrics and Gynaecology (Prof L A Magee MD), School of Nursing (A Pashaei MSc), University of British Columbia, Vancouver, BC, Canada; Department of Primary Care and Public Health (Prof A Majeed MD), Imperial College London, London, United Kingdom;

Rama Medical College Hospital and Research Centre, Uttar Pradesh, India (K Malhotra MBBS); Department of Electrical Engineering (I Malik PhD), Department of Health and Rehabilitation Sciences (Prof G Nambi PhD), Prince Sattam bin Abdulaziz University, Al Kharj, Saudi Arabia; Far Eastern University, Manila, Philippines (J C Maravilla PhD); Faculty of Public Health (Prof S Martini PhD), Universitas Airlangga (University of Airlangga), Surabaya, Indonesia; Indonesian Public Health Association, Surabaya, Indonesia (Prof S Martini PhD); Campus Fortaleza (F R Martins-Melo PhD), Federal Institute of Education, Science and Technology of Ceará, Fortaleza, Brazil; Department of Nutrition and Dietetics (M Martorell PhD), Centre for Healthy Living (M Martorell PhD), University of Concepción, Concepción, Chile; Department of Anatomy, Genetics and Biomedical Informatics (Y Mathangasinghe PhD), University of Colombo, Colombo, Sri Lanka; Department of Social Medicine (R Mattiello PhD), Federal University of Rio Grande do Sul, Porto Alegre, Brazil; Department of Social Medicine and Family (M Mazaheri PhD), Dezful University of Medical Sciences, Dezful, Iran; Department of Medical Oncology and Hematology (M A Mendez-Lopez PhD), Kantonsspital St. Gallen, St. Gallen, Switzerland; Universidad Nacional Mayor de San Marcos, Lima, Peru (W Mendoza MD); General Administration Department (A Meretoja MD), Comprehensive Cancer Center (T J Meretoja MD), Helsinki University Hospital, Helsinki, Finland; School of Health Sciences (A Meretoja MD), University of Melbourne, Melbourne, VIC, Australia; University of Helsinki, Helsinki, Finland (T J Meretoja MD); National Cancer Registry (I Michalek PhD), Department of Pathology (I Michalek PhD), Maria Sklodowska-Curie National Research Institute of Oncology, Warsaw, Poland; Faculty of Nursing and Midwifery (Prof M Mirghafourvand PhD), Social Determinants of Health Research Center (Prof S Mohammad-Alizadeh-Charandabi PhD), Midwifery Department (Prof S Mohammad-Alizadeh-Charandabi PhD), Tabriz University of Medical Sciences, Tabriz, Iran; Internal Medicine Programme (Prof E M Mirrakhimov PhD), Kyrgyz State Medical Academy, Bishkek, Kyrgyzstan; Department of Atherosclerosis and Coronary Heart Disease (Prof E M Mirrakhimov PhD), National Center of Cardiology and Internal Disease, Bishkek, Kyrgyzstan; Department of Hospital Administration (M Mirza MD), Department of Community Medicine and Family Medicine (S S Sahoo MD), Department of Radiodiagnosis (P Singh MD), All India Institute of Medical Sciences, Bathinda, India; Department of Epidemiology and Biostatistics (E Mishio Bawa MPHIL), University of South Carolina, Columbia, SC, United States of America; Molecular Biology Unit (N S Mohamed MSc), Bio-Statistical and Molecular Biology Department (N S Mohamed MSc), Sirius Training and Research Centre, Khartoum, Sudan; Obstetrics and Gynecology (G Mohammed FRCOG), University Hospital Sharjah, Sharjah, United Arab Emirates; Department of Pharmaceutical Sciences (S Mohammed PhD), Notre Dame of Maryland University, Baltimore, MD, United States of America; Department of Pharmacy (S Mohammed PhD), Mizan-Tepi University, Mizan, Ethiopia; Health Systems and Policy Research Unit (Prof S Mohammed PhD), Ahmadu Bello University, Zaria, Nigeria; Institute of Clinical Physiology (S Molinaro PhD), National Research Council, Pisa, Italy; Clinical Epidemiology and Public Health Research Unit (L Monasta DSc, L Ronfani PhD, G Zamagni MSc), Burlo Garofolo Institute for Maternal and Child Health, Trieste, Italy; AI & Cyber Futures Institute (M Moni PhD), Charles Sturt University, Bathurst, NSW, Australia; Computer, Electrical, and Mathematical Sciences and Engineering Division (P Moraga PhD), King Abdullah University of Science and Technology, Thuwal, Saudi Arabia; Clinical Research Development Unit (N Morovatdar MD), Biotechnology Research Center (Prof A

Sahebkar PhD), Mashhad University of Medical Sciences, Mashhad, Iran; Department of Clinical Biochemistry (A Mosapour PhD), Social Determinants of Health Research Center (S Mouodi PhD), Babol University of Medical Sciences, Babol, Iran; Department of Clinical Biochemistry (A Mosapour PhD), Tarbiat Modares University, Tehran, Iran; Federal Institute for Population Research, Wiesbaden, Germany (Prof U O Mueller MD); Center for Population and Health, Wiesbaden, Germany (Prof U O Mueller MD); School of Medicine (F Mughal FRCGP), Keele University, Keele, United Kingdom; Division of Psychology and Mental Health (F Mughal FRCGP), University of Manchester, Manchester, United Kingdom; Department of Medicine (A Mulita PhD), Democritus University of Thrace, Alexandroupolis, Greece; Department of Surgery (F Mulita PhD), General University Hospital of Patras, Patras, Greece; Faculty of Medicine (F Mulita PhD), University of Thessaly, Larissa, Greece; School of Economics (M K Muriithi PhD), University of Nairobi, Nairobi, Kenya; Department of Community Medicine (T S Nair MD), MOSC Medical College, Kolenchery, India; Department of Medical Laboratory Analysis (H H Najmuldeen PhD), Cihan University Sulaymaniya, Sulaymaniyah, Iraq; Suraj Eye Institute, Nagpur, India (V Nangia MD); National Dental Research Institute Singapore (G G Nascimento PhD), Duke-NUS Medical School, Singapore, Singapore; Department of Circulation and Medical Imaging (J Nauman PhD), Norwegian University of Science and Technology, Trondheim, Norway; Department of Public Health (G Nguefack-Tsague PhD), University of Yaoundé I, Yaoundé, Cameroon; Department of Biological Sciences (J W Ngunjiri PhD), University of Embu, Embu, Kenya; Department of Medical Engineering (D H Nguyen BS), University of South Florida, Tampa, FL, United States of America; Cardiovascular Research Department (H Q Nguyen MD), Methodist Hospital, Merrillville, IL, United States of America; Department of Surgery (P T Nguyen MD), Danang Family Hospital, Da Nang, Vietnam; International Islamic University Islamabad, Islamabad, Pakistan (R K Niazi PhD); Center for Public Health (L A Nnyanzi PhD), Teesside University, Middlesbrough, United Kingdom; Global Research Institute (Prof S Nomura PhD), Keio University, Tokyo, Japan; Department of Microbiology and Molecular Genetics (M Noreen PhD), The Women University Multan, Multan, Pakistan; Department of Public Health (D Nurrika PhD), Banten School of Health Science, South Tangerang, Indonesia; Ministry of Research, Technology and Higher Education (D Nurrika PhD), Higher Education Service Institutions (LL-DIKTI) Region IV, Bandung, Indonesia; Center of Excellence in Reproductive Health Innovation (CERHI) (C I Nzoputam MPH), University of Benin, Benin City, Nigeria; Department of Physiology (O J Nzoputam PhD), University of Benin, Edo, Nigeria; Department of Physiology (O J Nzoputam PhD), Benson Idahosa University, Benin City, Nigeria; Department of Applied Economics and Quantitative Analysis (Prof B Oancea PhD), University of Bucharest, Bucharest, Romania; School of Health Systems & Public Health (R E Ogunsakin PhD), University of Pretoria, Pretoria, South Africa; Department of Food and Nutrition (A P Okekunle PhD), Seoul National University, Seoul, South Korea; School of Pharmacy (O C Okonji MSc), University of the Western Cape, Cape Town, South Africa; Department of Medical Physiology (P G Okwute MSc), Department of Psychiatry (A T Olagunju MD), University of Lagos, Lagos, Nigeria; Department of Population and Community Health (B O Olakunde PhD), University of North Texas Health Science Center, Fort Worth, TX, United States of America; Department of Nursing Science (M I Olatubi PhD), Bowen University, Iwo, Nigeria; Executive Director (B O Olusanya PhD), Centre for Healthy Start Initiative, Lagos, Nigeria; Department of Pharmacotherapy and Pharmaceutical Care (M Ordak PhD), Department of Biochemistry

and Pharmacogenomics (M Zielińska MPharm), Medical University of Warsaw, Warsaw, Poland; Department of Biology (W M S Osman PhD), Khalifa University, Abu Dhabi, United Arab Emirates; School of Medicine (U L Osuagwu PhD), Western Sydney University, Bathurst, NSW, Australia; Department of Optometry and Vision Science (U L Osuagwu PhD), University of KwaZulu-Natal, KwaZulu-Natal, South Africa; Laboratory of Public Health Indicators Analysis and Health Digitalization (N Otstavnov BA, S S Otstavnov PhD), Moscow Institute of Physics and Technology, Dolgoprudny, Russia; Department of Project Management (S S Otstavnov PhD), Department of Health Care Administration and Economics (Prof V Vlassov MD), National Research University Higher School of Economics, Moscow, Russia; Faculty of Medicine (Prof A Ouyahia PhD), University Ferhat Abbas of Setif, Setif, Algeria; Division of Infectious Diseases (Prof A Ouyahia PhD), University Hospital of Setif, Setif, Algeria; National School of Public Health (A Padron-Monedero PhD), Institute of Health Carlos III, Madrid, Spain; Department of Public Health (A Pana PhD), Babes Bolyai University, Cluj Napoca, Romania; Department of Health Metrics (A Pana PhD), Center for Health Outcomes & Evaluation, Bucharest, Romania; Department of Community Medicine and Family Medicine (P P Parija MD), All India Institute of Medical Sciences, Jammu, India; Department of Epidemiology and Community Health (R R Parikh MD), University of Minnesota, Minneapolis, MN, United States of America; Department of Research and Training (S K Patel PhD), Population Council Institute, New Delhi, India; College of Dental Medicine (Prof S Patil PhD), Roseman University of Health Sciences, South Jordan, UT, United States of America; Centre of Molecular Medicine and Diagnostics (COMManD) (Prof S Patil PhD), Saveetha Dental College and Hospitals (K Rengasamy PhD, M Tovani-Palone PhD), Center for Global Health Research (Prof A Sahebkar PhD), Saveetha University, Chennai, India; Department of Genetics (S Pawar PhD), Yale University, New Haven, CT, United States of America; Clinical Research Department (P Pedersini MSc), IRCCS Fondazione Don Carlo Gnocchi, Milan, Italy; Center for Research and Innovation (V F Pepito MSc), Ateneo De Manila University, Pasig City, Philippines; School of Population Health (Prof G Pereira PhD), Curtin University, Bentley, WA, Australia; Centre for Fertility and Health (Prof G Pereira PhD), Norwegian Institute of Public Health, Oslo, Norway; Department of Orthopedics (J Pereira MS), Yenepoya Medical College, Mangalore, India; Department of Applied Nursing (Prof M O Pereira PhD), Federal University of Minas Gerais, Belo Horizonte, Brazil; Social and Economic Survey Research Institute (Prof A Perianayagam PhD), Qatar University, Doha, Qatar; Mario Negri Institute for Pharmacological Research, Bergamo, Italy (N Perico MD, Prof G Remuzzi MD); Facultad de Medicina (F E Petermann-Rocha PhD), Universidad Diego Portales (Diego Portales University), Santiago, Chile; School of Cardiovascular and Metabolic Health (F E Petermann-Rocha PhD), University of Glasgow, Glasgow, United Kingdom; Faculty of Medicine of Nam Can Tho University (M K Phan MD), University of Medicine, Nam Can Tho University, Vietnam; School of Pharmacy (A K Philip PhD), University of Nizwa, Nizwa, Oman; International Center of Medical Sciences Research, Islamabad, Pakistan (Z Z Piracha PhD); Medical College (V Podder HSC), Tairunnessa Memorial Medical College and Hospital, Gazipur, Bangladesh; School of Public Health (V Podder HSC), University of Adelaide, Adelaide, SA, Australia; College of Health Sciences (CHS) (Prof D Poddighe PhD), VinUniversity, Hanoi, Kazakhstan; Clinical Academic Department of Pediatrics (Prof D Poddighe PhD), University Medical Center (UMC), Astana, Kazakhstan; Department of Community Medicine (P M S Pradhan MD), Tribhuvan University, Kathmandu, Nepal;

Department of Population Science and Human Resource Development (Prof M Rahman DrPH), University of Rajshahi, Rajshahi, Bangladesh; Department of Public Health (V Rahmanian PhD), Torbat Jam Faculty of Medical Sciences, Torbat Jam, Iran; Department of Medical, Surgical and Experimental Sciences (I Raimondo MD), University of Sassari, Sassari, Italy; Gynecology and Breast Care Center (I Raimondo MD), Mater Olbia Hospital (Qatar Foundation Endowment and Policlinico Universitario Agostino Gemelli IRCCS Foundation), Olbia, Italy; Department of Radiology (S Ramasamy MD), Stanford University, Stanford, CA, United States of America; Department of Research (C L Ranabhat PhD), Eastern Scientific LLC, Richmond, KY, United States of America; Department of Health Promotion and Administration (C L Ranabhat PhD), Eastern Kentucky University, Richmond, KY, United States of America; Centre for Clinical Pharmacology (N Rancic PhD), University of Defence in Belgrade, Belgrade, Serbia; Centre for Clinical Pharmacology (N Rancic PhD), Medical College of Georgia at Augusta University, Belgrade, Serbia; Department of Oral Pathology, Microbiology and Forensic Odontology (S Rao Other), Sharavathi Dental College and Hospital, Shimogga, India; Barcelona Institute for Global Health, Barcelona, Spain (Prof D Rasella PhD); Department of Medicine (A M Rashid MD), Jinnah Sindh Medical University, Karachi, Pakistan; Baylor University, Dallas, TX, United States of America (A M Rashid MD); Department of Biological Sciences (Prof E M M Redwan PhD), King Abdulaziz University, Jeddah, Egypt; Department of Protein Research (Prof E M M Redwan PhD), Research and Academic Institution, Alexandria, Egypt; Centre for Excellence in Pharmaceutical Sciences (K Rengasamy PhD), North-West University, Potchefstroom, South Africa; School of Medicine (Prof A M N Renzaho PhD), Western Sydney University, Campbelltown, NSW, Australia; Translational Health Research Institute (Prof A M N Renzaho PhD), Burnet Institute, Campbelltown, NSW, Australia; Department of Clinical Research (Prof L Roever PhD), University of Sao Paulo, Ribeirão Preto, Brazil; Gilbert and Rose-Marie Chagoury School of Medicine (Prof L Roever PhD), Lebanese American University, Beirut, Lebanon; Center for Indigenous Health Research (P Rohloff MD), Wuqu' Kawoq Maya Health Alliance, Tecpan, Guatemala; Department of Internal Medicine (G M Rwegerera MD), University of Botswana, Gaborone, Botswana; Faculty of Medicine (Z Saadatian PhD), Infectious Diseases Research Center (Z Saadatian PhD), Gonabad University of Medical Sciences, Gonabad, Iran; Health Information Management (M Sadeghi PhD), Semnan University of Medical Sciences, Semnan, Iran; Department of Pharmaceutical Chemistry (Prof M Saeb PhD), International Medical University, Gdańsk, Poland; Clinical and Biomedical Research Center (Prof U Saeed PhD), Foundation University Islamabad, Islamabad, Pakistan; International Center of Medical Sciences Research (ICMSR), Islamabad, Pakistan (Prof U Saeed PhD); Department of Family and Generations (H Sahoo PhD), International Institute for Population Sciences, Mumbai, India; Public Health and Community Medicine Department (M R Salem MD), Cairo University, Giza, Egypt; Department of Entomology (A M Samy PhD), Medical Ain Shams Research Institute (MASRI) (A M Samy PhD), Ain Shams University, Cairo, Egypt; Department of Pediatrics (Prof R K Sanjeev MD), SRM University, Chennai, India; Department of Public Health (Y Sarikhani PhD), Jahrom University of Medical Sciences, Jahrom, Iran; Department of Oral Pathology and Microbiology (Prof S C Sarode PhD), Dr. D.Y. Patil Vidyapeeth, Pune, India; UGC Centre of Advanced Study in Psychology (M Satpathy PhD), Utkal University, Bhubaneswar, India; Udyam-Global Association for Sustainable Development, Bhubaneswar, India (M Satpathy PhD); Department of Public Health Sciences (M Sawhney

PhD), University of North Carolina at Charlotte, Charlotte, NC, United States of America; Department of Preventive and Social Medicine (G Saya MD), Jawaharlal Institute of Postgraduate Medical Education and Research, Puducherry, India; Psychiatry Clinic (M Saylan MD), Holy Savior Armenian Hospital, Istanbul, Turkiye; Dobney Hypertension Centre (Prof M P Schlaich MD), The University of Western Australia, Perth, WA, Australia; Hypertension and Kidney Disease Laboratory (Prof M P Schlaich MD), Baker Heart and Diabetes Institute, Melbourne, VIC, Australia; Department of Health Sciences (I J C Schneider PhD), Federal University of Santa Catarina, Araranguá, Brazil; Department of Cardiovascular Sciences (A Schuermans BSc, J Van den Eynde BSc), Katholieke Universiteit Leuven, Leuven, Belgium; Department of Biomedical Sciences (P Sengupta PhD), Gulf Medical University, Ajman, United Arab Emirates; Emergency Department (S Senthilkumaran PhD), Manian Medical Centre, Erode, India; Fourth Department of General Surgery (D Serban PhD), Emergency University Hospital Bucharest, Bucharest, Romania; National Heart, Lung, and Blood Institute (A Seylani BS), National Institutes of Health, Rockville, MD, United States of America; Ophthalmology (J Shah BS), Weill Cornell Medicine, New York, NY, United States of America; Department of Microbiology (P A Shah MBBS), Rajiv Gandhi University of Health Sciences, Bangalore, India; Independent Consultant, Karachi, Pakistan (M A Shaikh MD); Department of Pathology and Laboratory Medicine (S Sham MD), Northwell Health, New York, NY, United States of America; Amity Institute of Public Health (M Shannawaz PhD), Amity University, Noida, India; institute of public health (M M Sharew MPH), University of Gondar, Gondar, Ethiopia; Finnish Institute of Occupational Health, Helsinki, Finland (R Shiri PhD); Department of Medical-Surgical Nursing (S Shorofi PhD), Mazandaran University of Medical Sciences, Sari, Iran; Psychology Department (K Shuja MS), National University of Modern Languages, Islamabad, Pakistan; The Cooper Institute, Dallas, TX, United States of America (K Shuval PhD); School of Health (Prof C R Simpson PhD), Victoria University of Wellington, Wellington, New Zealand; Usher Institute (Prof C R Simpson PhD), College of Medicine and Veterinary Medicine (G Verras MD), University of Edinburgh, Edinburgh, United Kingdom; School of Medicine (Prof J A Singh MD), Baylor College of Medicine, Houston, TX, United States of America; Department of Medicine Service (Prof J A Singh MD), US Department of Veterans Affairs (VA), Houston, TX, United States of America; Global and European Health Education and Study Institute (N Skhvitaridze MBA), University of Georgia, Tbilisi, Georgia; NCDC (N Skhvitaridze MBA), National Center for Disease Control and Public Health, Tbilisi, Georgia; Department of Surgery (B Socea PhD), "Sf. Pantelimon" Emergency Clinical Hospital Bucharest, Bucharest, Romania; School of Health and Biomedical Science (A Sohag MSc), Royal Melbourne Institute of Technology (RMIT) University, Melbourne, VIC, Australia; Department of Nursing (Y Solomon MSc), Department of Public Health (Y M Tefera MPH), Dire Dawa University, Dire Dawa, Ethiopia; Department of Health Policy and Management (S Song PhD), University of Georgia College of Public Health, Athens, GA, United States of America; 3rd Department of Cardiology (M Spartalis PhD), University of Athens, Athens, Greece; Center for Biotechnology and Microbiology (M Suleman PhD), University of Swat, Swat, Pakistan; School of Life Sciences (M Suleman PhD), Xiamen University, Xiamen, China; Department of Maternal and Child Health (S Sultana MPH), Projahnmo Research Foundation, Dhaka, Bangladesh; Yusuf Hamied Department of Chemistry (H Z Sun PhD), University of Cambridge, Cambridge, United Kingdom; Institute of Integrated Intelligence and Systems (Prof J Sun PhD), Griffith University, Brisbane, QLD, Australia; Northwestern

University, Chicago, IL, United States of America (M D Szeto MS); Department of Medicine (Prof R Tabarés-Seisdedos PhD), University of Valencia, Valencia, Spain; Department of Biostatistics and Epidemiology (M Taheri Soodejani PhD), Shahid Sadoughi University of Medical Sciences, Yazd, Iran; Department of Epidemiology (J L J Tamuzi MSc), Department of Industrial Psychology (E Teye-Kwadjo PhD), Stellenbosch University, Cape Town, South Africa; Department of Medicine (J L J Tamuzi MSc), Northlands Medical Group, Omuthiya, Namibia; Department of Surgery (K Tan PhD), Saw Swee Hock School of Public Health (Prof S Yi PhD), National University of Singapore, Singapore, Singapore; National Research and Innovation Agency, Jakarta, Indonesia (I U Tarigan PhD); School of Nursing and Midwifery (B Taye MSc), Department of Pediatrics and Child Health Nursing (S S Yehualashet MSc), Debre Berhan University, Debre Berhan, Ethiopia; Pediatric Intensive Care Unit (Prof M Temsah MD), King Saud University, Riyadh, Saudi Arabia; Department of Epidemiology and Biostatistics (M Teramoto MD), Department of Bioengineering and Therapeutical Sciences (Prof M Zastrozhin PhD), University of California San Francisco, San Francisco, CA, United States of America; Department of Psychology (E Teye-Kwadjo PhD), University of Ghana, Legon, Ghana; Department of Endocrinology, Diabetes and Metabolism (Prof N Thomas PhD), Christian Medical College and Hospital (CMC), Vellore, India; Laboratory of Public Health Indicators Analysis and Health Digitalization (M V Titova PhD), Moscow Institute of Physics and Technology, Moscow, Russia; Institute of Public Health (R Topor-Madry PhD), Jagiellonian University Medical College, Kraków, Poland; Agency for Health Technology Assessment and Tariff System, Warsaw, Poland (R Topor-Madry PhD); Department of Community Medicine and Family Medicine (J P Tripathy MD), All India Institute of Medical Sciences, Nagpur, India; Department of Health Sciences (S J Tromans PhD), University of Leicester, Leicester, United Kingdom; Adult Learning Disability Service (S J Tromans PhD), Leicestershire Partnership National Health Service Trust, Leicester, United Kingdom; Department of Public Health (C S Ubah DrPH), Brody School of Medicine, Greenville, NC, United States of America; College of Public Health (C S Ubah DrPH), Temple University, Philadelphia, PA, United States of America; Medical Genomics Research Department (Prof M Umair PhD), King Abdullah International Medical Research Center, Riyadh, Saudi Arabia; Department of Life Sciences (Prof M Umair PhD), University of Management and Technology, Lahore, Pakistan; Department of Paraclinical Sciences (S Umakanthan MD), University of the West Indies, St. Augustine, Trinidad and Tobago; Department of Cardiovascular, Endocrine-metabolic Diseases and Aging (B Unim PhD), National Institute of Health, Rome, Italy; College of Health and Sport Sciences (A G Vaithinathan MSc), University of Bahrain, Zallaq, Bahrain; Clinical Cancer Research Center (S Valadan Tahbaz PhD), Milad General Hospital, Tehran, Iran; Department of Microbiology (S Valadan Tahbaz PhD), Islamic Azad University, Tehran, Iran; Department of Biomedical Sciences (M Valenti MD), Humanitas University, Milan, Italy; Dermatology Unit (M Valenti MD), IRCCS Humanitas Research Hospital, Milan, Italy; Department of Surgery (G Verras MD), University of Southampton, Southampton, United Kingdom; Department of Health Science and Public Health (L Villani MD), Università Cattolica del Sacro Cuore (Catholic University of Sacred Heart), Rome, Italy; Occupational Medicine Unit (Prof F S Violante MD), Sant'Orsola Malpighi Hospital, Bologna, Italy; School of Public Health (F Wang PhD), Xuzhou Medical University, Xuzhou, China; Department of Neurosurgery (S Wang MD), Capital Medical University, Beijing, China; Department of Neurosurgery (S Wang MD), Beijing Tiantan Hospital, Beijing, China; Department of Basic Biomedical Sciences (Y Wang

MD), Shandong University, Jinan, China; Department of Neuroscience (Y Wang MD), Mount Sinai Health System, New York, United States of America; Department of Human Nutrition and Food Sciences (E G Wassie MSc), Debre Markos University, Debre Markos, Ethiopia; Department of Parasitology (Prof K G Weerakoon PhD), Rajarata University of Sri Lanka, Anuradhapura, Sri Lanka; National Data Management Center for Health (NDMC) (A A Wolde MPH), Ethiopian Public Health Institute, Addis Ababa, Ethiopia; Cardiovascular Program (X Xu PhD), The George Institute for Global Health, Sydney, NSW, Australia; Environmental Health and Epidemiology (V Yadav MD), National Institute for Research in Environmental Health, Bhopal, India; Department of Cancer Epidemiology and Prevention Research (L Yang PhD), Alberta Health Services, Calgary, AB, Canada; Faculty of Medicine (Y Yano MD), Department of Public Health (Prof N Yonemoto PhD), Juntendo University, Tokyo, Japan; KHANA Center for Population Health Research, Phnom Penh, Cambodia (Prof S Yi PhD); Department of Health Management (A Yiğit PhD, V Yiğit PhD), Süleyman Demirel University, Isparta, Turkiye; Department of Biostatistics (Prof N Yonemoto PhD), University of Toyama, Toyama, Japan; Basic Sciences Department (B A Zaman PhD), University of Duhok, Duhok, Iraq; Department of Administration (Prof M Zastrozhin PhD), PGxAI, San Francisco, CA, United States of America; School of Public Health (Y Zhang PhD), Wuhan University of Science and Technology, Wuhan, China; School of Public Health (Prof Z Zhang PhD), Wuhan University, Wuhan, China; College of Traditional Chinese Medicine (H Zhao MD), Hebei University, Baoding, China; Department of Public Health (L Zuhriyah PhD), Universitas Brawijaya, Malang, Indonesia.

8.3 Authors' Contributions

8.3.1 Managing the overall research enterprise

Tahiya Alam, Rakhi Dandona, Ashley Harris, Simon I Hay, Nicholas J Kassebaum, Christopher J L Murray, Mohsen Naghavi, and Austin E Schumacher.

8.3.2 Writing the first draft of the manuscript

Haley Comfort, Rakhi Dandona, Nicholas J Kassebaum, and Theresa A McHugh.

8.3.3 Primary responsibility for applying analytical methods to produce estimates

Haley Comfort, William M Gardner, Erin A May, and Katherine R Paulson.

8.3.4 Primary responsibility for seeking, cataloguing, extracting, or cleaning data; designing or coding figures and tables

Rakhi Dandona, Meghan E Frisch, John E Fuller, Nicholas J Kassebaum, Andrew T Leever, Mohsen Naghavi, Heather Jean Taylor, Corey Teply, and Nicholas Alexander Verghese.

8.3.5 Providing data or critical feedback on data sources

Yohannes Habtegiorgis Abate, Hedayat Abbastabar, Samar Abd ElHafeez, Rizwan Suliankatchi Abdulkader, Mesfin Abebe, Aidin Abedi, Hassan Abidi, Richard Gyan Aboagye, Hassan Abolhassani, Niveen ME Abu-Rmeileh, Mesafint Molla Adane, Qorinah Estiningtyas Sakilah Adnani, Saira Afzal, Antonella Agodi, Bright Opoku Ahinkorah, Danish Ahmad, Ali

Ahmadi, Ayman Ahmed, Haroon Ahmed, Fahad Mashhour Alanezi, Turki M Alanzi, Mohammed Albashtawy, Abdelazeem M Algammal, Abid Ali, Liaqat Ali, Sheikh Mohammad Alif, Syed Mohamed Aljunid, Joseph Uy Almazan, Louay Almidani, Sami Almustanyir, Edward Kwabena Ameyaw, Davood Anvari, Razique Anwer, Anton A Artamonov, Zatollah Asemi, Akeza Awealom Asgedom, Tahira Ashraf, Alok Atreya, Madhu Sudhan Atteraya, Beatriz Paulina Ayala Quintanilla, Ahmed Y Azzam, Mahsa Babaei, Sara Bagherieh, Maciej Banach, Aduragbemi Banke-Thomas, Berihun Bantie Bantie, Martina Barchitta, Mainak Bardhan, Mojtaba Bayani, Nebiyou Simegnew Bayleyegn, Uzma Iqbal Belgaumi, Dinesh Bhandari, Sonu Bhaskar, Suraj Bhattarai, Dejana Braithwaite, Nadeem Shafique Butt, Francieli Cembranel, Pamela Roxana Chacón-Uscamaita, Vijay Kumar Chattu, Malizgani Paul Chavula, Abdulaal Chitheer, Dinh-Toi Chu, Haley Comfort, Natalia Cruz-Martins, Lalit Dandona, Rakhi Dandona, Aso Mohammad Darwesh, Saswati Das, Aklilu Tamire Debele, Msganaw Derese, Anteneh Mengist Dessie, Arkadeep Dhali, Vishal R Dhulipala, Haneil Larson Dsouza, Andre Rodrigues Duraes, Ebrahim Eini, Michael Ekholuenetale, Maysaa El Sayed Zaki, Mohammed Elshaer, Hawi Leul Esayas, Adeniyi Francis Fagbamigbe, Ildar Ravisovich Fakhradiyev, Andre Faro, Ali Fatehizadeh, Ginenus Fekadu, Artem Alekseevich Fomenkov, Meghan E Frisch, Takeshi Fukumoto, John E Fuller, Peter Andras Gaal, Yaseen Galali, Silvano Gallus, Balasankar Ganesan, Amanuel Tesfay Gebremedhin, Teferi Gebru Gebremeskel, Yohannes Fikadu Geda, Mika Gissler, Mahaveer Golechha, Pouya Goleij, Avirup Guha, Sapna Gupta, Veer Bala Gupta, Vivek Kumar Gupta, Rabih Halwani, Josep Maria Haro, Ashley Harris, Ali Hasanpour- Dehkordi, Soheil Hassanipour, Wen-Qiang He, Claudiu Herteliu, Md Mahbub Hossain, Mehdi Hosseinzadeh, Chengxi Hu, M Mamun Huda, Md Nazmul Huda, Hong-Han Huynh, Olayinka Stephen Ilesanmi, Farideh Iravanpour, Jalil Jaafari, Haitham Jahrami, Tahereh Javaheri, Sathish Kumar Jayapal, Charity Ehimwenma Joshua, Mikk Jürisson, Zubair Kabir, Nicholas J Kassebaum, Evie Shoshannah Kendal, Mohammad Keykhaei, Nauman Khalid, Faham Khamesipour, M Nuruzzaman Khan, Maseer Khan, Yusra H Khan, Khaled Khatab, Haitham Khatatbeh, Sorour Khateri, Min Seo Kim, Thanh V Kim, Yun Jin Kim, Adnan Kisa, Sezer Kisa, Sonali Kochhar, Soewarta Kosen, Vijay Krishnamoorthy, Barthelemy Kuate Defo, Raja Amir Hassan Kuchay, G Anil Kumar, Chandrakant Lahariya, Tri Laksono, Dharmesh Kumar Lal, Savita Lasrado, Kamaluddin Latief, Kaveh Latifinaibin, Thao Thi Thu Le, Munjae Lee, Sang-woong Lee, Andrew T Leever, Shanshan Li, Virendra S Ligade, Stephen S Lim, Gang Liu, Jue Liu, Xuefeng Liu, László Lorenzovici, Azeem Majeed, Kashish Malhotra, Tauqeer Hussain Mallhi, Joemer C Maravilla, Francisco Rogerlândio Rogerlândio Martins-Melo, Melvin Barrientos Marzan, Rita Mattiello, Andrea Maugeri, Maryam Mazaheri, Rishi P Mediratta, Walter Mendoza, Ritesh G Menezes, Atte Meretoja, Irmina Maria Michalek, Le Huu Nhat Minh, Erkin M Mirrakhimov, Salahuddin Mohammed, Shafiu Mohammed, Ali H Mokdad, Sara Momtazmanesh, Lorenzo Monasta, Mohammad Ali Moni, Ulrich Otto Mueller, Faraz Mughal, Admir Mulita, Francesk Mulita, Christopher J L Murray, Mohsen Naghavi, Josephine W Ngunjiri, Dang H Nguyen, Hien Quang Nguyen, Phat Tuan Nguyen, Robina Khan Niazi, Lawrence Achilles Nnyanzi, Shuhei Nomura, Dieta Nurrika, Chimezie Igwegbe Nzoputam, Ogochukwu Janet Nzoputam, Bogdan Oancea, Kehinde O Obamiro, Akinkunmi Paul Okekunle, Osaretin Christabel Okonji, Andrew T Olagunju, Matthew Idowu Olatubi, Bolajoko Olubukunola Olusanya, Wael M S Osman, Uchechukwu Levi Osuagwu, Adrian Otoiu, Amel Ouyahia, Mayowa O Owolabi, Jagadish Rao Padubidri, Adrian Pana, Romil R Parikh, Sangram Kishor Patel, Shankargouda Patil, Katherine R Paulson, Shrikant Pawar, Veincent Christian Filipino Pepito, Prince Peprah,

Gavin Pereira, Jeevan Pereira, Maria Odete Pereira, Arokiasamy Perianayagam, Konrad Pesudovs, Tom Pham, My Kieu Phan, Anil K Philip, Zahra Zahid Piracha, Vivek Podder, Hadi Raeisi Shahraki, Pankaja Raghav, Shakthi Kumaran Ramasamy, Chhabi Lal Ranabhat, Nemanja Rancic, Chythra R Rao, Sowmya J Rao, Ahmed Mustafa Rashid, Reza Rawassizadeh, Kannan RR Rengasamy, Andre M N Renzaho, Hannah Elizabeth Robinson-Oden, Leonardo Roever, Peter Rohloff, Luca Ronfani, Godfrey M Rwegerera, Aly M A Saad, Siamak Sabour, Basema Ahmad Saddik, Umar Saeed, Marwa Rashad Salem, Abdallah M Samy, Maheswar Satpathy, Monika Sawhney, Mete Saylan, Markus P Schlaich, Ione Jayce Ceola Schneider, Austin E Schumacher, Subramanian Senthilkumaran, Dragos Serban, Pritik A Shah, Samiah Shahid, Masood Ali Shaikh, Sunder Sham, Mohammed Shannawaz, Mequannent Melaku Sharew, Adithi Shetty, B Suresh Kumar Shetty, Sina Shool, Kanwar Hamza Shuja, Negussie Boti Sidamo, Jasvinder A Singh, Paramdeep Singh, Natia Skhvitaridze, Yonatan Solomon, Michael Spartalis, Chandrashekhar T Sreeramareddy, Muhammad Suleman, Haitong Zhe Sun, Mindy D Szeto, Rafael Tabarés-Seisdedos, Shima Tabatabai, Moslem Taheri Soodejani, Ker-Kan Tan, Heather Jean Taylor, Yibekal Manaye Tefera, Corey Teply, Nihal Thomas, Amir Tiyuri, Marcos Roberto Tovani-Palone, Samuel Joseph Tromans, Muhammad Umair, Srikanth Umakanthan, Sahel Valadan Tahbaz, Jef Van den Eynde, Shoban Babu Varthya, Nicholas Alexander Verghese, Georgios-Ioannis Verras, Vasily Vlassov, Mandaras Tariku Walde, Shu Wang, Yuichiro Yano, Sisay Shewasinad Yehualashet, Siyan Yi, Naohiro Yonemoto, Burhan Abdullah Zaman, Michael Zastrozhin, and Magdalena Zielińska.

8.3.6 Developing methods or computational machinery

Aleksandr Y Aravkin, Haley Comfort, William M Gardner, Simon I Hay, Nicholas J Kassebaum, Andrew T Leever, Ali H Mokdad, Christopher J L Murray, Mohsen Naghavi, Katherine R Paulson, Austin E Schumacher, Heather Jean Taylor, Corey Teply, and Nicholas Alexander Verghese.

8.3.7 Providing critical feedback on methods or results

Yohannes Habtegiorgis Abate, Hedayat Abbastabar, Samar Abd ElHafeez, Michael Abdelmasseh, Sherief Abd-Elsalam, Daba Abdissa, Meriem Abdoun, Rizwan Suliankatchi Abdulkader, Mesfin Abebe, Aidin Abedi, Hassan Abidi, Olumide Abiodun, Richard Gyan Aboagye, Hassan Abolhassani, Michael R M Abrigo, Eman Abu-Gharbieh, Mesafint Molla Adane, Isaac Yeboah Addo, Bulcha Guye Adema, Miracle Ayomikun Adesina, Daniel Adedayo Adeyinka, Qorinah Estiningtyas Sakilah Adnani, Saira Afzal, Suneth Buddhika Agampodi, Antonella Agodi, Williams Agyemang-Duah, Bright Opoku Ahinkorah, Aqeel Ahmad, Danish Ahmad, Ali Ahmadi, Ayman Ahmed, Haroon Ahmed, Luai A Ahmed, Karolina Akinosoglou, Syed Mahfuz Al Hasan, Ziyad Al-Aly, Khurshid Alam, Fahad Mashhour Alanezi, Turki M Alanzi, Mohammed Albashtawy, Sharifullah Alemi, Abdelazeem M Algammal, Adel Ali Saeed Al-Gheethi, Abid Ali, Liaqat Ali, Mohammed Usman Ali, Sheikh Mohammad Alif, Syed Mohamed Aljunid, Joseph Uy Almazan, Hesham M Al-Mekhlafi, Louay Almidani, Sami Almustanyir, Khalid A Altirkawi, Hany Aly, Safwat Aly, Reza Amani, Edward Kwabena Ameyaw, Abebe Feyissa Amhare, Tarek Tawfik Amin, Sohrab Amiri, Catalina Liliana Andrei, Tudorel Andrei, Amir Anoushiravani, Adnan Ansar, Davood Anvari, Razique Anwer, Francis Appiah, Morteza Arab-Zozani, Demelash Areda, Anton A Artamonov, Mulu Tiruneh Asemu, Akeza Awealom Asgedom, Tahira Ashraf, Melash Belachew Asresie, Daniel Atlaw, Maha

Moh'd Wahbi Atout, Alok Atreya, Madhu Sudhan Atteraya, Beatriz Paulina Ayala Quintanilla, Haleh Ayatollahi, Sina Azadnajafabad, Rui M S Azevedo, Ahmed Y Azzam, Darshan B B, Mahsa Babaei, Muhammad Badar, Ashish D Badiye, Nayereh Baghcheghi, Soroush Baghdadi, Nasser Bagheri, Sara Bagherieh, Farshad Bahrami Asl, Ruhai Bai, Ravleen Kaur Bakshi, Maciej Banach, Aduragbemi Banke-Thomas, Hansi Bansal, Berihun Bantie Bantie, Martina Barchitta, Mainak Bardhan, Pritish Baskaran, Kavita Batra, Mojtaba Bayani, Nebiyou Simegnew Bayleyegn, Tahmina Begum, Amir Hossein Behnoush, Uzma Iqbal Belgaumi, Amiel Nazer C Bermudez, Dinesh Bhandari, Nikha Bhardwaj, Pankaj Bhardwaj, Sonu Bhaskar, Suraj Bhattarai, Virginia Bodolica, Dejana Braithwaite, Hermann Brenner, Yasser Bustanji, Nadeem Shafique Butt, Zahid A Butt, Abdul Cadri, Ismael Campos-Nonato, Francieli Cembranel, Ester Cerin, Pamela Roxana Chacón-Uscamaita, Jaykaran Charan, Vijay Kumar Chattu, Dhun Chauhan, Gerald Chi, William C S Cho, Sonali Gajanan Choudhari, Dinh-Toi Chu, Haley Comfort, Natalia Cruz-Martins, Omid Dadras, Maxwell Ayindenaba Dalaba, Lalit Dandona, Rakhi Dandona, Aso Mohammad Darwesh, Jai K Das, Saswati Das, Nihar Ranjan Dash, Kairat Davletov, Berhanu Gidisa Debela, Aklilu Tamire Debele, Msganaw Derese, Kebede Deribe, Emina Dervišević, Anteneh Mengist Dessie, Arkadeep Dhali, Vishal R Dhulipala, M Ashworth Dirac, Haneil Larson Dsouza, Sulagna Dutta, Arkadiusz Marian Dziedzic, Abdelaziz Ed-Dra, Kristina Edvardsson, Ebrahim Eini, Michael Ekholuenetale, Maysaa El Sayed Zaki, Islam Y Elgendy, Muhammed Elhadi, Mohammed Elshaer, Ibrahim Elsohaby, Theophilus I Emeto, Luchuo Engelbert Bain, Hawi Leul Esayas, Babak Eshrati, Adeniyi Francis Fagbamigbe, Ildar Ravisovich Fakhradiyev, Ali Faramarzi, Andre Faro, Ali Fatehizadeh, Ginenus Fekadu, Florian Fischer, Artem Alekseevich Fomenkov, Takeshi Fukumoto, Peter Andras Gaal, Abhay Motiramji Gaidhane, Yaseen Galali, Balasankar Ganesan, William M Gardner, Federica Gazzelloni, Mesfin Gebrehiwot, Amanuel Tesfay Gebremedhin, Teferi Gebru Gebremeskel, Yohannes Fikadu Geda, Kebede Embaye Gezae, Ramy Mohamed Ghazy, Gloria Gheno, Alessandro Gialluisi, Mika Gissler, James C Glasbey, Logan M Glasstetter, Mahaveer Golechha, Davide Golinelli, Michal Grivna, Avirup Guha, Stefano Guicciardi, Hanbing Guo, Sapna Gupta, Veer Bala Gupta, Vivek Kumar Gupta, Rabih Halwani, Samer Hamidi, Nicholas Nathaniel Hartman, Taufiq Hasan, Ali Hasanpour- Dehkordi, Md Saquib Hasnain, Soheil Hassanipour, Simon I Hay, Wen-Qiang He, Mohammad Heidari, Brenda Yuliana Herrera-Serna, Claudiu Herteliu, Kamran Hessami, Kamal Hezam, Yuta Hiraike, Ramesh Holla, Md Mahbub Hossain, Hassan Hosseinzadeh, Mehdi Hosseinzadeh, Mihaela Hostiuc, Chengxi Hu, M Mamun Huda, Md Nazmul Huda, Hong-Han Huynh, Bing-Fang Hwang, Pulwasha Maria Iftikhar, Olayinka Stephen Ilesanmi, Irena M Ilic, Milena D Ilic, Mustapha Immurana, Arad Iranmehr, Farideh Iravanpour, Masao Iwagami, Chidozie Declan Iwu, Assefa N Iyasu, Jalil Jaafari, Haitham Jahrami, Nilofer Javadi, Tahereh Javaheri, Sathish Kumar Jayapal, Alelign Tasew Jema, Mohammad Jokar, Nitin Joseph, Charity Ehimwenma Joshua, Mikk Jürisson, Ali Kabir, Zubair Kabir, Ibraheem M Karaye, Hanie Karimi, Nicholas J Kassebaum, Joonas H Kauppila, Evie Shoshannah Kendal, Nauman Khalid, Faham Khamesipour, M Nuruzzaman Khan, Maseer Khan, Yusra H Khan, Khaled Khatab, Haitham Khatatbeh, Moawiah Mohammad Khatatbeh, Sorour Khateri, Moein Khormali, Min Seo Kim, Thanh V Kim, Yun Jin Kim, Ruth W Kimokoti, Adnan Kisa, Sezer Kisa, Sonali Kochhar, Ali-Asghar Kolahi, Farzad Kompani, Hamid Reza Koohestani, Ai Koyanagi, Kewal Krishan, Vijay Krishnamoorthy, Barthelemy Kuate Defo, Mohammed Kuddus, G Anil Kumar, Om P Kurmi, Carlo La Vecchia, Ben Lacey, Chandrakant Lahariya, Tri Laksono, Dharmesh Kumar Lal, Savita Lasrado, Kamaluddin

Latief, Kaveh Latifinaibin, Thao Thi Thu Le, Munjae Lee, Sang-woong Lee, Wei-Chen Lee, Yo Han Lee, Andrew T Leever, Jacopo Lenzi, Ming-Chieh Li, Shanshan Li, Virendra S Ligade, Stephen S Lim, Gang Liu, Jue Liu, Xuefeng Liu, László Lorenzovici, Ahmed M. Afifi, Áurea M Madureira-Carvalho, Laura A Magee, Azeem Majeed, Elaheh Malakan Rad, Kashish Malhotra, Ahmad Azam Malik, Iram Malik, Tauqeer Hussain Mallhi, Joemer C Maravilla, Santi Martini, Francisco Rogerlândio Rogerlândio Martins-Melo, Miquel Martorell, Melvin Barrientos Marzan, Yasith Mathangasinghe, Rita Mattiello, Andrea Maugeri, Erin A May, Mahsa Mayeli, Rishi P Mediratta, Kamran Mehrabani-Zeinabad, Gebrekiros Gebremichael Meles, Hadush Negash Meles, Max Alberto Mendez-Lopez, Walter Mendoza, Ritesh G Menezes, Atte Meretoja, Tuomo J Meretoja, Irmina Maria Michalek, Le Huu Nhat Minh, Reza Mirfakhraie, Andreea Mirica, Erkin M Mirrakhimov, Moonis Mirza, Eric Mishio Bawa, Sanjeev Misra, Nouh Saad Mohamed, Sakineh Mohammad-Alizadeh-Charandabi, Ghada Mohammed, Salahuddin Mohammed, Shafiu Mohammed, Ali H Mokdad, Sabrina Molinaro, Sara Momtazmanesh, Mohammad Ali Moni, AmirAli Moodi Ghalibaf, Paula Moraga, Negar Morovatdar, Simin Mouodi, Ulrich Otto Mueller, Faraz Mughal, Admir Mulita, Francesk Mulita, Moses K Muriithi, Christopher J L Murray, Mohsen Naghavi, Tapas Sadasivan Nair, Hastyar Hama Rashid Najmuldeen, Gopal Nambi, Vinay Nangia, Javaid Nauman, Seyed Aria Nejadghaderi, Georges Nguefack-Tsague, Josephine W Ngunjiri, Dang H Nguyen, Hau Thi Hien Nguyen, Hien Quang Nguyen, Phat Tuan Nguyen, Robina Khan Niazi, Ali Nikoobar, Lawrence Achilles Nnyanzi, Efaq Ali Noman, Mamoona Noreen, Dieta Nurrika, Chimezie Igwegbe Nzoputam, Ogochukwu Janet Nzoputam, Bogdan Oancea, Kehinde O Obamiro, Ropo Ebenezer Ogunsakin, Akinkunmi Paul Okekunle, Osaretin Christabel Okonji, Patrick Godwin Okwute, Andrew T Olagunju, Babayemi Oluwaseun Olakunde, Matthew Idowu Olatubi, Isaac Iyinoluwa Olufadewa, Bolajoko Olubukunola Olusanya, Michal Ordak, Doris V Ortega-Altamirano, Wael M S Osman, Uchechukwu Levi Osuagwu, Adrian Otoiu, Nikita Otstavnov, Stanislav S Otstavnov, Amel Ouyahia, Mayowa O Owolabi, Jagadish Rao Padubidri, Adrian Pana, Pragyan Paramita Parija, Romil R Parikh, Ava Pashaei, Sangram Kishor Patel, Shankargouda Patil, Katherine R Paulson, Shrikant Pawar, Paolo Pedersini, Veincent Christian Filipino Pepito, Prince Peprah, Gavin Pereira, Jeevan Pereira, Marcos Pereira, Maria Odete Pereira, Arokiasamy Perianayagam, Konrad Pesudovs, Ionela-Roxana Petcu, Fanny Emily Petermann-Rocha, Parmida Sadat Pezeshki, My Kieu Phan, Anil K Philip, Manon Pigeolet, Zahra Zahid Piracha, Vivek Podder, Pranil Man Singh Pradhan, Hadi Raeisi Shahraki, Pankaja Raghav, Mosiur Rahman, Vahid Rahmanian, Chhabi Lal Ranabhat, Nemanja Rancic, Chythra R Rao, Sowmya J Rao, Ahmed Mustafa Rashid, Reza Rawassizadeh, Elrashdy Moustafa Mohamed Redwan, Kannan RR Rengasamy, Andre M N Renzaho, Nazila Rezaei, Negar Rezaei, Mohsen Rezaeian, Leonardo Roever, Peter Rohloff, Godfrey M Rwegerera, Aly M A Saad, Zahra Saadatian, Siamak Sabour, Basema Ahmad Saddik, Malihe Sadeghi, Mohammad Reza Saeb, Umar Saeed, Amene Saghazadeh, Fatemeh Saheb Sharif-Askari, Narjes Saheb Sharif-Askari, Harihar Sahoo, Soumya Swaroop Sahoo, Mohamed A Saleh, Sana Salehi, Marwa Rashad Salem, Abdallah M Samy, Rama Krishna Sanjeev, Yaser Sarikhani, Sachin C Sarode, Maheswar Satpathy, Monika Sawhney, Ganesh Kumar Saya, Mete Saylan, Markus P Schlaich, Ione Jayce Ceola Schneider, Art Schuermans, Austin E Schumacher, Pallav Sengupta, Subramanian Senthilkumaran, Sadaf G Sepanlou, Dragos Serban, SeyedAhmad SeyedAlinaghi, Mahan Shafie, Jaffer Shah, Pritik A Shah, Samiah Shahid, Masood Ali Shaikh, Mohd Shanawaz, Mohammed Shannawaz, Mequannent Melaku Sharew, Rahman Shiri, Siddharudha Shivalli, Sina Shool, Seyed Afshin Shorofi,

Kanwar Hamza Shuja, Kerem Shuval, Migbar Mekonnen Sibhat, Negussie Boti Sidamo, Jasvinder A Singh, Paramdeep Singh, Natia Skhvitaridze, Abdullah Al Mamun Sohag, Hamidreza Soleimani, Yonatan Solomon, Yi Song, Michael Spartalis, Chandrashekhar T Sreeramareddy, Andy Stergachis, Muhammad Suleman, Saima Sultana, Haitong Zhe Sun, Jing Sun, Mindy D Szeto, Rafael Tabarés-Seisdedos, Shima Tabatabai, Mohammad Tabish, Majid Taheri, Jacques Lukenze Tamuzi, Ker-Kan Tan, Ingan Ukur Tarigan, Razieh Tavakoli Oliaee, Birhan Tsegaw Taye, Heather Jean Taylor, Yibekal Manaye Tefera, Mohamad-Hani Temsah, Corey Teply, Masayuki Teramoto, Wegen Beyene Tesfamariam, Enoch Teye-Kwadjo, Aravind Thavamani, Nihal Thomas, Mariya Vladimirovna Titova, Amir Tiyuri, Roman Topor-Madry, Marcos Roberto Tovani-Palone, Jaya Prasad Tripathy, Samuel Joseph Tromans, Chukwudi S Ubah, Muhammad Umair, Srikanth Umakanthan, Sahel Valadan Tahbaz, Rohollah Valizadeh, Jef Van den Eynde, Shoban Babu Varthya, Nicholas Alexander Verghese, Massimiliano Veroux, Georgios-Ioannis Verras, Leonardo Villani, Francesco S Violante, Fang Wang, Shu Wang, Yanqing Wang, Yanzhong Wang, Emebet Gashaw Wassie, Kosala Gayan Weerakoon, Asrat Arja Wolde, Xiaoyue Xu, Vikas Yadav, Sisay Shewasinad Yehualashet, Siyan Yi, Arzu Yiğit, Vahit Yiğit, Paul Yip, Naohiro Yonemoto, Nazar Zaki, Giulia Zamagni, Burhan Abdullah Zaman, Michael Zastrozhin, Haijun Zhang, Yunquan Zhang, Hanqing Zhao, Claire Chenwen Zhong, Magdalena Zielińska, and Lilik Zuhriyah.

8.3.8 Drafting the work or revising it critically for important intellectual content

Yohannes Habtegiorgis Abate, Samar Abd ElHafeez, Michael Abdelmasseh, Sherief Abd-Elsalam, Daba Abdissa, Mesfin Abebe, Aidin Abedi, Hassan Abidi, Olumide Abiodun, Hassan Abolhassani, Eman Abu-Gharbieh, Niveen ME Abu-Rmeileh, Mesafint Molla Adane, Isaac Yeboah Addo, Bulcha Guye Adema, Charles Oluwaseun Oluwaseun Adetunji, Daniel Adedayo Adeyinka, Qorinah Estiningtyas Sakilah Adnani, Saira Afzal, Suneth Buddhika Agampodi, Antonella Agodi, Bright Opoku Ahinkorah, Danish Ahmad, Ali Ahmadi, Ayman Ahmed, Haroon Ahmed, Luai A Ahmed, Marjan Ajami, Khurshid Alam, Mohammed Albashtawy, Abdelazeem M Algammal, Abid Ali, Liaqat Ali, Mohammed Usman Ali, Louay Almidani, Sami Almustanyir, Hany Aly, Safwat Aly, Reza Amani, Tarek Tawfik Amin, Sohrab Amiri, Amir Anoushiravani, Francis Appiah, Morteza Arab-Zozani, Brhane Berhe Aregawi, Umesh Raj Aryal, Mulu Tiruneh Asemu, Daniel Atlaw, Maha Moh'd Wahbi Atout, Alok Atreya, Avinash Aujayeb, Beatriz Paulina Ayala Quintanilla, Seyed Mohammad Ayyoubzadeh, Sina Azadnajafabad, Rui M S Azevedo, Ahmed Y Azzam, Mahsa Babaei, Muhammad Badar, Ashish D Badiye, Soroush Baghdadi, Sara Bagherieh, Ruhai Bai, Kiran Bam, Maciej Banach, Aduragbemi Banke-Thomas, Hansi Bansal, Berihun Bantie Bantie, Martina Barchitta, Azadeh Bashiri, Afisu Basiru, Pritish Baskaran, Mojtaba Bayani, Neeraj Bedi, Amir Hossein Behnoush, Uzma Iqbal Belgaumi, Kebede A Beyene, Bharti Bhandari Bhandari, Dinesh Bhandari, Sonu Bhaskar, Virginia Bodolica, Dejana Braithwaite, Hermann Brenner, Yasser Bustanji, Abdul Cadri, Ismael Campos-Nonato, Maria Sofia Cattaruzza, Francieli Cembranel, Ester Cerin, Pamela Roxana Chacón-Uscamaita, Vijay Kumar Chattu, Dhun Chauhan, Malizgani Paul Chavula, Simiao Chen, William C S Cho, Dinh-Toi Chu, Haley Comfort, Natalia Cruz-Martins, Nihar Ranjan Dash, Claudio Alberto Dávila-Cervantes, Aklilu Tamire Debele, Msganaw Derese, Kebede Deribe, Anteneh Mengist Dessie, Arkadeep Dhali, Vishal R Dhulipala, M Ashworth Dirac, Wanyue Dong, Bezabih Terefe Dora, Haneil Larson Dsouza, Arkadiusz Marian Dziedzic, Ebrahim Eini, Michael Ekholuenetale, Maysaa El Sayed Zaki, Islam Y Elgendy, Muhammed Elhadi, Mohammed Elshaer, Theophilus I Emeto,

Francesco Esposito, Adeniyi Francis Fagbamigbe, Ali Faramarzi, Andre Faro, Ali Fatehizadeh, Florian Fischer, Takeshi Fukumoto, Peter Andras Gaal, Márió Gajdács, Yaseen Galali, Silvano Gallus, Balasankar Ganesan, Federica Gazzelloni, Amanuel Tesfay Gebremedhin, Teferi Gebru Gebremeskel, Yohannes Fikadu Geda, Ramy Mohamed Ghazy, Alessandro Gialluisi, James C Glasbey, Davide Golinelli, Michal Grivna, Avirup Guha, Stefano Guicciardi, Sapna Gupta, Veer Bala Gupta, Vivek Kumar Gupta, Sebastian Haller, Rabih Halwani, Alexis J Handal, Josep Maria Haro, Ali Hasanpour- Dehkordi, Md Saquib Hasnain, Simon I Hay, Wen-Qiang He, Claudiu Herteliu, Kamran Hessami, Kamal Hezam, Yuta Hiraike, Ramesh Holla, Md Mahbub Hossain, Sorin Hostiuc, Junjie Huang, Md Nazmul Huda, Hong-Han Huynh, Pulwasha Maria Iftikhar, Olayinka Stephen Ilesanmi, Irena M Ilic, Milena D Ilic, Mustapha Immurana, Arad Iranmehr, Farideh Iravanpour, Chidozie Declan Iwu, Assefa N Iyasu, Abdollah Jafarzadeh, Haitham Jahrami, Manthan Dilipkumar Janodia, Nilofer Javadi, Sathish Kumar Jayapal, Alelign Tasew Jema, Nitin Joseph, Charity Ehimwenma Joshua, Mikk Jürisson, Ali Kabir, Hanie Karimi, Hengameh Kasraei, Nicholas J Kassebaum, Joonas H Kauppila, Evie Shoshannah Kendal, Nauman Khalid, M Nuruzzaman Khan, Maseer Khan, Yusra H Khan, Khaled Khatab, Haitham Khatatbeh, Moawiah Mohammad Khatatbeh, Hamid Reza Khayat Kashani, Min Seo Kim, Yun Jin Kim, Adnan Kisa, Sezer Kisa, Sonali Kochhar, Farzad Kompani, Ai Koyanagi, Kewal Krishan, Barthelemy Kuate Defo, Raja Amir Hassan Kuchay, Mohammed Kuddus, Om P Kurmi, Carlo La Vecchia, Chandrakant Lahariya, Savita Lasrado, Kamaluddin Latief, Kaveh Latifinaibin, Thao Thi Thu Le, Jacopo Lenzi, Jue Liu, Xuefeng Liu, László Lorenzovici, Masoud Lotfizadeh, Ahmed M. Afifi, Áurea M Madureira-Carvalho, Laura A Magee, Elaheh Malakan Rad, Kashish Malhotra, Ahmad Azam Malik, Tauqeer Hussain Mallhi, Francisco Rogerlândio Rogerlândio Martins-Melo, Miquel Martorell, Yasith Mathangasinghe, Andrea Maugeri, Mahsa Mayeli, Theresa A McHugh, Rishi P Mediratta, Gebrekiros Gebremichael Meles, Hadush Negash Meles, Max Alberto Mendez-Lopez, Walter Mendoza, Ritesh G Menezes, Atte Meretoja, Tuomo J Meretoja, Irmina Maria Michalek, Le Huu Nhat Minh, Reza Mirfakhraie, Mojgan Mirghafourvand, Moonis Mirza, Eric Mishio Bawa, Biru Abdissa Mizana, Nouh Saad Mohamed, Sakineh Mohammad-Alizadeh-Charandabi, Ghada Mohammed, Salahuddin Mohammed, Shafiu Mohammed, Ali H Mokdad, Sabrina Molinaro, Sara Momtazmanesh, Lorenzo Monasta, Mohammad Ali Moni, AmirAli Moodi Ghalibaf, Paula Moraga, Abbas Mosapour, Parsa Mousavi, Ulrich Otto Mueller, Faraz Mughal, Admir Mulita, Francesk Mulita, Christopher J L Murray, Tapas Sadasivan Nair, Gustavo G Nascimento, Javaid Nauman, Seyed Aria Nejadghaderi, Mohammad Hadi Nematollahi, Georges Nguefack-Tsague, Josephine W Ngunjiri, Dang H Nguyen, Hau Thi Hien Nguyen, Hien Quang Nguyen, Phat Tuan Nguyen, Robina Khan Niazi, Lawrence Achilles Nnyanzi, Mamoona Noreen, Dieta Nurrika, Chimezie Igwegbe Nzoputam, Ogochukwu Janet Nzoputam, Bogdan Oancea, Kehinde O Obamiro, Sylvester Reuben Okeke, Osaretin Christabel Okonji, Patrick Godwin Okwute, Andrew T Olagunju, Babayemi Oluwaseun Olakunde, Matthew Idowu Olatubi, Bolajoko Olubukunola Olusanya, Michal Ordak, Doris V Ortega-Altamirano, Wael M S Osman, Uchechukwu Levi Osuagwu, Adrian Otoiu, Nikita Otstavnov, Stanislav S Otstavnov, Amel Ouyahia, Mayowa O Owolabi, Alicia Padron-Monedero, Jagadish Rao Padubidri, Romil R Parikh, Shankargouda Patil, Shrikant Pawar, Paolo Pedersini, Veincent Christian Filipino Pepito, Gavin Pereira, Jeevan Pereira, Marcos Pereira, Arokiasamy Perianayagam, Norberto Perico, Konrad Pesudovs, Ionela-Roxana Petcu, Fanny Emily Petermann-Rocha, Zahra Zahid Piracha, Vivek Podder, Dimitri Poddighe, Pranil Man Singh Pradhan, Hadi Raeisi Shahraki, Pankaja Raghav, Ivano

Raimondo, Shakthi Kumaran Ramasamy, Chhabi Lal Ranabhat, Nemanja Rancic, Chythra R Rao, Sowmya J Rao, Davide Rasella, Ahmed Mustafa Rashid, Elrashdy Moustafa Mohamed Redwan, Giuseppe Remuzzi, Kannan RR Rengasamy, Andre M N Renzaho, Nazila Rezaei, Leonardo Roever, Peter Rohloff, Luca Ronfani, Godfrey M Rwegerera, Aly M A Saad, Siamak Sabour, Basema Ahmad Saddik, Umar Saeed, Dominic Sagoe, Fatemeh Saheb Sharif-Askari, Narjes Saheb Sharif-Askari, Amirhossein Sahebkar, Soumya Swaroop Sahoo, Marwa Rashad Salem, Abdallah M Samy, Yaser Sarikhani, Sachin C Sarode, Maheswar Satpathy, Ganesh Kumar Saya, Mete Saylan, Markus P Schlaich, Ione Jayce Ceola Schneider, Art Schuermans, Sadaf G Sepanlou, Dragos Serban, Allen Seylani, Mahan Shafie, Jaffer Shah, Pritik A Shah, Samiah Shahid, Mohd Shanawaz, Mohammed Shannawaz, Mequannent Melaku Sharew, Manoj Sharma, Pavanchand H Shetty, Reza Shirkoohi, Siddharudha Shivalli, Sina Shool, Seyed Afshin Shorofi, Migbar Mekonnen Sibhat, Negussie Boti Sidamo, João Pedro Silva, Colin R Simpson, Jasvinder A Singh, Paramdeep Singh, Surjit Singh, Natia Skhvitaridze, Bogdan Socea, Hamidreza Soleimani, Yonatan Solomon, Suhang Song, Yi Song, Michael Spartalis, Chandrashekhar T Sreeramareddy, Muhammad Suleman, Shima Tabatabai, Mohammad Tabish, Majid Taheri, Jacques Lukenze Tamuzi, Ker-Kan Tan, Razieh Tavakoli Oliaee, Birhan Tsegaw Taye, Yibekal Manaye Tefera, Mohamad-Hani Temsah, Masayuki Teramoto, Wegen Beyene Tesfamariam, Samar Tharwat, Nihal Thomas, Roman Topor-Madry, Marcos Roberto Tovani-Palone, Jaya Prasad Tripathy, Samuel Joseph Tromans, Chukwudi S Ubah, Muhammad Umair, Srikanth Umakanthan, Brigid Unim, Asokan Govindaraj Vaithinathan, Sahel Valadan Tahbaz, Mario Valenti, Jef Van den Eynde, Shoban Babu Varthya, Massimiliano Veroux, Georgios-Ioannis Verras, Leonardo Villani, Vasily Vlassov, Mandaras Tariku Walde, Fang Wang, Shu Wang, Yanzhong Wang, Emebet Gashaw Wassie, Kosala Gayan Weerakoon, Vikas Yadav, Lin Yang, Arzu Yiğit, Vahit Yiğit, Naohiro Yonemoto, Burhan Abdullah Zaman, Michael Zastrozhin, Haijun Zhang, Zhi-Jiang Zhang, Claire Chenwen Zhong, and Magdalena Zielińska.

8.3.9 Managing the estimation or publications process

Tahiya Alam, Haley Comfort, Simon I Hay, Nicholas J Kassebaum, Theresa A McHugh, Ali H Mokdad, Christopher J L Murray, and Mohsen Naghavi.
