# Supplementary material for: Global, regional, and national stillbirths at 20 weeks' gestation or longer in 204 countries and territories, 1990–2021: findings from the Global Burden of Disease Study 2021
Source: Lancet. 2024 Nov 16;404(10466):1955–88. doi: 10.1016/S0140-6736(24)01925-1 (PMC11694012; doi:10.1016/S0140-6736(24)01925-1)
Supplement: Supplementary appendix 2 [file mmc2.pdf]

THE LANCET

Supplementary appendix 2

This appendix formed part of the original submission and has been peer reviewed. We post it as supplied by the authors.

Supplement to: GBD 2021 Global Stillbirths Collaborators. Global, regional, and national stillbirths at 20 weeks' gestation or longer in 204 countries and territories, 1990–2021: findings from the Global Burden of Disease Study 2021. *Lancet* 2024; published online Nov 4. https://doi.org/10.1016/S0140-6736(24)01925-1.

Appendix 2 to Stillbirths GBD 2021

Table of Contents

1 Appendix 2 Figure S1: Regional trends in the number of stillbirths, stillbirth rate (SBR; stillbirths per 1000 births), SBR/NMR (neonatal deaths per 1000 live births), and annualised rate of change (AROC) of SBR, by threshold, 1990 to 2021 2

2 Appendix 2 Figure S2: Maps of stillbirth rate (SBR; stillbirths ≥20, ≥22, and ≥28 weeks' gestation per 1000 births), percentage of stillbirths between 20 and <28 weeks' gestation, and number of stillbirths between 20 and 28 weeks' gestation in 1990, 2000 2010, 2015, 2020, and 2021 2

3 Appendix 2 Figure S3: Comparison of SBR estimates to LSEIG 2015 estimates and IGME 2021 estimates 2

4 Appendix 2 Figure S4: Model results, source list, and final estimates of number of stillbirths, SBR, and SBR/NMR for both thresholds, by location 3

5 Appendix 2 Table S1A: Annual estimates of stillbirth (in thousands) for ≥20 weeks, by location 3

6 Appendix 2 Table S1B: Annual estimates of stillbirth (in thousands) for ≥22 weeks, by location 3

7 Appendix 2 Table S1C: Annual estimates of stillbirth (in thousands) for ≥28 weeks, by location 3

8 Appendix 2 Table S2A: Annual estimates of SBR (per 1000 births) for ≥20 weeks, by location 3

9 Appendix 2 Table S2B: Annual estimates of SBR (per 1000 births) for ≥22 weeks, by location 4

10 Appendix 2 Table S2C: Annual estimates of SBR (per 1000 births) for ≥28 weeks, by location 4

11 Appendix 2 Table S3A: Annual estimates of SBR/NMR for ≥20 weeks, by location 4

12 Appendix 2 Table S3B: Annual estimates of SBR/NMR for ≥22 weeks, by location 4

13 Appendix 2 Table S3C: Annual estimates of SBR/NMR for ≥28 weeks, by location 4

14 Appendix 2 Table S4: Distribution of countries based on stillbirth performance 4

15 Appendix 2 Table S5: Comparison of GBD v. LSEIG for 2014 and GBD v. IGME for 2021 4

1 Appendix 2 Figure S1: Regional trends in the number of stillbirths, stillbirth rate (SBR; stillbirths per 1000 births), SBR/NMR (neonatal deaths per 1000 live births), and annualised rate of change (AROC) of SBR, by threshold, 1990 to 2021

```
Caption: (A) The solid lines represent the estimates for the total number of
stillbirths (in millions) for the ≥20 weeks and ≥28 weeks thresholds and the
dashed lines indicate the 95% uncertainty interval (UI), specified by color,
between 1990 and 2021. (B) The solid lines represent the SBR estimates per 10
00 births for the ≥20 weeks and ≥28 weeks thresholds and the dashed lines ind
icate the 95% UI, specified by color, between 1990 and 2021. (C) The solid li
nes represent the SBR/NMR estimates for the ≥20 weeks and ≥28 weeks threshold
s and the dashed lines indicate the 95% UI, specified by color, between 1990
and 2021. (D) The solid lines represent the annualised rate of change of SBR
between adjacent years for the ≥20 weeks and ≥28 weeks thresholds and the das
hed lines indicate the 95% UI, between 1990 and 2021. SBR=stillbirth rate. NM
R=neonatal mortality rate. AROC=annualised rate of change.
```

2 Appendix 2 Figure S2: Maps of stillbirth rate (SBR; stillbirths ≥20, ≥22, and ≥28 weeks' gestation per 1000 births), percentage of stillbirths between 20 and <28 weeks' gestation, and number of stillbirths between 20 and 28 weeks' gestation in 1990, 2000, 2010, 2015, 2020, and 2021

```
Caption: (A) The map shows SBR (per 1000 births) in the specified year for th
e ≥20 weeks threshold. The color scale diverges at an SBR of 12 to indicate w
hether countries met the ENAP target of 12 or fewer stillbirths per 1000 birt
hs in the specified year. (B) The map shows SBR (per 1000 births) in the spec
ified year for the ≥28 weeks threshold. The color scale diverges at an SBR of
12 to indicate whether countries met the ENAP target of 12 or fewer stillbirt
hs per 1000 births in the specified year. (C) The map shows the percentage of
stillbirths that occurred between 20 and <28 weeks' gestation in the specifie
d year. Countries with a larger percentage of stillbirths between 20 and <28
weeks' are darker in color. (D) The map shows the number of stillbirths that
occurred between 20 and <28 weeks' gestation in the specified year. Countries
with a larger percentage of stillbirths between 20 and <28 weeks' are darker
in color. SBR=stillbirth rate. ENAP=Every Newborn Action Plan.
```

3 Appendix 2 Figure S3: Comparison of SBR estimates to LSEIG 2015 estimates and IGME 2021 estimates

```
Caption: The scatter compares the SBR (per 1000 births) estimates for the ≥28
weeks threshold between GBD versus LSEIG in 2015 and GBD versus IGME estimate
s in 2021 where color denotes the group which released the estimate and shape
indicates year. SBR=stillbirth rate. GBD=Global Burden of Diseases, Injuries,
```

and Risk Factors Study. LSEIG=Lancet Stillbirth Epidemiology Investigator Group. IGME=UN Inter-agency Group for Child Mortality Estimation.

4 Appendix 2 Figure S4: Model results, source list, and final estimates of number of stillbirths, SBR, and SBR/NMR for both thresholds, by location

Caption: Each page depicts the input data throughout the various adjustment steps and the results from each stage of the model for a given location between 1990 and 2021. The top two graphs show SBR/NMR for the ≥28 week threshold (left) and ≥20 weeks threshold (right). The bottom two graphs show SBR (per 1000 births) for the ≥28 week threshold (left) and ≥20 weeks threshold (right). On the far right, the top table lists the input data sources and the bottom table provides results for total stillbirths (thousands), SBR (per 1000 births), and SBR/NMR for each five-year period from 1990 to 2020 as well as 2021. SBR=stillbirth rate. NMR=neonatal mortality rate.

5 Appendix 2 Table S1A: Annual estimates of stillbirth (in thousands) for ≥20 weeks, by location

Caption: The table shows annual estimates of stillbirth (in thousands) for the ≥20 weeks gestation threshold.

6 Appendix 2 Table S1B: Annual estimates of stillbirth (in thousands) for ≥22 weeks, by location

Caption: The table shows annual estimates of stillbirth (in thousands) for the ≥22 weeks gestation threshold.

7 Appendix 2 Table S1C: Annual estimates of stillbirth (in thousands) for ≥28 weeks, by location

Caption: The table shows annual estimates of stillbirth (in thousands) for the ≥28 weeks gestation threshold.

8 Appendix 2 Table S2A: Annual estimates of SBR (per 1000 births) for ≥20 weeks, by location

Caption: The table shows annual estimates of SBR (per 1000 births) for the ≥20 weeks gestation threshold. SBR=stillbirth rate.

9 Appendix 2 Table S2B: Annual estimates of SBR (per 1000 births) for ≥22 weeks, by location

```
Caption: The table shows annual estimates of SBR (per 1000 births) for the ≥2
2 weeks gestation threshold. SBR=stillbirth rate.
```

10 Appendix 2 Table S2C: Annual estimates of SBR (per 1000 births) for ≥28 weeks, by location

```
Caption: The table shows annual estimates of SBR (per 1000 births) for the ≥2
8 weeks gestation threshold. SBR=stillbirth rate.
```

11 Appendix 2 Table S3A: Annual estimates of SBR/NMR for ≥20 weeks, by location

```
Caption: The table shows annual estimates of SBR/NMR for the ≥20 weeks gestat
ion threshold. SBR=stillbirth rate. NMR=neonatal mortality rate.
```

12 Appendix 2 Table S3B: Annual estimates of SBR/NMR for ≥22 weeks, by location

```
Caption: The table shows annual estimates of SBR/NMR for the ≥22 weeks gestat
ion threshold. SBR=stillbirth rate. NMR=neonatal mortality rate.
```

13 Appendix 2 Table S3C: Annual estimates of SBR/NMR for ≥28 weeks, by location

```
Caption: The table shows annual estimates of SBR/NMR for the ≥28 weeks gestat
ion threshold. SBR=stillbirth rate. NMR=neonatal mortality rate.
```

14 Appendix 2 Table S4: Distribution of countries based on stillbirth performance

```
Caption: The table identifies the countries present in each of the 25 groups
based on O:E ratio of SBR and delta NMR/SBR that are illustrated in Figure 4C
in the main text.
```

15 Appendix 2 Table S5: Comparison of GBD v. LSEIG for 2015 and GBD v. IGME for 2021

```
Caption: The table compares estimates of SBR (per 1000 births) between GBD, L
SEIG, and IGME. GBD=Global Burden of Diseases, Injuries, and Risk Factors Stu
dy. IGME=UN Inter-agency Group for Child Mortality Investigation. LSEIG=Lance
t Stillbirth Epidemiology Investigator Group. SBR=stillbirth rate.
```

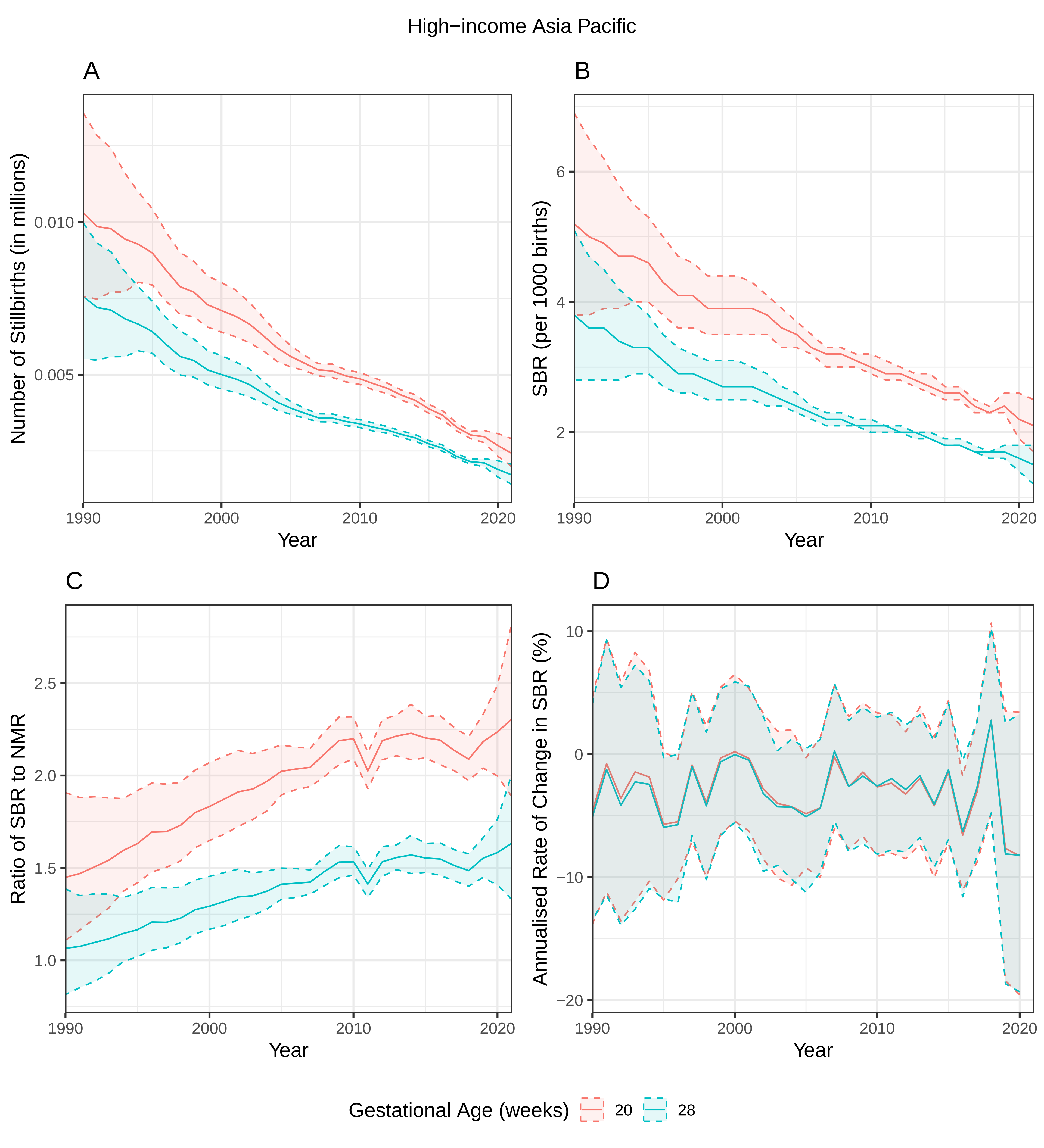

High−income Asia Pacific
A
Number of Stillbirths (in millions)
0.010
0.005
1990
2000
2010
2020
Year
B
SBR (per 1000 births)
6
4
2
1990
2000
2010
2020
Year
C
Ratio of SBR to NMR
2.5
2.0
1.5
1.0
1990
2000
2010
2020
Year
D
Annualised Rate of Change in SBR (%)
10
0
−10
−20
1990
2000
2010
2020
Year
Gestational Age (weeks)
20
28

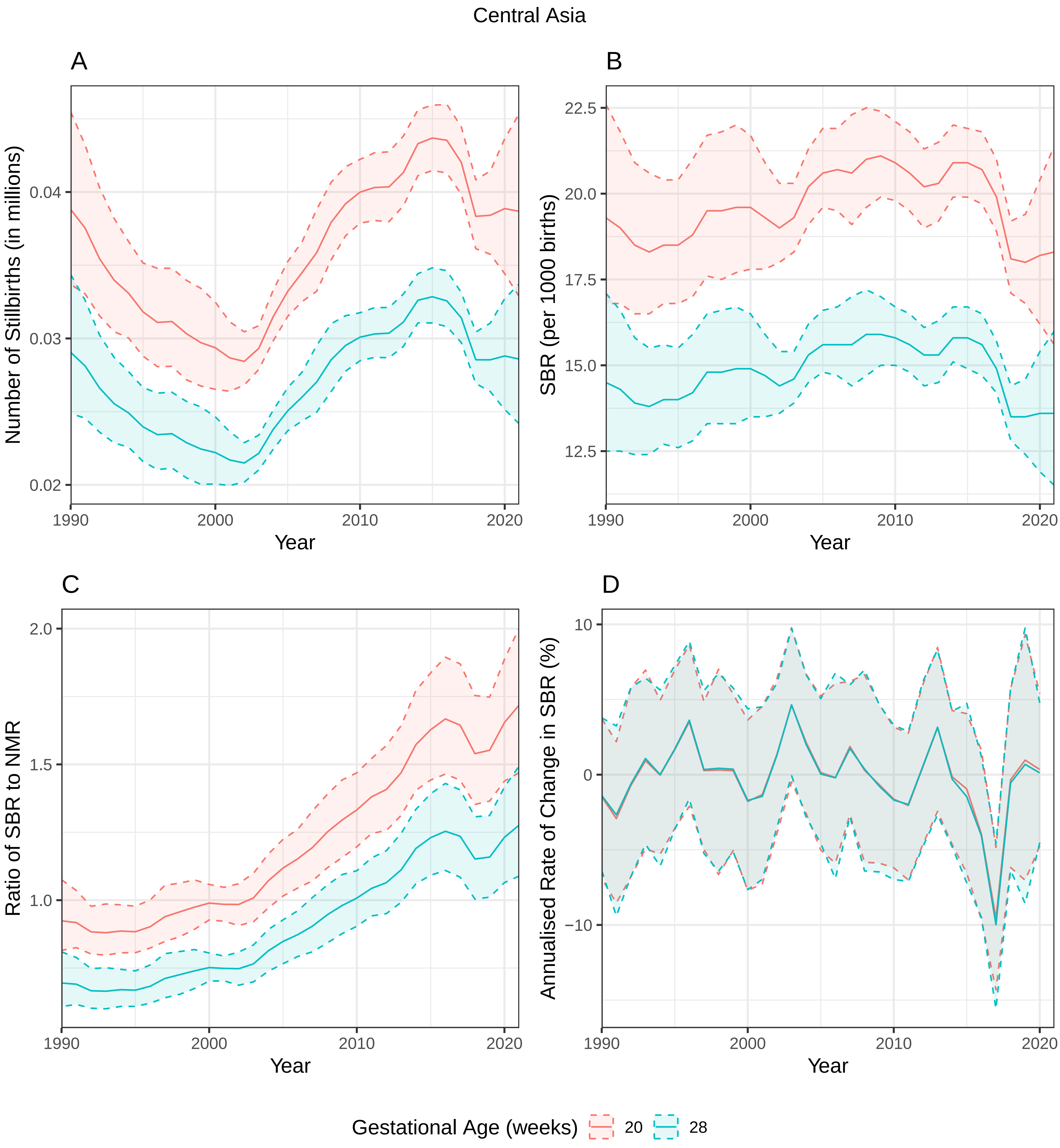
Central Asia
A
Number of Stillbirths (in millions)
0.04
0.03
0.02
1990
2000
2010
2020
Year
B
SBR (per 1000 births)
22.5
20.0
17.5
15.0
12.5
1990
2000
2010
2020
Year
C
Ratio of SBR to NMR
2.0
1.5
1.0
1990
2000
2010
2020
Year
D
Annualised Rate of Change in SBR (%)
10
0
−10
1990
2000
2010
2020
Year
Gestational Age (weeks)
20
28

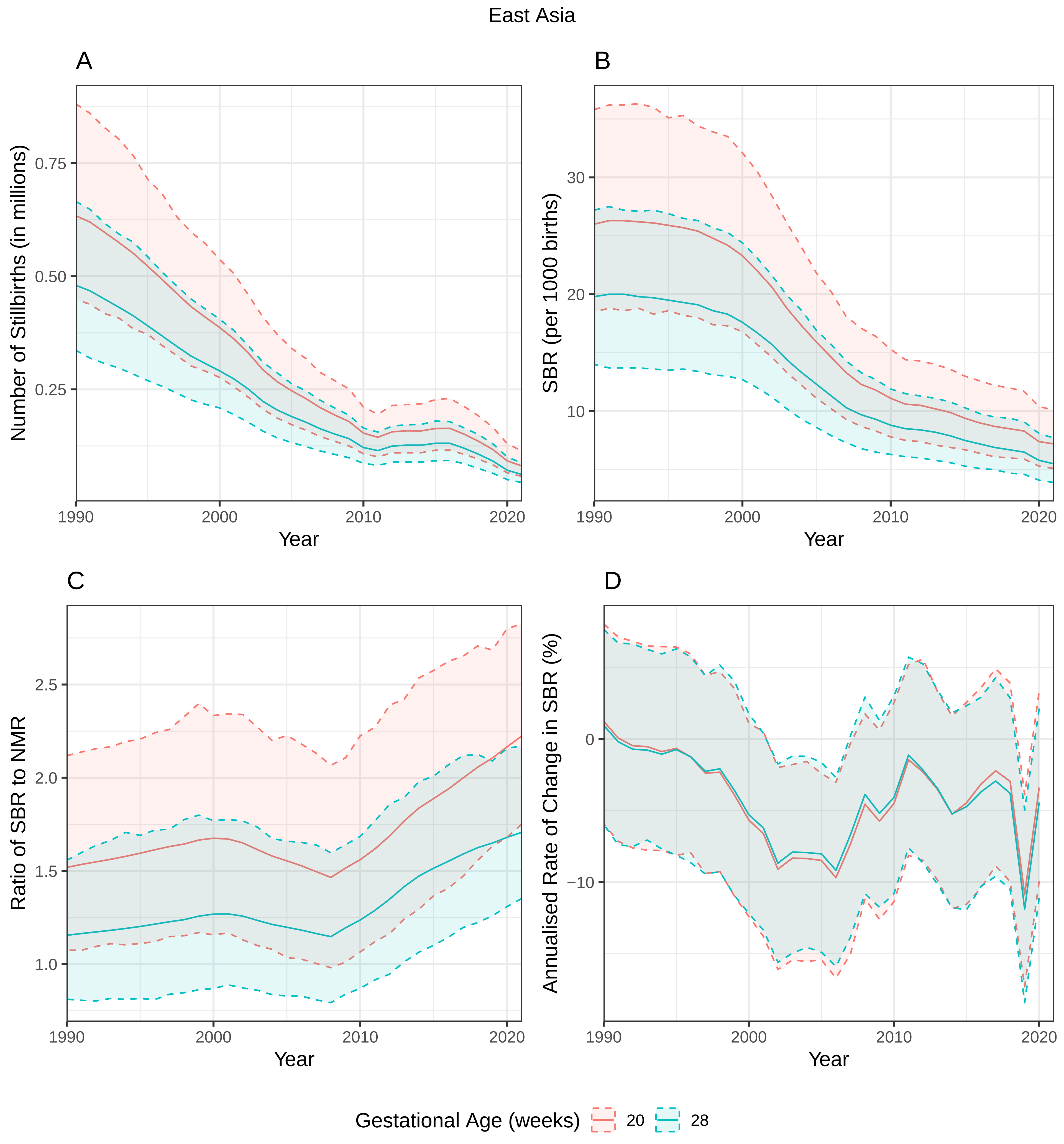

East Asia
A
Number of Stillbirths (in millions)
0.75
0.50
0.25
1990
2000
2010
2020
Year
B
SBR (per 1000 births)
30
20
10
1990
2000
2010
2020
Year
C
Ratio of SBR to NMR
2.5
2.0
1.5
1.0
1990
2000
2010
2020
Year
D
Annualised Rate of Change in SBR (%)
0
−10
1990
2000
2010
2020
Year
Gestational Age (weeks)
20
28

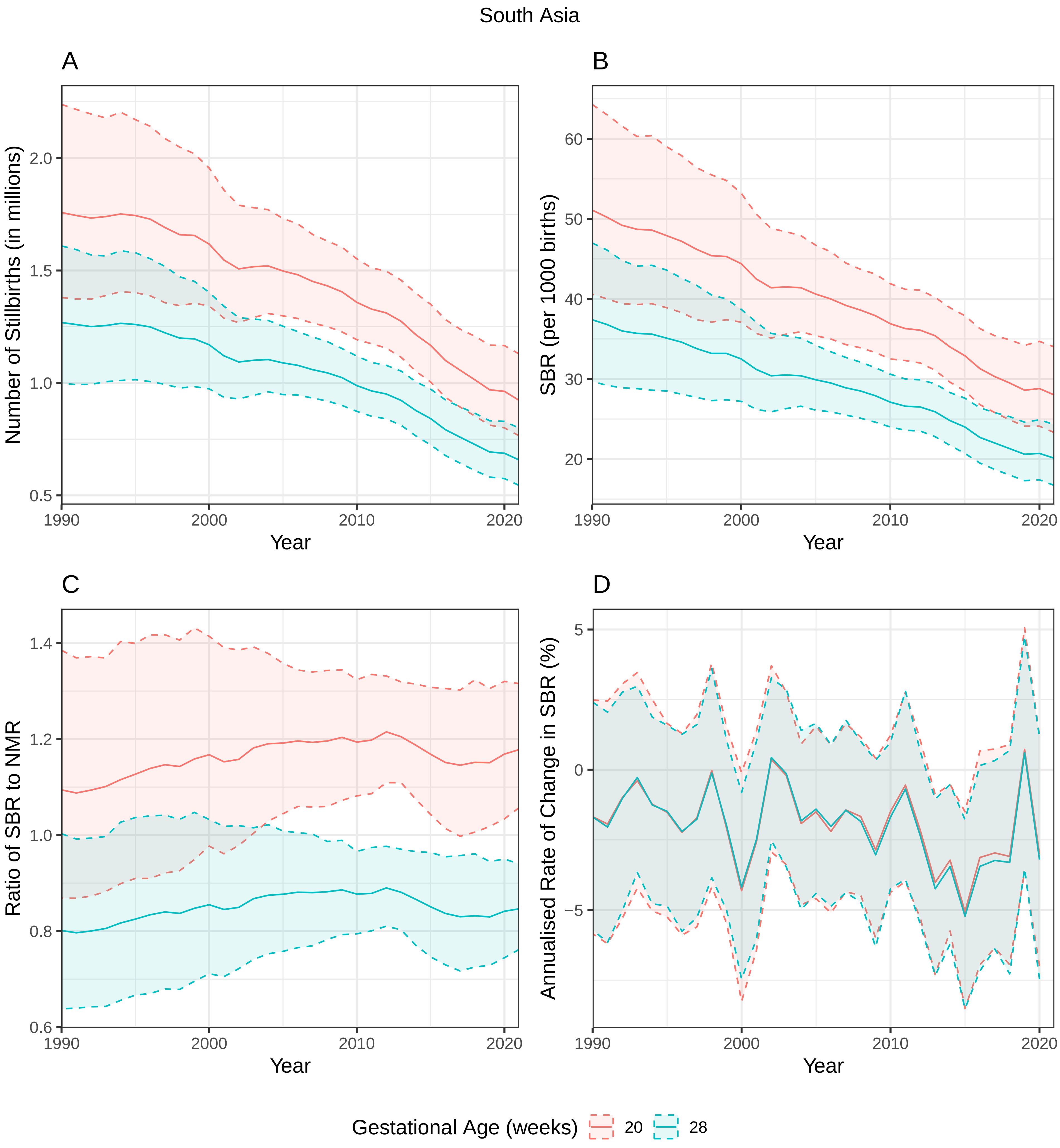

South Asia
A
Number of Stillbirths (in millions)
0.5
1.0
1.5
2.0
1990
2000
2010
2020
Year
B
SBR (per 1000 births)
20
30
40
50
60
1990
2000
2010
2020
Year
C
Ratio of SBR to NMR
0.6
0.8
1.0
1.2
1.4
1990
2000
2010
2020
Year
D
Annualised Rate of Change in SBR (%)
−5
0
5
1990
2000
2010
2020
Year
Gestational Age (weeks)
20
28

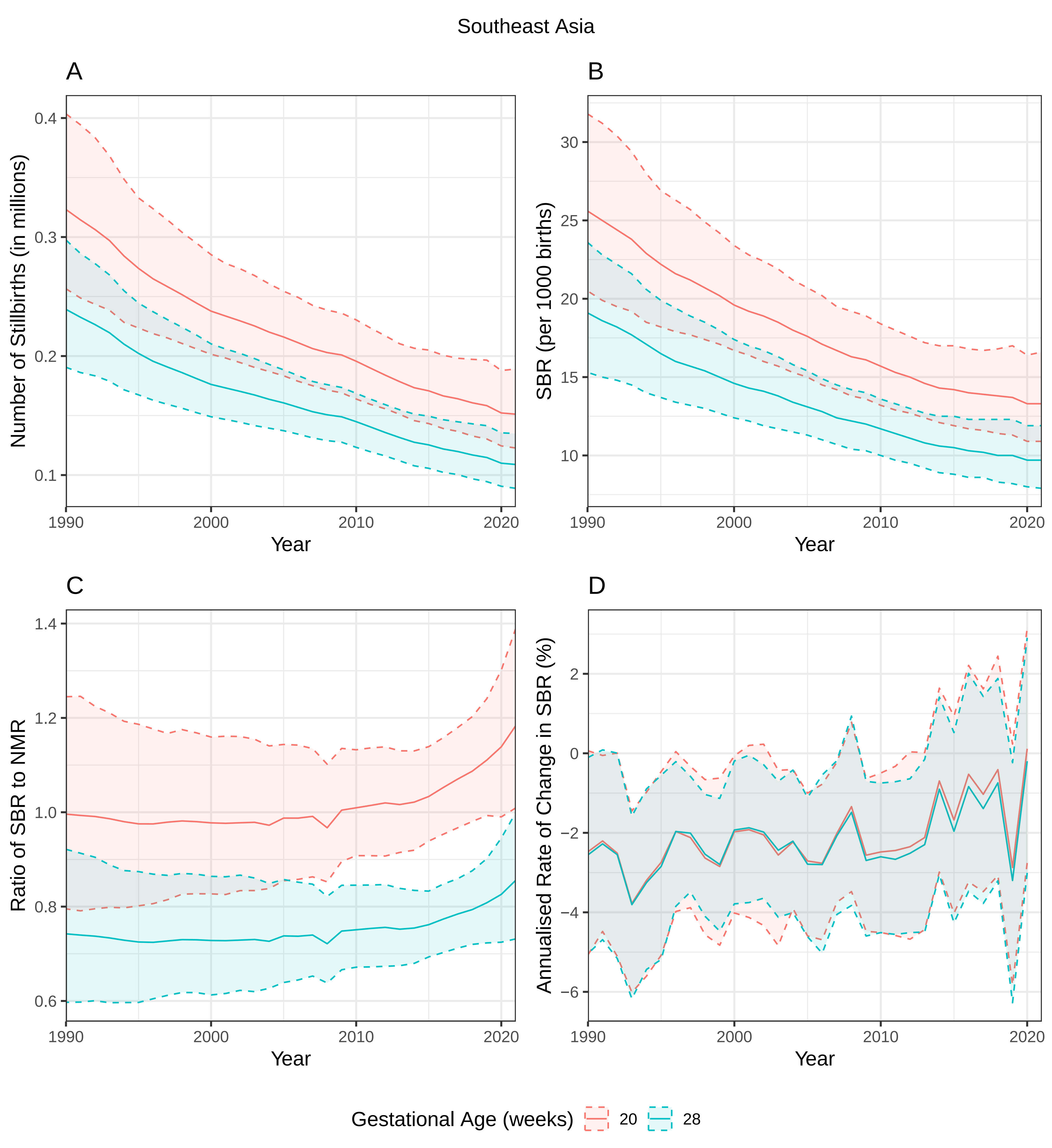
Southeast Asia
A
Number of Stillbirths (in millions)
0.1
0.2
0.3
0.4
1990
2000
2010
2020
Year
B
SBR (per 1000 births)
10
15
20
25
30
1990
2000
2010
2020
Year
C
Ratio of SBR to NMR
0.6
0.8
1.0
1.2
1.4
1990
2000
2010
2020
Year
D
Annualised Rate of Change in SBR (%)
−6
−4
−2
0
2
1990
2000
2010
2020
Year
Gestational Age (weeks)
20
28

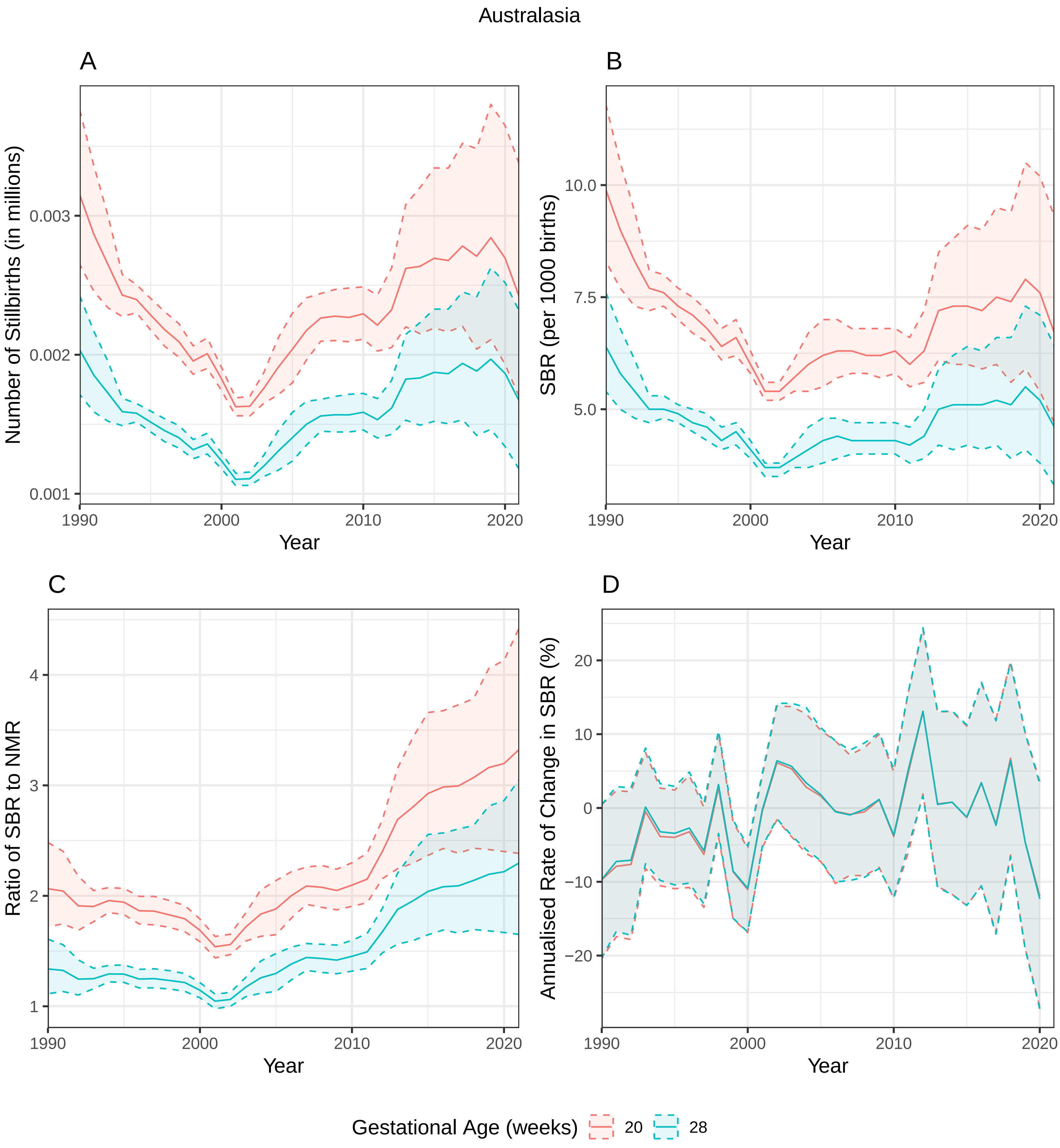
Australasia
A
Number of Stillbirths (in millions)
0.001
0.002
0.003
Year
1990
2000
2010
2020
B
SBR (per 1000 births)
5.0
7.5
10.0
Year
C
Ratio of SBR to NMR
1
2
3
4
Year
D
Annualised Rate of Change in SBR (%)
−20
−10
0
10
20
Year
Gestational Age (weeks)
20
28

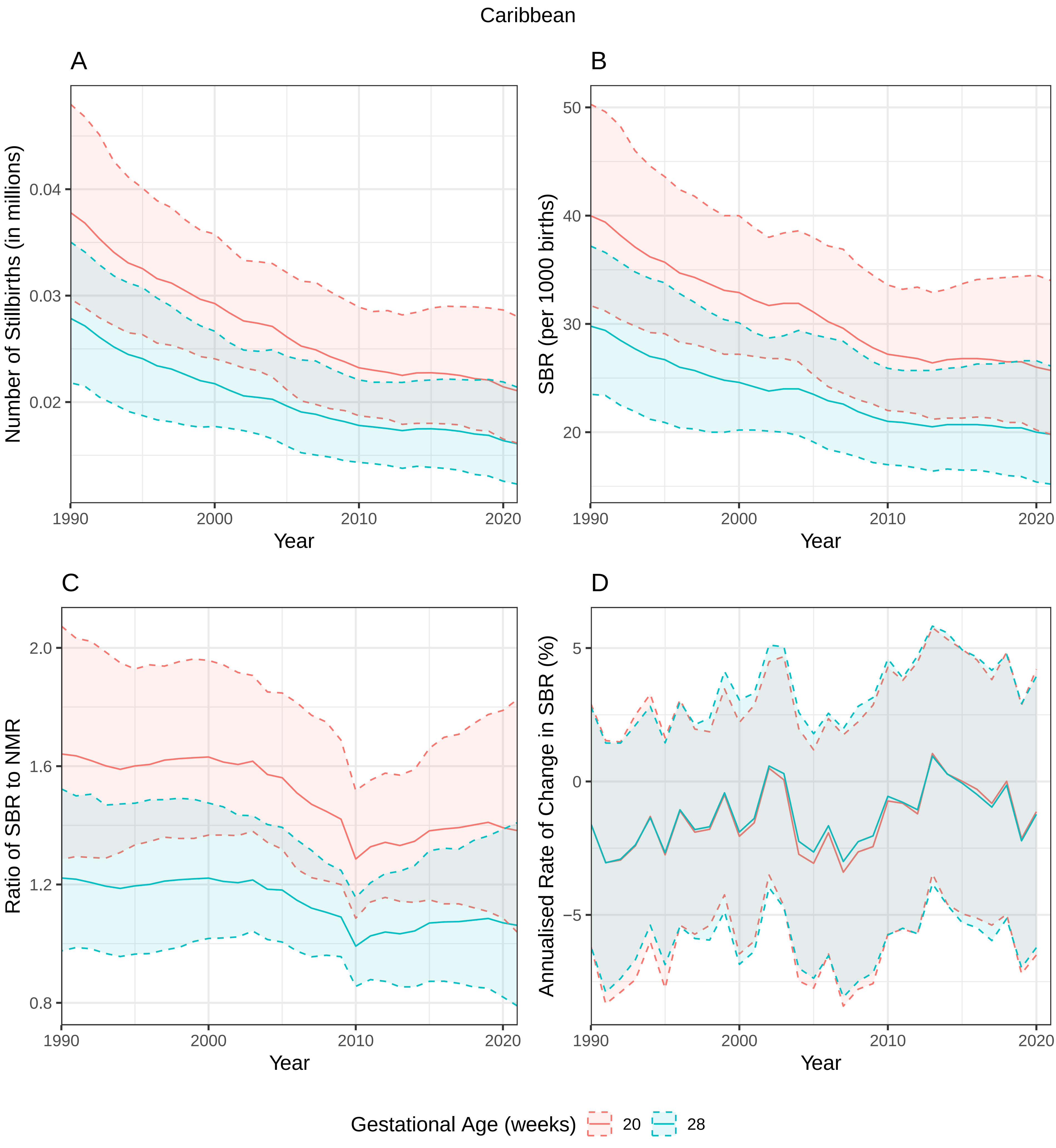

Caribbean
A
Number of Stillbirths (in millions)
0.04
0.03
0.02
1990
2000
2010
2020
Year
B
SBR (per 1000 births)
50
40
30
20
1990
2000
2010
2020
Year
C
Ratio of SBR to NMR
2.0
1.6
1.2
0.8
1990
2000
2010
2020
Year
D
Annualised Rate of Change in SBR (%)
5
0
−5
1990
2000
2010
2020
Year
Gestational Age (weeks)
20
28

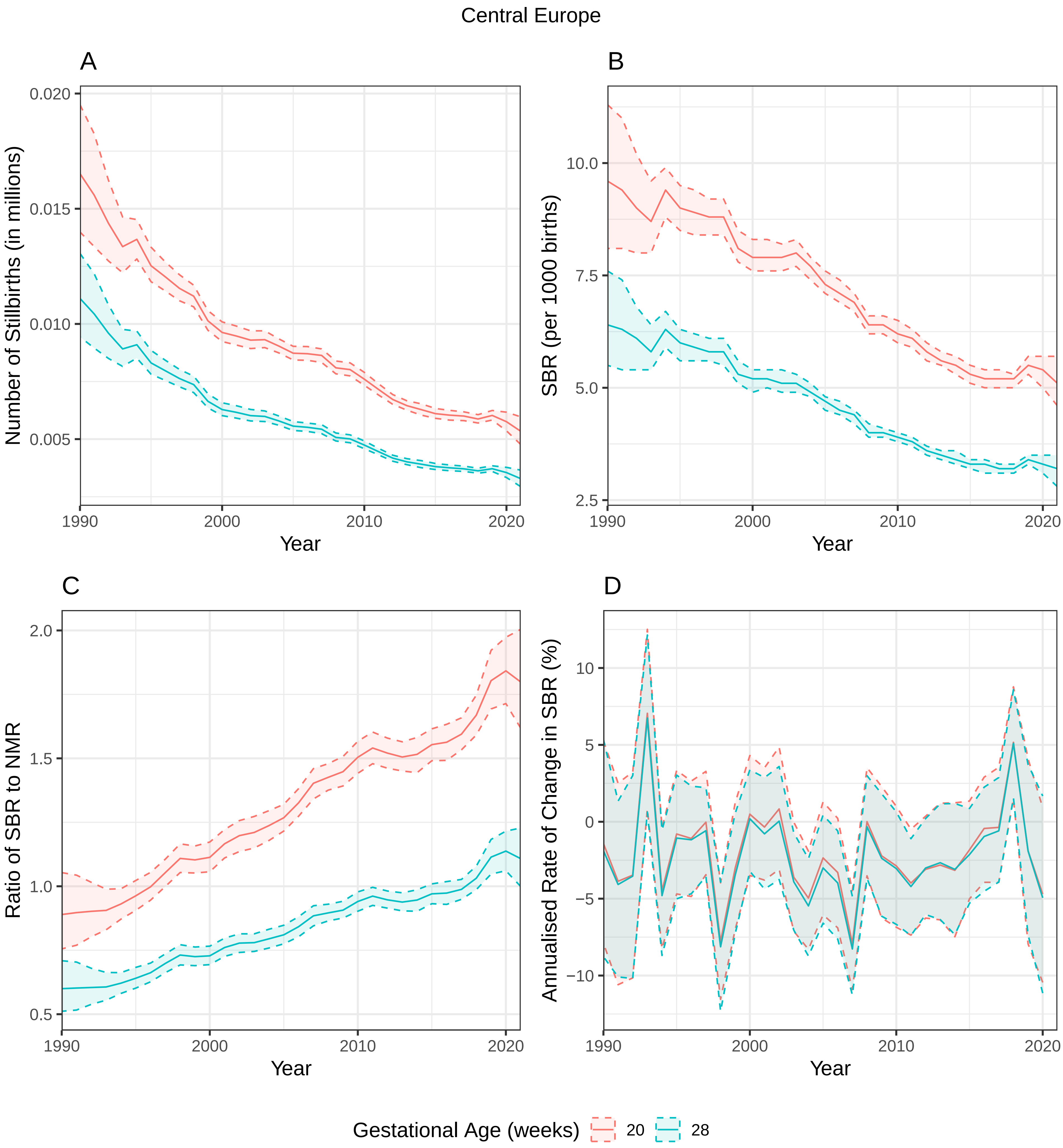

Central Europe
A
Number of Stillbirths (in millions)
0.020
0.015
0.010
0.005
1990
2000
2010
2020
Year
B
SBR (per 1000 births)
10.0
7.5
5.0
2.5
1990
2000
2010
2020
Year
C
Ratio of SBR to NMR
2.0
1.5
1.0
0.5
1990
2000
2010
2020
Year
D
Annualised Rate of Change in SBR (%)
10
5
0
−5
−10
1990
2000
2010
2020
Year
Gestational Age (weeks)
20
28

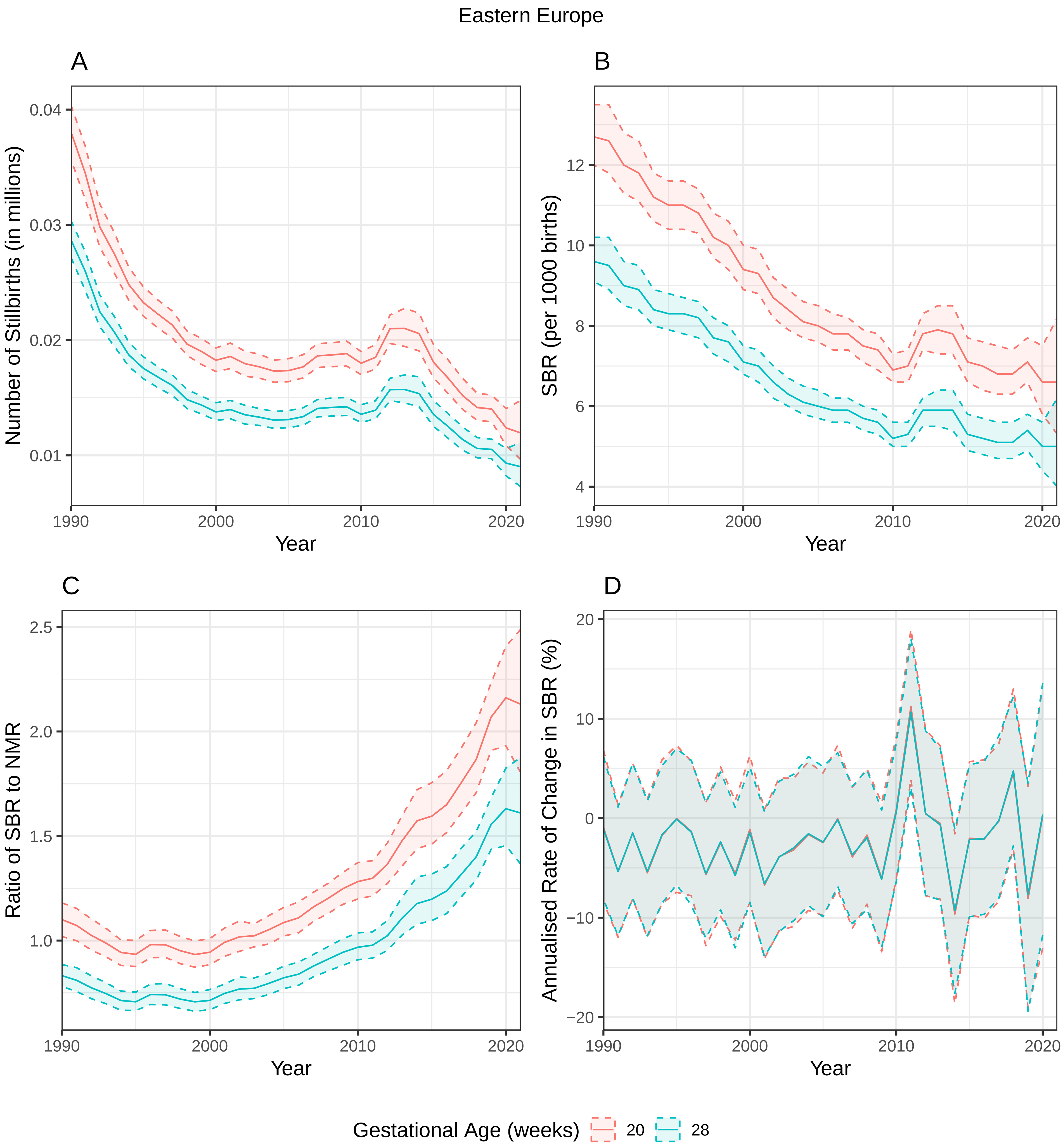

Eastern Europe
A
Number of Stillbirths (in millions)
0.04
0.03
0.02
0.01
1990
2000
2010
2020
Year
B
SBR (per 1000 births)
12
10
8
6
4
1990
2000
2010
2020
Year
C
Ratio of SBR to NMR
2.5
2.0
1.5
1.0
1990
2000
2010
2020
Year
D
Annualised Rate of Change in SBR (%)
20
10
0
−10
−20
1990
2000
2010
2020
Year
Gestational Age (weeks)
20
28

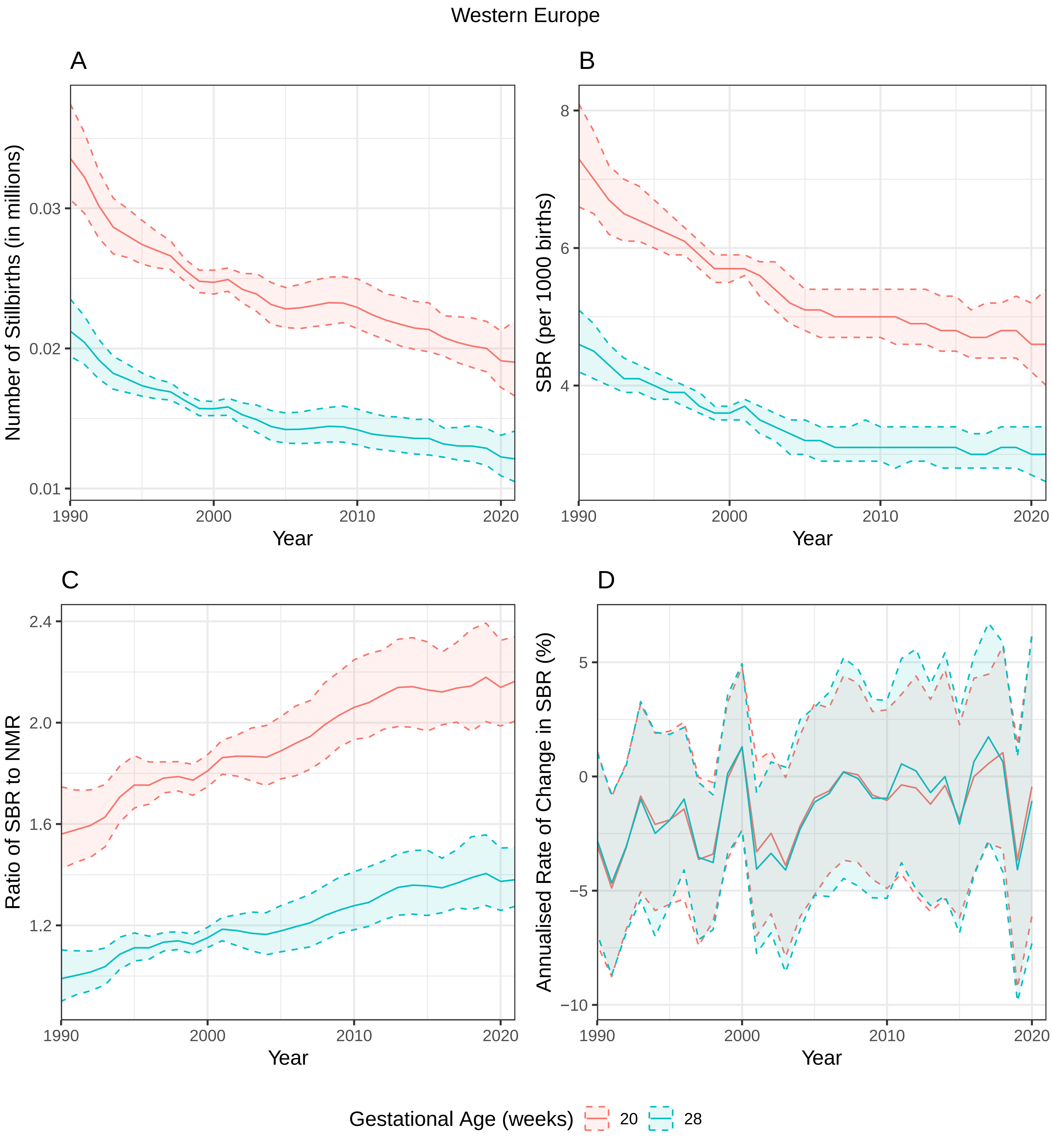

Western Europe
A
Number of Stillbirths (in millions)
0.01
0.02
0.03
1990
2000
2010
2020
Year
B
SBR (per 1000 births)
4
6
8
1990
2000
2010
2020
Year
C
Ratio of SBR to NMR
1.2
1.6
2.0
2.4
1990
2000
2010
2020
Year
D
Annualised Rate of Change in SBR (%)
−10
−5
0
5
1990
2000
2010
2020
Year
Gestational Age (weeks)
20
28

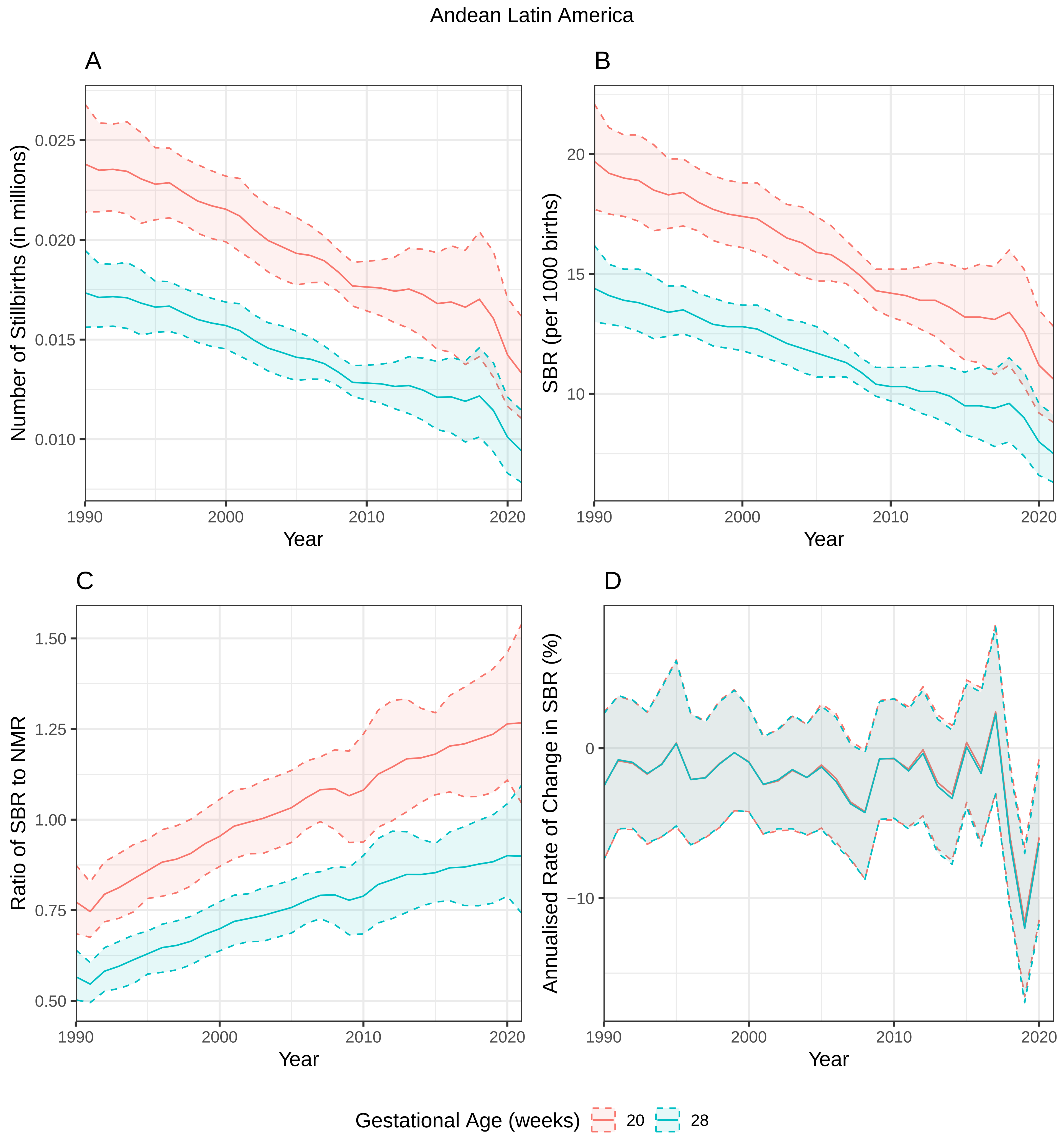

Andean Latin America
A
Number of Stillbirths (in millions)
0.025
0.020
0.015
0.010
1990
2000
2010
2020
Year
B
SBR (per 1000 births)
20
15
10
1990
2000
2010
2020
Year
C
Ratio of SBR to NMR
1.50
1.25
1.00
0.75
0.50
1990
2000
2010
2020
Year
D
Annualised Rate of Change in SBR (%)
0
−10
1990
2000
2010
2020
Year
Gestational Age (weeks)
20
28

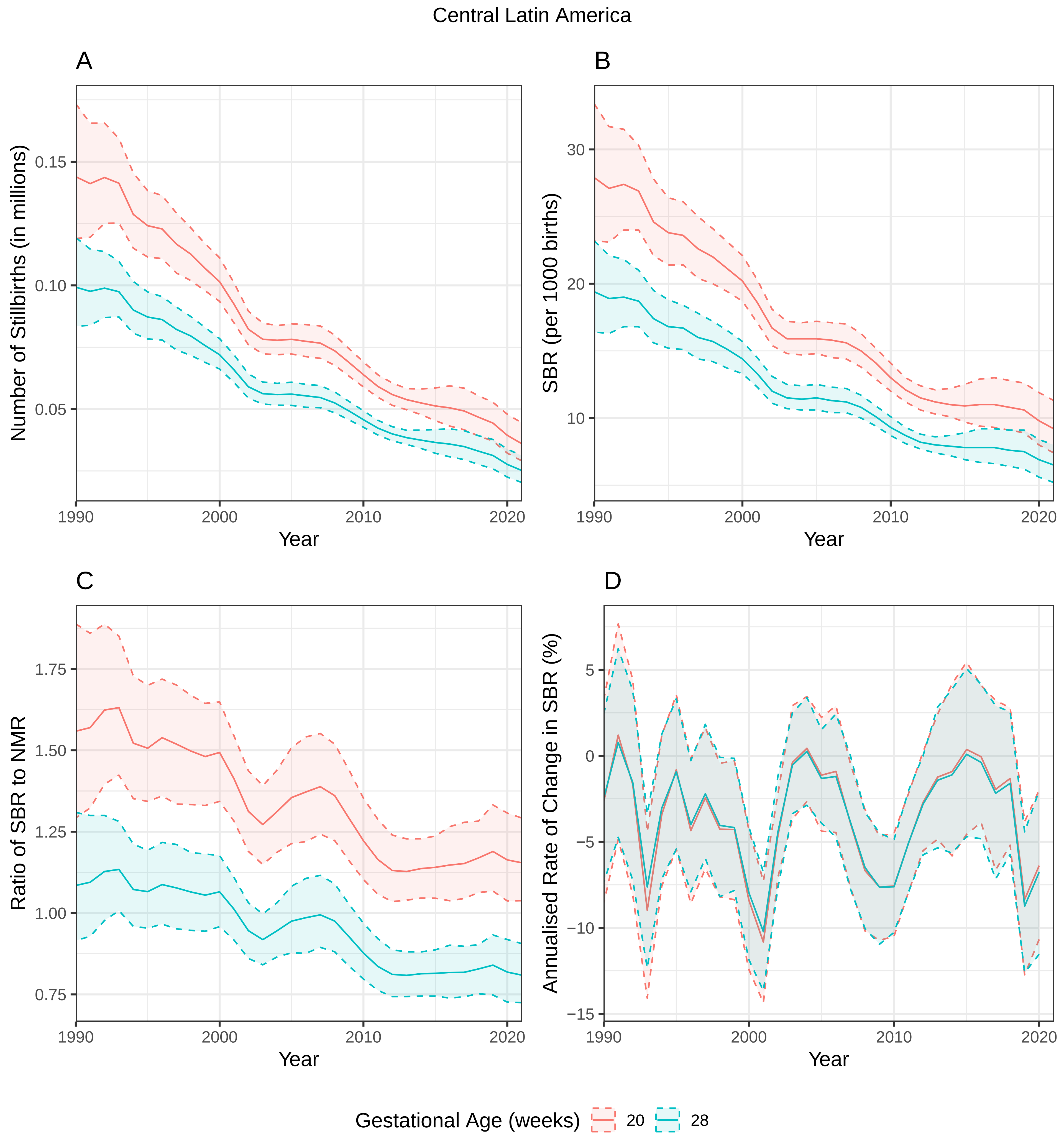
Central Latin America
A
Number of Stillbirths (in millions)
0.15
0.10
0.05
Year
B
SBR (per 1000 births)
30
20
10
C
Ratio of SBR to NMR
1.75
1.50
1.25
1.00
0.75
D
Annualised Rate of Change in SBR (%)
5
0
−5
−10
−15
1990
2000
2010
2020
Gestational Age (weeks)
20
28

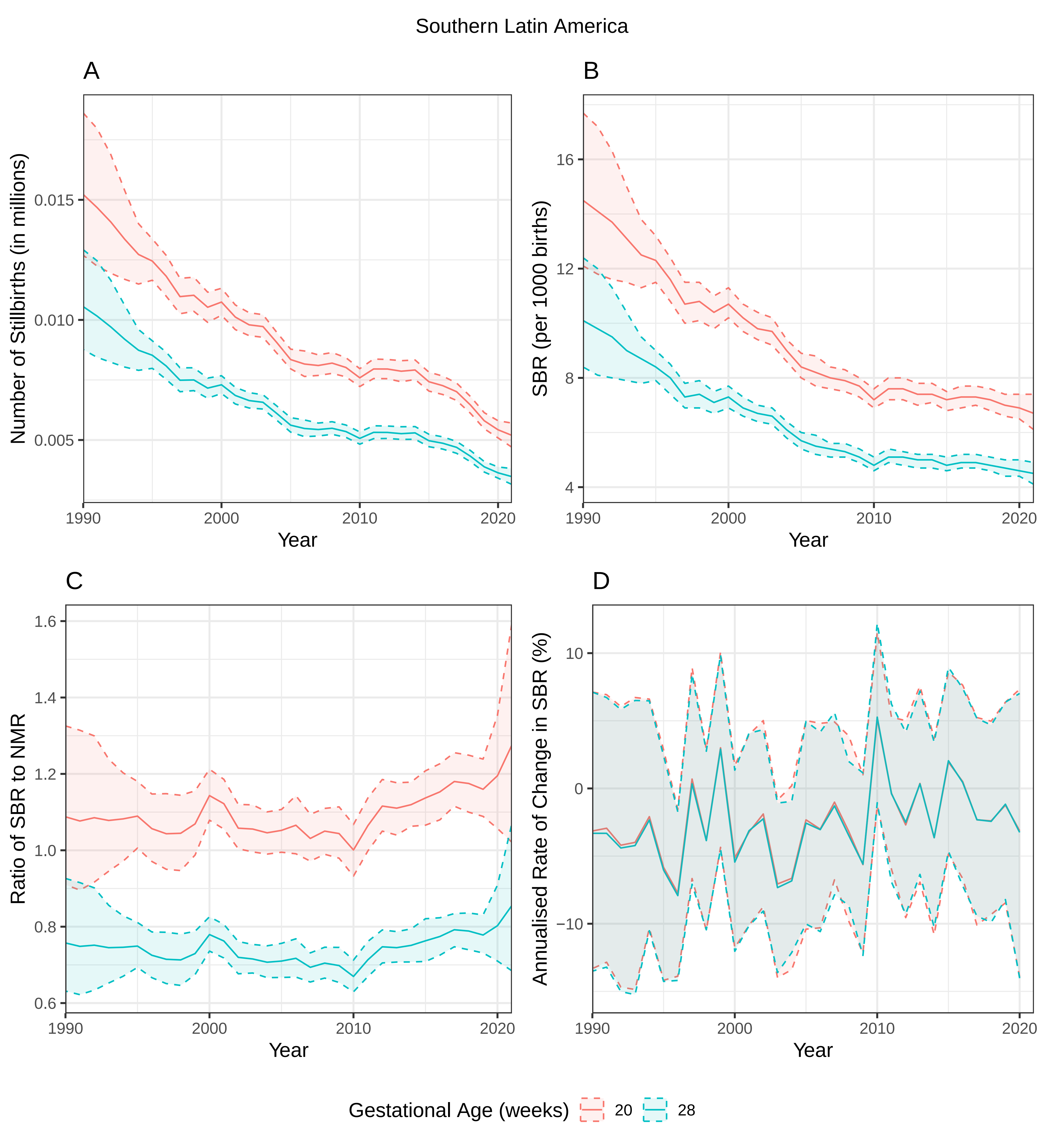

Southern Latin America
A
Number of Stillbirths (in millions)
0.015
0.010
0.005
1990
2000
2010
2020
Year
B
SBR (per 1000 births)
16
12
8
4
1990
2000
2010
2020
Year
C
Ratio of SBR to NMR
1.6
1.4
1.2
1.0
0.8
0.6
1990
2000
2010
2020
Year
D
Annualised Rate of Change in SBR (%)
10
0
-10
1990
2000
2010
2020
Year
Gestational Age (weeks)
20
28

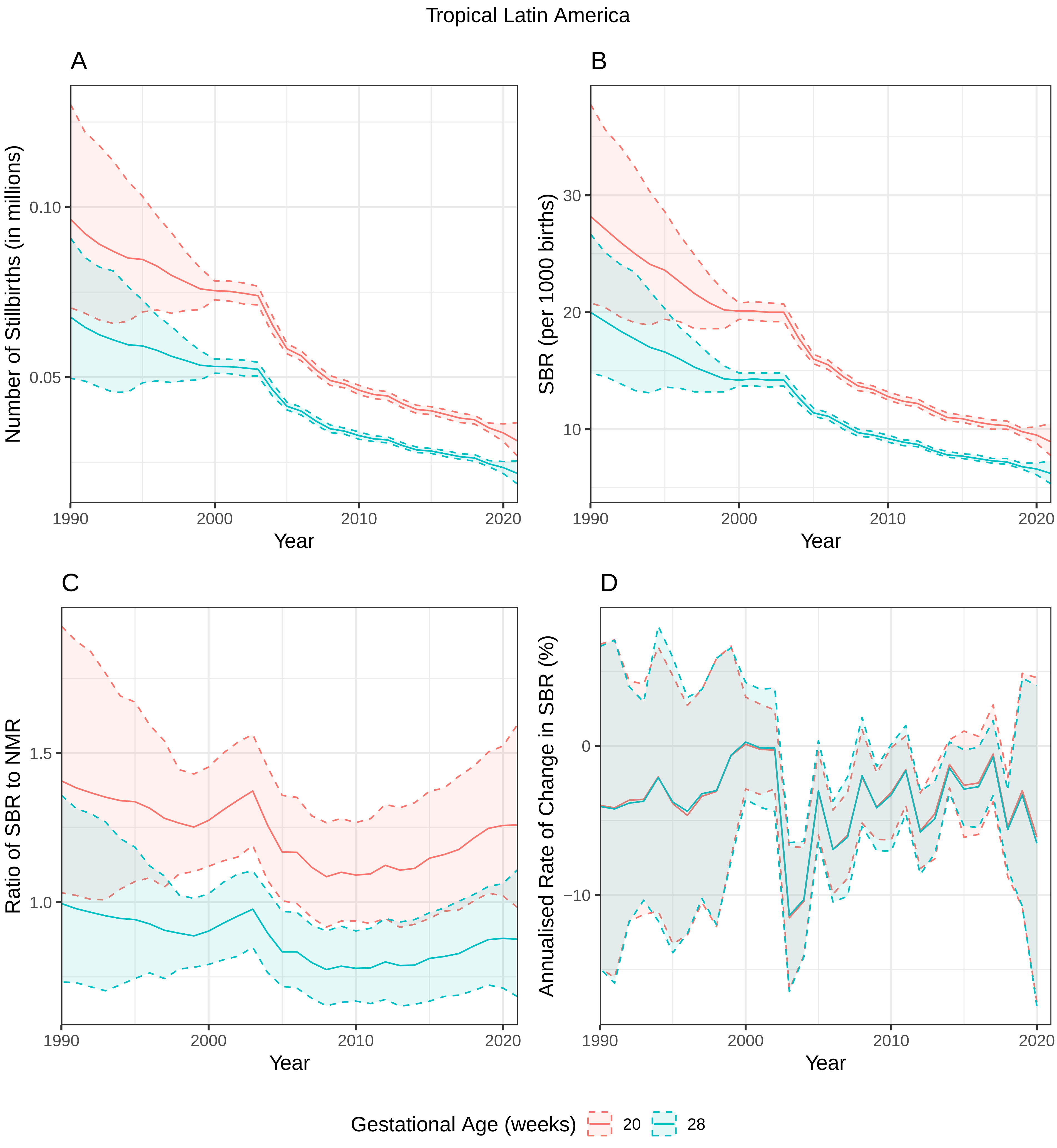

Tropical Latin America
A
Number of Stillbirths (in millions)
0.10
0.05
1990
2000
2010
2020
Year
B
SBR (per 1000 births)
30
20
10
1990
2000
2010
2020
Year
C
Ratio of SBR to NMR
1.5
1.0
1990
2000
2010
2020
Year
D
Annualised Rate of Change in SBR (%)
0
−10
1990
2000
2010
2020
Year
Gestational Age (weeks)
20
28

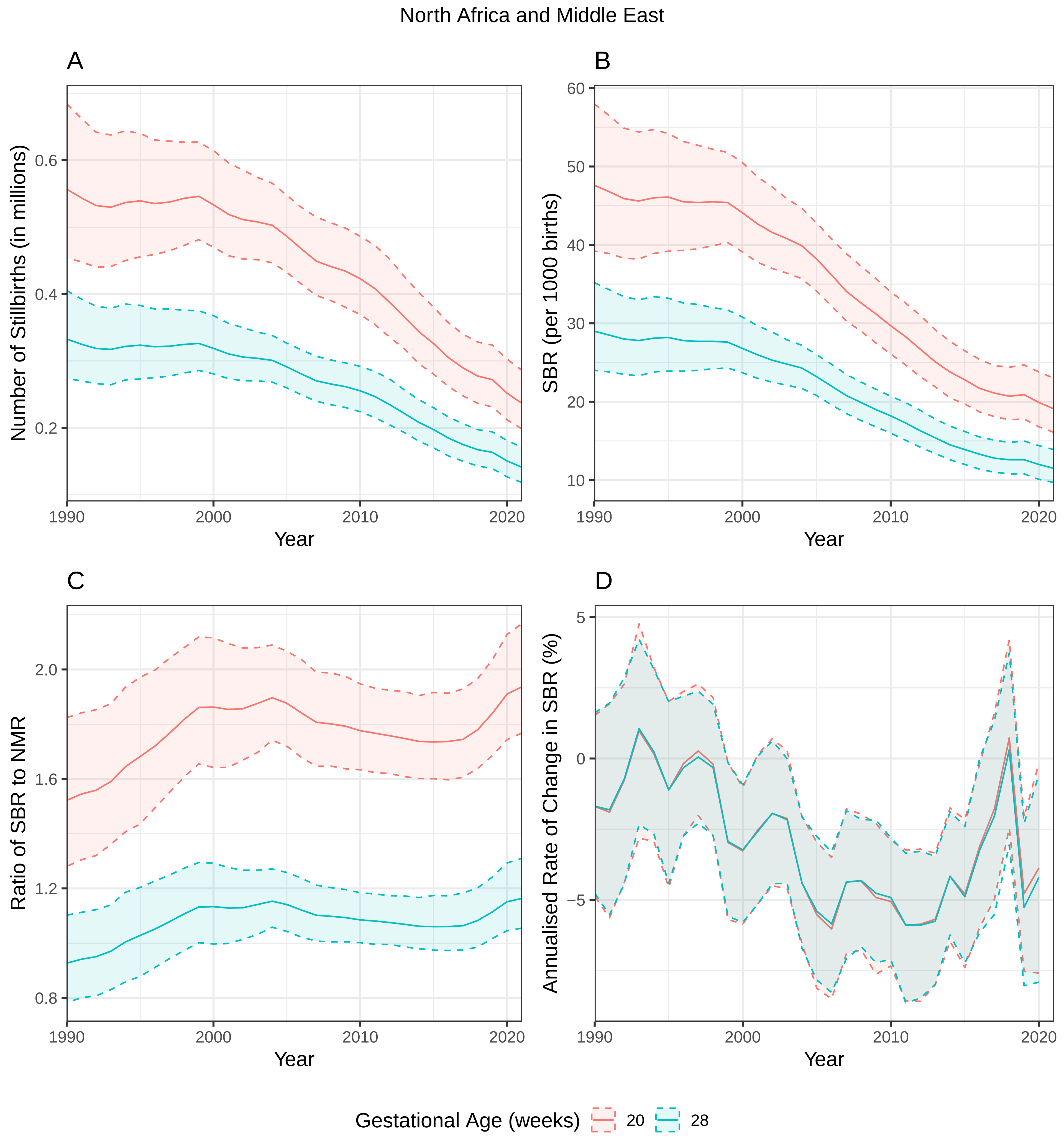
North Africa and Middle East
A
Number of Stillbirths (in millions)
0.6
0.4
0.2
1990
2000
2010
2020
Year
B
SBR (per 1000 births)
60
50
40
30
20
10
1990
2000
2010
2020
Year
C
Ratio of SBR to NMR
2.0
1.6
1.2
0.8
1990
2000
2010
2020
Year
D
Annualised Rate of Change in SBR (%)
5
0
-5
1990
2000
2010
2020
Year
Gestational Age (weeks)
20
28

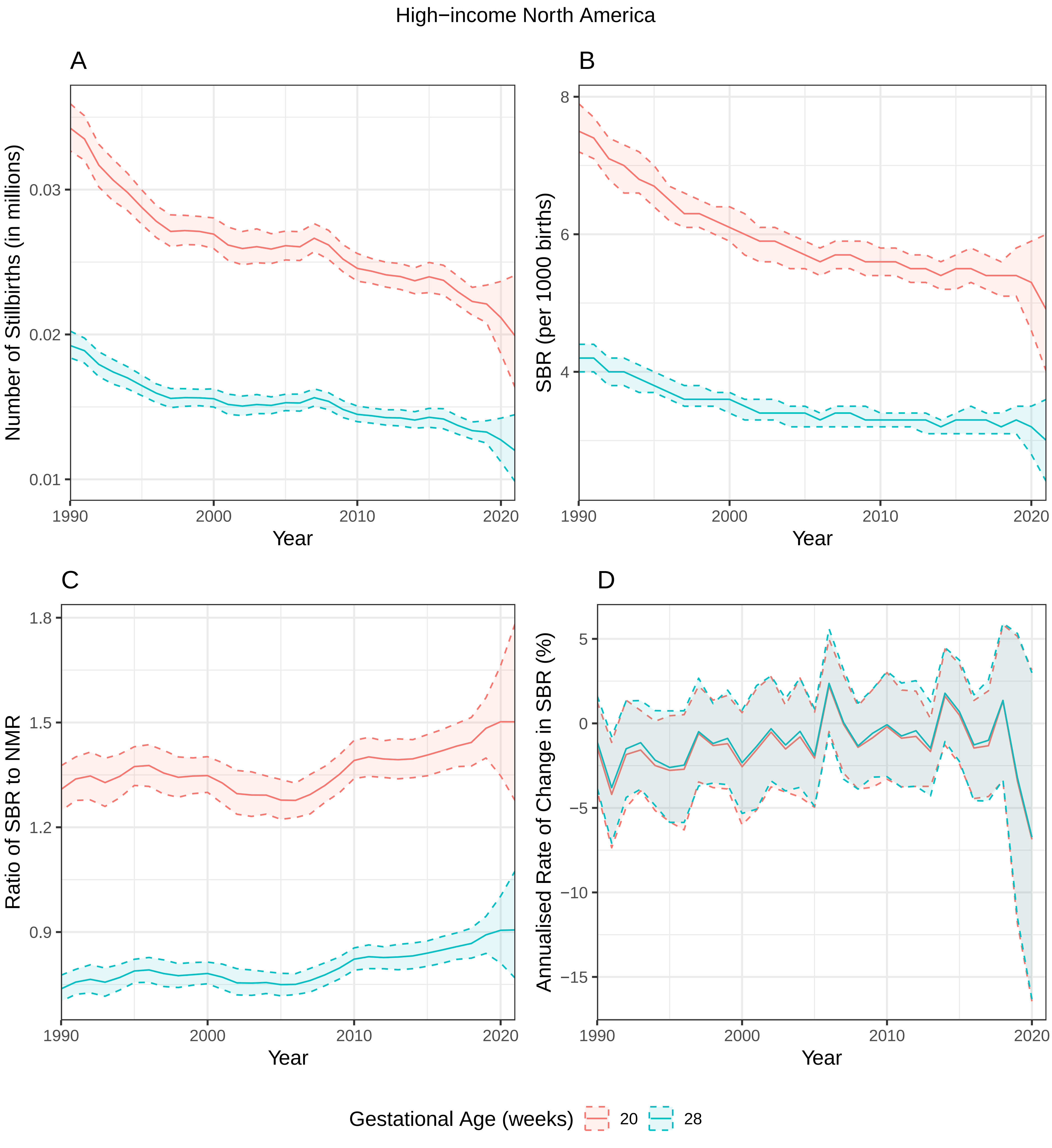
High−income North America
A
Number of Stillbirths (in millions)
0.03
0.02
0.01
1990
2000
2010
2020
Year
B
SBR (per 1000 births)
8
6
4
1990
2000
2010
2020
Year
C
Ratio of SBR to NMR
1.8
1.5
1.2
0.9
1990
2000
2010
2020
Year
D
Annualised Rate of Change in SBR (%)
5
0
−5
−10
−15
1990
2000
2010
2020
Year
Gestational Age (weeks)
20
28

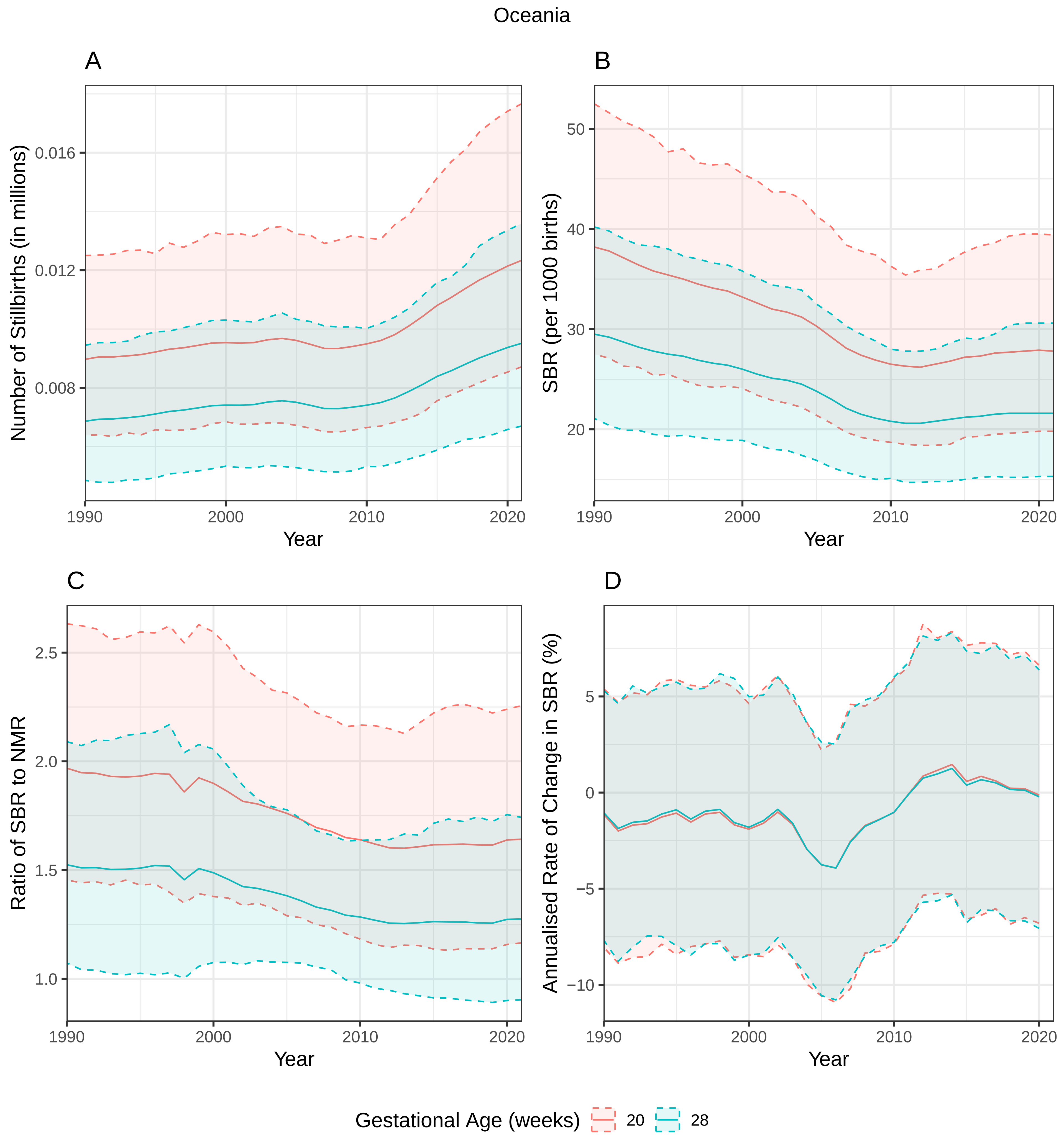
Oceania
A
Number of Stillbirths (in millions)
0.016
0.012
0.008
1990
2000
2010
2020
Year
B
SBR (per 1000 births)
50
40
30
20
1990
2000
2010
2020
Year
C
Ratio of SBR to NMR
2.5
2.0
1.5
1.0
1990
2000
2010
2020
Year
D
Annualised Rate of Change in SBR (%)
5
0
−5
−10
1990
2000
2010
2020
Year
Gestational Age (weeks)
20
28

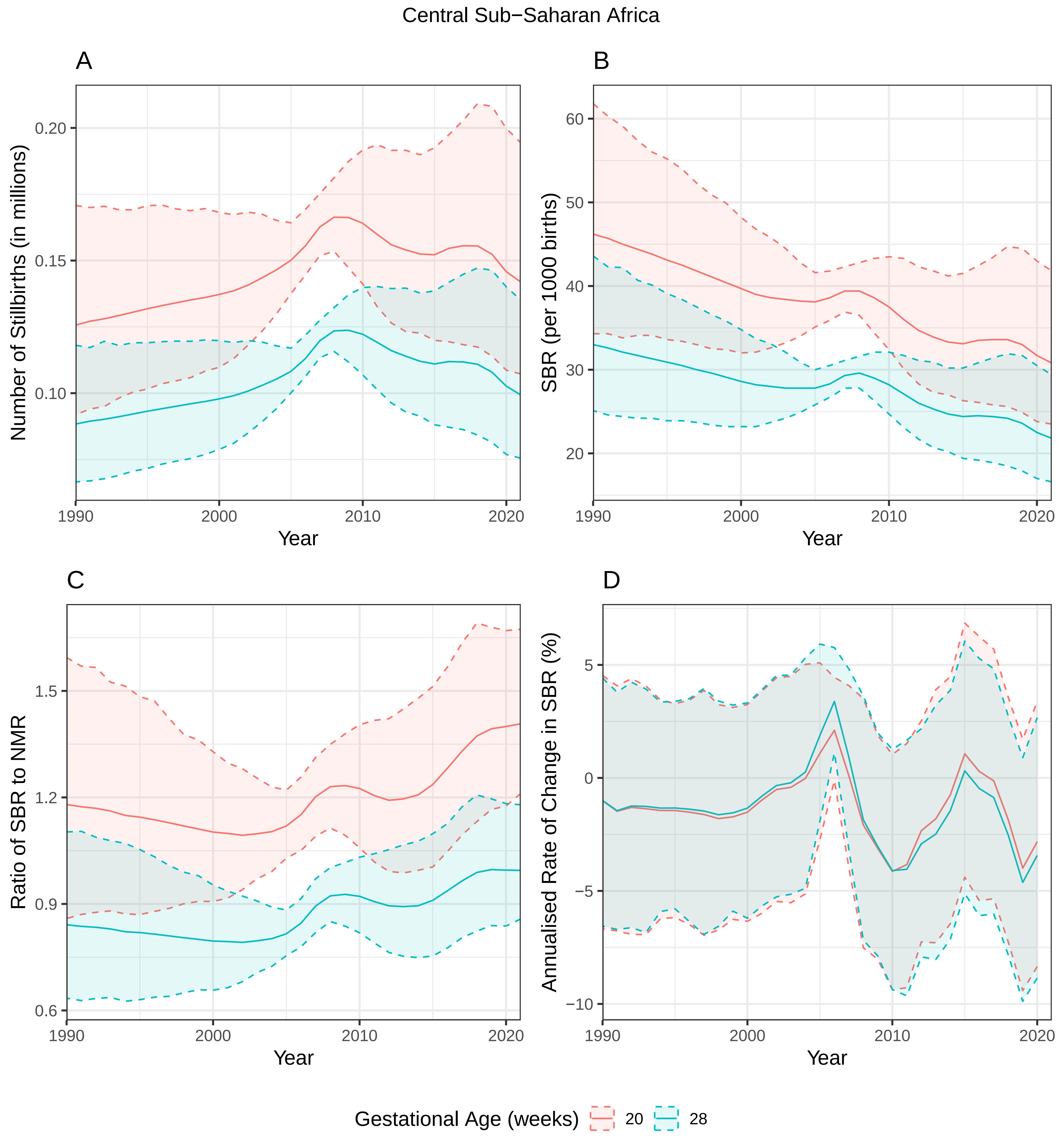

Central Sub−Saharan Africa
A
Number of Stillbirths (in millions)
0.20
0.15
0.10
1990
2000
2010
2020
Year
B
SBR (per 1000 births)
60
50
40
30
20
1990
2000
2010
2020
Year
C
Ratio of SBR to NMR
1.5
1.2
0.9
0.6
1990
2000
2010
2020
Year
D
Annualised Rate of Change in SBR (%)
5
0
−5
−10
1990
2000
2010
2020
Year
Gestational Age (weeks)
20
28

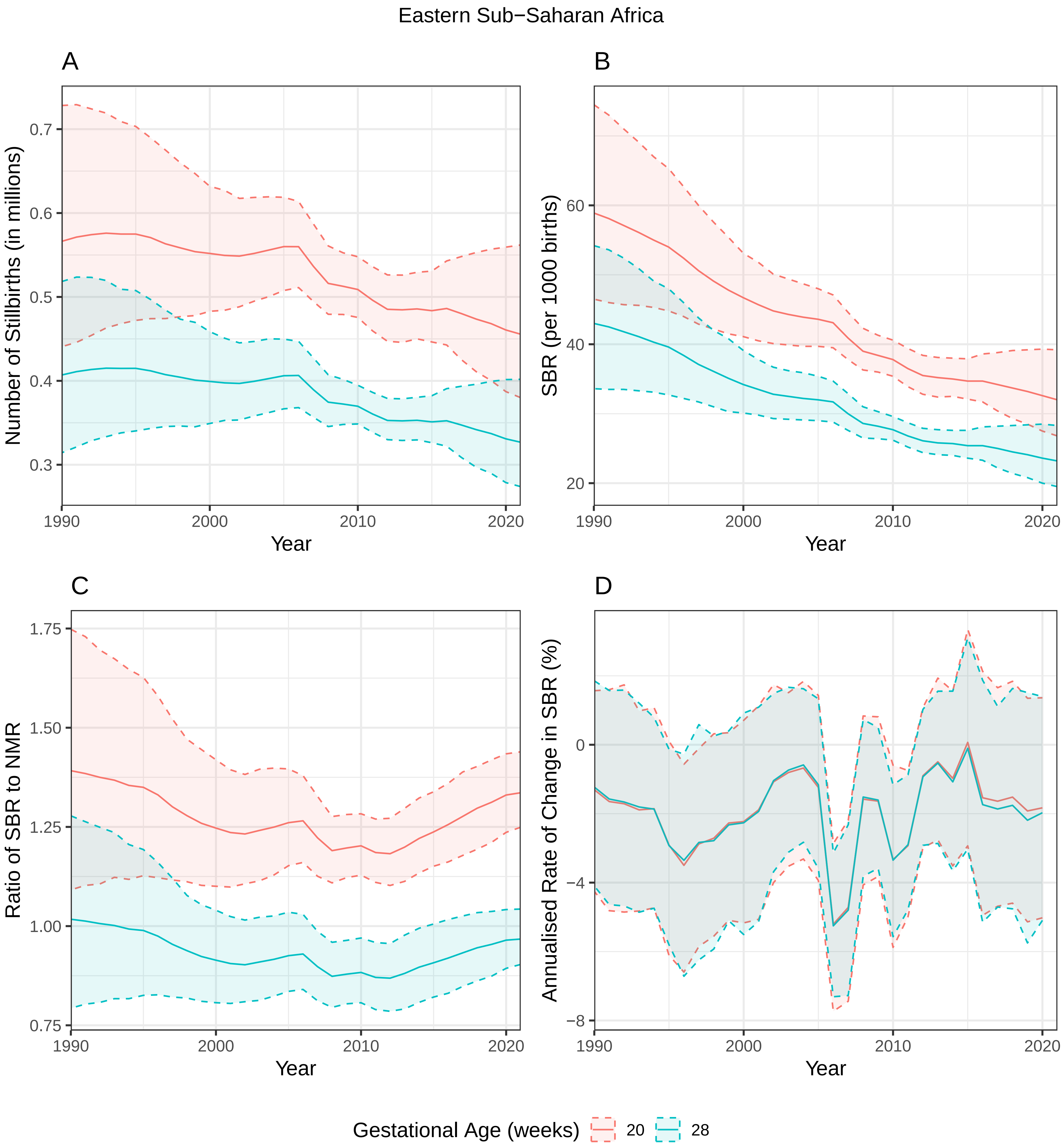

Eastern Sub−Saharan Africa
A
Number of Stillbirths (in millions)
0.3
0.4
0.5
0.6
0.7
1990
2000
2010
2020
Year
B
SBR (per 1000 births)
20
40
60
1990
2000
2010
2020
Year
C
Ratio of SBR to NMR
0.75
1.00
1.25
1.50
1.75
1990
2000
2010
2020
Year
D
Annualised Rate of Change in SBR (%)
−8
−4
0
1990
2000
2010
2020
Year
Gestational Age (weeks)
20
28

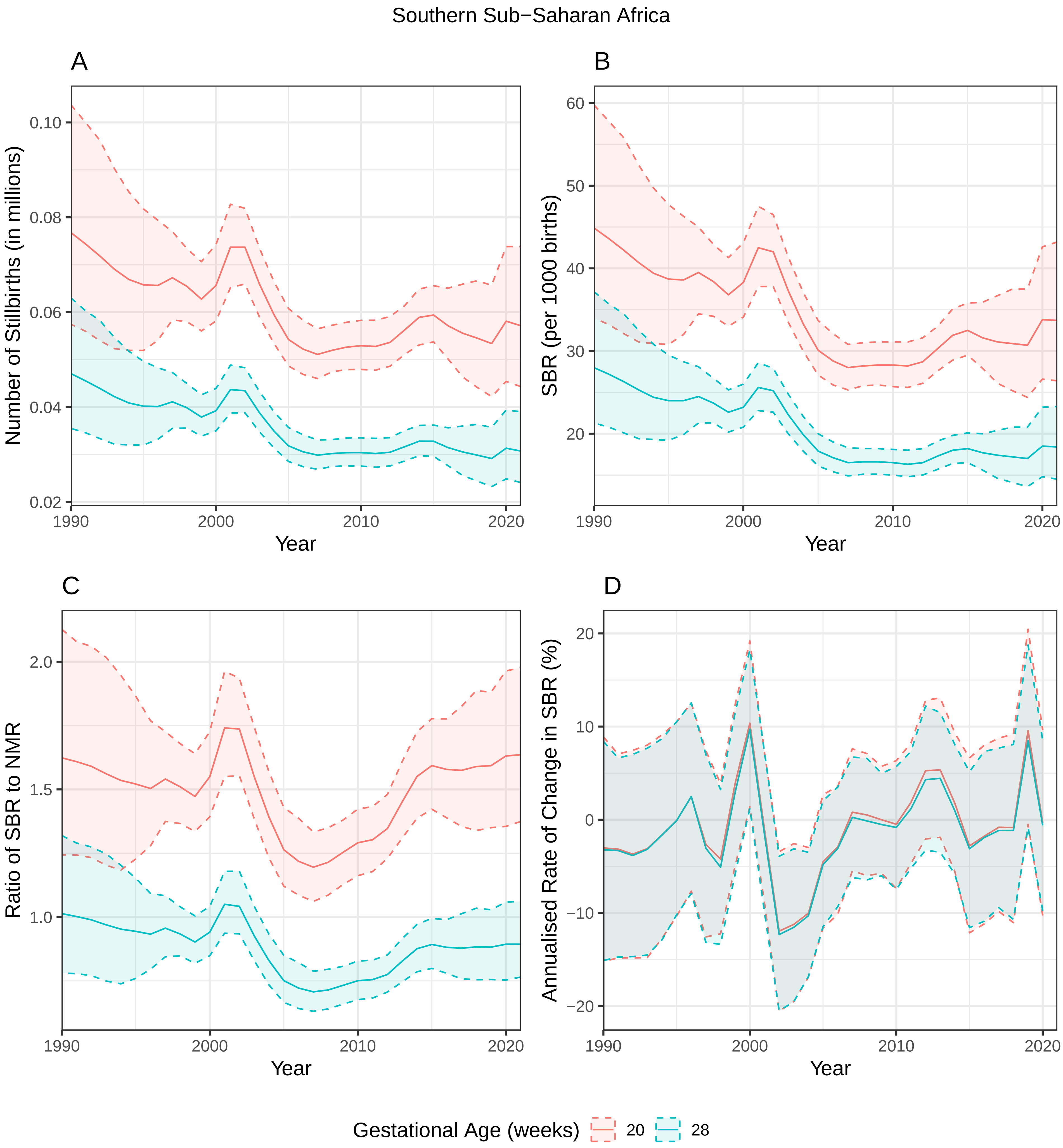

Southern Sub−Saharan Africa
A
Number of Stillbirths (in millions)
0.02
0.04
0.06
0.08
0.10
1990
2000
2010
2020
Year
B
SBR (per 1000 births)
20
30
40
50
60
1990
2000
2010
2020
Year
C
Ratio of SBR to NMR
1.0
1.5
2.0
1990
2000
2010
2020
Year
D
Annualised Rate of Change in SBR (%)
−20
−10
0
10
20
1990
2000
2010
2020
Year
Gestational Age (weeks)
20
28

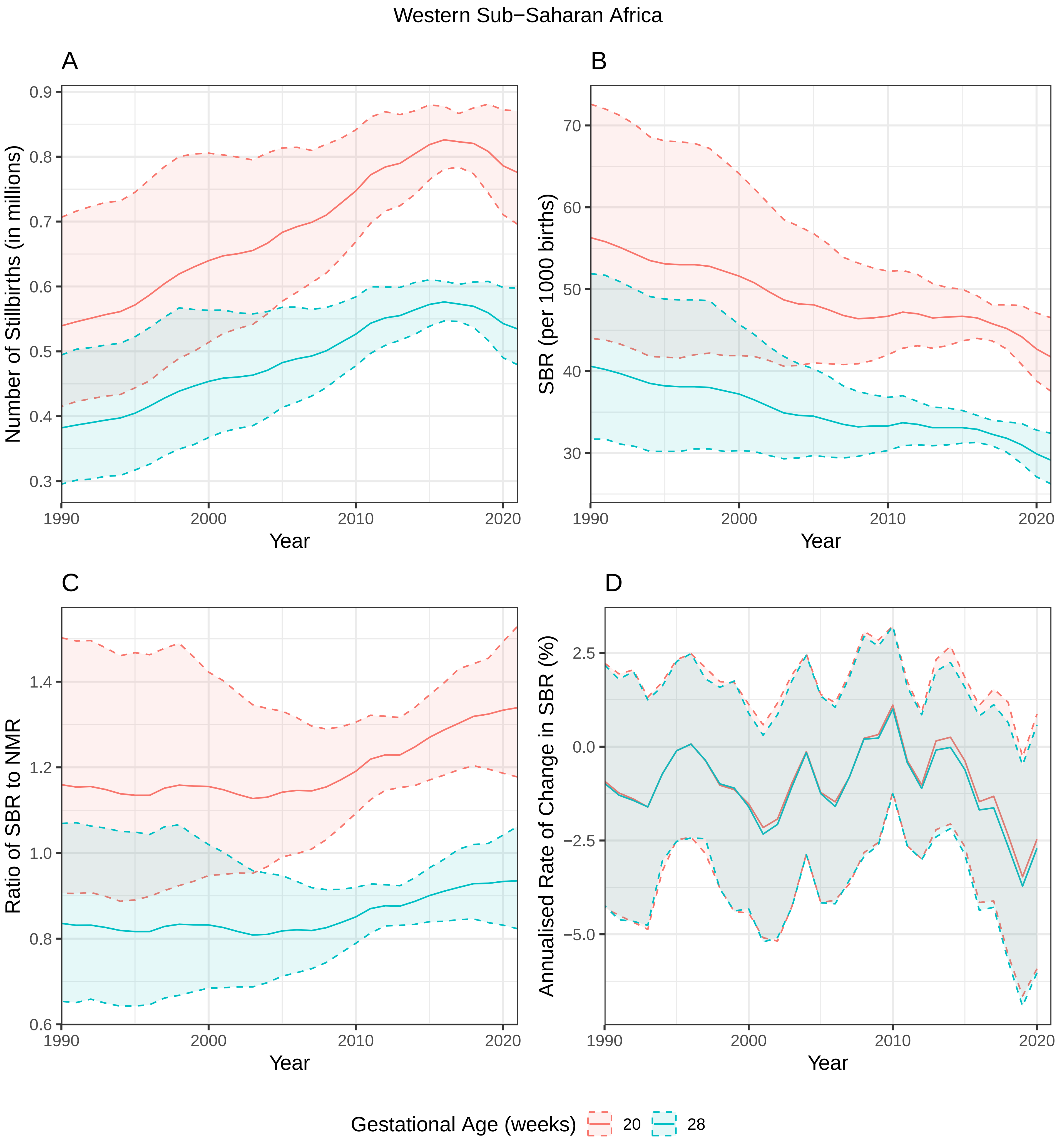
Western Sub−Saharan Africa
A
Number of Stillbirths (in millions)
0.3
0.4
0.5
0.6
0.7
0.8
0.9
1990
2000
2010
2020
Year
B
SBR (per 1000 births)
30
40
50
60
70
1990
2000
2010
2020
Year
C
Ratio of SBR to NMR
0.6
0.8
1.0
1.2
1.4
1990
2000
2010
2020
Year
D
Annualised Rate of Change in SBR (%)
−5.0
−2.5
0.0
2.5
1990
2000
2010
2020
Year
Gestational Age (weeks)
20
28

Figure 2A: SBR (per 1000 births) at 20 weeks' gestation in 1990

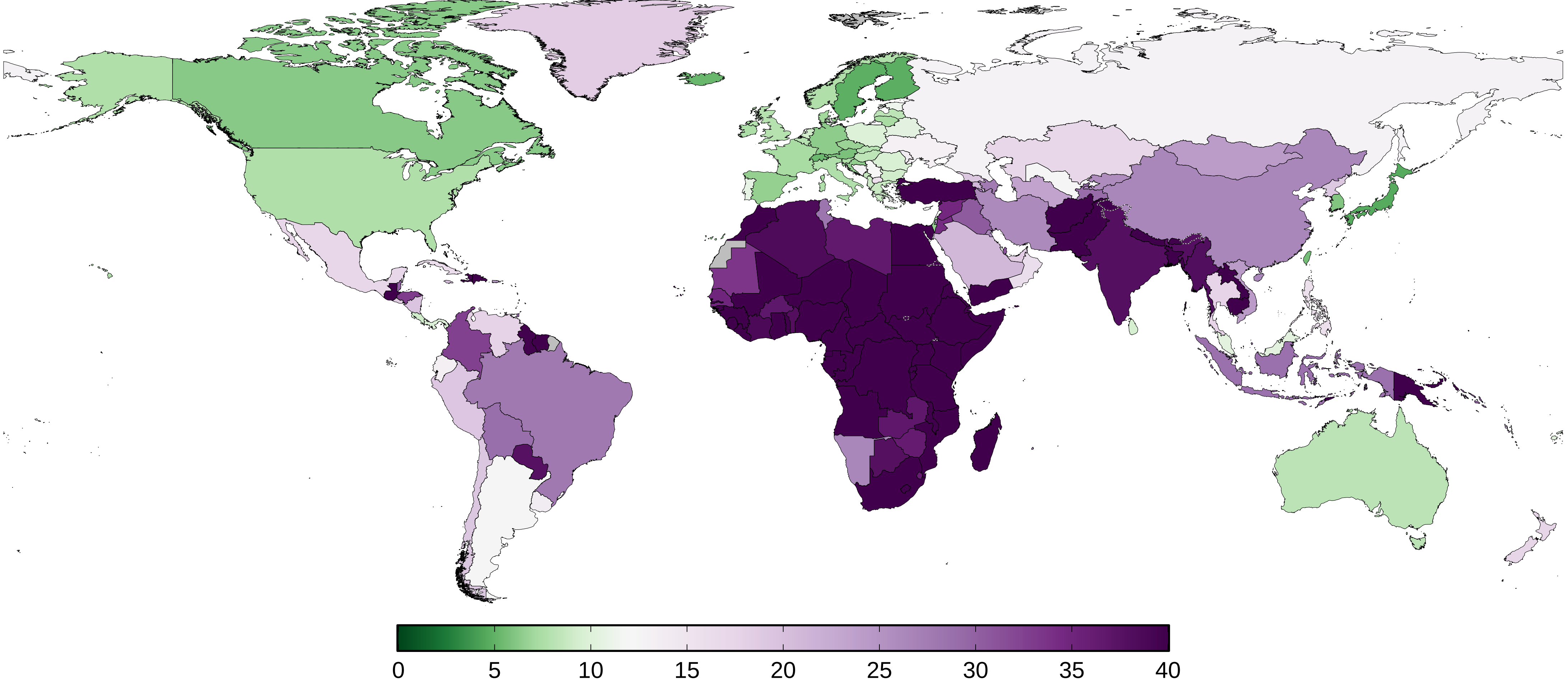

Figure 2A: SBR (per 1000 births) at 20 weeks' gestation in 2000

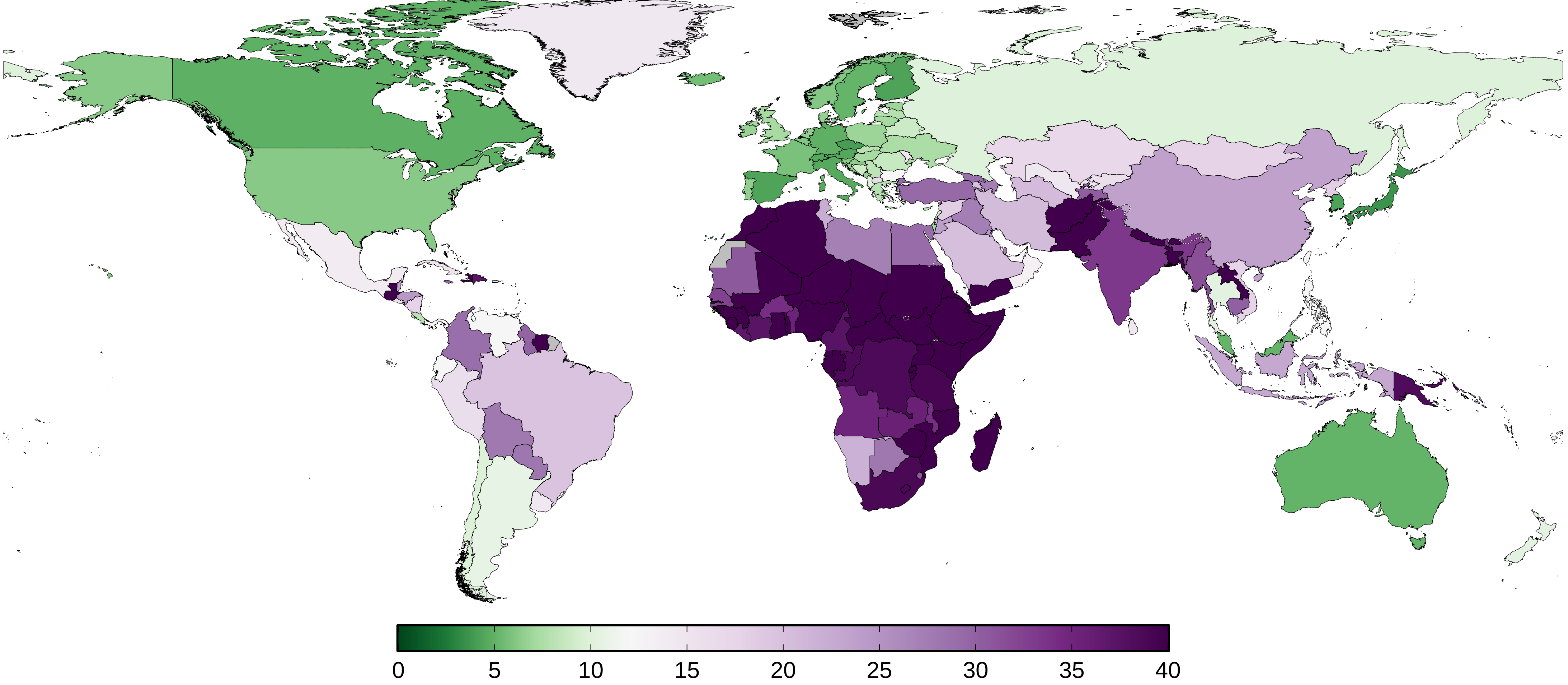

Figure 2A: SBR (per 1000 births) at 20 weeks' gestation in 2010

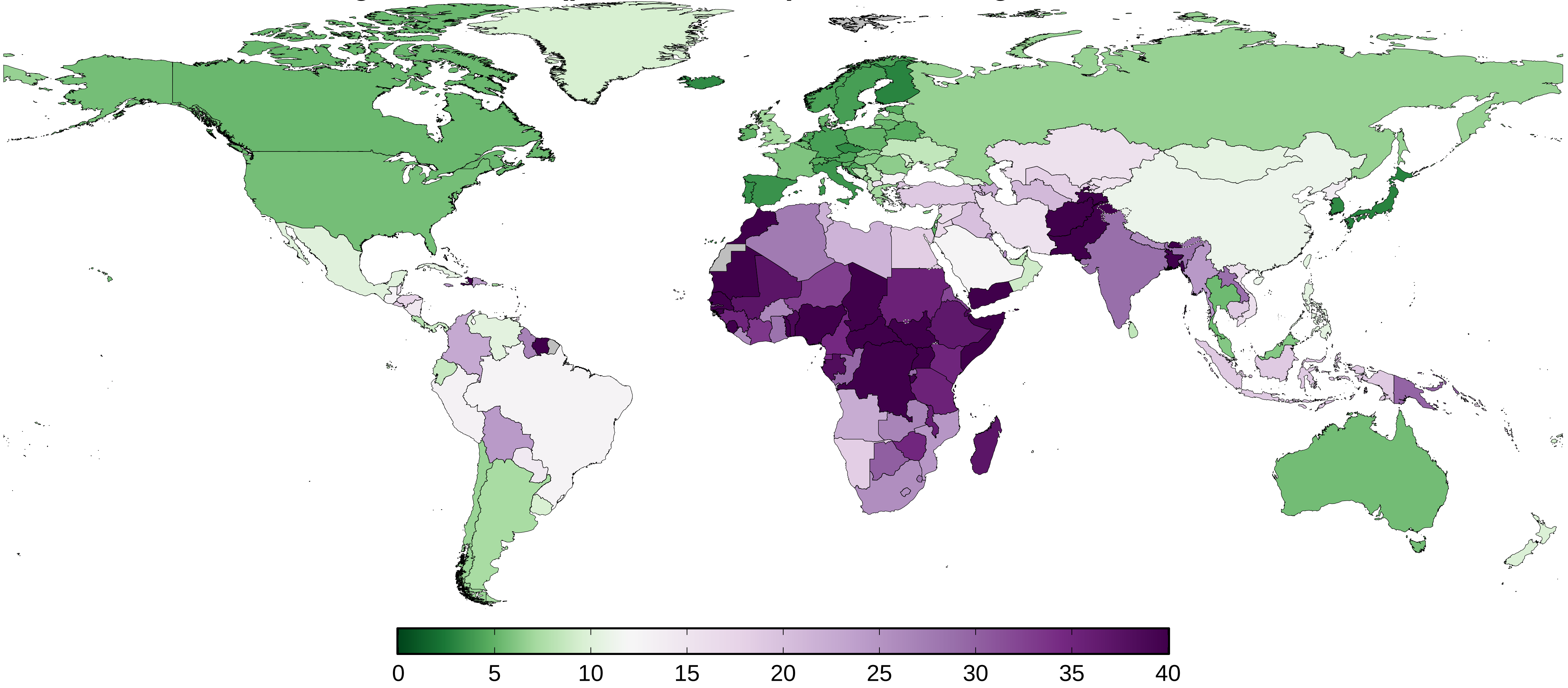

Figure 2A: SBR (per 1000 births) at 20 weeks' gestation in 2015

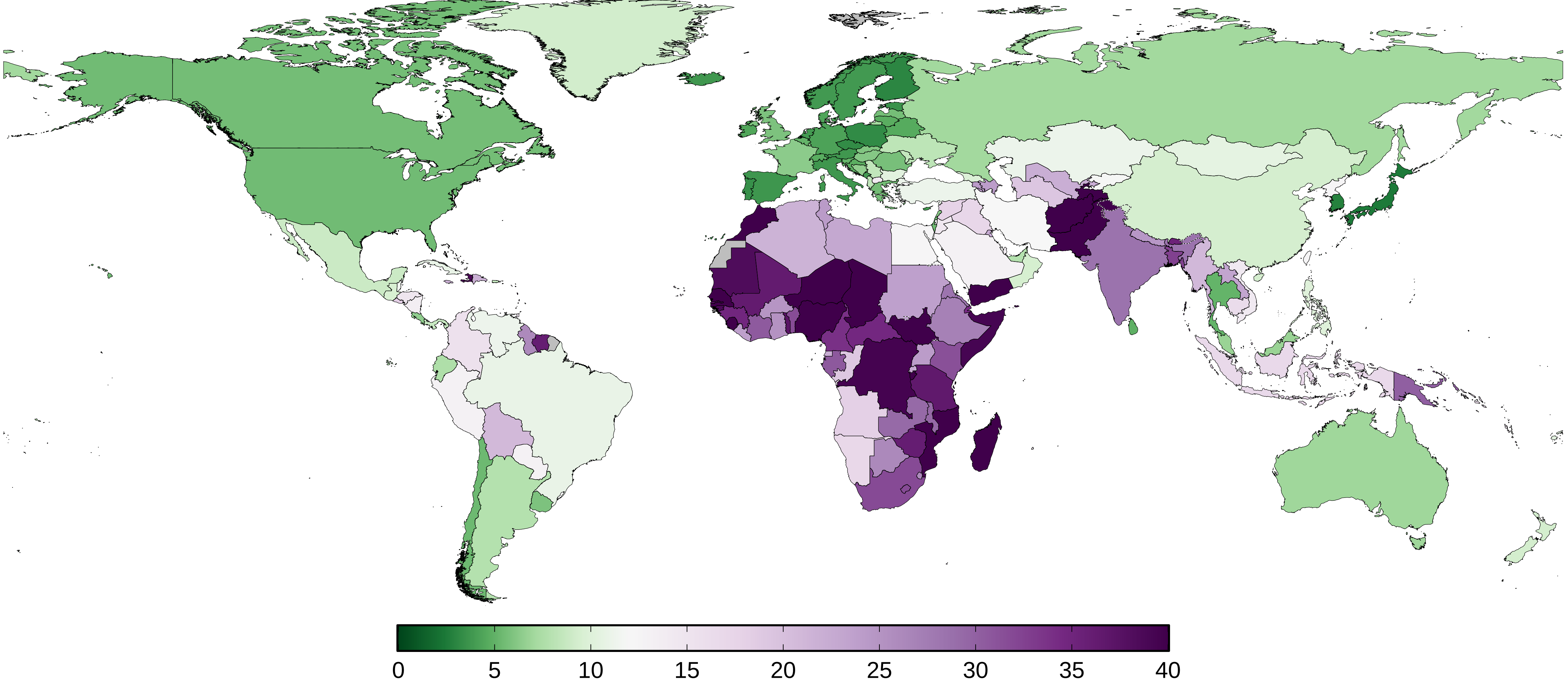

Figure 2A: SBR (per 1000 births) at 20 weeks' gestation in 2020

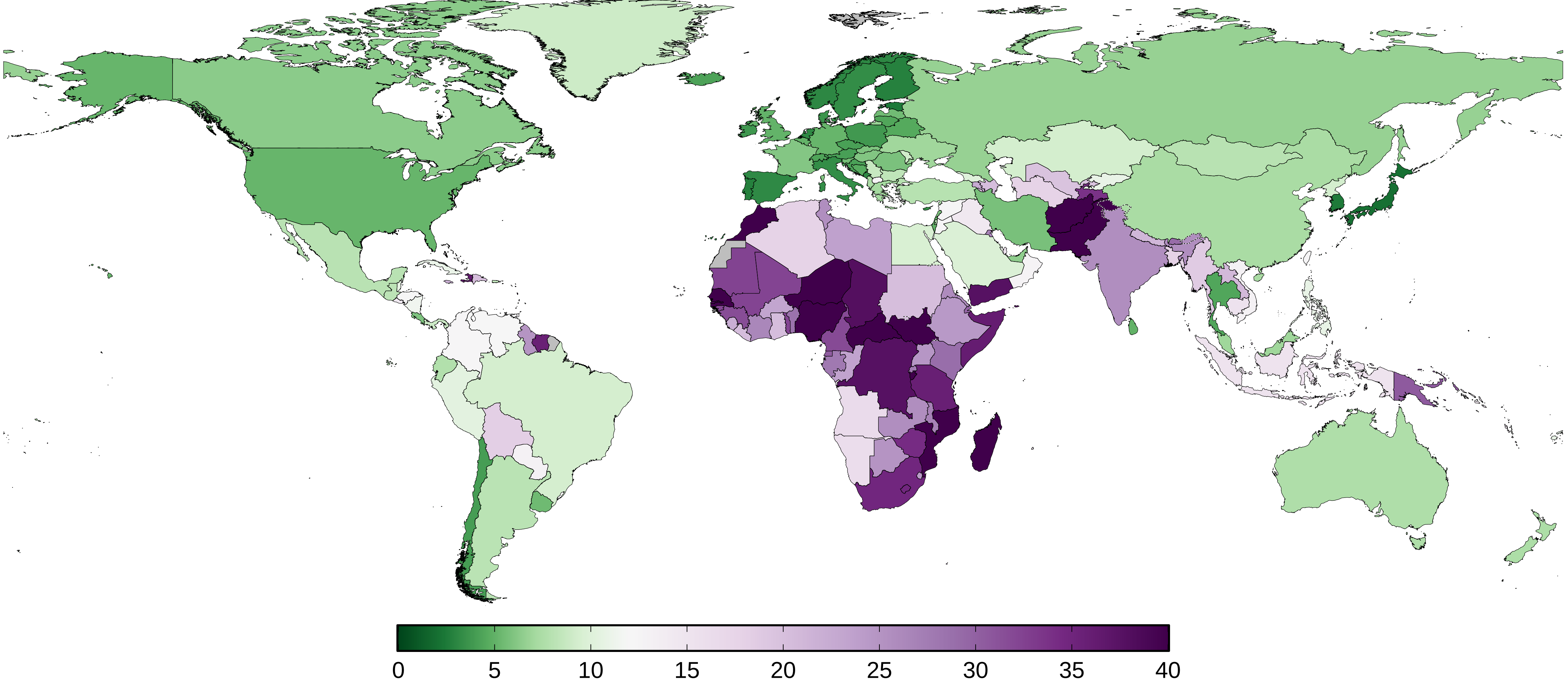

Figure 2A: SBR (per 1000 births) at 20 weeks' gestation in 2021

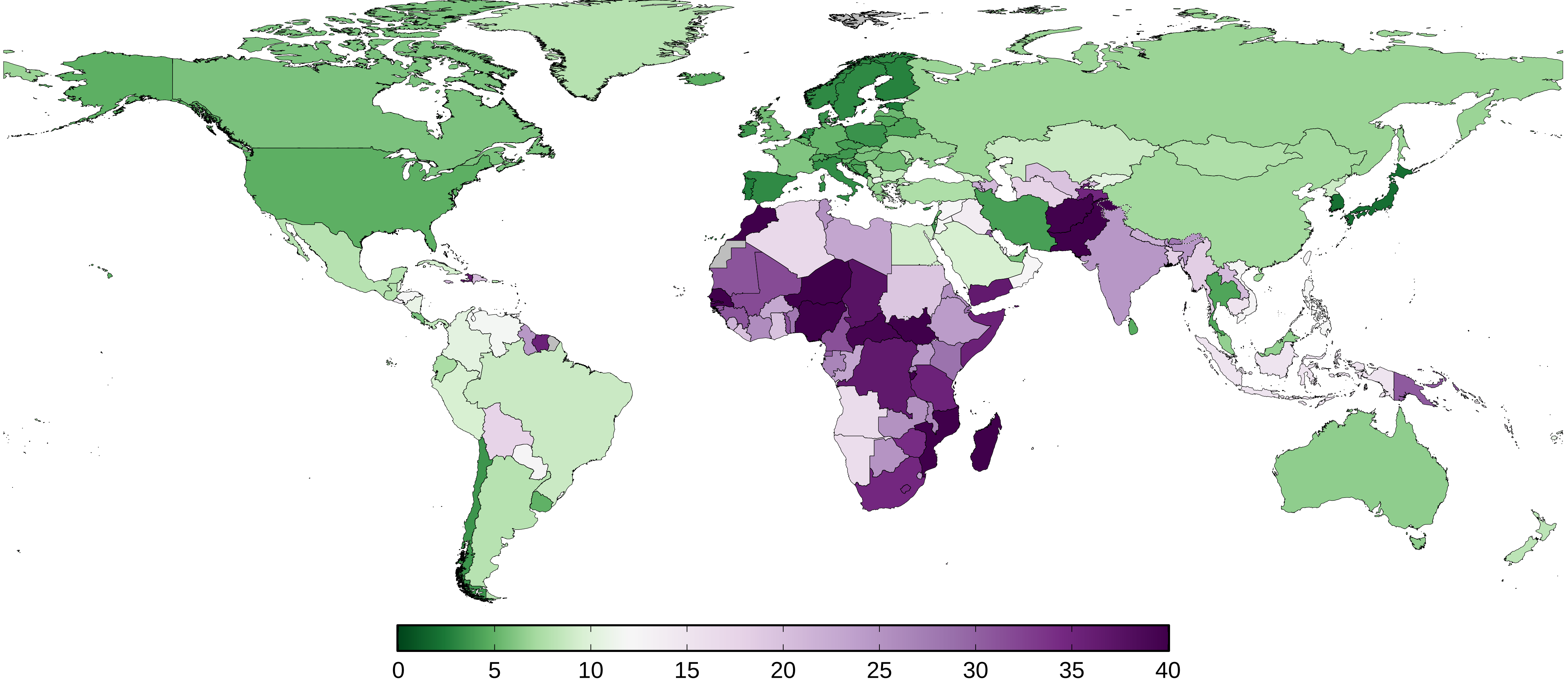

Figure 2B: SBR (per 1000 births) at 22 weeks' gestation in 1990

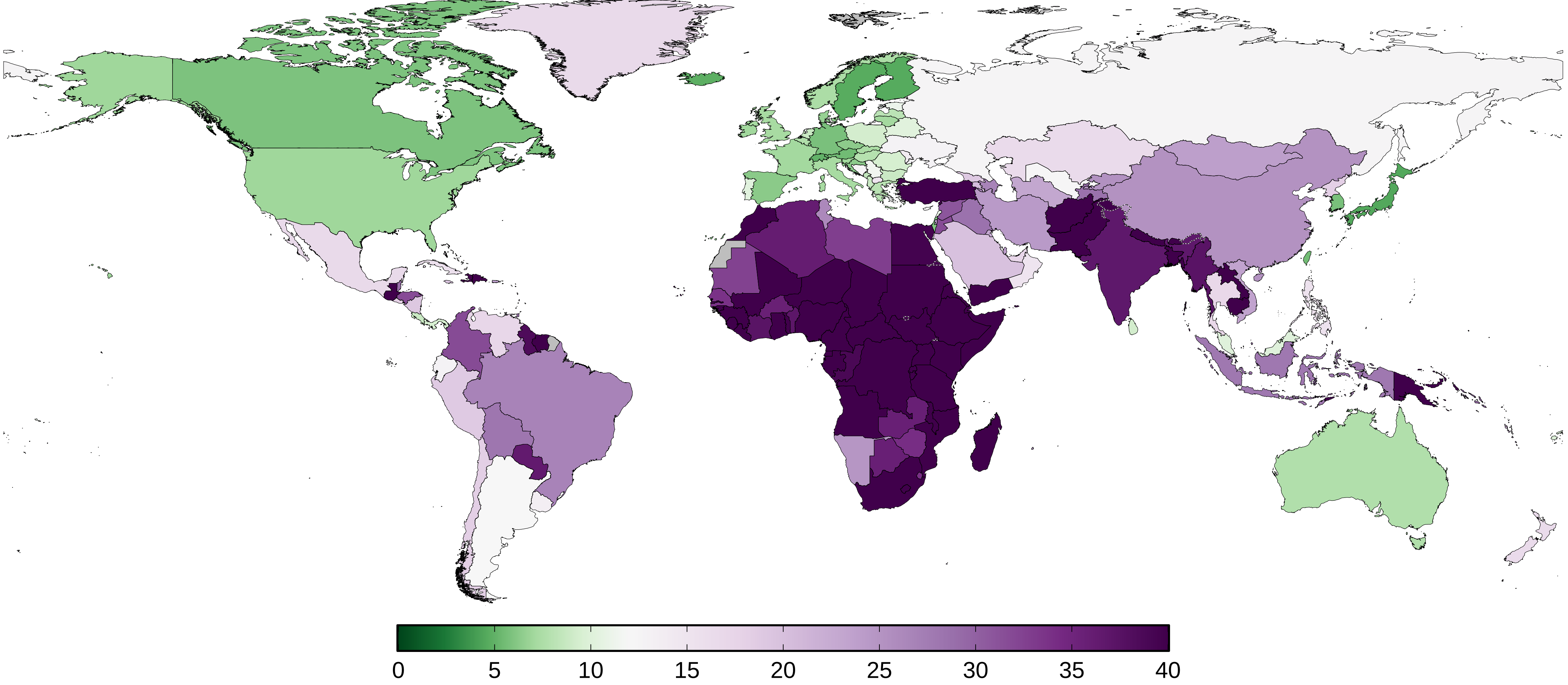

Figure 2B: SBR (per 1000 births) at 22 weeks' gestation in 2000

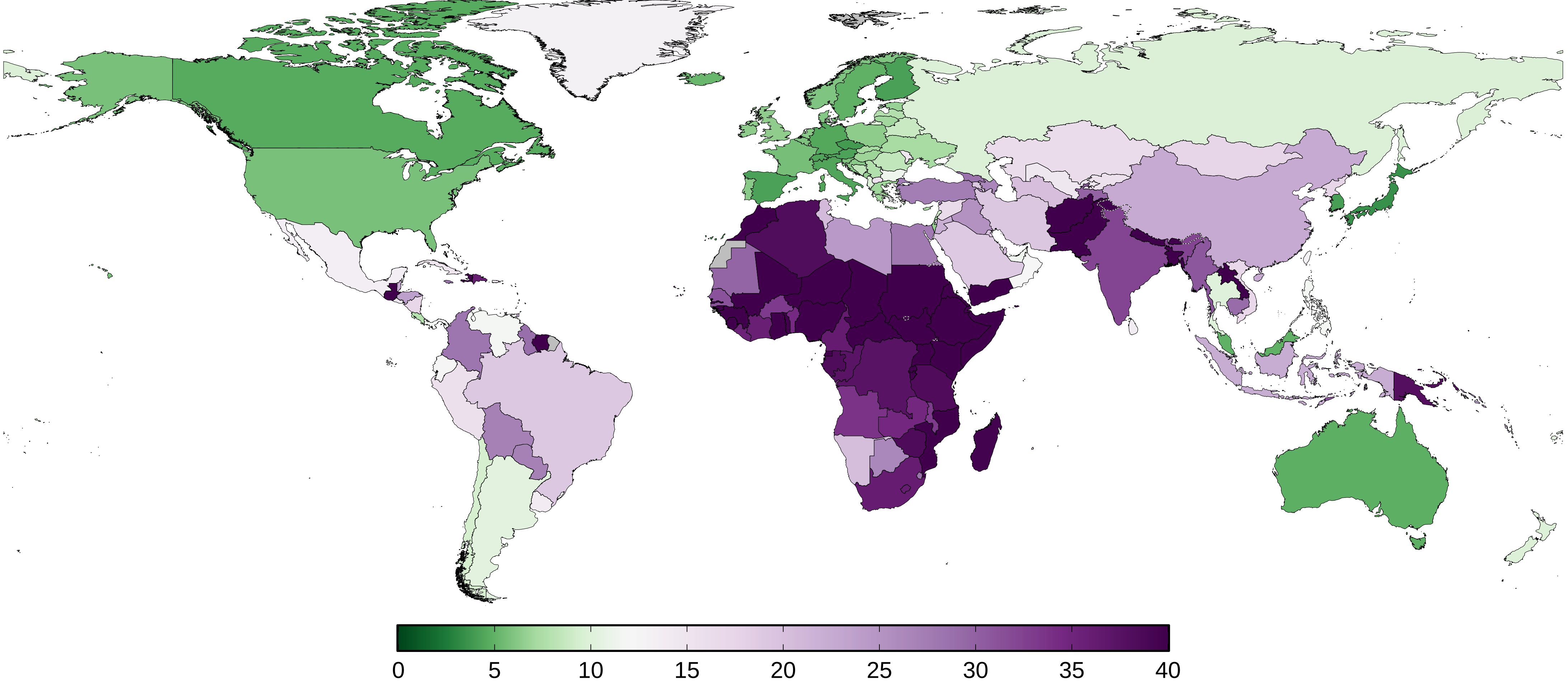

Figure 2B: SBR (per 1000 births) at 22 weeks' gestation in 2010

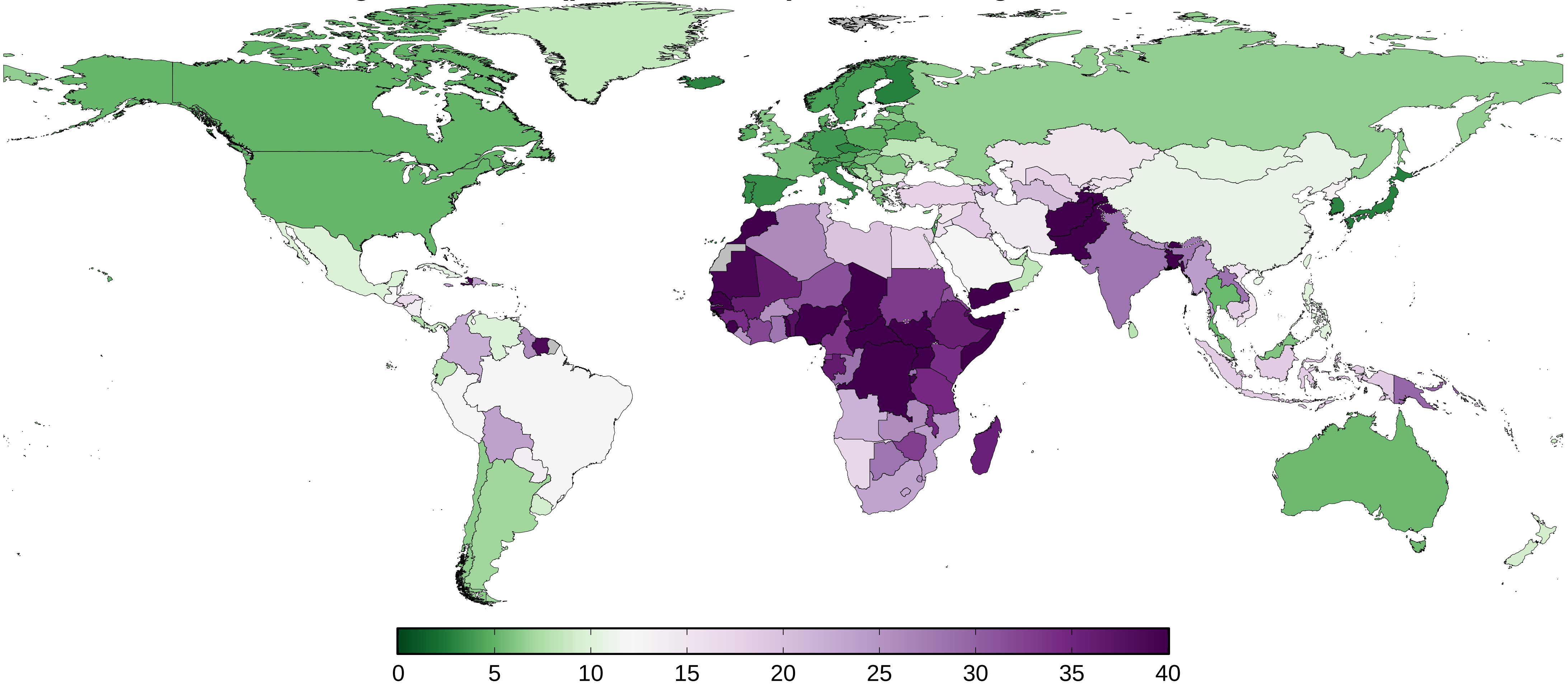

Figure 2B: SBR (per 1000 births) at 22 weeks' gestation in 2015

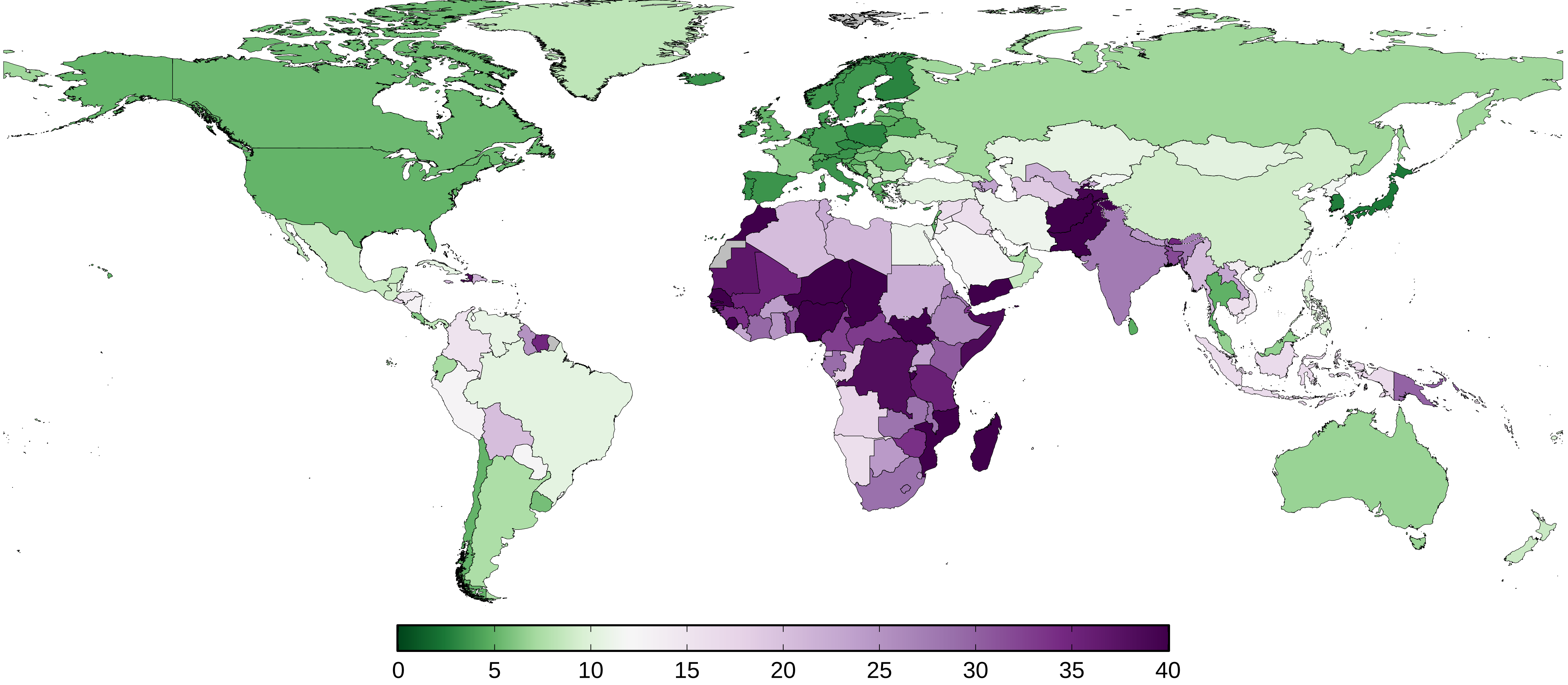

Figure 2B: SBR (per 1000 births) at 22 weeks' gestation in 2020

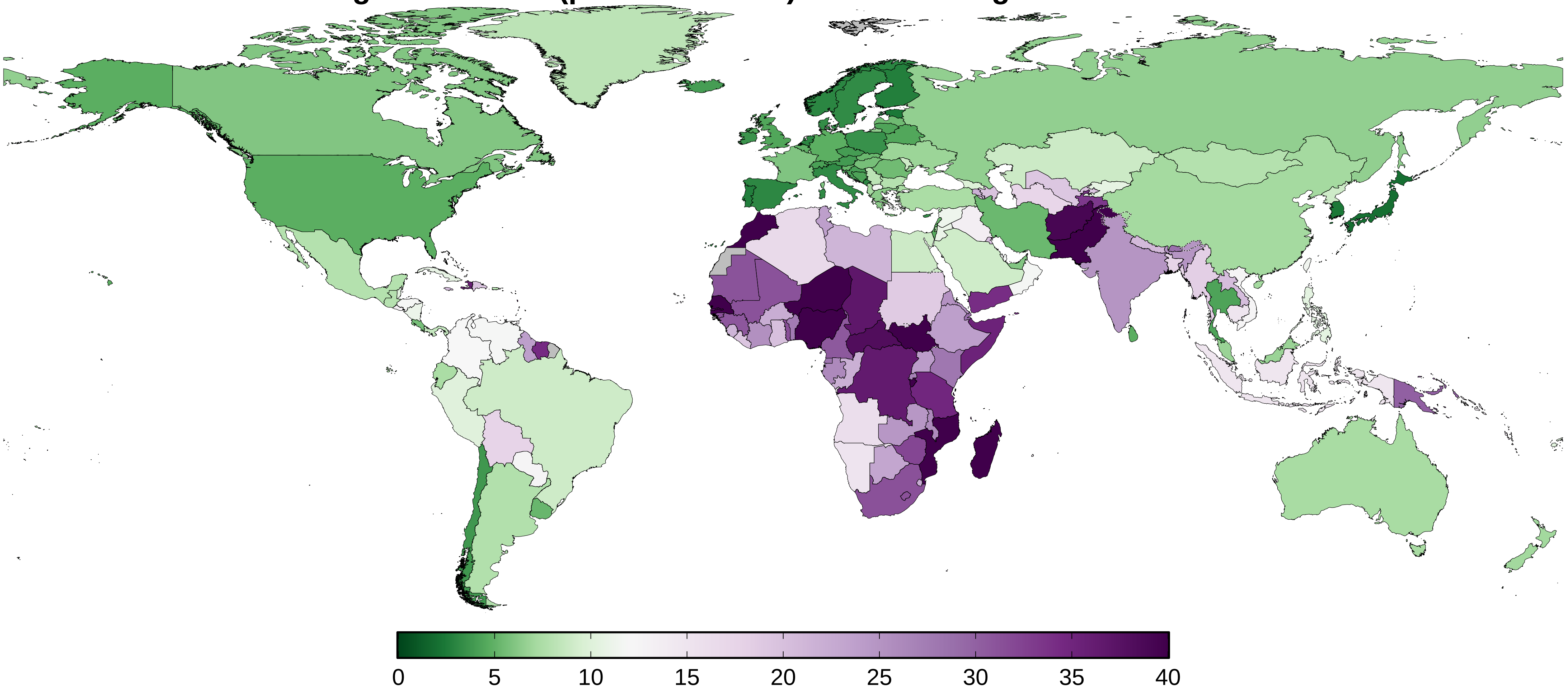

Figure 2B: SBR (per 1000 births) at 22 weeks' gestation in 2021

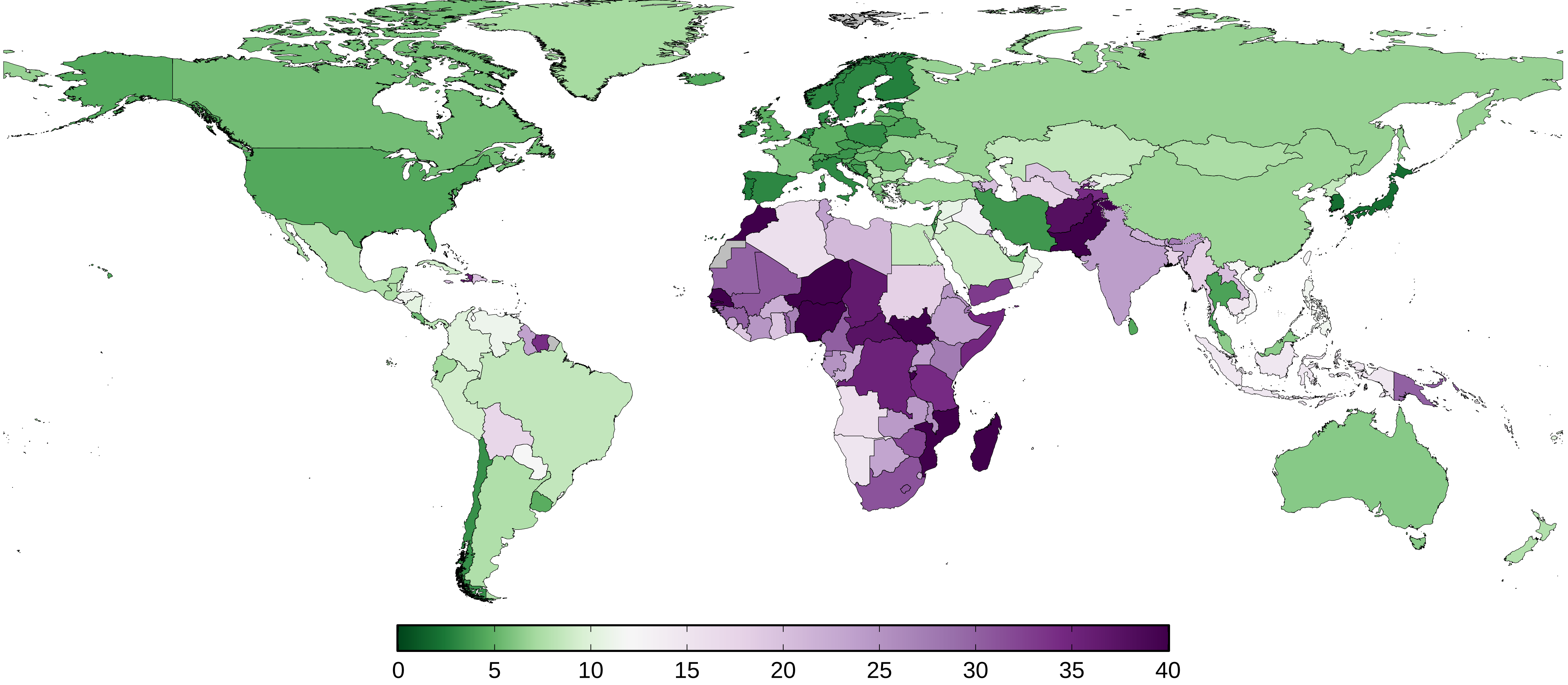

Figure 2C: SBR (per 1000 births) at 28 weeks' gestation in 1990

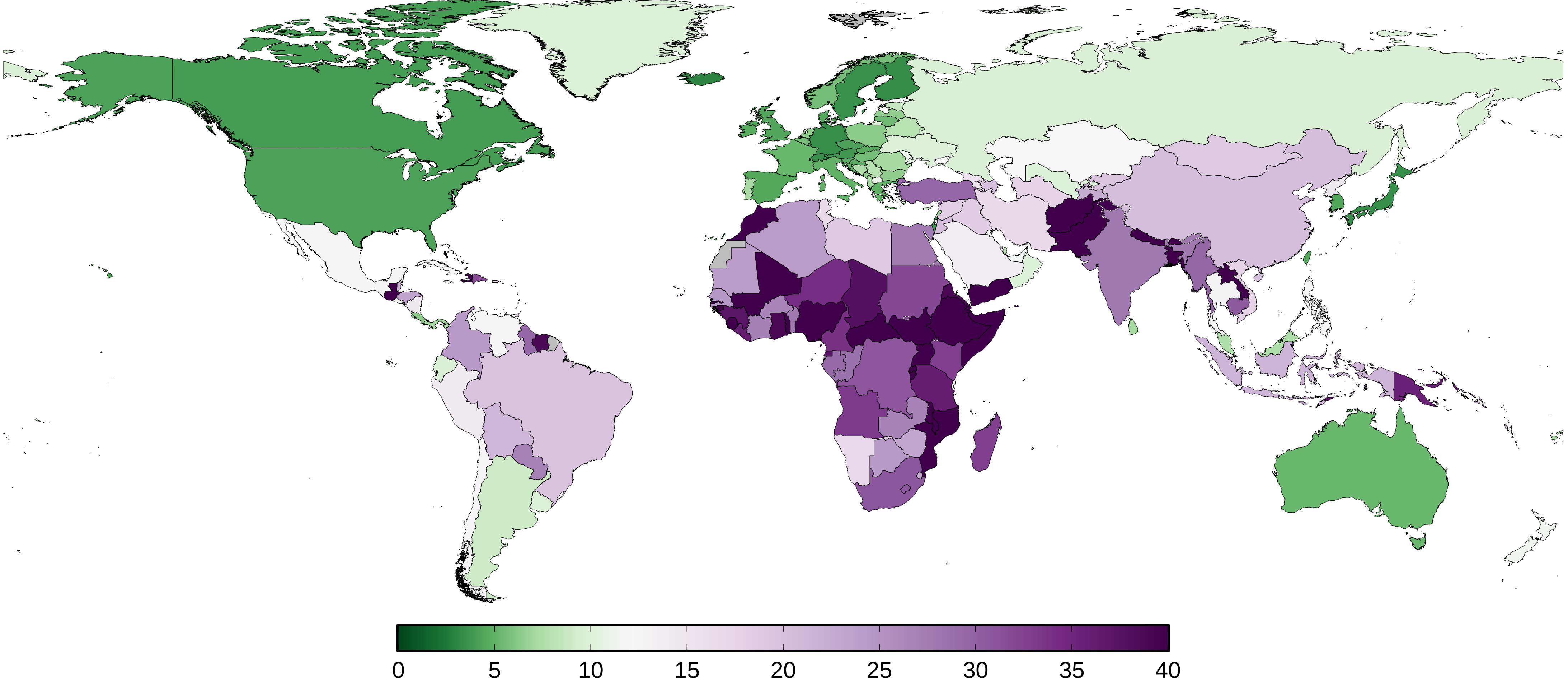

Figure 2C: SBR (per 1000 births) at 28 weeks' gestation in 2000

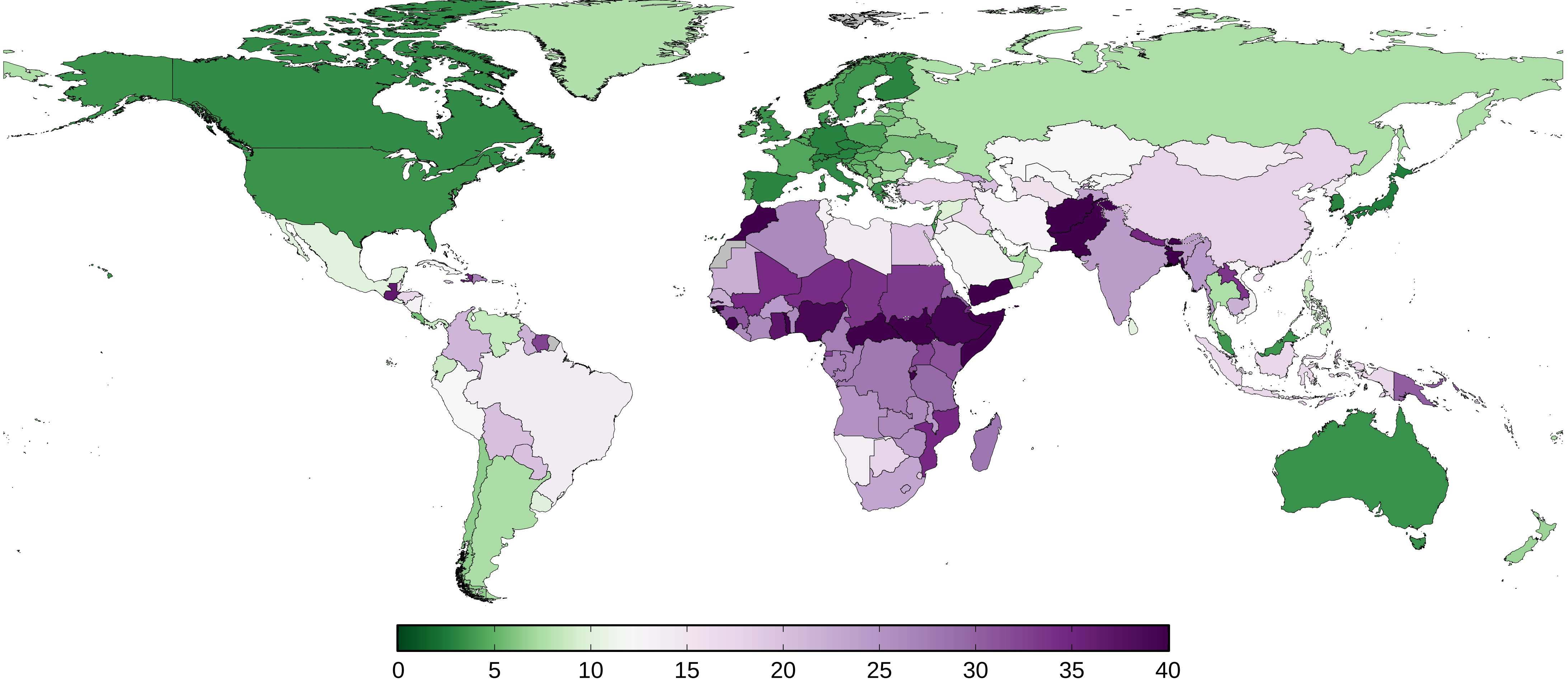

Figure 2C: SBR (per 1000 births) at 28 weeks' gestation in 2010

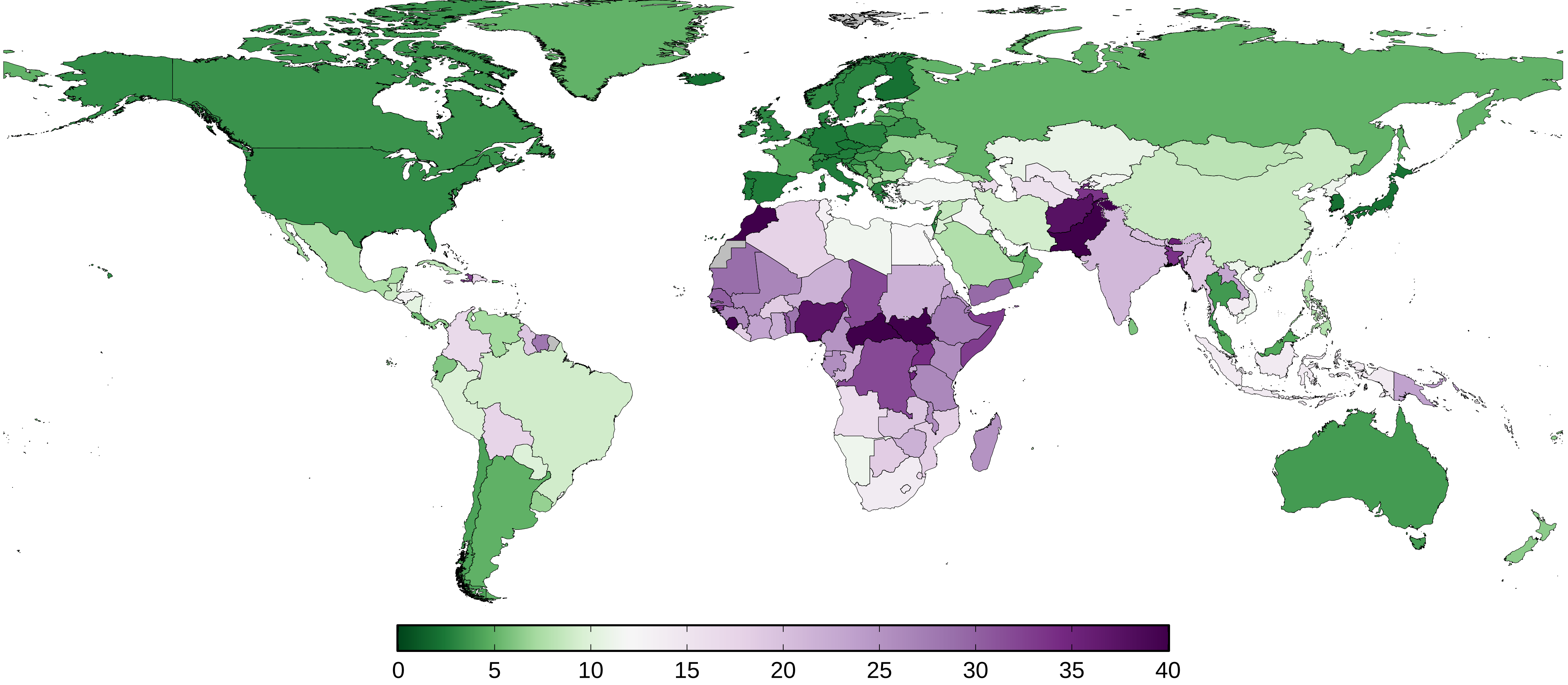

Figure 2C: SBR (per 1000 births) at 28 weeks' gestation in 2015

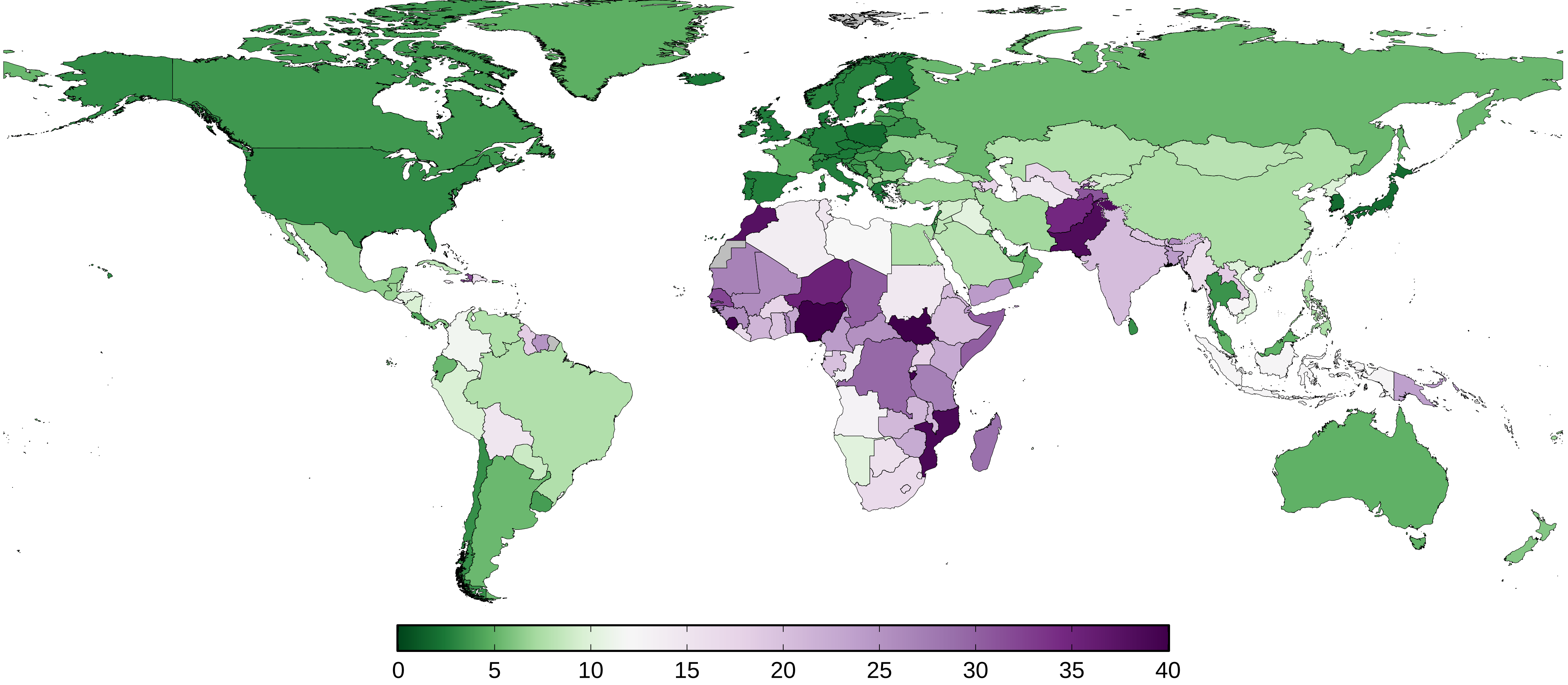

Figure 2C: SBR (per 1000 births) at 28 weeks' gestation in 2020

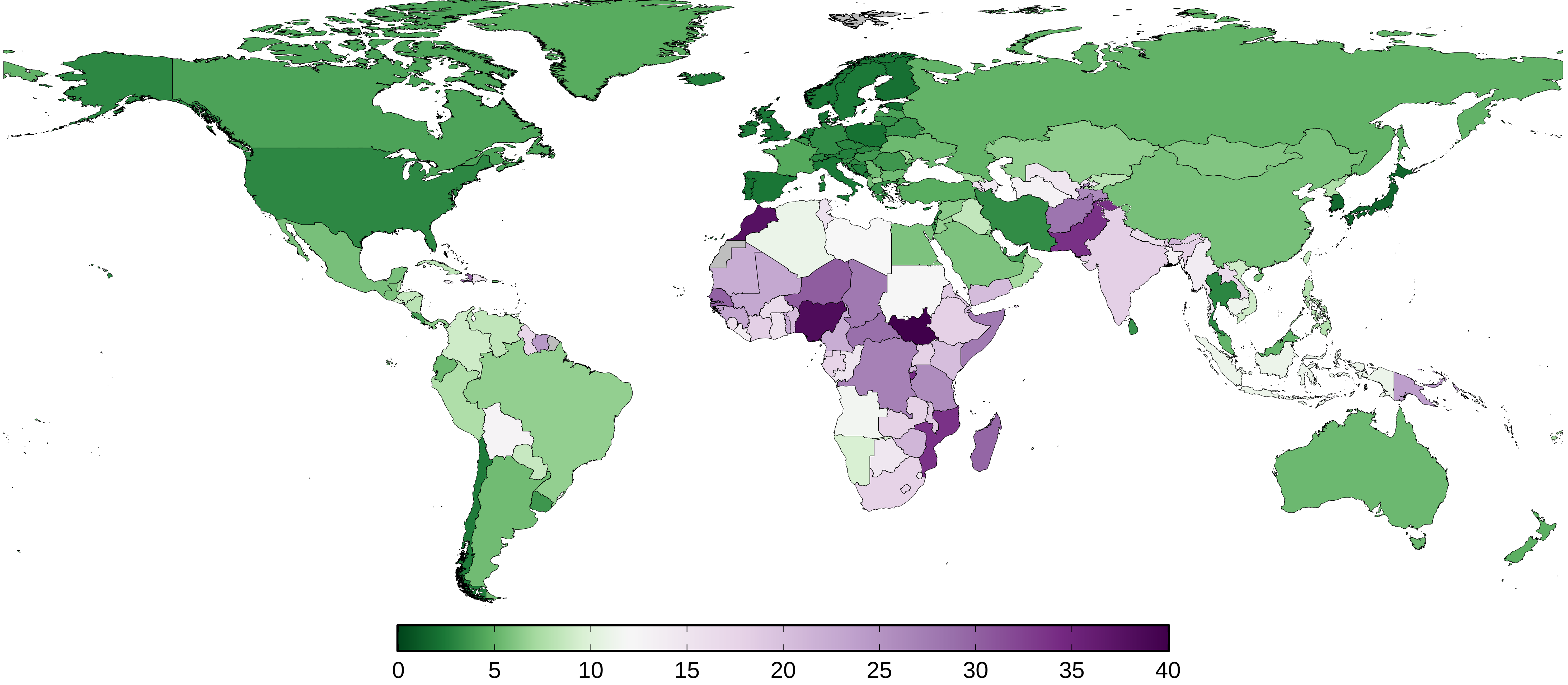

Figure 2C: SBR (per 1000 births) at 28 weeks' gestation in 2021

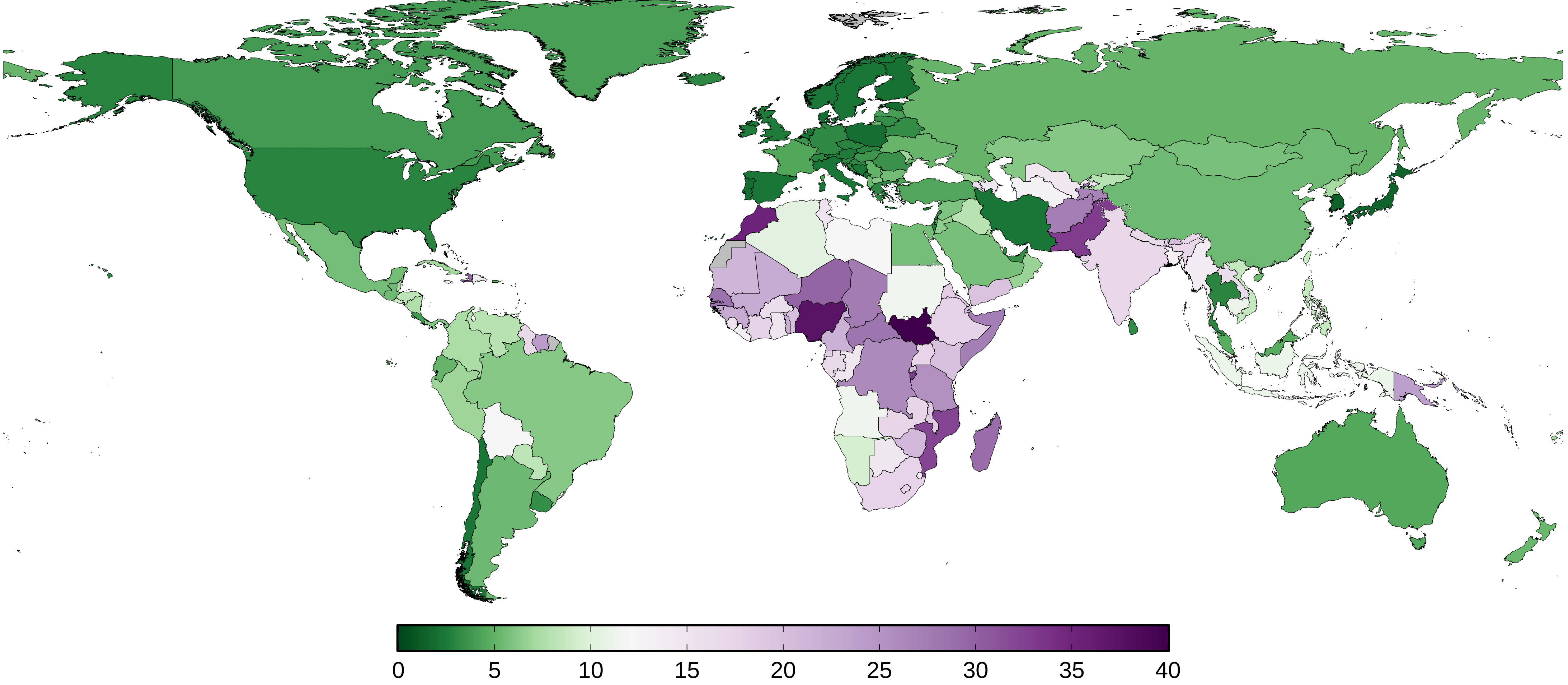

Figure 2D: Percentage of stillbirths between 20 and <28 weeks' gestation in 1990

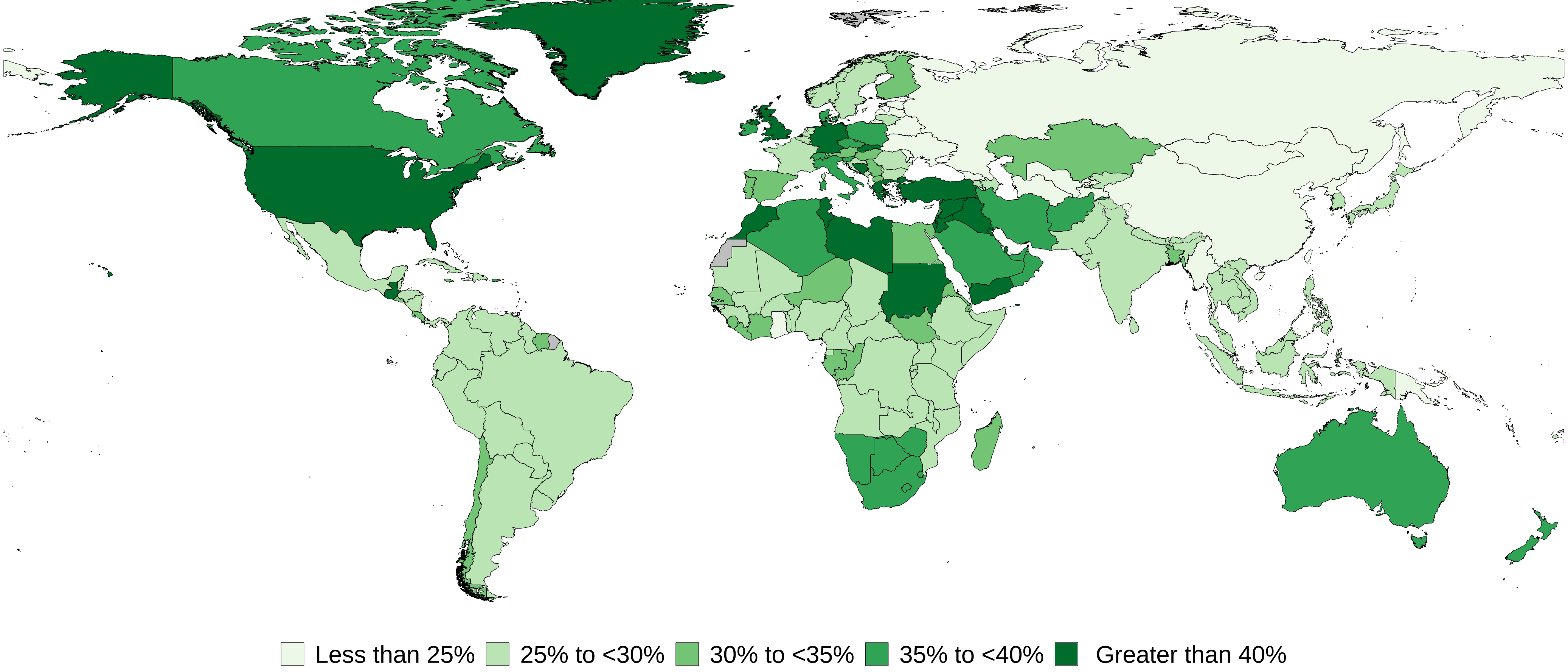

Figure 2D: Percentage of stillbirths between 20 and <28 weeks' gestation in 2000

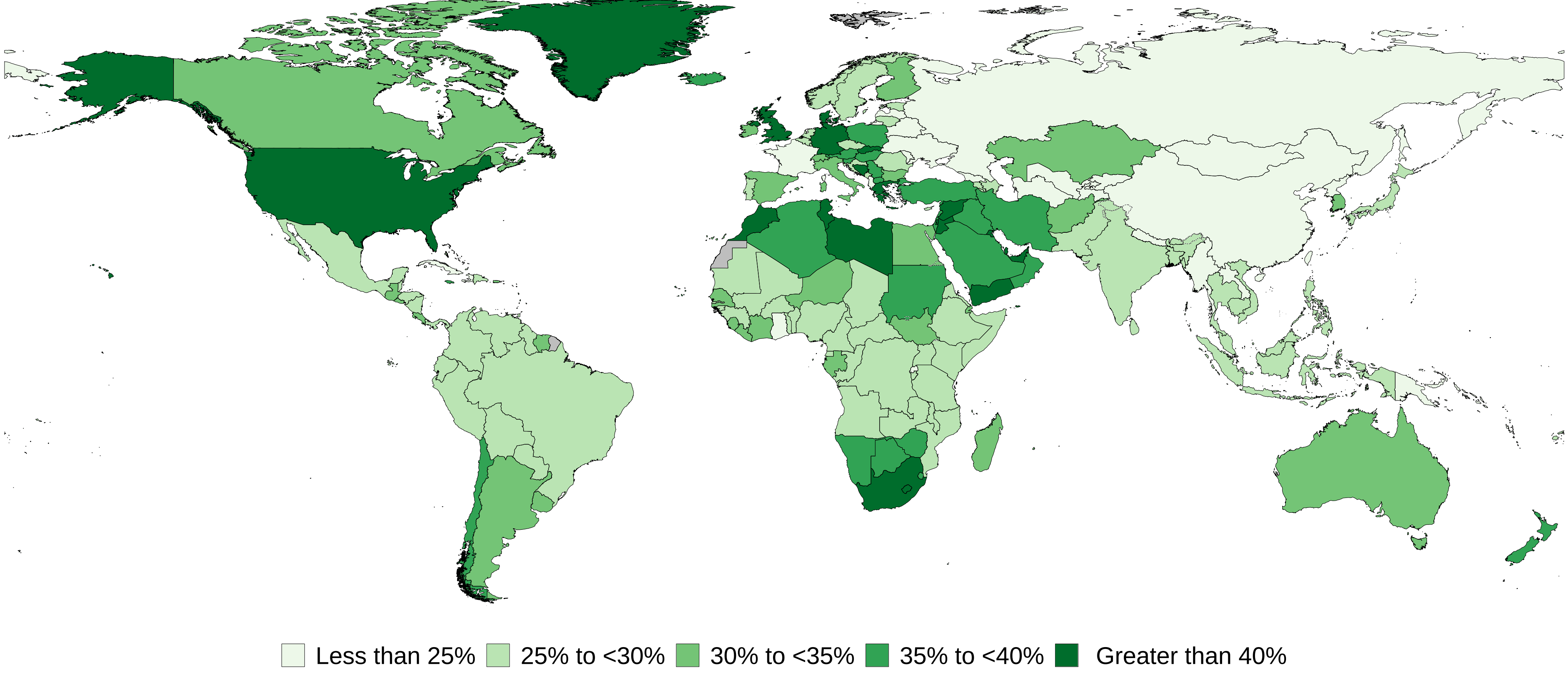

Figure 2D: Percentage of stillbirths between 20 and <28 weeks' gestation in 2010

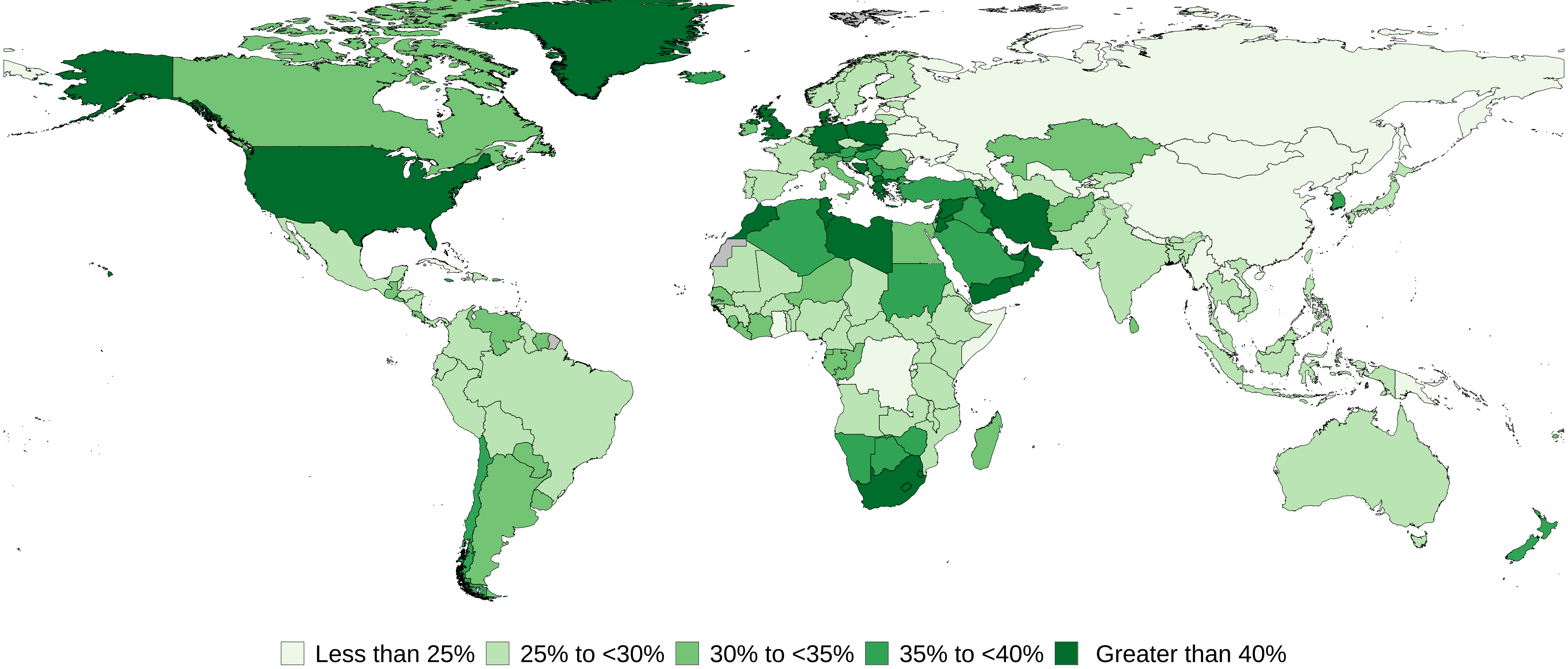

Figure 2D: Percentage of stillbirths between 20 and <28 weeks' gestation in 2015

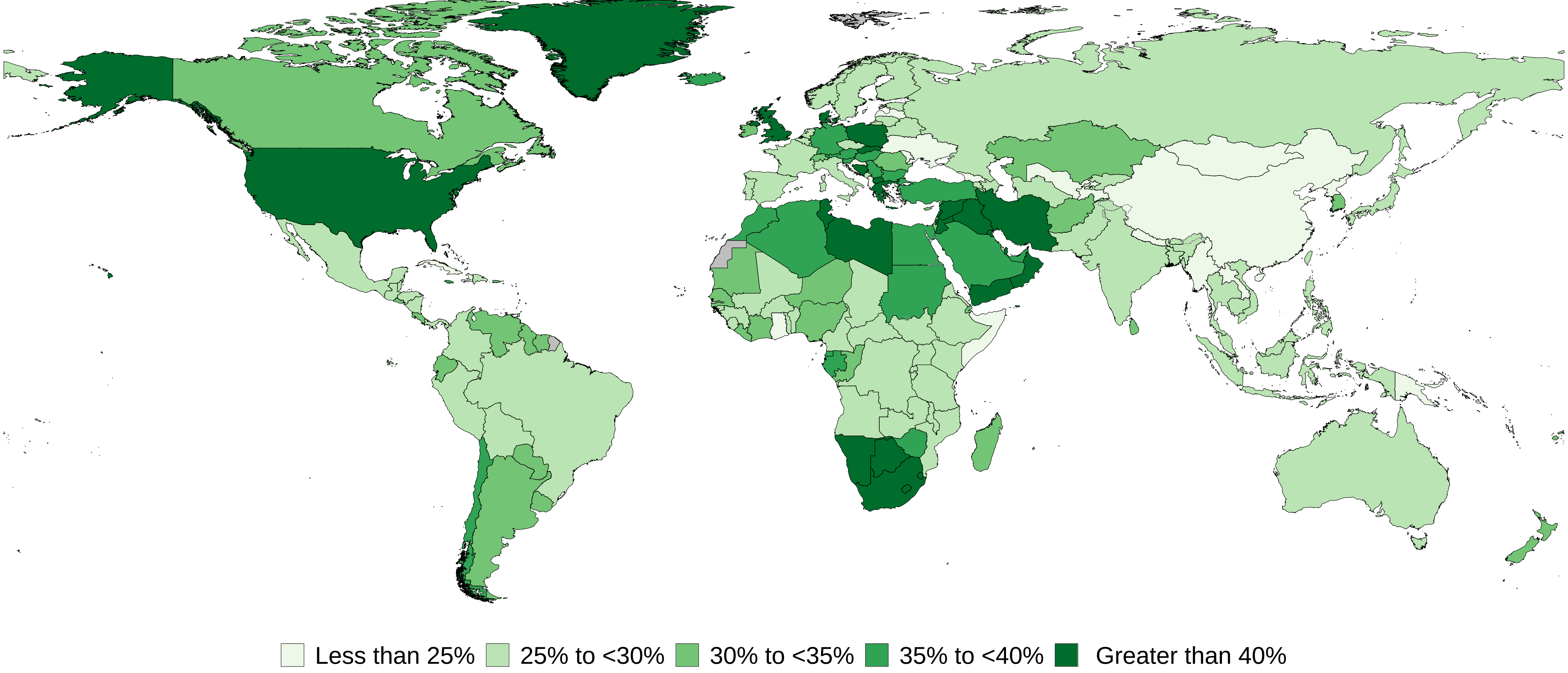

Figure 2D: Percentage of stillbirths between 20 and <28 weeks' gestation in 2020

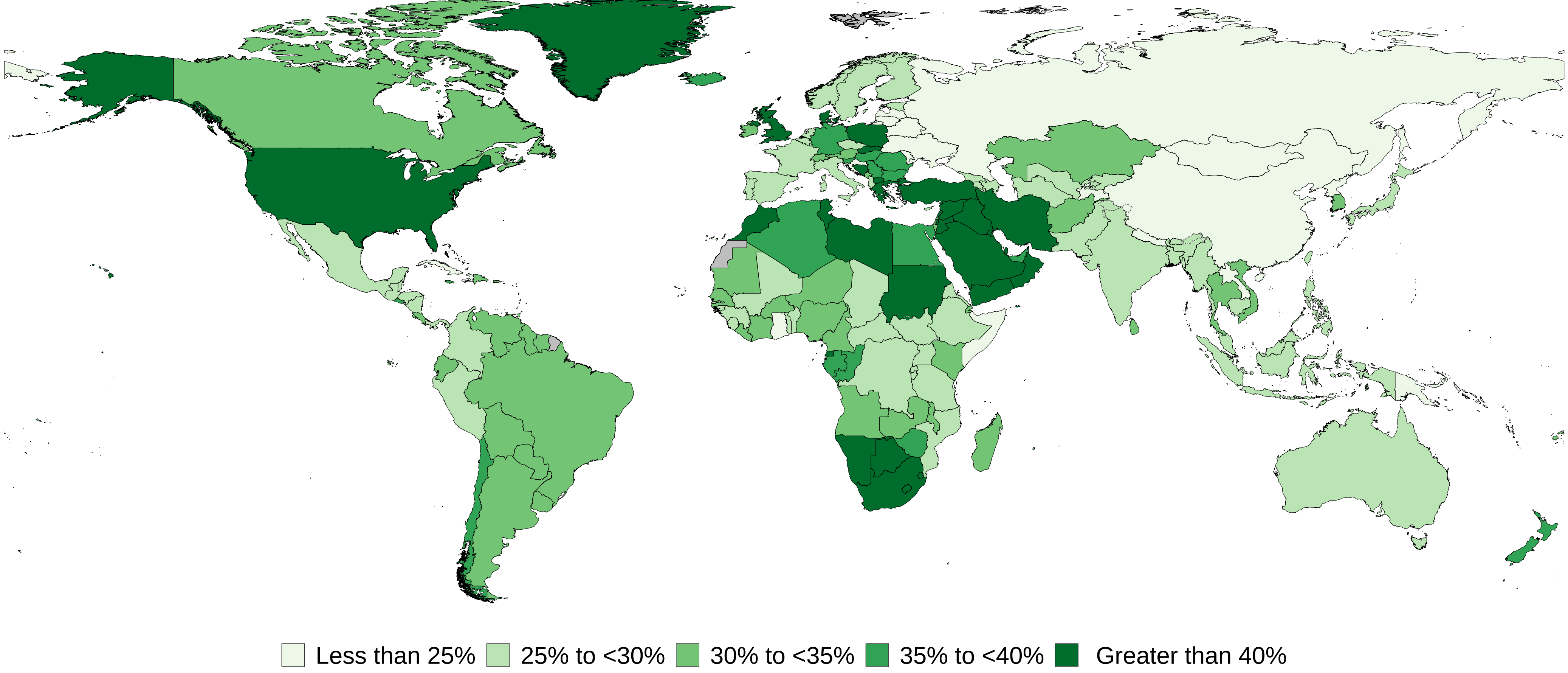

Figure 2D: Percentage of stillbirths between 20 and <28 weeks' gestation in 2021

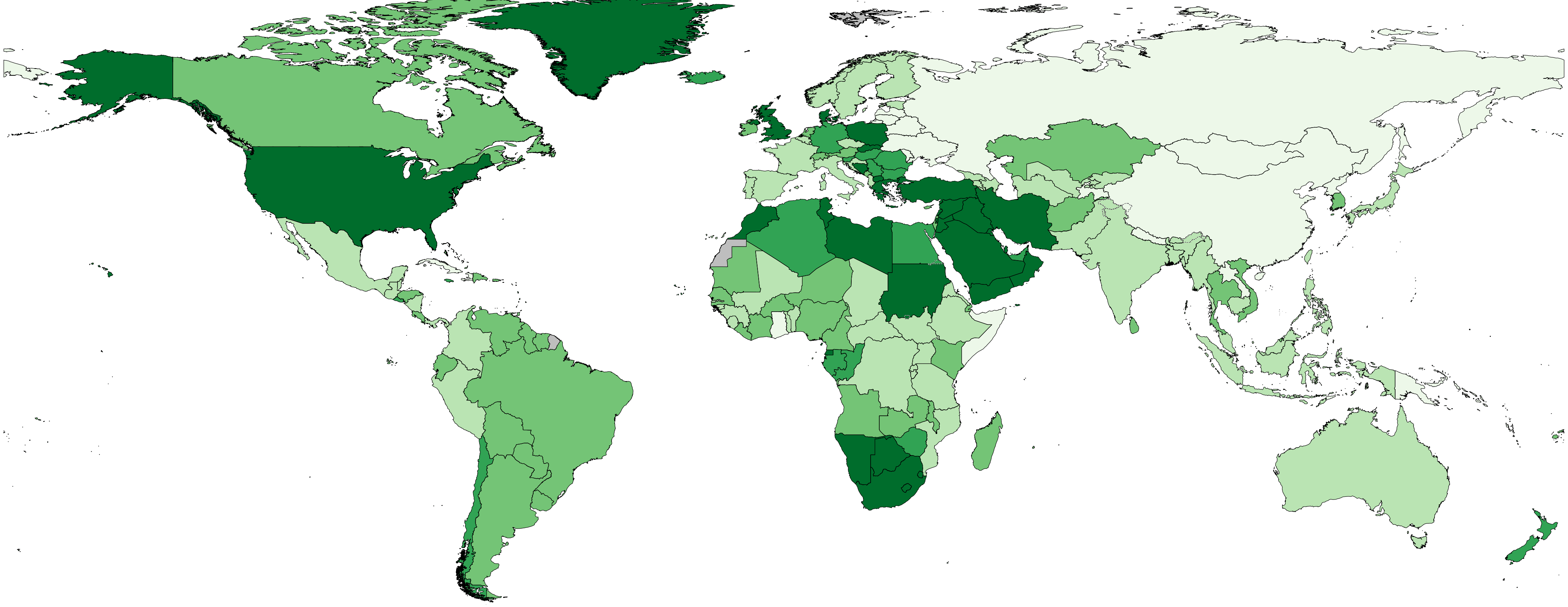

Less than 25% | 25% to <30% | 30% to <35% | 35% to <40% | Greater than 40%

Figure 2E: Number of stillbirths between 20 and <28 weeks' gestation in 1990

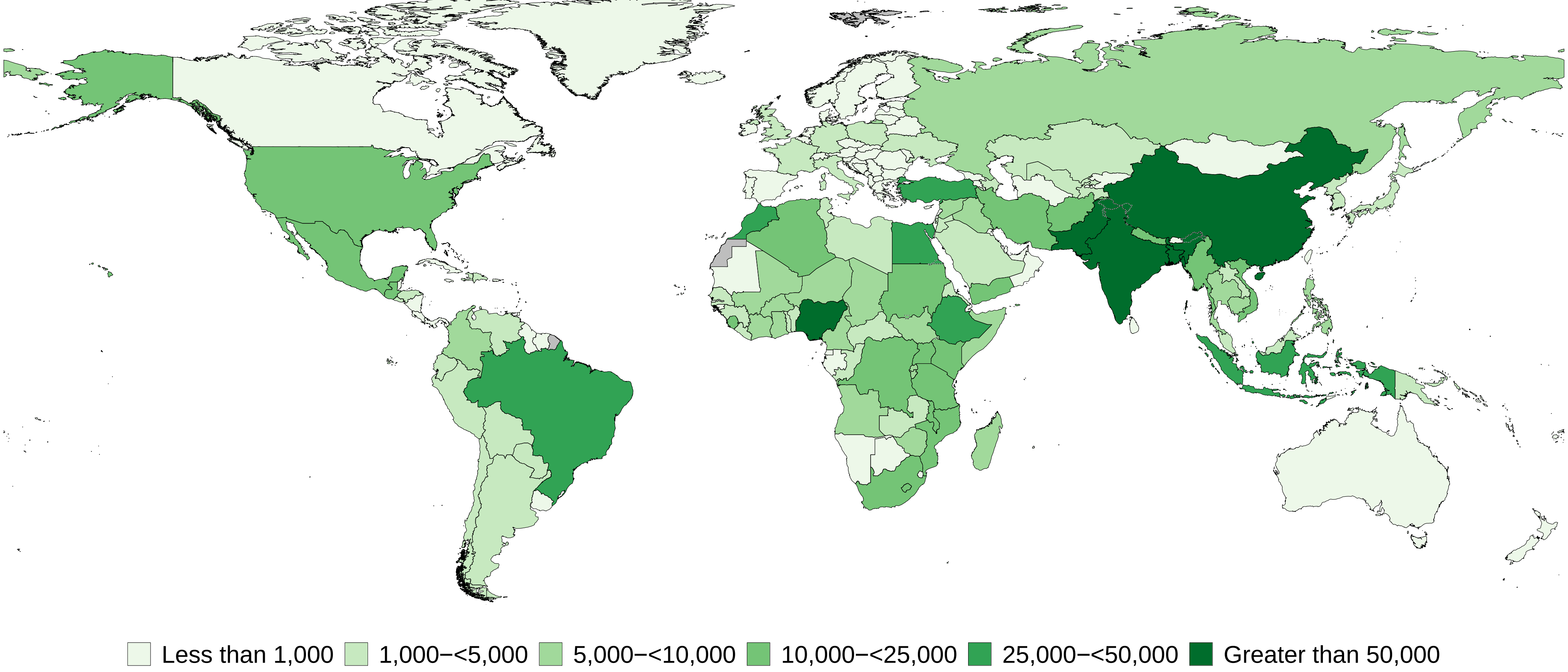

Figure 2E: Number of stillbirths between 20 and <28 weeks' gestation in 2000

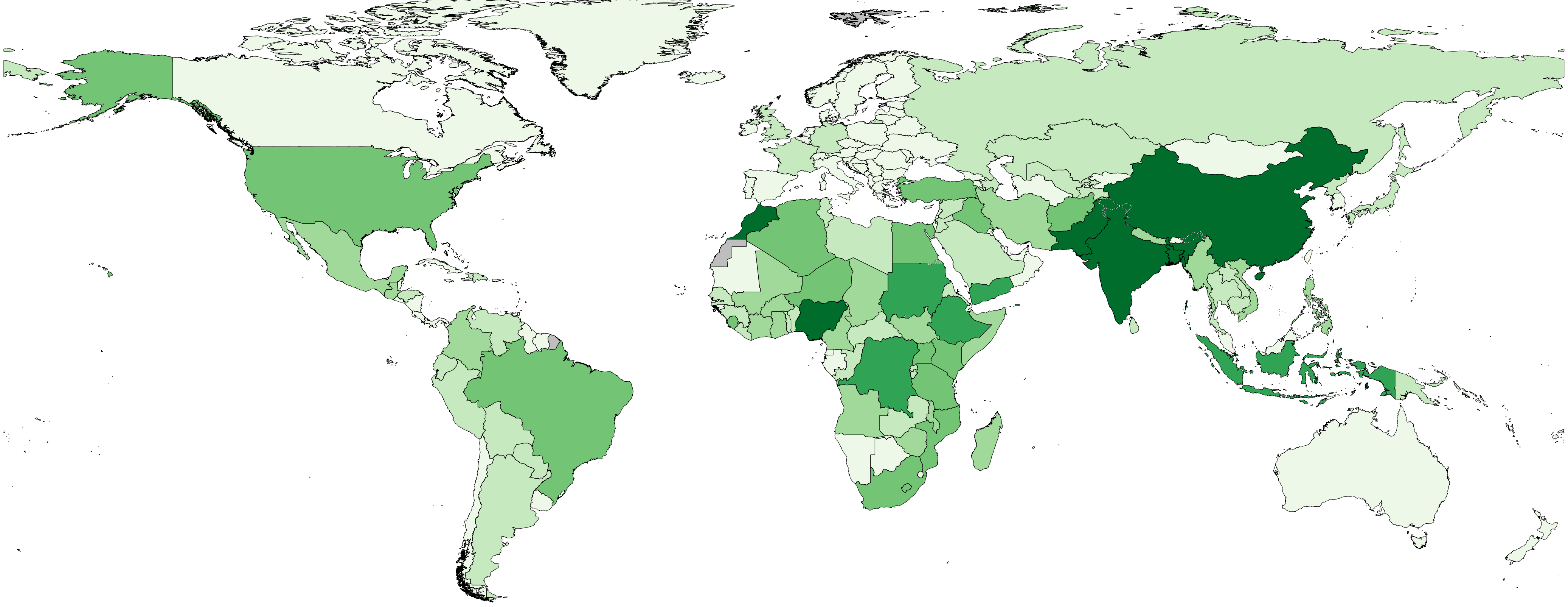

Less than 1,000 | 1,000−<5,000 | 5,000−<10,000 | 10,000−<25,000 | 25,000−<50,000 | Greater than 50,000

Figure 2E: Number of stillbirths between 20 and <28 weeks' gestation in 2010

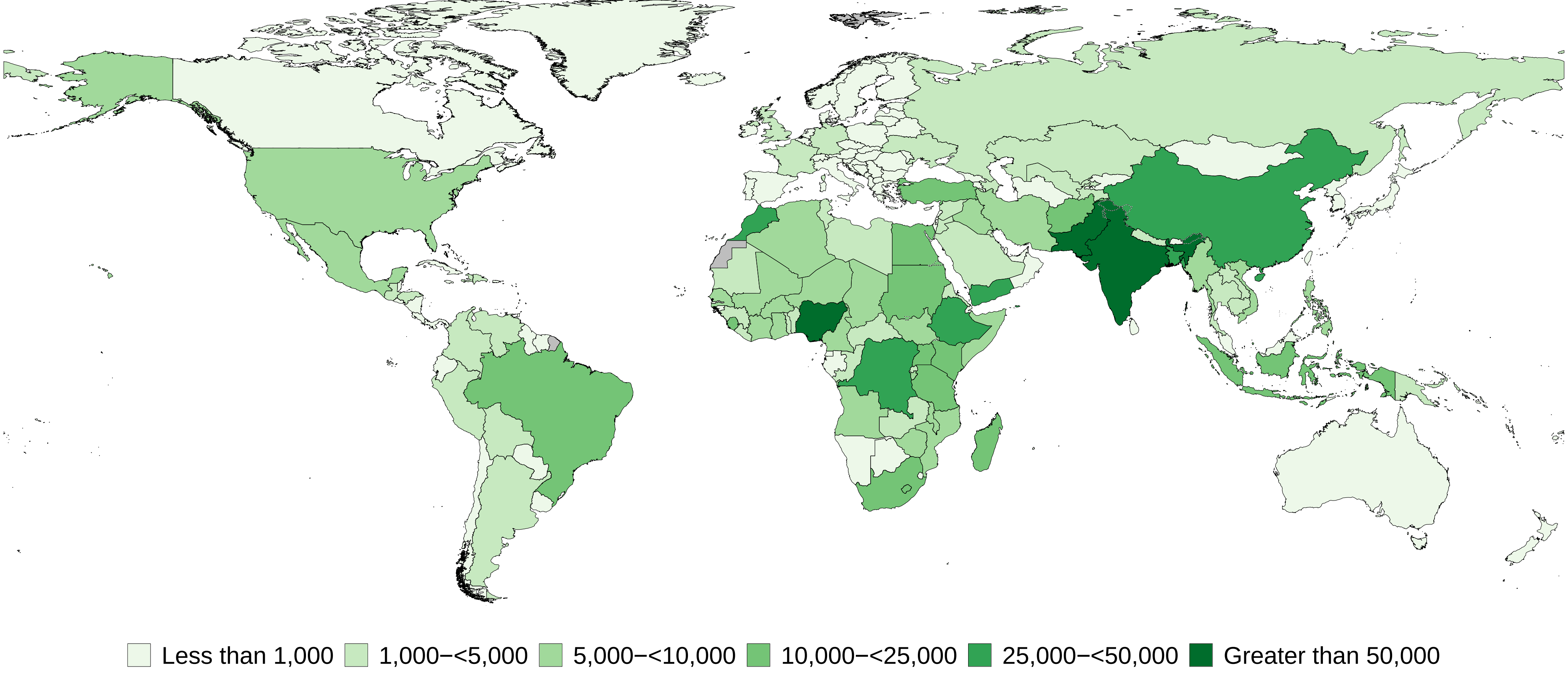

Figure 2E: Number of stillbirths between 20 and <28 weeks' gestation in 2015

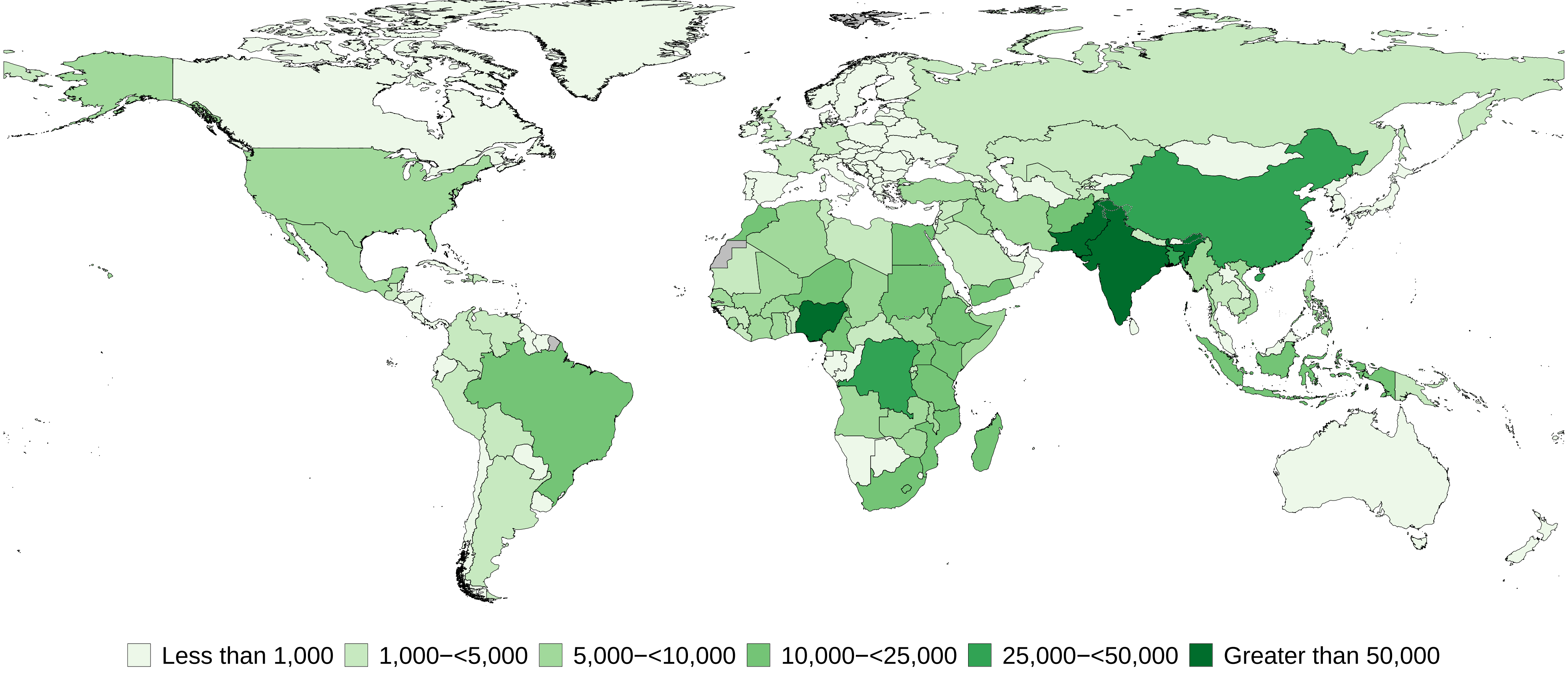

Figure 2E: Number of stillbirths between 20 and <28 weeks' gestation in 2020

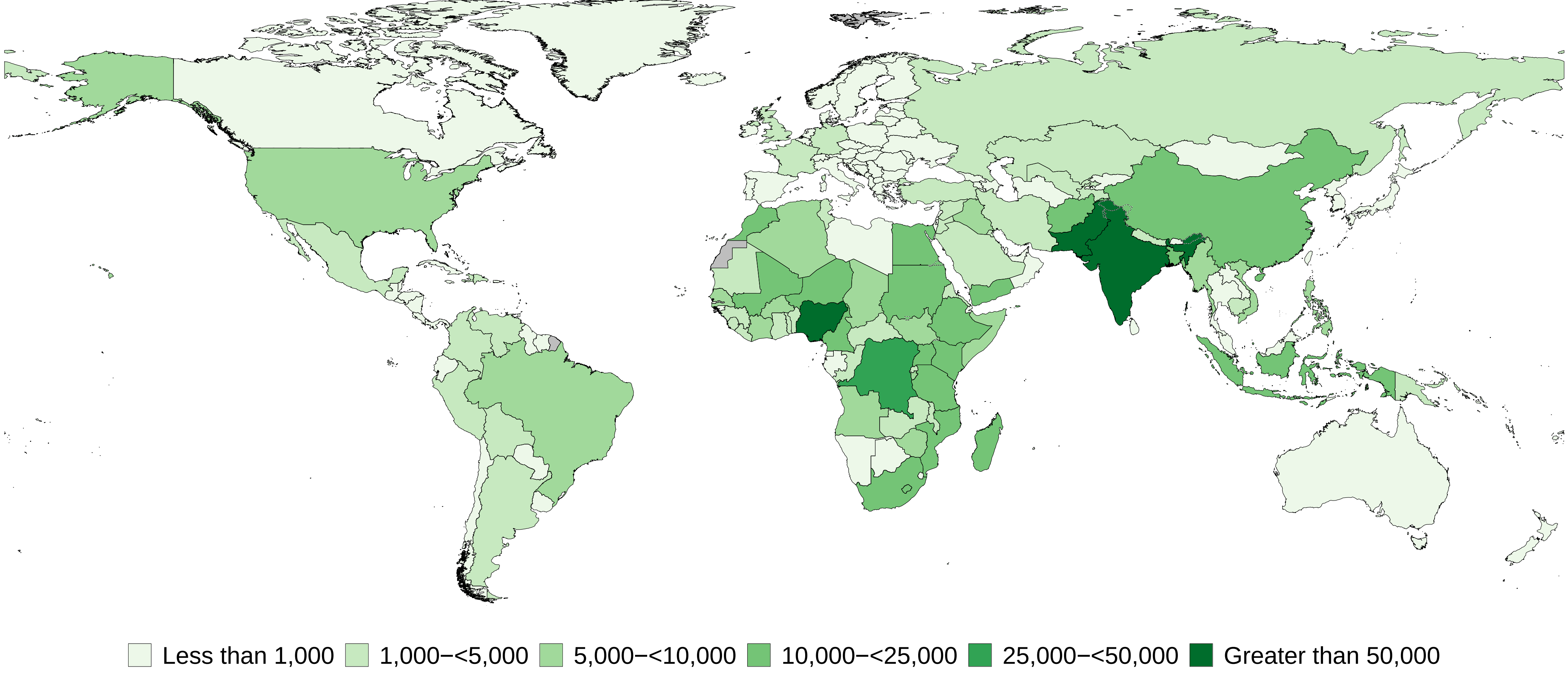

Figure 2E: Number of stillbirths between 20 and <28 weeks' gestation in 2021

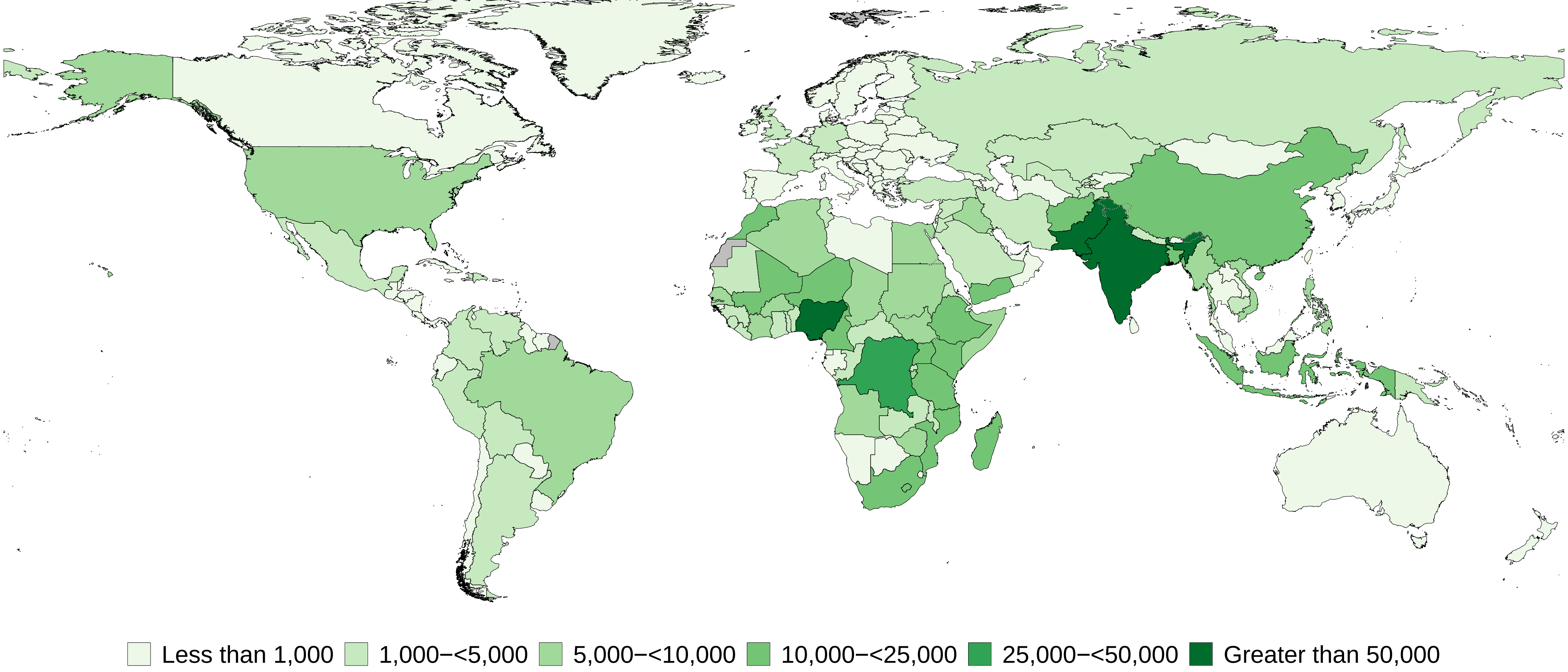

Figure 3: Comparison of SBR estimates to LSEIG 2015 estimates and IGME 2021 estimates

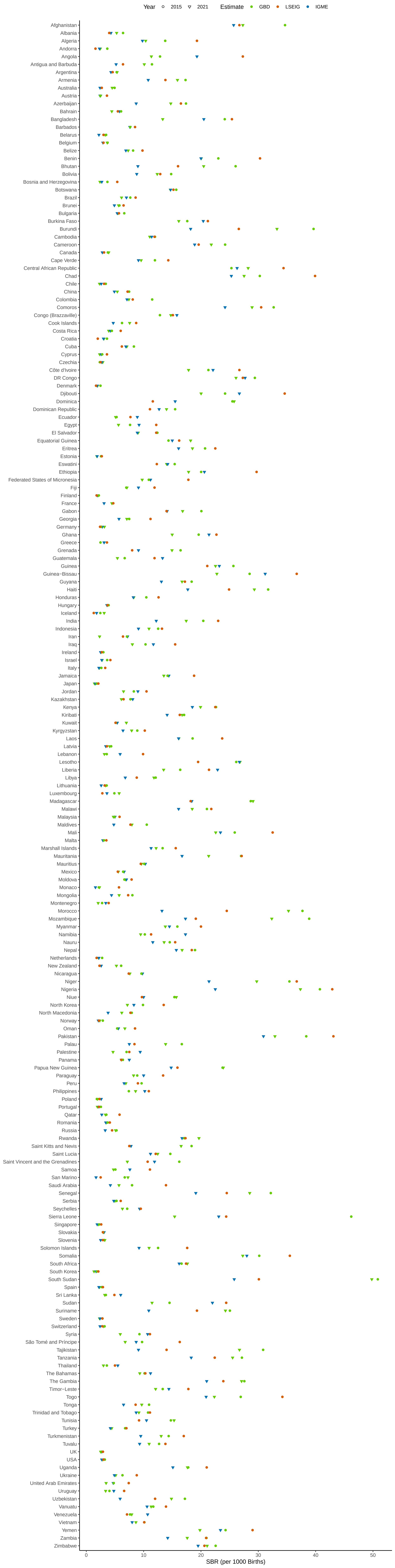

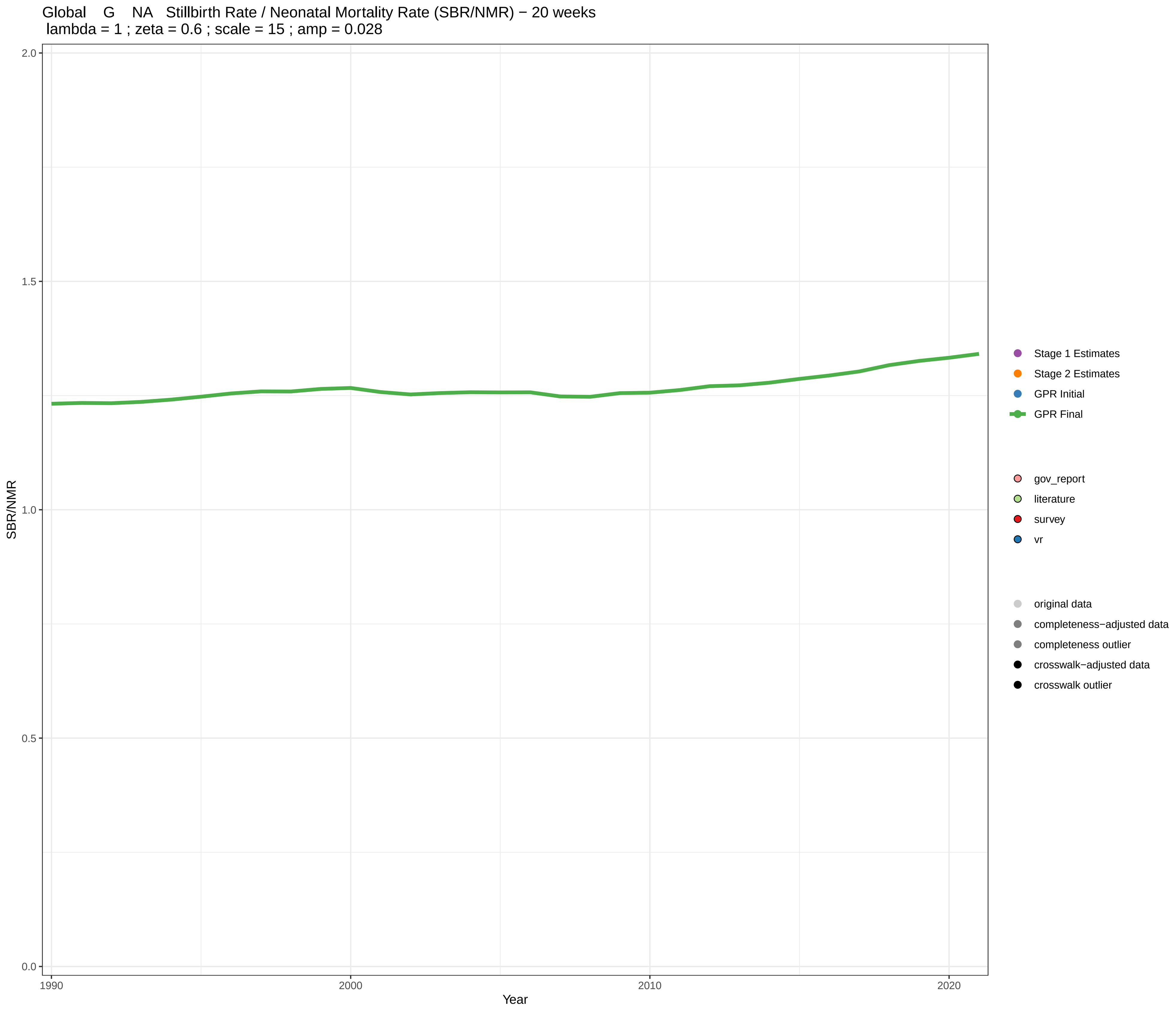

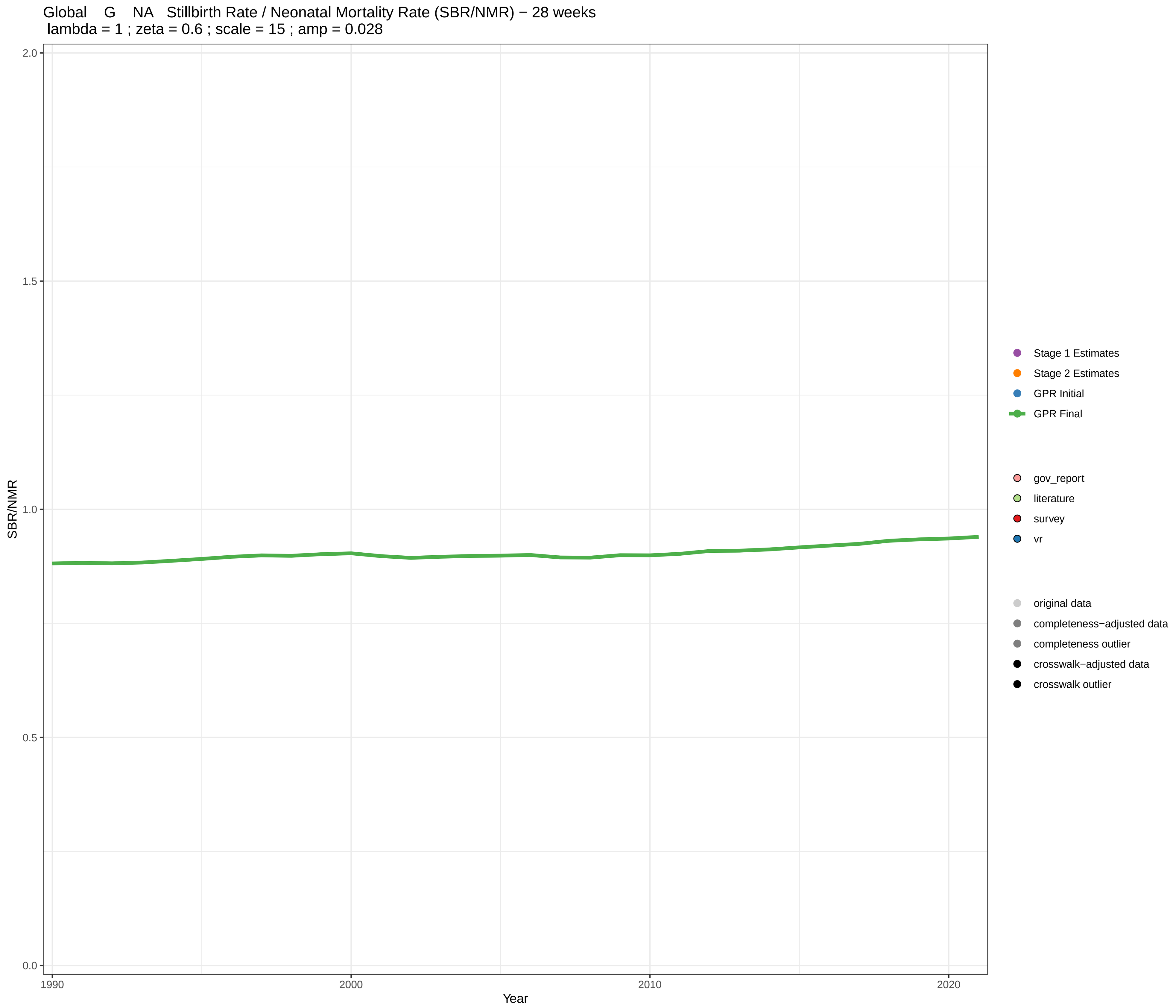

Definition	Years	Outlier	Source.Type	Title

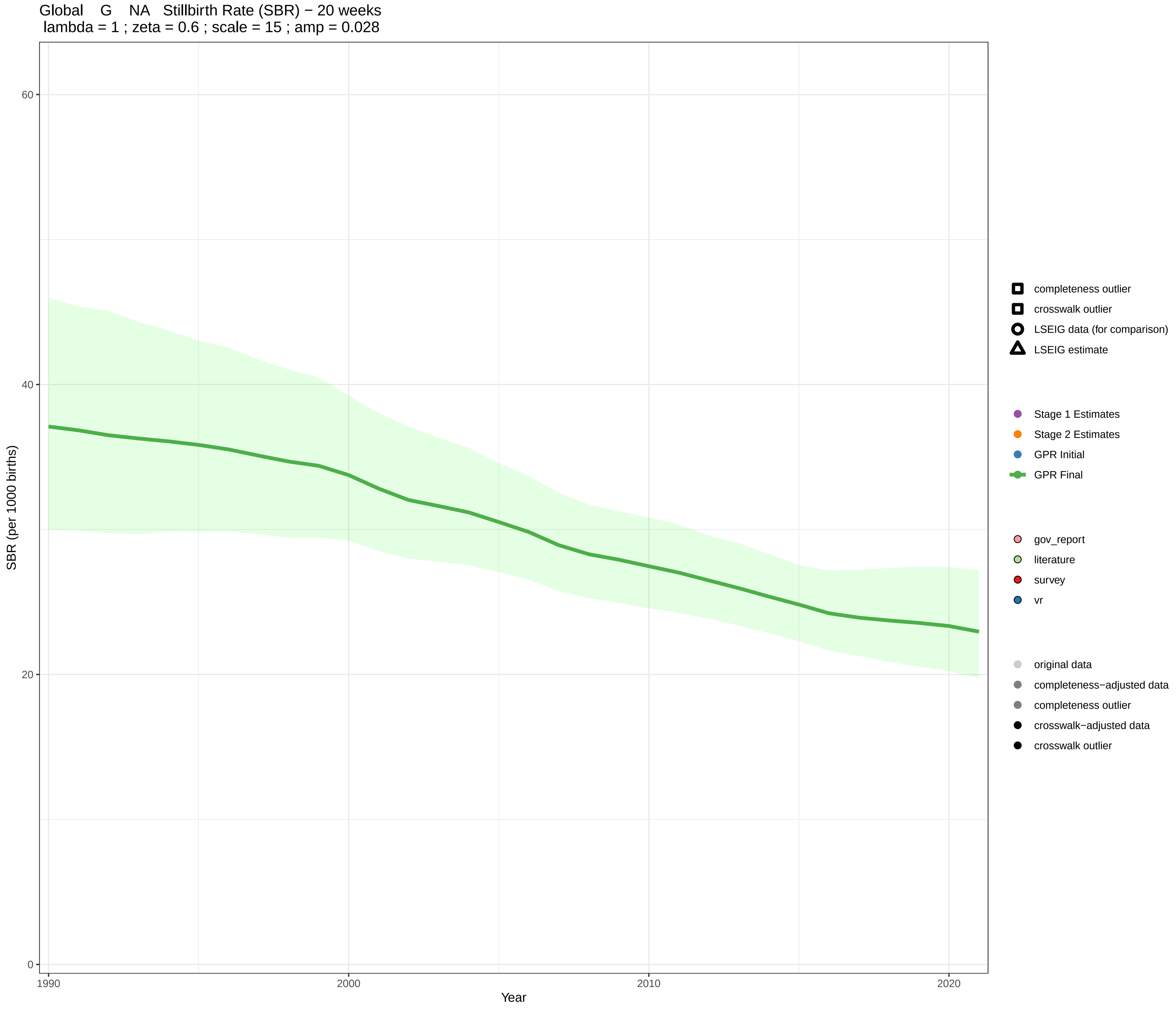

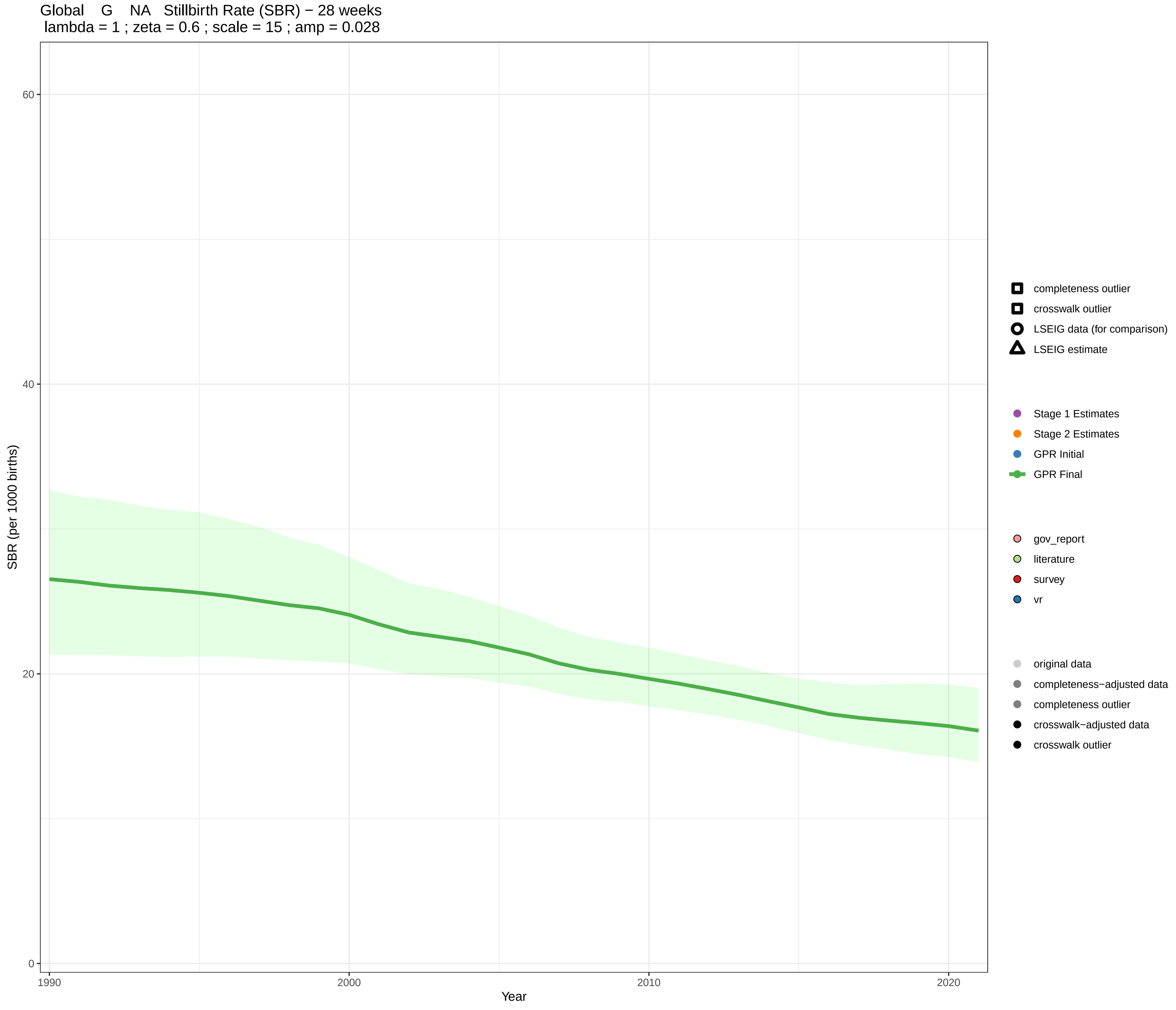

Year	Total Stillbirths (in thousands)	Total Stillbirths (in thousands)	SBR (per 1000 births)	SBR (per 1000 births)	SBR/NMR	SBR/NMR
	20 weeks	28 weeks	20 weeks	28 weeks	20 weeks	28 weeks
1990	5080.5<br>(4072.9 to 6349.7)	3593.7<br>(2868.9 to 4451.7)	37.1<br>(30.0 to 46.0)	26.5<br>(21.3 to 32.7)	1.23<br>(1.00 to 1.53)	0.88<br>(0.71 to 1.09)
1995	4844.5<br>(4009.5 to 5857.5)	3423.6<br>(2820.2 to 4190.5)	35.8<br>(29.9 to 43.0)	25.6<br>(21.2 to 31.2)	1.25<br>(1.04 to 1.50)	0.89<br>(0.74 to 1.08)
2000	4535.4<br>(3908.9 to 5299.9)	3202.6<br>(2746.5 to 3748.4)	33.8<br>(29.2 to 39.2)	24.1<br>(20.7 to 28.1)	1.27<br>(1.10 to 1.47)	0.90<br>(0.78 to 1.05)
2005	4210.5<br>(3718.2 to 4790.5)	2982.4<br>(2643.2 to 3385.1)	30.5<br>(27.0 to 34.6)	21.8<br>(19.4 to 24.7)	1.26<br>(1.11 to 1.42)	0.90<br>(0.80 to 1.02)
2010	3891.4<br>(3469.4 to 4383.5)	2762.1<br>(2487.5 to 3070.7)	27.5<br>(24.6 to 30.8)	19.7<br>(17.7 to 21.8)	1.26<br>(1.12 to 1.41)	0.90<br>(0.81 to 1.00)
2015	3609.7<br>(3234.1 to 4018.6)	2552.6<br>(2291.9 to 2844.9)	24.8<br>(22.3 to 27.6)	17.7<br>(15.9 to 19.7)	1.29<br>(1.16 to 1.43)	0.92<br>(0.82 to 1.02)
2020	3137.8<br>(2713.1 to 3699.9)	2187.6<br>(1898.7 to 2580.2)	23.3<br>(20.3 to 27.4)	16.4<br>(14.3 to 19.3)	1.33<br>(1.16 to 1.57)	0.94<br>(0.81 to 1.10)
2021	3039.9<br>(2606.7 to 3618.0)	2114.2<br>(1821.3 to 2508.2)	23.0<br>(19.7 to 27.2)	16.1<br>(13.9 to 19.0)	1.34<br>(1.15 to 1.59)	0.94<br>(0.81 to 1.11)

Central Europe, Eastern Europe, and Central Asia S2 NA Stillbirth Rate / Neonatal Mortality Rate (SBR/NMR) − 20 weeks
lambda = 1 ; zeta = 0.6 ; scale = 15 ; amp = 0.028

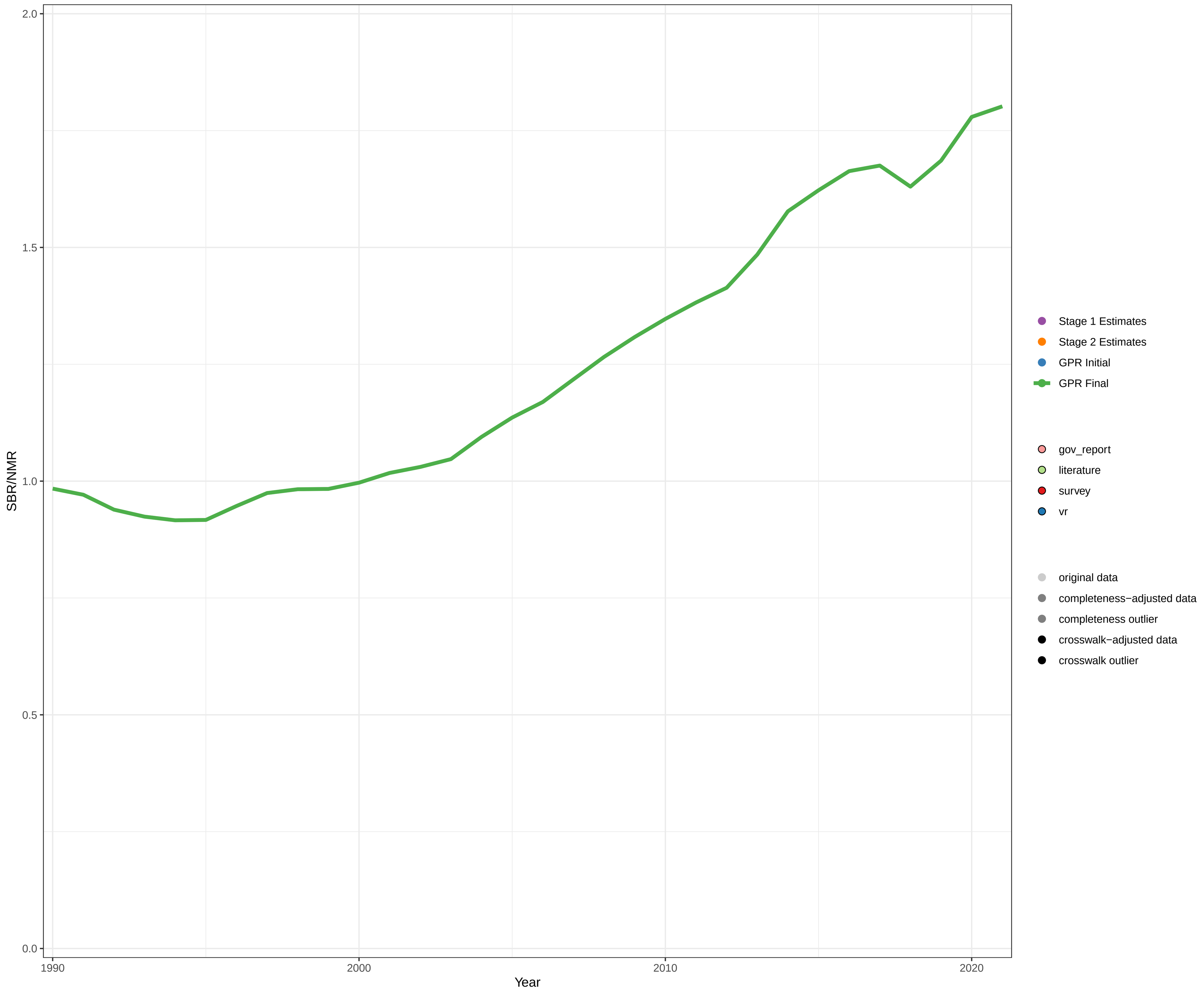

Central Europe, Eastern Europe, and Central Asia S2 NA Stillbirth Rate / Neonatal Mortality Rate (SBR/NMR) − 28 weeks
lambda = 1 ; zeta = 0.6 ; scale = 15 ; amp = 0.028

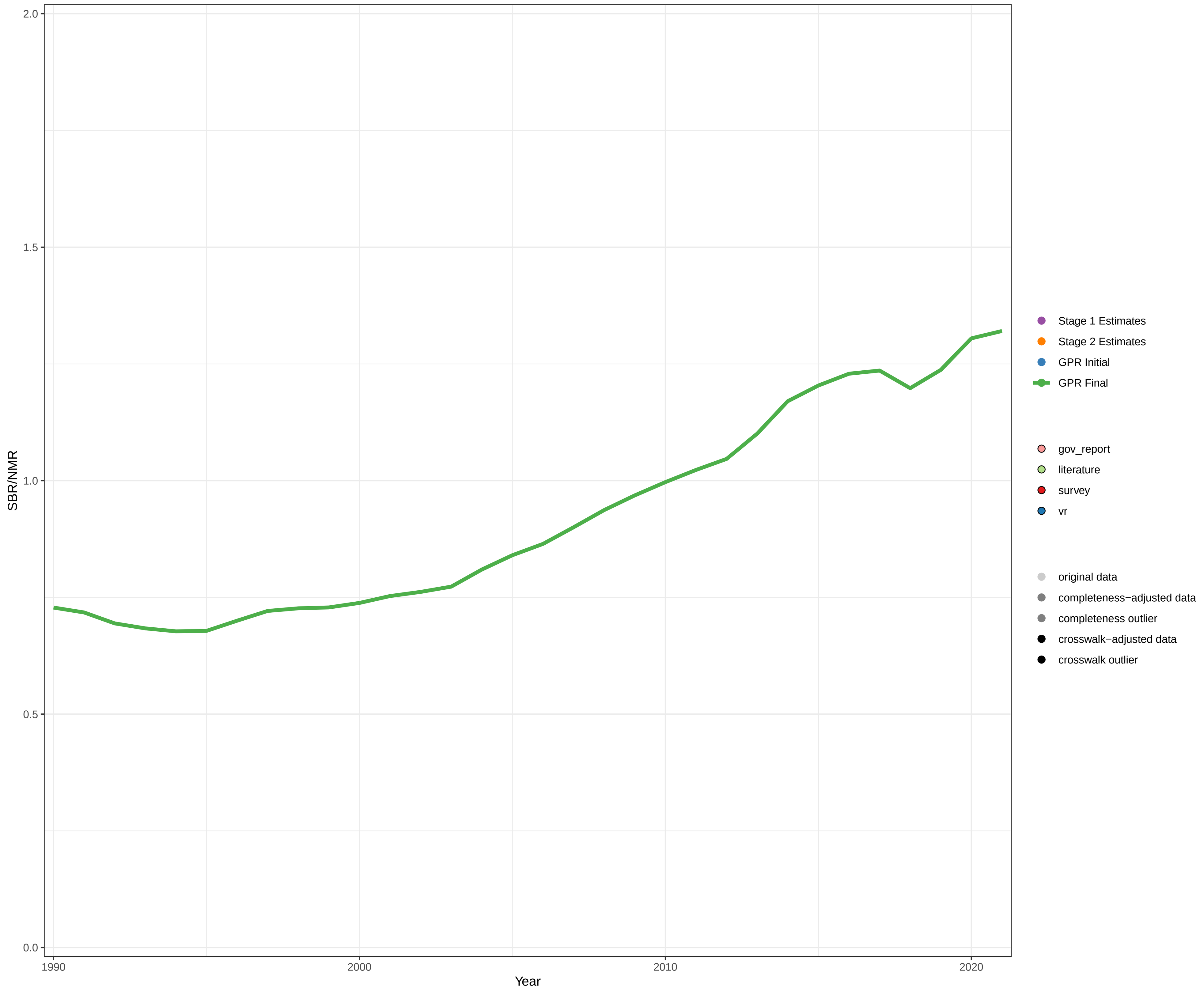

Definition	Years	Outlier	Source.Type	Title

Central Europe, Eastern Europe, and Central Asia S2 NA Stillbirth Rate (SBR) − 20 weeks
lambda = 1 ; zeta = 0.6 ; scale = 15 ; amp = 0.028

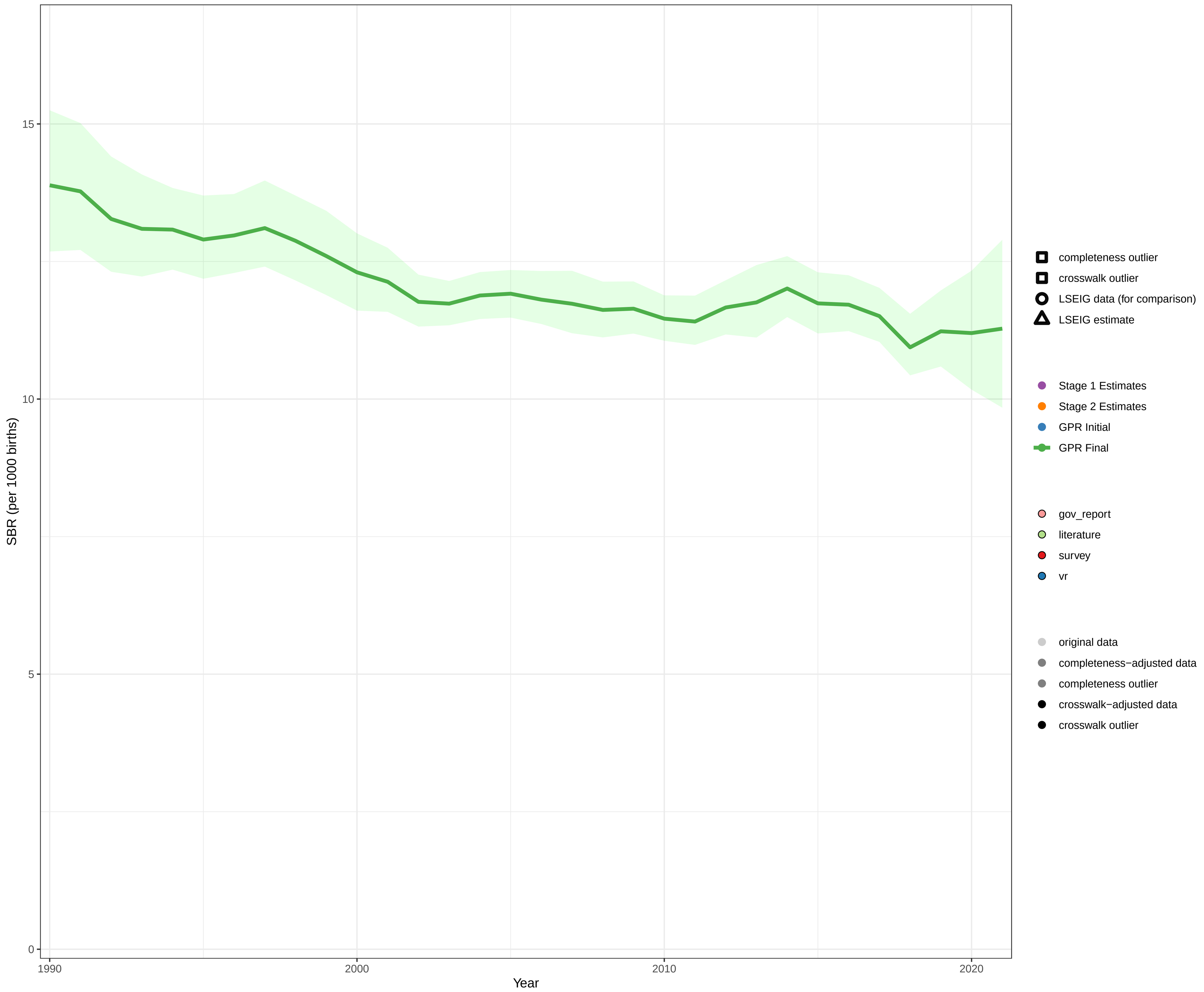

Central Europe, Eastern Europe, and Central Asia S2 NA Stillbirth Rate (SBR) − 28 weeks
lambda = 1 ; zeta = 0.6 ; scale = 15 ; amp = 0.028

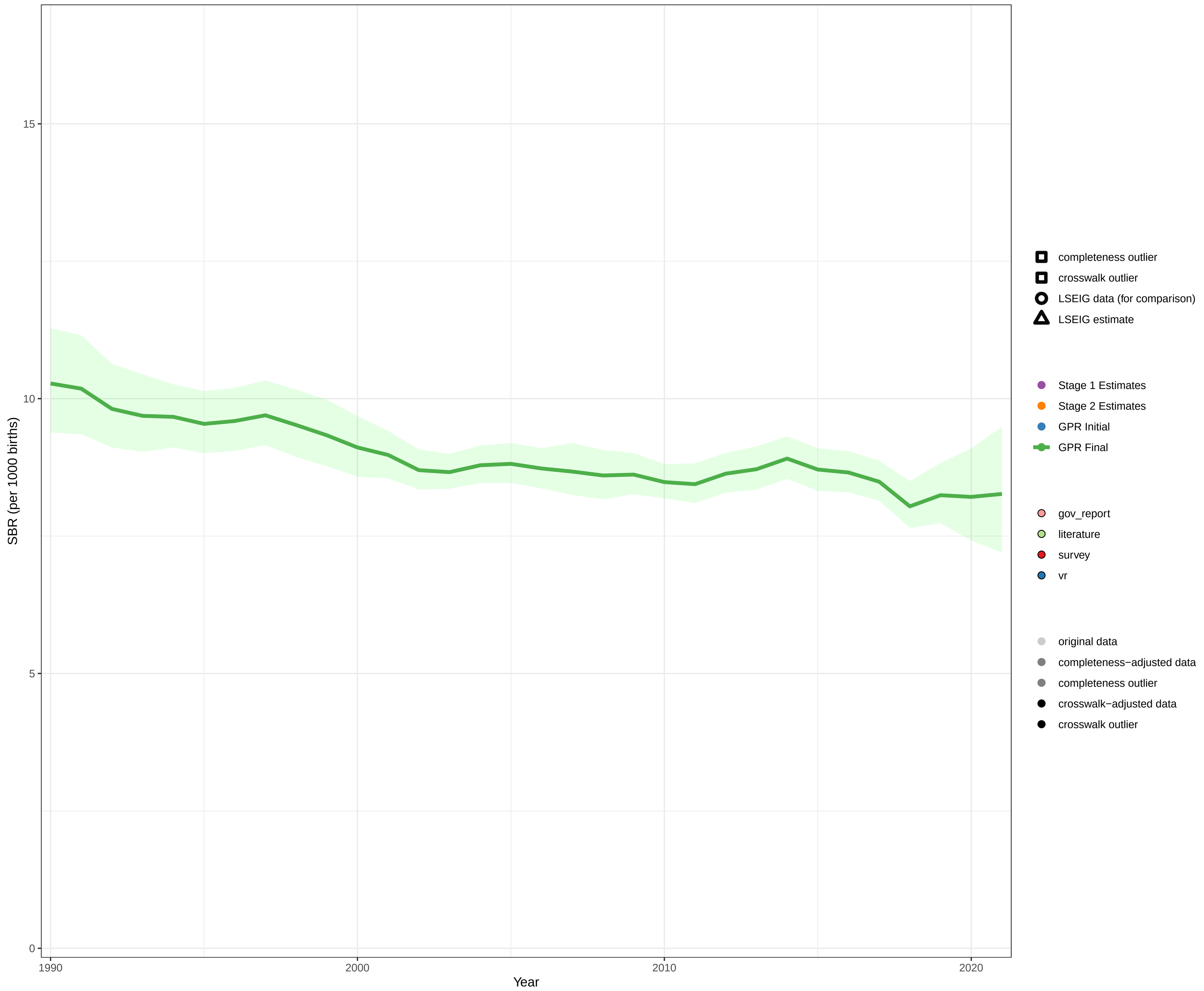

Year	Total Stillbirths (in thousands)	Total Stillbirths (in thousands)	SBR (per 1000 births)	SBR (per 1000 births)	SBR/NMR	SBR/NMR
	20 weeks	28 weeks	20 weeks	28 weeks	20 weeks	28 weeks
1990	93.4 (85.2 to 102.7)	68.9 (62.9 to 75.7)	13.9 (12.7 to 15.2)	10.3 (9.4 to 11.3)	0.98 (0.90 to 1.08)	0.73 (0.67 to 0.80)
1995	67.6 (63.8 to 71.8)	49.8 (47.0 to 53.0)	12.9 (12.2 to 13.7)	9.5 (9.0 to 10.1)	0.92 (0.87 to 0.97)	0.68 (0.64 to 0.72)
2000	57.3 (54.0 to 60.6)	42.3 (39.8 to 44.9)	12.3 (11.6 to 13.0)	9.1 (8.6 to 9.7)	1.00 (0.94 to 1.05)	0.74 (0.69 to 0.78)
2005	59.3 (57.1 to 61.5)	43.7 (42.0 to 45.6)	11.9 (11.5 to 12.3)	8.8 (8.5 to 9.2)	1.14 (1.09 to 1.18)	0.84 (0.81 to 0.88)
2010	65.6 (63.3 to 68.0)	48.4 (46.7 to 50.3)	11.5 (11.1 to 11.9)	8.5 (8.2 to 8.8)	1.35 (1.30 to 1.40)	1.00 (0.96 to 1.04)
2015	67.9 (64.7 to 71.2)	50.2 (47.9 to 52.5)	11.7 (11.2 to 12.3)	8.7 (8.3 to 9.1)	1.62 (1.55 to 1.70)	1.20 (1.15 to 1.26)
2020	57.0 (51.7 to 62.9)	41.7 (37.6 to 46.2)	11.2 (10.2 to 12.3)	8.2 (7.4 to 9.1)	1.78 (1.62 to 1.96)	1.30 (1.18 to 1.45)
2021	56.0 (48.8 to 64.1)	40.9 (35.6 to 47.0)	11.3 (9.8 to 12.9)	8.3 (7.2 to 9.5)	1.80 (1.57 to 2.06)	1.32 (1.15 to 1.52)

Central Asia R2 Central Asia Stillbirth Rate / Neonatal Mortality Rate (SBR/NMR) − 20 weeks
lambda = 1 ; zeta = 0.6 ; scale = 15 ; amp = 0.028

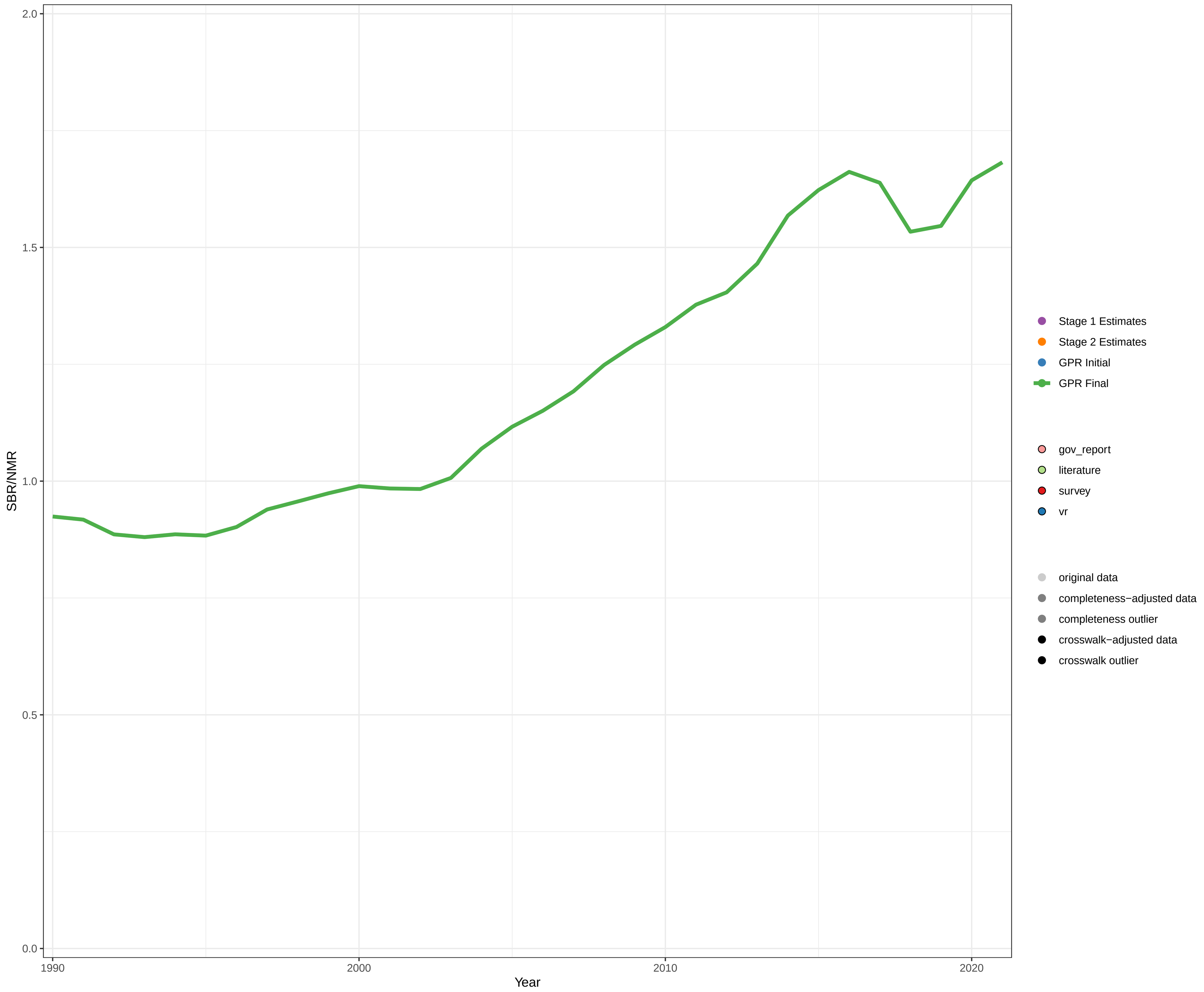

Central Asia R2 Central Asia Stillbirth Rate / Neonatal Mortality Rate (SBR/NMR) − 28 weeks
lambda = 1 ; zeta = 0.6 ; scale = 15 ; amp = 0.028

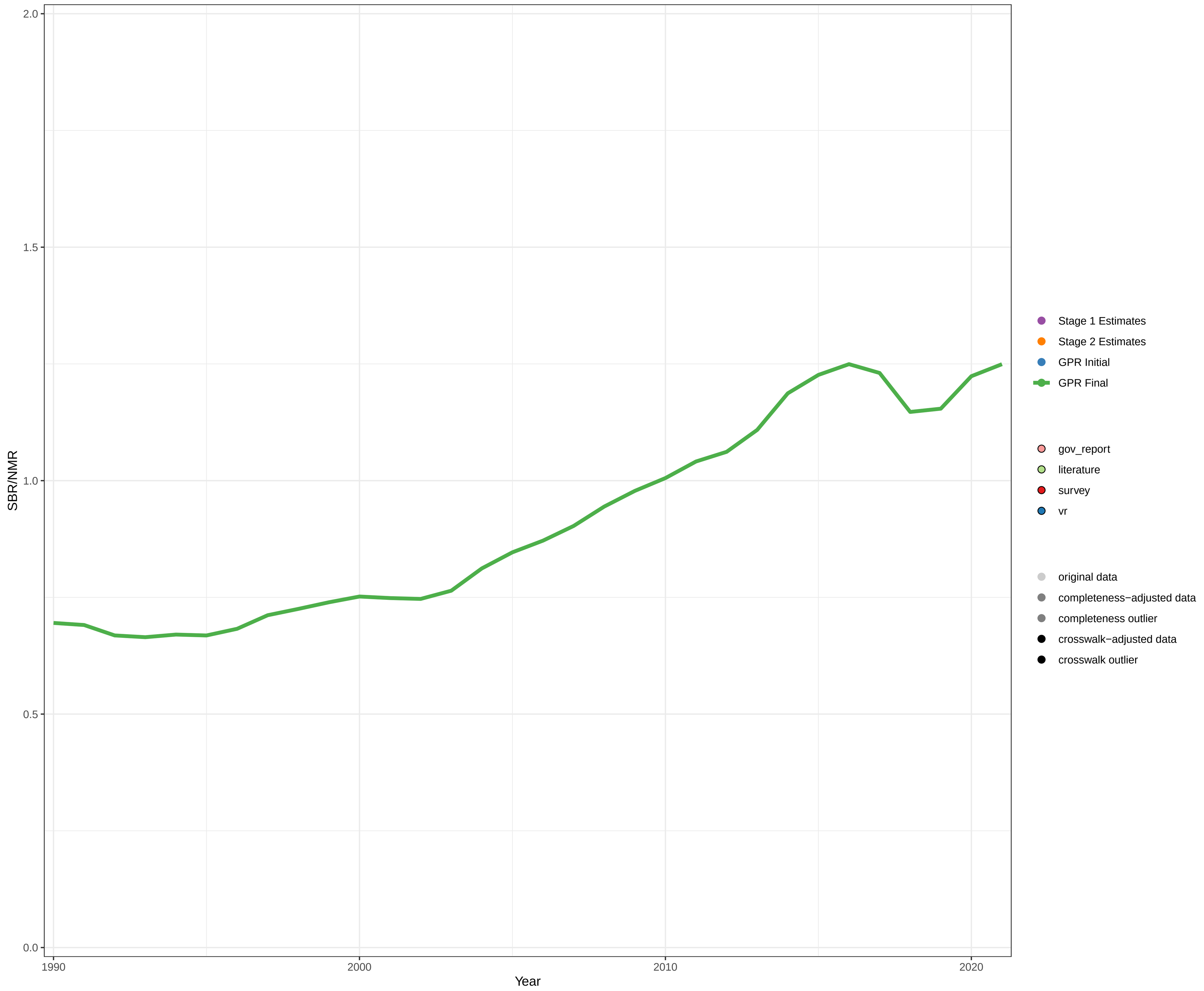

Definition	Years	Outlier	Source.Type	Title

Central Asia R2 Central Asia Stillbirth Rate (SBR) − 20 weeks
lambda = 1 ; zeta = 0.6 ; scale = 15 ; amp = 0.028

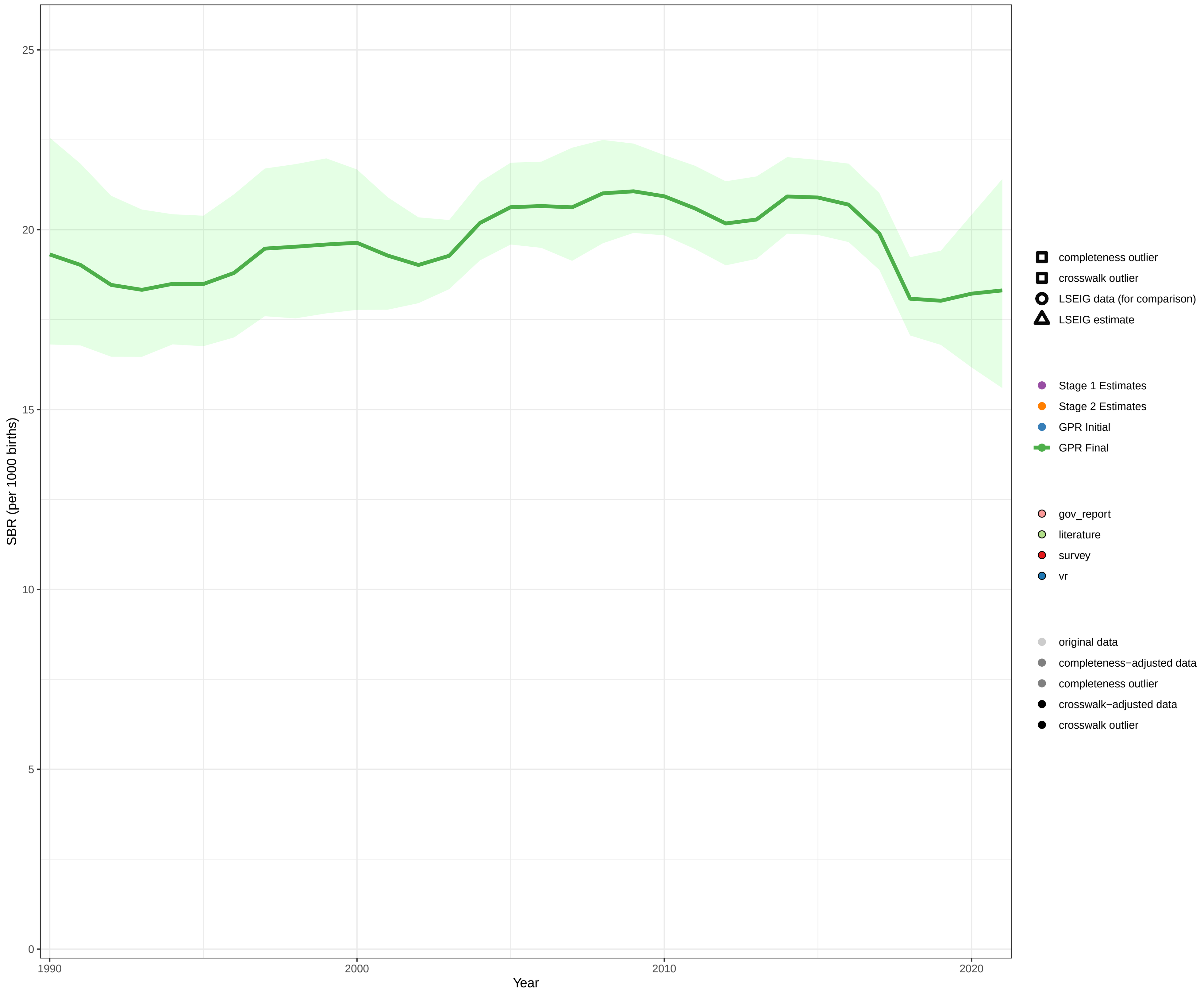

Central Asia R2 Central Asia Stillbirth Rate (SBR) − 28 weeks
lambda = 1 ; zeta = 0.6 ; scale = 15 ; amp = 0.028

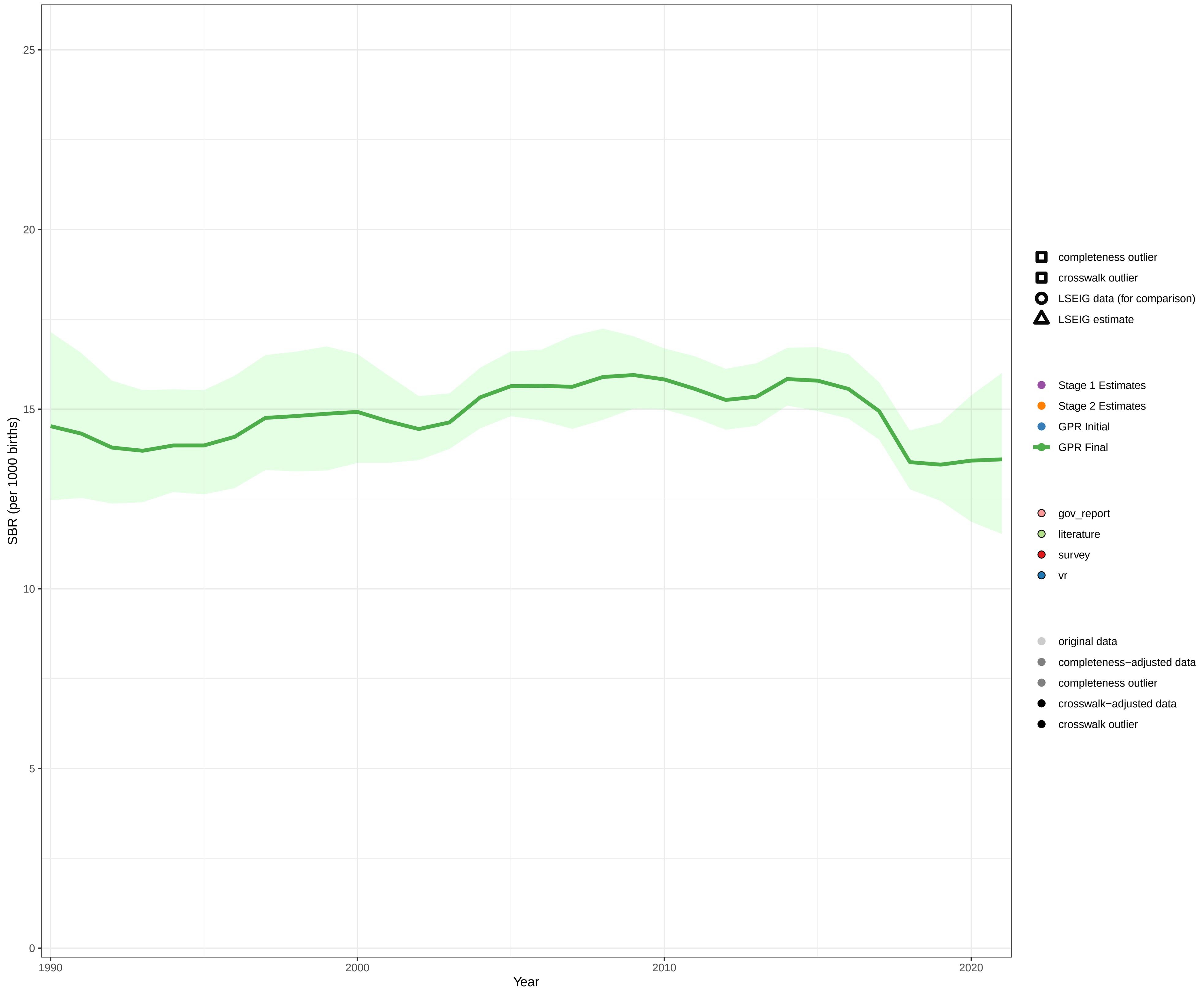

Year	Total Stillbirths (in thousands)	Total Stillbirths (in thousands)	SBR (per 1000 births)	SBR (per 1000 births)	SBR/NMR	SBR/NMR
	20 weeks	28 weeks	20 weeks	28 weeks	20 weeks	28 weeks
1990	38.8 (33.7 to 45.5)	29.1 (24.9 to 34.4)	19.3 (16.8 to 22.6)	14.5 (12.5 to 17.1)	0.92 (0.80 to 1.08)	0.70 (0.60 to 0.82)
1995	31.8 (28.8 to 35.2)	24.0 (21.6 to 26.6)	18.5 (16.8 to 20.4)	14.0 (12.6 to 15.5)	0.88 (0.80 to 0.97)	0.67 (0.60 to 0.74)
2000	29.4 (26.5 to 32.5)	22.2 (20.1 to 24.6)	19.6 (17.8 to 21.7)	14.9 (13.5 to 16.5)	0.99 (0.90 to 1.09)	0.75 (0.68 to 0.83)
2005	33.2 (31.5 to 35.2)	25.1 (23.7 to 26.6)	20.6 (19.6 to 21.9)	15.6 (14.8 to 16.6)	1.12 (1.06 to 1.18)	0.85 (0.80 to 0.90)
2010	40.0 (37.9 to 42.2)	30.1 (28.5 to 31.8)	20.9 (19.8 to 22.1)	15.8 (15.0 to 16.7)	1.33 (1.26 to 1.40)	1.01 (0.95 to 1.06)
2015	43.7 (41.5 to 45.9)	32.8 (31.1 to 34.8)	20.9 (19.9 to 21.9)	15.8 (14.9 to 16.7)	1.62 (1.54 to 1.70)	1.23 (1.16 to 1.30)
2020	38.9 (34.4 to 43.6)	28.8 (25.1 to 32.7)	18.2 (16.2 to 20.4)	13.6 (11.9 to 15.4)	1.64 (1.46 to 1.84)	1.22 (1.07 to 1.39)
2021	38.7 (32.8 to 45.4)	28.6 (24.2 to 33.7)	18.3 (15.6 to 21.4)	13.6 (11.5 to 16.0)	1.68 (1.43 to 1.97)	1.25 (1.06 to 1.47)

Armenia ARM Central Asia Stillbirth Rate / Neonatal Mortality Rate (SBR/NMR) − 20 weeks
lambda = 0.2 ; zeta = 0.99 ; scale = 5 ; amp = 0.028

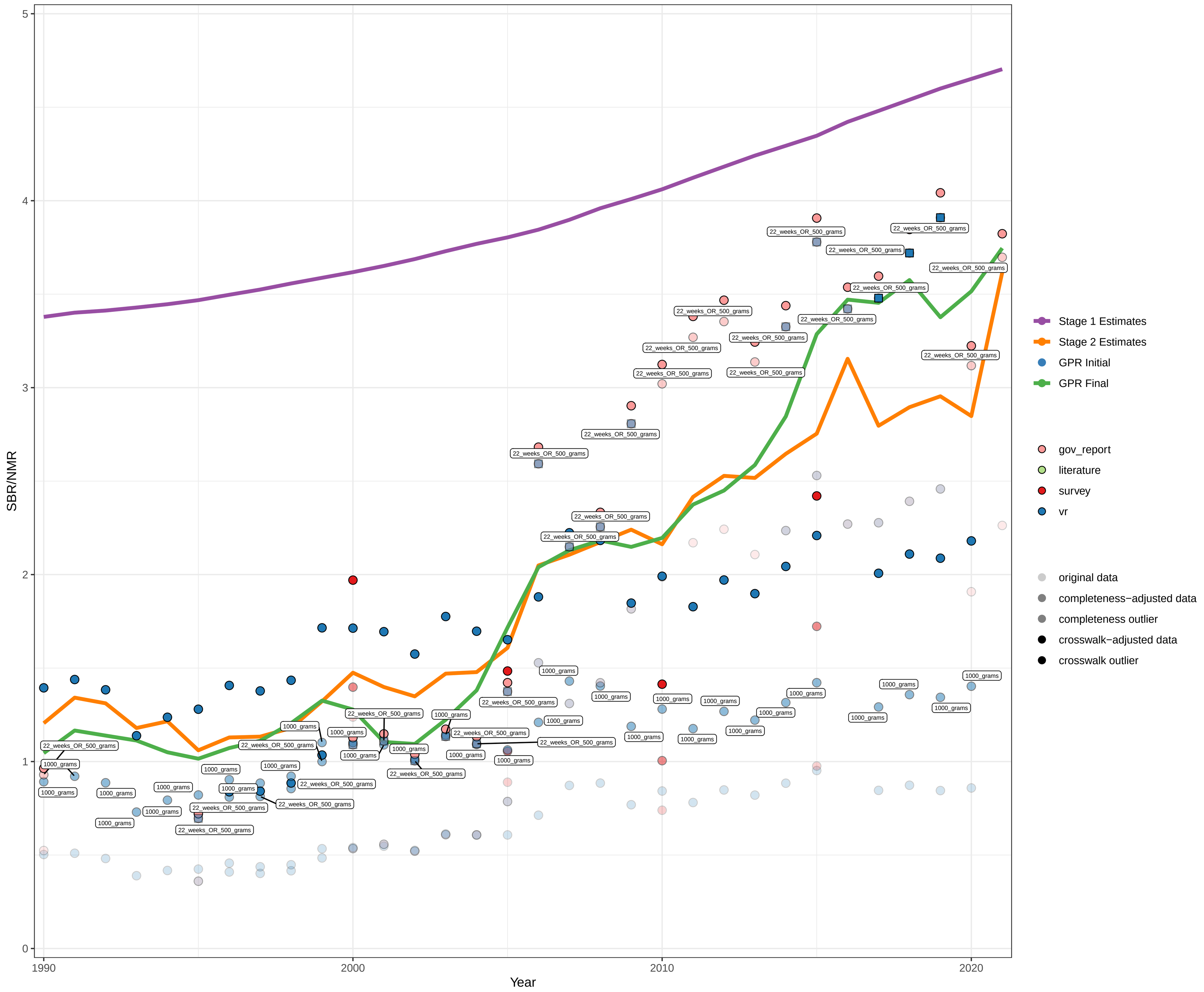

Armenia ARM Central Asia Stillbirth Rate / Neonatal Mortality Rate (SBR/NMR) − 28 weeks
lambda = 0.2 ; zeta = 0.99 ; scale = 5 ; amp = 0.028

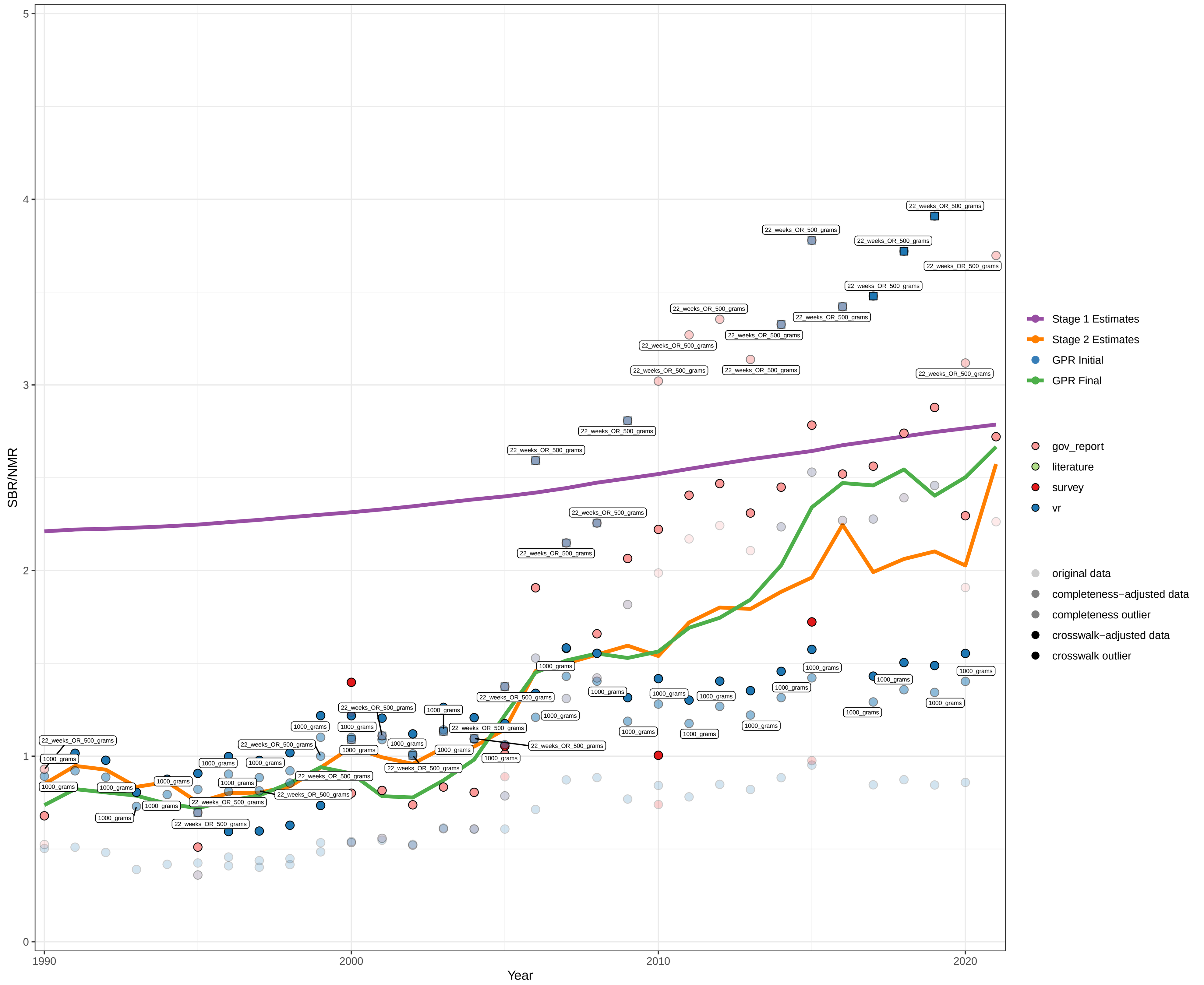

Definition	Years	Outlier	Source.Type	Title
1000_grams	1990−2015, 2017−2020	0	VR	European Health for All Database
22_weeks_OR_500_grams	1990, 1995, 2000−2021	0	Statistical Report	Armenia Demographic Handbook
22_weeks_OR_500_grams	1996−1999, 2017−2019	0	VR	United Nations Demographic Yearbook
28_weeks	2000	0	Survey	Armenia Demographic and Health Survey 2000
28_weeks	2005	0	Survey	Armenia Demographic and Health Survey 2005
28_weeks	2010	0	Survey	Armenia Demographic and Health Survey 2010
28_weeks	2015	0	Survey	Armenia Demographic and Health Survey 2015−2016
22_weeks_OR_500_grams	1995, 2000−2009, 2014−2016	1	VR	United Nations Demographic Yearbook

Armenia ARM Central Asia Stillbirth Rate (SBR) − 20 weeks
lambda = 0.2 ; zeta = 0.99 ; scale = 5 ; amp = 0.028

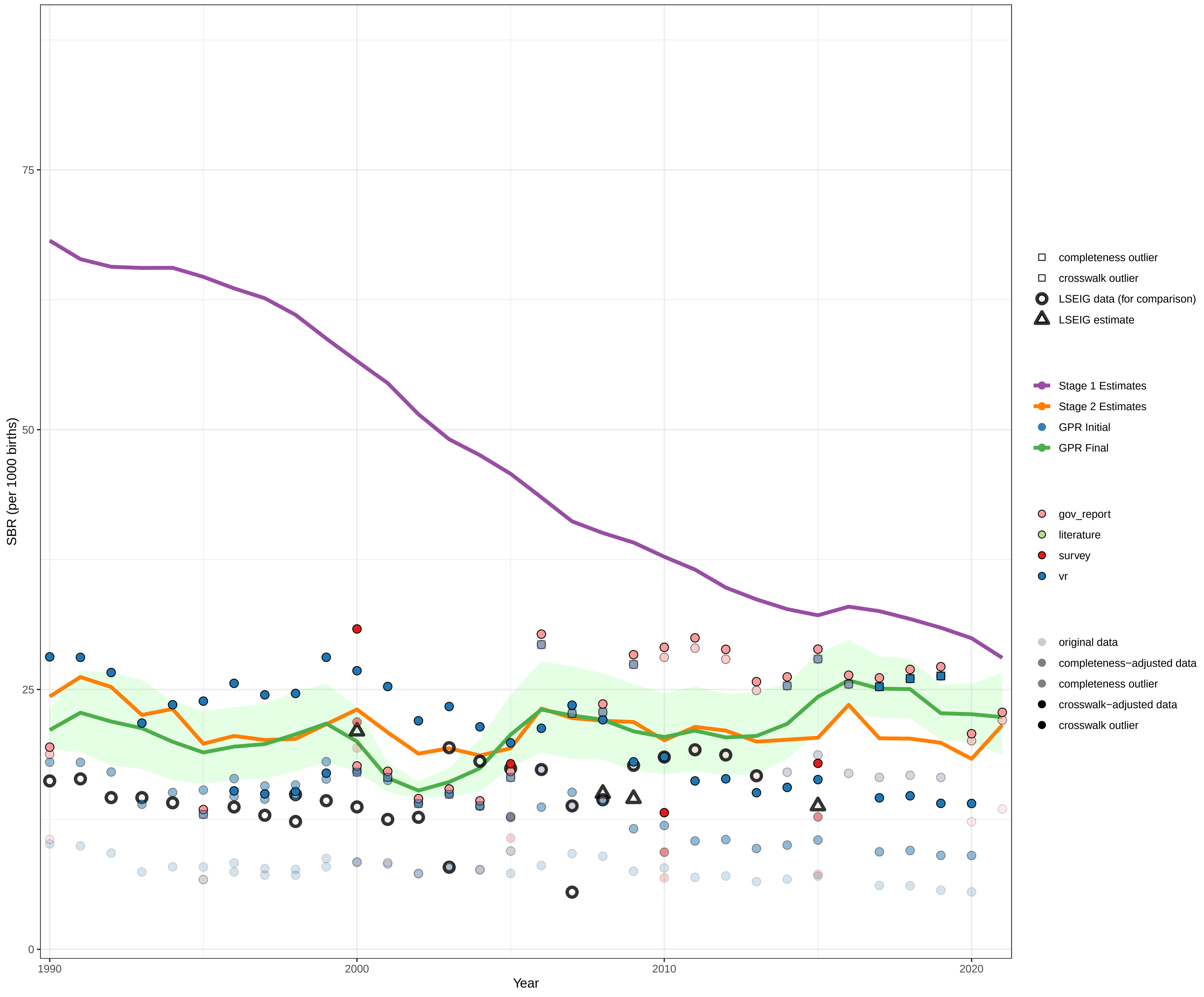

Armenia ARM Central Asia Stillbirth Rate (SBR) − 28 weeks
lambda = 0.2 ; zeta = 0.99 ; scale = 5 ; amp = 0.028

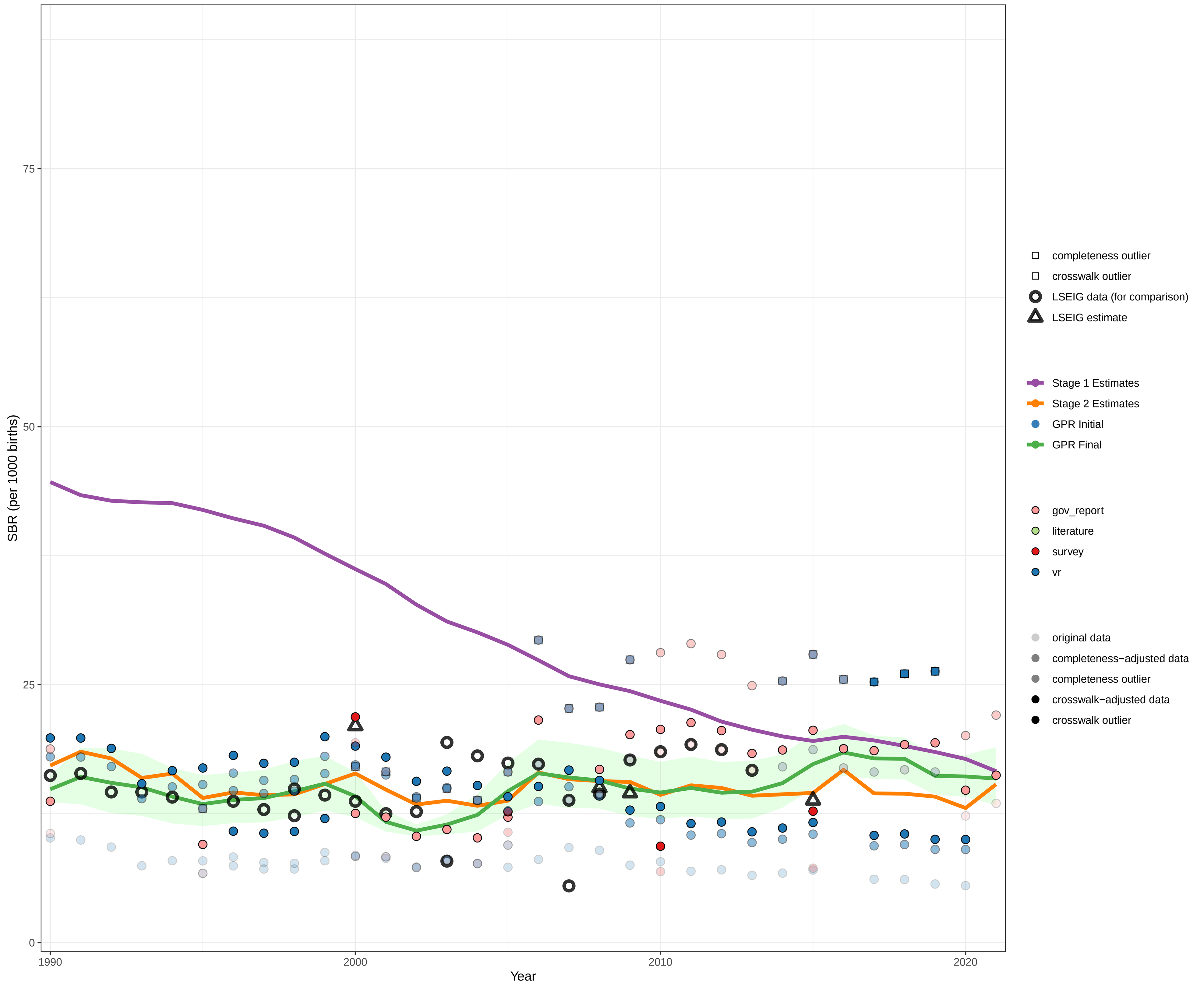

Year	Total Stillbirths (in thousands)	Total Stillbirths (in thousands)	SBR (per 1000 births)	SBR (per 1000 births)	SBR/NMR	SBR/NMR
	20 weeks	28 weeks	20 weeks	28 weeks	20 weeks	28 weeks
1990	1.7 (1.5 to 1.8)	1.2 (1.1 to 1.3)	21.1 (19.3 to 23.1)	14.9 (13.6 to 16.3)	1.05 (0.96 to 1.14)	0.74 (0.67 to 0.81)
1995	1.1 (0.9 to 1.3)	0.8 (0.6 to 0.9)	18.9 (15.9 to 22.9)	13.4 (11.3 to 16.2)	1.02 (0.85 to 1.23)	0.72 (0.61 to 0.87)
2000	0.9 (0.7 to 1.0)	0.6 (0.5 to 0.7)	20.0 (17.1 to 23.3)	14.2 (12.1 to 16.5)	1.28 (1.09 to 1.49)	0.91 (0.78 to 1.06)
2005	0.9 (0.8 to 1.1)	0.6 (0.5 to 0.8)	20.6 (17.4 to 24.5)	14.7 (12.4 to 17.4)	1.72 (1.45 to 2.04)	1.22 (1.03 to 1.45)
2010	1.0 (0.8 to 1.2)	0.7 (0.6 to 0.8)	20.4 (16.8 to 24.6)	14.5 (12.0 to 17.5)	2.20 (1.81 to 2.65)	1.56 (1.29 to 1.88)
2015	1.1 (0.9 to 1.3)	0.8 (0.7 to 0.9)	24.3 (20.9 to 28.5)	17.3 (14.9 to 20.3)	3.29 (2.83 to 3.85)	2.34 (2.02 to 2.74)
2020	0.8 (0.7 to 1.0)	0.6 (0.5 to 0.7)	22.6 (19.8 to 25.6)	16.1 (14.1 to 18.2)	3.52 (3.08 to 3.98)	2.50 (2.19 to 2.83)
2021	0.8 (0.7 to 1.0)	0.6 (0.5 to 0.7)	22.3 (18.7 to 26.6)	15.9 (13.3 to 18.9)	3.75 (3.14 to 4.47)	2.67 (2.23 to 3.18)

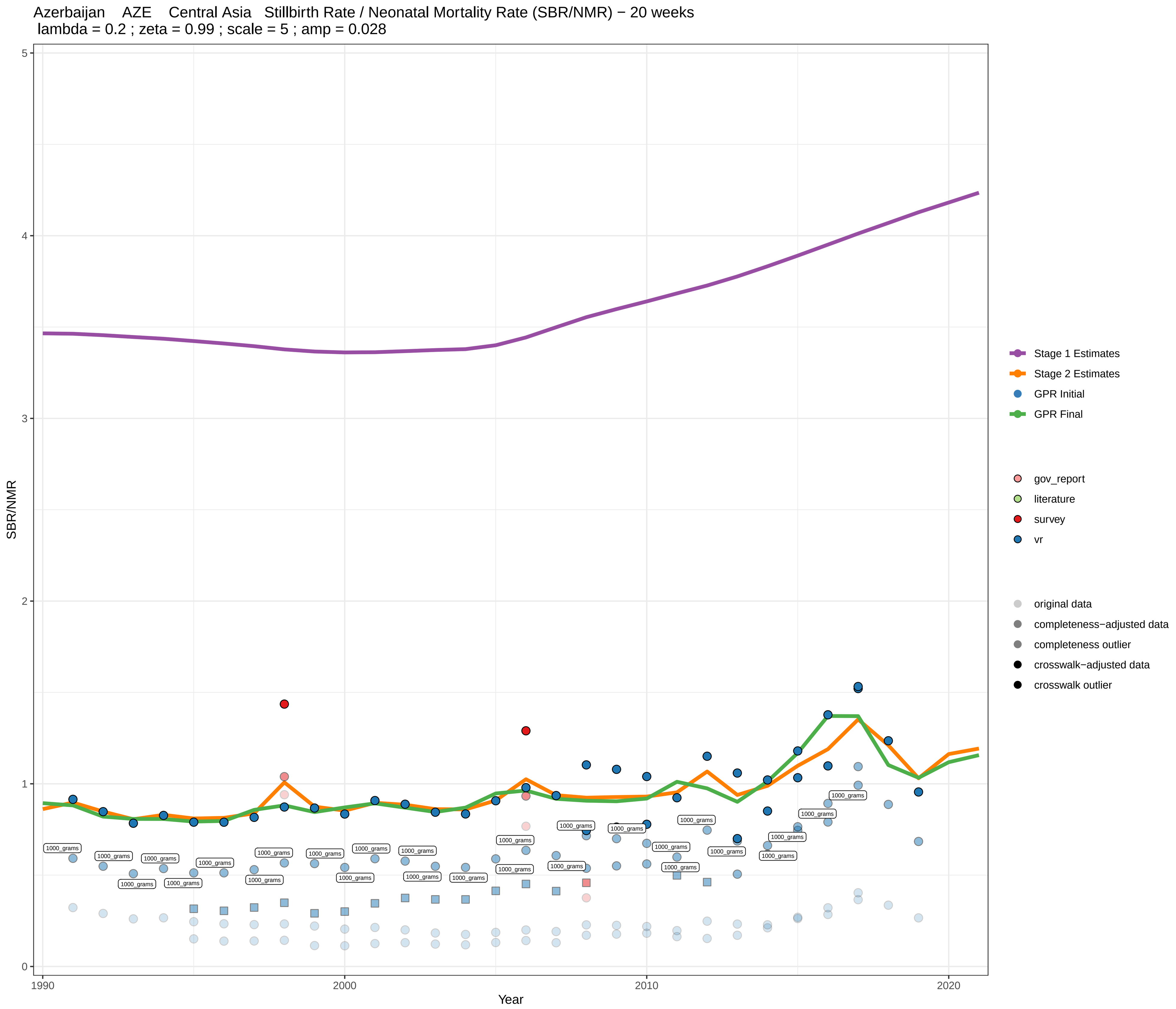

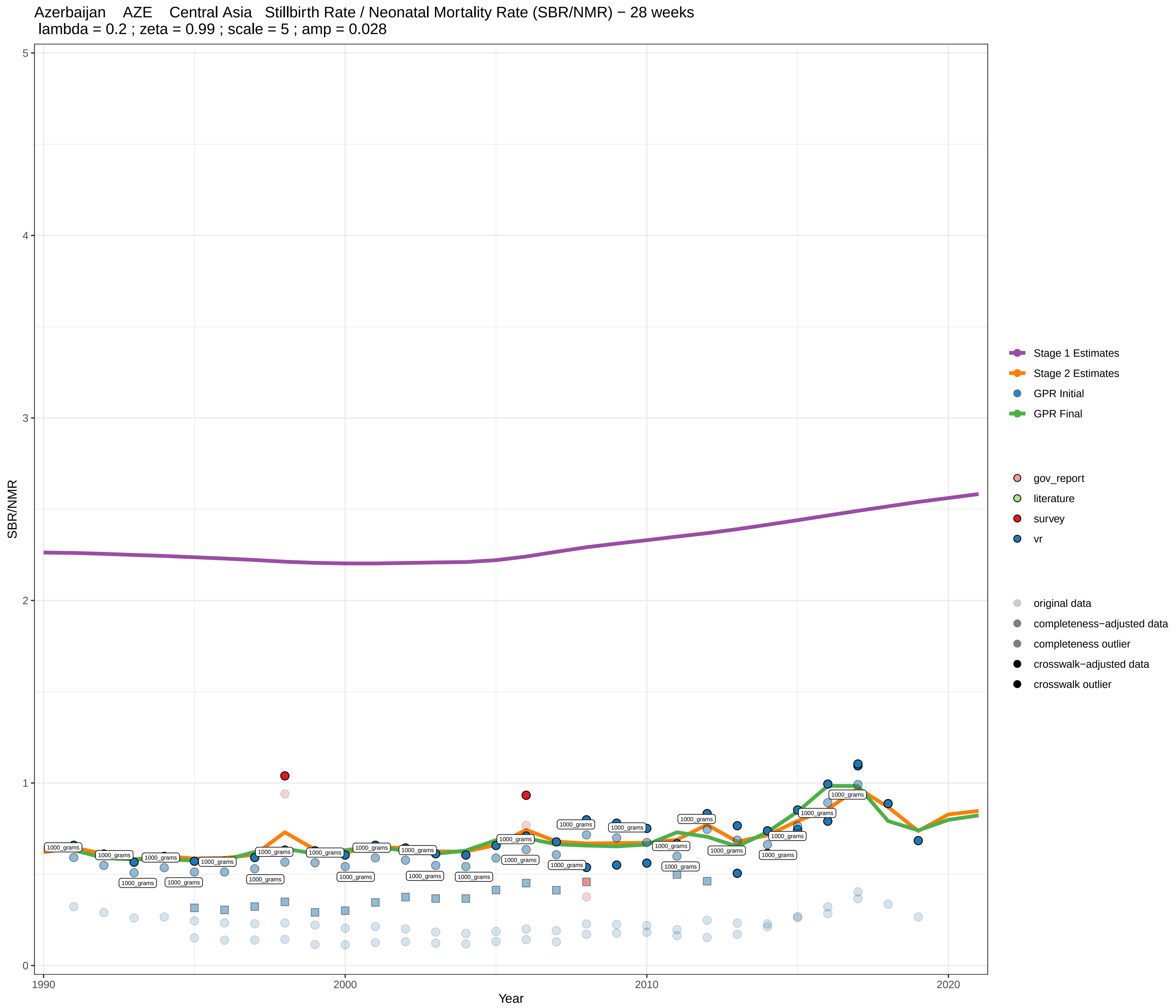

Definition	Years	Outlier	Source.Type	Title
1000_grams	1991−2017	0	VR	European Health for All Database
28_weeks	1998	0	Survey	Azerbaijan Reproductive Health Survey 2001
28_weeks	2006	0	Survey	Azerbaijan Demographic and Health Survey 2006
28_weeks	2008−2010, 2013−2019	0	VR	United Nations Demographic Yearbook
28_weeks	1995−2007, 2011, 2012	1	VR	United Nations Demographic Yearbook
28_weeks	2008	1	Survey	Azerbaijan Demographic and Health Survey 2011

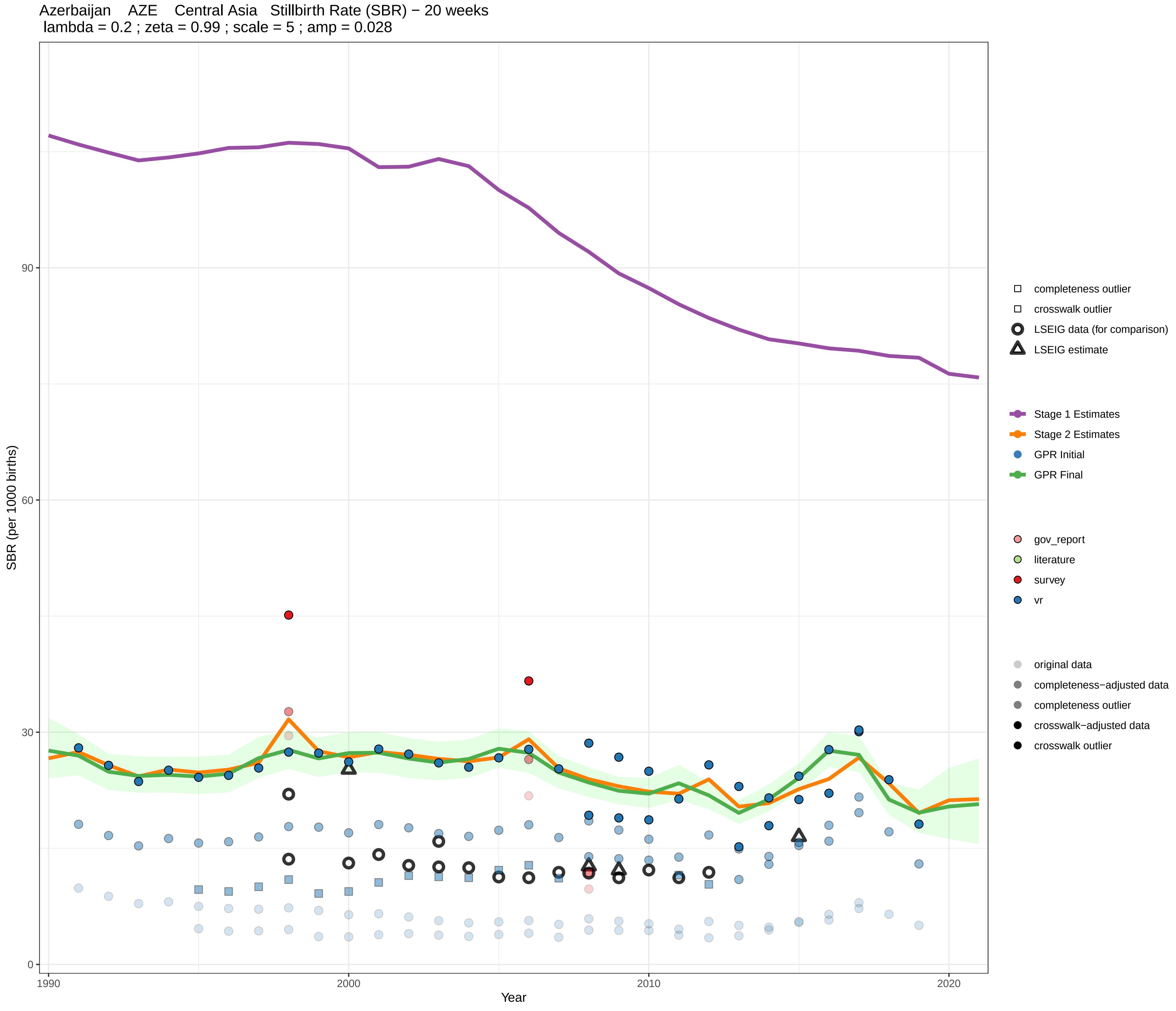

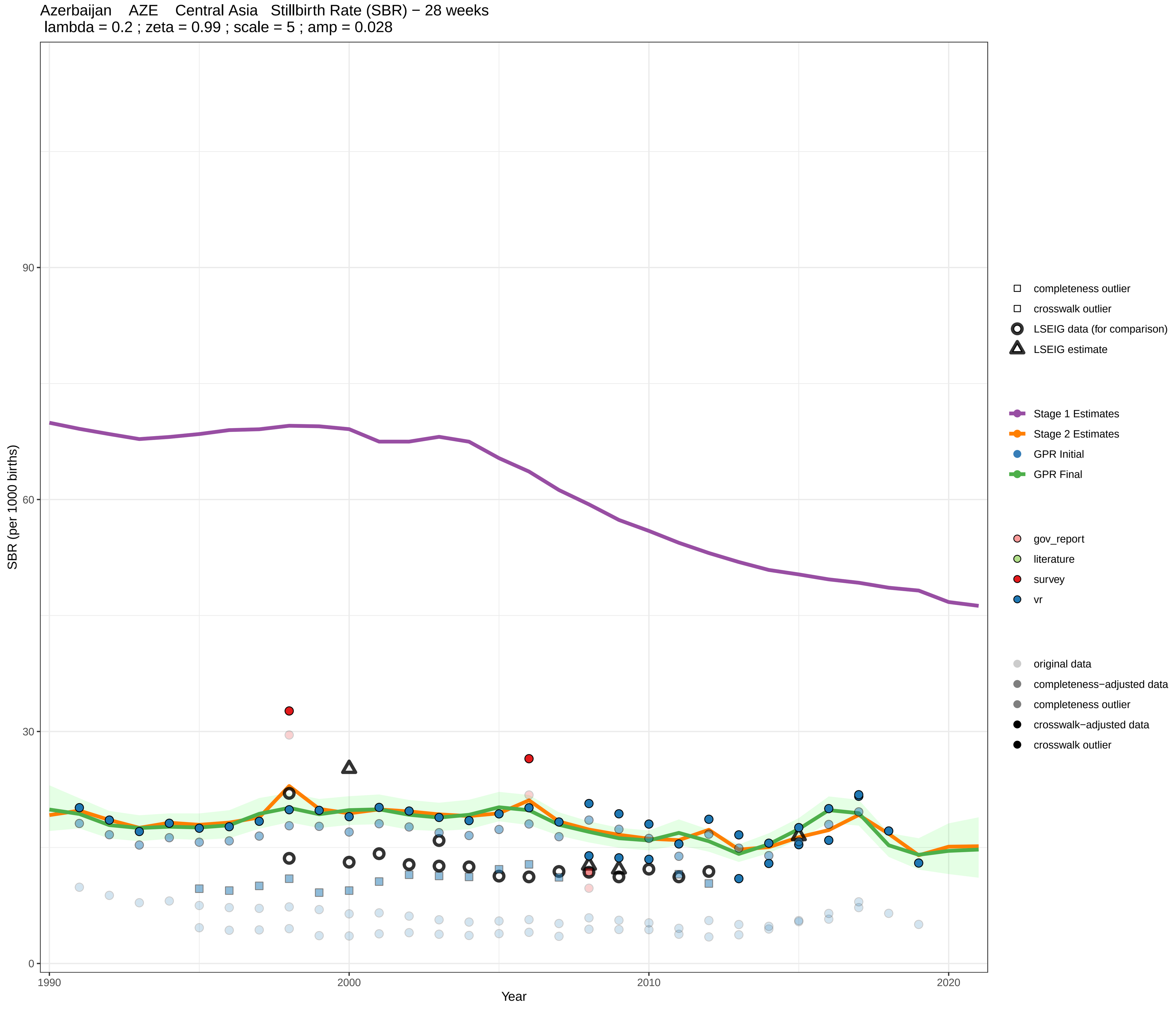

Year	Total Stillbirths (in thousands)	Total Stillbirths (in thousands)	SBR (per 1000 births)	SBR (per 1000 births)	SBR/NMR	SBR/NMR
	20 weeks	28 weeks	20 weeks	28 weeks	20 weeks	28 weeks
1990	5.4 (4.6 to 6.2)	3.8 (3.3 to 4.4)	27.6 (24.1 to 31.9)	19.9 (17.1 to 23.0)	0.89 (0.78 to 1.03)	0.64 (0.55 to 0.75)
1995	3.9 (3.6 to 4.4)	2.8 (2.6 to 3.1)	24.3 (22.0 to 26.8)	17.6 (16.0 to 19.4)	0.79 (0.72 to 0.88)	0.57 (0.52 to 0.63)
2000	3.8 (3.4 to 4.1)	2.7 (2.4 to 3.0)	27.3 (24.9 to 30.0)	19.8 (17.9 to 21.6)	0.87 (0.79 to 0.96)	0.63 (0.57 to 0.69)
2005	4.2 (3.9 to 4.7)	3.1 (2.8 to 3.4)	27.9 (25.4 to 30.5)	20.2 (18.4 to 22.2)	0.95 (0.86 to 1.04)	0.69 (0.63 to 0.75)
2010	3.9 (3.6 to 4.3)	2.8 (2.6 to 3.1)	22.1 (20.2 to 24.1)	15.9 (14.6 to 17.2)	0.92 (0.84 to 1.01)	0.66 (0.61 to 0.72)
2015	4.2 (3.9 to 4.6)	3.0 (2.8 to 3.3)	24.1 (22.2 to 26.0)	17.4 (16.0 to 18.8)	1.17 (1.08 to 1.26)	0.84 (0.78 to 0.91)
2020	3.0 (2.4 to 3.7)	2.1 (1.7 to 2.6)	20.4 (16.2 to 25.4)	14.6 (11.6 to 18.1)	1.12 (0.89 to 1.39)	0.80 (0.63 to 0.99)
2021	2.9 (2.2 to 3.8)	2.1 (1.6 to 2.7)	20.7 (15.6 to 26.6)	14.7 (11.1 to 18.9)	1.16 (0.87 to 1.49)	0.82 (0.62 to 1.06)

Georgia GEO Central Asia Stillbirth Rate / Neonatal Mortality Rate (SBR/NMR) − 20 weeks
lambda = 0.2 ; zeta = 0.99 ; scale = 5 ; amp = 0.028

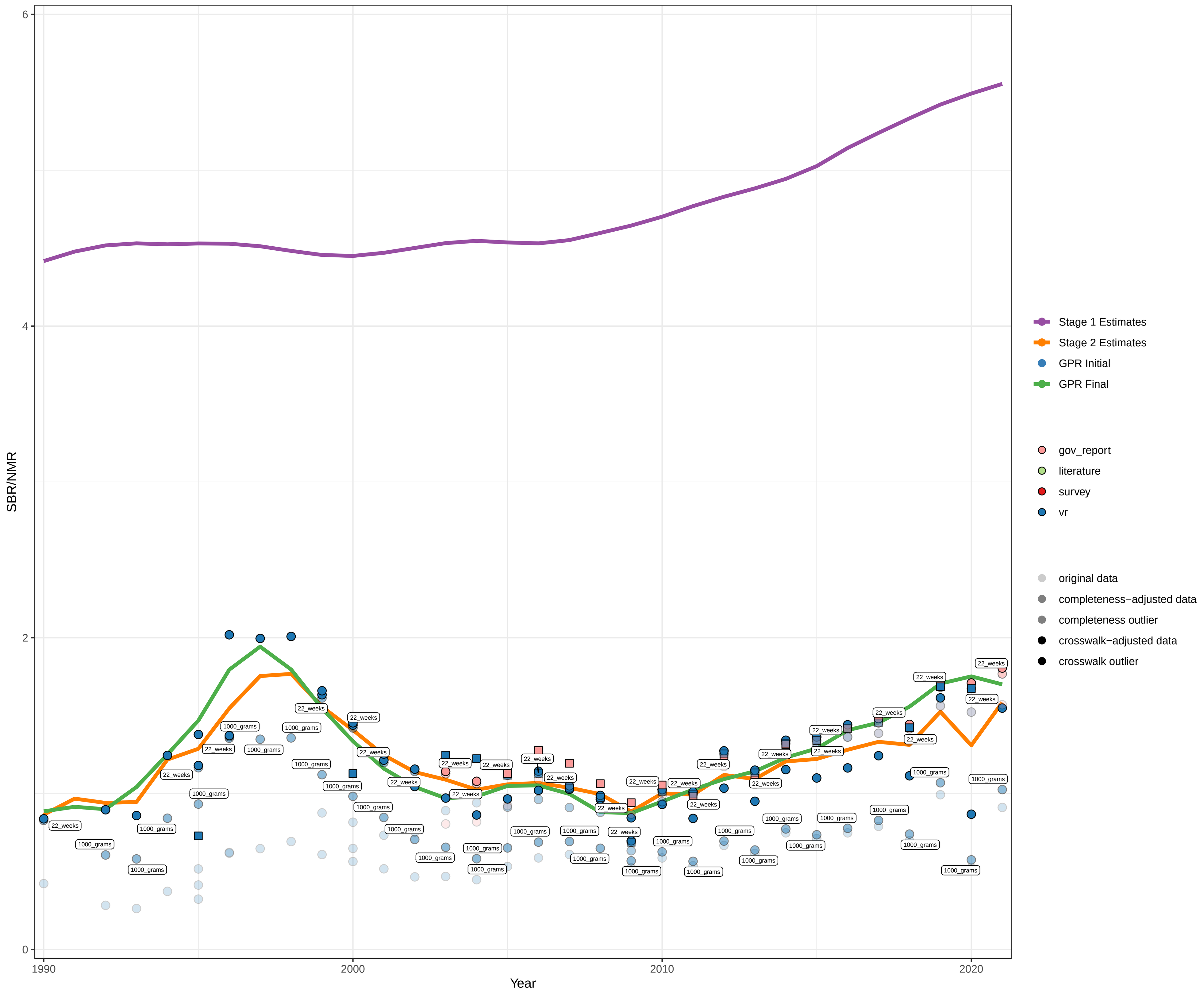

Georgia GEO Central Asia Stillbirth Rate / Neonatal Mortality Rate (SBR/NMR) − 28 weeks
lambda = 0.2 ; zeta = 0.99 ; scale = 5 ; amp = 0.028

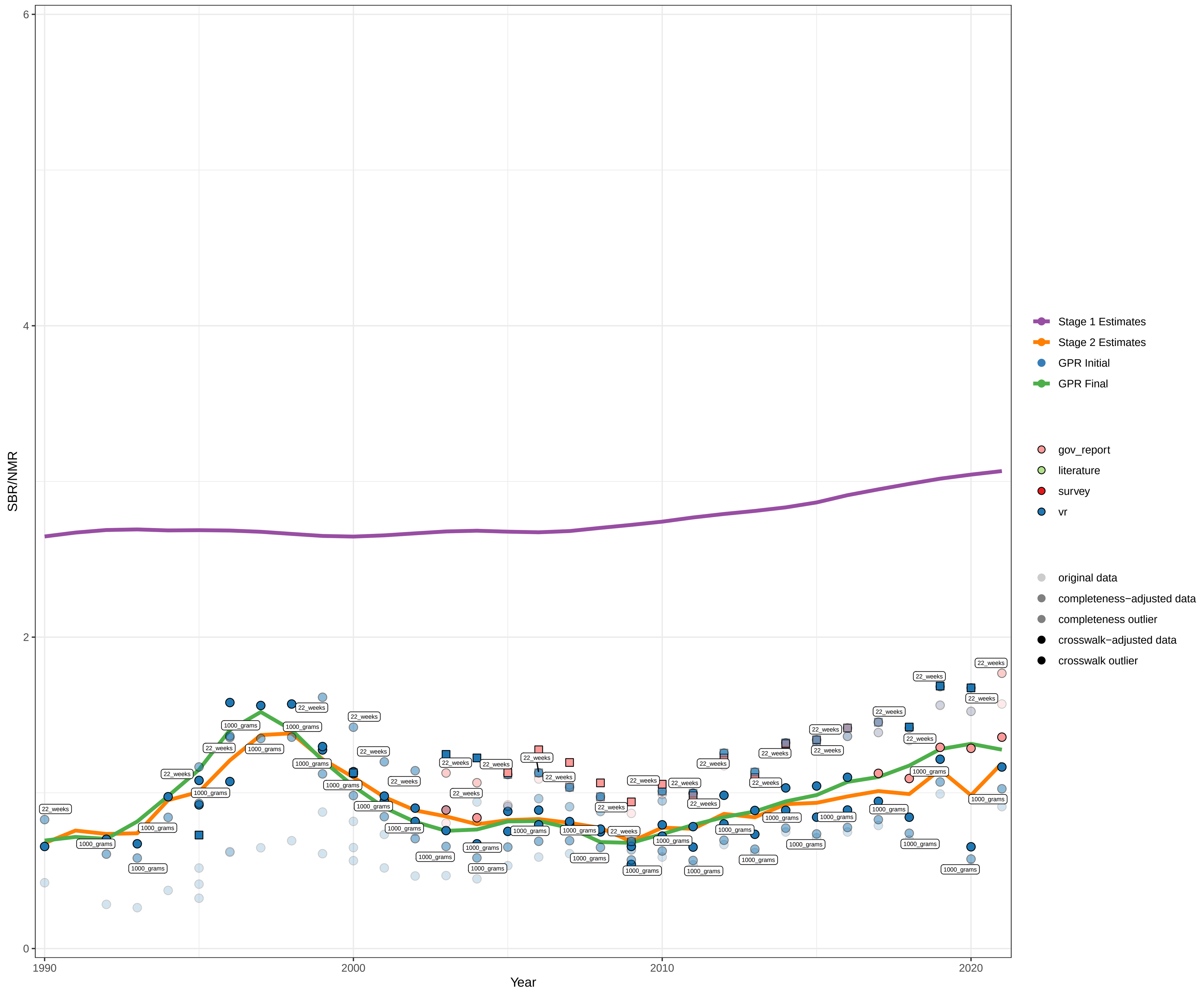

Definition	Years	Outlier	Source.Type	Title
1000_grams	1992−2021	0	VR	European Health for All Database
22_weeks	1990, 1995, 2000, 2005−2016	0	VR	Demographic Situation in Georgia
22_weeks	1995, 1996, 1999−2004, 2018−2020	0	VR	United Nations Demographic Yearbook
22_weeks	2003−2005	0	Statistical Report	Georgia Health Statistical Yearbook
22_weeks	2006−2014, 2017−2021	0	Statistical Report	Georgia Health Care Statistical Yearbook
22_weeks	2005−2017	1	VR	United Nations Demographic Yearbook
22_weeks	2015, 2016	1	Statistical Report	Georgia Health Care Statistical Yearbook

Georgia GEO Central Asia Stillbirth Rate (SBR) − 20 weeks
lambda = 0.2 ; zeta = 0.99 ; scale = 5 ; amp = 0.028

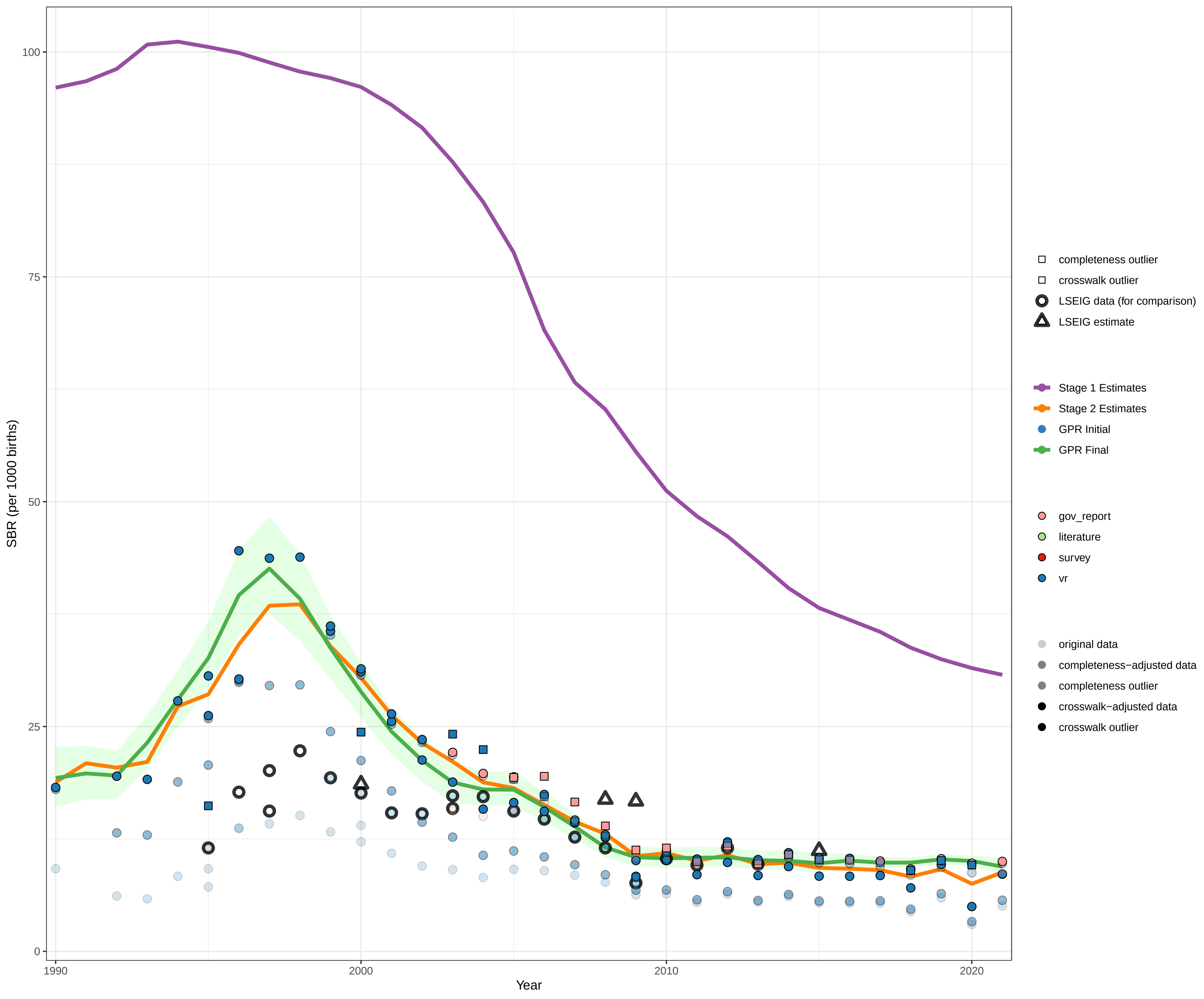

Georgia GEO Central Asia Stillbirth Rate (SBR) − 28 weeks
lambda = 0.2 ; zeta = 0.99 ; scale = 5 ; amp = 0.028

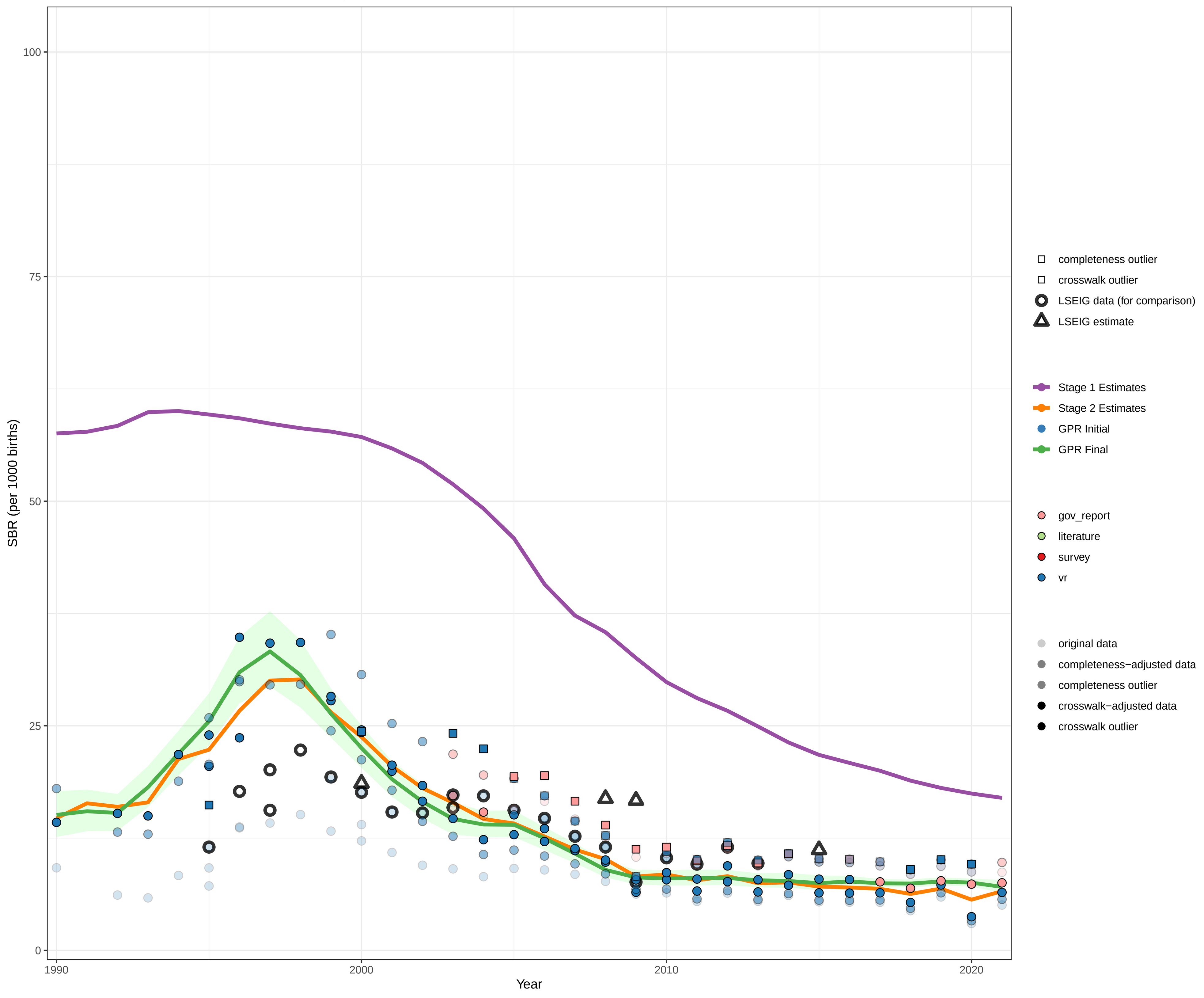

Year	Total Stillbirths (in thousands)	Total Stillbirths (in thousands)	SBR (per 1000 births)	SBR (per 1000 births)	SBR/NMR	SBR/NMR
	20 weeks	28 weeks	20 weeks	28 weeks	20 weeks	28 weeks
1990	1.7 (1.4 to 2.0)	1.3 (1.1 to 1.5)	19.3 (16.1 to 22.7)	15.1 (12.6 to 17.7)	0.89 (0.74 to 1.04)	0.69 (0.58 to 0.82)
1995	2.0 (1.8 to 2.2)	1.5 (1.4 to 1.7)	32.6 (29.2 to 36.6)	25.5 (22.8 to 28.6)	1.47 (1.31 to 1.65)	1.15 (1.03 to 1.29)
2000	1.5 (1.3 to 1.6)	1.1 (1.0 to 1.3)	28.9 (26.0 to 32.1)	22.5 (20.3 to 25.1)	1.34 (1.20 to 1.49)	1.04 (0.94 to 1.16)
2005	0.9 (0.8 to 1.0)	0.7 (0.6 to 0.8)	18.0 (16.2 to 20.0)	14.0 (12.6 to 15.6)	1.05 (0.95 to 1.17)	0.82 (0.74 to 0.91)
2010	0.6 (0.5 to 0.6)	0.4 (0.4 to 0.5)	10.3 (9.3 to 11.5)	8.0 (7.2 to 8.9)	0.95 (0.85 to 1.06)	0.74 (0.66 to 0.82)
2015	0.5 (0.5 to 0.6)	0.4 (0.4 to 0.5)	9.8 (8.8 to 10.9)	7.5 (6.7 to 8.3)	1.29 (1.16 to 1.44)	0.99 (0.88 to 1.10)
2020	0.5 (0.4 to 0.5)	0.4 (0.3 to 0.4)	10.1 (9.5 to 10.6)	7.5 (7.1 to 8.0)	1.75 (1.65 to 1.86)	1.31 (1.24 to 1.39)
2021	0.4 (0.4 to 0.5)	0.3 (0.3 to 0.4)	9.4 (8.5 to 10.4)	7.1 (6.4 to 7.8)	1.70 (1.54 to 1.88)	1.28 (1.16 to 1.42)

Kazakhstan KAZ Central Asia Stillbirth Rate / Neonatal Mortality Rate (SBR/NMR) − 20 weeks
lambda = 0.2 ; zeta = 0.99 ; scale = 5 ; amp = 0.028

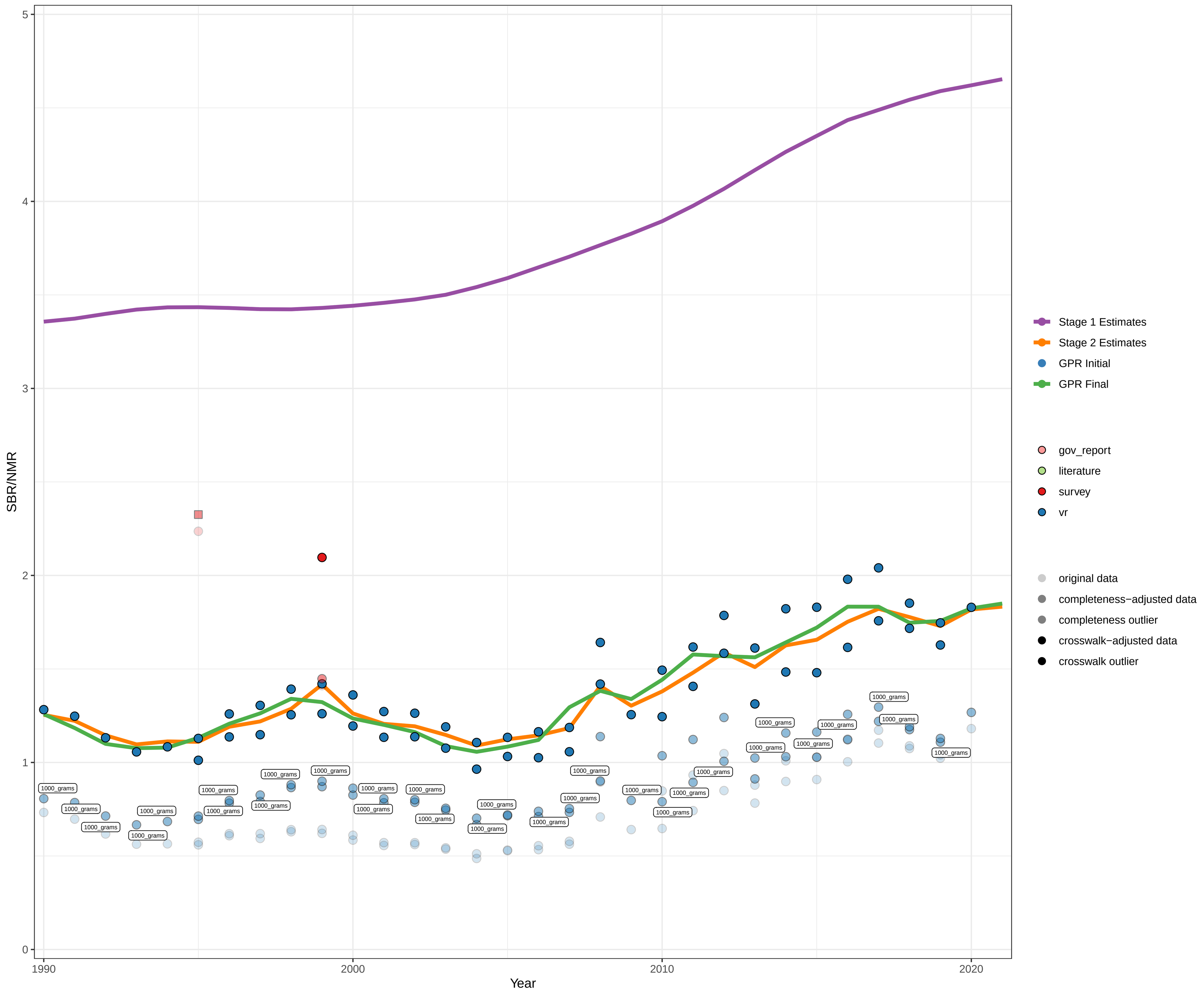

Kazakhstan KAZ Central Asia Stillbirth Rate / Neonatal Mortality Rate (SBR/NMR) − 28 weeks
lambda = 0.2 ; zeta = 0.99 ; scale = 5 ; amp = 0.028

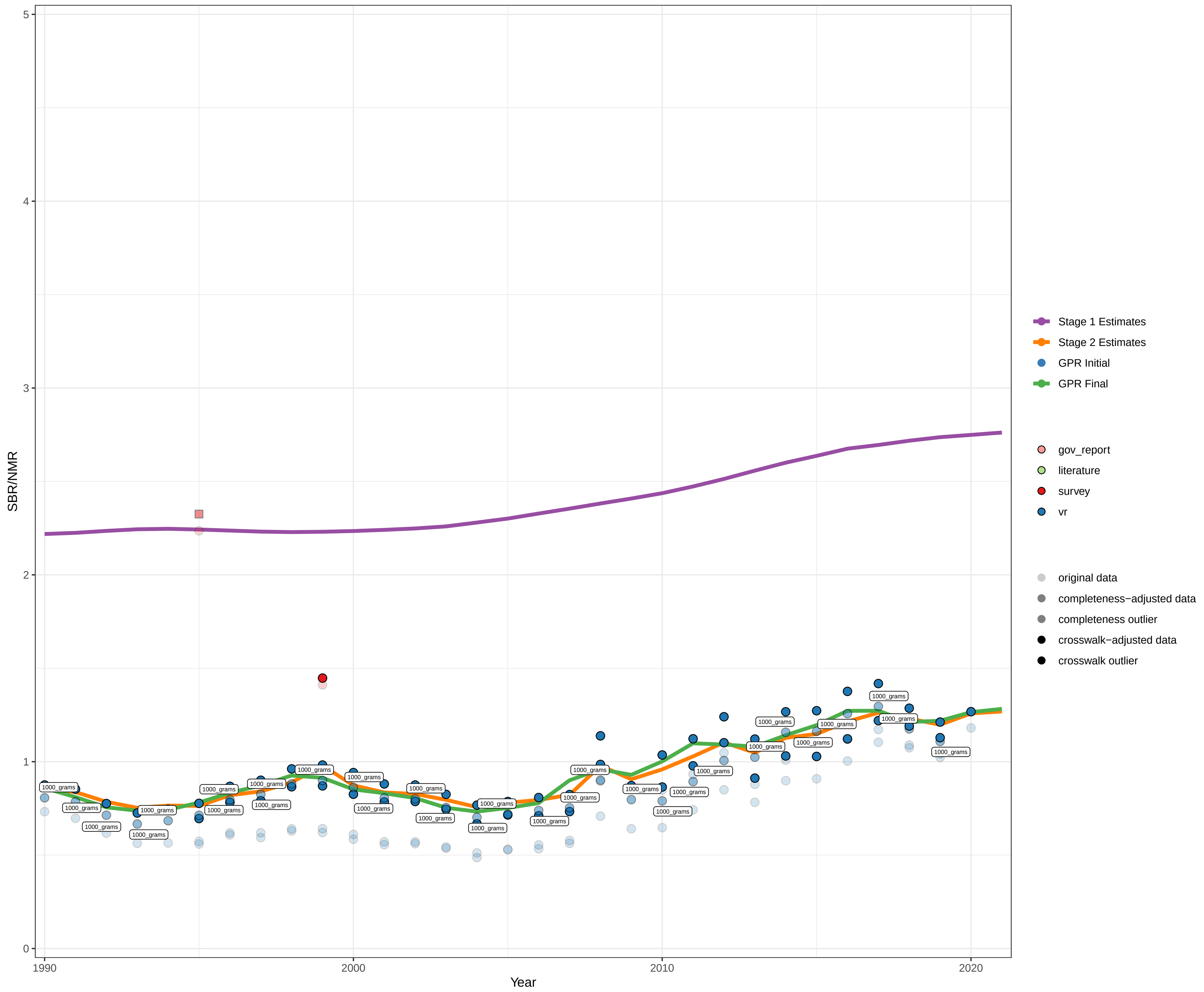

Definition	Years	Outlier	Source.Type	Title
1000_grams	1990−2019	0	VR	European Health for All Database
28_weeks	1995−2008, 2010−2020	0	VR	United Nations Demographic Yearbook
28_weeks	1999	0	Survey	Kazakhstan Demographic and Health Survey 1999
28_weeks	1995	1	Survey	Kazakhstan Demographic and Health Survey 1995

Kazakhstan KAZ Central Asia Stillbirth Rate (SBR) − 20 weeks
lambda = 0.2 ; zeta = 0.99 ; scale = 5 ; amp = 0.028

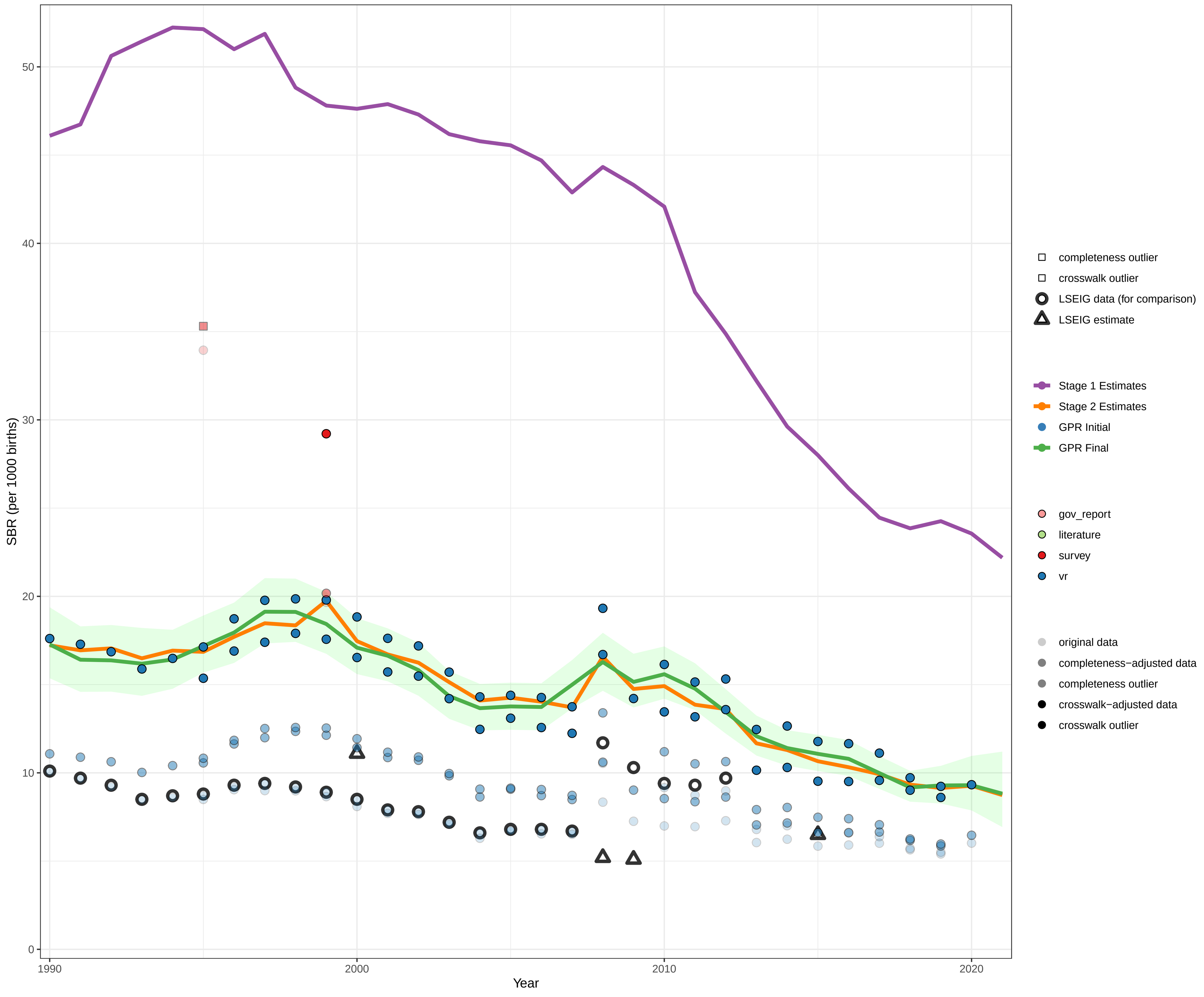

Kazakhstan KAZ Central Asia Stillbirth Rate (SBR) − 28 weeks
lambda = 0.2 ; zeta = 0.99 ; scale = 5 ; amp = 0.028

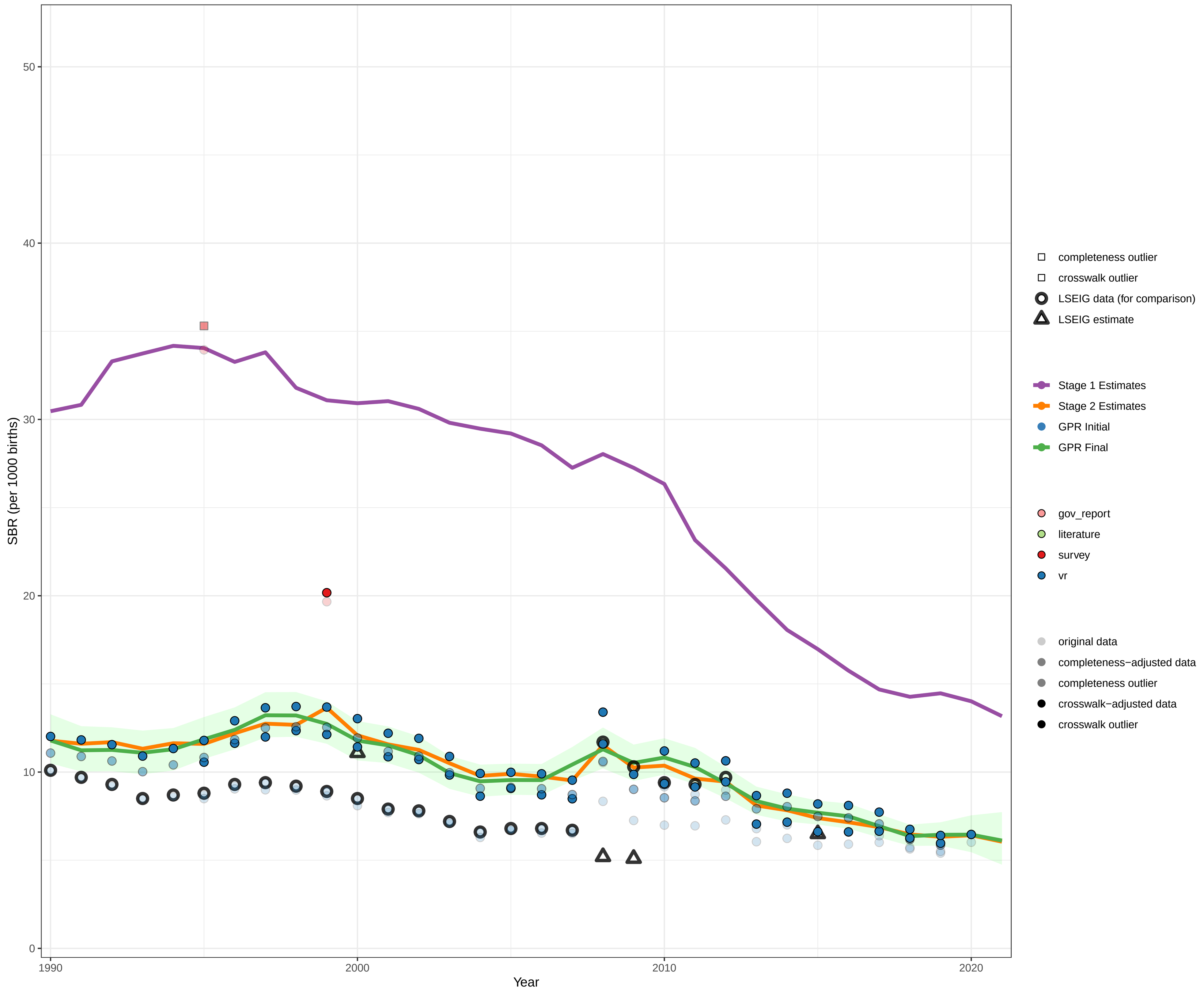

Year	Total Stillbirths (in thousands)	Total Stillbirths (in thousands)	SBR (per 1000 births)	SBR (per 1000 births)	SBR/NMR	SBR/NMR
	20 weeks	28 weeks	20 weeks	28 weeks	20 weeks	28 weeks
1990	6.6 (5.8 to 7.4)	4.5 (4.0 to 5.0)	17.3 (15.4 to 19.4)	11.8 (10.5 to 13.3)	1.26 (1.12 to 1.41)	0.86 (0.76 to 0.97)
1995	4.8 (4.4 to 5.3)	3.3 (3.0 to 3.6)	17.2 (15.7 to 18.9)	11.9 (10.8 to 13.1)	1.13 (1.03 to 1.25)	0.78 (0.71 to 0.86)
2000	3.9 (3.5 to 4.2)	2.6 (2.4 to 2.9)	17.1 (15.6 to 18.8)	11.8 (10.7 to 12.9)	1.24 (1.13 to 1.36)	0.85 (0.77 to 0.93)
2005	4.1 (3.7 to 4.5)	2.8 (2.6 to 3.1)	13.8 (12.4 to 15.1)	9.5 (8.7 to 10.5)	1.08 (0.98 to 1.19)	0.75 (0.69 to 0.83)
2010	5.8 (5.3 to 6.4)	4.0 (3.7 to 4.4)	15.6 (14.2 to 17.2)	10.8 (9.8 to 11.9)	1.44 (1.31 to 1.59)	1.00 (0.91 to 1.10)
2015	4.5 (4.1 to 4.9)	3.1 (2.8 to 3.4)	11.1 (10.1 to 12.2)	7.7 (7.0 to 8.4)	1.72 (1.57 to 1.89)	1.20 (1.09 to 1.30)
2020	4.0 (3.4 to 4.7)	2.8 (2.3 to 3.2)	9.3 (7.9 to 11.0)	6.5 (5.5 to 7.5)	1.82 (1.54 to 2.15)	1.27 (1.07 to 1.48)
2021	3.8 (3.0 to 4.8)	2.6 (2.0 to 3.3)	8.8 (6.9 to 11.2)	6.1 (4.7 to 7.7)	1.85 (1.45 to 2.35)	1.28 (1.00 to 1.62)

Kyrgyzstan KGZ Central Asia Stillbirth Rate / Neonatal Mortality Rate (SBR/NMR) − 20 weeks
lambda = 0.2 ; zeta = 0.99 ; scale = 5 ; amp = 0.028

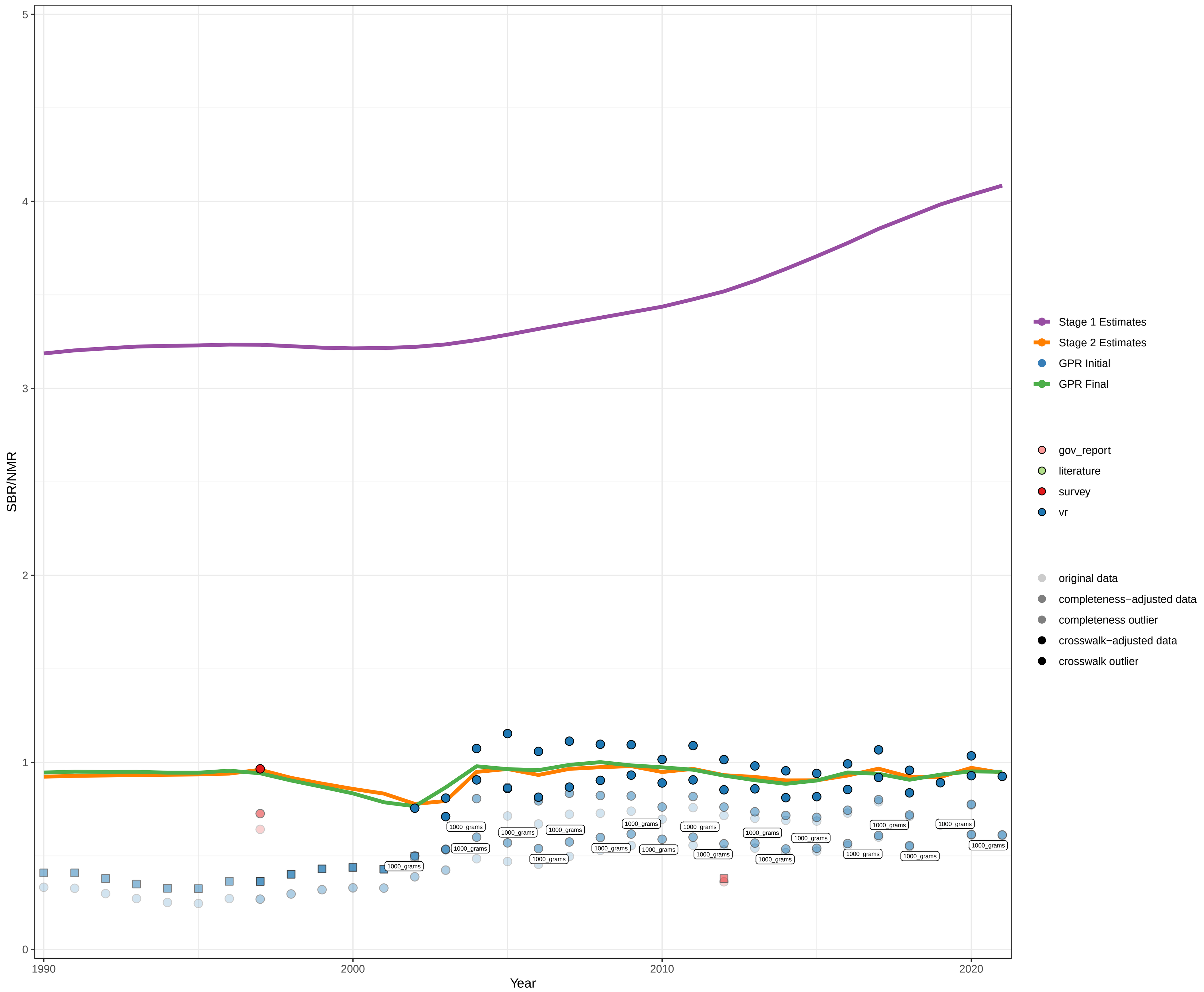

Kyrgyzstan KGZ Central Asia Stillbirth Rate / Neonatal Mortality Rate (SBR/NMR) − 28 weeks
lambda = 0.2 ; zeta = 0.99 ; scale = 5 ; amp = 0.028

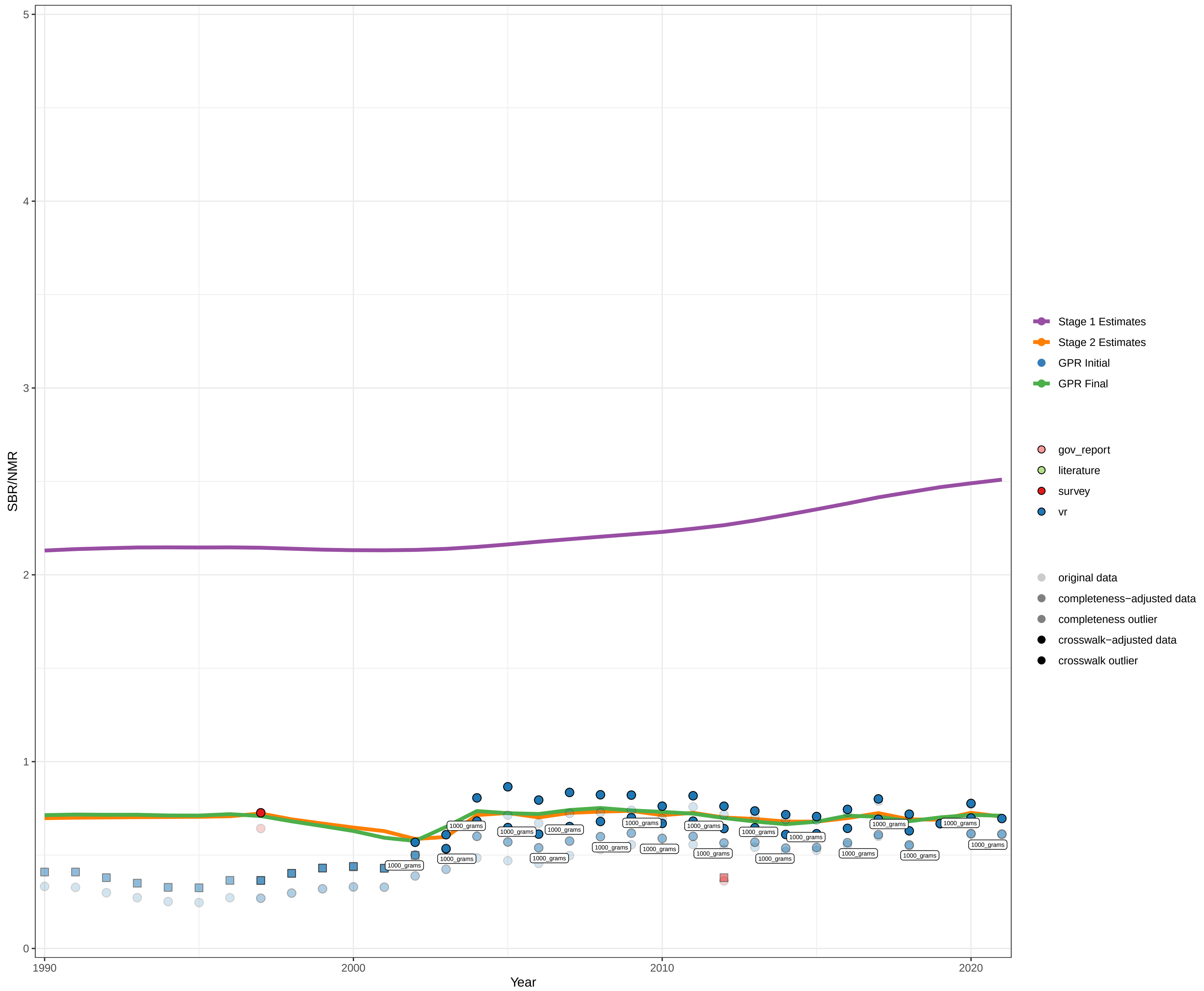

Definition	Years	Outlier	Source.Type	Title
1000_grams	2002−2018, 2020, 2021	0	VR	European Health for All Database
28_weeks	1997	0	Survey	Kyrgyzstan Demographic and Health Survey 1997
28_weeks	2003−2020	0	VR	United Nations Demographic Yearbook
1000_grams	1990−2001	1	VR	European Health for All Database
28_weeks	1997−2002	1	VR	United Nations Demographic Yearbook
28_weeks	2012	1	Survey	Kyrgyzstan Demographic and Health Survey 2012

Kyrgyzstan KGZ Central Asia Stillbirth Rate (SBR) − 20 weeks
lambda = 0.2 ; zeta = 0.99 ; scale = 5 ; amp = 0.028

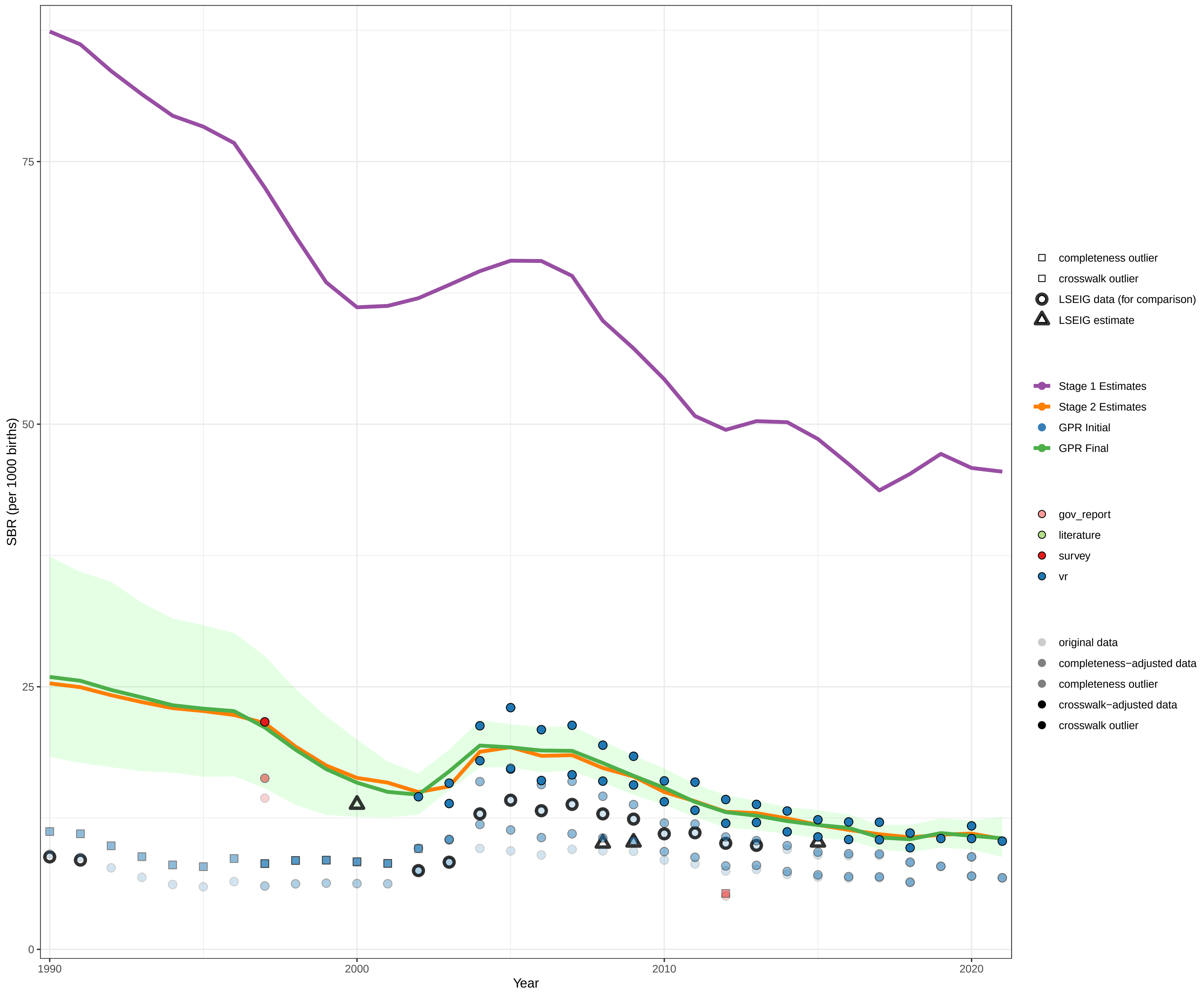

Kyrgyzstan KGZ Central Asia Stillbirth Rate (SBR) − 28 weeks
lambda = 0.2 ; zeta = 0.99 ; scale = 5 ; amp = 0.028

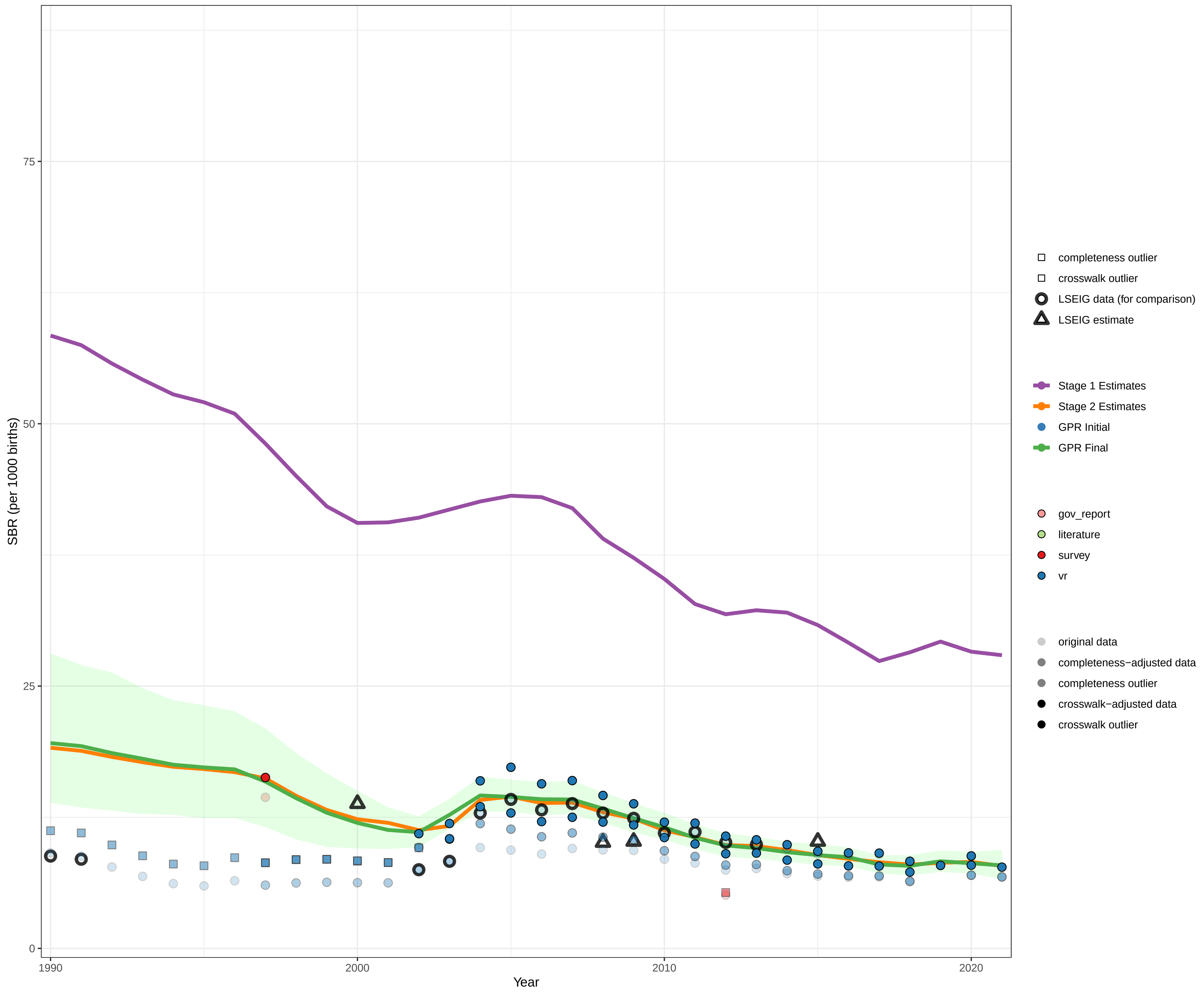

Year	Total Stillbirths (in thousands)	Total Stillbirths (in thousands)	SBR (per 1000 births)	SBR (per 1000 births)	SBR/NMR	SBR/NMR
	20 weeks	28 weeks	20 weeks	28 weeks	20 weeks	28 weeks
1990	3.5 (2.5 to 5.2)	2.7 (1.9 to 3.8)	25.9 (18.3 to 37.4)	19.6 (13.9 to 28.1)	0.95 (0.67 to 1.36)	0.71 (0.51 to 1.03)
1995	2.7 (1.9 to 3.7)	2.0 (1.5 to 2.8)	22.9 (16.4 to 30.9)	17.3 (12.4 to 23.2)	0.94 (0.68 to 1.27)	0.71 (0.51 to 0.96)
2000	1.7 (1.4 to 2.2)	1.3 (1.0 to 1.6)	15.9 (12.6 to 20.0)	12.0 (9.5 to 15.0)	0.83 (0.66 to 1.05)	0.63 (0.50 to 0.79)
2005	2.5 (2.2 to 2.8)	1.8 (1.7 to 2.1)	19.2 (17.3 to 21.4)	14.4 (13.0 to 16.1)	0.96 (0.87 to 1.07)	0.72 (0.65 to 0.81)
2010	2.5 (2.3 to 2.8)	1.9 (1.7 to 2.1)	15.4 (13.8 to 17.2)	11.5 (10.3 to 12.9)	0.97 (0.87 to 1.09)	0.73 (0.65 to 0.82)
2015	2.1 (1.9 to 2.3)	1.6 (1.4 to 1.7)	11.8 (10.6 to 13.3)	8.9 (8.0 to 9.9)	0.90 (0.81 to 1.01)	0.68 (0.61 to 0.76)
2020	1.8 (1.6 to 2.0)	1.3 (1.2 to 1.5)	10.8 (9.5 to 12.3)	8.1 (7.1 to 9.2)	0.95 (0.84 to 1.08)	0.71 (0.63 to 0.81)
2021	1.7 (1.4 to 2.0)	1.3 (1.1 to 1.5)	10.6 (8.8 to 12.6)	7.9 (6.6 to 9.4)	0.95 (0.79 to 1.13)	0.71 (0.59 to 0.85)

Mongolia MNG Central Asia Stillbirth Rate / Neonatal Mortality Rate (SBR/NMR) − 20 weeks
lambda = 0.2 ; zeta = 0.99 ; scale = 5 ; amp = 0.028

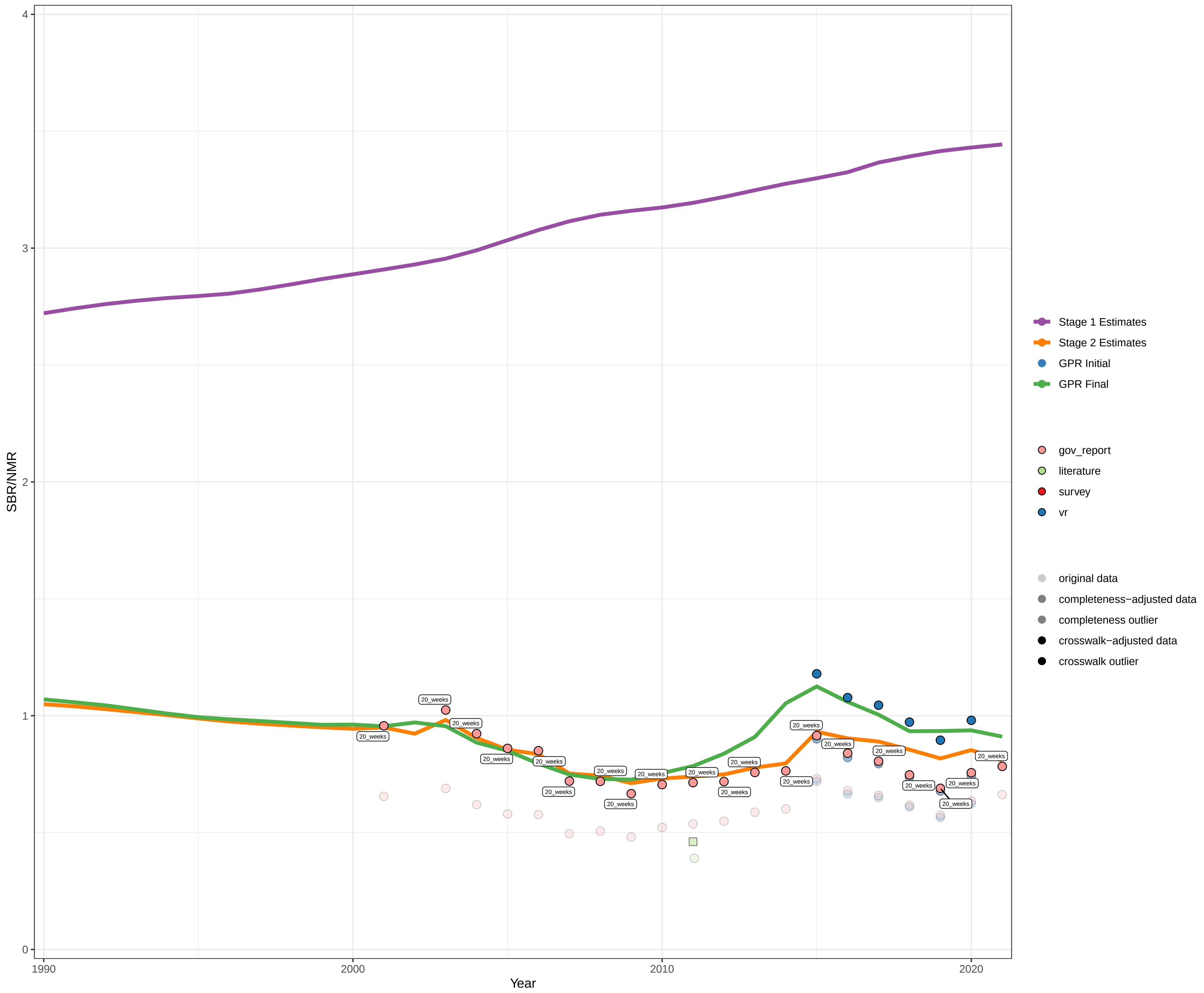

Mongolia MNG Central Asia Stillbirth Rate / Neonatal Mortality Rate (SBR/NMR) − 28 weeks
lambda = 0.2 ; zeta = 0.99 ; scale = 5 ; amp = 0.028

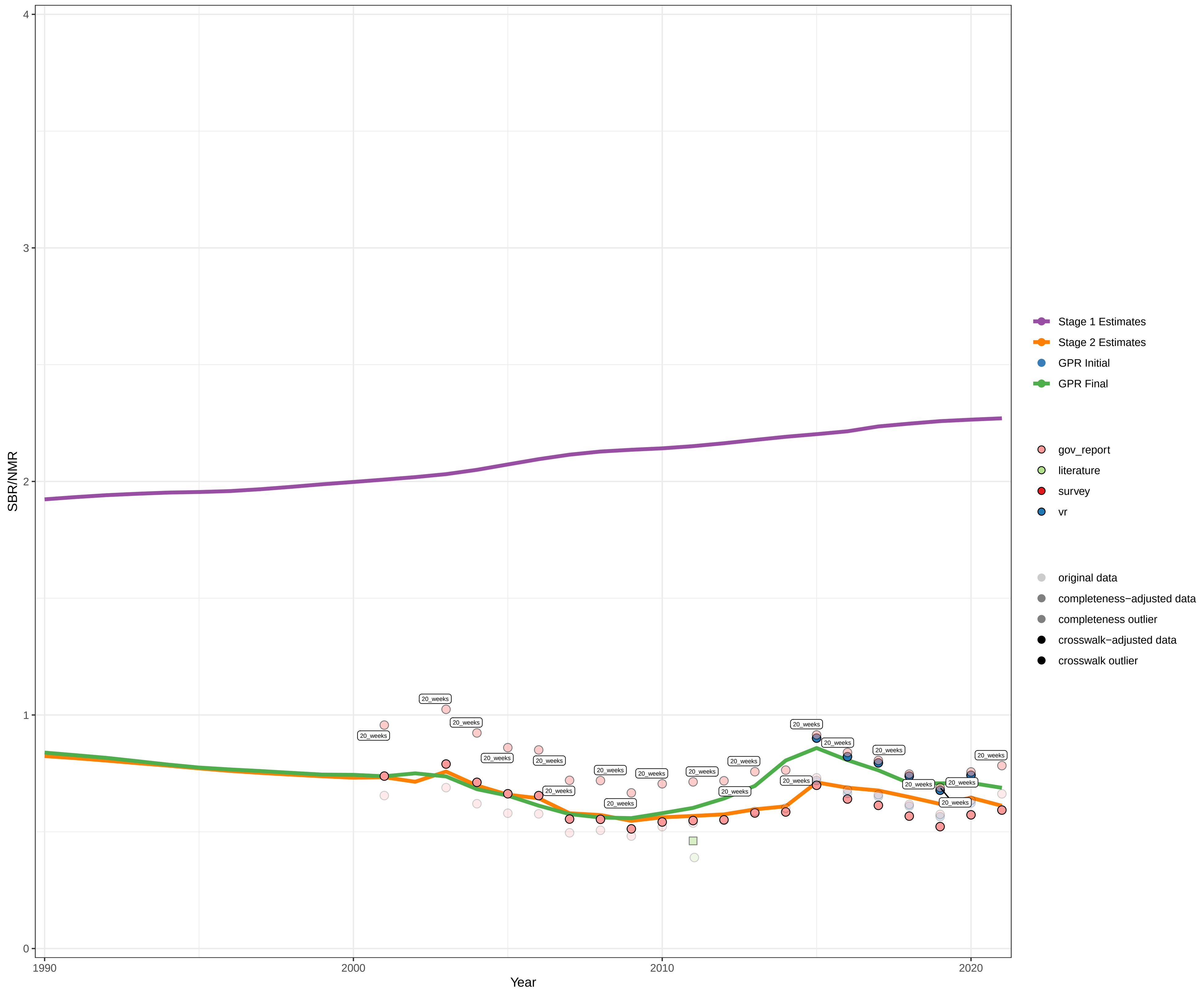

Definition	Years	Outlier	Source.Type	Title
20_weeks	2001, 2003−2021	0	Statistical Report	Mongolia Health Indicators
28_weeks	2015−2020	0	VR	United Nations Demographic Yearbook
28_weeks_OR_1000_grams	2011	1	Sci Lit	Maternal complications and perinatal mortality: findings of the World Health Organization Multicountry Survey on Maternal and Newborn Health

Mongolia MNG Central Asia Stillbirth Rate (SBR) − 20 weeks
lambda = 0.2 ; zeta = 0.99 ; scale = 5 ; amp = 0.028

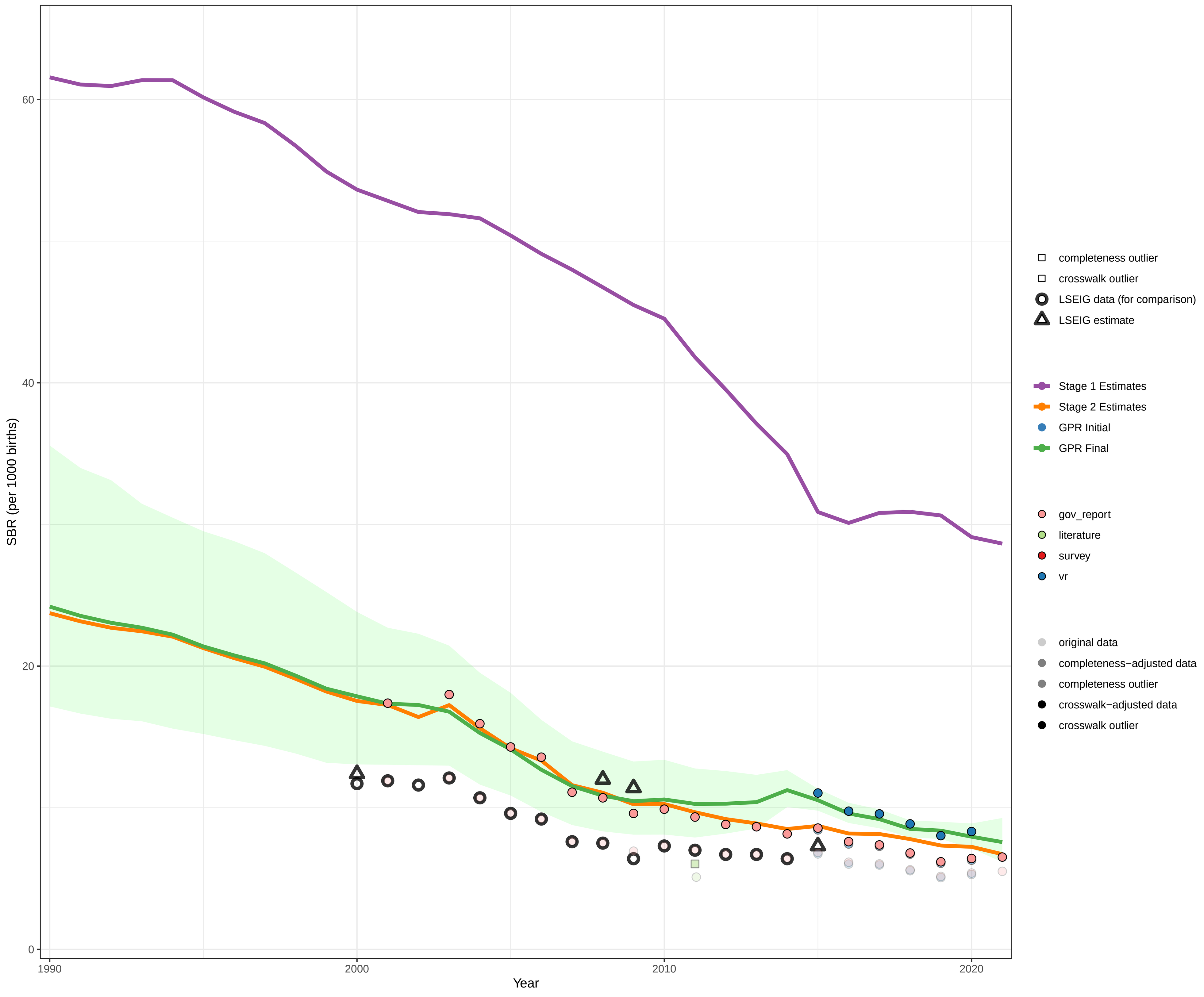

Mongolia MNG Central Asia Stillbirth Rate (SBR) − 28 weeks
lambda = 0.2 ; zeta = 0.99 ; scale = 5 ; amp = 0.028

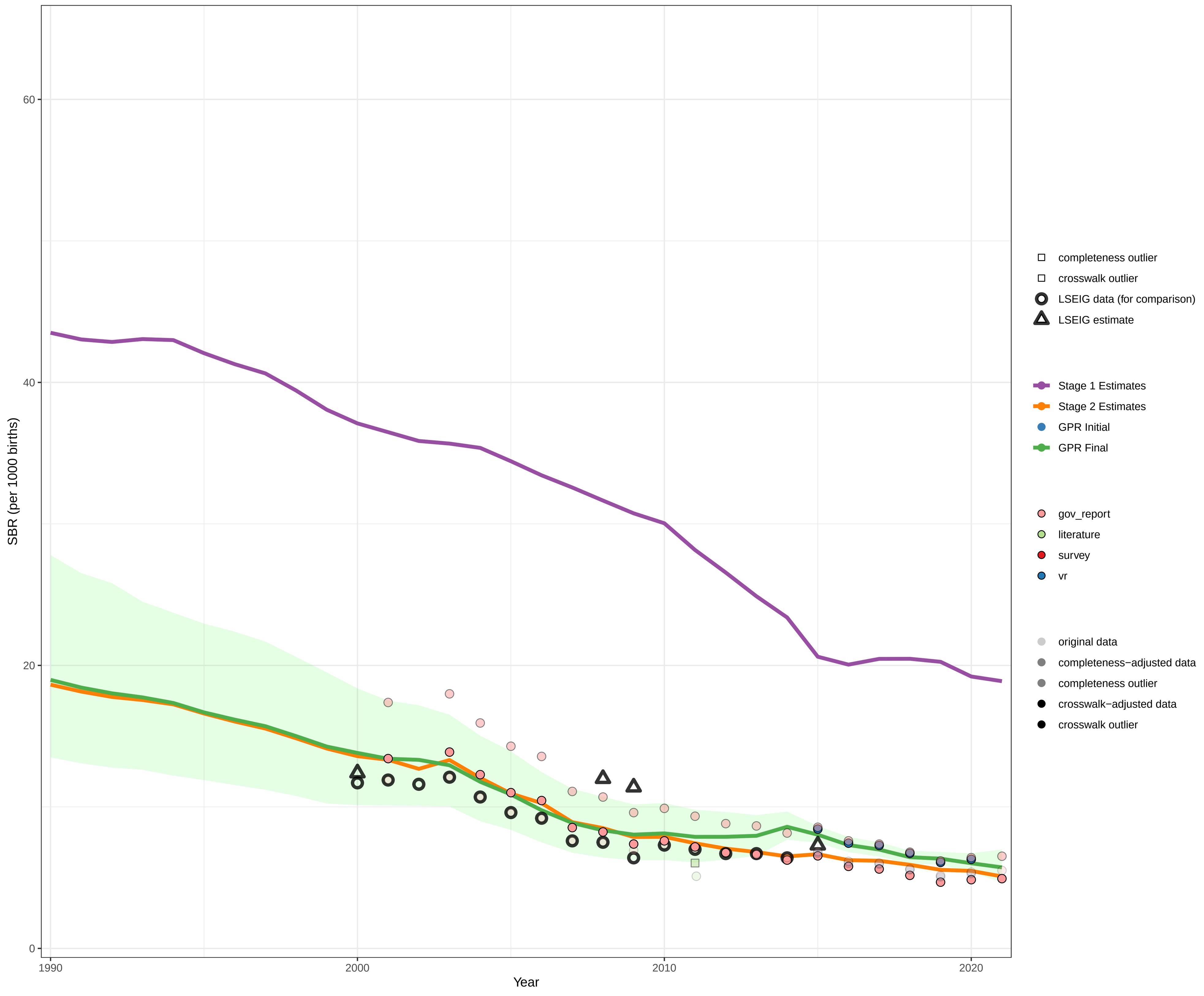

Year	Total Stillbirths (in thousands)	Total Stillbirths (in thousands)	SBR (per 1000 births)	SBR (per 1000 births)	SBR/NMR	SBR/NMR
	20 weeks	28 weeks	20 weeks	28 weeks	20 weeks	28 weeks
1990	1.8 (1.3 to 2.6)	1.4 (1.0 to 2.0)	24.2 (17.2 to 35.6)	19.0 (13.5 to 27.8)	1.07 (0.76 to 1.57)	0.84 (0.60 to 1.23)
1995	1.3 (0.9 to 1.8)	1.0 (0.7 to 1.4)	21.4 (15.2 to 29.5)	16.7 (11.9 to 23.0)	0.99 (0.71 to 1.37)	0.78 (0.55 to 1.07)
2000	1.0 (0.7 to 1.3)	0.7 (0.5 to 1.0)	17.9 (13.1 to 23.8)	13.8 (10.1 to 18.4)	0.96 (0.70 to 1.28)	0.74 (0.54 to 0.99)
2005	0.8 (0.6 to 1.0)	0.6 (0.5 to 0.8)	14.1 (10.9 to 18.1)	10.9 (8.4 to 13.9)	0.85 (0.65 to 1.09)	0.65 (0.50 to 0.84)
2010	0.8 (0.6 to 1.0)	0.6 (0.5 to 0.8)	10.6 (8.1 to 13.4)	8.1 (6.2 to 10.3)	0.75 (0.58 to 0.95)	0.58 (0.44 to 0.73)
2015	0.9 (0.8 to 1.0)	0.7 (0.6 to 0.7)	10.5 (9.8 to 11.3)	8.0 (7.5 to 8.7)	1.13 (1.05 to 1.21)	0.86 (0.80 to 0.92)
2020	0.7 (0.6 to 0.7)	0.5 (0.4 to 0.5)	8.0 (7.1 to 8.9)	6.0 (5.4 to 6.7)	0.94 (0.84 to 1.05)	0.71 (0.64 to 0.79)
2021	0.6 (0.5 to 0.7)	0.5 (0.4 to 0.6)	7.6 (6.2 to 9.3)	5.7 (4.7 to 7.0)	0.91 (0.74 to 1.12)	0.69 (0.56 to 0.84)

Tajikistan TJK Central Asia Stillbirth Rate / Neonatal Mortality Rate (SBR/NMR) − 20 weeks
lambda = 0.2 ; zeta = 0.99 ; scale = 5 ; amp = 0.028

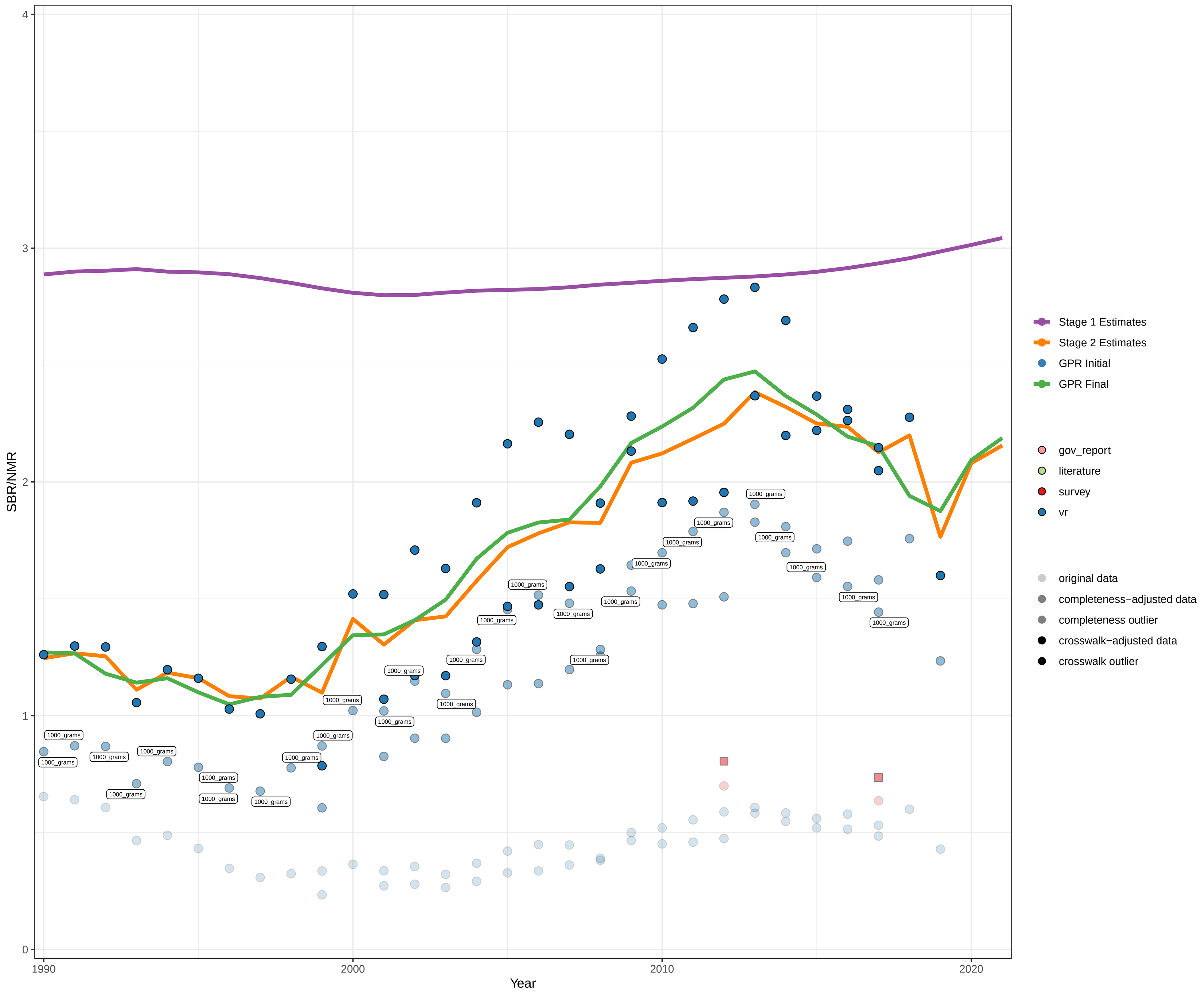

Tajikistan TJK Central Asia Stillbirth Rate / Neonatal Mortality Rate (SBR/NMR) − 28 weeks
lambda = 0.2 ; zeta = 0.99 ; scale = 5 ; amp = 0.028

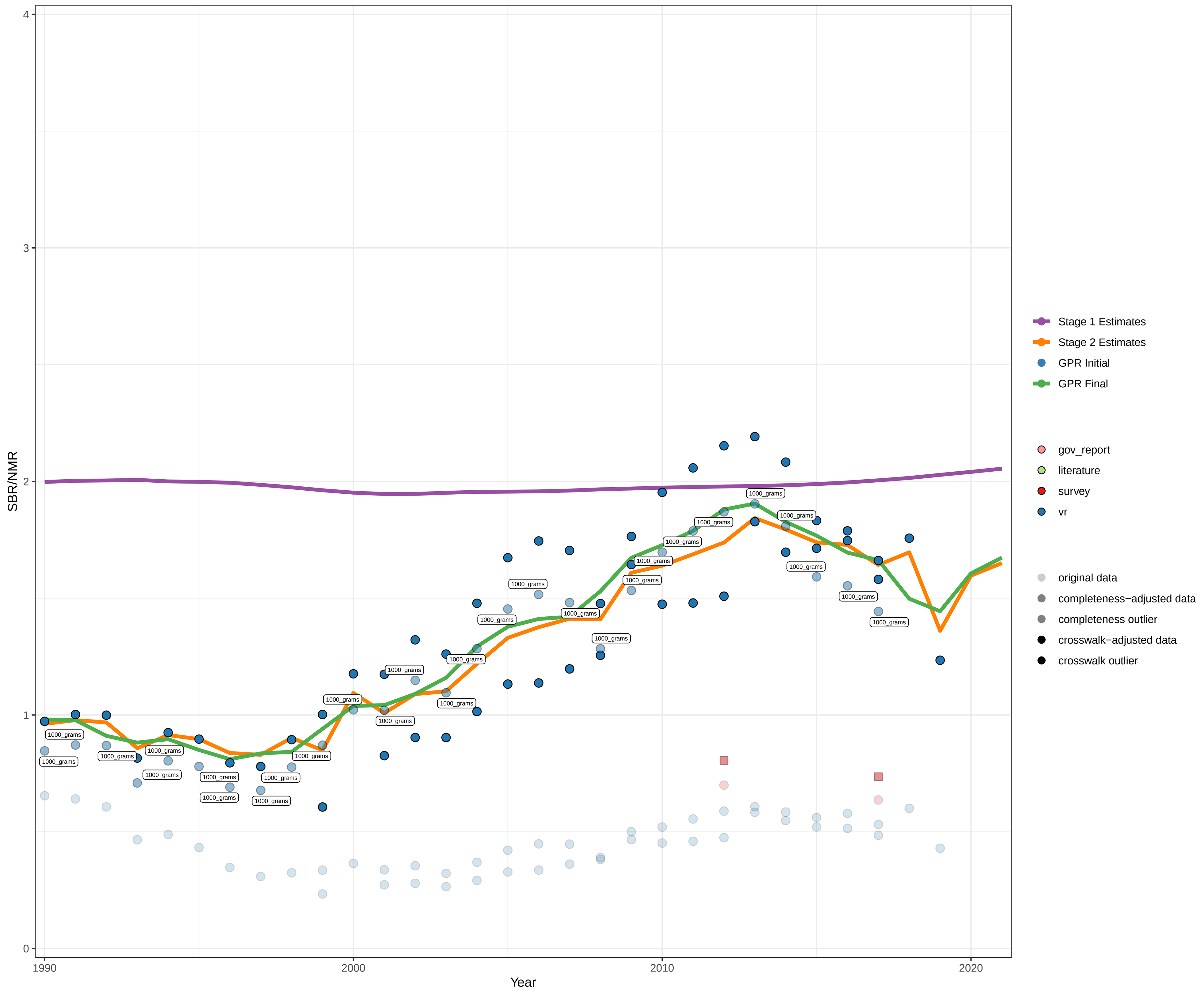

Definition	Years	Outlier	Source.Type	Title
1000_grams	1990−2017	0	VR	European Health for All Database
28_weeks	1999, 2001−2019	0	VR	United Nations Demographic Yearbook
28_weeks	2012	1	Survey	Tajikistan Demographic and Health Survey 2012
28_weeks	2017	1	Survey	Tajikistan Demographic and Health Survey 2017

Tajikistan TJK Central Asia Stillbirth Rate (SBR) − 20 weeks
lambda = 0.2 ; zeta = 0.99 ; scale = 5 ; amp = 0.028

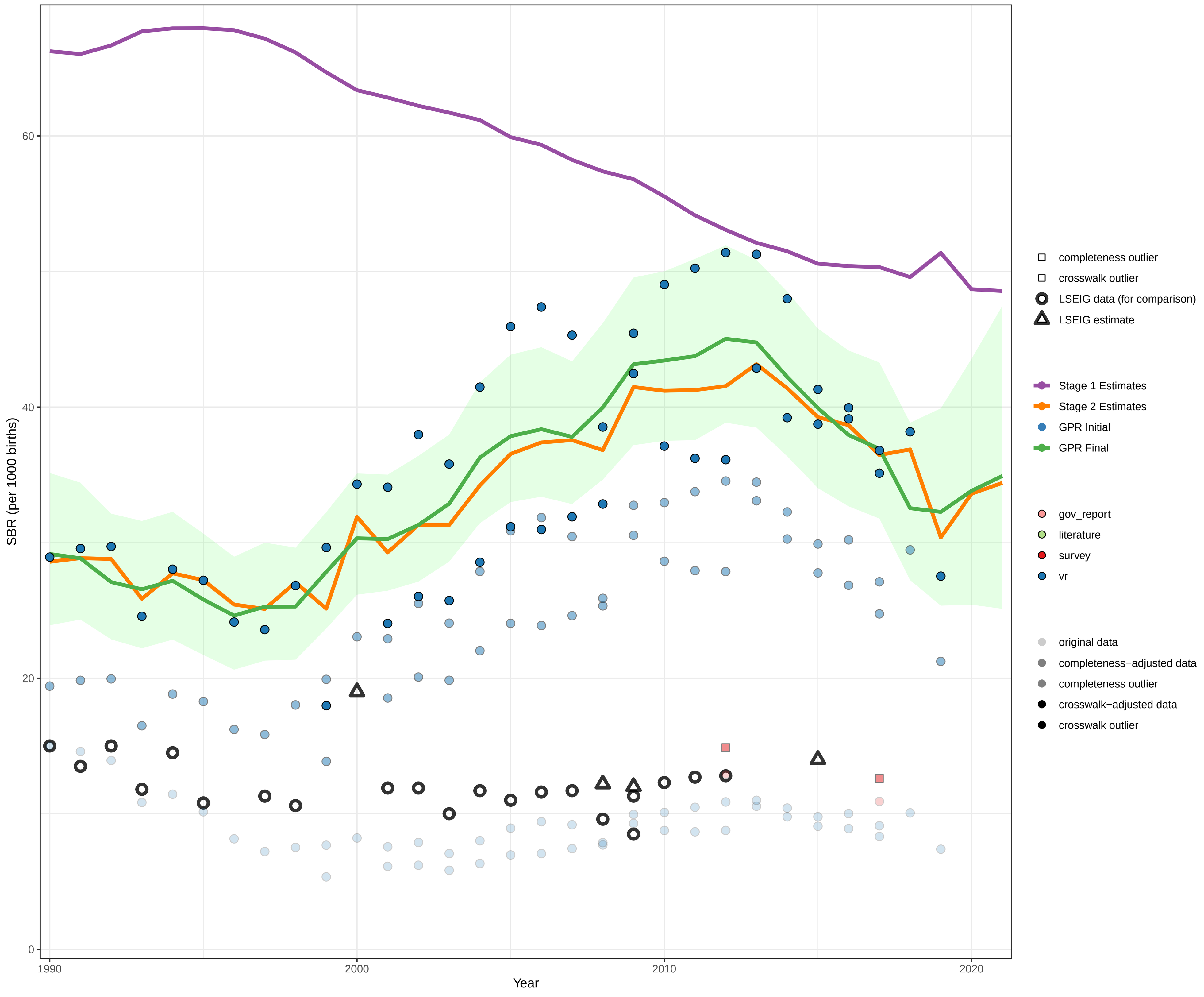

Tajikistan TJK Central Asia Stillbirth Rate (SBR) − 28 weeks
lambda = 0.2 ; zeta = 0.99 ; scale = 5 ; amp = 0.028

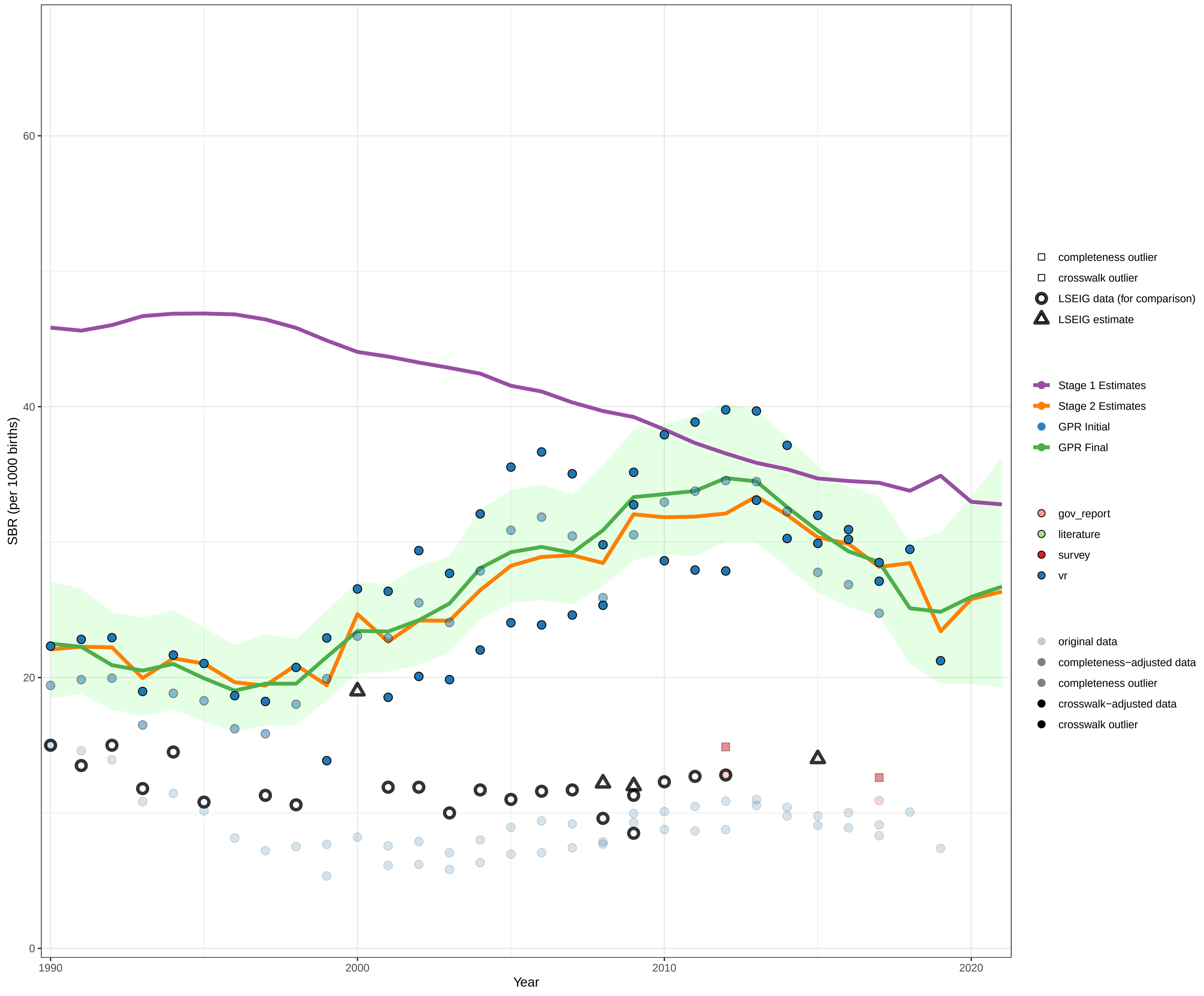

Year	Total Stillbirths (in thousands)	Total Stillbirths (in thousands)	SBR (per 1000 births)	SBR (per 1000 births)	SBR/NMR	SBR/NMR
	20 weeks	28 weeks	20 weeks	28 weeks	20 weeks	28 weeks
1990	6.4 (5.2 to 7.7)	4.9 (4.0 to 5.9)	29.2 (23.9 to 35.1)	22.5 (18.4 to 27.1)	1.27 (1.04 to 1.53)	0.98 (0.80 to 1.18)
1995	5.4 (4.6 to 6.5)	4.2 (3.5 to 5.0)	25.8 (21.7 to 30.7)	19.9 (16.8 to 23.7)	1.10 (0.93 to 1.31)	0.85 (0.72 to 1.01)
2000	6.2 (5.3 to 7.2)	4.7 (4.1 to 5.5)	30.3 (26.2 to 35.1)	23.4 (20.3 to 27.1)	1.34 (1.16 to 1.56)	1.04 (0.90 to 1.20)
2005	8.2 (7.1 to 9.6)	6.3 (5.5 to 7.3)	37.9 (33.0 to 43.9)	29.3 (25.5 to 33.9)	1.78 (1.55 to 2.07)	1.38 (1.20 to 1.59)
2010	11.3 (9.7 to 13.1)	8.6 (7.5 to 10.0)	43.4 (37.5 to 50.0)	33.5 (29.1 to 38.7)	2.24 (1.93 to 2.58)	1.73 (1.50 to 1.99)
2015	11.6 (9.9 to 13.4)	8.9 (7.6 to 10.3)	39.9 (34.0 to 45.8)	30.8 (26.3 to 35.6)	2.29 (1.95 to 2.62)	1.77 (1.51 to 2.04)
2020	10.1 (7.5 to 13.1)	7.7 (5.7 to 9.9)	33.8 (25.4 to 43.6)	26.0 (19.5 to 33.4)	2.09 (1.57 to 2.70)	1.61 (1.21 to 2.07)
2021	10.4 (7.4 to 14.3)	7.9 (5.6 to 10.8)	34.9 (25.1 to 47.5)	26.7 (19.3 to 36.2)	2.19 (1.57 to 2.97)	1.67 (1.21 to 2.27)

Turkmenistan TKM Central Asia Stillbirth Rate / Neonatal Mortality Rate (SBR/NMR) − 20 weeks
lambda = 0.6 ; zeta = 0.8 ; scale = 15 ; amp = 0.028

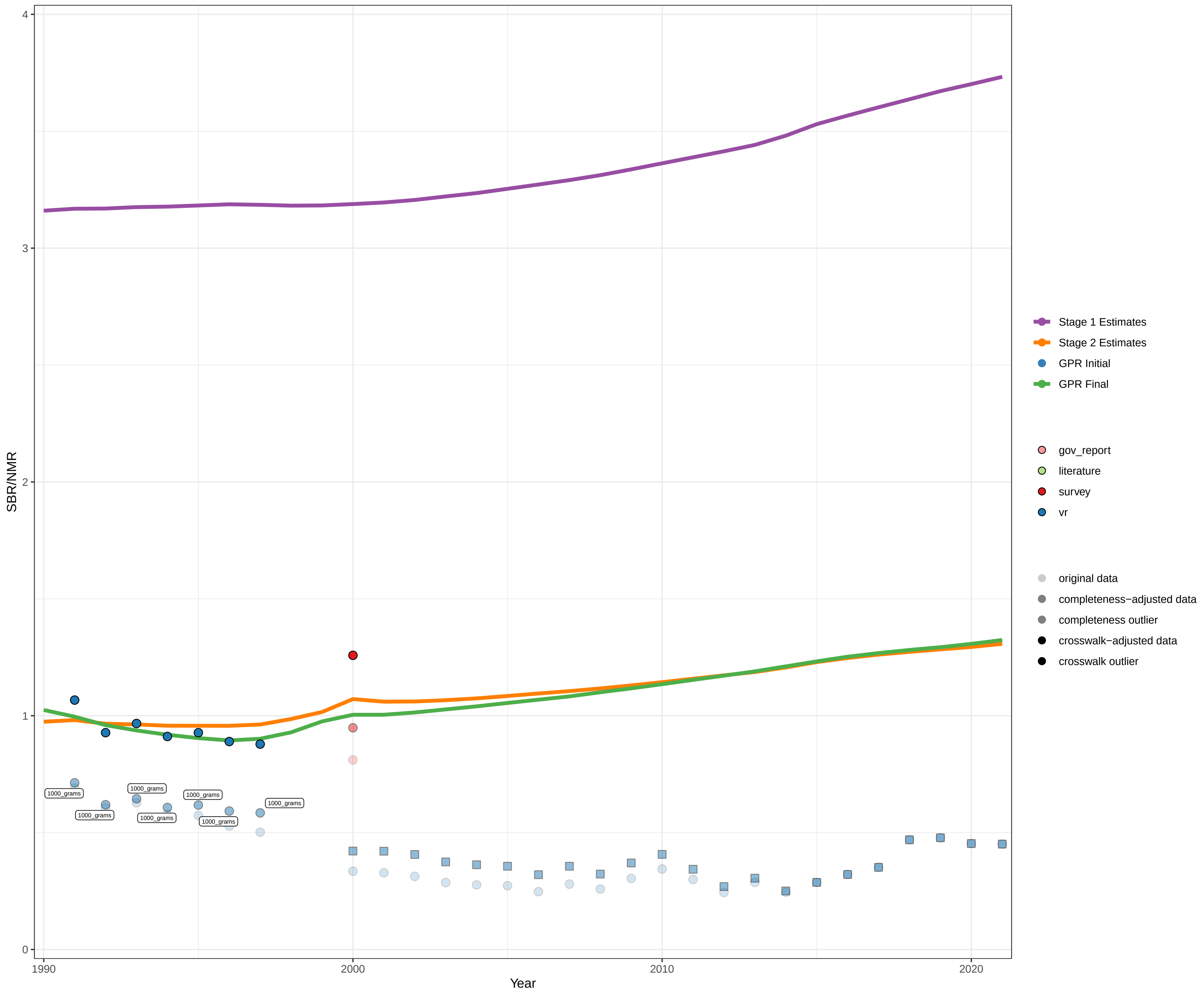

Turkmenistan TKM Central Asia Stillbirth Rate / Neonatal Mortality Rate (SBR/NMR) − 28 weeks
lambda = 0.6 ; zeta = 0.8 ; scale = 15 ; amp = 0.028

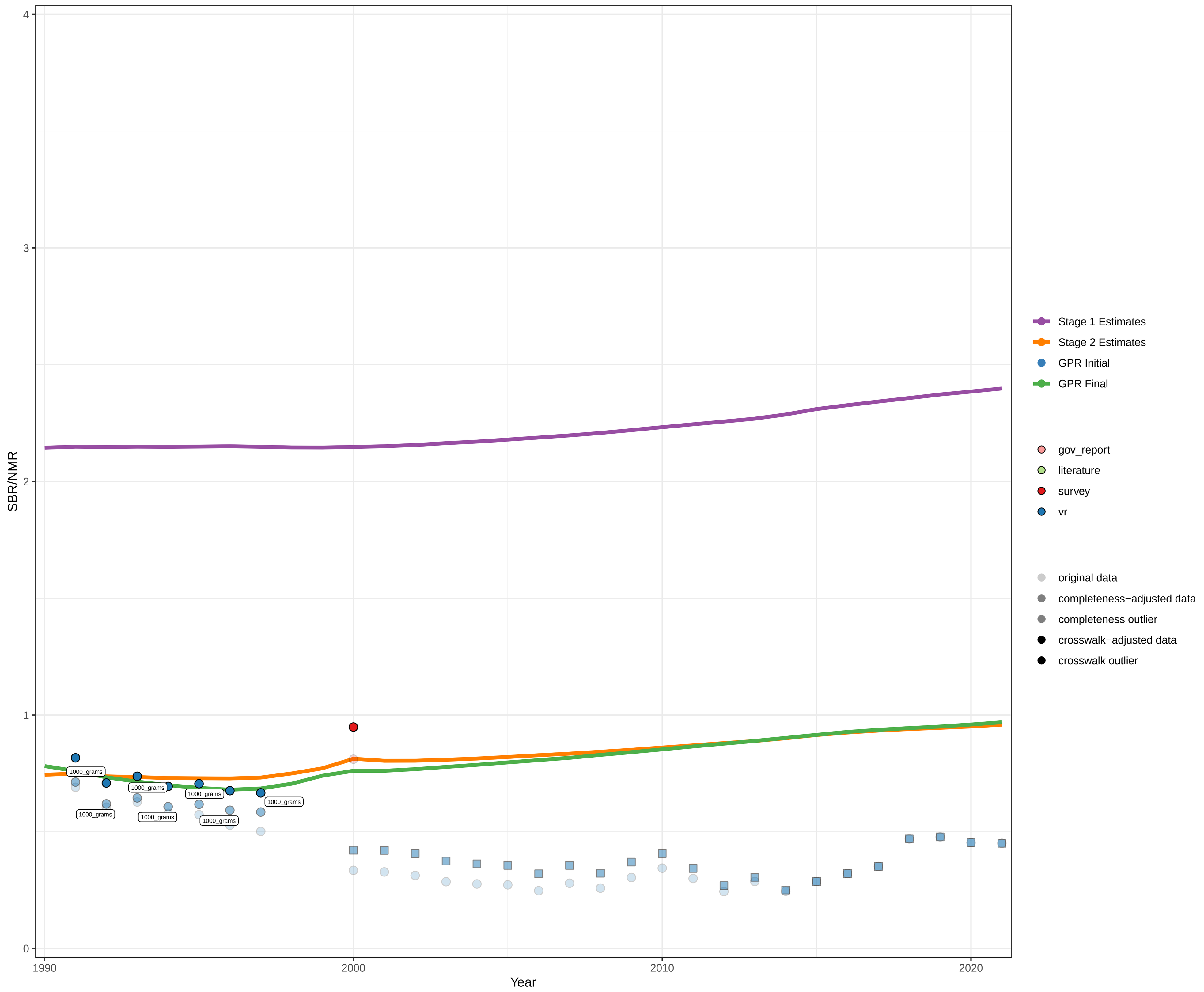

Definition	Years	Outlier	Source.Type	Title
1000_grams	1991−1997	0	VR	European Health for All Database
28_weeks	2000	0	Survey	Turkmenistan Demographic and Health Survey 2000
1000_grams	2000−2021	1	VR	European Health for All Database

Turkmenistan TKM Central Asia Stillbirth Rate (SBR) − 20 weeks
lambda = 0.6 ; zeta = 0.8 ; scale = 15 ; amp = 0.028

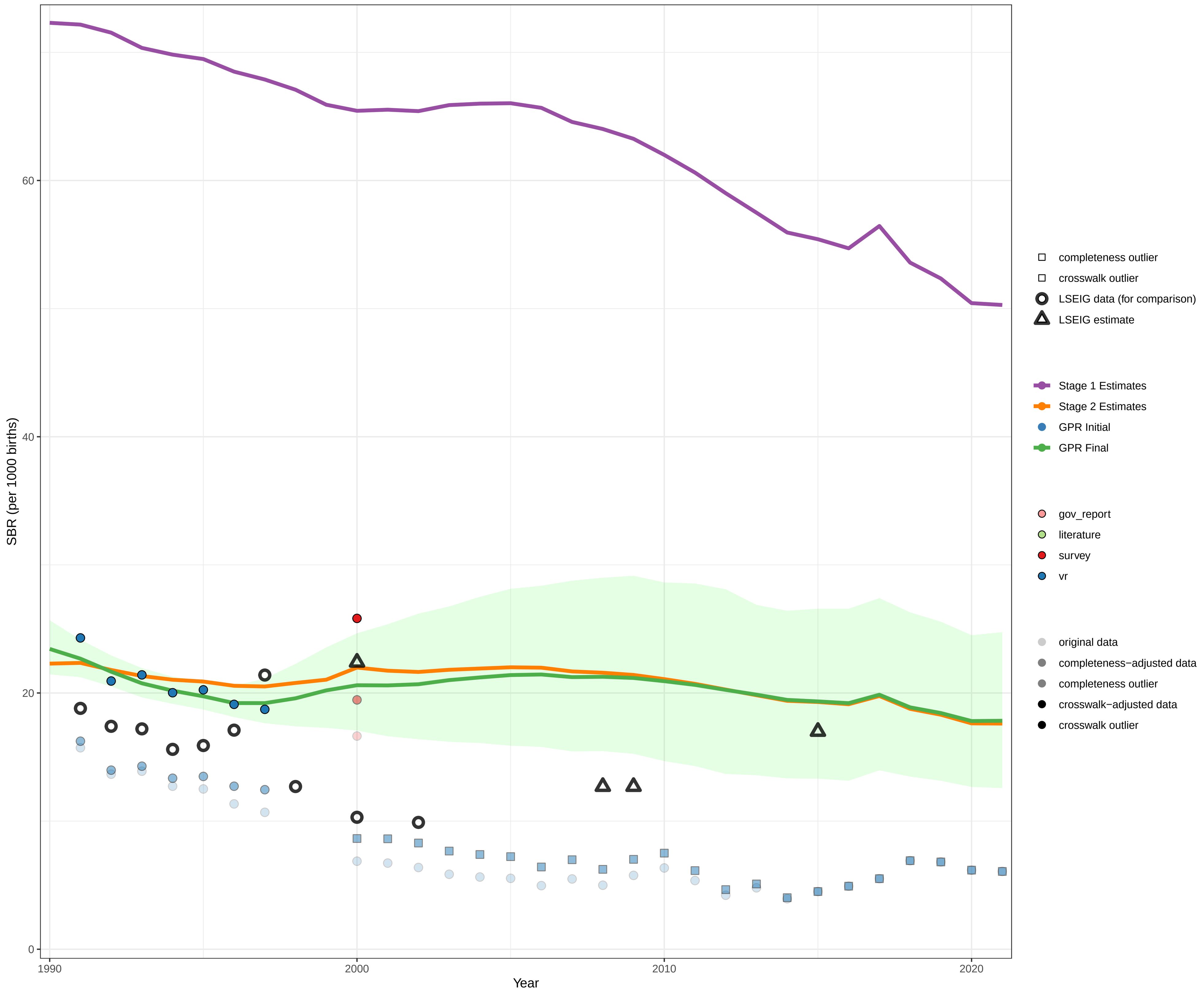

Turkmenistan TKM Central Asia Stillbirth Rate (SBR) − 28 weeks
lambda = 0.6 ; zeta = 0.8 ; scale = 15 ; amp = 0.028

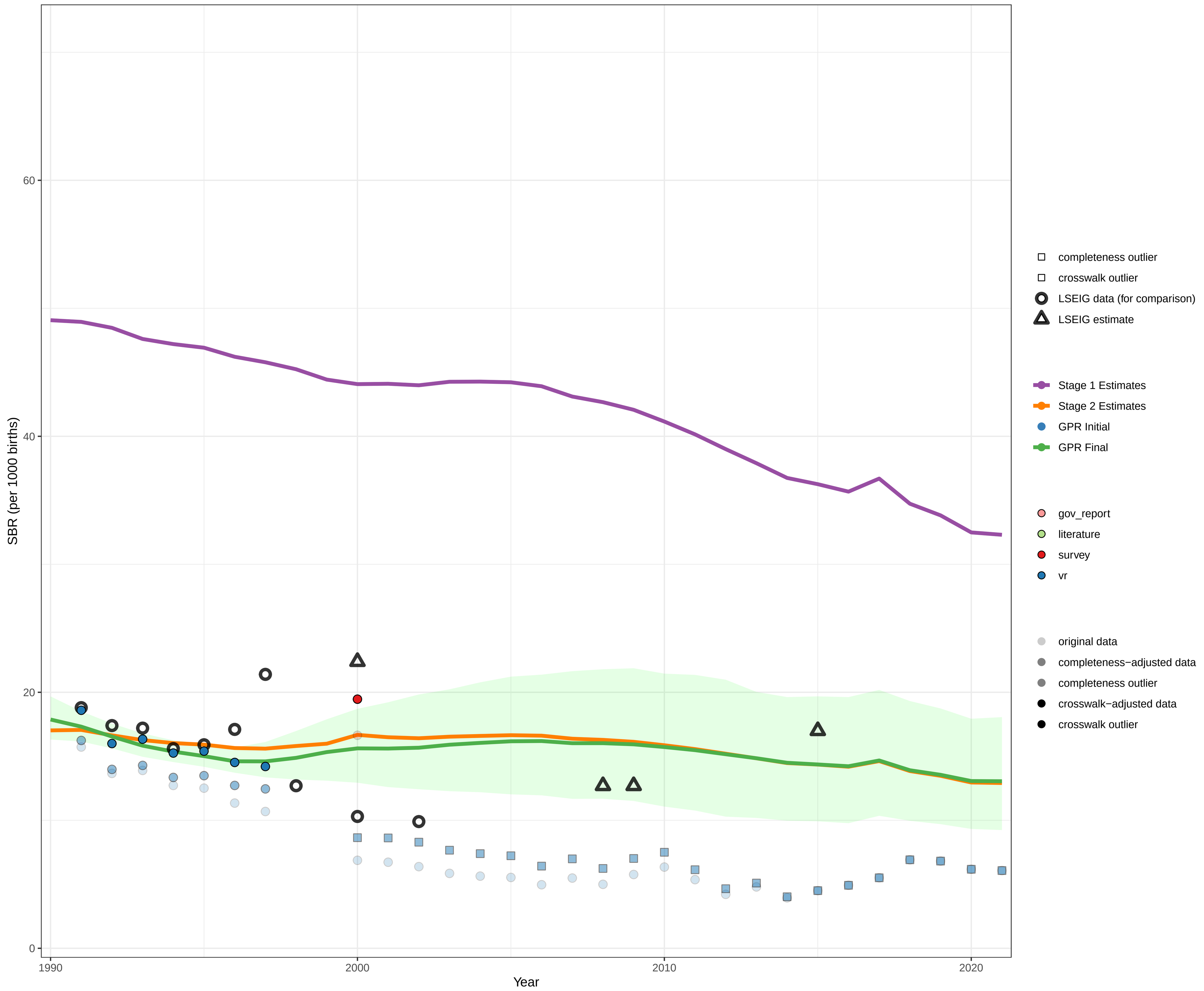

Year	Total Stillbirths (in thousands)	Total Stillbirths (in thousands)	SBR (per 1000 births)	SBR (per 1000 births)	SBR/NMR	SBR/NMR
	20 weeks	28 weeks	20 weeks	28 weeks	20 weeks	28 weeks
1990	3.0 (2.7 to 3.3)	2.3 (2.1 to 2.5)	23.4 (21.4 to 25.7)	17.9 (16.3 to 19.7)	1.02 (0.94 to 1.12)	0.78 (0.71 to 0.86)
1995	2.4 (2.3 to 2.6)	1.8 (1.7 to 1.9)	19.7 (18.7 to 20.9)	15.0 (14.2 to 16.0)	0.90 (0.86 to 0.96)	0.69 (0.65 to 0.73)
2000	2.3 (1.9 to 2.8)	1.8 (1.4 to 2.1)	20.6 (17.1 to 24.7)	15.6 (12.9 to 18.7)	1.00 (0.83 to 1.20)	0.76 (0.63 to 0.91)
2005	2.3 (1.7 to 3.1)	1.7 (1.3 to 2.3)	21.4 (15.9 to 28.1)	16.2 (12.0 to 21.2)	1.05 (0.78 to 1.39)	0.80 (0.59 to 1.05)
2010	2.3 (1.6 to 3.2)	1.7 (1.2 to 2.4)	20.9 (14.7 to 28.6)	15.7 (11.1 to 21.5)	1.13 (0.80 to 1.55)	0.85 (0.60 to 1.16)
2015	2.2 (1.5 to 3.1)	1.6 (1.1 to 2.3)	19.3 (13.3 to 26.6)	14.4 (9.9 to 19.7)	1.23 (0.85 to 1.69)	0.92 (0.63 to 1.25)
2020	2.0 (1.4 to 2.8)	1.5 (1.0 to 2.0)	17.8 (12.7 to 24.5)	13.1 (9.3 to 17.9)	1.31 (0.93 to 1.80)	0.96 (0.68 to 1.32)
2021	2.0 (1.4 to 2.8)	1.5 (1.0 to 2.0)	17.8 (12.6 to 24.7)	13.1 (9.2 to 18.1)	1.32 (0.93 to 1.84)	0.97 (0.69 to 1.34)

Uzbekistan UZB Central Asia Stillbirth Rate / Neonatal Mortality Rate (SBR/NMR) − 20 weeks
lambda = 0.2 ; zeta = 0.99 ; scale = 5 ; amp = 0.028

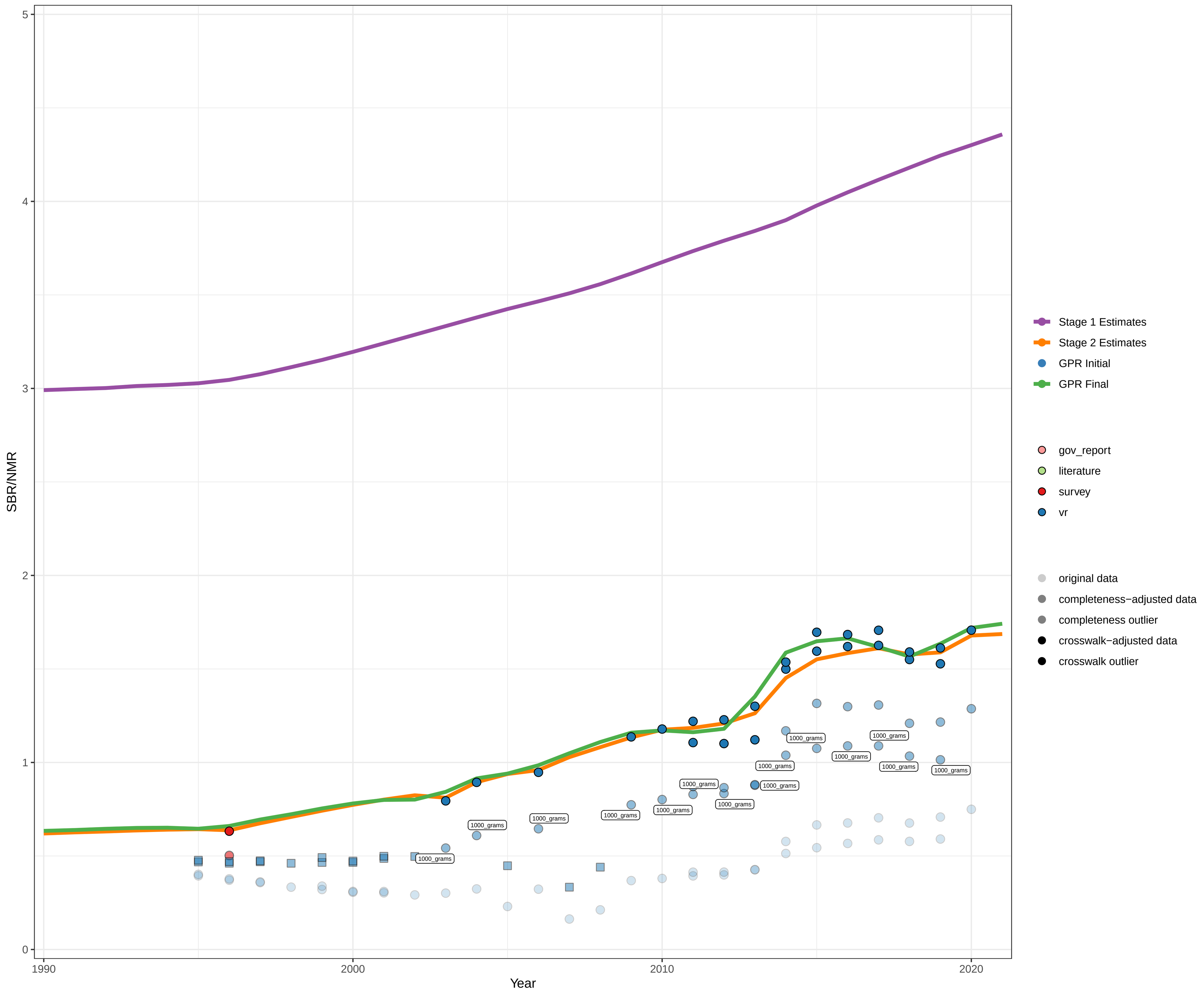

Uzbekistan UZB Central Asia Stillbirth Rate / Neonatal Mortality Rate (SBR/NMR) − 28 weeks
lambda = 0.2 ; zeta = 0.99 ; scale = 5 ; amp = 0.028

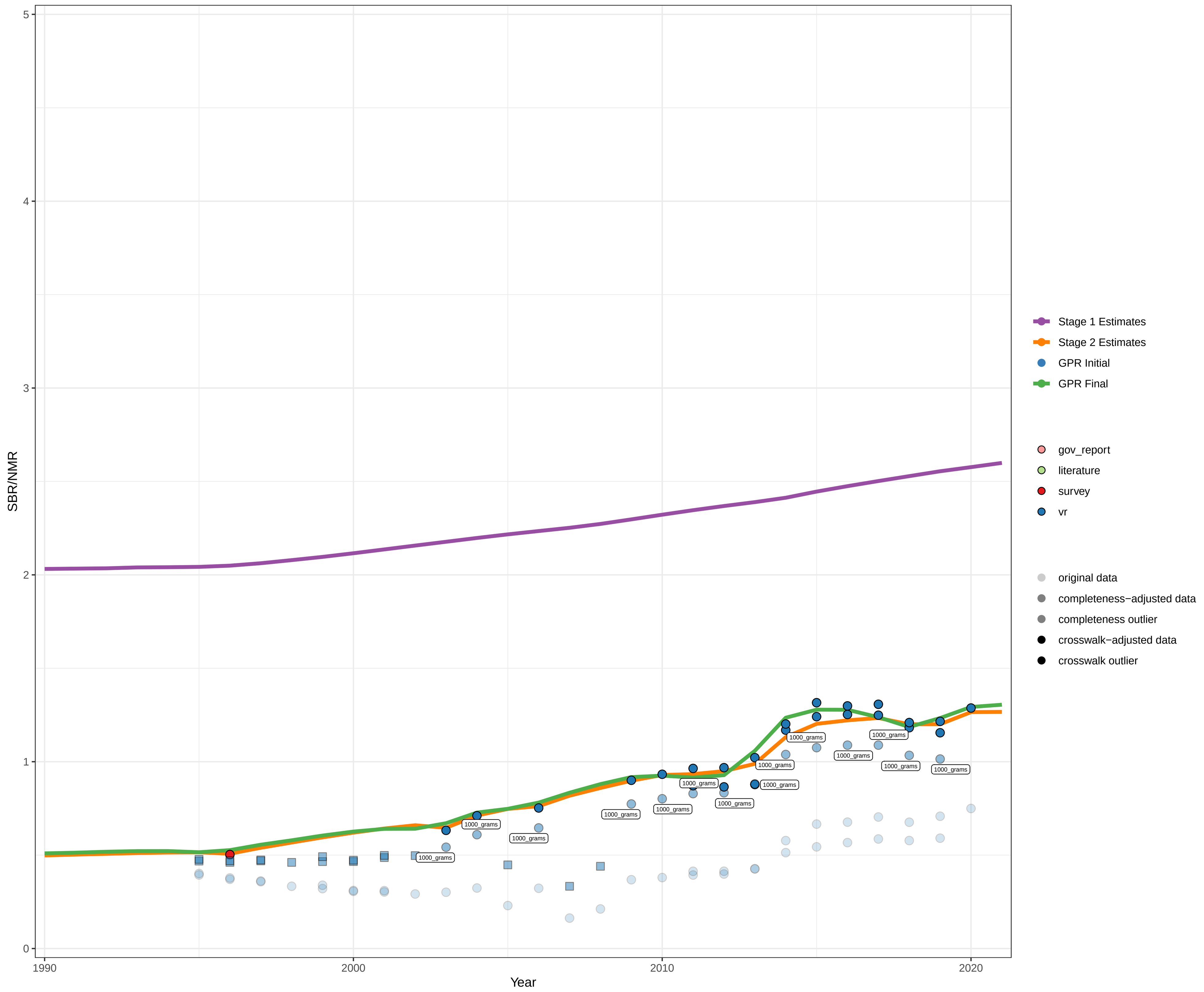

Definition	Years	Outlier	Source.Type	Title
1000_grams	2003, 2004, 2006, 2009−2019	0	VR	European Health for All Database
28_weeks	1996	0	Survey	Uzbekistan Demographic and Health Survey 1996
28_weeks	2011−2020	0	VR	United Nations Demographic Yearbook
1000_grams	1995−2002, 2005, 2007, 2008	1	VR	European Health for All Database
28_weeks	1995−1997, 1999−2001	1	VR	United Nations Demographic Yearbook

Uzbekistan UZB Central Asia Stillbirth Rate (SBR) − 20 weeks
lambda = 0.2 ; zeta = 0.99 ; scale = 5 ; amp = 0.028

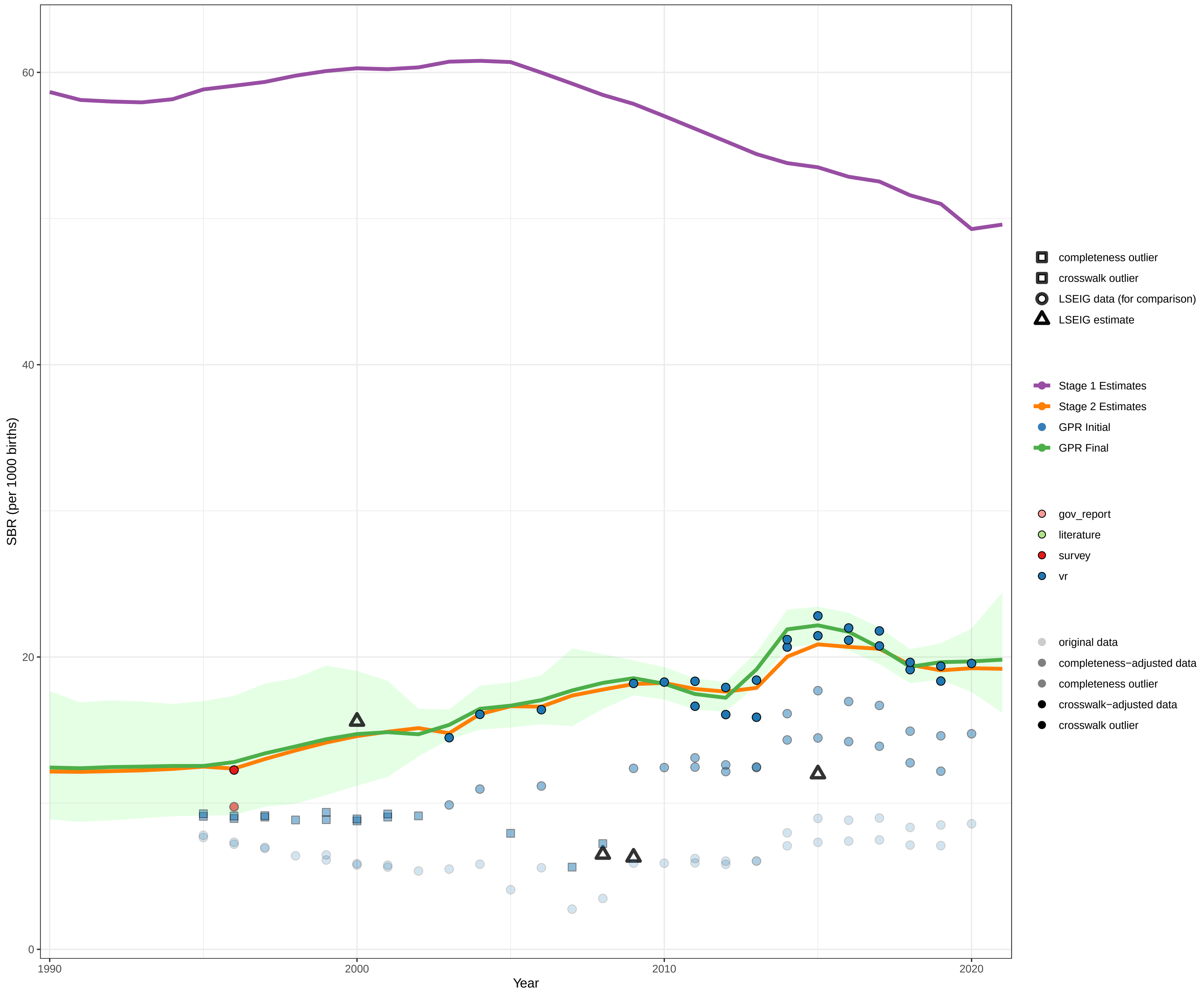

Uzbekistan UZB Central Asia Stillbirth Rate (SBR) − 28 weeks
lambda = 0.2 ; zeta = 0.99 ; scale = 5 ; amp = 0.028

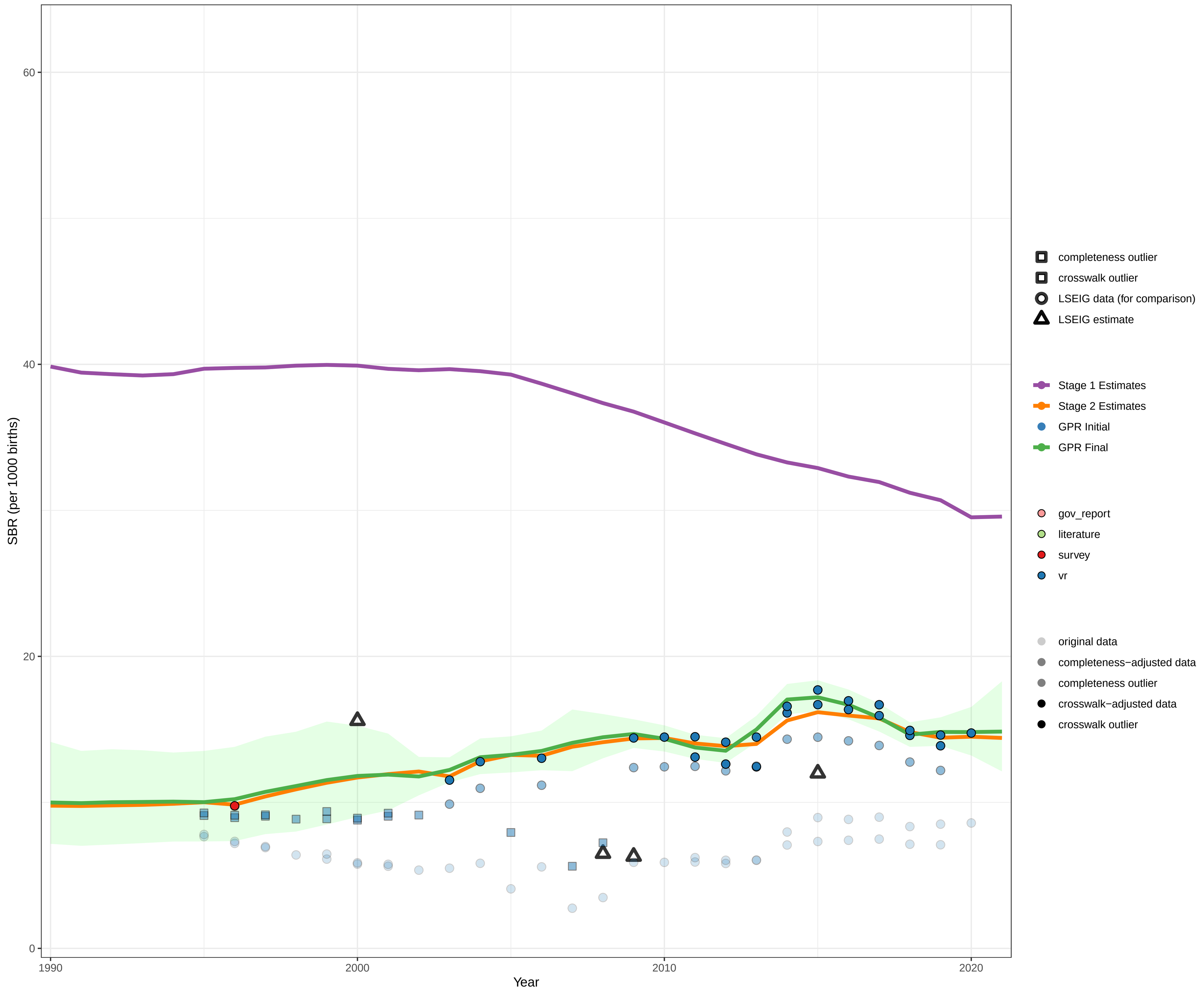

Year	Total Stillbirths (in thousands)	Total Stillbirths (in thousands)	SBR (per 1000 births)	SBR (per 1000 births)	SBR/NMR	SBR/NMR
	20 weeks	28 weeks	20 weeks	28 weeks	20 weeks	28 weeks
1990	8.9 (6.3 to 12.7)	7.1 (5.1 to 10.1)	12.4 (8.9 to 17.7)	10.0 (7.2 to 14.1)	0.63 (0.45 to 0.90)	0.51 (0.37 to 0.72)
1995	8.2 (5.9 to 11.1)	6.5 (4.7 to 8.8)	12.5 (9.1 to 17.0)	10.0 (7.3 to 13.5)	0.65 (0.47 to 0.87)	0.52 (0.38 to 0.70)
2000	8.2 (6.2 to 10.7)	6.6 (5.0 to 8.5)	14.7 (11.2 to 19.0)	11.8 (9.0 to 15.3)	0.78 (0.59 to 1.01)	0.63 (0.48 to 0.81)
2005	9.3 (8.4 to 10.2)	7.4 (6.7 to 8.1)	16.7 (15.2 to 18.3)	13.3 (12.1 to 14.5)	0.94 (0.86 to 1.03)	0.75 (0.68 to 0.82)
2010	11.7 (11.0 to 12.5)	9.2 (8.7 to 9.8)	18.2 (17.1 to 19.3)	14.4 (13.5 to 15.3)	1.17 (1.10 to 1.24)	0.93 (0.87 to 0.98)
2015	16.5 (15.6 to 17.5)	12.8 (12.0 to 13.6)	22.2 (20.9 to 23.4)	17.2 (16.2 to 18.4)	1.65 (1.56 to 1.74)	1.28 (1.21 to 1.37)
2020	16.0 (14.3 to 17.9)	12.0 (10.7 to 13.4)	19.7 (17.6 to 22.0)	14.8 (13.2 to 16.5)	1.72 (1.54 to 1.92)	1.29 (1.15 to 1.44)
2021	16.0 (13.0 to 19.9)	12.0 (9.7 to 14.8)	19.8 (16.2 to 24.4)	14.8 (12.1 to 18.3)	1.74 (1.42 to 2.15)	1.30 (1.07 to 1.61)

Central Europe R8 Central Europe Stillbirth Rate / Neonatal Mortality Rate (SBR/NMR) − 20 weeks
lambda = 1 ; zeta = 0.6 ; scale = 15 ; amp = 0.028

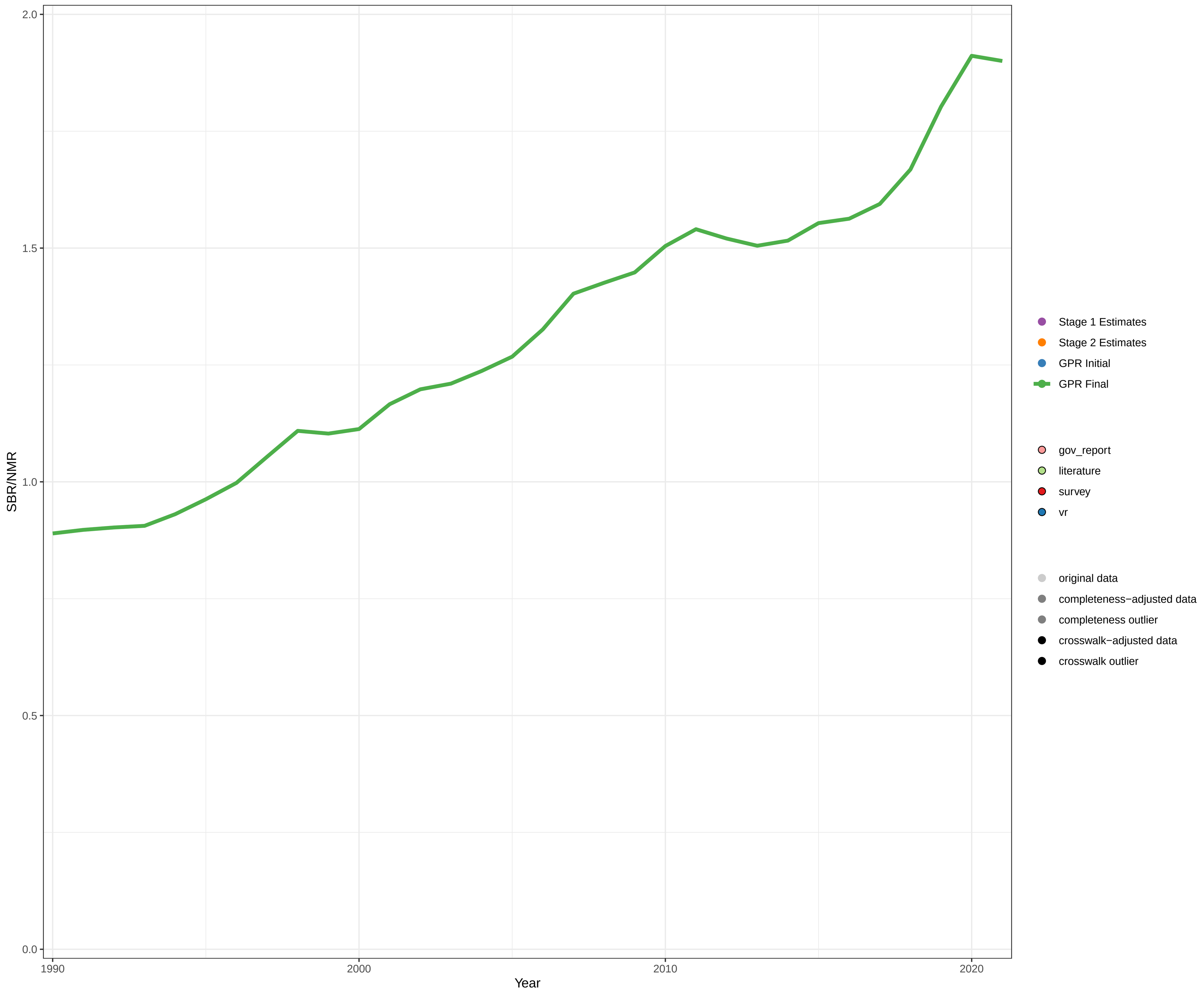

Central Europe R8 Central Europe Stillbirth Rate / Neonatal Mortality Rate (SBR/NMR) − 28 weeks
lambda = 1 ; zeta = 0.6 ; scale = 15 ; amp = 0.028

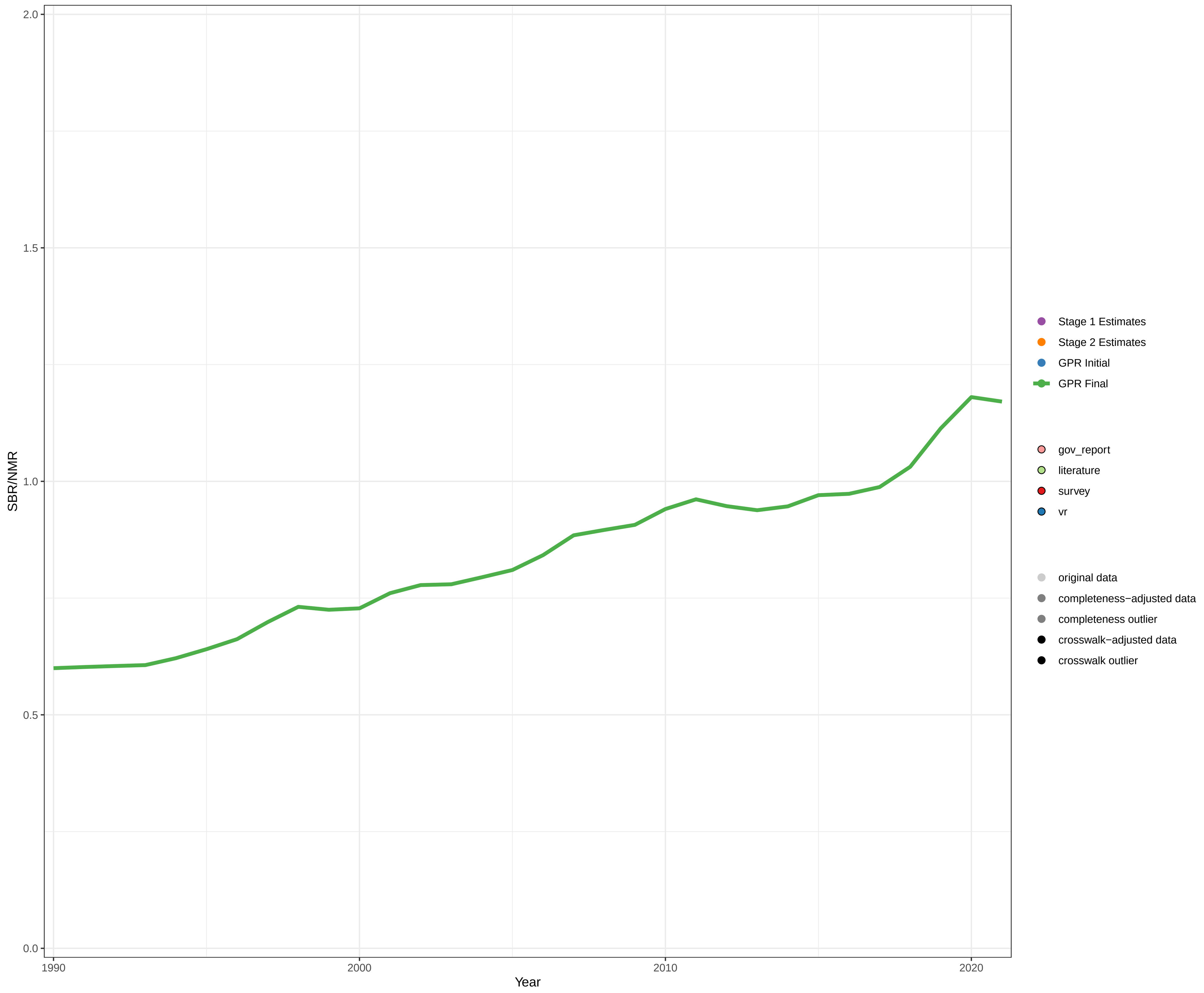

Definition	Years	Outlier	Source.Type	Title

Central Europe R8 Central Europe Stillbirth Rate (SBR) − 20 weeks
lambda = 1 ; zeta = 0.6 ; scale = 15 ; amp = 0.028

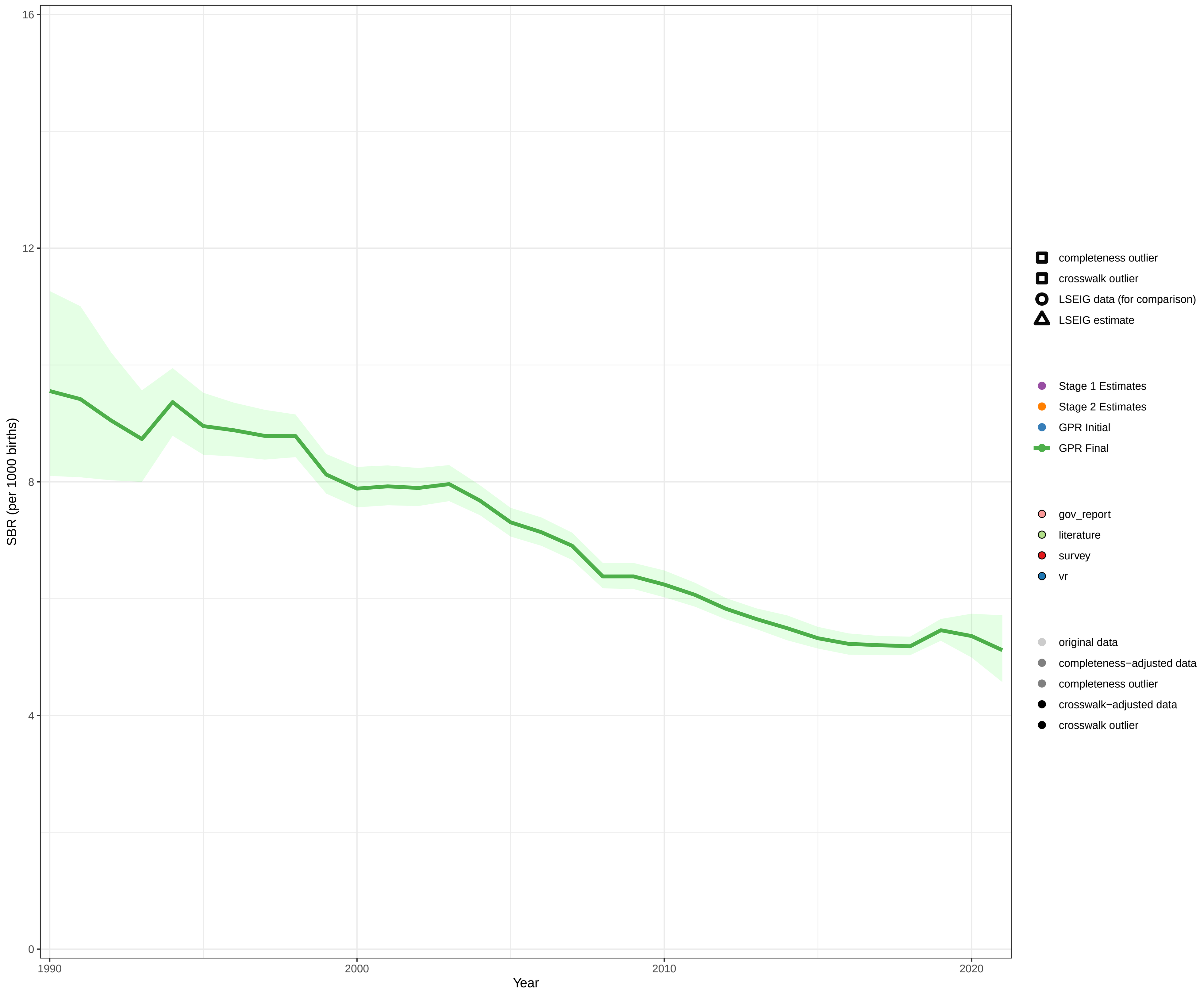

Central Europe R8 Central Europe Stillbirth Rate (SBR) − 28 weeks
lambda = 1 ; zeta = 0.6 ; scale = 15 ; amp = 0.028

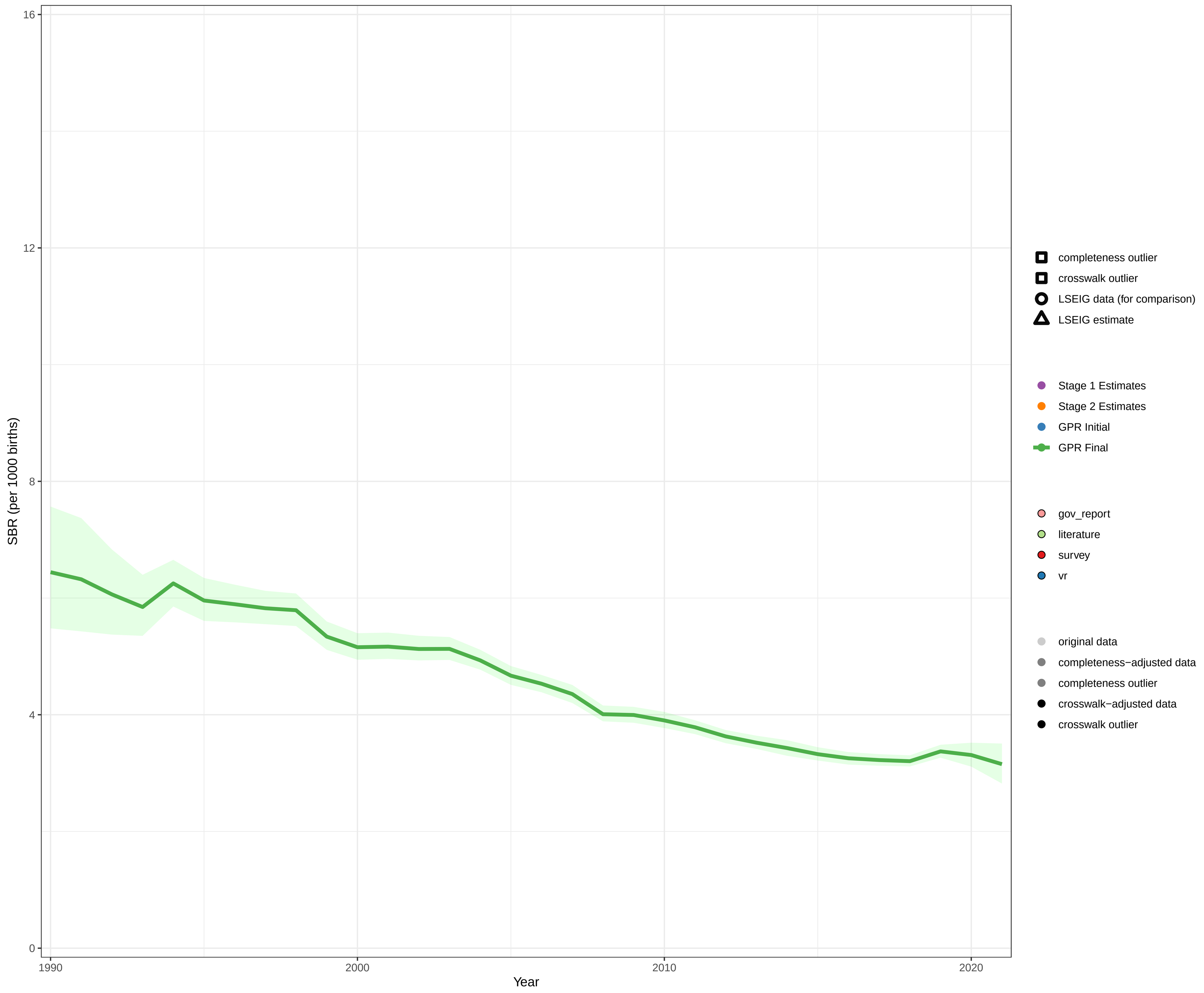

Year	Total Stillbirths (in thousands)	Total Stillbirths (in thousands)	SBR (per 1000 births)	SBR (per 1000 births)	SBR/NMR	SBR/NMR
	20 weeks	28 weeks	20 weeks	28 weeks	20 weeks	28 weeks
1990	16.5 (14.0 to 19.5)	11.1 (9.4 to 13.1)	9.6 (8.1 to 11.3)	6.4 (5.5 to 7.6)	0.89 (0.75 to 1.05)	0.60 (0.51 to 0.70)
1995	12.5 (11.8 to 13.3)	8.3 (7.8 to 8.9)	9.0 (8.5 to 9.5)	6.0 (5.6 to 6.3)	0.96 (0.91 to 1.02)	0.64 (0.60 to 0.68)
2000	9.6 (9.2 to 10.1)	6.3 (6.0 to 6.6)	7.9 (7.6 to 8.3)	5.2 (4.9 to 5.4)	1.11 (1.07 to 1.17)	0.73 (0.70 to 0.76)
2005	8.7 (8.4 to 9.0)	5.6 (5.4 to 5.8)	7.3 (7.1 to 7.6)	4.7 (4.5 to 4.8)	1.27 (1.23 to 1.31)	0.81 (0.78 to 0.84)
2010	7.6 (7.3 to 7.9)	4.7 (4.6 to 4.9)	6.2 (6.0 to 6.5)	3.9 (3.8 to 4.0)	1.50 (1.45 to 1.56)	0.94 (0.91 to 0.98)
2015	6.1 (5.9 to 6.3)	3.8 (3.7 to 3.9)	5.3 (5.1 to 5.5)	3.3 (3.2 to 3.4)	1.55 (1.50 to 1.61)	0.97 (0.94 to 1.01)
2020	5.8 (5.4 to 6.2)	3.5 (3.3 to 3.8)	5.4 (5.0 to 5.7)	3.3 (3.1 to 3.5)	1.91 (1.78 to 2.05)	1.18 (1.11 to 1.26)
2021	5.3 (4.8 to 6.0)	3.3 (2.9 to 3.7)	5.1 (4.6 to 5.7)	3.2 (2.8 to 3.5)	1.90 (1.70 to 2.12)	1.17 (1.05 to 1.30)

Albania ALB Central Europe Stillbirth Rate / Neonatal Mortality Rate (SBR/NMR) − 20 weeks
lambda = 0.6 ; zeta = 0.8 ; scale = 15 ; amp = 0.028

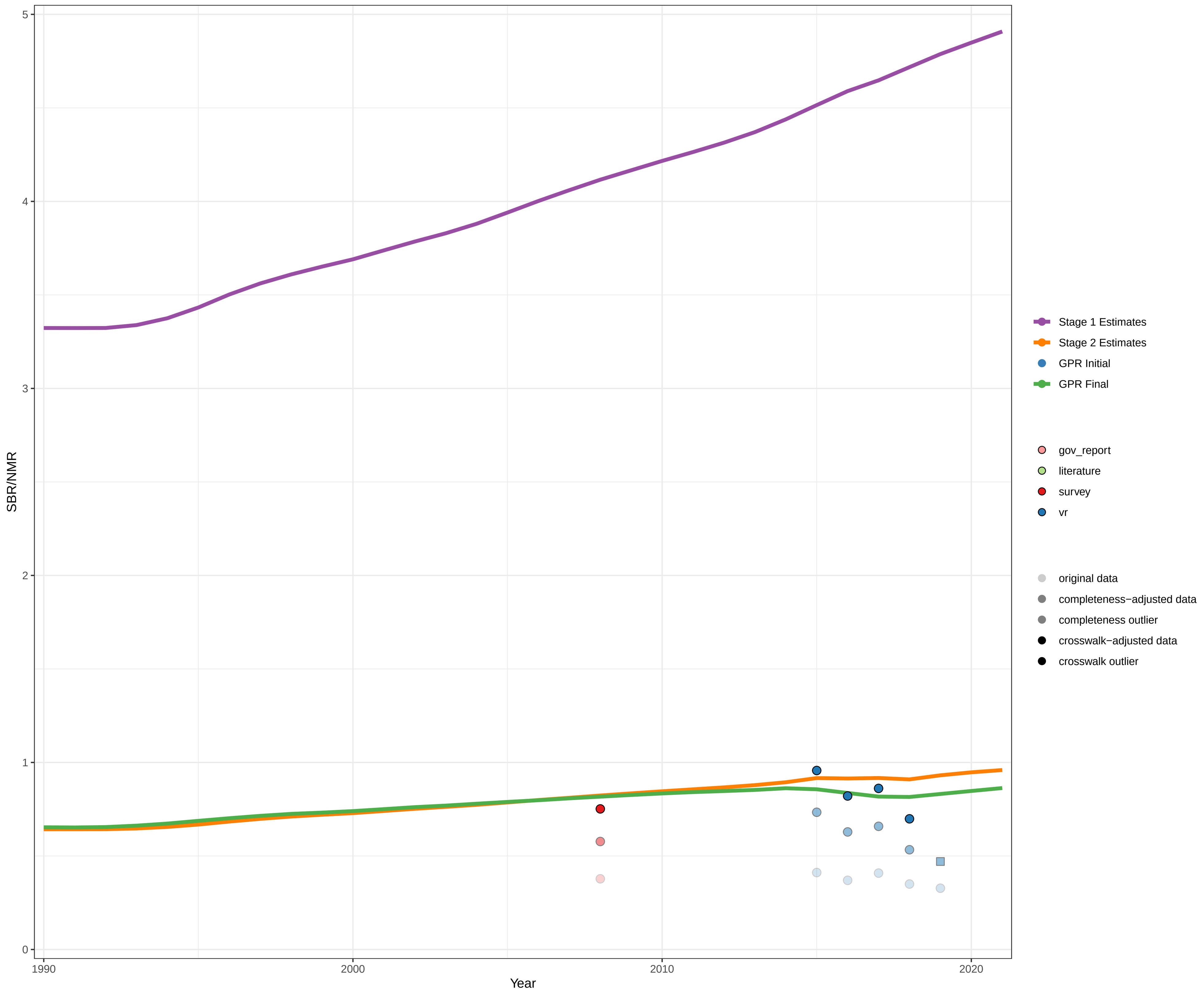

Albania ALB Central Europe Stillbirth Rate / Neonatal Mortality Rate (SBR/NMR) − 28 weeks
lambda = 0.6 ; zeta = 0.8 ; scale = 15 ; amp = 0.028

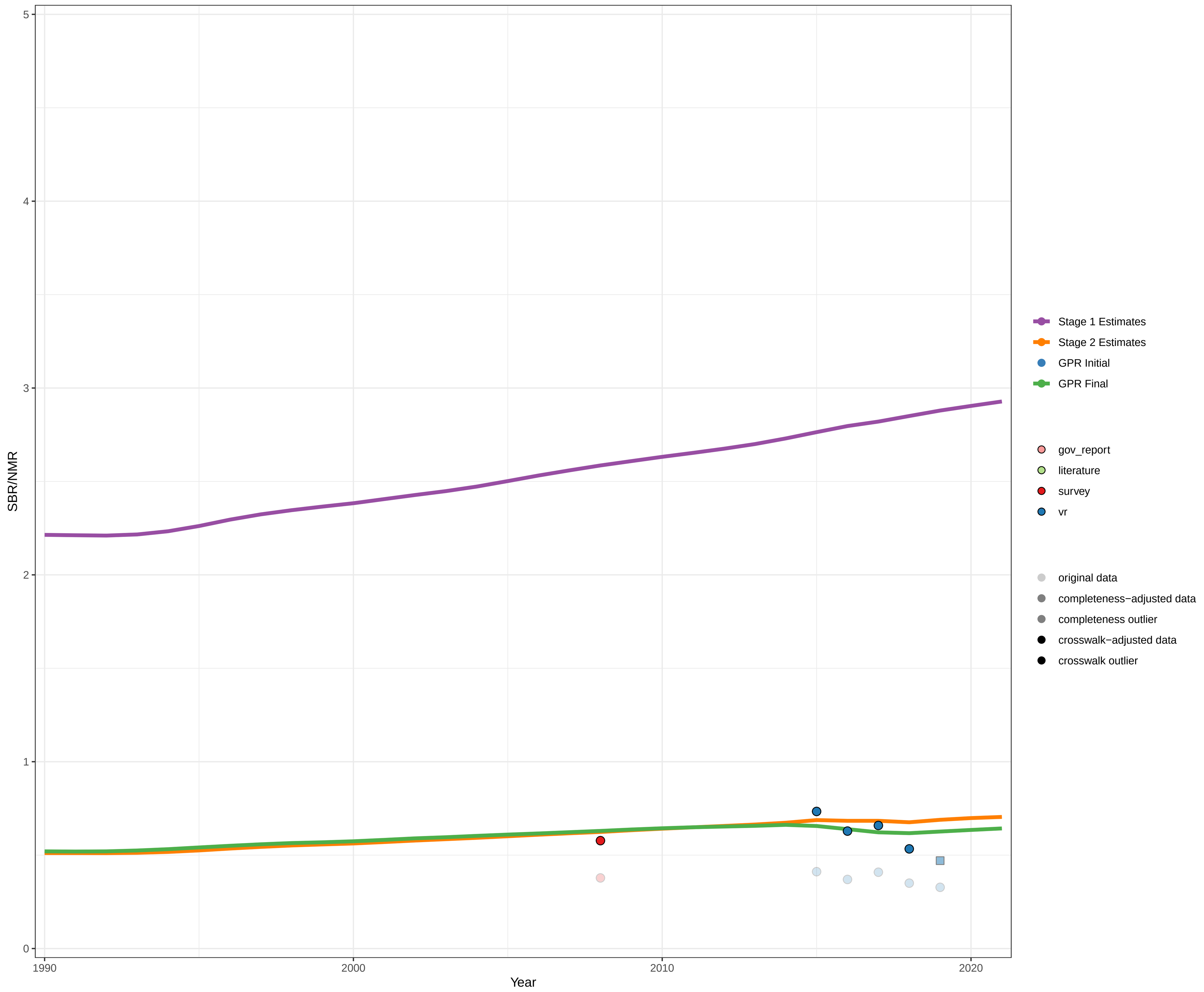

Definition	Years	Outlier	Source.Type	Title
28_weeks	2008	0	Survey	Albania Demographic and Health Survey 2008−2009
28_weeks	2015−2018	0	VR	United Nations Demographic Yearbook
28_weeks	2019	1	VR	United Nations Demographic Yearbook

Albania ALB Central Europe Stillbirth Rate (SBR) − 20 weeks
lambda = 0.6 ; zeta = 0.8 ; scale = 15 ; amp = 0.028

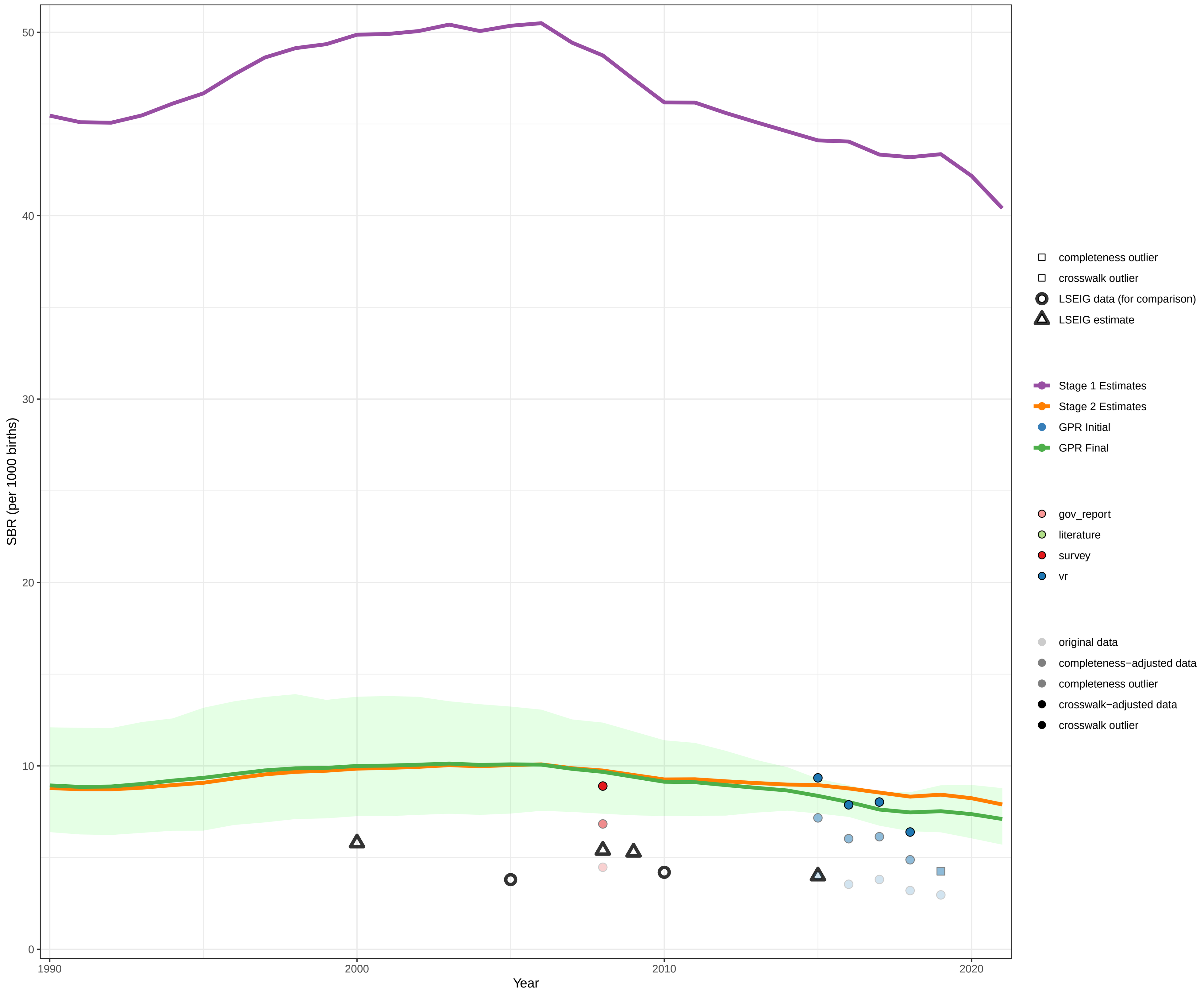

Albania ALB Central Europe Stillbirth Rate (SBR) − 28 weeks
lambda = 0.6 ; zeta = 0.8 ; scale = 15 ; amp = 0.028

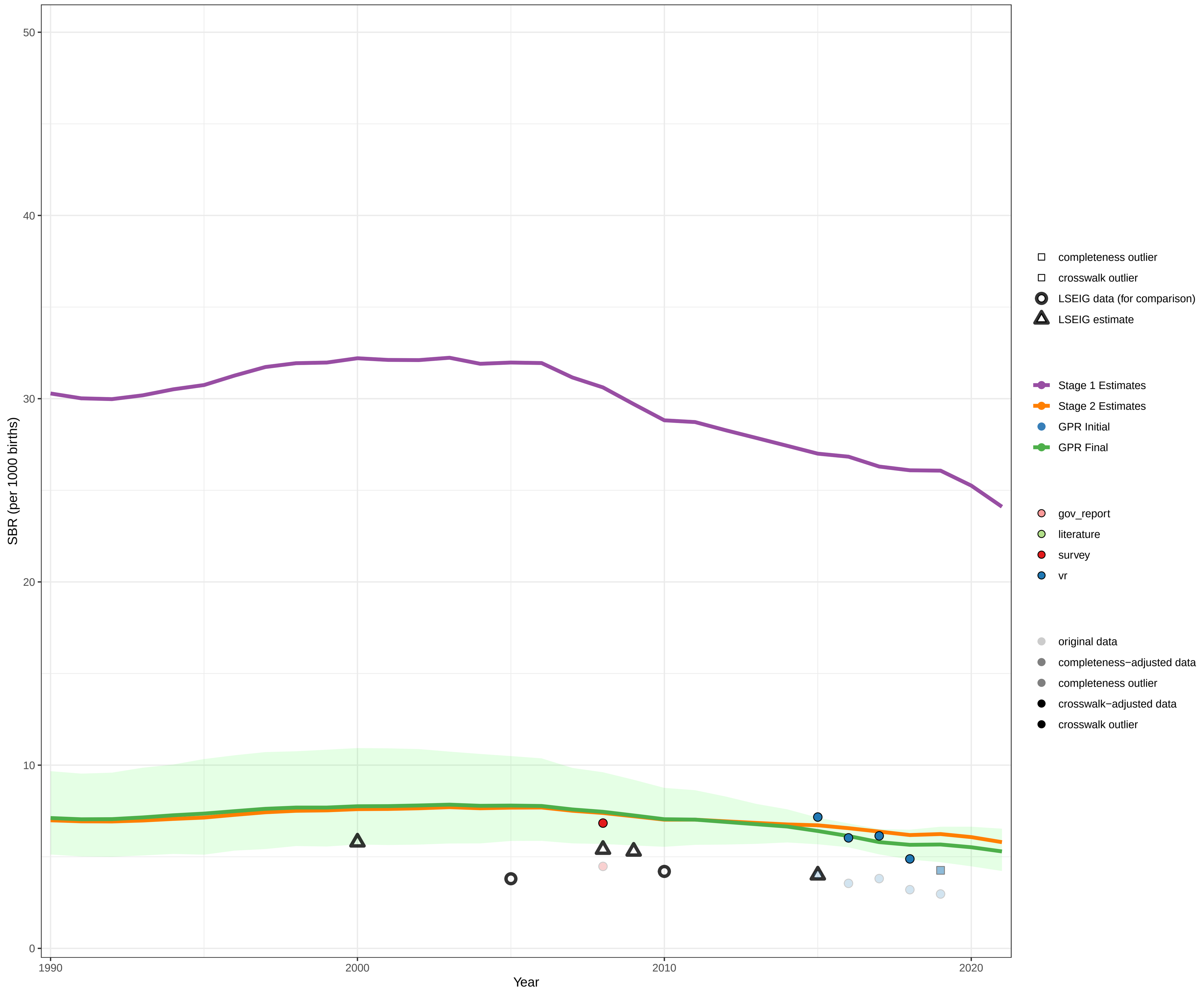

Year	Total Stillbirths (in thousands)	Total Stillbirths (in thousands)	SBR (per 1000 births)	SBR (per 1000 births)	SBR/NMR	SBR/NMR
	20 weeks	28 weeks	20 weeks	28 weeks	20 weeks	28 weeks
1990	0.7 (0.5 to 1.0)	0.6 (0.4 to 0.8)	8.9 (6.4 to 12.1)	7.1 (5.1 to 9.7)	0.65 (0.47 to 0.88)	0.52 (0.37 to 0.71)
1995	0.6 (0.4 to 0.9)	0.5 (0.4 to 0.7)	9.4 (6.5 to 13.2)	7.4 (5.1 to 10.3)	0.69 (0.48 to 0.97)	0.54 (0.38 to 0.76)
2000	0.5 (0.4 to 0.7)	0.4 (0.3 to 0.6)	10.0 (7.3 to 13.8)	7.8 (5.7 to 10.9)	0.74 (0.54 to 1.02)	0.57 (0.42 to 0.81)
2005	0.4 (0.3 to 0.5)	0.3 (0.2 to 0.4)	10.1 (7.4 to 13.2)	7.8 (5.9 to 10.5)	0.79 (0.58 to 1.04)	0.61 (0.46 to 0.82)
2010	0.3 (0.3 to 0.4)	0.2 (0.2 to 0.3)	9.1 (7.3 to 11.4)	7.0 (5.5 to 8.8)	0.83 (0.66 to 1.04)	0.64 (0.51 to 0.80)
2015	0.3 (0.2 to 0.3)	0.2 (0.2 to 0.2)	8.4 (7.4 to 9.3)	6.4 (5.7 to 7.1)	0.86 (0.76 to 0.95)	0.66 (0.58 to 0.73)
2020	0.2 (0.2 to 0.3)	0.2 (0.1 to 0.2)	7.4 (6.1 to 9.0)	5.5 (4.5 to 6.6)	0.85 (0.70 to 1.03)	0.63 (0.51 to 0.76)
2021	0.2 (0.2 to 0.2)	0.1 (0.1 to 0.2)	7.1 (5.7 to 8.8)	5.3 (4.2 to 6.5)	0.86 (0.69 to 1.07)	0.64 (0.51 to 0.79)

Bosnia and Herzegovina   BIH   Central Europe   Stillbirth Rate / Neonatal Mortality Rate (SBR/NMR) − 20 weeks
lambda = 0.4 ; zeta = 0.9 ; scale = 10 ; amp = 0.028

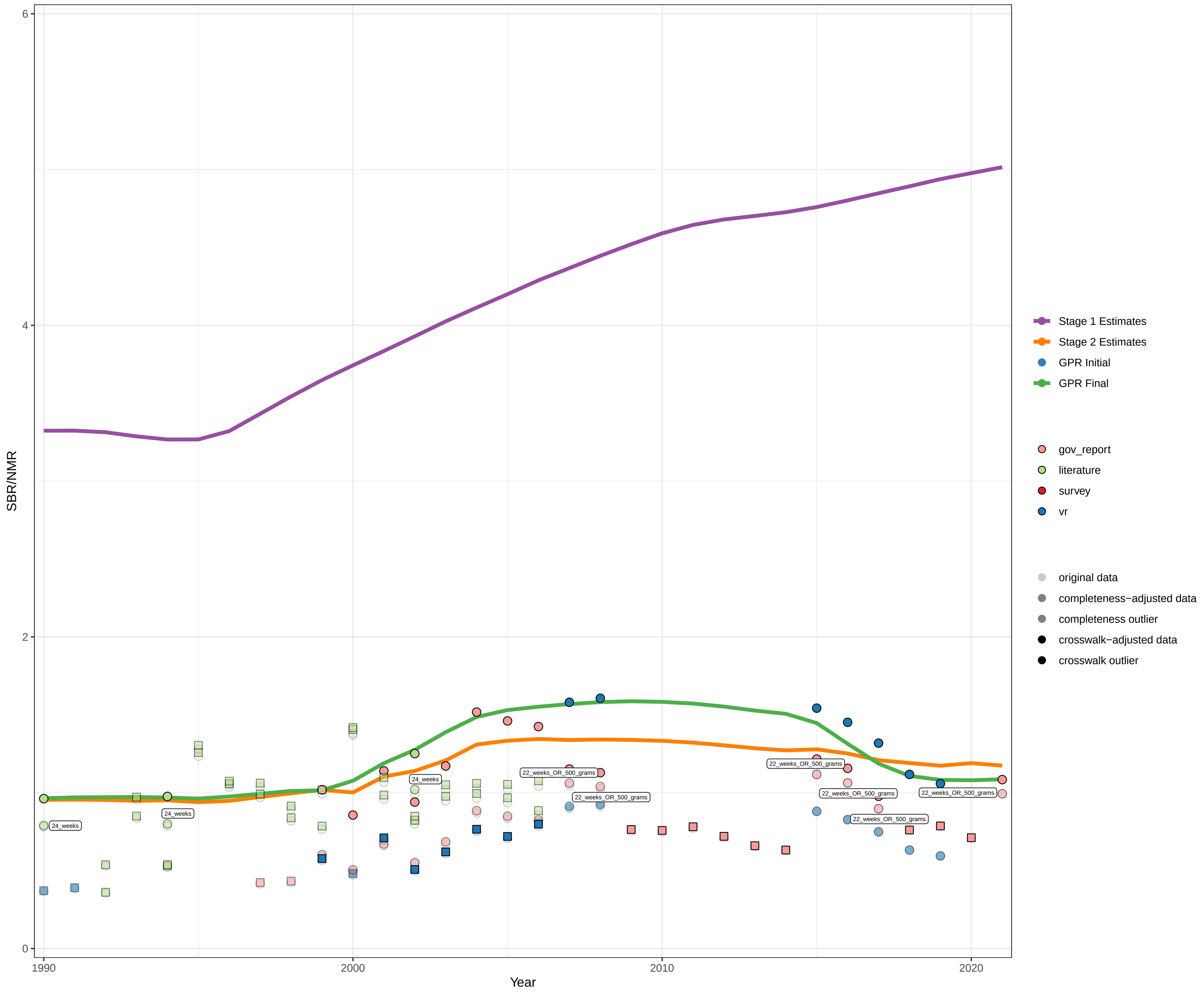

Bosnia and Herzegovina   BIH   Central Europe   Stillbirth Rate / Neonatal Mortality Rate (SBR/NMR) − 28 weeks
lambda = 0.4 ; zeta = 0.9 ; scale = 10 ; amp = 0.028

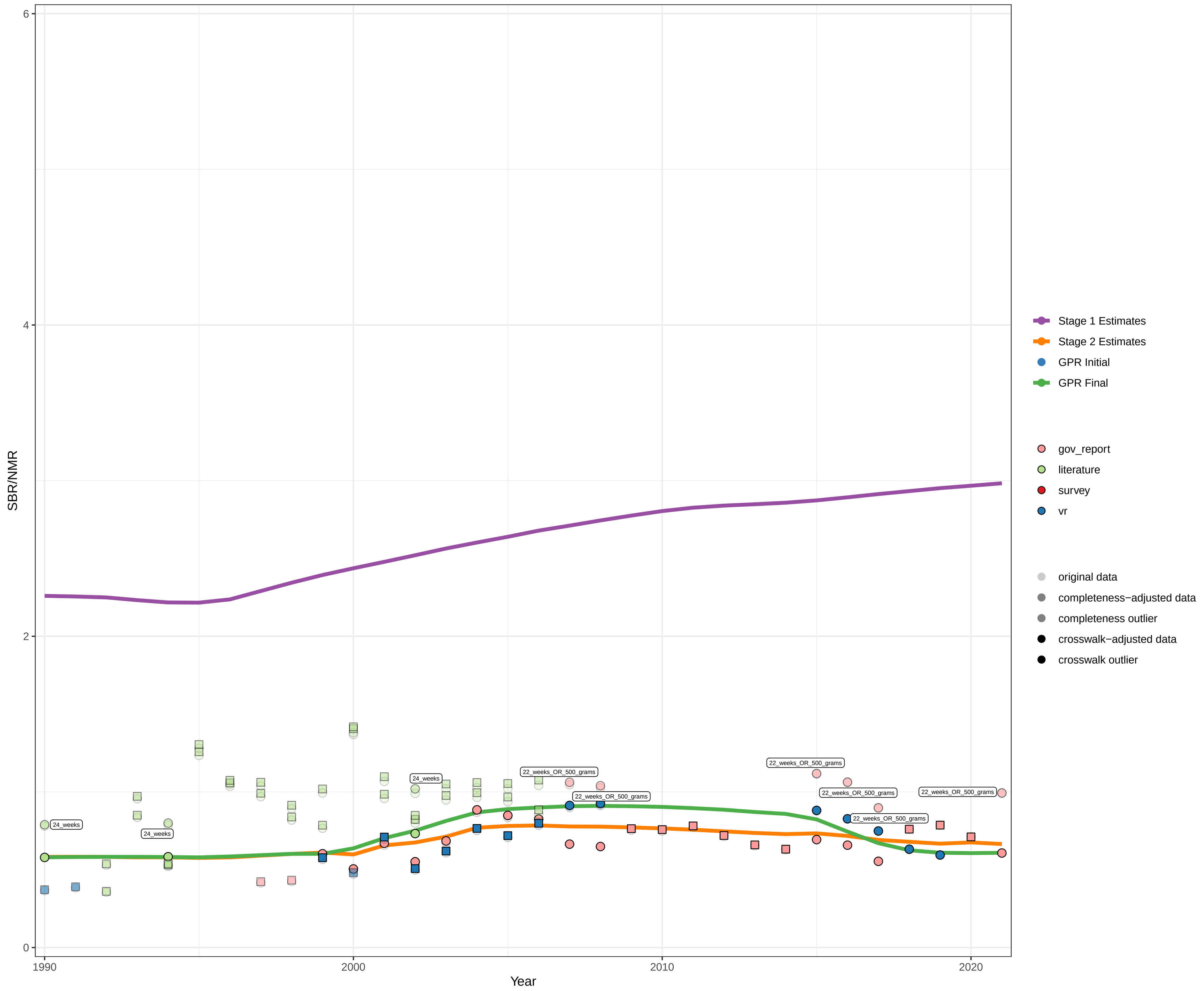

Definition	Years	Outlier	Source.Type	Title
22_weeks_OR_500_grams	2007−2016	0	Statistical Report	Bosnia and Herzegovina − Federation of Bosnia and Herzegovina Statistical Yearbook
22_weeks_OR_500_grams	2017−2021	0	Statistical Report	Federation of Bosnia and Herzegovina Statistical Yearbook
24_weeks	1990, 1994, 2002	0	Sci Lit	Perinatal and maternal outcomes in Tuzla Canton during 1992−1995 war in Bosnia and Herzegovina
28_weeks	1999−2006	0	Statistical Report	Bosnia and Herzegovina − Federation of Bosnia and Herzegovina Statistical Yearbook
28_weeks	2007, 2008, 2015−2019	0	VR	United Nations Demographic Yearbook
1000_grams	1990, 1991	1	VR	European Health for All Database
1000_grams, 500_grams	1992−2006	1	Sci Lit	Perinatal mortality during fifteen−year period in Tuzla Canton, Bosnia and Herzegovina
28_weeks	1997, 1998	1	Statistical Report	Bosnia and Herzegovina − Federation of Bosnia and Herzegovina Statistical Yearbook
28_weeks	2000	1	VR	United Nations Demographic Yearbook
28_weeks	1999, 2001−2006	2	VR	United Nations Demographic Yearbook

Bosnia and Herzegovina   BIH   Central Europe   Stillbirth Rate (SBR) − 20 weeks
lambda = 0.4 ; zeta = 0.9 ; scale = 10 ; amp = 0.028

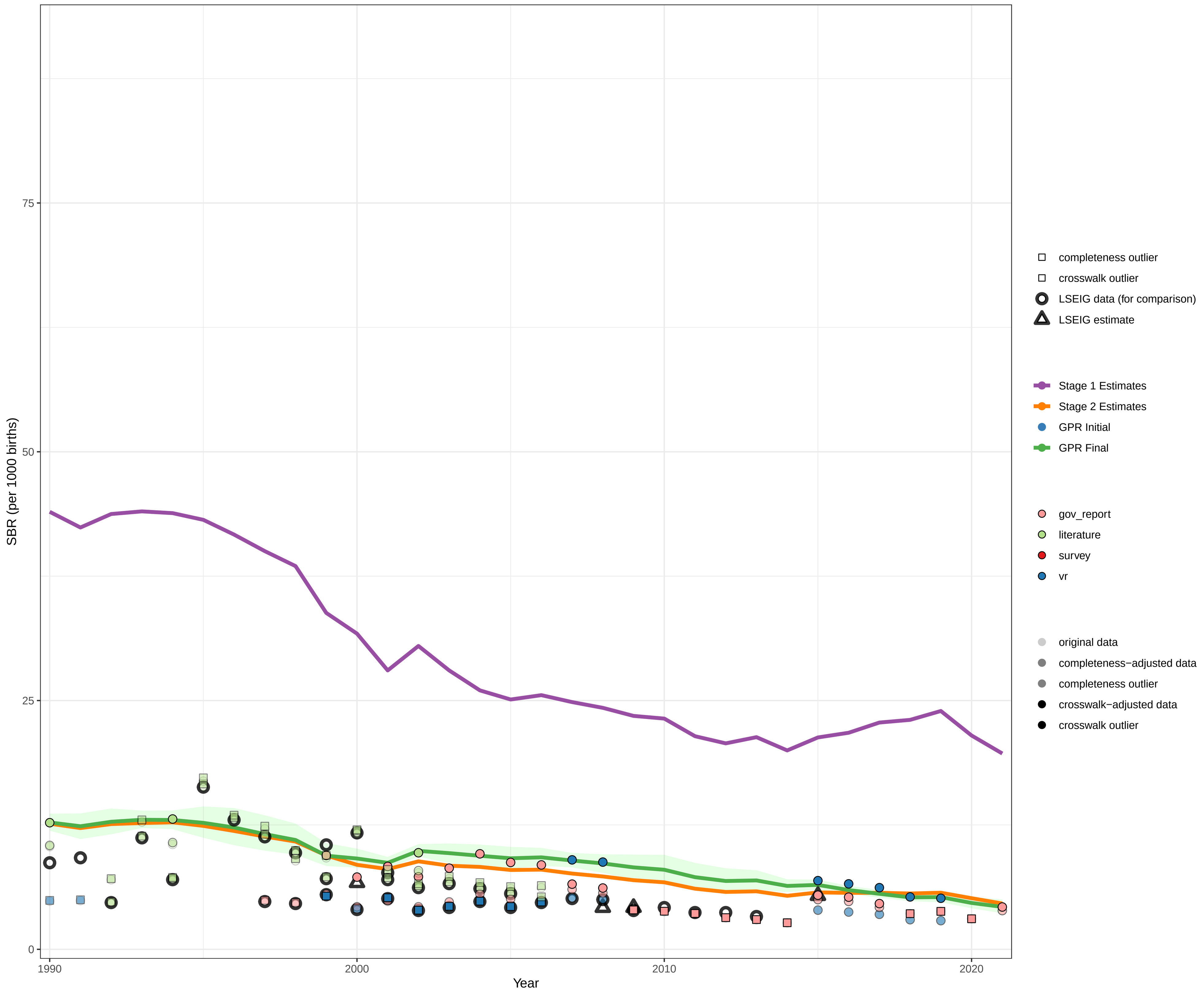

Bosnia and Herzegovina   BIH   Central Europe   Stillbirth Rate (SBR) − 28 weeks
lambda = 0.4 ; zeta = 0.9 ; scale = 10 ; amp = 0.028

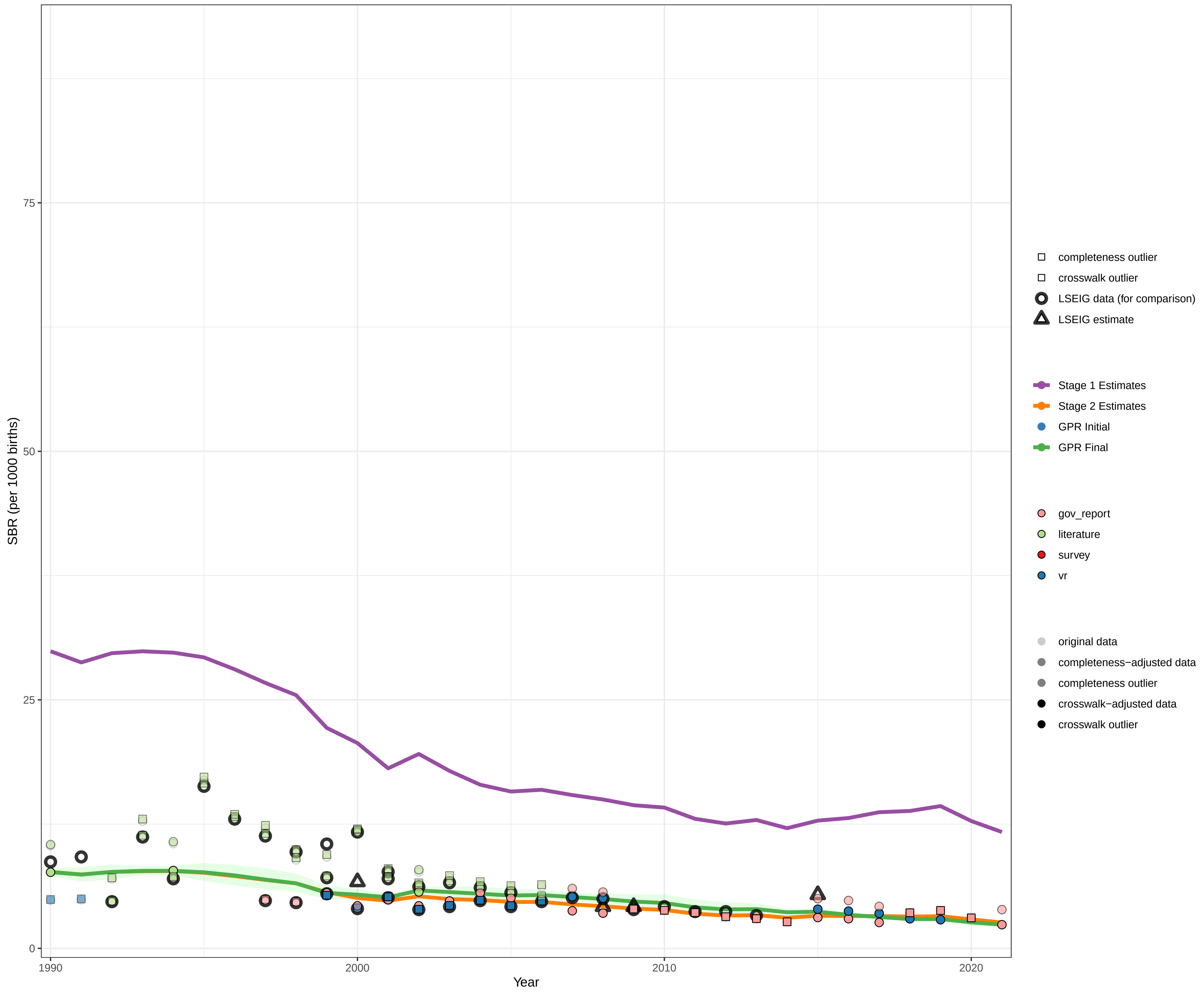

Year	Total Stillbirths (in thousands)	Total Stillbirths (in thousands)	SBR (per 1000 births)	SBR (per 1000 births)	SBR/NMR	SBR/NMR
	20 weeks	28 weeks	20 weeks	28 weeks	20 weeks	28 weeks
1990	0.9 (0.8 to 0.9)	0.5 (0.5 to 0.5)	12.8 (11.9 to 13.6)	7.7 (7.2 to 8.1)	0.96 (0.90 to 1.03)	0.58 (0.55 to 0.61)
1995	0.7 (0.6 to 0.7)	0.4 (0.4 to 0.4)	12.7 (11.2 to 14.4)	7.7 (6.8 to 8.6)	0.96 (0.85 to 1.09)	0.58 (0.51 to 0.65)
2000	0.4 (0.3 to 0.4)	0.2 (0.2 to 0.2)	9.1 (8.2 to 10.1)	5.4 (4.9 to 6.0)	1.08 (0.97 to 1.19)	0.64 (0.57 to 0.70)
2005	0.3 (0.3 to 0.4)	0.2 (0.2 to 0.2)	9.1 (8.1 to 10.3)	5.3 (4.7 to 6.0)	1.53 (1.35 to 1.72)	0.89 (0.79 to 1.00)
2010	0.3 (0.2 to 0.3)	0.2 (0.1 to 0.2)	8.0 (6.7 to 9.5)	4.6 (3.8 to 5.4)	1.58 (1.32 to 1.88)	0.90 (0.75 to 1.07)
2015	0.2 (0.2 to 0.2)	0.1 (0.1 to 0.1)	6.5 (6.0 to 7.0)	3.7 (3.4 to 4.0)	1.45 (1.34 to 1.57)	0.82 (0.76 to 0.89)
2020	0.1 (0.1 to 0.1)	0.1 (0.1 to 0.1)	4.7 (4.1 to 5.3)	2.6 (2.3 to 3.0)	1.08 (0.96 to 1.22)	0.61 (0.54 to 0.69)
2021	0.1 (0.1 to 0.1)	0.1 (0.1 to 0.1)	4.3 (3.6 to 5.0)	2.4 (2.0 to 2.8)	1.09 (0.92 to 1.28)	0.61 (0.52 to 0.71)

Bulgaria BGR Central Europe Stillbirth Rate / Neonatal Mortality Rate (SBR/NMR) − 20 weeks
lambda = 0.2 ; zeta = 0.99 ; scale = 5 ; amp = 0.028

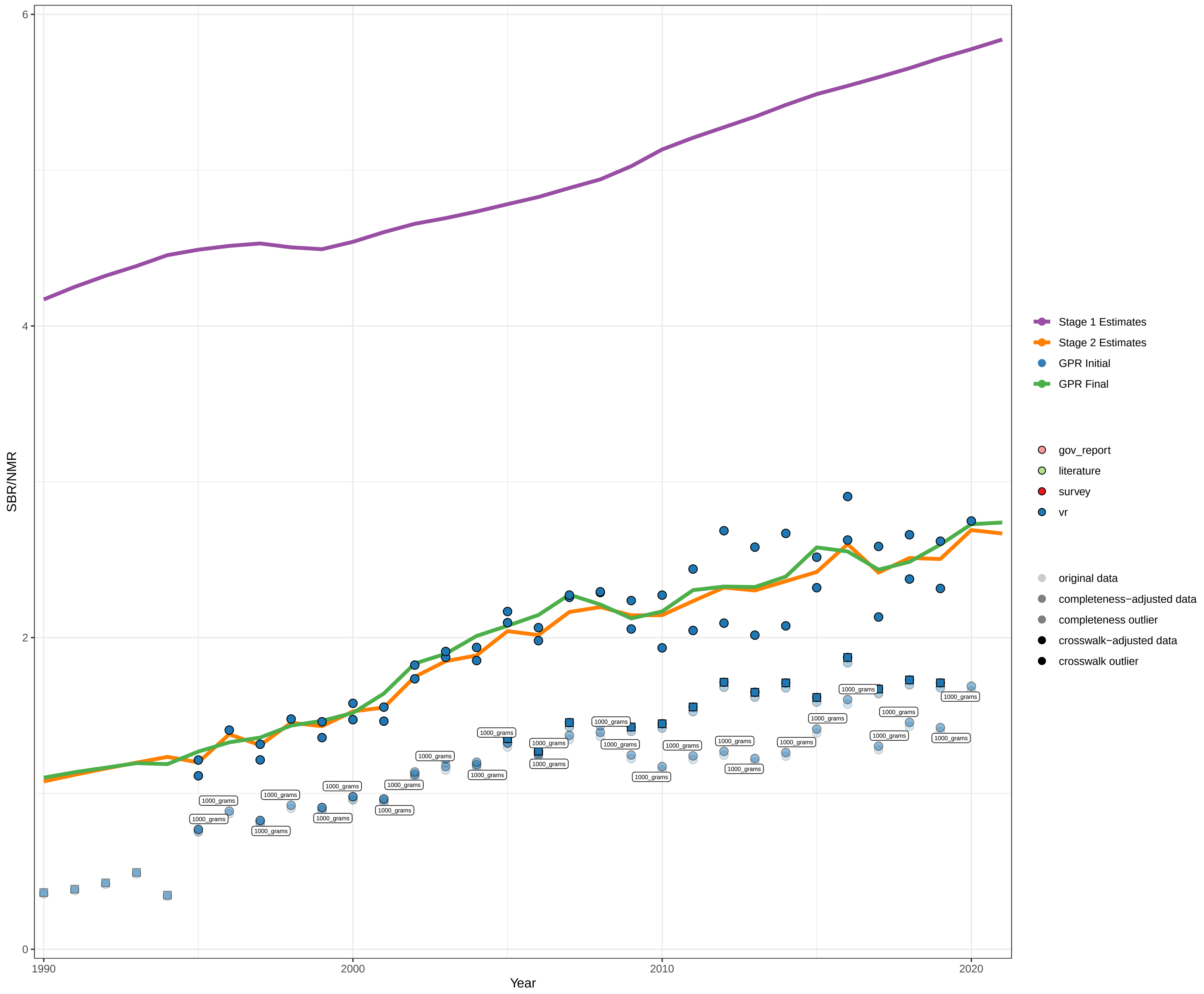

Bulgaria BGR Central Europe Stillbirth Rate / Neonatal Mortality Rate (SBR/NMR) − 28 weeks
lambda = 0.2 ; zeta = 0.99 ; scale = 5 ; amp = 0.028

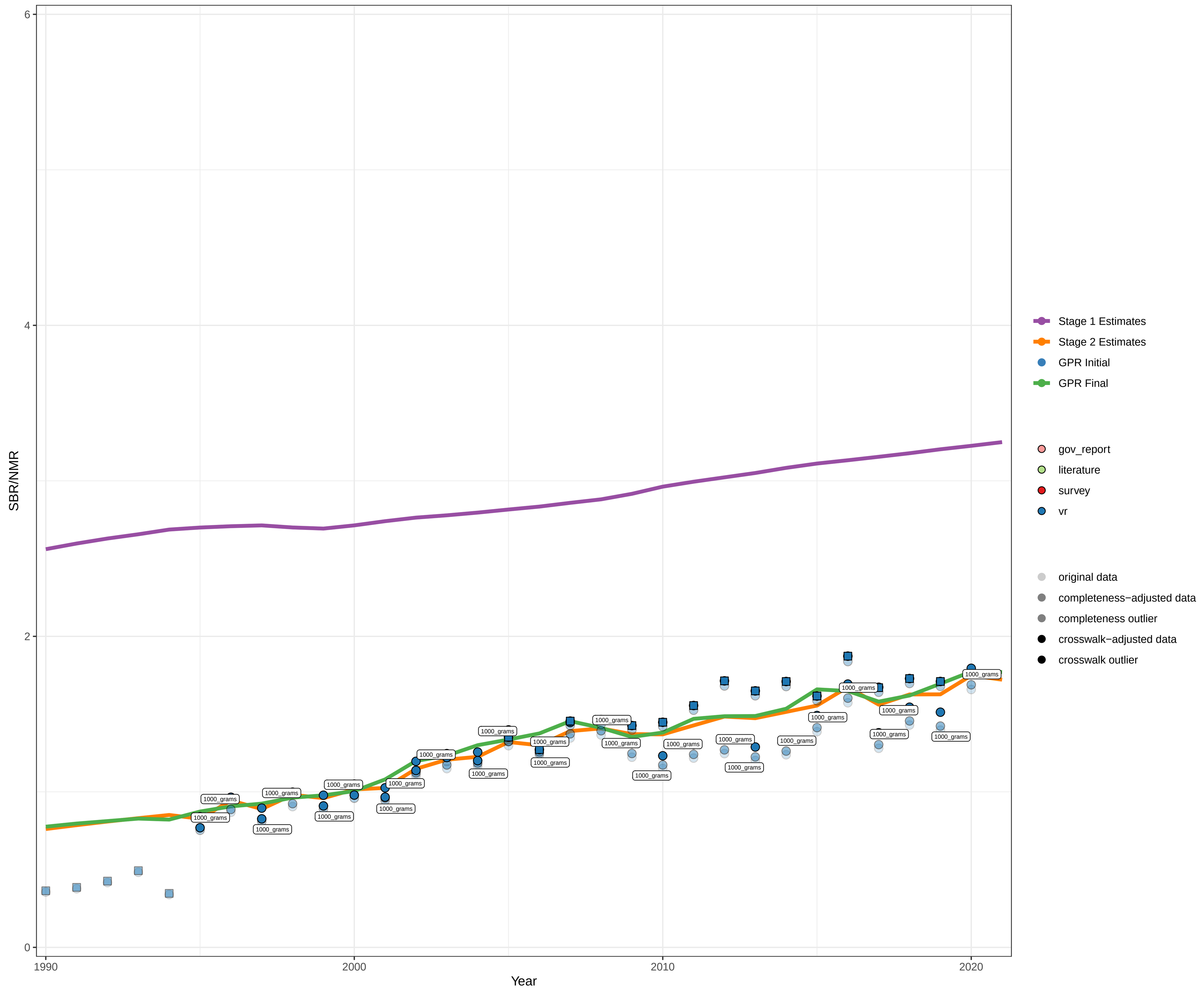

Definition	Years	Outlier	Source.Type	Title
1000_grams	1995−2020	0	VR	European Health for All Database
28_weeks	1995, 1997, 1999−2004, 2008	0	VR	United Nations Demographic Yearbook
28_weeks	2005−2019	0	VR	Bulgaria Still−births by Causes
1000_grams	1990−1994	1	VR	European Health for All Database
28_weeks	2005−2007, 2009−2019	2	VR	United Nations Demographic Yearbook

Bulgaria BGR Central Europe Stillbirth Rate (SBR) − 20 weeks
lambda = 0.2 ; zeta = 0.99 ; scale = 5 ; amp = 0.028

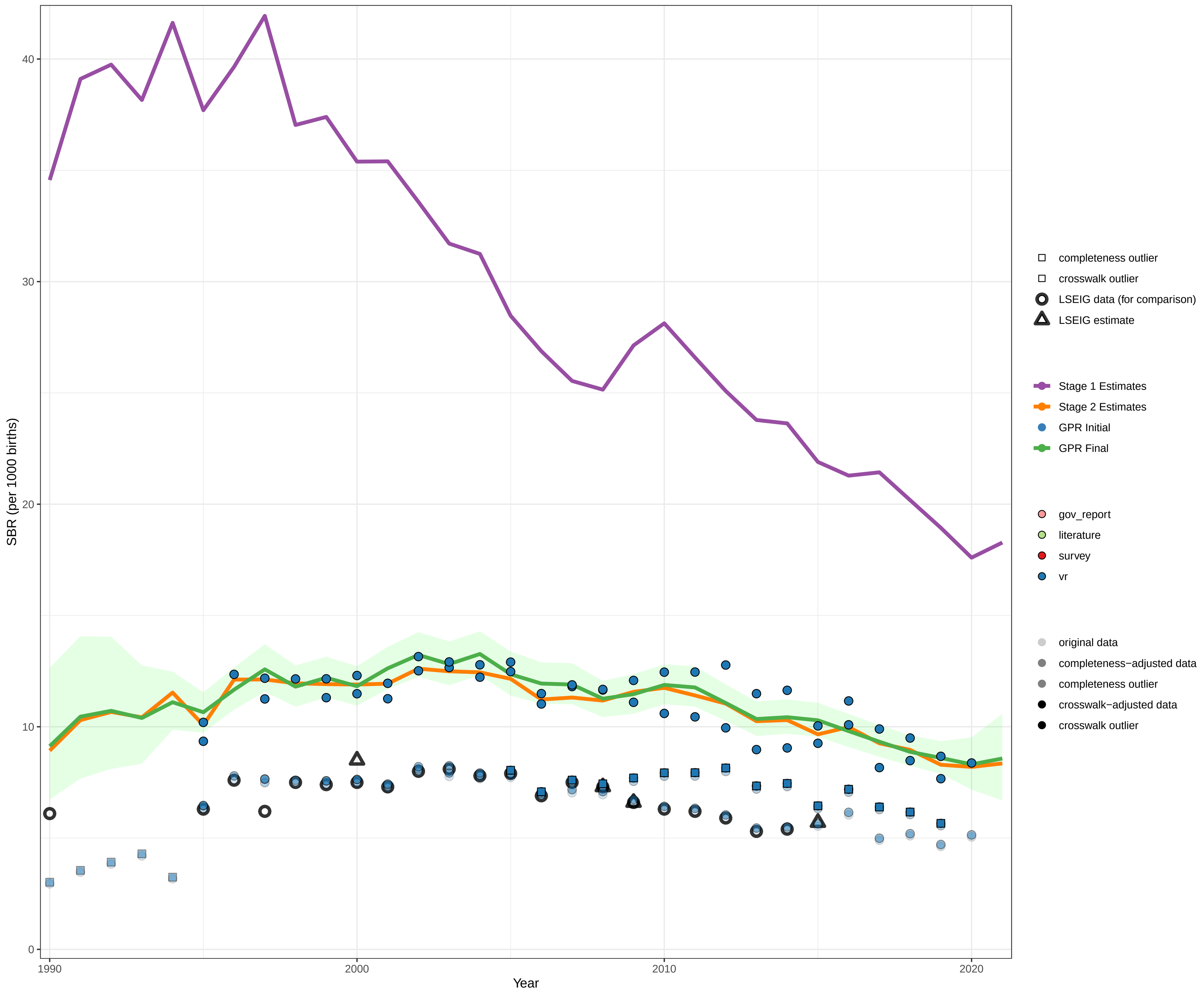

Bulgaria BGR Central Europe Stillbirth Rate (SBR) − 28 weeks
lambda = 0.2 ; zeta = 0.99 ; scale = 5 ; amp = 0.028

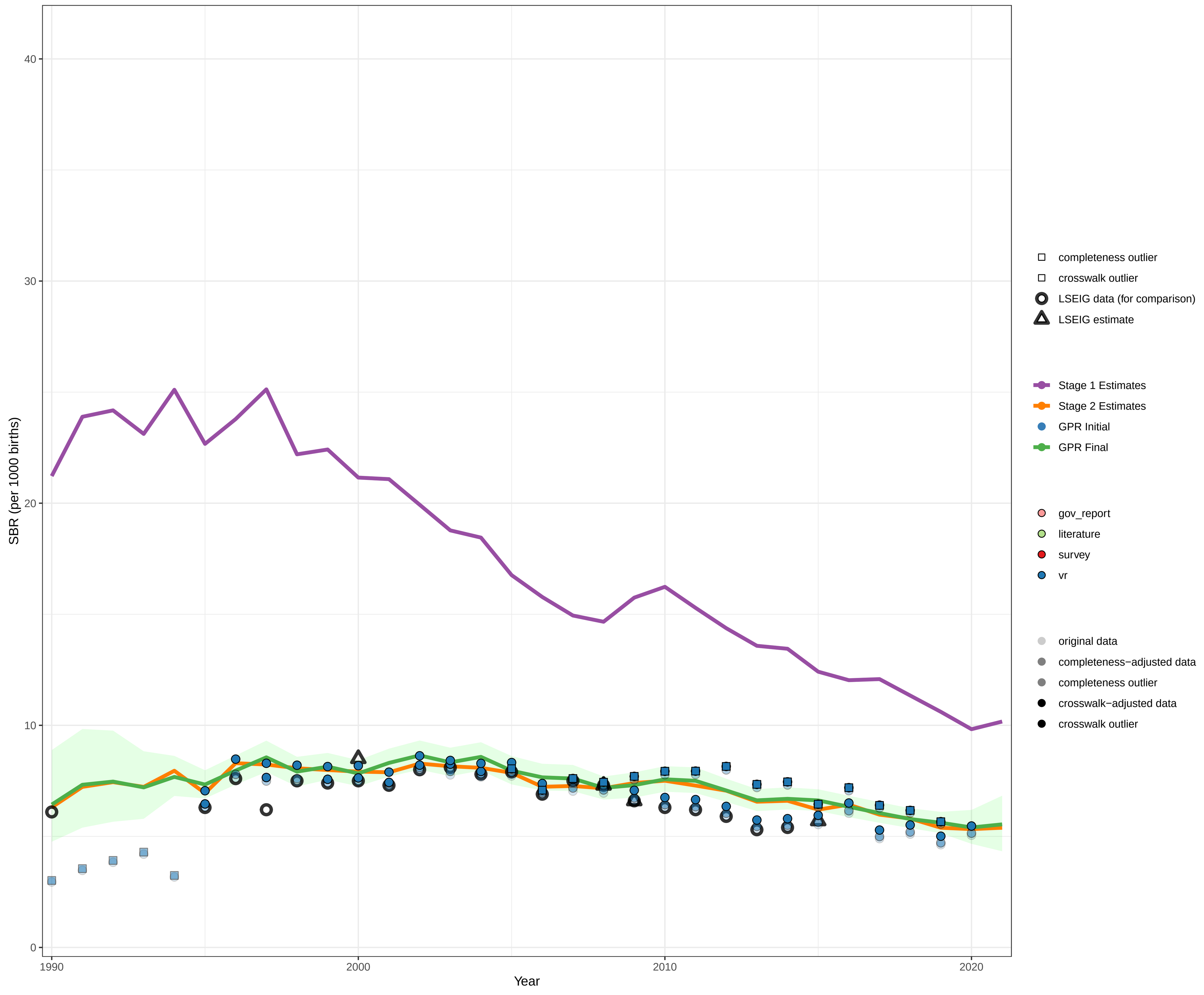

Year	Total Stillbirths (in thousands)	Total Stillbirths (in thousands)	SBR (per 1000 births)	SBR (per 1000 births)	SBR/NMR	SBR/NMR
	20 weeks	28 weeks	20 weeks	28 weeks	20 weeks	28 weeks
1990	0.9 (0.7 to 1.3)	0.7 (0.5 to 0.9)	9.1 (6.7 to 12.6)	6.4 (4.8 to 8.9)	1.10 (0.81 to 1.53)	0.78 (0.57 to 1.07)
1995	0.8 (0.7 to 0.8)	0.5 (0.5 to 0.6)	10.7 (9.7 to 11.5)	7.3 (6.7 to 8.0)	1.27 (1.16 to 1.38)	0.87 (0.80 to 0.95)
2000	0.8 (0.8 to 0.9)	0.6 (0.5 to 0.6)	11.8 (10.9 to 12.7)	7.8 (7.3 to 8.4)	1.52 (1.40 to 1.63)	1.01 (0.93 to 1.08)
2005	0.9 (0.8 to 1.0)	0.6 (0.5 to 0.6)	12.4 (11.4 to 13.4)	8.0 (7.3 to 8.6)	2.08 (1.92 to 2.25)	1.34 (1.23 to 1.45)
2010	0.9 (0.8 to 1.0)	0.6 (0.5 to 0.6)	11.9 (11.0 to 12.8)	7.6 (7.0 to 8.2)	2.17 (2.01 to 2.34)	1.38 (1.28 to 1.49)
2015	0.7 (0.6 to 0.7)	0.4 (0.4 to 0.5)	10.3 (9.6 to 11.1)	6.6 (6.1 to 7.1)	2.58 (2.39 to 2.78)	1.66 (1.54 to 1.78)
2020	0.5 (0.4 to 0.6)	0.3 (0.3 to 0.4)	8.3 (7.2 to 9.5)	5.4 (4.7 to 6.2)	2.73 (2.36 to 3.13)	1.77 (1.53 to 2.03)
2021	0.5 (0.4 to 0.6)	0.3 (0.3 to 0.4)	8.6 (6.7 to 10.6)	5.5 (4.3 to 6.8)	2.74 (2.14 to 3.38)	1.77 (1.38 to 2.18)

Croatia HRV Central Europe Stillbirth Rate / Neonatal Mortality Rate (SBR/NMR) − 20 weeks
lambda = 0.2 ; zeta = 0.99 ; scale = 5 ; amp = 0.028

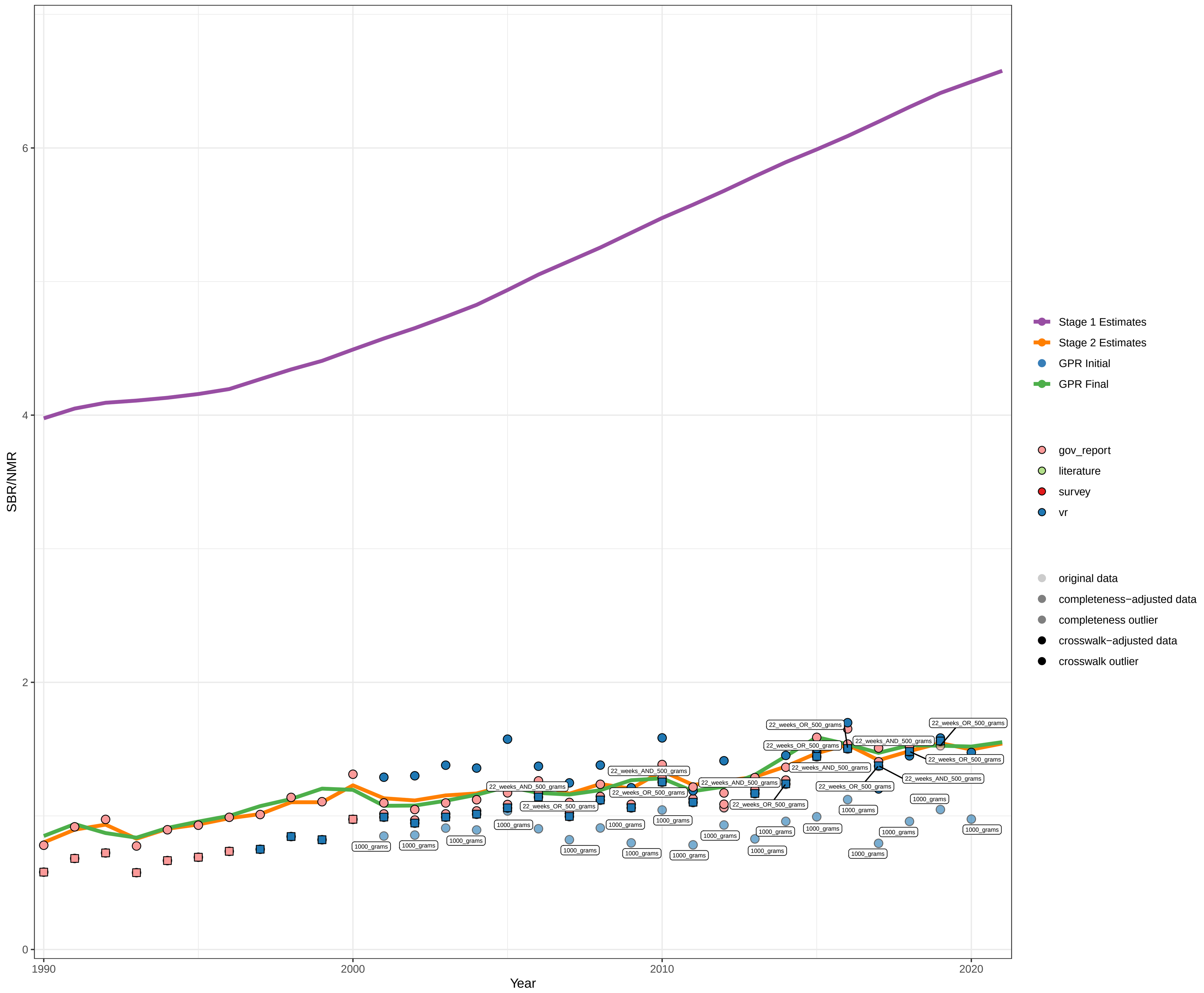

Croatia HRV Central Europe Stillbirth Rate / Neonatal Mortality Rate (SBR/NMR) − 28 weeks
lambda = 0.2 ; zeta = 0.99 ; scale = 5 ; amp = 0.028

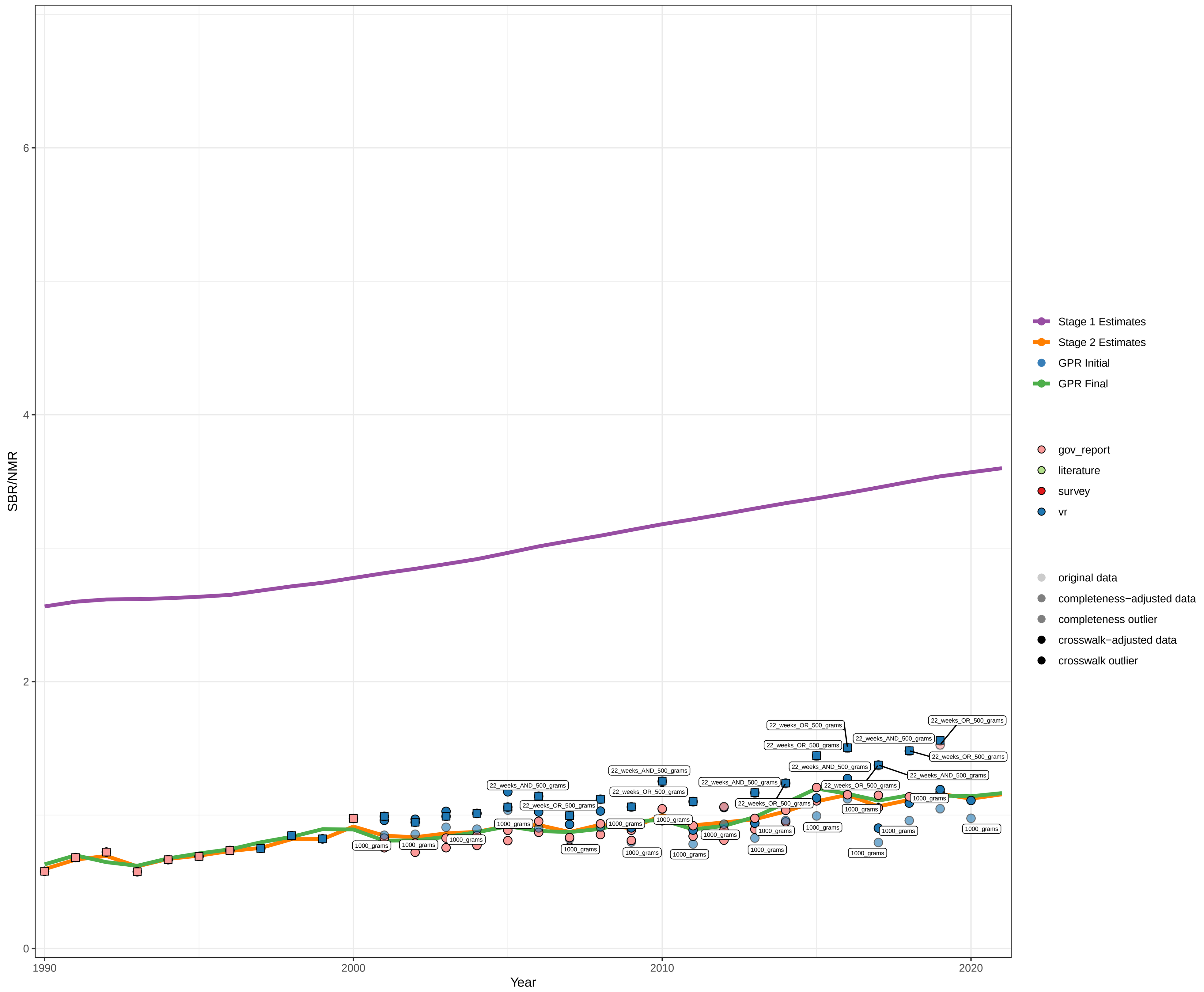

Definition	Years	Outlier	Source.Type	Title
1000_grams	2001−2020	0	VR	European Health for All Database
22_weeks_AND_500_grams	2001−2017	0	Statistical Report	Croatia Statistical Yearbook
22_weeks_OR_500_grams	2001−2019	0	Statistical Report	Croatia Health Statistics Yearbook
22_weeks_OR_500_grams	2001−2019	0	VR	United Nations Demographic Yearbook
28_weeks	1990−2000	0	Statistical Report	Croatia Statistical Yearbook
28_weeks	1990	2	Statistical Report	Croatia Health Service Yearbook
28_weeks	1991−2000	2	Statistical Report	Croatia Health Statistics Yearbook
28_weeks	1995−2000	2	VR	United Nations Demographic Yearbook

Croatia HRV Central Europe Stillbirth Rate (SBR) − 20 weeks
lambda = 0.2 ; zeta = 0.99 ; scale = 5 ; amp = 0.028

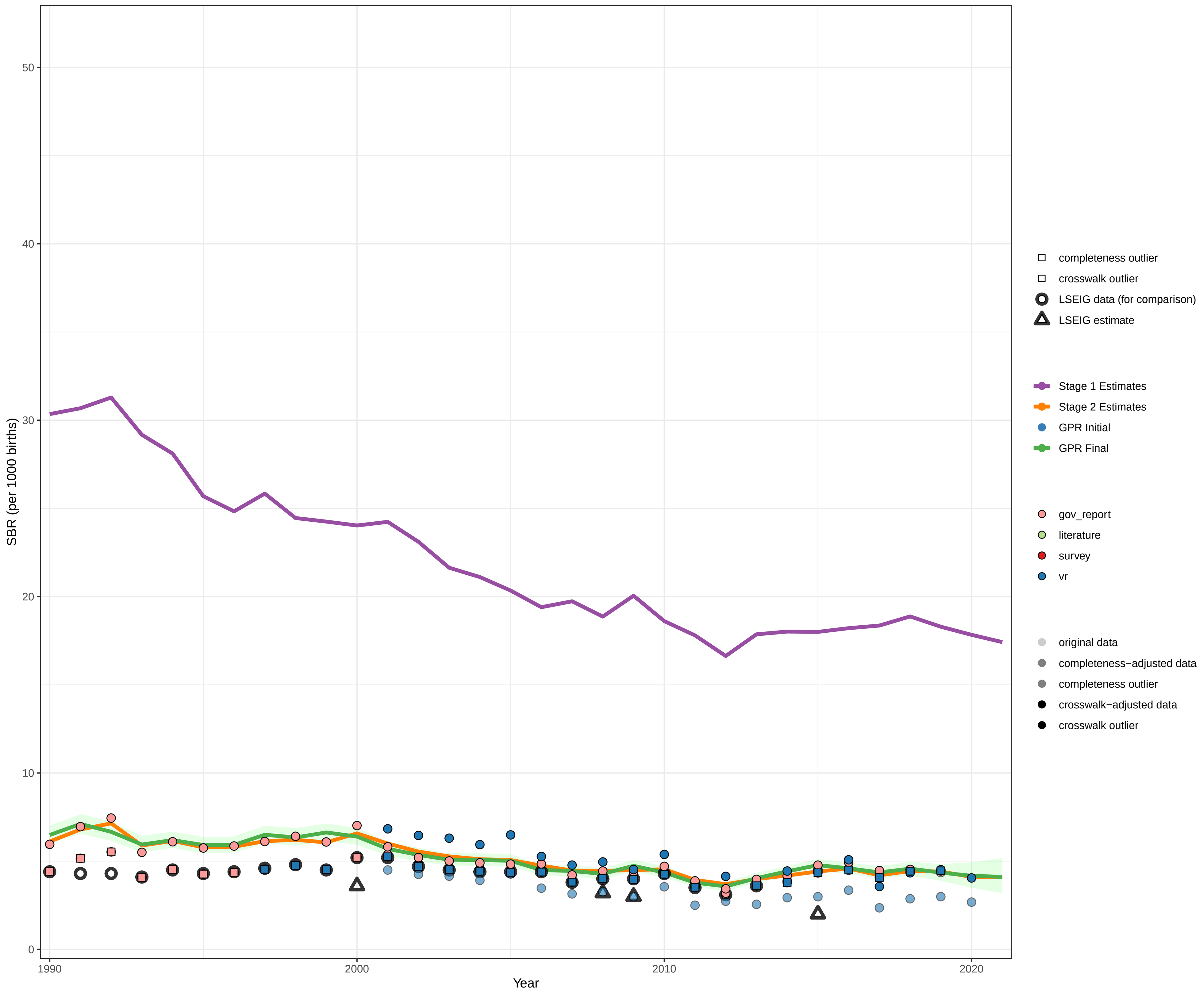

Croatia HRV Central Europe Stillbirth Rate (SBR) − 28 weeks
lambda = 0.2 ; zeta = 0.99 ; scale = 5 ; amp = 0.028

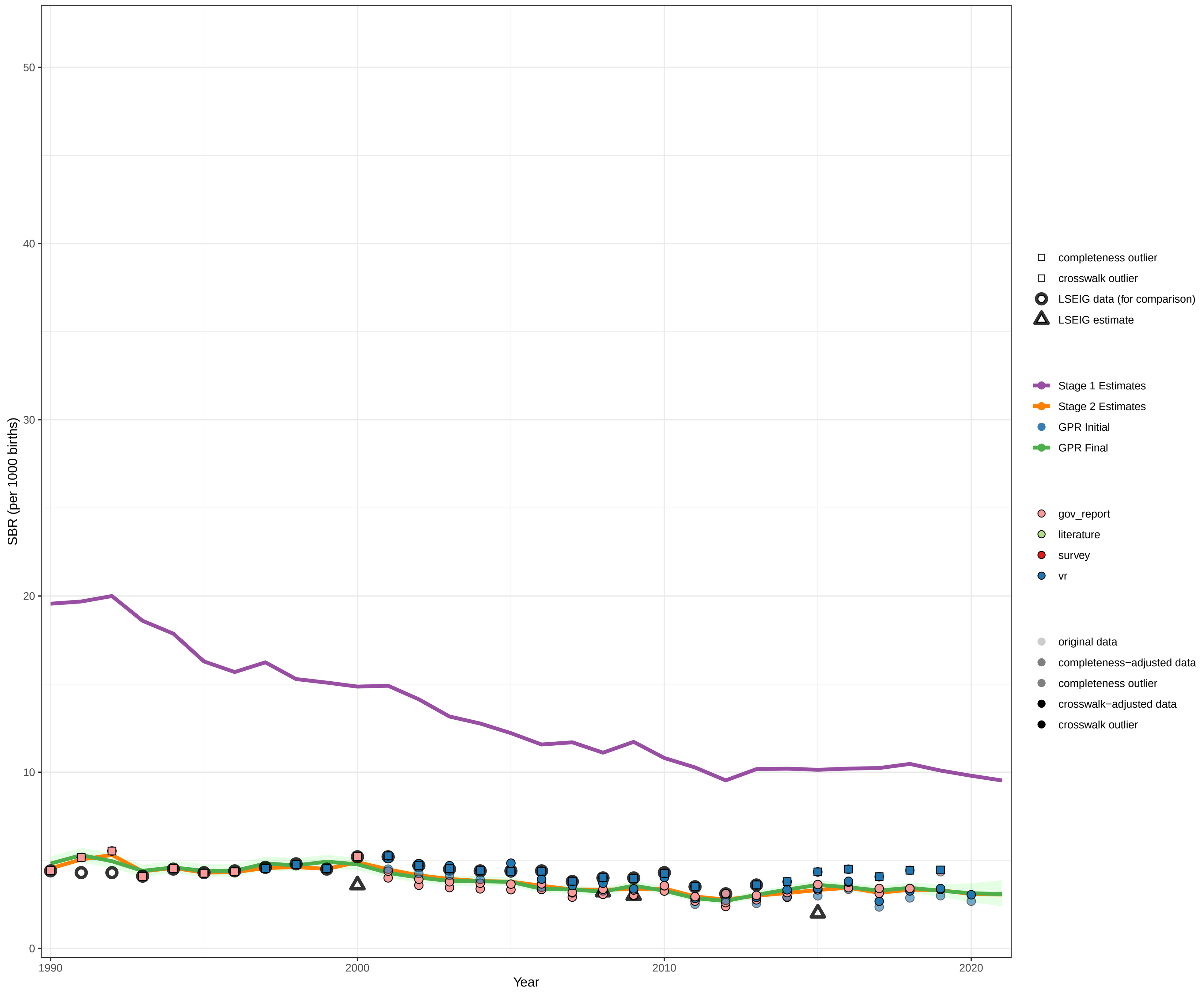

Year	Total Stillbirths (in thousands)	Total Stillbirths (in thousands)	SBR (per 1000 births)	SBR (per 1000 births)	SBR/NMR	SBR/NMR
	20 weeks	28 weeks	20 weeks	28 weeks	20 weeks	28 weeks
1990	0.3<br>(0.3 to 0.4)	0.3<br>(0.2 to 0.3)	6.5<br>(6.0 to 7.0)	4.8<br>(4.5 to 5.2)	0.85<br>(0.79 to 0.92)	0.63<br>(0.58 to 0.68)
1995	0.3<br>(0.3 to 0.3)	0.2<br>(0.2 to 0.3)	5.9<br>(5.4 to 6.4)	4.4<br>(4.1 to 4.8)	0.96<br>(0.88 to 1.03)	0.71<br>(0.66 to 0.78)
2000	0.3<br>(0.3 to 0.3)	0.2<br>(0.2 to 0.2)	6.4<br>(5.9 to 6.9)	4.8<br>(4.4 to 5.1)	1.20<br>(1.11 to 1.29)	0.89<br>(0.83 to 0.96)
2005	0.2<br>(0.2 to 0.2)	0.2<br>(0.1 to 0.2)	5.0<br>(4.7 to 5.4)	3.8<br>(3.5 to 4.0)	1.22<br>(1.14 to 1.31)	0.92<br>(0.85 to 0.98)
2010	0.2<br>(0.2 to 0.2)	0.1<br>(0.1 to 0.2)	4.4<br>(4.1 to 4.7)	3.3<br>(3.1 to 3.5)	1.28<br>(1.20 to 1.38)	0.96<br>(0.90 to 1.04)
2015	0.2<br>(0.2 to 0.2)	0.1<br>(0.1 to 0.1)	4.8<br>(4.4 to 5.1)	3.6<br>(3.3 to 3.9)	1.59<br>(1.48 to 1.71)	1.20<br>(1.11 to 1.29)
2020	0.1<br>(0.1 to 0.2)	0.1<br>(0.1 to 0.1)	4.2<br>(3.5 to 4.9)	3.1<br>(2.6 to 3.7)	1.52<br>(1.27 to 1.79)	1.14<br>(0.96 to 1.35)
2021	0.1<br>(0.1 to 0.2)	0.1<br>(0.1 to 0.1)	4.1<br>(3.2 to 5.2)	3.1<br>(2.4 to 3.9)	1.55<br>(1.20 to 1.95)	1.16<br>(0.90 to 1.46)

Czechia CZE Central Europe Stillbirth Rate / Neonatal Mortality Rate (SBR/NMR) − 20 weeks
lambda = 0.2 ; zeta = 0.99 ; scale = 5 ; amp = 0.028

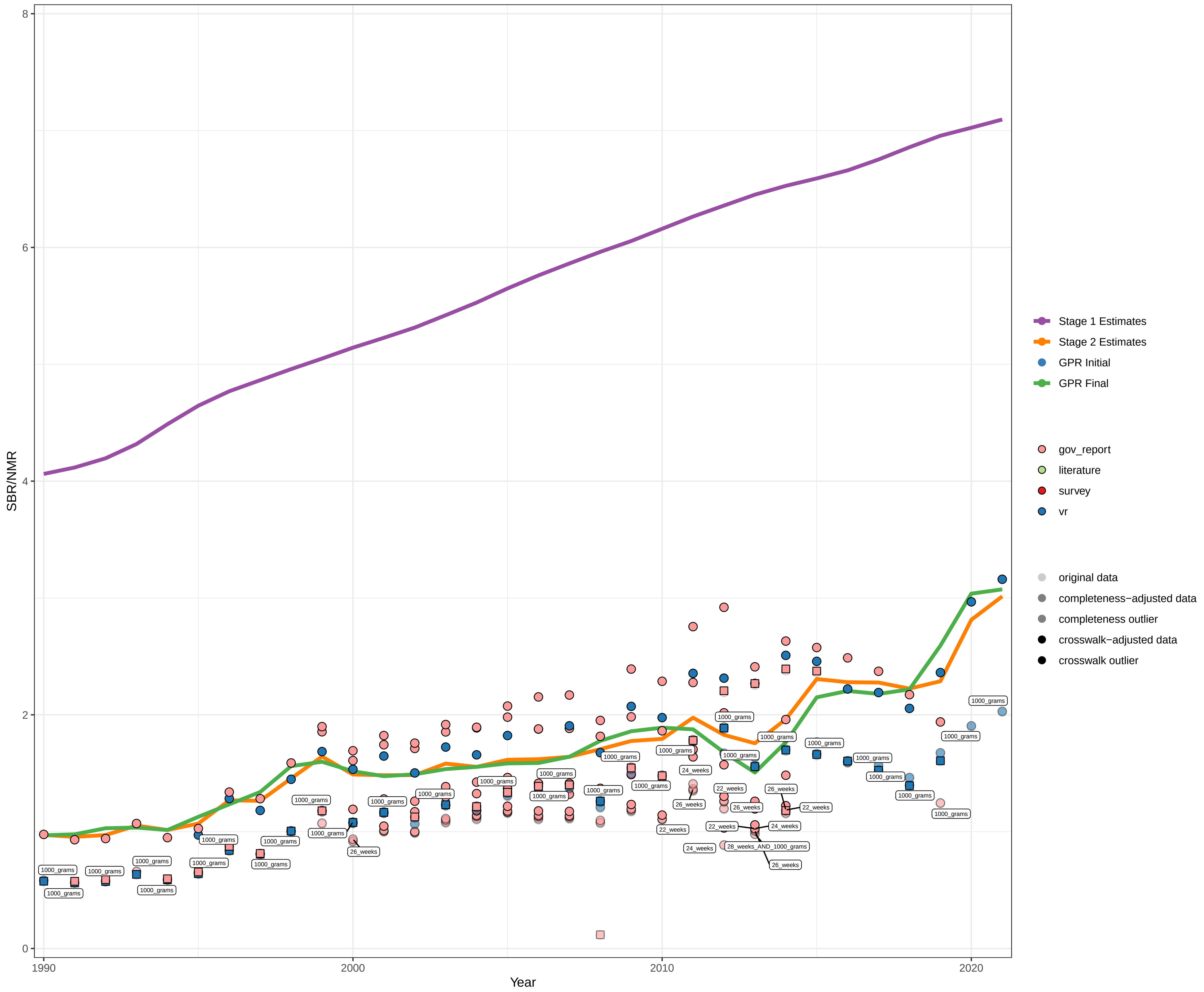

Czechia CZE Central Europe Stillbirth Rate / Neonatal Mortality Rate (SBR/NMR) − 28 weeks
lambda = 0.2 ; zeta = 0.99 ; scale = 5 ; amp = 0.028

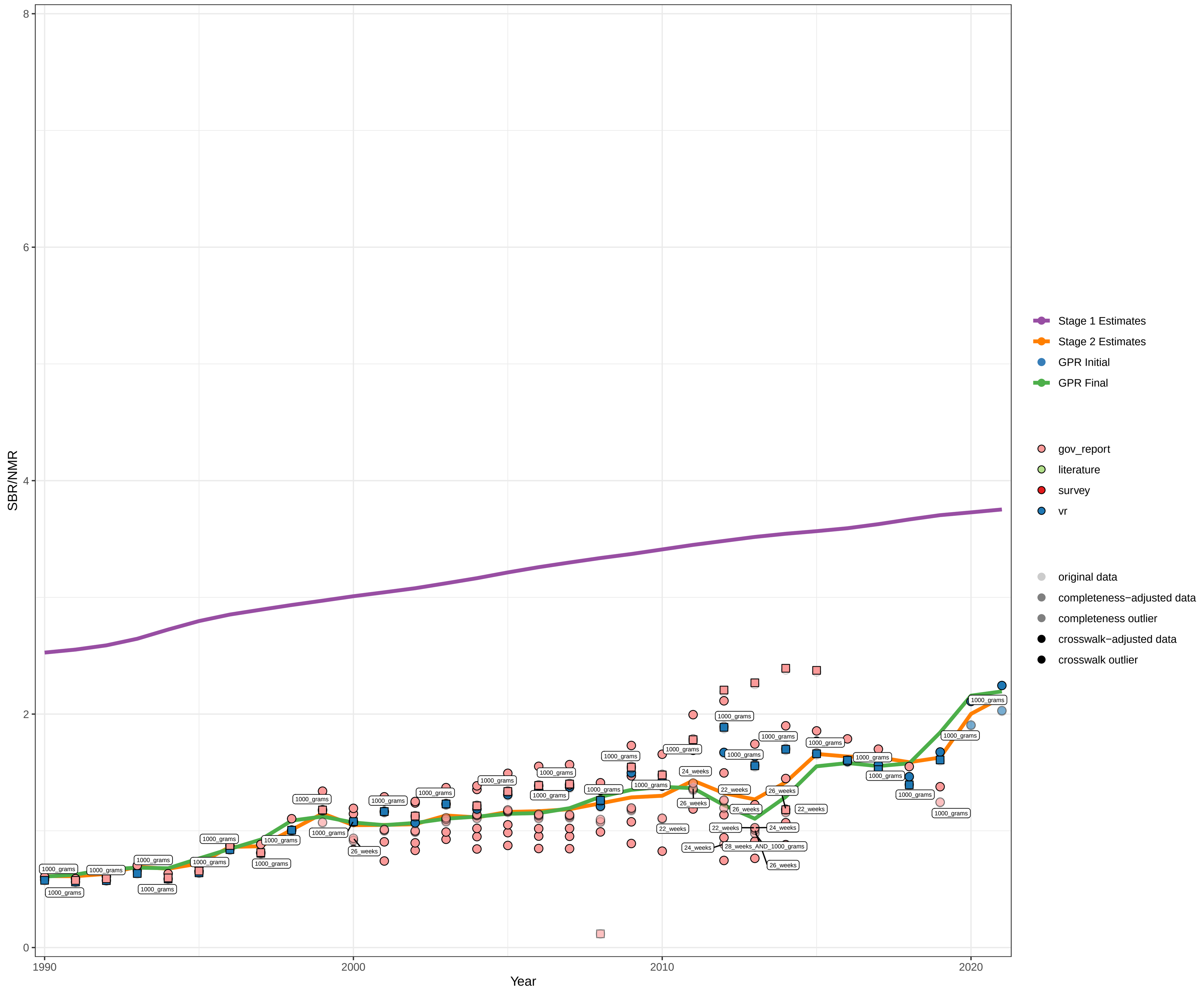

Definition	Years	Outlier	Source.Type	Title
1000_grams	1990−2015	0	Statistical Report	Czech Republic Mother and Newborn
1000_grams	1990−2021	0	VR	European Health for All Database
1000_grams	1991, 1992, 1994−2019	0	Statistical Report	Czech Republic Health Statistics Yearbook
22_weeks	2001, 2004−2007, 2009, 2010, 2012, 2013	0	Statistical Report	Czech Republic Mother and Newborn
22_weeks, 26_weeks, 28_weeks_AND_1000_grams	2014	0	Statistical Report	Czech Republic Mother and Newborn
24_weeks	2002−2007, 2011−2013	0	Statistical Report	Czech Republic Mother and Newborn
26_weeks	2000−2009, 2011−2013	0	Statistical Report	Czech Republic Mother and Newborn
28_weeks	1995−2019	0	VR	United Nations Demographic Yearbook
28_weeks_AND_1000_grams	1999−2013	0	Statistical Report	Czech Republic Mother and Newborn
22_weeks	2008	1	Statistical Report	Czech Republic Mother and Newborn

Czechia CZE Central Europe Stillbirth Rate (SBR) − 20 weeks
lambda = 0.2 ; zeta = 0.99 ; scale = 5 ; amp = 0.028

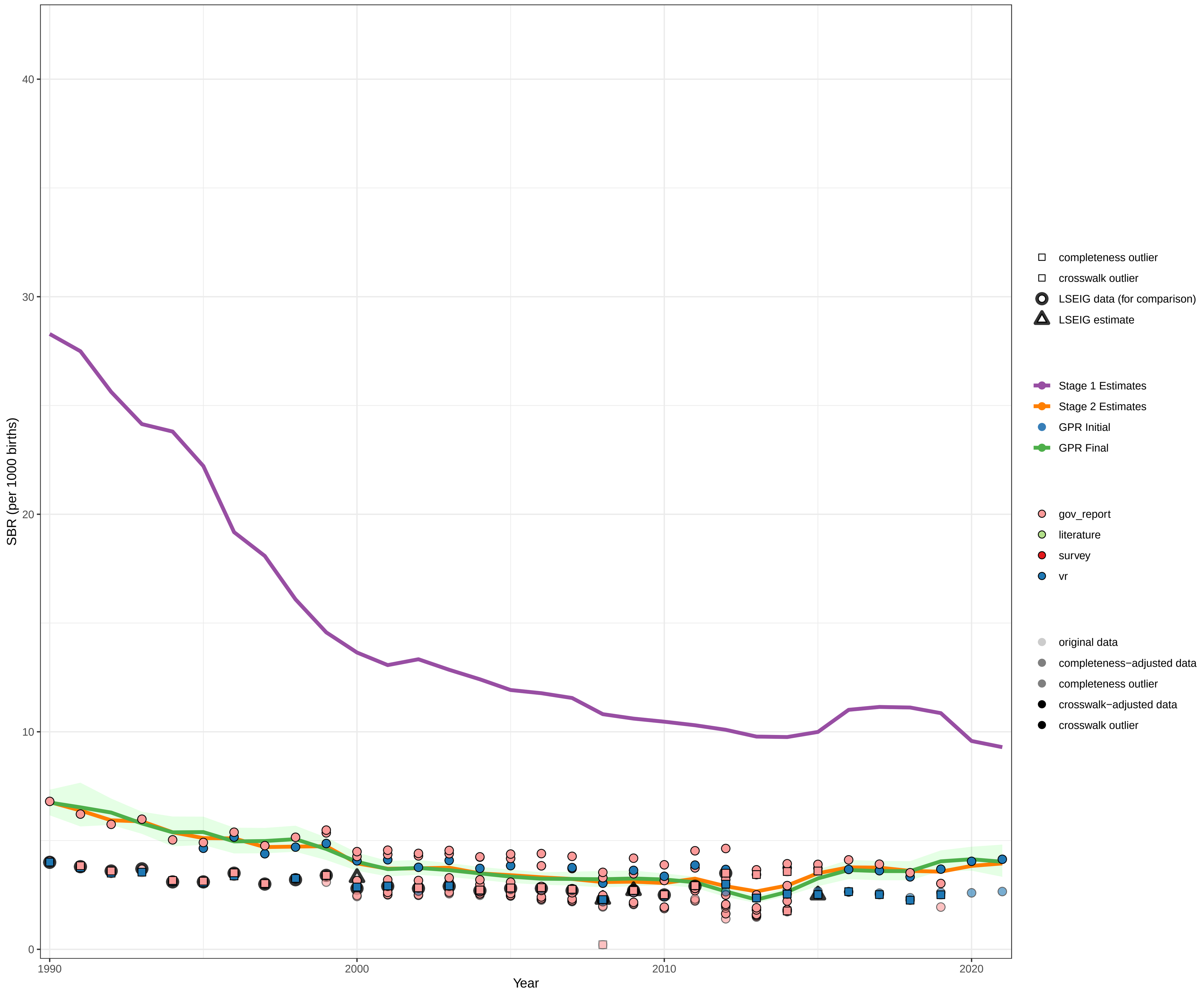

Czechia CZE Central Europe Stillbirth Rate (SBR) − 28 weeks
lambda = 0.2 ; zeta = 0.99 ; scale = 5 ; amp = 0.028

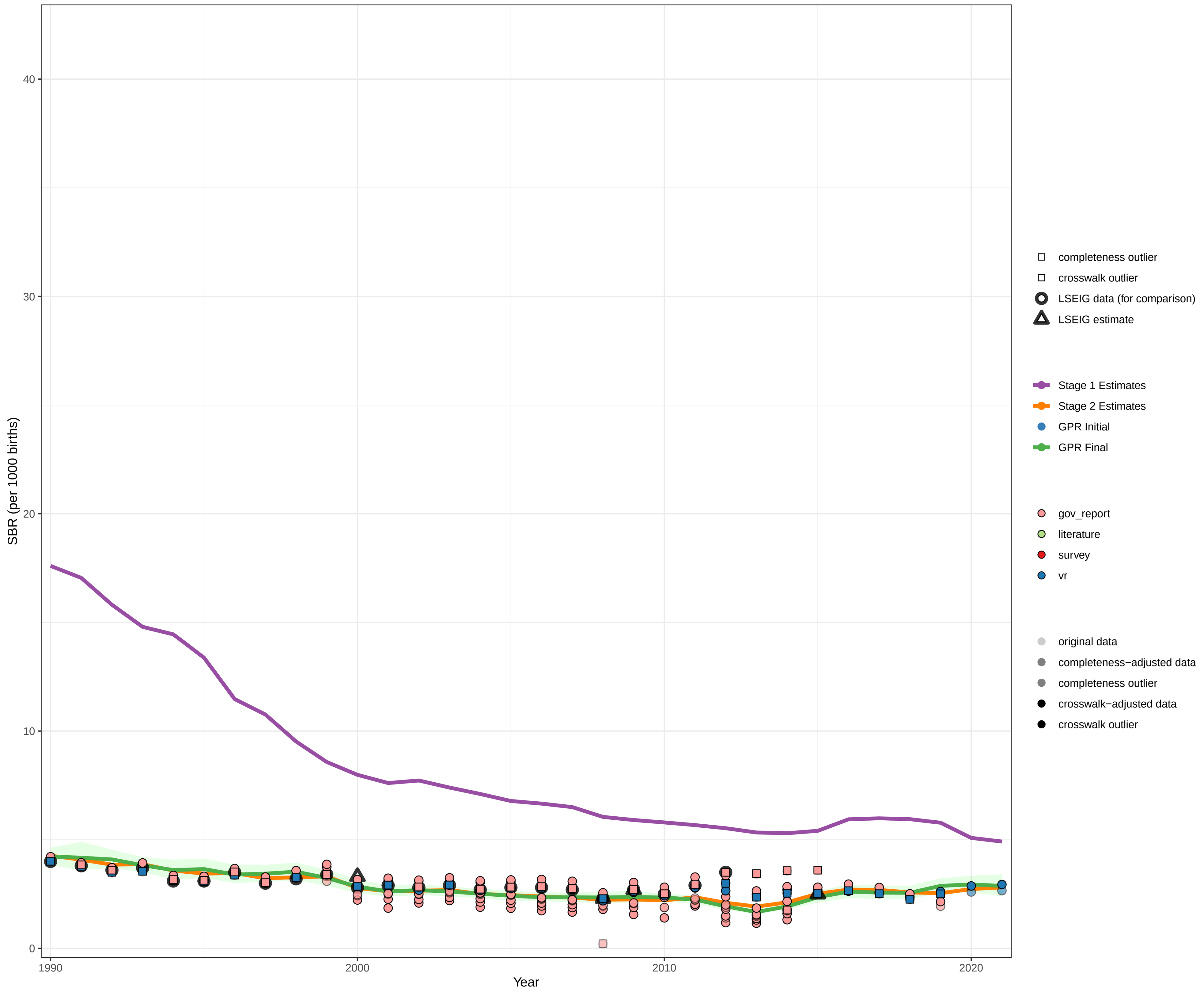

Year	Total Stillbirths (in thousands)	Total Stillbirths (in thousands)	SBR (per 1000 births)	SBR (per 1000 births)	SBR/NMR	SBR/NMR
	20 weeks	28 weeks	20 weeks	28 weeks	20 weeks	28 weeks
1990	0.9 (0.8 to 1.0)	0.5 (0.5 to 0.6)	6.7 (6.2 to 7.3)	4.2 (3.8 to 4.6)	0.97 (0.88 to 1.05)	0.61 (0.55 to 0.66)
1995	0.5 (0.4 to 0.6)	0.3 (0.3 to 0.4)	5.4 (4.8 to 6.1)	3.6 (3.2 to 4.1)	1.13 (1.00 to 1.28)	0.76 (0.68 to 0.86)
2000	0.4 (0.3 to 0.4)	0.3 (0.2 to 0.3)	4.0 (3.6 to 4.4)	2.8 (2.5 to 3.2)	1.52 (1.36 to 1.68)	1.07 (0.96 to 1.20)
2005	0.4 (0.3 to 0.4)	0.3 (0.2 to 0.3)	3.3 (3.1 to 3.7)	2.4 (2.2 to 2.7)	1.58 (1.45 to 1.74)	1.15 (1.04 to 1.26)
2010	0.4 (0.3 to 0.4)	0.3 (0.2 to 0.3)	3.2 (3.0 to 3.5)	2.3 (2.2 to 2.5)	1.89 (1.74 to 2.05)	1.38 (1.27 to 1.49)
2015	0.4 (0.3 to 0.4)	0.3 (0.2 to 0.3)	3.3 (2.9 to 3.7)	2.4 (2.1 to 2.6)	2.15 (1.91 to 2.42)	1.55 (1.38 to 1.74)
2020	0.5 (0.4 to 0.5)	0.3 (0.3 to 0.4)	4.1 (3.6 to 4.7)	2.9 (2.6 to 3.3)	3.04 (2.65 to 3.46)	2.16 (1.89 to 2.45)
2021	0.4 (0.4 to 0.5)	0.3 (0.3 to 0.4)	4.0 (3.3 to 4.8)	2.9 (2.4 to 3.4)	3.07 (2.54 to 3.68)	2.19 (1.84 to 2.60)

Hungary HUN Central Europe Stillbirth Rate / Neonatal Mortality Rate (SBR/NMR) − 20 weeks
lambda = 0.2 ; zeta = 0.99 ; scale = 5 ; amp = 0.028

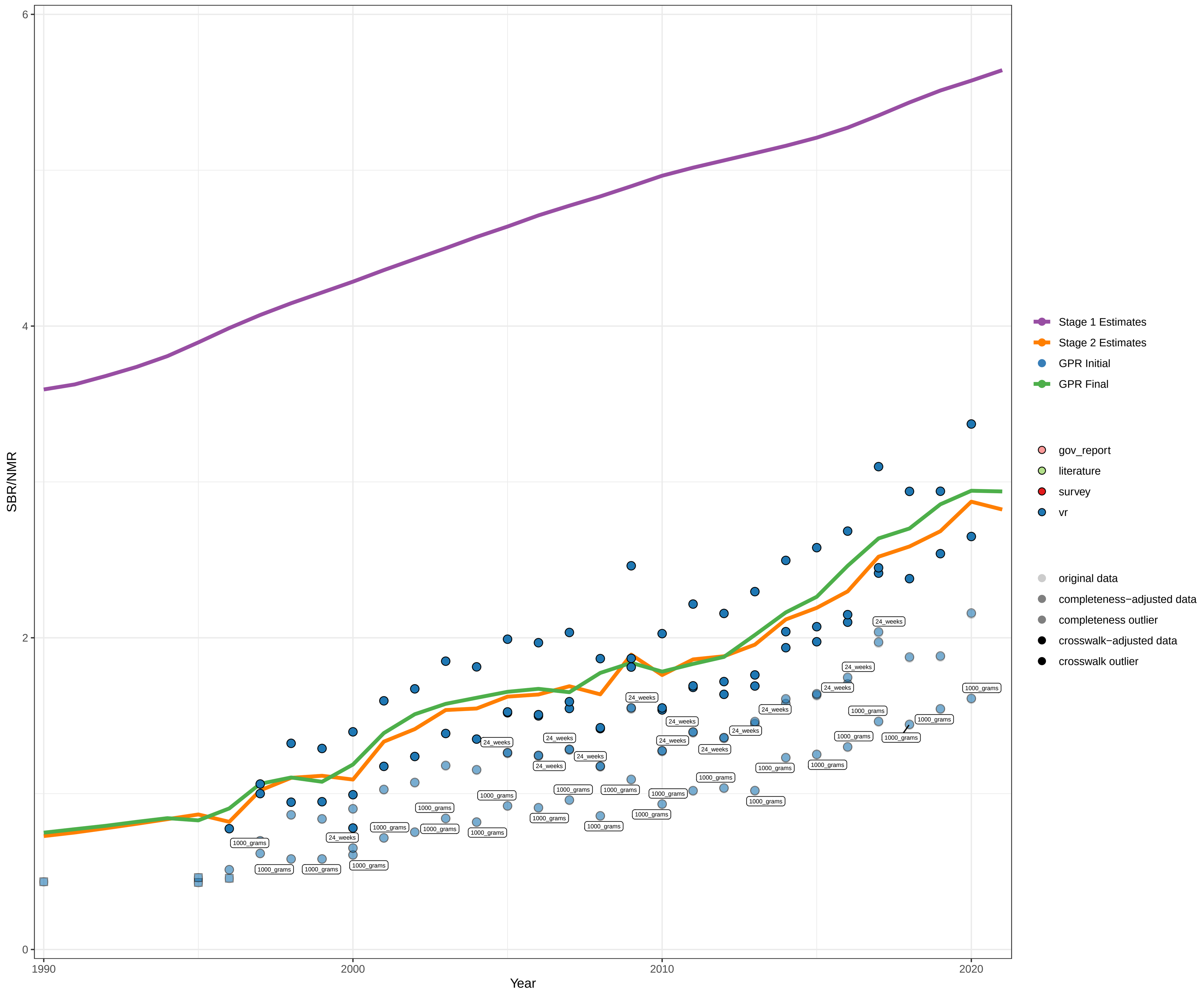

Hungary HUN Central Europe Stillbirth Rate / Neonatal Mortality Rate (SBR/NMR) − 28 weeks
lambda = 0.2 ; zeta = 0.99 ; scale = 5 ; amp = 0.028

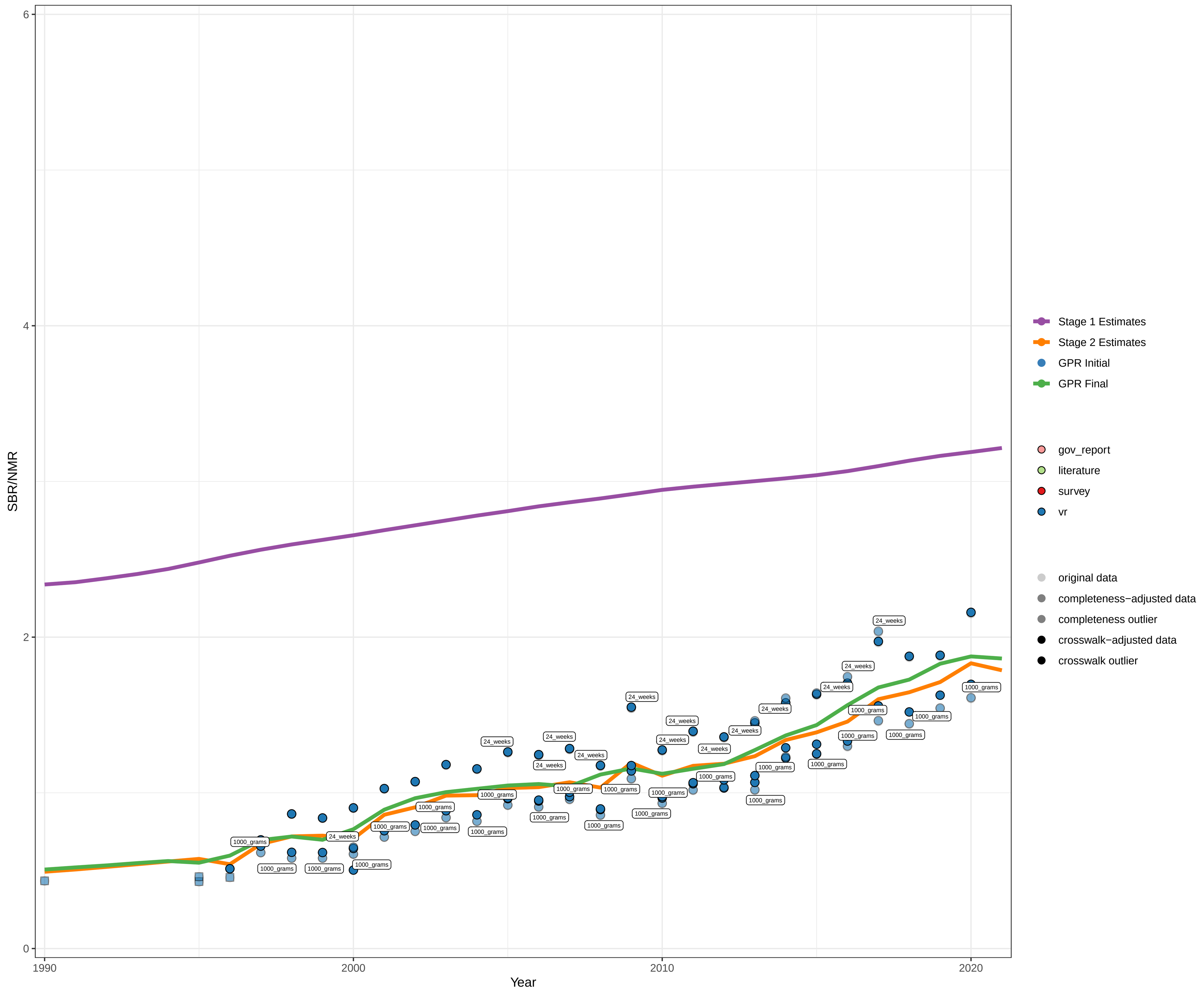

Definition	Years	Outlier	Source.Type	Title
1000_grams	1997−2020	0	VR	European Health for All Database
24_weeks	2000, 2005−2017	0	VR	Hungary Demographic Yearbook
28_weeks	1996−2020	0	VR	United Nations Demographic Yearbook
1000_grams	1995, 1996	1	VR	European Health for All Database
28_weeks	1990	1	VR	Hungary Demographic Yearbook
28_weeks	1995	1	VR	United Nations Demographic Yearbook

Hungary HUN Central Europe Stillbirth Rate (SBR) − 20 weeks
lambda = 0.2 ; zeta = 0.99 ; scale = 5 ; amp = 0.028

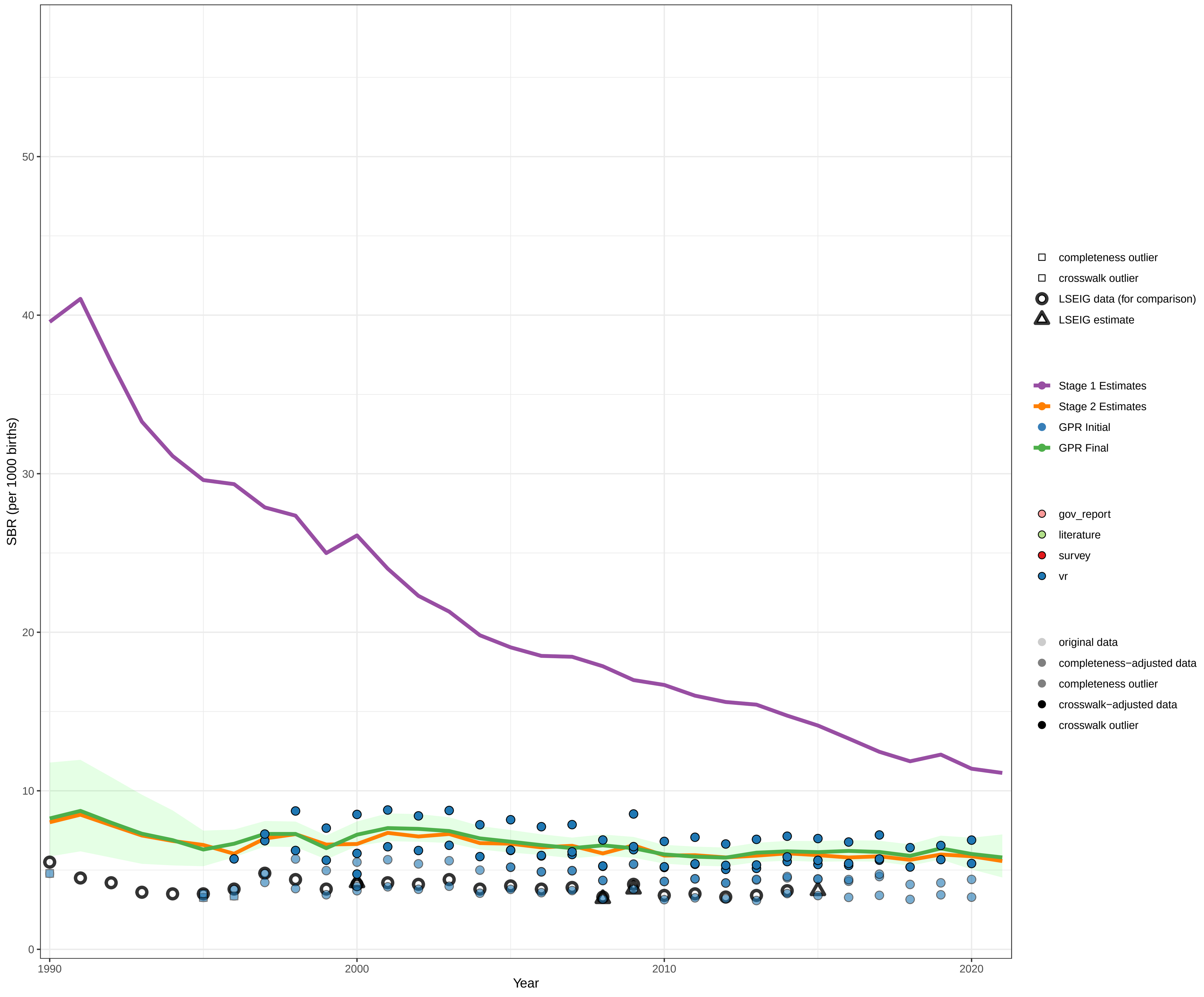

Hungary HUN Central Europe Stillbirth Rate (SBR) − 28 weeks
lambda = 0.2 ; zeta = 0.99 ; scale = 5 ; amp = 0.028

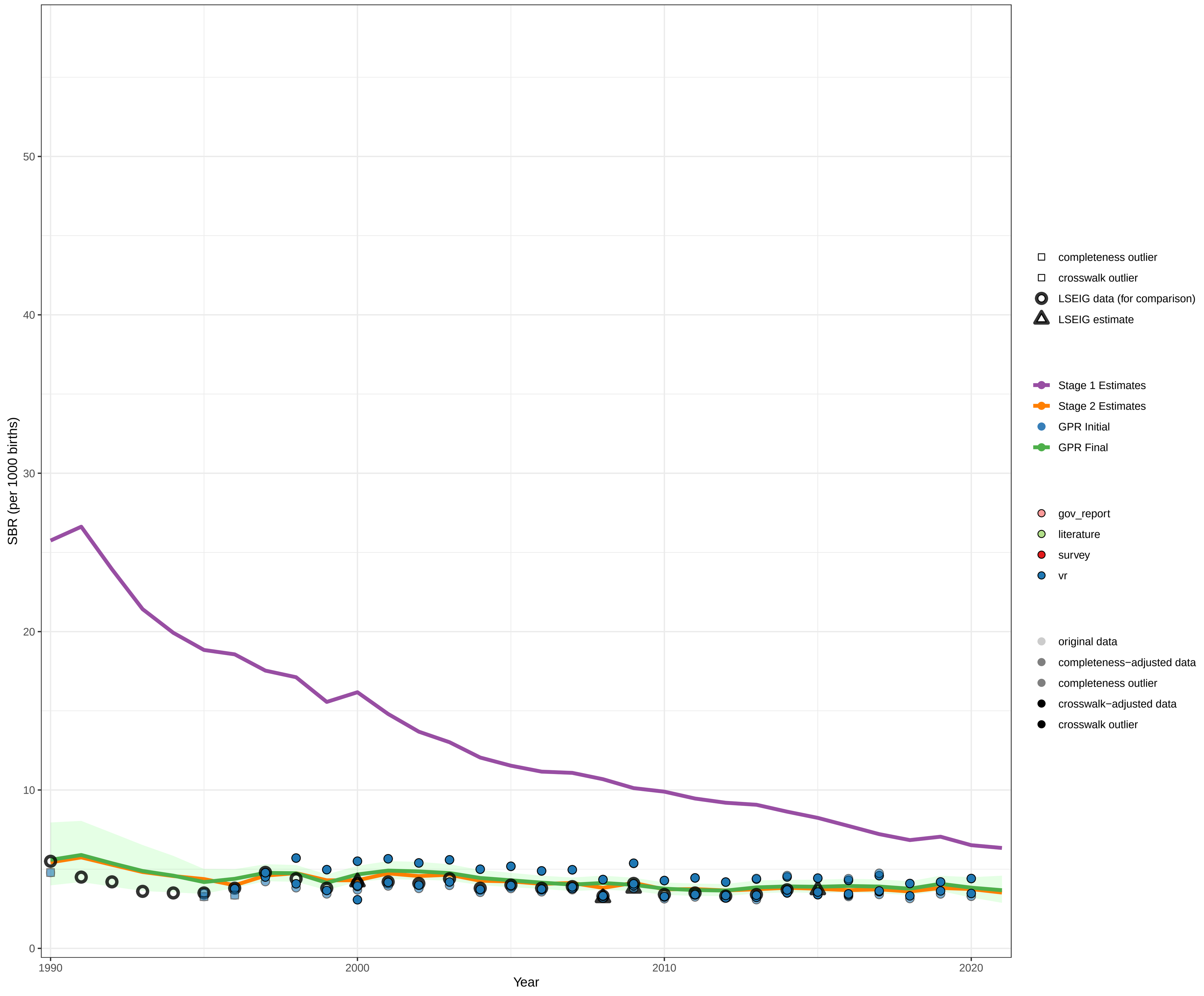

Year	Total Stillbirths (in thousands)	Total Stillbirths (in thousands)	SBR (per 1000 births)	SBR (per 1000 births)	SBR/NMR	SBR/NMR
	20 weeks	28 weeks	20 weeks	28 weeks	20 weeks	28 weeks
1990	1.0<br>(0.7 to 1.5)	0.7<br>(0.5 to 1.0)	8.3<br>(5.9 to 11.8)	5.6<br>(4.0 to 8.0)	0.75<br>(0.53 to 1.07)	0.51<br>(0.36 to 0.72)
1995	0.7<br>(0.6 to 0.8)	0.5<br>(0.4 to 0.5)	6.3<br>(5.2 to 7.5)	4.2<br>(3.4 to 5.0)	0.83<br>(0.69 to 0.99)	0.55<br>(0.45 to 0.66)
2000	0.7<br>(0.6 to 0.8)	0.5<br>(0.4 to 0.5)	7.2<br>(6.5 to 8.1)	4.7<br>(4.2 to 5.2)	1.19<br>(1.07 to 1.32)	0.77<br>(0.69 to 0.86)
2005	0.7<br>(0.6 to 0.7)	0.4<br>(0.4 to 0.5)	6.8<br>(6.1 to 7.5)	4.3<br>(3.9 to 4.8)	1.65<br>(1.49 to 1.83)	1.05<br>(0.94 to 1.16)
2010	0.5<br>(0.5 to 0.6)	0.3<br>(0.3 to 0.4)	6.0<br>(5.4 to 6.6)	3.8<br>(3.4 to 4.2)	1.78<br>(1.61 to 1.96)	1.12<br>(1.01 to 1.24)
2015	0.6<br>(0.5 to 0.6)	0.4<br>(0.3 to 0.4)	6.1<br>(5.5 to 6.8)	3.9<br>(3.5 to 4.3)	2.26<br>(2.04 to 2.52)	1.44<br>(1.29 to 1.60)
2020	0.5<br>(0.5 to 0.6)	0.3<br>(0.3 to 0.4)	6.0<br>(5.0 to 7.0)	3.8<br>(3.2 to 4.5)	2.94<br>(2.47 to 3.45)	1.88<br>(1.57 to 2.20)
2021	0.5<br>(0.4 to 0.6)	0.3<br>(0.3 to 0.4)	5.8<br>(4.5 to 7.2)	3.7<br>(2.9 to 4.6)	2.94<br>(2.30 to 3.67)	1.86<br>(1.46 to 2.33)

Montenegro   MNE   Central Europe   Stillbirth Rate / Neonatal Mortality Rate (SBR/NMR) − 20 weeks
lambda = 0.6 ; zeta = 0.8 ; scale = 15 ; amp = 0.028

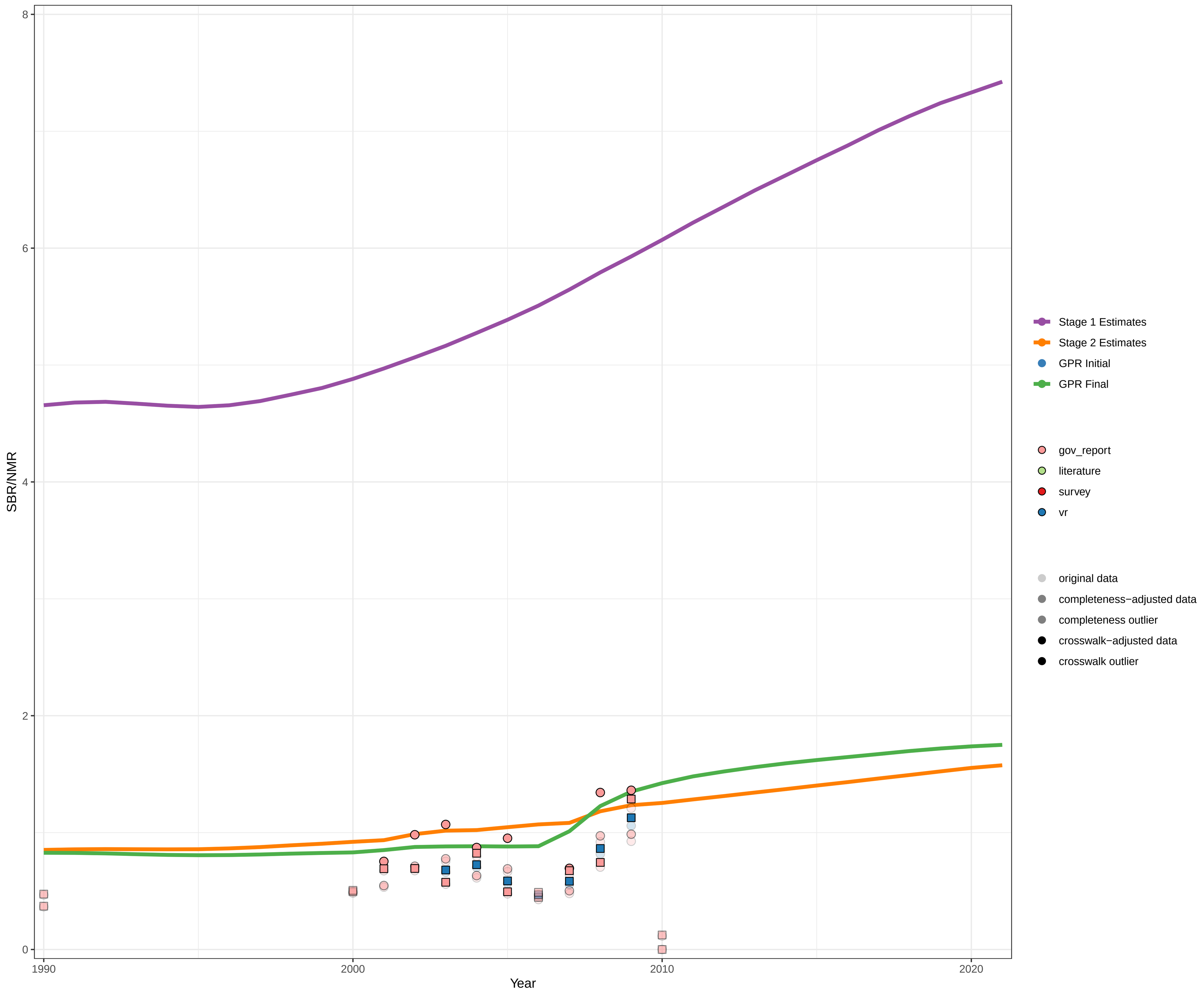

Montenegro   MNE   Central Europe   Stillbirth Rate / Neonatal Mortality Rate (SBR/NMR) − 28 weeks
lambda = 0.6 ; zeta = 0.8 ; scale = 15 ; amp = 0.028

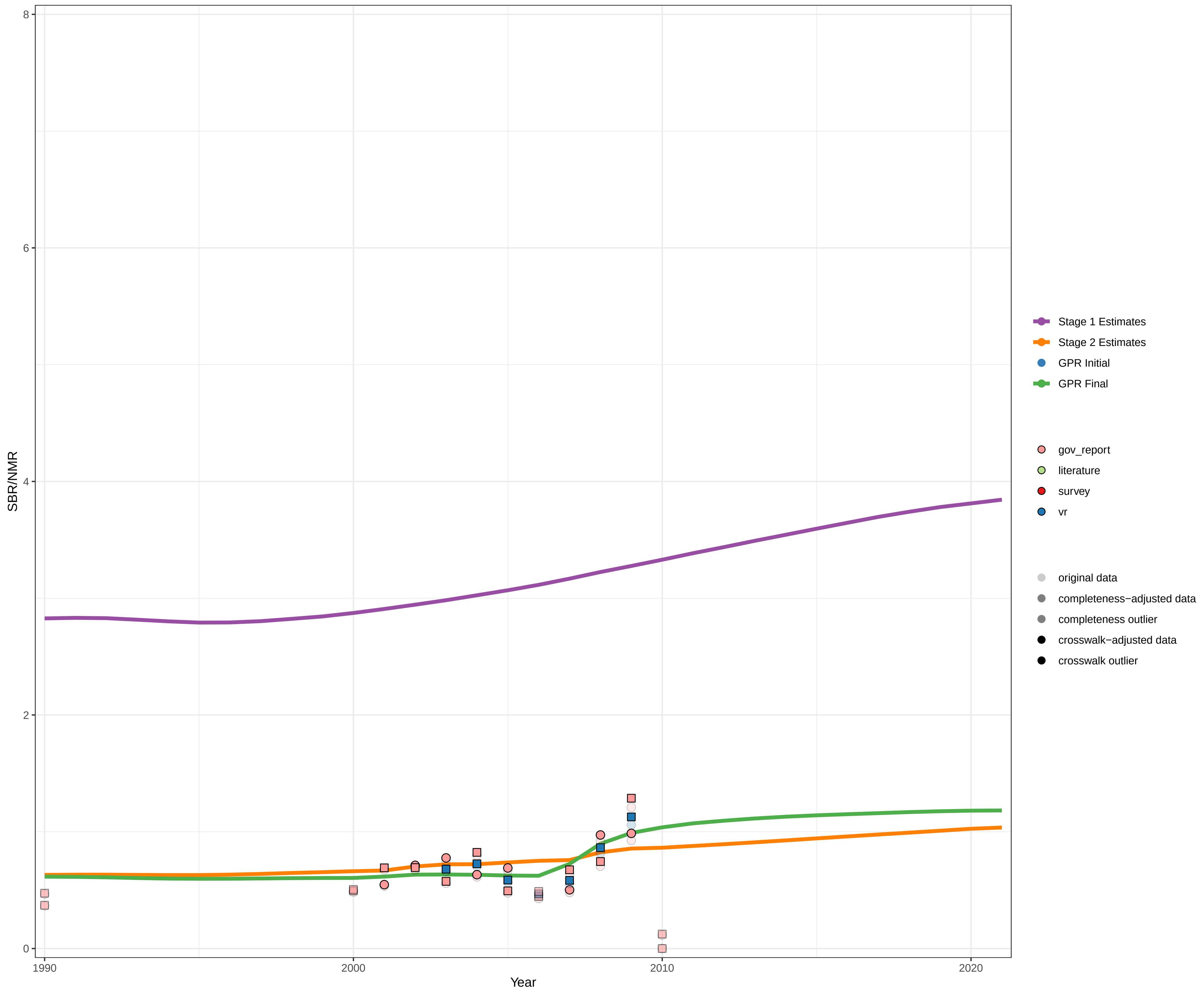

Definition	Years	Outlier	Source.Type	Title
28_weeks	2001−2005, 2007−2009	0	Statistical Report	Montenegro Statistical Yearbook
28_weeks	2004	0	VR	United Nations Demographic Yearbook
28_weeks	1990, 2000, 2006, 2010	1	Statistical Report	Montenegro Statistical Yearbook
28_weeks	2006	1	VR	United Nations Demographic Yearbook
28_weeks	2001−2005, 2007−2009	2	Statistical Report	Montenegro Statistical Yearbook
28_weeks	2003, 2005, 2007−2009	2	VR	United Nations Demographic Yearbook

Montenegro   MNE   Central Europe   Stillbirth Rate (SBR) − 20 weeks
lambda = 0.6 ; zeta = 0.8 ; scale = 15 ; amp = 0.028

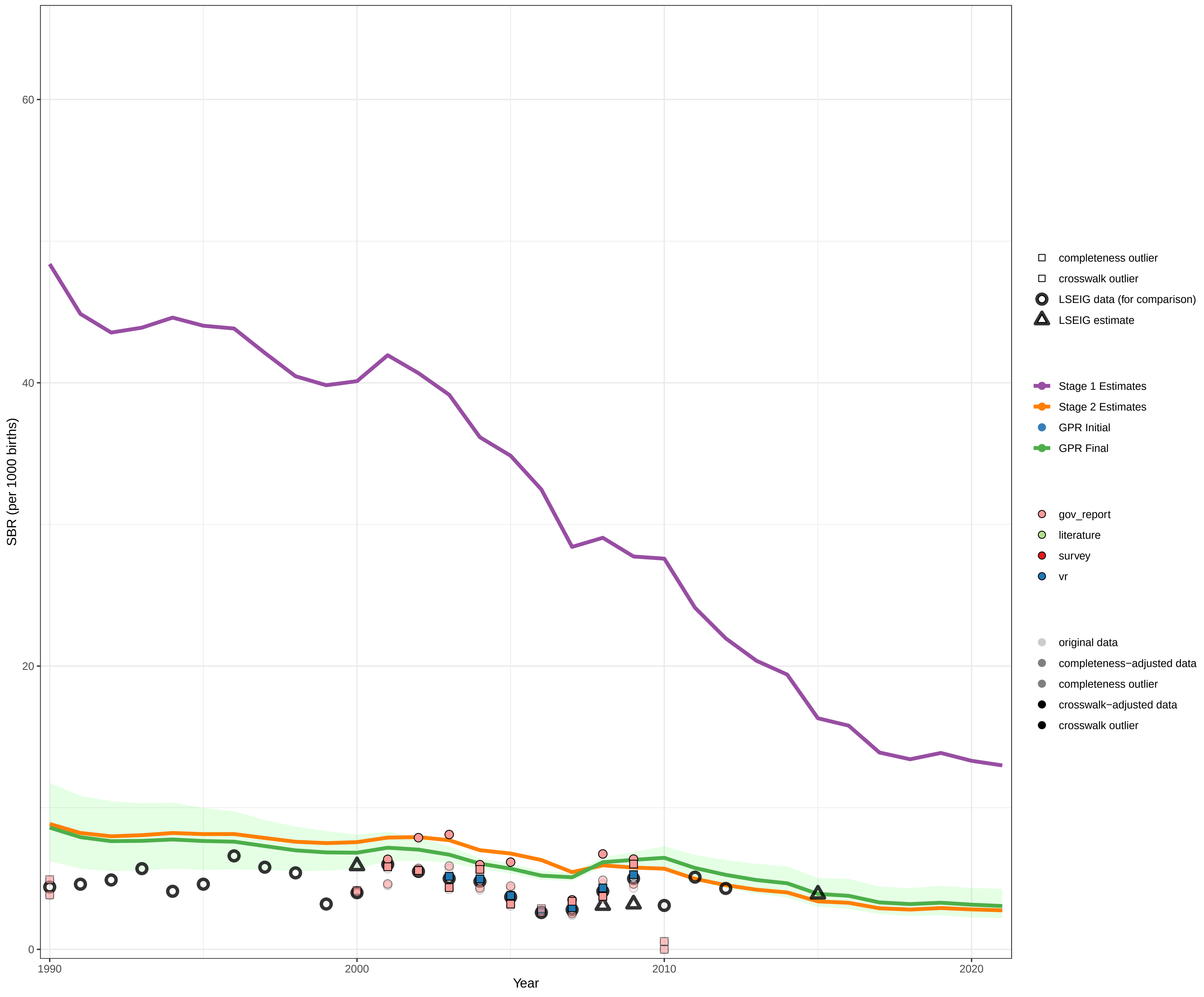

Montenegro   MNE   Central Europe   Stillbirth Rate (SBR) − 28 weeks
lambda = 0.6 ; zeta = 0.8 ; scale = 15 ; amp = 0.028

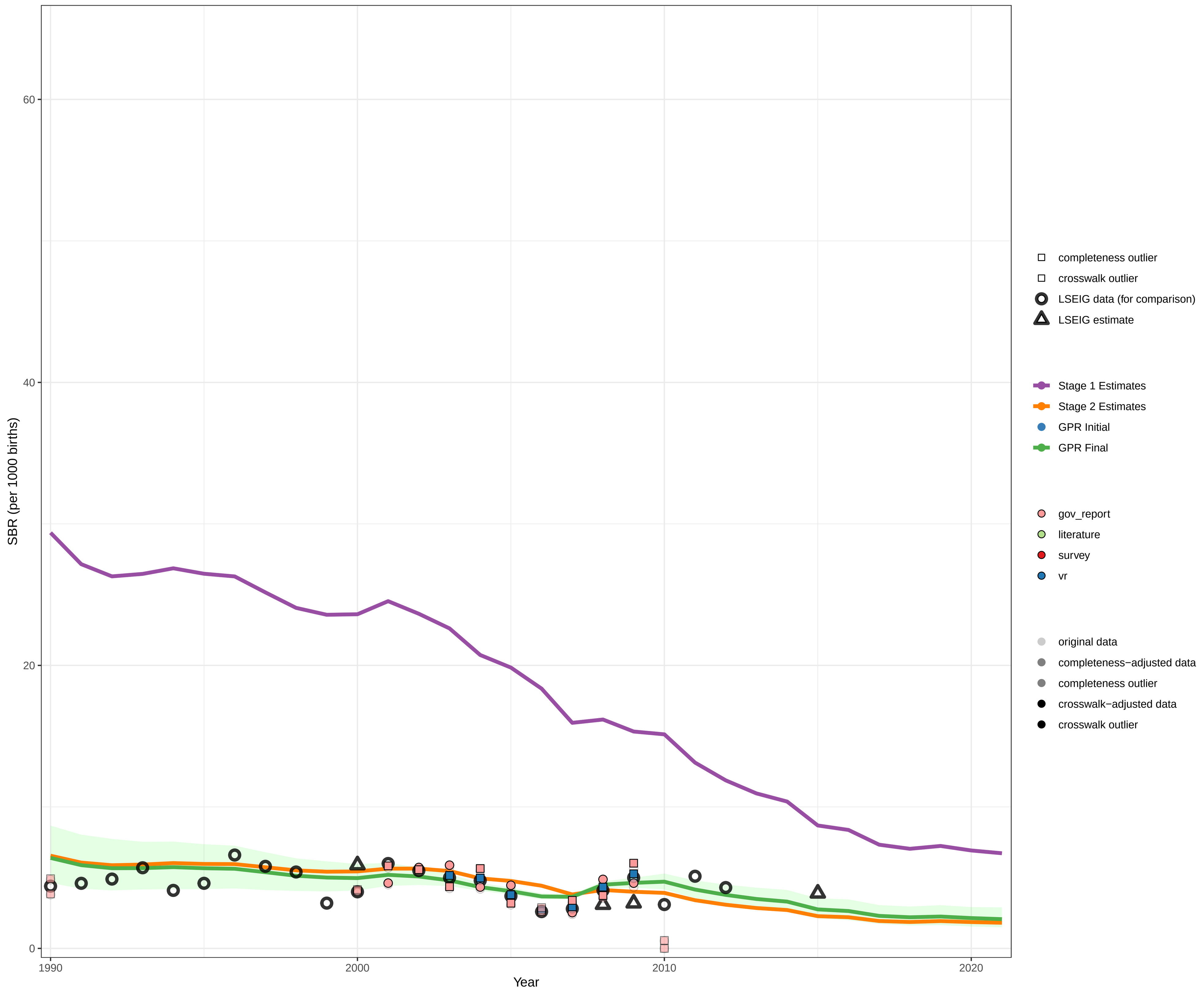

Year	Total Stillbirths (in thousands)	Total Stillbirths (in thousands)	SBR (per 1000 births)	SBR (per 1000 births)	SBR/NMR	SBR/NMR
	20 weeks	28 weeks	20 weeks	28 weeks	20 weeks	28 weeks
1990	0.1<br>(0.1 to 0.1)	0.1<br>(0.0 to 0.1)	8.6<br>(6.2 to 11.8)	6.4<br>(4.6 to 8.7)	0.83<br>(0.60 to 1.13)	0.62<br>(0.45 to 0.84)
1995	0.1<br>(0.1 to 0.1)	0.1<br>(0.0 to 0.1)	7.6<br>(5.6 to 10.0)	5.7<br>(4.2 to 7.4)	0.81<br>(0.59 to 1.05)	0.60<br>(0.44 to 0.78)
2000	0.1<br>(0.0 to 0.1)	0.0<br>(0.0 to 0.1)	6.8<br>(5.7 to 8.1)	5.0<br>(4.1 to 6.0)	0.83<br>(0.69 to 0.99)	0.60<br>(0.50 to 0.73)
2005	0.0<br>(0.0 to 0.0)	0.0<br>(0.0 to 0.0)	5.7<br>(5.4 to 6.1)	4.0<br>(3.8 to 4.3)	0.88<br>(0.83 to 0.94)	0.63<br>(0.59 to 0.66)
2010	0.0<br>(0.0 to 0.1)	0.0<br>(0.0 to 0.0)	6.5<br>(5.7 to 7.3)	4.7<br>(4.2 to 5.3)	1.42<br>(1.26 to 1.60)	1.04<br>(0.92 to 1.16)
2015	0.0<br>(0.0 to 0.0)	0.0<br>(0.0 to 0.0)	3.9<br>(3.0 to 5.0)	2.8<br>(2.1 to 3.5)	1.62<br>(1.25 to 2.09)	1.14<br>(0.88 to 1.46)
2020	0.0<br>(0.0 to 0.0)	0.0<br>(0.0 to 0.0)	3.2<br>(2.3 to 4.3)	2.1<br>(1.5 to 2.9)	1.74<br>(1.24 to 2.39)	1.18<br>(0.85 to 1.61)
2021	0.0<br>(0.0 to 0.0)	0.0<br>(0.0 to 0.0)	3.1<br>(2.2 to 4.3)	2.1<br>(1.5 to 2.9)	1.75<br>(1.25 to 2.44)	1.18<br>(0.85 to 1.66)

North Macedonia   MKD   Central Europe   Stillbirth Rate / Neonatal Mortality Rate (SBR/NMR) − 20 weeks
lambda = 0.2 ; zeta = 0.99 ; scale = 5 ; amp = 0.028

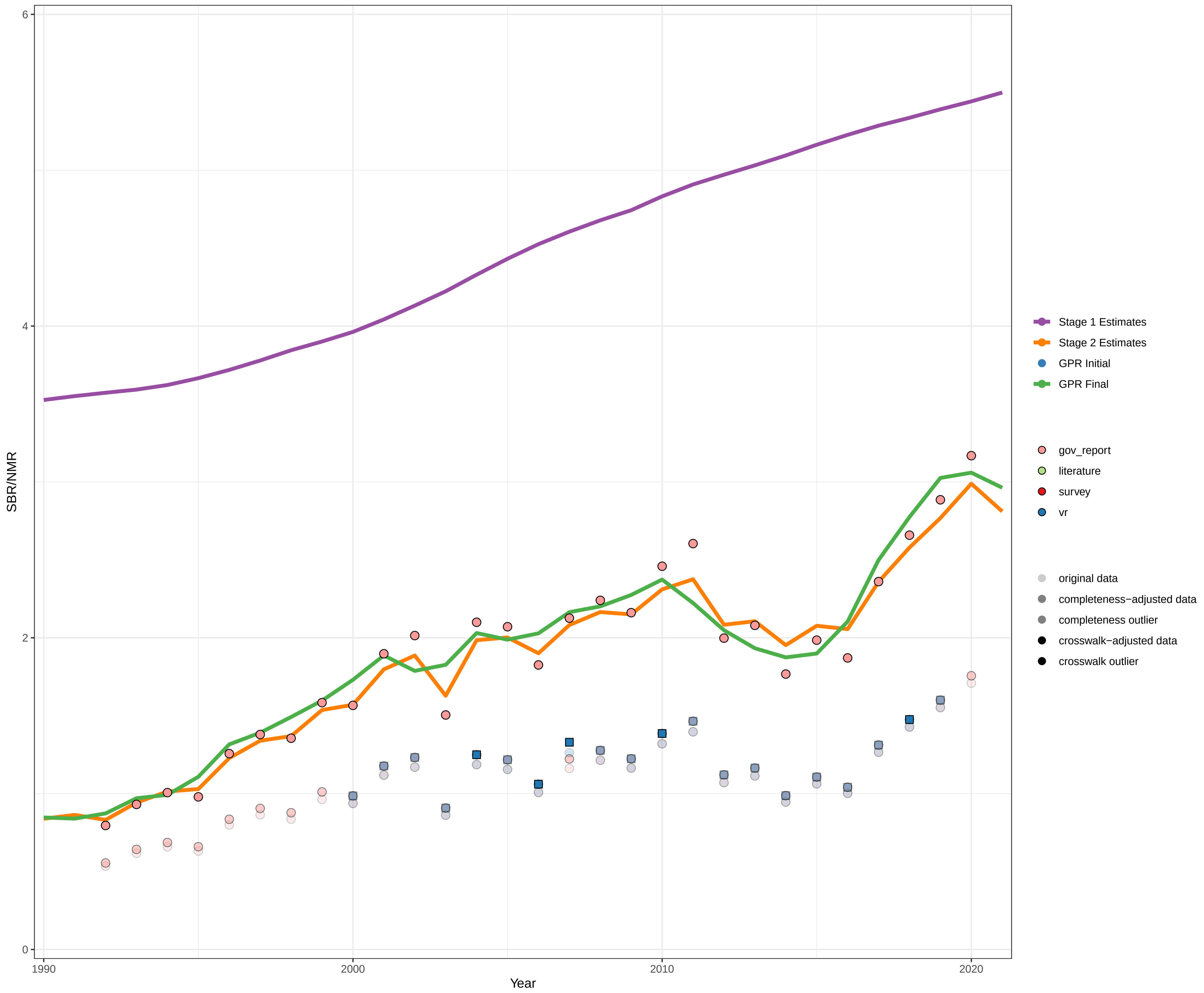

North Macedonia   MKD   Central Europe   Stillbirth Rate / Neonatal Mortality Rate (SBR/NMR) − 28 weeks
lambda = 0.2 ; zeta = 0.99 ; scale = 5 ; amp = 0.028

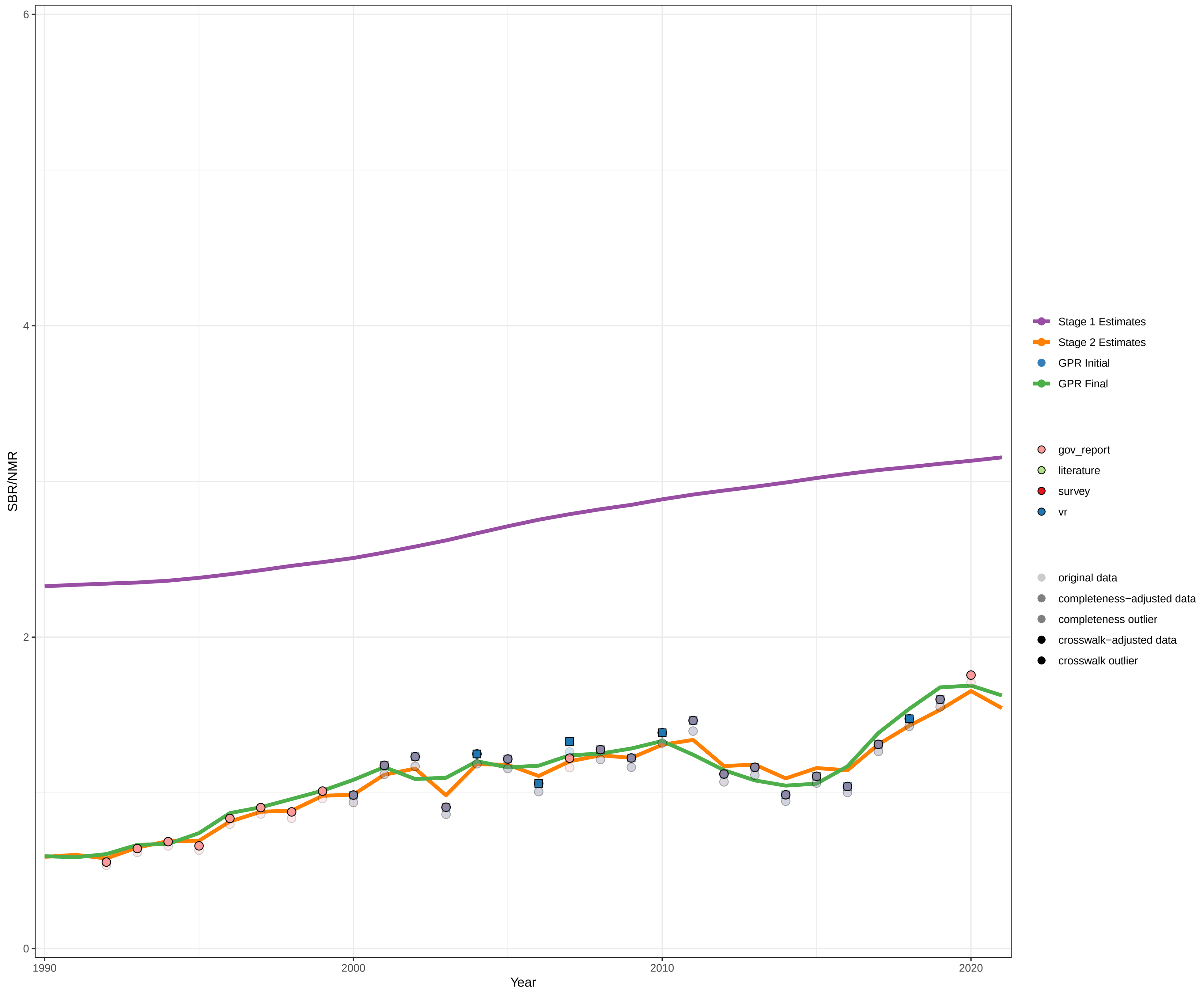

Definition	Years	Outlier	Source.Type	Title
28_weeks	1992−2020	0	Statistical Report	Macedonia Statistical Yearbook
28_weeks	2010	0	VR	United Nations Demographic Yearbook
28_weeks	2000−2003, 2005, 2008, 2009, 2011−2017, 2019	1	VR	United Nations Demographic Yearbook
28_weeks	2004, 2006, 2007, 2018	2	VR	United Nations Demographic Yearbook

North Macedonia   MKD   Central Europe   Stillbirth Rate (SBR) − 20 weeks
lambda = 0.2 ; zeta = 0.99 ; scale = 5 ; amp = 0.028

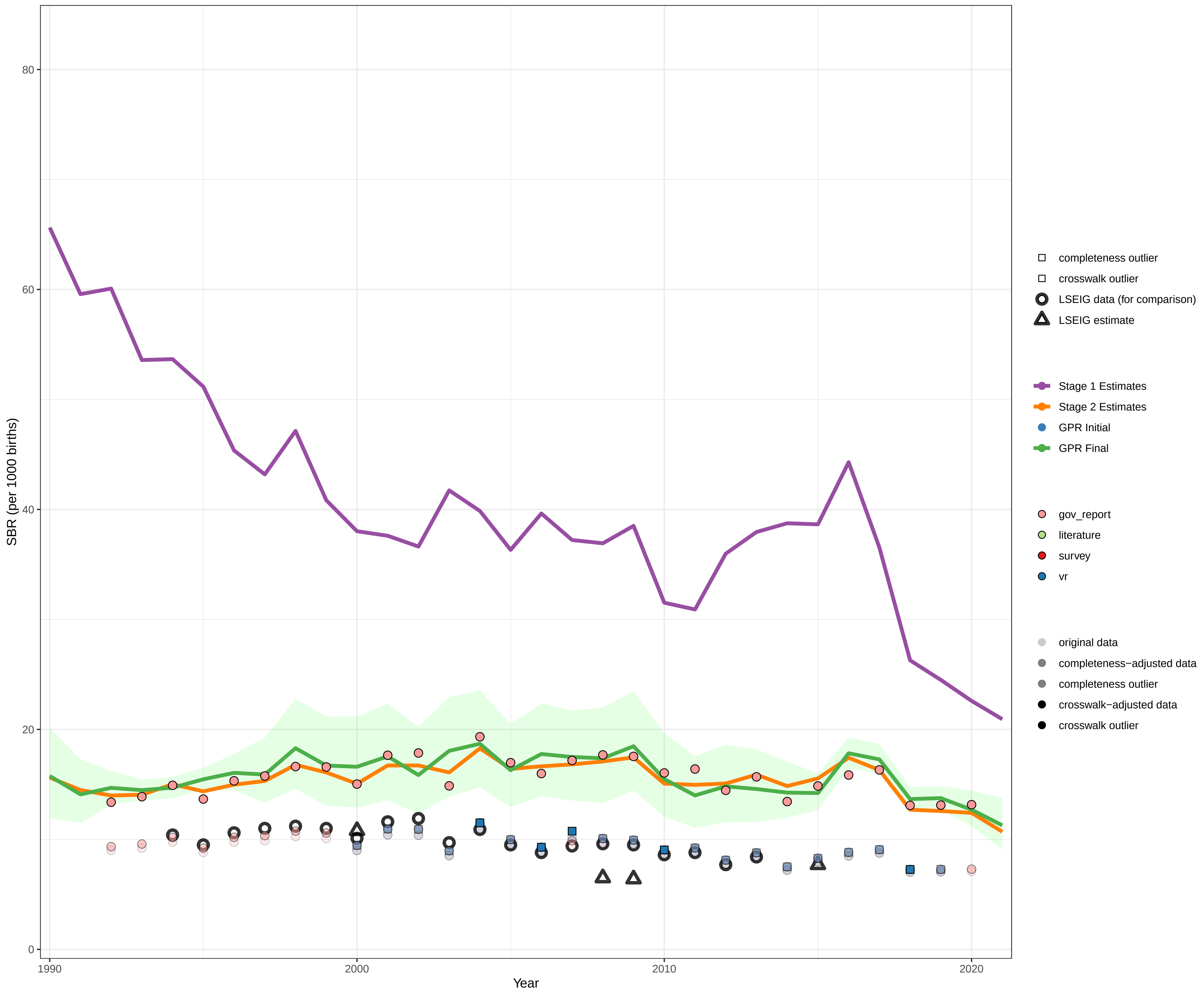

North Macedonia   MKD   Central Europe   Stillbirth Rate (SBR) − 28 weeks
lambda = 0.2 ; zeta = 0.99 ; scale = 5 ; amp = 0.028

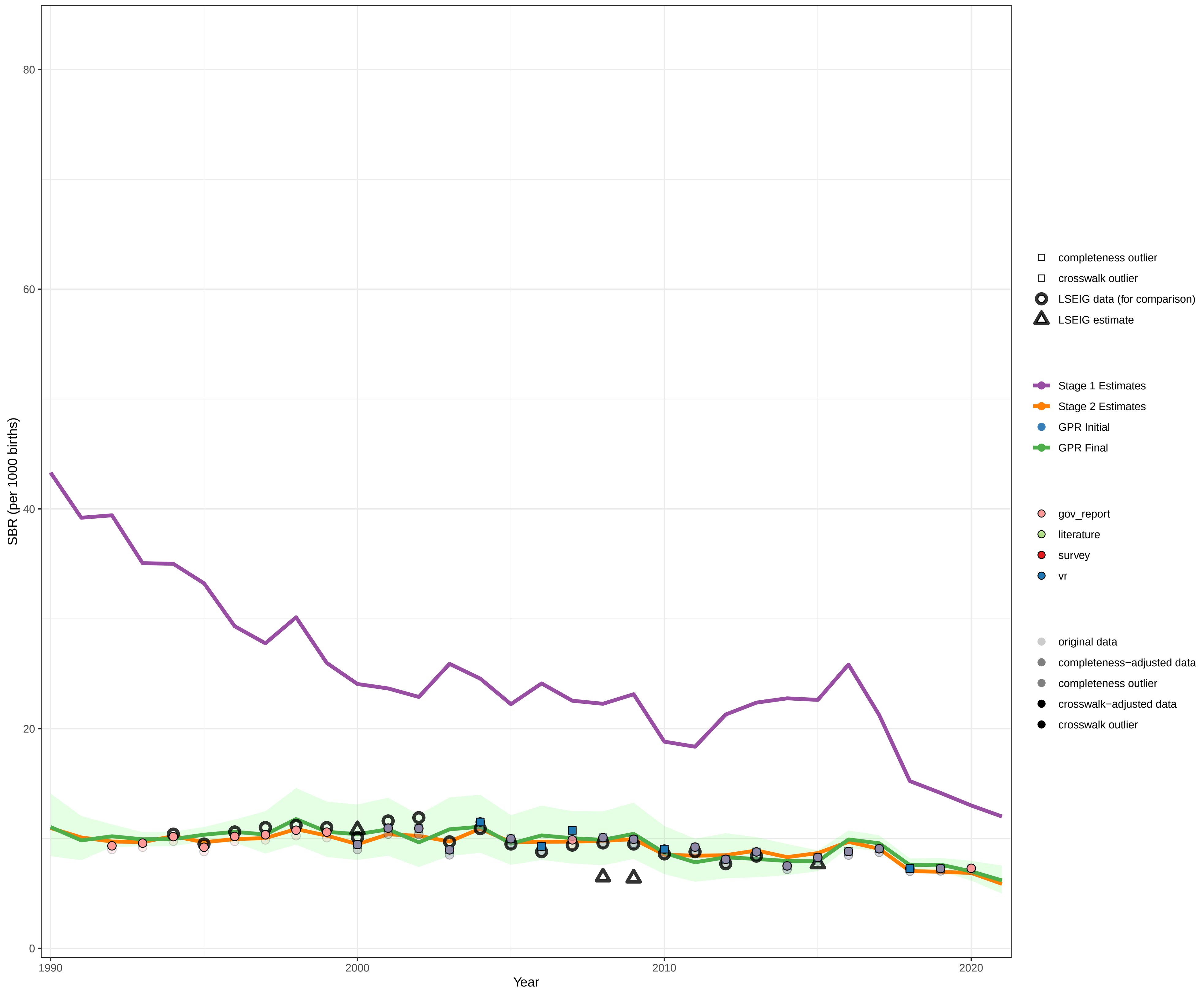

Year	Total Stillbirths (in thousands)	Total Stillbirths (in thousands)	SBR (per 1000 births)	SBR (per 1000 births)	SBR/NMR	SBR/NMR
	20 weeks	28 weeks	20 weeks	28 weeks	20 weeks	28 weeks
1990	0.6 (0.4 to 0.7)	0.4 (0.3 to 0.5)	15.8 (12.0 to 20.2)	11.0 (8.4 to 14.1)	0.85 (0.64 to 1.08)	0.59 (0.45 to 0.76)
1995	0.5 (0.5 to 0.5)	0.3 (0.3 to 0.4)	15.5 (14.5 to 16.5)	10.3 (9.7 to 11.1)	1.11 (1.04 to 1.18)	0.74 (0.70 to 0.79)
2000	0.5 (0.4 to 0.6)	0.3 (0.2 to 0.4)	16.6 (12.9 to 21.2)	10.4 (8.0 to 13.1)	1.73 (1.34 to 2.21)	1.08 (0.84 to 1.37)
2005	0.4 (0.3 to 0.5)	0.2 (0.2 to 0.3)	16.3 (12.9 to 20.5)	9.5 (7.6 to 12.1)	1.99 (1.58 to 2.50)	1.16 (0.93 to 1.48)
2010	0.4 (0.3 to 0.5)	0.2 (0.2 to 0.3)	15.5 (12.1 to 19.6)	8.7 (6.8 to 11.1)	2.37 (1.85 to 3.01)	1.33 (1.04 to 1.71)
2015	0.3 (0.3 to 0.4)	0.2 (0.2 to 0.2)	14.2 (12.6 to 16.0)	7.9 (7.0 to 8.9)	1.90 (1.69 to 2.14)	1.06 (0.94 to 1.19)
2020	0.2 (0.2 to 0.3)	0.1 (0.1 to 0.2)	12.7 (11.2 to 14.4)	7.0 (6.2 to 8.0)	3.06 (2.70 to 3.48)	1.69 (1.49 to 1.92)
2021	0.2 (0.2 to 0.3)	0.1 (0.1 to 0.1)	11.3 (9.1 to 13.8)	6.2 (5.0 to 7.5)	2.96 (2.38 to 3.62)	1.63 (1.31 to 1.98)

Poland POL Central Europe Stillbirth Rate / Neonatal Mortality Rate (SBR/NMR) − 20 weeks
lambda = 0.2 ; zeta = 0.99 ; scale = 5 ; amp = 0.028

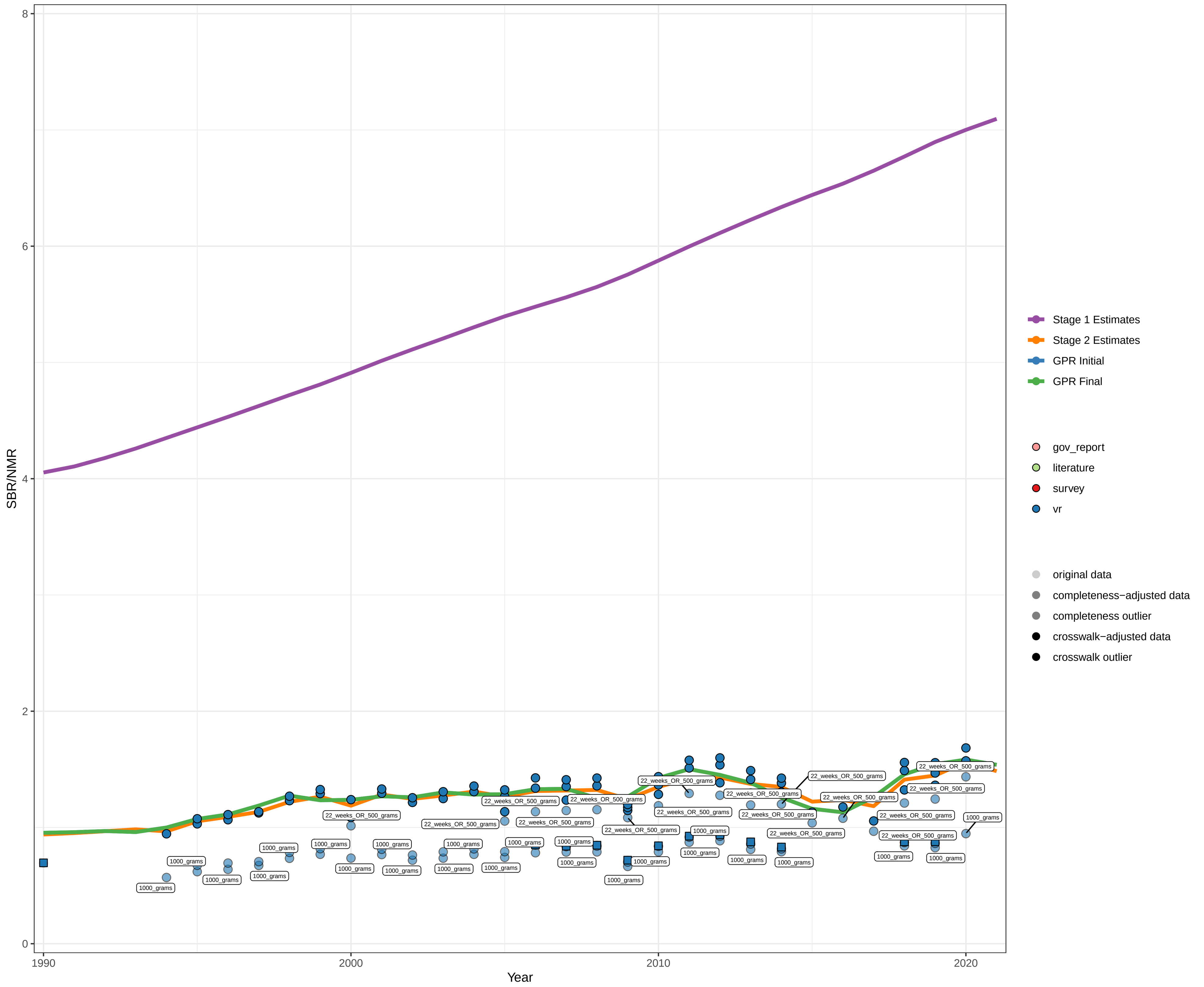

Poland POL Central Europe Stillbirth Rate / Neonatal Mortality Rate (SBR/NMR) − 28 weeks
lambda = 0.2 ; zeta = 0.99 ; scale = 5 ; amp = 0.028

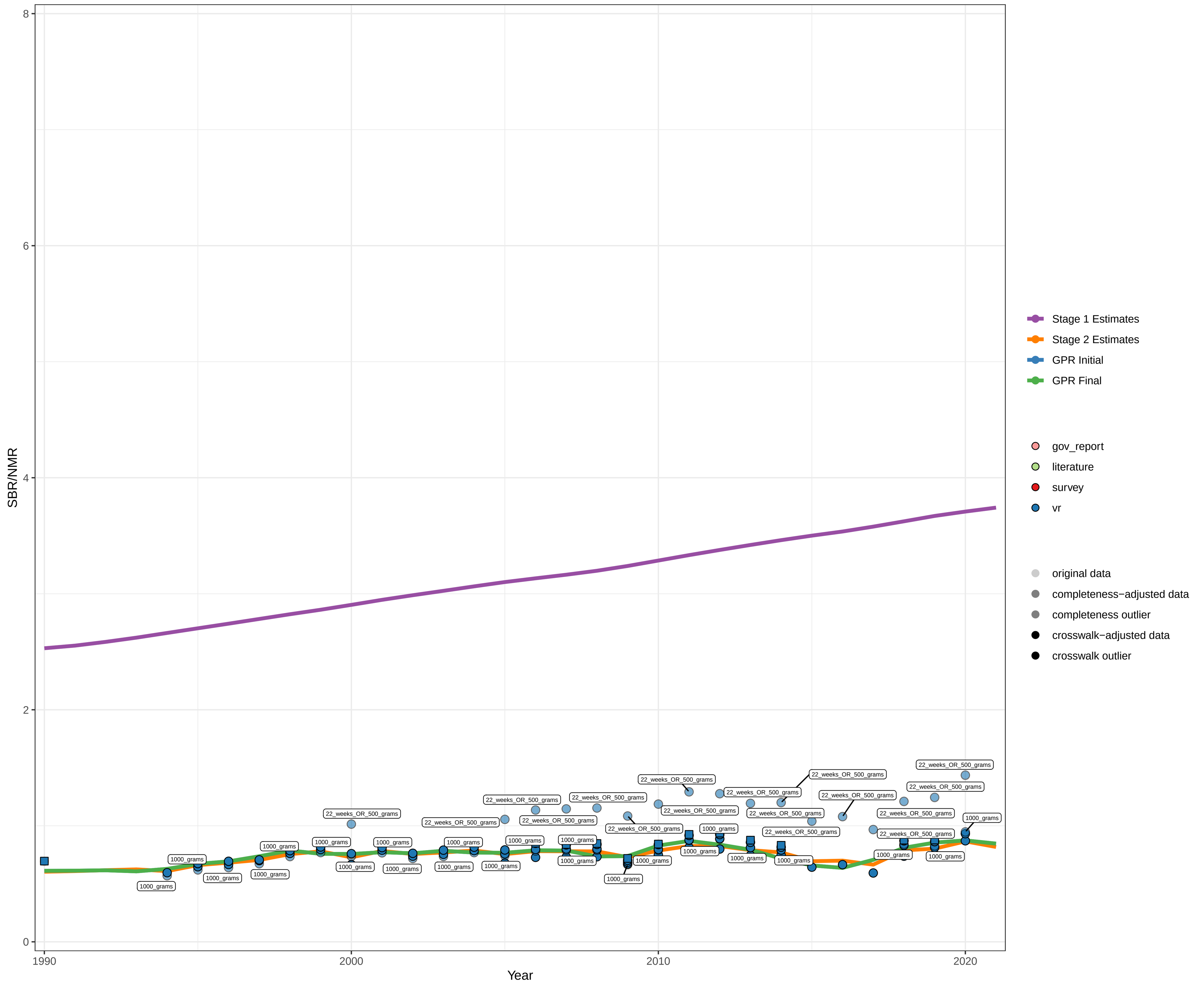

Definition	Years	Outlier	Source.Type	Title
1000_grams	1994−2014, 2018−2020	0	VR	European Health for All Database
22_weeks_OR_500_grams	1990, 2000, 2005−2020	0	VR	Poland Demographic Yearbook
28_weeks	1995−1999, 2001−2005	0	VR	United Nations Demographic Yearbook
28_weeks	2006−2014, 2018, 2019	0	VR	Poland Demographic Yearbook
28_weeks	2006−2014, 2018, 2019	2	VR	United Nations Demographic Yearbook

Poland POL Central Europe Stillbirth Rate (SBR) − 20 weeks
lambda = 0.2 ; zeta = 0.99 ; scale = 5 ; amp = 0.028

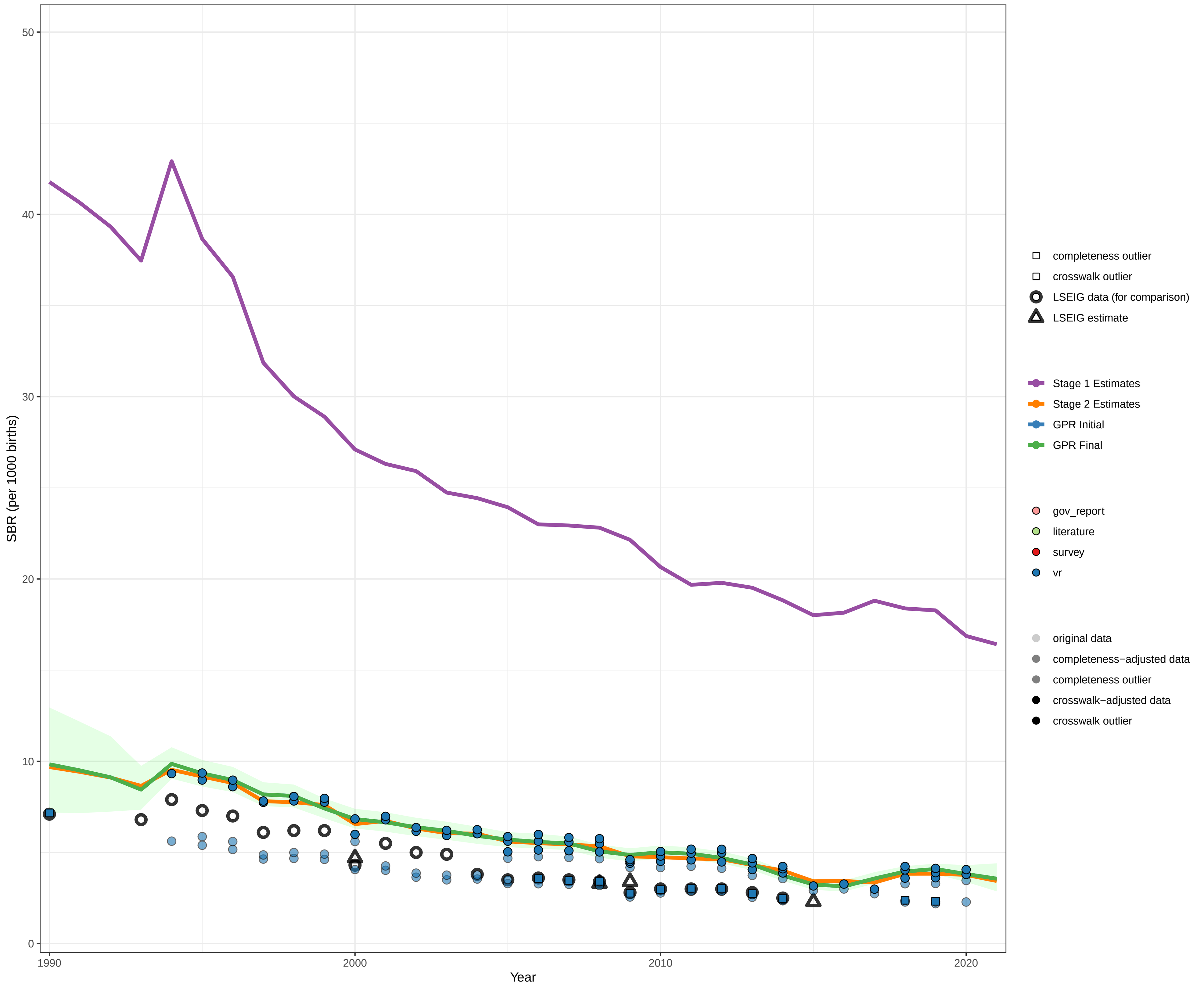

Poland POL Central Europe Stillbirth Rate (SBR) − 28 weeks
lambda = 0.2 ; zeta = 0.99 ; scale = 5 ; amp = 0.028

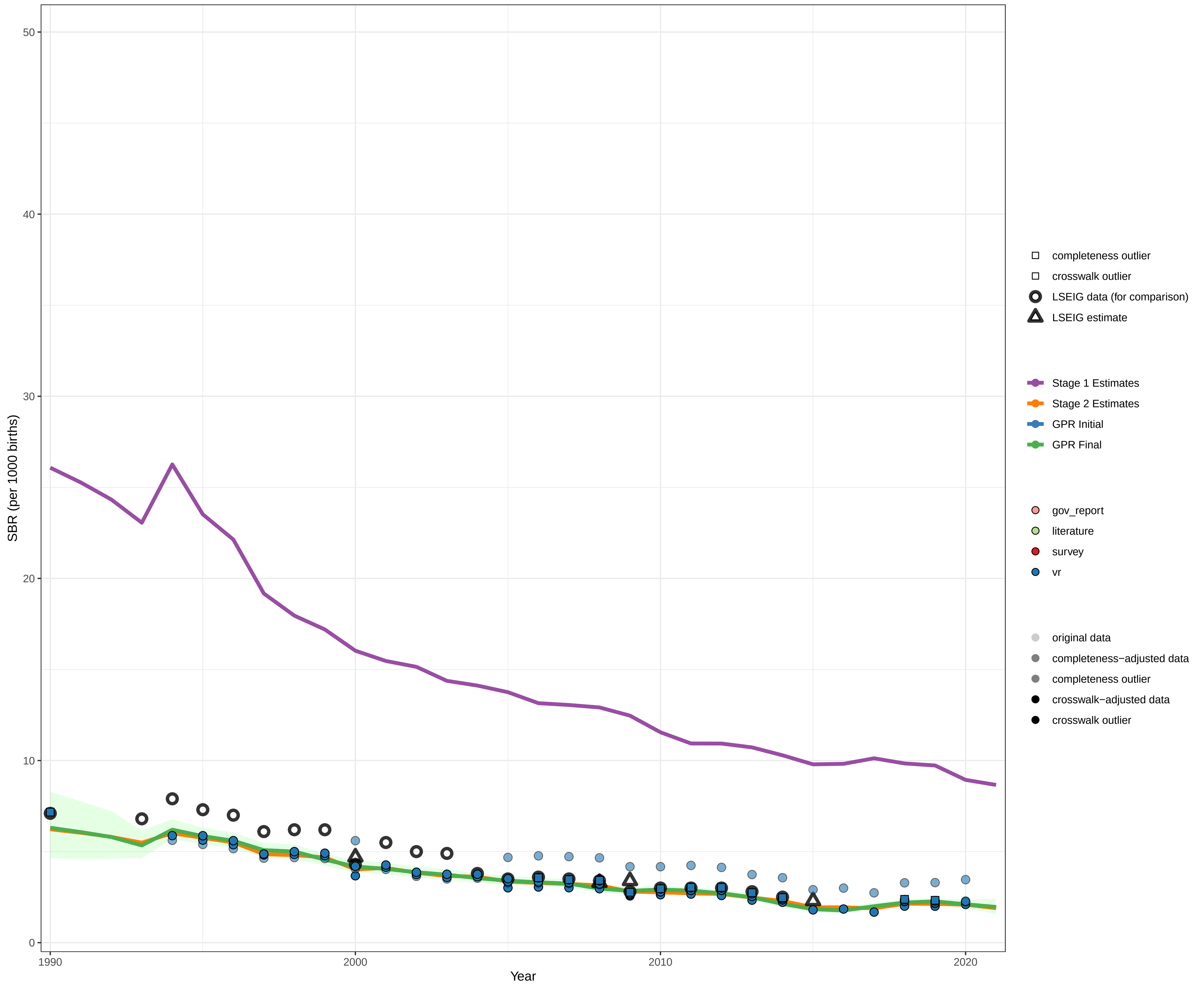

Year	Total Stillbirths (in thousands)	Total Stillbirths (in thousands)	SBR (per 1000 births)	SBR (per 1000 births)	SBR/NMR	SBR/NMR
	20 weeks	28 weeks	20 weeks	28 weeks	20 weeks	28 weeks
1990	5.4 (4.0 to 7.2)	3.5 (2.5 to 4.6)	9.8 (7.2 to 13.0)	6.3 (4.6 to 8.3)	0.95 (0.70 to 1.26)	0.61 (0.45 to 0.80)
1995	4.1 (3.7 to 4.4)	2.5 (2.3 to 2.7)	9.3 (8.6 to 10.1)	5.8 (5.4 to 6.3)	1.07 (0.99 to 1.16)	0.67 (0.62 to 0.72)
2000	2.5 (2.3 to 2.8)	1.5 (1.4 to 1.7)	6.8 (6.3 to 7.4)	4.2 (3.9 to 4.5)	1.24 (1.14 to 1.34)	0.76 (0.70 to 0.82)
2005	2.1 (2.0 to 2.3)	1.3 (1.2 to 1.3)	5.7 (5.3 to 6.1)	3.4 (3.2 to 3.6)	1.29 (1.19 to 1.38)	0.77 (0.71 to 0.82)
2010	2.0 (1.9 to 2.2)	1.2 (1.1 to 1.3)	5.0 (4.7 to 5.4)	2.9 (2.7 to 3.1)	1.43 (1.33 to 1.53)	0.83 (0.77 to 0.89)
2015	1.2 (1.1 to 1.4)	0.7 (0.6 to 0.8)	3.2 (3.0 to 3.6)	1.8 (1.7 to 2.0)	1.16 (1.06 to 1.27)	0.66 (0.60 to 0.72)
2020	1.4 (1.2 to 1.5)	0.8 (0.7 to 0.8)	3.8 (3.4 to 4.3)	2.1 (1.9 to 2.4)	1.58 (1.40 to 1.79)	0.87 (0.78 to 0.98)
2021	1.2 (1.0 to 1.5)	0.7 (0.5 to 0.8)	3.6 (2.9 to 4.4)	2.0 (1.6 to 2.4)	1.54 (1.24 to 1.90)	0.85 (0.68 to 1.04)

Romania ROU Central Europe Stillbirth Rate / Neonatal Mortality Rate (SBR/NMR) − 20 weeks
lambda = 0.2 ; zeta = 0.99 ; scale = 5 ; amp = 0.028

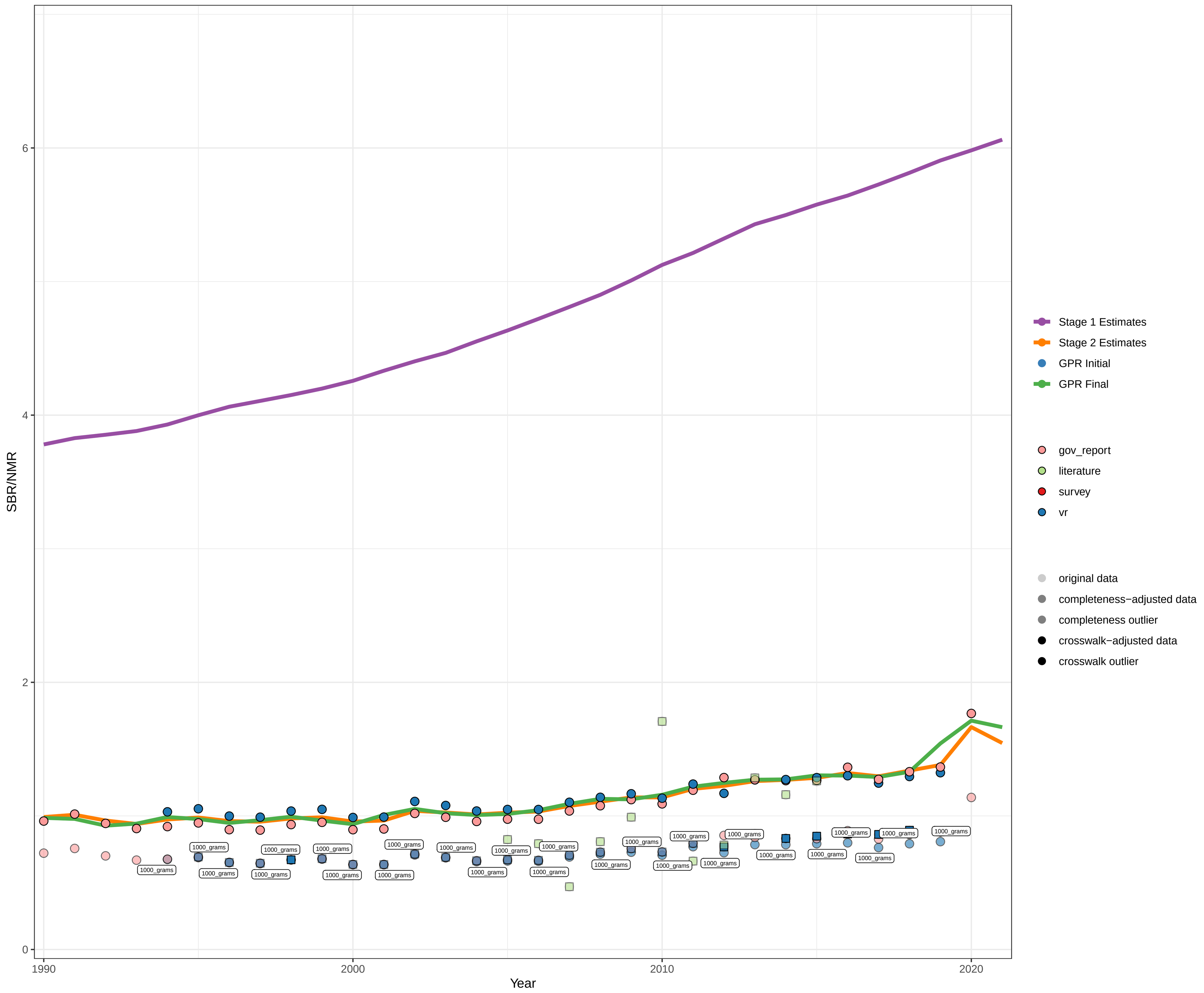

Romania ROU Central Europe Stillbirth Rate / Neonatal Mortality Rate (SBR/NMR) − 28 weeks
lambda = 0.2 ; zeta = 0.99 ; scale = 5 ; amp = 0.028

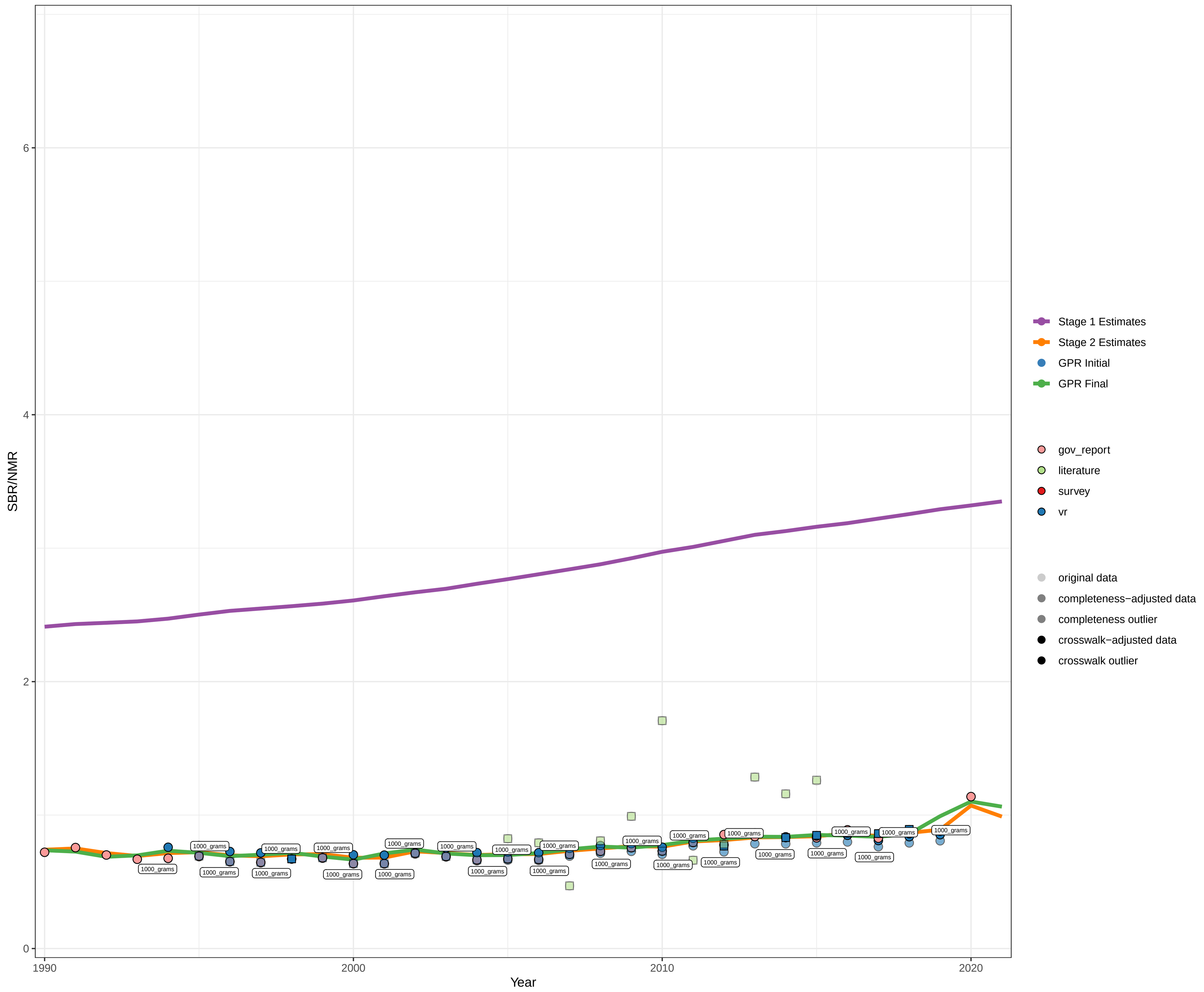

Definition	Years	Outlier	Source.Type	Title
1000_grams	1994−2019	0	VR	European Health for All Database
28_weeks	1990−2020	0	Statistical Report	Romania Statistical Yearbook
28_weeks	1995−1997, 1999−2011	1	VR	United Nations Demographic Yearbook
28_weeks	2005−2015	1	Sci Lit	Mode of Delivery in Stillbirth
28_weeks	1998, 2012−2019	2	VR	United Nations Demographic Yearbook

Romania ROU Central Europe Stillbirth Rate (SBR) − 20 weeks
lambda = 0.2 ; zeta = 0.99 ; scale = 5 ; amp = 0.028

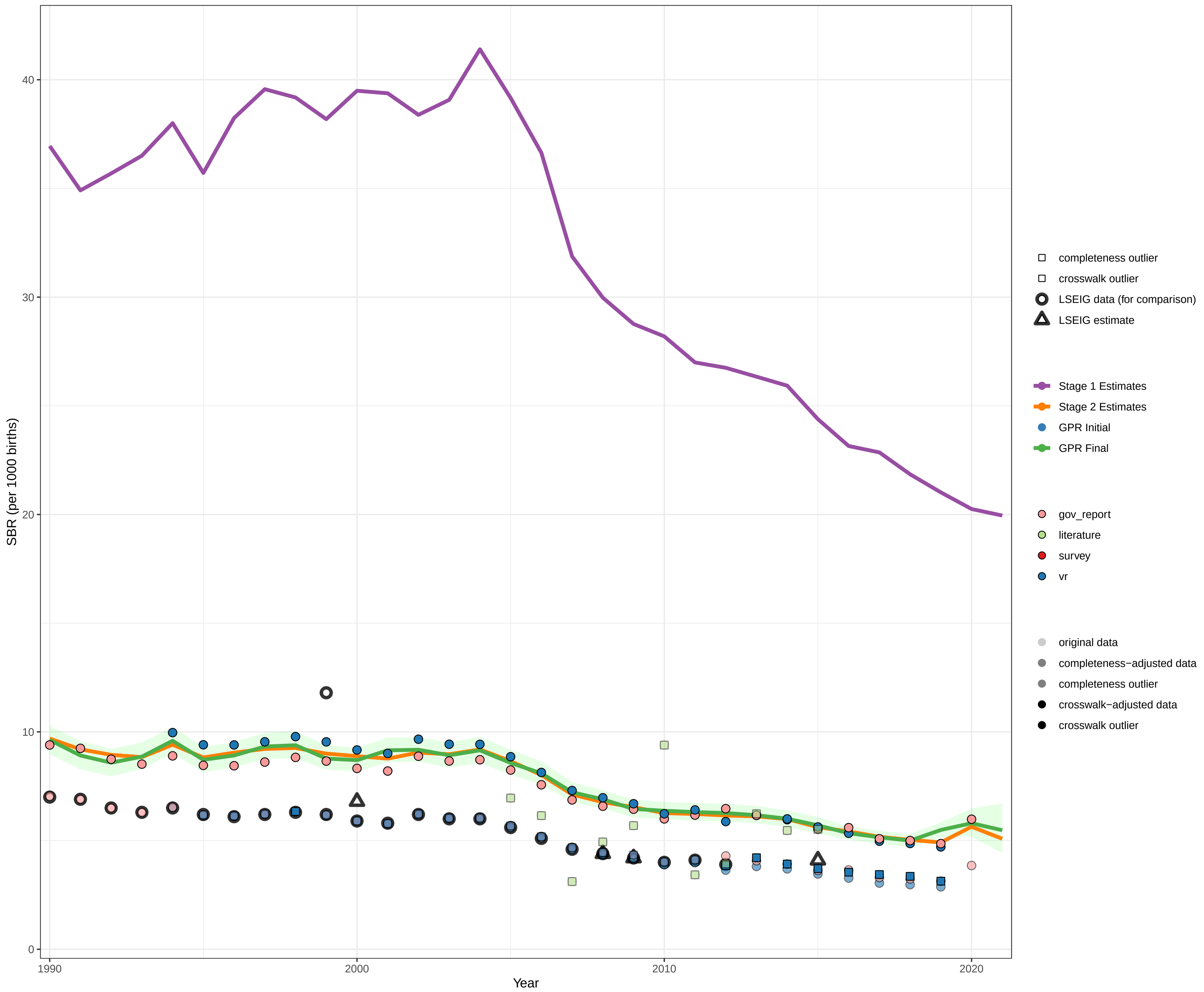

Romania ROU Central Europe Stillbirth Rate (SBR) − 28 weeks
lambda = 0.2 ; zeta = 0.99 ; scale = 5 ; amp = 0.028

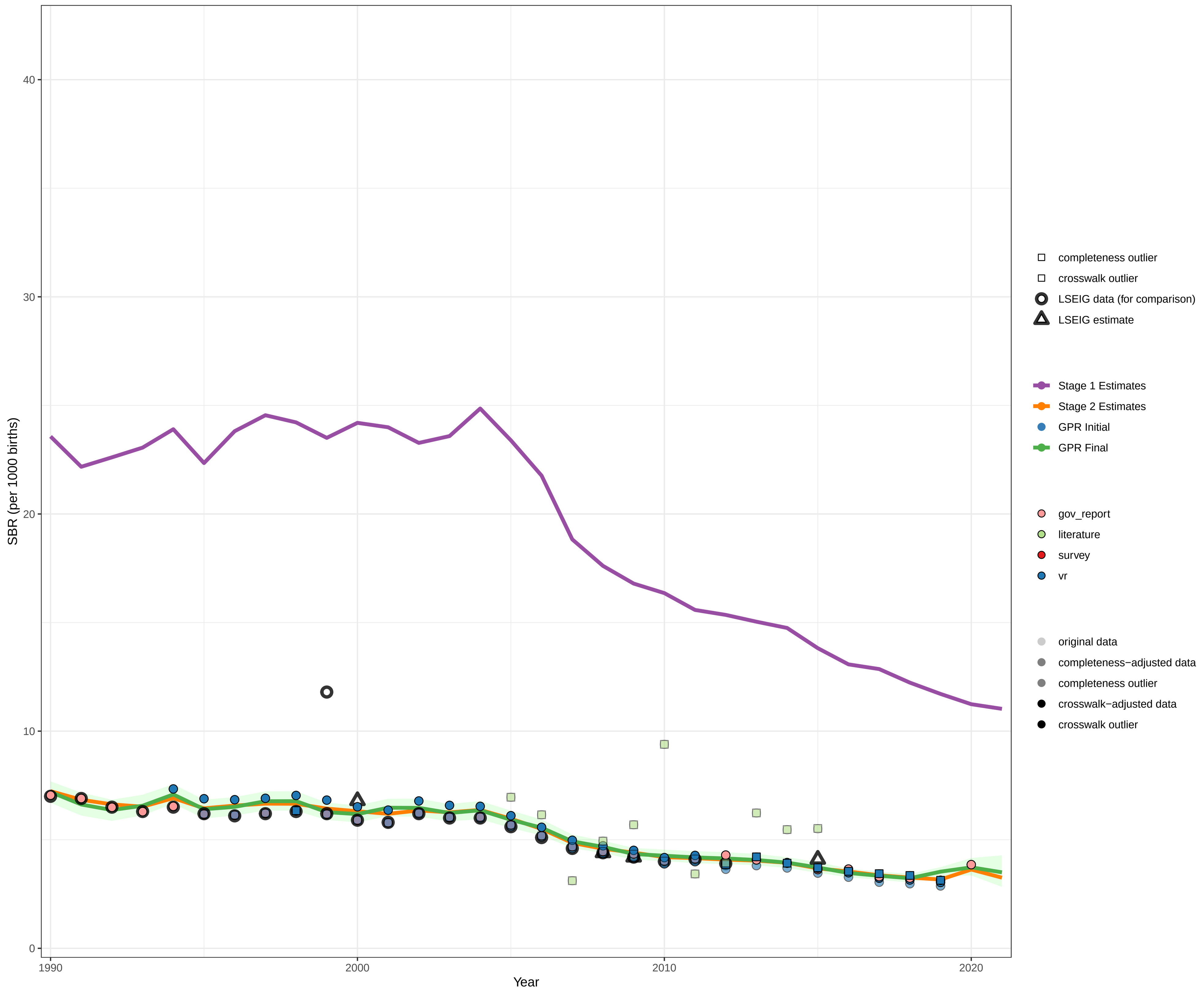

Year	Total Stillbirths (in thousands)	Total Stillbirths (in thousands)	SBR (per 1000 births)	SBR (per 1000 births)	SBR/NMR	SBR/NMR
	20 weeks	28 weeks	20 weeks	28 weeks	20 weeks	28 weeks
1990	2.9 (2.7 to 3.1)	2.2 (2.0 to 2.3)	9.6 (9.0 to 10.3)	7.2 (6.7 to 7.7)	0.99 (0.92 to 1.05)	0.74 (0.69 to 0.79)
1995	2.1 (1.9 to 2.2)	1.5 (1.4 to 1.6)	8.7 (8.1 to 9.3)	6.4 (6.0 to 6.8)	0.98 (0.91 to 1.04)	0.72 (0.67 to 0.77)
2000	1.9 (1.8 to 2.1)	1.4 (1.3 to 1.5)	8.7 (8.2 to 9.3)	6.2 (5.8 to 6.6)	0.94 (0.88 to 1.00)	0.67 (0.63 to 0.71)
2005	1.8 (1.7 to 2.0)	1.3 (1.2 to 1.3)	8.6 (8.0 to 9.2)	5.9 (5.5 to 6.3)	1.01 (0.95 to 1.09)	0.70 (0.66 to 0.75)
2010	1.3 (1.3 to 1.4)	0.9 (0.8 to 1.0)	6.4 (6.0 to 6.8)	4.3 (4.0 to 4.5)	1.16 (1.09 to 1.23)	0.78 (0.73 to 0.83)
2015	1.1 (1.0 to 1.2)	0.7 (0.7 to 0.8)	5.7 (5.3 to 6.1)	3.7 (3.5 to 4.0)	1.30 (1.22 to 1.39)	0.85 (0.80 to 0.91)
2020	1.1 (0.9 to 1.2)	0.7 (0.6 to 0.8)	5.8 (5.2 to 6.5)	3.7 (3.4 to 4.2)	1.71 (1.53 to 1.91)	1.10 (0.99 to 1.23)
2021	1.0 (0.8 to 1.2)	0.6 (0.5 to 0.8)	5.5 (4.4 to 6.7)	3.5 (2.8 to 4.3)	1.66 (1.34 to 2.04)	1.06 (0.86 to 1.30)

Serbia SRB Central Europe Stillbirth Rate / Neonatal Mortality Rate (SBR/NMR) − 20 weeks
lambda = 0.4 ; zeta = 0.9 ; scale = 10 ; amp = 0.028

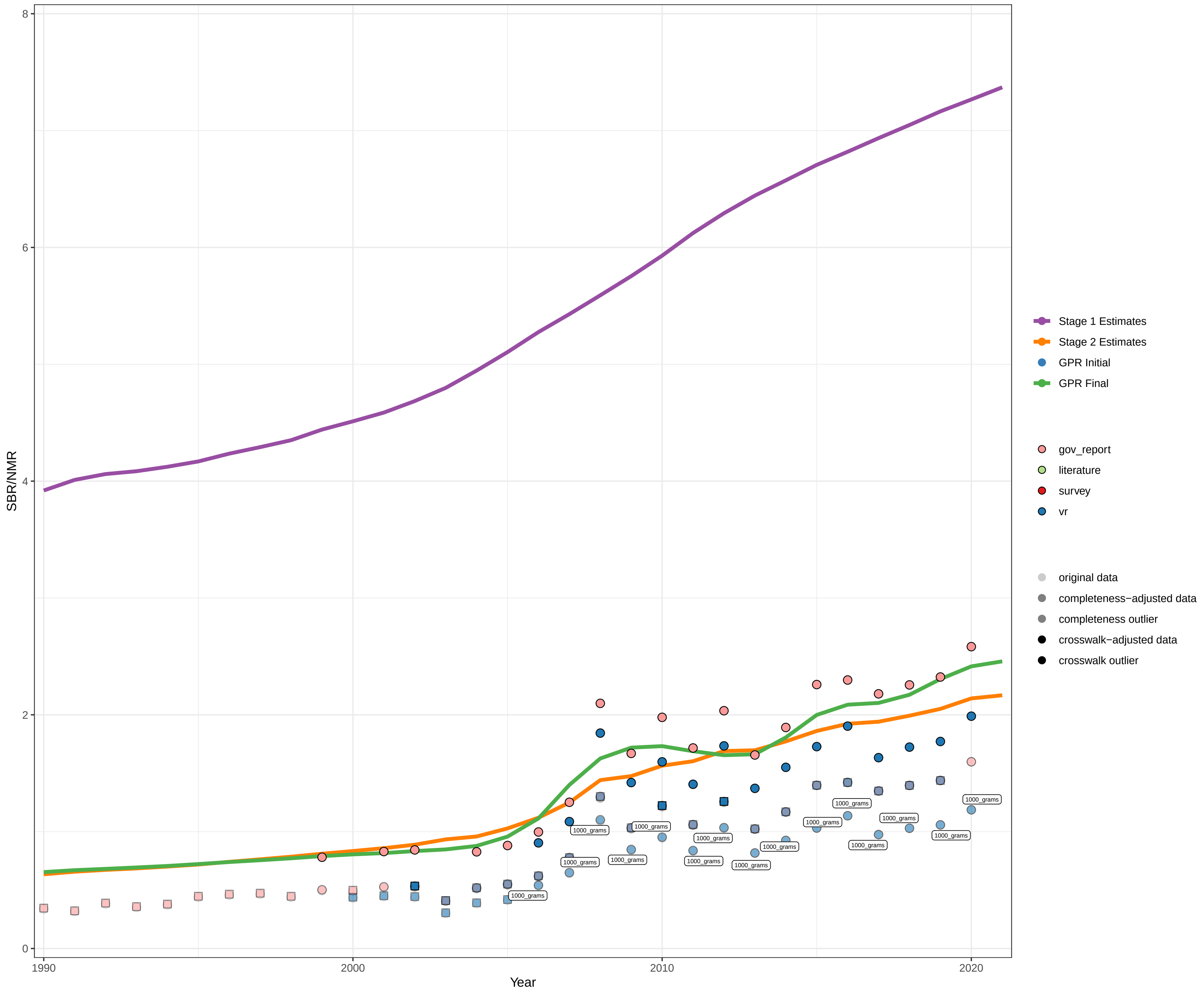

Serbia SRB Central Europe Stillbirth Rate / Neonatal Mortality Rate (SBR/NMR) − 28 weeks
lambda = 0.4 ; zeta = 0.9 ; scale = 10 ; amp = 0.028

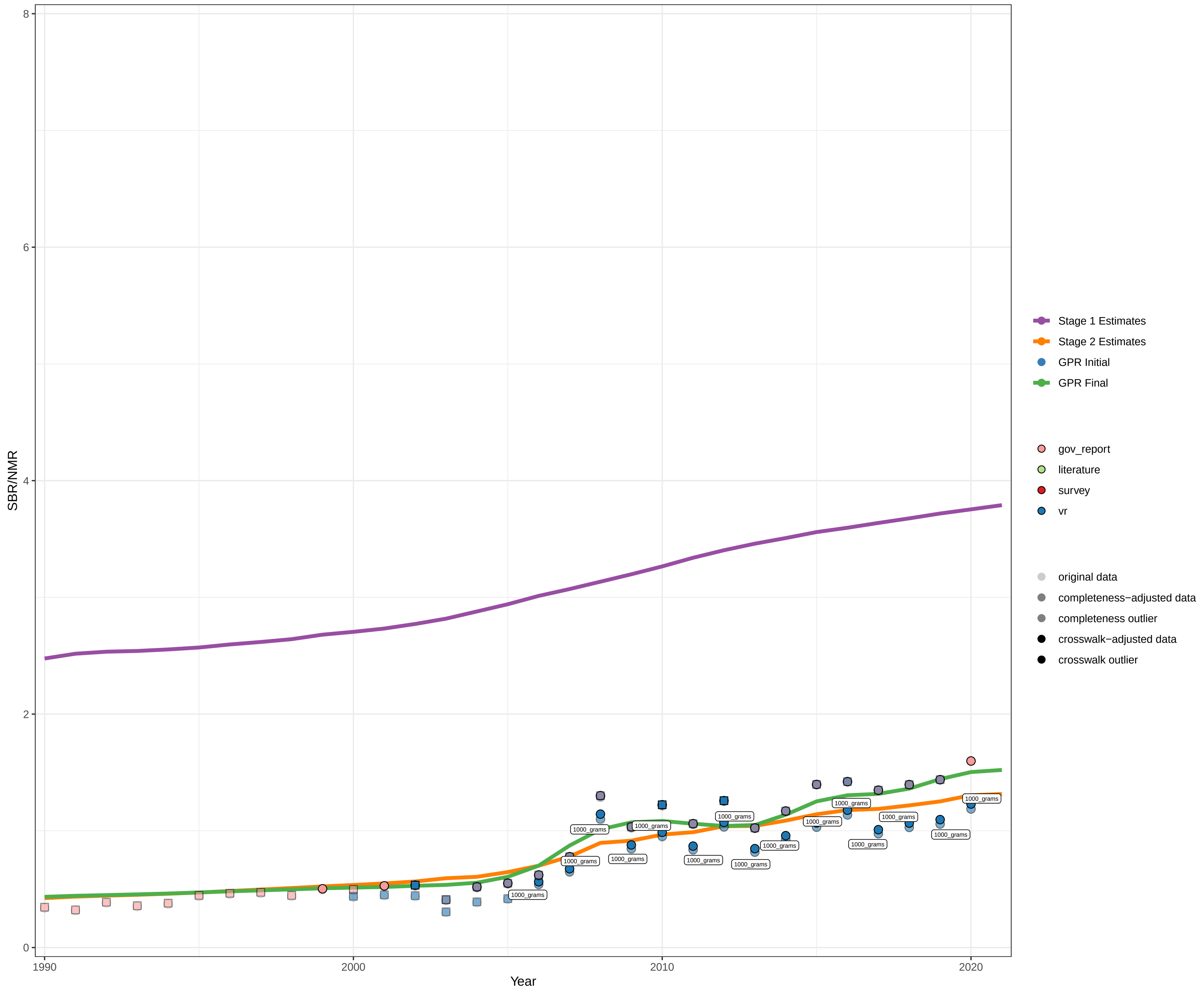

Definition	Years	Outlier	Source.Type	Title
1000_grams	2006−2020	0	VR	European Health for All Database
28_weeks	1999, 2001, 2002, 2004−2020	0	Statistical Report	Serbia Demographic Yearbook
1000_grams	2000−2005	1	VR	European Health for All Database
28_weeks	1990−1998, 2000, 2003	1	Statistical Report	Serbia Demographic Yearbook
28_weeks	2003−2009, 2011, 2013−2019	1	VR	United Nations Demographic Yearbook
28_weeks	2002, 2010, 2012	2	VR	United Nations Demographic Yearbook

Serbia SRB Central Europe Stillbirth Rate (SBR) − 20 weeks
lambda = 0.4 ; zeta = 0.9 ; scale = 10 ; amp = 0.028

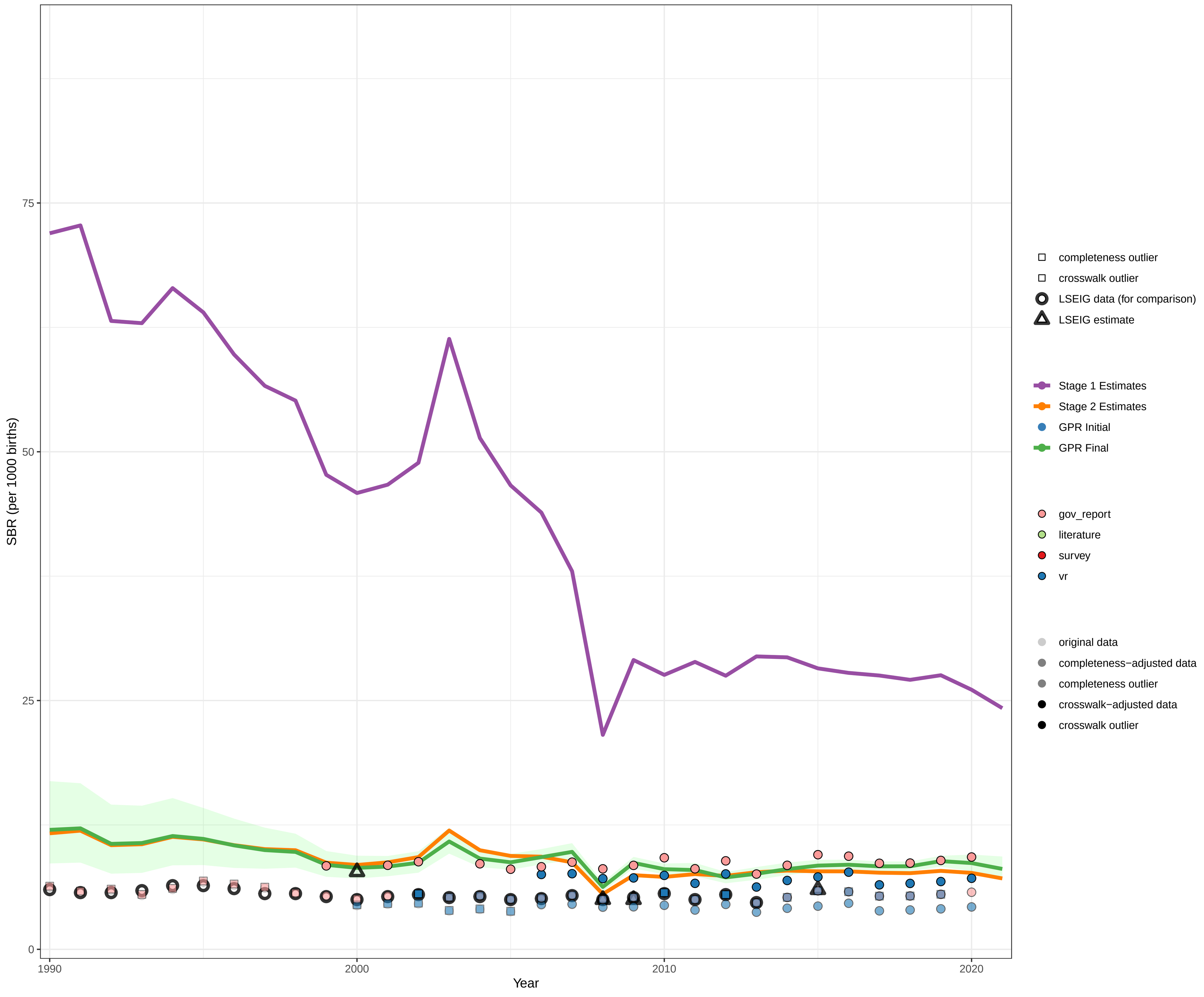

Serbia SRB Central Europe Stillbirth Rate (SBR) − 28 weeks
lambda = 0.4 ; zeta = 0.9 ; scale = 10 ; amp = 0.028

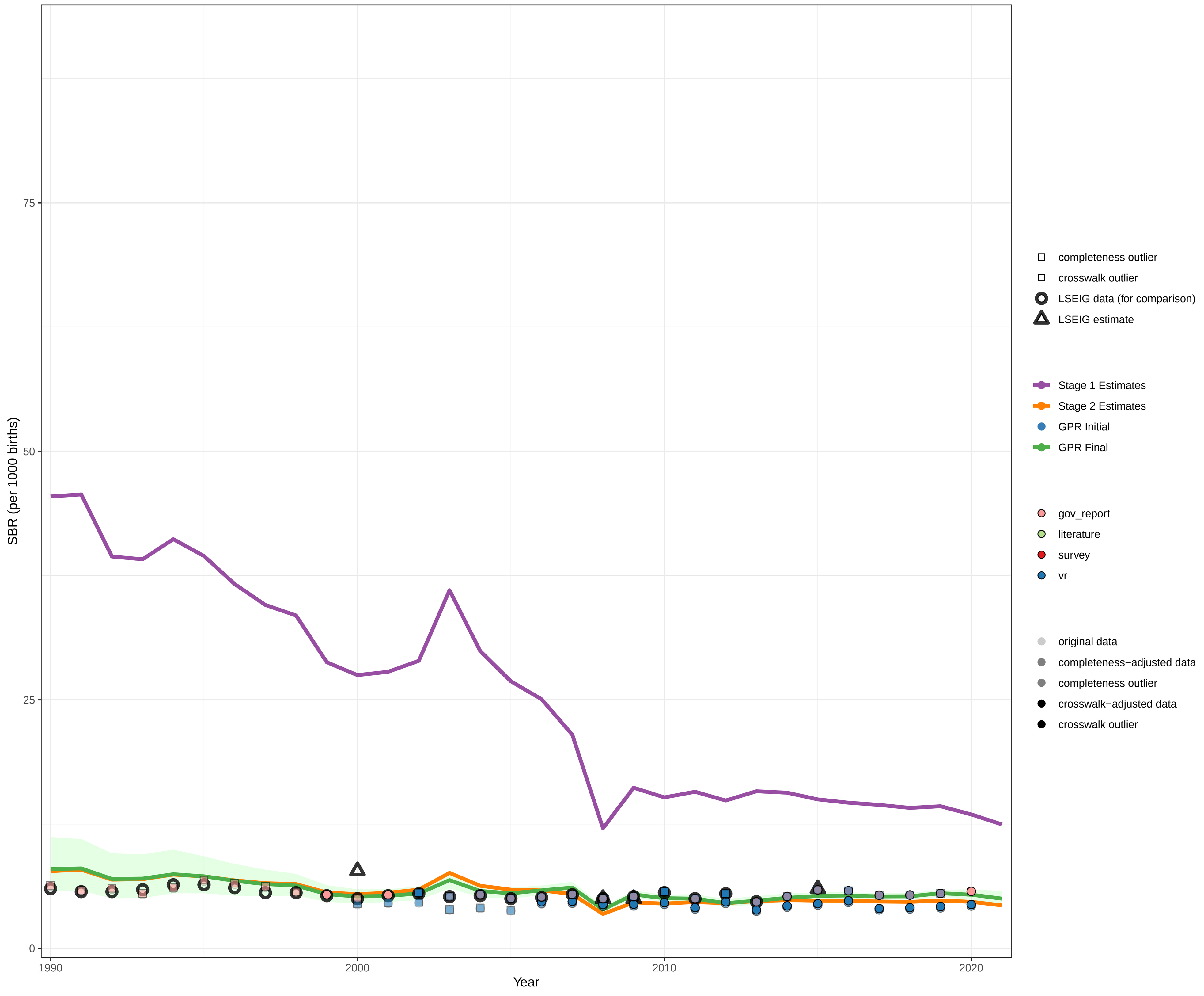

Year	Total Stillbirths (in thousands)	Total Stillbirths (in thousands)	SBR (per 1000 births)	SBR (per 1000 births)	SBR/NMR	SBR/NMR
	20 weeks	28 weeks	20 weeks	28 weeks	20 weeks	28 weeks
1990	1.7 (1.2 to 2.5)	1.1 (0.8 to 1.6)	12.0 (8.6 to 16.9)	8.0 (5.7 to 11.2)	0.65 (0.47 to 0.92)	0.43 (0.31 to 0.61)
1995	1.5 (1.1 to 1.9)	1.0 (0.7 to 1.2)	11.1 (8.5 to 14.2)	7.2 (5.5 to 9.3)	0.72 (0.55 to 0.93)	0.47 (0.36 to 0.60)
2000	0.9 (0.7 to 1.0)	0.5 (0.5 to 0.6)	8.2 (7.1 to 9.4)	5.2 (4.5 to 5.9)	0.81 (0.70 to 0.93)	0.51 (0.45 to 0.58)
2005	0.8 (0.8 to 0.9)	0.5 (0.5 to 0.6)	8.7 (8.0 to 9.6)	5.5 (5.0 to 6.1)	0.96 (0.88 to 1.05)	0.61 (0.55 to 0.67)
2010	0.6 (0.6 to 0.7)	0.4 (0.4 to 0.4)	8.1 (7.5 to 8.6)	5.0 (4.7 to 5.4)	1.73 (1.62 to 1.85)	1.08 (1.01 to 1.16)
2015	0.6 (0.5 to 0.6)	0.3 (0.3 to 0.4)	8.4 (7.8 to 9.0)	5.3 (4.9 to 5.6)	2.00 (1.86 to 2.14)	1.25 (1.16 to 1.34)
2020	0.5 (0.5 to 0.6)	0.3 (0.3 to 0.4)	8.7 (7.9 to 9.5)	5.4 (4.9 to 5.9)	2.41 (2.19 to 2.65)	1.50 (1.36 to 1.65)
2021	0.5 (0.4 to 0.6)	0.3 (0.3 to 0.4)	8.1 (7.0 to 9.3)	5.0 (4.3 to 5.8)	2.46 (2.12 to 2.84)	1.52 (1.31 to 1.76)

Slovakia SVK Central Europe Stillbirth Rate / Neonatal Mortality Rate (SBR/NMR) − 20 weeks
lambda = 0.2 ; zeta = 0.99 ; scale = 5 ; amp = 0.028

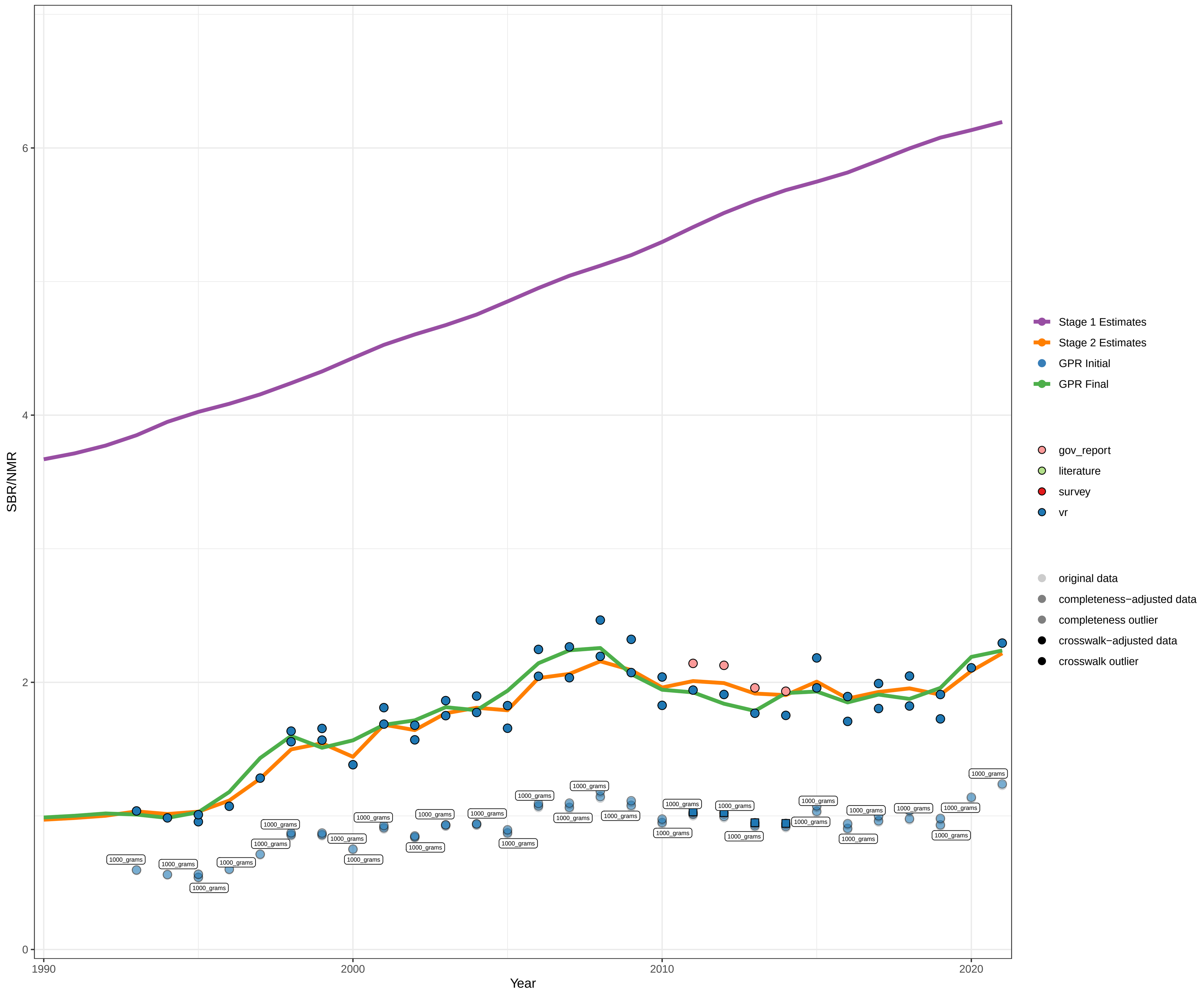

Slovakia SVK Central Europe Stillbirth Rate / Neonatal Mortality Rate (SBR/NMR) − 28 weeks
lambda = 0.2 ; zeta = 0.99 ; scale = 5 ; amp = 0.028

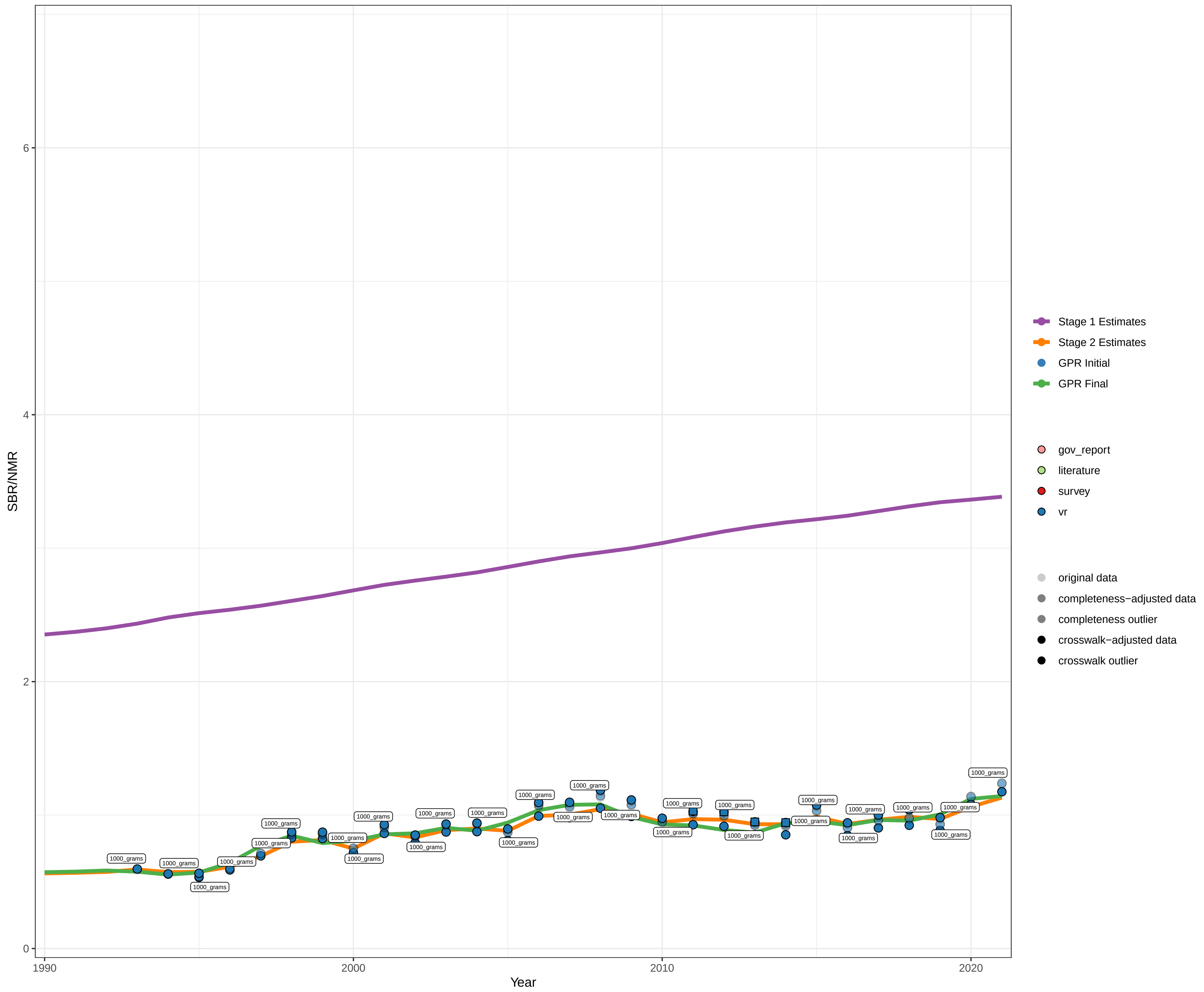

Definition	Years	Outlier	Source.Type	Title
1000_grams	1993−2021	0	VR	European Health for All Database
28_weeks	1995, 1998, 1999, 2001−2010, 2015−2019	0	VR	United Nations Demographic Yearbook
28_weeks	2011−2014	0	Statistical Report	Population Change in the Slovak Republic
28_weeks	2011−2014	2	VR	United Nations Demographic Yearbook

Slovakia SVK Central Europe Stillbirth Rate (SBR) − 20 weeks
lambda = 0.2 ; zeta = 0.99 ; scale = 5 ; amp = 0.028

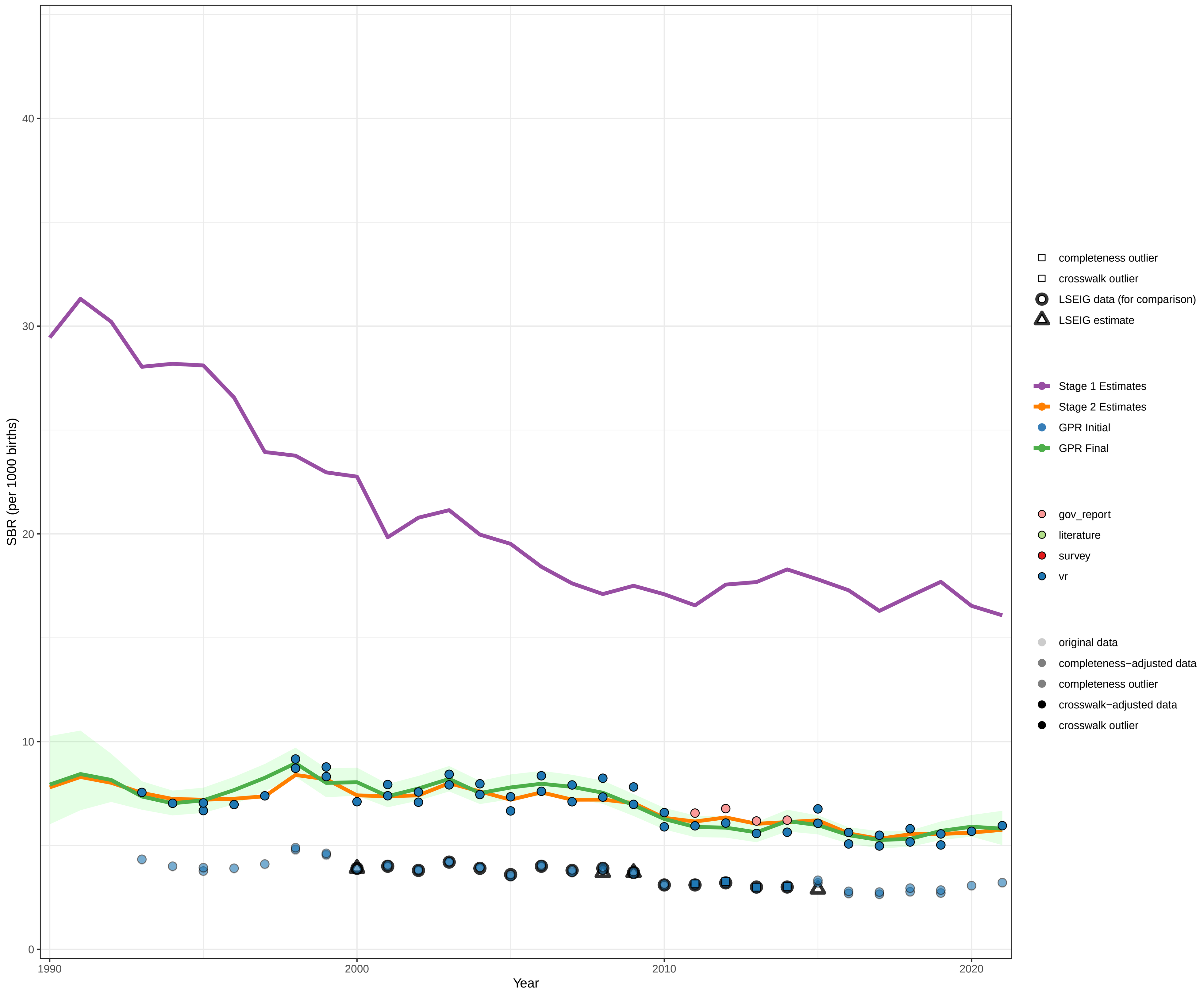

Slovakia SVK Central Europe Stillbirth Rate (SBR) − 28 weeks
lambda = 0.2 ; zeta = 0.99 ; scale = 5 ; amp = 0.028

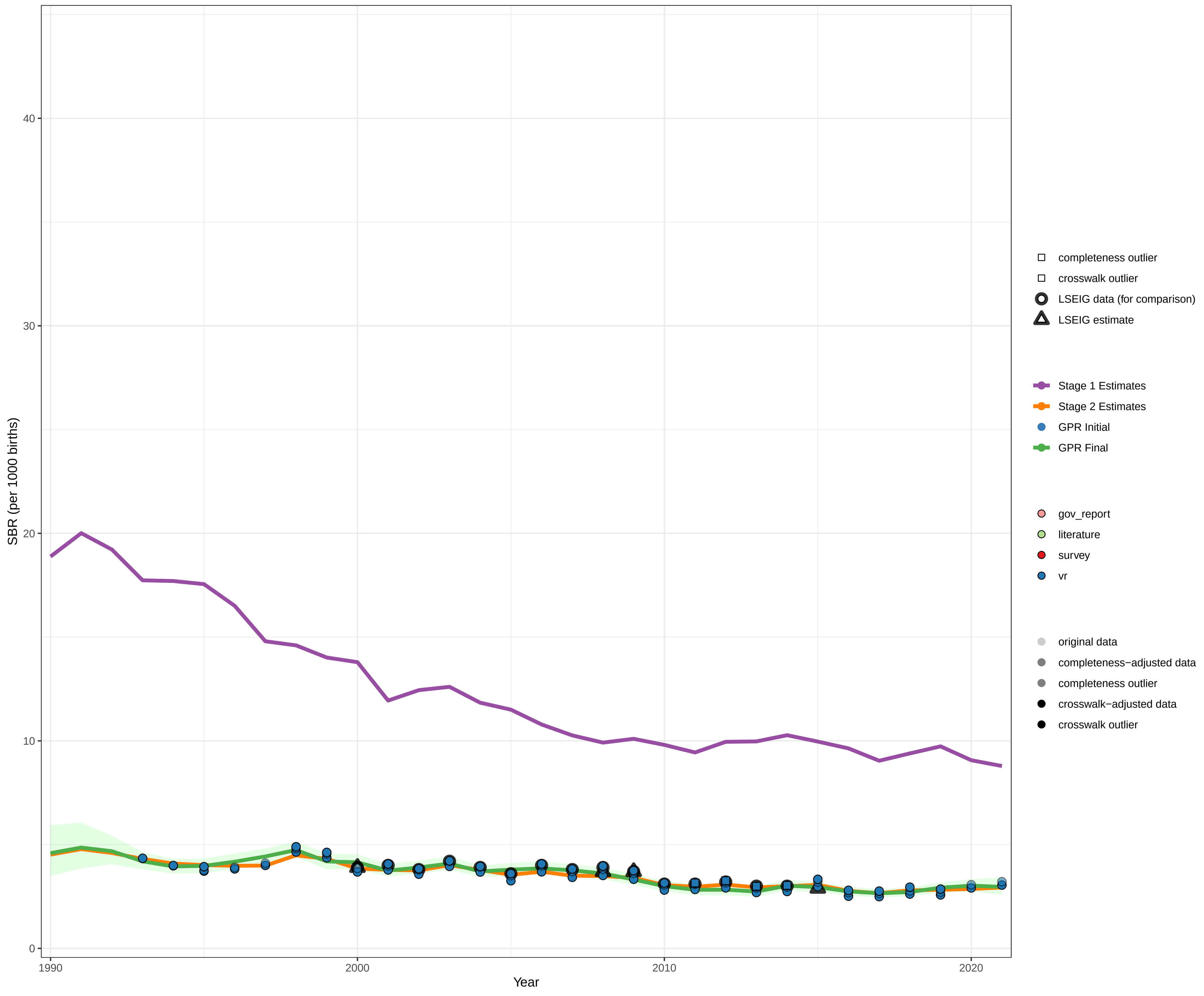

Year	Total Stillbirths (in thousands)	Total Stillbirths (in thousands)	SBR (per 1000 births)	SBR (per 1000 births)	SBR/NMR	SBR/NMR
	20 weeks	28 weeks	20 weeks	28 weeks	20 weeks	28 weeks
1990	0.6 (0.5 to 0.8)	0.4 (0.3 to 0.5)	7.9 (6.0 to 10.3)	4.6 (3.5 to 5.9)	0.99 (0.75 to 1.28)	0.57 (0.43 to 0.74)
1995	0.4 (0.4 to 0.5)	0.2 (0.2 to 0.3)	7.2 (6.6 to 7.8)	4.0 (3.6 to 4.3)	1.03 (0.94 to 1.11)	0.57 (0.52 to 0.62)
2000	0.4 (0.4 to 0.5)	0.2 (0.2 to 0.2)	8.0 (7.4 to 8.7)	4.1 (3.8 to 4.5)	1.57 (1.44 to 1.70)	0.81 (0.74 to 0.88)
2005	0.4 (0.4 to 0.5)	0.2 (0.2 to 0.2)	7.8 (7.2 to 8.4)	3.8 (3.5 to 4.1)	1.94 (1.80 to 2.09)	0.94 (0.87 to 1.02)
2010	0.4 (0.4 to 0.4)	0.2 (0.2 to 0.2)	6.3 (5.8 to 6.8)	3.0 (2.8 to 3.3)	1.94 (1.79 to 2.11)	0.93 (0.85 to 1.01)
2015	0.3 (0.3 to 0.4)	0.2 (0.2 to 0.2)	6.0 (5.5 to 6.5)	3.0 (2.7 to 3.2)	1.93 (1.79 to 2.08)	0.95 (0.88 to 1.03)
2020	0.3 (0.3 to 0.4)	0.2 (0.2 to 0.2)	5.9 (5.4 to 6.5)	3.0 (2.7 to 3.3)	2.19 (2.01 to 2.40)	1.12 (1.02 to 1.24)
2021	0.3 (0.3 to 0.4)	0.2 (0.1 to 0.2)	5.8 (5.0 to 6.7)	3.0 (2.6 to 3.4)	2.24 (1.93 to 2.57)	1.14 (0.99 to 1.32)

Slovenia SVN Central Europe Stillbirth Rate / Neonatal Mortality Rate (SBR/NMR) − 20 weeks
lambda = 0.2 ; zeta = 0.99 ; scale = 5 ; amp = 0.028

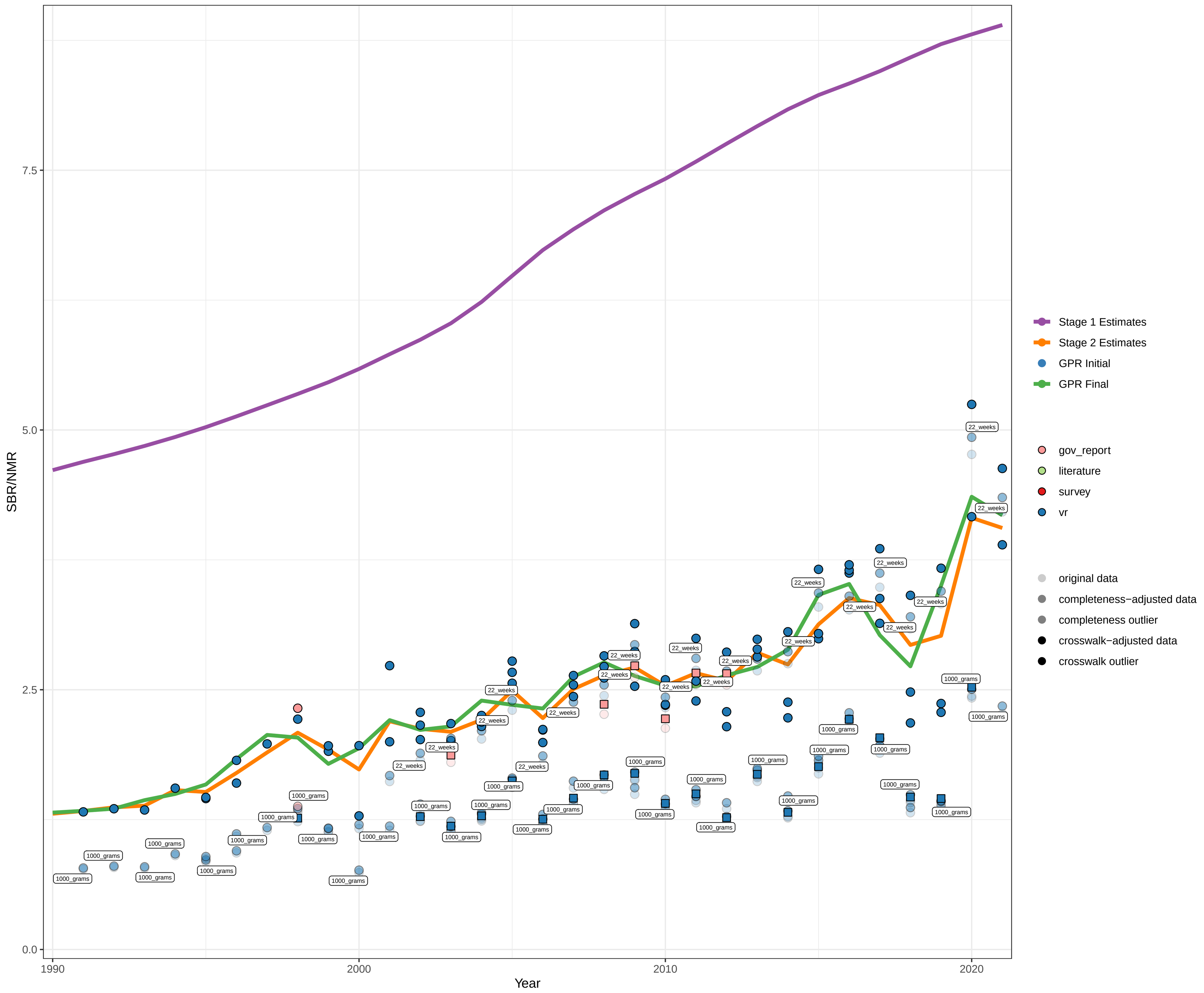

Slovenia SVN Central Europe Stillbirth Rate / Neonatal Mortality Rate (SBR/NMR) − 28 weeks
lambda = 0.2 ; zeta = 0.99 ; scale = 5 ; amp = 0.028

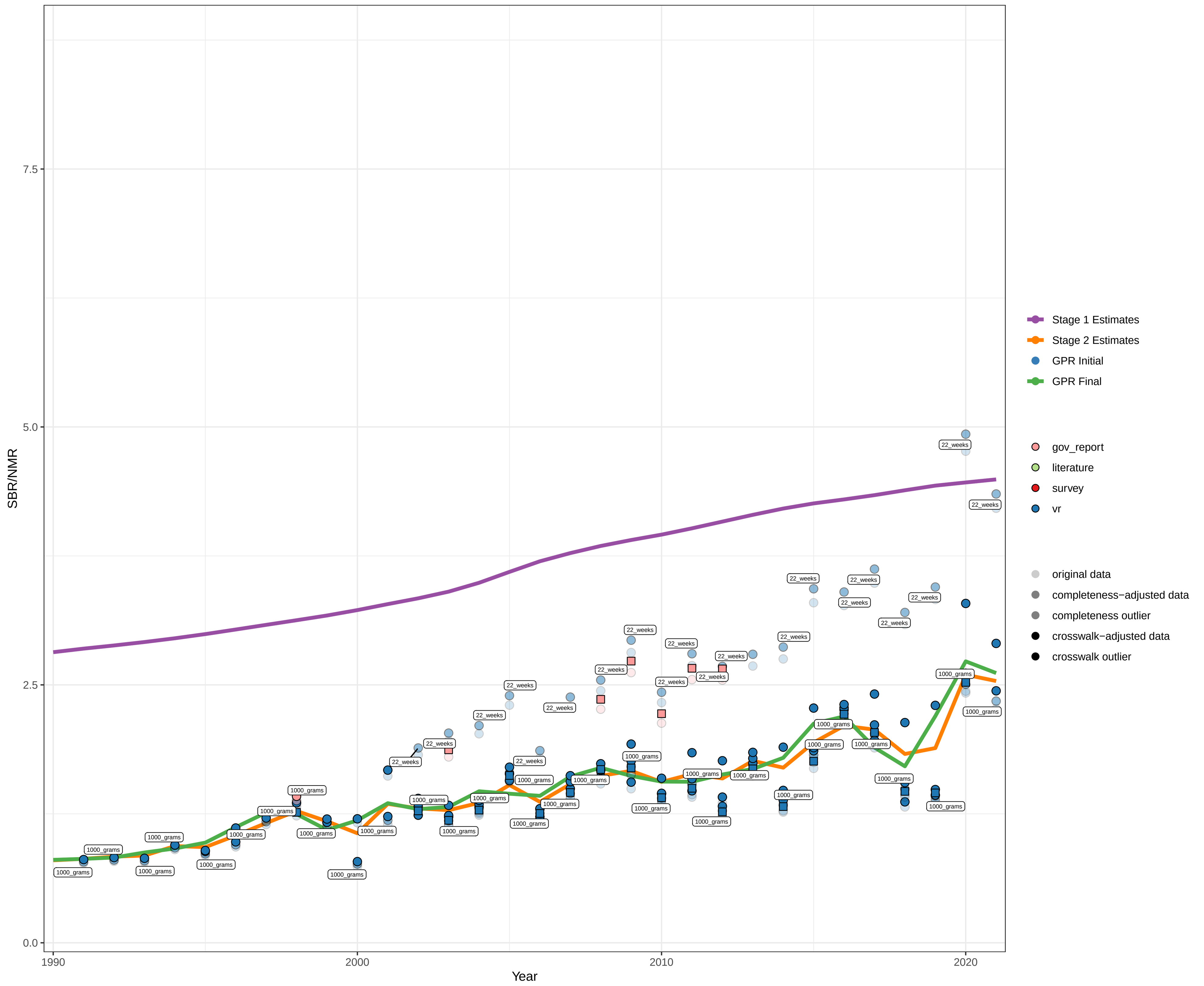

Definition	Years	Outlier	Source.Type	Title
1000_grams	1991−2020	0	VR	European Health for All Database
1000_grams	1998, 2003, 2008−2012	0	Statistical Report	Slovenia Health Statistical Yearbook
1000_grams, 22_weeks	2002−2021	0	VR	Slovenia Stillbirths per 1000 Births by Region
28_weeks	1995, 1996, 1998−2019	0	VR	United Nations Demographic Yearbook

Slovenia SVN Central Europe Stillbirth Rate (SBR) − 20 weeks
lambda = 0.2 ; zeta = 0.99 ; scale = 5 ; amp = 0.028

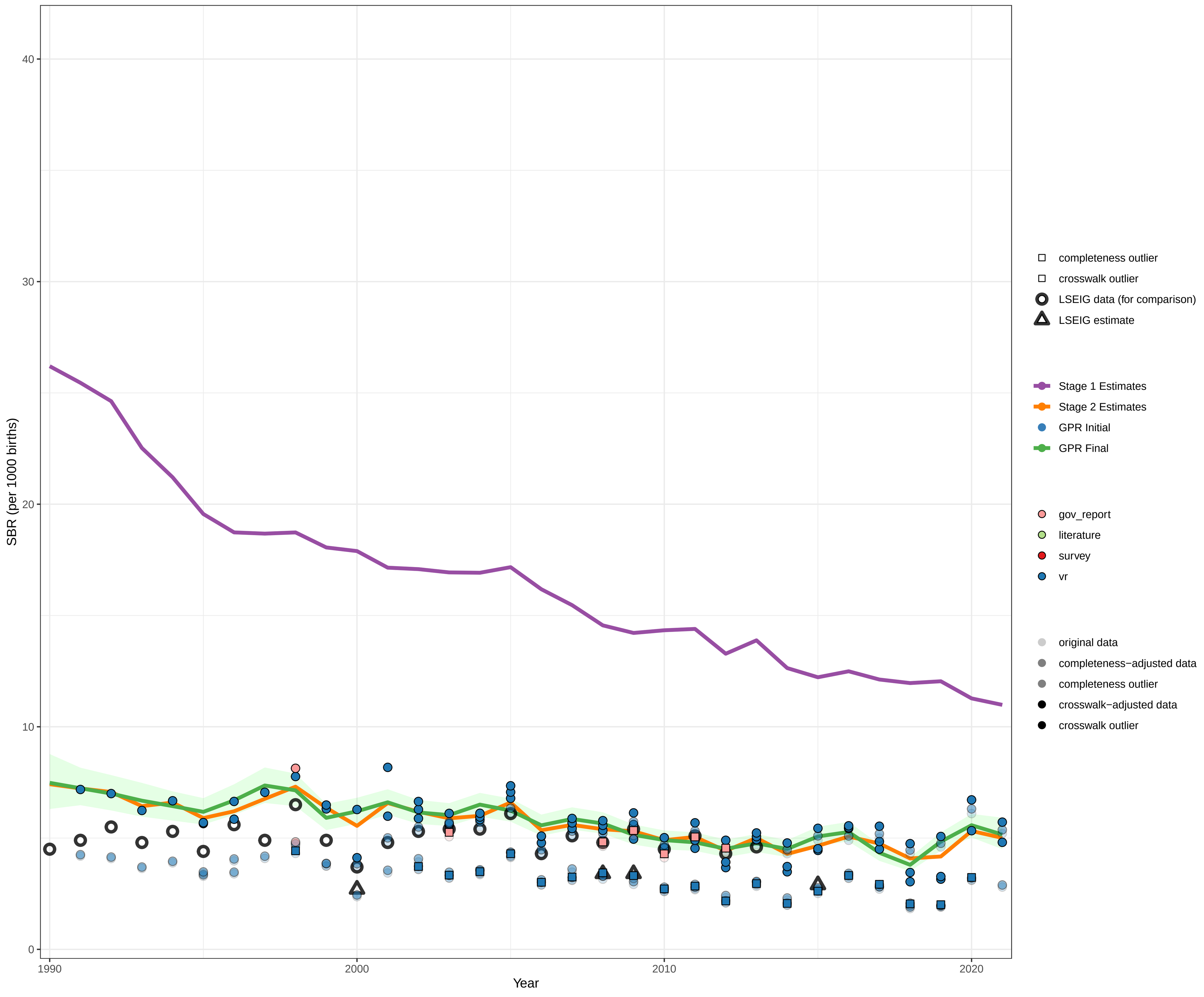

Slovenia SVN Central Europe Stillbirth Rate (SBR) − 28 weeks
lambda = 0.2 ; zeta = 0.99 ; scale = 5 ; amp = 0.028

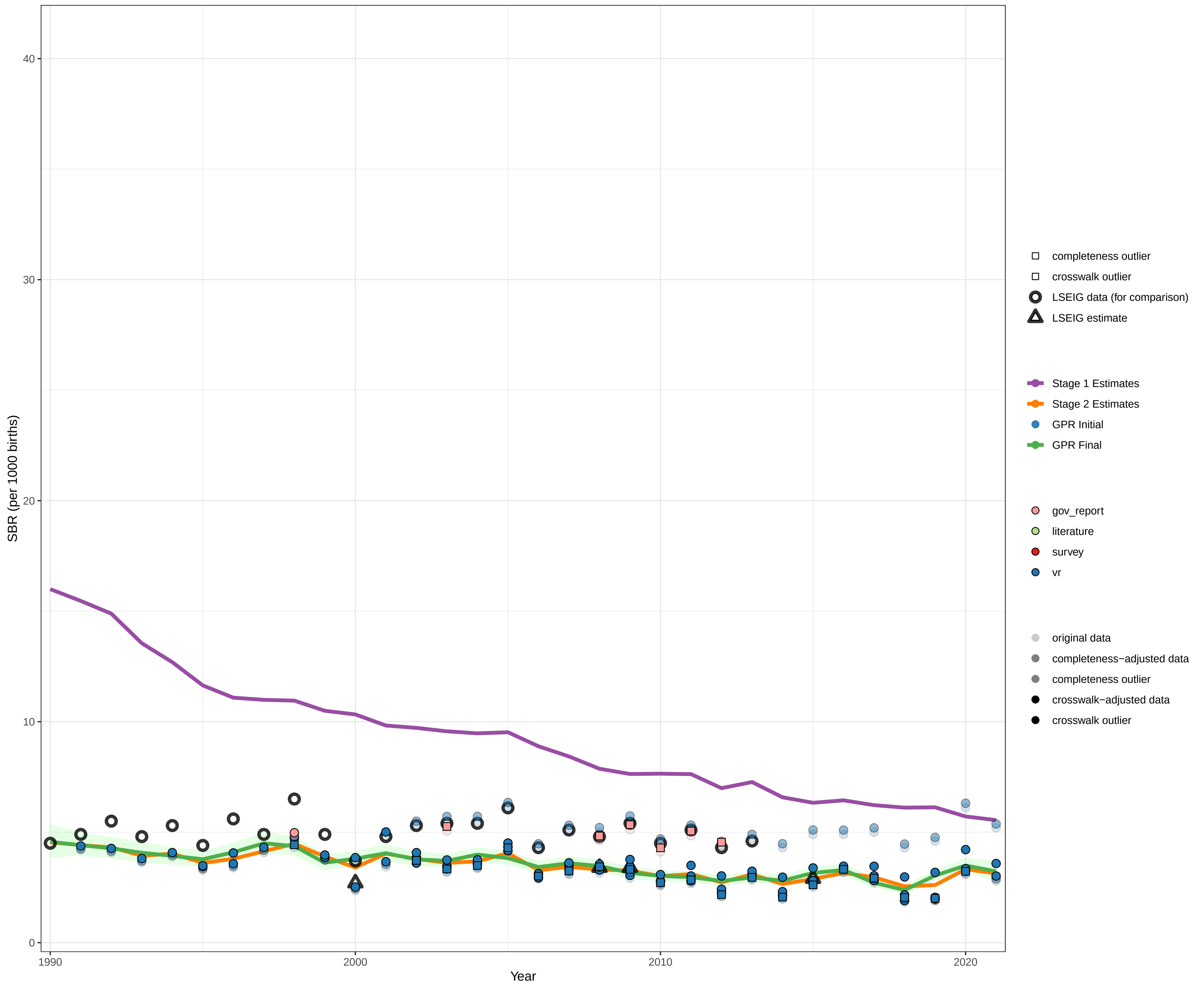

Year	Total Stillbirths (in thousands)	Total Stillbirths (in thousands)	SBR (per 1000 births)	SBR (per 1000 births)	SBR/NMR	SBR/NMR
	20 weeks	28 weeks	20 weeks	28 weeks	20 weeks	28 weeks
1990	0.2 (0.1 to 0.2)	0.1 (0.1 to 0.1)	7.5 (6.3 to 8.8)	4.6 (3.8 to 5.4)	1.32 (1.11 to 1.55)	0.80 (0.68 to 0.94)
1995	0.1 (0.1 to 0.1)	0.1 (0.1 to 0.1)	6.2 (5.6 to 6.8)	3.8 (3.4 to 4.2)	1.59 (1.44 to 1.75)	0.97 (0.88 to 1.07)
2000	0.1 (0.1 to 0.1)	0.1 (0.1 to 0.1)	6.2 (5.6 to 6.8)	3.8 (3.5 to 4.2)	1.94 (1.76 to 2.13)	1.19 (1.08 to 1.30)
2005	0.1 (0.1 to 0.1)	0.1 (0.1 to 0.1)	6.2 (5.7 to 6.8)	3.8 (3.5 to 4.2)	2.35 (2.17 to 2.56)	1.44 (1.33 to 1.57)
2010	0.1 (0.1 to 0.1)	0.1 (0.1 to 0.1)	4.9 (4.5 to 5.3)	3.0 (2.8 to 3.3)	2.54 (2.32 to 2.76)	1.56 (1.43 to 1.70)
2015	0.1 (0.1 to 0.1)	0.1 (0.1 to 0.1)	5.1 (4.7 to 5.5)	3.2 (2.9 to 3.4)	3.41 (3.14 to 3.71)	2.12 (1.95 to 2.31)
2020	0.1 (0.1 to 0.1)	0.1 (0.1 to 0.1)	5.6 (5.0 to 6.1)	3.5 (3.2 to 3.8)	4.36 (3.93 to 4.75)	2.73 (2.48 to 3.00)
2021	0.1 (0.1 to 0.1)	0.1 (0.1 to 0.1)	5.2 (4.5 to 5.9)	3.2 (2.8 to 3.7)	4.18 (3.65 to 4.79)	2.61 (2.27 to 3.01)

Eastern Europe R9 Eastern Europe Stillbirth Rate / Neonatal Mortality Rate (SBR/NMR) − 20 weeks
lambda = 1 ; zeta = 0.6 ; scale = 15 ; amp = 0.028

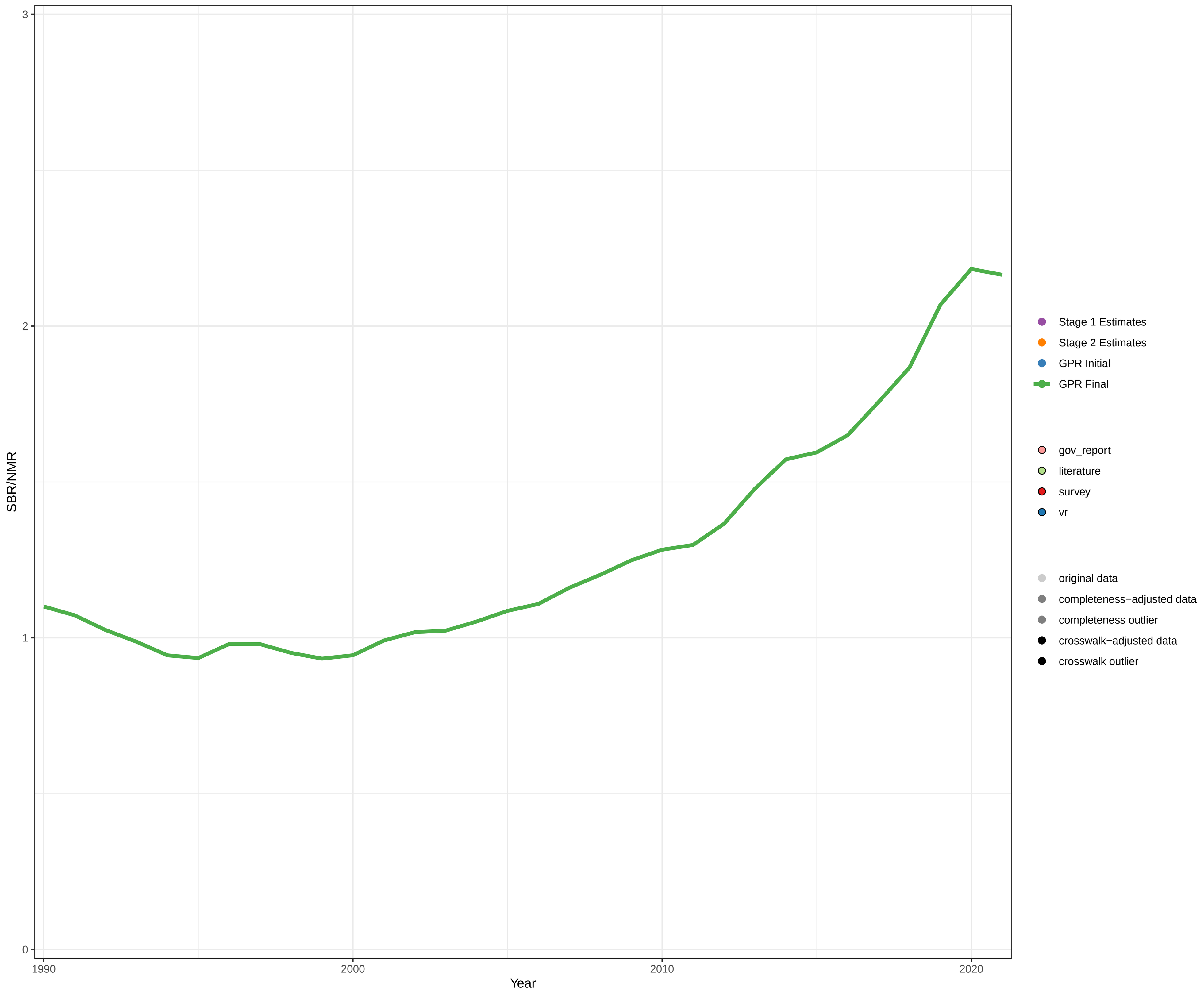

Eastern Europe R9 Eastern Europe Stillbirth Rate / Neonatal Mortality Rate (SBR/NMR) − 28 weeks
lambda = 1 ; zeta = 0.6 ; scale = 15 ; amp = 0.028

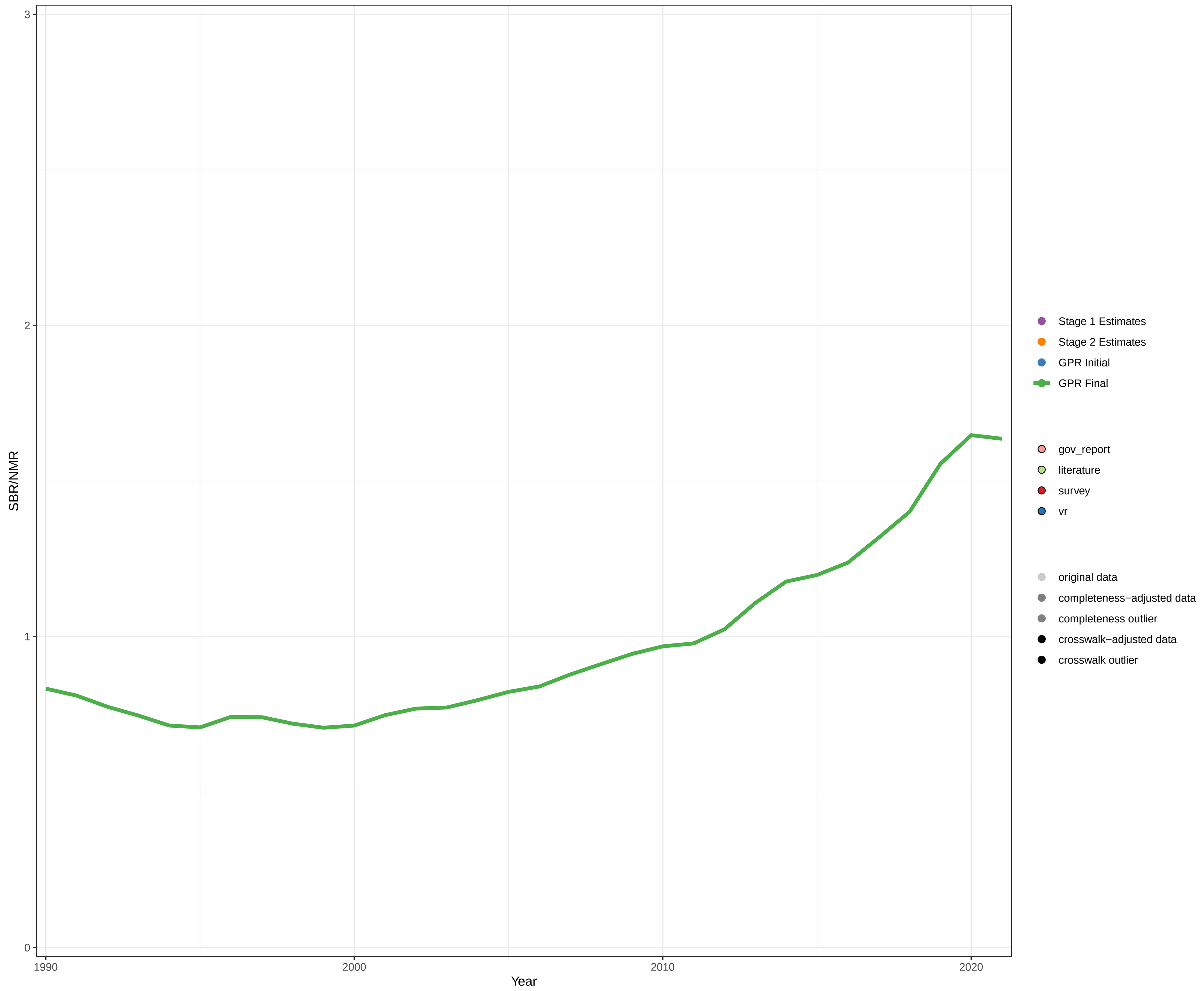

Definition	Years	Outlier	Source.Type	Title

Eastern Europe R9 Eastern Europe Stillbirth Rate (SBR) − 20 weeks
lambda = 1 ; zeta = 0.6 ; scale = 15 ; amp = 0.028

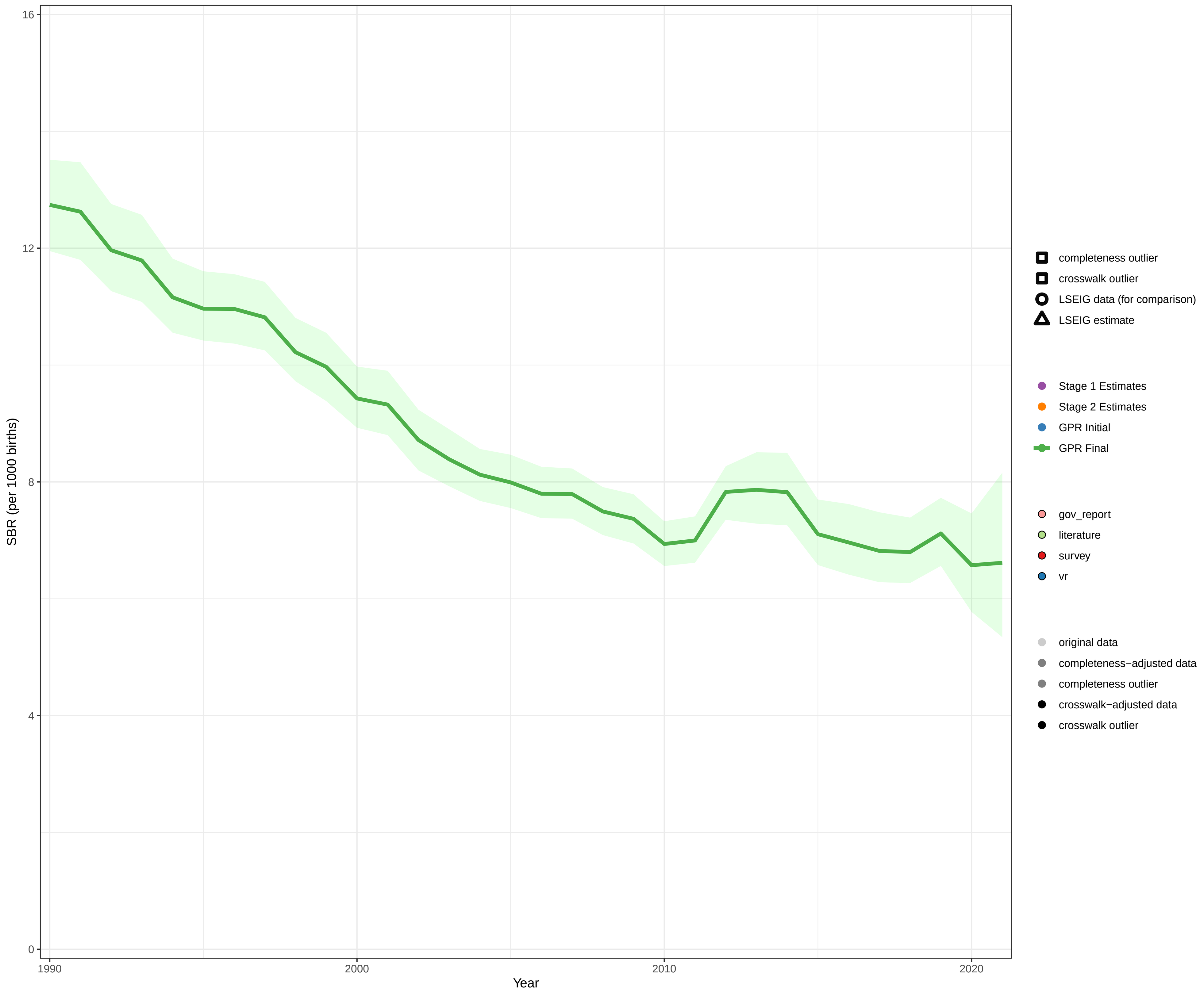

Eastern Europe R9 Eastern Europe Stillbirth Rate (SBR) − 28 weeks
lambda = 1 ; zeta = 0.6 ; scale = 15 ; amp = 0.028

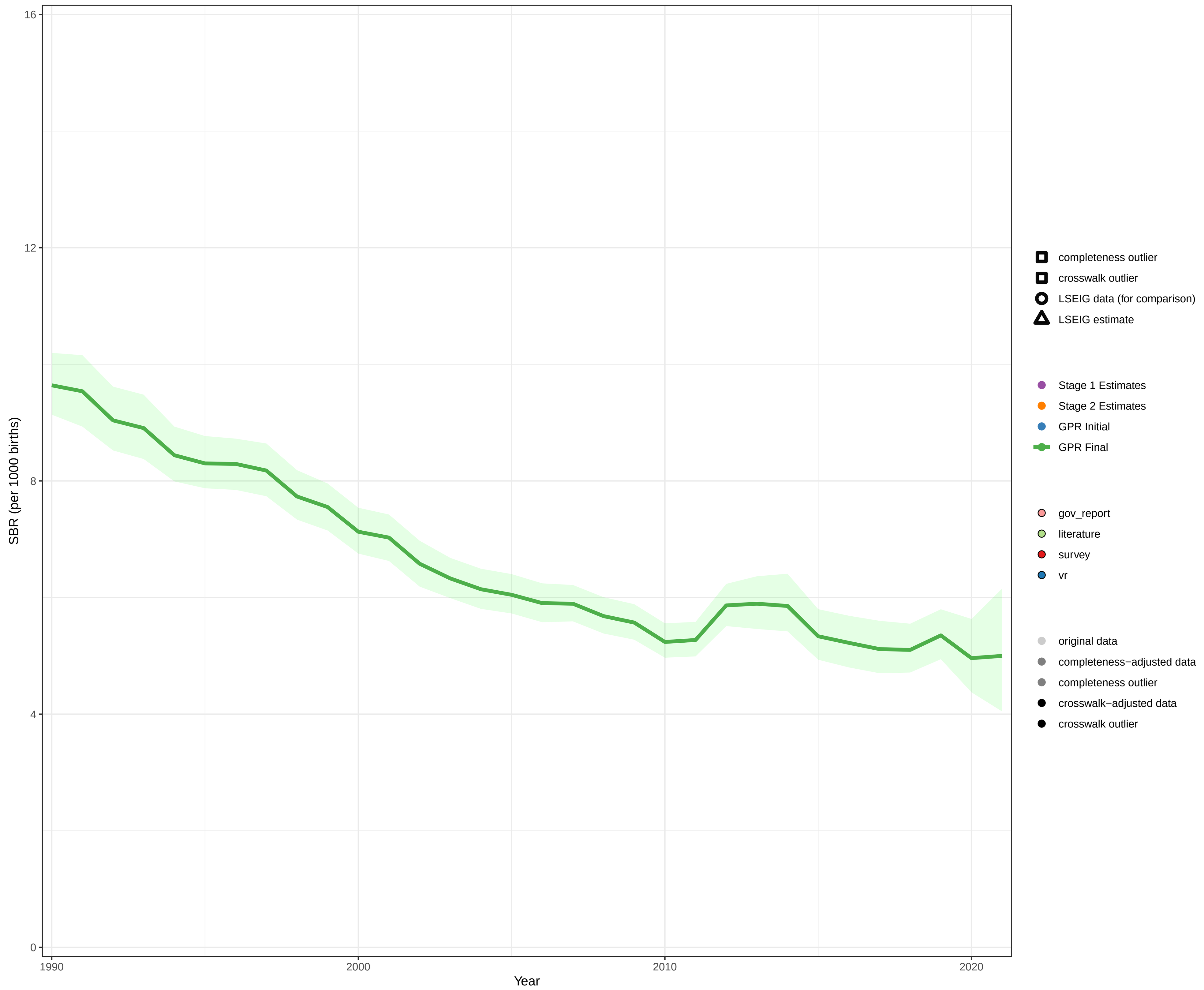

Year	Total Stillbirths (in thousands)	Total Stillbirths (in thousands)	SBR (per 1000 births)	SBR (per 1000 births)	SBR/NMR	SBR/NMR
	20 weeks	28 weeks	20 weeks	28 weeks	20 weeks	28 weeks
1990	38.1 (35.7 to 40.4)	28.7 (27.2 to 30.4)	12.7 (12.0 to 13.5)	9.6 (9.1 to 10.2)	1.10 (1.03 to 1.17)	0.83 (0.79 to 0.88)
1995	23.3 (22.1 to 24.6)	17.6 (16.6 to 18.6)	11.0 (10.4 to 11.6)	8.3 (7.9 to 8.8)	0.94 (0.89 to 0.99)	0.71 (0.67 to 0.75)
2000	18.3 (17.3 to 19.3)	13.8 (13.0 to 14.6)	9.4 (8.9 to 10.0)	7.1 (6.8 to 7.5)	0.94 (0.89 to 1.00)	0.71 (0.68 to 0.75)
2005	17.4 (16.4 to 18.4)	13.1 (12.4 to 13.9)	8.0 (7.6 to 8.5)	6.0 (5.7 to 6.4)	1.09 (1.03 to 1.15)	0.82 (0.78 to 0.87)
2010	18.0 (17.0 to 19.0)	13.6 (12.9 to 14.4)	6.9 (6.6 to 7.3)	5.2 (5.0 to 5.6)	1.28 (1.21 to 1.35)	0.97 (0.92 to 1.03)
2015	18.1 (16.7 to 19.6)	13.5 (12.5 to 14.7)	7.1 (6.6 to 7.7)	5.3 (4.9 to 5.8)	1.59 (1.48 to 1.73)	1.20 (1.11 to 1.30)
2020	12.4 (10.9 to 14.1)	9.3 (8.2 to 10.6)	6.6 (5.8 to 7.5)	5.0 (4.4 to 5.6)	2.18 (1.92 to 2.48)	1.65 (1.45 to 1.87)
2021	12.0 (9.6 to 14.8)	9.0 (7.3 to 11.1)	6.6 (5.3 to 8.2)	5.0 (4.0 to 6.2)	2.16 (1.75 to 2.67)	1.64 (1.32 to 2.01)

Belarus BLR Eastern Europe Stillbirth Rate / Neonatal Mortality Rate (SBR/NMR) − 20 weeks
lambda = 0.2 ; zeta = 0.99 ; scale = 5 ; amp = 0.028

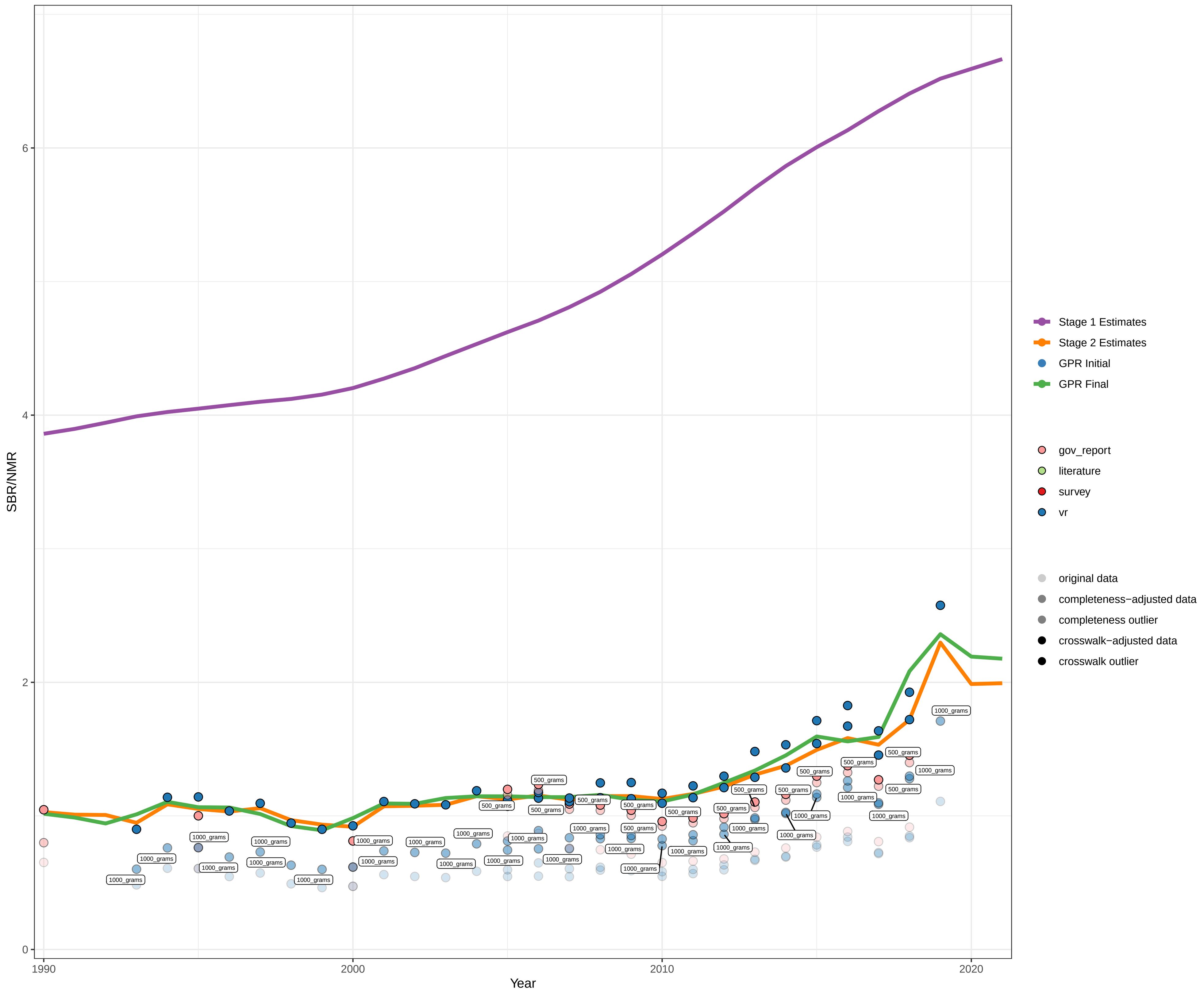

Belarus BLR Eastern Europe Stillbirth Rate / Neonatal Mortality Rate (SBR/NMR) − 28 weeks
lambda = 0.2 ; zeta = 0.99 ; scale = 5 ; amp = 0.028

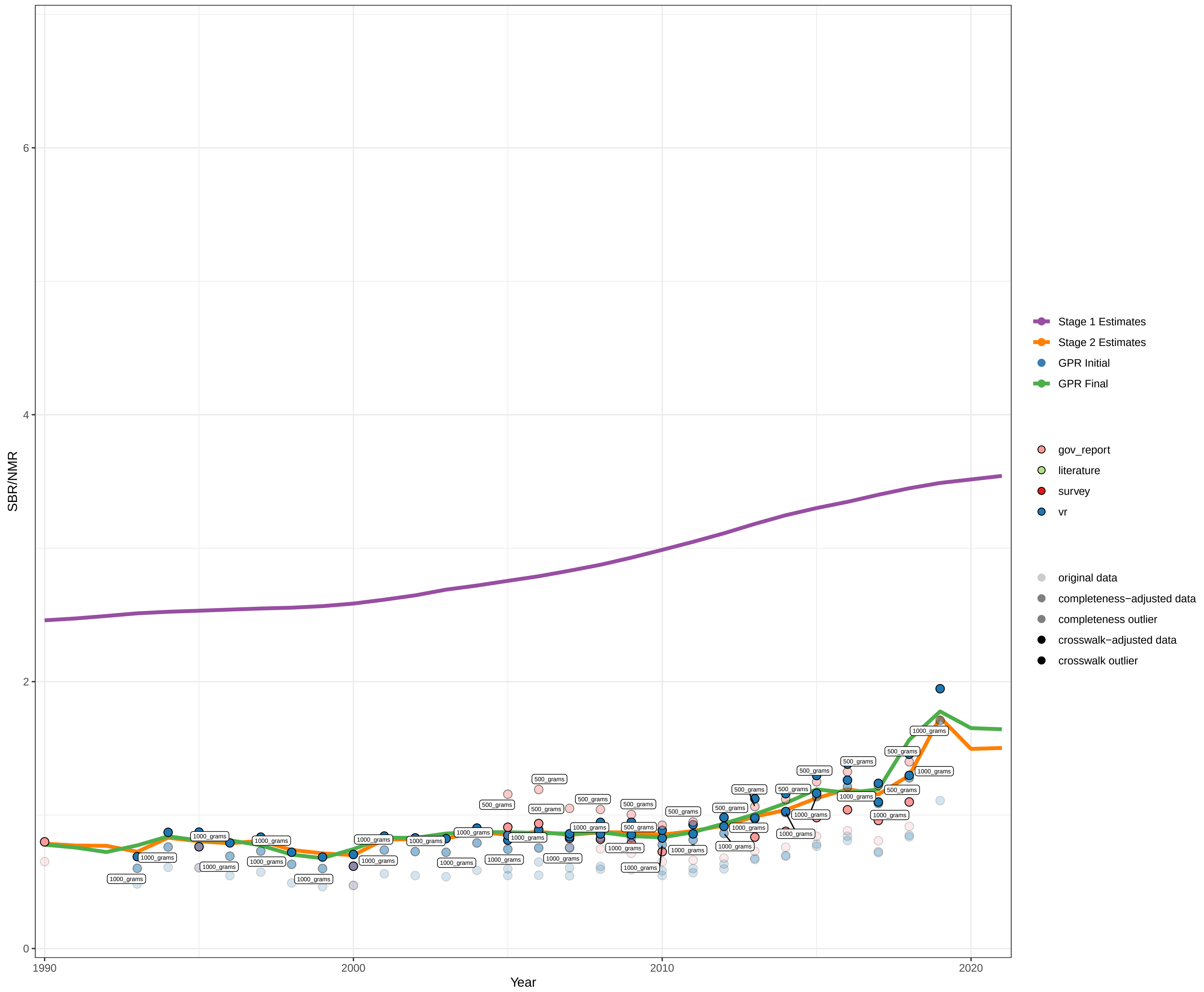

Definition	Years	Outlier	Source.Type	Title
1000_grams	1993−2019	0	VR	European Health for All Database
28_weeks	1990, 1995, 2000	0	Statistical Report	Belarus Demographic Yearbook
28_weeks	2005−2018	0	VR	United Nations Demographic Yearbook
500_grams	2005−2018	0	Statistical Report	Belarus Demographic Yearbook

Belarus BLR Eastern Europe Stillbirth Rate (SBR) − 20 weeks
lambda = 0.2 ; zeta = 0.99 ; scale = 5 ; amp = 0.028

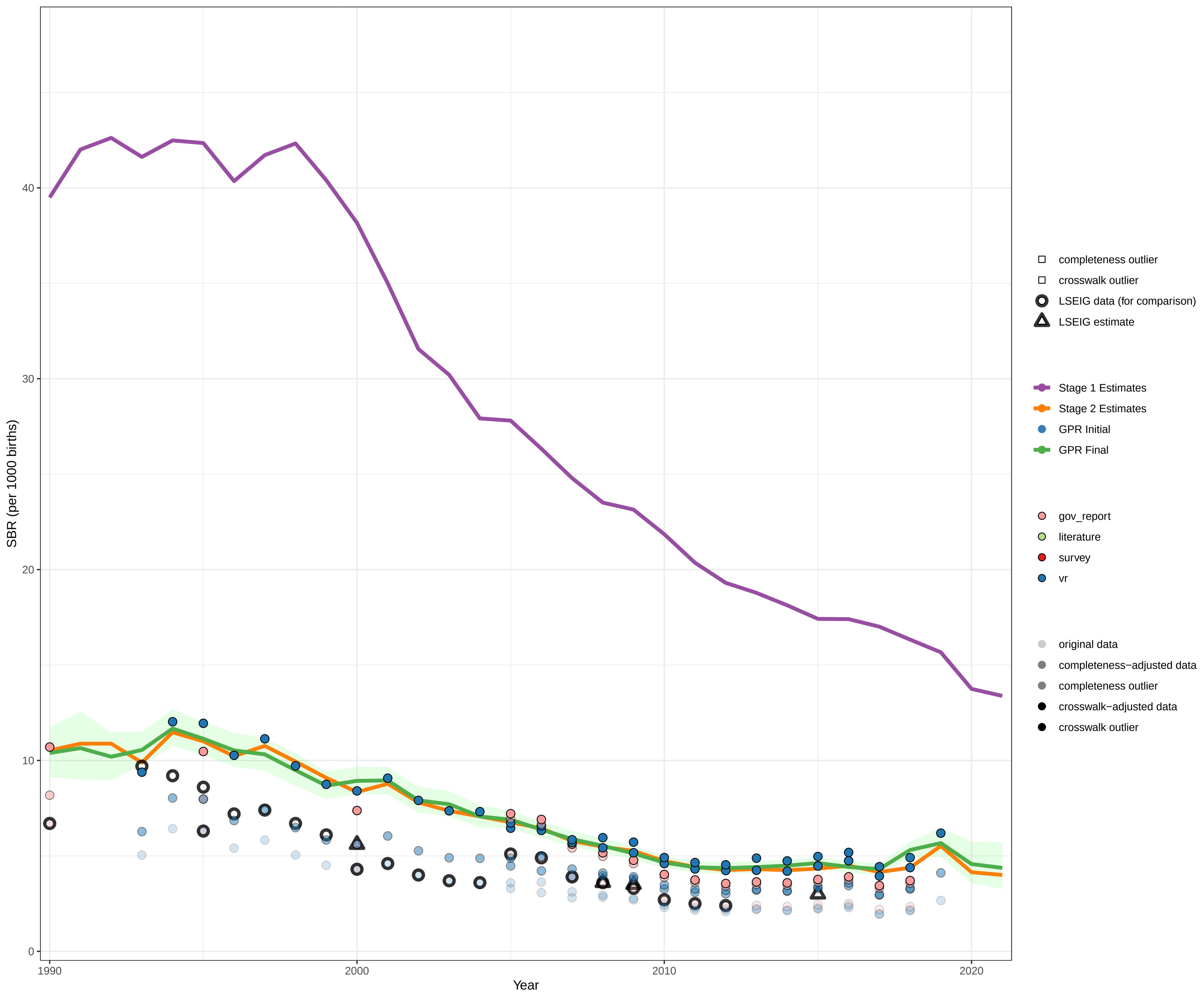

Belarus BLR Eastern Europe Stillbirth Rate (SBR) − 28 weeks
lambda = 0.2 ; zeta = 0.99 ; scale = 5 ; amp = 0.028

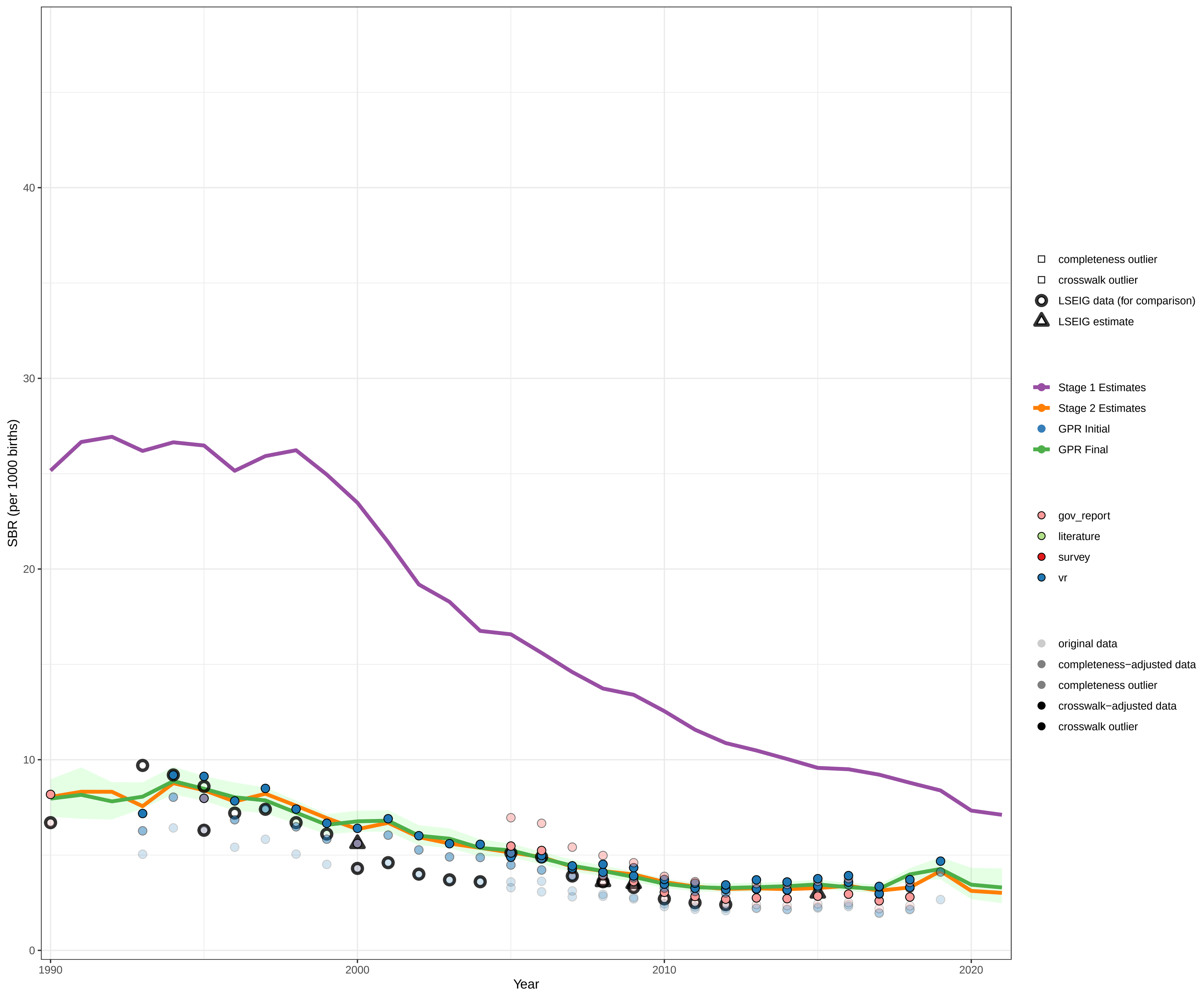

Year	Total Stillbirths (in thousands)	Total Stillbirths (in thousands)	SBR (per 1000 births)	SBR (per 1000 births)	SBR/NMR	SBR/NMR
	20 weeks	28 weeks	20 weeks	28 weeks	20 weeks	28 weeks
1990	1.5 (1.3 to 1.7)	1.1 (1.0 to 1.3)	10.4 (9.1 to 11.8)	8.0 (7.0 to 9.0)	1.02 (0.89 to 1.15)	0.78 (0.69 to 0.88)
1995	1.1 (1.0 to 1.2)	0.9 (0.8 to 0.9)	11.1 (10.3 to 12.0)	8.5 (7.8 to 9.1)	1.06 (0.98 to 1.15)	0.81 (0.75 to 0.87)
2000	0.8 (0.8 to 0.9)	0.6 (0.6 to 0.7)	8.9 (8.2 to 9.7)	6.8 (6.2 to 7.3)	0.98 (0.90 to 1.06)	0.75 (0.68 to 0.81)
2005	0.7 (0.6 to 0.7)	0.5 (0.5 to 0.5)	6.9 (6.4 to 7.4)	5.2 (4.9 to 5.6)	1.15 (1.07 to 1.22)	0.87 (0.81 to 0.93)
2010	0.5 (0.5 to 0.6)	0.4 (0.4 to 0.4)	4.7 (4.4 to 5.0)	3.5 (3.3 to 3.8)	1.11 (1.04 to 1.19)	0.83 (0.78 to 0.90)
2015	0.5 (0.5 to 0.6)	0.4 (0.4 to 0.4)	4.6 (4.3 to 4.9)	3.5 (3.2 to 3.7)	1.59 (1.49 to 1.71)	1.19 (1.12 to 1.28)
2020	0.4 (0.3 to 0.5)	0.3 (0.2 to 0.4)	4.6 (3.6 to 5.8)	3.4 (2.7 to 4.3)	2.19 (1.71 to 2.76)	1.65 (1.29 to 2.08)
2021	0.4 (0.3 to 0.5)	0.3 (0.2 to 0.4)	4.4 (3.3 to 5.7)	3.3 (2.5 to 4.3)	2.18 (1.63 to 2.84)	1.64 (1.23 to 2.14)

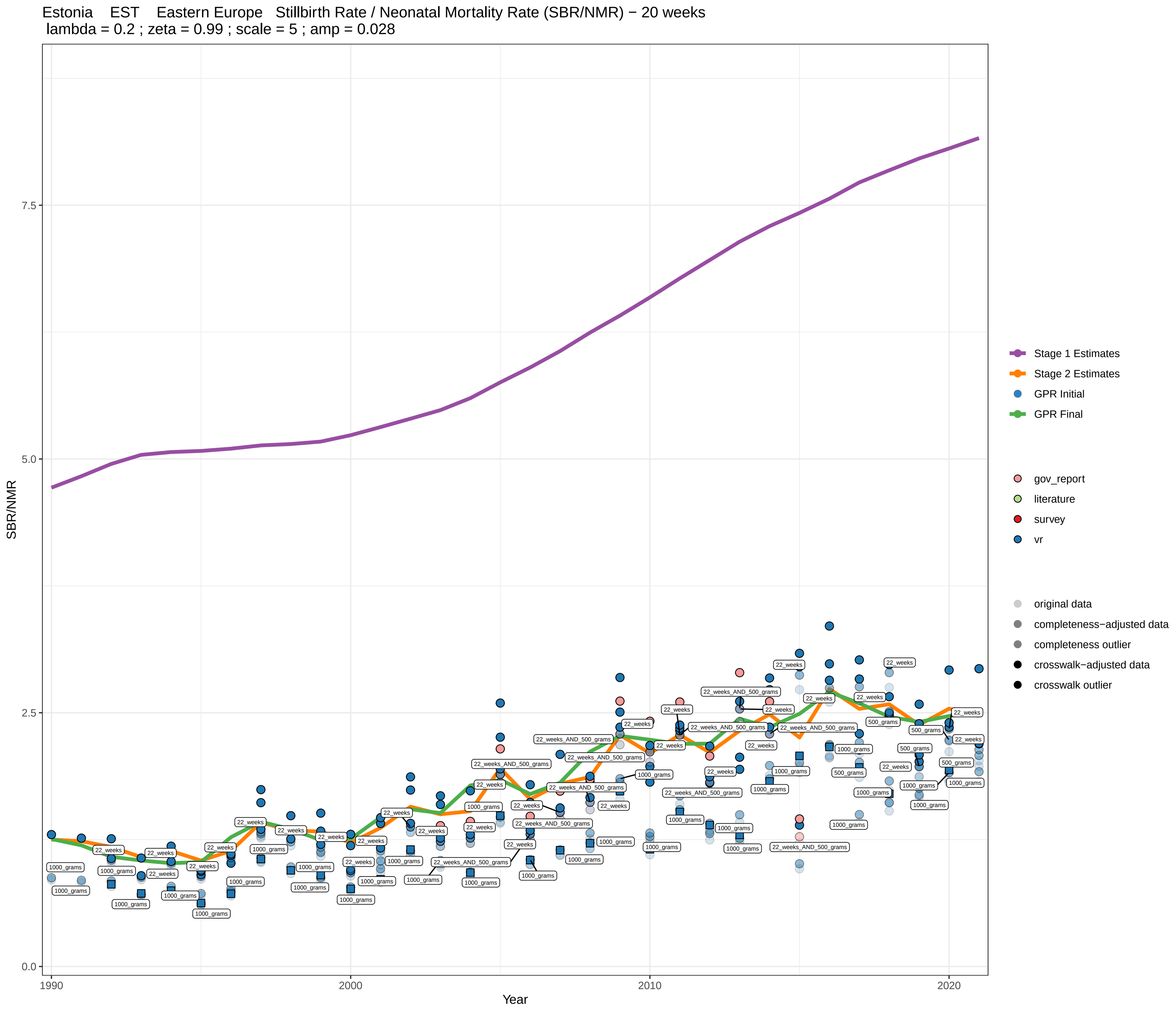

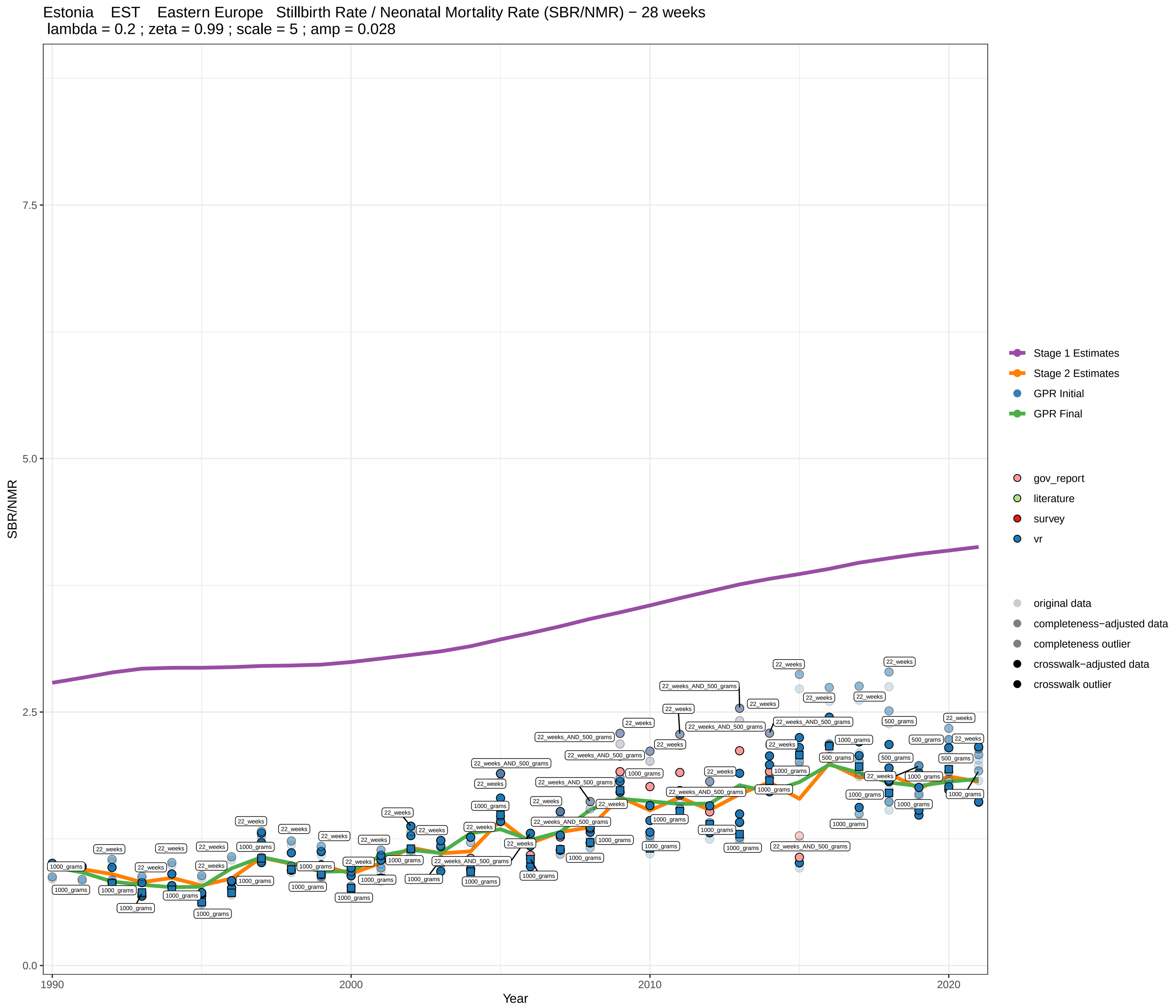

Definition	Years	Outlier	Source.Type	Title
1000_grams	1990−2020	0	VR	European Health for All Database
1000_grams	1992−2021	0	VR	Estonia Medical Birth Registry − Perinatal Mortality by Birth Weight
22_weeks	1992−2021	0	VR	Estonia Medical Birth Registry − Perinatal Mortality
22_weeks_AND_500_grams	2003−2015	0	Statistical Report	Estonia Statistical Yearbook
28_weeks	1995−1997, 1999−2019	0	VR	United Nations Demographic Yearbook
500_grams	2017−2021	0	VR	Estonia Medical Birth Registry − Perinatal Mortality by Birth Weight

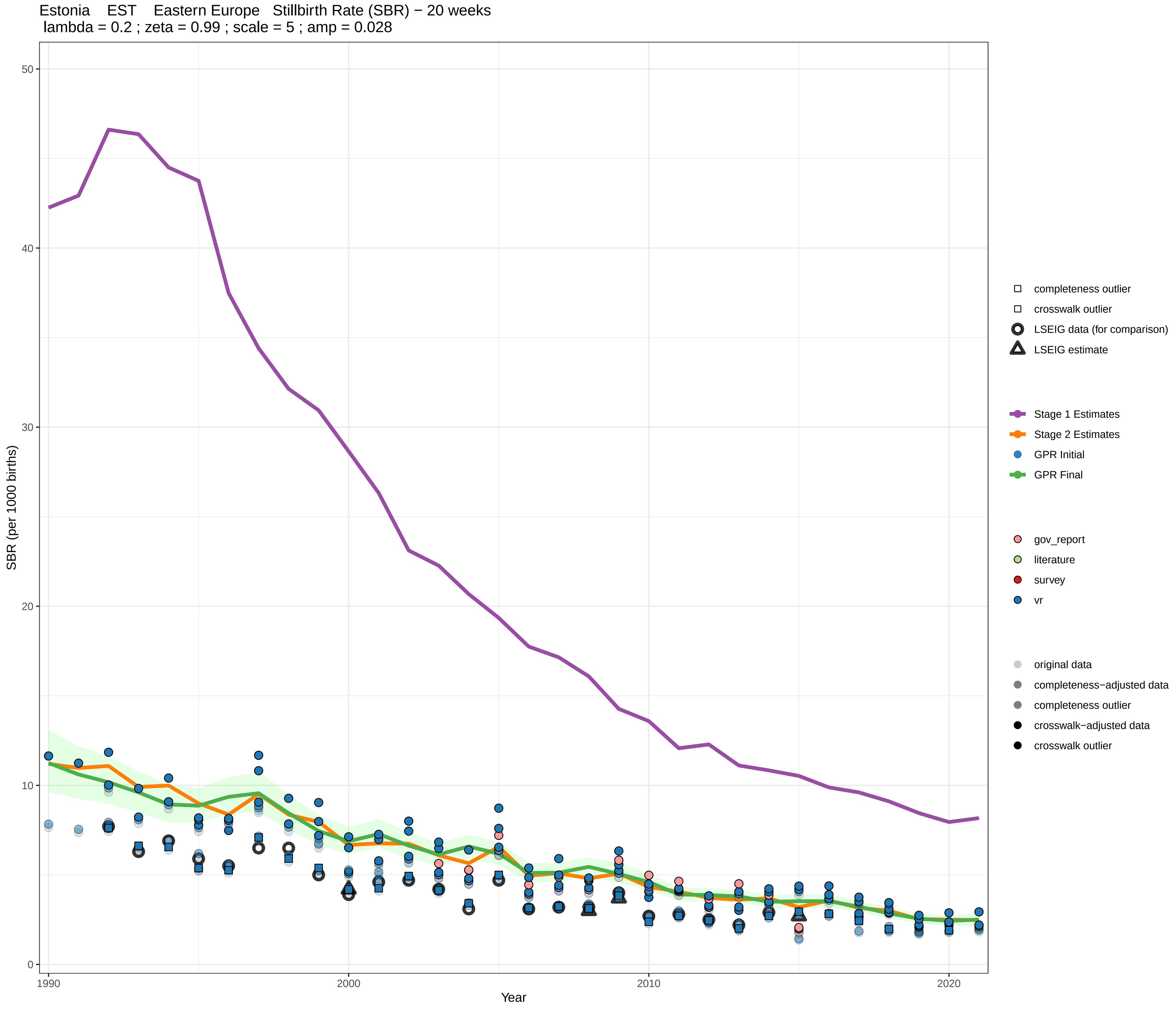

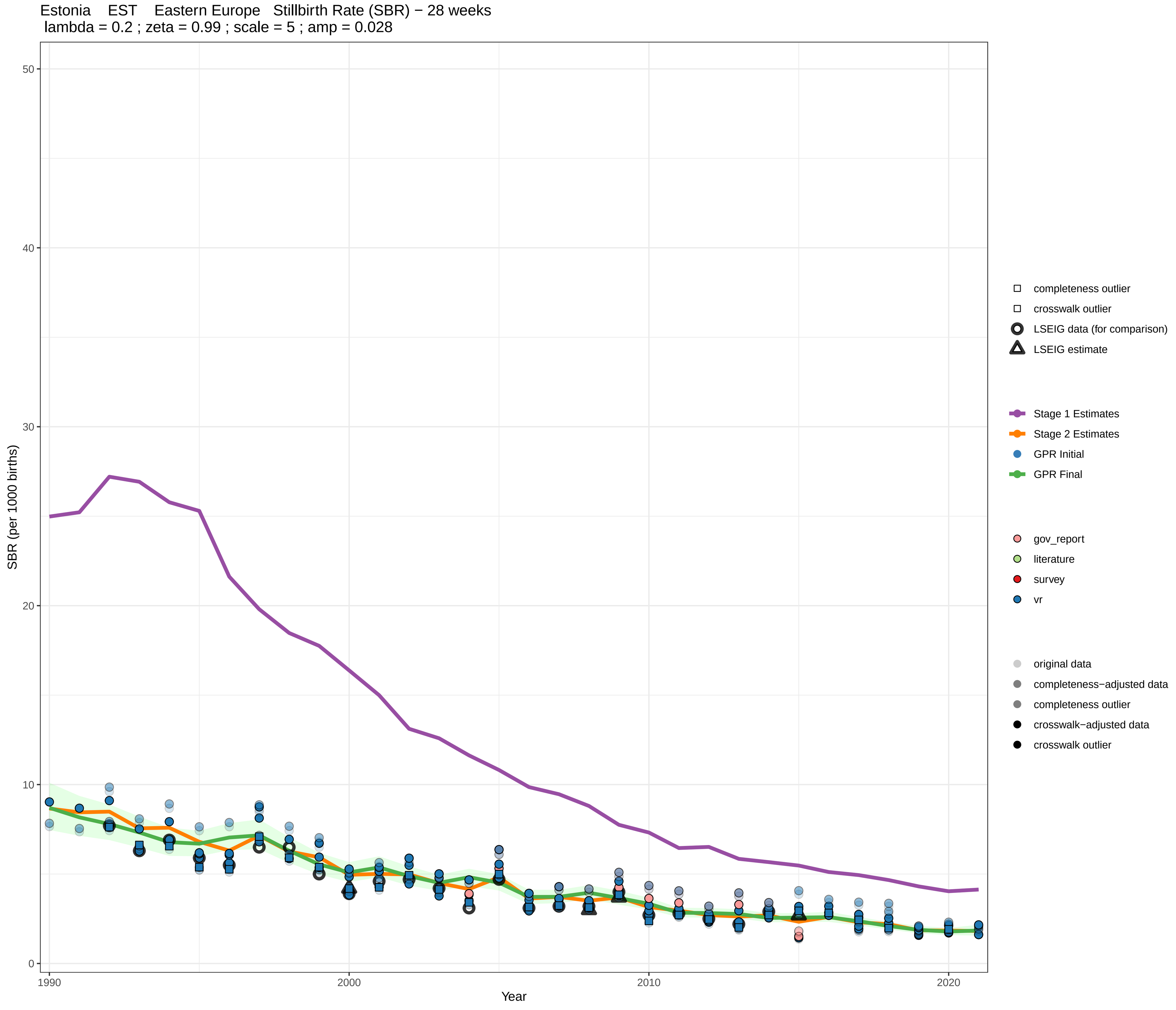

Year	Total Stillbirths (in thousands)	Total Stillbirths (in thousands)	SBR (per 1000 births)	SBR (per 1000 births)	SBR/NMR	SBR/NMR
	20 weeks	28 weeks	20 weeks	28 weeks	20 weeks	28 weeks
1990	0.2 (0.2 to 0.3)	0.2 (0.2 to 0.2)	11.2 (9.6 to 13.1)	8.7 (7.5 to 10.1)	1.26 (1.07 to 1.46)	0.97 (0.83 to 1.13)
1995	0.1 (0.1 to 0.1)	0.1 (0.1 to 0.1)	8.9 (7.9 to 9.8)	6.7 (6.0 to 7.4)	1.03 (0.92 to 1.14)	0.78 (0.70 to 0.86)
2000	0.1 (0.1 to 0.1)	0.1 (0.1 to 0.1)	6.9 (6.1 to 7.7)	5.1 (4.6 to 5.7)	1.26 (1.12 to 1.40)	0.93 (0.83 to 1.04)
2005	0.1 (0.1 to 0.1)	0.1 (0.1 to 0.1)	6.2 (5.6 to 6.9)	4.5 (4.1 to 5.0)	1.84 (1.66 to 2.04)	1.34 (1.21 to 1.49)
2010	0.1 (0.1 to 0.1)	0.1 (0.0 to 0.1)	4.6 (4.2 to 5.1)	3.3 (3.0 to 3.7)	2.23 (2.02 to 2.47)	1.62 (1.46 to 1.78)
2015	0.0 (0.0 to 0.1)	0.0 (0.0 to 0.0)	3.5 (3.2 to 3.9)	2.6 (2.3 to 2.8)	2.49 (2.24 to 2.77)	1.81 (1.63 to 1.99)
2020	0.0 (0.0 to 0.0)	0.0 (0.0 to 0.0)	2.4 (2.2 to 2.7)	1.8 (1.6 to 2.0)	2.47 (2.19 to 2.75)	1.81 (1.61 to 2.02)
2021	0.0 (0.0 to 0.0)	0.0 (0.0 to 0.0)	2.5 (2.1 to 2.9)	1.8 (1.6 to 2.2)	2.50 (2.11 to 2.94)	1.84 (1.56 to 2.16)

Latvia LVA Eastern Europe Stillbirth Rate / Neonatal Mortality Rate (SBR/NMR) − 20 weeks
lambda = 0.2 ; zeta = 0.99 ; scale = 5 ; amp = 0.028

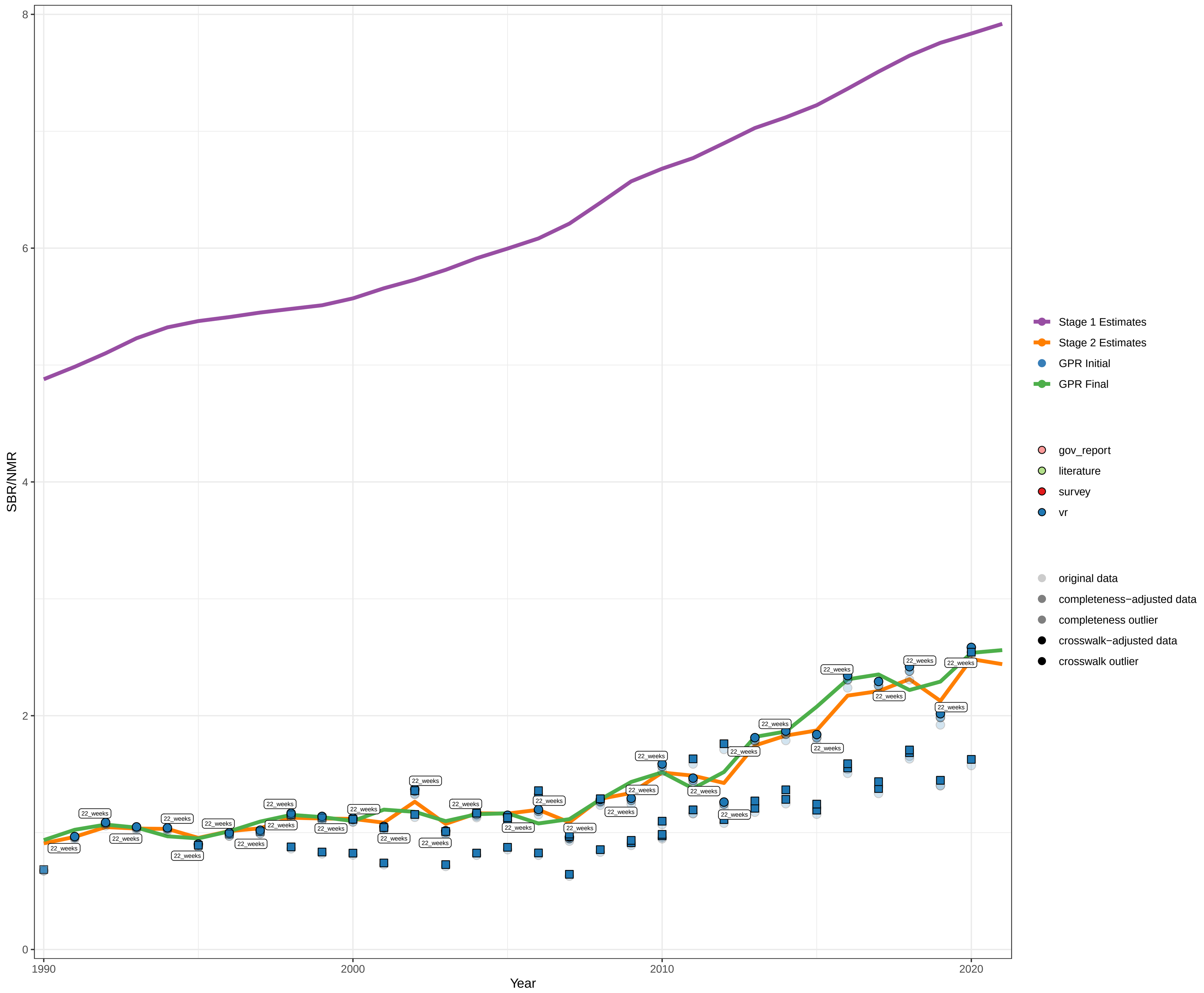

Latvia LVA Eastern Europe Stillbirth Rate / Neonatal Mortality Rate (SBR/NMR) − 28 weeks
lambda = 0.2 ; zeta = 0.99 ; scale = 5 ; amp = 0.028

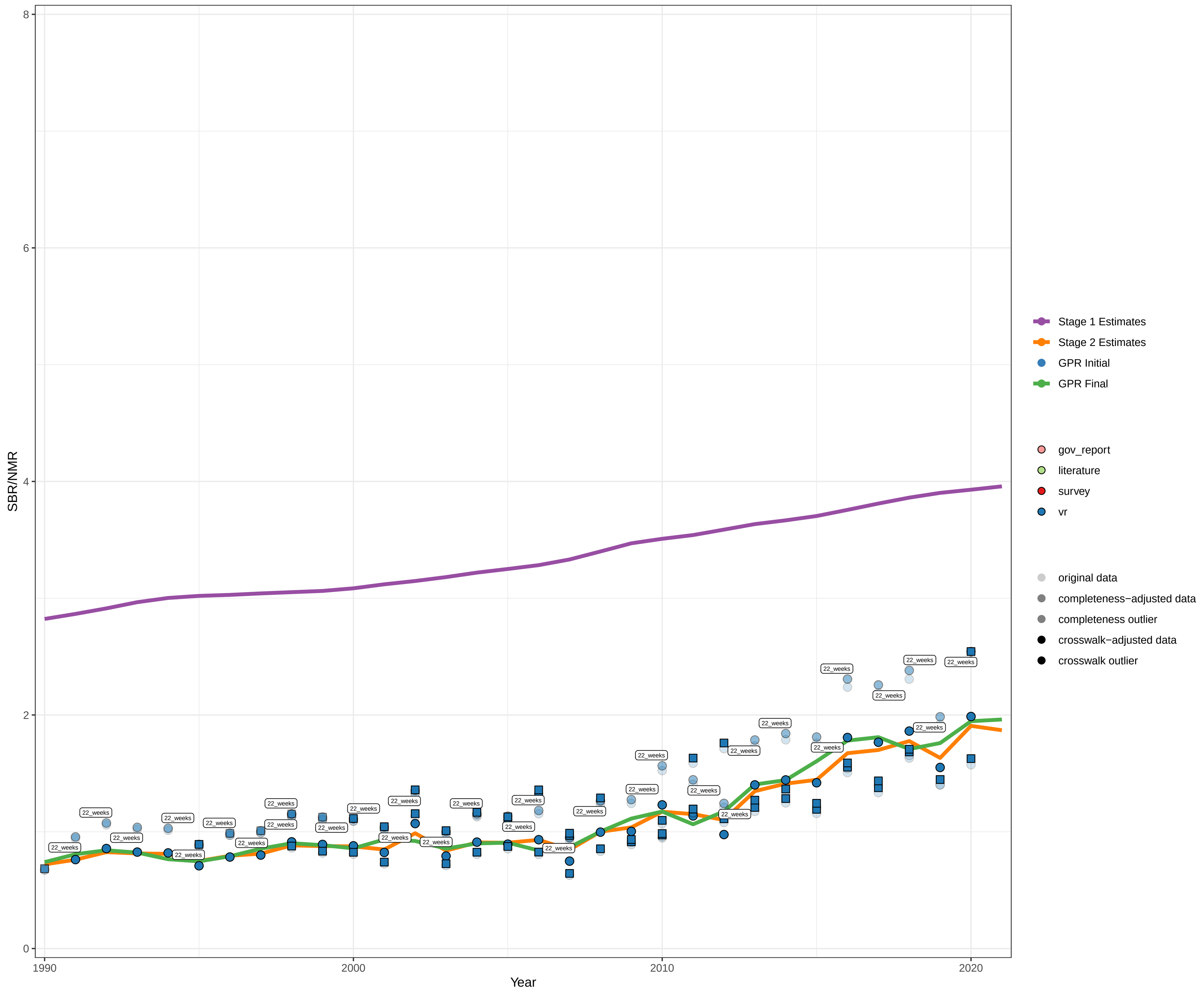

Definition	Years	Outlier	Source.Type	Title
22_weeks	1991−2020	0	VR	Latvia Number of Live Births and Stillbirths by Sex
22_weeks	1995, 2000−2020	0	VR	United Nations Demographic Yearbook
22_weeks	1998−2018, 2020	0	VR	European Health for All Database
22_weeks	1990	1	VR	Latvia Number of Live Births and Stillbirths by Sex
22_weeks	1990, 2019	1	VR	European Health for All Database
22_weeks	1996−1999	1	VR	United Nations Demographic Yearbook

Latvia LVA Eastern Europe Stillbirth Rate (SBR) − 20 weeks
lambda = 0.2 ; zeta = 0.99 ; scale = 5 ; amp = 0.028

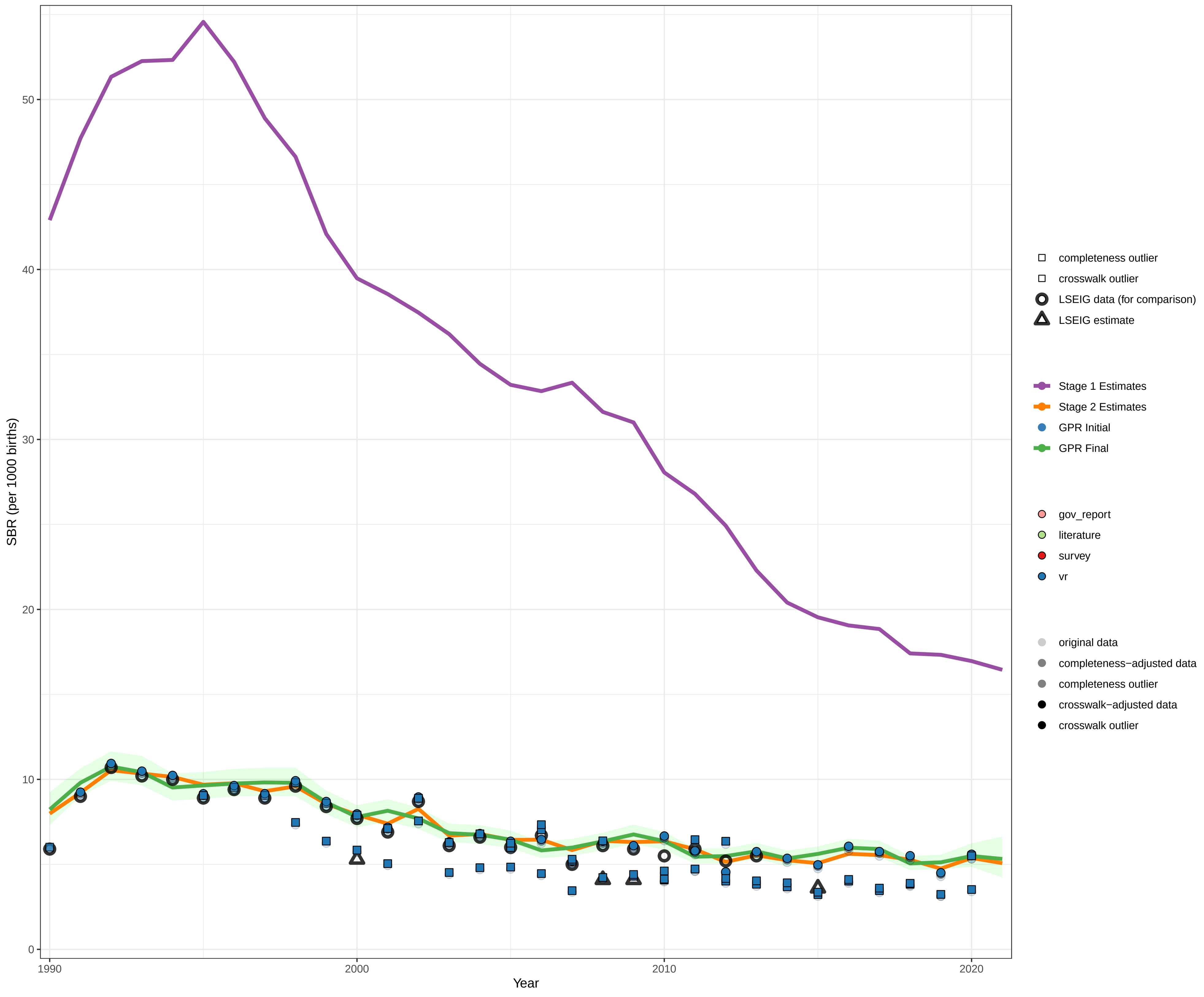

Latvia LVA Eastern Europe Stillbirth Rate (SBR) − 28 weeks
lambda = 0.2 ; zeta = 0.99 ; scale = 5 ; amp = 0.028

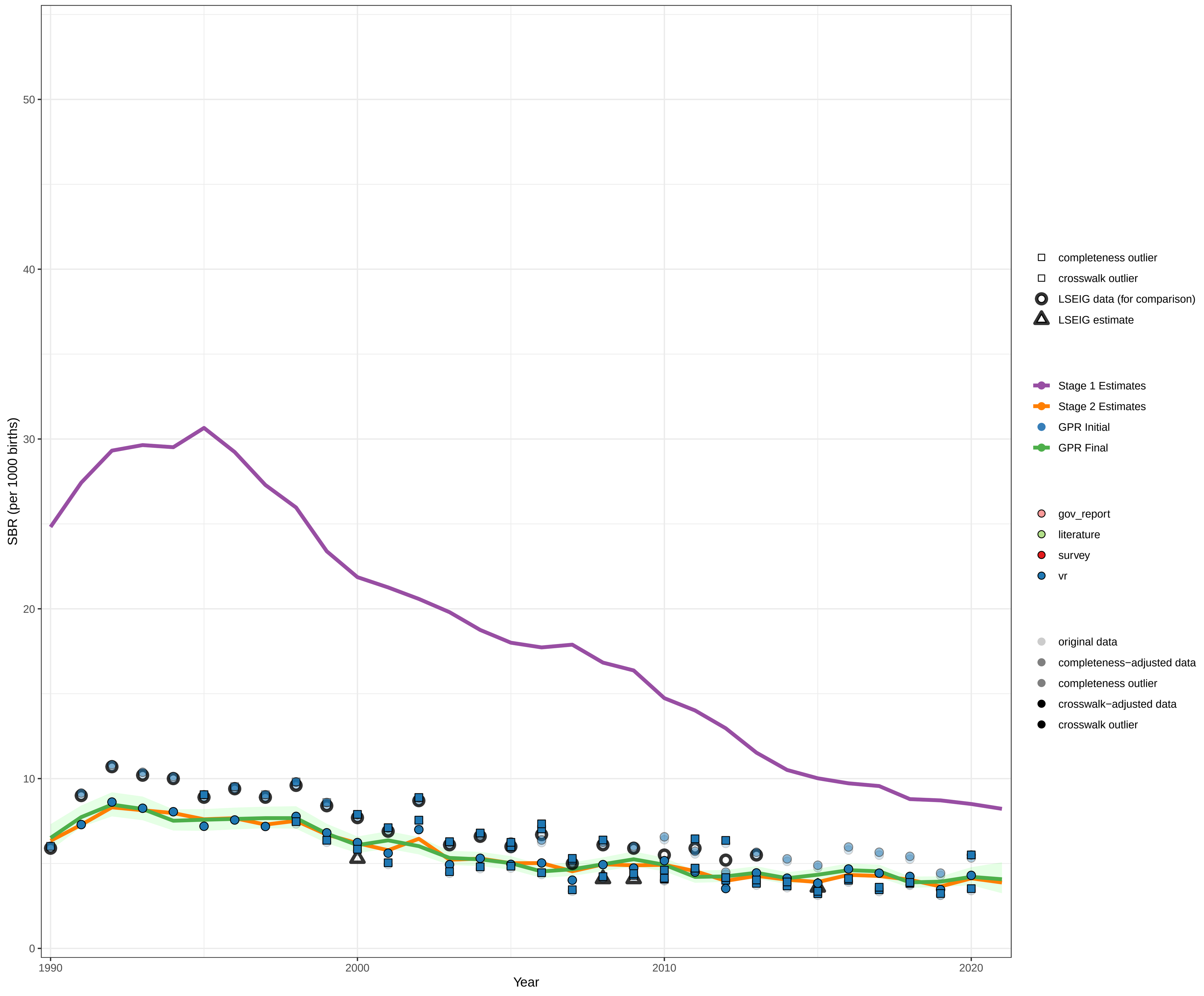

Year	Total Stillbirths (in thousands)	Total Stillbirths (in thousands)	SBR (per 1000 births)	SBR (per 1000 births)	SBR/NMR	SBR/NMR
	20 weeks	28 weeks	20 weeks	28 weeks	20 weeks	28 weeks
1990	0.3 (0.3 to 0.3)	0.2 (0.2 to 0.3)	8.2 (7.3 to 9.3)	6.5 (5.8 to 7.3)	0.94 (0.83 to 1.05)	0.74 (0.66 to 0.83)
1995	0.2 (0.2 to 0.2)	0.2 (0.2 to 0.2)	9.6 (8.9 to 10.4)	7.6 (6.9 to 8.2)	0.95 (0.87 to 1.03)	0.75 (0.68 to 0.81)
2000	0.2 (0.1 to 0.2)	0.1 (0.1 to 0.1)	7.8 (7.2 to 8.5)	6.1 (5.6 to 6.6)	1.10 (1.01 to 1.20)	0.86 (0.79 to 0.93)
2005	0.1 (0.1 to 0.1)	0.1 (0.1 to 0.1)	6.5 (6.0 to 7.0)	5.0 (4.6 to 5.4)	1.17 (1.08 to 1.26)	0.91 (0.84 to 0.98)
2010	0.1 (0.1 to 0.1)	0.1 (0.1 to 0.1)	6.4 (5.9 to 6.9)	4.9 (4.5 to 5.3)	1.52 (1.40 to 1.64)	1.17 (1.08 to 1.27)
2015	0.1 (0.1 to 0.1)	0.1 (0.1 to 0.1)	5.6 (5.2 to 6.1)	4.3 (4.0 to 4.7)	2.08 (1.92 to 2.24)	1.60 (1.48 to 1.73)
2020	0.1 (0.1 to 0.1)	0.1 (0.1 to 0.1)	5.5 (4.9 to 6.2)	4.2 (3.7 to 4.8)	2.54 (2.24 to 2.88)	1.95 (1.72 to 2.21)
2021	0.1 (0.1 to 0.1)	0.1 (0.1 to 0.1)	5.3 (4.2 to 6.6)	4.1 (3.2 to 5.1)	2.56 (2.04 to 3.19)	1.96 (1.56 to 2.44)

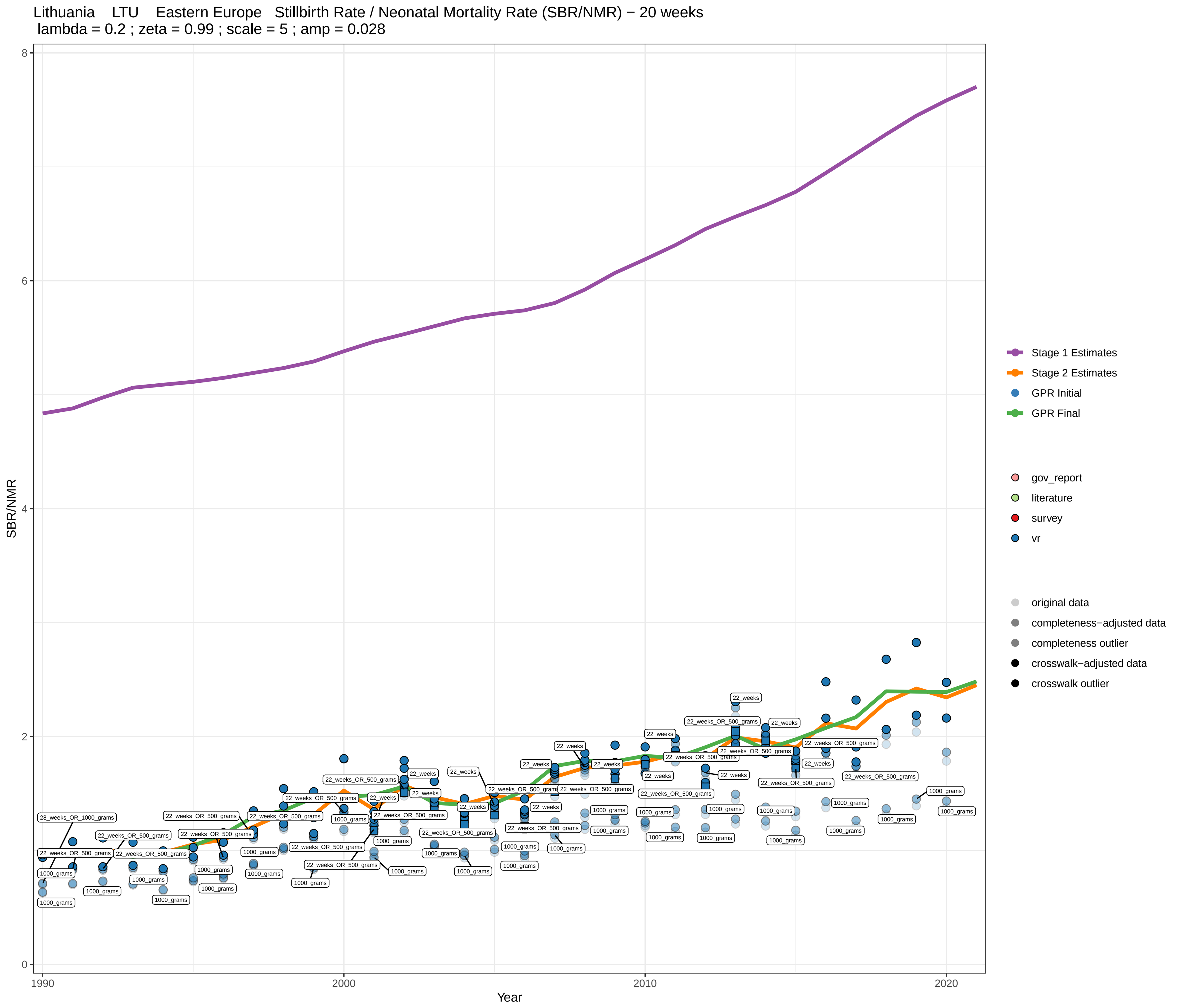

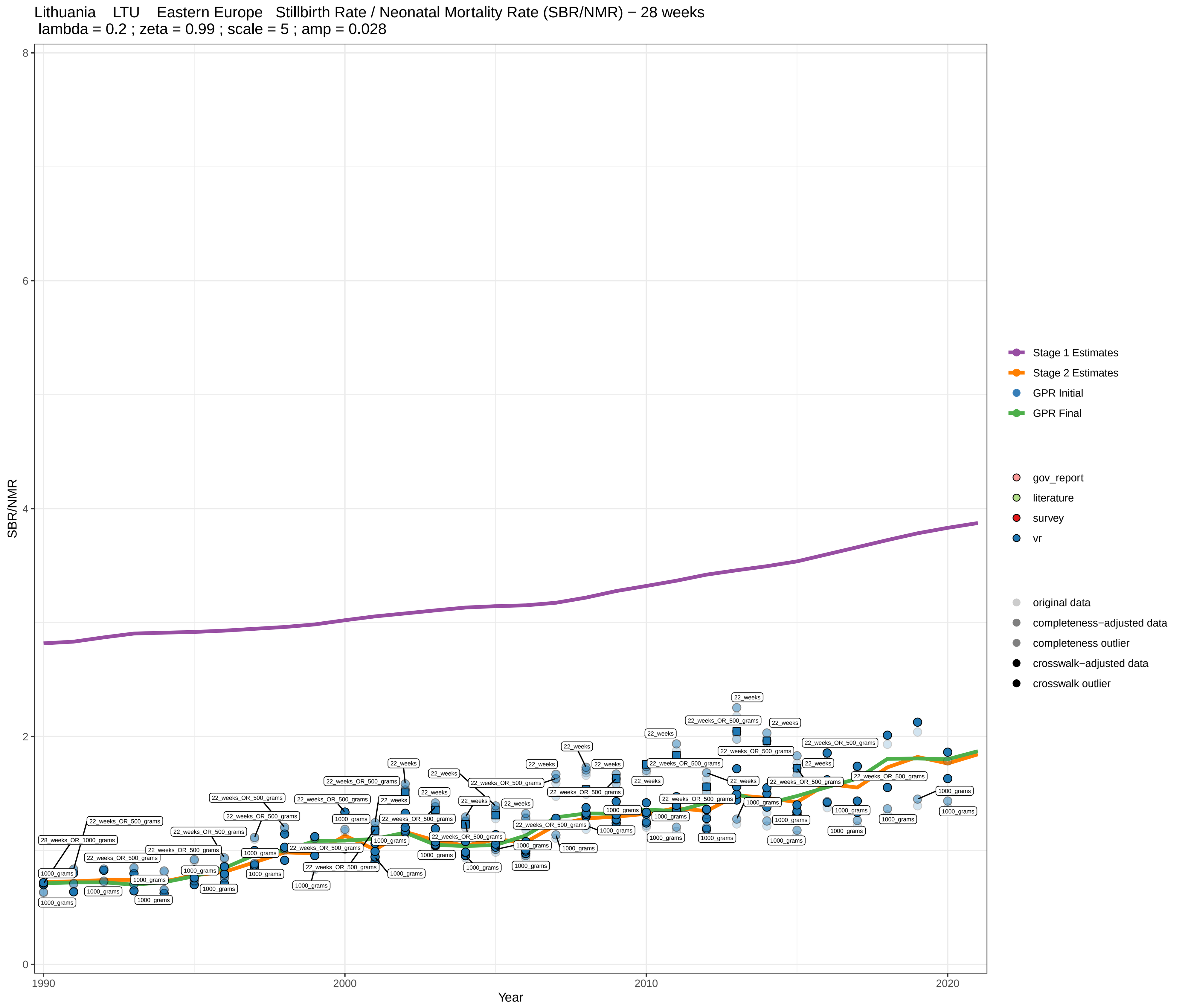

Definition	Years	Outlier	Source.Type	Title
1000_grams	1990−2020	0	VR	European Health for All Database
22_weeks, 28_weeks	2001−2015	0	VR	Lithuania Medical Birth Register Report
22_weeks_OR_500_grams	1991−2017	0	VR	Lithuania Demographic Yearbook
28_weeks	1995−2000, 2016−2020	0	VR	United Nations Demographic Yearbook
28_weeks_OR_1000_grams	1990	0	VR	Lithuania Demographic Yearbook
28_weeks	2001−2015	2	VR	United Nations Demographic Yearbook

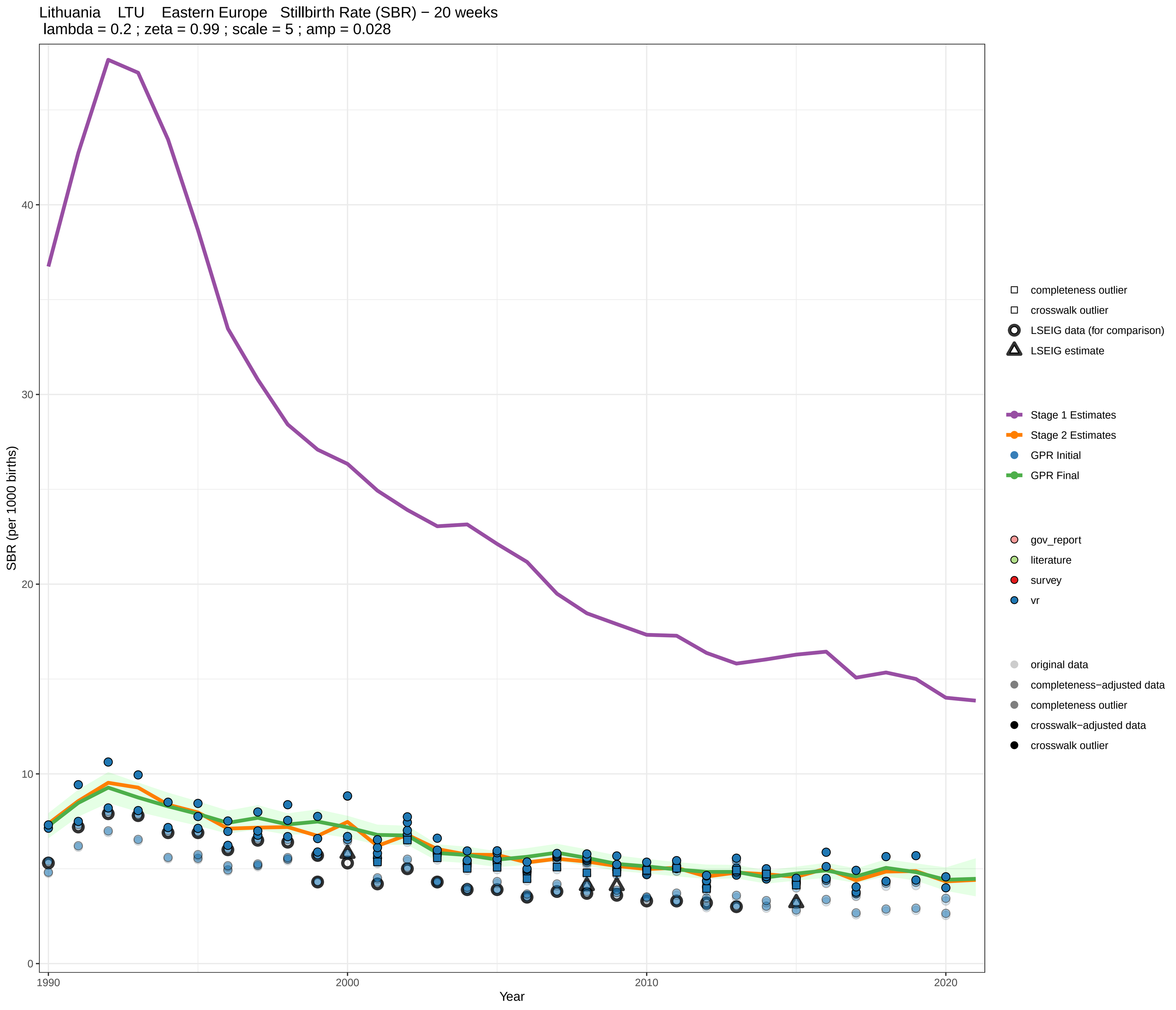

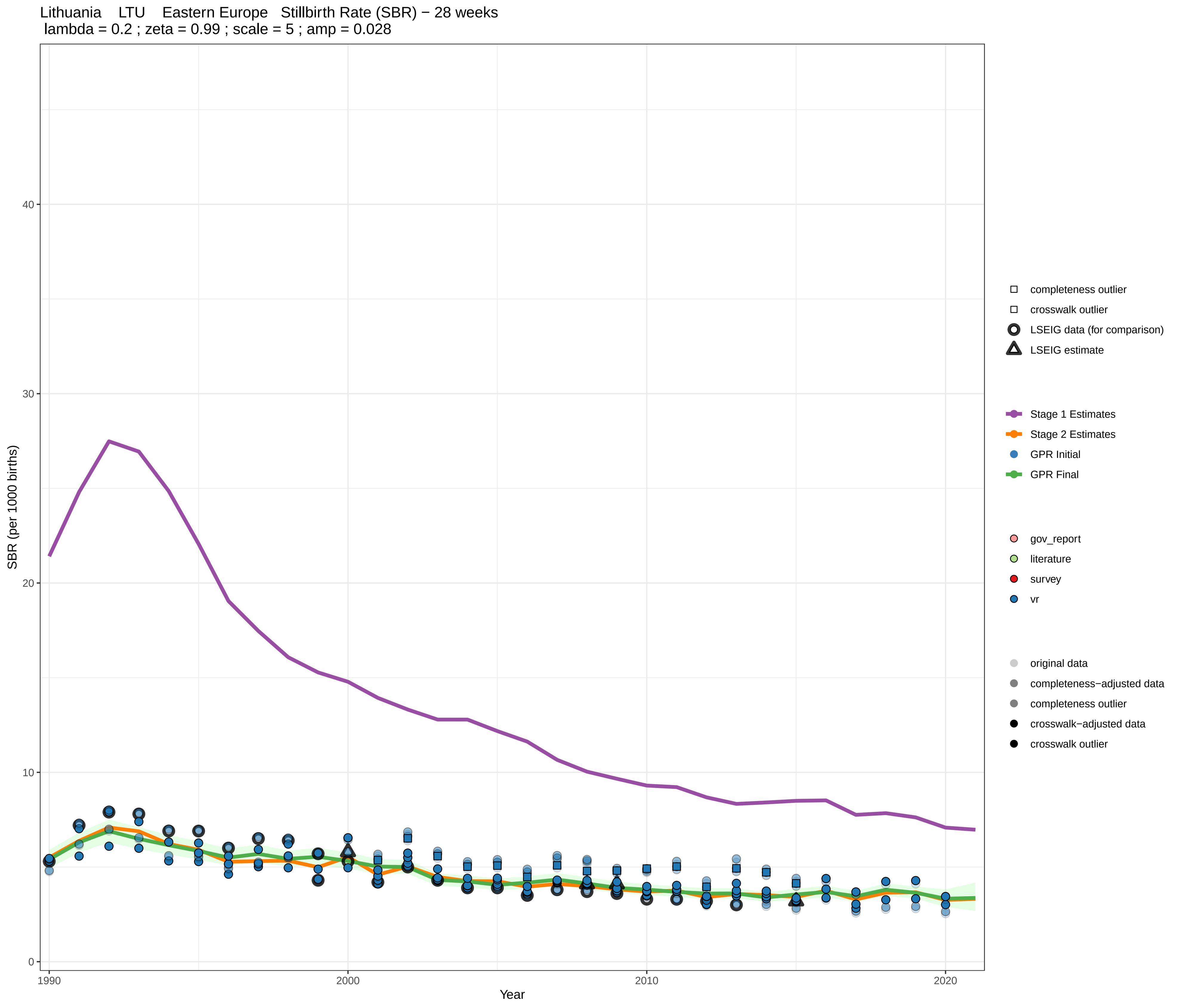

Year	Total Stillbirths (in thousands)	Total Stillbirths (in thousands)	SBR (per 1000 births)	SBR (per 1000 births)	SBR/NMR	SBR/NMR
	20 weeks	28 weeks	20 weeks	28 weeks	20 weeks	28 weeks
1990	0.4 (0.4 to 0.4)	0.3 (0.3 to 0.3)	7.3 (6.6 to 7.9)	5.4 (4.9 to 5.9)	0.95 (0.87 to 1.04)	0.71 (0.65 to 0.78)
1995	0.3 (0.3 to 0.3)	0.2 (0.2 to 0.3)	7.9 (7.3 to 8.6)	5.8 (5.4 to 6.3)	1.04 (0.96 to 1.13)	0.77 (0.71 to 0.84)
2000	0.2 (0.2 to 0.3)	0.2 (0.2 to 0.2)	7.2 (6.6 to 7.8)	5.3 (4.9 to 5.8)	1.47 (1.35 to 1.59)	1.09 (1.00 to 1.18)
2005	0.2 (0.2 to 0.2)	0.1 (0.1 to 0.1)	5.5 (5.1 to 5.9)	4.1 (3.8 to 4.4)	1.41 (1.31 to 1.53)	1.05 (0.97 to 1.13)
2010	0.2 (0.2 to 0.2)	0.1 (0.1 to 0.1)	5.1 (4.8 to 5.5)	3.8 (3.5 to 4.1)	1.83 (1.70 to 1.97)	1.36 (1.26 to 1.46)
2015	0.1 (0.1 to 0.2)	0.1 (0.1 to 0.1)	4.7 (4.4 to 5.1)	3.5 (3.3 to 3.9)	1.97 (1.82 to 2.13)	1.48 (1.36 to 1.61)
2020	0.1 (0.1 to 0.1)	0.1 (0.1 to 0.1)	4.4 (3.8 to 5.1)	3.3 (2.9 to 3.8)	2.39 (2.07 to 2.74)	1.80 (1.56 to 2.06)
2021	0.1 (0.1 to 0.1)	0.1 (0.1 to 0.1)	4.5 (3.5 to 5.6)	3.4 (2.7 to 4.2)	2.48 (1.97 to 3.08)	1.87 (1.49 to 2.32)

Republic of Moldova MDA Eastern Europe Stillbirth Rate / Neonatal Mortality Rate (SBR/NMR) − 20 weeks
lambda = 0.2 ; zeta = 0.99 ; scale = 5 ; amp = 0.028

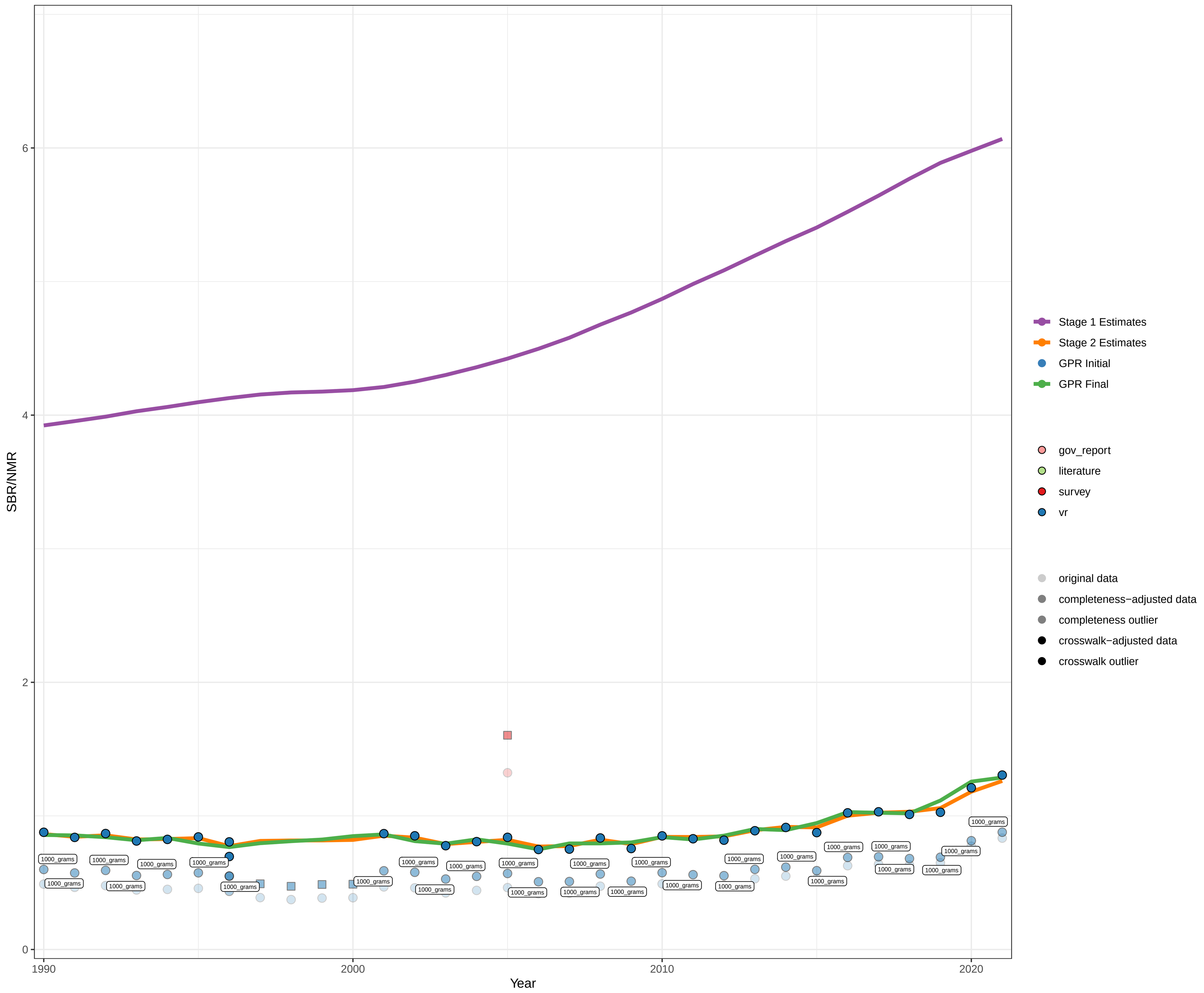

Republic of Moldova MDA Eastern Europe Stillbirth Rate / Neonatal Mortality Rate (SBR/NMR) − 28 weeks
lambda = 0.2 ; zeta = 0.99 ; scale = 5 ; amp = 0.028

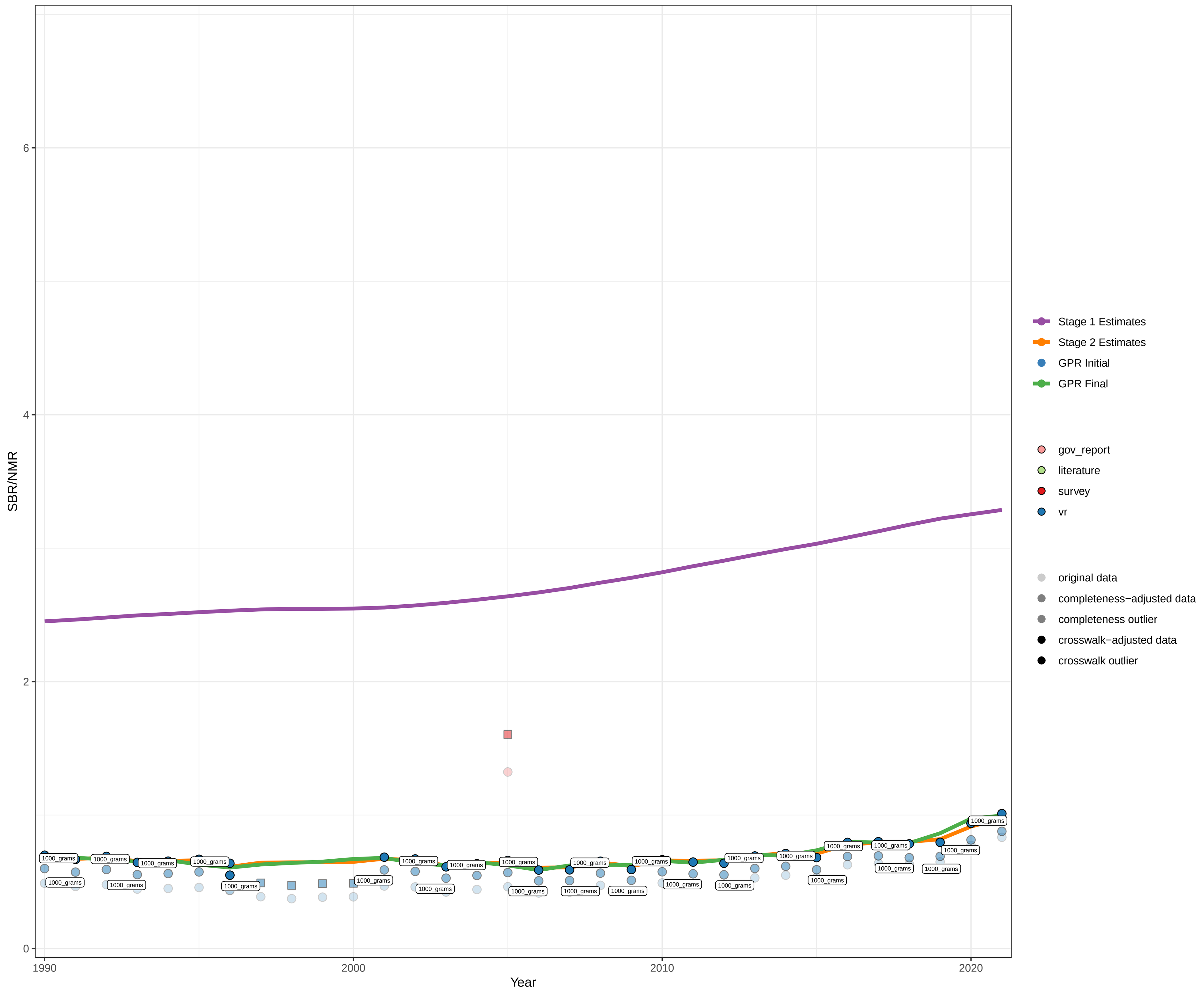

Definition	Years	Outlier	Source.Type	Title
1000_grams	1990−1996, 2001−2021	0	VR	European Health for All Database
28_weeks	1996	0	VR	United Nations Demographic Yearbook
1000_grams	1997−2000	1	VR	European Health for All Database
28_weeks	2005	1	Survey	Moldova Demographic and Health Survey 2005

Republic of Moldova MDA Eastern Europe Stillbirth Rate (SBR) − 20 weeks
lambda = 0.2 ; zeta = 0.99 ; scale = 5 ; amp = 0.028

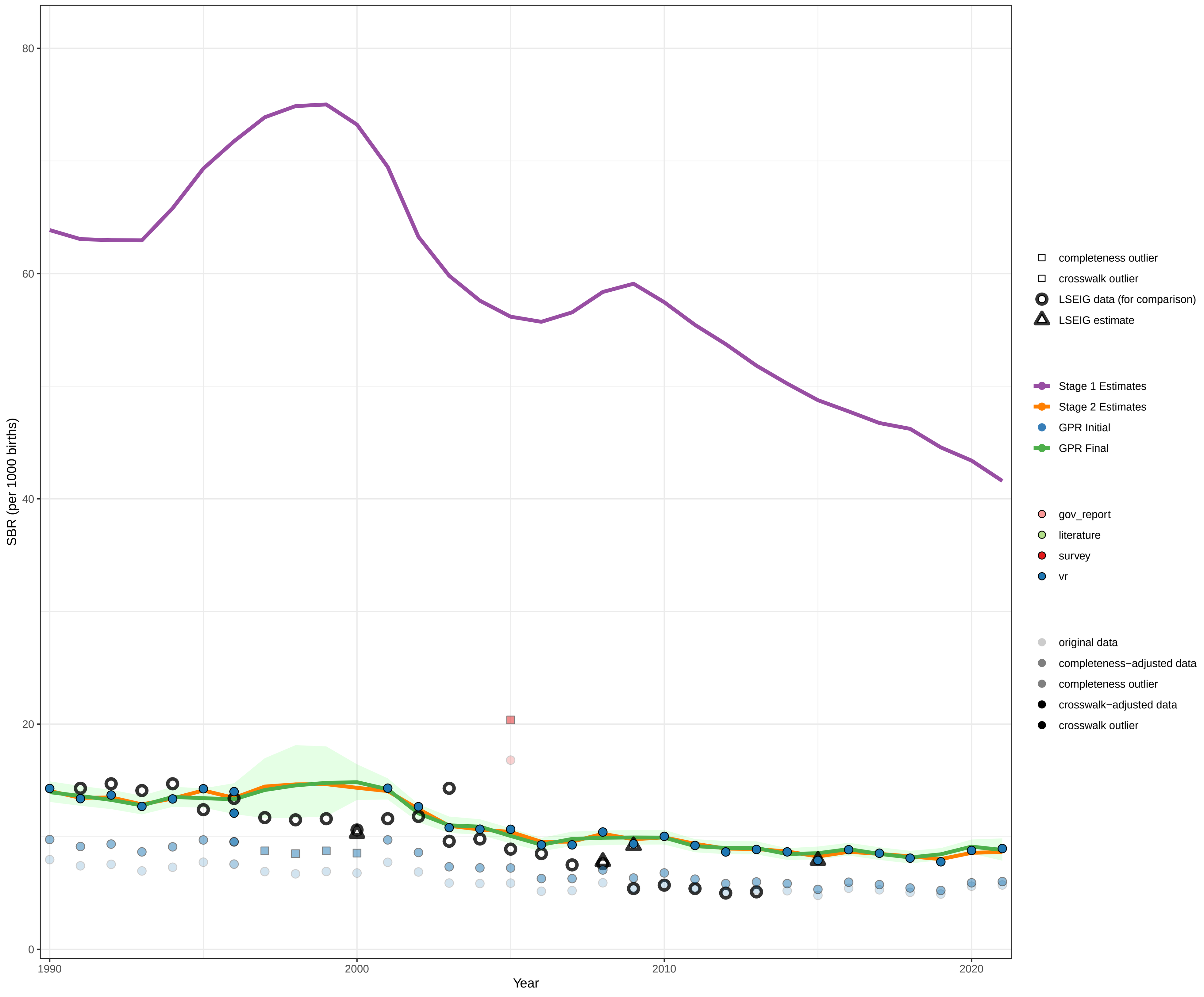

Republic of Moldova MDA Eastern Europe Stillbirth Rate (SBR) − 28 weeks
lambda = 0.2 ; zeta = 0.99 ; scale = 5 ; amp = 0.028

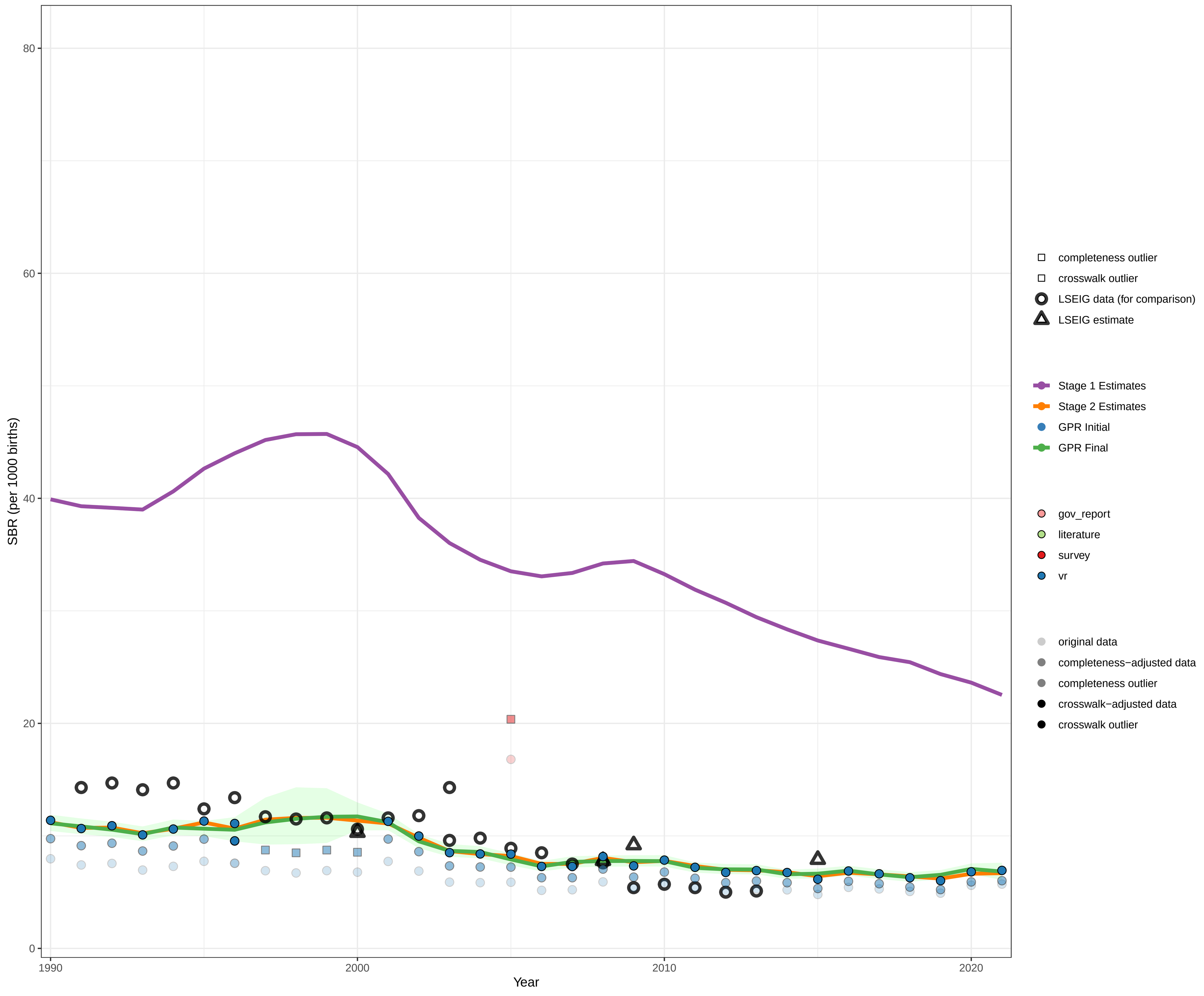

Year	Total Stillbirths (in thousands)	Total Stillbirths (in thousands)	SBR (per 1000 births)	SBR (per 1000 births)	SBR/NMR	SBR/NMR
	20 weeks	28 weeks	20 weeks	28 weeks	20 weeks	28 weeks
1990	1.1<br>(1.1 to 1.2)	0.9<br>(0.8 to 1.0)	14.0<br>(13.1 to 14.9)	11.1<br>(10.4 to 11.9)	0.86<br>(0.80 to 0.92)	0.68<br>(0.64 to 0.73)
1995	0.8<br>(0.7 to 0.8)	0.6<br>(0.6 to 0.6)	13.4<br>(12.6 to 14.3)	10.6<br>(10.0 to 11.3)	0.79<br>(0.74 to 0.85)	0.63<br>(0.59 to 0.67)
2000	0.6<br>(0.6 to 0.7)	0.5<br>(0.4 to 0.5)	14.8<br>(13.3 to 16.4)	11.7<br>(10.5 to 13.0)	0.85<br>(0.76 to 0.94)	0.67<br>(0.60 to 0.74)
2005	0.4<br>(0.4 to 0.4)	0.3<br>(0.3 to 0.3)	10.1<br>(9.4 to 10.8)	7.9<br>(7.4 to 8.5)	0.79<br>(0.74 to 0.85)	0.62<br>(0.58 to 0.67)
2010	0.4<br>(0.4 to 0.4)	0.3<br>(0.3 to 0.3)	9.9<br>(9.3 to 10.6)	7.8<br>(7.3 to 8.3)	0.84<br>(0.79 to 0.90)	0.66<br>(0.62 to 0.70)
2015	0.3<br>(0.3 to 0.3)	0.2<br>(0.2 to 0.3)	8.5<br>(8.0 to 9.1)	6.6<br>(6.2 to 7.1)	0.95<br>(0.89 to 1.01)	0.74<br>(0.69 to 0.79)
2020	0.3<br>(0.3 to 0.3)	0.2<br>(0.2 to 0.2)	9.1<br>(8.5 to 9.8)	7.1<br>(6.6 to 7.5)	1.26<br>(1.17 to 1.34)	0.97<br>(0.91 to 1.04)
2021	0.3<br>(0.2 to 0.3)	0.2<br>(0.2 to 0.2)	8.8<br>(7.9 to 9.9)	6.8<br>(6.1 to 7.6)	1.29<br>(1.15 to 1.44)	1.00<br>(0.89 to 1.11)

Russian Federation RUS Eastern Europe Stillbirth Rate / Neonatal Mortality Rate (SBR/NMR) − 20 weeks
lambda = 0.2 ; zeta = 0.99 ; scale = 5 ; amp = 0.028

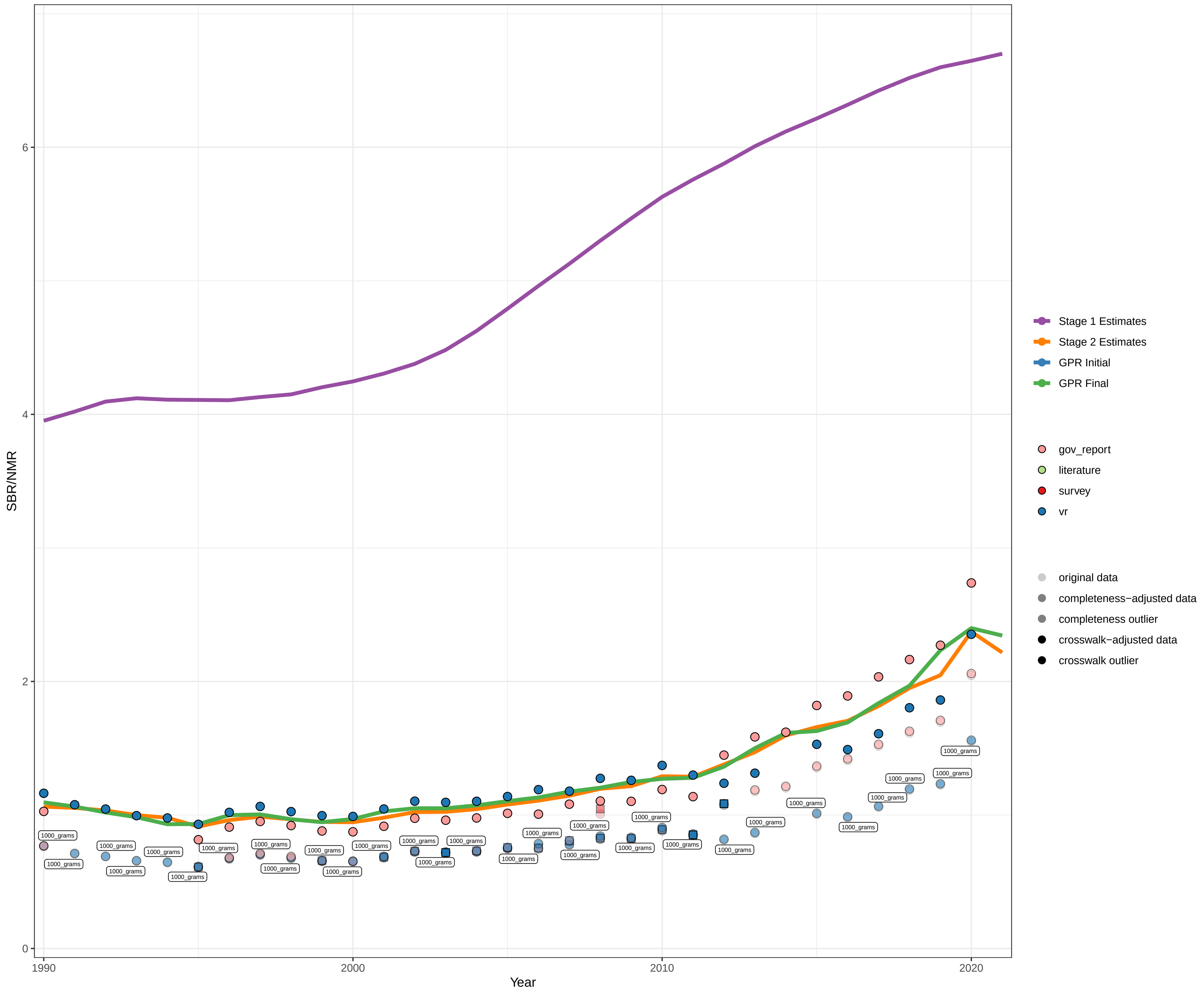

Russian Federation RUS Eastern Europe Stillbirth Rate / Neonatal Mortality Rate (SBR/NMR) − 28 weeks
lambda = 0.2 ; zeta = 0.99 ; scale = 5 ; amp = 0.028

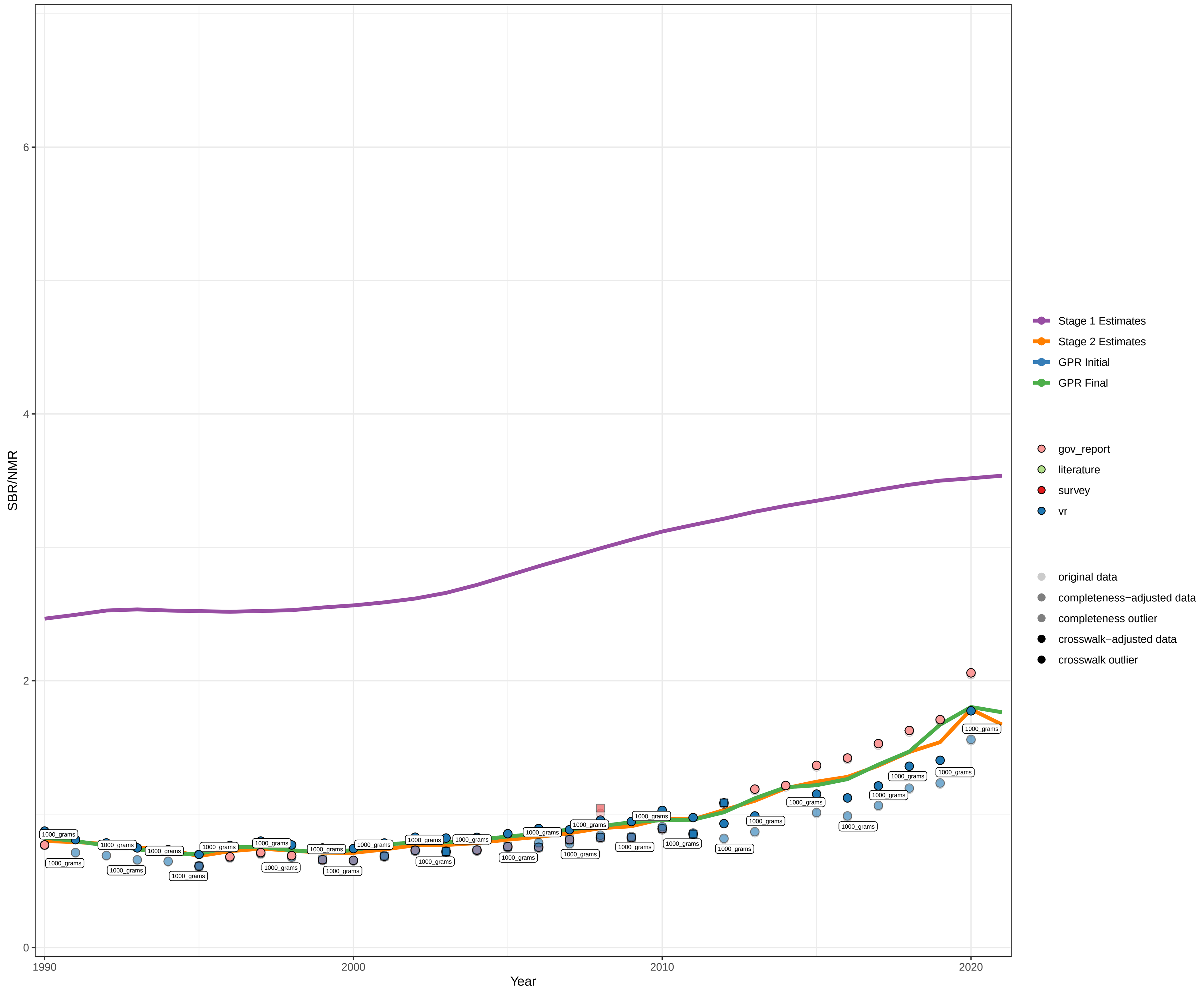

Definition	Years	Outlier	Source.Type	Title
1000_grams	1990−2013, 2015−2020	0	VR	European Health for All Database
28_weeks	1990, 1995−2020	0	Statistical Report	Russia Demographic Yearbook
28_weeks	1995, 1999, 2001, 2002, 2004−2010	1	VR	United Nations Demographic Yearbook
other	2008	1	Survey	Russia Reproductive Health Survey 2011
28_weeks	2003, 2011, 2012	2	VR	United Nations Demographic Yearbook

Russian Federation RUS Eastern Europe Stillbirth Rate (SBR) − 20 weeks
lambda = 0.2 ; zeta = 0.99 ; scale = 5 ; amp = 0.028

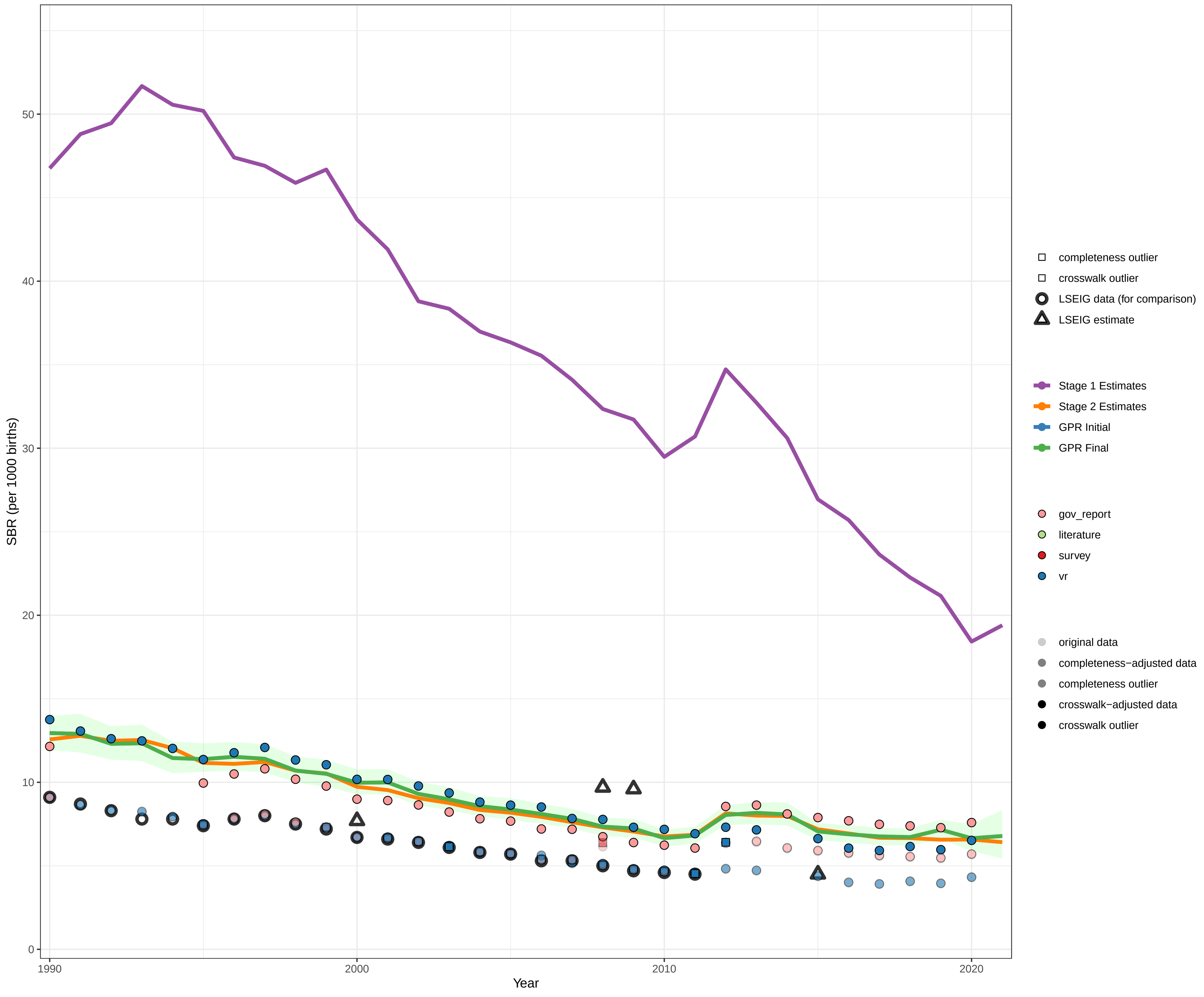

Russian Federation RUS Eastern Europe Stillbirth Rate (SBR) − 28 weeks
lambda = 0.2 ; zeta = 0.99 ; scale = 5 ; amp = 0.028

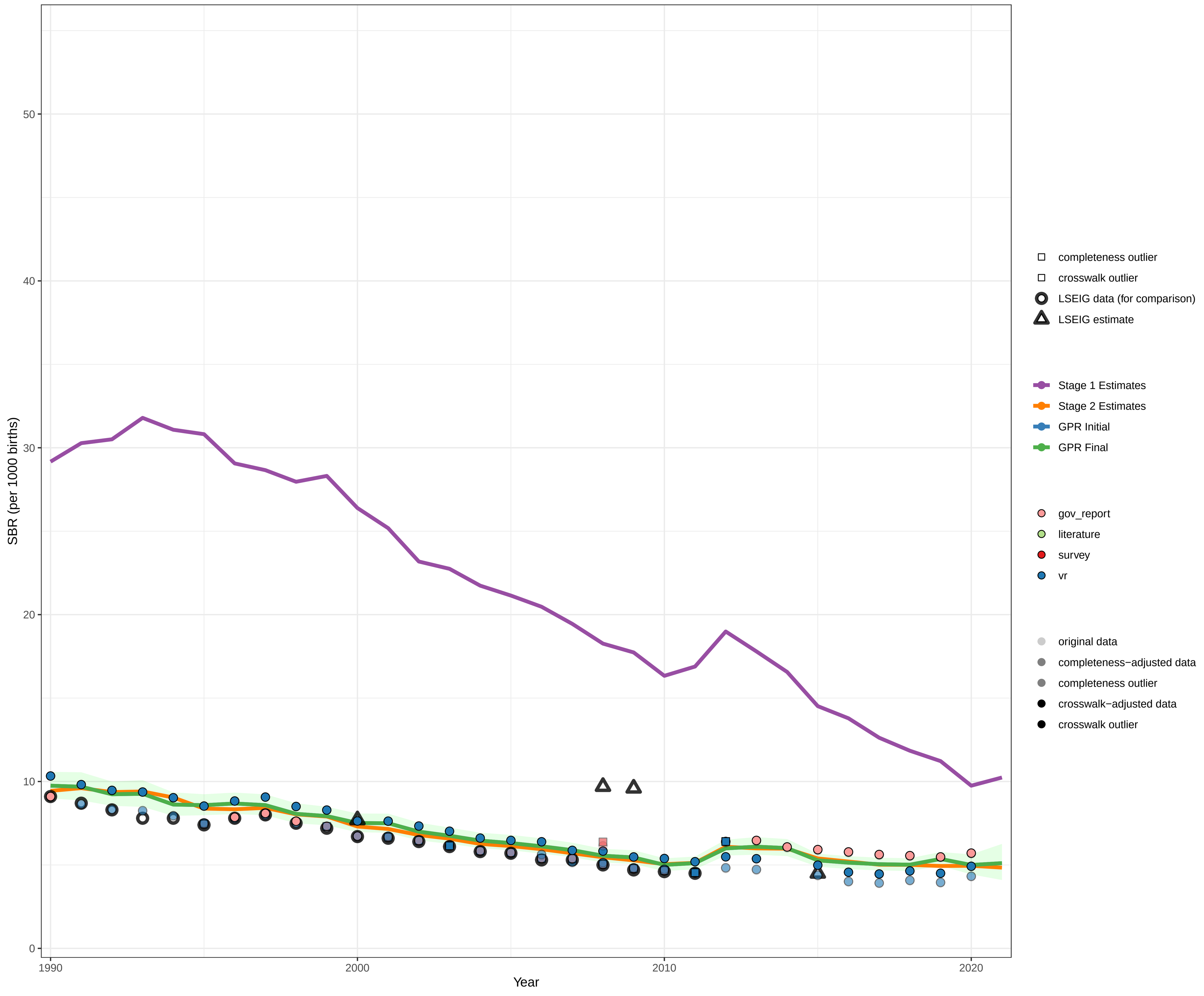

Year	Total Stillbirths (in thousands)	Total Stillbirths (in thousands)	SBR (per 1000 births)	SBR (per 1000 births)	SBR/NMR	SBR/NMR
	20 weeks	28 weeks	20 weeks	28 weeks	20 weeks	28 weeks
1990	25.7 (23.6 to 27.8)	19.3 (17.7 to 20.9)	12.9 (11.9 to 14.0)	9.7 (9.0 to 10.6)	1.09 (1.01 to 1.18)	0.82 (0.76 to 0.89)
1995	15.9 (14.9 to 17.3)	12.0 (11.1 to 12.9)	11.4 (10.6 to 12.3)	8.6 (8.0 to 9.2)	0.93 (0.87 to 1.01)	0.70 (0.65 to 0.76)
2000	13.5 (12.5 to 14.5)	10.1 (9.4 to 10.9)	10.0 (9.3 to 10.8)	7.5 (7.0 to 8.1)	0.97 (0.90 to 1.05)	0.73 (0.68 to 0.79)
2005	12.7 (11.8 to 13.8)	9.6 (8.9 to 10.3)	8.4 (7.7 to 9.1)	6.3 (5.9 to 6.8)	1.10 (1.02 to 1.19)	0.83 (0.78 to 0.90)
2010	12.4 (11.5 to 13.4)	9.3 (8.6 to 10.0)	6.7 (6.2 to 7.2)	5.0 (4.6 to 5.4)	1.27 (1.18 to 1.37)	0.96 (0.88 to 1.03)
2015	13.5 (12.5 to 14.4)	10.0 (9.3 to 10.8)	7.1 (6.5 to 7.6)	5.3 (4.9 to 5.7)	1.63 (1.51 to 1.74)	1.22 (1.13 to 1.31)
2020	9.4 (8.3 to 10.6)	7.1 (6.3 to 8.0)	6.6 (5.9 to 7.5)	5.0 (4.4 to 5.6)	2.40 (2.12 to 2.70)	1.80 (1.60 to 2.03)
2021	9.2 (7.4 to 11.4)	6.9 (5.6 to 8.5)	6.8 (5.4 to 8.3)	5.1 (4.1 to 6.3)	2.34 (1.88 to 2.88)	1.76 (1.42 to 2.16)

Ukraine UKR Eastern Europe Stillbirth Rate / Neonatal Mortality Rate (SBR/NMR) − 20 weeks
lambda = 0.2 ; zeta = 0.99 ; scale = 5 ; amp = 0.028

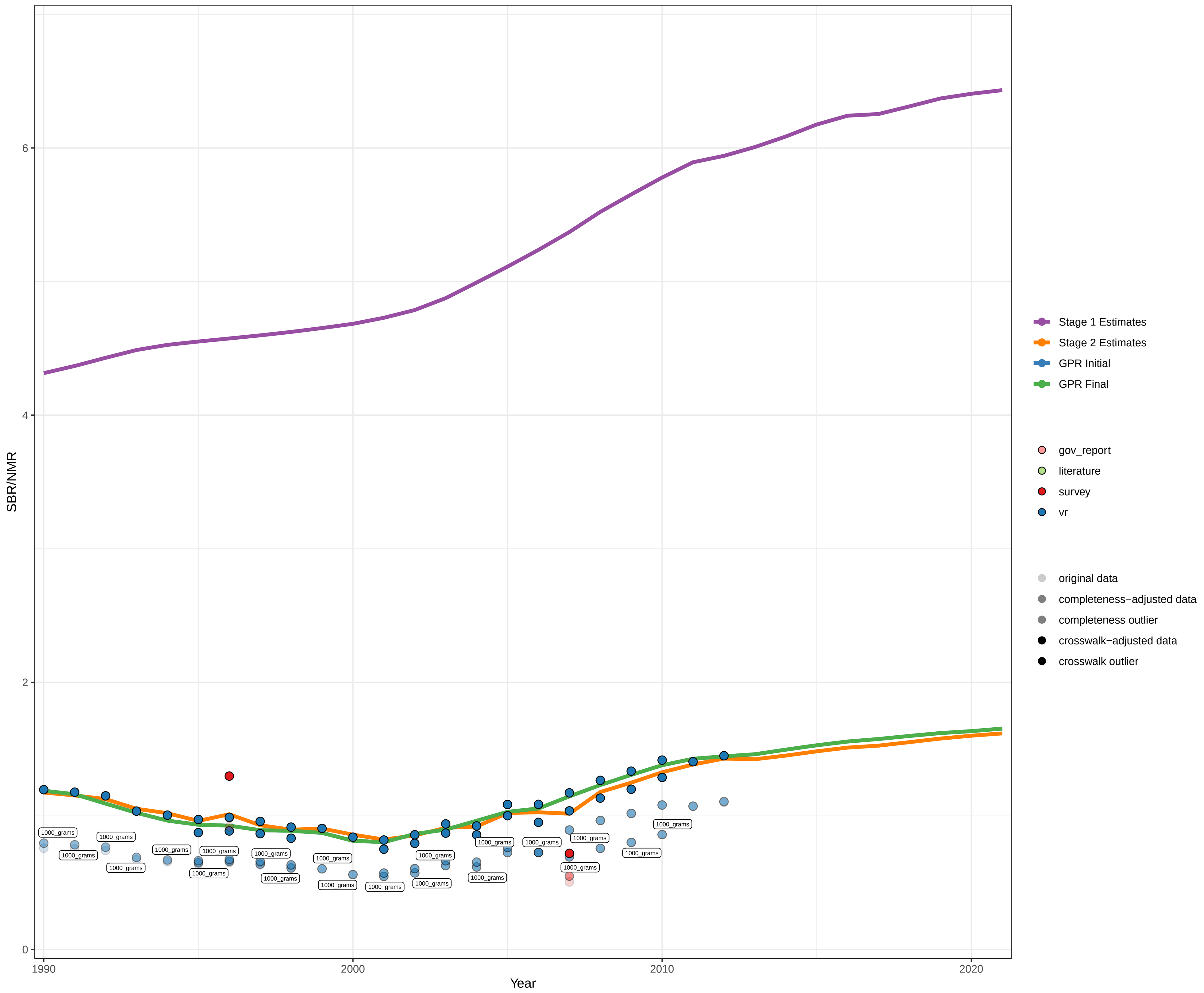

Ukraine UKR Eastern Europe Stillbirth Rate / Neonatal Mortality Rate (SBR/NMR) − 28 weeks
lambda = 0.2 ; zeta = 0.99 ; scale = 5 ; amp = 0.028

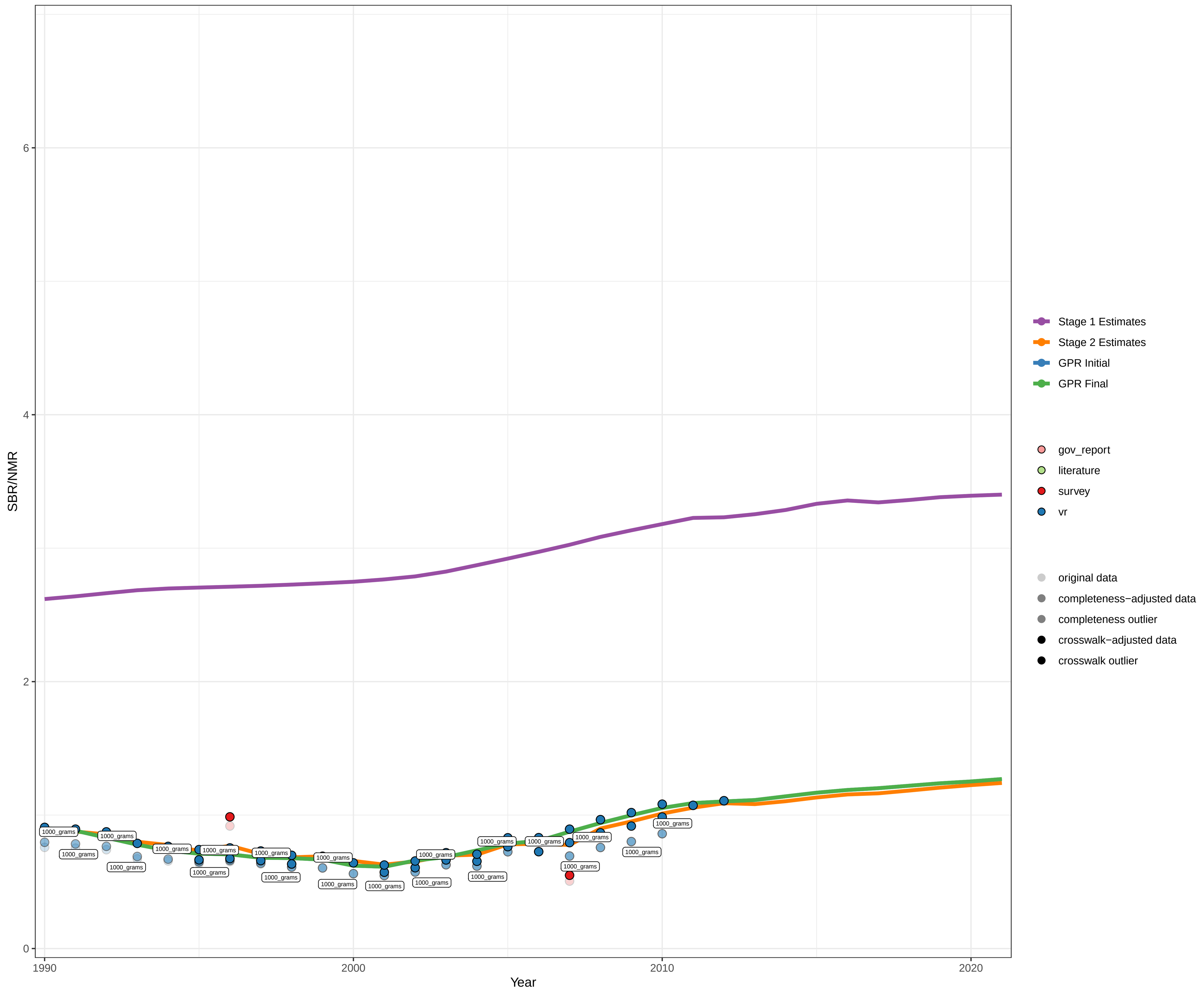

Definition	Years	Outlier	Source.Type	Title
1000_grams	1990−2010	0	VR	European Health for All Database
28_weeks	1995−1998, 2001−2012	0	VR	United Nations Demographic Yearbook
28_weeks	1996	0	Survey	Ukraine Reproductive Health Survey 1999
28_weeks	2007	0	Survey	Ukraine Demographic and Health Survey 2007

Ukraine UKR Eastern Europe Stillbirth Rate (SBR) − 20 weeks
lambda = 0.2 ; zeta = 0.99 ; scale = 5 ; amp = 0.028

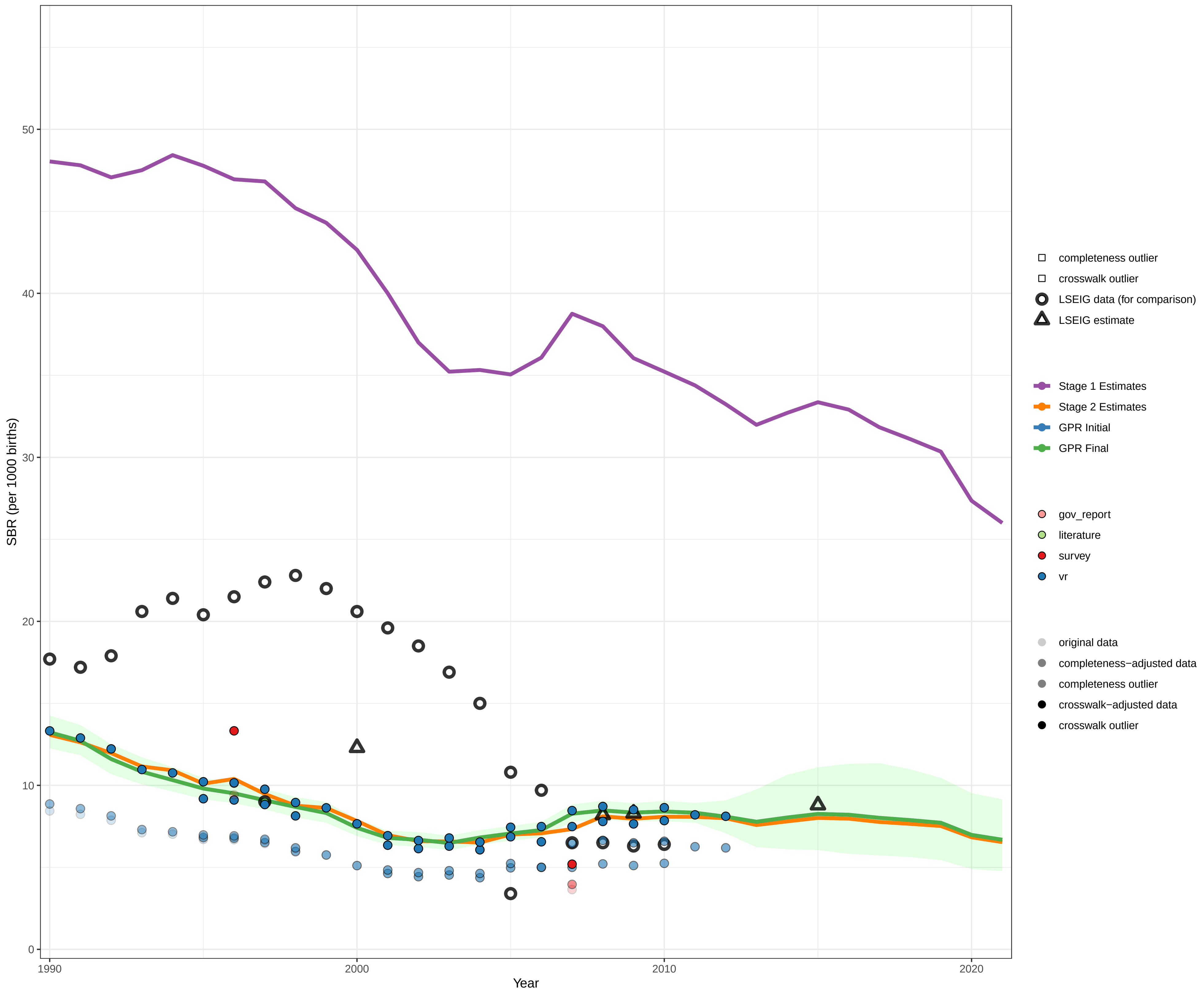

Ukraine UKR Eastern Europe Stillbirth Rate (SBR) − 28 weeks
lambda = 0.2 ; zeta = 0.99 ; scale = 5 ; amp = 0.028

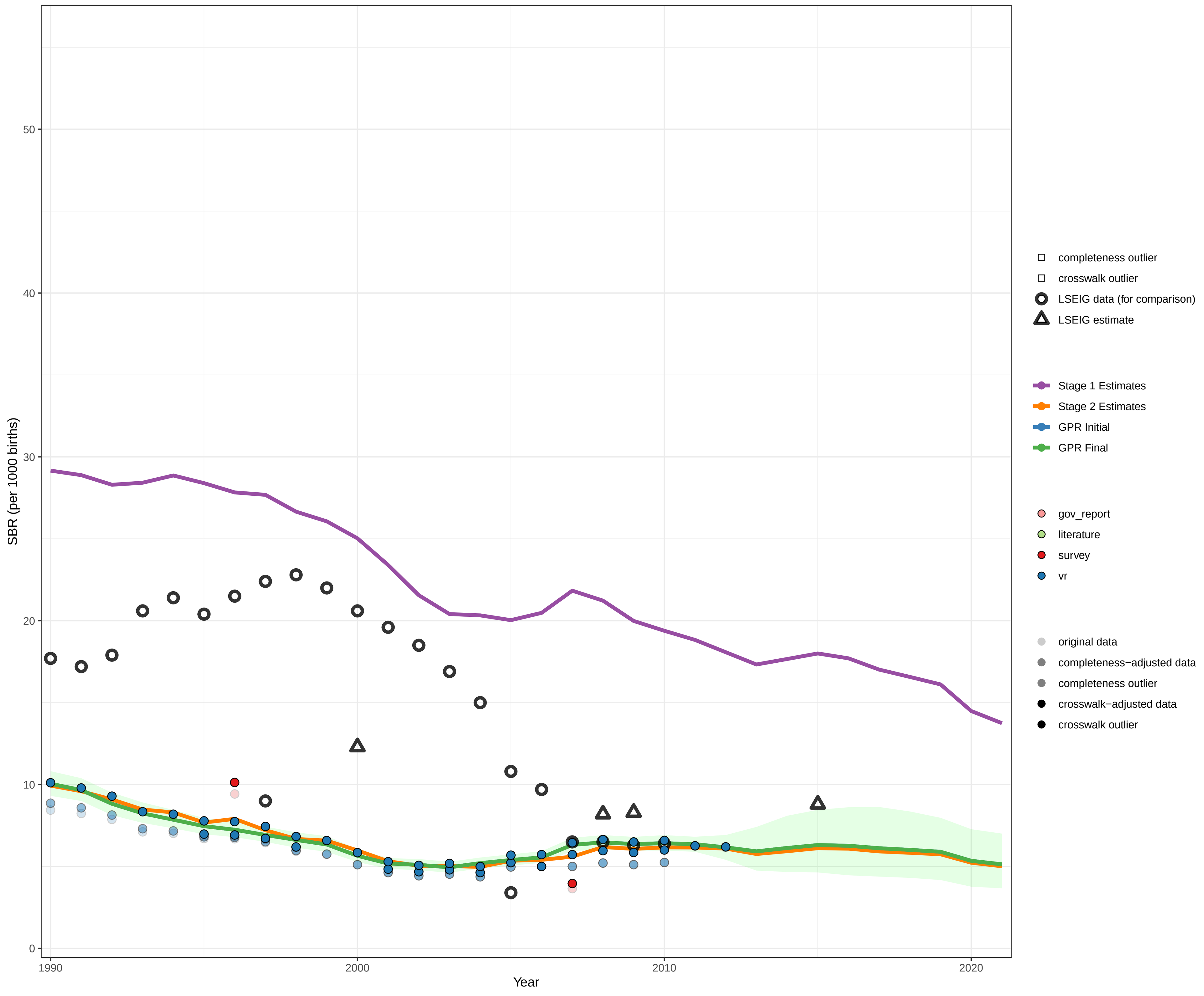

Year	Total Stillbirths (in thousands)	Total Stillbirths (in thousands)	SBR (per 1000 births)	SBR (per 1000 births)	SBR/NMR	SBR/NMR
	20 weeks	28 weeks	20 weeks	28 weeks	20 weeks	28 weeks
1990	8.9 (8.2 to 9.6)	6.7 (6.2 to 7.2)	13.2 (12.3 to 14.3)	10.0 (9.3 to 10.8)	1.19 (1.10 to 1.28)	0.90 (0.84 to 0.97)
1995	4.8 (4.5 to 5.1)	3.6 (3.4 to 3.9)	9.8 (9.2 to 10.5)	7.5 (7.0 to 8.0)	0.93 (0.87 to 1.00)	0.71 (0.66 to 0.76)
2000	2.9 (2.7 to 3.1)	2.2 (2.0 to 2.3)	7.4 (6.9 to 8.0)	5.7 (5.3 to 6.1)	0.81 (0.76 to 0.87)	0.62 (0.58 to 0.67)
2005	3.2 (3.0 to 3.4)	2.4 (2.3 to 2.6)	7.1 (6.6 to 7.5)	5.4 (5.1 to 5.7)	1.03 (0.97 to 1.10)	0.79 (0.74 to 0.84)
2010	4.3 (4.0 to 4.7)	3.3 (3.1 to 3.6)	8.4 (7.8 to 9.1)	6.4 (6.0 to 6.9)	1.38 (1.28 to 1.49)	1.05 (0.98 to 1.13)
2015	3.4 (2.5 to 4.6)	2.6 (1.9 to 3.5)	8.3 (6.1 to 11.1)	6.3 (4.6 to 8.5)	1.53 (1.12 to 2.06)	1.17 (0.86 to 1.57)
2020	2.1 (1.4 to 2.8)	1.6 (1.1 to 2.1)	7.0 (4.9 to 9.5)	5.3 (3.8 to 7.3)	1.63 (1.15 to 2.23)	1.25 (0.88 to 1.70)
2021	1.9 (1.3 to 2.6)	1.4 (1.0 to 2.0)	6.7 (4.8 to 9.1)	5.1 (3.7 to 7.0)	1.65 (1.18 to 2.26)	1.27 (0.91 to 1.73)

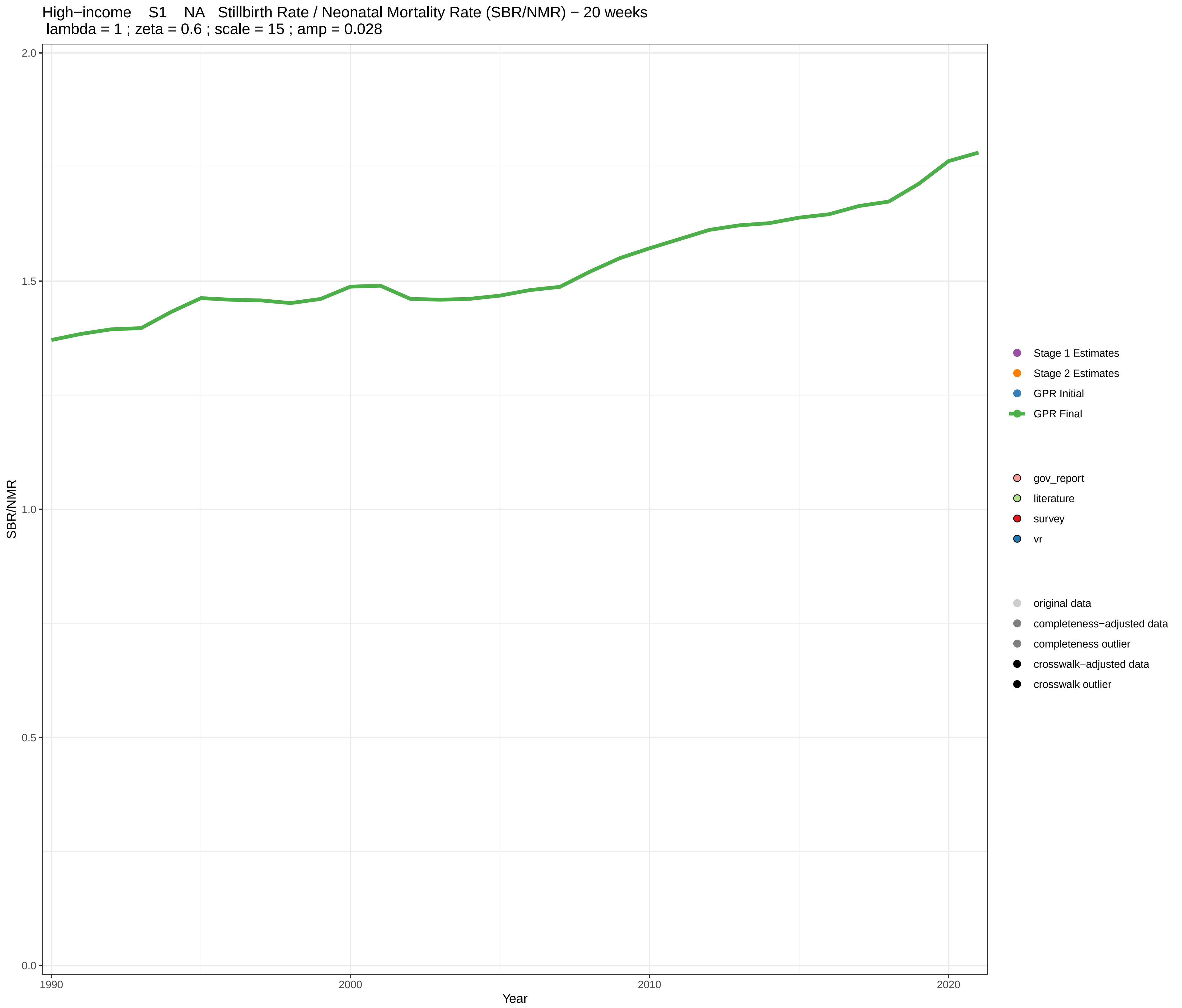

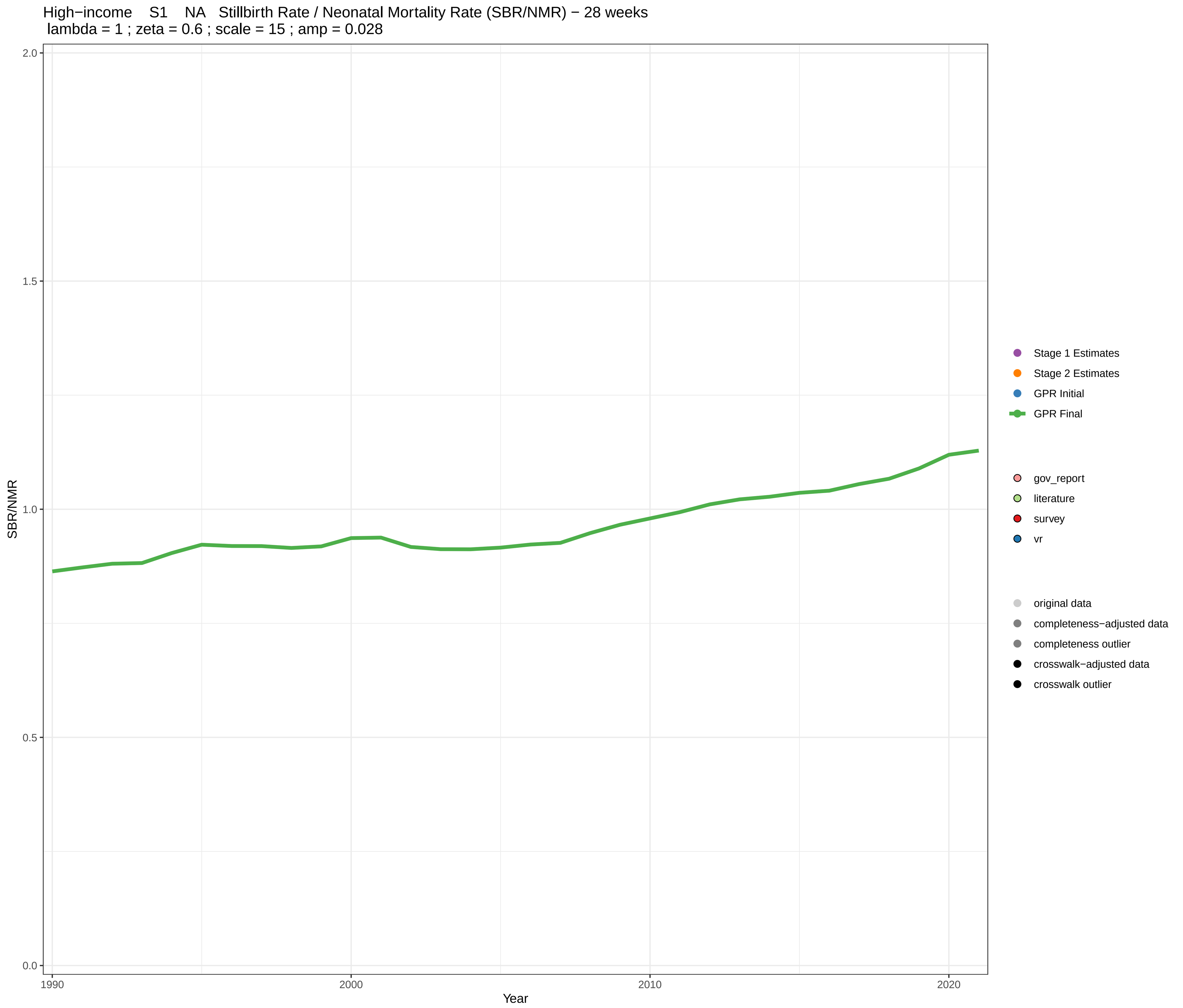

Definition	Years	Outlier	Source.Type	Title

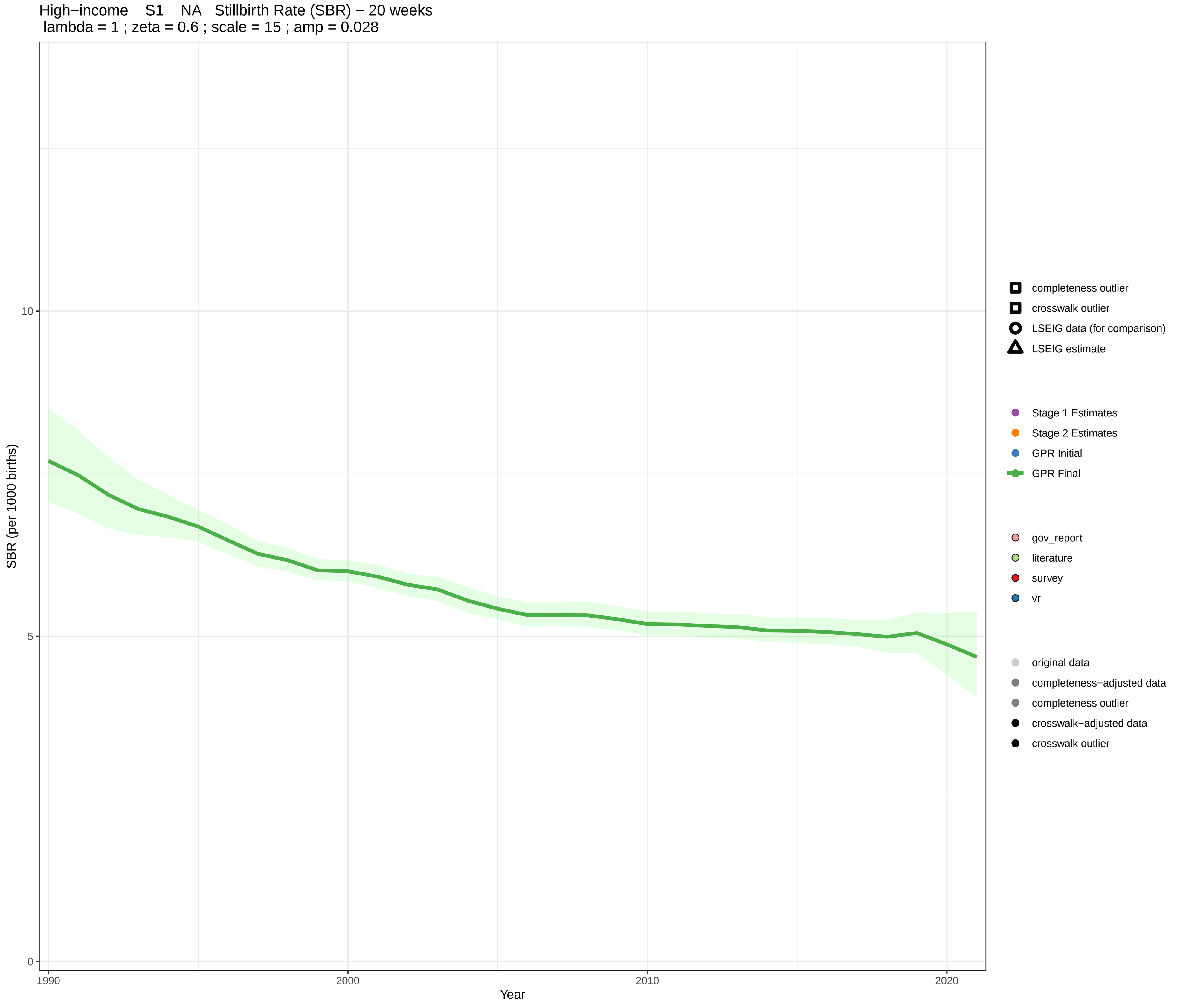

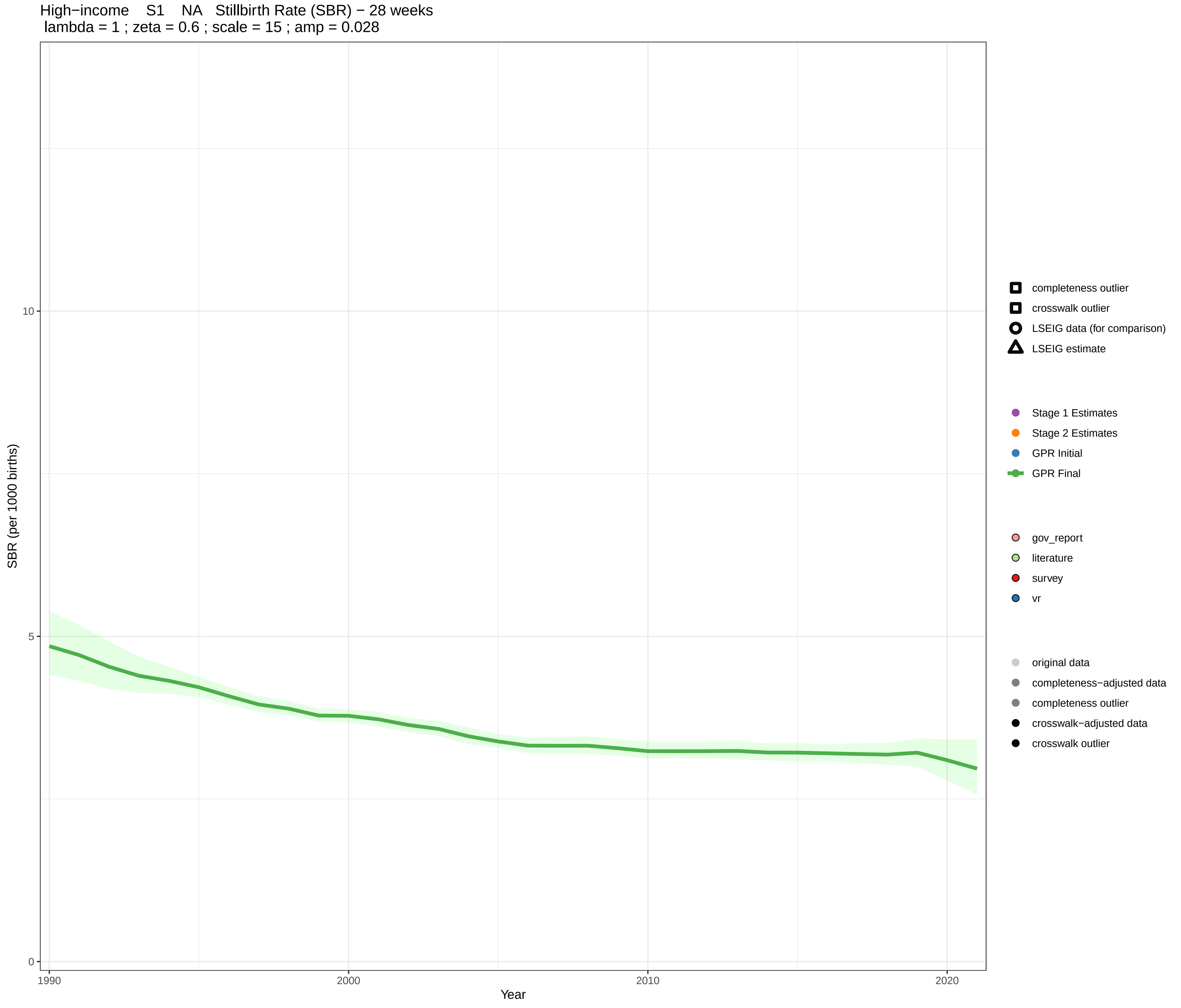

Year	Total Stillbirths (in thousands)	Total Stillbirths (in thousands)	SBR (per 1000 births)	SBR (per 1000 births)	SBR/NMR	SBR/NMR
	20 weeks	28 weeks	20 weeks	28 weeks	20 weeks	28 weeks
1990	96.5<br>(88.6 to 106.8)	60.6<br>(55.1 to 67.4)	7.7<br>(7.1 to 8.5)	4.8<br>(4.4 to 5.4)	1.37<br>(1.26 to 1.52)	0.86<br>(0.79 to 0.96)
1995	79.9<br>(77.0 to 82.9)	50.3<br>(48.5 to 52.2)	6.7<br>(6.4 to 6.9)	4.2<br>(4.1 to 4.4)	1.46<br>(1.41 to 1.52)	0.92<br>(0.89 to 0.96)
2000	71.3<br>(69.5 to 73.3)	44.8<br>(43.6 to 46.0)	6.0<br>(5.8 to 6.2)	3.8<br>(3.7 to 3.9)	1.49<br>(1.45 to 1.53)	0.94<br>(0.91 to 0.96)
2005	64.9<br>(63.0 to 67.3)	40.4<br>(39.2 to 41.8)	5.4<br>(5.3 to 5.6)	3.4<br>(3.3 to 3.5)	1.47<br>(1.42 to 1.52)	0.92<br>(0.89 to 0.95)
2010	62.2<br>(60.1 to 64.5)	38.7<br>(37.3 to 40.4)	5.2<br>(5.0 to 5.4)	3.2<br>(3.1 to 3.4)	1.57<br>(1.52 to 1.63)	0.98<br>(0.94 to 1.02)
2015	59.3<br>(57.2 to 61.8)	37.4<br>(35.8 to 39.1)	5.1<br>(4.9 to 5.3)	3.2<br>(3.1 to 3.4)	1.64<br>(1.58 to 1.71)	1.04<br>(0.99 to 1.08)
2020	51.1<br>(46.2 to 56.1)	32.4<br>(29.1 to 35.7)	4.9<br>(4.4 to 5.4)	3.1<br>(2.8 to 3.4)	1.76<br>(1.60 to 1.94)	1.12<br>(1.01 to 1.23)
2021	48.9<br>(42.4 to 56.3)	30.9<br>(26.8 to 35.7)	4.7<br>(4.1 to 5.4)	3.0<br>(2.6 to 3.4)	1.78<br>(1.54 to 2.05)	1.13<br>(0.98 to 1.30)

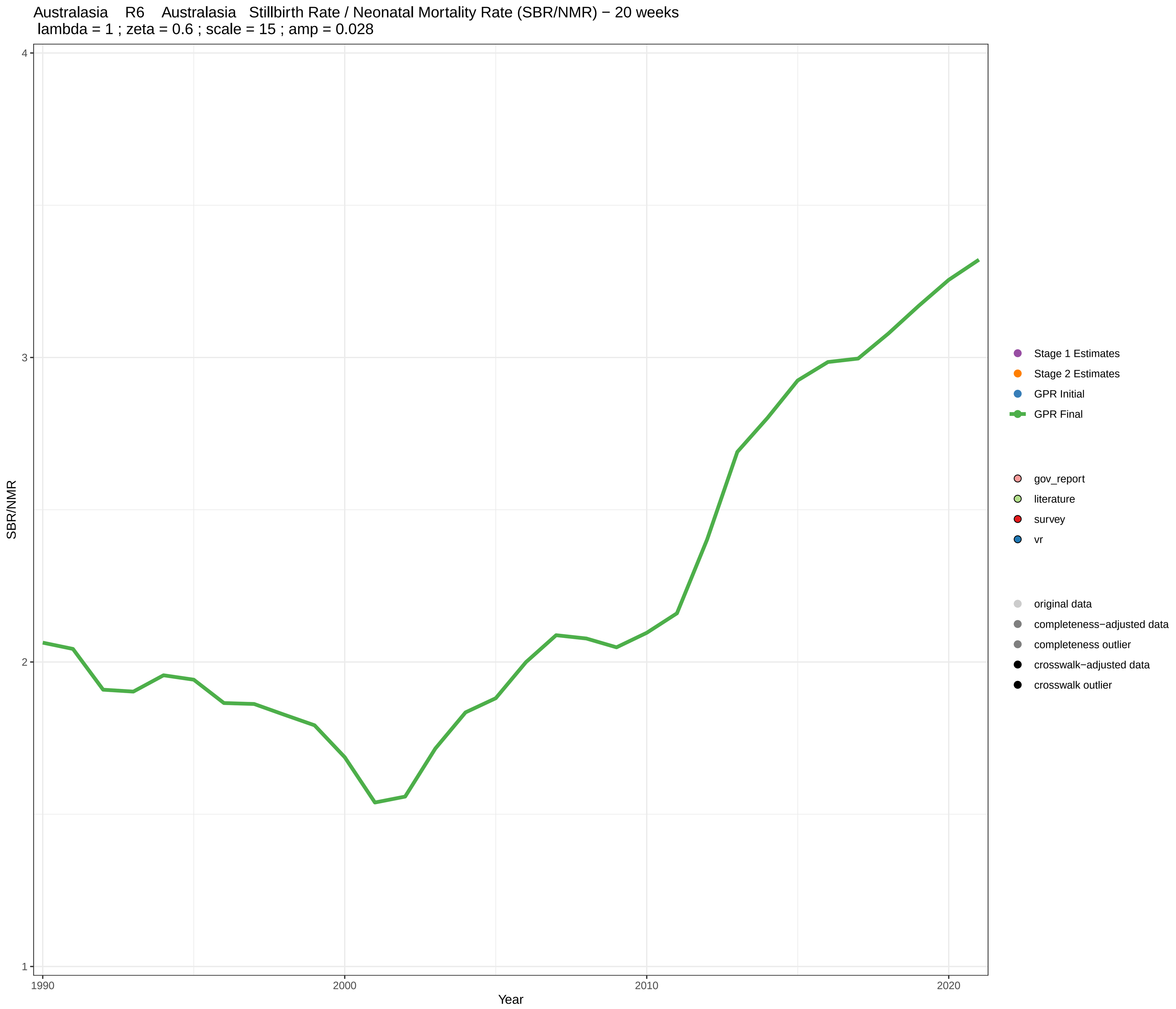

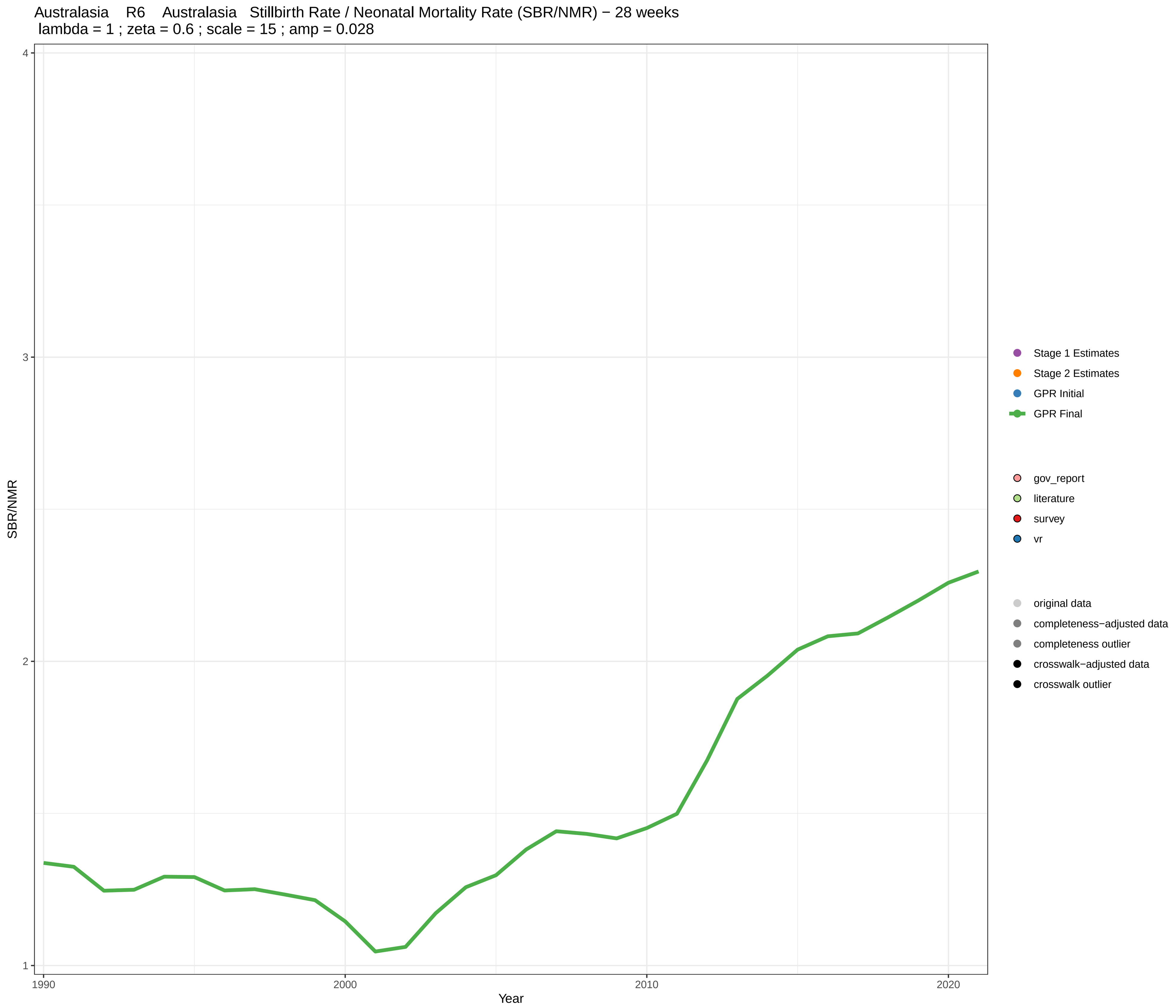

Definition	Years	Outlier	Source.Type	Title

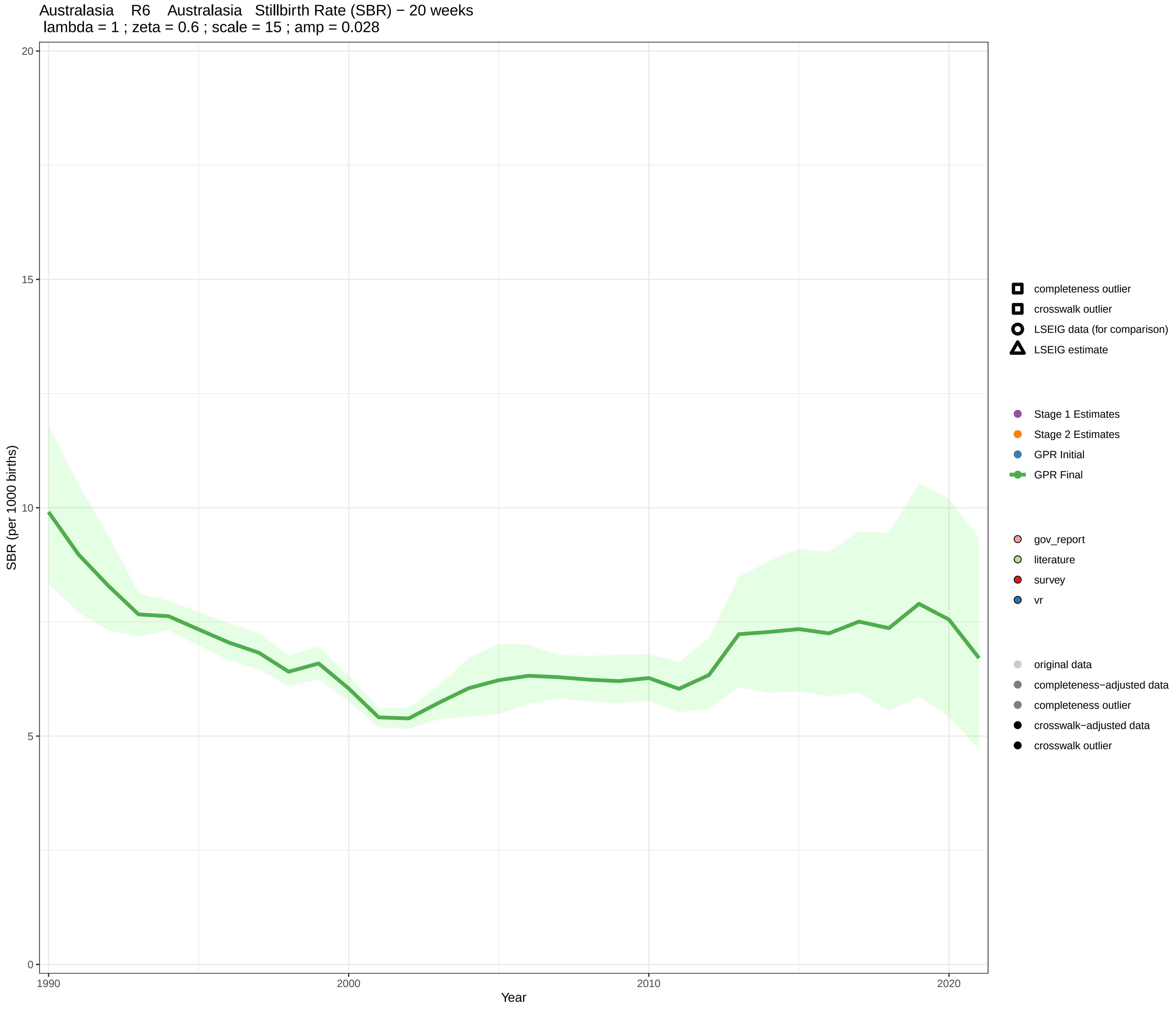

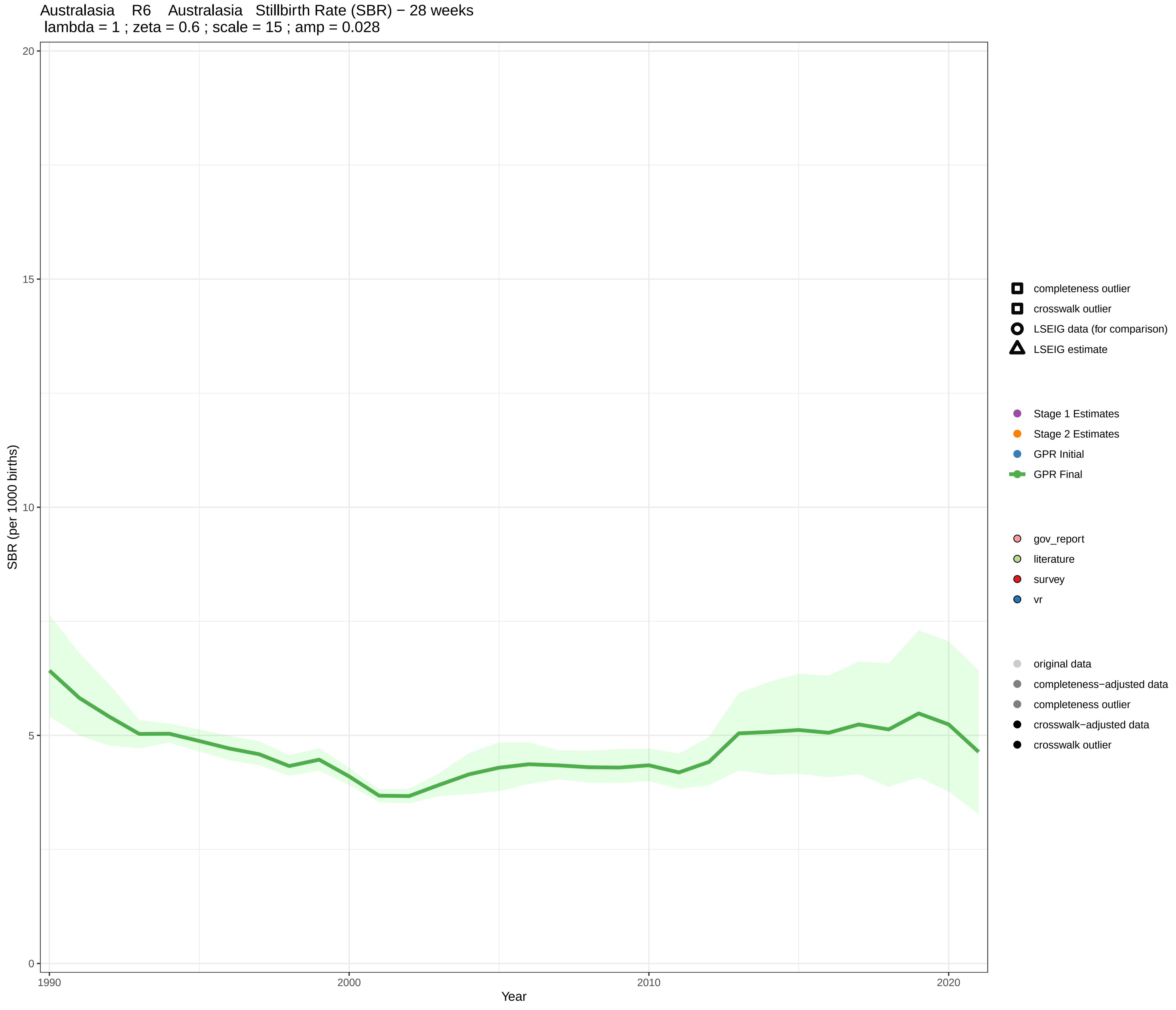

Year	Total Stillbirths (in thousands)	Total Stillbirths (in thousands)	SBR (per 1000 births)	SBR (per 1000 births)	SBR/NMR	SBR/NMR
	20 weeks	28 weeks	20 weeks	28 weeks	20 weeks	28 weeks
1990	3.2<br>(2.6 to 3.8)	2.0<br>(1.7 to 2.4)	9.9<br>(8.3 to 11.8)	6.4<br>(5.4 to 7.6)	2.06<br>(1.74 to 2.46)	1.34<br>(1.13 to 1.59)
1995	2.3<br>(2.2 to 2.4)	1.5<br>(1.4 to 1.6)	7.3<br>(7.0 to 7.7)	4.9<br>(4.7 to 5.1)	1.94<br>(1.85 to 2.04)	1.29<br>(1.23 to 1.36)
2000	1.8<br>(1.7 to 1.9)	1.2<br>(1.2 to 1.3)	6.0<br>(5.8 to 6.3)	4.1<br>(3.9 to 4.3)	1.69<br>(1.61 to 1.76)	1.15<br>(1.09 to 1.20)
2005	2.0<br>(1.8 to 2.3)	1.4<br>(1.2 to 1.6)	6.2<br>(5.5 to 7.0)	4.3<br>(3.8 to 4.8)	1.88<br>(1.66 to 2.12)	1.30<br>(1.14 to 1.46)
2010	2.3<br>(2.1 to 2.5)	1.6<br>(1.5 to 1.7)	6.3<br>(5.8 to 6.8)	4.3<br>(4.0 to 4.7)	2.10<br>(1.93 to 2.27)	1.45<br>(1.34 to 1.58)
2015	2.7<br>(2.2 to 3.3)	1.9<br>(1.5 to 2.3)	7.3<br>(6.0 to 9.1)	5.1<br>(4.2 to 6.4)	2.92<br>(2.38 to 3.62)	2.04<br>(1.66 to 2.53)
2020	2.7<br>(1.9 to 3.6)	1.9<br>(1.3 to 2.5)	7.6<br>(5.4 to 10.2)	5.2<br>(3.8 to 7.1)	3.25<br>(2.34 to 4.40)	2.26<br>(1.62 to 3.04)
2021	2.4<br>(1.7 to 3.4)	1.7<br>(1.2 to 2.3)	6.7<br>(4.7 to 9.3)	4.6<br>(3.3 to 6.4)	3.32<br>(2.34 to 4.62)	2.30<br>(1.62 to 3.18)

Australia AUS Australasia Stillbirth Rate / Neonatal Mortality Rate (SBR/NMR) − 20 weeks
lambda = 0.2 ; zeta = 0.99 ; scale = 5 ; amp = 0.028

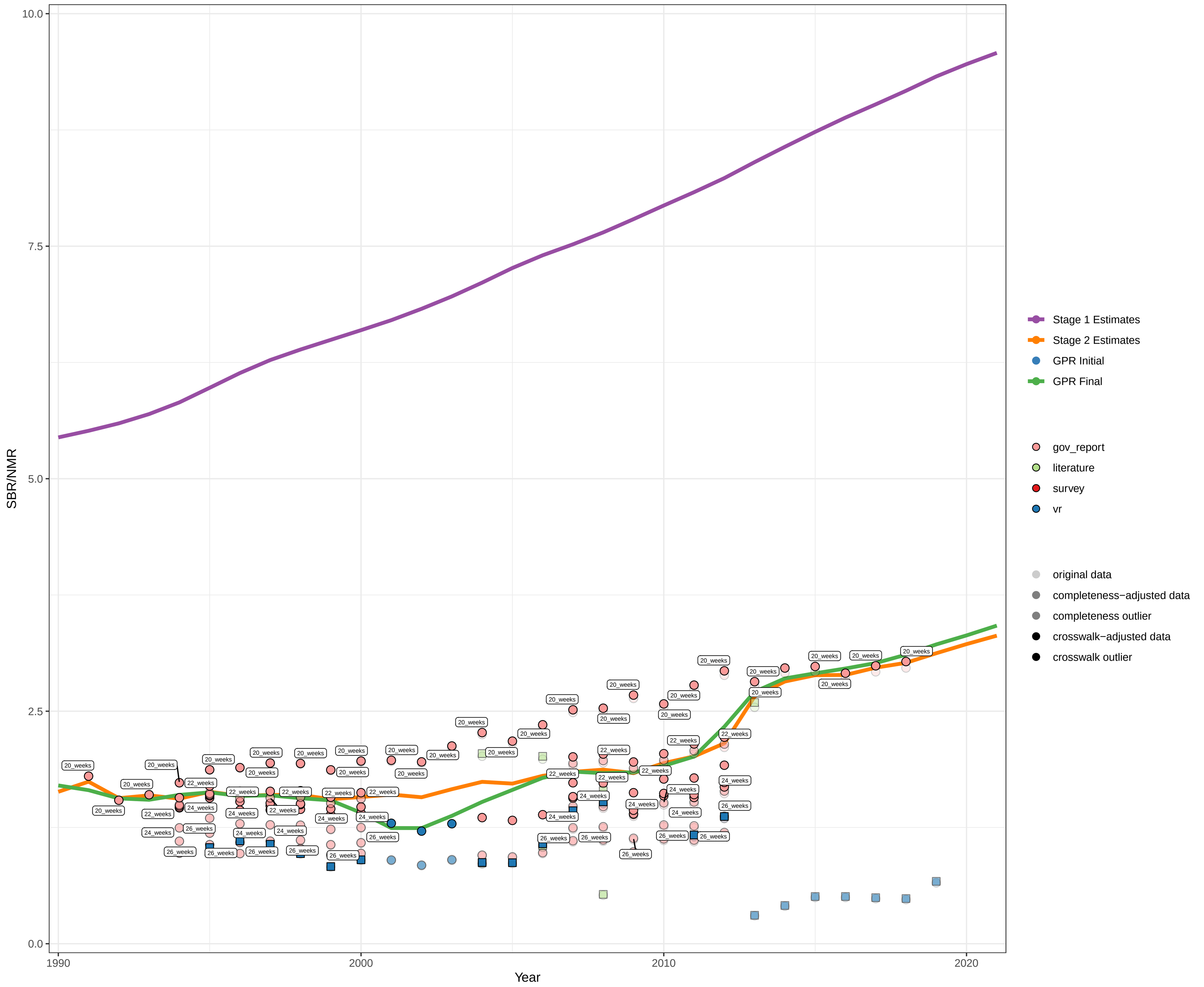

Australia AUS Australasia Stillbirth Rate / Neonatal Mortality Rate (SBR/NMR) − 28 weeks
lambda = 0.2 ; zeta = 0.99 ; scale = 5 ; amp = 0.028

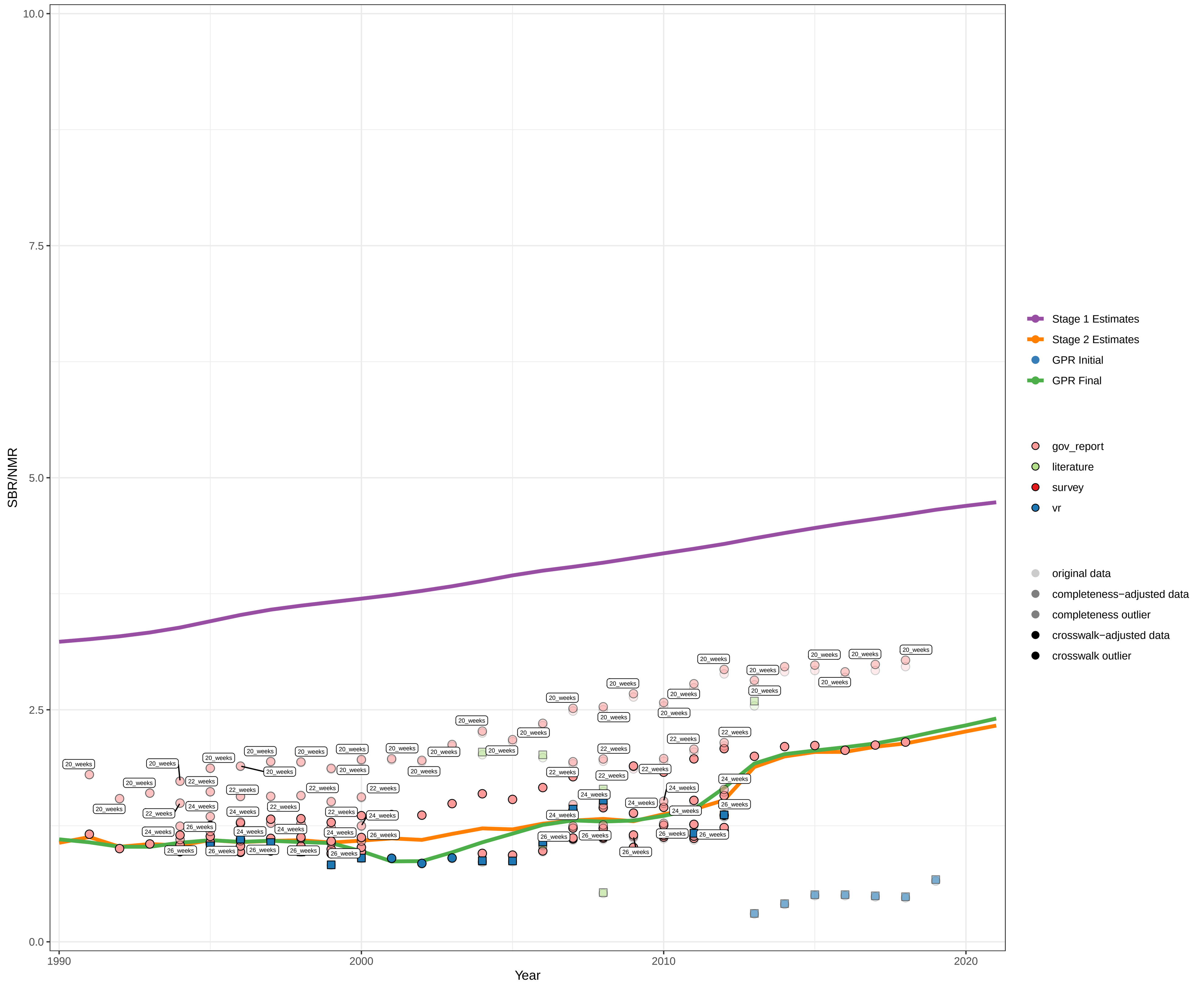

Definition	Years	Outlier	Source.Type	Title
20_weeks	1991−2012	0	Statistical Report	Australia's Mothers and Babies
20_weeks	2013−2018	0	Statistical Report	Australia's Mothers and Babies In Brief
22_weeks, 24_weeks, 26_weeks	1994−2000, 2007−2012	0	Statistical Report	Australia's Mothers and Babies
28_weeks	1994−2000, 2004−2012	0	Statistical Report	Australia's Mothers and Babies
28_weeks	2001−2003	0	VR	United Nations Demographic Yearbook
20_weeks	2006	1	Sci Lit	Perinatal mortality disparities between public care and private obstetrician−led care: a propensity score analysis
20_weeks	2008	1	Sci Lit	Planned private homebirth in Victoria 2000−2015: a retrospective cohort study of Victorian perinatal data
20_weeks	2013	1	Sci Lit	Seasonal Trivalent Influenza Vaccination During Pregnancy and the Incidence of Stillbirth: Population−Based Retrospective Cohort Study
20_weeks, 28_weeks	2004	1	Sci Lit	Maternal ethnicity, stillbirth and neonatal death risk in Western Australia 1998−2010
22_weeks	2008	1	Sci Lit	The burden of adverse obstetric and perinatal outcomes from maternal smoking in an Australian cohort
24_weeks	2006	1	Sci Lit	Maternal region of birth and stillbirth in Victoria, Australia 2000−2011: A retrospective cohort study of Victorian perinatal data
28_weeks	2013−2019	1	VR	United Nations Demographic Yearbook
28_weeks	1995−2000, 2004−2008, 2011, 2012	2	VR	United Nations Demographic Yearbook

Australia AUS Australasia Stillbirth Rate (SBR) − 20 weeks
lambda = 0.2 ; zeta = 0.99 ; scale = 5 ; amp = 0.028

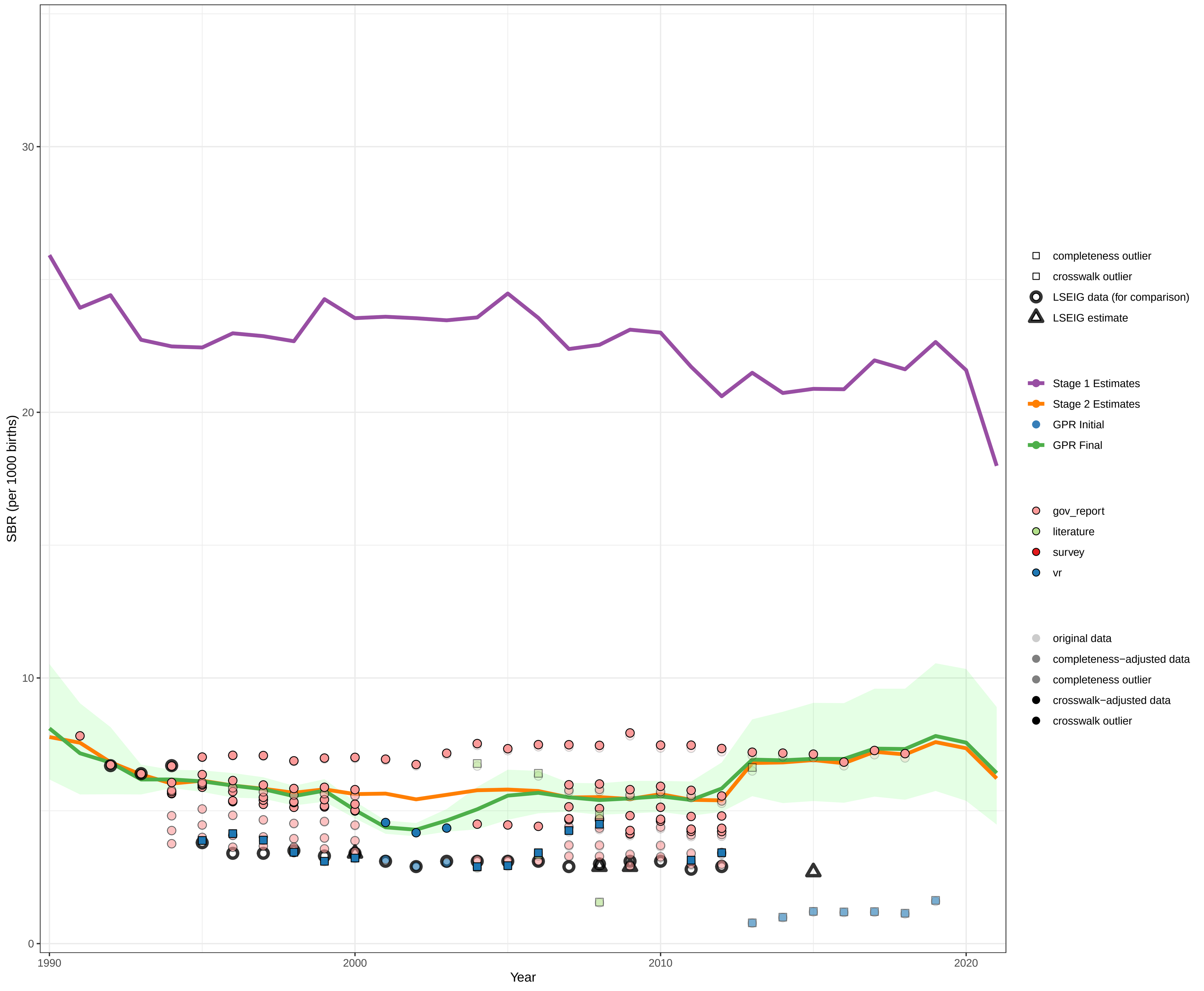

Australia AUS Australasia Stillbirth Rate (SBR) − 28 weeks
lambda = 0.2 ; zeta = 0.99 ; scale = 5 ; amp = 0.028

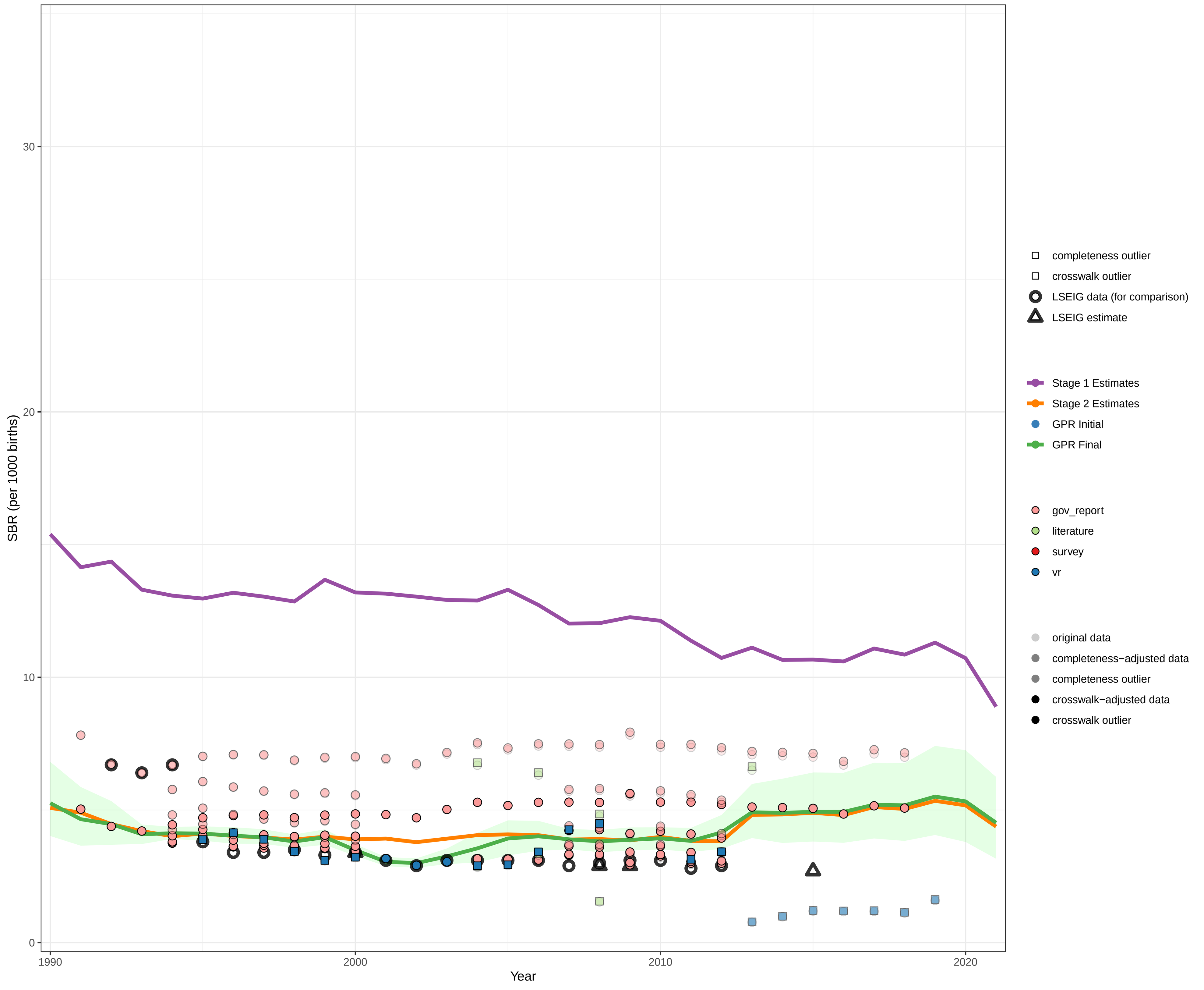

Year	Total Stillbirths (in thousands)	Total Stillbirths (in thousands)	SBR (per 1000 births)	SBR (per 1000 births)	SBR/NMR	SBR/NMR
	20 weeks	28 weeks	20 weeks	28 weeks	20 weeks	28 weeks
1990	2.1 (1.6 to 2.7)	1.3 (1.0 to 1.8)	8.1 (6.2 to 10.5)	5.3 (4.0 to 6.8)	1.70 (1.30 to 2.21)	1.10 (0.84 to 1.43)
1995	1.5 (1.4 to 1.7)	1.0 (1.0 to 1.1)	6.1 (5.7 to 6.5)	4.1 (3.8 to 4.4)	1.63 (1.52 to 1.74)	1.09 (1.02 to 1.17)
2000	1.2 (1.2 to 1.3)	0.9 (0.8 to 0.9)	5.0 (4.7 to 5.4)	3.5 (3.3 to 3.7)	1.41 (1.32 to 1.50)	0.98 (0.92 to 1.04)
2005	1.5 (1.2 to 1.8)	1.0 (0.9 to 1.2)	5.6 (4.7 to 6.5)	3.9 (3.3 to 4.6)	1.65 (1.38 to 1.94)	1.17 (0.98 to 1.37)
2010	1.7 (1.5 to 1.9)	1.2 (1.1 to 1.3)	5.5 (5.0 to 6.1)	3.9 (3.5 to 4.3)	1.91 (1.71 to 2.11)	1.36 (1.21 to 1.50)
2015	2.1 (1.6 to 2.8)	1.5 (1.2 to 2.0)	7.0 (5.4 to 9.1)	4.9 (3.8 to 6.4)	2.91 (2.24 to 3.79)	2.06 (1.59 to 2.68)
2020	2.3 (1.6 to 3.1)	1.6 (1.1 to 2.2)	7.6 (5.4 to 10.3)	5.3 (3.8 to 7.3)	3.32 (2.35 to 4.53)	2.33 (1.66 to 3.18)
2021	1.9 (1.3 to 2.7)	1.4 (0.9 to 1.9)	6.4 (4.5 to 8.9)	4.5 (3.2 to 6.2)	3.42 (2.38 to 4.74)	2.41 (1.68 to 3.33)

New Zealand NZL Australasia Stillbirth Rate / Neonatal Mortality Rate (SBR/NMR) − 20 weeks
lambda = 0.2 ; zeta = 0.99 ; scale = 5 ; amp = 0.028

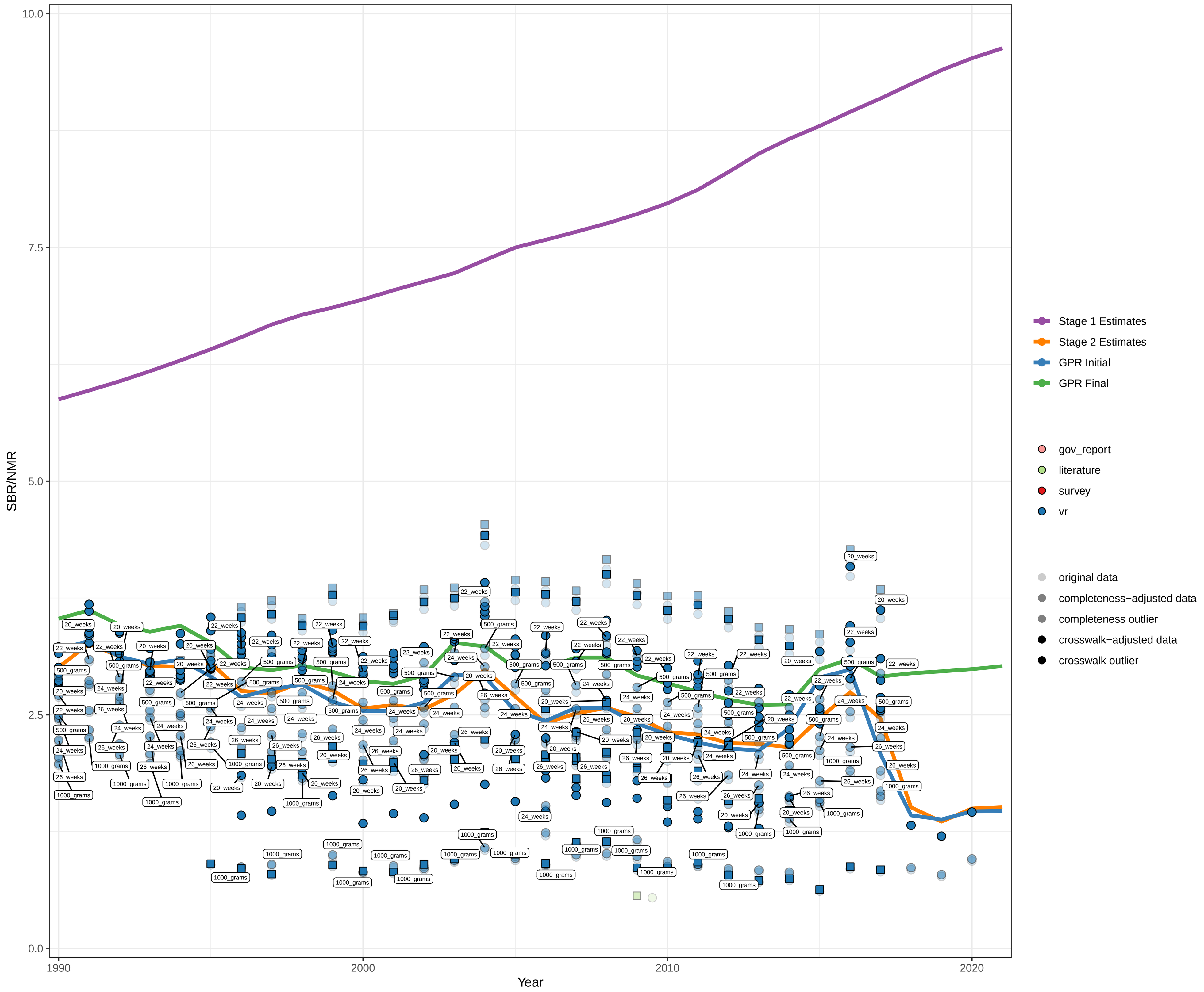

New Zealand NZL Australasia Stillbirth Rate / Neonatal Mortality Rate (SBR/NMR) − 28 weeks
lambda = 0.2 ; zeta = 0.99 ; scale = 5 ; amp = 0.028

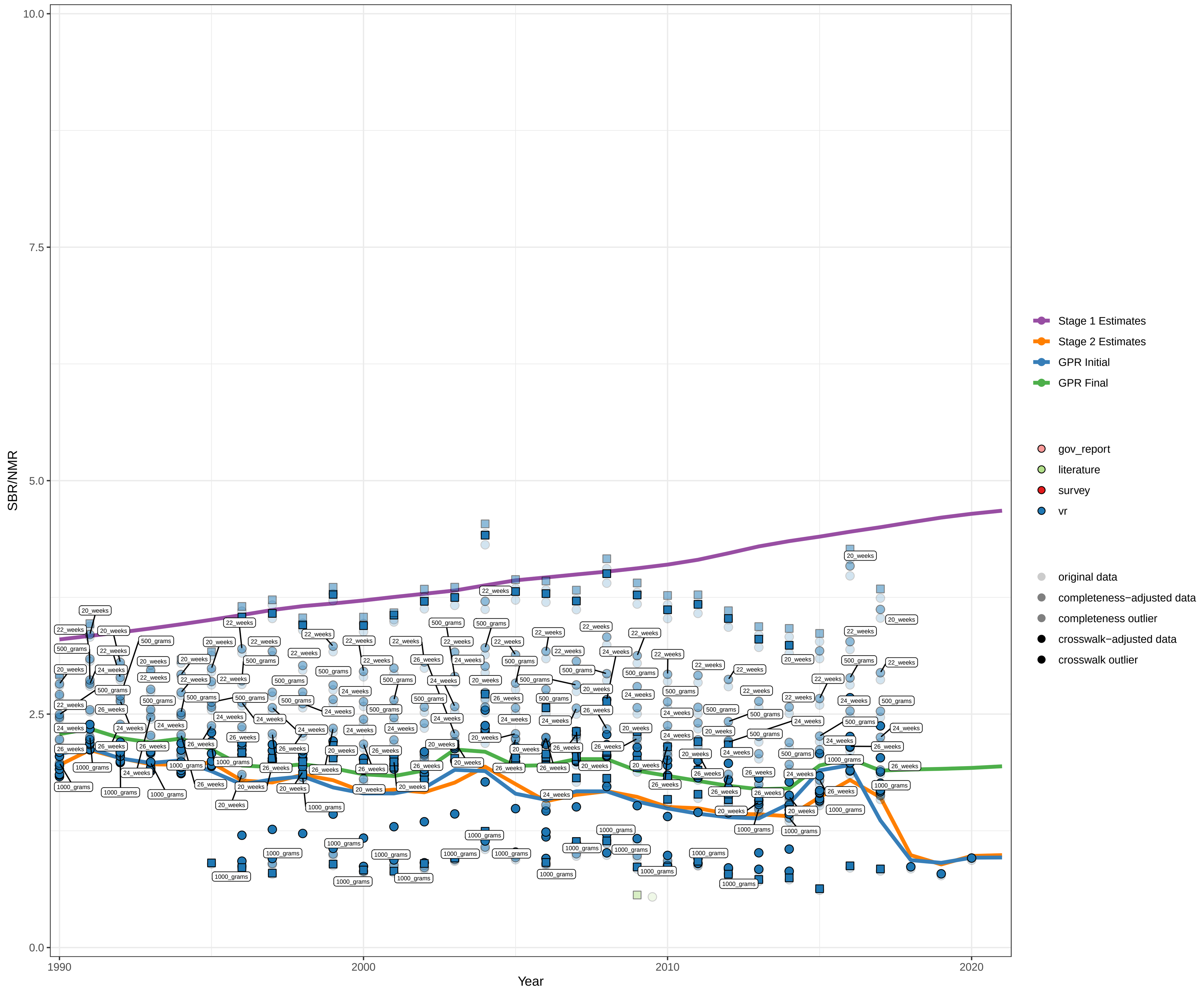

Definition	Years	Outlier	Source.Type	Title
1000_grams	1990−2017	0	VR	New Zealand Fetal and Infant Deaths
1000_grams, 22_weeks, 24_weeks, 26_weeks, 500_grams	2003, 2004, 2008, 2009	0	VR	New Zealand Fetal and Infant Deaths
20_weeks	1990−2012, 2015−2017	0	VR	New Zealand Fetal and Infant Deaths
20_weeks	2013, 2014	0	VR	New Zealand Tenth Annual Report of the Perinatal and Maternal Mortality Review Committee: Reporting Mortality
22_weeks, 24_weeks, 26_weeks, 500_grams	1990−2002, 2005−2007, 2010−2017	0	VR	New Zealand Fetal and Infant Deaths
24_weeks, 28_weeks	2006	0	VR	New Zealand Perinatal and Maternal Mortality Review Committee: Second Report to the Minister of Health: July to June
28_weeks	1990−2002, 2005, 2015−2017	0	VR	New Zealand Fetal and Infant Deaths
28_weeks	2003, 2004	0	VR	New Zealand Fetal and Infant Deaths
28_weeks	2007−2014	0	VR	New Zealand Tenth Annual Report of the Perinatal and Maternal Mortality Review Committee: Reporting Mortality
28_weeks	2018−2020	0	VR	United Nations Demographic Yearbook
other	1990−2002, 2005−2007, 2010−2017	1	VR	New Zealand Fetal and Infant Deaths
other	2003, 2004, 2008, 2009	1	VR	New Zealand Fetal and Infant Deaths
other	2009	1	Sci Lit	INTERGROWTH−21st vs customized birthweight standards for<a0>identification of perinatal mortality and morbidity
20_weeks	1996−2014	2	VR	New Zealand Fetal and Infant Deaths
20_weeks	2003, 2004, 2008, 2009	2	VR	New Zealand Fetal and Infant Deaths
20_weeks	2006	2	VR	New Zealand Perinatal and Maternal Mortality Review Committee: Second Report to the Minister of Health: July to June
20_weeks	2007−2012	2	VR	New Zealand Tenth Annual Report of the Perinatal and Maternal Mortality Review Committee: Reporting Mortality
28_weeks	1995−1997, 1999−2017	2	VR	United Nations Demographic Yearbook
28_weeks	2006, 2007, 2010−2014	2	VR	New Zealand Fetal and Infant Deaths
28_weeks	2008, 2009	2	VR	New Zealand Fetal and Infant Deaths

New Zealand NZL Australasia Stillbirth Rate (SBR) − 20 weeks
lambda = 0.2 ; zeta = 0.99 ; scale = 5 ; amp = 0.028

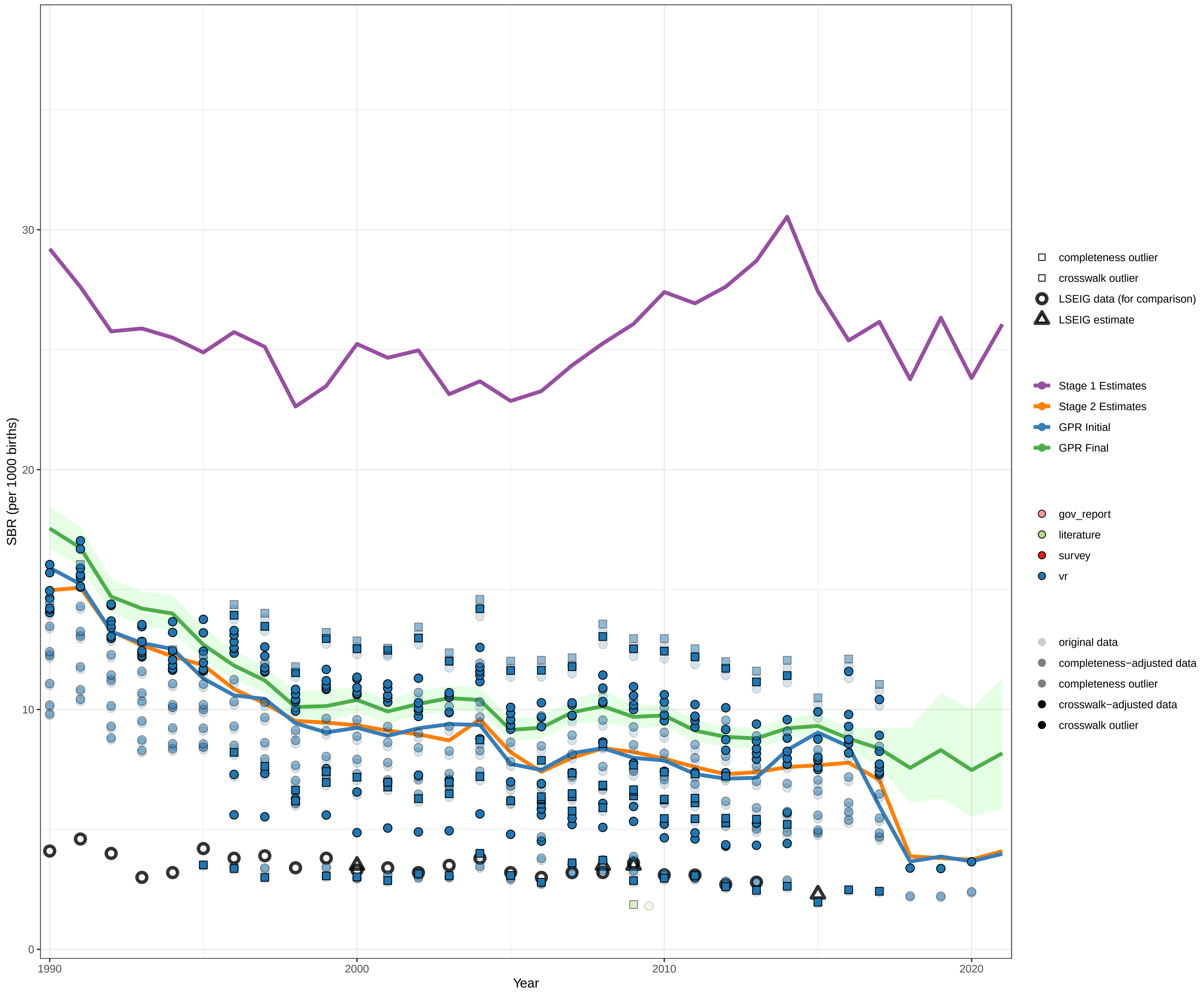

New Zealand NZL Australasia Stillbirth Rate (SBR) − 28 weeks
lambda = 0.2 ; zeta = 0.99 ; scale = 5 ; amp = 0.028

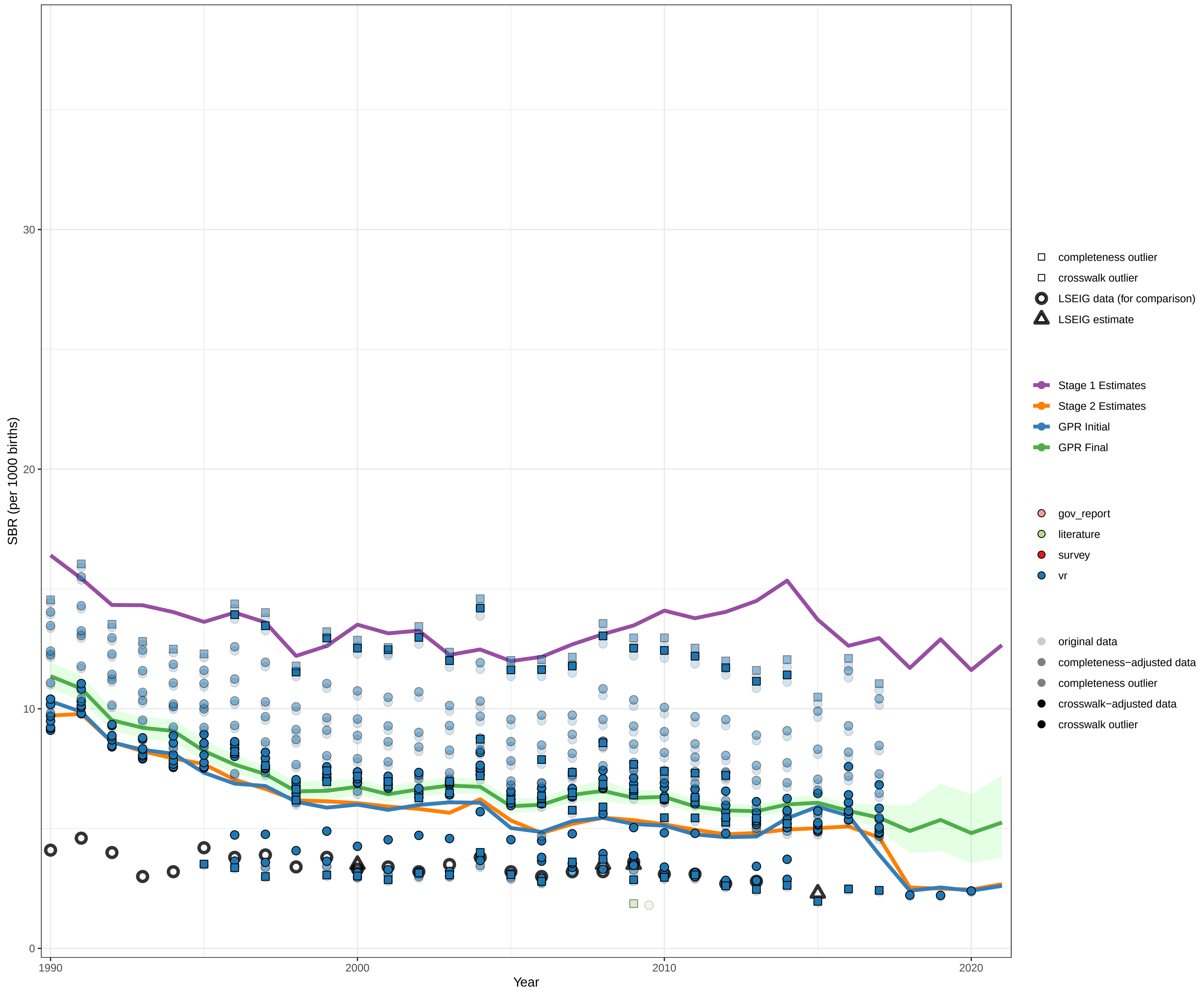

Year	Total Stillbirths (in thousands)	Total Stillbirths (in thousands)	SBR (per 1000 births)	SBR (per 1000 births)	SBR/NMR	SBR/NMR
	20 weeks	28 weeks	20 weeks	28 weeks	20 weeks	28 weeks
1990	1.1<br>(1.0 to 1.1)	0.7<br>(0.7 to 0.7)	17.5<br>(16.7 to 18.4)	11.4<br>(10.8 to 11.9)	3.53<br>(3.37 to 3.71)	2.29<br>(2.18 to 2.40)
1995	0.7<br>(0.7 to 0.8)	0.5<br>(0.5 to 0.5)	12.7<br>(12.0 to 13.4)	8.2<br>(7.8 to 8.7)	3.27<br>(3.10 to 3.46)	2.12<br>(2.01 to 2.24)
2000	0.6<br>(0.6 to 0.6)	0.4<br>(0.4 to 0.4)	10.4<br>(9.9 to 10.9)	6.7<br>(6.4 to 7.1)	2.86<br>(2.71 to 3.00)	1.86<br>(1.76 to 1.95)
2005	0.5<br>(0.5 to 0.6)	0.4<br>(0.3 to 0.4)	9.1<br>(8.7 to 9.6)	5.9<br>(5.6 to 6.2)	3.00<br>(2.85 to 3.15)	1.95<br>(1.85 to 2.04)
2010	0.6<br>(0.6 to 0.6)	0.4<br>(0.4 to 0.4)	9.7<br>(9.3 to 10.2)	6.3<br>(6.0 to 6.6)	2.84<br>(2.70 to 2.97)	1.84<br>(1.75 to 1.93)
2015	0.6<br>(0.5 to 0.6)	0.4<br>(0.3 to 0.4)	9.3<br>(8.8 to 9.8)	6.1<br>(5.8 to 6.4)	2.99<br>(2.84 to 3.15)	1.95<br>(1.85 to 2.05)
2020	0.4<br>(0.3 to 0.6)	0.3<br>(0.2 to 0.4)	7.5<br>(5.5 to 10.0)	4.8<br>(3.6 to 6.4)	2.99<br>(2.21 to 4.01)	1.92<br>(1.43 to 2.57)
2021	0.5<br>(0.3 to 0.7)	0.3<br>(0.2 to 0.4)	8.2<br>(5.8 to 11.3)	5.3<br>(3.8 to 7.2)	3.02<br>(2.16 to 4.18)	1.94<br>(1.39 to 2.68)

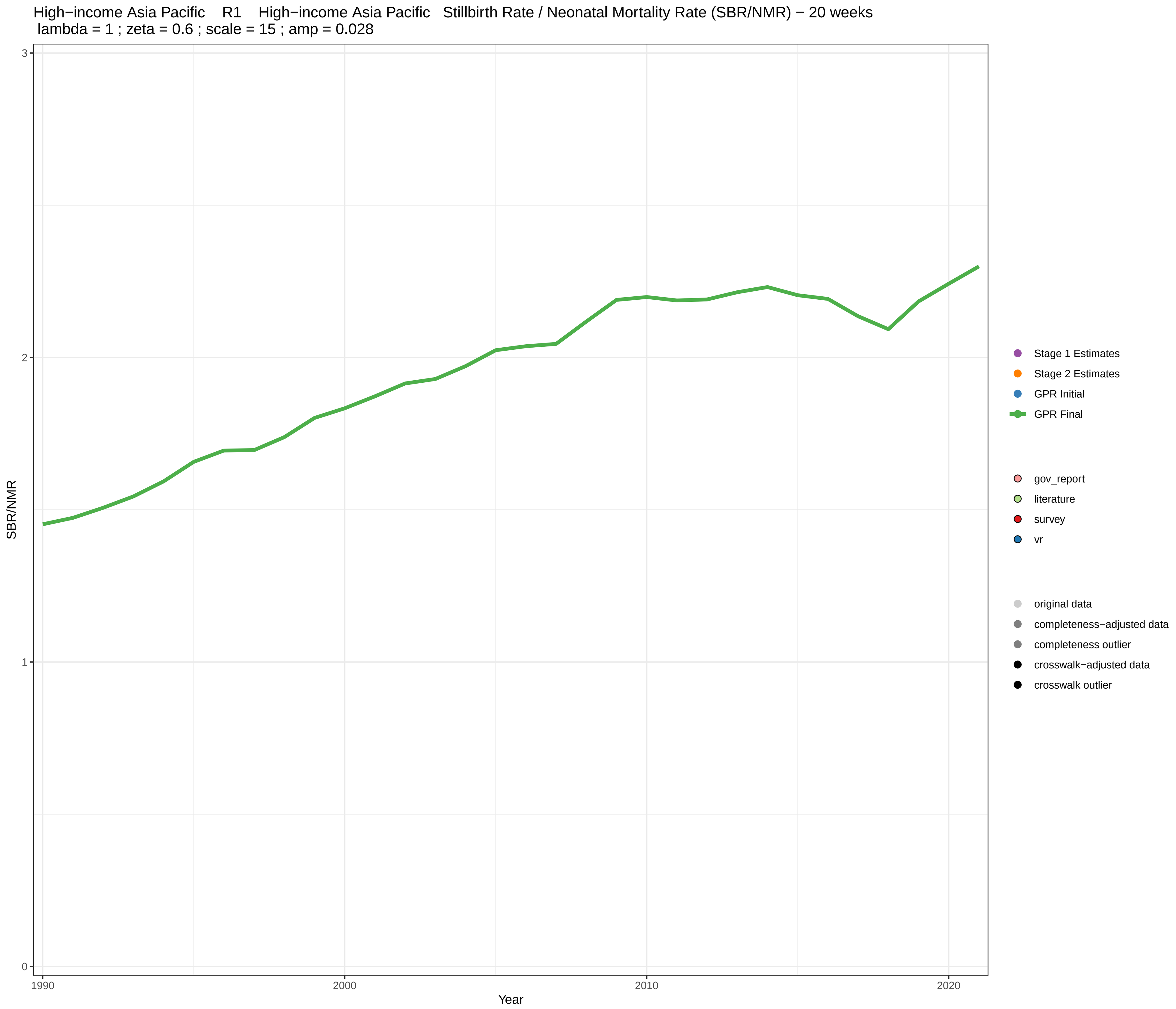

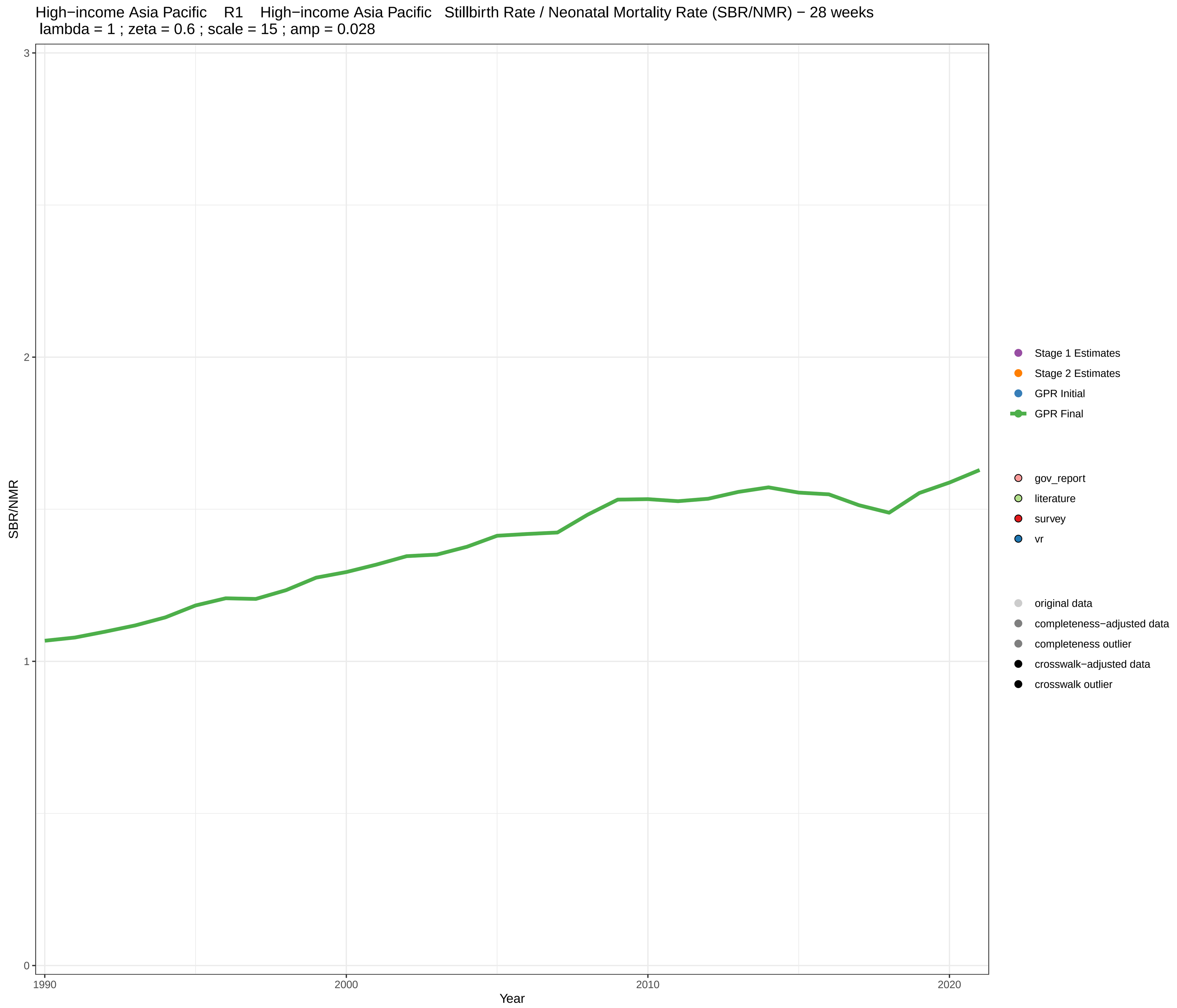

Definition	Years	Outlier	Source.Type	Title

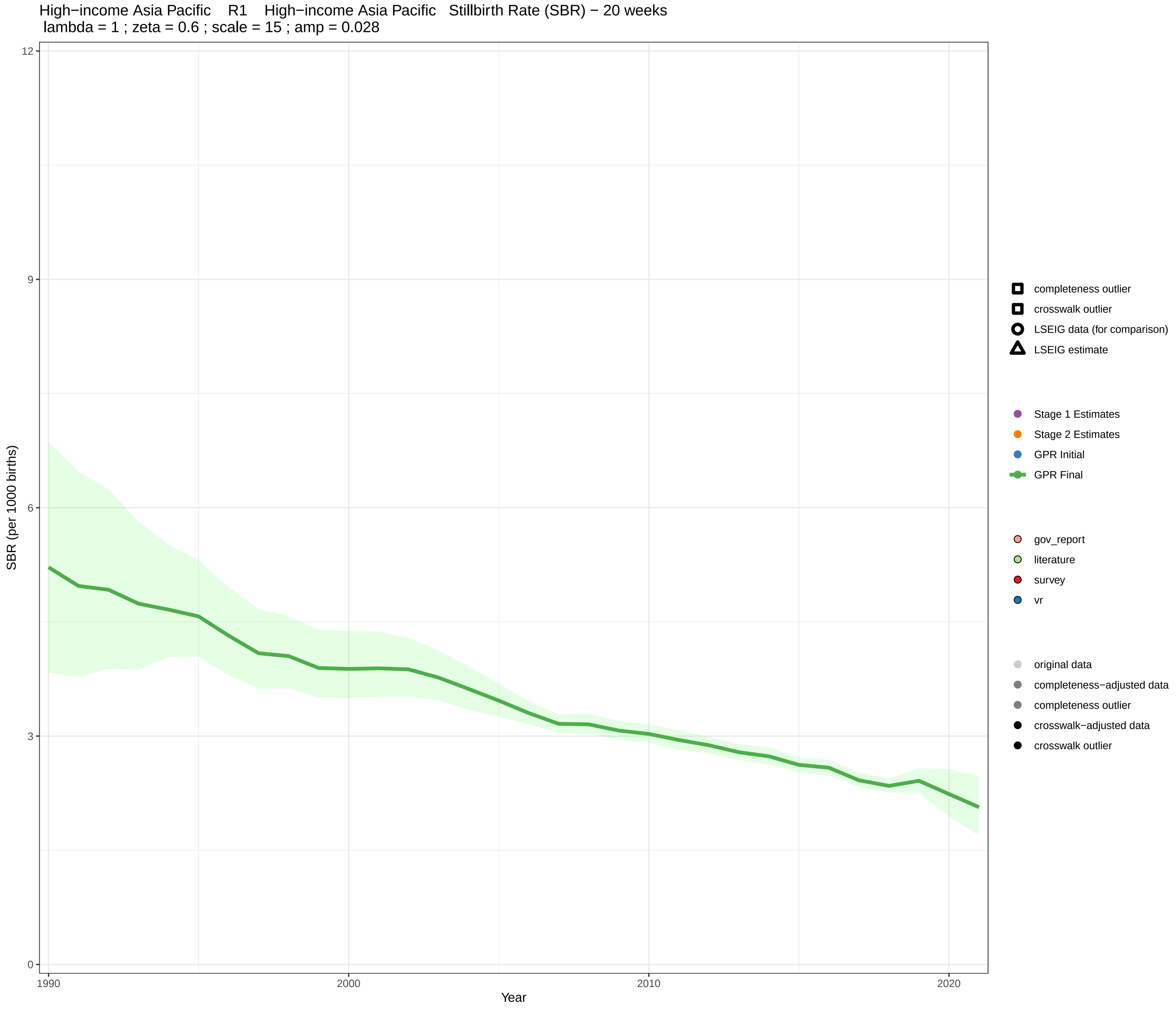

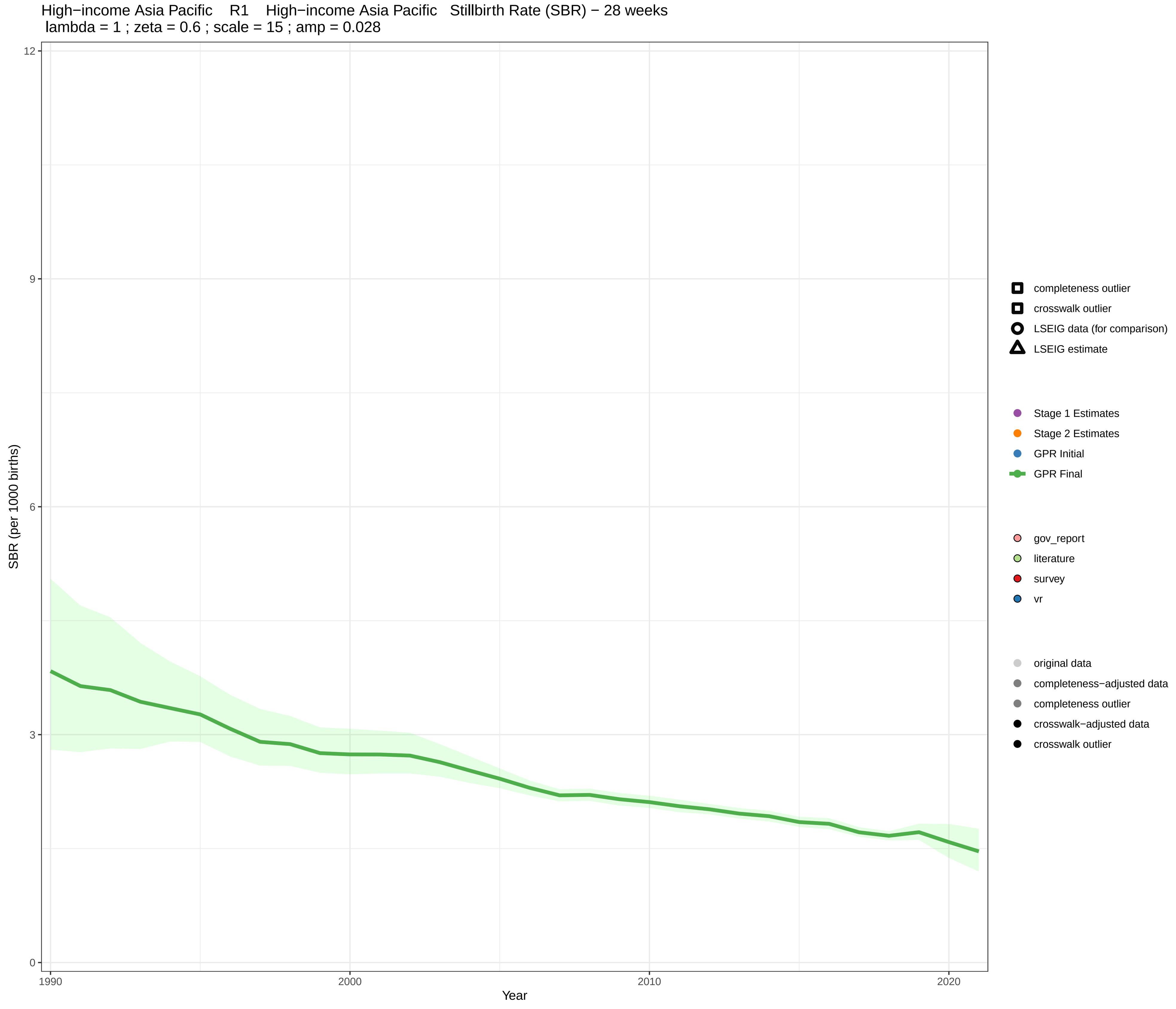

Year	Total Stillbirths (in thousands)	Total Stillbirths (in thousands)	SBR (per 1000 births)	SBR (per 1000 births)	SBR/NMR	SBR/NMR
	20 weeks	28 weeks	20 weeks	28 weeks	20 weeks	28 weeks
1990	10.3 (7.6 to 13.6)	7.6 (5.5 to 10.0)	5.2 (3.8 to 6.9)	3.8 (2.8 to 5.1)	1.45 (1.07 to 1.91)	1.07 (0.78 to 1.41)
1995	9.0 (7.9 to 10.4)	6.4 (5.7 to 7.4)	4.6 (4.0 to 5.3)	3.3 (2.9 to 3.8)	1.66 (1.46 to 1.92)	1.18 (1.05 to 1.37)
2000	7.1 (6.4 to 8.0)	5.0 (4.5 to 5.6)	3.9 (3.5 to 4.4)	2.7 (2.5 to 3.1)	1.83 (1.65 to 2.07)	1.29 (1.17 to 1.45)
2005	5.6 (5.3 to 6.0)	3.9 (3.7 to 4.1)	3.5 (3.3 to 3.7)	2.4 (2.3 to 2.6)	2.02 (1.90 to 2.15)	1.41 (1.34 to 1.49)
2010	4.9 (4.7 to 5.1)	3.4 (3.3 to 3.5)	3.0 (2.9 to 3.2)	2.1 (2.0 to 2.2)	2.20 (2.11 to 2.29)	1.53 (1.48 to 1.59)
2015	3.9 (3.7 to 4.0)	2.7 (2.6 to 2.8)	2.6 (2.5 to 2.7)	1.8 (1.8 to 1.9)	2.20 (2.12 to 2.29)	1.55 (1.50 to 1.61)
2020	2.7 (2.3 to 3.1)	1.9 (1.6 to 2.2)	2.2 (1.9 to 2.6)	1.6 (1.4 to 1.8)	2.24 (1.95 to 2.57)	1.59 (1.38 to 1.83)
2021	2.4 (2.0 to 2.9)	1.7 (1.4 to 2.1)	2.1 (1.7 to 2.5)	1.5 (1.2 to 1.8)	2.30 (1.88 to 2.75)	1.63 (1.33 to 1.96)

Brunei Darussalam BRN High−income Asia Pacific Stillbirth Rate / Neonatal Mortality Rate (SBR/NMR) − 20 weeks
lambda = 0.4 ; zeta = 0.9 ; scale = 10 ; amp = 0.028

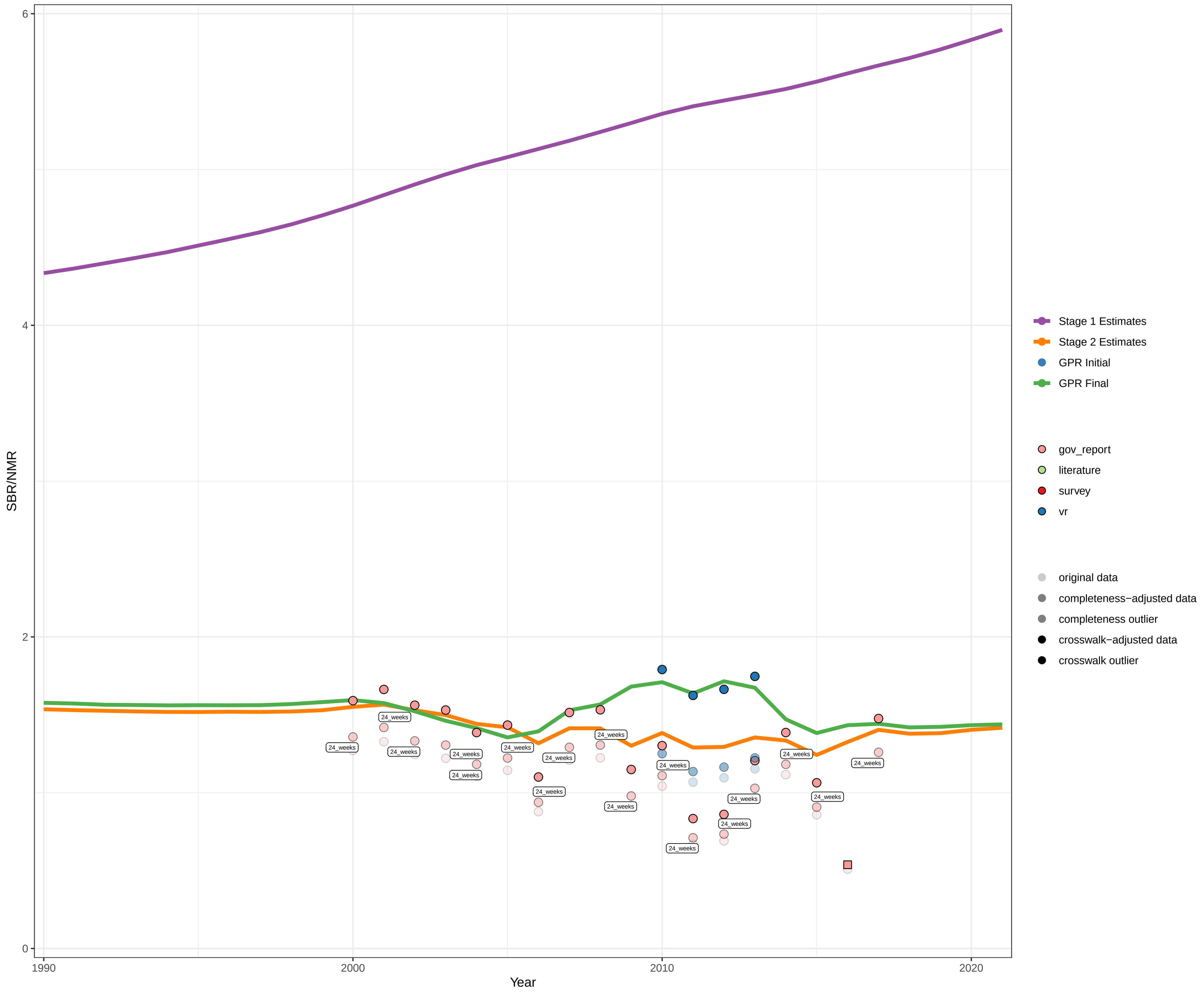

Brunei Darussalam BRN High−income Asia Pacific Stillbirth Rate / Neonatal Mortality Rate (SBR/NMR) − 28 weeks
lambda = 0.4 ; zeta = 0.9 ; scale = 10 ; amp = 0.028

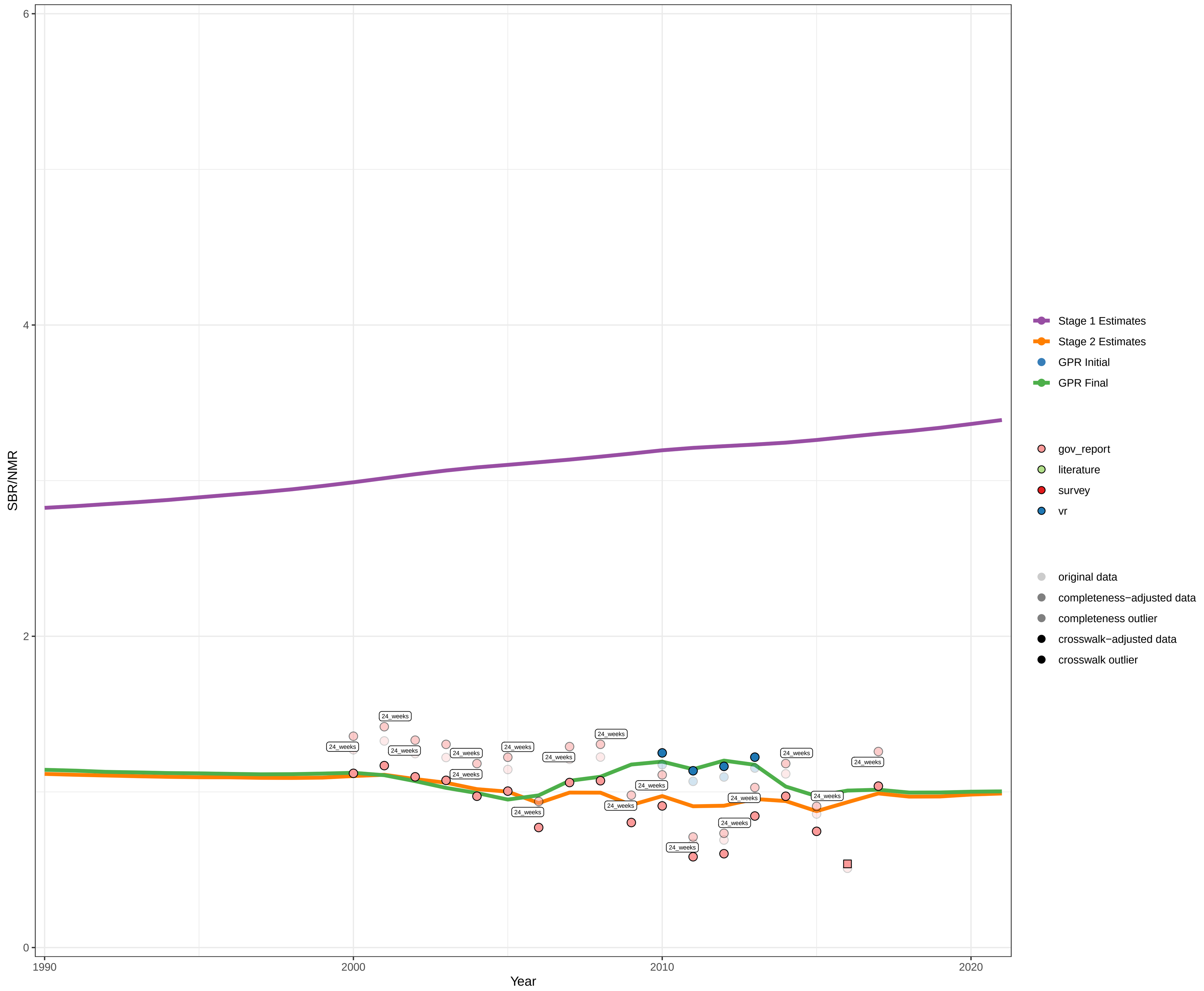

Definition	Years	Outlier	Source.Type	Title
24_weeks	2000−2017	0	Statistical Report	Brunei Health Information Booklet
28_weeks	2010−2013	0	VR	United Nations Demographic Yearbook

Brunei Darussalam BRN High−income Asia Pacific Stillbirth Rate (SBR) − 20 weeks
lambda = 0.4 ; zeta = 0.9 ; scale = 10 ; amp = 0.028

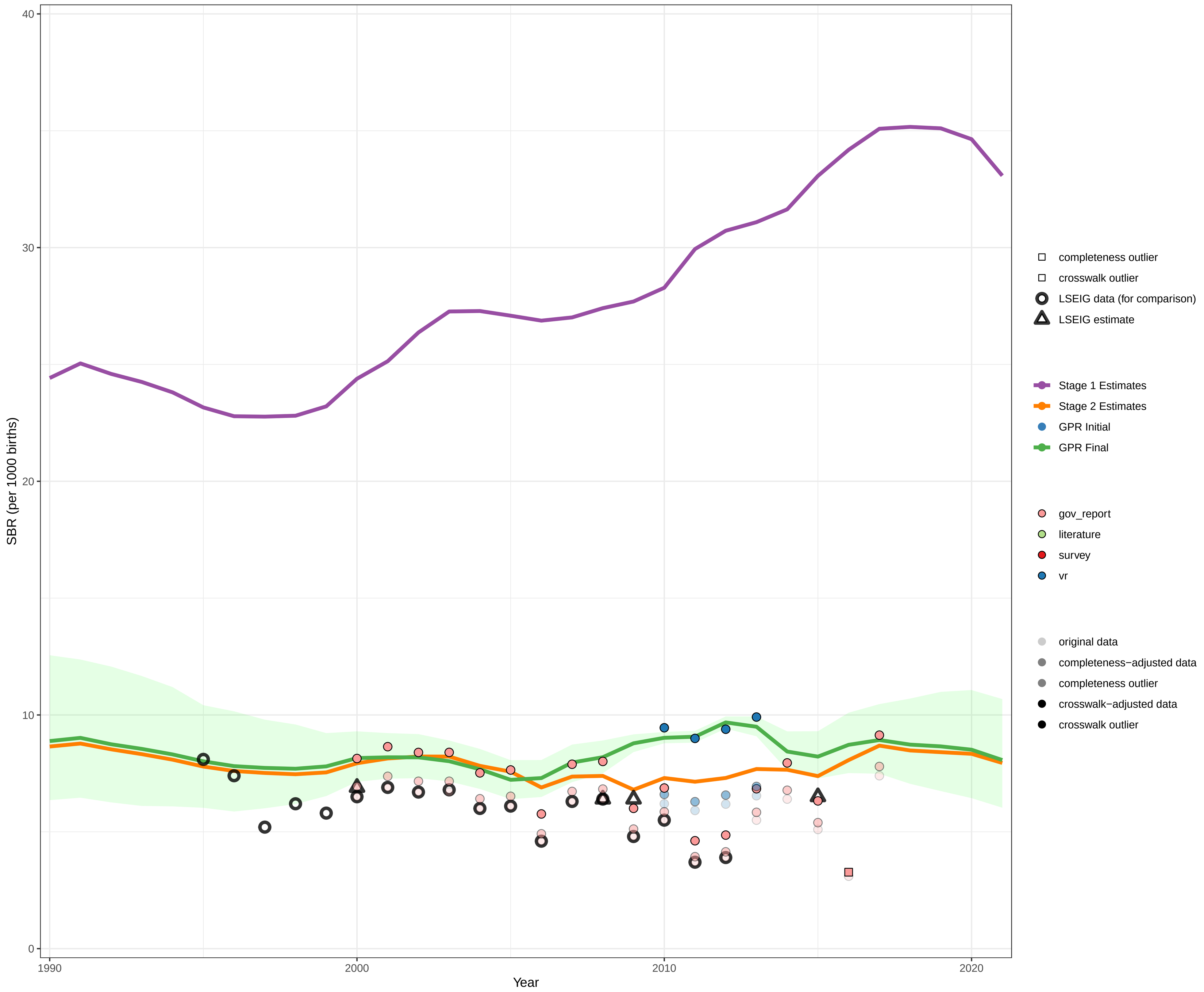

Brunei Darussalam BRN High−income Asia Pacific Stillbirth Rate (SBR) − 28 weeks
lambda = 0.4 ; zeta = 0.9 ; scale = 10 ; amp = 0.028

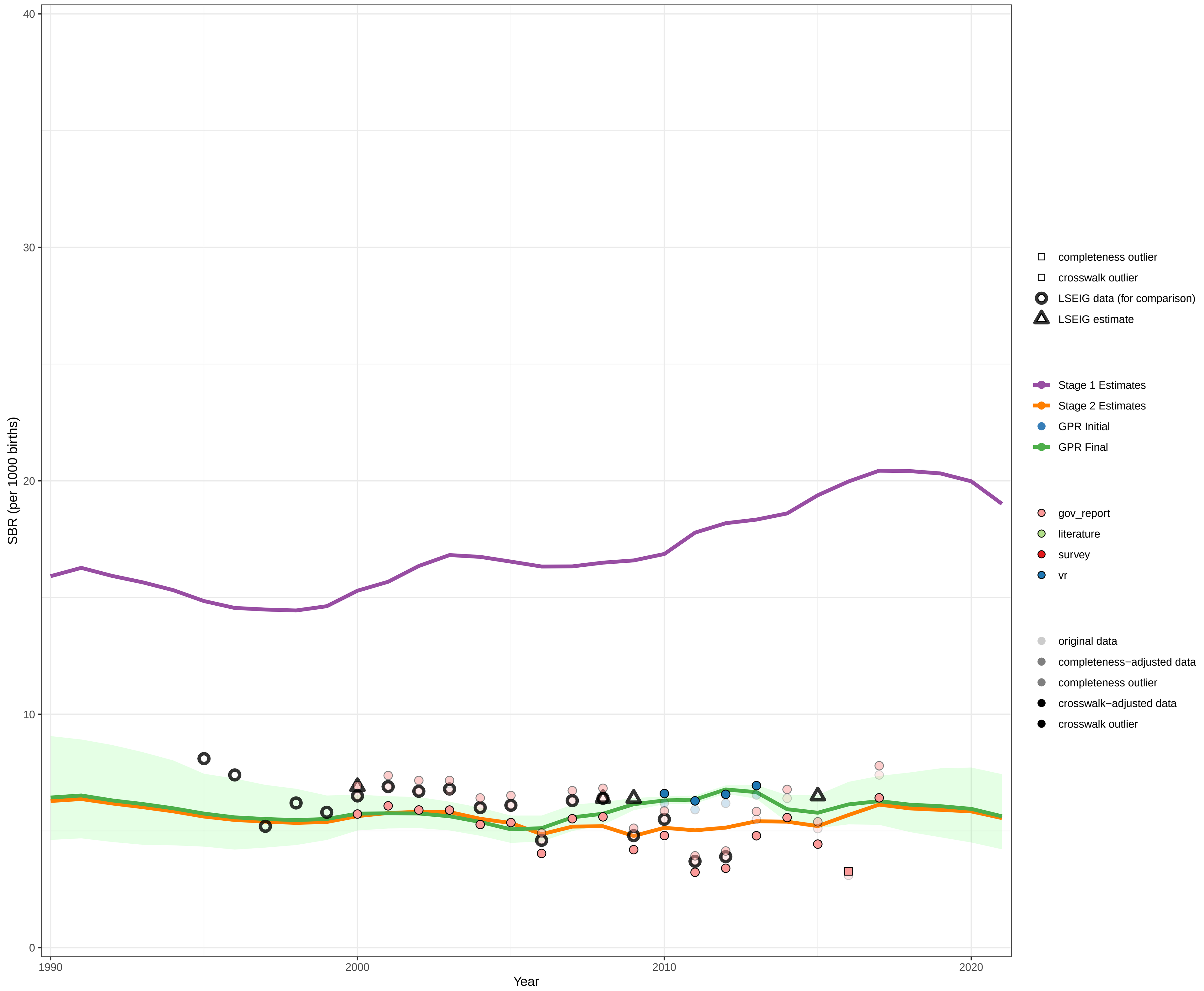

Year	Total Stillbirths (in thousands)	Total Stillbirths (in thousands)	SBR (per 1000 births)	SBR (per 1000 births)	SBR/NMR	SBR/NMR
	20 weeks	28 weeks	20 weeks	28 weeks	20 weeks	28 weeks
1990	0.1 (0.0 to 0.1)	0.0 (0.0 to 0.1)	8.9 (6.4 to 12.6)	6.4 (4.6 to 9.1)	1.58 (1.13 to 2.23)	1.14 (0.82 to 1.61)
1995	0.1 (0.0 to 0.1)	0.0 (0.0 to 0.1)	8.0 (6.0 to 10.4)	5.7 (4.3 to 7.5)	1.56 (1.17 to 2.03)	1.12 (0.84 to 1.45)
2000	0.1 (0.1 to 0.1)	0.0 (0.0 to 0.0)	8.2 (7.1 to 9.3)	5.7 (5.0 to 6.6)	1.59 (1.40 to 1.82)	1.12 (0.98 to 1.28)
2005	0.0 (0.0 to 0.1)	0.0 (0.0 to 0.0)	7.2 (6.4 to 8.1)	5.1 (4.5 to 5.7)	1.35 (1.20 to 1.51)	0.95 (0.84 to 1.06)
2010	0.1 (0.1 to 0.1)	0.0 (0.0 to 0.0)	9.0 (8.8 to 9.3)	6.3 (6.1 to 6.5)	1.71 (1.66 to 1.76)	1.19 (1.16 to 1.23)
2015	0.1 (0.0 to 0.1)	0.0 (0.0 to 0.0)	8.2 (7.3 to 9.3)	5.8 (5.1 to 6.5)	1.38 (1.23 to 1.57)	0.97 (0.86 to 1.10)
2020	0.1 (0.0 to 0.1)	0.0 (0.0 to 0.0)	8.5 (6.4 to 11.1)	5.9 (4.5 to 7.7)	1.43 (1.08 to 1.86)	1.00 (0.76 to 1.30)
2021	0.1 (0.0 to 0.1)	0.0 (0.0 to 0.0)	8.1 (6.0 to 10.7)	5.6 (4.2 to 7.4)	1.44 (1.08 to 1.90)	1.00 (0.75 to 1.33)

Japan JPN High−income Asia Pacific Stillbirth Rate / Neonatal Mortality Rate (SBR/NMR) − 20 weeks
lambda = 0.2 ; zeta = 0.99 ; scale = 5 ; amp = 0.028

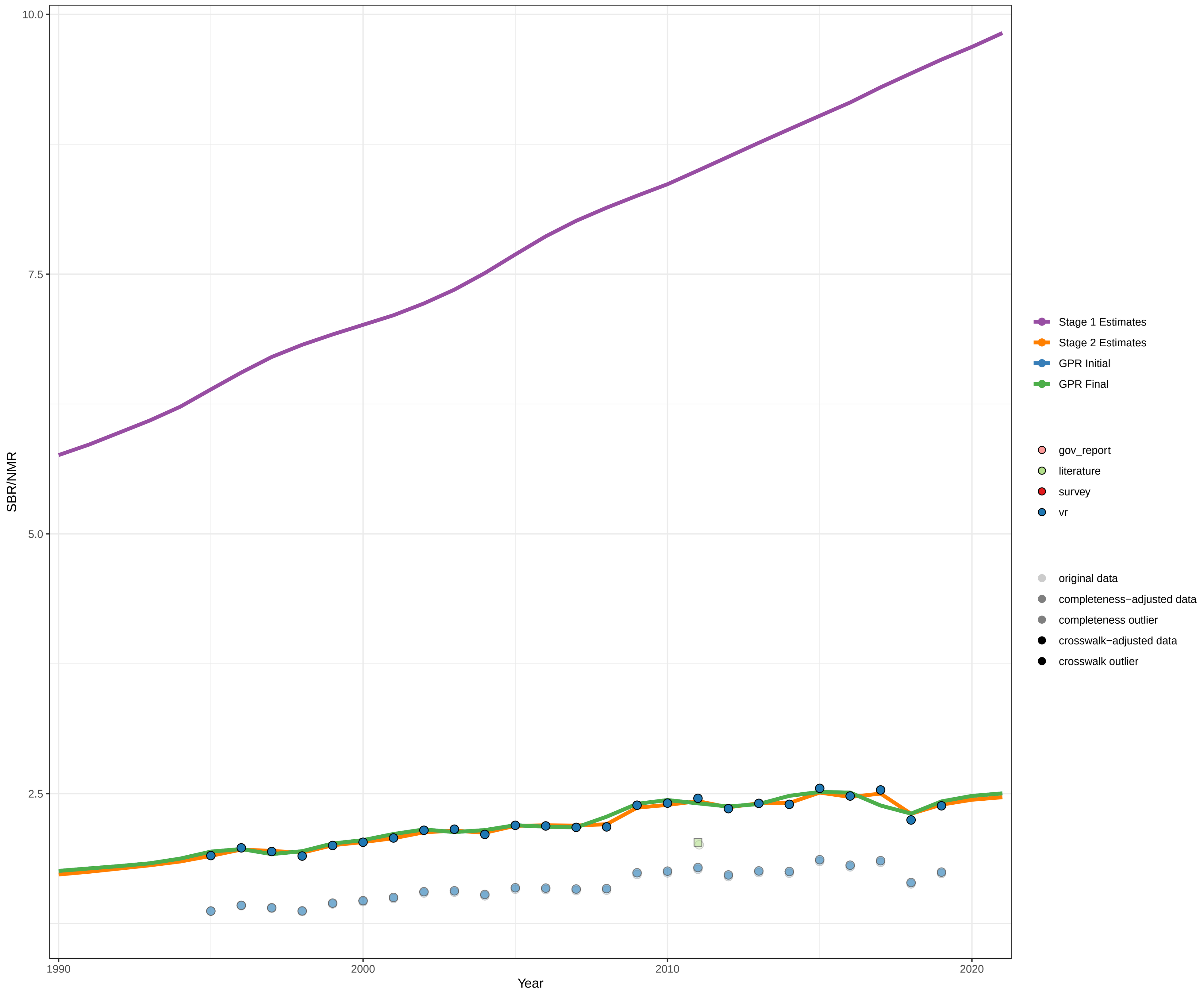

Japan JPN High−income Asia Pacific Stillbirth Rate / Neonatal Mortality Rate (SBR/NMR) − 28 weeks
lambda = 0.2 ; zeta = 0.99 ; scale = 5 ; amp = 0.028

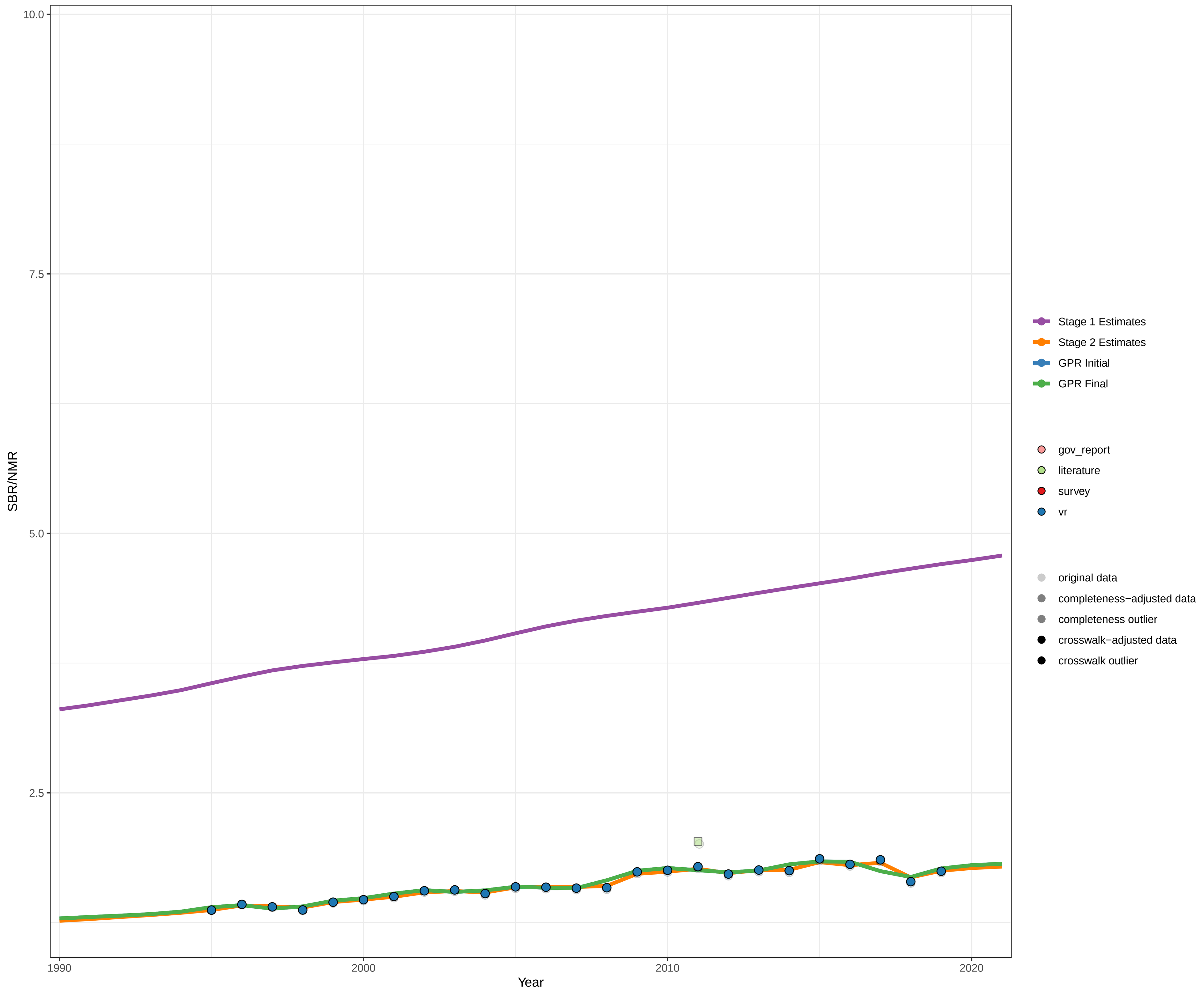

Definition	Years	Outlier	Source.Type	Title
28_weeks	1995−2019	0	VR	United Nations Demographic Yearbook
28_weeks_OR_1000_grams	2011	1	Sci Lit	Maternal complications and perinatal mortality: findings of the World Health Organization Multicountry Survey on Maternal and Newborn Health

Japan JPN High−income Asia Pacific Stillbirth Rate (SBR) − 20 weeks
lambda = 0.2 ; zeta = 0.99 ; scale = 5 ; amp = 0.028

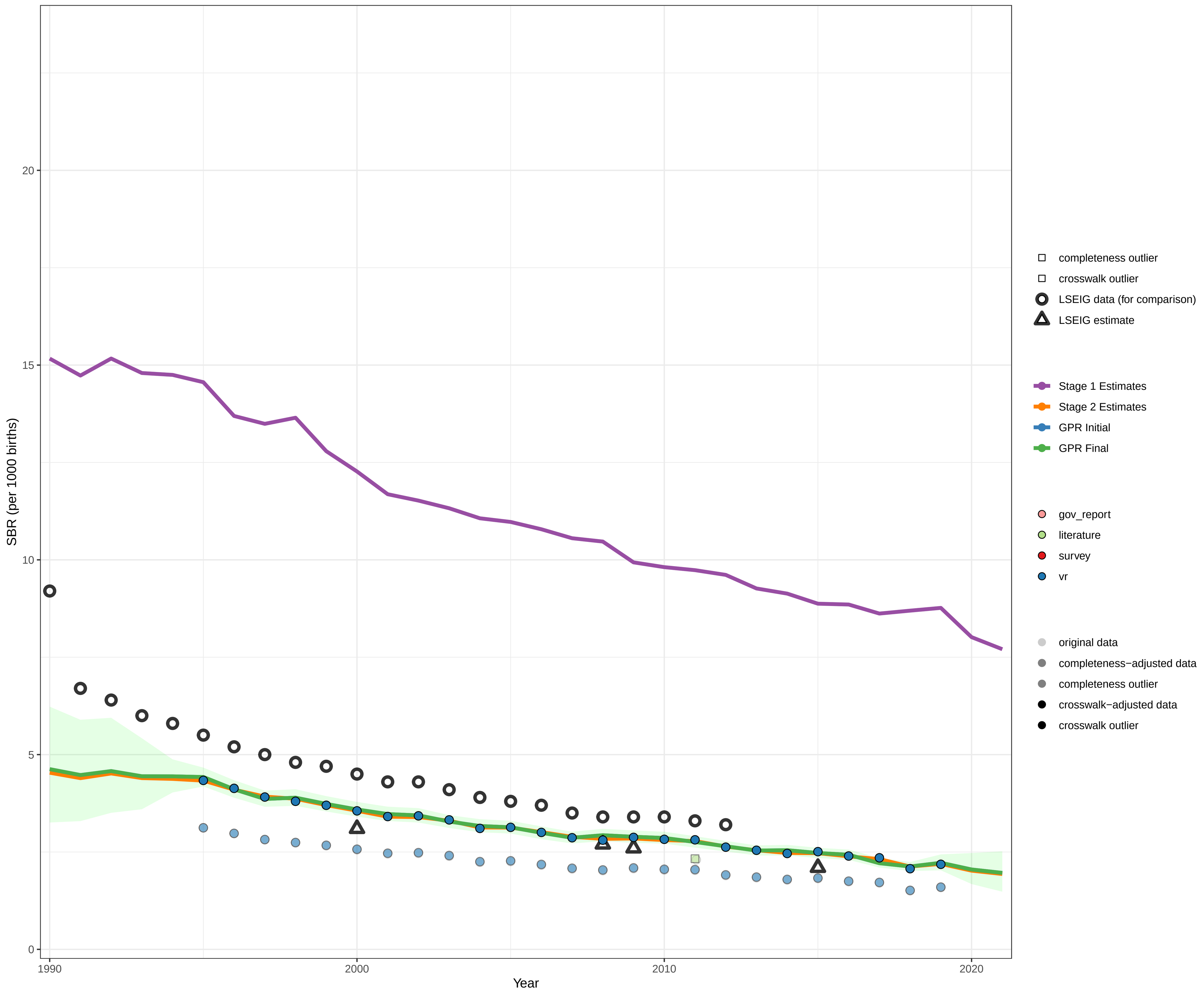

Japan JPN High−income Asia Pacific Stillbirth Rate (SBR) − 28 weeks
lambda = 0.2 ; zeta = 0.99 ; scale = 5 ; amp = 0.028

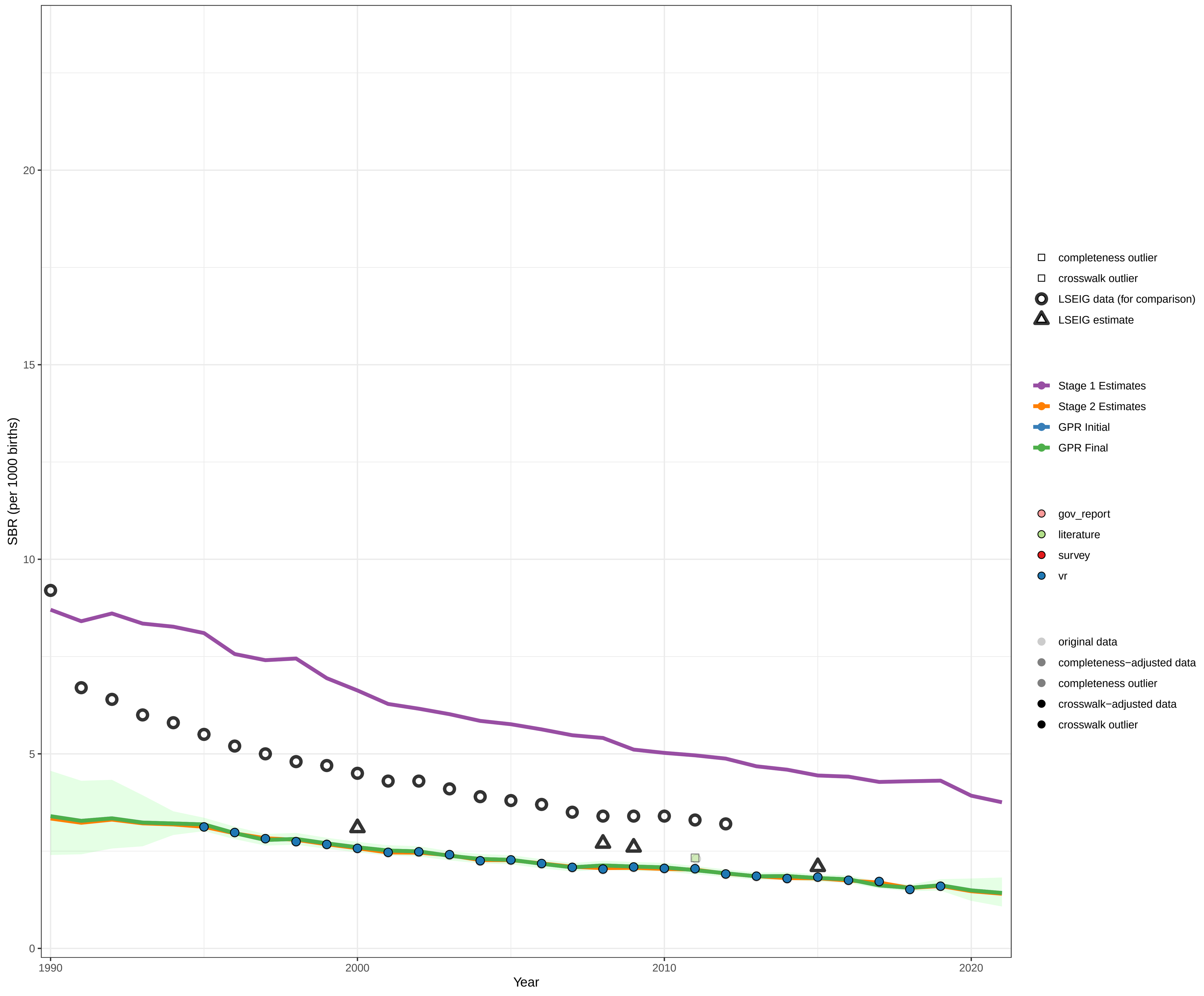

Year	Total Stillbirths (in thousands)	Total Stillbirths (in thousands)	SBR (per 1000 births)	SBR (per 1000 births)	SBR/NMR	SBR/NMR
	20 weeks	28 weeks	20 weeks	28 weeks	20 weeks	28 weeks
1990	5.7 (4.0 to 7.7)	4.2 (2.9 to 5.6)	4.6 (3.3 to 6.2)	3.4 (2.4 to 4.6)	1.76 (1.24 to 2.37)	1.29 (0.91 to 1.73)
1995	5.3 (5.0 to 5.6)	3.8 (3.6 to 4.0)	4.4 (4.2 to 4.7)	3.2 (3.0 to 3.4)	1.94 (1.84 to 2.05)	1.40 (1.32 to 1.47)
2000	4.2 (4.0 to 4.5)	3.1 (2.9 to 3.2)	3.6 (3.4 to 3.8)	2.6 (2.5 to 2.7)	2.05 (1.95 to 2.16)	1.49 (1.41 to 1.56)
2005	3.4 (3.2 to 3.6)	2.5 (2.4 to 2.6)	3.1 (3.0 to 3.3)	2.3 (2.2 to 2.4)	2.20 (2.08 to 2.31)	1.60 (1.51 to 1.68)
2010	3.1 (2.9 to 3.2)	2.2 (2.1 to 2.4)	2.9 (2.7 to 3.0)	2.1 (2.0 to 2.2)	2.44 (2.31 to 2.57)	1.78 (1.69 to 1.87)
2015	2.5 (2.3 to 2.6)	1.8 (1.7 to 1.9)	2.5 (2.3 to 2.6)	1.8 (1.7 to 1.9)	2.52 (2.38 to 2.65)	1.84 (1.74 to 1.94)
2020	1.7 (1.4 to 2.1)	1.3 (1.0 to 1.5)	2.1 (1.7 to 2.5)	1.5 (1.2 to 1.8)	2.48 (2.03 to 2.99)	1.80 (1.48 to 2.17)
2021	1.6 (1.2 to 2.1)	1.2 (0.9 to 1.5)	2.0 (1.5 to 2.5)	1.4 (1.1 to 1.8)	2.50 (1.89 to 3.21)	1.82 (1.37 to 2.32)

Aichi JPN_35446 High−income Asia Pacific Stillbirth Rate / Neonatal Mortality Rate (SBR/NMR) − 20 weeks
lambda = 1 ; zeta = 0.6 ; scale = 15 ; amp = 0.028

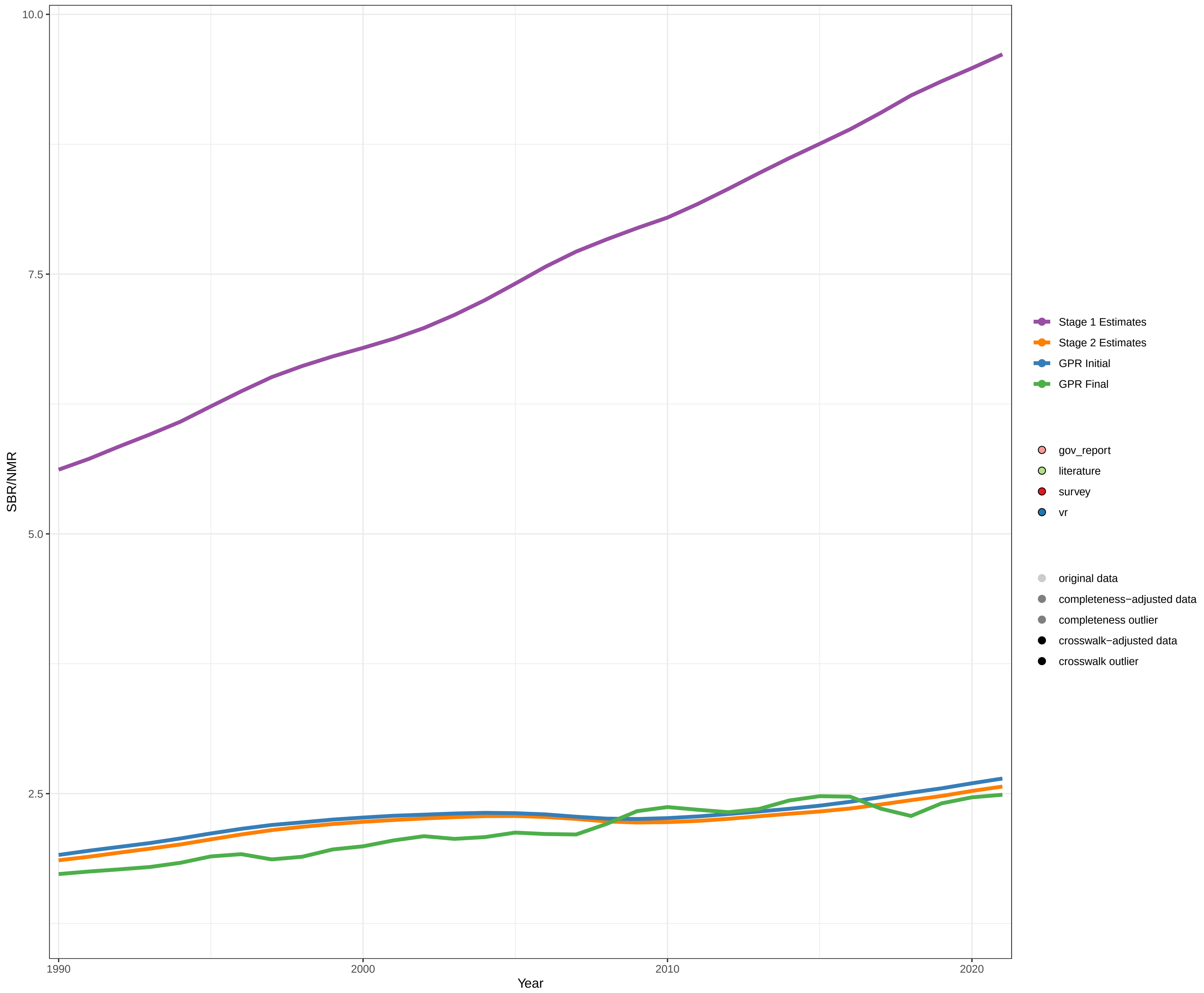

Aichi JPN_35446 High−income Asia Pacific Stillbirth Rate / Neonatal Mortality Rate (SBR/NMR) − 28 weeks
lambda = 1 ; zeta = 0.6 ; scale = 15 ; amp = 0.028

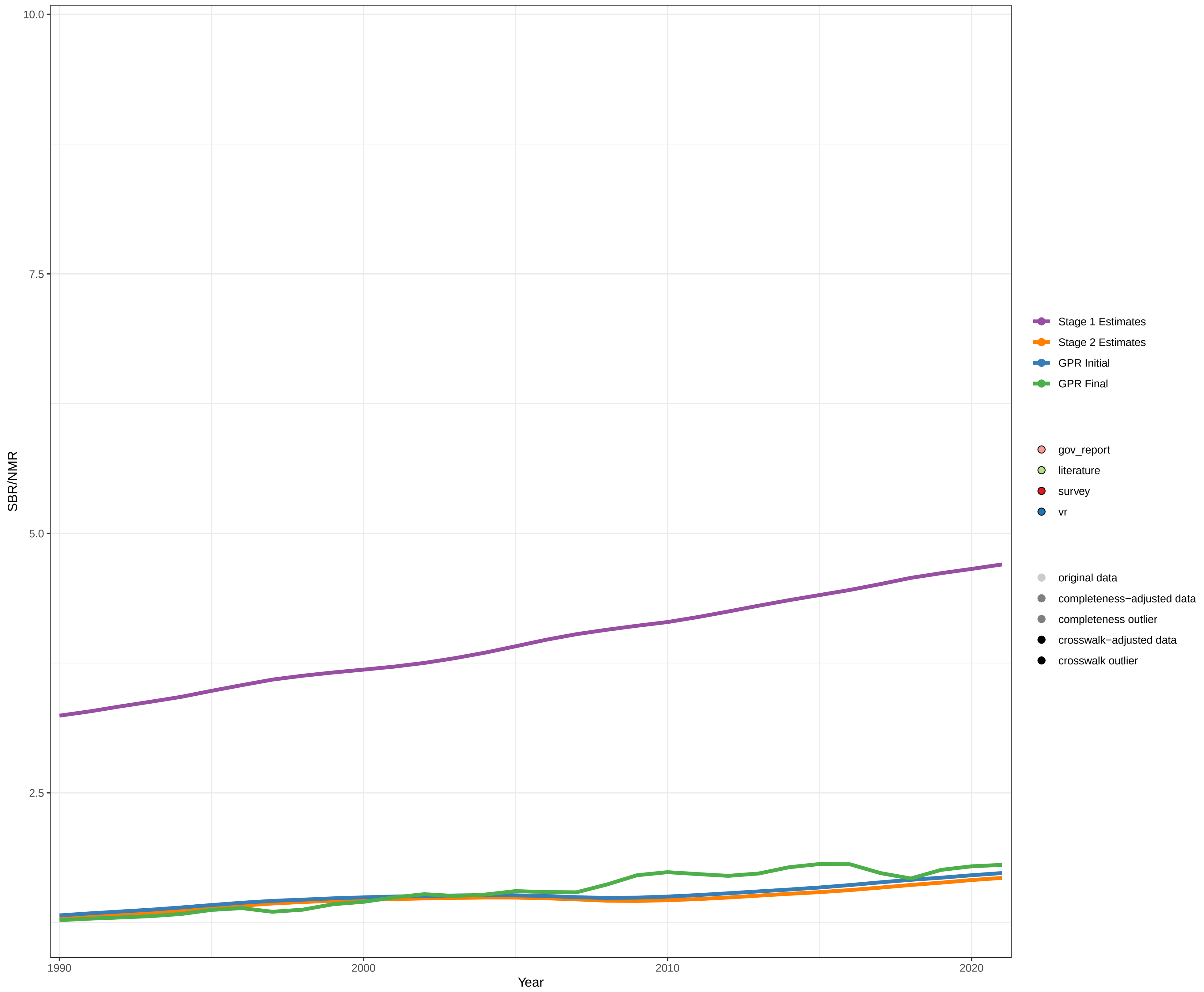

Definition	Years	Outlier	Source.Type	Title

Aichi JPN_35446 High−income Asia Pacific Stillbirth Rate (SBR) − 20 weeks
lambda = 1 ; zeta = 0.6 ; scale = 15 ; amp = 0.028

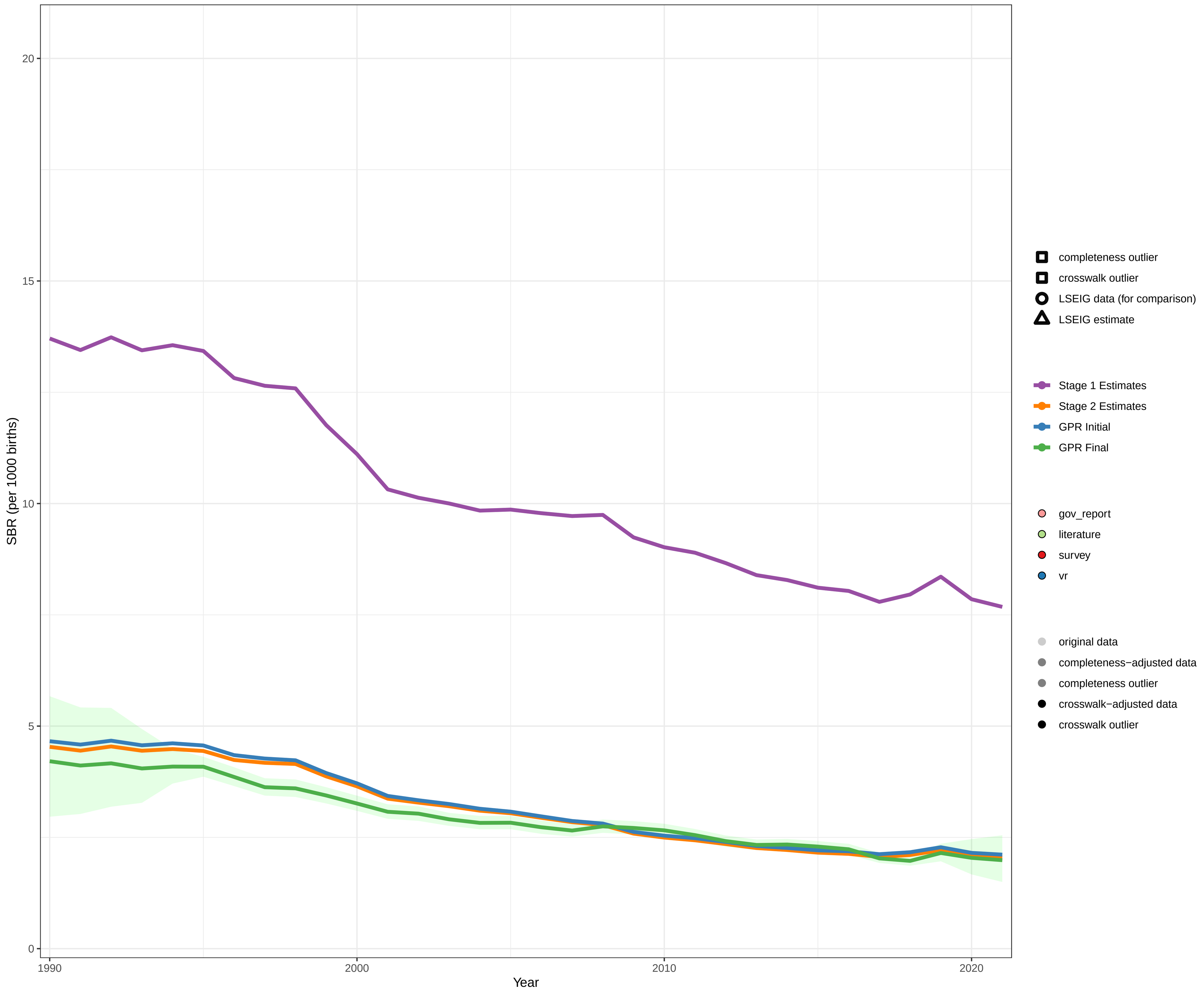

Aichi JPN_35446 High−income Asia Pacific Stillbirth Rate (SBR) − 28 weeks
lambda = 1 ; zeta = 0.6 ; scale = 15 ; amp = 0.028

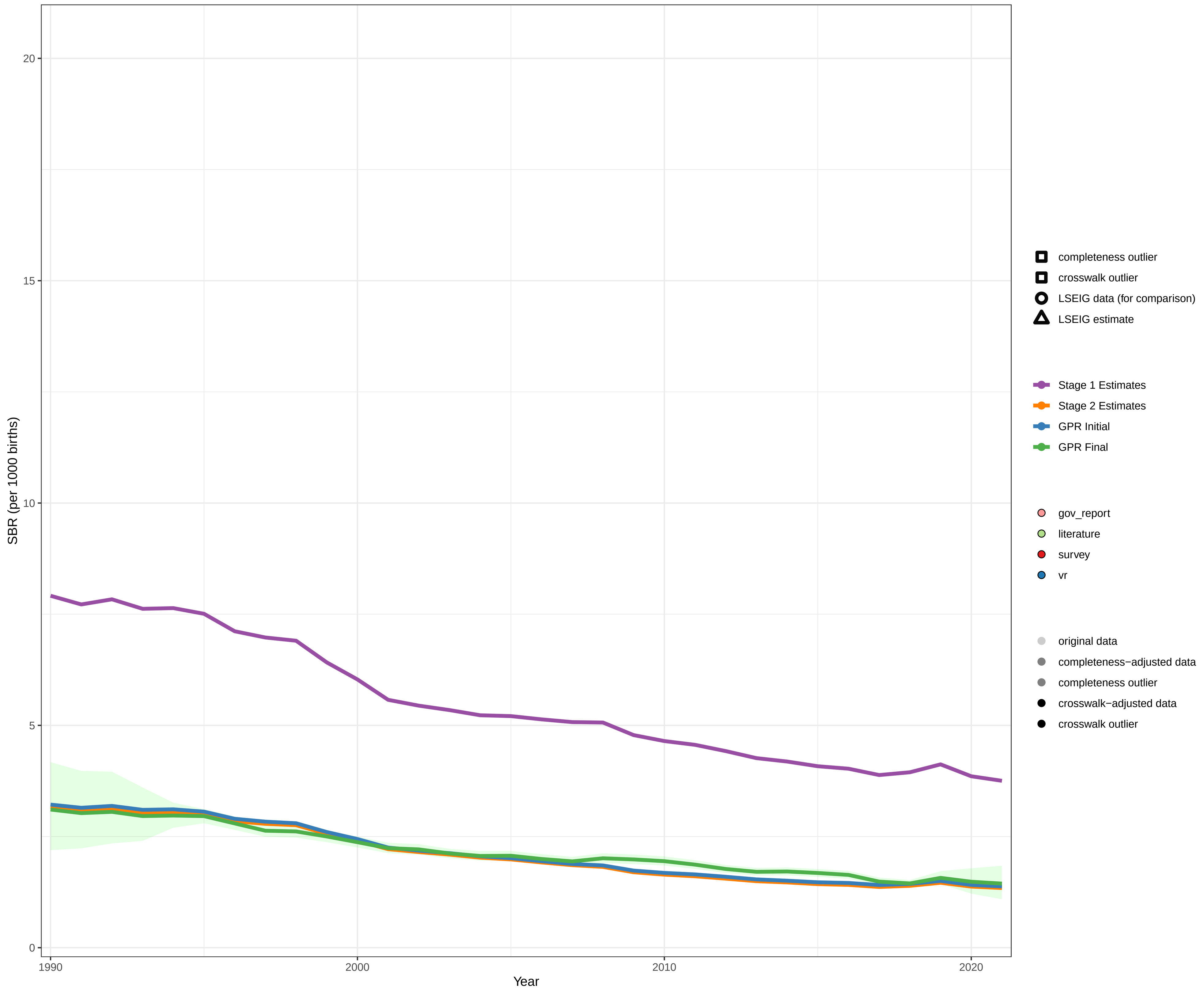

Year	Total Stillbirths (in thousands)	Total Stillbirths (in thousands)	SBR (per 1000 births)	SBR (per 1000 births)	SBR/NMR	SBR/NMR
	20 weeks	28 weeks	20 weeks	28 weeks	20 weeks	28 weeks
1990	0.3 (0.2 to 0.4)	0.2 (0.2 to 0.3)	4.2 (3.0 to 5.7)	3.1 (2.2 to 4.2)	1.73 (1.21 to 2.32)	1.27 (0.90 to 1.71)
1995	0.3 (0.3 to 0.3)	0.2 (0.2 to 0.2)	4.1 (3.9 to 4.3)	3.0 (2.8 to 3.1)	1.90 (1.79 to 2.00)	1.37 (1.30 to 1.45)
2000	0.2 (0.2 to 0.3)	0.2 (0.2 to 0.2)	3.3 (3.1 to 3.4)	2.4 (2.3 to 2.5)	1.99 (1.89 to 2.10)	1.45 (1.38 to 1.53)
2005	0.2 (0.2 to 0.2)	0.1 (0.1 to 0.2)	2.8 (2.7 to 3.0)	2.1 (2.0 to 2.2)	2.13 (2.02 to 2.24)	1.55 (1.47 to 1.64)
2010	0.2 (0.2 to 0.2)	0.1 (0.1 to 0.1)	2.7 (2.5 to 2.8)	1.9 (1.8 to 2.1)	2.37 (2.25 to 2.50)	1.74 (1.65 to 1.83)
2015	0.1 (0.1 to 0.2)	0.1 (0.1 to 0.1)	2.3 (2.2 to 2.4)	1.7 (1.6 to 1.8)	2.48 (2.35 to 2.61)	1.81 (1.72 to 1.91)
2020	0.1 (0.1 to 0.1)	0.1 (0.1 to 0.1)	2.0 (1.7 to 2.5)	1.5 (1.2 to 1.8)	2.46 (2.01 to 2.97)	1.79 (1.47 to 2.16)
2021	0.1 (0.1 to 0.1)	0.1 (0.1 to 0.1)	2.0 (1.5 to 2.5)	1.4 (1.1 to 1.8)	2.49 (1.88 to 3.19)	1.81 (1.37 to 2.31)

Akita JPN_35428 High−income Asia Pacific Stillbirth Rate / Neonatal Mortality Rate (SBR/NMR) − 20 weeks
lambda = 1 ; zeta = 0.6 ; scale = 15 ; amp = 0.028

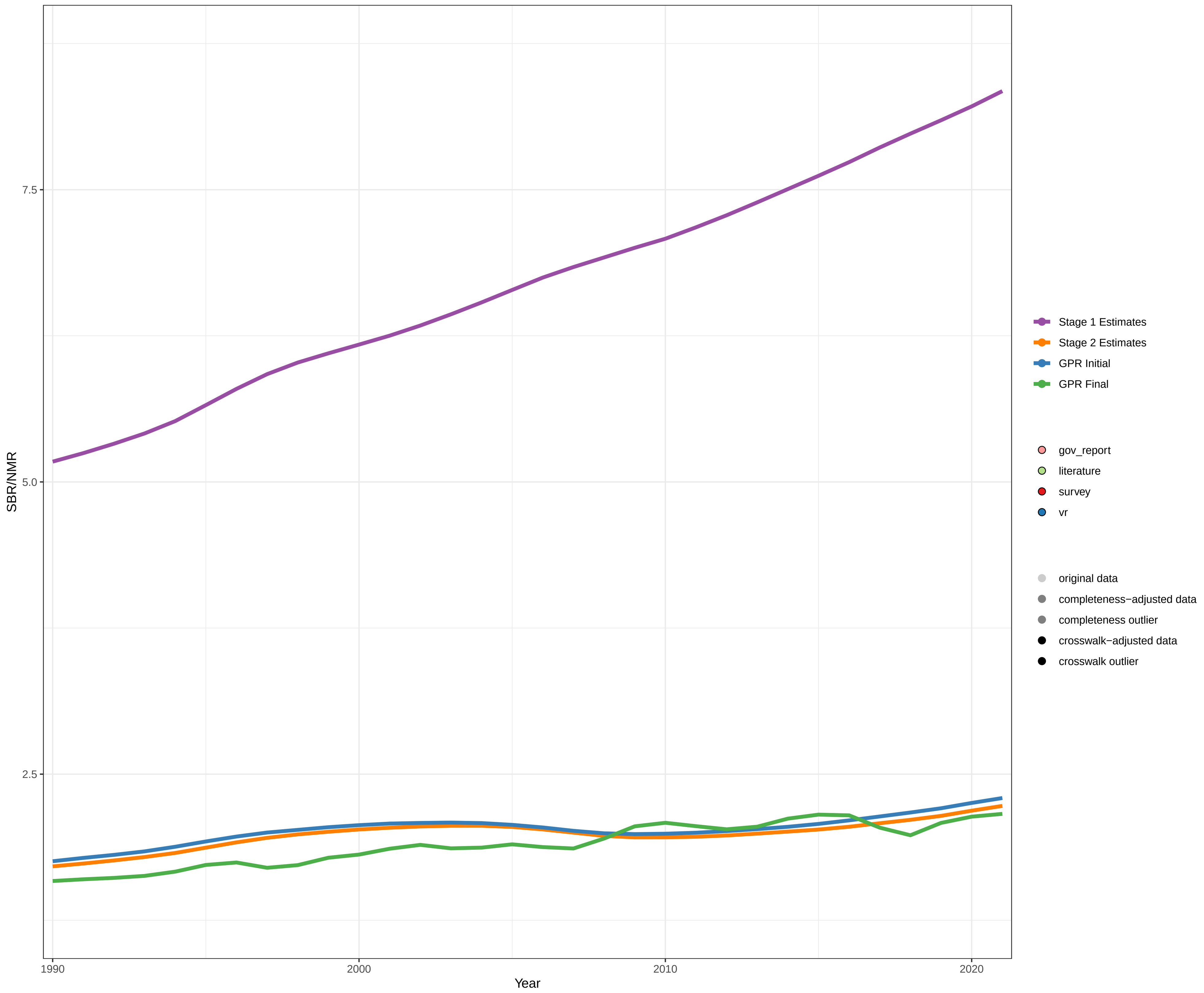

Akita JPN_35428 High−income Asia Pacific Stillbirth Rate / Neonatal Mortality Rate (SBR/NMR) − 28 weeks
lambda = 1 ; zeta = 0.6 ; scale = 15 ; amp = 0.028

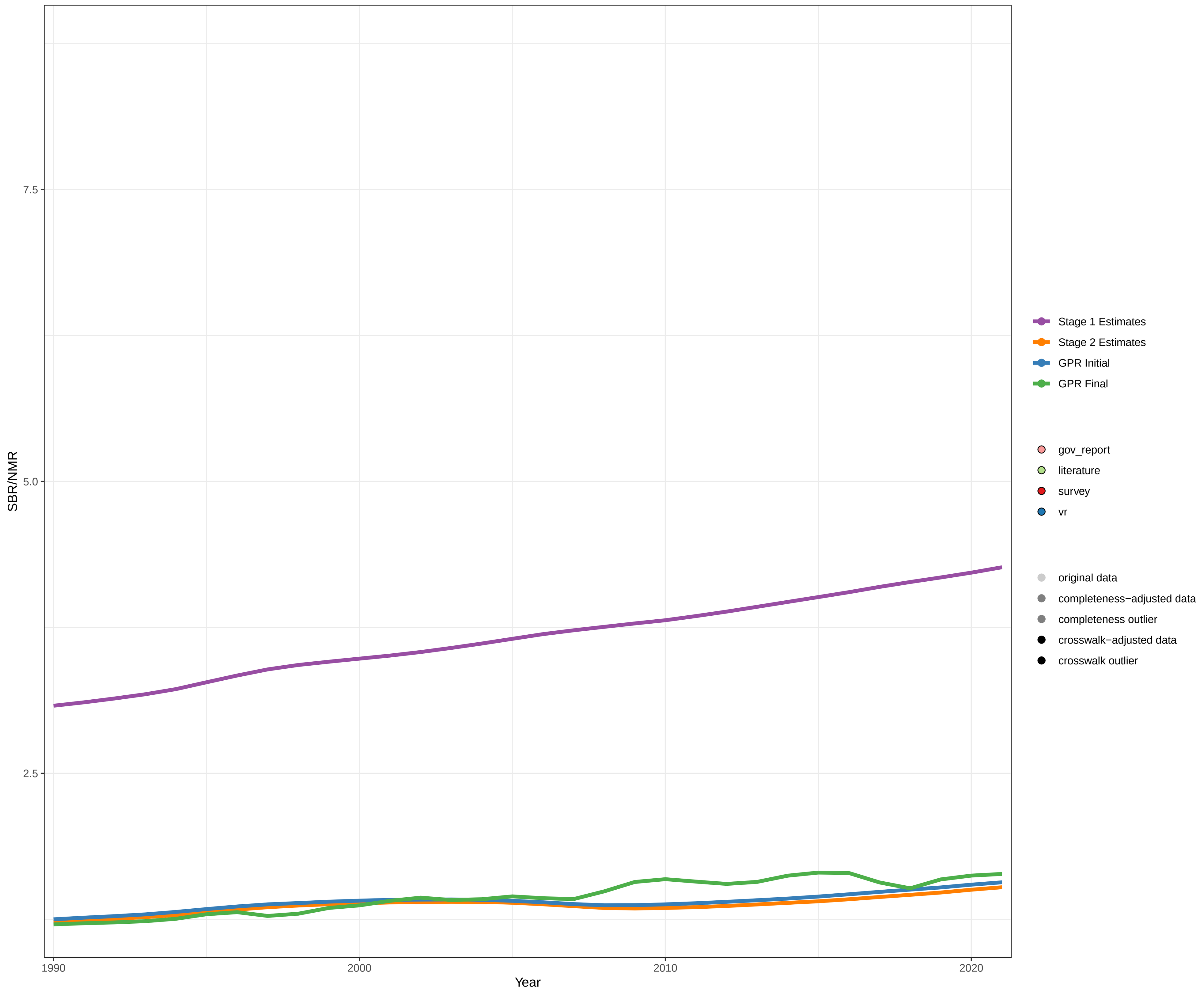

Definition	Years	Outlier	Source.Type	Title

Akita JPN_35428 High−income Asia Pacific Stillbirth Rate (SBR) − 20 weeks
lambda = 1 ; zeta = 0.6 ; scale = 15 ; amp = 0.028

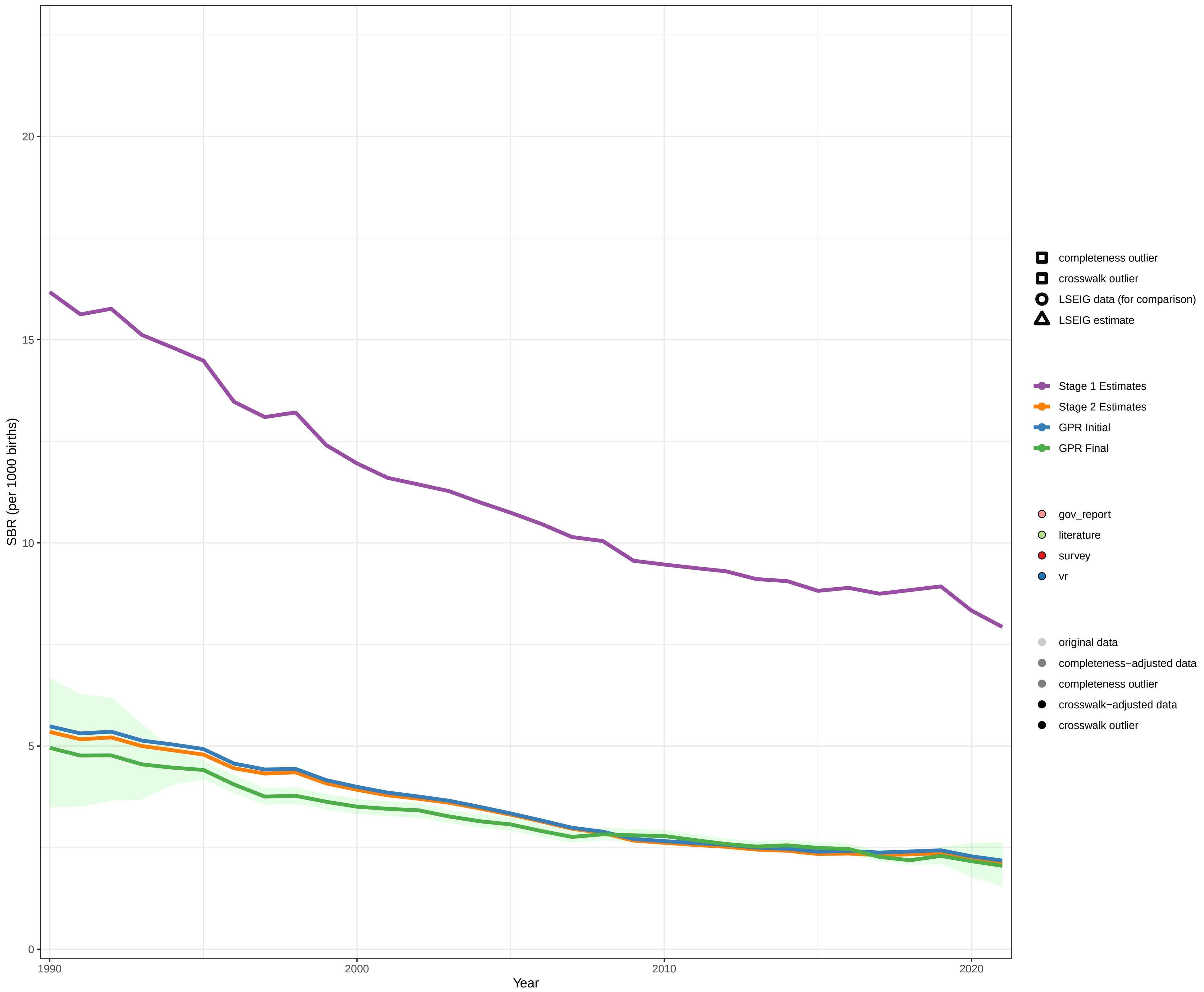

Akita JPN_35428 High−income Asia Pacific Stillbirth Rate (SBR) − 28 weeks
lambda = 1 ; zeta = 0.6 ; scale = 15 ; amp = 0.028

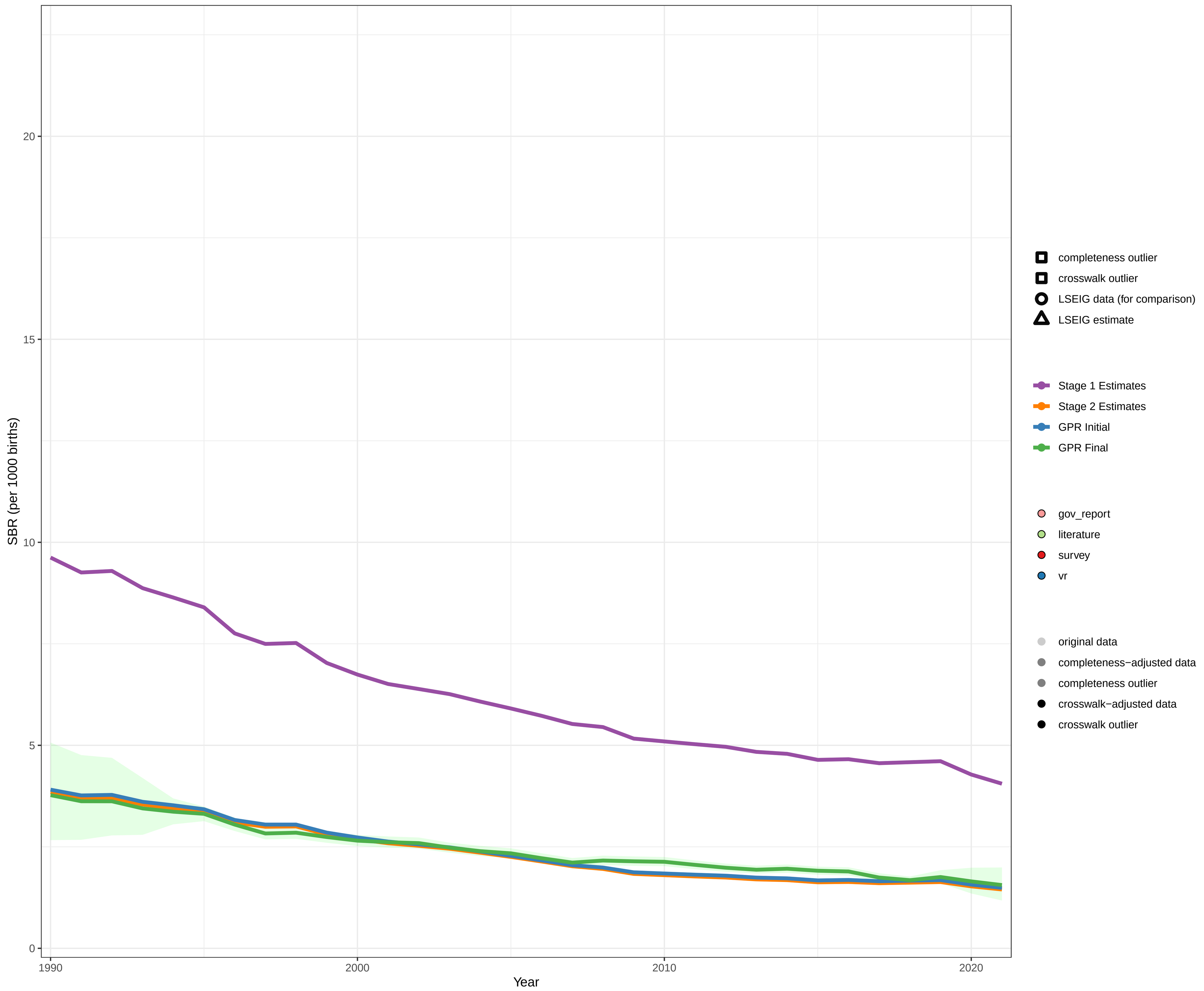

Year	Total Stillbirths (in thousands)	Total Stillbirths (in thousands)	SBR (per 1000 births)	SBR (per 1000 births)	SBR/NMR	SBR/NMR
	20 weeks	28 weeks	20 weeks	28 weeks	20 weeks	28 weeks
1990	0.1 (0.0 to 0.1)	0.0 (0.0 to 0.1)	5.0 (3.5 to 6.7)	3.8 (2.7 to 5.1)	1.59 (1.12 to 2.14)	1.21 (0.85 to 1.62)
1995	0.0 (0.0 to 0.0)	0.0 (0.0 to 0.0)	4.4 (4.2 to 4.7)	3.3 (3.1 to 3.5)	1.72 (1.63 to 1.82)	1.29 (1.22 to 1.36)
2000	0.0 (0.0 to 0.0)	0.0 (0.0 to 0.0)	3.5 (3.3 to 3.7)	2.7 (2.5 to 2.8)	1.81 (1.72 to 1.91)	1.37 (1.30 to 1.44)
2005	0.0 (0.0 to 0.0)	0.0 (0.0 to 0.0)	3.1 (2.9 to 3.2)	2.3 (2.2 to 2.5)	1.90 (1.80 to 2.00)	1.45 (1.37 to 1.52)
2010	0.0 (0.0 to 0.0)	0.0 (0.0 to 0.0)	2.8 (2.6 to 2.9)	2.1 (2.0 to 2.2)	2.08 (1.98 to 2.20)	1.59 (1.51 to 1.68)
2015	0.0 (0.0 to 0.0)	0.0 (0.0 to 0.0)	2.5 (2.4 to 2.6)	1.9 (1.8 to 2.0)	2.15 (2.04 to 2.27)	1.65 (1.57 to 1.74)
2020	0.0 (0.0 to 0.0)	0.0 (0.0 to 0.0)	2.2 (1.8 to 2.6)	1.6 (1.4 to 2.0)	2.14 (1.75 to 2.58)	1.62 (1.33 to 1.96)
2021	0.0 (0.0 to 0.0)	0.0 (0.0 to 0.0)	2.1 (1.5 to 2.6)	1.6 (1.2 to 2.0)	2.16 (1.63 to 2.77)	1.64 (1.24 to 2.09)

Aomori JPN_35425 High−income Asia Pacific Stillbirth Rate / Neonatal Mortality Rate (SBR/NMR) − 20 weeks
lambda = 1 ; zeta = 0.6 ; scale = 15 ; amp = 0.028

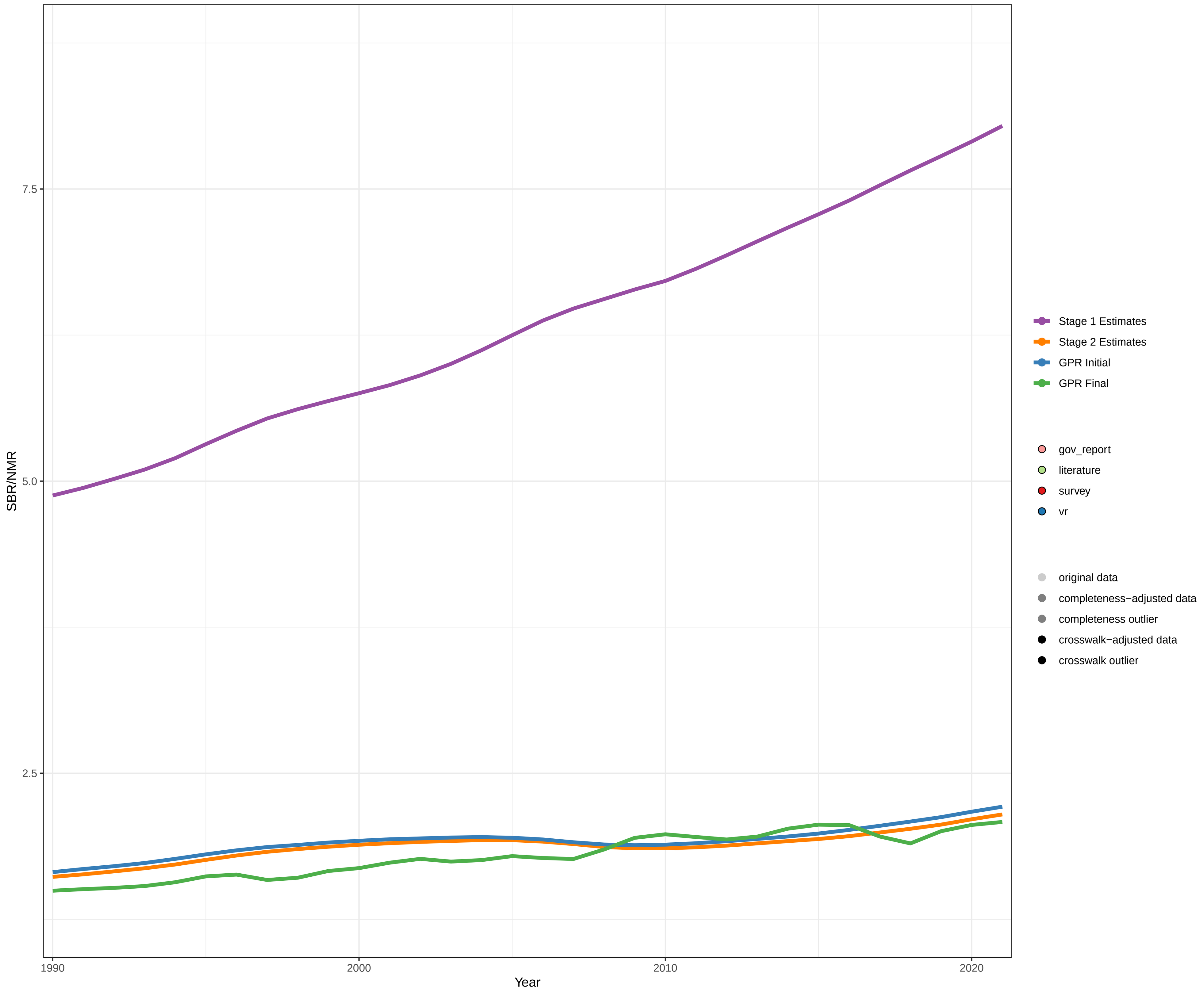

Aomori JPN_35425 High−income Asia Pacific Stillbirth Rate / Neonatal Mortality Rate (SBR/NMR) − 28 weeks
lambda = 1 ; zeta = 0.6 ; scale = 15 ; amp = 0.028

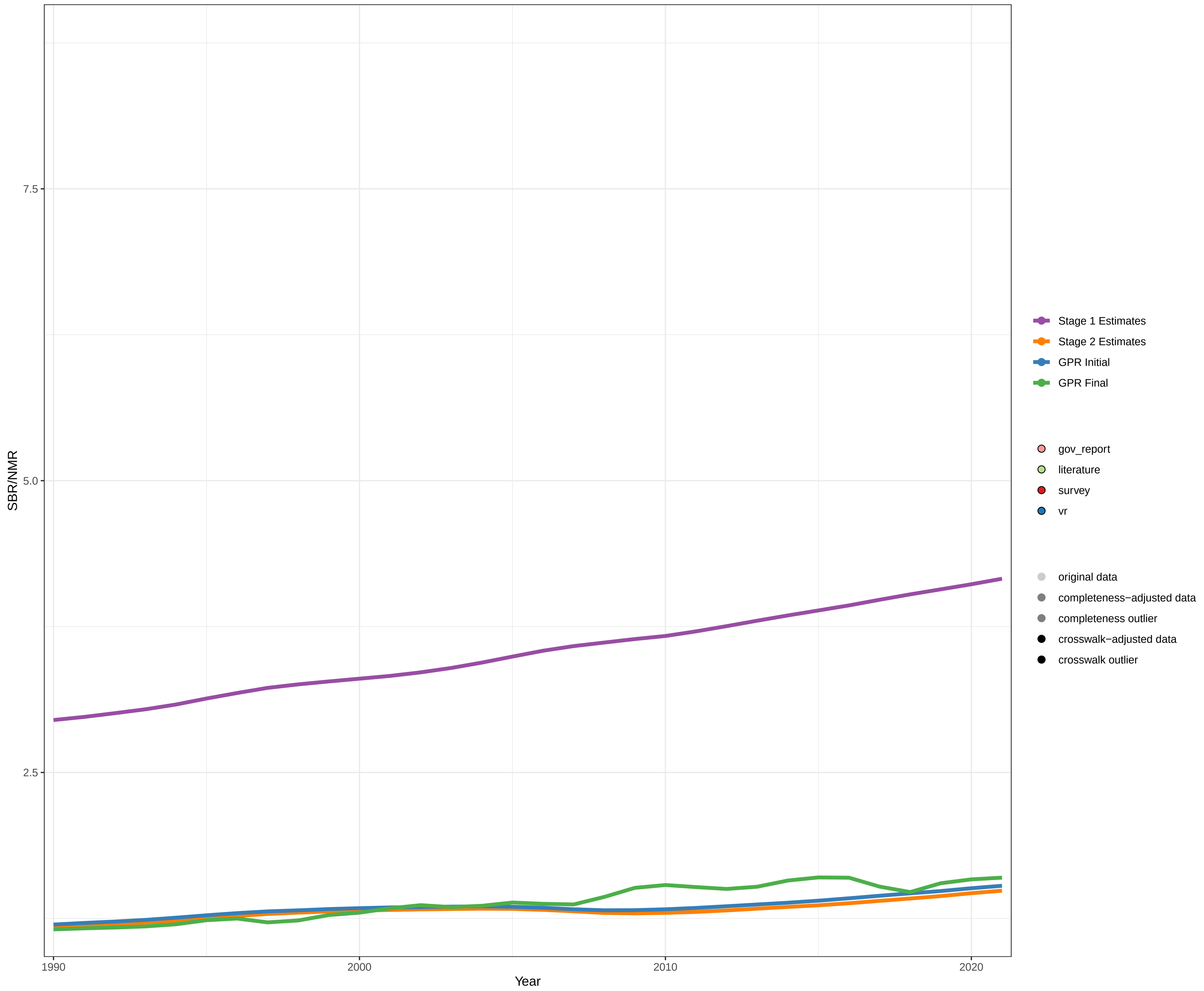

Definition	Years	Outlier	Source.Type	Title

Aomori JPN_35425 High−income Asia Pacific Stillbirth Rate (SBR) − 20 weeks
lambda = 1 ; zeta = 0.6 ; scale = 15 ; amp = 0.028

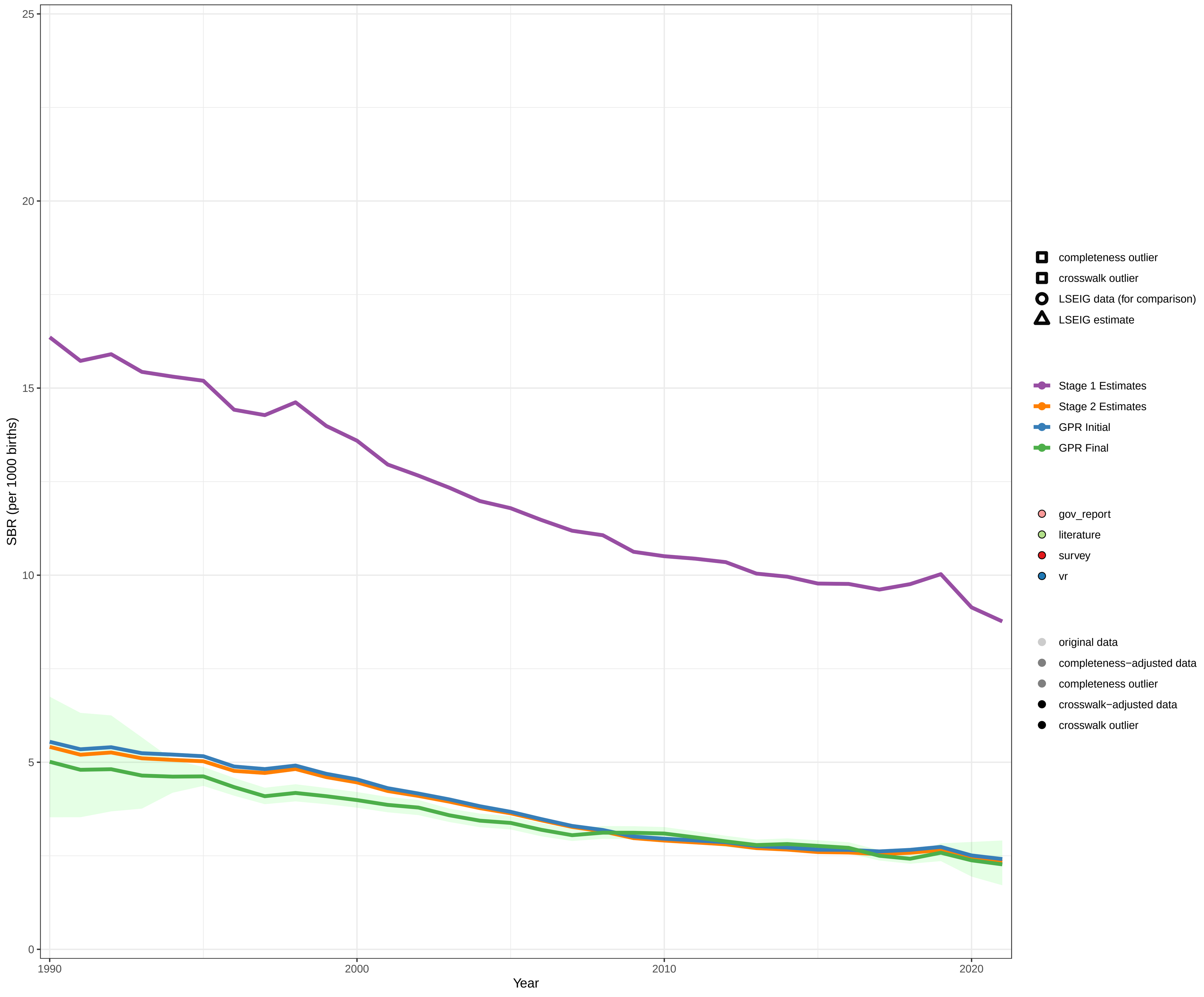

Aomori JPN_35425 High−income Asia Pacific Stillbirth Rate (SBR) − 28 weeks
lambda = 1 ; zeta = 0.6 ; scale = 15 ; amp = 0.028

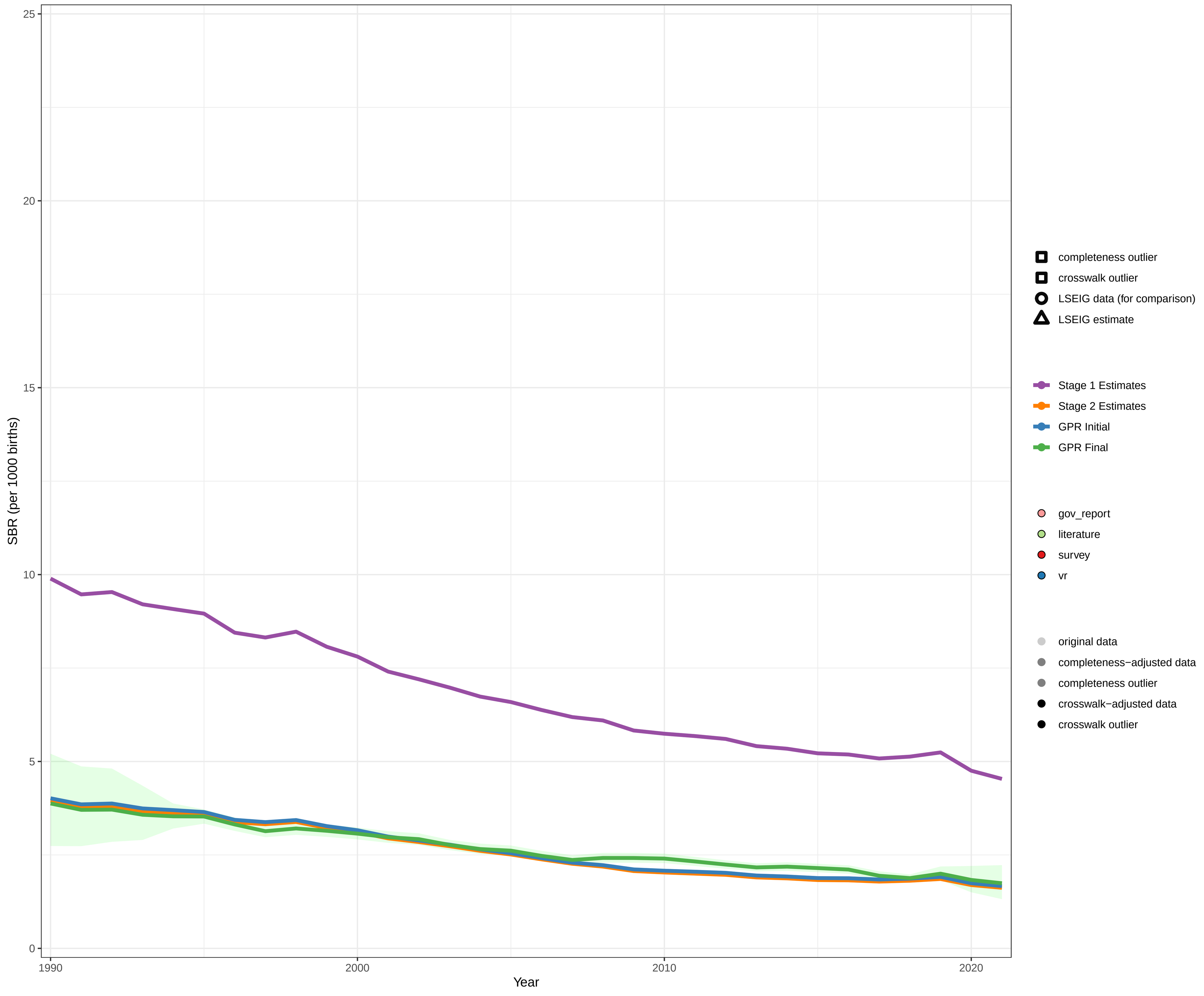

Year	Total Stillbirths (in thousands)	Total Stillbirths (in thousands)	SBR (per 1000 births)	SBR (per 1000 births)	SBR/NMR	SBR/NMR
	20 weeks	28 weeks	20 weeks	28 weeks	20 weeks	28 weeks
1990	0.1<br>(0.1 to 0.1)	0.1<br>(0.0 to 0.1)	5.0<br>(3.5 to 6.8)	3.9<br>(2.7 to 5.2)	1.49<br>(1.05 to 2.01)	1.16<br>(0.82 to 1.55)
1995	0.1<br>(0.1 to 0.1)	0.0<br>(0.0 to 0.1)	4.6<br>(4.4 to 4.9)	3.5<br>(3.3 to 3.7)	1.62<br>(1.53 to 1.71)	1.23<br>(1.17 to 1.30)
2000	0.1<br>(0.0 to 0.1)	0.0<br>(0.0 to 0.0)	4.0<br>(3.8 to 4.2)	3.1<br>(2.9 to 3.2)	1.69<br>(1.60 to 1.78)	1.30<br>(1.23 to 1.37)
2005	0.0<br>(0.0 to 0.0)	0.0<br>(0.0 to 0.0)	3.4<br>(3.2 to 3.6)	2.6<br>(2.5 to 2.8)	1.79<br>(1.70 to 1.89)	1.39<br>(1.32 to 1.46)
2010	0.0<br>(0.0 to 0.0)	0.0<br>(0.0 to 0.0)	3.1<br>(2.9 to 3.3)	2.4<br>(2.3 to 2.5)	1.98<br>(1.88 to 2.09)	1.54<br>(1.46 to 1.62)
2015	0.0<br>(0.0 to 0.0)	0.0<br>(0.0 to 0.0)	2.8<br>(2.6 to 2.9)	2.1<br>(2.0 to 2.3)	2.06<br>(1.95 to 2.17)	1.60<br>(1.52 to 1.69)
2020	0.0<br>(0.0 to 0.0)	0.0<br>(0.0 to 0.0)	2.4<br>(1.9 to 2.9)	1.8<br>(1.5 to 2.2)	2.06<br>(1.68 to 2.48)	1.58<br>(1.30 to 1.91)
2021	0.0<br>(0.0 to 0.0)	0.0<br>(0.0 to 0.0)	2.3<br>(1.7 to 2.9)	1.7<br>(1.3 to 2.2)	2.08<br>(1.57 to 2.67)	1.60<br>(1.21 to 2.04)

Chiba JPN_35435 High−income Asia Pacific Stillbirth Rate / Neonatal Mortality Rate (SBR/NMR) − 20 weeks
lambda = 1 ; zeta = 0.6 ; scale = 15 ; amp = 0.028

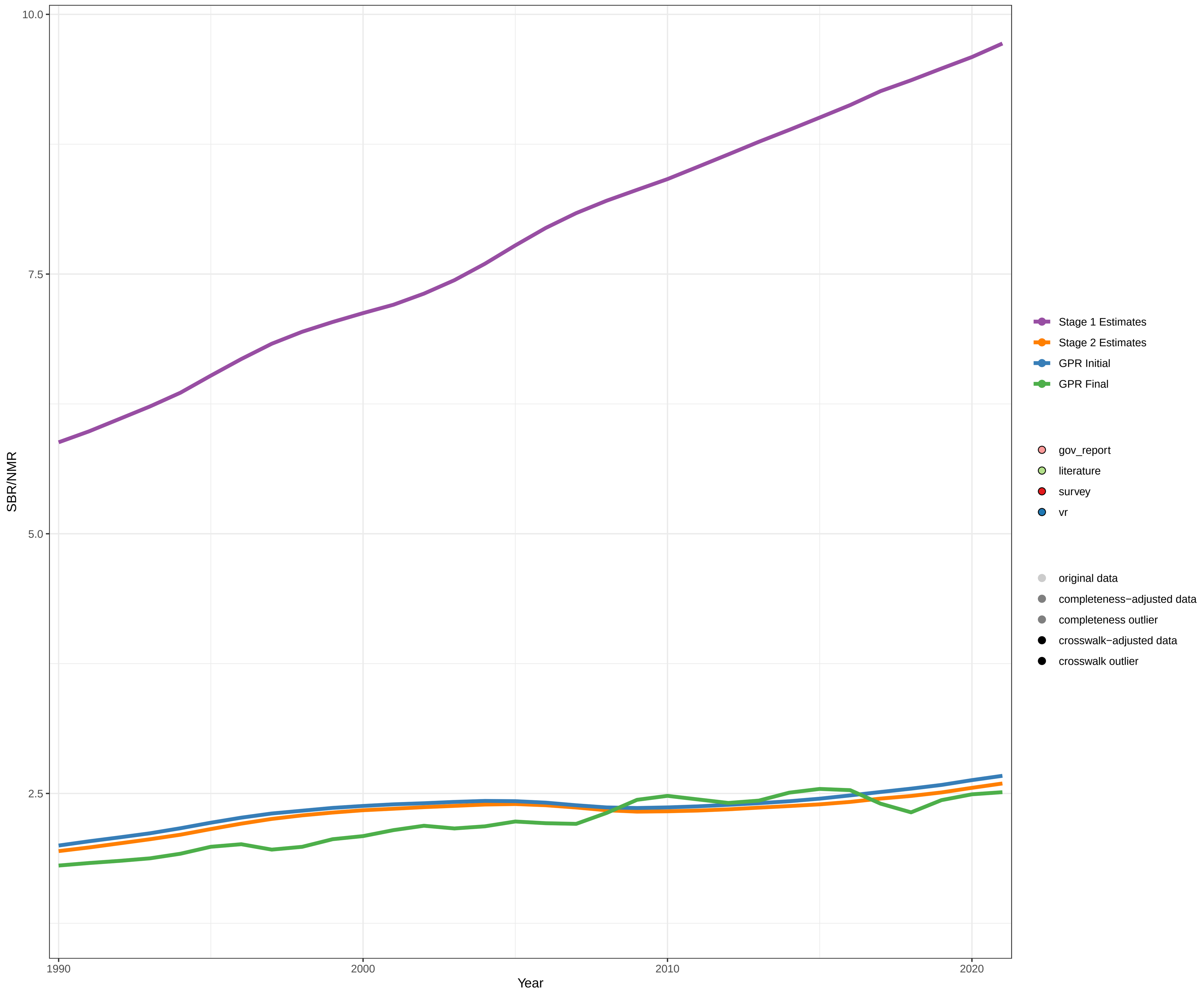

Chiba JPN_35435 High−income Asia Pacific Stillbirth Rate / Neonatal Mortality Rate (SBR/NMR) − 28 weeks
lambda = 1 ; zeta = 0.6 ; scale = 15 ; amp = 0.028

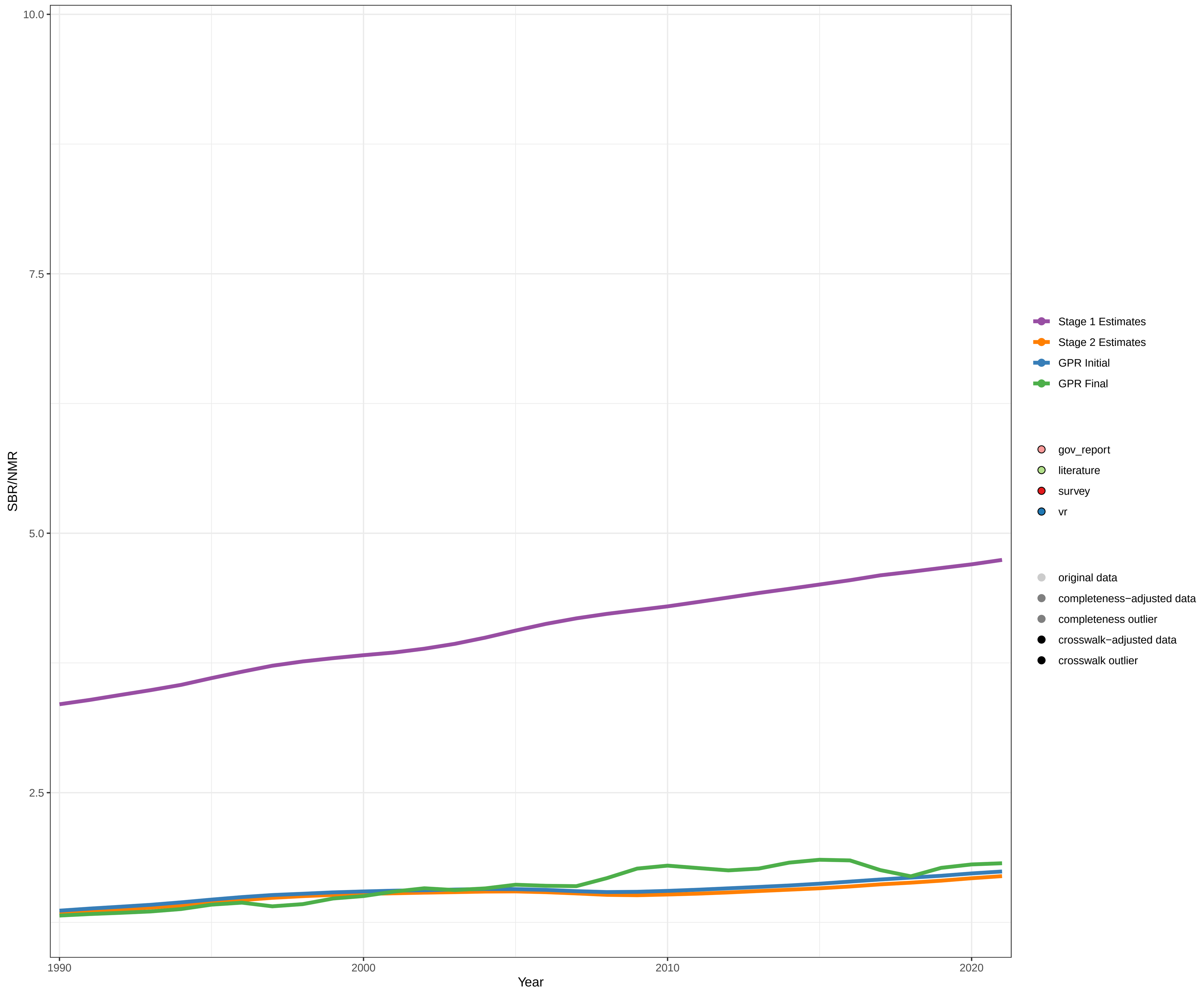

Definition	Years	Outlier	Source.Type	Title

Chiba JPN_35435 High−income Asia Pacific Stillbirth Rate (SBR) − 20 weeks
lambda = 1 ; zeta = 0.6 ; scale = 15 ; amp = 0.028

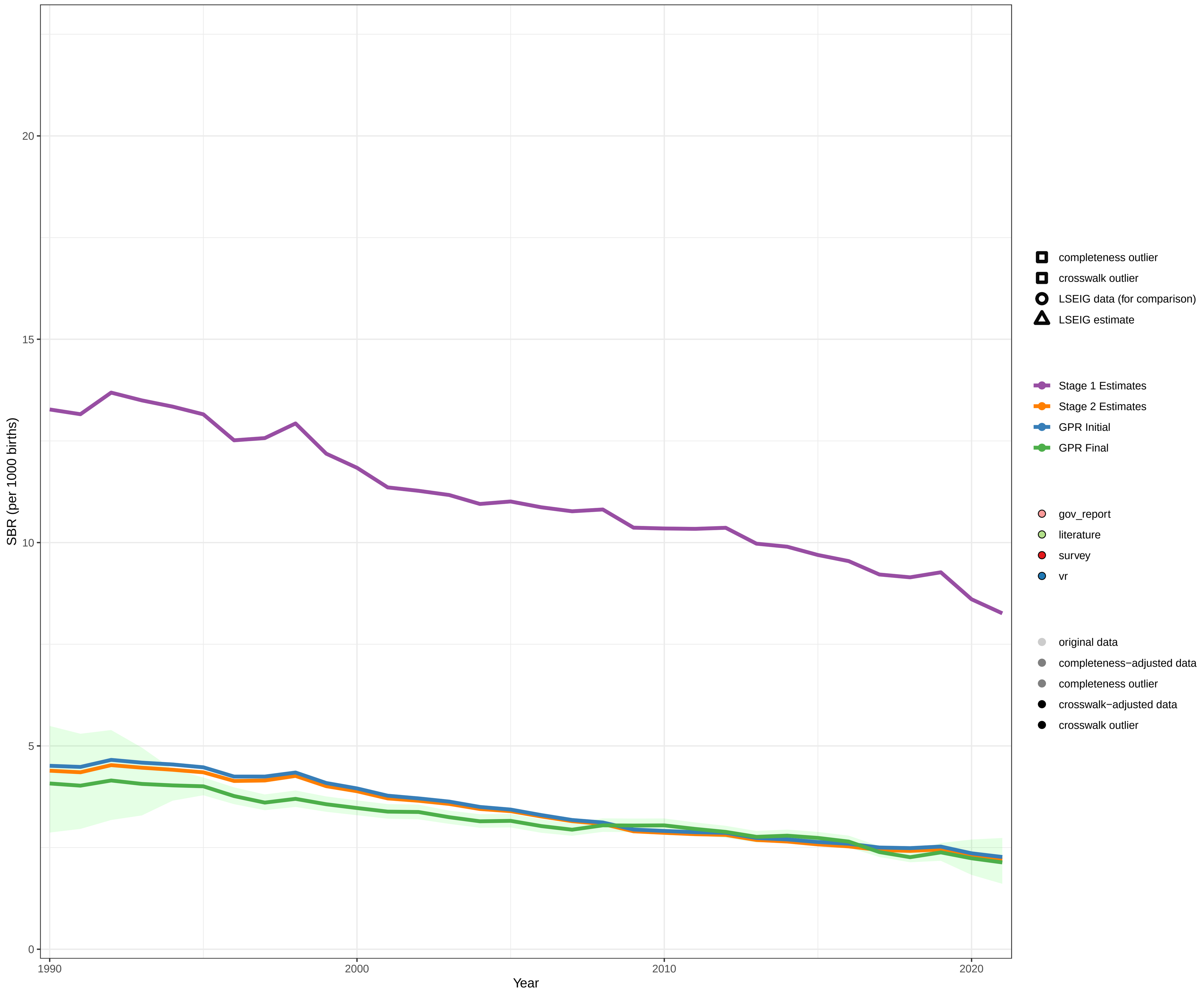

Chiba JPN_35435 High−income Asia Pacific Stillbirth Rate (SBR) − 28 weeks
lambda = 1 ; zeta = 0.6 ; scale = 15 ; amp = 0.028

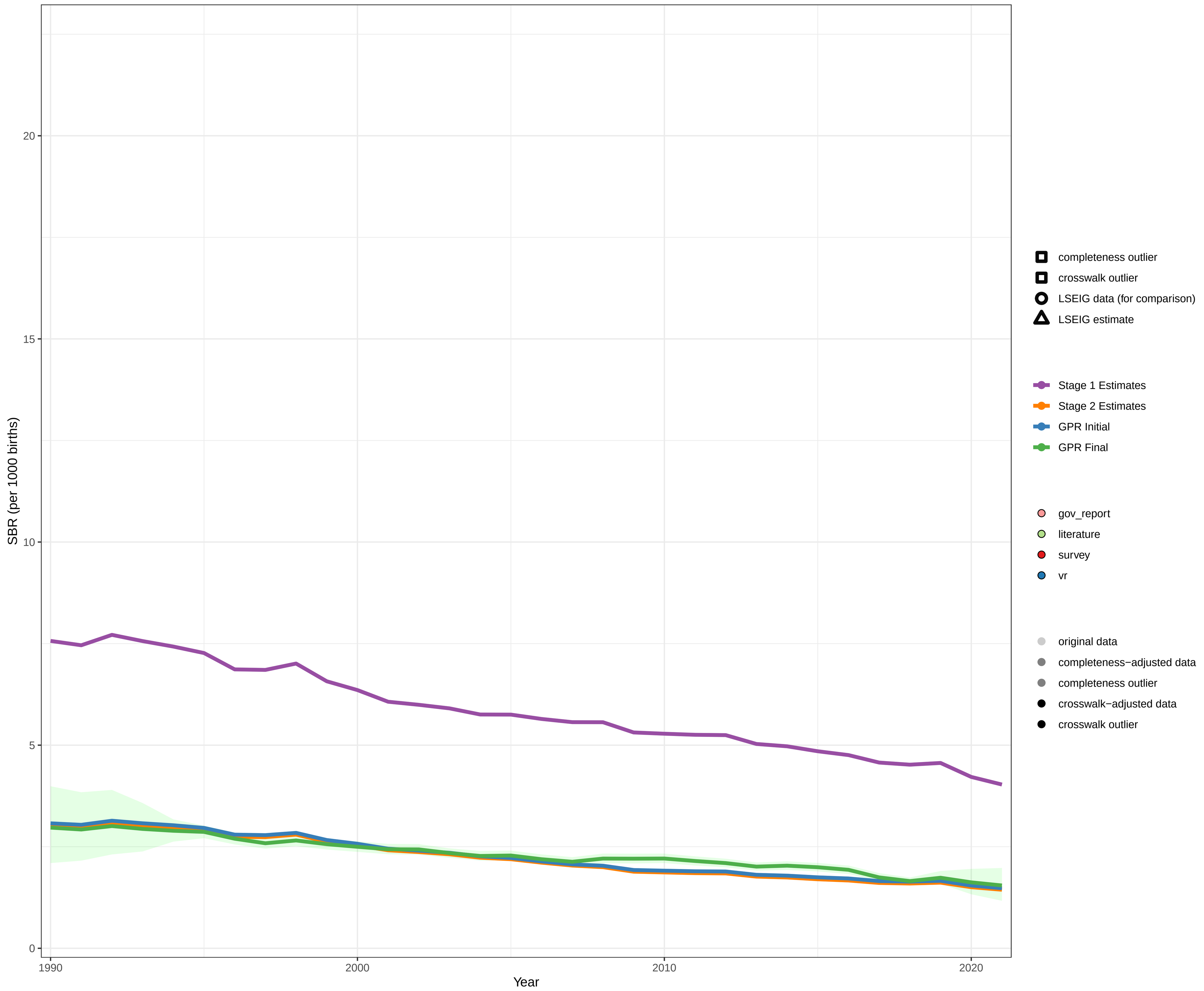

Year	Total Stillbirths (in thousands)	Total Stillbirths (in thousands)	SBR (per 1000 births)	SBR (per 1000 births)	SBR/NMR	SBR/NMR
	20 weeks	28 weeks	20 weeks	28 weeks	20 weeks	28 weeks
1990	0.2<br>(0.2 to 0.3)	0.2<br>(0.1 to 0.2)	4.1<br>(2.9 to 5.5)	3.0<br>(2.1 to 4.0)	1.81<br>(1.27 to 2.43)	1.32<br>(0.93 to 1.77)
1995	0.2<br>(0.2 to 0.2)	0.2<br>(0.1 to 0.2)	4.0<br>(3.8 to 4.2)	2.9<br>(2.7 to 3.0)	1.99<br>(1.88 to 2.09)	1.42<br>(1.34 to 1.50)
2000	0.2<br>(0.2 to 0.2)	0.1<br>(0.1 to 0.1)	3.5<br>(3.3 to 3.7)	2.5<br>(2.4 to 2.6)	2.09<br>(1.98 to 2.20)	1.50<br>(1.43 to 1.58)
2005	0.2<br>(0.2 to 0.2)	0.1<br>(0.1 to 0.1)	3.2<br>(3.0 to 3.3)	2.3<br>(2.2 to 2.4)	2.23<br>(2.12 to 2.35)	1.61<br>(1.53 to 1.70)
2010	0.2<br>(0.1 to 0.2)	0.1<br>(0.1 to 0.1)	3.0<br>(2.9 to 3.2)	2.2<br>(2.1 to 2.3)	2.48<br>(2.35 to 2.62)	1.80<br>(1.71 to 1.90)
2015	0.1<br>(0.1 to 0.1)	0.1<br>(0.1 to 0.1)	2.7<br>(2.6 to 2.9)	2.0<br>(1.9 to 2.1)	2.54<br>(2.41 to 2.68)	1.85<br>(1.76 to 1.95)
2020	0.1<br>(0.1 to 0.1)	0.1<br>(0.1 to 0.1)	2.2<br>(1.8 to 2.7)	1.6<br>(1.3 to 2.0)	2.49<br>(2.04 to 3.01)	1.81<br>(1.48 to 2.18)
2021	0.1<br>(0.1 to 0.1)	0.1<br>(0.0 to 0.1)	2.1<br>(1.6 to 2.7)	1.5<br>(1.2 to 2.0)	2.51<br>(1.89 to 3.22)	1.82<br>(1.38 to 2.33)

Ehime JPN_35461 High−income Asia Pacific Stillbirth Rate / Neonatal Mortality Rate (SBR/NMR) − 20 weeks
lambda = 1 ; zeta = 0.6 ; scale = 15 ; amp = 0.028

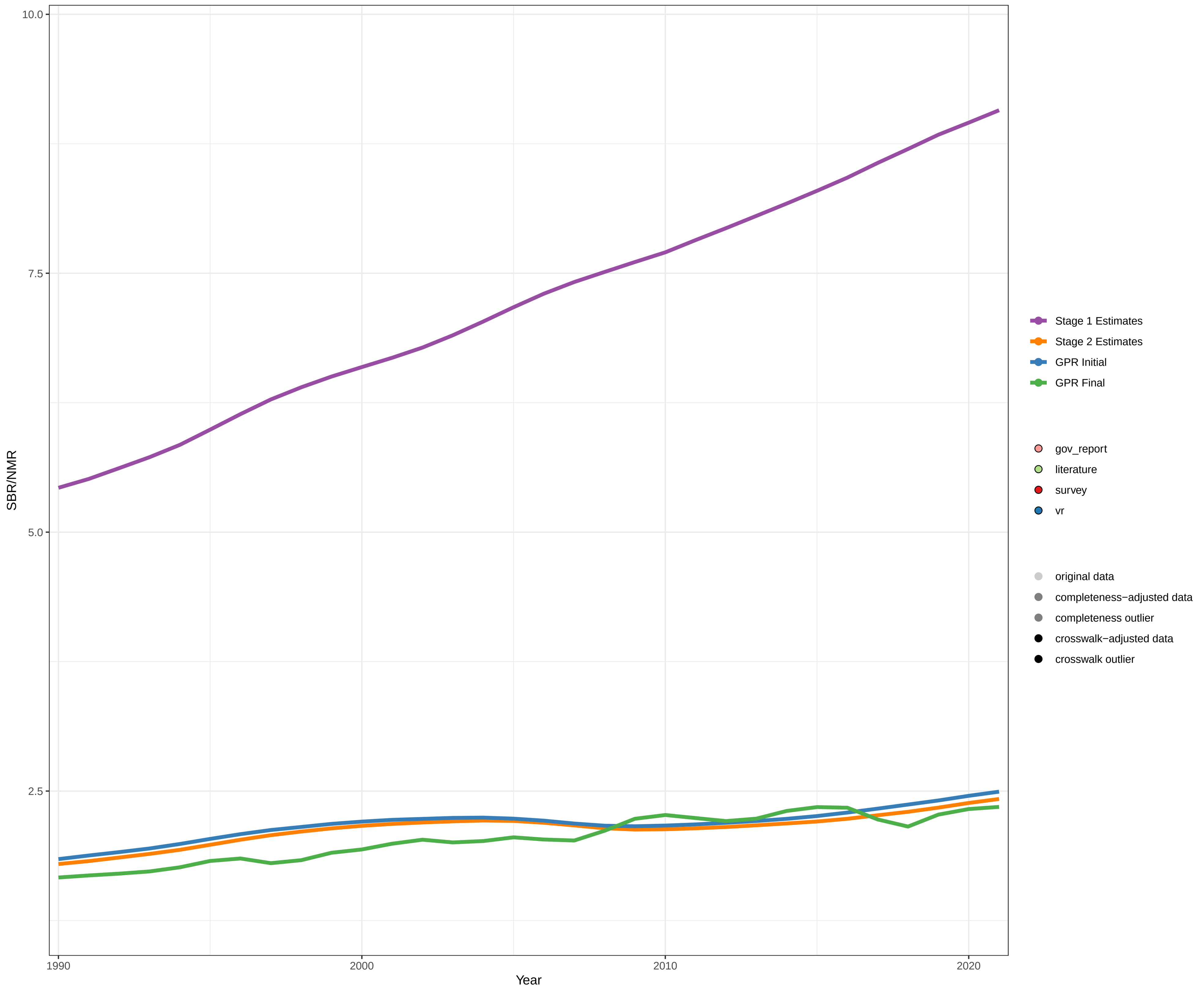

Ehime JPN_35461 High−income Asia Pacific Stillbirth Rate / Neonatal Mortality Rate (SBR/NMR) − 28 weeks
lambda = 1 ; zeta = 0.6 ; scale = 15 ; amp = 0.028

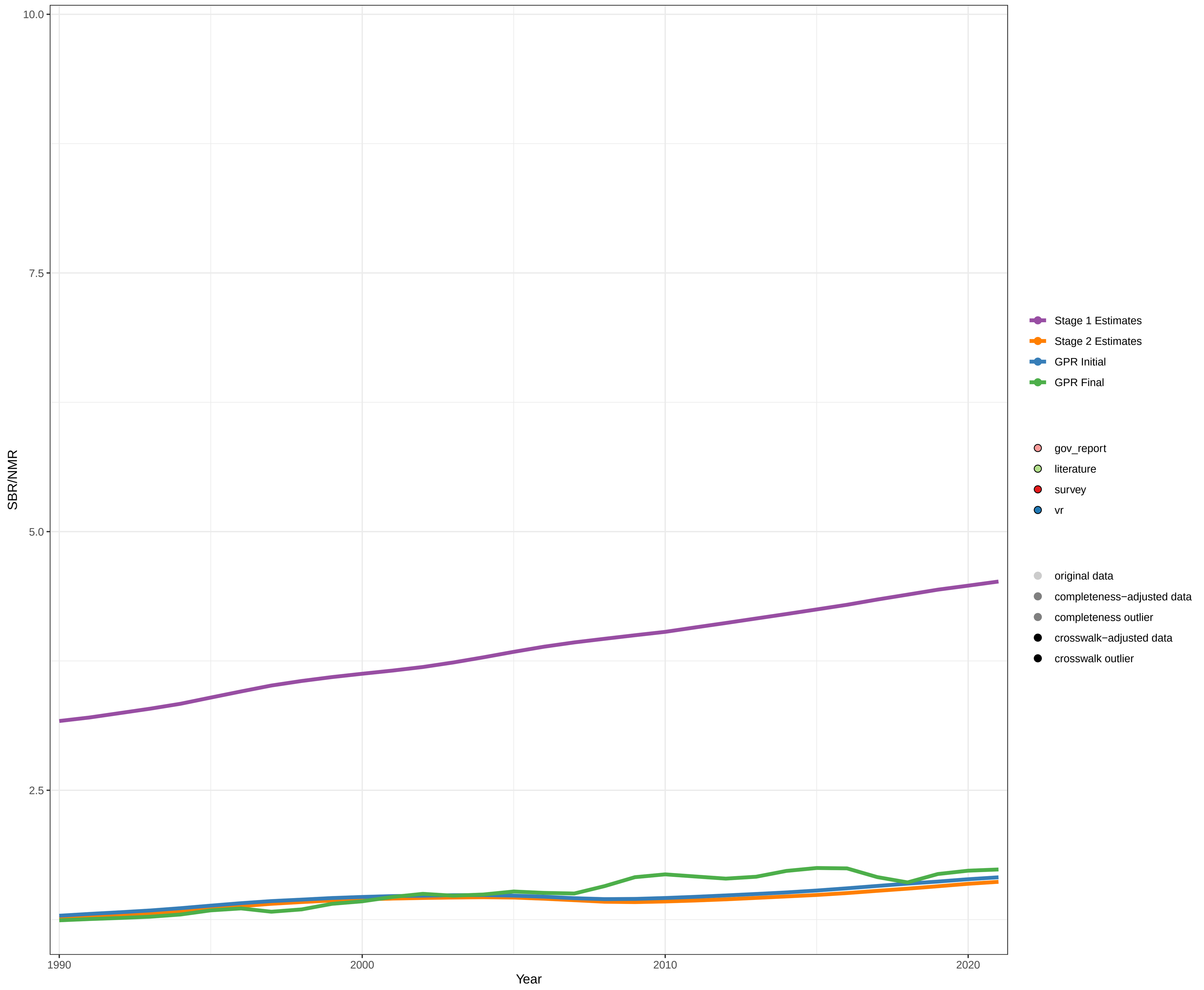

Definition	Years	Outlier	Source.Type	Title

Ehime JPN_35461 High−income Asia Pacific Stillbirth Rate (SBR) − 20 weeks
lambda = 1 ; zeta = 0.6 ; scale = 15 ; amp = 0.028

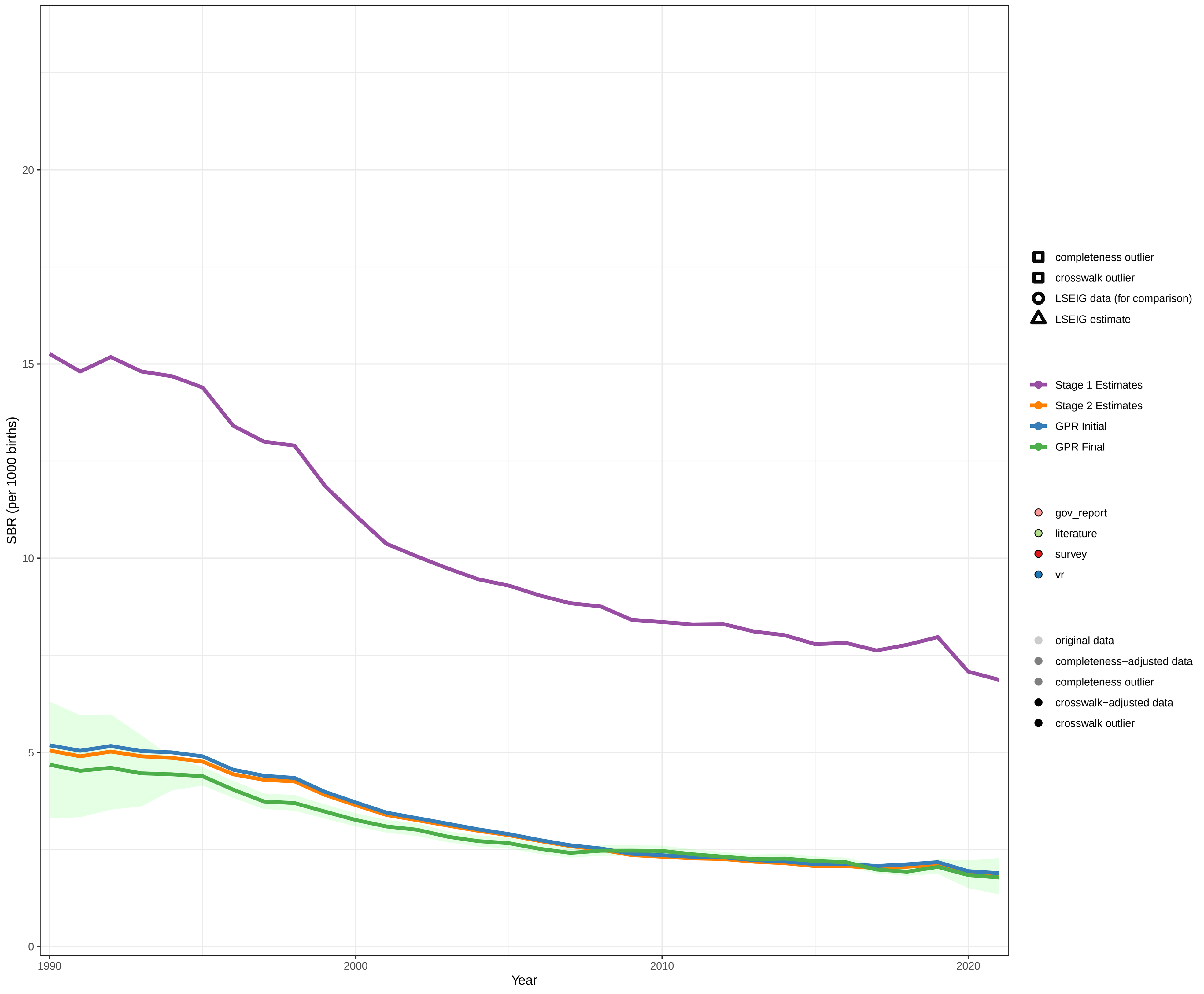

Ehime JPN_35461 High−income Asia Pacific Stillbirth Rate (SBR) − 28 weeks
lambda = 1 ; zeta = 0.6 ; scale = 15 ; amp = 0.028

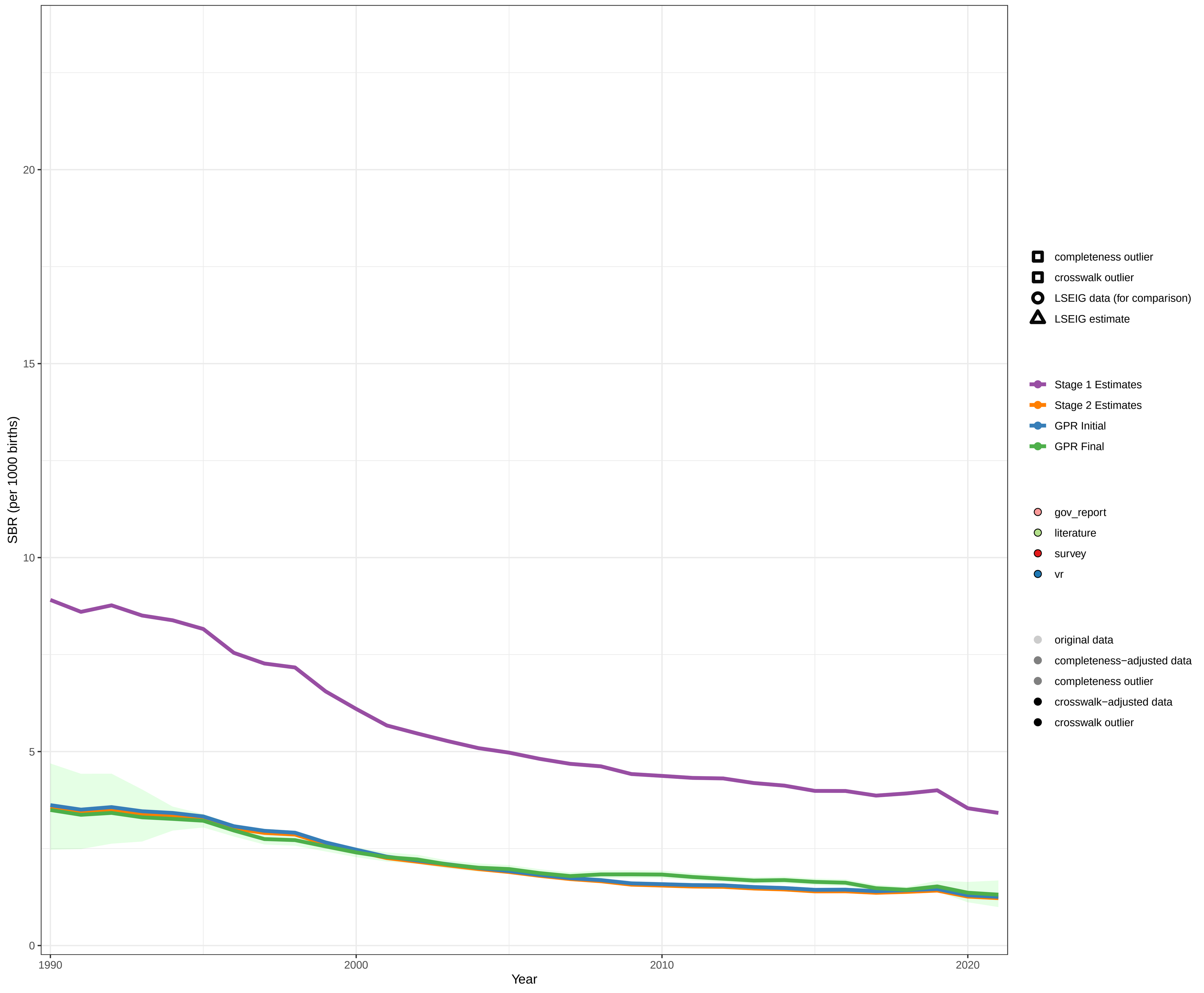

Year	Total Stillbirths (in thousands)	Total Stillbirths (in thousands)	SBR (per 1000 births)	SBR (per 1000 births)	SBR/NMR	SBR/NMR
	20 weeks	28 weeks	20 weeks	28 weeks	20 weeks	28 weeks
1990	0.1 (0.0 to 0.1)	0.1 (0.0 to 0.1)	4.7 (3.3 to 6.3)	3.5 (2.5 to 4.7)	1.67 (1.17 to 2.24)	1.24 (0.88 to 1.67)
1995	0.1 (0.1 to 0.1)	0.0 (0.0 to 0.0)	4.4 (4.1 to 4.6)	3.2 (3.0 to 3.4)	1.83 (1.72 to 1.92)	1.34 (1.27 to 1.41)
2000	0.0 (0.0 to 0.0)	0.0 (0.0 to 0.0)	3.3 (3.1 to 3.4)	2.4 (2.3 to 2.5)	1.94 (1.84 to 2.04)	1.43 (1.35 to 1.50)
2005	0.0 (0.0 to 0.0)	0.0 (0.0 to 0.0)	2.7 (2.5 to 2.8)	2.0 (1.9 to 2.1)	2.05 (1.95 to 2.16)	1.52 (1.44 to 1.60)
2010	0.0 (0.0 to 0.0)	0.0 (0.0 to 0.0)	2.5 (2.3 to 2.6)	1.8 (1.7 to 1.9)	2.27 (2.15 to 2.40)	1.69 (1.60 to 1.78)
2015	0.0 (0.0 to 0.0)	0.0 (0.0 to 0.0)	2.2 (2.1 to 2.3)	1.6 (1.6 to 1.7)	2.35 (2.22 to 2.47)	1.75 (1.66 to 1.84)
2020	0.0 (0.0 to 0.0)	0.0 (0.0 to 0.0)	1.8 (1.5 to 2.2)	1.4 (1.1 to 1.6)	2.33 (1.90 to 2.81)	1.72 (1.41 to 2.07)
2021	0.0 (0.0 to 0.0)	0.0 (0.0 to 0.0)	1.8 (1.3 to 2.3)	1.3 (1.0 to 1.7)	2.35 (1.77 to 3.01)	1.73 (1.31 to 2.22)

Fukui JPN_35441 High−income Asia Pacific Stillbirth Rate / Neonatal Mortality Rate (SBR/NMR) − 20 weeks
lambda = 1 ; zeta = 0.6 ; scale = 15 ; amp = 0.028

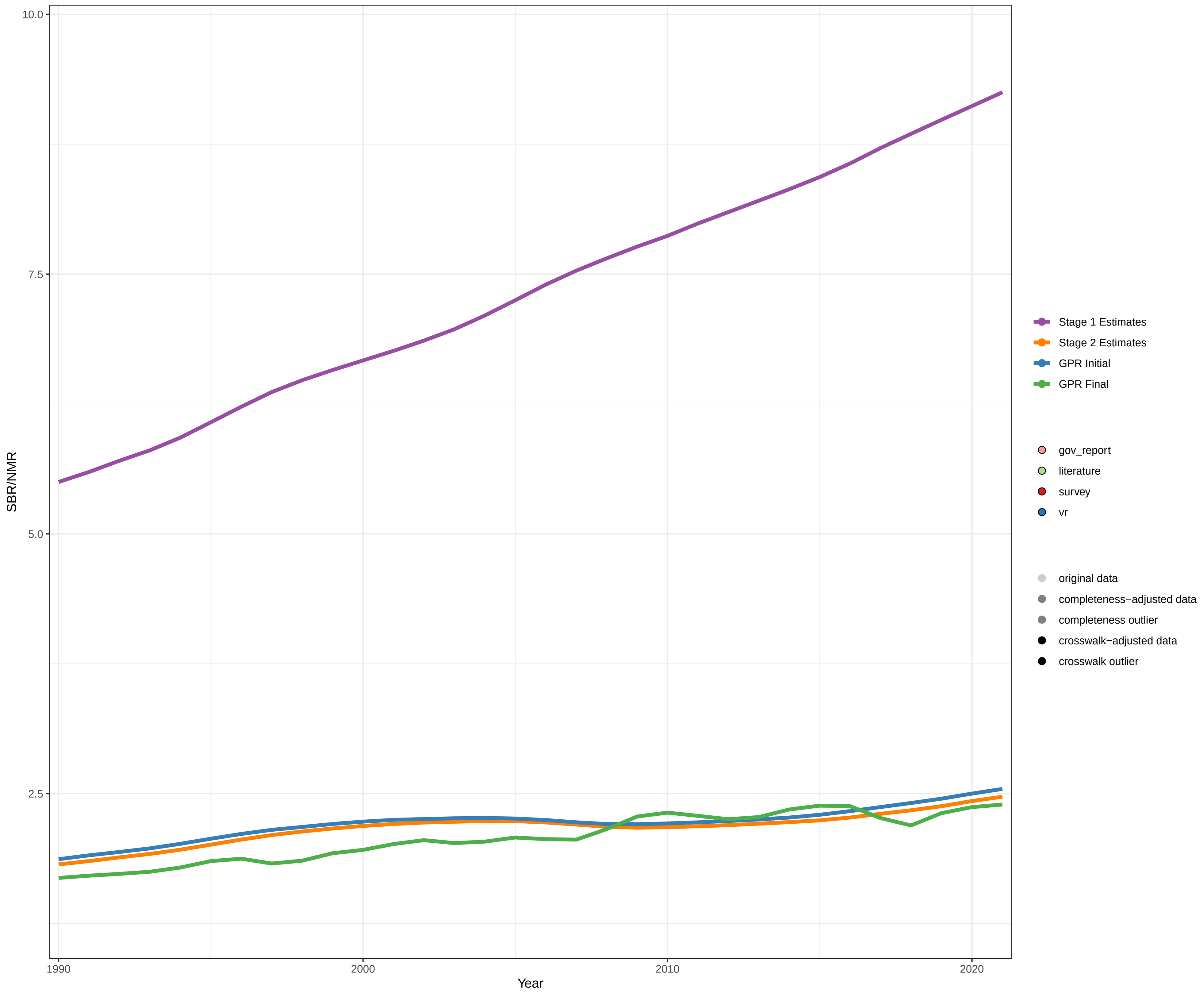

Fukui JPN_35441 High−income Asia Pacific Stillbirth Rate / Neonatal Mortality Rate (SBR/NMR) − 28 weeks
lambda = 1 ; zeta = 0.6 ; scale = 15 ; amp = 0.028

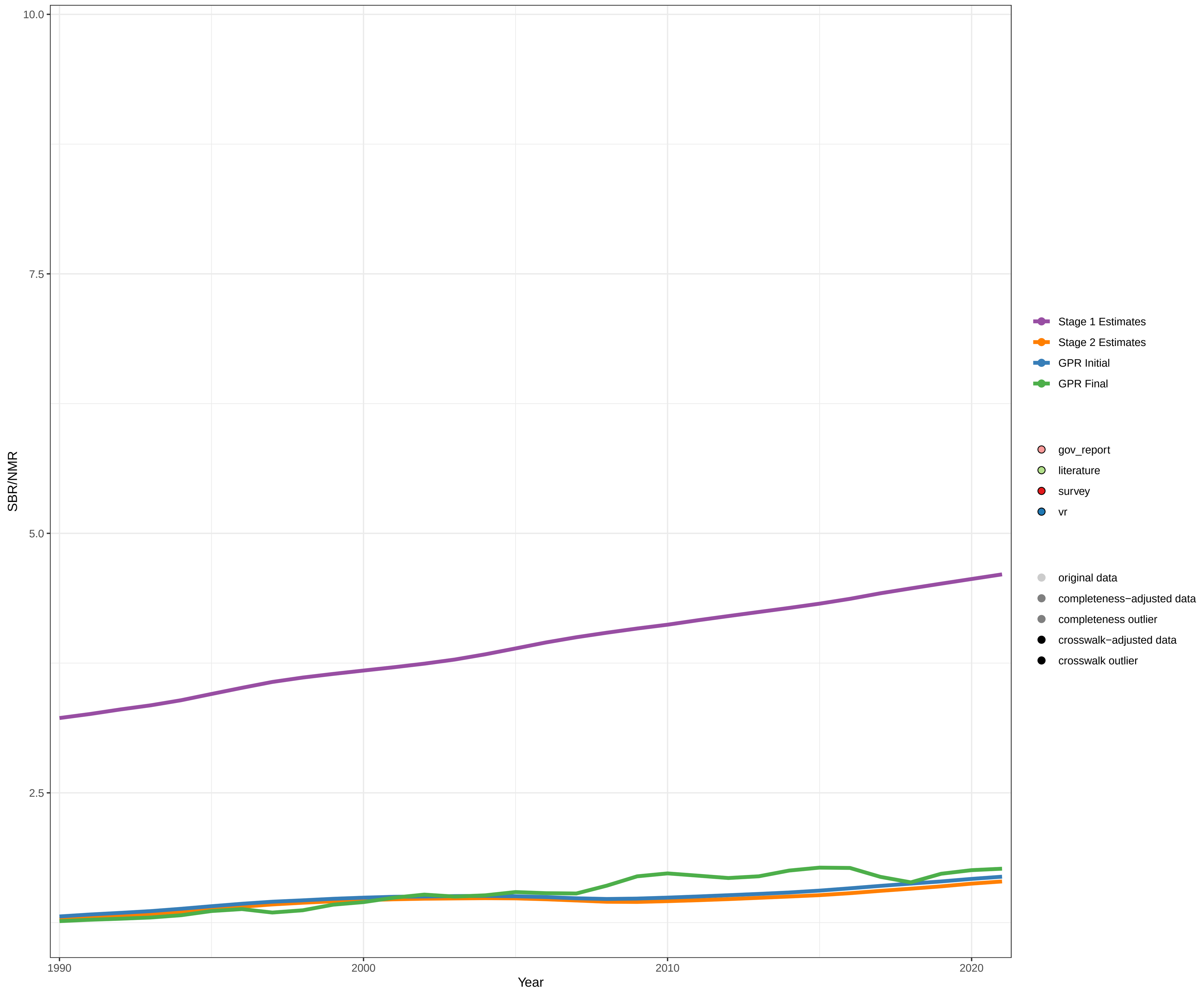

Definition	Years	Outlier	Source.Type	Title

Fukui JPN_35441 High−income Asia Pacific Stillbirth Rate (SBR) − 20 weeks
lambda = 1 ; zeta = 0.6 ; scale = 15 ; amp = 0.028

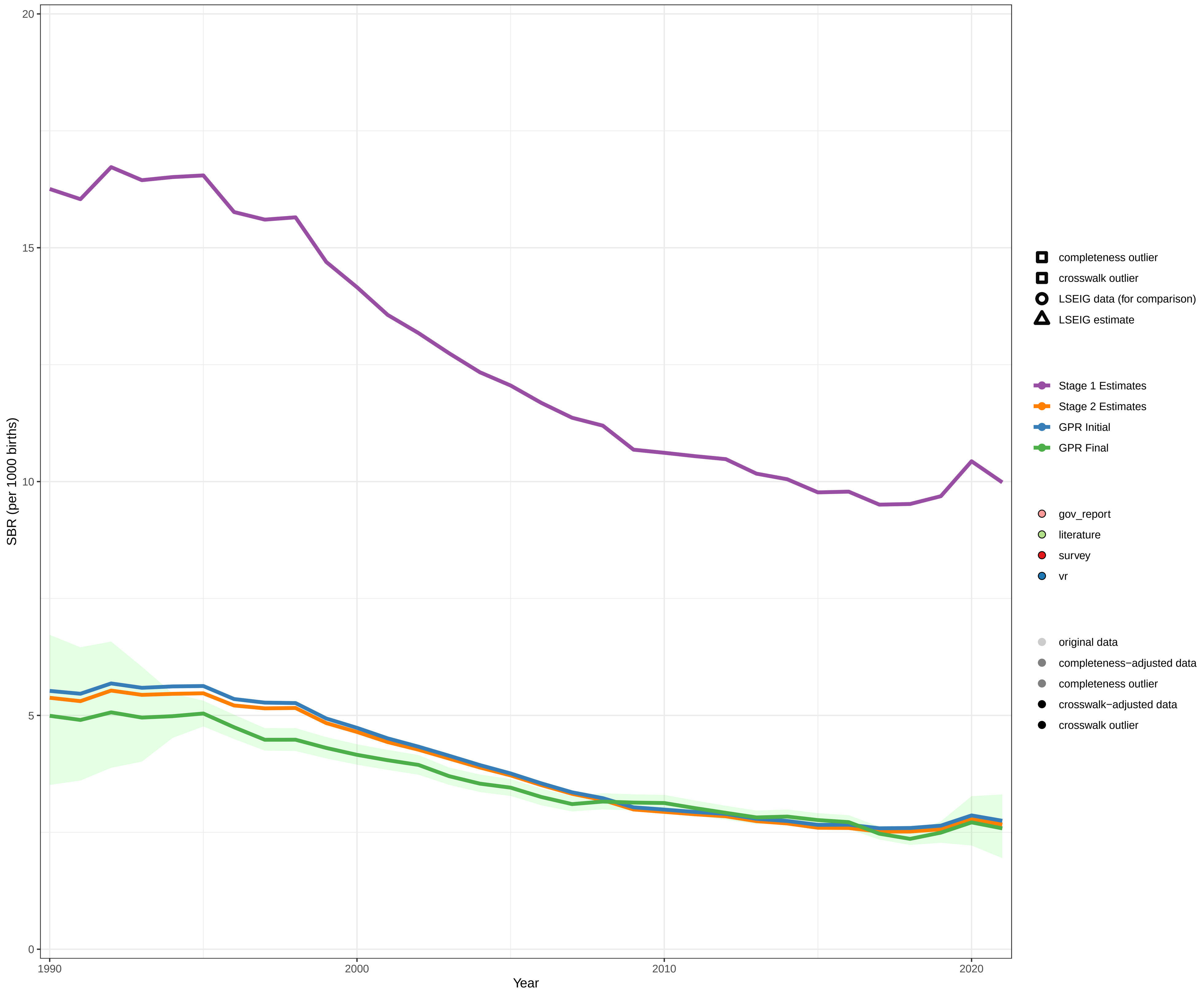

Fukui JPN_35441 High−income Asia Pacific Stillbirth Rate (SBR) − 28 weeks
lambda = 1 ; zeta = 0.6 ; scale = 15 ; amp = 0.028

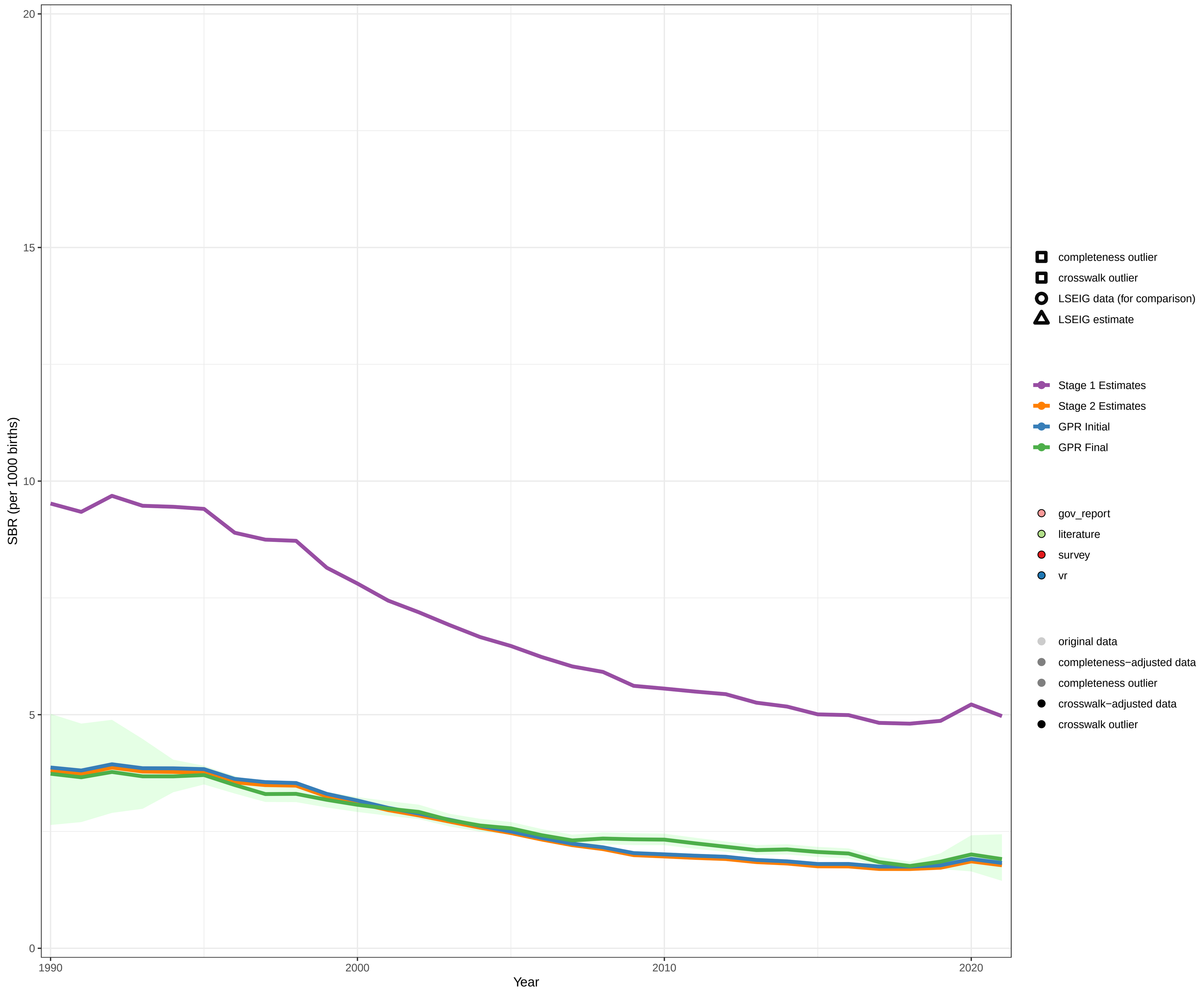

Year	Total Stillbirths (in thousands)	Total Stillbirths (in thousands)	SBR (per 1000 births)	SBR (per 1000 births)	SBR/NMR	SBR/NMR
	20 weeks	28 weeks	20 weeks	28 weeks	20 weeks	28 weeks
1990	0.0 (0.0 to 0.1)	0.0 (0.0 to 0.0)	5.0 (3.5 to 6.7)	3.7 (2.6 to 5.0)	1.69 (1.19 to 2.27)	1.26 (0.89 to 1.70)
1995	0.0 (0.0 to 0.0)	0.0 (0.0 to 0.0)	5.0 (4.8 to 5.3)	3.7 (3.5 to 3.9)	1.85 (1.75 to 1.95)	1.36 (1.29 to 1.43)
2000	0.0 (0.0 to 0.0)	0.0 (0.0 to 0.0)	4.2 (3.9 to 4.4)	3.1 (2.9 to 3.2)	1.96 (1.86 to 2.06)	1.45 (1.37 to 1.52)
2005	0.0 (0.0 to 0.0)	0.0 (0.0 to 0.0)	3.5 (3.3 to 3.6)	2.6 (2.4 to 2.7)	2.08 (1.97 to 2.19)	1.54 (1.47 to 1.63)
2010	0.0 (0.0 to 0.0)	0.0 (0.0 to 0.0)	3.1 (3.0 to 3.3)	2.3 (2.2 to 2.5)	2.32 (2.20 to 2.45)	1.72 (1.64 to 1.82)
2015	0.0 (0.0 to 0.0)	0.0 (0.0 to 0.0)	2.8 (2.6 to 2.9)	2.1 (2.0 to 2.2)	2.38 (2.26 to 2.51)	1.78 (1.69 to 1.87)
2020	0.0 (0.0 to 0.0)	0.0 (0.0 to 0.0)	2.7 (2.2 to 3.3)	2.0 (1.6 to 2.4)	2.37 (1.94 to 2.86)	1.76 (1.44 to 2.11)
2021	0.0 (0.0 to 0.0)	0.0 (0.0 to 0.0)	2.6 (1.9 to 3.3)	1.9 (1.4 to 2.4)	2.40 (1.81 to 3.07)	1.77 (1.34 to 2.26)

Fukuoka JPN_35463 High−income Asia Pacific Stillbirth Rate / Neonatal Mortality Rate (SBR/NMR) − 20 weeks
lambda = 1 ; zeta = 0.6 ; scale = 15 ; amp = 0.028

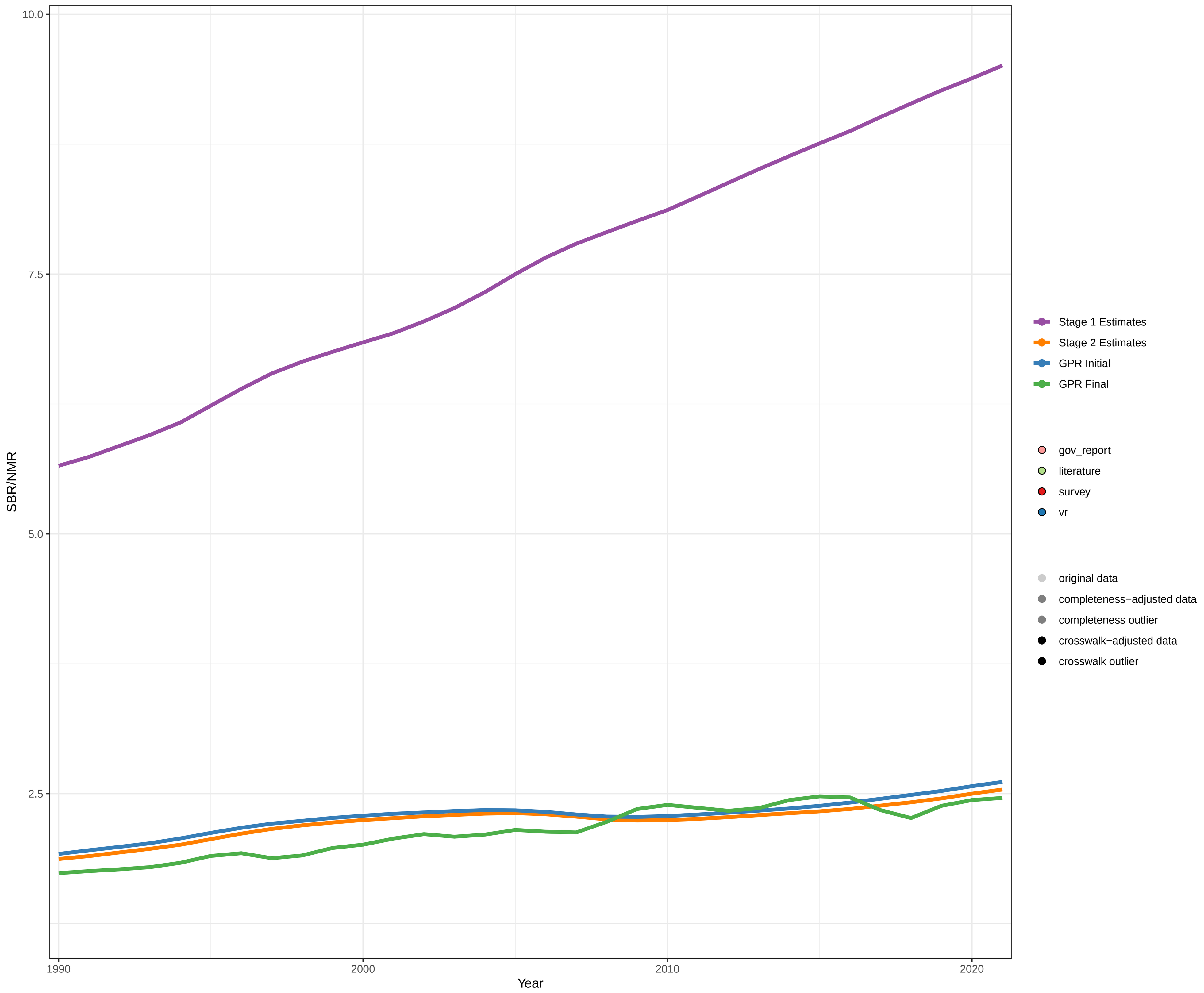

Fukuoka JPN_35463 High−income Asia Pacific Stillbirth Rate / Neonatal Mortality Rate (SBR/NMR) − 28 weeks
lambda = 1 ; zeta = 0.6 ; scale = 15 ; amp = 0.028

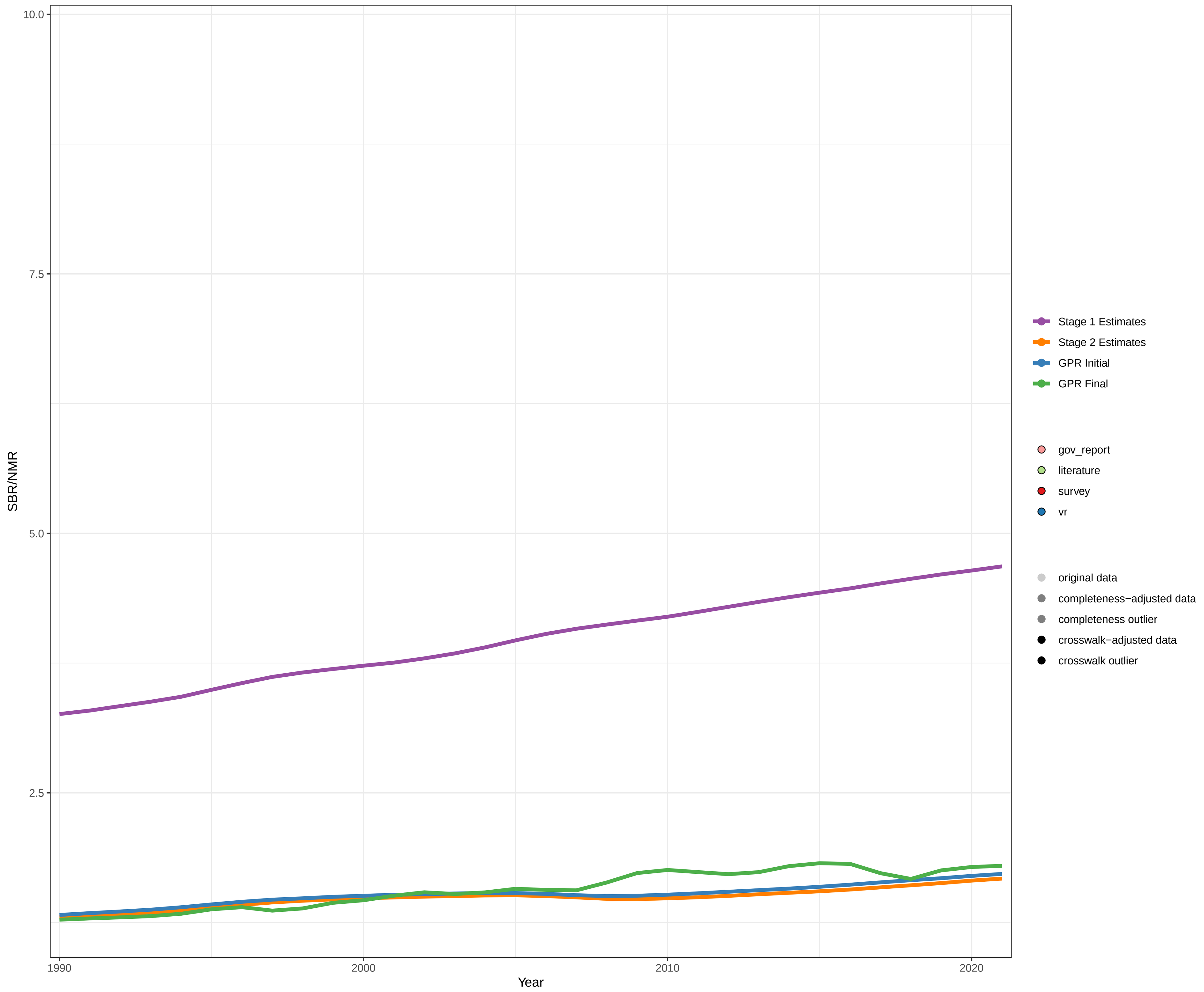

Definition	Years	Outlier	Source.Type	Title

Fukuoka JPN_35463 High−income Asia Pacific Stillbirth Rate (SBR) − 20 weeks
lambda = 1 ; zeta = 0.6 ; scale = 15 ; amp = 0.028

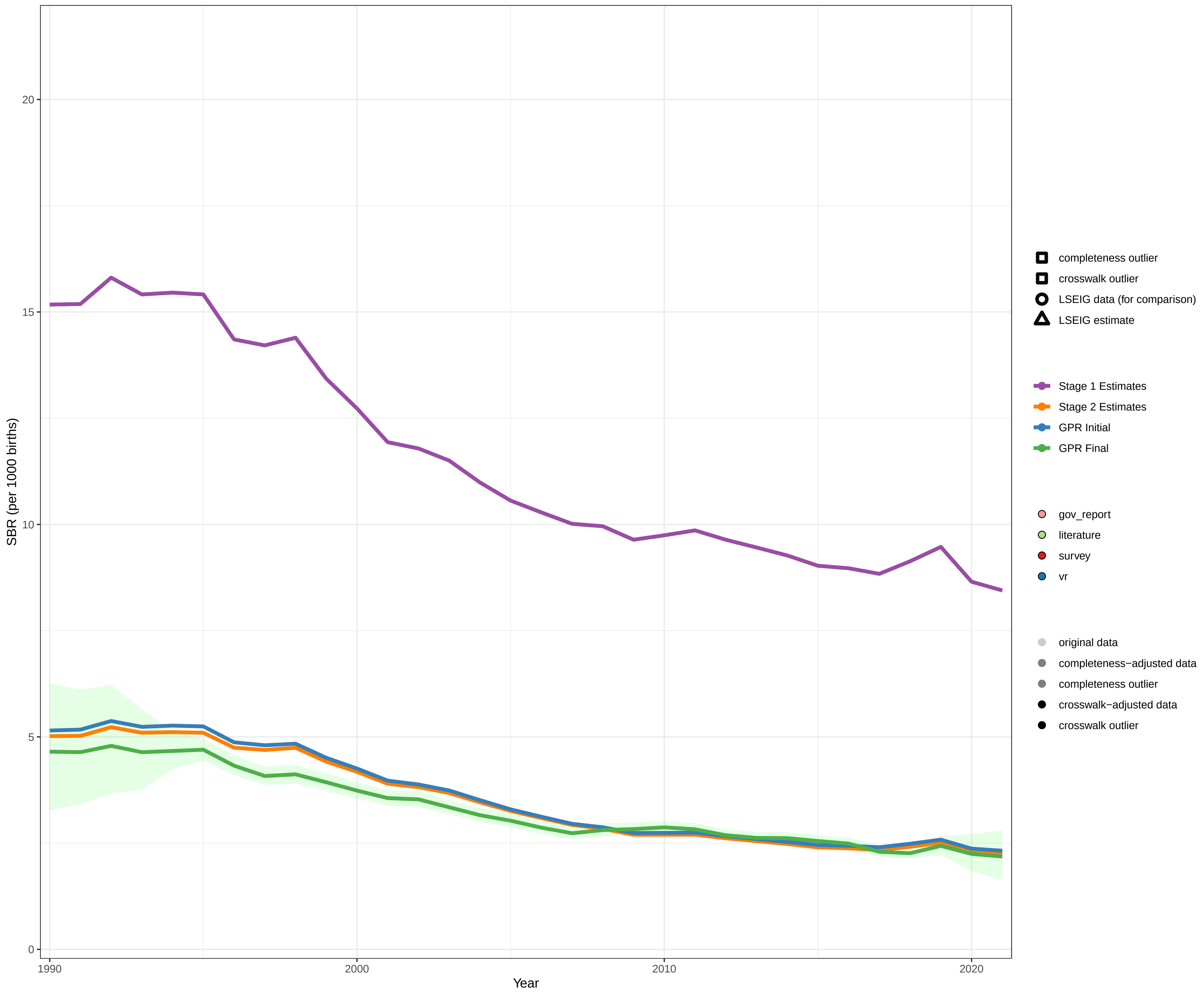

Fukuoka JPN_35463 High−income Asia Pacific Stillbirth Rate (SBR) − 28 weeks
lambda = 1 ; zeta = 0.6 ; scale = 15 ; amp = 0.028

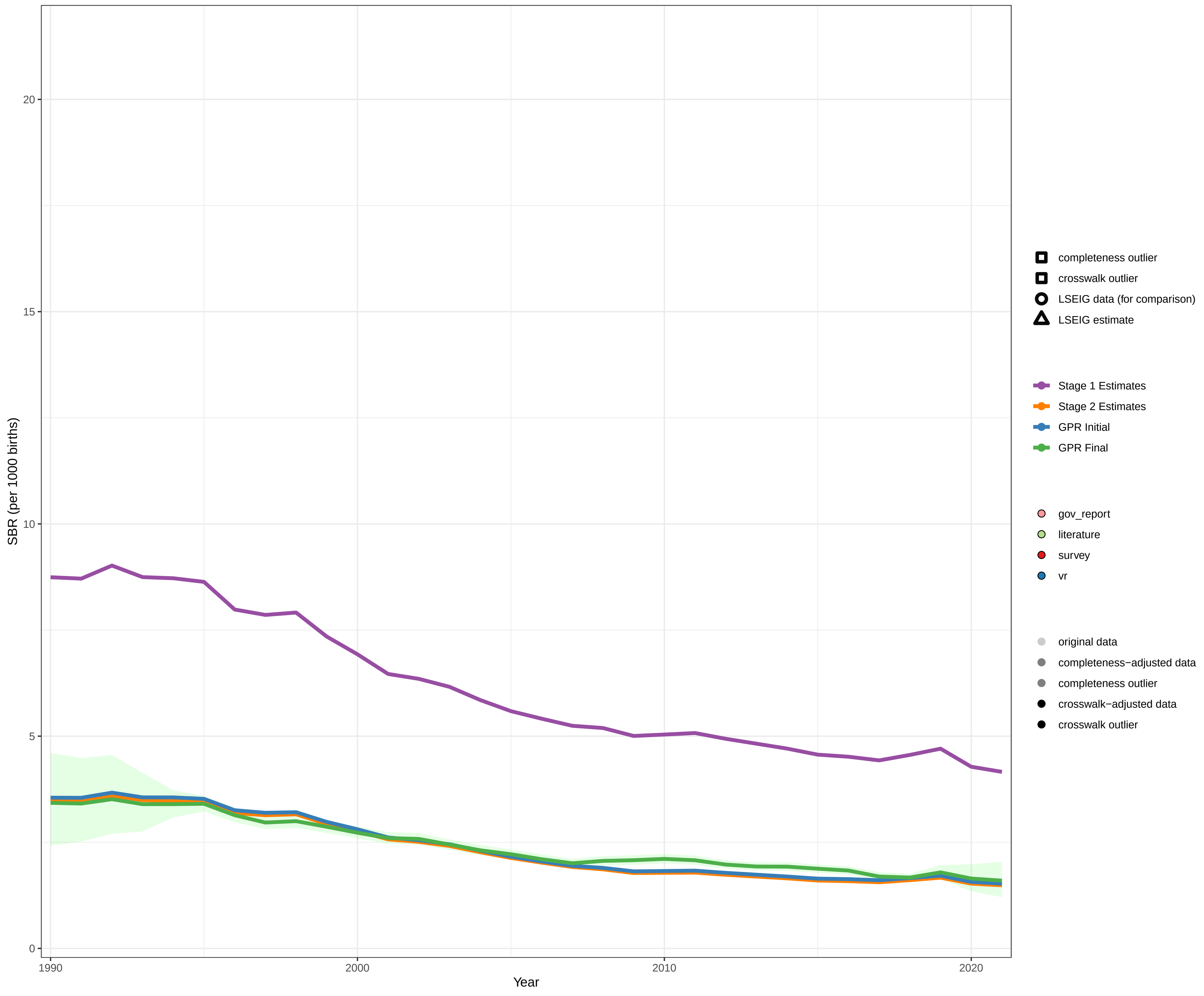

Year	Total Stillbirths (in thousands)	Total Stillbirths (in thousands)	SBR (per 1000 births)	SBR (per 1000 births)	SBR/NMR	SBR/NMR
	20 weeks	28 weeks	20 weeks	28 weeks	20 weeks	28 weeks
1990	0.2 (0.2 to 0.3)	0.2 (0.1 to 0.2)	4.7 (3.3 to 6.3)	3.4 (2.4 to 4.6)	1.73 (1.22 to 2.34)	1.28 (0.90 to 1.72)
1995	0.2 (0.2 to 0.2)	0.2 (0.2 to 0.2)	4.7 (4.4 to 5.0)	3.4 (3.2 to 3.6)	1.90 (1.80 to 2.00)	1.38 (1.30 to 1.45)
2000	0.2 (0.2 to 0.2)	0.1 (0.1 to 0.1)	3.7 (3.5 to 3.9)	2.7 (2.6 to 2.9)	2.01 (1.91 to 2.12)	1.47 (1.39 to 1.54)
2005	0.1 (0.1 to 0.1)	0.1 (0.1 to 0.1)	3.0 (2.9 to 3.2)	2.2 (2.1 to 2.3)	2.15 (2.04 to 2.27)	1.58 (1.50 to 1.66)
2010	0.1 (0.1 to 0.1)	0.1 (0.1 to 0.1)	2.9 (2.7 to 3.0)	2.1 (2.0 to 2.2)	2.39 (2.27 to 2.53)	1.76 (1.67 to 1.85)
2015	0.1 (0.1 to 0.1)	0.1 (0.1 to 0.1)	2.5 (2.4 to 2.7)	1.9 (1.8 to 2.0)	2.47 (2.34 to 2.61)	1.82 (1.73 to 1.92)
2020	0.1 (0.1 to 0.1)	0.1 (0.1 to 0.1)	2.2 (1.8 to 2.7)	1.6 (1.3 to 2.0)	2.44 (1.99 to 2.94)	1.79 (1.46 to 2.15)
2021	0.1 (0.1 to 0.1)	0.1 (0.0 to 0.1)	2.2 (1.6 to 2.8)	1.6 (1.2 to 2.0)	2.46 (1.85 to 3.15)	1.80 (1.36 to 2.30)

Fukushima JPN_35430 High−income Asia Pacific Stillbirth Rate / Neonatal Mortality Rate (SBR/NMR) − 20 weeks
lambda = 1 ; zeta = 0.6 ; scale = 15 ; amp = 0.028

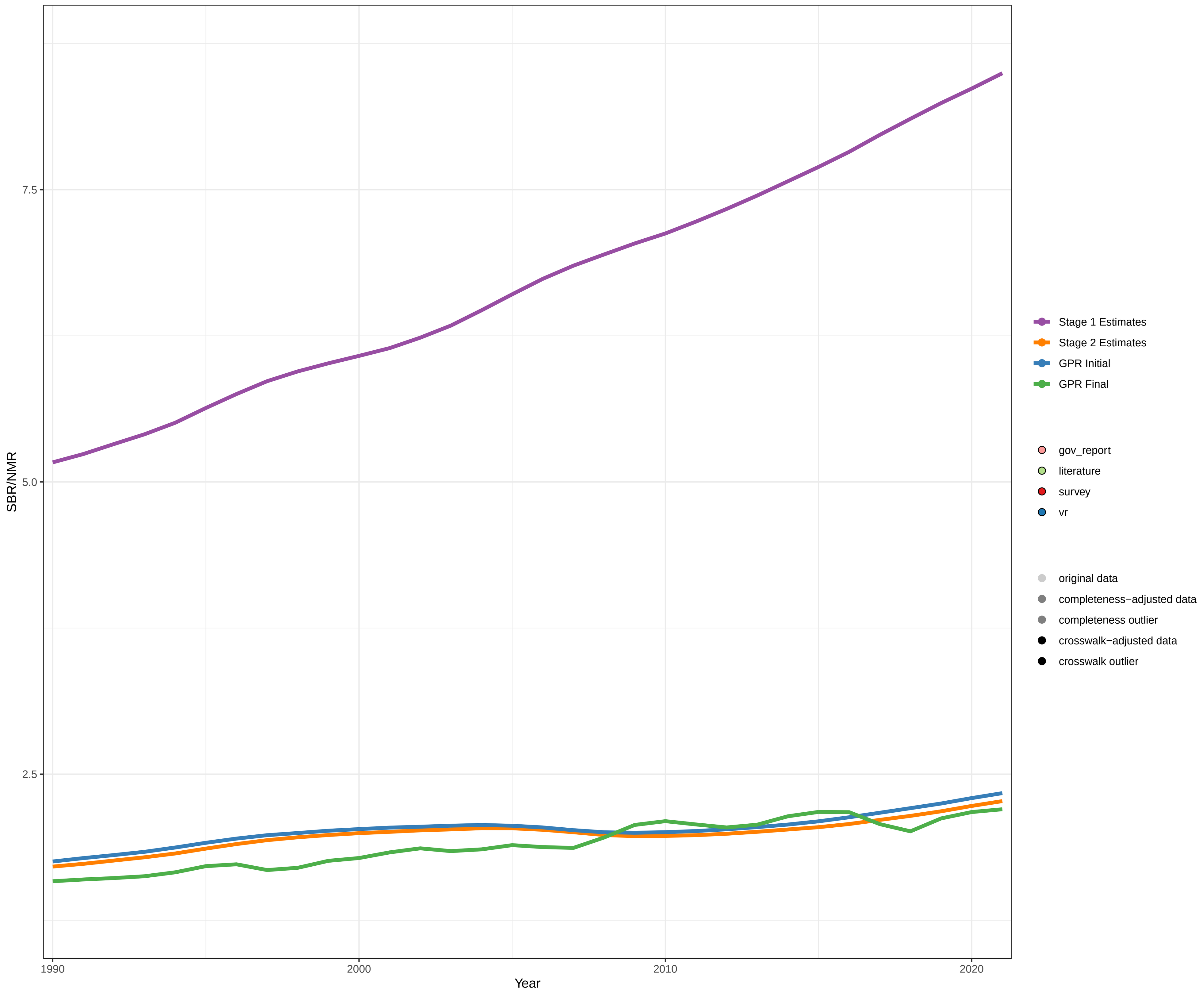

Fukushima JPN_35430 High−income Asia Pacific Stillbirth Rate / Neonatal Mortality Rate (SBR/NMR) − 28 weeks
lambda = 1 ; zeta = 0.6 ; scale = 15 ; amp = 0.028

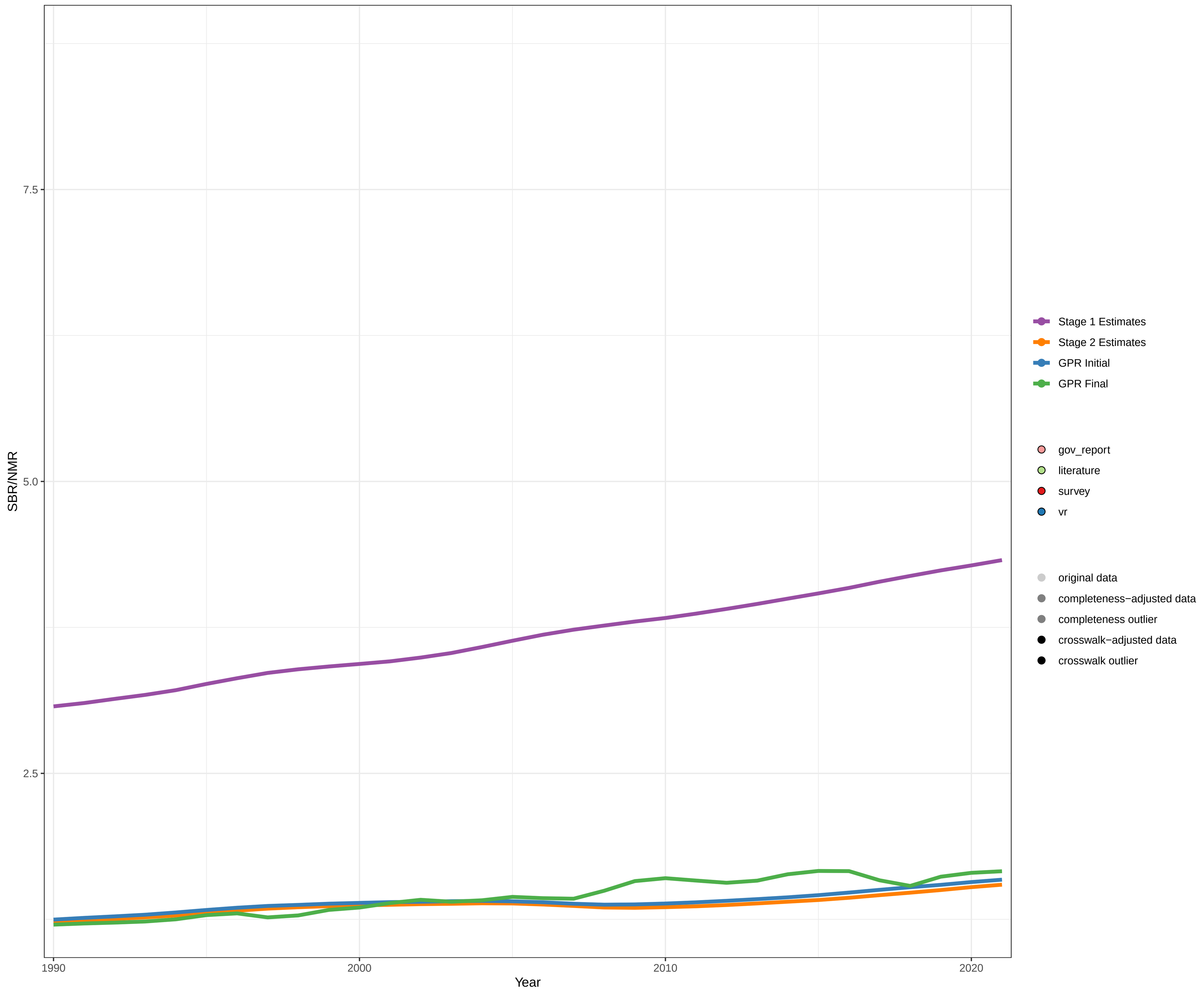

Definition	Years	Outlier	Source.Type	Title

Fukushima JPN_35430 High−income Asia Pacific Stillbirth Rate (SBR) − 20 weeks
lambda = 1 ; zeta = 0.6 ; scale = 15 ; amp = 0.028

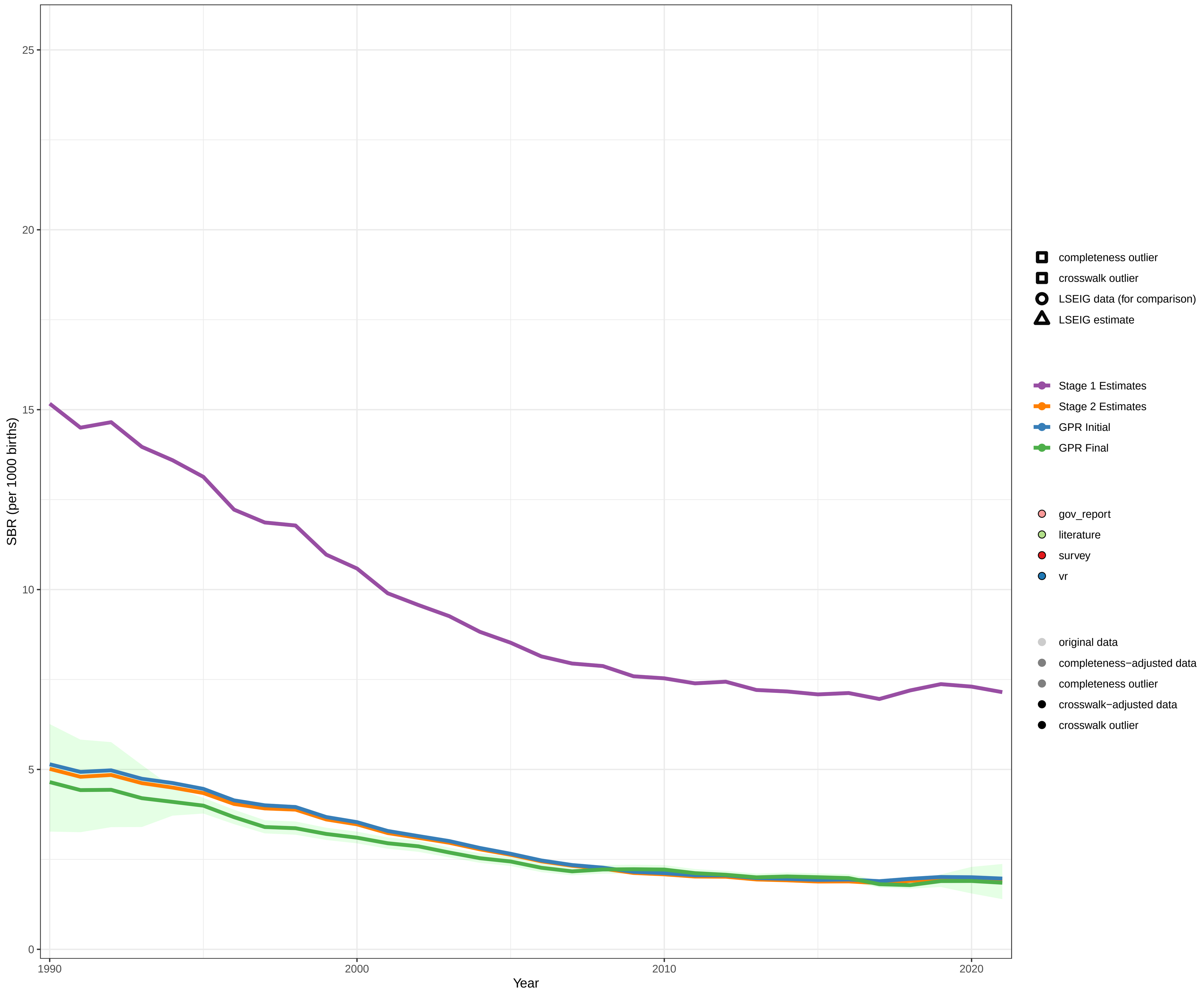

Fukushima JPN_35430 High−income Asia Pacific Stillbirth Rate (SBR) − 28 weeks
lambda = 1 ; zeta = 0.6 ; scale = 15 ; amp = 0.028

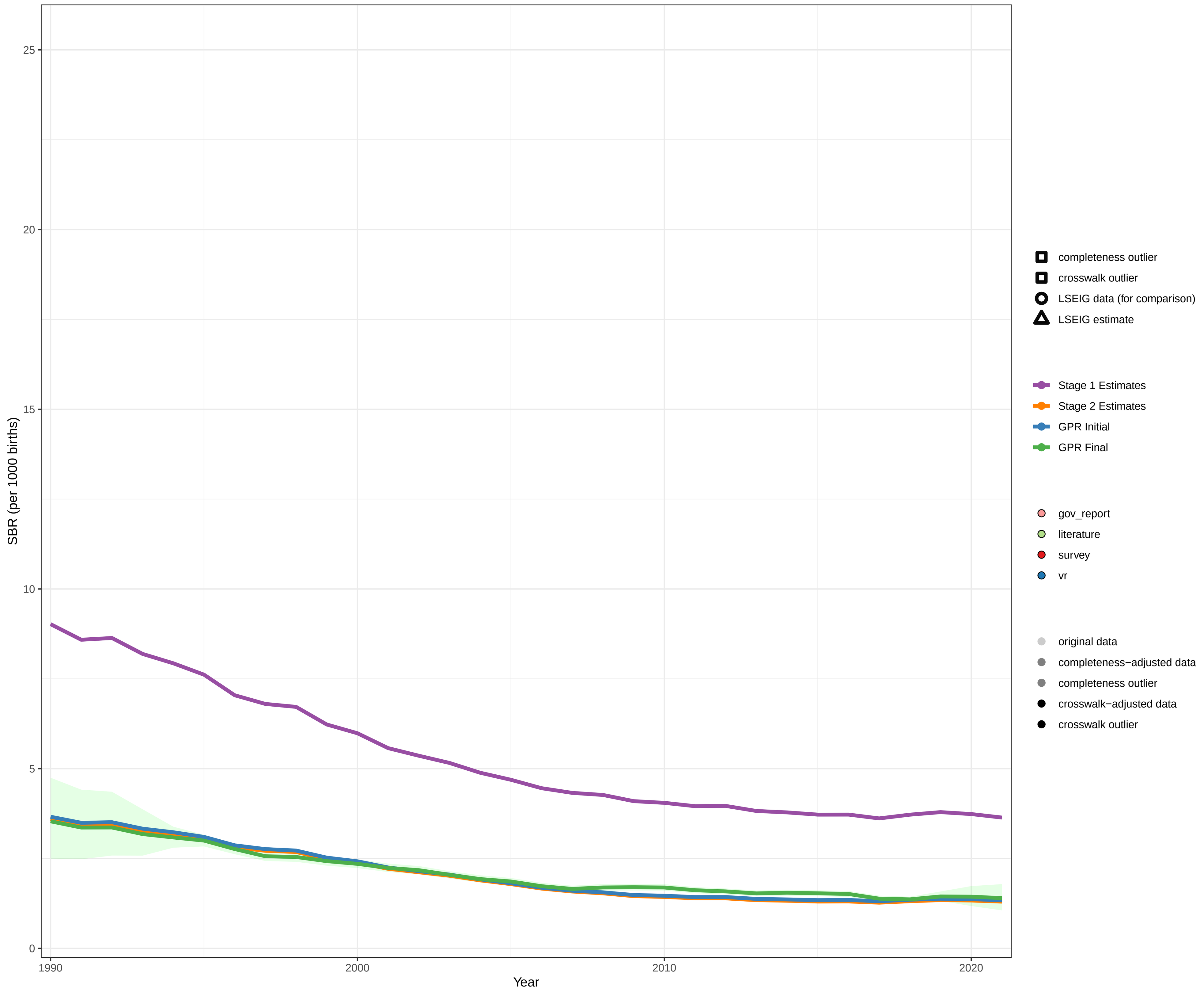

Year	Total Stillbirths (in thousands)	Total Stillbirths (in thousands)	SBR (per 1000 births)	SBR (per 1000 births)	SBR/NMR	SBR/NMR
	20 weeks	28 weeks	20 weeks	28 weeks	20 weeks	28 weeks
1990	0.1 (0.1 to 0.1)	0.1 (0.1 to 0.1)	4.6 (3.3 to 6.3)	3.5 (2.5 to 4.7)	1.58 (1.11 to 2.13)	1.20 (0.85 to 1.62)
1995	0.1 (0.1 to 0.1)	0.1 (0.1 to 0.1)	4.0 (3.8 to 4.2)	3.0 (2.8 to 3.2)	1.71 (1.62 to 1.81)	1.29 (1.22 to 1.36)
2000	0.1 (0.1 to 0.1)	0.0 (0.0 to 0.1)	3.1 (2.9 to 3.3)	2.4 (2.2 to 2.5)	1.78 (1.69 to 1.88)	1.35 (1.28 to 1.42)
2005	0.0 (0.0 to 0.0)	0.0 (0.0 to 0.0)	2.4 (2.3 to 2.6)	1.9 (1.8 to 2.0)	1.89 (1.80 to 1.99)	1.44 (1.37 to 1.52)
2010	0.0 (0.0 to 0.0)	0.0 (0.0 to 0.0)	2.2 (2.1 to 2.3)	1.7 (1.6 to 1.8)	2.10 (1.99 to 2.22)	1.60 (1.52 to 1.69)
2015	0.0 (0.0 to 0.0)	0.0 (0.0 to 0.0)	2.0 (1.9 to 2.1)	1.5 (1.5 to 1.6)	2.18 (2.06 to 2.29)	1.66 (1.58 to 1.75)
2020	0.0 (0.0 to 0.0)	0.0 (0.0 to 0.0)	1.9 (1.6 to 2.3)	1.4 (1.2 to 1.7)	2.18 (1.78 to 2.63)	1.65 (1.35 to 1.99)
2021	0.0 (0.0 to 0.0)	0.0 (0.0 to 0.0)	1.9 (1.4 to 2.4)	1.4 (1.1 to 1.8)	2.20 (1.66 to 2.82)	1.66 (1.26 to 2.12)

Gifu JPN_35444 High−income Asia Pacific Stillbirth Rate / Neonatal Mortality Rate (SBR/NMR) − 20 weeks
lambda = 1 ; zeta = 0.6 ; scale = 15 ; amp = 0.028

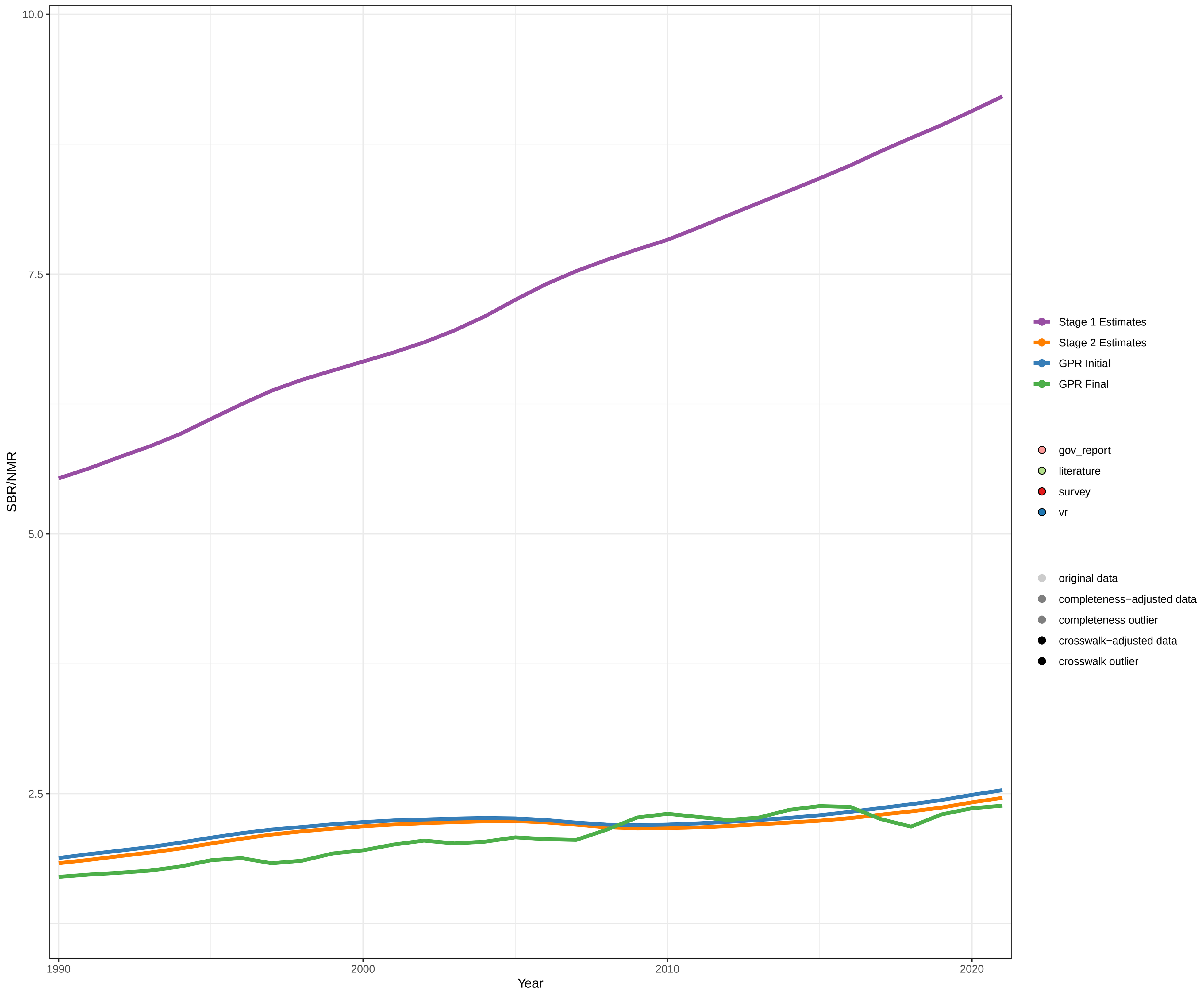

Gifu JPN_35444 High−income Asia Pacific Stillbirth Rate / Neonatal Mortality Rate (SBR/NMR) − 28 weeks
lambda = 1 ; zeta = 0.6 ; scale = 15 ; amp = 0.028

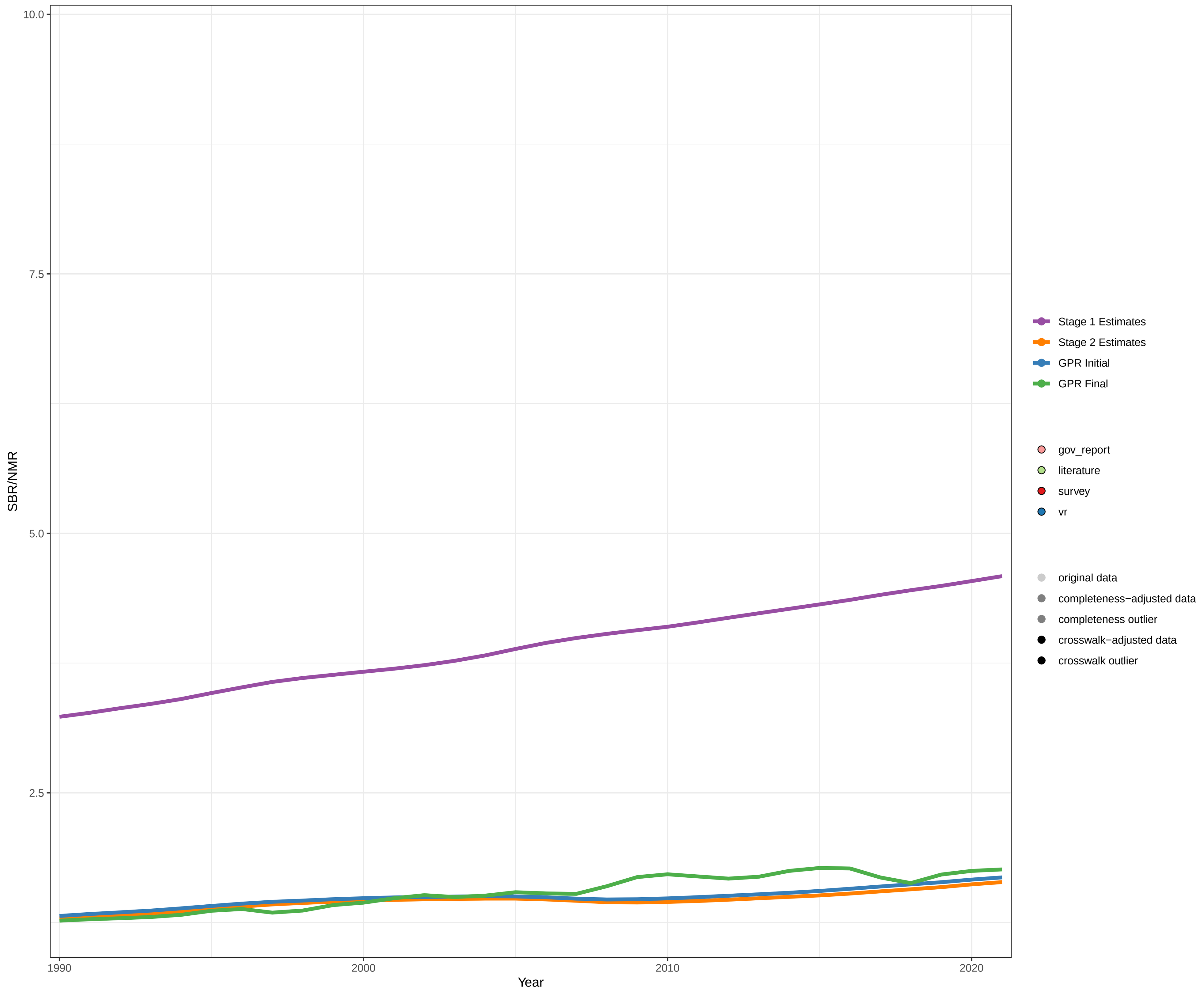

Definition	Years	Outlier	Source.Type	Title

Gifu JPN_35444 High−income Asia Pacific Stillbirth Rate (SBR) − 20 weeks
lambda = 1 ; zeta = 0.6 ; scale = 15 ; amp = 0.028

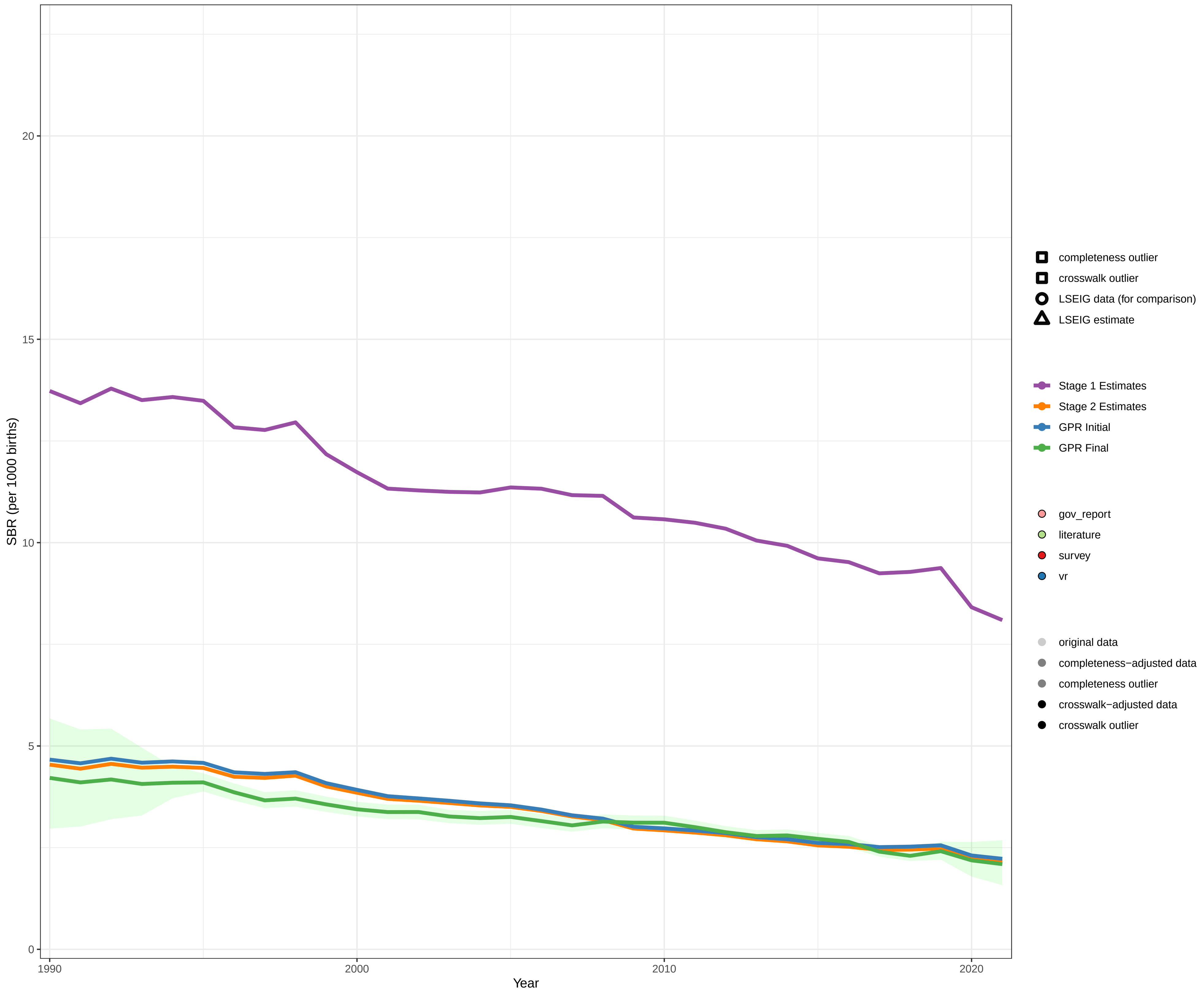

Gifu JPN_35444 High−income Asia Pacific Stillbirth Rate (SBR) − 28 weeks
lambda = 1 ; zeta = 0.6 ; scale = 15 ; amp = 0.028

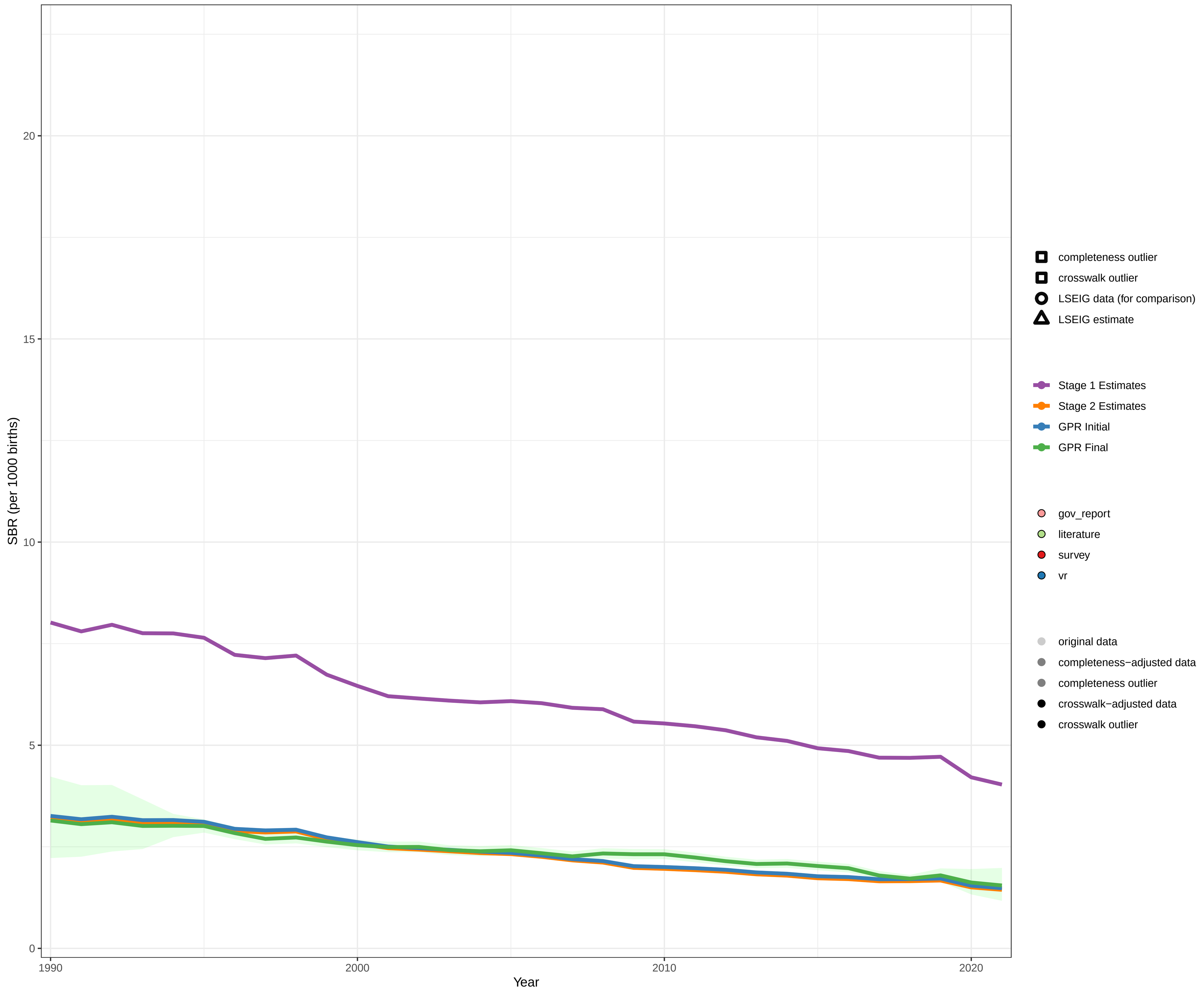

Year	Total Stillbirths (in thousands)	Total Stillbirths (in thousands)	SBR (per 1000 births)	SBR (per 1000 births)	SBR/NMR	SBR/NMR
	20 weeks	28 weeks	20 weeks	28 weeks	20 weeks	28 weeks
1990	0.1<br>(0.1 to 0.1)	0.1<br>(0.0 to 0.1)	4.2<br>(3.0 to 5.7)	3.1<br>(2.2 to 4.2)	1.70<br>(1.20 to 2.29)	1.27<br>(0.90 to 1.70)
1995	0.1<br>(0.1 to 0.1)	0.1<br>(0.1 to 0.1)	4.1<br>(3.9 to 4.3)	3.0<br>(2.8 to 3.2)	1.86<br>(1.76 to 1.96)	1.36<br>(1.29 to 1.44)
2000	0.1<br>(0.1 to 0.1)	0.1<br>(0.0 to 0.1)	3.4<br>(3.3 to 3.6)	2.5<br>(2.4 to 2.7)	1.95<br>(1.86 to 2.06)	1.44<br>(1.37 to 1.52)
2005	0.1<br>(0.1 to 0.1)	0.0<br>(0.0 to 0.0)	3.3<br>(3.1 to 3.4)	2.4<br>(2.3 to 2.5)	2.08<br>(1.97 to 2.19)	1.54<br>(1.46 to 1.62)
2010	0.1<br>(0.1 to 0.1)	0.0<br>(0.0 to 0.0)	3.1<br>(3.0 to 3.3)	2.3<br>(2.2 to 2.4)	2.31<br>(2.19 to 2.43)	1.72<br>(1.63 to 1.81)
2015	0.0<br>(0.0 to 0.0)	0.0<br>(0.0 to 0.0)	2.7<br>(2.6 to 2.9)	2.0<br>(1.9 to 2.1)	2.38<br>(2.26 to 2.51)	1.78<br>(1.68 to 1.87)
2020	0.0<br>(0.0 to 0.0)	0.0<br>(0.0 to 0.0)	2.2<br>(1.8 to 2.6)	1.6<br>(1.3 to 2.0)	2.36<br>(1.93 to 2.85)	1.75<br>(1.43 to 2.11)
2021	0.0<br>(0.0 to 0.0)	0.0<br>(0.0 to 0.0)	2.1<br>(1.6 to 2.7)	1.5<br>(1.2 to 2.0)	2.38<br>(1.80 to 3.05)	1.76<br>(1.33 to 2.25)

Gunma JPN_35433 High−income Asia Pacific Stillbirth Rate / Neonatal Mortality Rate (SBR/NMR) − 20 weeks
lambda = 1 ; zeta = 0.6 ; scale = 15 ; amp = 0.028

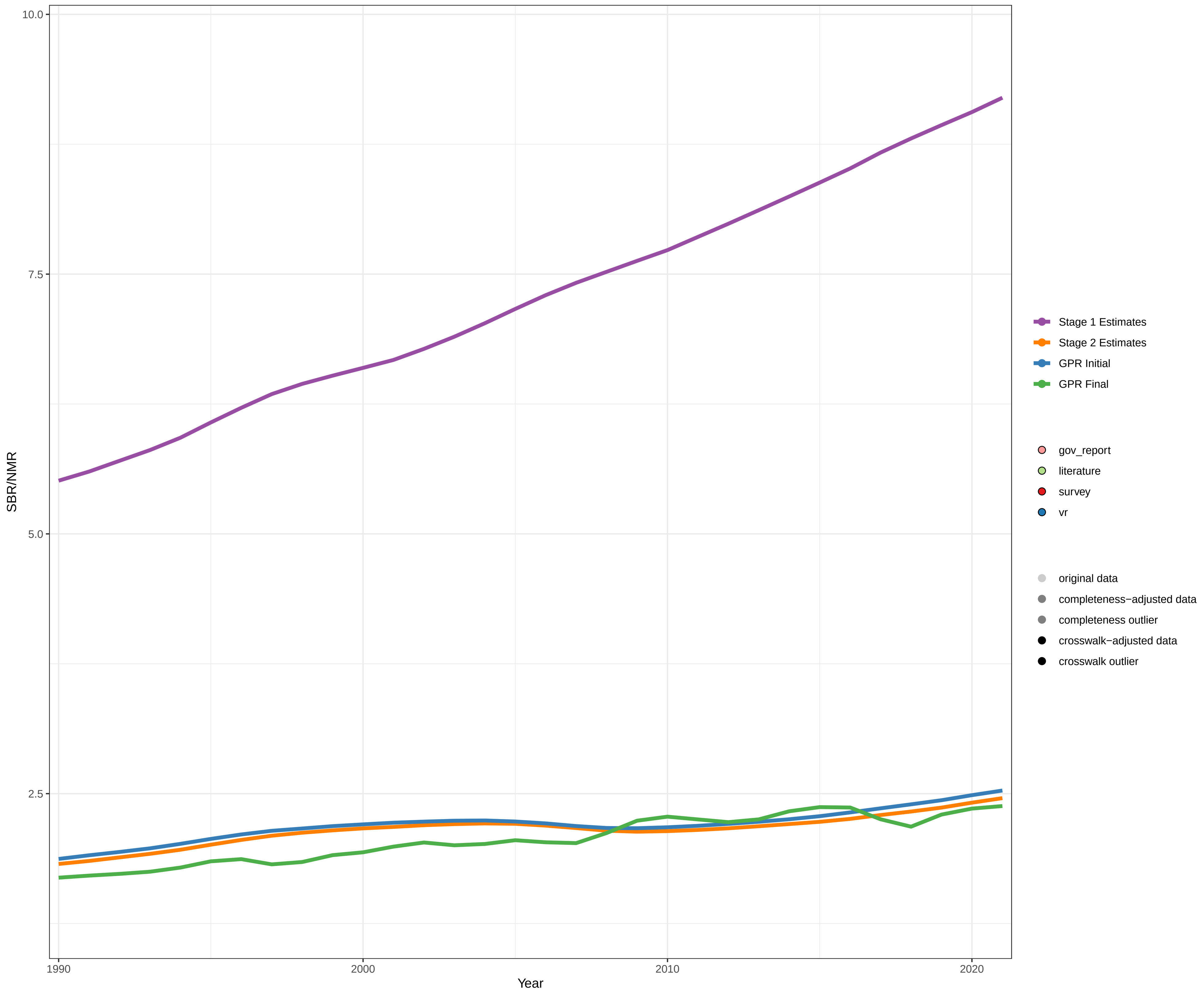

Gunma JPN_35433 High−income Asia Pacific Stillbirth Rate / Neonatal Mortality Rate (SBR/NMR) − 28 weeks
lambda = 1 ; zeta = 0.6 ; scale = 15 ; amp = 0.028

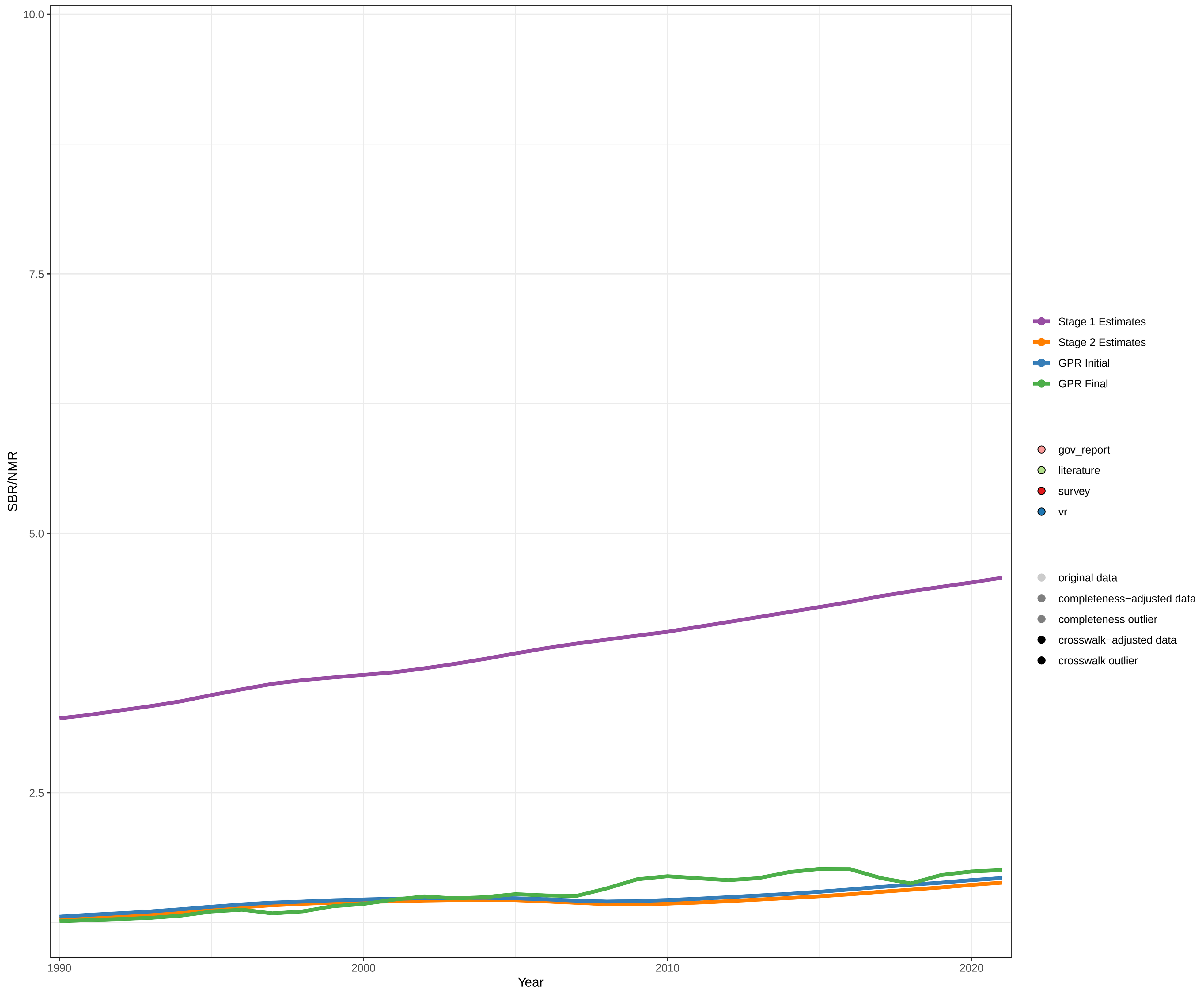

Definition	Years	Outlier	Source.Type	Title

Gunma JPN_35433 High−income Asia Pacific Stillbirth Rate (SBR) − 20 weeks
lambda = 1 ; zeta = 0.6 ; scale = 15 ; amp = 0.028

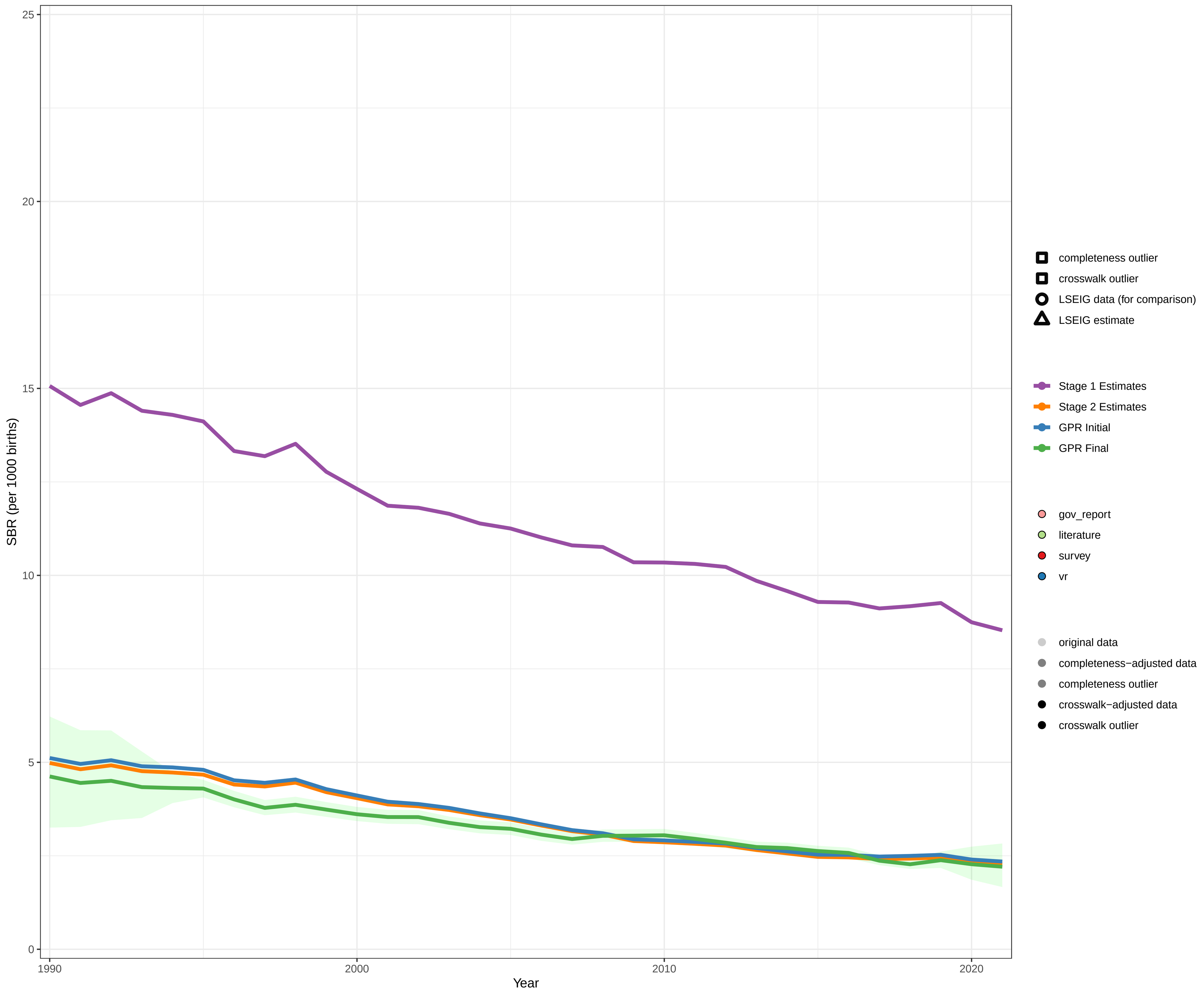

Gunma JPN_35433 High−income Asia Pacific Stillbirth Rate (SBR) − 28 weeks
lambda = 1 ; zeta = 0.6 ; scale = 15 ; amp = 0.028

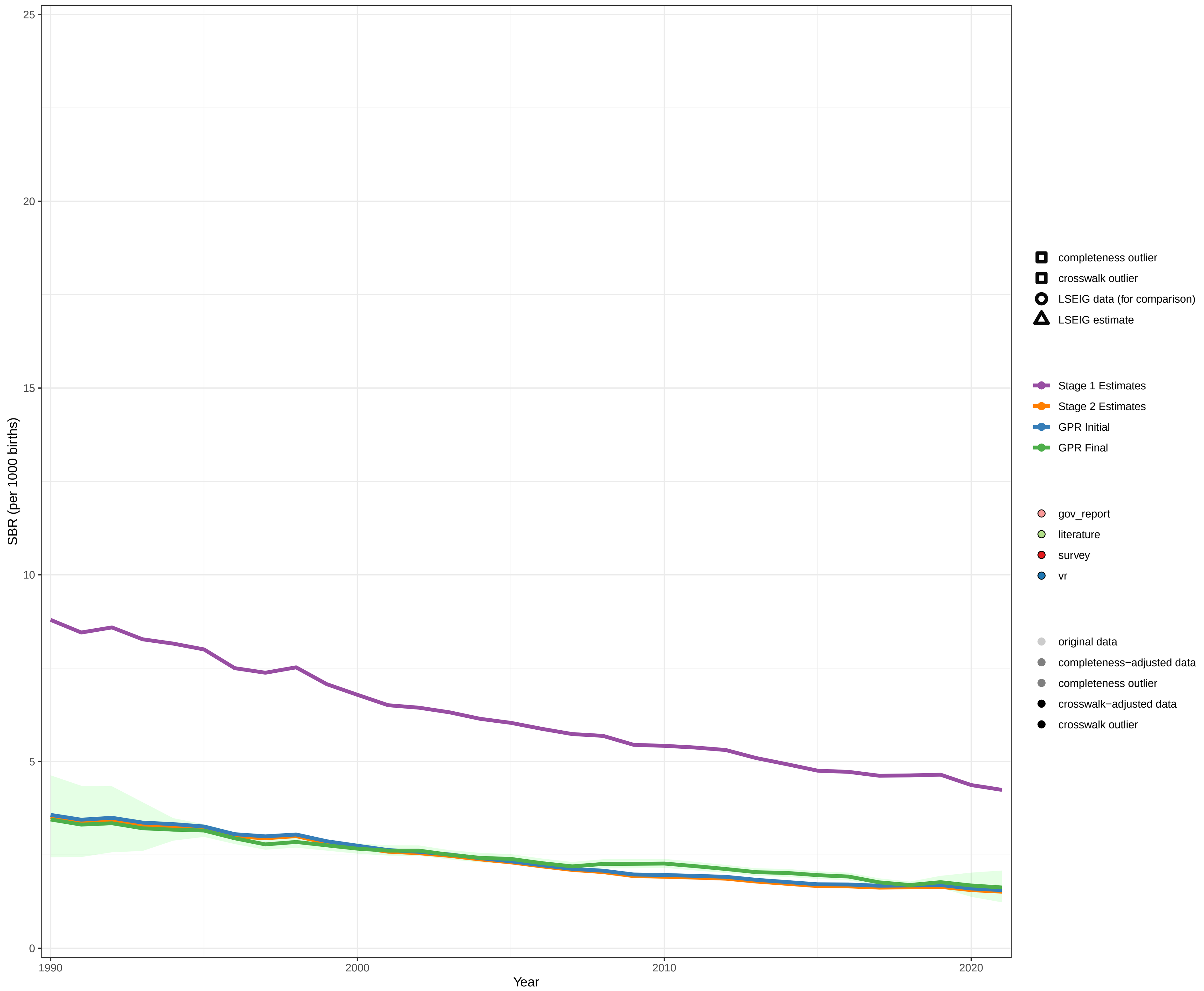

Year	Total Stillbirths (in thousands)	Total Stillbirths (in thousands)	SBR (per 1000 births)	SBR (per 1000 births)	SBR/NMR	SBR/NMR
	20 weeks	28 weeks	20 weeks	28 weeks	20 weeks	28 weeks
1990	0.1 (0.1 to 0.1)	0.1 (0.0 to 0.1)	4.6 (3.3 to 6.2)	3.4 (2.4 to 4.6)	1.69 (1.19 to 2.28)	1.26 (0.89 to 1.69)
1995	0.1 (0.1 to 0.1)	0.1 (0.1 to 0.1)	4.3 (4.1 to 4.5)	3.2 (3.0 to 3.3)	1.85 (1.75 to 1.95)	1.36 (1.28 to 1.43)
2000	0.1 (0.1 to 0.1)	0.1 (0.0 to 0.1)	3.6 (3.4 to 3.8)	2.7 (2.5 to 2.8)	1.94 (1.84 to 2.04)	1.43 (1.36 to 1.51)
2005	0.1 (0.1 to 0.1)	0.0 (0.0 to 0.0)	3.2 (3.1 to 3.4)	2.4 (2.3 to 2.5)	2.05 (1.95 to 2.16)	1.52 (1.45 to 1.60)
2010	0.0 (0.0 to 0.1)	0.0 (0.0 to 0.0)	3.0 (2.9 to 3.2)	2.3 (2.2 to 2.4)	2.28 (2.16 to 2.41)	1.70 (1.61 to 1.79)
2015	0.0 (0.0 to 0.0)	0.0 (0.0 to 0.0)	2.6 (2.5 to 2.8)	2.0 (1.9 to 2.1)	2.37 (2.25 to 2.50)	1.77 (1.68 to 1.86)
2020	0.0 (0.0 to 0.0)	0.0 (0.0 to 0.0)	2.3 (1.9 to 2.7)	1.7 (1.4 to 2.0)	2.36 (1.93 to 2.84)	1.74 (1.43 to 2.10)
2021	0.0 (0.0 to 0.0)	0.0 (0.0 to 0.0)	2.2 (1.7 to 2.8)	1.6 (1.2 to 2.1)	2.38 (1.79 to 3.05)	1.76 (1.33 to 2.25)

Hiroshima JPN_35457 High−income Asia Pacific Stillbirth Rate / Neonatal Mortality Rate (SBR/NMR) − 20 weeks
lambda = 1 ; zeta = 0.6 ; scale = 15 ; amp = 0.028

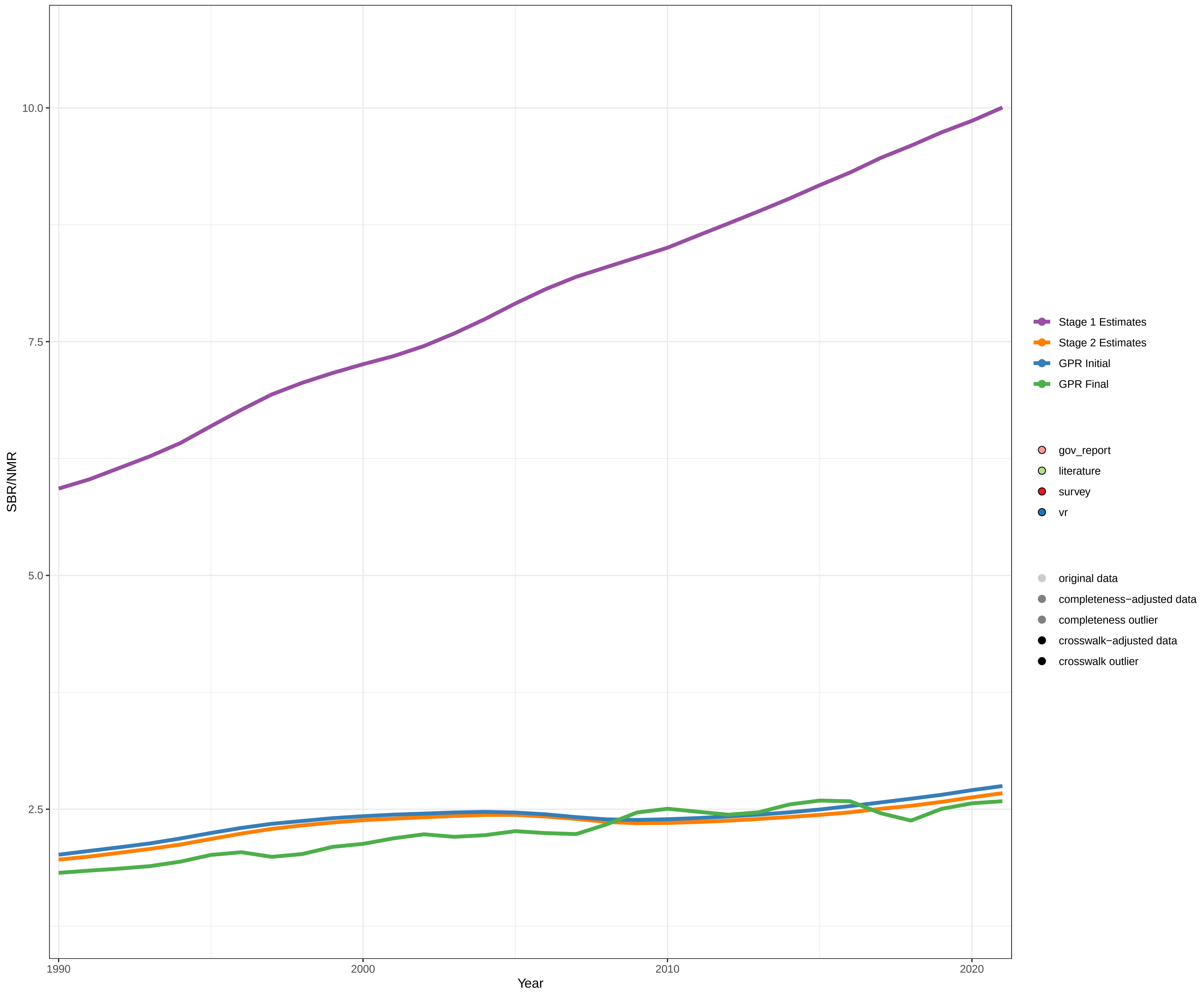

Hiroshima JPN_35457 High−income Asia Pacific Stillbirth Rate / Neonatal Mortality Rate (SBR/NMR) − 28 weeks
lambda = 1 ; zeta = 0.6 ; scale = 15 ; amp = 0.028

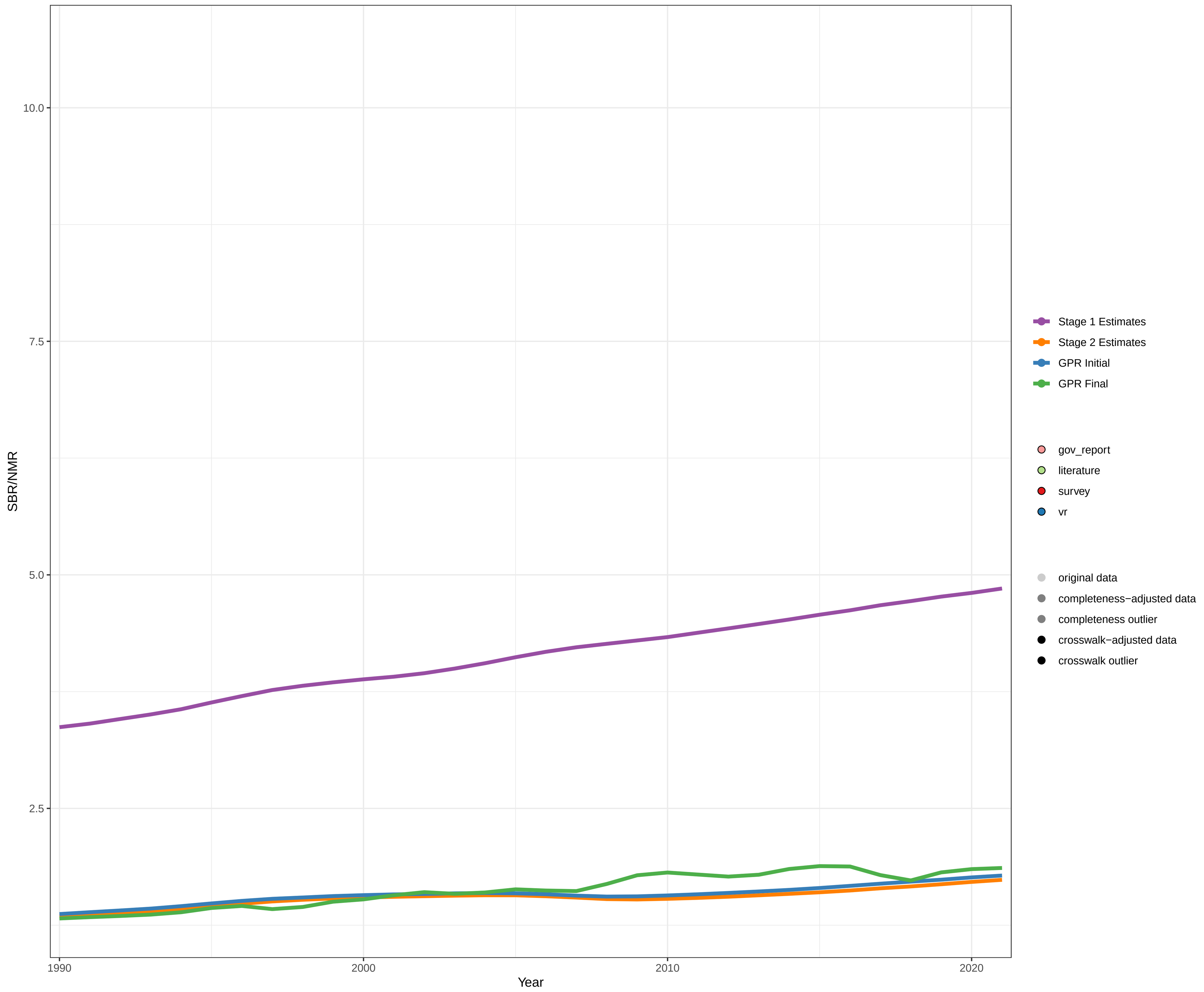

Definition	Years	Outlier	Source.Type	Title

Hiroshima JPN_35457 High−income Asia Pacific Stillbirth Rate (SBR) − 20 weeks
lambda = 1 ; zeta = 0.6 ; scale = 15 ; amp = 0.028

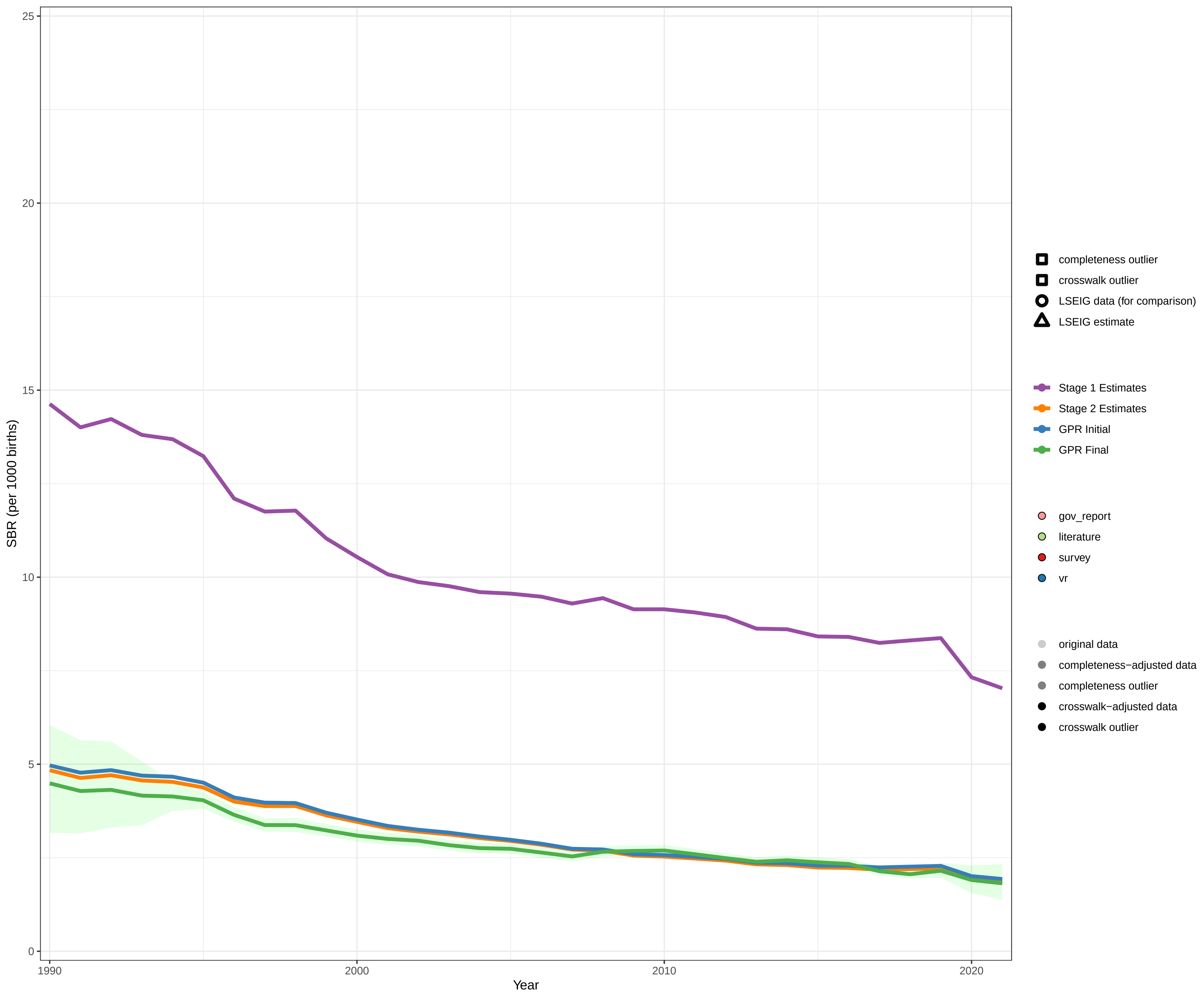

Hiroshima JPN_35457 High−income Asia Pacific Stillbirth Rate (SBR) − 28 weeks
lambda = 1 ; zeta = 0.6 ; scale = 15 ; amp = 0.028

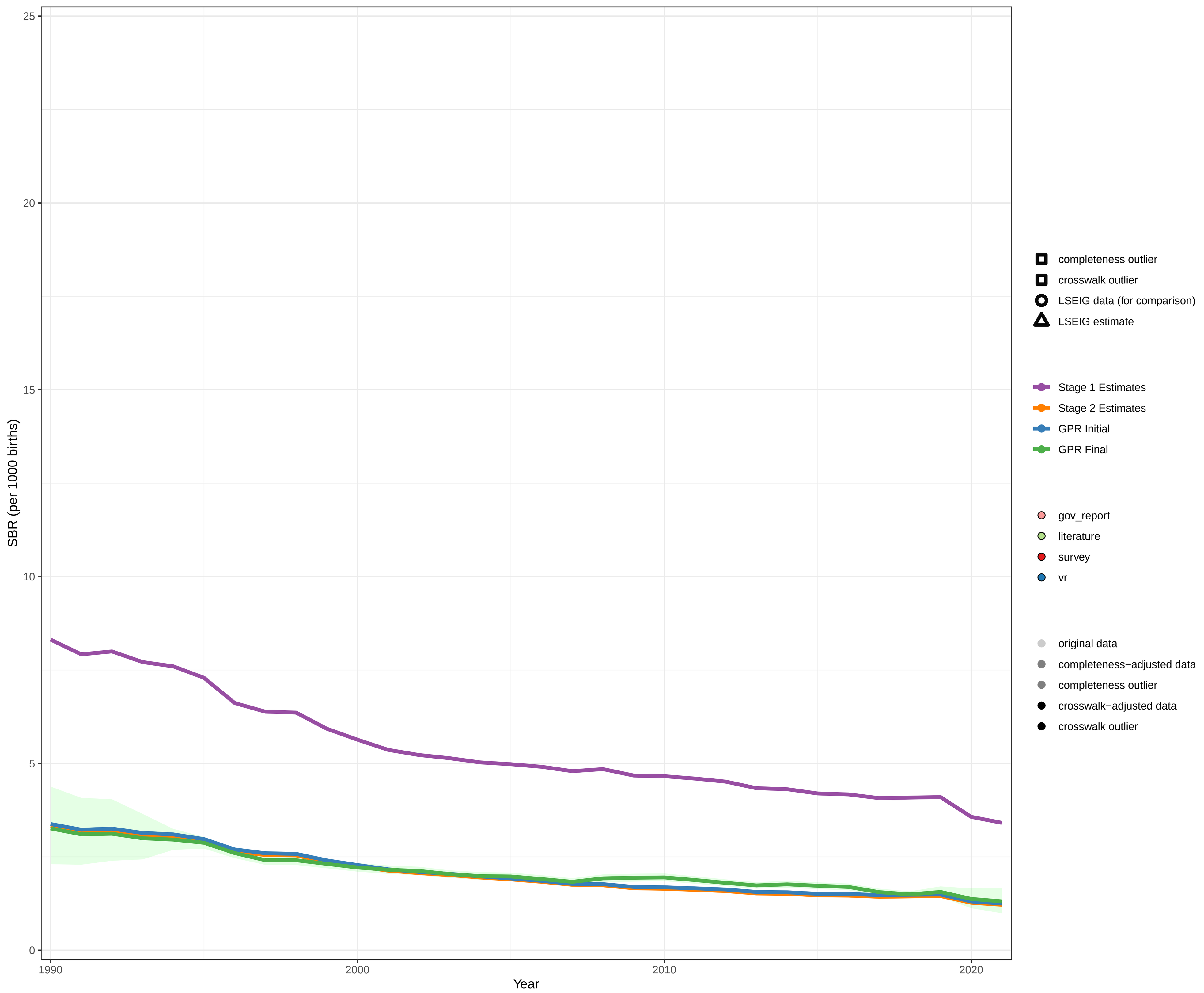

Year	Total Stillbirths (in thousands)	Total Stillbirths (in thousands)	SBR (per 1000 births)	SBR (per 1000 births)	SBR/NMR	SBR/NMR
	20 weeks	28 weeks	20 weeks	28 weeks	20 weeks	28 weeks
1990	0.1<br>(0.1 to 0.2)	0.1<br>(0.1 to 0.1)	4.5<br>(3.2 to 6.0)	3.3<br>(2.3 to 4.4)	1.82<br>(1.28 to 2.45)	1.32<br>(0.93 to 1.78)
1995	0.1<br>(0.1 to 0.1)	0.1<br>(0.1 to 0.1)	4.0<br>(3.8 to 4.3)	2.9<br>(2.7 to 3.0)	2.01<br>(1.90 to 2.12)	1.43<br>(1.36 to 1.51)
2000	0.1<br>(0.1 to 0.1)	0.1<br>(0.1 to 0.1)	3.1<br>(2.9 to 3.3)	2.2<br>(2.1 to 2.3)	2.13<br>(2.02 to 2.24)	1.53<br>(1.45 to 1.61)
2005	0.1<br>(0.1 to 0.1)	0.1<br>(0.0 to 0.1)	2.7<br>(2.6 to 2.9)	2.0<br>(1.9 to 2.1)	2.27<br>(2.15 to 2.39)	1.63<br>(1.55 to 1.72)
2010	0.1<br>(0.1 to 0.1)	0.0<br>(0.0 to 0.1)	2.7<br>(2.6 to 2.8)	1.9<br>(1.9 to 2.1)	2.51<br>(2.38 to 2.65)	1.81<br>(1.72 to 1.91)
2015	0.1<br>(0.1 to 0.1)	0.0<br>(0.0 to 0.0)	2.4<br>(2.3 to 2.5)	1.7<br>(1.6 to 1.8)	2.59<br>(2.46 to 2.73)	1.88<br>(1.79 to 1.98)
2020	0.0<br>(0.0 to 0.0)	0.0<br>(0.0 to 0.0)	1.9<br>(1.6 to 2.3)	1.4<br>(1.1 to 1.7)	2.56<br>(2.10 to 3.09)	1.85<br>(1.52 to 2.23)
2021	0.0<br>(0.0 to 0.0)	0.0<br>(0.0 to 0.0)	1.8<br>(1.4 to 2.3)	1.3<br>(1.0 to 1.7)	2.59<br>(1.95 to 3.31)	1.86<br>(1.41 to 2.38)

Hokkaido JPN_35424 High−income Asia Pacific Stillbirth Rate / Neonatal Mortality Rate (SBR/NMR) − 20 weeks
lambda = 1 ; zeta = 0.6 ; scale = 15 ; amp = 0.028

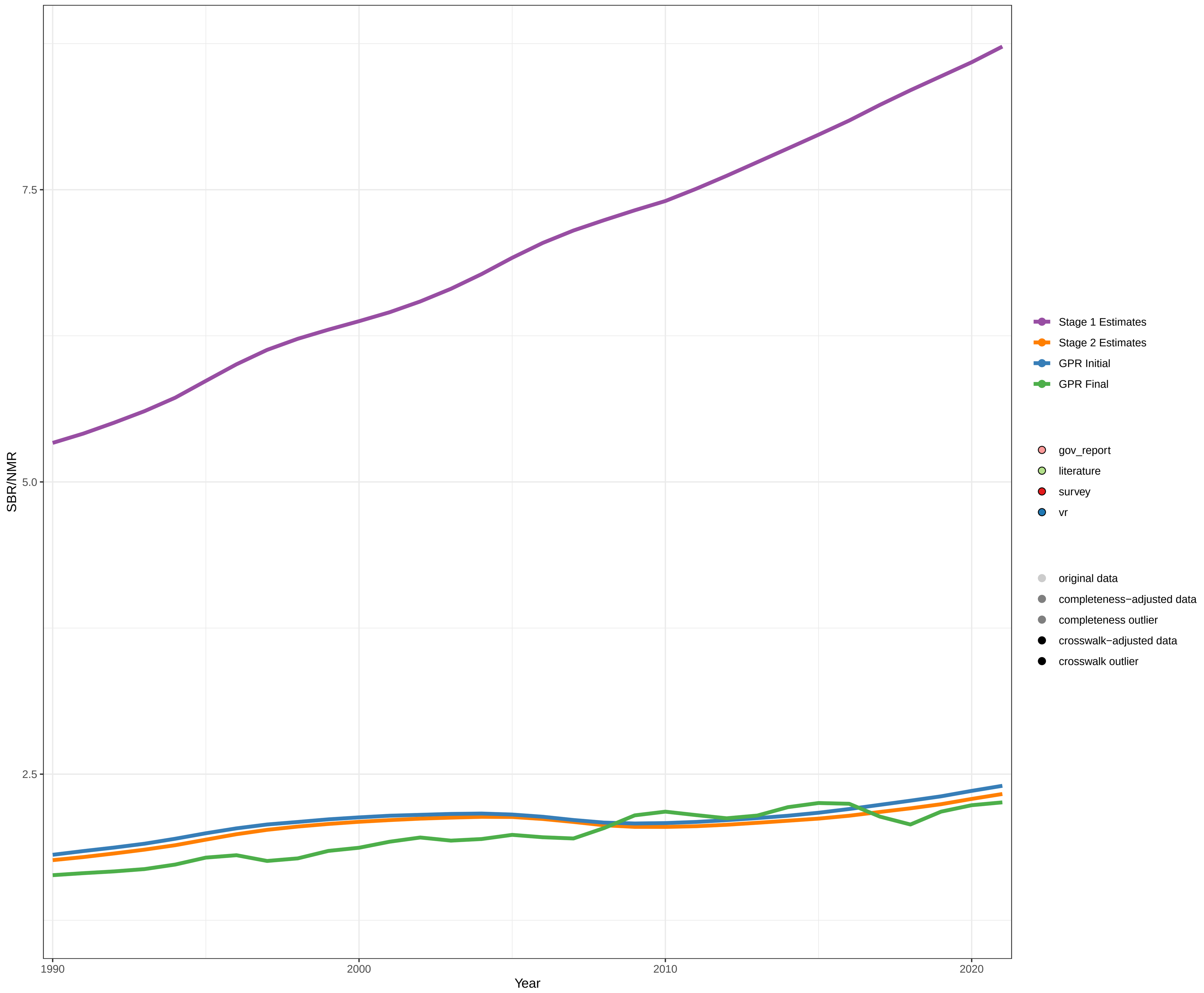

Hokkaido JPN_35424 High−income Asia Pacific Stillbirth Rate / Neonatal Mortality Rate (SBR/NMR) − 28 weeks
lambda = 1 ; zeta = 0.6 ; scale = 15 ; amp = 0.028

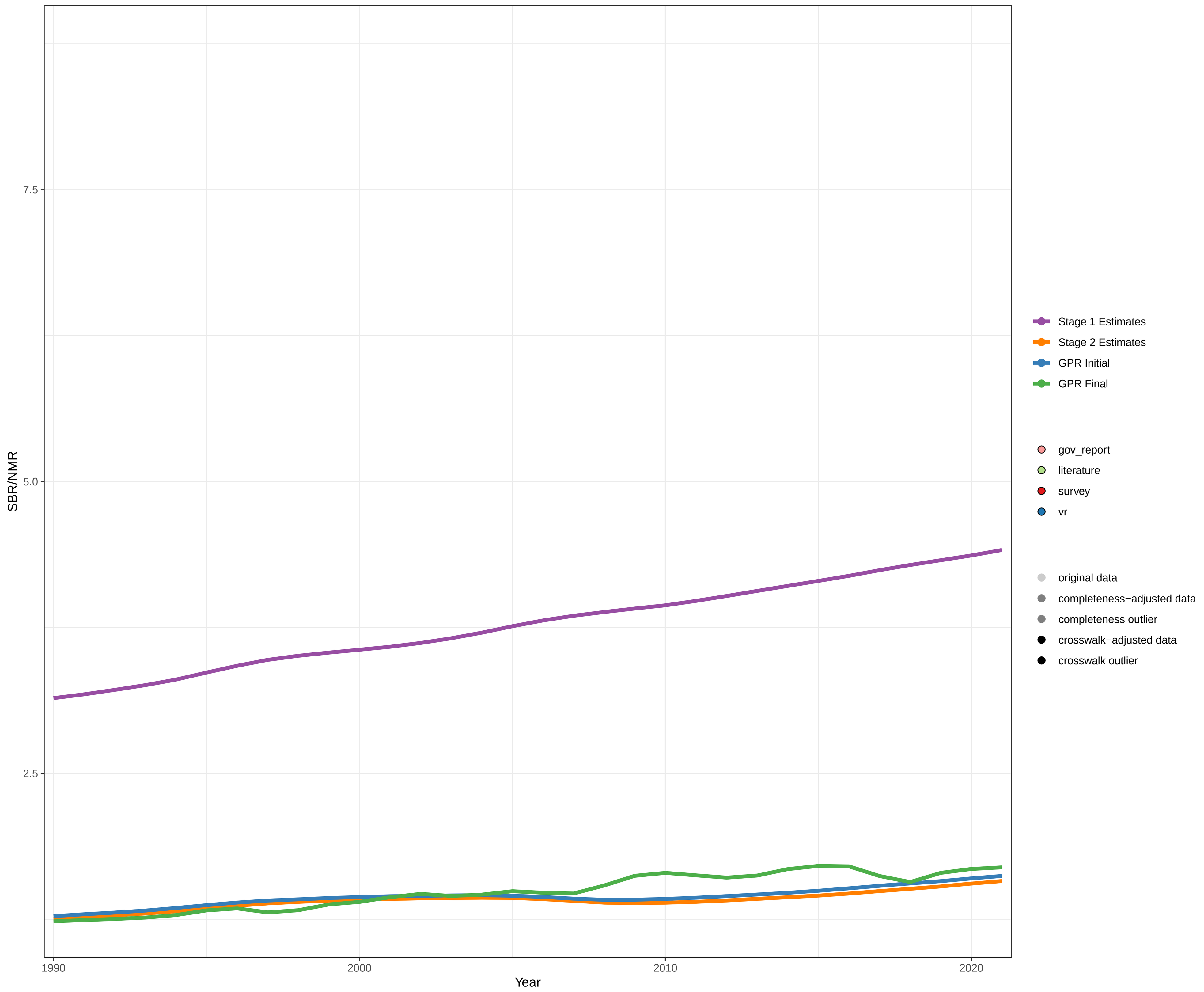

Definition	Years	Outlier	Source.Type	Title

Hokkaido JPN_35424 High−income Asia Pacific Stillbirth Rate (SBR) − 20 weeks
lambda = 1 ; zeta = 0.6 ; scale = 15 ; amp = 0.028

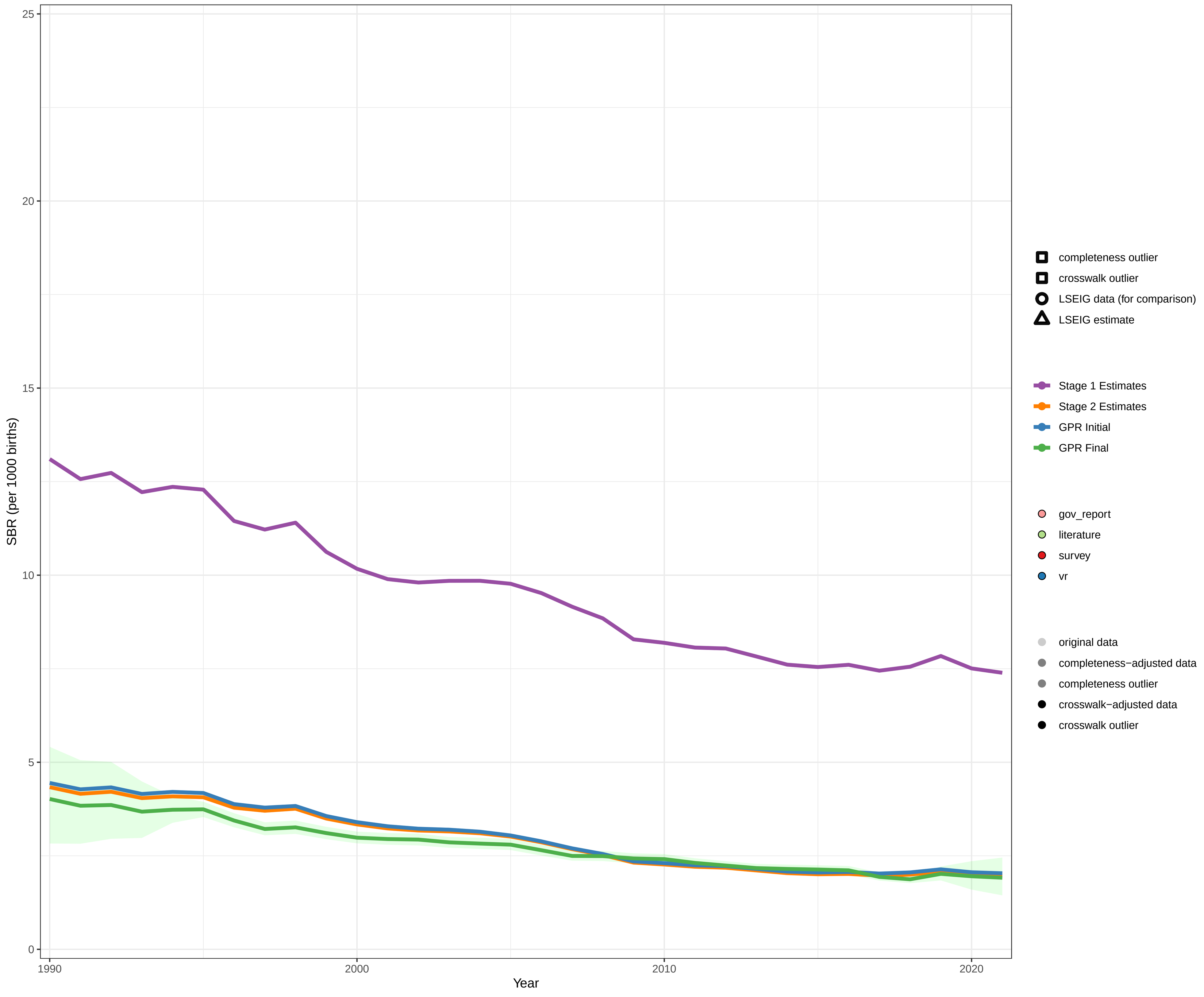

Hokkaido JPN_35424 High−income Asia Pacific Stillbirth Rate (SBR) − 28 weeks
lambda = 1 ; zeta = 0.6 ; scale = 15 ; amp = 0.028

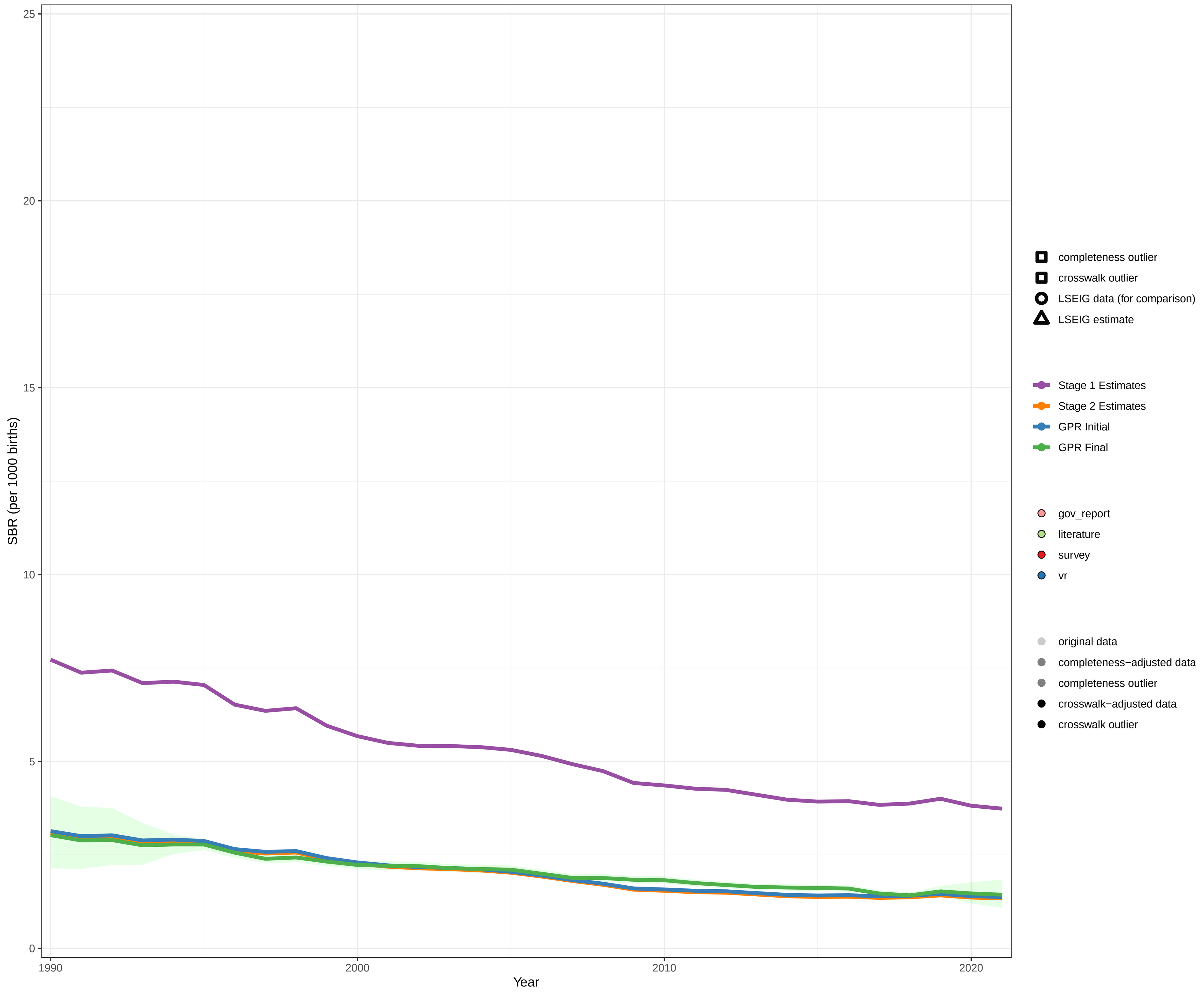

Year	Total Stillbirths (in thousands)	Total Stillbirths (in thousands)	SBR (per 1000 births)	SBR (per 1000 births)	SBR/NMR	SBR/NMR
	20 weeks	28 weeks	20 weeks	28 weeks	20 weeks	28 weeks
1990	0.2<br>(0.2 to 0.3)	0.2<br>(0.1 to 0.2)	4.0<br>(2.8 to 5.4)	3.0<br>(2.1 to 4.1)	1.64<br>(1.15 to 2.20)	1.23<br>(0.87 to 1.66)
1995	0.2<br>(0.2 to 0.2)	0.1<br>(0.1 to 0.1)	3.7<br>(3.5 to 3.9)	2.8<br>(2.6 to 2.9)	1.79<br>(1.69 to 1.88)	1.33<br>(1.25 to 1.40)
2000	0.1<br>(0.1 to 0.1)	0.1<br>(0.1 to 0.1)	3.0<br>(2.8 to 3.1)	2.2<br>(2.1 to 2.4)	1.87<br>(1.78 to 1.97)	1.40<br>(1.33 to 1.47)
2005	0.1<br>(0.1 to 0.1)	0.1<br>(0.1 to 0.1)	2.8<br>(2.7 to 2.9)	2.1<br>(2.0 to 2.2)	1.98<br>(1.88 to 2.09)	1.49<br>(1.42 to 1.57)
2010	0.1<br>(0.1 to 0.1)	0.1<br>(0.1 to 0.1)	2.4<br>(2.3 to 2.5)	1.8<br>(1.7 to 1.9)	2.18<br>(2.07 to 2.30)	1.65<br>(1.56 to 1.74)
2015	0.1<br>(0.1 to 0.1)	0.1<br>(0.1 to 0.1)	2.1<br>(2.0 to 2.2)	1.6<br>(1.5 to 1.7)	2.25<br>(2.14 to 2.37)	1.71<br>(1.62 to 1.80)
2020	0.1<br>(0.0 to 0.1)	0.0<br>(0.0 to 0.1)	2.0<br>(1.6 to 2.4)	1.5<br>(1.2 to 1.8)	2.23<br>(1.83 to 2.70)	1.68<br>(1.38 to 2.02)
2021	0.1<br>(0.0 to 0.1)	0.0<br>(0.0 to 0.1)	1.9<br>(1.4 to 2.5)	1.4<br>(1.1 to 1.8)	2.26<br>(1.70 to 2.89)	1.70<br>(1.28 to 2.17)

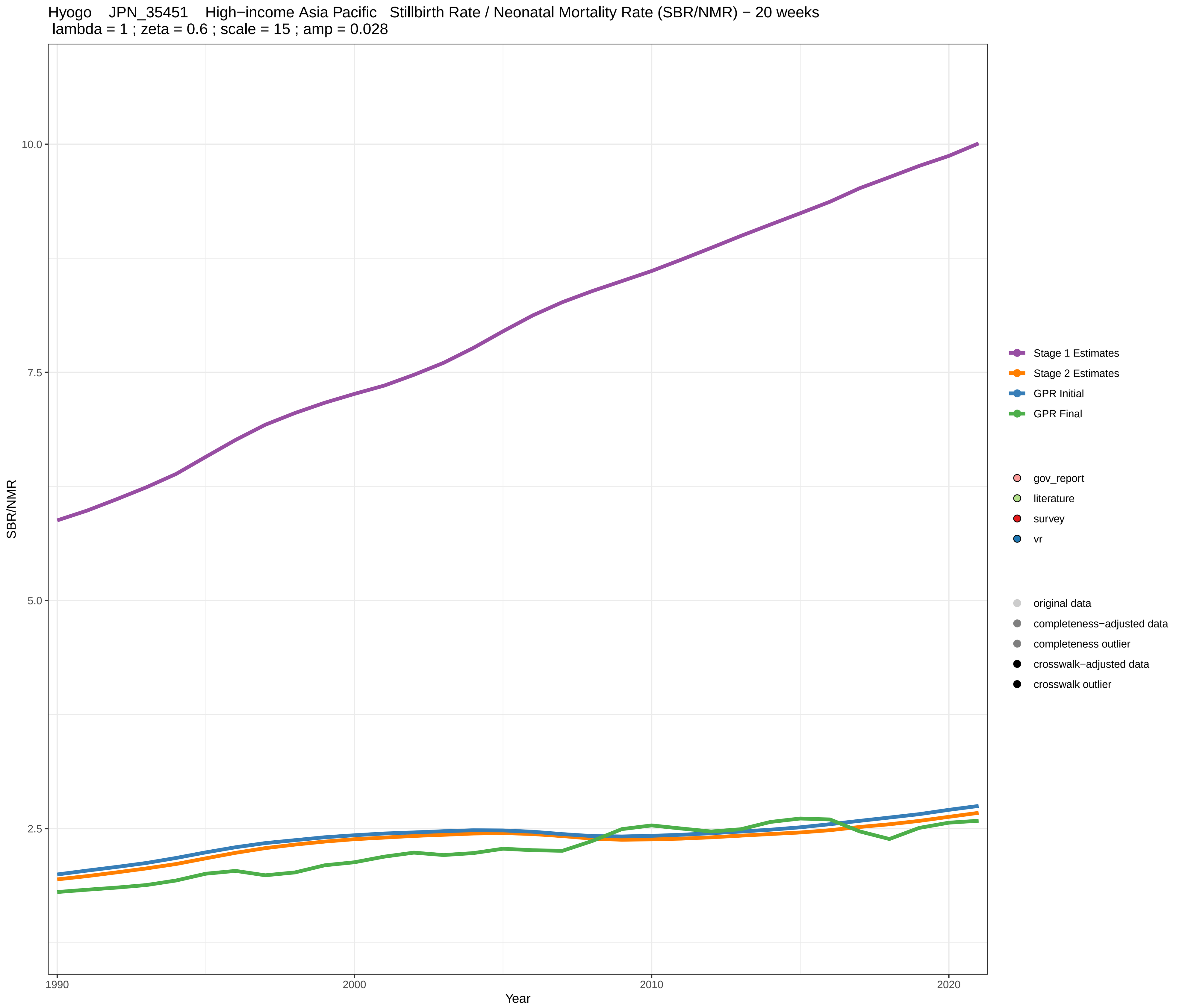

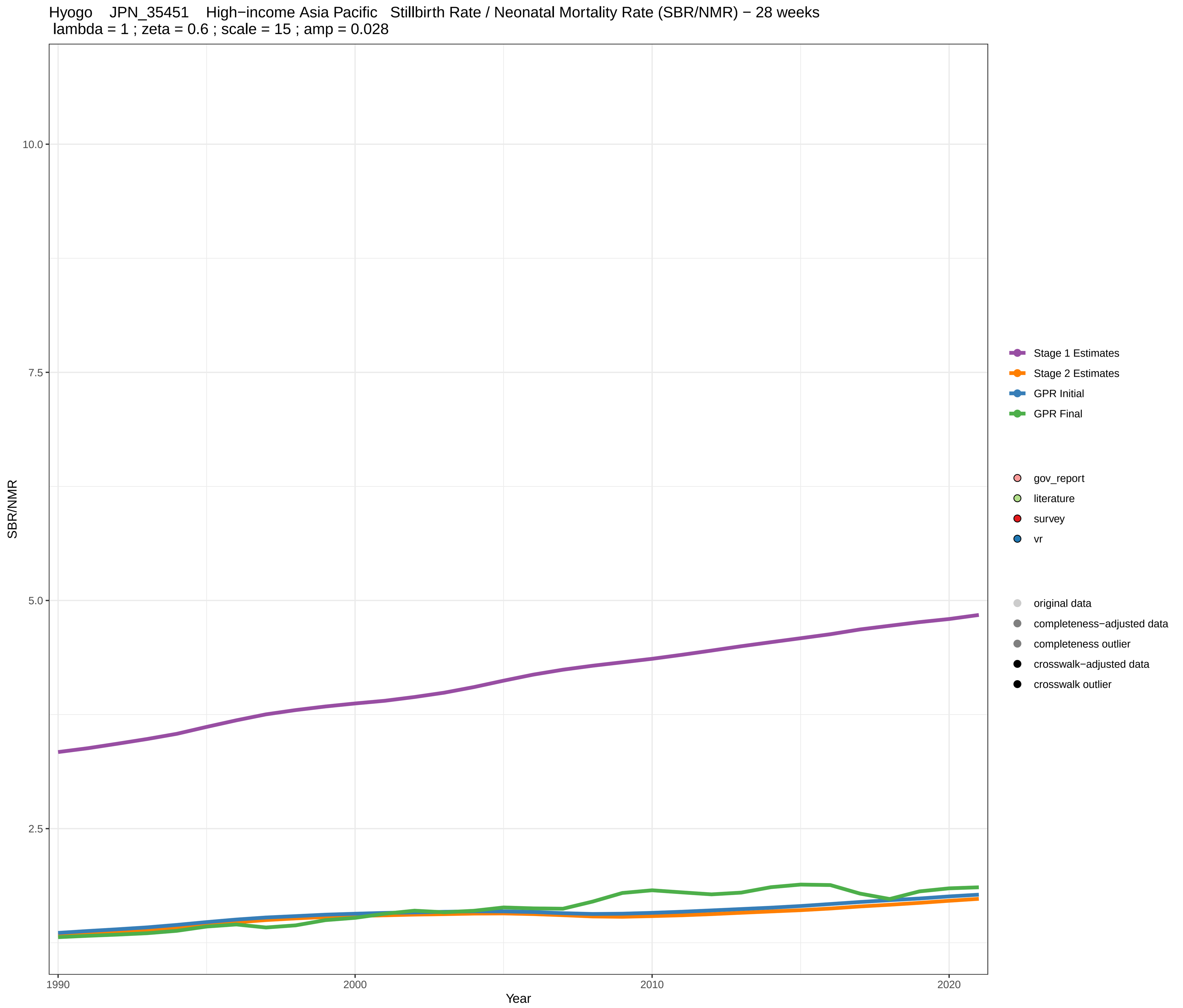

Definition	Years	Outlier	Source.Type	Title

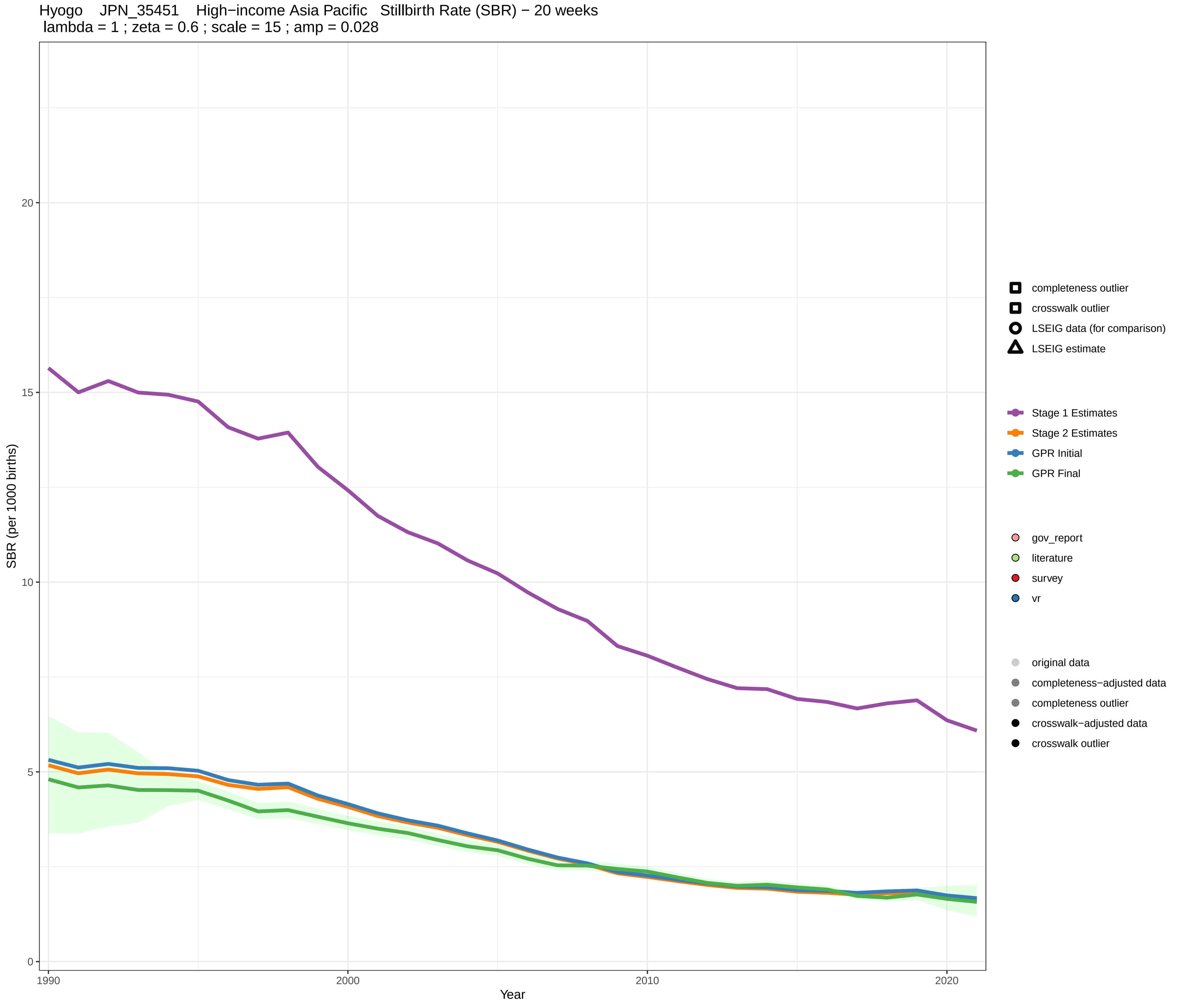

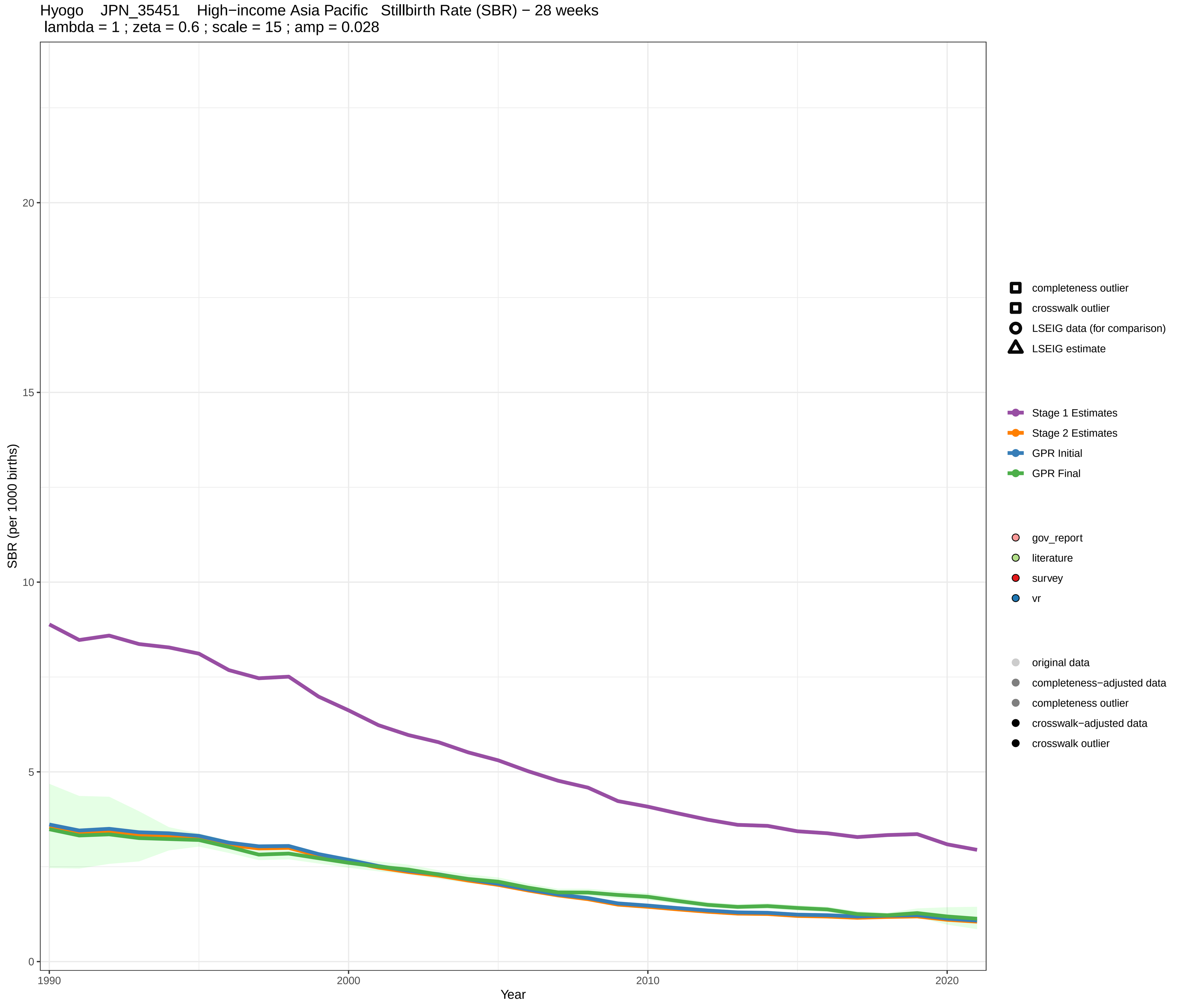

Year	Total Stillbirths (in thousands)	Total Stillbirths (in thousands)	SBR (per 1000 births)	SBR (per 1000 births)	SBR/NMR	SBR/NMR
	20 weeks	28 weeks	20 weeks	28 weeks	20 weeks	28 weeks
1990	0.3 (0.2 to 0.3)	0.2 (0.1 to 0.3)	4.8 (3.4 to 6.5)	3.5 (2.5 to 4.7)	1.81 (1.27 to 2.43)	1.31 (0.93 to 1.76)
1995	0.2 (0.2 to 0.3)	0.2 (0.2 to 0.2)	4.5 (4.3 to 4.7)	3.2 (3.0 to 3.4)	2.01 (1.90 to 2.12)	1.43 (1.35 to 1.50)
2000	0.2 (0.2 to 0.2)	0.1 (0.1 to 0.1)	3.6 (3.5 to 3.8)	2.6 (2.5 to 2.7)	2.13 (2.02 to 2.25)	1.52 (1.45 to 1.60)
2005	0.1 (0.1 to 0.2)	0.1 (0.1 to 0.1)	2.9 (2.8 to 3.1)	2.1 (2.0 to 2.2)	2.28 (2.16 to 2.40)	1.64 (1.55 to 1.72)
2010	0.1 (0.1 to 0.1)	0.1 (0.1 to 0.1)	2.4 (2.3 to 2.5)	1.7 (1.6 to 1.8)	2.54 (2.41 to 2.68)	1.82 (1.73 to 1.93)
2015	0.1 (0.1 to 0.1)	0.1 (0.1 to 0.1)	2.0 (1.9 to 2.1)	1.4 (1.3 to 1.5)	2.61 (2.47 to 2.75)	1.89 (1.79 to 1.99)
2020	0.1 (0.1 to 0.1)	0.0 (0.0 to 0.1)	1.7 (1.4 to 2.0)	1.2 (1.0 to 1.4)	2.57 (2.10 to 3.10)	1.85 (1.51 to 2.22)
2021	0.1 (0.0 to 0.1)	0.0 (0.0 to 0.1)	1.6 (1.2 to 2.0)	1.1 (0.9 to 1.4)	2.59 (1.95 to 3.32)	1.86 (1.40 to 2.38)

Ibaraki JPN_35431 High−income Asia Pacific Stillbirth Rate / Neonatal Mortality Rate (SBR/NMR) − 20 weeks
lambda = 1 ; zeta = 0.6 ; scale = 15 ; amp = 0.028

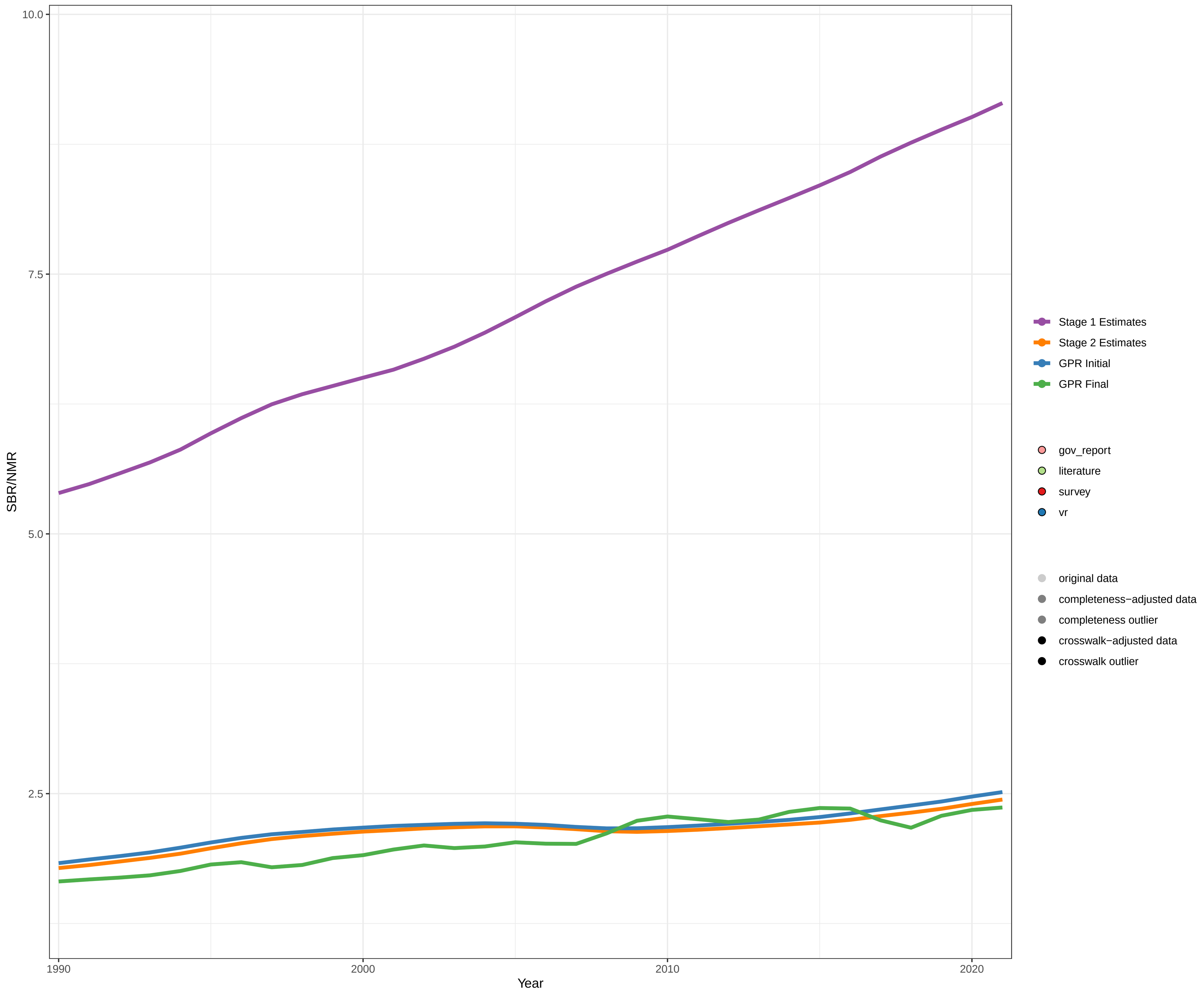

Ibaraki JPN_35431 High−income Asia Pacific Stillbirth Rate / Neonatal Mortality Rate (SBR/NMR) − 28 weeks
lambda = 1 ; zeta = 0.6 ; scale = 15 ; amp = 0.028

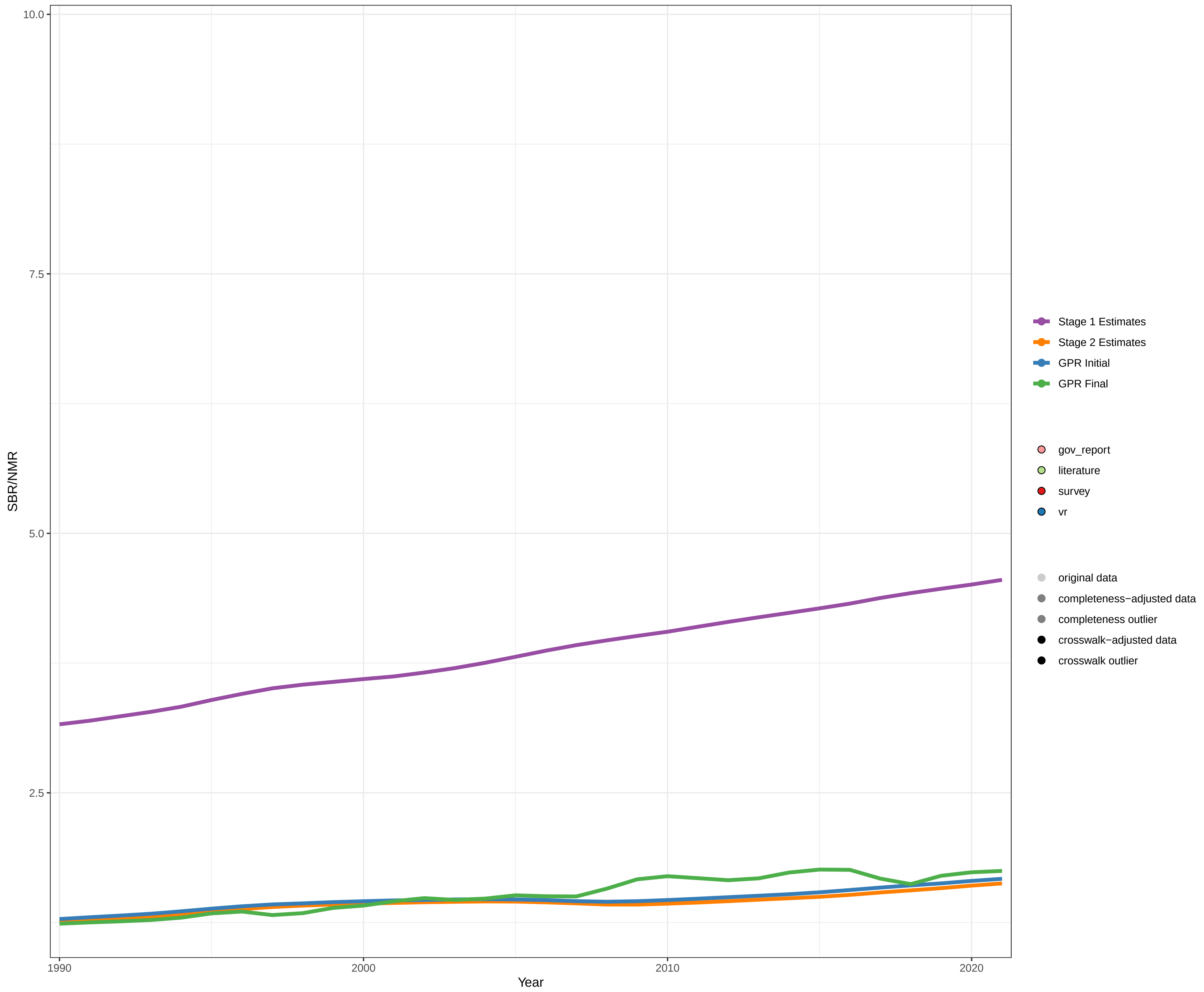

Definition	Years	Outlier	Source.Type	Title

Ibaraki JPN_35431 High−income Asia Pacific Stillbirth Rate (SBR) − 20 weeks
lambda = 1 ; zeta = 0.6 ; scale = 15 ; amp = 0.028

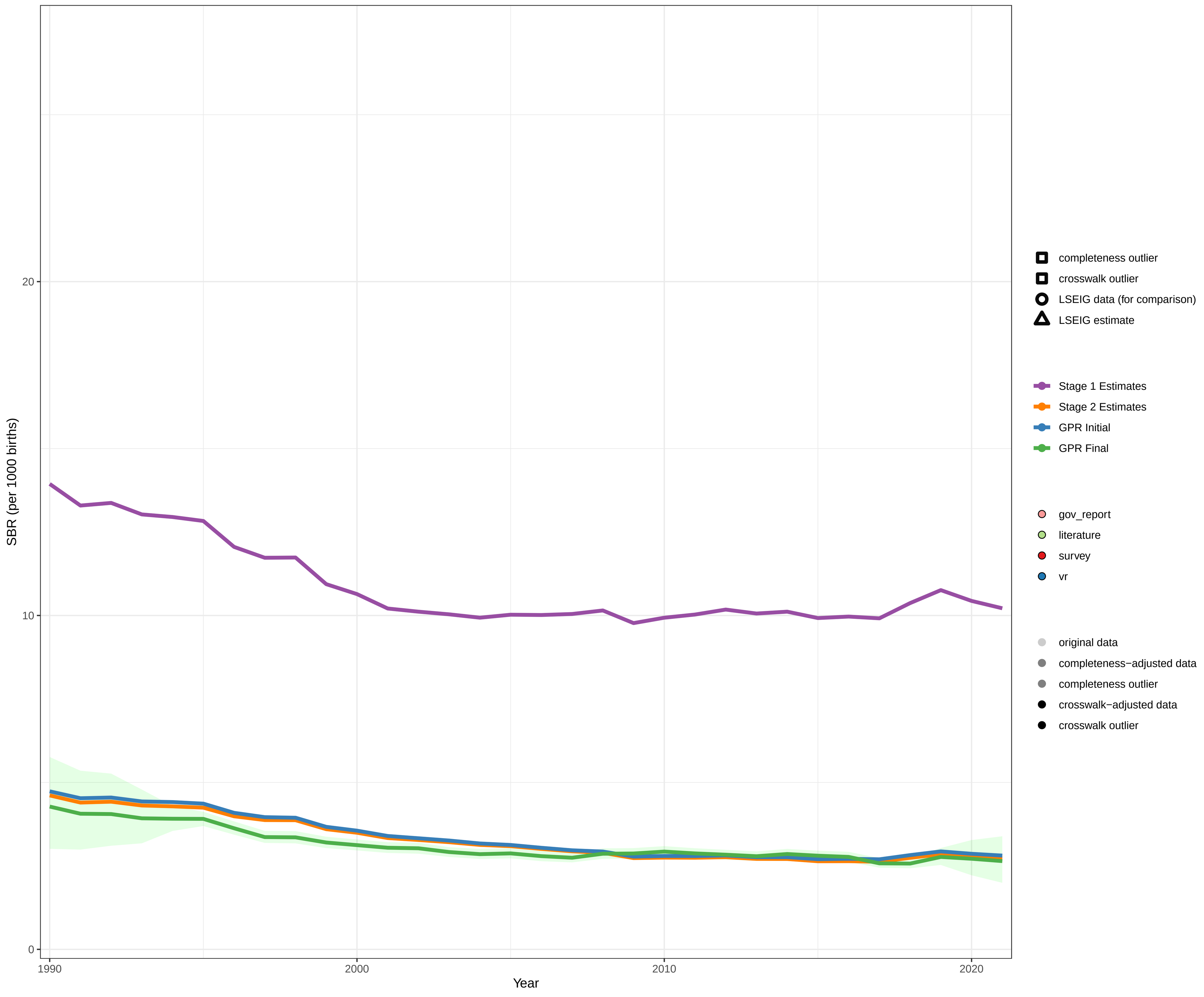

Ibaraki JPN_35431 High−income Asia Pacific Stillbirth Rate (SBR) − 28 weeks
lambda = 1 ; zeta = 0.6 ; scale = 15 ; amp = 0.028

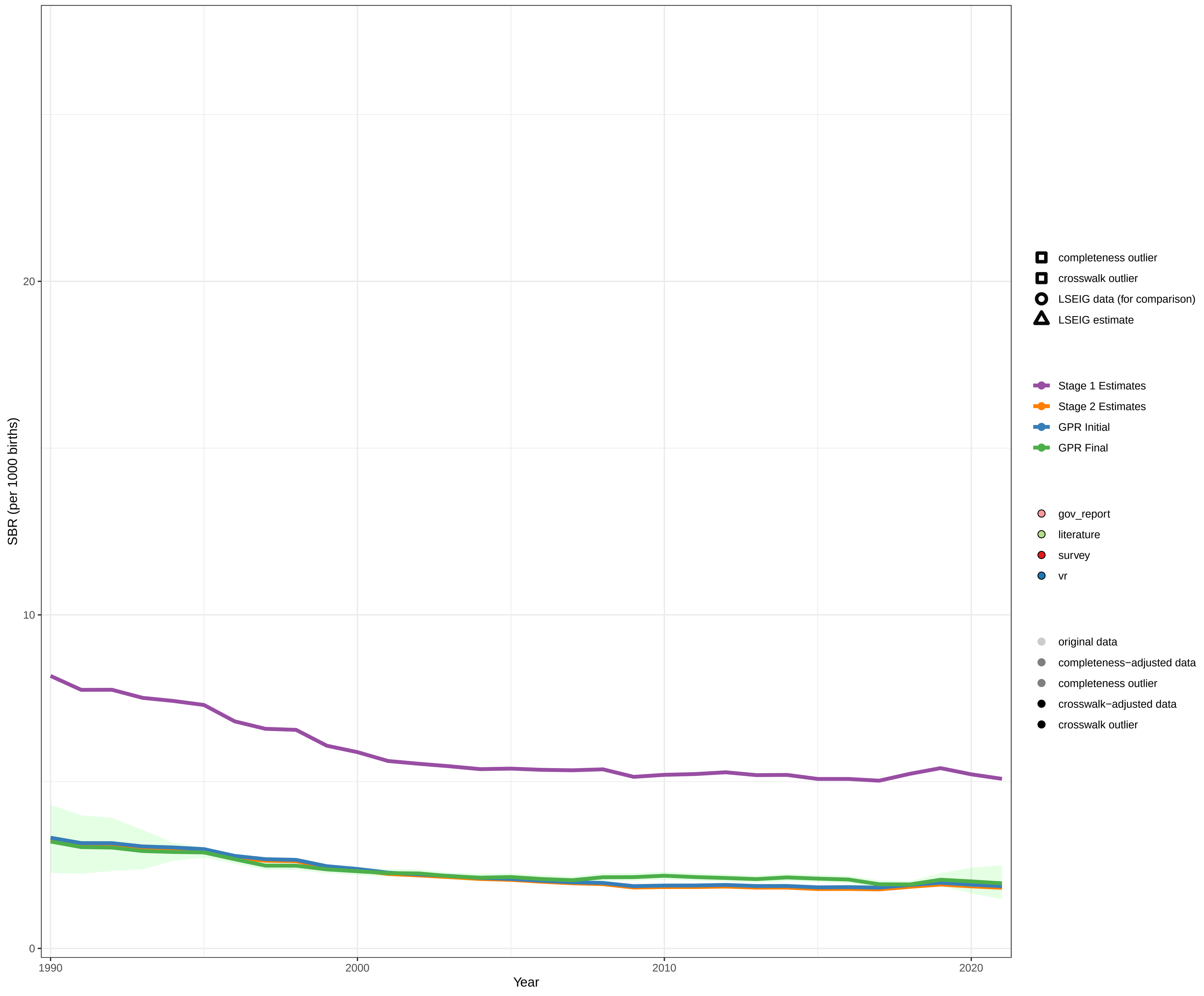

Year	Total Stillbirths (in thousands)	Total Stillbirths (in thousands)	SBR (per 1000 births)	SBR (per 1000 births)	SBR/NMR	SBR/NMR
	20 weeks	28 weeks	20 weeks	28 weeks	20 weeks	28 weeks
1990	0.1 (0.1 to 0.2)	0.1 (0.1 to 0.1)	4.3 (3.0 to 5.8)	3.2 (2.3 to 4.3)	1.65 (1.16 to 2.23)	1.24 (0.88 to 1.67)
1995	0.1 (0.1 to 0.1)	0.1 (0.1 to 0.1)	3.9 (3.7 to 4.1)	2.9 (2.7 to 3.0)	1.82 (1.72 to 1.92)	1.34 (1.27 to 1.41)
2000	0.1 (0.1 to 0.1)	0.1 (0.1 to 0.1)	3.1 (3.0 to 3.3)	2.3 (2.2 to 2.4)	1.91 (1.81 to 2.01)	1.41 (1.34 to 1.49)
2005	0.1 (0.1 to 0.1)	0.1 (0.1 to 0.1)	2.9 (2.7 to 3.0)	2.1 (2.0 to 2.3)	2.03 (1.93 to 2.14)	1.51 (1.44 to 1.59)
2010	0.1 (0.1 to 0.1)	0.1 (0.0 to 0.1)	2.9 (2.8 to 3.1)	2.2 (2.1 to 2.3)	2.28 (2.16 to 2.41)	1.70 (1.61 to 1.79)
2015	0.1 (0.1 to 0.1)	0.0 (0.0 to 0.0)	2.8 (2.7 to 3.0)	2.1 (2.0 to 2.2)	2.36 (2.24 to 2.49)	1.76 (1.67 to 1.85)
2020	0.0 (0.0 to 0.1)	0.0 (0.0 to 0.0)	2.7 (2.2 to 3.3)	2.0 (1.6 to 2.4)	2.34 (1.92 to 2.83)	1.73 (1.42 to 2.09)
2021	0.0 (0.0 to 0.1)	0.0 (0.0 to 0.0)	2.6 (2.0 to 3.4)	2.0 (1.5 to 2.5)	2.37 (1.78 to 3.03)	1.75 (1.32 to 2.23)

Ishikawa JPN_35440 High−income Asia Pacific Stillbirth Rate / Neonatal Mortality Rate (SBR/NMR) − 20 weeks
lambda = 1 ; zeta = 0.6 ; scale = 15 ; amp = 0.028

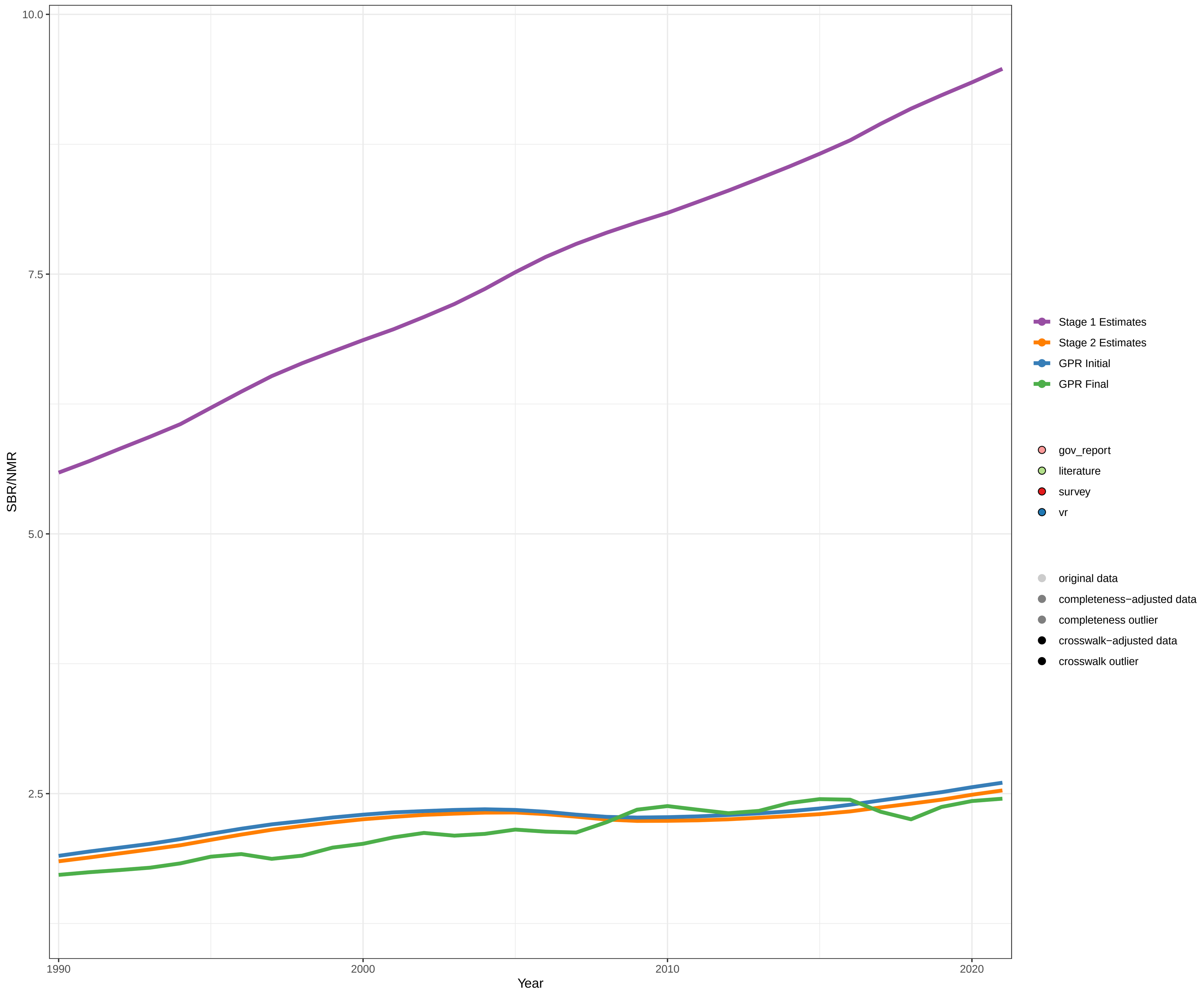

Ishikawa JPN_35440 High−income Asia Pacific Stillbirth Rate / Neonatal Mortality Rate (SBR/NMR) − 28 weeks
lambda = 1 ; zeta = 0.6 ; scale = 15 ; amp = 0.028

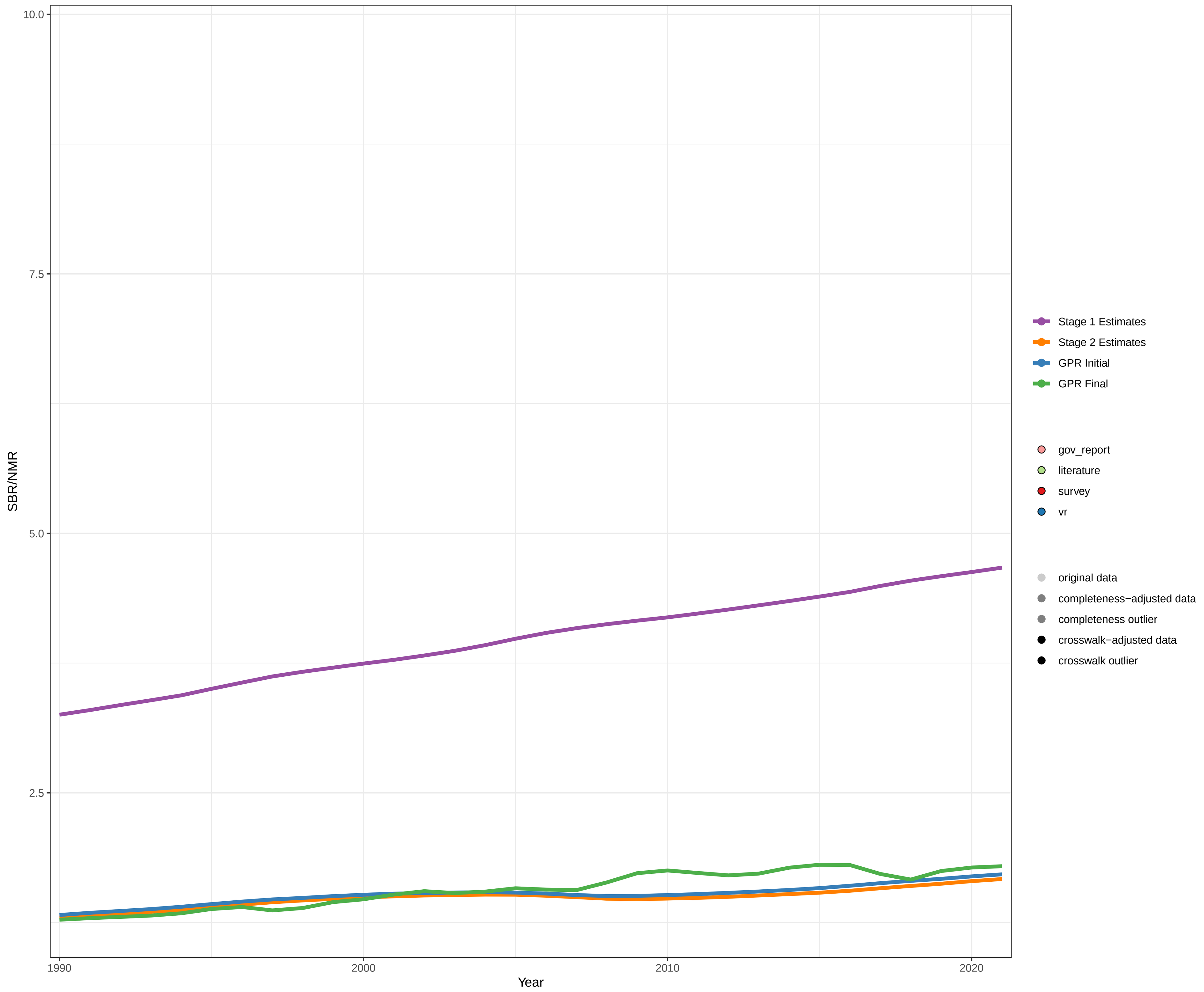

Definition	Years	Outlier	Source.Type	Title

Ishikawa JPN_35440 High−income Asia Pacific Stillbirth Rate (SBR) − 20 weeks
lambda = 1 ; zeta = 0.6 ; scale = 15 ; amp = 0.028

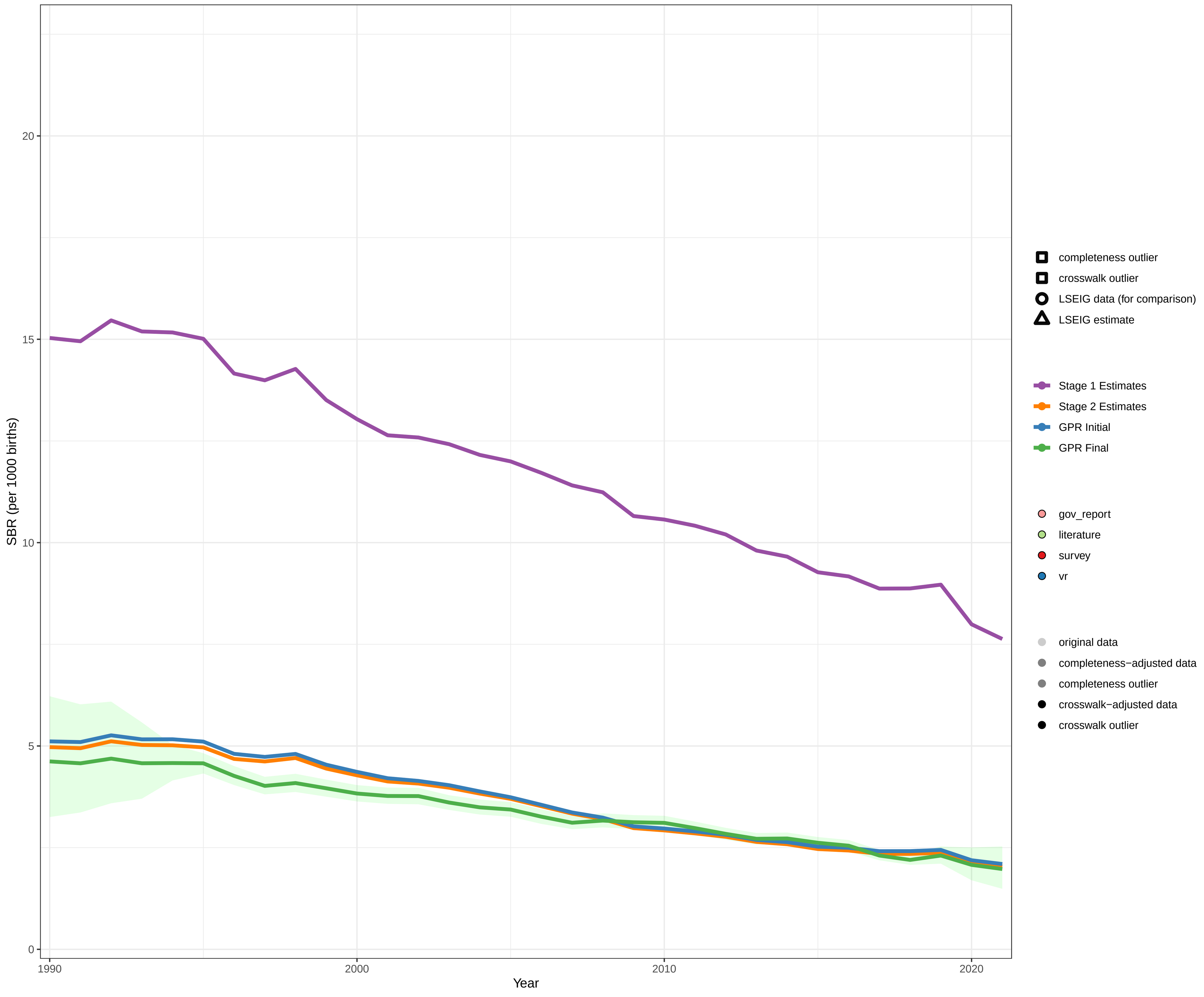

Ishikawa JPN_35440 High−income Asia Pacific Stillbirth Rate (SBR) − 28 weeks
lambda = 1 ; zeta = 0.6 ; scale = 15 ; amp = 0.028

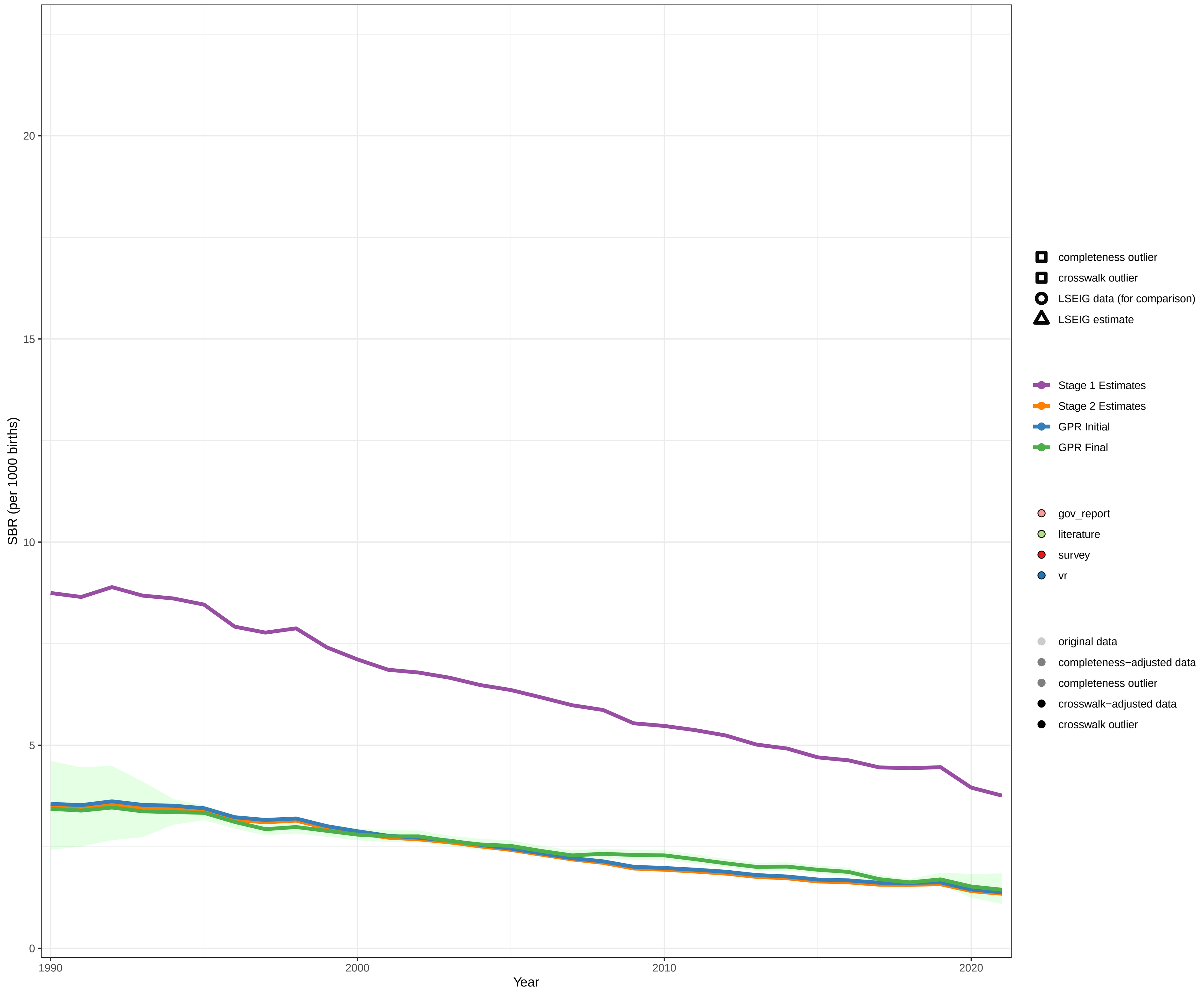

Year	Total Stillbirths (in thousands)	Total Stillbirths (in thousands)	SBR (per 1000 births)	SBR (per 1000 births)	SBR/NMR	SBR/NMR
	20 weeks	28 weeks	20 weeks	28 weeks	20 weeks	28 weeks
1990	0.1 (0.0 to 0.1)	0.0 (0.0 to 0.1)	4.6 (3.3 to 6.2)	3.4 (2.4 to 4.6)	1.72 (1.21 to 2.31)	1.28 (0.90 to 1.72)
1995	0.1 (0.0 to 0.1)	0.0 (0.0 to 0.0)	4.6 (4.3 to 4.8)	3.3 (3.2 to 3.5)	1.89 (1.79 to 2.00)	1.38 (1.31 to 1.46)
2000	0.0 (0.0 to 0.0)	0.0 (0.0 to 0.0)	3.8 (3.6 to 4.0)	2.8 (2.7 to 2.9)	2.02 (1.91 to 2.13)	1.47 (1.40 to 1.55)
2005	0.0 (0.0 to 0.0)	0.0 (0.0 to 0.0)	3.4 (3.3 to 3.6)	2.5 (2.4 to 2.7)	2.15 (2.04 to 2.27)	1.58 (1.50 to 1.66)
2010	0.0 (0.0 to 0.0)	0.0 (0.0 to 0.0)	3.1 (3.0 to 3.3)	2.3 (2.2 to 2.4)	2.38 (2.26 to 2.51)	1.75 (1.66 to 1.85)
2015	0.0 (0.0 to 0.0)	0.0 (0.0 to 0.0)	2.6 (2.5 to 2.8)	1.9 (1.8 to 2.0)	2.45 (2.32 to 2.58)	1.81 (1.71 to 1.90)
2020	0.0 (0.0 to 0.0)	0.0 (0.0 to 0.0)	2.1 (1.7 to 2.5)	1.5 (1.2 to 1.8)	2.43 (1.99 to 2.93)	1.78 (1.46 to 2.14)
2021	0.0 (0.0 to 0.0)	0.0 (0.0 to 0.0)	2.0 (1.5 to 2.5)	1.4 (1.1 to 1.8)	2.45 (1.85 to 3.14)	1.79 (1.36 to 2.29)

Iwate JPN_35426 High−income Asia Pacific Stillbirth Rate / Neonatal Mortality Rate (SBR/NMR) − 20 weeks
lambda = 1 ; zeta = 0.6 ; scale = 15 ; amp = 0.028

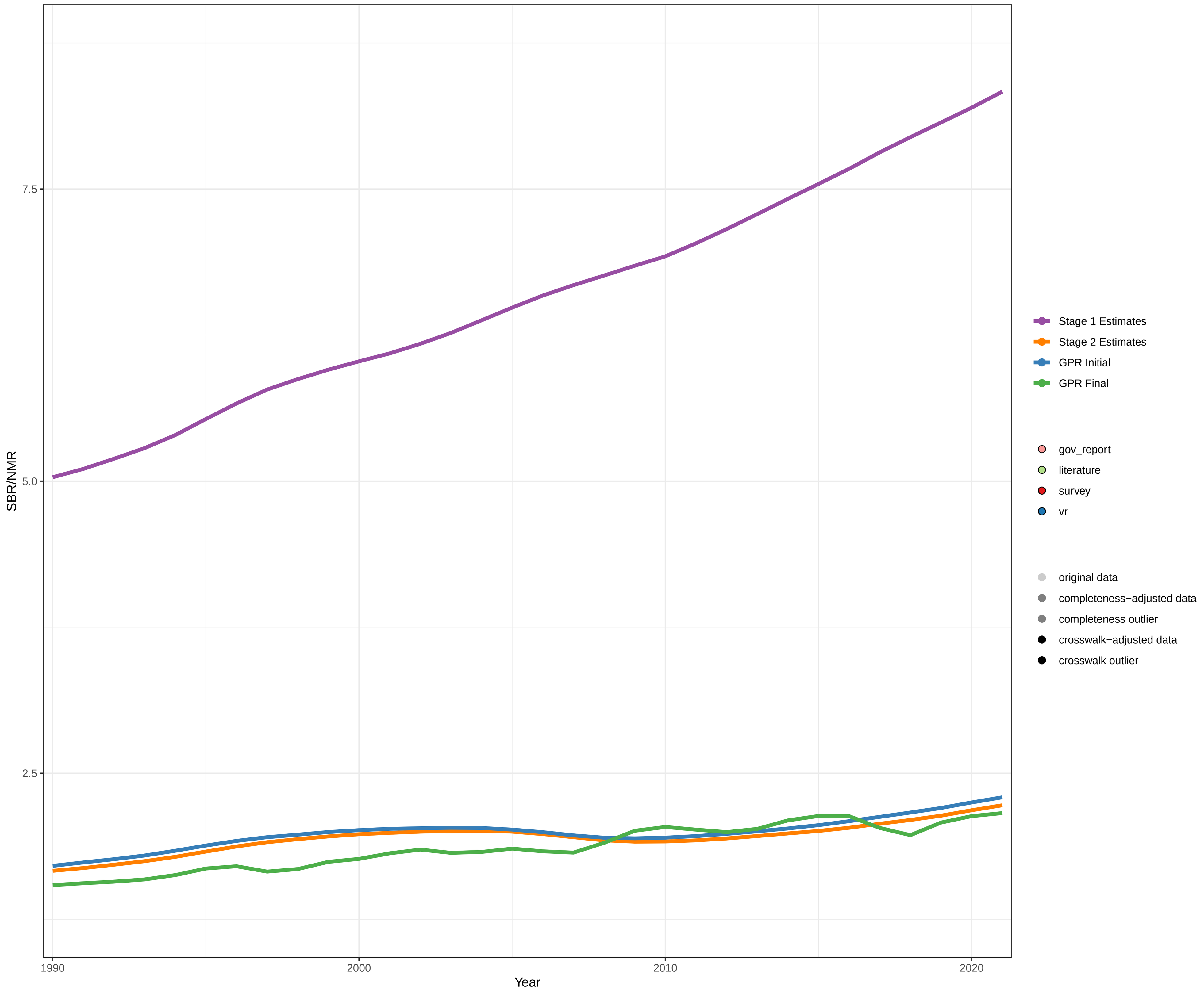

Iwate JPN_35426 High−income Asia Pacific Stillbirth Rate / Neonatal Mortality Rate (SBR/NMR) − 28 weeks
lambda = 1 ; zeta = 0.6 ; scale = 15 ; amp = 0.028

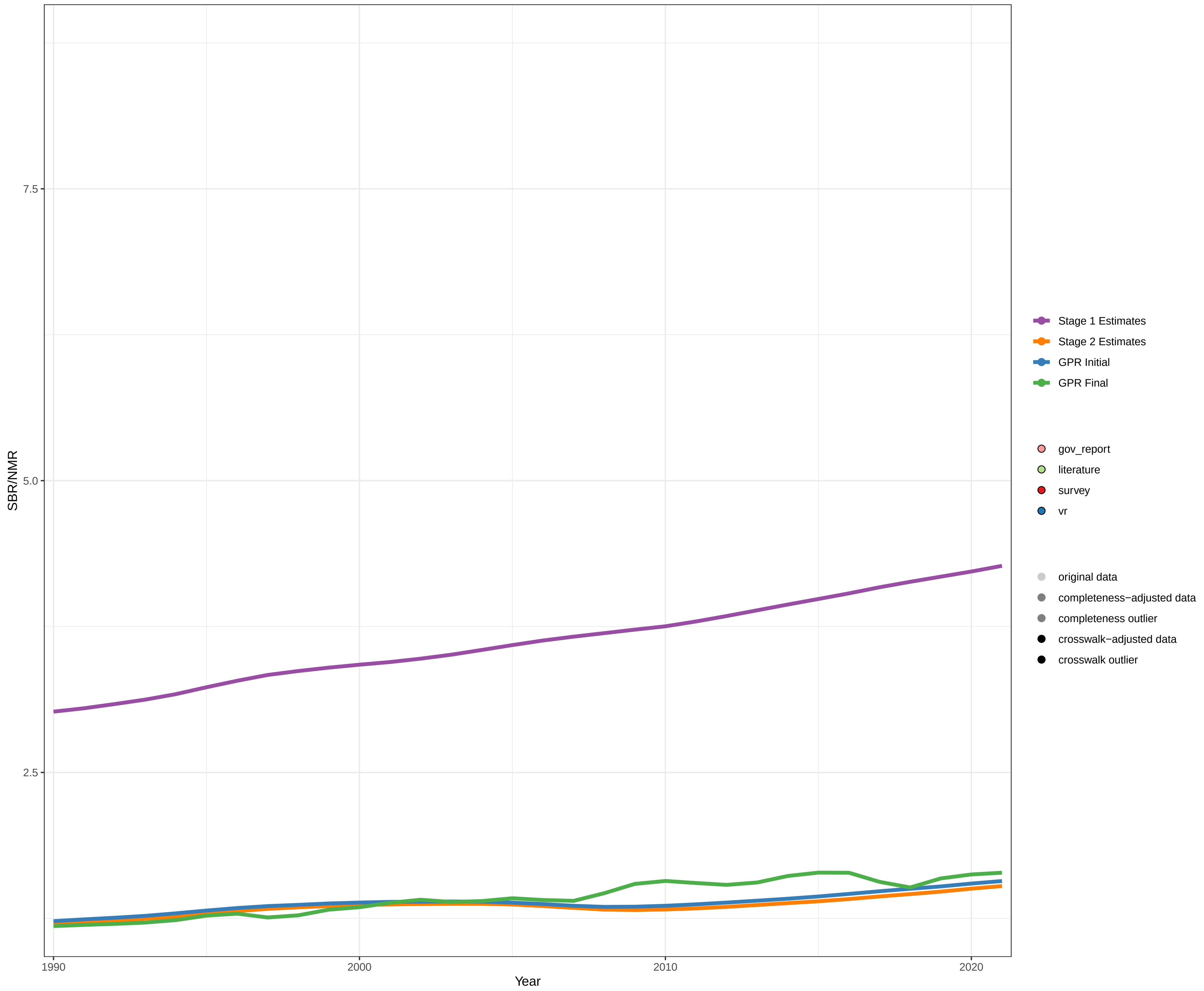

Definition	Years	Outlier	Source.Type	Title

Iwate JPN_35426 High−income Asia Pacific Stillbirth Rate (SBR) − 20 weeks
lambda = 1 ; zeta = 0.6 ; scale = 15 ; amp = 0.028

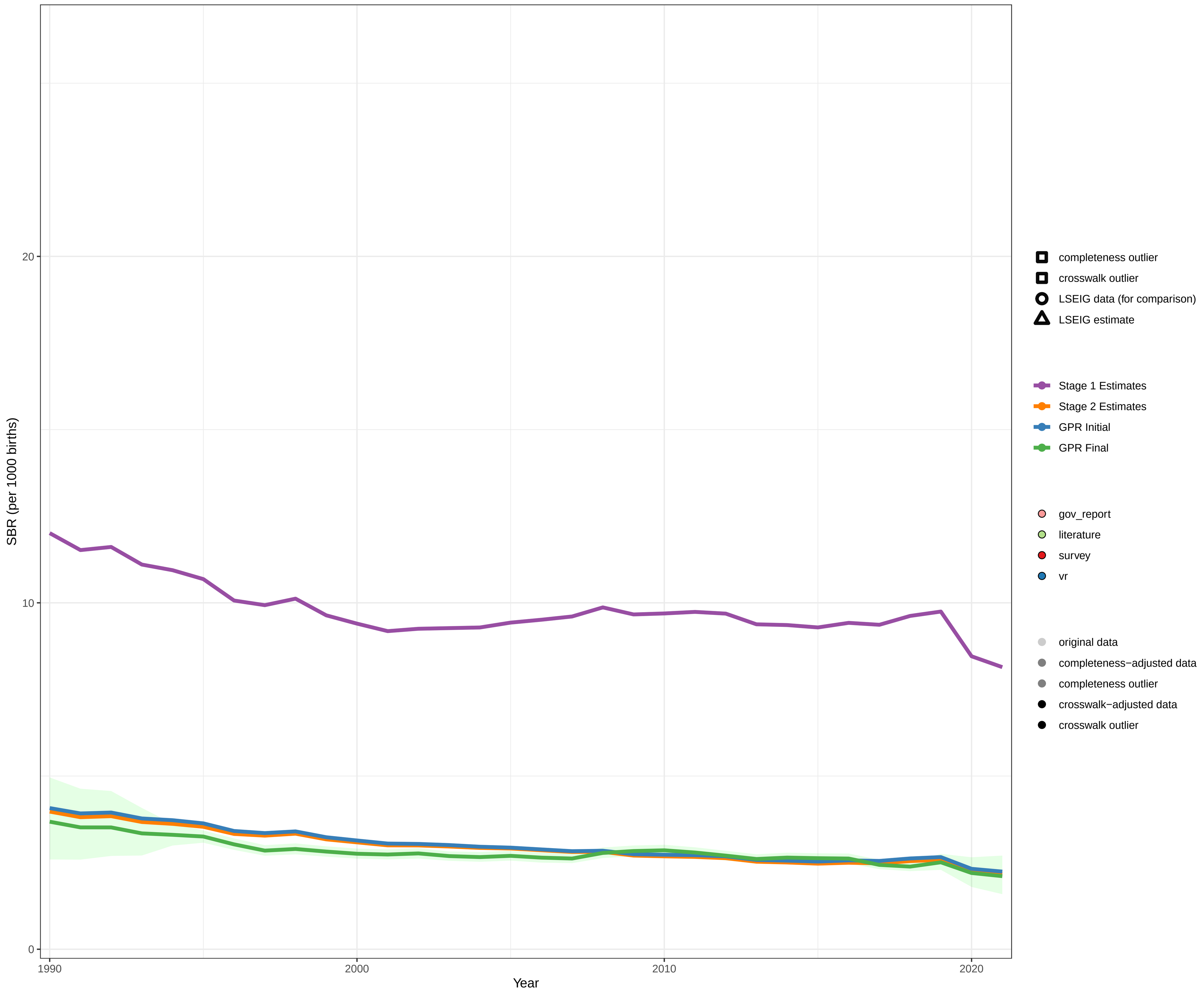

Iwate JPN_35426 High−income Asia Pacific Stillbirth Rate (SBR) − 28 weeks
lambda = 1 ; zeta = 0.6 ; scale = 15 ; amp = 0.028

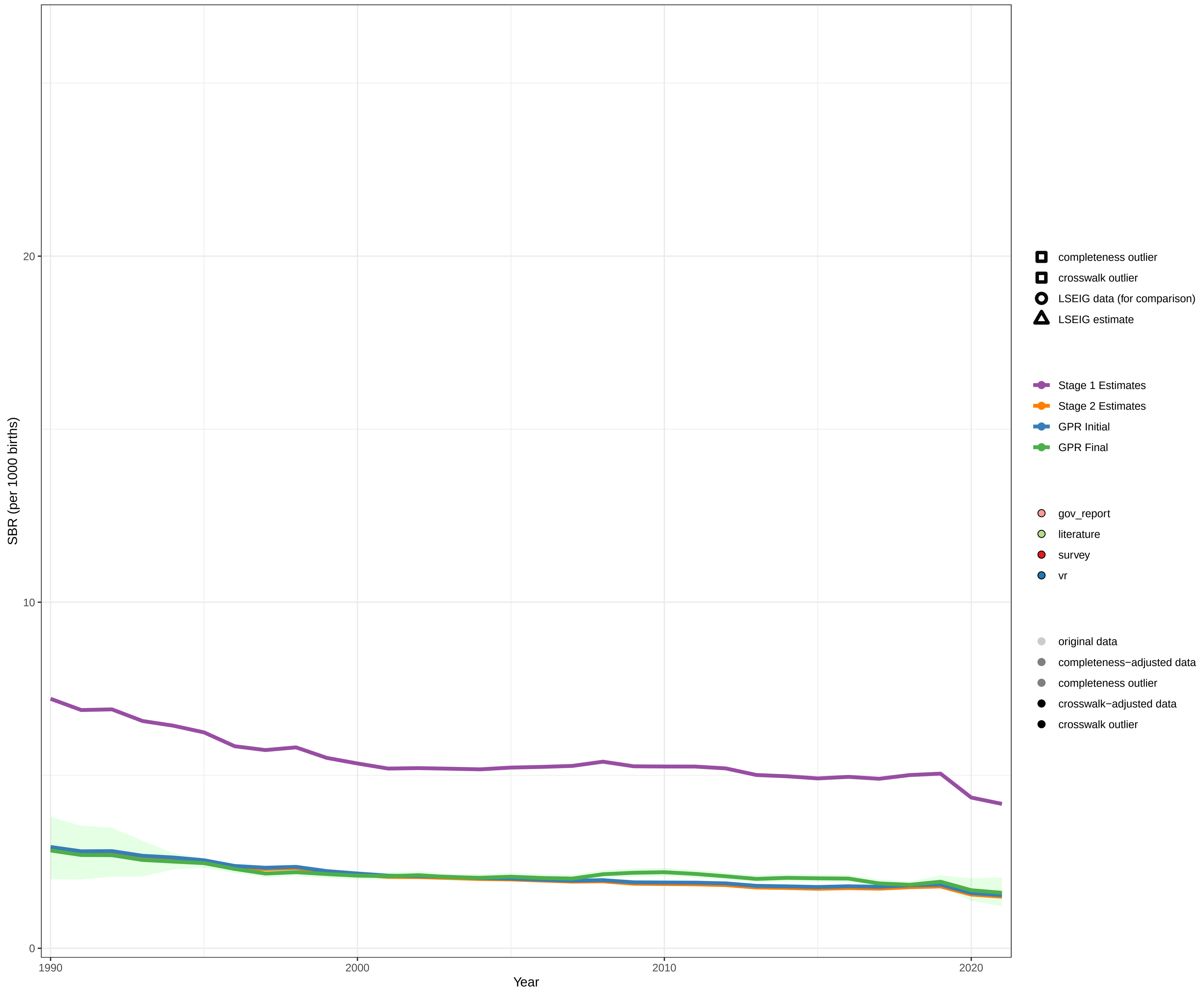

Year	Total Stillbirths (in thousands)	Total Stillbirths (in thousands)	SBR (per 1000 births)	SBR (per 1000 births)	SBR/NMR	SBR/NMR
	20 weeks	28 weeks	20 weeks	28 weeks	20 weeks	28 weeks
1990	0.1 (0.0 to 0.1)	0.0 (0.0 to 0.1)	3.7 (2.6 to 5.0)	2.8 (2.0 to 3.8)	1.54 (1.09 to 2.08)	1.18 (0.84 to 1.59)
1995	0.0 (0.0 to 0.0)	0.0 (0.0 to 0.0)	3.3 (3.1 to 3.4)	2.5 (2.3 to 2.6)	1.68 (1.59 to 1.78)	1.27 (1.20 to 1.34)
2000	0.0 (0.0 to 0.0)	0.0 (0.0 to 0.0)	2.8 (2.6 to 2.9)	2.1 (2.0 to 2.2)	1.77 (1.68 to 1.86)	1.35 (1.28 to 1.42)
2005	0.0 (0.0 to 0.0)	0.0 (0.0 to 0.0)	2.7 (2.6 to 2.8)	2.1 (2.0 to 2.2)	1.85 (1.76 to 1.95)	1.42 (1.35 to 1.50)
2010	0.0 (0.0 to 0.0)	0.0 (0.0 to 0.0)	2.9 (2.7 to 3.0)	2.2 (2.1 to 2.3)	2.04 (1.94 to 2.15)	1.57 (1.49 to 1.66)
2015	0.0 (0.0 to 0.0)	0.0 (0.0 to 0.0)	2.6 (2.5 to 2.8)	2.0 (1.9 to 2.1)	2.13 (2.02 to 2.25)	1.64 (1.56 to 1.73)
2020	0.0 (0.0 to 0.0)	0.0 (0.0 to 0.0)	2.2 (1.8 to 2.7)	1.7 (1.4 to 2.0)	2.13 (1.74 to 2.57)	1.63 (1.33 to 1.96)
2021	0.0 (0.0 to 0.0)	0.0 (0.0 to 0.0)	2.1 (1.6 to 2.7)	1.6 (1.2 to 2.1)	2.16 (1.63 to 2.77)	1.64 (1.24 to 2.10)

Kagawa JPN_35460 High−income Asia Pacific Stillbirth Rate / Neonatal Mortality Rate (SBR/NMR) − 20 weeks
lambda = 1 ; zeta = 0.6 ; scale = 15 ; amp = 0.028

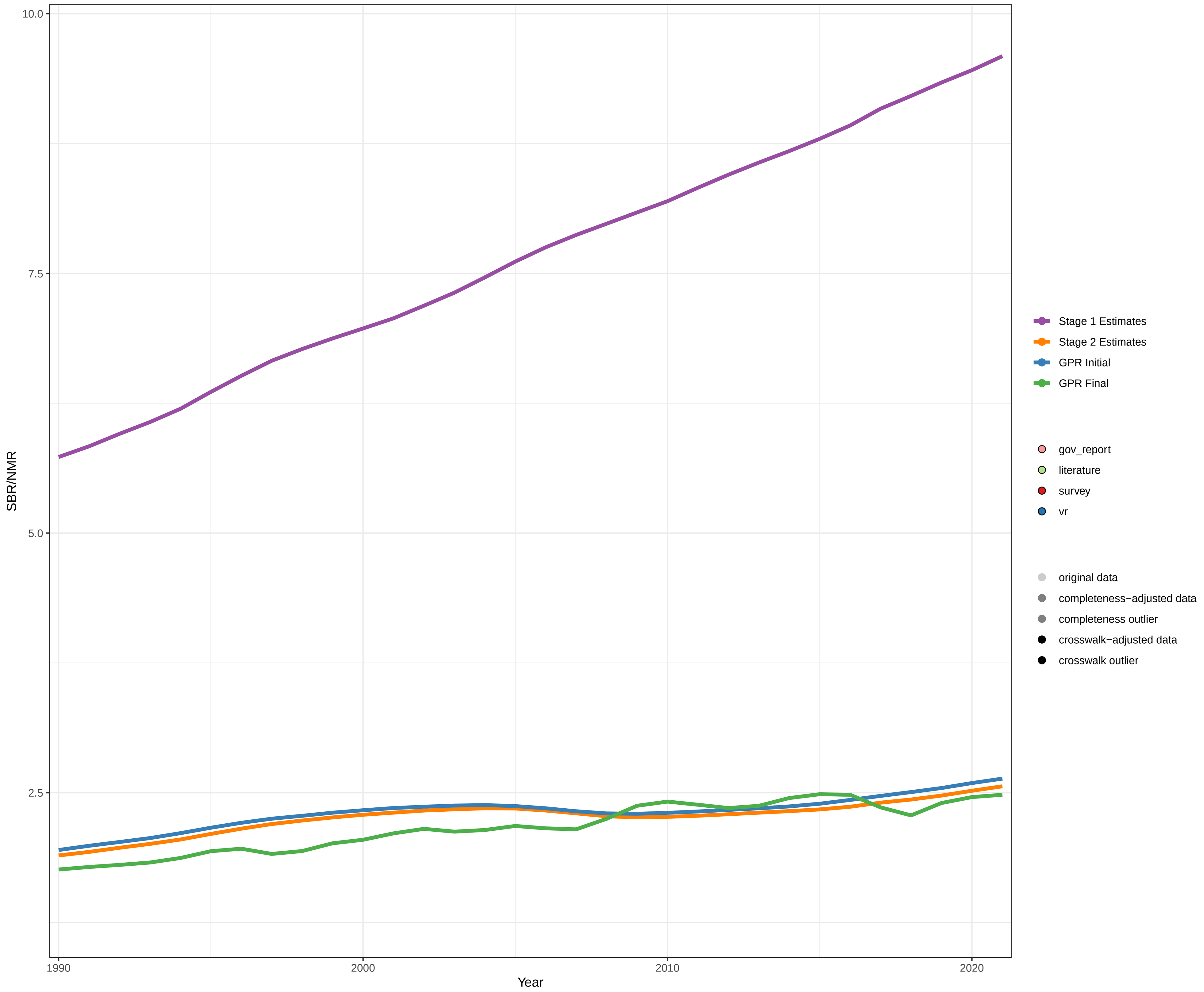

Kagawa JPN_35460 High−income Asia Pacific Stillbirth Rate / Neonatal Mortality Rate (SBR/NMR) − 28 weeks
lambda = 1 ; zeta = 0.6 ; scale = 15 ; amp = 0.028

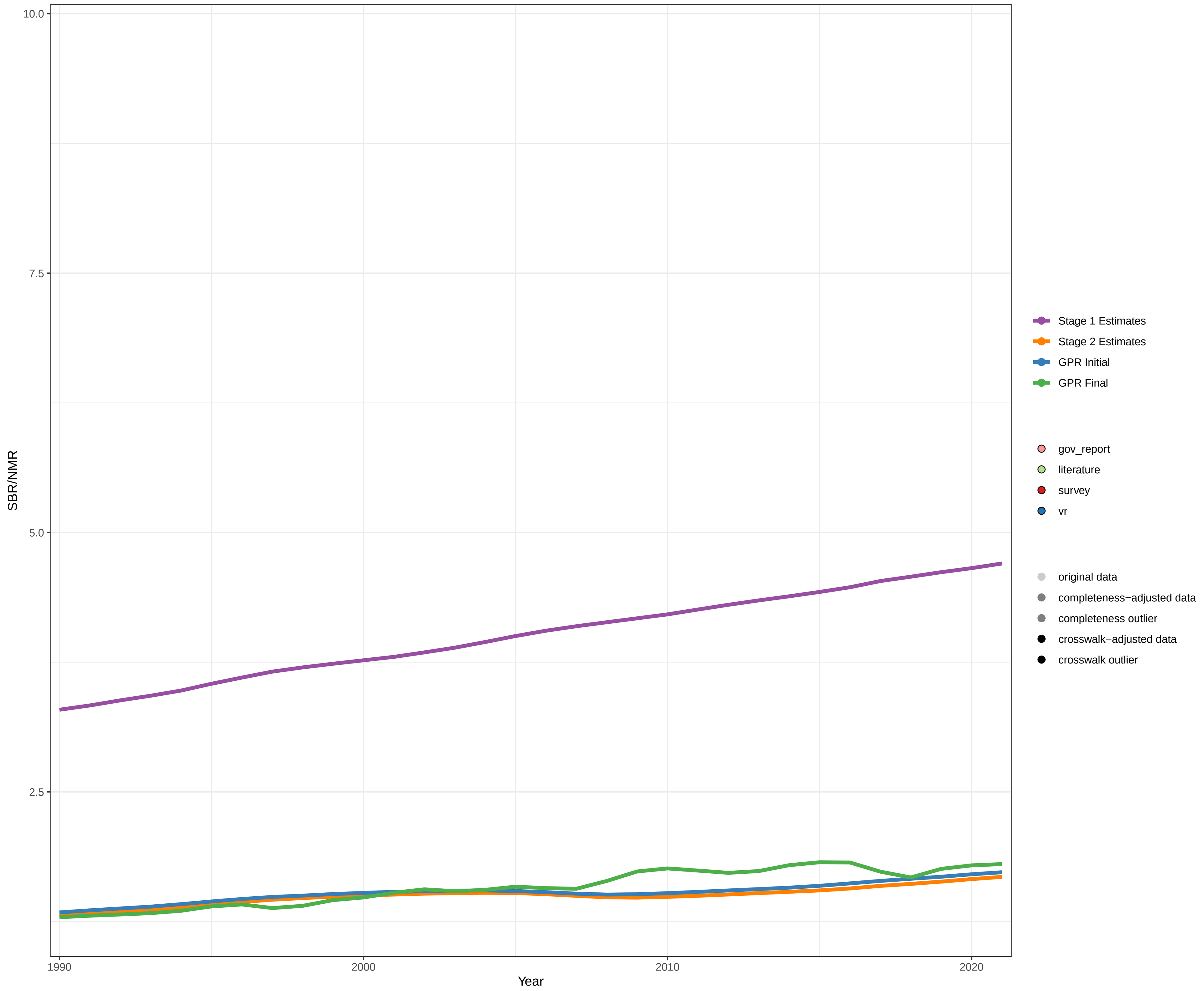

Definition	Years	Outlier	Source.Type	Title

Kagawa JPN_35460 High−income Asia Pacific Stillbirth Rate (SBR) − 20 weeks
lambda = 1 ; zeta = 0.6 ; scale = 15 ; amp = 0.028

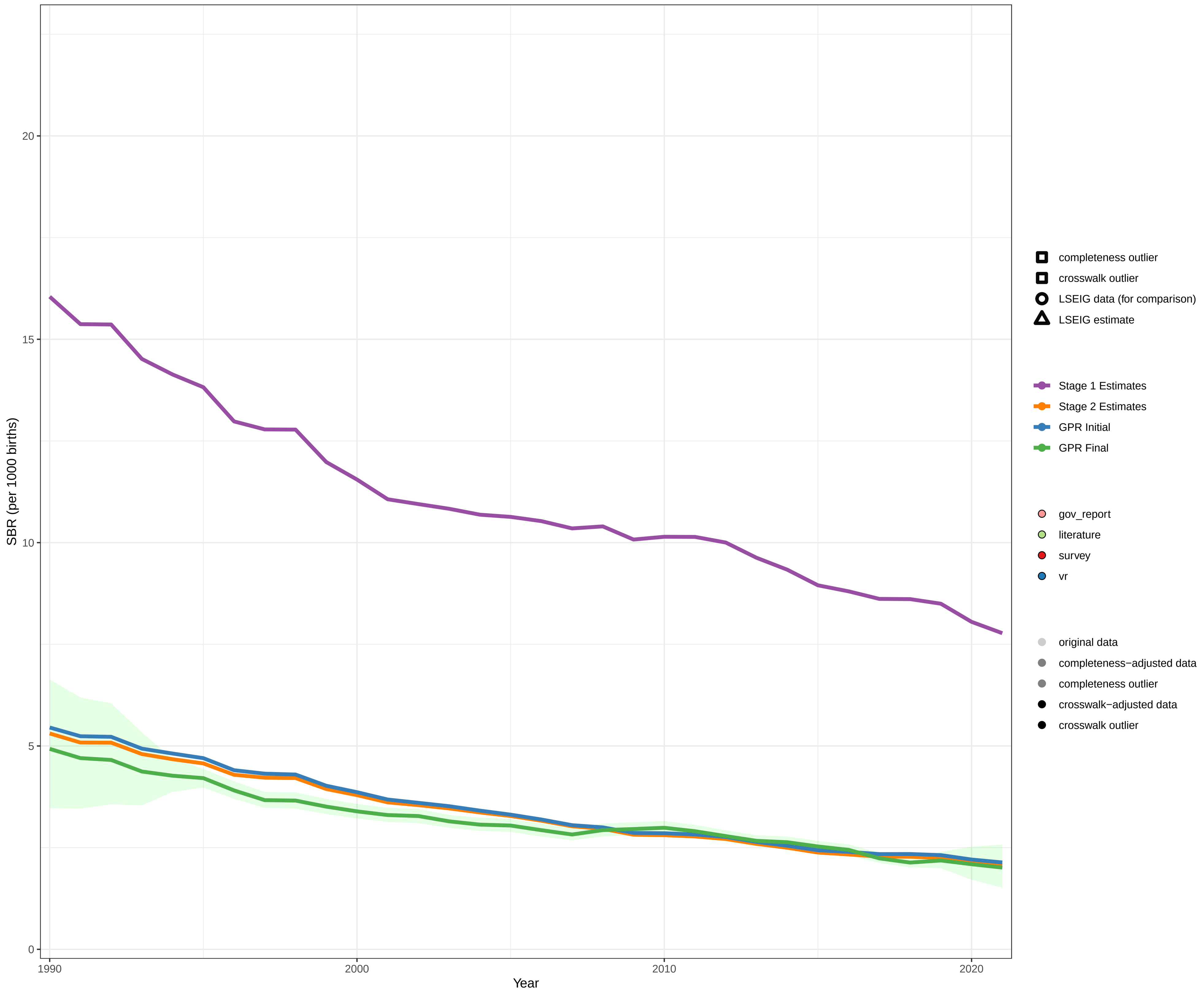

Kagawa JPN_35460 High−income Asia Pacific Stillbirth Rate (SBR) − 28 weeks
lambda = 1 ; zeta = 0.6 ; scale = 15 ; amp = 0.028

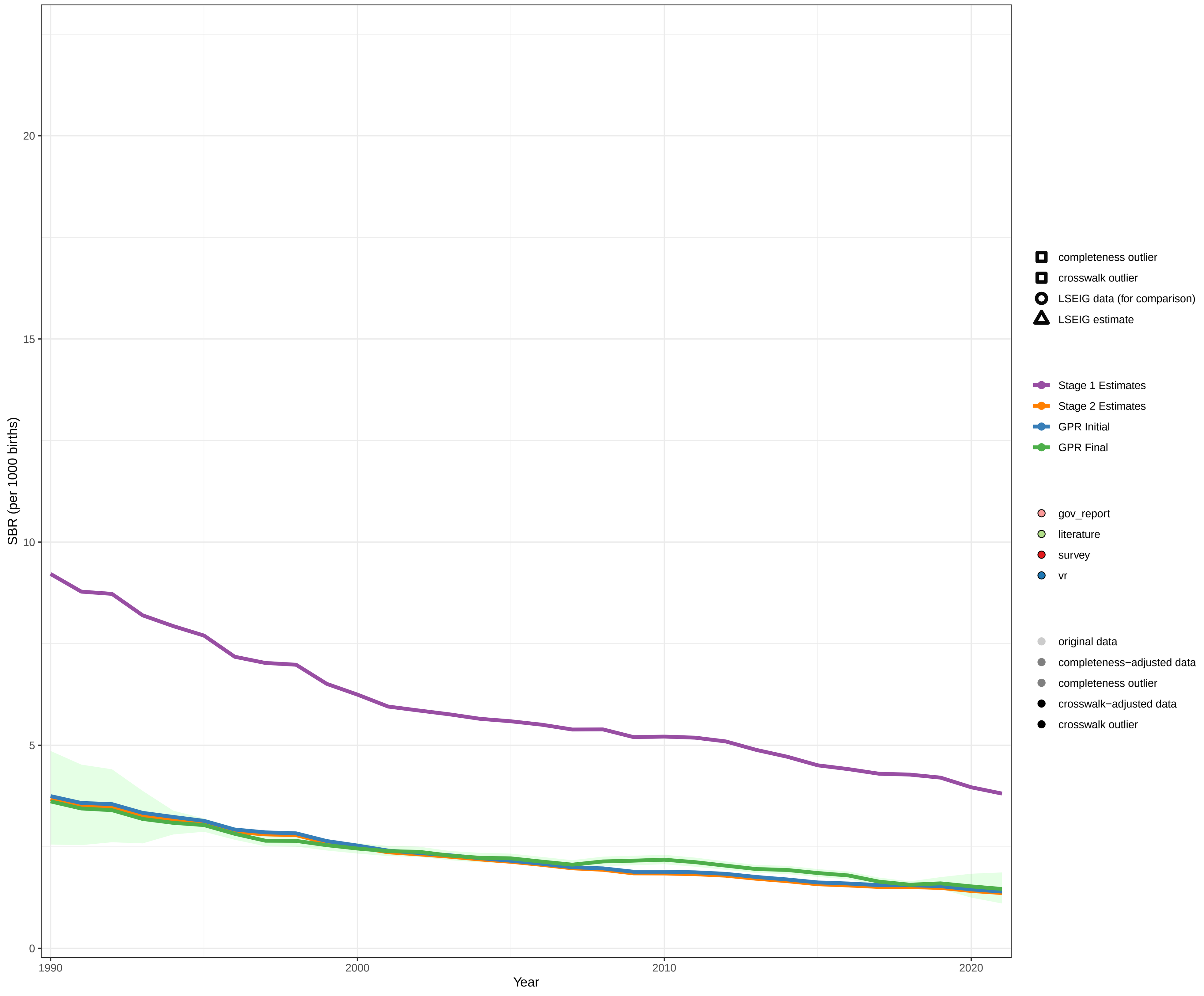

Year	Total Stillbirths (in thousands)	Total Stillbirths (in thousands)	SBR (per 1000 births)	SBR (per 1000 births)	SBR/NMR	SBR/NMR
	20 weeks	28 weeks	20 weeks	28 weeks	20 weeks	28 weeks
1990	0.0 (0.0 to 0.1)	0.0 (0.0 to 0.0)	4.9 (3.5 to 6.6)	3.6 (2.6 to 4.9)	1.76 (1.24 to 2.37)	1.29 (0.91 to 1.74)
1995	0.0 (0.0 to 0.0)	0.0 (0.0 to 0.0)	4.2 (4.0 to 4.4)	3.0 (2.9 to 3.2)	1.94 (1.83 to 2.04)	1.40 (1.32 to 1.47)
2000	0.0 (0.0 to 0.0)	0.0 (0.0 to 0.0)	3.4 (3.2 to 3.6)	2.5 (2.3 to 2.6)	2.05 (1.94 to 2.16)	1.48 (1.41 to 1.56)
2005	0.0 (0.0 to 0.0)	0.0 (0.0 to 0.0)	3.0 (2.9 to 3.2)	2.2 (2.1 to 2.3)	2.18 (2.07 to 2.30)	1.59 (1.51 to 1.67)
2010	0.0 (0.0 to 0.0)	0.0 (0.0 to 0.0)	3.0 (2.8 to 3.2)	2.2 (2.1 to 2.3)	2.41 (2.29 to 2.55)	1.76 (1.67 to 1.86)
2015	0.0 (0.0 to 0.0)	0.0 (0.0 to 0.0)	2.5 (2.4 to 2.7)	1.9 (1.8 to 2.0)	2.49 (2.36 to 2.62)	1.82 (1.73 to 1.92)
2020	0.0 (0.0 to 0.0)	0.0 (0.0 to 0.0)	2.1 (1.7 to 2.5)	1.5 (1.2 to 1.8)	2.46 (2.01 to 2.97)	1.79 (1.47 to 2.16)
2021	0.0 (0.0 to 0.0)	0.0 (0.0 to 0.0)	2.0 (1.5 to 2.6)	1.5 (1.1 to 1.9)	2.48 (1.87 to 3.18)	1.80 (1.36 to 2.31)

Kagoshima JPN_35469 High−income Asia Pacific Stillbirth Rate / Neonatal Mortality Rate (SBR/NMR) − 20 weeks
lambda = 1 ; zeta = 0.6 ; scale = 15 ; amp = 0.028

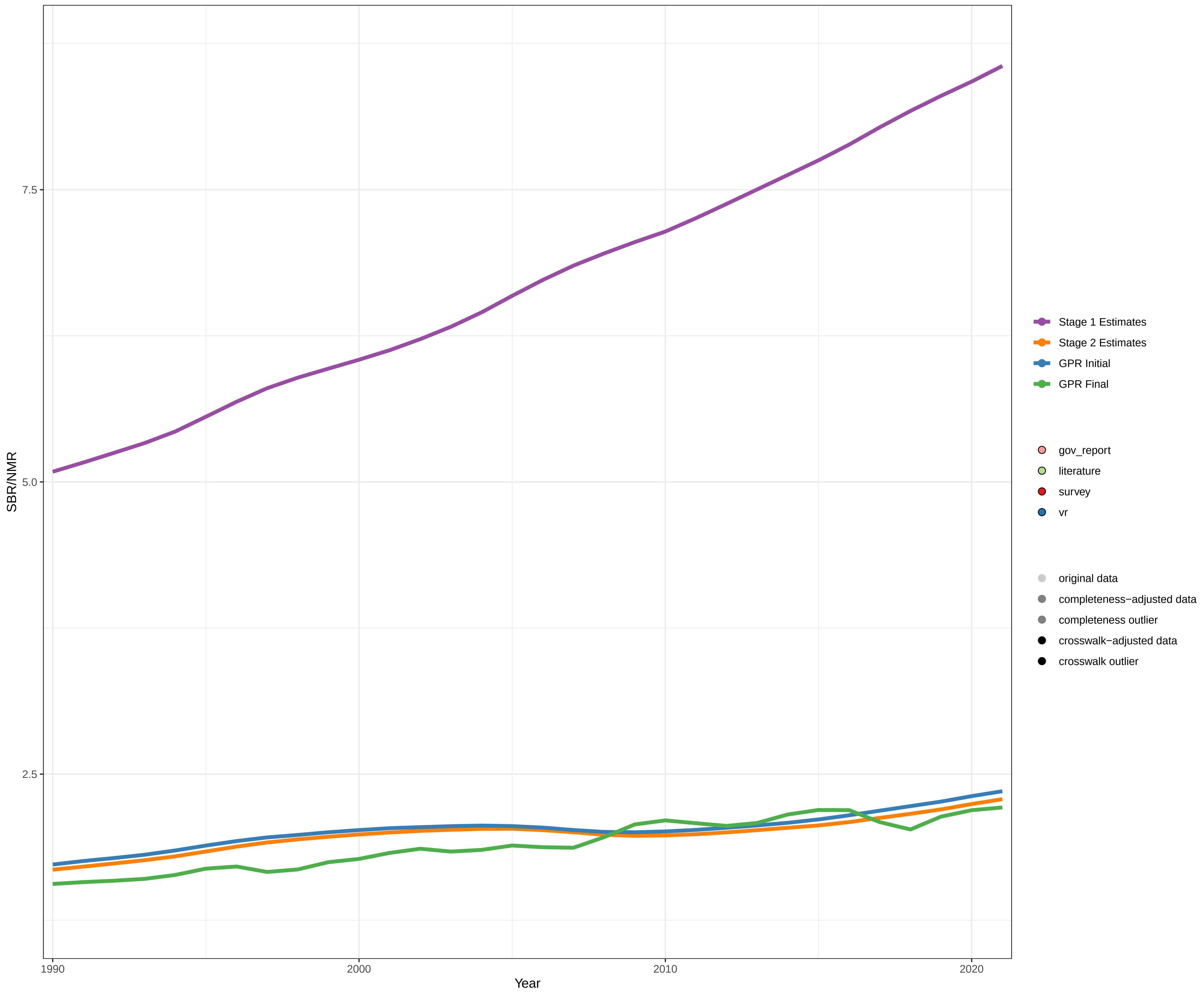

Kagoshima JPN_35469 High−income Asia Pacific Stillbirth Rate / Neonatal Mortality Rate (SBR/NMR) − 28 weeks
lambda = 1 ; zeta = 0.6 ; scale = 15 ; amp = 0.028

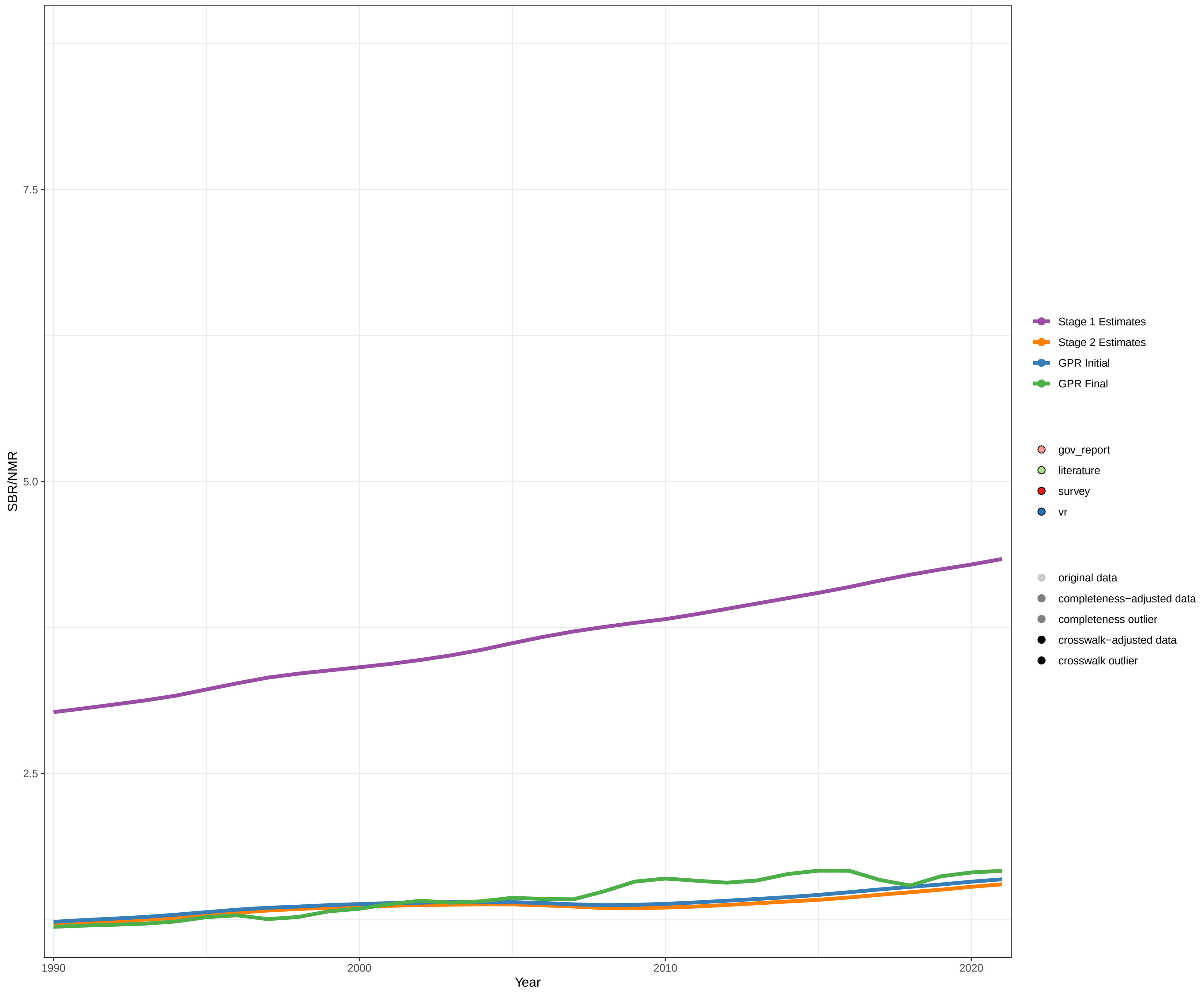

Definition	Years	Outlier	Source.Type	Title

Kagoshima JPN_35469 High−income Asia Pacific Stillbirth Rate (SBR) − 20 weeks
lambda = 1 ; zeta = 0.6 ; scale = 15 ; amp = 0.028

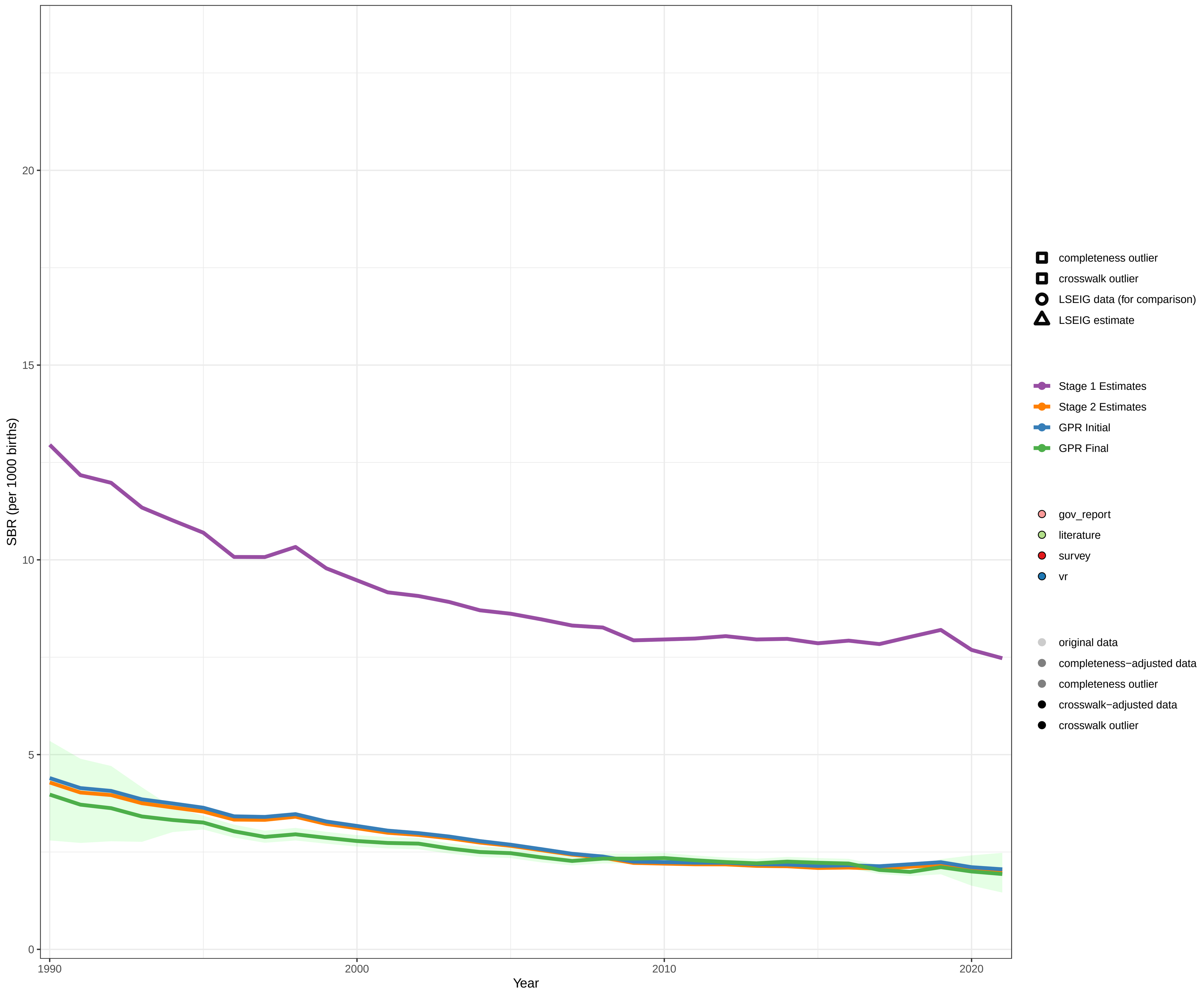

Kagoshima JPN_35469 High−income Asia Pacific Stillbirth Rate (SBR) − 28 weeks
lambda = 1 ; zeta = 0.6 ; scale = 15 ; amp = 0.028

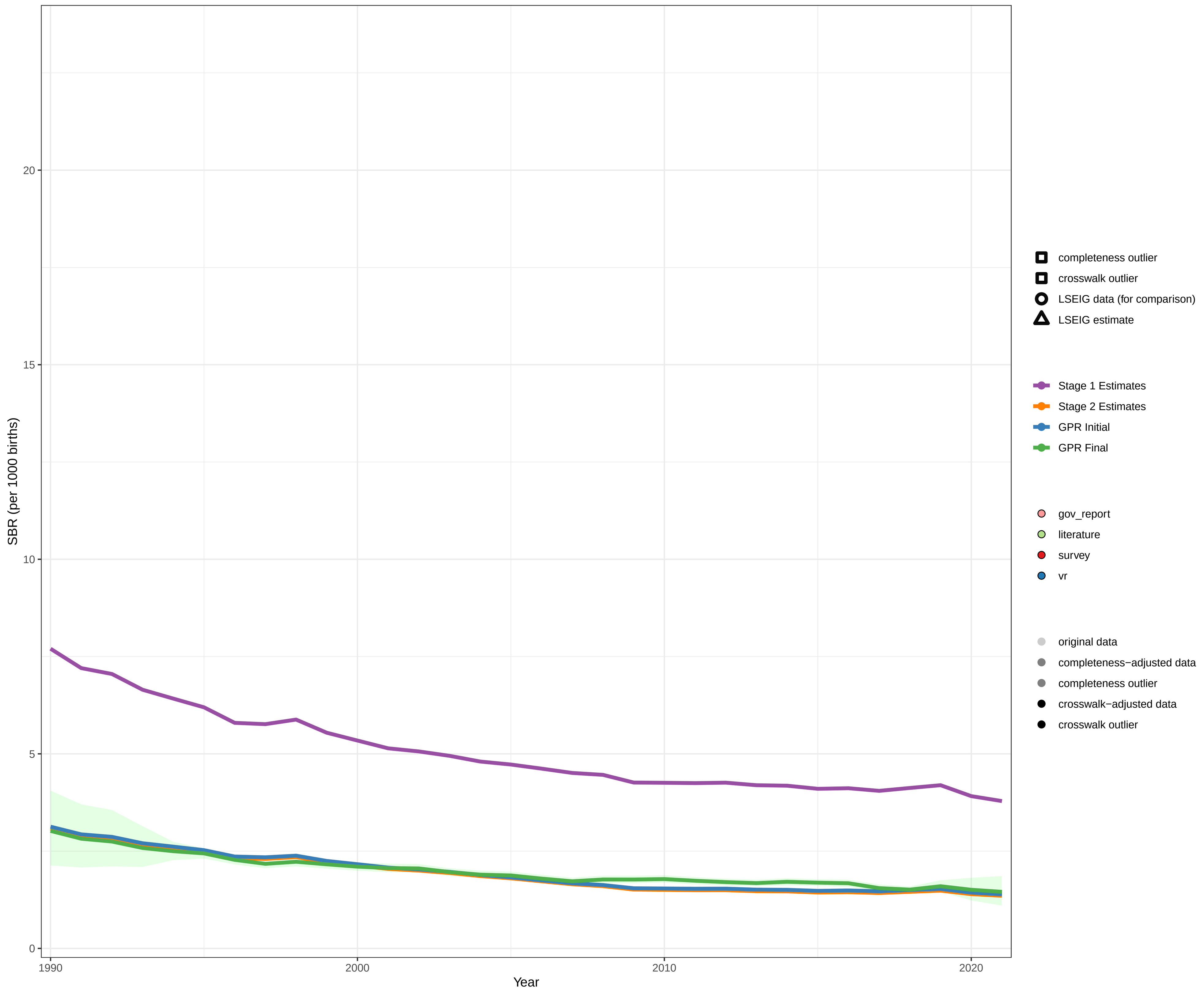

Year	Total Stillbirths (in thousands)	Total Stillbirths (in thousands)	SBR (per 1000 births)	SBR (per 1000 births)	SBR/NMR	SBR/NMR
	20 weeks	28 weeks	20 weeks	28 weeks	20 weeks	28 weeks
1990	0.1 (0.1 to 0.1)	0.1 (0.0 to 0.1)	4.0 (2.8 to 5.4)	3.0 (2.1 to 4.1)	1.56 (1.10 to 2.10)	1.19 (0.84 to 1.59)
1995	0.1 (0.1 to 0.1)	0.0 (0.0 to 0.0)	3.3 (3.1 to 3.4)	2.4 (2.3 to 2.6)	1.69 (1.60 to 1.78)	1.27 (1.20 to 1.34)
2000	0.0 (0.0 to 0.0)	0.0 (0.0 to 0.0)	2.8 (2.6 to 2.9)	2.1 (2.0 to 2.2)	1.77 (1.68 to 1.87)	1.34 (1.27 to 1.41)
2005	0.0 (0.0 to 0.0)	0.0 (0.0 to 0.0)	2.5 (2.3 to 2.6)	1.9 (1.8 to 2.0)	1.89 (1.79 to 1.99)	1.43 (1.36 to 1.51)
2010	0.0 (0.0 to 0.0)	0.0 (0.0 to 0.0)	2.3 (2.2 to 2.5)	1.8 (1.7 to 1.9)	2.10 (2.00 to 2.22)	1.60 (1.52 to 1.69)
2015	0.0 (0.0 to 0.0)	0.0 (0.0 to 0.0)	2.2 (2.1 to 2.3)	1.7 (1.6 to 1.8)	2.19 (2.08 to 2.31)	1.67 (1.58 to 1.76)
2020	0.0 (0.0 to 0.0)	0.0 (0.0 to 0.0)	2.0 (1.6 to 2.4)	1.5 (1.2 to 1.8)	2.19 (1.79 to 2.64)	1.65 (1.35 to 1.99)
2021	0.0 (0.0 to 0.0)	0.0 (0.0 to 0.0)	1.9 (1.5 to 2.5)	1.5 (1.1 to 1.9)	2.22 (1.67 to 2.84)	1.67 (1.26 to 2.13)

Kanagawa JPN_35437 High−income Asia Pacific Stillbirth Rate / Neonatal Mortality Rate (SBR/NMR) − 20 weeks
lambda = 1 ; zeta = 0.6 ; scale = 15 ; amp = 0.028

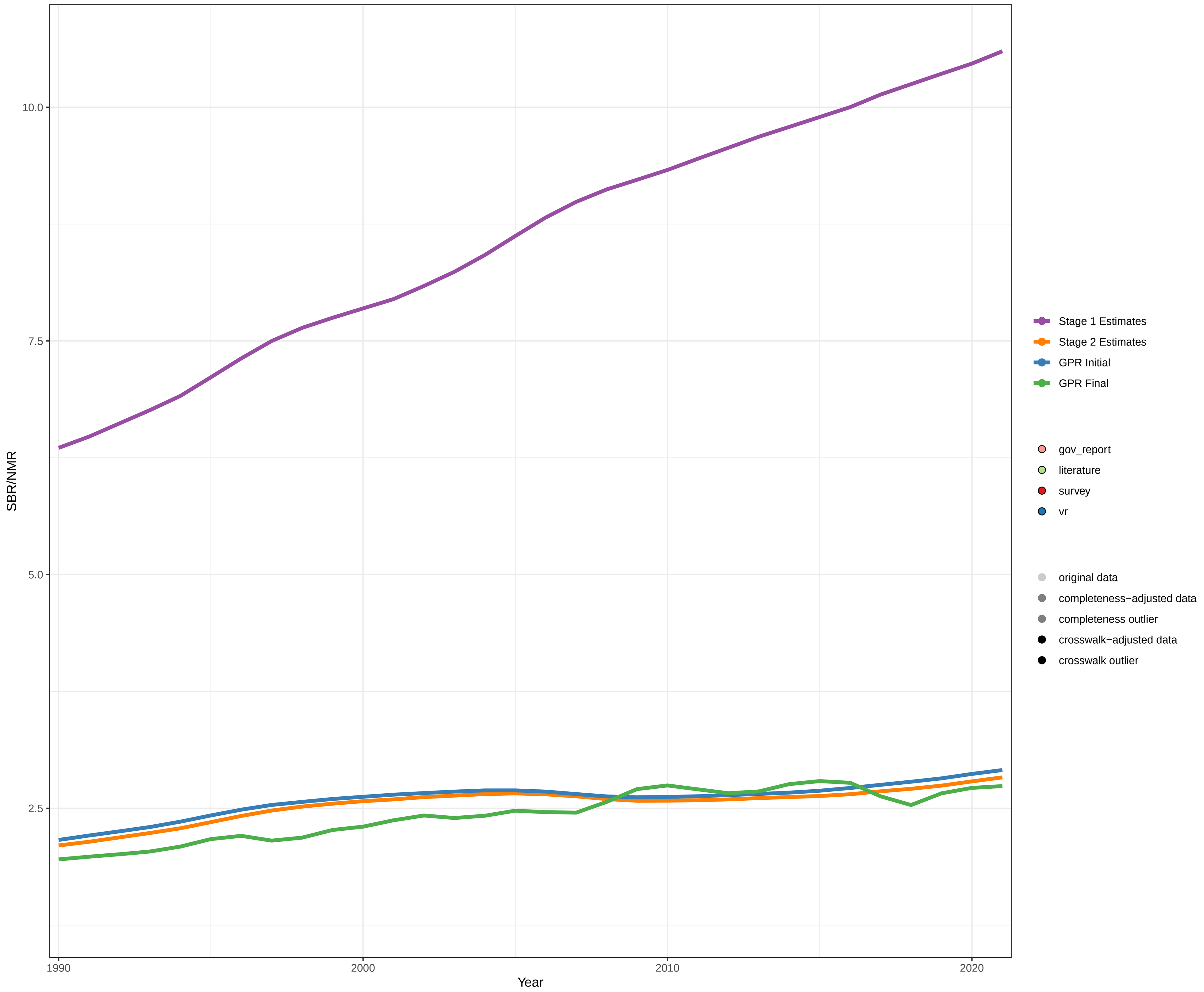

Kanagawa JPN_35437 High−income Asia Pacific Stillbirth Rate / Neonatal Mortality Rate (SBR/NMR) − 28 weeks
lambda = 1 ; zeta = 0.6 ; scale = 15 ; amp = 0.028

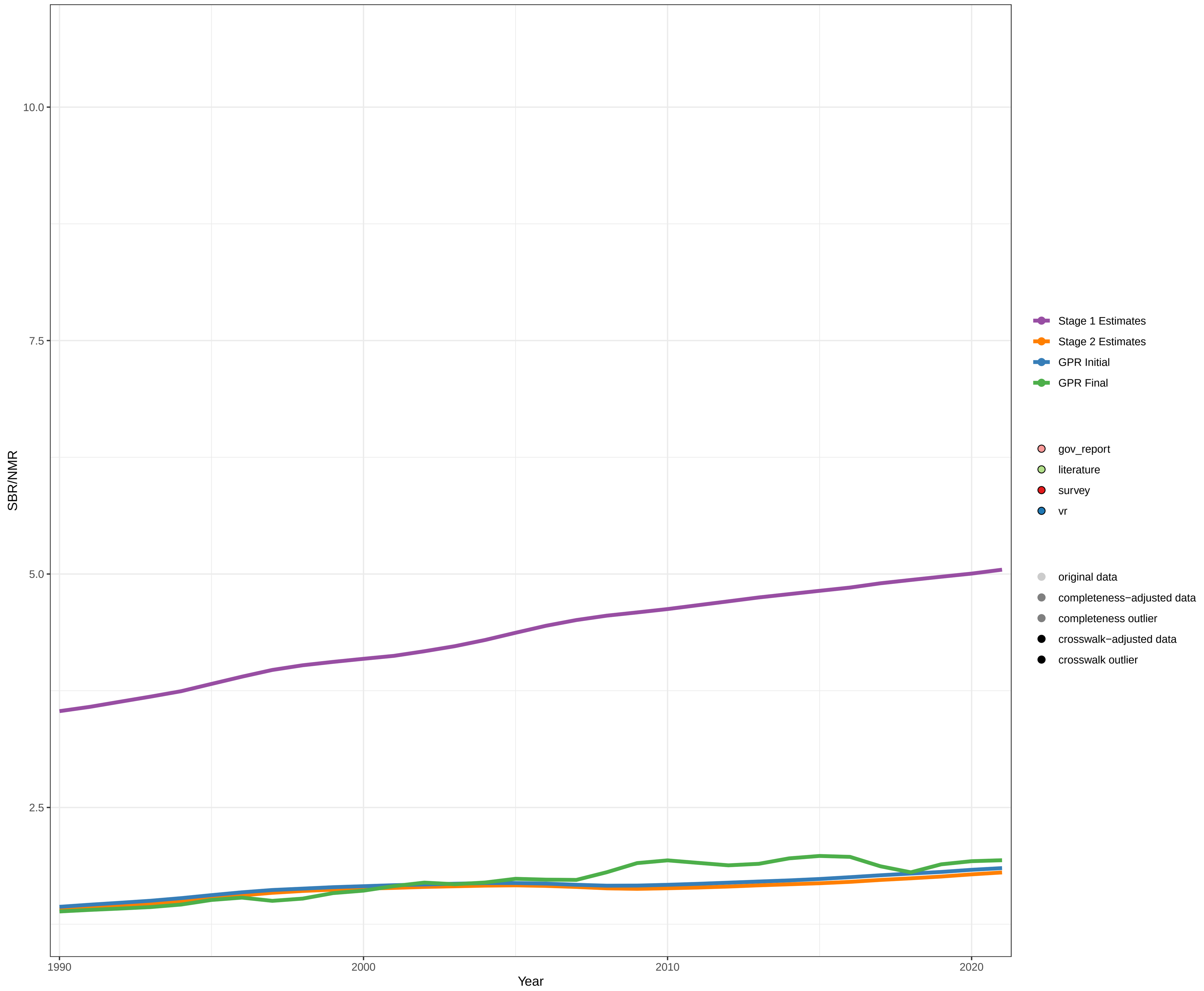

Definition	Years	Outlier	Source.Type	Title

Kanagawa JPN_35437 High−income Asia Pacific Stillbirth Rate (SBR) − 20 weeks
lambda = 1 ; zeta = 0.6 ; scale = 15 ; amp = 0.028

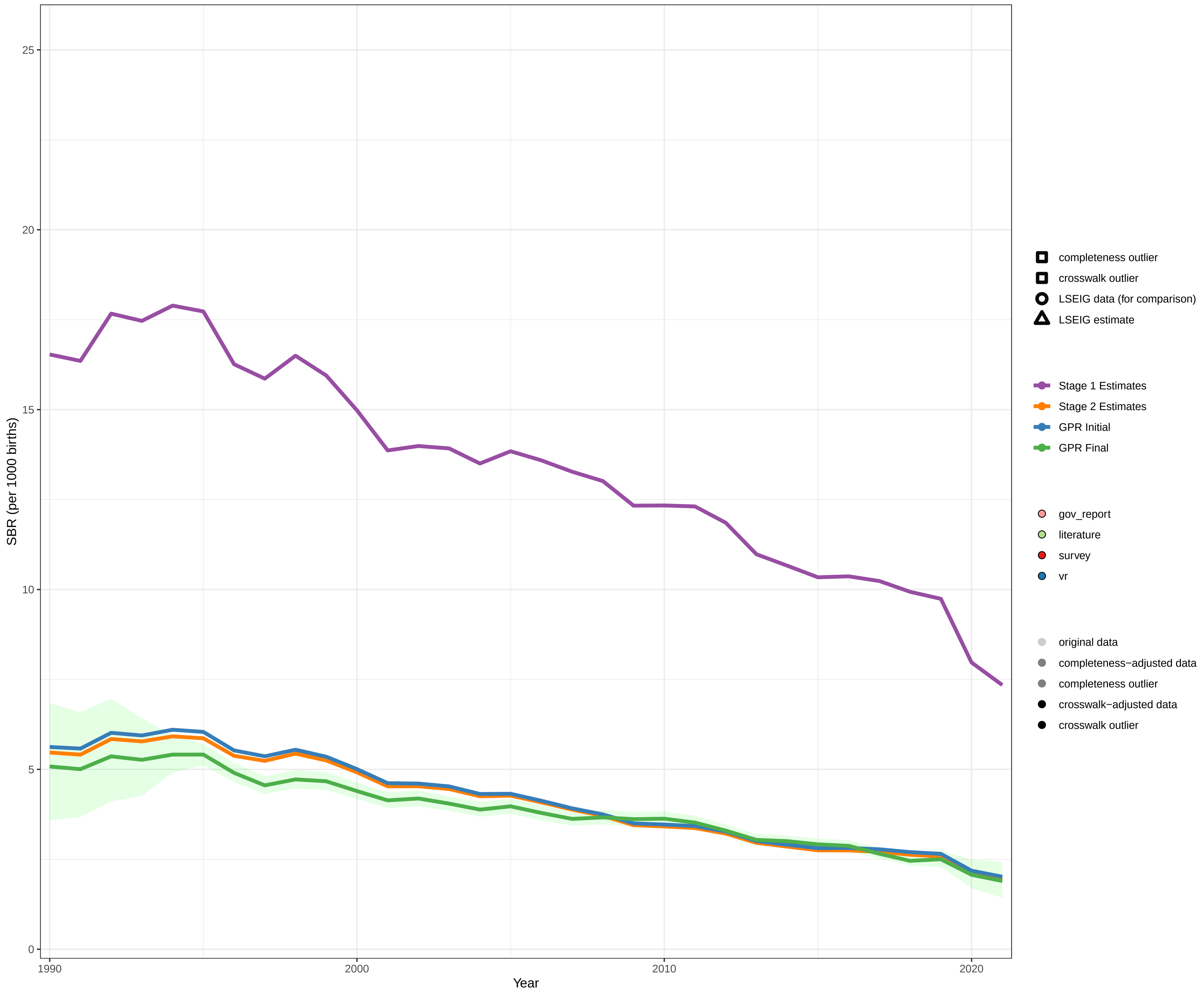

Kanagawa JPN_35437 High−income Asia Pacific Stillbirth Rate (SBR) − 28 weeks
lambda = 1 ; zeta = 0.6 ; scale = 15 ; amp = 0.028

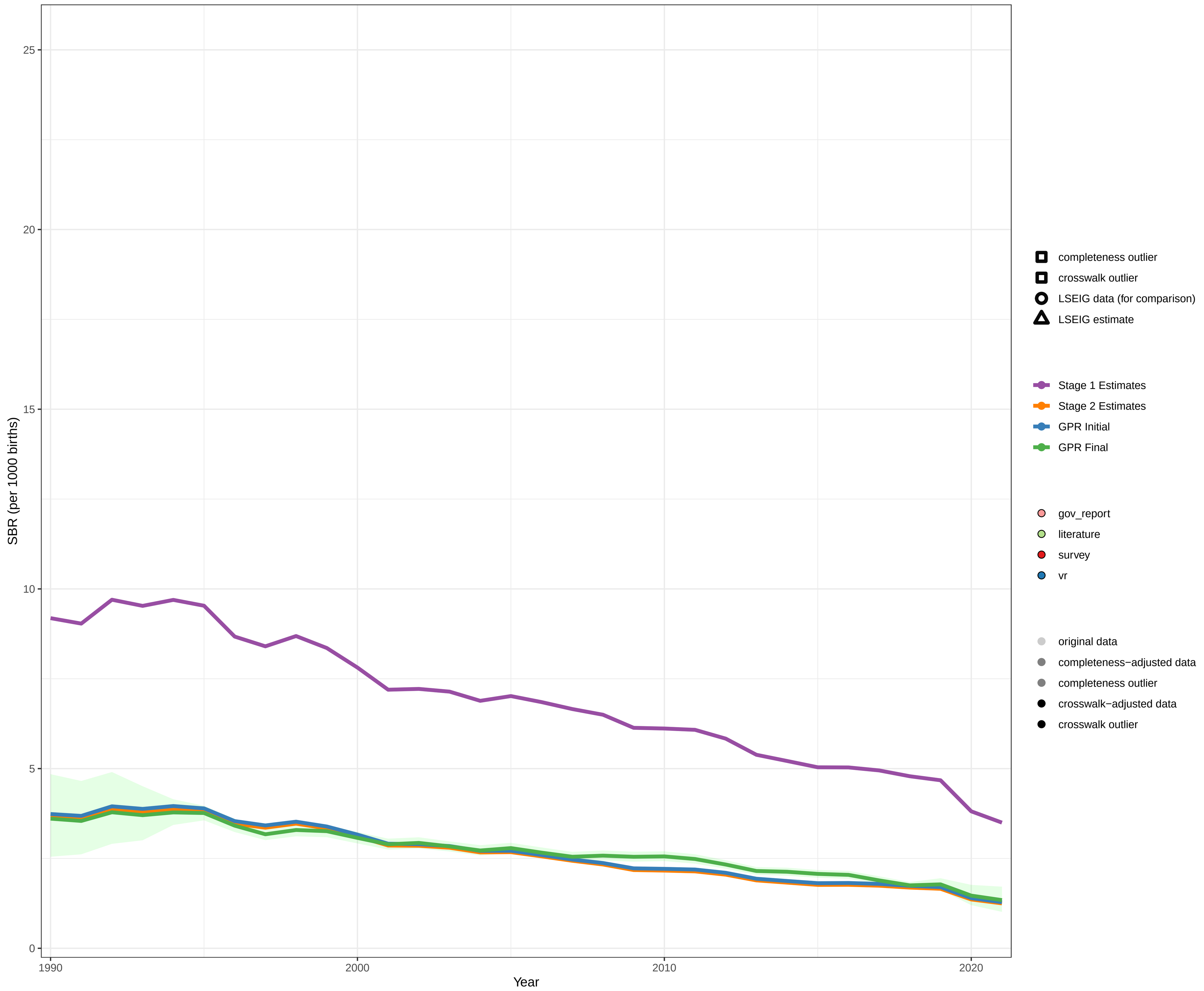

Year	Total Stillbirths (in thousands)	Total Stillbirths (in thousands)	SBR (per 1000 births)	SBR (per 1000 births)	SBR/NMR	SBR/NMR
	20 weeks	28 weeks	20 weeks	28 weeks	20 weeks	28 weeks
1990	0.4 (0.3 to 0.5)	0.3 (0.2 to 0.4)	5.1 (3.6 to 6.8)	3.6 (2.5 to 4.8)	1.95 (1.37 to 2.63)	1.39 (0.98 to 1.86)
1995	0.4 (0.4 to 0.5)	0.3 (0.3 to 0.3)	5.4 (5.1 to 5.7)	3.8 (3.6 to 4.0)	2.17 (2.05 to 2.29)	1.51 (1.43 to 1.59)
2000	0.4 (0.3 to 0.4)	0.3 (0.2 to 0.3)	4.4 (4.2 to 4.6)	3.1 (2.9 to 3.2)	2.30 (2.19 to 2.43)	1.61 (1.53 to 1.70)
2005	0.3 (0.3 to 0.3)	0.2 (0.2 to 0.2)	4.0 (3.8 to 4.2)	2.8 (2.6 to 2.9)	2.47 (2.35 to 2.61)	1.74 (1.65 to 1.83)
2010	0.3 (0.3 to 0.3)	0.2 (0.2 to 0.2)	3.6 (3.4 to 3.8)	2.6 (2.4 to 2.7)	2.74 (2.60 to 2.90)	1.93 (1.84 to 2.04)
2015	0.2 (0.2 to 0.2)	0.2 (0.1 to 0.2)	2.9 (2.8 to 3.1)	2.1 (2.0 to 2.2)	2.79 (2.65 to 2.94)	1.98 (1.88 to 2.09)
2020	0.1 (0.1 to 0.2)	0.1 (0.1 to 0.1)	2.1 (1.7 to 2.5)	1.5 (1.2 to 1.8)	2.72 (2.22 to 3.28)	1.92 (1.58 to 2.32)
2021	0.1 (0.1 to 0.1)	0.1 (0.1 to 0.1)	1.9 (1.4 to 2.4)	1.3 (1.0 to 1.7)	2.74 (2.06 to 3.51)	1.94 (1.46 to 2.47)

Kochi JPN_35462 High−income Asia Pacific Stillbirth Rate / Neonatal Mortality Rate (SBR/NMR) − 20 weeks
lambda = 1 ; zeta = 0.6 ; scale = 15 ; amp = 0.028

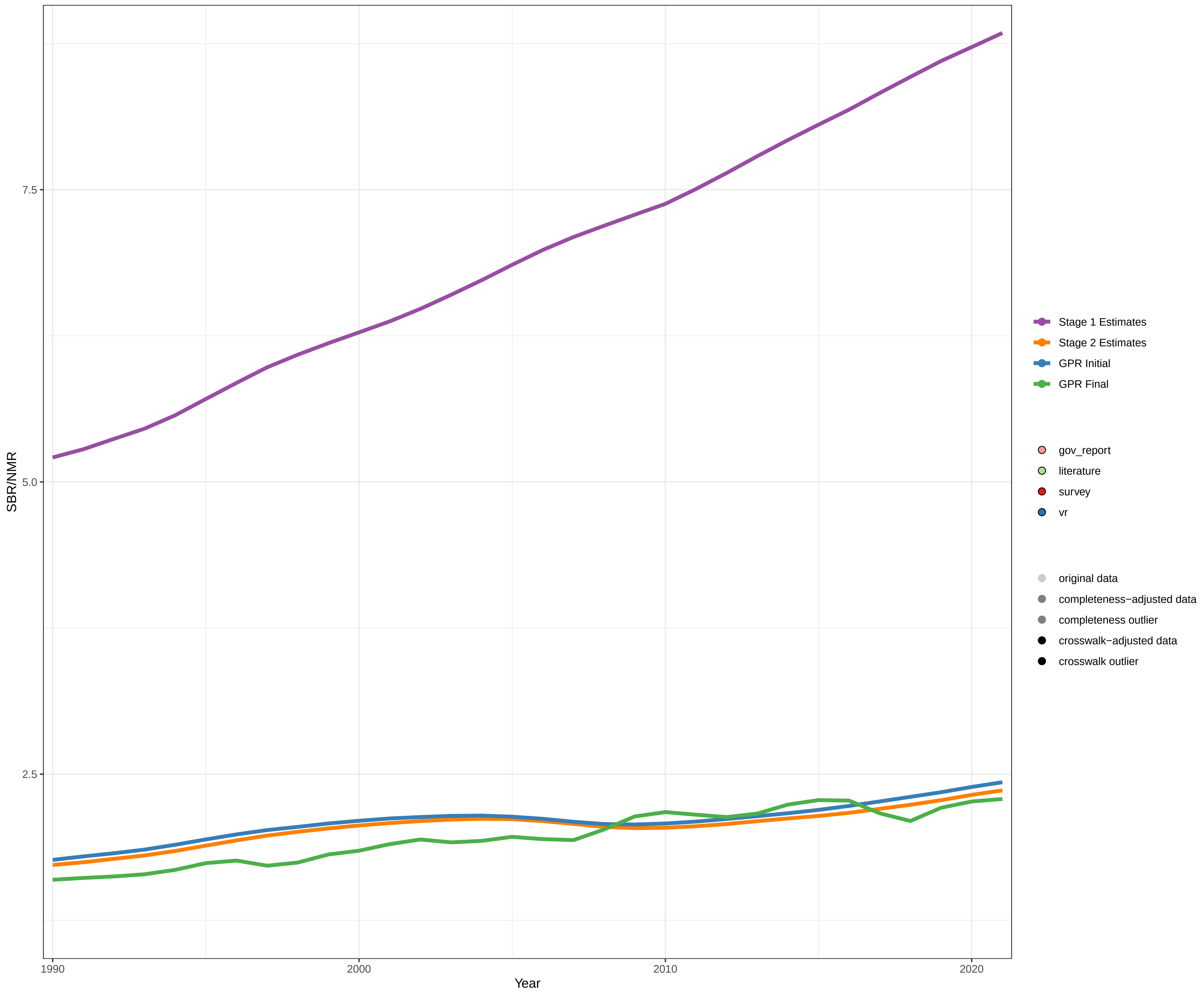

Kochi JPN_35462 High−income Asia Pacific Stillbirth Rate / Neonatal Mortality Rate (SBR/NMR) − 28 weeks
lambda = 1 ; zeta = 0.6 ; scale = 15 ; amp = 0.028

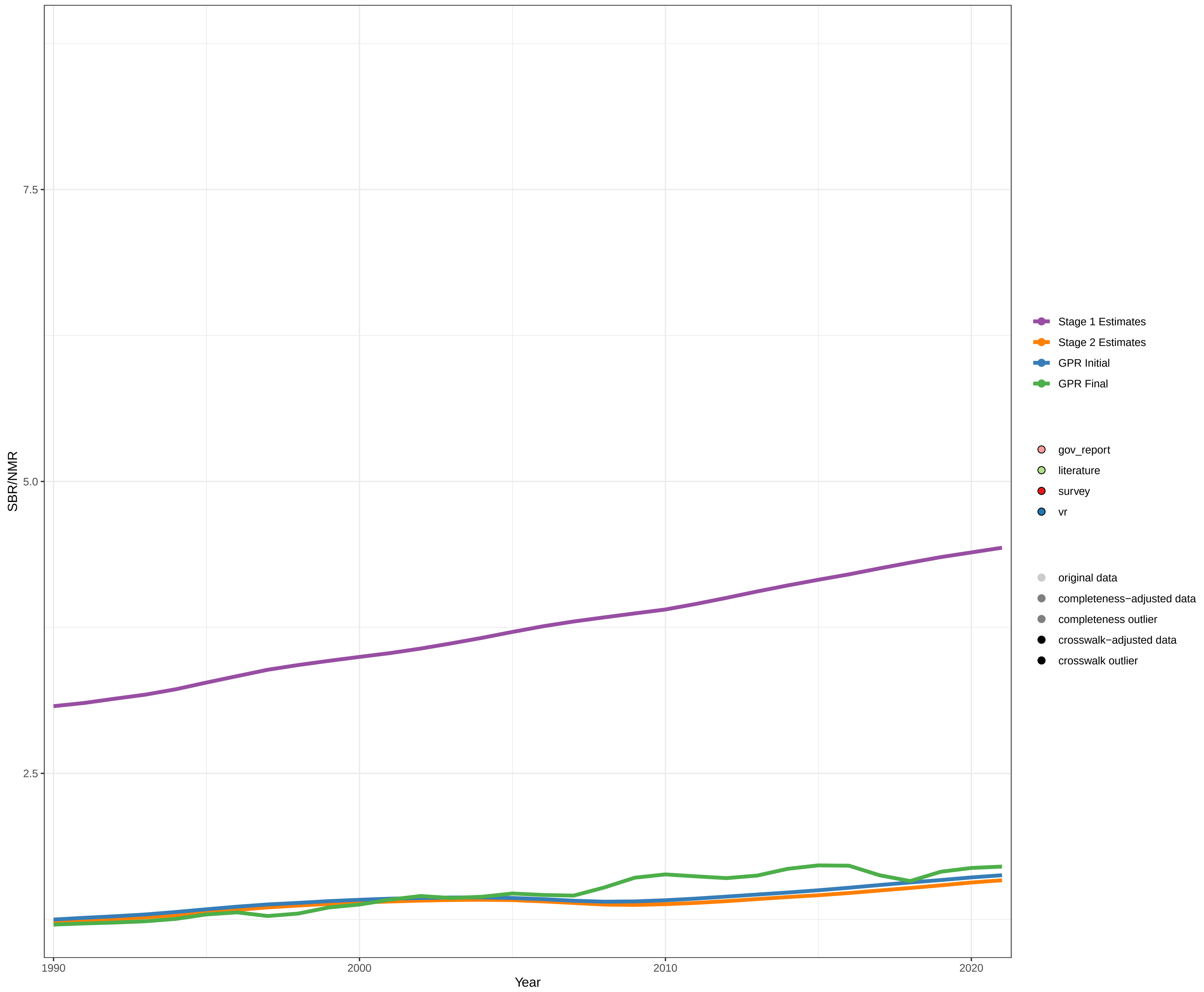

Definition	Years	Outlier	Source.Type	Title

Kochi JPN_35462 High−income Asia Pacific Stillbirth Rate (SBR) − 20 weeks
lambda = 1 ; zeta = 0.6 ; scale = 15 ; amp = 0.028

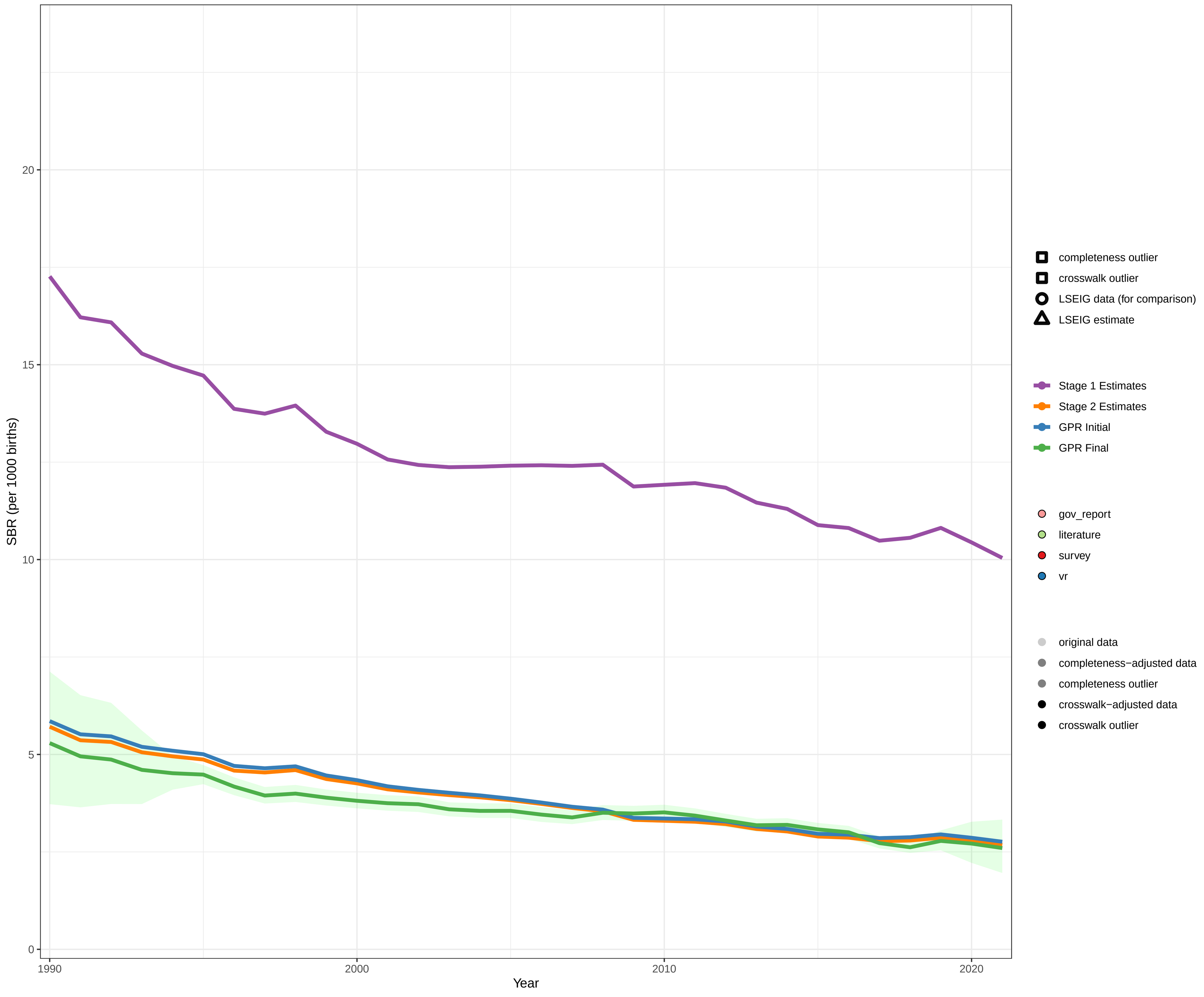

Kochi JPN_35462 High−income Asia Pacific Stillbirth Rate (SBR) − 28 weeks
lambda = 1 ; zeta = 0.6 ; scale = 15 ; amp = 0.028

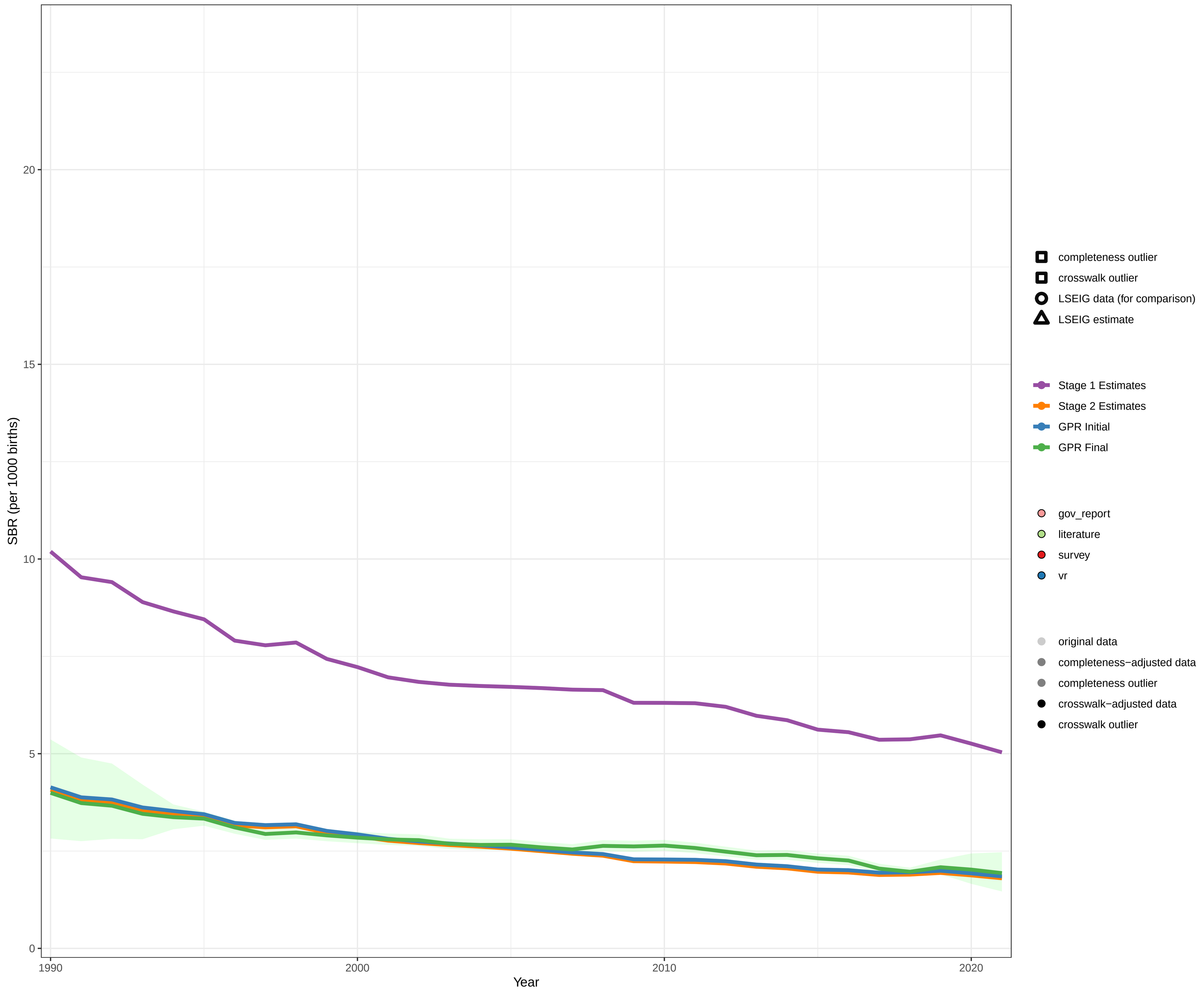

Year	Total Stillbirths (in thousands)	Total Stillbirths (in thousands)	SBR (per 1000 births)	SBR (per 1000 births)	SBR/NMR	SBR/NMR
	20 weeks	28 weeks	20 weeks	28 weeks	20 weeks	28 weeks
1990	0.0 (0.0 to 0.1)	0.0 (0.0 to 0.0)	5.3 (3.7 to 7.1)	4.0 (2.8 to 5.4)	1.60 (1.12 to 2.15)	1.21 (0.85 to 1.62)
1995	0.0 (0.0 to 0.0)	0.0 (0.0 to 0.0)	4.5 (4.2 to 4.7)	3.3 (3.2 to 3.5)	1.74 (1.64 to 1.83)	1.29 (1.22 to 1.36)
2000	0.0 (0.0 to 0.0)	0.0 (0.0 to 0.0)	3.8 (3.6 to 4.0)	2.8 (2.7 to 3.0)	1.85 (1.75 to 1.95)	1.38 (1.31 to 1.45)
2005	0.0 (0.0 to 0.0)	0.0 (0.0 to 0.0)	3.6 (3.4 to 3.7)	2.7 (2.5 to 2.8)	1.96 (1.86 to 2.07)	1.47 (1.40 to 1.55)
2010	0.0 (0.0 to 0.0)	0.0 (0.0 to 0.0)	3.5 (3.3 to 3.7)	2.6 (2.5 to 2.8)	2.18 (2.06 to 2.30)	1.63 (1.55 to 1.72)
2015	0.0 (0.0 to 0.0)	0.0 (0.0 to 0.0)	3.1 (2.9 to 3.2)	2.3 (2.2 to 2.4)	2.28 (2.16 to 2.40)	1.71 (1.62 to 1.80)
2020	0.0 (0.0 to 0.0)	0.0 (0.0 to 0.0)	2.7 (2.2 to 3.3)	2.0 (1.7 to 2.4)	2.27 (1.85 to 2.73)	1.69 (1.38 to 2.04)
2021	0.0 (0.0 to 0.0)	0.0 (0.0 to 0.0)	2.6 (2.0 to 3.3)	1.9 (1.5 to 2.5)	2.29 (1.72 to 2.93)	1.70 (1.29 to 2.18)

Kumamoto JPN_35466 High−income Asia Pacific Stillbirth Rate / Neonatal Mortality Rate (SBR/NMR) − 20 weeks
lambda = 1 ; zeta = 0.6 ; scale = 15 ; amp = 0.028

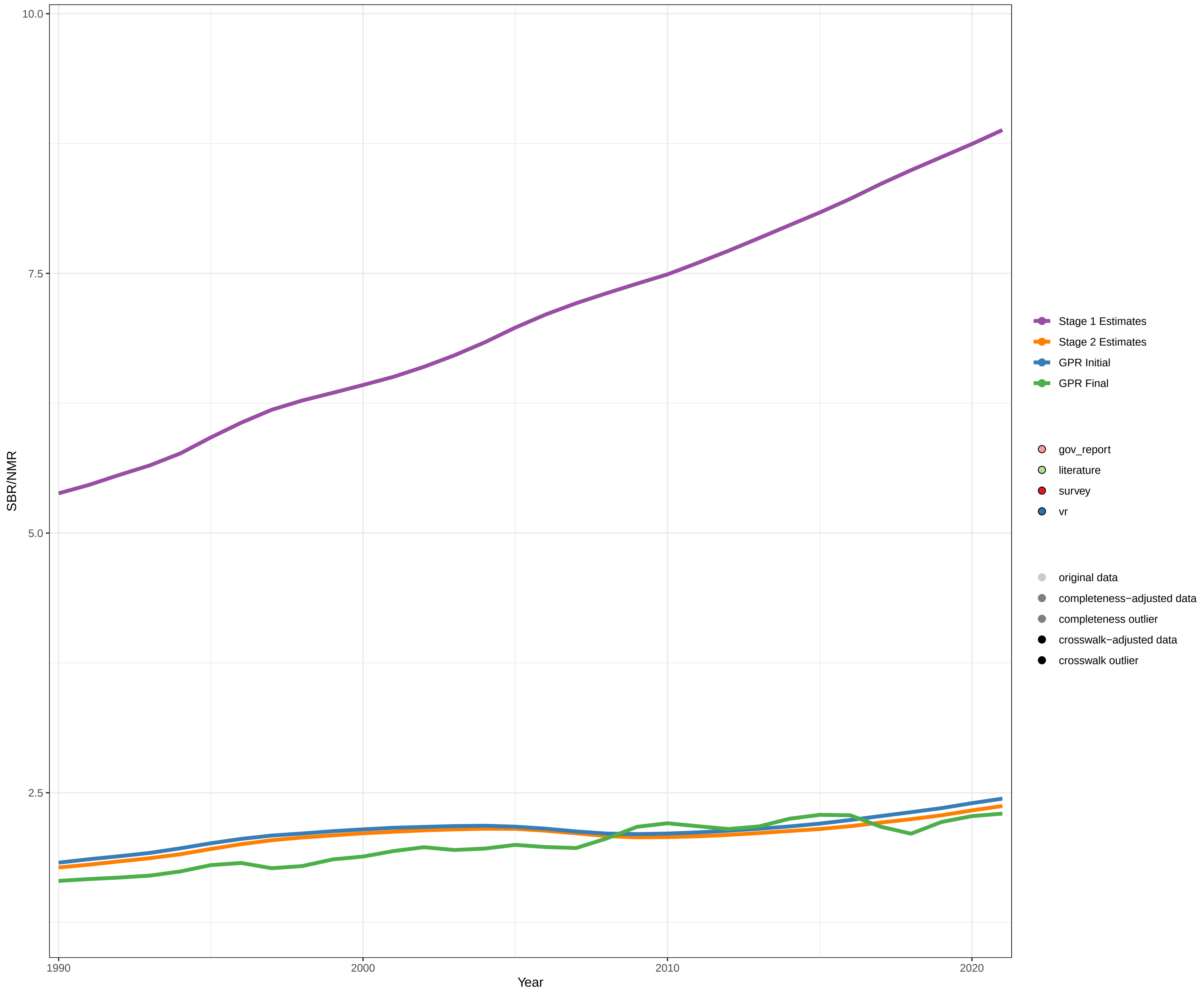

Kumamoto JPN_35466 High−income Asia Pacific Stillbirth Rate / Neonatal Mortality Rate (SBR/NMR) − 28 weeks
lambda = 1 ; zeta = 0.6 ; scale = 15 ; amp = 0.028

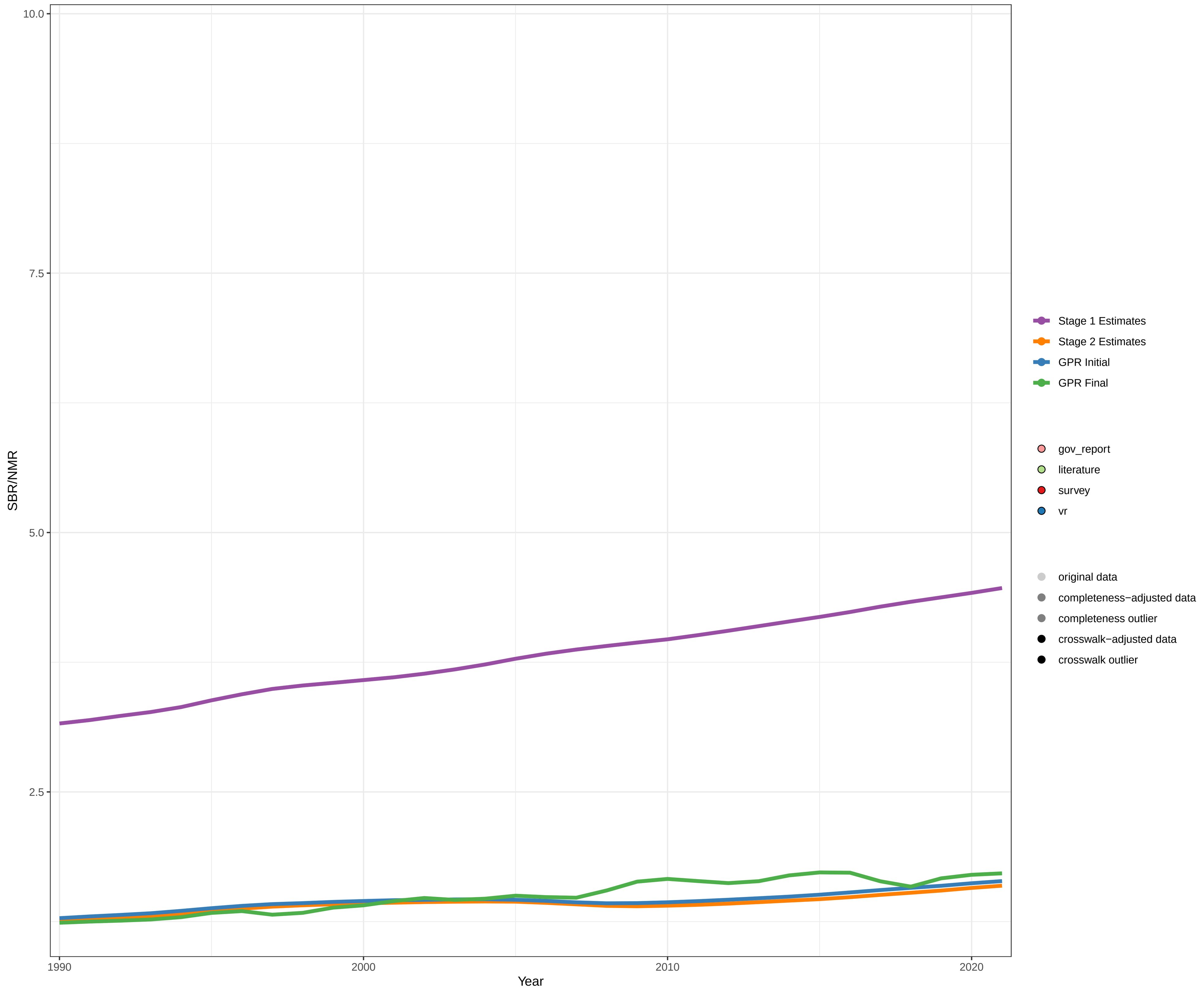

Definition	Years	Outlier	Source.Type	Title

Kumamoto JPN_35466 High−income Asia Pacific Stillbirth Rate (SBR) − 20 weeks
lambda = 1 ; zeta = 0.6 ; scale = 15 ; amp = 0.028

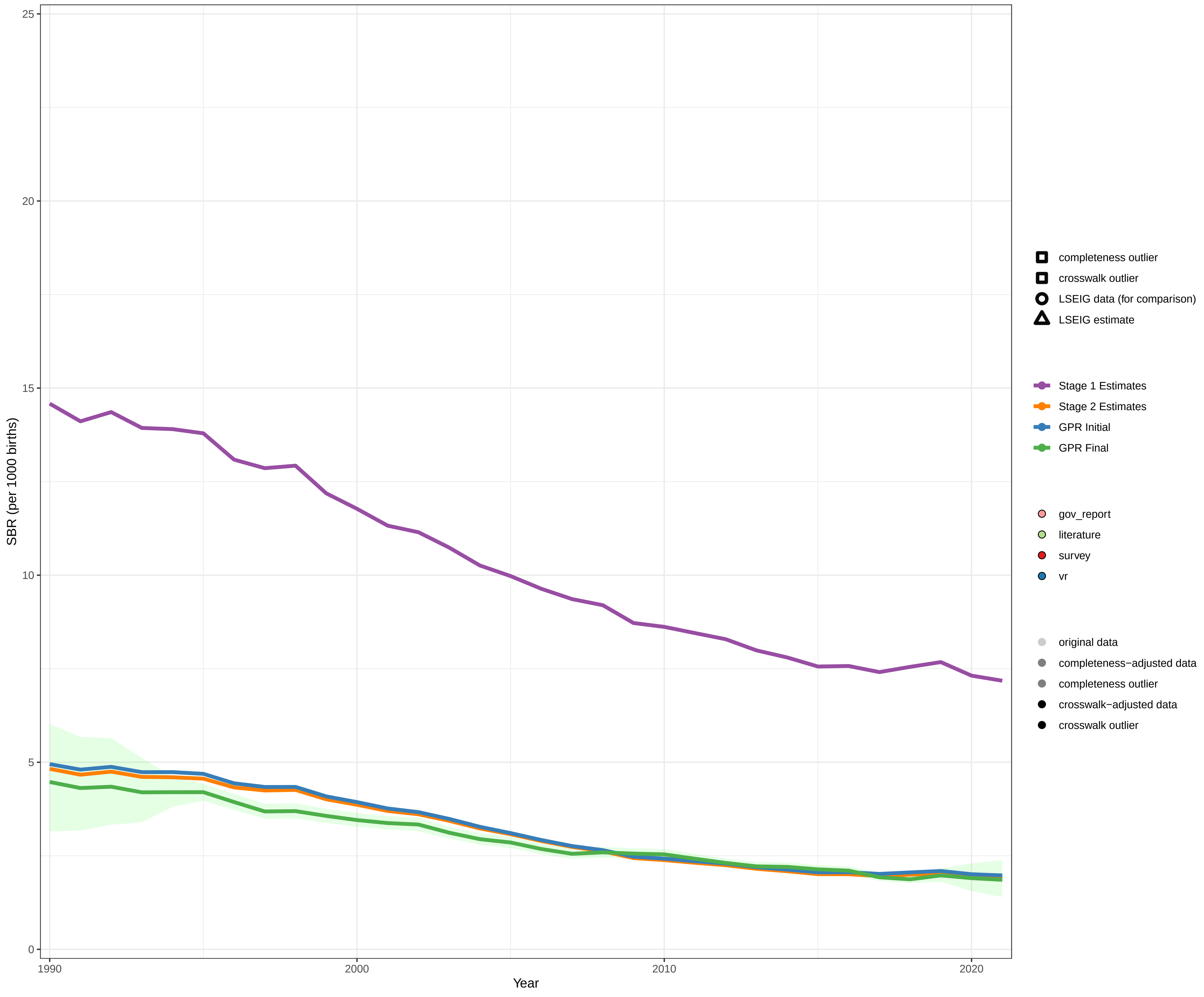

Kumamoto JPN_35466 High−income Asia Pacific Stillbirth Rate (SBR) − 28 weeks
lambda = 1 ; zeta = 0.6 ; scale = 15 ; amp = 0.028

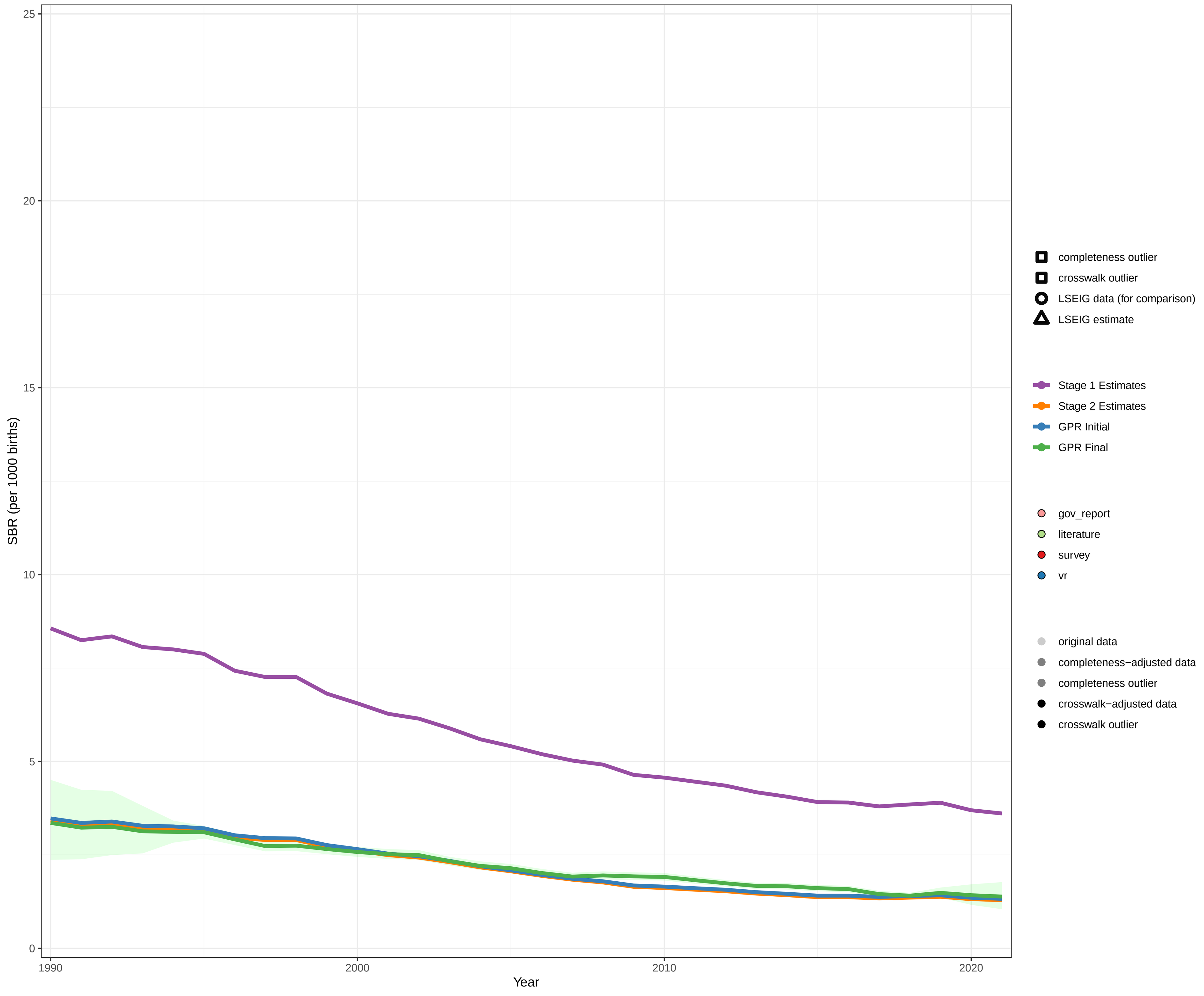

Year	Total Stillbirths (in thousands)	Total Stillbirths (in thousands)	SBR (per 1000 births)	SBR (per 1000 births)	SBR/NMR	SBR/NMR
	20 weeks	28 weeks	20 weeks	28 weeks	20 weeks	28 weeks
1990	0.1 (0.1 to 0.1)	0.1 (0.0 to 0.1)	4.5 (3.1 to 6.0)	3.4 (2.4 to 4.5)	1.65 (1.16 to 2.22)	1.24 (0.88 to 1.66)
1995	0.1 (0.1 to 0.1)	0.1 (0.1 to 0.1)	4.2 (4.0 to 4.4)	3.1 (2.9 to 3.3)	1.80 (1.70 to 1.90)	1.33 (1.26 to 1.41)
2000	0.1 (0.1 to 0.1)	0.0 (0.0 to 0.0)	3.5 (3.3 to 3.6)	2.6 (2.4 to 2.7)	1.89 (1.79 to 1.99)	1.41 (1.34 to 1.48)
2005	0.0 (0.0 to 0.0)	0.0 (0.0 to 0.0)	2.9 (2.7 to 3.0)	2.1 (2.0 to 2.3)	2.00 (1.89 to 2.10)	1.50 (1.42 to 1.58)
2010	0.0 (0.0 to 0.0)	0.0 (0.0 to 0.0)	2.5 (2.4 to 2.7)	1.9 (1.8 to 2.0)	2.21 (2.09 to 2.33)	1.66 (1.58 to 1.75)
2015	0.0 (0.0 to 0.0)	0.0 (0.0 to 0.0)	2.1 (2.0 to 2.3)	1.6 (1.5 to 1.7)	2.29 (2.17 to 2.41)	1.72 (1.64 to 1.82)
2020	0.0 (0.0 to 0.0)	0.0 (0.0 to 0.0)	1.9 (1.6 to 2.3)	1.4 (1.2 to 1.7)	2.27 (1.86 to 2.74)	1.70 (1.39 to 2.05)
2021	0.0 (0.0 to 0.0)	0.0 (0.0 to 0.0)	1.9 (1.4 to 2.4)	1.4 (1.0 to 1.8)	2.30 (1.73 to 2.95)	1.72 (1.30 to 2.19)

Kyoto JPN_35449 High−income Asia Pacific Stillbirth Rate / Neonatal Mortality Rate (SBR/NMR) − 20 weeks
lambda = 1 ; zeta = 0.6 ; scale = 15 ; amp = 0.028

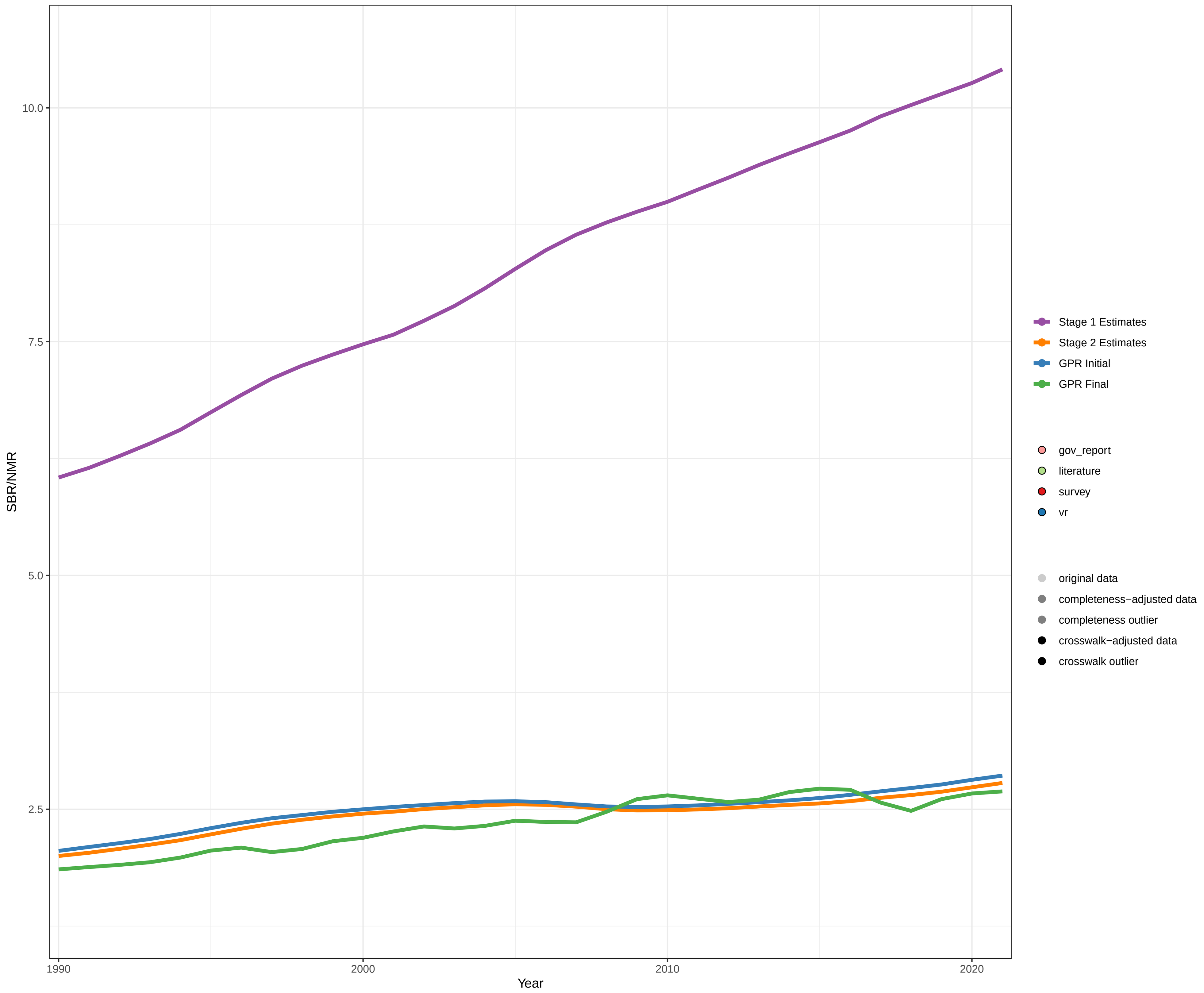

Kyoto JPN_35449 High−income Asia Pacific Stillbirth Rate / Neonatal Mortality Rate (SBR/NMR) − 28 weeks
lambda = 1 ; zeta = 0.6 ; scale = 15 ; amp = 0.028

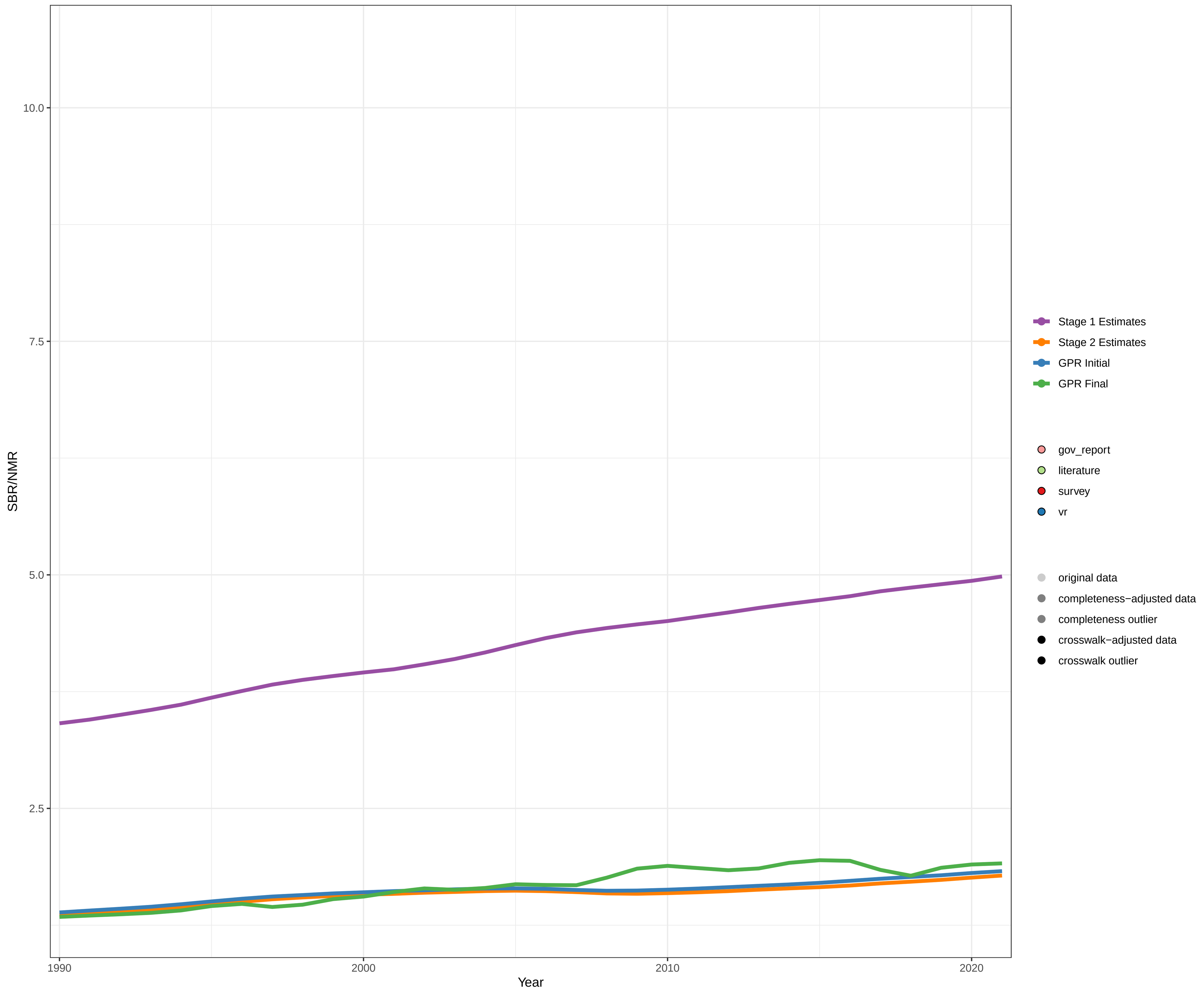

Definition	Years	Outlier	Source.Type	Title

Kyoto JPN_35449 High−income Asia Pacific Stillbirth Rate (SBR) − 20 weeks
lambda = 1 ; zeta = 0.6 ; scale = 15 ; amp = 0.028

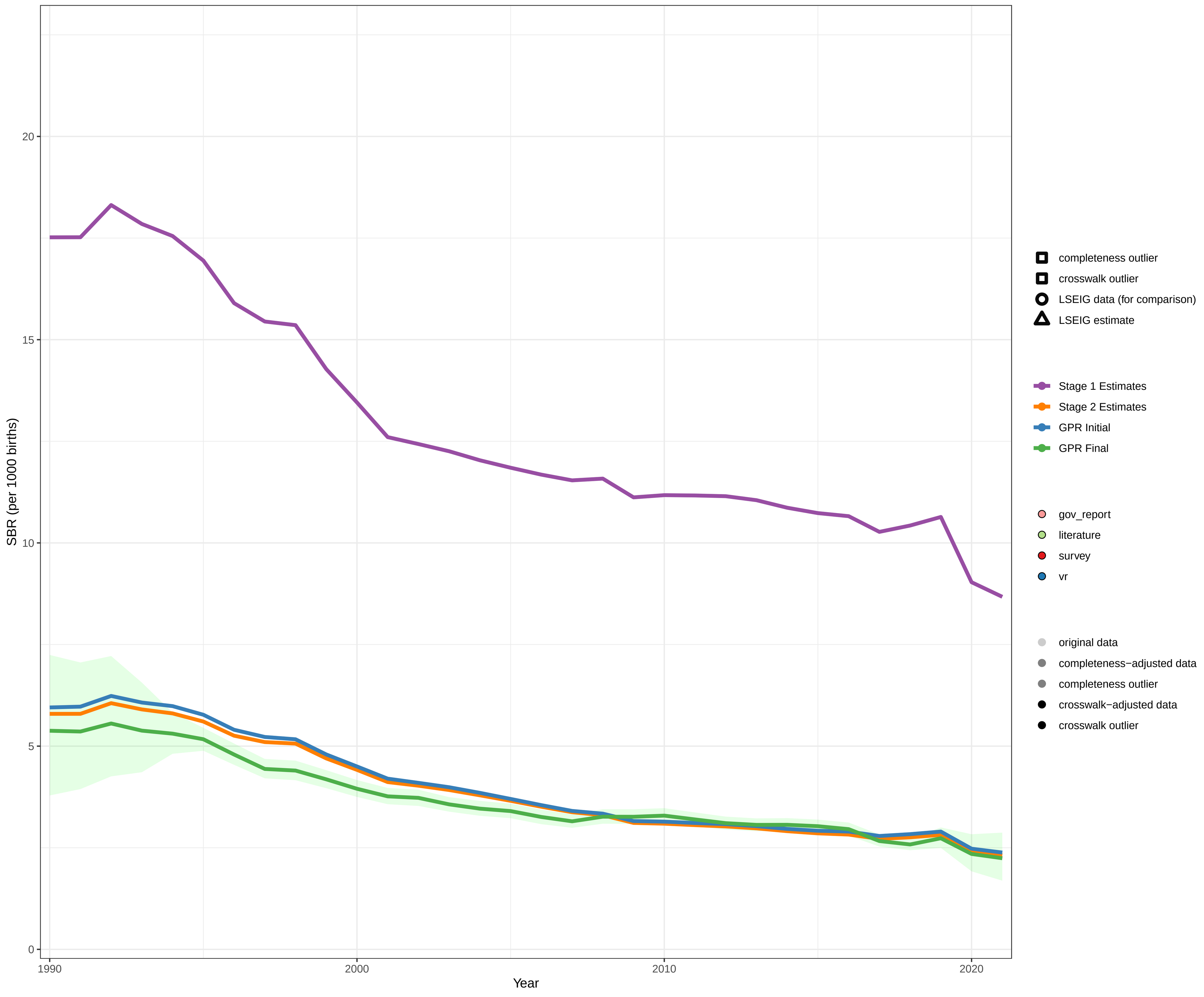

Kyoto JPN_35449 High−income Asia Pacific Stillbirth Rate (SBR) − 28 weeks
lambda = 1 ; zeta = 0.6 ; scale = 15 ; amp = 0.028

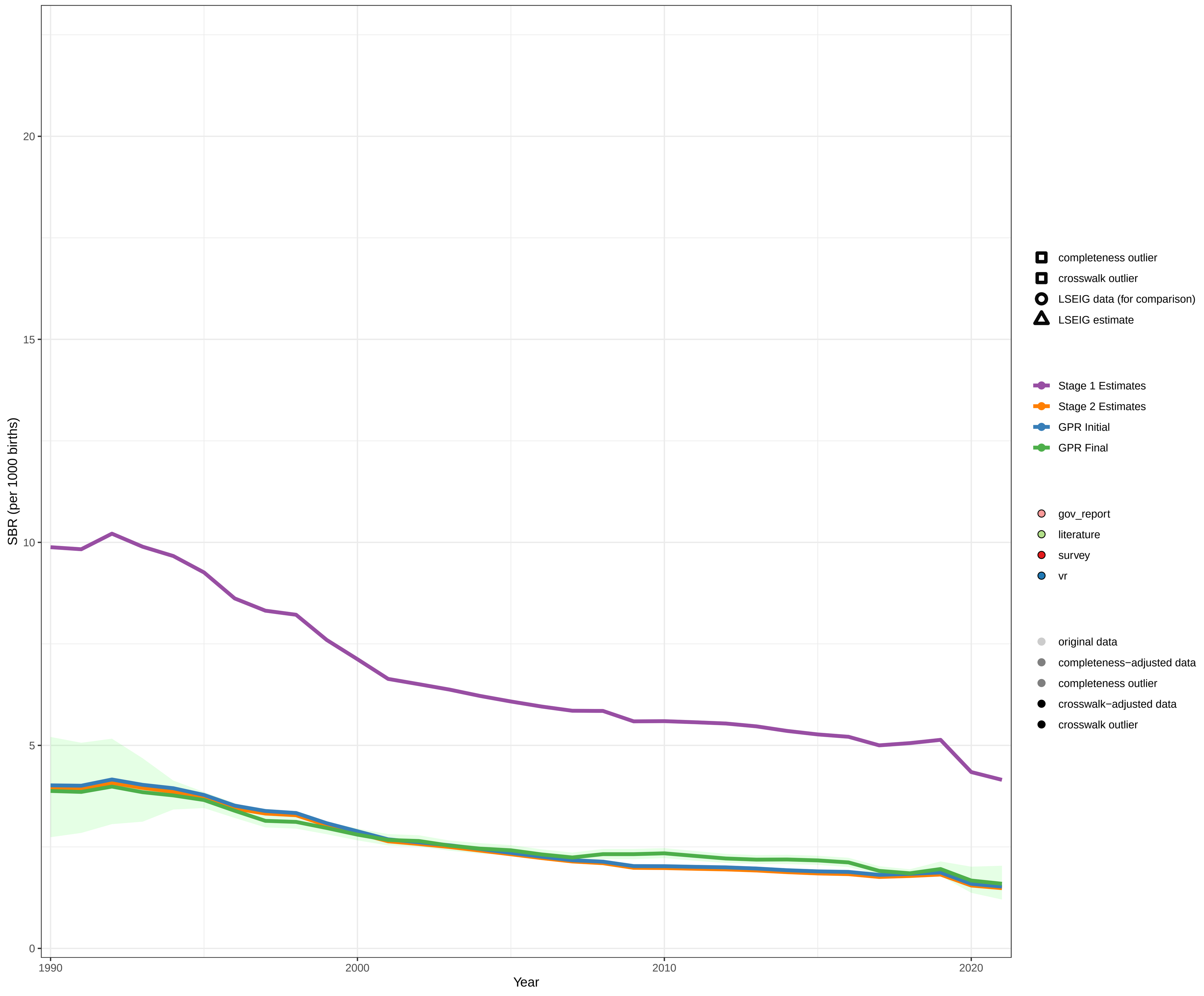

Year	Total Stillbirths (in thousands)	Total Stillbirths (in thousands)	SBR (per 1000 births)	SBR (per 1000 births)	SBR/NMR	SBR/NMR
	20 weeks	28 weeks	20 weeks	28 weeks	20 weeks	28 weeks
1990	0.1 (0.1 to 0.2)	0.1 (0.1 to 0.1)	5.4 (3.8 to 7.2)	3.9 (2.7 to 5.2)	1.86 (1.31 to 2.50)	1.34 (0.95 to 1.80)
1995	0.1 (0.1 to 0.1)	0.1 (0.1 to 0.1)	5.2 (4.9 to 5.5)	3.7 (3.5 to 3.9)	2.06 (1.94 to 2.17)	1.45 (1.38 to 1.53)
2000	0.1 (0.1 to 0.1)	0.1 (0.1 to 0.1)	4.0 (3.8 to 4.2)	2.8 (2.7 to 3.0)	2.19 (2.08 to 2.31)	1.56 (1.48 to 1.64)
2005	0.1 (0.1 to 0.1)	0.1 (0.1 to 0.1)	3.4 (3.2 to 3.6)	2.4 (2.3 to 2.5)	2.38 (2.25 to 2.50)	1.69 (1.60 to 1.78)
2010	0.1 (0.1 to 0.1)	0.0 (0.0 to 0.1)	3.3 (3.1 to 3.5)	2.3 (2.2 to 2.5)	2.65 (2.51 to 2.80)	1.89 (1.79 to 1.99)
2015	0.1 (0.1 to 0.1)	0.0 (0.0 to 0.0)	3.0 (2.9 to 3.2)	2.2 (2.1 to 2.3)	2.72 (2.58 to 2.87)	1.95 (1.85 to 2.05)
2020	0.0 (0.0 to 0.0)	0.0 (0.0 to 0.0)	2.3 (1.9 to 2.8)	1.7 (1.4 to 2.0)	2.67 (2.18 to 3.22)	1.90 (1.56 to 2.29)
2021	0.0 (0.0 to 0.0)	0.0 (0.0 to 0.0)	2.2 (1.7 to 2.9)	1.6 (1.2 to 2.0)	2.69 (2.03 to 3.45)	1.91 (1.45 to 2.44)

Mie JPN_35447 High−income Asia Pacific Stillbirth Rate / Neonatal Mortality Rate (SBR/NMR) − 20 weeks
lambda = 1 ; zeta = 0.6 ; scale = 15 ; amp = 0.028

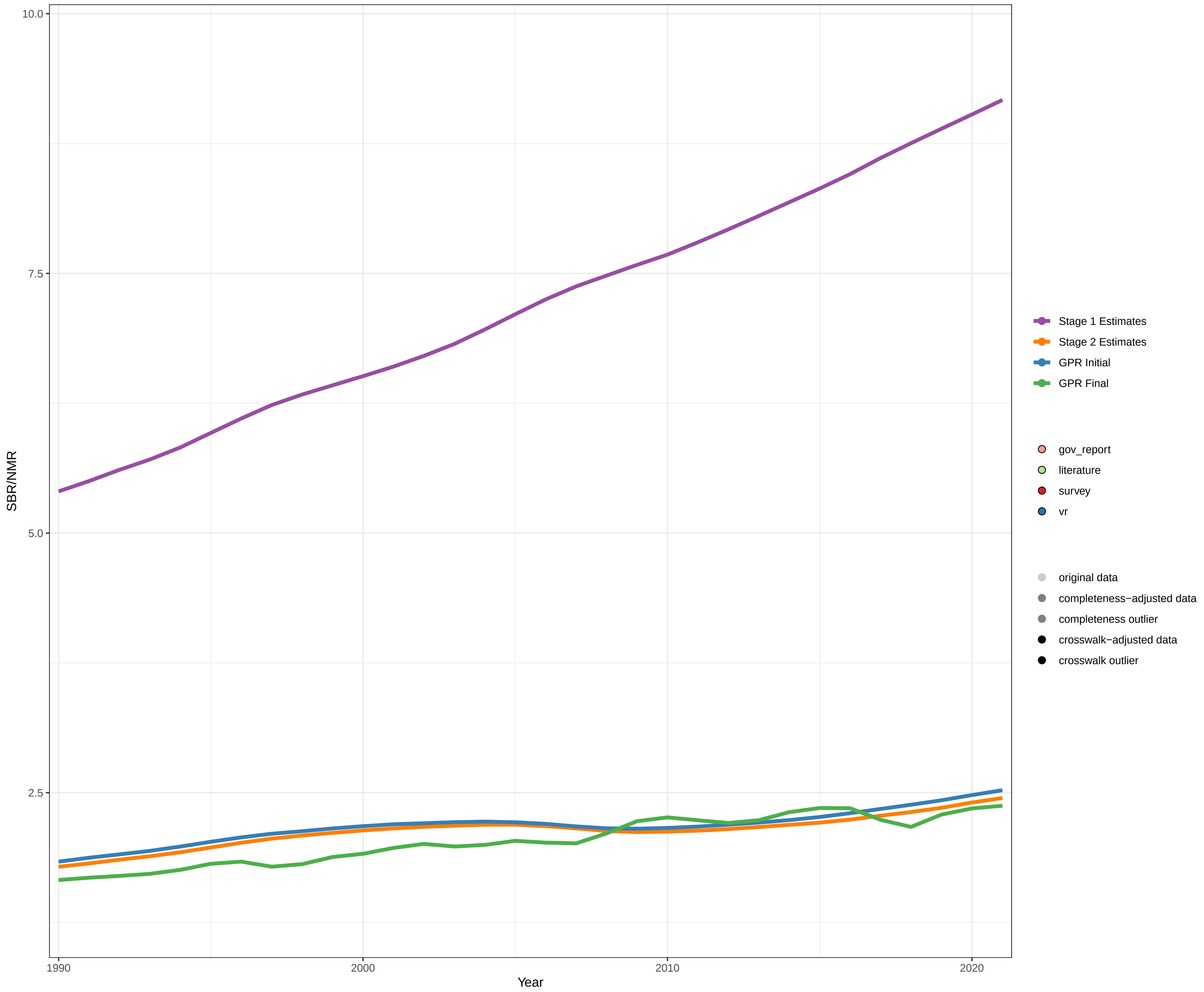

Mie JPN_35447 High−income Asia Pacific Stillbirth Rate / Neonatal Mortality Rate (SBR/NMR) − 28 weeks
lambda = 1 ; zeta = 0.6 ; scale = 15 ; amp = 0.028

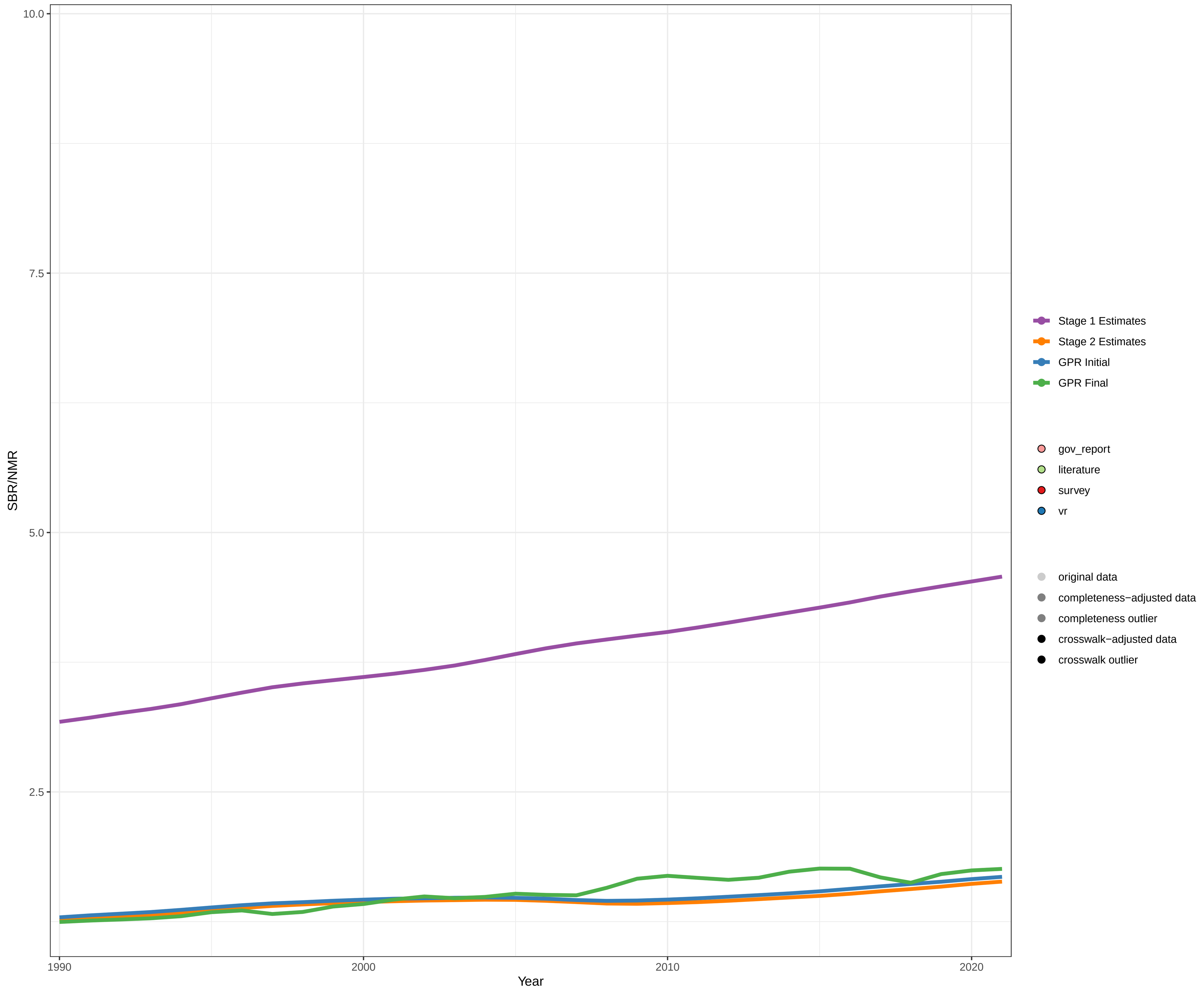

Definition	Years	Outlier	Source.Type	Title

Mie JPN_35447 High−income Asia Pacific Stillbirth Rate (SBR) − 20 weeks
lambda = 1 ; zeta = 0.6 ; scale = 15 ; amp = 0.028

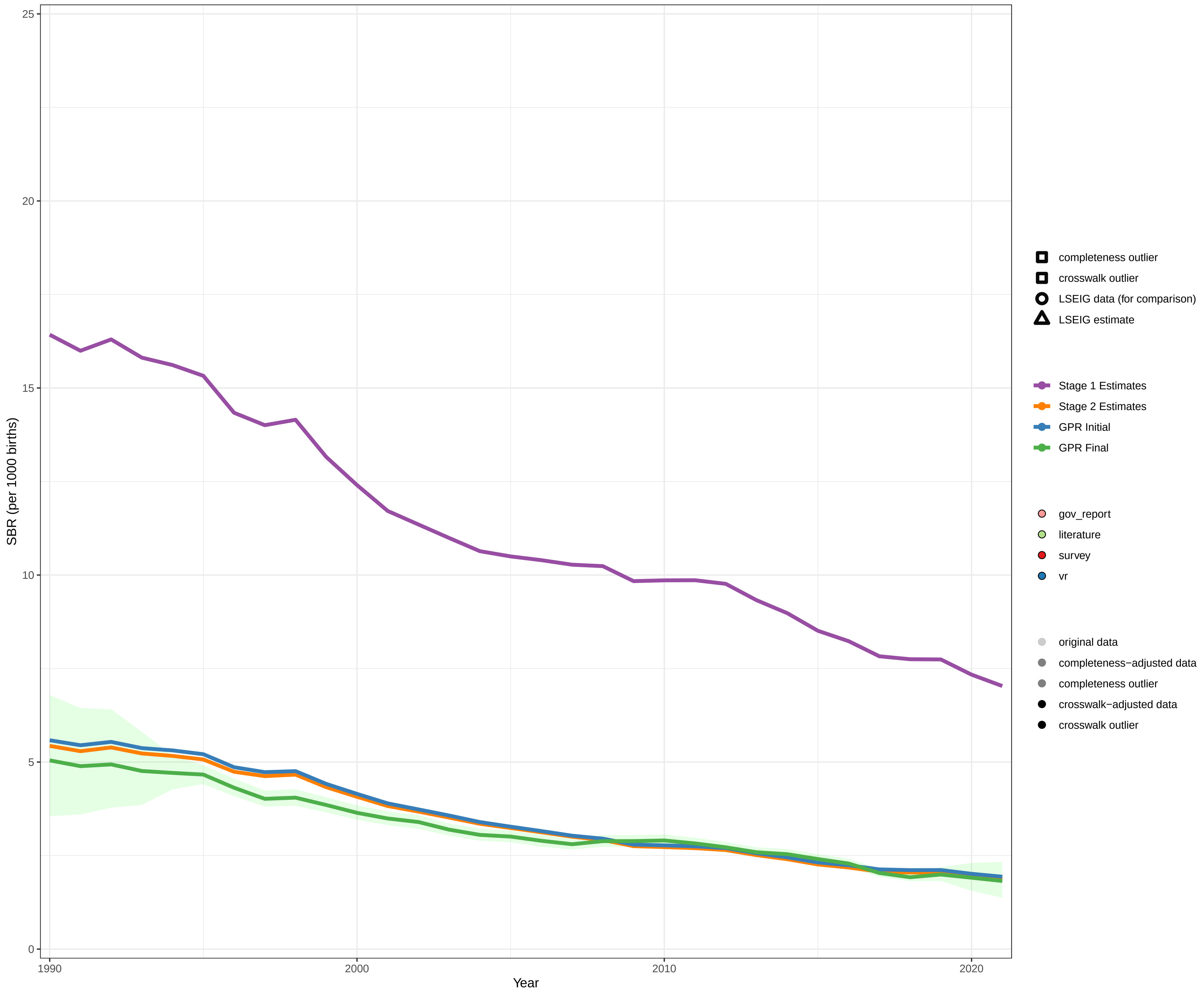

Mie JPN_35447 High−income Asia Pacific Stillbirth Rate (SBR) − 28 weeks
lambda = 1 ; zeta = 0.6 ; scale = 15 ; amp = 0.028

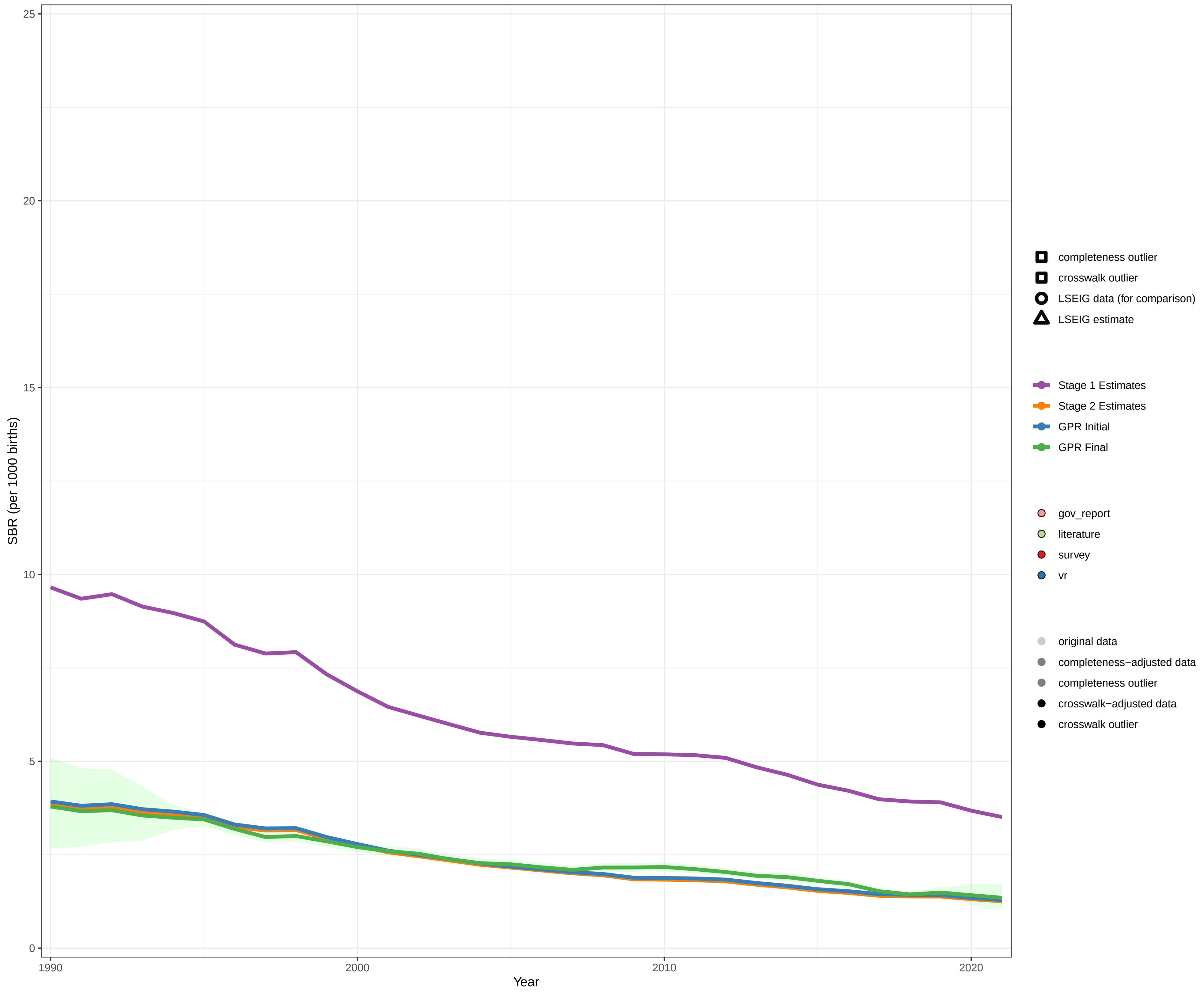

Year	Total Stillbirths (in thousands)	Total Stillbirths (in thousands)	SBR (per 1000 births)	SBR (per 1000 births)	SBR/NMR	SBR/NMR
	20 weeks	28 weeks	20 weeks	28 weeks	20 weeks	28 weeks
1990	0.1 (0.1 to 0.1)	0.1 (0.0 to 0.1)	5.0 (3.6 to 6.8)	3.8 (2.7 to 5.1)	1.66 (1.17 to 2.23)	1.25 (0.88 to 1.67)
1995	0.1 (0.1 to 0.1)	0.1 (0.1 to 0.1)	4.7 (4.4 to 4.9)	3.4 (3.3 to 3.6)	1.82 (1.72 to 1.91)	1.34 (1.27 to 1.41)
2000	0.1 (0.1 to 0.1)	0.0 (0.0 to 0.0)	3.6 (3.5 to 3.8)	2.7 (2.6 to 2.8)	1.91 (1.81 to 2.02)	1.42 (1.35 to 1.50)
2005	0.0 (0.0 to 0.1)	0.0 (0.0 to 0.0)	3.0 (2.9 to 3.2)	2.2 (2.1 to 2.4)	2.04 (1.93 to 2.15)	1.52 (1.44 to 1.60)
2010	0.0 (0.0 to 0.0)	0.0 (0.0 to 0.0)	2.9 (2.8 to 3.1)	2.2 (2.1 to 2.3)	2.26 (2.15 to 2.39)	1.69 (1.61 to 1.78)
2015	0.0 (0.0 to 0.0)	0.0 (0.0 to 0.0)	2.4 (2.3 to 2.5)	1.8 (1.7 to 1.9)	2.35 (2.23 to 2.48)	1.76 (1.67 to 1.85)
2020	0.0 (0.0 to 0.0)	0.0 (0.0 to 0.0)	1.9 (1.6 to 2.3)	1.4 (1.2 to 1.7)	2.35 (1.92 to 2.83)	1.74 (1.43 to 2.10)
2021	0.0 (0.0 to 0.0)	0.0 (0.0 to 0.0)	1.8 (1.4 to 2.3)	1.3 (1.0 to 1.7)	2.37 (1.79 to 3.04)	1.76 (1.33 to 2.25)

Miyagi JPN_35427 High−income Asia Pacific Stillbirth Rate / Neonatal Mortality Rate (SBR/NMR) − 20 weeks
lambda = 1 ; zeta = 0.6 ; scale = 15 ; amp = 0.028

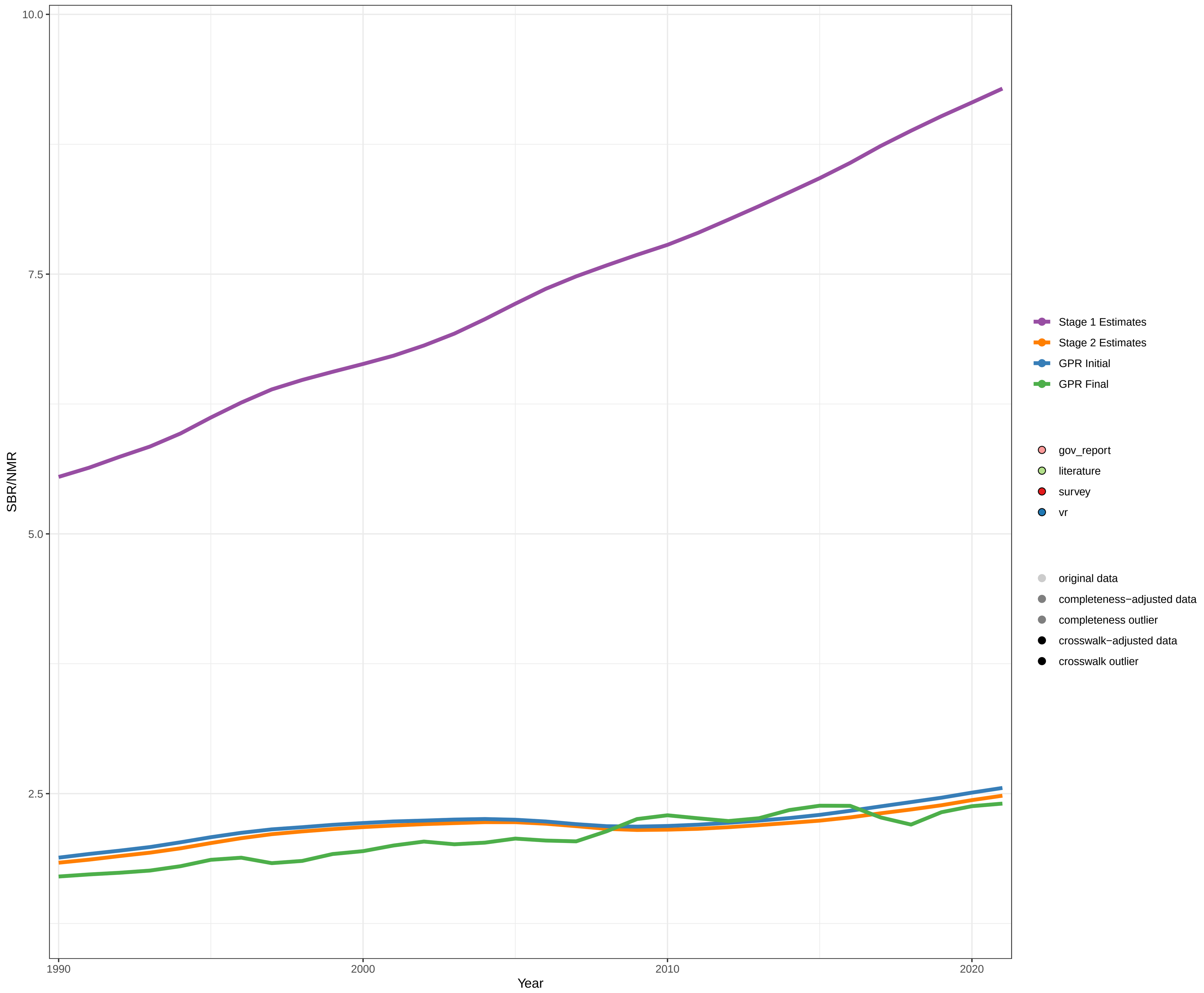

Miyagi JPN_35427 High−income Asia Pacific Stillbirth Rate / Neonatal Mortality Rate (SBR/NMR) − 28 weeks
lambda = 1 ; zeta = 0.6 ; scale = 15 ; amp = 0.028

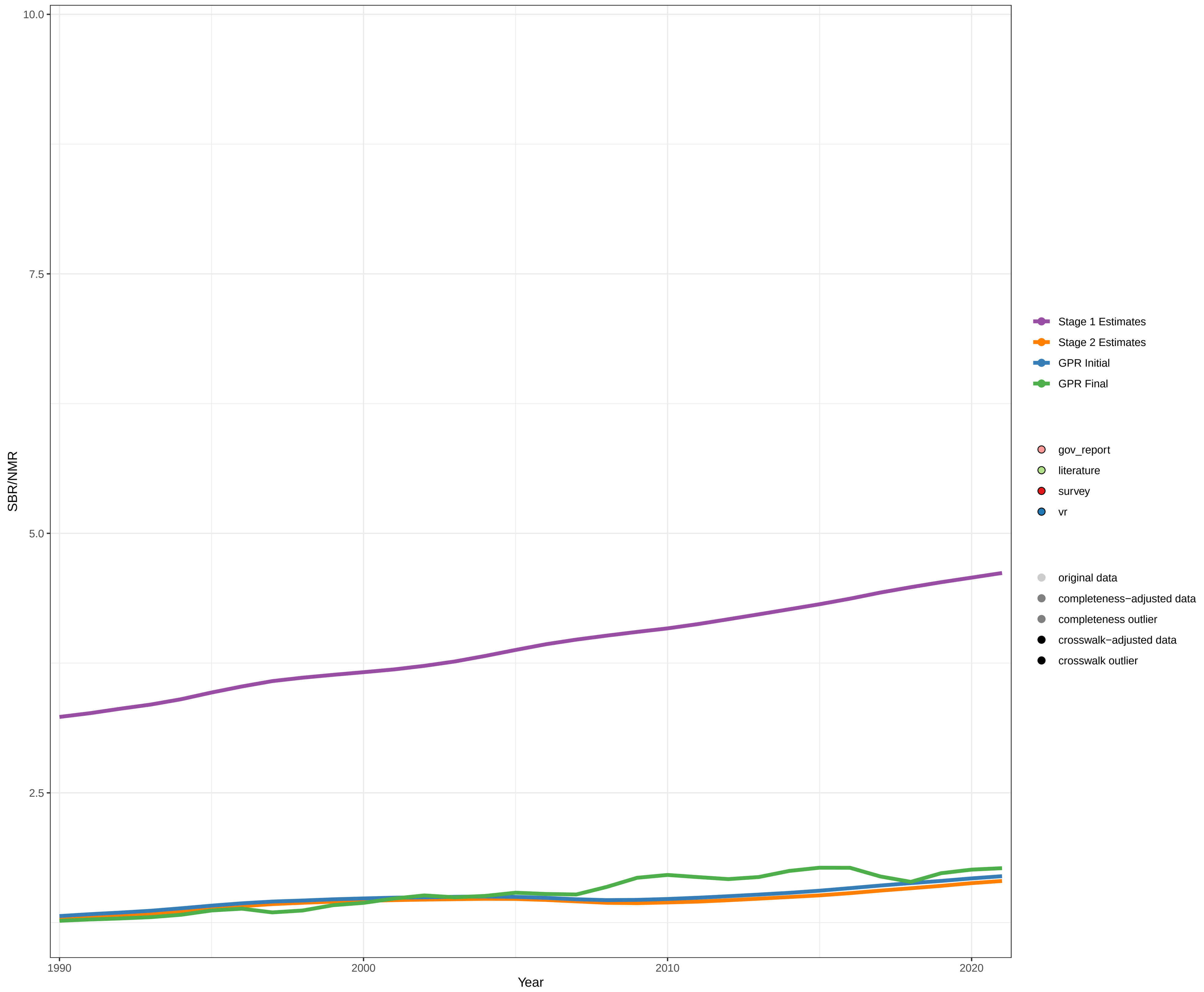

Definition	Years	Outlier	Source.Type	Title

Miyagi JPN_35427 High−income Asia Pacific Stillbirth Rate (SBR) − 20 weeks
lambda = 1 ; zeta = 0.6 ; scale = 15 ; amp = 0.028

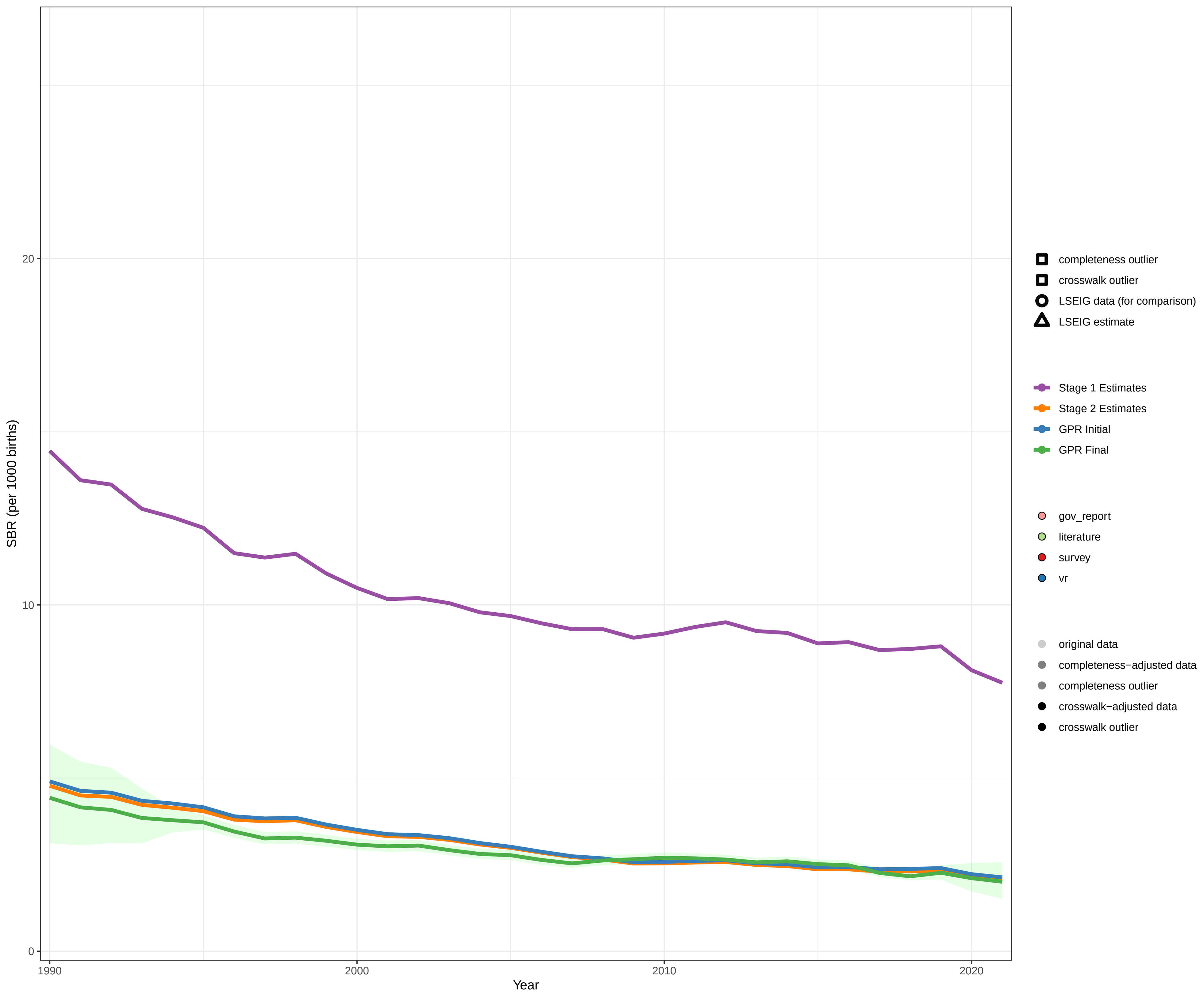

Miyagi JPN_35427 High−income Asia Pacific Stillbirth Rate (SBR) − 28 weeks
lambda = 1 ; zeta = 0.6 ; scale = 15 ; amp = 0.028

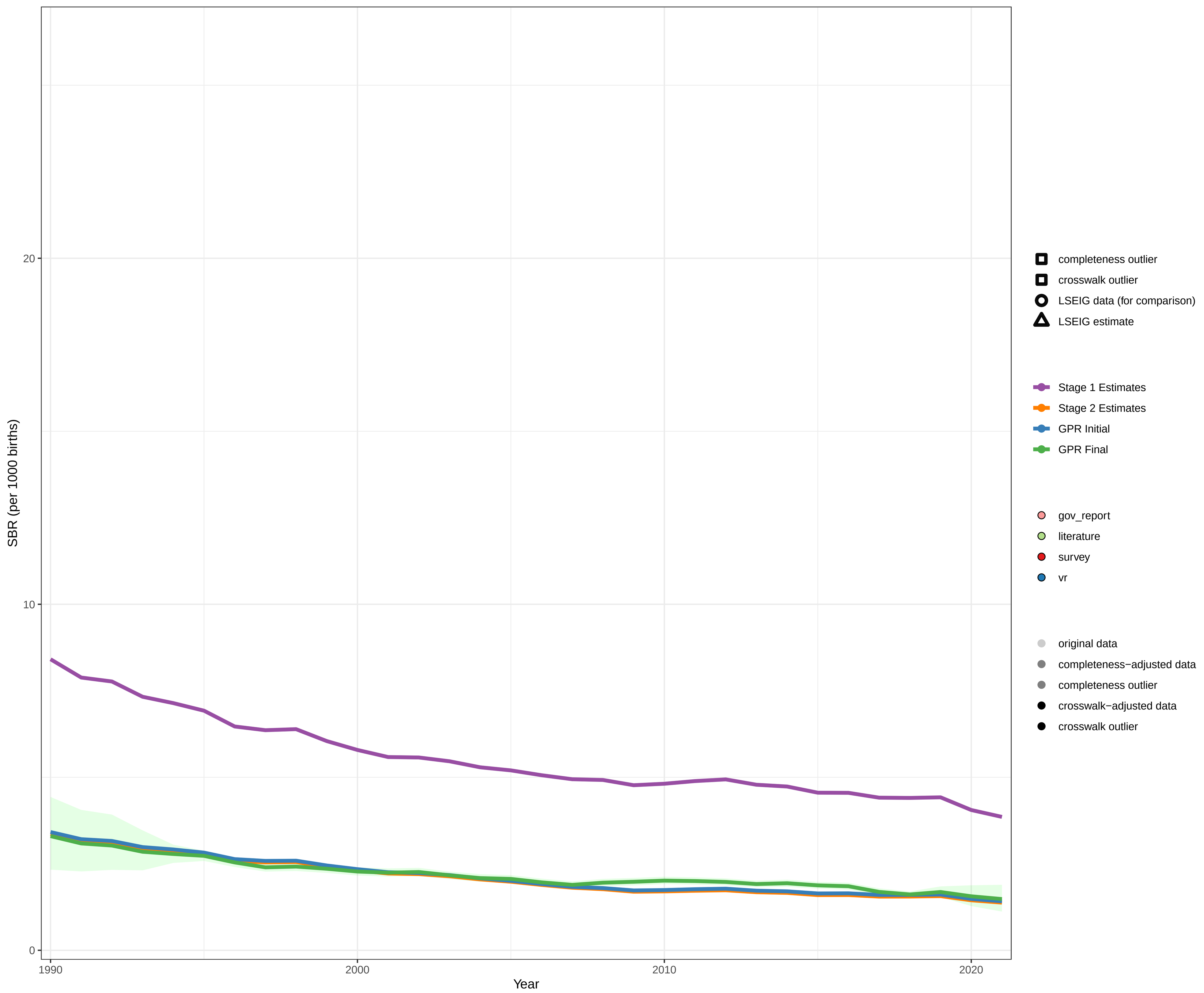

Year	Total Stillbirths (in thousands)	Total Stillbirths (in thousands)	SBR (per 1000 births)	SBR (per 1000 births)	SBR/NMR	SBR/NMR
	20 weeks	28 weeks	20 weeks	28 weeks	20 weeks	28 weeks
1990	0.1 (0.1 to 0.1)	0.1 (0.1 to 0.1)	4.4 (3.1 to 6.0)	3.3 (2.3 to 4.4)	1.70 (1.20 to 2.29)	1.27 (0.90 to 1.70)
1995	0.1 (0.1 to 0.1)	0.1 (0.1 to 0.1)	3.7 (3.5 to 3.9)	2.7 (2.6 to 2.9)	1.86 (1.76 to 1.96)	1.37 (1.29 to 1.44)
2000	0.1 (0.1 to 0.1)	0.0 (0.0 to 0.1)	3.1 (2.9 to 3.2)	2.3 (2.2 to 2.4)	1.95 (1.85 to 2.05)	1.44 (1.37 to 1.52)
2005	0.1 (0.1 to 0.1)	0.0 (0.0 to 0.0)	2.8 (2.6 to 2.9)	2.1 (2.0 to 2.2)	2.07 (1.96 to 2.18)	1.54 (1.46 to 1.62)
2010	0.1 (0.0 to 0.1)	0.0 (0.0 to 0.0)	2.7 (2.6 to 2.9)	2.0 (1.9 to 2.1)	2.29 (2.17 to 2.42)	1.71 (1.62 to 1.80)
2015	0.0 (0.0 to 0.0)	0.0 (0.0 to 0.0)	2.5 (2.4 to 2.7)	1.9 (1.8 to 2.0)	2.38 (2.26 to 2.51)	1.78 (1.69 to 1.87)
2020	0.0 (0.0 to 0.0)	0.0 (0.0 to 0.0)	2.1 (1.7 to 2.5)	1.6 (1.3 to 1.9)	2.38 (1.94 to 2.87)	1.76 (1.44 to 2.12)
2021	0.0 (0.0 to 0.0)	0.0 (0.0 to 0.0)	2.0 (1.5 to 2.6)	1.5 (1.1 to 1.9)	2.40 (1.81 to 3.08)	1.77 (1.34 to 2.27)

Miyazaki JPN_35468 High−income Asia Pacific Stillbirth Rate / Neonatal Mortality Rate (SBR/NMR) − 20 weeks
lambda = 1 ; zeta = 0.6 ; scale = 15 ; amp = 0.028

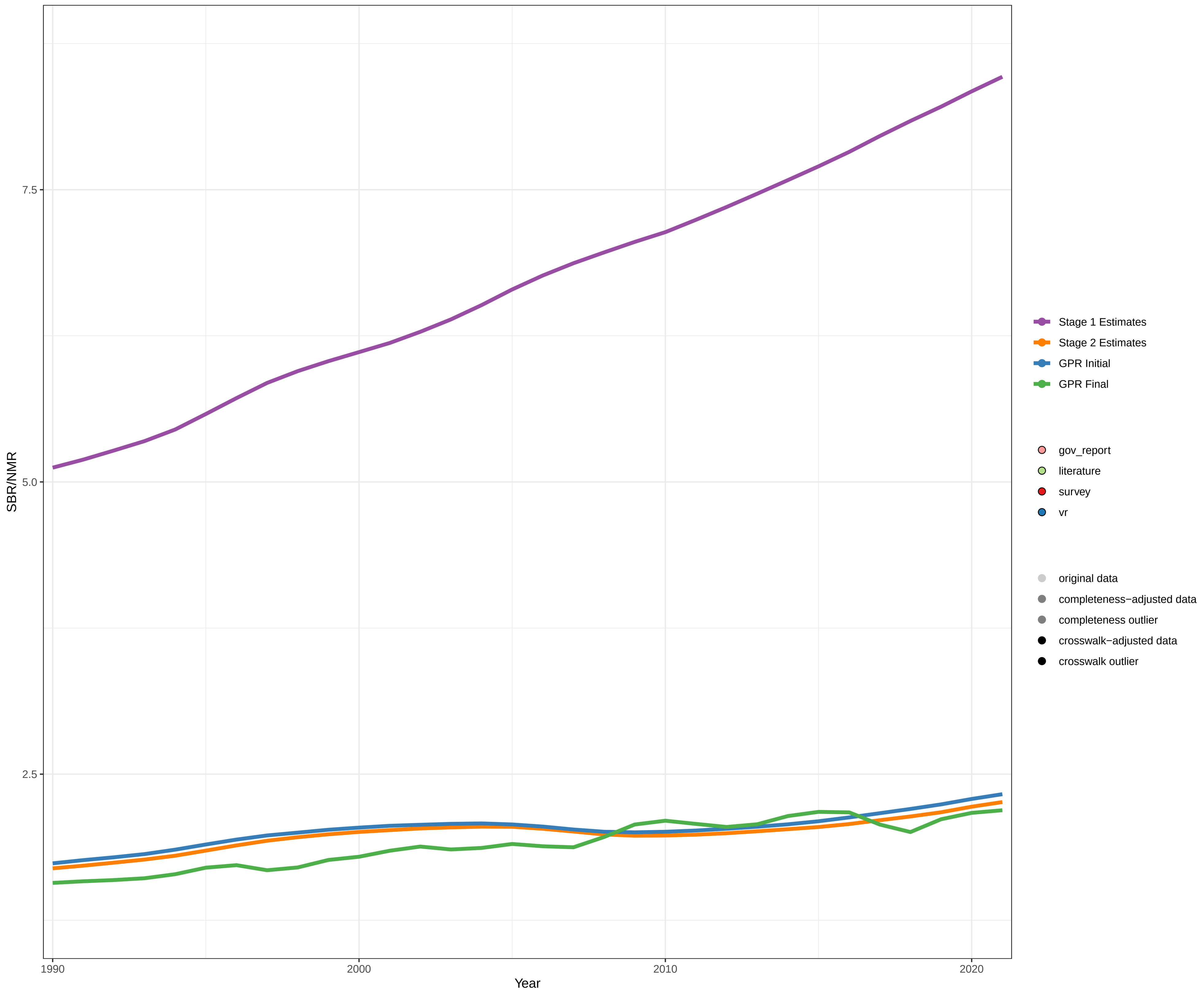

Miyazaki JPN_35468 High−income Asia Pacific Stillbirth Rate / Neonatal Mortality Rate (SBR/NMR) − 28 weeks
lambda = 1 ; zeta = 0.6 ; scale = 15 ; amp = 0.028

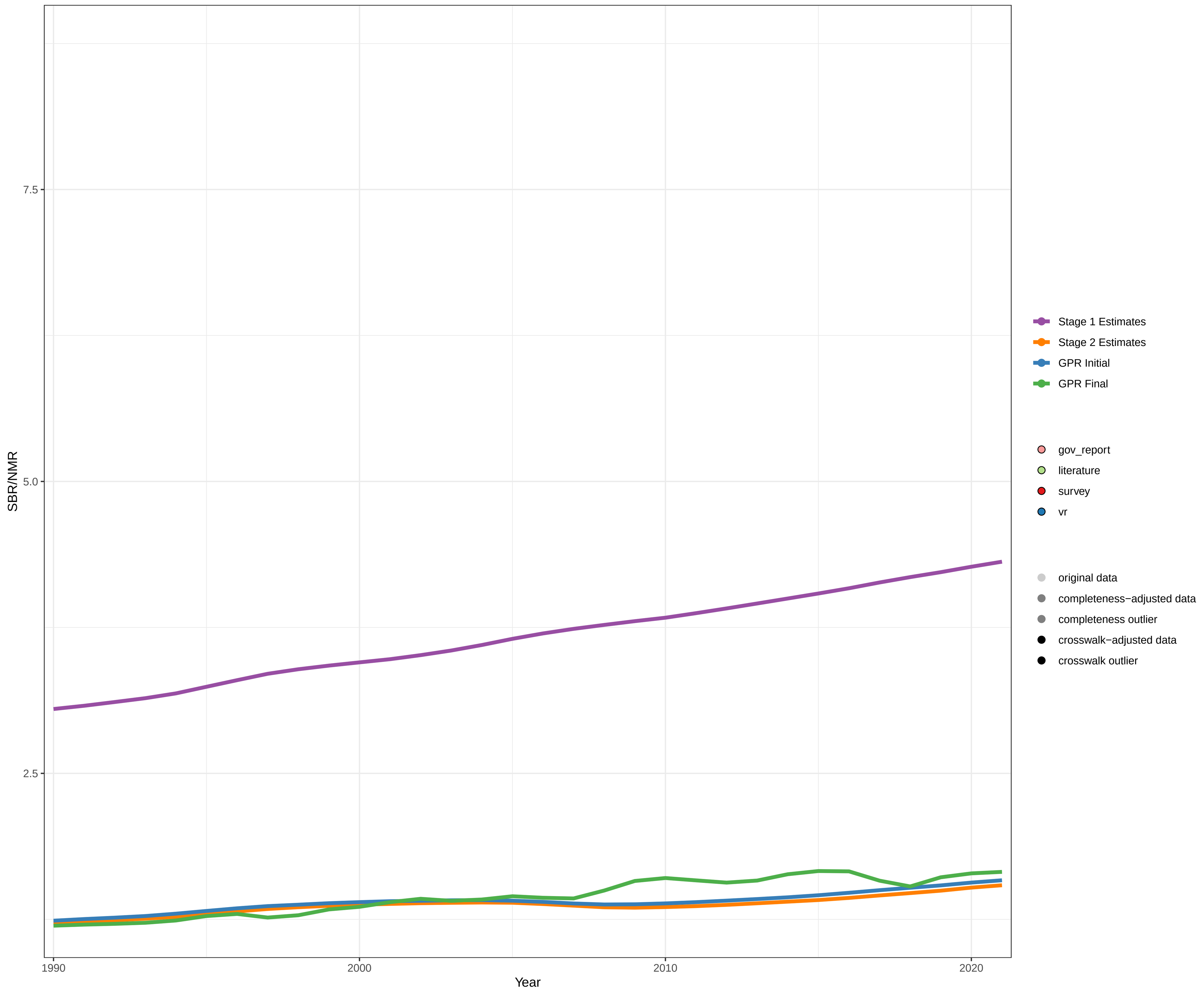

Definition	Years	Outlier	Source.Type	Title

Miyazaki JPN_35468 High−income Asia Pacific Stillbirth Rate (SBR) − 20 weeks
lambda = 1 ; zeta = 0.6 ; scale = 15 ; amp = 0.028

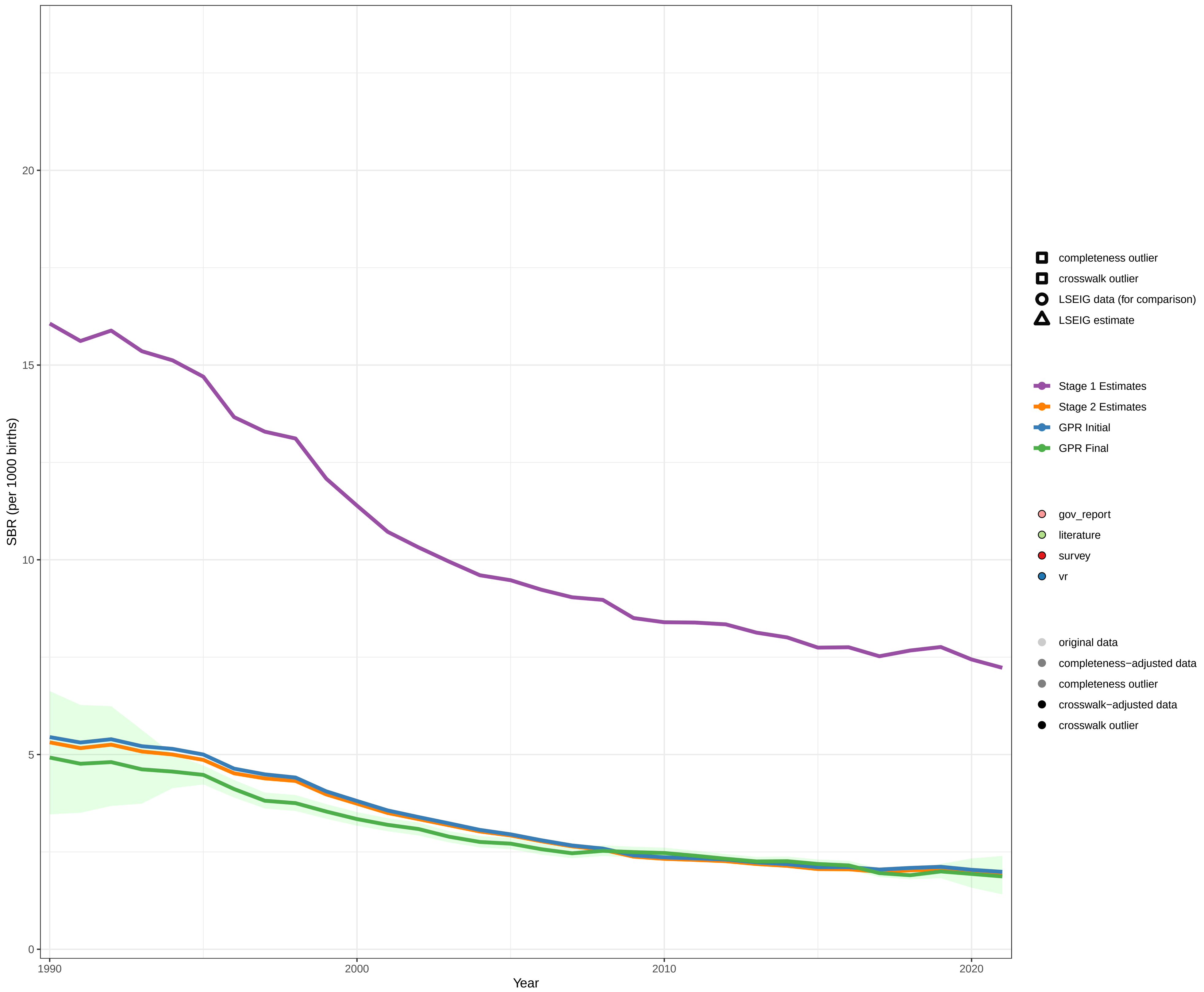

Miyazaki JPN_35468 High−income Asia Pacific Stillbirth Rate (SBR) − 28 weeks
lambda = 1 ; zeta = 0.6 ; scale = 15 ; amp = 0.028

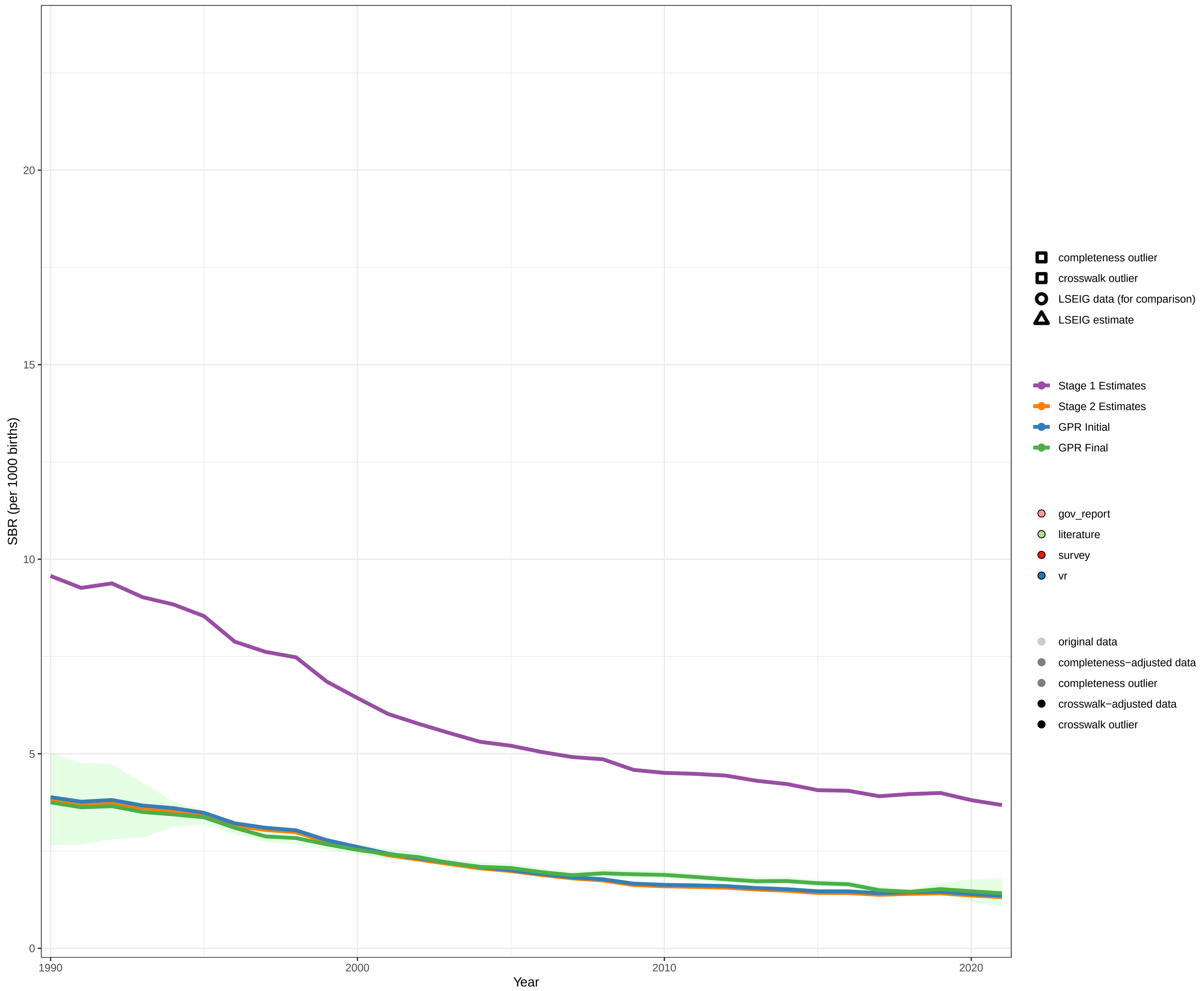

Year	Total Stillbirths (in thousands)	Total Stillbirths (in thousands)	SBR (per 1000 births)	SBR (per 1000 births)	SBR/NMR	SBR/NMR
	20 weeks	28 weeks	20 weeks	28 weeks	20 weeks	28 weeks
1990	0.1 (0.0 to 0.1)	0.0 (0.0 to 0.1)	4.9 (3.5 to 6.6)	3.8 (2.6 to 5.0)	1.57 (1.10 to 2.11)	1.20 (0.84 to 1.61)
1995	0.1 (0.0 to 0.1)	0.0 (0.0 to 0.0)	4.5 (4.2 to 4.7)	3.4 (3.2 to 3.5)	1.70 (1.61 to 1.79)	1.28 (1.21 to 1.35)
2000	0.0 (0.0 to 0.0)	0.0 (0.0 to 0.0)	3.3 (3.2 to 3.5)	2.5 (2.4 to 2.7)	1.79 (1.70 to 1.89)	1.36 (1.29 to 1.43)
2005	0.0 (0.0 to 0.0)	0.0 (0.0 to 0.0)	2.7 (2.6 to 2.9)	2.1 (2.0 to 2.2)	1.90 (1.80 to 2.00)	1.45 (1.37 to 1.52)
2010	0.0 (0.0 to 0.0)	0.0 (0.0 to 0.0)	2.5 (2.3 to 2.6)	1.9 (1.8 to 2.0)	2.10 (1.99 to 2.22)	1.60 (1.52 to 1.69)
2015	0.0 (0.0 to 0.0)	0.0 (0.0 to 0.0)	2.2 (2.1 to 2.3)	1.7 (1.6 to 1.8)	2.18 (2.06 to 2.29)	1.66 (1.58 to 1.75)
2020	0.0 (0.0 to 0.0)	0.0 (0.0 to 0.0)	1.9 (1.6 to 2.3)	1.5 (1.2 to 1.8)	2.17 (1.77 to 2.62)	1.64 (1.35 to 1.98)
2021	0.0 (0.0 to 0.0)	0.0 (0.0 to 0.0)	1.9 (1.4 to 2.4)	1.4 (1.1 to 1.8)	2.19 (1.65 to 2.81)	1.66 (1.25 to 2.12)

Nagano JPN_35443 High−income Asia Pacific Stillbirth Rate / Neonatal Mortality Rate (SBR/NMR) − 20 weeks
lambda = 1 ; zeta = 0.6 ; scale = 15 ; amp = 0.028

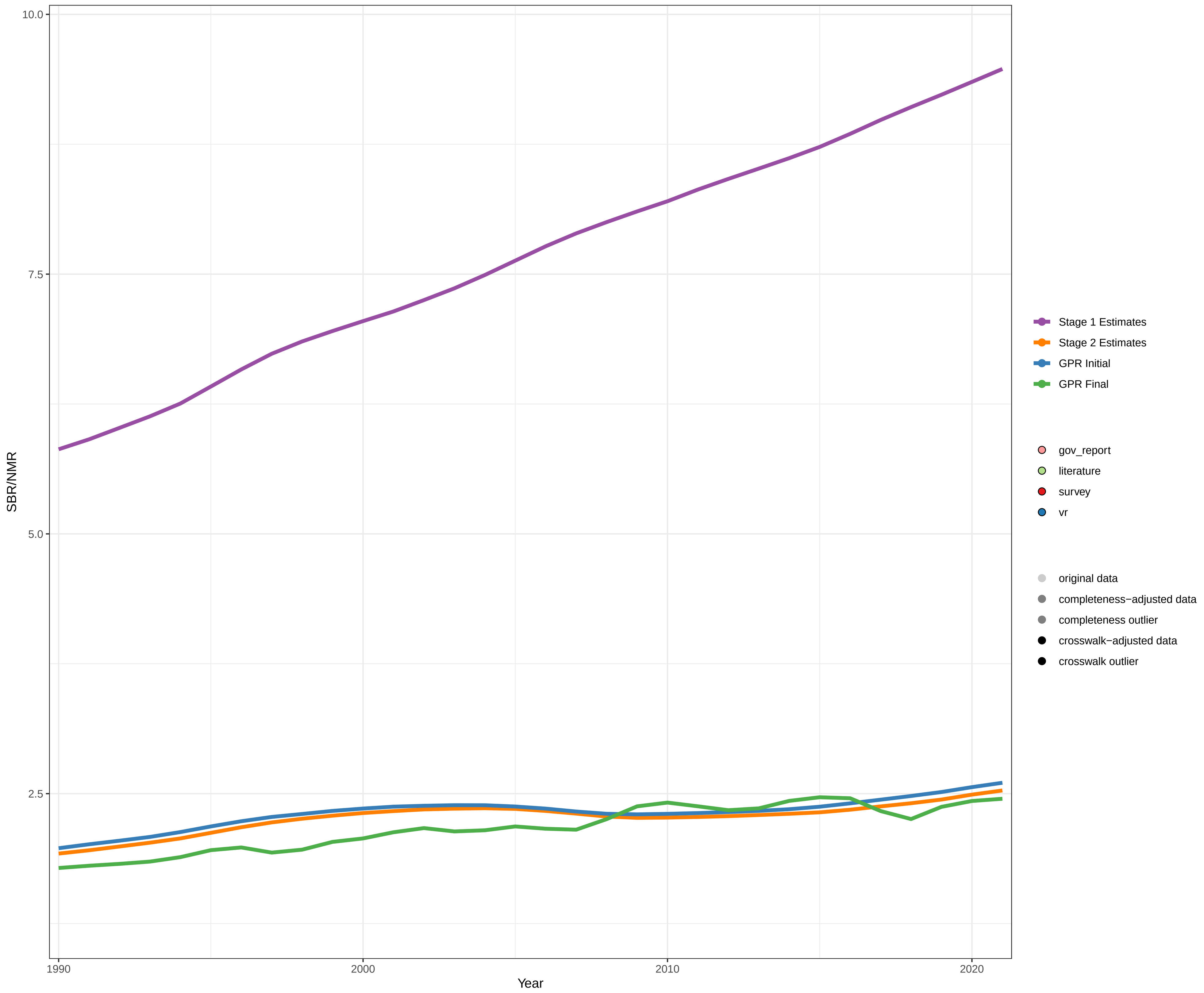

Nagano JPN_35443 High−income Asia Pacific Stillbirth Rate / Neonatal Mortality Rate (SBR/NMR) − 28 weeks
lambda = 1 ; zeta = 0.6 ; scale = 15 ; amp = 0.028

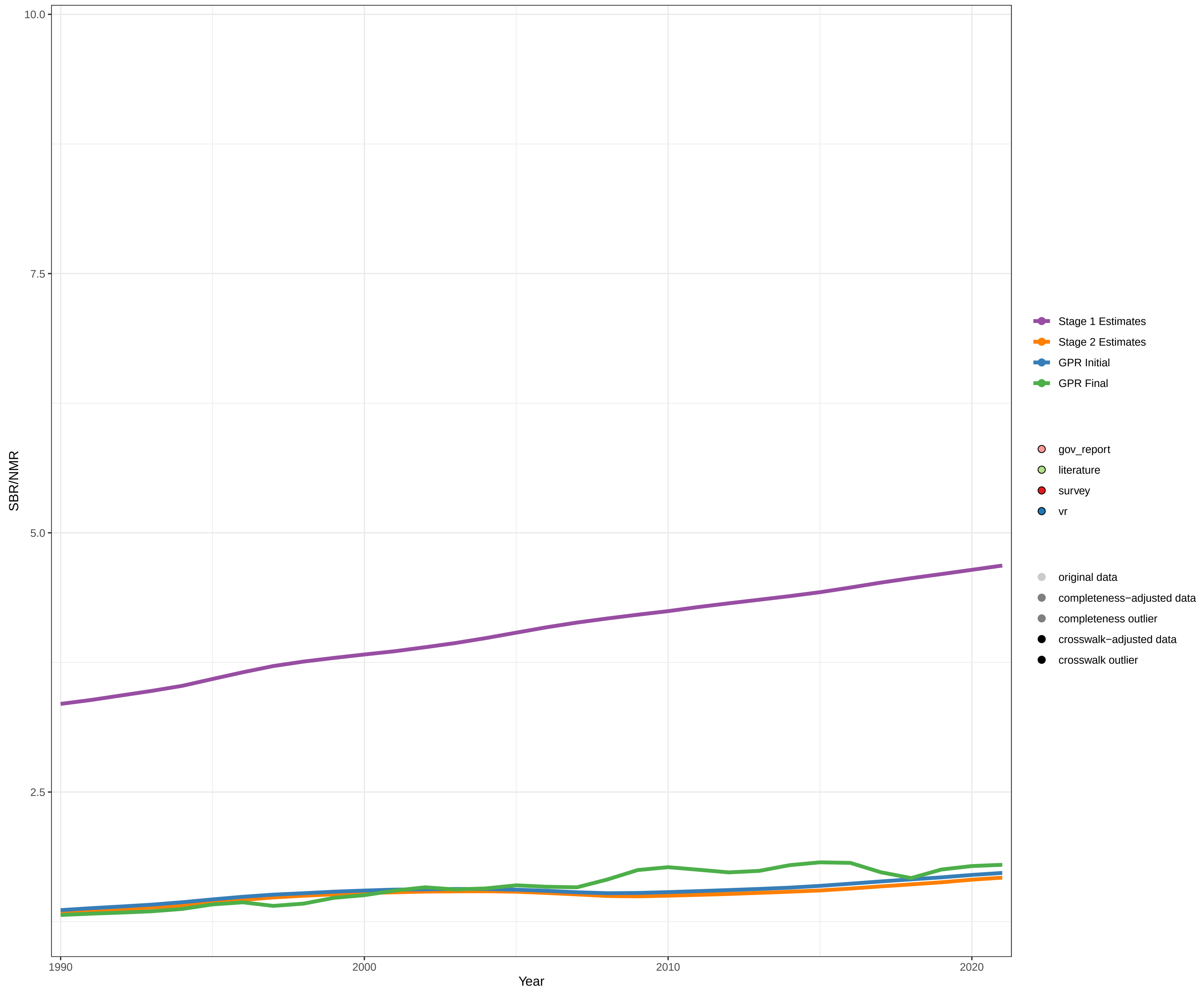

Definition	Years	Outlier	Source.Type	Title

Nagano JPN_35443 High−income Asia Pacific Stillbirth Rate (SBR) − 20 weeks
lambda = 1 ; zeta = 0.6 ; scale = 15 ; amp = 0.028

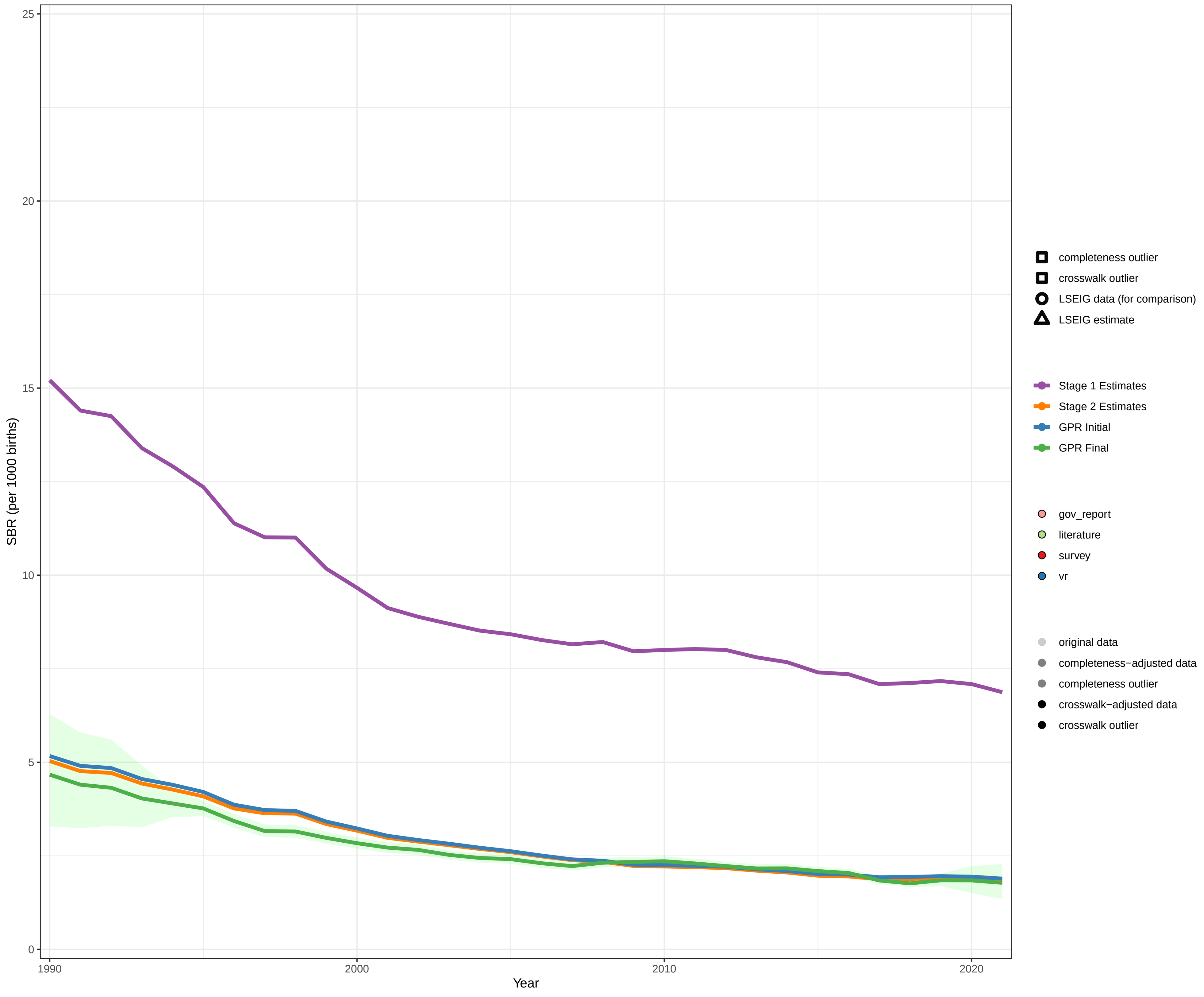

Nagano JPN_35443 High−income Asia Pacific Stillbirth Rate (SBR) − 28 weeks
lambda = 1 ; zeta = 0.6 ; scale = 15 ; amp = 0.028

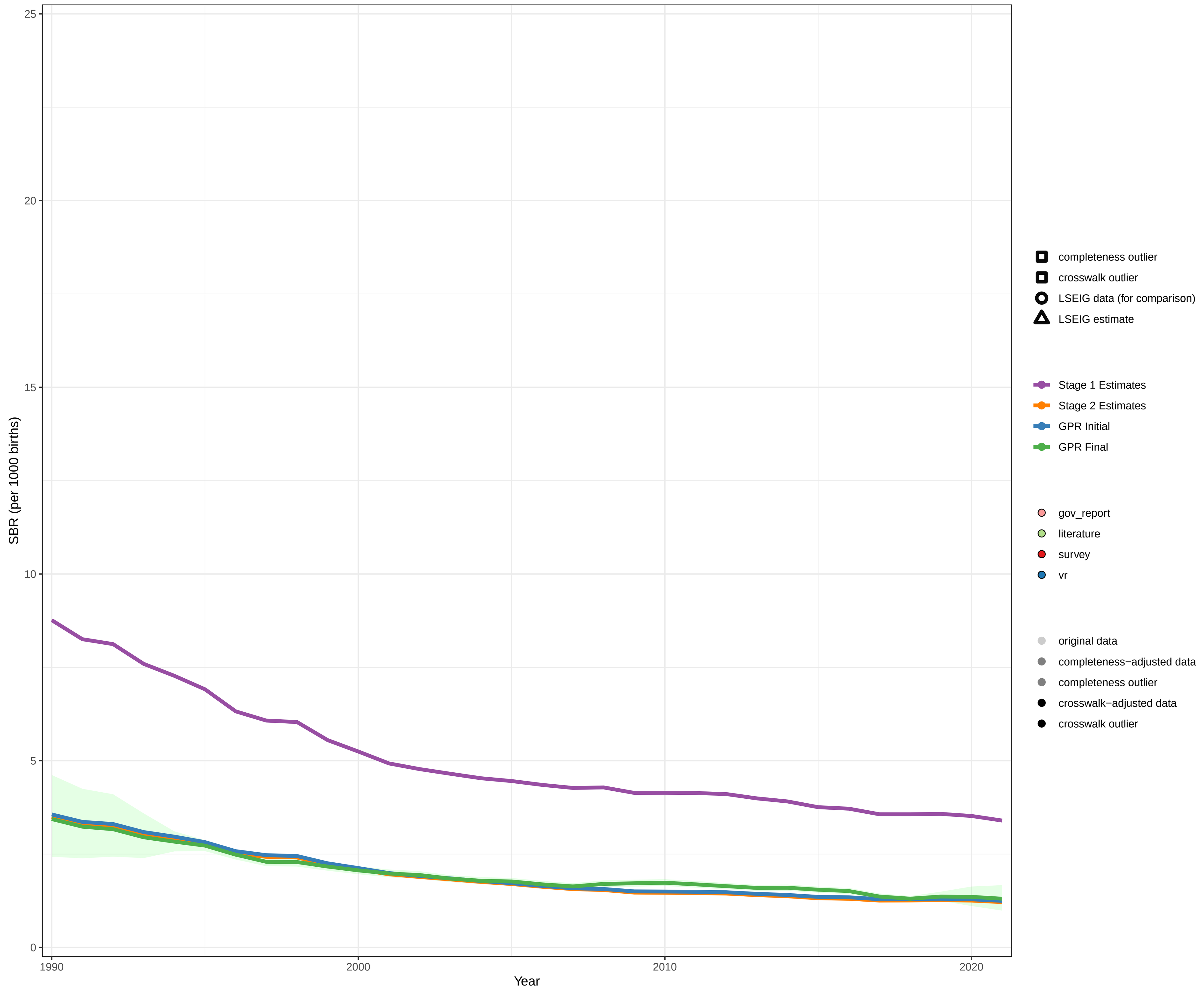

Year	Total Stillbirths (in thousands)	Total Stillbirths (in thousands)	SBR (per 1000 births)	SBR (per 1000 births)	SBR/NMR	SBR/NMR
	20 weeks	28 weeks	20 weeks	28 weeks	20 weeks	28 weeks
1990	0.1 (0.1 to 0.1)	0.1 (0.1 to 0.1)	4.7 (3.3 to 6.3)	3.4 (2.4 to 4.6)	1.78 (1.26 to 2.40)	1.31 (0.93 to 1.77)
1995	0.1 (0.1 to 0.1)	0.1 (0.1 to 0.1)	3.8 (3.6 to 4.0)	2.7 (2.6 to 2.9)	1.96 (1.85 to 2.06)	1.42 (1.34 to 1.49)
2000	0.1 (0.1 to 0.1)	0.0 (0.0 to 0.0)	2.8 (2.7 to 3.0)	2.1 (2.0 to 2.2)	2.07 (1.96 to 2.18)	1.51 (1.43 to 1.59)
2005	0.0 (0.0 to 0.0)	0.0 (0.0 to 0.0)	2.4 (2.3 to 2.5)	1.8 (1.7 to 1.9)	2.18 (2.07 to 2.30)	1.60 (1.52 to 1.69)
2010	0.0 (0.0 to 0.0)	0.0 (0.0 to 0.0)	2.4 (2.2 to 2.5)	1.7 (1.6 to 1.8)	2.41 (2.29 to 2.55)	1.78 (1.69 to 1.87)
2015	0.0 (0.0 to 0.0)	0.0 (0.0 to 0.0)	2.1 (2.0 to 2.2)	1.5 (1.5 to 1.6)	2.47 (2.34 to 2.60)	1.82 (1.73 to 1.92)
2020	0.0 (0.0 to 0.0)	0.0 (0.0 to 0.0)	1.8 (1.5 to 2.2)	1.4 (1.1 to 1.6)	2.43 (1.99 to 2.93)	1.79 (1.46 to 2.15)
2021	0.0 (0.0 to 0.0)	0.0 (0.0 to 0.0)	1.8 (1.3 to 2.3)	1.3 (1.0 to 1.7)	2.45 (1.85 to 3.14)	1.80 (1.36 to 2.30)

Nagasaki JPN_35465 High−income Asia Pacific Stillbirth Rate / Neonatal Mortality Rate (SBR/NMR) − 20 weeks
lambda = 1 ; zeta = 0.6 ; scale = 15 ; amp = 0.028

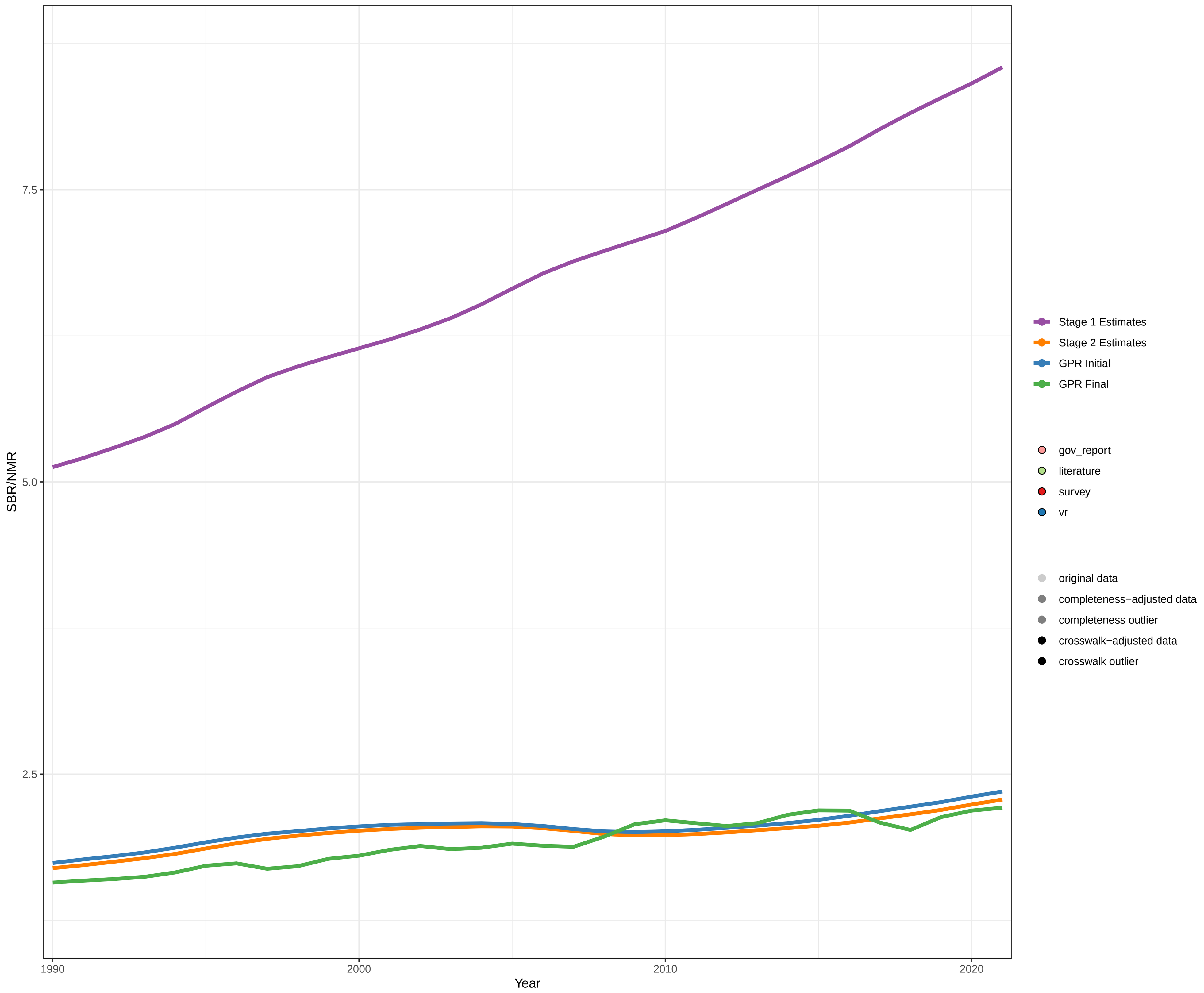

Nagasaki JPN_35465 High−income Asia Pacific Stillbirth Rate / Neonatal Mortality Rate (SBR/NMR) − 28 weeks
lambda = 1 ; zeta = 0.6 ; scale = 15 ; amp = 0.028

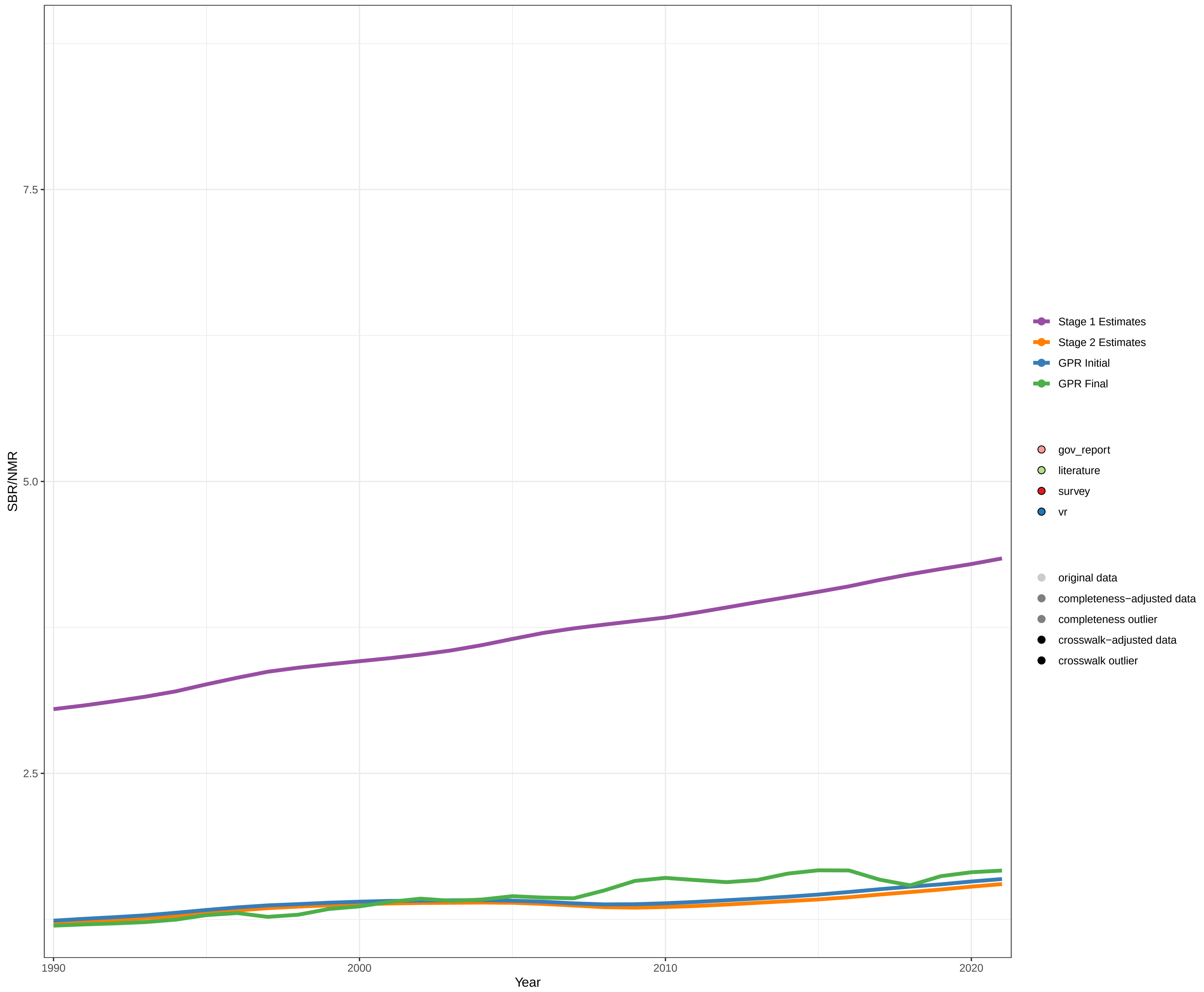

Definition	Years	Outlier	Source.Type	Title

Nagasaki JPN_35465 High−income Asia Pacific Stillbirth Rate (SBR) − 20 weeks
lambda = 1 ; zeta = 0.6 ; scale = 15 ; amp = 0.028

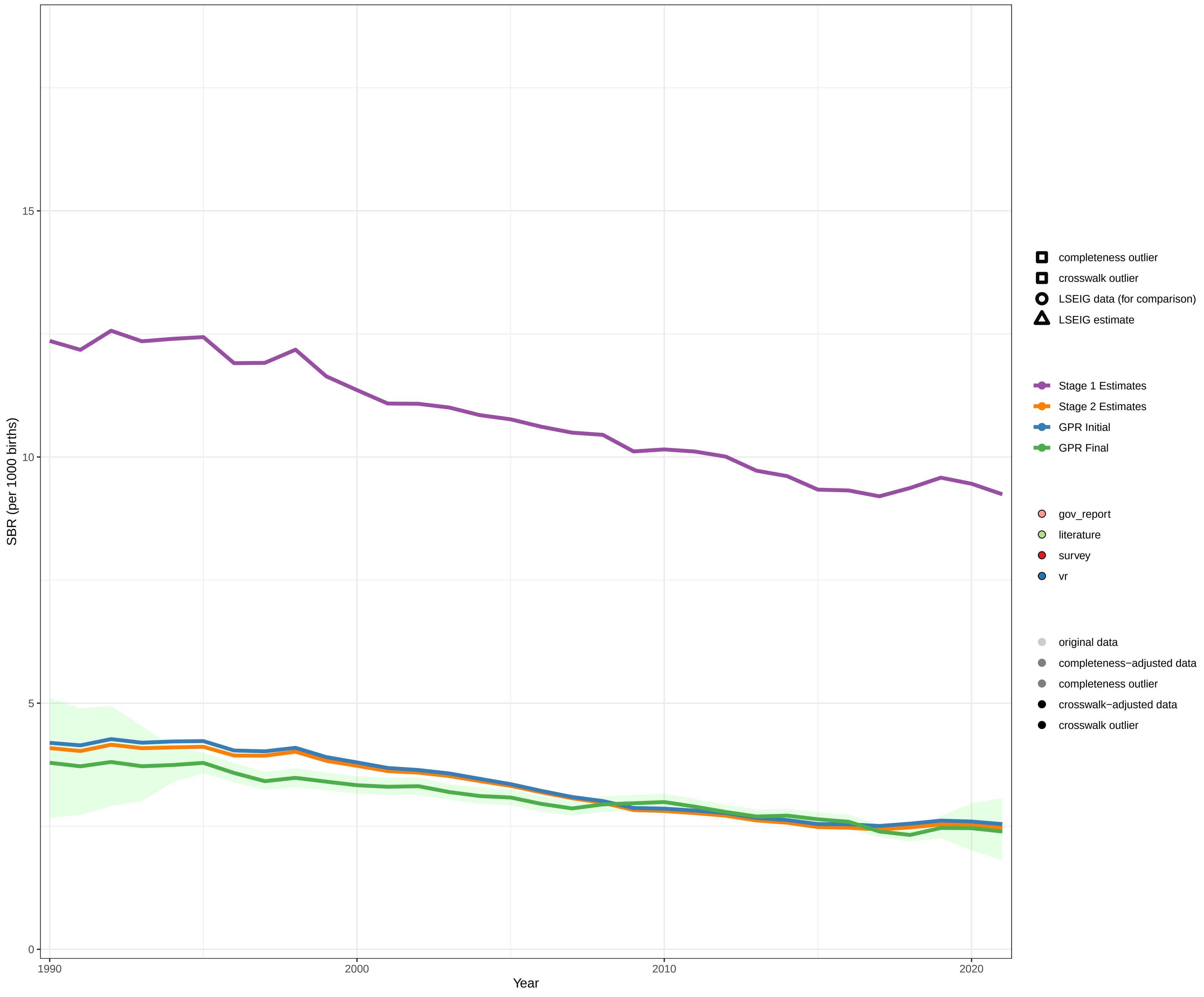

Nagasaki JPN_35465 High−income Asia Pacific Stillbirth Rate (SBR) − 28 weeks
lambda = 1 ; zeta = 0.6 ; scale = 15 ; amp = 0.028

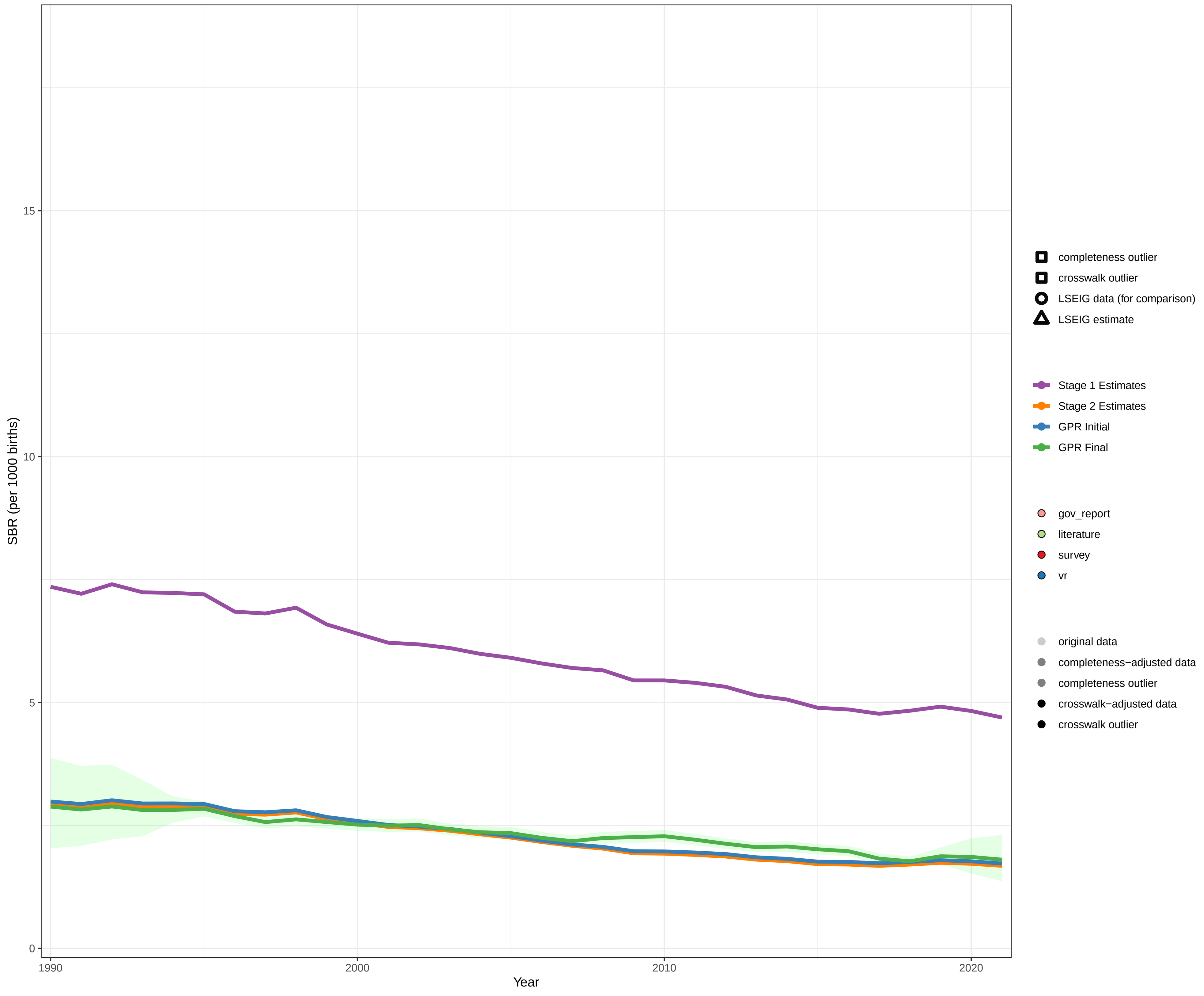

Year	Total Stillbirths (in thousands)	Total Stillbirths (in thousands)	SBR (per 1000 births)	SBR (per 1000 births)	SBR/NMR	SBR/NMR
	20 weeks	28 weeks	20 weeks	28 weeks	20 weeks	28 weeks
1990	0.1 (0.0 to 0.1)	0.0 (0.0 to 0.1)	3.8 (2.7 to 5.1)	2.9 (2.0 to 3.9)	1.57 (1.11 to 2.12)	1.20 (0.84 to 1.61)
1995	0.1 (0.1 to 0.1)	0.0 (0.0 to 0.0)	3.8 (3.6 to 4.0)	2.8 (2.7 to 3.0)	1.72 (1.62 to 1.81)	1.29 (1.22 to 1.36)
2000	0.0 (0.0 to 0.0)	0.0 (0.0 to 0.0)	3.3 (3.2 to 3.5)	2.5 (2.4 to 2.7)	1.80 (1.71 to 1.90)	1.36 (1.29 to 1.43)
2005	0.0 (0.0 to 0.0)	0.0 (0.0 to 0.0)	3.1 (2.9 to 3.2)	2.3 (2.2 to 2.5)	1.91 (1.81 to 2.01)	1.45 (1.37 to 1.52)
2010	0.0 (0.0 to 0.0)	0.0 (0.0 to 0.0)	3.0 (2.8 to 3.2)	2.3 (2.2 to 2.4)	2.11 (2.00 to 2.22)	1.60 (1.52 to 1.69)
2015	0.0 (0.0 to 0.0)	0.0 (0.0 to 0.0)	2.6 (2.5 to 2.8)	2.0 (1.9 to 2.1)	2.19 (2.08 to 2.31)	1.67 (1.58 to 1.76)
2020	0.0 (0.0 to 0.0)	0.0 (0.0 to 0.0)	2.5 (2.0 to 3.0)	1.9 (1.5 to 2.2)	2.19 (1.79 to 2.64)	1.65 (1.35 to 1.99)
2021	0.0 (0.0 to 0.0)	0.0 (0.0 to 0.0)	2.4 (1.8 to 3.1)	1.8 (1.4 to 2.3)	2.21 (1.67 to 2.84)	1.67 (1.26 to 2.13)

Nara JPN_35452 High−income Asia Pacific Stillbirth Rate / Neonatal Mortality Rate (SBR/NMR) − 20 weeks
lambda = 1 ; zeta = 0.6 ; scale = 15 ; amp = 0.028

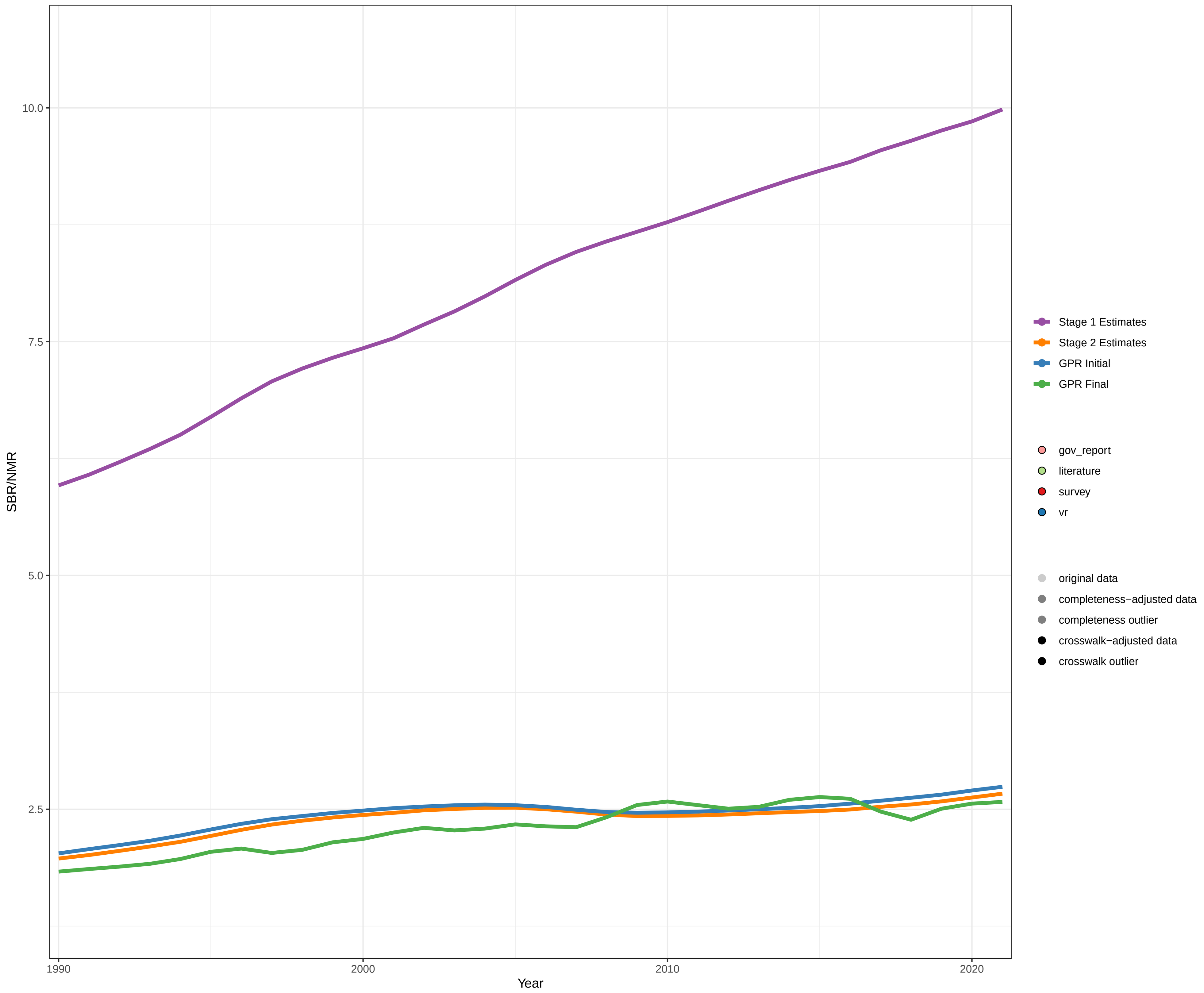

Nara JPN_35452 High−income Asia Pacific Stillbirth Rate / Neonatal Mortality Rate (SBR/NMR) − 28 weeks
lambda = 1 ; zeta = 0.6 ; scale = 15 ; amp = 0.028

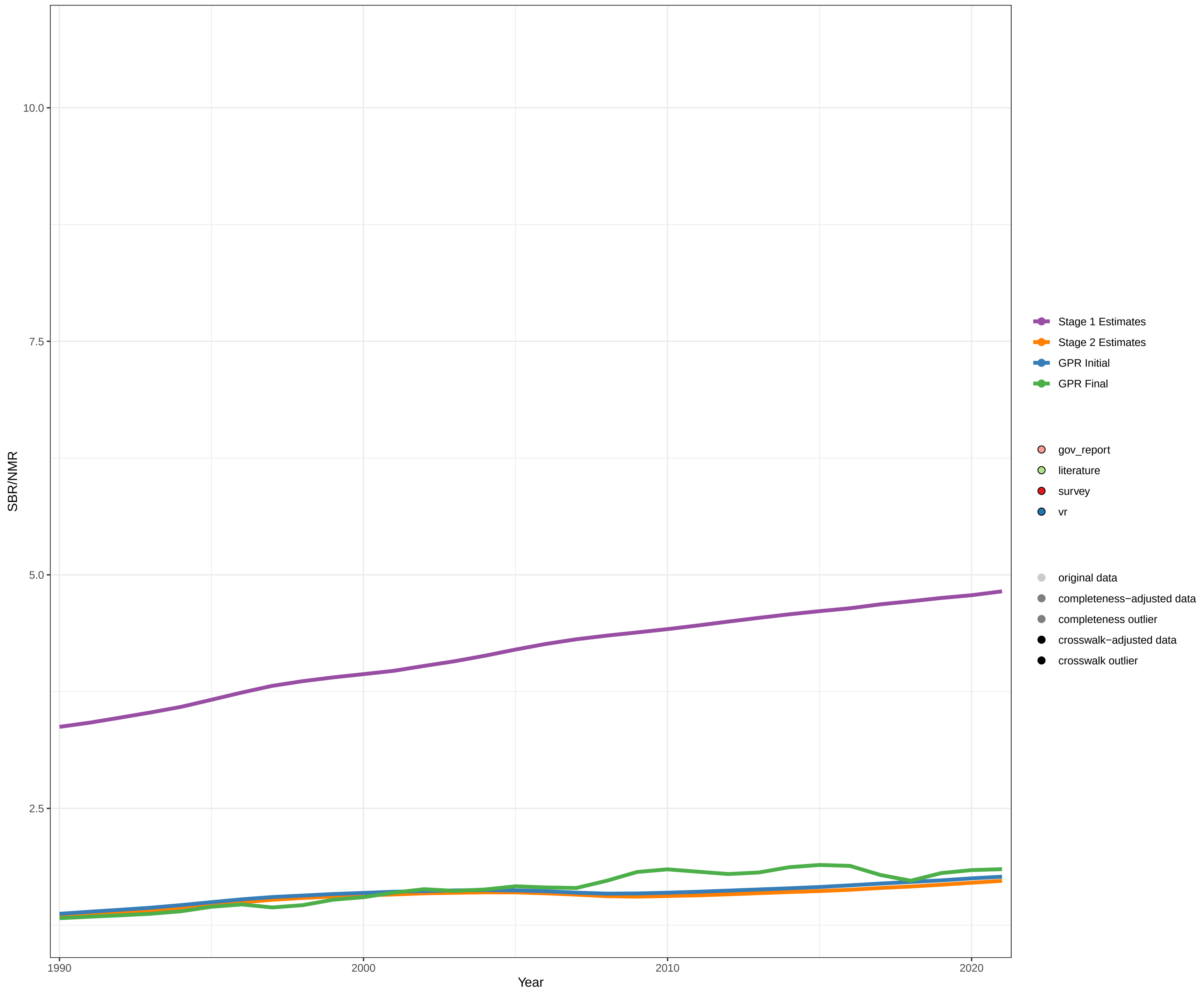

Definition	Years	Outlier	Source.Type	Title

Nara JPN_35452 High−income Asia Pacific Stillbirth Rate (SBR) − 20 weeks
lambda = 1 ; zeta = 0.6 ; scale = 15 ; amp = 0.028

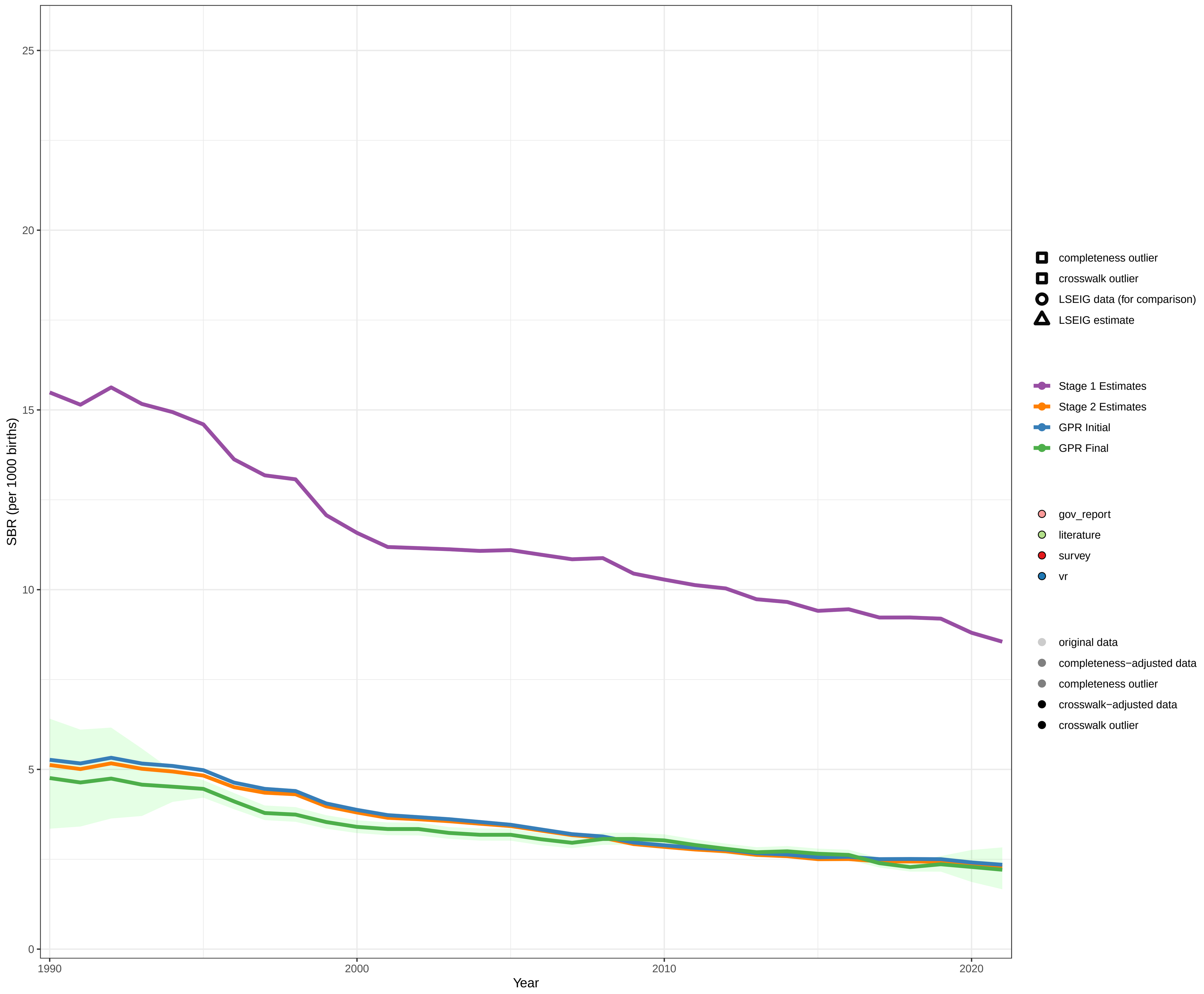

Nara JPN_35452 High−income Asia Pacific Stillbirth Rate (SBR) − 28 weeks
lambda = 1 ; zeta = 0.6 ; scale = 15 ; amp = 0.028

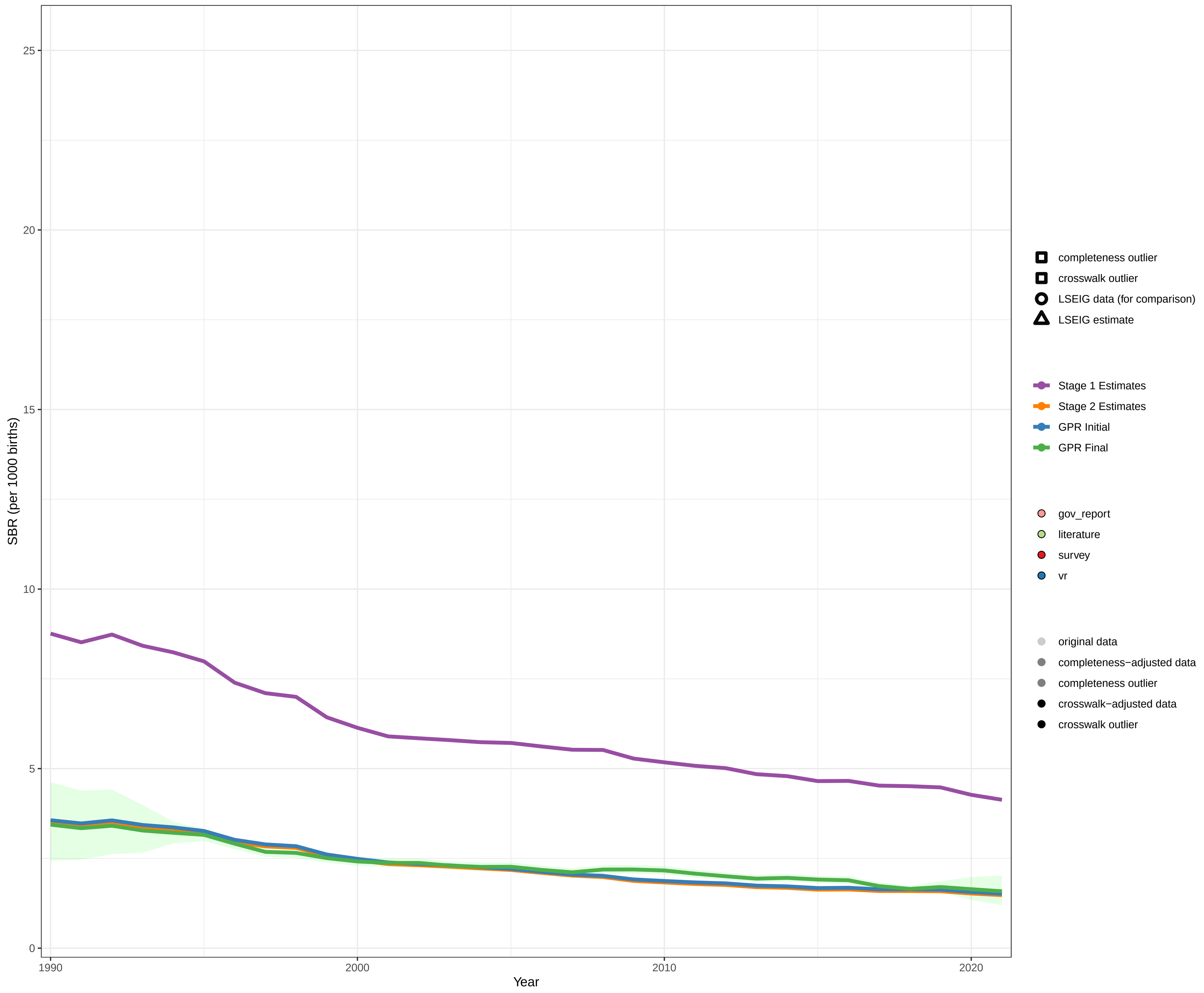

Year	Total Stillbirths (in thousands)	Total Stillbirths (in thousands)	SBR (per 1000 births)	SBR (per 1000 births)	SBR/NMR	SBR/NMR
	20 weeks	28 weeks	20 weeks	28 weeks	20 weeks	28 weeks
1990	0.1 (0.0 to 0.1)	0.0 (0.0 to 0.1)	4.8 (3.4 to 6.4)	3.4 (2.4 to 4.6)	1.83 (1.29 to 2.47)	1.32 (0.94 to 1.78)
1995	0.1 (0.1 to 0.1)	0.0 (0.0 to 0.0)	4.5 (4.2 to 4.7)	3.2 (3.0 to 3.3)	2.04 (1.93 to 2.16)	1.45 (1.37 to 1.53)
2000	0.0 (0.0 to 0.0)	0.0 (0.0 to 0.0)	3.4 (3.2 to 3.6)	2.4 (2.3 to 2.5)	2.18 (2.07 to 2.30)	1.55 (1.47 to 1.63)
2005	0.0 (0.0 to 0.0)	0.0 (0.0 to 0.0)	3.2 (3.0 to 3.4)	2.3 (2.2 to 2.4)	2.34 (2.22 to 2.46)	1.67 (1.58 to 1.75)
2010	0.0 (0.0 to 0.0)	0.0 (0.0 to 0.0)	3.0 (2.9 to 3.2)	2.2 (2.1 to 2.3)	2.58 (2.45 to 2.73)	1.85 (1.75 to 1.95)
2015	0.0 (0.0 to 0.0)	0.0 (0.0 to 0.0)	2.7 (2.5 to 2.8)	1.9 (1.8 to 2.0)	2.63 (2.49 to 2.77)	1.89 (1.80 to 2.00)
2020	0.0 (0.0 to 0.0)	0.0 (0.0 to 0.0)	2.3 (1.9 to 2.8)	1.6 (1.3 to 2.0)	2.56 (2.09 to 3.09)	1.84 (1.51 to 2.22)
2021	0.0 (0.0 to 0.0)	0.0 (0.0 to 0.0)	2.2 (1.7 to 2.8)	1.6 (1.2 to 2.0)	2.58 (1.94 to 3.30)	1.85 (1.40 to 2.36)

Niigata JPN_35438 High−income Asia Pacific Stillbirth Rate / Neonatal Mortality Rate (SBR/NMR) − 20 weeks
lambda = 1 ; zeta = 0.6 ; scale = 15 ; amp = 0.028

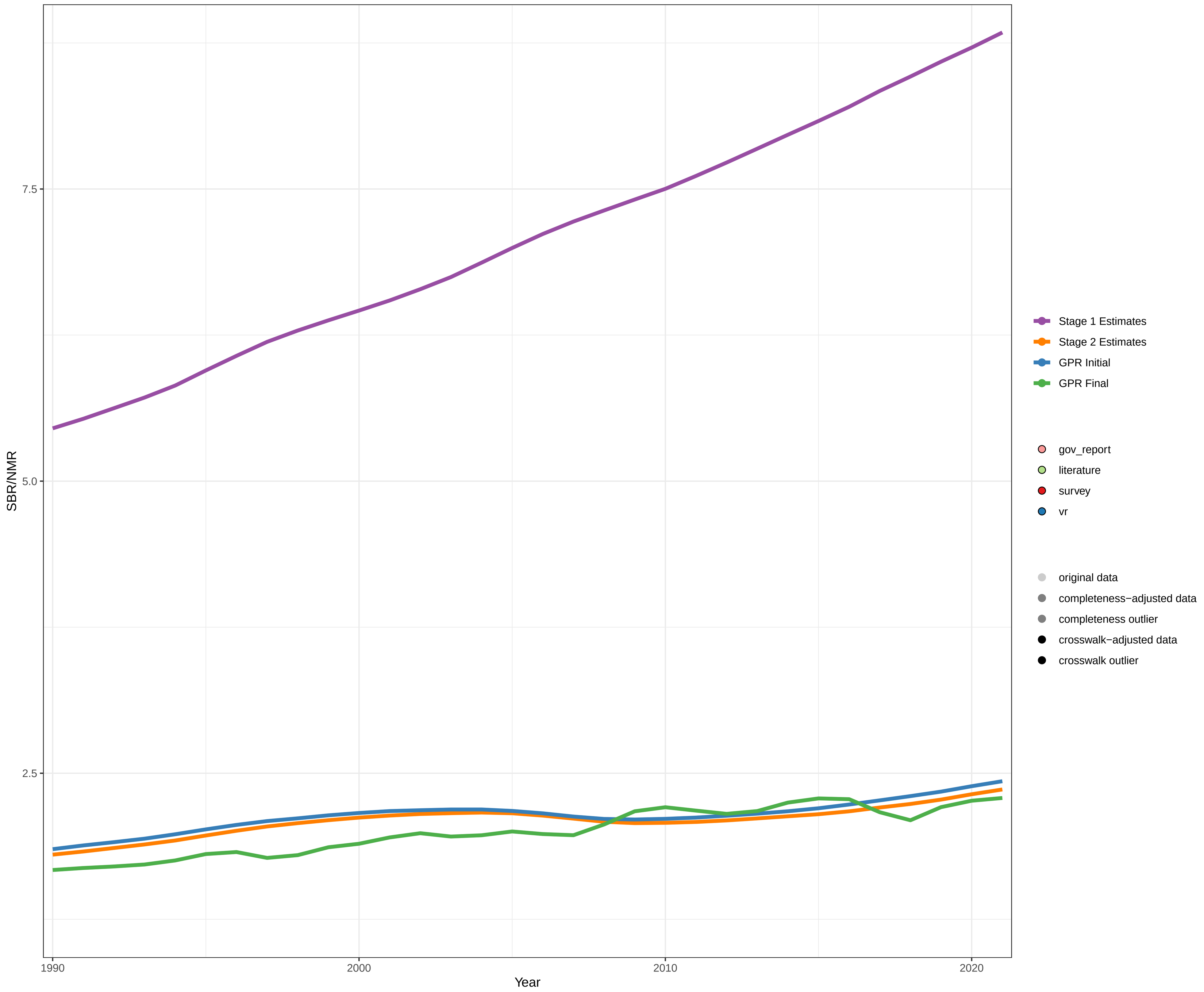

Niigata JPN_35438 High−income Asia Pacific Stillbirth Rate / Neonatal Mortality Rate (SBR/NMR) − 28 weeks
lambda = 1 ; zeta = 0.6 ; scale = 15 ; amp = 0.028

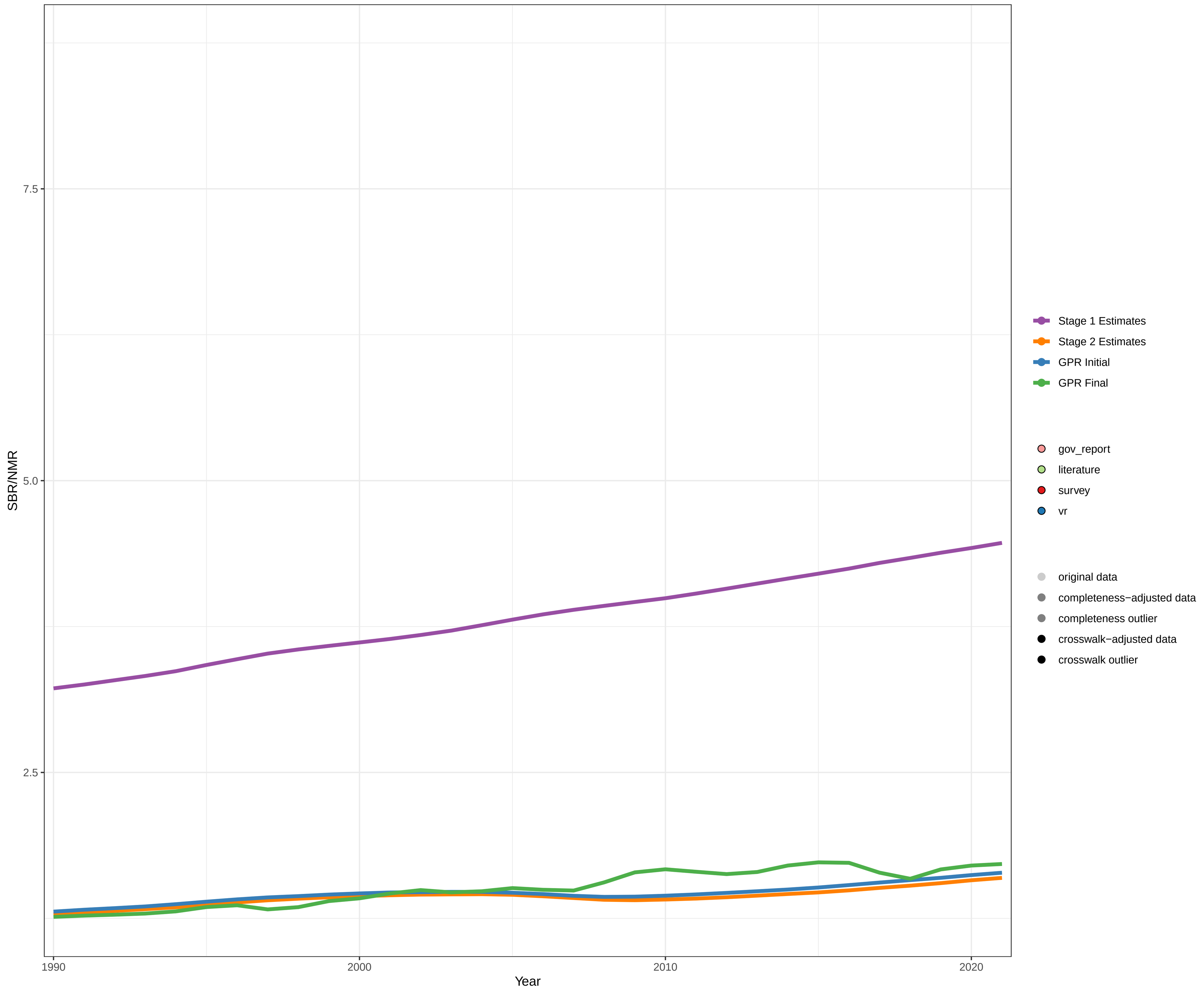

Definition	Years	Outlier	Source.Type	Title

Niigata JPN_35438 High−income Asia Pacific Stillbirth Rate (SBR) − 20 weeks
lambda = 1 ; zeta = 0.6 ; scale = 15 ; amp = 0.028

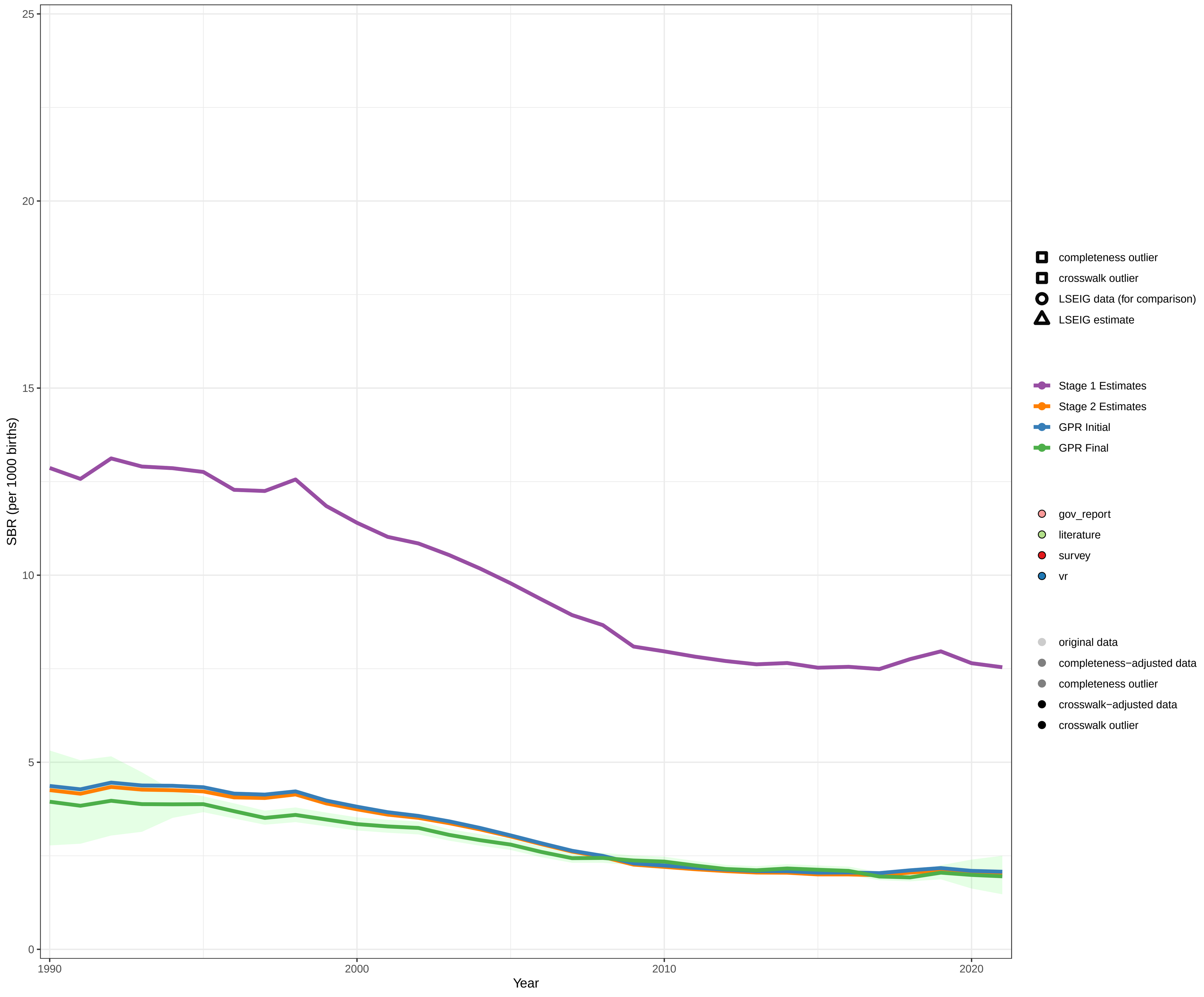

Niigata JPN_35438 High−income Asia Pacific Stillbirth Rate (SBR) − 28 weeks
lambda = 1 ; zeta = 0.6 ; scale = 15 ; amp = 0.028

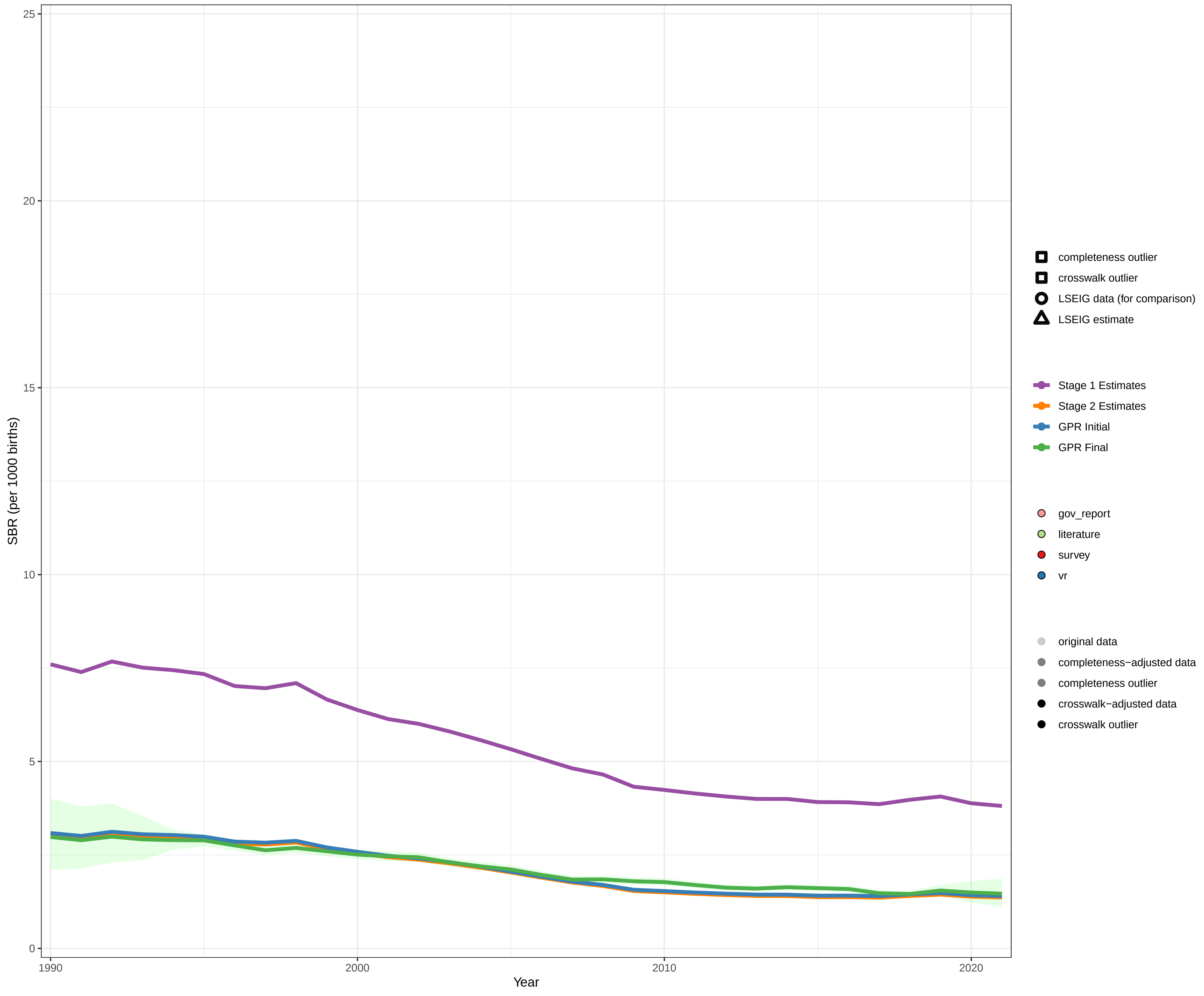

Year	Total Stillbirths (in thousands)	Total Stillbirths (in thousands)	SBR (per 1000 births)	SBR (per 1000 births)	SBR/NMR	SBR/NMR
	20 weeks	28 weeks	20 weeks	28 weeks	20 weeks	28 weeks
1990	0.1 (0.1 to 0.1)	0.1 (0.1 to 0.1)	3.9 (2.8 to 5.3)	3.0 (2.1 to 4.0)	1.67 (1.18 to 2.25)	1.26 (0.89 to 1.70)
1995	0.1 (0.1 to 0.1)	0.1 (0.1 to 0.1)	3.9 (3.7 to 4.1)	2.9 (2.7 to 3.0)	1.81 (1.71 to 1.91)	1.35 (1.27 to 1.42)
2000	0.1 (0.1 to 0.1)	0.1 (0.1 to 0.1)	3.3 (3.2 to 3.5)	2.5 (2.4 to 2.6)	1.90 (1.80 to 2.00)	1.42 (1.35 to 1.50)
2005	0.1 (0.1 to 0.1)	0.0 (0.0 to 0.0)	2.8 (2.7 to 2.9)	2.1 (2.0 to 2.2)	2.00 (1.90 to 2.11)	1.51 (1.43 to 1.59)
2010	0.0 (0.0 to 0.0)	0.0 (0.0 to 0.0)	2.3 (2.2 to 2.5)	1.8 (1.7 to 1.9)	2.21 (2.10 to 2.33)	1.67 (1.59 to 1.76)
2015	0.0 (0.0 to 0.0)	0.0 (0.0 to 0.0)	2.1 (2.0 to 2.2)	1.6 (1.5 to 1.7)	2.28 (2.17 to 2.41)	1.73 (1.64 to 1.82)
2020	0.0 (0.0 to 0.0)	0.0 (0.0 to 0.0)	2.0 (1.6 to 2.4)	1.5 (1.2 to 1.8)	2.26 (1.85 to 2.73)	1.70 (1.39 to 2.05)
2021	0.0 (0.0 to 0.0)	0.0 (0.0 to 0.0)	2.0 (1.5 to 2.5)	1.5 (1.1 to 1.9)	2.29 (1.72 to 2.93)	1.72 (1.30 to 2.19)

Oita JPN_35467 High−income Asia Pacific Stillbirth Rate / Neonatal Mortality Rate (SBR/NMR) − 20 weeks
lambda = 1 ; zeta = 0.6 ; scale = 15 ; amp = 0.028

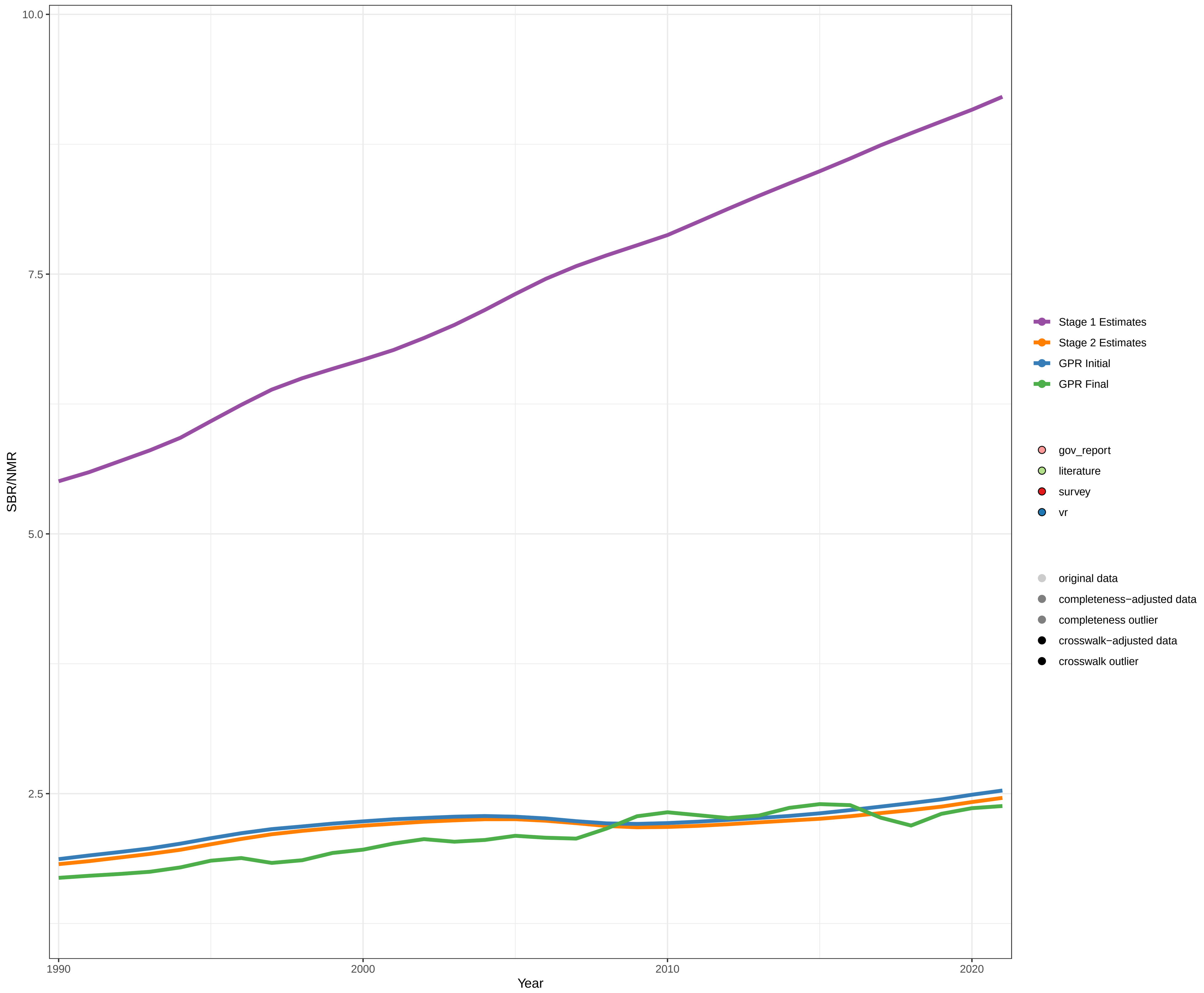

Oita JPN_35467 High−income Asia Pacific Stillbirth Rate / Neonatal Mortality Rate (SBR/NMR) − 28 weeks
lambda = 1 ; zeta = 0.6 ; scale = 15 ; amp = 0.028

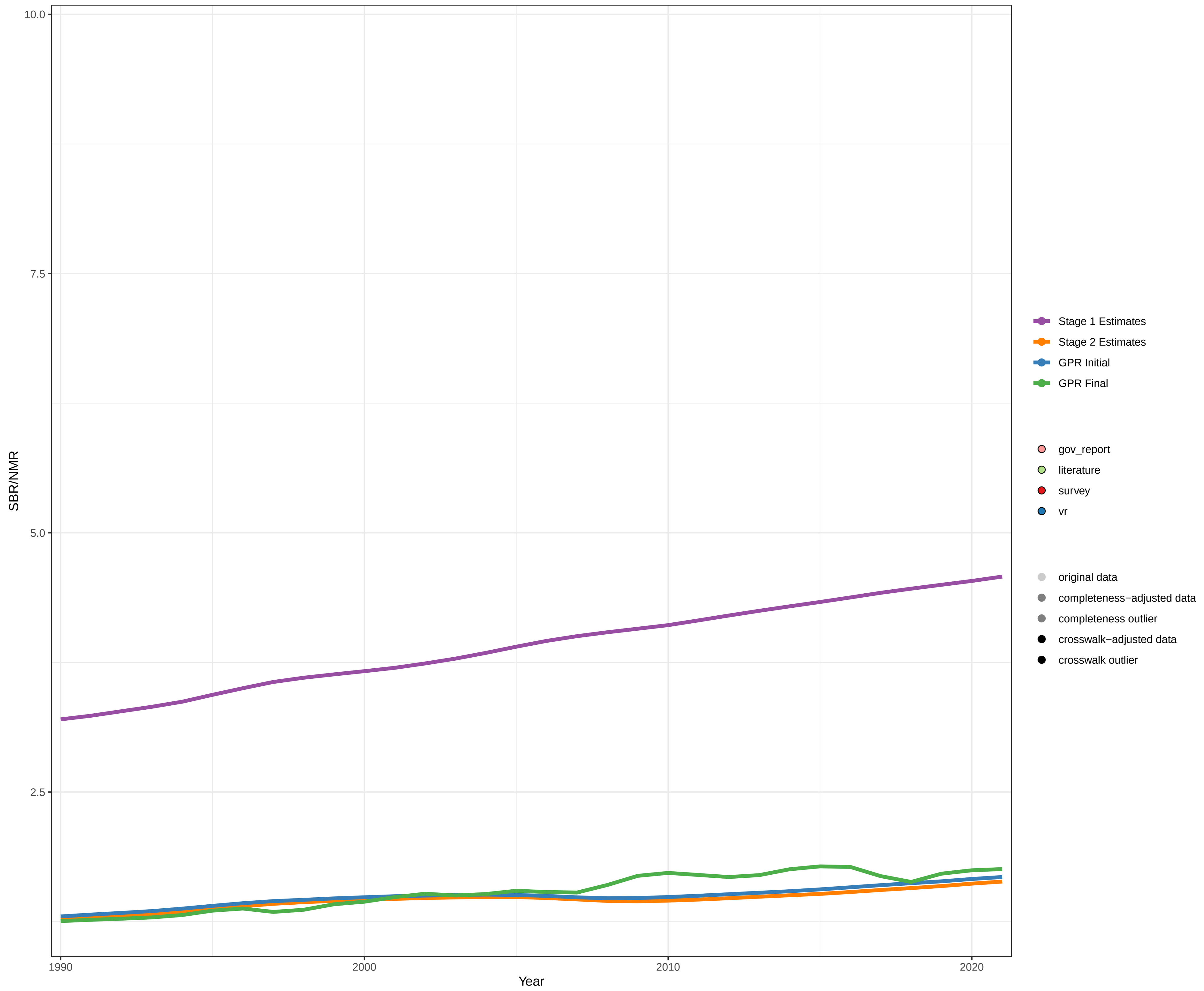

Definition	Years	Outlier	Source.Type	Title

Oita JPN_35467 High−income Asia Pacific Stillbirth Rate (SBR) − 20 weeks
lambda = 1 ; zeta = 0.6 ; scale = 15 ; amp = 0.028

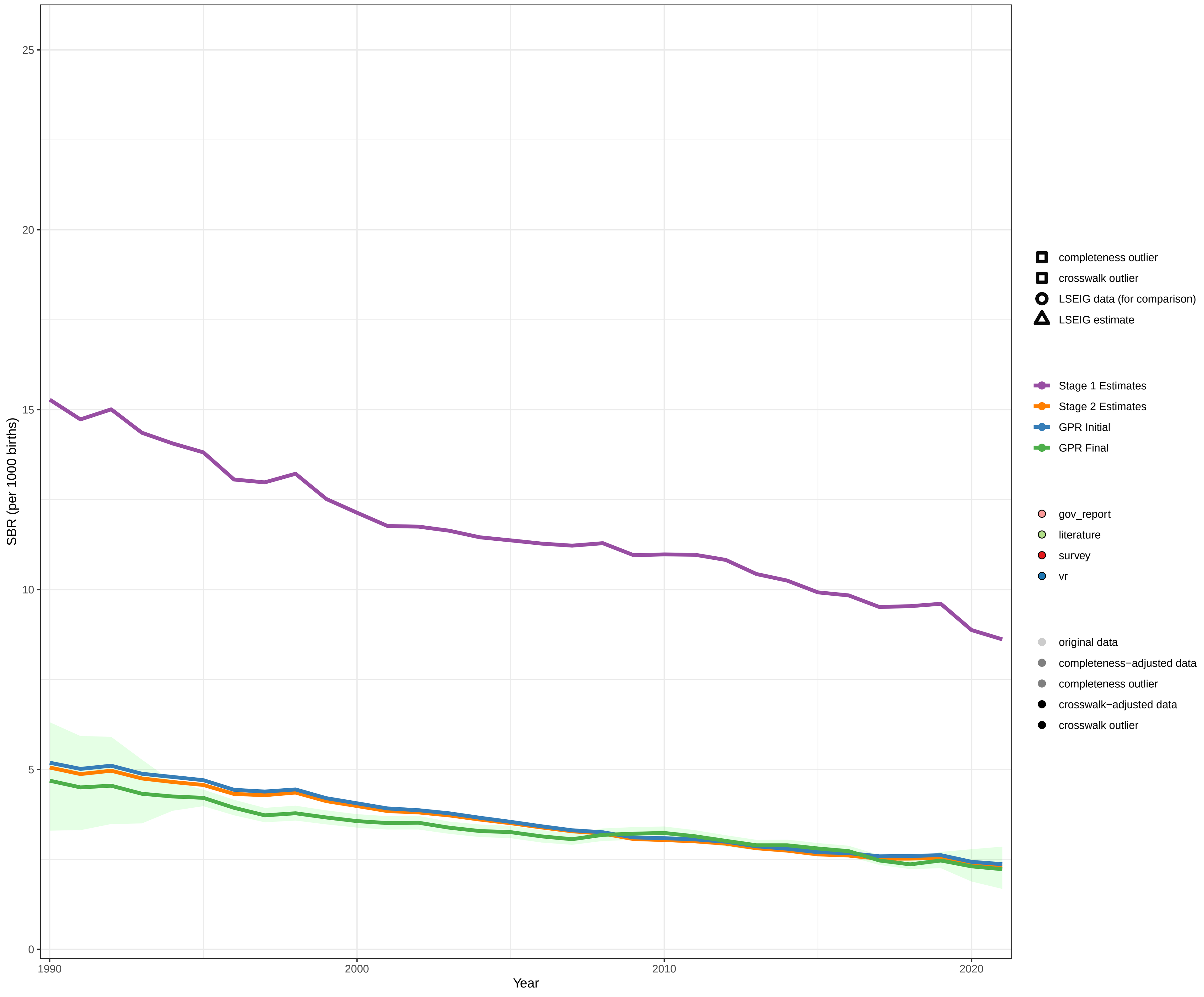

Oita JPN_35467 High−income Asia Pacific Stillbirth Rate (SBR) − 28 weeks
lambda = 1 ; zeta = 0.6 ; scale = 15 ; amp = 0.028

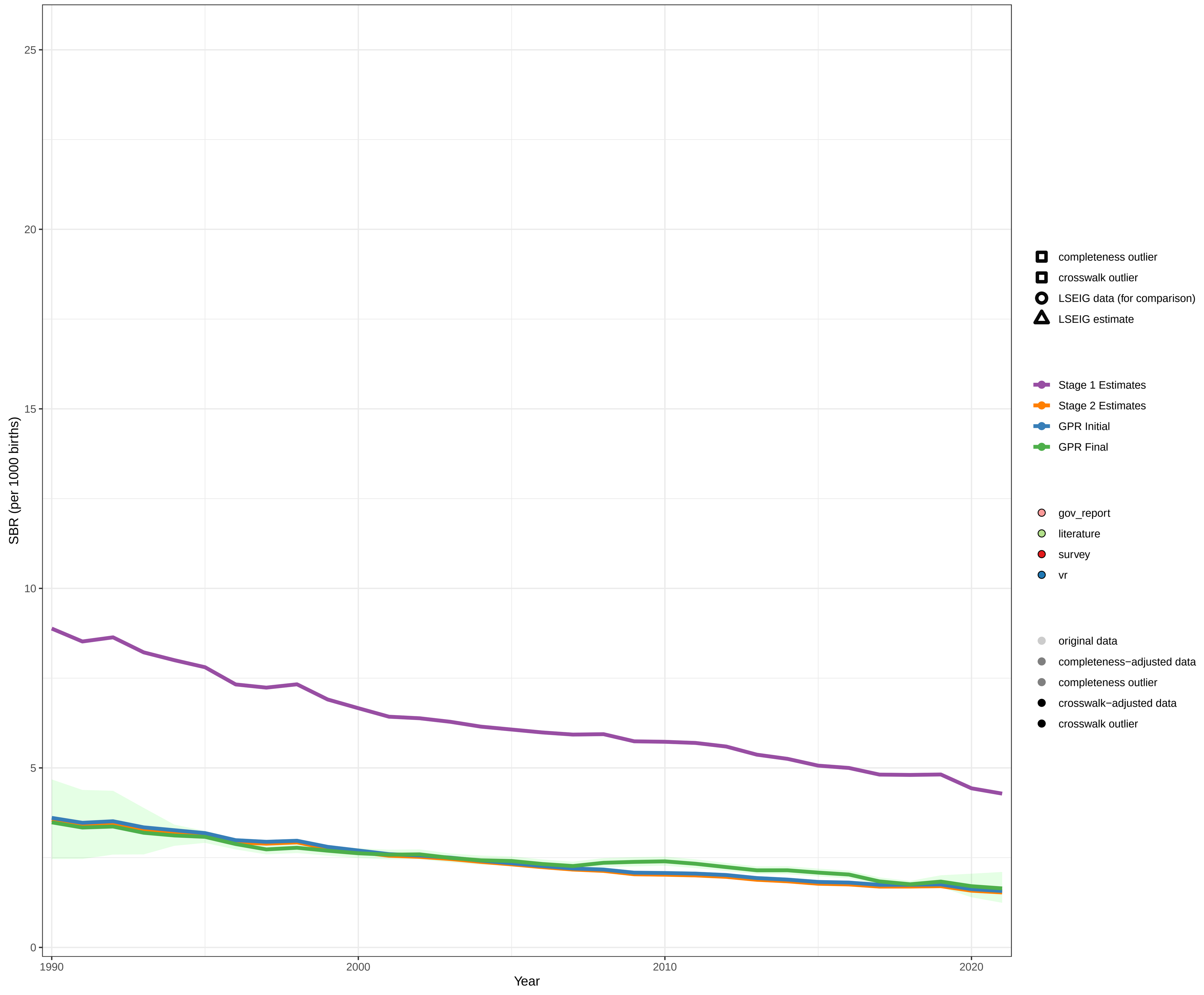

Year	Total Stillbirths (in thousands)	Total Stillbirths (in thousands)	SBR (per 1000 births)	SBR (per 1000 births)	SBR/NMR	SBR/NMR
	20 weeks	28 weeks	20 weeks	28 weeks	20 weeks	28 weeks
1990	0.1 (0.0 to 0.1)	0.0 (0.0 to 0.1)	4.7 (3.3 to 6.3)	3.5 (2.5 to 4.7)	1.69 (1.19 to 2.28)	1.26 (0.89 to 1.69)
1995	0.0 (0.0 to 0.1)	0.0 (0.0 to 0.0)	4.2 (4.0 to 4.4)	3.1 (2.9 to 3.2)	1.85 (1.75 to 1.96)	1.36 (1.28 to 1.43)
2000	0.0 (0.0 to 0.0)	0.0 (0.0 to 0.0)	3.6 (3.4 to 3.8)	2.6 (2.5 to 2.8)	1.96 (1.86 to 2.07)	1.44 (1.37 to 1.52)
2005	0.0 (0.0 to 0.0)	0.0 (0.0 to 0.0)	3.3 (3.1 to 3.4)	2.4 (2.3 to 2.5)	2.09 (1.99 to 2.21)	1.55 (1.47 to 1.63)
2010	0.0 (0.0 to 0.0)	0.0 (0.0 to 0.0)	3.2 (3.1 to 3.4)	2.4 (2.3 to 2.5)	2.32 (2.20 to 2.45)	1.72 (1.63 to 1.81)
2015	0.0 (0.0 to 0.0)	0.0 (0.0 to 0.0)	2.8 (2.7 to 3.0)	2.1 (2.0 to 2.2)	2.40 (2.27 to 2.53)	1.78 (1.69 to 1.88)
2020	0.0 (0.0 to 0.0)	0.0 (0.0 to 0.0)	2.3 (1.9 to 2.8)	1.7 (1.4 to 2.1)	2.36 (1.93 to 2.85)	1.75 (1.43 to 2.10)
2021	0.0 (0.0 to 0.0)	0.0 (0.0 to 0.0)	2.2 (1.7 to 2.9)	1.6 (1.2 to 2.1)	2.38 (1.79 to 3.05)	1.76 (1.33 to 2.25)

Okayama JPN_35456 High−income Asia Pacific Stillbirth Rate / Neonatal Mortality Rate (SBR/NMR) − 20 weeks
lambda = 1 ; zeta = 0.6 ; scale = 15 ; amp = 0.028

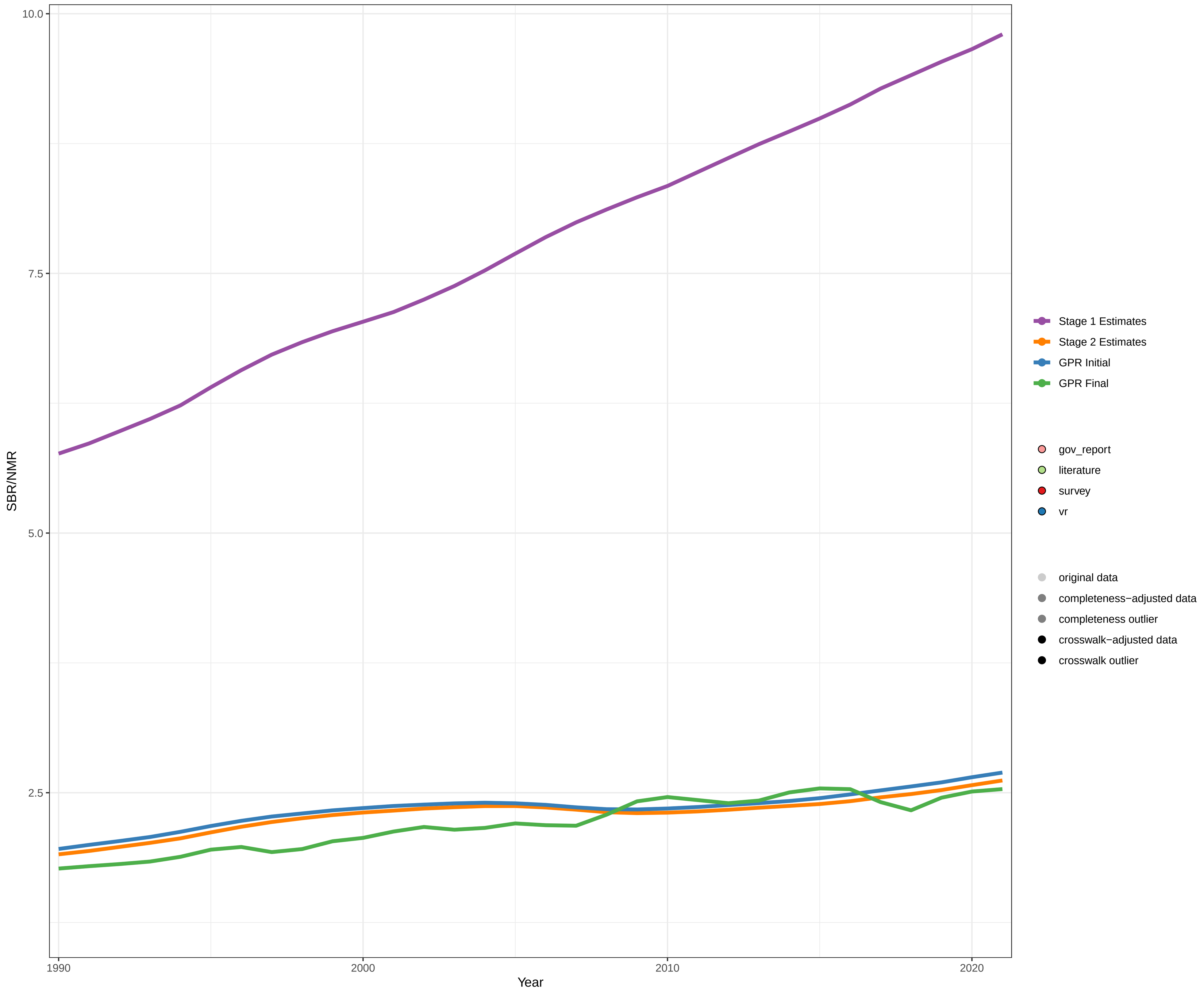

Okayama JPN_35456 High−income Asia Pacific Stillbirth Rate / Neonatal Mortality Rate (SBR/NMR) − 28 weeks
lambda = 1 ; zeta = 0.6 ; scale = 15 ; amp = 0.028

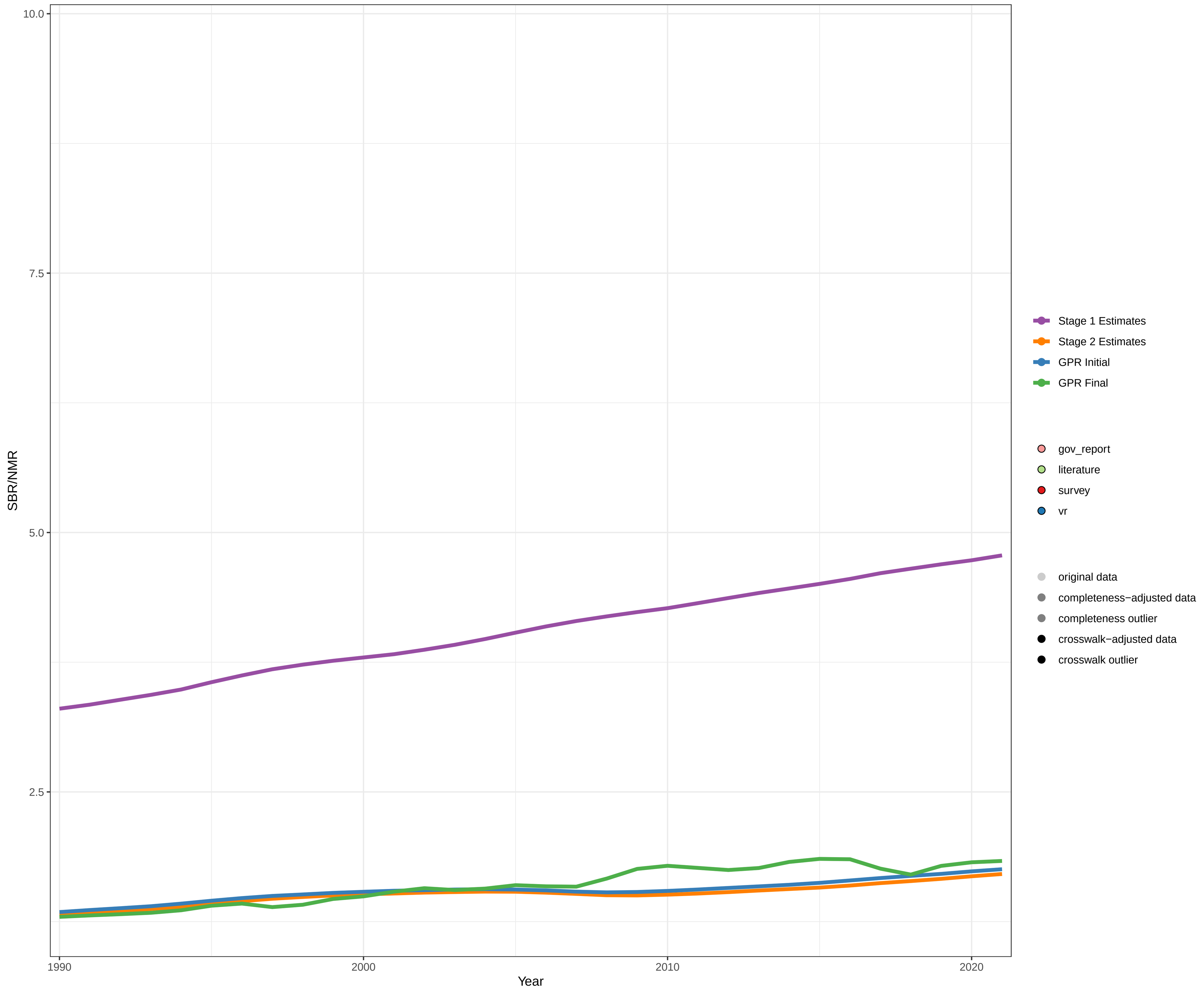

Definition	Years	Outlier	Source.Type	Title

Okayama JPN_35456 High−income Asia Pacific Stillbirth Rate (SBR) − 20 weeks
lambda = 1 ; zeta = 0.6 ; scale = 15 ; amp = 0.028

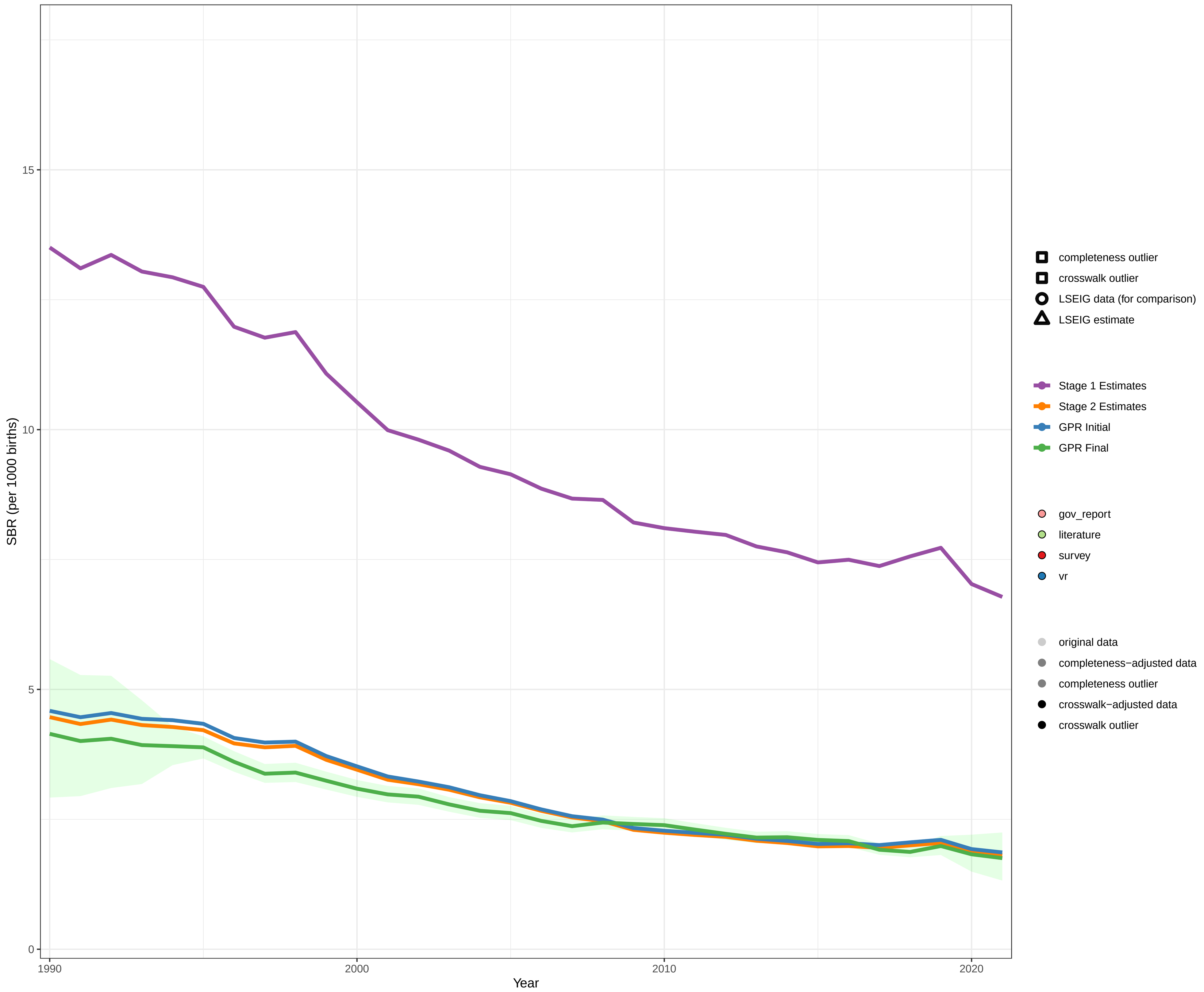

Okayama JPN_35456 High−income Asia Pacific Stillbirth Rate (SBR) − 28 weeks
lambda = 1 ; zeta = 0.6 ; scale = 15 ; amp = 0.028

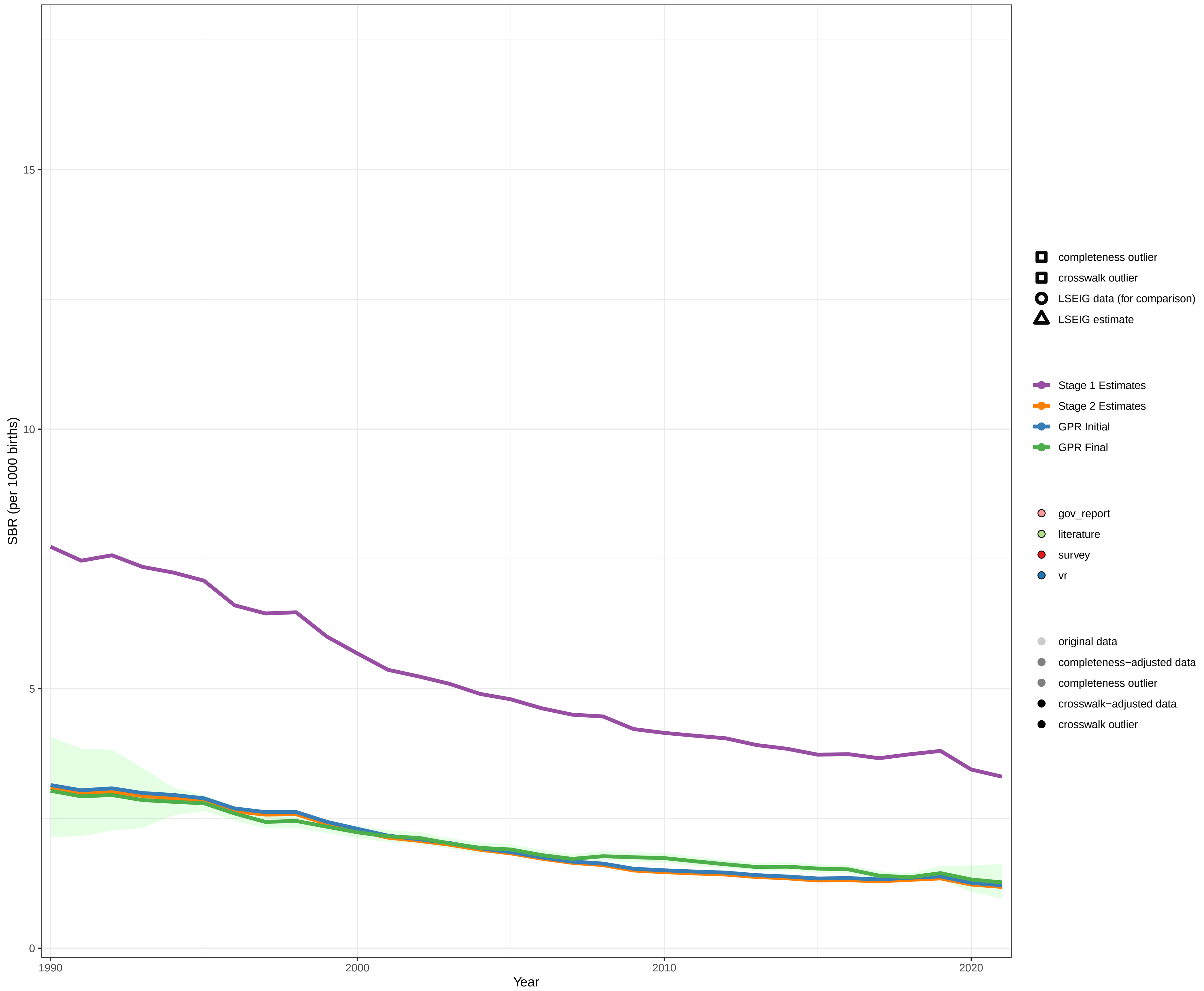

Year	Total Stillbirths (in thousands)	Total Stillbirths (in thousands)	SBR (per 1000 births)	SBR (per 1000 births)	SBR/NMR	SBR/NMR
	20 weeks	28 weeks	20 weeks	28 weeks	20 weeks	28 weeks
1990	0.1 (0.1 to 0.1)	0.1 (0.0 to 0.1)	4.1 (2.9 to 5.6)	3.0 (2.1 to 4.1)	1.77 (1.25 to 2.38)	1.30 (0.92 to 1.74)
1995	0.1 (0.1 to 0.1)	0.1 (0.0 to 0.1)	3.9 (3.7 to 4.1)	2.8 (2.6 to 2.9)	1.95 (1.84 to 2.06)	1.40 (1.33 to 1.48)
2000	0.1 (0.1 to 0.1)	0.0 (0.0 to 0.0)	3.1 (2.9 to 3.3)	2.2 (2.1 to 2.4)	2.07 (1.96 to 2.18)	1.49 (1.42 to 1.57)
2005	0.0 (0.0 to 0.0)	0.0 (0.0 to 0.0)	2.6 (2.5 to 2.8)	1.9 (1.8 to 2.0)	2.20 (2.09 to 2.32)	1.60 (1.52 to 1.69)
2010	0.0 (0.0 to 0.0)	0.0 (0.0 to 0.0)	2.4 (2.3 to 2.5)	1.7 (1.6 to 1.8)	2.46 (2.33 to 2.60)	1.79 (1.70 to 1.89)
2015	0.0 (0.0 to 0.0)	0.0 (0.0 to 0.0)	2.1 (2.0 to 2.2)	1.5 (1.5 to 1.6)	2.54 (2.41 to 2.68)	1.85 (1.76 to 1.95)
2020	0.0 (0.0 to 0.0)	0.0 (0.0 to 0.0)	1.8 (1.5 to 2.2)	1.3 (1.1 to 1.6)	2.51 (2.05 to 3.03)	1.82 (1.49 to 2.19)
2021	0.0 (0.0 to 0.0)	0.0 (0.0 to 0.0)	1.8 (1.3 to 2.2)	1.3 (1.0 to 1.6)	2.53 (1.91 to 3.25)	1.83 (1.39 to 2.35)

Okinawa JPN_35470 High−income Asia Pacific Stillbirth Rate / Neonatal Mortality Rate (SBR/NMR) − 20 weeks
lambda = 1 ; zeta = 0.6 ; scale = 15 ; amp = 0.028

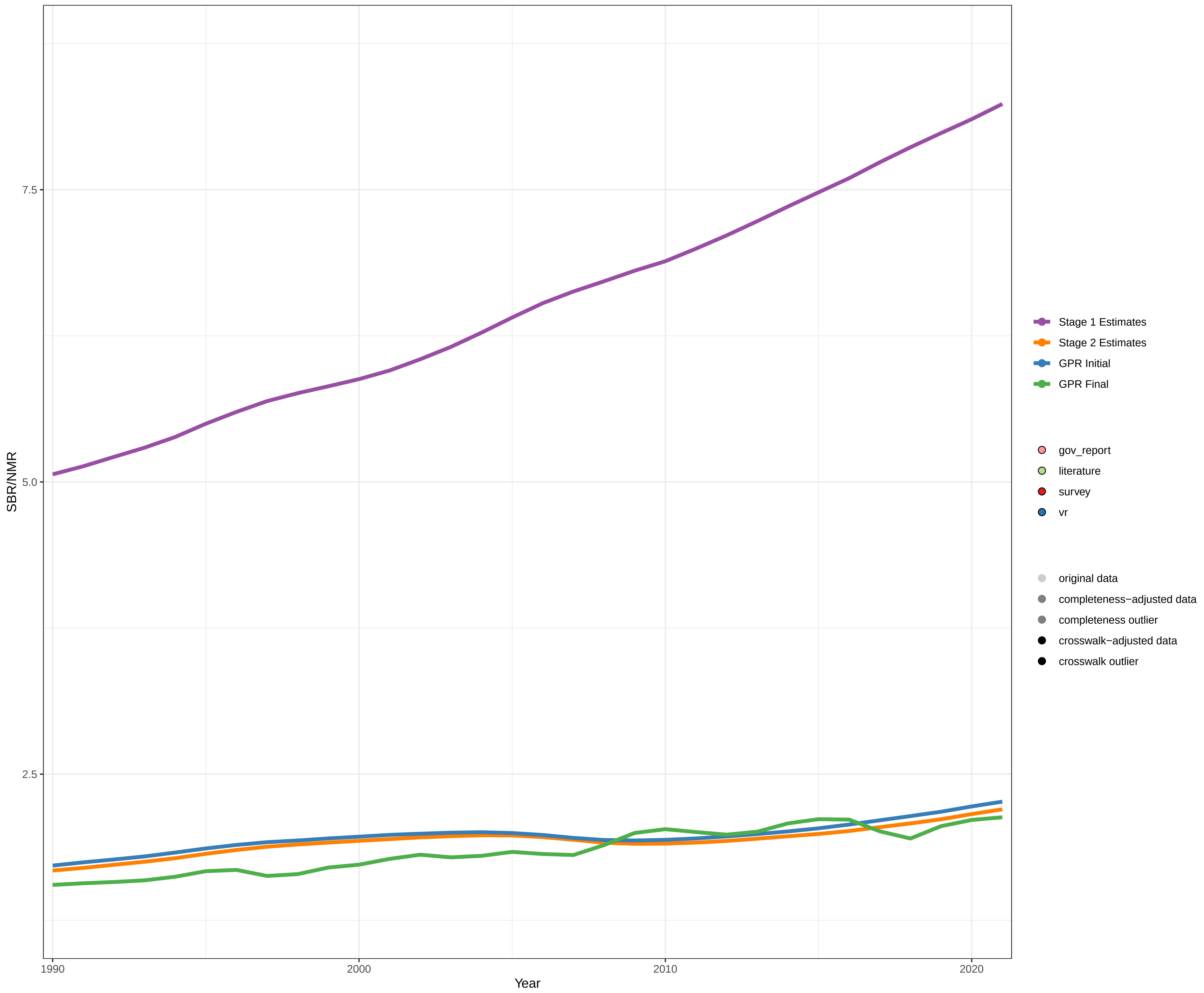

Okinawa JPN_35470 High−income Asia Pacific Stillbirth Rate / Neonatal Mortality Rate (SBR/NMR) − 28 weeks
lambda = 1 ; zeta = 0.6 ; scale = 15 ; amp = 0.028

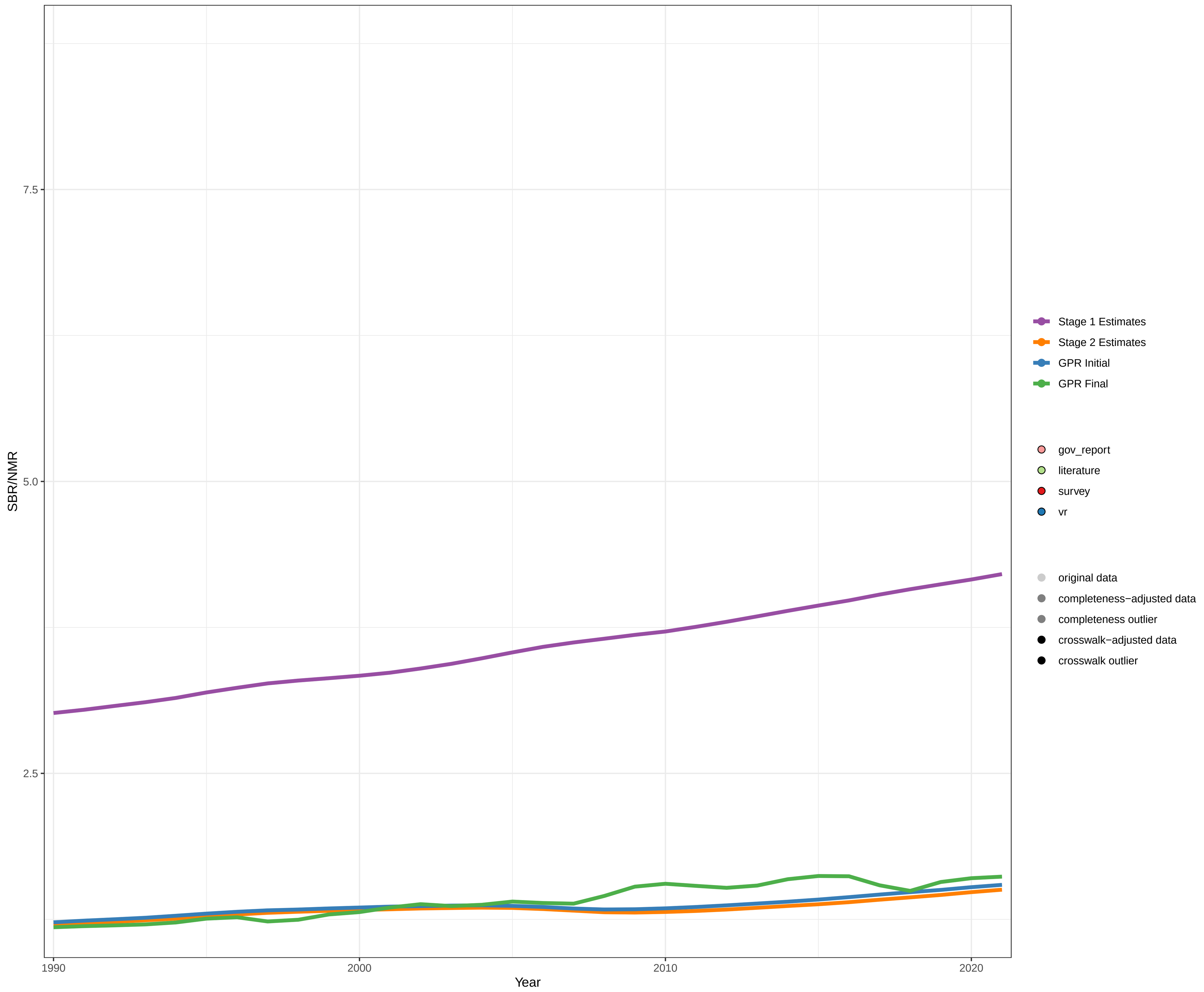

Definition	Years	Outlier	Source.Type	Title

Okinawa JPN_35470 High−income Asia Pacific Stillbirth Rate (SBR) − 20 weeks
lambda = 1 ; zeta = 0.6 ; scale = 15 ; amp = 0.028

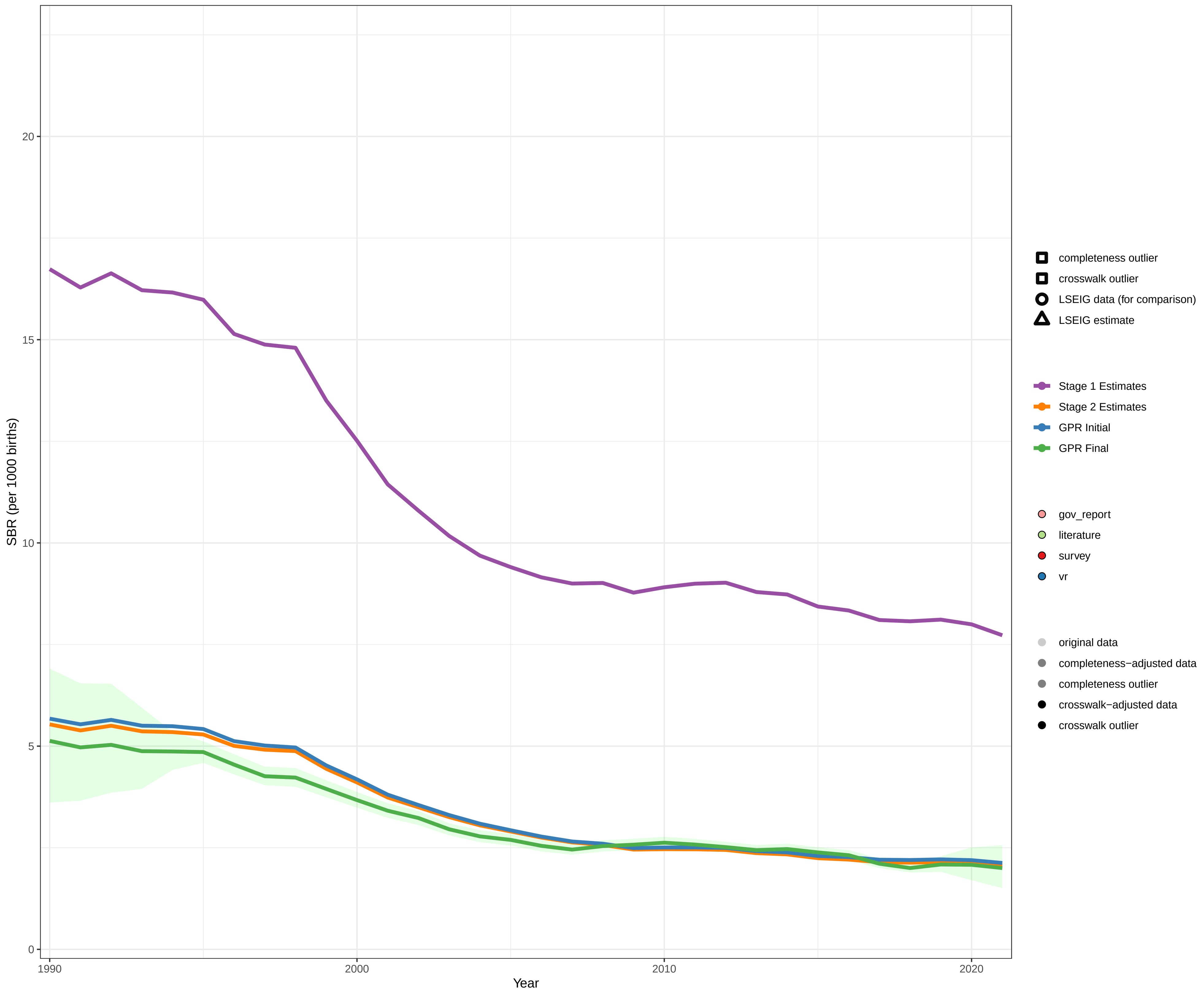

Okinawa JPN_35470 High−income Asia Pacific Stillbirth Rate (SBR) − 28 weeks
lambda = 1 ; zeta = 0.6 ; scale = 15 ; amp = 0.028

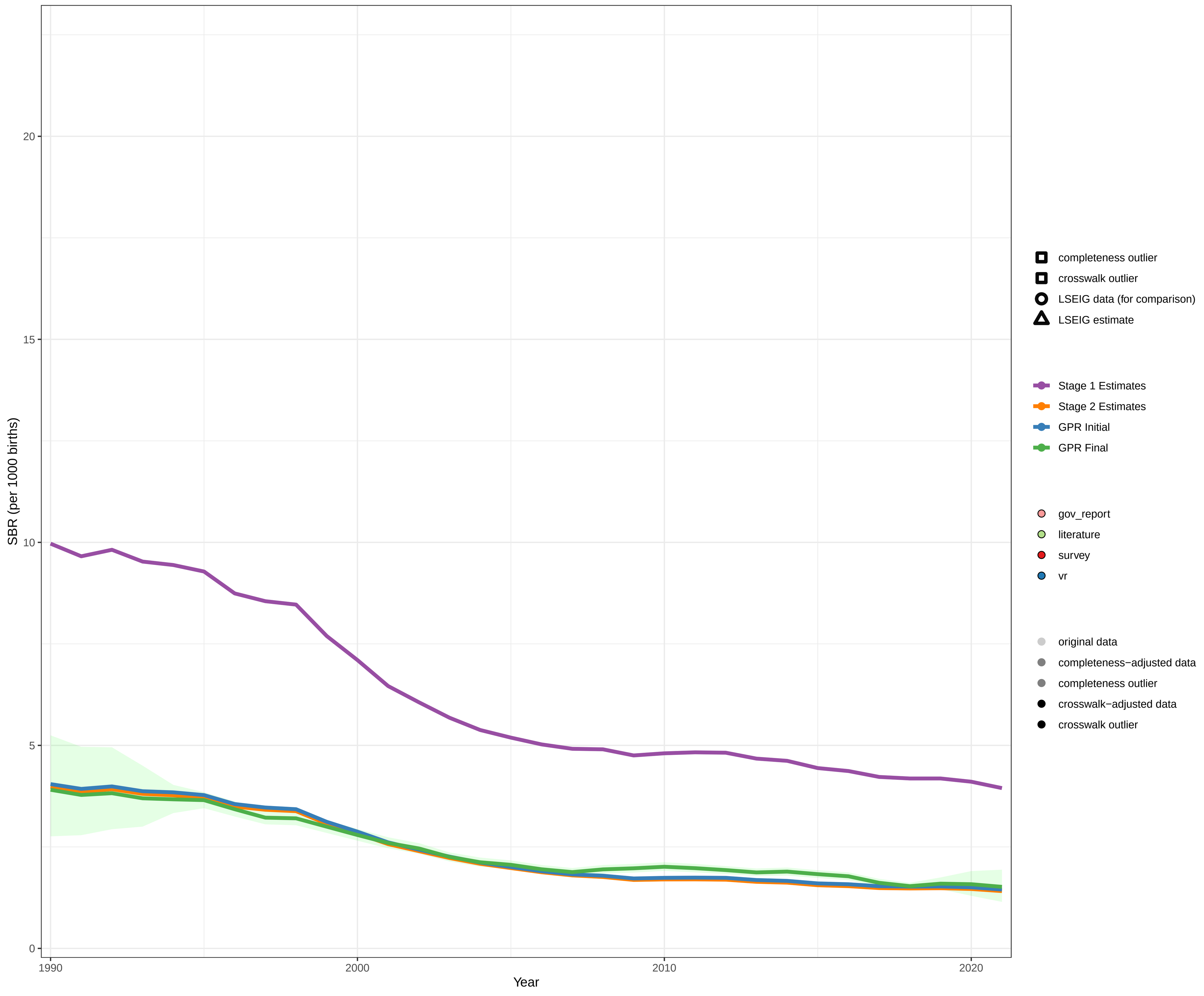

Year	Total Stillbirths (in thousands)	Total Stillbirths (in thousands)	SBR (per 1000 births)	SBR (per 1000 births)	SBR/NMR	SBR/NMR
	20 weeks	28 weeks	20 weeks	28 weeks	20 weeks	28 weeks
1990	0.1 (0.1 to 0.1)	0.1 (0.0 to 0.1)	5.1 (3.6 to 6.9)	3.9 (2.8 to 5.2)	1.55 (1.09 to 2.09)	1.18 (0.84 to 1.59)
1995	0.1 (0.1 to 0.1)	0.1 (0.1 to 0.1)	4.9 (4.6 to 5.1)	3.7 (3.5 to 3.8)	1.67 (1.58 to 1.76)	1.26 (1.19 to 1.32)
2000	0.1 (0.1 to 0.1)	0.0 (0.0 to 0.0)	3.7 (3.5 to 3.9)	2.8 (2.7 to 2.9)	1.73 (1.64 to 1.82)	1.31 (1.25 to 1.38)
2005	0.0 (0.0 to 0.0)	0.0 (0.0 to 0.0)	2.7 (2.6 to 2.8)	2.1 (2.0 to 2.2)	1.83 (1.74 to 1.93)	1.40 (1.33 to 1.48)
2010	0.0 (0.0 to 0.0)	0.0 (0.0 to 0.0)	2.6 (2.5 to 2.8)	2.0 (1.9 to 2.1)	2.03 (1.93 to 2.14)	1.55 (1.48 to 1.64)
2015	0.0 (0.0 to 0.0)	0.0 (0.0 to 0.0)	2.4 (2.3 to 2.5)	1.8 (1.7 to 1.9)	2.11 (2.00 to 2.23)	1.62 (1.54 to 1.71)
2020	0.0 (0.0 to 0.0)	0.0 (0.0 to 0.0)	2.1 (1.7 to 2.5)	1.6 (1.3 to 1.9)	2.11 (1.72 to 2.54)	1.60 (1.31 to 1.93)
2021	0.0 (0.0 to 0.0)	0.0 (0.0 to 0.0)	2.0 (1.5 to 2.6)	1.5 (1.1 to 1.9)	2.13 (1.61 to 2.73)	1.62 (1.22 to 2.07)

Osaka JPN_35450 High−income Asia Pacific Stillbirth Rate / Neonatal Mortality Rate (SBR/NMR) − 20 weeks
lambda = 1 ; zeta = 0.6 ; scale = 15 ; amp = 0.028

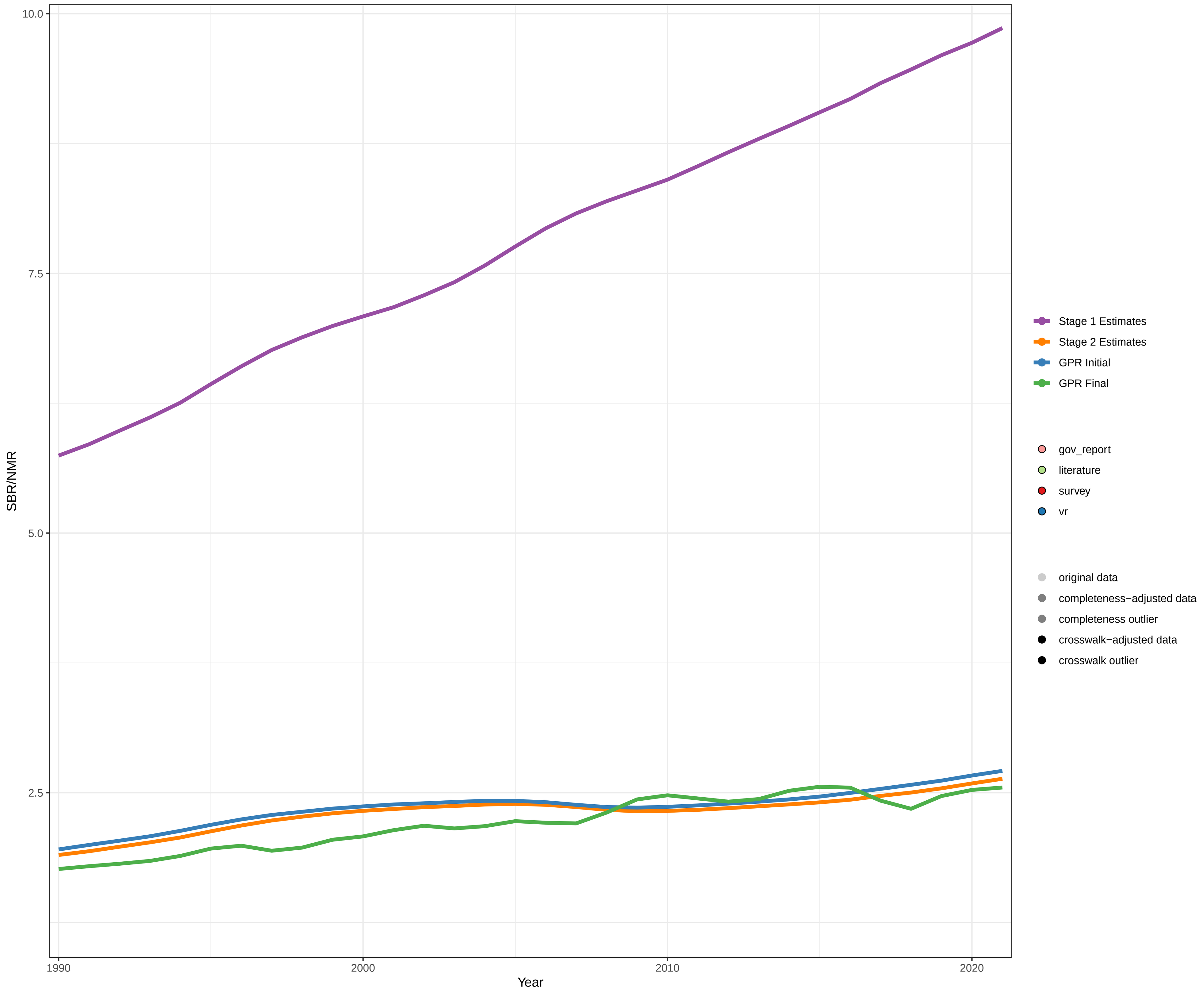

Osaka JPN_35450 High−income Asia Pacific Stillbirth Rate / Neonatal Mortality Rate (SBR/NMR) − 28 weeks
lambda = 1 ; zeta = 0.6 ; scale = 15 ; amp = 0.028

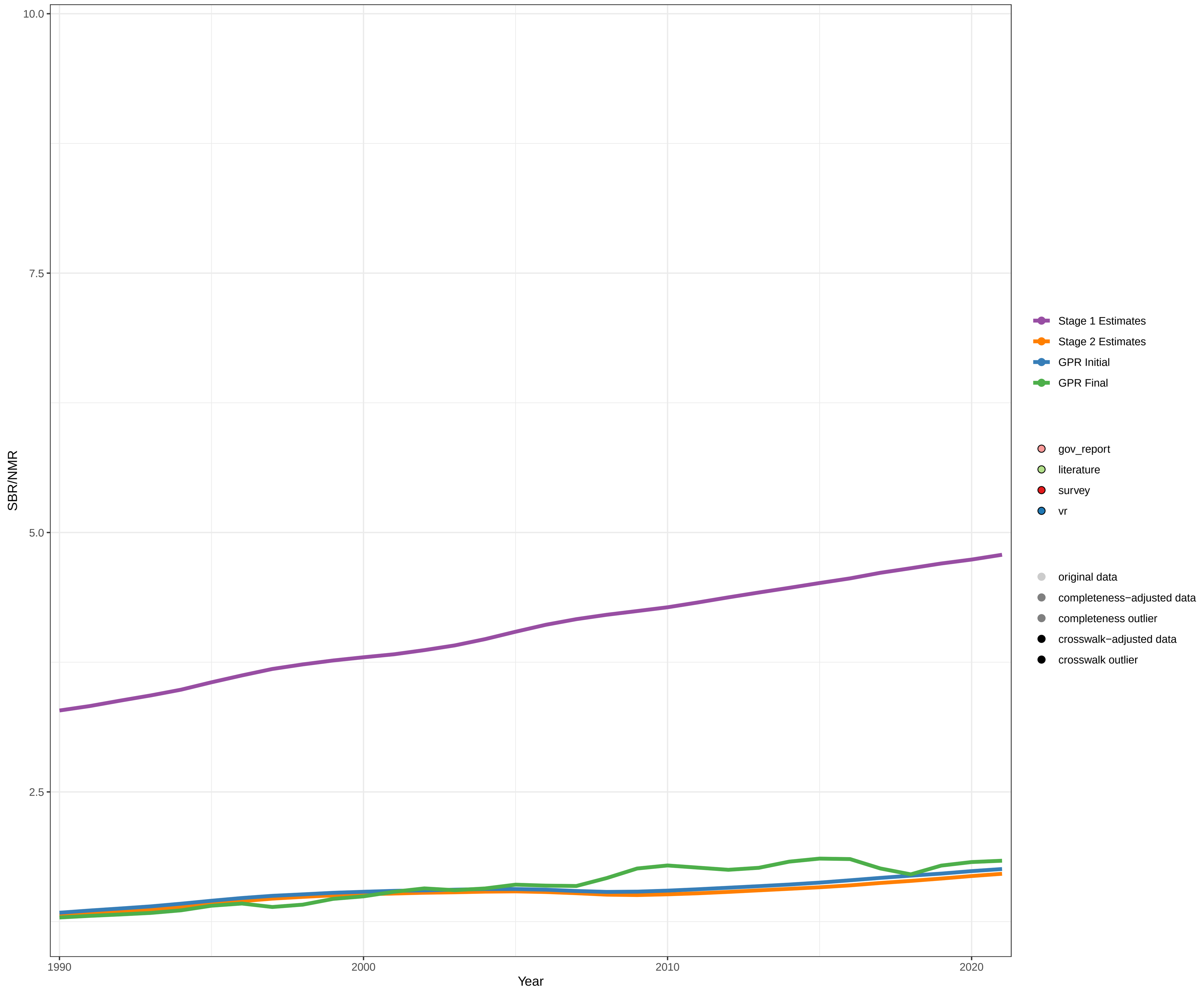

Definition	Years	Outlier	Source.Type	Title

Osaka JPN_35450 High−income Asia Pacific Stillbirth Rate (SBR) − 20 weeks
lambda = 1 ; zeta = 0.6 ; scale = 15 ; amp = 0.028

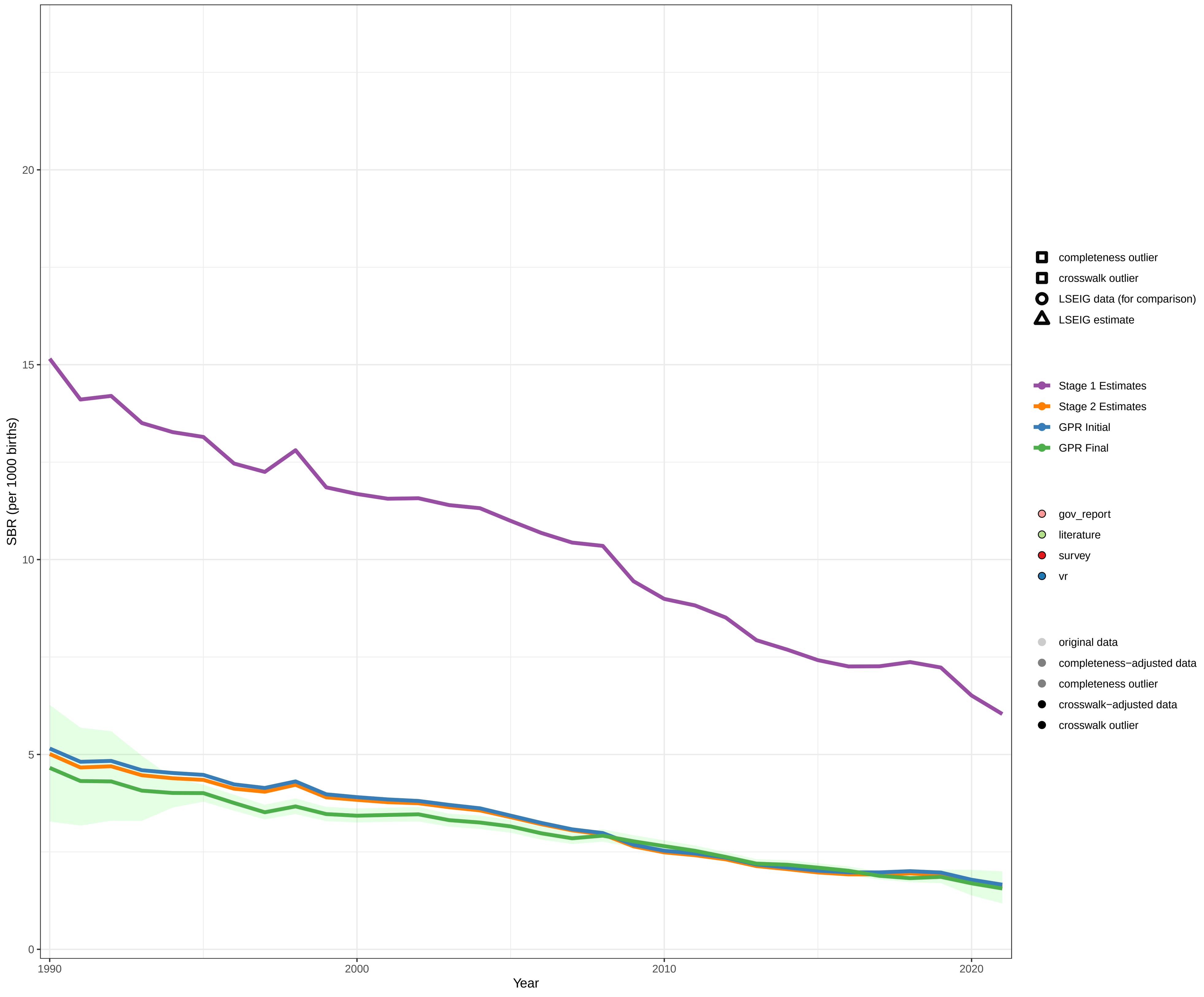

Osaka JPN_35450 High−income Asia Pacific Stillbirth Rate (SBR) − 28 weeks
lambda = 1 ; zeta = 0.6 ; scale = 15 ; amp = 0.028

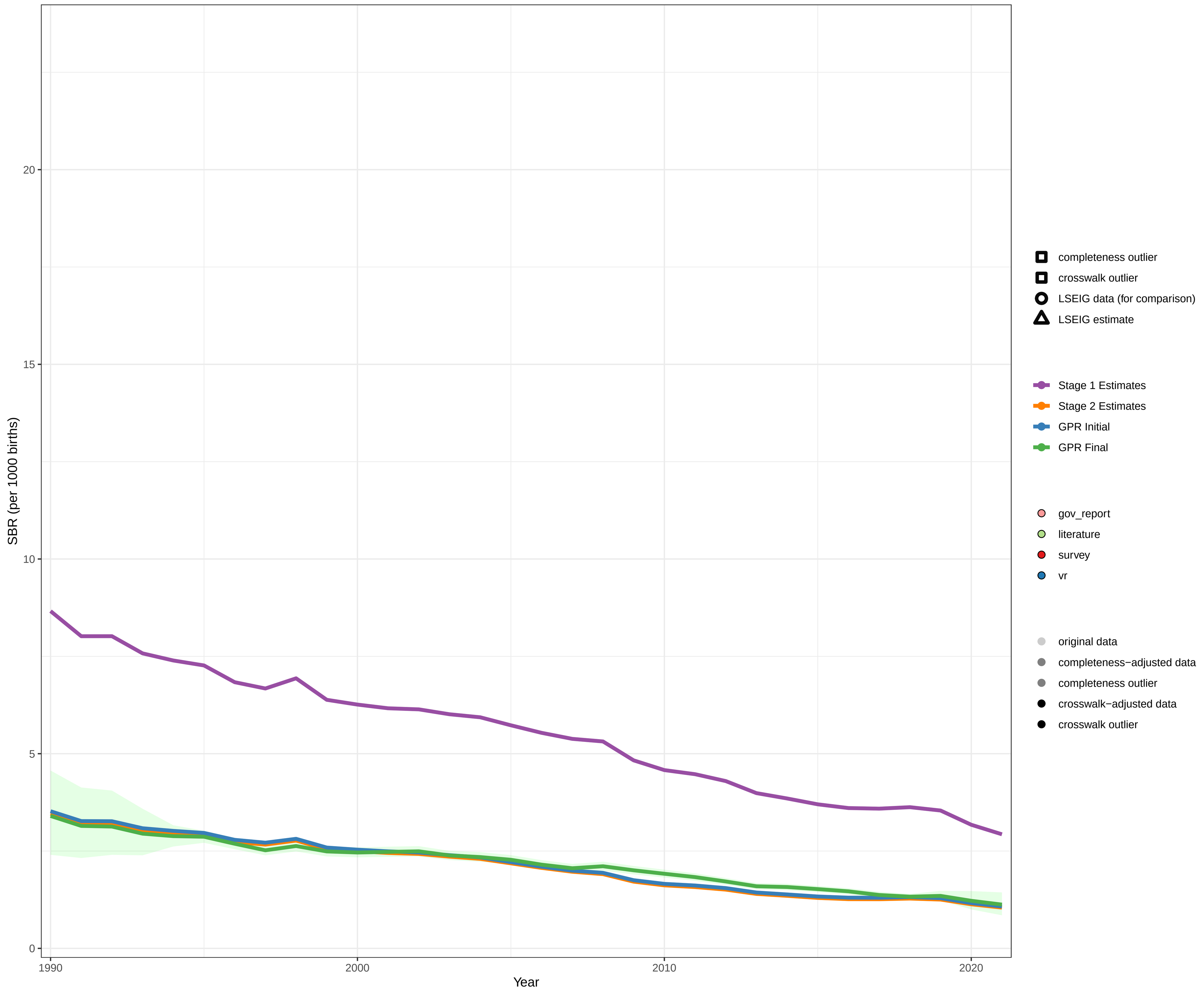

Year	Total Stillbirths (in thousands)	Total Stillbirths (in thousands)	SBR (per 1000 births)	SBR (per 1000 births)	SBR/NMR	SBR/NMR
	20 weeks	28 weeks	20 weeks	28 weeks	20 weeks	28 weeks
1990	0.4 (0.3 to 0.5)	0.3 (0.2 to 0.4)	4.7 (3.3 to 6.3)	3.4 (2.4 to 4.6)	1.77 (1.24 to 2.38)	1.29 (0.91 to 1.73)
1995	0.4 (0.3 to 0.4)	0.3 (0.2 to 0.3)	4.0 (3.8 to 4.2)	2.9 (2.7 to 3.0)	1.96 (1.85 to 2.07)	1.40 (1.33 to 1.48)
2000	0.3 (0.3 to 0.3)	0.2 (0.2 to 0.2)	3.4 (3.3 to 3.6)	2.5 (2.3 to 2.6)	2.08 (1.97 to 2.19)	1.49 (1.42 to 1.57)
2005	0.2 (0.2 to 0.3)	0.2 (0.2 to 0.2)	3.2 (3.0 to 3.3)	2.3 (2.2 to 2.4)	2.23 (2.11 to 2.35)	1.61 (1.52 to 1.69)
2010	0.2 (0.2 to 0.2)	0.1 (0.1 to 0.2)	2.6 (2.5 to 2.8)	1.9 (1.8 to 2.0)	2.48 (2.35 to 2.61)	1.79 (1.70 to 1.89)
2015	0.1 (0.1 to 0.2)	0.1 (0.1 to 0.1)	2.1 (2.0 to 2.2)	1.5 (1.4 to 1.6)	2.56 (2.42 to 2.69)	1.86 (1.76 to 1.96)
2020	0.1 (0.1 to 0.1)	0.1 (0.1 to 0.1)	1.7 (1.4 to 2.0)	1.2 (1.0 to 1.5)	2.53 (2.07 to 3.05)	1.82 (1.49 to 2.20)
2021	0.1 (0.1 to 0.1)	0.1 (0.1 to 0.1)	1.6 (1.2 to 2.0)	1.1 (0.9 to 1.4)	2.55 (1.92 to 3.27)	1.84 (1.39 to 2.35)

Saga JPN_35464 High−income Asia Pacific Stillbirth Rate / Neonatal Mortality Rate (SBR/NMR) − 20 weeks
lambda = 1 ; zeta = 0.6 ; scale = 15 ; amp = 0.028

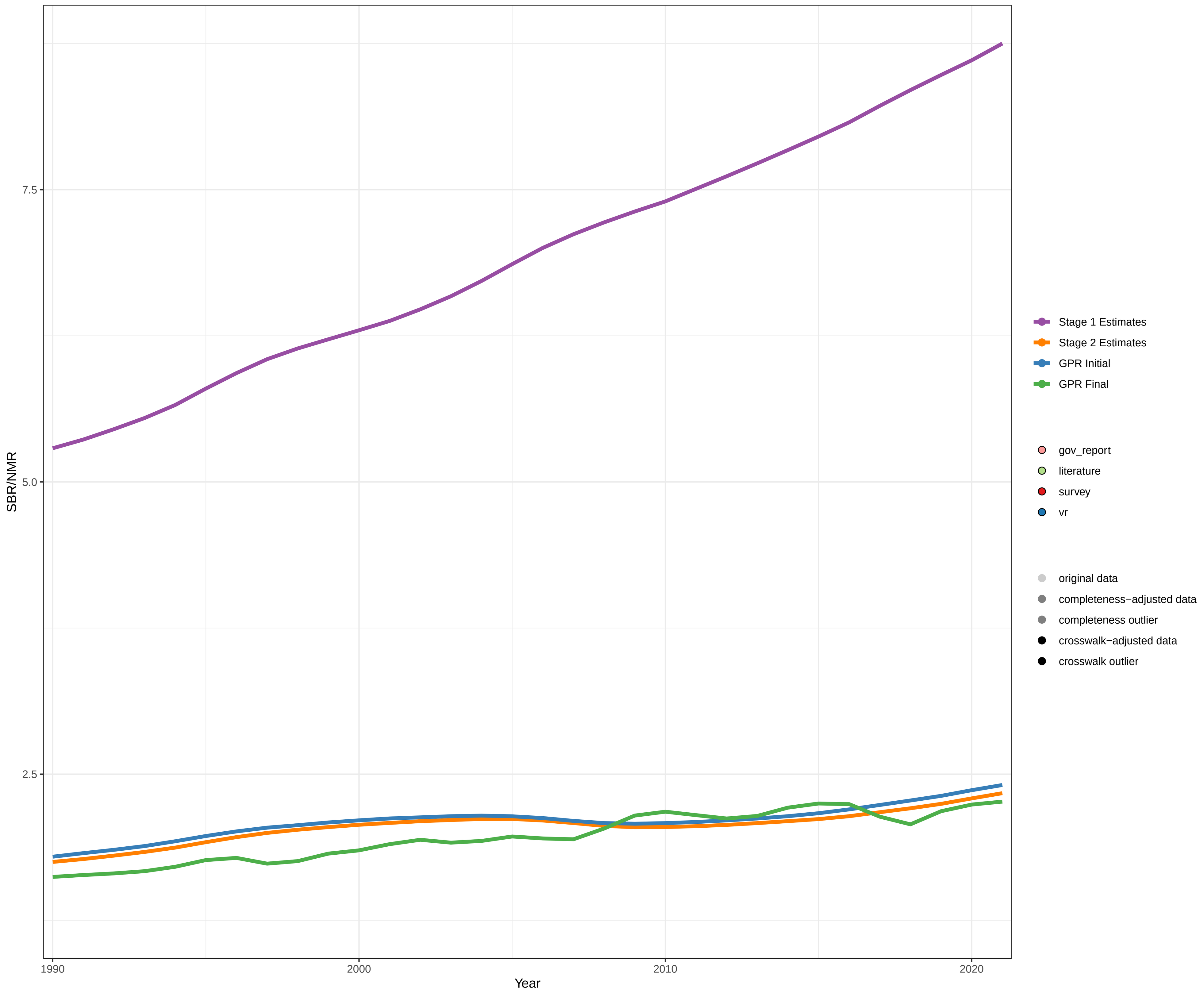

Saga JPN_35464 High−income Asia Pacific Stillbirth Rate / Neonatal Mortality Rate (SBR/NMR) − 28 weeks
lambda = 1 ; zeta = 0.6 ; scale = 15 ; amp = 0.028

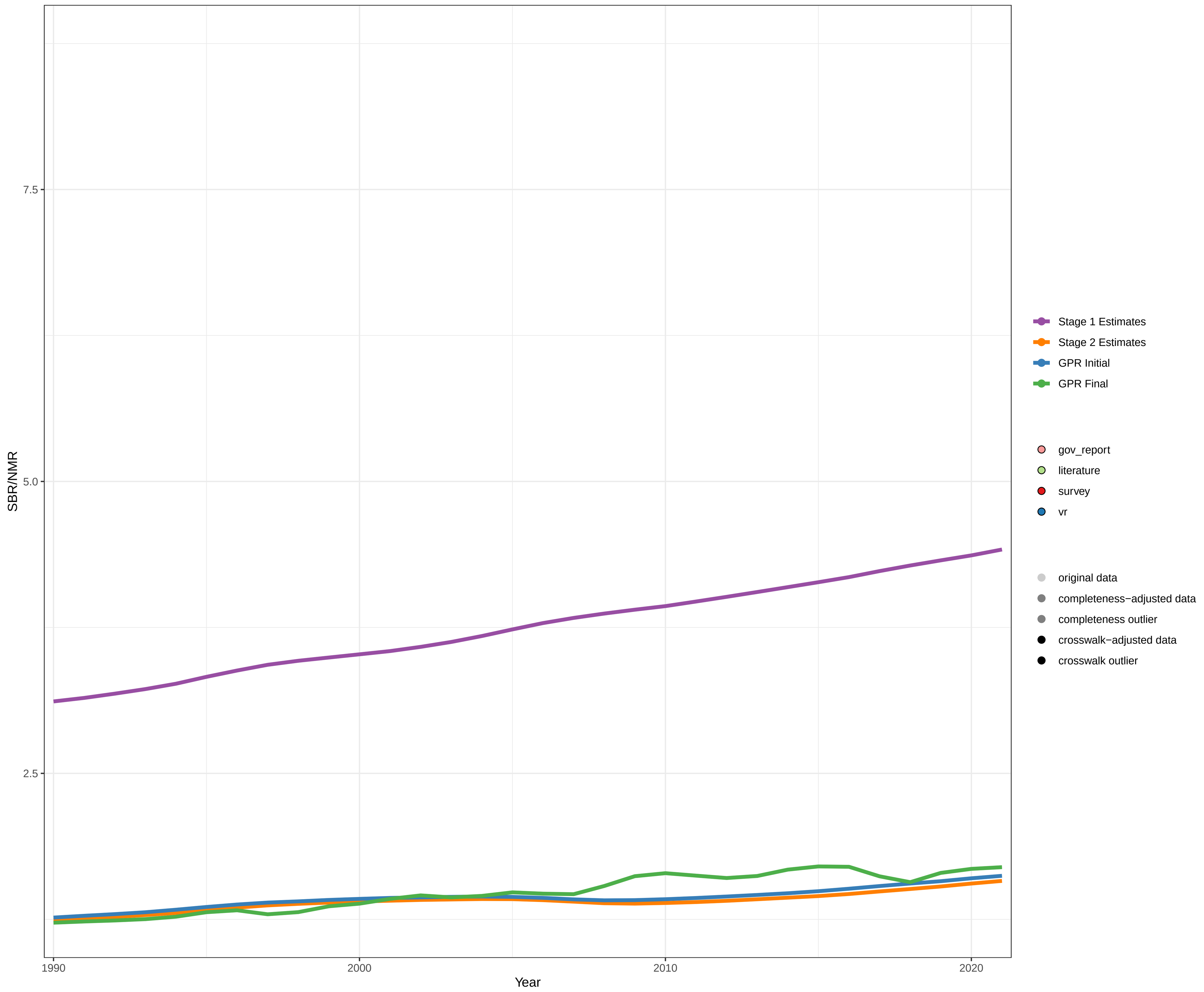

Definition	Years	Outlier	Source.Type	Title

Saga JPN_35464 High−income Asia Pacific Stillbirth Rate (SBR) − 20 weeks
lambda = 1 ; zeta = 0.6 ; scale = 15 ; amp = 0.028

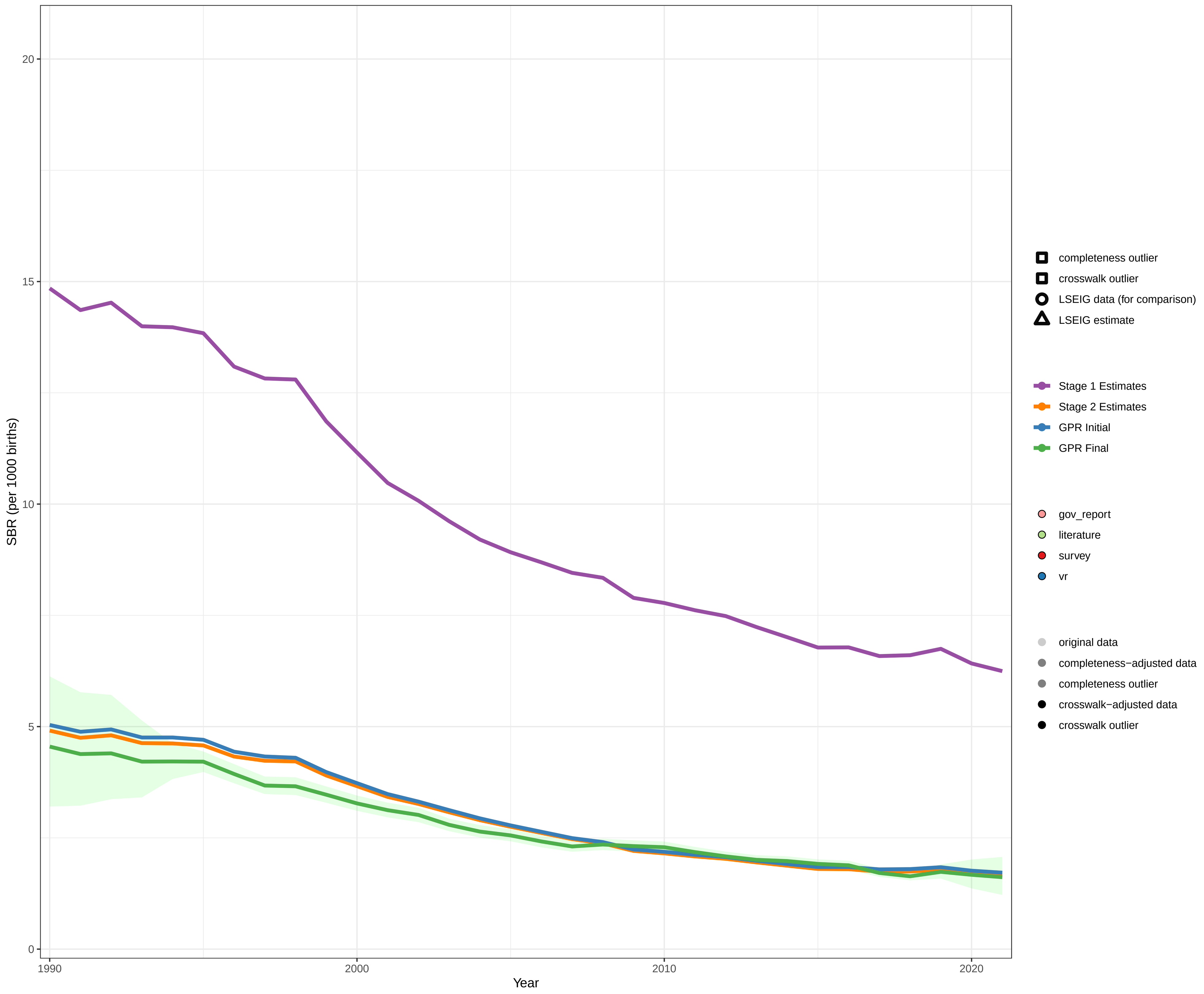

Saga JPN_35464 High−income Asia Pacific Stillbirth Rate (SBR) − 28 weeks
lambda = 1 ; zeta = 0.6 ; scale = 15 ; amp = 0.028

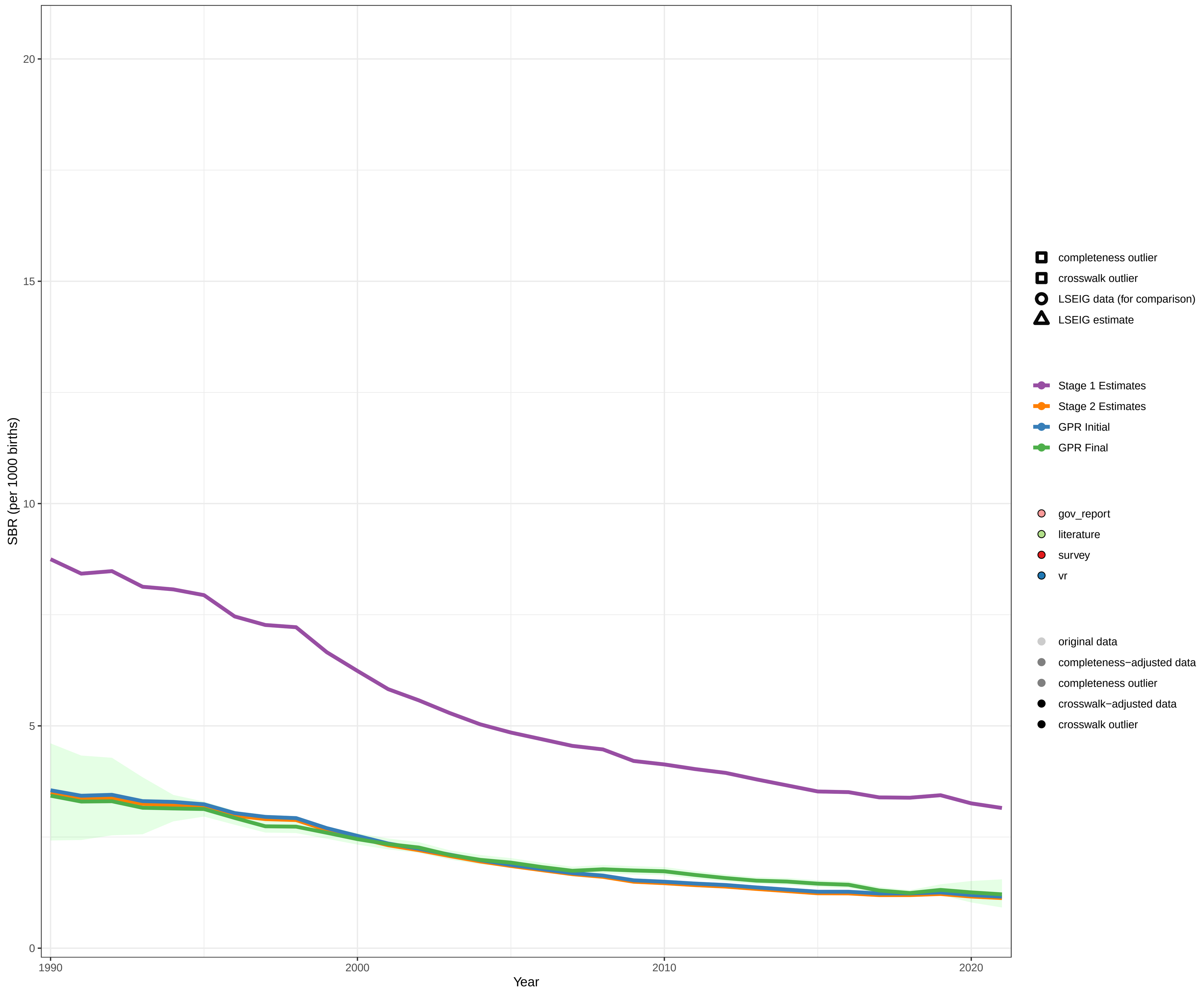

Year	Total Stillbirths (in thousands)	Total Stillbirths (in thousands)	SBR (per 1000 births)	SBR (per 1000 births)	SBR/NMR	SBR/NMR
	20 weeks	28 weeks	20 weeks	28 weeks	20 weeks	28 weeks
1990	0.0 (0.0 to 0.1)	0.0 (0.0 to 0.0)	4.6 (3.2 to 6.1)	3.4 (2.4 to 4.6)	1.62 (1.14 to 2.18)	1.22 (0.86 to 1.64)
1995	0.0 (0.0 to 0.0)	0.0 (0.0 to 0.0)	4.2 (4.0 to 4.4)	3.1 (3.0 to 3.3)	1.76 (1.67 to 1.86)	1.31 (1.24 to 1.38)
2000	0.0 (0.0 to 0.0)	0.0 (0.0 to 0.0)	3.3 (3.1 to 3.5)	2.5 (2.3 to 2.6)	1.85 (1.75 to 1.95)	1.38 (1.31 to 1.46)
2005	0.0 (0.0 to 0.0)	0.0 (0.0 to 0.0)	2.6 (2.4 to 2.7)	1.9 (1.8 to 2.0)	1.97 (1.87 to 2.07)	1.48 (1.41 to 1.56)
2010	0.0 (0.0 to 0.0)	0.0 (0.0 to 0.0)	2.3 (2.2 to 2.4)	1.7 (1.6 to 1.8)	2.18 (2.07 to 2.30)	1.65 (1.56 to 1.74)
2015	0.0 (0.0 to 0.0)	0.0 (0.0 to 0.0)	1.9 (1.8 to 2.0)	1.5 (1.4 to 1.5)	2.25 (2.13 to 2.37)	1.70 (1.62 to 1.79)
2020	0.0 (0.0 to 0.0)	0.0 (0.0 to 0.0)	1.7 (1.4 to 2.0)	1.3 (1.0 to 1.5)	2.24 (1.83 to 2.70)	1.68 (1.38 to 2.03)
2021	0.0 (0.0 to 0.0)	0.0 (0.0 to 0.0)	1.6 (1.2 to 2.1)	1.2 (0.9 to 1.5)	2.27 (1.71 to 2.90)	1.70 (1.28 to 2.17)

Saitama JPN_35434 High−income Asia Pacific Stillbirth Rate / Neonatal Mortality Rate (SBR/NMR) − 20 weeks
lambda = 1 ; zeta = 0.6 ; scale = 15 ; amp = 0.028

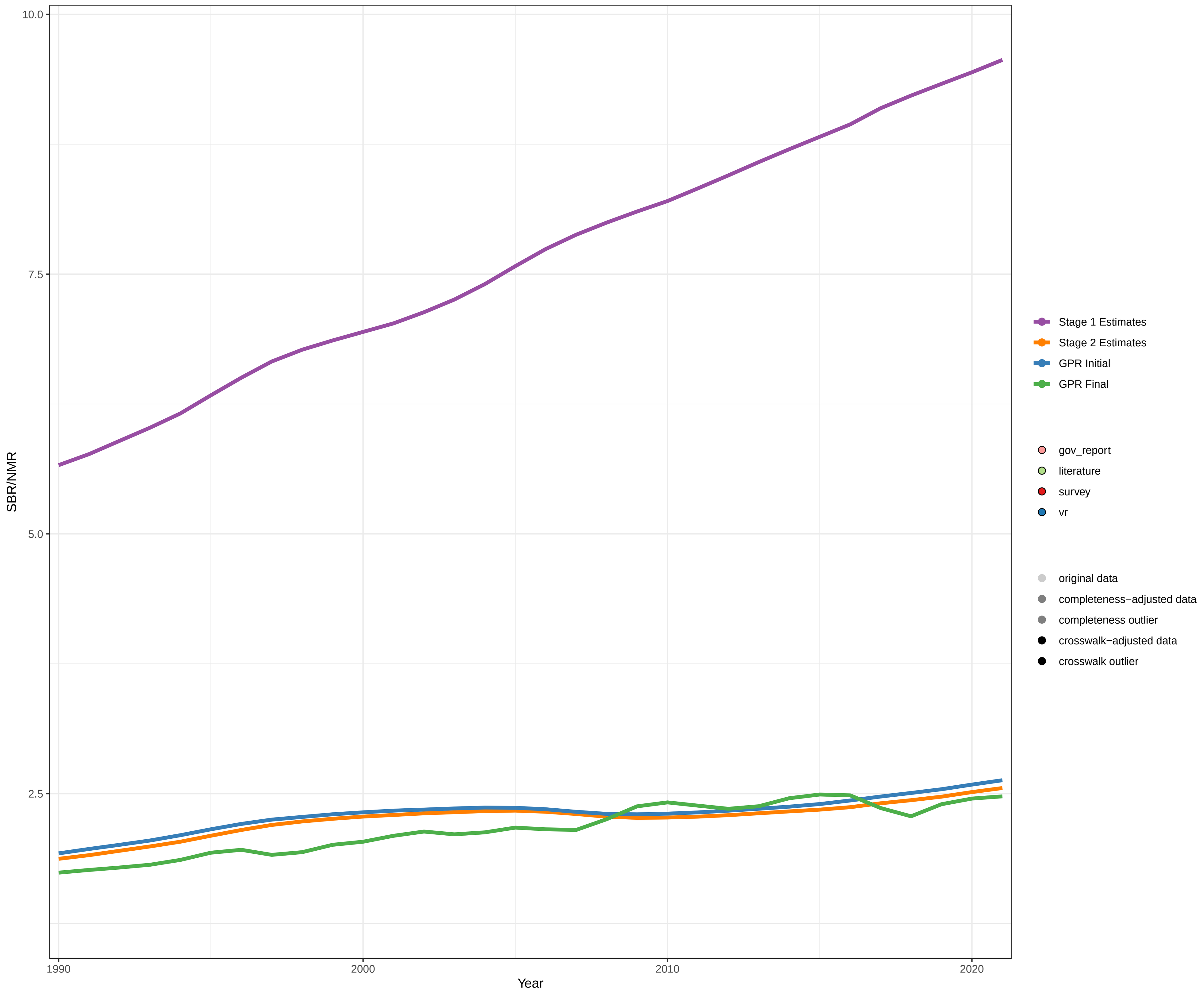

Saitama JPN_35434 High−income Asia Pacific Stillbirth Rate / Neonatal Mortality Rate (SBR/NMR) − 28 weeks
lambda = 1 ; zeta = 0.6 ; scale = 15 ; amp = 0.028

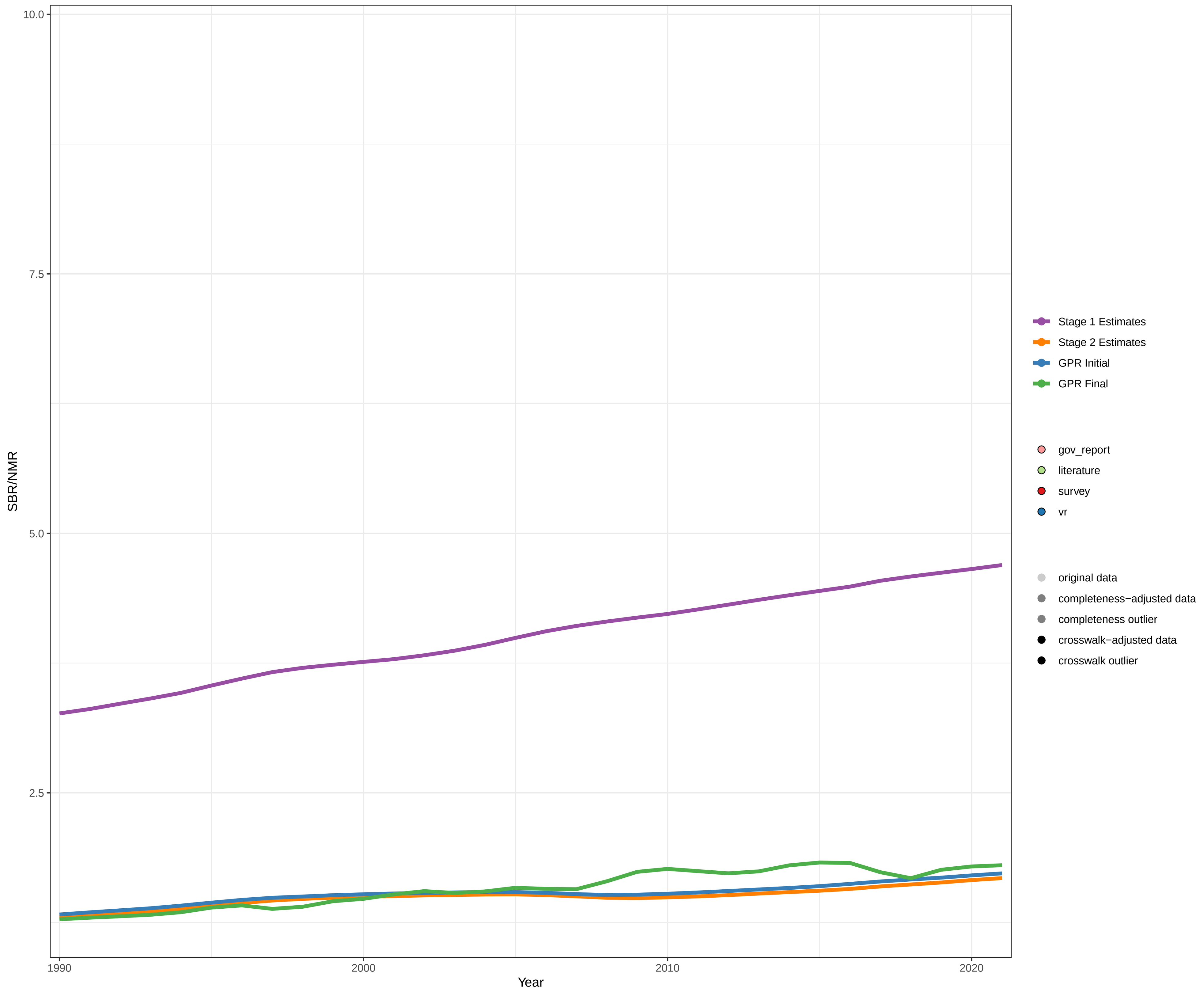

Definition	Years	Outlier	Source.Type	Title

Saitama JPN_35434 High−income Asia Pacific Stillbirth Rate (SBR) − 20 weeks
lambda = 1 ; zeta = 0.6 ; scale = 15 ; amp = 0.028

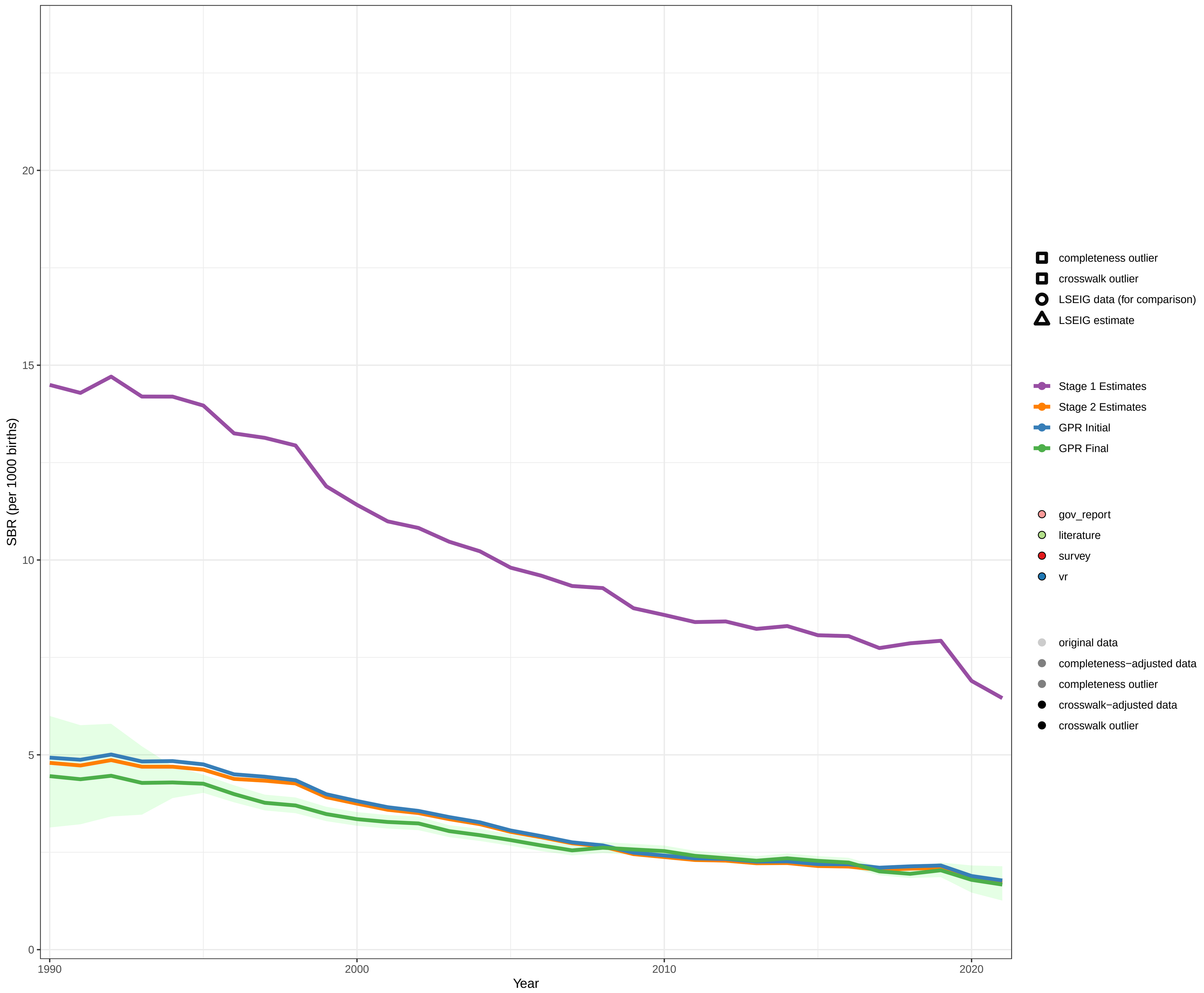

Saitama JPN_35434 High−income Asia Pacific Stillbirth Rate (SBR) − 28 weeks
lambda = 1 ; zeta = 0.6 ; scale = 15 ; amp = 0.028

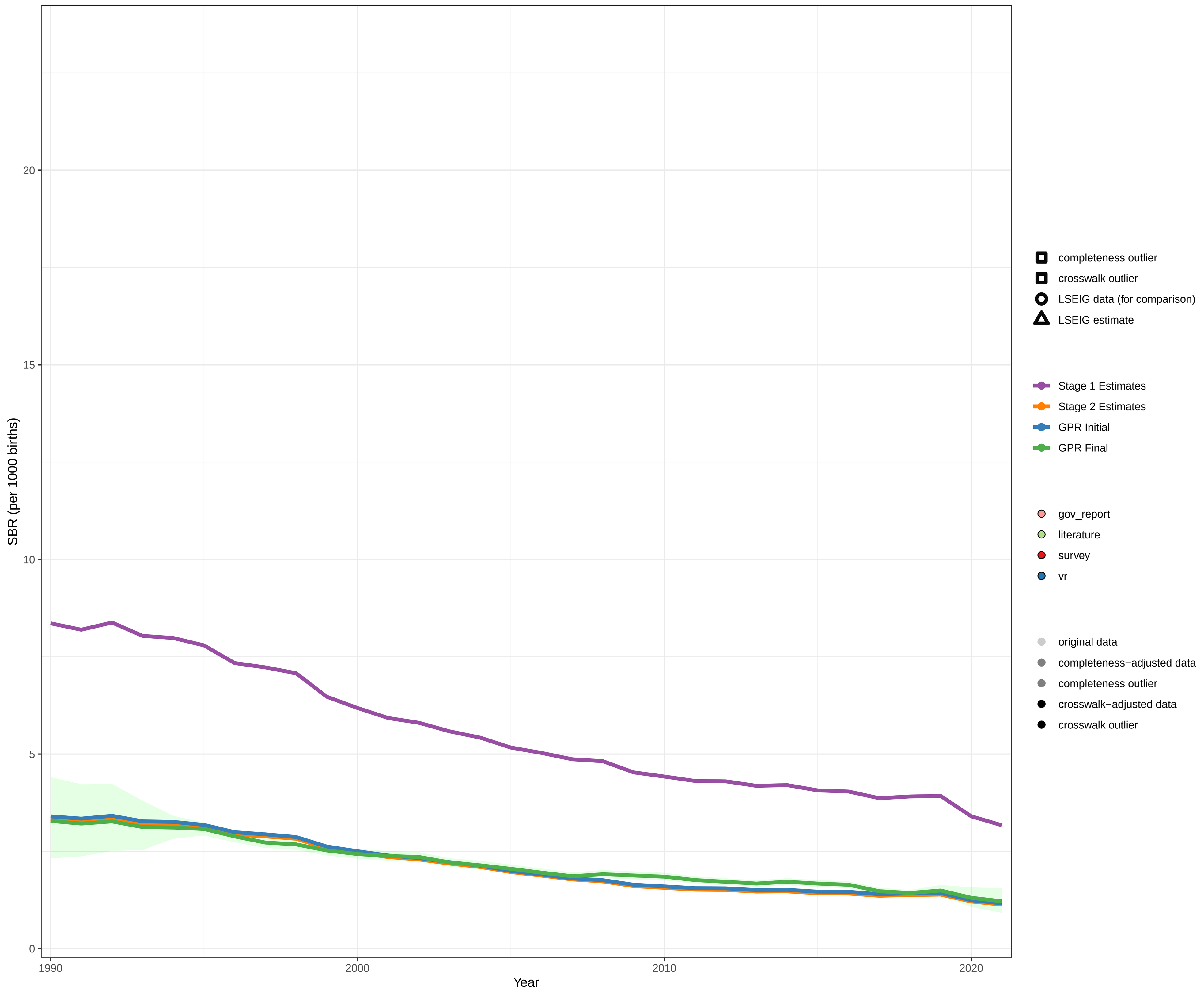

Year	Total Stillbirths (in thousands)	Total Stillbirths (in thousands)	SBR (per 1000 births)	SBR (per 1000 births)	SBR/NMR	SBR/NMR
	20 weeks	28 weeks	20 weeks	28 weeks	20 weeks	28 weeks
1990	0.3 (0.2 to 0.4)	0.2 (0.1 to 0.3)	4.5 (3.1 to 6.0)	3.3 (2.3 to 4.4)	1.74 (1.22 to 2.34)	1.28 (0.91 to 1.72)
1995	0.3 (0.3 to 0.3)	0.2 (0.2 to 0.2)	4.3 (4.0 to 4.5)	3.1 (2.9 to 3.2)	1.93 (1.83 to 2.04)	1.39 (1.32 to 1.47)
2000	0.2 (0.2 to 0.2)	0.2 (0.2 to 0.2)	3.3 (3.2 to 3.5)	2.4 (2.3 to 2.6)	2.04 (1.93 to 2.15)	1.48 (1.40 to 1.56)
2005	0.2 (0.2 to 0.2)	0.1 (0.1 to 0.1)	2.8 (2.7 to 3.0)	2.1 (1.9 to 2.2)	2.17 (2.06 to 2.29)	1.59 (1.50 to 1.67)
2010	0.2 (0.1 to 0.2)	0.1 (0.1 to 0.1)	2.5 (2.4 to 2.7)	1.9 (1.8 to 2.0)	2.42 (2.29 to 2.55)	1.77 (1.68 to 1.86)
2015	0.1 (0.1 to 0.1)	0.1 (0.1 to 0.1)	2.3 (2.2 to 2.4)	1.7 (1.6 to 1.8)	2.49 (2.36 to 2.63)	1.83 (1.73 to 1.93)
2020	0.1 (0.1 to 0.1)	0.1 (0.1 to 0.1)	1.8 (1.5 to 2.2)	1.3 (1.1 to 1.6)	2.45 (2.00 to 2.96)	1.79 (1.47 to 2.16)
2021	0.1 (0.1 to 0.1)	0.1 (0.0 to 0.1)	1.7 (1.3 to 2.1)	1.2 (0.9 to 1.6)	2.47 (1.86 to 3.17)	1.80 (1.36 to 2.30)

Shiga JPN_35448 High−income Asia Pacific Stillbirth Rate / Neonatal Mortality Rate (SBR/NMR) − 20 weeks
lambda = 1 ; zeta = 0.6 ; scale = 15 ; amp = 0.028

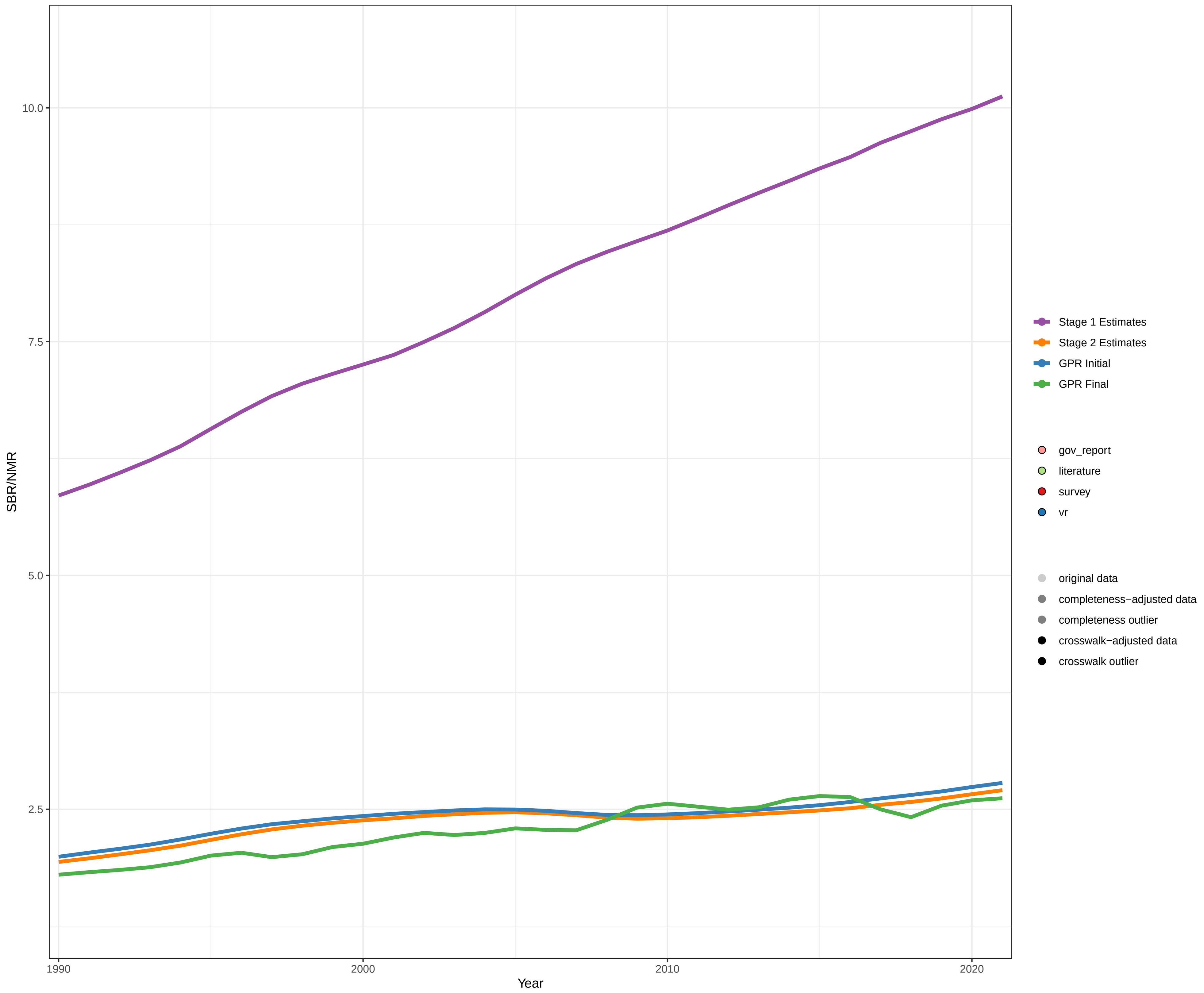

Shiga JPN_35448 High−income Asia Pacific Stillbirth Rate / Neonatal Mortality Rate (SBR/NMR) − 28 weeks
lambda = 1 ; zeta = 0.6 ; scale = 15 ; amp = 0.028

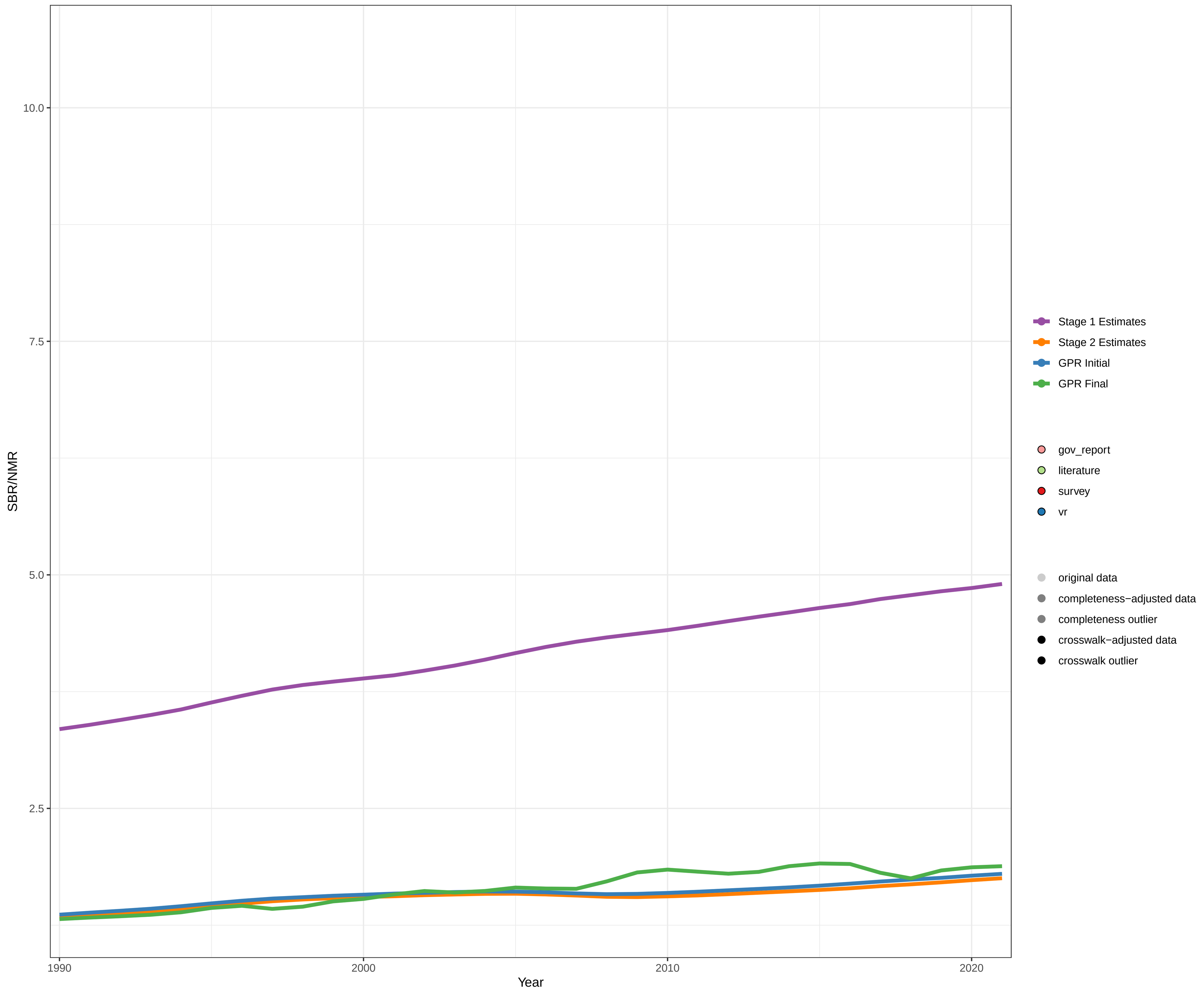

Definition	Years	Outlier	Source.Type	Title

Shiga JPN_35448 High−income Asia Pacific Stillbirth Rate (SBR) − 20 weeks
lambda = 1 ; zeta = 0.6 ; scale = 15 ; amp = 0.028

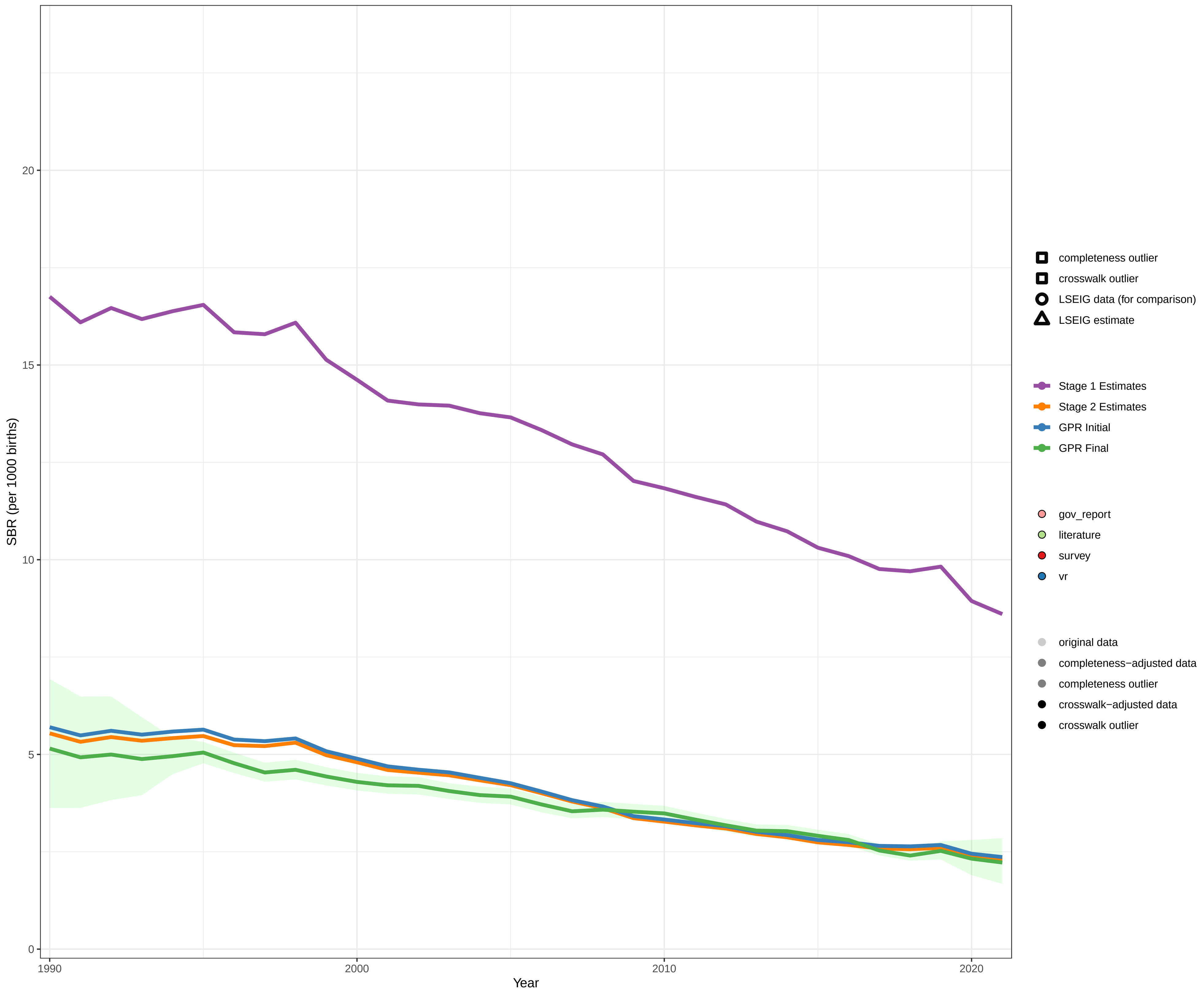

Shiga JPN_35448 High−income Asia Pacific Stillbirth Rate (SBR) − 28 weeks
lambda = 1 ; zeta = 0.6 ; scale = 15 ; amp = 0.028

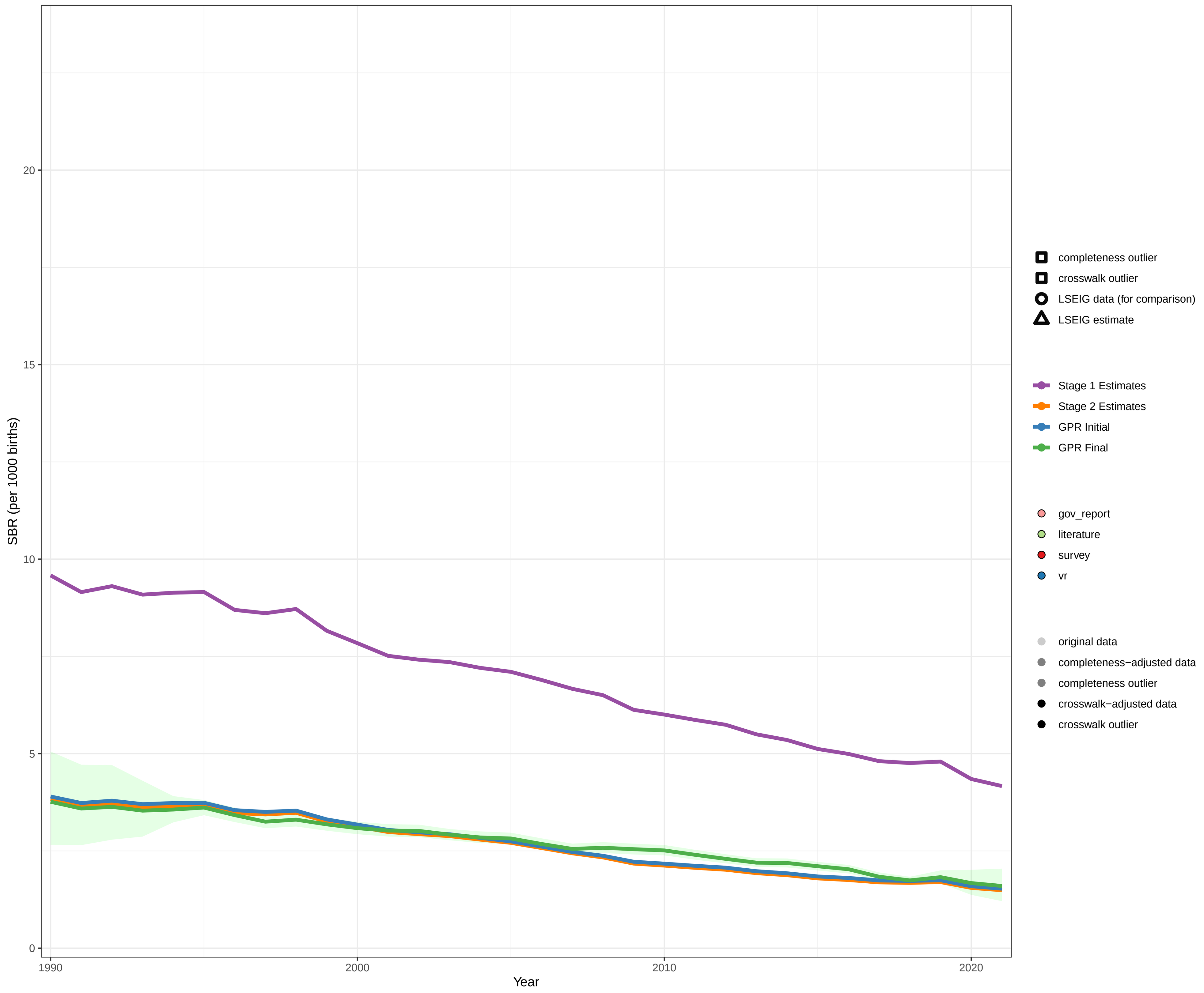

Year	Total Stillbirths (in thousands)	Total Stillbirths (in thousands)	SBR (per 1000 births)	SBR (per 1000 births)	SBR/NMR	SBR/NMR
	20 weeks	28 weeks	20 weeks	28 weeks	20 weeks	28 weeks
1990	0.1 (0.0 to 0.1)	0.1 (0.0 to 0.1)	5.2 (3.6 to 6.9)	3.8 (2.7 to 5.1)	1.80 (1.27 to 2.42)	1.32 (0.93 to 1.77)
1995	0.1 (0.1 to 0.1)	0.0 (0.0 to 0.1)	5.0 (4.8 to 5.3)	3.6 (3.4 to 3.8)	2.00 (1.89 to 2.11)	1.43 (1.36 to 1.51)
2000	0.1 (0.1 to 0.1)	0.0 (0.0 to 0.0)	4.3 (4.1 to 4.5)	3.1 (2.9 to 3.3)	2.13 (2.02 to 2.25)	1.53 (1.45 to 1.61)
2005	0.1 (0.0 to 0.1)	0.0 (0.0 to 0.0)	3.9 (3.7 to 4.1)	2.8 (2.7 to 3.0)	2.30 (2.18 to 2.42)	1.65 (1.57 to 1.74)
2010	0.0 (0.0 to 0.0)	0.0 (0.0 to 0.0)	3.5 (3.3 to 3.7)	2.5 (2.4 to 2.7)	2.56 (2.43 to 2.70)	1.84 (1.75 to 1.95)
2015	0.0 (0.0 to 0.0)	0.0 (0.0 to 0.0)	2.9 (2.8 to 3.1)	2.1 (2.0 to 2.2)	2.64 (2.50 to 2.78)	1.91 (1.81 to 2.01)
2020	0.0 (0.0 to 0.0)	0.0 (0.0 to 0.0)	2.3 (1.9 to 2.8)	1.7 (1.4 to 2.0)	2.60 (2.12 to 3.13)	1.87 (1.53 to 2.25)
2021	0.0 (0.0 to 0.0)	0.0 (0.0 to 0.0)	2.2 (1.7 to 2.9)	1.6 (1.2 to 2.0)	2.62 (1.97 to 3.35)	1.88 (1.42 to 2.41)

Shimane JPN_35455 High−income Asia Pacific Stillbirth Rate / Neonatal Mortality Rate (SBR/NMR) − 20 weeks
lambda = 1 ; zeta = 0.6 ; scale = 15 ; amp = 0.028

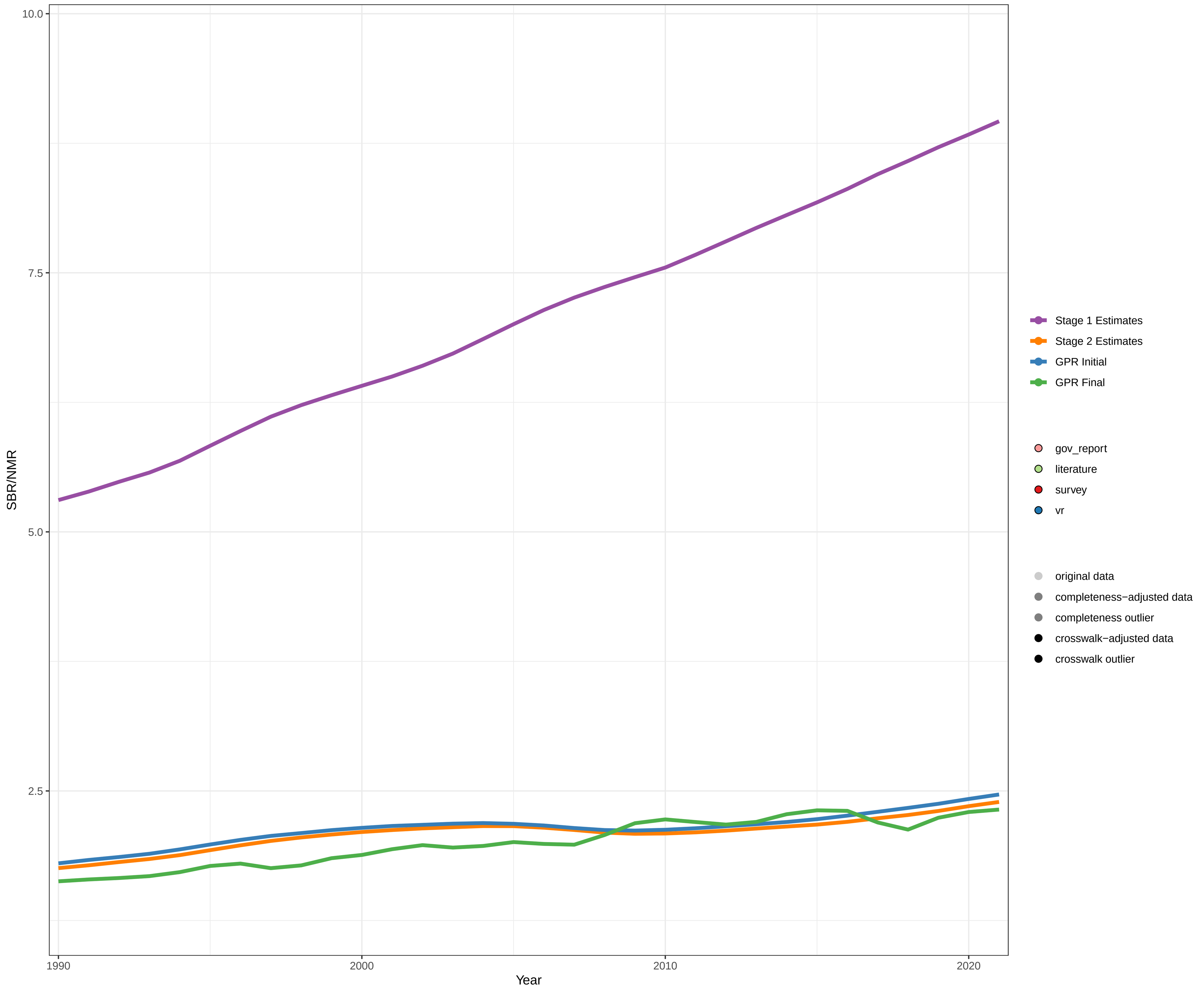

Shimane JPN_35455 High−income Asia Pacific Stillbirth Rate / Neonatal Mortality Rate (SBR/NMR) − 28 weeks
lambda = 1 ; zeta = 0.6 ; scale = 15 ; amp = 0.028

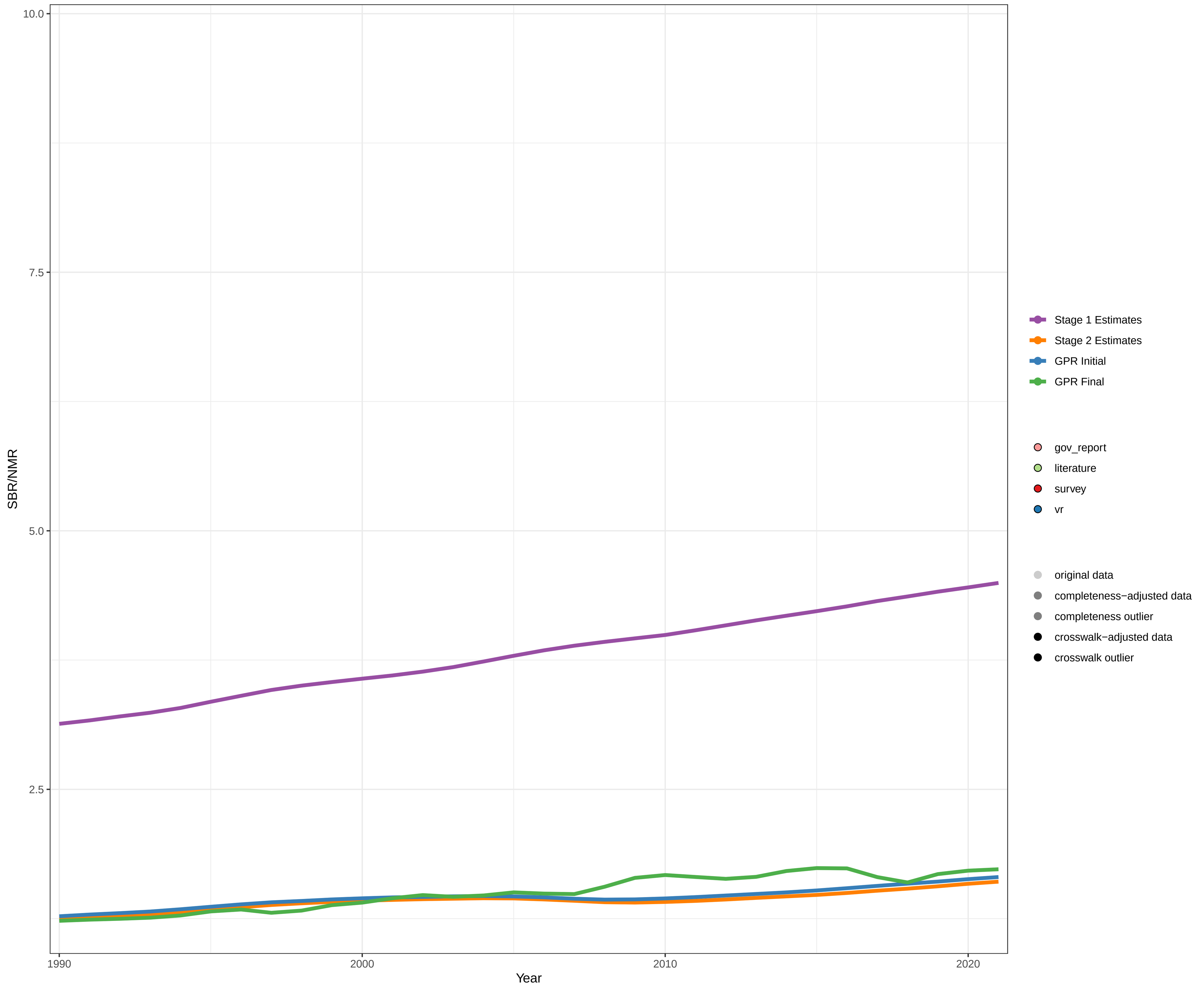

Definition	Years	Outlier	Source.Type	Title

Shimane JPN_35455 High−income Asia Pacific Stillbirth Rate (SBR) − 20 weeks
lambda = 1 ; zeta = 0.6 ; scale = 15 ; amp = 0.028

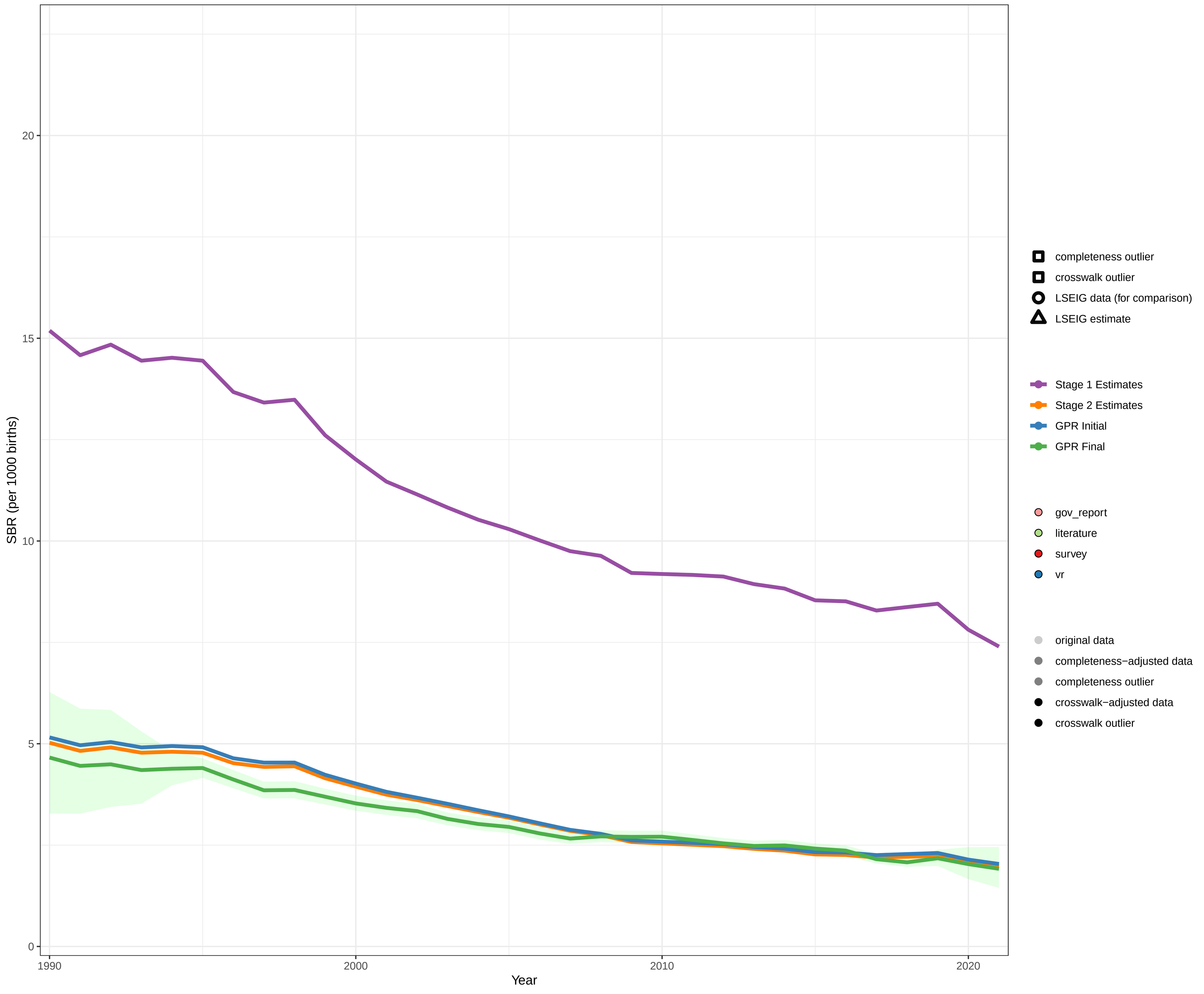

Shimane JPN_35455 High−income Asia Pacific Stillbirth Rate (SBR) − 28 weeks
lambda = 1 ; zeta = 0.6 ; scale = 15 ; amp = 0.028

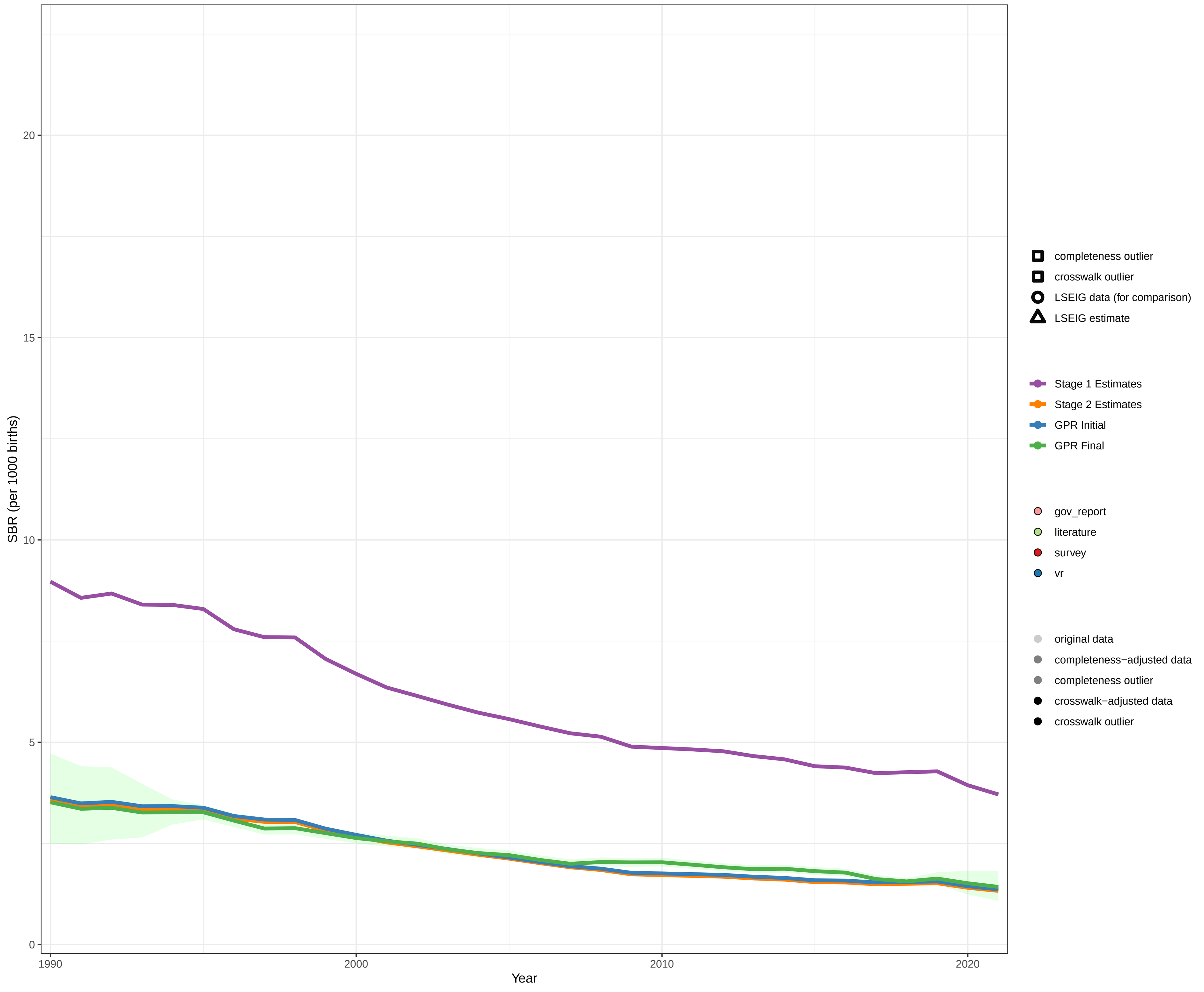

Year	Total Stillbirths (in thousands)	Total Stillbirths (in thousands)	SBR (per 1000 births)	SBR (per 1000 births)	SBR/NMR	SBR/NMR
	20 weeks	28 weeks	20 weeks	28 weeks	20 weeks	28 weeks
1990	0.0 (0.0 to 0.0)	0.0 (0.0 to 0.0)	4.7 (3.3 to 6.3)	3.5 (2.5 to 4.7)	1.63 (1.15 to 2.19)	1.23 (0.87 to 1.65)
1995	0.0 (0.0 to 0.0)	0.0 (0.0 to 0.0)	4.4 (4.2 to 4.6)	3.3 (3.1 to 3.4)	1.78 (1.68 to 1.87)	1.32 (1.25 to 1.39)
2000	0.0 (0.0 to 0.0)	0.0 (0.0 to 0.0)	3.5 (3.3 to 3.7)	2.6 (2.5 to 2.8)	1.88 (1.79 to 1.98)	1.40 (1.33 to 1.48)
2005	0.0 (0.0 to 0.0)	0.0 (0.0 to 0.0)	2.9 (2.8 to 3.1)	2.2 (2.1 to 2.3)	2.01 (1.90 to 2.11)	1.50 (1.43 to 1.58)
2010	0.0 (0.0 to 0.0)	0.0 (0.0 to 0.0)	2.7 (2.6 to 2.9)	2.0 (1.9 to 2.1)	2.23 (2.11 to 2.35)	1.67 (1.59 to 1.76)
2015	0.0 (0.0 to 0.0)	0.0 (0.0 to 0.0)	2.4 (2.3 to 2.5)	1.8 (1.7 to 1.9)	2.31 (2.19 to 2.44)	1.74 (1.65 to 1.83)
2020	0.0 (0.0 to 0.0)	0.0 (0.0 to 0.0)	2.0 (1.7 to 2.5)	1.5 (1.2 to 1.8)	2.30 (1.88 to 2.77)	1.71 (1.40 to 2.06)
2021	0.0 (0.0 to 0.0)	0.0 (0.0 to 0.0)	1.9 (1.4 to 2.5)	1.4 (1.1 to 1.8)	2.32 (1.75 to 2.97)	1.73 (1.31 to 2.21)

Shizuoka   JPN_35445   High−income Asia Pacific   Stillbirth Rate / Neonatal Mortality Rate (SBR/NMR) − 20 weeks
lambda = 1 ; zeta = 0.6 ; scale = 15 ; amp = 0.028

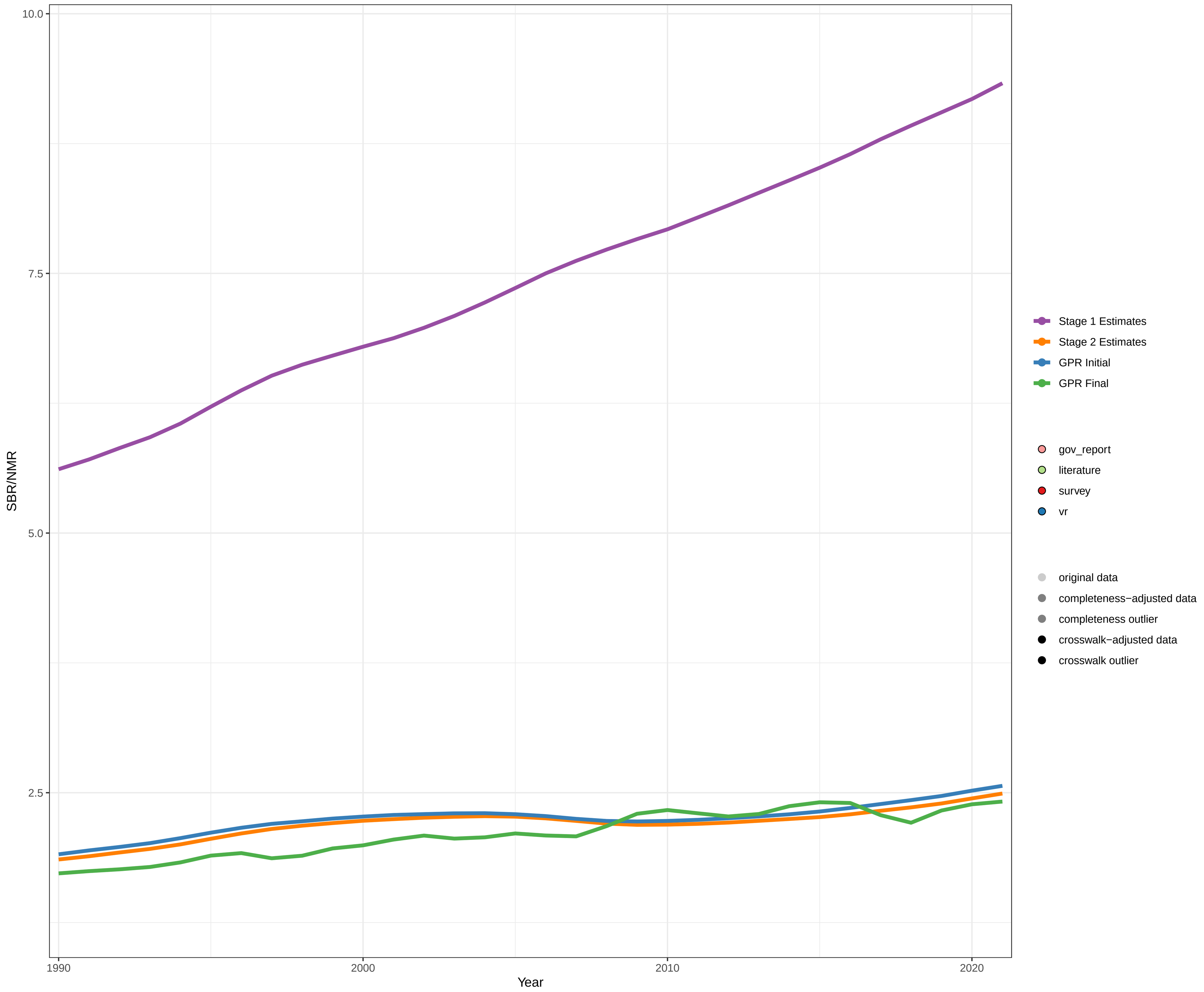

Shizuoka   JPN_35445   High−income Asia Pacific   Stillbirth Rate / Neonatal Mortality Rate (SBR/NMR) − 28 weeks
lambda = 1 ; zeta = 0.6 ; scale = 15 ; amp = 0.028

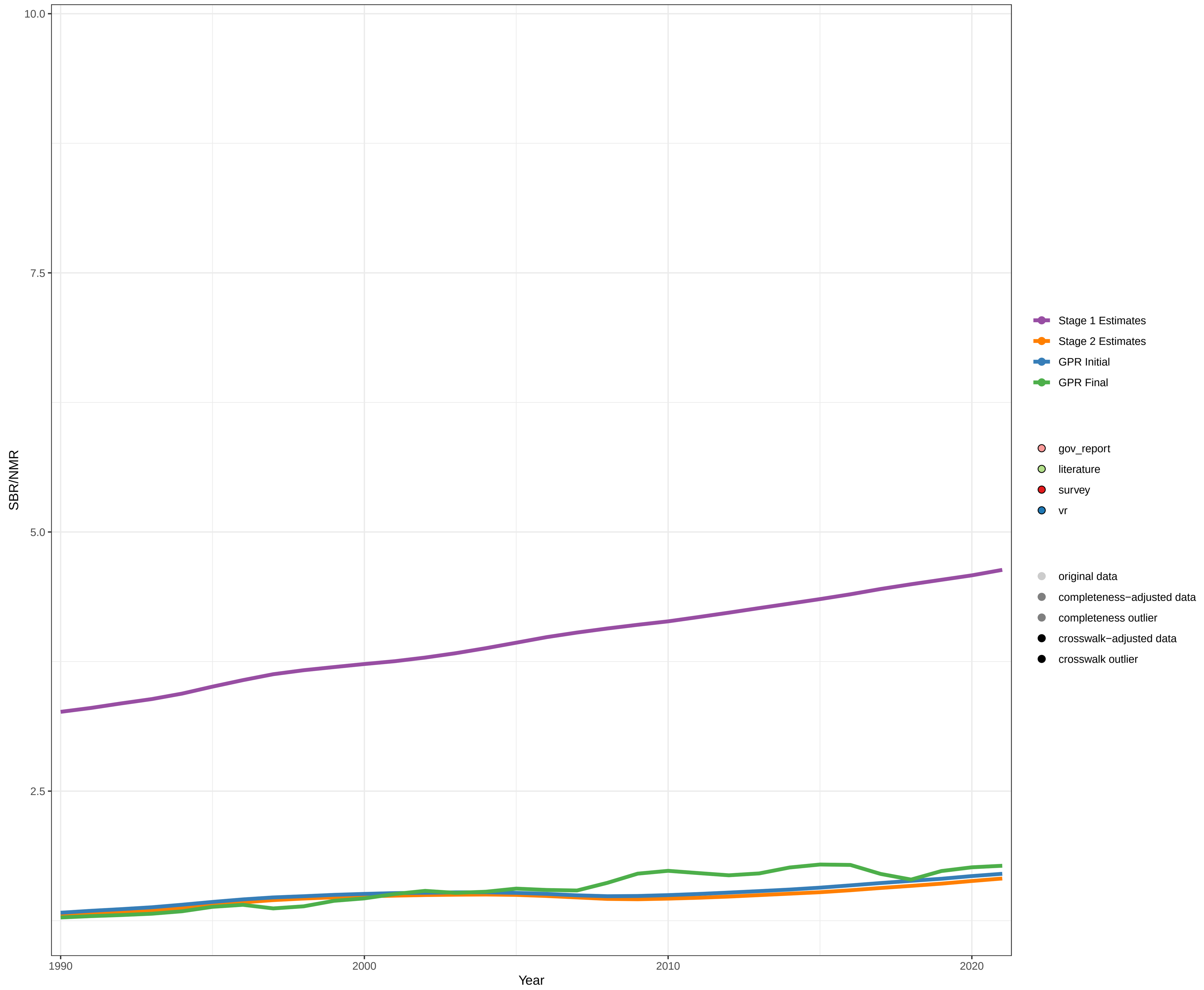

Definition	Years	Outlier	Source.Type	Title

Shizuoka   JPN_35445   High−income Asia Pacific   Stillbirth Rate (SBR) − 20 weeks
lambda = 1 ; zeta = 0.6 ; scale = 15 ; amp = 0.028

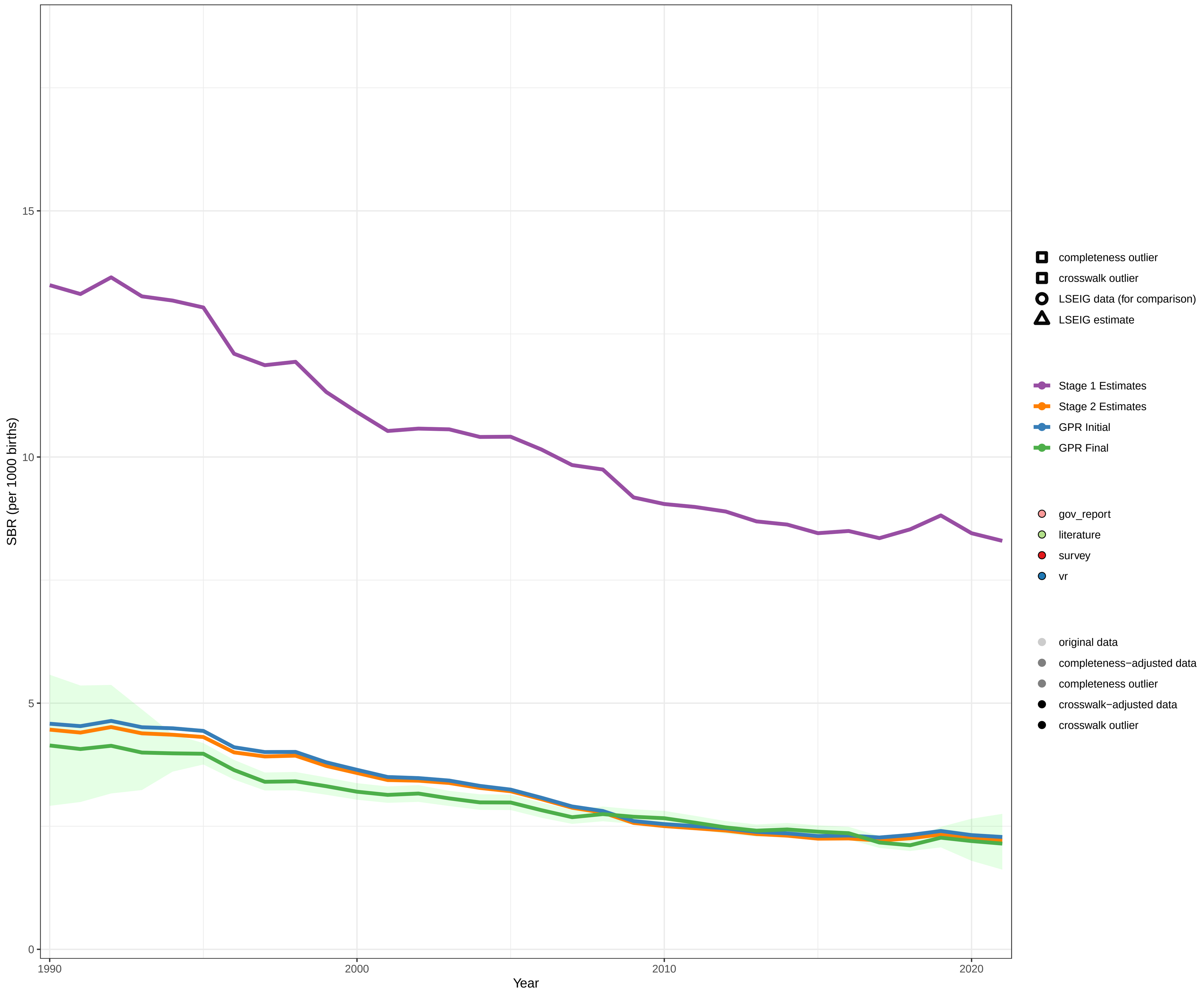

Shizuoka   JPN_35445   High−income Asia Pacific   Stillbirth Rate (SBR) − 28 weeks
lambda = 1 ; zeta = 0.6 ; scale = 15 ; amp = 0.028

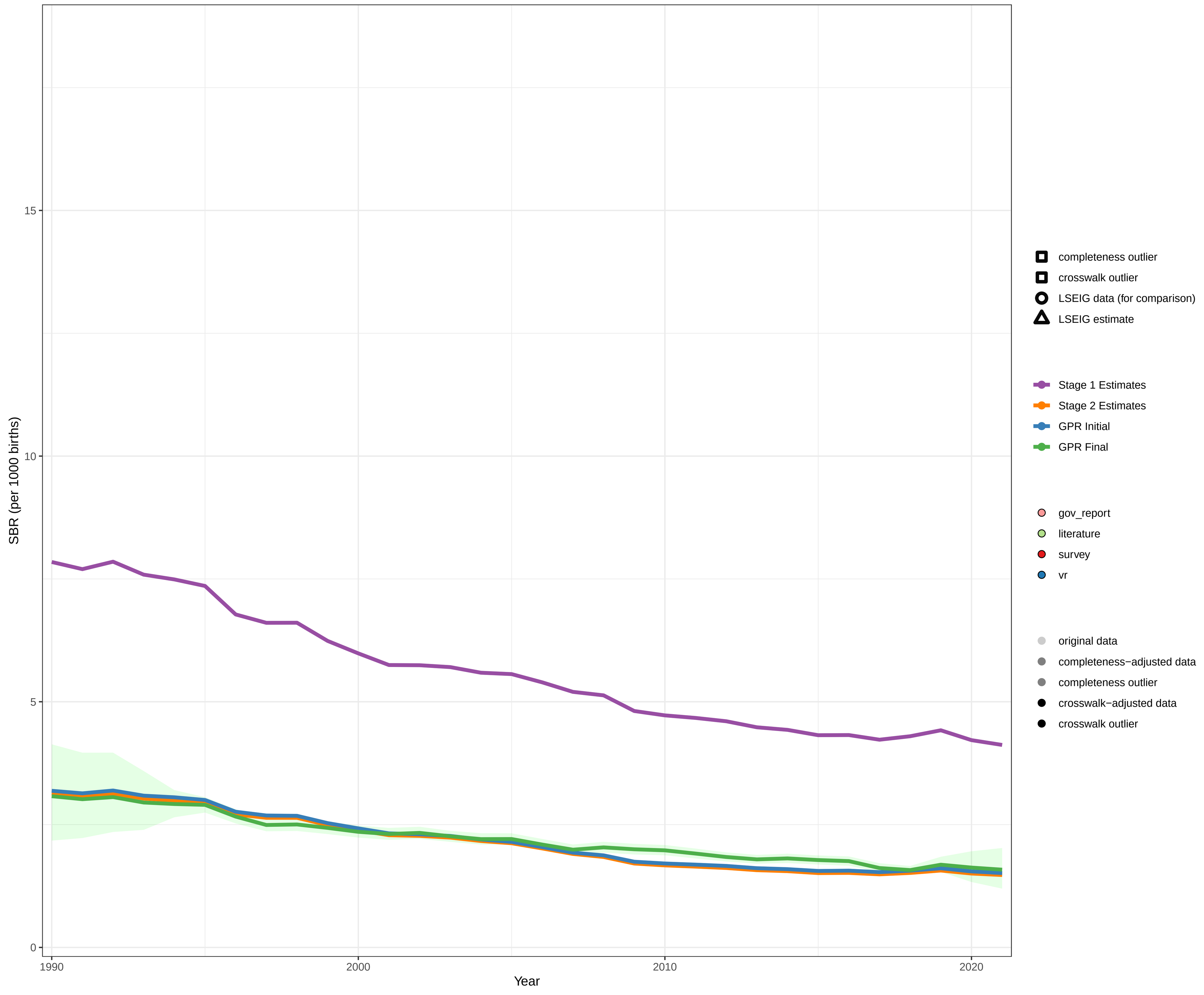

Year	Total Stillbirths (in thousands)	Total Stillbirths (in thousands)	SBR (per 1000 births)	SBR (per 1000 births)	SBR/NMR	SBR/NMR
	20 weeks	28 weeks	20 weeks	28 weeks	20 weeks	28 weeks
1990	0.2 (0.1 to 0.2)	0.1 (0.1 to 0.2)	4.1 (2.9 to 5.6)	3.1 (2.2 to 4.1)	1.72 (1.21 to 2.32)	1.28 (0.90 to 1.72)
1995	0.1 (0.1 to 0.2)	0.1 (0.1 to 0.1)	4.0 (3.8 to 4.2)	2.9 (2.7 to 3.1)	1.89 (1.79 to 2.00)	1.38 (1.31 to 1.46)
2000	0.1 (0.1 to 0.1)	0.1 (0.1 to 0.1)	3.2 (3.0 to 3.4)	2.4 (2.2 to 2.5)	1.99 (1.89 to 2.10)	1.47 (1.39 to 1.54)
2005	0.1 (0.1 to 0.1)	0.1 (0.1 to 0.1)	3.0 (2.8 to 3.1)	2.2 (2.1 to 2.3)	2.11 (2.00 to 2.22)	1.56 (1.48 to 1.64)
2010	0.1 (0.1 to 0.1)	0.1 (0.1 to 0.1)	2.7 (2.5 to 2.8)	2.0 (1.9 to 2.1)	2.33 (2.21 to 2.46)	1.73 (1.64 to 1.83)
2015	0.1 (0.1 to 0.1)	0.1 (0.0 to 0.1)	2.4 (2.3 to 2.5)	1.8 (1.7 to 1.9)	2.41 (2.28 to 2.54)	1.79 (1.70 to 1.89)
2020	0.1 (0.0 to 0.1)	0.0 (0.0 to 0.0)	2.2 (1.8 to 2.7)	1.6 (1.3 to 2.0)	2.39 (1.95 to 2.88)	1.76 (1.45 to 2.13)
2021	0.0 (0.0 to 0.1)	0.0 (0.0 to 0.0)	2.1 (1.6 to 2.8)	1.6 (1.2 to 2.0)	2.41 (1.82 to 3.09)	1.78 (1.35 to 2.28)

Tochigi JPN_35432 High−income Asia Pacific Stillbirth Rate / Neonatal Mortality Rate (SBR/NMR) − 20 weeks
lambda = 1 ; zeta = 0.6 ; scale = 15 ; amp = 0.028

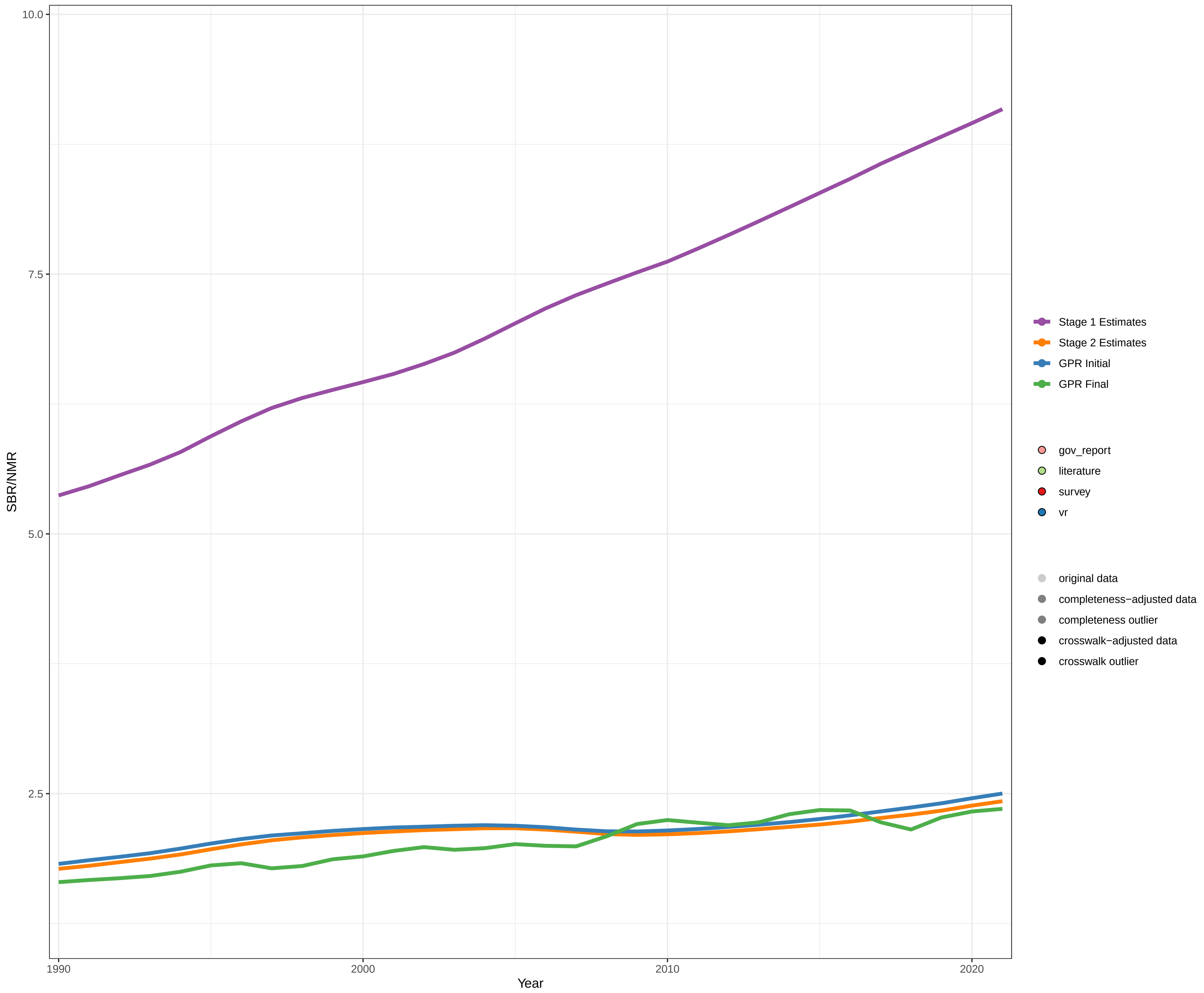

Tochigi JPN_35432 High−income Asia Pacific Stillbirth Rate / Neonatal Mortality Rate (SBR/NMR) − 28 weeks
lambda = 1 ; zeta = 0.6 ; scale = 15 ; amp = 0.028

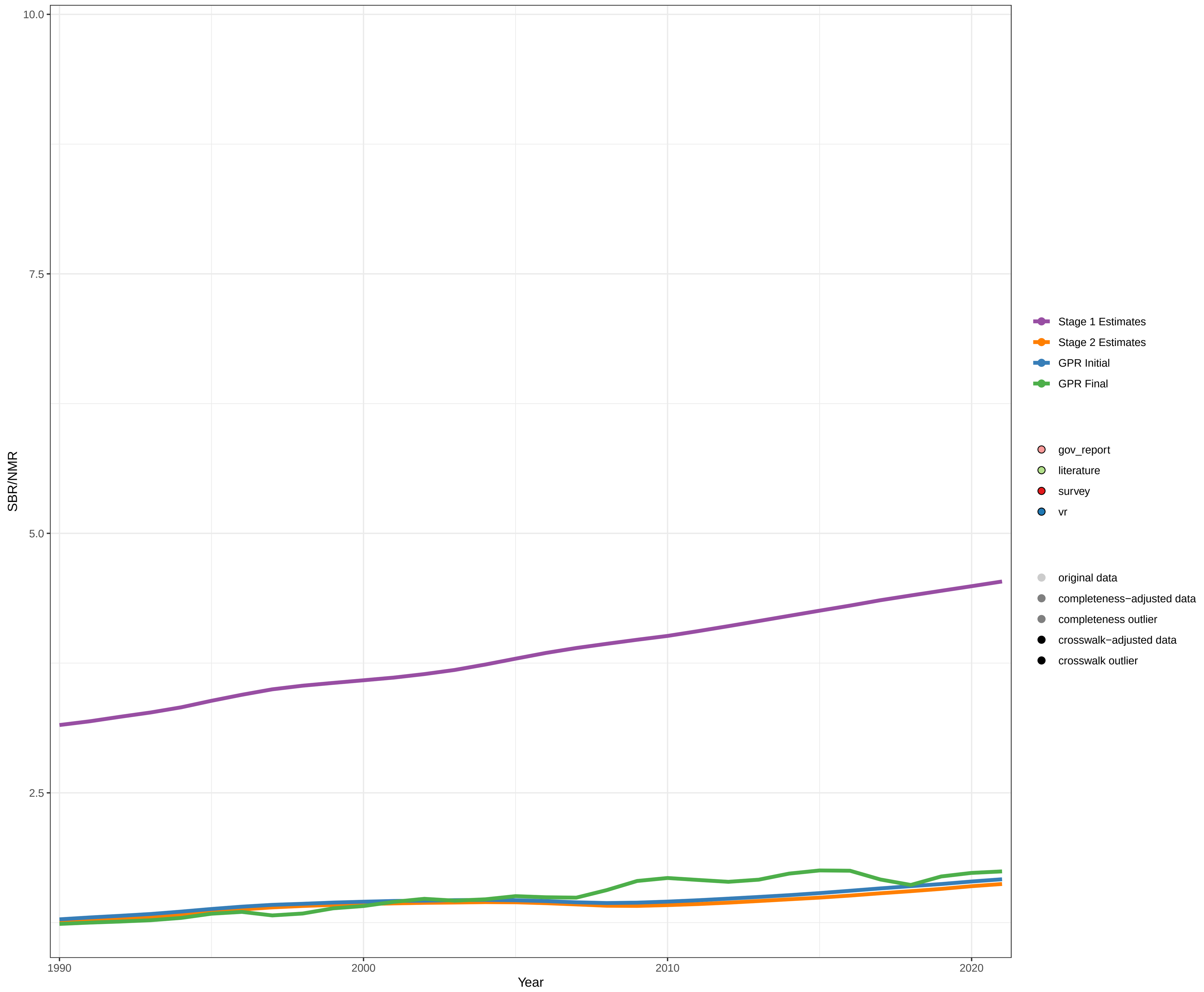

Definition	Years	Outlier	Source.Type	Title

Tochigi JPN_35432 High−income Asia Pacific Stillbirth Rate (SBR) − 20 weeks
lambda = 1 ; zeta = 0.6 ; scale = 15 ; amp = 0.028

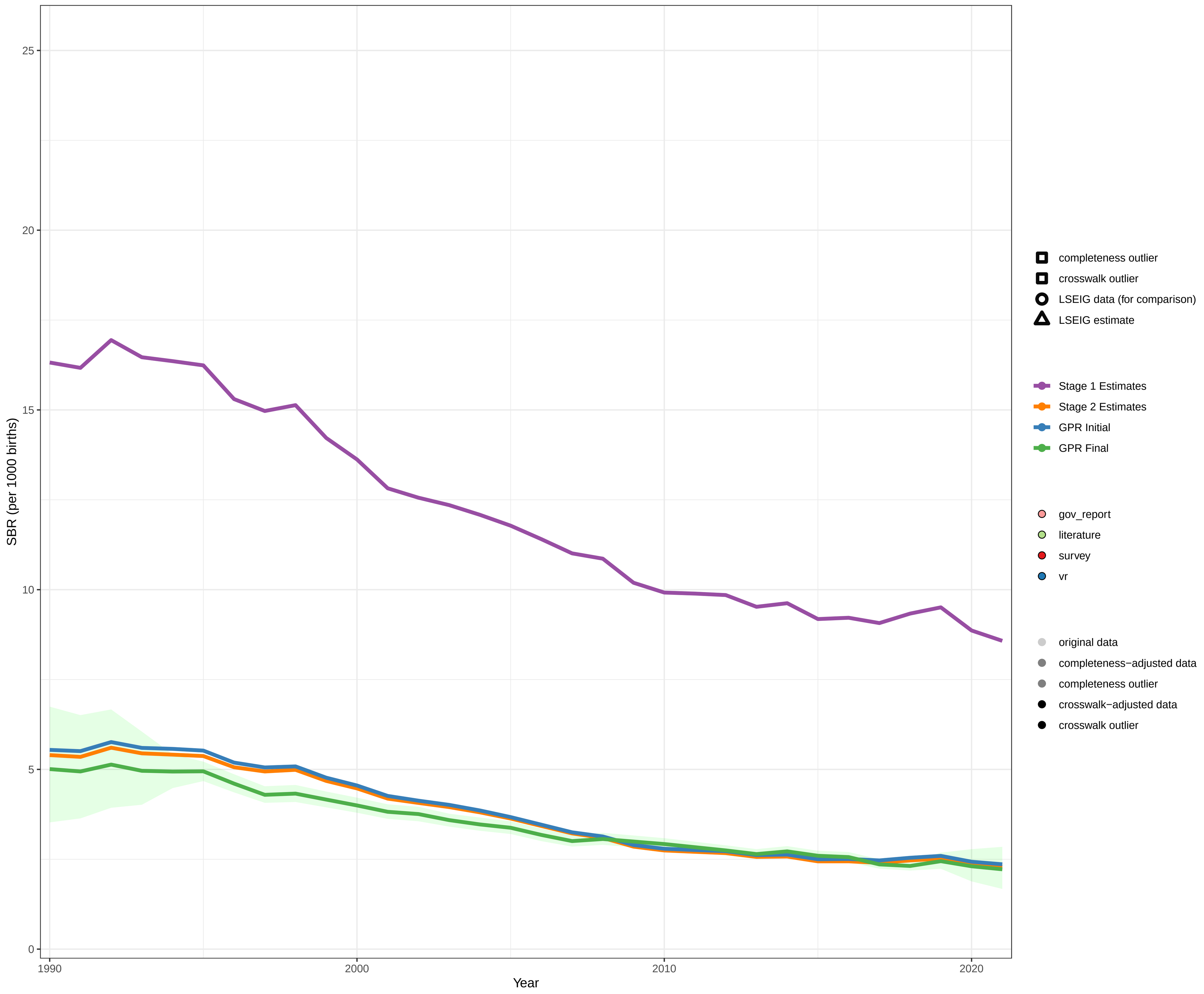

Tochigi JPN_35432 High−income Asia Pacific Stillbirth Rate (SBR) − 28 weeks
lambda = 1 ; zeta = 0.6 ; scale = 15 ; amp = 0.028

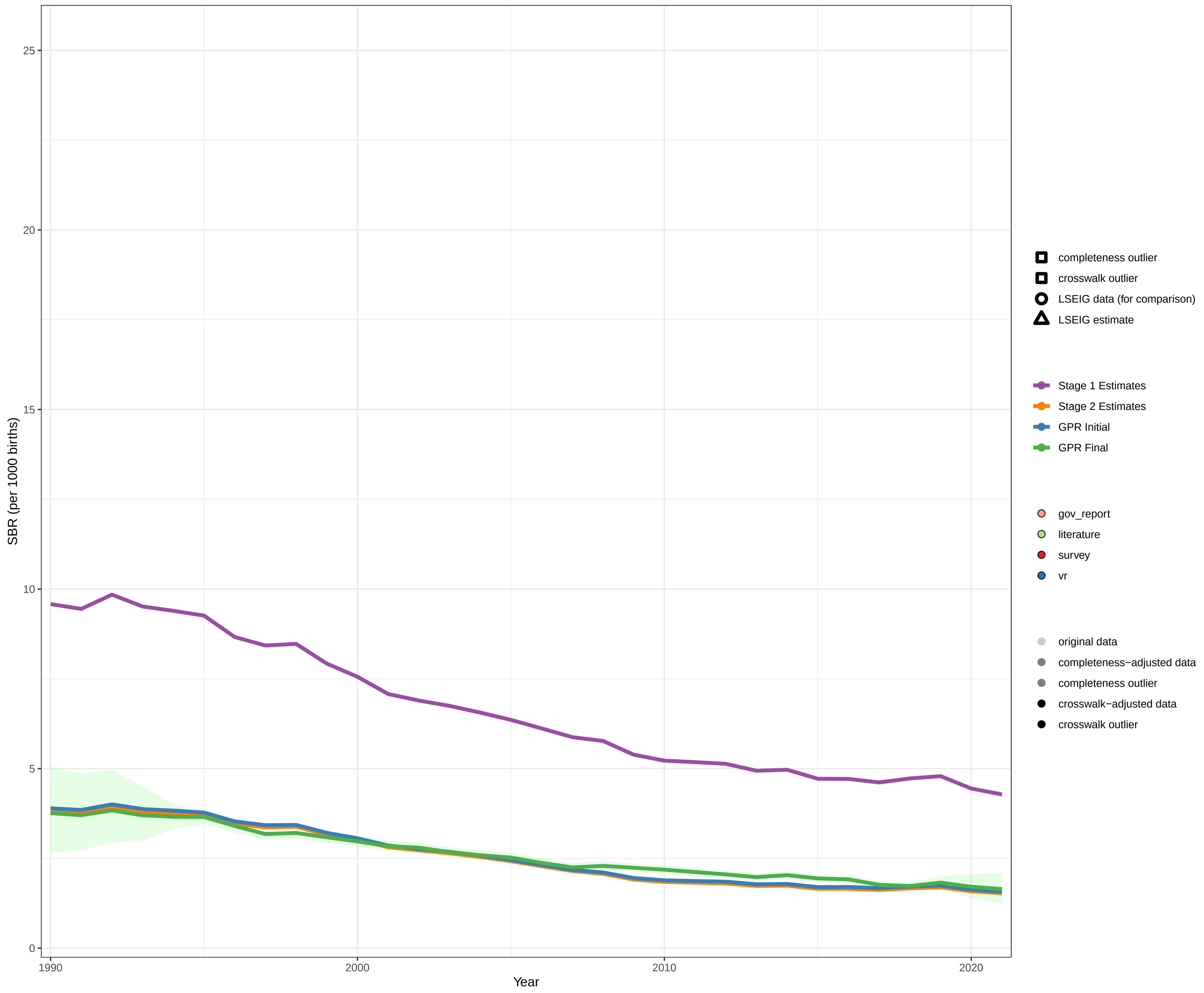

Year	Total Stillbirths (in thousands)	Total Stillbirths (in thousands)	SBR (per 1000 births)	SBR (per 1000 births)	SBR/NMR	SBR/NMR
	20 weeks	28 weeks	20 weeks	28 weeks	20 weeks	28 weeks
1990	0.1 (0.1 to 0.1)	0.1 (0.1 to 0.1)	5.0 (3.5 to 6.7)	3.8 (2.7 to 5.0)	1.65 (1.16 to 2.22)	1.24 (0.87 to 1.66)
1995	0.1 (0.1 to 0.1)	0.1 (0.1 to 0.1)	4.9 (4.7 to 5.2)	3.7 (3.5 to 3.8)	1.81 (1.71 to 1.91)	1.34 (1.26 to 1.41)
2000	0.1 (0.1 to 0.1)	0.1 (0.1 to 0.1)	4.0 (3.8 to 4.2)	3.0 (2.8 to 3.1)	1.90 (1.80 to 2.00)	1.41 (1.34 to 1.48)
2005	0.1 (0.1 to 0.1)	0.0 (0.0 to 0.0)	3.4 (3.2 to 3.6)	2.5 (2.4 to 2.7)	2.01 (1.91 to 2.12)	1.50 (1.43 to 1.58)
2010	0.0 (0.0 to 0.1)	0.0 (0.0 to 0.0)	2.9 (2.8 to 3.1)	2.2 (2.1 to 2.3)	2.25 (2.13 to 2.37)	1.68 (1.59 to 1.77)
2015	0.0 (0.0 to 0.0)	0.0 (0.0 to 0.0)	2.6 (2.5 to 2.7)	1.9 (1.8 to 2.0)	2.34 (2.22 to 2.47)	1.75 (1.66 to 1.85)
2020	0.0 (0.0 to 0.0)	0.0 (0.0 to 0.0)	2.3 (1.9 to 2.8)	1.7 (1.4 to 2.1)	2.33 (1.90 to 2.81)	1.73 (1.42 to 2.08)
2021	0.0 (0.0 to 0.0)	0.0 (0.0 to 0.0)	2.2 (1.7 to 2.8)	1.6 (1.2 to 2.1)	2.35 (1.77 to 3.02)	1.74 (1.32 to 2.23)

Tokushima JPN_35459 High−income Asia Pacific Stillbirth Rate / Neonatal Mortality Rate (SBR/NMR) − 20 weeks
lambda = 1 ; zeta = 0.6 ; scale = 15 ; amp = 0.028

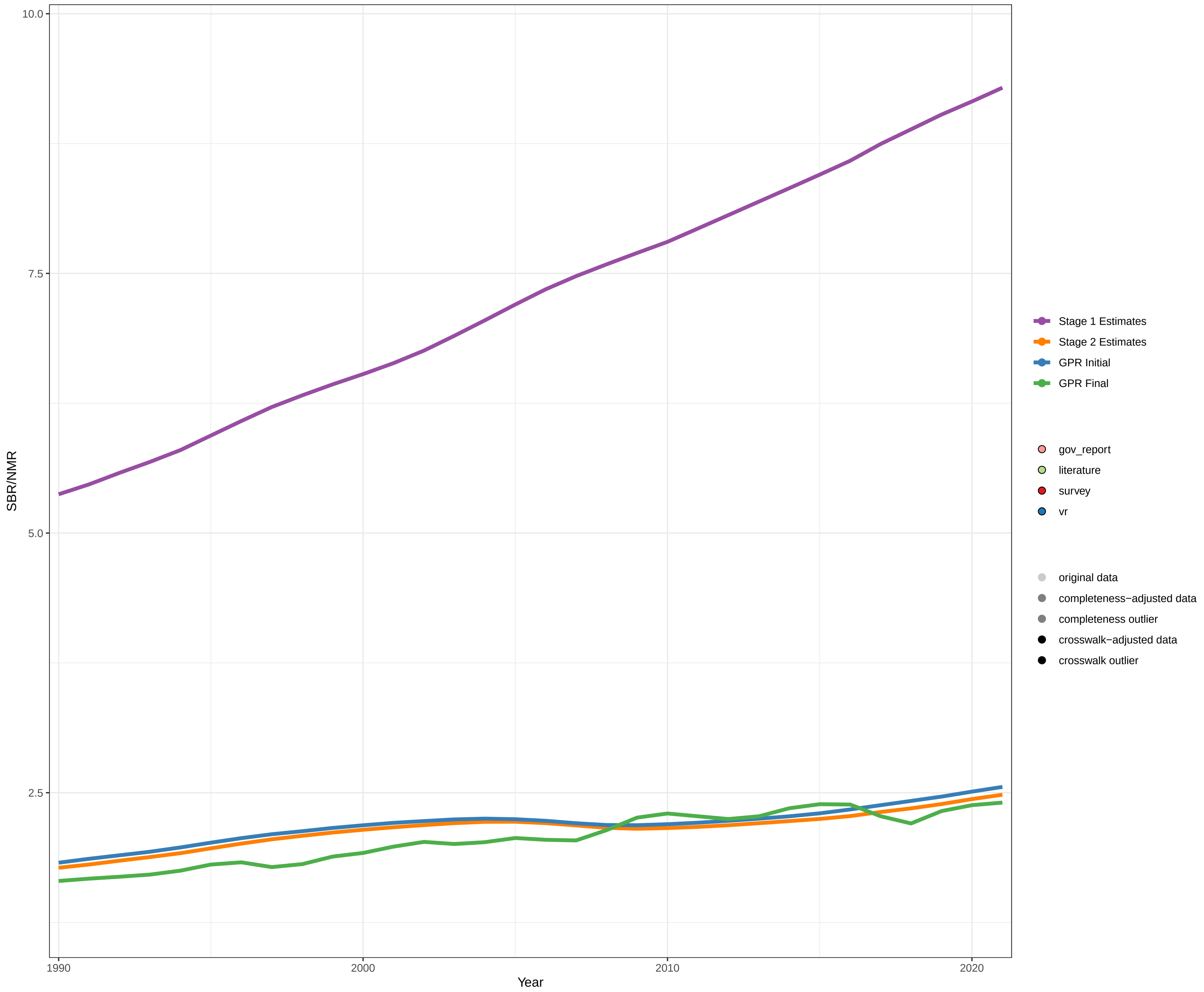

Tokushima JPN_35459 High−income Asia Pacific Stillbirth Rate / Neonatal Mortality Rate (SBR/NMR) − 28 weeks
lambda = 1 ; zeta = 0.6 ; scale = 15 ; amp = 0.028

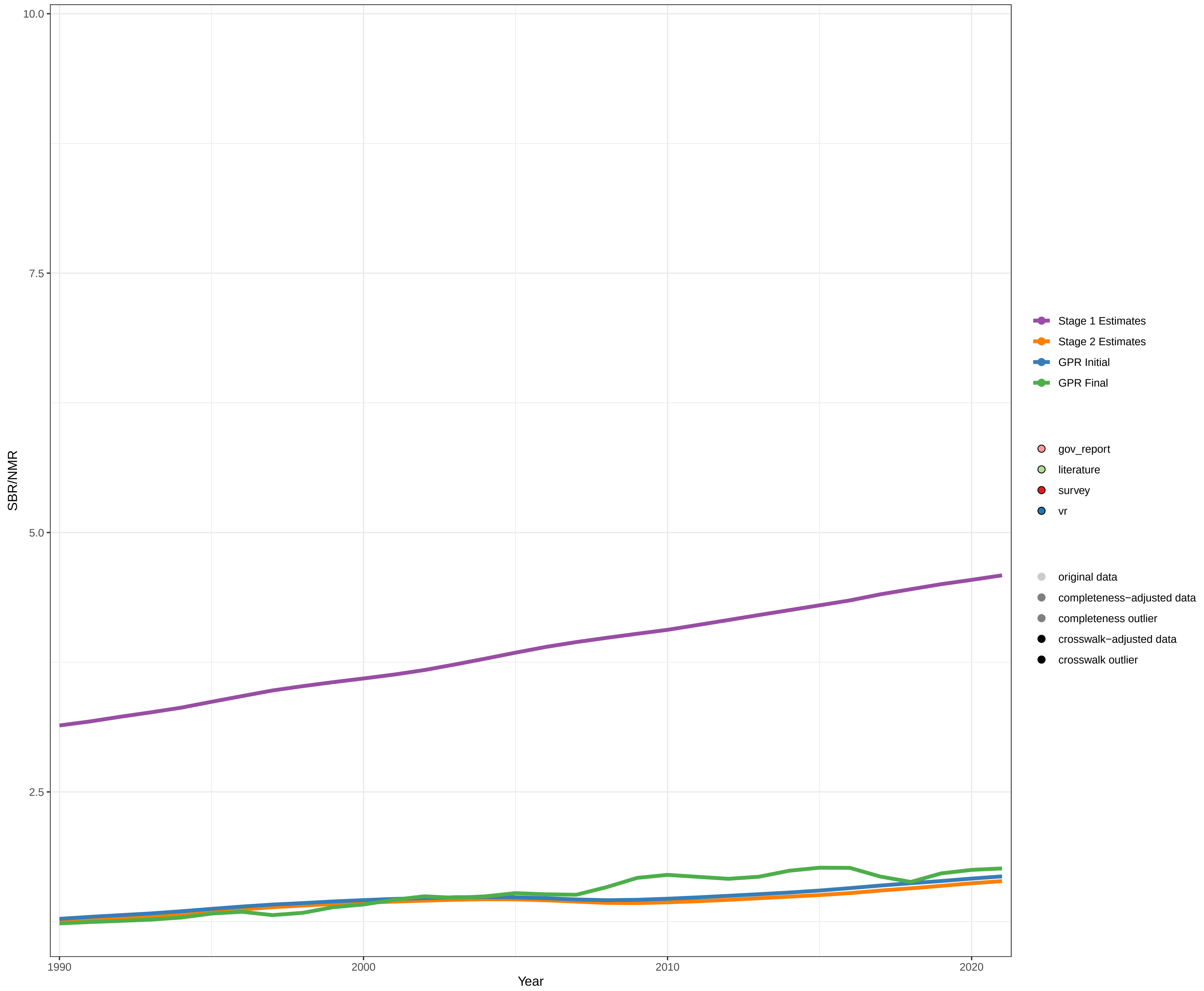

Definition	Years	Outlier	Source.Type	Title

Tokushima JPN_35459 High−income Asia Pacific Stillbirth Rate (SBR) − 20 weeks
lambda = 1 ; zeta = 0.6 ; scale = 15 ; amp = 0.028

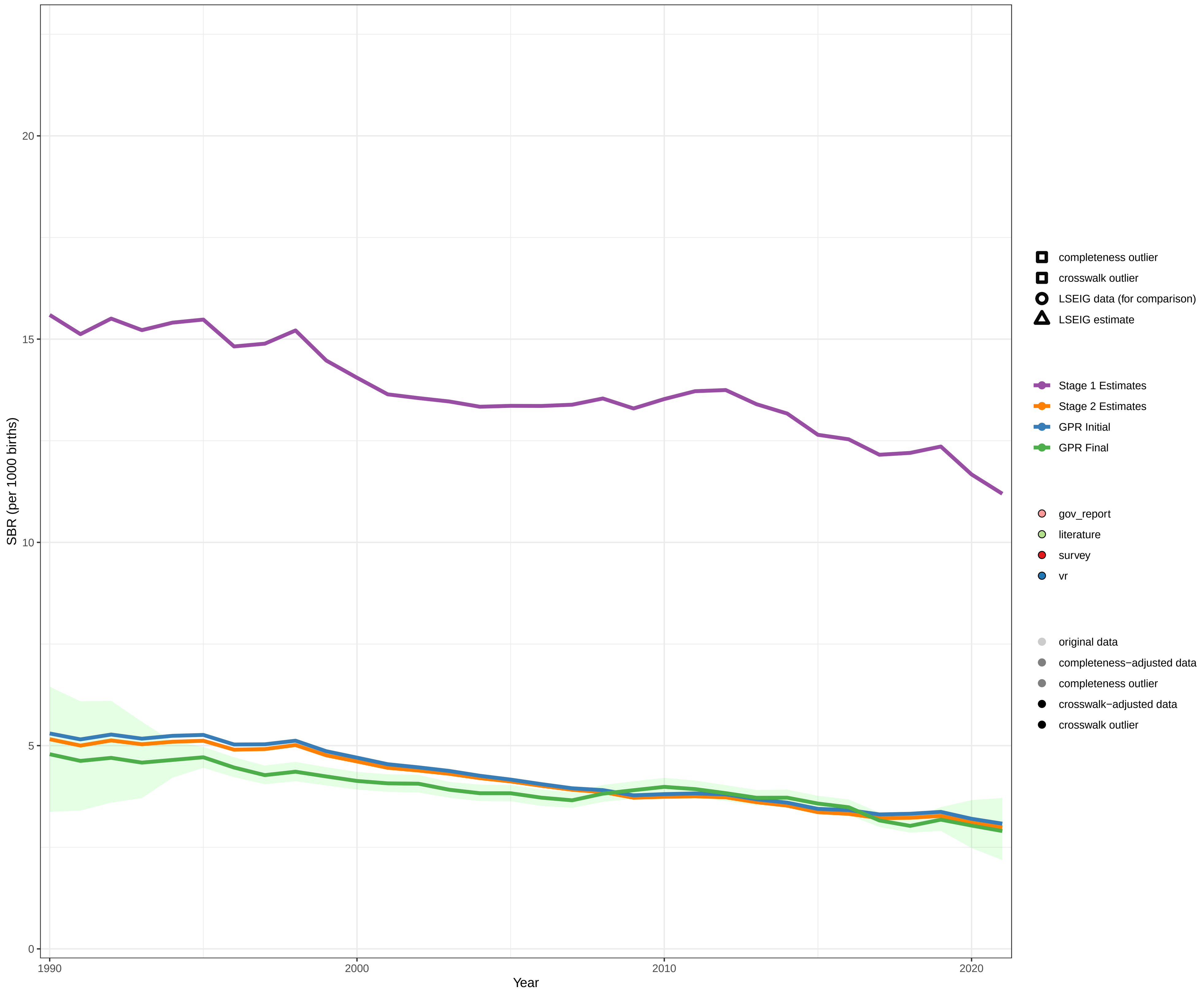

Tokushima JPN_35459 High−income Asia Pacific Stillbirth Rate (SBR) − 28 weeks
lambda = 1 ; zeta = 0.6 ; scale = 15 ; amp = 0.028

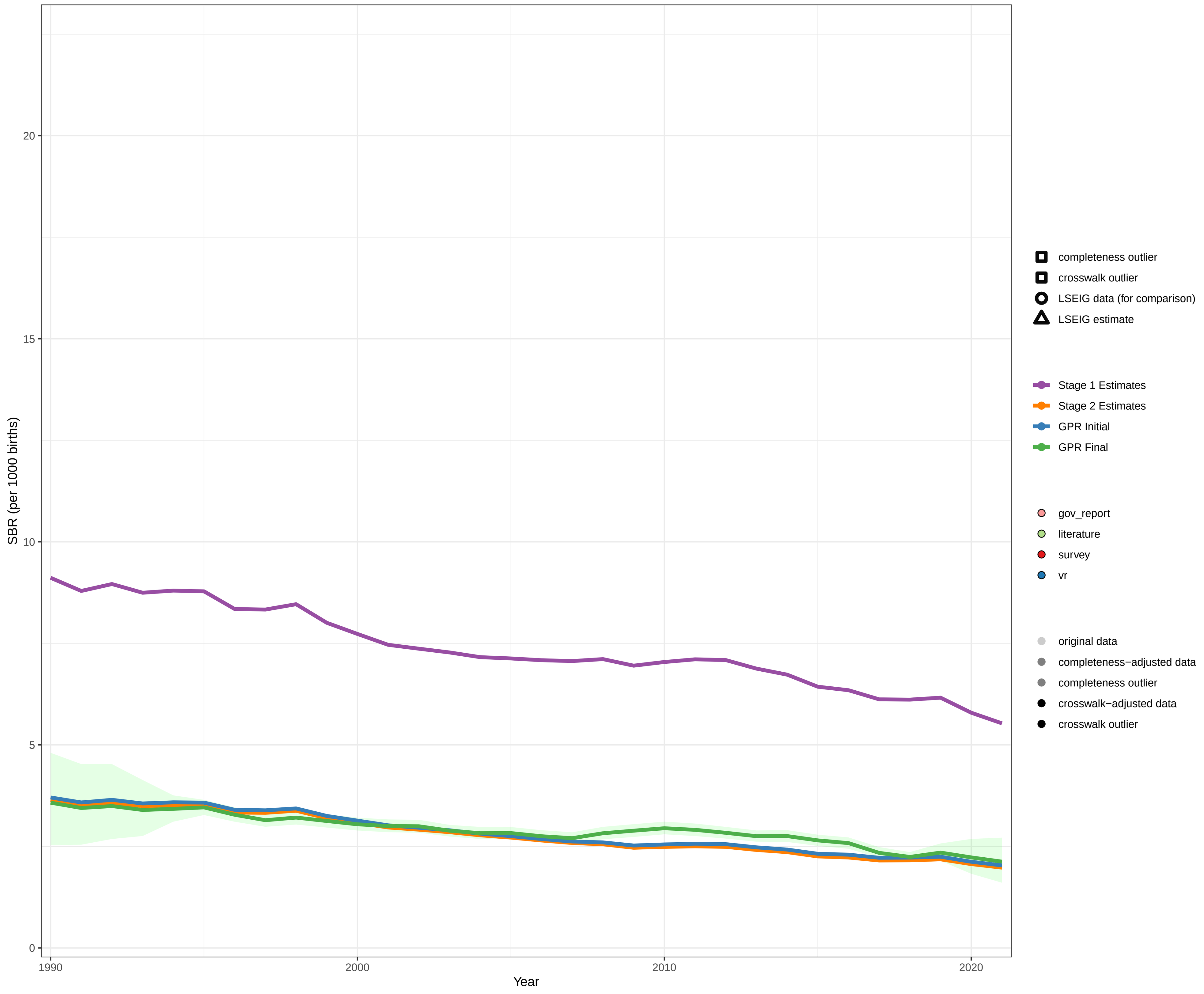

Year	Total Stillbirths (in thousands)	Total Stillbirths (in thousands)	SBR (per 1000 births)	SBR (per 1000 births)	SBR/NMR	SBR/NMR
	20 weeks	28 weeks	20 weeks	28 weeks	20 weeks	28 weeks
1990	0.0 (0.0 to 0.1)	0.0 (0.0 to 0.0)	4.8 (3.4 to 6.5)	3.6 (2.5 to 4.8)	1.65 (1.16 to 2.22)	1.23 (0.87 to 1.66)
1995	0.0 (0.0 to 0.0)	0.0 (0.0 to 0.0)	4.7 (4.5 to 5.0)	3.5 (3.3 to 3.6)	1.81 (1.71 to 1.91)	1.33 (1.26 to 1.40)
2000	0.0 (0.0 to 0.0)	0.0 (0.0 to 0.0)	4.1 (3.9 to 4.4)	3.0 (2.9 to 3.2)	1.92 (1.82 to 2.02)	1.42 (1.34 to 1.49)
2005	0.0 (0.0 to 0.0)	0.0 (0.0 to 0.0)	3.8 (3.6 to 4.0)	2.8 (2.7 to 3.0)	2.06 (1.96 to 2.17)	1.52 (1.45 to 1.60)
2010	0.0 (0.0 to 0.0)	0.0 (0.0 to 0.0)	4.0 (3.8 to 4.2)	2.9 (2.8 to 3.1)	2.30 (2.18 to 2.43)	1.70 (1.61 to 1.79)
2015	0.0 (0.0 to 0.0)	0.0 (0.0 to 0.0)	3.6 (3.4 to 3.8)	2.6 (2.5 to 2.8)	2.39 (2.26 to 2.52)	1.77 (1.68 to 1.86)
2020	0.0 (0.0 to 0.0)	0.0 (0.0 to 0.0)	3.0 (2.5 to 3.7)	2.2 (1.8 to 2.7)	2.38 (1.95 to 2.87)	1.75 (1.43 to 2.11)
2021	0.0 (0.0 to 0.0)	0.0 (0.0 to 0.0)	2.9 (2.2 to 3.7)	2.1 (1.6 to 2.7)	2.41 (1.81 to 3.08)	1.76 (1.33 to 2.25)

Tokyo JPN_35436 High−income Asia Pacific Stillbirth Rate / Neonatal Mortality Rate (SBR/NMR) − 20 weeks
lambda = 1 ; zeta = 0.6 ; scale = 15 ; amp = 0.028

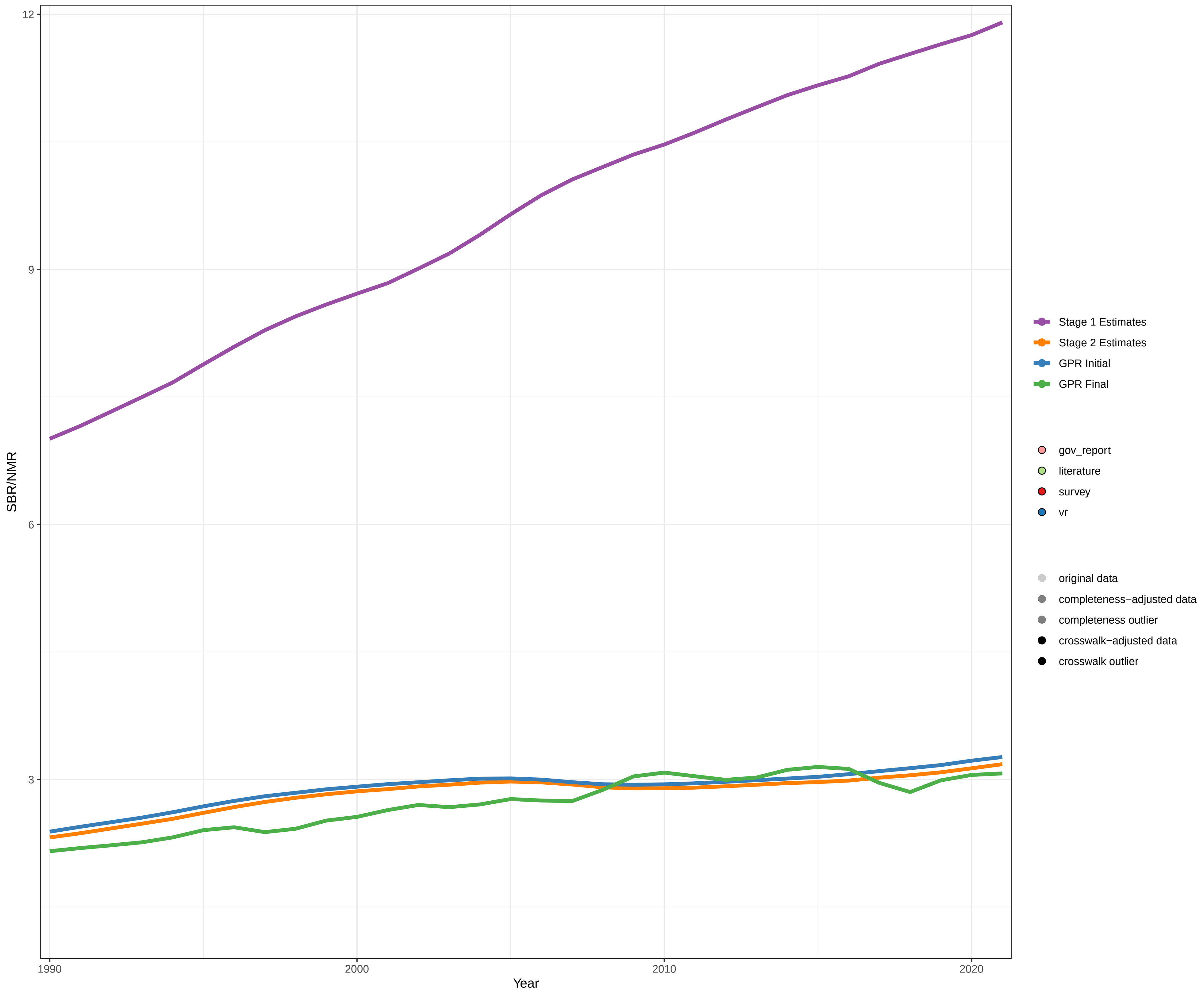

Tokyo JPN_35436 High−income Asia Pacific Stillbirth Rate / Neonatal Mortality Rate (SBR/NMR) − 28 weeks
lambda = 1 ; zeta = 0.6 ; scale = 15 ; amp = 0.028

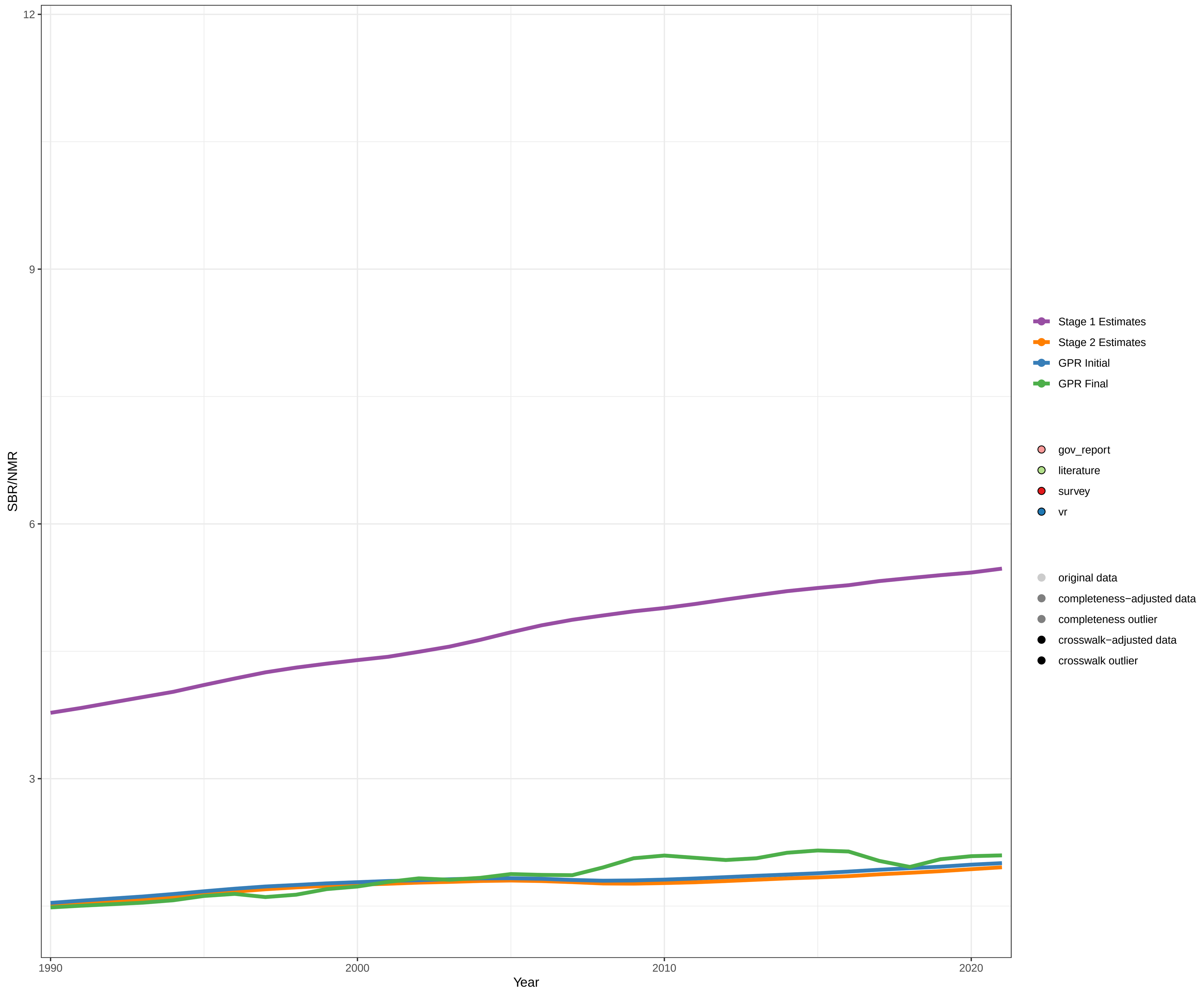

Definition	Years	Outlier	Source.Type	Title

Tokyo JPN_35436 High−income Asia Pacific Stillbirth Rate (SBR) − 20 weeks
lambda = 1 ; zeta = 0.6 ; scale = 15 ; amp = 0.028

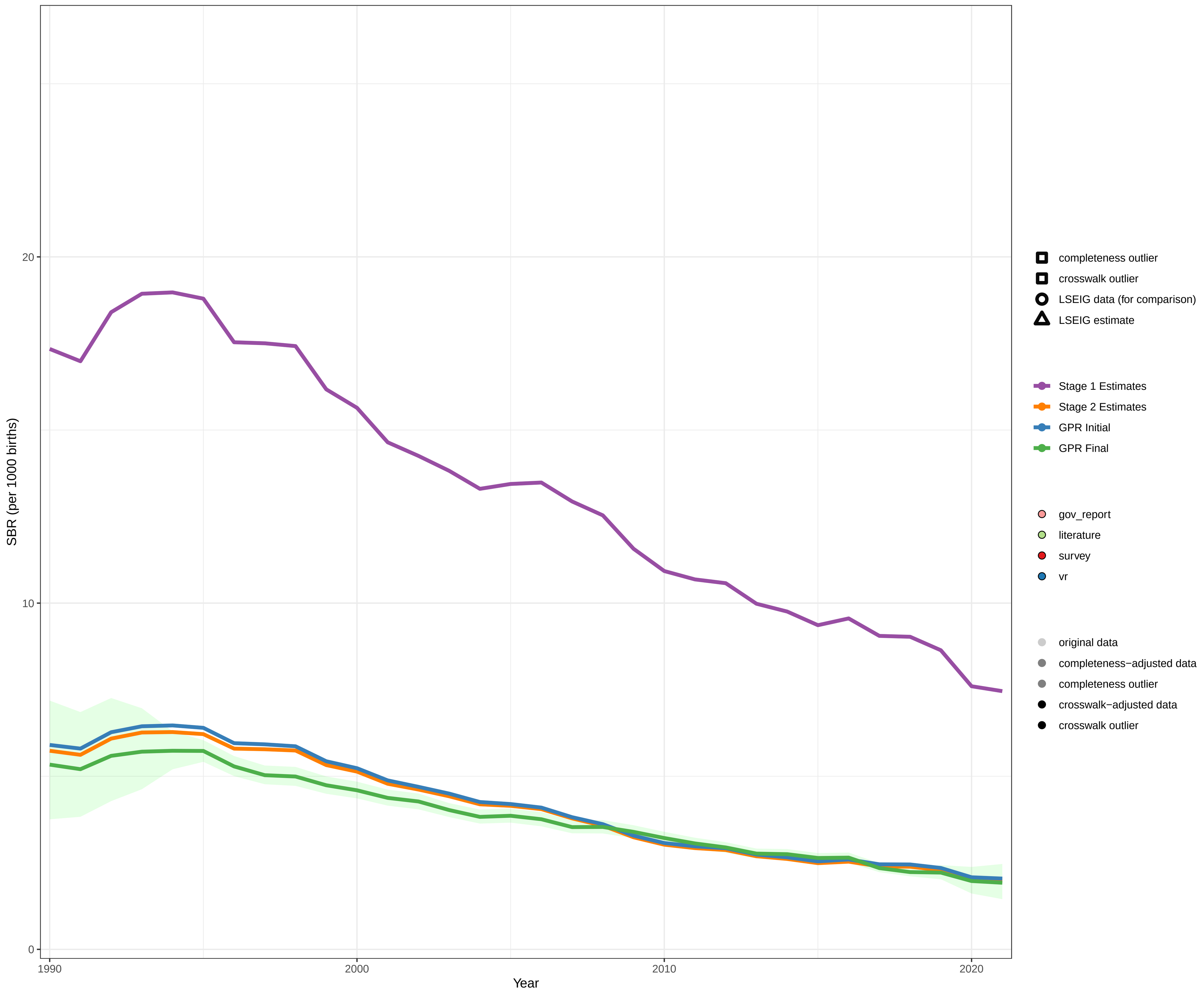

Tokyo JPN_35436 High−income Asia Pacific Stillbirth Rate (SBR) − 28 weeks
lambda = 1 ; zeta = 0.6 ; scale = 15 ; amp = 0.028

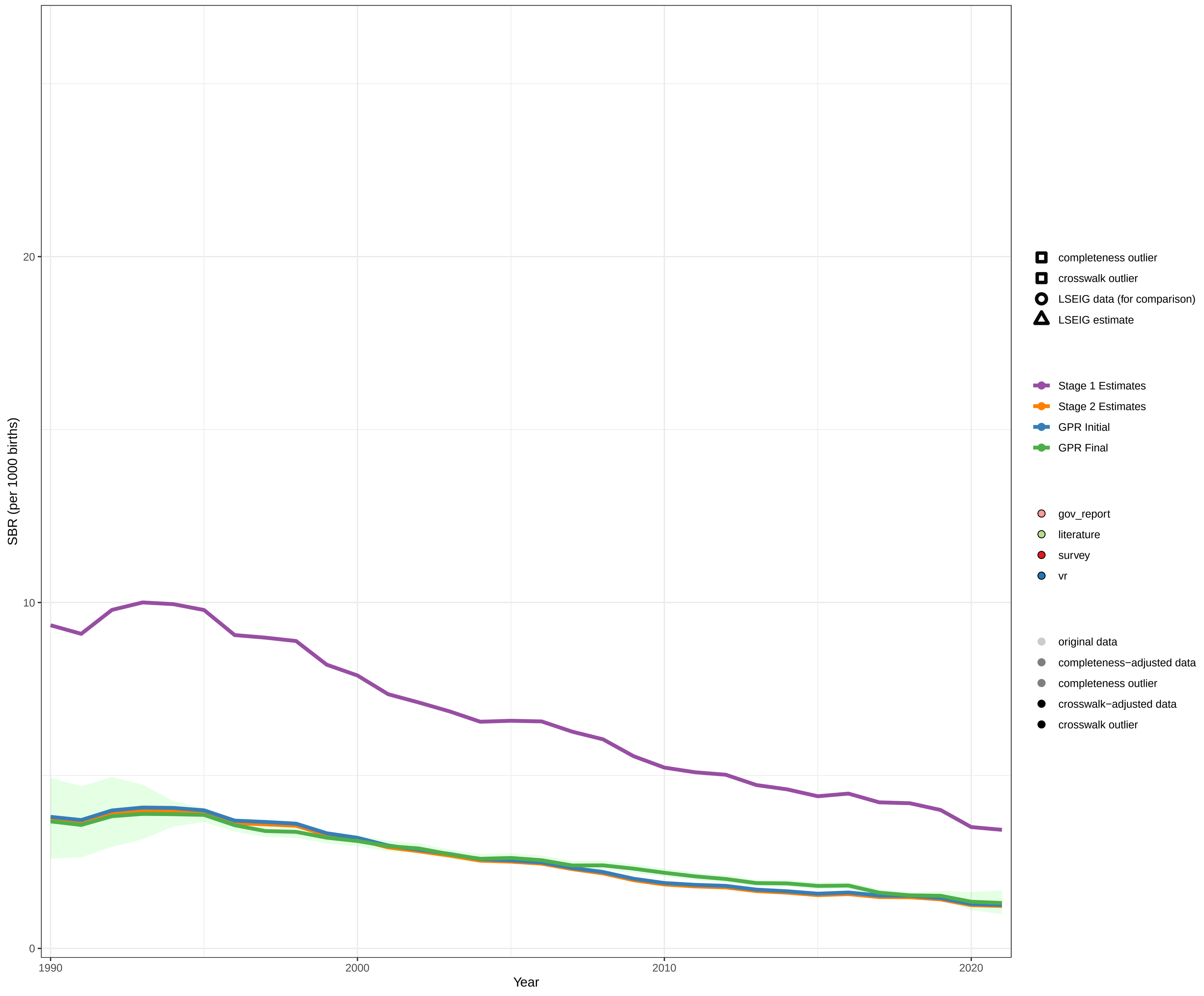

Year	Total Stillbirths (in thousands)	Total Stillbirths (in thousands)	SBR (per 1000 births)	SBR (per 1000 births)	SBR/NMR	SBR/NMR
	20 weeks	28 weeks	20 weeks	28 weeks	20 weeks	28 weeks
1990	0.6 (0.4 to 0.8)	0.4 (0.3 to 0.5)	5.3 (3.8 to 7.2)	3.7 (2.6 to 4.9)	2.16 (1.52 to 2.90)	1.48 (1.05 to 1.99)
1995	0.6 (0.5 to 0.6)	0.4 (0.4 to 0.4)	5.7 (5.4 to 6.0)	3.9 (3.7 to 4.1)	2.40 (2.27 to 2.53)	1.62 (1.53 to 1.71)
2000	0.5 (0.4 to 0.5)	0.3 (0.3 to 0.3)	4.6 (4.4 to 4.8)	3.1 (2.9 to 3.3)	2.56 (2.43 to 2.70)	1.73 (1.64 to 1.82)
2005	0.4 (0.4 to 0.4)	0.3 (0.2 to 0.3)	3.9 (3.7 to 4.1)	2.6 (2.5 to 2.8)	2.77 (2.63 to 2.92)	1.88 (1.78 to 1.98)
2010	0.3 (0.3 to 0.4)	0.2 (0.2 to 0.2)	3.2 (3.1 to 3.4)	2.2 (2.1 to 2.3)	3.08 (2.92 to 3.25)	2.10 (1.99 to 2.21)
2015	0.3 (0.3 to 0.3)	0.2 (0.2 to 0.2)	2.6 (2.5 to 2.8)	1.8 (1.7 to 1.9)	3.15 (2.98 to 3.32)	2.15 (2.04 to 2.27)
2020	0.2 (0.2 to 0.2)	0.1 (0.1 to 0.2)	2.0 (1.6 to 2.4)	1.3 (1.1 to 1.6)	3.05 (2.50 to 3.68)	2.09 (1.71 to 2.51)
2021	0.2 (0.1 to 0.3)	0.1 (0.1 to 0.2)	1.9 (1.5 to 2.5)	1.3 (1.0 to 1.7)	3.07 (2.32 to 3.94)	2.10 (1.59 to 2.68)

Tottori   JPN_35454   High−income Asia Pacific   Stillbirth Rate / Neonatal Mortality Rate (SBR/NMR) − 20 weeks
lambda = 1 ; zeta = 0.6 ; scale = 15 ; amp = 0.028

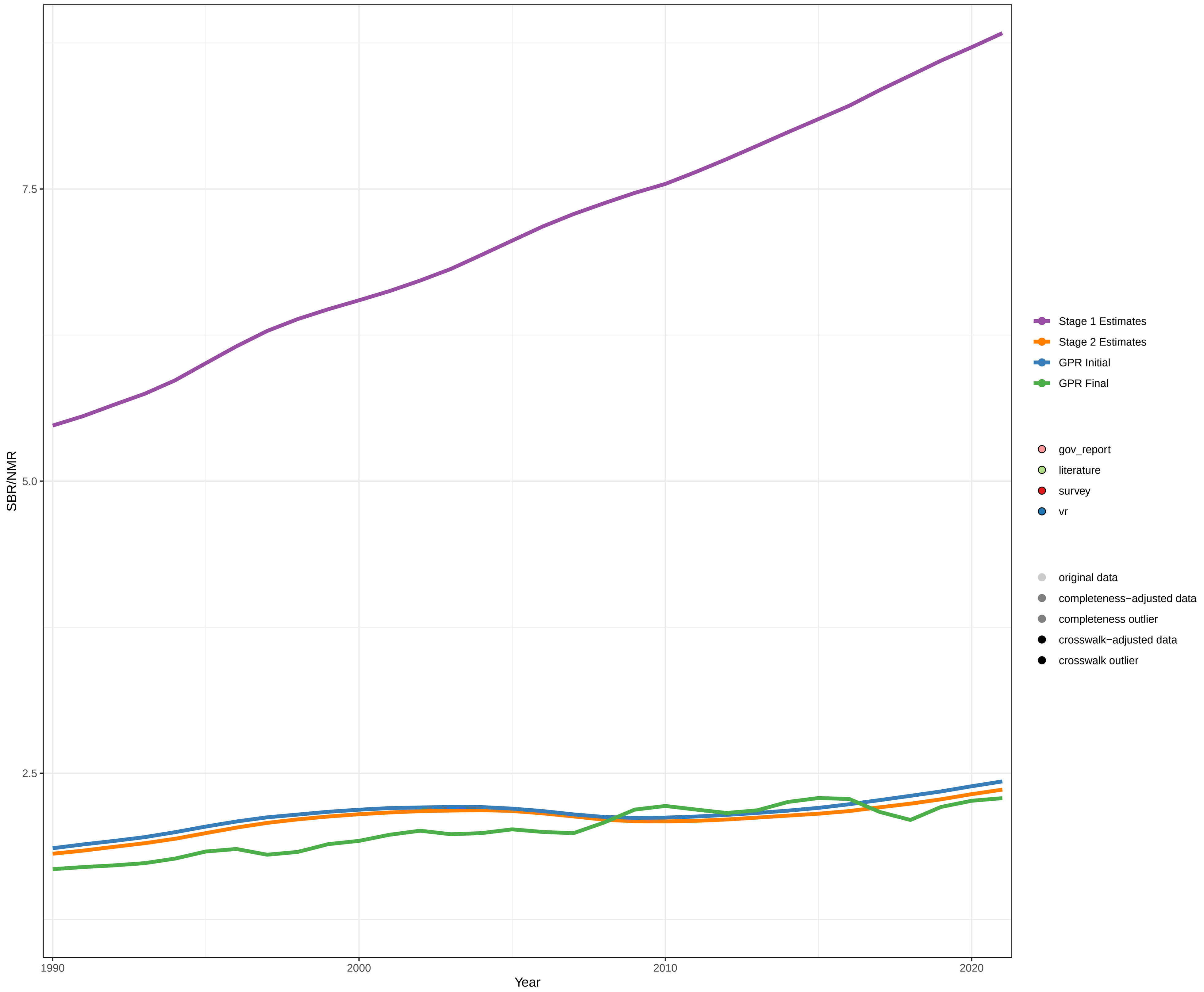

Tottori   JPN_35454   High−income Asia Pacific   Stillbirth Rate / Neonatal Mortality Rate (SBR/NMR) − 28 weeks
lambda = 1 ; zeta = 0.6 ; scale = 15 ; amp = 0.028

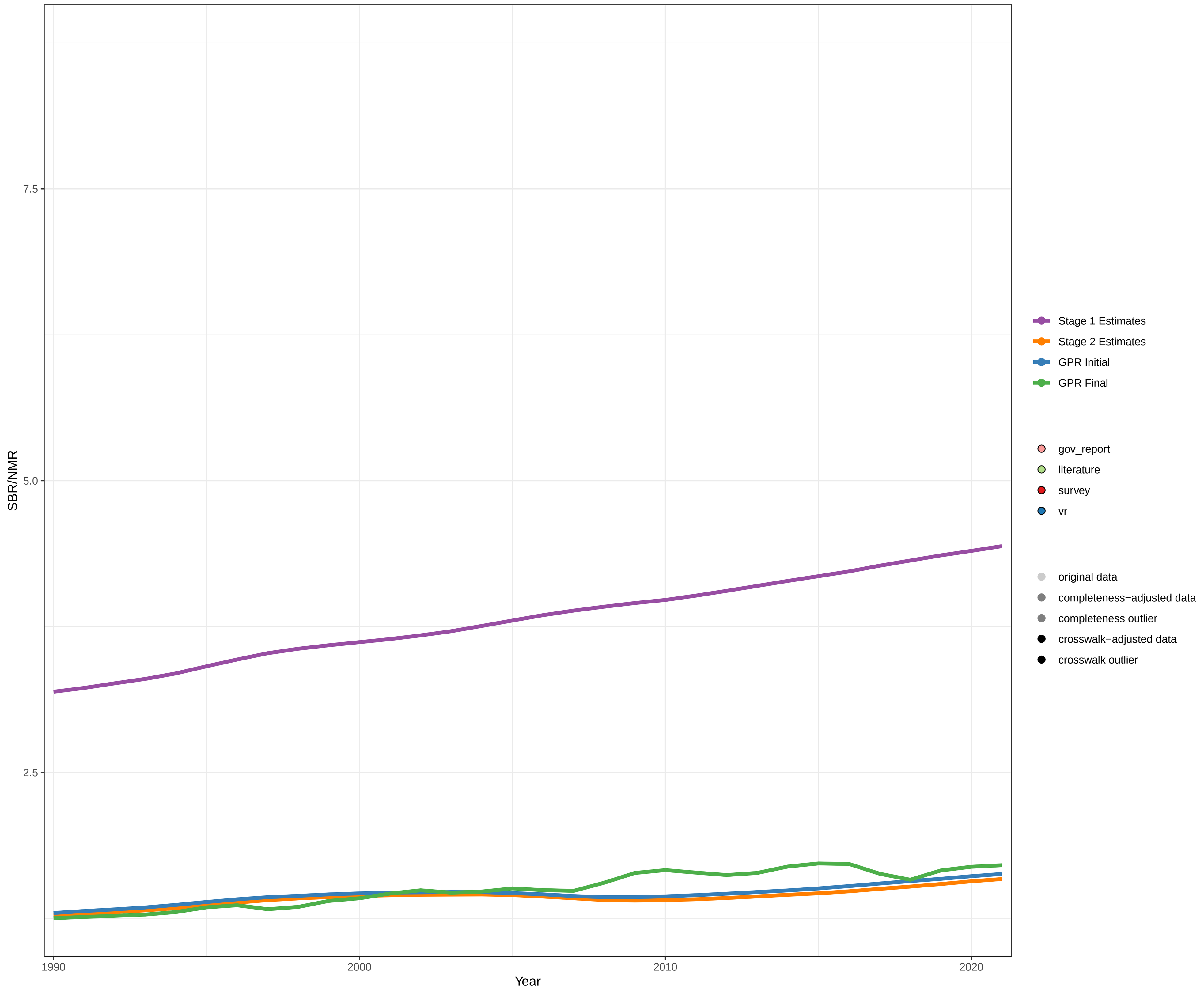

Definition	Years	Outlier	Source.Type	Title

Tottori   JPN_35454   High−income Asia Pacific   Stillbirth Rate (SBR) − 20 weeks
lambda = 1 ; zeta = 0.6 ; scale = 15 ; amp = 0.028

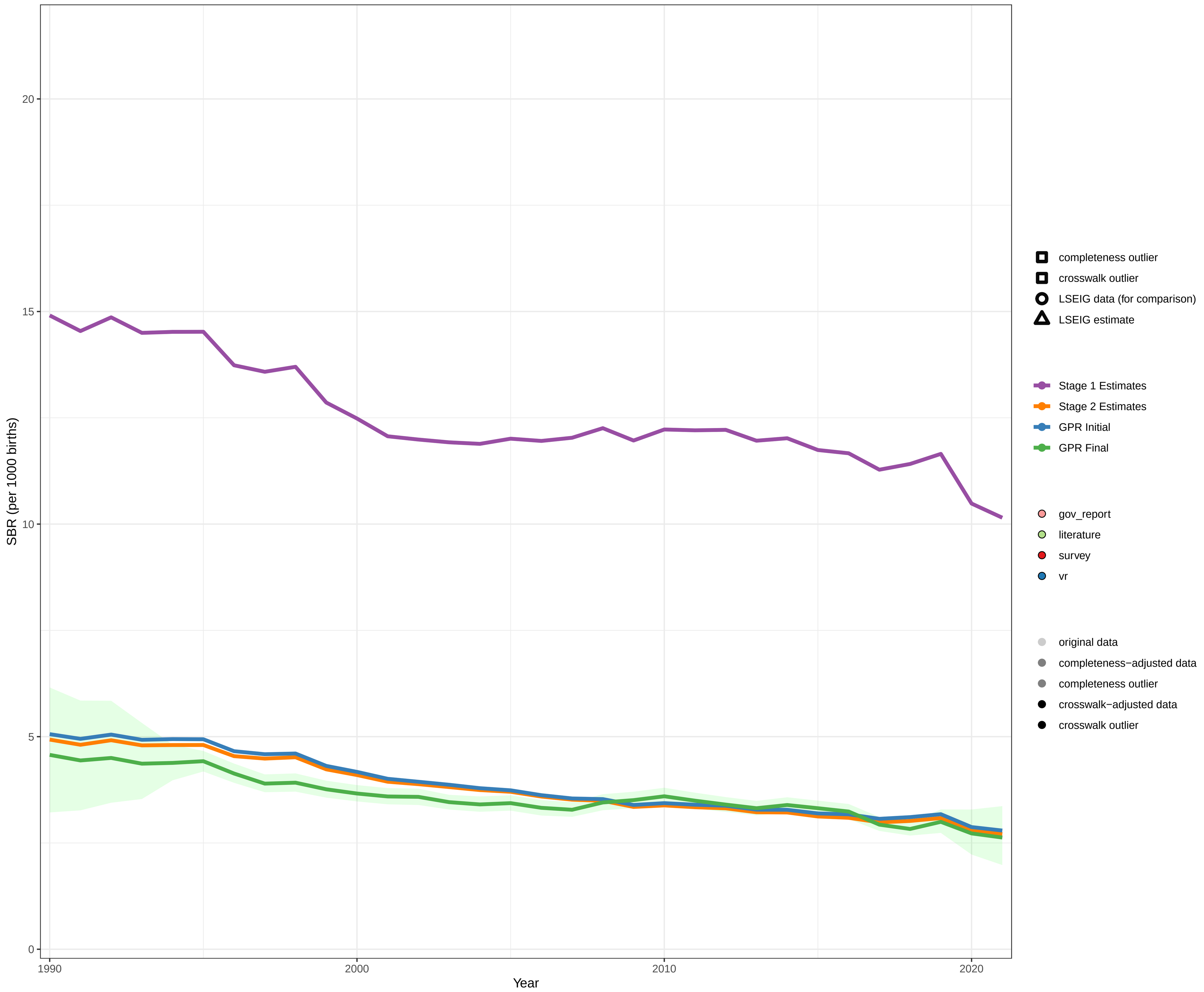

Tottori   JPN_35454   High−income Asia Pacific   Stillbirth Rate (SBR) − 28 weeks
lambda = 1 ; zeta = 0.6 ; scale = 15 ; amp = 0.028

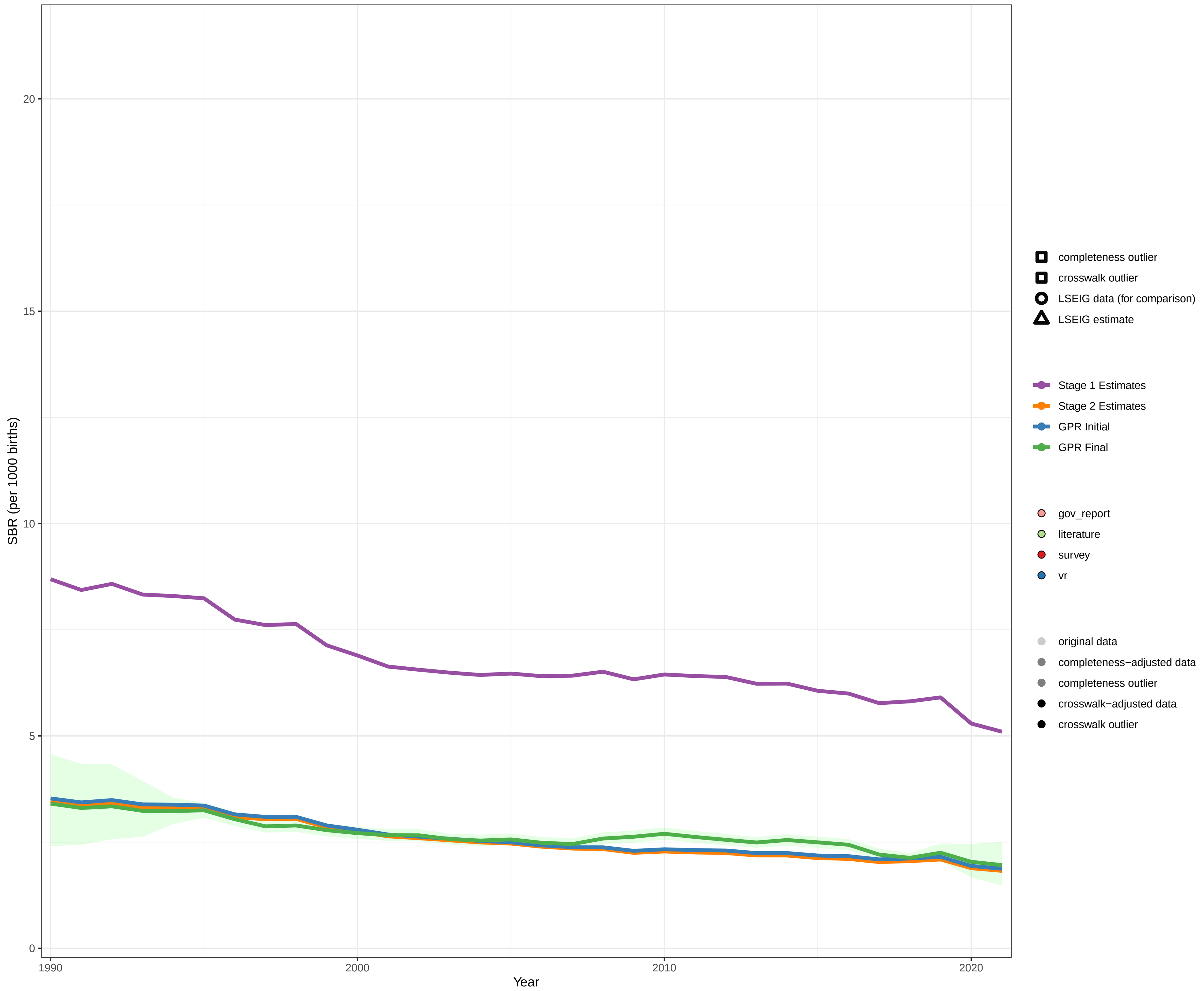

Year	Total Stillbirths (in thousands)	Total Stillbirths (in thousands)	SBR (per 1000 births)	SBR (per 1000 births)	SBR/NMR	SBR/NMR
	20 weeks	28 weeks	20 weeks	28 weeks	20 weeks	28 weeks
1990	0.0 (0.0 to 0.0)	0.0 (0.0 to 0.0)	4.6 (3.2 to 6.2)	3.4 (2.4 to 4.6)	1.68 (1.18 to 2.26)	1.25 (0.88 to 1.68)
1995	0.0 (0.0 to 0.0)	0.0 (0.0 to 0.0)	4.4 (4.2 to 4.7)	3.2 (3.1 to 3.4)	1.83 (1.73 to 1.93)	1.34 (1.27 to 1.42)
2000	0.0 (0.0 to 0.0)	0.0 (0.0 to 0.0)	3.7 (3.5 to 3.9)	2.7 (2.6 to 2.9)	1.92 (1.82 to 2.03)	1.42 (1.35 to 1.50)
2005	0.0 (0.0 to 0.0)	0.0 (0.0 to 0.0)	3.4 (3.3 to 3.6)	2.6 (2.4 to 2.7)	2.02 (1.92 to 2.13)	1.51 (1.43 to 1.59)
2010	0.0 (0.0 to 0.0)	0.0 (0.0 to 0.0)	3.6 (3.4 to 3.8)	2.7 (2.6 to 2.8)	2.22 (2.11 to 2.34)	1.66 (1.58 to 1.76)
2015	0.0 (0.0 to 0.0)	0.0 (0.0 to 0.0)	3.3 (3.1 to 3.5)	2.5 (2.4 to 2.6)	2.29 (2.17 to 2.41)	1.72 (1.63 to 1.81)
2020	0.0 (0.0 to 0.0)	0.0 (0.0 to 0.0)	2.7 (2.2 to 3.3)	2.0 (1.7 to 2.5)	2.26 (1.85 to 2.73)	1.69 (1.39 to 2.04)
2021	0.0 (0.0 to 0.0)	0.0 (0.0 to 0.0)	2.6 (2.0 to 3.4)	2.0 (1.5 to 2.5)	2.29 (1.72 to 2.93)	1.71 (1.29 to 2.18)

Toyama JPN_35439 High−income Asia Pacific Stillbirth Rate / Neonatal Mortality Rate (SBR/NMR) − 20 weeks
lambda = 1 ; zeta = 0.6 ; scale = 15 ; amp = 0.028

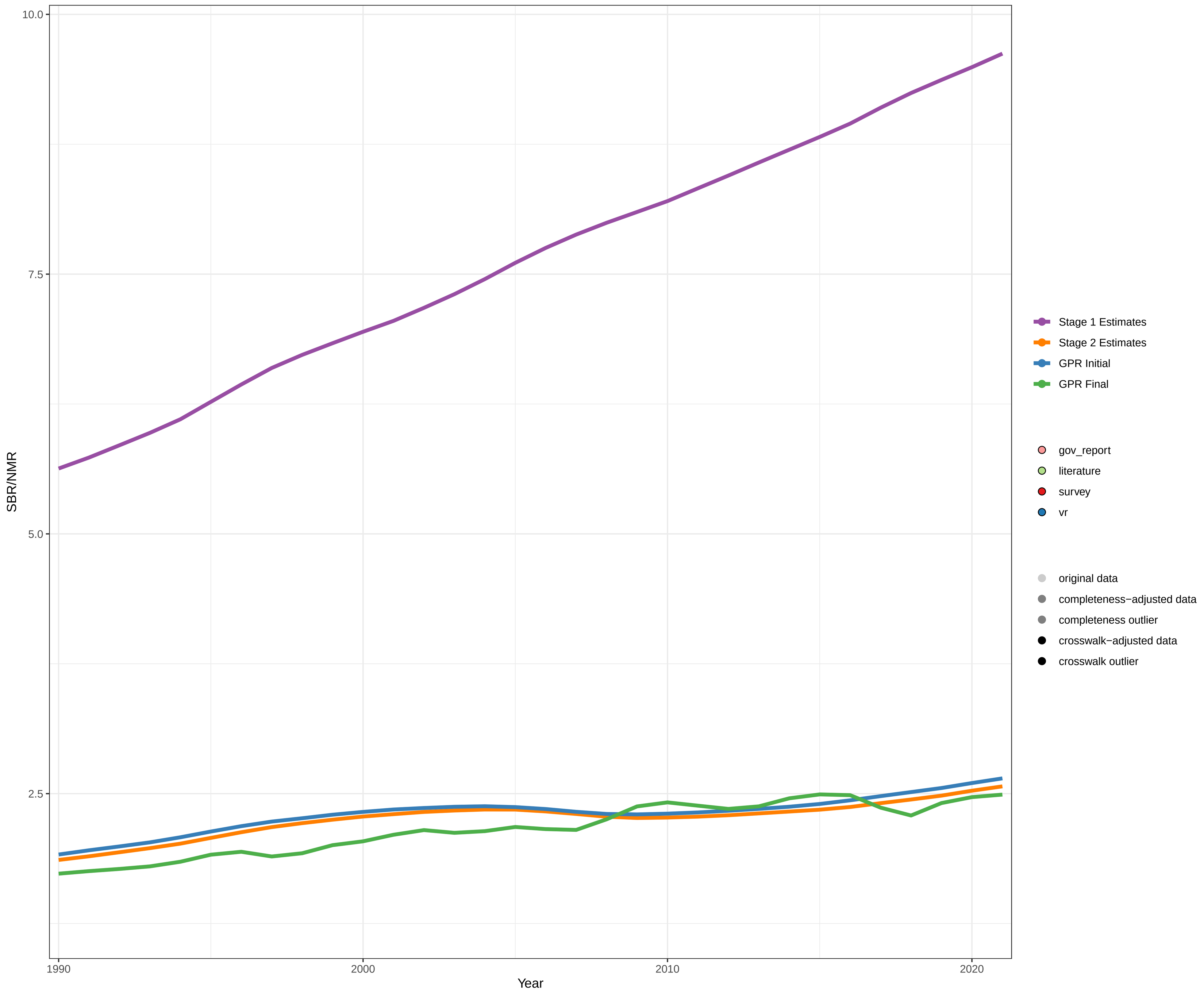

Toyama JPN_35439 High−income Asia Pacific Stillbirth Rate / Neonatal Mortality Rate (SBR/NMR) − 28 weeks
lambda = 1 ; zeta = 0.6 ; scale = 15 ; amp = 0.028

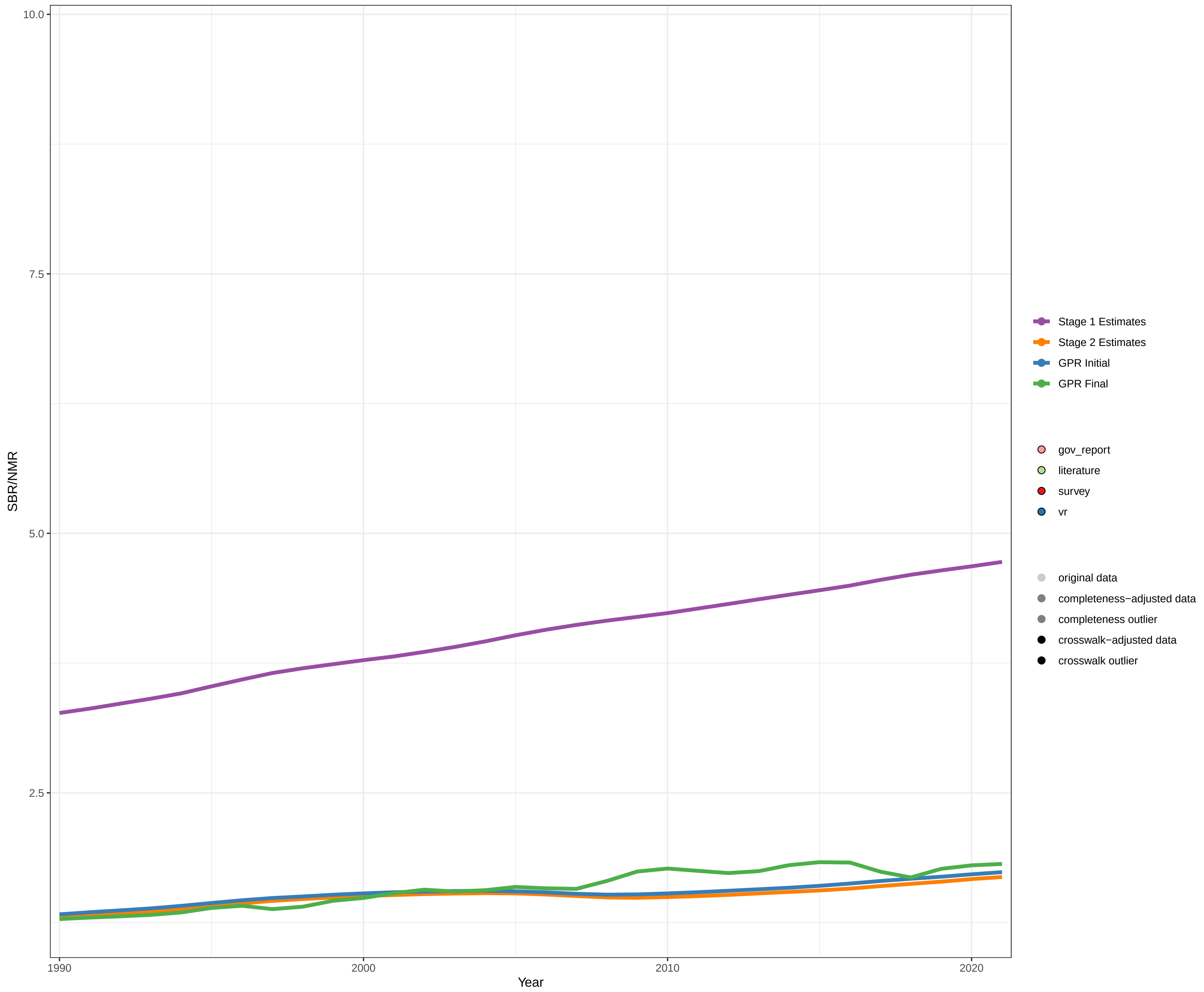

Definition	Years	Outlier	Source.Type	Title

Toyama JPN_35439 High−income Asia Pacific Stillbirth Rate (SBR) − 20 weeks
lambda = 1 ; zeta = 0.6 ; scale = 15 ; amp = 0.028

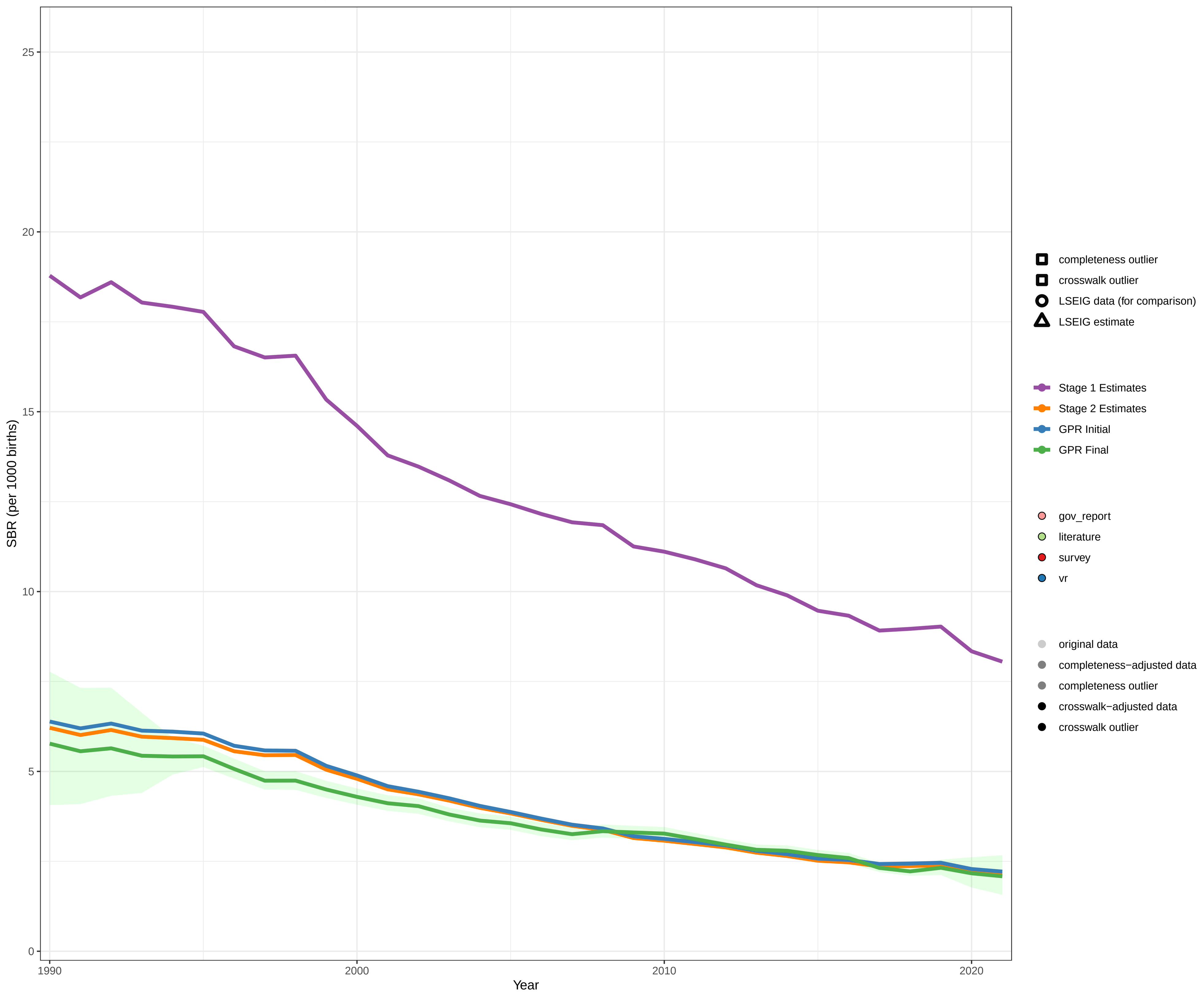

Toyama JPN_35439 High−income Asia Pacific Stillbirth Rate (SBR) − 28 weeks
lambda = 1 ; zeta = 0.6 ; scale = 15 ; amp = 0.028

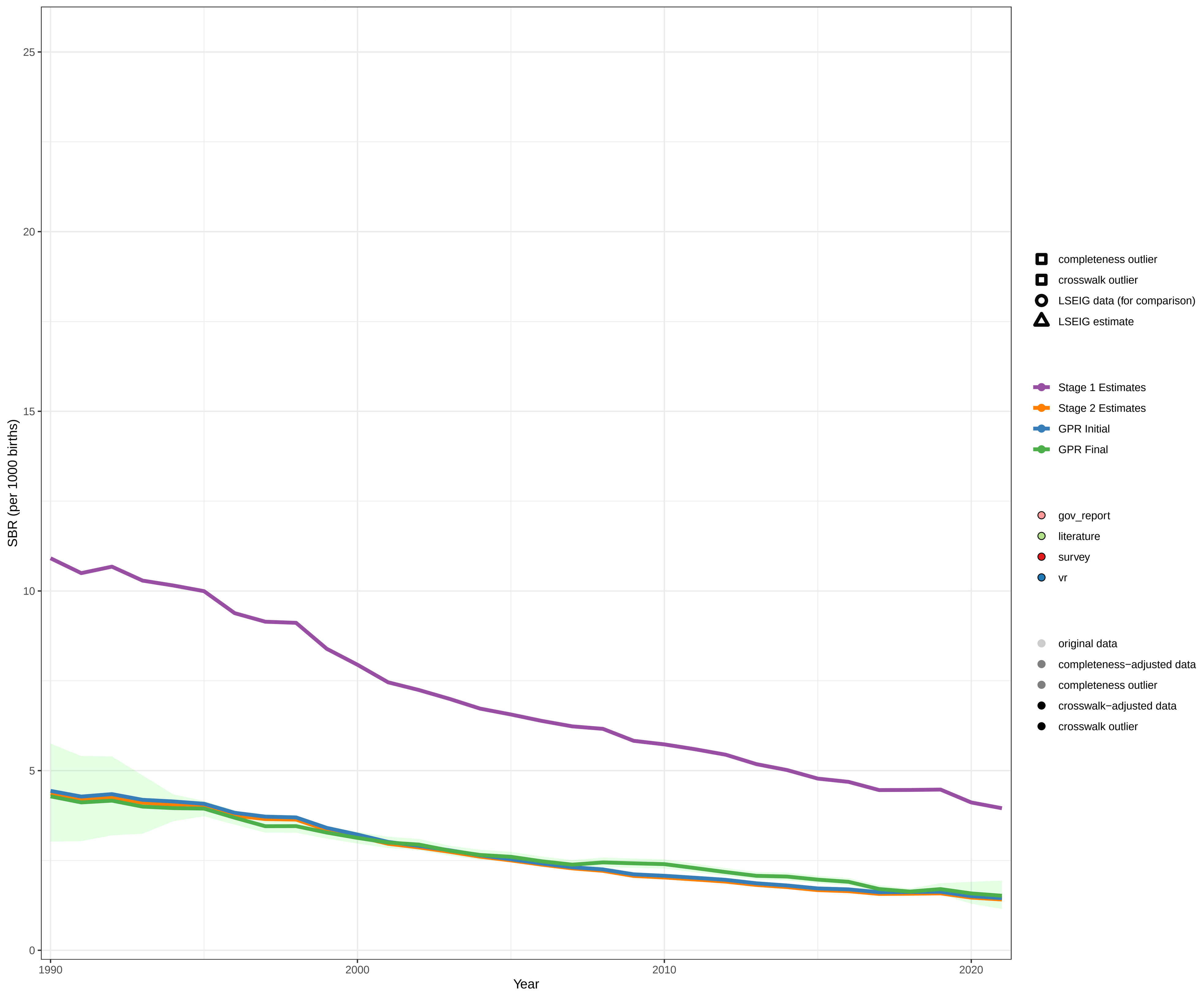

Year	Total Stillbirths (in thousands)	Total Stillbirths (in thousands)	SBR (per 1000 births)	SBR (per 1000 births)	SBR/NMR	SBR/NMR
	20 weeks	28 weeks	20 weeks	28 weeks	20 weeks	28 weeks
1990	0.1 (0.0 to 0.1)	0.0 (0.0 to 0.1)	5.8 (4.1 to 7.8)	4.3 (3.0 to 5.8)	1.73 (1.22 to 2.33)	1.28 (0.91 to 1.72)
1995	0.1 (0.1 to 0.1)	0.0 (0.0 to 0.0)	5.4 (5.1 to 5.7)	3.9 (3.7 to 4.2)	1.91 (1.81 to 2.02)	1.39 (1.32 to 1.47)
2000	0.0 (0.0 to 0.0)	0.0 (0.0 to 0.0)	4.3 (4.1 to 4.5)	3.1 (3.0 to 3.3)	2.04 (1.94 to 2.15)	1.49 (1.41 to 1.57)
2005	0.0 (0.0 to 0.0)	0.0 (0.0 to 0.0)	3.6 (3.4 to 3.7)	2.6 (2.5 to 2.7)	2.18 (2.07 to 2.30)	1.59 (1.51 to 1.68)
2010	0.0 (0.0 to 0.0)	0.0 (0.0 to 0.0)	3.3 (3.1 to 3.5)	2.4 (2.3 to 2.5)	2.42 (2.29 to 2.55)	1.77 (1.68 to 1.87)
2015	0.0 (0.0 to 0.0)	0.0 (0.0 to 0.0)	2.7 (2.5 to 2.8)	2.0 (1.9 to 2.1)	2.49 (2.36 to 2.63)	1.83 (1.74 to 1.93)
2020	0.0 (0.0 to 0.0)	0.0 (0.0 to 0.0)	2.2 (1.8 to 2.6)	1.6 (1.3 to 1.9)	2.47 (2.02 to 2.98)	1.80 (1.48 to 2.17)
2021	0.0 (0.0 to 0.0)	0.0 (0.0 to 0.0)	2.1 (1.6 to 2.7)	1.5 (1.1 to 1.9)	2.49 (1.88 to 3.19)	1.81 (1.37 to 2.32)

Wakayama JPN_35453 High−income Asia Pacific Stillbirth Rate / Neonatal Mortality Rate (SBR/NMR) − 20 weeks
lambda = 1 ; zeta = 0.6 ; scale = 15 ; amp = 0.028

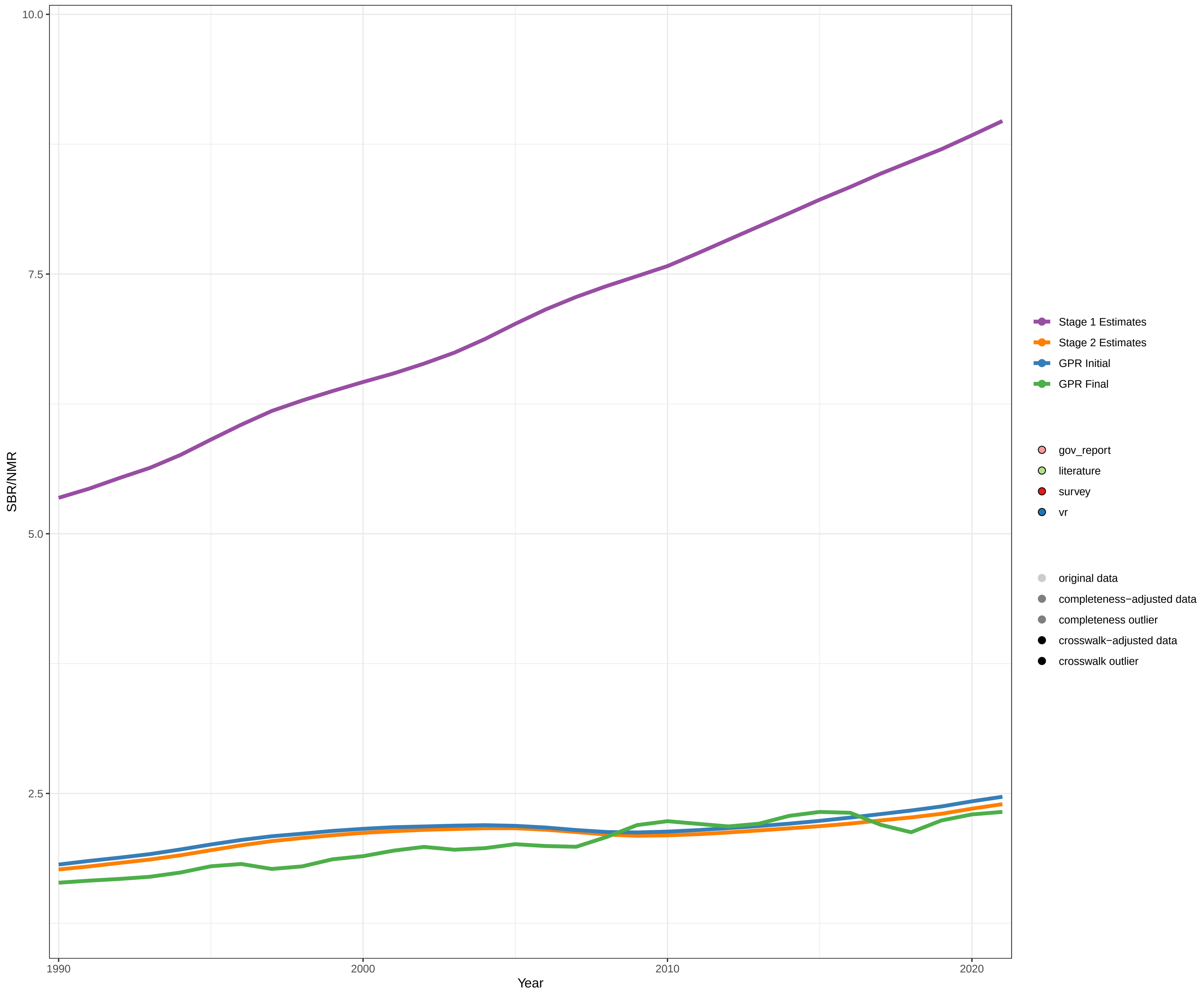

Wakayama JPN_35453 High−income Asia Pacific Stillbirth Rate / Neonatal Mortality Rate (SBR/NMR) − 28 weeks
lambda = 1 ; zeta = 0.6 ; scale = 15 ; amp = 0.028

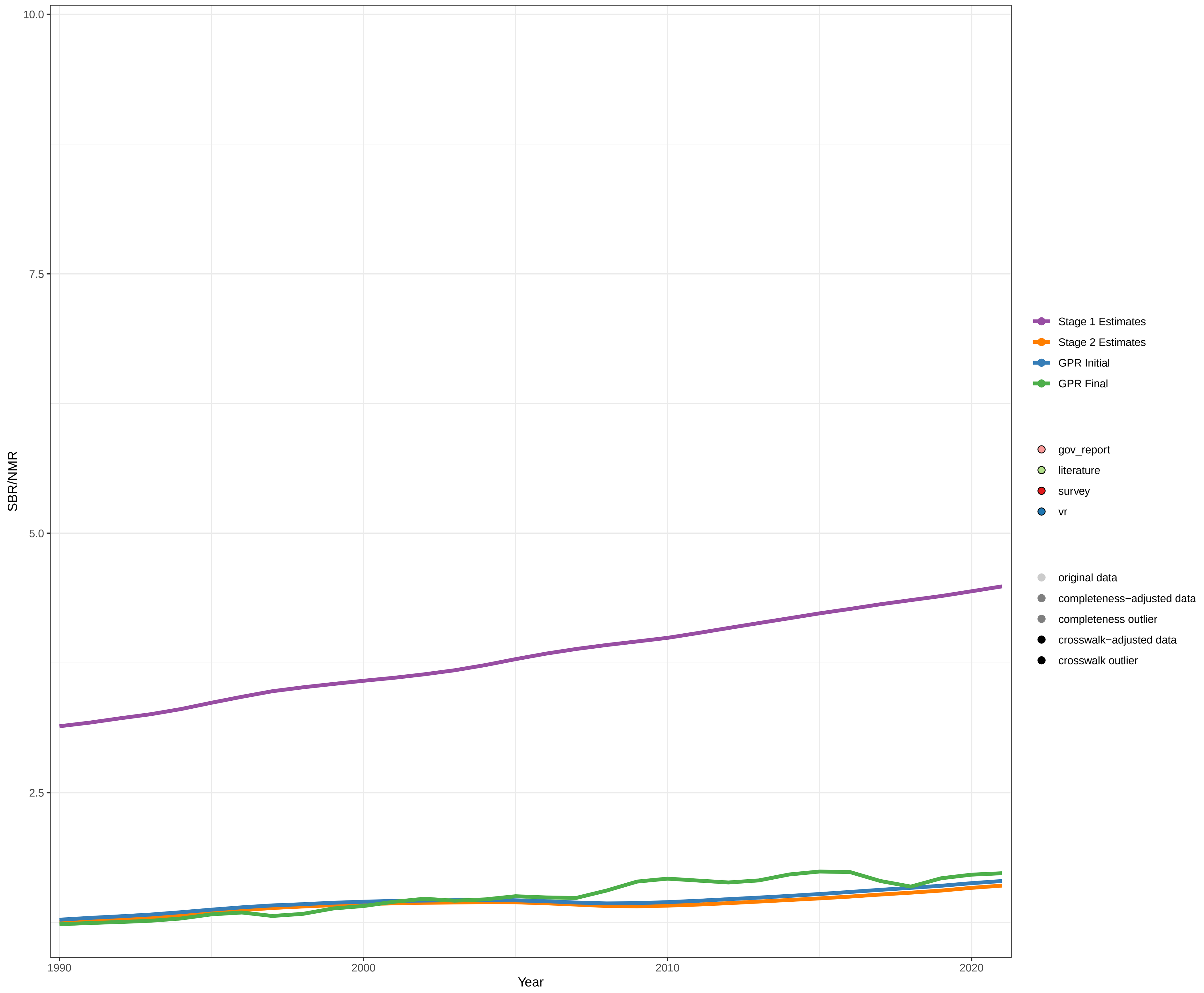

Definition	Years	Outlier	Source.Type	Title

Wakayama JPN_35453 High−income Asia Pacific Stillbirth Rate (SBR) − 20 weeks
lambda = 1 ; zeta = 0.6 ; scale = 15 ; amp = 0.028

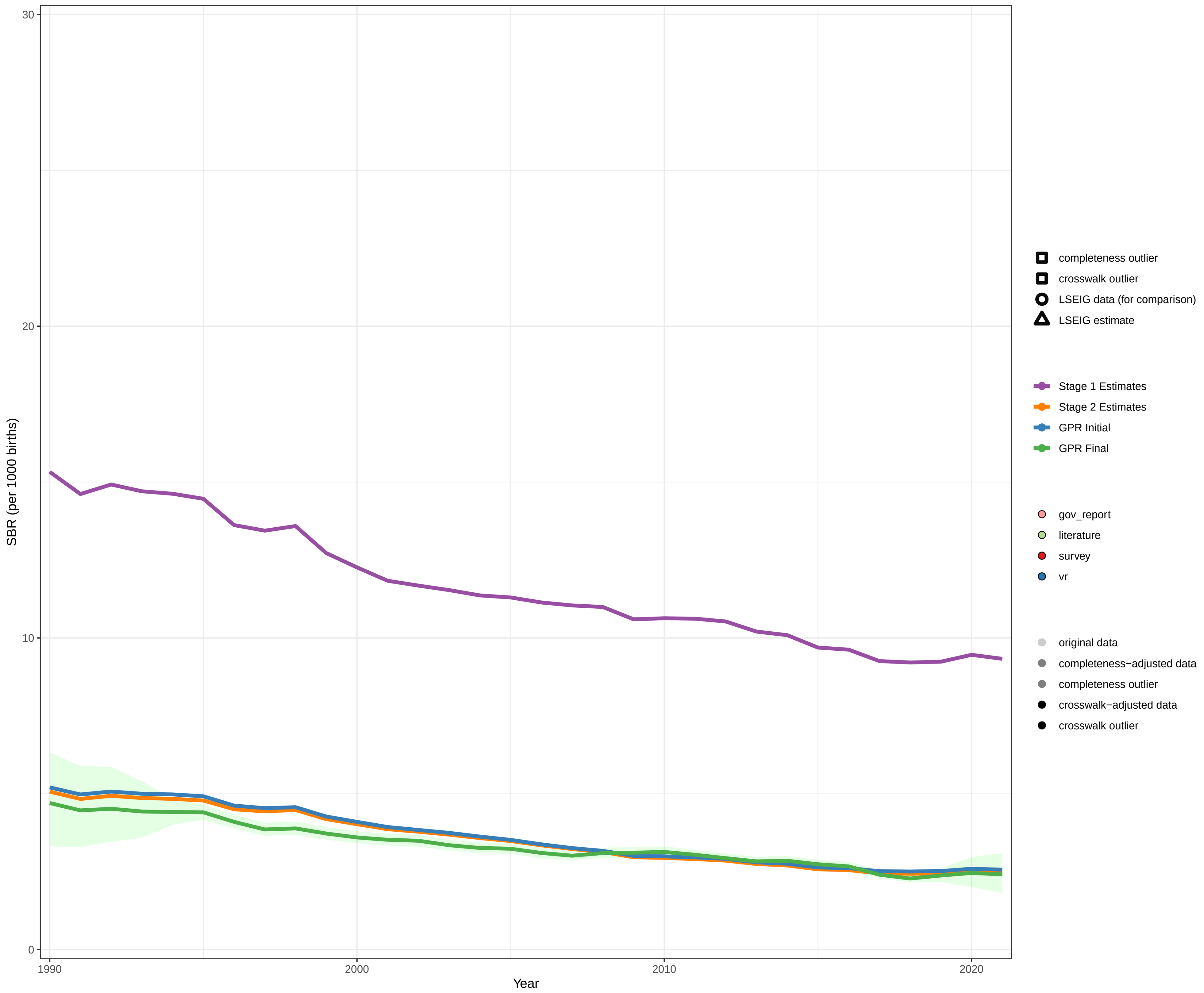

Wakayama JPN_35453 High−income Asia Pacific Stillbirth Rate (SBR) − 28 weeks
lambda = 1 ; zeta = 0.6 ; scale = 15 ; amp = 0.028

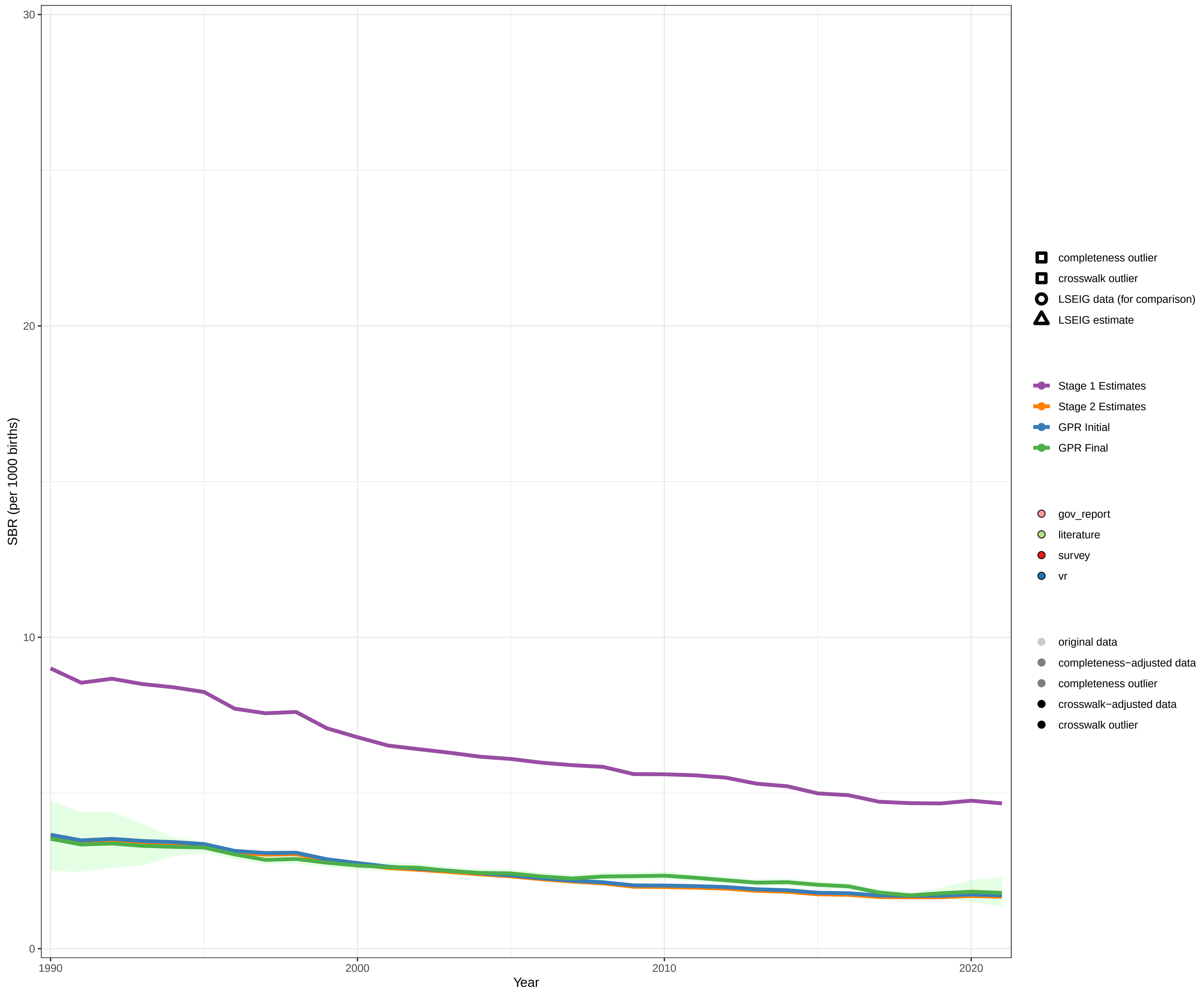

Year	Total Stillbirths (in thousands)	Total Stillbirths (in thousands)	SBR (per 1000 births)	SBR (per 1000 births)	SBR/NMR	SBR/NMR
	20 weeks	28 weeks	20 weeks	28 weeks	20 weeks	28 weeks
1990	0.0 (0.0 to 0.1)	0.0 (0.0 to 0.0)	4.7 (3.3 to 6.3)	3.5 (2.5 to 4.7)	1.64 (1.15 to 2.21)	1.23 (0.87 to 1.65)
1995	0.0 (0.0 to 0.0)	0.0 (0.0 to 0.0)	4.4 (4.2 to 4.6)	3.3 (3.1 to 3.4)	1.80 (1.70 to 1.90)	1.33 (1.26 to 1.40)
2000	0.0 (0.0 to 0.0)	0.0 (0.0 to 0.0)	3.6 (3.4 to 3.8)	2.7 (2.5 to 2.8)	1.90 (1.80 to 2.00)	1.41 (1.34 to 1.48)
2005	0.0 (0.0 to 0.0)	0.0 (0.0 to 0.0)	3.2 (3.1 to 3.4)	2.4 (2.3 to 2.5)	2.01 (1.91 to 2.12)	1.50 (1.43 to 1.58)
2010	0.0 (0.0 to 0.0)	0.0 (0.0 to 0.0)	3.1 (3.0 to 3.3)	2.3 (2.2 to 2.5)	2.23 (2.12 to 2.36)	1.67 (1.59 to 1.76)
2015	0.0 (0.0 to 0.0)	0.0 (0.0 to 0.0)	2.7 (2.6 to 2.9)	2.1 (1.9 to 2.2)	2.32 (2.20 to 2.45)	1.74 (1.65 to 1.83)
2020	0.0 (0.0 to 0.0)	0.0 (0.0 to 0.0)	2.5 (2.0 to 3.0)	1.8 (1.5 to 2.2)	2.30 (1.88 to 2.77)	1.71 (1.40 to 2.06)
2021	0.0 (0.0 to 0.0)	0.0 (0.0 to 0.0)	2.4 (1.8 to 3.1)	1.8 (1.4 to 2.3)	2.32 (1.75 to 2.98)	1.72 (1.30 to 2.20)

Yamagata JPN_35429 High−income Asia Pacific Stillbirth Rate / Neonatal Mortality Rate (SBR/NMR) − 20 weeks
lambda = 1 ; zeta = 0.6 ; scale = 15 ; amp = 0.028

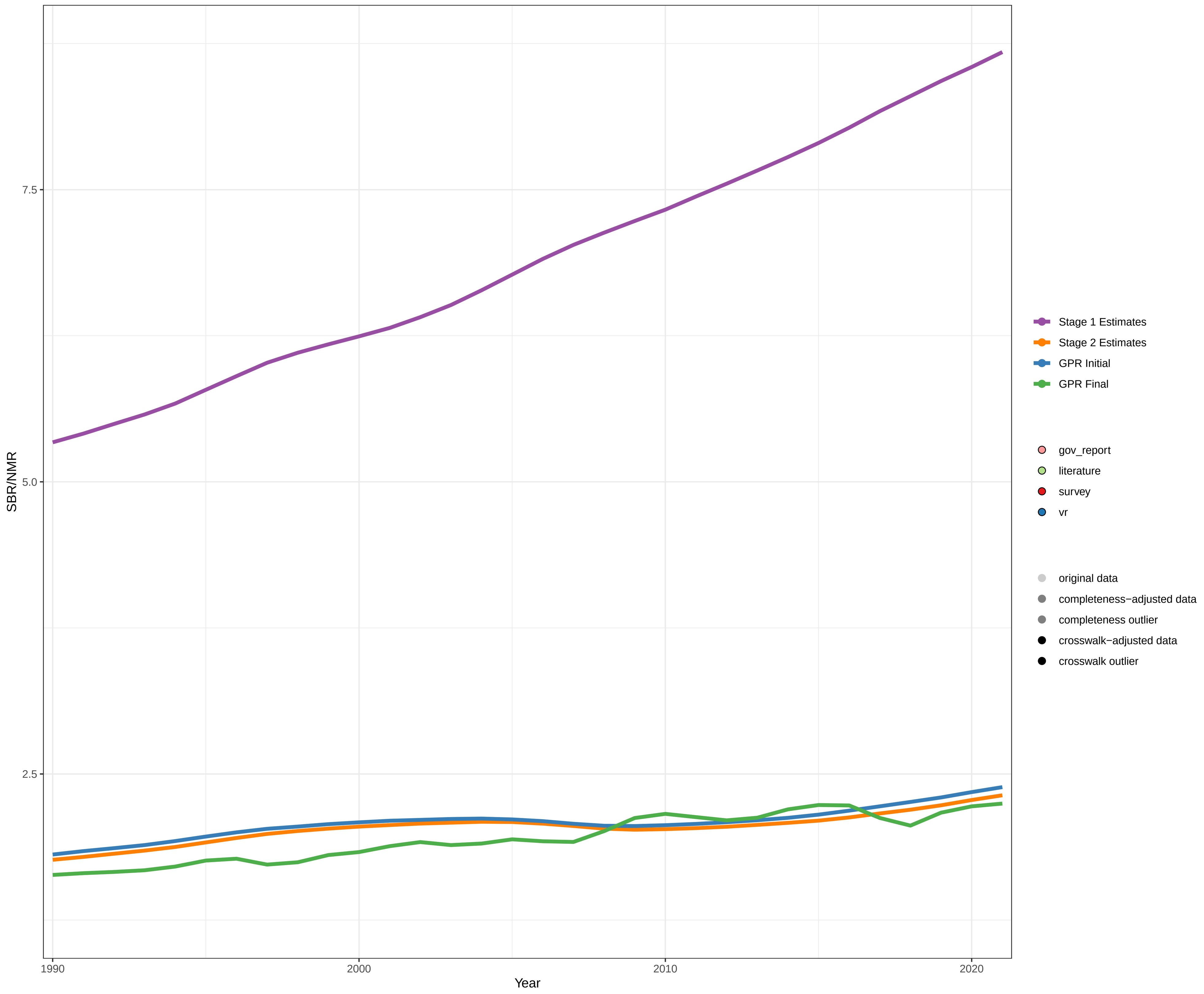

Yamagata JPN_35429 High−income Asia Pacific Stillbirth Rate / Neonatal Mortality Rate (SBR/NMR) − 28 weeks
lambda = 1 ; zeta = 0.6 ; scale = 15 ; amp = 0.028

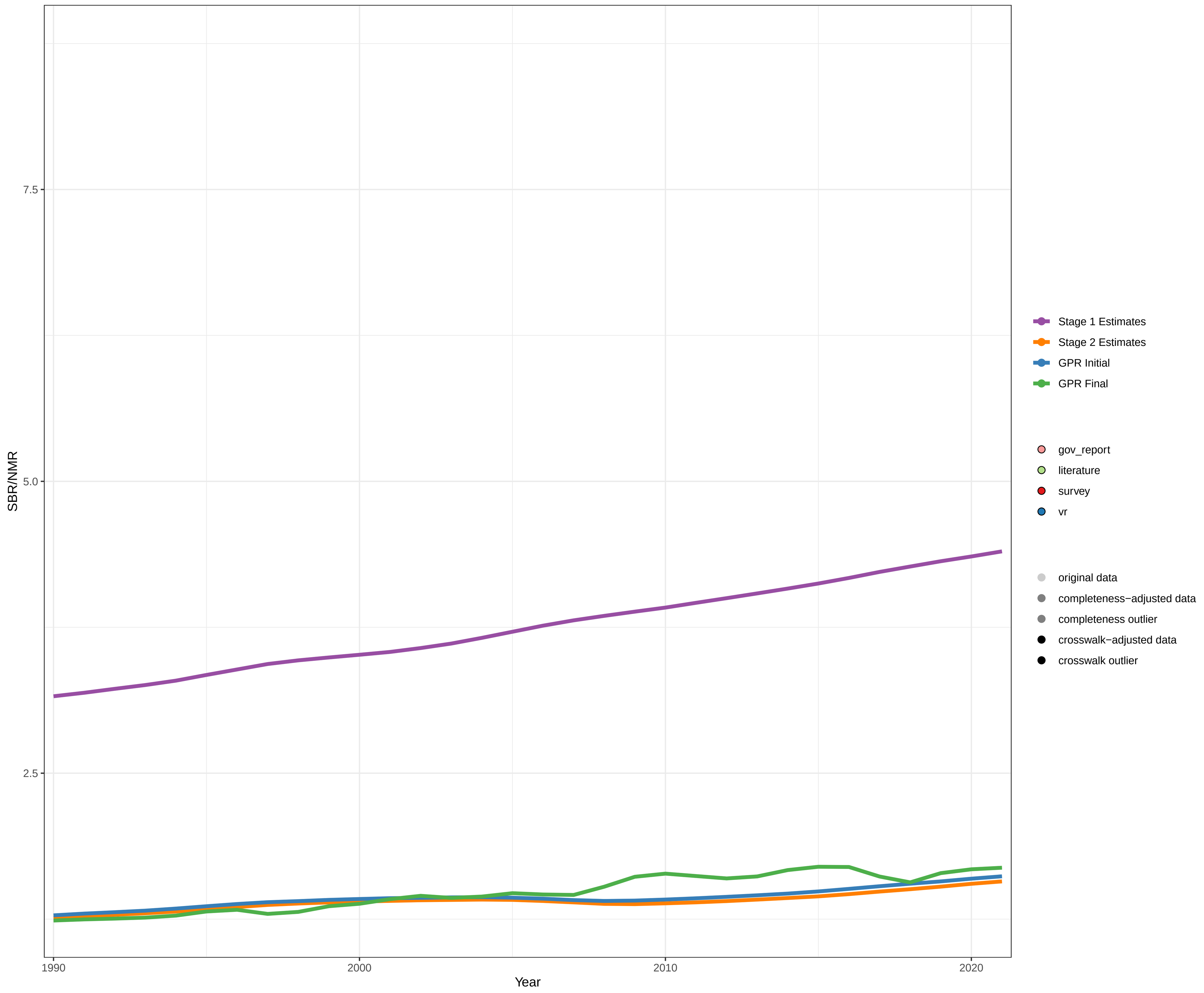

Definition	Years	Outlier	Source.Type	Title

Yamagata JPN_35429 High−income Asia Pacific Stillbirth Rate (SBR) − 20 weeks
lambda = 1 ; zeta = 0.6 ; scale = 15 ; amp = 0.028

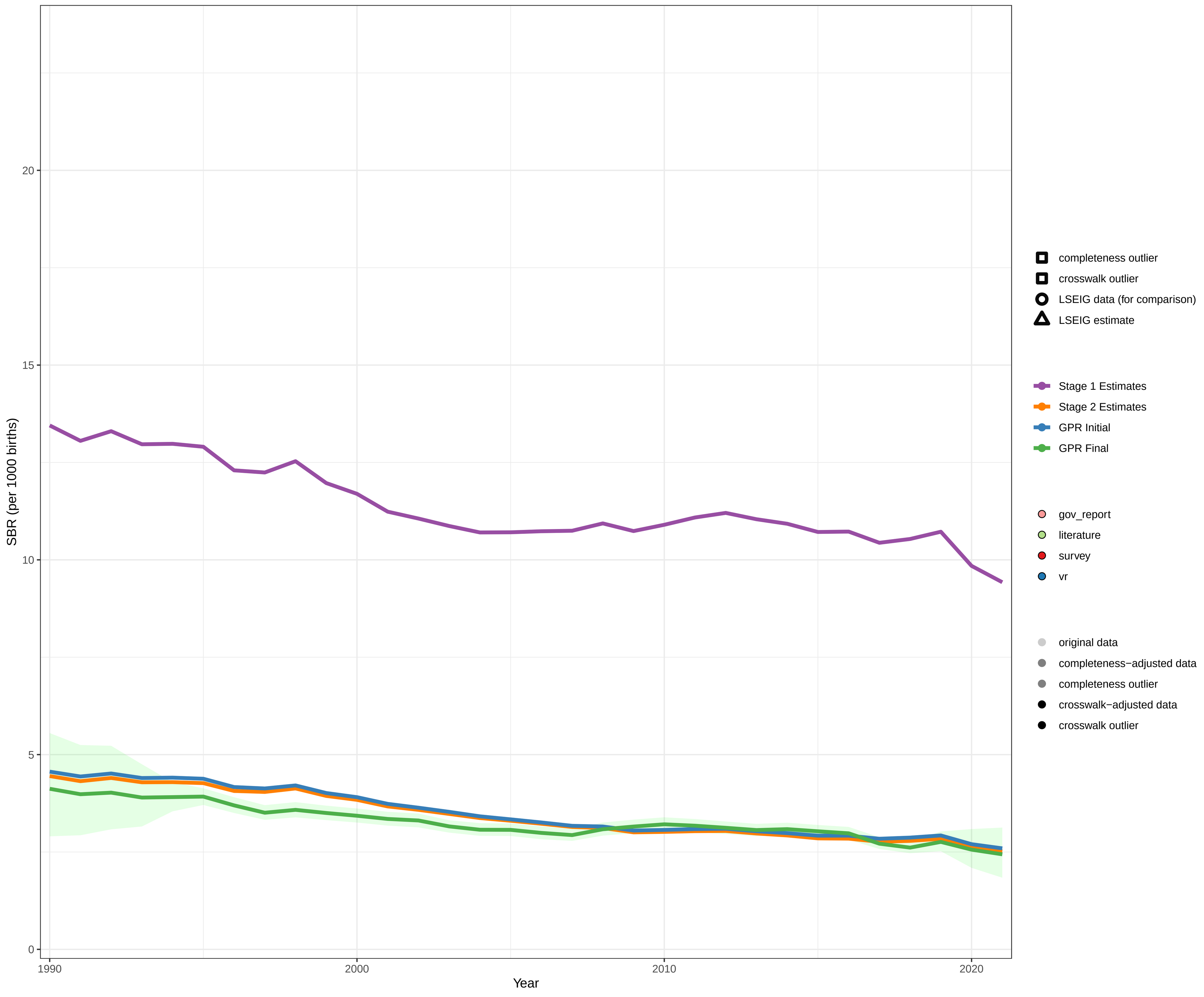

Yamagata JPN_35429 High−income Asia Pacific Stillbirth Rate (SBR) − 28 weeks
lambda = 1 ; zeta = 0.6 ; scale = 15 ; amp = 0.028

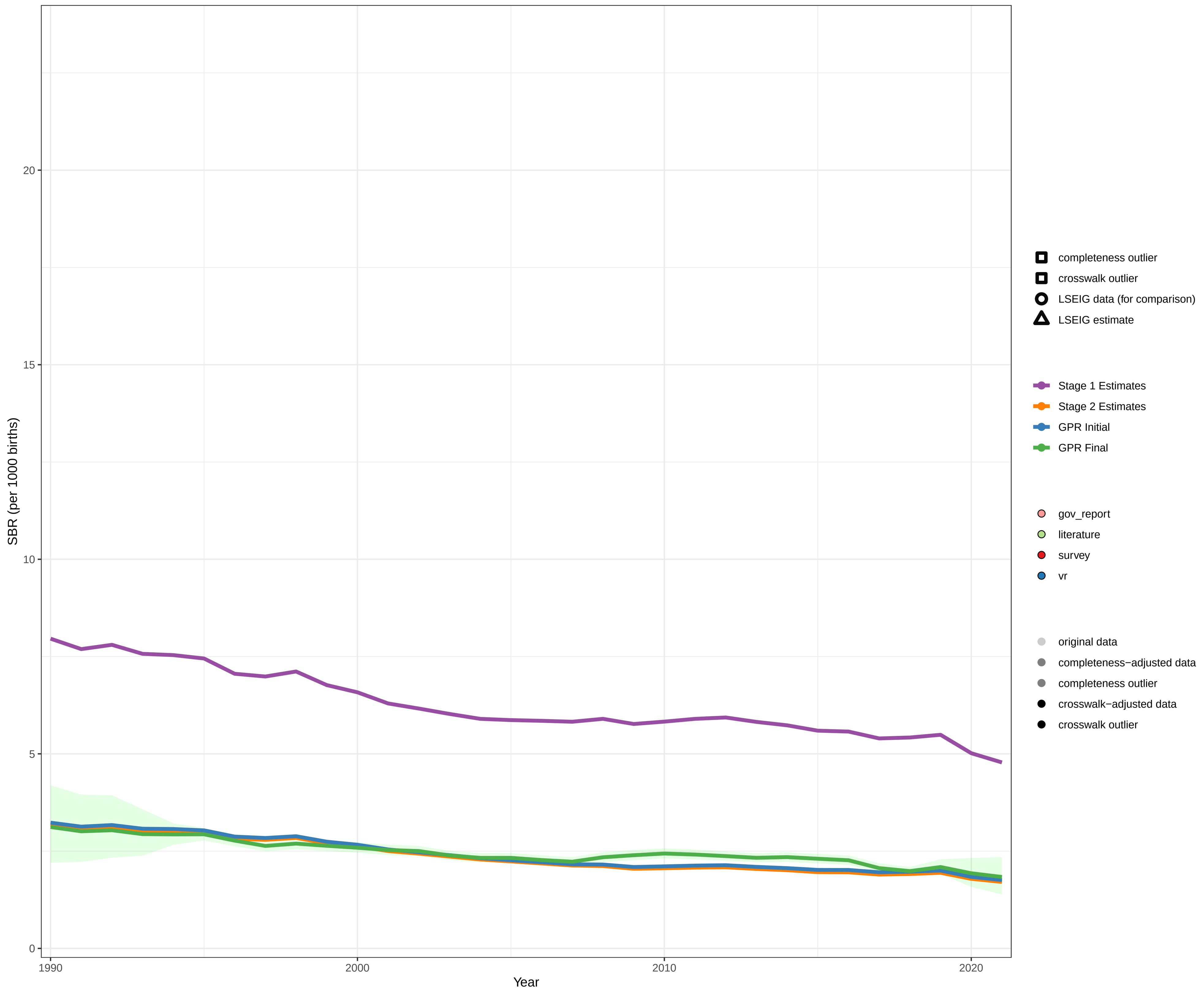

Year	Total Stillbirths (in thousands)	Total Stillbirths (in thousands)	SBR (per 1000 births)	SBR (per 1000 births)	SBR/NMR	SBR/NMR
	20 weeks	28 weeks	20 weeks	28 weeks	20 weeks	28 weeks
1990	0.1 (0.0 to 0.1)	0.0 (0.0 to 0.1)	4.1 (2.9 to 5.6)	3.1 (2.2 to 4.2)	1.64 (1.15 to 2.20)	1.24 (0.87 to 1.66)
1995	0.0 (0.0 to 0.0)	0.0 (0.0 to 0.0)	3.9 (3.7 to 4.1)	2.9 (2.8 to 3.1)	1.76 (1.66 to 1.85)	1.32 (1.24 to 1.39)
2000	0.0 (0.0 to 0.0)	0.0 (0.0 to 0.0)	3.4 (3.3 to 3.6)	2.6 (2.5 to 2.7)	1.83 (1.74 to 1.93)	1.38 (1.31 to 1.46)
2005	0.0 (0.0 to 0.0)	0.0 (0.0 to 0.0)	3.1 (2.9 to 3.2)	2.3 (2.2 to 2.5)	1.94 (1.84 to 2.04)	1.47 (1.40 to 1.55)
2010	0.0 (0.0 to 0.0)	0.0 (0.0 to 0.0)	3.2 (3.0 to 3.4)	2.4 (2.3 to 2.6)	2.16 (2.05 to 2.28)	1.64 (1.56 to 1.73)
2015	0.0 (0.0 to 0.0)	0.0 (0.0 to 0.0)	3.0 (2.9 to 3.2)	2.3 (2.2 to 2.4)	2.23 (2.12 to 2.35)	1.70 (1.61 to 1.79)
2020	0.0 (0.0 to 0.0)	0.0 (0.0 to 0.0)	2.6 (2.1 to 3.1)	1.9 (1.6 to 2.3)	2.22 (1.82 to 2.68)	1.68 (1.37 to 2.02)
2021	0.0 (0.0 to 0.0)	0.0 (0.0 to 0.0)	2.4 (1.8 to 3.1)	1.8 (1.4 to 2.3)	2.25 (1.69 to 2.88)	1.69 (1.28 to 2.16)

Yamaguchi   JPN_35458   High−income Asia Pacific   Stillbirth Rate / Neonatal Mortality Rate (SBR/NMR) − 20 weeks
lambda = 1 ; zeta = 0.6 ; scale = 15 ; amp = 0.028

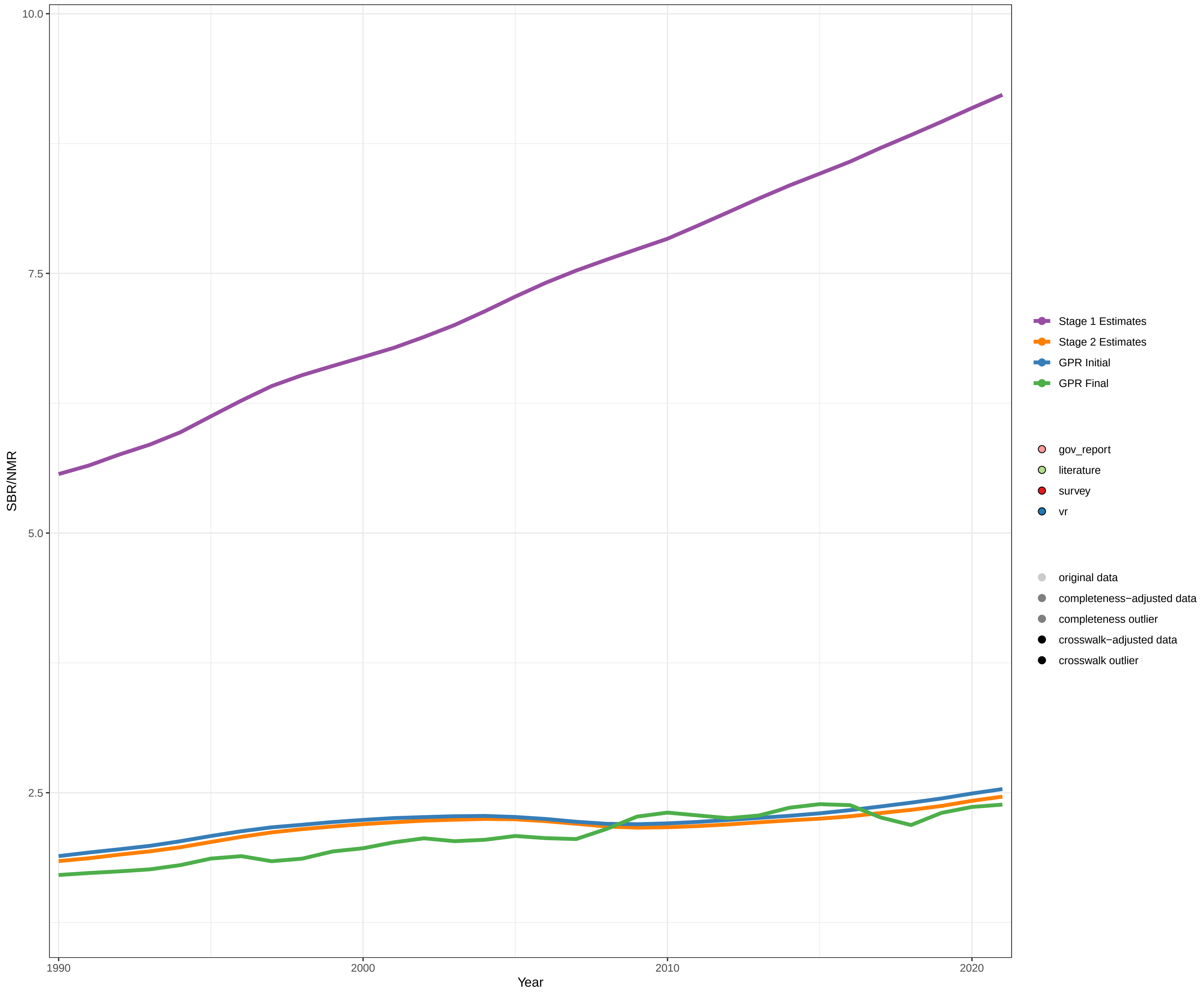

Yamaguchi   JPN_35458   High−income Asia Pacific   Stillbirth Rate / Neonatal Mortality Rate (SBR/NMR) − 28 weeks
lambda = 1 ; zeta = 0.6 ; scale = 15 ; amp = 0.028

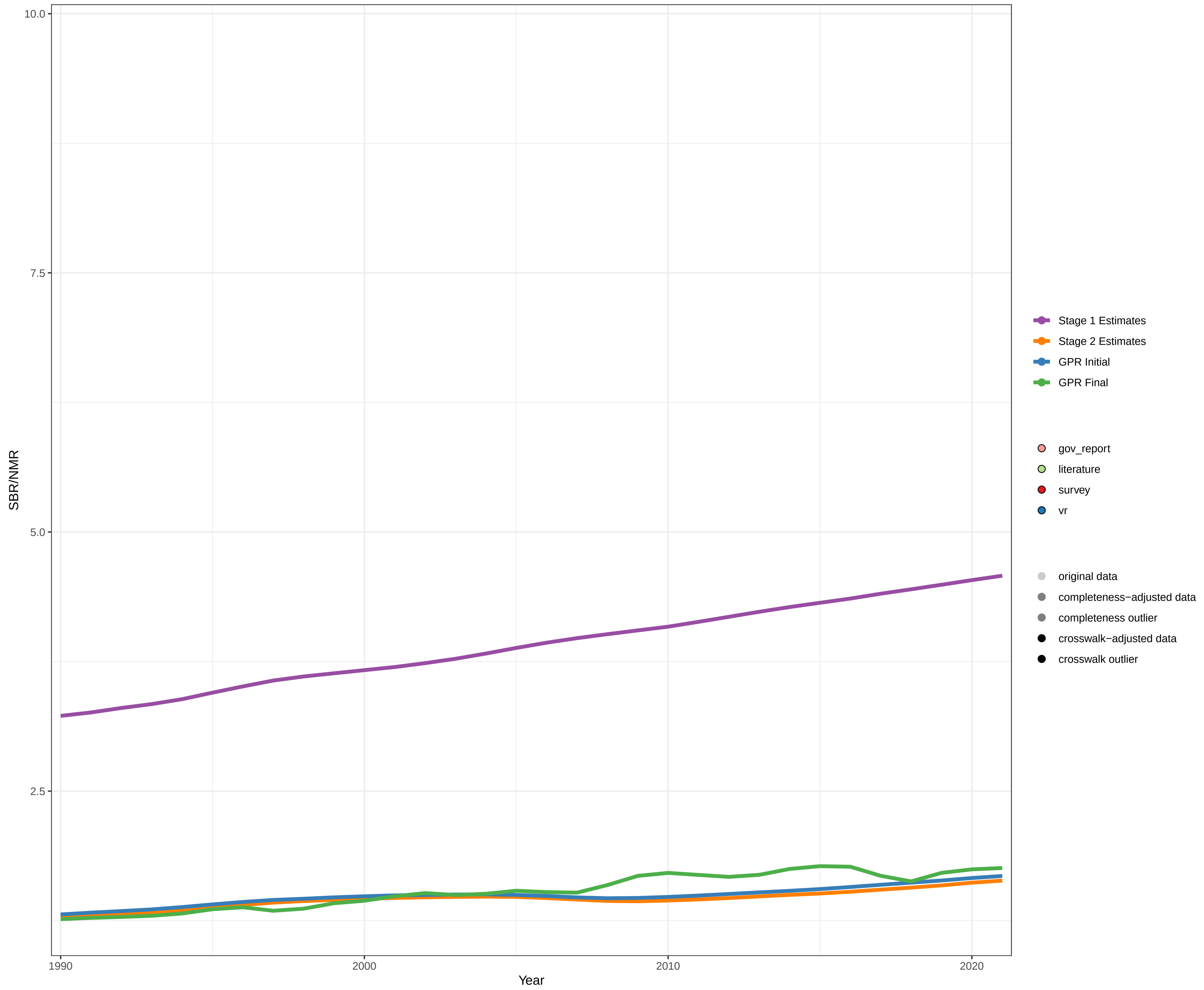

Definition	Years	Outlier	Source.Type	Title

Yamaguchi   JPN_35458   High−income Asia Pacific   Stillbirth Rate (SBR) − 20 weeks
lambda = 1 ; zeta = 0.6 ; scale = 15 ; amp = 0.028

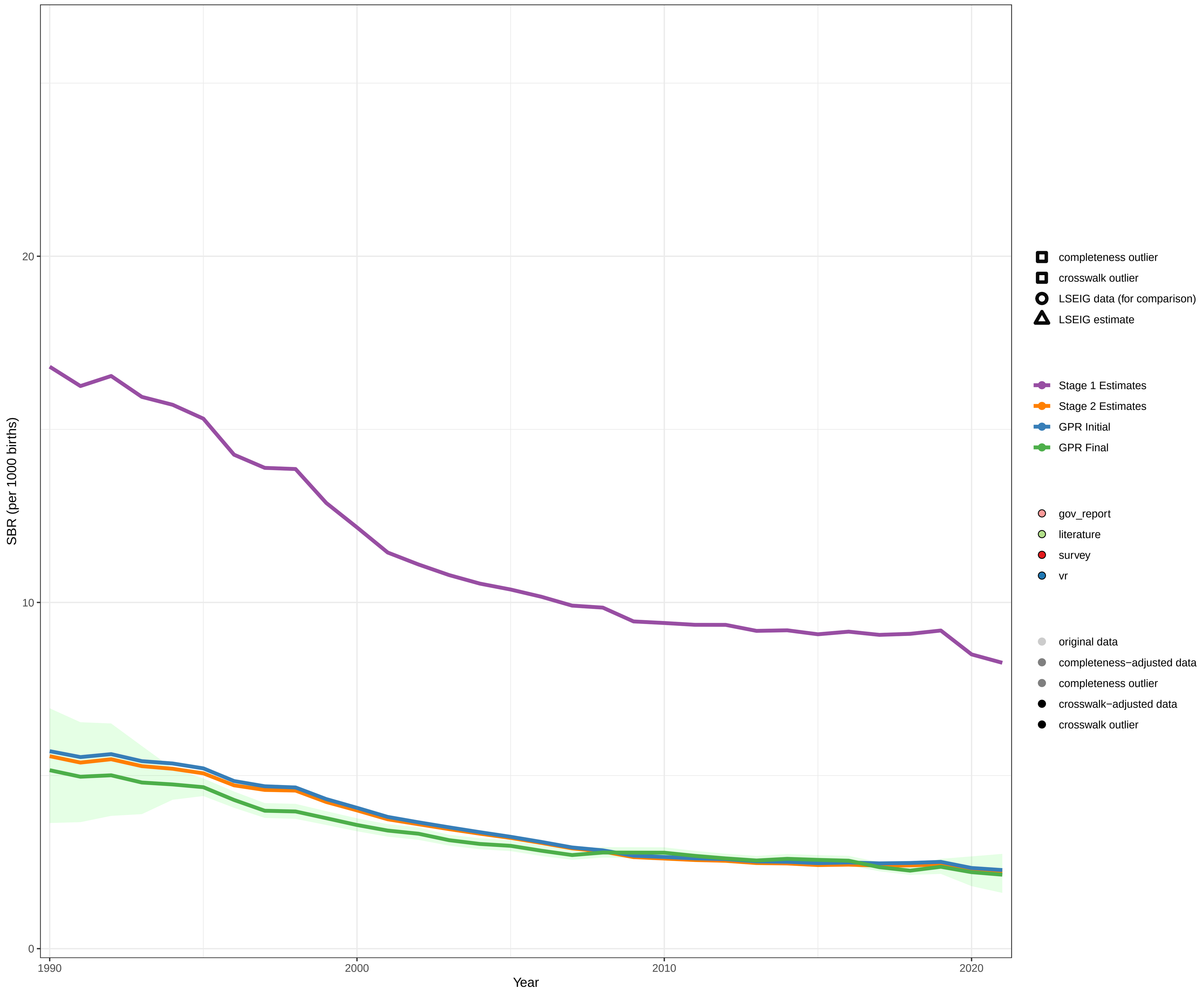

Yamaguchi   JPN_35458   High−income Asia Pacific   Stillbirth Rate (SBR) − 28 weeks
lambda = 1 ; zeta = 0.6 ; scale = 15 ; amp = 0.028

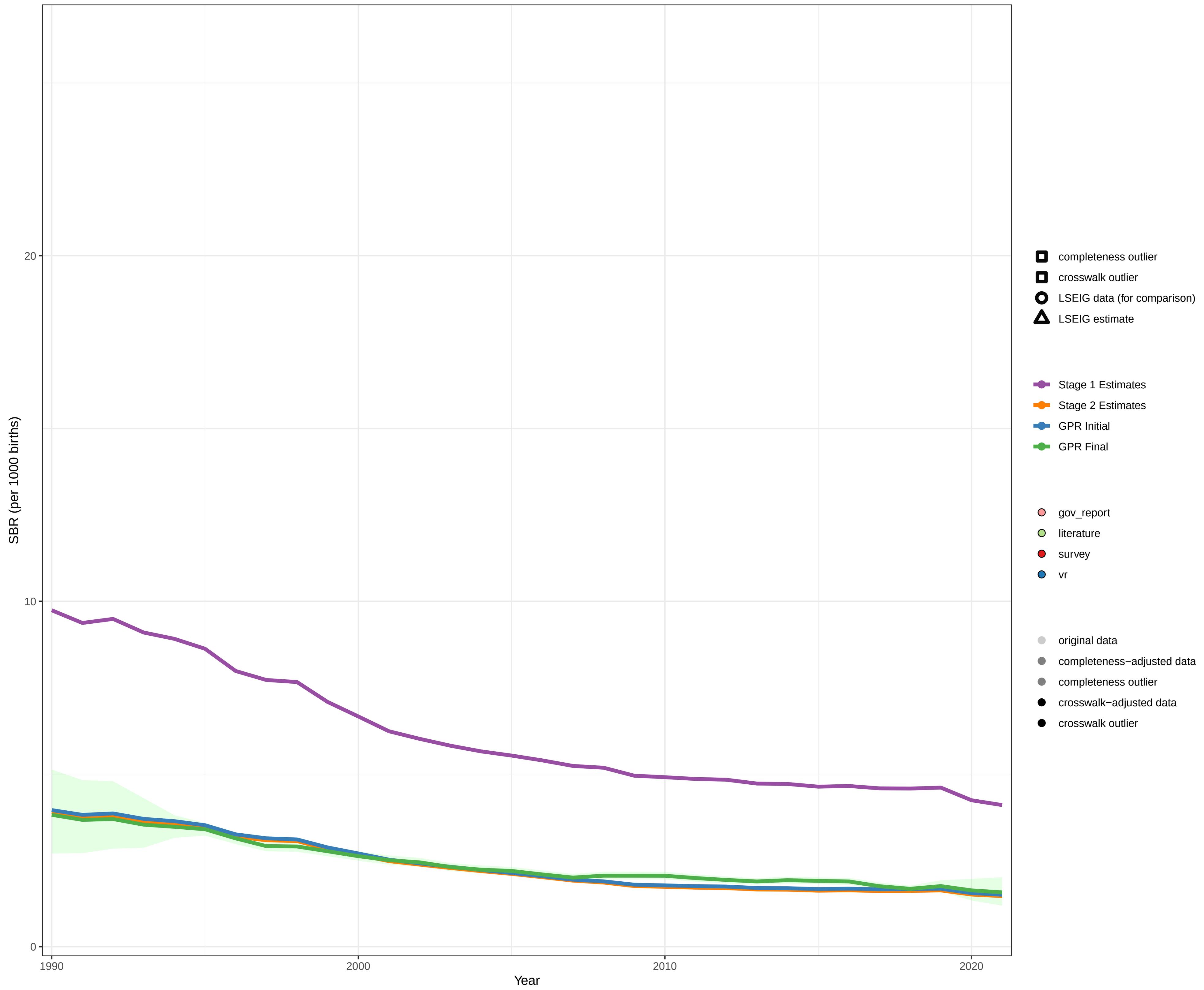

Year	Total Stillbirths (in thousands)	Total Stillbirths (in thousands)	SBR (per 1000 births)	SBR (per 1000 births)	SBR/NMR	SBR/NMR
	20 weeks	28 weeks	20 weeks	28 weeks	20 weeks	28 weeks
1990	0.1 (0.1 to 0.1)	0.1 (0.0 to 0.1)	5.2 (3.6 to 6.9)	3.8 (2.7 to 5.1)	1.71 (1.20 to 2.30)	1.26 (0.89 to 1.70)
1995	0.1 (0.1 to 0.1)	0.0 (0.0 to 0.0)	4.7 (4.4 to 4.9)	3.4 (3.2 to 3.6)	1.87 (1.76 to 1.97)	1.36 (1.29 to 1.43)
2000	0.0 (0.0 to 0.0)	0.0 (0.0 to 0.0)	3.6 (3.4 to 3.8)	2.6 (2.5 to 2.8)	1.97 (1.87 to 2.07)	1.44 (1.37 to 1.52)
2005	0.0 (0.0 to 0.0)	0.0 (0.0 to 0.0)	3.0 (2.8 to 3.1)	2.2 (2.1 to 2.3)	2.08 (1.98 to 2.19)	1.54 (1.46 to 1.62)
2010	0.0 (0.0 to 0.0)	0.0 (0.0 to 0.0)	2.8 (2.6 to 2.9)	2.1 (2.0 to 2.2)	2.31 (2.19 to 2.44)	1.71 (1.62 to 1.81)
2015	0.0 (0.0 to 0.0)	0.0 (0.0 to 0.0)	2.6 (2.4 to 2.7)	1.9 (1.8 to 2.0)	2.39 (2.26 to 2.52)	1.78 (1.68 to 1.87)
2020	0.0 (0.0 to 0.0)	0.0 (0.0 to 0.0)	2.2 (1.8 to 2.7)	1.6 (1.3 to 2.0)	2.36 (1.93 to 2.85)	1.75 (1.43 to 2.10)
2021	0.0 (0.0 to 0.0)	0.0 (0.0 to 0.0)	2.1 (1.6 to 2.7)	1.6 (1.2 to 2.0)	2.39 (1.80 to 3.06)	1.76 (1.33 to 2.25)

Yamanashi JPN_35442 High−income Asia Pacific Stillbirth Rate / Neonatal Mortality Rate (SBR/NMR) − 20 weeks
lambda = 1 ; zeta = 0.6 ; scale = 15 ; amp = 0.028

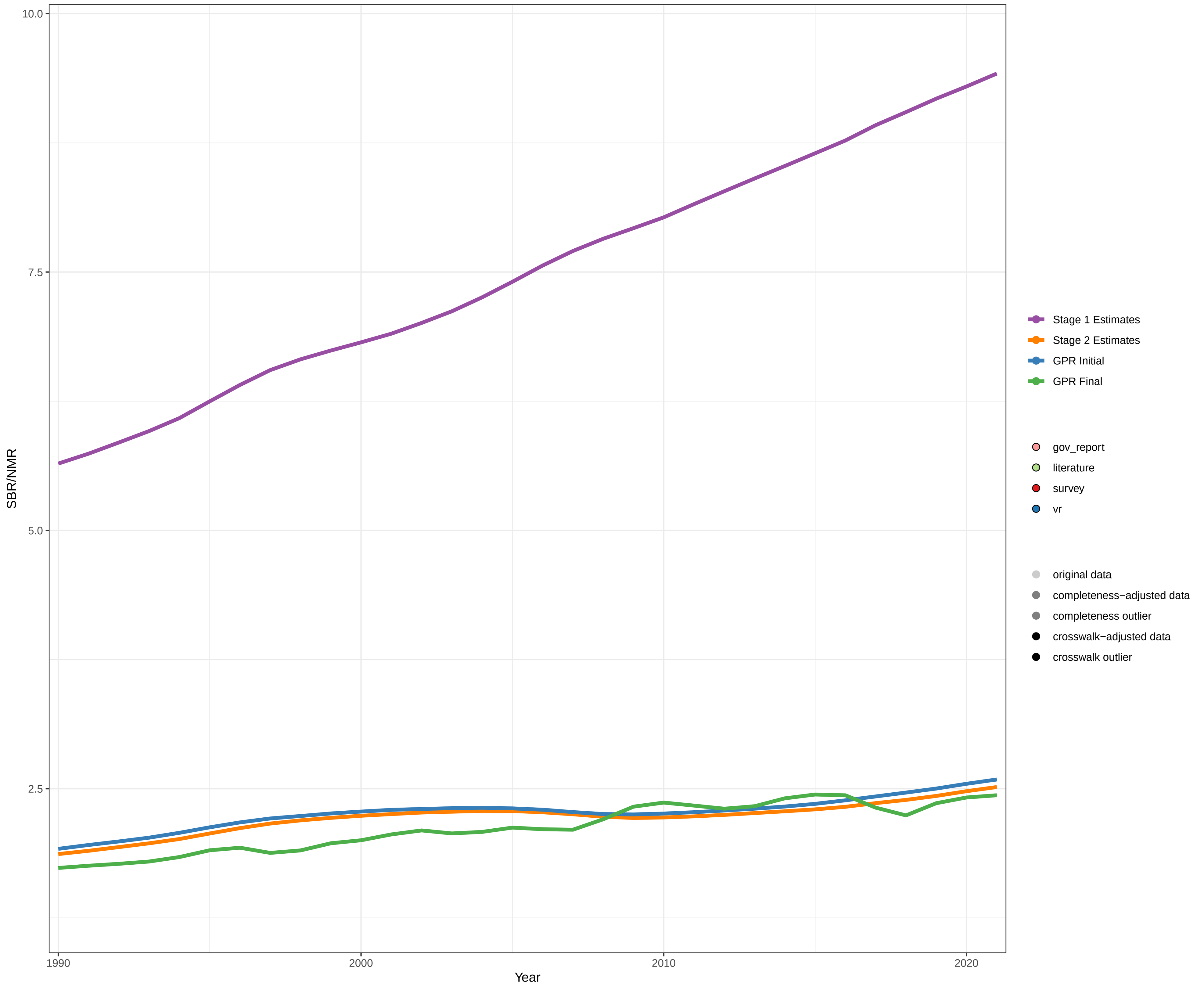

Yamanashi JPN_35442 High−income Asia Pacific Stillbirth Rate / Neonatal Mortality Rate (SBR/NMR) − 28 weeks
lambda = 1 ; zeta = 0.6 ; scale = 15 ; amp = 0.028

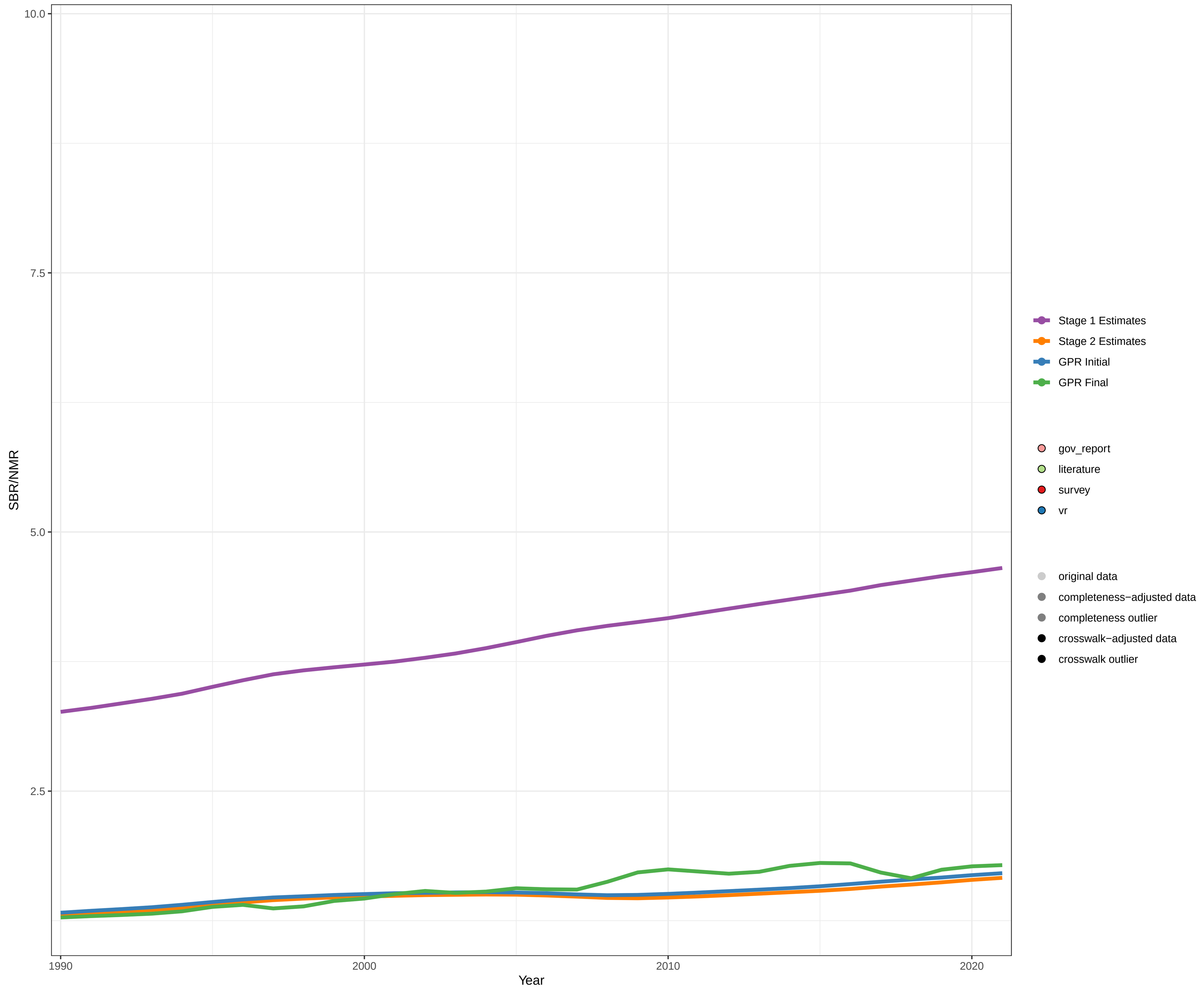

Definition	Years	Outlier	Source.Type	Title

Yamanashi JPN_35442 High−income Asia Pacific Stillbirth Rate (SBR) − 20 weeks
lambda = 1 ; zeta = 0.6 ; scale = 15 ; amp = 0.028

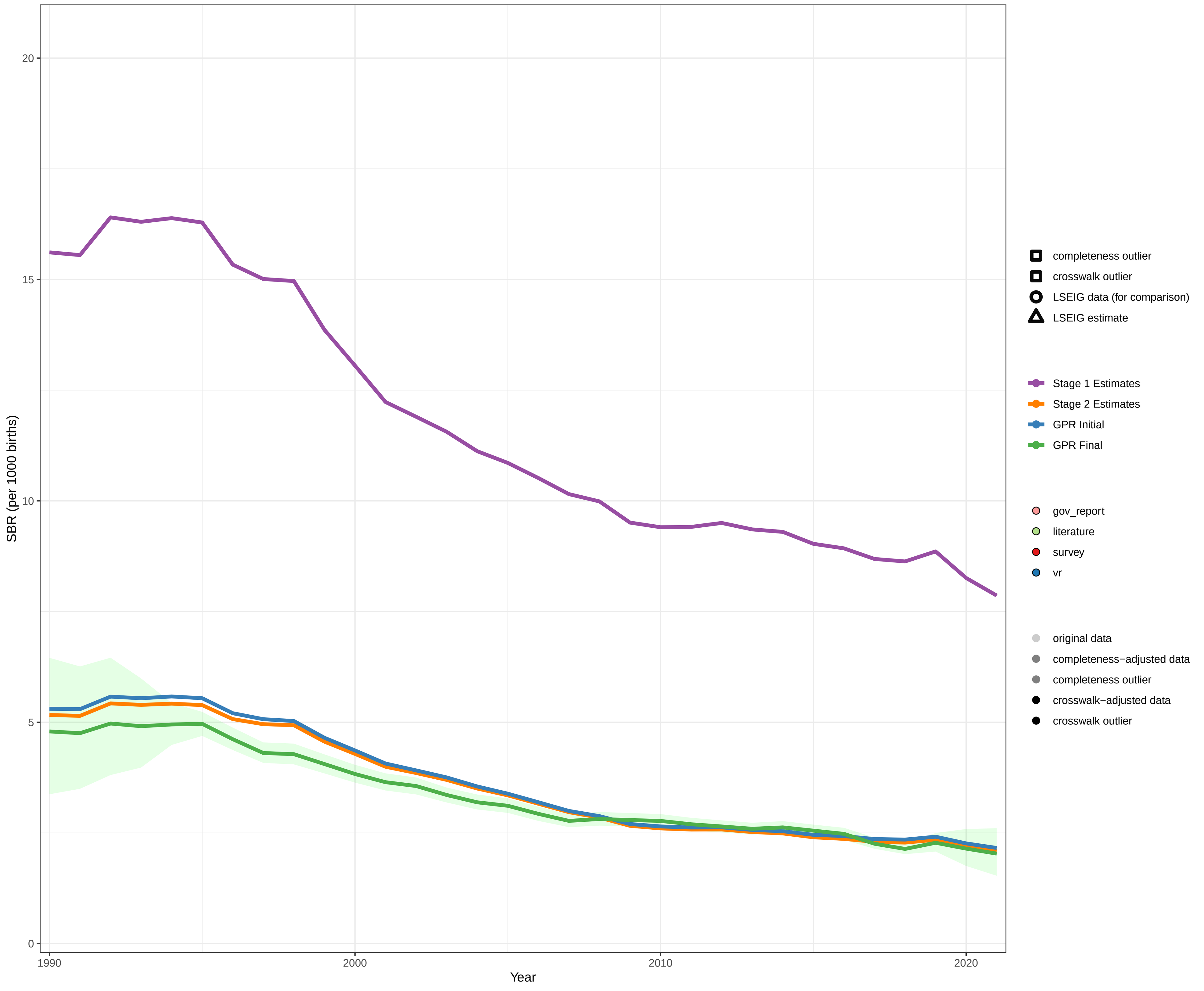

Yamanashi JPN_35442 High−income Asia Pacific Stillbirth Rate (SBR) − 28 weeks
lambda = 1 ; zeta = 0.6 ; scale = 15 ; amp = 0.028

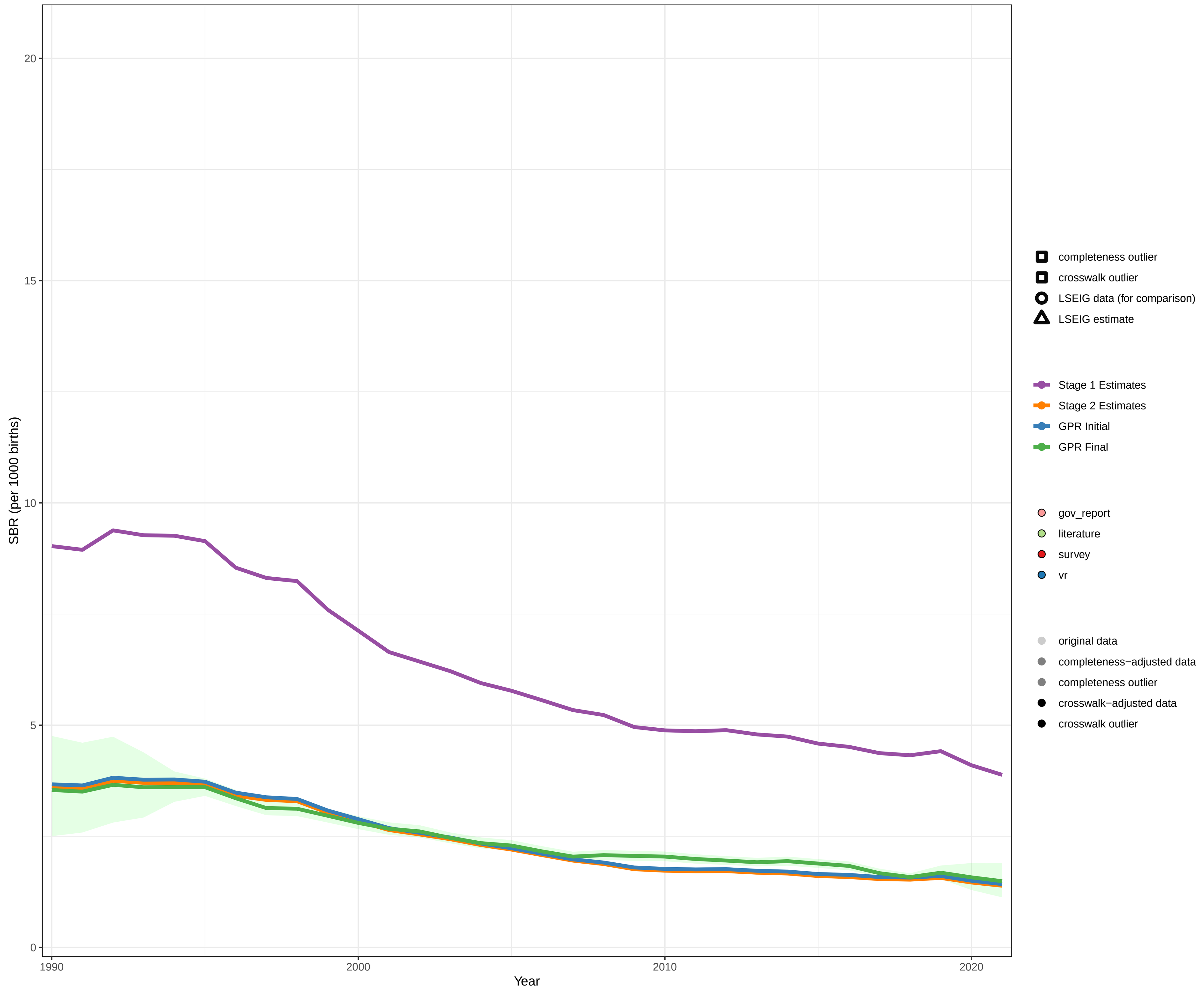

Year	Total Stillbirths (in thousands)	Total Stillbirths (in thousands)	SBR (per 1000 births)	SBR (per 1000 births)	SBR/NMR	SBR/NMR
	20 weeks	28 weeks	20 weeks	28 weeks	20 weeks	28 weeks
1990	0.0 (0.0 to 0.1)	0.0 (0.0 to 0.0)	4.8 (3.4 to 6.5)	3.5 (2.5 to 4.8)	1.73 (1.22 to 2.33)	1.28 (0.90 to 1.72)
1995	0.0 (0.0 to 0.0)	0.0 (0.0 to 0.0)	5.0 (4.7 to 5.2)	3.6 (3.4 to 3.8)	1.90 (1.80 to 2.01)	1.38 (1.31 to 1.46)
2000	0.0 (0.0 to 0.0)	0.0 (0.0 to 0.0)	3.8 (3.6 to 4.0)	2.8 (2.7 to 3.0)	2.00 (1.90 to 2.11)	1.46 (1.39 to 1.54)
2005	0.0 (0.0 to 0.0)	0.0 (0.0 to 0.0)	3.1 (3.0 to 3.3)	2.3 (2.2 to 2.4)	2.12 (2.01 to 2.24)	1.56 (1.48 to 1.65)
2010	0.0 (0.0 to 0.0)	0.0 (0.0 to 0.0)	2.8 (2.6 to 2.9)	2.0 (1.9 to 2.2)	2.37 (2.24 to 2.50)	1.74 (1.66 to 1.84)
2015	0.0 (0.0 to 0.0)	0.0 (0.0 to 0.0)	2.6 (2.4 to 2.7)	1.9 (1.8 to 2.0)	2.44 (2.32 to 2.58)	1.81 (1.71 to 1.90)
2020	0.0 (0.0 to 0.0)	0.0 (0.0 to 0.0)	2.1 (1.8 to 2.6)	1.6 (1.3 to 1.9)	2.42 (1.97 to 2.91)	1.77 (1.45 to 2.14)
2021	0.0 (0.0 to 0.0)	0.0 (0.0 to 0.0)	2.0 (1.5 to 2.6)	1.5 (1.1 to 1.9)	2.44 (1.84 to 3.12)	1.79 (1.35 to 2.28)

Republic of Korea KOR High−income Asia Pacific Stillbirth Rate / Neonatal Mortality Rate (SBR/NMR) − 20 weeks
lambda = 0.4 ; zeta = 0.9 ; scale = 10 ; amp = 0.028

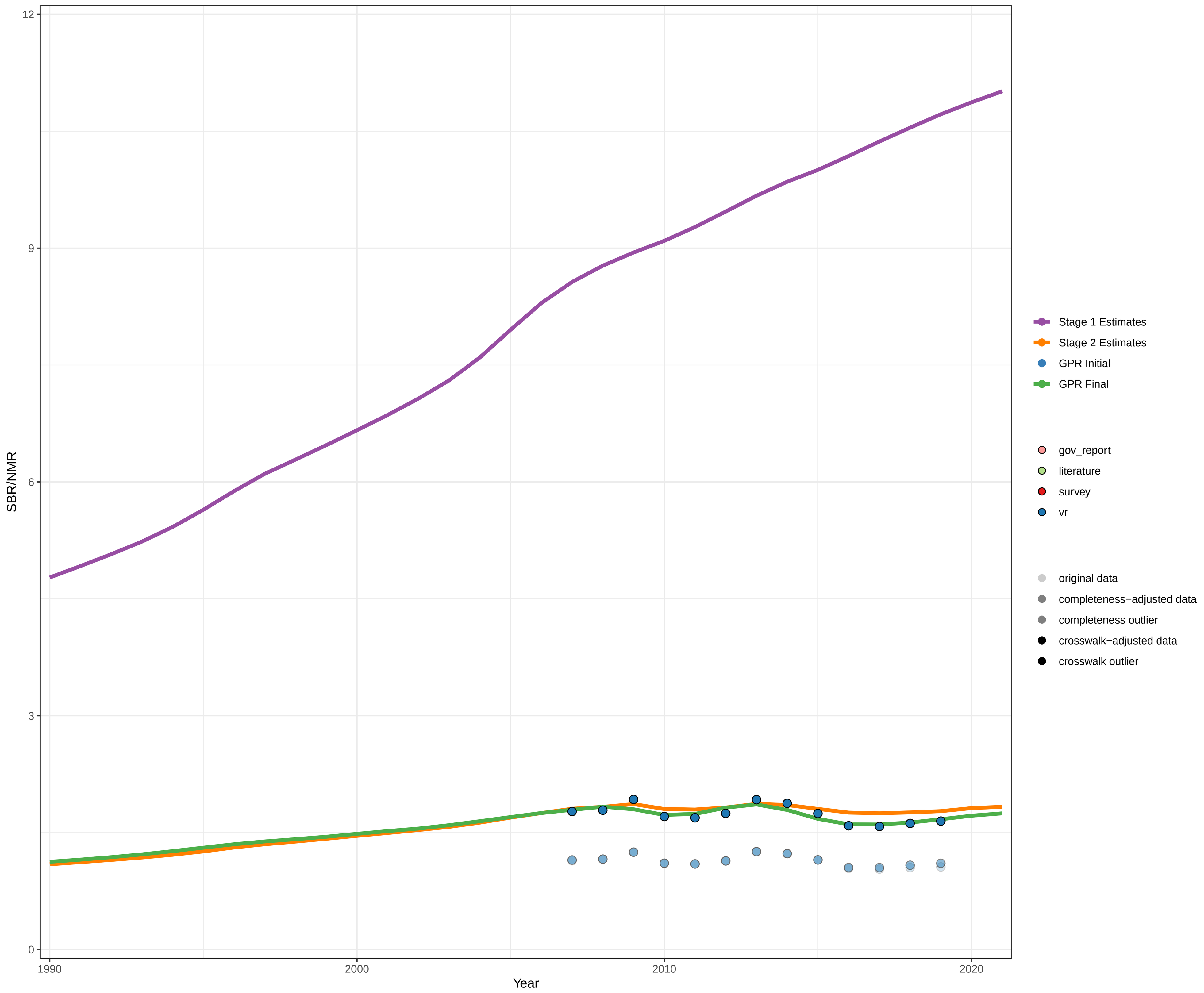

Republic of Korea KOR High−income Asia Pacific Stillbirth Rate / Neonatal Mortality Rate (SBR/NMR) − 28 weeks
lambda = 0.4 ; zeta = 0.9 ; scale = 10 ; amp = 0.028

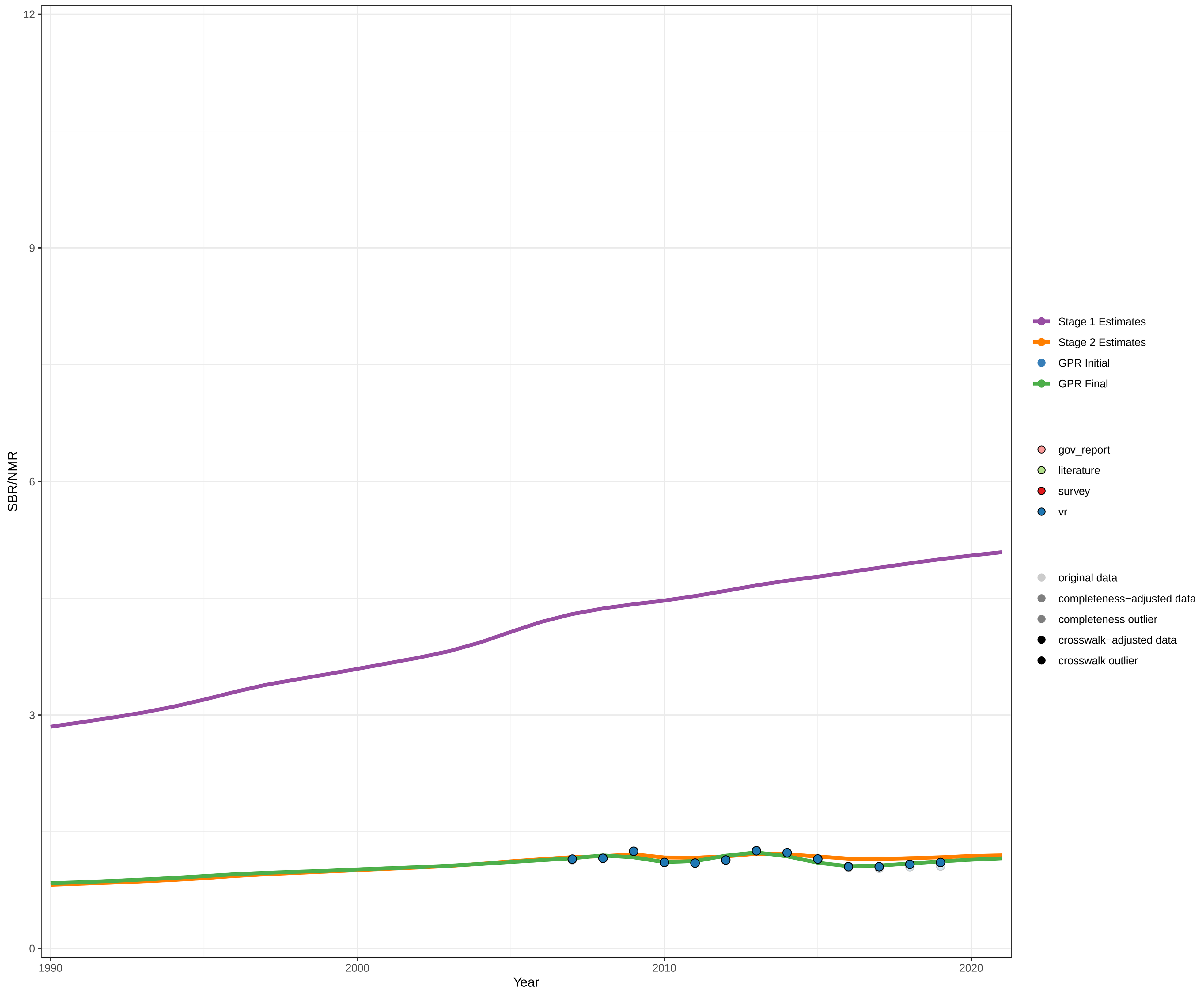

Definition	Years	Outlier	Source.Type	Title
28_weeks	2007−2019	0	VR	United Nations Demographic Yearbook

Republic of Korea KOR High−income Asia Pacific Stillbirth Rate (SBR) − 20 weeks
lambda = 0.4 ; zeta = 0.9 ; scale = 10 ; amp = 0.028

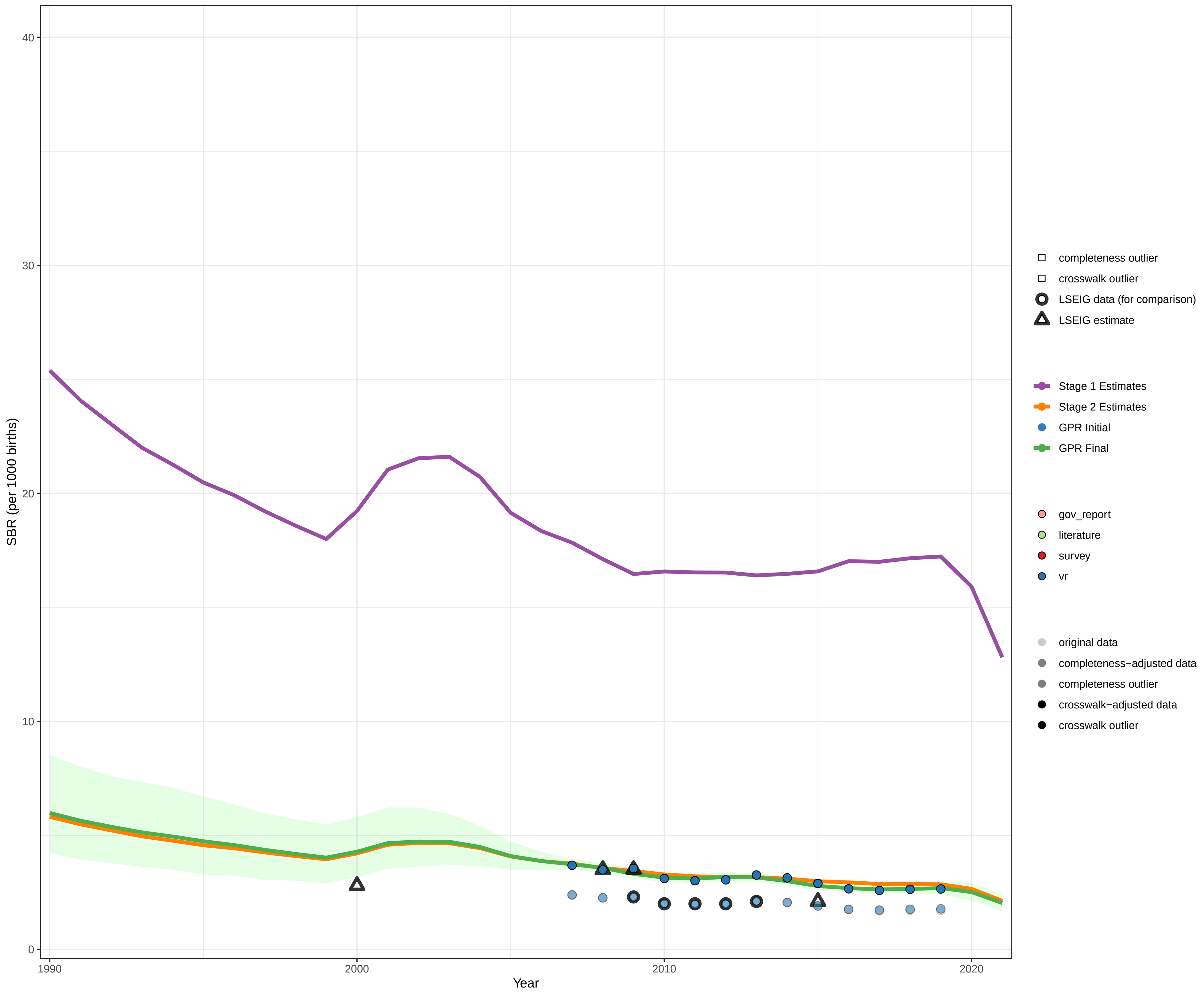

Republic of Korea KOR High−income Asia Pacific Stillbirth Rate (SBR) − 28 weeks
lambda = 0.4 ; zeta = 0.9 ; scale = 10 ; amp = 0.028

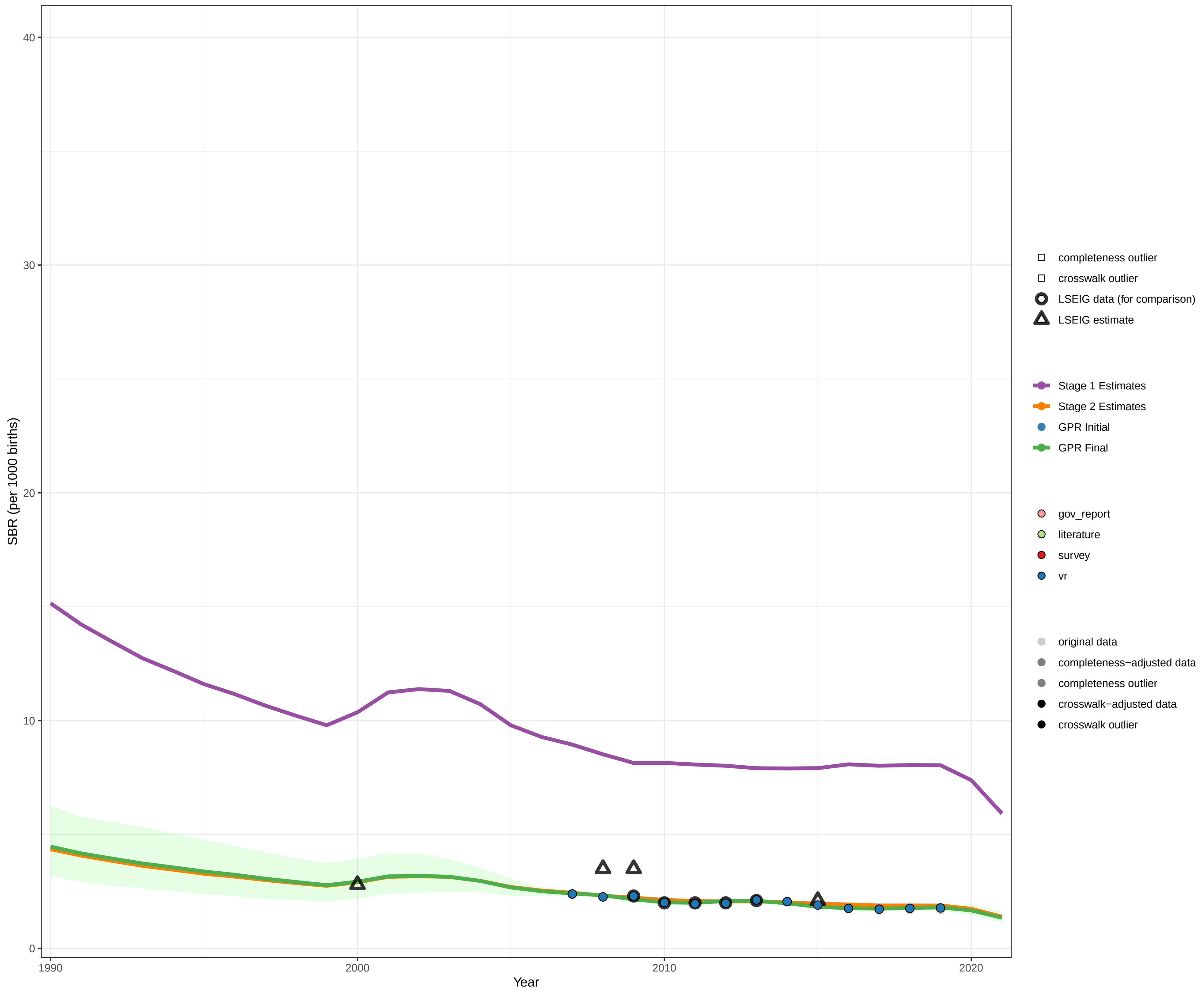

Year	Total Stillbirths (in thousands)	Total Stillbirths (in thousands)	SBR (per 1000 births)	SBR (per 1000 births)	SBR/NMR	SBR/NMR
	20 weeks	28 weeks	20 weeks	28 weeks	20 weeks	28 weeks
1990	4.1 (2.9 to 5.8)	3.1 (2.2 to 4.3)	6.0 (4.2 to 8.5)	4.5 (3.2 to 6.3)	1.12 (0.79 to 1.60)	0.84 (0.60 to 1.18)
1995	3.3 (2.3 to 4.7)	2.4 (1.7 to 3.4)	4.7 (3.3 to 6.7)	3.4 (2.4 to 4.8)	1.31 (0.90 to 1.85)	0.93 (0.66 to 1.32)
2000	2.5 (1.8 to 3.4)	1.7 (1.3 to 2.3)	4.3 (3.2 to 5.8)	2.9 (2.2 to 3.9)	1.48 (1.10 to 2.01)	1.02 (0.76 to 1.36)
2005	1.9 (1.6 to 2.2)	1.2 (1.1 to 1.4)	4.1 (3.5 to 4.7)	2.7 (2.3 to 3.1)	1.70 (1.45 to 1.96)	1.11 (0.96 to 1.27)
2010	1.5 (1.4 to 1.6)	0.9 (0.9 to 1.0)	3.1 (2.9 to 3.4)	2.0 (1.9 to 2.1)	1.73 (1.62 to 1.84)	1.11 (1.06 to 1.16)
2015	1.1 (1.1 to 1.2)	0.8 (0.7 to 0.8)	2.8 (2.6 to 3.0)	1.8 (1.7 to 1.9)	1.68 (1.57 to 1.79)	1.10 (1.05 to 1.15)
2020	0.7 (0.6 to 0.8)	0.5 (0.4 to 0.5)	2.5 (2.1 to 2.9)	1.7 (1.5 to 1.9)	1.72 (1.46 to 1.99)	1.14 (1.00 to 1.31)
2021	0.5 (0.5 to 0.7)	0.4 (0.3 to 0.4)	2.0 (1.7 to 2.4)	1.3 (1.1 to 1.6)	1.75 (1.44 to 2.10)	1.16 (0.97 to 1.38)

Singapore SGP High−income Asia Pacific Stillbirth Rate / Neonatal Mortality Rate (SBR/NMR) − 20 weeks
lambda = 0.2 ; zeta = 0.99 ; scale = 5 ; amp = 0.028

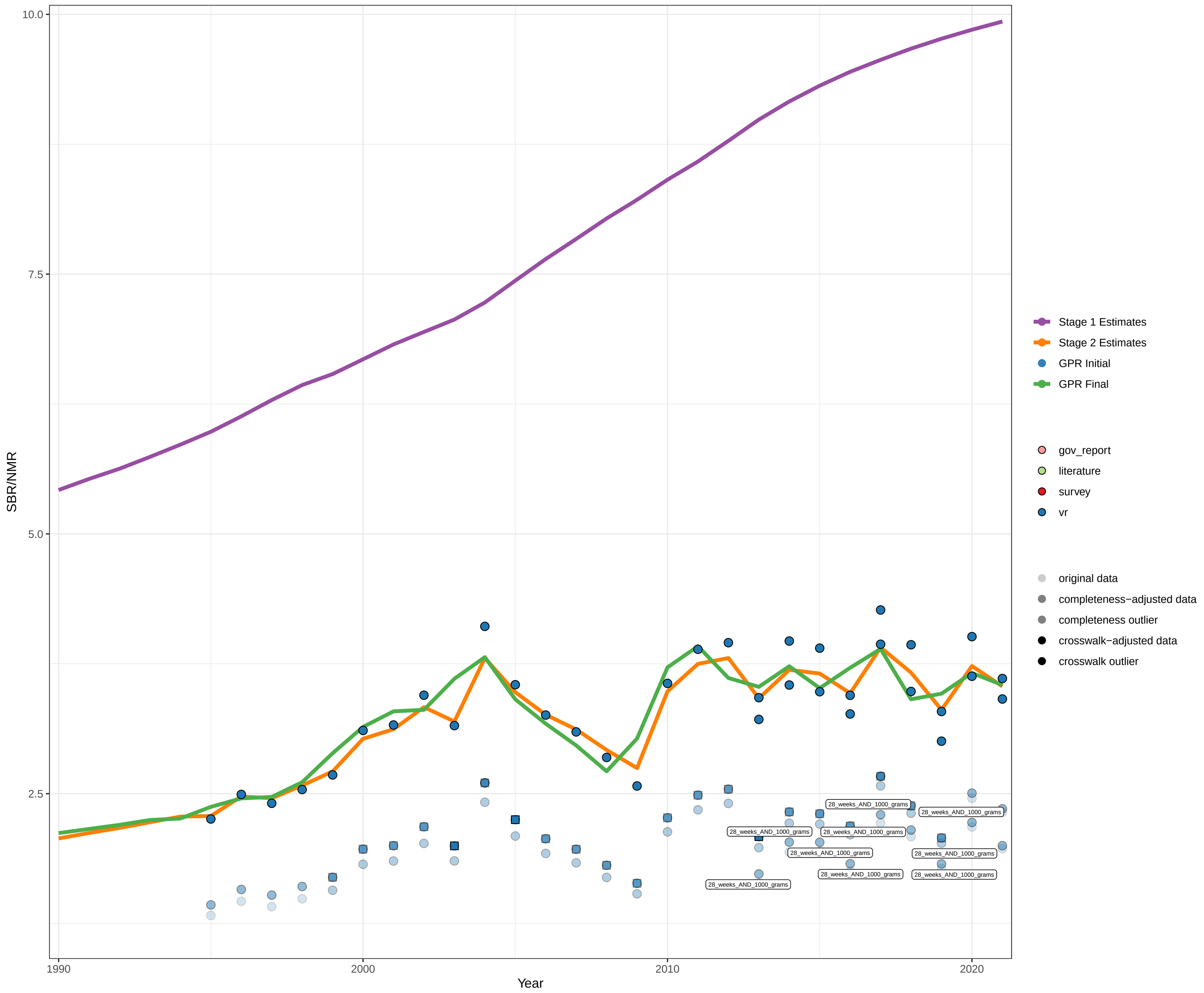

Singapore SGP High−income Asia Pacific Stillbirth Rate / Neonatal Mortality Rate (SBR/NMR) − 28 weeks
lambda = 0.2 ; zeta = 0.99 ; scale = 5 ; amp = 0.028

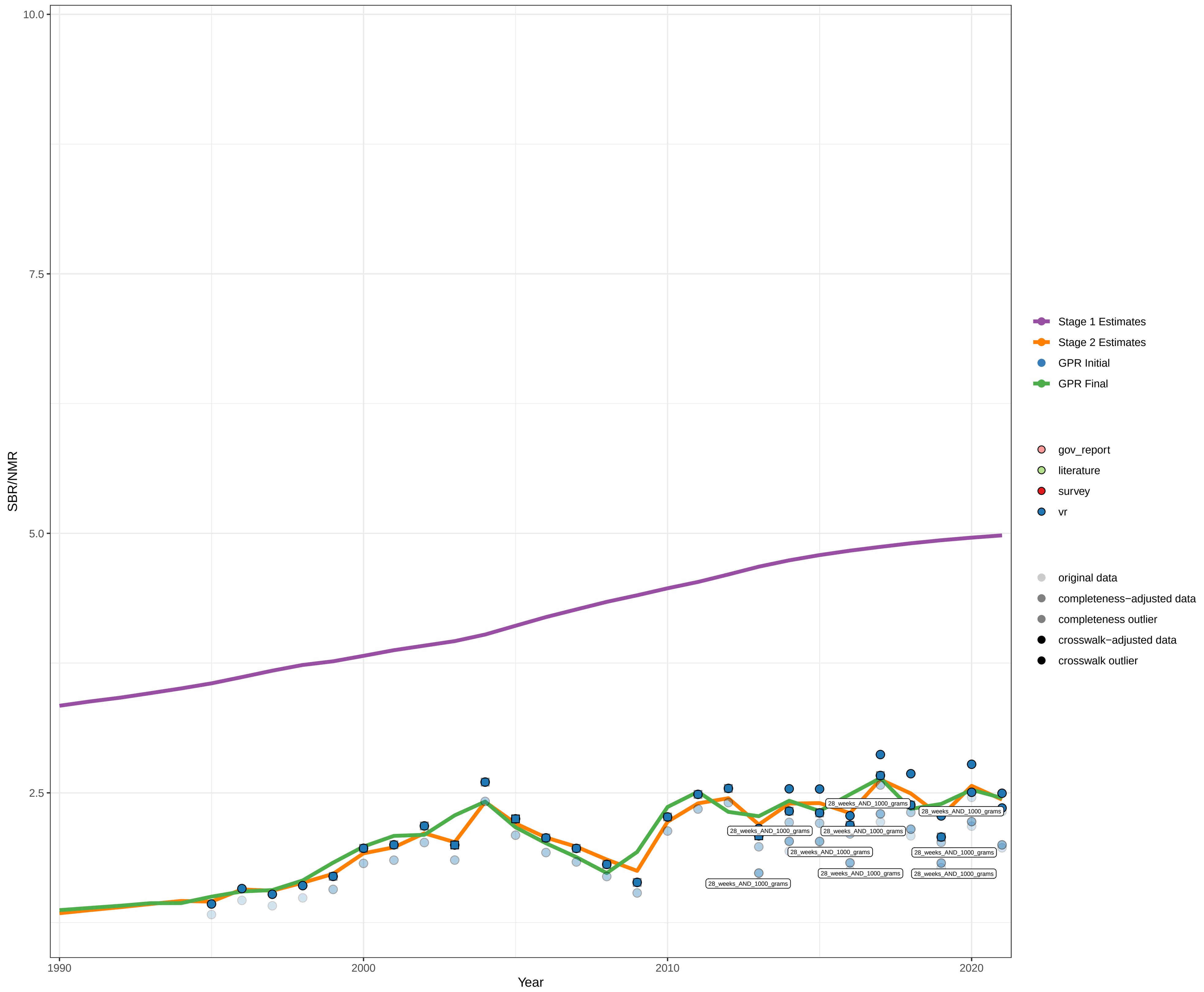

Definition	Years	Outlier	Source.Type	Title
28_weeks	1995−1998, 2003	0	VR	United Nations Demographic Yearbook
28_weeks	1999−2021	0	VR	Singapore Report on Registration of Births and Deaths
28_weeks_AND_1000_grams	2013−2021	0	VR	Singapore Report on Registration of Births and Deaths
28_weeks	1999−2002, 2004, 2006−2012, 2014−2019	1	VR	United Nations Demographic Yearbook
28_weeks	2005, 2013	2	VR	United Nations Demographic Yearbook

Singapore SGP High−income Asia Pacific Stillbirth Rate (SBR) − 20 weeks
lambda = 0.2 ; zeta = 0.99 ; scale = 5 ; amp = 0.028

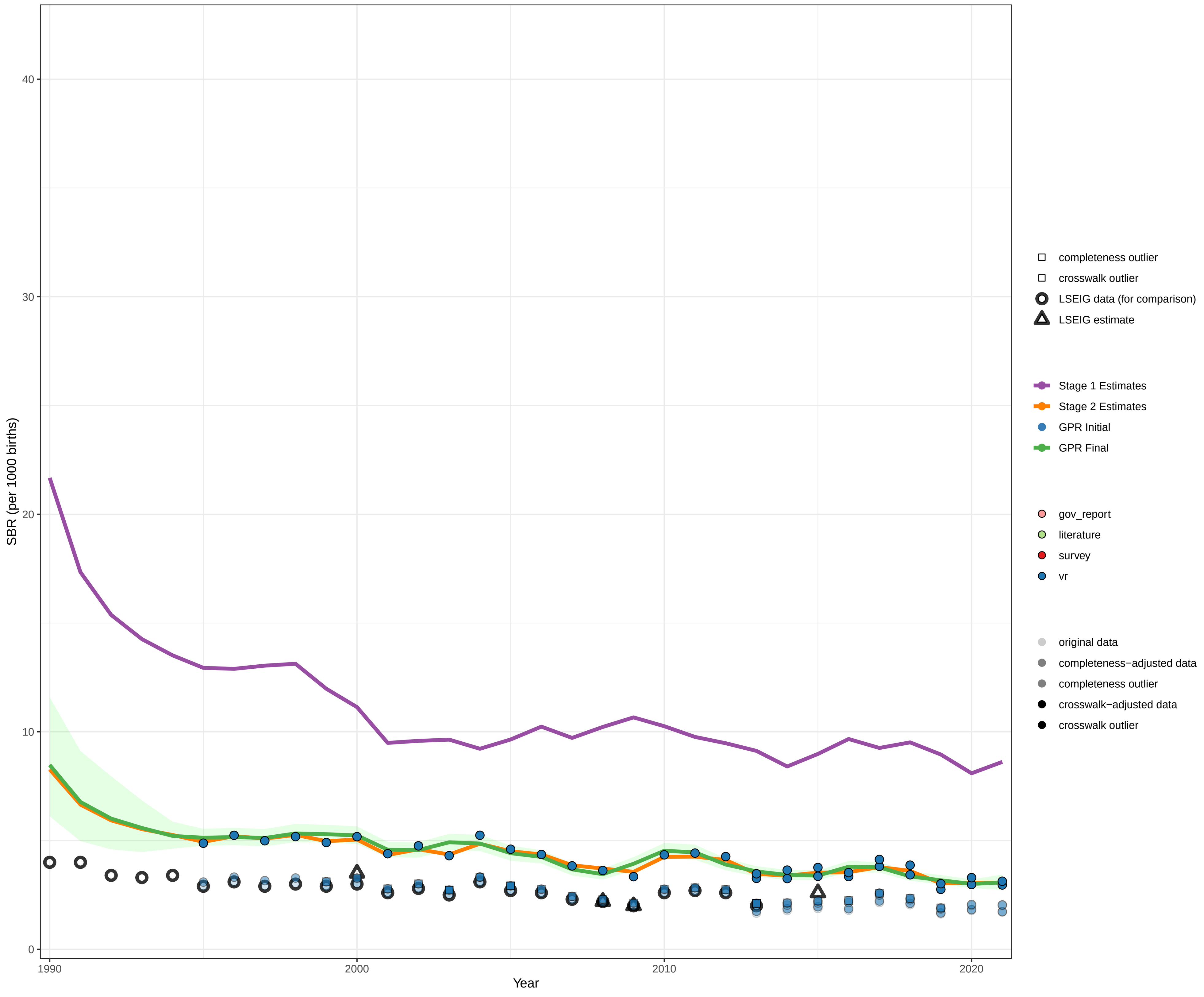

Singapore SGP High−income Asia Pacific Stillbirth Rate (SBR) − 28 weeks
lambda = 0.2 ; zeta = 0.99 ; scale = 5 ; amp = 0.028

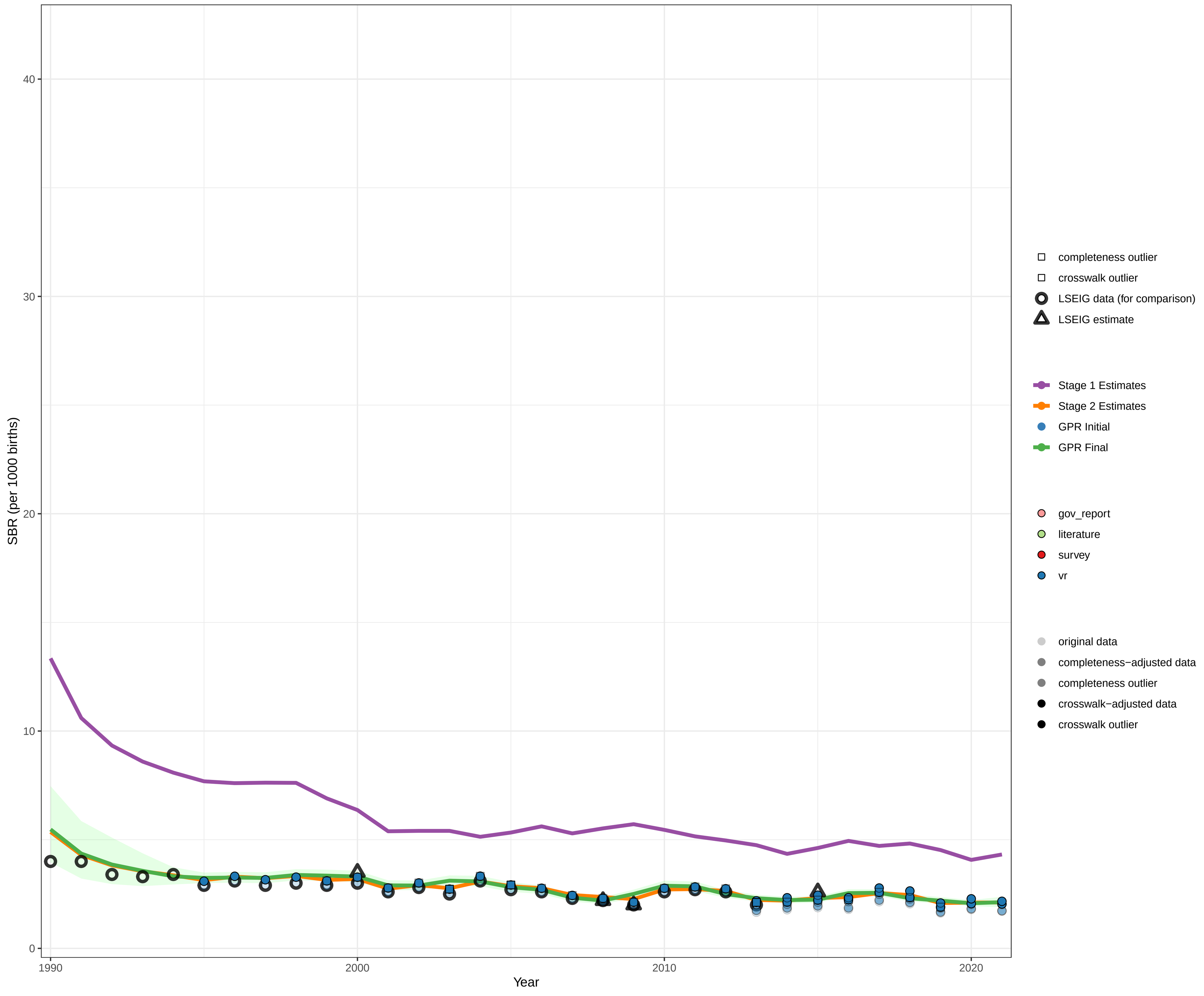

Year	Total Stillbirths (in thousands)	Total Stillbirths (in thousands)	SBR (per 1000 births)	SBR (per 1000 births)	SBR/NMR	SBR/NMR
	20 weeks	28 weeks	20 weeks	28 weeks	20 weeks	28 weeks
1990	0.5 (0.3 to 0.6)	0.3 (0.2 to 0.4)	8.5 (6.1 to 11.6)	5.5 (4.0 to 7.5)	2.12 (1.53 to 2.90)	1.37 (0.99 to 1.87)
1995	0.3 (0.3 to 0.3)	0.2 (0.2 to 0.2)	5.1 (4.8 to 5.5)	3.2 (3.0 to 3.5)	2.37 (2.20 to 2.56)	1.50 (1.39 to 1.61)
2000	0.3 (0.3 to 0.3)	0.2 (0.2 to 0.2)	5.2 (4.8 to 5.7)	3.3 (3.0 to 3.6)	3.14 (2.90 to 3.39)	1.98 (1.83 to 2.14)
2005	0.2 (0.2 to 0.2)	0.1 (0.1 to 0.2)	4.4 (4.1 to 4.8)	2.8 (2.6 to 3.0)	3.41 (3.14 to 3.68)	2.17 (2.01 to 2.35)
2010	0.3 (0.2 to 0.3)	0.2 (0.2 to 0.2)	4.5 (4.2 to 4.9)	2.9 (2.7 to 3.1)	3.72 (3.43 to 4.01)	2.36 (2.18 to 2.56)
2015	0.2 (0.2 to 0.2)	0.1 (0.1 to 0.1)	3.4 (3.2 to 3.6)	2.2 (2.1 to 2.4)	3.52 (3.30 to 3.76)	2.32 (2.18 to 2.49)
2020	0.2 (0.2 to 0.2)	0.1 (0.1 to 0.1)	3.0 (2.8 to 3.2)	2.1 (1.9 to 2.2)	3.66 (3.43 to 3.91)	2.53 (2.37 to 2.70)
2021	0.2 (0.2 to 0.2)	0.1 (0.1 to 0.1)	3.1 (2.7 to 3.4)	2.1 (1.9 to 2.4)	3.55 (3.16 to 3.96)	2.45 (2.18 to 2.74)

High−income North America   R16   High−income North America   Stillbirth Rate / Neonatal Mortality Rate (SBR/NMR) − 20 weeks
lambda = 1 ; zeta = 0.6 ; scale = 15 ; amp = 0.028

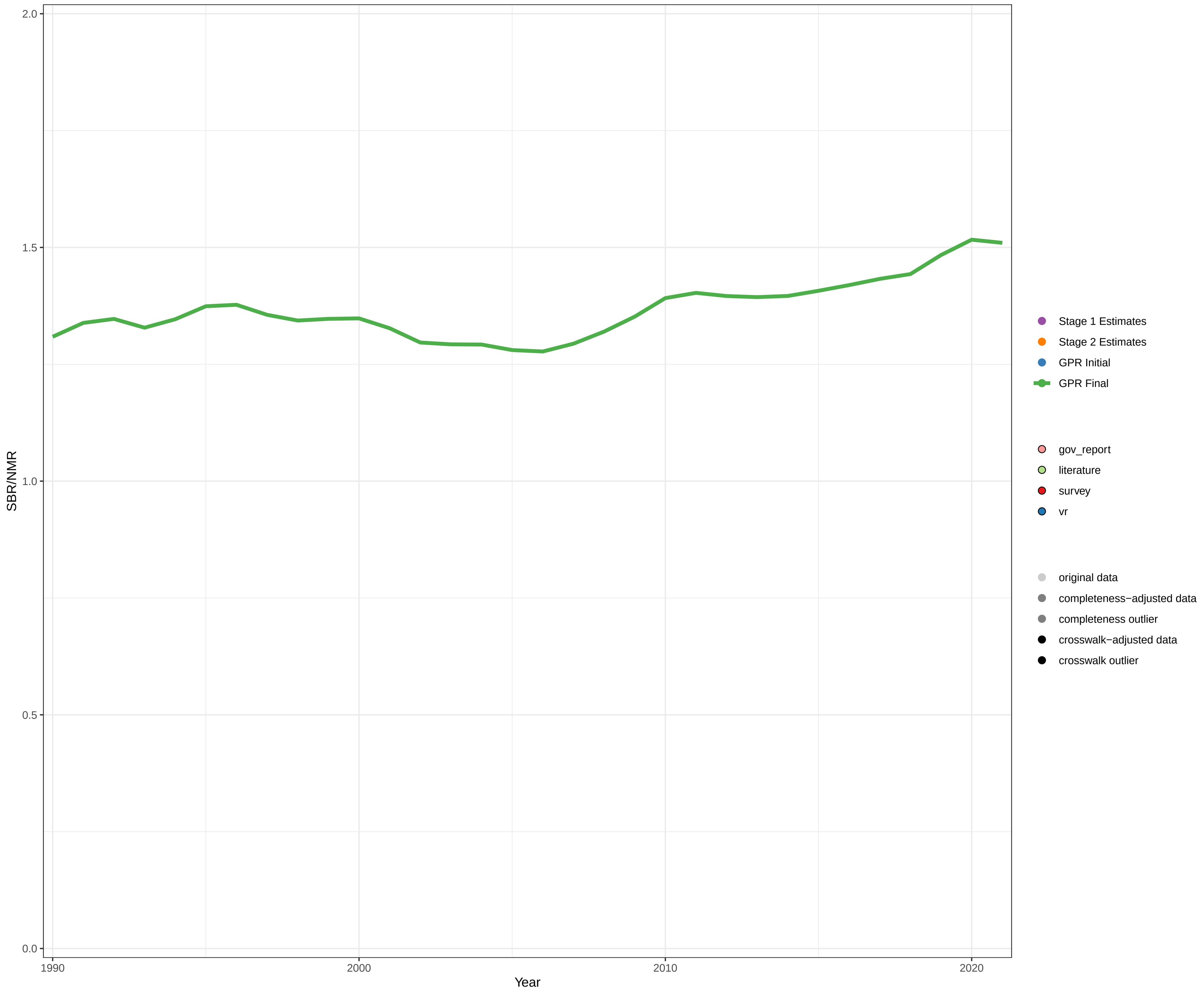

High−income North America   R16   High−income North America   Stillbirth Rate / Neonatal Mortality Rate (SBR/NMR) − 28 weeks
lambda = 1 ; zeta = 0.6 ; scale = 15 ; amp = 0.028

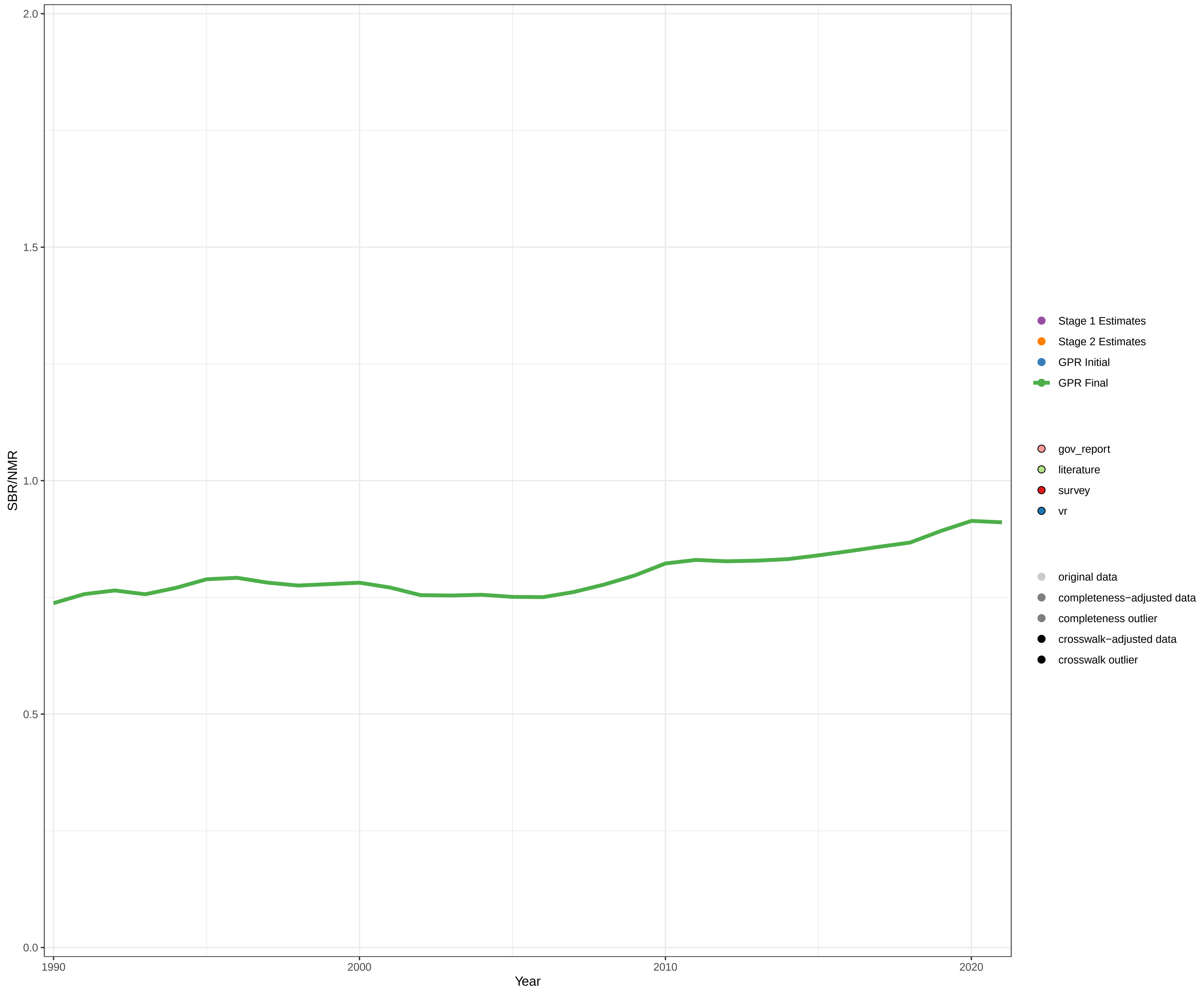

Definition	Years	Outlier	Source.Type	Title

High−income North America   R16   High−income North America   Stillbirth Rate (SBR) − 20 weeks
lambda = 1 ; zeta = 0.6 ; scale = 15 ; amp = 0.028

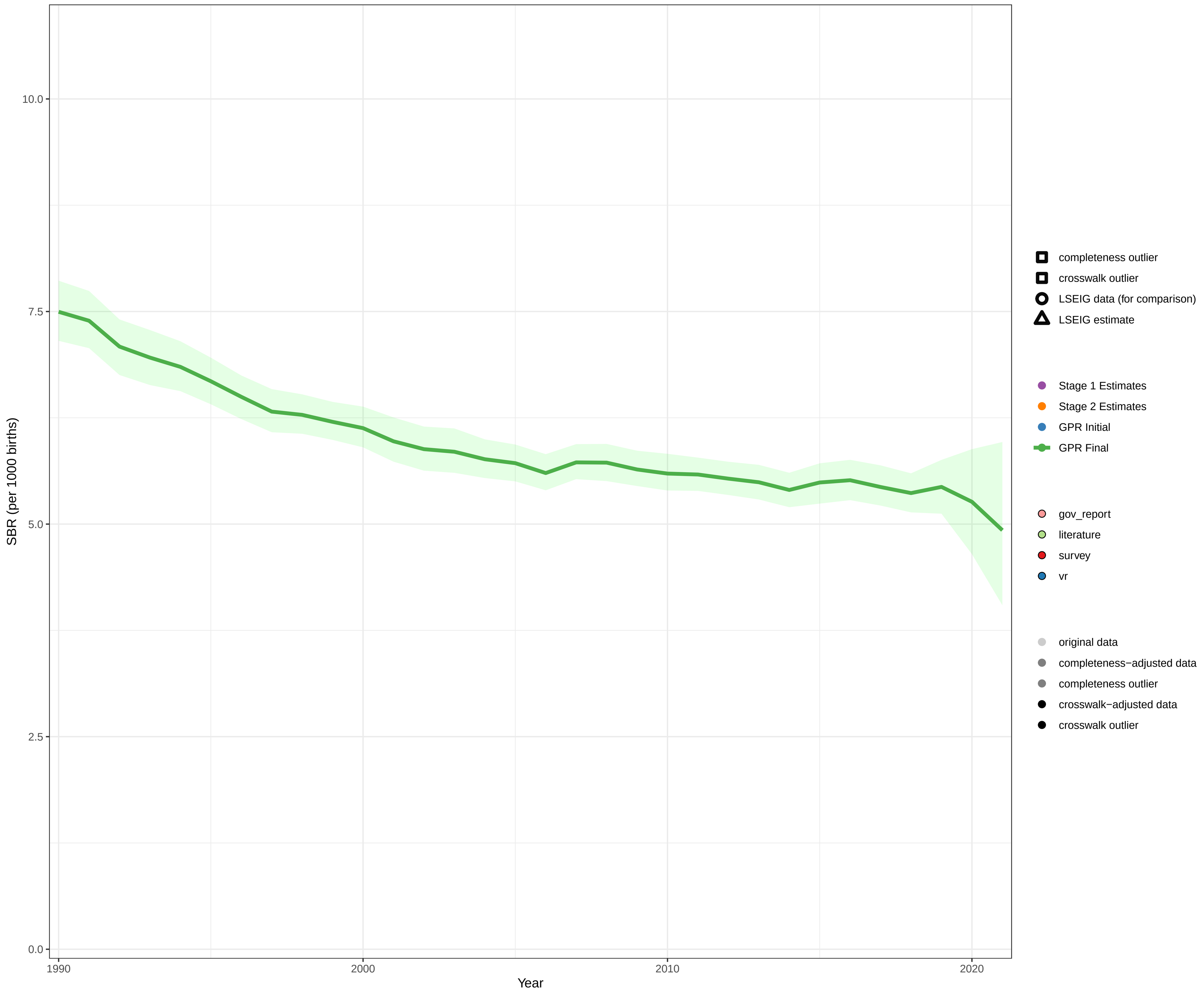

High−income North America   R16   High−income North America   Stillbirth Rate (SBR) − 28 weeks
lambda = 1 ; zeta = 0.6 ; scale = 15 ; amp = 0.028

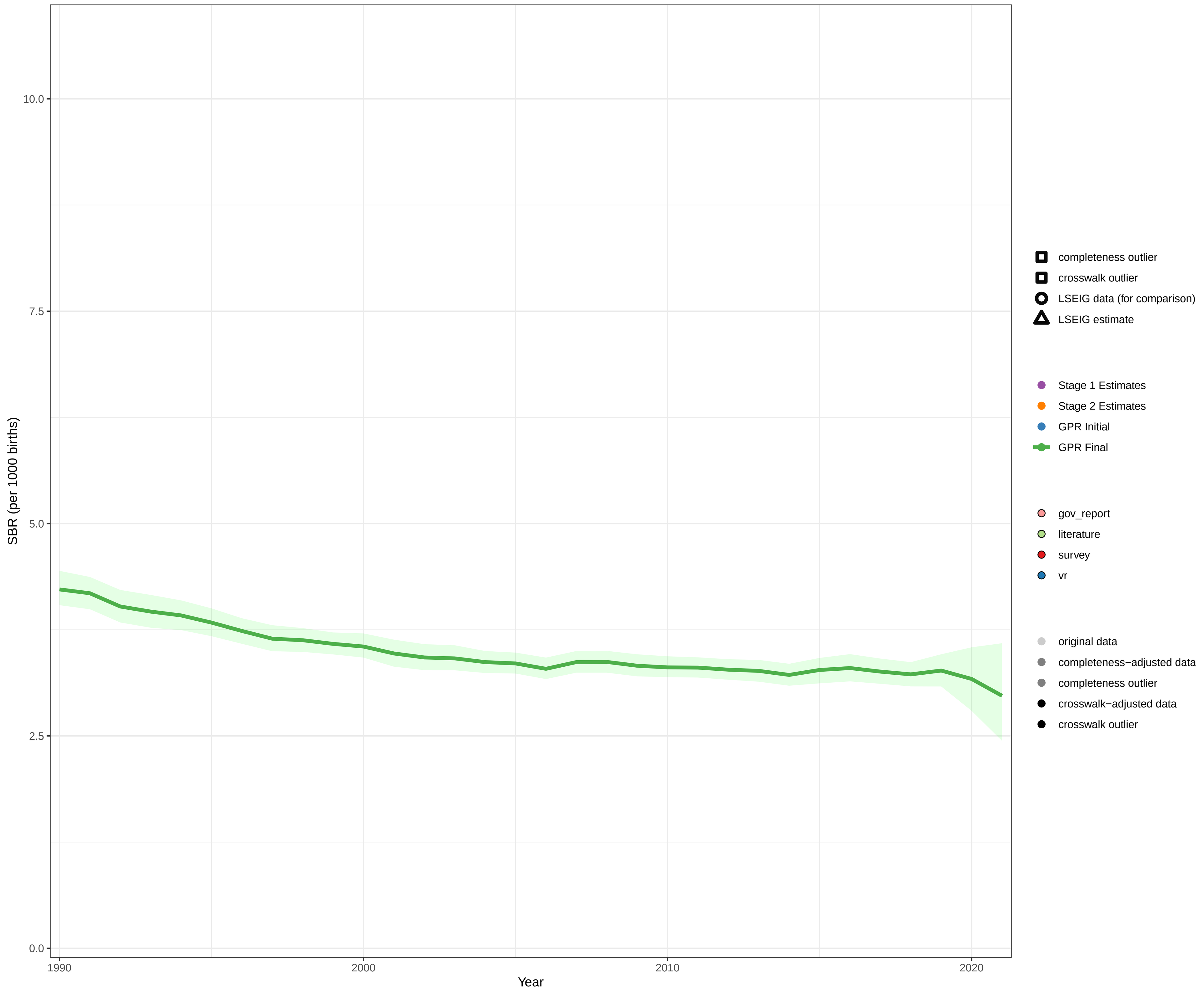

Year	Total Stillbirths (in thousands)	Total Stillbirths (in thousands)	SBR (per 1000 births)	SBR (per 1000 births)	SBR/NMR	SBR/NMR
	20 weeks	28 weeks	20 weeks	28 weeks	20 weeks	28 weeks
1990	34.3 (32.7 to 35.9)	19.2 (18.4 to 20.2)	7.5 (7.2 to 7.9)	4.2 (4.0 to 4.4)	1.31 (1.25 to 1.37)	0.74 (0.71 to 0.78)
1995	28.8 (27.6 to 30.0)	16.5 (15.8 to 17.2)	6.7 (6.4 to 7.0)	3.8 (3.7 to 4.0)	1.37 (1.32 to 1.43)	0.79 (0.76 to 0.82)
2000	26.9 (25.9 to 28.0)	15.6 (15.0 to 16.2)	6.1 (5.9 to 6.4)	3.6 (3.4 to 3.7)	1.35 (1.30 to 1.40)	0.78 (0.75 to 0.82)
2005	26.1 (25.1 to 27.1)	15.3 (14.7 to 15.9)	5.7 (5.5 to 5.9)	3.4 (3.2 to 3.5)	1.28 (1.23 to 1.33)	0.75 (0.72 to 0.78)
2010	24.6 (23.7 to 25.6)	14.5 (14.0 to 15.1)	5.6 (5.4 to 5.8)	3.3 (3.2 to 3.4)	1.39 (1.34 to 1.45)	0.82 (0.79 to 0.86)
2015	24.0 (22.9 to 25.0)	14.3 (13.6 to 14.9)	5.5 (5.2 to 5.7)	3.3 (3.1 to 3.4)	1.41 (1.34 to 1.47)	0.84 (0.80 to 0.88)
2020	21.2 (18.7 to 23.7)	12.7 (11.2 to 14.2)	5.3 (4.6 to 5.9)	3.2 (2.8 to 3.5)	1.52 (1.34 to 1.69)	0.91 (0.81 to 1.02)
2021	19.9 (16.3 to 24.1)	12.0 (9.8 to 14.5)	4.9 (4.0 to 6.0)	3.0 (2.4 to 3.6)	1.51 (1.24 to 1.83)	0.91 (0.75 to 1.10)

Canada CAN High−income North America Stillbirth Rate / Neonatal Mortality Rate (SBR/NMR) − 20 weeks
lambda = 0.2 ; zeta = 0.99 ; scale = 5 ; amp = 0.028

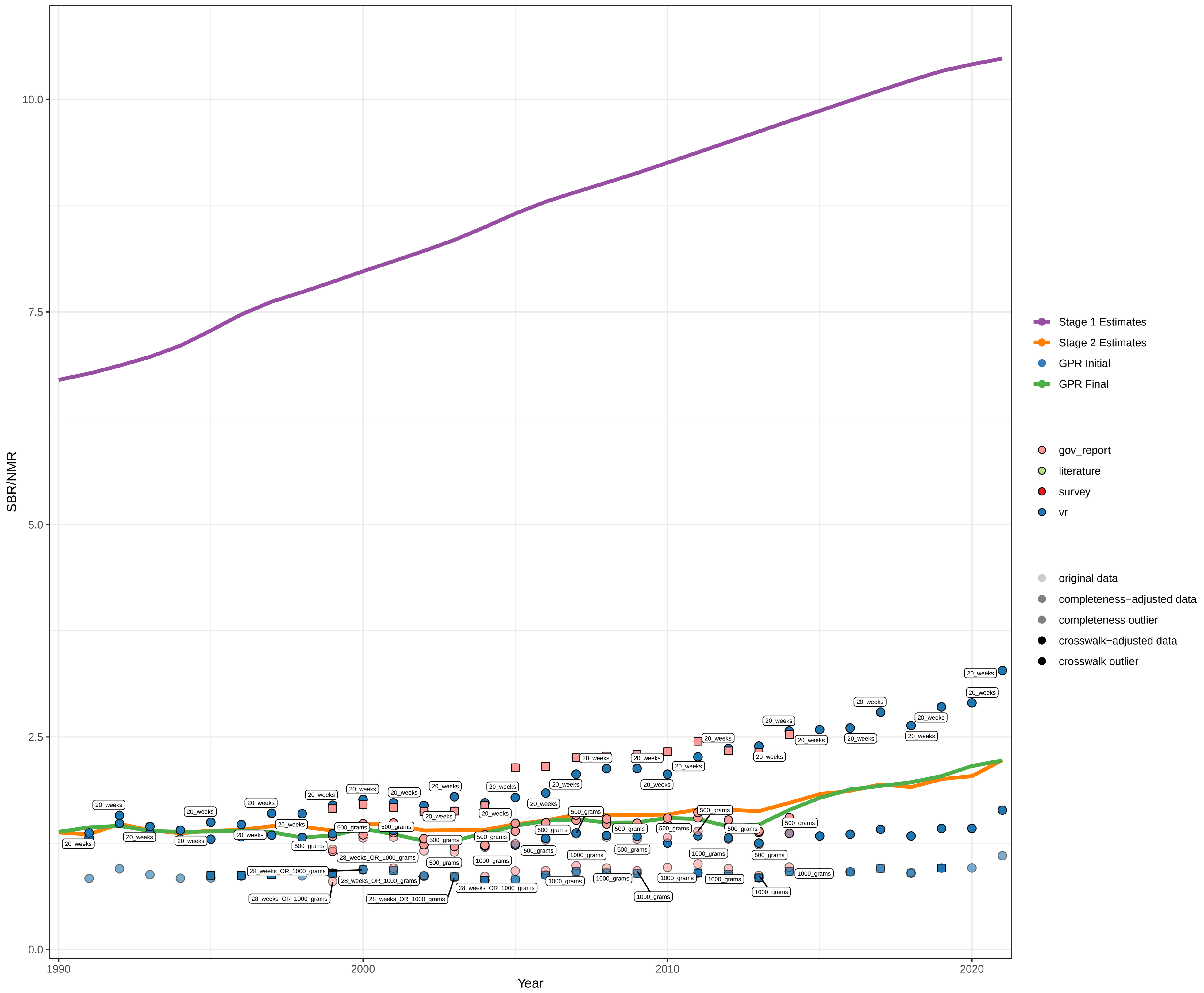

Canada CAN High−income North America Stillbirth Rate / Neonatal Mortality Rate (SBR/NMR) − 28 weeks
lambda = 0.2 ; zeta = 0.99 ; scale = 5 ; amp = 0.028

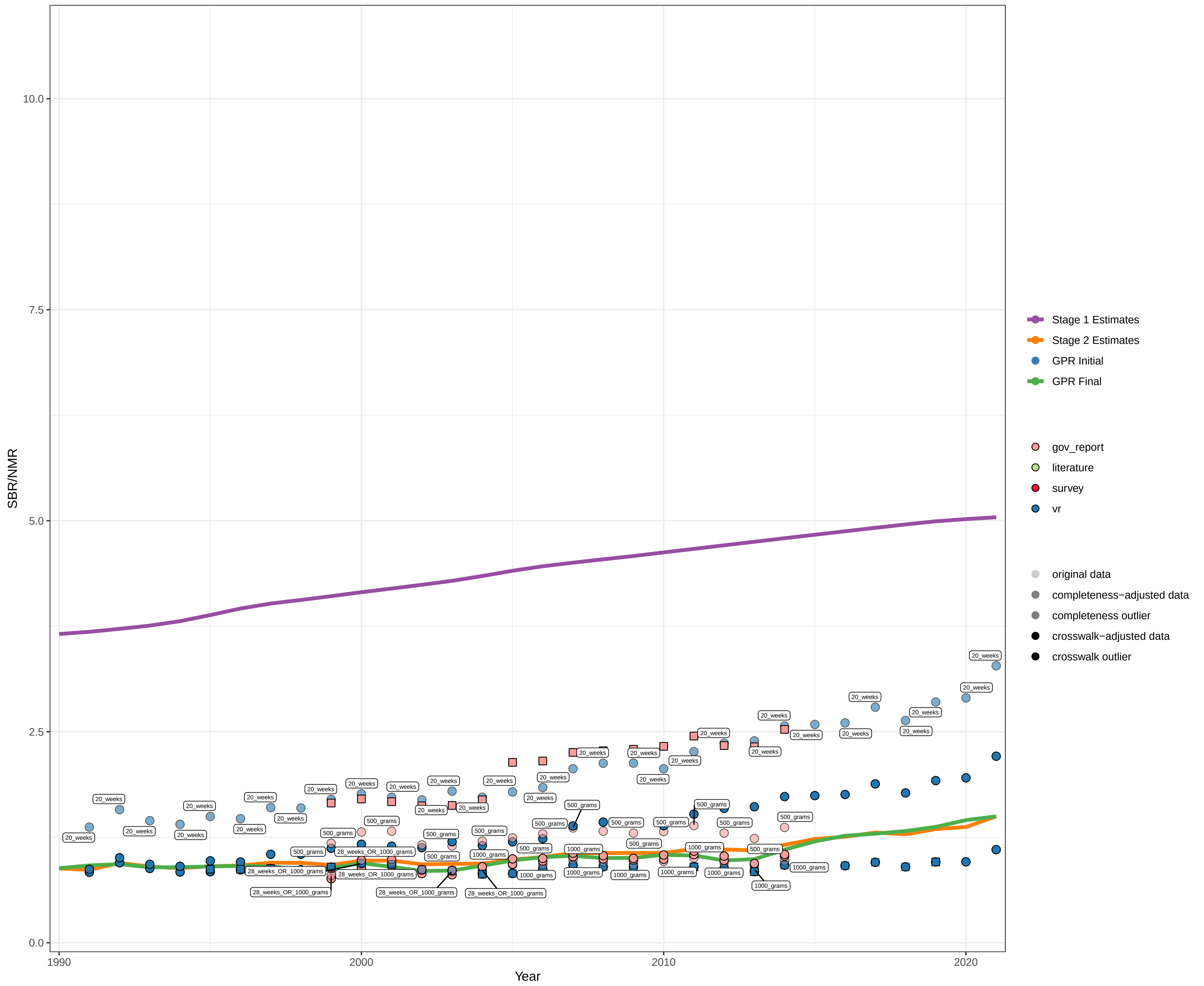

Definition	Years	Outlier	Source.Type	Title
1000_grams	2005−2014	0	Statistical Report	Perinatal Health Indicators for Canada : A Report from the Canadian Perinatal Surveillance System
20_weeks	2004	0	Statistical Report	Perinatal Health Indicators for Canada : A Report from the Canadian Perinatal Surveillance System
20_weeks, 28_weeks	1991−2021	0	VR	Canada CANSIM Database − Fetal Deaths (20 Weeks or More of Gestation) and Late Fetal Deaths (28 Weeks or More of Gestation)
28_weeks_OR_1000_grams	2001−2004	0	Statistical Report	Perinatal Health Indicators for Canada : A Report from the Canadian Perinatal Surveillance System
28_weeks_OR_1000_grams, 500_grams	1999, 2000	0	Statistical Report	Perinatal Health Indicators for Canada
500_grams	2001−2014	0	Statistical Report	Perinatal Health Indicators for Canada : A Report from the Canadian Perinatal Surveillance System
28_weeks	2000−2003, 2005−2009, 2012, 2014−2018	1	VR	United Nations Demographic Yearbook
20_weeks	1999, 2000	2	Statistical Report	Perinatal Health Indicators for Canada
20_weeks	2001−2003, 2005−2014	2	Statistical Report	Perinatal Health Indicators for Canada : A Report from the Canadian Perinatal Surveillance System
28_weeks	1995−1997, 1999, 2004, 2010, 2011, 2013, 2019	2	VR	United Nations Demographic Yearbook

Canada CAN High−income North America Stillbirth Rate (SBR) − 20 weeks
lambda = 0.2 ; zeta = 0.99 ; scale = 5 ; amp = 0.028

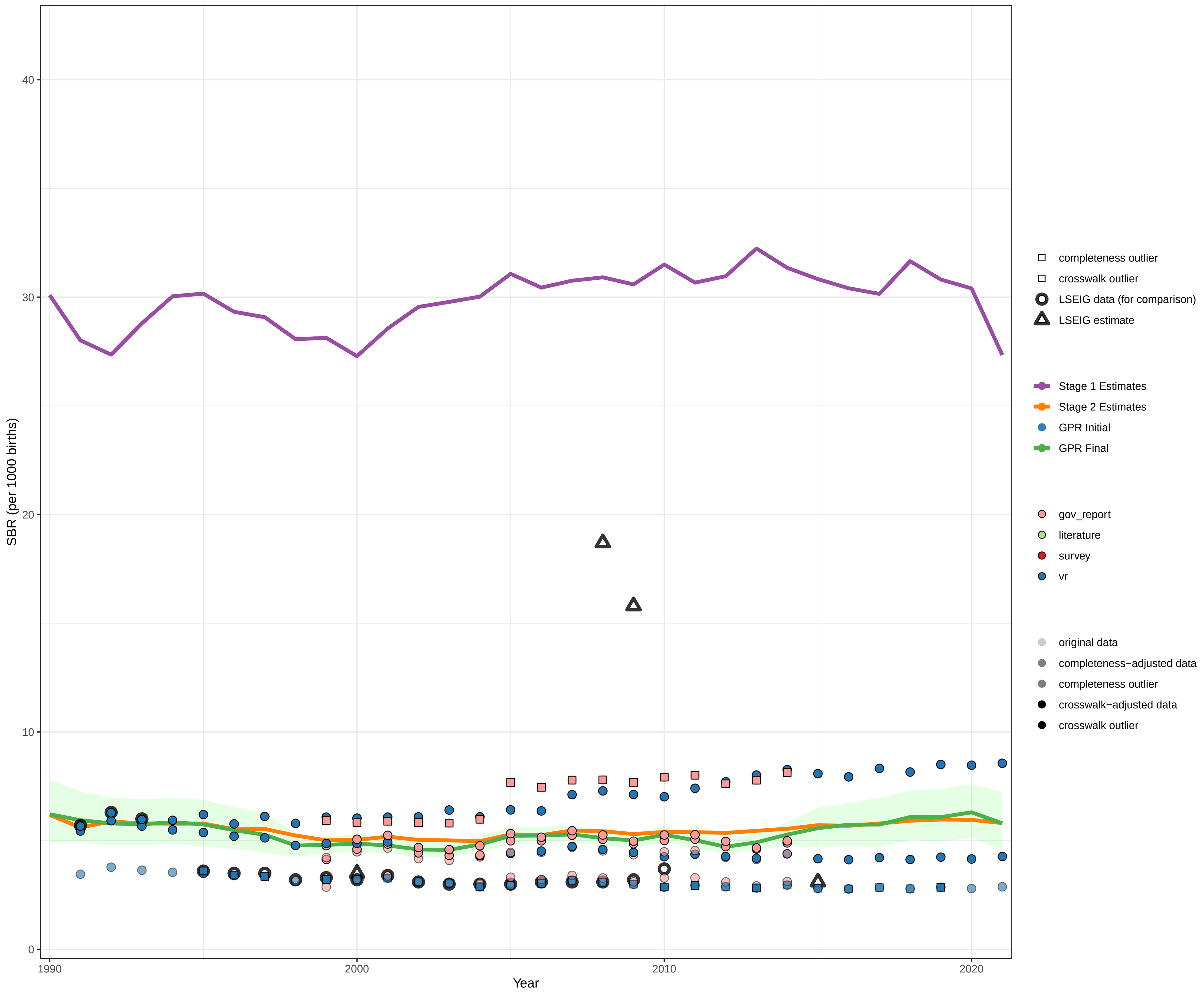

Canada CAN High−income North America Stillbirth Rate (SBR) − 28 weeks
lambda = 0.2 ; zeta = 0.99 ; scale = 5 ; amp = 0.028

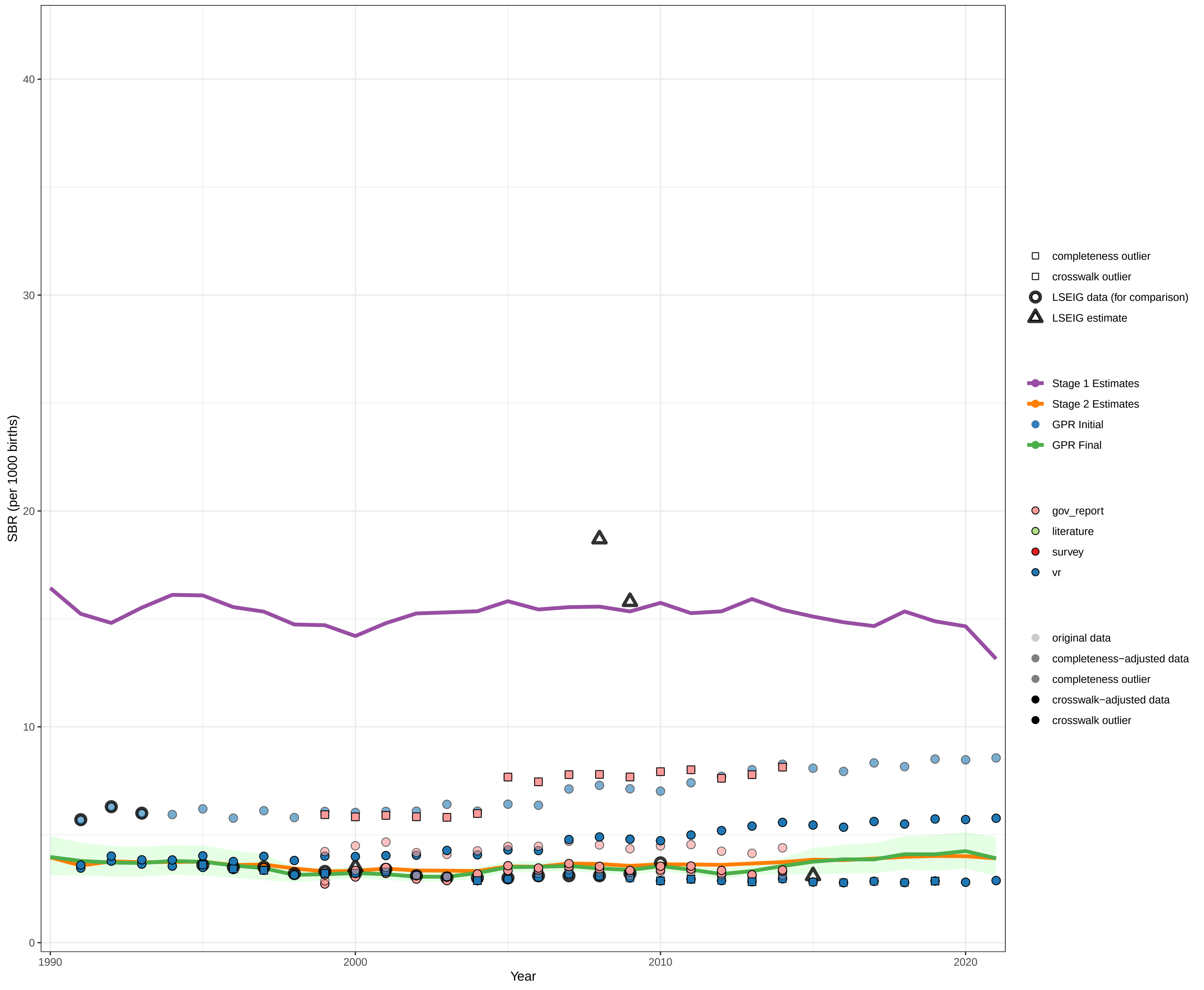

Year	Total Stillbirths (in thousands)	Total Stillbirths (in thousands)	SBR (per 1000 births)	SBR (per 1000 births)	SBR/NMR	SBR/NMR
	20 weeks	28 weeks	20 weeks	28 weeks	20 weeks	28 weeks
1990	2.5 (2.0 to 3.2)	1.6 (1.3 to 2.0)	6.2 (4.9 to 7.8)	4.0 (3.1 to 4.9)	1.38 (1.09 to 1.74)	0.88 (0.70 to 1.10)
1995	2.2 (1.8 to 2.6)	1.4 (1.2 to 1.7)	5.7 (4.7 to 6.9)	3.7 (3.1 to 4.5)	1.39 (1.14 to 1.66)	0.91 (0.75 to 1.09)
2000	1.6 (1.5 to 1.8)	1.1 (1.0 to 1.2)	4.9 (4.5 to 5.3)	3.2 (3.0 to 3.5)	1.42 (1.31 to 1.55)	0.94 (0.87 to 1.02)
2005	1.8 (1.7 to 2.0)	1.2 (1.1 to 1.3)	5.2 (4.8 to 5.6)	3.5 (3.2 to 3.8)	1.45 (1.35 to 1.57)	0.98 (0.90 to 1.05)
2010	2.0 (1.9 to 2.1)	1.3 (1.2 to 1.4)	5.3 (4.9 to 5.7)	3.5 (3.3 to 3.8)	1.55 (1.44 to 1.66)	1.04 (0.97 to 1.12)
2015	2.1 (1.8 to 2.5)	1.4 (1.2 to 1.7)	5.6 (4.7 to 6.5)	3.8 (3.2 to 4.4)	1.78 (1.50 to 2.08)	1.20 (1.01 to 1.40)
2020	2.3 (1.9 to 2.8)	1.5 (1.2 to 1.9)	6.3 (5.1 to 7.6)	4.2 (3.4 to 5.1)	2.16 (1.75 to 2.60)	1.45 (1.18 to 1.76)
2021	2.1 (1.7 to 2.6)	1.4 (1.1 to 1.8)	5.8 (4.6 to 7.2)	3.9 (3.1 to 4.9)	2.22 (1.75 to 2.78)	1.50 (1.18 to 1.87)

Greenland GRL High−income North America Stillbirth Rate / Neonatal Mortality Rate (SBR/NMR) − 20 weeks
lambda = 0.8 ; zeta = 0.7 ; scale = 15 ; amp = 0.028

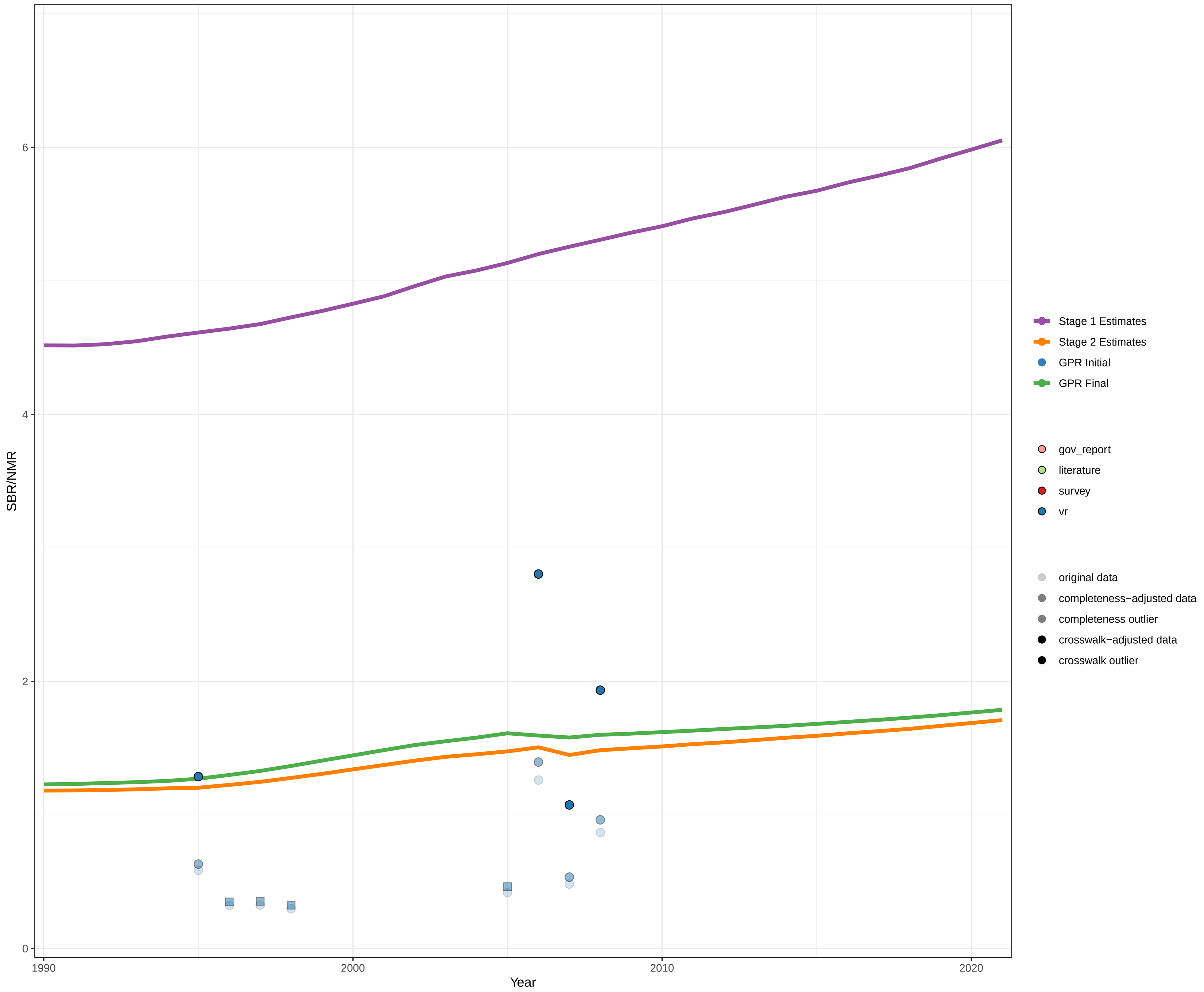

Greenland GRL High−income North America Stillbirth Rate / Neonatal Mortality Rate (SBR/NMR) − 28 weeks
lambda = 0.8 ; zeta = 0.7 ; scale = 15 ; amp = 0.028

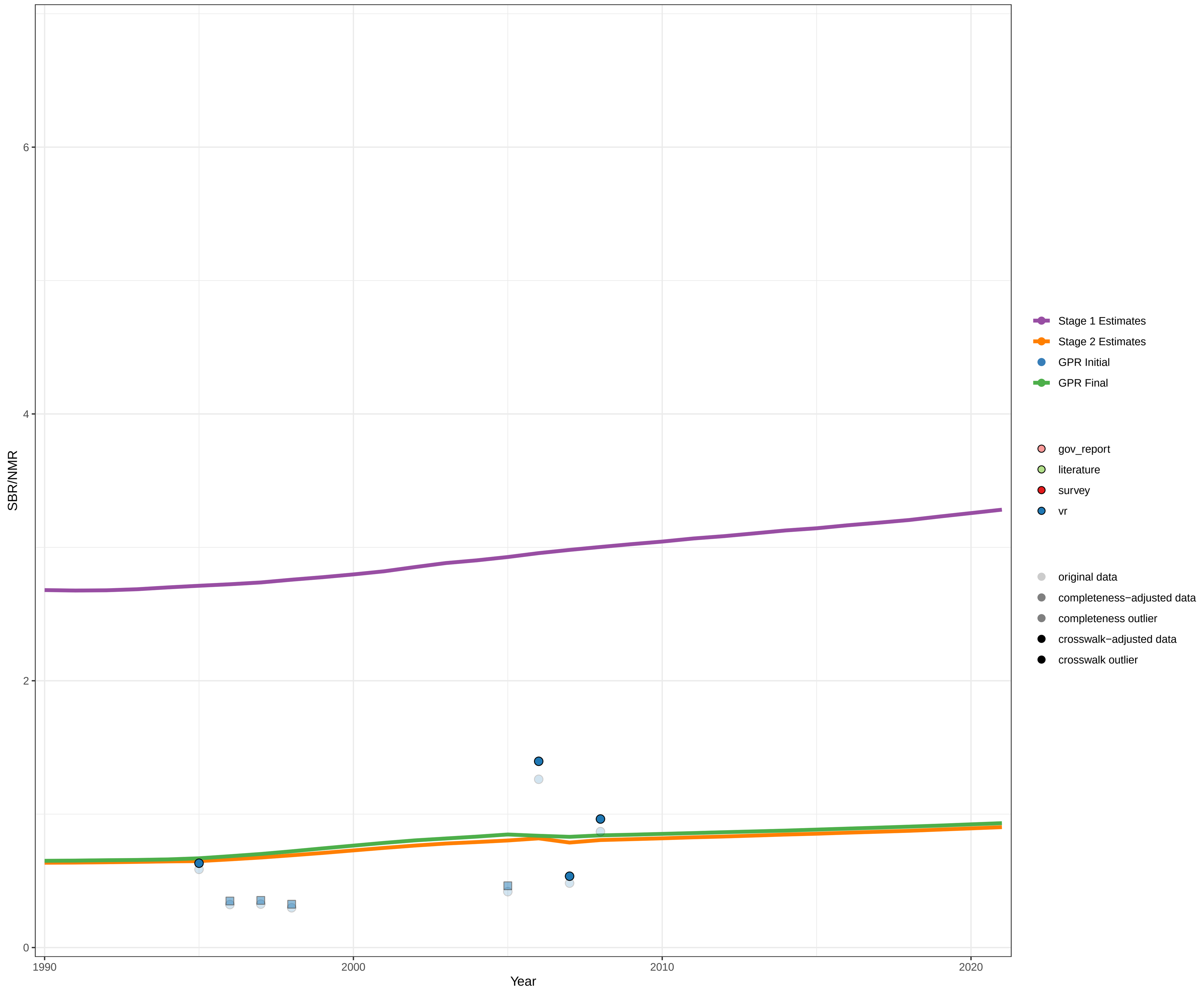

Definition	Years	Outlier	Source.Type	Title
28_weeks	1995, 2006−2008	0	VR	United Nations Demographic Yearbook
28_weeks	1996−1998, 2005	1	VR	United Nations Demographic Yearbook

Greenland GRL High−income North America Stillbirth Rate (SBR) − 20 weeks
lambda = 0.8 ; zeta = 0.7 ; scale = 15 ; amp = 0.028

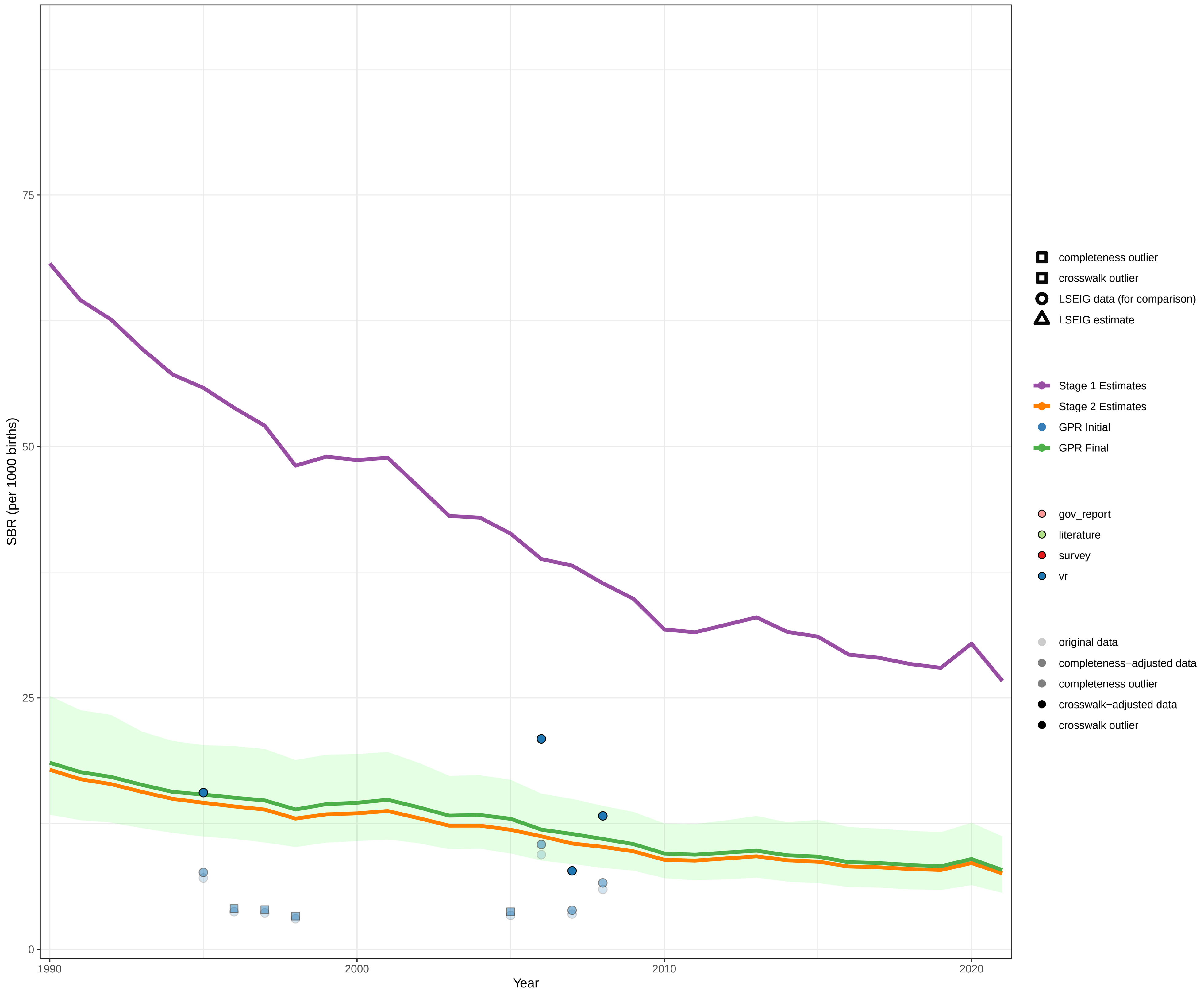

Greenland GRL High−income North America Stillbirth Rate (SBR) − 28 weeks
lambda = 0.8 ; zeta = 0.7 ; scale = 15 ; amp = 0.028

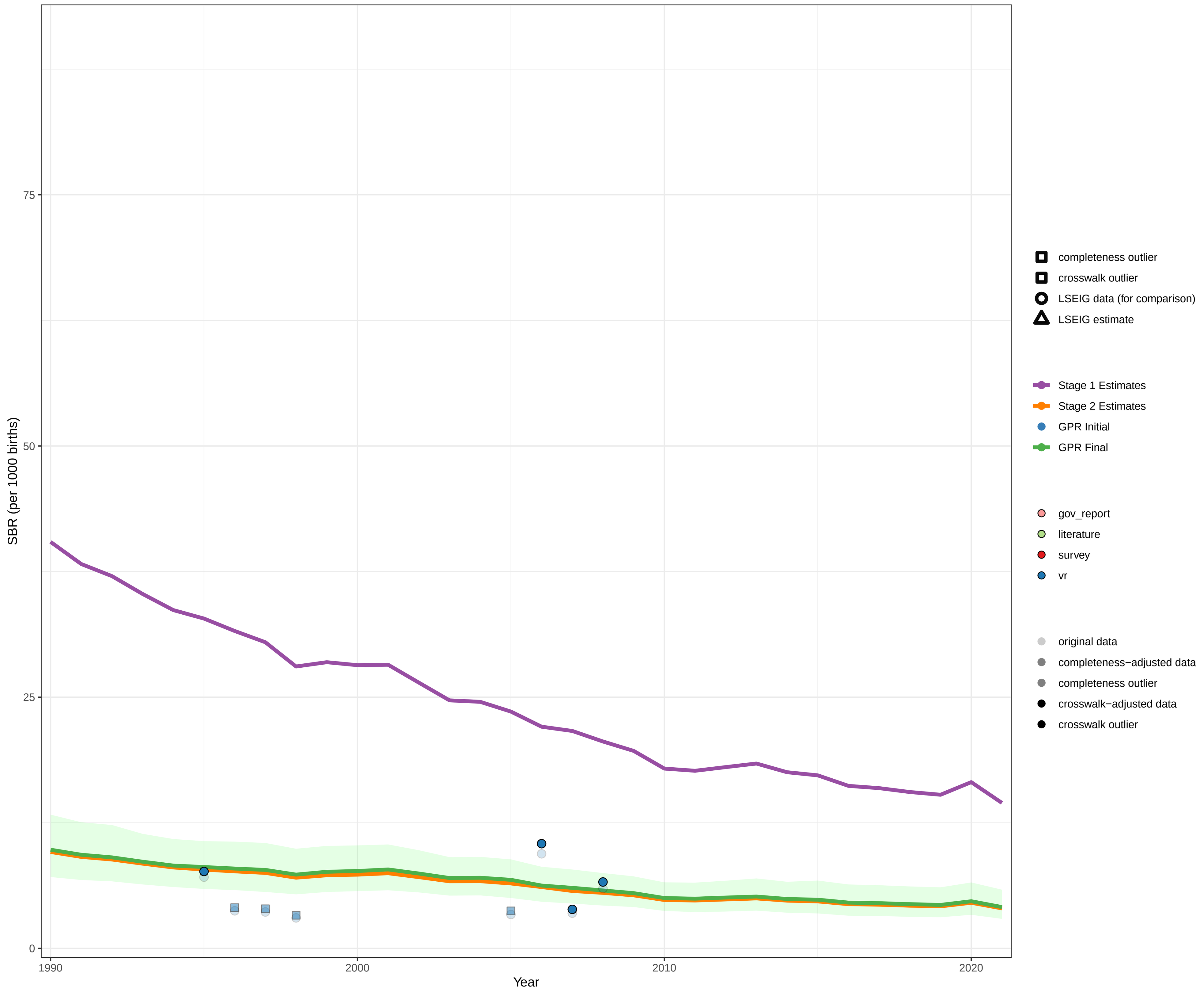

Year	Total Stillbirths (in thousands)	Total Stillbirths (in thousands)	SBR (per 1000 births)	SBR (per 1000 births)	SBR/NMR	SBR/NMR
	20 weeks	28 weeks	20 weeks	28 weeks	20 weeks	28 weeks
1990	0.0 (0.0 to 0.0)	0.0 (0.0 to 0.0)	18.6 (13.4 to 25.2)	9.8 (7.1 to 13.3)	1.23 (0.89 to 1.67)	0.65 (0.47 to 0.88)
1995	0.0 (0.0 to 0.0)	0.0 (0.0 to 0.0)	15.4 (11.2 to 20.3)	8.1 (5.9 to 10.7)	1.27 (0.93 to 1.68)	0.67 (0.49 to 0.88)
2000	0.0 (0.0 to 0.0)	0.0 (0.0 to 0.0)	14.6 (10.8 to 19.4)	7.7 (5.7 to 10.2)	1.45 (1.07 to 1.93)	0.76 (0.57 to 1.02)
2005	0.0 (0.0 to 0.0)	0.0 (0.0 to 0.0)	13.0 (9.5 to 16.9)	6.8 (5.0 to 8.9)	1.61 (1.18 to 2.09)	0.85 (0.62 to 1.10)
2010	0.0 (0.0 to 0.0)	0.0 (0.0 to 0.0)	9.5 (7.1 to 12.5)	5.0 (3.7 to 6.6)	1.62 (1.20 to 2.13)	0.85 (0.63 to 1.12)
2015	0.0 (0.0 to 0.0)	0.0 (0.0 to 0.0)	9.2 (6.6 to 12.9)	4.8 (3.5 to 6.8)	1.68 (1.20 to 2.35)	0.88 (0.63 to 1.23)
2020	0.0 (0.0 to 0.0)	0.0 (0.0 to 0.0)	9.0 (6.4 to 12.6)	4.7 (3.3 to 6.6)	1.77 (1.26 to 2.48)	0.92 (0.66 to 1.29)
2021	0.0 (0.0 to 0.0)	0.0 (0.0 to 0.0)	7.9 (5.6 to 11.2)	4.1 (2.9 to 5.8)	1.79 (1.27 to 2.55)	0.93 (0.67 to 1.32)

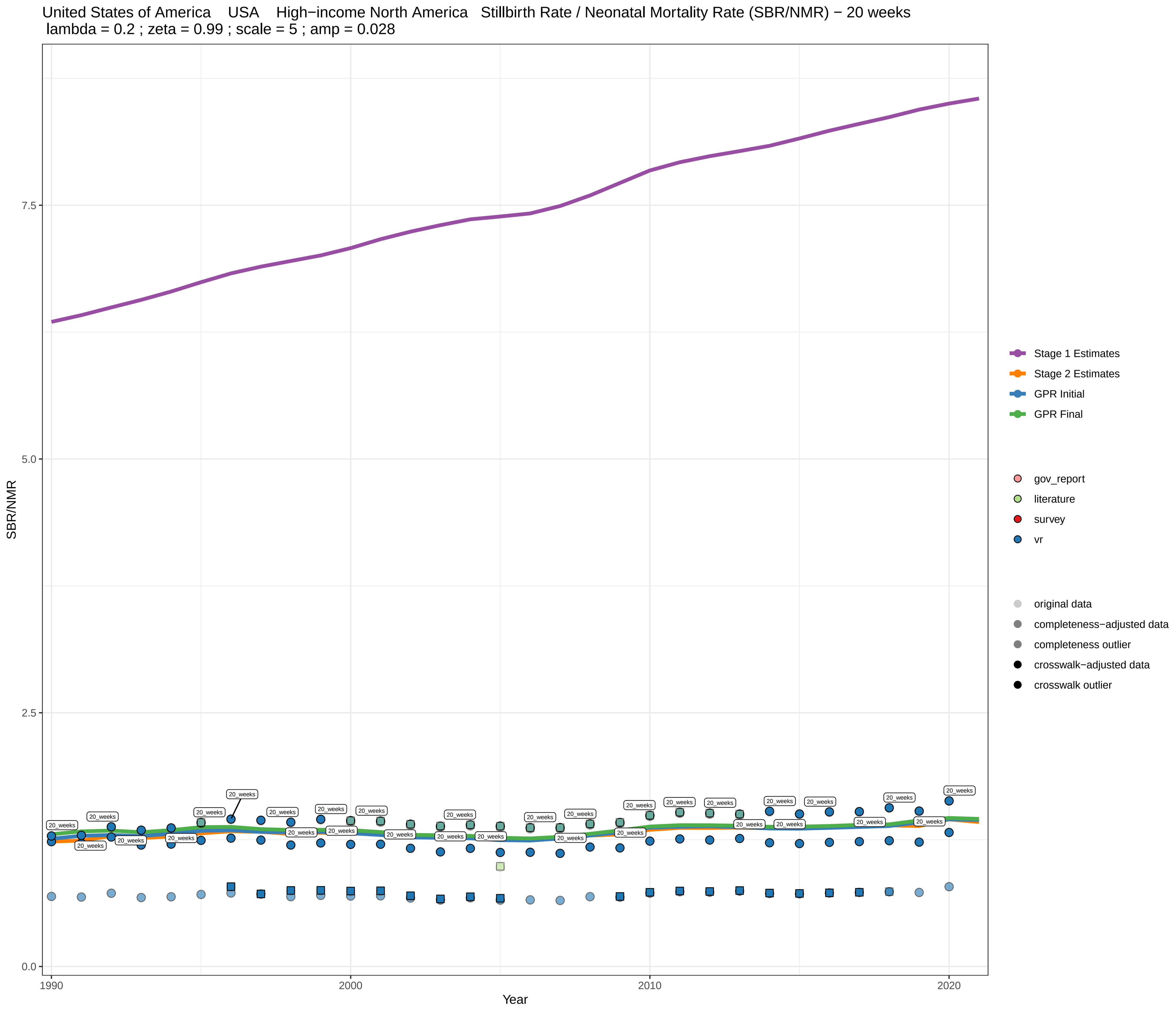

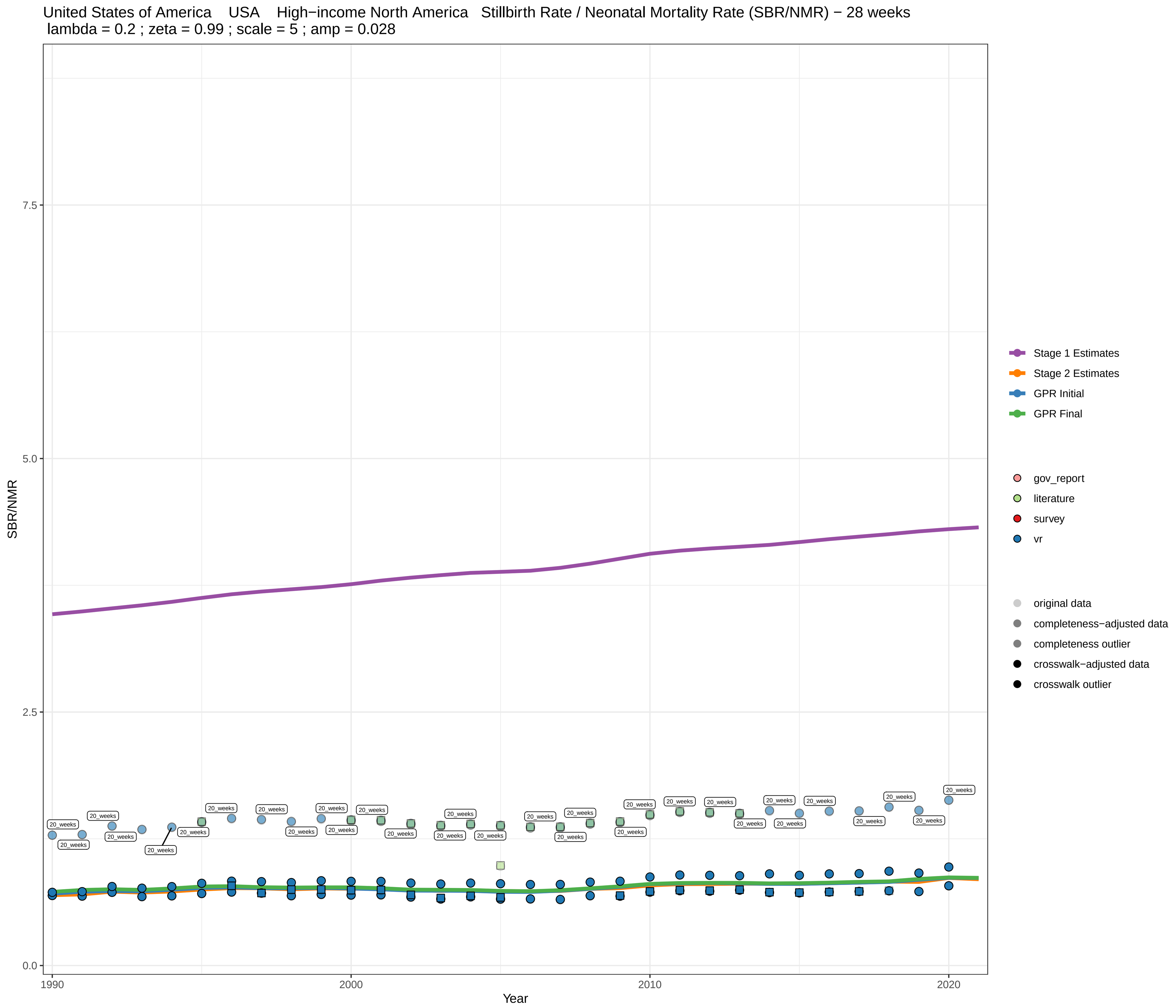

Definition	Years	Outlier	Source.Type	Title
20_weeks, 28_weeks	1990−2020	0	VR	United States NVSS Custom Fetal Death Data
20_weeks	1995, 2000−2013	1	Sci Lit	United States Fetal and Perinatal Mortality 2013
22_weeks	2005	1	Sci Lit	Ambient Temperature and Stillbirth: A Multi−Center Retrospective Cohort Study
28_weeks	2018	1	VR	United Nations Demographic Yearbook
28_weeks	1996−2005, 2009−2017	2	VR	United Nations Demographic Yearbook

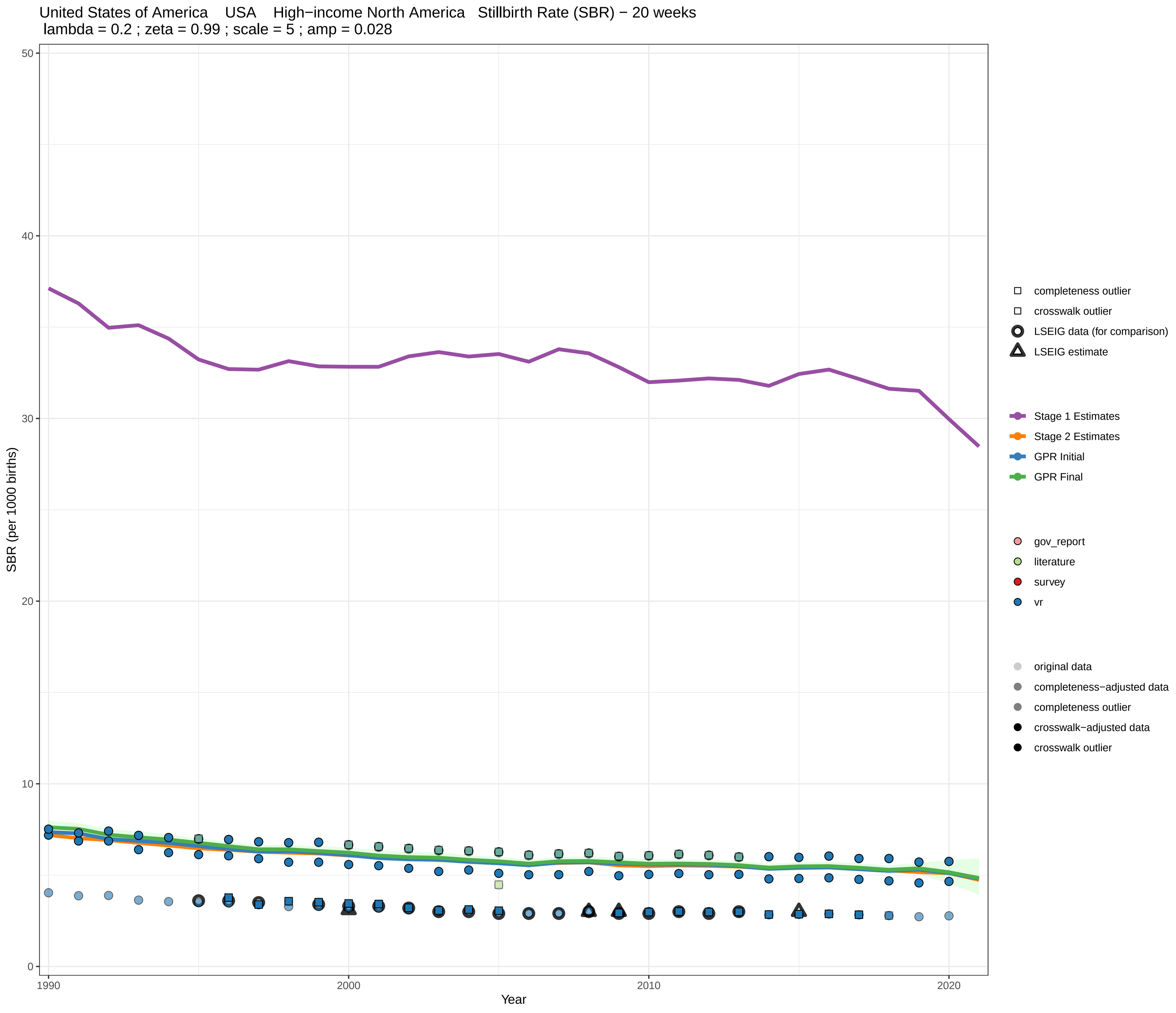

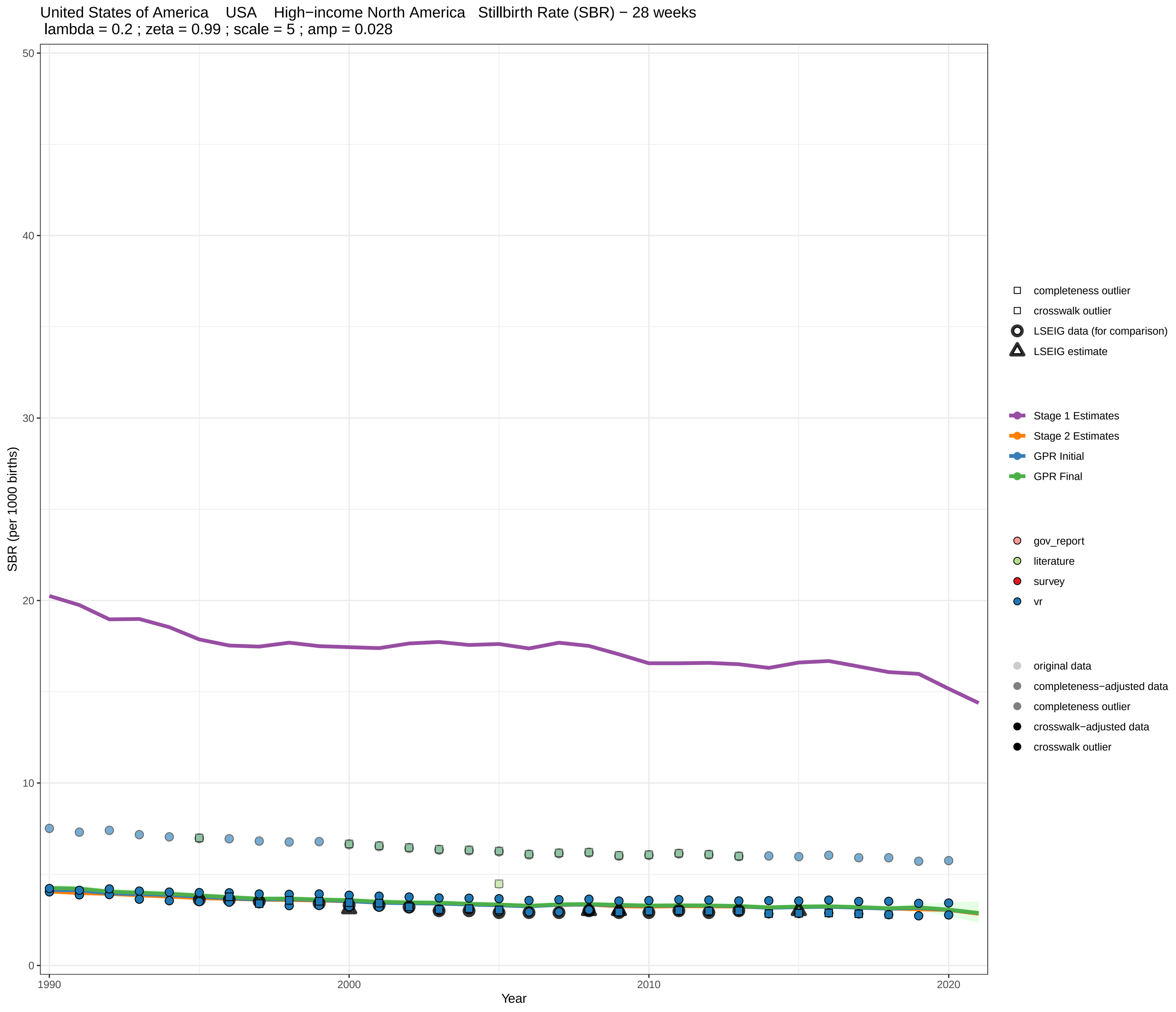

Year	Total Stillbirths (in thousands)	Total Stillbirths (in thousands)	SBR (per 1000 births)	SBR (per 1000 births)	SBR/NMR	SBR/NMR
	20 weeks	28 weeks	20 weeks	28 weeks	20 weeks	28 weeks
1990	31.7 (30.3 to 33.2)	17.6 (16.9 to 18.4)	7.6 (7.3 to 8.0)	4.2 (4.1 to 4.4)	1.30 (1.25 to 1.36)	0.73 (0.70 to 0.76)
1995	26.6 (25.6 to 27.5)	15.0 (14.5 to 15.6)	6.8 (6.5 to 7.0)	3.8 (3.7 to 4.0)	1.37 (1.32 to 1.42)	0.78 (0.75 to 0.81)
2000	25.3 (24.3 to 26.4)	14.5 (13.9 to 15.2)	6.2 (6.0 to 6.5)	3.6 (3.4 to 3.7)	1.34 (1.29 to 1.40)	0.77 (0.74 to 0.81)
2005	24.3 (23.3 to 25.3)	14.1 (13.5 to 14.6)	5.8 (5.5 to 6.0)	3.3 (3.2 to 3.5)	1.27 (1.22 to 1.32)	0.74 (0.71 to 0.77)
2010	22.6 (21.7 to 23.6)	13.1 (12.6 to 13.7)	5.6 (5.4 to 5.9)	3.3 (3.2 to 3.4)	1.38 (1.33 to 1.44)	0.81 (0.78 to 0.84)
2015	21.8 (20.9 to 22.7)	12.8 (12.2 to 13.4)	5.5 (5.2 to 5.7)	3.2 (3.1 to 3.4)	1.38 (1.32 to 1.43)	0.81 (0.77 to 0.85)
2020	18.9 (16.6 to 21.3)	11.2 (9.8 to 12.6)	5.2 (4.5 to 5.8)	3.1 (2.7 to 3.5)	1.46 (1.29 to 1.65)	0.87 (0.76 to 0.98)
2021	17.8 (14.4 to 21.7)	10.5 (8.5 to 12.9)	4.8 (3.9 to 5.9)	2.9 (2.3 to 3.5)	1.45 (1.18 to 1.78)	0.87 (0.70 to 1.06)

Alabama USA_523 High−income North America Stillbirth Rate / Neonatal Mortality Rate (SBR/NMR) − 20 weeks
lambda = 0.2 ; zeta = 0.99 ; scale = 5 ; amp = 0.028

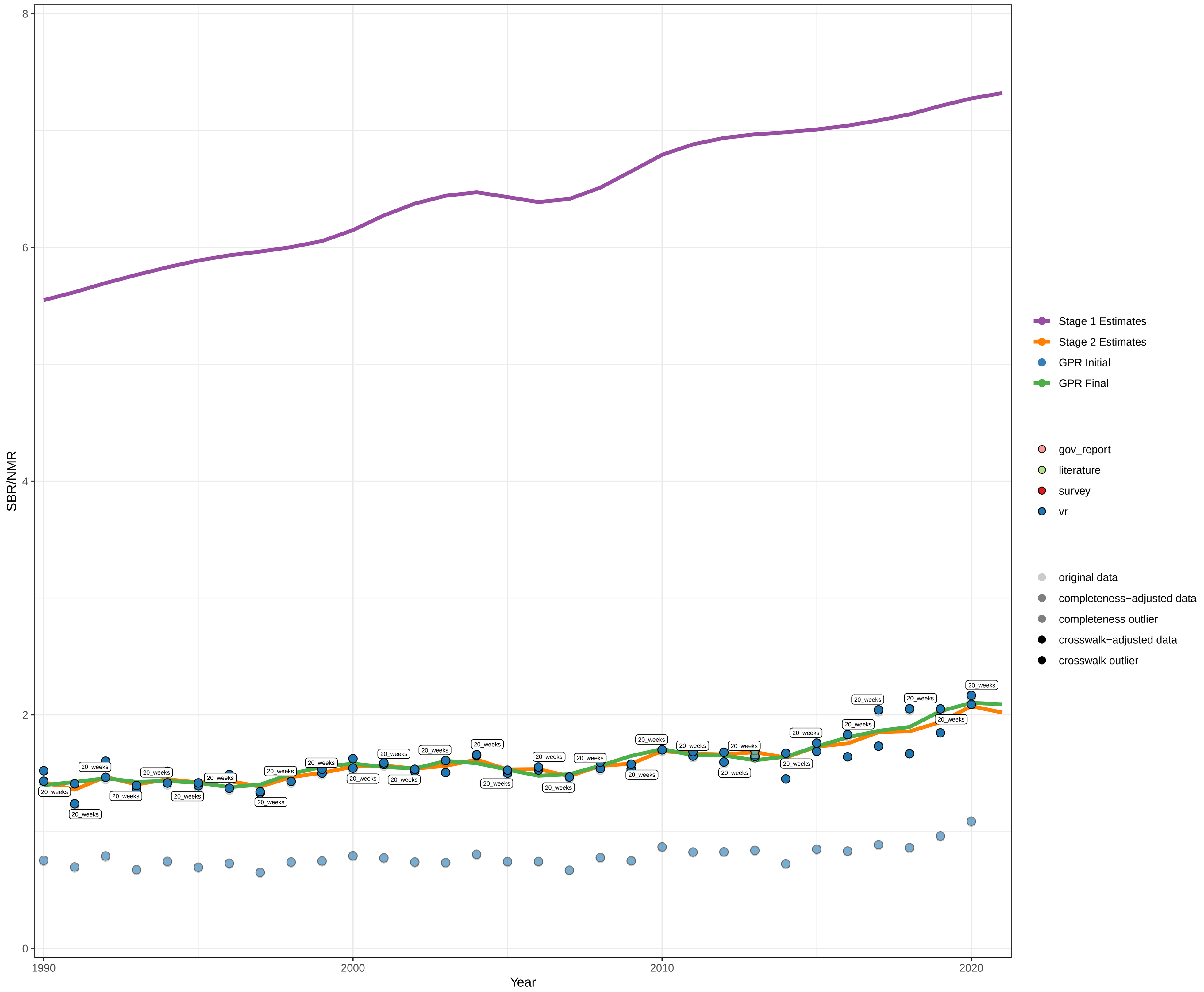

Alabama USA_523 High−income North America Stillbirth Rate / Neonatal Mortality Rate (SBR/NMR) − 28 weeks
lambda = 0.2 ; zeta = 0.99 ; scale = 5 ; amp = 0.028

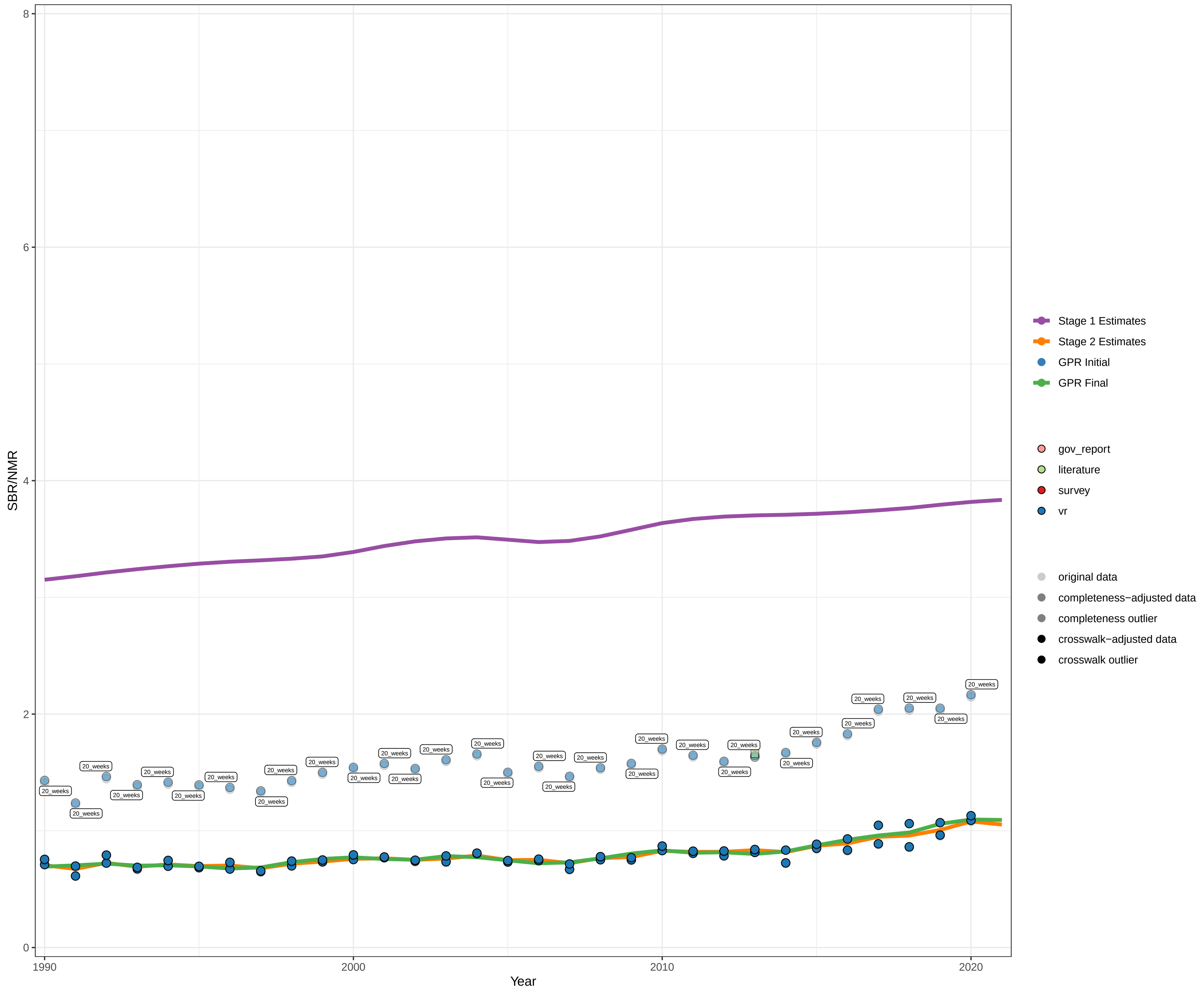

Definition	Years	Outlier	Source.Type	Title
20_weeks, 28_weeks	1990−2020	0	VR	United States NVSS Custom Fetal Death Data
20_weeks	2013	1	Sci Lit	United States Fetal and Perinatal Mortality 2013

Alabama USA_523 High−income North America Stillbirth Rate (SBR) − 20 weeks
lambda = 0.2 ; zeta = 0.99 ; scale = 5 ; amp = 0.028

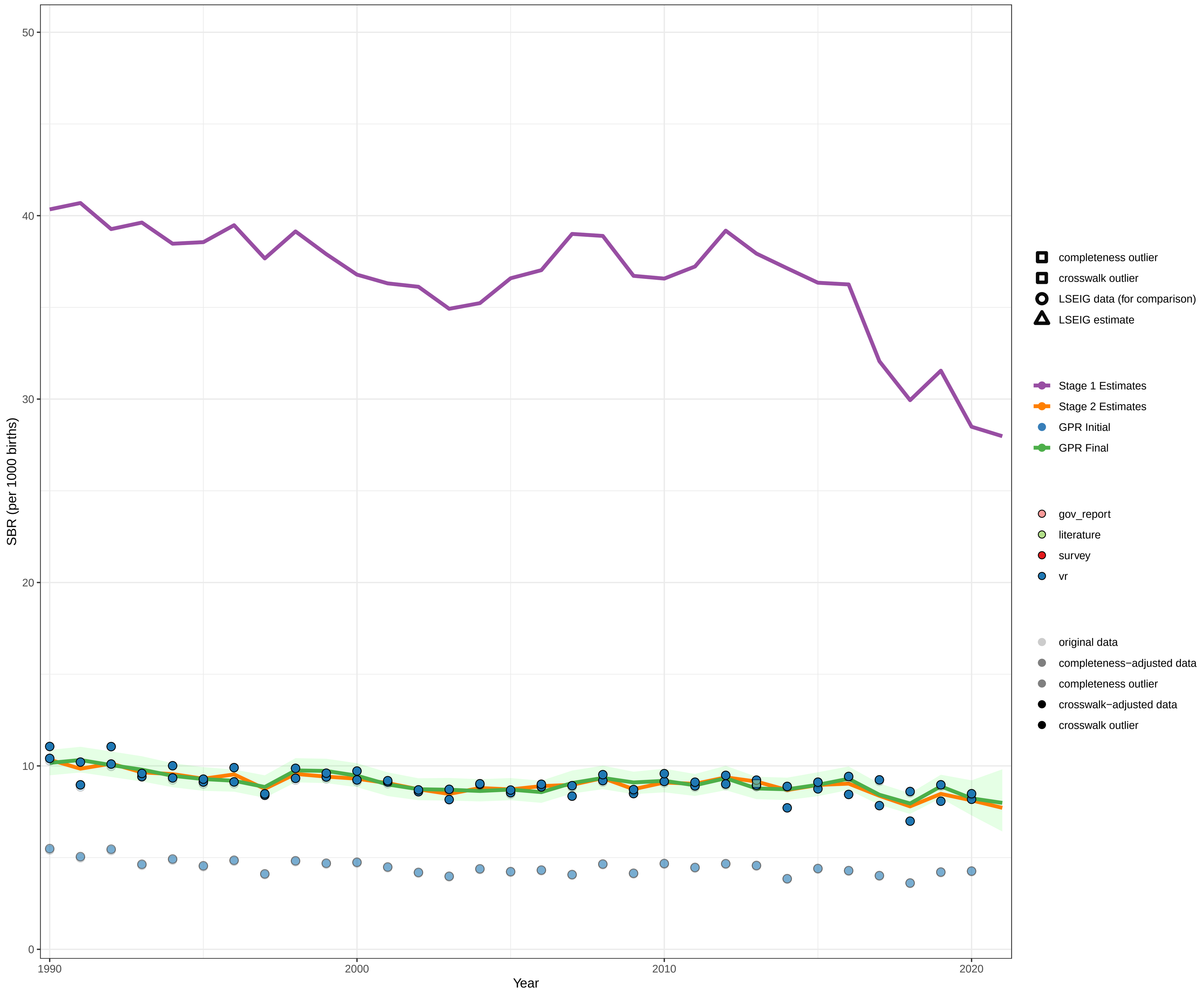

Alabama USA_523 High−income North America Stillbirth Rate (SBR) − 28 weeks
lambda = 0.2 ; zeta = 0.99 ; scale = 5 ; amp = 0.028

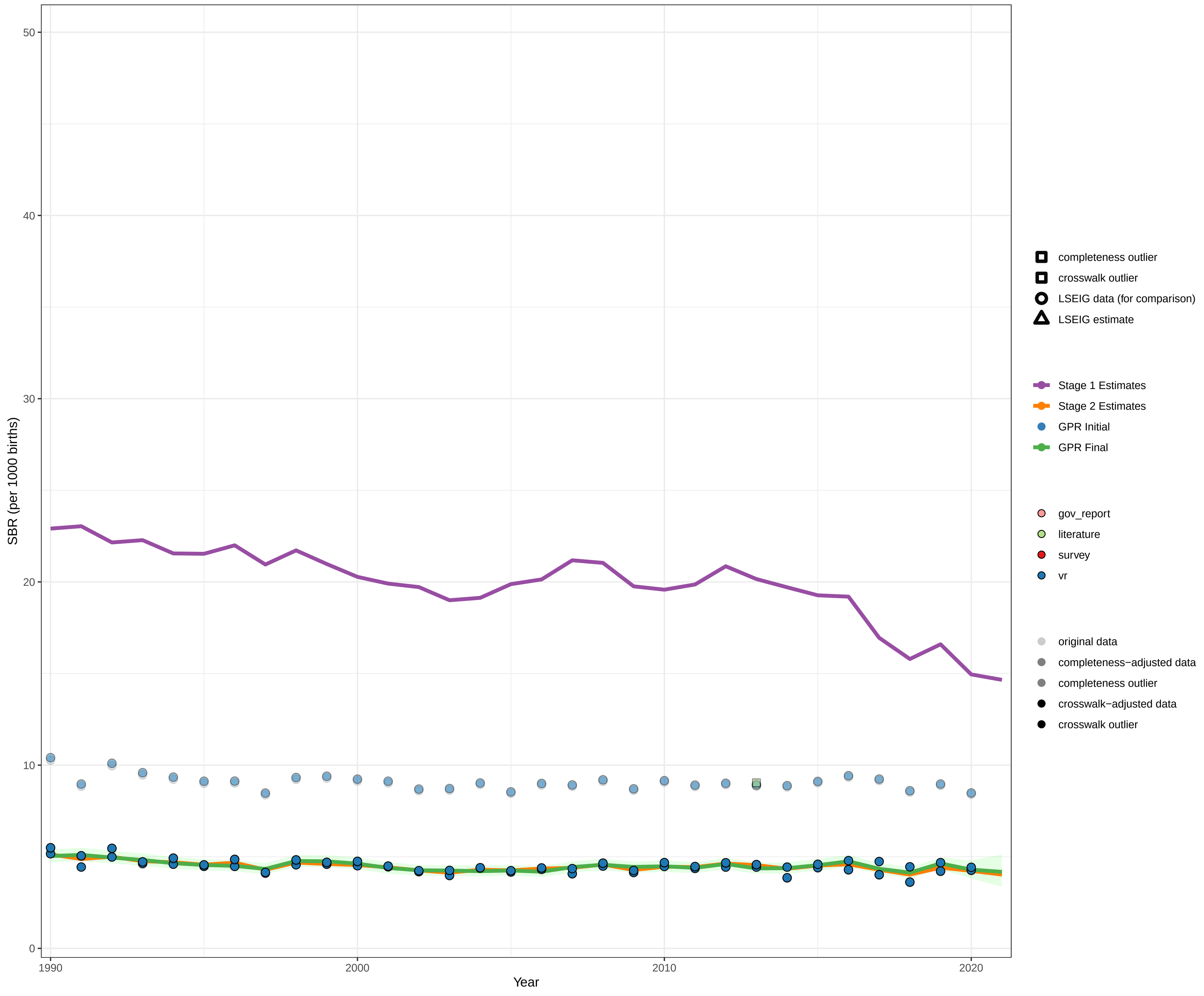

Year	Total Stillbirths (in thousands)	Total Stillbirths (in thousands)	SBR (per 1000 births)	SBR (per 1000 births)	SBR/NMR	SBR/NMR
	20 weeks	28 weeks	20 weeks	28 weeks	20 weeks	28 weeks
1990	0.7 (0.6 to 0.7)	0.3 (0.3 to 0.3)	10.2 (9.5 to 10.9)	5.0 (4.7 to 5.4)	1.40 (1.31 to 1.50)	0.69 (0.65 to 0.74)
1995	0.6 (0.5 to 0.6)	0.3 (0.3 to 0.3)	9.3 (8.6 to 9.9)	4.5 (4.3 to 4.9)	1.42 (1.32 to 1.52)	0.69 (0.65 to 0.74)
2000	0.6 (0.6 to 0.6)	0.3 (0.3 to 0.3)	9.5 (8.8 to 10.1)	4.6 (4.3 to 5.0)	1.58 (1.48 to 1.70)	0.77 (0.72 to 0.83)
2005	0.5 (0.5 to 0.6)	0.3 (0.2 to 0.3)	8.7 (8.1 to 9.3)	4.3 (4.0 to 4.6)	1.53 (1.43 to 1.64)	0.75 (0.70 to 0.80)
2010	0.5 (0.5 to 0.6)	0.3 (0.2 to 0.3)	9.2 (8.6 to 9.8)	4.5 (4.2 to 4.8)	1.71 (1.59 to 1.83)	0.83 (0.77 to 0.89)
2015	0.5 (0.5 to 0.6)	0.3 (0.3 to 0.3)	9.0 (8.4 to 9.7)	4.5 (4.2 to 4.9)	1.73 (1.61 to 1.86)	0.88 (0.82 to 0.94)
2020	0.5 (0.4 to 0.5)	0.3 (0.2 to 0.3)	8.2 (7.3 to 9.2)	4.3 (3.8 to 4.8)	2.10 (1.86 to 2.35)	1.10 (0.97 to 1.23)
2021	0.5 (0.4 to 0.6)	0.2 (0.2 to 0.3)	8.0 (6.4 to 9.8)	4.2 (3.4 to 5.1)	2.09 (1.68 to 2.57)	1.09 (0.88 to 1.34)

Alaska USA_524 High−income North America Stillbirth Rate / Neonatal Mortality Rate (SBR/NMR) − 20 weeks
lambda = 0.2 ; zeta = 0.99 ; scale = 5 ; amp = 0.028

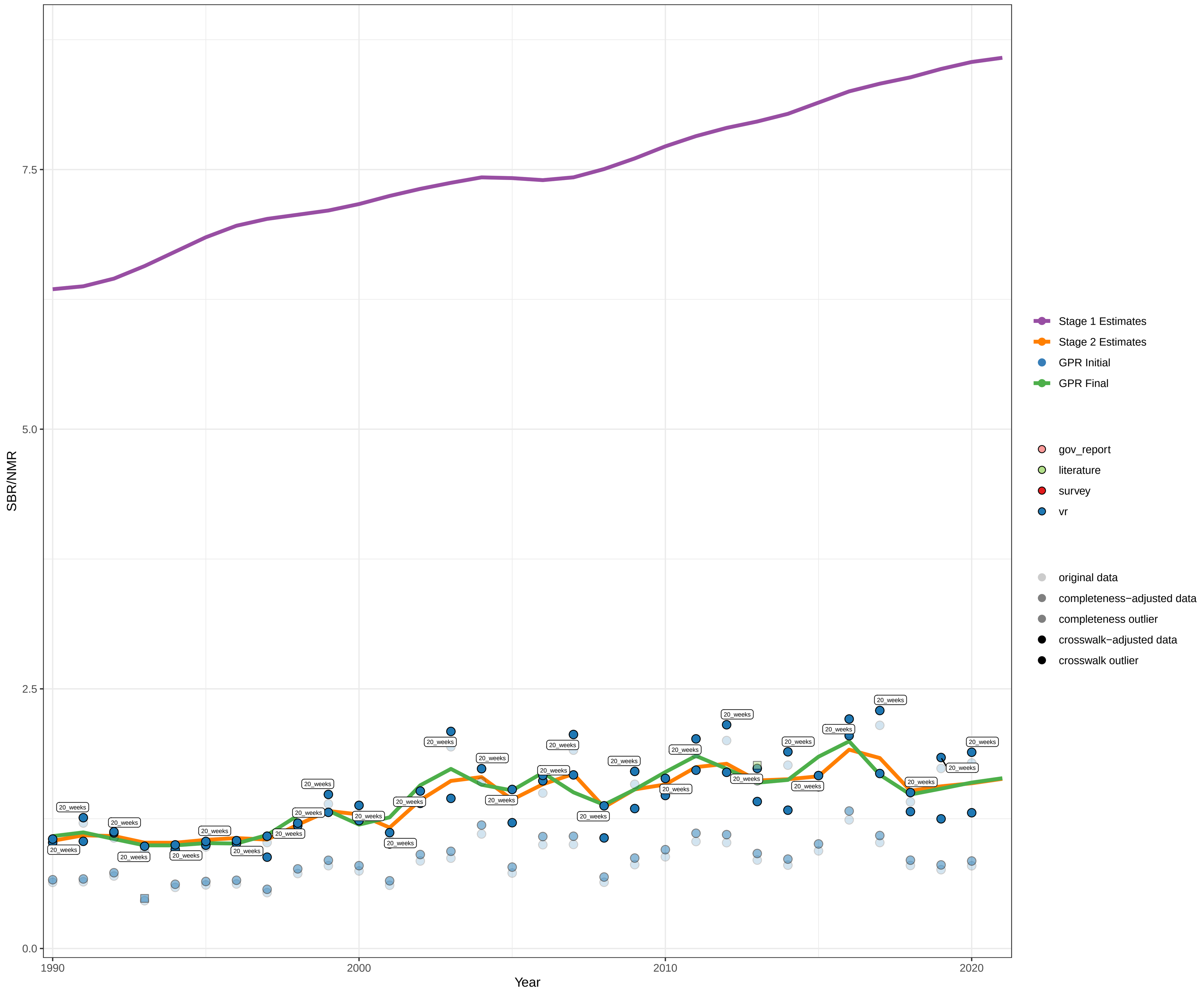

Alaska USA_524 High−income North America Stillbirth Rate / Neonatal Mortality Rate (SBR/NMR) − 28 weeks
lambda = 0.2 ; zeta = 0.99 ; scale = 5 ; amp = 0.028

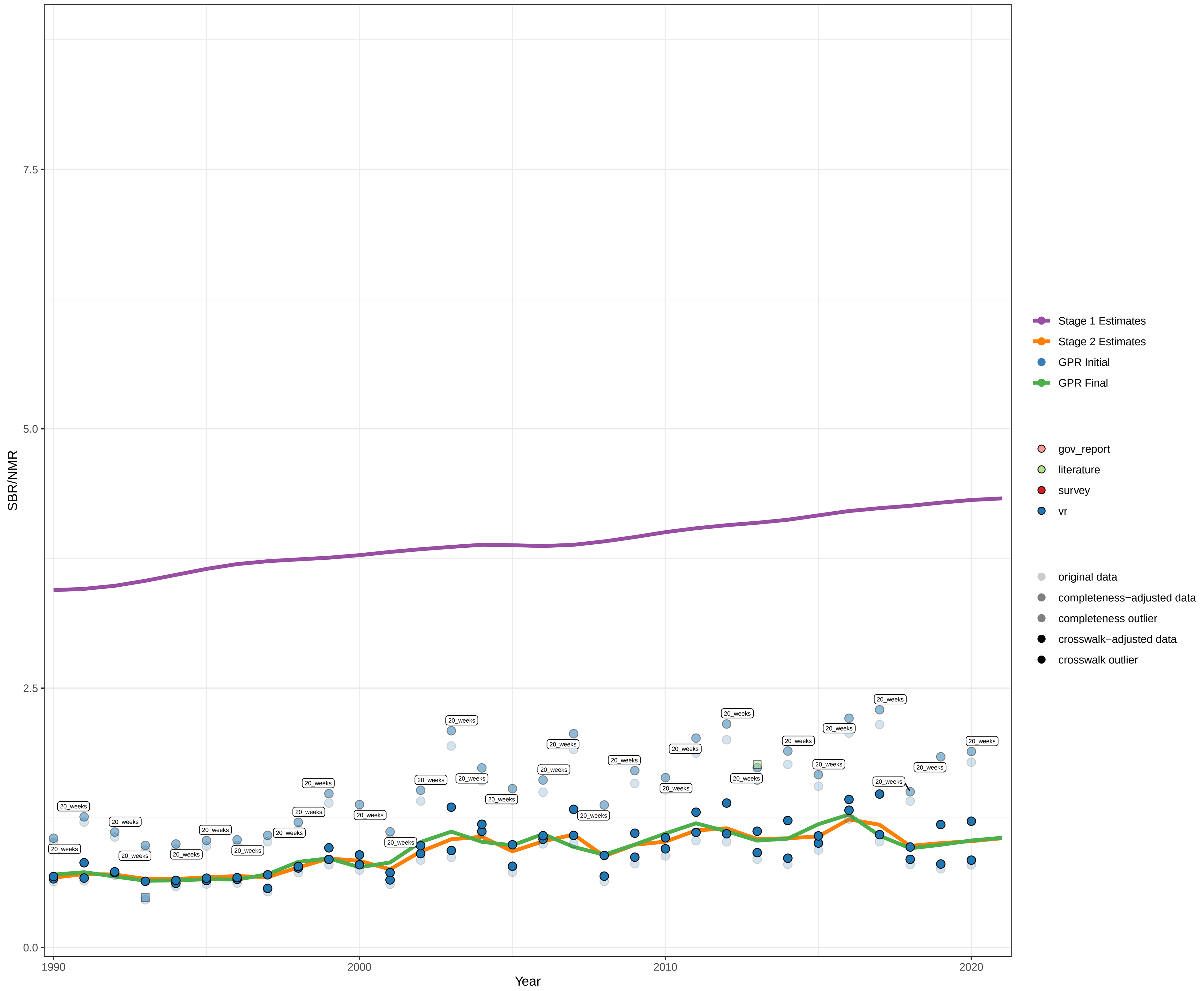

Definition	Years	Outlier	Source.Type	Title
20_weeks	1990−2020	0	VR	United States NVSS Custom Fetal Death Data
28_weeks	1990−1992, 1994−2020	0	VR	United States NVSS Custom Fetal Death Data
20_weeks	2013	1	Sci Lit	United States Fetal and Perinatal Mortality 2013
28_weeks	1993	1	VR	United States NVSS Custom Fetal Death Data

Alaska USA_524 High−income North America Stillbirth Rate (SBR) − 20 weeks
lambda = 0.2 ; zeta = 0.99 ; scale = 5 ; amp = 0.028

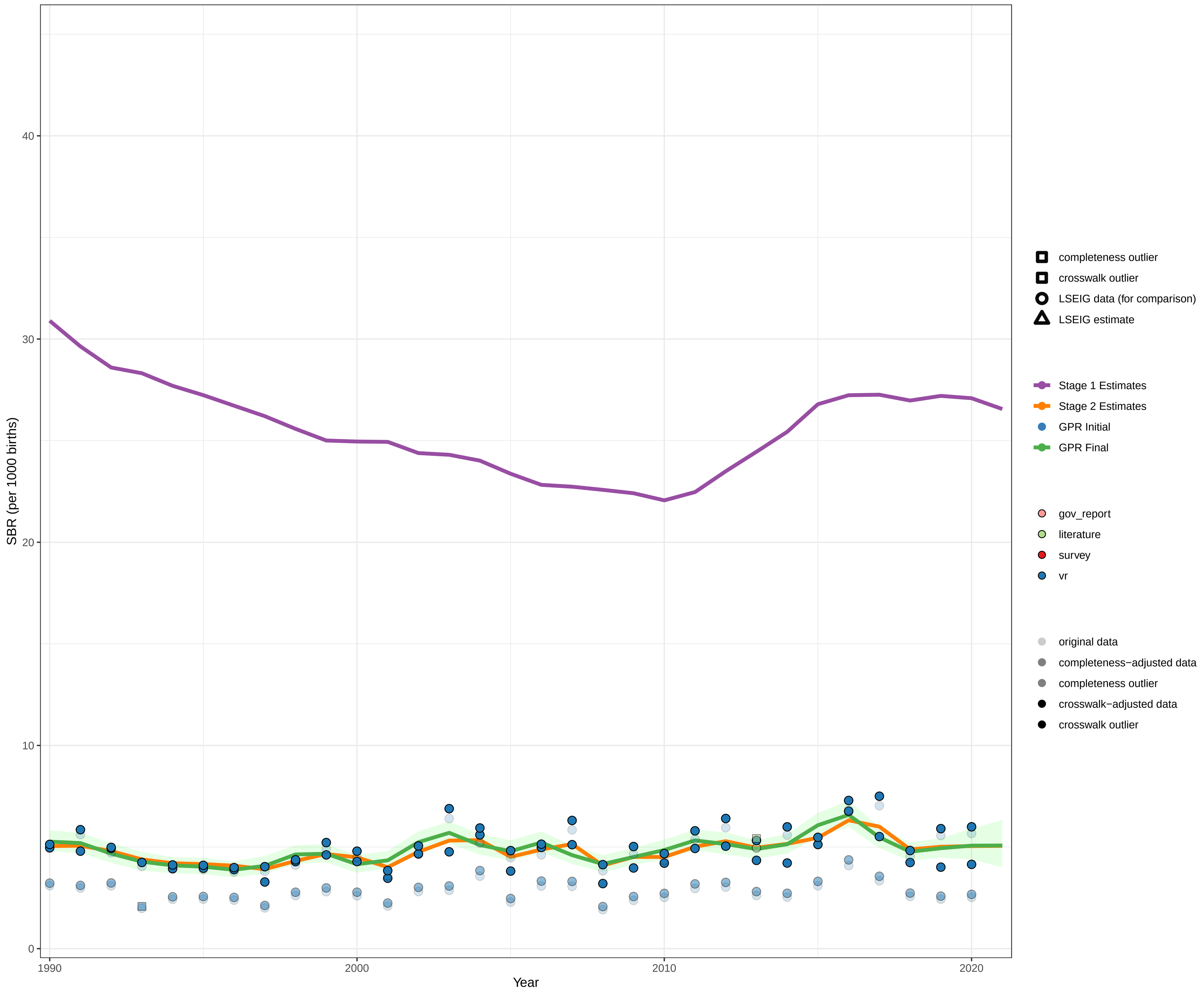

Alaska USA_524 High−income North America Stillbirth Rate (SBR) − 28 weeks
lambda = 0.2 ; zeta = 0.99 ; scale = 5 ; amp = 0.028

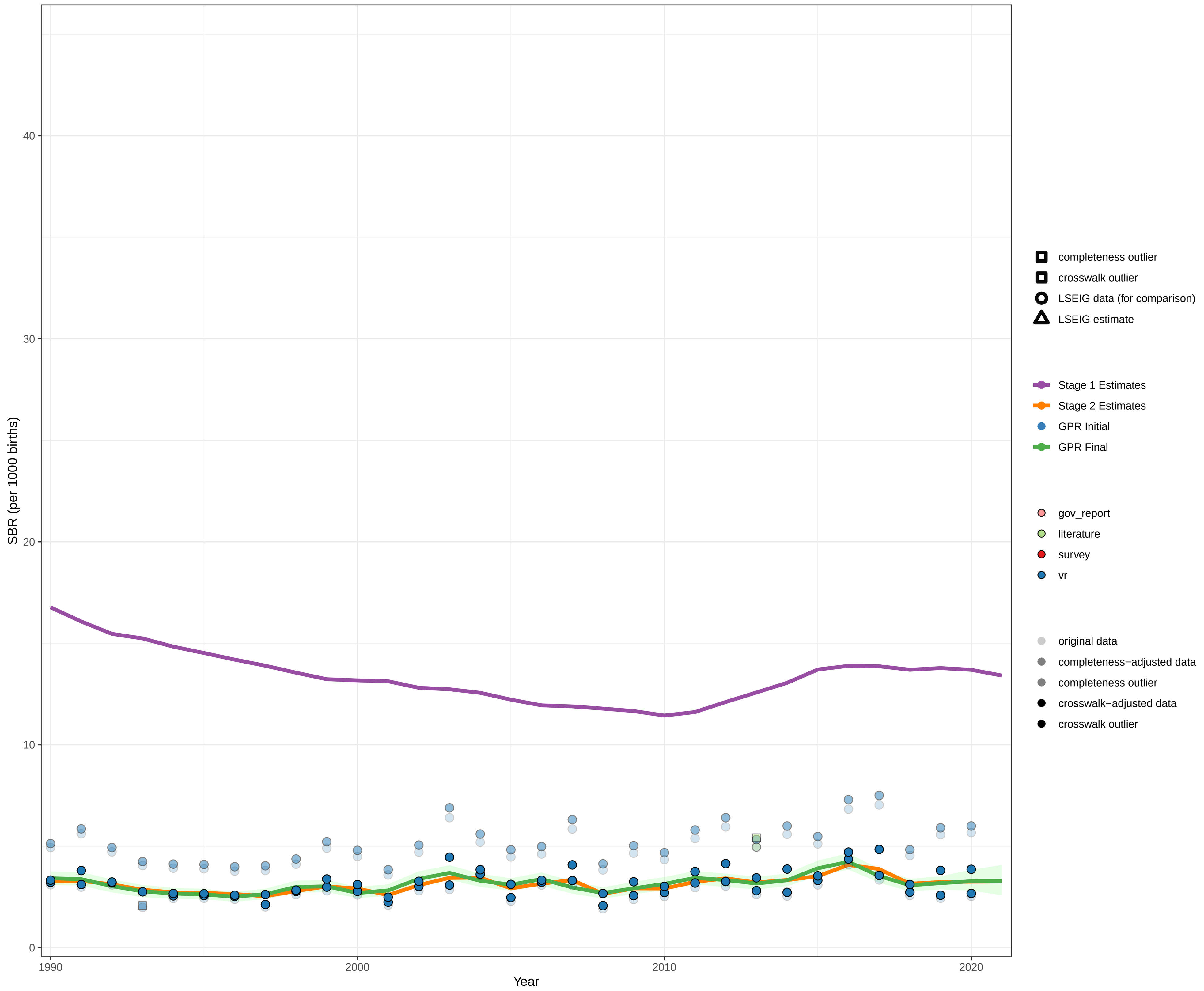

Year	Total Stillbirths (in thousands)	Total Stillbirths (in thousands)	SBR (per 1000 births)	SBR (per 1000 births)	SBR/NMR	SBR/NMR
	20 weeks	28 weeks	20 weeks	28 weeks	20 weeks	28 weeks
1990	0.1 (0.1 to 0.1)	0.0 (0.0 to 0.0)	5.3 (4.8 to 5.8)	3.4 (3.1 to 3.8)	1.08 (0.98 to 1.20)	0.70 (0.64 to 0.77)
1995	0.0 (0.0 to 0.0)	0.0 (0.0 to 0.0)	4.0 (3.7 to 4.5)	2.6 (2.4 to 2.9)	1.01 (0.92 to 1.12)	0.66 (0.60 to 0.73)
2000	0.0 (0.0 to 0.0)	0.0 (0.0 to 0.0)	4.2 (3.7 to 4.6)	2.7 (2.4 to 3.0)	1.19 (1.07 to 1.33)	0.77 (0.70 to 0.86)
2005	0.1 (0.0 to 0.1)	0.0 (0.0 to 0.0)	4.8 (4.3 to 5.3)	3.1 (2.8 to 3.4)	1.52 (1.38 to 1.69)	0.99 (0.89 to 1.08)
2010	0.1 (0.0 to 0.1)	0.0 (0.0 to 0.0)	4.9 (4.4 to 5.4)	3.1 (2.8 to 3.5)	1.70 (1.54 to 1.88)	1.10 (0.99 to 1.22)
2015	0.1 (0.1 to 0.1)	0.0 (0.0 to 0.0)	6.1 (5.5 to 6.7)	3.9 (3.5 to 4.3)	1.85 (1.67 to 2.03)	1.19 (1.07 to 1.31)
2020	0.0 (0.0 to 0.1)	0.0 (0.0 to 0.0)	5.1 (4.4 to 5.9)	3.3 (2.8 to 3.8)	1.60 (1.38 to 1.86)	1.03 (0.89 to 1.21)
2021	0.0 (0.0 to 0.1)	0.0 (0.0 to 0.0)	5.1 (4.0 to 6.3)	3.3 (2.6 to 4.1)	1.64 (1.29 to 2.05)	1.06 (0.84 to 1.32)

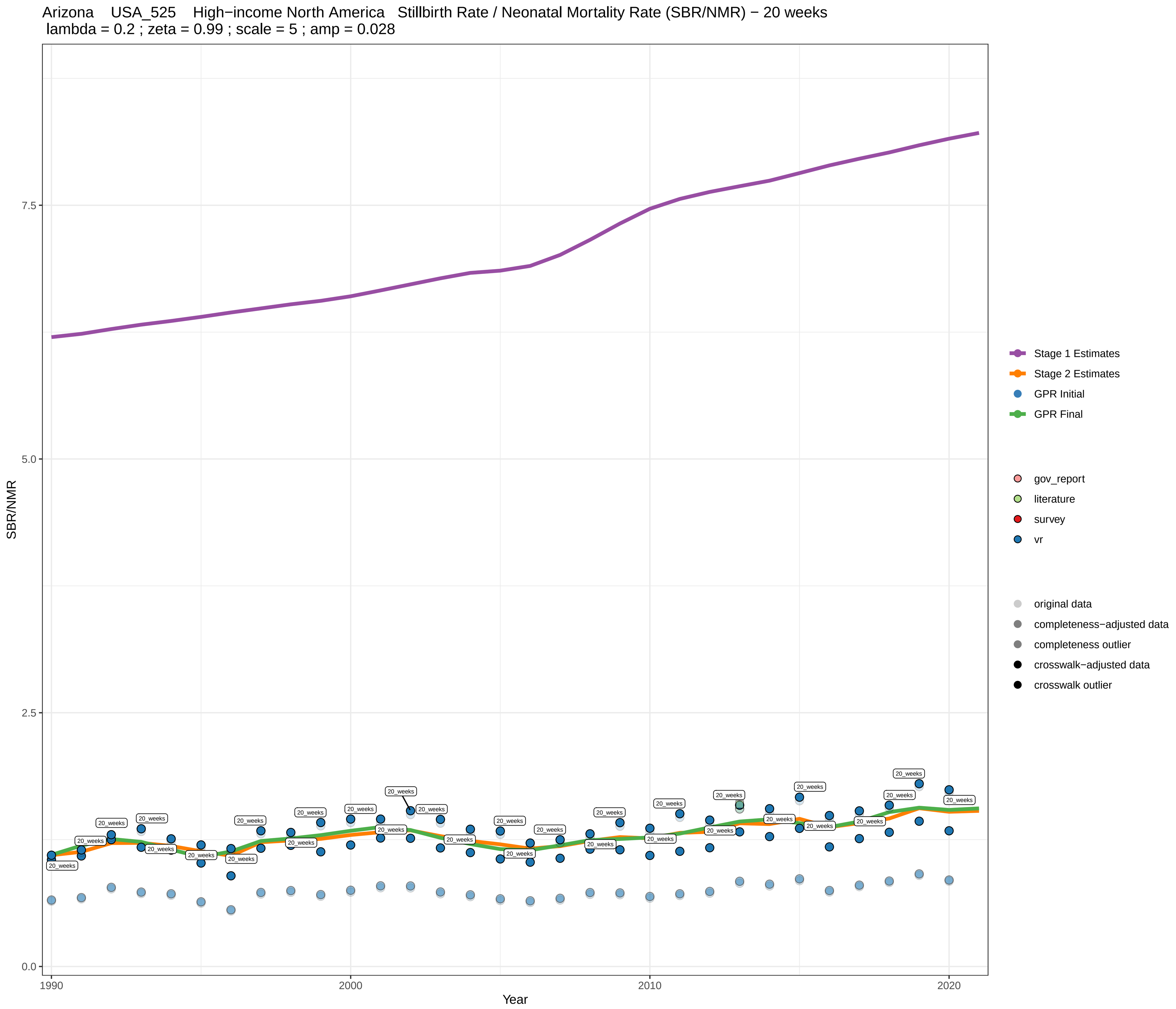

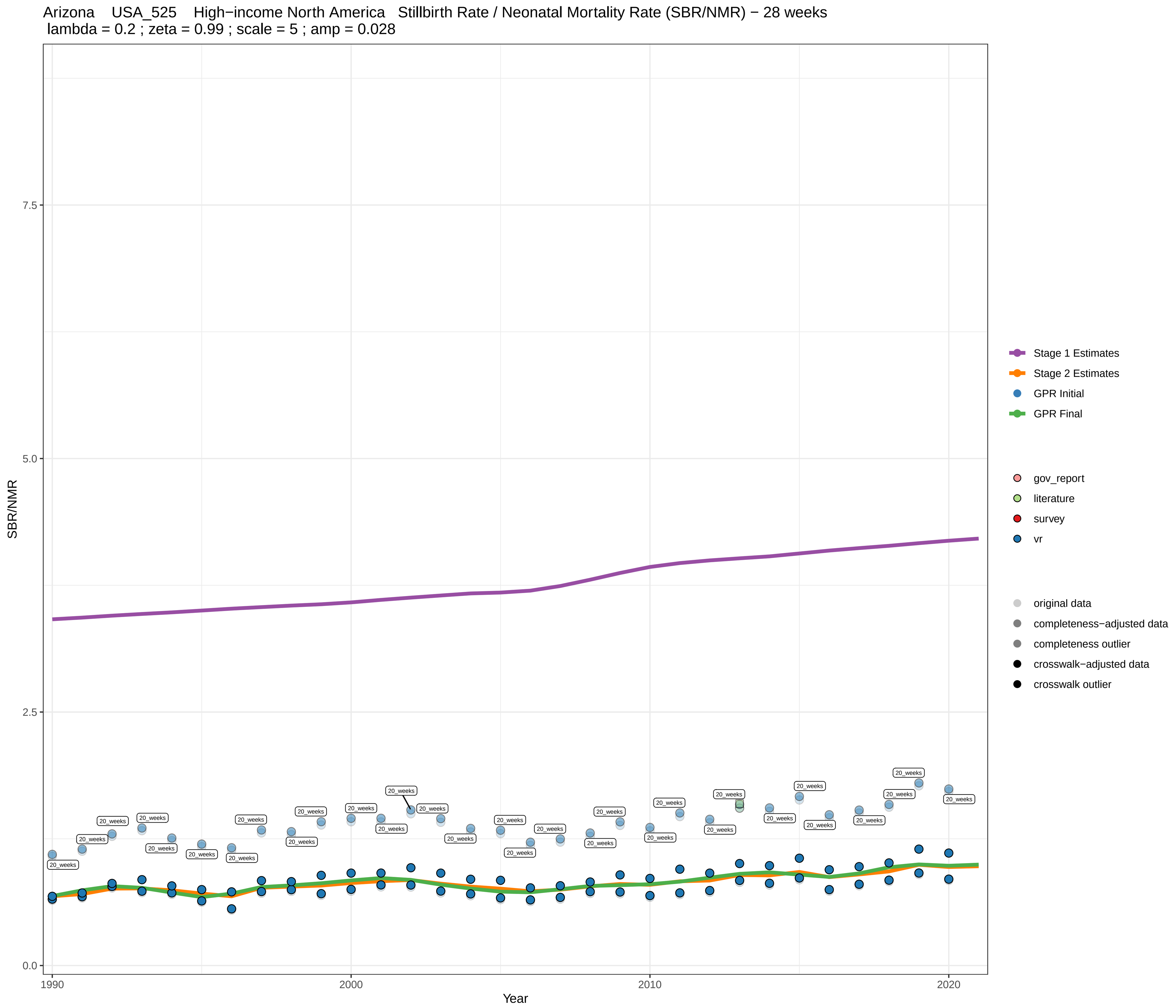

Definition	Years	Outlier	Source.Type	Title
20_weeks, 28_weeks	1990−2020	0	VR	United States NVSS Custom Fetal Death Data
20_weeks	2013	1	Sci Lit	United States Fetal and Perinatal Mortality 2013

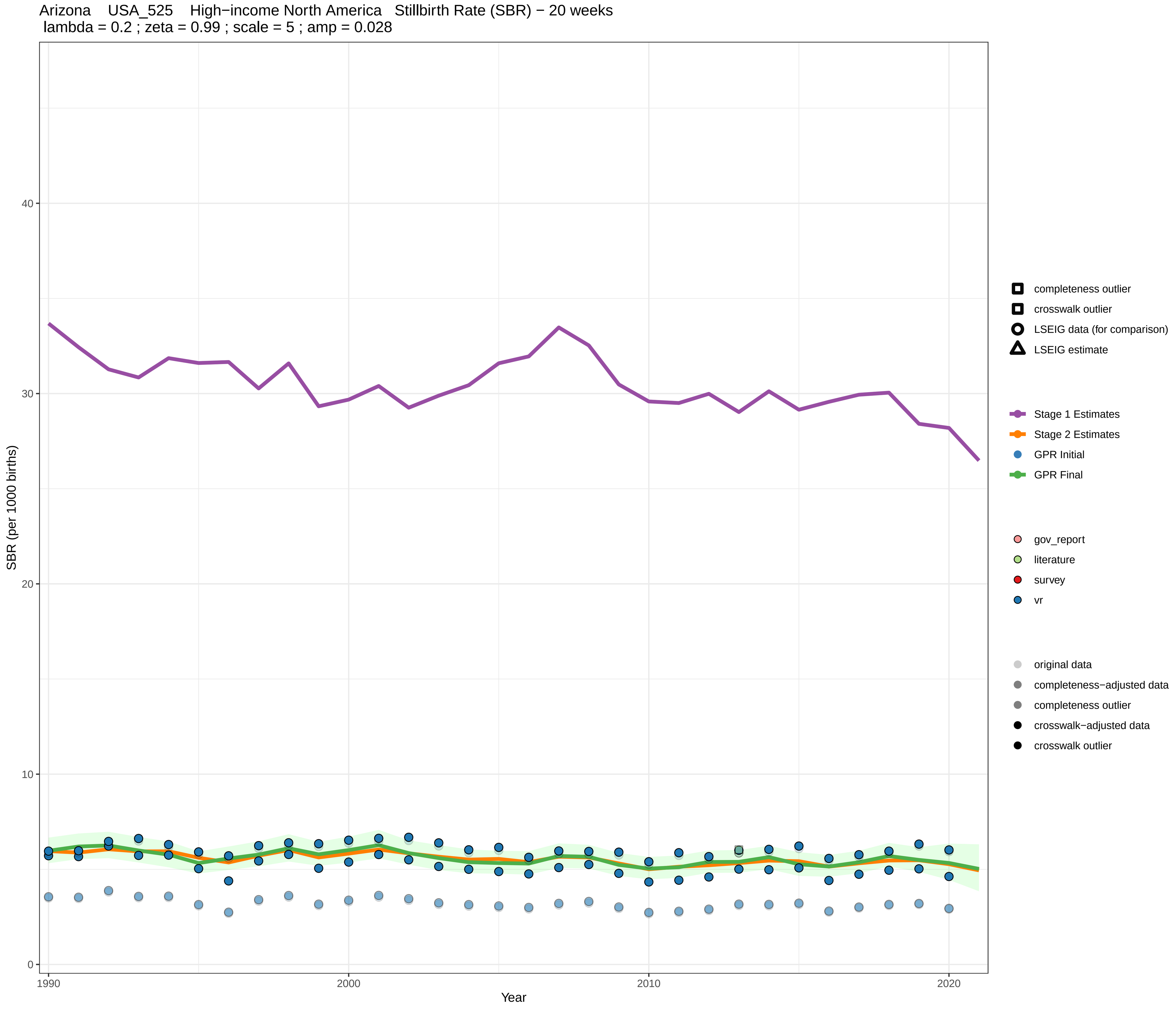

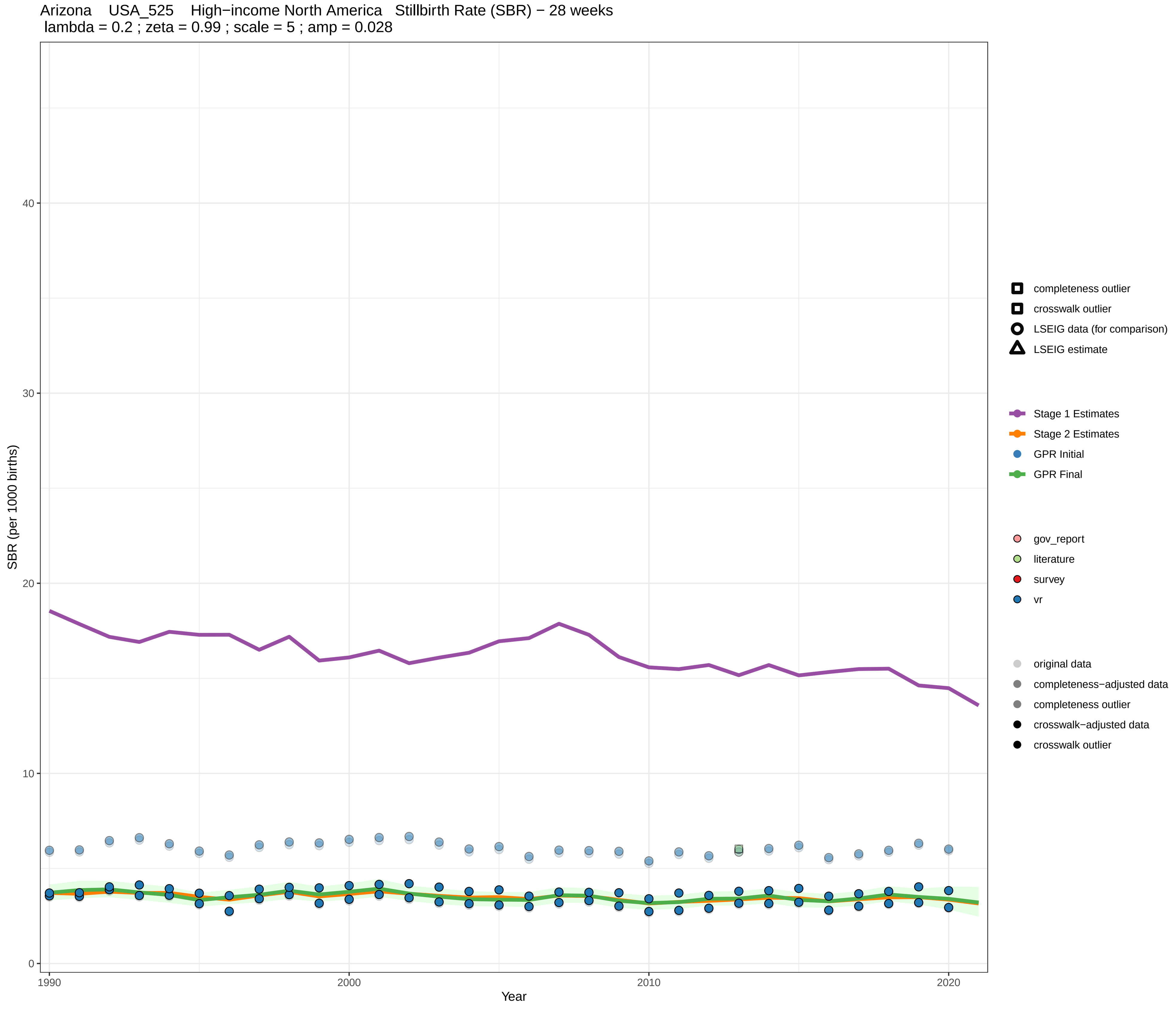

Year	Total Stillbirths (in thousands)	Total Stillbirths (in thousands)	SBR (per 1000 births)	SBR (per 1000 births)	SBR/NMR	SBR/NMR
	20 weeks	28 weeks	20 weeks	28 weeks	20 weeks	28 weeks
1990	0.4 (0.4 to 0.5)	0.3 (0.2 to 0.3)	6.0 (5.3 to 6.7)	3.7 (3.3 to 4.2)	1.10 (0.98 to 1.23)	0.69 (0.61 to 0.76)
1995	0.4 (0.3 to 0.4)	0.2 (0.2 to 0.3)	5.3 (4.8 to 5.9)	3.3 (3.0 to 3.7)	1.08 (0.97 to 1.20)	0.68 (0.61 to 0.75)
2000	0.5 (0.5 to 0.6)	0.3 (0.3 to 0.4)	6.0 (5.4 to 6.7)	3.8 (3.4 to 4.2)	1.34 (1.19 to 1.49)	0.84 (0.75 to 0.94)
2005	0.5 (0.5 to 0.6)	0.3 (0.3 to 0.4)	5.3 (4.8 to 6.0)	3.4 (3.0 to 3.8)	1.16 (1.03 to 1.30)	0.73 (0.65 to 0.82)
2010	0.4 (0.4 to 0.5)	0.3 (0.3 to 0.3)	5.0 (4.5 to 5.7)	3.2 (2.8 to 3.6)	1.27 (1.13 to 1.43)	0.80 (0.71 to 0.90)
2015	0.4 (0.4 to 0.5)	0.3 (0.3 to 0.3)	5.3 (4.7 to 5.9)	3.3 (3.0 to 3.7)	1.41 (1.25 to 1.58)	0.90 (0.79 to 1.00)
2020	0.4 (0.3 to 0.5)	0.3 (0.2 to 0.3)	5.3 (4.4 to 6.4)	3.4 (2.8 to 4.1)	1.54 (1.29 to 1.84)	0.98 (0.82 to 1.17)
2021	0.4 (0.3 to 0.5)	0.2 (0.2 to 0.3)	5.0 (3.8 to 6.3)	3.2 (2.5 to 4.0)	1.56 (1.19 to 1.96)	1.00 (0.76 to 1.25)

Arkansas   USA_526   High−income North America   Stillbirth Rate / Neonatal Mortality Rate (SBR/NMR) − 20 weeks
lambda = 0.2 ; zeta = 0.99 ; scale = 5 ; amp = 0.028

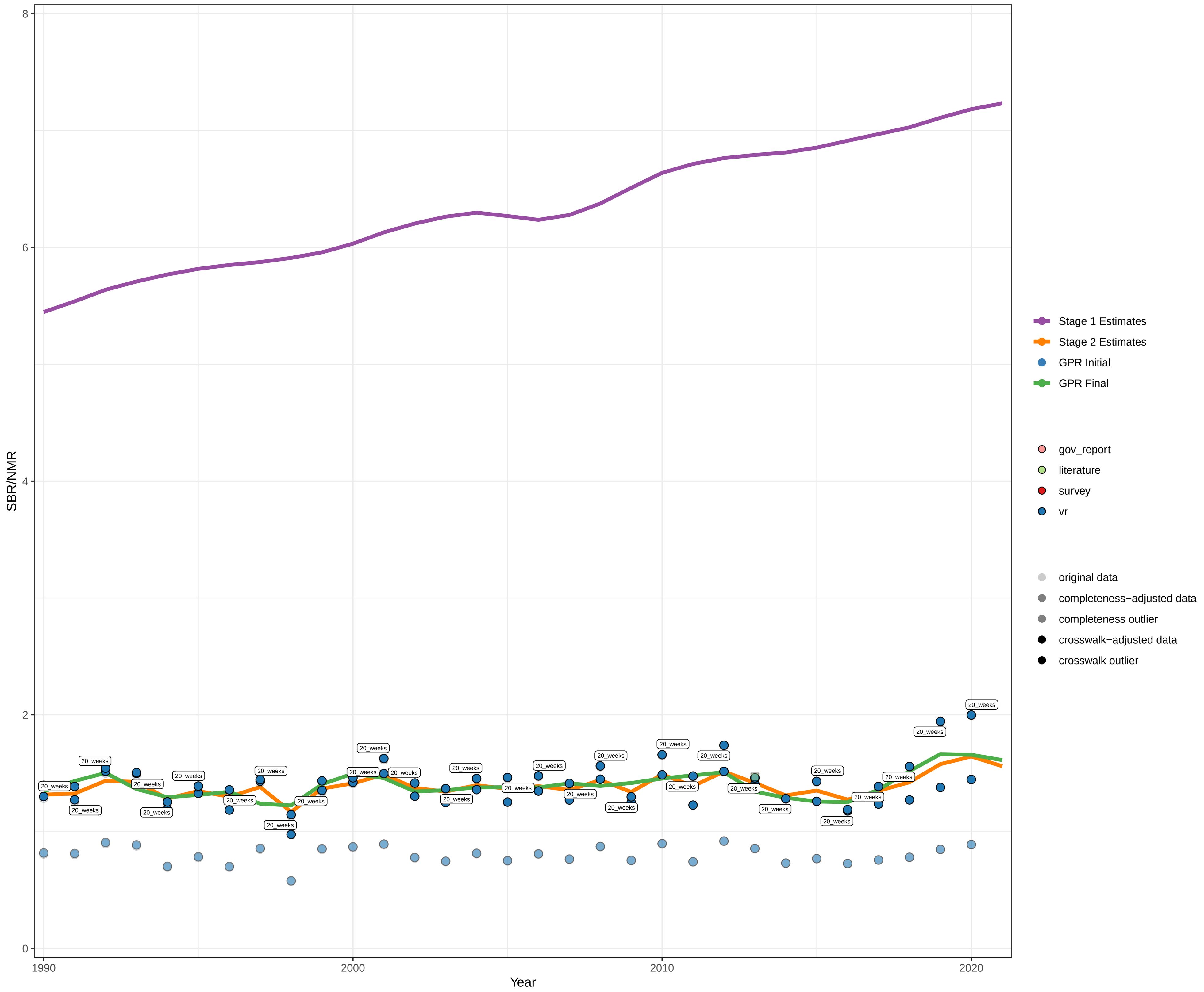

Arkansas   USA_526   High−income North America   Stillbirth Rate / Neonatal Mortality Rate (SBR/NMR) − 28 weeks
lambda = 0.2 ; zeta = 0.99 ; scale = 5 ; amp = 0.028

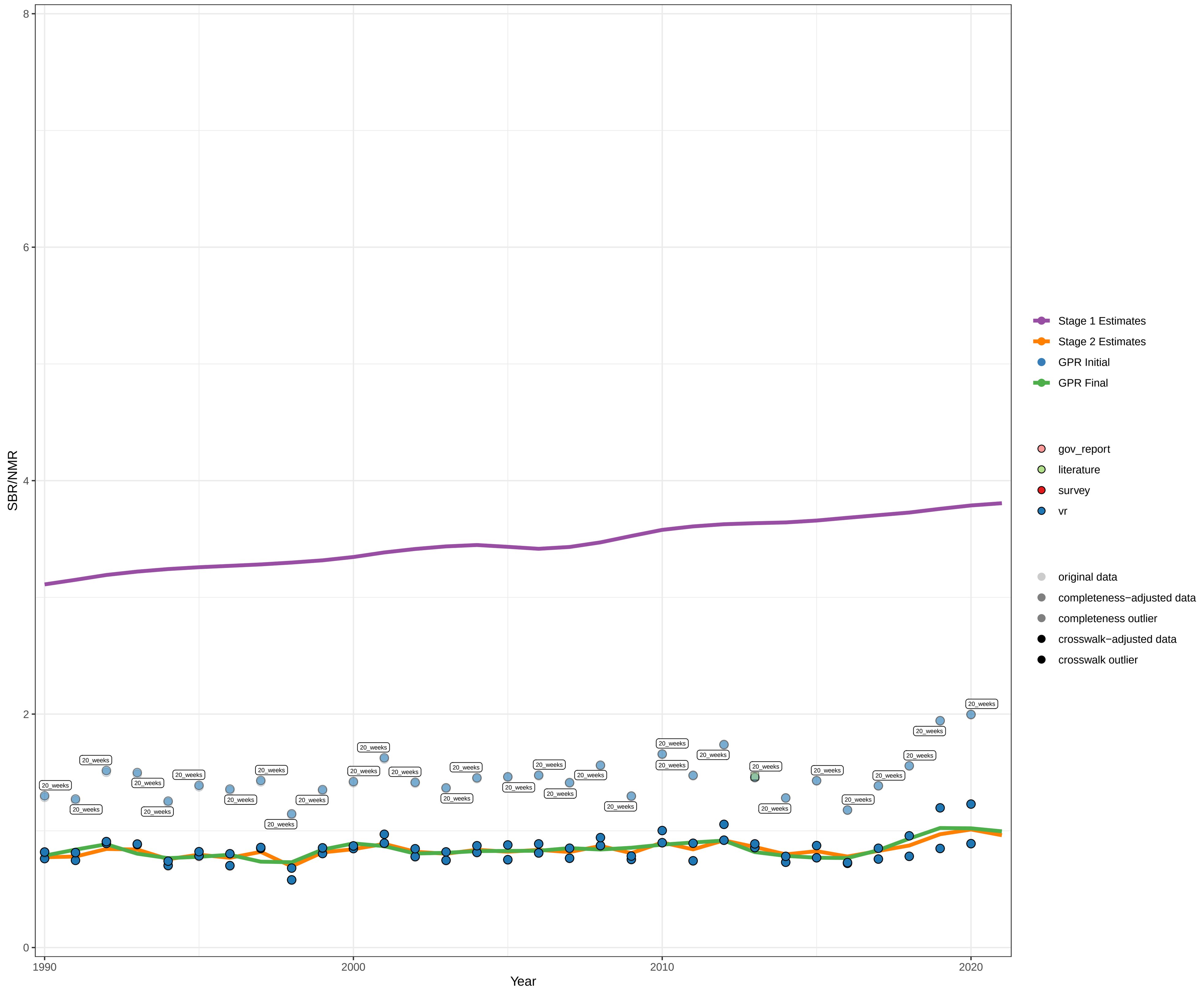

Definition	Years	Outlier	Source.Type	Title
20_weeks, 28_weeks	1990−2020	0	VR	United States NVSS Custom Fetal Death Data
20_weeks	2013	1	Sci Lit	United States Fetal and Perinatal Mortality 2013

Arkansas   USA_526   High−income North America   Stillbirth Rate (SBR) − 20 weeks
lambda = 0.2 ; zeta = 0.99 ; scale = 5 ; amp = 0.028

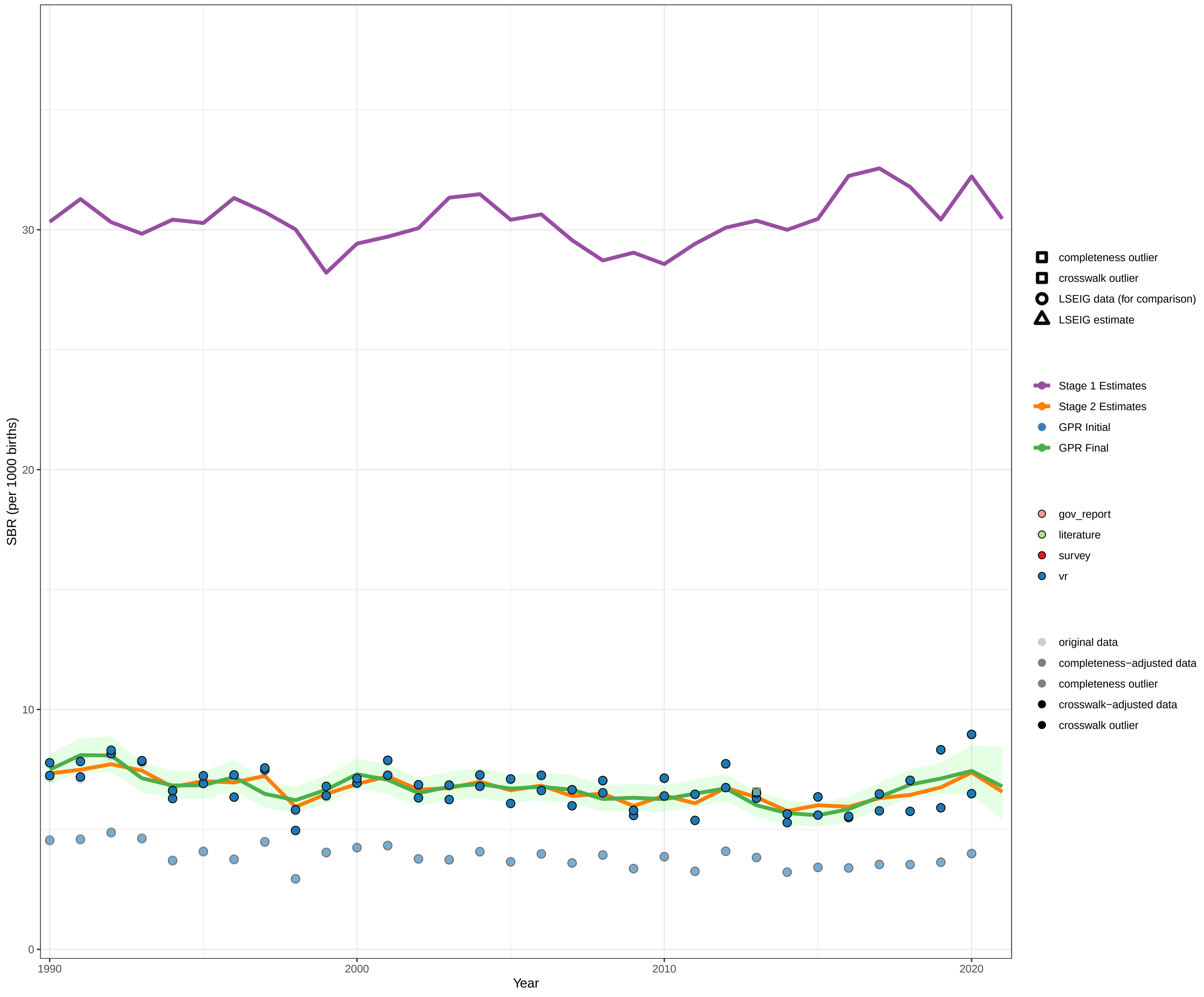

Arkansas   USA_526   High−income North America   Stillbirth Rate (SBR) − 28 weeks
lambda = 0.2 ; zeta = 0.99 ; scale = 5 ; amp = 0.028

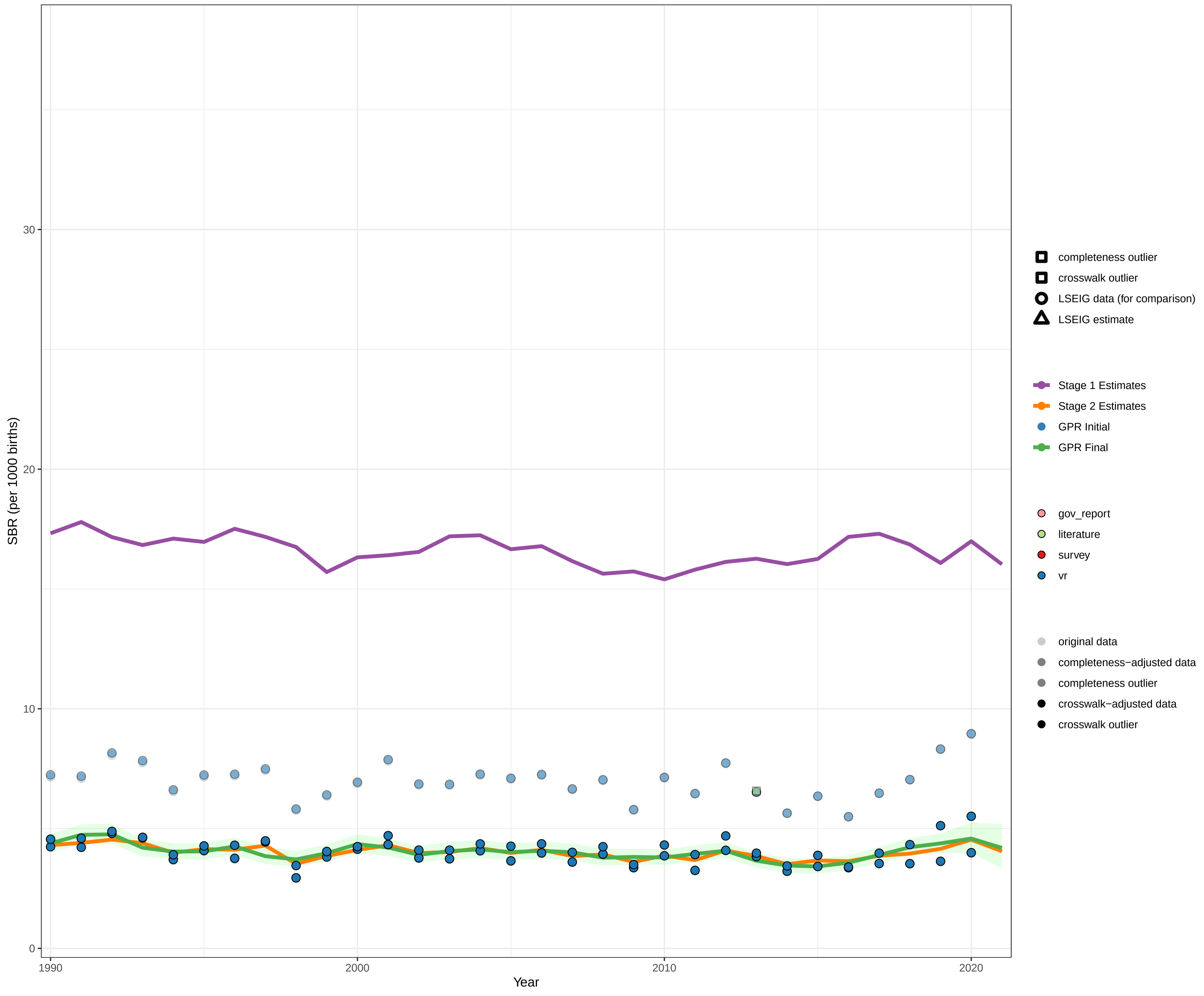

Year	Total Stillbirths (in thousands)	Total Stillbirths (in thousands)	SBR (per 1000 births)	SBR (per 1000 births)	SBR/NMR	SBR/NMR
	20 weeks	28 weeks	20 weeks	28 weeks	20 weeks	28 weeks
1990	0.3 (0.3 to 0.3)	0.2 (0.1 to 0.2)	7.5 (6.9 to 8.1)	4.4 (4.0 to 4.8)	1.35 (1.23 to 1.46)	0.79 (0.72 to 0.86)
1995	0.2 (0.2 to 0.3)	0.1 (0.1 to 0.2)	6.8 (6.3 to 7.4)	4.0 (3.7 to 4.4)	1.31 (1.20 to 1.43)	0.78 (0.71 to 0.85)
2000	0.3 (0.3 to 0.3)	0.2 (0.1 to 0.2)	7.3 (6.7 to 7.9)	4.4 (4.0 to 4.8)	1.50 (1.37 to 1.63)	0.89 (0.82 to 0.98)
2005	0.3 (0.2 to 0.3)	0.2 (0.1 to 0.2)	6.7 (6.1 to 7.3)	4.0 (3.7 to 4.4)	1.38 (1.26 to 1.50)	0.83 (0.76 to 0.90)
2010	0.2 (0.2 to 0.3)	0.1 (0.1 to 0.2)	6.3 (5.7 to 6.8)	3.8 (3.5 to 4.1)	1.45 (1.33 to 1.59)	0.88 (0.81 to 0.96)
2015	0.2 (0.2 to 0.2)	0.1 (0.1 to 0.1)	5.6 (5.1 to 6.1)	3.4 (3.1 to 3.7)	1.26 (1.15 to 1.37)	0.77 (0.70 to 0.84)
2020	0.3 (0.2 to 0.3)	0.2 (0.1 to 0.2)	7.4 (6.4 to 8.5)	4.6 (4.0 to 5.2)	1.66 (1.44 to 1.90)	1.02 (0.88 to 1.17)
2021	0.2 (0.2 to 0.3)	0.2 (0.1 to 0.2)	6.8 (5.4 to 8.4)	4.2 (3.3 to 5.2)	1.61 (1.28 to 2.00)	1.00 (0.79 to 1.23)

California   USA_527   High−income North America   Stillbirth Rate / Neonatal Mortality Rate (SBR/NMR) − 20 weeks
lambda = 0.2 ; zeta = 0.99 ; scale = 5 ; amp = 0.028

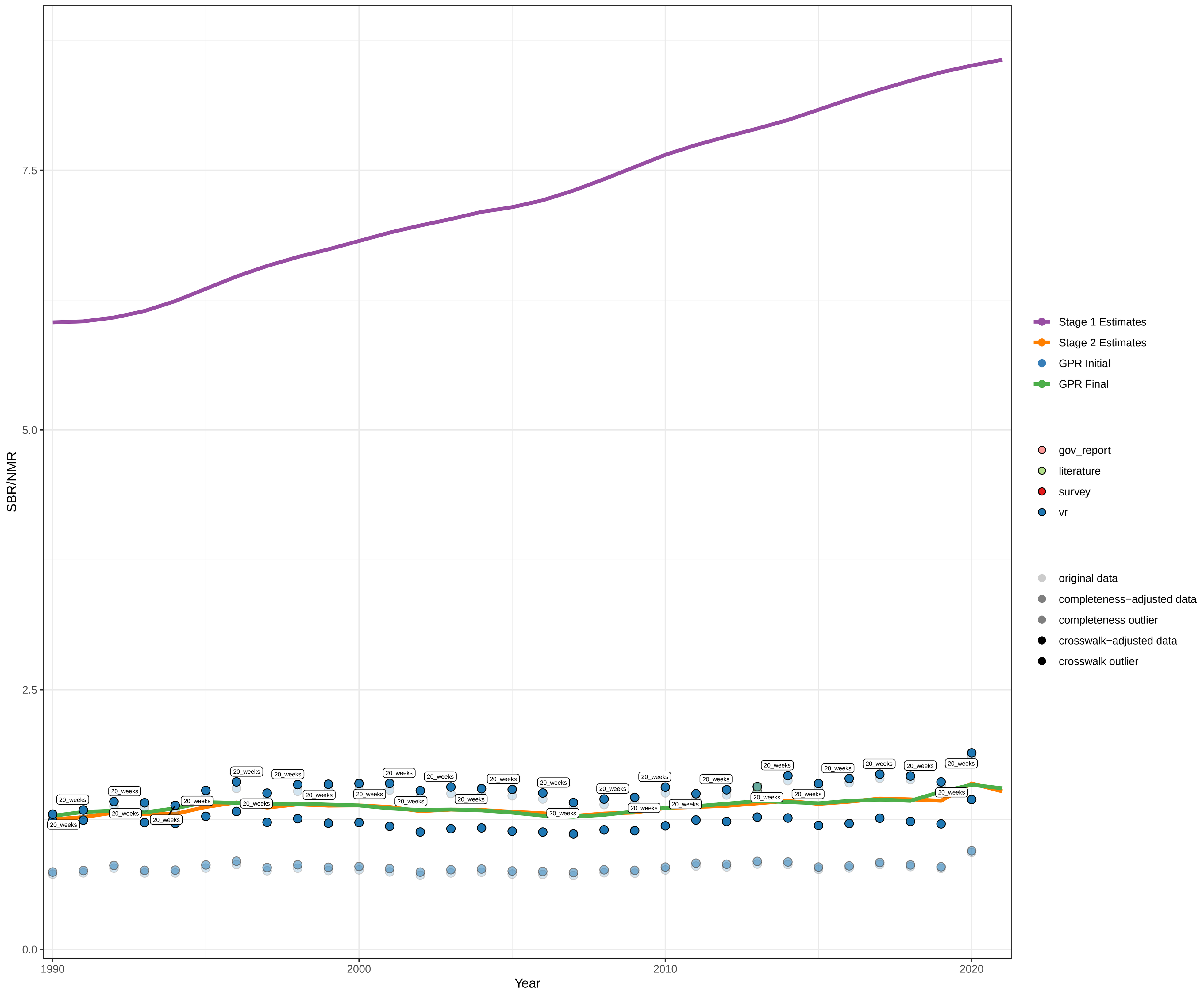

California   USA_527   High−income North America   Stillbirth Rate / Neonatal Mortality Rate (SBR/NMR) − 28 weeks
lambda = 0.2 ; zeta = 0.99 ; scale = 5 ; amp = 0.028

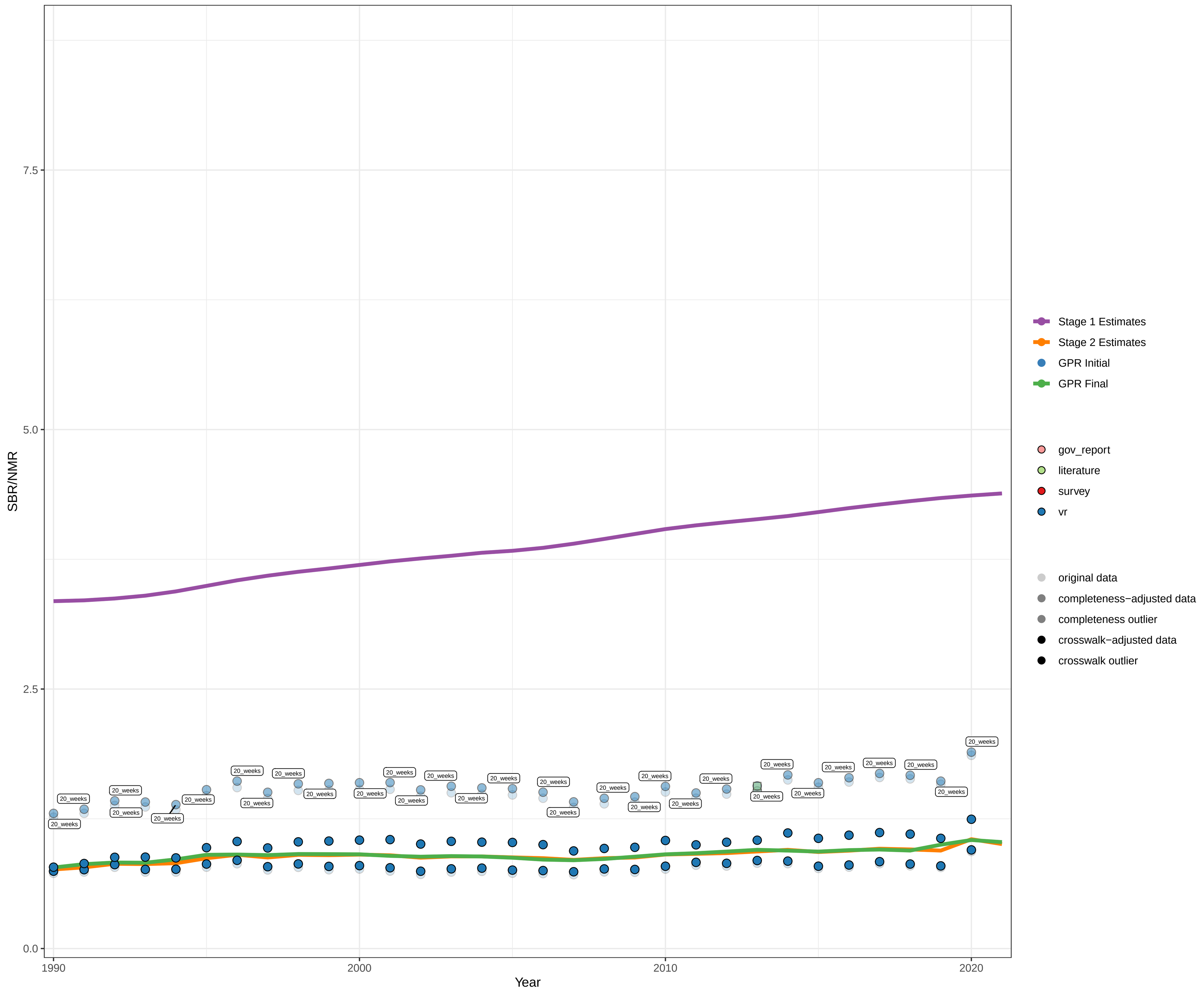

Definition	Years	Outlier	Source.Type	Title
20_weeks, 28_weeks	1990−2020	0	VR	United States NVSS Custom Fetal Death Data
20_weeks	2013	1	Sci Lit	United States Fetal and Perinatal Mortality 2013

California   USA_527   High−income North America   Stillbirth Rate (SBR) − 20 weeks
lambda = 0.2 ; zeta = 0.99 ; scale = 5 ; amp = 0.028

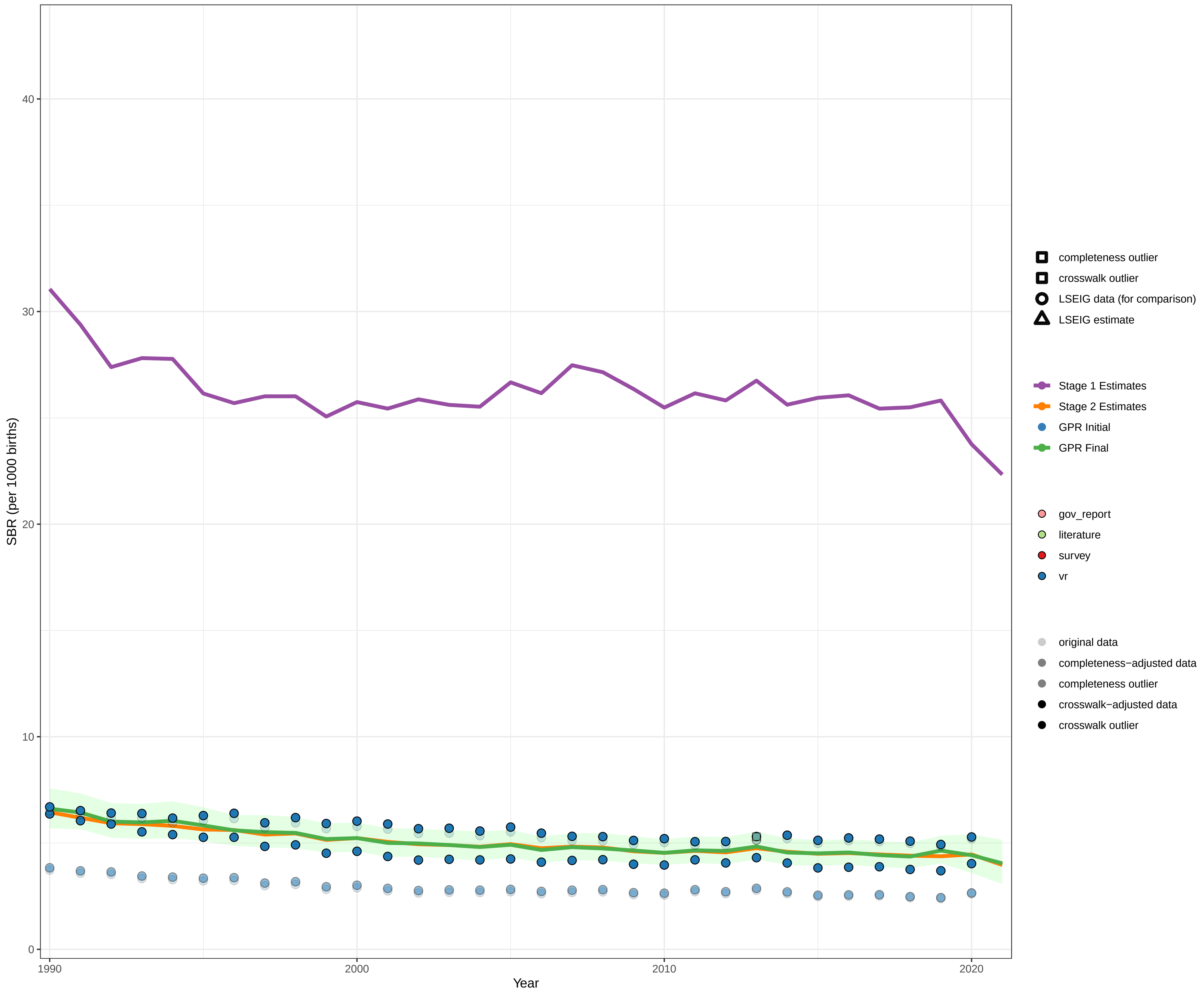

California   USA_527   High−income North America   Stillbirth Rate (SBR) − 28 weeks
lambda = 0.2 ; zeta = 0.99 ; scale = 5 ; amp = 0.028

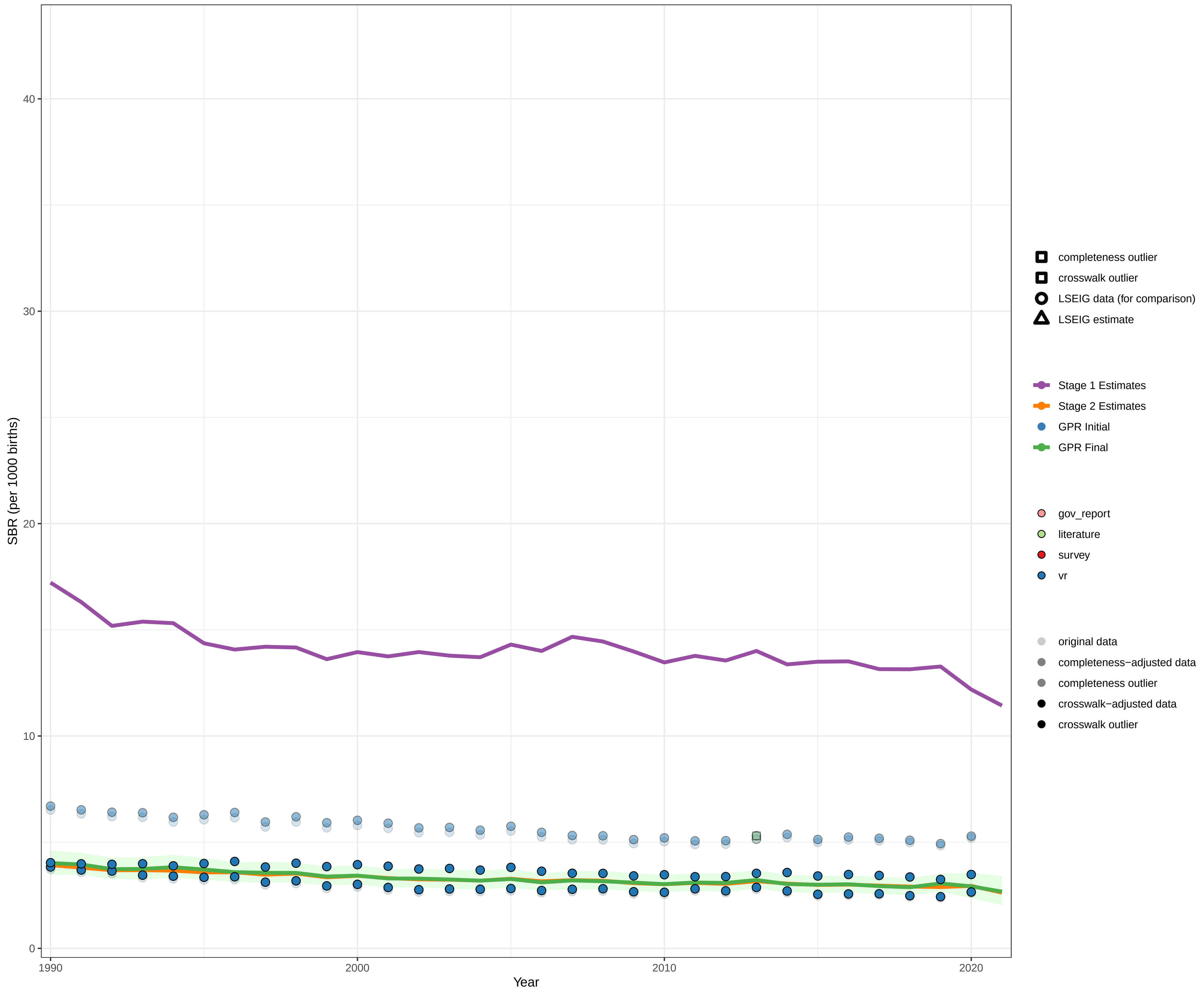

Year	Total Stillbirths (in thousands)	Total Stillbirths (in thousands)	SBR (per 1000 births)	SBR (per 1000 births)	SBR/NMR	SBR/NMR
	20 weeks	28 weeks	20 weeks	28 weeks	20 weeks	28 weeks
1990	4.0 (3.4 to 4.6)	2.4 (2.1 to 2.8)	6.6 (5.7 to 7.6)	4.0 (3.5 to 4.6)	1.29 (1.11 to 1.47)	0.78 (0.67 to 0.89)
1995	3.2 (2.8 to 3.7)	2.0 (1.8 to 2.4)	5.8 (5.1 to 6.7)	3.7 (3.2 to 4.3)	1.42 (1.24 to 1.63)	0.90 (0.79 to 1.04)
2000	2.8 (2.5 to 3.2)	1.8 (1.6 to 2.1)	5.2 (4.6 to 6.0)	3.4 (3.0 to 3.9)	1.39 (1.22 to 1.58)	0.91 (0.80 to 1.03)
2005	2.7 (2.4 to 3.1)	1.8 (1.6 to 2.1)	4.9 (4.3 to 5.6)	3.3 (2.9 to 3.7)	1.32 (1.16 to 1.50)	0.87 (0.77 to 1.00)
2010	2.3 (2.1 to 2.7)	1.6 (1.4 to 1.8)	4.5 (4.0 to 5.2)	3.0 (2.6 to 3.5)	1.36 (1.19 to 1.55)	0.91 (0.79 to 1.04)
2015	2.2 (1.9 to 2.5)	1.5 (1.3 to 1.7)	4.5 (3.9 to 5.1)	3.0 (2.6 to 3.4)	1.41 (1.22 to 1.60)	0.93 (0.81 to 1.06)
2020	1.9 (1.5 to 2.3)	1.2 (1.0 to 1.5)	4.4 (3.6 to 5.4)	2.9 (2.4 to 3.6)	1.59 (1.29 to 1.94)	1.05 (0.85 to 1.28)
2021	1.7 (1.3 to 2.2)	1.1 (0.9 to 1.4)	4.0 (3.1 to 5.2)	2.7 (2.0 to 3.4)	1.55 (1.18 to 1.98)	1.03 (0.78 to 1.30)

Colorado USA_528 High−income North America Stillbirth Rate / Neonatal Mortality Rate (SBR/NMR) − 20 weeks
lambda = 0.2 ; zeta = 0.99 ; scale = 5 ; amp = 0.028

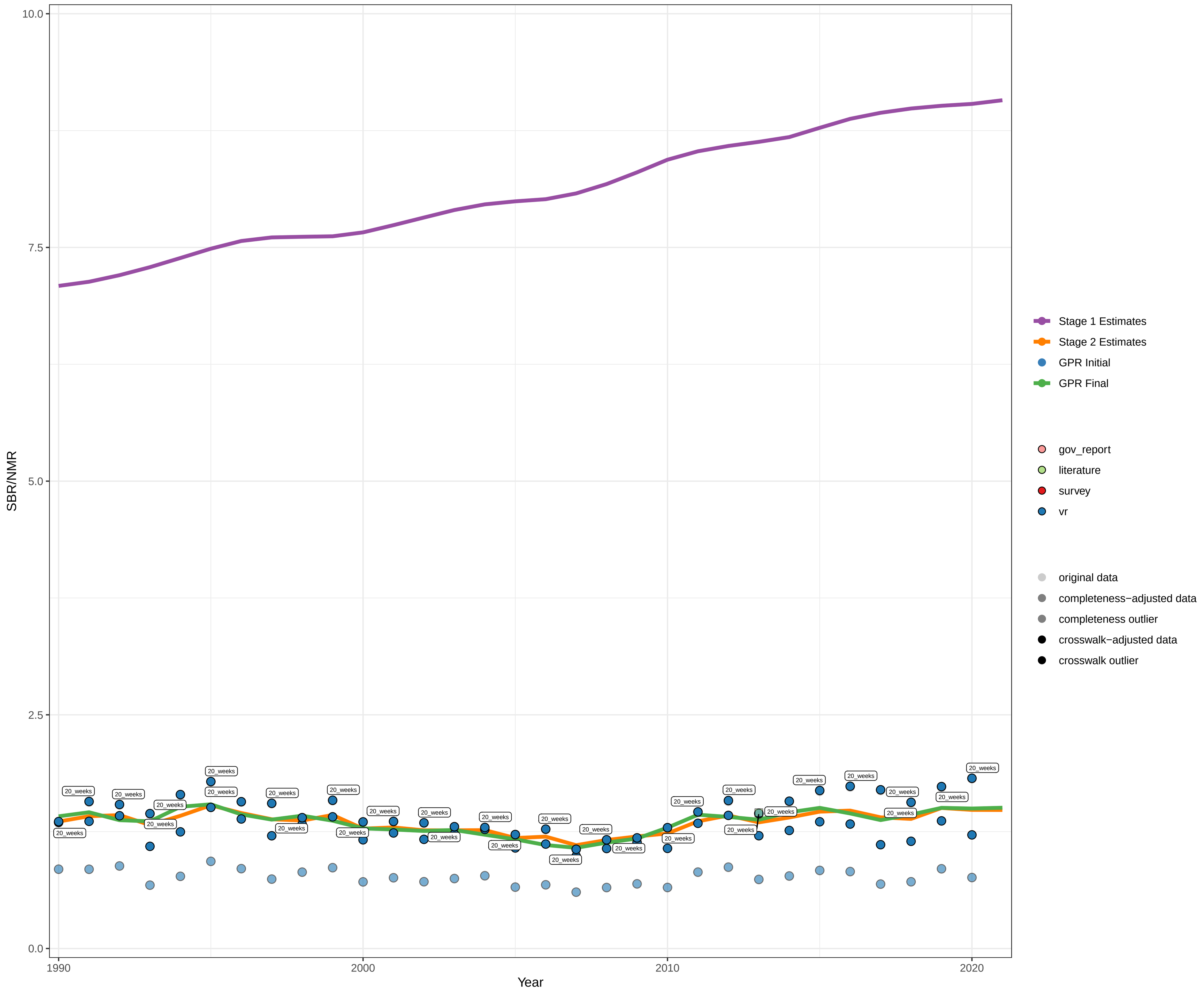

Colorado USA_528 High−income North America Stillbirth Rate / Neonatal Mortality Rate (SBR/NMR) − 28 weeks
lambda = 0.2 ; zeta = 0.99 ; scale = 5 ; amp = 0.028

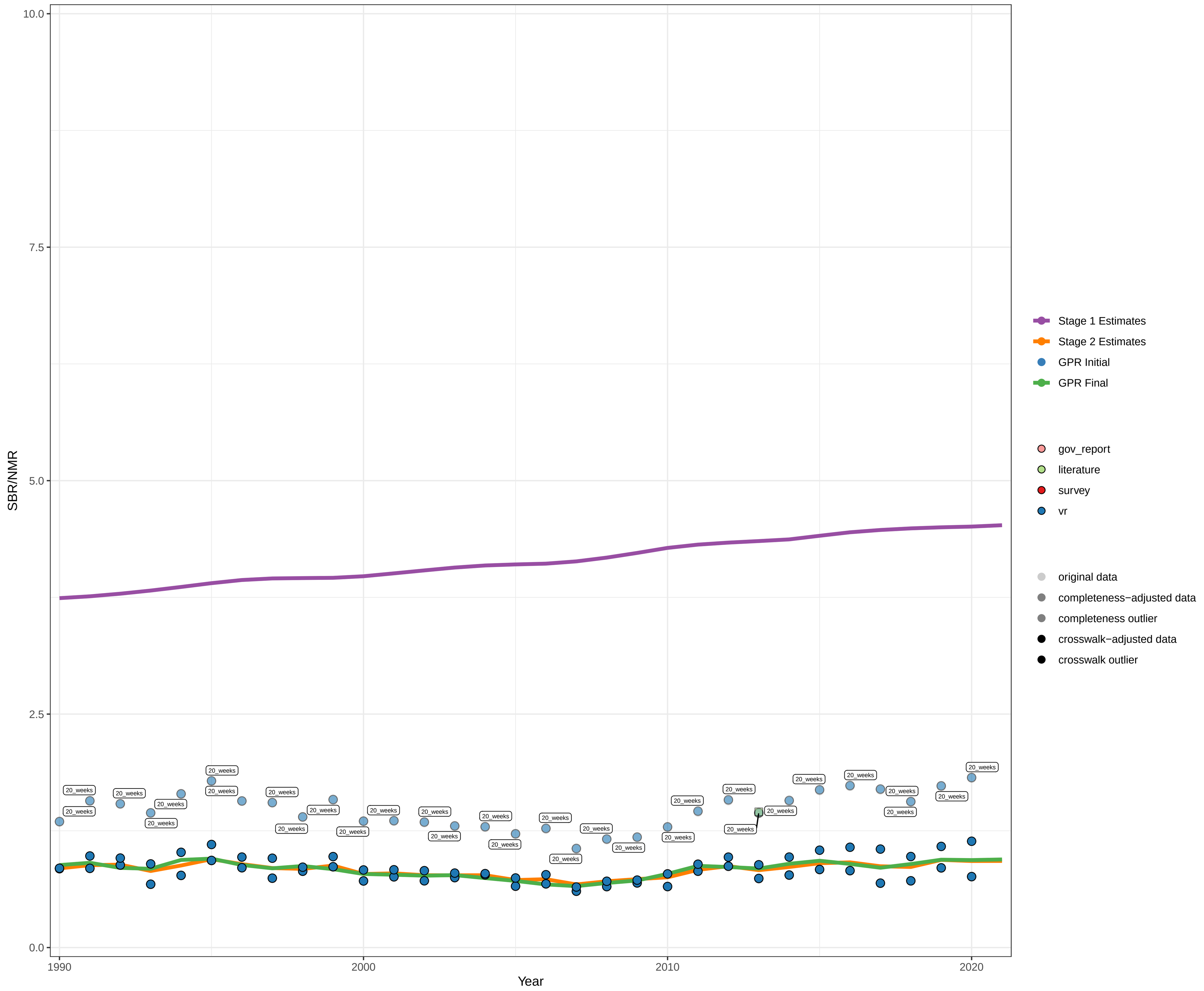

Definition	Years	Outlier	Source.Type	Title
20_weeks, 28_weeks	1990−2020	0	VR	United States NVSS Custom Fetal Death Data
20_weeks	2013	1	Sci Lit	United States Fetal and Perinatal Mortality 2013

Colorado USA_528 High−income North America Stillbirth Rate (SBR) − 20 weeks
lambda = 0.2 ; zeta = 0.99 ; scale = 5 ; amp = 0.028

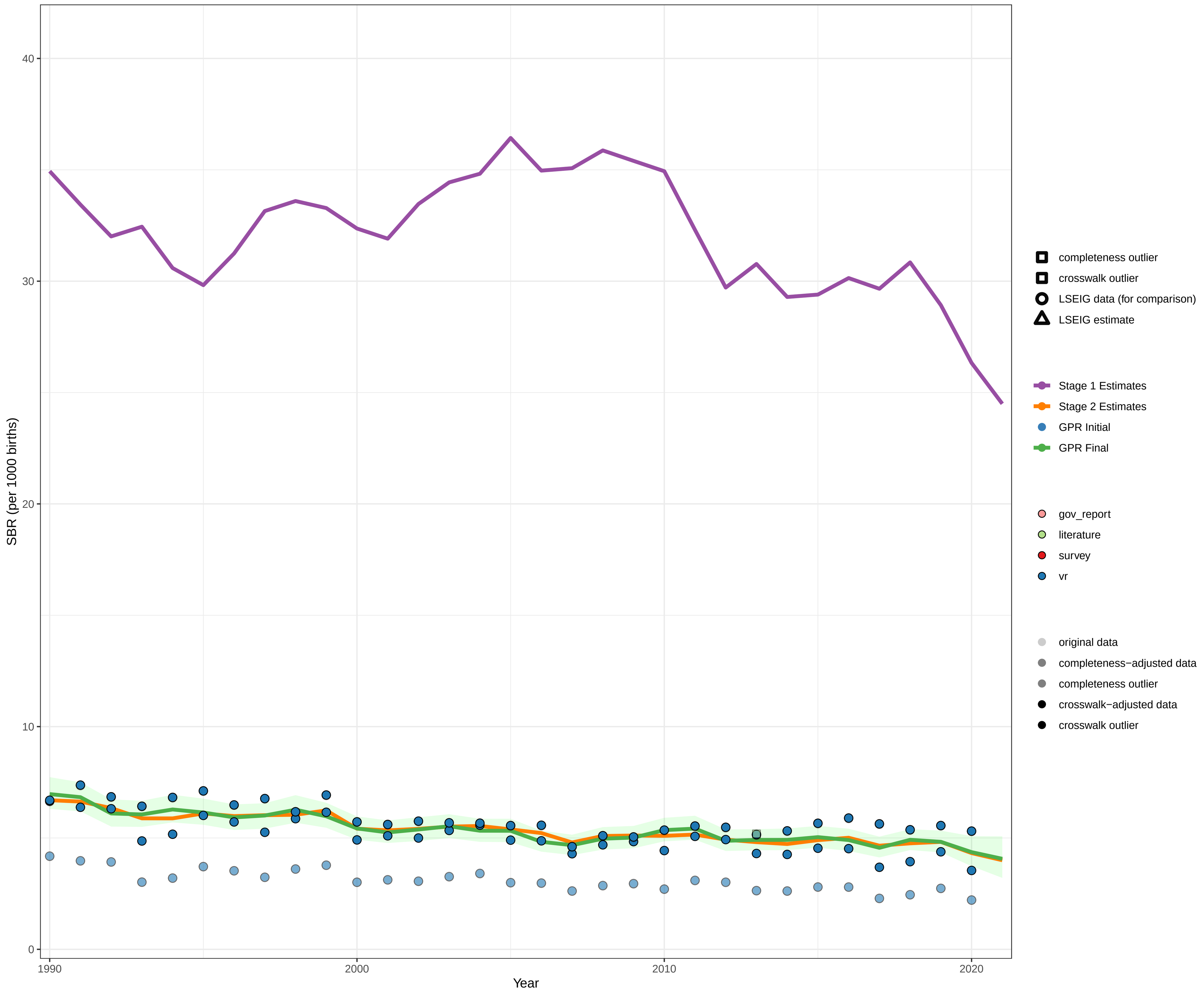

Colorado USA_528 High−income North America Stillbirth Rate (SBR) − 28 weeks
lambda = 0.2 ; zeta = 0.99 ; scale = 5 ; amp = 0.028

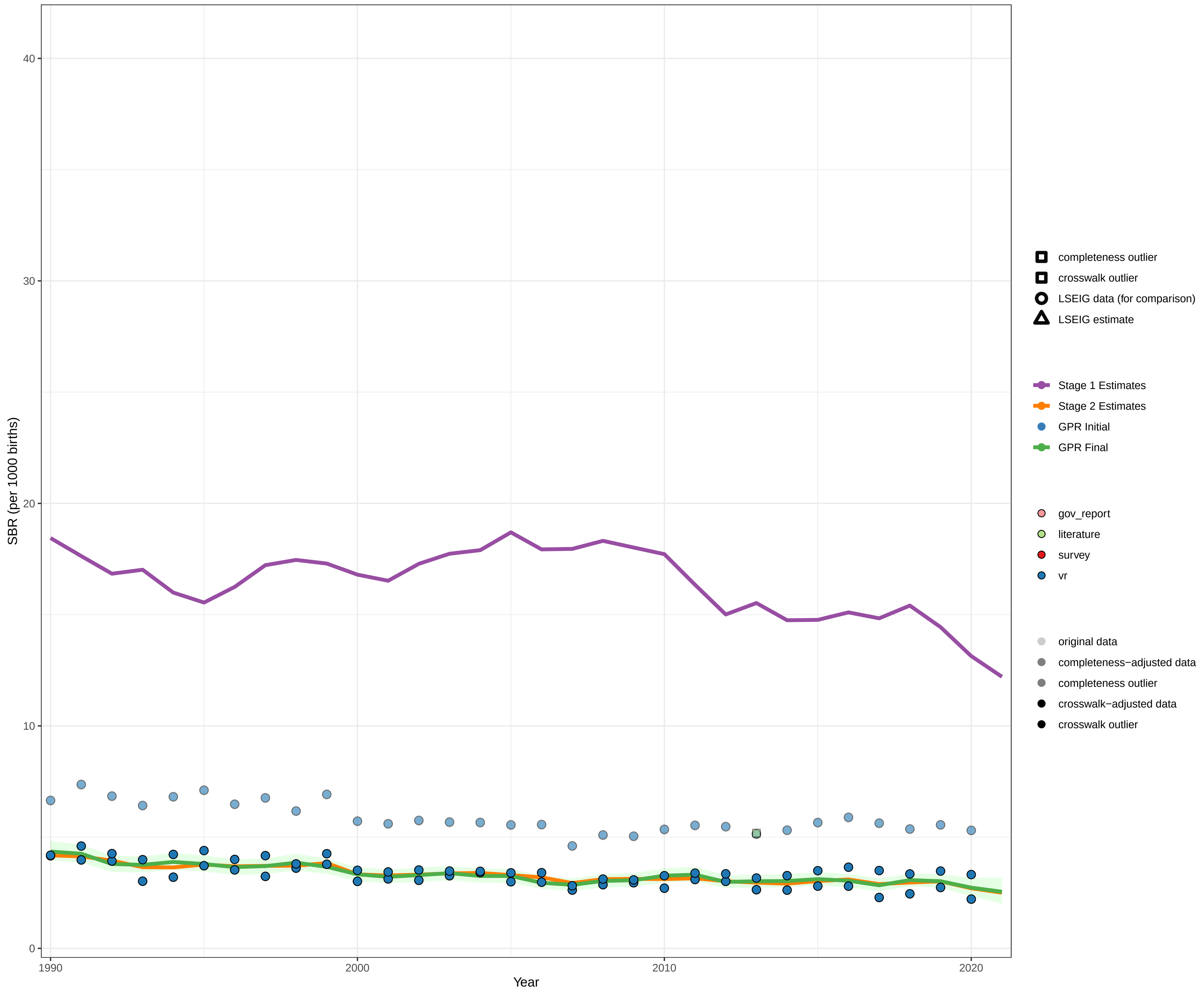

Year	Total Stillbirths (in thousands)	Total Stillbirths (in thousands)	SBR (per 1000 births)	SBR (per 1000 births)	SBR/NMR	SBR/NMR
	20 weeks	28 weeks	20 weeks	28 weeks	20 weeks	28 weeks
1990	0.4 (0.3 to 0.4)	0.2 (0.2 to 0.3)	7.0 (6.3 to 7.7)	4.4 (3.9 to 4.8)	1.41 (1.28 to 1.57)	0.88 (0.80 to 0.98)
1995	0.3 (0.3 to 0.4)	0.2 (0.2 to 0.2)	6.1 (5.6 to 6.8)	3.8 (3.5 to 4.2)	1.54 (1.40 to 1.70)	0.95 (0.87 to 1.05)
2000	0.4 (0.3 to 0.4)	0.2 (0.2 to 0.2)	5.4 (4.9 to 6.0)	3.3 (3.0 to 3.7)	1.28 (1.16 to 1.41)	0.79 (0.71 to 0.87)
2005	0.4 (0.3 to 0.4)	0.2 (0.2 to 0.2)	5.3 (4.8 to 5.9)	3.3 (2.9 to 3.6)	1.17 (1.06 to 1.29)	0.71 (0.65 to 0.79)
2010	0.4 (0.3 to 0.4)	0.2 (0.2 to 0.2)	5.4 (4.8 to 5.9)	3.3 (3.0 to 3.6)	1.29 (1.17 to 1.43)	0.79 (0.72 to 0.87)
2015	0.3 (0.3 to 0.4)	0.2 (0.2 to 0.2)	5.0 (4.6 to 5.5)	3.1 (2.8 to 3.4)	1.50 (1.36 to 1.66)	0.93 (0.84 to 1.02)
2020	0.3 (0.2 to 0.3)	0.2 (0.1 to 0.2)	4.4 (3.7 to 5.1)	2.7 (2.3 to 3.2)	1.50 (1.28 to 1.74)	0.94 (0.80 to 1.09)
2021	0.3 (0.2 to 0.3)	0.2 (0.1 to 0.2)	4.1 (3.2 to 5.1)	2.5 (2.0 to 3.2)	1.51 (1.19 to 1.88)	0.94 (0.75 to 1.17)

Connecticut USA_529 High−income North America Stillbirth Rate / Neonatal Mortality Rate (SBR/NMR) − 20 weeks
lambda = 0.2 ; zeta = 0.99 ; scale = 5 ; amp = 0.028

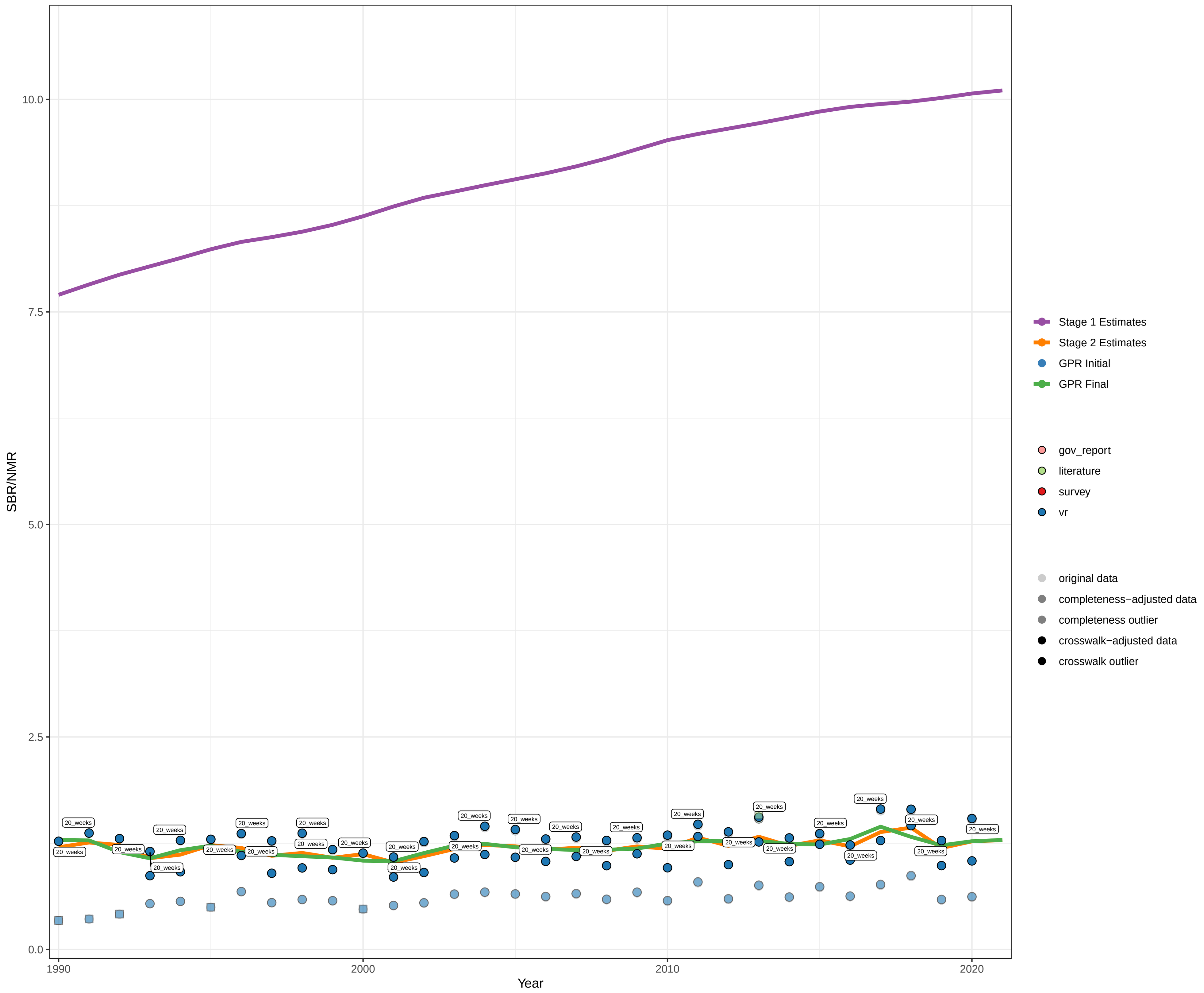

Connecticut USA_529 High−income North America Stillbirth Rate / Neonatal Mortality Rate (SBR/NMR) − 28 weeks
lambda = 0.2 ; zeta = 0.99 ; scale = 5 ; amp = 0.028

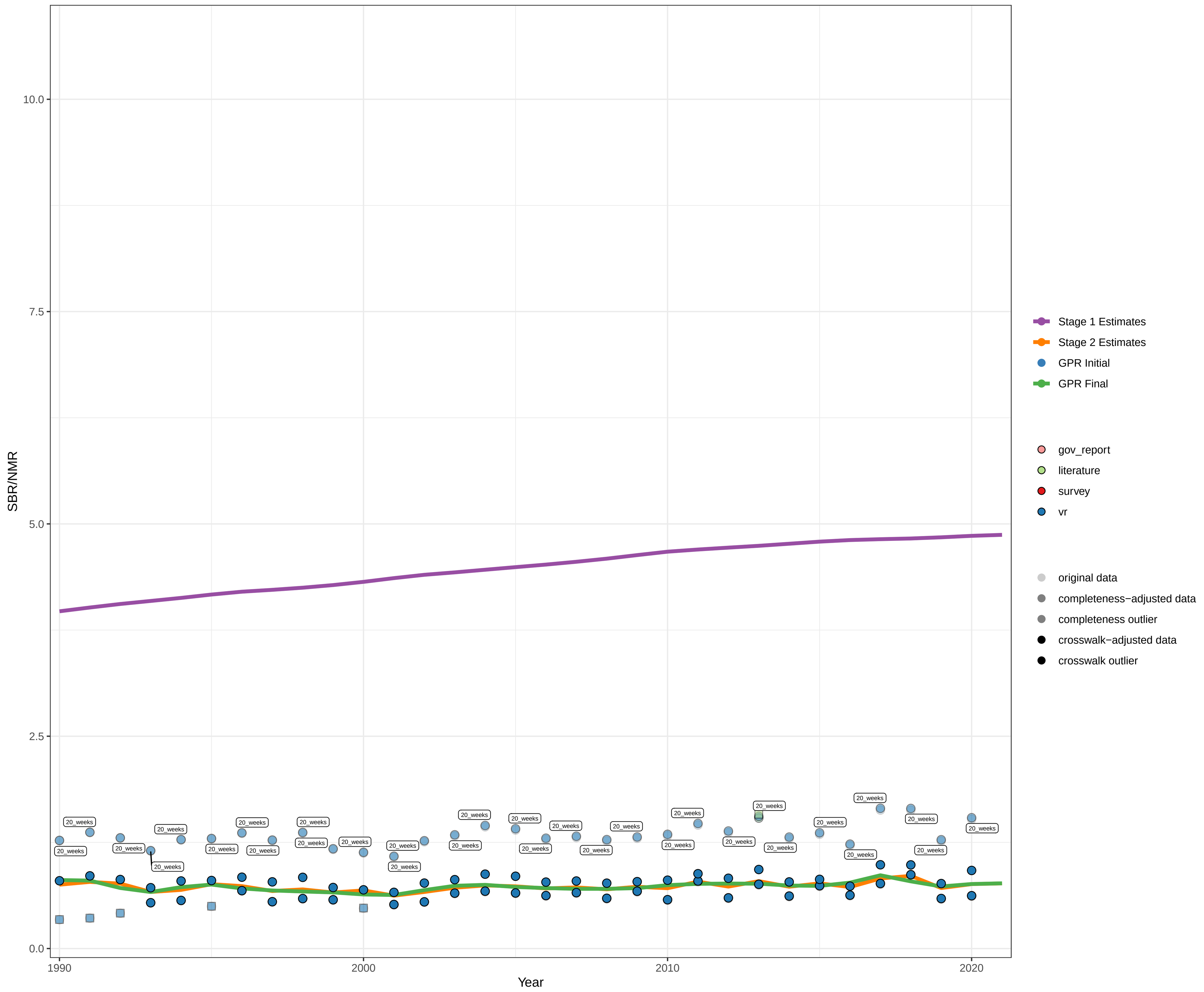

Definition	Years	Outlier	Source.Type	Title
20_weeks	1990−2020	0	VR	United States NVSS Custom Fetal Death Data
28_weeks	1993, 1994, 1996−1999, 2001−2020	0	VR	United States NVSS Custom Fetal Death Data
20_weeks	2013	1	Sci Lit	United States Fetal and Perinatal Mortality 2013
28_weeks	1990−1992, 1995, 2000	1	VR	United States NVSS Custom Fetal Death Data

Connecticut USA_529 High−income North America Stillbirth Rate (SBR) − 20 weeks
lambda = 0.2 ; zeta = 0.99 ; scale = 5 ; amp = 0.028

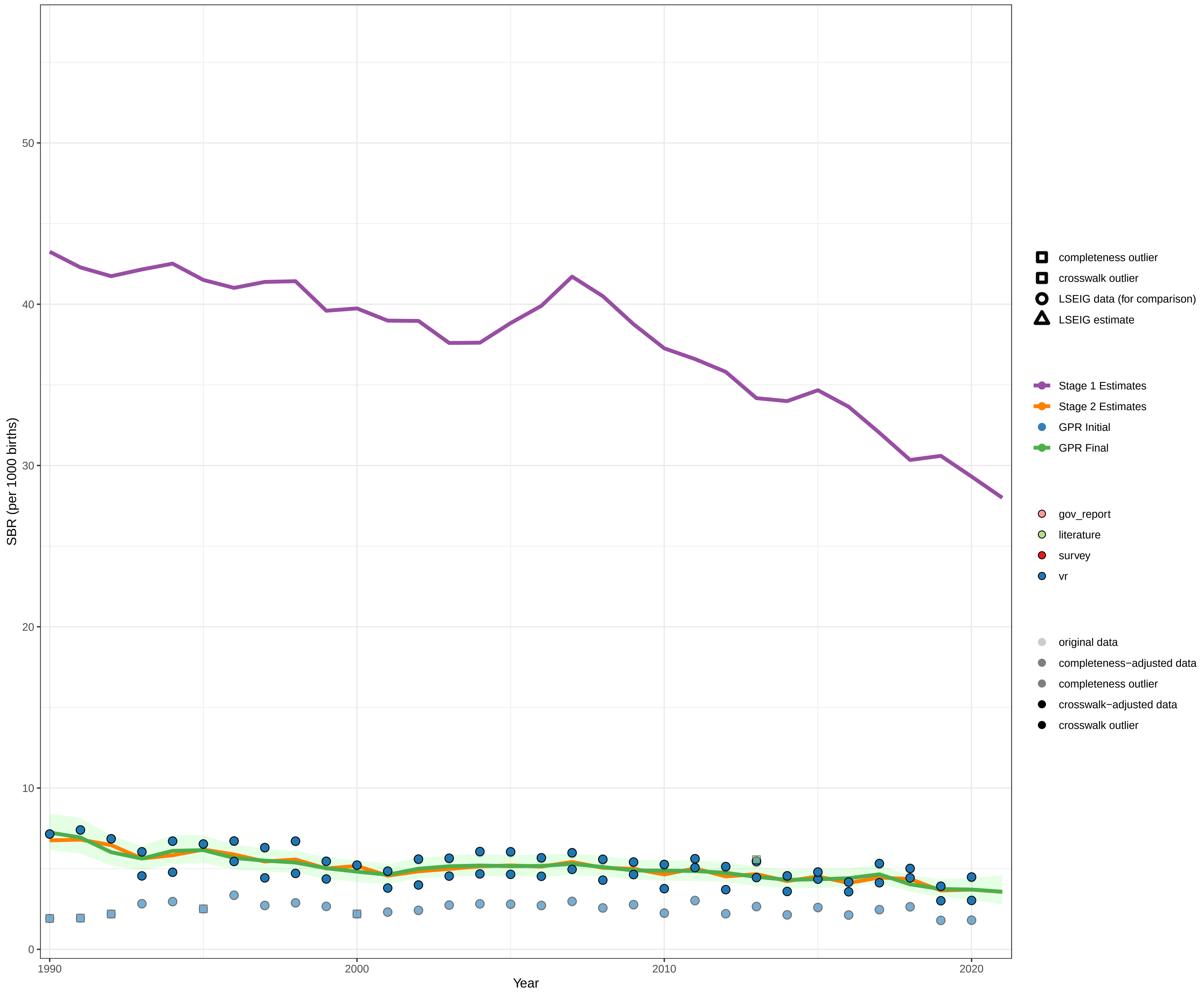

Connecticut USA_529 High−income North America Stillbirth Rate (SBR) − 28 weeks
lambda = 0.2 ; zeta = 0.99 ; scale = 5 ; amp = 0.028

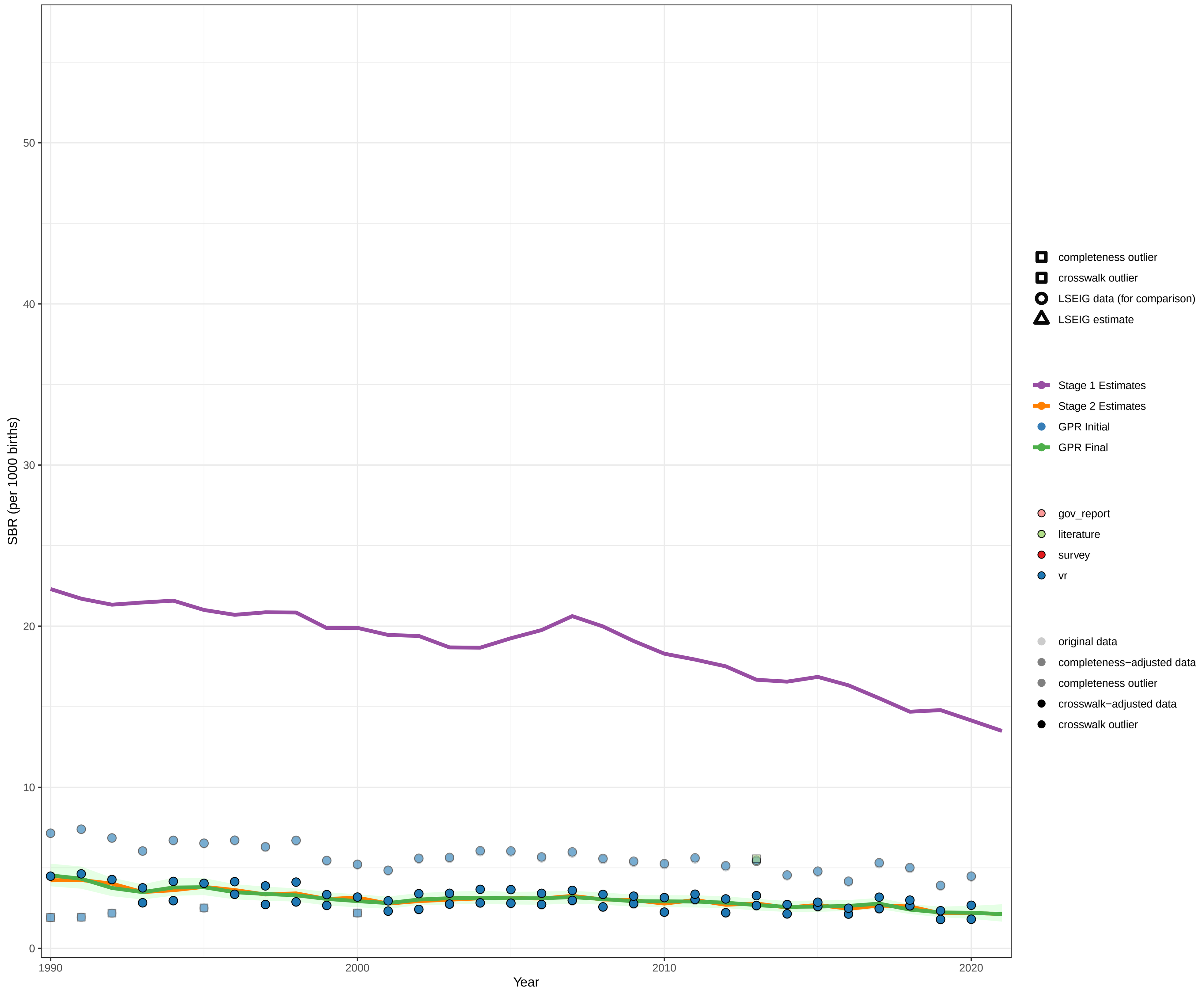

Year	Total Stillbirths (in thousands)	Total Stillbirths (in thousands)	SBR (per 1000 births)	SBR (per 1000 births)	SBR/NMR	SBR/NMR
	20 weeks	28 weeks	20 weeks	28 weeks	20 weeks	28 weeks
1990	0.4 (0.3 to 0.4)	0.2 (0.2 to 0.3)	7.2 (6.2 to 8.4)	4.5 (3.9 to 5.3)	1.29 (1.10 to 1.50)	0.81 (0.69 to 0.94)
1995	0.3 (0.2 to 0.3)	0.2 (0.1 to 0.2)	6.2 (5.4 to 7.1)	3.8 (3.3 to 4.4)	1.22 (1.06 to 1.40)	0.75 (0.66 to 0.87)
2000	0.2 (0.2 to 0.2)	0.1 (0.1 to 0.1)	4.8 (4.2 to 5.5)	2.9 (2.5 to 3.4)	1.05 (0.91 to 1.20)	0.64 (0.55 to 0.73)
2005	0.2 (0.2 to 0.2)	0.1 (0.1 to 0.1)	5.2 (4.5 to 5.8)	3.1 (2.7 to 3.5)	1.20 (1.05 to 1.36)	0.72 (0.64 to 0.82)
2010	0.2 (0.2 to 0.2)	0.1 (0.1 to 0.1)	4.9 (4.3 to 5.5)	2.9 (2.6 to 3.3)	1.25 (1.09 to 1.41)	0.75 (0.65 to 0.84)
2015	0.2 (0.1 to 0.2)	0.1 (0.1 to 0.1)	4.3 (3.8 to 5.0)	2.6 (2.3 to 3.0)	1.23 (1.08 to 1.42)	0.74 (0.65 to 0.85)
2020	0.1 (0.1 to 0.2)	0.1 (0.1 to 0.1)	3.7 (3.1 to 4.4)	2.2 (1.8 to 2.6)	1.27 (1.05 to 1.52)	0.76 (0.63 to 0.91)
2021	0.1 (0.1 to 0.2)	0.1 (0.1 to 0.1)	3.6 (2.8 to 4.6)	2.1 (1.7 to 2.7)	1.29 (1.00 to 1.66)	0.77 (0.60 to 0.99)

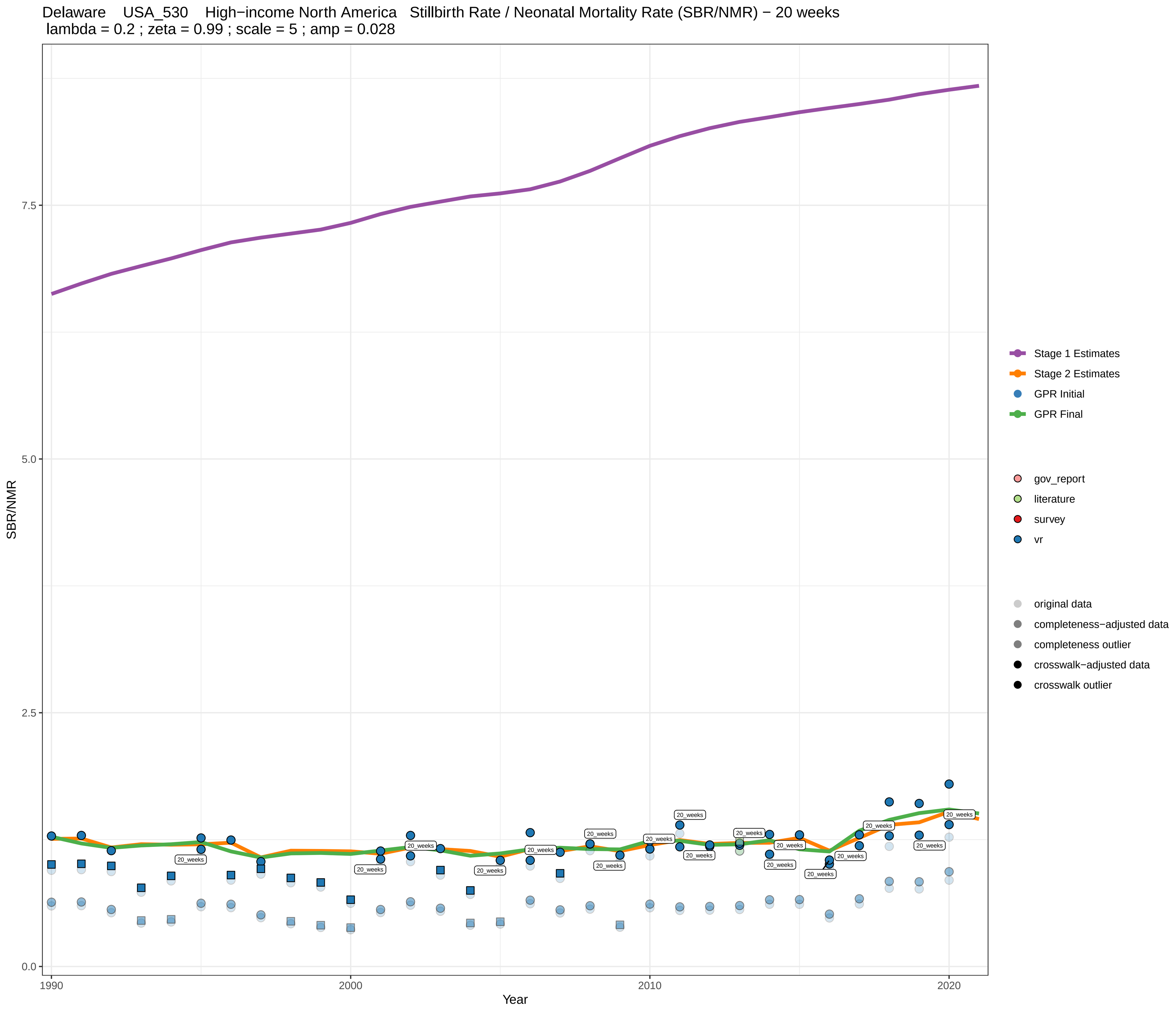

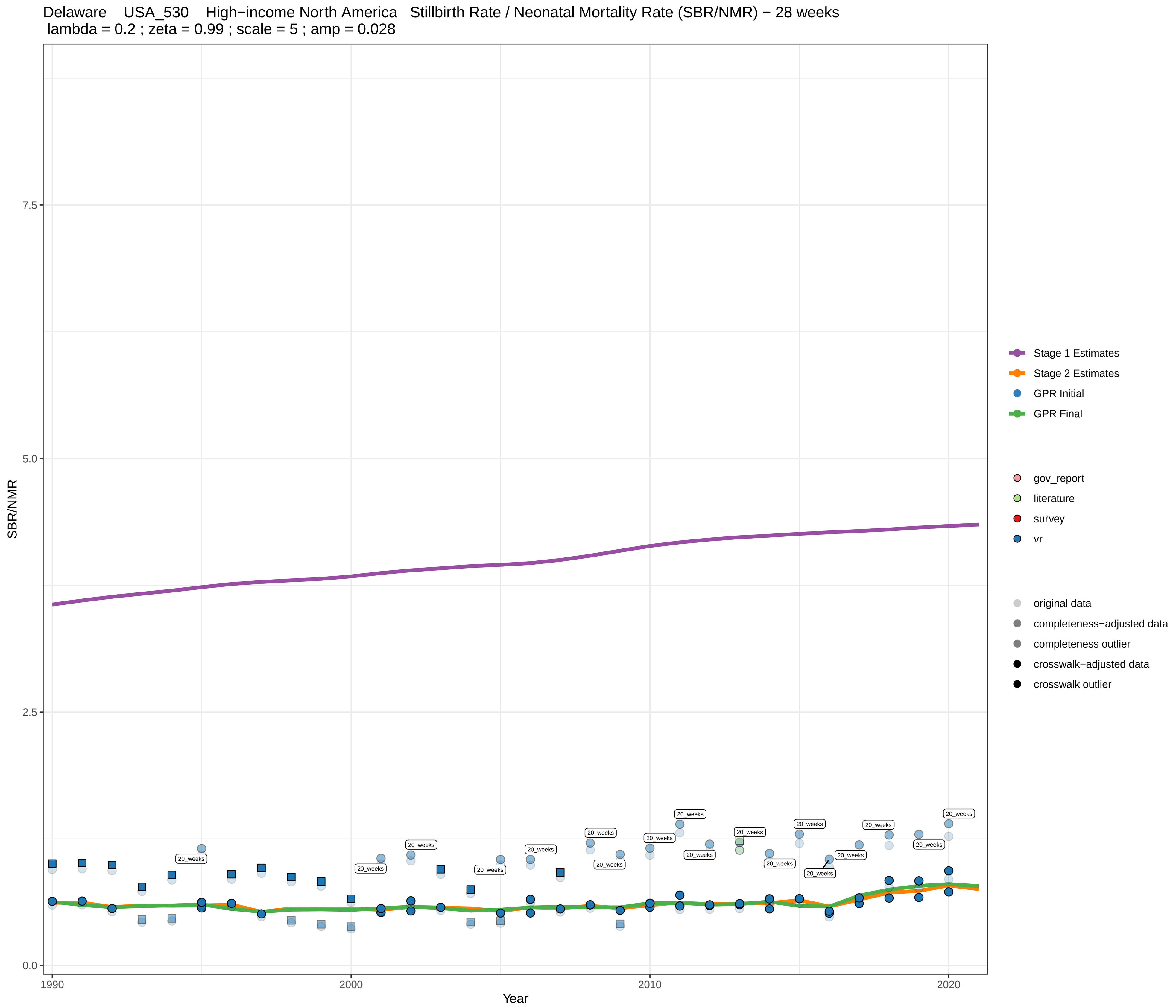

Definition	Years	Outlier	Source.Type	Title
20_weeks	1995, 2001, 2002, 2005, 2006, 2008−2020	0	VR	United States NVSS Custom Fetal Death Data
28_weeks	1990−1992, 1995−1997, 2001−2003, 2006−2008, 2010−2020	0	VR	United States NVSS Custom Fetal Death Data
20_weeks	2013	1	Sci Lit	United States Fetal and Perinatal Mortality 2013
28_weeks	1993, 1994, 1998−2000, 2004, 2005, 2009	1	VR	United States NVSS Custom Fetal Death Data
20_weeks	1990−1994, 1996−2000, 2003, 2004, 2007	2	VR	United States NVSS Custom Fetal Death Data

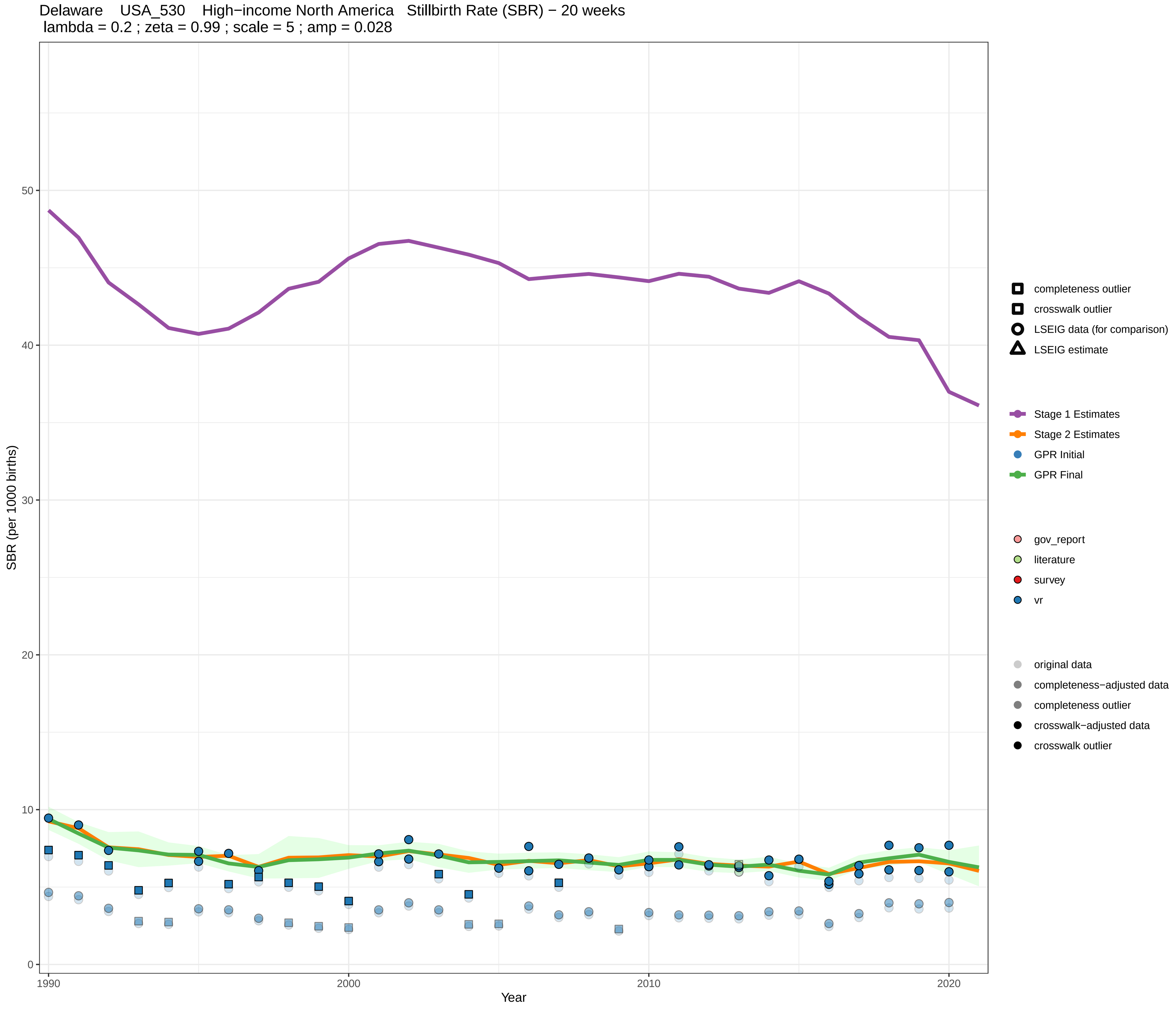

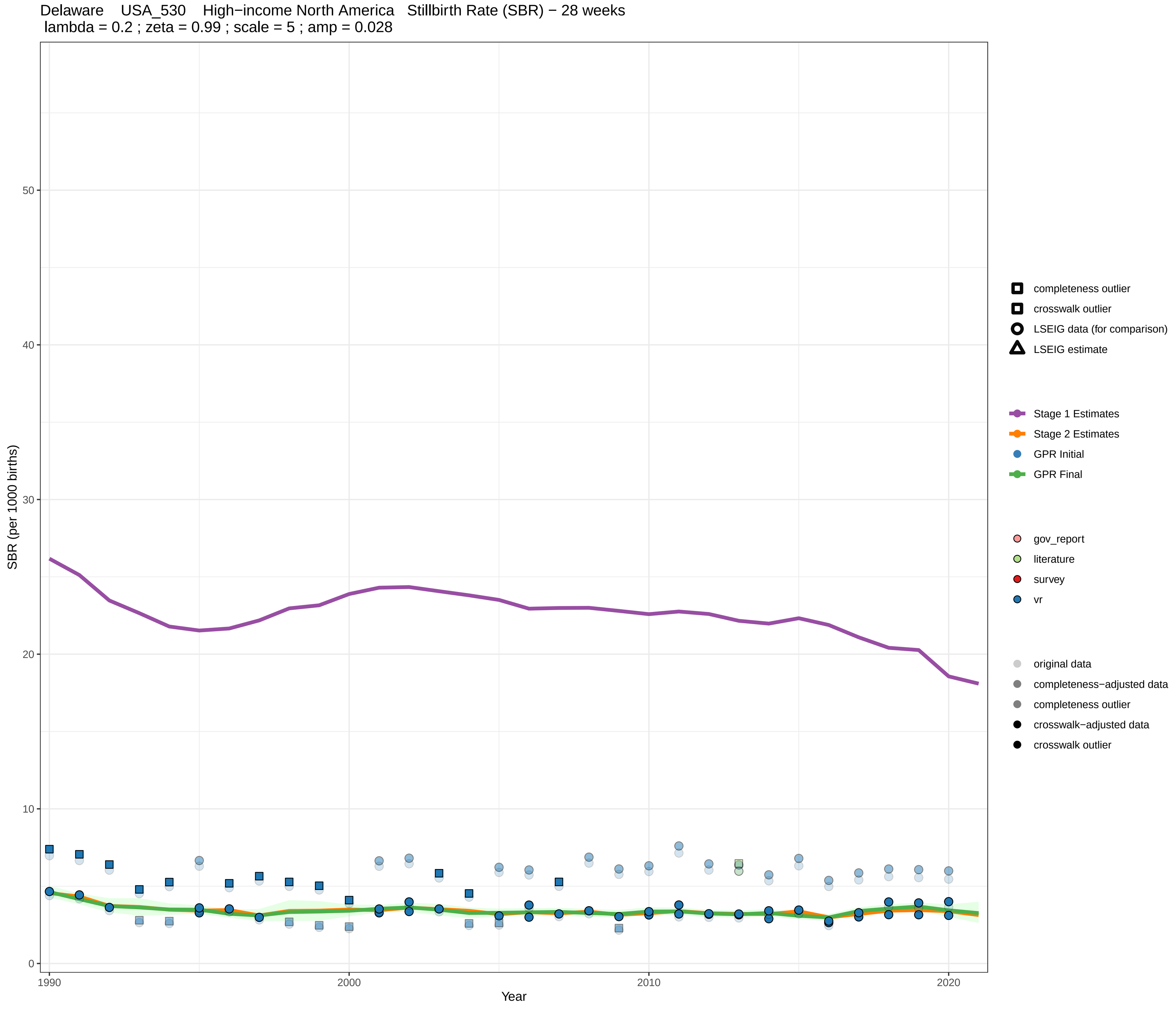

Year	Total Stillbirths (in thousands)	Total Stillbirths (in thousands)	SBR (per 1000 births)	SBR (per 1000 births)	SBR/NMR	SBR/NMR
	20 weeks	28 weeks	20 weeks	28 weeks	20 weeks	28 weeks
1990	0.1 (0.1 to 0.1)	0.1 (0.0 to 0.1)	9.4 (8.7 to 10.2)	4.6 (4.3 to 5.0)	1.28 (1.18 to 1.39)	0.63 (0.58 to 0.68)
1995	0.1 (0.1 to 0.1)	0.0 (0.0 to 0.0)	7.1 (6.5 to 7.6)	3.5 (3.2 to 3.8)	1.23 (1.13 to 1.33)	0.60 (0.56 to 0.65)
2000	0.1 (0.1 to 0.1)	0.0 (0.0 to 0.0)	6.9 (6.2 to 7.7)	3.4 (3.0 to 3.8)	1.11 (0.99 to 1.24)	0.55 (0.49 to 0.61)
2005	0.1 (0.1 to 0.1)	0.0 (0.0 to 0.0)	6.6 (6.1 to 7.2)	3.3 (3.0 to 3.6)	1.11 (1.03 to 1.20)	0.55 (0.51 to 0.60)
2010	0.1 (0.1 to 0.1)	0.0 (0.0 to 0.0)	6.8 (6.3 to 7.3)	3.4 (3.1 to 3.6)	1.24 (1.16 to 1.34)	0.62 (0.57 to 0.67)
2015	0.1 (0.1 to 0.1)	0.0 (0.0 to 0.0)	6.1 (5.6 to 6.5)	3.1 (2.9 to 3.3)	1.15 (1.07 to 1.24)	0.59 (0.55 to 0.63)
2020	0.1 (0.1 to 0.1)	0.0 (0.0 to 0.0)	6.6 (5.8 to 7.4)	3.4 (3.0 to 3.8)	1.55 (1.36 to 1.72)	0.80 (0.70 to 0.89)
2021	0.1 (0.1 to 0.1)	0.0 (0.0 to 0.0)	6.3 (5.0 to 7.7)	3.3 (2.6 to 4.0)	1.51 (1.21 to 1.85)	0.78 (0.63 to 0.96)

District of Columbia USA_531 High−income North America Stillbirth Rate / Neonatal Mortality Rate (SBR/NMR) − 20 weeks
lambda = 0.2 ; zeta = 0.99 ; scale = 5 ; amp = 0.028

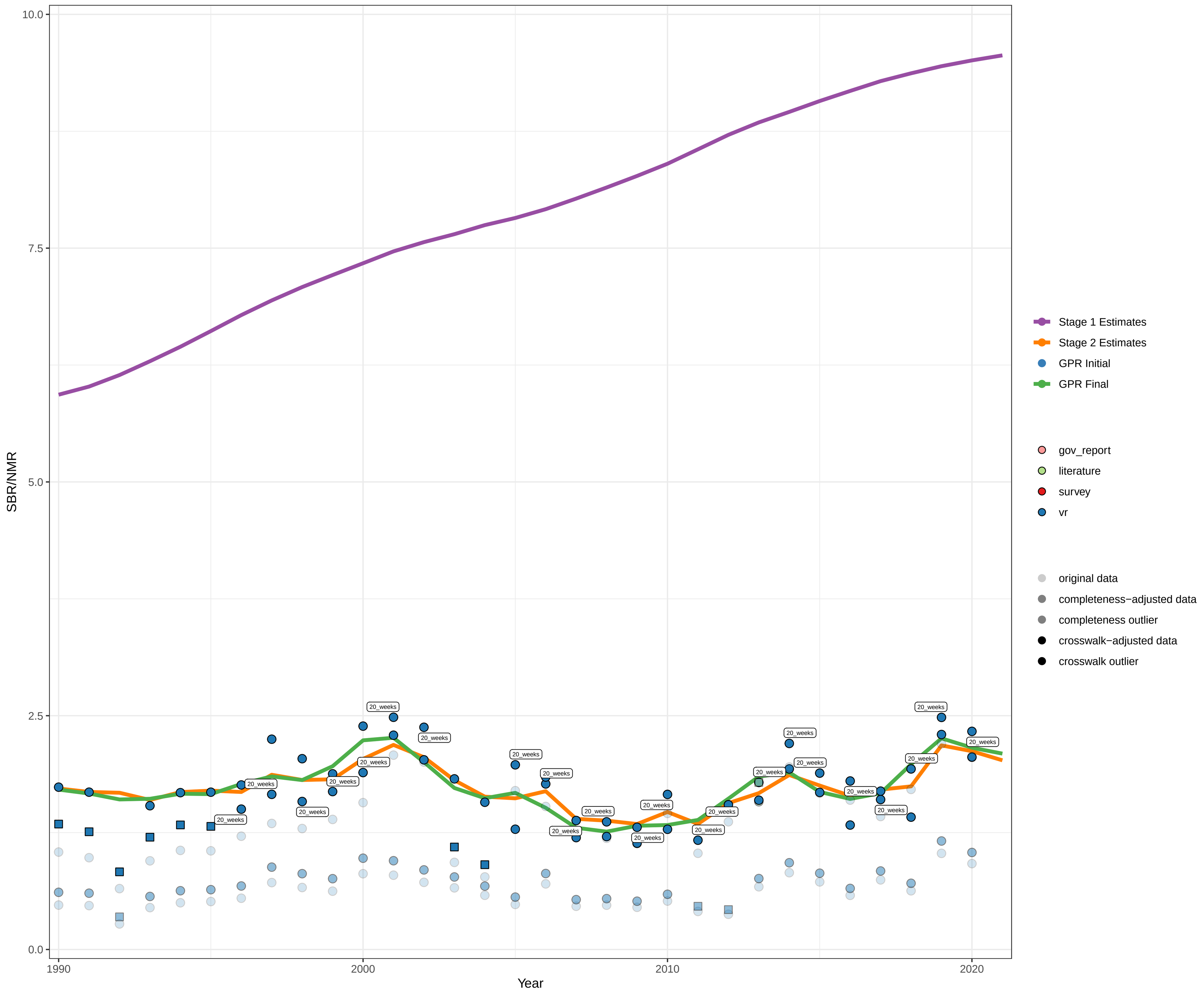

District of Columbia USA_531 High−income North America Stillbirth Rate / Neonatal Mortality Rate (SBR/NMR) − 28 weeks
lambda = 0.2 ; zeta = 0.99 ; scale = 5 ; amp = 0.028

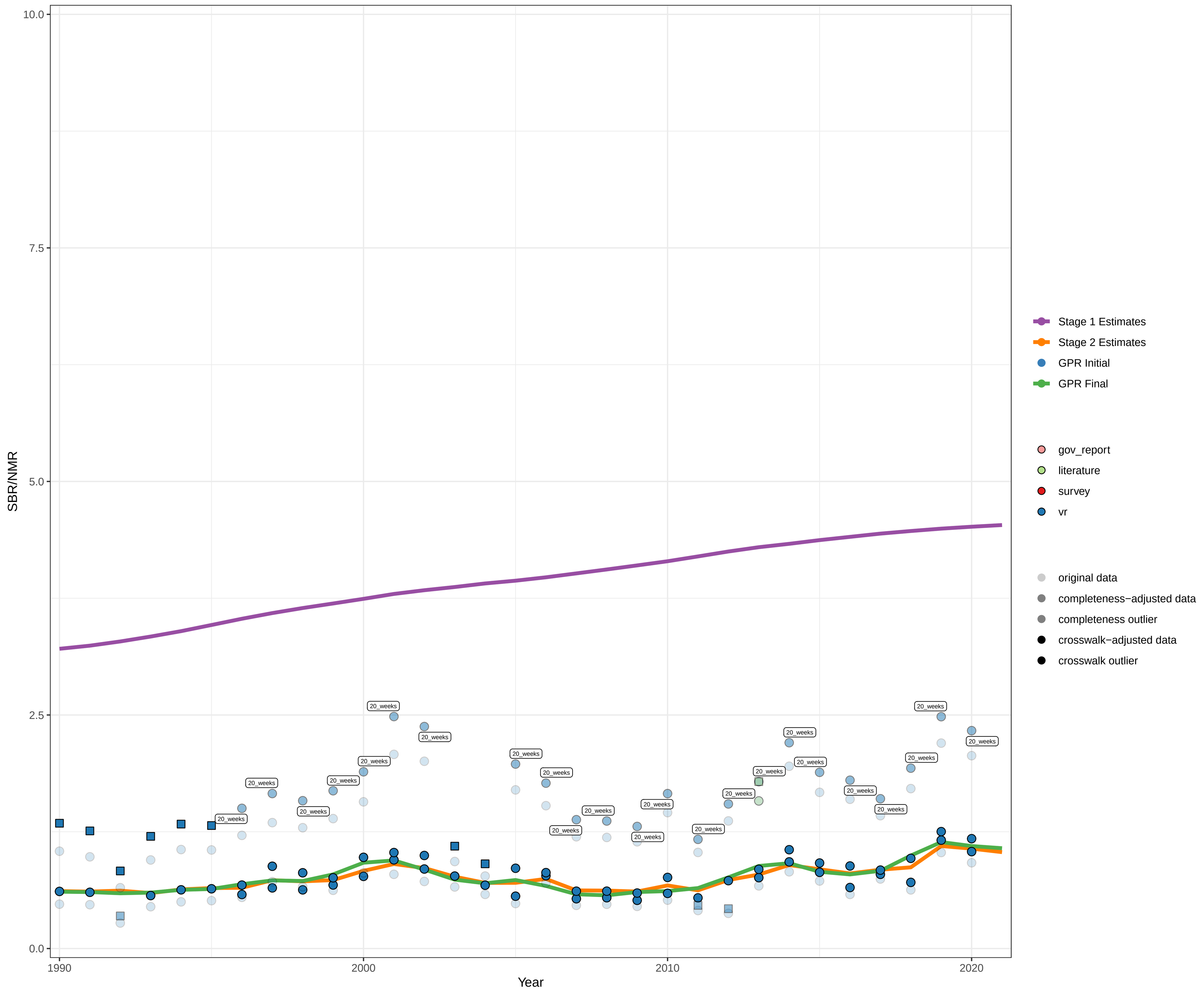

Definition	Years	Outlier	Source.Type	Title
20_weeks	1996−2002, 2005−2020	0	VR	United States NVSS Custom Fetal Death Data
28_weeks	1990, 1991, 1993−2010, 2013−2020	0	VR	United States NVSS Custom Fetal Death Data
20_weeks	2013	1	Sci Lit	United States Fetal and Perinatal Mortality 2013
28_weeks	1992, 2011, 2012	1	VR	United States NVSS Custom Fetal Death Data
20_weeks	1990−1995, 2003, 2004	2	VR	United States NVSS Custom Fetal Death Data

District of Columbia USA_531 High−income North America Stillbirth Rate (SBR) − 20 weeks
lambda = 0.2 ; zeta = 0.99 ; scale = 5 ; amp = 0.028

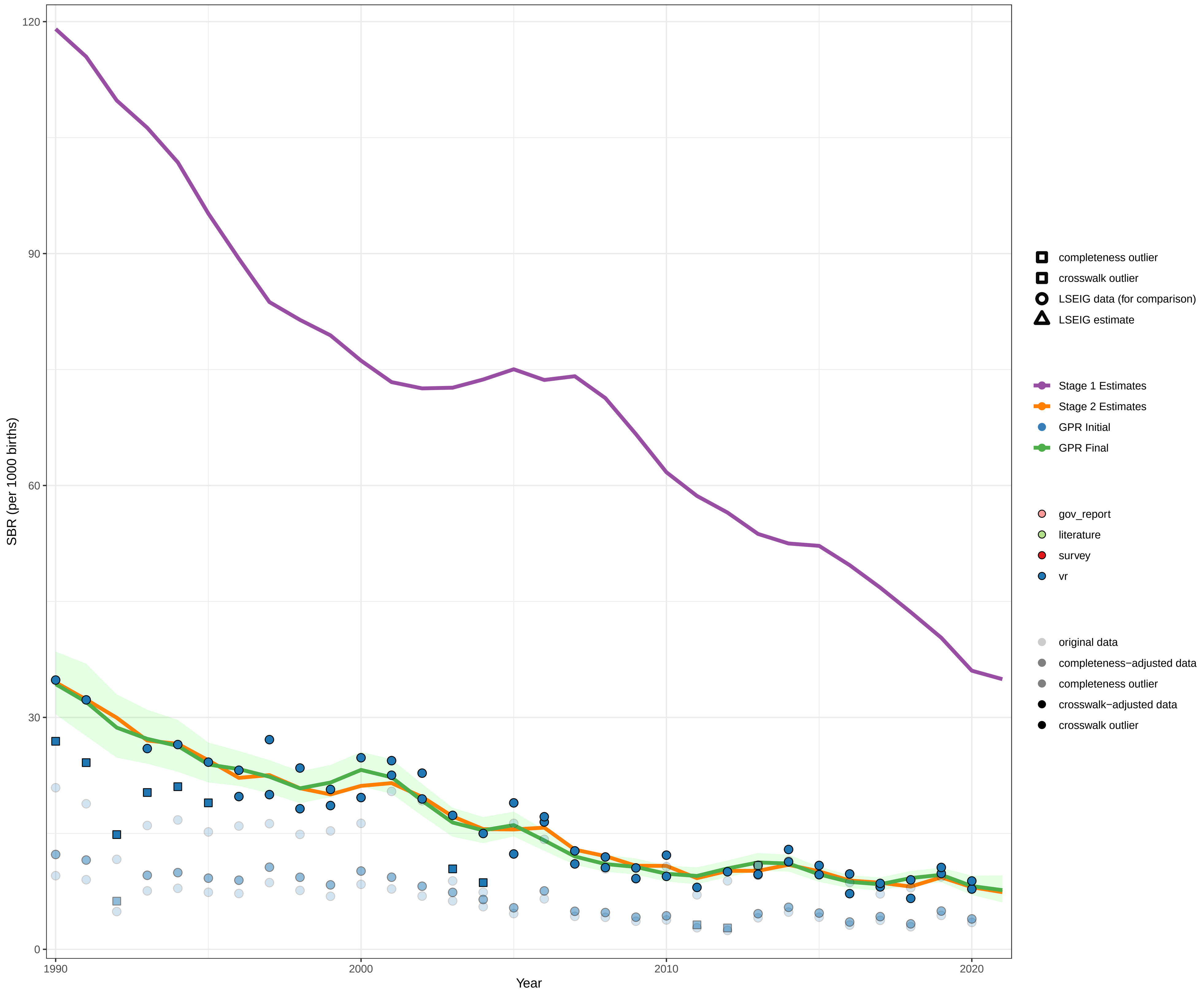

District of Columbia USA_531 High−income North America Stillbirth Rate (SBR) − 28 weeks
lambda = 0.2 ; zeta = 0.99 ; scale = 5 ; amp = 0.028

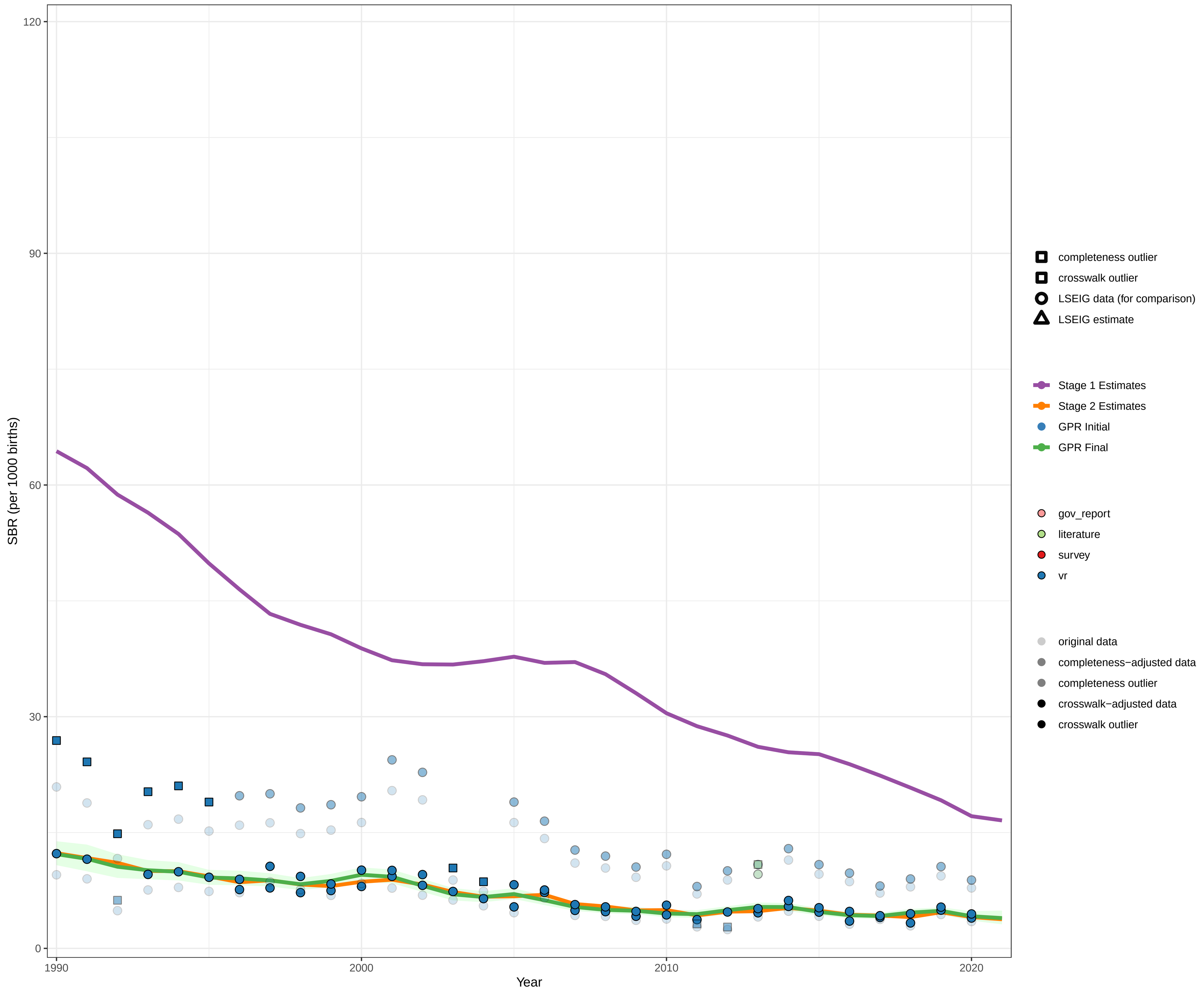

Year	Total Stillbirths (in thousands)	Total Stillbirths (in thousands)	SBR (per 1000 births)	SBR (per 1000 births)	SBR/NMR	SBR/NMR
	20 weeks	28 weeks	20 weeks	28 weeks	20 weeks	28 weeks
1990	0.4 (0.4 to 0.5)	0.1 (0.1 to 0.2)	34.3 (30.4 to 38.5)	12.2 (10.8 to 13.9)	1.71 (1.51 to 1.92)	0.61 (0.54 to 0.69)
1995	0.2 (0.2 to 0.3)	0.1 (0.1 to 0.1)	23.9 (21.6 to 26.8)	9.2 (8.2 to 10.2)	1.66 (1.50 to 1.86)	0.64 (0.57 to 0.71)
2000	0.2 (0.2 to 0.2)	0.1 (0.1 to 0.1)	23.2 (21.1 to 25.5)	9.5 (8.7 to 10.5)	2.24 (2.03 to 2.46)	0.92 (0.83 to 1.01)
2005	0.1 (0.1 to 0.1)	0.1 (0.1 to 0.1)	16.1 (14.6 to 17.8)	7.0 (6.4 to 7.8)	1.68 (1.52 to 1.86)	0.73 (0.67 to 0.81)
2010	0.1 (0.1 to 0.1)	0.0 (0.0 to 0.0)	9.8 (8.8 to 10.8)	4.5 (4.0 to 5.0)	1.33 (1.19 to 1.47)	0.61 (0.55 to 0.68)
2015	0.1 (0.1 to 0.1)	0.0 (0.0 to 0.0)	9.7 (8.7 to 10.7)	4.7 (4.3 to 5.2)	1.69 (1.52 to 1.86)	0.82 (0.74 to 0.90)
2020	0.1 (0.1 to 0.1)	0.0 (0.0 to 0.0)	8.2 (7.0 to 9.5)	4.2 (3.6 to 4.8)	2.16 (1.85 to 2.51)	1.10 (0.95 to 1.27)
2021	0.1 (0.1 to 0.1)	0.0 (0.0 to 0.0)	7.7 (6.1 to 9.6)	3.9 (3.1 to 4.9)	2.09 (1.66 to 2.62)	1.07 (0.85 to 1.34)

Florida USA_532 High−income North America Stillbirth Rate / Neonatal Mortality Rate (SBR/NMR) − 20 weeks
lambda = 0.2 ; zeta = 0.99 ; scale = 5 ; amp = 0.028

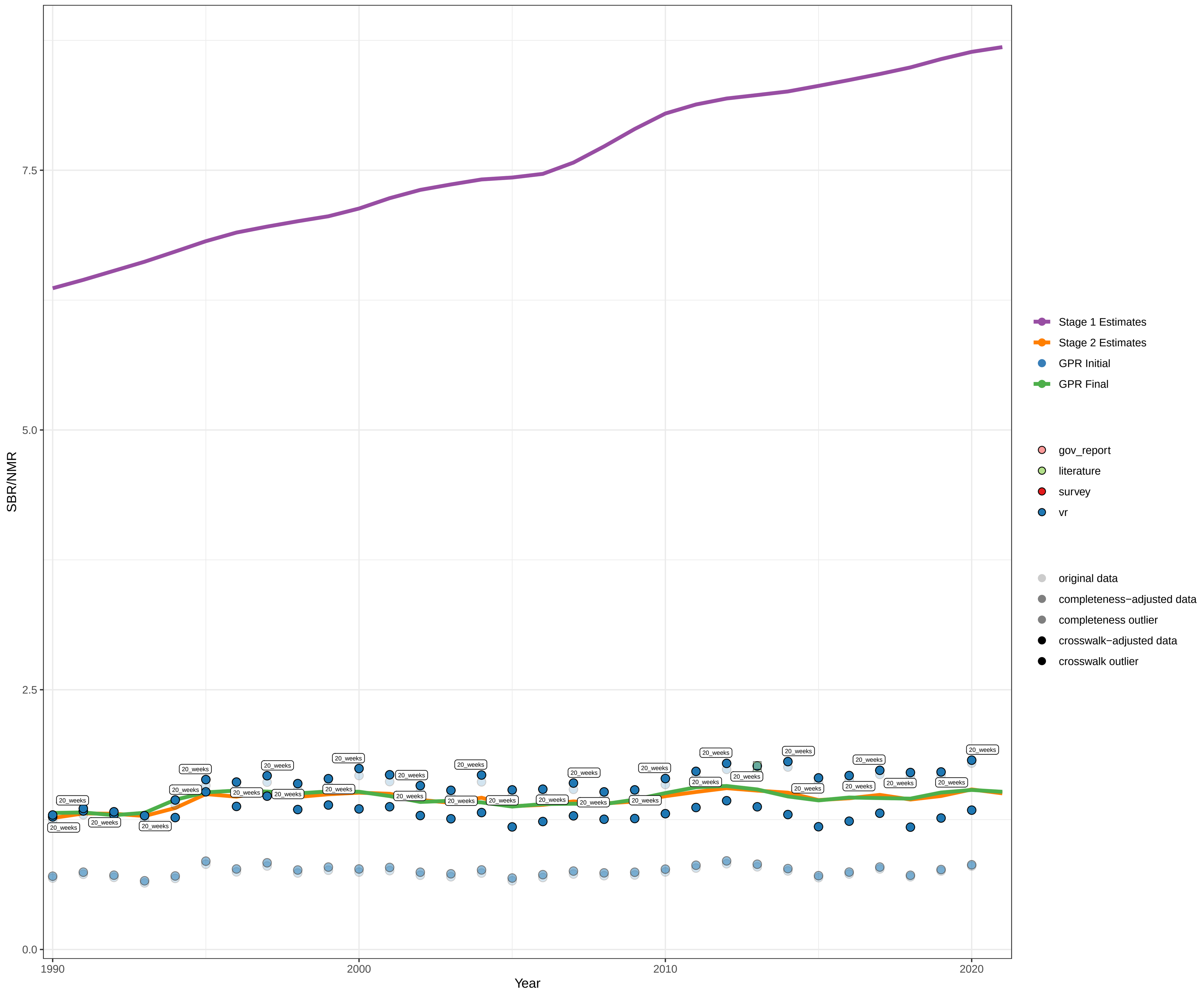

Florida USA_532 High−income North America Stillbirth Rate / Neonatal Mortality Rate (SBR/NMR) − 28 weeks
lambda = 0.2 ; zeta = 0.99 ; scale = 5 ; amp = 0.028

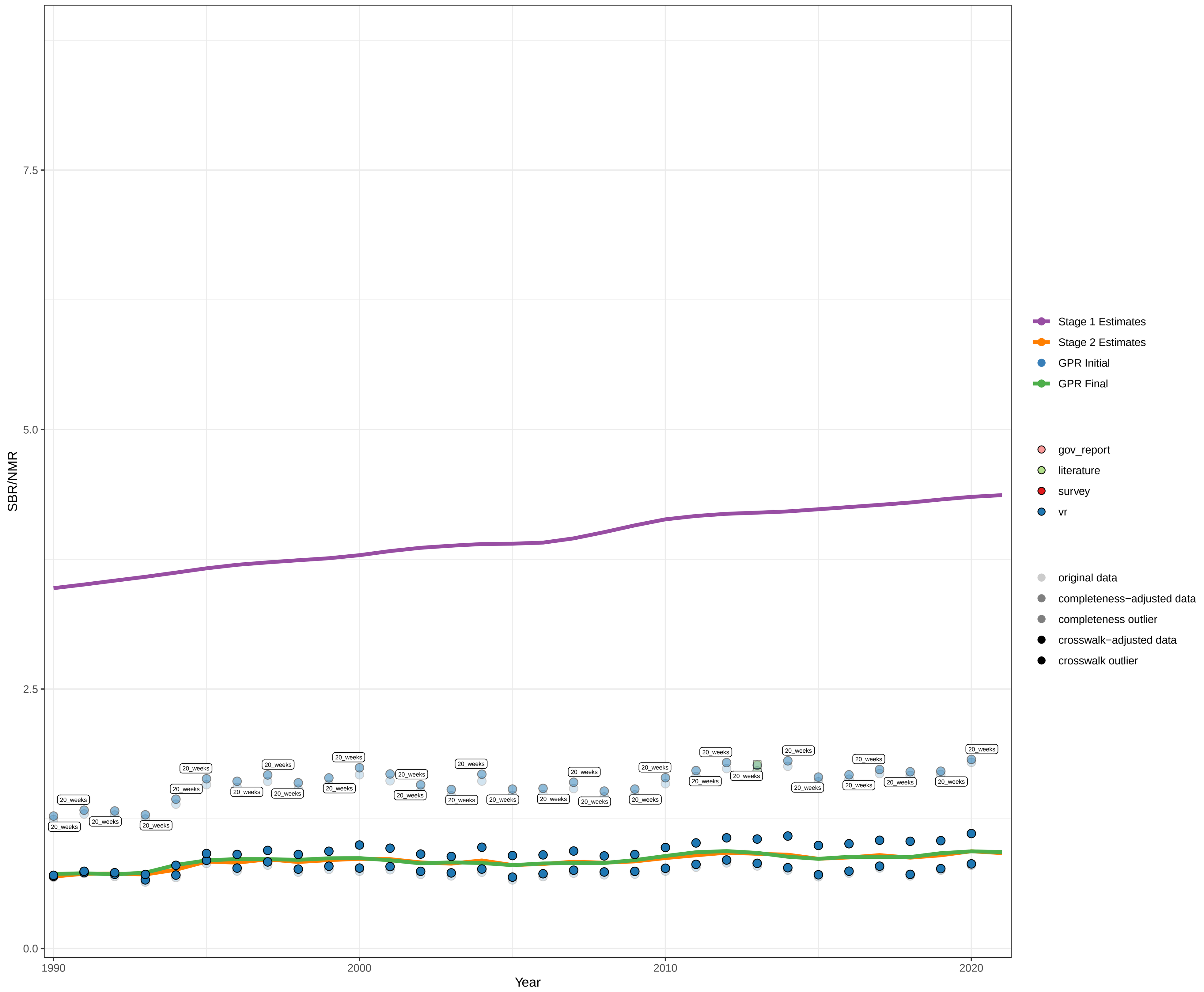

Definition	Years	Outlier	Source.Type	Title
20_weeks, 28_weeks	1990−2020	0	VR	United States NVSS Custom Fetal Death Data
20_weeks	2013	1	Sci Lit	United States Fetal and Perinatal Mortality 2013

Florida USA_532 High−income North America Stillbirth Rate (SBR) − 20 weeks
lambda = 0.2 ; zeta = 0.99 ; scale = 5 ; amp = 0.028

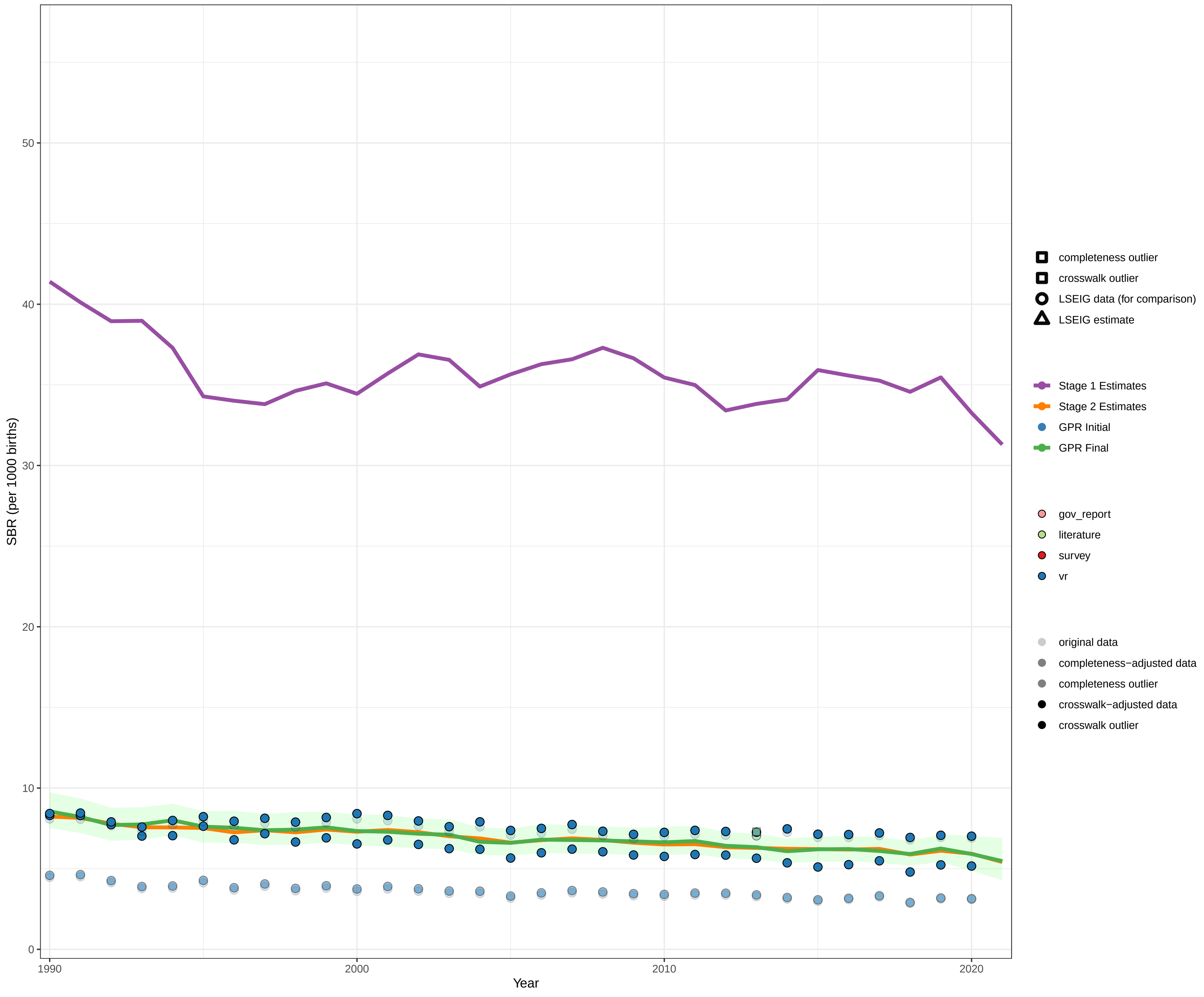

Florida USA_532 High−income North America Stillbirth Rate (SBR) − 28 weeks
lambda = 0.2 ; zeta = 0.99 ; scale = 5 ; amp = 0.028

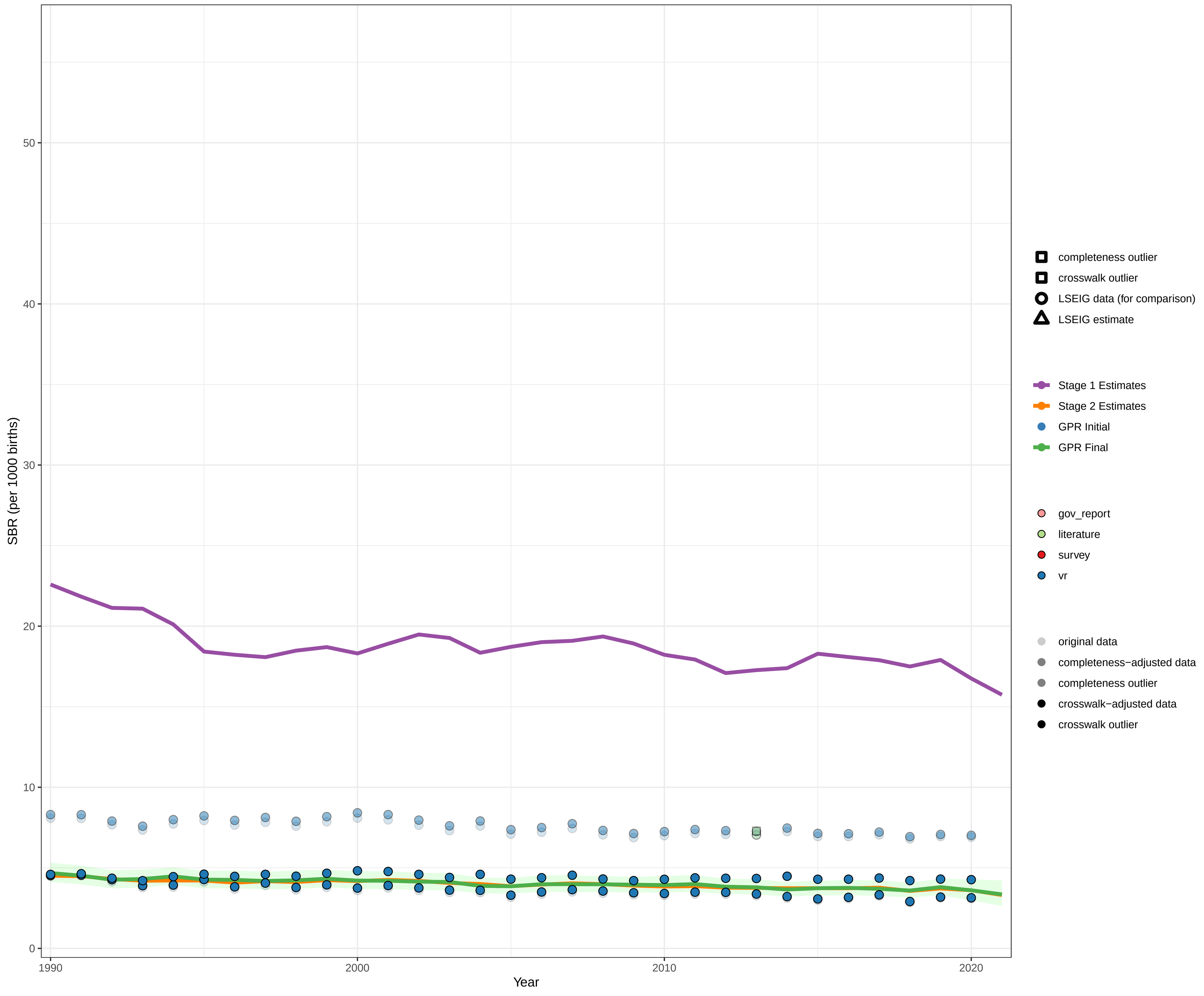

Year	Total Stillbirths (in thousands)	Total Stillbirths (in thousands)	SBR (per 1000 births)	SBR (per 1000 births)	SBR/NMR	SBR/NMR
	20 weeks	28 weeks	20 weeks	28 weeks	20 weeks	28 weeks
1990	1.7 (1.5 to 1.9)	0.9 (0.8 to 1.0)	8.5 (7.5 to 9.7)	4.7 (4.1 to 5.3)	1.31 (1.16 to 1.50)	0.72 (0.63 to 0.82)
1995	1.4 (1.3 to 1.6)	0.8 (0.7 to 0.9)	7.6 (6.6 to 8.6)	4.3 (3.7 to 4.8)	1.51 (1.32 to 1.70)	0.85 (0.74 to 0.96)
2000	1.5 (1.3 to 1.7)	0.9 (0.8 to 1.0)	7.3 (6.4 to 8.4)	4.2 (3.7 to 4.8)	1.52 (1.33 to 1.73)	0.87 (0.77 to 0.99)
2005	1.5 (1.3 to 1.7)	0.9 (0.8 to 1.0)	6.6 (5.8 to 7.5)	3.9 (3.4 to 4.4)	1.38 (1.21 to 1.56)	0.80 (0.71 to 0.91)
2010	1.4 (1.3 to 1.6)	0.8 (0.7 to 1.0)	6.6 (5.8 to 7.6)	3.9 (3.5 to 4.5)	1.51 (1.32 to 1.72)	0.89 (0.78 to 1.02)
2015	1.4 (1.2 to 1.6)	0.9 (0.7 to 1.0)	6.2 (5.4 to 7.0)	3.7 (3.3 to 4.2)	1.43 (1.26 to 1.62)	0.86 (0.76 to 0.97)
2020	1.3 (1.1 to 1.5)	0.8 (0.6 to 0.9)	5.9 (4.9 to 7.0)	3.6 (3.0 to 4.3)	1.54 (1.26 to 1.83)	0.94 (0.77 to 1.11)
2021	1.2 (0.9 to 1.5)	0.7 (0.6 to 0.9)	5.5 (4.3 to 6.9)	3.4 (2.6 to 4.2)	1.52 (1.19 to 1.91)	0.93 (0.73 to 1.17)

Georgia USA_533 High−income North America Stillbirth Rate / Neonatal Mortality Rate (SBR/NMR) − 20 weeks
lambda = 0.2 ; zeta = 0.99 ; scale = 5 ; amp = 0.028

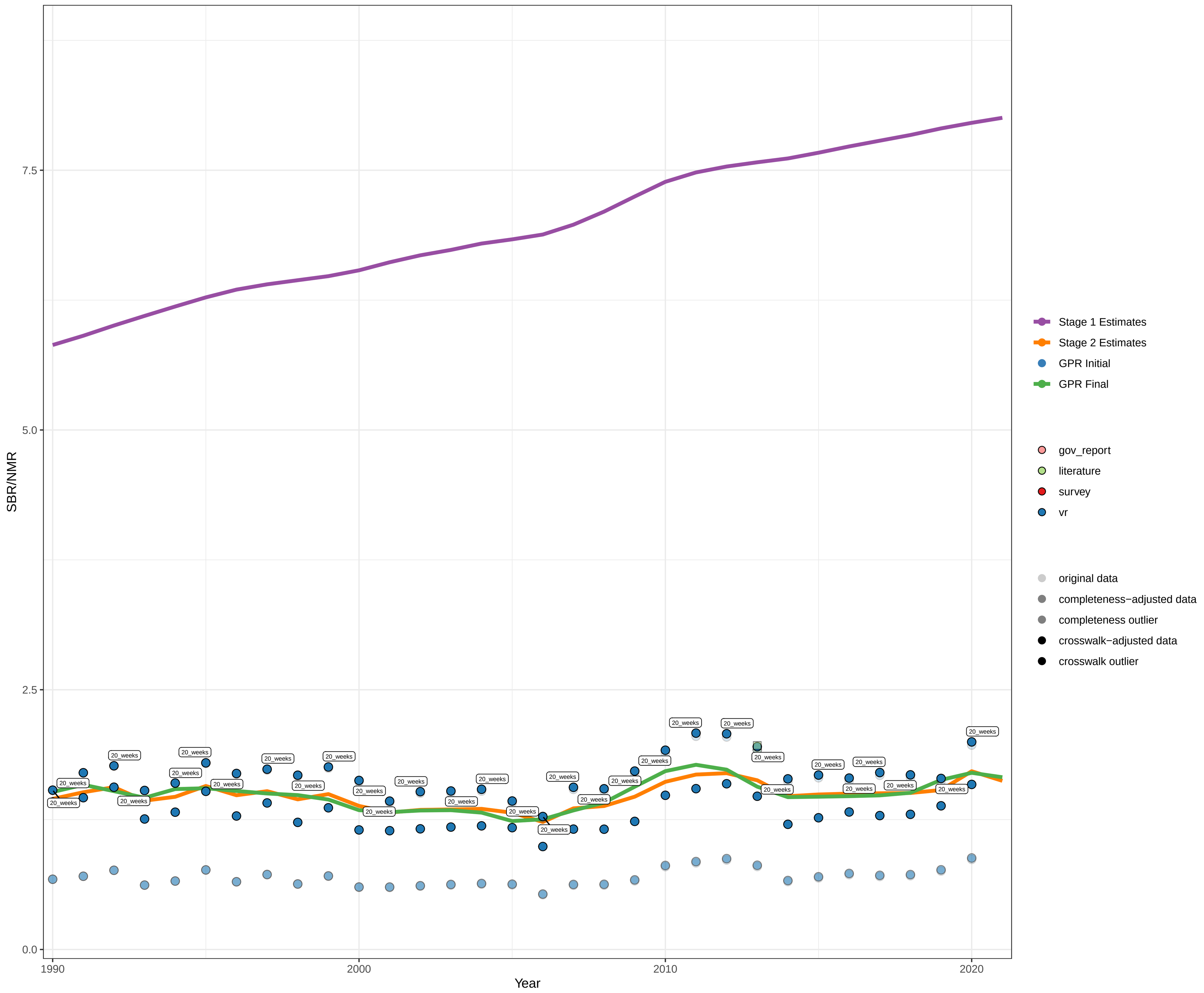

Georgia USA_533 High−income North America Stillbirth Rate / Neonatal Mortality Rate (SBR/NMR) − 28 weeks
lambda = 0.2 ; zeta = 0.99 ; scale = 5 ; amp = 0.028

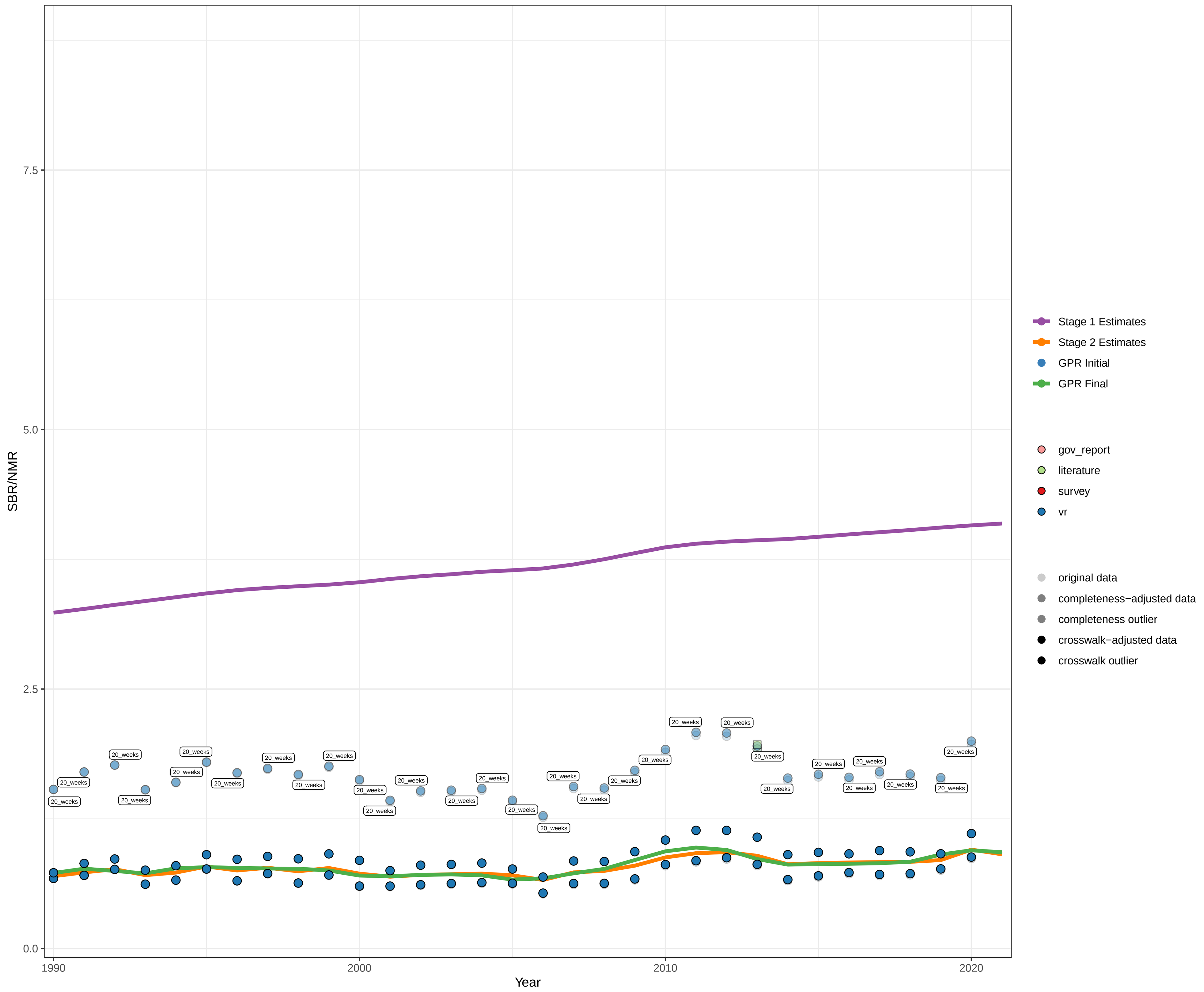

Definition	Years	Outlier	Source.Type	Title
20_weeks, 28_weeks	1990−2020	0	VR	United States NVSS Custom Fetal Death Data
20_weeks	2013	1	Sci Lit	United States Fetal and Perinatal Mortality 2013

Georgia USA_533 High−income North America Stillbirth Rate (SBR) − 20 weeks
lambda = 0.2 ; zeta = 0.99 ; scale = 5 ; amp = 0.028

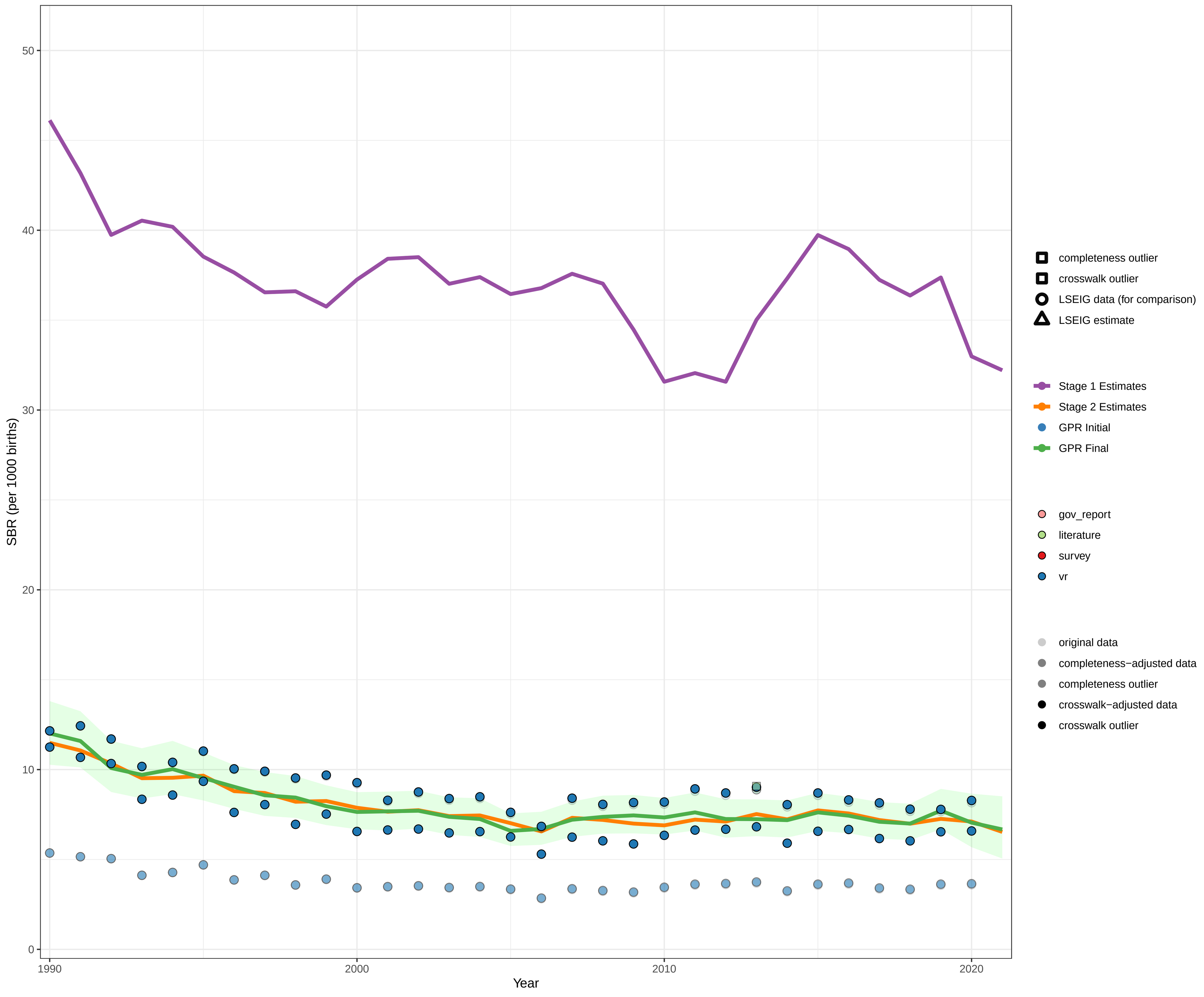

Georgia USA_533 High−income North America Stillbirth Rate (SBR) − 28 weeks
lambda = 0.2 ; zeta = 0.99 ; scale = 5 ; amp = 0.028

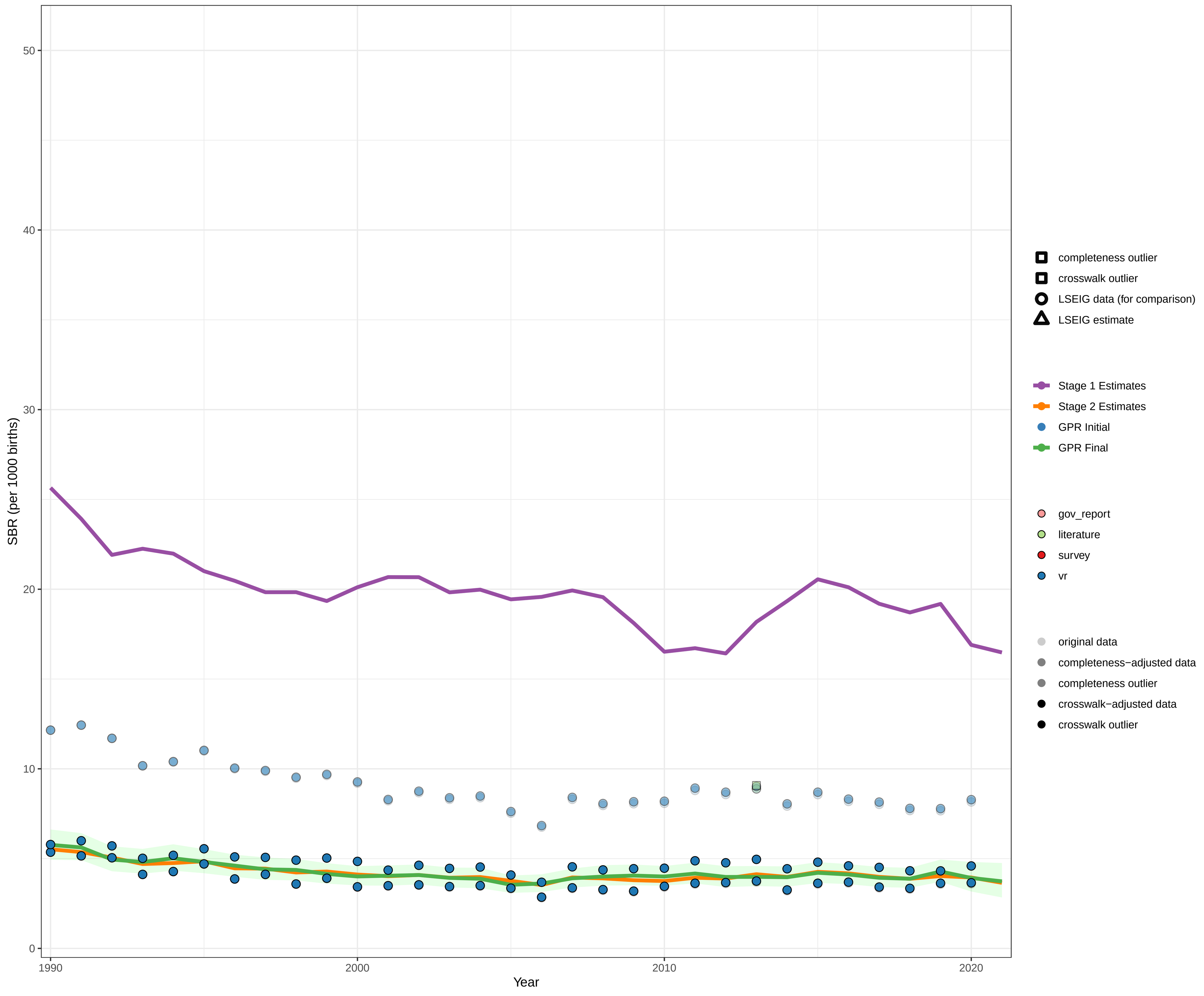

Year	Total Stillbirths (in thousands)	Total Stillbirths (in thousands)	SBR (per 1000 births)	SBR (per 1000 births)	SBR/NMR	SBR/NMR
	20 weeks	28 weeks	20 weeks	28 weeks	20 weeks	28 weeks
1990	1.3 (1.1 to 1.5)	0.6 (0.5 to 0.7)	12.0 (10.3 to 13.8)	5.8 (4.9 to 6.6)	1.52 (1.30 to 1.74)	0.73 (0.62 to 0.84)
1995	1.1 (1.0 to 1.3)	0.6 (0.5 to 0.6)	9.5 (8.3 to 11.0)	4.8 (4.2 to 5.5)	1.55 (1.35 to 1.78)	0.78 (0.68 to 0.90)
2000	1.0 (0.9 to 1.2)	0.5 (0.5 to 0.6)	7.6 (6.7 to 8.7)	4.0 (3.5 to 4.6)	1.34 (1.17 to 1.53)	0.70 (0.62 to 0.80)
2005	1.0 (0.9 to 1.1)	0.5 (0.5 to 0.6)	6.6 (5.8 to 7.6)	3.5 (3.1 to 4.1)	1.24 (1.08 to 1.42)	0.66 (0.58 to 0.76)
2010	1.0 (0.9 to 1.2)	0.6 (0.5 to 0.6)	7.3 (6.4 to 8.4)	4.0 (3.5 to 4.6)	1.72 (1.49 to 1.97)	0.94 (0.82 to 1.07)
2015	1.0 (0.9 to 1.2)	0.6 (0.5 to 0.6)	7.6 (6.6 to 8.7)	4.2 (3.6 to 4.8)	1.47 (1.27 to 1.68)	0.81 (0.70 to 0.93)
2020	0.9 (0.7 to 1.1)	0.5 (0.4 to 0.6)	7.0 (5.7 to 8.7)	3.9 (3.2 to 4.8)	1.70 (1.37 to 2.09)	0.95 (0.76 to 1.16)
2021	0.8 (0.6 to 1.1)	0.5 (0.4 to 0.6)	6.7 (5.1 to 8.5)	3.7 (2.8 to 4.8)	1.66 (1.26 to 2.12)	0.93 (0.70 to 1.18)

Hawaii USA_534 High−income North America Stillbirth Rate / Neonatal Mortality Rate (SBR/NMR) − 20 weeks
lambda = 0.2 ; zeta = 0.99 ; scale = 5 ; amp = 0.028

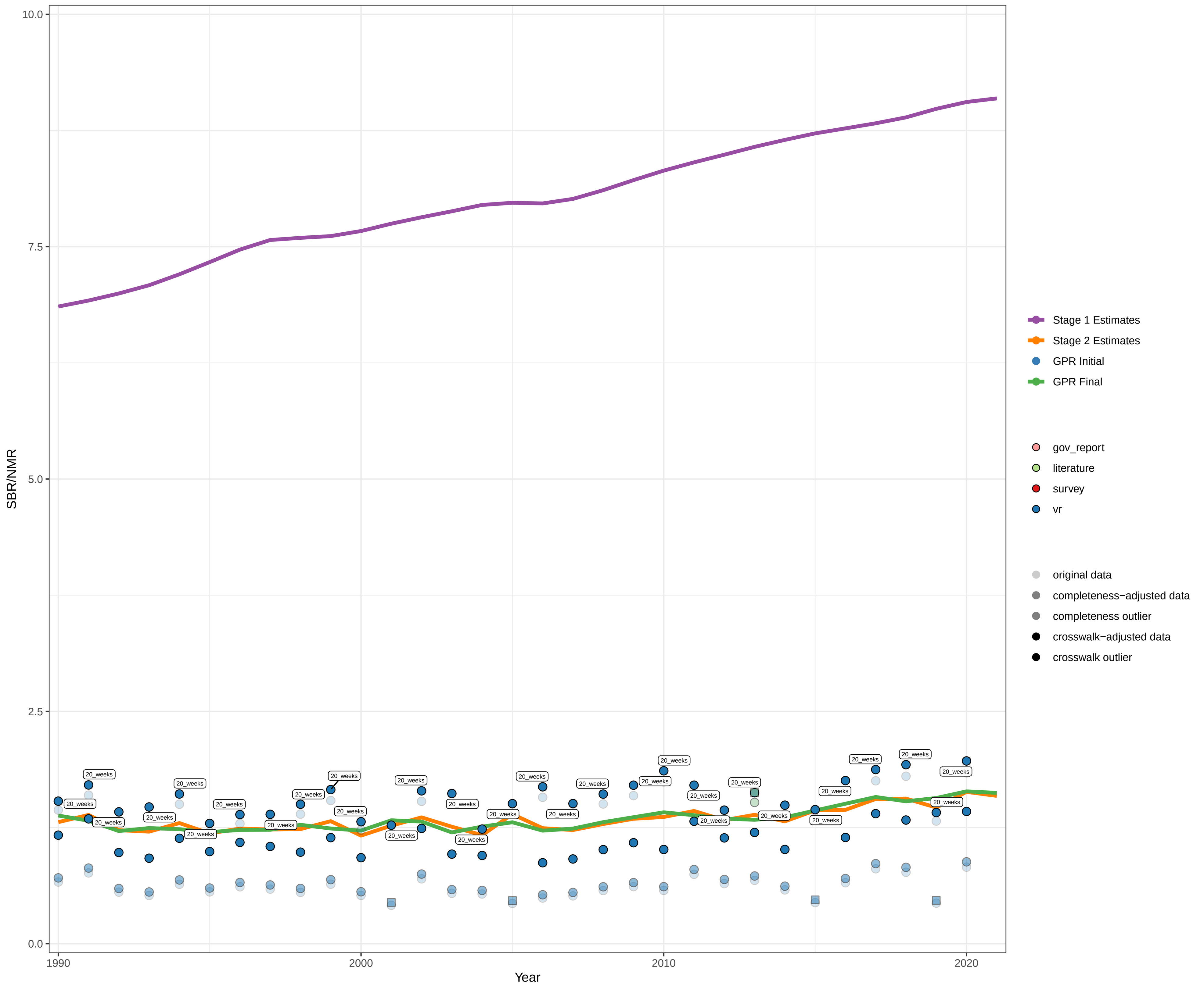

Hawaii USA_534 High−income North America Stillbirth Rate / Neonatal Mortality Rate (SBR/NMR) − 28 weeks
lambda = 0.2 ; zeta = 0.99 ; scale = 5 ; amp = 0.028

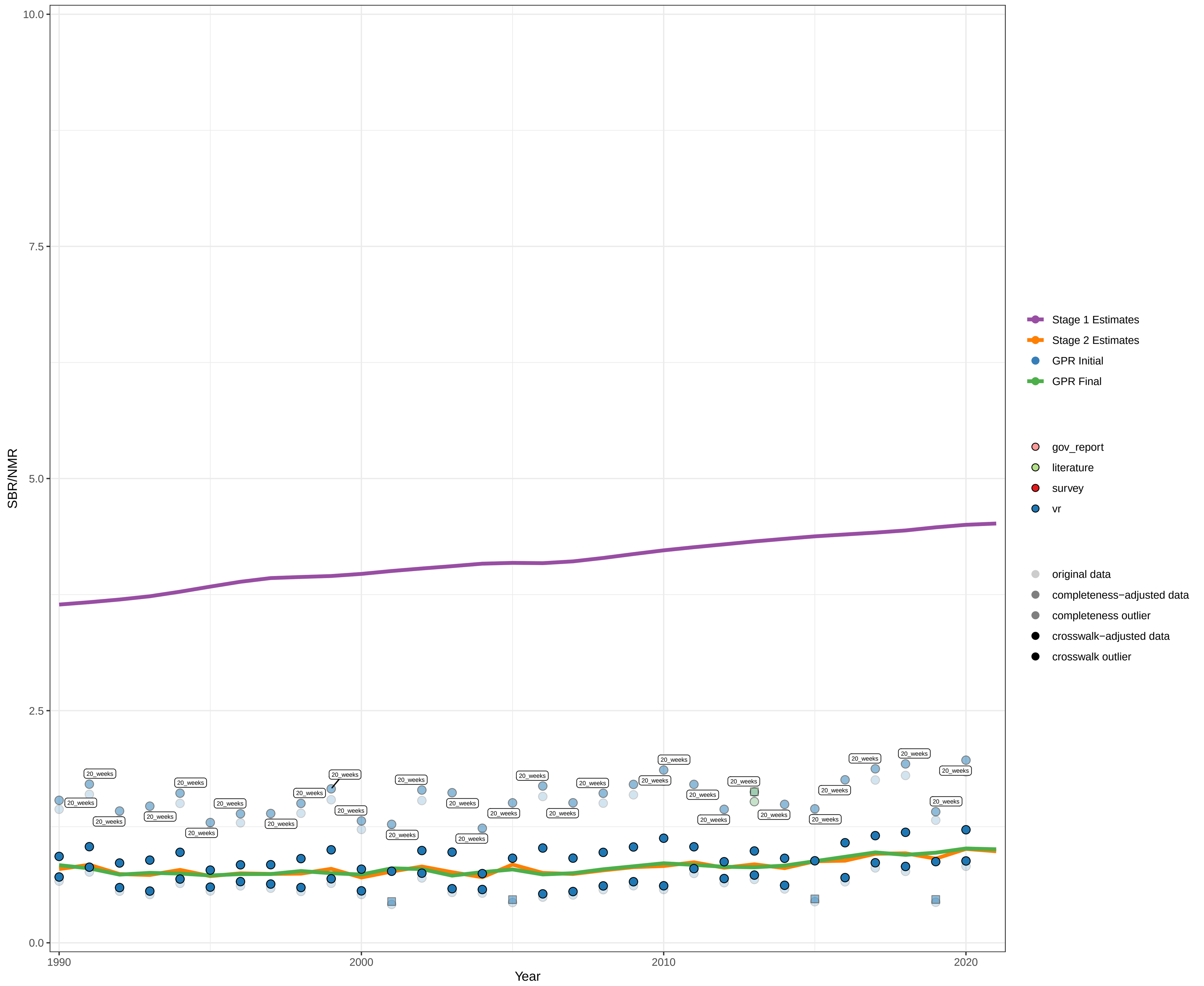

Definition	Years	Outlier	Source.Type	Title
20_weeks	1990−2020	0	VR	United States NVSS Custom Fetal Death Data
28_weeks	1990−2000, 2002−2004, 2006−2014, 2016−2018, 2020	0	VR	United States NVSS Custom Fetal Death Data
20_weeks	2013	1	Sci Lit	United States Fetal and Perinatal Mortality 2013
28_weeks	2001, 2005, 2015, 2019	1	VR	United States NVSS Custom Fetal Death Data

Hawaii USA_534 High−income North America Stillbirth Rate (SBR) − 20 weeks
lambda = 0.2 ; zeta = 0.99 ; scale = 5 ; amp = 0.028

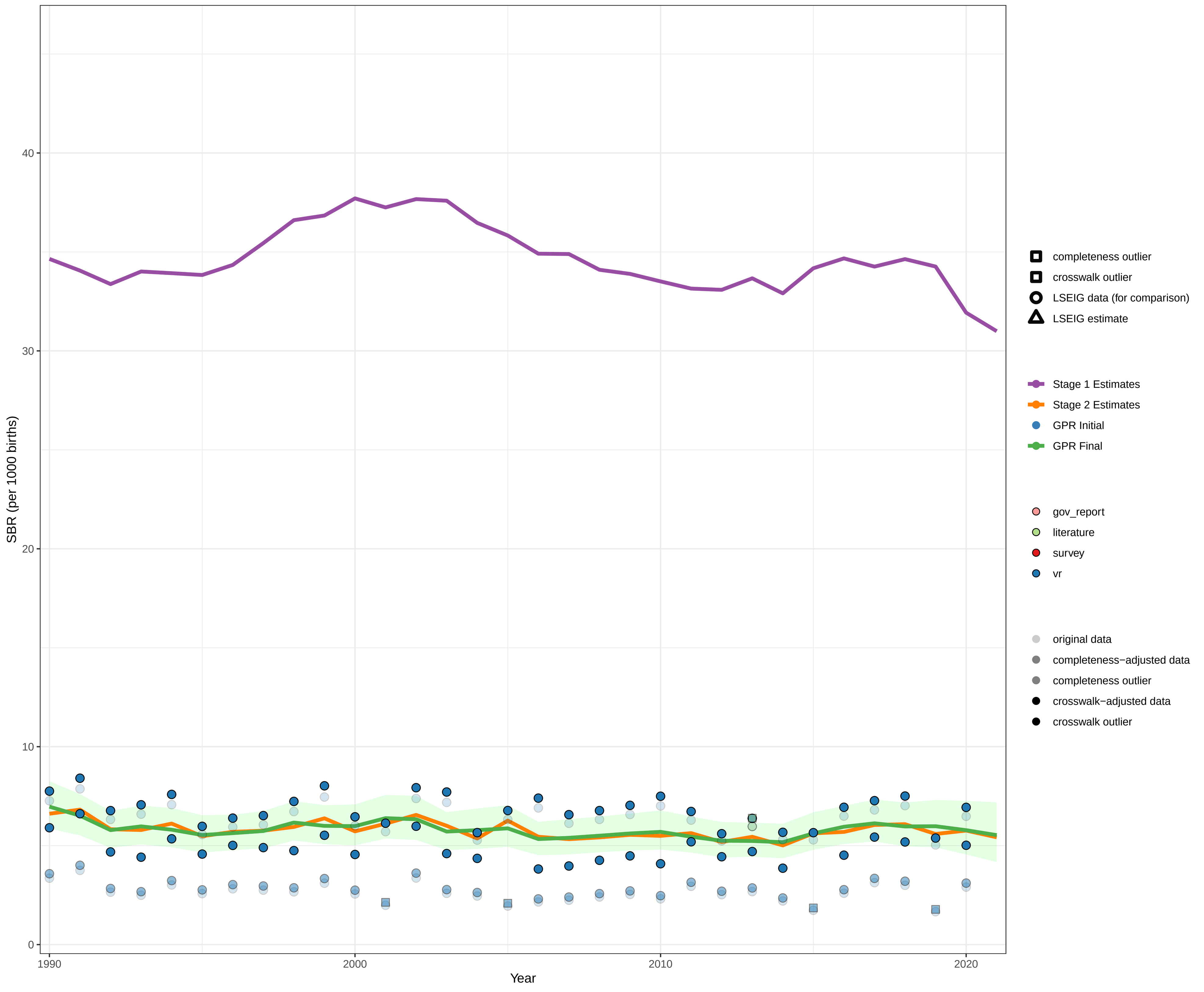

Hawaii USA_534 High−income North America Stillbirth Rate (SBR) − 28 weeks
lambda = 0.2 ; zeta = 0.99 ; scale = 5 ; amp = 0.028

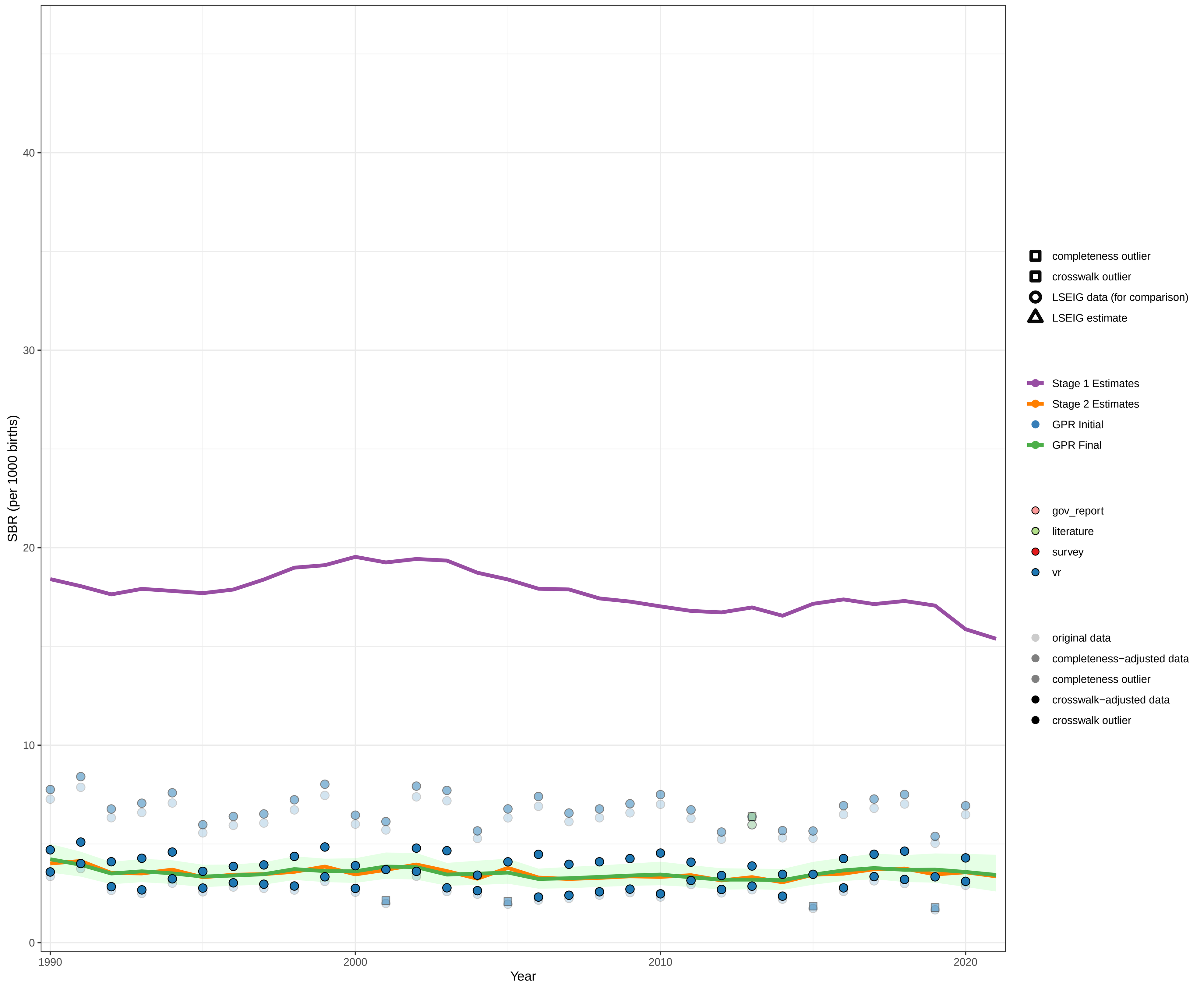

Year	Total Stillbirths (in thousands)	Total Stillbirths (in thousands)	SBR (per 1000 births)	SBR (per 1000 births)	SBR/NMR	SBR/NMR
	20 weeks	28 weeks	20 weeks	28 weeks	20 weeks	28 weeks
1990	0.1 (0.1 to 0.2)	0.1 (0.1 to 0.1)	7.0 (5.9 to 8.3)	4.2 (3.6 to 5.0)	1.38 (1.16 to 1.63)	0.84 (0.70 to 0.99)
1995	0.1 (0.1 to 0.1)	0.1 (0.1 to 0.1)	5.5 (4.7 to 6.5)	3.3 (2.8 to 3.9)	1.20 (1.01 to 1.42)	0.73 (0.61 to 0.86)
2000	0.1 (0.1 to 0.1)	0.1 (0.1 to 0.1)	6.0 (5.0 to 7.1)	3.6 (3.0 to 4.3)	1.22 (1.02 to 1.44)	0.74 (0.62 to 0.87)
2005	0.1 (0.1 to 0.1)	0.1 (0.1 to 0.1)	5.9 (4.9 to 7.0)	3.6 (3.0 to 4.3)	1.31 (1.10 to 1.57)	0.79 (0.66 to 0.95)
2010	0.1 (0.1 to 0.1)	0.1 (0.1 to 0.1)	5.7 (4.8 to 6.8)	3.5 (2.9 to 4.1)	1.41 (1.19 to 1.68)	0.86 (0.72 to 1.02)
2015	0.1 (0.1 to 0.1)	0.1 (0.1 to 0.1)	5.6 (4.8 to 6.7)	3.4 (2.9 to 4.1)	1.43 (1.22 to 1.70)	0.88 (0.75 to 1.04)
2020	0.1 (0.1 to 0.1)	0.1 (0.0 to 0.1)	5.8 (4.6 to 7.3)	3.6 (2.8 to 4.5)	1.64 (1.29 to 2.06)	1.02 (0.80 to 1.28)
2021	0.1 (0.1 to 0.1)	0.1 (0.0 to 0.1)	5.5 (4.2 to 7.2)	3.4 (2.6 to 4.5)	1.62 (1.22 to 2.11)	1.01 (0.76 to 1.31)

Idaho USA_535 High−income North America Stillbirth Rate / Neonatal Mortality Rate (SBR/NMR) − 20 weeks
lambda = 0.2 ; zeta = 0.99 ; scale = 5 ; amp = 0.028

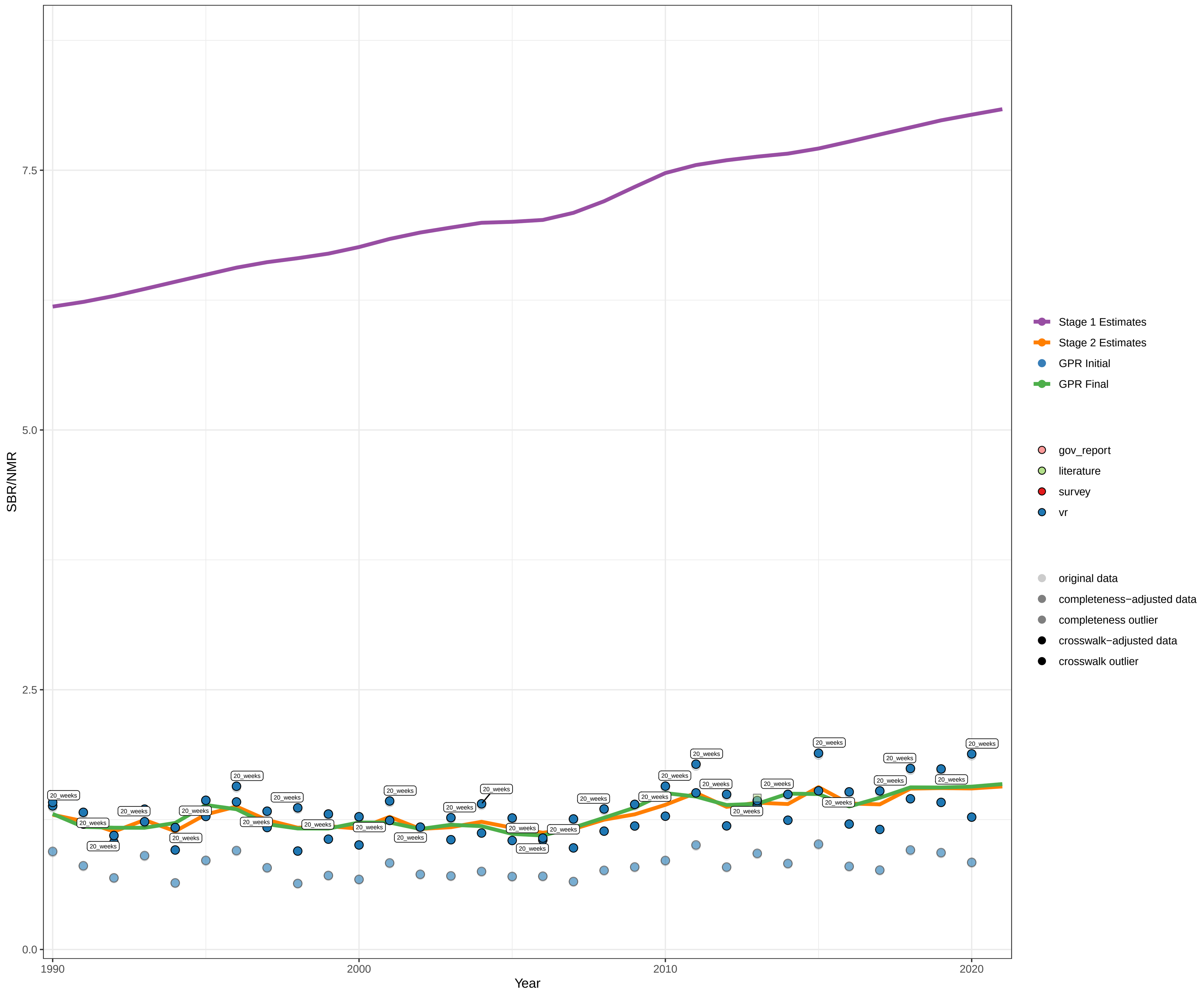

Idaho USA_535 High−income North America Stillbirth Rate / Neonatal Mortality Rate (SBR/NMR) − 28 weeks
lambda = 0.2 ; zeta = 0.99 ; scale = 5 ; amp = 0.028

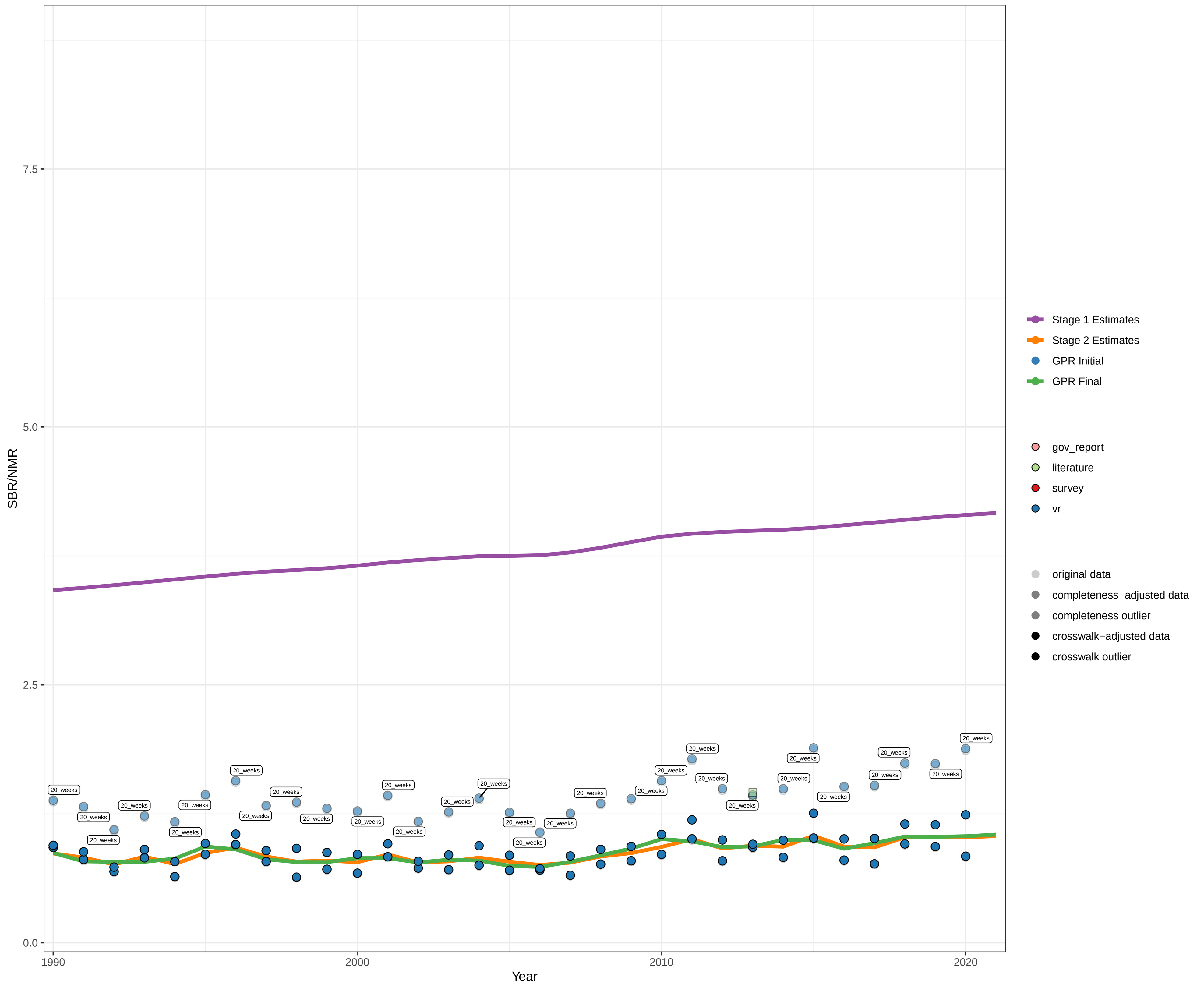

Definition	Years	Outlier	Source.Type	Title
20_weeks, 28_weeks	1990−2020	0	VR	United States NVSS Custom Fetal Death Data
20_weeks	2013	1	Sci Lit	United States Fetal and Perinatal Mortality 2013

Idaho USA_535 High−income North America Stillbirth Rate (SBR) − 20 weeks
lambda = 0.2 ; zeta = 0.99 ; scale = 5 ; amp = 0.028

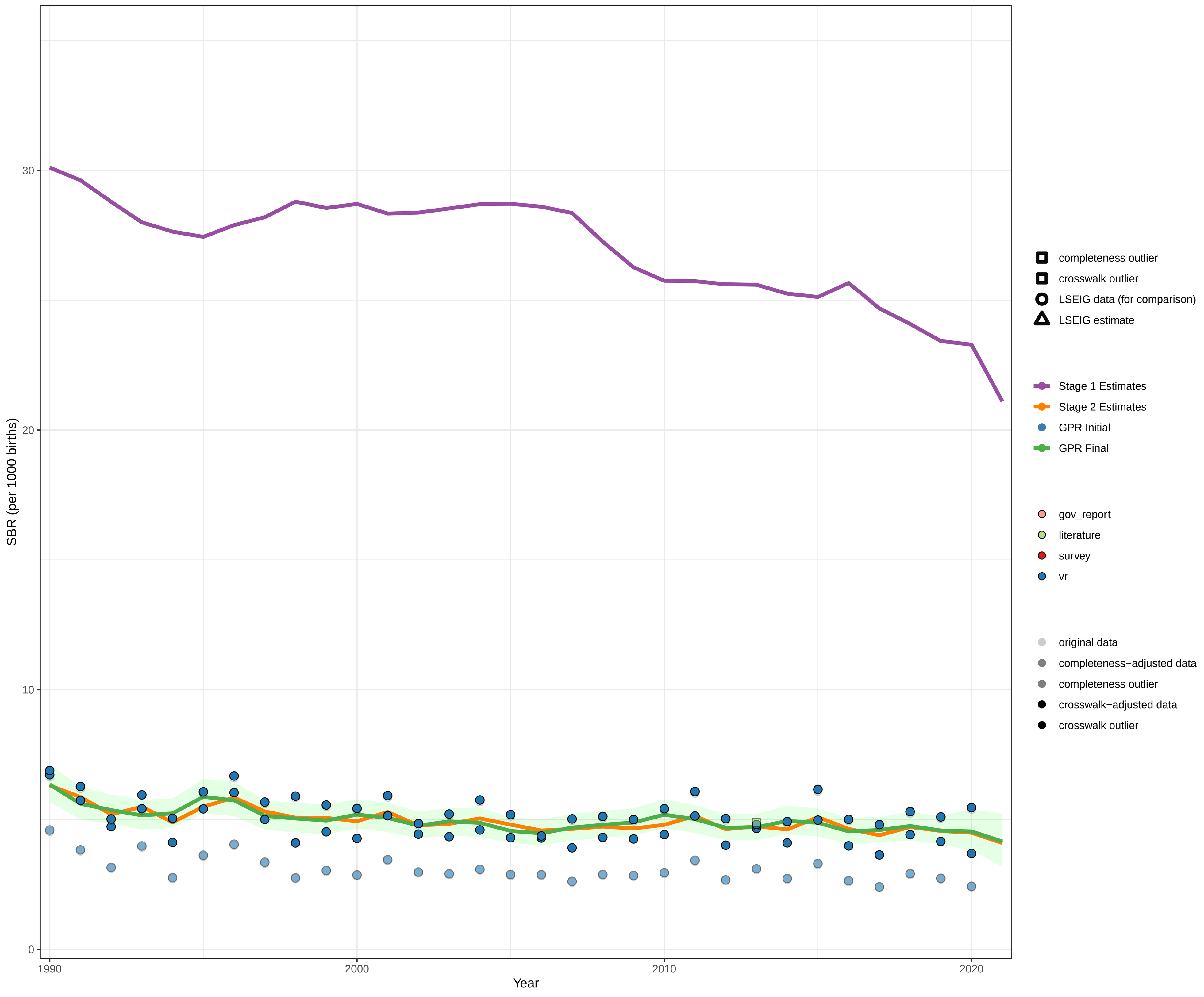

Idaho USA_535 High−income North America Stillbirth Rate (SBR) − 28 weeks
lambda = 0.2 ; zeta = 0.99 ; scale = 5 ; amp = 0.028

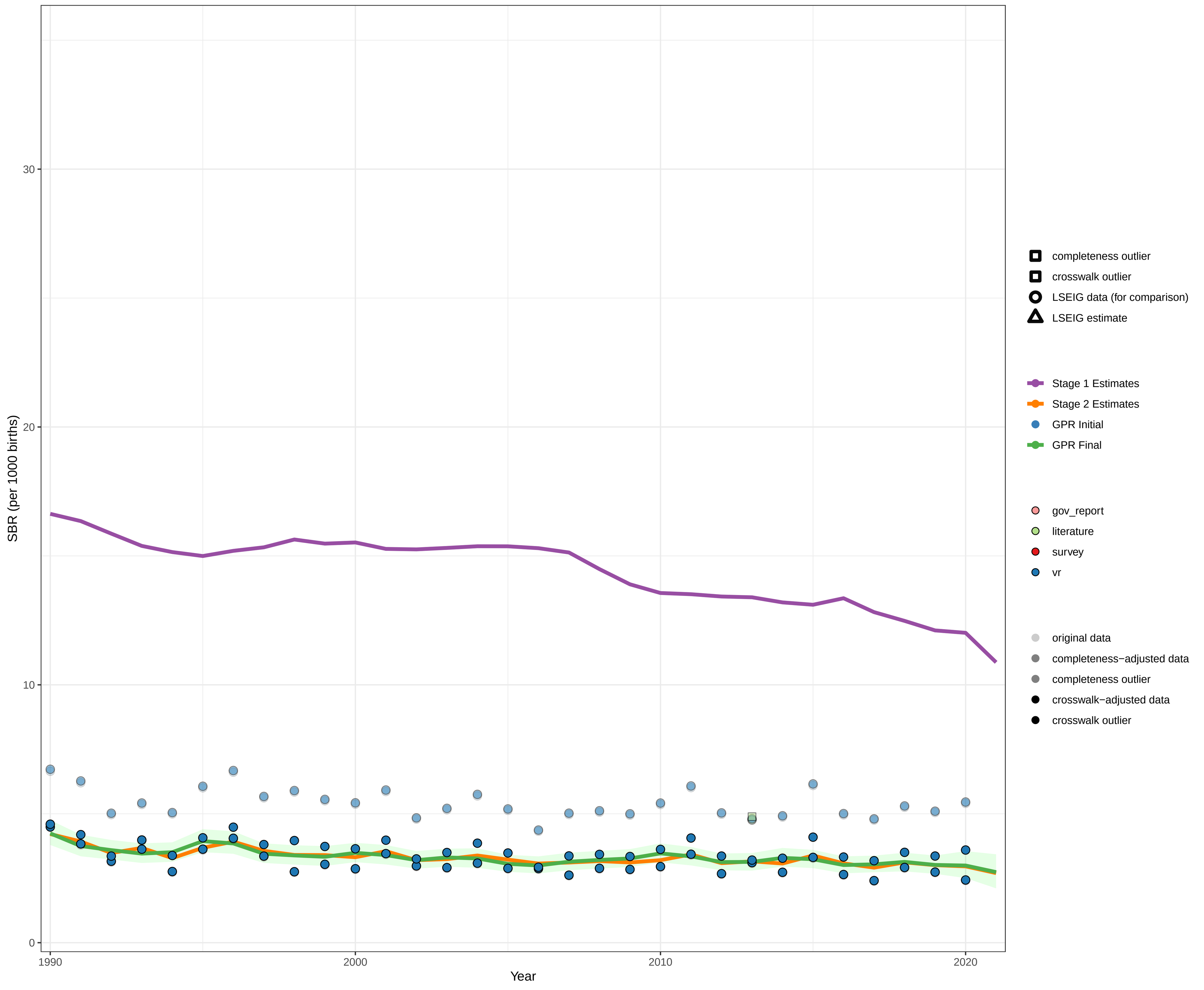

Year	Total Stillbirths (in thousands)	Total Stillbirths (in thousands)	SBR (per 1000 births)	SBR (per 1000 births)	SBR/NMR	SBR/NMR
	20 weeks	28 weeks	20 weeks	28 weeks	20 weeks	28 weeks
1990	0.1 (0.1 to 0.1)	0.1 (0.1 to 0.1)	6.3 (5.7 to 7.1)	4.2 (3.8 to 4.8)	1.31 (1.17 to 1.47)	0.87 (0.78 to 0.98)
1995	0.1 (0.1 to 0.1)	0.1 (0.1 to 0.1)	5.9 (5.2 to 6.6)	3.9 (3.5 to 4.4)	1.39 (1.24 to 1.56)	0.93 (0.83 to 1.04)
2000	0.1 (0.1 to 0.1)	0.1 (0.1 to 0.1)	5.2 (4.6 to 5.8)	3.5 (3.1 to 3.9)	1.22 (1.09 to 1.36)	0.82 (0.74 to 0.91)
2005	0.1 (0.1 to 0.1)	0.1 (0.1 to 0.1)	4.6 (4.1 to 5.1)	3.1 (2.7 to 3.4)	1.11 (1.00 to 1.24)	0.75 (0.67 to 0.83)
2010	0.1 (0.1 to 0.1)	0.1 (0.1 to 0.1)	5.2 (4.7 to 5.8)	3.5 (3.1 to 3.9)	1.51 (1.35 to 1.68)	1.01 (0.90 to 1.12)
2015	0.1 (0.1 to 0.1)	0.1 (0.1 to 0.1)	4.9 (4.4 to 5.4)	3.2 (2.9 to 3.6)	1.50 (1.34 to 1.67)	0.99 (0.89 to 1.10)
2020	0.1 (0.1 to 0.1)	0.1 (0.1 to 0.1)	4.5 (3.8 to 5.4)	3.0 (2.5 to 3.6)	1.57 (1.31 to 1.86)	1.03 (0.86 to 1.23)
2021	0.1 (0.1 to 0.1)	0.1 (0.0 to 0.1)	4.2 (3.2 to 5.2)	2.7 (2.1 to 3.4)	1.59 (1.22 to 2.00)	1.05 (0.81 to 1.31)

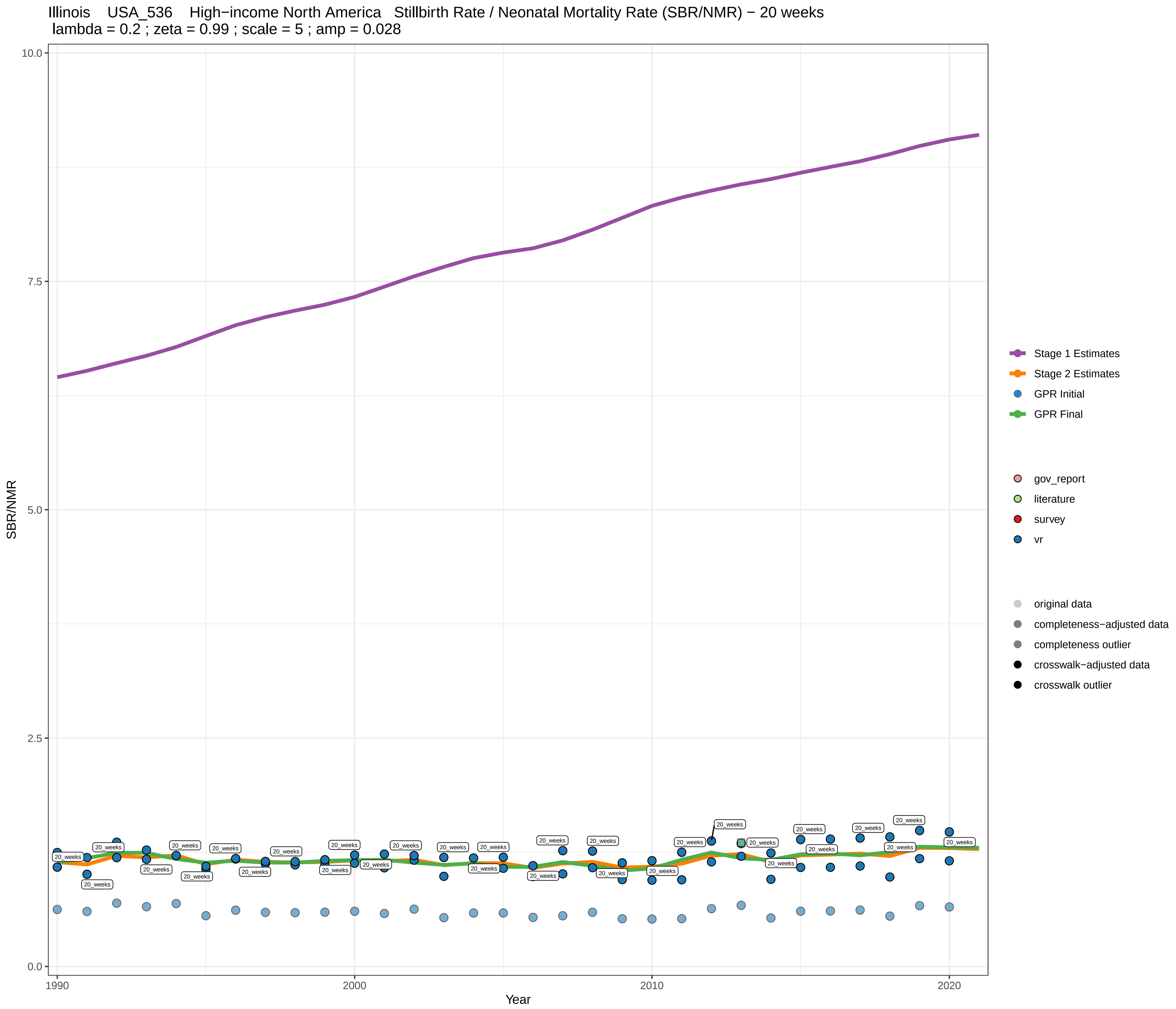

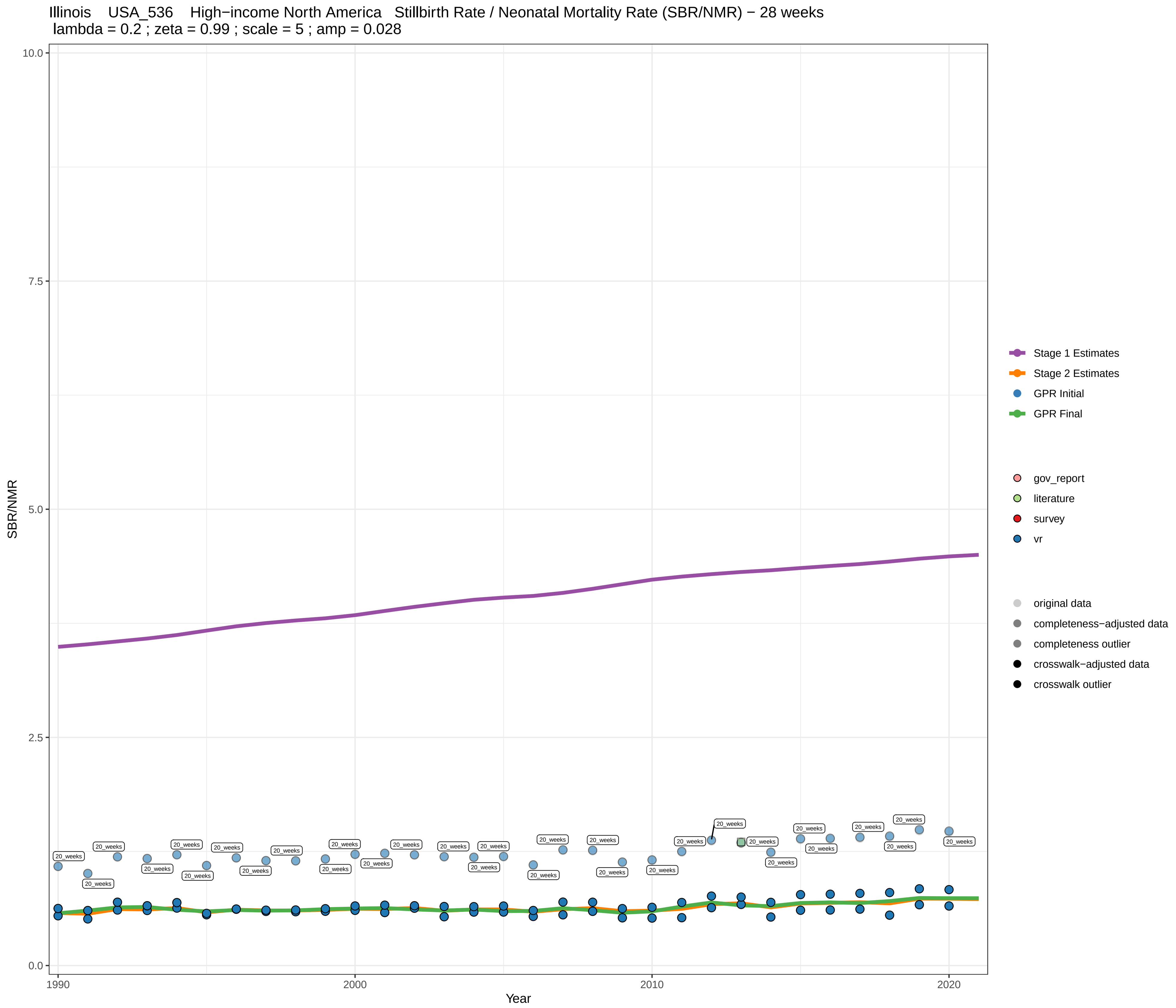

Definition	Years	Outlier	Source.Type	Title
20_weeks, 28_weeks	1990−2020	0	VR	United States NVSS Custom Fetal Death Data
20_weeks	2013	1	Sci Lit	United States Fetal and Perinatal Mortality 2013

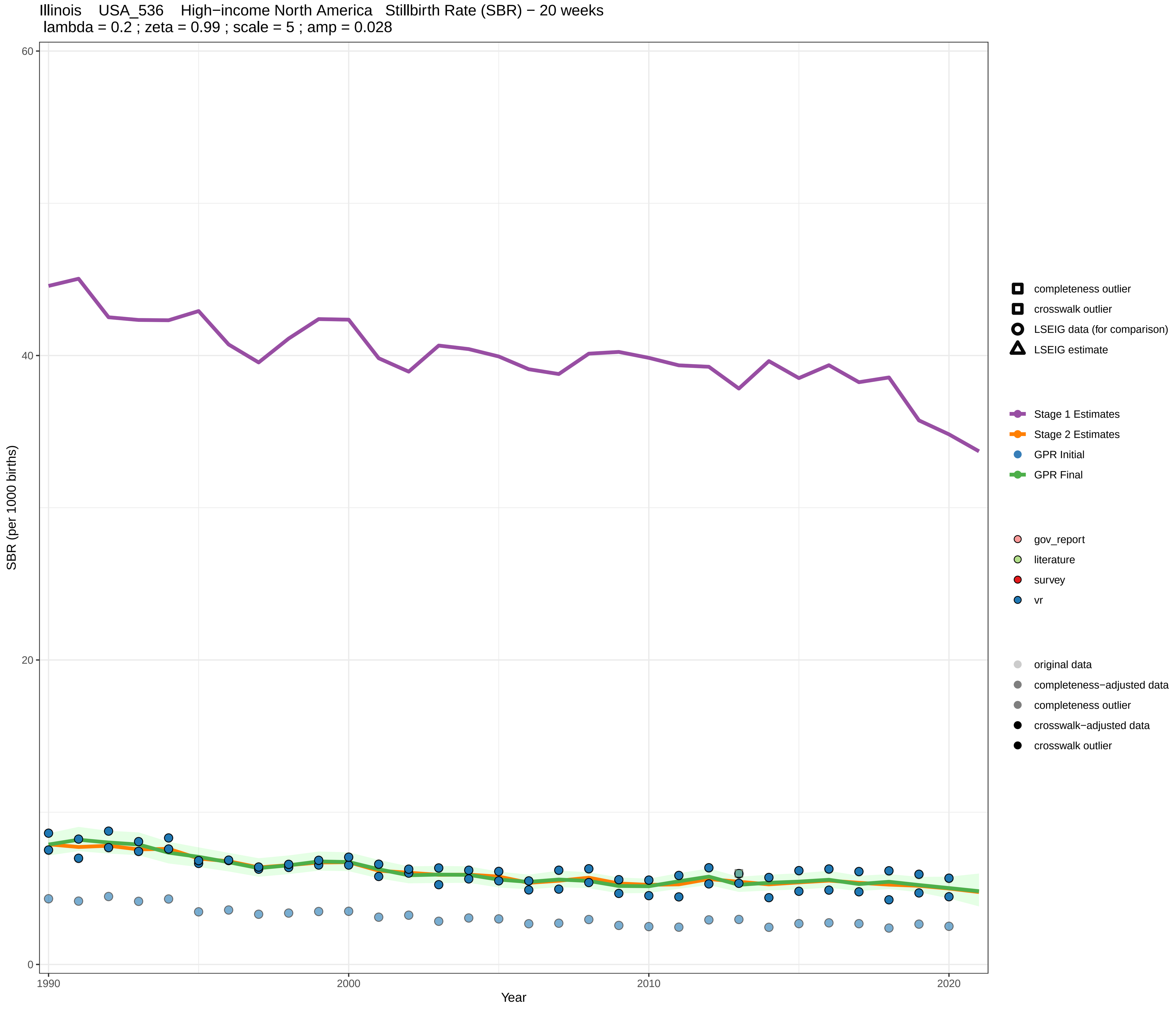

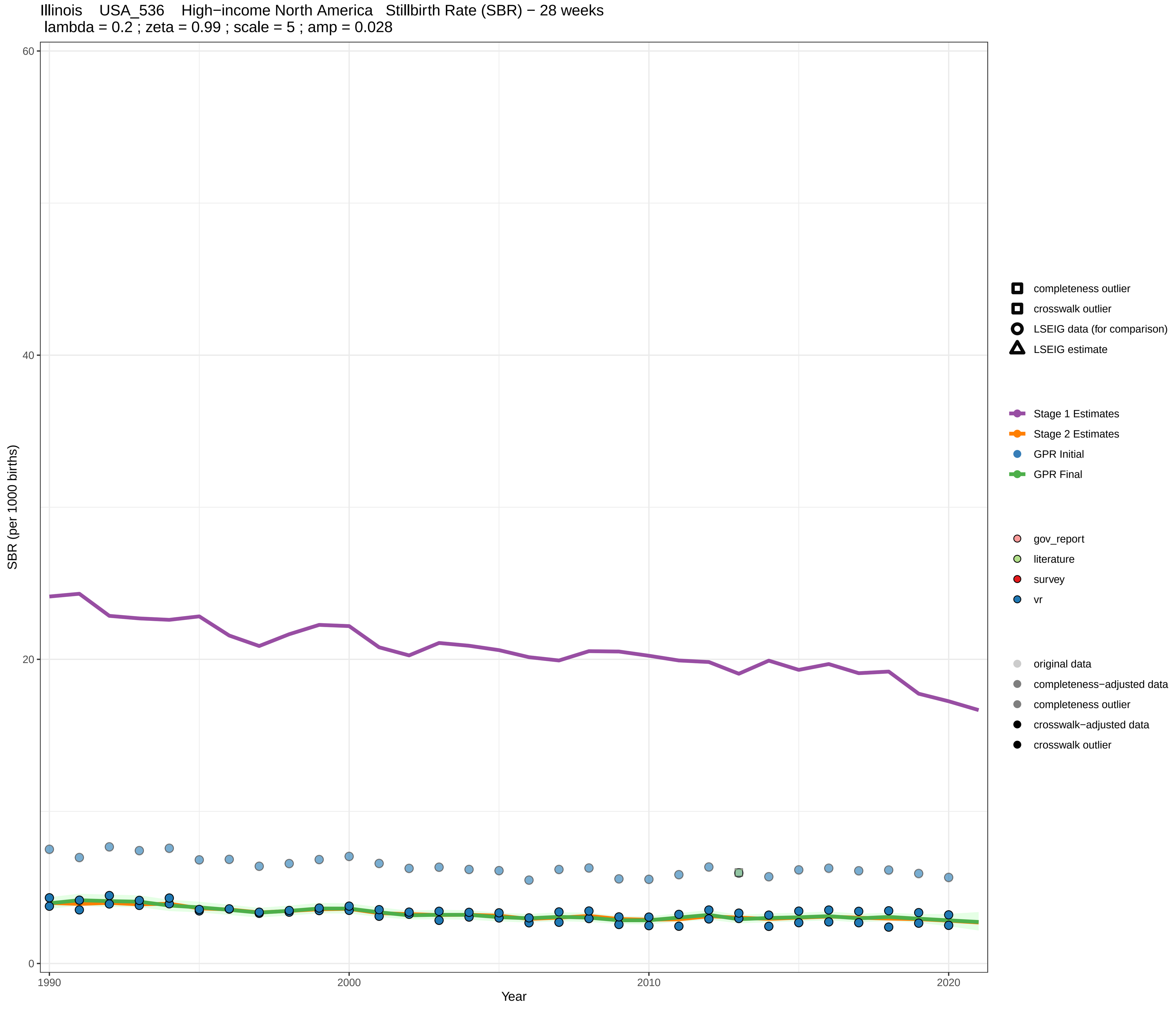

Year	Total Stillbirths (in thousands)	Total Stillbirths (in thousands)	SBR (per 1000 births)	SBR (per 1000 births)	SBR/NMR	SBR/NMR
	20 weeks	28 weeks	20 weeks	28 weeks	20 weeks	28 weeks
1990	1.6<br>(1.4 to 1.7)	0.8<br>(0.7 to 0.9)	7.9<br>(7.2 to 8.6)	4.0<br>(3.6 to 4.4)	1.14<br>(1.04 to 1.25)	0.57<br>(0.52 to 0.63)
1995	1.3<br>(1.2 to 1.4)	0.7<br>(0.6 to 0.7)	7.1<br>(6.4 to 7.7)	3.7<br>(3.4 to 4.1)	1.14<br>(1.03 to 1.24)	0.59<br>(0.54 to 0.65)
2000	1.2<br>(1.1 to 1.4)	0.7<br>(0.6 to 0.7)	6.7<br>(6.1 to 7.4)	3.6<br>(3.3 to 4.0)	1.16<br>(1.06 to 1.28)	0.63<br>(0.57 to 0.69)
2005	1.0<br>(0.9 to 1.1)	0.5<br>(0.5 to 0.6)	5.6<br>(5.1 to 6.1)	3.0<br>(2.8 to 3.3)	1.09<br>(0.99 to 1.19)	0.60<br>(0.54 to 0.65)
2010	0.8<br>(0.8 to 0.9)	0.5<br>(0.4 to 0.5)	5.1<br>(4.7 to 5.6)	2.8<br>(2.6 to 3.1)	1.07<br>(0.98 to 1.18)	0.59<br>(0.54 to 0.65)
2015	0.8<br>(0.8 to 0.9)	0.5<br>(0.4 to 0.5)	5.4<br>(4.9 to 6.0)	3.0<br>(2.8 to 3.3)	1.23<br>(1.11 to 1.35)	0.69<br>(0.62 to 0.75)
2020	0.7<br>(0.6 to 0.8)	0.4<br>(0.3 to 0.4)	5.0<br>(4.3 to 5.8)	2.8<br>(2.5 to 3.3)	1.30<br>(1.13 to 1.50)	0.74<br>(0.64 to 0.85)
2021	0.6<br>(0.5 to 0.8)	0.4<br>(0.3 to 0.4)	4.8<br>(3.8 to 6.0)	2.7<br>(2.2 to 3.4)	1.30<br>(1.03 to 1.61)	0.74<br>(0.59 to 0.91)

Indiana USA_537 High−income North America Stillbirth Rate / Neonatal Mortality Rate (SBR/NMR) − 20 weeks
lambda = 0.2 ; zeta = 0.99 ; scale = 5 ; amp = 0.028

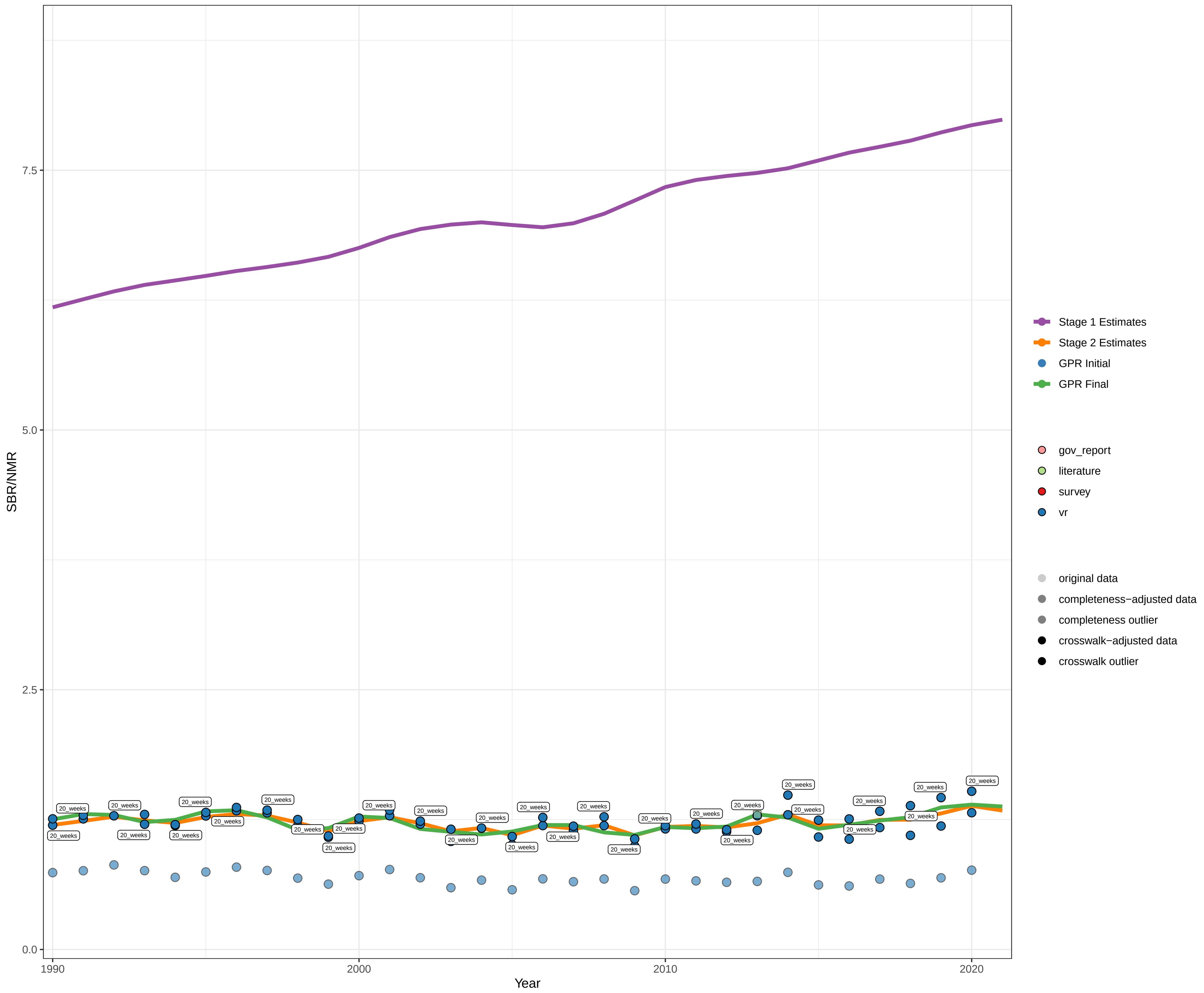

Indiana USA_537 High−income North America Stillbirth Rate / Neonatal Mortality Rate (SBR/NMR) − 28 weeks
lambda = 0.2 ; zeta = 0.99 ; scale = 5 ; amp = 0.028

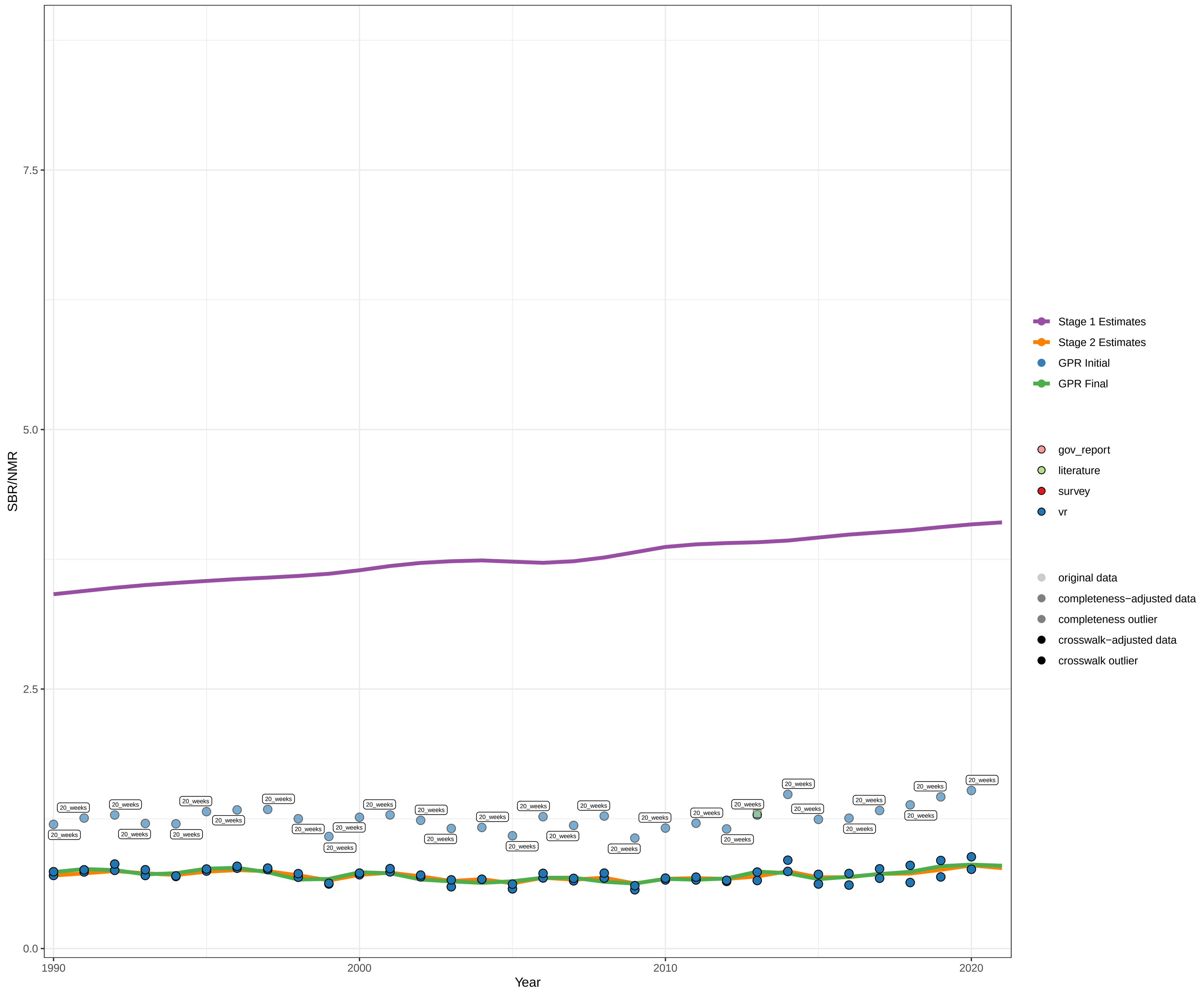

Definition	Years	Outlier	Source.Type	Title
20_weeks, 28_weeks	1990−2020	0	VR	United States NVSS Custom Fetal Death Data
20_weeks	2013	1	Sci Lit	United States Fetal and Perinatal Mortality 2013

Indiana USA_537 High−income North America Stillbirth Rate (SBR) − 20 weeks
lambda = 0.2 ; zeta = 0.99 ; scale = 5 ; amp = 0.028

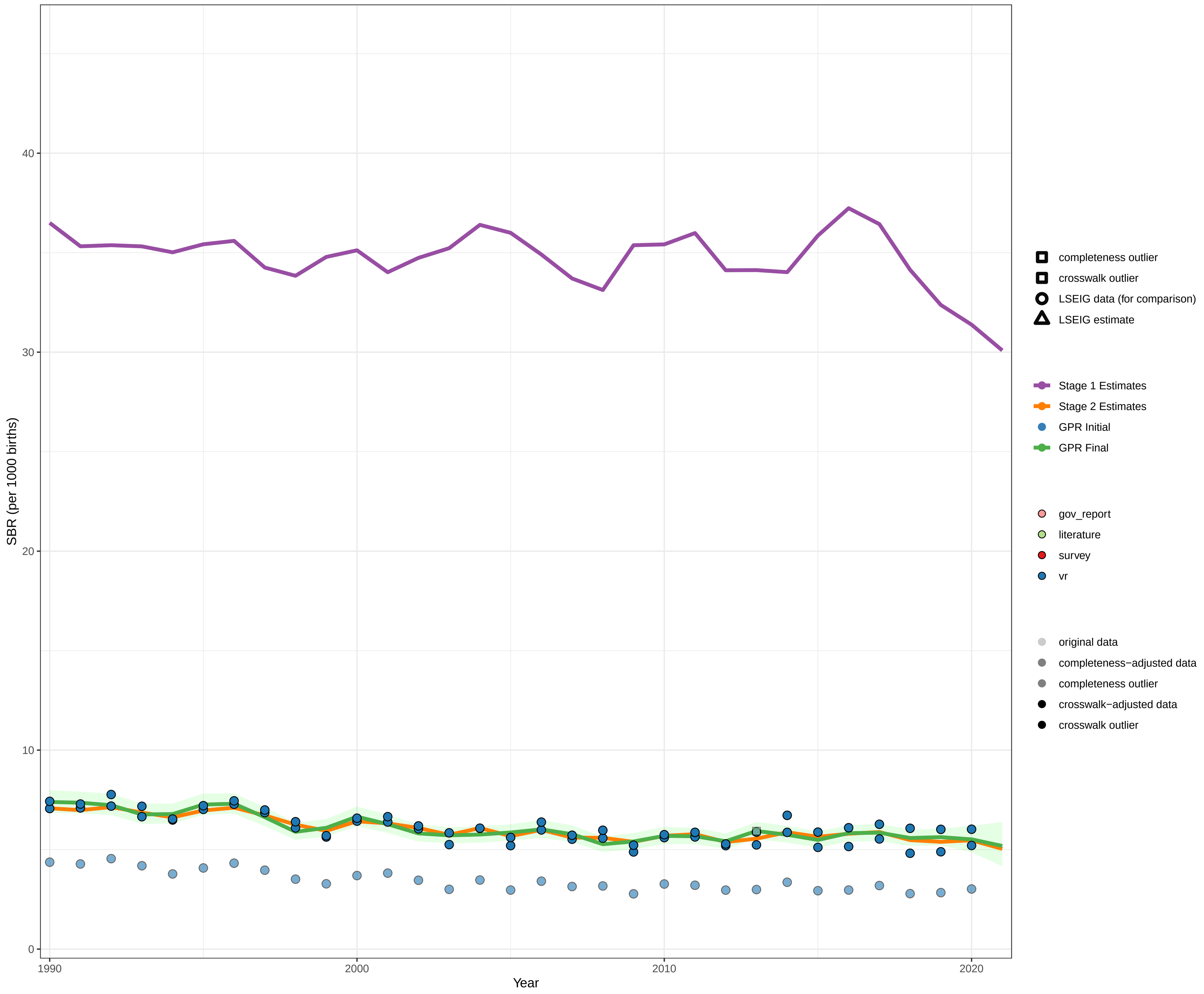

Indiana USA_537 High−income North America Stillbirth Rate (SBR) − 28 weeks
lambda = 0.2 ; zeta = 0.99 ; scale = 5 ; amp = 0.028

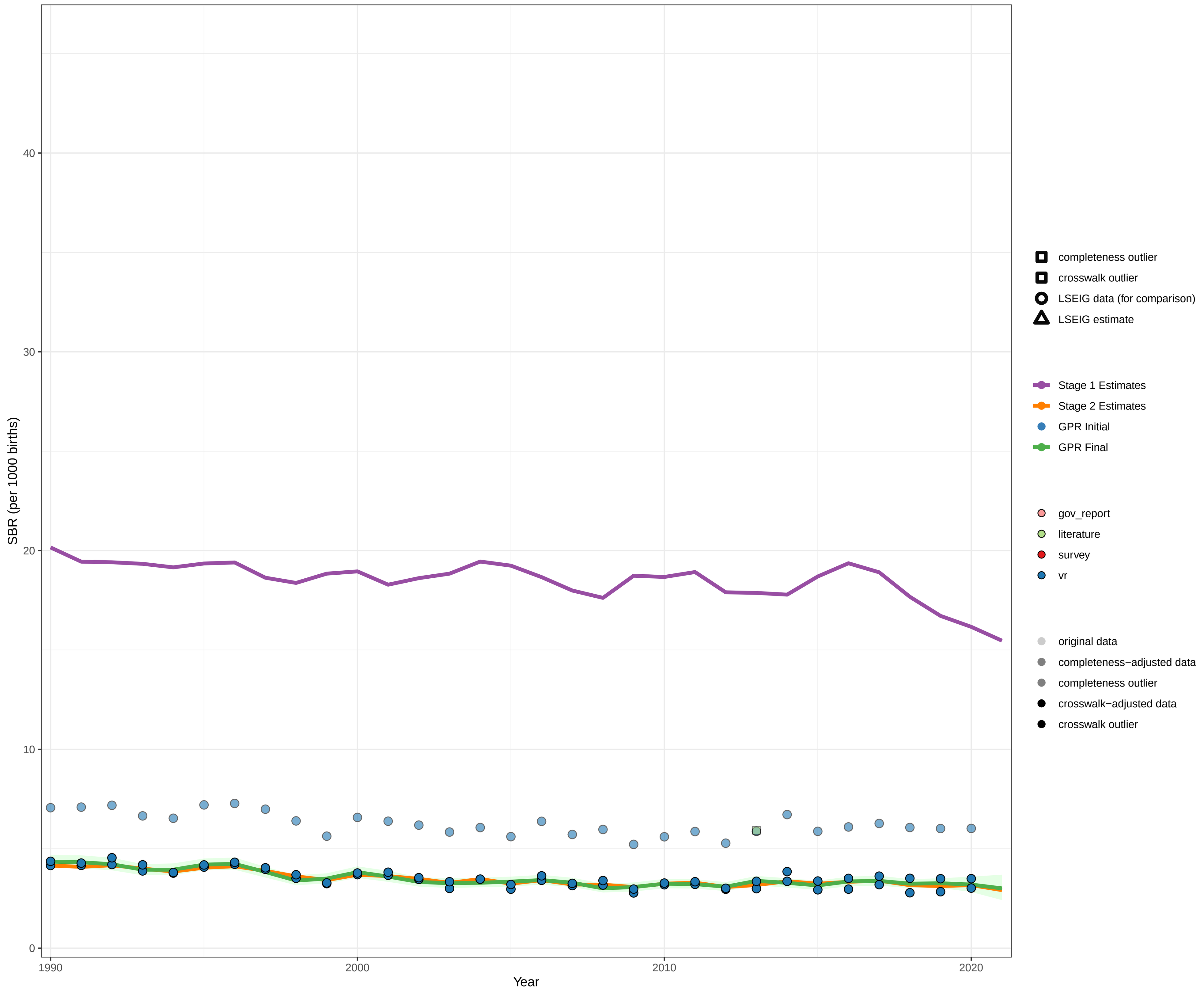

Year	Total Stillbirths (in thousands)	Total Stillbirths (in thousands)	SBR (per 1000 births)	SBR (per 1000 births)	SBR/NMR	SBR/NMR
	20 weeks	28 weeks	20 weeks	28 weeks	20 weeks	28 weeks
1990	0.6 (0.6 to 0.7)	0.4 (0.4 to 0.4)	7.4 (6.8 to 8.0)	4.4 (4.1 to 4.7)	1.25 (1.16 to 1.35)	0.74 (0.69 to 0.79)
1995	0.6 (0.6 to 0.7)	0.3 (0.3 to 0.4)	7.3 (6.8 to 7.8)	4.2 (3.9 to 4.5)	1.33 (1.24 to 1.43)	0.77 (0.72 to 0.83)
2000	0.6 (0.5 to 0.6)	0.3 (0.3 to 0.4)	6.7 (6.2 to 7.2)	3.8 (3.5 to 4.1)	1.28 (1.19 to 1.38)	0.74 (0.68 to 0.79)
2005	0.5 (0.5 to 0.6)	0.3 (0.3 to 0.3)	5.9 (5.5 to 6.3)	3.3 (3.1 to 3.6)	1.14 (1.06 to 1.22)	0.65 (0.60 to 0.70)
2010	0.5 (0.4 to 0.5)	0.3 (0.3 to 0.3)	5.7 (5.3 to 6.1)	3.2 (3.0 to 3.5)	1.18 (1.09 to 1.27)	0.67 (0.62 to 0.72)
2015	0.5 (0.4 to 0.5)	0.3 (0.2 to 0.3)	5.5 (5.1 to 5.9)	3.2 (2.9 to 3.4)	1.16 (1.08 to 1.25)	0.67 (0.62 to 0.72)
2020	0.4 (0.4 to 0.5)	0.3 (0.2 to 0.3)	5.5 (4.9 to 6.2)	3.2 (2.8 to 3.6)	1.39 (1.23 to 1.57)	0.81 (0.71 to 0.91)
2021	0.4 (0.3 to 0.5)	0.2 (0.2 to 0.3)	5.2 (4.2 to 6.4)	3.0 (2.4 to 3.7)	1.38 (1.10 to 1.69)	0.80 (0.64 to 0.98)

Iowa USA_538 High−income North America Stillbirth Rate / Neonatal Mortality Rate (SBR/NMR) − 20 weeks
lambda = 0.2 ; zeta = 0.99 ; scale = 5 ; amp = 0.028

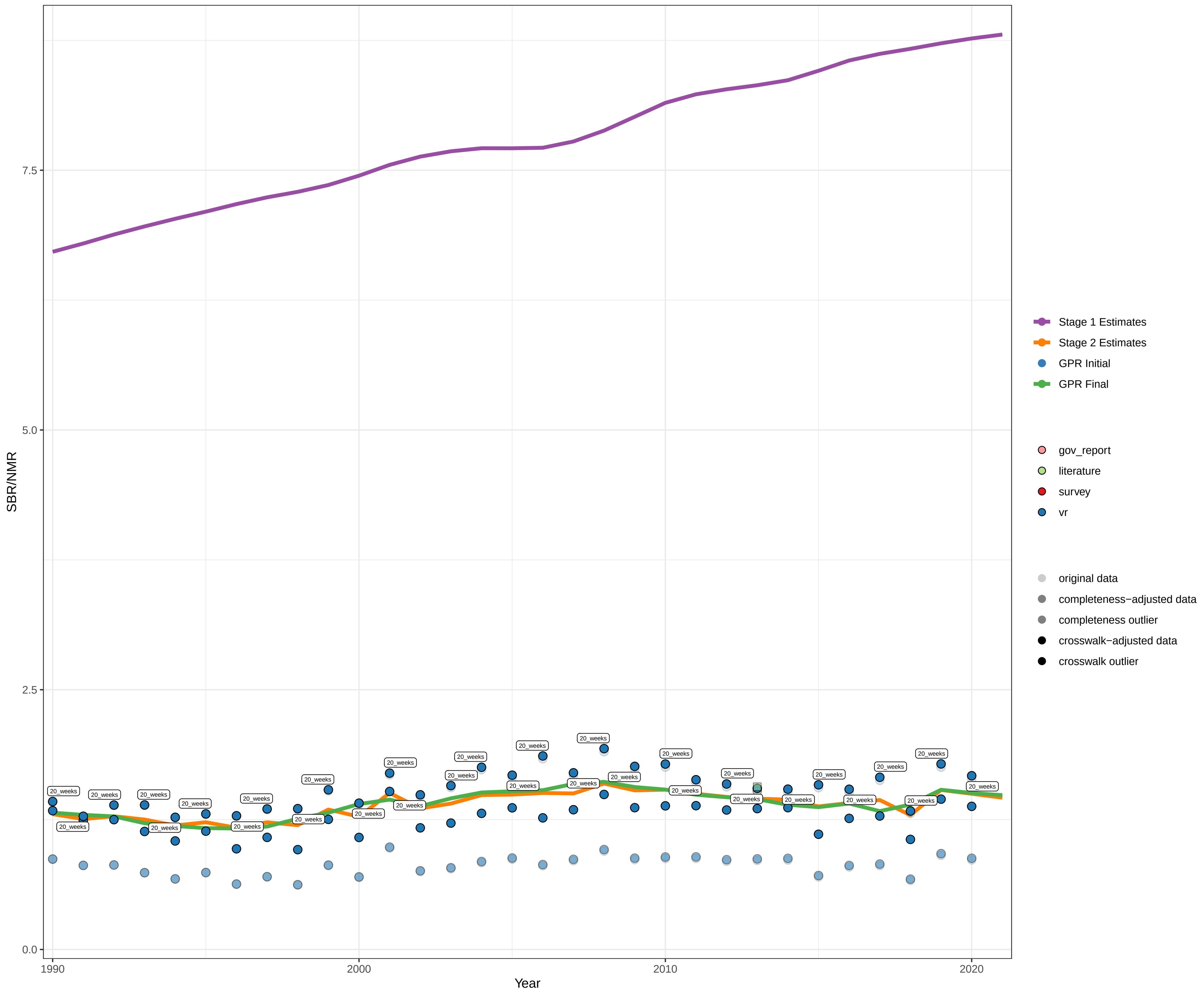

Iowa USA_538 High−income North America Stillbirth Rate / Neonatal Mortality Rate (SBR/NMR) − 28 weeks
lambda = 0.2 ; zeta = 0.99 ; scale = 5 ; amp = 0.028

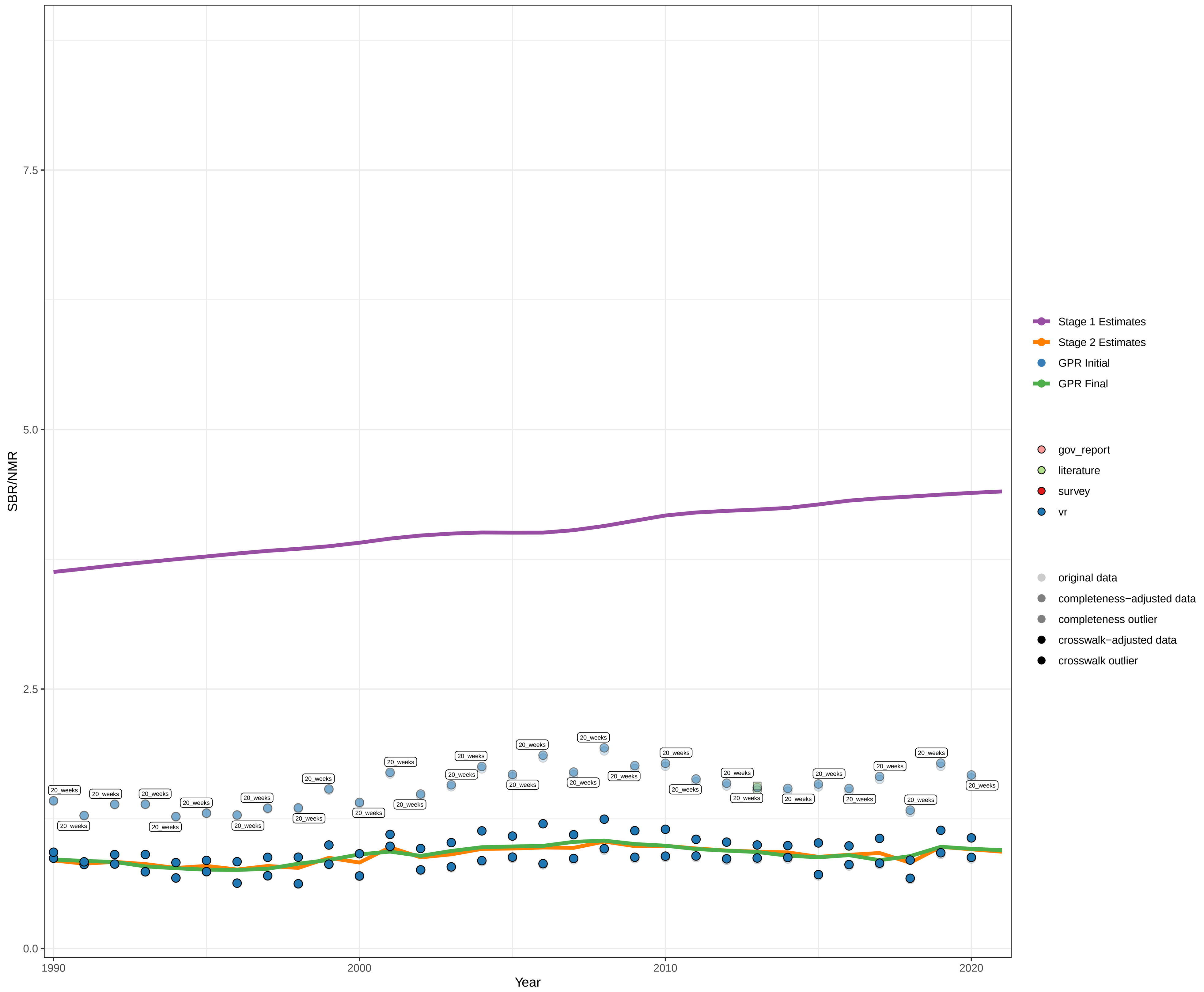

Definition	Years	Outlier	Source.Type	Title
20_weeks, 28_weeks	1990−2020	0	VR	United States NVSS Custom Fetal Death Data
20_weeks	2013	1	Sci Lit	United States Fetal and Perinatal Mortality 2013

Iowa USA_538 High−income North America Stillbirth Rate (SBR) − 20 weeks
lambda = 0.2 ; zeta = 0.99 ; scale = 5 ; amp = 0.028

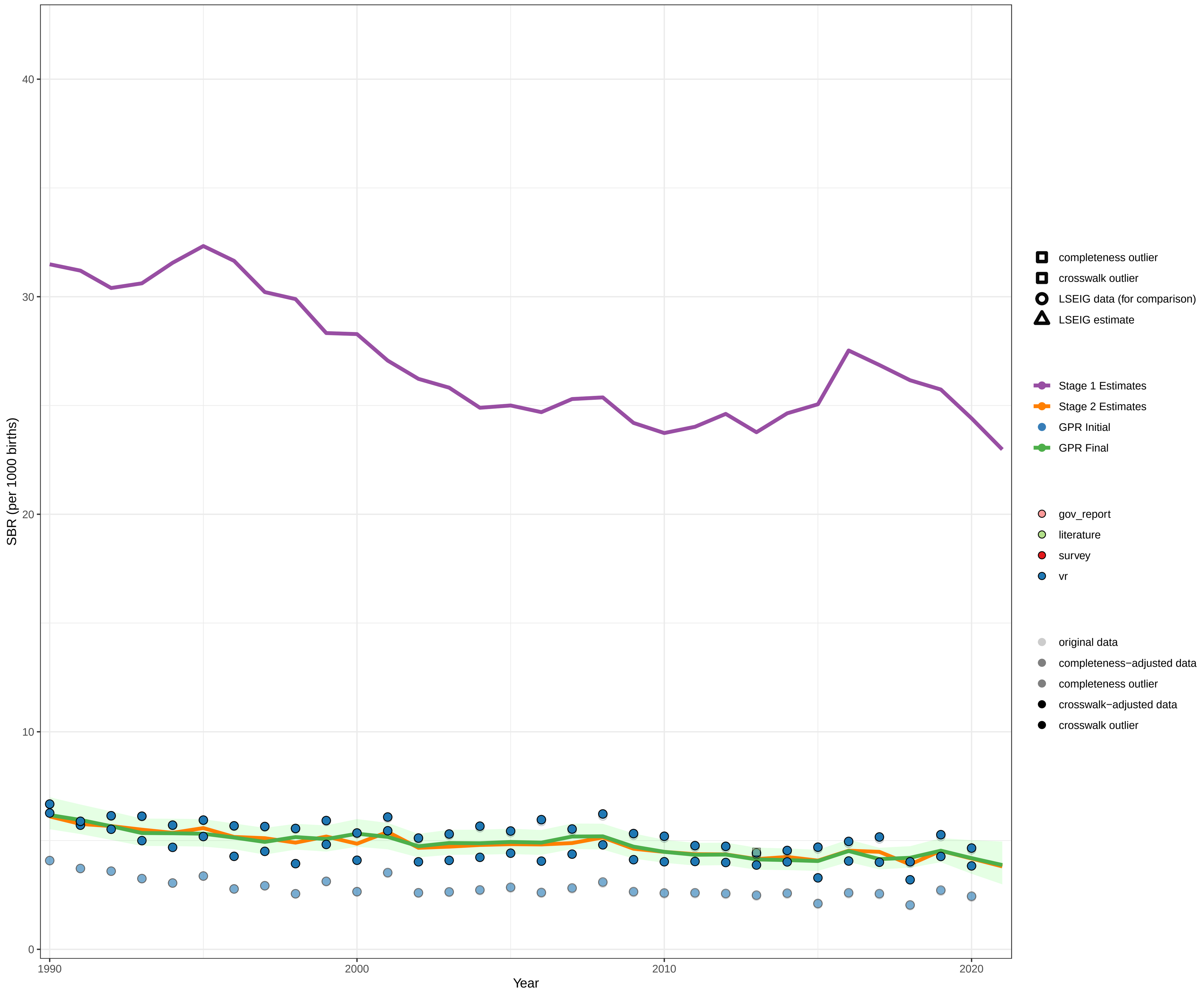

Iowa USA_538 High−income North America Stillbirth Rate (SBR) − 28 weeks
lambda = 0.2 ; zeta = 0.99 ; scale = 5 ; amp = 0.028

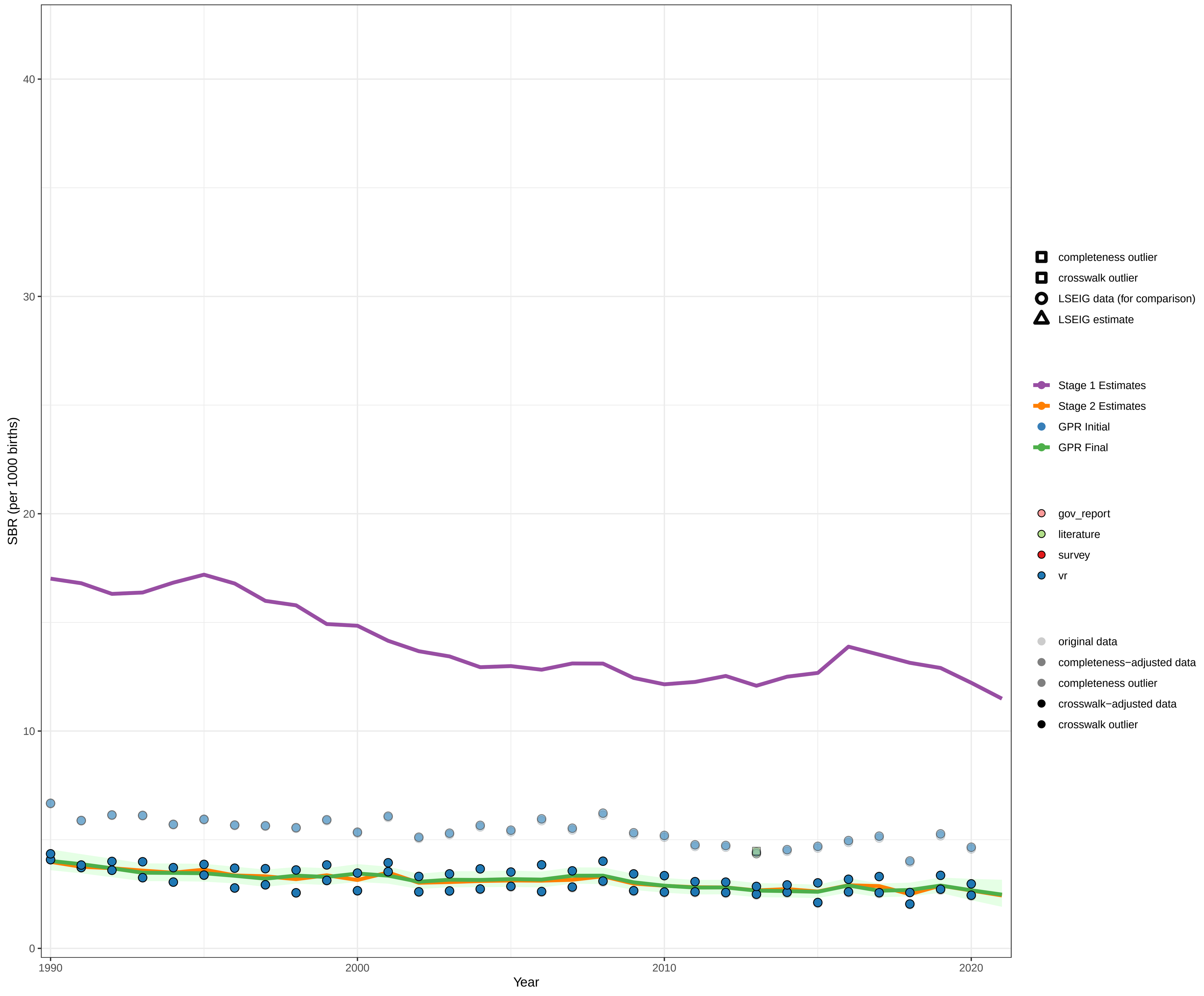

Year	Total Stillbirths (in thousands)	Total Stillbirths (in thousands)	SBR (per 1000 births)	SBR (per 1000 births)	SBR/NMR	SBR/NMR
	20 weeks	28 weeks	20 weeks	28 weeks	20 weeks	28 weeks
1990	0.2 (0.2 to 0.3)	0.2 (0.1 to 0.2)	6.2 (5.5 to 7.0)	4.0 (3.6 to 4.6)	1.32 (1.18 to 1.49)	0.86 (0.77 to 0.97)
1995	0.2 (0.2 to 0.2)	0.1 (0.1 to 0.1)	5.3 (4.7 to 6.0)	3.5 (3.1 to 3.9)	1.17 (1.04 to 1.31)	0.76 (0.67 to 0.85)
2000	0.2 (0.2 to 0.2)	0.1 (0.1 to 0.1)	5.3 (4.7 to 6.0)	3.4 (3.1 to 3.9)	1.40 (1.24 to 1.58)	0.91 (0.80 to 1.02)
2005	0.2 (0.2 to 0.2)	0.1 (0.1 to 0.1)	4.9 (4.4 to 5.5)	3.2 (2.8 to 3.6)	1.52 (1.35 to 1.71)	0.98 (0.87 to 1.10)
2010	0.2 (0.2 to 0.2)	0.1 (0.1 to 0.1)	4.5 (4.0 to 5.0)	2.9 (2.6 to 3.2)	1.54 (1.37 to 1.73)	0.99 (0.88 to 1.11)
2015	0.2 (0.1 to 0.2)	0.1 (0.1 to 0.1)	4.1 (3.6 to 4.6)	2.6 (2.3 to 2.9)	1.37 (1.22 to 1.54)	0.88 (0.78 to 0.99)
2020	0.2 (0.1 to 0.2)	0.1 (0.1 to 0.1)	4.2 (3.5 to 5.0)	2.7 (2.2 to 3.2)	1.51 (1.25 to 1.80)	0.96 (0.80 to 1.15)
2021	0.1 (0.1 to 0.2)	0.1 (0.1 to 0.1)	3.9 (3.0 to 5.0)	2.5 (1.9 to 3.2)	1.49 (1.15 to 1.90)	0.95 (0.73 to 1.21)

Kansas USA_539 High−income North America Stillbirth Rate / Neonatal Mortality Rate (SBR/NMR) − 20 weeks
lambda = 0.2 ; zeta = 0.99 ; scale = 5 ; amp = 0.028

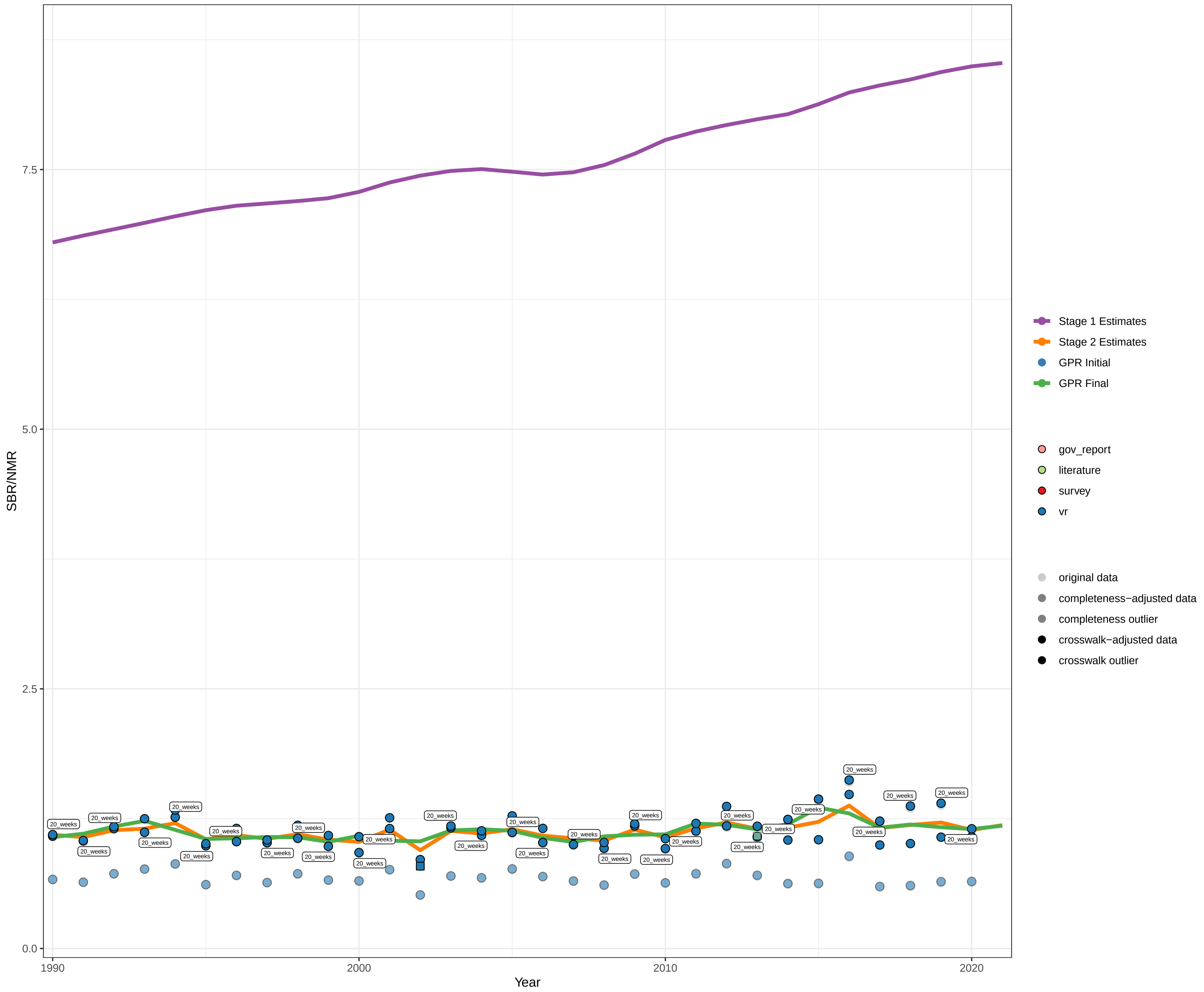

Kansas USA_539 High−income North America Stillbirth Rate / Neonatal Mortality Rate (SBR/NMR) − 28 weeks
lambda = 0.2 ; zeta = 0.99 ; scale = 5 ; amp = 0.028

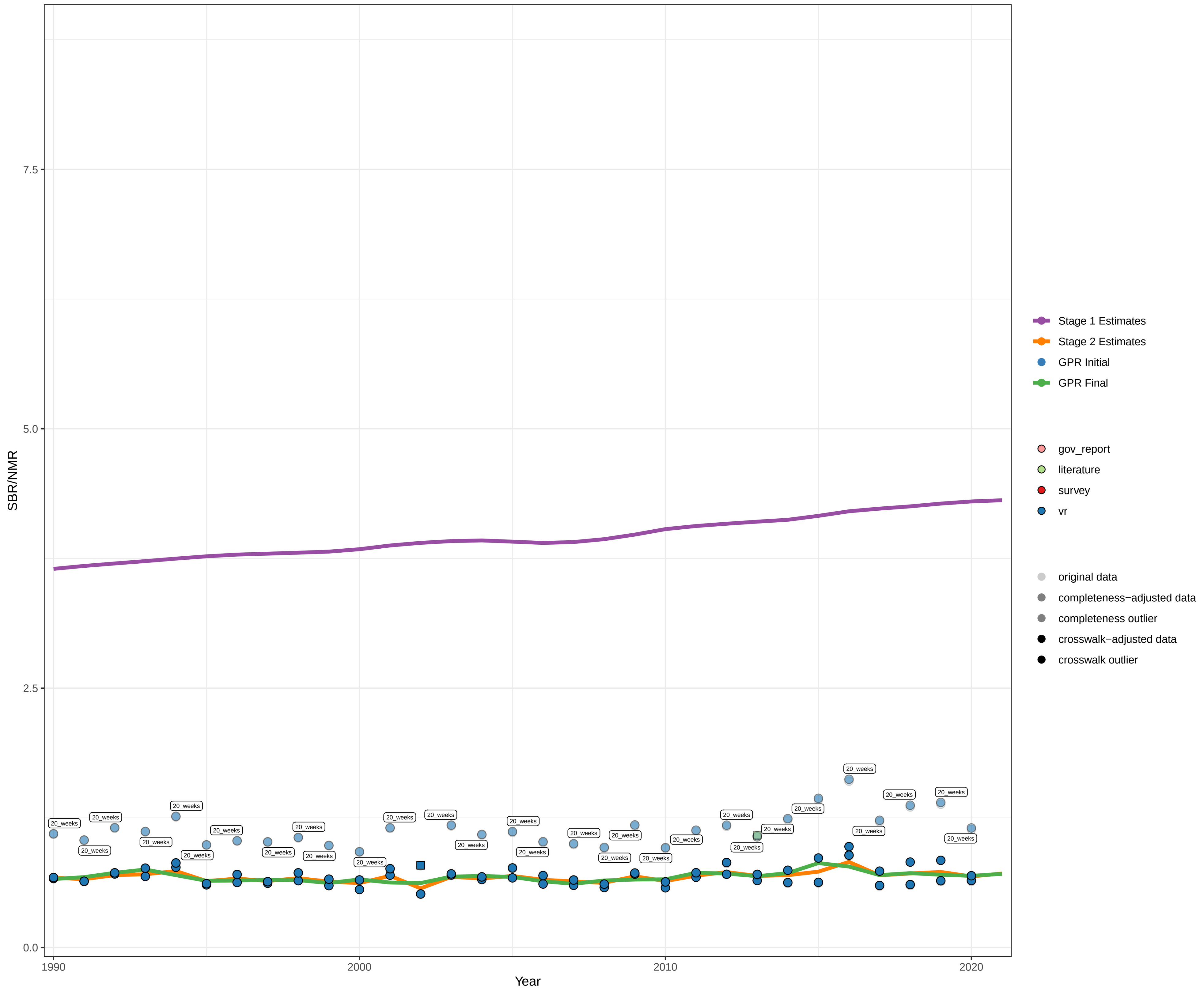

Definition	Years	Outlier	Source.Type	Title
20_weeks	1990−2001, 2003−2020	0	VR	United States NVSS Custom Fetal Death Data
28_weeks	1990−2020	0	VR	United States NVSS Custom Fetal Death Data
20_weeks	2013	1	Sci Lit	United States Fetal and Perinatal Mortality 2013
20_weeks	2002	2	VR	United States NVSS Custom Fetal Death Data

Kansas USA_539 High−income North America Stillbirth Rate (SBR) − 20 weeks
lambda = 0.2 ; zeta = 0.99 ; scale = 5 ; amp = 0.028

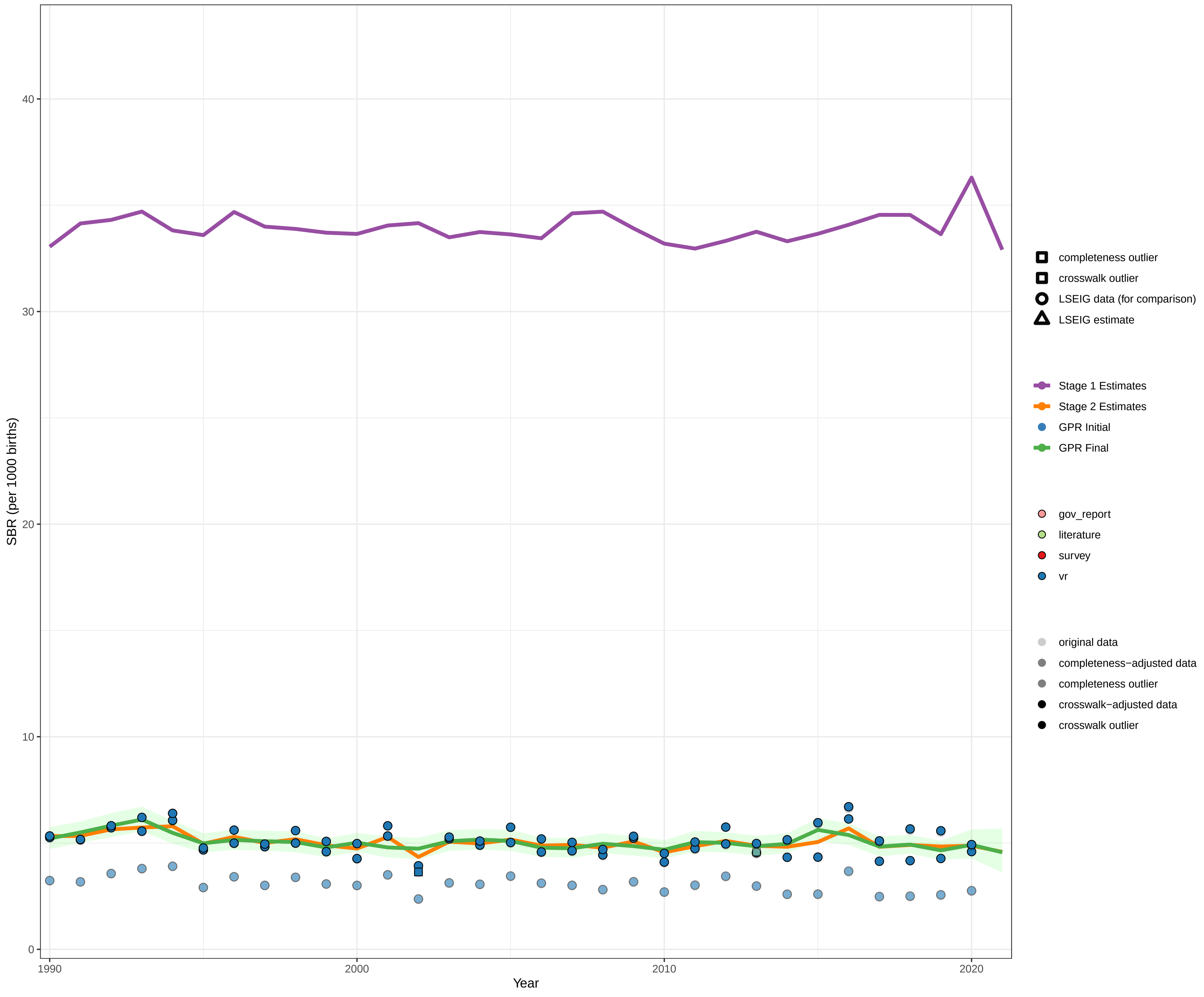

Kansas USA_539 High−income North America Stillbirth Rate (SBR) − 28 weeks
lambda = 0.2 ; zeta = 0.99 ; scale = 5 ; amp = 0.028

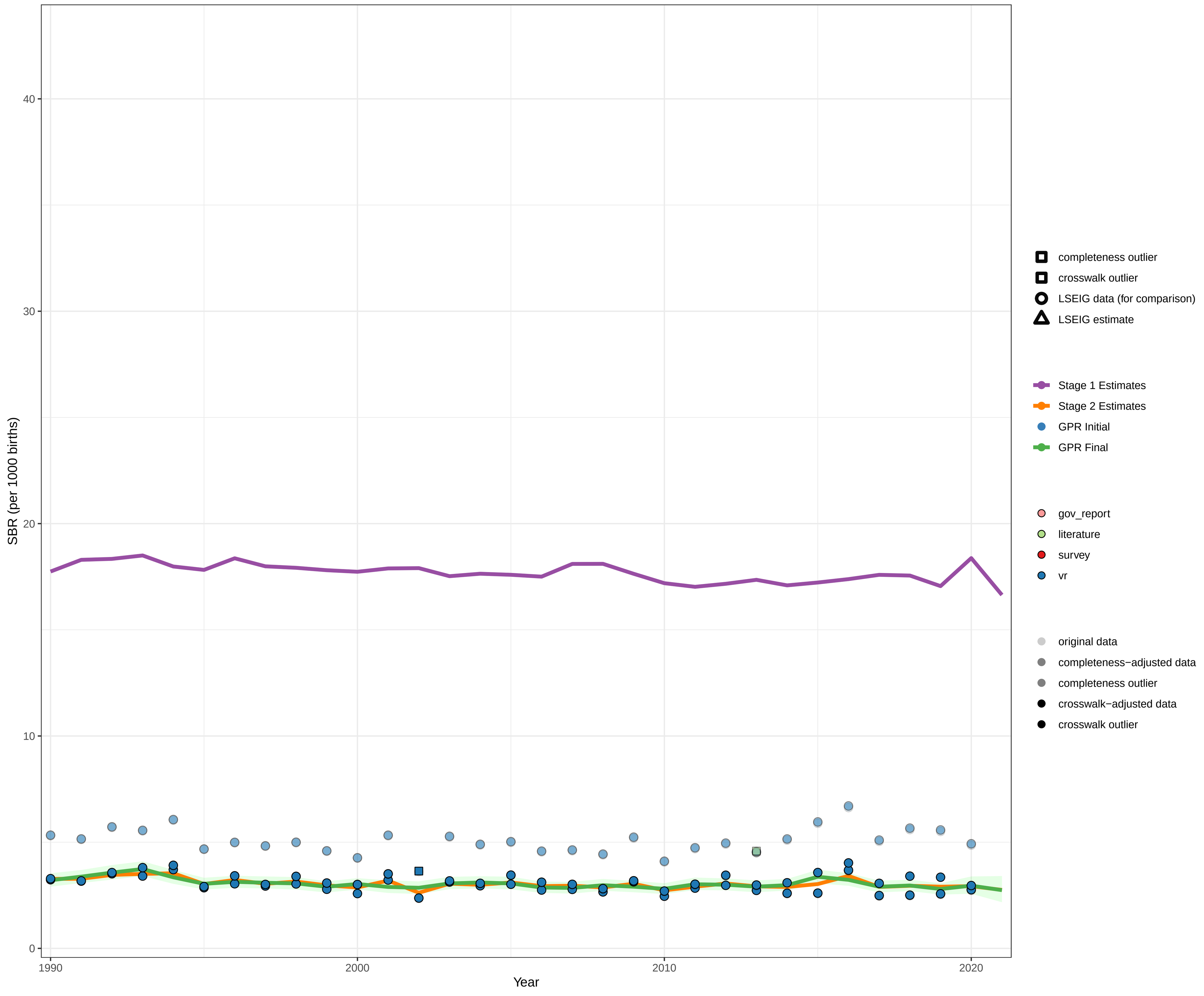

Year	Total Stillbirths (in thousands)	Total Stillbirths (in thousands)	SBR (per 1000 births)	SBR (per 1000 births)	SBR/NMR	SBR/NMR
	20 weeks	28 weeks	20 weeks	28 weeks	20 weeks	28 weeks
1990	0.2 (0.2 to 0.2)	0.1 (0.1 to 0.1)	5.2 (4.7 to 5.8)	3.2 (2.9 to 3.5)	1.07 (0.97 to 1.18)	0.66 (0.60 to 0.73)
1995	0.2 (0.2 to 0.2)	0.1 (0.1 to 0.1)	5.0 (4.5 to 5.5)	3.0 (2.8 to 3.3)	1.06 (0.96 to 1.16)	0.64 (0.59 to 0.70)
2000	0.2 (0.2 to 0.2)	0.1 (0.1 to 0.1)	5.0 (4.6 to 5.5)	3.0 (2.8 to 3.3)	1.09 (0.99 to 1.19)	0.66 (0.60 to 0.72)
2005	0.2 (0.2 to 0.2)	0.1 (0.1 to 0.1)	5.1 (4.6 to 5.6)	3.1 (2.8 to 3.4)	1.13 (1.03 to 1.25)	0.68 (0.62 to 0.75)
2010	0.2 (0.2 to 0.2)	0.1 (0.1 to 0.1)	4.7 (4.3 to 5.1)	2.8 (2.6 to 3.1)	1.10 (1.00 to 1.20)	0.66 (0.60 to 0.72)
2015	0.2 (0.2 to 0.2)	0.1 (0.1 to 0.1)	5.6 (5.1 to 6.2)	3.4 (3.1 to 3.7)	1.36 (1.23 to 1.49)	0.81 (0.74 to 0.89)
2020	0.2 (0.1 to 0.2)	0.1 (0.1 to 0.1)	4.9 (4.3 to 5.6)	3.0 (2.6 to 3.4)	1.15 (1.00 to 1.32)	0.69 (0.60 to 0.79)
2021	0.2 (0.1 to 0.2)	0.1 (0.1 to 0.1)	4.6 (3.6 to 5.7)	2.7 (2.2 to 3.4)	1.18 (0.94 to 1.47)	0.71 (0.56 to 0.88)

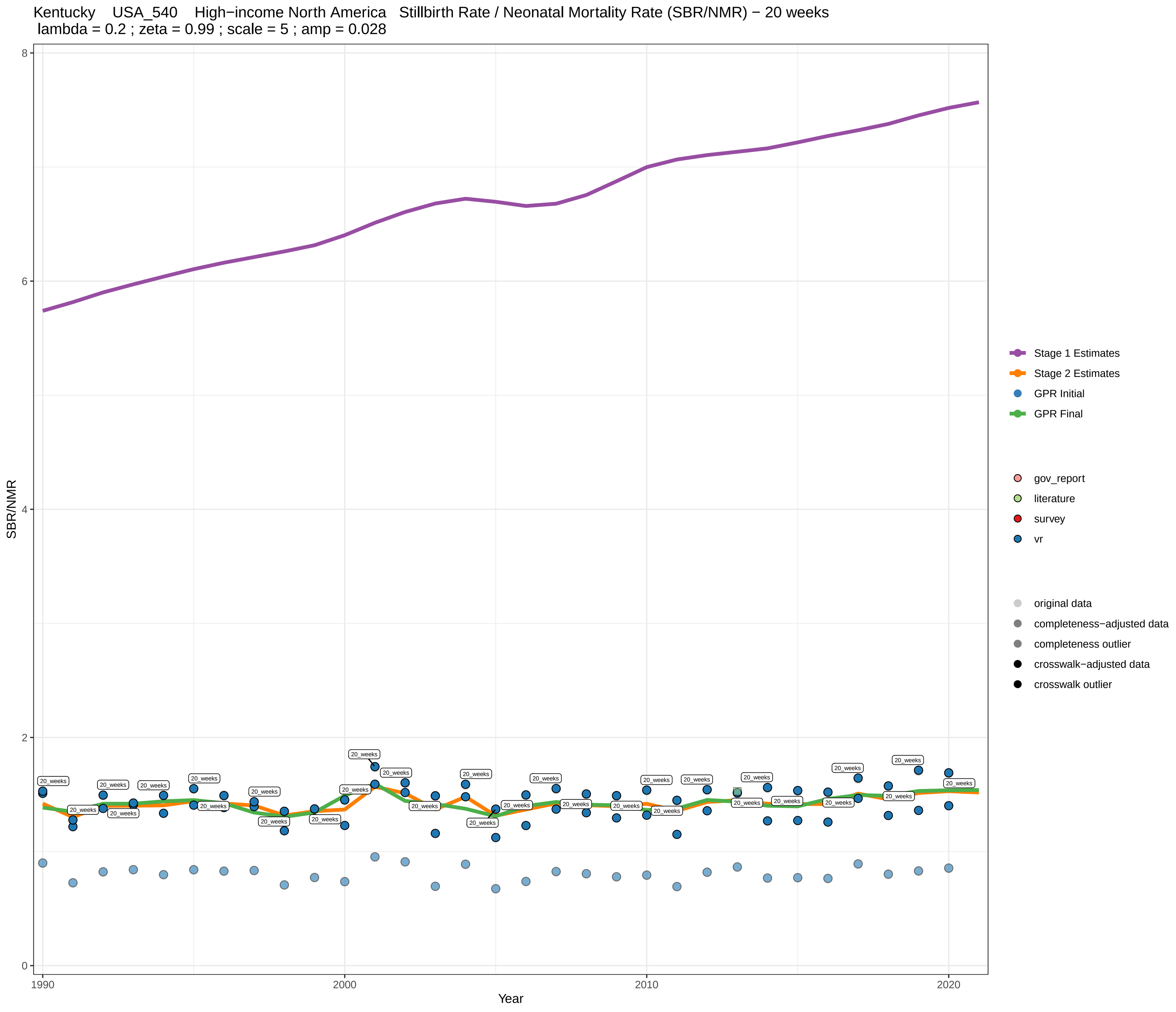

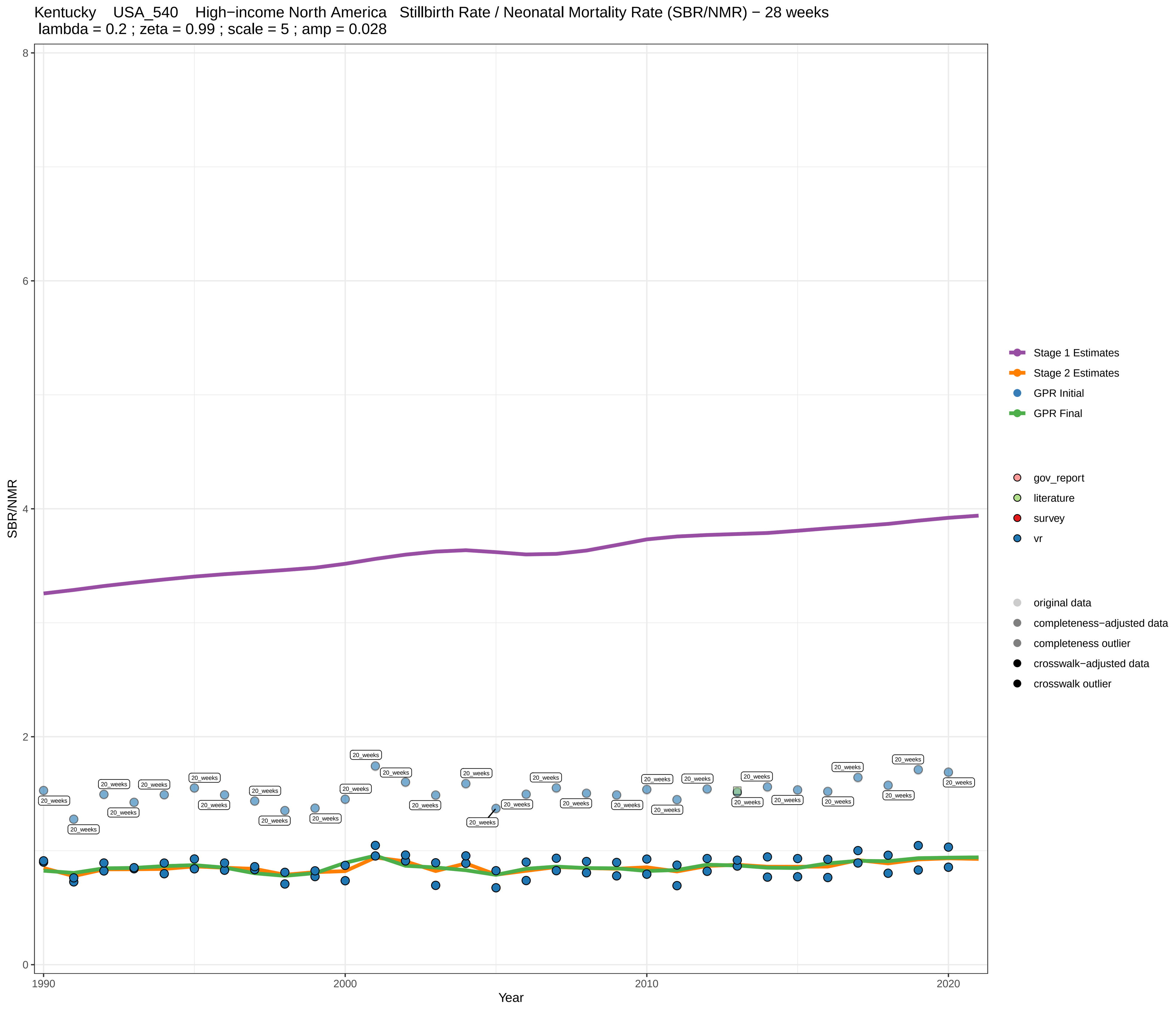

Definition	Years	Outlier	Source.Type	Title
20_weeks, 28_weeks	1990−2020	0	VR	United States NVSS Custom Fetal Death Data
20_weeks	2013	1	Sci Lit	United States Fetal and Perinatal Mortality 2013

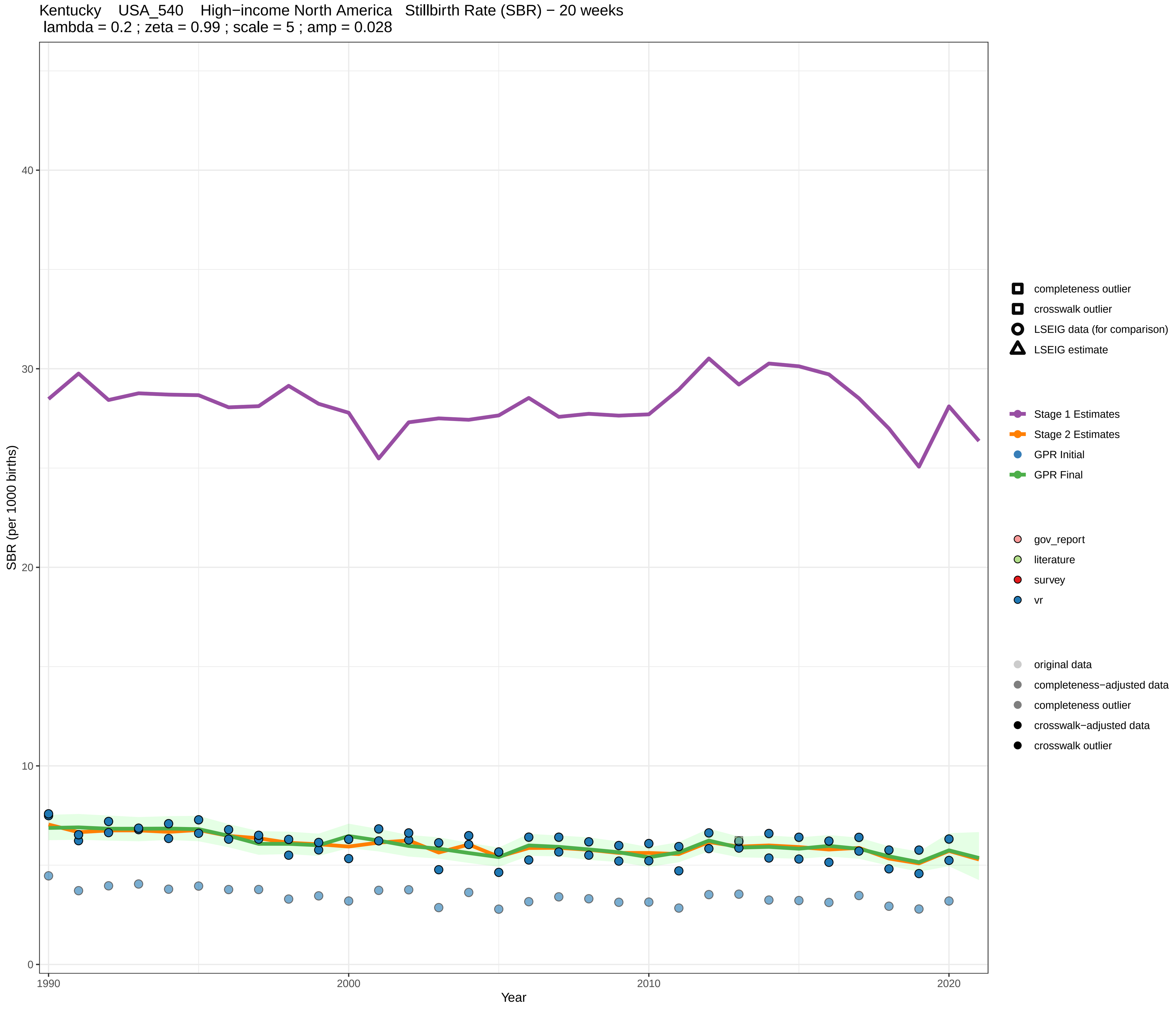

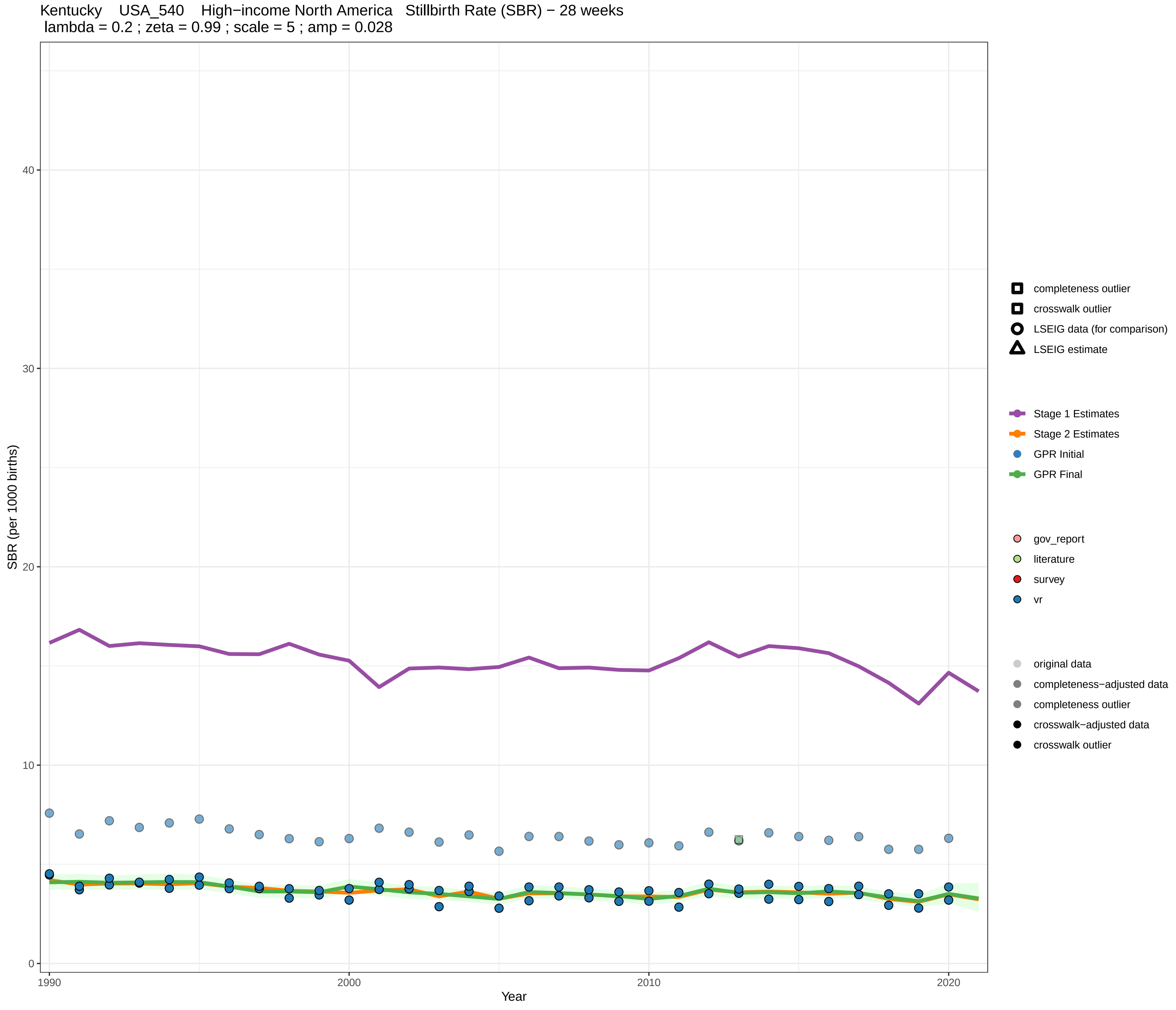

Year	Total Stillbirths (in thousands)	Total Stillbirths (in thousands)	SBR (per 1000 births)	SBR (per 1000 births)	SBR/NMR	SBR/NMR
	20 weeks	28 weeks	20 weeks	28 weeks	20 weeks	28 weeks
1990	0.4 (0.3 to 0.4)	0.2 (0.2 to 0.2)	6.9 (6.3 to 7.5)	4.1 (3.7 to 4.5)	1.39 (1.26 to 1.52)	0.82 (0.75 to 0.90)
1995	0.4 (0.3 to 0.4)	0.2 (0.2 to 0.2)	6.8 (6.2 to 7.5)	4.1 (3.7 to 4.5)	1.45 (1.32 to 1.59)	0.87 (0.80 to 0.95)
2000	0.4 (0.3 to 0.4)	0.2 (0.2 to 0.2)	6.5 (5.9 to 7.1)	3.9 (3.5 to 4.3)	1.49 (1.35 to 1.63)	0.89 (0.82 to 0.98)
2005	0.3 (0.3 to 0.3)	0.2 (0.2 to 0.2)	5.4 (4.9 to 5.9)	3.3 (3.0 to 3.6)	1.31 (1.19 to 1.43)	0.79 (0.72 to 0.86)
2010	0.3 (0.3 to 0.3)	0.2 (0.2 to 0.2)	5.4 (4.9 to 5.9)	3.3 (3.0 to 3.6)	1.36 (1.24 to 1.49)	0.82 (0.75 to 0.90)
2015	0.3 (0.3 to 0.4)	0.2 (0.2 to 0.2)	5.8 (5.3 to 6.4)	3.5 (3.2 to 3.9)	1.40 (1.27 to 1.53)	0.85 (0.78 to 0.93)
2020	0.3 (0.3 to 0.3)	0.2 (0.2 to 0.2)	5.7 (4.9 to 6.6)	3.5 (3.0 to 4.0)	1.54 (1.32 to 1.77)	0.94 (0.81 to 1.08)
2021	0.3 (0.2 to 0.3)	0.2 (0.1 to 0.2)	5.4 (4.2 to 6.7)	3.3 (2.6 to 4.1)	1.54 (1.22 to 1.91)	0.94 (0.75 to 1.17)

Louisiana USA_541 High−income North America Stillbirth Rate / Neonatal Mortality Rate (SBR/NMR) − 20 weeks
lambda = 0.2 ; zeta = 0.99 ; scale = 5 ; amp = 0.028

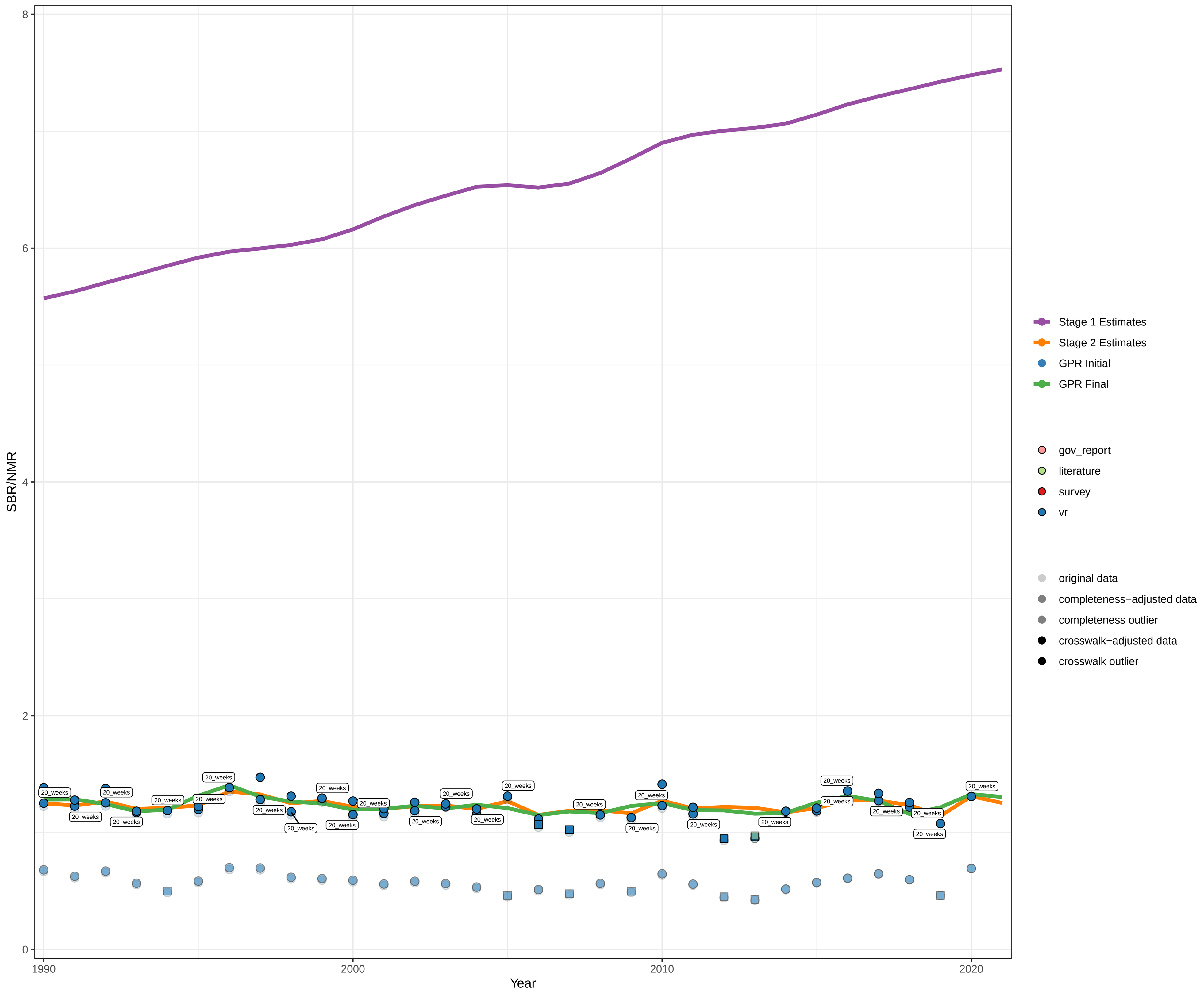

Louisiana USA_541 High−income North America Stillbirth Rate / Neonatal Mortality Rate (SBR/NMR) − 28 weeks
lambda = 0.2 ; zeta = 0.99 ; scale = 5 ; amp = 0.028

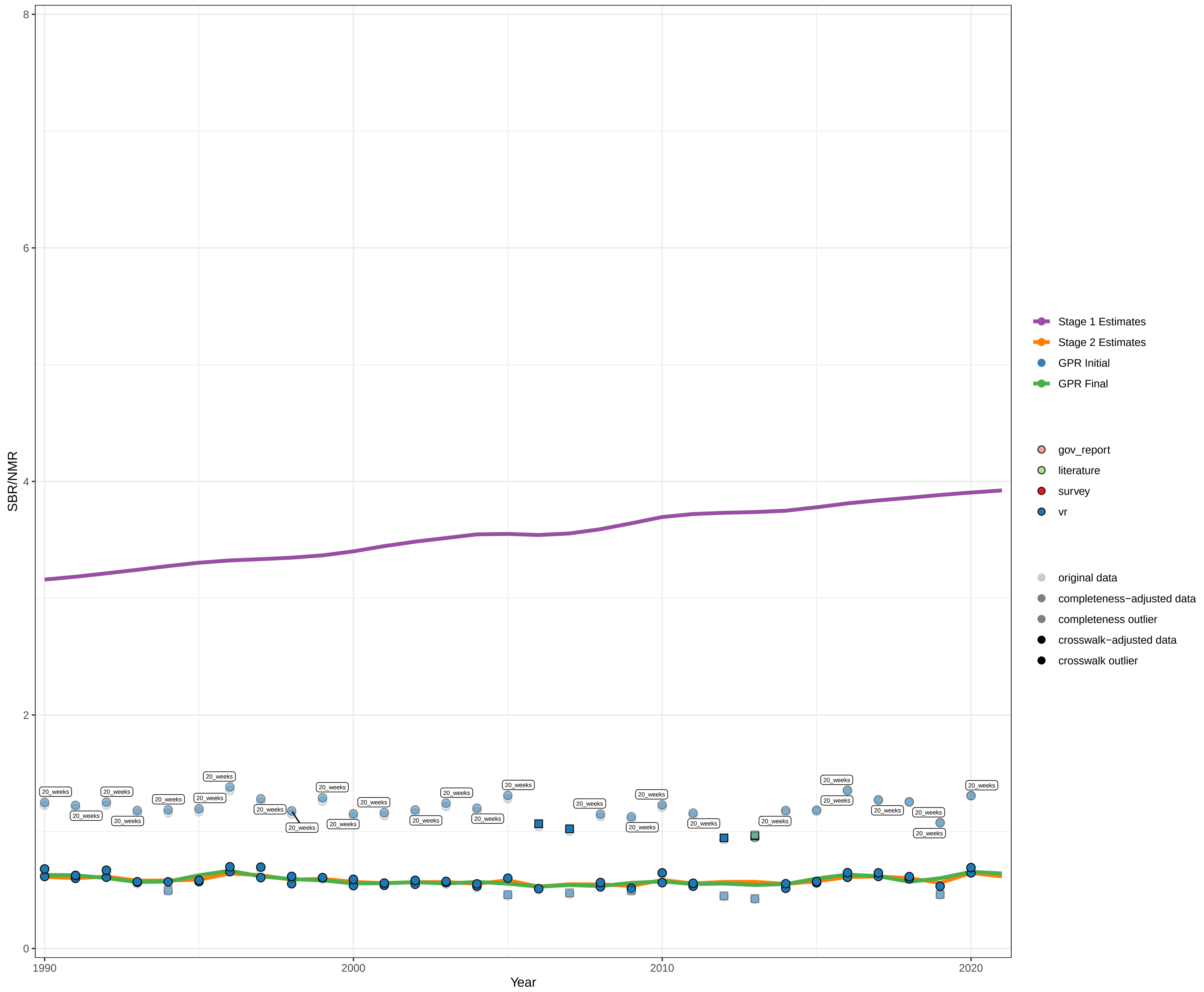

Definition	Years	Outlier	Source.Type	Title
20_weeks	1990−2005, 2008−2011, 2014−2020	0	VR	United States NVSS Custom Fetal Death Data
28_weeks	1990−1993, 1995−2004, 2006, 2008, 2010, 2011, 2014−2018, 2020	0	VR	United States NVSS Custom Fetal Death Data
20_weeks	2013	1	Sci Lit	United States Fetal and Perinatal Mortality 2013
28_weeks	1994, 2005, 2007, 2009, 2012, 2013, 2019	1	VR	United States NVSS Custom Fetal Death Data
20_weeks	2006, 2007, 2012, 2013	2	VR	United States NVSS Custom Fetal Death Data

Louisiana USA_541 High−income North America Stillbirth Rate (SBR) − 20 weeks
lambda = 0.2 ; zeta = 0.99 ; scale = 5 ; amp = 0.028

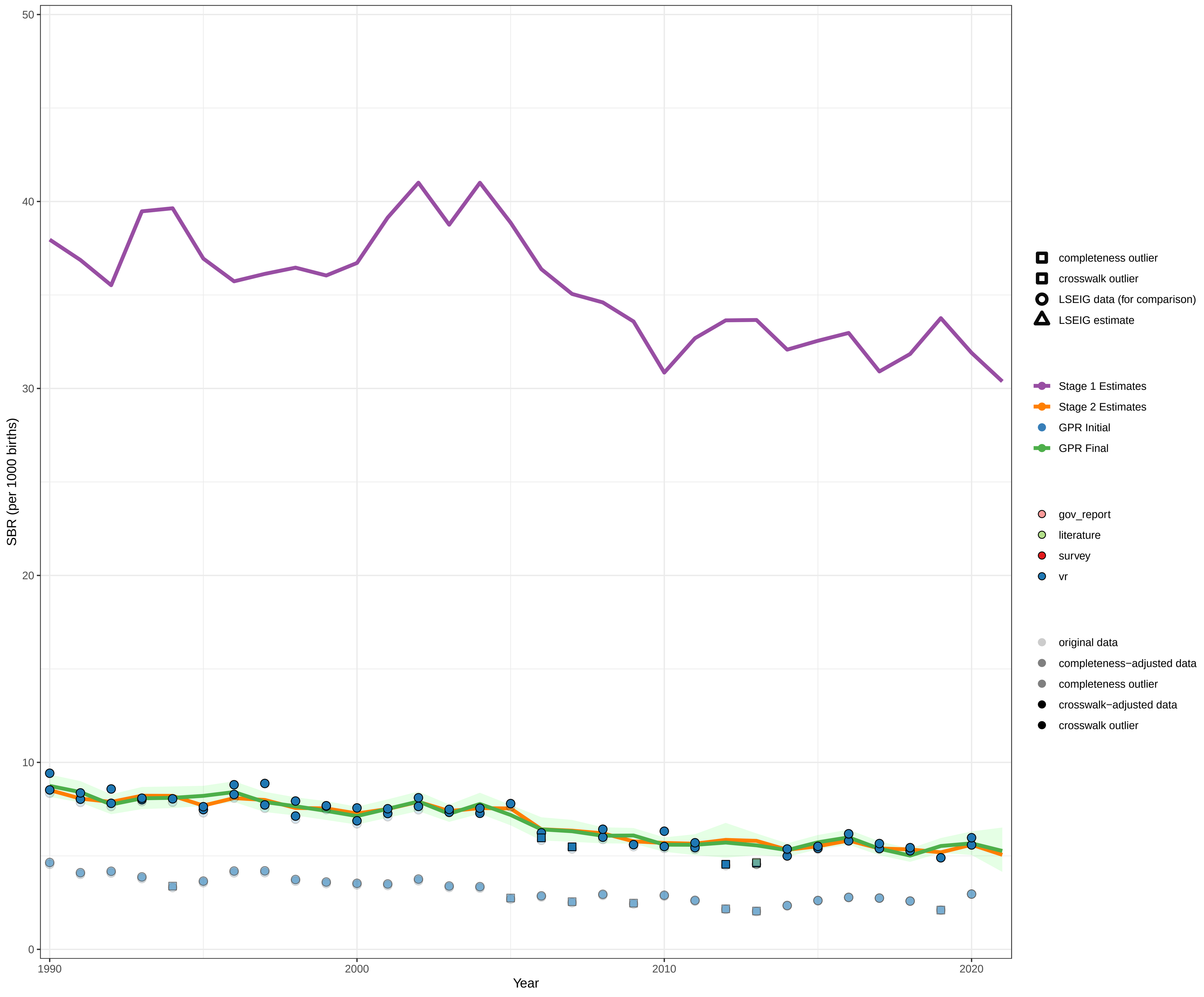

Louisiana USA_541 High−income North America Stillbirth Rate (SBR) − 28 weeks
lambda = 0.2 ; zeta = 0.99 ; scale = 5 ; amp = 0.028

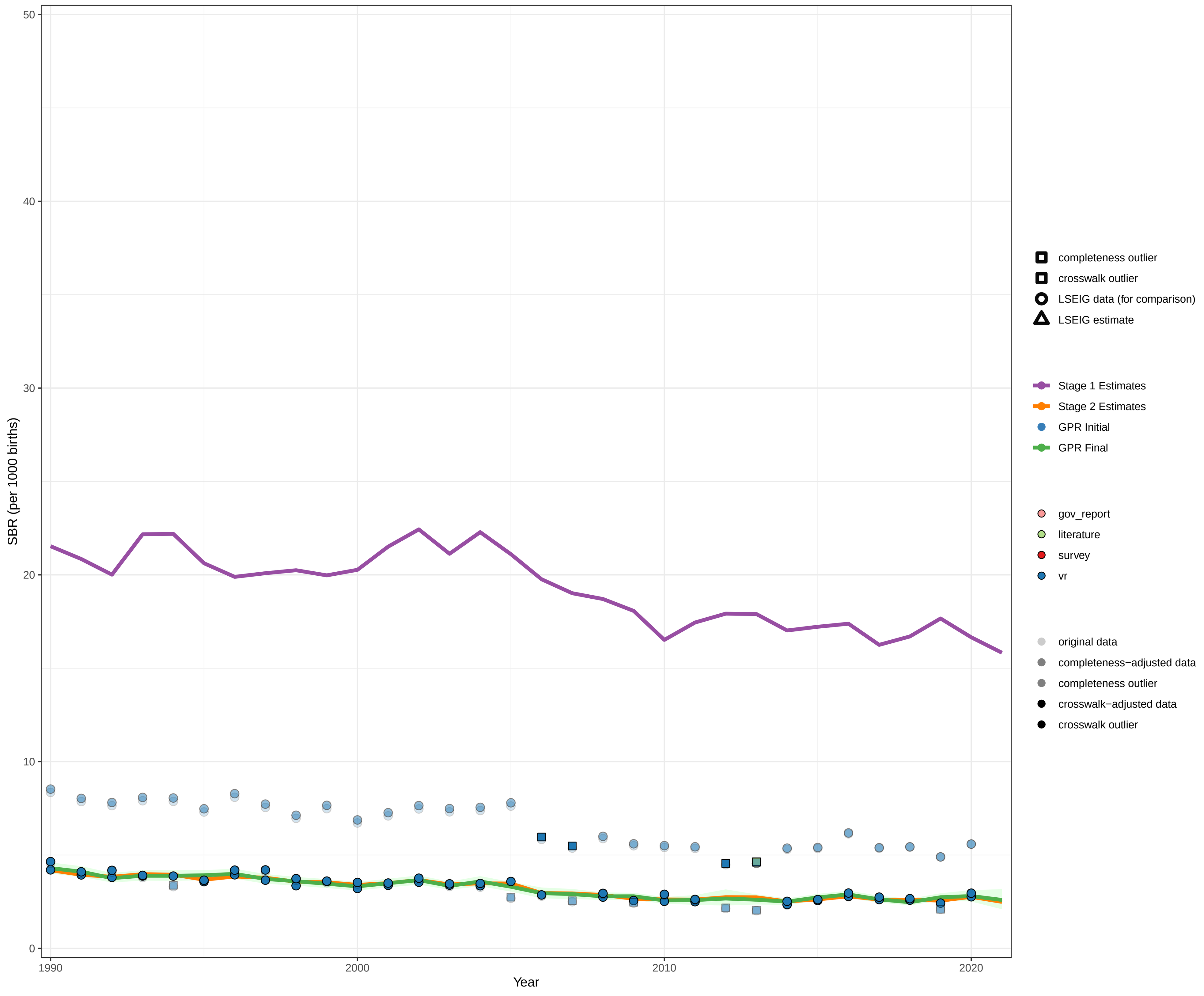

Year	Total Stillbirths (in thousands)	Total Stillbirths (in thousands)	SBR (per 1000 births)	SBR (per 1000 births)	SBR/NMR	SBR/NMR
	20 weeks	28 weeks	20 weeks	28 weeks	20 weeks	28 weeks
1990	0.6 (0.6 to 0.7)	0.3 (0.3 to 0.3)	8.7 (8.2 to 9.4)	4.3 (4.0 to 4.6)	1.28 (1.20 to 1.37)	0.63 (0.59 to 0.68)
1995	0.5 (0.5 to 0.6)	0.3 (0.2 to 0.3)	8.2 (7.7 to 8.7)	3.9 (3.6 to 4.2)	1.32 (1.23 to 1.40)	0.63 (0.58 to 0.67)
2000	0.5 (0.4 to 0.5)	0.2 (0.2 to 0.2)	7.1 (6.7 to 7.6)	3.3 (3.1 to 3.6)	1.20 (1.12 to 1.28)	0.56 (0.52 to 0.60)
2005	0.5 (0.4 to 0.5)	0.2 (0.2 to 0.2)	7.2 (6.6 to 7.7)	3.3 (3.0 to 3.6)	1.21 (1.12 to 1.30)	0.56 (0.51 to 0.60)
2010	0.4 (0.3 to 0.4)	0.2 (0.2 to 0.2)	5.6 (5.2 to 6.0)	2.6 (2.4 to 2.8)	1.25 (1.17 to 1.34)	0.57 (0.54 to 0.62)
2015	0.4 (0.3 to 0.4)	0.2 (0.2 to 0.2)	5.7 (5.4 to 6.1)	2.7 (2.6 to 2.9)	1.26 (1.17 to 1.34)	0.60 (0.56 to 0.64)
2020	0.3 (0.3 to 0.4)	0.2 (0.1 to 0.2)	5.7 (5.0 to 6.3)	2.8 (2.5 to 3.1)	1.33 (1.18 to 1.48)	0.66 (0.59 to 0.73)
2021	0.3 (0.2 to 0.4)	0.1 (0.1 to 0.2)	5.3 (4.1 to 6.5)	2.6 (2.1 to 3.2)	1.30 (1.03 to 1.61)	0.64 (0.52 to 0.79)

Maine USA_542 High−income North America Stillbirth Rate / Neonatal Mortality Rate (SBR/NMR) − 20 weeks
lambda = 0.2 ; zeta = 0.99 ; scale = 5 ; amp = 0.028

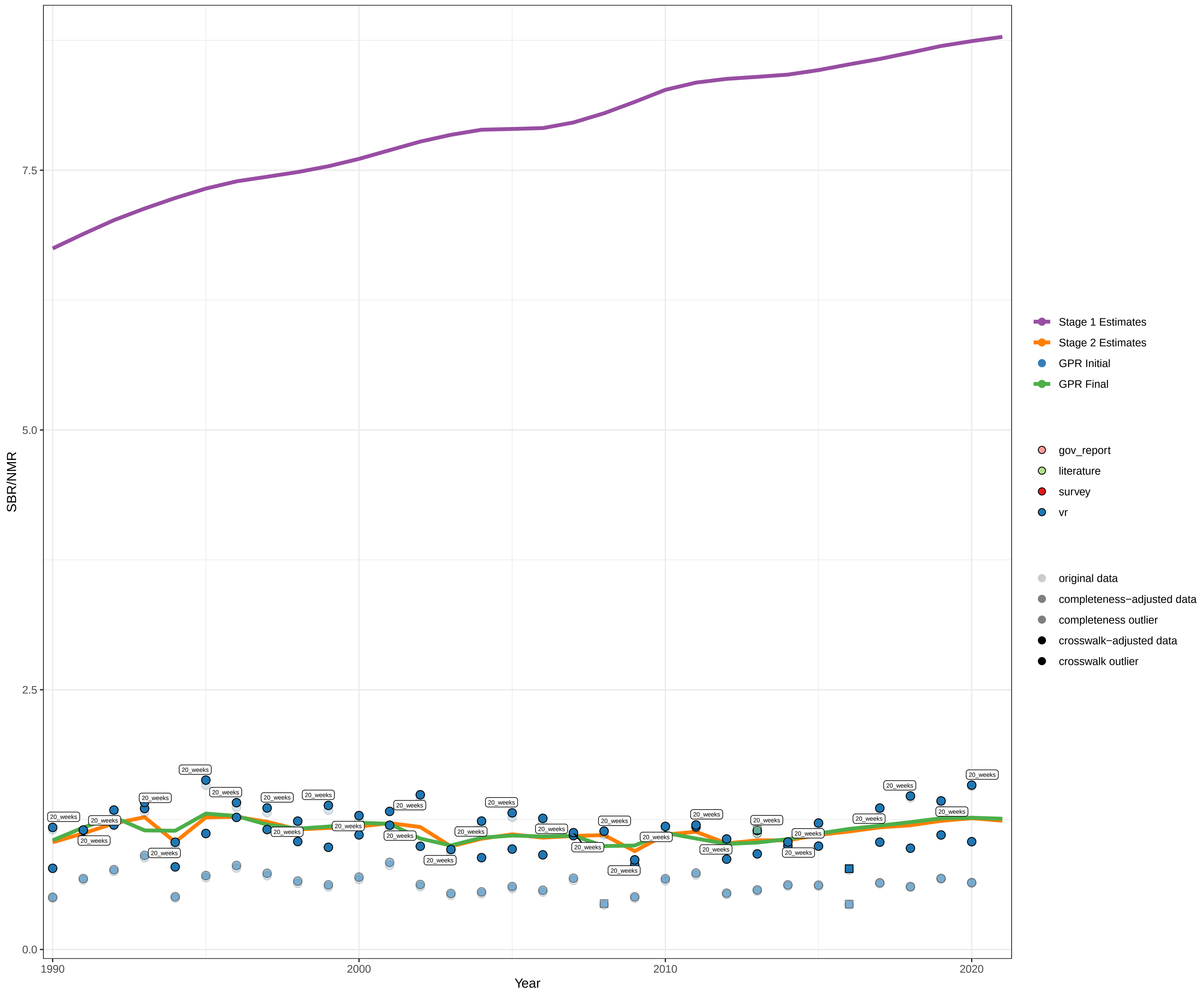

Maine USA_542 High−income North America Stillbirth Rate / Neonatal Mortality Rate (SBR/NMR) − 28 weeks
lambda = 0.2 ; zeta = 0.99 ; scale = 5 ; amp = 0.028

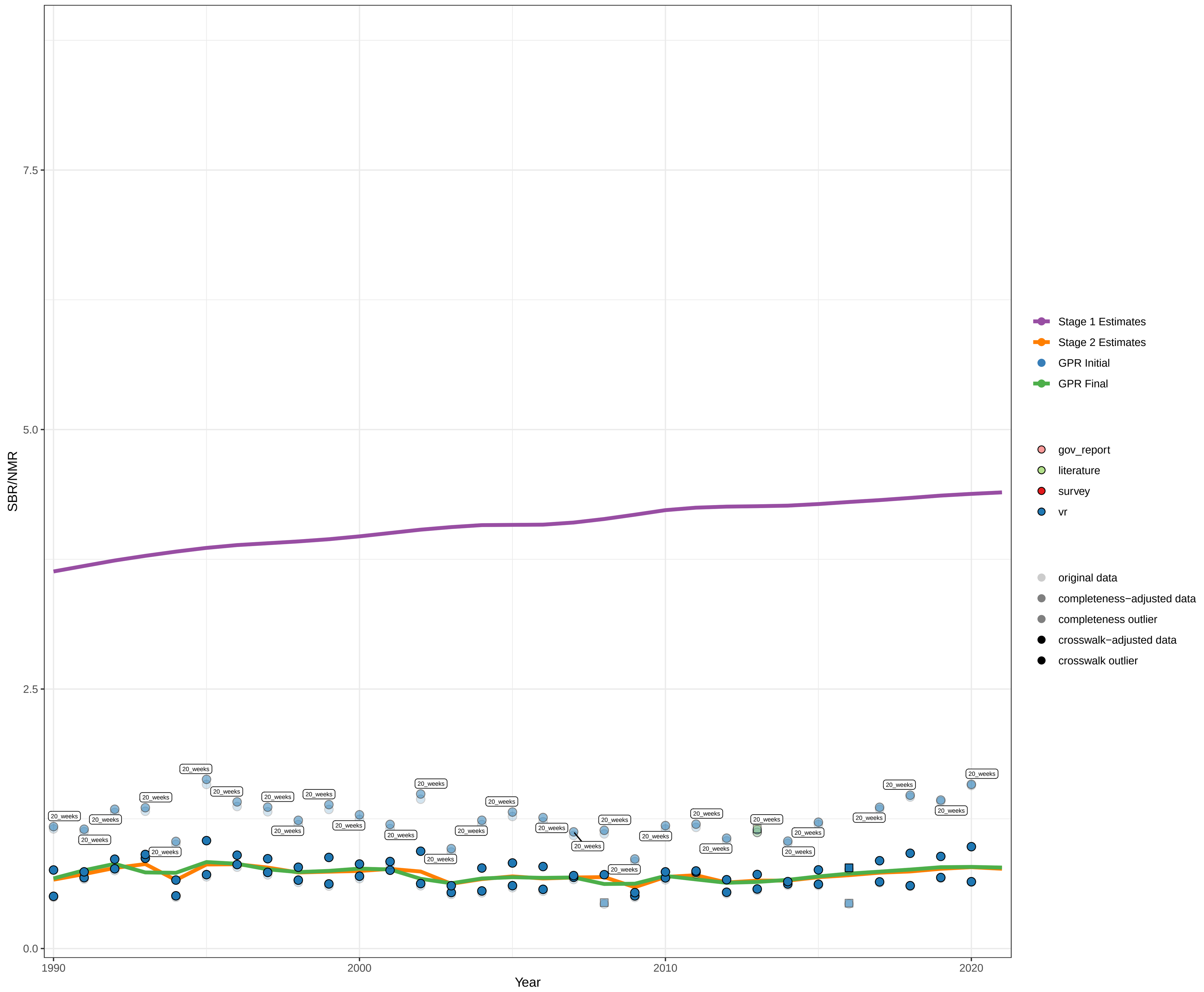

Definition	Years	Outlier	Source.Type	Title
20_weeks	1990−2015, 2017−2020	0	VR	United States NVSS Custom Fetal Death Data
28_weeks	1990−2007, 2009−2015, 2017−2020	0	VR	United States NVSS Custom Fetal Death Data
20_weeks	2013	1	Sci Lit	United States Fetal and Perinatal Mortality 2013
28_weeks	2008, 2016	1	VR	United States NVSS Custom Fetal Death Data
20_weeks	2016	2	VR	United States NVSS Custom Fetal Death Data

Maine USA_542 High−income North America Stillbirth Rate (SBR) − 20 weeks
lambda = 0.2 ; zeta = 0.99 ; scale = 5 ; amp = 0.028

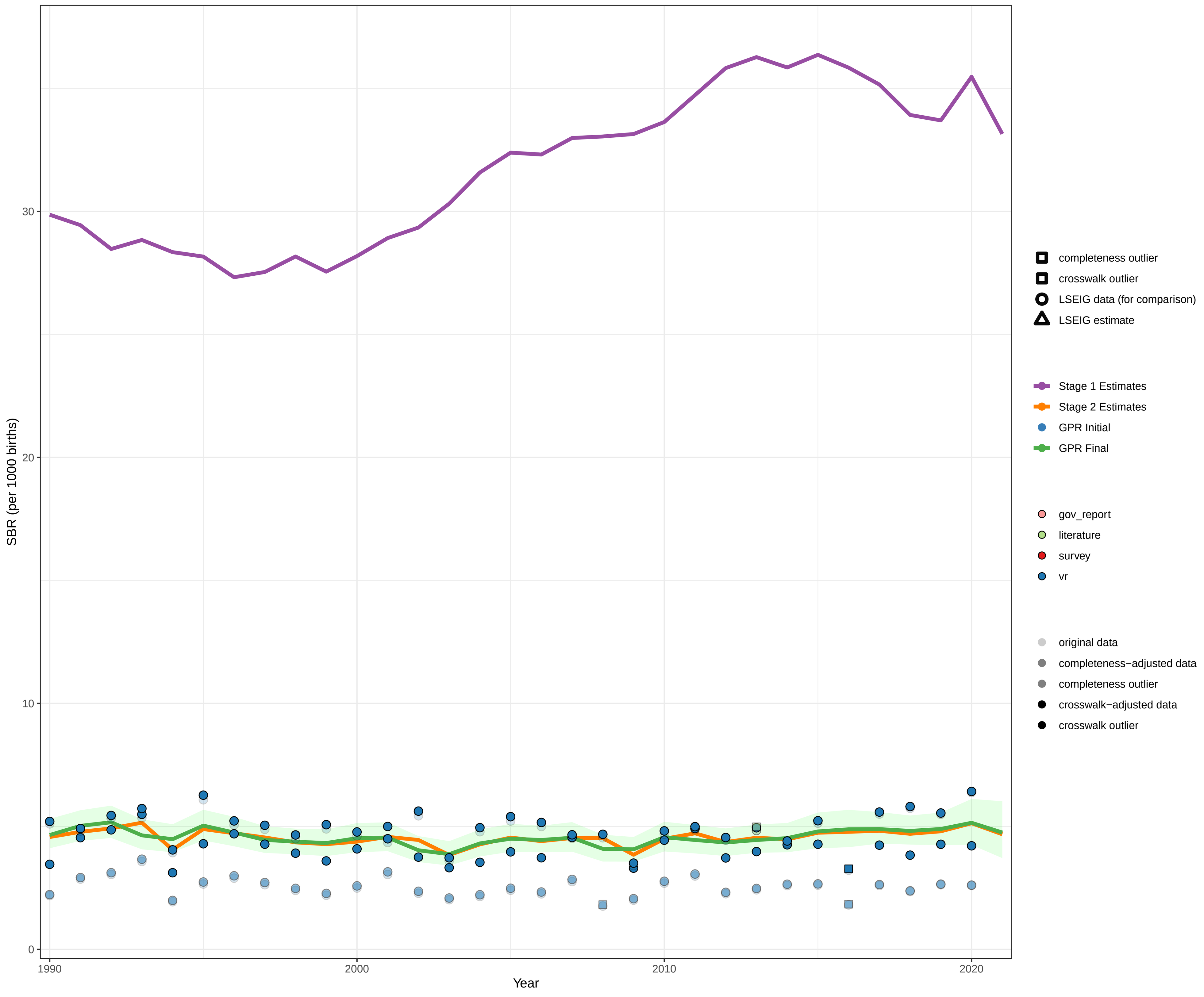

Maine USA_542 High−income North America Stillbirth Rate (SBR) − 28 weeks
lambda = 0.2 ; zeta = 0.99 ; scale = 5 ; amp = 0.028

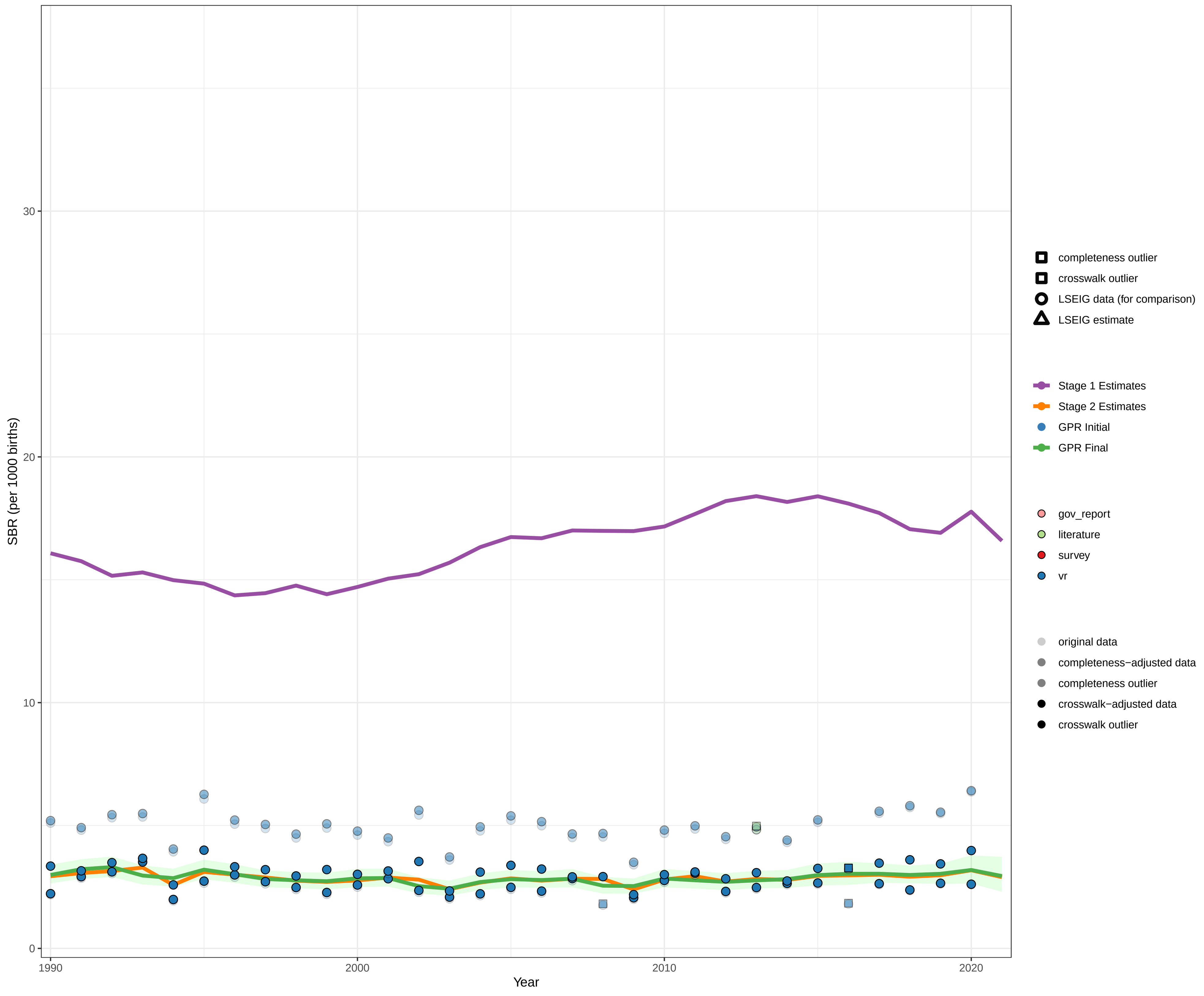

Year	Total Stillbirths (in thousands)	Total Stillbirths (in thousands)	SBR (per 1000 births)	SBR (per 1000 births)	SBR/NMR	SBR/NMR
	20 weeks	28 weeks	20 weeks	28 weeks	20 weeks	28 weeks
1990	0.1 (0.1 to 0.1)	0.1 (0.0 to 0.1)	4.6 (4.1 to 5.3)	3.0 (2.6 to 3.4)	1.05 (0.93 to 1.20)	0.68 (0.60 to 0.77)
1995	0.1 (0.1 to 0.1)	0.0 (0.0 to 0.1)	5.0 (4.4 to 5.7)	3.2 (2.8 to 3.6)	1.31 (1.15 to 1.48)	0.83 (0.73 to 0.94)
2000	0.1 (0.1 to 0.1)	0.0 (0.0 to 0.0)	4.5 (4.0 to 5.1)	2.9 (2.5 to 3.2)	1.22 (1.07 to 1.39)	0.77 (0.67 to 0.87)
2005	0.1 (0.1 to 0.1)	0.0 (0.0 to 0.0)	4.5 (4.0 to 5.1)	2.8 (2.5 to 3.2)	1.10 (0.97 to 1.24)	0.69 (0.61 to 0.78)
2010	0.1 (0.1 to 0.1)	0.0 (0.0 to 0.0)	4.6 (4.0 to 5.2)	2.8 (2.5 to 3.2)	1.12 (0.98 to 1.28)	0.70 (0.61 to 0.80)
2015	0.1 (0.1 to 0.1)	0.0 (0.0 to 0.0)	4.8 (4.1 to 5.6)	3.0 (2.6 to 3.5)	1.12 (0.96 to 1.29)	0.69 (0.60 to 0.81)
2020	0.1 (0.0 to 0.1)	0.0 (0.0 to 0.0)	5.1 (4.2 to 6.1)	3.2 (2.6 to 3.8)	1.27 (1.05 to 1.51)	0.79 (0.65 to 0.93)
2021	0.1 (0.0 to 0.1)	0.0 (0.0 to 0.0)	4.7 (3.7 to 6.0)	2.9 (2.3 to 3.7)	1.26 (0.98 to 1.60)	0.78 (0.61 to 0.99)

Maryland USA_543 High−income North America Stillbirth Rate / Neonatal Mortality Rate (SBR/NMR) − 20 weeks
lambda = 0.2 ; zeta = 0.99 ; scale = 5 ; amp = 0.028

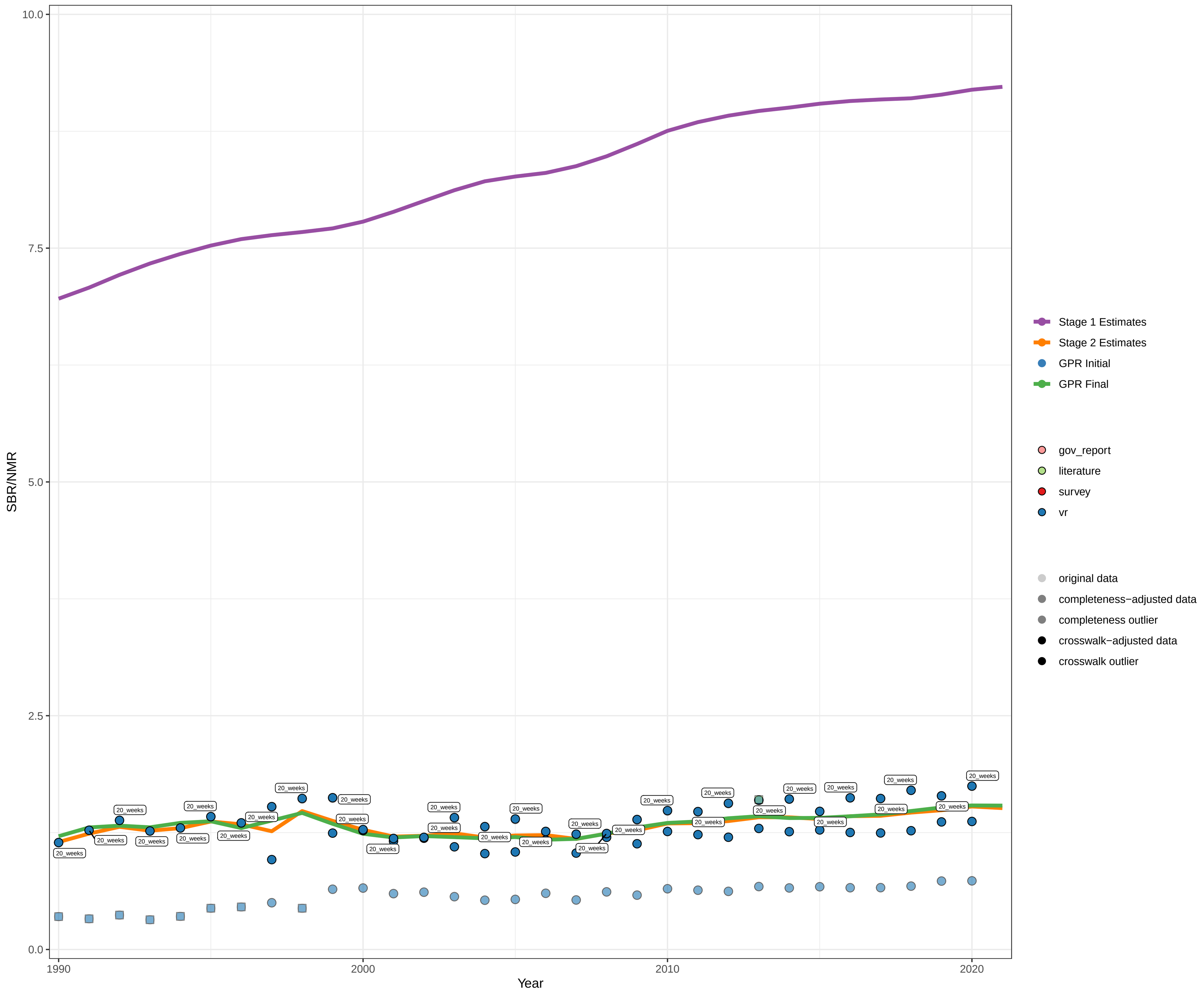

Maryland USA_543 High−income North America Stillbirth Rate / Neonatal Mortality Rate (SBR/NMR) − 28 weeks
lambda = 0.2 ; zeta = 0.99 ; scale = 5 ; amp = 0.028

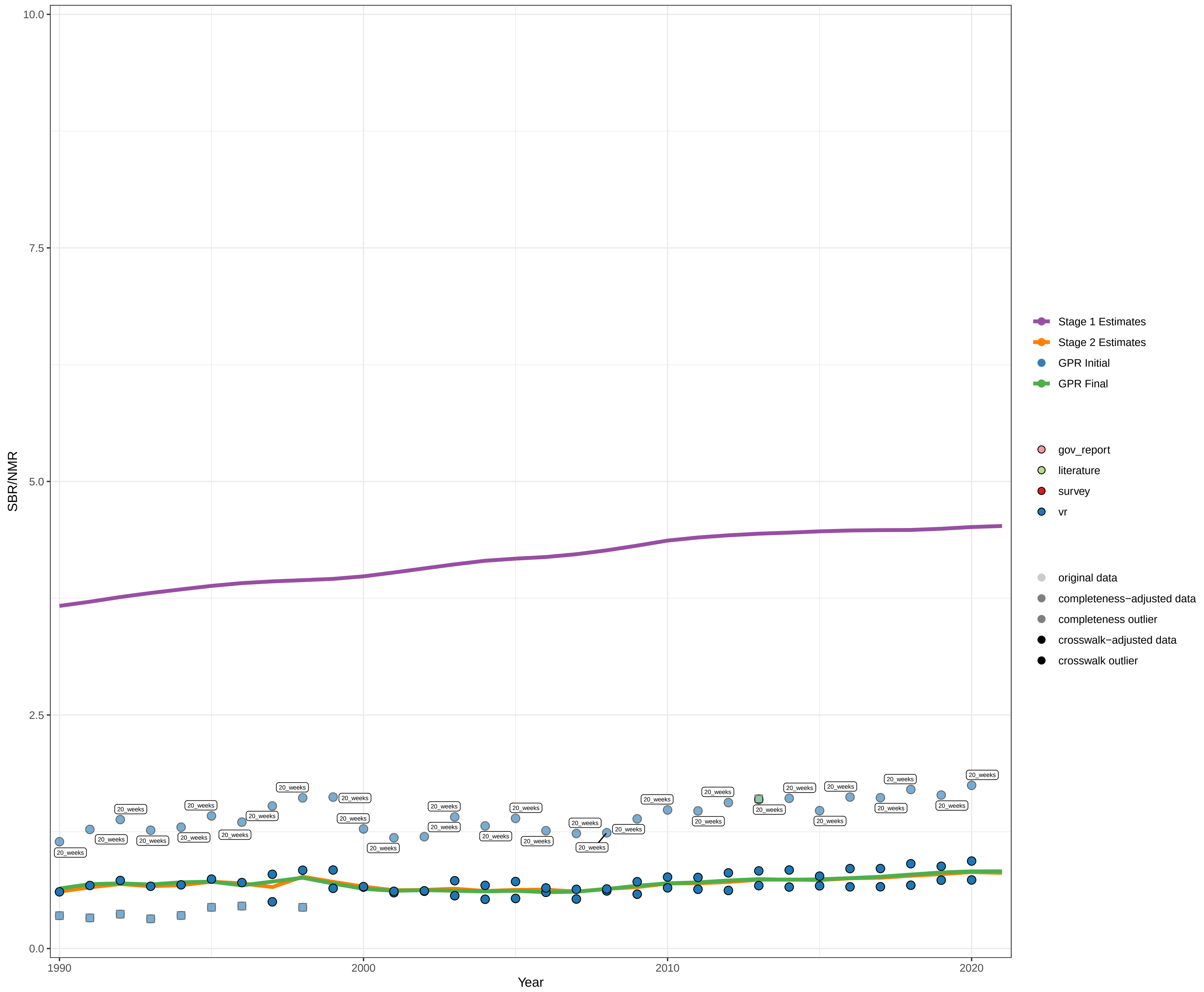

Definition	Years	Outlier	Source.Type	Title
20_weeks	1990−2020	0	VR	United States NVSS Custom Fetal Death Data
28_weeks	1997, 1999−2020	0	VR	United States NVSS Custom Fetal Death Data
20_weeks	2013	1	Sci Lit	United States Fetal and Perinatal Mortality 2013
28_weeks	1990−1996, 1998	1	VR	United States NVSS Custom Fetal Death Data

Maryland USA_543 High−income North America Stillbirth Rate (SBR) − 20 weeks
lambda = 0.2 ; zeta = 0.99 ; scale = 5 ; amp = 0.028

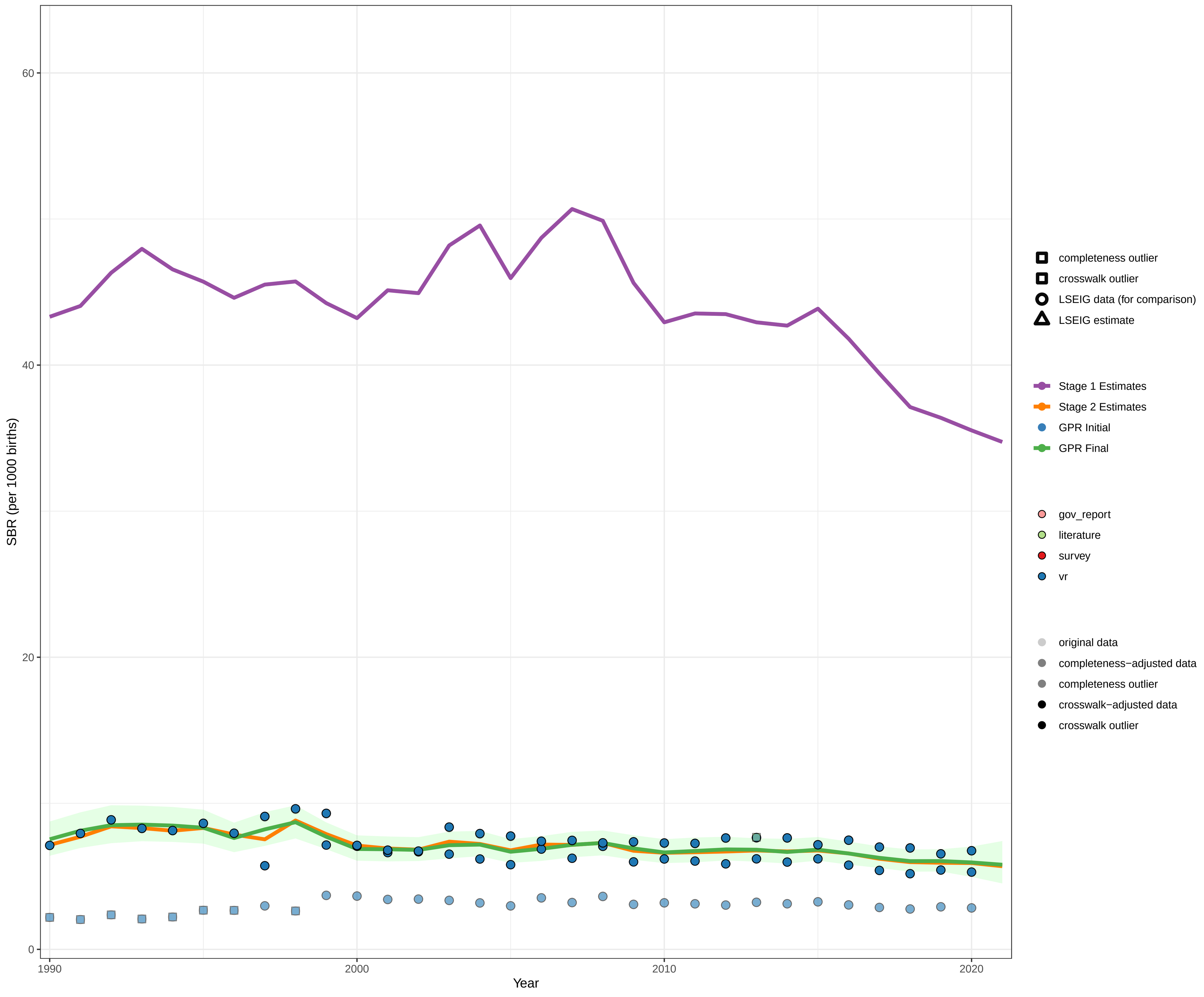

Maryland USA_543 High−income North America Stillbirth Rate (SBR) − 28 weeks
lambda = 0.2 ; zeta = 0.99 ; scale = 5 ; amp = 0.028

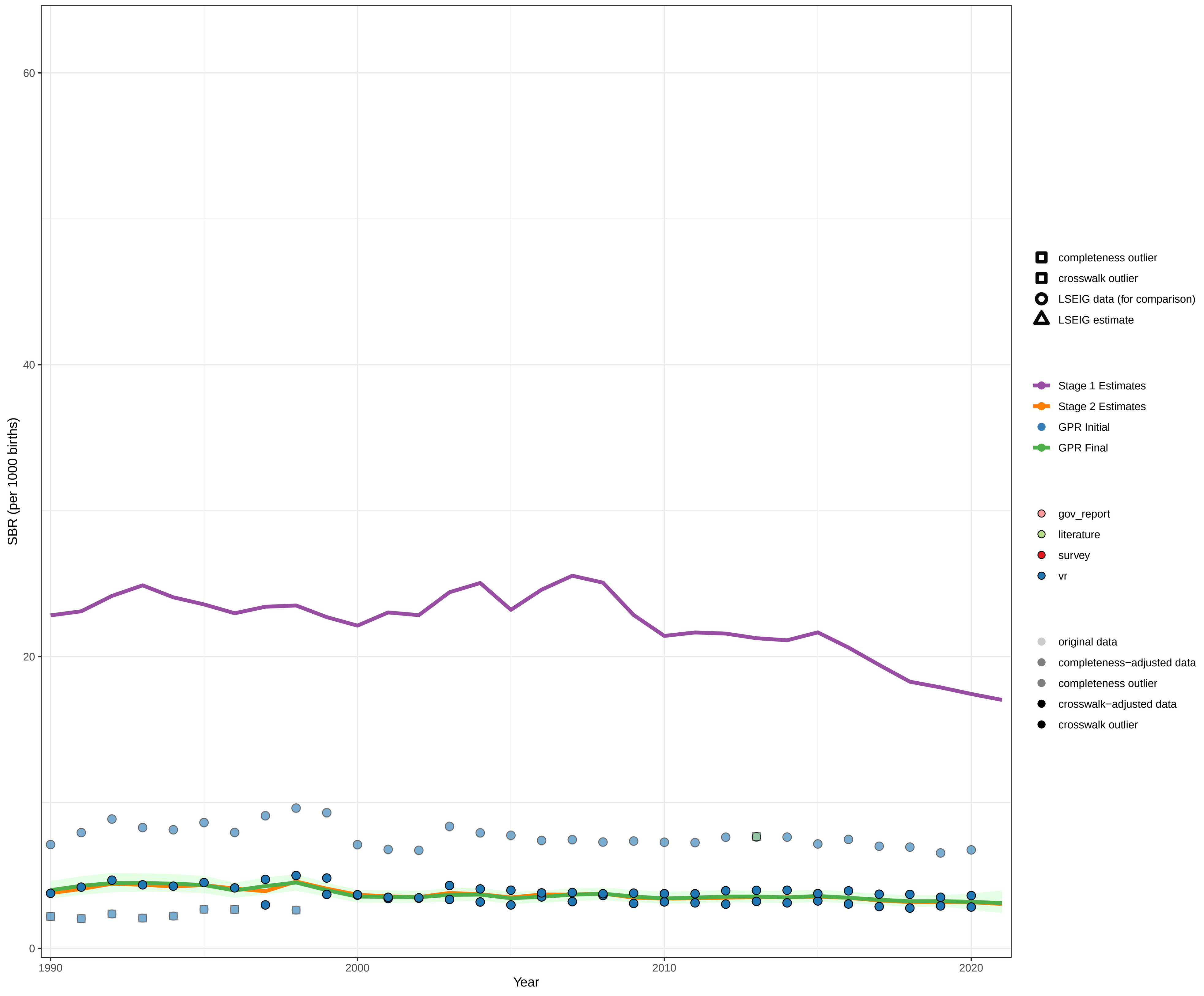

Year	Total Stillbirths (in thousands)	Total Stillbirths (in thousands)	SBR (per 1000 births)	SBR (per 1000 births)	SBR/NMR	SBR/NMR
	20 weeks	28 weeks	20 weeks	28 weeks	20 weeks	28 weeks
1990	0.6<br>(0.5 to 0.7)	0.3<br>(0.3 to 0.4)	7.5<br>(6.4 to 8.8)	4.0<br>(3.4 to 4.6)	1.21<br>(1.03 to 1.41)	0.64<br>(0.55 to 0.74)
1995	0.6<br>(0.5 to 0.7)	0.3<br>(0.3 to 0.4)	8.3<br>(7.2 to 9.6)	4.3<br>(3.8 to 5.0)	1.37<br>(1.19 to 1.58)	0.72<br>(0.62 to 0.82)
2000	0.5<br>(0.5 to 0.6)	0.3<br>(0.2 to 0.3)	6.9<br>(6.1 to 7.8)	3.5<br>(3.1 to 4.0)	1.24<br>(1.09 to 1.41)	0.64<br>(0.57 to 0.72)
2005	0.5<br>(0.4 to 0.6)	0.3<br>(0.2 to 0.3)	6.7<br>(5.9 to 7.5)	3.4<br>(3.1 to 3.9)	1.20<br>(1.06 to 1.36)	0.62<br>(0.55 to 0.70)
2010	0.5<br>(0.4 to 0.5)	0.2<br>(0.2 to 0.3)	6.6<br>(5.9 to 7.5)	3.4<br>(3.1 to 3.9)	1.35<br>(1.20 to 1.54)	0.70<br>(0.62 to 0.79)
2015	0.5<br>(0.4 to 0.6)	0.3<br>(0.2 to 0.3)	6.8<br>(6.1 to 7.7)	3.6<br>(3.2 to 4.0)	1.41<br>(1.25 to 1.59)	0.74<br>(0.66 to 0.83)
2020	0.4<br>(0.3 to 0.5)	0.2<br>(0.2 to 0.3)	6.0<br>(5.0 to 7.0)	3.2<br>(2.7 to 3.8)	1.54<br>(1.28 to 1.82)	0.83<br>(0.69 to 0.97)
2021	0.4<br>(0.3 to 0.5)	0.2<br>(0.2 to 0.3)	5.8<br>(4.5 to 7.4)	3.1<br>(2.4 to 4.0)	1.54<br>(1.20 to 1.97)	0.83<br>(0.65 to 1.05)

Massachusetts USA_544 High−income North America Stillbirth Rate / Neonatal Mortality Rate (SBR/NMR) − 20 weeks
lambda = 0.2 ; zeta = 0.99 ; scale = 5 ; amp = 0.028

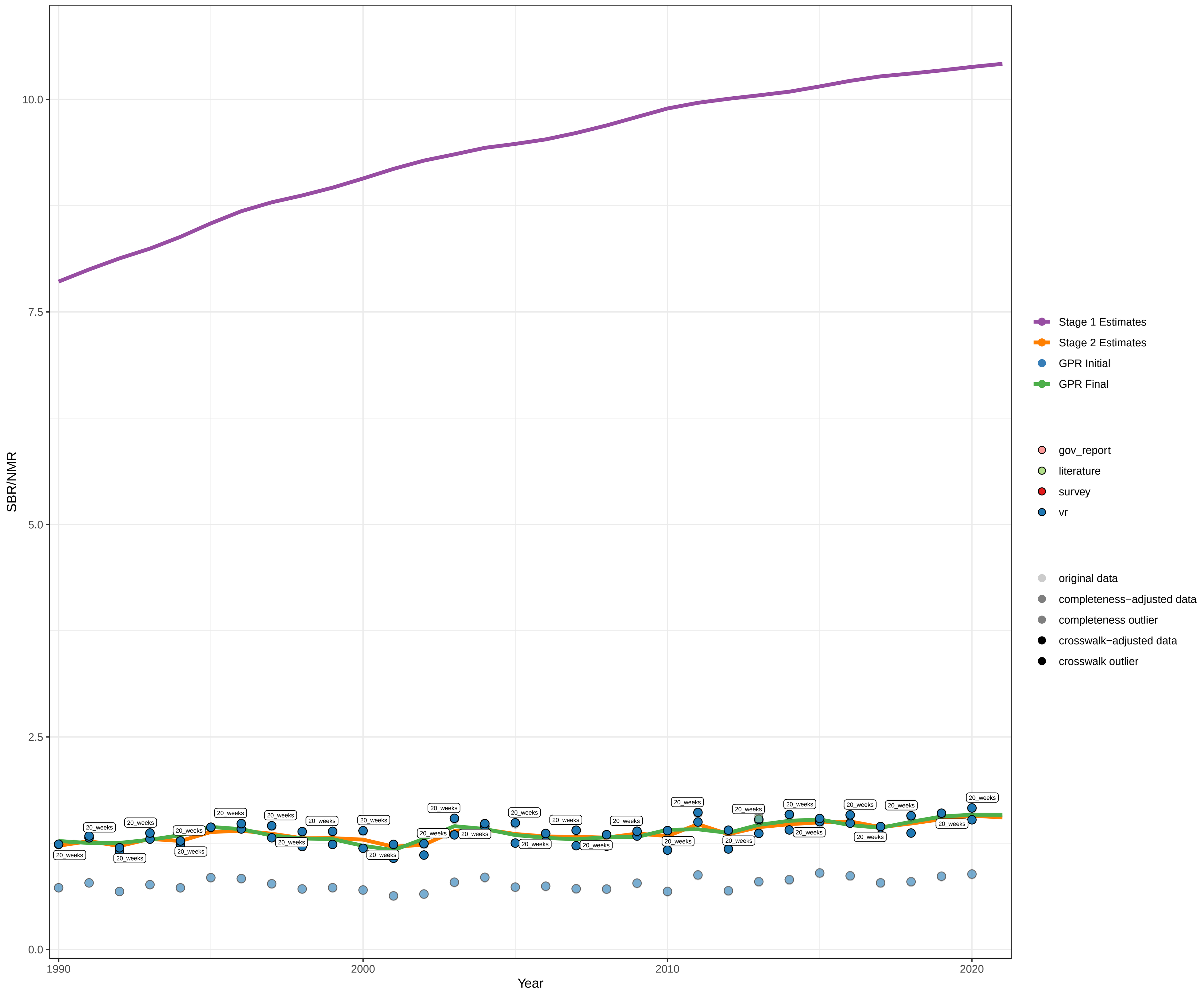

Massachusetts USA_544 High−income North America Stillbirth Rate / Neonatal Mortality Rate (SBR/NMR) − 28 weeks
lambda = 0.2 ; zeta = 0.99 ; scale = 5 ; amp = 0.028

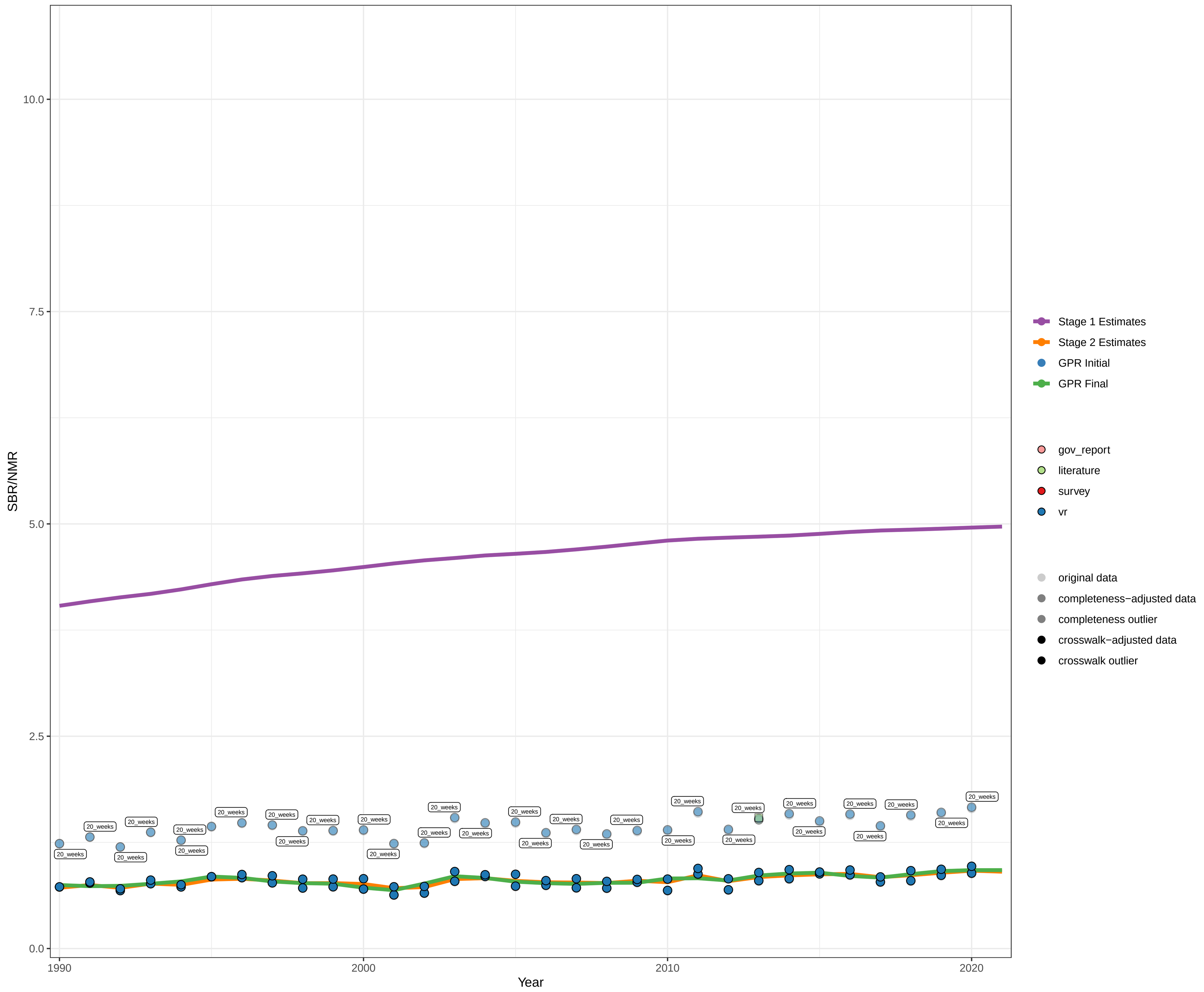

Definition	Years	Outlier	Source.Type	Title
20_weeks, 28_weeks	1990−2020	0	VR	United States NVSS Custom Fetal Death Data
20_weeks	2013	1	Sci Lit	United States Fetal and Perinatal Mortality 2013

Massachusetts USA_544 High−income North America Stillbirth Rate (SBR) − 20 weeks
lambda = 0.2 ; zeta = 0.99 ; scale = 5 ; amp = 0.028

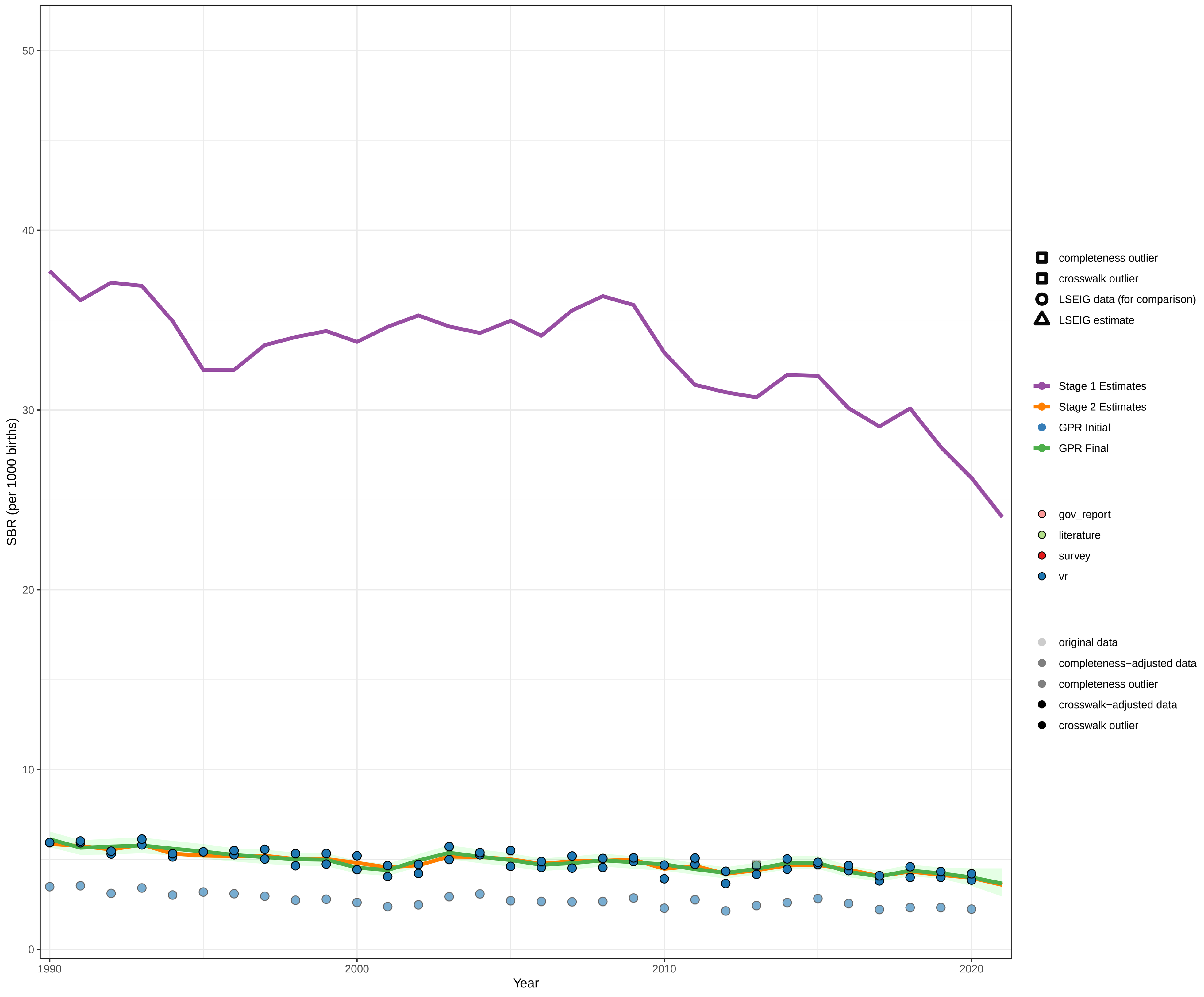

Massachusetts USA_544 High−income North America Stillbirth Rate (SBR) − 28 weeks
lambda = 0.2 ; zeta = 0.99 ; scale = 5 ; amp = 0.028

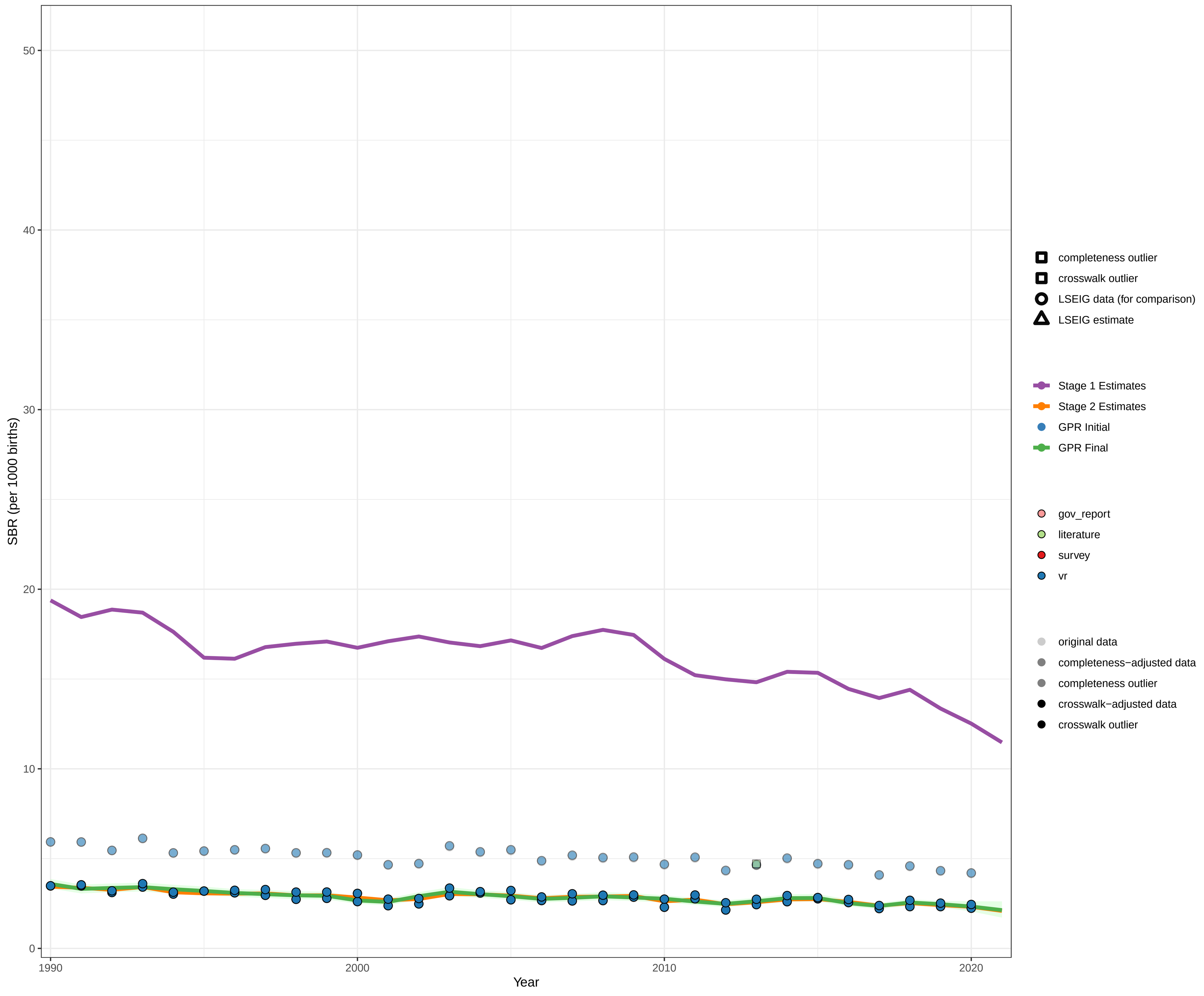

Year	Total Stillbirths (in thousands)	Total Stillbirths (in thousands)	SBR (per 1000 births)	SBR (per 1000 births)	SBR/NMR	SBR/NMR
	20 weeks	28 weeks	20 weeks	28 weeks	20 weeks	28 weeks
1990	0.6 (0.5 to 0.6)	0.3 (0.3 to 0.4)	6.1 (5.7 to 6.6)	3.6 (3.3 to 3.9)	1.28 (1.18 to 1.37)	0.75 (0.69 to 0.81)
1995	0.4 (0.4 to 0.5)	0.3 (0.2 to 0.3)	5.4 (5.0 to 5.9)	3.2 (3.0 to 3.4)	1.44 (1.34 to 1.55)	0.85 (0.79 to 0.91)
2000	0.4 (0.3 to 0.4)	0.2 (0.2 to 0.2)	4.5 (4.2 to 4.9)	2.7 (2.5 to 2.9)	1.22 (1.13 to 1.32)	0.72 (0.67 to 0.77)
2005	0.4 (0.4 to 0.4)	0.2 (0.2 to 0.2)	5.0 (4.6 to 5.3)	2.9 (2.7 to 3.1)	1.35 (1.25 to 1.45)	0.79 (0.73 to 0.85)
2010	0.3 (0.3 to 0.4)	0.2 (0.2 to 0.2)	4.7 (4.4 to 5.1)	2.8 (2.6 to 3.0)	1.41 (1.30 to 1.52)	0.82 (0.76 to 0.88)
2015	0.4 (0.3 to 0.4)	0.2 (0.2 to 0.2)	4.8 (4.5 to 5.2)	2.8 (2.6 to 3.0)	1.53 (1.42 to 1.65)	0.89 (0.83 to 0.96)
2020	0.3 (0.2 to 0.3)	0.2 (0.1 to 0.2)	4.0 (3.5 to 4.5)	2.3 (2.1 to 2.6)	1.59 (1.40 to 1.78)	0.92 (0.81 to 1.04)
2021	0.3 (0.2 to 0.3)	0.1 (0.1 to 0.2)	3.7 (2.9 to 4.5)	2.1 (1.7 to 2.6)	1.59 (1.27 to 1.95)	0.92 (0.74 to 1.14)

Michigan USA_545 High−income North America Stillbirth Rate / Neonatal Mortality Rate (SBR/NMR) − 20 weeks
lambda = 0.2 ; zeta = 0.99 ; scale = 5 ; amp = 0.028

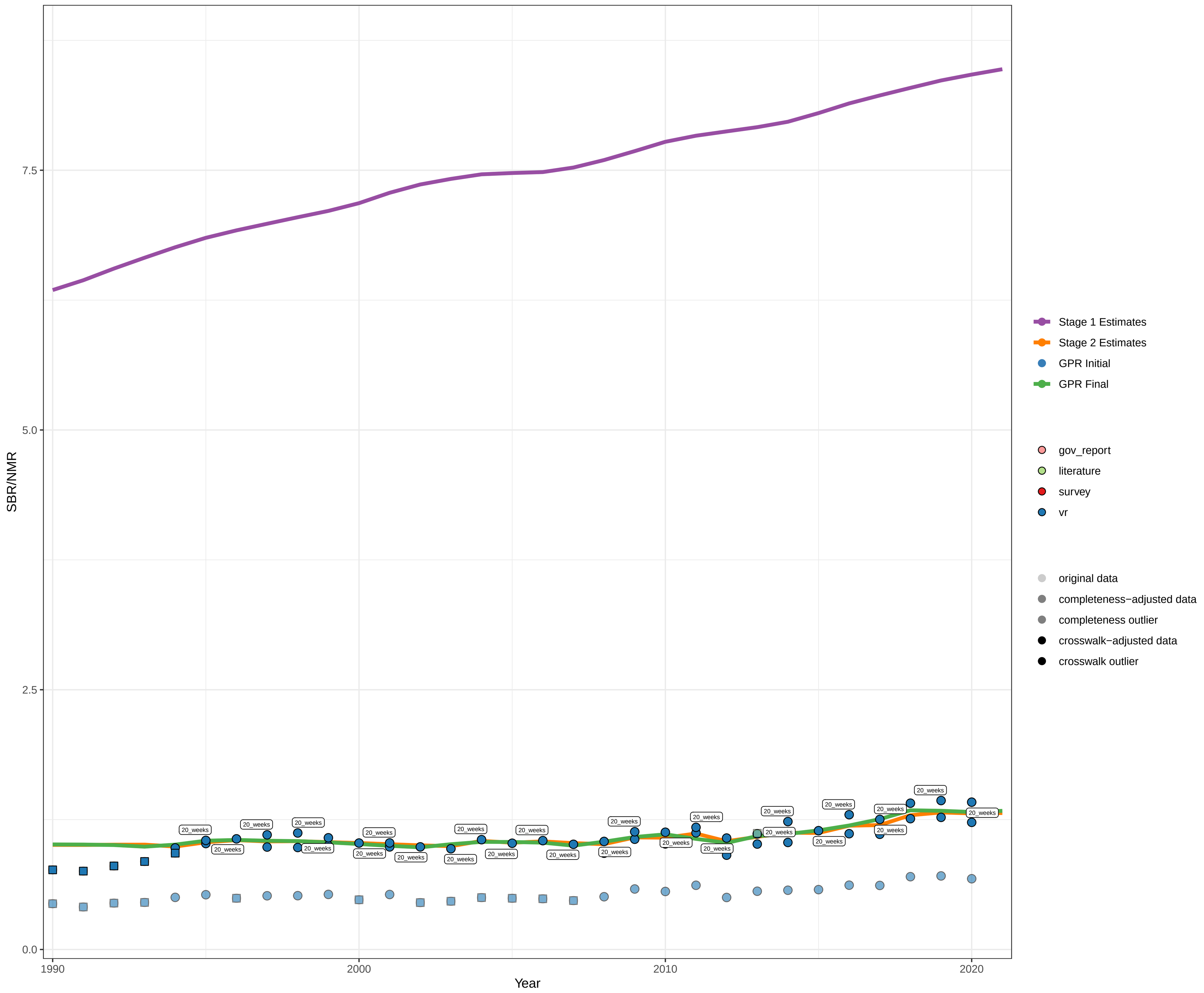

Michigan USA_545 High−income North America Stillbirth Rate / Neonatal Mortality Rate (SBR/NMR) − 28 weeks
lambda = 0.2 ; zeta = 0.99 ; scale = 5 ; amp = 0.028

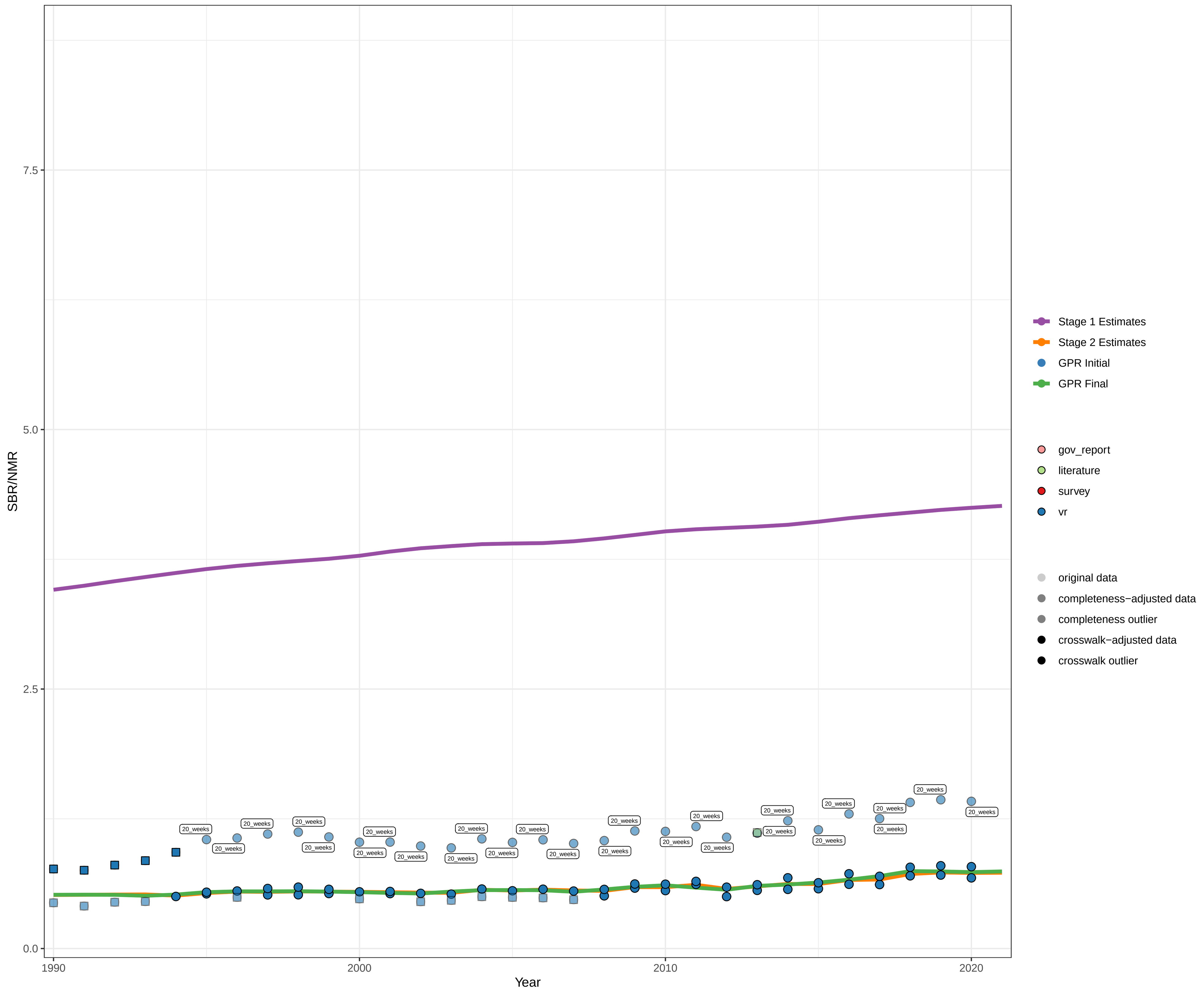

Definition	Years	Outlier	Source.Type	Title
20_weeks	1995−2020	0	VR	United States NVSS Custom Fetal Death Data
28_weeks	1994, 1995, 1997−1999, 2001, 2008−2020	0	VR	United States NVSS Custom Fetal Death Data
20_weeks	2013	1	Sci Lit	United States Fetal and Perinatal Mortality 2013
28_weeks	1990−1993, 1996, 2000, 2002−2007	1	VR	United States NVSS Custom Fetal Death Data
20_weeks	1990−1994	2	VR	United States NVSS Custom Fetal Death Data

Michigan USA_545 High−income North America Stillbirth Rate (SBR) − 20 weeks
lambda = 0.2 ; zeta = 0.99 ; scale = 5 ; amp = 0.028

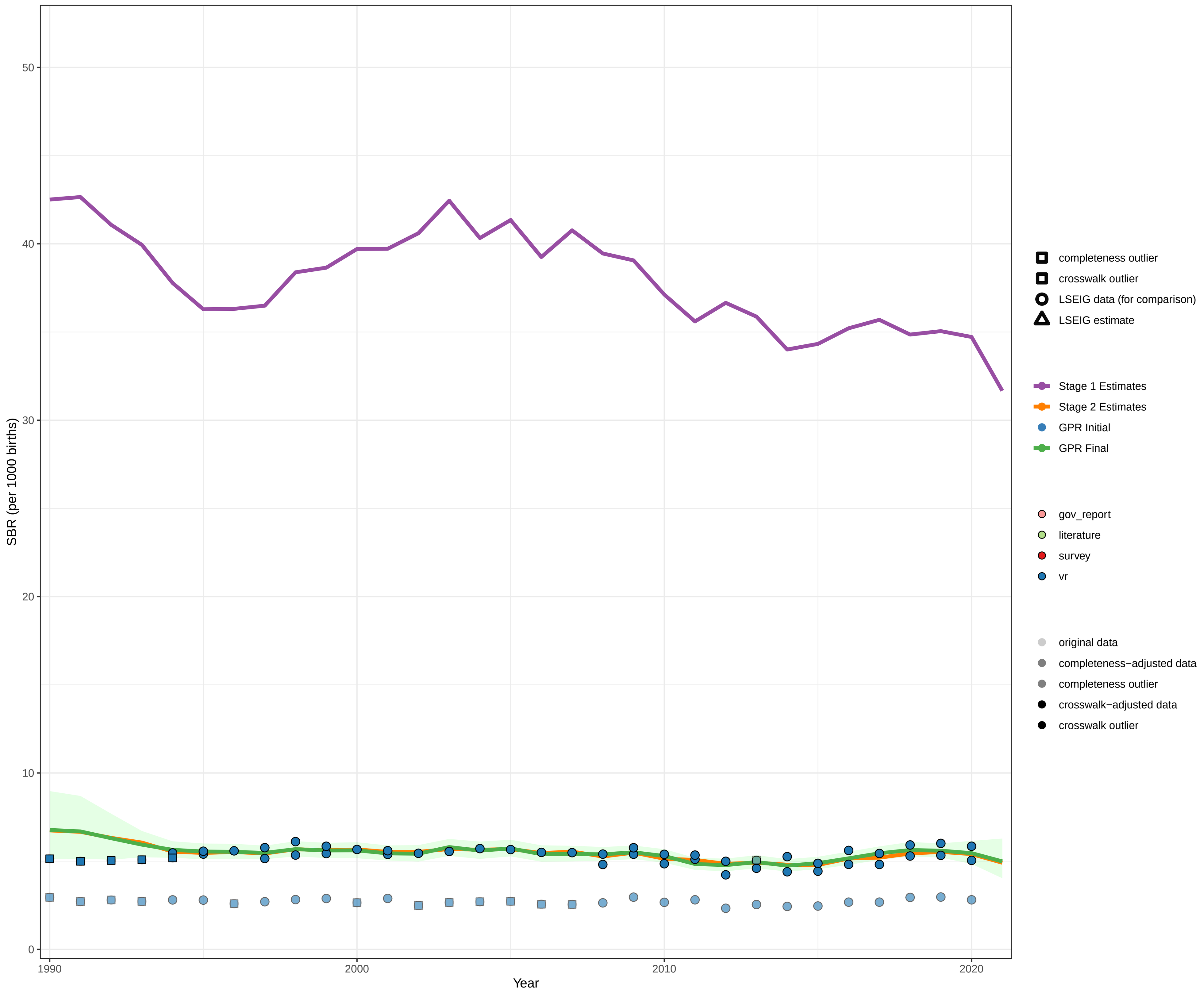

Michigan USA_545 High−income North America Stillbirth Rate (SBR) − 28 weeks
lambda = 0.2 ; zeta = 0.99 ; scale = 5 ; amp = 0.028

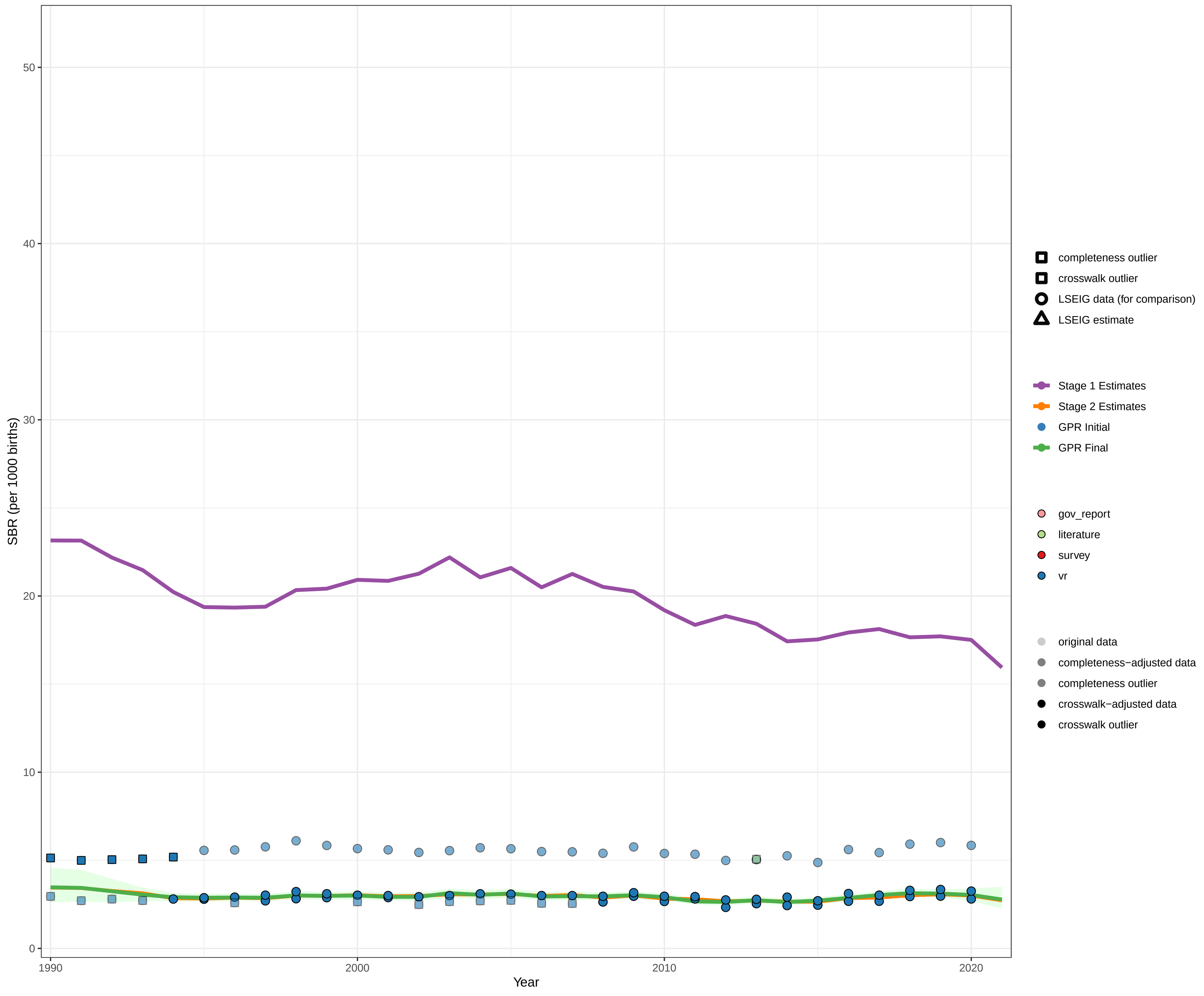

Year	Total Stillbirths (in thousands)	Total Stillbirths (in thousands)	SBR (per 1000 births)	SBR (per 1000 births)	SBR/NMR	SBR/NMR
	20 weeks	28 weeks	20 weeks	28 weeks	20 weeks	28 weeks
1990	1.0 (0.8 to 1.4)	0.5 (0.4 to 0.7)	6.8 (5.1 to 9.0)	3.5 (2.6 to 4.6)	1.01 (0.76 to 1.34)	0.52 (0.39 to 0.68)
1995	0.7 (0.7 to 0.8)	0.4 (0.4 to 0.4)	5.5 (5.1 to 6.0)	2.9 (2.7 to 3.1)	1.05 (0.96 to 1.13)	0.54 (0.50 to 0.59)
2000	0.8 (0.7 to 0.8)	0.4 (0.4 to 0.4)	5.6 (5.2 to 6.1)	3.0 (2.8 to 3.2)	1.02 (0.93 to 1.10)	0.54 (0.50 to 0.59)
2005	0.7 (0.7 to 0.8)	0.4 (0.4 to 0.4)	5.7 (5.3 to 6.2)	3.1 (2.9 to 3.4)	1.03 (0.95 to 1.12)	0.56 (0.52 to 0.61)
2010	0.6 (0.6 to 0.6)	0.3 (0.3 to 0.4)	5.3 (4.9 to 5.7)	2.9 (2.7 to 3.1)	1.11 (1.03 to 1.19)	0.61 (0.57 to 0.65)
2015	0.6 (0.5 to 0.6)	0.3 (0.3 to 0.3)	4.9 (4.5 to 5.2)	2.7 (2.5 to 2.9)	1.15 (1.07 to 1.23)	0.64 (0.59 to 0.68)
2020	0.6 (0.5 to 0.6)	0.3 (0.3 to 0.4)	5.5 (4.8 to 6.1)	3.0 (2.7 to 3.4)	1.32 (1.17 to 1.49)	0.74 (0.65 to 0.83)
2021	0.5 (0.4 to 0.7)	0.3 (0.2 to 0.4)	5.0 (4.0 to 6.3)	2.8 (2.2 to 3.5)	1.33 (1.08 to 1.68)	0.74 (0.60 to 0.93)

Minnesota USA_546 High−income North America Stillbirth Rate / Neonatal Mortality Rate (SBR/NMR) − 20 weeks
lambda = 0.2 ; zeta = 0.99 ; scale = 5 ; amp = 0.028

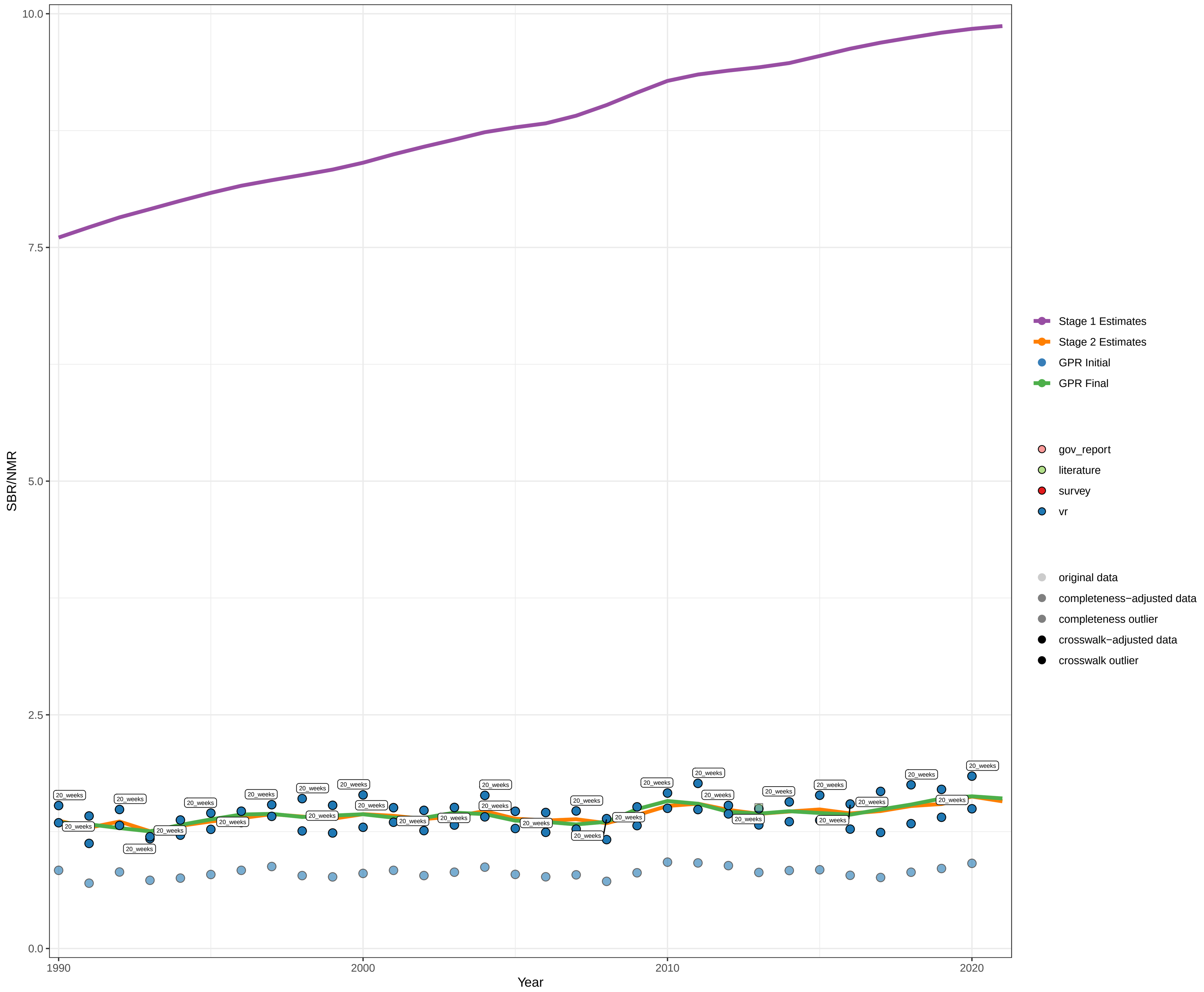

Minnesota USA_546 High−income North America Stillbirth Rate / Neonatal Mortality Rate (SBR/NMR) − 28 weeks
lambda = 0.2 ; zeta = 0.99 ; scale = 5 ; amp = 0.028

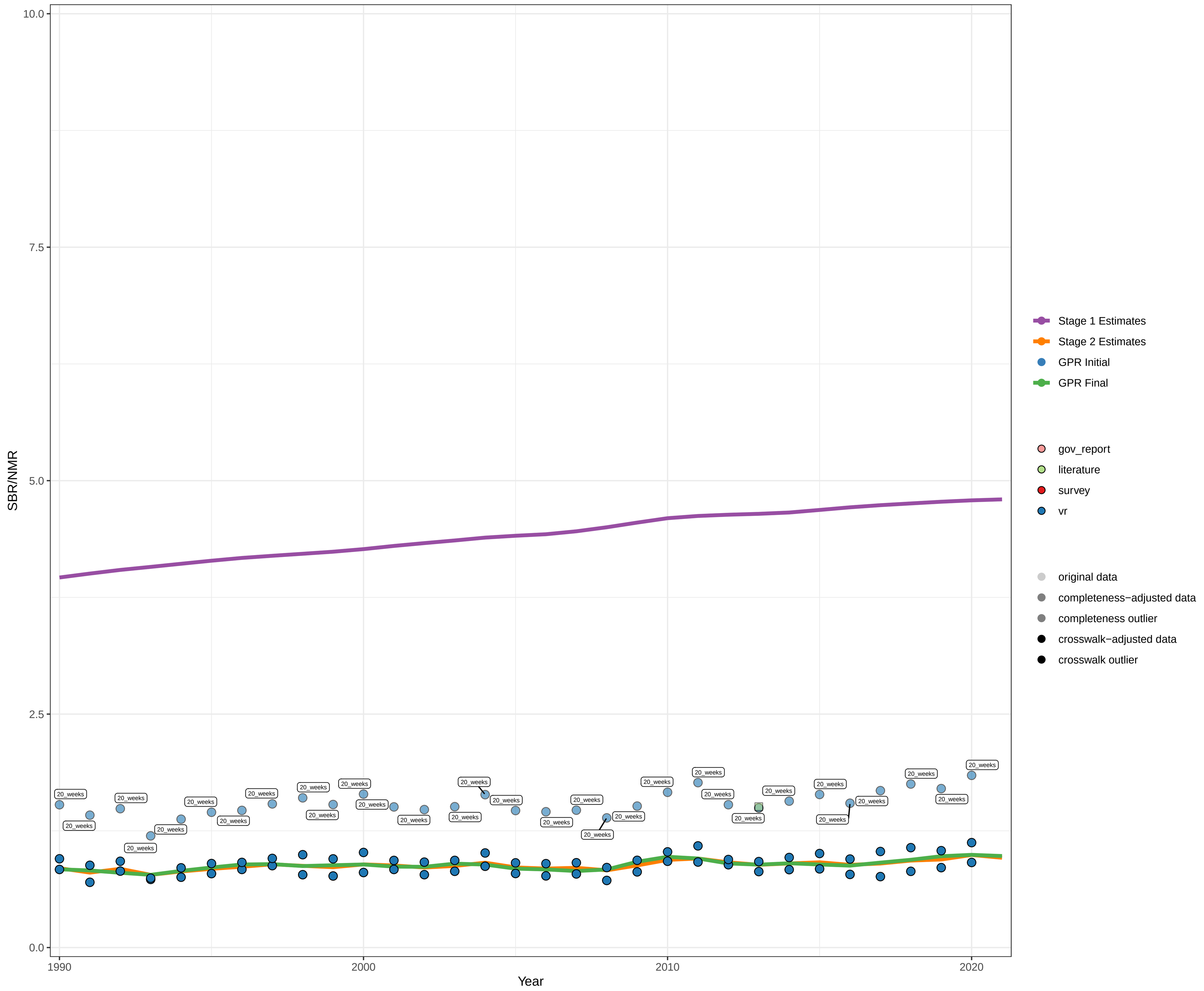

Definition	Years	Outlier	Source.Type	Title
20_weeks, 28_weeks	1990−2020	0	VR	United States NVSS Custom Fetal Death Data
20_weeks	2013	1	Sci Lit	United States Fetal and Perinatal Mortality 2013

Minnesota USA_546 High−income North America Stillbirth Rate (SBR) − 20 weeks
lambda = 0.2 ; zeta = 0.99 ; scale = 5 ; amp = 0.028

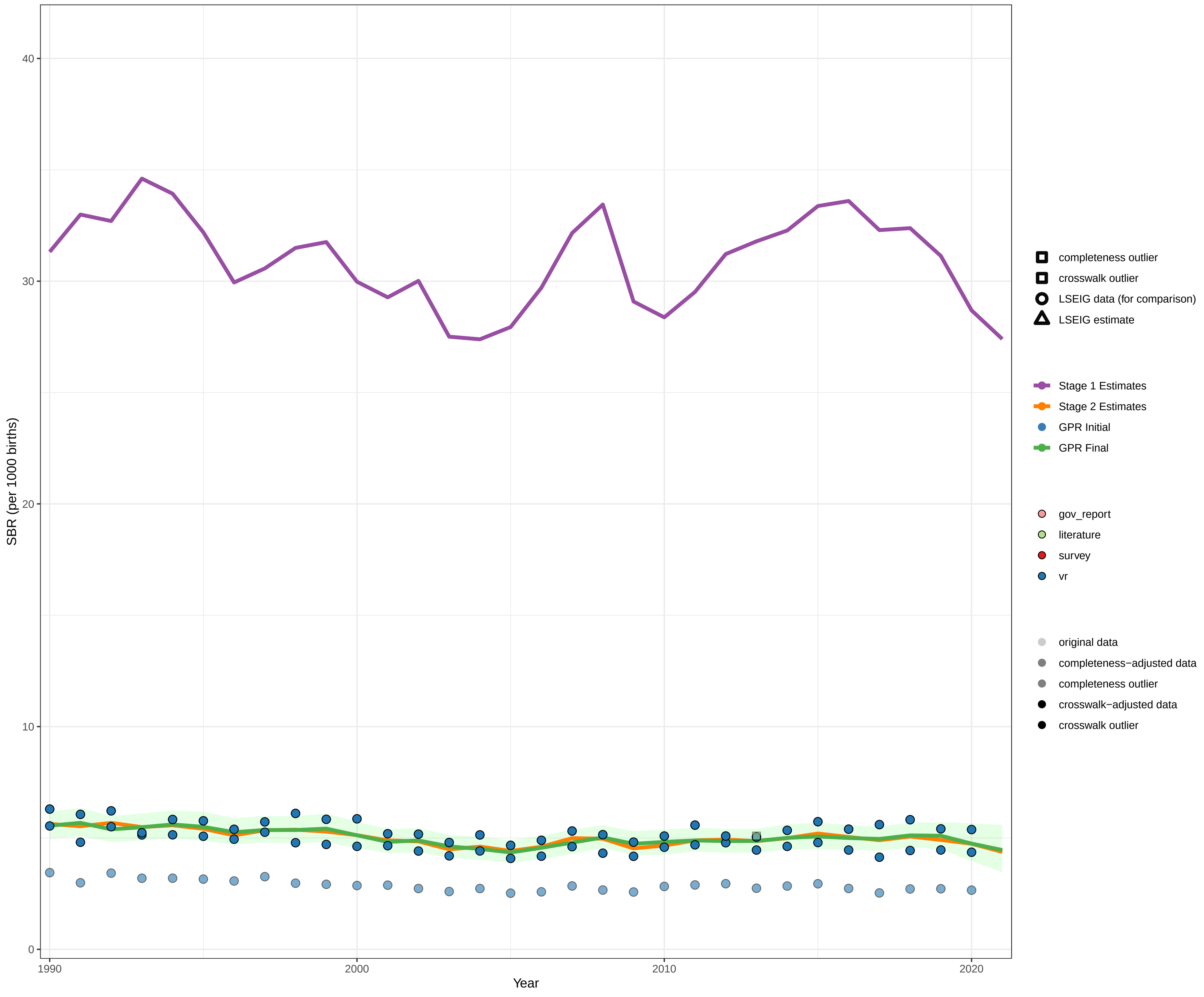

Minnesota USA_546 High−income North America Stillbirth Rate (SBR) − 28 weeks
lambda = 0.2 ; zeta = 0.99 ; scale = 5 ; amp = 0.028

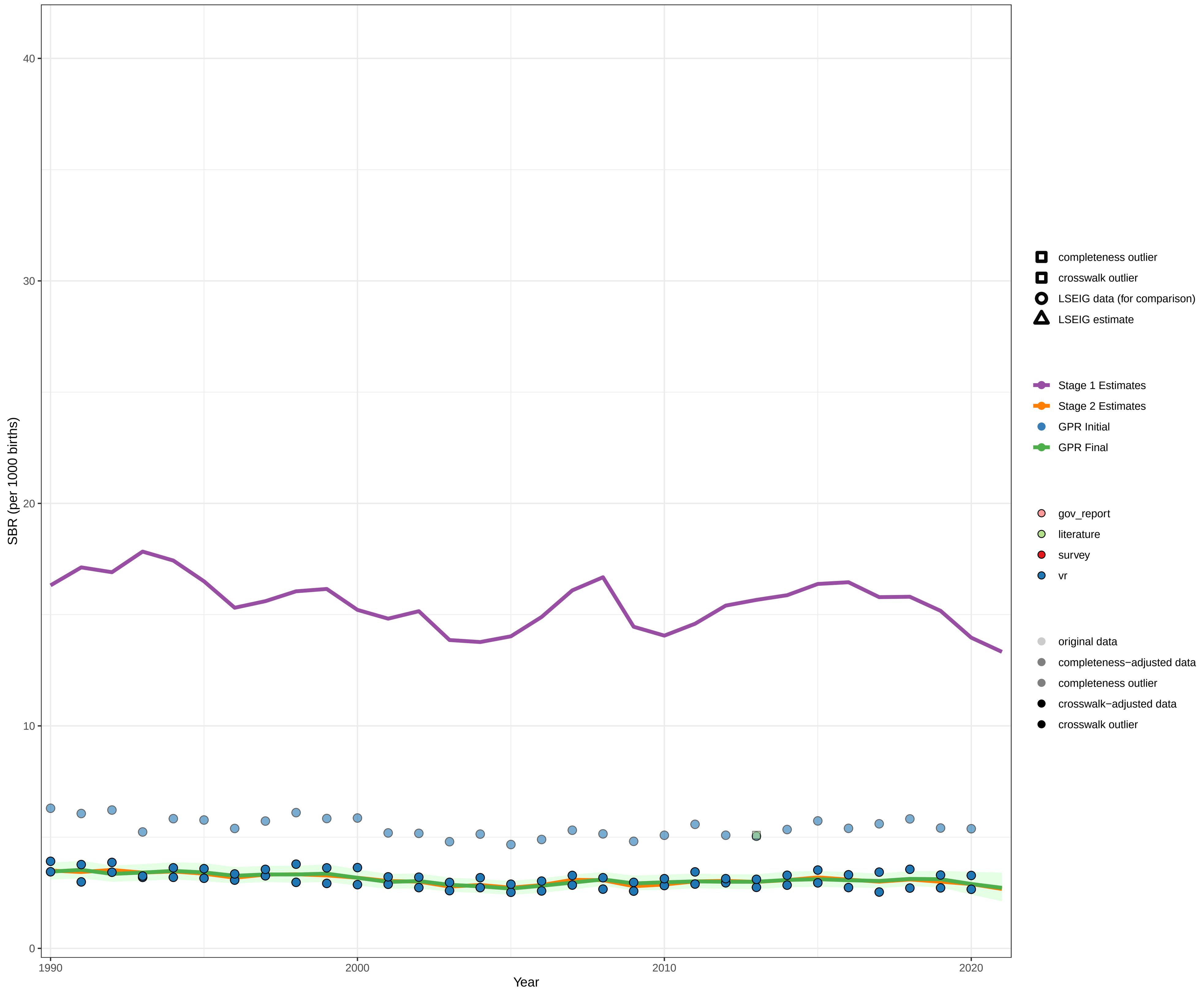

Year	Total Stillbirths (in thousands)	Total Stillbirths (in thousands)	SBR (per 1000 births)	SBR (per 1000 births)	SBR/NMR	SBR/NMR
	20 weeks	28 weeks	20 weeks	28 weeks	20 weeks	28 weeks
1990	0.4 (0.3 to 0.4)	0.2 (0.2 to 0.3)	5.6 (5.0 to 6.2)	3.5 (3.1 to 3.8)	1.35 (1.21 to 1.50)	0.84 (0.75 to 0.93)
1995	0.3 (0.3 to 0.4)	0.2 (0.2 to 0.2)	5.5 (4.9 to 6.2)	3.4 (3.0 to 3.8)	1.38 (1.22 to 1.55)	0.86 (0.76 to 0.96)
2000	0.3 (0.3 to 0.4)	0.2 (0.2 to 0.2)	5.1 (4.6 to 5.7)	3.2 (2.8 to 3.5)	1.44 (1.28 to 1.61)	0.89 (0.79 to 1.00)
2005	0.3 (0.3 to 0.3)	0.2 (0.2 to 0.2)	4.4 (3.9 to 4.9)	2.7 (2.4 to 3.0)	1.37 (1.23 to 1.54)	0.85 (0.76 to 0.95)
2010	0.3 (0.3 to 0.4)	0.2 (0.2 to 0.2)	4.8 (4.3 to 5.4)	3.0 (2.6 to 3.3)	1.58 (1.39 to 1.76)	0.97 (0.86 to 1.08)
2015	0.4 (0.3 to 0.4)	0.2 (0.2 to 0.2)	5.1 (4.5 to 5.7)	3.1 (2.8 to 3.5)	1.45 (1.29 to 1.63)	0.89 (0.79 to 1.00)
2020	0.3 (0.3 to 0.4)	0.2 (0.2 to 0.2)	4.7 (4.0 to 5.7)	2.9 (2.4 to 3.4)	1.63 (1.36 to 1.94)	0.99 (0.83 to 1.18)
2021	0.3 (0.2 to 0.4)	0.2 (0.1 to 0.2)	4.5 (3.5 to 5.6)	2.7 (2.1 to 3.4)	1.61 (1.25 to 2.01)	0.98 (0.76 to 1.23)

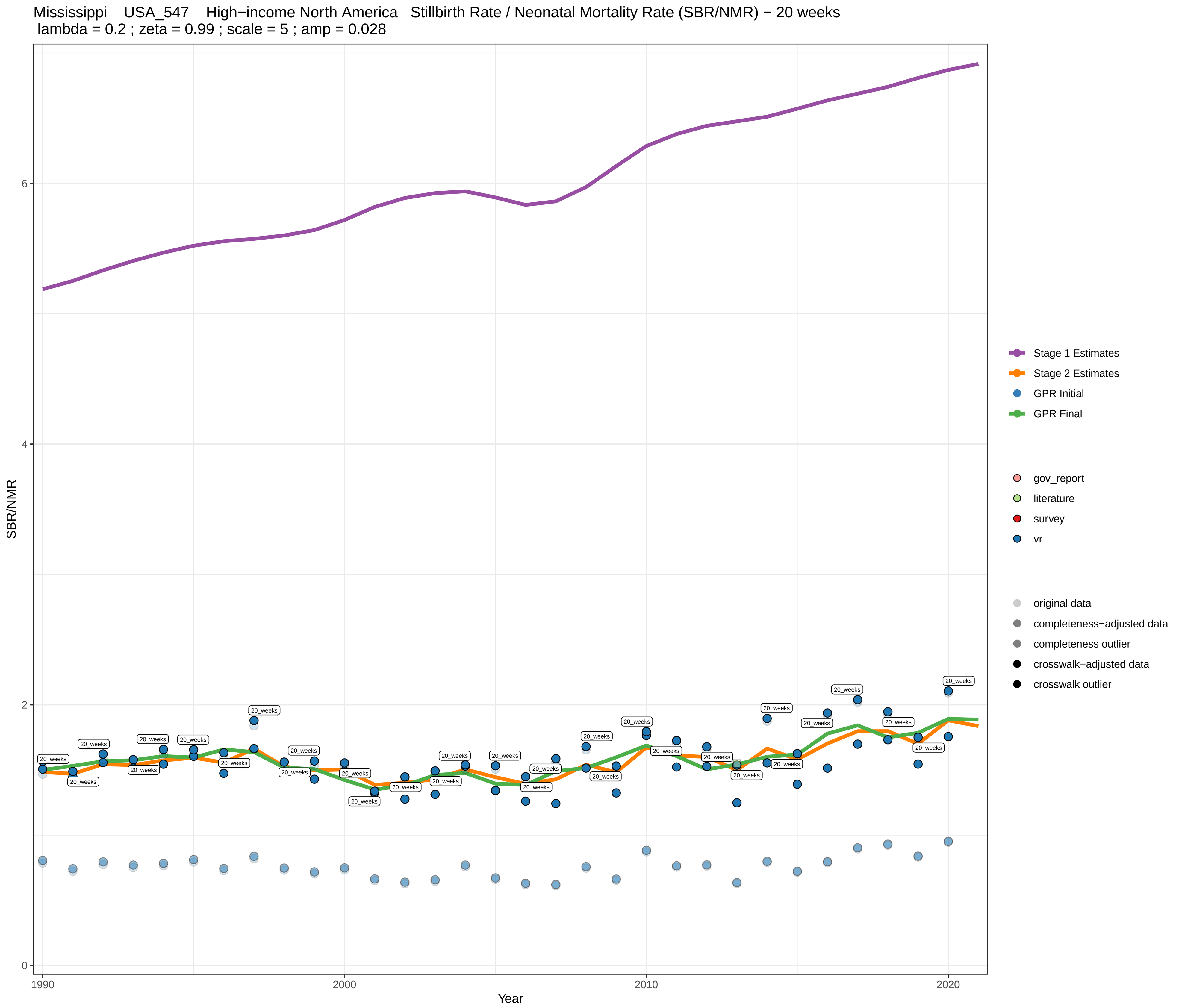

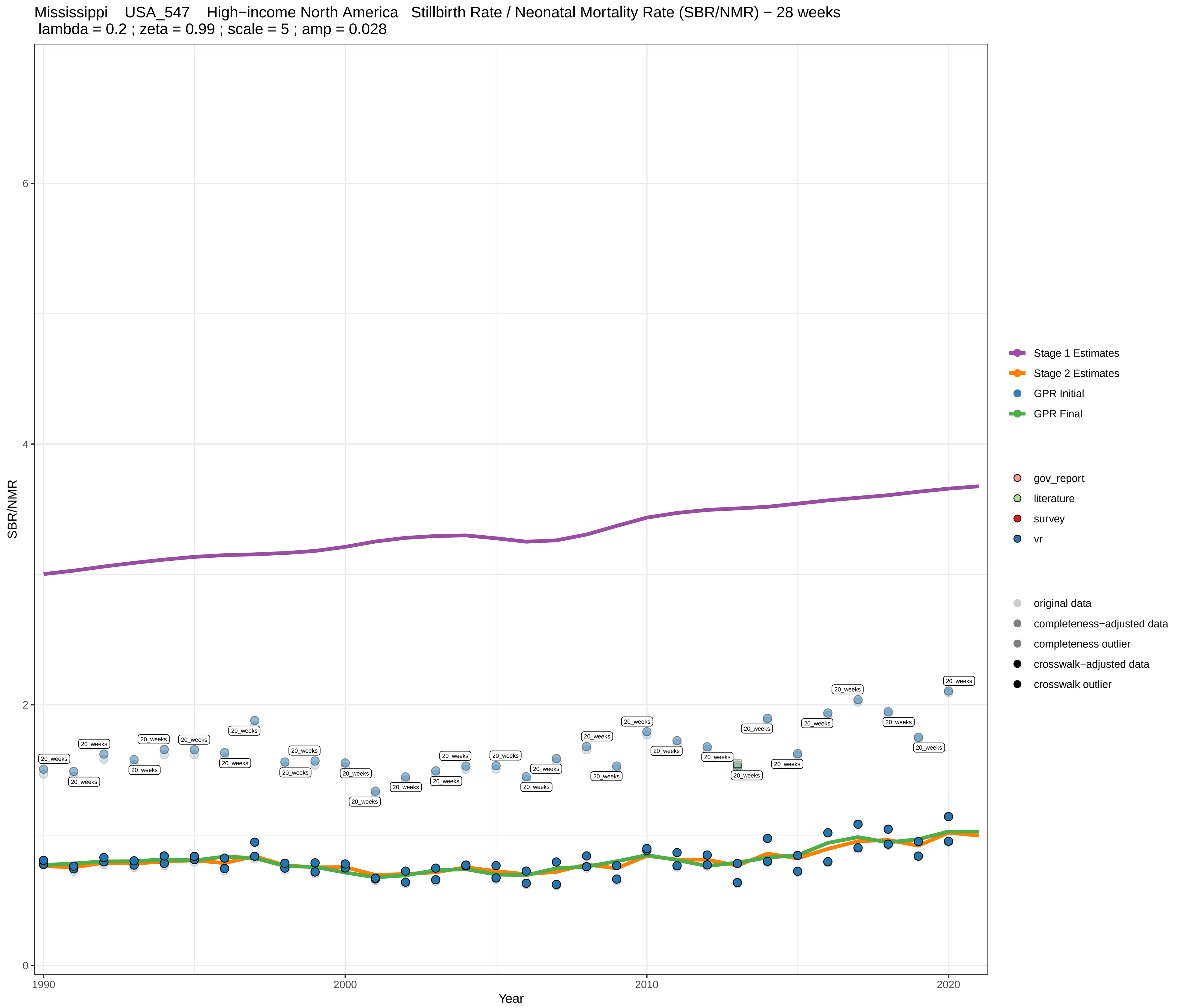

Definition	Years	Outlier	Source.Type	Title
20_weeks, 28_weeks	1990−2020	0	VR	United States NVSS Custom Fetal Death Data
20_weeks	2013	1	Sci Lit	United States Fetal and Perinatal Mortality 2013

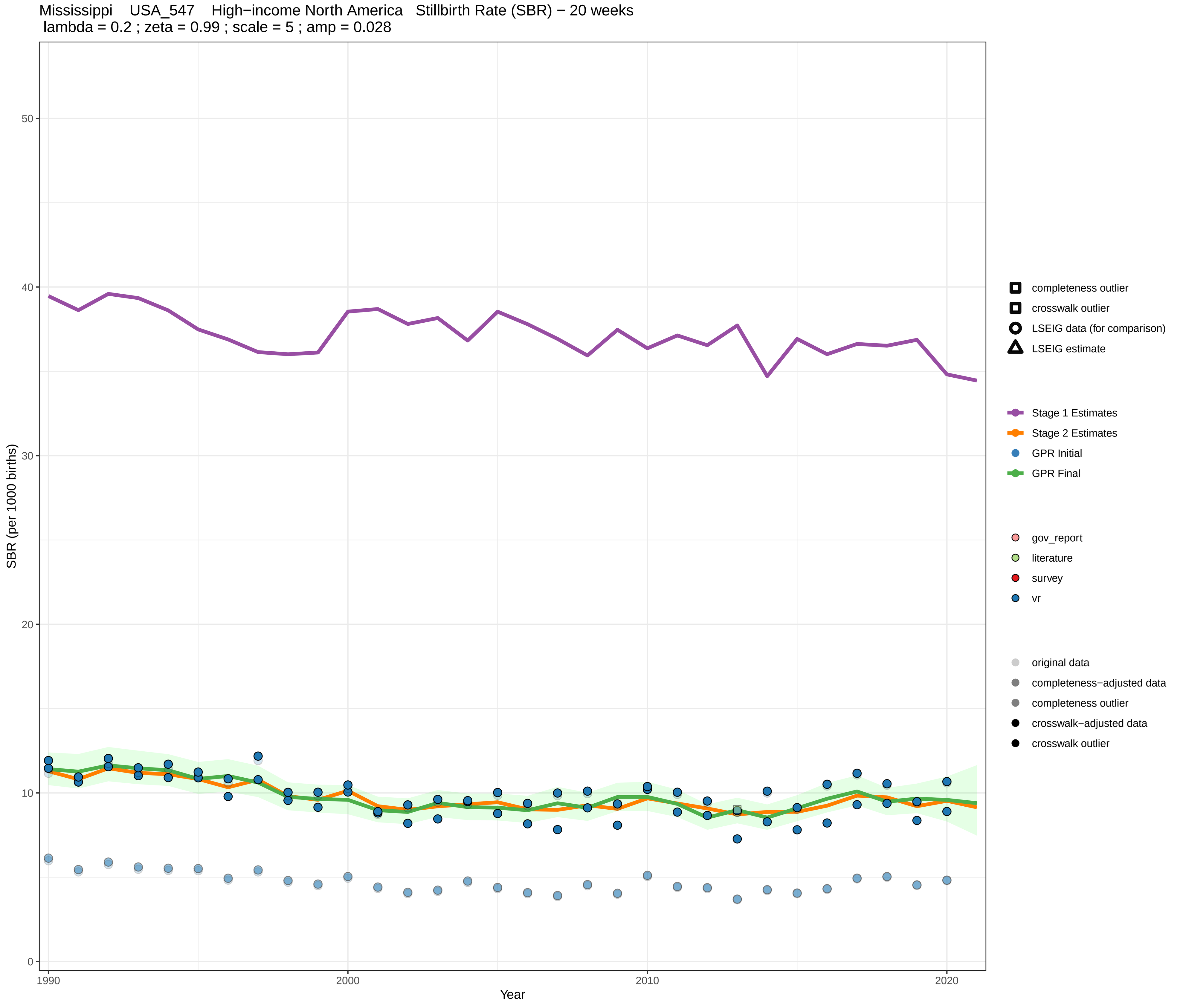

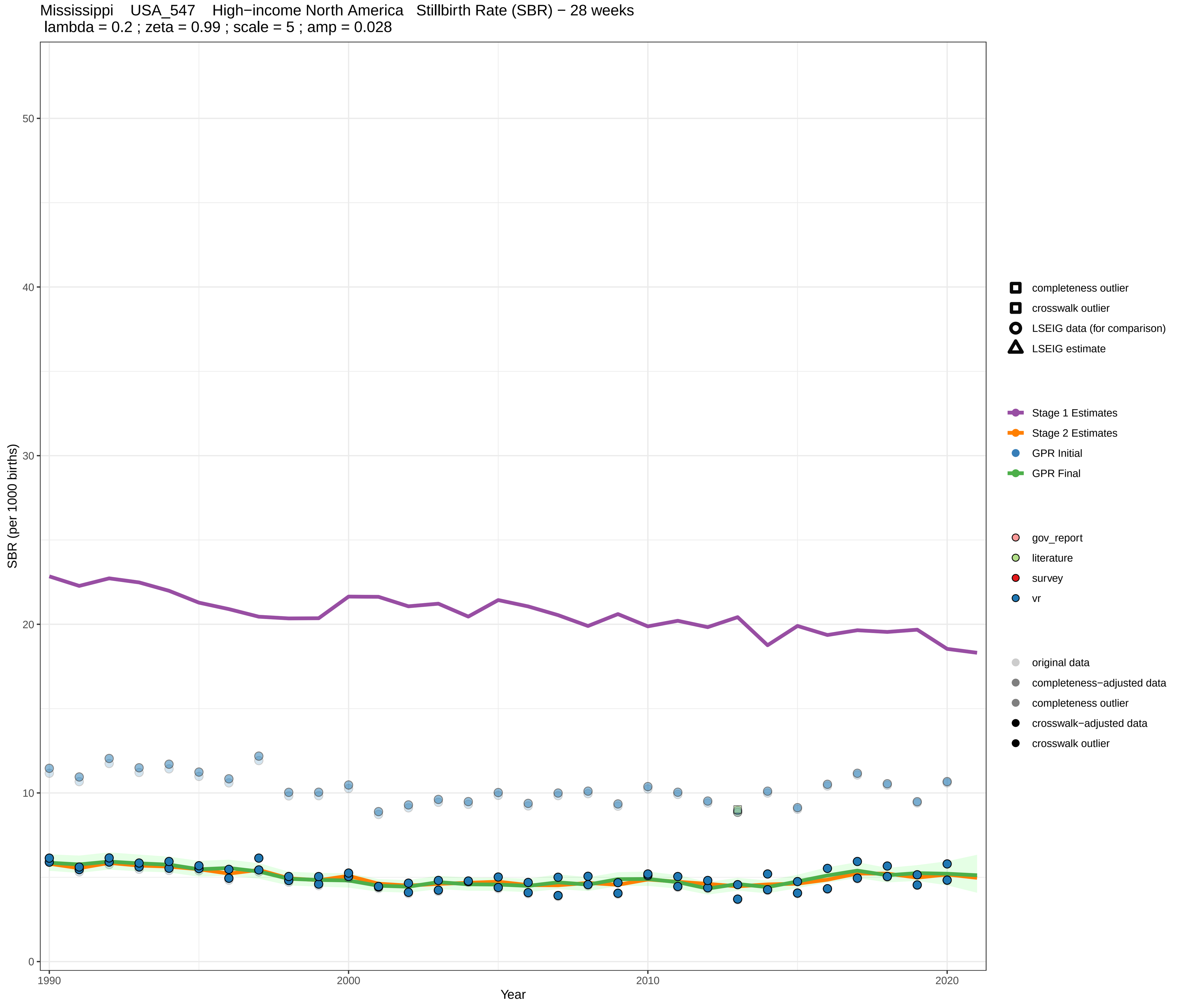

Year	Total Stillbirths (in thousands)	Total Stillbirths (in thousands)	SBR (per 1000 births)	SBR (per 1000 births)	SBR/NMR	SBR/NMR
	20 weeks	28 weeks	20 weeks	28 weeks	20 weeks	28 weeks
1990	0.5 (0.5 to 0.6)	0.3 (0.2 to 0.3)	11.4 (10.5 to 12.4)	5.9 (5.4 to 6.4)	1.50 (1.38 to 1.63)	0.77 (0.71 to 0.84)
1995	0.5 (0.4 to 0.5)	0.2 (0.2 to 0.2)	10.8 (9.9 to 11.8)	5.5 (5.0 to 6.0)	1.60 (1.46 to 1.74)	0.81 (0.74 to 0.88)
2000	0.4 (0.4 to 0.5)	0.2 (0.2 to 0.2)	9.6 (8.7 to 10.5)	4.8 (4.4 to 5.2)	1.42 (1.30 to 1.55)	0.71 (0.65 to 0.78)
2005	0.4 (0.4 to 0.4)	0.2 (0.2 to 0.2)	9.1 (8.4 to 10.0)	4.6 (4.2 to 5.0)	1.40 (1.28 to 1.52)	0.70 (0.64 to 0.76)
2010	0.4 (0.4 to 0.4)	0.2 (0.2 to 0.2)	9.8 (8.9 to 10.7)	4.9 (4.5 to 5.3)	1.69 (1.54 to 1.84)	0.85 (0.78 to 0.92)
2015	0.3 (0.3 to 0.4)	0.2 (0.2 to 0.2)	9.1 (8.3 to 9.9)	4.8 (4.4 to 5.2)	1.62 (1.48 to 1.76)	0.85 (0.78 to 0.92)
2020	0.3 (0.3 to 0.4)	0.2 (0.2 to 0.2)	9.6 (8.3 to 11.0)	5.2 (4.5 to 6.0)	1.89 (1.64 to 2.16)	1.03 (0.89 to 1.17)
2021	0.3 (0.3 to 0.4)	0.2 (0.1 to 0.2)	9.4 (7.5 to 11.6)	5.1 (4.1 to 6.3)	1.89 (1.50 to 2.34)	1.03 (0.82 to 1.27)

Missouri   USA_548   High−income North America   Stillbirth Rate / Neonatal Mortality Rate (SBR/NMR) − 20 weeks
lambda = 0.2 ; zeta = 0.99 ; scale = 5 ; amp = 0.028

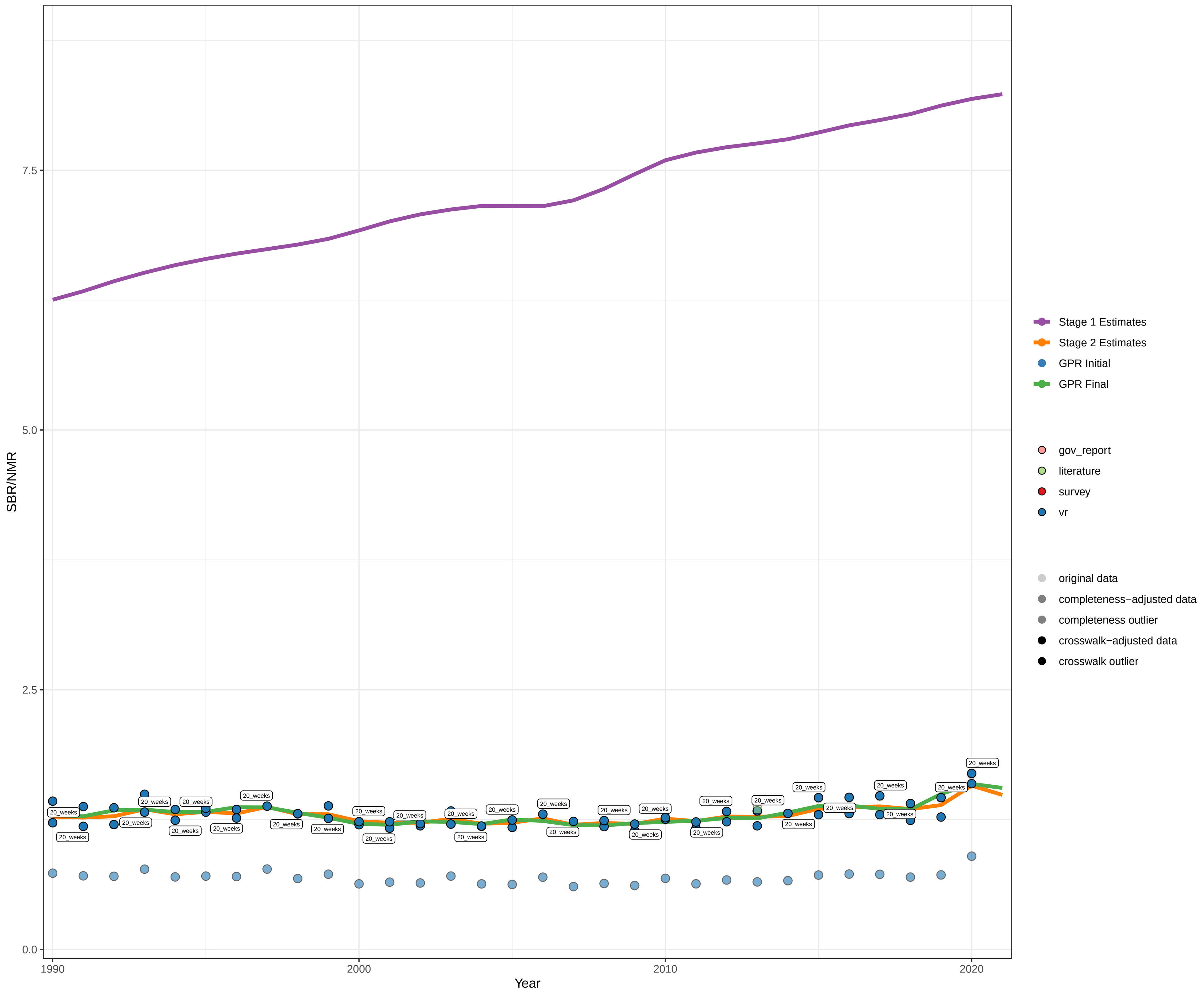

Missouri   USA_548   High−income North America   Stillbirth Rate / Neonatal Mortality Rate (SBR/NMR) − 28 weeks
lambda = 0.2 ; zeta = 0.99 ; scale = 5 ; amp = 0.028

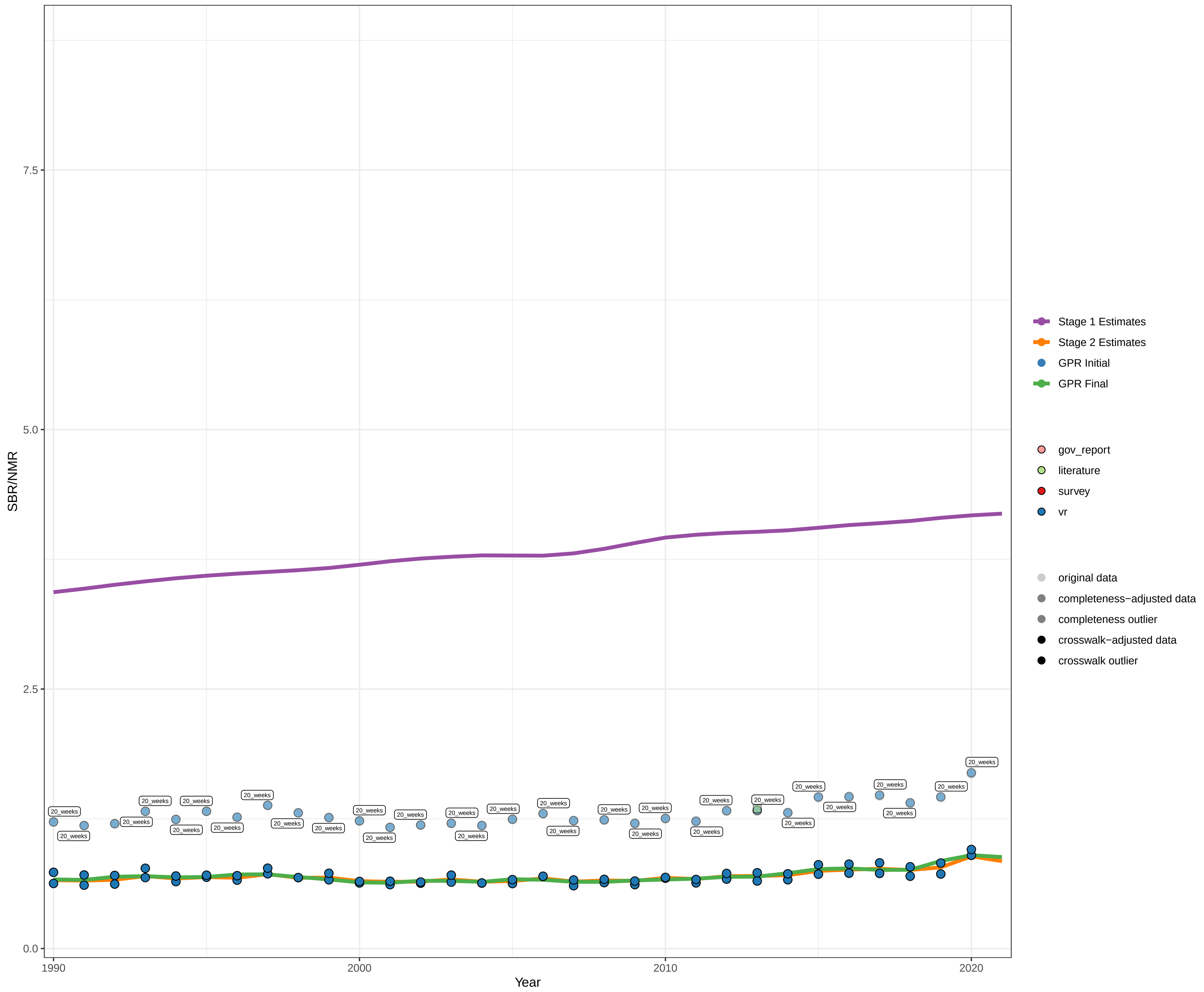

Definition	Years	Outlier	Source.Type	Title
20_weeks, 28_weeks	1990−2020	0	VR	United States NVSS Custom Fetal Death Data
20_weeks	2013	1	Sci Lit	United States Fetal and Perinatal Mortality 2013

Missouri   USA_548   High−income North America   Stillbirth Rate (SBR) − 20 weeks
lambda = 0.2 ; zeta = 0.99 ; scale = 5 ; amp = 0.028

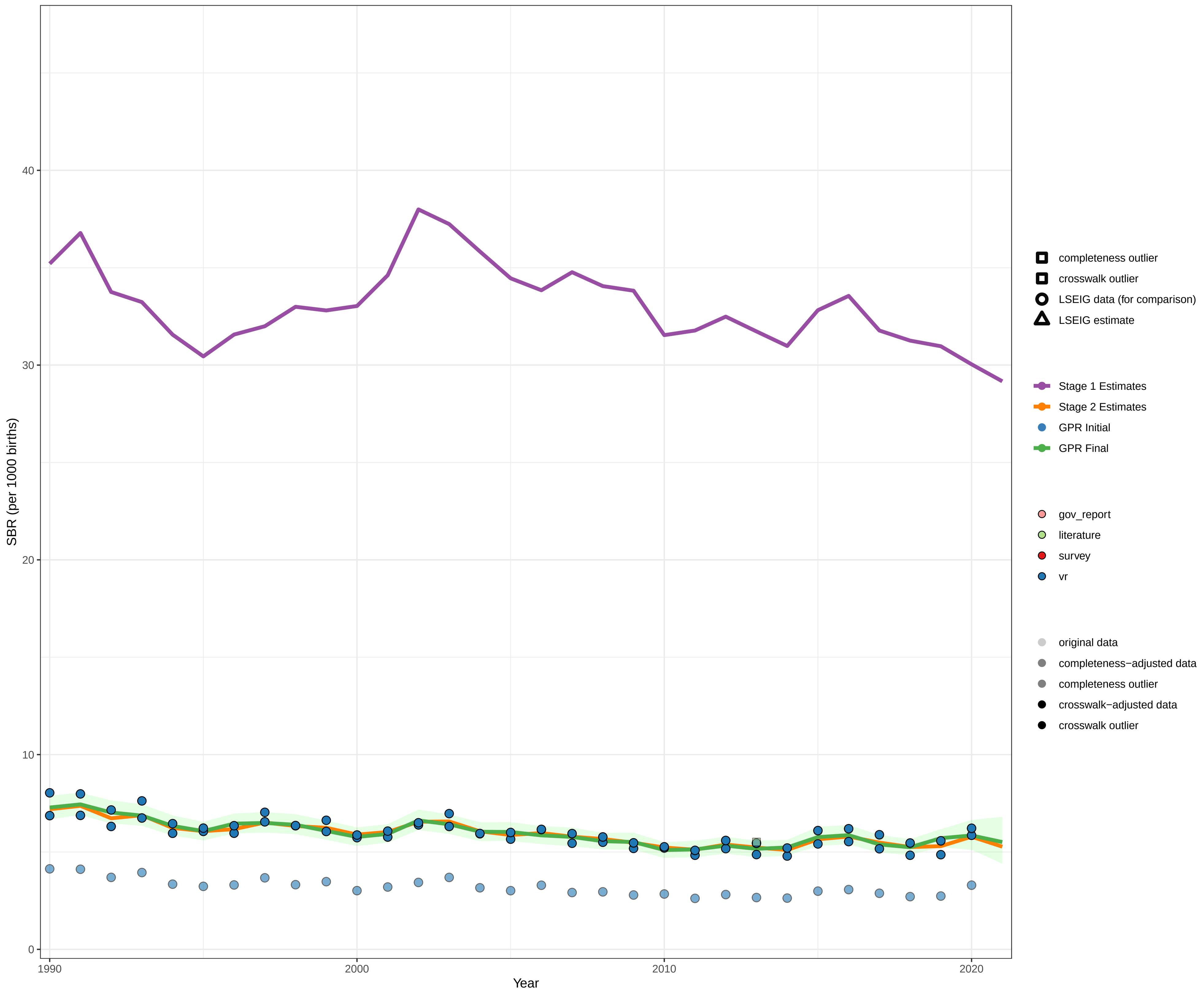

Missouri   USA_548   High−income North America   Stillbirth Rate (SBR) − 28 weeks
lambda = 0.2 ; zeta = 0.99 ; scale = 5 ; amp = 0.028

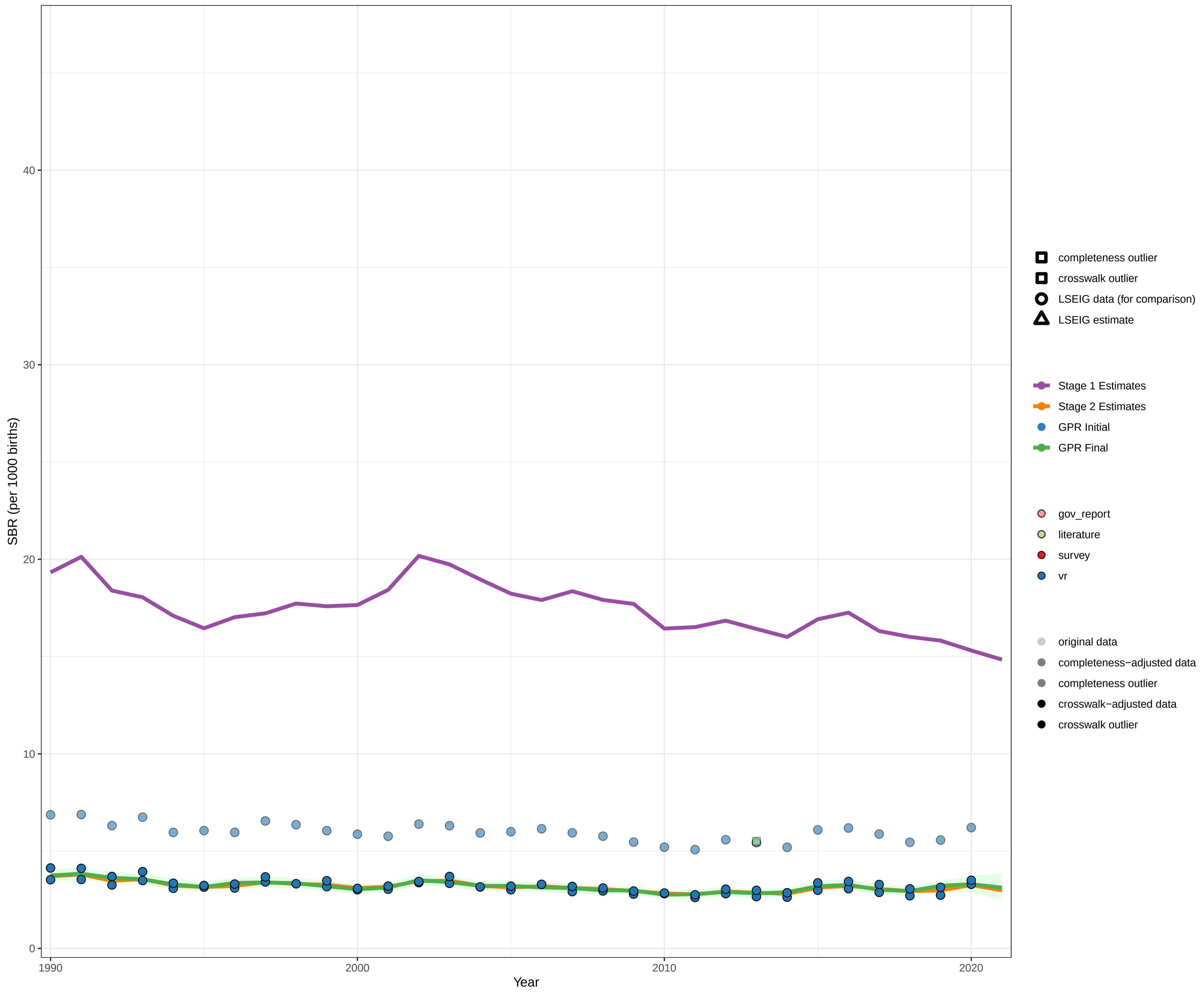

Year	Total Stillbirths (in thousands)	Total Stillbirths (in thousands)	SBR (per 1000 births)	SBR (per 1000 births)	SBR/NMR	SBR/NMR
	20 weeks	28 weeks	20 weeks	28 weeks	20 weeks	28 weeks
1990	0.6 (0.5 to 0.6)	0.3 (0.3 to 0.3)	7.3 (6.7 to 7.9)	3.7 (3.4 to 4.1)	1.29 (1.19 to 1.40)	0.67 (0.61 to 0.72)
1995	0.4 (0.4 to 0.5)	0.2 (0.2 to 0.3)	6.1 (5.6 to 6.5)	3.2 (2.9 to 3.4)	1.33 (1.22 to 1.43)	0.69 (0.64 to 0.74)
2000	0.4 (0.4 to 0.5)	0.2 (0.2 to 0.2)	5.8 (5.3 to 6.3)	3.0 (2.8 to 3.3)	1.21 (1.11 to 1.31)	0.64 (0.59 to 0.69)
2005	0.5 (0.4 to 0.5)	0.3 (0.2 to 0.3)	6.0 (5.6 to 6.5)	3.2 (3.0 to 3.5)	1.25 (1.16 to 1.36)	0.67 (0.62 to 0.72)
2010	0.4 (0.4 to 0.4)	0.2 (0.2 to 0.2)	5.1 (4.7 to 5.5)	2.8 (2.5 to 3.0)	1.23 (1.13 to 1.33)	0.66 (0.61 to 0.72)
2015	0.4 (0.4 to 0.5)	0.2 (0.2 to 0.3)	5.8 (5.3 to 6.3)	3.2 (2.9 to 3.5)	1.38 (1.27 to 1.51)	0.77 (0.70 to 0.83)
2020	0.4 (0.4 to 0.5)	0.2 (0.2 to 0.3)	5.8 (5.1 to 6.6)	3.3 (2.9 to 3.7)	1.59 (1.39 to 1.81)	0.90 (0.79 to 1.02)
2021	0.4 (0.3 to 0.5)	0.2 (0.2 to 0.3)	5.5 (4.4 to 6.8)	3.1 (2.5 to 3.9)	1.56 (1.24 to 1.92)	0.88 (0.71 to 1.09)

Montana USA_549 High−income North America Stillbirth Rate / Neonatal Mortality Rate (SBR/NMR) − 20 weeks
lambda = 0.2 ; zeta = 0.99 ; scale = 5 ; amp = 0.028

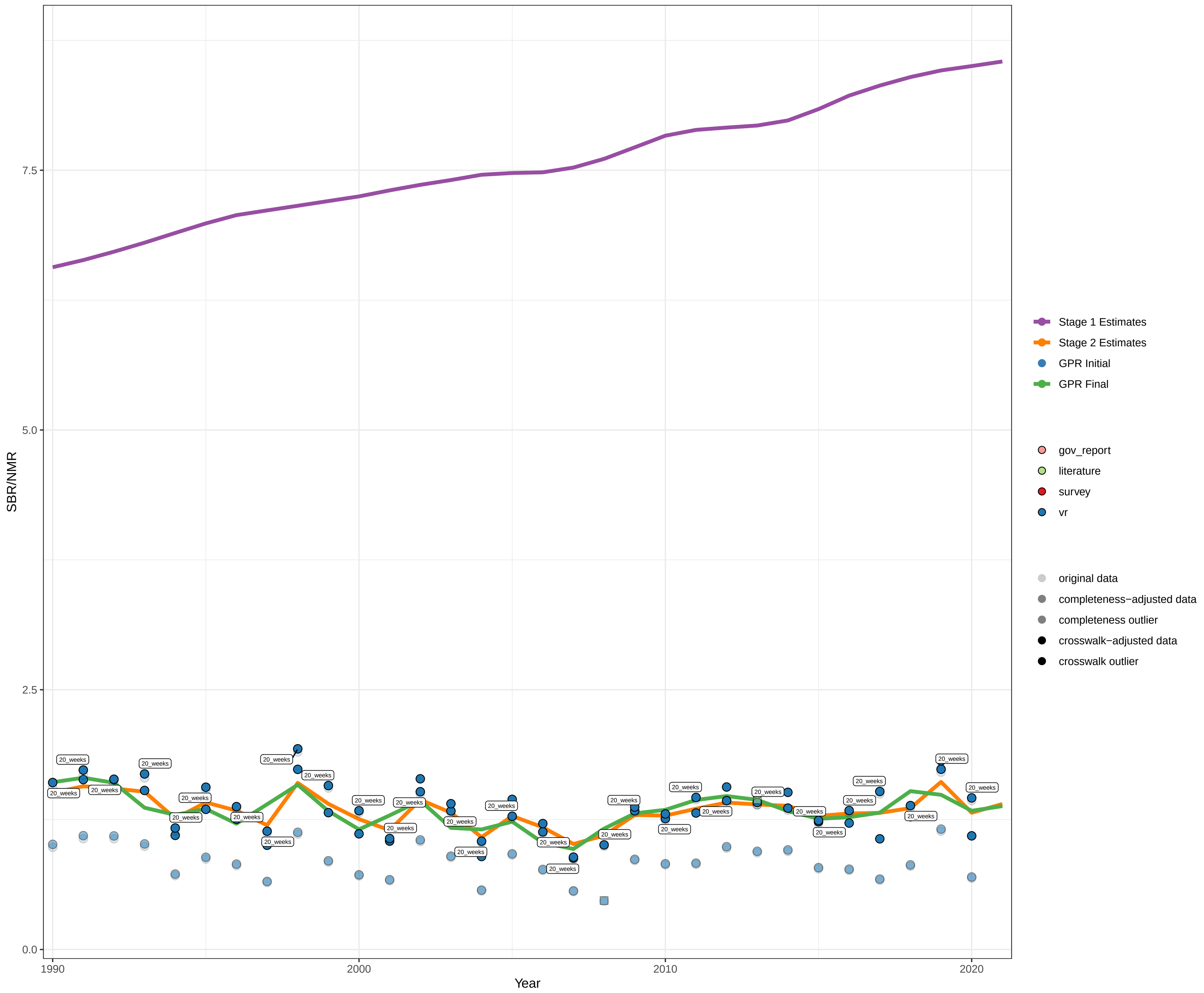

Montana USA_549 High−income North America Stillbirth Rate / Neonatal Mortality Rate (SBR/NMR) − 28 weeks
lambda = 0.2 ; zeta = 0.99 ; scale = 5 ; amp = 0.028

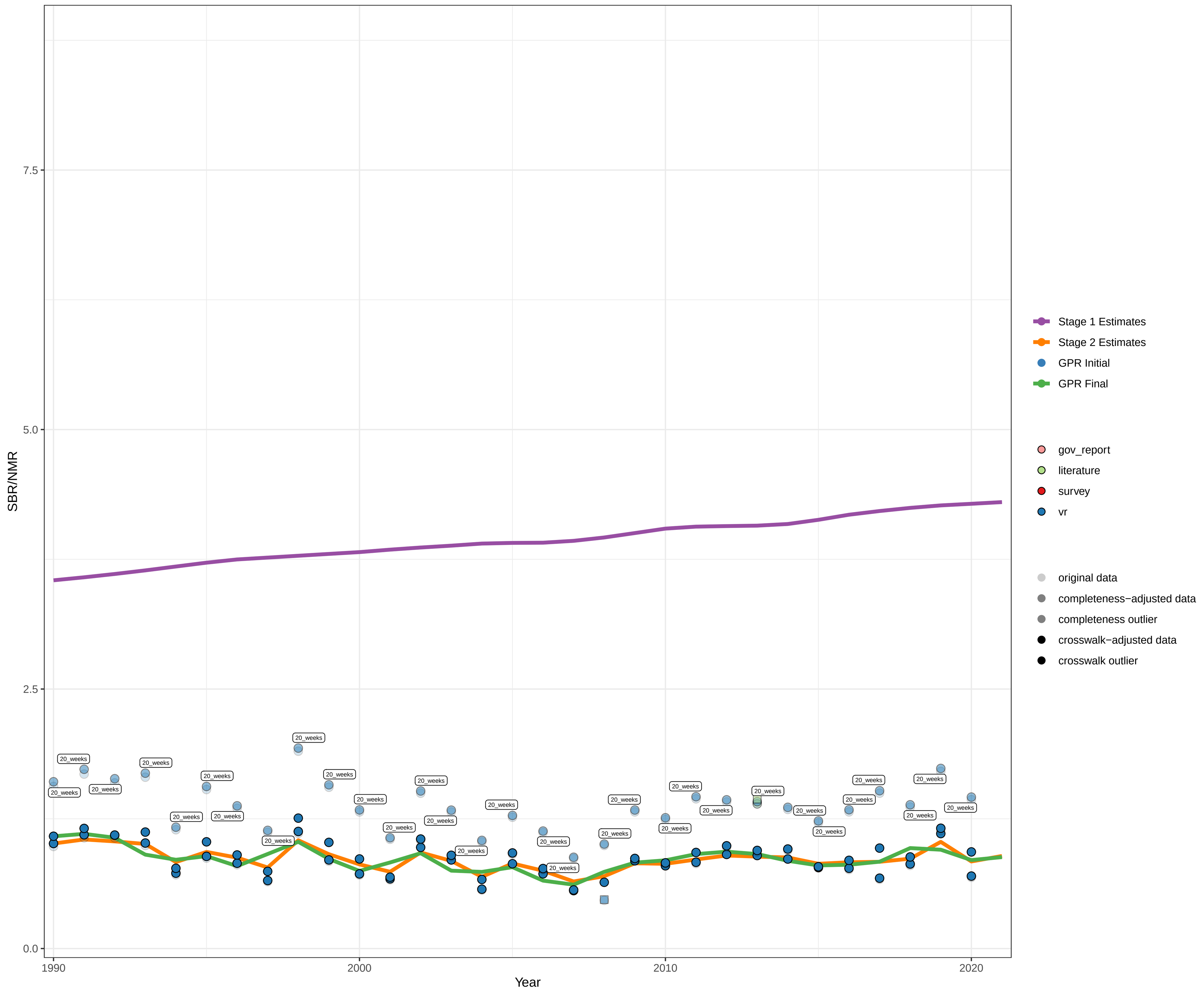

Definition	Years	Outlier	Source.Type	Title
20_weeks	1990−2020	0	VR	United States NVSS Custom Fetal Death Data
28_weeks	1990−2007, 2009−2020	0	VR	United States NVSS Custom Fetal Death Data
20_weeks	2013	1	Sci Lit	United States Fetal and Perinatal Mortality 2013
28_weeks	2008	1	VR	United States NVSS Custom Fetal Death Data

Montana USA_549 High−income North America Stillbirth Rate (SBR) − 20 weeks
lambda = 0.2 ; zeta = 0.99 ; scale = 5 ; amp = 0.028

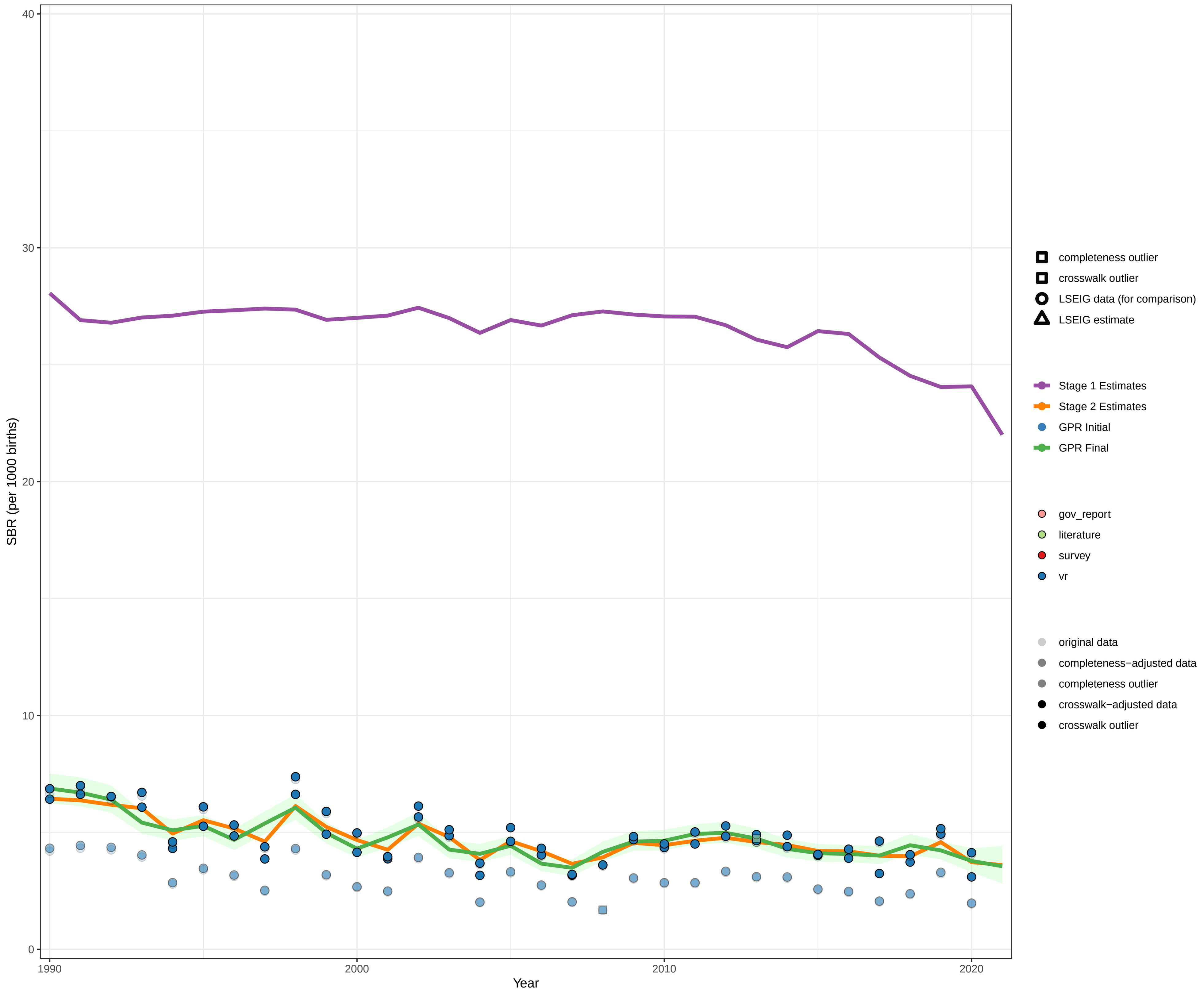

Montana USA_549 High−income North America Stillbirth Rate (SBR) − 28 weeks
lambda = 0.2 ; zeta = 0.99 ; scale = 5 ; amp = 0.028

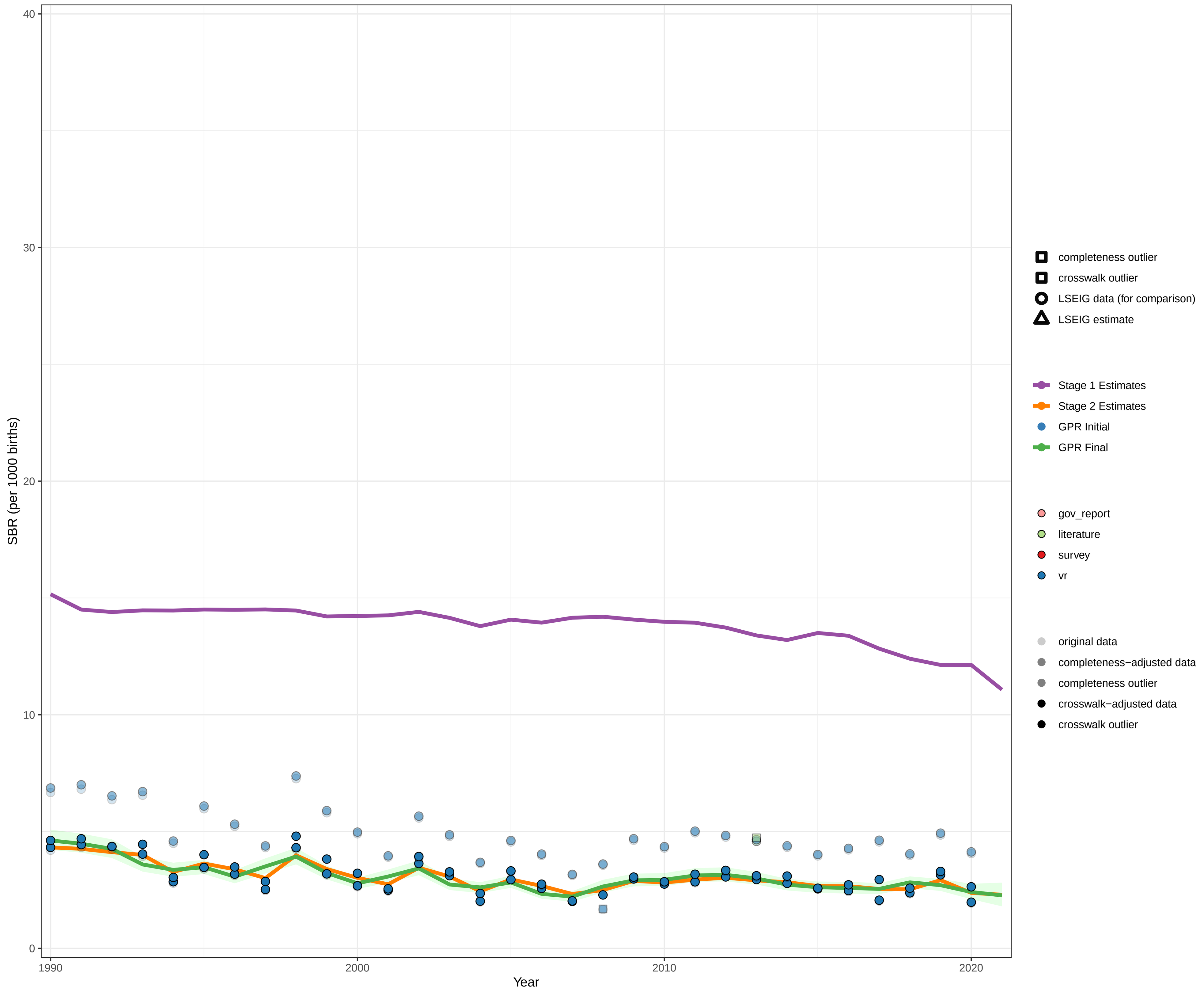

Year	Total Stillbirths (in thousands)	Total Stillbirths (in thousands)	SBR (per 1000 births)	SBR (per 1000 births)	SBR/NMR	SBR/NMR
	20 weeks	28 weeks	20 weeks	28 weeks	20 weeks	28 weeks
1990	0.1 (0.1 to 0.1)	0.1 (0.0 to 0.1)	6.9 (6.2 to 7.5)	4.6 (4.2 to 5.1)	1.61 (1.46 to 1.76)	1.08 (0.99 to 1.19)
1995	0.1 (0.1 to 0.1)	0.0 (0.0 to 0.0)	5.3 (4.8 to 5.7)	3.5 (3.2 to 3.8)	1.35 (1.24 to 1.47)	0.89 (0.82 to 0.97)
2000	0.0 (0.0 to 0.1)	0.0 (0.0 to 0.0)	4.3 (3.9 to 4.7)	2.8 (2.5 to 3.0)	1.16 (1.05 to 1.26)	0.75 (0.68 to 0.81)
2005	0.1 (0.0 to 0.1)	0.0 (0.0 to 0.0)	4.4 (4.0 to 4.9)	2.8 (2.6 to 3.1)	1.23 (1.12 to 1.35)	0.78 (0.72 to 0.86)
2010	0.1 (0.1 to 0.1)	0.0 (0.0 to 0.0)	4.6 (4.2 to 5.1)	2.9 (2.7 to 3.2)	1.34 (1.23 to 1.48)	0.85 (0.77 to 0.93)
2015	0.1 (0.0 to 0.1)	0.0 (0.0 to 0.0)	4.1 (3.8 to 4.5)	2.6 (2.4 to 2.9)	1.26 (1.15 to 1.38)	0.80 (0.73 to 0.88)
2020	0.0 (0.0 to 0.0)	0.0 (0.0 to 0.0)	3.8 (3.3 to 4.3)	2.4 (2.1 to 2.8)	1.33 (1.16 to 1.53)	0.85 (0.74 to 0.98)
2021	0.0 (0.0 to 0.0)	0.0 (0.0 to 0.0)	3.6 (2.8 to 4.4)	2.3 (1.8 to 2.8)	1.38 (1.09 to 1.71)	0.88 (0.70 to 1.09)

Nebraska USA_550 High−income North America Stillbirth Rate / Neonatal Mortality Rate (SBR/NMR) − 20 weeks
lambda = 0.2 ; zeta = 0.99 ; scale = 5 ; amp = 0.028

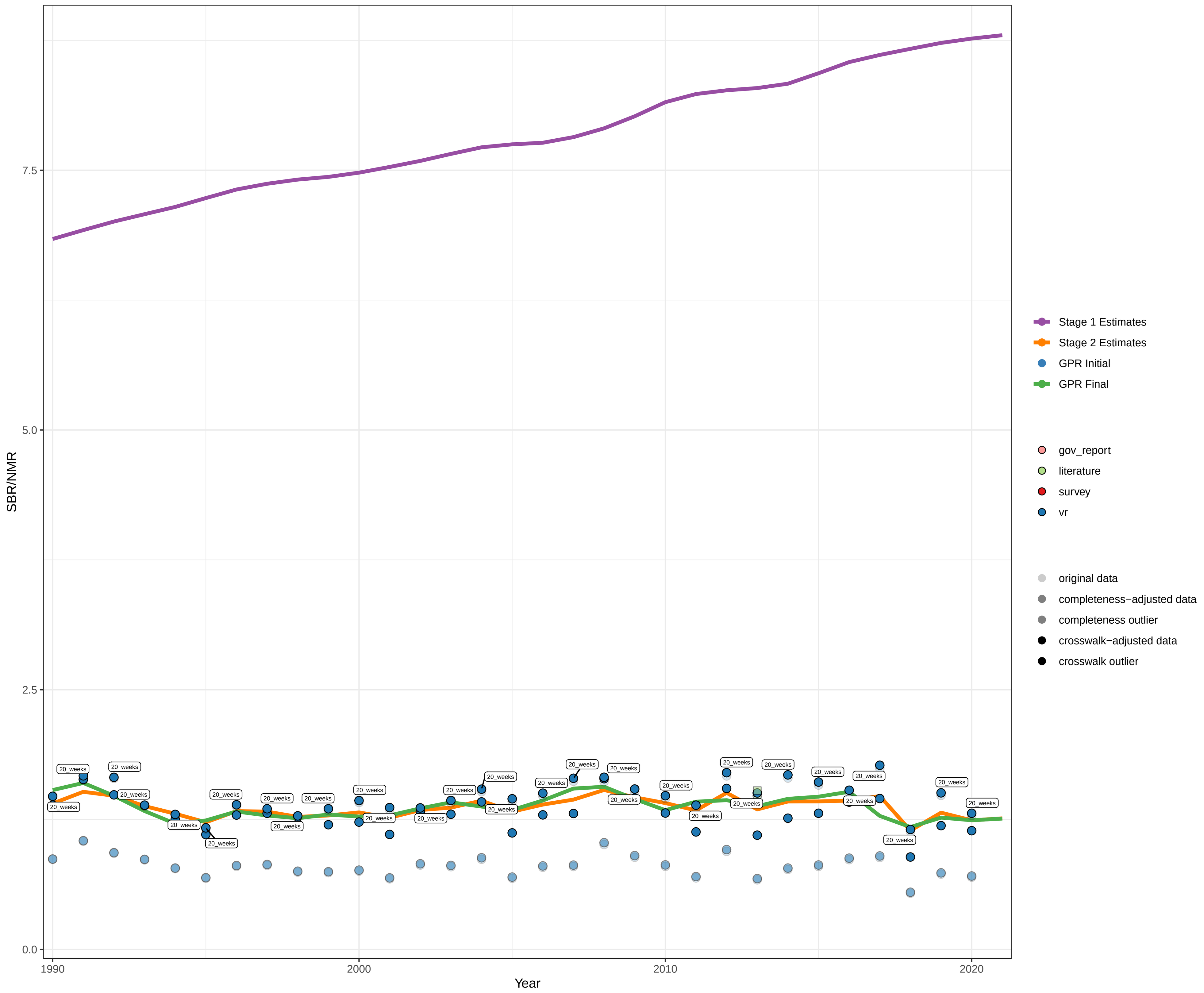

Nebraska USA_550 High−income North America Stillbirth Rate / Neonatal Mortality Rate (SBR/NMR) − 28 weeks
lambda = 0.2 ; zeta = 0.99 ; scale = 5 ; amp = 0.028

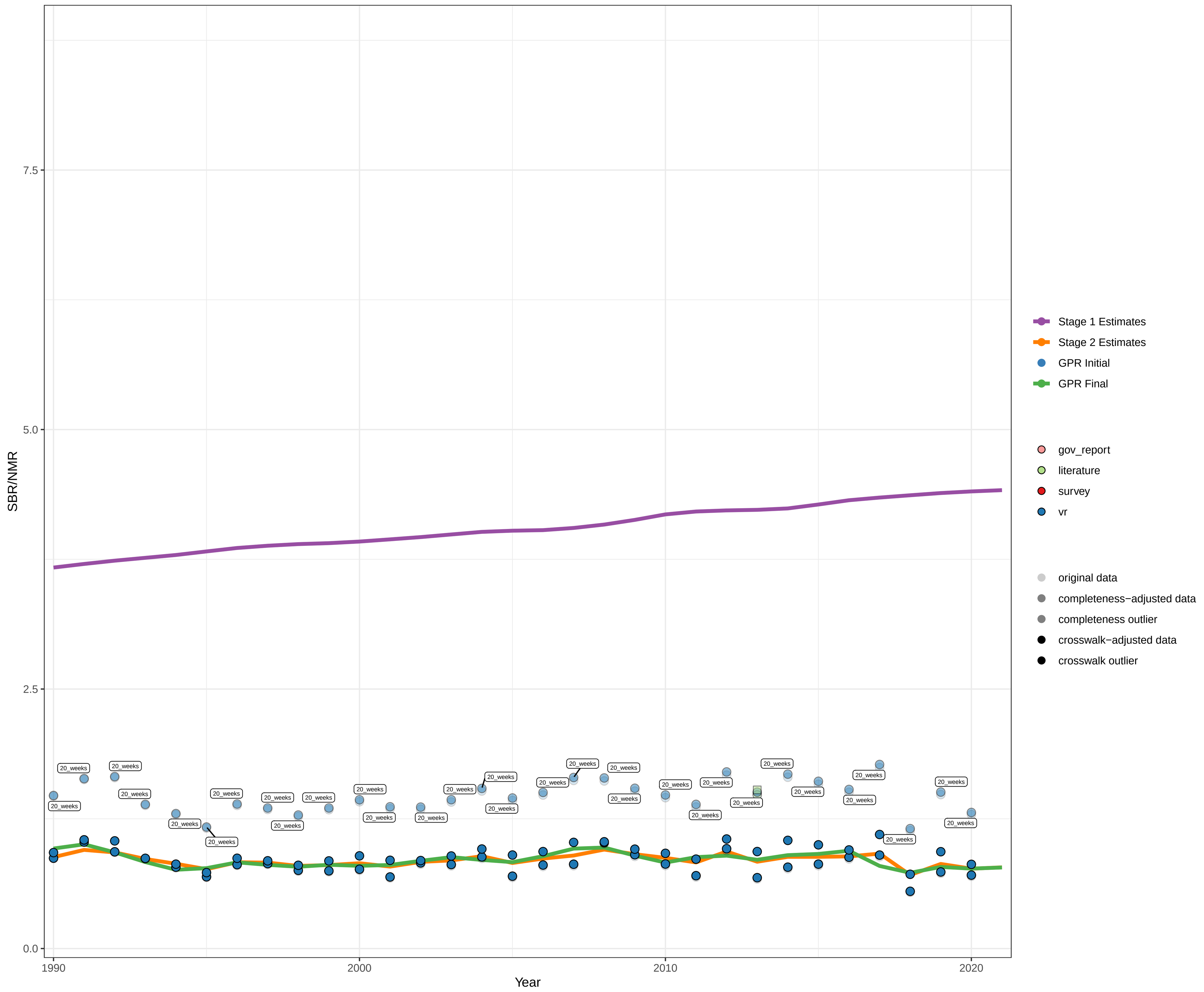

Definition	Years	Outlier	Source.Type	Title
20_weeks, 28_weeks	1990−2020	0	VR	United States NVSS Custom Fetal Death Data
20_weeks	2013	1	Sci Lit	United States Fetal and Perinatal Mortality 2013

Nebraska USA_550 High−income North America Stillbirth Rate (SBR) − 20 weeks
lambda = 0.2 ; zeta = 0.99 ; scale = 5 ; amp = 0.028

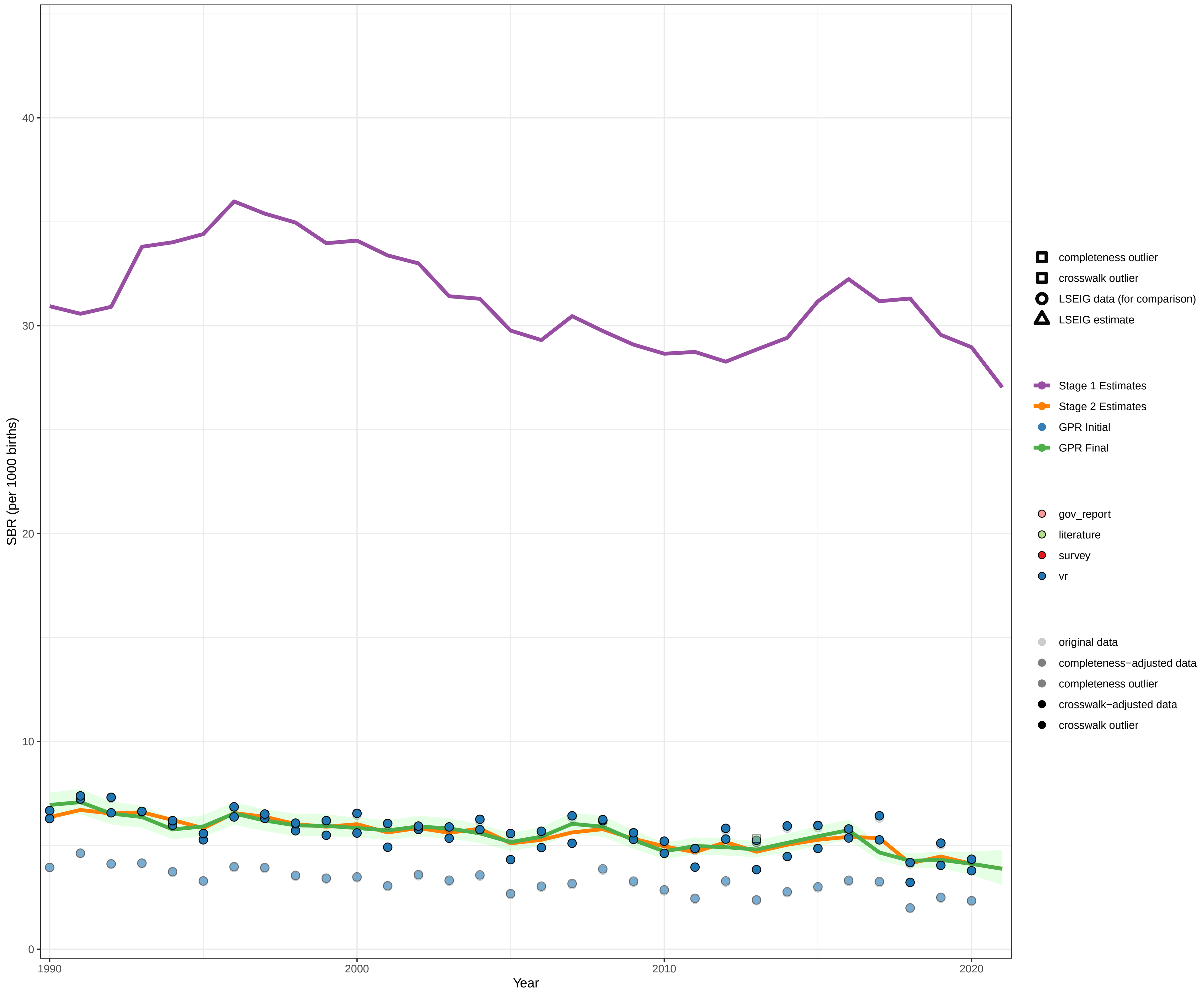

Nebraska USA_550 High−income North America Stillbirth Rate (SBR) − 28 weeks
lambda = 0.2 ; zeta = 0.99 ; scale = 5 ; amp = 0.028

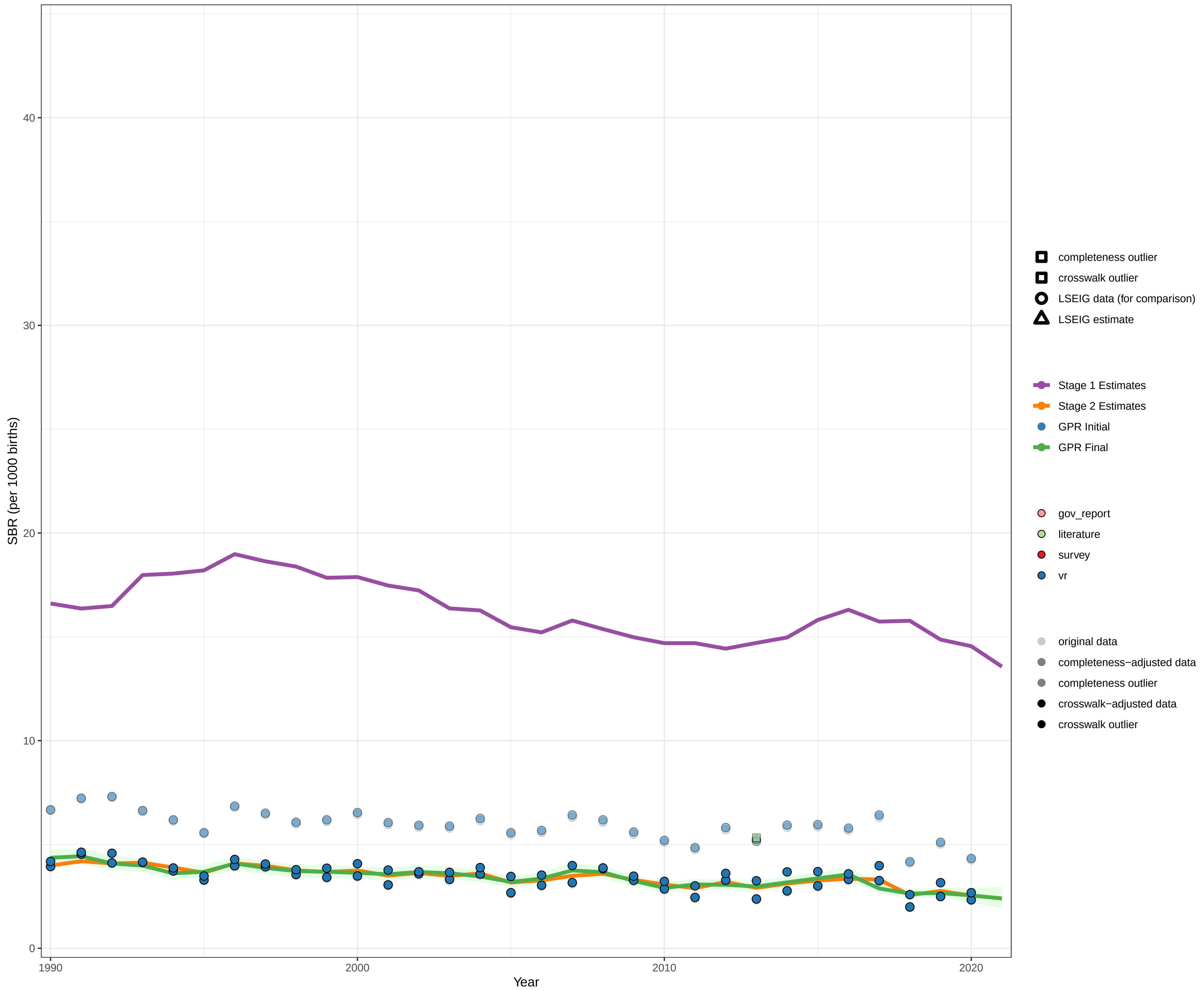

Year	Total Stillbirths (in thousands)	Total Stillbirths (in thousands)	SBR (per 1000 births)	SBR (per 1000 births)	SBR/NMR	SBR/NMR
	20 weeks	28 weeks	20 weeks	28 weeks	20 weeks	28 weeks
1990	0.2 (0.2 to 0.2)	0.1 (0.1 to 0.1)	6.9 (6.4 to 7.5)	4.4 (4.0 to 4.7)	1.53 (1.41 to 1.67)	0.96 (0.89 to 1.04)
1995	0.1 (0.1 to 0.2)	0.1 (0.1 to 0.1)	5.9 (5.4 to 6.4)	3.7 (3.4 to 4.0)	1.24 (1.14 to 1.35)	0.77 (0.71 to 0.84)
2000	0.1 (0.1 to 0.2)	0.1 (0.1 to 0.1)	5.8 (5.4 to 6.3)	3.6 (3.3 to 3.9)	1.28 (1.18 to 1.39)	0.80 (0.73 to 0.86)
2005	0.1 (0.1 to 0.1)	0.1 (0.1 to 0.1)	5.2 (4.7 to 5.6)	3.2 (2.9 to 3.5)	1.34 (1.23 to 1.46)	0.83 (0.76 to 0.90)
2010	0.1 (0.1 to 0.1)	0.1 (0.1 to 0.1)	4.7 (4.3 to 5.1)	2.9 (2.7 to 3.2)	1.34 (1.23 to 1.46)	0.83 (0.76 to 0.90)
2015	0.1 (0.1 to 0.2)	0.1 (0.1 to 0.1)	5.4 (5.0 to 5.9)	3.4 (3.1 to 3.7)	1.47 (1.34 to 1.60)	0.91 (0.84 to 0.99)
2020	0.1 (0.1 to 0.1)	0.1 (0.1 to 0.1)	4.1 (3.6 to 4.7)	2.5 (2.2 to 2.9)	1.24 (1.08 to 1.42)	0.77 (0.67 to 0.88)
2021	0.1 (0.1 to 0.1)	0.1 (0.0 to 0.1)	3.9 (3.1 to 4.8)	2.4 (1.9 to 3.0)	1.26 (1.00 to 1.56)	0.78 (0.62 to 0.96)

Nevada USA_551 High−income North America Stillbirth Rate / Neonatal Mortality Rate (SBR/NMR) − 20 weeks
lambda = 0.2 ; zeta = 0.99 ; scale = 5 ; amp = 0.028

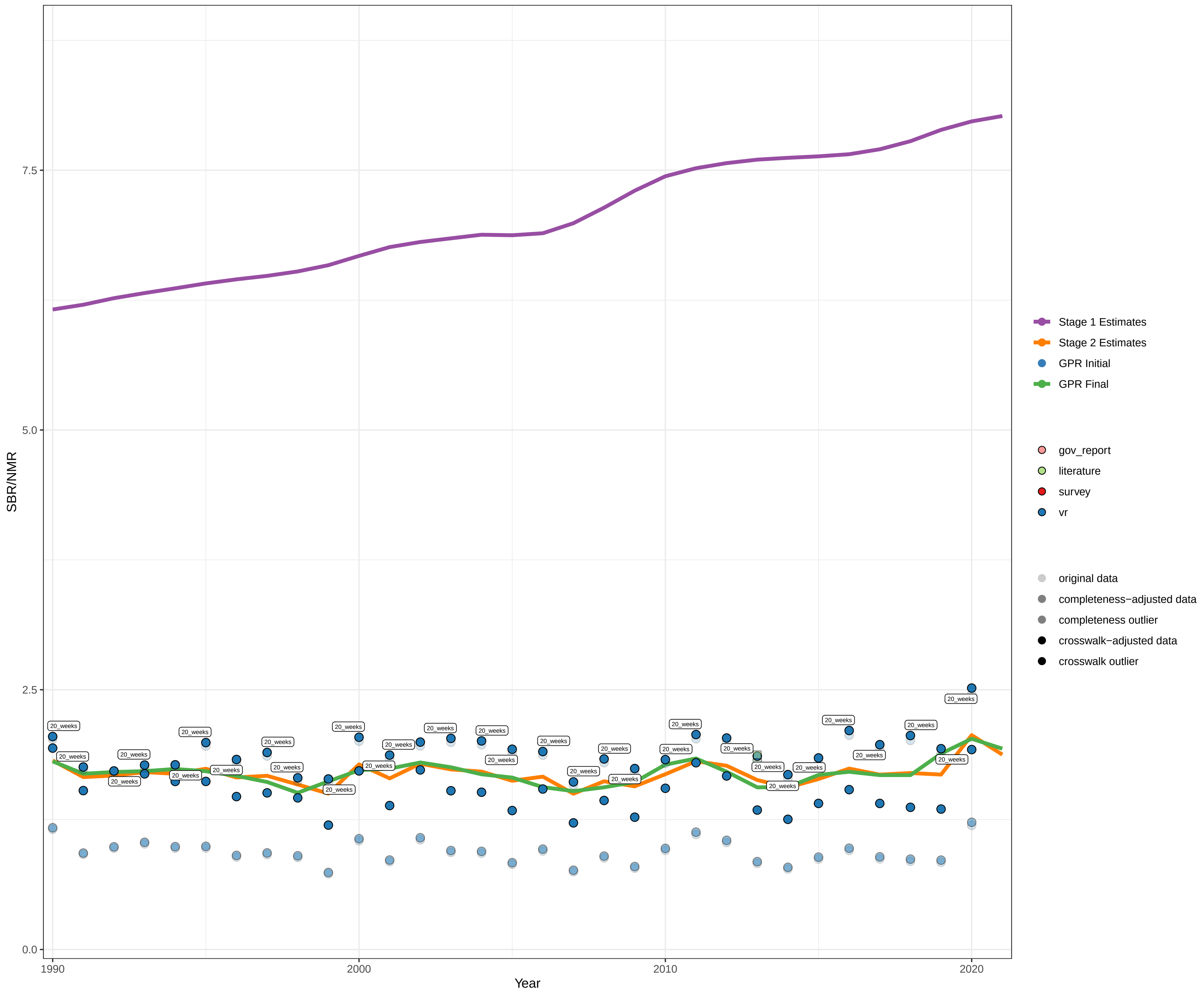

Nevada USA_551 High−income North America Stillbirth Rate / Neonatal Mortality Rate (SBR/NMR) − 28 weeks
lambda = 0.2 ; zeta = 0.99 ; scale = 5 ; amp = 0.028

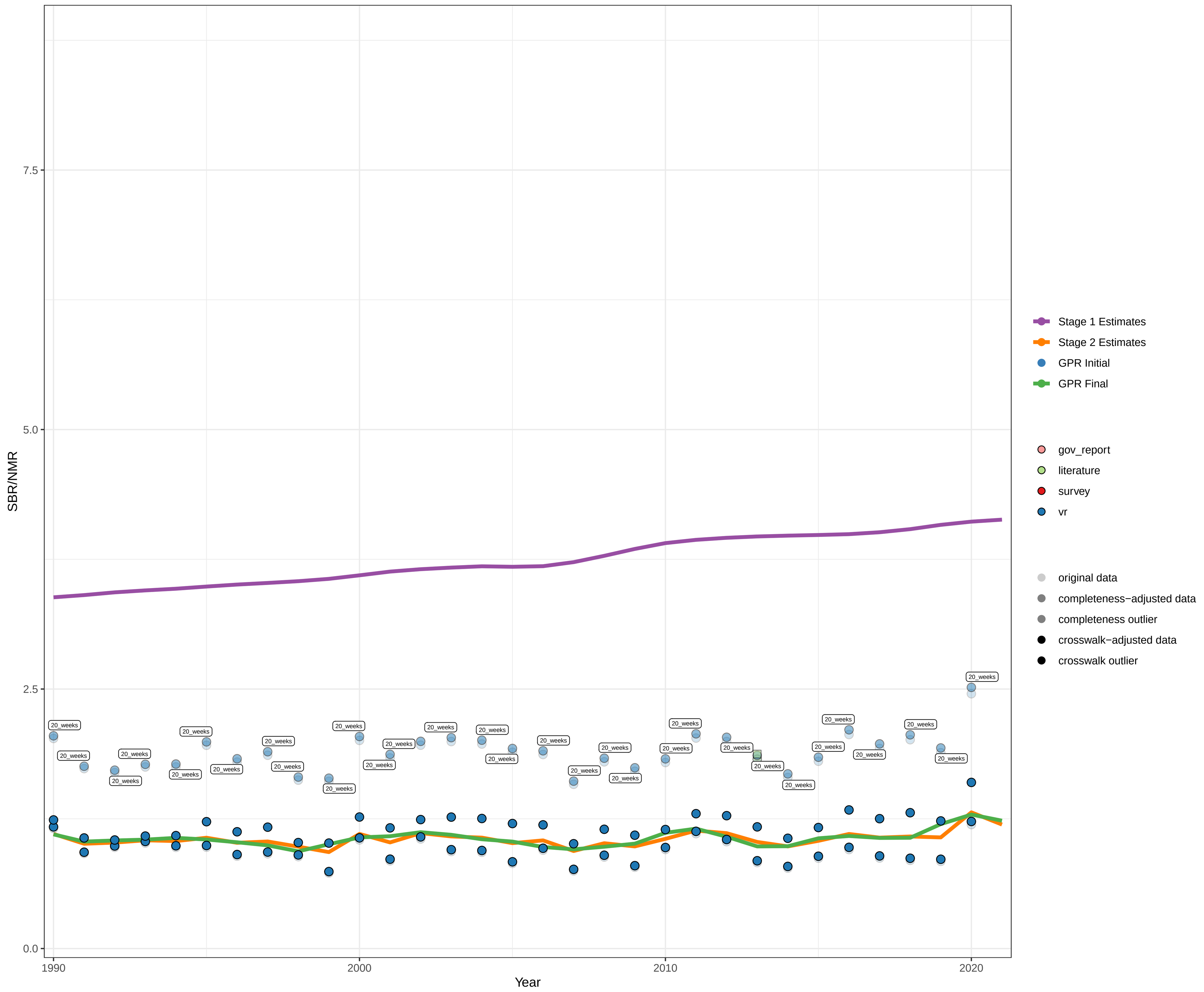

Definition	Years	Outlier	Source.Type	Title
20_weeks, 28_weeks	1990−2020	0	VR	United States NVSS Custom Fetal Death Data
20_weeks	2013	1	Sci Lit	United States Fetal and Perinatal Mortality 2013

Nevada USA_551 High−income North America Stillbirth Rate (SBR) − 20 weeks
lambda = 0.2 ; zeta = 0.99 ; scale = 5 ; amp = 0.028

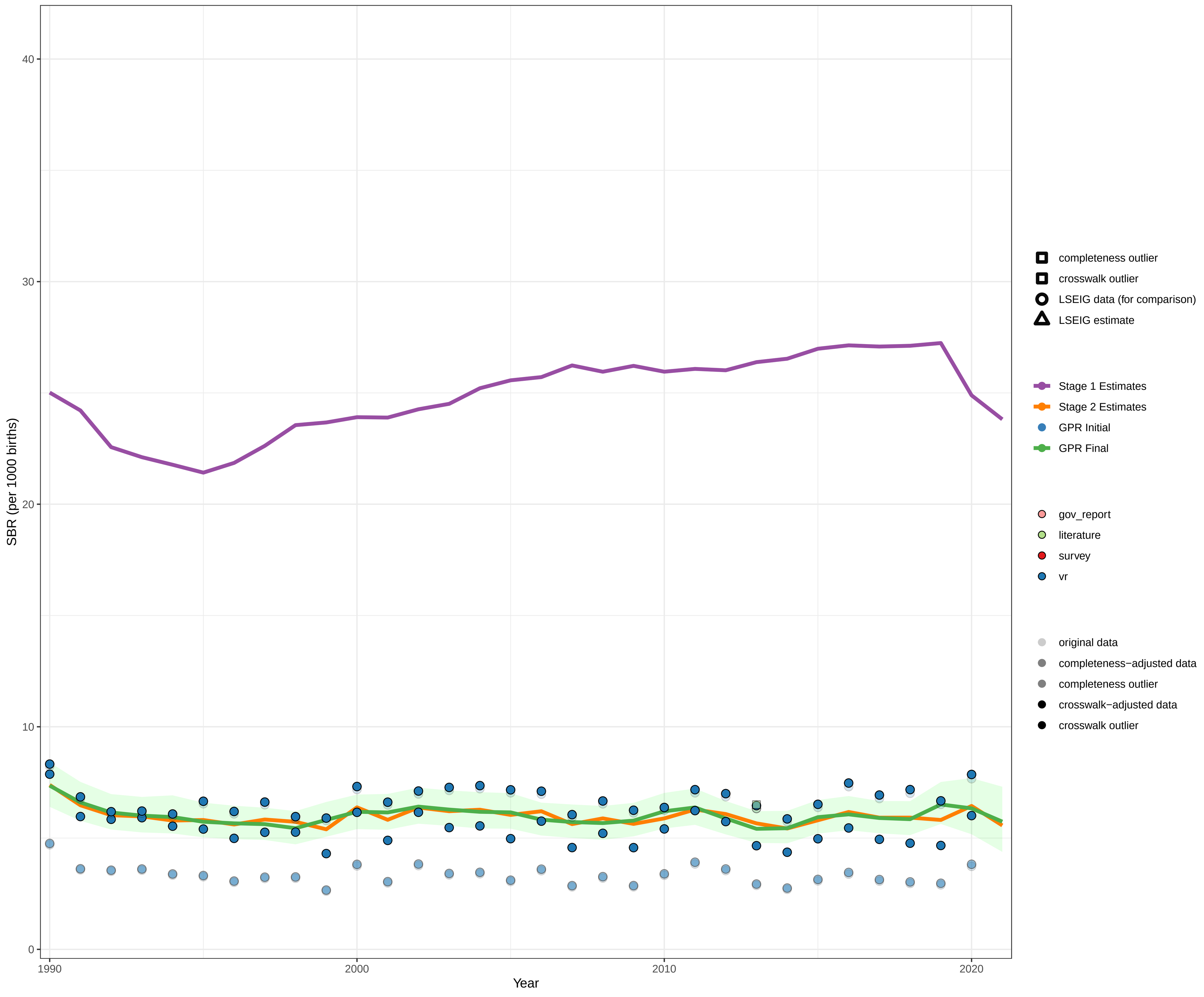

Nevada USA_551 High−income North America Stillbirth Rate (SBR) − 28 weeks
lambda = 0.2 ; zeta = 0.99 ; scale = 5 ; amp = 0.028

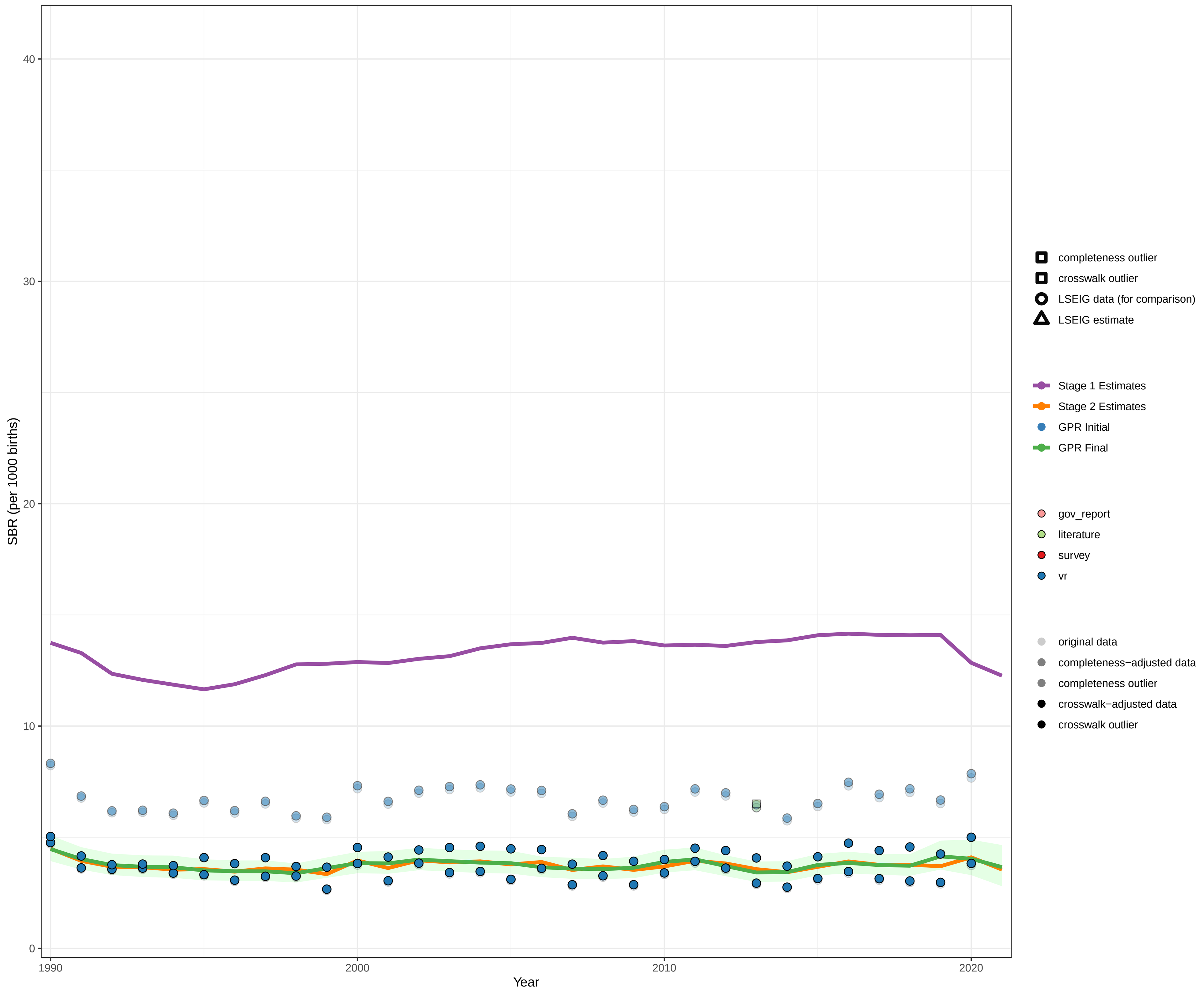

Year	Total Stillbirths (in thousands)	Total Stillbirths (in thousands)	SBR (per 1000 births)	SBR (per 1000 births)	SBR/NMR	SBR/NMR
	20 weeks	28 weeks	20 weeks	28 weeks	20 weeks	28 weeks
1990	0.2 (0.1 to 0.2)	0.1 (0.1 to 0.1)	7.4 (6.4 to 8.4)	4.5 (3.9 to 5.1)	1.81 (1.58 to 2.07)	1.10 (0.97 to 1.25)
1995	0.1 (0.1 to 0.2)	0.1 (0.1 to 0.1)	5.7 (5.0 to 6.6)	3.5 (3.1 to 4.0)	1.72 (1.51 to 1.97)	1.05 (0.92 to 1.20)
2000	0.2 (0.2 to 0.2)	0.1 (0.1 to 0.1)	6.2 (5.4 to 7.0)	3.8 (3.4 to 4.3)	1.73 (1.51 to 1.94)	1.07 (0.94 to 1.21)
2005	0.2 (0.2 to 0.3)	0.1 (0.1 to 0.2)	6.2 (5.4 to 7.0)	3.8 (3.4 to 4.4)	1.65 (1.46 to 1.89)	1.03 (0.91 to 1.18)
2010	0.2 (0.2 to 0.2)	0.1 (0.1 to 0.2)	6.2 (5.4 to 7.0)	3.9 (3.4 to 4.4)	1.78 (1.56 to 2.02)	1.11 (0.98 to 1.27)
2015	0.2 (0.2 to 0.2)	0.1 (0.1 to 0.2)	5.9 (5.2 to 6.7)	3.8 (3.3 to 4.2)	1.68 (1.47 to 1.90)	1.06 (0.93 to 1.20)
2020	0.2 (0.2 to 0.3)	0.1 (0.1 to 0.2)	6.3 (5.2 to 7.7)	4.0 (3.3 to 4.9)	2.03 (1.66 to 2.46)	1.29 (1.05 to 1.57)
2021	0.2 (0.1 to 0.2)	0.1 (0.1 to 0.2)	5.7 (4.4 to 7.3)	3.7 (2.8 to 4.6)	1.93 (1.47 to 2.46)	1.23 (0.94 to 1.57)

New Hampshire USA_552 High−income North America Stillbirth Rate / Neonatal Mortality Rate (SBR/NMR) − 20 weeks
lambda = 0.2 ; zeta = 0.99 ; scale = 5 ; amp = 0.028

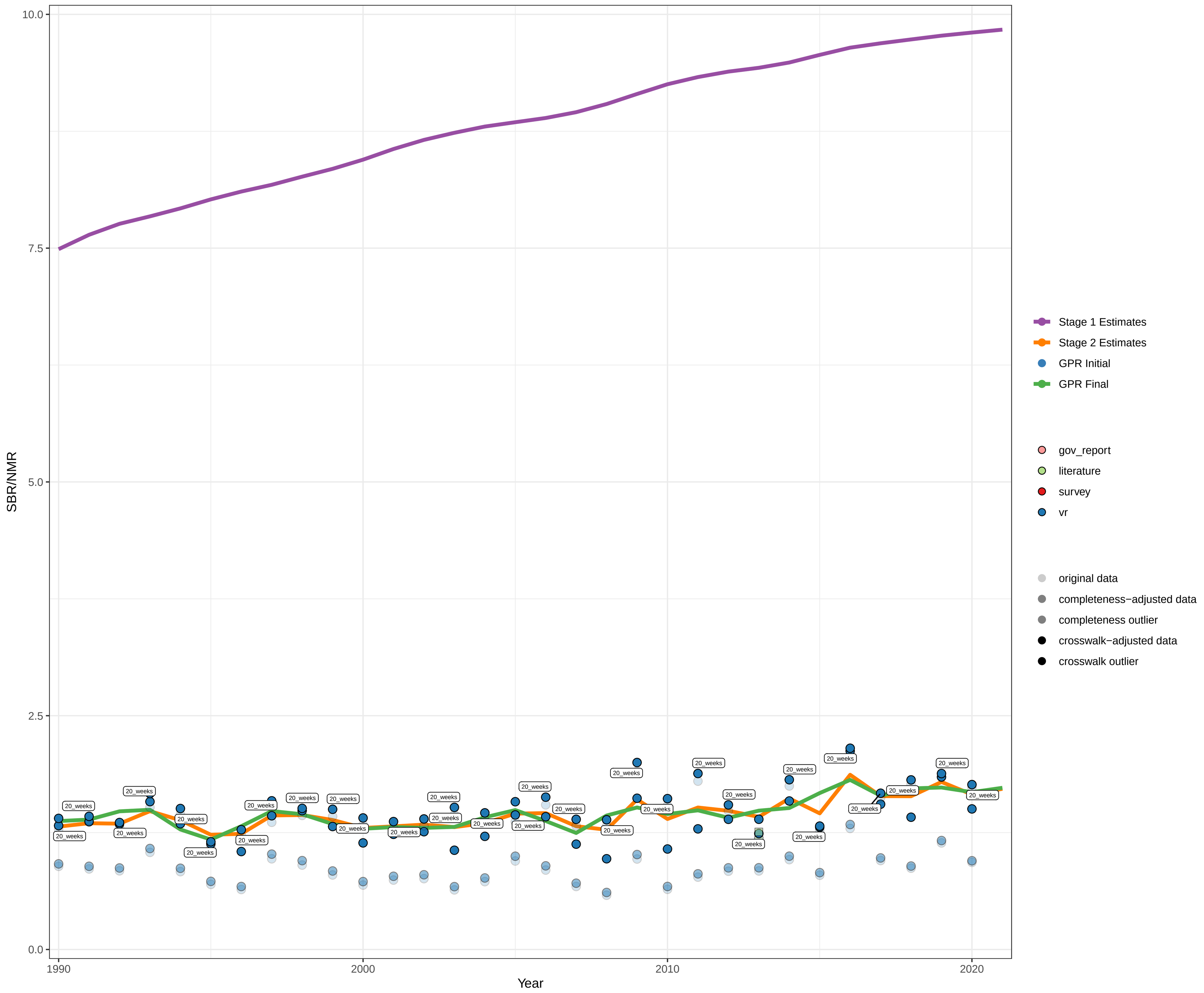

New Hampshire USA_552 High−income North America Stillbirth Rate / Neonatal Mortality Rate (SBR/NMR) − 28 weeks
lambda = 0.2 ; zeta = 0.99 ; scale = 5 ; amp = 0.028

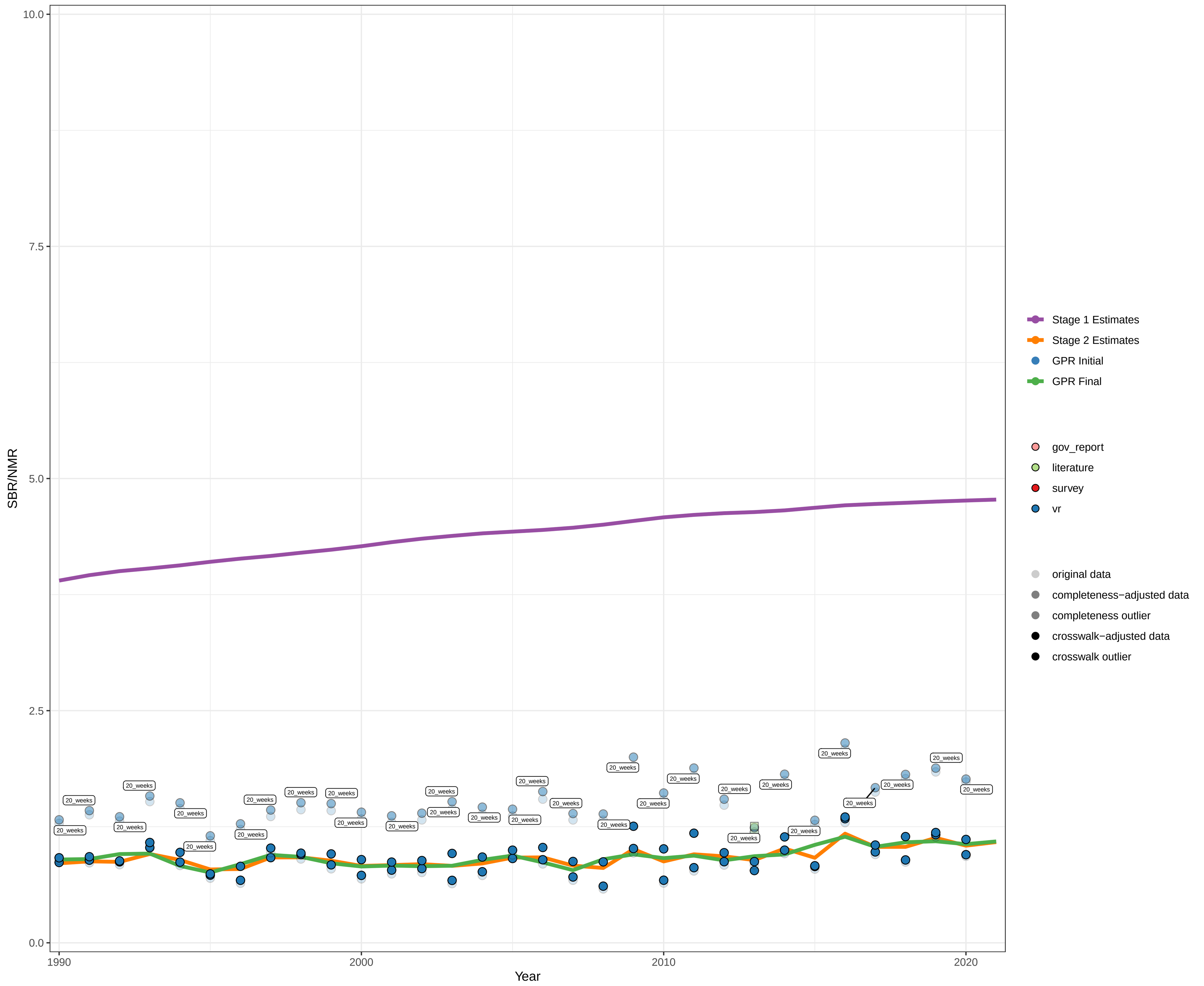

Definition	Years	Outlier	Source.Type	Title
20_weeks, 28_weeks	1990−2020	0	VR	United States NVSS Custom Fetal Death Data
20_weeks	2013	1	Sci Lit	United States Fetal and Perinatal Mortality 2013

New Hampshire USA_552 High−income North America Stillbirth Rate (SBR) − 20 weeks
lambda = 0.2 ; zeta = 0.99 ; scale = 5 ; amp = 0.028

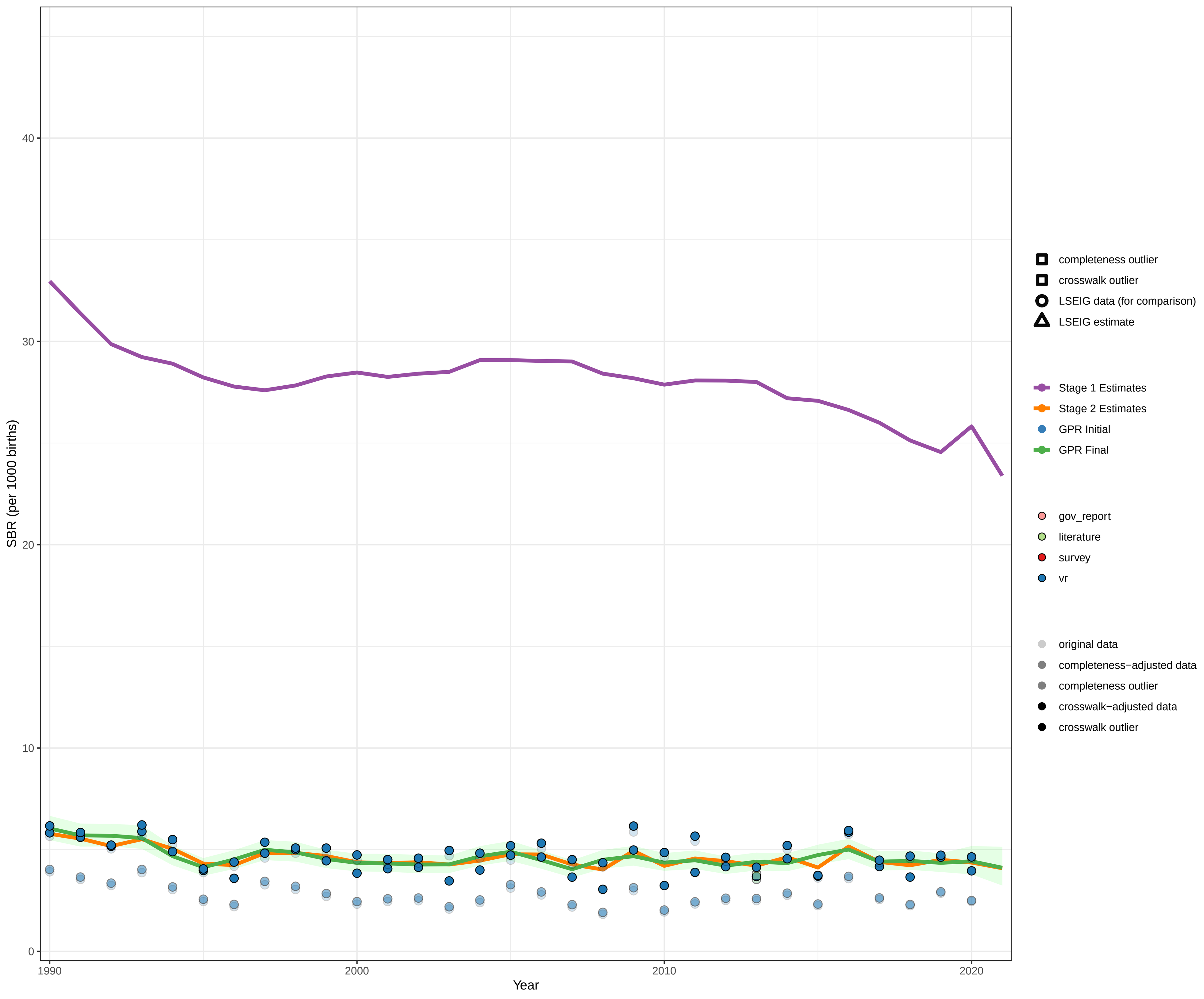

New Hampshire USA_552 High−income North America Stillbirth Rate (SBR) − 28 weeks
lambda = 0.2 ; zeta = 0.99 ; scale = 5 ; amp = 0.028

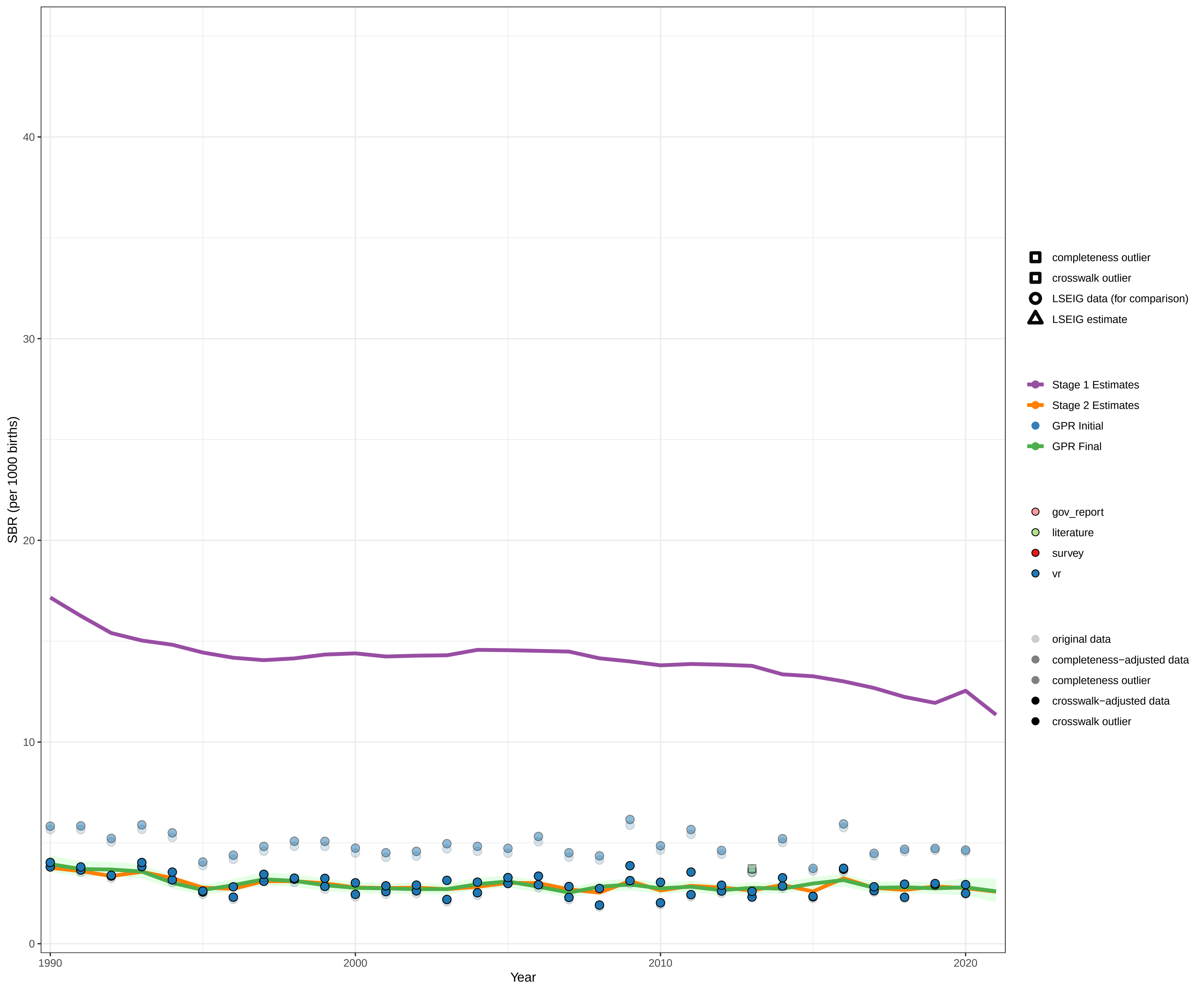

Year	Total Stillbirths (in thousands)	Total Stillbirths (in thousands)	SBR (per 1000 births)	SBR (per 1000 births)	SBR/NMR	SBR/NMR
	20 weeks	28 weeks	20 weeks	28 weeks	20 weeks	28 weeks
1990	0.1 (0.1 to 0.1)	0.1 (0.1 to 0.1)	6.0 (5.5 to 6.7)	3.9 (3.6 to 4.4)	1.37 (1.24 to 1.51)	0.90 (0.81 to 0.99)
1995	0.1 (0.1 to 0.1)	0.0 (0.0 to 0.0)	4.1 (3.7 to 4.6)	2.7 (2.4 to 2.9)	1.18 (1.06 to 1.30)	0.76 (0.68 to 0.83)
2000	0.1 (0.1 to 0.1)	0.0 (0.0 to 0.0)	4.4 (3.9 to 4.8)	2.8 (2.5 to 3.1)	1.29 (1.17 to 1.42)	0.82 (0.74 to 0.91)
2005	0.1 (0.1 to 0.1)	0.0 (0.0 to 0.0)	4.9 (4.4 to 5.4)	3.1 (2.8 to 3.4)	1.49 (1.35 to 1.65)	0.94 (0.85 to 1.03)
2010	0.1 (0.1 to 0.1)	0.0 (0.0 to 0.0)	4.4 (4.0 to 4.8)	2.7 (2.5 to 3.0)	1.45 (1.31 to 1.60)	0.91 (0.82 to 1.00)
2015	0.1 (0.1 to 0.1)	0.0 (0.0 to 0.0)	4.7 (4.3 to 5.2)	3.0 (2.7 to 3.3)	1.67 (1.51 to 1.85)	1.05 (0.95 to 1.17)
2020	0.1 (0.0 to 0.1)	0.0 (0.0 to 0.0)	4.4 (3.8 to 5.2)	2.8 (2.4 to 3.3)	1.68 (1.44 to 1.97)	1.06 (0.91 to 1.24)
2021	0.1 (0.0 to 0.1)	0.0 (0.0 to 0.0)	4.1 (3.2 to 5.1)	2.6 (2.0 to 3.2)	1.73 (1.36 to 2.16)	1.09 (0.86 to 1.36)

New Jersey USA_553 High−income North America Stillbirth Rate / Neonatal Mortality Rate (SBR/NMR) − 20 weeks
lambda = 0.2 ; zeta = 0.99 ; scale = 5 ; amp = 0.028

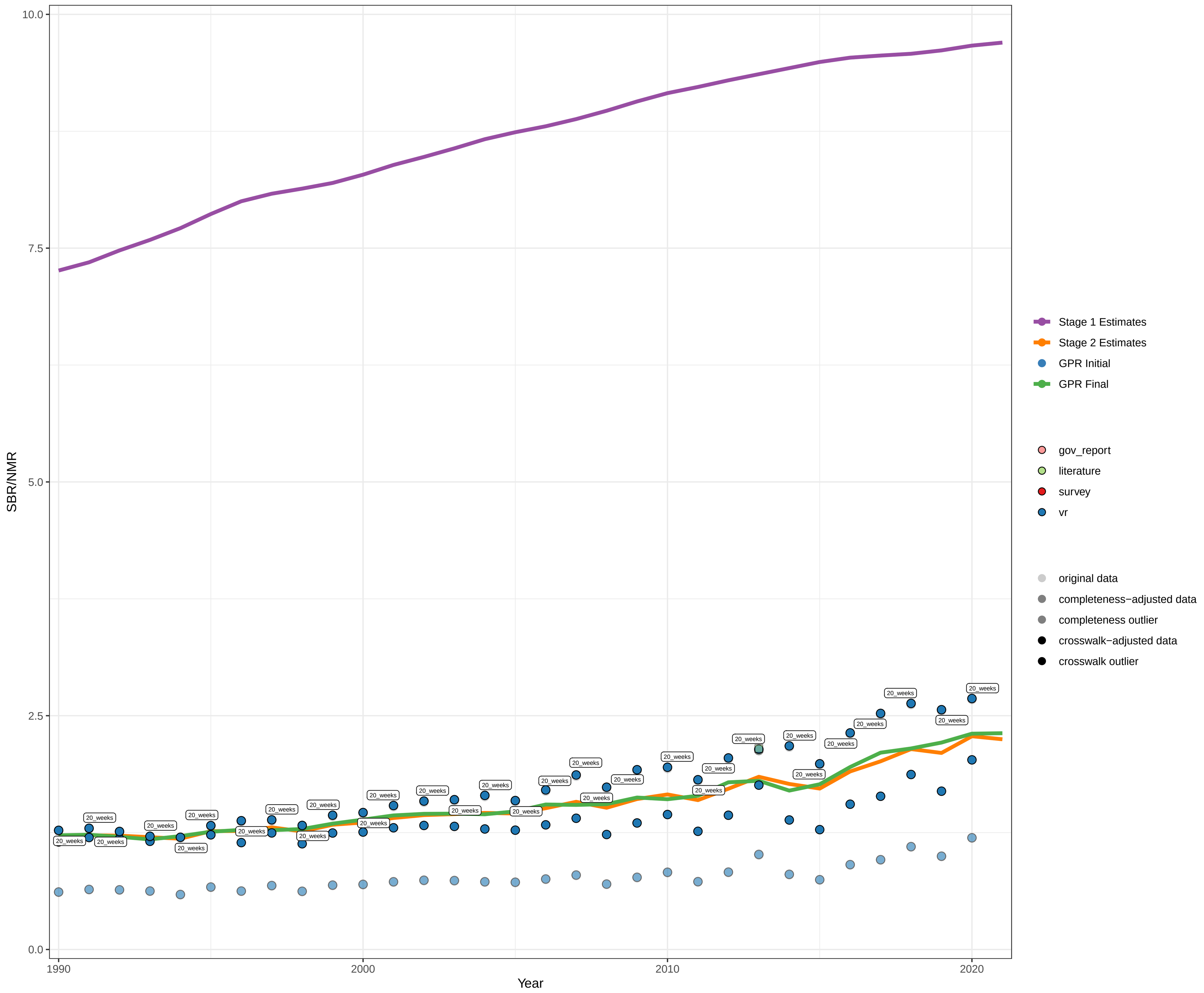

New Jersey USA_553 High−income North America Stillbirth Rate / Neonatal Mortality Rate (SBR/NMR) − 28 weeks
lambda = 0.2 ; zeta = 0.99 ; scale = 5 ; amp = 0.028

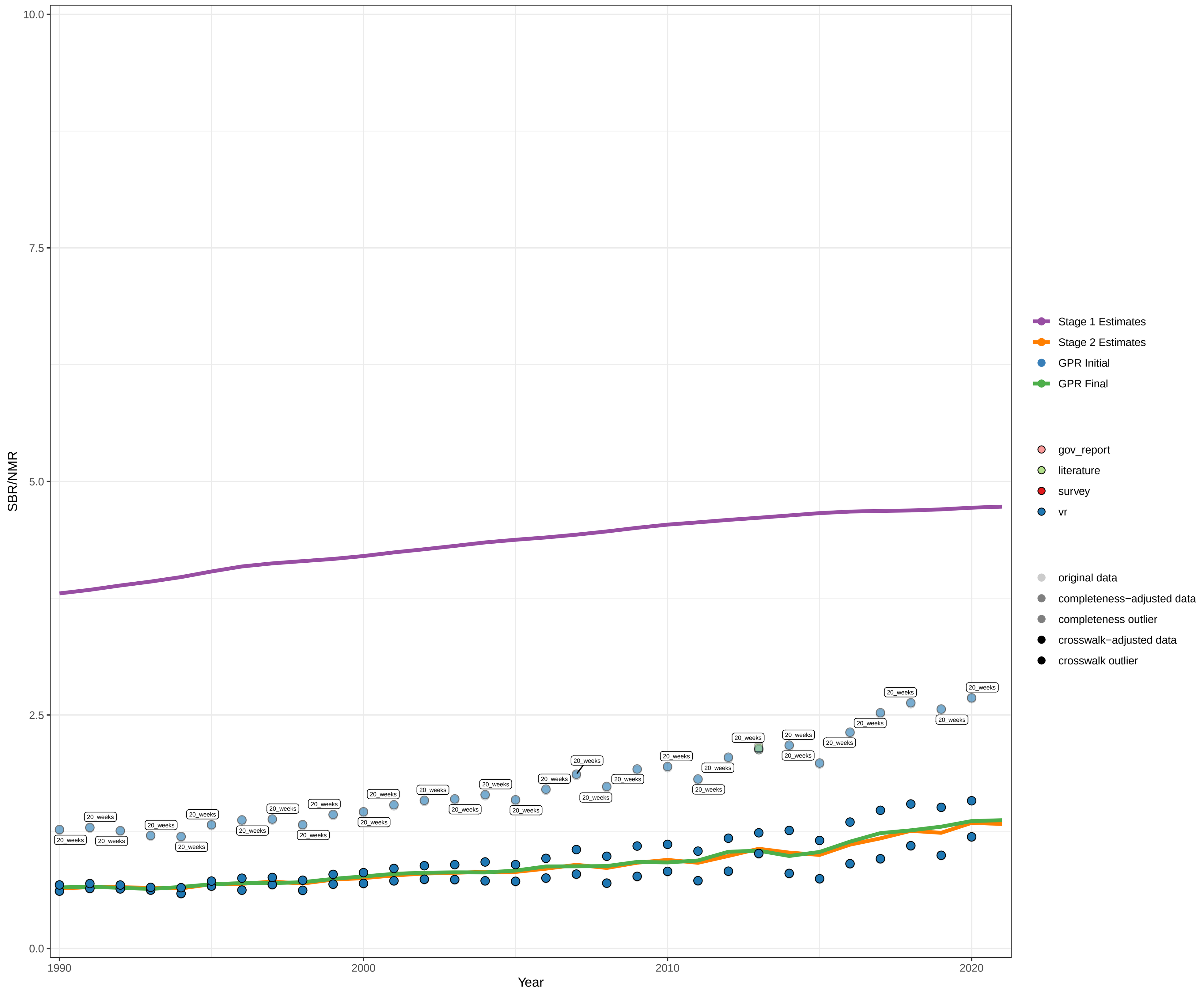

Definition	Years	Outlier	Source.Type	Title
20_weeks, 28_weeks	1990−2020	0	VR	United States NVSS Custom Fetal Death Data
20_weeks	2013	1	Sci Lit	United States Fetal and Perinatal Mortality 2013

New Jersey USA_553 High−income North America Stillbirth Rate (SBR) − 20 weeks
lambda = 0.2 ; zeta = 0.99 ; scale = 5 ; amp = 0.028

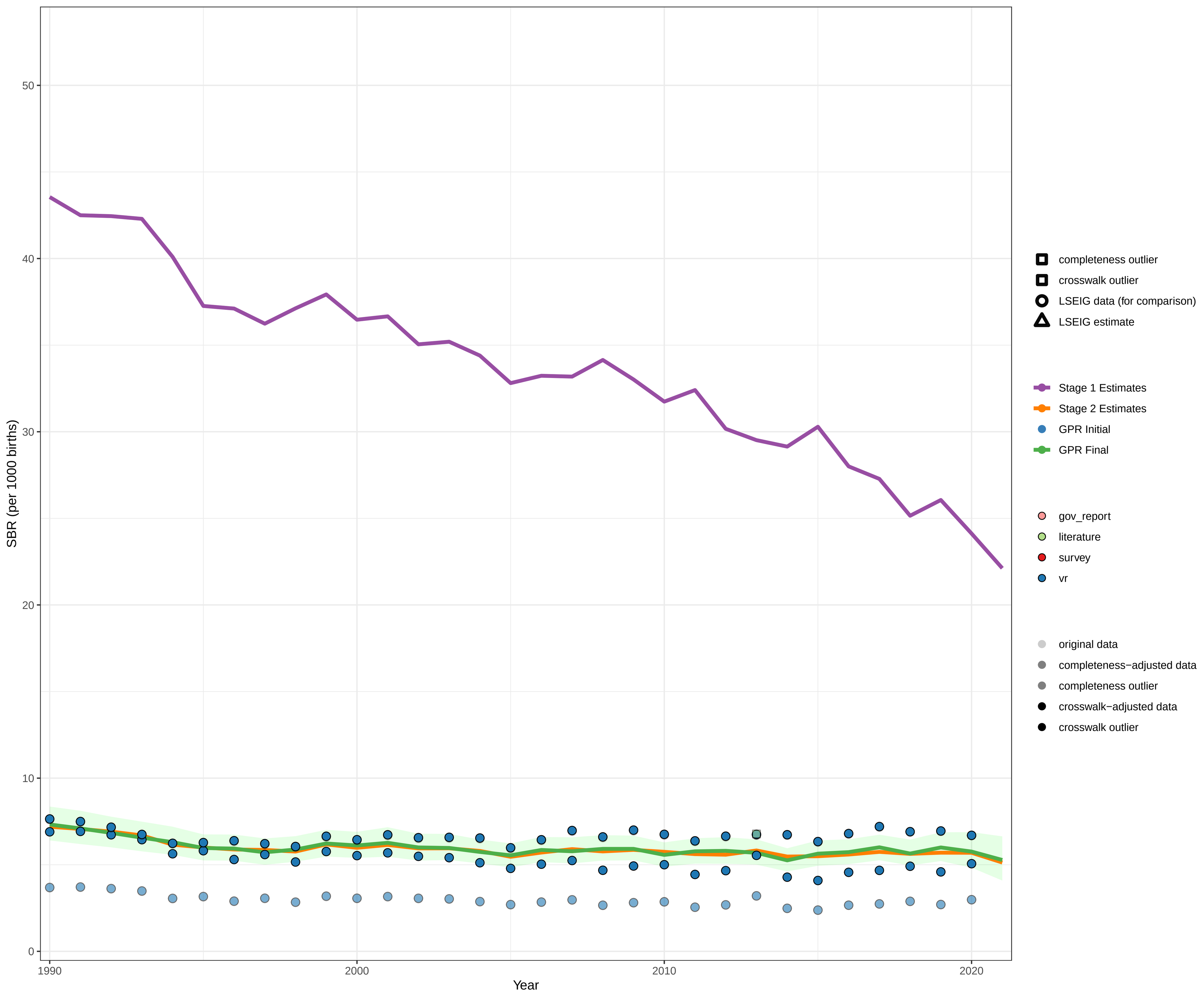

New Jersey USA_553 High−income North America Stillbirth Rate (SBR) − 28 weeks
lambda = 0.2 ; zeta = 0.99 ; scale = 5 ; amp = 0.028

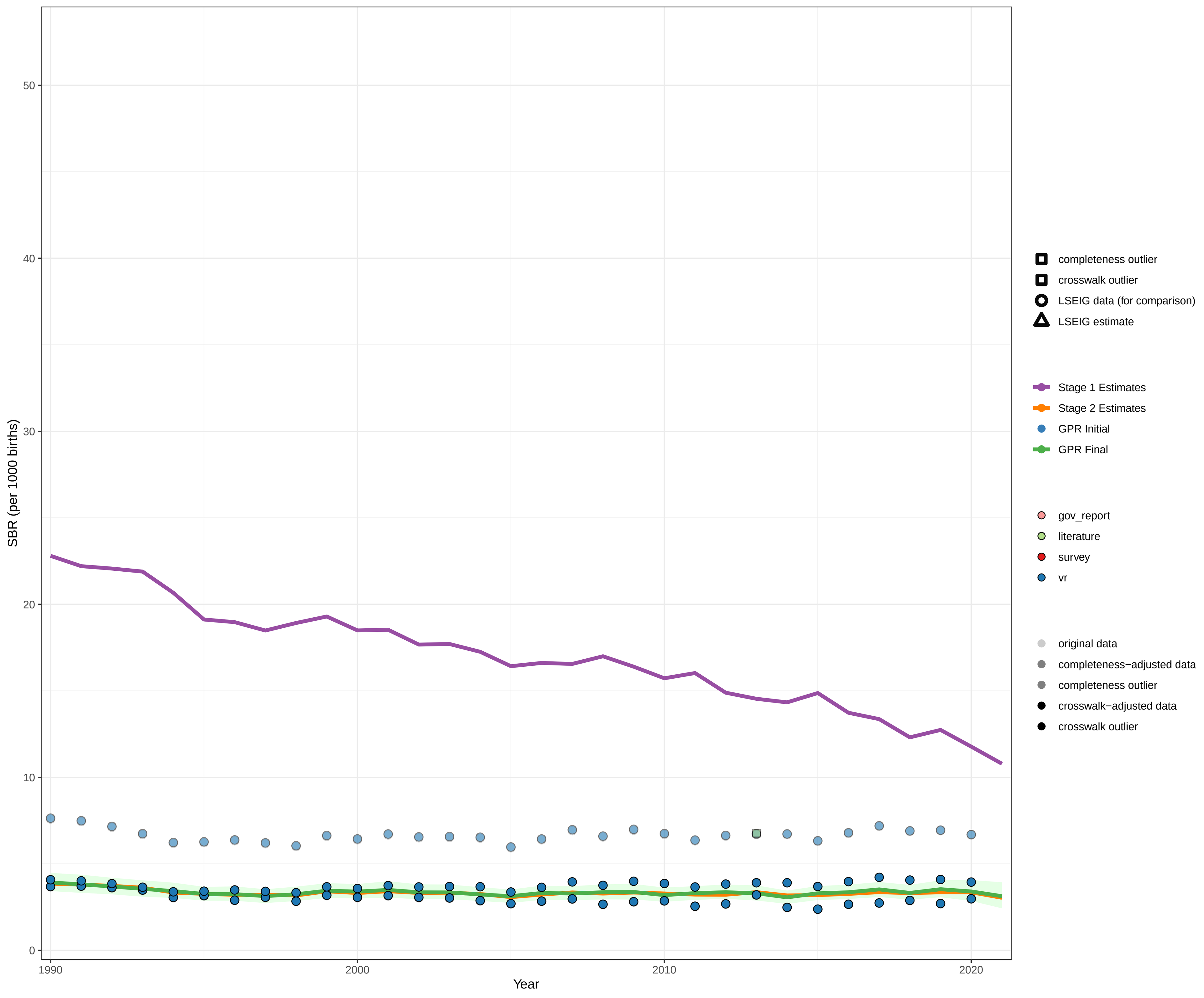

Year	Total Stillbirths (in thousands)	Total Stillbirths (in thousands)	SBR (per 1000 births)	SBR (per 1000 births)	SBR/NMR	SBR/NMR
	20 weeks	28 weeks	20 weeks	28 weeks	20 weeks	28 weeks
1990	0.9<br>(0.8 to 1.0)	0.5<br>(0.4 to 0.6)	7.3<br>(6.4 to 8.4)	3.9<br>(3.4 to 4.5)	1.22<br>(1.07 to 1.39)	0.65<br>(0.57 to 0.75)
1995	0.7<br>(0.6 to 0.8)	0.4<br>(0.3 to 0.4)	6.0<br>(5.2 to 6.8)	3.3<br>(2.9 to 3.7)	1.26<br>(1.11 to 1.43)	0.69<br>(0.61 to 0.78)
2000	0.7<br>(0.6 to 0.8)	0.4<br>(0.3 to 0.4)	6.1<br>(5.4 to 6.9)	3.4<br>(3.0 to 3.8)	1.39<br>(1.23 to 1.57)	0.77<br>(0.68 to 0.87)
2005	0.6<br>(0.5 to 0.7)	0.3<br>(0.3 to 0.4)	5.5<br>(4.8 to 6.2)	3.1<br>(2.7 to 3.5)	1.48<br>(1.29 to 1.66)	0.83<br>(0.73 to 0.94)
2010	0.6<br>(0.5 to 0.7)	0.3<br>(0.3 to 0.4)	5.6<br>(4.9 to 6.3)	3.2<br>(2.8 to 3.6)	1.61<br>(1.41 to 1.81)	0.92<br>(0.81 to 1.05)
2015	0.6<br>(0.5 to 0.7)	0.3<br>(0.3 to 0.4)	5.6<br>(4.9 to 6.4)	3.3<br>(2.9 to 3.7)	1.77<br>(1.55 to 2.01)	1.03<br>(0.91 to 1.17)
2020	0.6<br>(0.5 to 0.7)	0.3<br>(0.3 to 0.4)	5.8<br>(4.8 to 6.9)	3.4<br>(2.9 to 4.1)	2.31<br>(1.94 to 2.76)	1.36<br>(1.14 to 1.63)
2021	0.5<br>(0.4 to 0.7)	0.3<br>(0.2 to 0.4)	5.3<br>(4.1 to 6.6)	3.1<br>(2.4 to 3.9)	2.31<br>(1.79 to 2.91)	1.37<br>(1.07 to 1.73)

New Mexico   USA_554   High−income North America   Stillbirth Rate / Neonatal Mortality Rate (SBR/NMR) − 20 weeks
lambda = 0.2 ; zeta = 0.99 ; scale = 5 ; amp = 0.028

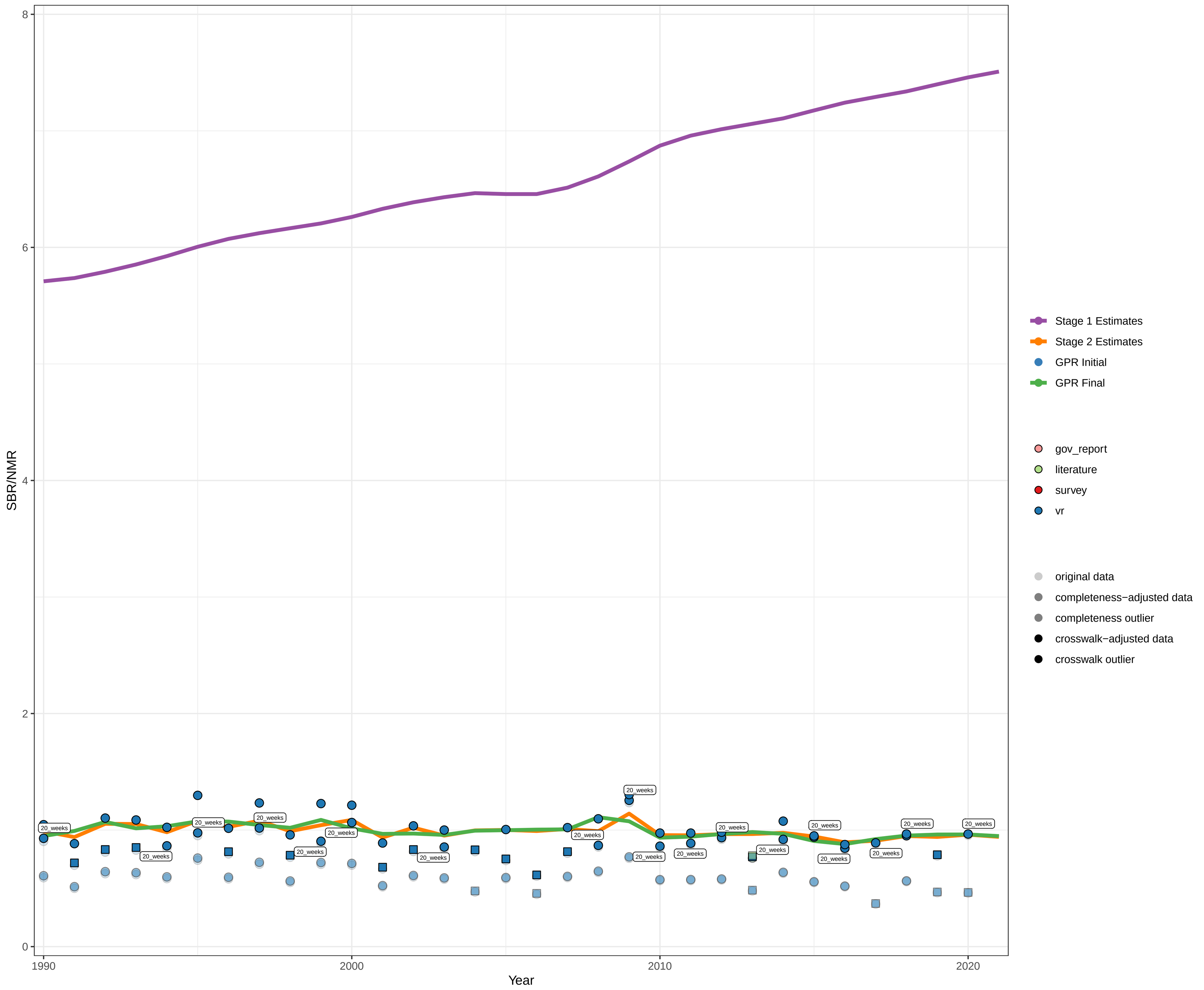

New Mexico   USA_554   High−income North America   Stillbirth Rate / Neonatal Mortality Rate (SBR/NMR) − 28 weeks
lambda = 0.2 ; zeta = 0.99 ; scale = 5 ; amp = 0.028

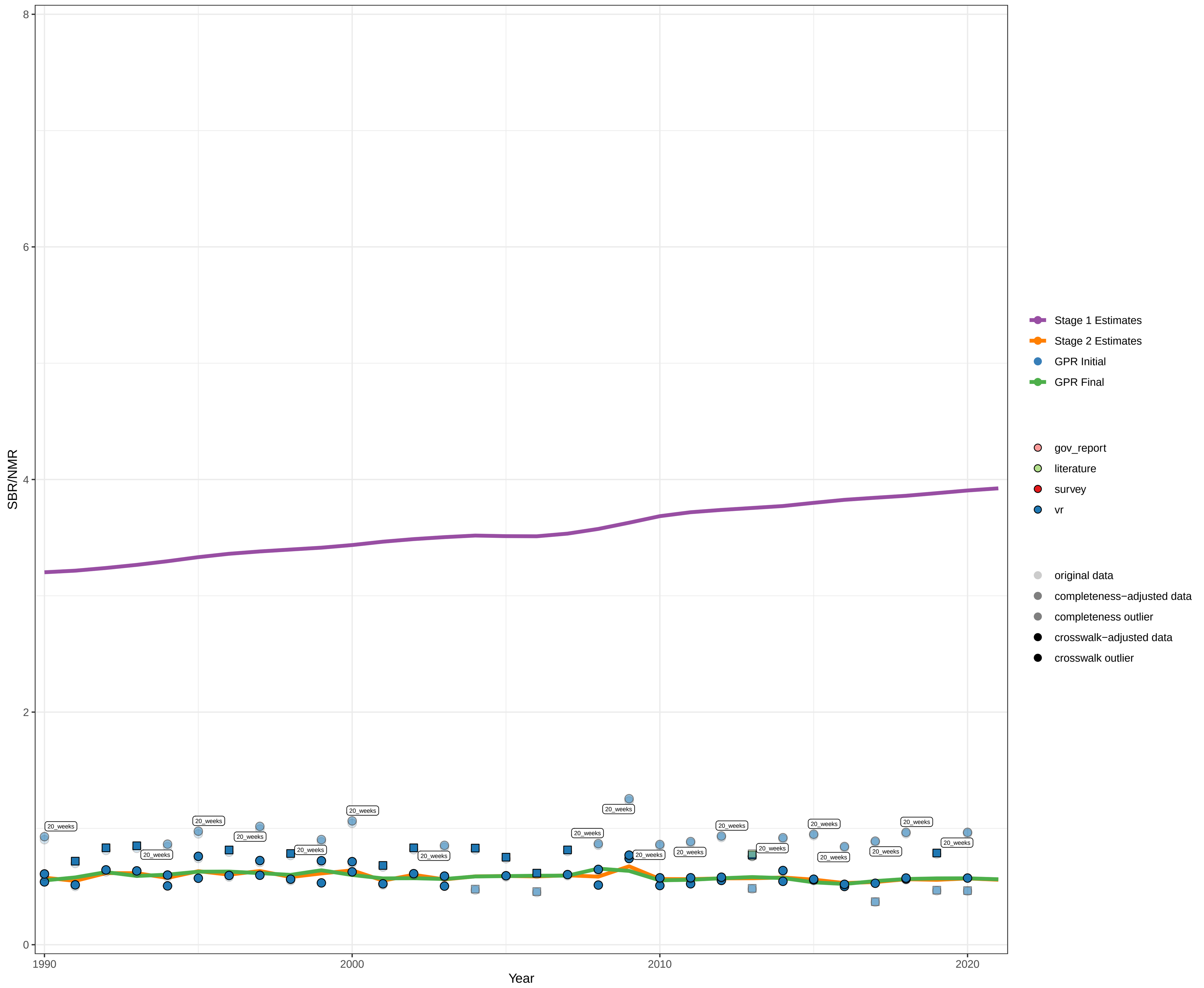

Definition	Years	Outlier	Source.Type	Title
20_weeks	1990, 1994, 1995, 1997, 1999, 2000, 2003, 2008−2012, 2014−2018, 2020	0	VR	United States NVSS Custom Fetal Death Data
28_weeks	1990−2003, 2005, 2007−2012, 2014−2016, 2018	0	VR	United States NVSS Custom Fetal Death Data
20_weeks	2013	1	Sci Lit	United States Fetal and Perinatal Mortality 2013
28_weeks	2004, 2006, 2013, 2017, 2019, 2020	1	VR	United States NVSS Custom Fetal Death Data
20_weeks	1991−1993, 1996, 1998, 2001, 2002, 2004−2007, 2013, 2019	2	VR	United States NVSS Custom Fetal Death Data

New Mexico   USA_554   High−income North America   Stillbirth Rate (SBR) − 20 weeks
lambda = 0.2 ; zeta = 0.99 ; scale = 5 ; amp = 0.028

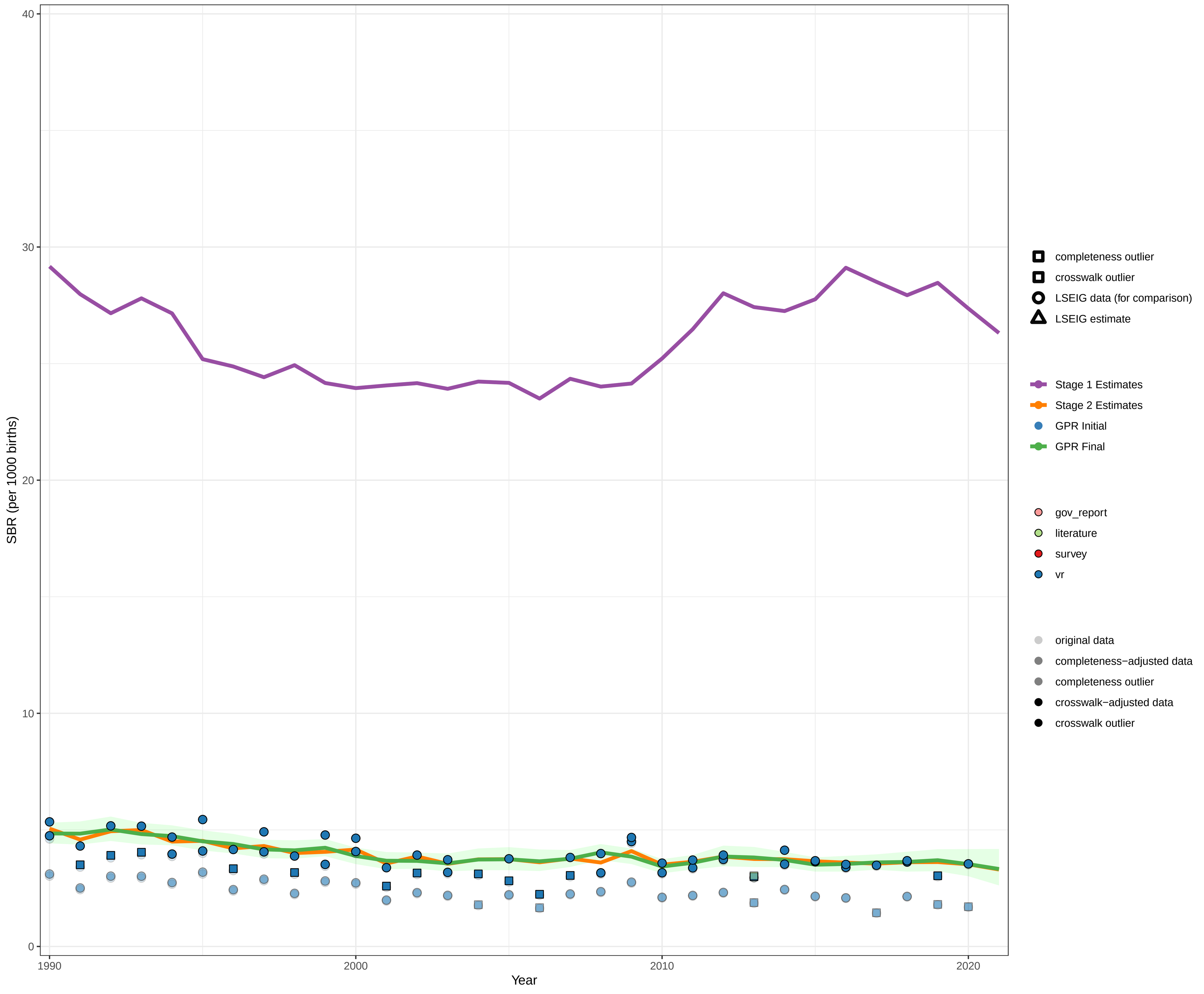

New Mexico   USA_554   High−income North America   Stillbirth Rate (SBR) − 28 weeks
lambda = 0.2 ; zeta = 0.99 ; scale = 5 ; amp = 0.028

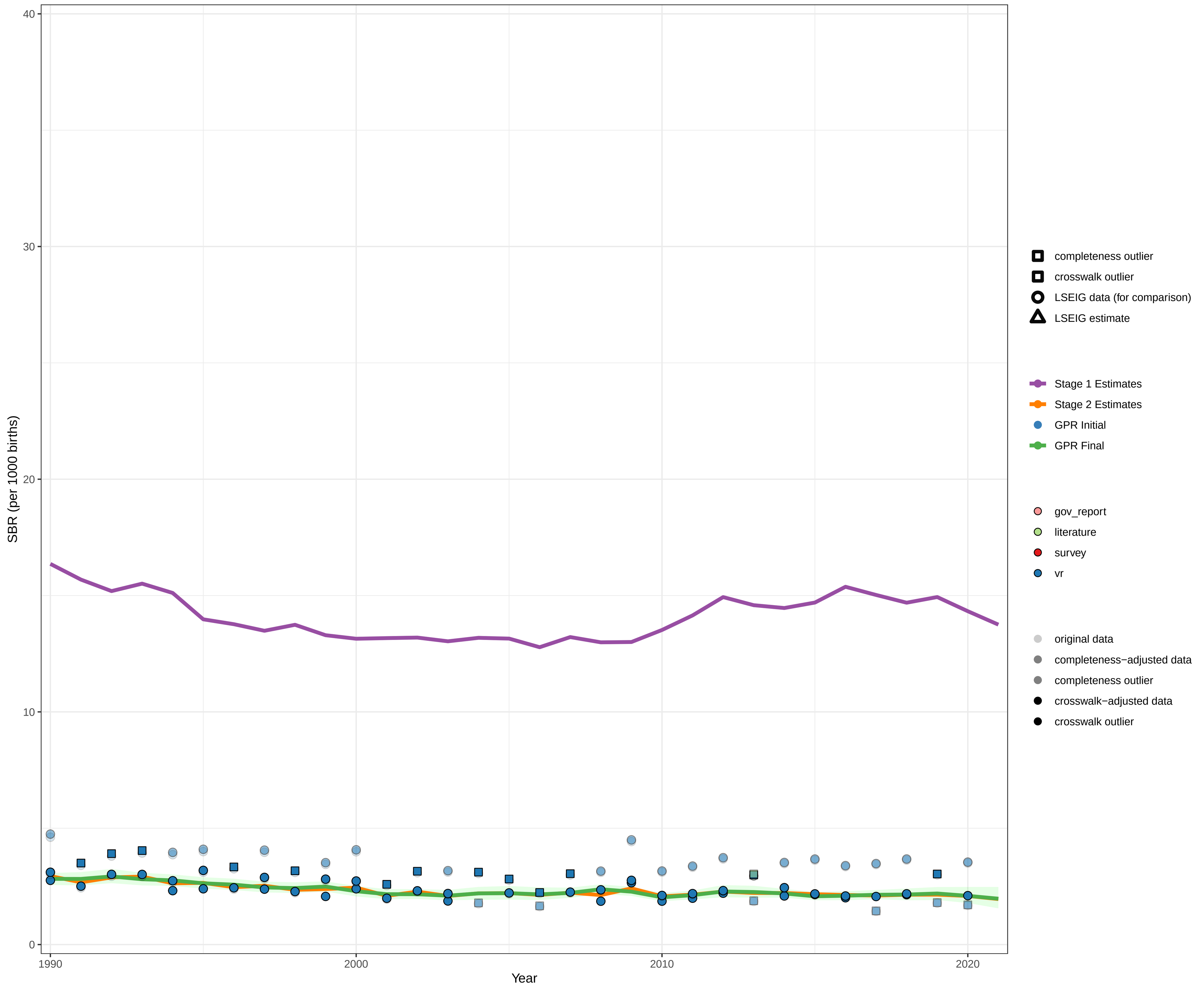

Year	Total Stillbirths (in thousands)	Total Stillbirths (in thousands)	SBR (per 1000 births)	SBR (per 1000 births)	SBR/NMR	SBR/NMR
	20 weeks	28 weeks	20 weeks	28 weeks	20 weeks	28 weeks
1990	0.1 (0.1 to 0.1)	0.1 (0.1 to 0.1)	4.8 (4.4 to 5.3)	2.8 (2.6 to 3.1)	0.95 (0.87 to 1.04)	0.55 (0.50 to 0.60)
1995	0.1 (0.1 to 0.1)	0.1 (0.1 to 0.1)	4.5 (4.1 to 5.0)	2.6 (2.4 to 2.9)	1.08 (0.98 to 1.19)	0.63 (0.57 to 0.69)
2000	0.1 (0.1 to 0.1)	0.1 (0.1 to 0.1)	3.9 (3.5 to 4.2)	2.3 (2.1 to 2.5)	1.01 (0.92 to 1.11)	0.60 (0.54 to 0.66)
2005	0.1 (0.1 to 0.1)	0.1 (0.1 to 0.1)	3.7 (3.3 to 4.3)	2.2 (1.9 to 2.5)	1.00 (0.87 to 1.14)	0.59 (0.52 to 0.67)
2010	0.1 (0.1 to 0.1)	0.1 (0.1 to 0.1)	3.4 (3.1 to 3.7)	2.0 (1.9 to 2.2)	0.93 (0.86 to 1.02)	0.55 (0.51 to 0.60)
2015	0.1 (0.1 to 0.1)	0.1 (0.0 to 0.1)	3.5 (3.2 to 3.8)	2.1 (1.9 to 2.3)	0.91 (0.83 to 0.99)	0.54 (0.49 to 0.58)
2020	0.1 (0.1 to 0.1)	0.0 (0.0 to 0.1)	3.5 (3.0 to 4.2)	2.1 (1.8 to 2.5)	0.96 (0.82 to 1.14)	0.57 (0.49 to 0.68)
2021	0.1 (0.1 to 0.1)	0.0 (0.0 to 0.1)	3.3 (2.6 to 4.2)	2.0 (1.6 to 2.5)	0.95 (0.75 to 1.19)	0.56 (0.44 to 0.71)

New York USA_555 High−income North America Stillbirth Rate / Neonatal Mortality Rate (SBR/NMR) − 20 weeks
lambda = 0.2 ; zeta = 0.99 ; scale = 5 ; amp = 0.028

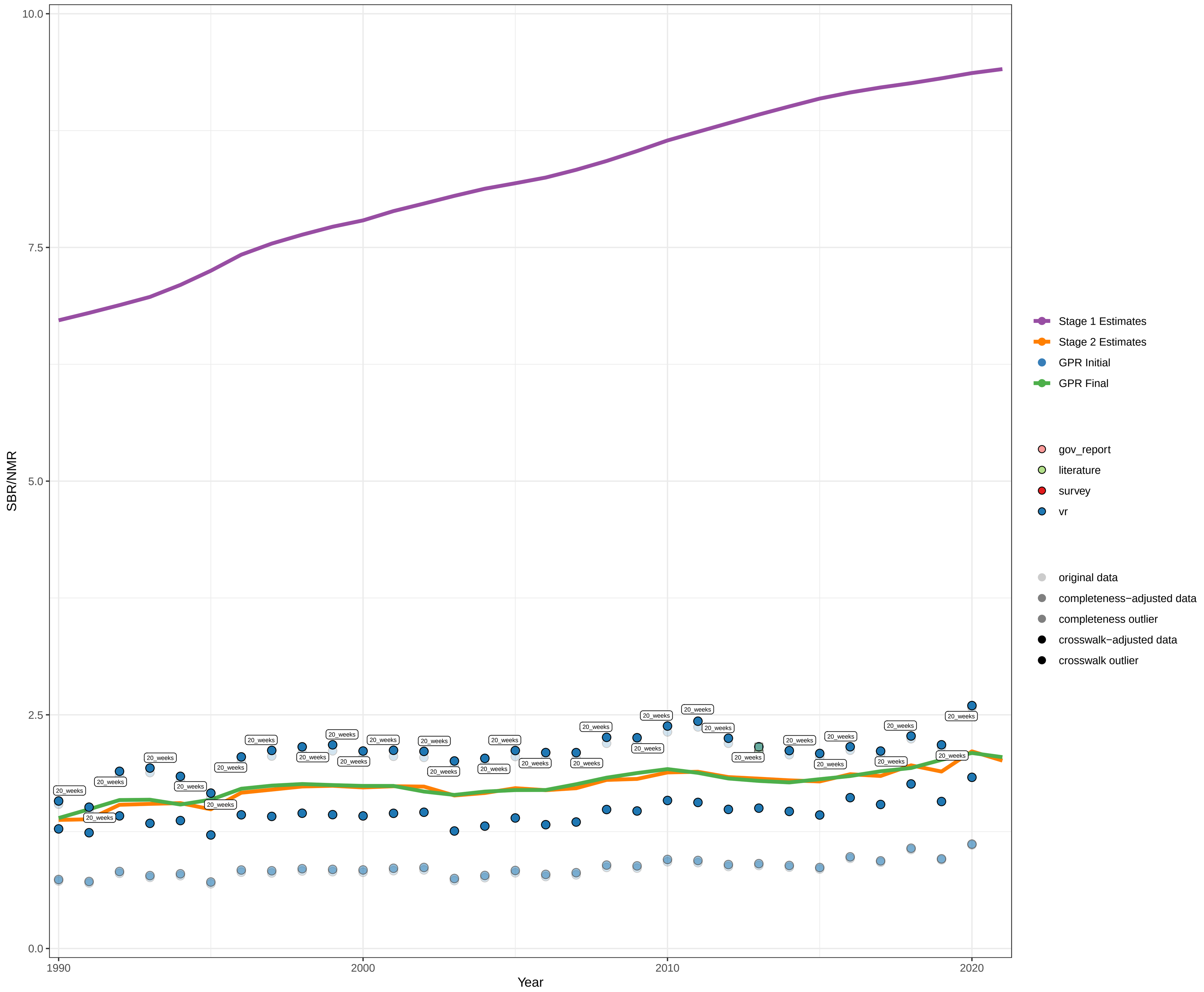

New York USA_555 High−income North America Stillbirth Rate / Neonatal Mortality Rate (SBR/NMR) − 28 weeks
lambda = 0.2 ; zeta = 0.99 ; scale = 5 ; amp = 0.028

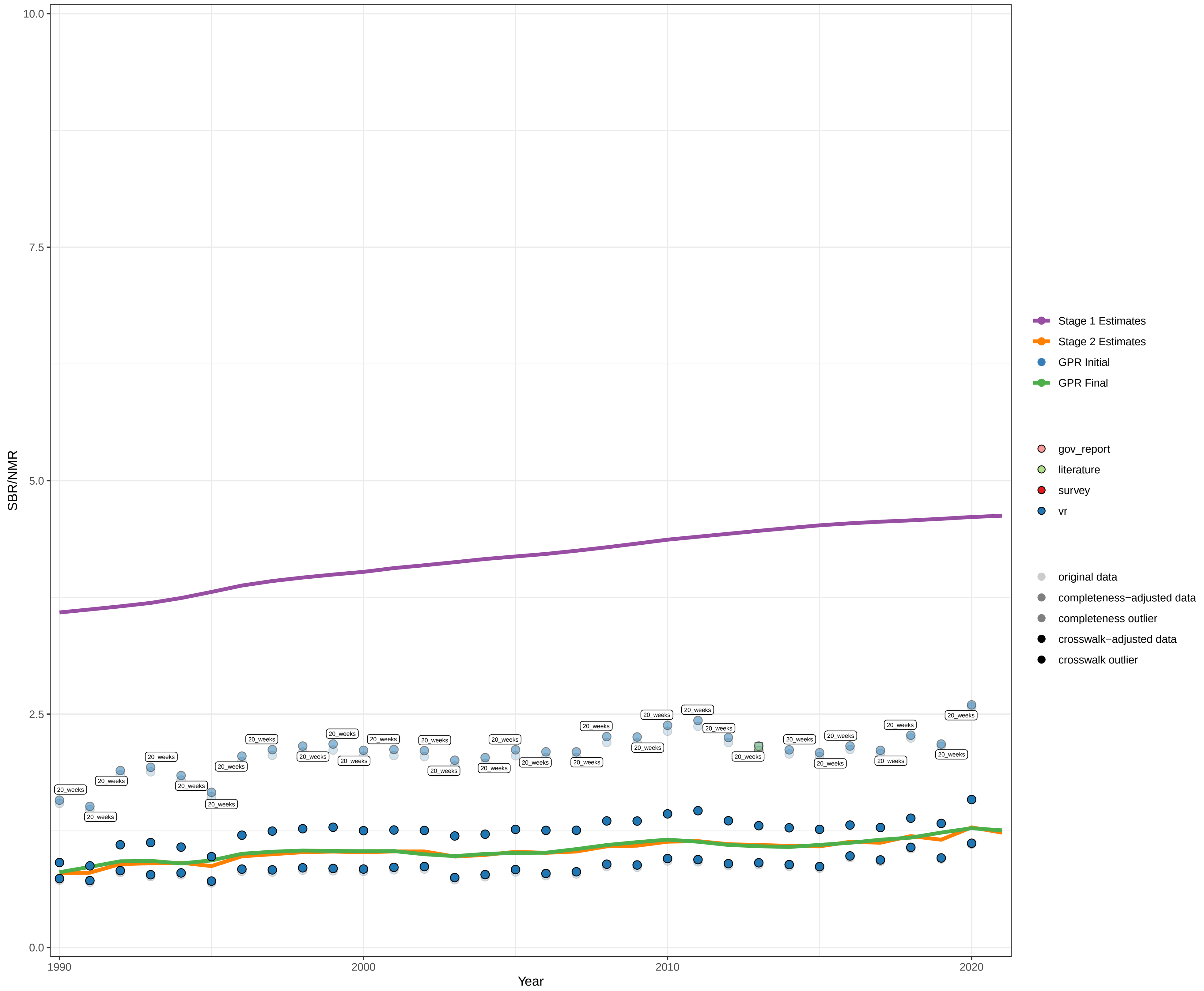

Definition	Years	Outlier	Source.Type	Title
20_weeks, 28_weeks	1990−2020	0	VR	United States NVSS Custom Fetal Death Data
20_weeks	2013	1	Sci Lit	United States Fetal and Perinatal Mortality 2013

New York USA_555 High−income North America Stillbirth Rate (SBR) − 20 weeks
lambda = 0.2 ; zeta = 0.99 ; scale = 5 ; amp = 0.028

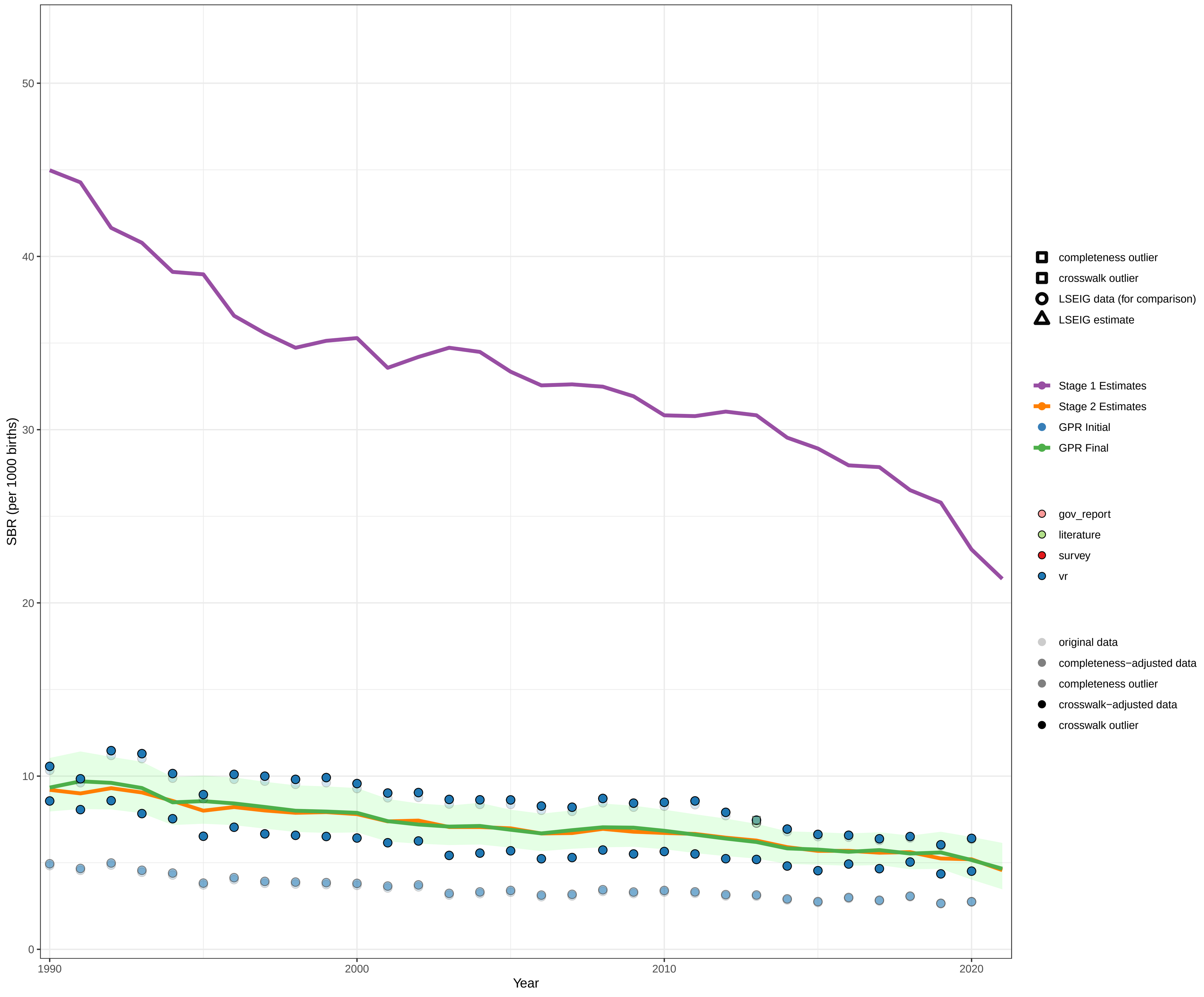

New York USA_555 High−income North America Stillbirth Rate (SBR) − 28 weeks
lambda = 0.2 ; zeta = 0.99 ; scale = 5 ; amp = 0.028

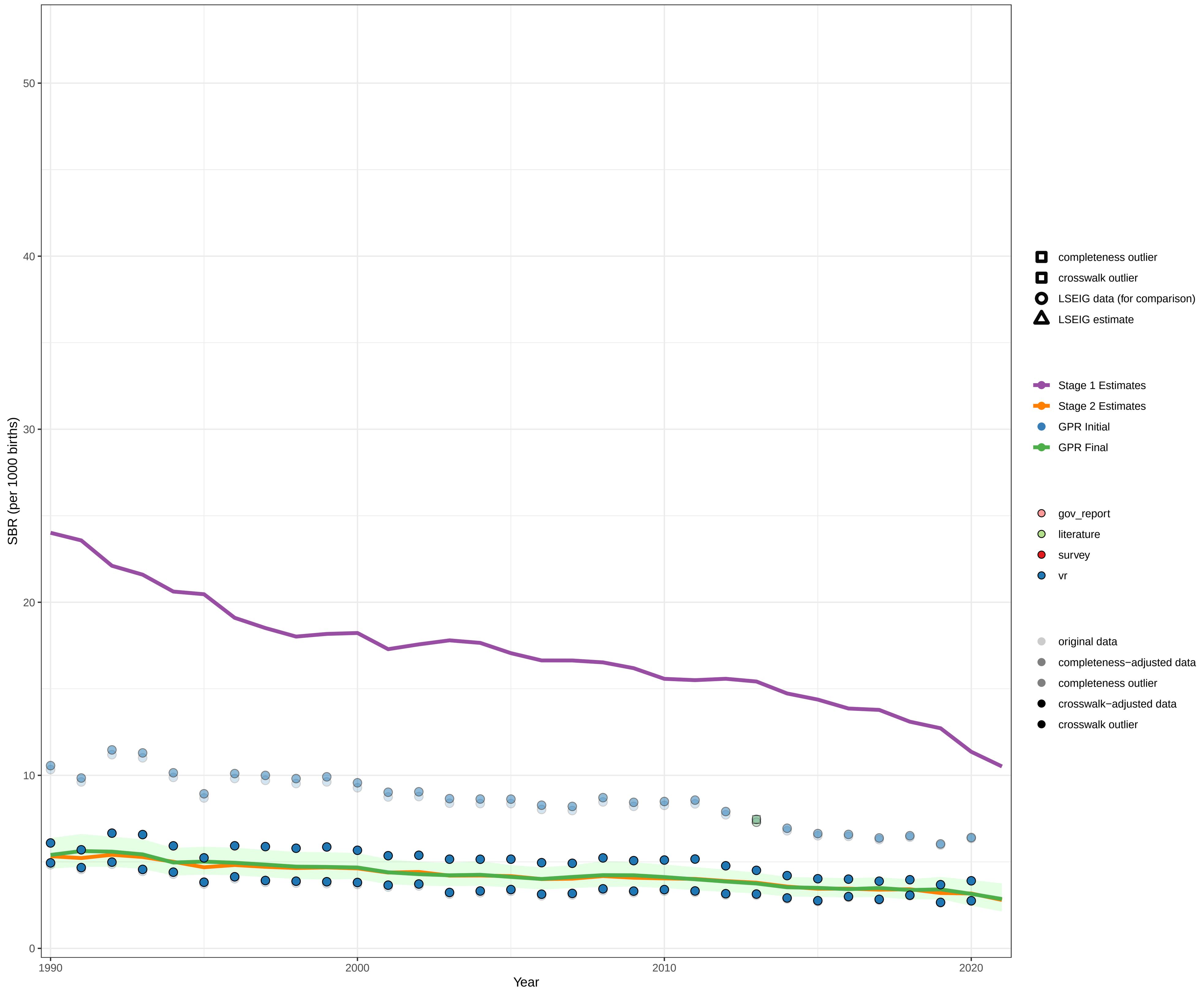

Year	Total Stillbirths (in thousands)	Total Stillbirths (in thousands)	SBR (per 1000 births)	SBR (per 1000 births)	SBR/NMR	SBR/NMR
	20 weeks	28 weeks	20 weeks	28 weeks	20 weeks	28 weeks
1990	2.8 (2.4 to 3.3)	1.6 (1.4 to 1.9)	9.3 (8.0 to 11.0)	5.4 (4.6 to 6.4)	1.40 (1.19 to 1.65)	0.81 (0.69 to 0.95)
1995	2.3 (2.0 to 2.7)	1.4 (1.2 to 1.6)	8.6 (7.3 to 10.0)	5.0 (4.3 to 5.9)	1.59 (1.35 to 1.87)	0.93 (0.79 to 1.09)
2000	2.0 (1.8 to 2.4)	1.2 (1.0 to 1.4)	7.9 (6.7 to 9.3)	4.7 (4.0 to 5.5)	1.74 (1.49 to 2.06)	1.03 (0.89 to 1.22)
2005	1.7 (1.5 to 2.0)	1.0 (0.9 to 1.2)	6.9 (5.9 to 8.1)	4.1 (3.5 to 4.8)	1.69 (1.44 to 1.98)	1.01 (0.86 to 1.18)
2010	1.7 (1.4 to 2.0)	1.0 (0.8 to 1.2)	6.8 (5.8 to 8.1)	4.1 (3.5 to 4.8)	1.92 (1.62 to 2.26)	1.16 (0.98 to 1.36)
2015	1.4 (1.2 to 1.6)	0.8 (0.7 to 1.0)	5.8 (4.9 to 6.8)	3.5 (3.0 to 4.1)	1.81 (1.54 to 2.13)	1.10 (0.93 to 1.29)
2020	1.1 (0.9 to 1.4)	0.7 (0.5 to 0.9)	5.2 (4.0 to 6.5)	3.1 (2.5 to 4.0)	2.09 (1.63 to 2.63)	1.28 (1.00 to 1.60)
2021	1.0 (0.7 to 1.3)	0.6 (0.4 to 0.8)	4.7 (3.5 to 6.1)	2.9 (2.1 to 3.8)	2.05 (1.52 to 2.70)	1.25 (0.94 to 1.65)

North Carolina   USA_556   High−income North America   Stillbirth Rate / Neonatal Mortality Rate (SBR/NMR) − 20 weeks
lambda = 0.2 ; zeta = 0.99 ; scale = 5 ; amp = 0.028

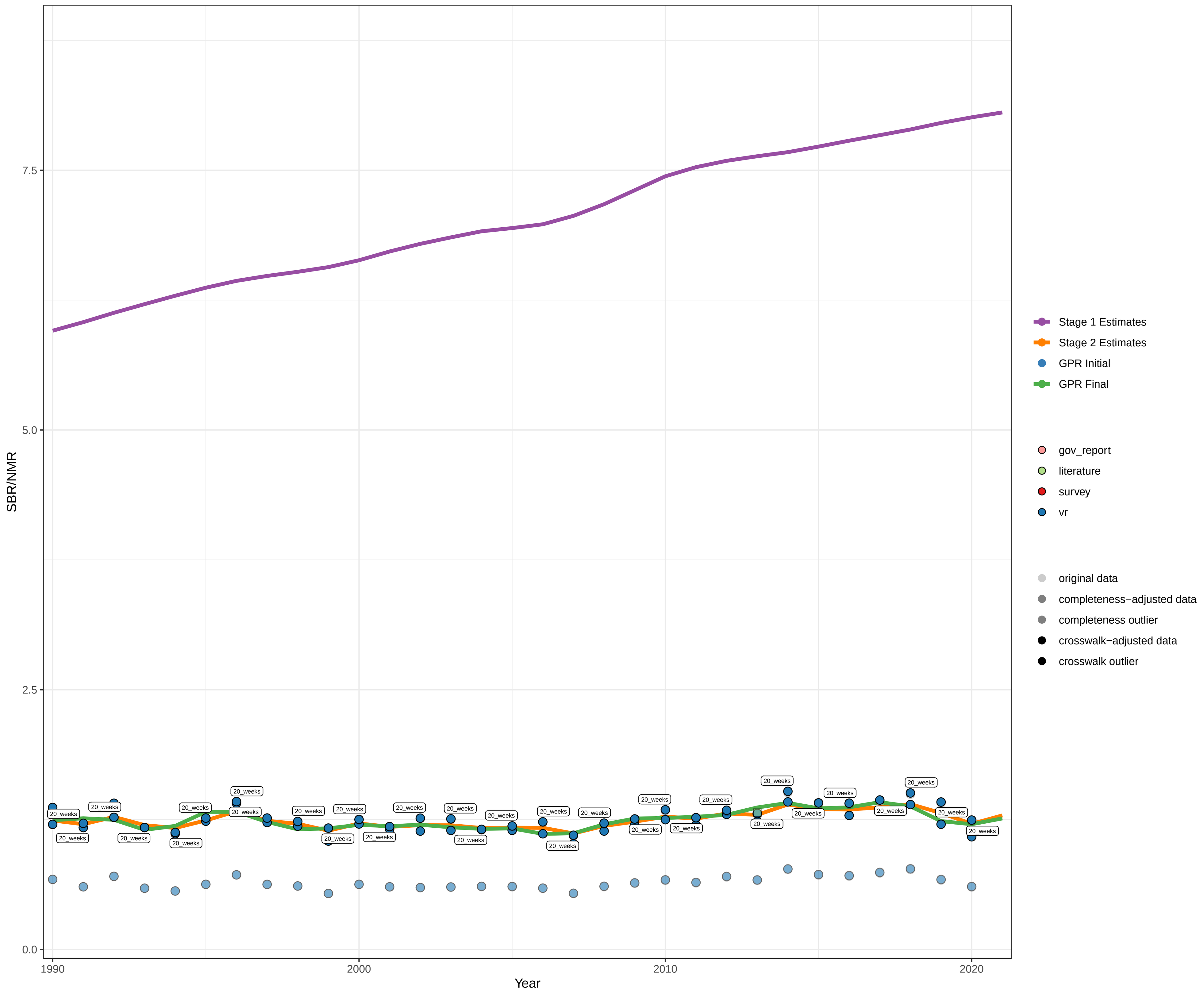

North Carolina   USA_556   High−income North America   Stillbirth Rate / Neonatal Mortality Rate (SBR/NMR) − 28 weeks
lambda = 0.2 ; zeta = 0.99 ; scale = 5 ; amp = 0.028

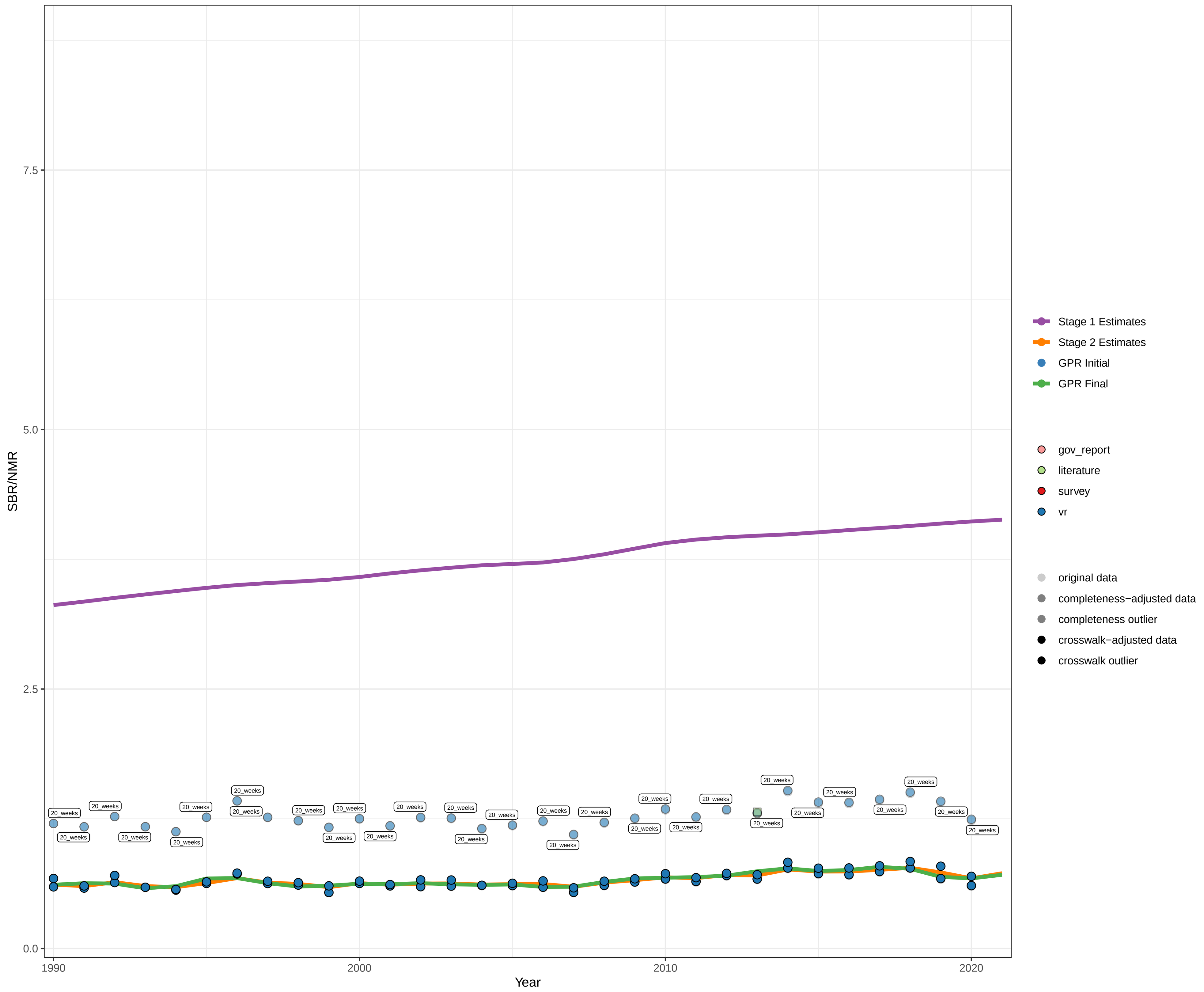

Definition	Years	Outlier	Source.Type	Title
20_weeks, 28_weeks	1990−2020	0	VR	United States NVSS Custom Fetal Death Data
20_weeks	2013	1	Sci Lit	United States Fetal and Perinatal Mortality 2013

North Carolina   USA_556   High−income North America   Stillbirth Rate (SBR) − 20 weeks
lambda = 0.2 ; zeta = 0.99 ; scale = 5 ; amp = 0.028

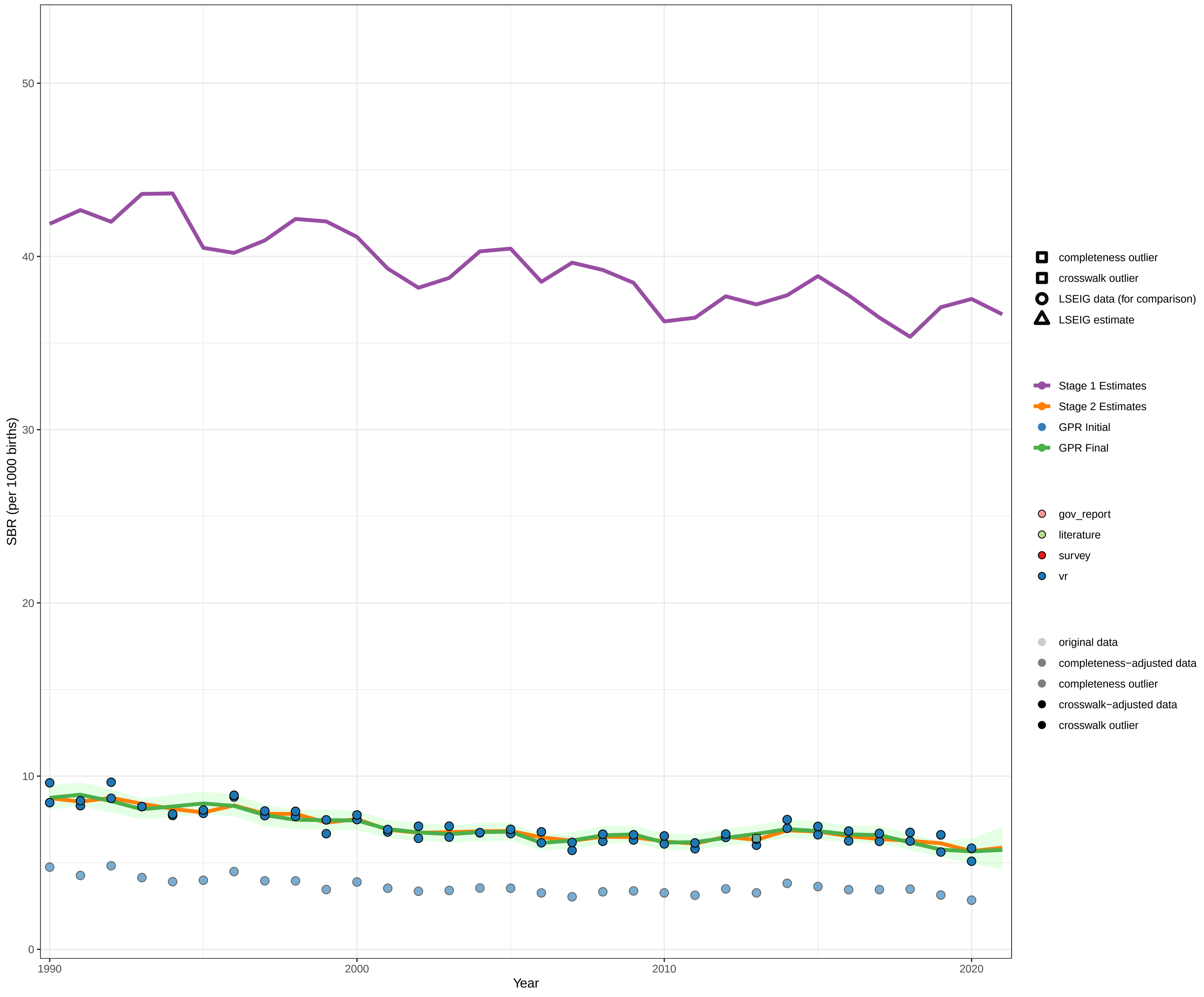

North Carolina   USA_556   High−income North America   Stillbirth Rate (SBR) − 28 weeks
lambda = 0.2 ; zeta = 0.99 ; scale = 5 ; amp = 0.028

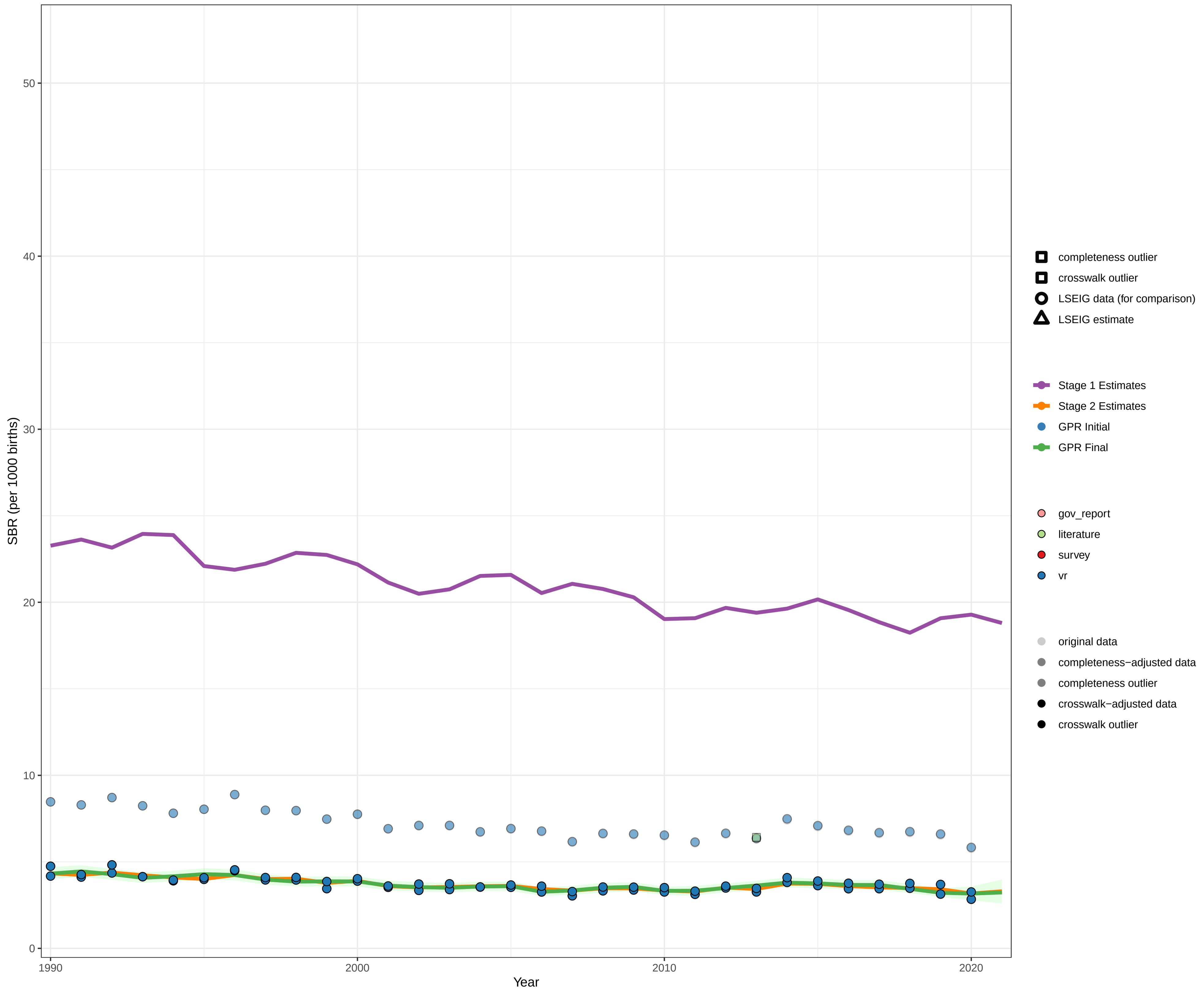

Year	Total Stillbirths (in thousands)	Total Stillbirths (in thousands)	SBR (per 1000 births)	SBR (per 1000 births)	SBR/NMR	SBR/NMR
	20 weeks	28 weeks	20 weeks	28 weeks	20 weeks	28 weeks
1990	0.9 (0.8 to 1.0)	0.5 (0.4 to 0.5)	8.7 (8.1 to 9.4)	4.3 (4.0 to 4.7)	1.24 (1.15 to 1.34)	0.61 (0.57 to 0.67)
1995	0.9 (0.8 to 0.9)	0.4 (0.4 to 0.5)	8.4 (7.8 to 9.1)	4.3 (4.0 to 4.6)	1.32 (1.23 to 1.43)	0.67 (0.63 to 0.73)
2000	0.9 (0.8 to 1.0)	0.5 (0.4 to 0.5)	7.4 (6.9 to 8.0)	3.9 (3.6 to 4.2)	1.20 (1.11 to 1.29)	0.62 (0.58 to 0.67)
2005	0.9 (0.8 to 0.9)	0.5 (0.4 to 0.5)	6.8 (6.3 to 7.3)	3.6 (3.3 to 3.9)	1.17 (1.08 to 1.25)	0.62 (0.57 to 0.66)
2010	0.8 (0.7 to 0.8)	0.4 (0.4 to 0.4)	6.2 (5.7 to 6.7)	3.3 (3.1 to 3.6)	1.27 (1.17 to 1.38)	0.68 (0.63 to 0.73)
2015	0.8 (0.8 to 0.9)	0.5 (0.4 to 0.5)	6.8 (6.3 to 7.4)	3.8 (3.5 to 4.0)	1.36 (1.26 to 1.47)	0.75 (0.69 to 0.80)
2020	0.7 (0.6 to 0.8)	0.4 (0.3 to 0.4)	5.7 (5.0 to 6.4)	3.2 (2.8 to 3.6)	1.21 (1.06 to 1.36)	0.68 (0.59 to 0.76)
2021	0.7 (0.6 to 0.9)	0.4 (0.3 to 0.5)	5.7 (4.6 to 7.1)	3.2 (2.6 to 4.0)	1.26 (1.01 to 1.56)	0.71 (0.57 to 0.87)

North Dakota USA_557 High−income North America Stillbirth Rate / Neonatal Mortality Rate (SBR/NMR) − 20 weeks
lambda = 0.2 ; zeta = 0.99 ; scale = 5 ; amp = 0.028

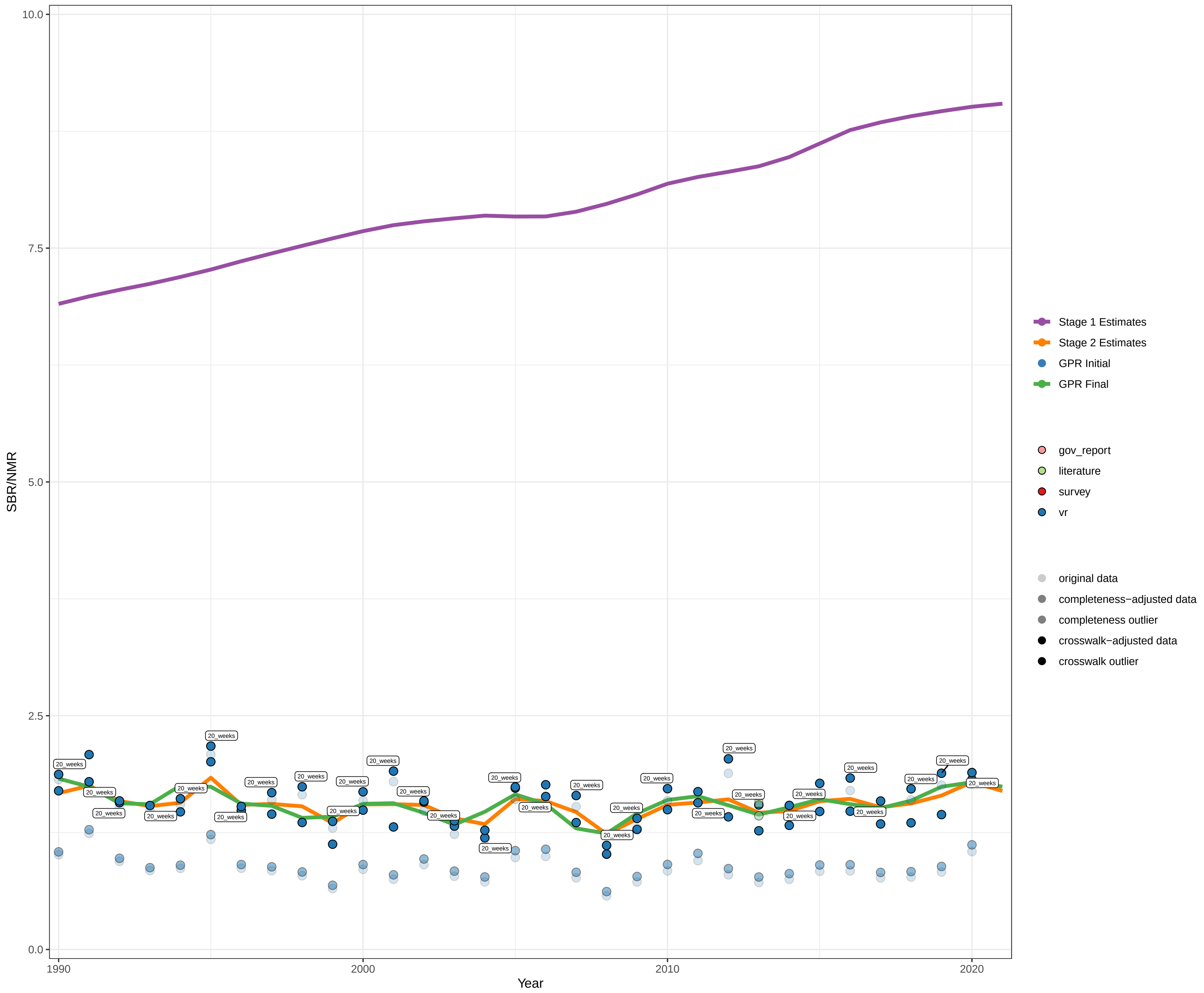

North Dakota USA_557 High−income North America Stillbirth Rate / Neonatal Mortality Rate (SBR/NMR) − 28 weeks
lambda = 0.2 ; zeta = 0.99 ; scale = 5 ; amp = 0.028

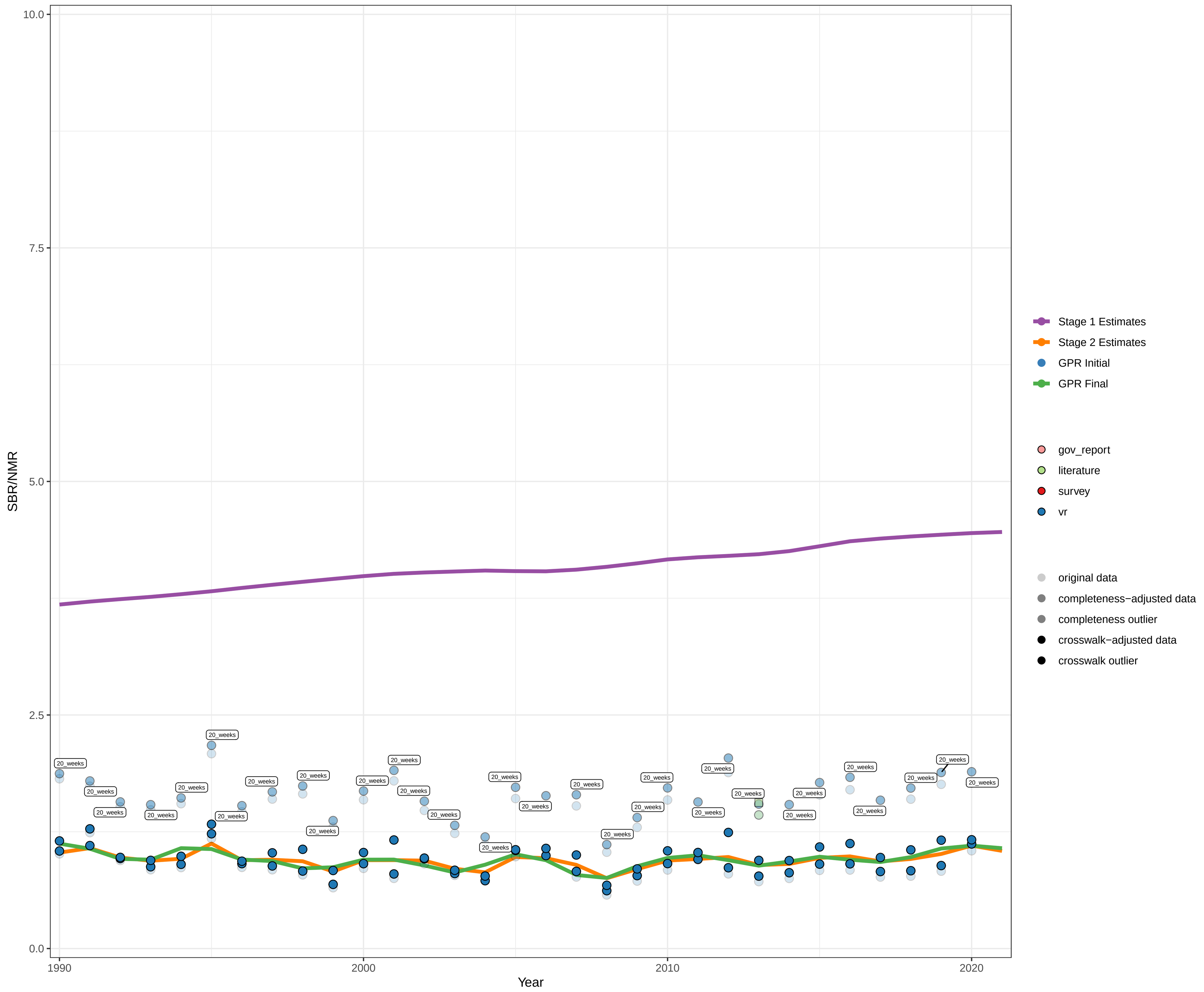

Definition	Years	Outlier	Source.Type	Title
20_weeks, 28_weeks	1990−2020	0	VR	United States NVSS Custom Fetal Death Data
20_weeks	2013	1	Sci Lit	United States Fetal and Perinatal Mortality 2013

North Dakota USA_557 High−income North America Stillbirth Rate (SBR) − 20 weeks
lambda = 0.2 ; zeta = 0.99 ; scale = 5 ; amp = 0.028

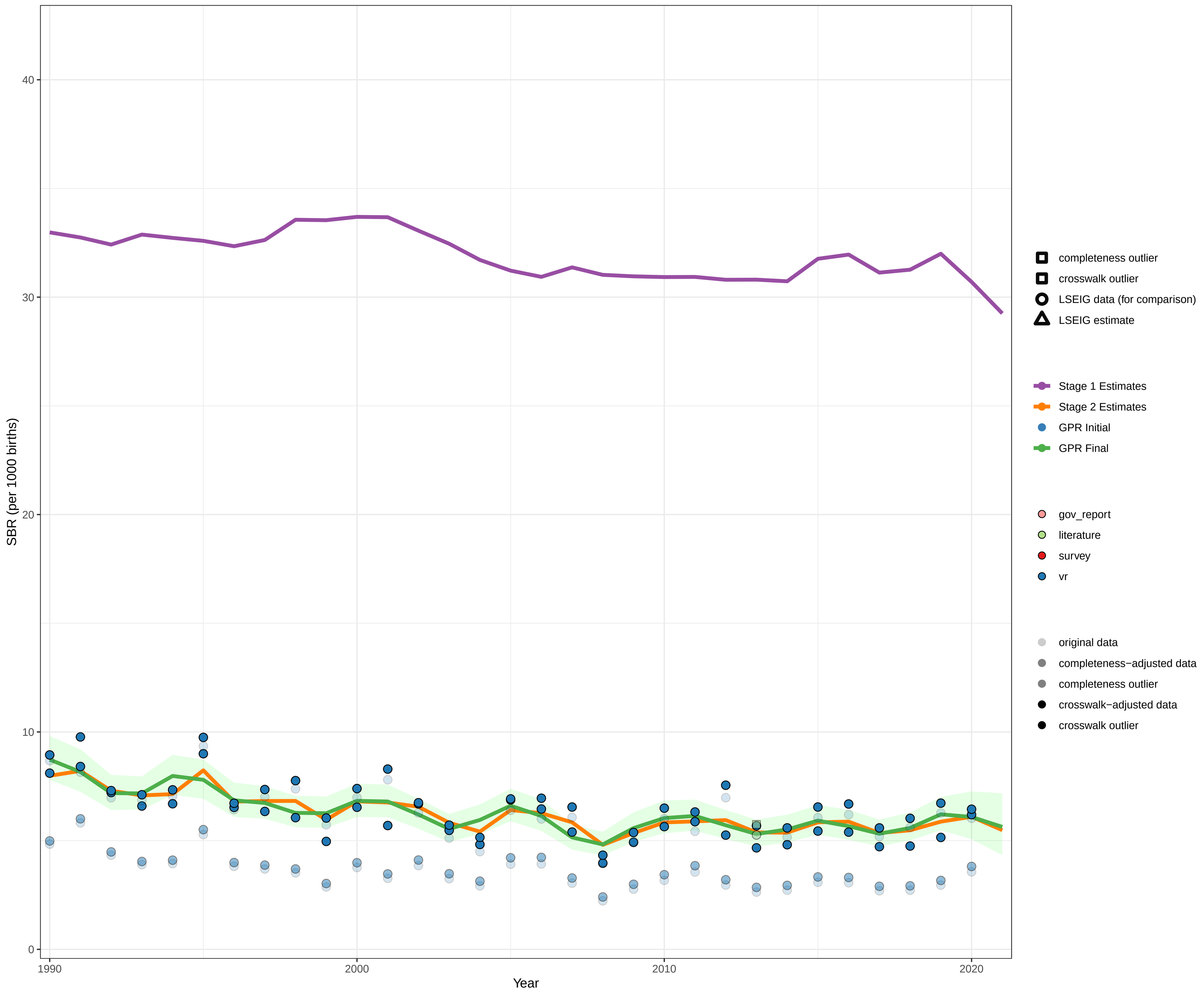

North Dakota USA_557 High−income North America Stillbirth Rate (SBR) − 28 weeks
lambda = 0.2 ; zeta = 0.99 ; scale = 5 ; amp = 0.028

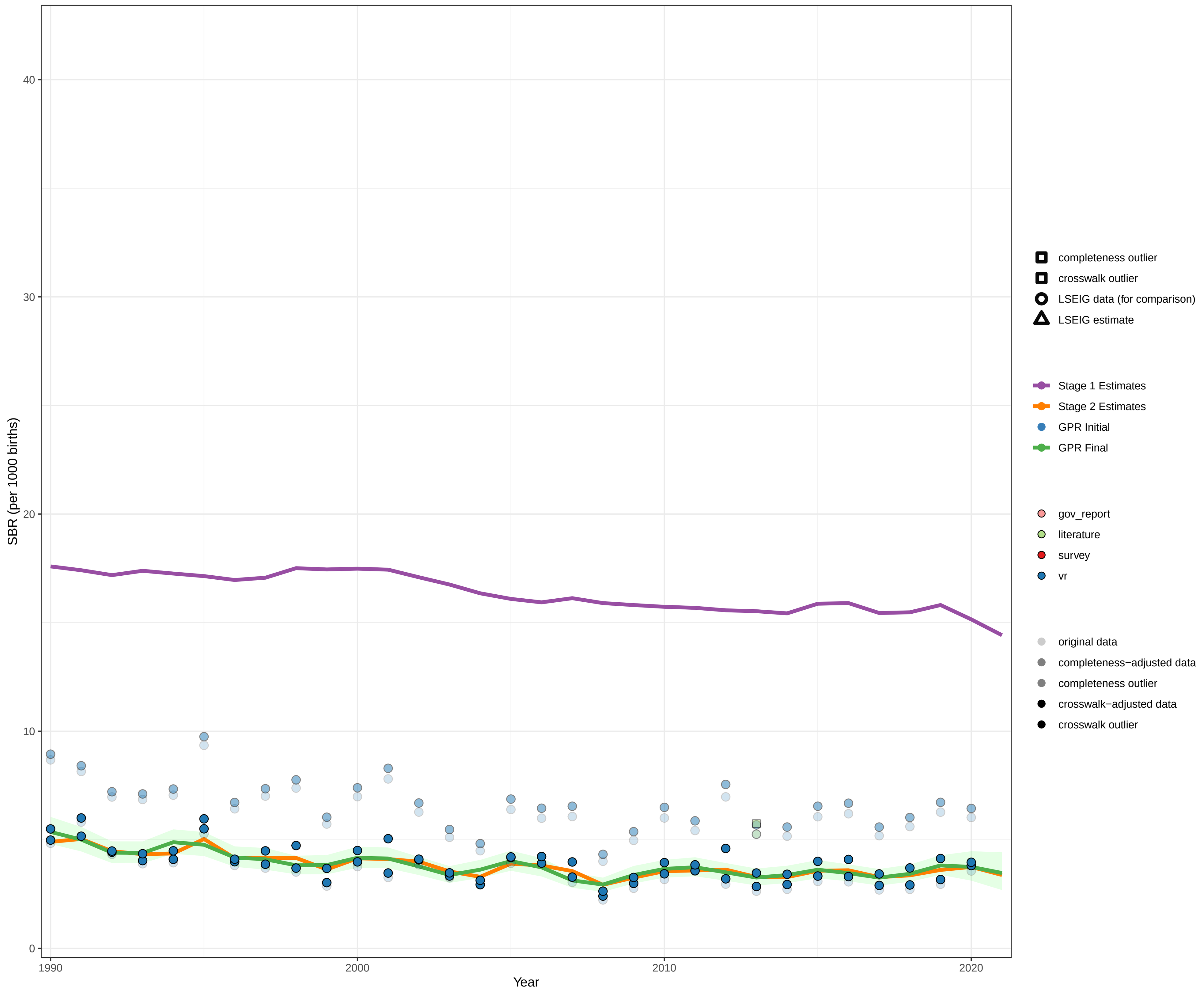

Year	Total Stillbirths (in thousands)	Total Stillbirths (in thousands)	SBR (per 1000 births)	SBR (per 1000 births)	SBR/NMR	SBR/NMR
	20 weeks	28 weeks	20 weeks	28 weeks	20 weeks	28 weeks
1990	0.1 (0.1 to 0.1)	0.1 (0.0 to 0.1)	8.7 (7.8 to 9.8)	5.4 (4.8 to 6.1)	1.83 (1.63 to 2.05)	1.12 (1.01 to 1.27)
1995	0.1 (0.1 to 0.1)	0.0 (0.0 to 0.0)	7.8 (6.9 to 8.7)	4.8 (4.3 to 5.4)	1.74 (1.55 to 1.95)	1.06 (0.95 to 1.20)
2000	0.1 (0.0 to 0.1)	0.0 (0.0 to 0.0)	6.8 (6.1 to 7.6)	4.2 (3.7 to 4.7)	1.56 (1.39 to 1.74)	0.95 (0.85 to 1.07)
2005	0.1 (0.1 to 0.1)	0.0 (0.0 to 0.0)	6.6 (5.9 to 7.4)	4.0 (3.6 to 4.5)	1.66 (1.48 to 1.86)	1.01 (0.90 to 1.13)
2010	0.1 (0.1 to 0.1)	0.0 (0.0 to 0.0)	6.0 (5.3 to 6.9)	3.7 (3.3 to 4.1)	1.60 (1.41 to 1.81)	0.97 (0.86 to 1.08)
2015	0.1 (0.1 to 0.1)	0.0 (0.0 to 0.0)	5.9 (5.3 to 6.6)	3.6 (3.2 to 4.1)	1.61 (1.43 to 1.80)	0.98 (0.88 to 1.10)
2020	0.1 (0.1 to 0.1)	0.0 (0.0 to 0.0)	6.1 (5.1 to 7.3)	3.8 (3.1 to 4.5)	1.79 (1.49 to 2.13)	1.10 (0.92 to 1.31)
2021	0.1 (0.0 to 0.1)	0.0 (0.0 to 0.0)	5.6 (4.3 to 7.2)	3.5 (2.7 to 4.4)	1.74 (1.34 to 2.22)	1.07 (0.83 to 1.37)

Ohio   USA_558   High−income North America   Stillbirth Rate / Neonatal Mortality Rate (SBR/NMR) − 20 weeks
lambda = 0.2 ; zeta = 0.99 ; scale = 5 ; amp = 0.028

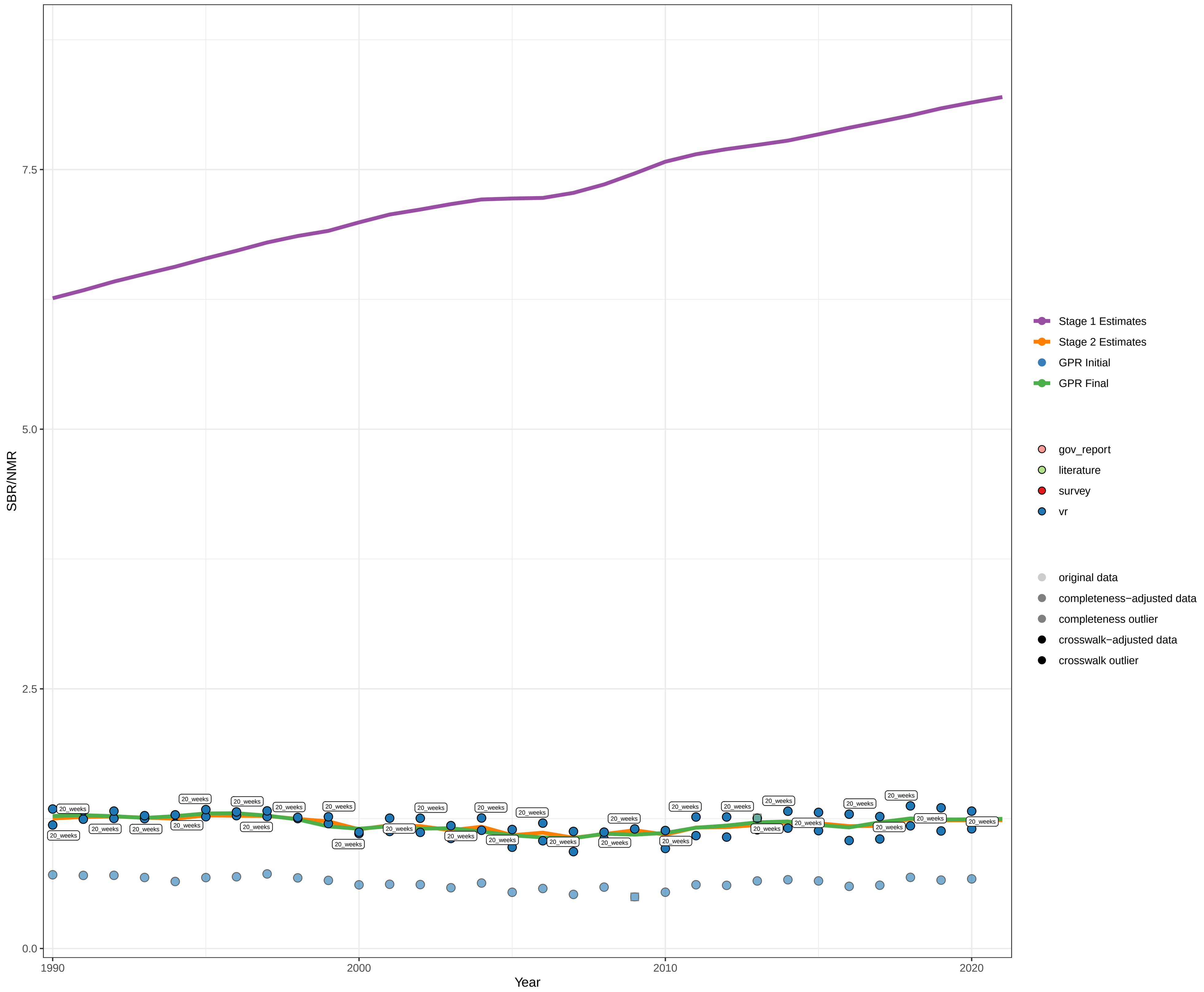

Ohio   USA_558   High−income North America   Stillbirth Rate / Neonatal Mortality Rate (SBR/NMR) − 28 weeks
lambda = 0.2 ; zeta = 0.99 ; scale = 5 ; amp = 0.028

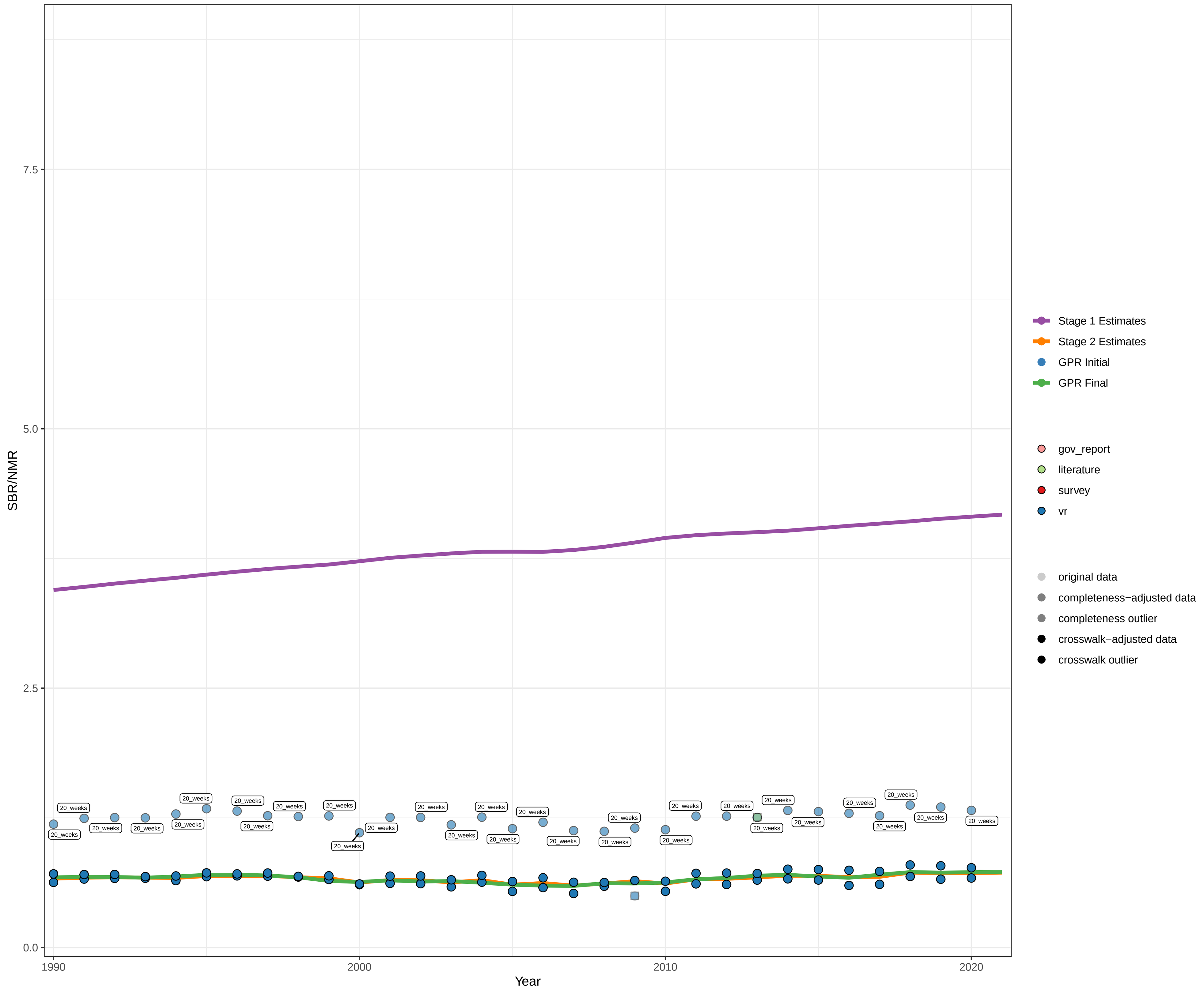

Definition	Years	Outlier	Source.Type	Title
20_weeks	1990−2020	0	VR	United States NVSS Custom Fetal Death Data
28_weeks	1990−2008, 2010−2020	0	VR	United States NVSS Custom Fetal Death Data
20_weeks	2013	1	Sci Lit	United States Fetal and Perinatal Mortality 2013
28_weeks	2009	1	VR	United States NVSS Custom Fetal Death Data

Ohio   USA_558   High−income North America   Stillbirth Rate (SBR) − 20 weeks
lambda = 0.2 ; zeta = 0.99 ; scale = 5 ; amp = 0.028

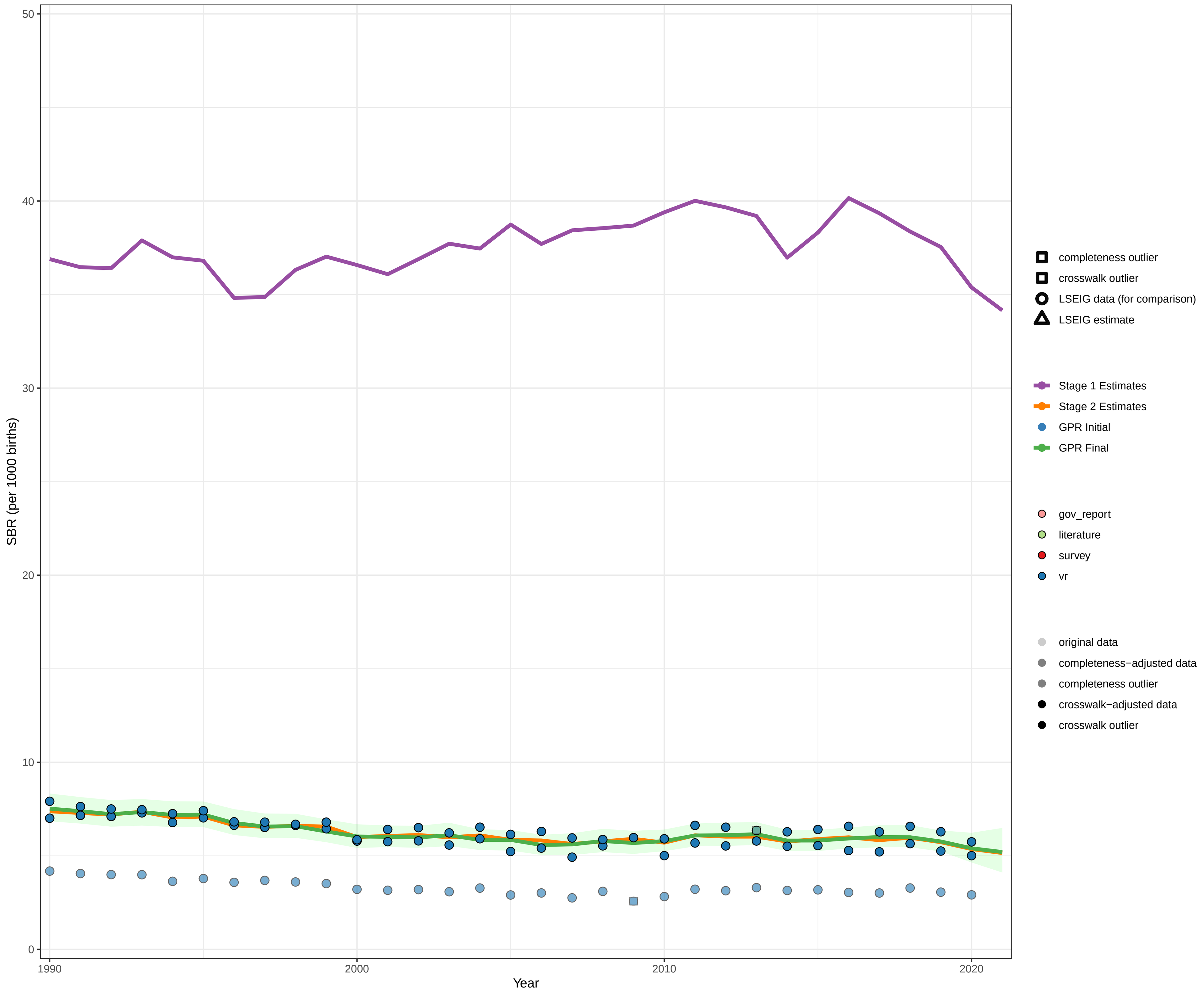

Ohio   USA_558   High−income North America   Stillbirth Rate (SBR) − 28 weeks
lambda = 0.2 ; zeta = 0.99 ; scale = 5 ; amp = 0.028

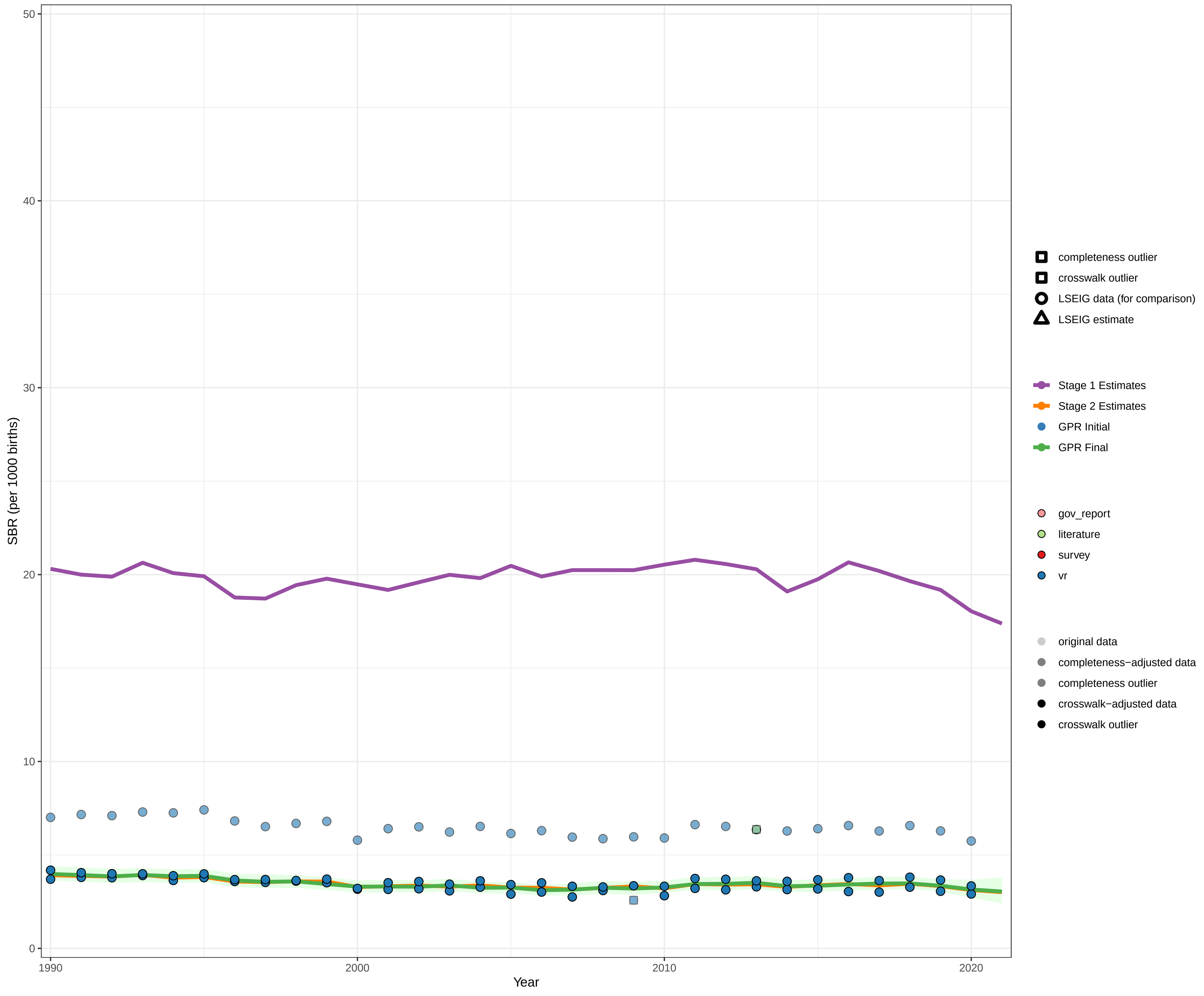

Year	Total Stillbirths (in thousands)	Total Stillbirths (in thousands)	SBR (per 1000 births)	SBR (per 1000 births)	SBR/NMR	SBR/NMR
	20 weeks	28 weeks	20 weeks	28 weeks	20 weeks	28 weeks
1990	1.3 (1.2 to 1.4)	0.7 (0.6 to 0.7)	7.5 (6.8 to 8.3)	4.0 (3.6 to 4.4)	1.28 (1.16 to 1.41)	0.68 (0.62 to 0.75)
1995	1.1 (1.0 to 1.2)	0.6 (0.5 to 0.7)	7.2 (6.5 to 7.9)	3.9 (3.5 to 4.3)	1.30 (1.18 to 1.43)	0.70 (0.64 to 0.77)
2000	0.9 (0.8 to 1.0)	0.5 (0.5 to 0.6)	6.0 (5.4 to 6.7)	3.3 (3.0 to 3.6)	1.15 (1.04 to 1.28)	0.63 (0.57 to 0.69)
2005	0.9 (0.8 to 1.0)	0.5 (0.4 to 0.5)	5.8 (5.3 to 6.4)	3.3 (2.9 to 3.5)	1.09 (0.98 to 1.19)	0.61 (0.55 to 0.66)
2010	0.8 (0.7 to 0.9)	0.5 (0.4 to 0.5)	5.8 (5.2 to 6.4)	3.3 (3.0 to 3.6)	1.11 (1.01 to 1.23)	0.63 (0.57 to 0.69)
2015	0.8 (0.7 to 0.9)	0.5 (0.4 to 0.5)	5.8 (5.3 to 6.4)	3.3 (3.0 to 3.7)	1.19 (1.08 to 1.31)	0.69 (0.62 to 0.75)
2020	0.7 (0.6 to 0.8)	0.4 (0.3 to 0.5)	5.4 (4.6 to 6.2)	3.2 (2.7 to 3.7)	1.24 (1.07 to 1.43)	0.73 (0.62 to 0.85)
2021	0.7 (0.5 to 0.8)	0.4 (0.3 to 0.5)	5.2 (4.1 to 6.5)	3.0 (2.4 to 3.8)	1.25 (0.99 to 1.56)	0.73 (0.58 to 0.91)

Oklahoma   USA_559   High−income North America   Stillbirth Rate / Neonatal Mortality Rate (SBR/NMR) − 20 weeks
lambda = 0.2 ; zeta = 0.99 ; scale = 5 ; amp = 0.028

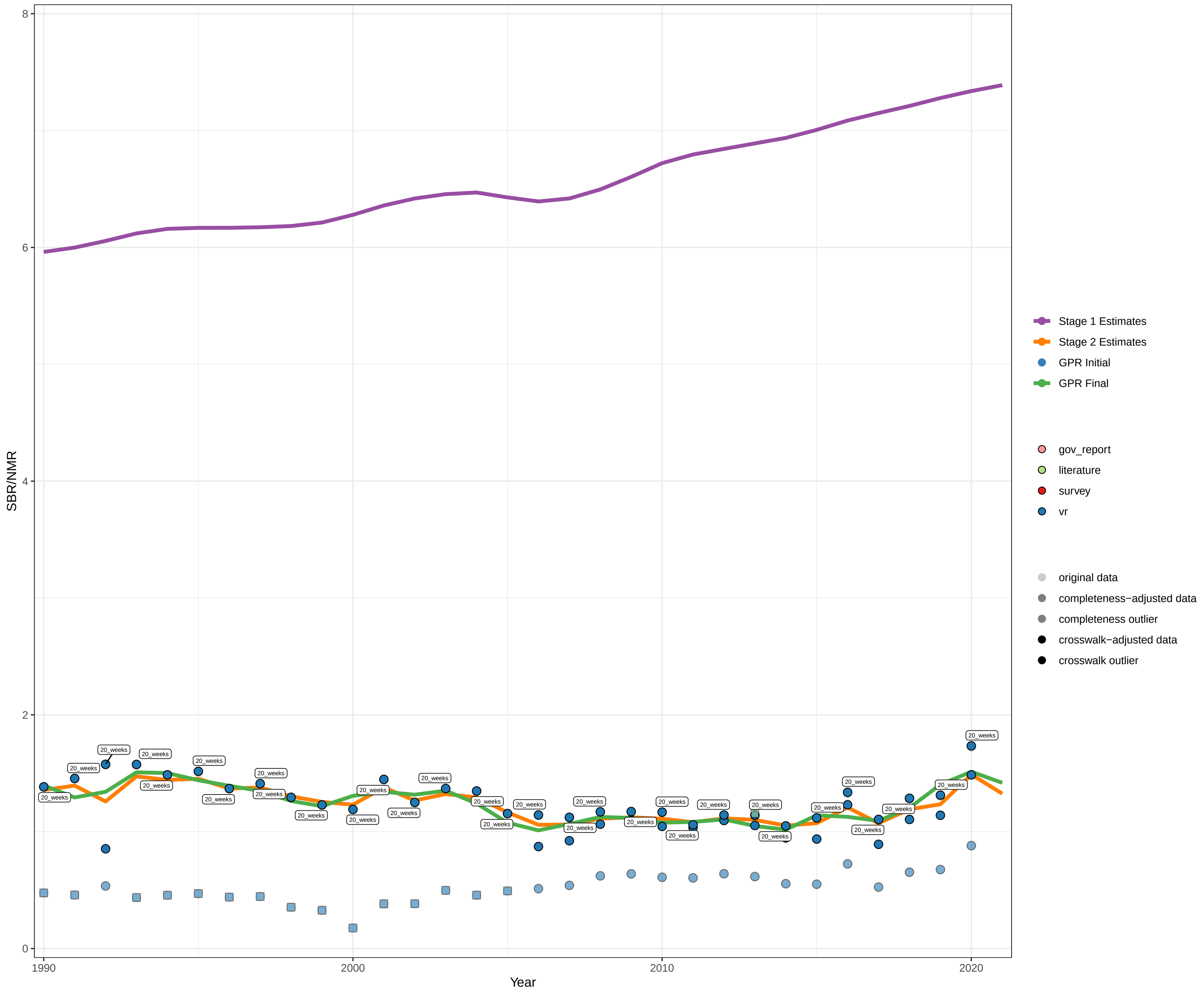

Oklahoma   USA_559   High−income North America   Stillbirth Rate / Neonatal Mortality Rate (SBR/NMR) − 28 weeks
lambda = 0.2 ; zeta = 0.99 ; scale = 5 ; amp = 0.028

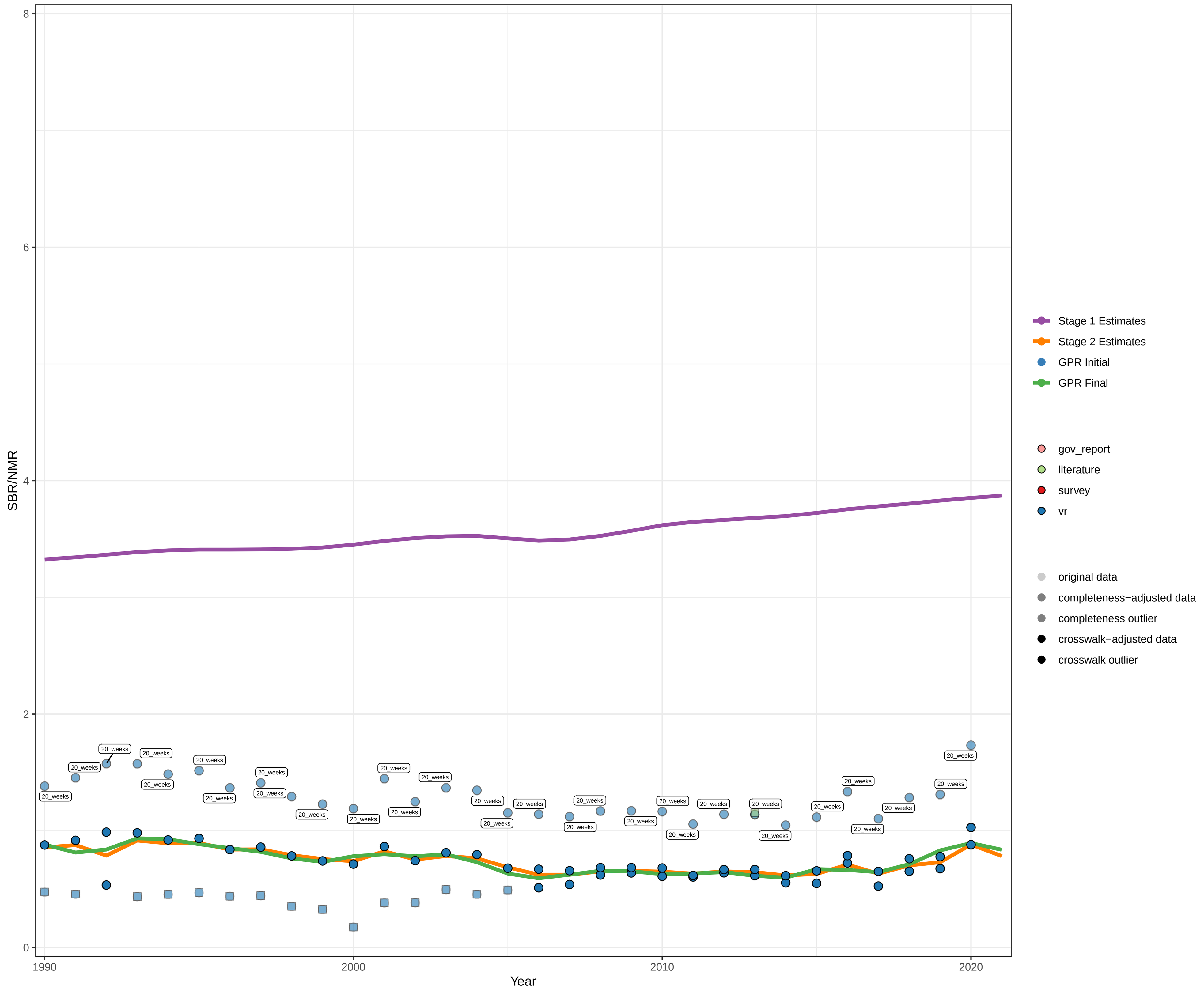

Definition	Years	Outlier	Source.Type	Title
20_weeks	1990−2020	0	VR	United States NVSS Custom Fetal Death Data
28_weeks	1992, 2006−2020	0	VR	United States NVSS Custom Fetal Death Data
20_weeks	2013	1	Sci Lit	United States Fetal and Perinatal Mortality 2013
28_weeks	1990, 1991, 1993−2005	1	VR	United States NVSS Custom Fetal Death Data

Oklahoma   USA_559   High−income North America   Stillbirth Rate (SBR) − 20 weeks
lambda = 0.2 ; zeta = 0.99 ; scale = 5 ; amp = 0.028

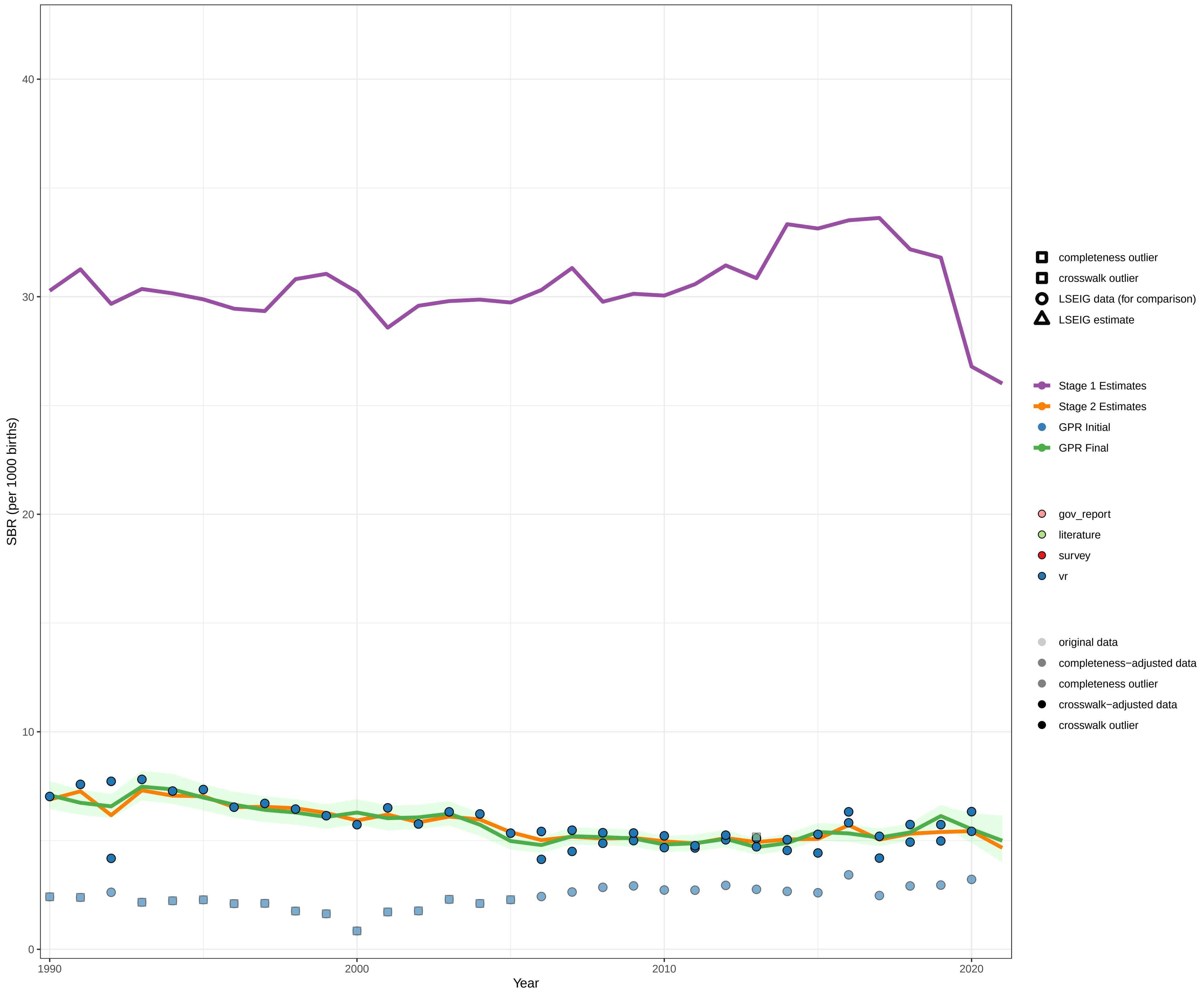

Oklahoma   USA_559   High−income North America   Stillbirth Rate (SBR) − 28 weeks
lambda = 0.2 ; zeta = 0.99 ; scale = 5 ; amp = 0.028

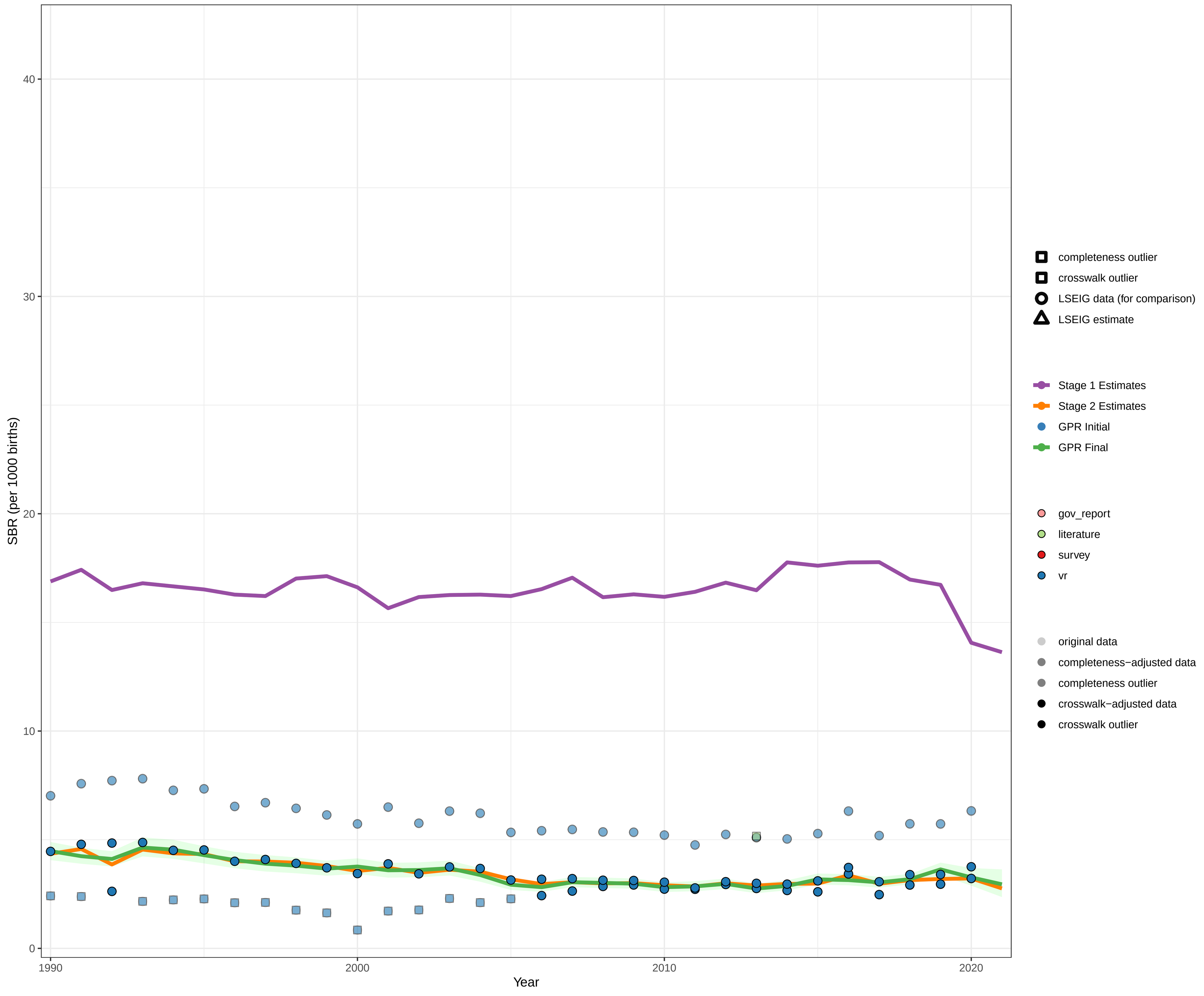

Year	Total Stillbirths (in thousands)	Total Stillbirths (in thousands)	SBR (per 1000 births)	SBR (per 1000 births)	SBR/NMR	SBR/NMR
	20 weeks	28 weeks	20 weeks	28 weeks	20 weeks	28 weeks
1990	0.3 (0.3 to 0.4)	0.2 (0.2 to 0.2)	7.1 (6.4 to 7.7)	4.5 (4.1 to 4.9)	1.40 (1.27 to 1.52)	0.88 (0.80 to 0.96)
1995	0.3 (0.3 to 0.4)	0.2 (0.2 to 0.2)	7.0 (6.4 to 7.6)	4.3 (3.9 to 4.7)	1.44 (1.32 to 1.57)	0.89 (0.81 to 0.97)
2000	0.3 (0.3 to 0.3)	0.2 (0.2 to 0.2)	6.3 (5.7 to 6.9)	3.8 (3.4 to 4.1)	1.31 (1.19 to 1.43)	0.78 (0.71 to 0.86)
2005	0.3 (0.2 to 0.3)	0.2 (0.1 to 0.2)	5.0 (4.6 to 5.4)	2.9 (2.7 to 3.2)	1.08 (0.99 to 1.17)	0.63 (0.58 to 0.69)
2010	0.2 (0.2 to 0.3)	0.1 (0.1 to 0.2)	4.8 (4.5 to 5.2)	2.8 (2.6 to 3.0)	1.08 (1.00 to 1.17)	0.63 (0.58 to 0.68)
2015	0.3 (0.3 to 0.3)	0.2 (0.2 to 0.2)	5.4 (5.0 to 5.8)	3.2 (2.9 to 3.4)	1.14 (1.06 to 1.23)	0.67 (0.62 to 0.73)
2020	0.3 (0.2 to 0.3)	0.2 (0.1 to 0.2)	5.5 (4.9 to 6.2)	3.3 (2.9 to 3.7)	1.51 (1.34 to 1.71)	0.89 (0.79 to 1.01)
2021	0.2 (0.2 to 0.3)	0.1 (0.1 to 0.2)	5.0 (4.0 to 6.2)	2.9 (2.4 to 3.6)	1.42 (1.14 to 1.75)	0.84 (0.67 to 1.03)

Oregon USA_560 High−income North America Stillbirth Rate / Neonatal Mortality Rate (SBR/NMR) − 20 weeks
lambda = 0.2 ; zeta = 0.99 ; scale = 5 ; amp = 0.028

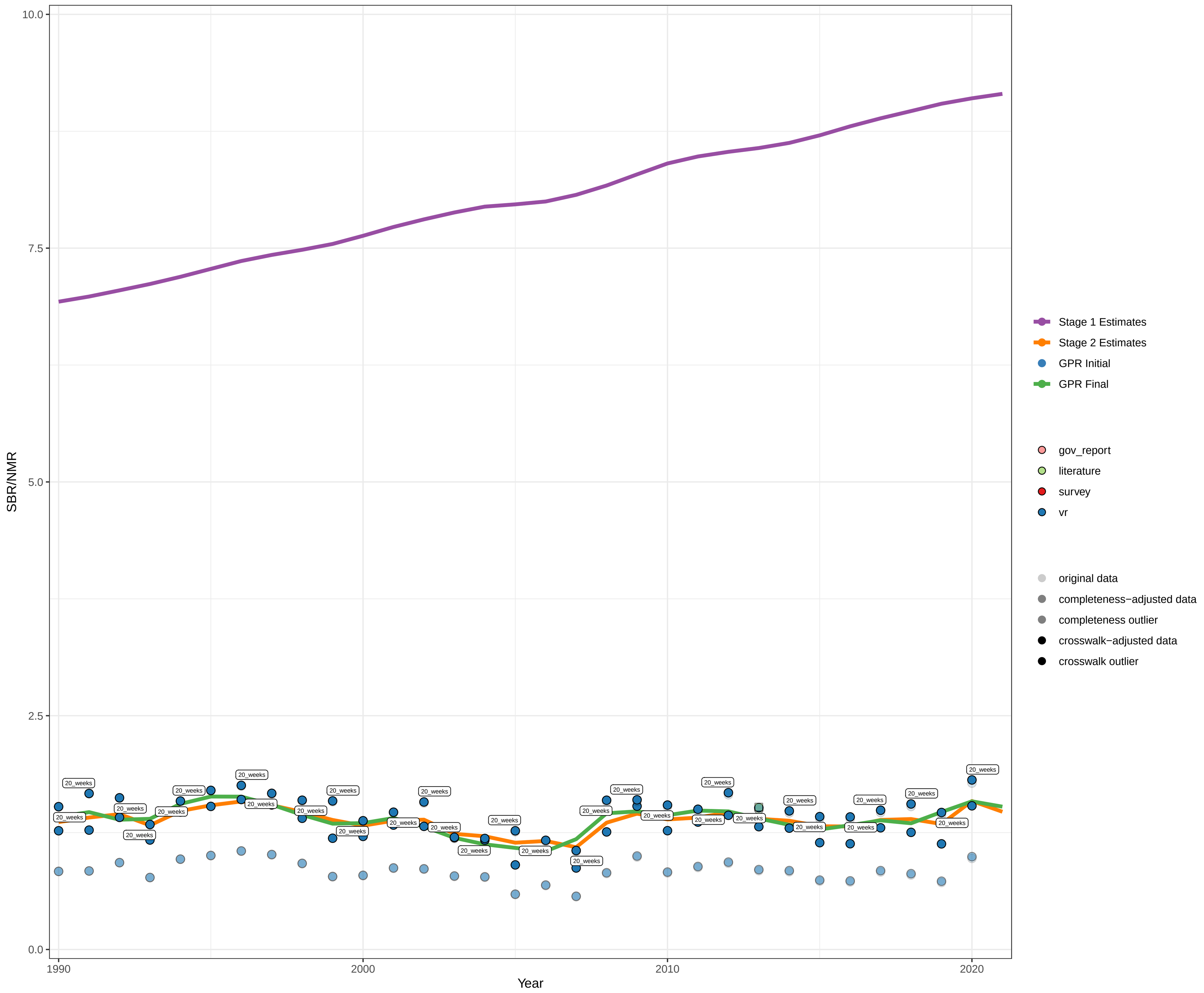

Oregon USA_560 High−income North America Stillbirth Rate / Neonatal Mortality Rate (SBR/NMR) − 28 weeks
lambda = 0.2 ; zeta = 0.99 ; scale = 5 ; amp = 0.028

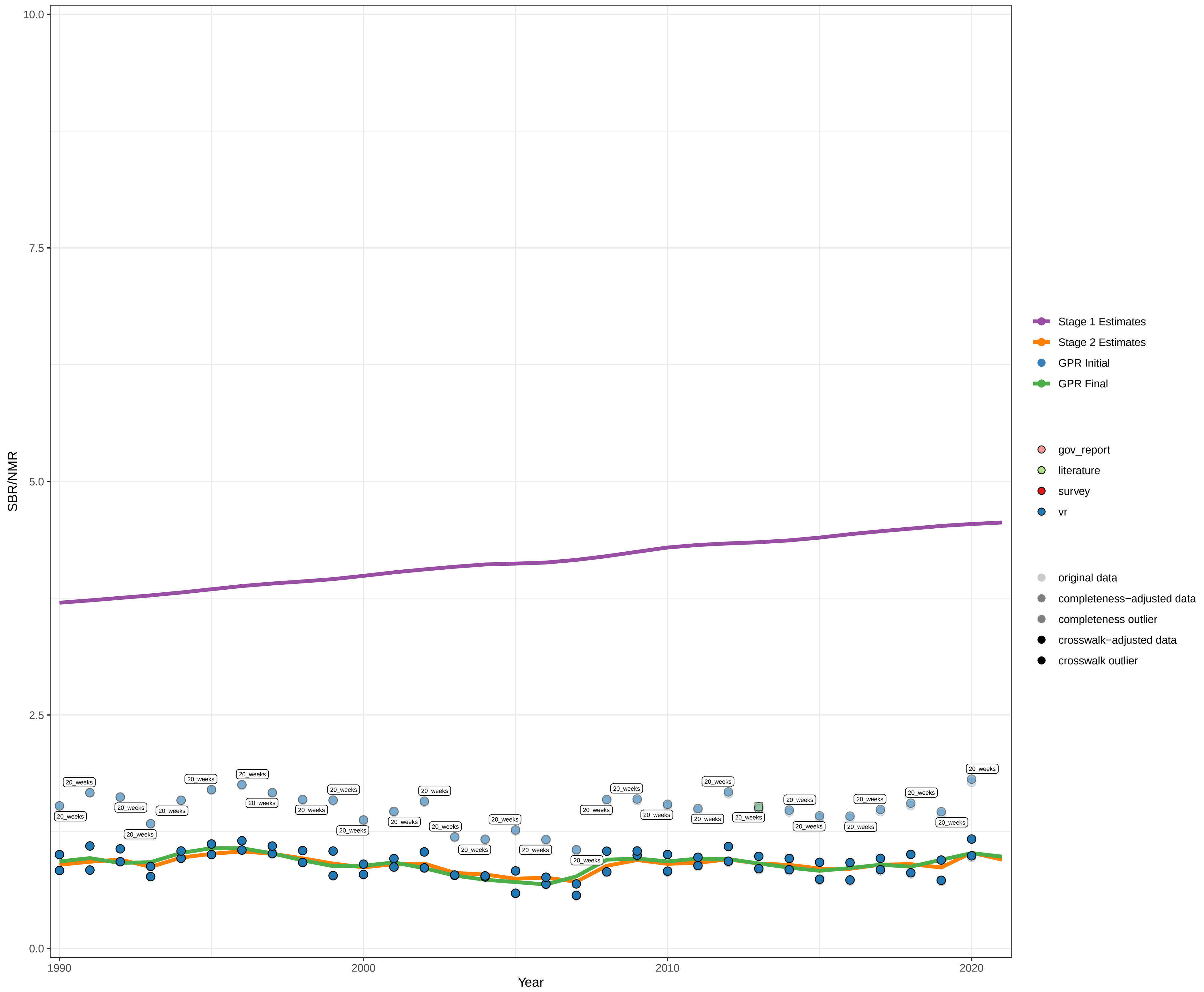

Definition	Years	Outlier	Source.Type	Title
20_weeks, 28_weeks	1990−2020	0	VR	United States NVSS Custom Fetal Death Data
20_weeks	2013	1	Sci Lit	United States Fetal and Perinatal Mortality 2013

Oregon USA_560 High−income North America Stillbirth Rate (SBR) − 20 weeks
lambda = 0.2 ; zeta = 0.99 ; scale = 5 ; amp = 0.028

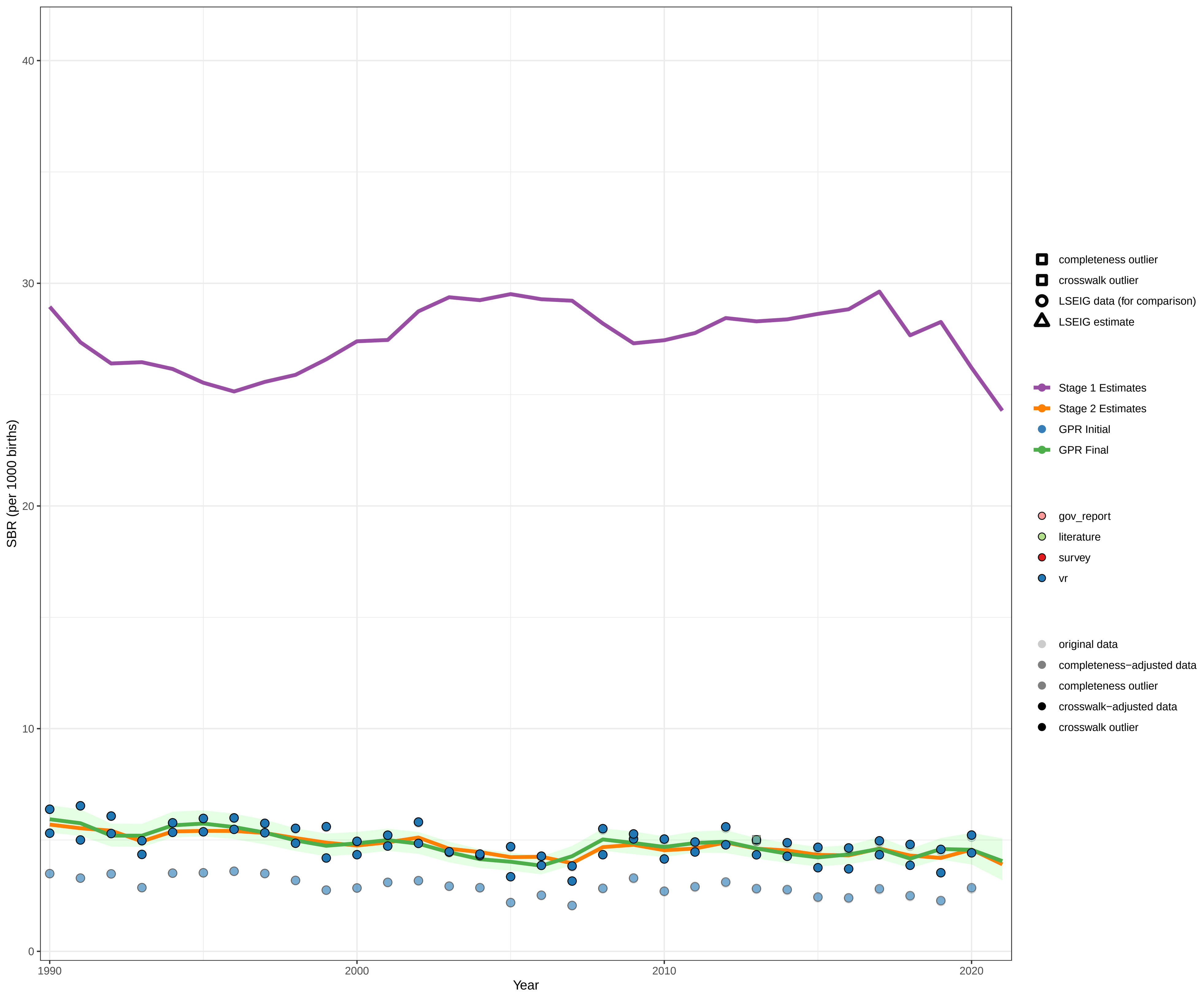

Oregon USA_560 High−income North America Stillbirth Rate (SBR) − 28 weeks
lambda = 0.2 ; zeta = 0.99 ; scale = 5 ; amp = 0.028

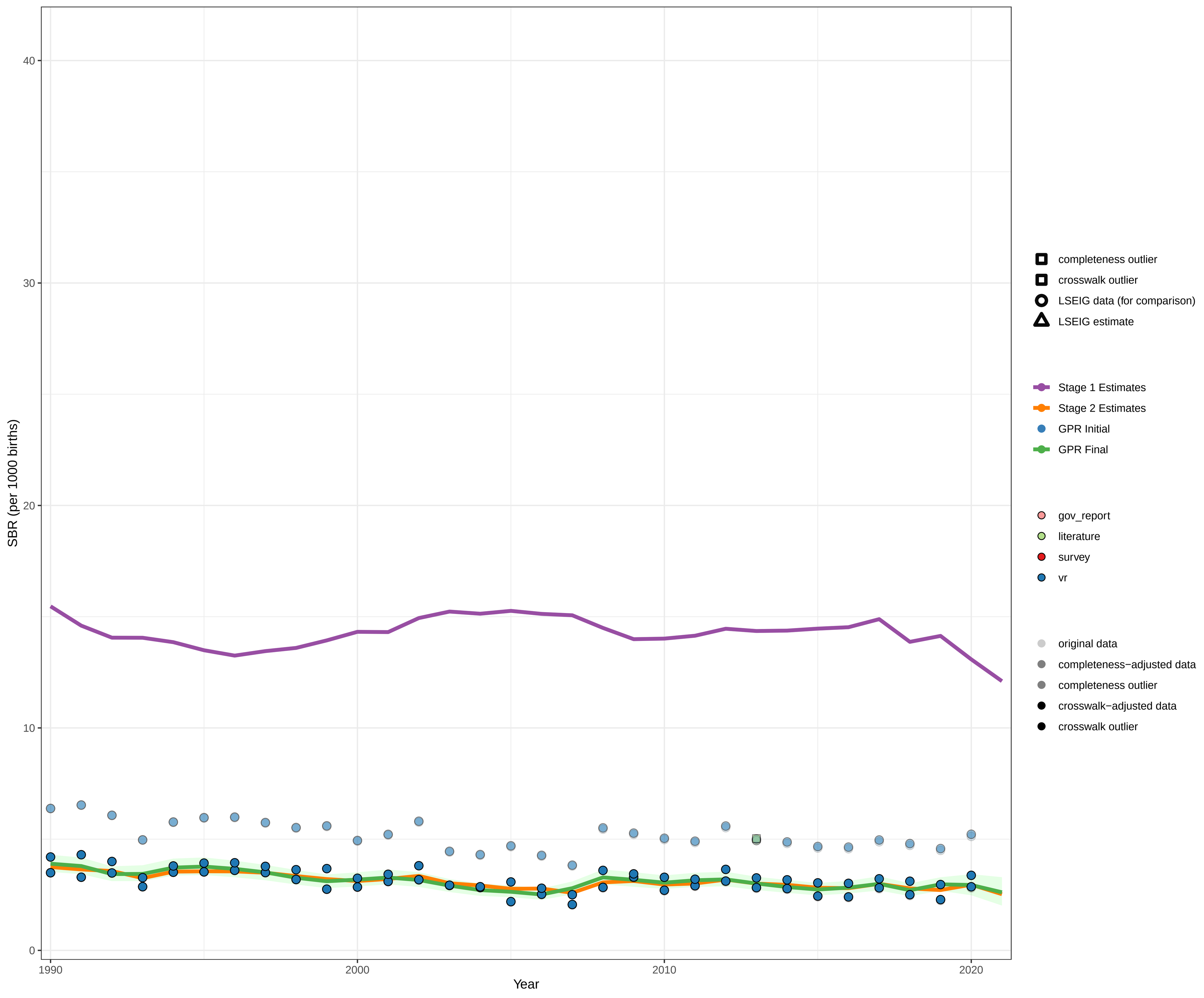

Year	Total Stillbirths (in thousands)	Total Stillbirths (in thousands)	SBR (per 1000 births)	SBR (per 1000 births)	SBR/NMR	SBR/NMR
	20 weeks	28 weeks	20 weeks	28 weeks	20 weeks	28 weeks
1990	0.3 (0.2 to 0.3)	0.2 (0.2 to 0.2)	5.9 (5.3 to 6.6)	3.9 (3.5 to 4.3)	1.42 (1.28 to 1.57)	0.93 (0.84 to 1.03)
1995	0.2 (0.2 to 0.3)	0.2 (0.1 to 0.2)	5.7 (5.2 to 6.3)	3.8 (3.4 to 4.2)	1.64 (1.47 to 1.81)	1.07 (0.97 to 1.19)
2000	0.2 (0.2 to 0.2)	0.1 (0.1 to 0.2)	4.9 (4.4 to 5.4)	3.2 (2.9 to 3.5)	1.35 (1.22 to 1.49)	0.89 (0.80 to 0.98)
2005	0.2 (0.2 to 0.2)	0.1 (0.1 to 0.1)	4.0 (3.6 to 4.4)	2.6 (2.4 to 2.9)	1.09 (0.98 to 1.20)	0.71 (0.65 to 0.79)
2010	0.2 (0.2 to 0.2)	0.1 (0.1 to 0.2)	4.7 (4.2 to 5.2)	3.0 (2.8 to 3.4)	1.44 (1.30 to 1.58)	0.93 (0.85 to 1.03)
2015	0.2 (0.2 to 0.2)	0.1 (0.1 to 0.1)	4.2 (3.8 to 4.7)	2.7 (2.5 to 3.0)	1.28 (1.16 to 1.42)	0.83 (0.75 to 0.92)
2020	0.2 (0.2 to 0.2)	0.1 (0.1 to 0.1)	4.6 (3.9 to 5.3)	2.9 (2.5 to 3.4)	1.58 (1.36 to 1.85)	1.02 (0.86 to 1.19)
2021	0.2 (0.1 to 0.2)	0.1 (0.1 to 0.1)	4.1 (3.2 to 5.1)	2.6 (2.0 to 3.3)	1.53 (1.20 to 1.91)	0.98 (0.76 to 1.24)

Pennsylvania USA_561 High−income North America Stillbirth Rate / Neonatal Mortality Rate (SBR/NMR) − 20 weeks
lambda = 0.2 ; zeta = 0.99 ; scale = 5 ; amp = 0.028

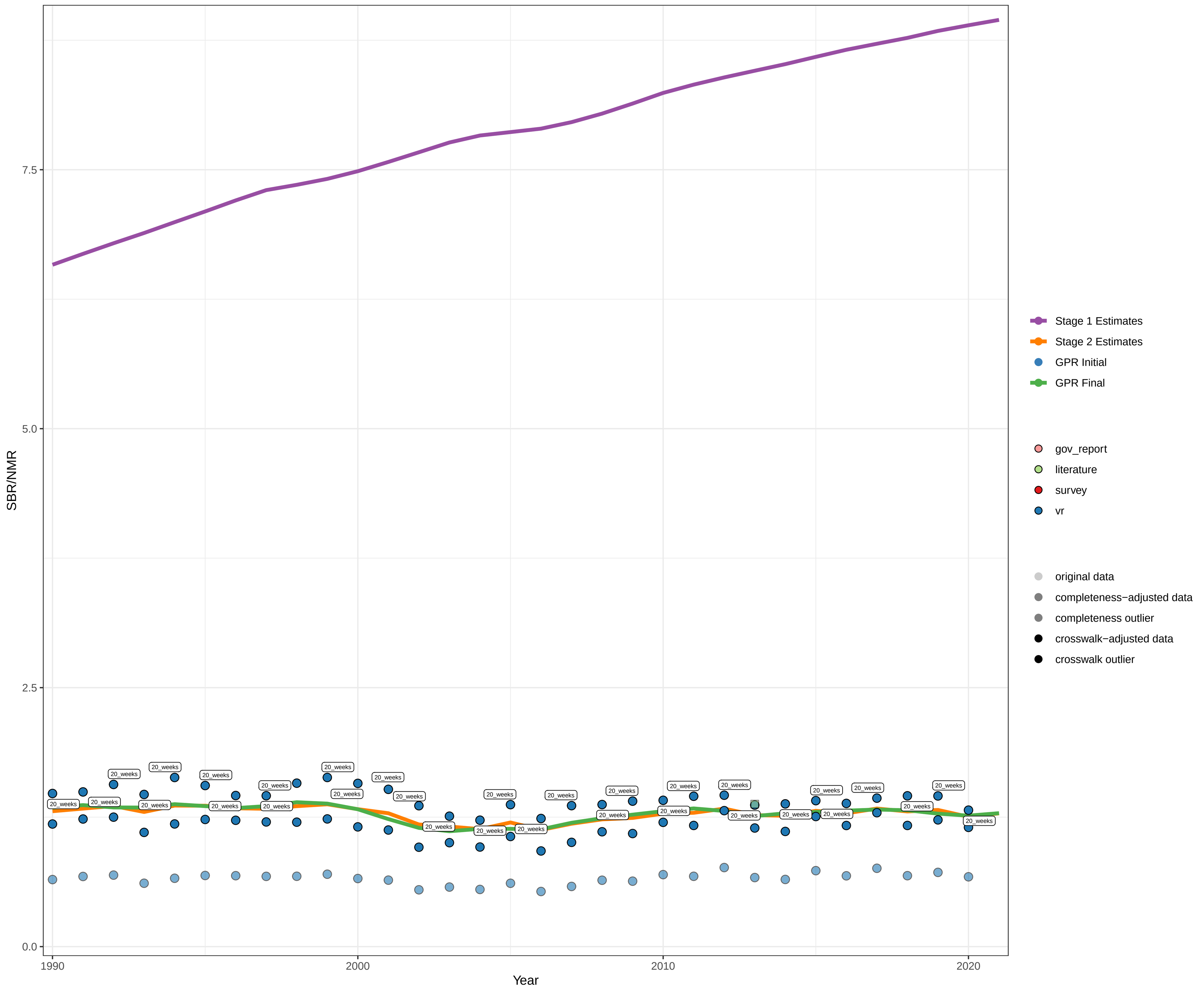

Pennsylvania USA_561 High−income North America Stillbirth Rate / Neonatal Mortality Rate (SBR/NMR) − 28 weeks
lambda = 0.2 ; zeta = 0.99 ; scale = 5 ; amp = 0.028

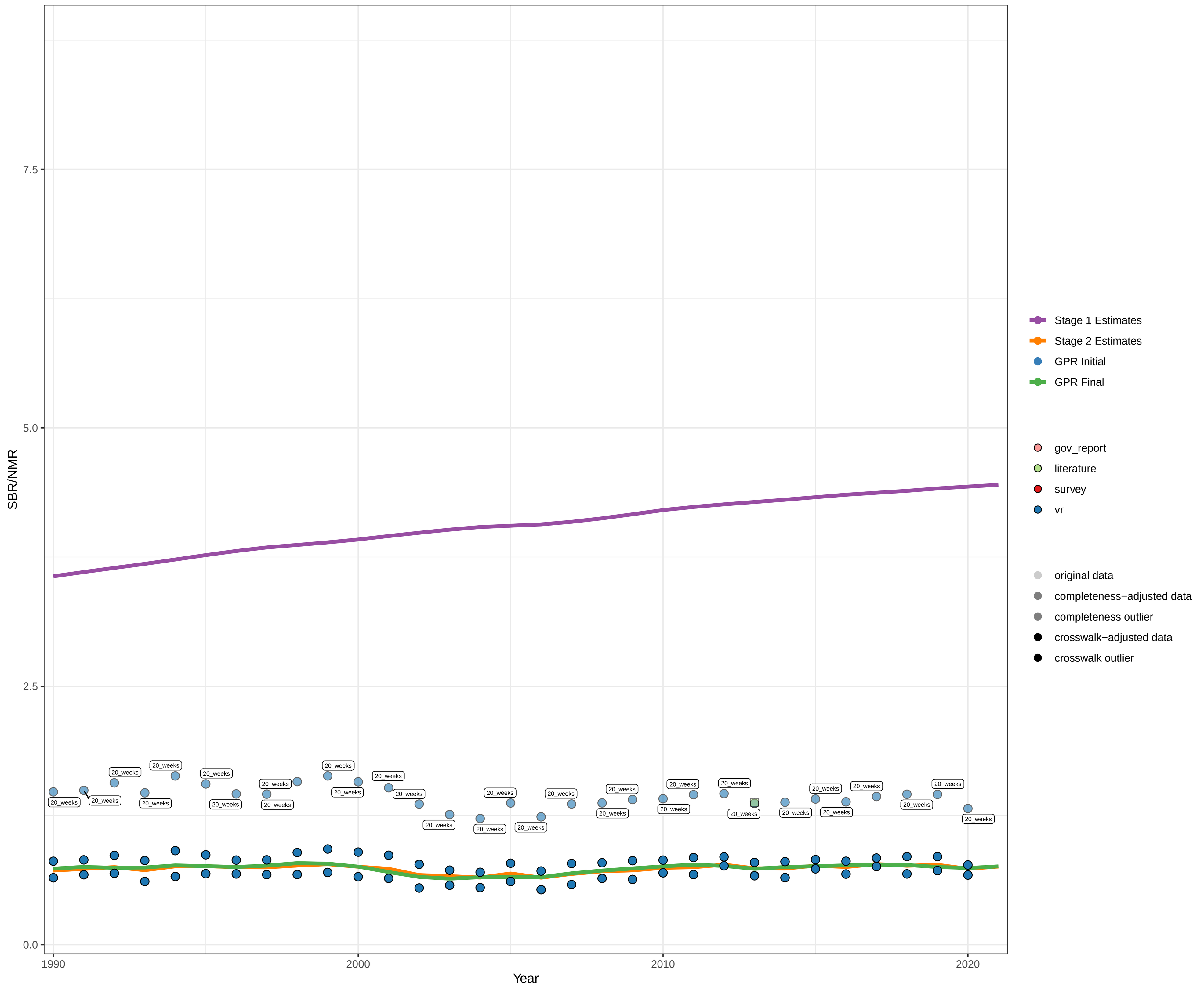

Definition	Years	Outlier	Source.Type	Title
20_weeks, 28_weeks	1990−2020	0	VR	United States NVSS Custom Fetal Death Data
20_weeks	2013	1	Sci Lit	United States Fetal and Perinatal Mortality 2013

Pennsylvania USA_561 High−income North America Stillbirth Rate (SBR) − 20 weeks
lambda = 0.2 ; zeta = 0.99 ; scale = 5 ; amp = 0.028

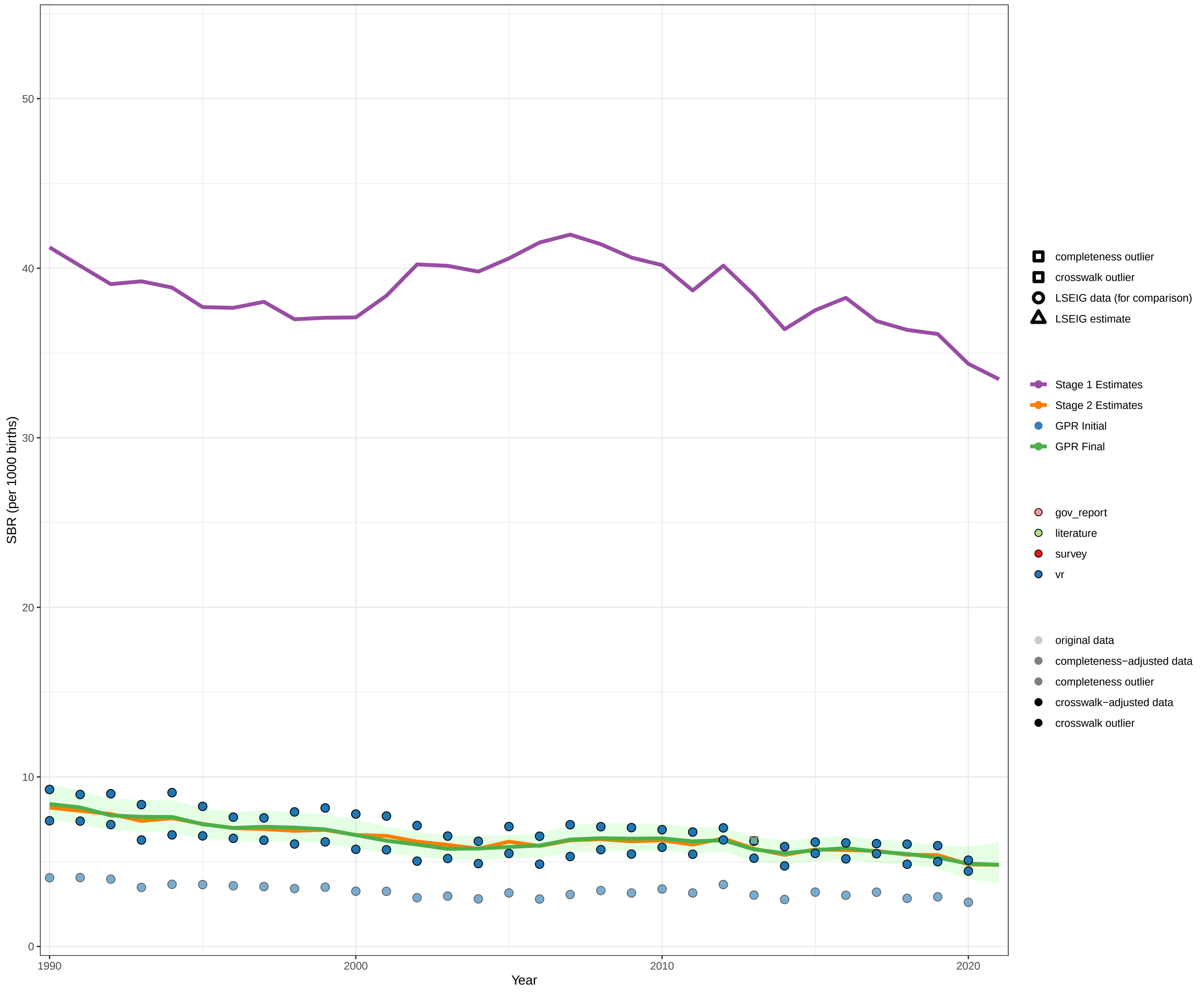

Pennsylvania USA_561 High−income North America Stillbirth Rate (SBR) − 28 weeks
lambda = 0.2 ; zeta = 0.99 ; scale = 5 ; amp = 0.028

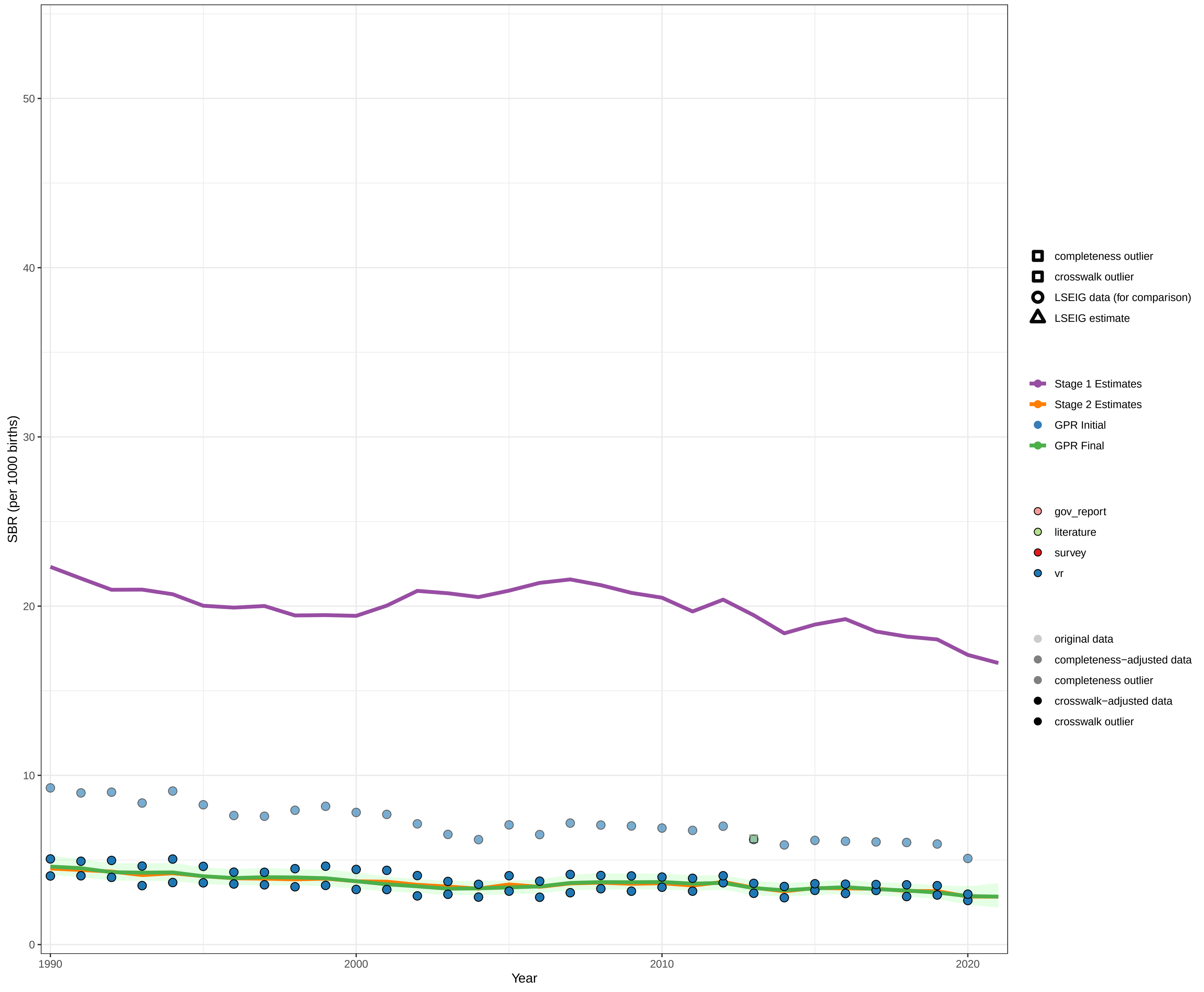

Year	Total Stillbirths (in thousands)	Total Stillbirths (in thousands)	SBR (per 1000 births)	SBR (per 1000 births)	SBR/NMR	SBR/NMR
	20 weeks	28 weeks	20 weeks	28 weeks	20 weeks	28 weeks
1990	1.5 (1.3 to 1.7)	0.8 (0.7 to 0.9)	8.4 (7.5 to 9.6)	4.6 (4.1 to 5.2)	1.34 (1.19 to 1.53)	0.74 (0.65 to 0.84)
1995	1.1 (1.0 to 1.2)	0.6 (0.5 to 0.7)	7.2 (6.4 to 8.2)	4.0 (3.6 to 4.6)	1.36 (1.20 to 1.54)	0.76 (0.67 to 0.86)
2000	1.0 (0.8 to 1.1)	0.5 (0.5 to 0.6)	6.6 (5.8 to 7.5)	3.7 (3.3 to 4.3)	1.33 (1.17 to 1.51)	0.75 (0.66 to 0.86)
2005	0.9 (0.8 to 1.0)	0.5 (0.4 to 0.6)	5.9 (5.2 to 6.6)	3.4 (3.0 to 3.8)	1.14 (1.00 to 1.27)	0.65 (0.58 to 0.73)
2010	0.9 (0.8 to 1.0)	0.5 (0.5 to 0.6)	6.4 (5.6 to 7.2)	3.7 (3.3 to 4.2)	1.31 (1.15 to 1.49)	0.76 (0.67 to 0.86)
2015	0.8 (0.7 to 0.9)	0.5 (0.4 to 0.5)	5.7 (5.0 to 6.4)	3.3 (2.9 to 3.7)	1.30 (1.14 to 1.46)	0.76 (0.67 to 0.85)
2020	0.6 (0.5 to 0.8)	0.4 (0.3 to 0.5)	4.9 (4.0 to 5.9)	2.9 (2.4 to 3.5)	1.26 (1.04 to 1.52)	0.74 (0.61 to 0.89)
2021	0.6 (0.5 to 0.8)	0.4 (0.3 to 0.5)	4.8 (3.7 to 6.2)	2.8 (2.2 to 3.6)	1.29 (0.99 to 1.65)	0.76 (0.58 to 0.97)

Rhode Island USA_562 High−income North America Stillbirth Rate / Neonatal Mortality Rate (SBR/NMR) − 20 weeks
lambda = 0.2 ; zeta = 0.99 ; scale = 5 ; amp = 0.028

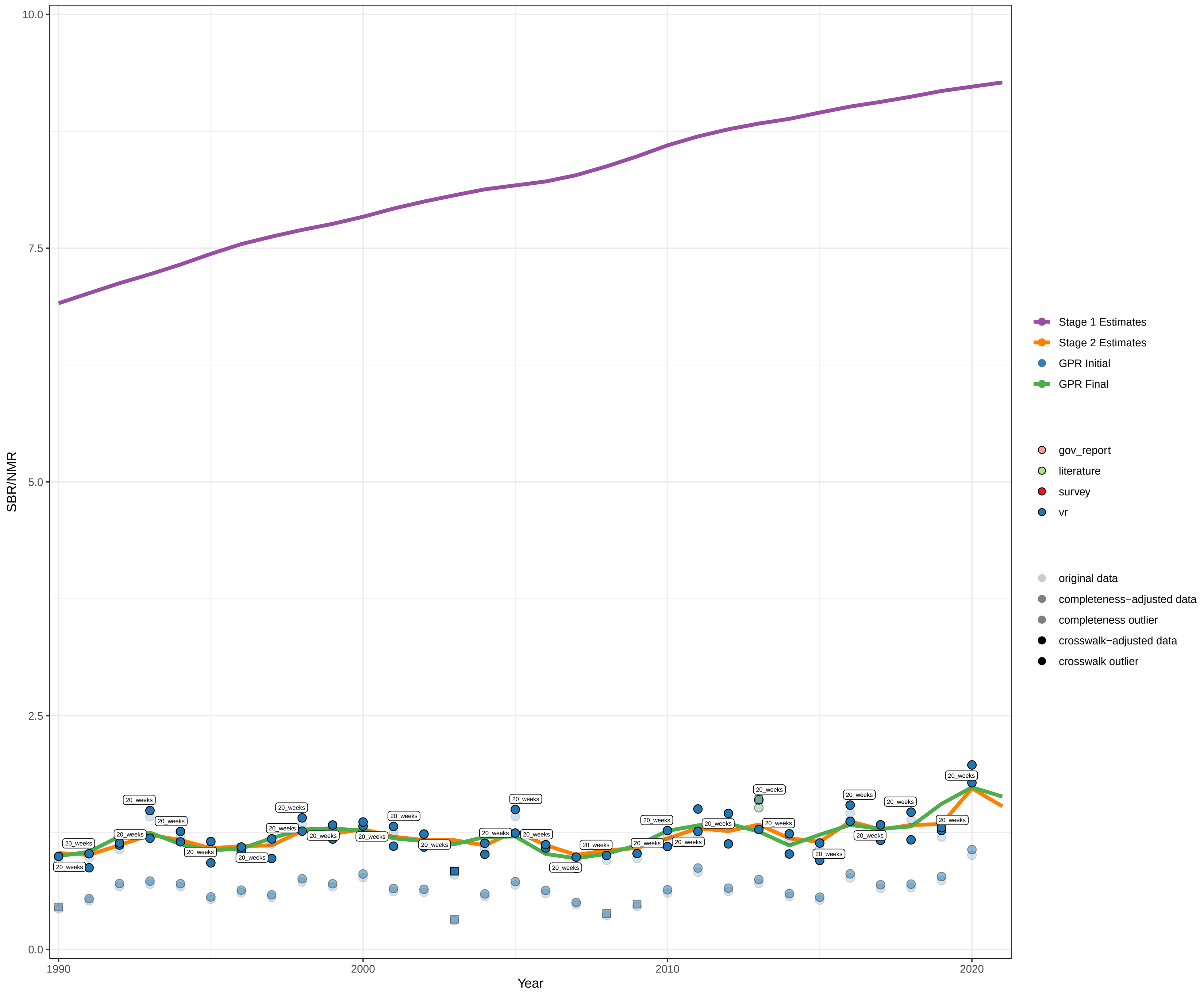

Rhode Island USA_562 High−income North America Stillbirth Rate / Neonatal Mortality Rate (SBR/NMR) − 28 weeks
lambda = 0.2 ; zeta = 0.99 ; scale = 5 ; amp = 0.028

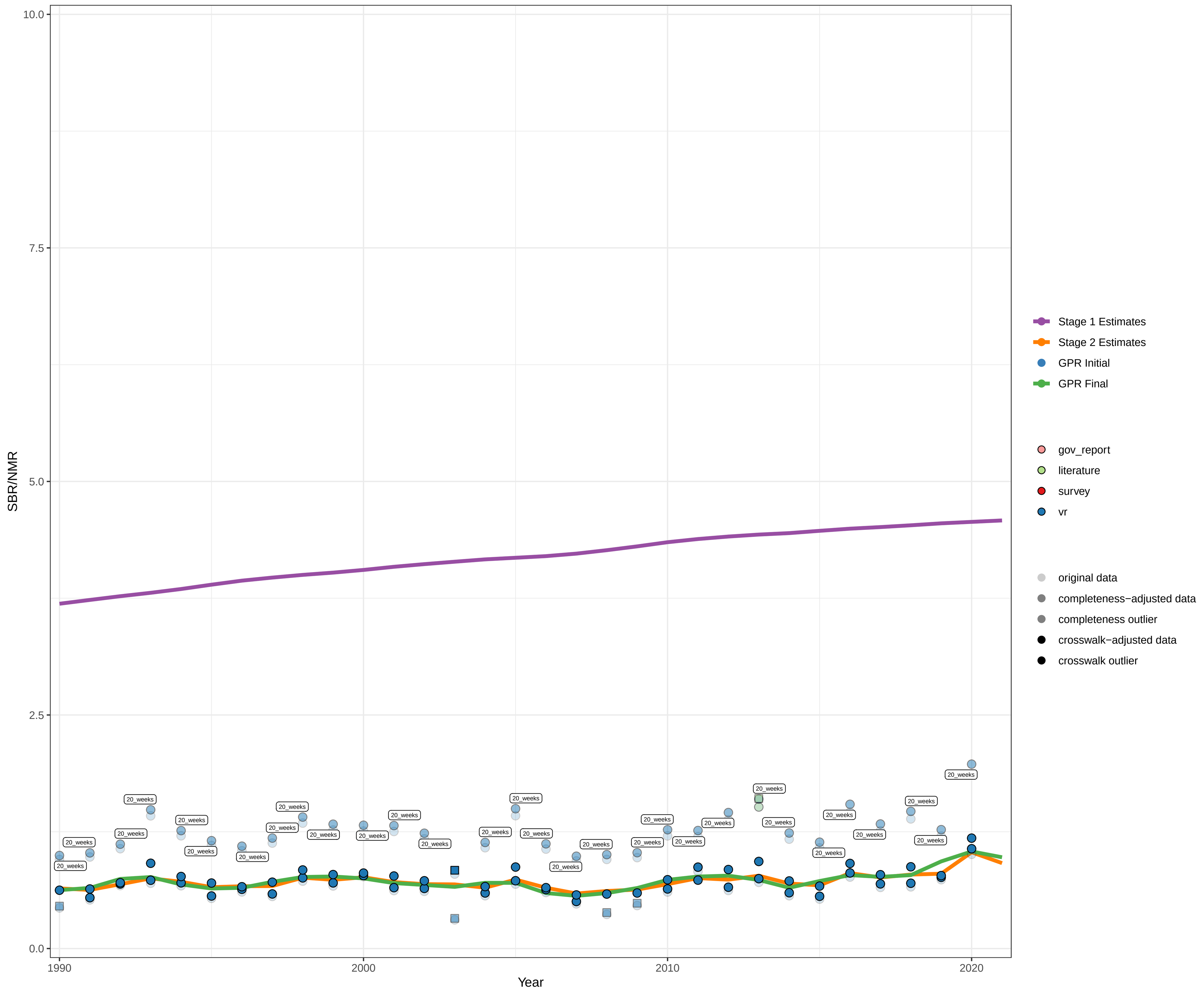

Definition	Years	Outlier	Source.Type	Title
20_weeks	1990−2002, 2004−2020	0	VR	United States NVSS Custom Fetal Death Data
28_weeks	1991−2002, 2004−2007, 2010−2020	0	VR	United States NVSS Custom Fetal Death Data
20_weeks	2013	1	Sci Lit	United States Fetal and Perinatal Mortality 2013
28_weeks	1990, 2003, 2008, 2009	1	VR	United States NVSS Custom Fetal Death Data
20_weeks	2003	2	VR	United States NVSS Custom Fetal Death Data

Rhode Island USA_562 High−income North America Stillbirth Rate (SBR) − 20 weeks
lambda = 0.2 ; zeta = 0.99 ; scale = 5 ; amp = 0.028

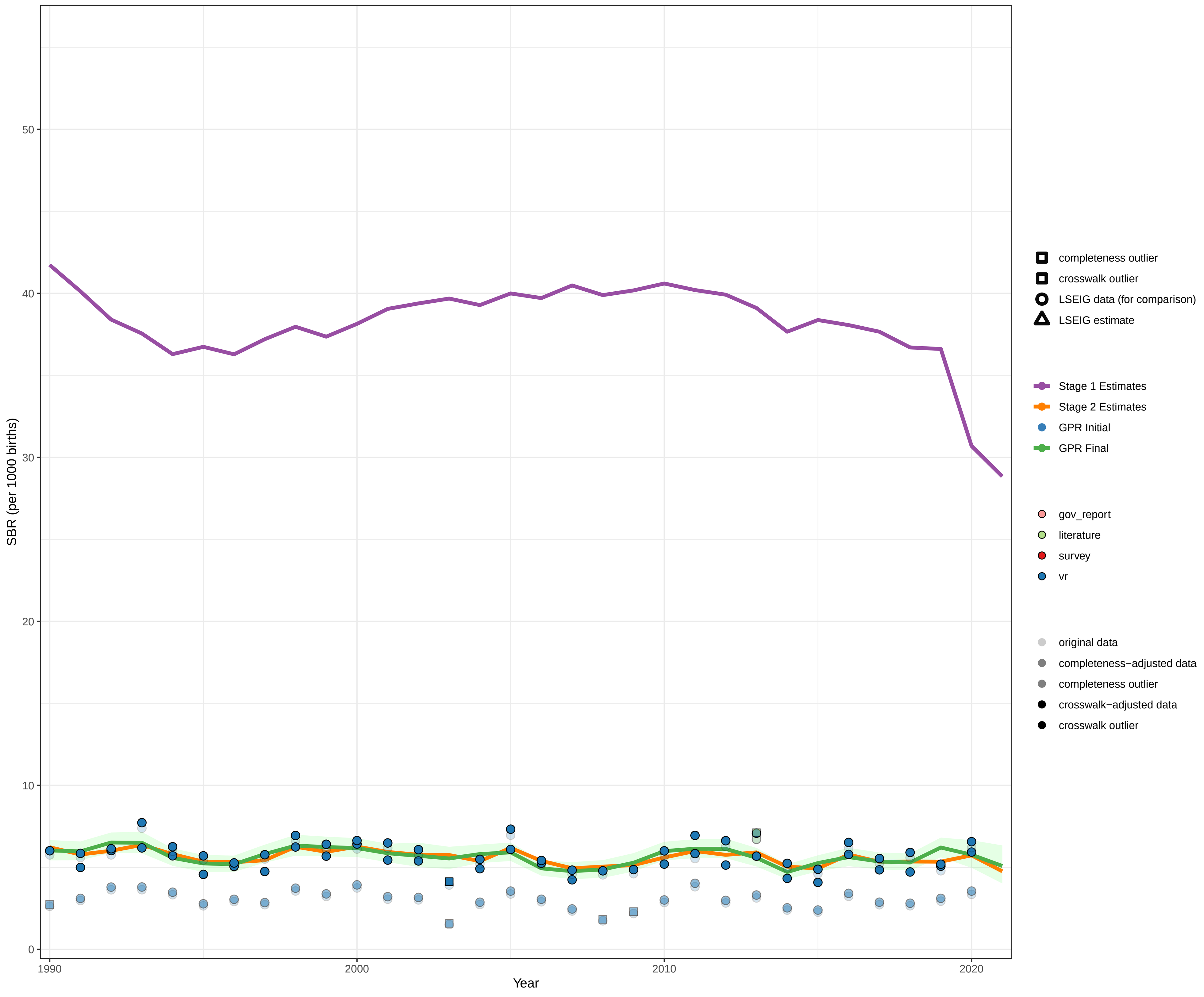

Rhode Island USA_562 High−income North America Stillbirth Rate (SBR) − 28 weeks
lambda = 0.2 ; zeta = 0.99 ; scale = 5 ; amp = 0.028

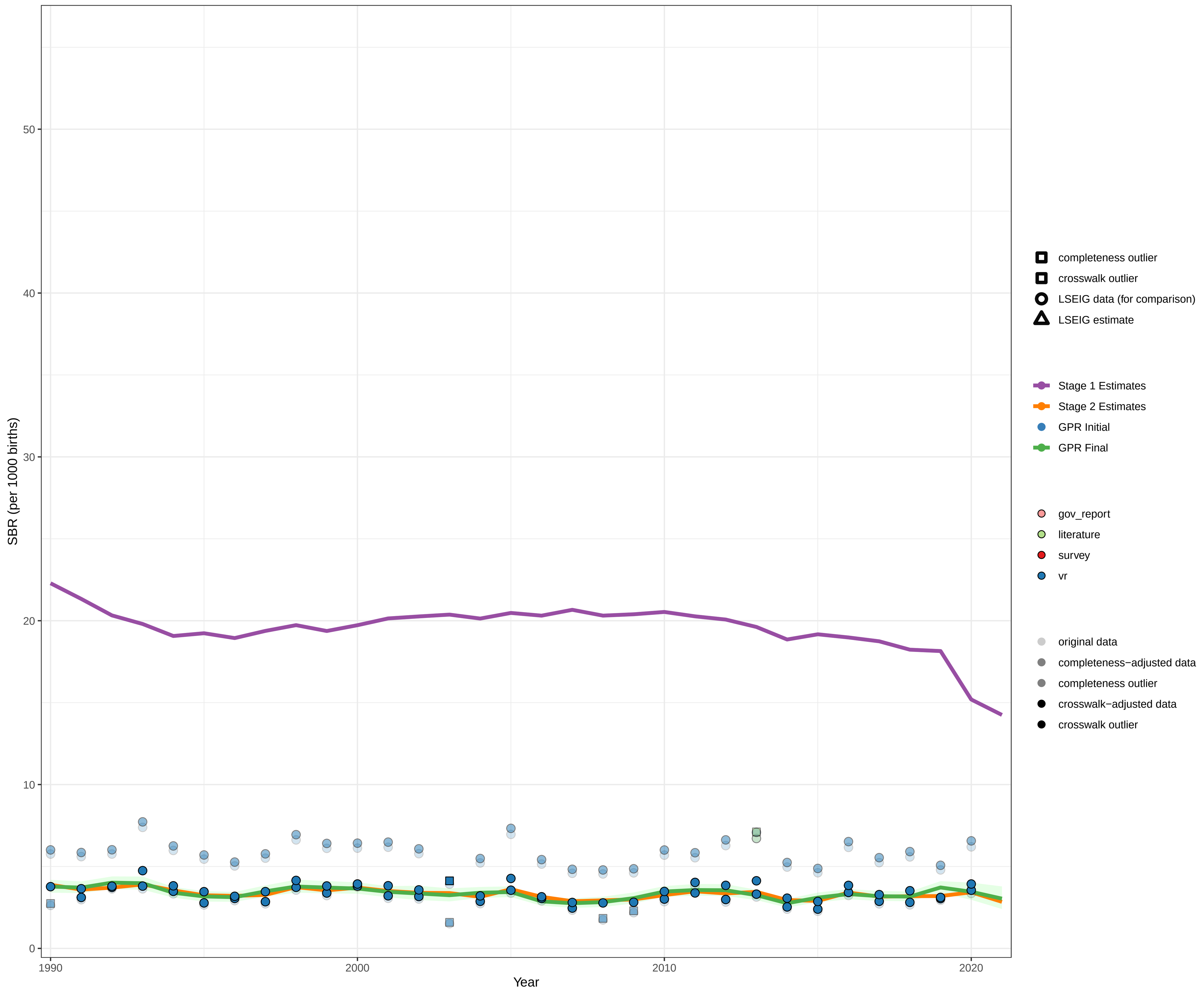

Year	Total Stillbirths (in thousands)	Total Stillbirths (in thousands)	SBR (per 1000 births)	SBR (per 1000 births)	SBR/NMR	SBR/NMR
	20 weeks	28 weeks	20 weeks	28 weeks	20 weeks	28 weeks
1990	0.1 (0.1 to 0.1)	0.1 (0.1 to 0.1)	6.0 (5.4 to 6.7)	3.8 (3.4 to 4.2)	1.00 (0.90 to 1.10)	0.63 (0.56 to 0.69)
1995	0.1 (0.1 to 0.1)	0.0 (0.0 to 0.0)	5.2 (4.7 to 5.8)	3.2 (2.9 to 3.5)	1.06 (0.96 to 1.17)	0.64 (0.58 to 0.71)
2000	0.1 (0.1 to 0.1)	0.0 (0.0 to 0.1)	6.2 (5.6 to 6.8)	3.7 (3.3 to 4.0)	1.27 (1.16 to 1.39)	0.75 (0.69 to 0.83)
2005	0.1 (0.1 to 0.1)	0.0 (0.0 to 0.1)	5.9 (5.4 to 6.5)	3.4 (3.1 to 3.8)	1.21 (1.10 to 1.33)	0.70 (0.64 to 0.77)
2010	0.1 (0.1 to 0.1)	0.0 (0.0 to 0.0)	6.0 (5.4 to 6.6)	3.5 (3.1 to 3.8)	1.27 (1.15 to 1.39)	0.74 (0.67 to 0.81)
2015	0.1 (0.1 to 0.1)	0.0 (0.0 to 0.0)	5.3 (4.8 to 5.7)	3.1 (2.8 to 3.4)	1.23 (1.11 to 1.34)	0.72 (0.66 to 0.79)
2020	0.1 (0.1 to 0.1)	0.0 (0.0 to 0.0)	5.8 (5.0 to 6.7)	3.5 (3.0 to 4.0)	1.73 (1.49 to 2.00)	1.04 (0.89 to 1.21)
2021	0.1 (0.0 to 0.1)	0.0 (0.0 to 0.0)	5.1 (4.0 to 6.3)	3.0 (2.4 to 3.8)	1.64 (1.29 to 2.04)	0.98 (0.77 to 1.22)

South Carolina USA_563 High−income North America Stillbirth Rate / Neonatal Mortality Rate (SBR/NMR) − 20 weeks
lambda = 0.2 ; zeta = 0.99 ; scale = 5 ; amp = 0.028

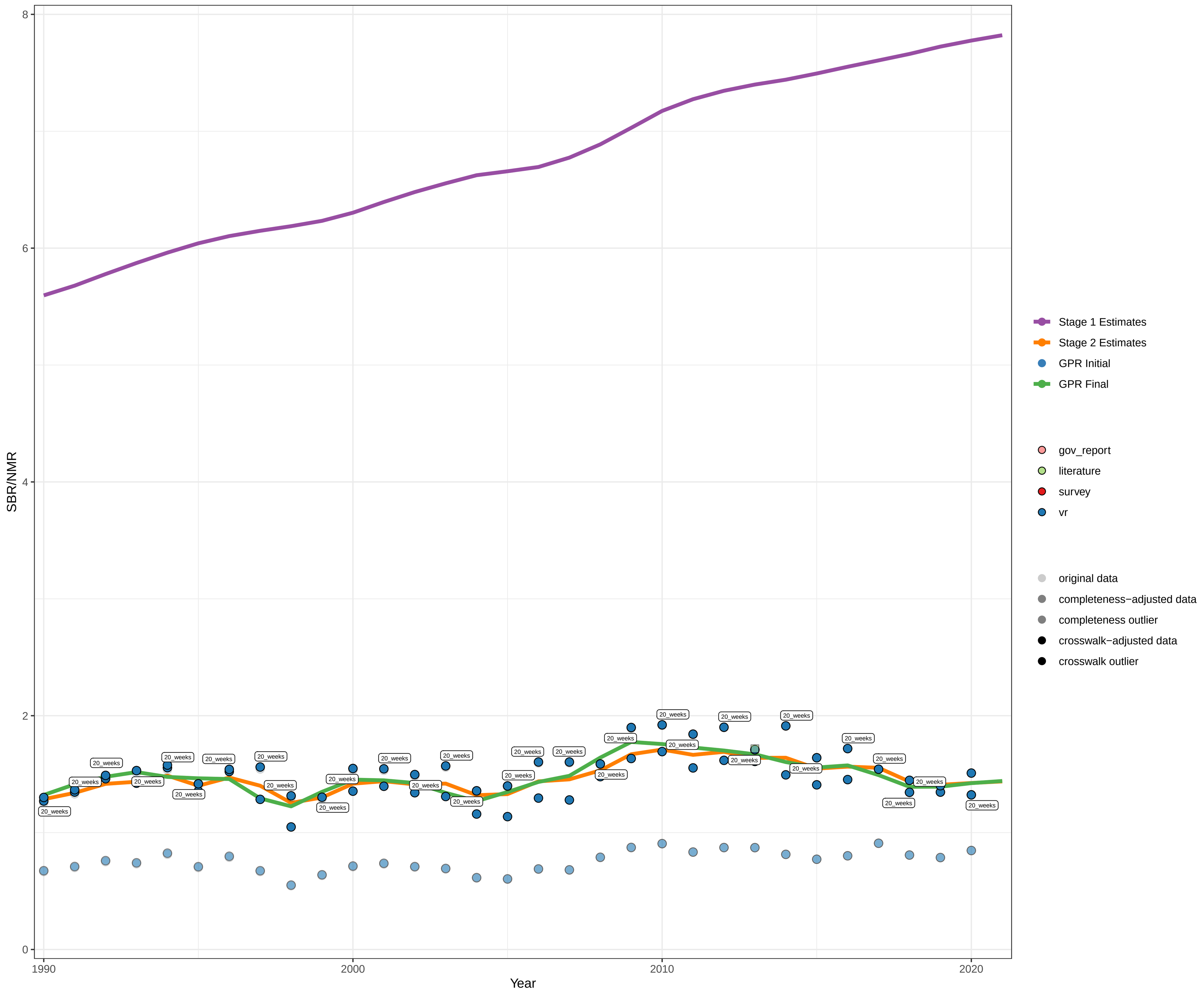

South Carolina USA_563 High−income North America Stillbirth Rate / Neonatal Mortality Rate (SBR/NMR) − 28 weeks
lambda = 0.2 ; zeta = 0.99 ; scale = 5 ; amp = 0.028

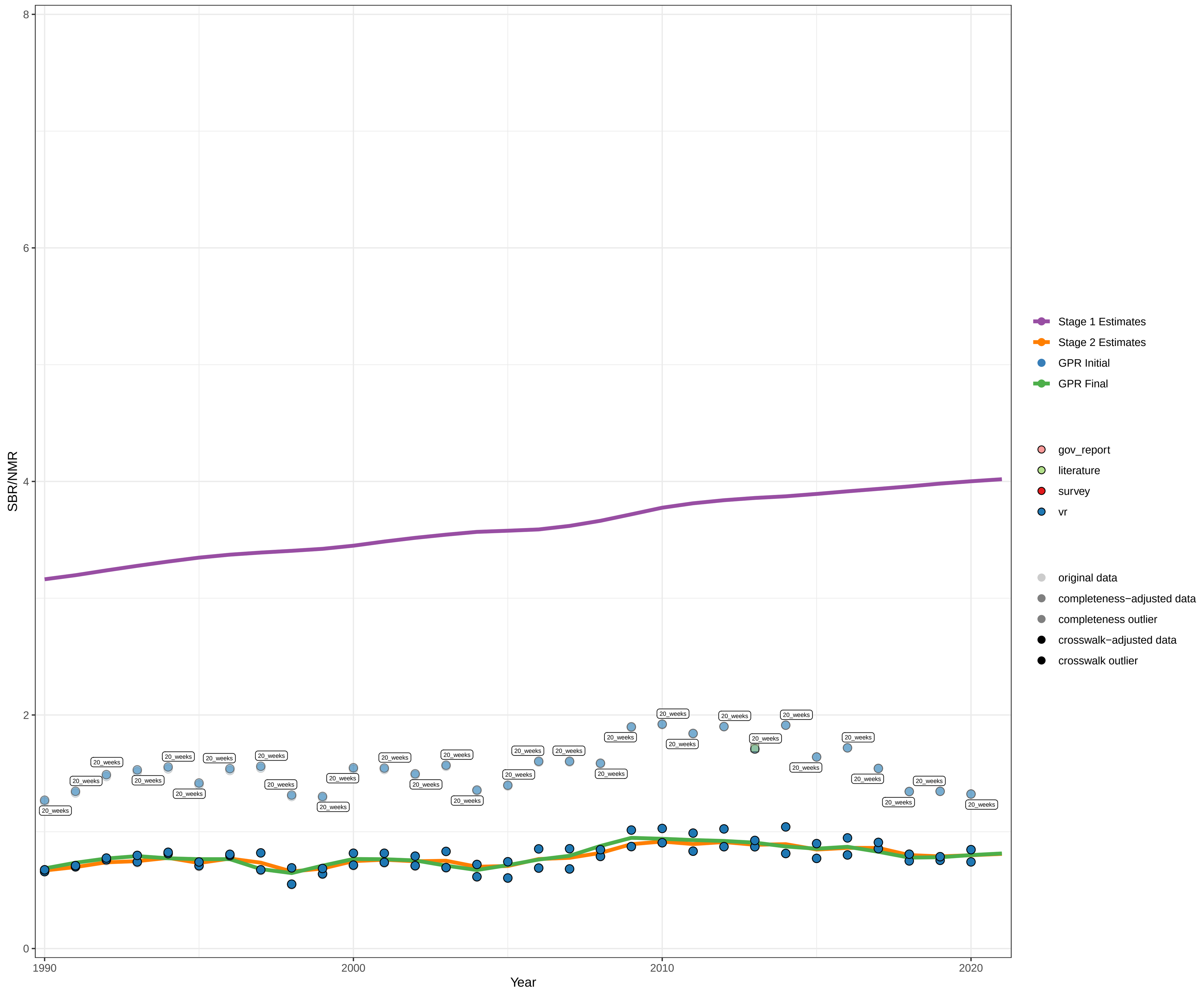

Definition	Years	Outlier	Source.Type	Title
20_weeks, 28_weeks	1990−2020	0	VR	United States NVSS Custom Fetal Death Data
20_weeks	2013	1	Sci Lit	United States Fetal and Perinatal Mortality 2013

South Carolina USA_563 High−income North America Stillbirth Rate (SBR) − 20 weeks
lambda = 0.2 ; zeta = 0.99 ; scale = 5 ; amp = 0.028

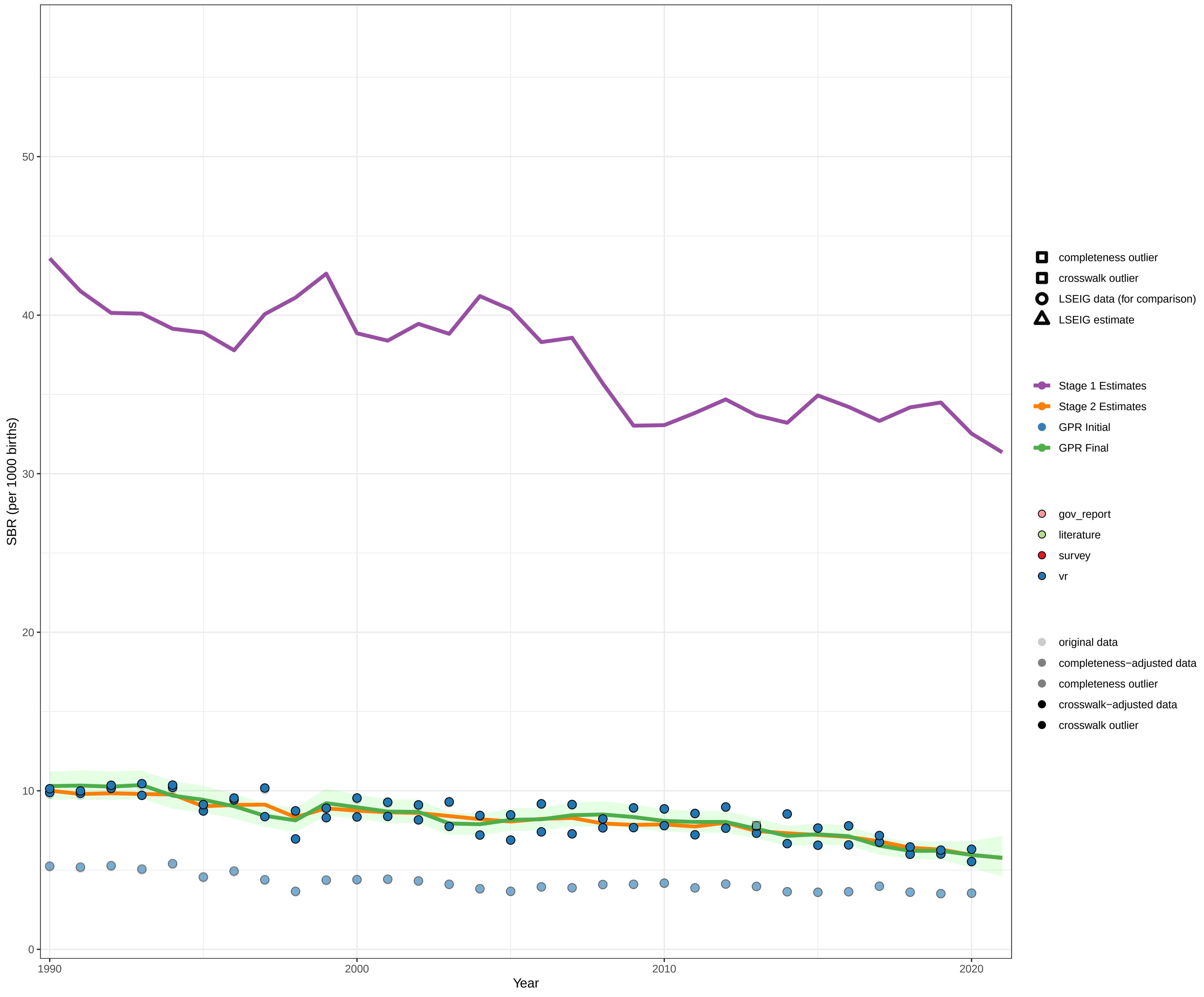

South Carolina USA_563 High−income North America Stillbirth Rate (SBR) − 28 weeks
lambda = 0.2 ; zeta = 0.99 ; scale = 5 ; amp = 0.028

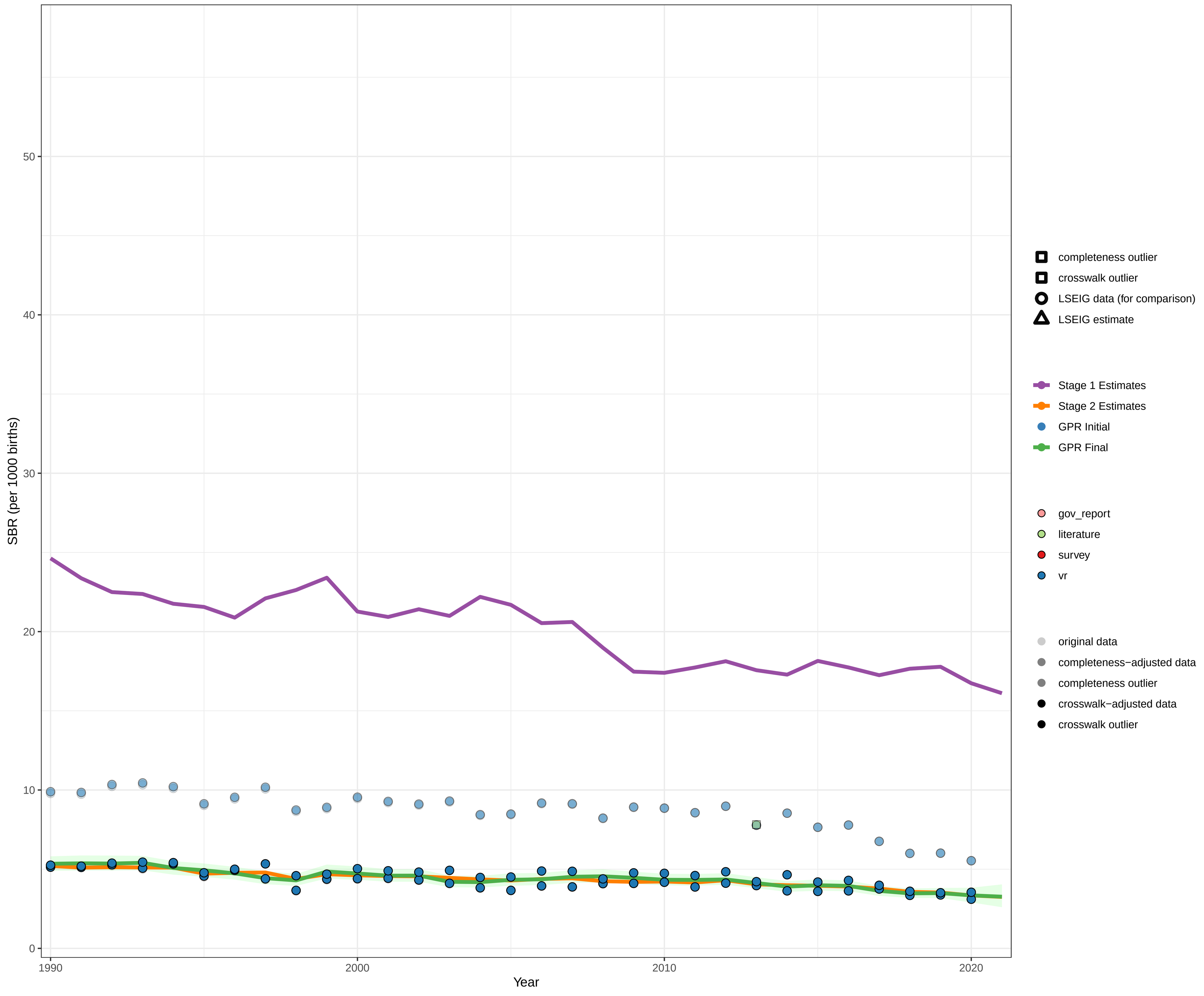

Year	Total Stillbirths (in thousands)	Total Stillbirths (in thousands)	SBR (per 1000 births)	SBR (per 1000 births)	SBR/NMR	SBR/NMR
	20 weeks	28 weeks	20 weeks	28 weeks	20 weeks	28 weeks
1990	0.6 (0.6 to 0.7)	0.3 (0.3 to 0.3)	10.3 (9.4 to 11.2)	5.3 (4.9 to 5.8)	1.32 (1.21 to 1.44)	0.69 (0.63 to 0.75)
1995	0.5 (0.4 to 0.5)	0.3 (0.2 to 0.3)	9.4 (8.6 to 10.3)	4.9 (4.5 to 5.4)	1.46 (1.34 to 1.60)	0.77 (0.70 to 0.83)
2000	0.5 (0.5 to 0.5)	0.3 (0.2 to 0.3)	9.0 (8.2 to 9.8)	4.7 (4.3 to 5.2)	1.45 (1.34 to 1.58)	0.77 (0.70 to 0.84)
2005	0.5 (0.4 to 0.5)	0.2 (0.2 to 0.3)	8.2 (7.5 to 8.9)	4.3 (4.0 to 4.7)	1.35 (1.23 to 1.47)	0.71 (0.65 to 0.78)
2010	0.5 (0.4 to 0.5)	0.2 (0.2 to 0.3)	8.1 (7.4 to 8.8)	4.3 (4.0 to 4.7)	1.76 (1.61 to 1.92)	0.94 (0.86 to 1.02)
2015	0.4 (0.4 to 0.4)	0.2 (0.2 to 0.2)	7.3 (6.6 to 7.9)	4.0 (3.6 to 4.3)	1.56 (1.42 to 1.70)	0.86 (0.78 to 0.93)
2020	0.3 (0.3 to 0.4)	0.2 (0.2 to 0.2)	6.0 (5.2 to 6.8)	3.3 (2.9 to 3.8)	1.42 (1.23 to 1.63)	0.80 (0.69 to 0.92)
2021	0.3 (0.3 to 0.4)	0.2 (0.1 to 0.2)	5.8 (4.6 to 7.2)	3.3 (2.6 to 4.0)	1.44 (1.15 to 1.79)	0.81 (0.65 to 1.01)

South Dakota   USA_564   High−income North America   Stillbirth Rate / Neonatal Mortality Rate (SBR/NMR) − 20 weeks
lambda = 0.2 ; zeta = 0.99 ; scale = 5 ; amp = 0.028

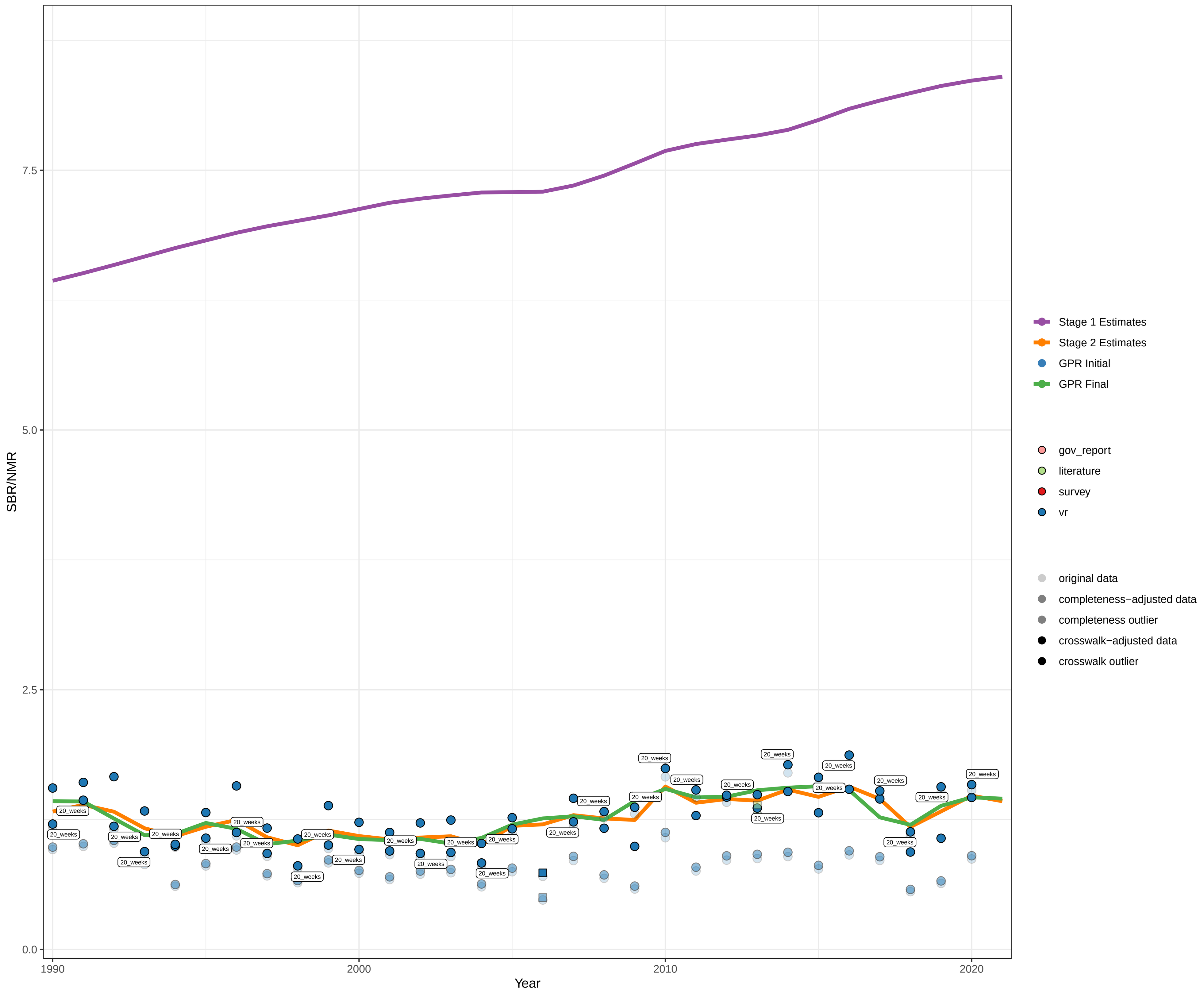

South Dakota   USA_564   High−income North America   Stillbirth Rate / Neonatal Mortality Rate (SBR/NMR) − 28 weeks
lambda = 0.2 ; zeta = 0.99 ; scale = 5 ; amp = 0.028

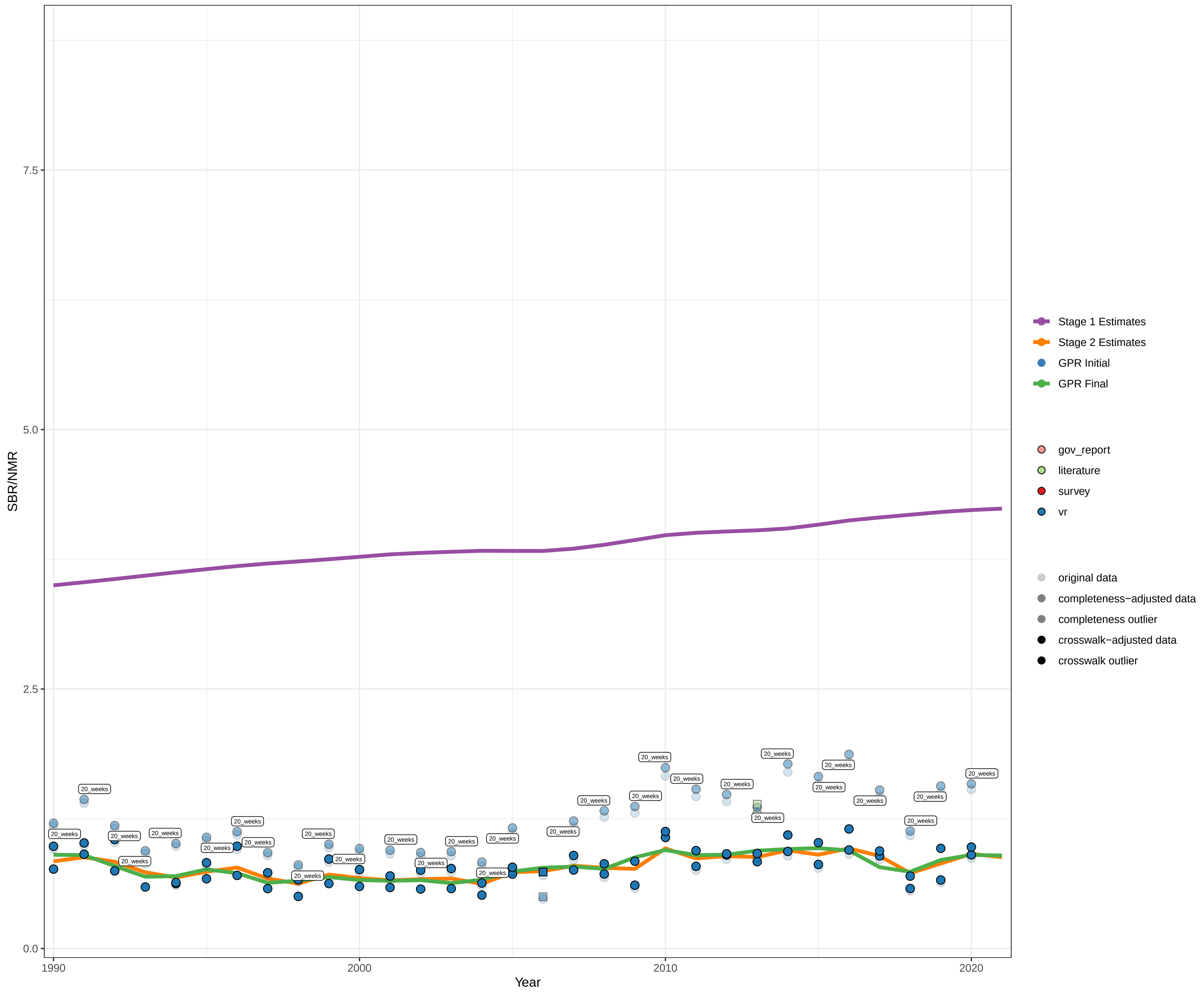

Definition	Years	Outlier	Source.Type	Title
20_weeks, 28_weeks	1990−2005, 2007−2020	0	VR	United States NVSS Custom Fetal Death Data
20_weeks	2013	1	Sci Lit	United States Fetal and Perinatal Mortality 2013
28_weeks	2006	1	VR	United States NVSS Custom Fetal Death Data
20_weeks	2006	2	VR	United States NVSS Custom Fetal Death Data

South Dakota   USA_564   High−income North America   Stillbirth Rate (SBR) − 20 weeks
lambda = 0.2 ; zeta = 0.99 ; scale = 5 ; amp = 0.028

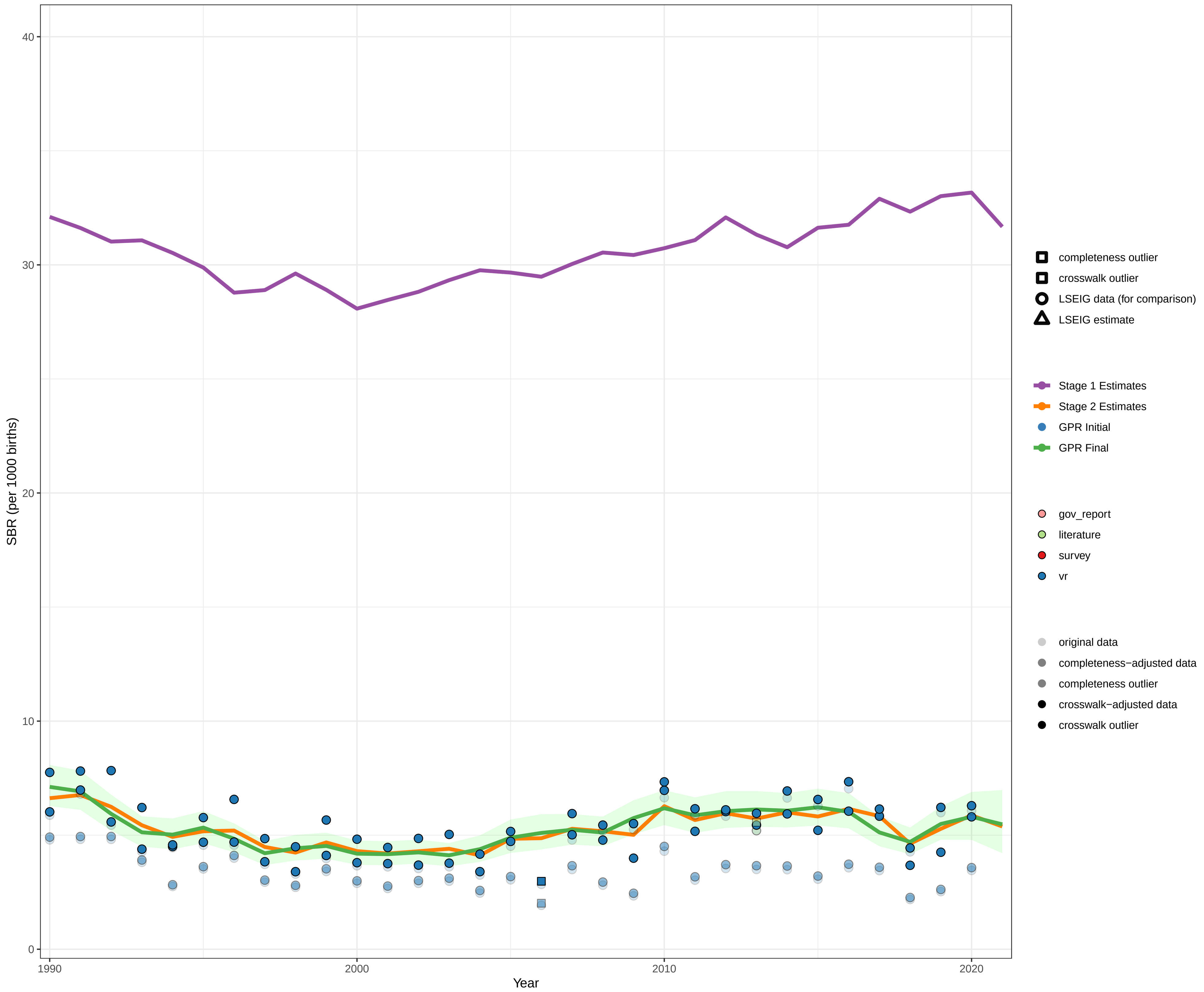

South Dakota   USA_564   High−income North America   Stillbirth Rate (SBR) − 28 weeks
lambda = 0.2 ; zeta = 0.99 ; scale = 5 ; amp = 0.028

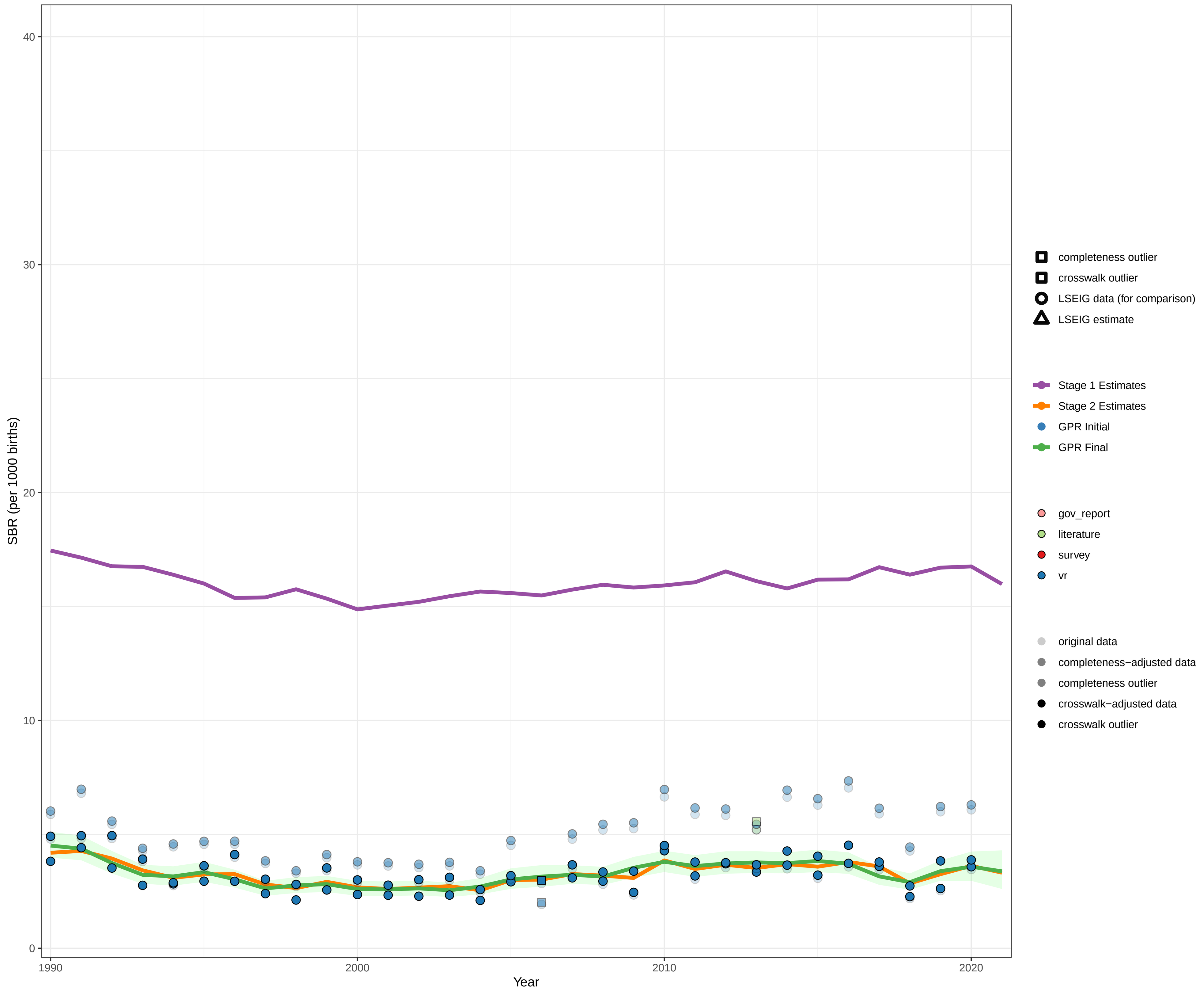

Year	Total Stillbirths (in thousands)	Total Stillbirths (in thousands)	SBR (per 1000 births)	SBR (per 1000 births)	SBR/NMR	SBR/NMR
	20 weeks	28 weeks	20 weeks	28 weeks	20 weeks	28 weeks
1990	0.1<br>(0.1 to 0.1)	0.1<br>(0.0 to 0.1)	7.1<br>(6.3 to 8.1)	4.5<br>(4.0 to 5.1)	1.43<br>(1.26 to 1.62)	0.90<br>(0.80 to 1.02)
1995	0.1<br>(0.0 to 0.1)	0.0<br>(0.0 to 0.0)	5.3<br>(4.6 to 6.1)	3.3<br>(2.9 to 3.8)	1.22<br>(1.06 to 1.38)	0.76<br>(0.67 to 0.87)
2000	0.0<br>(0.0 to 0.0)	0.0<br>(0.0 to 0.0)	4.2<br>(3.7 to 4.8)	2.6<br>(2.3 to 3.0)	1.06<br>(0.94 to 1.21)	0.66<br>(0.58 to 0.75)
2005	0.1<br>(0.1 to 0.1)	0.0<br>(0.0 to 0.0)	4.9<br>(4.2 to 5.7)	3.0<br>(2.6 to 3.5)	1.20<br>(1.04 to 1.40)	0.74<br>(0.64 to 0.86)
2010	0.1<br>(0.1 to 0.1)	0.0<br>(0.0 to 0.1)	6.2<br>(5.4 to 7.0)	3.8<br>(3.3 to 4.3)	1.55<br>(1.36 to 1.74)	0.95<br>(0.84 to 1.07)
2015	0.1<br>(0.1 to 0.1)	0.0<br>(0.0 to 0.1)	6.2<br>(5.4 to 7.0)	3.8<br>(3.3 to 4.3)	1.57<br>(1.37 to 1.78)	0.97<br>(0.84 to 1.09)
2020	0.1<br>(0.1 to 0.1)	0.0<br>(0.0 to 0.0)	5.8<br>(4.8 to 6.9)	3.6<br>(3.0 to 4.2)	1.46<br>(1.21 to 1.74)	0.90<br>(0.75 to 1.07)
2021	0.1<br>(0.0 to 0.1)	0.0<br>(0.0 to 0.0)	5.5<br>(4.2 to 7.0)	3.4<br>(2.6 to 4.3)	1.45<br>(1.12 to 1.85)	0.90<br>(0.69 to 1.14)

Tennessee USA_565 High−income North America Stillbirth Rate / Neonatal Mortality Rate (SBR/NMR) − 20 weeks
lambda = 0.2 ; zeta = 0.99 ; scale = 5 ; amp = 0.028

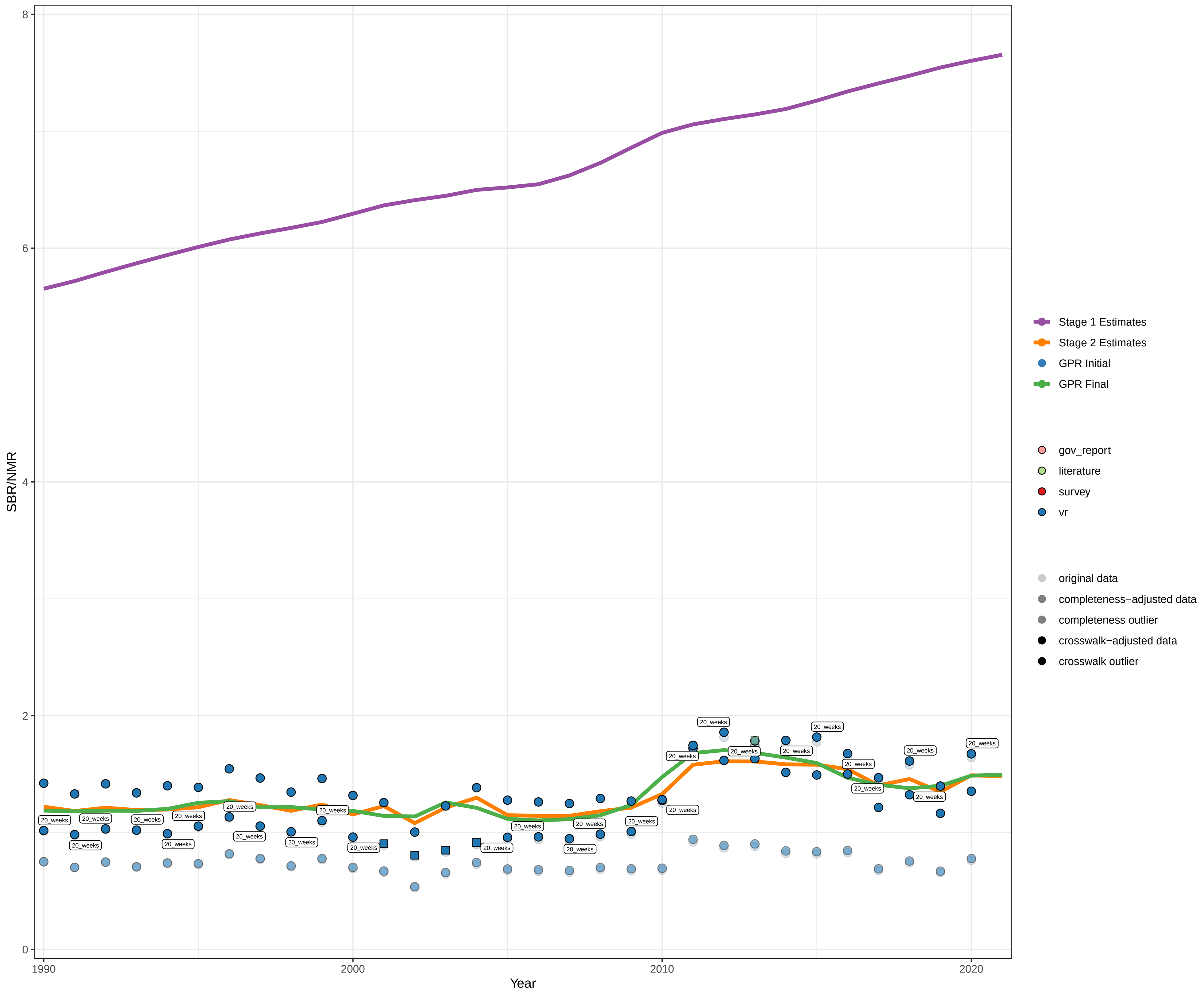

Tennessee USA_565 High−income North America Stillbirth Rate / Neonatal Mortality Rate (SBR/NMR) − 28 weeks
lambda = 0.2 ; zeta = 0.99 ; scale = 5 ; amp = 0.028

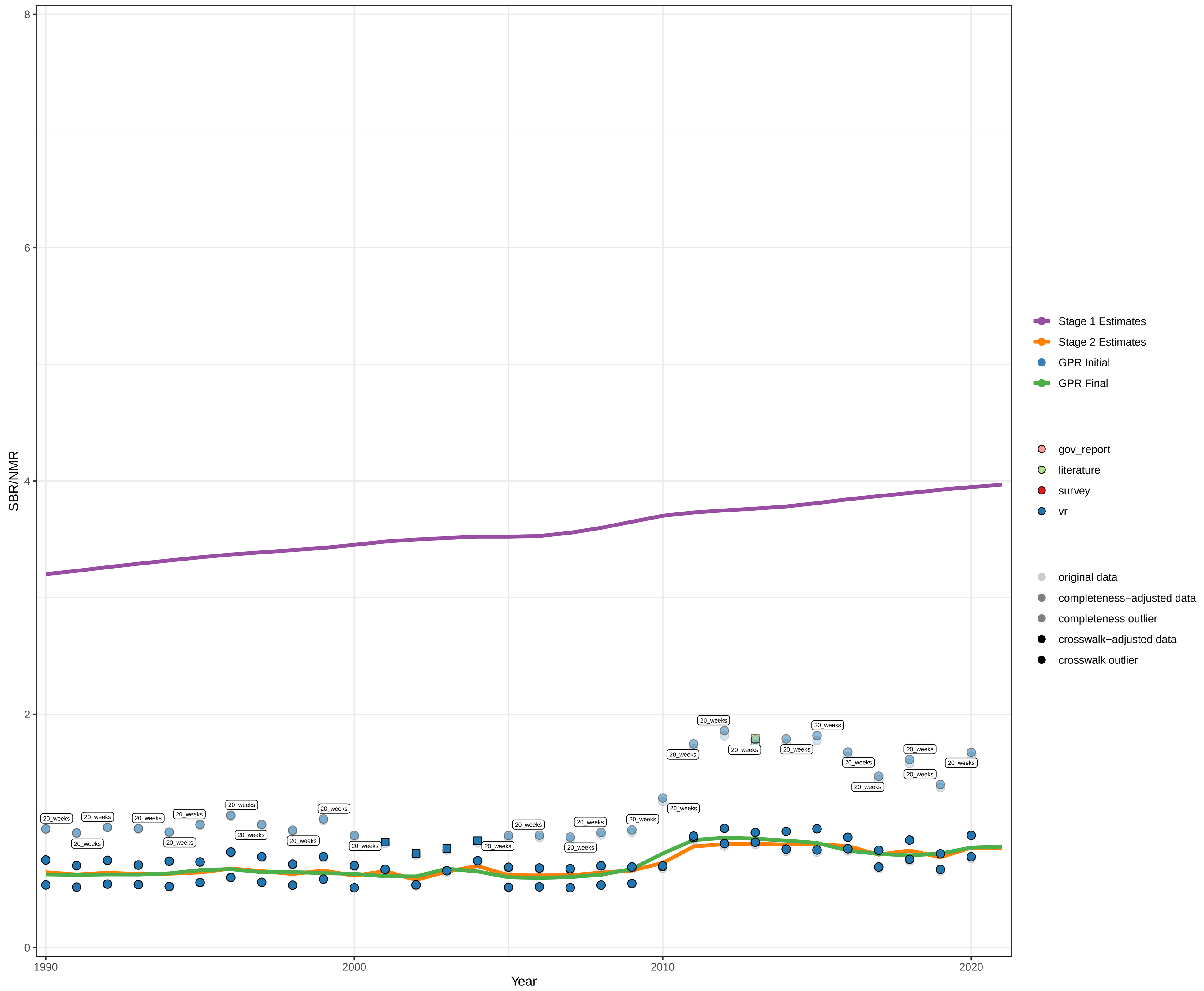

Definition	Years	Outlier	Source.Type	Title
20_weeks	1990−2000, 2005−2020	0	VR	United States NVSS Custom Fetal Death Data
28_weeks	1990−2020	0	VR	United States NVSS Custom Fetal Death Data
20_weeks	2013	1	Sci Lit	United States Fetal and Perinatal Mortality 2013
20_weeks	2001−2004	2	VR	United States NVSS Custom Fetal Death Data

Tennessee USA_565 High−income North America Stillbirth Rate (SBR) − 20 weeks
lambda = 0.2 ; zeta = 0.99 ; scale = 5 ; amp = 0.028

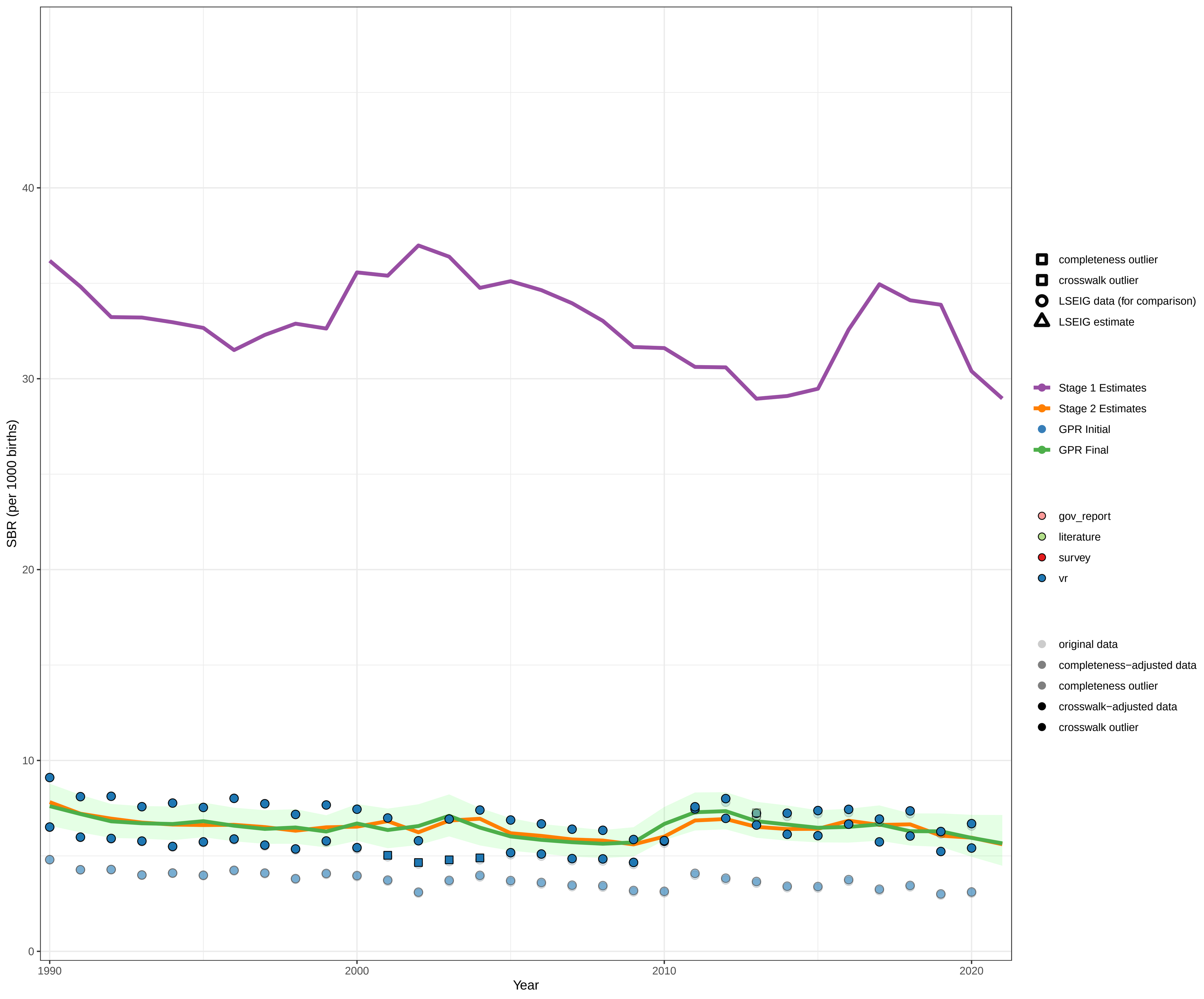

Tennessee USA_565 High−income North America Stillbirth Rate (SBR) − 28 weeks
lambda = 0.2 ; zeta = 0.99 ; scale = 5 ; amp = 0.028

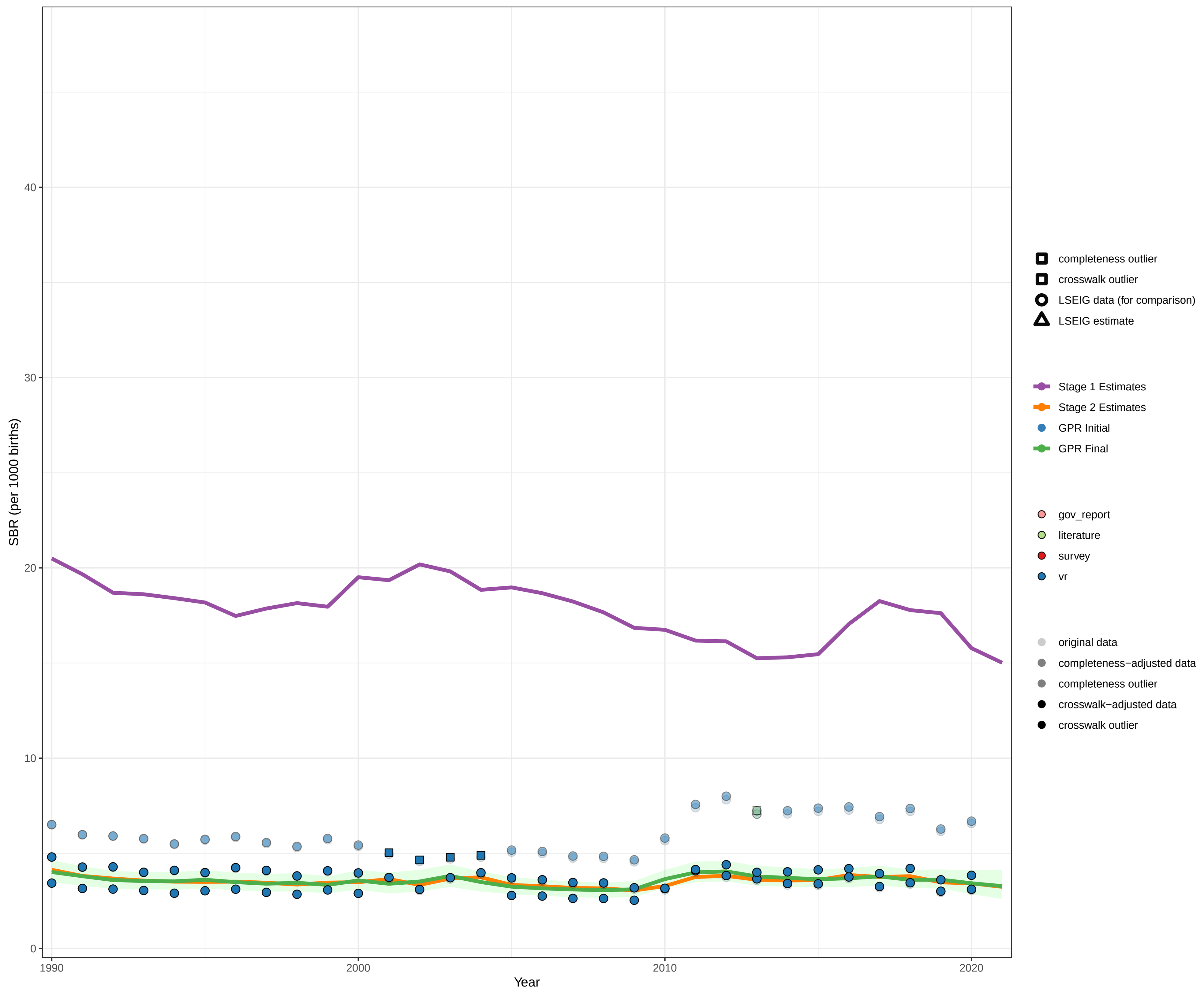

Year	Total Stillbirths (in thousands)	Total Stillbirths (in thousands)	SBR (per 1000 births)	SBR (per 1000 births)	SBR/NMR	SBR/NMR
	20 weeks	28 weeks	20 weeks	28 weeks	20 weeks	28 weeks
1990	0.6 (0.5 to 0.7)	0.3 (0.3 to 0.4)	7.6 (6.6 to 8.8)	4.0 (3.5 to 4.6)	1.19 (1.03 to 1.37)	0.63 (0.54 to 0.72)
1995	0.5 (0.4 to 0.6)	0.3 (0.2 to 0.3)	6.8 (6.0 to 7.8)	3.6 (3.2 to 4.1)	1.25 (1.10 to 1.43)	0.66 (0.58 to 0.76)
2000	0.5 (0.5 to 0.6)	0.3 (0.2 to 0.3)	6.7 (5.8 to 7.7)	3.6 (3.1 to 4.1)	1.19 (1.03 to 1.37)	0.63 (0.55 to 0.73)
2005	0.5 (0.5 to 0.6)	0.3 (0.3 to 0.3)	6.0 (5.3 to 7.0)	3.2 (2.8 to 3.8)	1.12 (0.98 to 1.30)	0.60 (0.53 to 0.70)
2010	0.6 (0.5 to 0.6)	0.3 (0.3 to 0.4)	6.7 (5.8 to 7.6)	3.6 (3.2 to 4.1)	1.48 (1.29 to 1.67)	0.81 (0.70 to 0.91)
2015	0.6 (0.5 to 0.6)	0.3 (0.3 to 0.4)	6.5 (5.7 to 7.4)	3.6 (3.2 to 4.2)	1.60 (1.41 to 1.82)	0.90 (0.79 to 1.02)
2020	0.5 (0.4 to 0.6)	0.3 (0.2 to 0.3)	5.9 (5.0 to 7.2)	3.4 (2.9 to 4.1)	1.49 (1.24 to 1.79)	0.86 (0.71 to 1.03)
2021	0.5 (0.4 to 0.6)	0.3 (0.2 to 0.3)	5.7 (4.5 to 7.1)	3.3 (2.6 to 4.1)	1.50 (1.19 to 1.89)	0.87 (0.69 to 1.09)

Texas USA_566 High−income North America Stillbirth Rate / Neonatal Mortality Rate (SBR/NMR) − 20 weeks
lambda = 0.2 ; zeta = 0.99 ; scale = 5 ; amp = 0.028

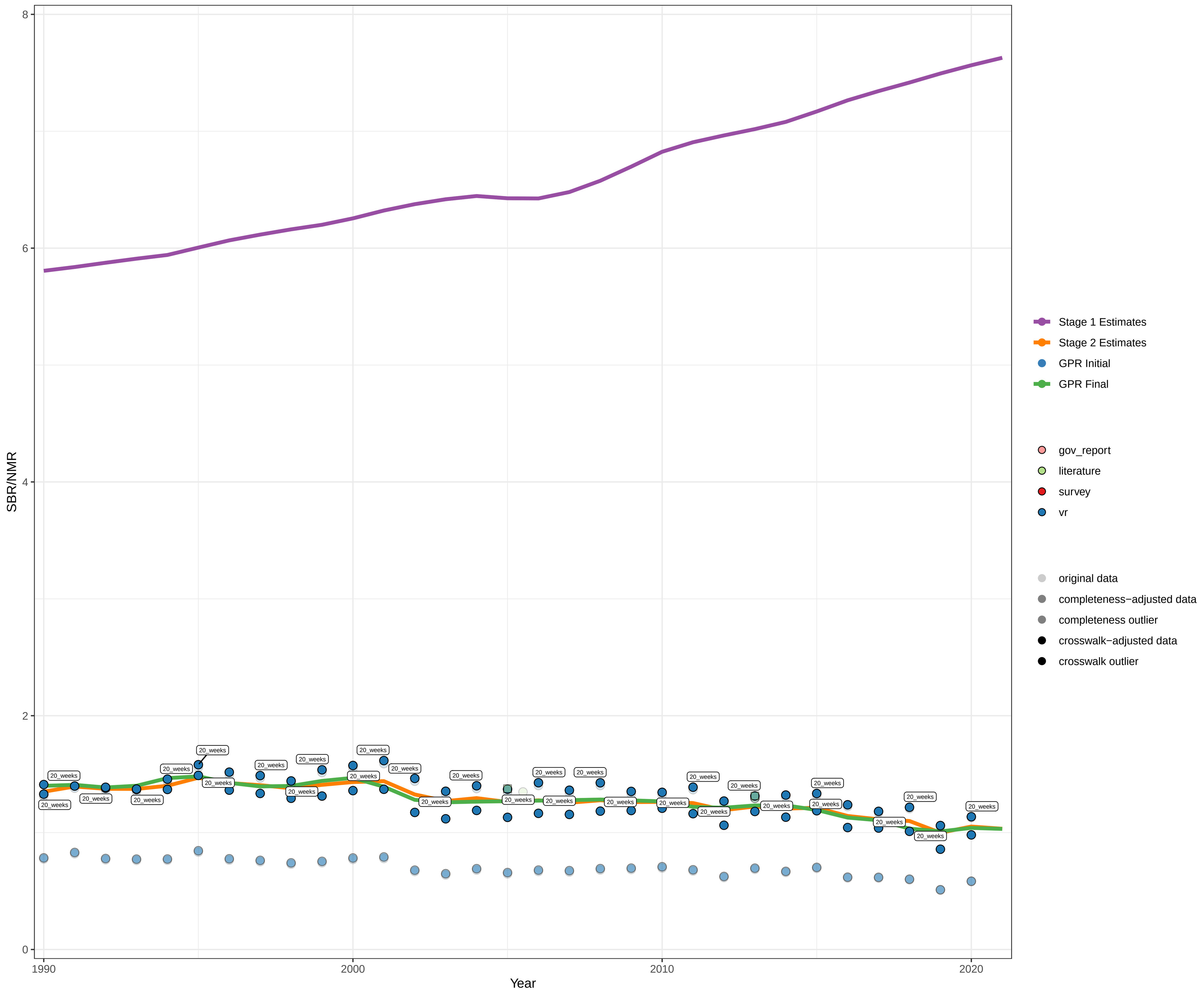

Texas USA_566 High−income North America Stillbirth Rate / Neonatal Mortality Rate (SBR/NMR) − 28 weeks
lambda = 0.2 ; zeta = 0.99 ; scale = 5 ; amp = 0.028

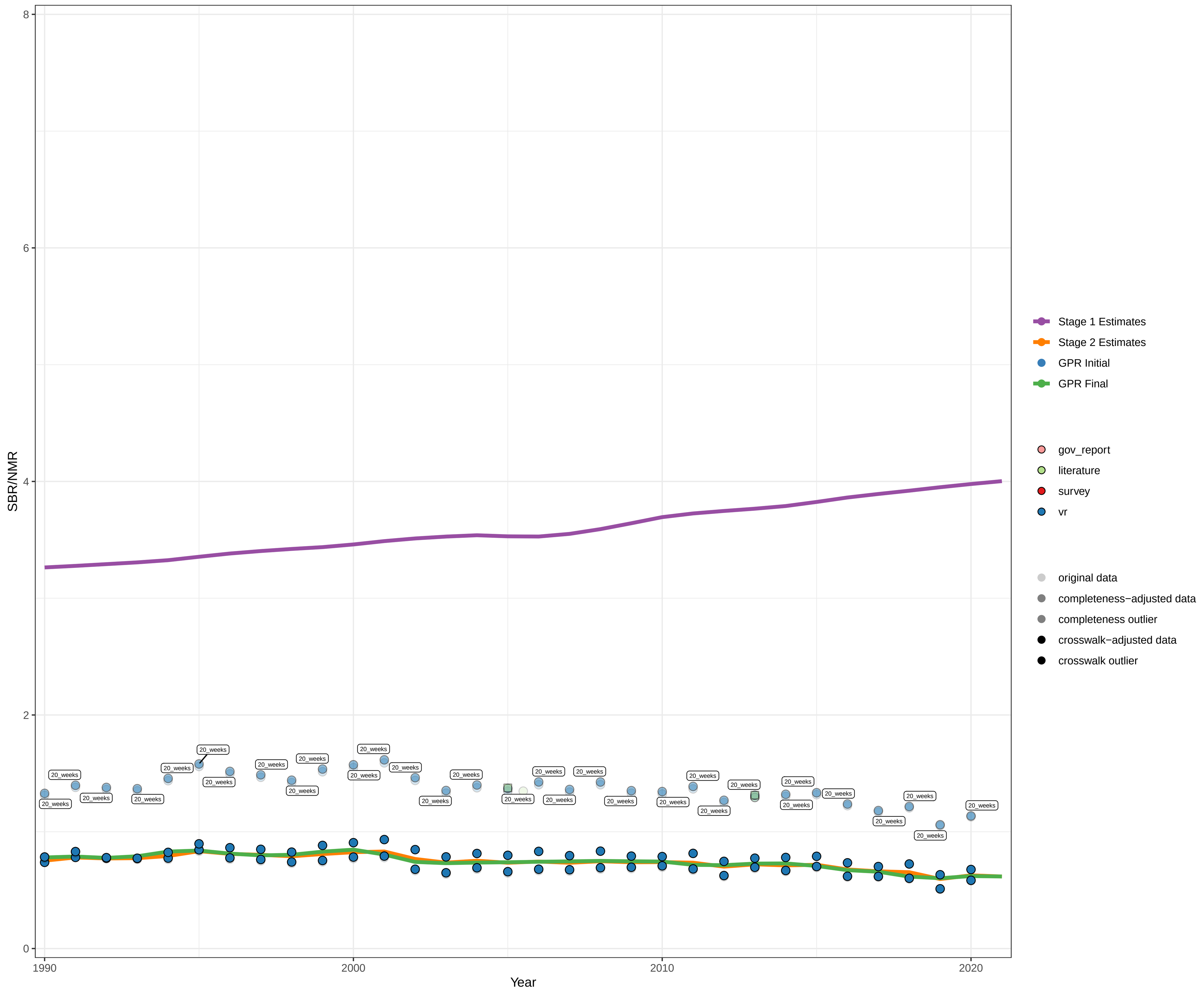

Definition	Years	Outlier	Source.Type	Title
20_weeks, 28_weeks	1990−2020	0	VR	United States NVSS Custom Fetal Death Data
20_weeks	2005	1	Sci Lit	Association of influenza vaccination on decreased stillbirth rate
20_weeks	2013	1	Sci Lit	United States Fetal and Perinatal Mortality 2013

Texas USA_566 High−income North America Stillbirth Rate (SBR) − 20 weeks
lambda = 0.2 ; zeta = 0.99 ; scale = 5 ; amp = 0.028

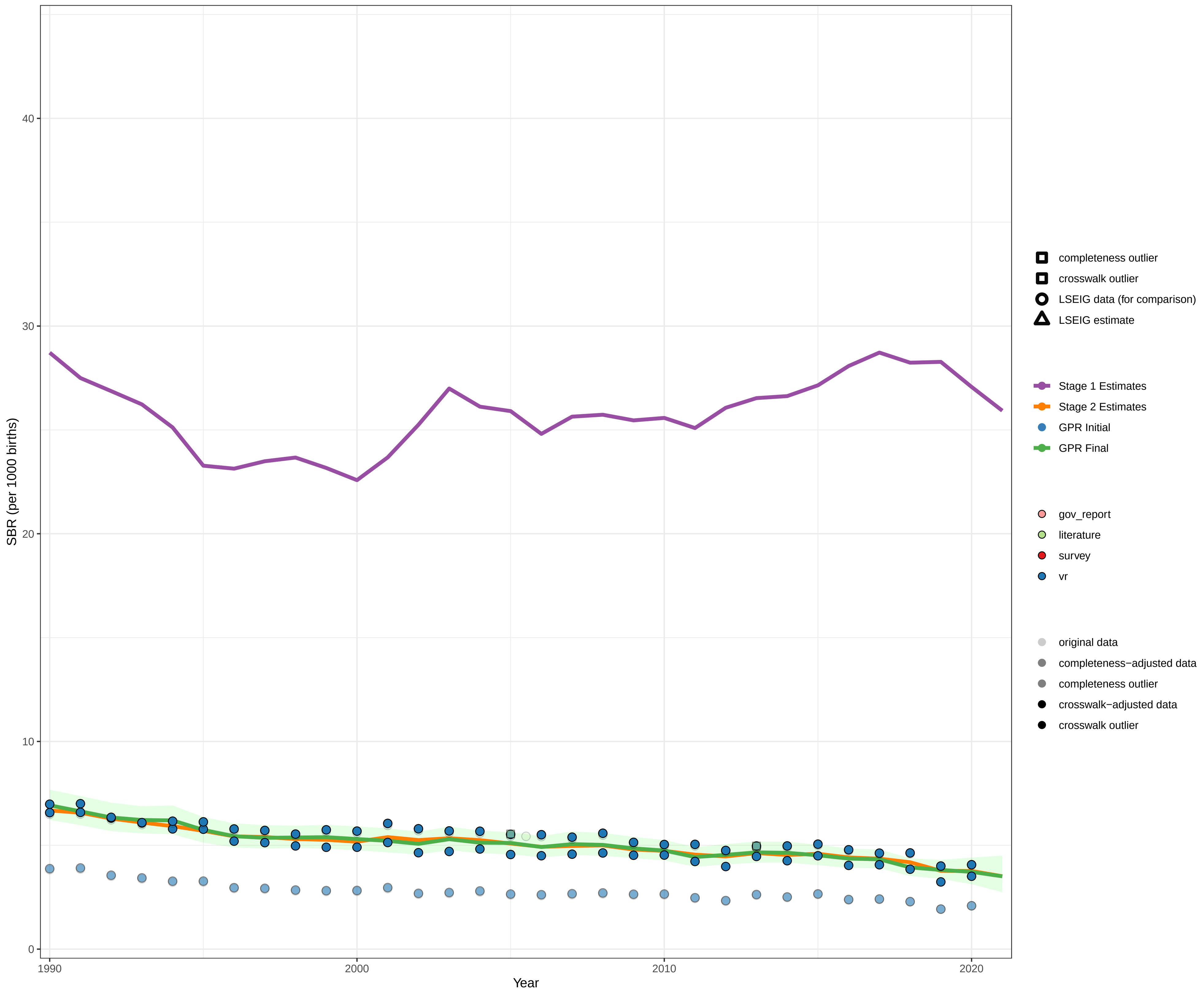

Texas USA_566 High−income North America Stillbirth Rate (SBR) − 28 weeks
lambda = 0.2 ; zeta = 0.99 ; scale = 5 ; amp = 0.028

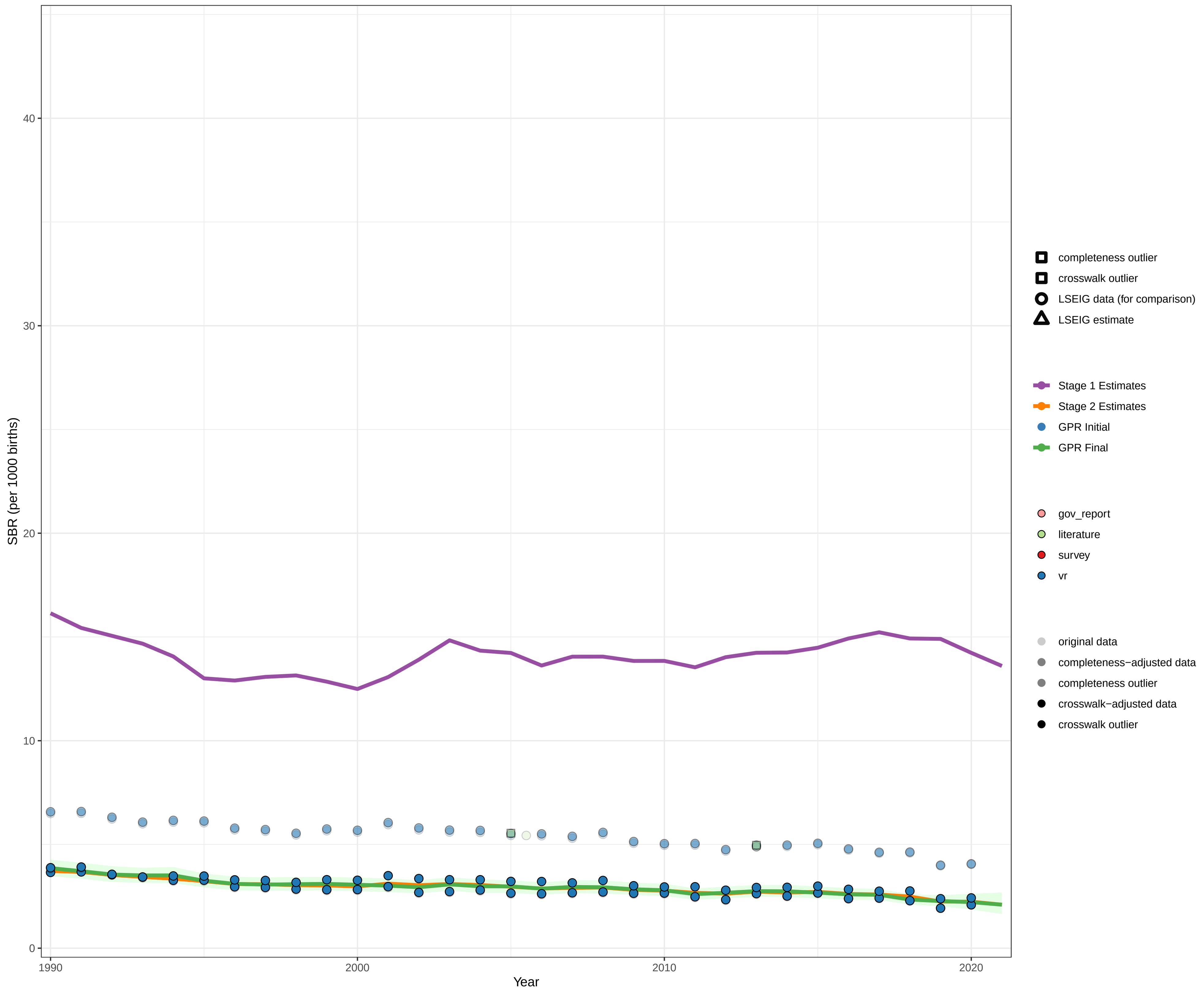

Year	Total Stillbirths (in thousands)	Total Stillbirths (in thousands)	SBR (per 1000 births)	SBR (per 1000 births)	SBR/NMR	SBR/NMR
	20 weeks	28 weeks	20 weeks	28 weeks	20 weeks	28 weeks
1990	2.2 (2.0 to 2.5)	1.2 (1.1 to 1.4)	6.9 (6.2 to 7.7)	3.9 (3.5 to 4.3)	1.40 (1.26 to 1.55)	0.78 (0.70 to 0.86)
1995	1.9 (1.7 to 2.1)	1.1 (1.0 to 1.2)	5.7 (5.1 to 6.4)	3.3 (2.9 to 3.6)	1.48 (1.32 to 1.64)	0.84 (0.76 to 0.93)
2000	1.9 (1.7 to 2.2)	1.1 (1.0 to 1.2)	5.3 (4.8 to 5.9)	3.1 (2.7 to 3.4)	1.47 (1.32 to 1.64)	0.85 (0.76 to 0.94)
2005	2.1 (1.8 to 2.3)	1.2 (1.1 to 1.3)	5.1 (4.6 to 5.6)	3.0 (2.7 to 3.3)	1.27 (1.13 to 1.40)	0.74 (0.66 to 0.81)
2010	1.9 (1.7 to 2.1)	1.1 (1.0 to 1.2)	4.7 (4.3 to 5.3)	2.8 (2.5 to 3.1)	1.27 (1.14 to 1.40)	0.75 (0.67 to 0.83)
2015	1.8 (1.6 to 2.0)	1.1 (1.0 to 1.2)	4.5 (4.0 to 5.0)	2.7 (2.4 to 3.0)	1.19 (1.07 to 1.33)	0.71 (0.63 to 0.79)
2020	1.4 (1.1 to 1.6)	0.8 (0.7 to 1.0)	3.7 (3.1 to 4.4)	2.2 (1.9 to 2.6)	1.04 (0.87 to 1.23)	0.62 (0.52 to 0.73)
2021	1.3 (1.0 to 1.7)	0.8 (0.6 to 1.0)	3.5 (2.7 to 4.5)	2.1 (1.6 to 2.7)	1.03 (0.81 to 1.32)	0.62 (0.48 to 0.79)

Utah USA_567 High−income North America Stillbirth Rate / Neonatal Mortality Rate (SBR/NMR) − 20 weeks
lambda = 0.2 ; zeta = 0.99 ; scale = 5 ; amp = 0.028

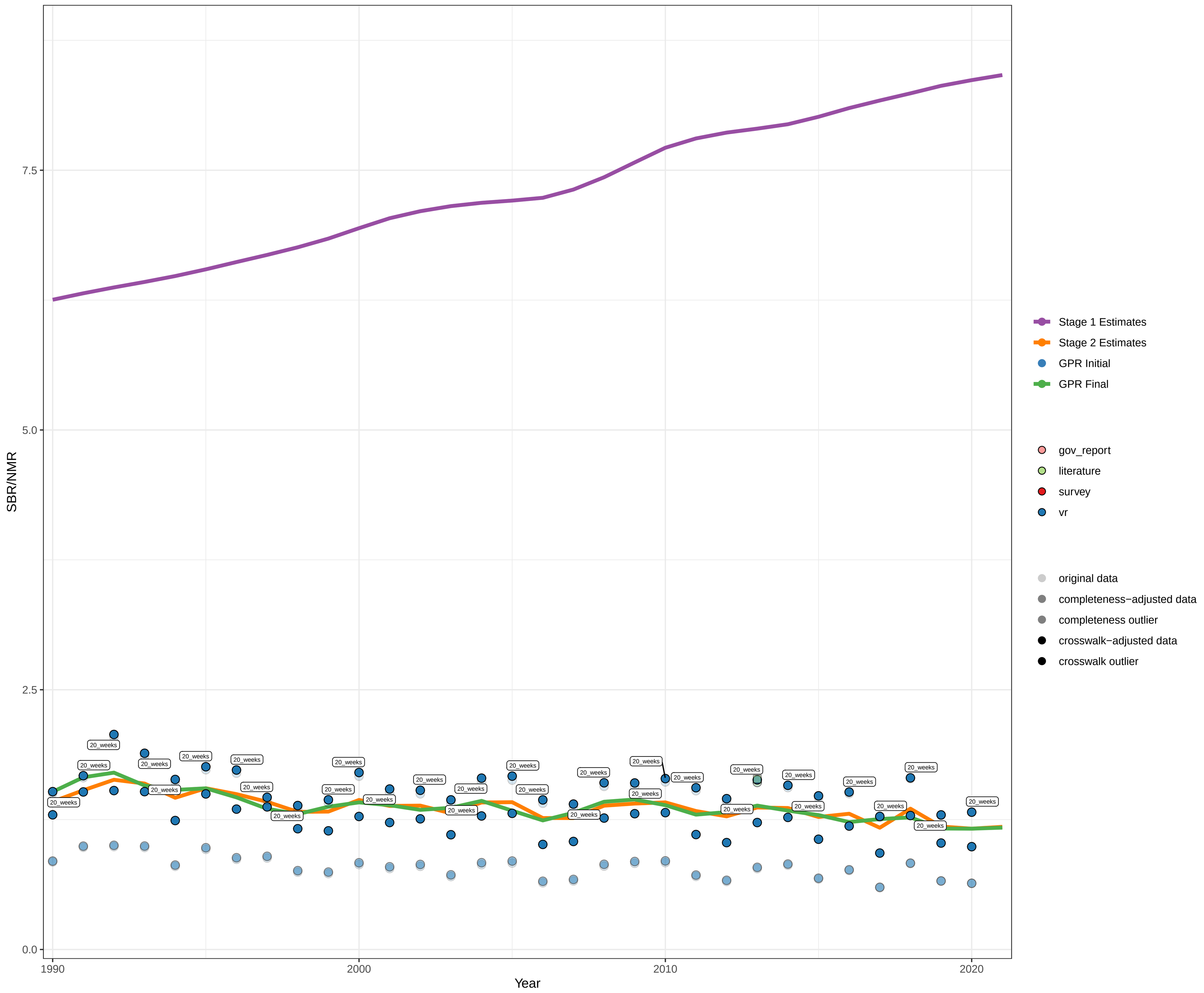

Utah USA_567 High−income North America Stillbirth Rate / Neonatal Mortality Rate (SBR/NMR) − 28 weeks
lambda = 0.2 ; zeta = 0.99 ; scale = 5 ; amp = 0.028

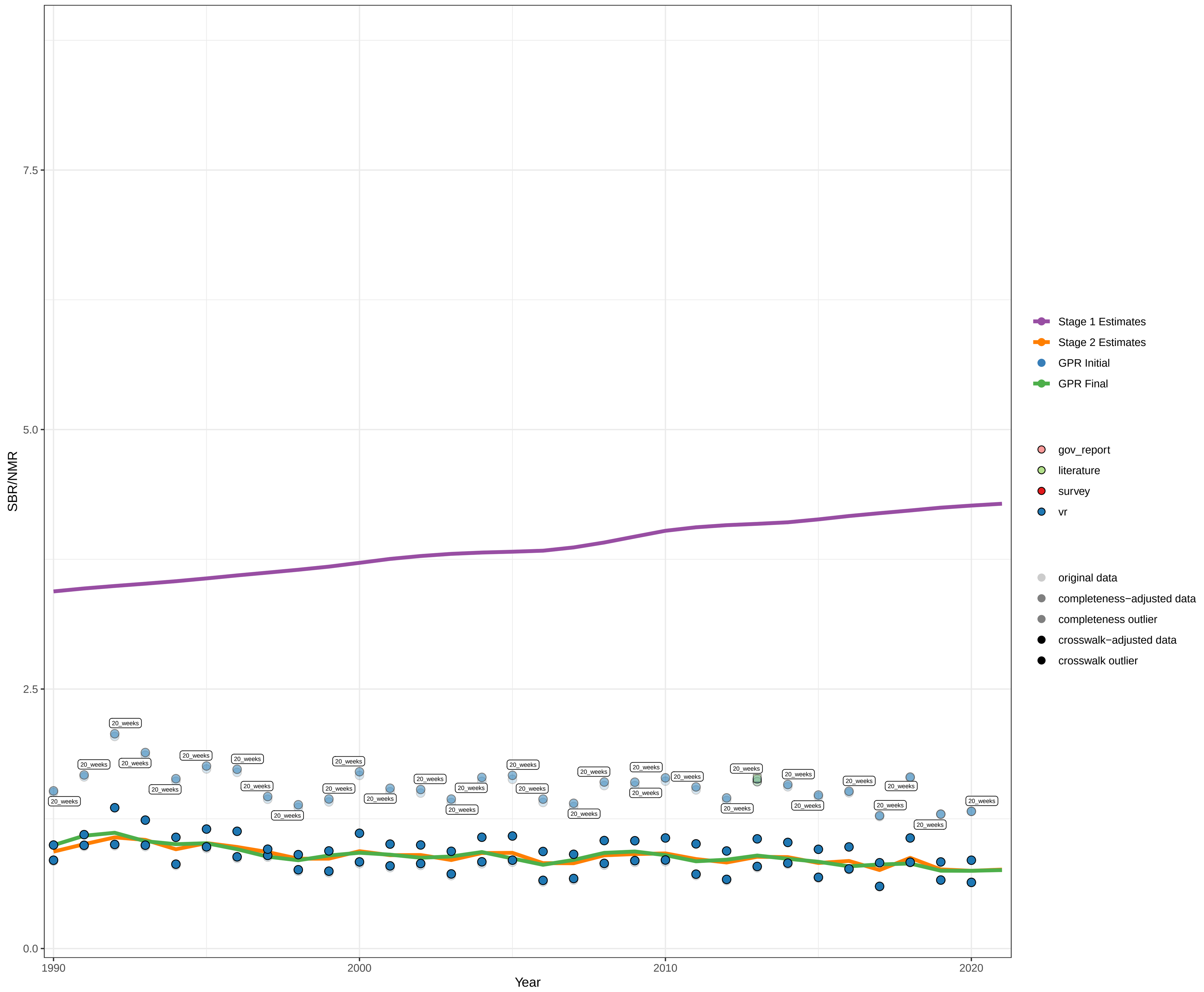

Definition	Years	Outlier	Source.Type	Title
20_weeks, 28_weeks	1990−2020	0	VR	United States NVSS Custom Fetal Death Data
20_weeks	2013	1	Sci Lit	United States Fetal and Perinatal Mortality 2013

Utah USA_567 High−income North America Stillbirth Rate (SBR) − 20 weeks
lambda = 0.2 ; zeta = 0.99 ; scale = 5 ; amp = 0.028

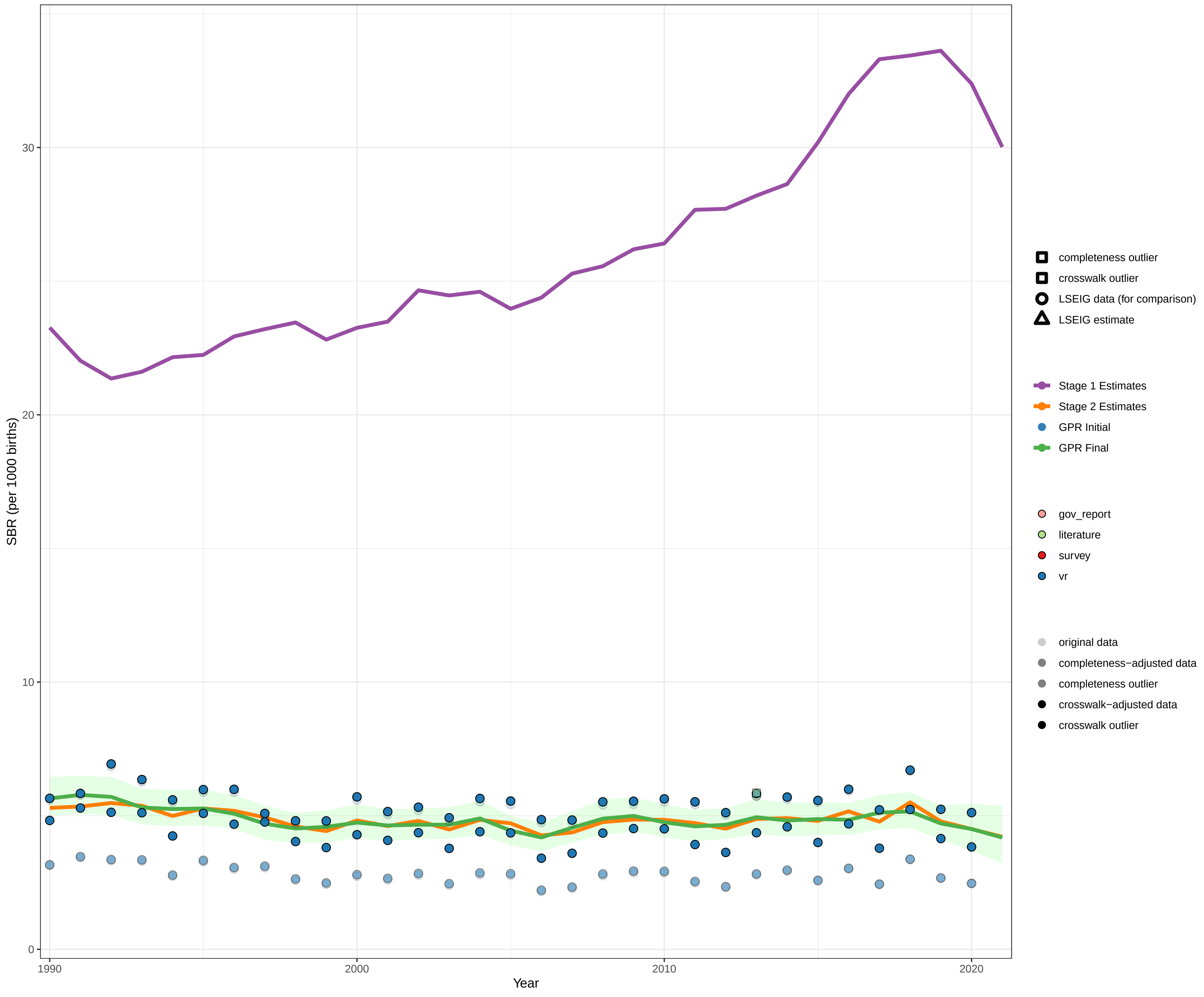

Utah USA_567 High−income North America Stillbirth Rate (SBR) − 28 weeks
lambda = 0.2 ; zeta = 0.99 ; scale = 5 ; amp = 0.028

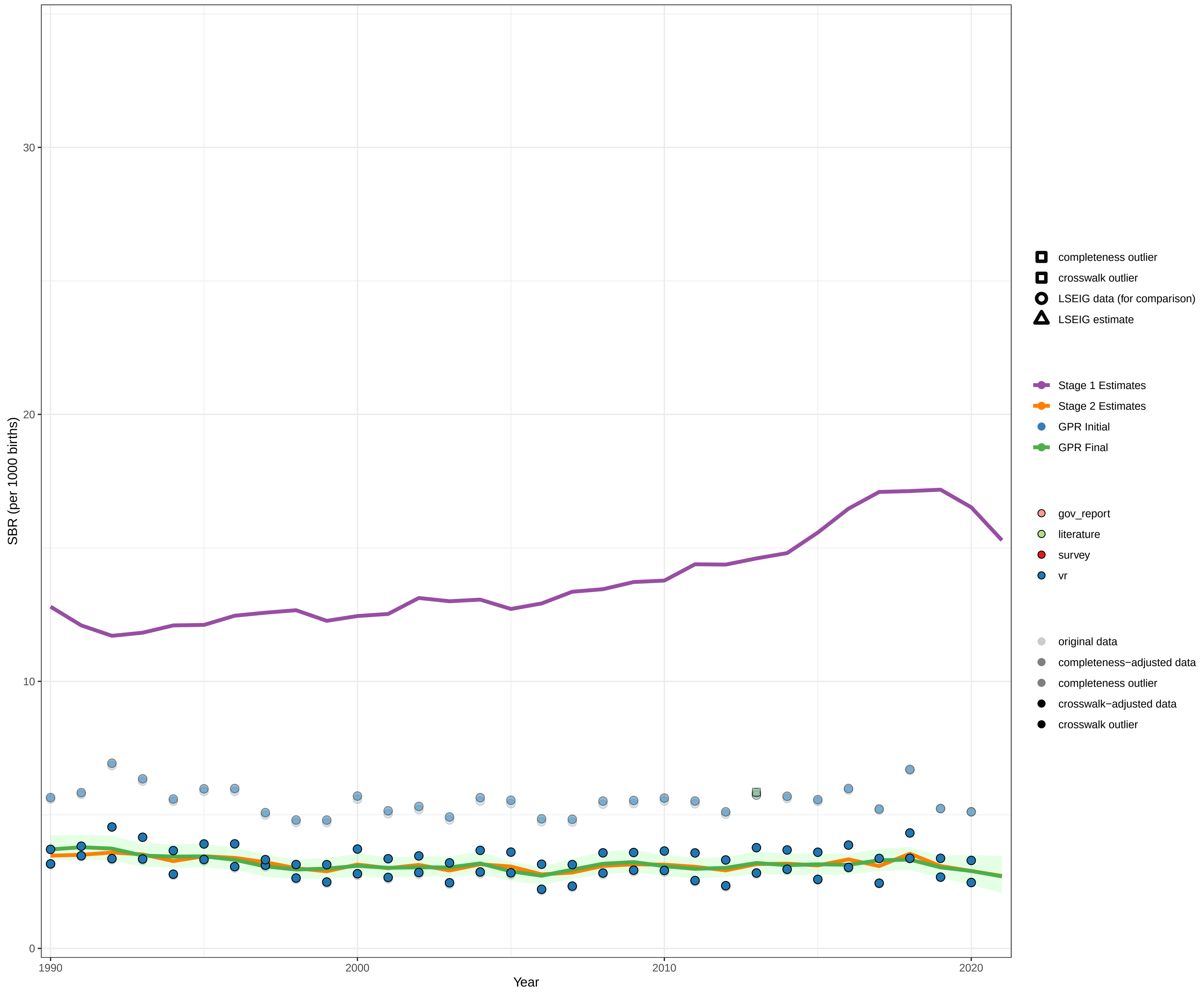

Year	Total Stillbirths (in thousands)	Total Stillbirths (in thousands)	SBR (per 1000 births)	SBR (per 1000 births)	SBR/NMR	SBR/NMR
	20 weeks	28 weeks	20 weeks	28 weeks	20 weeks	28 weeks
1990	0.2 (0.2 to 0.2)	0.1 (0.1 to 0.2)	5.6 (5.0 to 6.5)	3.7 (3.3 to 4.2)	1.52 (1.34 to 1.74)	1.00 (0.88 to 1.14)
1995	0.2 (0.2 to 0.2)	0.1 (0.1 to 0.2)	5.3 (4.6 to 6.0)	3.4 (3.0 to 3.9)	1.55 (1.36 to 1.76)	1.01 (0.89 to 1.15)
2000	0.2 (0.2 to 0.3)	0.1 (0.1 to 0.2)	4.7 (4.2 to 5.4)	3.1 (2.7 to 3.5)	1.42 (1.24 to 1.62)	0.92 (0.81 to 1.06)
2005	0.2 (0.2 to 0.3)	0.2 (0.1 to 0.2)	4.4 (3.9 to 5.0)	2.9 (2.5 to 3.2)	1.34 (1.17 to 1.50)	0.87 (0.76 to 0.97)
2010	0.3 (0.2 to 0.3)	0.2 (0.1 to 0.2)	4.8 (4.2 to 5.4)	3.1 (2.7 to 3.5)	1.39 (1.22 to 1.58)	0.90 (0.79 to 1.03)
2015	0.2 (0.2 to 0.3)	0.2 (0.1 to 0.2)	4.9 (4.3 to 5.5)	3.1 (2.8 to 3.6)	1.29 (1.13 to 1.46)	0.84 (0.73 to 0.94)
2020	0.2 (0.2 to 0.2)	0.1 (0.1 to 0.2)	4.5 (3.7 to 5.4)	2.9 (2.4 to 3.5)	1.16 (0.95 to 1.41)	0.75 (0.61 to 0.91)
2021	0.2 (0.1 to 0.2)	0.1 (0.1 to 0.2)	4.2 (3.2 to 5.4)	2.7 (2.1 to 3.5)	1.17 (0.90 to 1.51)	0.76 (0.58 to 0.97)

Vermont   USA_568   High−income North America   Stillbirth Rate / Neonatal Mortality Rate (SBR/NMR) − 20 weeks
lambda = 0.2 ; zeta = 0.99 ; scale = 5 ; amp = 0.028

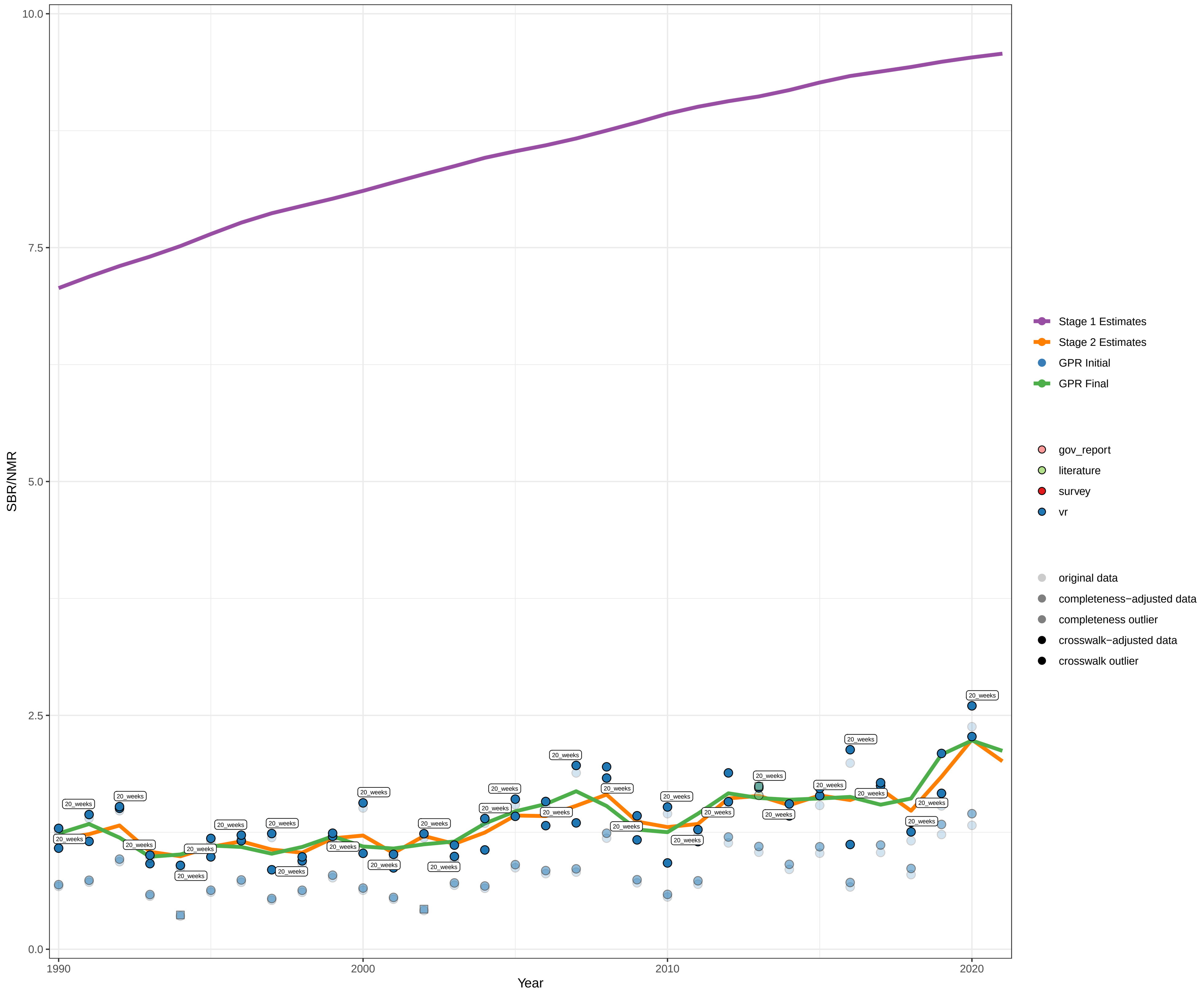

Vermont   USA_568   High−income North America   Stillbirth Rate / Neonatal Mortality Rate (SBR/NMR) − 28 weeks
lambda = 0.2 ; zeta = 0.99 ; scale = 5 ; amp = 0.028

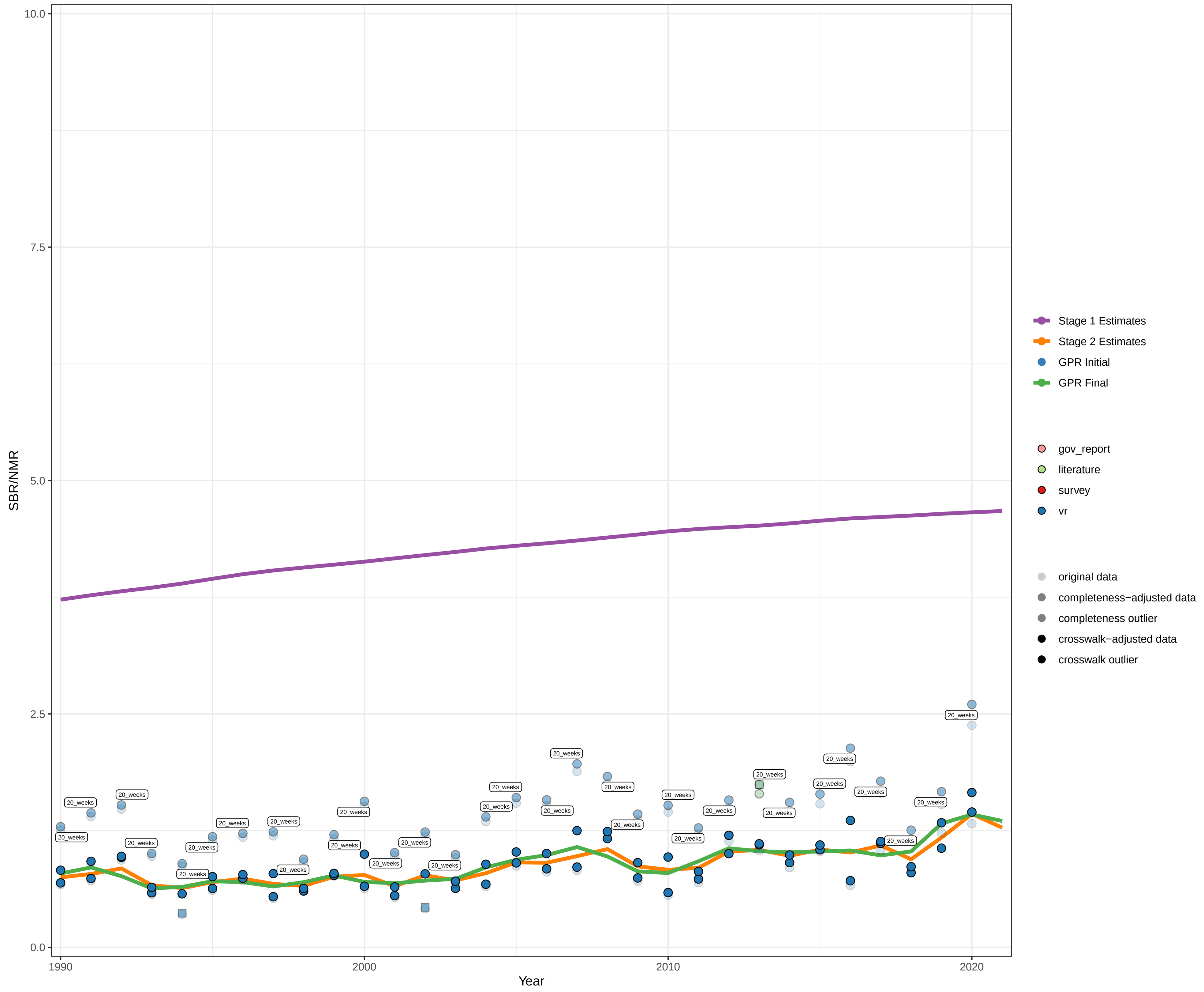

Definition	Years	Outlier	Source.Type	Title
20_weeks	1990−2020	0	VR	United States NVSS Custom Fetal Death Data
28_weeks	1990−1993, 1995−2001, 2003−2020	0	VR	United States NVSS Custom Fetal Death Data
20_weeks	2013	1	Sci Lit	United States Fetal and Perinatal Mortality 2013
28_weeks	1994, 2002	1	VR	United States NVSS Custom Fetal Death Data

Vermont   USA_568   High−income North America   Stillbirth Rate (SBR) − 20 weeks
lambda = 0.2 ; zeta = 0.99 ; scale = 5 ; amp = 0.028

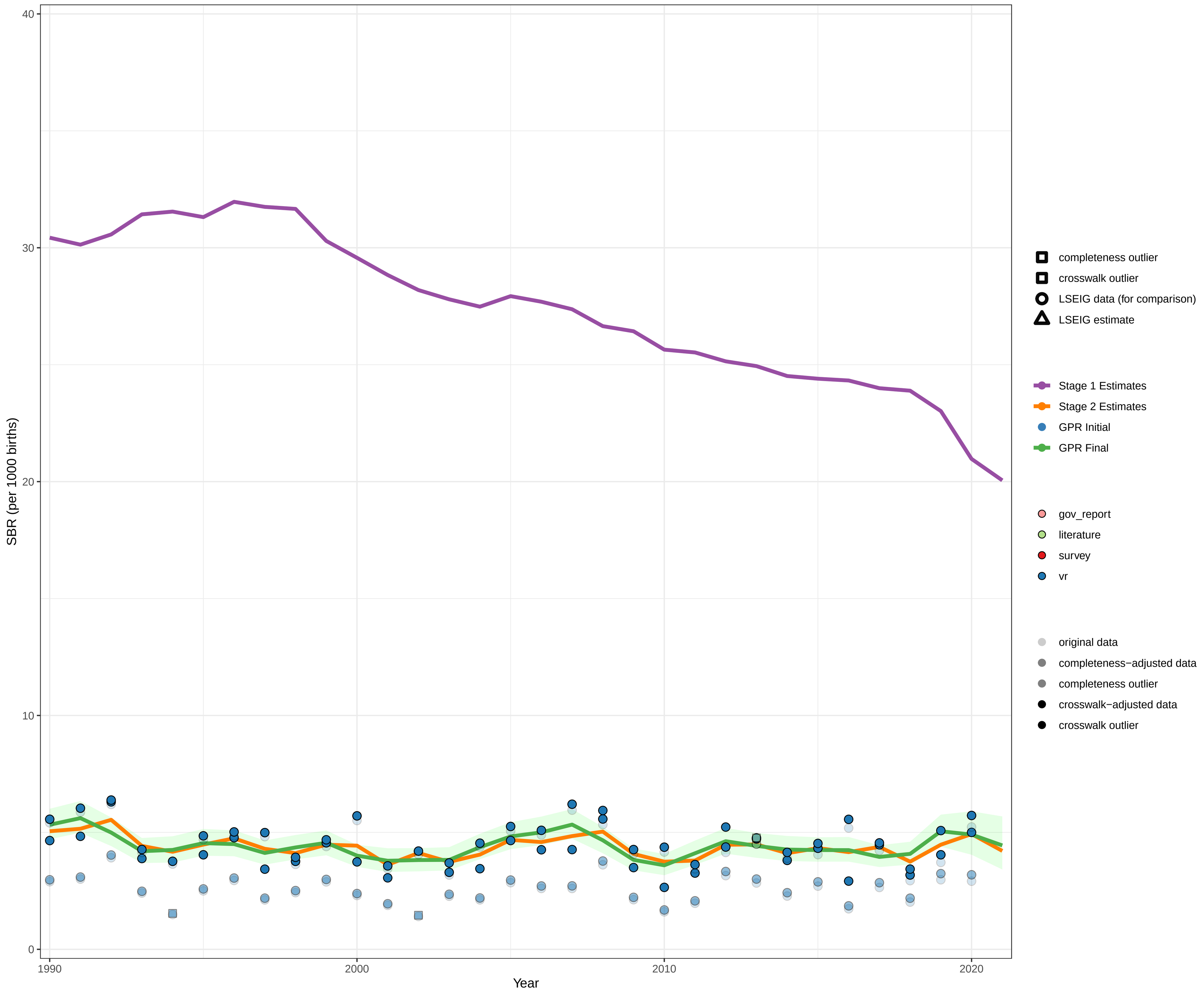

Vermont   USA_568   High−income North America   Stillbirth Rate (SBR) − 28 weeks
lambda = 0.2 ; zeta = 0.99 ; scale = 5 ; amp = 0.028

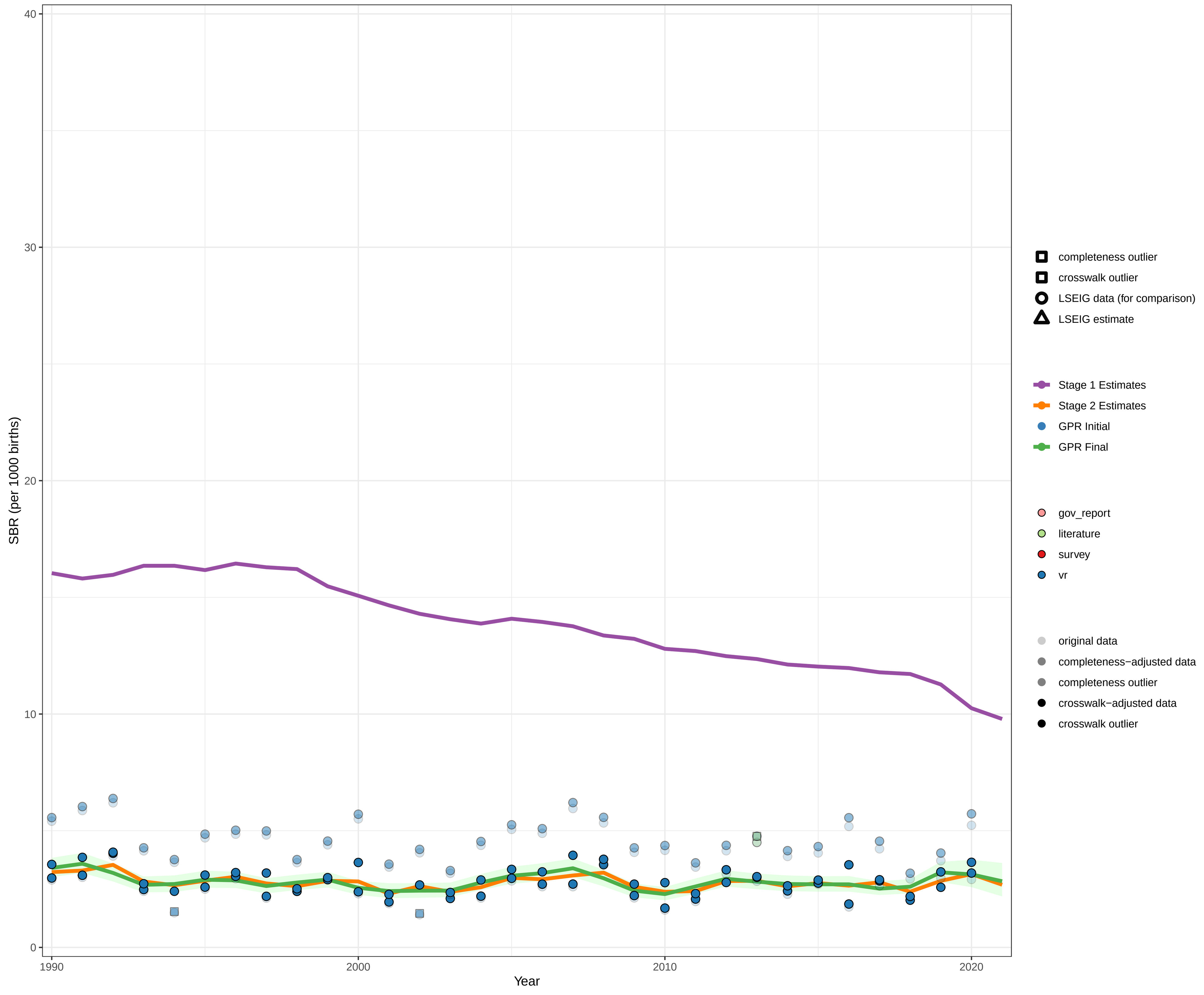

Year	Total Stillbirths (in thousands)	Total Stillbirths (in thousands)	SBR (per 1000 births)	SBR (per 1000 births)	SBR/NMR	SBR/NMR
	20 weeks	28 weeks	20 weeks	28 weeks	20 weeks	28 weeks
1990	0.0 (0.0 to 0.0)	0.0 (0.0 to 0.0)	5.3 (4.7 to 6.0)	3.4 (3.0 to 3.8)	1.24 (1.09 to 1.40)	0.79 (0.70 to 0.89)
1995	0.0 (0.0 to 0.0)	0.0 (0.0 to 0.0)	4.5 (4.0 to 5.1)	2.9 (2.6 to 3.3)	1.11 (0.98 to 1.26)	0.71 (0.62 to 0.80)
2000	0.0 (0.0 to 0.0)	0.0 (0.0 to 0.0)	4.0 (3.5 to 4.5)	2.6 (2.3 to 2.8)	1.10 (0.97 to 1.23)	0.70 (0.62 to 0.78)
2005	0.0 (0.0 to 0.0)	0.0 (0.0 to 0.0)	4.8 (4.3 to 5.4)	3.1 (2.7 to 3.5)	1.47 (1.31 to 1.66)	0.94 (0.83 to 1.06)
2010	0.0 (0.0 to 0.0)	0.0 (0.0 to 0.0)	3.6 (3.2 to 4.1)	2.3 (2.0 to 2.6)	1.25 (1.10 to 1.42)	0.80 (0.70 to 0.90)
2015	0.0 (0.0 to 0.0)	0.0 (0.0 to 0.0)	4.2 (3.7 to 4.8)	2.7 (2.4 to 3.0)	1.61 (1.42 to 1.82)	1.03 (0.91 to 1.16)
2020	0.0 (0.0 to 0.0)	0.0 (0.0 to 0.0)	4.9 (4.0 to 5.9)	3.1 (2.6 to 3.8)	2.23 (1.84 to 2.68)	1.42 (1.17 to 1.71)
2021	0.0 (0.0 to 0.0)	0.0 (0.0 to 0.0)	4.4 (3.4 to 5.7)	2.8 (2.2 to 3.6)	2.12 (1.63 to 2.71)	1.35 (1.04 to 1.73)

Virginia USA_569 High−income North America Stillbirth Rate / Neonatal Mortality Rate (SBR/NMR) − 20 weeks
lambda = 0.2 ; zeta = 0.99 ; scale = 5 ; amp = 0.028

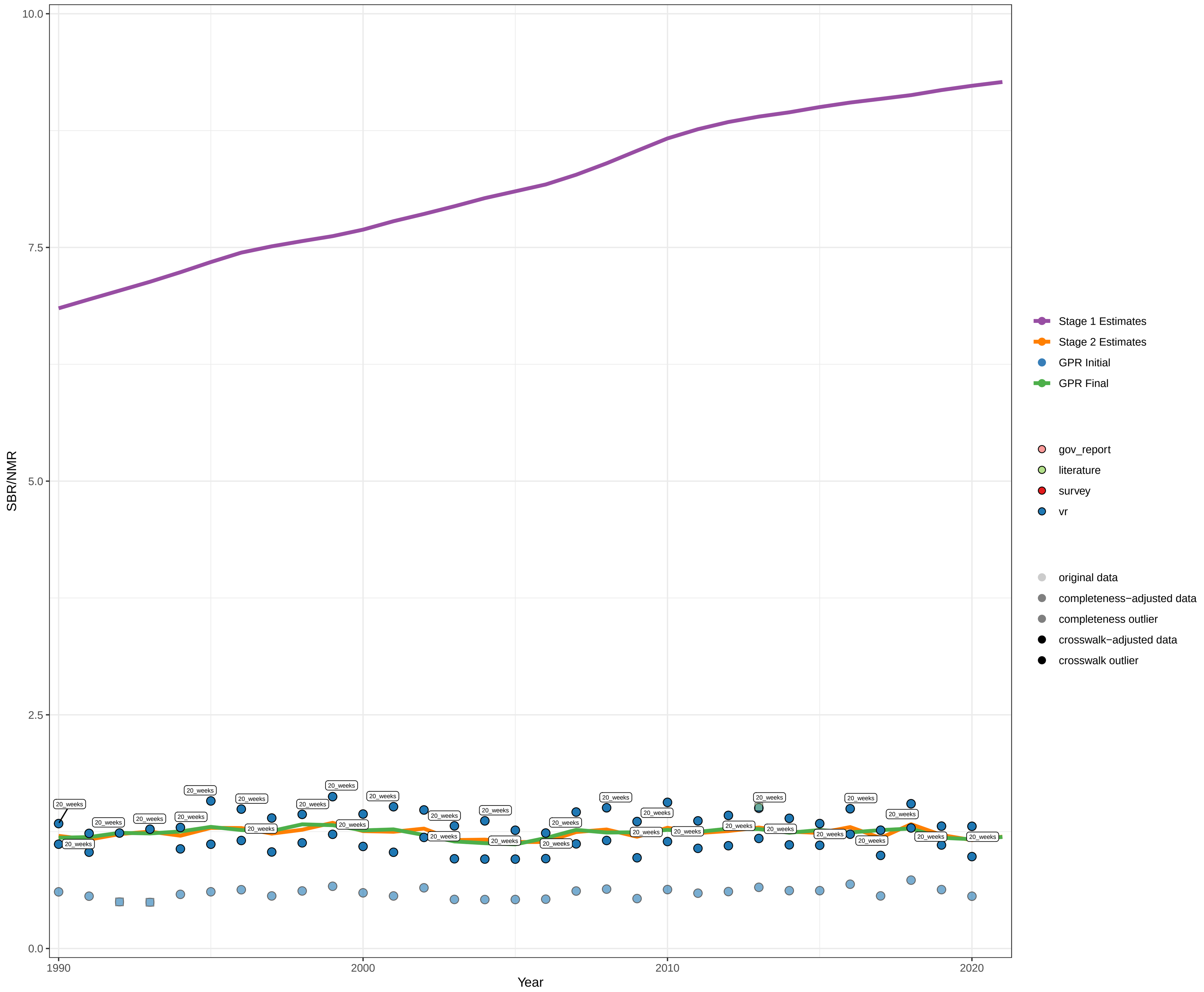

Virginia USA_569 High−income North America Stillbirth Rate / Neonatal Mortality Rate (SBR/NMR) − 28 weeks
lambda = 0.2 ; zeta = 0.99 ; scale = 5 ; amp = 0.028

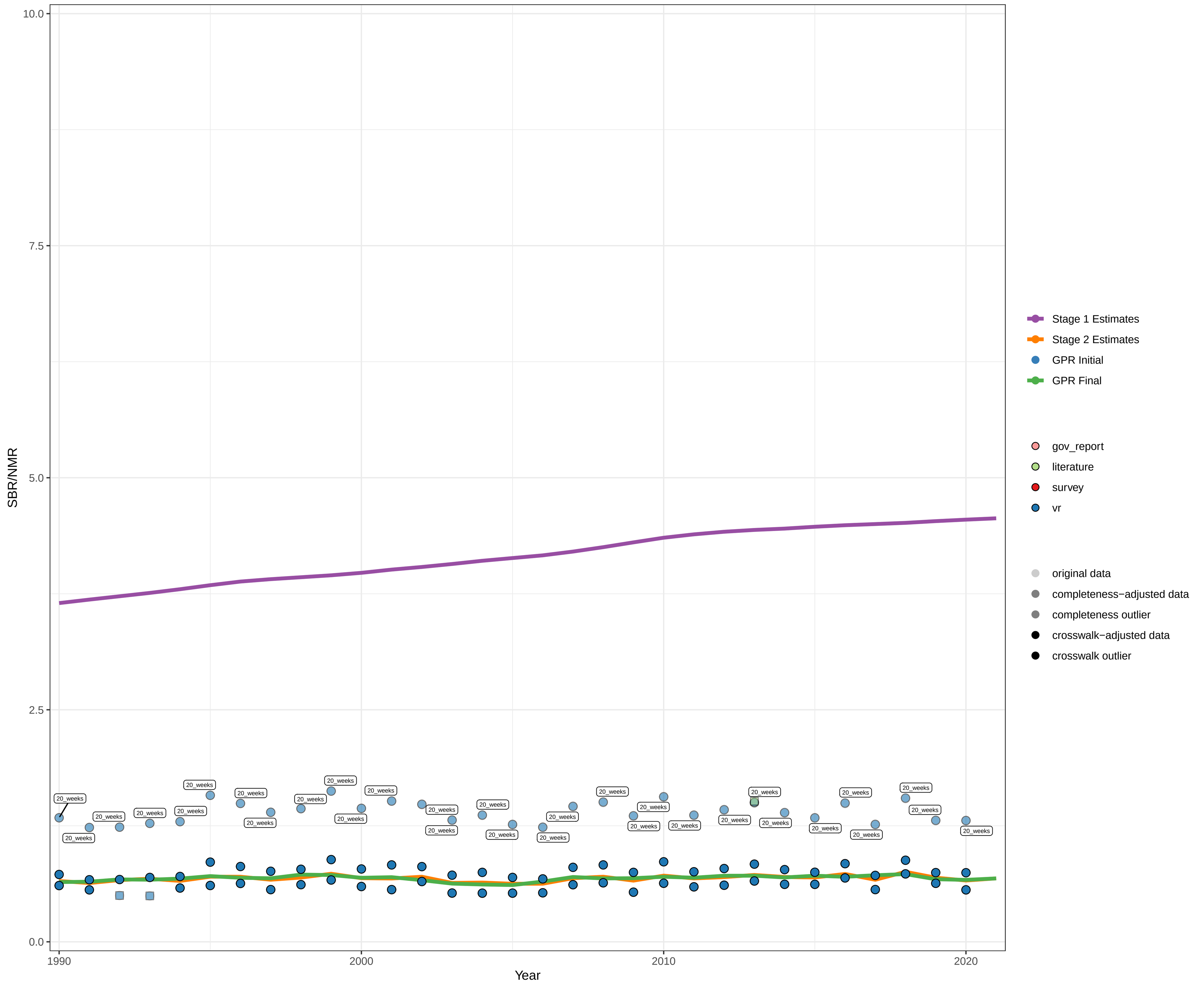

Definition	Years	Outlier	Source.Type	Title
20_weeks	1990−2020	0	VR	United States NVSS Custom Fetal Death Data
28_weeks	1990, 1991, 1994−2020	0	VR	United States NVSS Custom Fetal Death Data
20_weeks	2013	1	Sci Lit	United States Fetal and Perinatal Mortality 2013
28_weeks	1992, 1993	1	VR	United States NVSS Custom Fetal Death Data

Virginia USA_569 High−income North America Stillbirth Rate (SBR) − 20 weeks
lambda = 0.2 ; zeta = 0.99 ; scale = 5 ; amp = 0.028

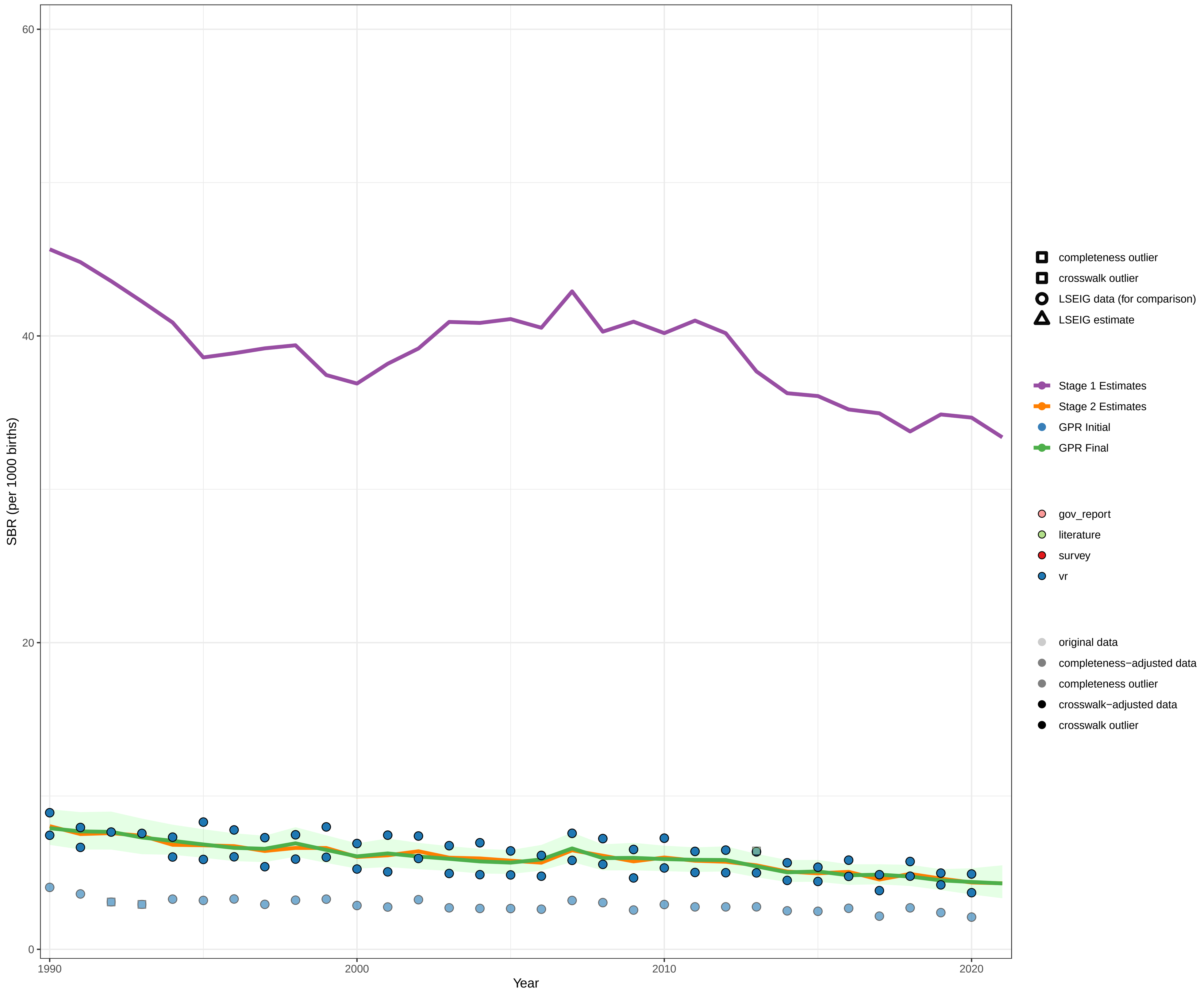

Virginia USA_569 High−income North America Stillbirth Rate (SBR) − 28 weeks
lambda = 0.2 ; zeta = 0.99 ; scale = 5 ; amp = 0.028

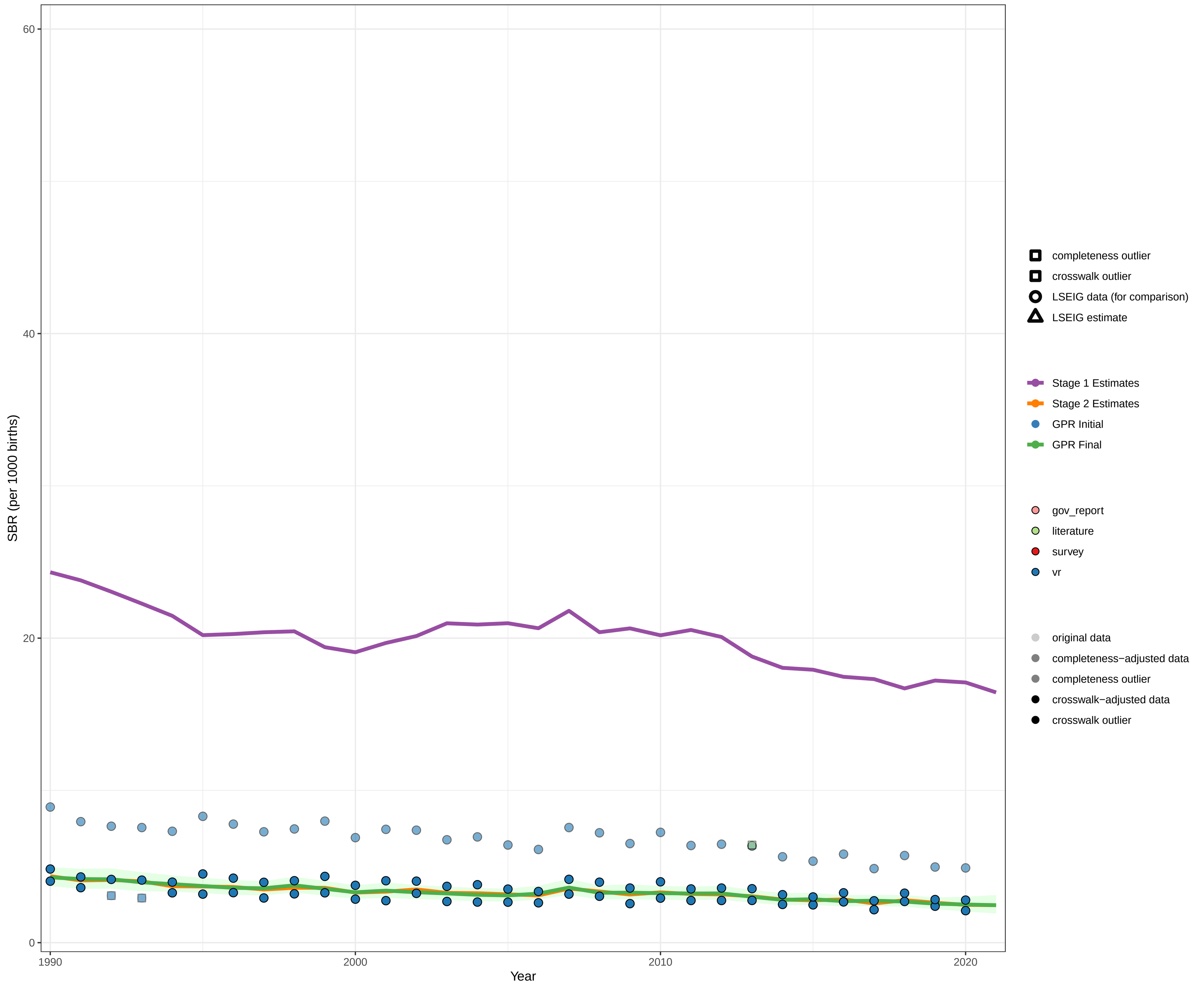

Year	Total Stillbirths (in thousands)	Total Stillbirths (in thousands)	SBR (per 1000 births)	SBR (per 1000 births)	SBR/NMR	SBR/NMR
	20 weeks	28 weeks	20 weeks	28 weeks	20 weeks	28 weeks
1990	0.8 (0.7 to 0.9)	0.4 (0.4 to 0.5)	7.9 (6.8 to 9.1)	4.3 (3.7 to 5.0)	1.18 (1.02 to 1.37)	0.64 (0.56 to 0.74)
1995	0.6 (0.6 to 0.7)	0.3 (0.3 to 0.4)	6.8 (6.0 to 7.8)	3.7 (3.3 to 4.3)	1.30 (1.14 to 1.49)	0.71 (0.62 to 0.81)
2000	0.6 (0.5 to 0.7)	0.3 (0.3 to 0.4)	6.1 (5.3 to 6.9)	3.3 (2.9 to 3.8)	1.26 (1.10 to 1.44)	0.69 (0.60 to 0.79)
2005	0.6 (0.5 to 0.7)	0.3 (0.3 to 0.4)	5.7 (4.9 to 6.5)	3.1 (2.7 to 3.6)	1.11 (0.97 to 1.28)	0.61 (0.53 to 0.70)
2010	0.6 (0.5 to 0.7)	0.3 (0.3 to 0.4)	5.9 (5.1 to 6.8)	3.2 (2.8 to 3.7)	1.27 (1.10 to 1.46)	0.70 (0.61 to 0.80)
2015	0.5 (0.5 to 0.6)	0.3 (0.3 to 0.3)	5.1 (4.4 to 5.8)	2.9 (2.5 to 3.3)	1.27 (1.10 to 1.45)	0.71 (0.62 to 0.82)
2020	0.4 (0.3 to 0.5)	0.2 (0.2 to 0.3)	4.4 (3.6 to 5.3)	2.5 (2.0 to 3.0)	1.17 (0.95 to 1.41)	0.67 (0.54 to 0.80)
2021	0.4 (0.3 to 0.5)	0.2 (0.2 to 0.3)	4.3 (3.3 to 5.5)	2.5 (1.9 to 3.1)	1.19 (0.92 to 1.52)	0.68 (0.53 to 0.87)

Washington USA_570 High−income North America Stillbirth Rate / Neonatal Mortality Rate (SBR/NMR) − 20 weeks
lambda = 0.2 ; zeta = 0.99 ; scale = 5 ; amp = 0.028

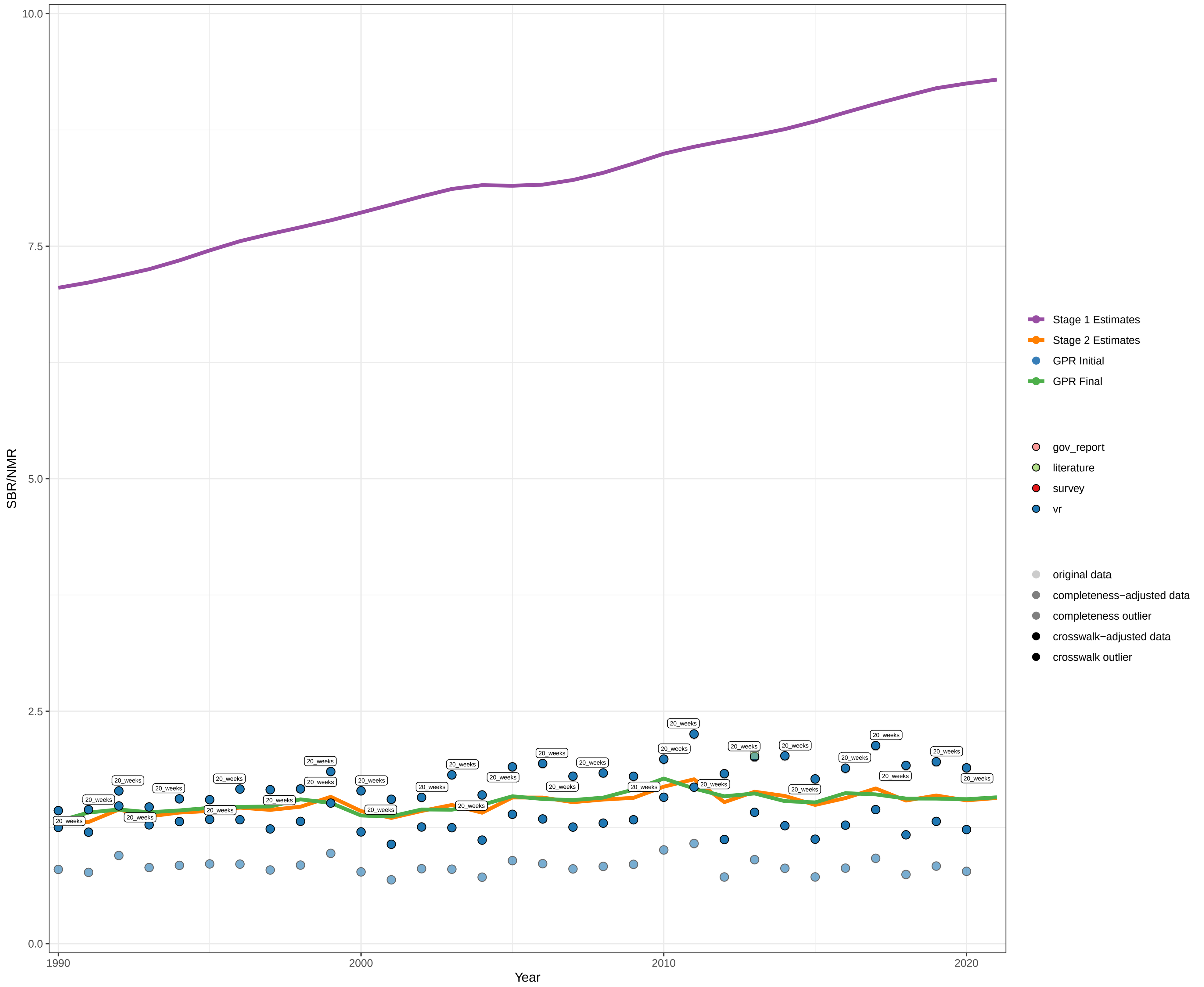

Washington USA_570 High−income North America Stillbirth Rate / Neonatal Mortality Rate (SBR/NMR) − 28 weeks
lambda = 0.2 ; zeta = 0.99 ; scale = 5 ; amp = 0.028

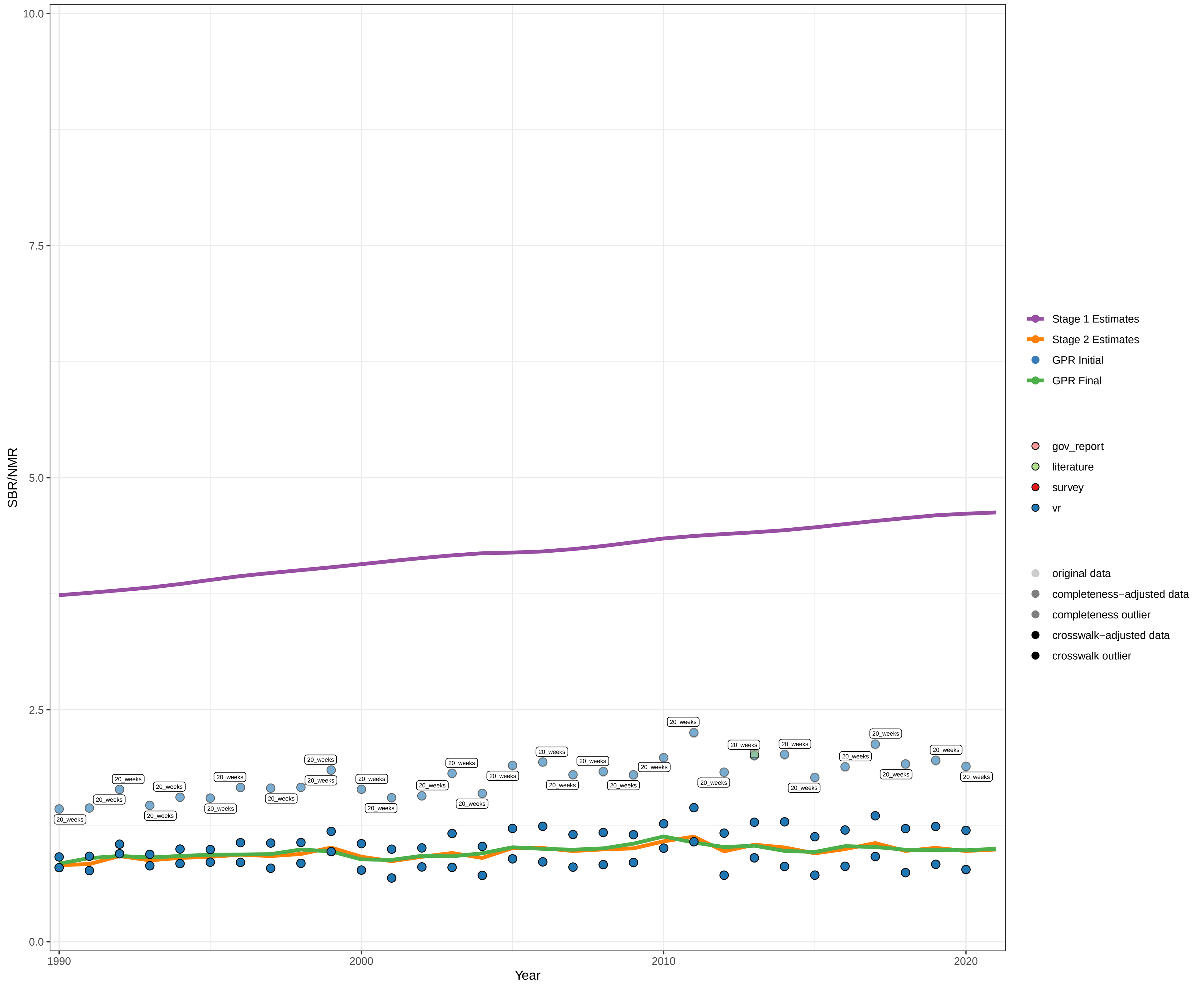

Definition	Years	Outlier	Source.Type	Title
20_weeks, 28_weeks	1990−2020	0	VR	United States NVSS Custom Fetal Death Data
20_weeks	2013	1	Sci Lit	United States Fetal and Perinatal Mortality 2013

Washington USA_570 High−income North America Stillbirth Rate (SBR) − 20 weeks
lambda = 0.2 ; zeta = 0.99 ; scale = 5 ; amp = 0.028

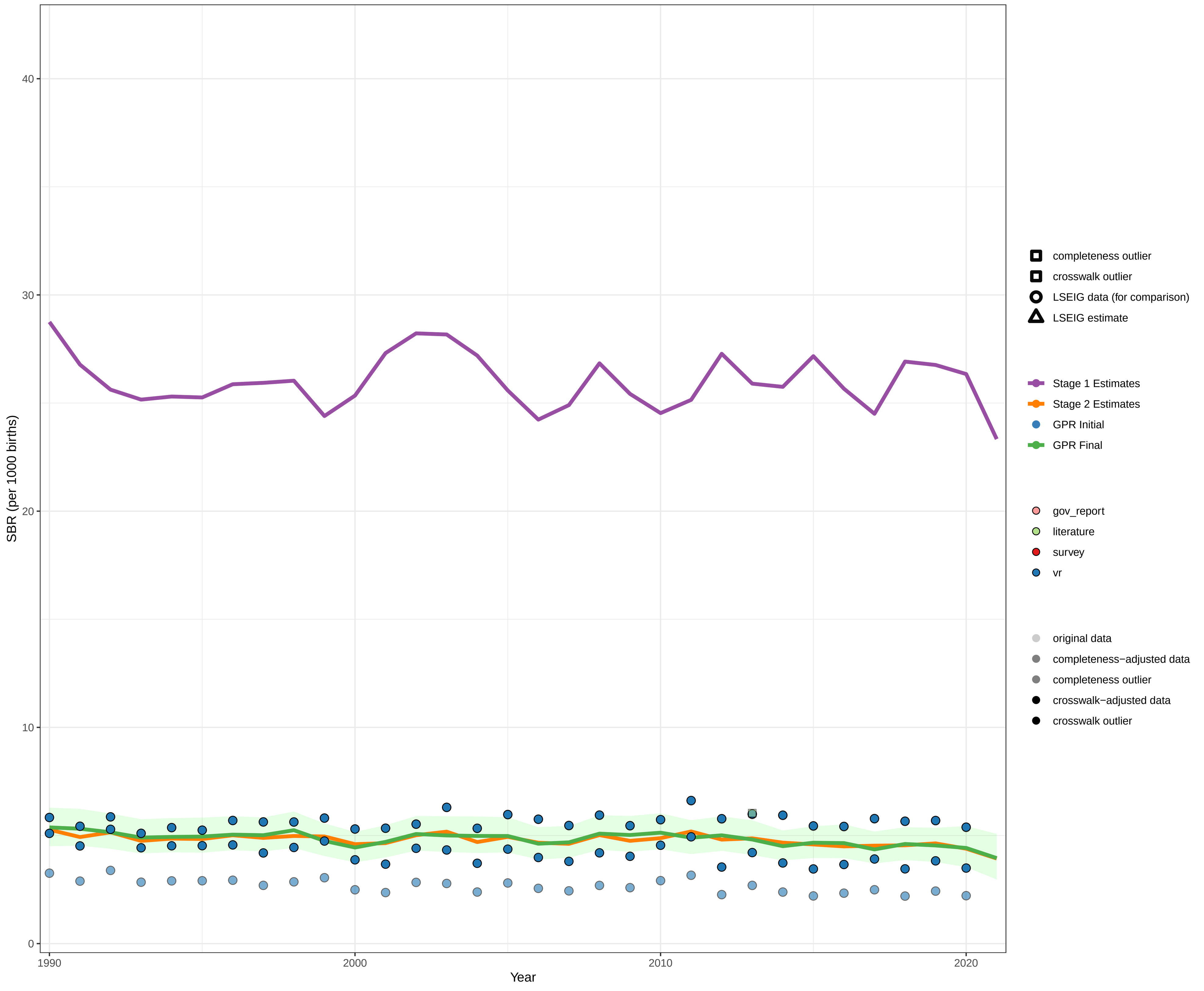

Washington USA_570 High−income North America Stillbirth Rate (SBR) − 28 weeks
lambda = 0.2 ; zeta = 0.99 ; scale = 5 ; amp = 0.028

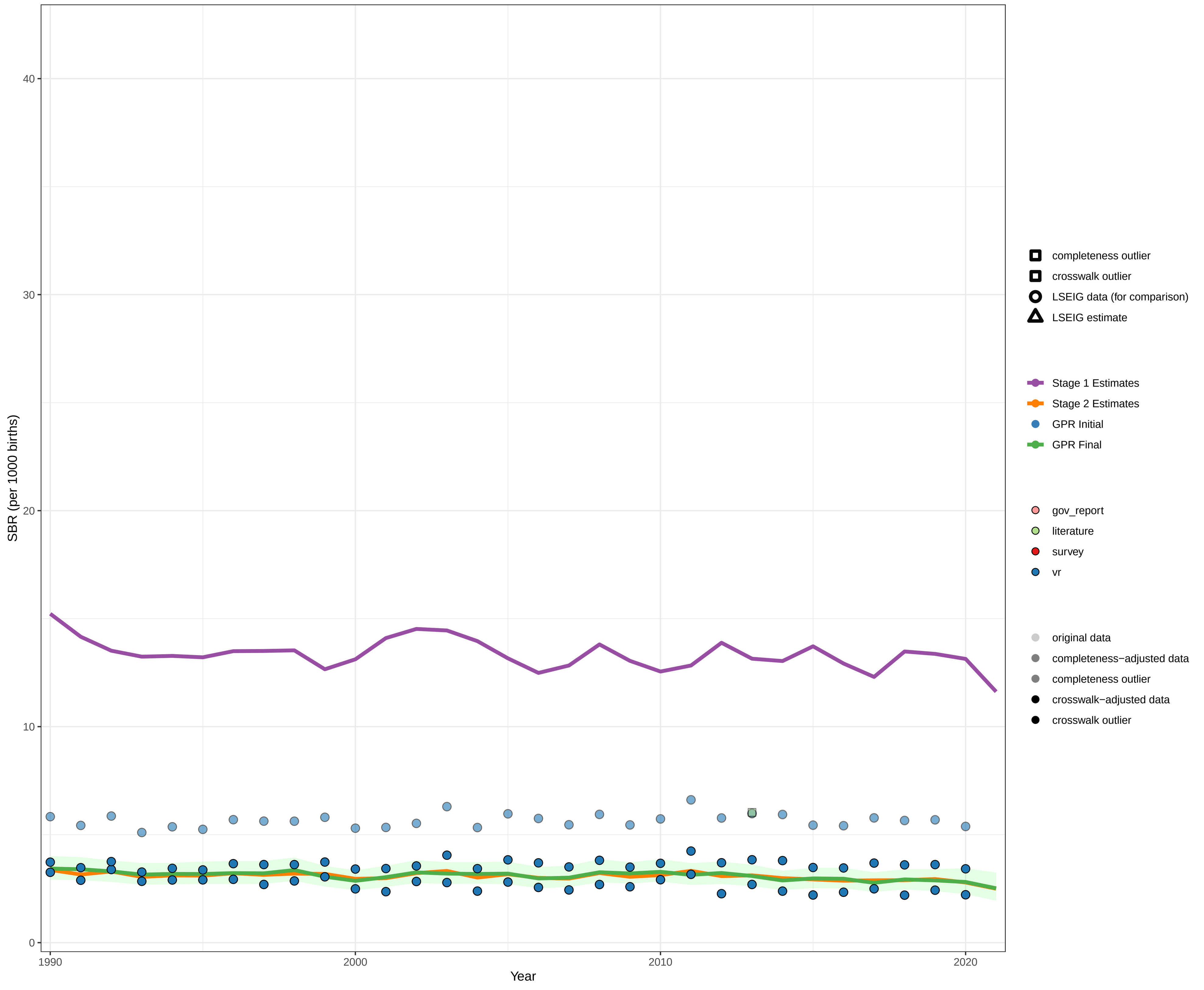

Year	Total Stillbirths (in thousands)	Total Stillbirths (in thousands)	SBR (per 1000 births)	SBR (per 1000 births)	SBR/NMR	SBR/NMR
	20 weeks	28 weeks	20 weeks	28 weeks	20 weeks	28 weeks
1990	0.4<br>(0.4 to 0.5)	0.3<br>(0.2 to 0.3)	5.4<br>(4.5 to 6.3)	3.4<br>(2.9 to 4.0)	1.32<br>(1.10 to 1.54)	0.84<br>(0.71 to 0.98)
1995	0.4<br>(0.3 to 0.5)	0.2<br>(0.2 to 0.3)	5.0<br>(4.2 to 5.8)	3.2<br>(2.7 to 3.8)	1.46<br>(1.24 to 1.72)	0.94<br>(0.80 to 1.11)
2000	0.4<br>(0.3 to 0.4)	0.2<br>(0.2 to 0.3)	4.4<br>(3.8 to 5.2)	2.9<br>(2.4 to 3.4)	1.38<br>(1.17 to 1.60)	0.89<br>(0.75 to 1.04)
2005	0.4<br>(0.4 to 0.5)	0.3<br>(0.2 to 0.3)	5.0<br>(4.2 to 5.8)	3.2<br>(2.7 to 3.8)	1.59<br>(1.34 to 1.86)	1.02<br>(0.86 to 1.20)
2010	0.4<br>(0.4 to 0.5)	0.3<br>(0.2 to 0.3)	5.1<br>(4.4 to 6.0)	3.3<br>(2.8 to 3.9)	1.78<br>(1.51 to 2.09)	1.14<br>(0.98 to 1.34)
2015	0.4<br>(0.4 to 0.5)	0.3<br>(0.2 to 0.3)	4.7<br>(4.0 to 5.4)	3.0<br>(2.5 to 3.5)	1.52<br>(1.29 to 1.77)	0.97<br>(0.82 to 1.13)
2020	0.4<br>(0.3 to 0.4)	0.2<br>(0.2 to 0.3)	4.4<br>(3.5 to 5.4)	2.8<br>(2.2 to 3.4)	1.55<br>(1.24 to 1.91)	0.99<br>(0.79 to 1.21)
2021	0.3<br>(0.2 to 0.4)	0.2<br>(0.2 to 0.3)	4.0<br>(3.0 to 5.1)	2.5<br>(1.9 to 3.2)	1.57<br>(1.18 to 2.02)	1.00<br>(0.77 to 1.29)

West Virginia USA_571 High−income North America Stillbirth Rate / Neonatal Mortality Rate (SBR/NMR) − 20 weeks
lambda = 0.2 ; zeta = 0.99 ; scale = 5 ; amp = 0.028

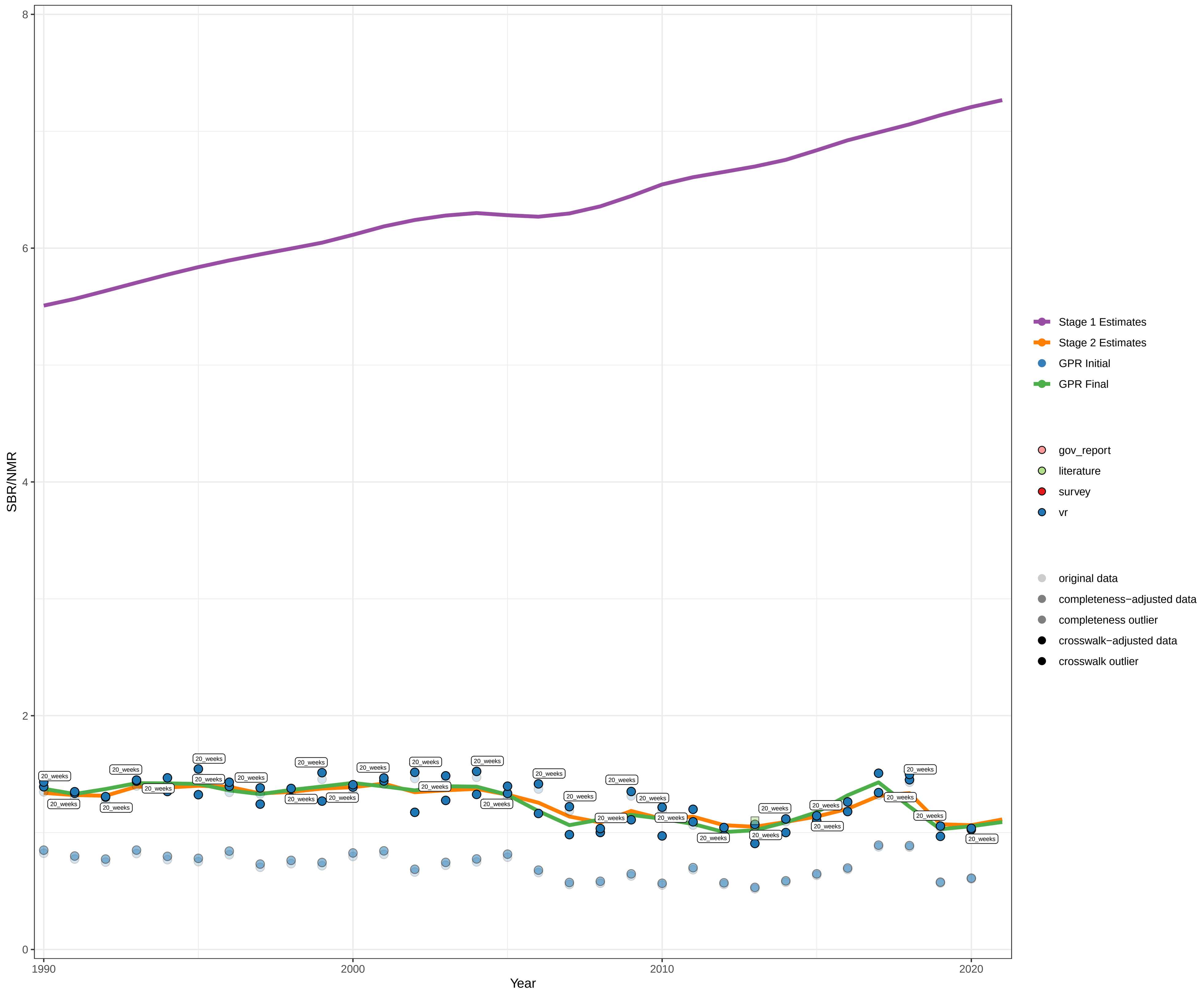

West Virginia USA_571 High−income North America Stillbirth Rate / Neonatal Mortality Rate (SBR/NMR) − 28 weeks
lambda = 0.2 ; zeta = 0.99 ; scale = 5 ; amp = 0.028

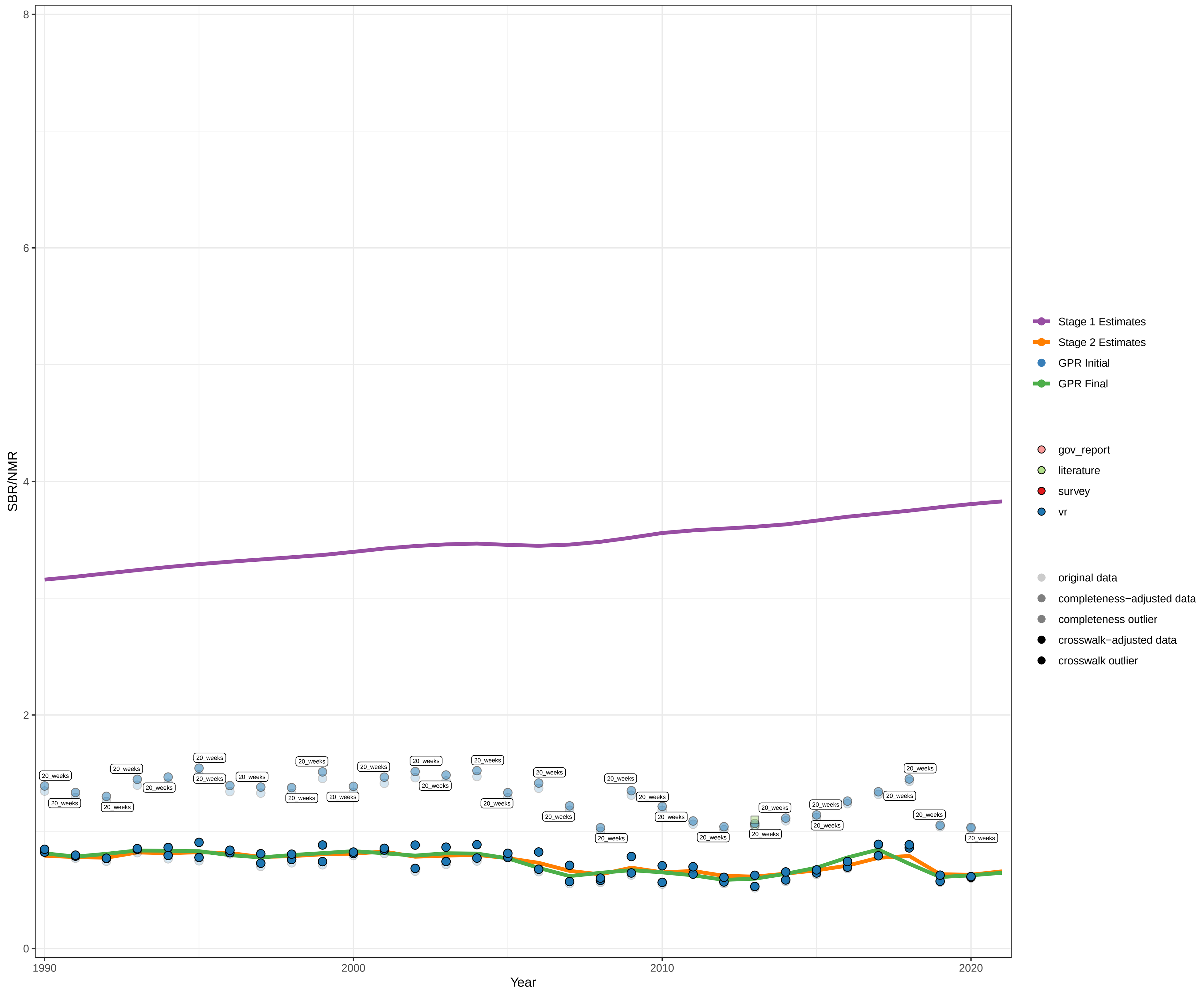

Definition	Years	Outlier	Source.Type	Title
20_weeks, 28_weeks	1990−2020	0	VR	United States NVSS Custom Fetal Death Data
20_weeks	2013	1	Sci Lit	United States Fetal and Perinatal Mortality 2013

West Virginia USA_571 High−income North America Stillbirth Rate (SBR) − 20 weeks
lambda = 0.2 ; zeta = 0.99 ; scale = 5 ; amp = 0.028

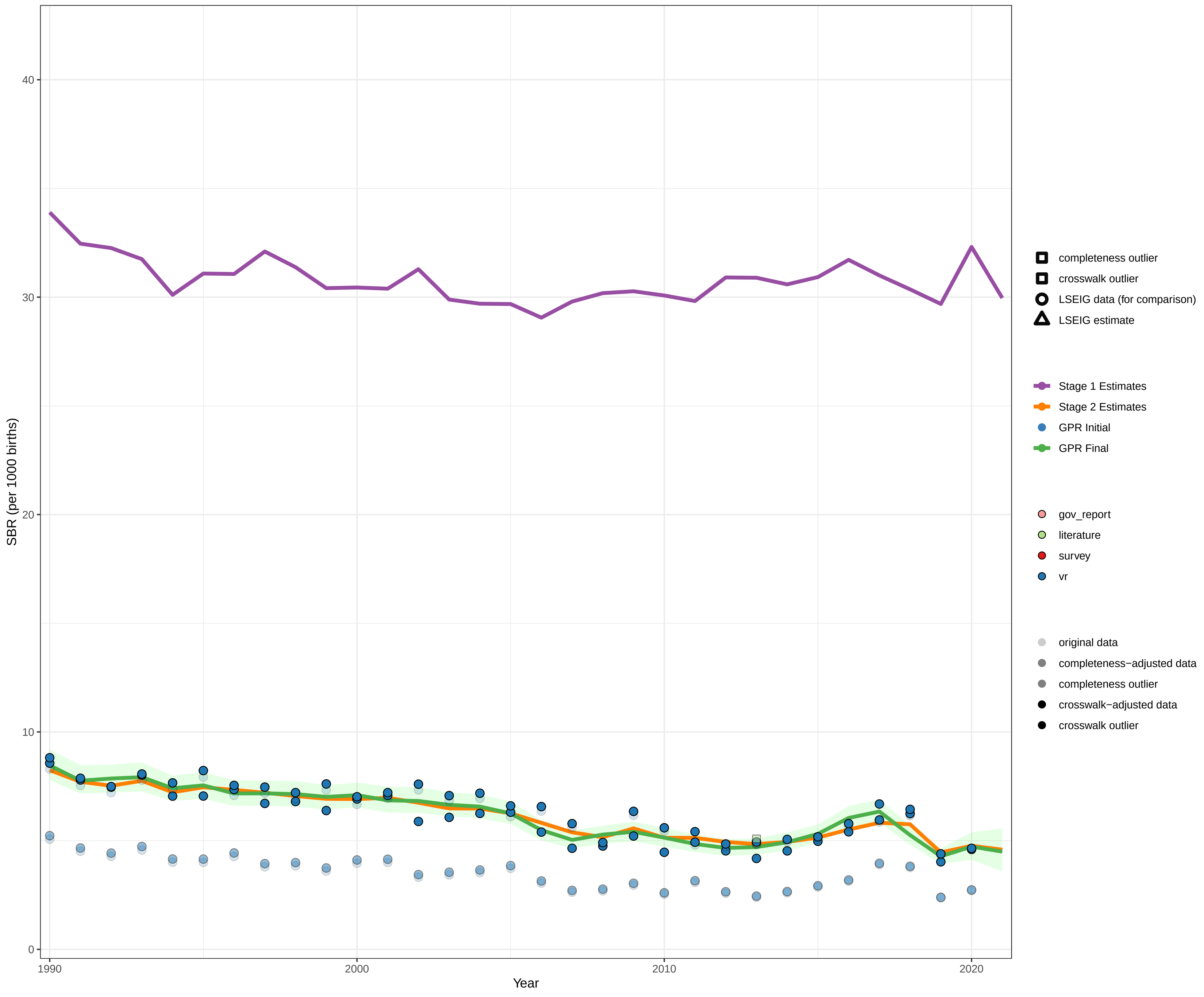

West Virginia USA_571 High−income North America Stillbirth Rate (SBR) − 28 weeks
lambda = 0.2 ; zeta = 0.99 ; scale = 5 ; amp = 0.028

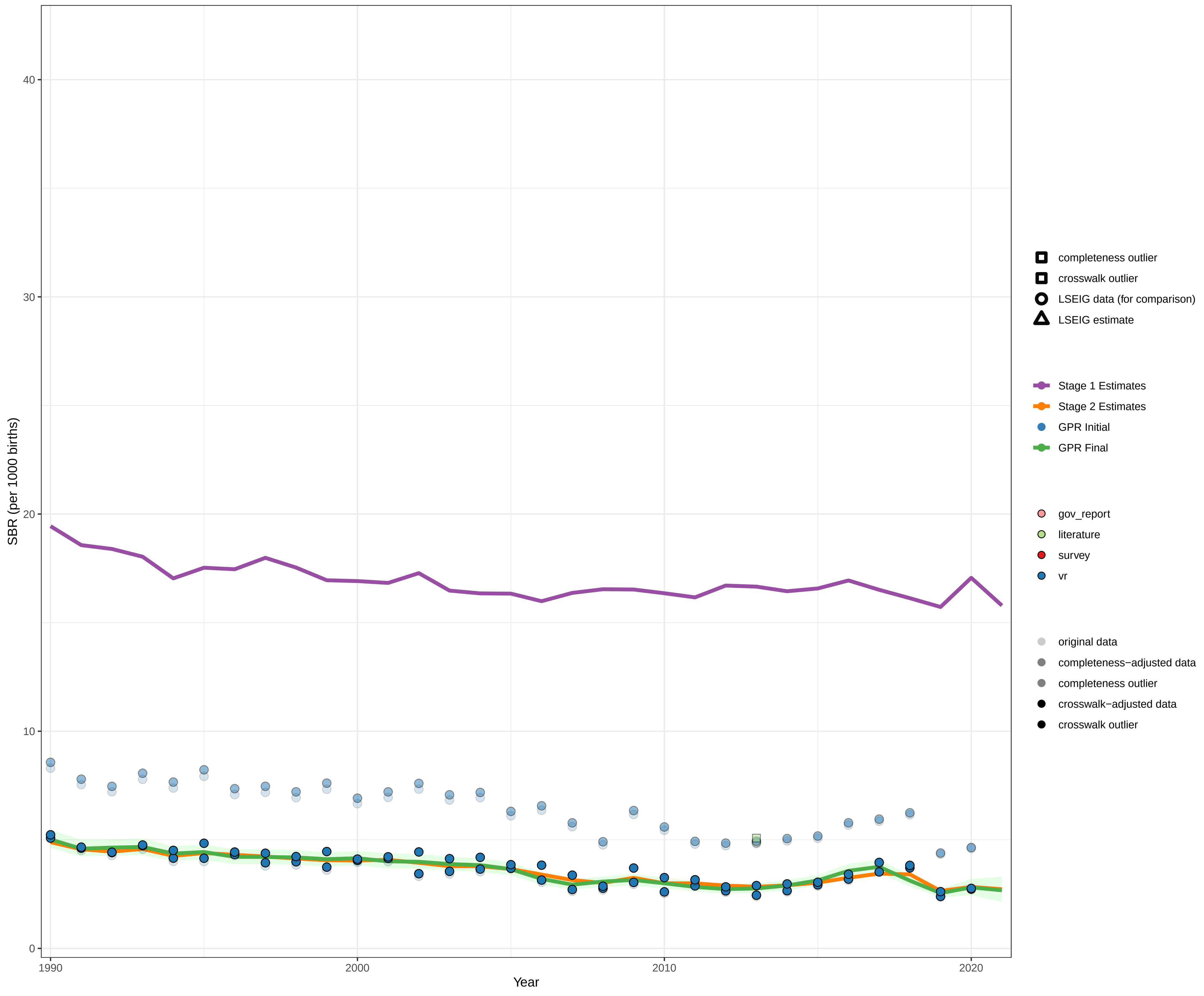

Year	Total Stillbirths (in thousands)	Total Stillbirths (in thousands)	SBR (per 1000 births)	SBR (per 1000 births)	SBR/NMR	SBR/NMR
	20 weeks	28 weeks	20 weeks	28 weeks	20 weeks	28 weeks
1990	0.2 (0.2 to 0.2)	0.1 (0.1 to 0.1)	8.5 (7.8 to 9.2)	5.0 (4.6 to 5.4)	1.38 (1.27 to 1.49)	0.81 (0.75 to 0.89)
1995	0.2 (0.1 to 0.2)	0.1 (0.1 to 0.1)	7.5 (6.9 to 8.2)	4.4 (4.1 to 4.8)	1.42 (1.30 to 1.53)	0.83 (0.77 to 0.90)
2000	0.1 (0.1 to 0.2)	0.1 (0.1 to 0.1)	7.1 (6.5 to 7.7)	4.2 (3.8 to 4.5)	1.42 (1.31 to 1.54)	0.83 (0.77 to 0.90)
2005	0.1 (0.1 to 0.1)	0.1 (0.1 to 0.1)	6.3 (5.8 to 6.8)	3.6 (3.4 to 4.0)	1.32 (1.22 to 1.44)	0.77 (0.71 to 0.84)
2010	0.1 (0.1 to 0.1)	0.1 (0.1 to 0.1)	5.1 (4.7 to 5.6)	3.0 (2.8 to 3.3)	1.12 (1.03 to 1.22)	0.65 (0.60 to 0.71)
2015	0.1 (0.1 to 0.1)	0.1 (0.1 to 0.1)	5.3 (4.9 to 5.7)	3.1 (2.9 to 3.4)	1.17 (1.08 to 1.27)	0.69 (0.64 to 0.75)
2020	0.1 (0.1 to 0.1)	0.0 (0.0 to 0.1)	4.7 (4.1 to 5.4)	2.8 (2.5 to 3.2)	1.05 (0.92 to 1.20)	0.63 (0.55 to 0.71)
2021	0.1 (0.1 to 0.1)	0.0 (0.0 to 0.1)	4.5 (3.6 to 5.6)	2.7 (2.1 to 3.3)	1.09 (0.87 to 1.35)	0.65 (0.52 to 0.80)

Wisconsin USA_572 High−income North America Stillbirth Rate / Neonatal Mortality Rate (SBR/NMR) − 20 weeks
lambda = 0.2 ; zeta = 0.99 ; scale = 5 ; amp = 0.028

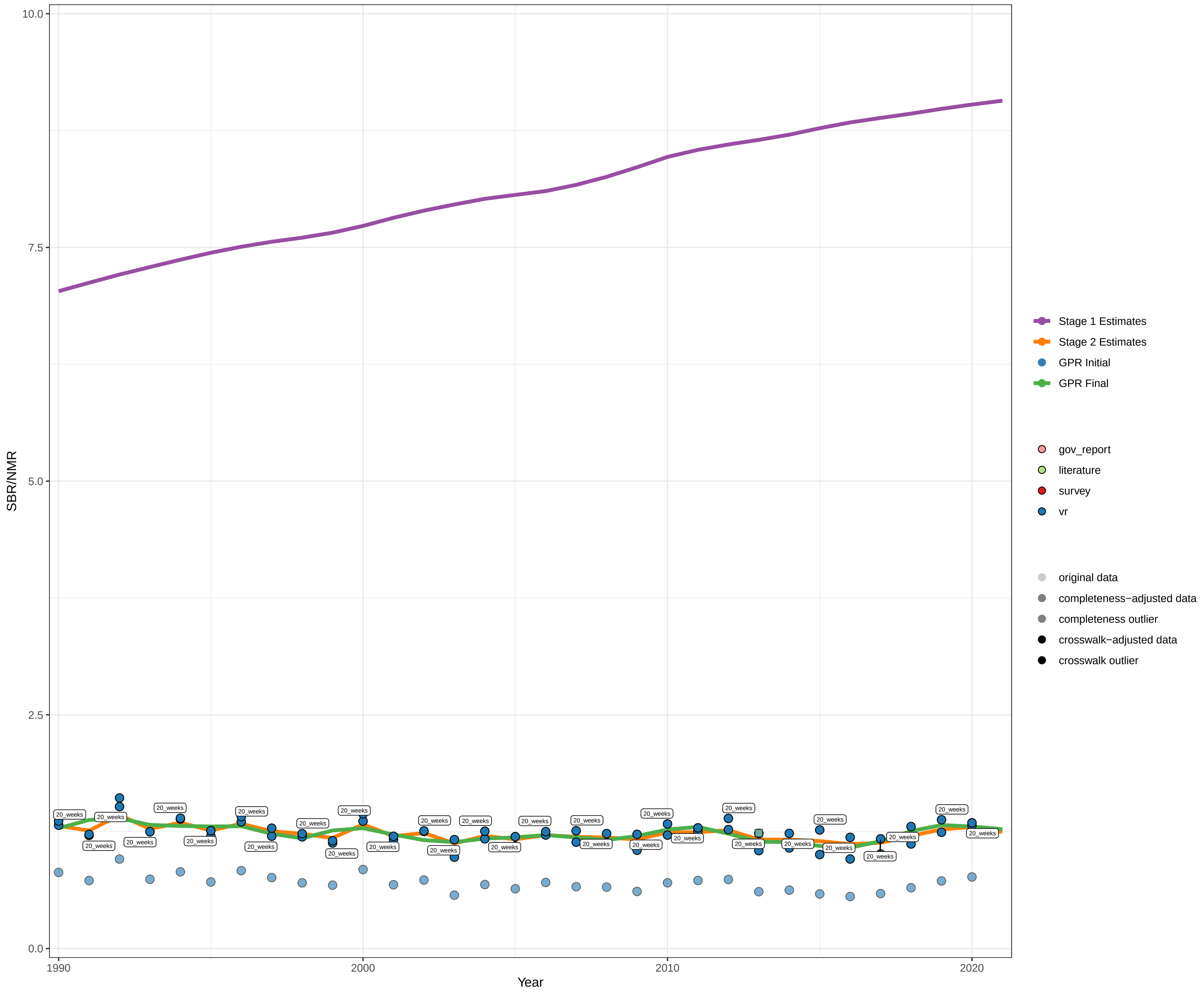

Wisconsin USA_572 High−income North America Stillbirth Rate / Neonatal Mortality Rate (SBR/NMR) − 28 weeks
lambda = 0.2 ; zeta = 0.99 ; scale = 5 ; amp = 0.028

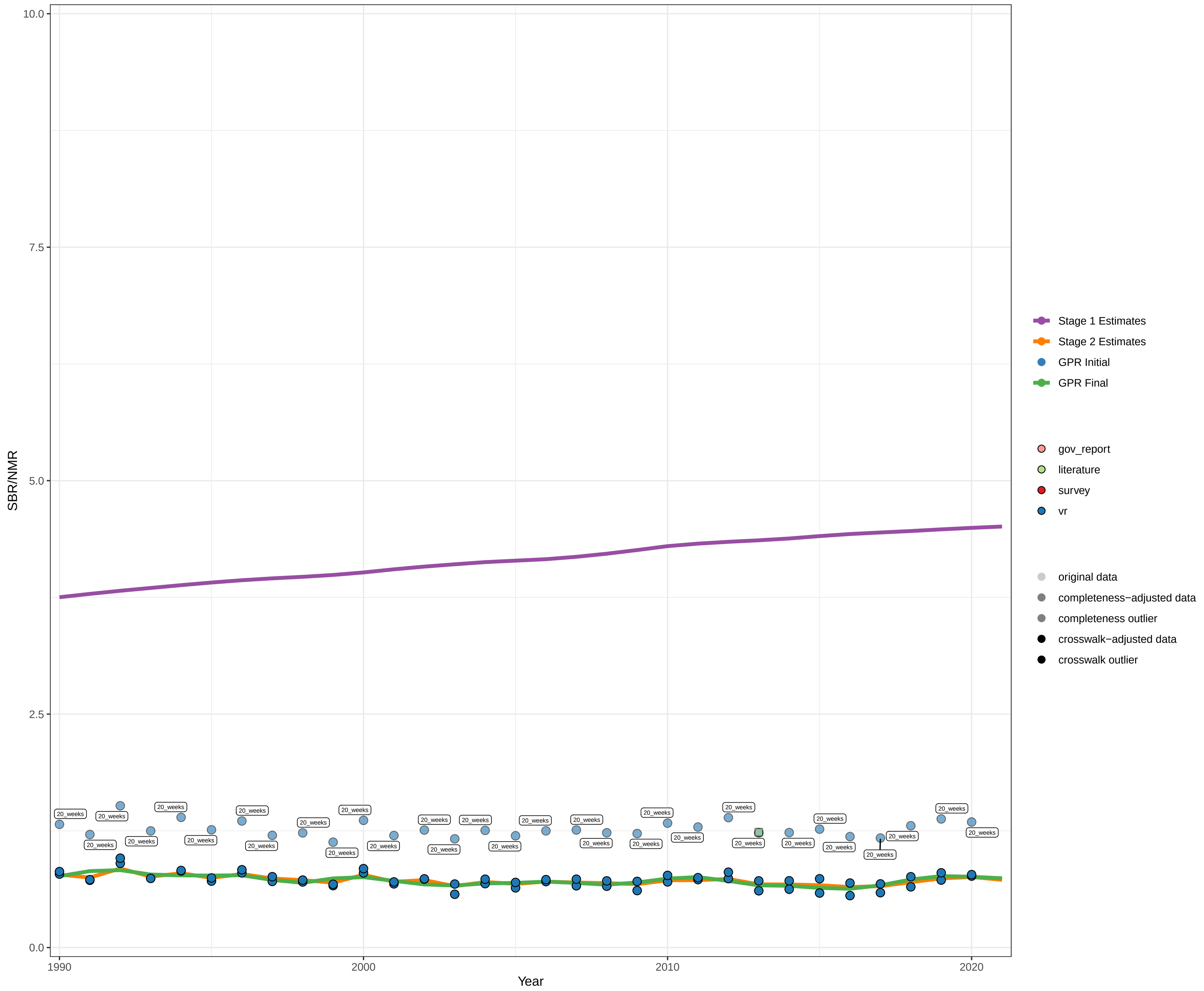

Definition	Years	Outlier	Source.Type	Title
20_weeks, 28_weeks	1990−2020	0	VR	United States NVSS Custom Fetal Death Data
20_weeks	2013	1	Sci Lit	United States Fetal and Perinatal Mortality 2013

Wisconsin USA_572 High−income North America Stillbirth Rate (SBR) − 20 weeks
lambda = 0.2 ; zeta = 0.99 ; scale = 5 ; amp = 0.028

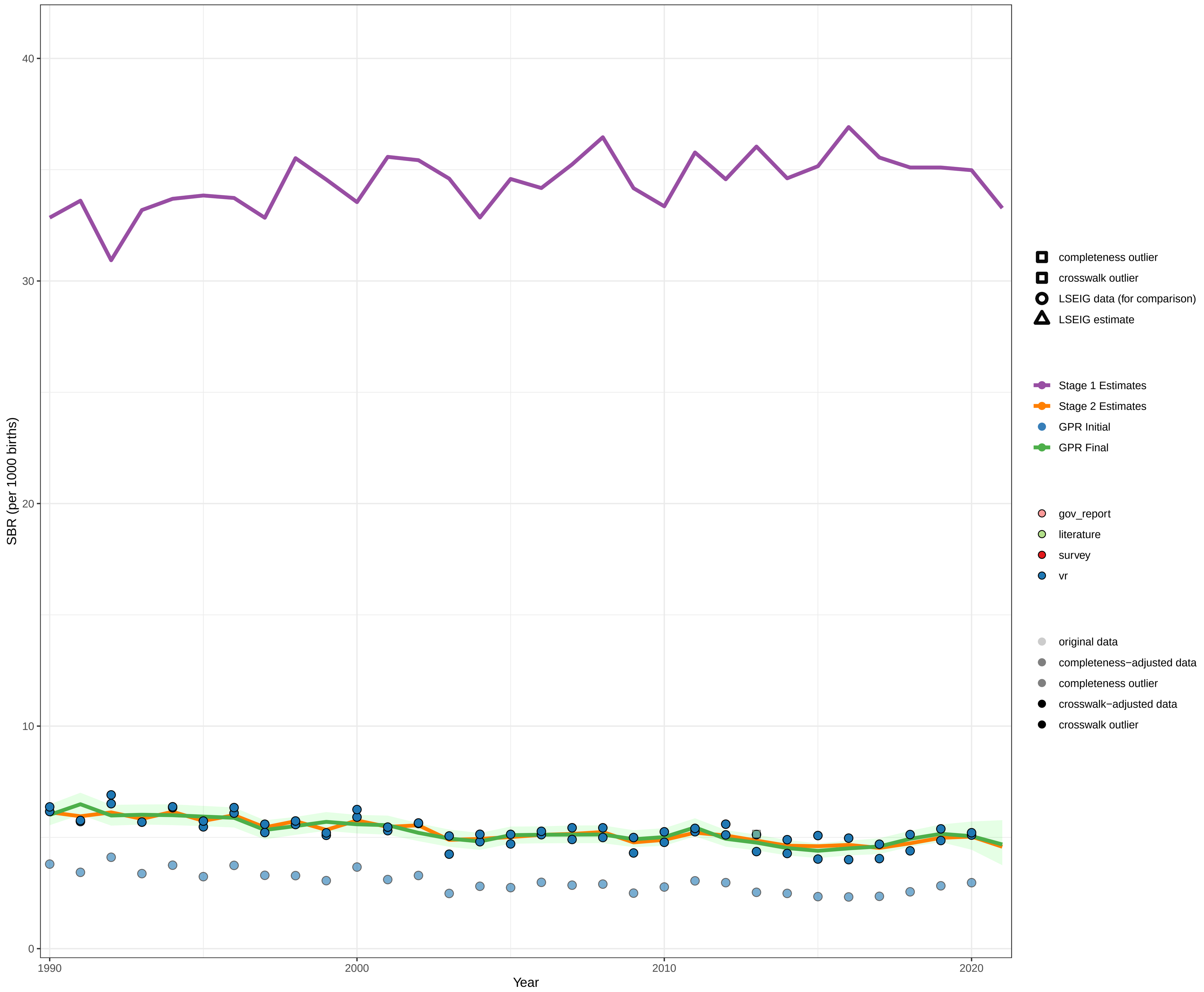

Wisconsin USA_572 High−income North America Stillbirth Rate (SBR) − 28 weeks
lambda = 0.2 ; zeta = 0.99 ; scale = 5 ; amp = 0.028

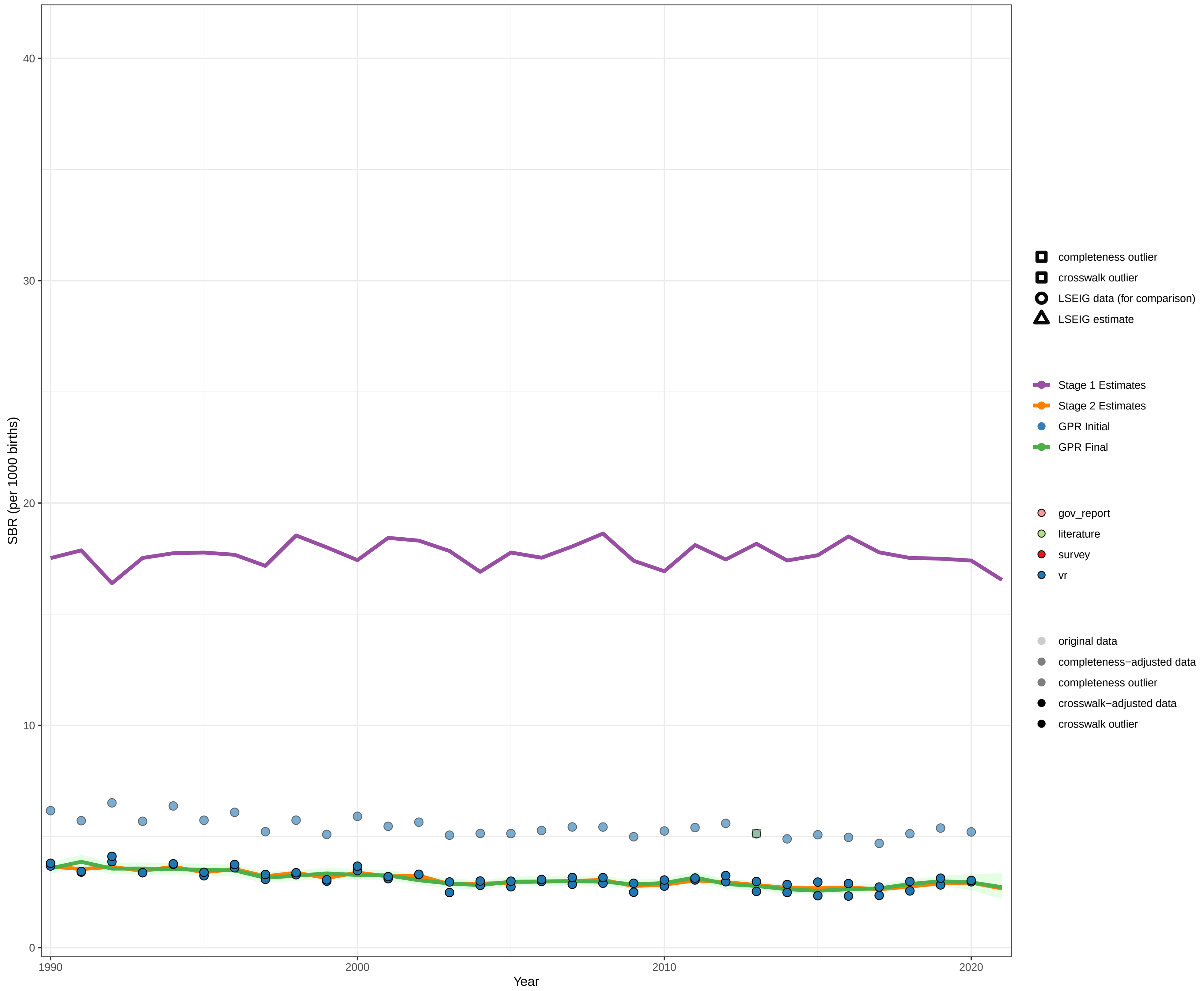

Year	Total Stillbirths (in thousands)	Total Stillbirths (in thousands)	SBR (per 1000 births)	SBR (per 1000 births)	SBR/NMR	SBR/NMR
	20 weeks	28 weeks	20 weeks	28 weeks	20 weeks	28 weeks
1990	0.4 (0.4 to 0.5)	0.3 (0.2 to 0.3)	6.0 (5.5 to 6.5)	3.6 (3.3 to 3.8)	1.29 (1.19 to 1.39)	0.76 (0.71 to 0.82)
1995	0.4 (0.4 to 0.4)	0.2 (0.2 to 0.3)	5.9 (5.5 to 6.4)	3.5 (3.3 to 3.8)	1.31 (1.21 to 1.41)	0.77 (0.72 to 0.83)
2000	0.4 (0.4 to 0.4)	0.2 (0.2 to 0.2)	5.6 (5.2 to 6.0)	3.3 (3.1 to 3.5)	1.29 (1.19 to 1.38)	0.75 (0.70 to 0.81)
2005	0.4 (0.3 to 0.4)	0.2 (0.2 to 0.2)	5.1 (4.7 to 5.5)	3.0 (2.8 to 3.2)	1.19 (1.10 to 1.28)	0.69 (0.64 to 0.75)
2010	0.3 (0.3 to 0.4)	0.2 (0.2 to 0.2)	5.0 (4.6 to 5.4)	2.9 (2.7 to 3.1)	1.27 (1.17 to 1.37)	0.74 (0.68 to 0.79)
2015	0.3 (0.3 to 0.3)	0.2 (0.2 to 0.2)	4.4 (4.1 to 4.7)	2.6 (2.4 to 2.8)	1.10 (1.02 to 1.18)	0.64 (0.59 to 0.69)
2020	0.3 (0.3 to 0.4)	0.2 (0.2 to 0.2)	5.1 (4.4 to 5.7)	2.9 (2.6 to 3.3)	1.31 (1.15 to 1.47)	0.76 (0.67 to 0.86)
2021	0.3 (0.2 to 0.4)	0.2 (0.1 to 0.2)	4.7 (3.7 to 5.8)	2.7 (2.2 to 3.3)	1.28 (1.02 to 1.57)	0.74 (0.60 to 0.91)

Wyoming USA_573 High−income North America Stillbirth Rate / Neonatal Mortality Rate (SBR/NMR) − 20 weeks
lambda = 0.2 ; zeta = 0.99 ; scale = 5 ; amp = 0.028

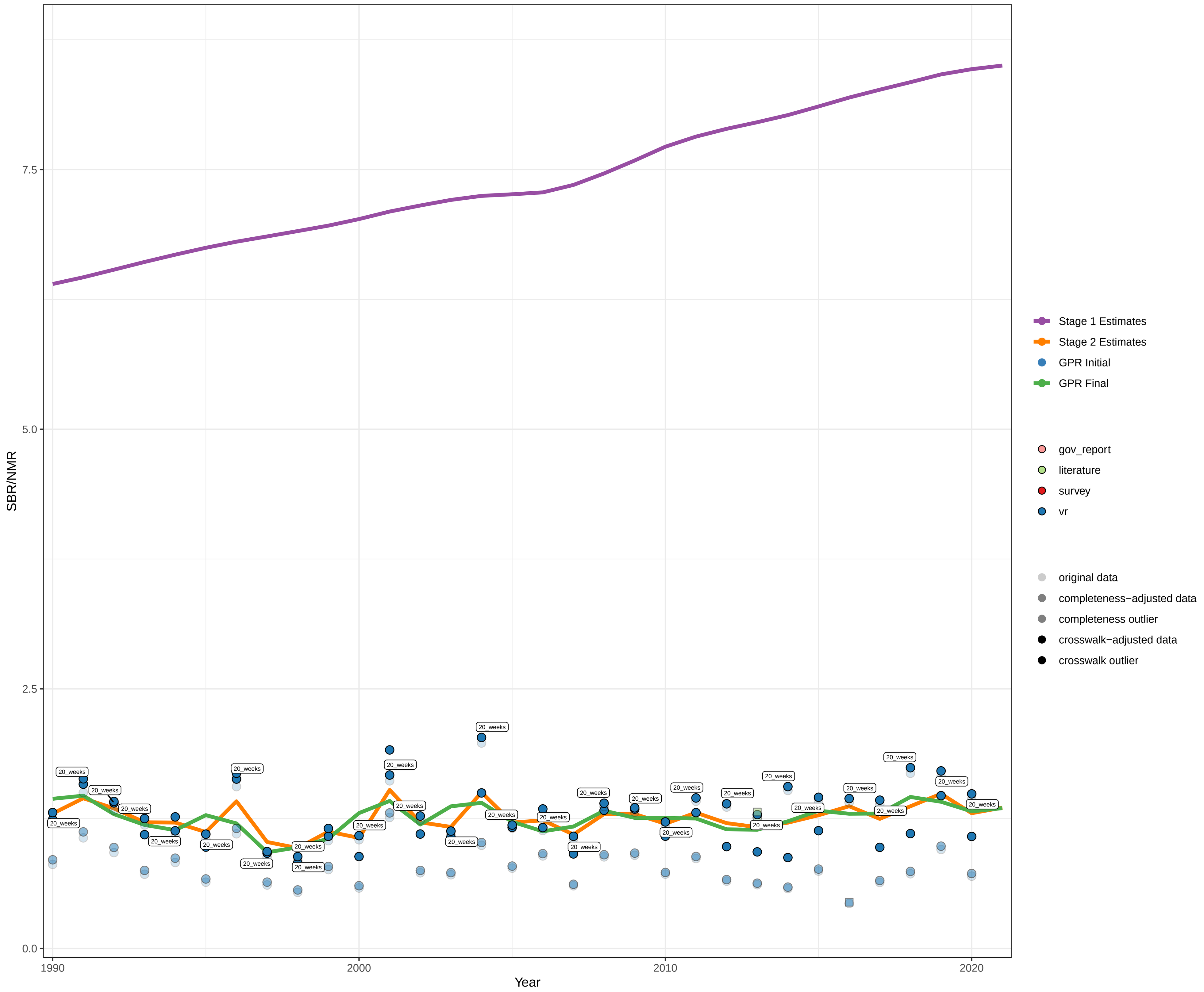

Wyoming USA_573 High−income North America Stillbirth Rate / Neonatal Mortality Rate (SBR/NMR) − 28 weeks
lambda = 0.2 ; zeta = 0.99 ; scale = 5 ; amp = 0.028

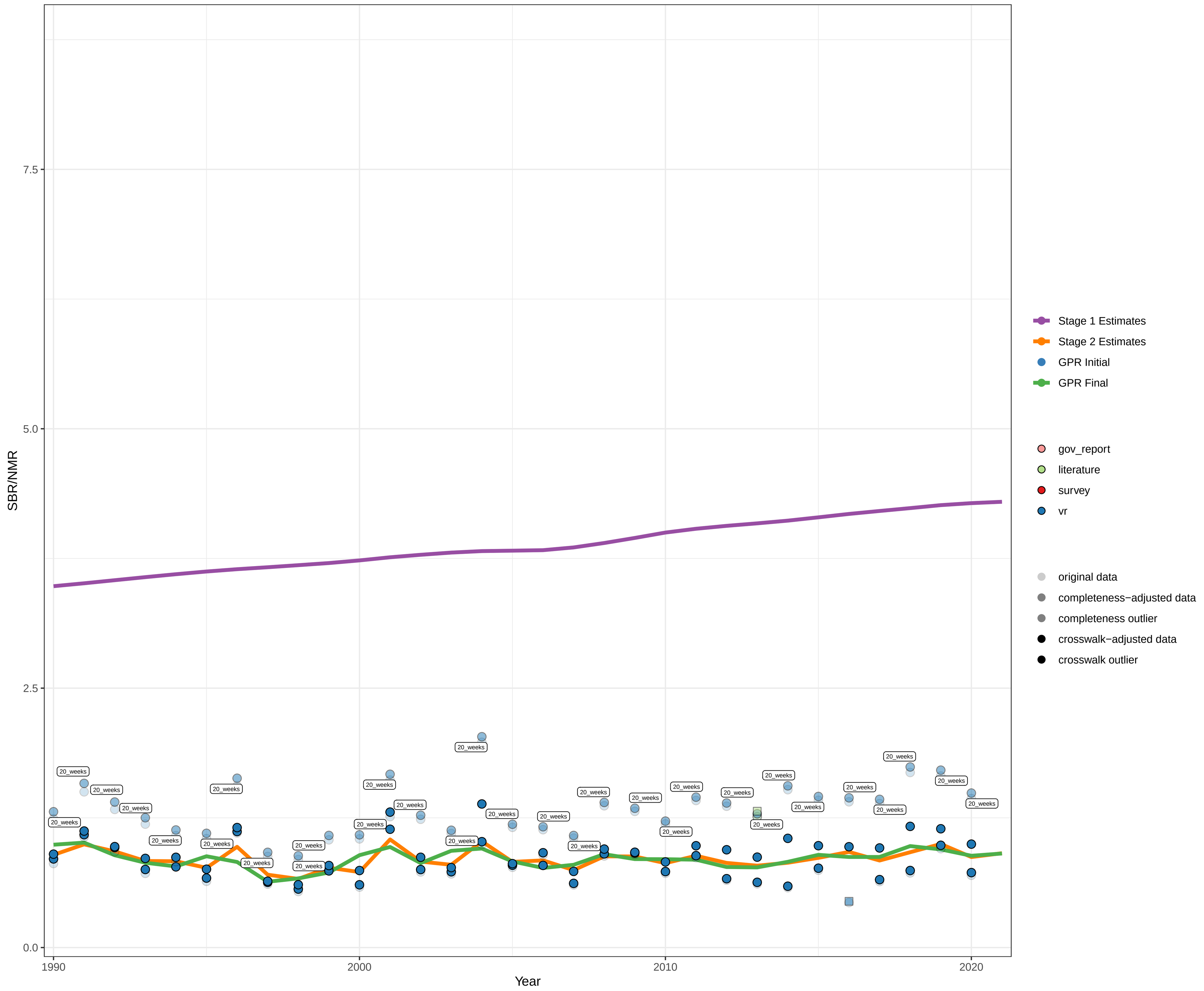

Definition	Years	Outlier	Source.Type	Title
20_weeks	1990−2020	0	VR	United States NVSS Custom Fetal Death Data
28_weeks	1990−2015, 2017−2020	0	VR	United States NVSS Custom Fetal Death Data
20_weeks	2013	1	Sci Lit	United States Fetal and Perinatal Mortality 2013
28_weeks	2016	1	VR	United States NVSS Custom Fetal Death Data

Wyoming USA_573 High−income North America Stillbirth Rate (SBR) − 20 weeks
lambda = 0.2 ; zeta = 0.99 ; scale = 5 ; amp = 0.028

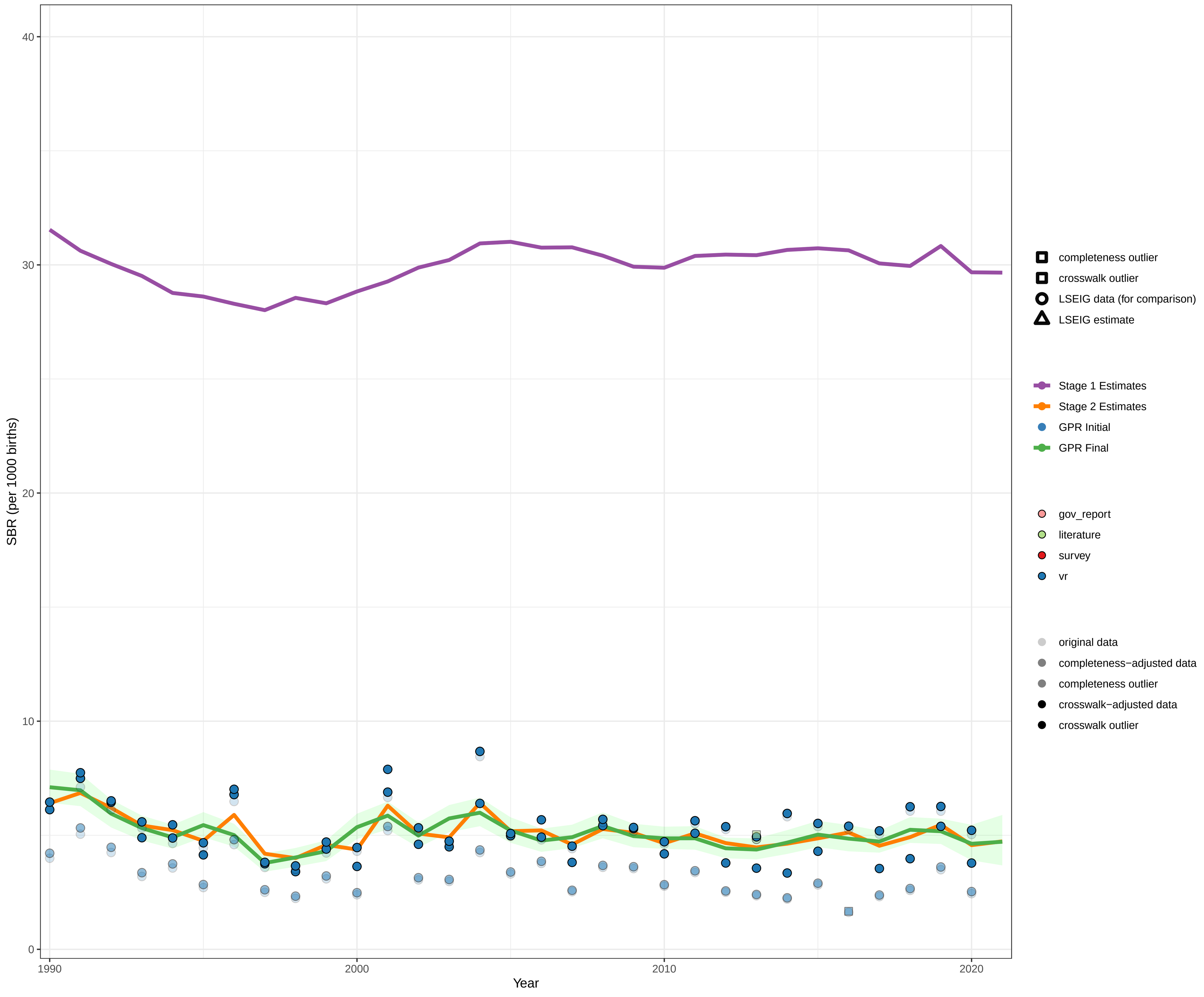

Wyoming USA_573 High−income North America Stillbirth Rate (SBR) − 28 weeks
lambda = 0.2 ; zeta = 0.99 ; scale = 5 ; amp = 0.028

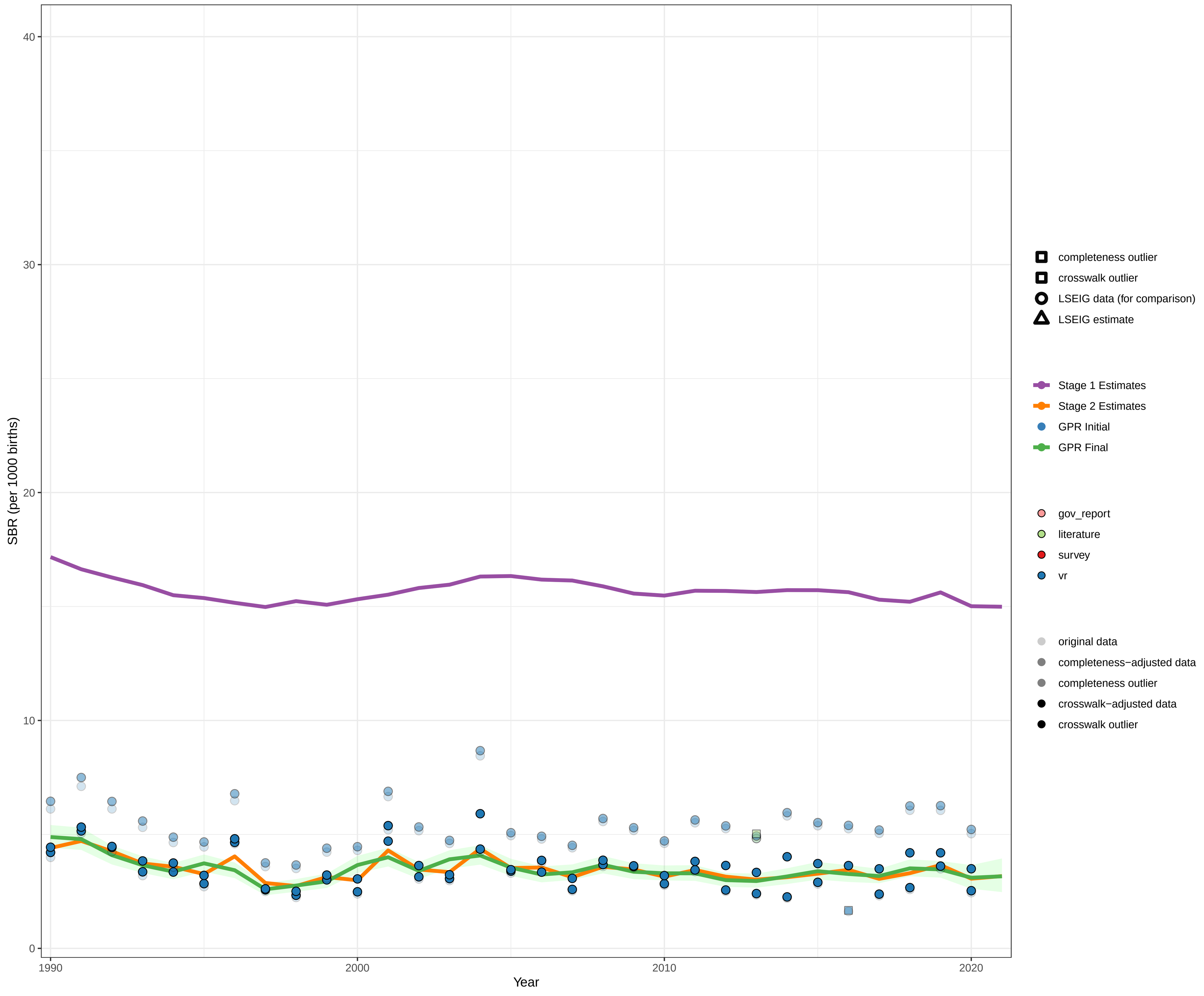

Year	Total Stillbirths (in thousands)	Total Stillbirths (in thousands)	SBR (per 1000 births)	SBR (per 1000 births)	SBR/NMR	SBR/NMR
	20 weeks	28 weeks	20 weeks	28 weeks	20 weeks	28 weeks
1990	0.0 (0.0 to 0.1)	0.0 (0.0 to 0.0)	7.1 (6.4 to 7.9)	4.9 (4.4 to 5.4)	1.44 (1.30 to 1.60)	0.99 (0.90 to 1.10)
1995	0.0 (0.0 to 0.0)	0.0 (0.0 to 0.0)	5.4 (4.9 to 6.0)	3.7 (3.4 to 4.1)	1.28 (1.16 to 1.42)	0.88 (0.79 to 0.97)
2000	0.0 (0.0 to 0.0)	0.0 (0.0 to 0.0)	5.4 (4.8 to 5.9)	3.7 (3.3 to 4.1)	1.30 (1.18 to 1.45)	0.89 (0.80 to 0.99)
2005	0.0 (0.0 to 0.0)	0.0 (0.0 to 0.0)	5.2 (4.7 to 5.8)	3.5 (3.2 to 3.9)	1.22 (1.09 to 1.35)	0.83 (0.75 to 0.92)
2010	0.0 (0.0 to 0.0)	0.0 (0.0 to 0.0)	4.9 (4.4 to 5.4)	3.3 (3.0 to 3.6)	1.26 (1.14 to 1.39)	0.85 (0.77 to 0.94)
2015	0.0 (0.0 to 0.0)	0.0 (0.0 to 0.0)	5.0 (4.5 to 5.6)	3.4 (3.0 to 3.8)	1.33 (1.18 to 1.49)	0.89 (0.80 to 1.00)
2020	0.0 (0.0 to 0.0)	0.0 (0.0 to 0.0)	4.6 (3.9 to 5.5)	3.1 (2.6 to 3.7)	1.32 (1.11 to 1.56)	0.88 (0.75 to 1.04)
2021	0.0 (0.0 to 0.0)	0.0 (0.0 to 0.0)	4.7 (3.7 to 5.9)	3.2 (2.5 to 3.9)	1.35 (1.05 to 1.69)	0.91 (0.71 to 1.13)

Southern Latin America R13 Southern Latin America Stillbirth Rate / Neonatal Mortality Rate (SBR/NMR) − 20 weeks
lambda = 1 ; zeta = 0.6 ; scale = 15 ; amp = 0.028

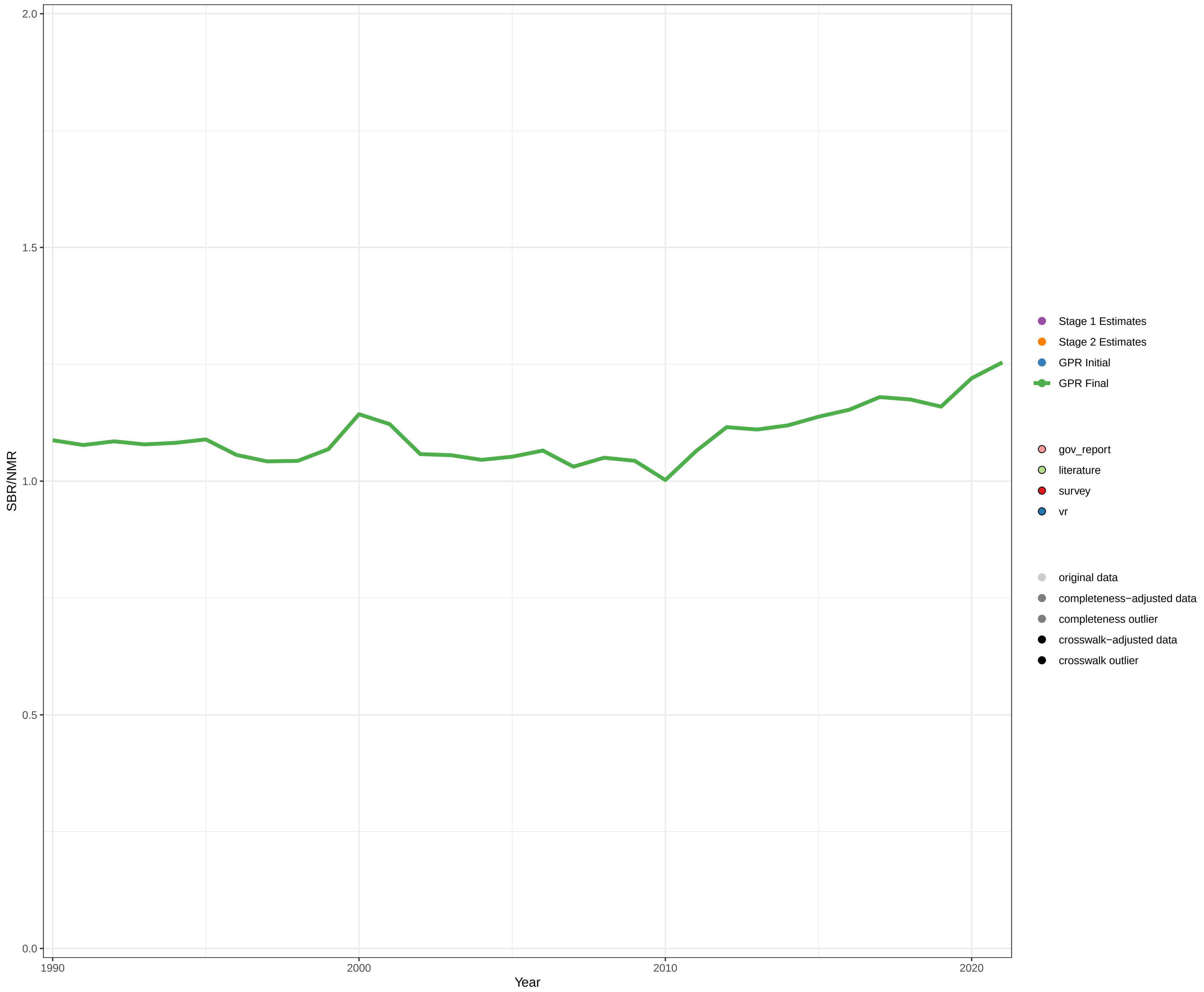

Southern Latin America R13 Southern Latin America Stillbirth Rate / Neonatal Mortality Rate (SBR/NMR) − 28 weeks
lambda = 1 ; zeta = 0.6 ; scale = 15 ; amp = 0.028

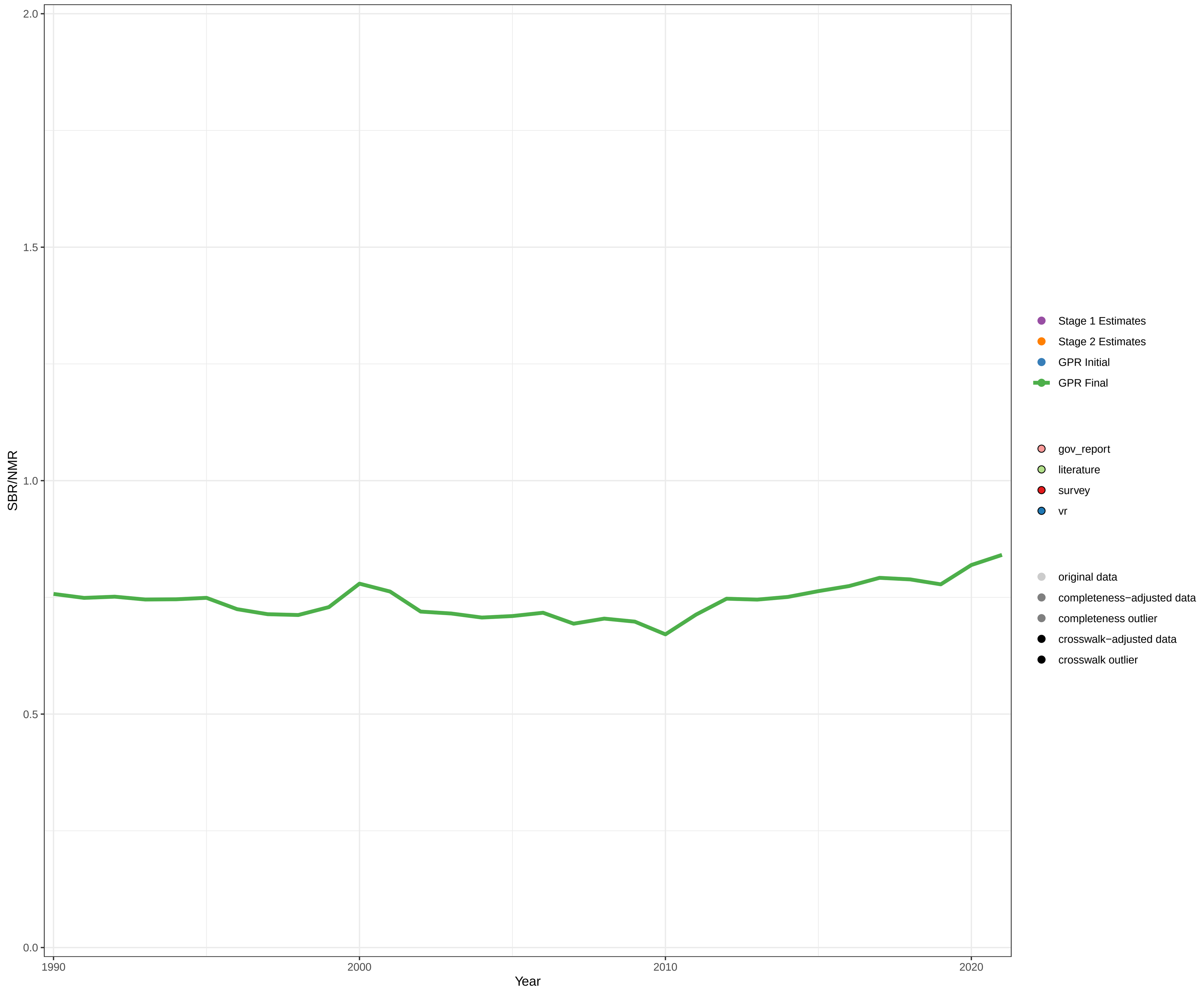

Definition	Years	Outlier	Source.Type	Title

Southern Latin America R13 Southern Latin America Stillbirth Rate (SBR) − 20 weeks
lambda = 1 ; zeta = 0.6 ; scale = 15 ; amp = 0.028

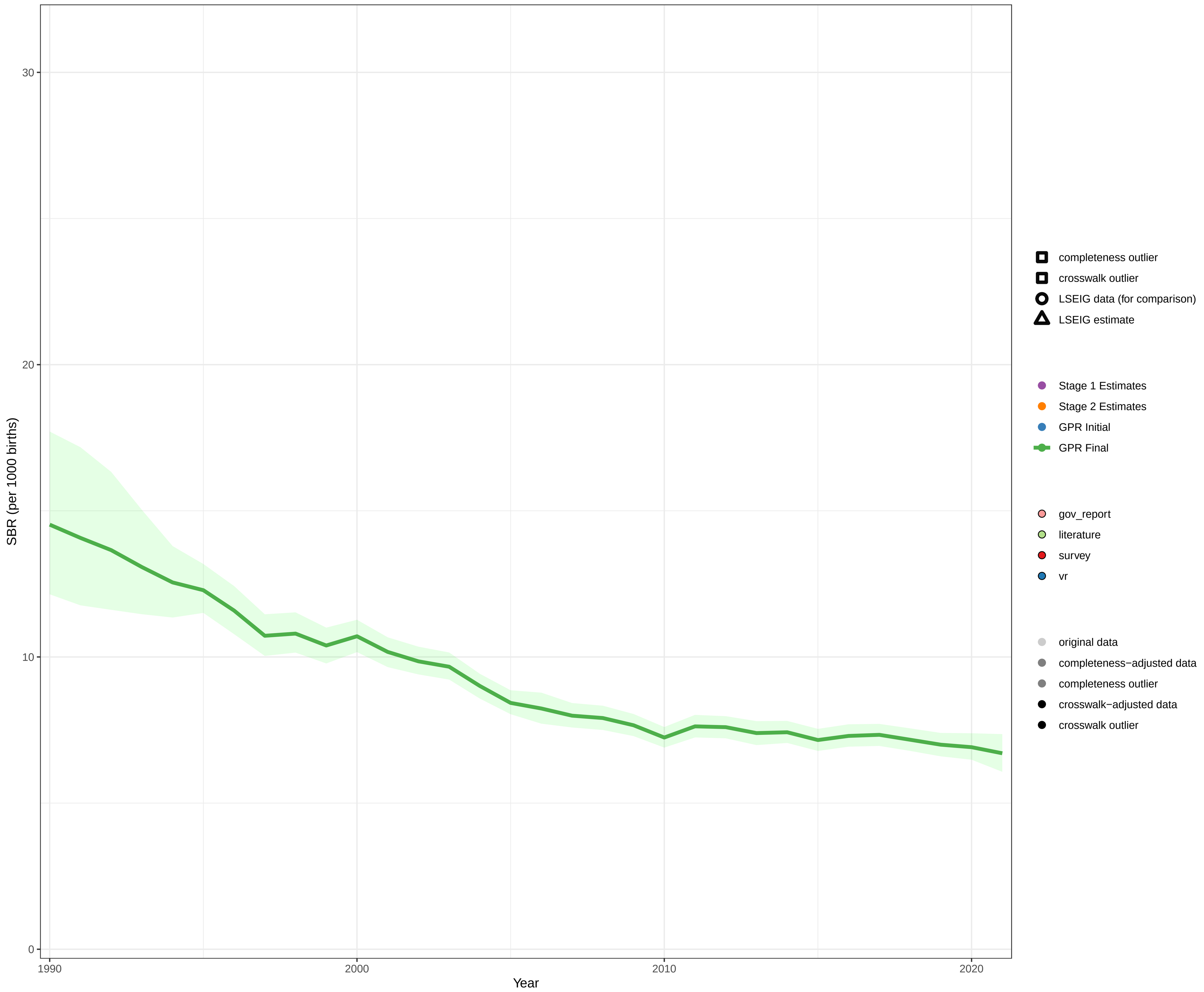

Southern Latin America R13 Southern Latin America Stillbirth Rate (SBR) − 28 weeks
lambda = 1 ; zeta = 0.6 ; scale = 15 ; amp = 0.028

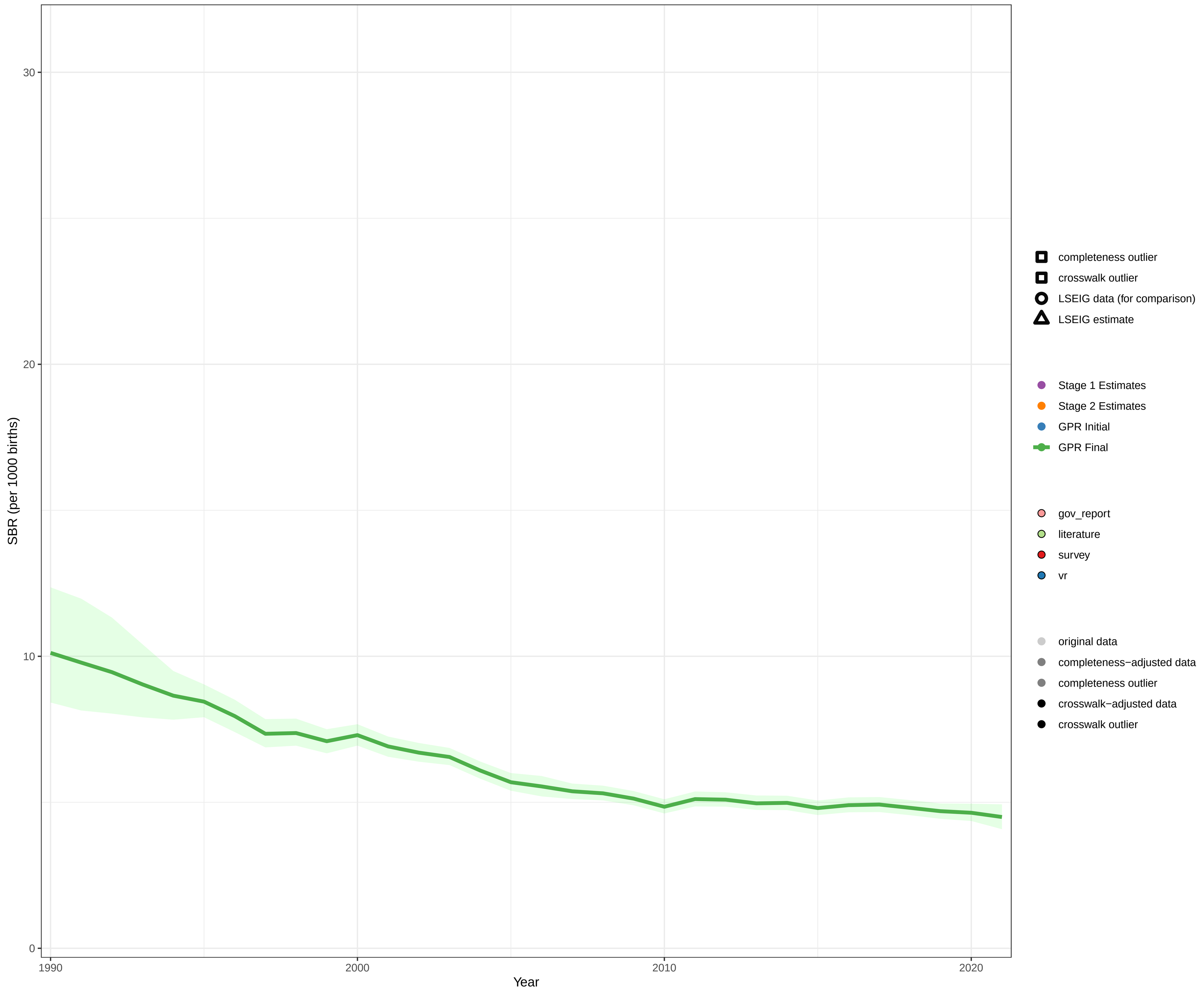

Year	Total Stillbirths (in thousands)	Total Stillbirths (in thousands)	SBR (per 1000 births)	SBR (per 1000 births)	SBR/NMR	SBR/NMR
	20 weeks	28 weeks	20 weeks	28 weeks	20 weeks	28 weeks
1990	15.2 (12.7 to 18.6)	10.6 (8.8 to 12.9)	14.5 (12.1 to 17.7)	10.1 (8.4 to 12.4)	1.09 (0.91 to 1.33)	0.76 (0.63 to 0.93)
1995	12.4 (11.7 to 13.4)	8.5 (8.0 to 9.1)	12.3 (11.5 to 13.2)	8.4 (7.9 to 9.0)	1.09 (1.02 to 1.17)	0.75 (0.70 to 0.80)
2000	10.7 (10.2 to 11.3)	7.3 (6.9 to 7.7)	10.7 (10.2 to 11.3)	7.3 (6.9 to 7.7)	1.14 (1.09 to 1.20)	0.78 (0.74 to 0.82)
2005	8.3 (8.0 to 8.8)	5.6 (5.3 to 5.9)	8.4 (8.0 to 8.9)	5.7 (5.4 to 6.0)	1.05 (1.00 to 1.11)	0.71 (0.67 to 0.75)
2010	7.6 (7.2 to 8.0)	5.1 (4.8 to 5.3)	7.2 (6.9 to 7.6)	4.8 (4.6 to 5.1)	1.00 (0.95 to 1.05)	0.67 (0.64 to 0.71)
2015	7.4 (7.0 to 7.8)	5.0 (4.7 to 5.2)	7.2 (6.8 to 7.5)	4.8 (4.6 to 5.1)	1.14 (1.08 to 1.20)	0.76 (0.73 to 0.80)
2020	5.4 (5.1 to 5.8)	3.6 (3.4 to 3.9)	6.9 (6.5 to 7.4)	4.6 (4.4 to 5.0)	1.22 (1.14 to 1.30)	0.82 (0.77 to 0.87)
2021	5.2 (4.7 to 5.7)	3.5 (3.2 to 3.8)	6.7 (6.1 to 7.4)	4.5 (4.1 to 4.9)	1.25 (1.14 to 1.38)	0.84 (0.76 to 0.92)

Argentina ARG Southern Latin America Stillbirth Rate / Neonatal Mortality Rate (SBR/NMR) − 20 weeks
lambda = 0.2 ; zeta = 0.99 ; scale = 5 ; amp = 0.028

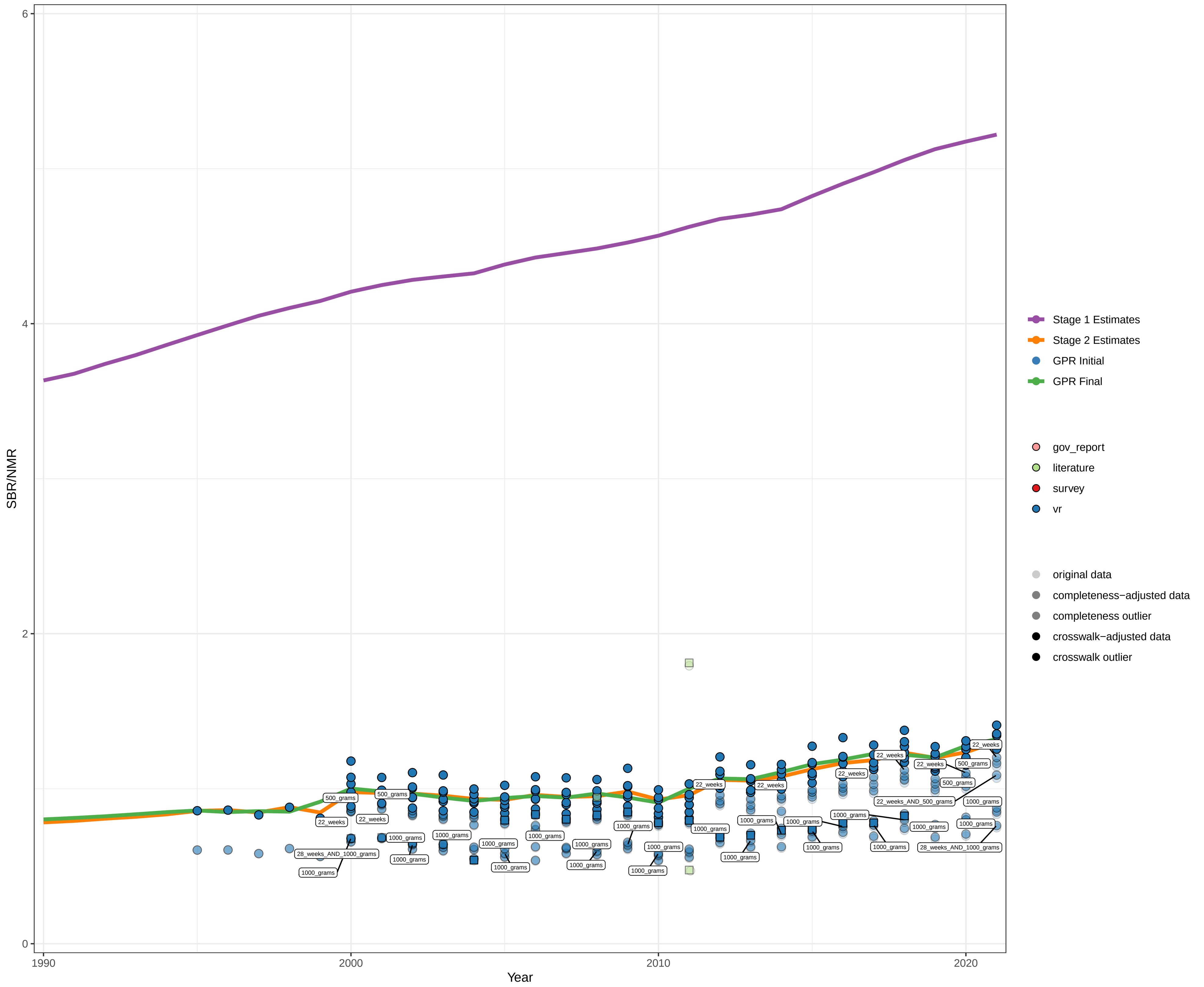

Argentina ARG Southern Latin America Stillbirth Rate / Neonatal Mortality Rate (SBR/NMR) − 28 weeks
lambda = 0.2 ; zeta = 0.99 ; scale = 5 ; amp = 0.028

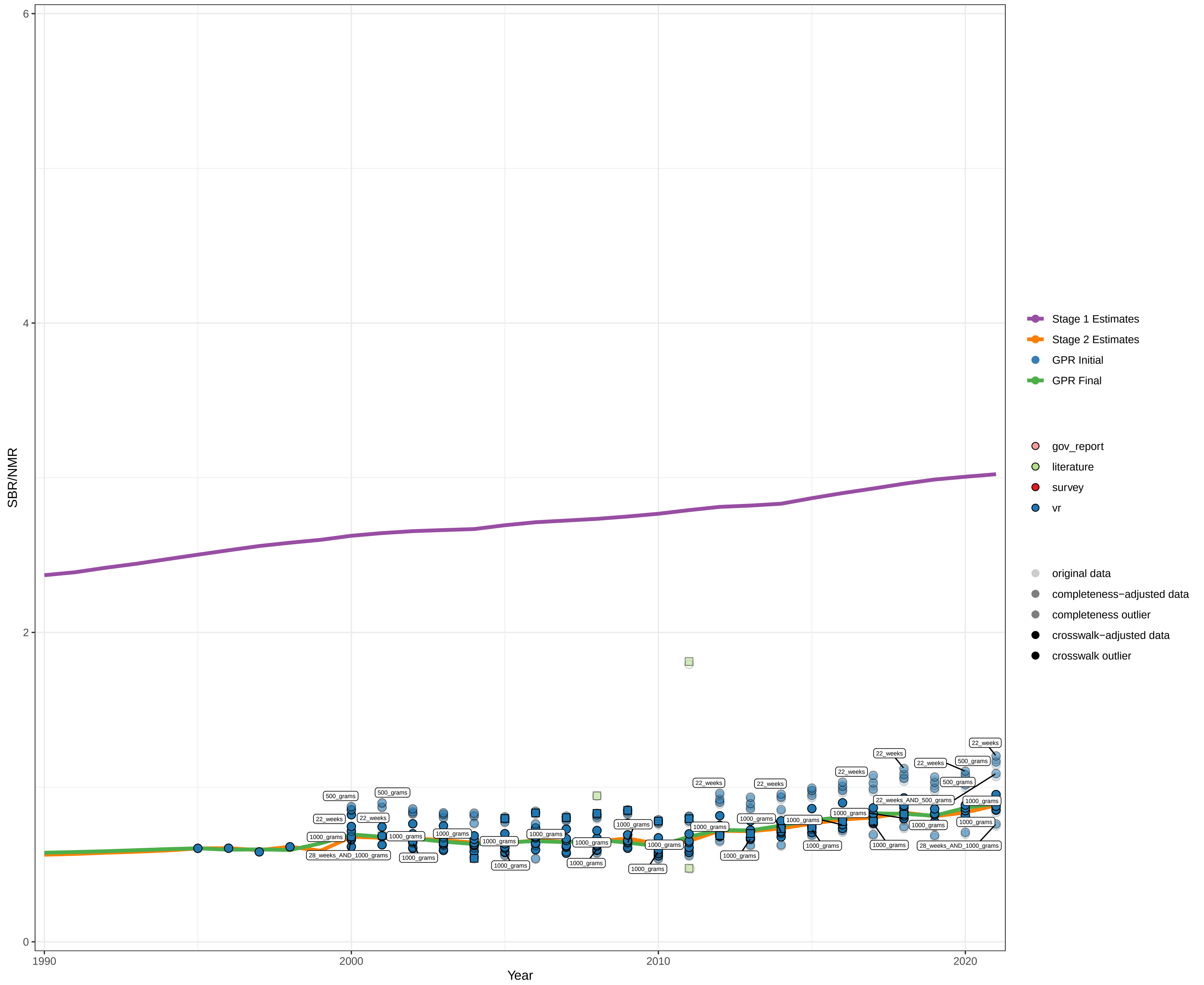

Definition	Years	Outlier	Source.Type	Title
1000_grams, 22_weeks, 28_weeks, 500_grams	2000−2021	0	VR	Argentina Vital Statistics
22_weeks_AND_500_grams, 28_weeks_AND_1000_grams	2000, 2002−2021	0	VR	Argentina Vital Statistics
28_weeks	1995−1999	0	VR	United Nations Demographic Yearbook
20_weeks	2011	1	Sci Lit	A prospective study of maternal, fetal and neonatal deaths in low− and middle−income countries
28_weeks	2000−2003	1	VR	United Nations Demographic Yearbook
28_weeks_AND_1000_grams	2008	1	Sci Lit	Epidemiology of stillbirth in low−middle income countries: A Global Network Study
28_weeks_OR_1000_grams	2011	1	Sci Lit	Maternal complications and perinatal mortality: findings of the World Health Organization Multicountry Survey on Maternal and Newborn Health
28_weeks	2004−2019	2	VR	United Nations Demographic Yearbook

Argentina ARG Southern Latin America Stillbirth Rate (SBR) − 20 weeks
lambda = 0.2 ; zeta = 0.99 ; scale = 5 ; amp = 0.028

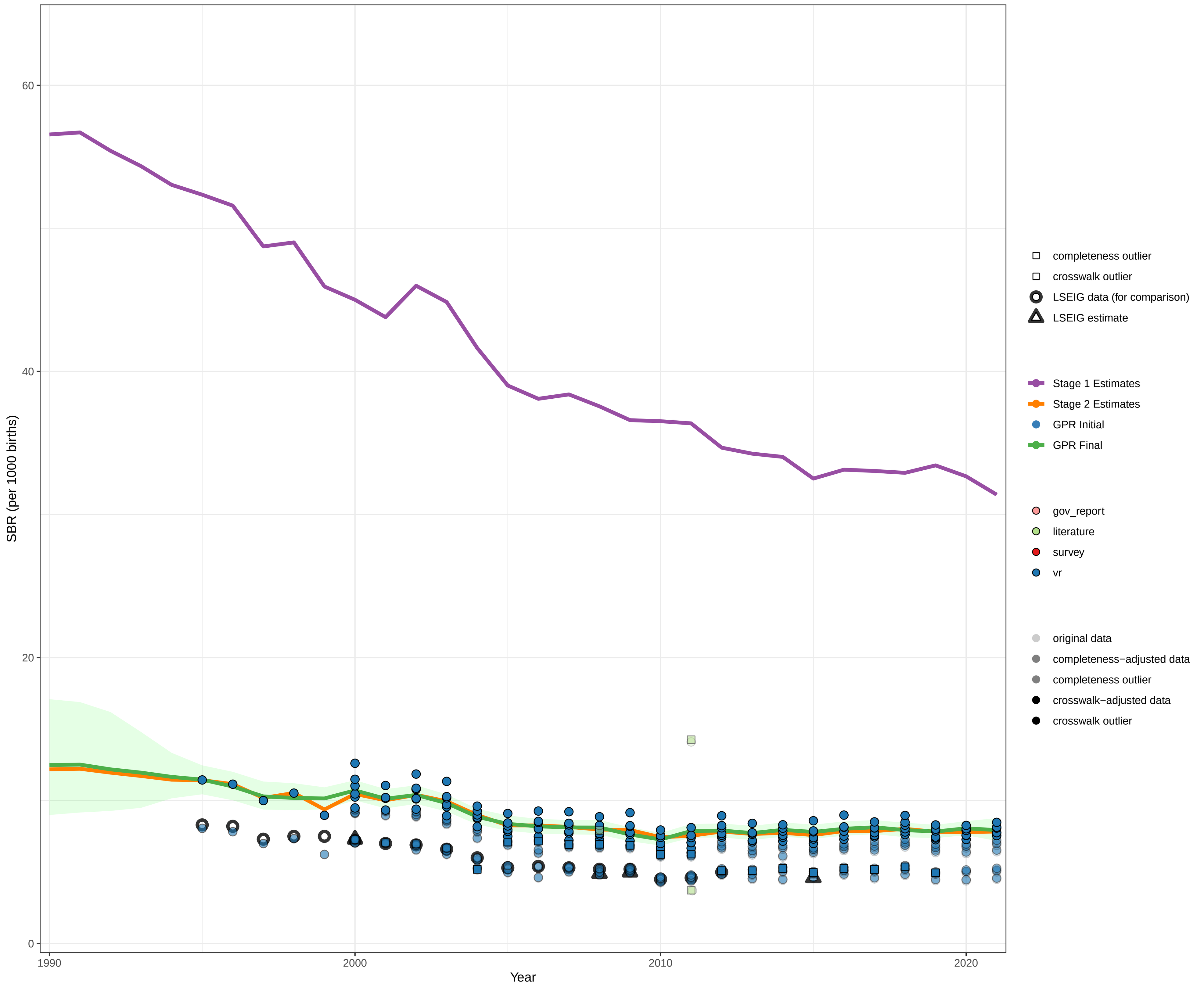

Argentina ARG Southern Latin America Stillbirth Rate (SBR) − 28 weeks
lambda = 0.2 ; zeta = 0.99 ; scale = 5 ; amp = 0.028

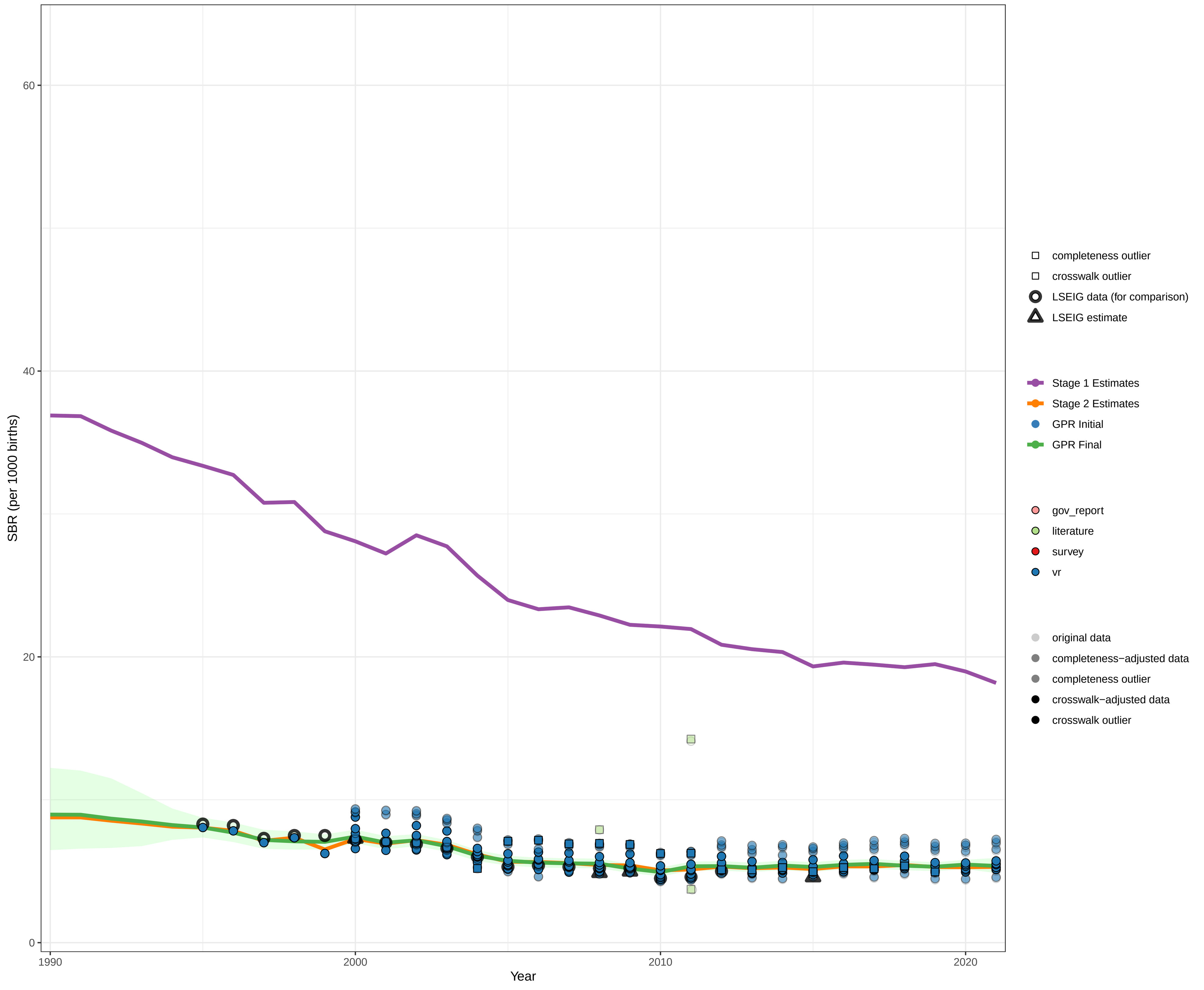

Year	Total Stillbirths (in thousands)	Total Stillbirths (in thousands)	SBR (per 1000 births)	SBR (per 1000 births)	SBR/NMR	SBR/NMR
	20 weeks	28 weeks	20 weeks	28 weeks	20 weeks	28 weeks
1990	8.6 (6.2 to 11.8)	6.1 (4.4 to 8.4)	12.5 (9.0 to 17.1)	9.0 (6.5 to 12.2)	0.80 (0.58 to 1.10)	0.58 (0.42 to 0.79)
1995	7.7 (7.0 to 8.4)	5.4 (4.9 to 5.9)	11.5 (10.4 to 12.5)	8.1 (7.4 to 8.7)	0.86 (0.78 to 0.93)	0.60 (0.55 to 0.66)
2000	7.4 (7.0 to 7.9)	5.1 (4.8 to 5.5)	10.7 (10.0 to 11.4)	7.4 (7.0 to 7.9)	1.00 (0.94 to 1.07)	0.69 (0.65 to 0.74)
2005	5.9 (5.6 to 6.3)	4.0 (3.8 to 4.3)	8.4 (7.9 to 9.0)	5.7 (5.4 to 6.1)	0.94 (0.89 to 1.01)	0.64 (0.61 to 0.68)
2010	5.5 (5.2 to 5.8)	3.7 (3.5 to 4.0)	7.3 (6.9 to 7.7)	4.9 (4.6 to 5.3)	0.91 (0.86 to 0.97)	0.62 (0.58 to 0.66)
2015	5.9 (5.5 to 6.3)	4.0 (3.7 to 4.2)	7.8 (7.3 to 8.3)	5.3 (5.0 to 5.6)	1.16 (1.09 to 1.23)	0.79 (0.74 to 0.84)
2020	4.4 (4.2 to 4.7)	3.0 (2.8 to 3.2)	8.1 (7.6 to 8.6)	5.5 (5.1 to 5.8)	1.28 (1.20 to 1.36)	0.87 (0.82 to 0.92)
2021	4.3 (3.9 to 4.8)	2.9 (2.6 to 3.2)	7.9 (7.2 to 8.8)	5.4 (4.9 to 5.9)	1.32 (1.19 to 1.46)	0.89 (0.81 to 0.99)

Chile CHL Southern Latin America Stillbirth Rate / Neonatal Mortality Rate (SBR/NMR) − 20 weeks
lambda = 0.2 ; zeta = 0.99 ; scale = 5 ; amp = 0.028

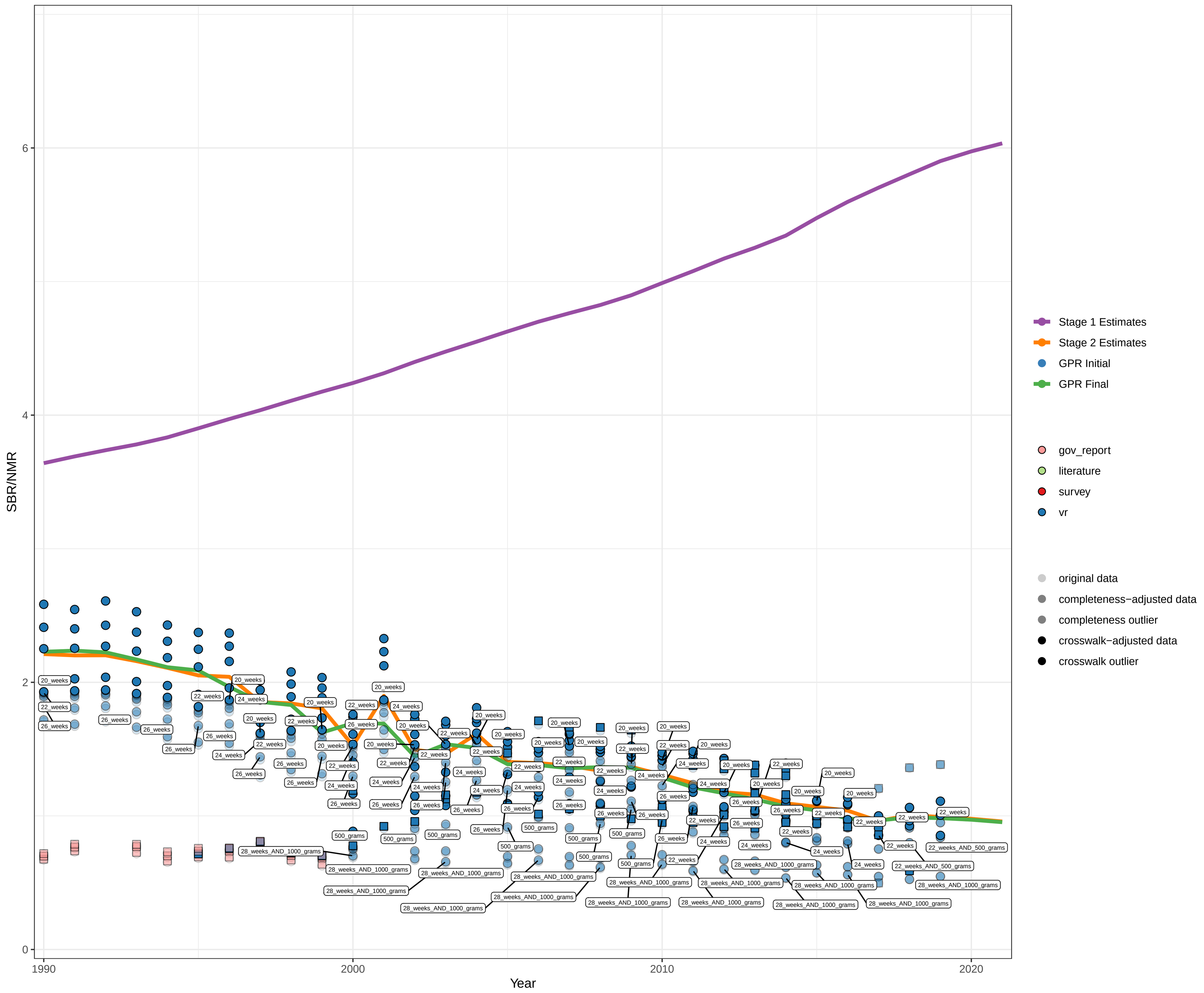

Chile CHL Southern Latin America Stillbirth Rate / Neonatal Mortality Rate (SBR/NMR) − 28 weeks
lambda = 0.2 ; zeta = 0.99 ; scale = 5 ; amp = 0.028

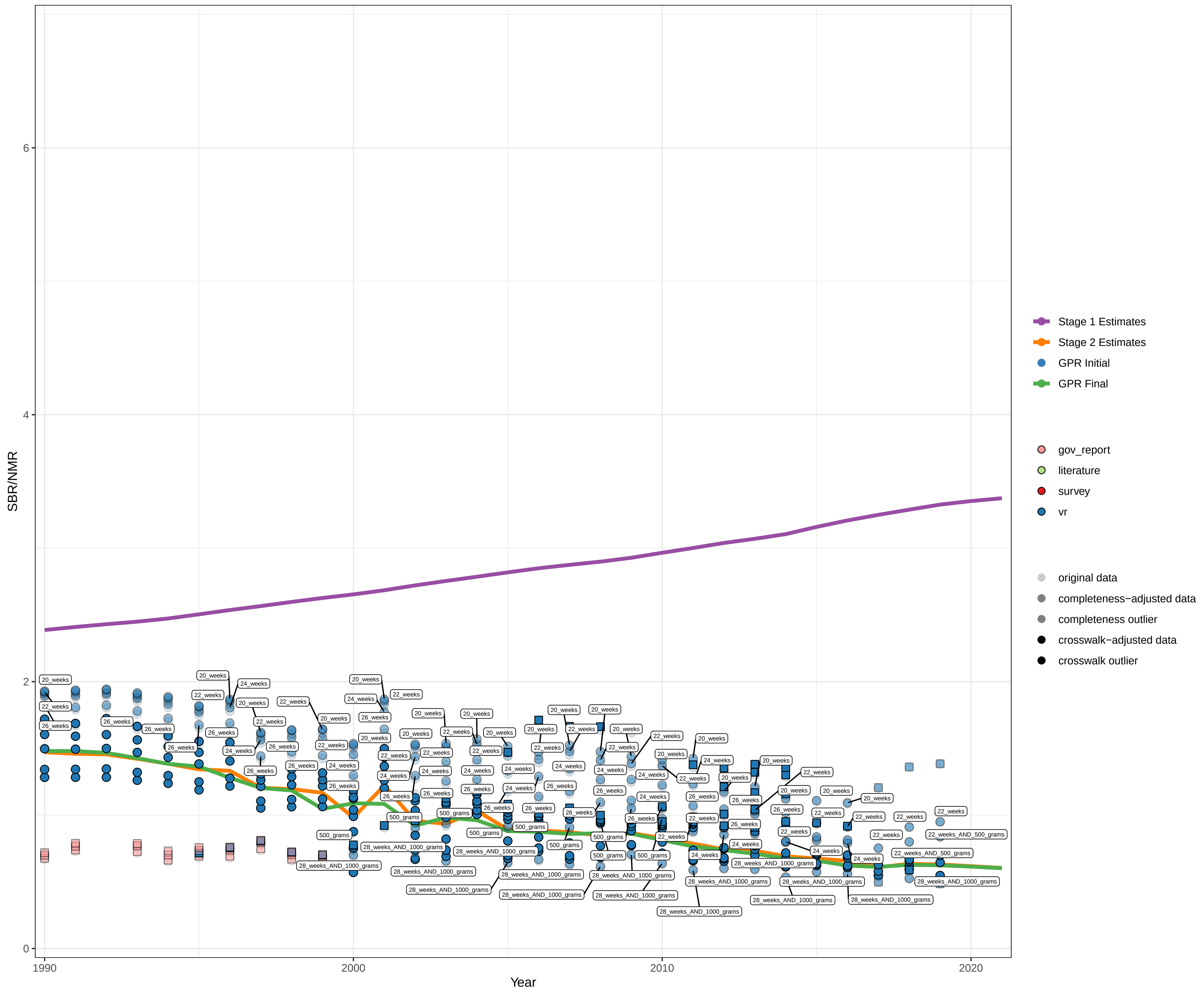

Definition	Years	Outlier	Source.Type	Title
20_weeks	1990−2011	0	VR	Chile Vital Statistics − Deaths
20_weeks, 24_weeks	2012−2016	0	VR	Chile Vital Statistics Yearbook
22_weeks, 22_weeks_AND_500_grams	2011−2019	0	VR	Chile Vital Statistics Yearbook
22_weeks, 24_weeks, 26_weeks	1990−2014	0	VR	Chile Vital Statistics − Deaths
28_weeks	1990−1999, 2001, 2002, 2004	0	VR	Chile Vital Statistics − Deaths
28_weeks	2000, 2002, 2003, 2005−2019	0	VR	Chile Vital Statistics Yearbook
28_weeks_AND_1000_grams	2000, 2002, 2003, 2005−2016, 2018	0	VR	Chile Vital Statistics Yearbook
500_grams	2000, 2002, 2003, 2005−2010	0	VR	Chile Vital Statistics Yearbook
28_weeks, 28_weeks_AND_1000_grams, 500_grams	1990, 1991, 1993−1999	1	Statistical Report	Chile Demographic Yearbook
28_weeks_AND_1000_grams	2017, 2019	1	VR	Chile Vital Statistics Yearbook
other	2017−2019	1	VR	Chile Vital Statistics Yearbook
20_weeks	2012−2014	2	VR	Chile Vital Statistics − Deaths
28_weeks	1995−2018	2	VR	United Nations Demographic Yearbook
28_weeks	2000, 2003, 2005−2014	2	VR	Chile Vital Statistics − Deaths

Chile CHL Southern Latin America Stillbirth Rate (SBR) − 20 weeks
lambda = 0.2 ; zeta = 0.99 ; scale = 5 ; amp = 0.028

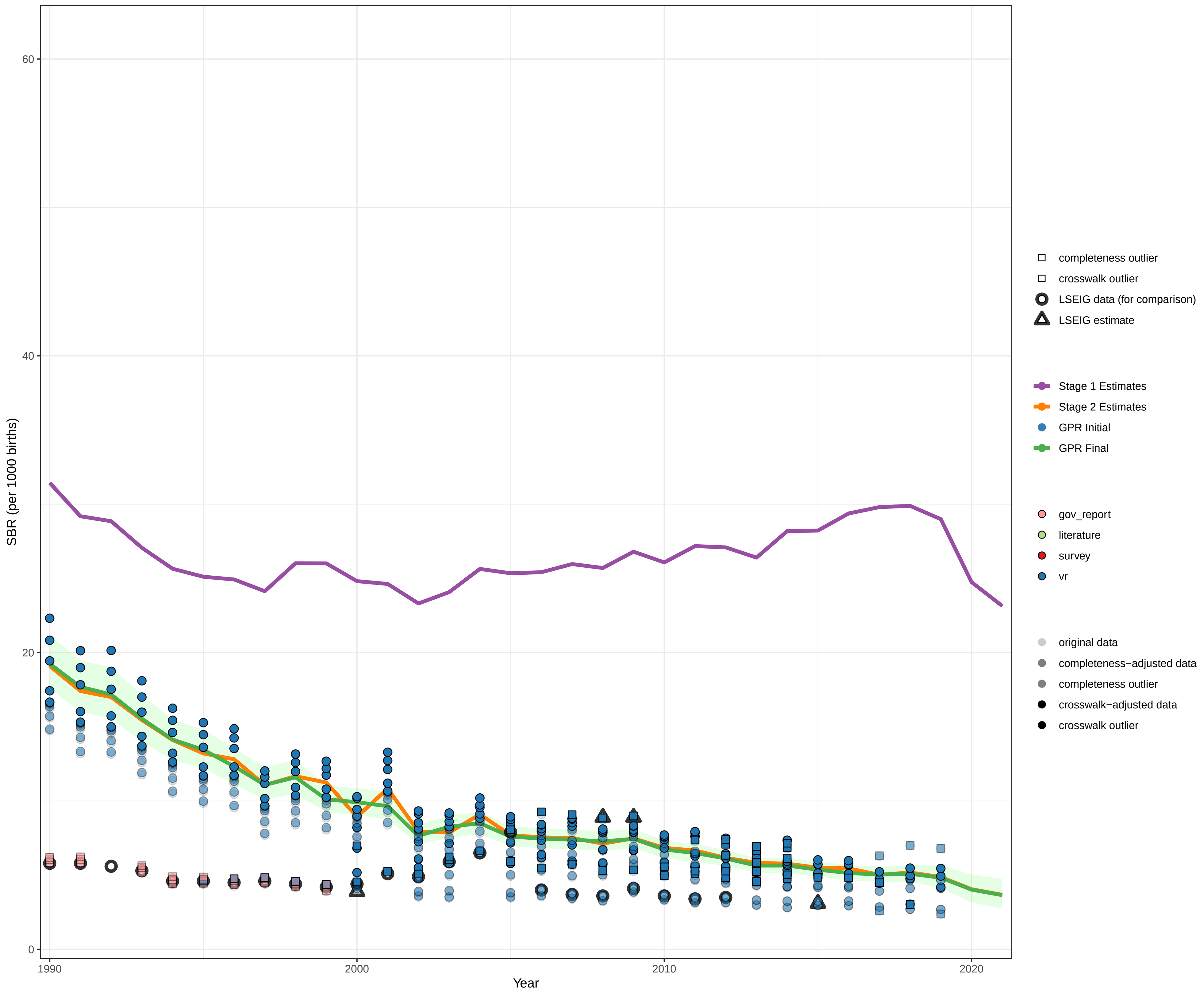

Chile CHL Southern Latin America Stillbirth Rate (SBR) − 28 weeks
lambda = 0.2 ; zeta = 0.99 ; scale = 5 ; amp = 0.028

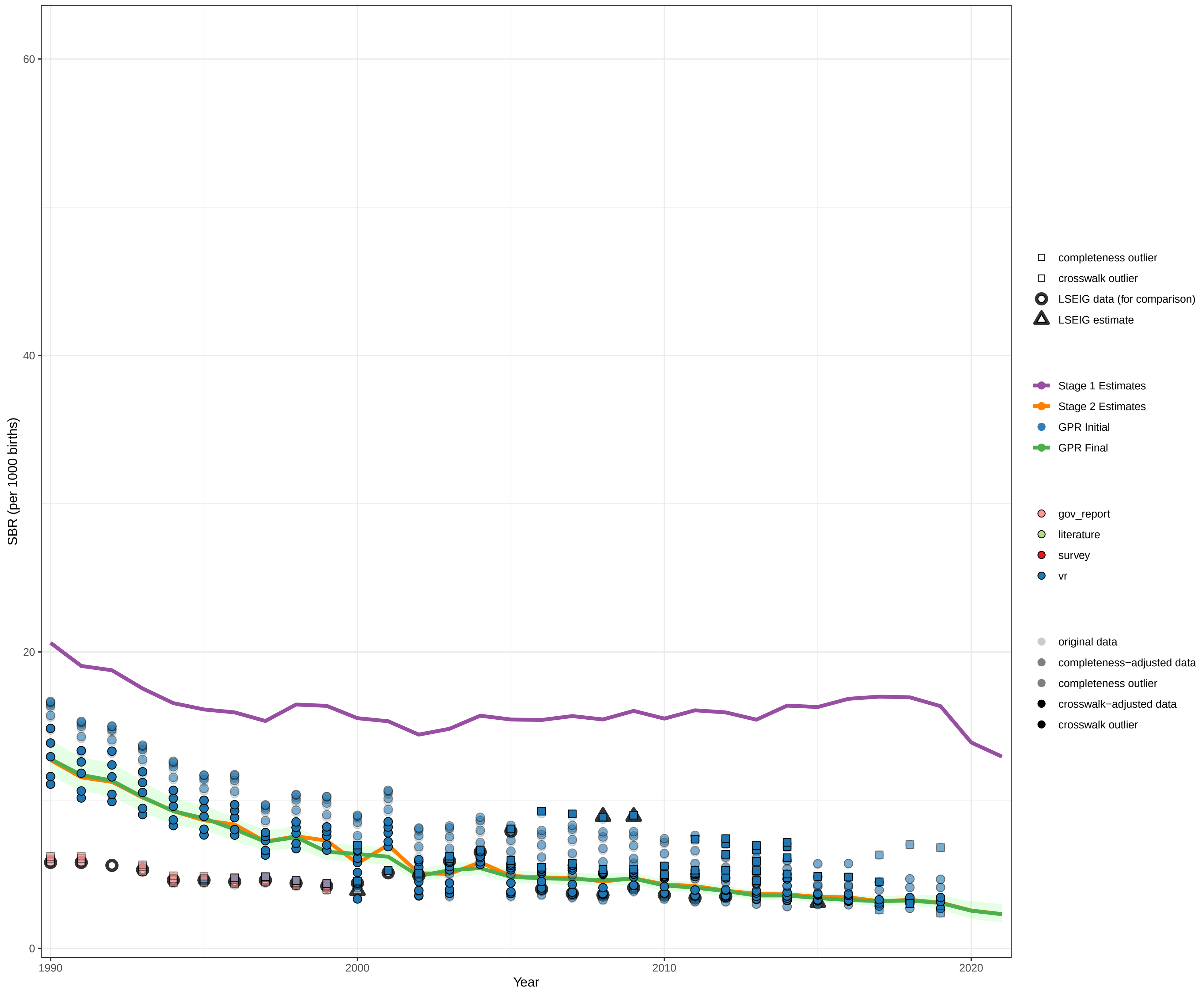

Year	Total Stillbirths (in thousands)	Total Stillbirths (in thousands)	SBR (per 1000 births)	SBR (per 1000 births)	SBR/NMR	SBR/NMR
	20 weeks	28 weeks	20 weeks	28 weeks	20 weeks	28 weeks
1990	5.8<br>(5.3 to 6.4)	3.9<br>(3.5 to 4.2)	19.2<br>(17.5 to 21.2)	12.8<br>(11.6 to 14.1)	2.23<br>(2.03 to 2.45)	1.48<br>(1.35 to 1.63)
1995	3.8<br>(3.5 to 4.2)	2.5<br>(2.2 to 2.7)	13.4<br>(12.2 to 14.8)	8.8<br>(8.0 to 9.7)	2.09<br>(1.90 to 2.30)	1.36<br>(1.24 to 1.50)
2000	2.5<br>(2.3 to 2.8)	1.6<br>(1.5 to 1.8)	9.9<br>(9.0 to 10.9)	6.4<br>(5.8 to 7.0)	1.69<br>(1.55 to 1.86)	1.09<br>(0.99 to 1.19)
2005	1.8<br>(1.6 to 1.9)	1.1<br>(1.0 to 1.2)	7.6<br>(7.0 to 8.3)	4.8<br>(4.4 to 5.3)	1.39<br>(1.27 to 1.52)	0.88<br>(0.81 to 0.96)
2010	1.7<br>(1.5 to 1.8)	1.1<br>(1.0 to 1.2)	6.7<br>(6.1 to 7.3)	4.2<br>(3.9 to 4.6)	1.29<br>(1.17 to 1.40)	0.81<br>(0.74 to 0.88)
2015	1.3<br>(1.2 to 1.4)	0.8<br>(0.7 to 0.9)	5.4<br>(4.9 to 5.9)	3.4<br>(3.1 to 3.7)	1.04<br>(0.95 to 1.15)	0.66<br>(0.60 to 0.72)
2020	0.8<br>(0.6 to 1.0)	0.5<br>(0.4 to 0.6)	4.0<br>(3.2 to 5.1)	2.5<br>(2.0 to 3.2)	0.97<br>(0.76 to 1.22)	0.61<br>(0.48 to 0.77)
2021	0.7<br>(0.5 to 0.9)	0.5<br>(0.3 to 0.6)	3.7<br>(2.7 to 4.7)	2.3<br>(1.7 to 3.0)	0.95<br>(0.71 to 1.24)	0.60<br>(0.45 to 0.78)

Uruguay URY Southern Latin America Stillbirth Rate / Neonatal Mortality Rate (SBR/NMR) − 20 weeks
lambda = 0.2 ; zeta = 0.99 ; scale = 5 ; amp = 0.028

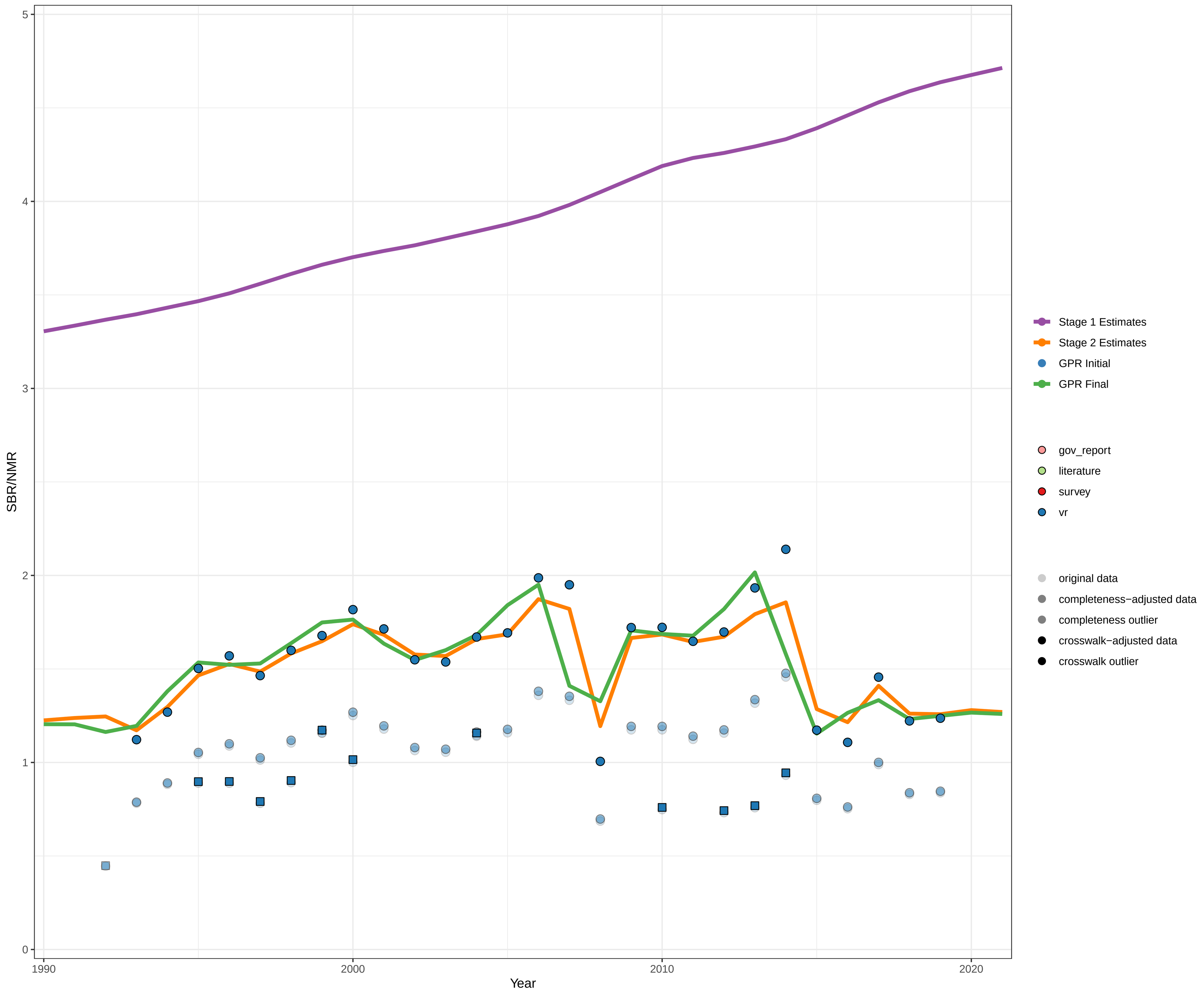

Uruguay URY Southern Latin America Stillbirth Rate / Neonatal Mortality Rate (SBR/NMR) − 28 weeks
lambda = 0.2 ; zeta = 0.99 ; scale = 5 ; amp = 0.028

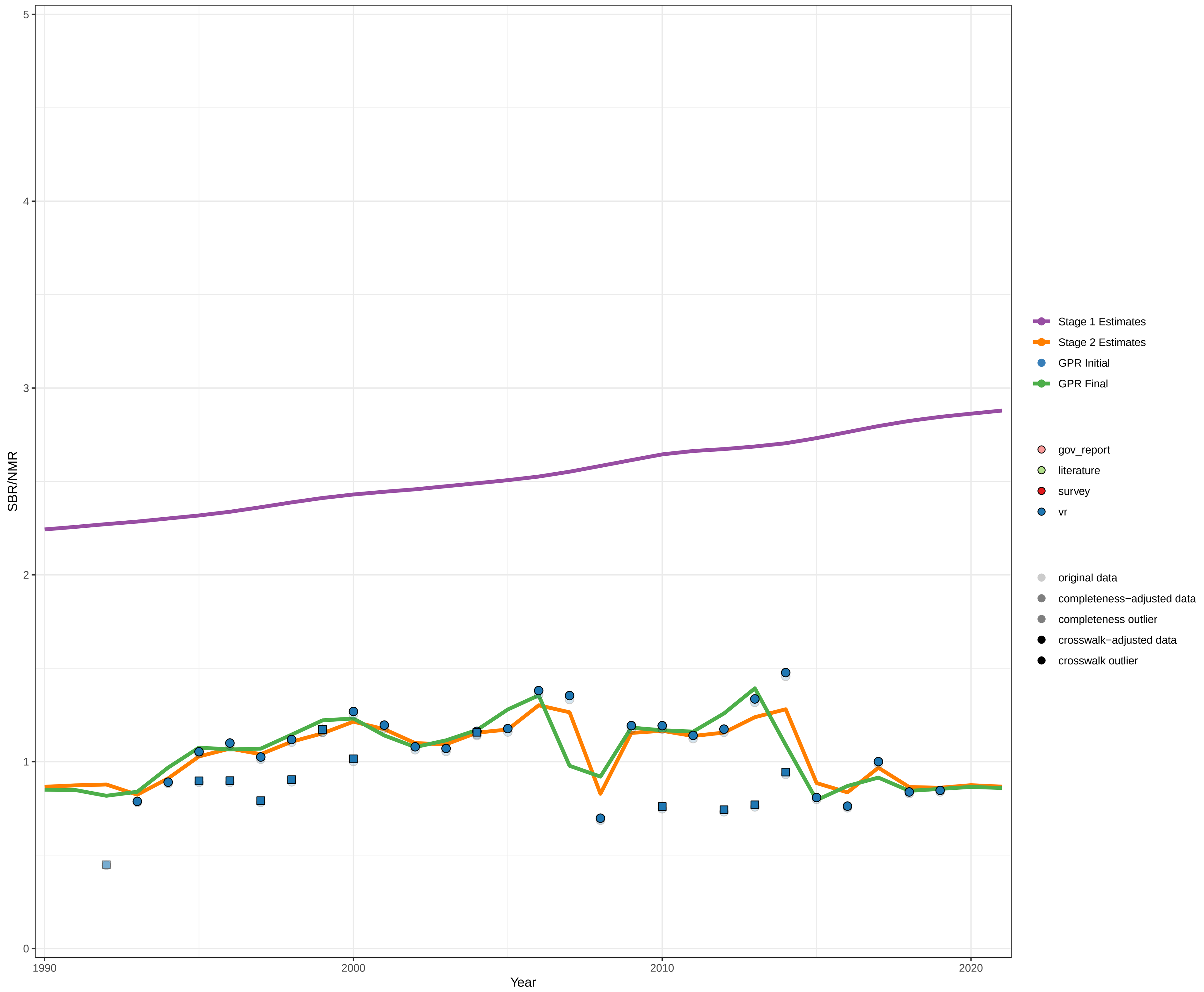

Definition	Years	Outlier	Source.Type	Title
28_weeks	1993−2014	0	VR	Uruguay Total Births and Deaths (All Ages Under 1 year, Children Under 5 Years and Fetal) 1875−
28_weeks	2015−2019	0	VR	United Nations Demographic Yearbook
28_weeks	1992	1	VR	Uruguay Total Births and Deaths (All Ages Under 1 year, Children Under 5 Years and Fetal) 1875−
28_weeks	1995−2000, 2004, 2010, 2012−2014	2	VR	United Nations Demographic Yearbook

Uruguay URY Southern Latin America Stillbirth Rate (SBR) − 20 weeks
lambda = 0.2 ; zeta = 0.99 ; scale = 5 ; amp = 0.028

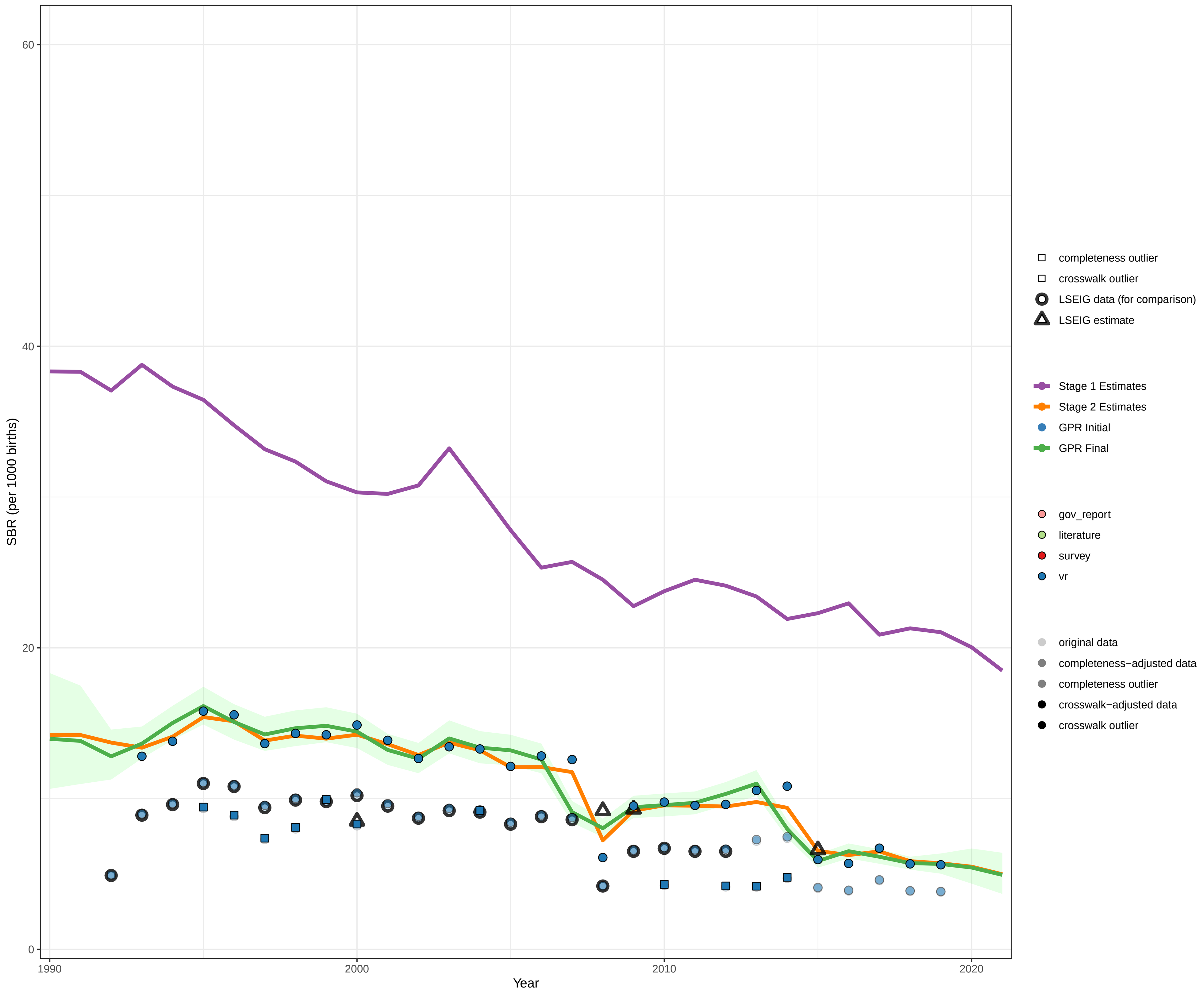

Uruguay URY Southern Latin America Stillbirth Rate (SBR) − 28 weeks
lambda = 0.2 ; zeta = 0.99 ; scale = 5 ; amp = 0.028

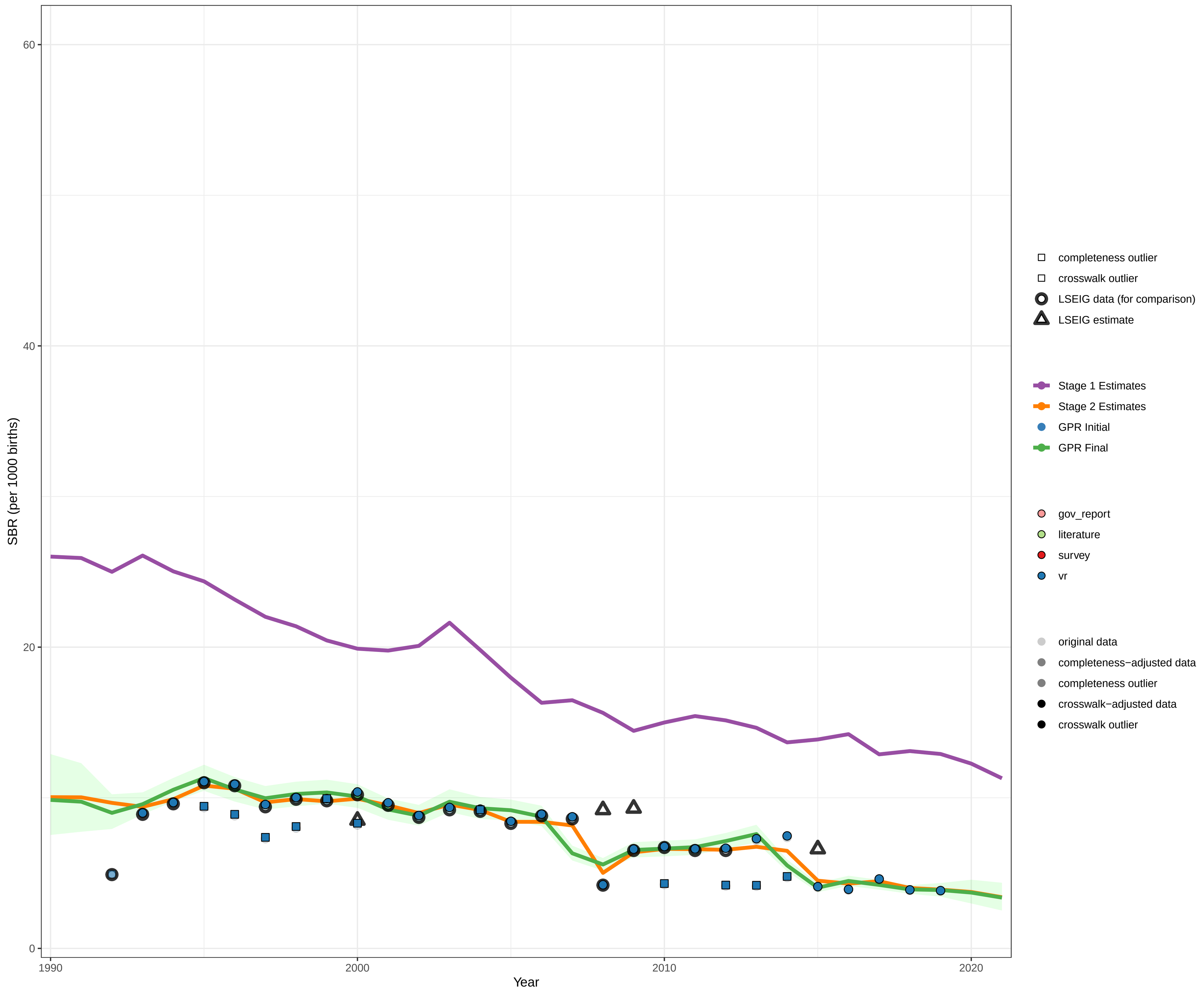

Year	Total Stillbirths (in thousands)	Total Stillbirths (in thousands)	SBR (per 1000 births)	SBR (per 1000 births)	SBR/NMR	SBR/NMR
	20 weeks	28 weeks	20 weeks	28 weeks	20 weeks	28 weeks
1990	0.8 (0.6 to 1.0)	0.6 (0.4 to 0.7)	14.0 (10.6 to 18.3)	9.9 (7.5 to 12.9)	1.20 (0.92 to 1.58)	0.85 (0.65 to 1.11)
1995	0.9 (0.9 to 1.0)	0.7 (0.6 to 0.7)	16.1 (14.9 to 17.4)	11.3 (10.5 to 12.2)	1.53 (1.42 to 1.66)	1.08 (1.00 to 1.16)
2000	0.8 (0.7 to 0.8)	0.5 (0.5 to 0.6)	14.4 (13.3 to 15.6)	10.1 (9.3 to 10.9)	1.76 (1.63 to 1.91)	1.23 (1.14 to 1.33)
2005	0.7 (0.6 to 0.7)	0.5 (0.4 to 0.5)	13.2 (12.2 to 14.2)	9.2 (8.5 to 9.9)	1.84 (1.70 to 1.99)	1.28 (1.18 to 1.38)
2010	0.4 (0.4 to 0.5)	0.3 (0.3 to 0.3)	9.6 (8.8 to 10.3)	6.6 (6.1 to 7.2)	1.69 (1.55 to 1.82)	1.17 (1.08 to 1.26)
2015	0.3 (0.3 to 0.3)	0.2 (0.2 to 0.2)	5.9 (5.4 to 6.3)	4.0 (3.7 to 4.4)	1.15 (1.07 to 1.25)	0.79 (0.74 to 0.86)
2020	0.2 (0.2 to 0.2)	0.1 (0.1 to 0.2)	5.4 (4.4 to 6.7)	3.7 (3.0 to 4.6)	1.27 (1.02 to 1.56)	0.86 (0.70 to 1.06)
2021	0.2 (0.1 to 0.2)	0.1 (0.1 to 0.2)	4.9 (3.7 to 6.4)	3.4 (2.5 to 4.4)	1.26 (0.94 to 1.63)	0.86 (0.64 to 1.11)

Western Europe R10 Western Europe Stillbirth Rate / Neonatal Mortality Rate (SBR/NMR) − 20 weeks
lambda = 1 ; zeta = 0.6 ; scale = 15 ; amp = 0.028

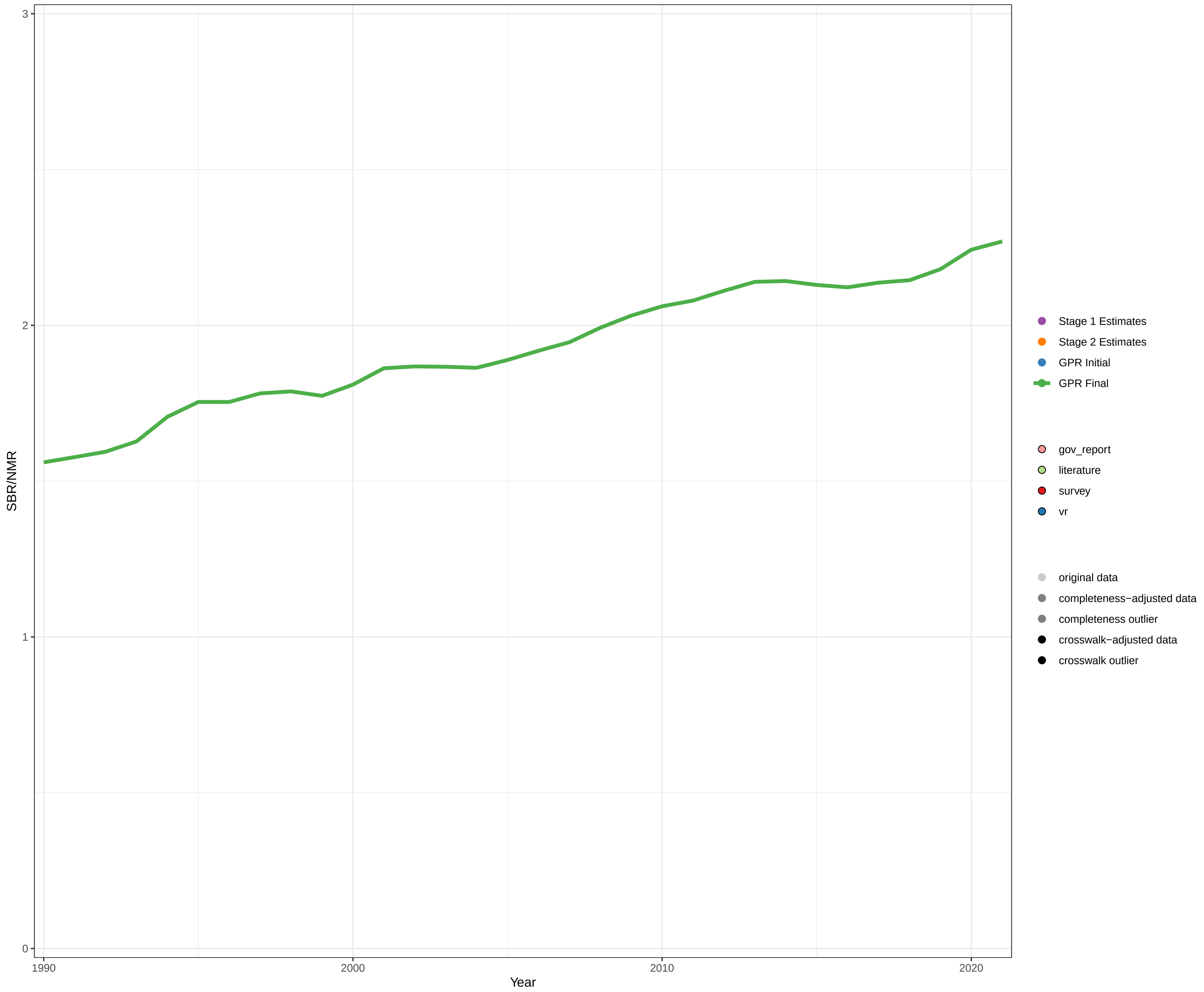

Western Europe R10 Western Europe Stillbirth Rate / Neonatal Mortality Rate (SBR/NMR) − 28 weeks
lambda = 1 ; zeta = 0.6 ; scale = 15 ; amp = 0.028

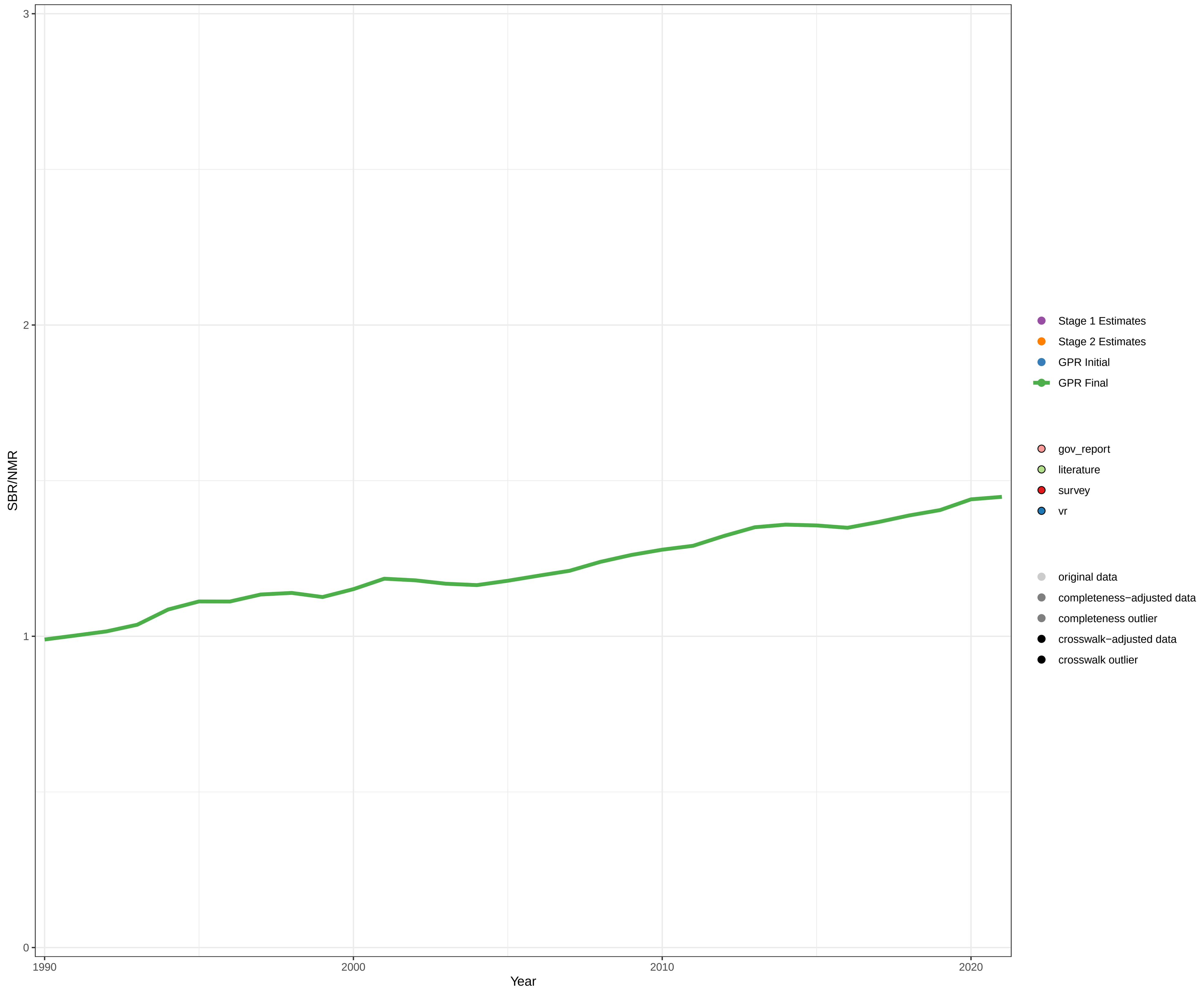

Definition	Years	Outlier	Source.Type	Title

Western Europe R10 Western Europe Stillbirth Rate (SBR) − 20 weeks
lambda = 1 ; zeta = 0.6 ; scale = 15 ; amp = 0.028

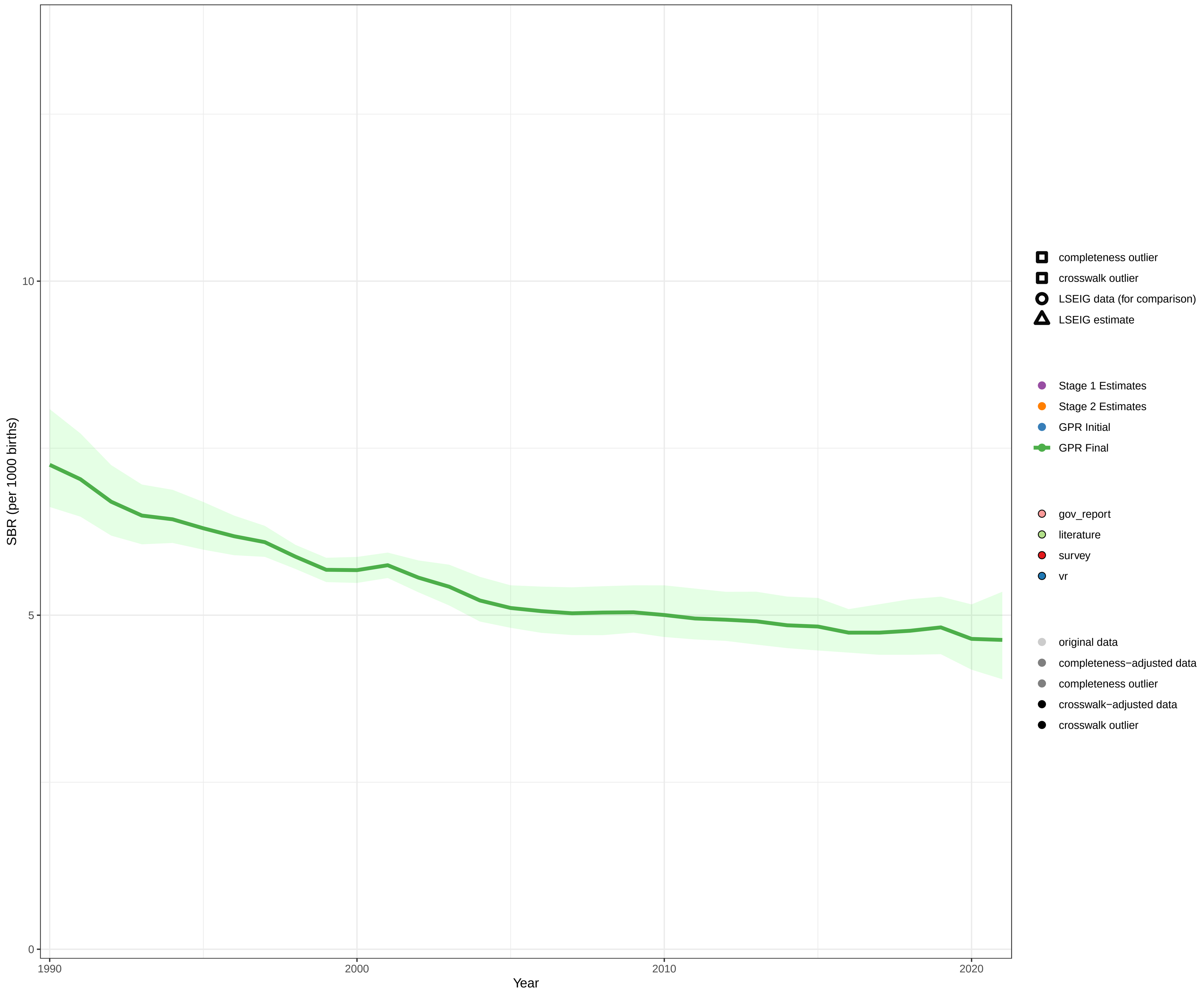

Western Europe R10 Western Europe Stillbirth Rate (SBR) − 28 weeks
lambda = 1 ; zeta = 0.6 ; scale = 15 ; amp = 0.028

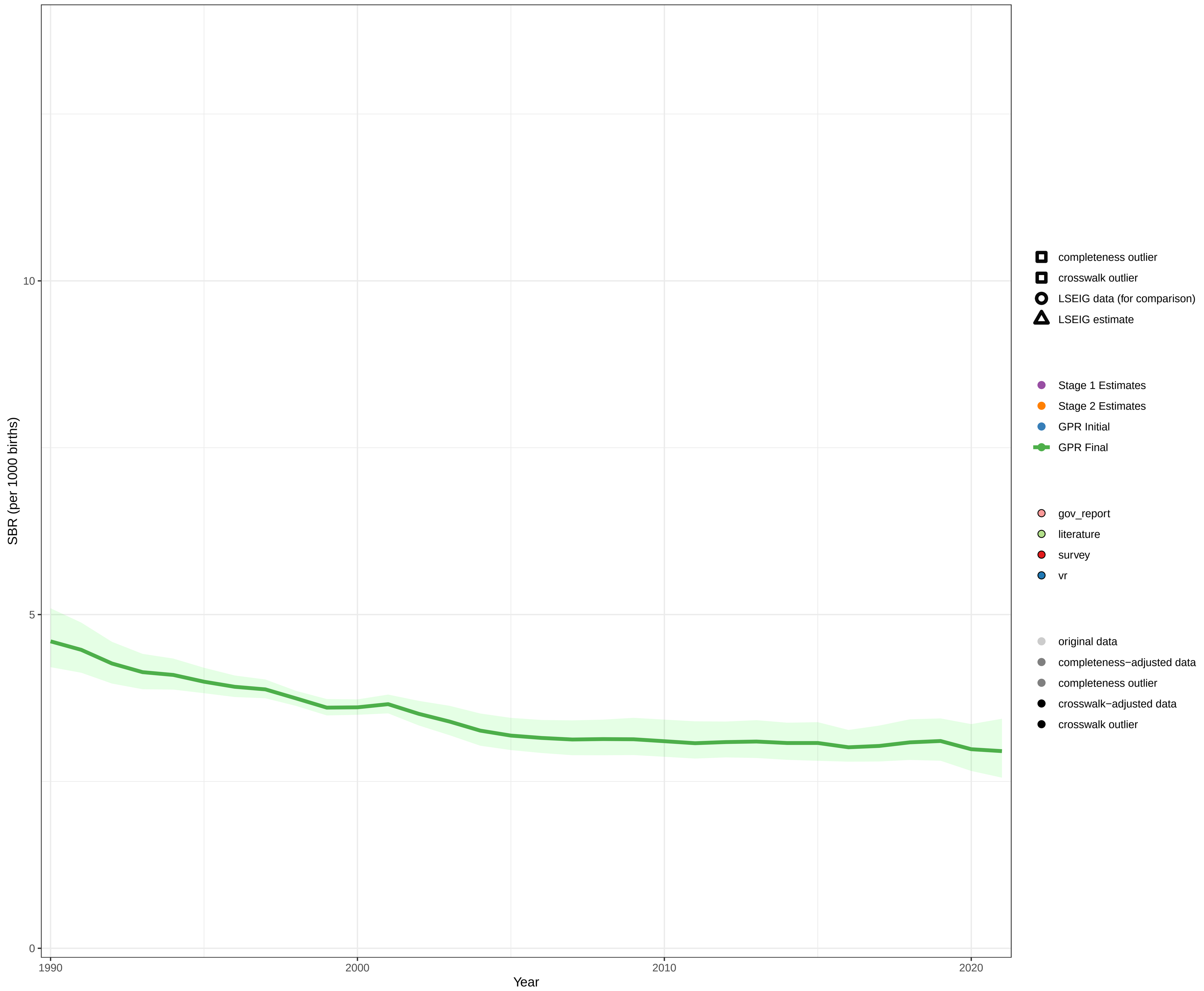

Year	Total Stillbirths (in thousands)	Total Stillbirths (in thousands)	SBR (per 1000 births)	SBR (per 1000 births)	SBR/NMR	SBR/NMR
	20 weeks	28 weeks	20 weeks	28 weeks	20 weeks	28 weeks
1990	33.6 (30.6 to 37.5)	21.2 (19.4 to 23.5)	7.3 (6.6 to 8.1)	4.6 (4.2 to 5.1)	1.56 (1.42 to 1.74)	0.99 (0.91 to 1.10)
1995	27.4 (26.0 to 29.1)	17.3 (16.6 to 18.3)	6.3 (6.0 to 6.7)	4.0 (3.8 to 4.2)	1.75 (1.66 to 1.86)	1.11 (1.06 to 1.17)
2000	24.7 (23.9 to 25.6)	15.7 (15.2 to 16.2)	5.7 (5.5 to 5.9)	3.6 (3.5 to 3.7)	1.81 (1.75 to 1.87)	1.15 (1.12 to 1.19)
2005	22.8 (21.5 to 24.3)	14.2 (13.2 to 15.4)	5.1 (4.8 to 5.4)	3.2 (3.0 to 3.5)	1.89 (1.78 to 2.01)	1.18 (1.10 to 1.28)
2010	22.9 (21.4 to 25.0)	14.2 (13.1 to 15.7)	5.0 (4.7 to 5.4)	3.1 (2.9 to 3.4)	2.06 (1.93 to 2.24)	1.28 (1.18 to 1.41)
2015	21.4 (19.8 to 23.3)	13.6 (12.4 to 15.0)	4.8 (4.5 to 5.3)	3.1 (2.8 to 3.4)	2.13 (1.97 to 2.32)	1.36 (1.24 to 1.49)
2020	19.1 (17.2 to 21.3)	12.3 (10.9 to 13.8)	4.6 (4.2 to 5.2)	3.0 (2.7 to 3.4)	2.24 (2.02 to 2.49)	1.44 (1.28 to 1.62)
2021	19.0 (16.6 to 22.0)	12.1 (10.5 to 14.1)	4.6 (4.0 to 5.4)	3.0 (2.6 to 3.4)	2.27 (1.98 to 2.62)	1.45 (1.25 to 1.69)

Andorra AND Western Europe Stillbirth Rate / Neonatal Mortality Rate (SBR/NMR) − 20 weeks
lambda = 0.2 ; zeta = 0.99 ; scale = 5 ; amp = 0.028

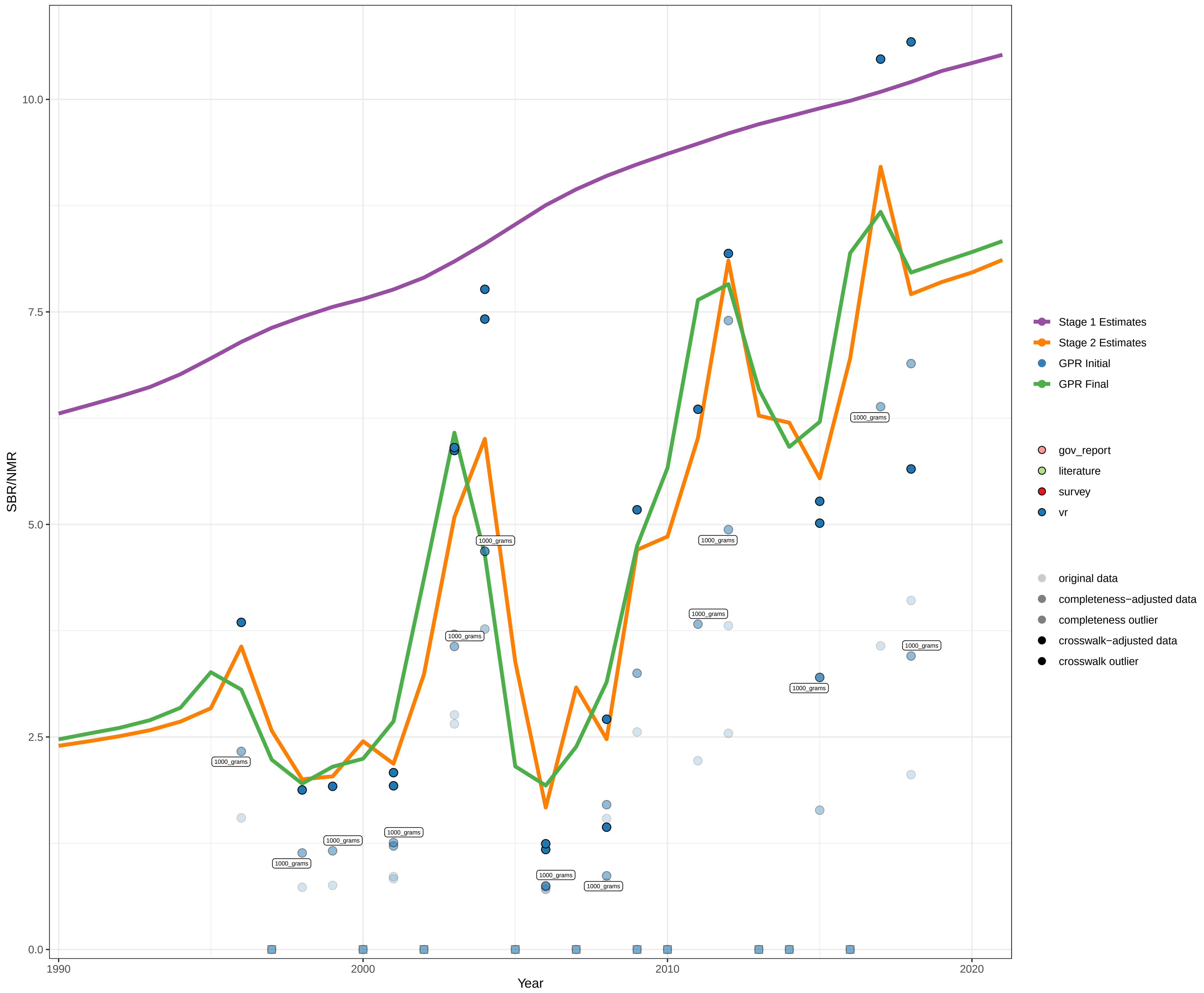

Andorra AND Western Europe Stillbirth Rate / Neonatal Mortality Rate (SBR/NMR) − 28 weeks
lambda = 0.2 ; zeta = 0.99 ; scale = 5 ; amp = 0.028

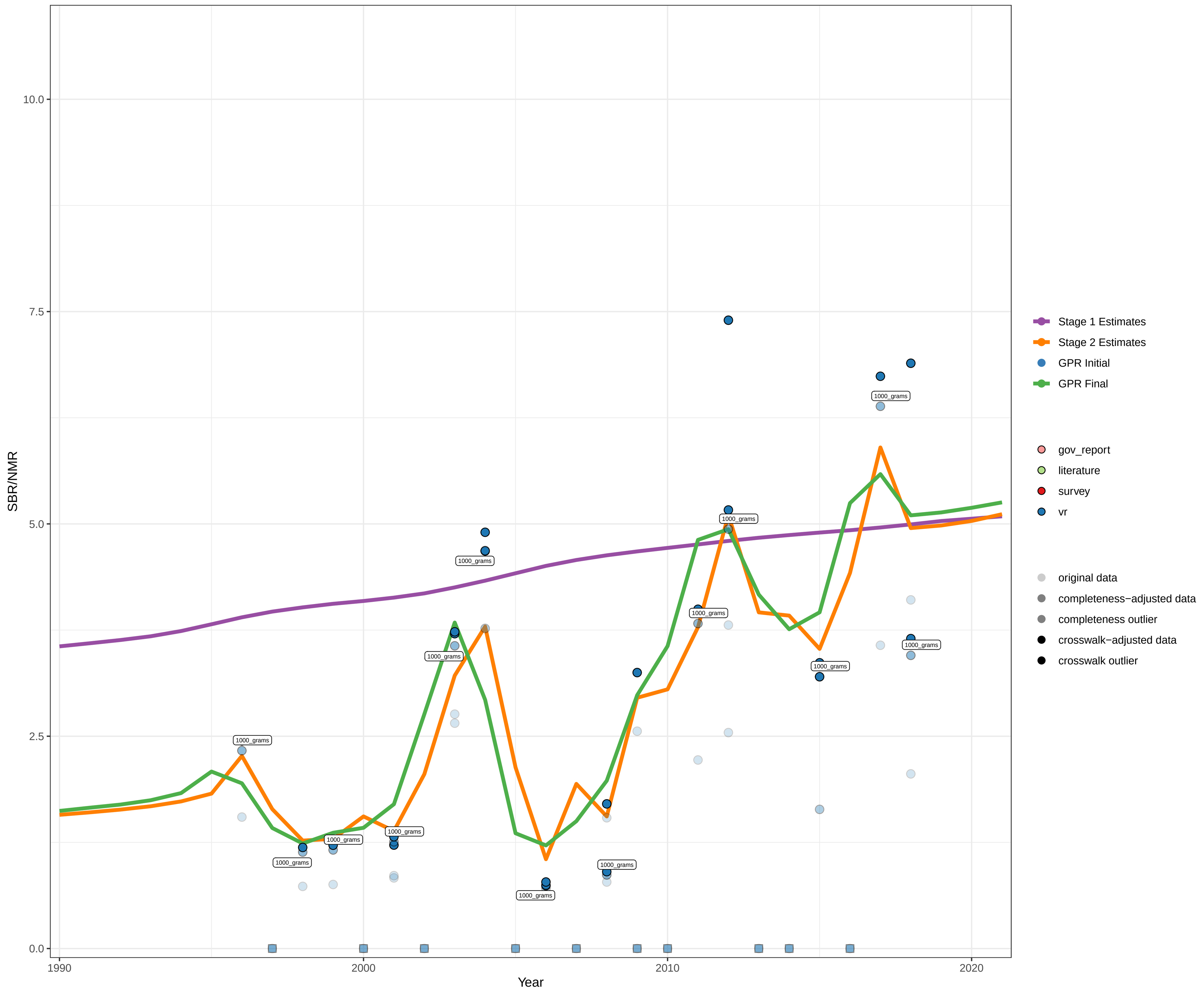

Definition	Years	Outlier	Source.Type	Title
1000_grams	1996, 1998, 1999, 2001, 2003, 2004, 2006, 2008, 2011, 2012, 2015, 2017, 2018	0	VR	European Health for All Database
28_weeks	2001, 2003, 2004, 2006, 2008, 2009, 2012, 2015, 2018	0	VR	United Nations Demographic Yearbook
1000_grams	1997, 2000, 2002, 2005, 2007, 2009, 2010, 2013, 2014, 2016	1	VR	European Health for All Database

Andorra AND Western Europe Stillbirth Rate (SBR) − 20 weeks
lambda = 0.2 ; zeta = 0.99 ; scale = 5 ; amp = 0.028

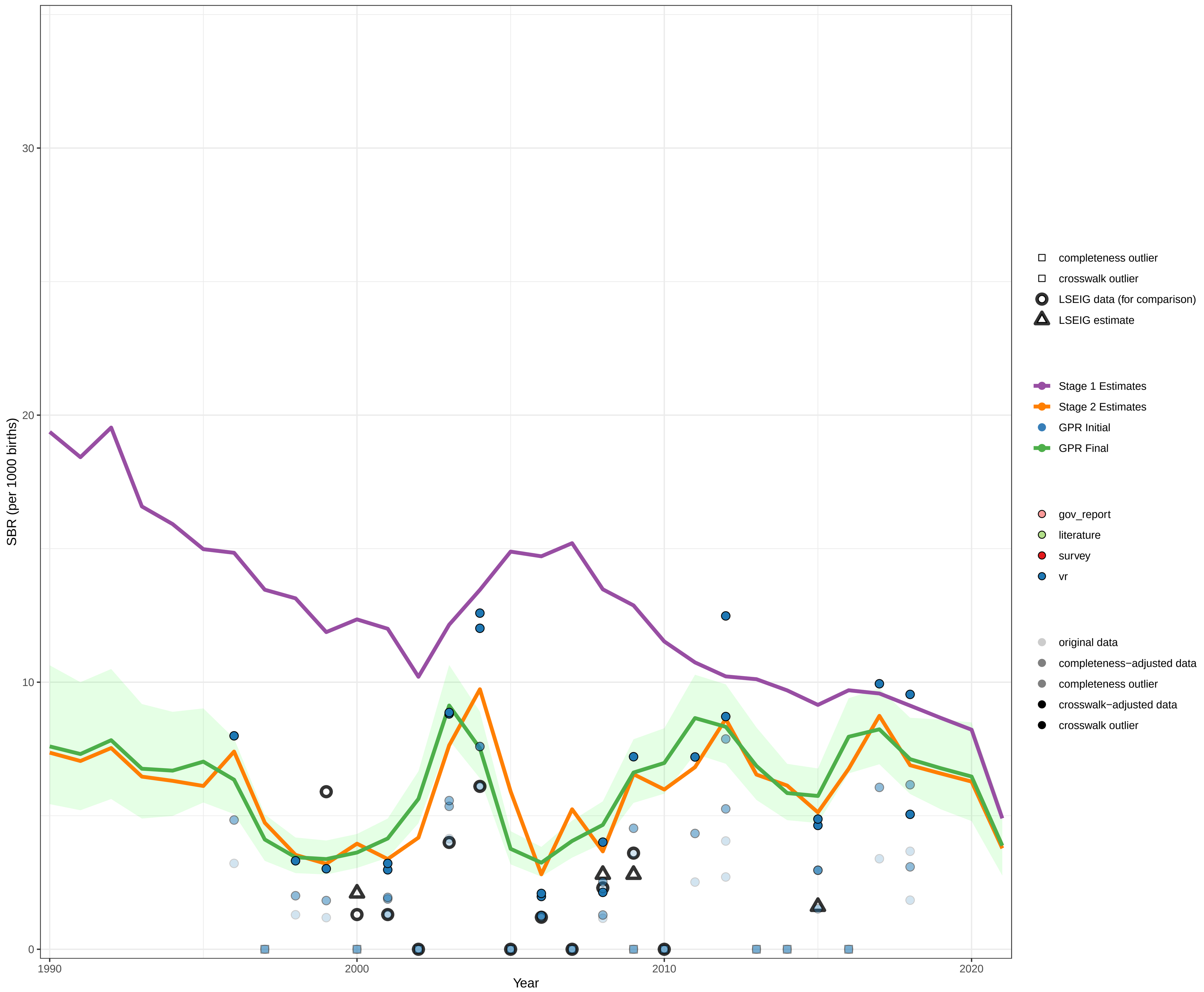

Andorra AND Western Europe Stillbirth Rate (SBR) − 28 weeks
lambda = 0.2 ; zeta = 0.99 ; scale = 5 ; amp = 0.028

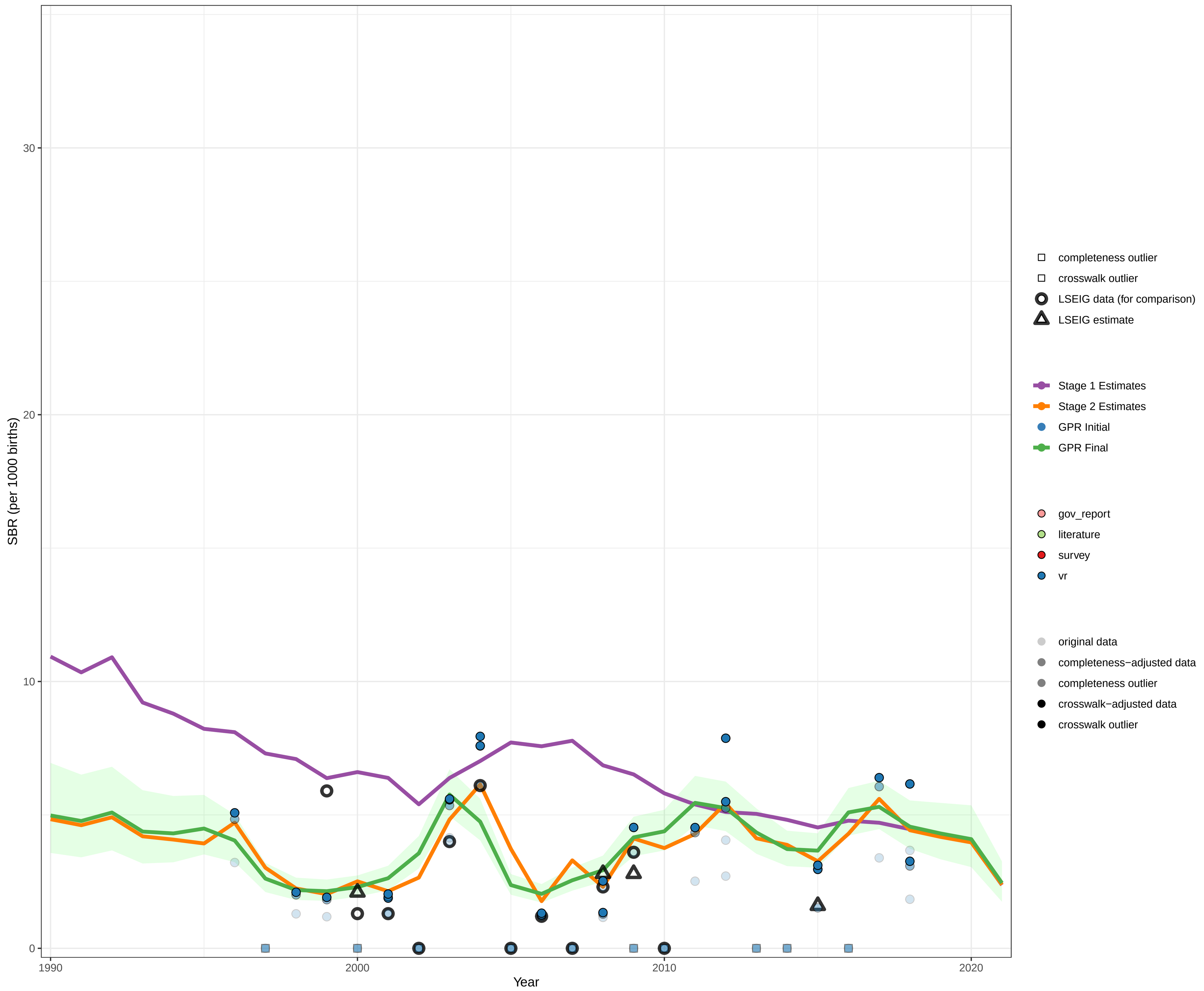

Year	Total Stillbirths (in thousands)	Total Stillbirths (in thousands)	SBR (per 1000 births)	SBR (per 1000 births)	SBR/NMR	SBR/NMR
	20 weeks	28 weeks	20 weeks	28 weeks	20 weeks	28 weeks
1990	0.0 (0.0 to 0.0)	0.0 (0.0 to 0.0)	7.6 (5.4 to 10.6)	5.0 (3.6 to 6.9)	2.47 (1.77 to 3.46)	1.62 (1.16 to 2.26)
1995	0.0 (0.0 to 0.0)	0.0 (0.0 to 0.0)	7.0 (5.5 to 9.0)	4.5 (3.5 to 5.7)	3.26 (2.55 to 4.19)	2.08 (1.63 to 2.67)
2000	0.0 (0.0 to 0.0)	0.0 (0.0 to 0.0)	3.6 (3.0 to 4.3)	2.3 (1.9 to 2.7)	2.24 (1.89 to 2.67)	1.42 (1.20 to 1.69)
2005	0.0 (0.0 to 0.0)	0.0 (0.0 to 0.0)	3.8 (3.2 to 4.4)	2.4 (2.0 to 2.8)	2.15 (1.82 to 2.53)	1.36 (1.15 to 1.59)
2010	0.0 (0.0 to 0.0)	0.0 (0.0 to 0.0)	7.0 (5.8 to 8.3)	4.4 (3.7 to 5.2)	5.66 (4.73 to 6.72)	3.56 (2.98 to 4.22)
2015	0.0 (0.0 to 0.0)	0.0 (0.0 to 0.0)	5.7 (4.7 to 6.8)	3.7 (3.0 to 4.3)	6.21 (5.11 to 7.32)	3.96 (3.27 to 4.66)
2020	0.0 (0.0 to 0.0)	0.0 (0.0 to 0.0)	6.5 (4.8 to 8.5)	4.1 (3.0 to 5.4)	8.20 (6.09 to 10.77)	5.19 (3.86 to 6.80)
2021	0.0 (0.0 to 0.0)	0.0 (0.0 to 0.0)	3.9 (2.8 to 5.2)	2.4 (1.7 to 3.3)	8.33 (5.93 to 11.13)	5.25 (3.75 to 7.00)

Austria AUT Western Europe Stillbirth Rate / Neonatal Mortality Rate (SBR/NMR) − 20 weeks
lambda = 0.2 ; zeta = 0.99 ; scale = 5 ; amp = 0.028

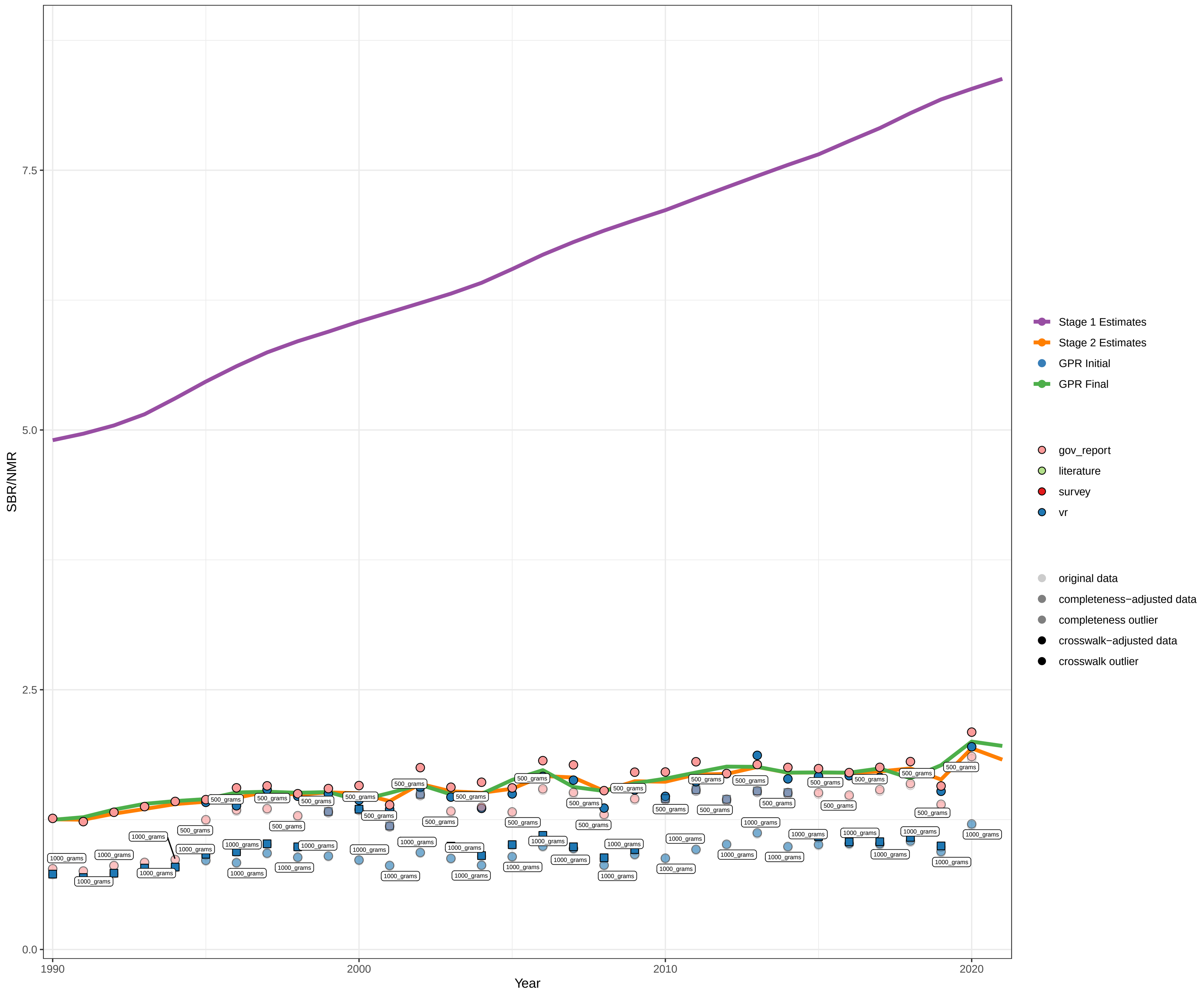

Austria AUT Western Europe Stillbirth Rate / Neonatal Mortality Rate (SBR/NMR) − 28 weeks
lambda = 0.2 ; zeta = 0.99 ; scale = 5 ; amp = 0.028

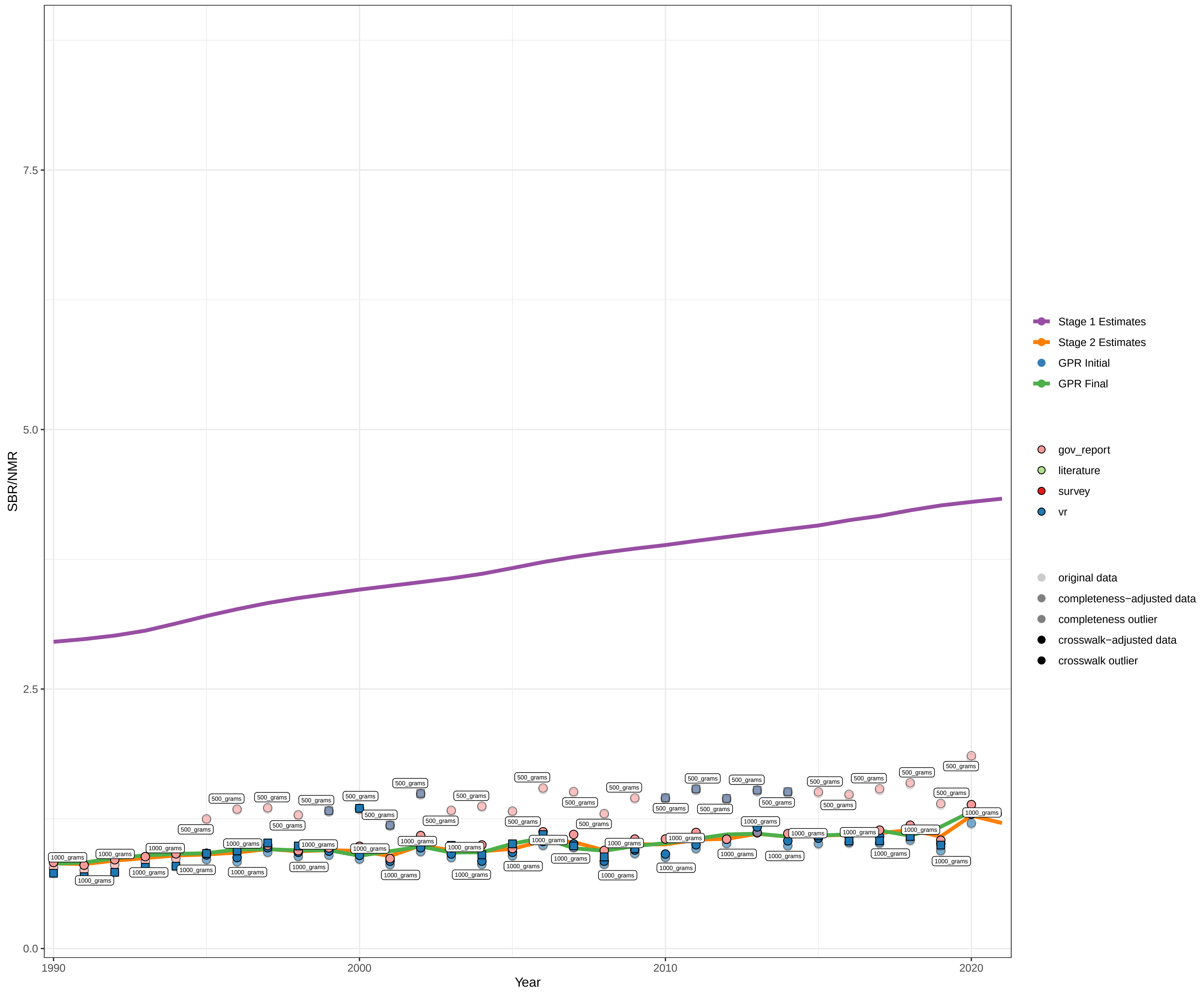

Definition	Years	Outlier	Source.Type	Title
1000_grams	1990−1994	0	Statistical Report	Austria Health Statistics Yearbook
1000_grams	1990−2020	0	VR	European Health for All Database
500_grams	1995−1998, 2000, 2003−2009, 2015−2019	0	VR	United Nations Demographic Yearbook
500_grams	1995−2020	0	Statistical Report	Austria Health Statistics Yearbook
500_grams	1999, 2001, 2002, 2010−2014	1	VR	United Nations Demographic Yearbook

Austria AUT Western Europe Stillbirth Rate (SBR) − 20 weeks
lambda = 0.2 ; zeta = 0.99 ; scale = 5 ; amp = 0.028

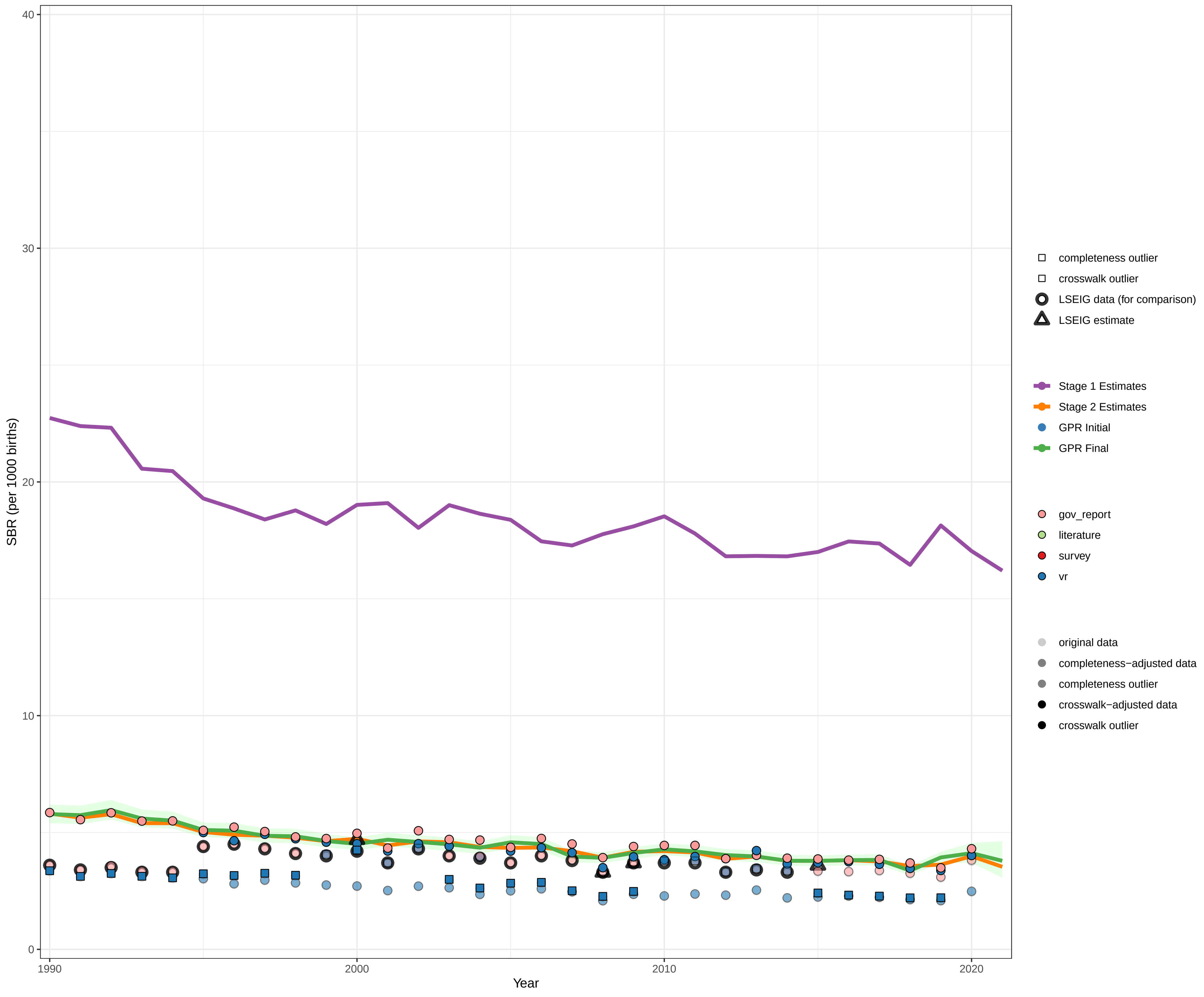

Austria AUT Western Europe Stillbirth Rate (SBR) − 28 weeks
lambda = 0.2 ; zeta = 0.99 ; scale = 5 ; amp = 0.028

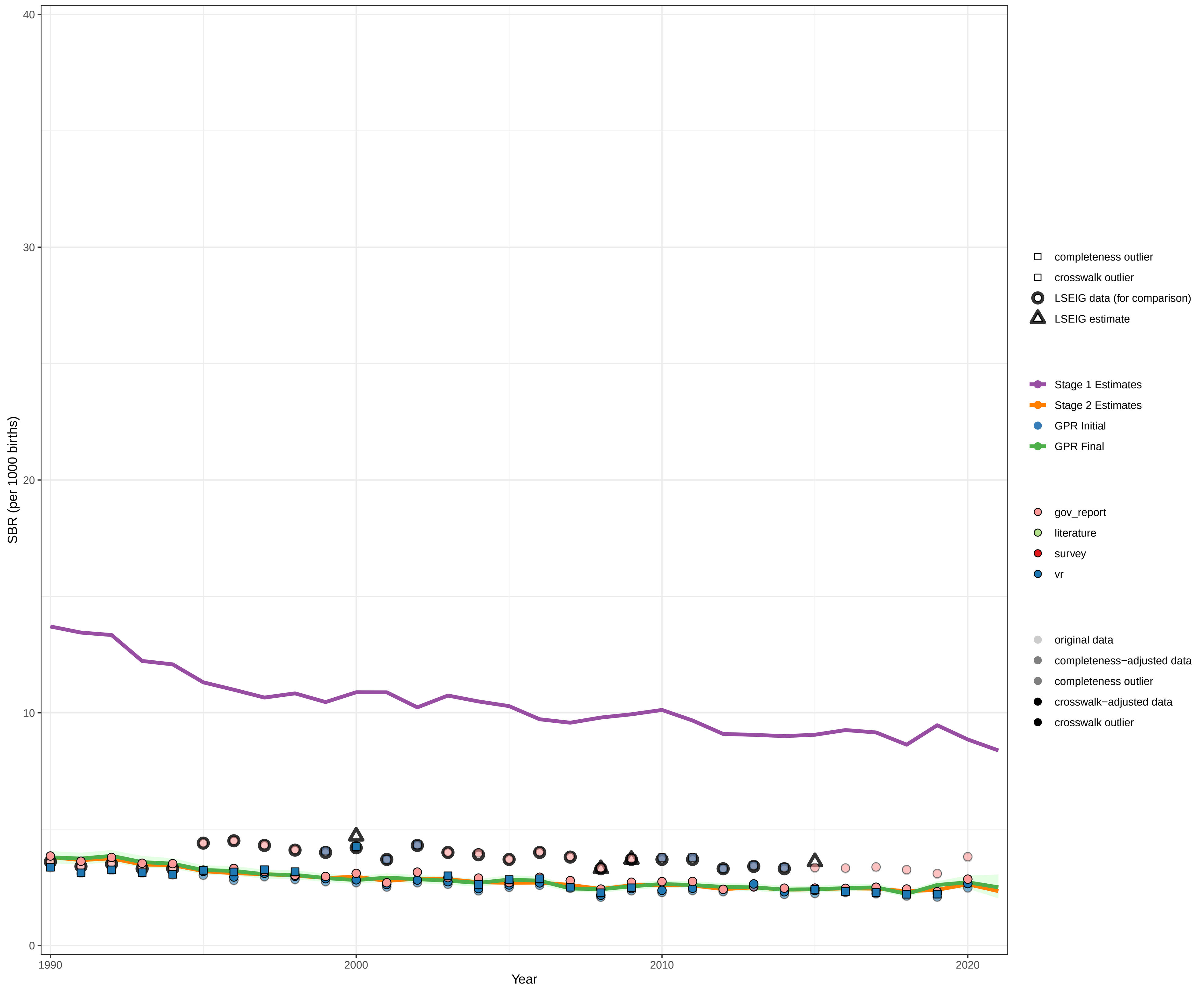

Year	Total Stillbirths (in thousands)	Total Stillbirths (in thousands)	SBR (per 1000 births)	SBR (per 1000 births)	SBR/NMR	SBR/NMR
	20 weeks	28 weeks	20 weeks	28 weeks	20 weeks	28 weeks
1990	0.5 (0.5 to 0.6)	0.4 (0.3 to 0.4)	5.8 (5.4 to 6.2)	3.8 (3.5 to 4.1)	1.25 (1.16 to 1.34)	0.82 (0.76 to 0.87)
1995	0.5 (0.4 to 0.5)	0.3 (0.3 to 0.3)	5.1 (4.8 to 5.4)	3.2 (3.1 to 3.4)	1.45 (1.36 to 1.54)	0.92 (0.86 to 0.98)
2000	0.4 (0.3 to 0.4)	0.2 (0.2 to 0.2)	4.5 (4.2 to 4.8)	2.8 (2.7 to 3.0)	1.43 (1.35 to 1.52)	0.89 (0.84 to 0.95)
2005	0.4 (0.3 to 0.4)	0.2 (0.2 to 0.2)	4.6 (4.3 to 4.9)	2.8 (2.7 to 3.0)	1.63 (1.54 to 1.74)	1.01 (0.95 to 1.08)
2010	0.3 (0.3 to 0.4)	0.2 (0.2 to 0.2)	4.3 (4.0 to 4.5)	2.6 (2.5 to 2.8)	1.64 (1.55 to 1.74)	1.02 (0.96 to 1.07)
2015	0.3 (0.3 to 0.3)	0.2 (0.2 to 0.2)	3.8 (3.6 to 4.0)	2.4 (2.3 to 2.6)	1.70 (1.60 to 1.81)	1.09 (1.02 to 1.16)
2020	0.4 (0.3 to 0.4)	0.2 (0.2 to 0.3)	4.1 (3.7 to 4.6)	2.7 (2.4 to 3.0)	2.00 (1.80 to 2.22)	1.32 (1.19 to 1.46)
2021	0.3 (0.3 to 0.4)	0.2 (0.2 to 0.3)	3.8 (3.1 to 4.6)	2.5 (2.0 to 3.1)	1.96 (1.59 to 2.39)	1.29 (1.05 to 1.58)

Belgium BEL Western Europe Stillbirth Rate / Neonatal Mortality Rate (SBR/NMR) − 20 weeks
lambda = 0.2 ; zeta = 0.99 ; scale = 5 ; amp = 0.028

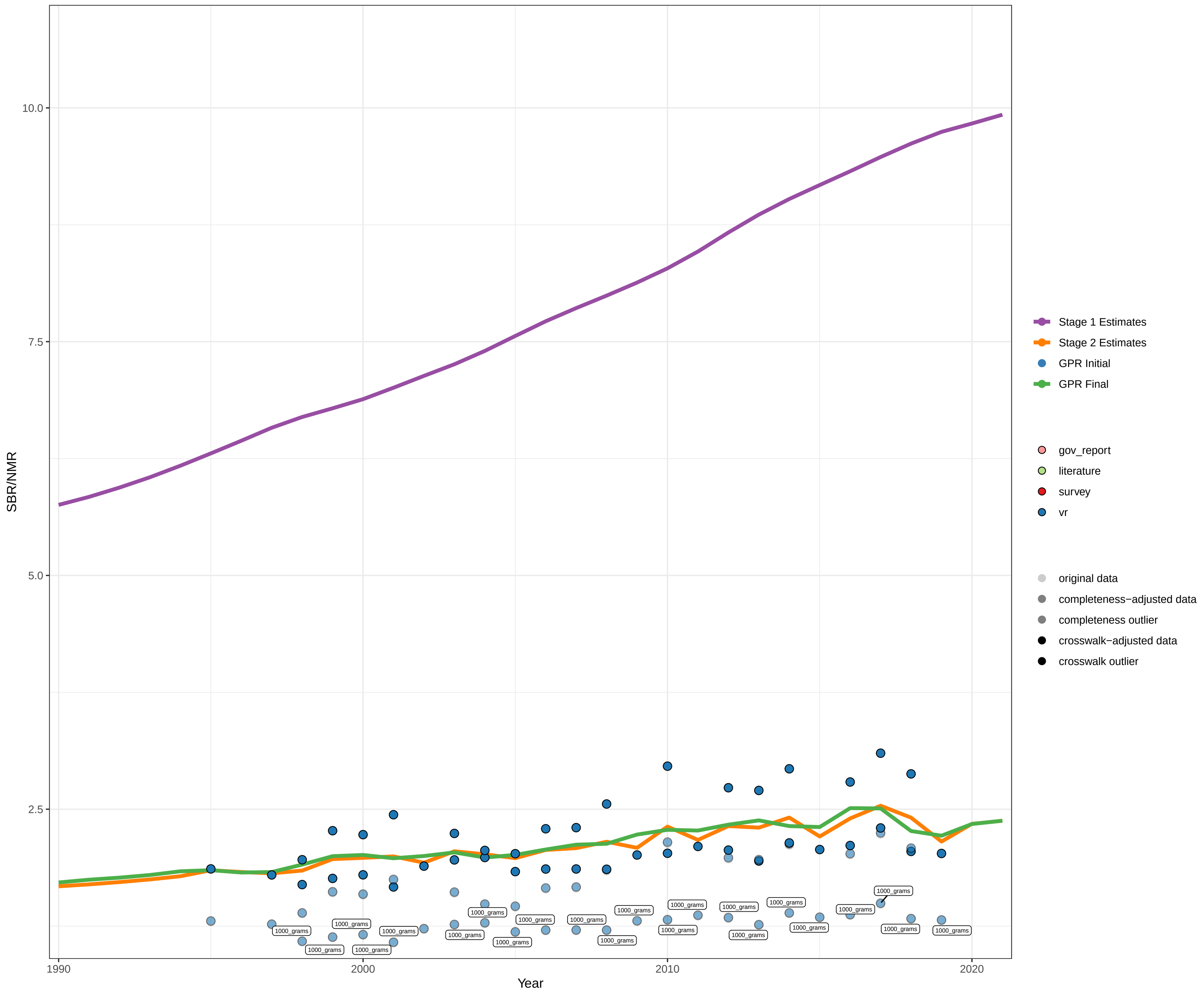

Belgium BEL Western Europe Stillbirth Rate / Neonatal Mortality Rate (SBR/NMR) − 28 weeks
lambda = 0.2 ; zeta = 0.99 ; scale = 5 ; amp = 0.028

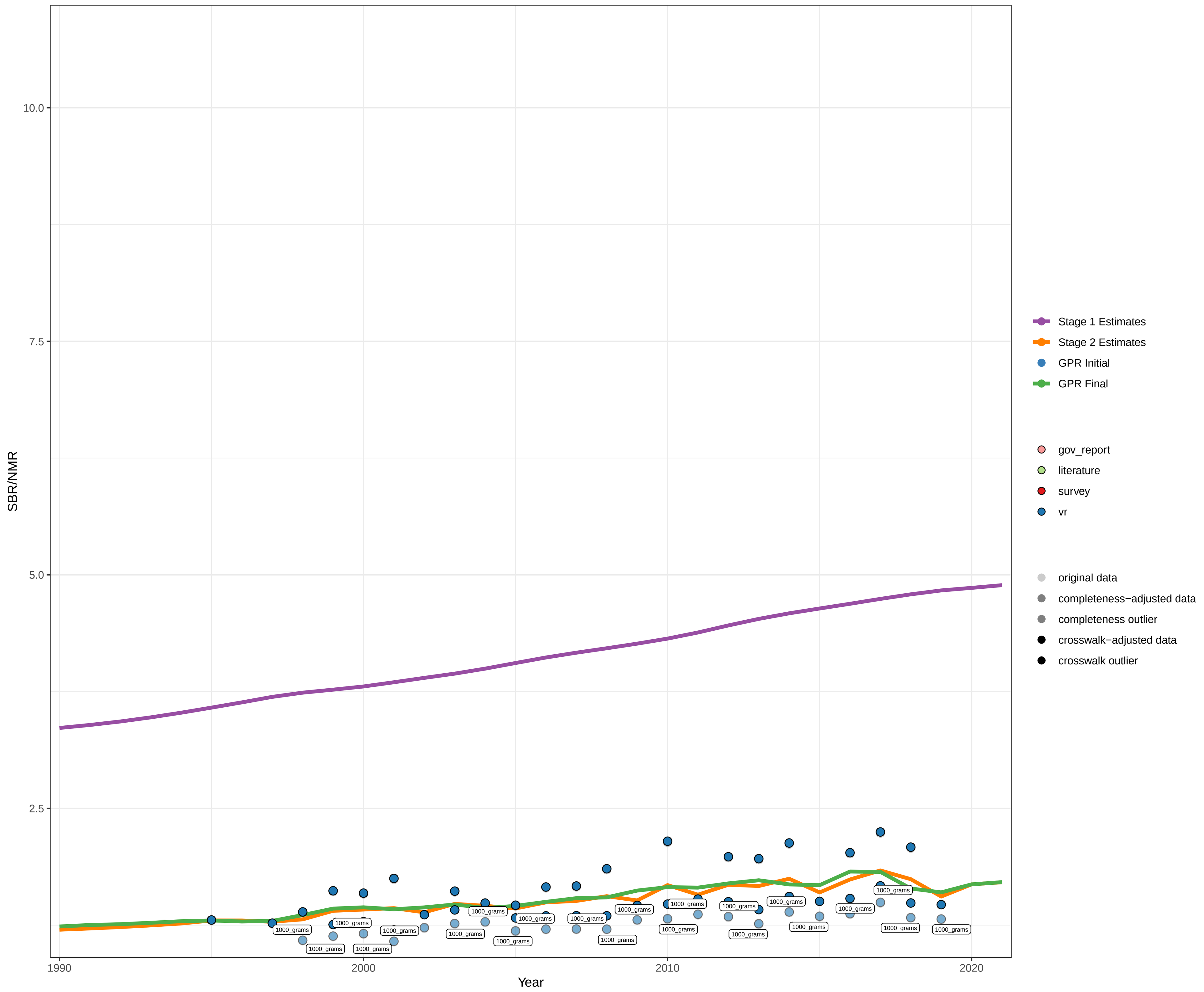

Definition	Years	Outlier	Source.Type	Title
1000_grams	1998−2019	0	VR	European Health for All Database
28_weeks	1995, 1997−2001, 2003−2008, 2010, 2012−2014, 2016−2018	0	VR	United Nations Demographic Yearbook

Belgium BEL Western Europe Stillbirth Rate (SBR) − 20 weeks
lambda = 0.2 ; zeta = 0.99 ; scale = 5 ; amp = 0.028

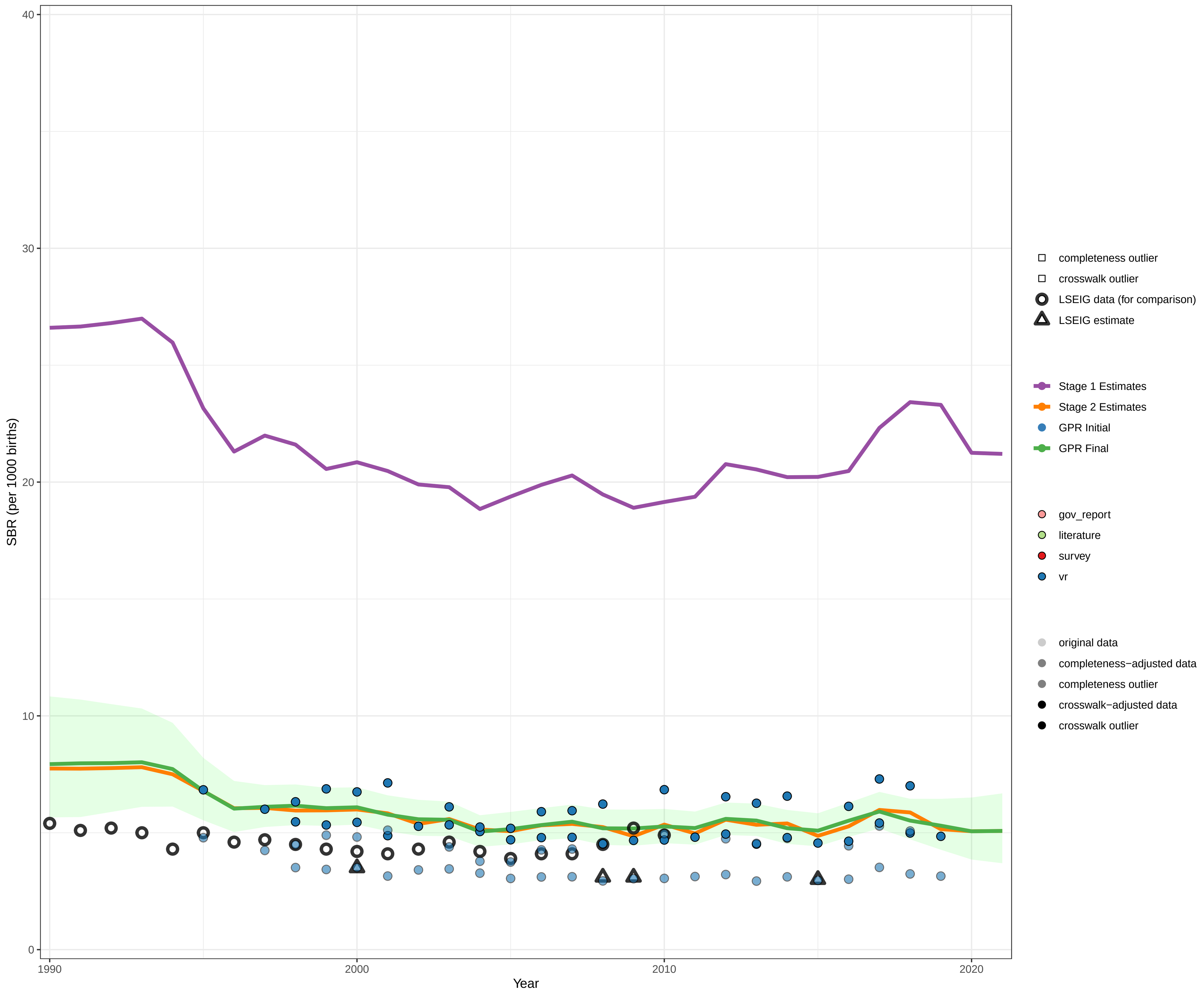

Belgium BEL Western Europe Stillbirth Rate (SBR) − 28 weeks
lambda = 0.2 ; zeta = 0.99 ; scale = 5 ; amp = 0.028

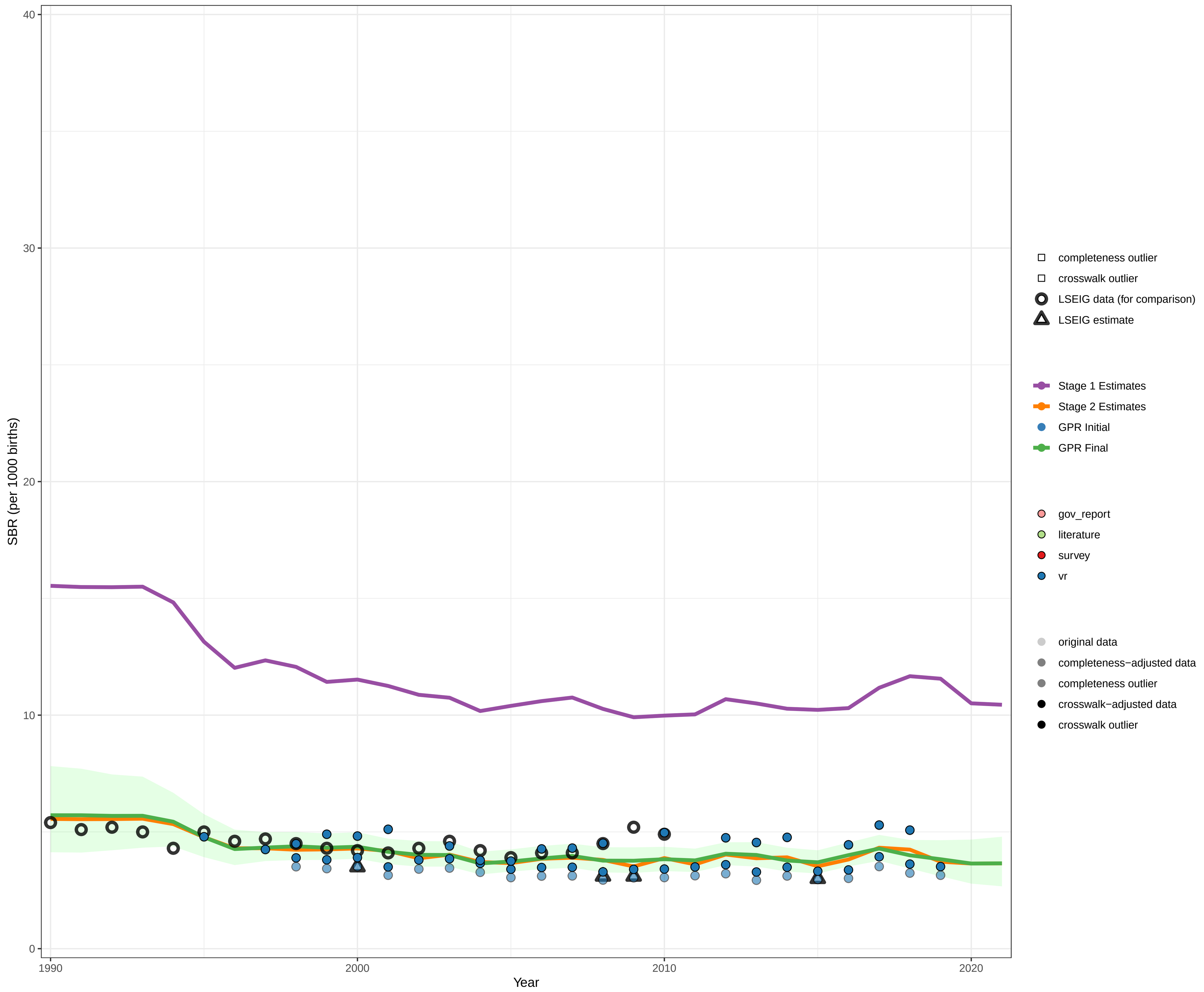

Year	Total Stillbirths (in thousands)	Total Stillbirths (in thousands)	SBR (per 1000 births)	SBR (per 1000 births)	SBR/NMR	SBR/NMR
	20 weeks	28 weeks	20 weeks	28 weeks	20 weeks	28 weeks
1990	1.0 (0.7 to 1.4)	0.7 (0.5 to 1.0)	7.9 (5.7 to 10.8)	5.7 (4.1 to 7.8)	1.72 (1.22 to 2.34)	1.24 (0.89 to 1.69)
1995	0.8 (0.6 to 1.0)	0.6 (0.5 to 0.7)	6.8 (5.5 to 8.2)	4.8 (3.9 to 5.8)	1.85 (1.51 to 2.23)	1.30 (1.07 to 1.57)
2000	0.7 (0.6 to 0.8)	0.5 (0.4 to 0.6)	6.1 (5.3 to 6.9)	4.4 (3.9 to 5.0)	2.01 (1.77 to 2.29)	1.44 (1.27 to 1.65)
2005	0.6 (0.5 to 0.7)	0.5 (0.4 to 0.5)	5.2 (4.5 to 5.9)	3.7 (3.3 to 4.3)	2.01 (1.76 to 2.30)	1.46 (1.29 to 1.66)
2010	0.7 (0.6 to 0.8)	0.5 (0.4 to 0.6)	5.3 (4.6 to 6.0)	3.8 (3.3 to 4.4)	2.28 (1.97 to 2.60)	1.66 (1.44 to 1.89)
2015	0.6 (0.5 to 0.7)	0.5 (0.4 to 0.5)	5.1 (4.4 to 5.8)	3.7 (3.2 to 4.2)	2.31 (2.00 to 2.65)	1.68 (1.46 to 1.91)
2020	0.6 (0.4 to 0.7)	0.4 (0.3 to 0.5)	5.1 (3.9 to 6.5)	3.6 (2.8 to 4.7)	2.34 (1.78 to 3.01)	1.69 (1.29 to 2.16)
2021	0.6 (0.4 to 0.8)	0.4 (0.3 to 0.5)	5.1 (3.7 to 6.7)	3.7 (2.7 to 4.8)	2.38 (1.73 to 3.13)	1.71 (1.25 to 2.24)

Cyprus CYP Western Europe Stillbirth Rate / Neonatal Mortality Rate (SBR/NMR) − 20 weeks
lambda = 0.4 ; zeta = 0.9 ; scale = 10 ; amp = 0.028

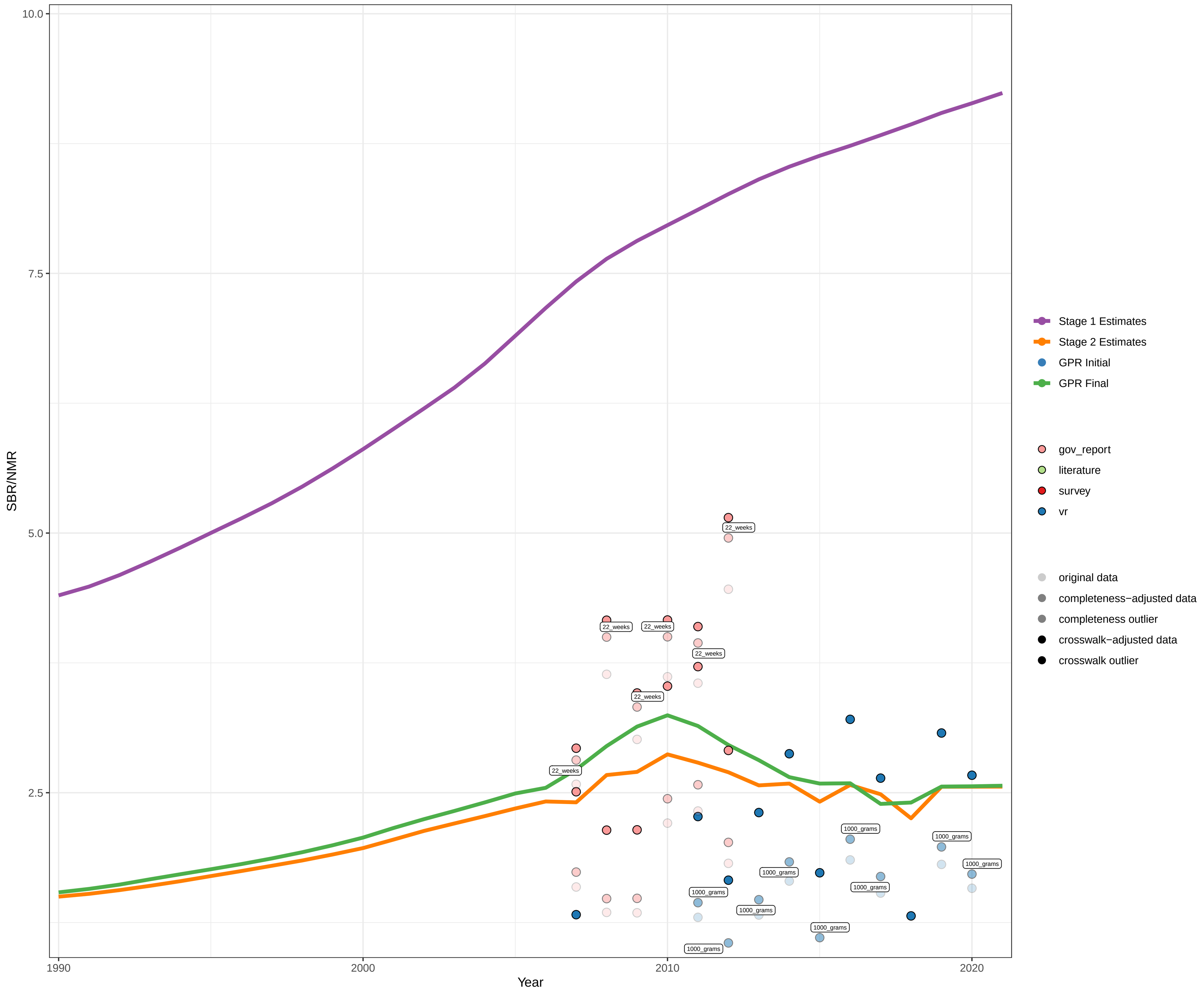

Cyprus CYP Western Europe Stillbirth Rate / Neonatal Mortality Rate (SBR/NMR) − 28 weeks
lambda = 0.4 ; zeta = 0.9 ; scale = 10 ; amp = 0.028

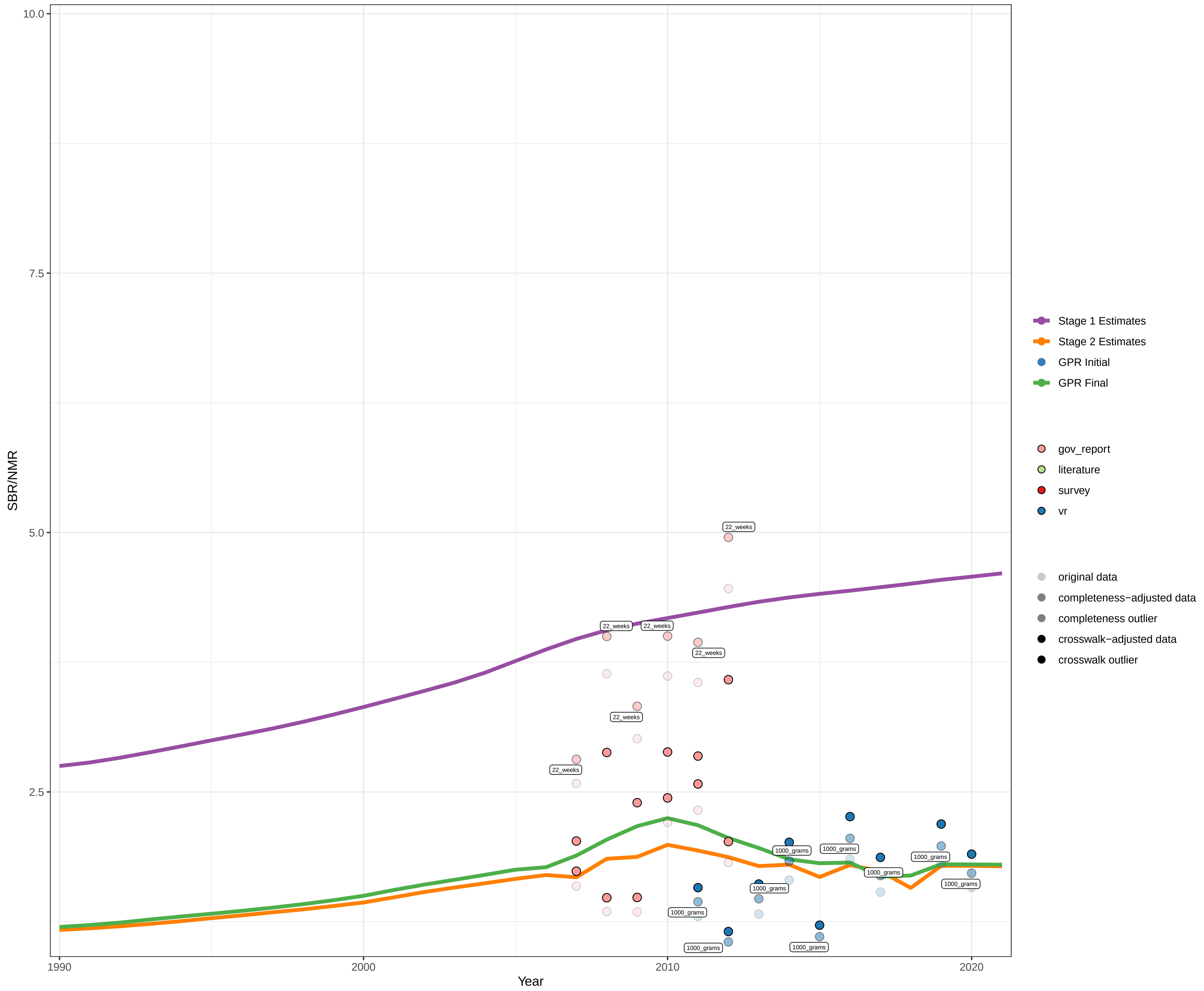

Definition	Years	Outlier	Source.Type	Title
1000_grams	2007, 2011−2020	0	VR	European Health for All Database
22_weeks, 28_weeks	2007−2012	0	Statistical Report	Cyprus Public Maternity Units Perinatal Health Indicators

Cyprus CYP Western Europe Stillbirth Rate (SBR) − 20 weeks
lambda = 0.4 ; zeta = 0.9 ; scale = 10 ; amp = 0.028

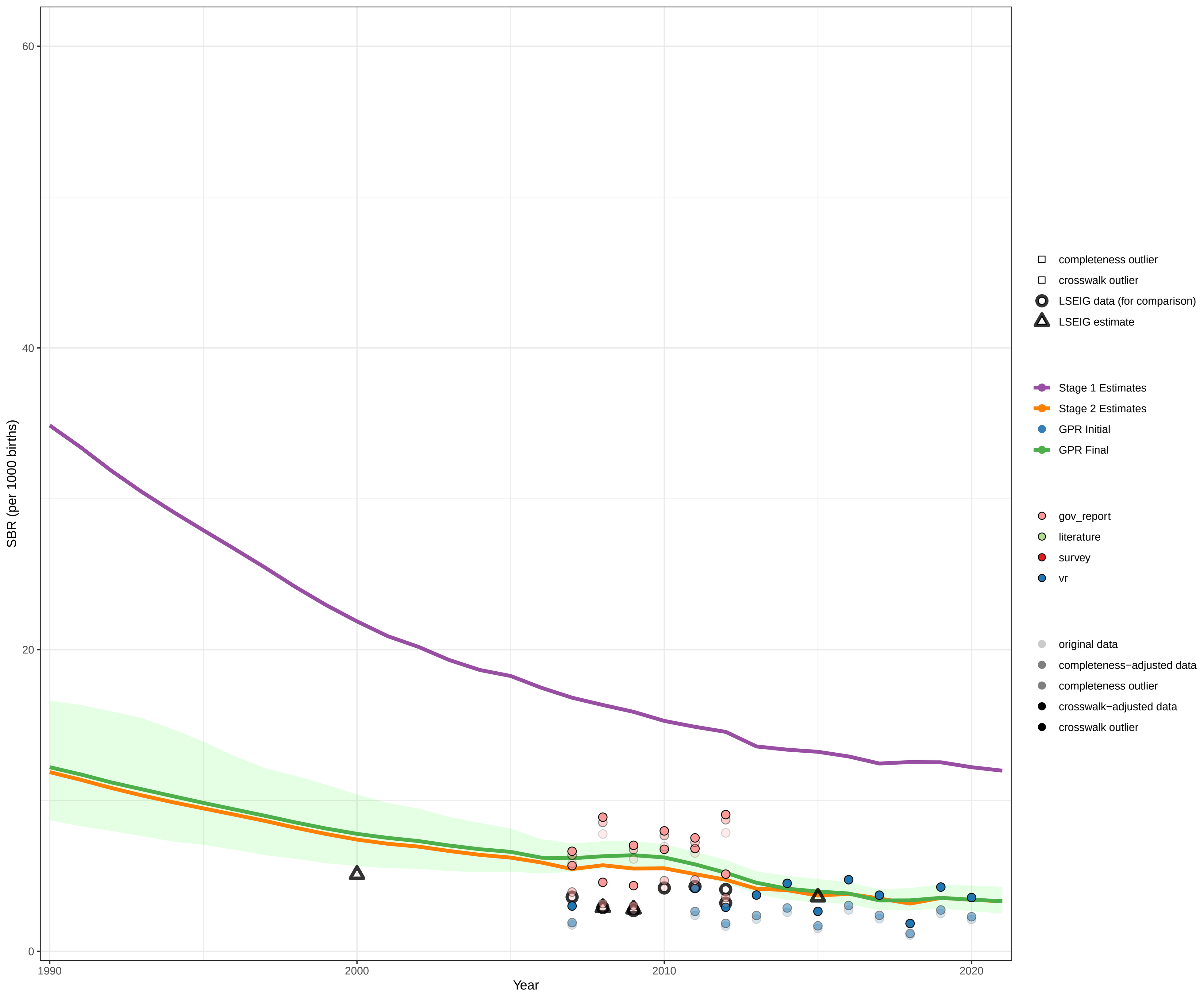

Cyprus CYP Western Europe Stillbirth Rate (SBR) − 28 weeks
lambda = 0.4 ; zeta = 0.9 ; scale = 10 ; amp = 0.028

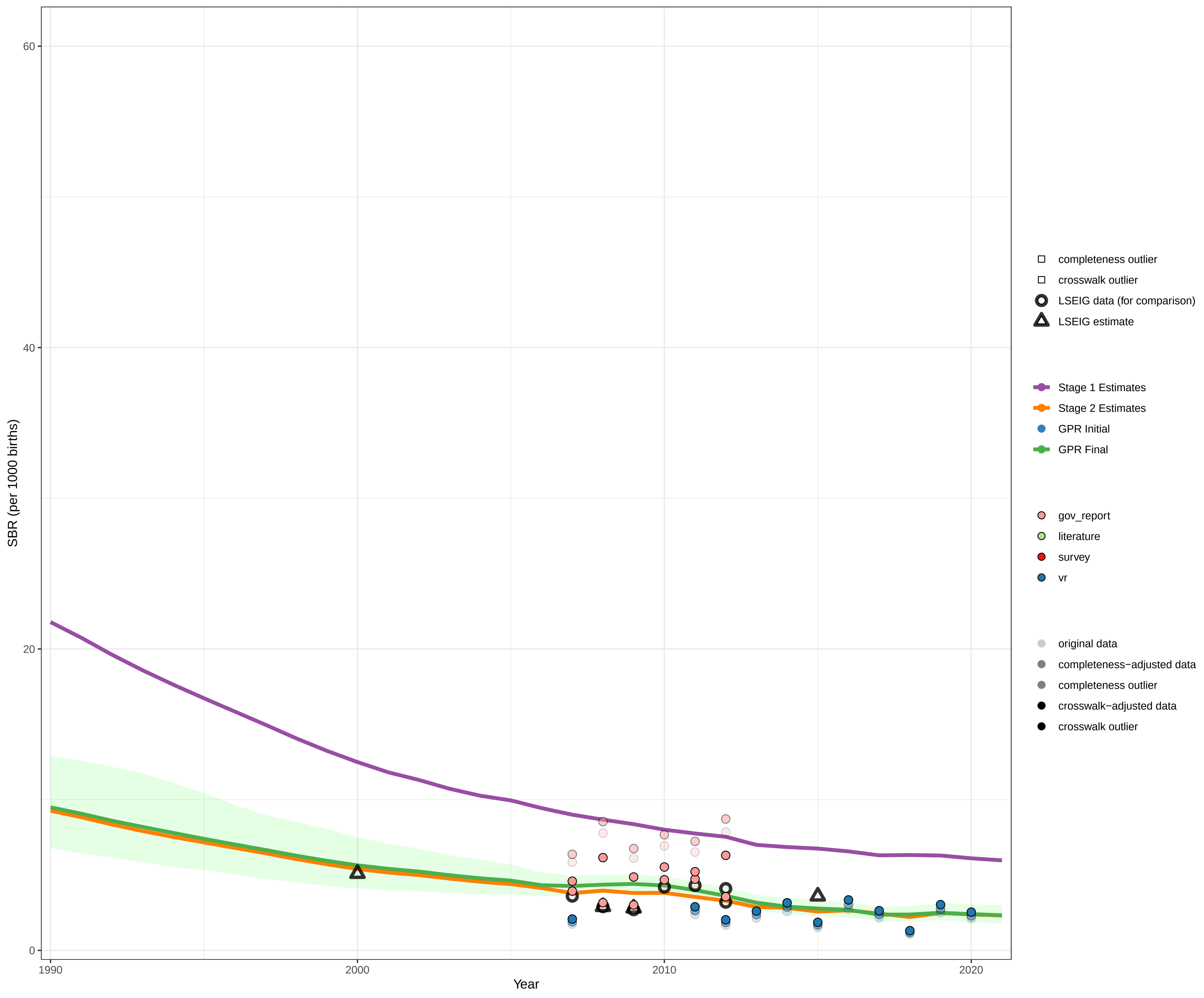

Year	Total Stillbirths (in thousands)	Total Stillbirths (in thousands)	SBR (per 1000 births)	SBR (per 1000 births)	SBR/NMR	SBR/NMR
	20 weeks	28 weeks	20 weeks	28 weeks	20 weeks	28 weeks
1990	0.2 (0.1 to 0.2)	0.1 (0.1 to 0.2)	12.2 (8.7 to 16.6)	9.5 (6.8 to 12.9)	1.54 (1.10 to 2.10)	1.20 (0.86 to 1.63)
1995	0.1 (0.1 to 0.2)	0.1 (0.1 to 0.1)	9.8 (7.1 to 13.9)	7.4 (5.3 to 10.4)	1.76 (1.27 to 2.49)	1.33 (0.95 to 1.87)
2000	0.1 (0.1 to 0.1)	0.1 (0.0 to 0.1)	7.8 (5.7 to 10.4)	5.6 (4.1 to 7.5)	2.07 (1.50 to 2.76)	1.50 (1.09 to 2.00)
2005	0.1 (0.1 to 0.1)	0.1 (0.0 to 0.1)	6.6 (5.3 to 8.1)	4.6 (3.7 to 5.7)	2.49 (2.00 to 3.08)	1.75 (1.41 to 2.16)
2010	0.1 (0.1 to 0.1)	0.1 (0.1 to 0.1)	6.2 (5.5 to 7.1)	4.3 (3.8 to 4.9)	3.25 (2.85 to 3.70)	2.25 (1.97 to 2.56)
2015	0.1 (0.0 to 0.1)	0.0 (0.0 to 0.0)	4.0 (3.2 to 4.8)	2.8 (2.3 to 3.3)	2.59 (2.11 to 3.13)	1.81 (1.48 to 2.18)
2020	0.1 (0.0 to 0.1)	0.0 (0.0 to 0.0)	3.4 (2.7 to 4.4)	2.4 (1.9 to 3.1)	2.56 (1.99 to 3.26)	1.80 (1.40 to 2.29)
2021	0.1 (0.0 to 0.1)	0.0 (0.0 to 0.0)	3.3 (2.5 to 4.3)	2.3 (1.8 to 3.0)	2.57 (1.94 to 3.30)	1.80 (1.36 to 2.30)

Denmark DNK Western Europe Stillbirth Rate / Neonatal Mortality Rate (SBR/NMR) − 20 weeks
lambda = 0.2 ; zeta = 0.99 ; scale = 5 ; amp = 0.028

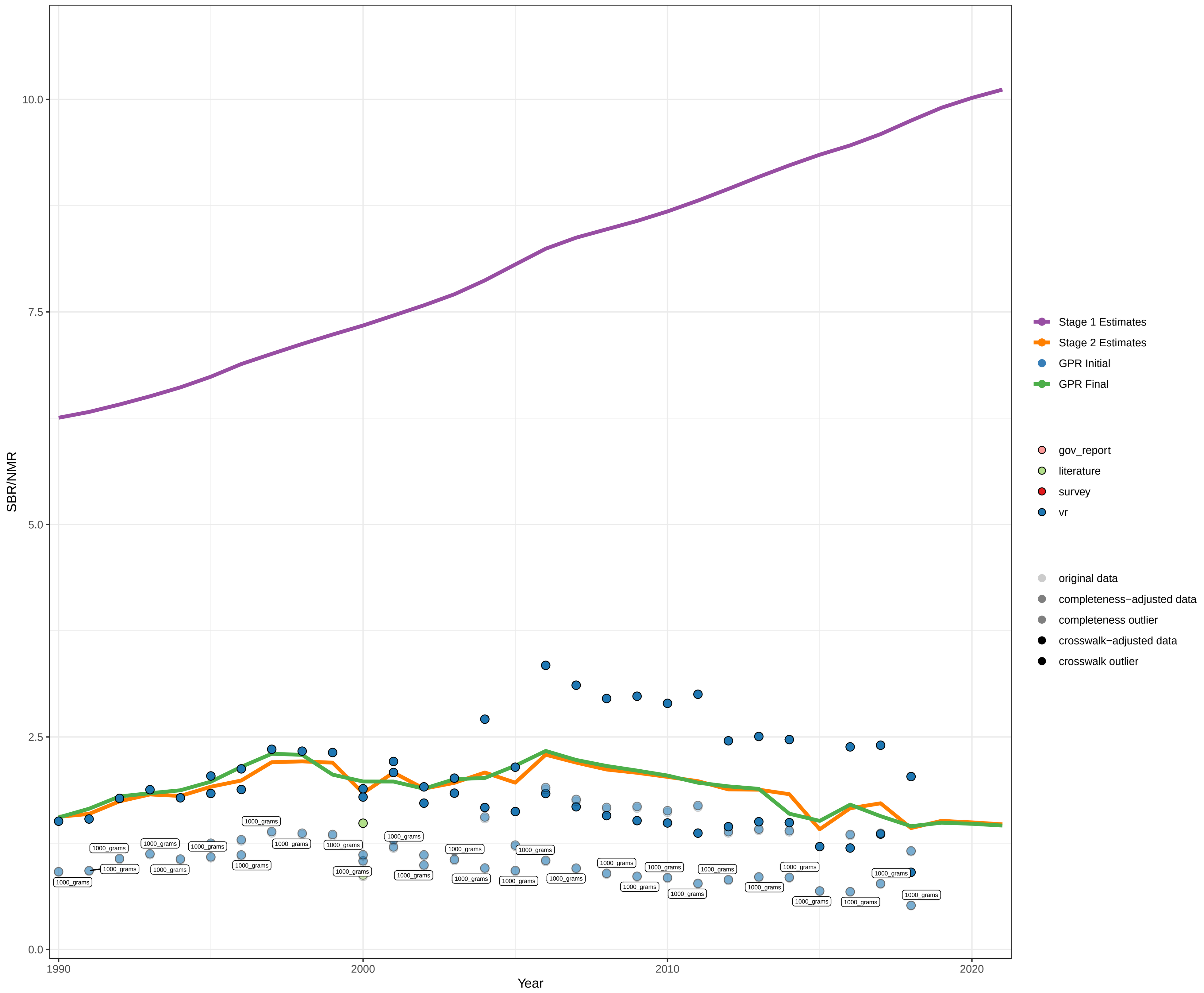

Denmark DNK Western Europe Stillbirth Rate / Neonatal Mortality Rate (SBR/NMR) − 28 weeks
lambda = 0.2 ; zeta = 0.99 ; scale = 5 ; amp = 0.028

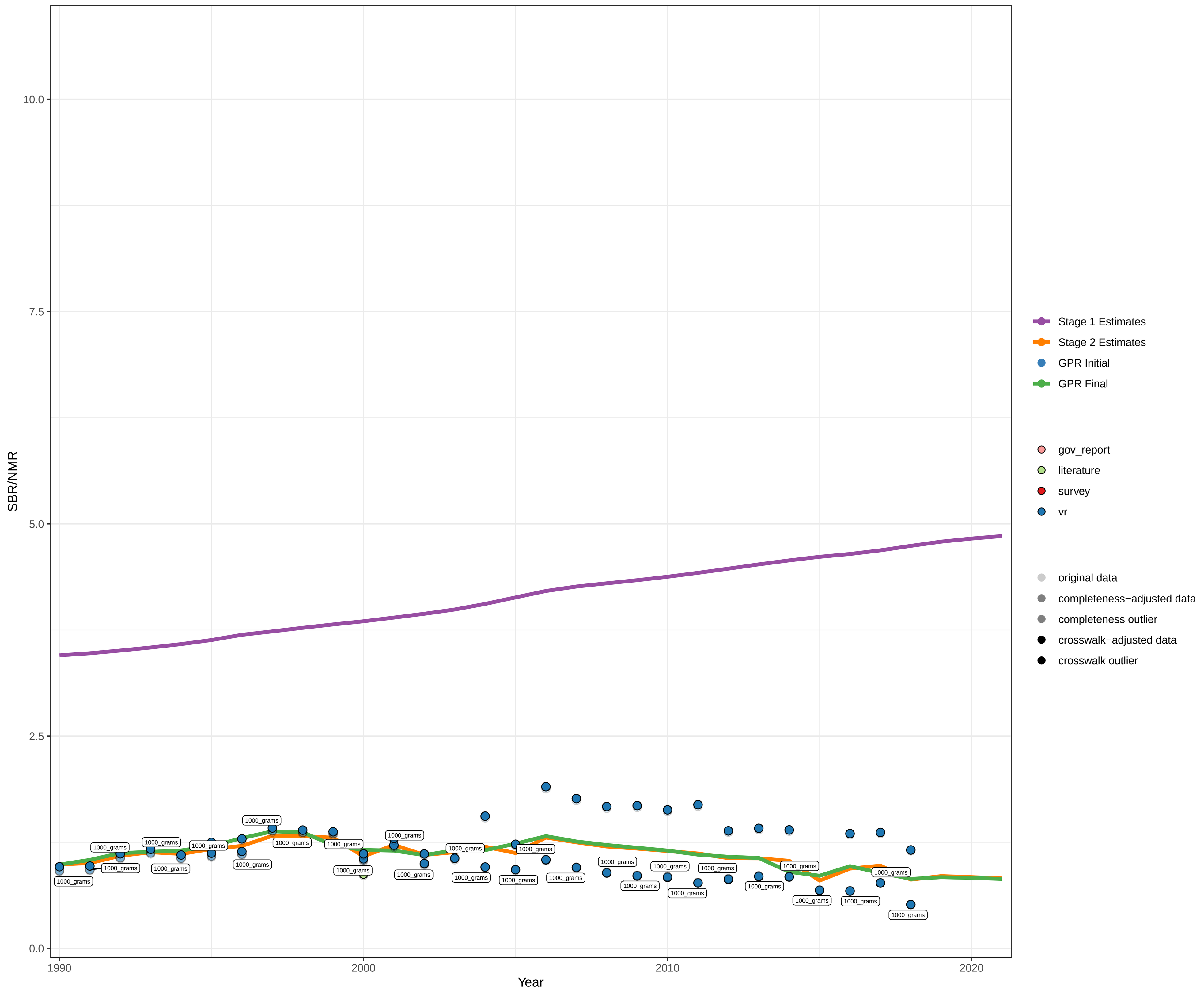

Definition	Years	Outlier	Source.Type	Title
1000_grams	1990−2018	0	VR	European Health for All Database
28_weeks	1995, 1996, 2000−2014, 2016−2018	0	VR	United Nations Demographic Yearbook
28_weeks	2000	0	Sci Lit	Prepregnancy obesity and fetal death: a study within the Danish National Birth Cohort

Denmark DNK Western Europe Stillbirth Rate (SBR) − 20 weeks
lambda = 0.2 ; zeta = 0.99 ; scale = 5 ; amp = 0.028

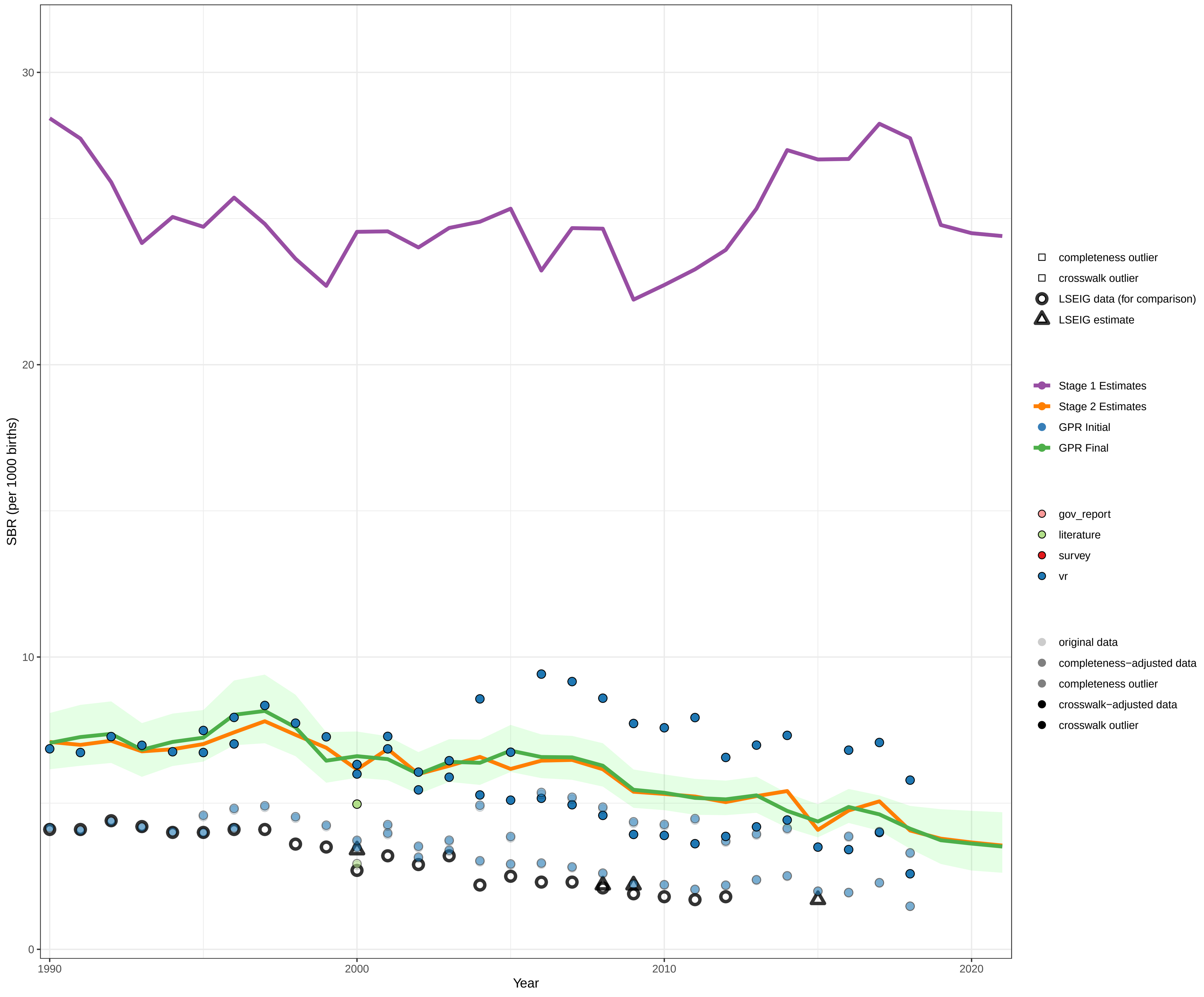

Denmark DNK Western Europe Stillbirth Rate (SBR) − 28 weeks
lambda = 0.2 ; zeta = 0.99 ; scale = 5 ; amp = 0.028

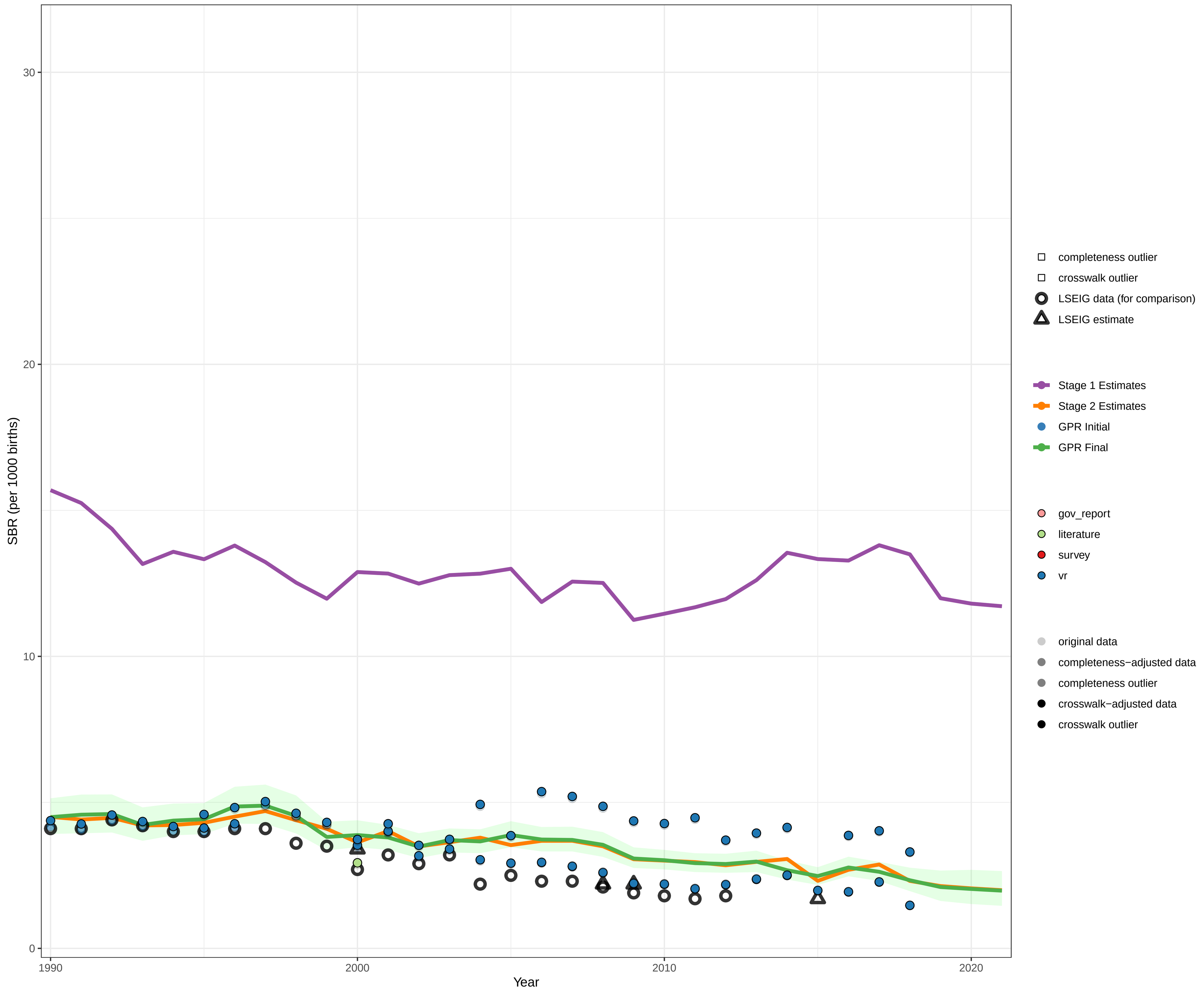

Year	Total Stillbirths (in thousands)	Total Stillbirths (in thousands)	SBR (per 1000 births)	SBR (per 1000 births)	SBR/NMR	SBR/NMR
	20 weeks	28 weeks	20 weeks	28 weeks	20 weeks	28 weeks
1990	0.5 (0.4 to 0.5)	0.3 (0.3 to 0.3)	7.1 (6.2 to 8.1)	4.5 (3.9 to 5.1)	1.55 (1.36 to 1.78)	0.99 (0.86 to 1.13)
1995	0.5 (0.4 to 0.6)	0.3 (0.3 to 0.3)	7.2 (6.4 to 8.2)	4.4 (3.9 to 5.0)	1.97 (1.75 to 2.23)	1.21 (1.07 to 1.36)
2000	0.4 (0.4 to 0.5)	0.3 (0.2 to 0.3)	6.6 (5.9 to 7.5)	3.9 (3.5 to 4.4)	1.98 (1.76 to 2.23)	1.16 (1.04 to 1.31)
2005	0.4 (0.4 to 0.5)	0.3 (0.2 to 0.3)	6.8 (6.1 to 7.7)	3.9 (3.5 to 4.4)	2.16 (1.93 to 2.44)	1.23 (1.10 to 1.39)
2010	0.3 (0.3 to 0.4)	0.2 (0.2 to 0.2)	5.4 (4.8 to 6.0)	3.0 (2.7 to 3.4)	2.05 (1.82 to 2.29)	1.15 (1.03 to 1.29)
2015	0.3 (0.2 to 0.3)	0.1 (0.1 to 0.2)	4.4 (3.8 to 5.0)	2.5 (2.2 to 2.8)	1.51 (1.33 to 1.72)	0.86 (0.75 to 0.96)
2020	0.2 (0.2 to 0.3)	0.1 (0.1 to 0.2)	3.6 (2.7 to 4.7)	2.0 (1.5 to 2.7)	1.48 (1.10 to 1.94)	0.83 (0.62 to 1.10)
2021	0.2 (0.2 to 0.3)	0.1 (0.1 to 0.2)	3.5 (2.6 to 4.7)	2.0 (1.5 to 2.6)	1.46 (1.09 to 1.94)	0.82 (0.60 to 1.10)

Finland FIN Western Europe Stillbirth Rate / Neonatal Mortality Rate (SBR/NMR) − 20 weeks
lambda = 0.2 ; zeta = 0.99 ; scale = 5 ; amp = 0.028

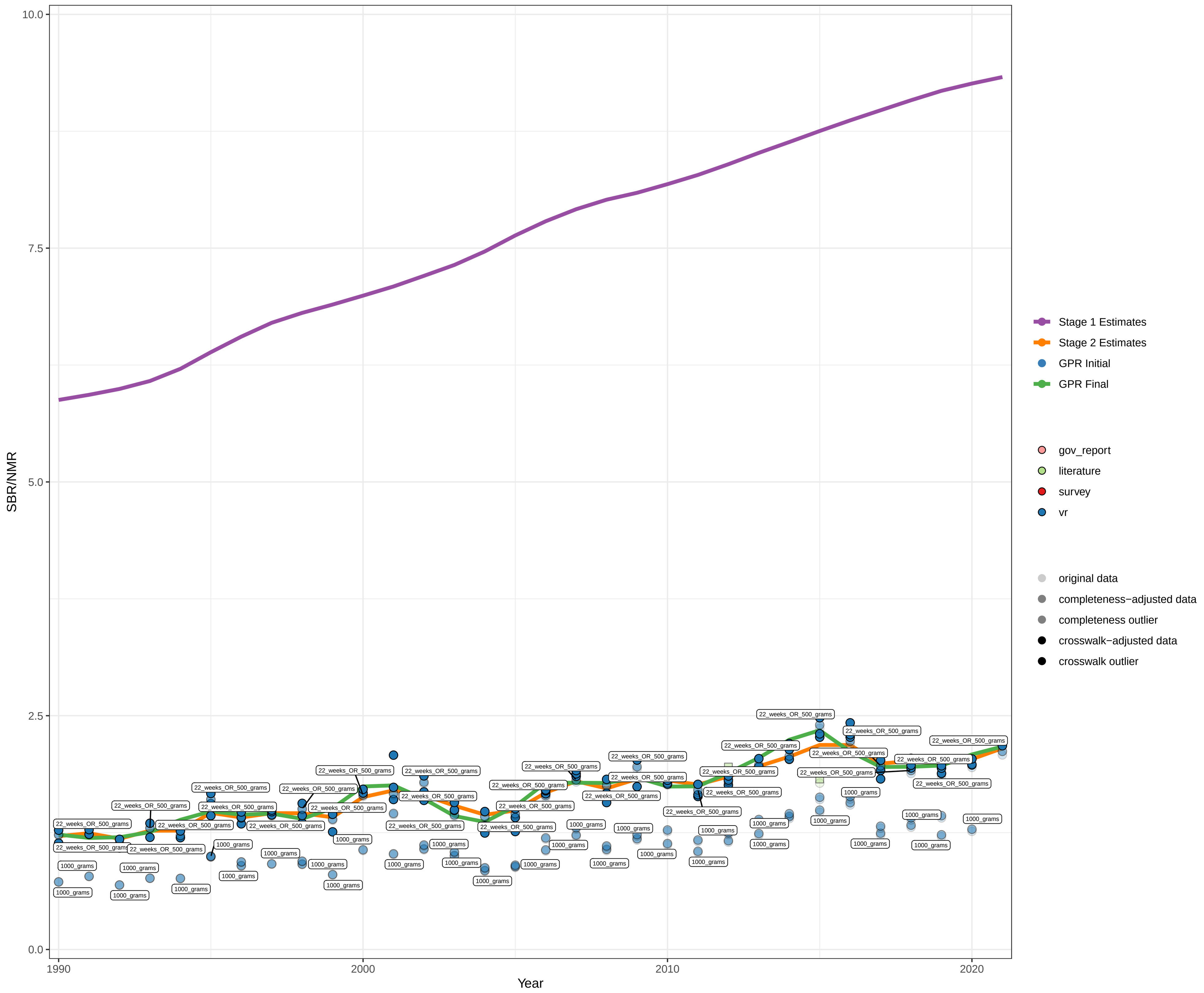

Finland FIN Western Europe Stillbirth Rate / Neonatal Mortality Rate (SBR/NMR) − 28 weeks
lambda = 0.2 ; zeta = 0.99 ; scale = 5 ; amp = 0.028

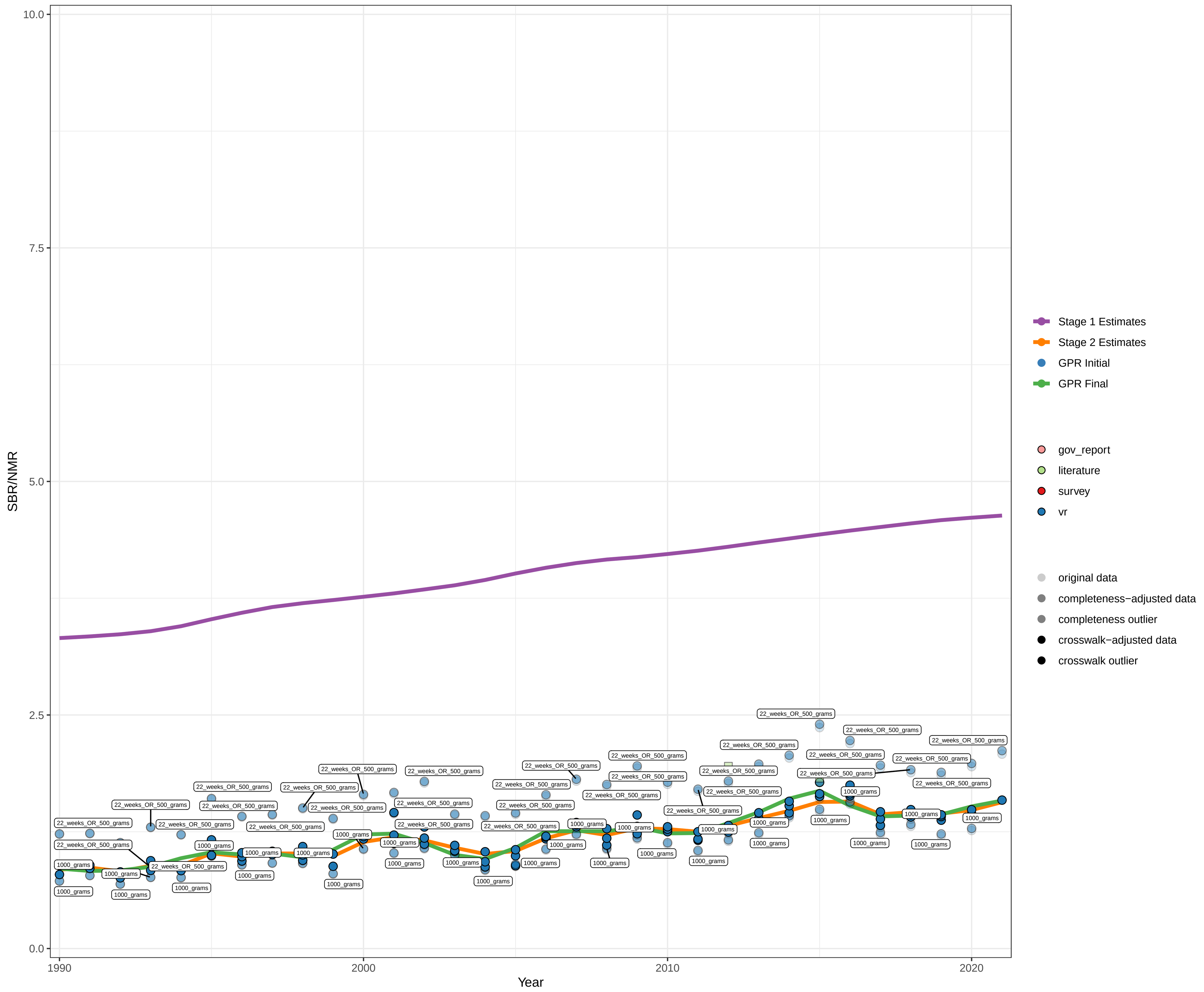

Definition	Years	Outlier	Source.Type	Title
1000_grams	1990−2020	0	VR	European Health for All Database
22_weeks_OR_500_grams	1990, 1995, 2000, 2005, 2010, 2015−2021	0	VR	Finland Perinatal Statistics: Parturients, Deliveries and Newborns (Preliminary)
22_weeks_OR_500_grams	1991	0	VR	Finland Parturients, Births and Newborns
22_weeks_OR_500_grams	1992, 1994, 1996−1999, 2001, 2002, 2004, 2006−2009, 2011−2014	0	VR	Finland Perinatal Statistics: Parturients, Deliveries and Newborns
22_weeks_OR_500_grams	1993, 2003	0	VR	Finland Perinatal Statistics
28_weeks	1995−1998, 2001−2019	0	VR	United Nations Demographic Yearbook
22_weeks	2012, 2015	1	Sci Lit	The impact of increased number of low−risk deliveries on maternal and neonatal outcomes: A retrospective cohort study in Finland in 2011−2015

Finland FIN Western Europe Stillbirth Rate (SBR) − 20 weeks
lambda = 0.2 ; zeta = 0.99 ; scale = 5 ; amp = 0.028

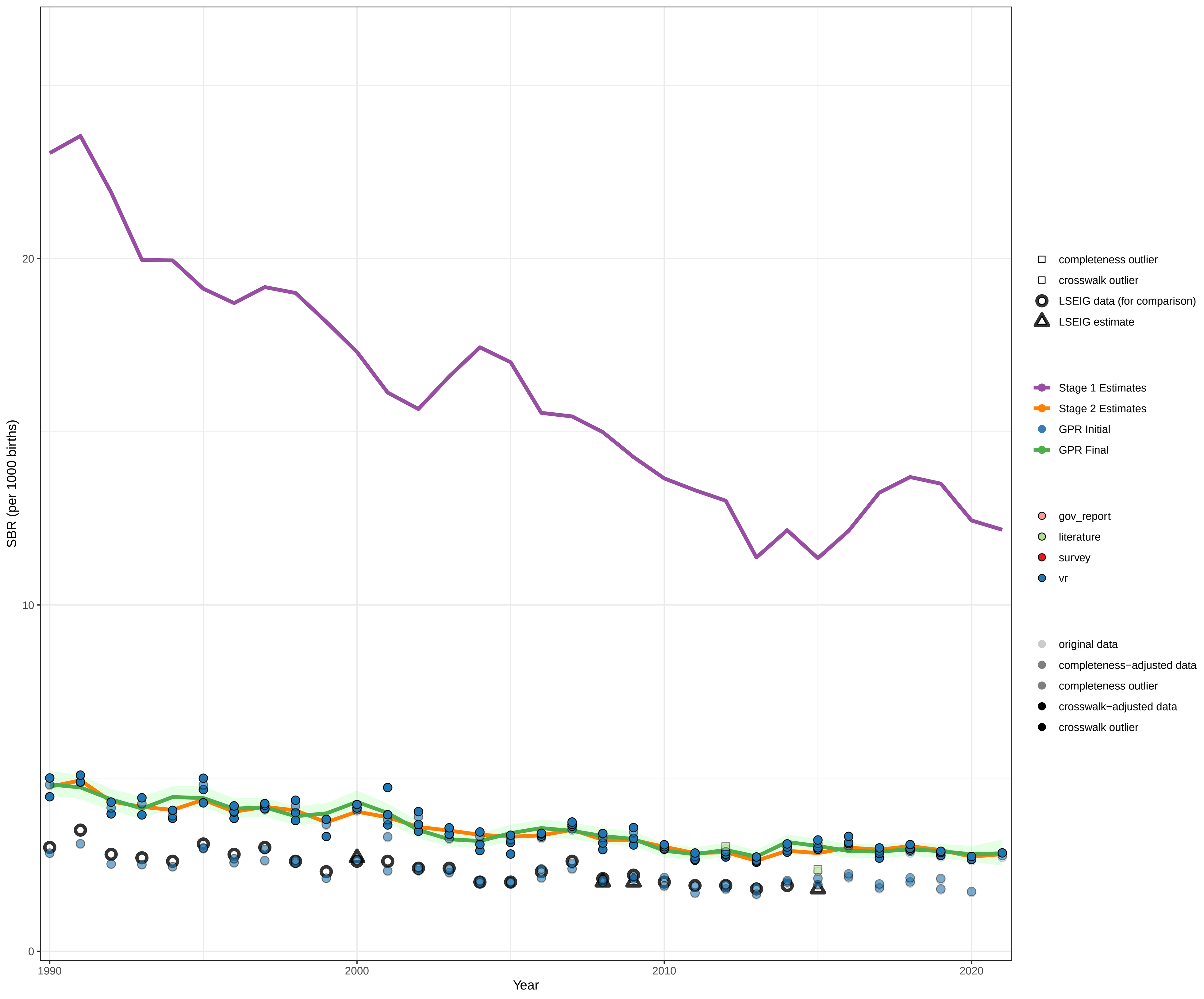

Finland FIN Western Europe Stillbirth Rate (SBR) − 28 weeks
lambda = 0.2 ; zeta = 0.99 ; scale = 5 ; amp = 0.028

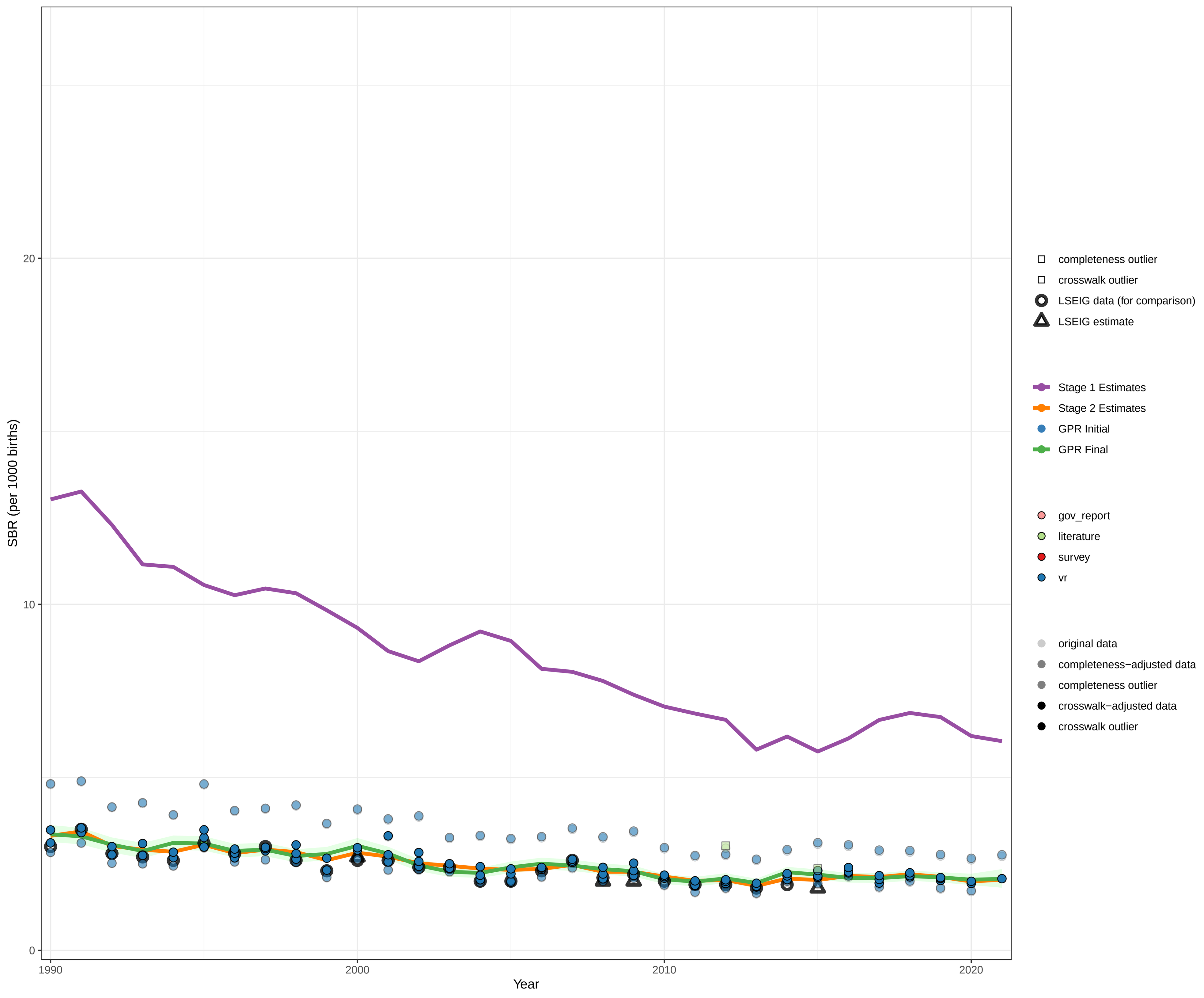

Year	Total Stillbirths (in thousands)	Total Stillbirths (in thousands)	SBR (per 1000 births)	SBR (per 1000 births)	SBR/NMR	SBR/NMR
	20 weeks	28 weeks	20 weeks	28 weeks	20 weeks	28 weeks
1990	0.3 (0.3 to 0.3)	0.2 (0.2 to 0.2)	4.8 (4.5 to 5.2)	3.4 (3.1 to 3.6)	1.23 (1.15 to 1.33)	0.86 (0.80 to 0.92)
1995	0.3 (0.3 to 0.3)	0.2 (0.2 to 0.2)	4.4 (4.1 to 4.8)	3.1 (2.9 to 3.3)	1.48 (1.38 to 1.59)	1.03 (0.96 to 1.10)
2000	0.2 (0.2 to 0.3)	0.2 (0.2 to 0.2)	4.3 (4.0 to 4.6)	3.0 (2.8 to 3.2)	1.74 (1.63 to 1.88)	1.22 (1.14 to 1.31)
2005	0.2 (0.2 to 0.2)	0.1 (0.1 to 0.1)	3.4 (3.2 to 3.7)	2.4 (2.2 to 2.6)	1.53 (1.43 to 1.64)	1.08 (1.00 to 1.15)
2010	0.2 (0.2 to 0.2)	0.1 (0.1 to 0.1)	2.9 (2.7 to 3.1)	2.1 (1.9 to 2.2)	1.74 (1.62 to 1.87)	1.23 (1.15 to 1.32)
2015	0.2 (0.2 to 0.2)	0.1 (0.1 to 0.1)	3.0 (2.8 to 3.2)	2.2 (2.0 to 2.3)	2.34 (2.19 to 2.51)	1.69 (1.58 to 1.80)
2020	0.1 (0.1 to 0.1)	0.1 (0.1 to 0.1)	2.8 (2.6 to 3.0)	2.0 (1.9 to 2.2)	2.09 (1.92 to 2.26)	1.52 (1.41 to 1.65)
2021	0.1 (0.1 to 0.2)	0.1 (0.1 to 0.1)	2.8 (2.5 to 3.2)	2.1 (1.8 to 2.3)	2.17 (1.90 to 2.47)	1.58 (1.39 to 1.80)

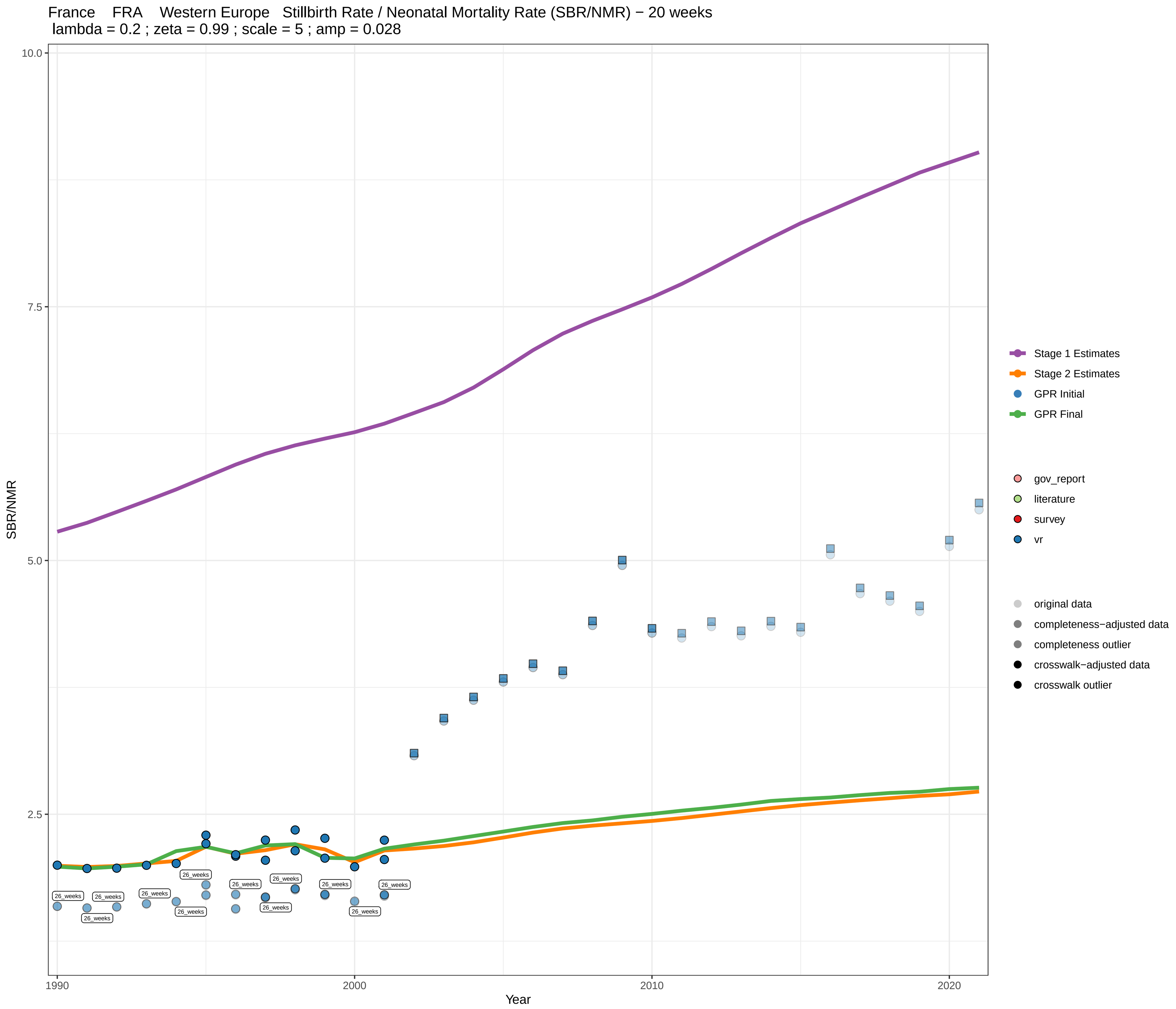

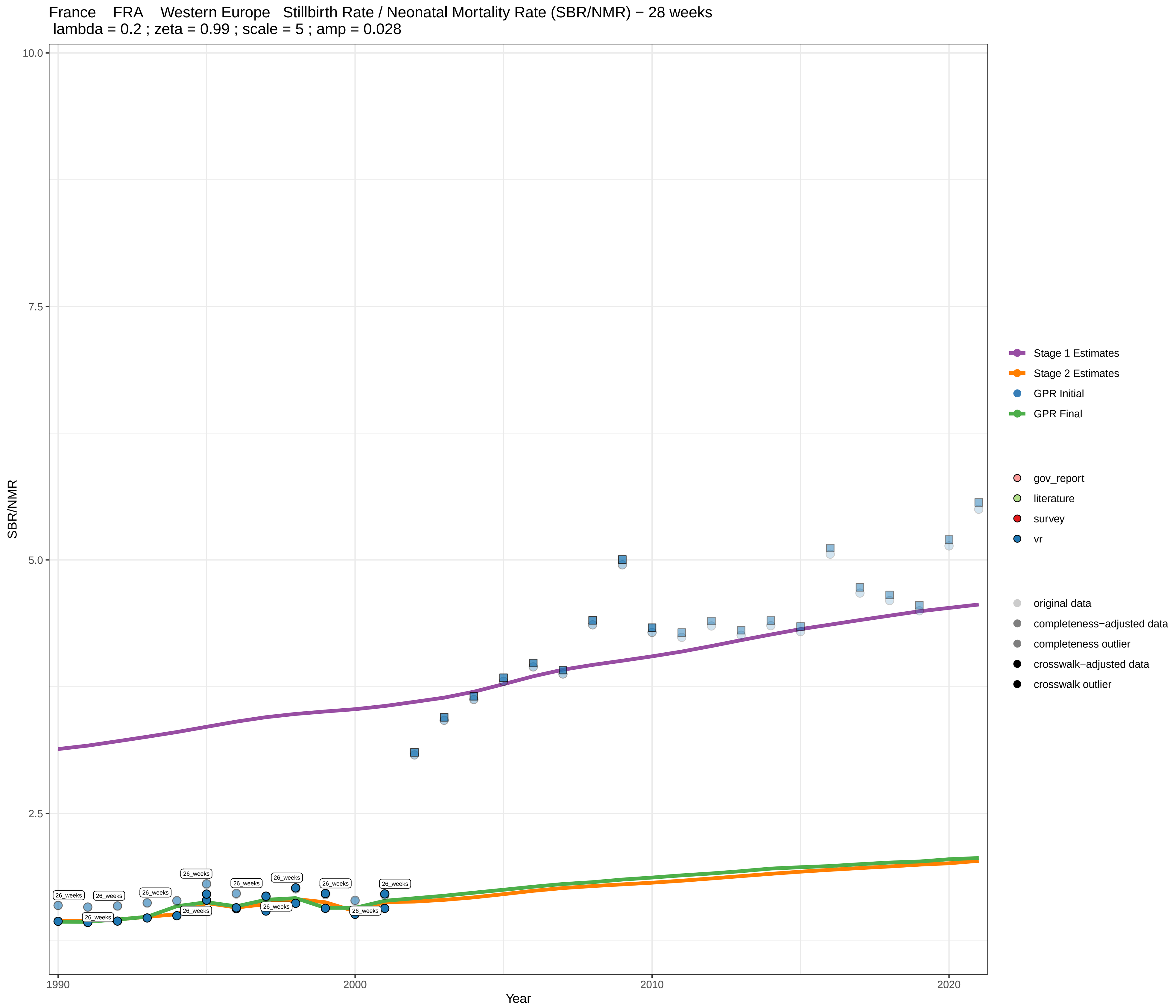

Definition	Years	Outlier	Source.Type	Title
26_weeks	1990−2001	0	VR	France Births and Stillbirths According to Child Sex
28_weeks	1995−1999, 2001	0	VR	United Nations Demographic Yearbook
22_weeks_OR_500_grams	2002−2008	1	VR	France Births and Stillbirths According to Child Sex
500_grams	2002−2010	1	VR	United Nations Demographic Yearbook
other	2009−2021	1	VR	France Births and Stillbirths According to Child Sex

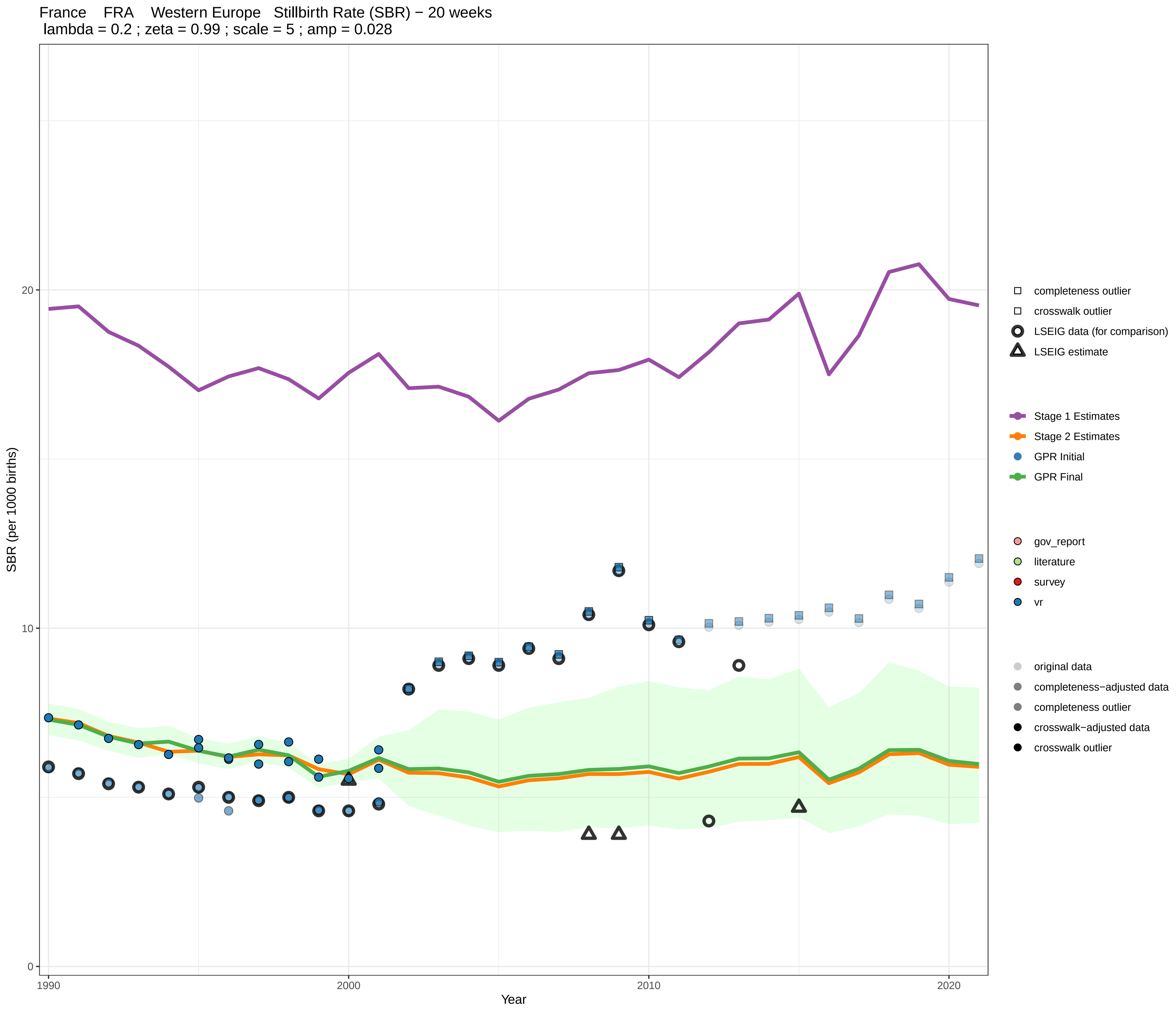

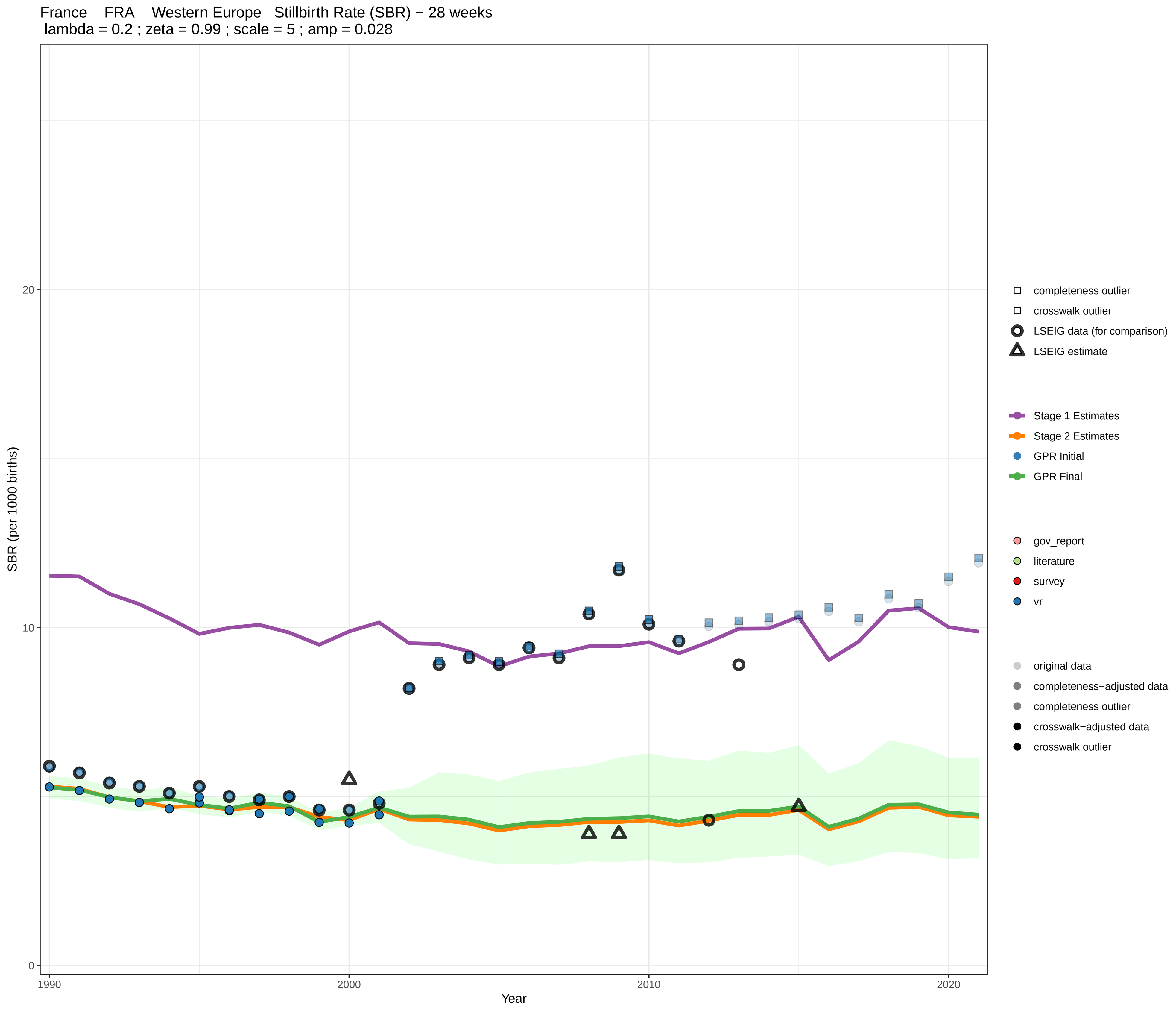

Year	Total Stillbirths (in thousands)	Total Stillbirths (in thousands)	SBR (per 1000 births)	SBR (per 1000 births)	SBR/NMR	SBR/NMR
	20 weeks	28 weeks	20 weeks	28 weeks	20 weeks	28 weeks
1990	5.6 (5.2 to 6.0)	4.0 (3.8 to 4.3)	7.3 (6.8 to 7.8)	5.3 (4.9 to 5.6)	1.98 (1.86 to 2.11)	1.43 (1.34 to 1.53)
1995	4.7 (4.4 to 5.0)	3.5 (3.3 to 3.7)	6.4 (6.0 to 6.8)	4.8 (4.5 to 5.0)	2.18 (2.05 to 2.31)	1.62 (1.53 to 1.72)
2000	4.5 (4.2 to 4.8)	3.4 (3.2 to 3.6)	5.8 (5.4 to 6.1)	4.4 (4.1 to 4.7)	2.06 (1.94 to 2.19)	1.57 (1.47 to 1.67)
2005	4.3 (3.1 to 5.8)	3.2 (2.4 to 4.3)	5.5 (4.0 to 7.3)	4.1 (3.0 to 5.5)	2.33 (1.69 to 3.11)	1.75 (1.27 to 2.33)
2010	4.8 (3.3 to 6.8)	3.5 (2.5 to 5.0)	5.9 (4.2 to 8.4)	4.4 (3.1 to 6.3)	2.50 (1.76 to 3.57)	1.87 (1.32 to 2.66)
2015	4.8 (3.3 to 6.7)	3.6 (2.5 to 5.0)	6.3 (4.4 to 8.8)	4.7 (3.3 to 6.5)	2.65 (1.84 to 3.68)	1.97 (1.37 to 2.73)
2020	4.3 (2.9 to 5.8)	3.2 (2.2 to 4.3)	6.1 (4.2 to 8.3)	4.5 (3.1 to 6.1)	2.75 (1.90 to 3.74)	2.05 (1.42 to 2.78)
2021	4.2 (3.0 to 5.8)	3.1 (2.2 to 4.3)	6.0 (4.2 to 8.2)	4.5 (3.2 to 6.1)	2.76 (1.96 to 3.81)	2.06 (1.47 to 2.83)

Germany DEU Western Europe Stillbirth Rate / Neonatal Mortality Rate (SBR/NMR) − 20 weeks
lambda = 0.2 ; zeta = 0.99 ; scale = 5 ; amp = 0.028

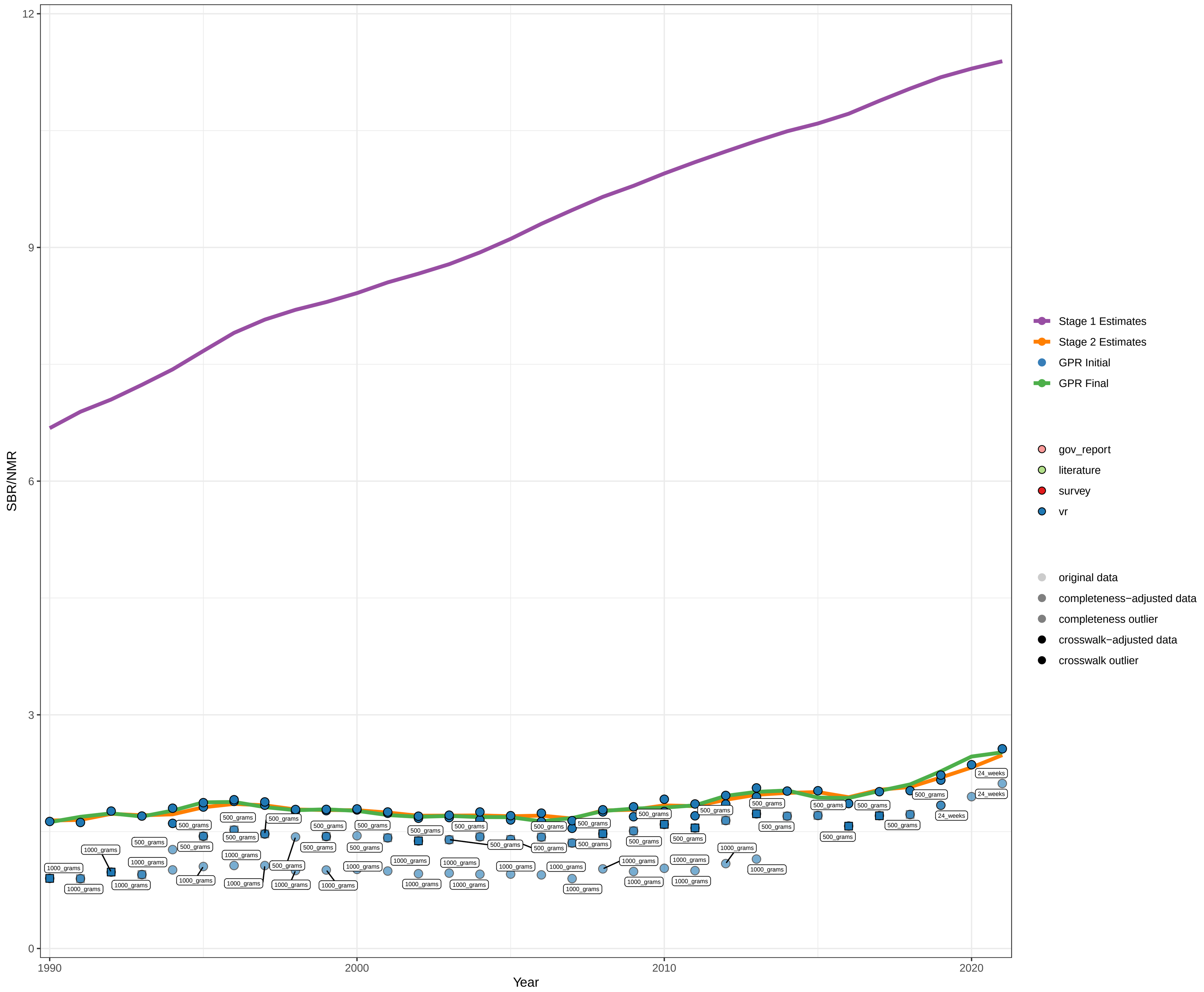

Germany DEU Western Europe Stillbirth Rate / Neonatal Mortality Rate (SBR/NMR) − 28 weeks
lambda = 0.2 ; zeta = 0.99 ; scale = 5 ; amp = 0.028

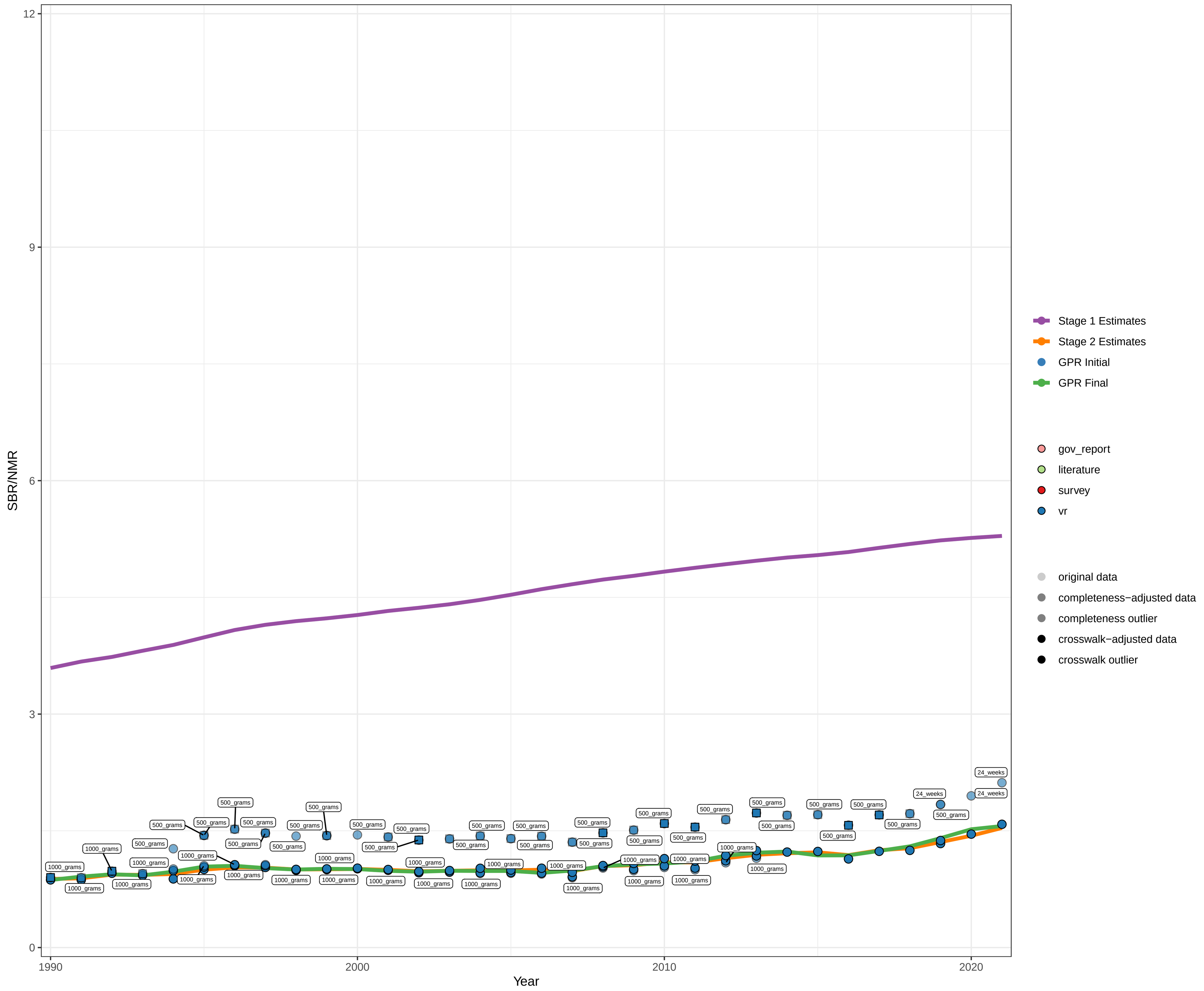

Definition	Years	Outlier	Source.Type	Title
1000_grams	1990, 1992, 1994−2013	0	VR	European Health for All Database
1000_grams	1990−1993	0	VR	Germany Stillbirths per 1000 Live and Stillborn
24_weeks	2019−2021	0	VR	Germany Stillbirths per 1000 Live and Stillborn
500_grams	1994−2018	0	VR	Germany Stillbirths per 1000 Live and Stillborn
500_grams	2002, 2008, 2010, 2011, 2013, 2016, 2017, 2019	0	VR	United Nations Demographic Yearbook
1000_grams	1991, 1993	1	VR	European Health for All Database
500_grams	1995−1997, 1999, 2001, 2003−2007, 2009, 2012, 2014, 2015, 2018	1	VR	United Nations Demographic Yearbook

Germany DEU Western Europe Stillbirth Rate (SBR) − 20 weeks
lambda = 0.2 ; zeta = 0.99 ; scale = 5 ; amp = 0.028

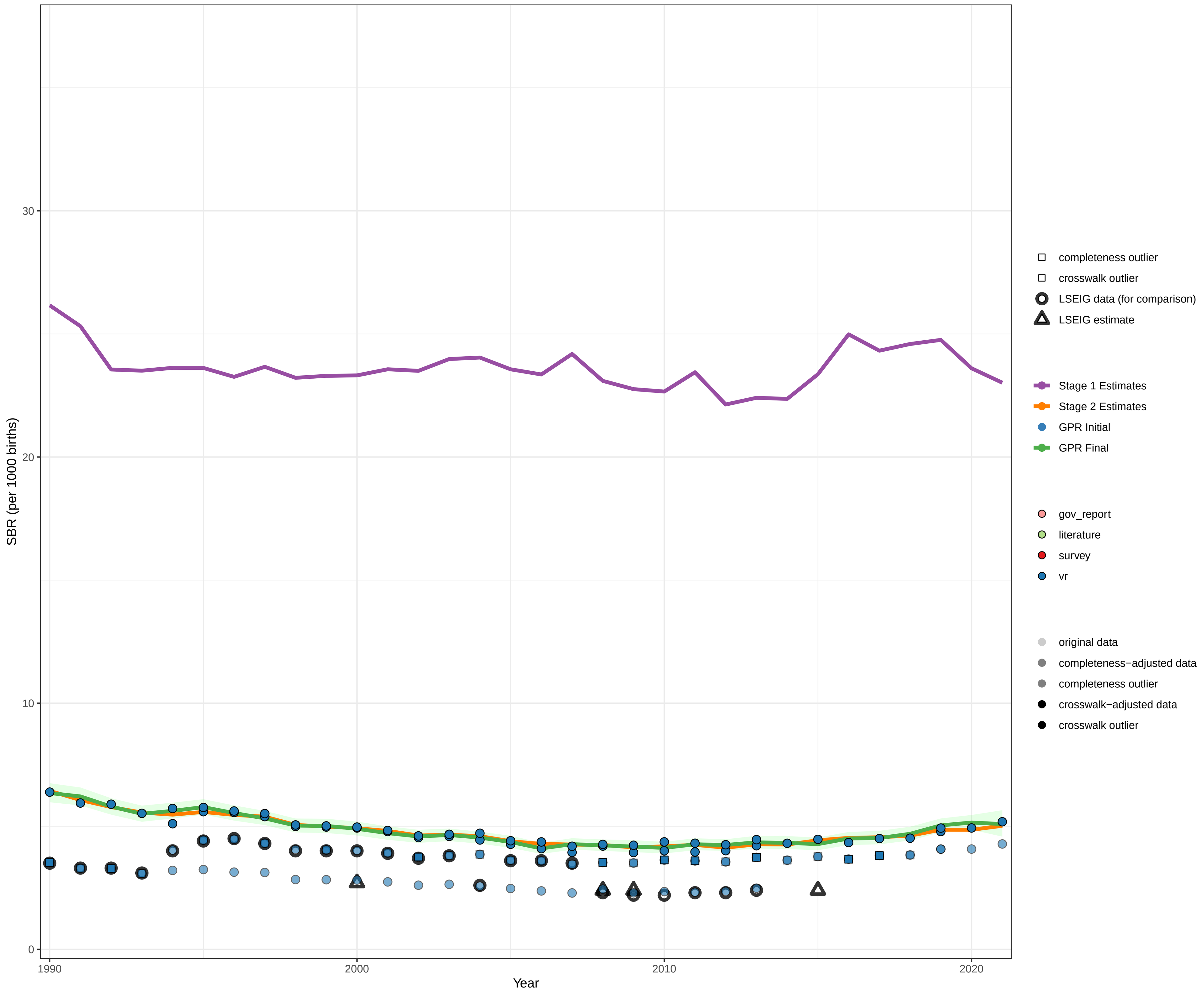

Germany DEU Western Europe Stillbirth Rate (SBR) − 28 weeks
lambda = 0.2 ; zeta = 0.99 ; scale = 5 ; amp = 0.028

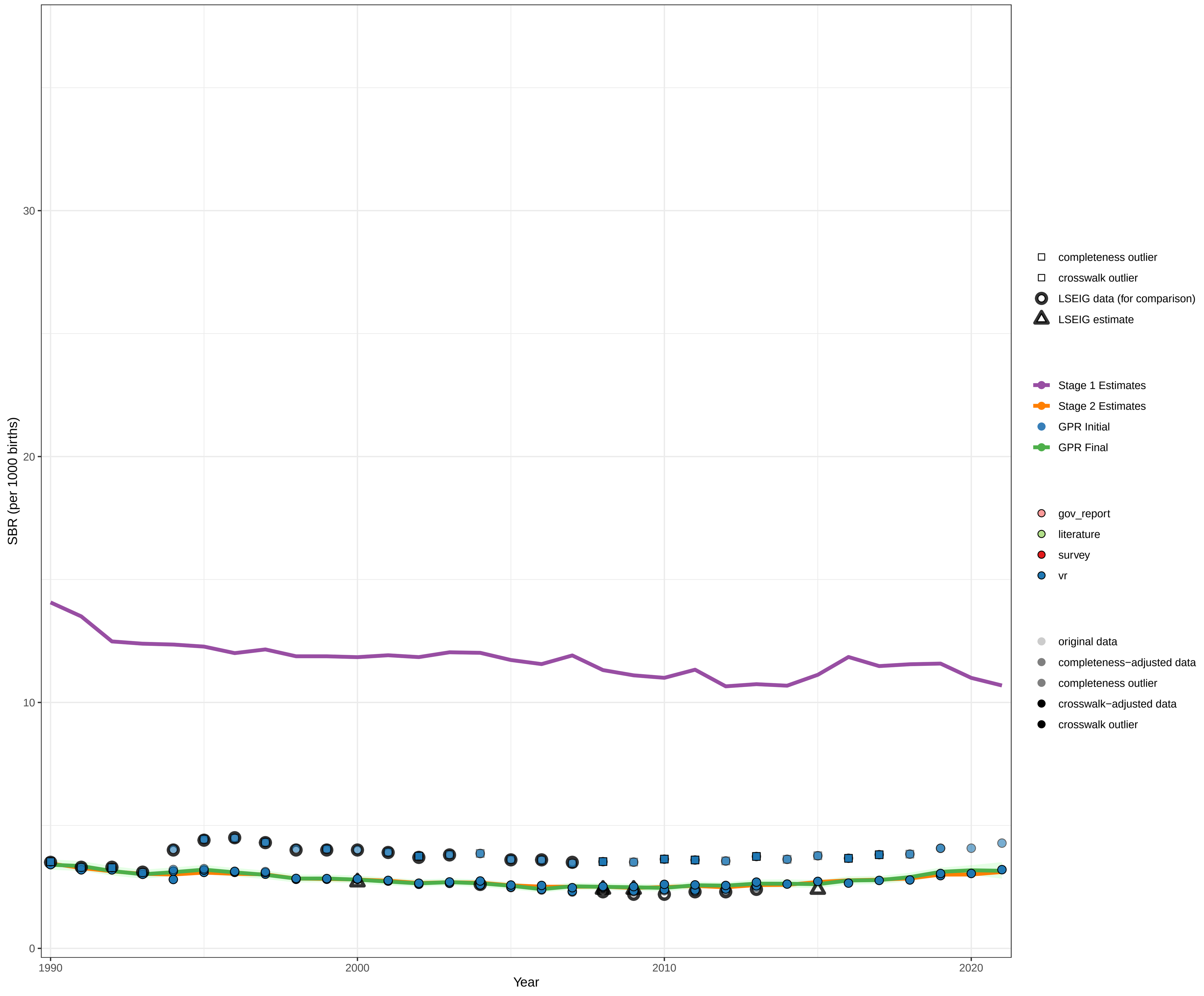

Year	Total Stillbirths (in thousands)	Total Stillbirths (in thousands)	SBR (per 1000 births)	SBR (per 1000 births)	SBR/NMR	SBR/NMR
	20 weeks	28 weeks	20 weeks	28 weeks	20 weeks	28 weeks
1990	5.5<br>(5.2 to 5.9)	2.9<br>(2.8 to 3.1)	6.4<br>(6.0 to 6.7)	3.4<br>(3.2 to 3.6)	1.62<br>(1.53 to 1.72)	0.87<br>(0.82 to 0.92)
1995	4.6<br>(4.3 to 4.8)	2.5<br>(2.4 to 2.7)	5.8<br>(5.5 to 6.1)	3.2<br>(3.0 to 3.4)	1.88<br>(1.78 to 1.98)	1.04<br>(0.98 to 1.10)
2000	3.7<br>(3.5 to 3.9)	2.1<br>(2.0 to 2.2)	4.9<br>(4.6 to 5.2)	2.8<br>(2.6 to 3.0)	1.77<br>(1.67 to 1.87)	1.01<br>(0.95 to 1.07)
2005	3.0<br>(2.8 to 3.2)	1.8<br>(1.7 to 1.9)	4.4<br>(4.1 to 4.6)	2.5<br>(2.4 to 2.7)	1.69<br>(1.59 to 1.78)	0.98<br>(0.93 to 1.04)
2010	2.8<br>(2.6 to 2.9)	1.7<br>(1.6 to 1.8)	4.1<br>(3.9 to 4.4)	2.5<br>(2.3 to 2.6)	1.81<br>(1.71 to 1.92)	1.08<br>(1.02 to 1.15)
2015	3.3<br>(3.1 to 3.5)	2.0<br>(1.9 to 2.1)	4.3<br>(4.0 to 4.5)	2.6<br>(2.5 to 2.8)	1.94<br>(1.83 to 2.06)	1.18<br>(1.11 to 1.26)
2020	4.1<br>(3.8 to 4.3)	2.5<br>(2.3 to 2.7)	5.1<br>(4.9 to 5.5)	3.2<br>(3.0 to 3.4)	2.46<br>(2.33 to 2.61)	1.52<br>(1.43 to 1.62)
2021	4.0<br>(3.6 to 4.5)	2.5<br>(2.2 to 2.8)	5.1<br>(4.6 to 5.7)	3.2<br>(2.8 to 3.5)	2.52<br>(2.27 to 2.80)	1.56<br>(1.40 to 1.74)

Greece GRC Western Europe Stillbirth Rate / Neonatal Mortality Rate (SBR/NMR) − 20 weeks
lambda = 0.2 ; zeta = 0.99 ; scale = 5 ; amp = 0.028

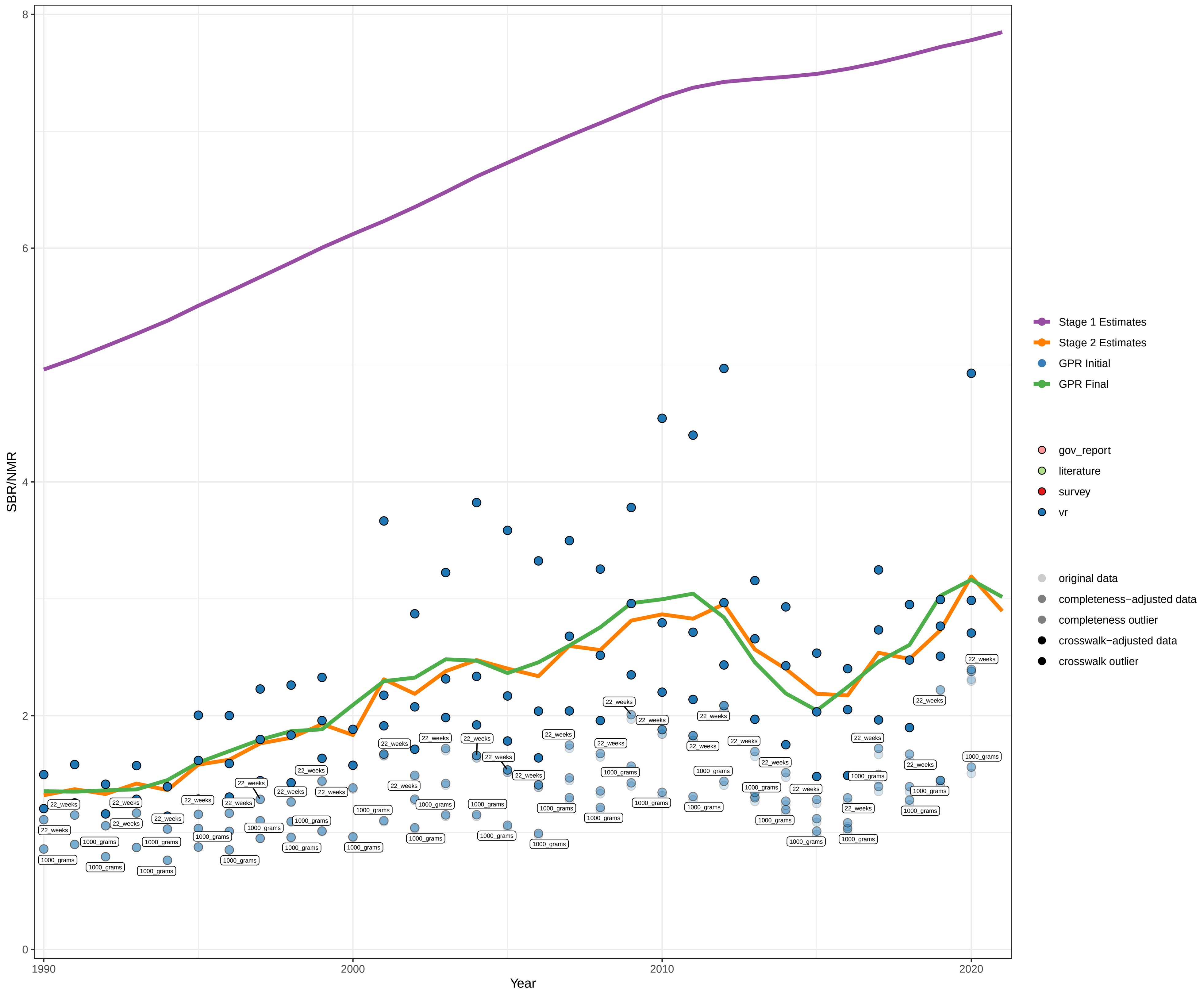

Greece GRC Western Europe Stillbirth Rate / Neonatal Mortality Rate (SBR/NMR) − 28 weeks
lambda = 0.2 ; zeta = 0.99 ; scale = 5 ; amp = 0.028

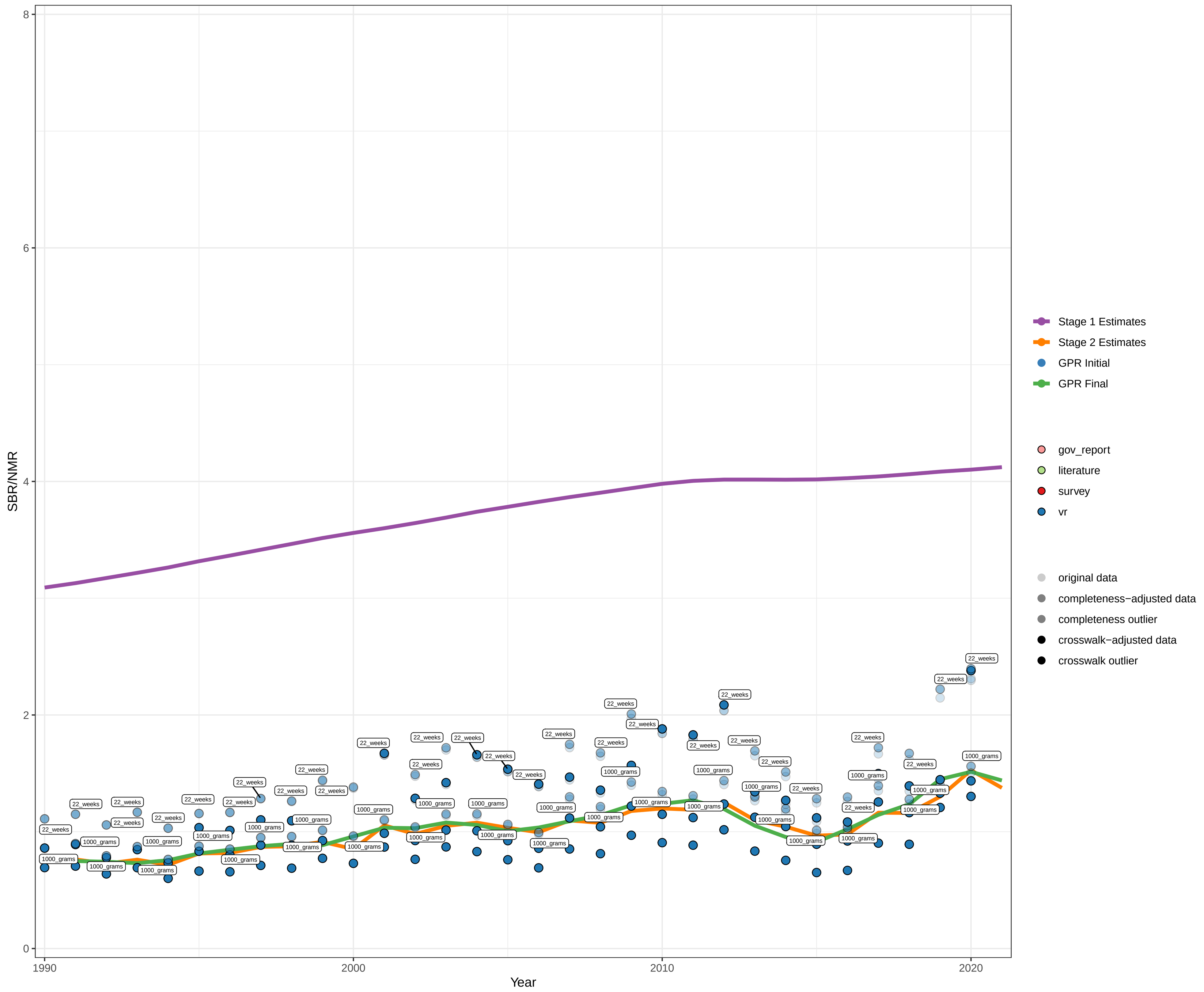

Definition	Years	Outlier	Source.Type	Title
1000_grams	1990−2020	0	VR	European Health for All Database
22_weeks	1990−2020	0	VR	Greece Infant Deaths and Stillbirths 1956−
28_weeks	1995−1999, 2001−2020	0	VR	United Nations Demographic Yearbook

Greece GRC Western Europe Stillbirth Rate (SBR) − 20 weeks
lambda = 0.2 ; zeta = 0.99 ; scale = 5 ; amp = 0.028

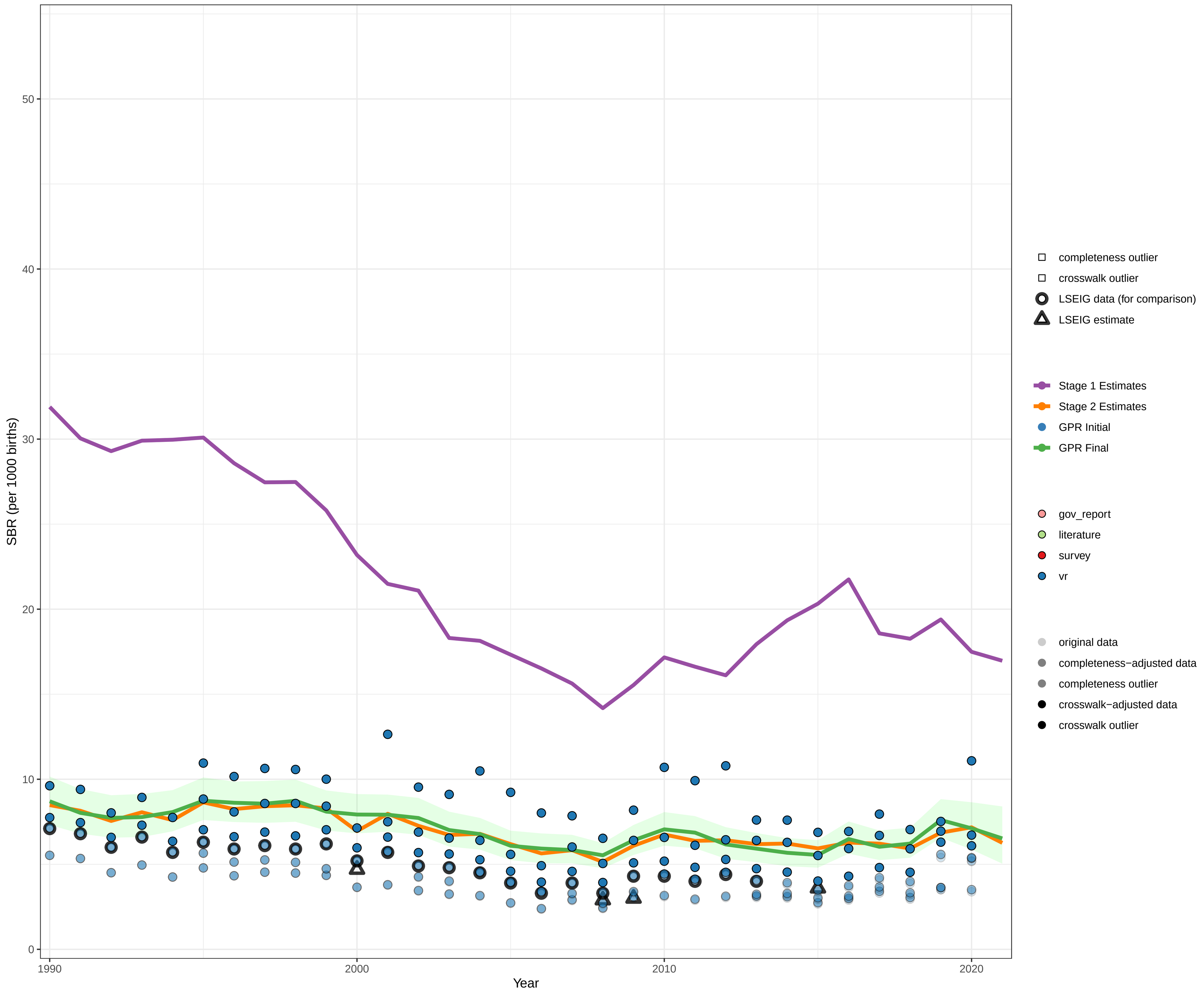

Greece GRC Western Europe Stillbirth Rate (SBR) − 28 weeks
lambda = 0.2 ; zeta = 0.99 ; scale = 5 ; amp = 0.028

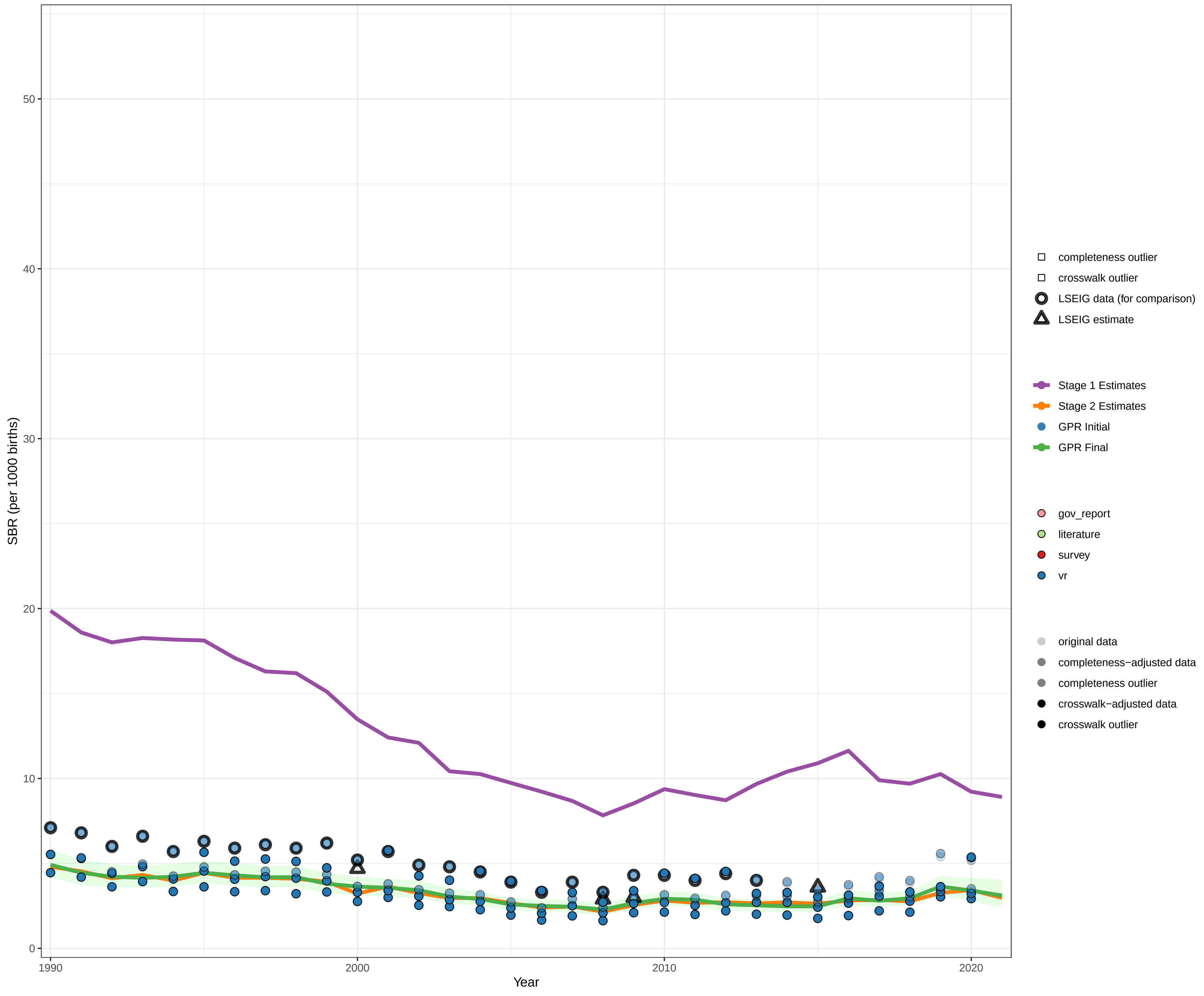

Year	Total Stillbirths (in thousands)	Total Stillbirths (in thousands)	SBR (per 1000 births)	SBR (per 1000 births)	SBR/NMR	SBR/NMR
	20 weeks	28 weeks	20 weeks	28 weeks	20 weeks	28 weeks
1990	0.9 (0.8 to 1.0)	0.5 (0.4 to 0.6)	8.7 (7.3 to 10.2)	4.9 (4.2 to 5.8)	1.35 (1.14 to 1.58)	0.77 (0.65 to 0.90)
1995	0.9 (0.8 to 1.0)	0.5 (0.4 to 0.5)	8.7 (7.6 to 10.1)	4.4 (3.8 to 5.2)	1.60 (1.39 to 1.85)	0.81 (0.70 to 0.94)
2000	0.8 (0.7 to 0.9)	0.4 (0.3 to 0.4)	7.9 (6.8 to 9.1)	3.6 (3.1 to 4.2)	2.09 (1.80 to 2.41)	0.96 (0.82 to 1.11)
2005	0.7 (0.6 to 0.8)	0.3 (0.2 to 0.3)	6.1 (5.2 to 7.0)	2.6 (2.2 to 3.0)	2.36 (2.04 to 2.71)	1.00 (0.86 to 1.15)
2010	0.8 (0.7 to 0.9)	0.3 (0.3 to 0.4)	7.1 (6.1 to 8.1)	2.9 (2.5 to 3.3)	3.00 (2.59 to 3.43)	1.24 (1.07 to 1.42)
2015	0.5 (0.4 to 0.6)	0.2 (0.2 to 0.3)	5.5 (4.8 to 6.4)	2.5 (2.1 to 2.9)	2.04 (1.77 to 2.37)	0.91 (0.79 to 1.06)
2020	0.6 (0.5 to 0.7)	0.3 (0.2 to 0.3)	7.1 (5.9 to 8.6)	3.4 (2.8 to 4.1)	3.16 (2.61 to 3.85)	1.51 (1.23 to 1.84)
2021	0.5 (0.4 to 0.7)	0.3 (0.2 to 0.3)	6.5 (5.0 to 8.4)	3.1 (2.4 to 4.0)	3.02 (2.33 to 3.89)	1.44 (1.11 to 1.87)

Iceland ISL Western Europe Stillbirth Rate / Neonatal Mortality Rate (SBR/NMR) − 20 weeks
lambda = 0.2 ; zeta = 0.99 ; scale = 5 ; amp = 0.028

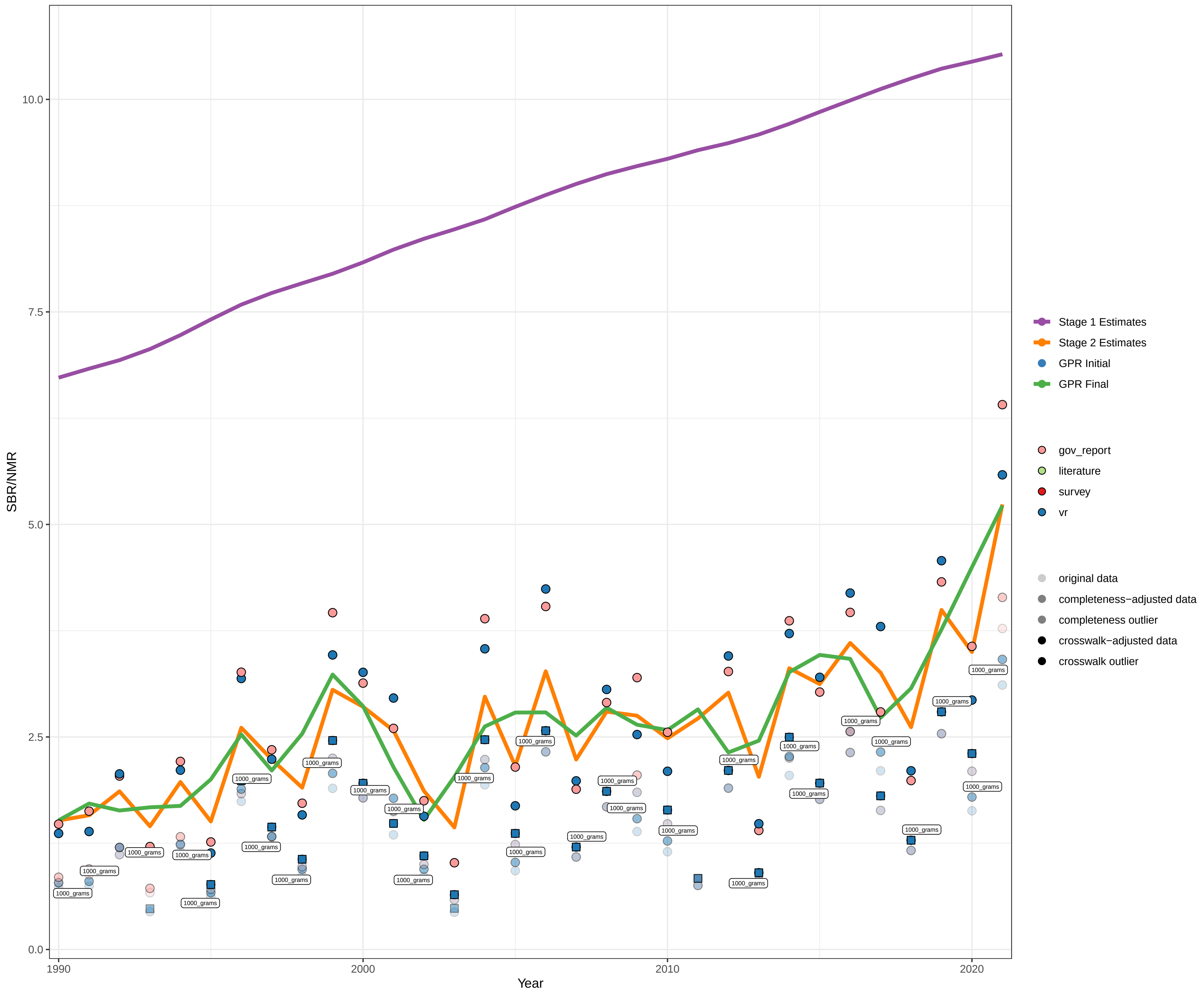

Iceland ISL Western Europe Stillbirth Rate / Neonatal Mortality Rate (SBR/NMR) − 28 weeks
lambda = 0.2 ; zeta = 0.99 ; scale = 5 ; amp = 0.028

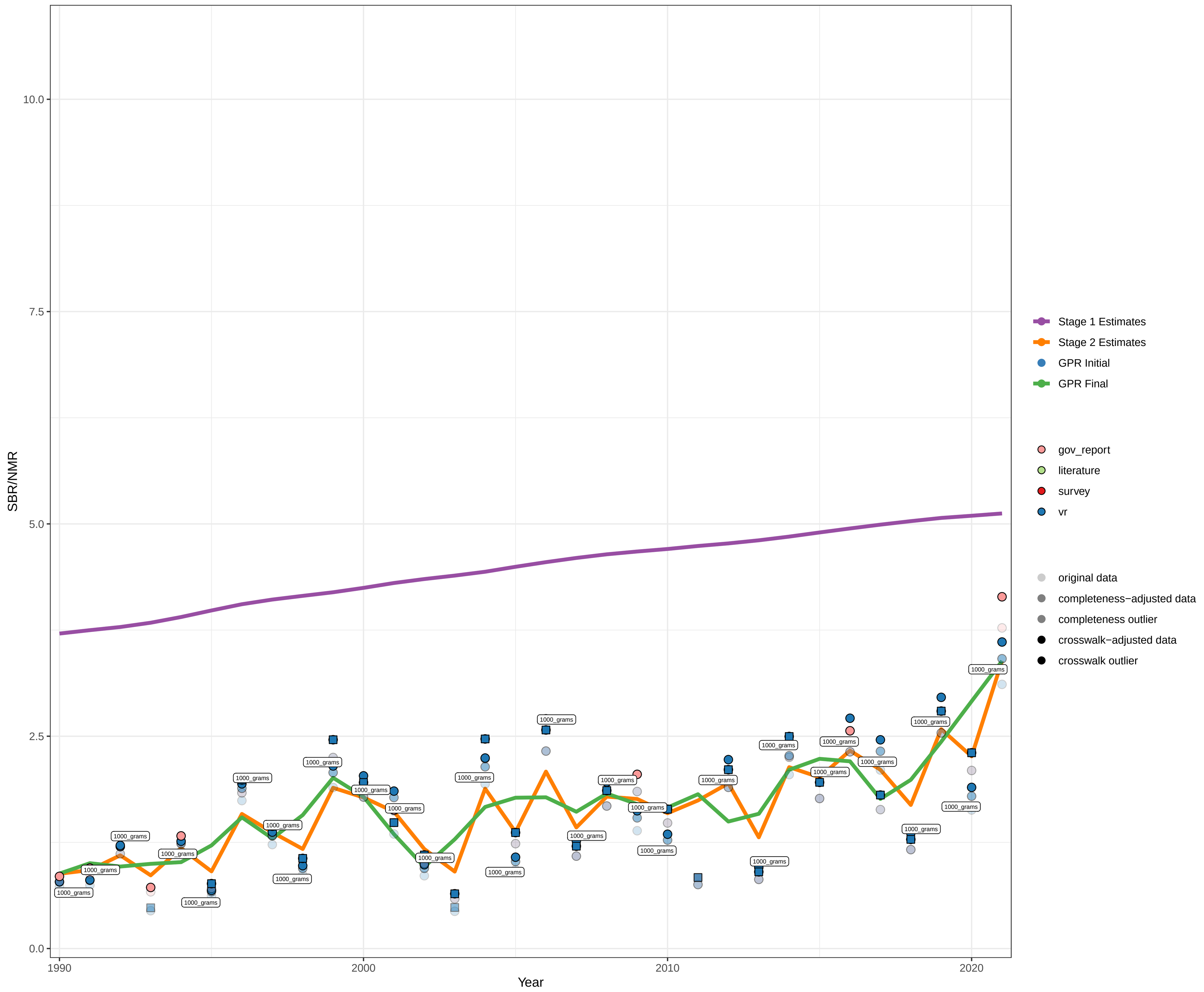

Definition	Years	Outlier	Source.Type	Title
1000_grams	1990−1992, 1994−2002, 2004−2010, 2012−2021	0	VR	European Health for All Database
28_weeks	1990−2010, 2012−2021	0	Statistical Report	Iceland Infant Mortality and Late Fetal Deaths
28_weeks	2009, 2016	0	VR	United Nations Demographic Yearbook
1000_grams	1993, 2003, 2011	1	VR	European Health for All Database
28_weeks	2011	1	Statistical Report	Iceland Infant Mortality and Late Fetal Deaths
28_weeks	2011	1	VR	United Nations Demographic Yearbook
28_weeks	1995−2008, 2010, 2012−2015, 2017−2020	2	VR	United Nations Demographic Yearbook

Iceland ISL Western Europe Stillbirth Rate (SBR) − 20 weeks
lambda = 0.2 ; zeta = 0.99 ; scale = 5 ; amp = 0.028

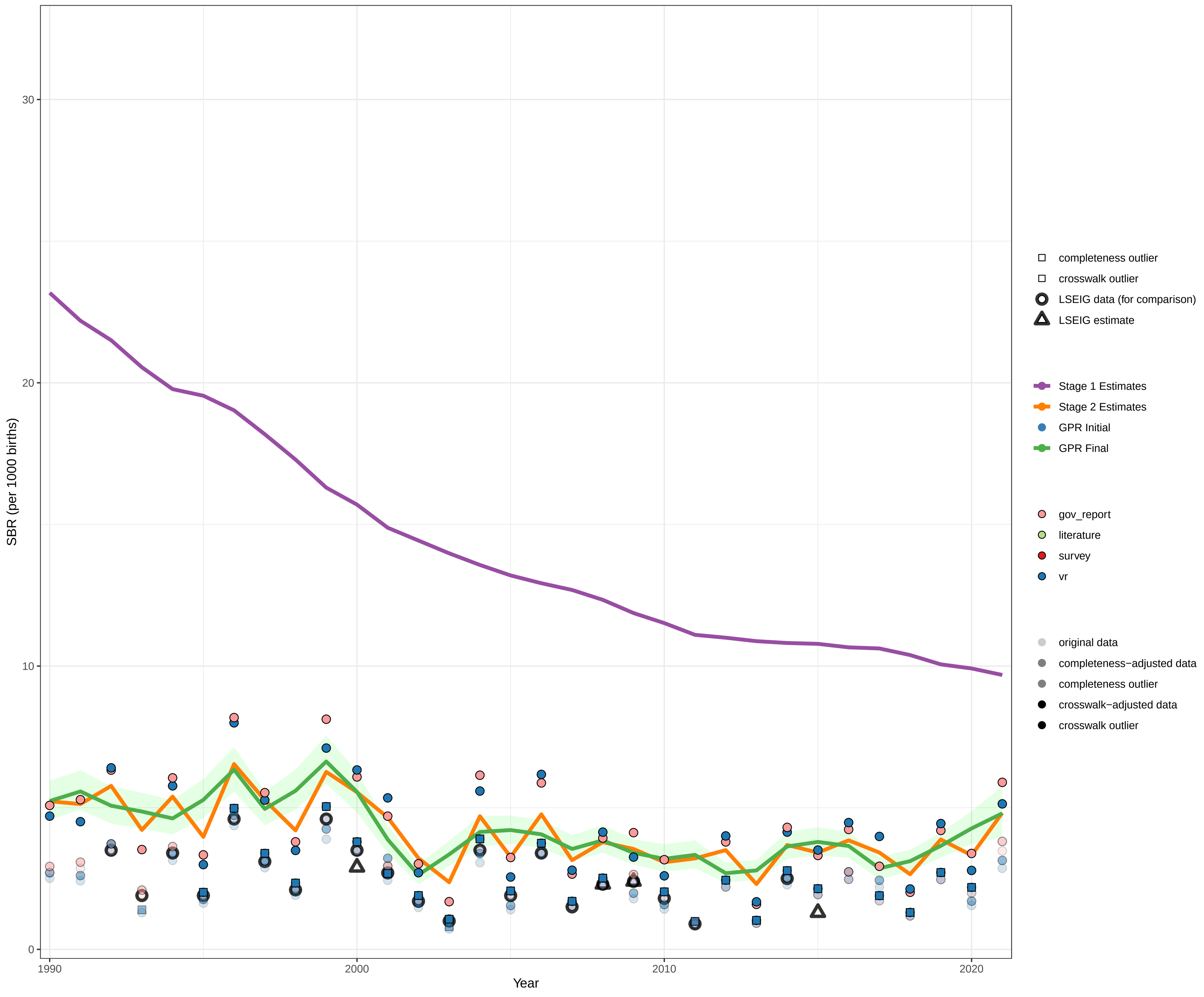

Iceland ISL Western Europe Stillbirth Rate (SBR) − 28 weeks
lambda = 0.2 ; zeta = 0.99 ; scale = 5 ; amp = 0.028

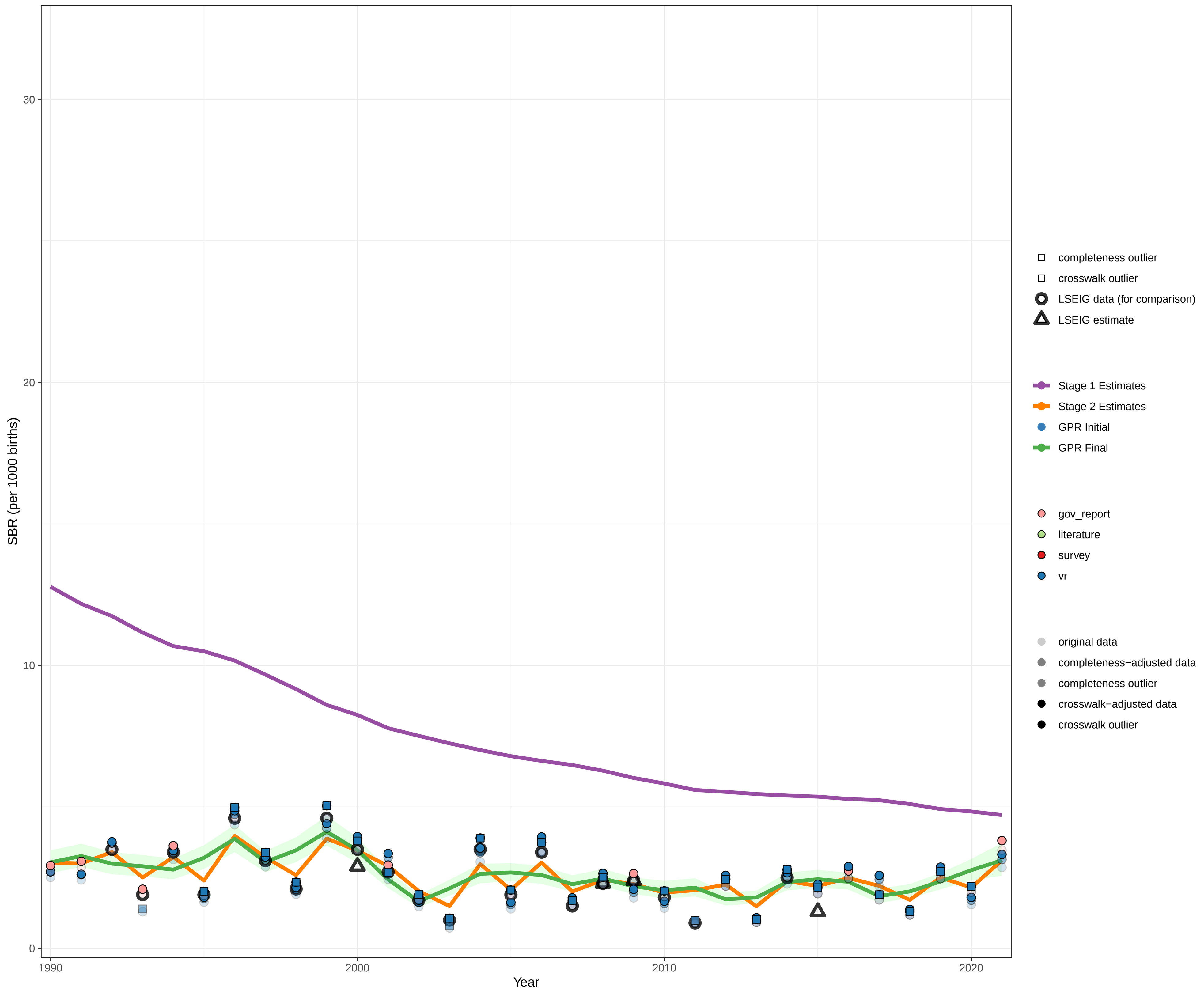

Year	Total Stillbirths (in thousands)	Total Stillbirths (in thousands)	SBR (per 1000 births)	SBR (per 1000 births)	SBR/NMR	SBR/NMR
	20 weeks	28 weeks	20 weeks	28 weeks	20 weeks	28 weeks
1990	0.0 (0.0 to 0.0)	0.0 (0.0 to 0.0)	5.2 (4.6 to 6.0)	3.0 (2.7 to 3.5)	1.52 (1.33 to 1.73)	0.88 (0.77 to 1.01)
1995	0.0 (0.0 to 0.0)	0.0 (0.0 to 0.0)	5.3 (4.6 to 6.0)	3.2 (2.8 to 3.6)	2.00 (1.76 to 2.28)	1.21 (1.07 to 1.38)
2000	0.0 (0.0 to 0.0)	0.0 (0.0 to 0.0)	5.6 (4.8 to 6.3)	3.5 (3.0 to 3.9)	2.86 (2.49 to 3.23)	1.79 (1.55 to 2.02)
2005	0.0 (0.0 to 0.0)	0.0 (0.0 to 0.0)	4.2 (3.7 to 4.7)	2.7 (2.4 to 3.0)	2.79 (2.47 to 3.13)	1.78 (1.57 to 1.99)
2010	0.0 (0.0 to 0.0)	0.0 (0.0 to 0.0)	3.2 (2.7 to 3.7)	2.1 (1.8 to 2.4)	2.58 (2.21 to 3.00)	1.66 (1.42 to 1.93)
2015	0.0 (0.0 to 0.0)	0.0 (0.0 to 0.0)	3.8 (3.3 to 4.3)	2.4 (2.1 to 2.8)	3.46 (3.03 to 3.94)	2.23 (1.95 to 2.54)
2020	0.0 (0.0 to 0.0)	0.0 (0.0 to 0.0)	4.3 (3.7 to 4.9)	2.8 (2.4 to 3.2)	4.50 (3.91 to 5.14)	2.91 (2.53 to 3.33)
2021	0.0 (0.0 to 0.0)	0.0 (0.0 to 0.0)	4.8 (4.0 to 5.7)	3.1 (2.6 to 3.7)	5.22 (4.31 to 6.25)	3.39 (2.80 to 4.05)

Ireland IRL Western Europe Stillbirth Rate / Neonatal Mortality Rate (SBR/NMR) − 20 weeks
lambda = 0.2 ; zeta = 0.99 ; scale = 5 ; amp = 0.028

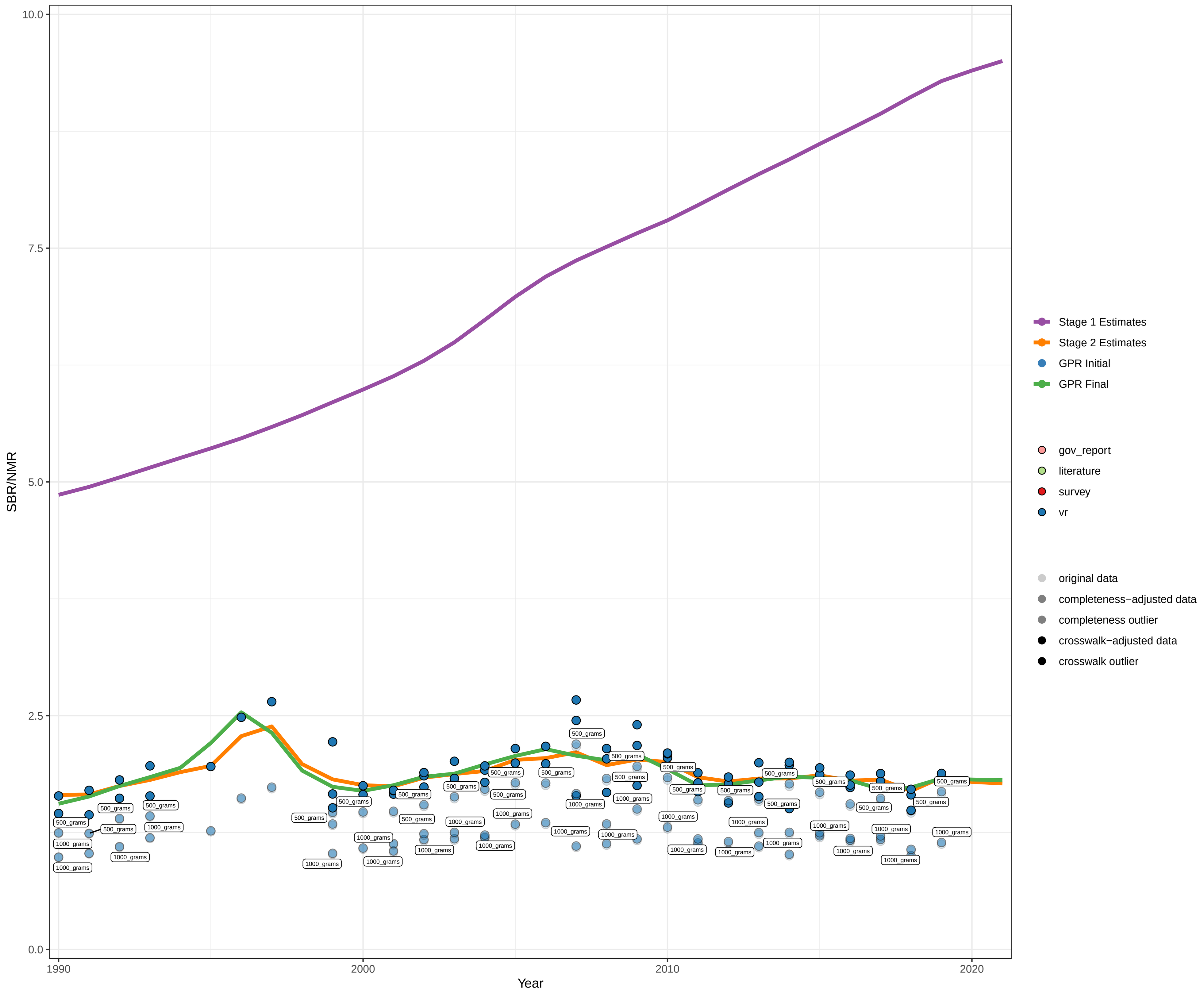

Ireland IRL Western Europe Stillbirth Rate / Neonatal Mortality Rate (SBR/NMR) − 28 weeks
lambda = 0.2 ; zeta = 0.99 ; scale = 5 ; amp = 0.028

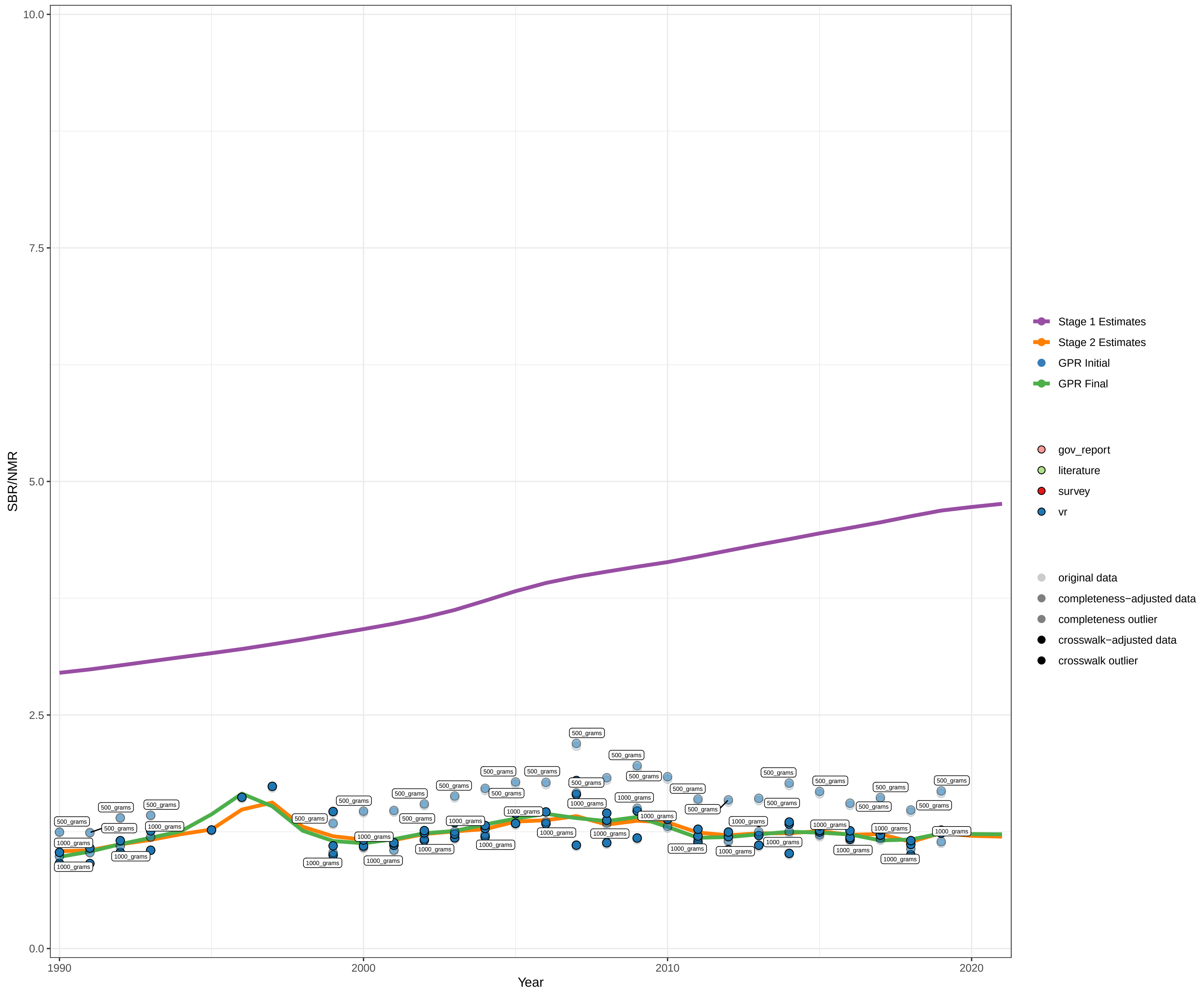

Definition	Years	Outlier	Source.Type	Title
1000_grams	1990−1993, 1999−2019	0	VR	European Health for All Database
28_weeks	1995−1997, 1999, 2001−2004, 2007−2018	0	VR	United Nations Demographic Yearbook
500_grams	1990−1993, 1999−2019	0	VR	Ireland Perinatal Statistics Report

Ireland IRL Western Europe Stillbirth Rate (SBR) − 20 weeks
lambda = 0.2 ; zeta = 0.99 ; scale = 5 ; amp = 0.028

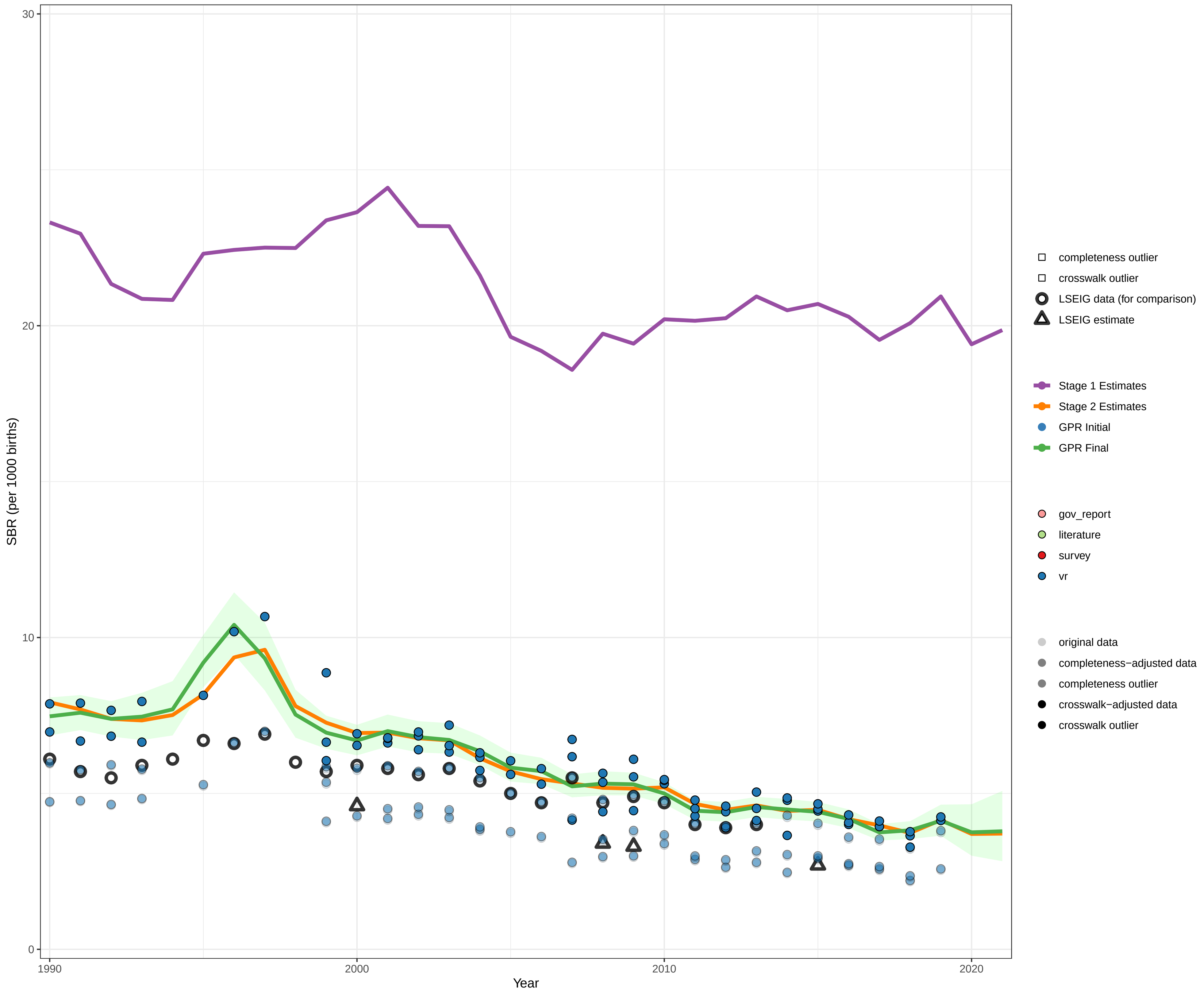

Ireland IRL Western Europe Stillbirth Rate (SBR) − 28 weeks
lambda = 0.2 ; zeta = 0.99 ; scale = 5 ; amp = 0.028

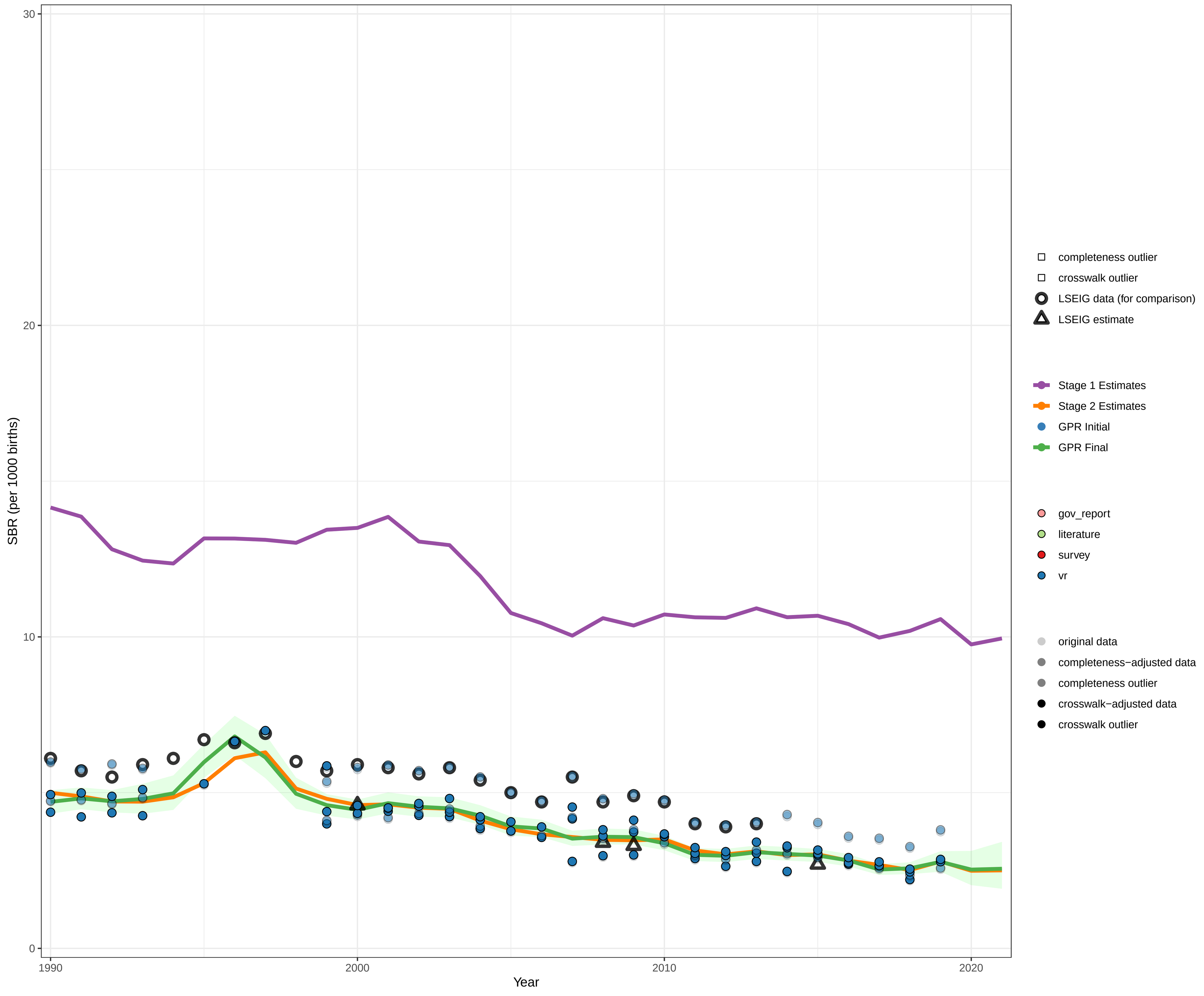

Year	Total Stillbirths (in thousands)	Total Stillbirths (in thousands)	SBR (per 1000 births)	SBR (per 1000 births)	SBR/NMR	SBR/NMR
	20 weeks	28 weeks	20 weeks	28 weeks	20 weeks	28 weeks
1990	0.4 (0.4 to 0.4)	0.3 (0.2 to 0.3)	7.5 (6.9 to 8.1)	4.7 (4.3 to 5.1)	1.56 (1.43 to 1.68)	0.98 (0.90 to 1.06)
1995	0.5 (0.4 to 0.5)	0.3 (0.3 to 0.3)	9.2 (8.4 to 10.1)	6.0 (5.5 to 6.6)	2.21 (2.02 to 2.42)	1.44 (1.31 to 1.57)
2000	0.4 (0.4 to 0.4)	0.3 (0.2 to 0.3)	6.7 (6.2 to 7.2)	4.4 (4.1 to 4.8)	1.70 (1.58 to 1.82)	1.13 (1.05 to 1.21)
2005	0.4 (0.3 to 0.4)	0.2 (0.2 to 0.3)	5.8 (5.4 to 6.3)	3.9 (3.6 to 4.2)	2.07 (1.91 to 2.24)	1.39 (1.29 to 1.50)
2010	0.4 (0.3 to 0.4)	0.3 (0.2 to 0.3)	5.0 (4.7 to 5.4)	3.4 (3.2 to 3.6)	1.93 (1.80 to 2.07)	1.30 (1.22 to 1.39)
2015	0.3 (0.3 to 0.3)	0.2 (0.2 to 0.2)	4.4 (4.1 to 4.7)	3.0 (2.8 to 3.2)	1.84 (1.71 to 1.97)	1.24 (1.15 to 1.33)
2020	0.2 (0.2 to 0.3)	0.1 (0.1 to 0.2)	3.8 (3.0 to 4.7)	2.5 (2.0 to 3.1)	1.82 (1.45 to 2.25)	1.23 (0.98 to 1.52)
2021	0.2 (0.2 to 0.3)	0.1 (0.1 to 0.2)	3.8 (2.8 to 5.1)	2.6 (1.9 to 3.4)	1.81 (1.35 to 2.43)	1.22 (0.92 to 1.64)

Israel ISR Western Europe Stillbirth Rate / Neonatal Mortality Rate (SBR/NMR) − 20 weeks
lambda = 0.2 ; zeta = 0.99 ; scale = 5 ; amp = 0.028

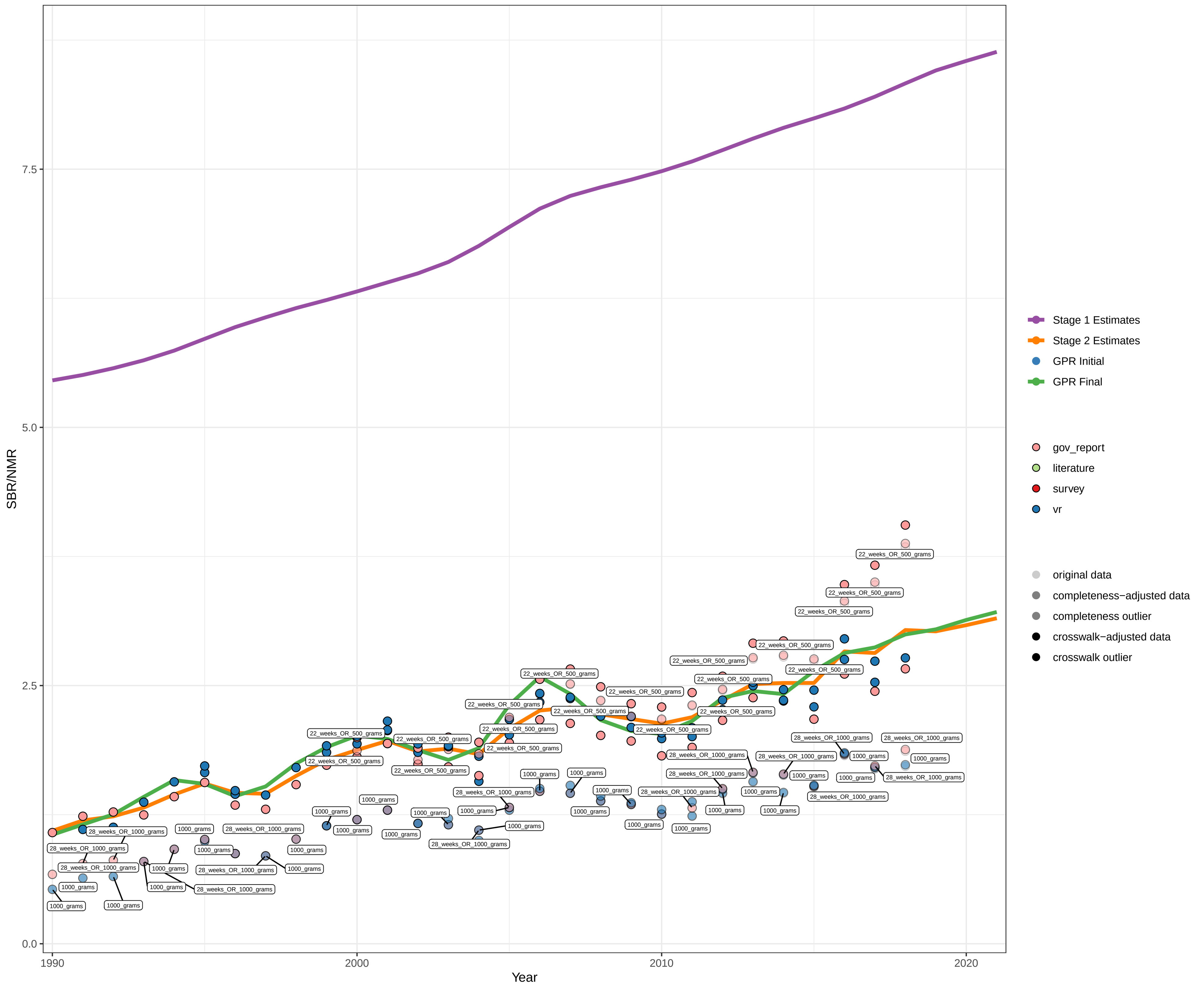

Israel ISR Western Europe Stillbirth Rate / Neonatal Mortality Rate (SBR/NMR) − 28 weeks
lambda = 0.2 ; zeta = 0.99 ; scale = 5 ; amp = 0.028

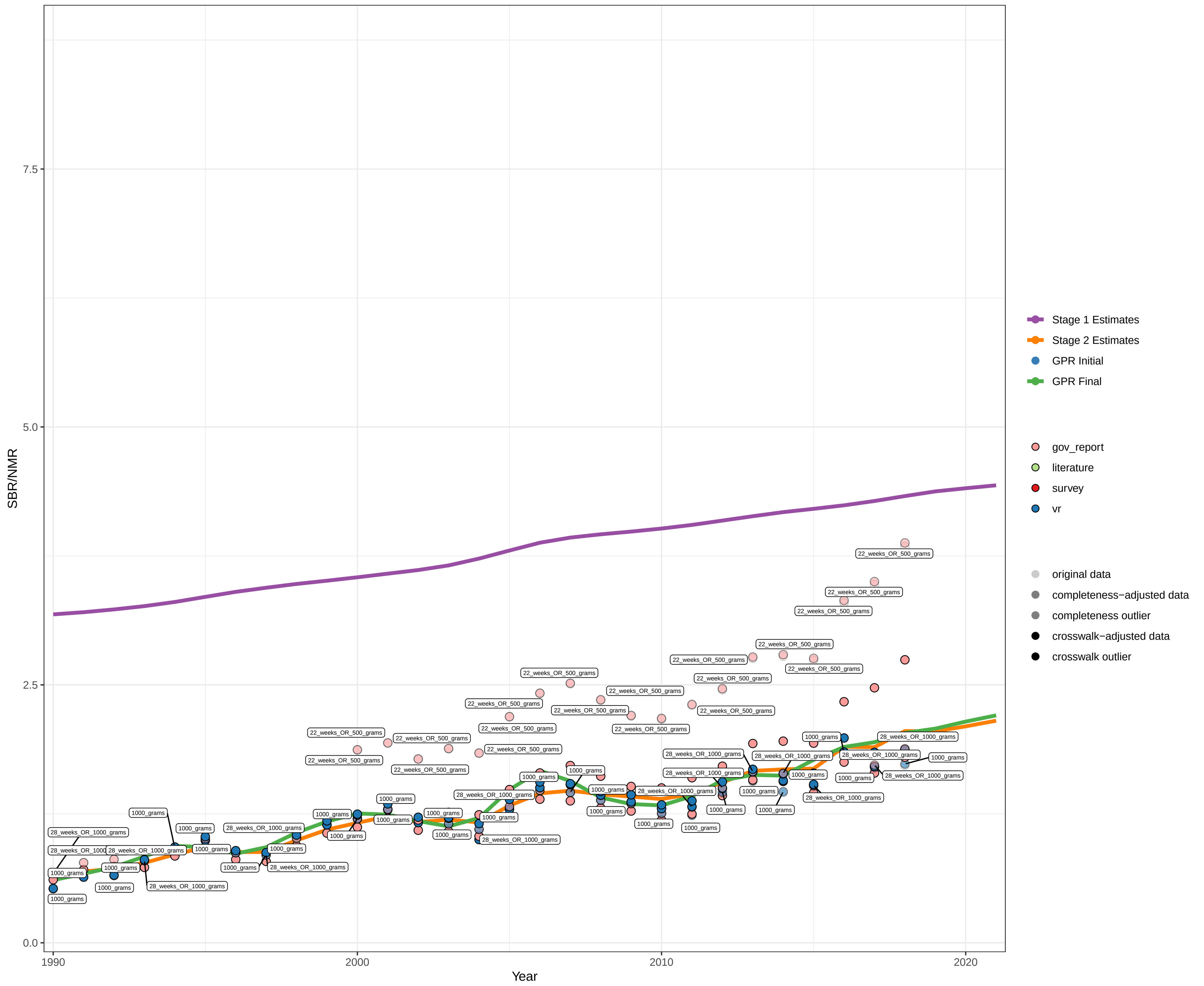

Definition	Years	Outlier	Source.Type	Title
1000_grams	1990−2018	0	VR	European Health for All Database
22_weeks_OR_500_grams	2000−2018	0	Statistical Report	Israel Statistical Abstract
28_weeks	1995, 1996, 1999−2017	0	VR	United Nations Demographic Yearbook
28_weeks_OR_1000_grams	1990−2018	0	Statistical Report	Israel Statistical Abstract

Israel ISR Western Europe Stillbirth Rate (SBR) − 20 weeks
lambda = 0.2 ; zeta = 0.99 ; scale = 5 ; amp = 0.028

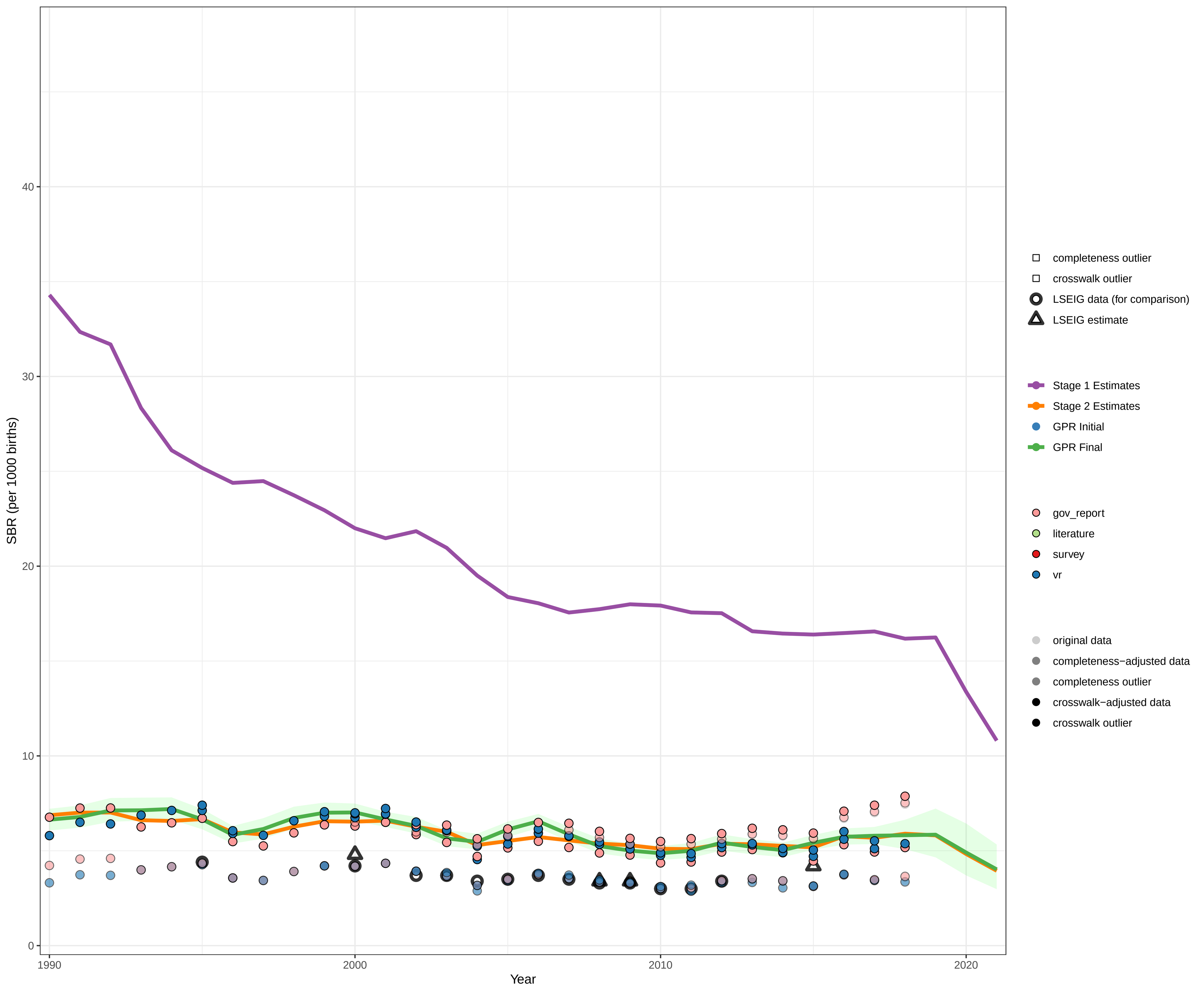

Israel ISR Western Europe Stillbirth Rate (SBR) − 28 weeks
lambda = 0.2 ; zeta = 0.99 ; scale = 5 ; amp = 0.028

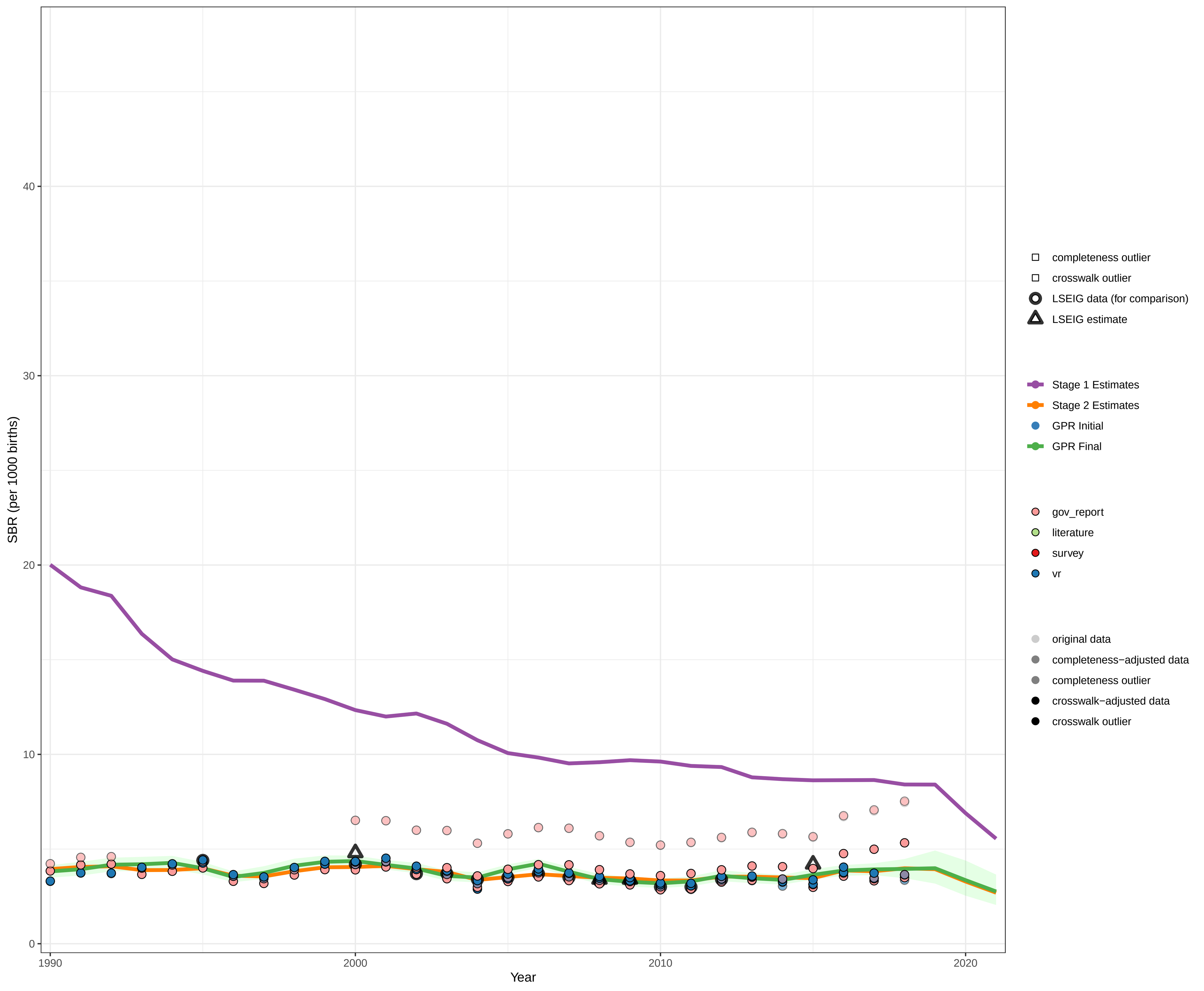

Year	Total Stillbirths (in thousands)	Total Stillbirths (in thousands)	SBR (per 1000 births)	SBR (per 1000 births)	SBR/NMR	SBR/NMR
	20 weeks	28 weeks	20 weeks	28 weeks	20 weeks	28 weeks
1990	0.7 (0.6 to 0.8)	0.4 (0.4 to 0.4)	6.6 (6.1 to 7.2)	3.8 (3.5 to 4.2)	1.06 (0.97 to 1.15)	0.61 (0.56 to 0.66)
1995	0.8 (0.7 to 0.9)	0.5 (0.4 to 0.5)	6.7 (6.1 to 7.2)	4.0 (3.7 to 4.3)	1.55 (1.43 to 1.68)	0.93 (0.85 to 1.00)
2000	1.0 (0.9 to 1.0)	0.6 (0.6 to 0.6)	7.0 (6.6 to 7.5)	4.4 (4.1 to 4.7)	2.02 (1.89 to 2.15)	1.25 (1.17 to 1.34)
2005	0.9 (0.8 to 1.0)	0.6 (0.5 to 0.6)	6.1 (5.7 to 6.5)	3.9 (3.7 to 4.2)	2.31 (2.16 to 2.47)	1.48 (1.39 to 1.58)
2010	0.8 (0.7 to 0.9)	0.5 (0.5 to 0.6)	4.9 (4.5 to 5.2)	3.2 (2.9 to 3.4)	2.03 (1.88 to 2.19)	1.33 (1.23 to 1.44)
2015	1.0 (0.9 to 1.1)	0.7 (0.6 to 0.7)	5.4 (5.1 to 5.8)	3.6 (3.4 to 3.9)	2.64 (2.46 to 2.84)	1.77 (1.65 to 1.92)
2020	0.9 (0.7 to 1.2)	0.6 (0.5 to 0.8)	4.9 (3.7 to 6.4)	3.4 (2.5 to 4.4)	3.14 (2.36 to 4.11)	2.14 (1.62 to 2.81)
2021	0.7 (0.5 to 1.0)	0.5 (0.4 to 0.7)	4.0 (3.0 to 5.3)	2.8 (2.0 to 3.7)	3.21 (2.38 to 4.26)	2.20 (1.64 to 2.92)

Italy ITA Western Europe Stillbirth Rate / Neonatal Mortality Rate (SBR/NMR) − 20 weeks
lambda = 0.2 ; zeta = 0.99 ; scale = 5 ; amp = 0.028

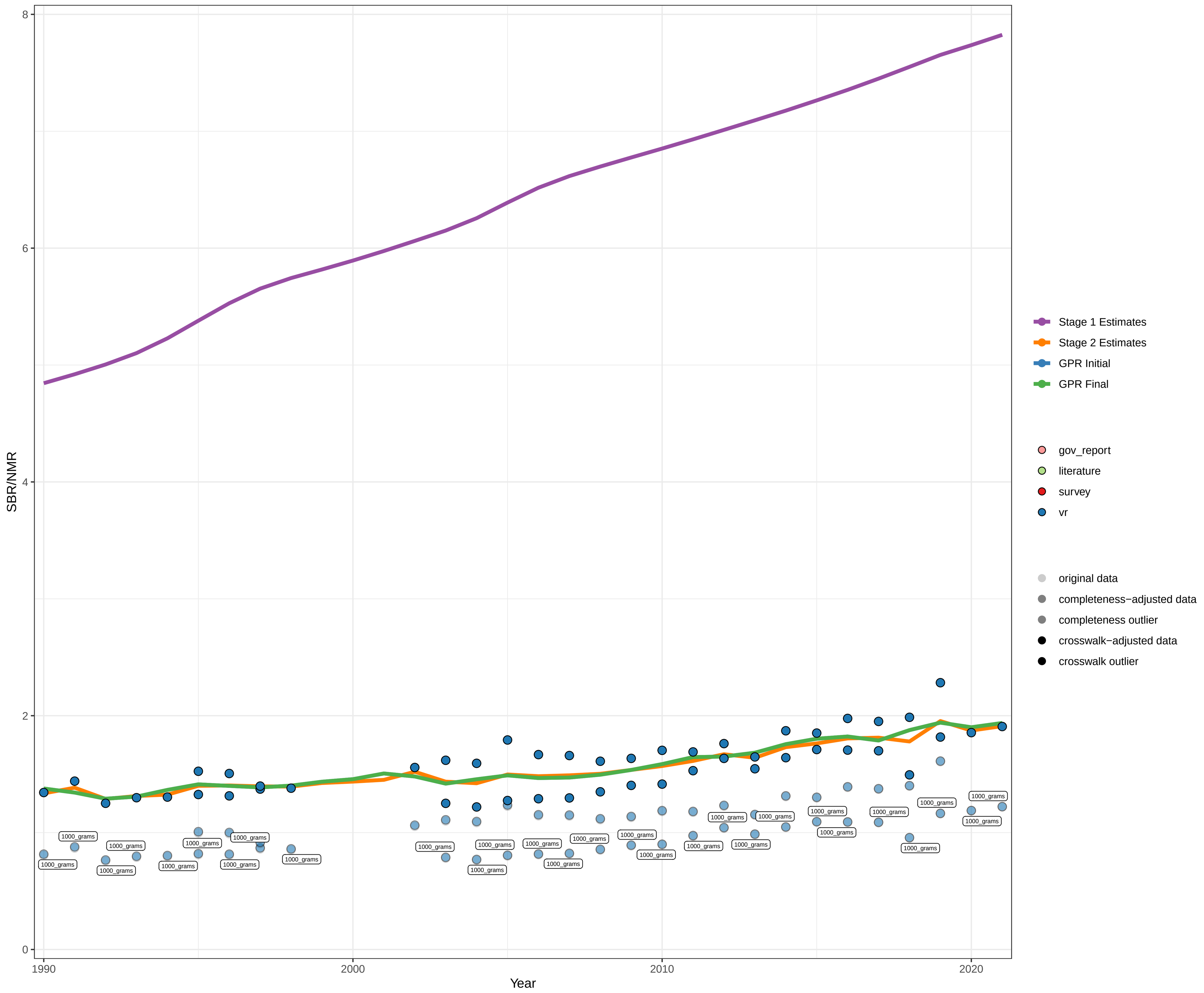

Italy ITA Western Europe Stillbirth Rate / Neonatal Mortality Rate (SBR/NMR) − 28 weeks
lambda = 0.2 ; zeta = 0.99 ; scale = 5 ; amp = 0.028

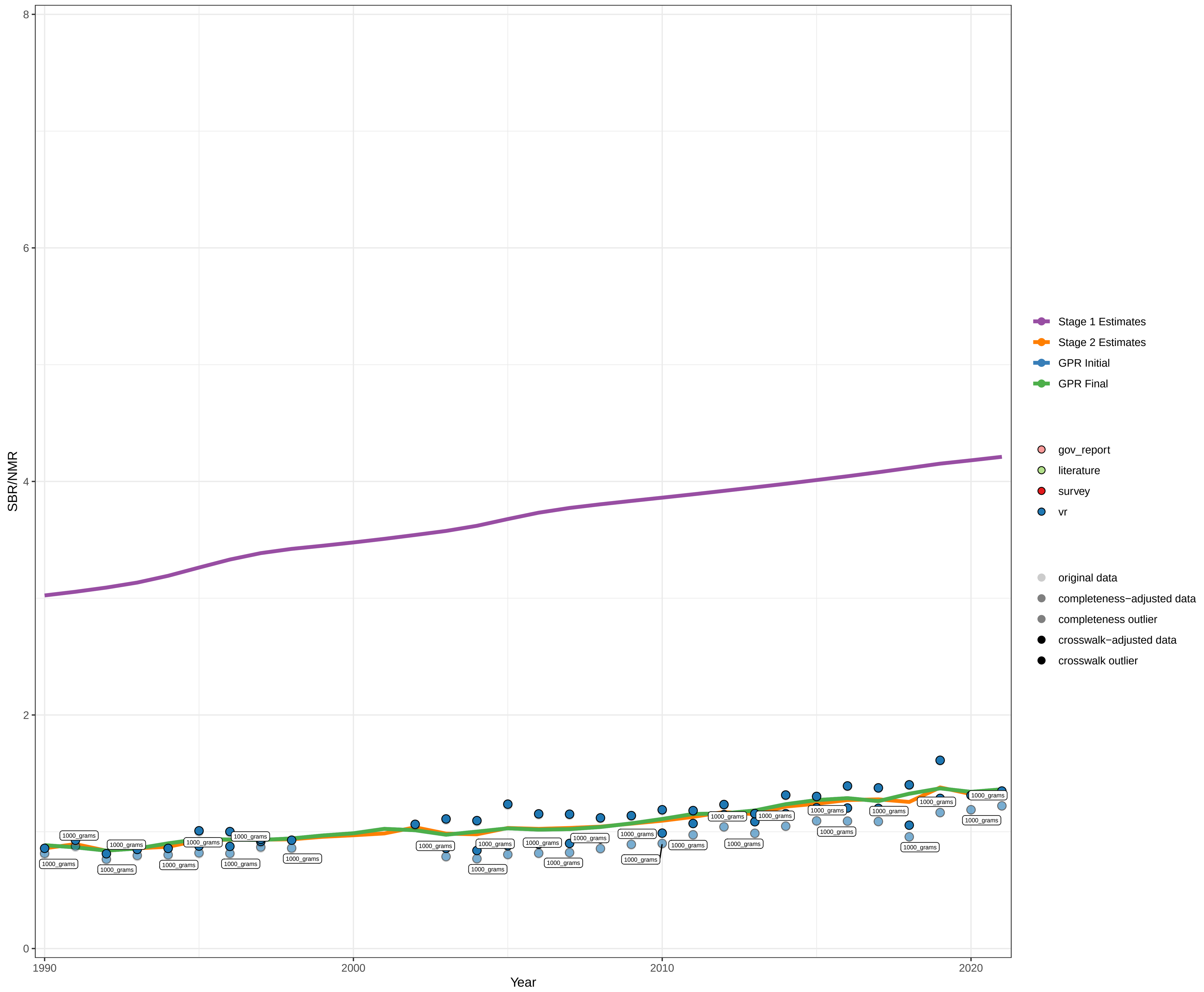

Definition	Years	Outlier	Source.Type	Title
1000_grams	1990−1998, 2003−2021	0	VR	European Health for All Database
28_weeks	1995−1997, 2002−2019	0	VR	United Nations Demographic Yearbook

Italy ITA Western Europe Stillbirth Rate (SBR) − 20 weeks
lambda = 0.2 ; zeta = 0.99 ; scale = 5 ; amp = 0.028

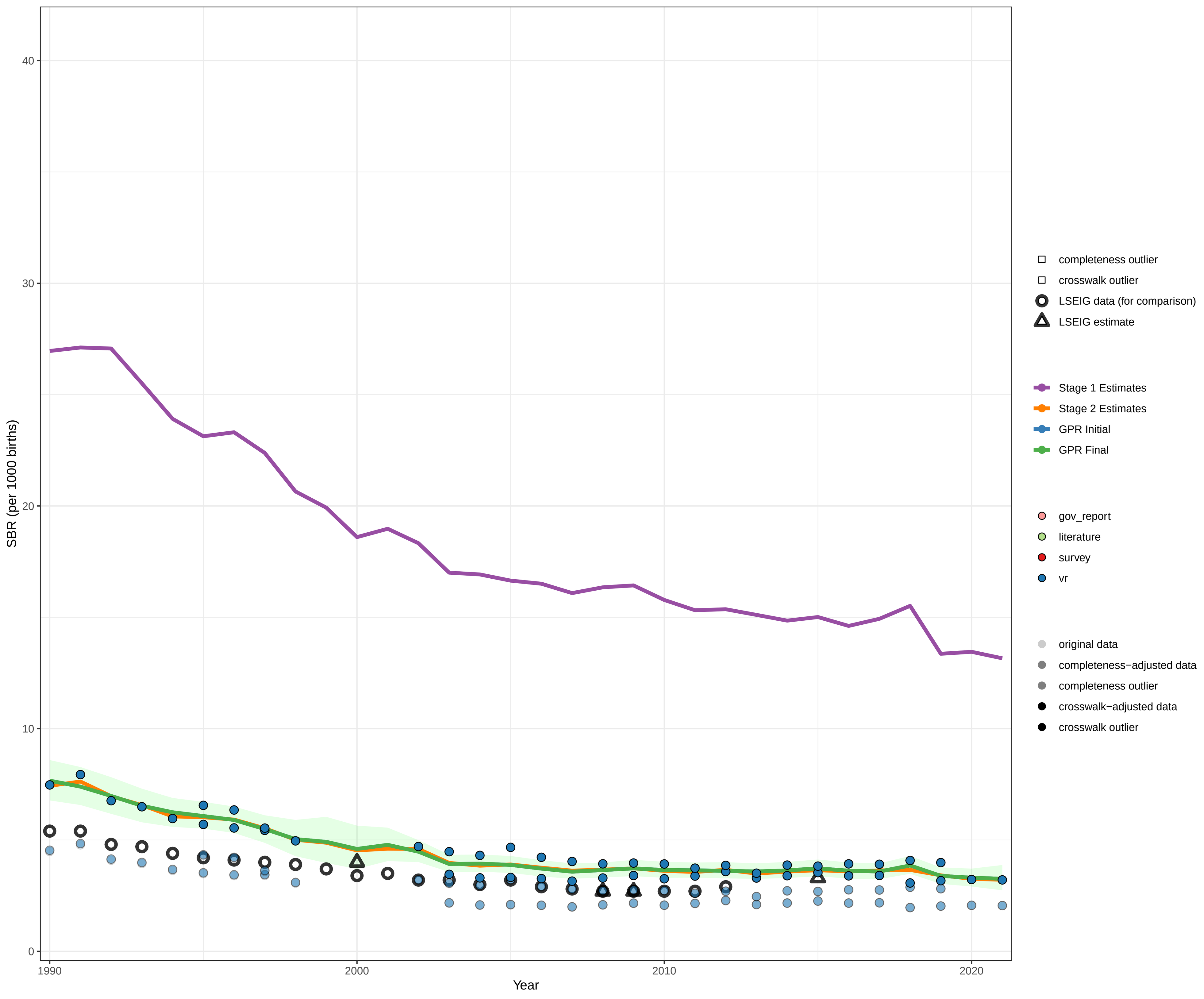

Italy ITA Western Europe Stillbirth Rate (SBR) − 28 weeks
lambda = 0.2 ; zeta = 0.99 ; scale = 5 ; amp = 0.028

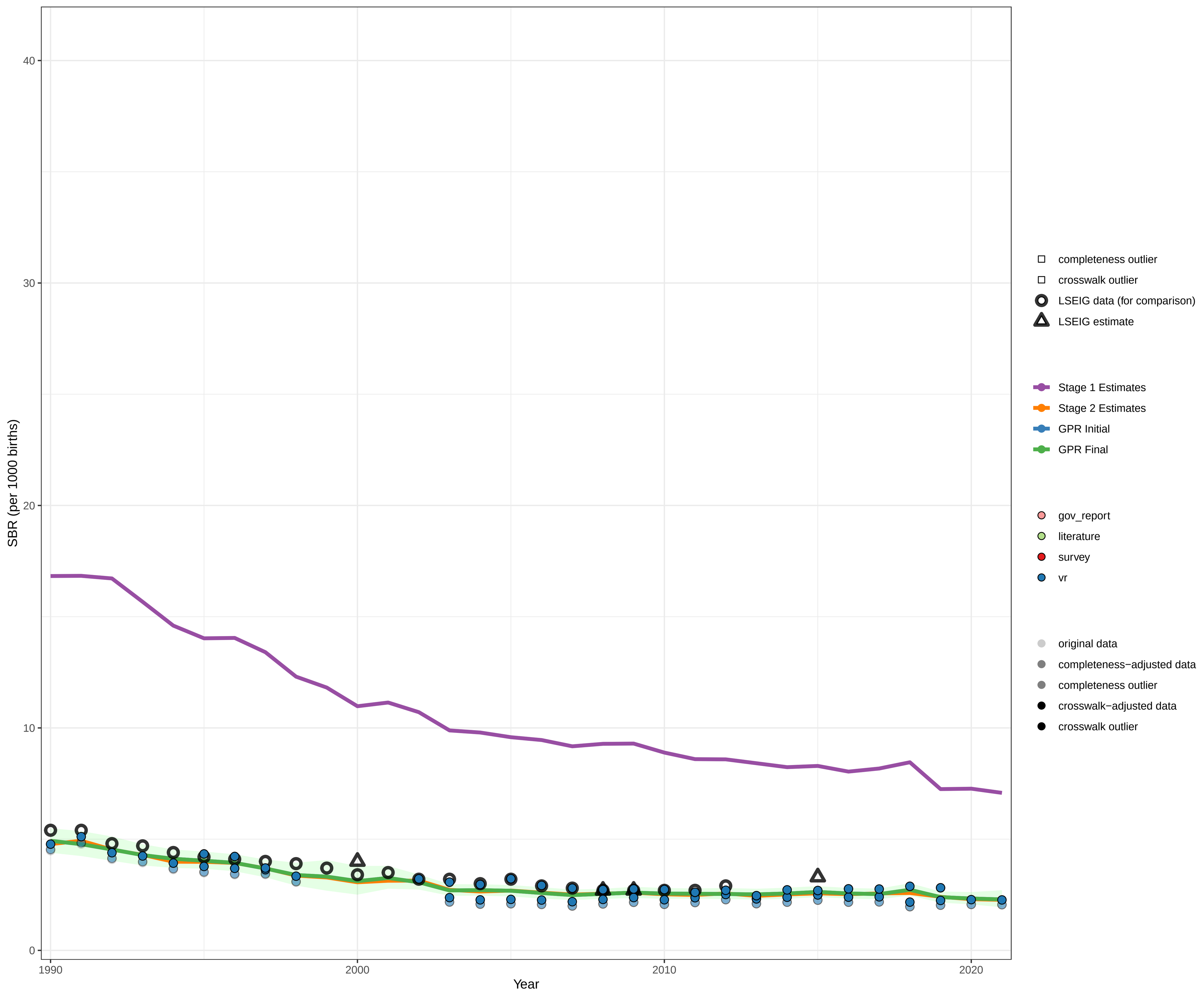

Year	Total Stillbirths (in thousands)	Total Stillbirths (in thousands)	SBR (per 1000 births)	SBR (per 1000 births)	SBR/NMR	SBR/NMR
	20 weeks	28 weeks	20 weeks	28 weeks	20 weeks	28 weeks
1990	4.4 (3.9 to 4.9)	2.8 (2.5 to 3.1)	7.7 (6.8 to 8.6)	4.9 (4.4 to 5.5)	1.38 (1.22 to 1.54)	0.89 (0.79 to 0.99)
1995	3.2 (2.9 to 3.6)	2.1 (1.9 to 2.4)	6.1 (5.5 to 6.7)	4.0 (3.7 to 4.4)	1.41 (1.28 to 1.56)	0.94 (0.85 to 1.03)
2000	2.5 (2.0 to 3.1)	1.7 (1.4 to 2.1)	4.6 (3.7 to 5.6)	3.1 (2.5 to 3.8)	1.46 (1.17 to 1.79)	0.99 (0.79 to 1.20)
2005	2.2 (2.0 to 2.4)	1.5 (1.4 to 1.6)	3.9 (3.5 to 4.3)	2.7 (2.4 to 2.9)	1.49 (1.35 to 1.64)	1.03 (0.93 to 1.13)
2010	2.0 (1.9 to 2.3)	1.4 (1.3 to 1.6)	3.7 (3.3 to 4.0)	2.6 (2.3 to 2.8)	1.59 (1.43 to 1.75)	1.11 (1.01 to 1.22)
2015	1.8 (1.6 to 2.0)	1.3 (1.2 to 1.4)	3.7 (3.4 to 4.1)	2.6 (2.4 to 2.9)	1.80 (1.63 to 2.00)	1.27 (1.16 to 1.40)
2020	1.3 (1.2 to 1.5)	0.9 (0.8 to 1.1)	3.3 (2.9 to 3.7)	2.3 (2.1 to 2.6)	1.90 (1.67 to 2.14)	1.34 (1.19 to 1.51)
2021	1.3 (1.1 to 1.5)	0.9 (0.8 to 1.1)	3.3 (2.7 to 3.9)	2.3 (1.9 to 2.7)	1.94 (1.63 to 2.30)	1.36 (1.15 to 1.61)

Luxembourg LUX Western Europe Stillbirth Rate / Neonatal Mortality Rate (SBR/NMR) − 20 weeks
lambda = 0.2 ; zeta = 0.99 ; scale = 5 ; amp = 0.028

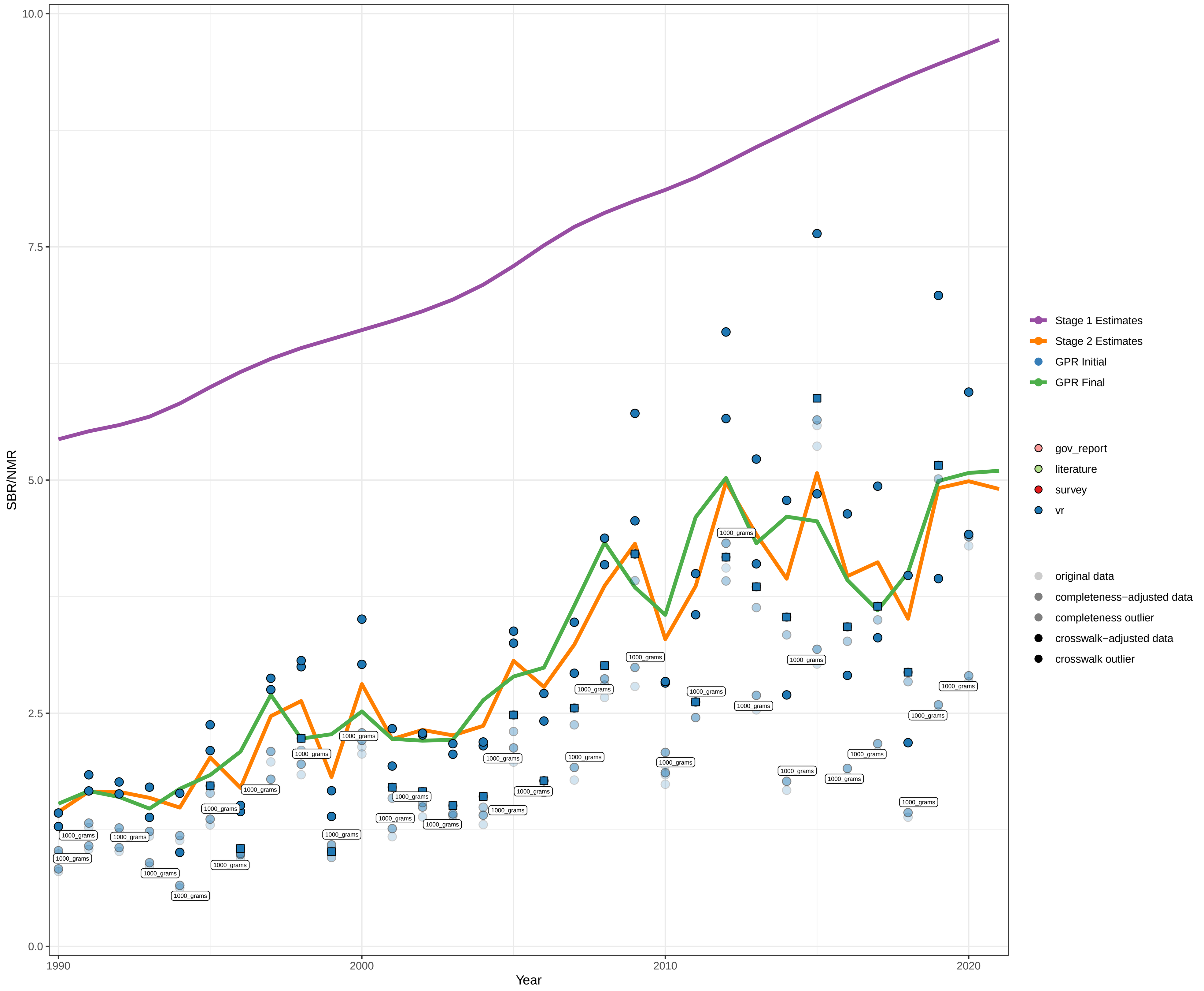

Luxembourg LUX Western Europe Stillbirth Rate / Neonatal Mortality Rate (SBR/NMR) − 28 weeks
lambda = 0.2 ; zeta = 0.99 ; scale = 5 ; amp = 0.028

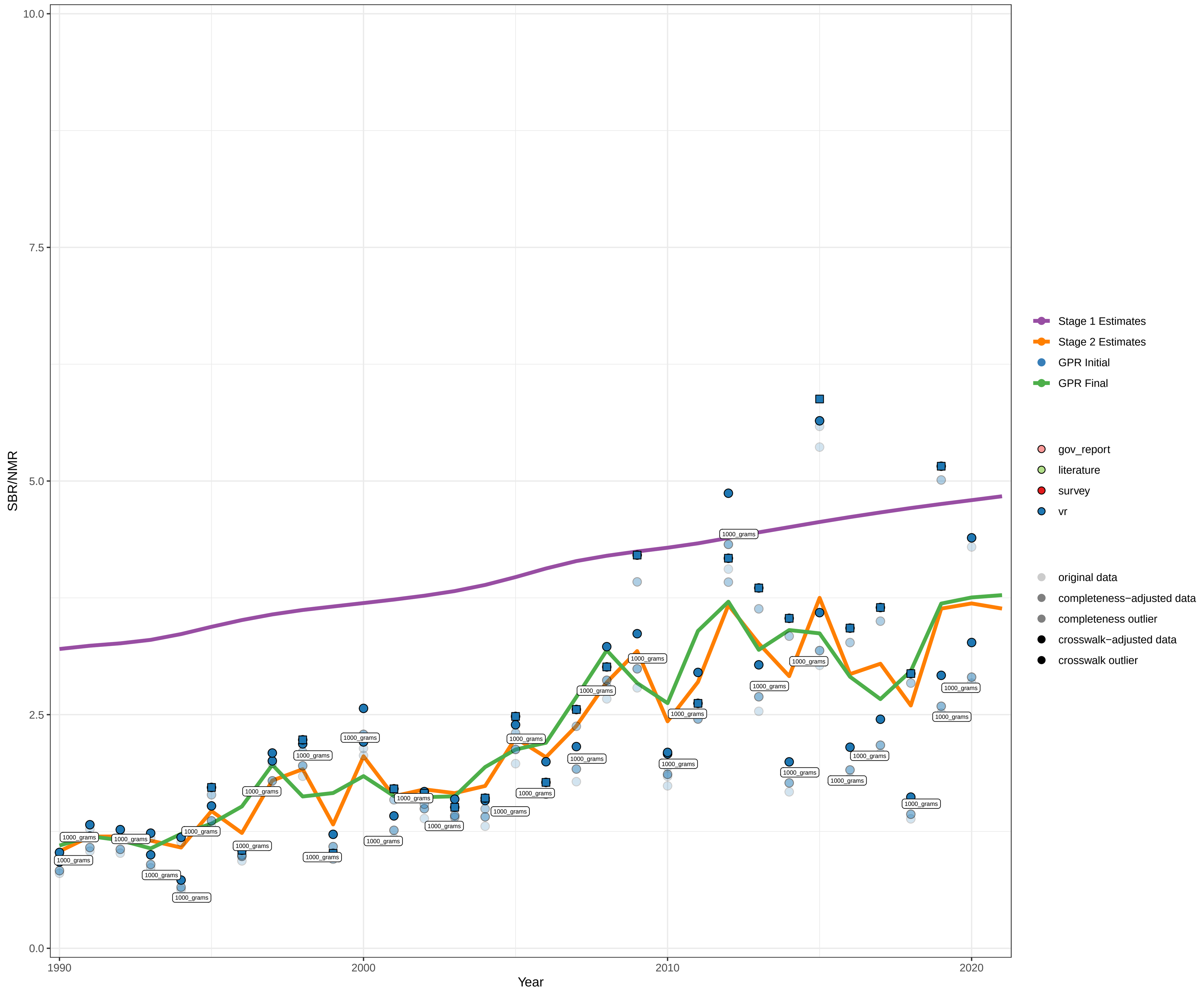

Definition	Years	Outlier	Source.Type	Title
1000_grams	1990−2020	0	VR	European Health for All Database
28_weeks	1990−2020	0	VR	Luxembourg Births by Viability, Sex and Situation of the Couple
28_weeks	1995, 1996, 1998, 1999, 2001−2019	2	VR	United Nations Demographic Yearbook

Luxembourg LUX Western Europe Stillbirth Rate (SBR) − 20 weeks
lambda = 0.2 ; zeta = 0.99 ; scale = 5 ; amp = 0.028

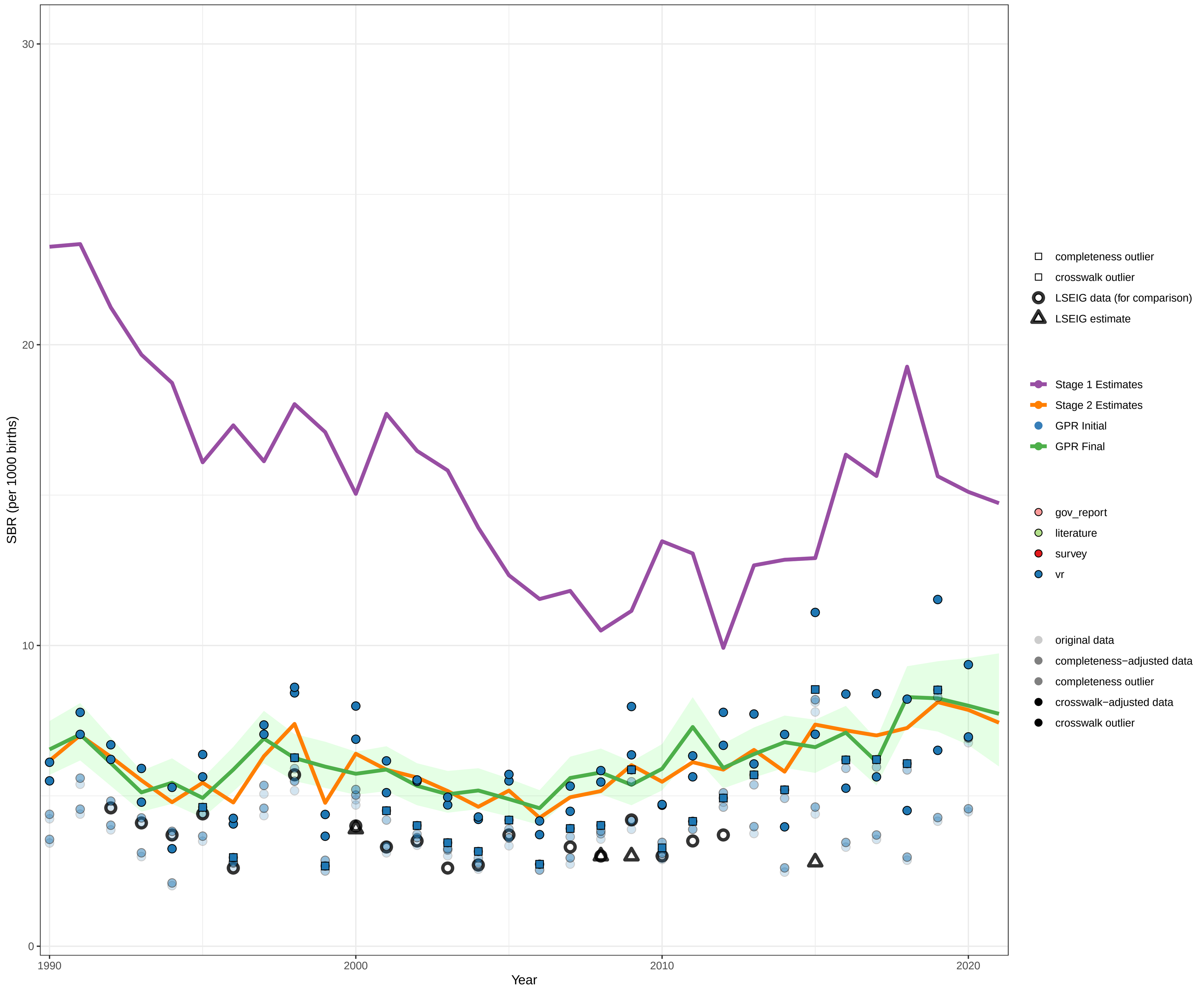

Luxembourg LUX Western Europe Stillbirth Rate (SBR) − 28 weeks
lambda = 0.2 ; zeta = 0.99 ; scale = 5 ; amp = 0.028

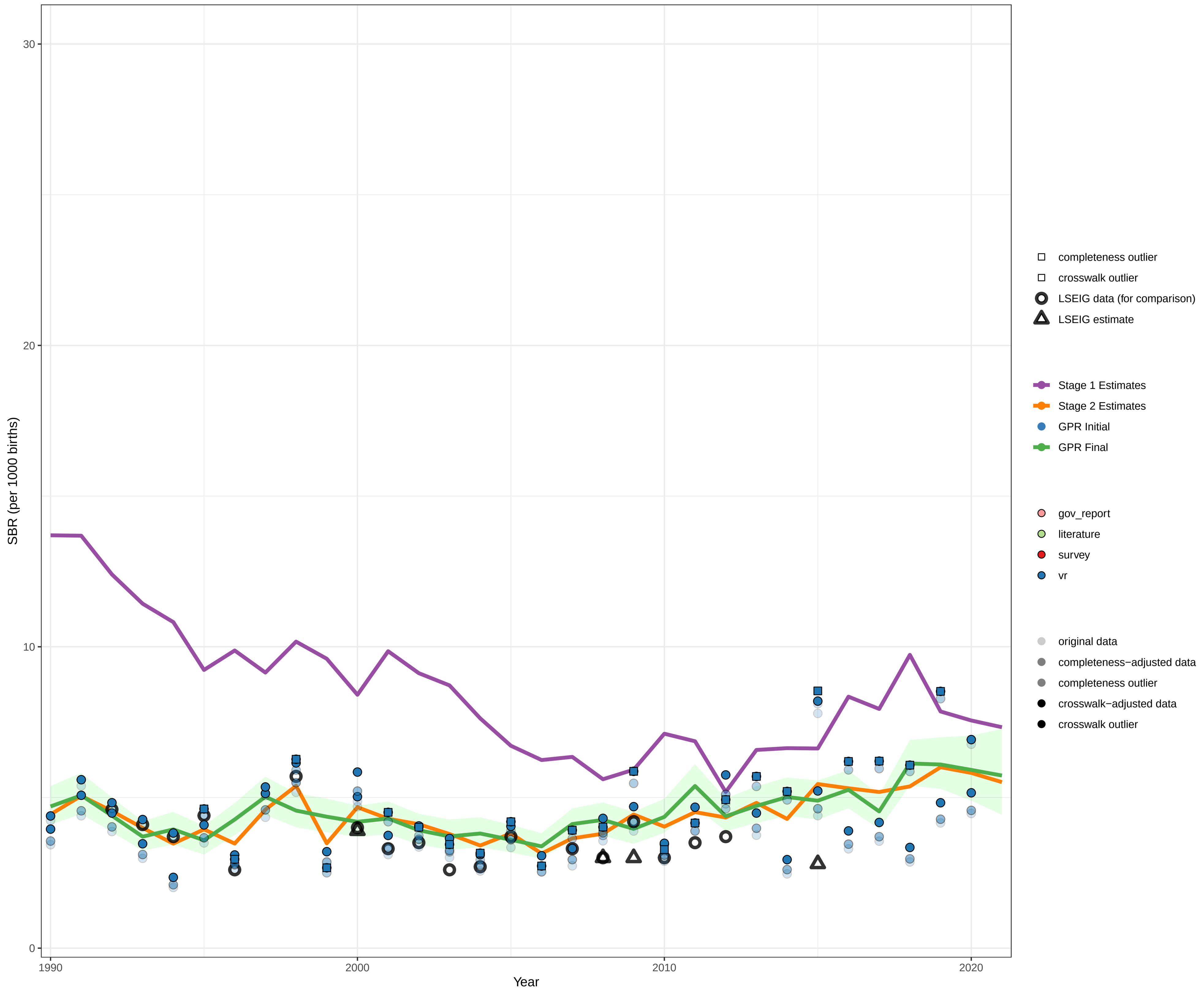

Year	Total Stillbirths (in thousands)	Total Stillbirths (in thousands)	SBR (per 1000 births)	SBR (per 1000 births)	SBR/NMR	SBR/NMR
	20 weeks	28 weeks	20 weeks	28 weeks	20 weeks	28 weeks
1990	0.0 (0.0 to 0.0)	0.0 (0.0 to 0.0)	6.5 (5.7 to 7.5)	4.7 (4.1 to 5.4)	1.53 (1.33 to 1.75)	1.10 (0.96 to 1.26)
1995	0.0 (0.0 to 0.0)	0.0 (0.0 to 0.0)	4.9 (4.3 to 5.6)	3.6 (3.1 to 4.1)	1.84 (1.59 to 2.08)	1.33 (1.16 to 1.51)
2000	0.0 (0.0 to 0.0)	0.0 (0.0 to 0.0)	5.7 (5.0 to 6.5)	4.2 (3.7 to 4.7)	2.52 (2.21 to 2.84)	1.84 (1.62 to 2.08)
2005	0.0 (0.0 to 0.0)	0.0 (0.0 to 0.0)	4.9 (4.3 to 5.6)	3.6 (3.2 to 4.1)	2.89 (2.55 to 3.30)	2.13 (1.88 to 2.42)
2010	0.0 (0.0 to 0.0)	0.0 (0.0 to 0.0)	5.9 (5.2 to 6.7)	4.4 (3.8 to 5.0)	3.55 (3.12 to 4.05)	2.62 (2.30 to 2.99)
2015	0.0 (0.0 to 0.0)	0.0 (0.0 to 0.0)	6.6 (5.8 to 7.5)	4.9 (4.3 to 5.6)	4.56 (3.97 to 5.19)	3.37 (2.94 to 3.83)
2020	0.1 (0.0 to 0.1)	0.0 (0.0 to 0.0)	8.0 (6.7 to 9.6)	5.9 (4.9 to 7.1)	5.08 (4.25 to 6.08)	3.75 (3.11 to 4.48)
2021	0.1 (0.0 to 0.1)	0.0 (0.0 to 0.0)	7.7 (6.0 to 9.7)	5.7 (4.4 to 7.3)	5.10 (3.94 to 6.42)	3.78 (2.92 to 4.80)

Malta MLT Western Europe Stillbirth Rate / Neonatal Mortality Rate (SBR/NMR) − 20 weeks
lambda = 0.2 ; zeta = 0.99 ; scale = 5 ; amp = 0.028

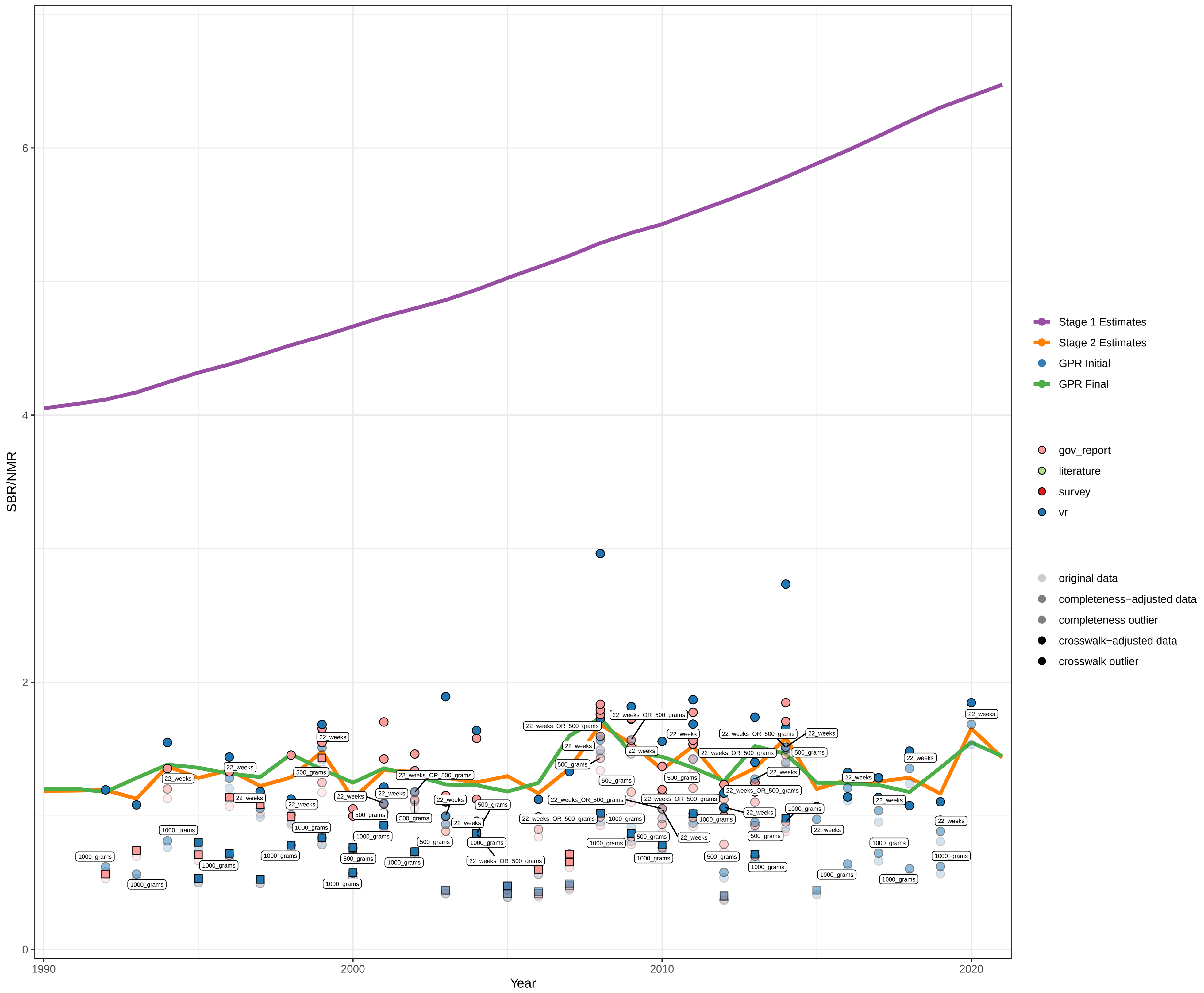

Malta MLT Western Europe Stillbirth Rate / Neonatal Mortality Rate (SBR/NMR) − 28 weeks
lambda = 0.2 ; zeta = 0.99 ; scale = 5 ; amp = 0.028

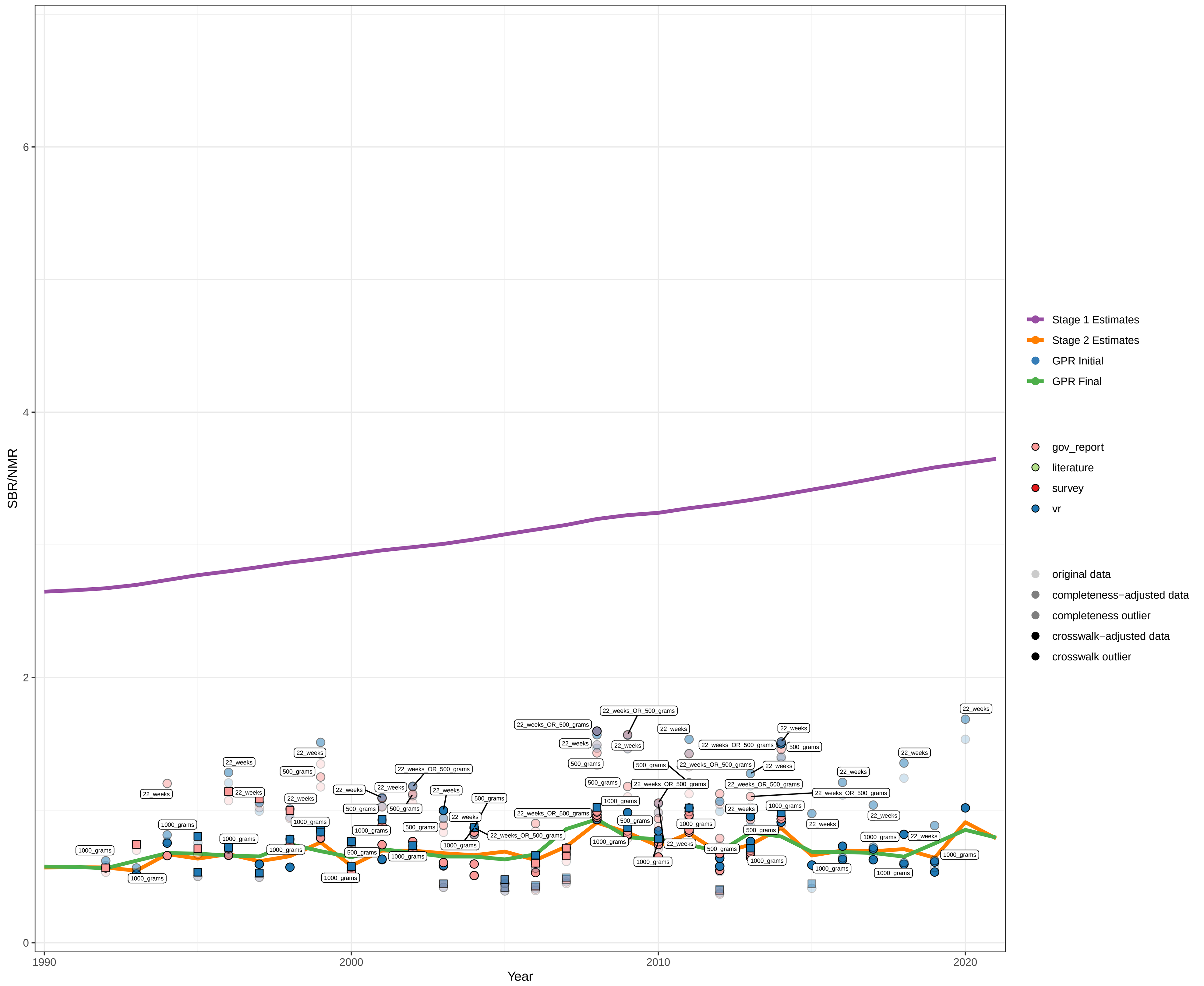

Definition	Years	Outlier	Source.Type	Title
1000_grams	1992−2002, 2004, 2008−2011, 2013, 2014, 2016−2019	0	VR	European Health for All Database
1000_grams	1995−2002, 2004, 2008−2011, 2013, 2014	0	Statistical Report	Malta Demographic Review
22_weeks	1992−1999	0	Statistical Report	Malta Demographic Review
22_weeks	1995−2004, 2006−2020	0	VR	Malta National Obstetric Information System Annual Report
22_weeks_OR_500_grams	2000−2004, 2006−2014	0	Statistical Report	Malta Demographic Review
28_weeks	2003, 2004, 2006−2014	0	VR	United Nations Demographic Yearbook
500_grams	1999−2004, 2006−2014	0	Statistical Report	Malta Demographic Review
1000_grams	2003, 2005−2007, 2012	1	Statistical Report	Malta Demographic Review
1000_grams	2003, 2005−2007, 2012, 2015	1	VR	European Health for All Database
22_weeks	2005	1	VR	Malta National Obstetric Information System Annual Report
22_weeks_OR_500_grams, 500_grams	2005	1	Statistical Report	Malta Demographic Review
28_weeks	2005	1	VR	United Nations Demographic Yearbook

Malta MLT Western Europe Stillbirth Rate (SBR) − 20 weeks
lambda = 0.2 ; zeta = 0.99 ; scale = 5 ; amp = 0.028

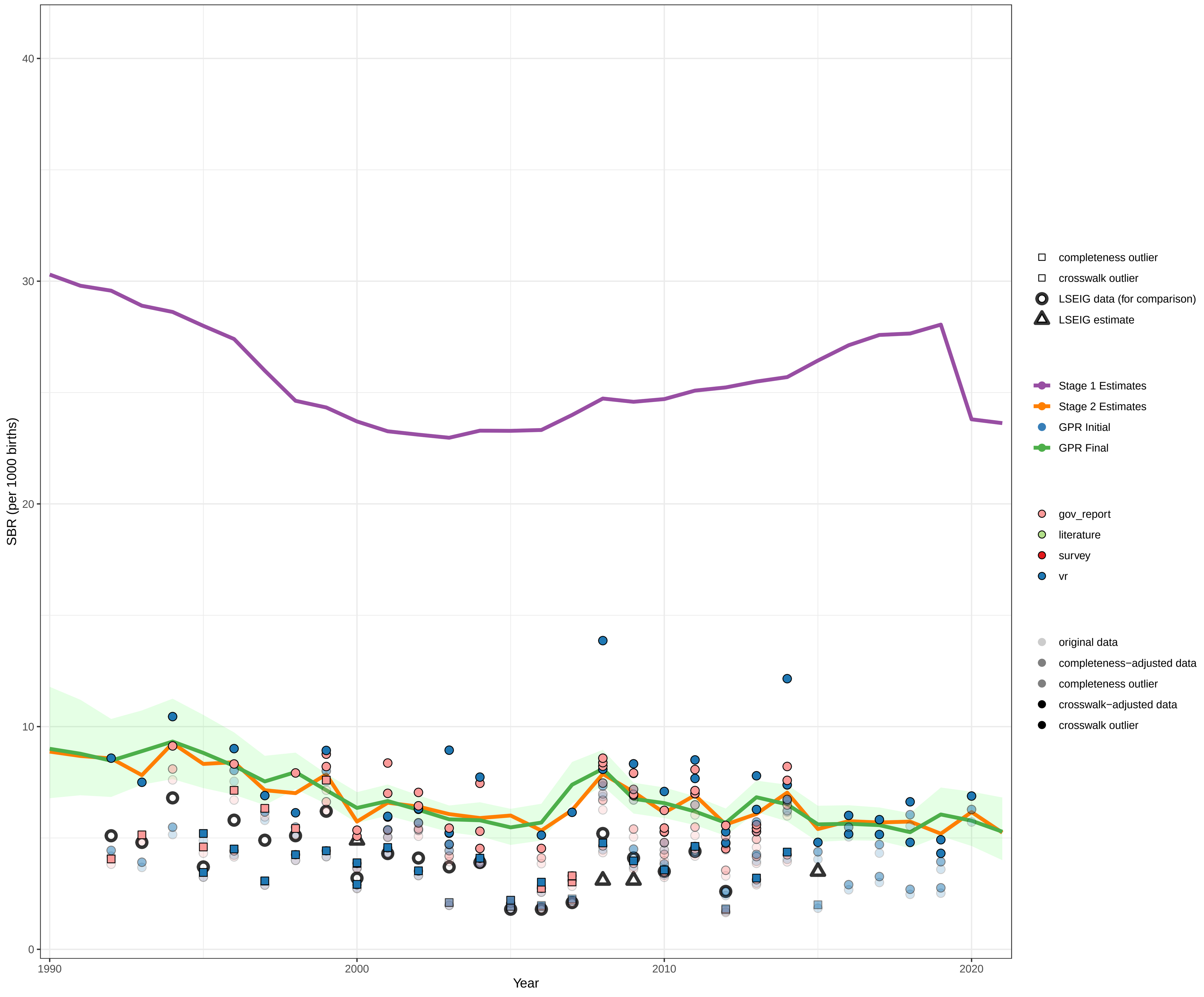

Malta MLT Western Europe Stillbirth Rate (SBR) − 28 weeks
lambda = 0.2 ; zeta = 0.99 ; scale = 5 ; amp = 0.028

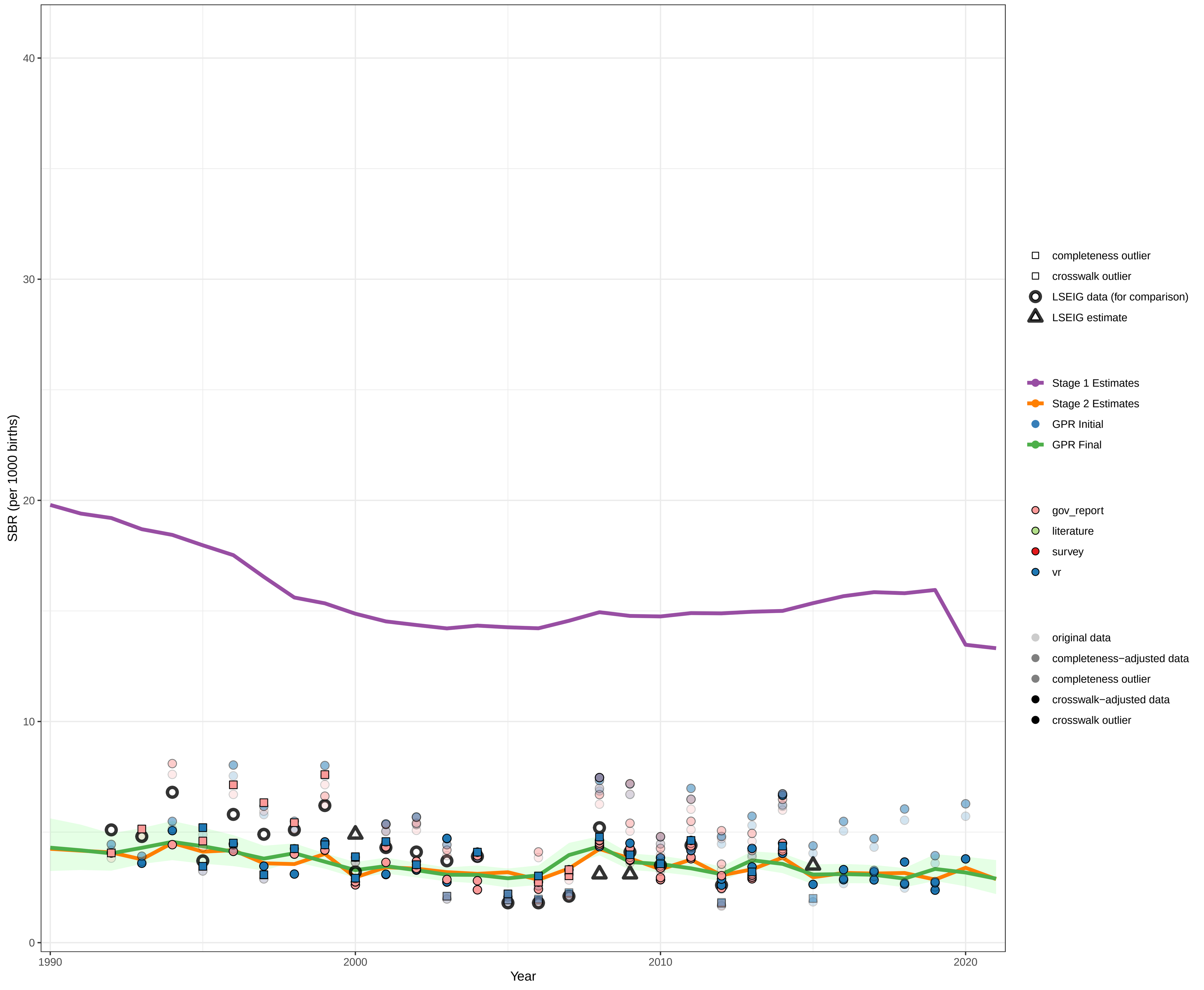

Year	Total Stillbirths (in thousands)	Total Stillbirths (in thousands)	SBR (per 1000 births)	SBR (per 1000 births)	SBR/NMR	SBR/NMR
	20 weeks	28 weeks	20 weeks	28 weeks	20 weeks	28 weeks
1990	0.0<br>(0.0 to 0.1)	0.0<br>(0.0 to 0.0)	9.0<br>(6.8 to 11.8)	4.3<br>(3.2 to 5.6)	1.20<br>(0.91 to 1.58)	0.57<br>(0.43 to 0.75)
1995	0.0<br>(0.0 to 0.1)	0.0<br>(0.0 to 0.0)	8.8<br>(7.2 to 10.5)	4.4<br>(3.6 to 5.2)	1.36<br>(1.12 to 1.62)	0.67<br>(0.55 to 0.80)
2000	0.0<br>(0.0 to 0.0)	0.0<br>(0.0 to 0.0)	6.3<br>(5.7 to 7.1)	3.3<br>(2.9 to 3.6)	1.25<br>(1.12 to 1.39)	0.65<br>(0.58 to 0.72)
2005	0.0<br>(0.0 to 0.0)	0.0<br>(0.0 to 0.0)	5.5<br>(4.7 to 6.3)	2.9<br>(2.5 to 3.4)	1.18<br>(1.01 to 1.36)	0.63<br>(0.54 to 0.72)
2010	0.0<br>(0.0 to 0.0)	0.0<br>(0.0 to 0.0)	6.6<br>(5.9 to 7.3)	3.6<br>(3.2 to 3.9)	1.44<br>(1.30 to 1.60)	0.78<br>(0.70 to 0.86)
2015	0.0<br>(0.0 to 0.0)	0.0<br>(0.0 to 0.0)	5.6<br>(4.8 to 6.4)	3.1<br>(2.6 to 3.5)	1.25<br>(1.07 to 1.43)	0.69<br>(0.59 to 0.79)
2020	0.0<br>(0.0 to 0.0)	0.0<br>(0.0 to 0.0)	5.8<br>(4.6 to 7.1)	3.2<br>(2.5 to 3.9)	1.55<br>(1.25 to 1.90)	0.85<br>(0.68 to 1.04)
2021	0.0<br>(0.0 to 0.0)	0.0<br>(0.0 to 0.0)	5.3<br>(4.0 to 6.8)	2.9<br>(2.2 to 3.7)	1.45<br>(1.10 to 1.87)	0.79<br>(0.60 to 1.02)

Monaco MCO Western Europe Stillbirth Rate / Neonatal Mortality Rate (SBR/NMR) − 20 weeks
lambda = 0.8 ; zeta = 0.7 ; scale = 15 ; amp = 0.028

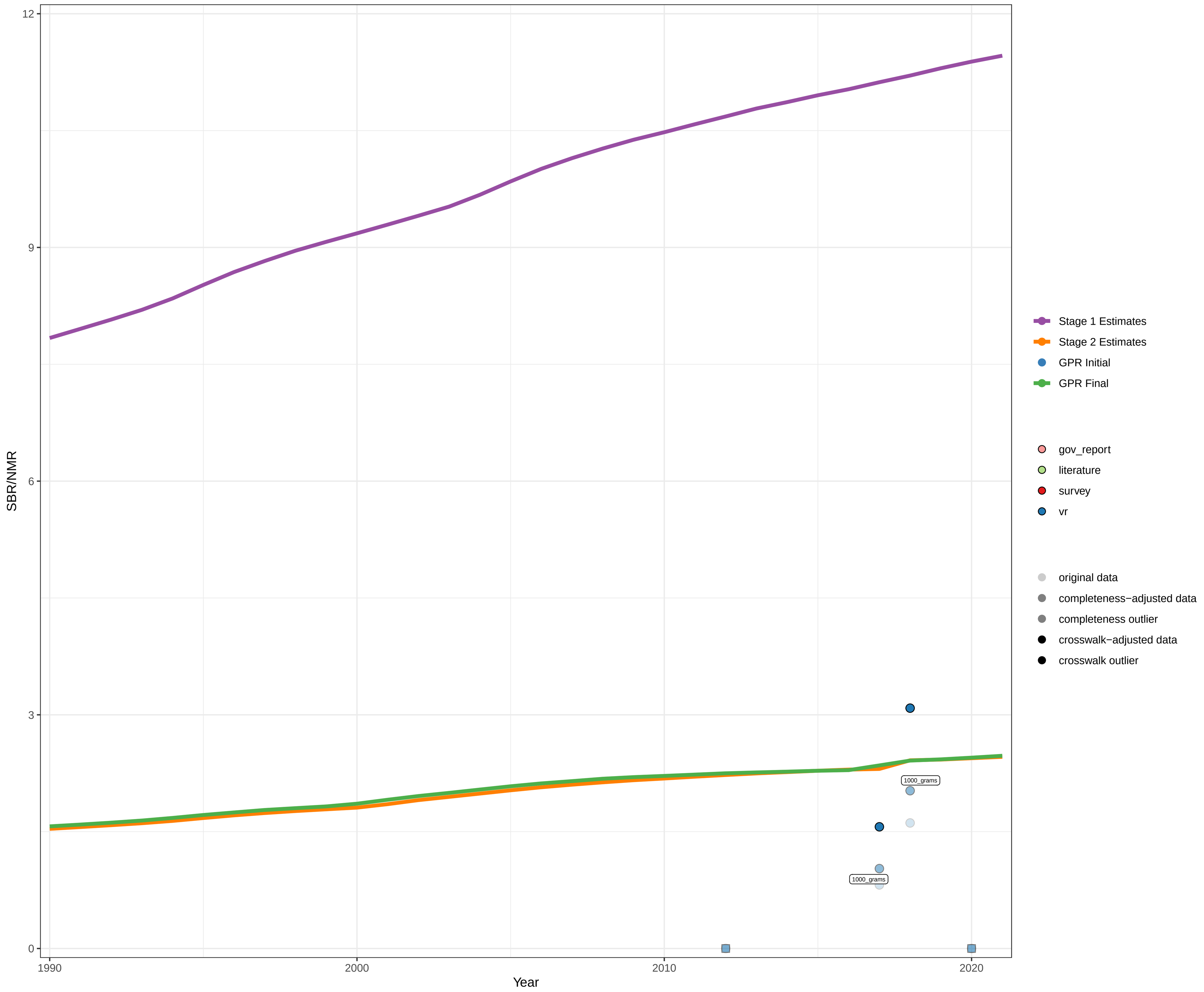

Monaco MCO Western Europe Stillbirth Rate / Neonatal Mortality Rate (SBR/NMR) − 28 weeks
lambda = 0.8 ; zeta = 0.7 ; scale = 15 ; amp = 0.028

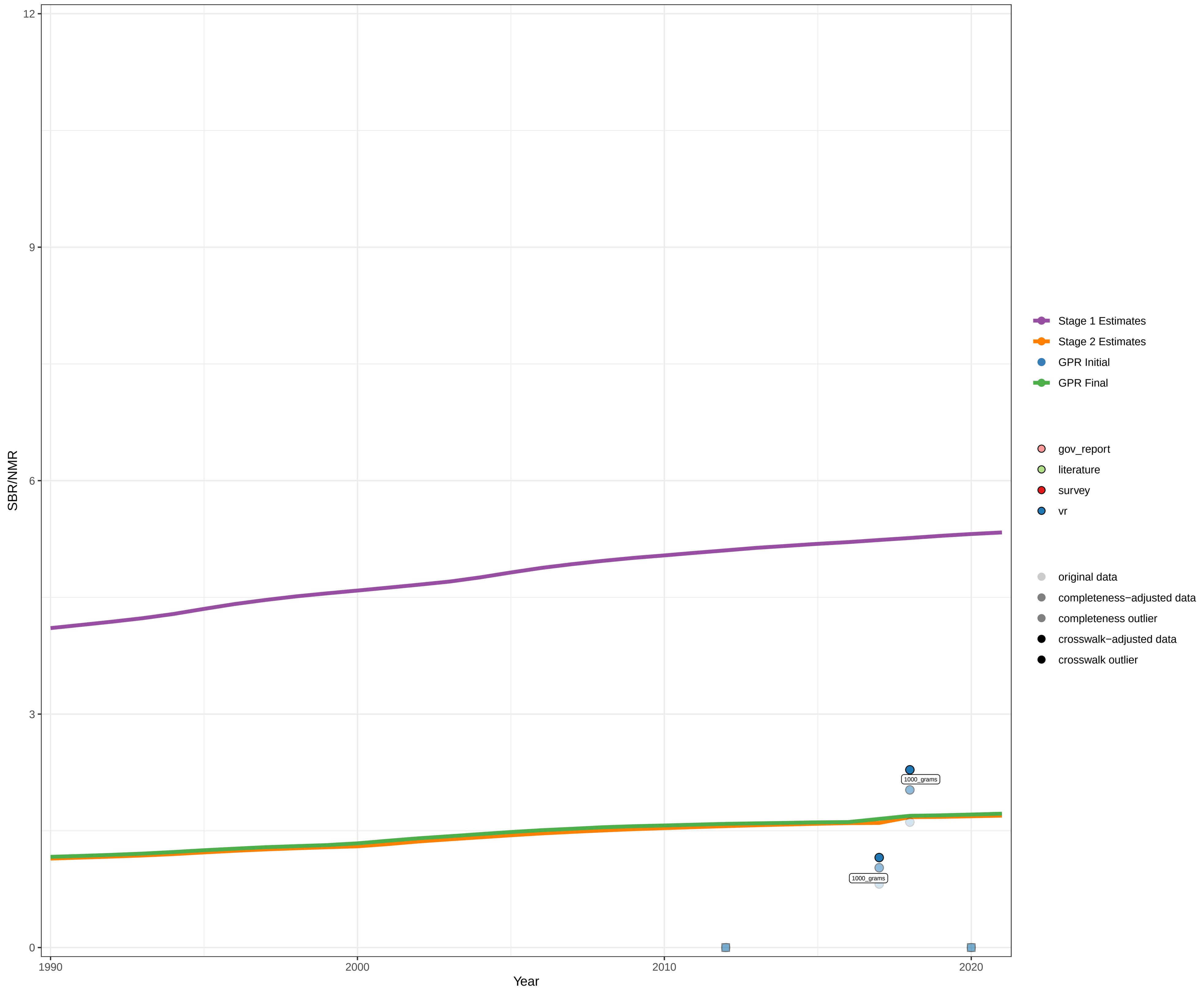

Definition	Years	Outlier	Source.Type	Title
1000_grams	2017, 2018	0	VR	European Health for All Database
1000_grams	2012, 2020	1	VR	European Health for All Database

Monaco MCO Western Europe Stillbirth Rate (SBR) − 20 weeks
lambda = 0.8 ; zeta = 0.7 ; scale = 15 ; amp = 0.028

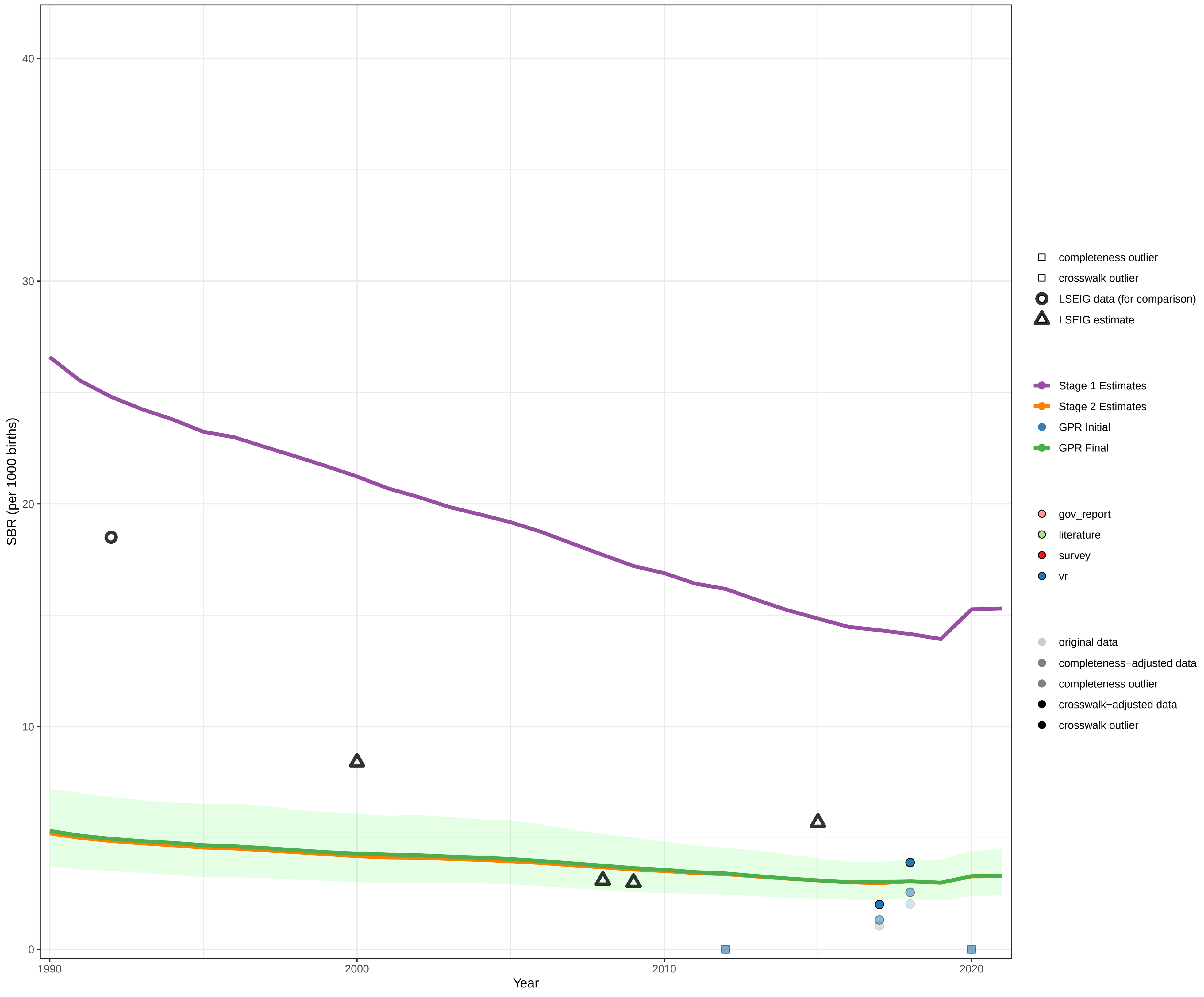

Monaco MCO Western Europe Stillbirth Rate (SBR) − 28 weeks
lambda = 0.8 ; zeta = 0.7 ; scale = 15 ; amp = 0.028

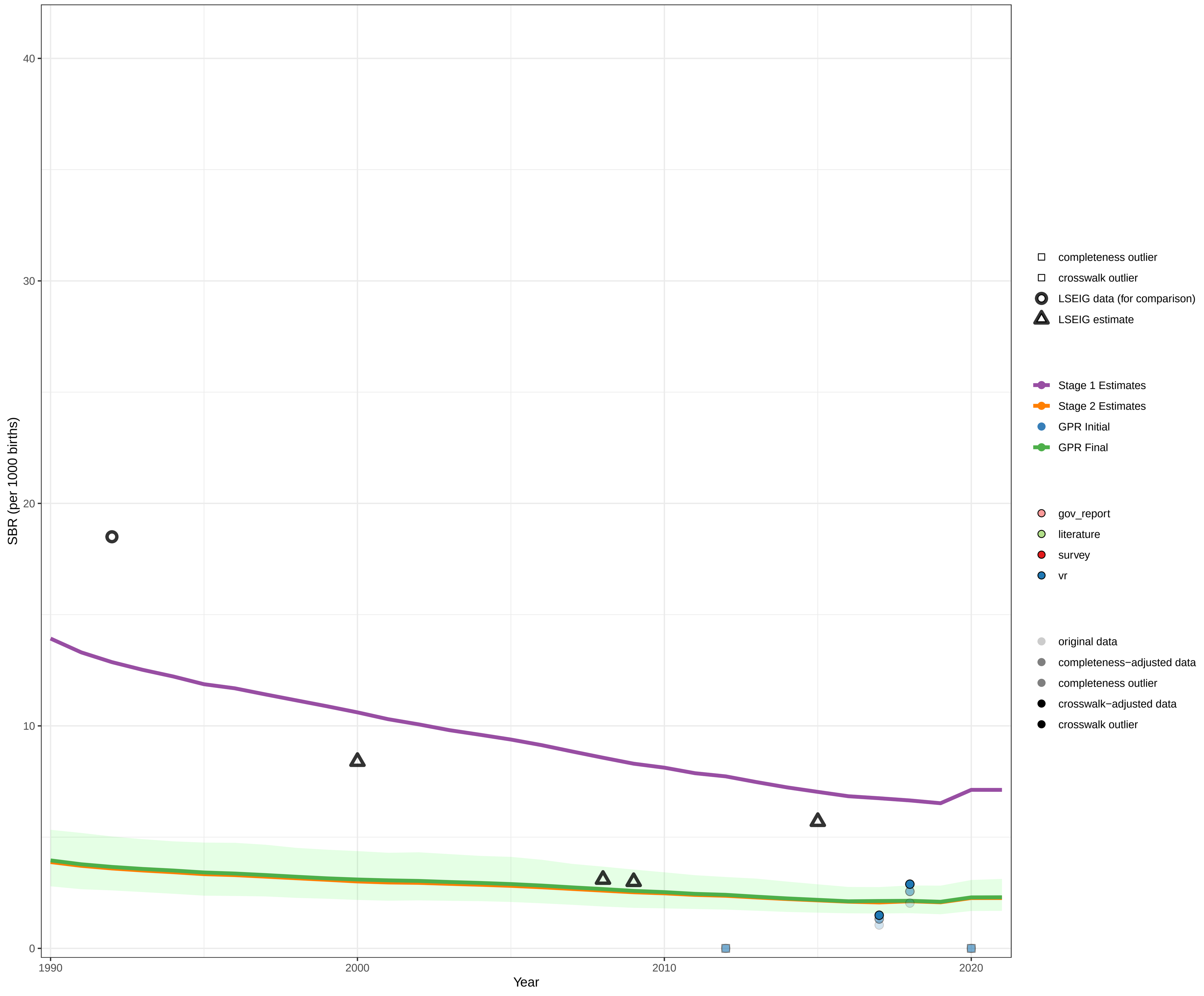

Year	Total Stillbirths (in thousands)	Total Stillbirths (in thousands)	SBR (per 1000 births)	SBR (per 1000 births)	SBR/NMR	SBR/NMR
	20 weeks	28 weeks	20 weeks	28 weeks	20 weeks	28 weeks
1990	0.0 (0.0 to 0.0)	0.0 (0.0 to 0.0)	5.3 (3.7 to 7.2)	4.0 (2.8 to 5.3)	1.57 (1.10 to 2.12)	1.17 (0.82 to 1.57)
1995	0.0 (0.0 to 0.0)	0.0 (0.0 to 0.0)	4.7 (3.2 to 6.5)	3.4 (2.4 to 4.8)	1.71 (1.19 to 2.40)	1.25 (0.87 to 1.74)
2000	0.0 (0.0 to 0.0)	0.0 (0.0 to 0.0)	4.3 (3.0 to 6.1)	3.1 (2.2 to 4.4)	1.86 (1.30 to 2.63)	1.34 (0.94 to 1.89)
2005	0.0 (0.0 to 0.0)	0.0 (0.0 to 0.0)	4.1 (2.9 to 5.8)	2.9 (2.1 to 4.1)	2.08 (1.50 to 2.97)	1.48 (1.07 to 2.11)
2010	0.0 (0.0 to 0.0)	0.0 (0.0 to 0.0)	3.6 (2.5 to 4.8)	2.5 (1.8 to 3.4)	2.22 (1.57 to 3.00)	1.57 (1.12 to 2.12)
2015	0.0 (0.0 to 0.0)	0.0 (0.0 to 0.0)	3.1 (2.3 to 4.1)	2.2 (1.6 to 2.9)	2.28 (1.67 to 3.02)	1.61 (1.18 to 2.12)
2020	0.0 (0.0 to 0.0)	0.0 (0.0 to 0.0)	3.3 (2.4 to 4.4)	2.3 (1.7 to 3.1)	2.45 (1.79 to 3.29)	1.71 (1.25 to 2.29)
2021	0.0 (0.0 to 0.0)	0.0 (0.0 to 0.0)	3.3 (2.4 to 4.5)	2.3 (1.7 to 3.1)	2.47 (1.81 to 3.37)	1.72 (1.26 to 2.34)

Netherlands NLD Western Europe Stillbirth Rate / Neonatal Mortality Rate (SBR/NMR) − 20 weeks
lambda = 0.2 ; zeta = 0.99 ; scale = 5 ; amp = 0.028

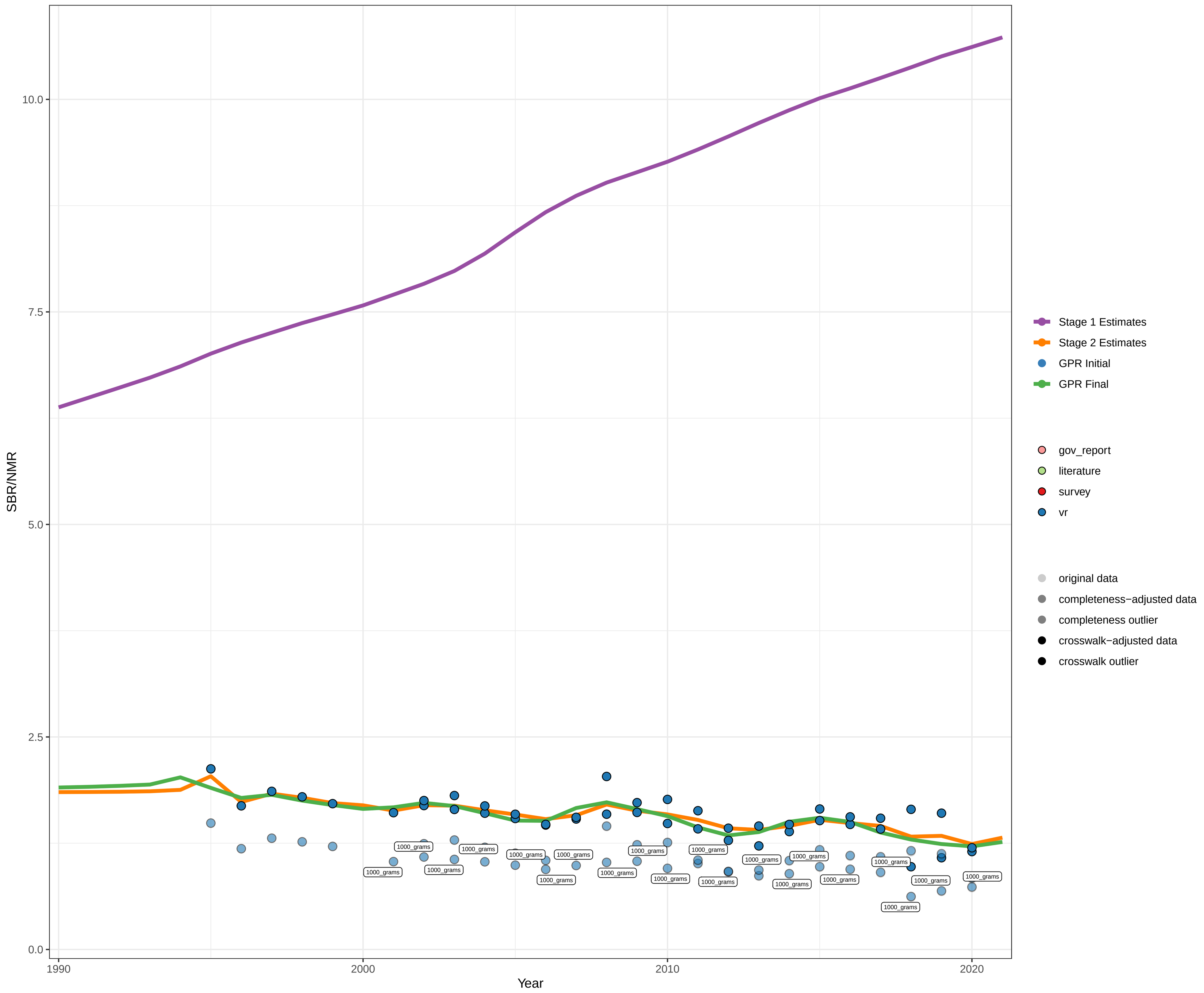

Netherlands NLD Western Europe Stillbirth Rate / Neonatal Mortality Rate (SBR/NMR) − 28 weeks
lambda = 0.2 ; zeta = 0.99 ; scale = 5 ; amp = 0.028

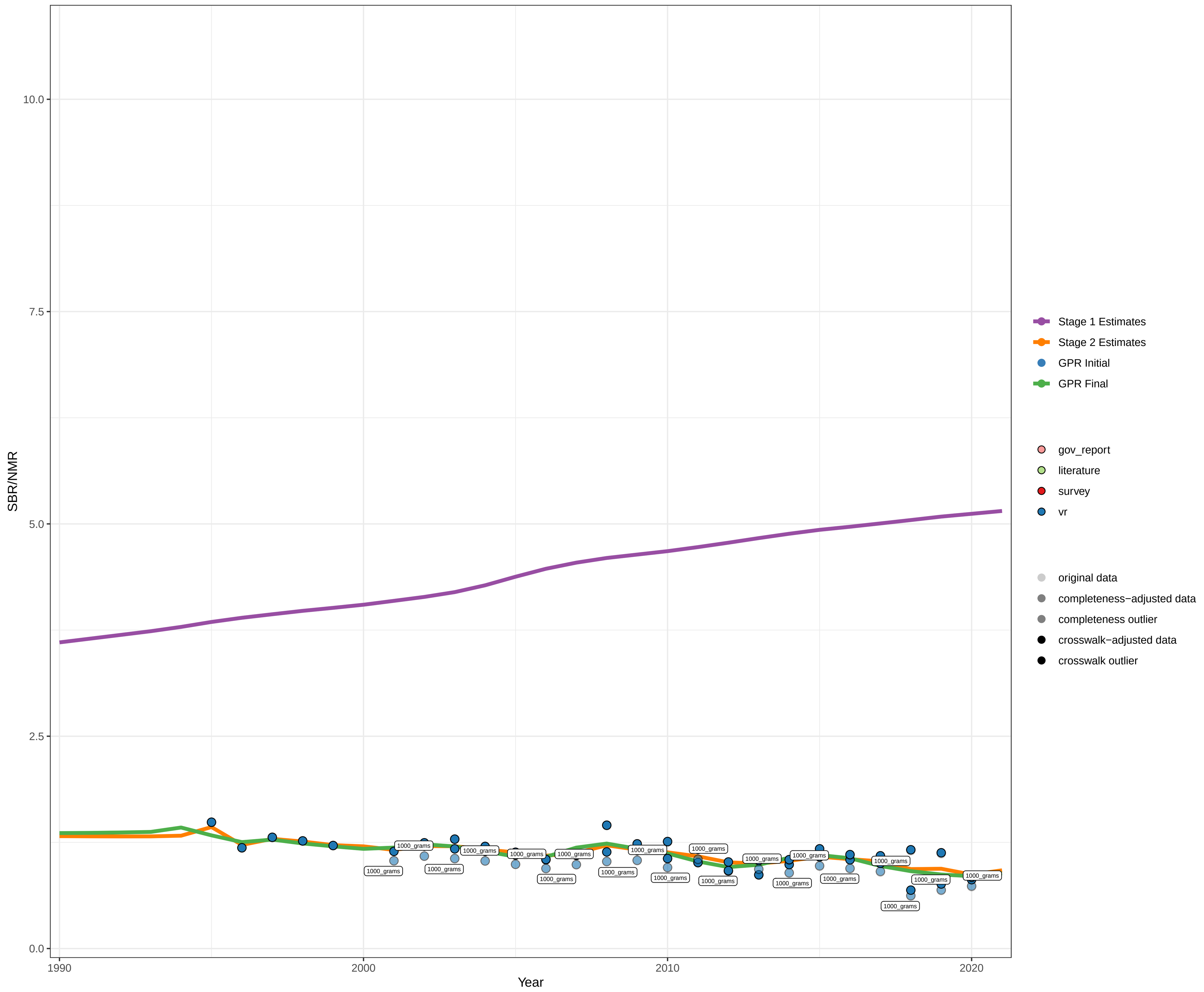

Definition	Years	Outlier	Source.Type	Title
1000_grams	2001−2020	0	VR	European Health for All Database
28_weeks	1995−1999, 2002−2020	0	VR	United Nations Demographic Yearbook

Netherlands NLD Western Europe Stillbirth Rate (SBR) − 20 weeks
lambda = 0.2 ; zeta = 0.99 ; scale = 5 ; amp = 0.028

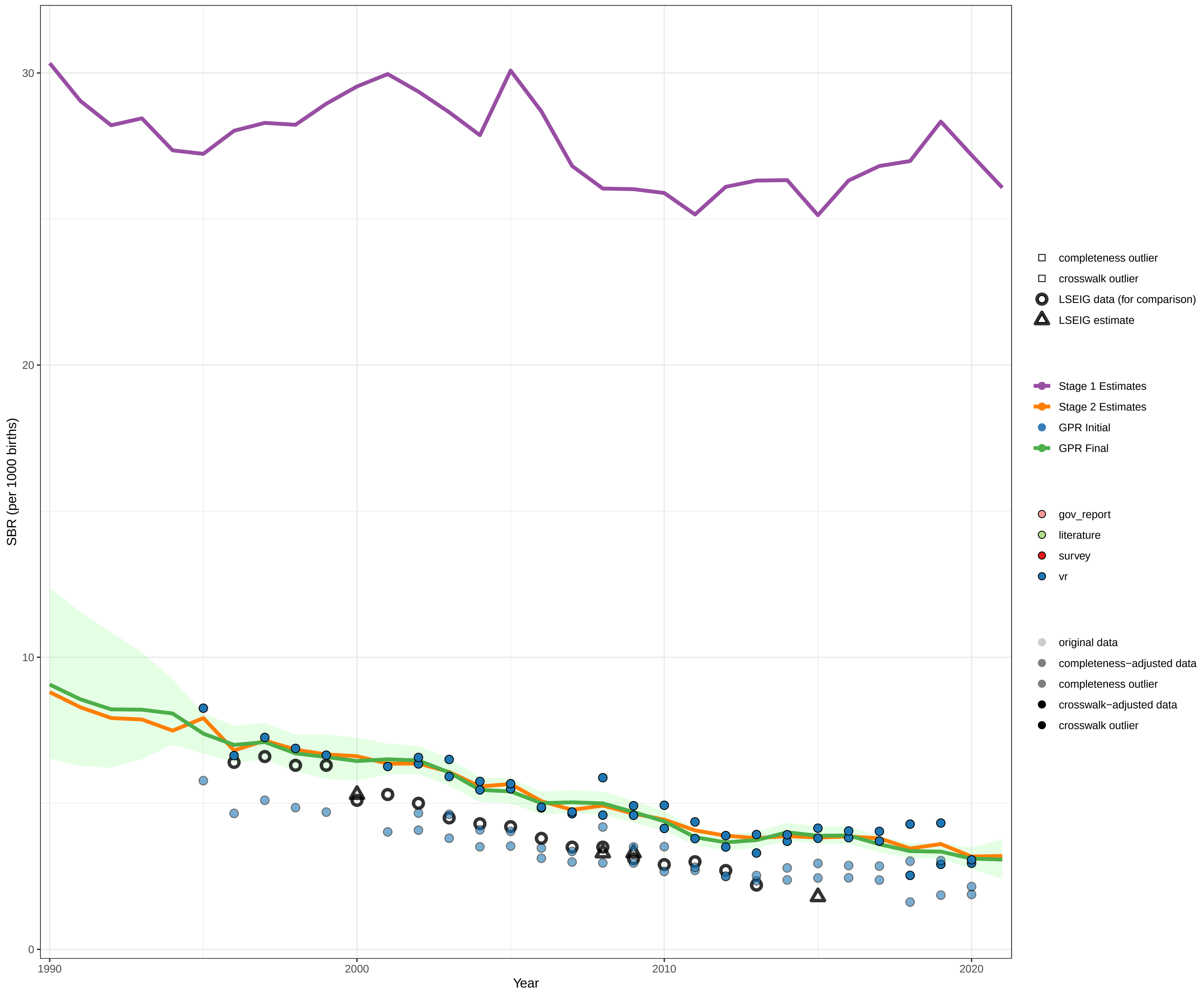

Netherlands NLD Western Europe Stillbirth Rate (SBR) − 28 weeks
lambda = 0.2 ; zeta = 0.99 ; scale = 5 ; amp = 0.028

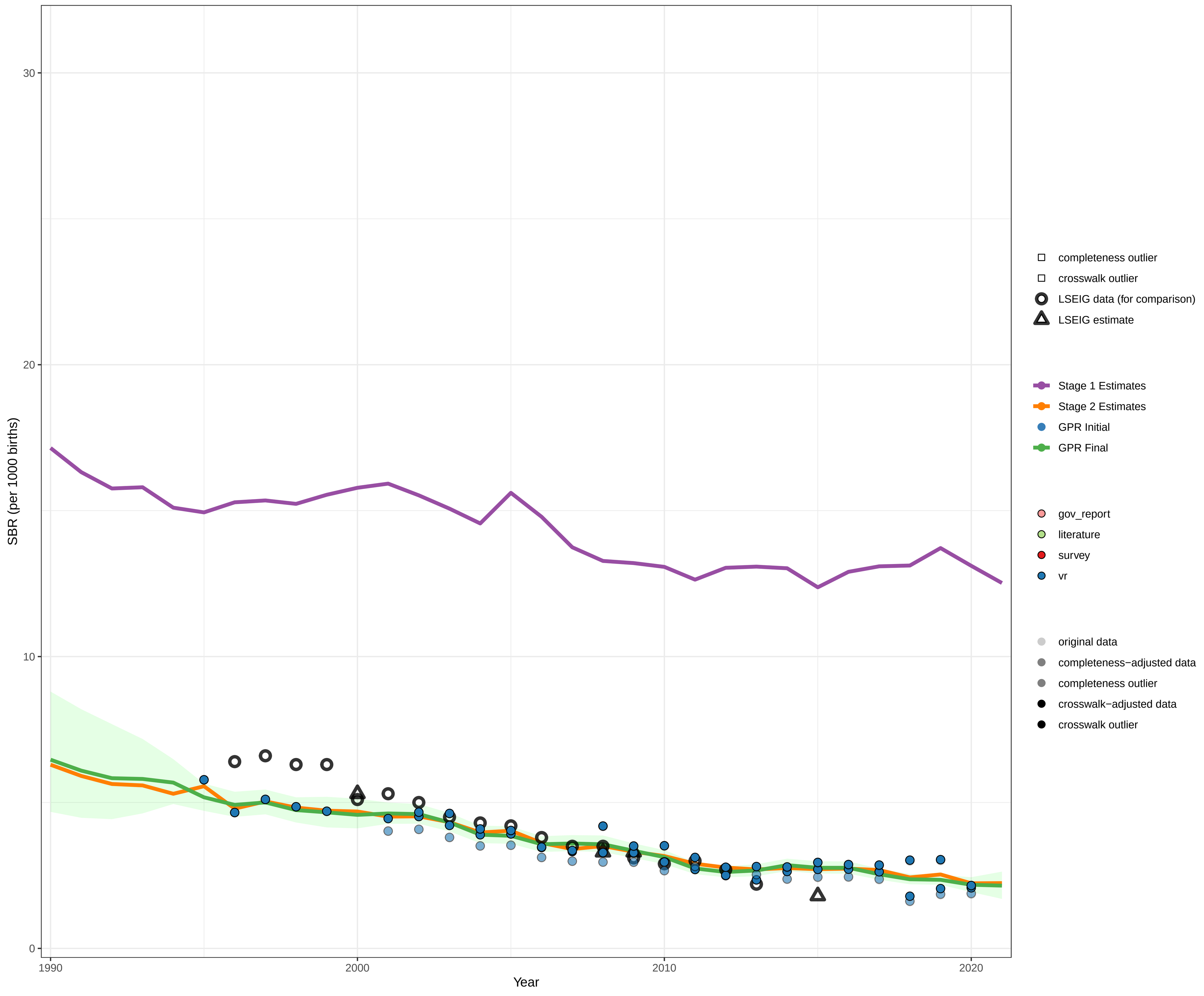

Year	Total Stillbirths (in thousands)	Total Stillbirths (in thousands)	SBR (per 1000 births)	SBR (per 1000 births)	SBR/NMR	SBR/NMR
	20 weeks	28 weeks	20 weeks	28 weeks	20 weeks	28 weeks
1990	1.8 (1.3 to 2.5)	1.3 (0.9 to 1.8)	9.1 (6.5 to 12.4)	6.5 (4.7 to 8.8)	1.91 (1.37 to 2.60)	1.36 (0.98 to 1.85)
1995	1.4 (1.3 to 1.6)	1.0 (0.9 to 1.1)	7.4 (6.7 to 8.1)	5.2 (4.7 to 5.6)	1.90 (1.73 to 2.08)	1.33 (1.21 to 1.45)
2000	1.3 (1.2 to 1.5)	0.9 (0.8 to 1.1)	6.4 (5.8 to 7.3)	4.6 (4.1 to 5.1)	1.65 (1.48 to 1.86)	1.17 (1.06 to 1.32)
2005	1.0 (0.9 to 1.1)	0.7 (0.7 to 0.8)	5.4 (5.0 to 5.9)	3.9 (3.6 to 4.2)	1.52 (1.40 to 1.64)	1.08 (1.01 to 1.17)
2010	0.8 (0.7 to 0.9)	0.6 (0.5 to 0.6)	4.4 (4.0 to 4.7)	3.1 (2.9 to 3.4)	1.57 (1.45 to 1.69)	1.12 (1.03 to 1.21)
2015	0.7 (0.6 to 0.7)	0.5 (0.4 to 0.5)	3.9 (3.6 to 4.2)	2.8 (2.6 to 3.0)	1.55 (1.44 to 1.68)	1.10 (1.02 to 1.19)
2020	0.5 (0.5 to 0.6)	0.4 (0.3 to 0.4)	3.1 (2.7 to 3.5)	2.2 (1.9 to 2.4)	1.21 (1.07 to 1.36)	0.85 (0.75 to 0.95)
2021	0.5 (0.4 to 0.7)	0.4 (0.3 to 0.5)	3.1 (2.4 to 3.8)	2.2 (1.7 to 2.6)	1.26 (0.99 to 1.55)	0.89 (0.70 to 1.08)

Norway NOR Western Europe Stillbirth Rate / Neonatal Mortality Rate (SBR/NMR) − 20 weeks
lambda = 0.2 ; zeta = 0.99 ; scale = 5 ; amp = 0.028

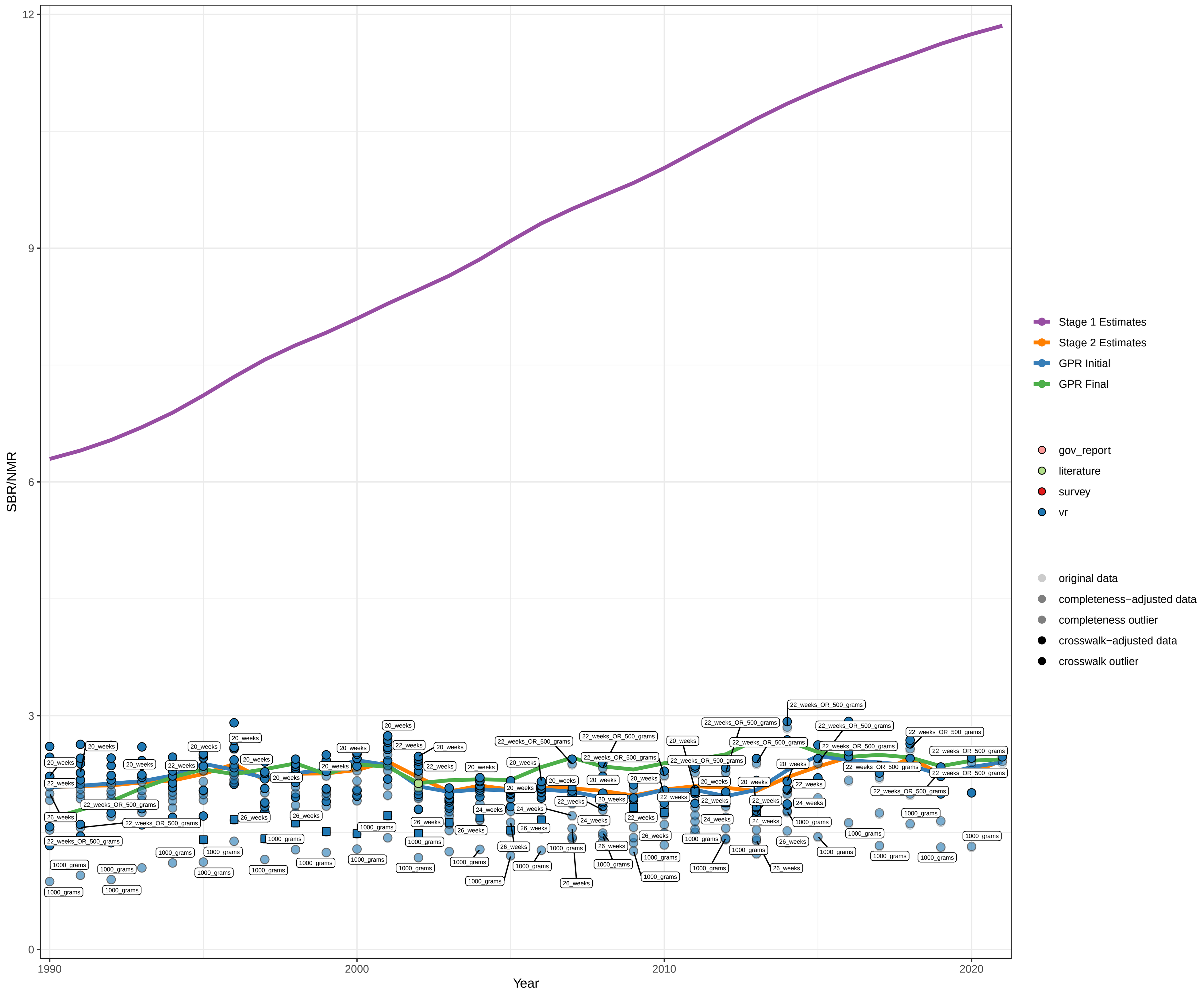

Norway NOR Western Europe Stillbirth Rate / Neonatal Mortality Rate (SBR/NMR) − 28 weeks
lambda = 0.2 ; zeta = 0.99 ; scale = 5 ; amp = 0.028

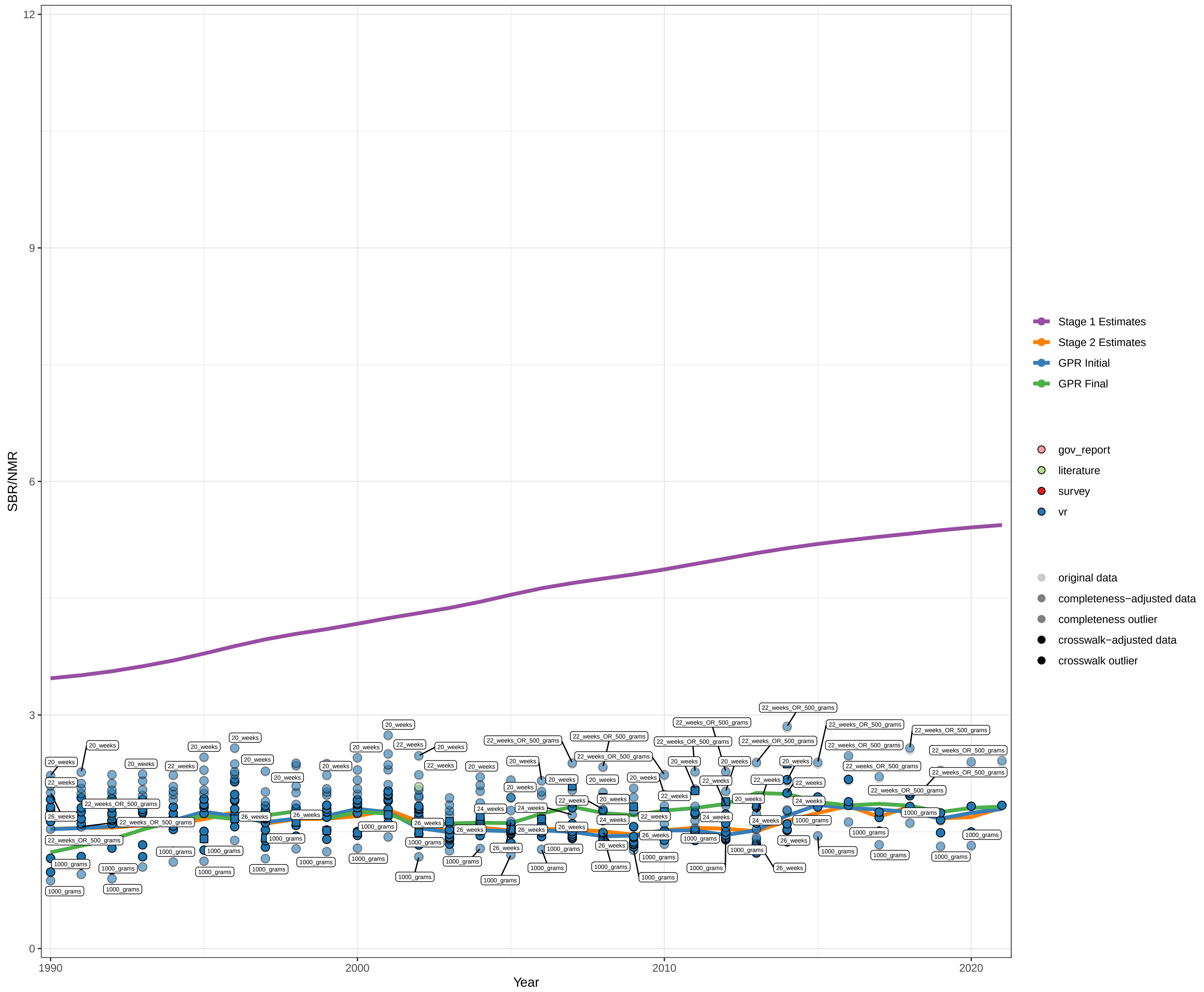

Definition	Years	Outlier	Source.Type	Title
1000_grams	1990−2020	0	VR	European Health for All Database
20_weeks, 22_weeks, 24_weeks, 26_weeks, 28_weeks	1990−2014	0	VR	Norway Medical Birth Registry
22_weeks_OR_500_grams	1990−2021	0	VR	Norway Medical Birth Registry
22_weeks_OR_500_grams	2002	0	Sci Lit	Stillbirth in relation to maternal country of birth and other migration related factors: a population−based study in Norway
28_weeks	2015−2019	0	VR	United Nations Demographic Yearbook
28_weeks	1995−2014	2	VR	United Nations Demographic Yearbook

Norway NOR Western Europe Stillbirth Rate (SBR) − 20 weeks
lambda = 0.2 ; zeta = 0.99 ; scale = 5 ; amp = 0.028

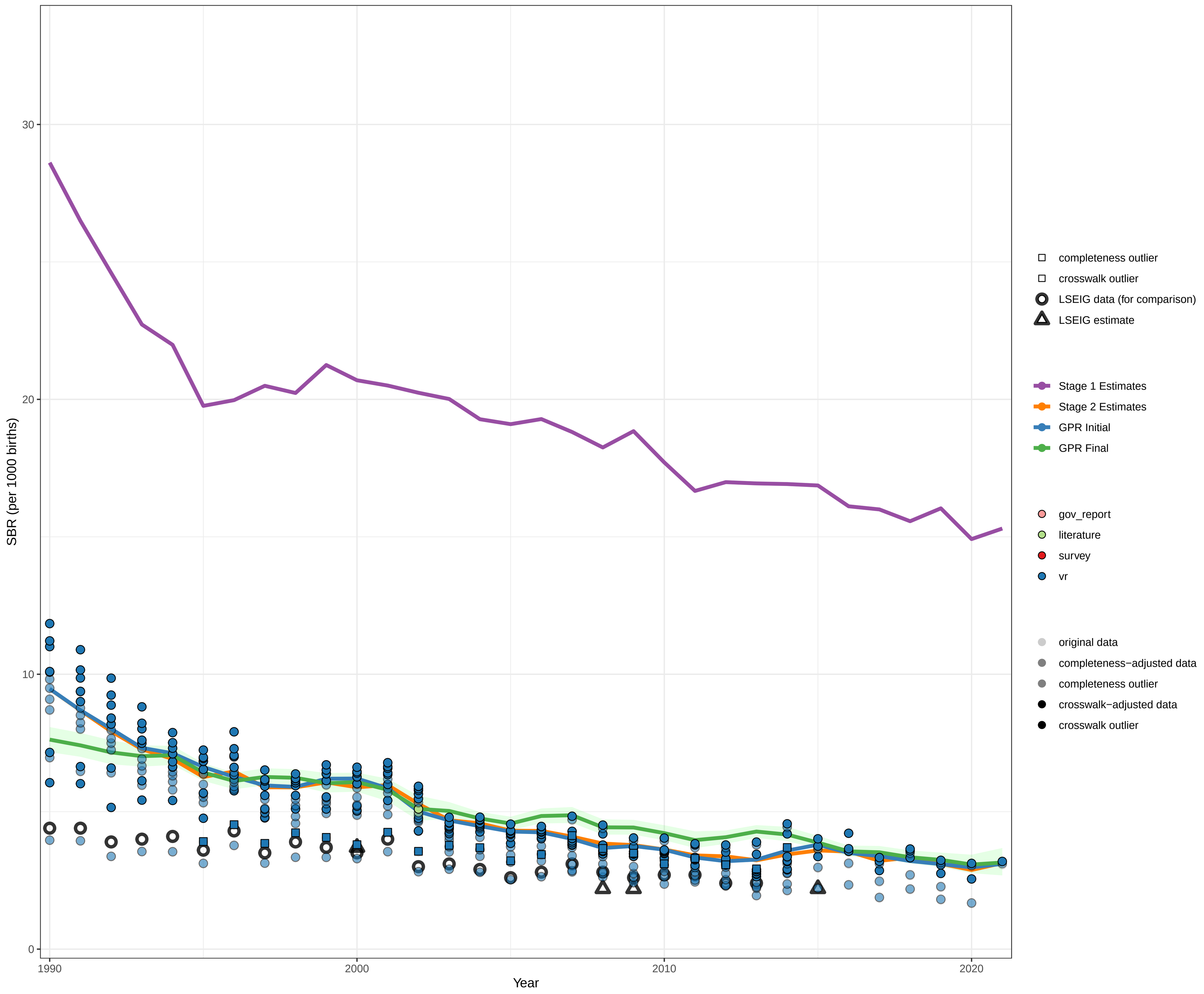

Norway NOR Western Europe Stillbirth Rate (SBR) − 28 weeks
lambda = 0.2 ; zeta = 0.99 ; scale = 5 ; amp = 0.028

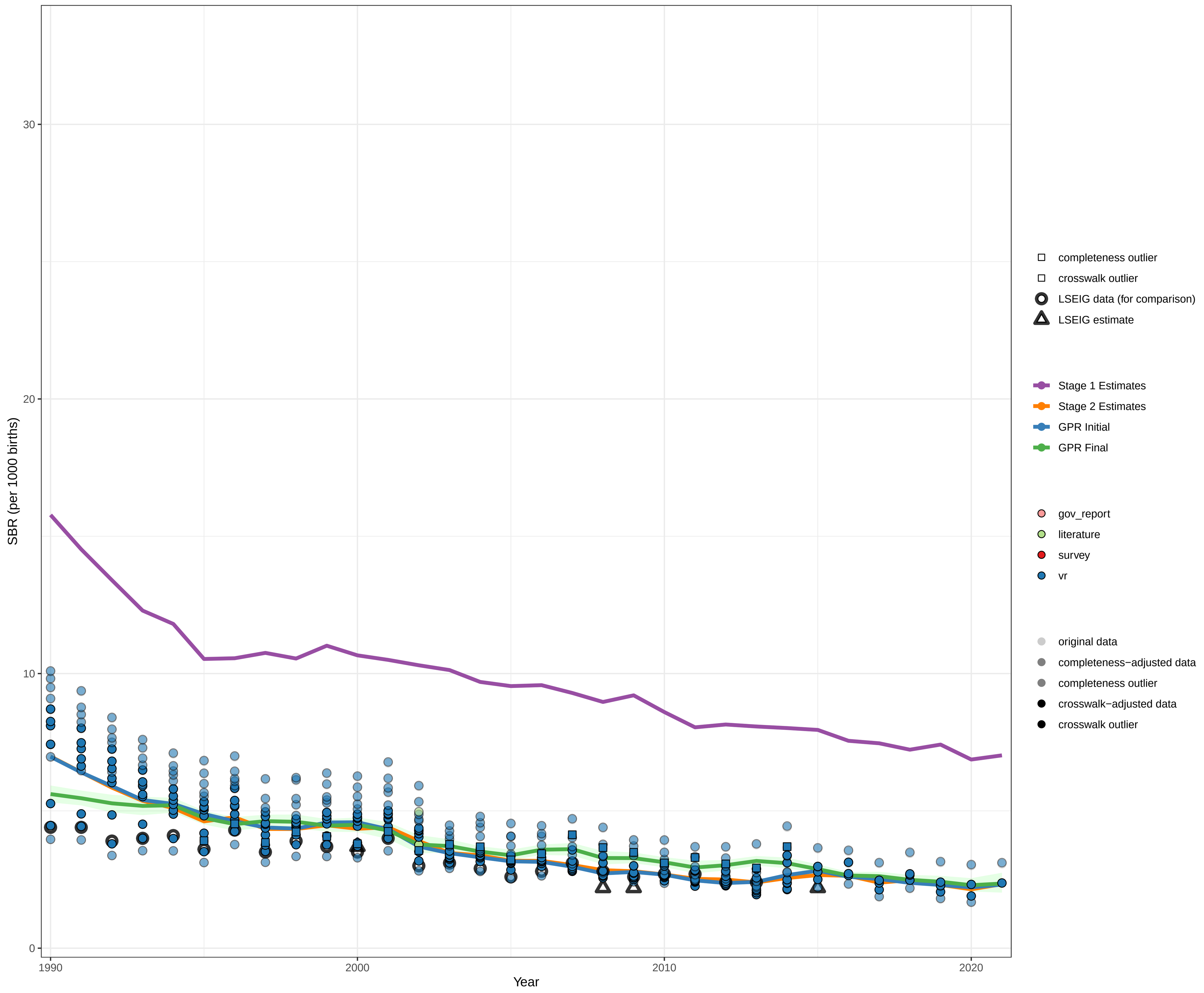

Year	Total Stillbirths (in thousands)	Total Stillbirths (in thousands)	SBR (per 1000 births)	SBR (per 1000 births)	SBR/NMR	SBR/NMR
	20 weeks	28 weeks	20 weeks	28 weeks	20 weeks	28 weeks
1990	0.4 (0.4 to 0.5)	0.3 (0.3 to 0.3)	7.6 (7.2 to 8.1)	5.6 (5.3 to 5.9)	1.68 (1.58 to 1.78)	1.23 (1.17 to 1.30)
1995	0.4 (0.4 to 0.4)	0.3 (0.3 to 0.3)	6.4 (6.1 to 6.8)	4.7 (4.5 to 5.0)	2.31 (2.20 to 2.43)	1.71 (1.62 to 1.80)
2000	0.4 (0.3 to 0.4)	0.3 (0.2 to 0.3)	6.1 (5.7 to 6.4)	4.5 (4.2 to 4.8)	2.38 (2.24 to 2.51)	1.76 (1.65 to 1.86)
2005	0.3 (0.3 to 0.3)	0.2 (0.2 to 0.2)	4.6 (4.3 to 4.8)	3.4 (3.2 to 3.6)	2.18 (2.06 to 2.29)	1.61 (1.53 to 1.71)
2010	0.3 (0.2 to 0.3)	0.2 (0.2 to 0.2)	4.2 (4.0 to 4.5)	3.1 (2.9 to 3.3)	2.39 (2.24 to 2.55)	1.77 (1.67 to 1.88)
2015	0.2 (0.2 to 0.2)	0.2 (0.2 to 0.2)	3.9 (3.7 to 4.1)	2.9 (2.7 to 3.1)	2.53 (2.40 to 2.70)	1.88 (1.78 to 1.99)
2020	0.2 (0.2 to 0.2)	0.1 (0.1 to 0.1)	3.1 (2.8 to 3.4)	2.3 (2.1 to 2.5)	2.42 (2.17 to 2.71)	1.81 (1.62 to 2.01)
2021	0.2 (0.2 to 0.2)	0.1 (0.1 to 0.2)	3.2 (2.7 to 3.7)	2.4 (2.0 to 2.7)	2.44 (2.08 to 2.85)	1.82 (1.57 to 2.13)

Agder NOR_60133 Western Europe Stillbirth Rate / Neonatal Mortality Rate (SBR/NMR) − 20 weeks
lambda = 0.2 ; zeta = 0.99 ; scale = 5 ; amp = 0.028

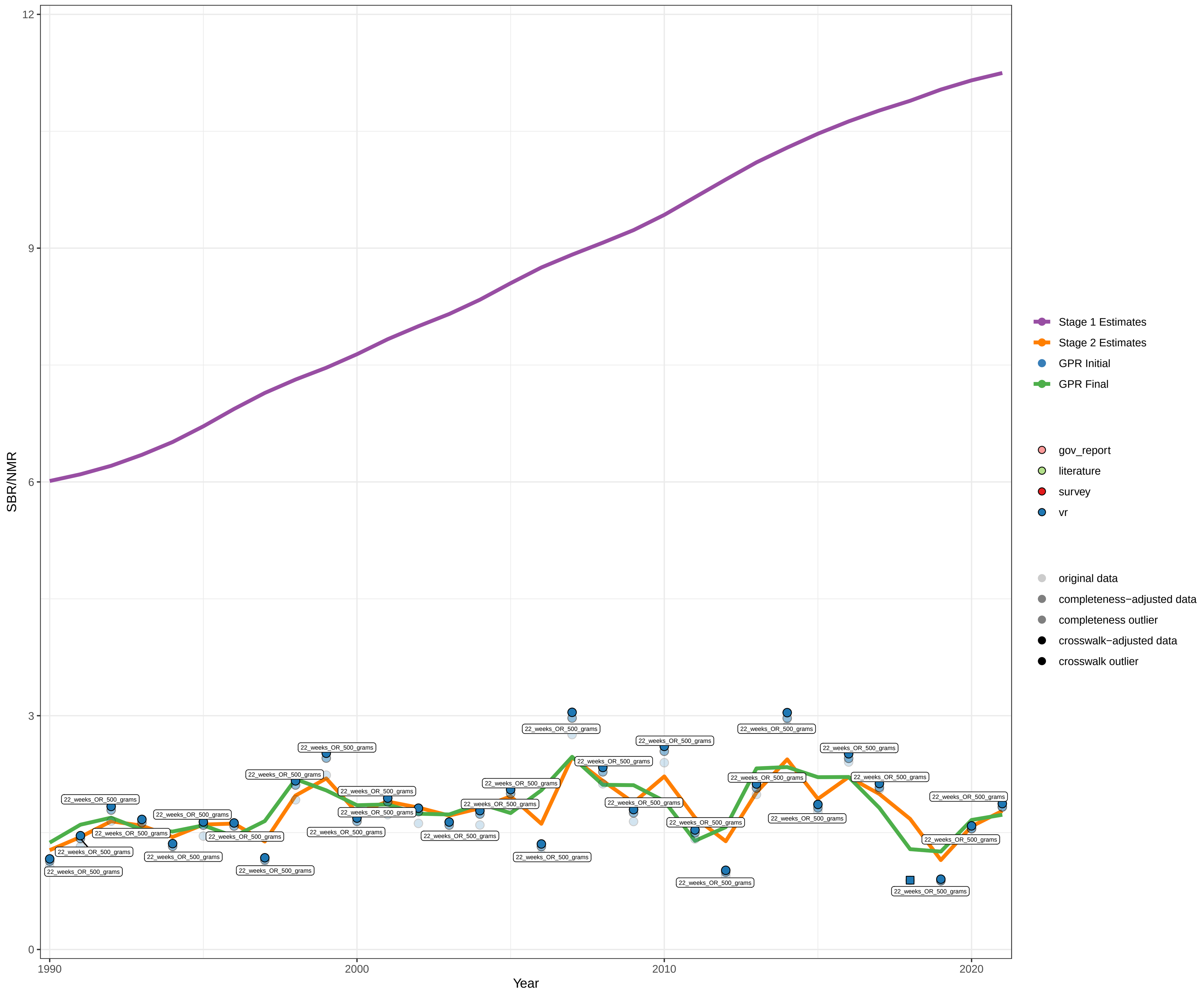

Agder NOR_60133 Western Europe Stillbirth Rate / Neonatal Mortality Rate (SBR/NMR) − 28 weeks
lambda = 0.2 ; zeta = 0.99 ; scale = 5 ; amp = 0.028

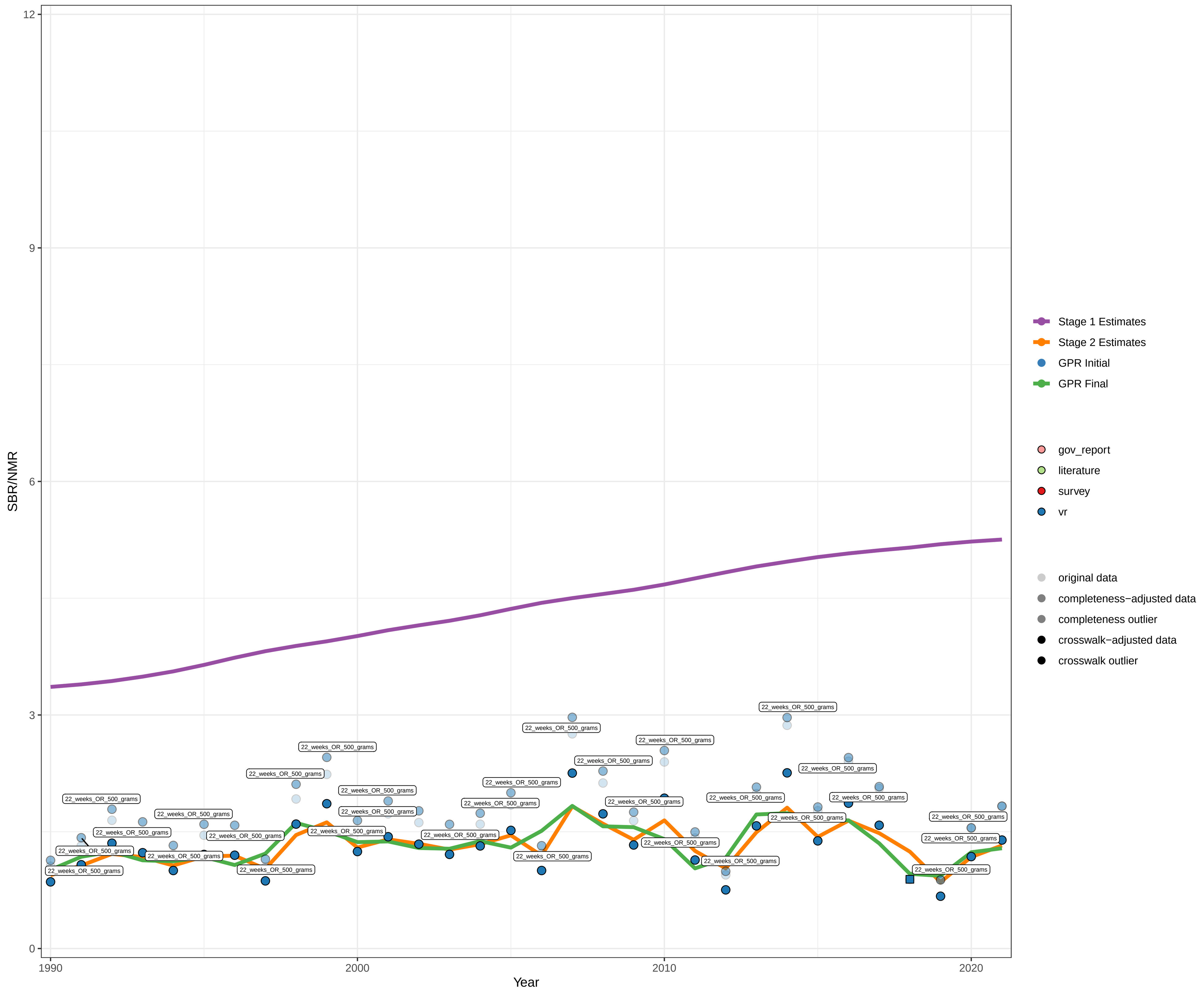

Definition	Years	Outlier	Source.Type	Title
22_weeks_OR_500_grams	1990−2021	0	VR	Norway Medical Birth Registry

Agder NOR_60133 Western Europe Stillbirth Rate (SBR) − 20 weeks
lambda = 0.2 ; zeta = 0.99 ; scale = 5 ; amp = 0.028

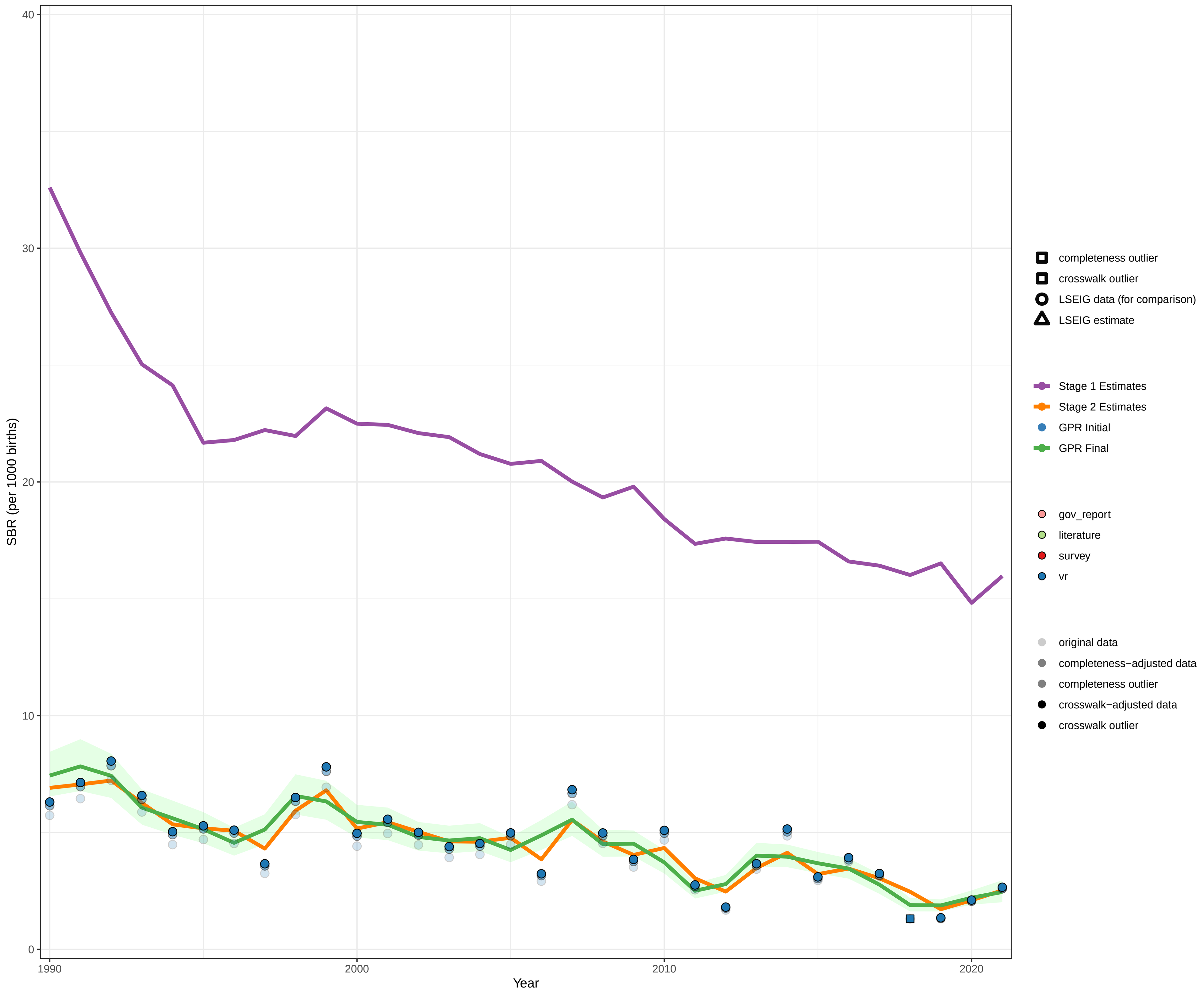

Agder NOR_60133 Western Europe Stillbirth Rate (SBR) − 28 weeks
lambda = 0.2 ; zeta = 0.99 ; scale = 5 ; amp = 0.028

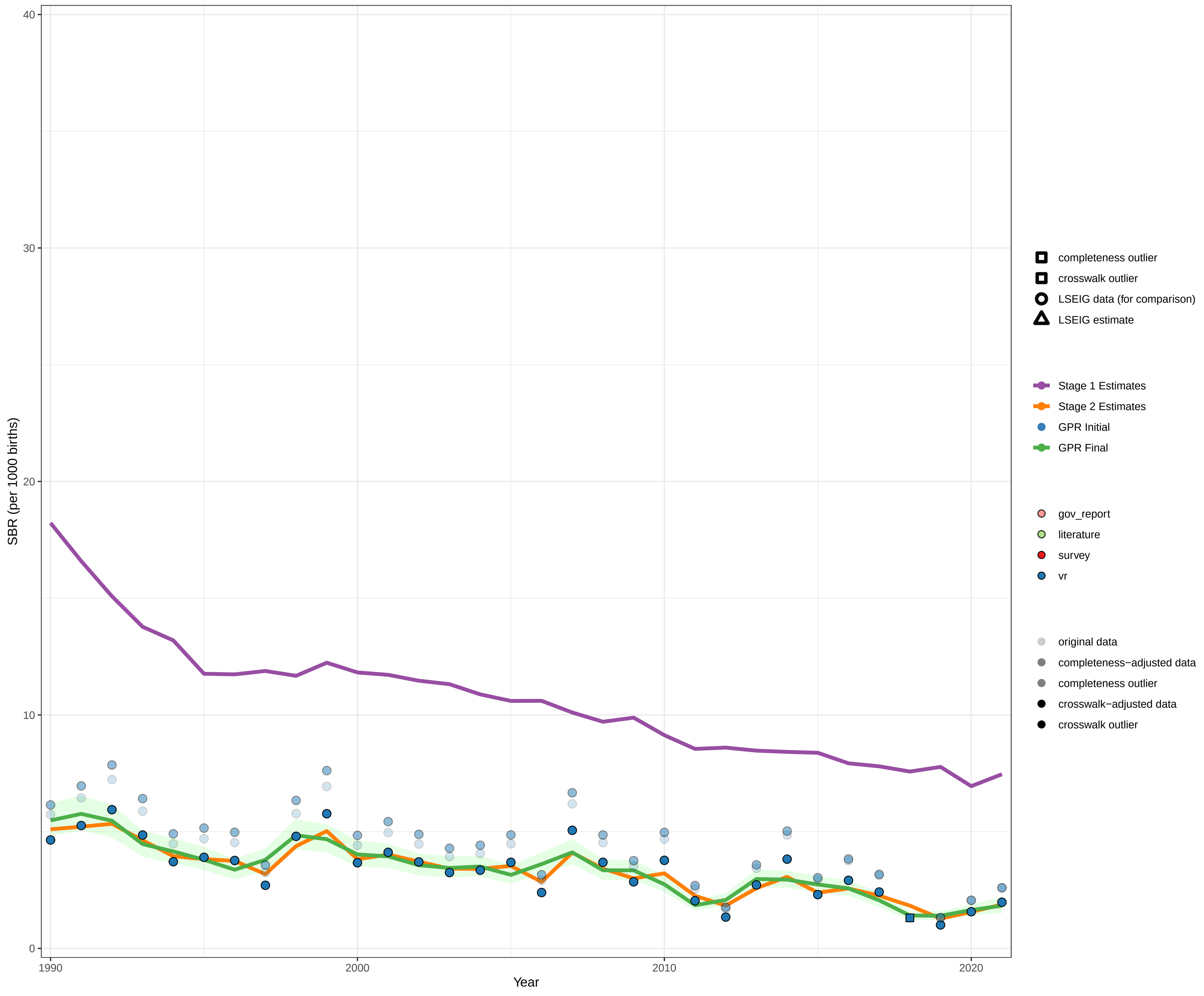

Year	Total Stillbirths (in thousands)	Total Stillbirths (in thousands)	SBR (per 1000 births)	SBR (per 1000 births)	SBR/NMR	SBR/NMR
	20 weeks	28 weeks	20 weeks	28 weeks	20 weeks	28 weeks
1990	0.0 (0.0 to 0.0)	0.0 (0.0 to 0.0)	7.4 (6.5 to 8.5)	5.5 (4.8 to 6.2)	1.37 (1.21 to 1.56)	1.01 (0.89 to 1.15)
1995	0.0 (0.0 to 0.0)	0.0 (0.0 to 0.0)	5.1 (4.5 to 5.9)	3.8 (3.4 to 4.3)	1.59 (1.41 to 1.82)	1.18 (1.04 to 1.34)
2000	0.0 (0.0 to 0.0)	0.0 (0.0 to 0.0)	5.5 (4.8 to 6.2)	4.0 (3.5 to 4.6)	1.85 (1.62 to 2.10)	1.37 (1.19 to 1.57)
2005	0.0 (0.0 to 0.0)	0.0 (0.0 to 0.0)	4.3 (3.7 to 4.8)	3.1 (2.8 to 3.6)	1.75 (1.53 to 1.98)	1.30 (1.14 to 1.47)
2010	0.0 (0.0 to 0.0)	0.0 (0.0 to 0.0)	3.7 (3.2 to 4.3)	2.7 (2.4 to 3.1)	1.91 (1.66 to 2.18)	1.41 (1.24 to 1.60)
2015	0.0 (0.0 to 0.0)	0.0 (0.0 to 0.0)	3.7 (3.3 to 4.2)	2.7 (2.4 to 3.1)	2.21 (1.95 to 2.50)	1.64 (1.45 to 1.86)
2020	0.0 (0.0 to 0.0)	0.0 (0.0 to 0.0)	2.2 (1.9 to 2.5)	1.6 (1.4 to 1.9)	1.66 (1.44 to 1.90)	1.24 (1.07 to 1.41)
2021	0.0 (0.0 to 0.0)	0.0 (0.0 to 0.0)	2.5 (2.0 to 3.0)	1.8 (1.5 to 2.2)	1.73 (1.42 to 2.08)	1.29 (1.06 to 1.55)

Innlandet   NOR_60135   Western Europe   Stillbirth Rate / Neonatal Mortality Rate (SBR/NMR) − 20 weeks
lambda = 0.2 ; zeta = 0.99 ; scale = 5 ; amp = 0.028

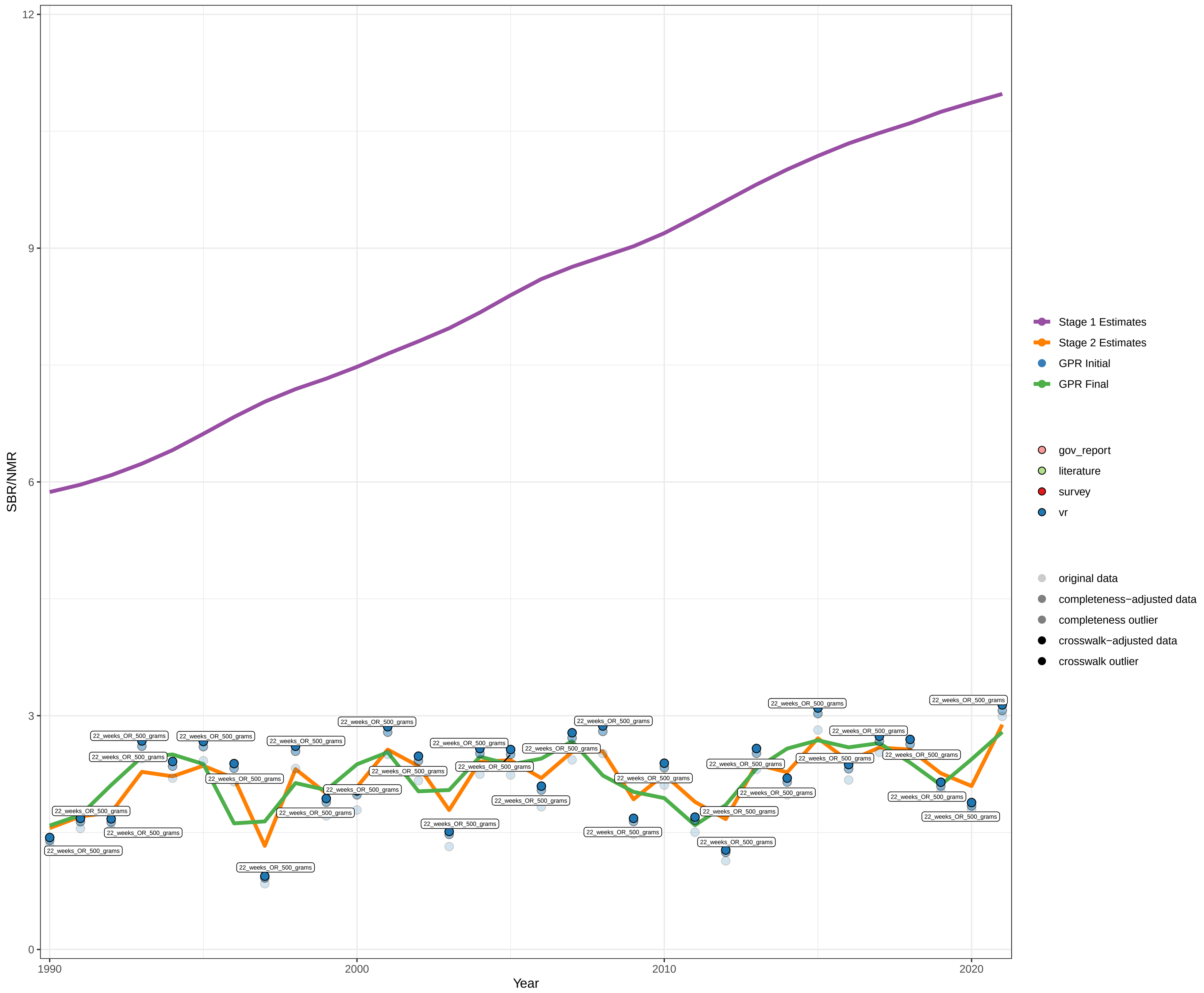

Innlandet   NOR_60135   Western Europe   Stillbirth Rate / Neonatal Mortality Rate (SBR/NMR) − 28 weeks
lambda = 0.2 ; zeta = 0.99 ; scale = 5 ; amp = 0.028

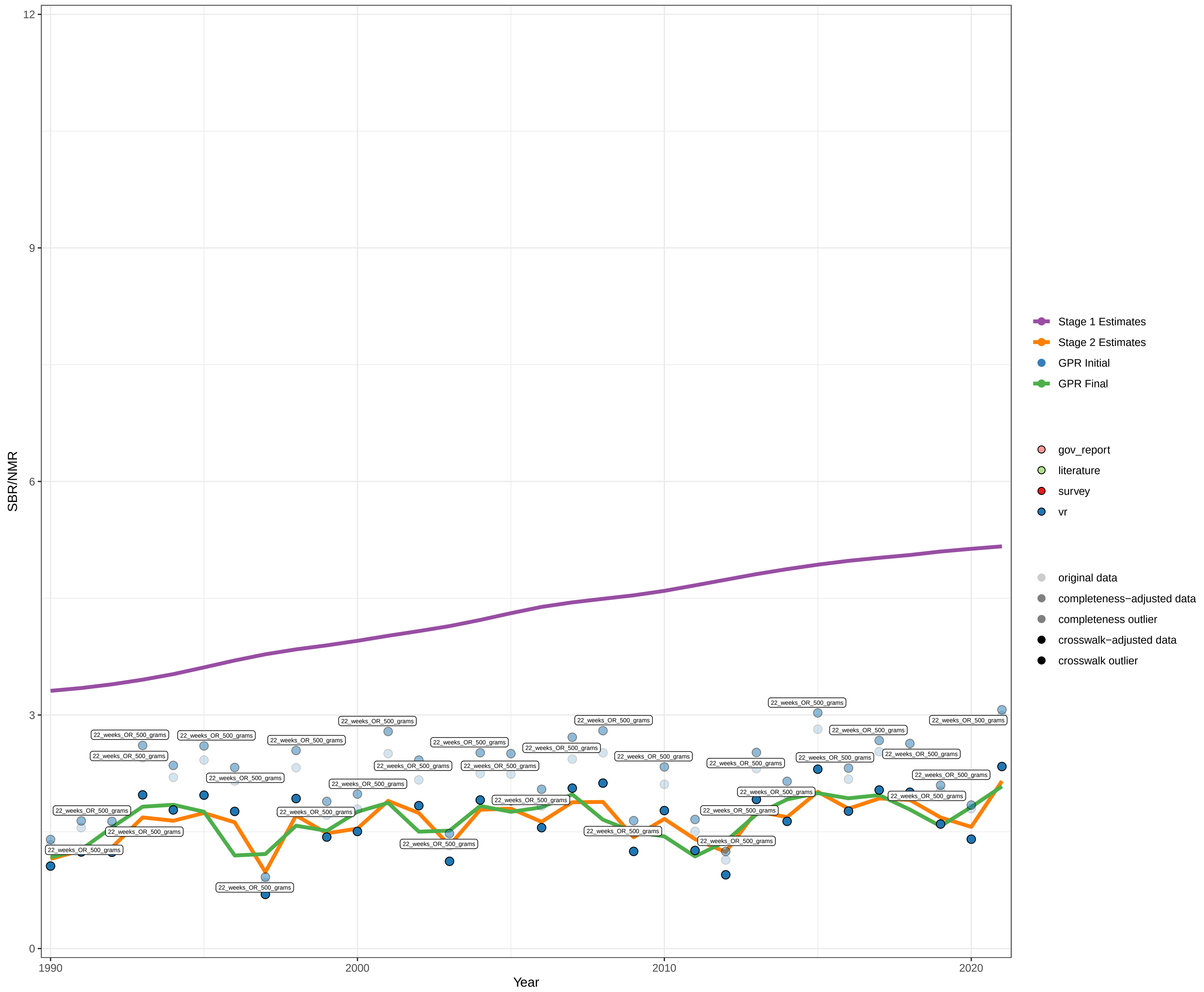

Definition	Years	Outlier	Source.Type	Title
22_weeks_OR_500_grams	1990−2021	0	VR	Norway Medical Birth Registry

Innlandet   NOR_60135   Western Europe   Stillbirth Rate (SBR) − 20 weeks
lambda = 0.2 ; zeta = 0.99 ; scale = 5 ; amp = 0.028

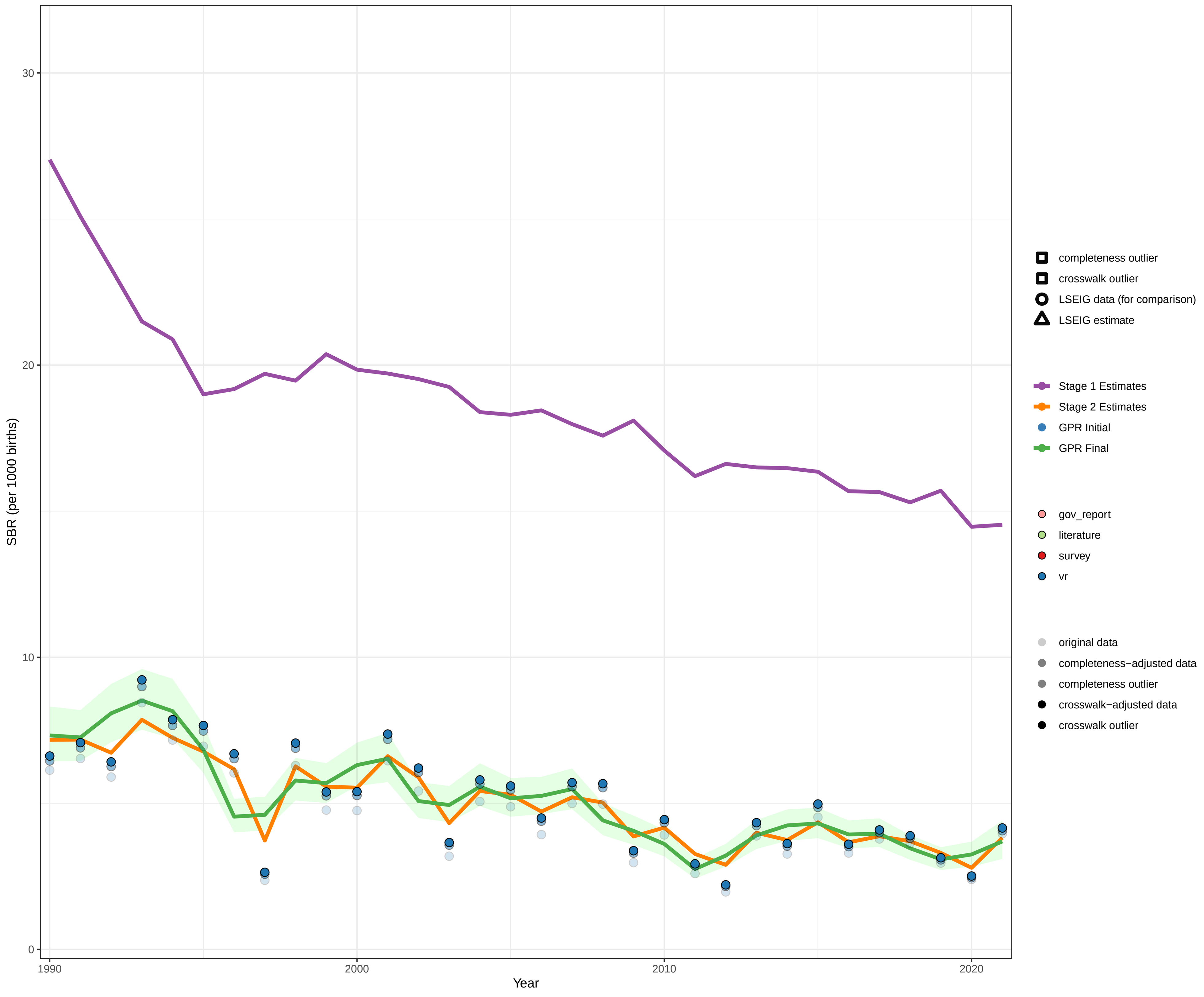

Innlandet   NOR_60135   Western Europe   Stillbirth Rate (SBR) − 28 weeks
lambda = 0.2 ; zeta = 0.99 ; scale = 5 ; amp = 0.028

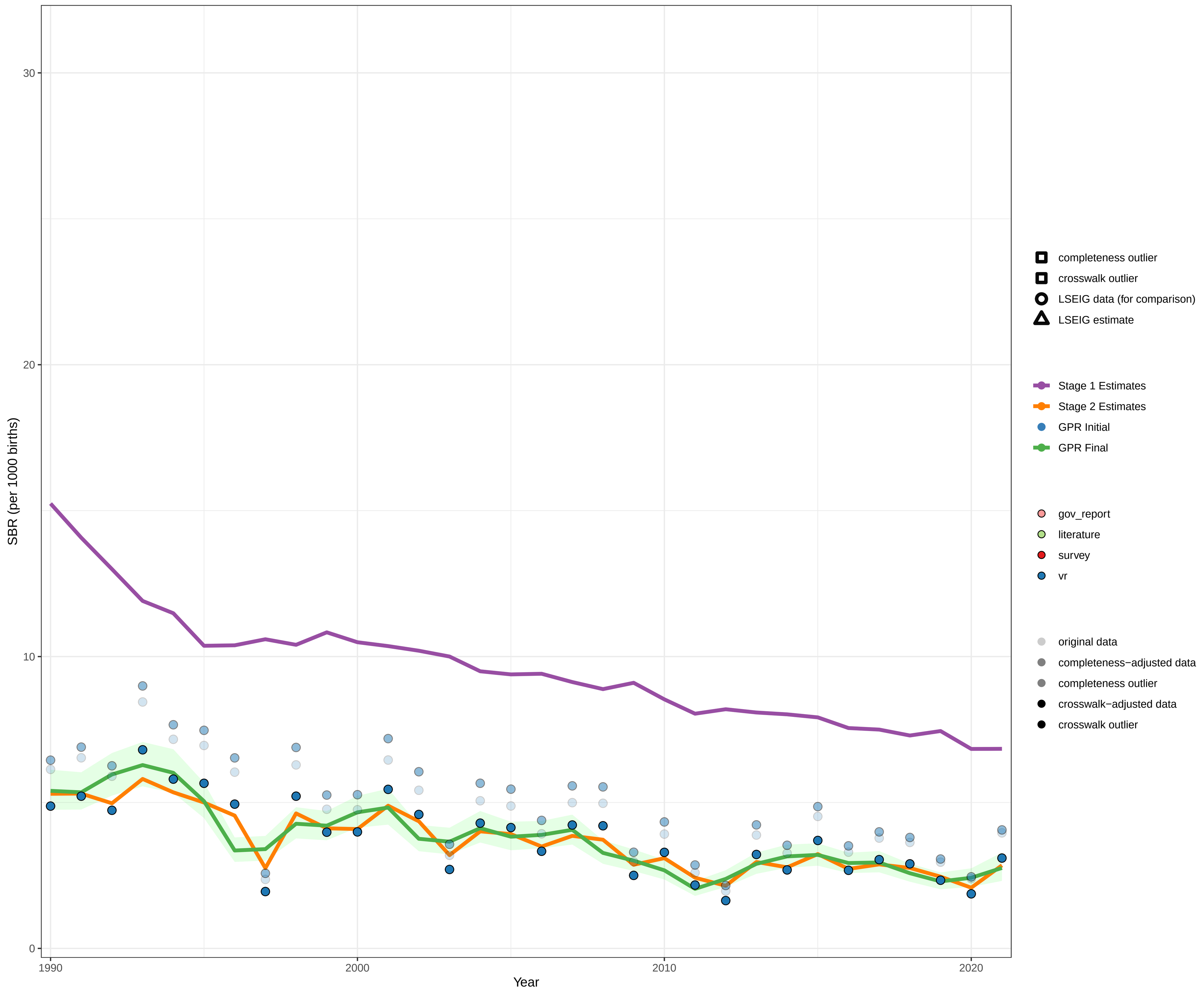

Year	Total Stillbirths (in thousands)	Total Stillbirths (in thousands)	SBR (per 1000 births)	SBR (per 1000 births)	SBR/NMR	SBR/NMR
	20 weeks	28 weeks	20 weeks	28 weeks	20 weeks	28 weeks
1990	0.0 (0.0 to 0.0)	0.0 (0.0 to 0.0)	7.3 (6.4 to 8.3)	5.4 (4.7 to 6.1)	1.59 (1.40 to 1.81)	1.17 (1.03 to 1.33)
1995	0.0 (0.0 to 0.0)	0.0 (0.0 to 0.0)	6.8 (6.0 to 7.7)	5.0 (4.5 to 5.7)	2.38 (2.10 to 2.68)	1.76 (1.56 to 1.98)
2000	0.0 (0.0 to 0.0)	0.0 (0.0 to 0.0)	6.3 (5.6 to 7.1)	4.7 (4.1 to 5.2)	2.38 (2.11 to 2.67)	1.76 (1.56 to 1.97)
2005	0.0 (0.0 to 0.0)	0.0 (0.0 to 0.0)	5.2 (4.5 to 5.9)	3.8 (3.4 to 4.3)	2.37 (2.08 to 2.69)	1.76 (1.54 to 1.99)
2010	0.0 (0.0 to 0.0)	0.0 (0.0 to 0.0)	3.6 (3.2 to 4.1)	2.7 (2.4 to 3.0)	1.94 (1.71 to 2.21)	1.44 (1.27 to 1.63)
2015	0.0 (0.0 to 0.0)	0.0 (0.0 to 0.0)	4.3 (3.8 to 4.8)	3.2 (2.8 to 3.6)	2.68 (2.37 to 3.02)	2.00 (1.76 to 2.24)
2020	0.0 (0.0 to 0.0)	0.0 (0.0 to 0.0)	3.2 (2.8 to 3.7)	2.4 (2.1 to 2.7)	2.44 (2.13 to 2.77)	1.82 (1.59 to 2.06)
2021	0.0 (0.0 to 0.0)	0.0 (0.0 to 0.0)	3.7 (3.1 to 4.4)	2.8 (2.3 to 3.3)	2.79 (2.34 to 3.33)	2.08 (1.75 to 2.48)

More og Romsdal   NOR_4923   Western Europe   Stillbirth Rate / Neonatal Mortality Rate (SBR/NMR) − 20 weeks
lambda = 0.2 ; zeta = 0.99 ; scale = 5 ; amp = 0.028

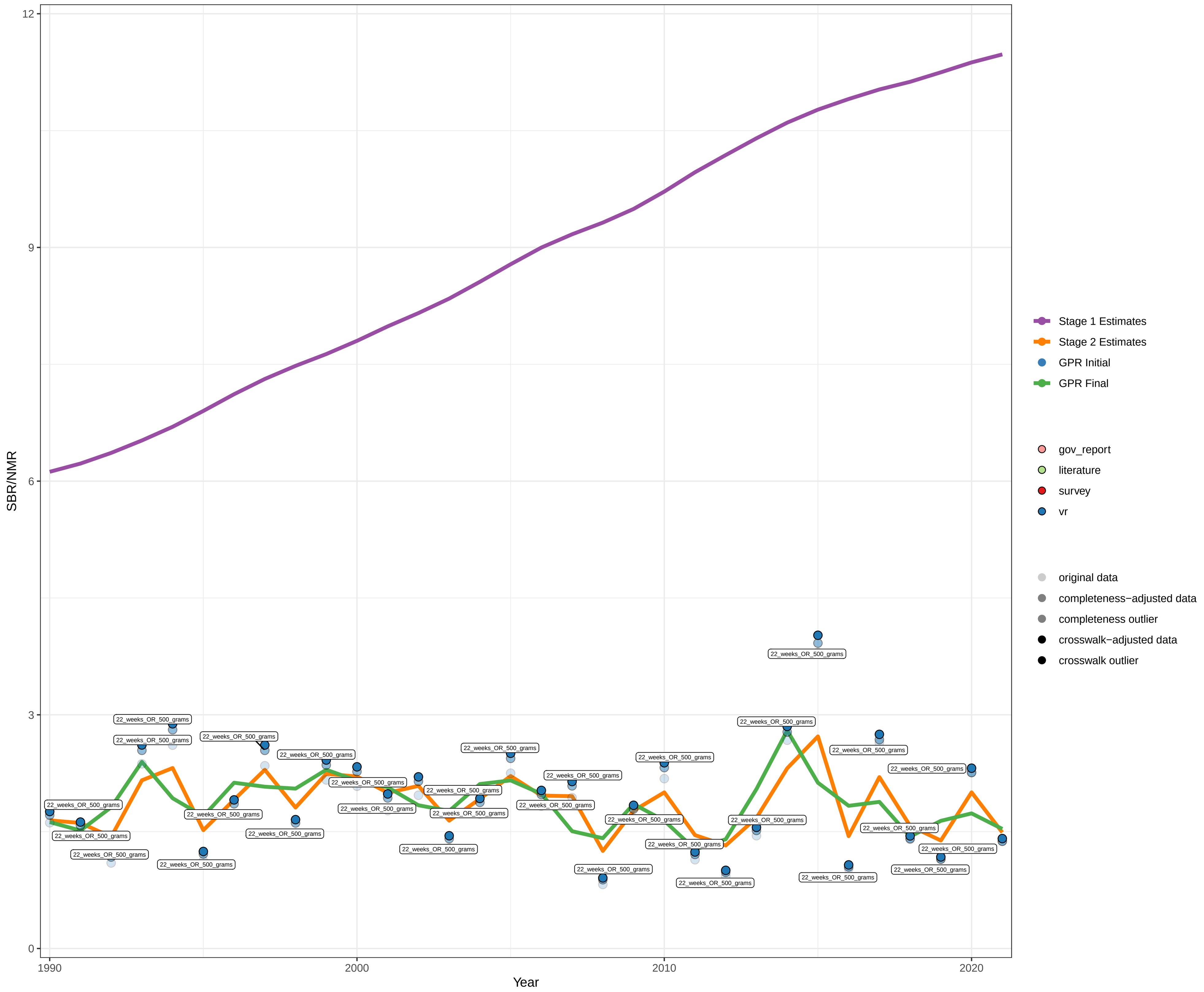

More og Romsdal   NOR_4923   Western Europe   Stillbirth Rate / Neonatal Mortality Rate (SBR/NMR) − 28 weeks
lambda = 0.2 ; zeta = 0.99 ; scale = 5 ; amp = 0.028

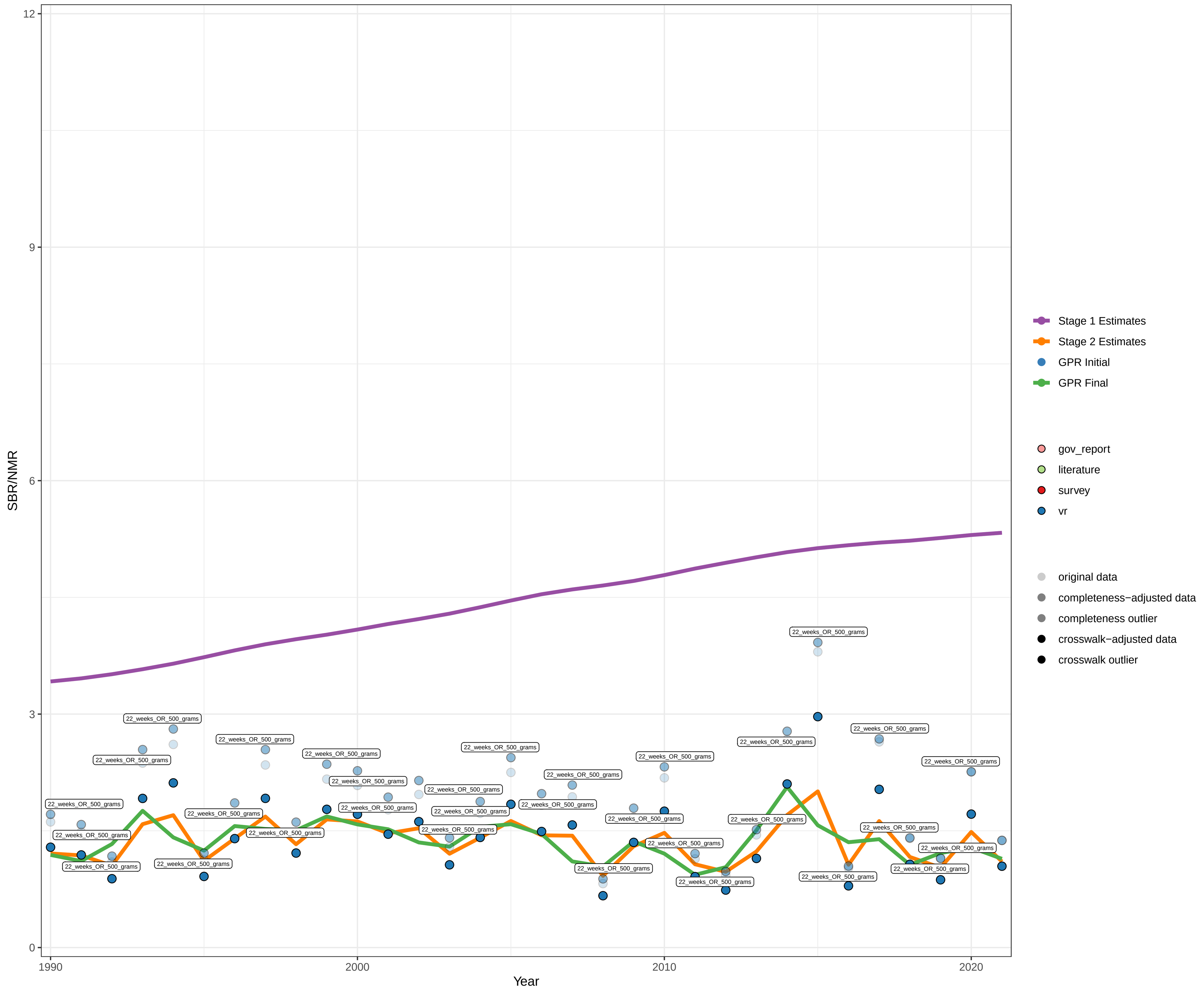

Definition	Years	Outlier	Source.Type	Title
22_weeks_OR_500_grams	1990−2021	0	VR	Norway Medical Birth Registry

More og Romsdal   NOR_4923   Western Europe   Stillbirth Rate (SBR) − 20 weeks
lambda = 0.2 ; zeta = 0.99 ; scale = 5 ; amp = 0.028

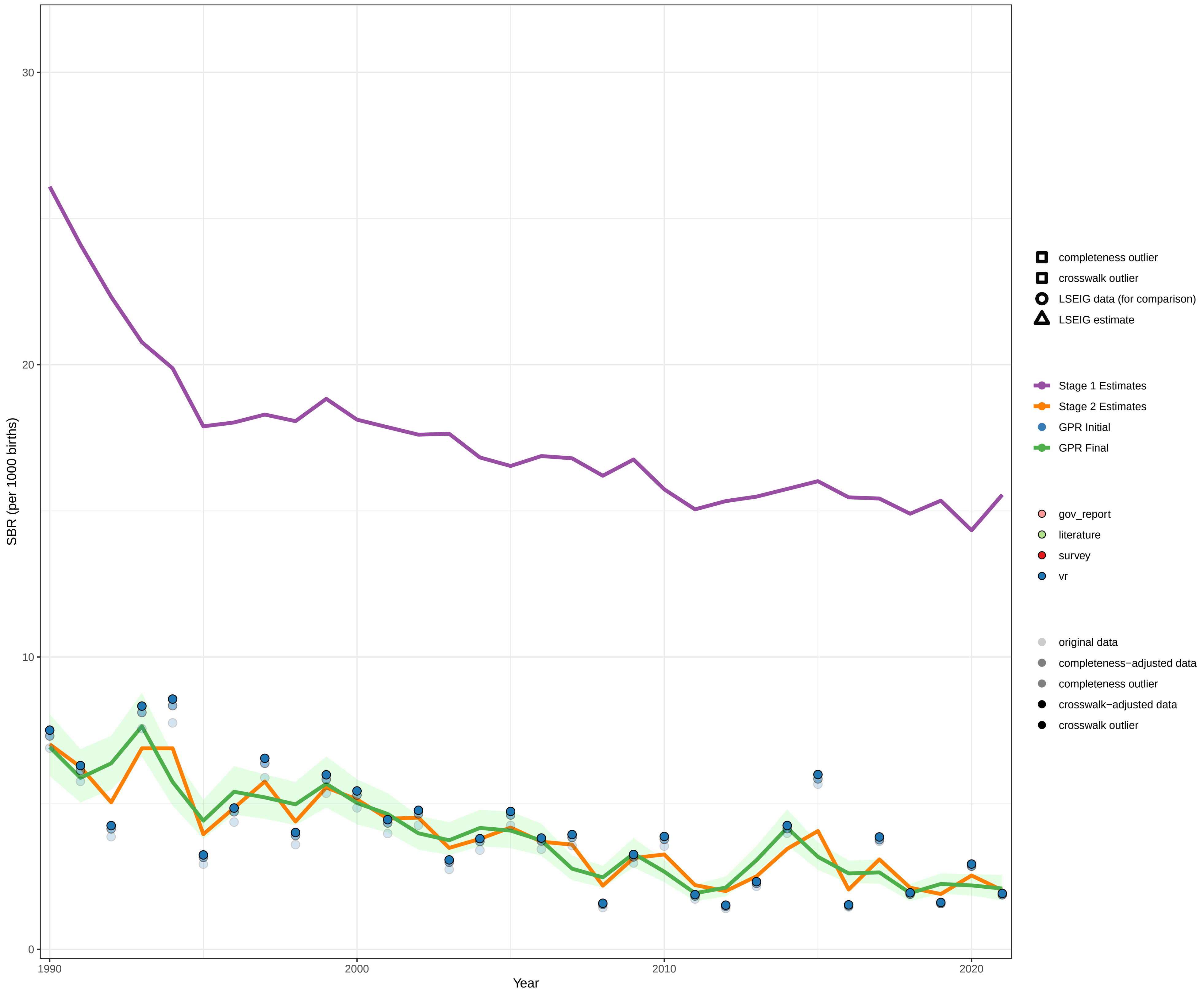

More og Romsdal   NOR_4923   Western Europe   Stillbirth Rate (SBR) − 28 weeks
lambda = 0.2 ; zeta = 0.99 ; scale = 5 ; amp = 0.028

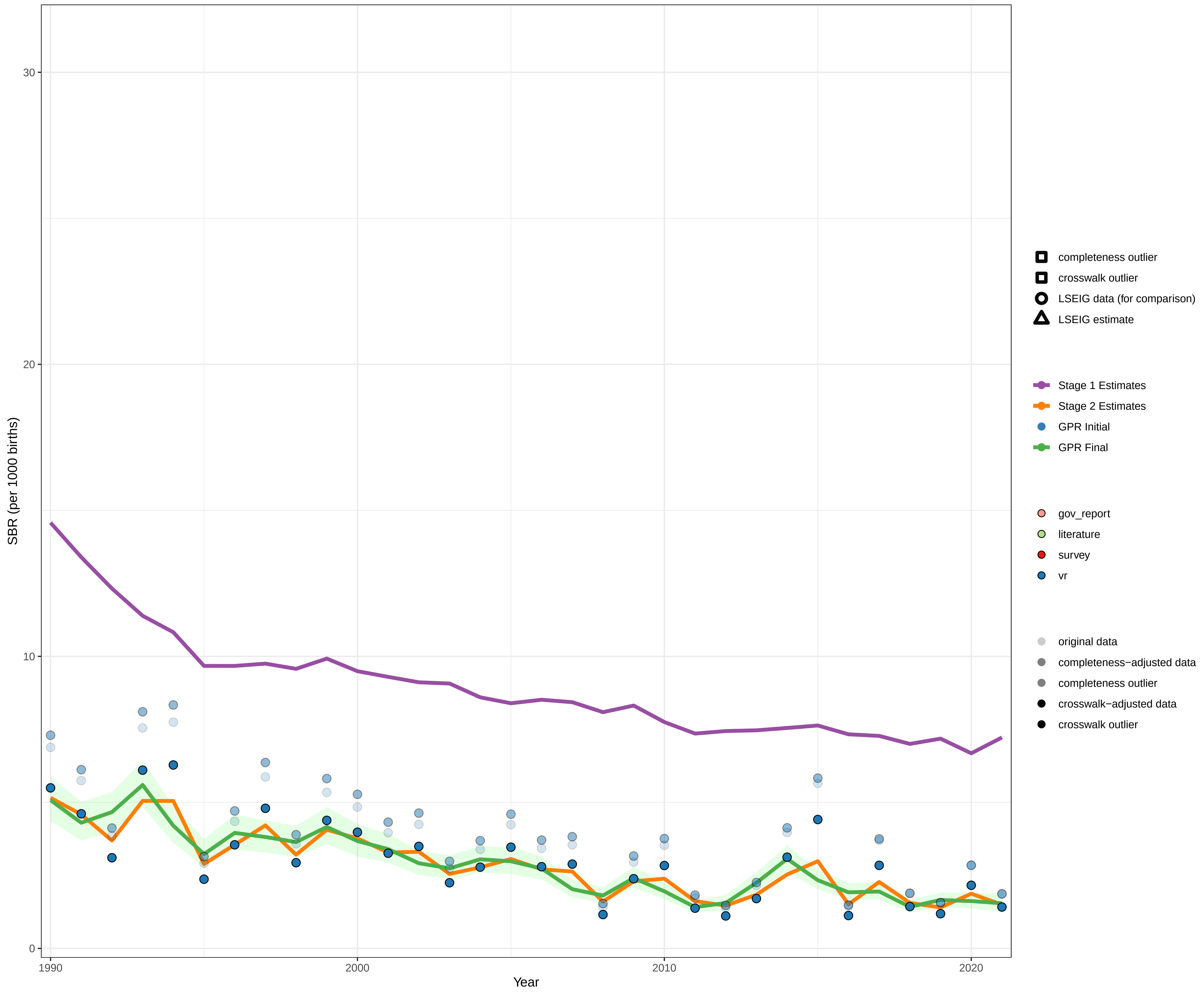

Year	Total Stillbirths (in thousands)	Total Stillbirths (in thousands)	SBR (per 1000 births)	SBR (per 1000 births)	SBR/NMR	SBR/NMR
	20 weeks	28 weeks	20 weeks	28 weeks	20 weeks	28 weeks
1990	0.0 (0.0 to 0.0)	0.0 (0.0 to 0.0)	6.9 (5.9 to 8.0)	5.1 (4.4 to 5.9)	1.62 (1.39 to 1.89)	1.19 (1.02 to 1.38)
1995	0.0 (0.0 to 0.0)	0.0 (0.0 to 0.0)	4.4 (3.8 to 5.1)	3.2 (2.8 to 3.8)	1.70 (1.46 to 1.97)	1.25 (1.07 to 1.45)
2000	0.0 (0.0 to 0.0)	0.0 (0.0 to 0.0)	5.0 (4.3 to 5.8)	3.7 (3.1 to 4.3)	2.15 (1.84 to 2.50)	1.58 (1.35 to 1.84)
2005	0.0 (0.0 to 0.0)	0.0 (0.0 to 0.0)	4.1 (3.5 to 4.7)	3.0 (2.5 to 3.5)	2.16 (1.84 to 2.50)	1.58 (1.35 to 1.84)
2010	0.0 (0.0 to 0.0)	0.0 (0.0 to 0.0)	2.7 (2.3 to 3.1)	2.0 (1.7 to 2.2)	1.64 (1.42 to 1.89)	1.21 (1.04 to 1.39)
2015	0.0 (0.0 to 0.0)	0.0 (0.0 to 0.0)	3.2 (2.7 to 3.7)	2.3 (2.0 to 2.7)	2.13 (1.83 to 2.46)	1.57 (1.35 to 1.81)
2020	0.0 (0.0 to 0.0)	0.0 (0.0 to 0.0)	2.2 (1.8 to 2.6)	1.6 (1.4 to 1.9)	1.73 (1.46 to 2.04)	1.29 (1.08 to 1.51)
2021	0.0 (0.0 to 0.0)	0.0 (0.0 to 0.0)	2.1 (1.7 to 2.5)	1.5 (1.2 to 1.9)	1.54 (1.23 to 1.88)	1.14 (0.92 to 1.39)

Nordland NOR_4926 Western Europe Stillbirth Rate / Neonatal Mortality Rate (SBR/NMR) − 20 weeks
lambda = 0.2 ; zeta = 0.99 ; scale = 5 ; amp = 0.028

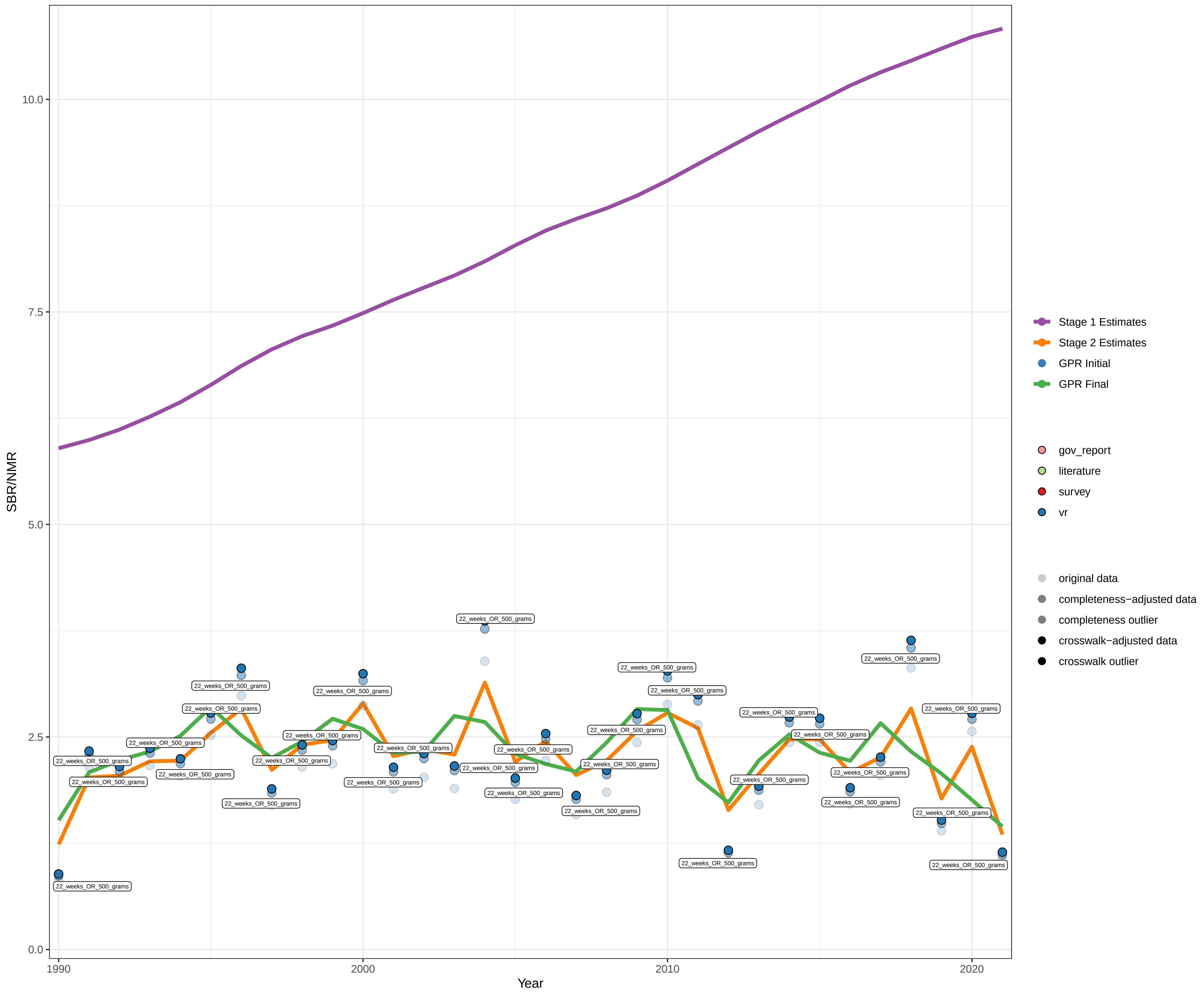

Nordland NOR_4926 Western Europe Stillbirth Rate / Neonatal Mortality Rate (SBR/NMR) − 28 weeks
lambda = 0.2 ; zeta = 0.99 ; scale = 5 ; amp = 0.028

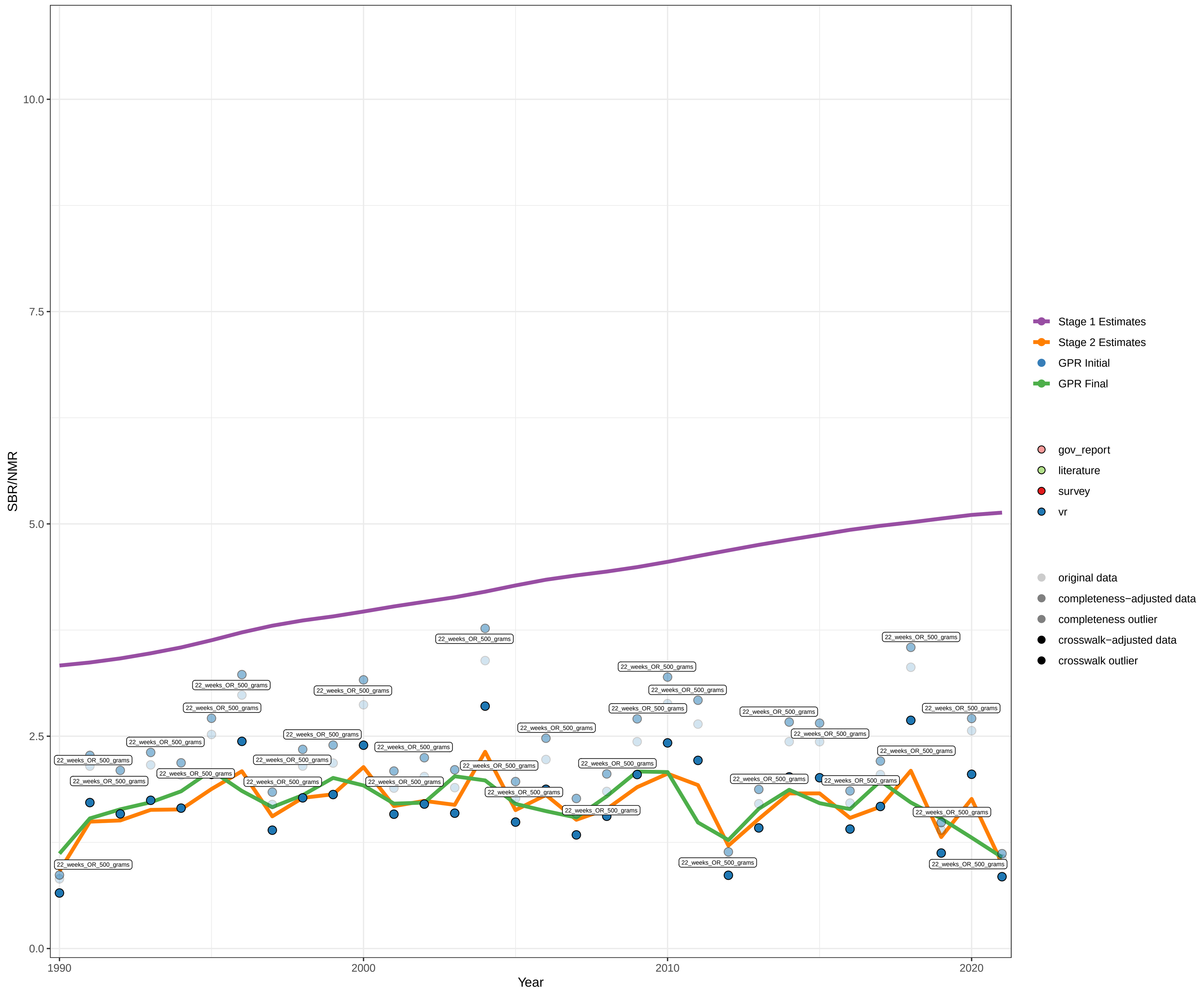

Definition	Years	Outlier	Source.Type	Title
22_weeks_OR_500_grams	1990−2021	0	VR	Norway Medical Birth Registry

Nordland NOR_4926 Western Europe Stillbirth Rate (SBR) − 20 weeks
lambda = 0.2 ; zeta = 0.99 ; scale = 5 ; amp = 0.028

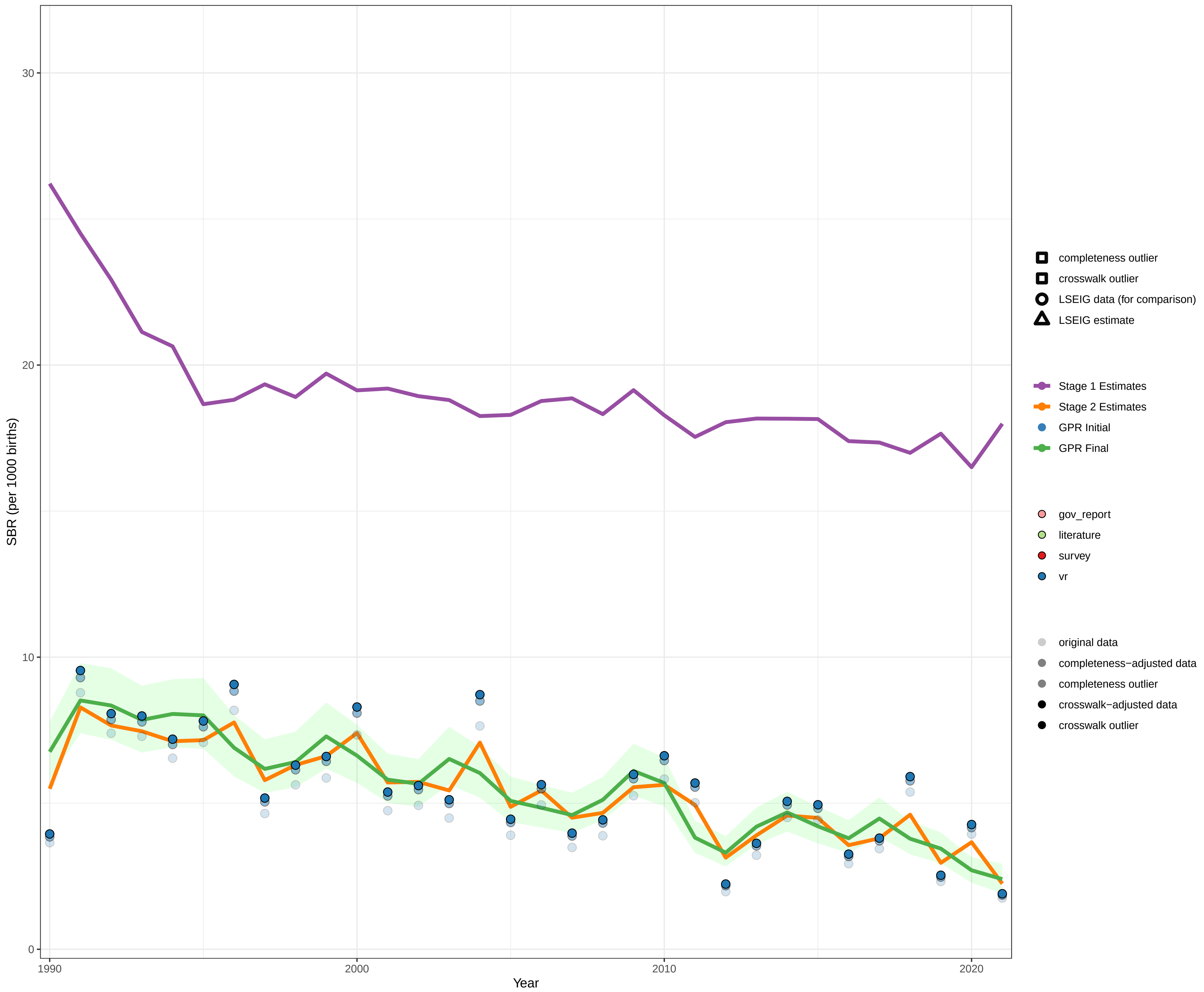

Nordland NOR_4926 Western Europe Stillbirth Rate (SBR) − 28 weeks
lambda = 0.2 ; zeta = 0.99 ; scale = 5 ; amp = 0.028

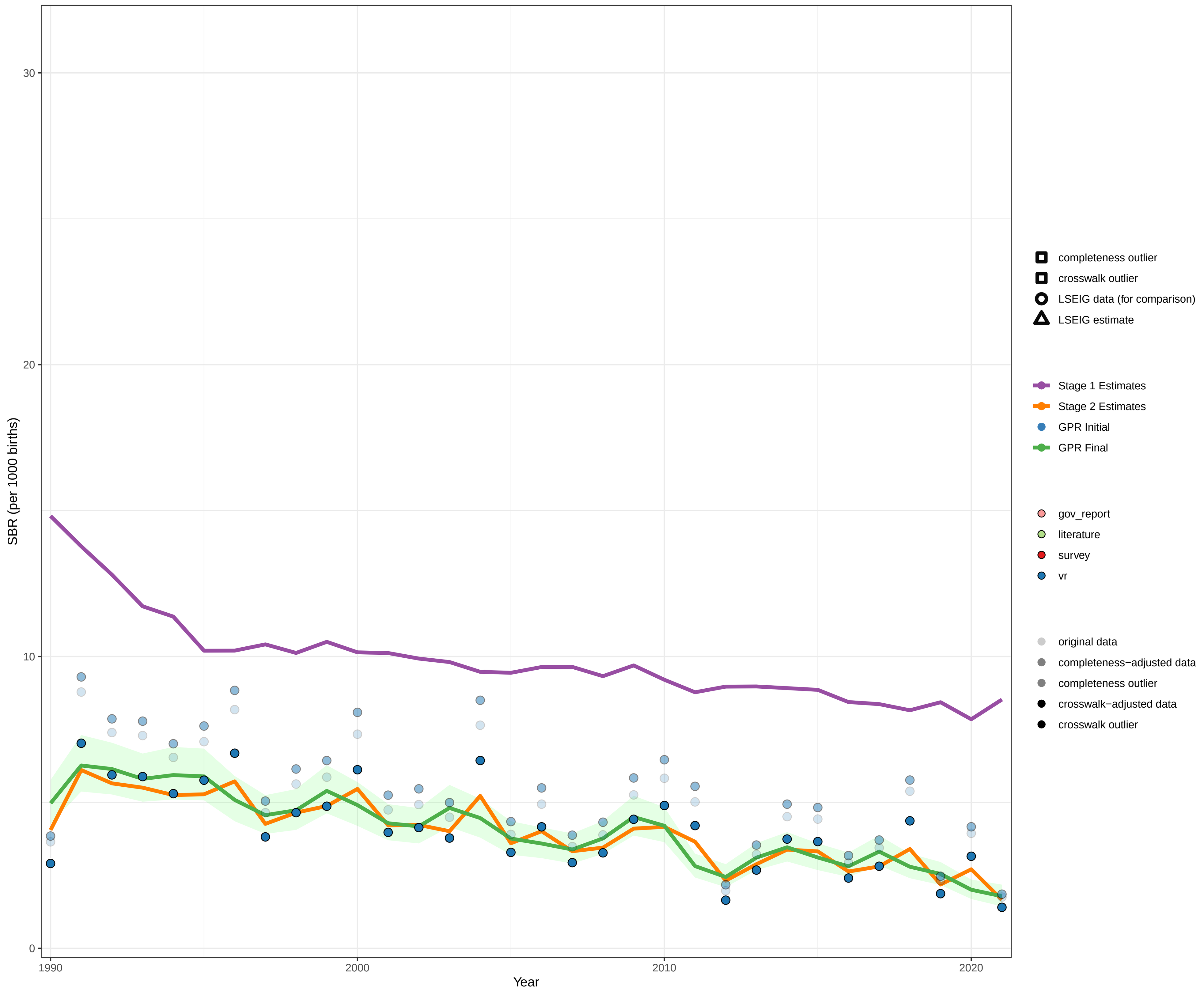

Year	Total Stillbirths (in thousands)	Total Stillbirths (in thousands)	SBR (per 1000 births)	SBR (per 1000 births)	SBR/NMR	SBR/NMR
	20 weeks	28 weeks	20 weeks	28 weeks	20 weeks	28 weeks
1990	0.0 (0.0 to 0.0)	0.0 (0.0 to 0.0)	6.8 (5.8 to 7.8)	5.0 (4.3 to 5.8)	1.52 (1.31 to 1.75)	1.12 (0.96 to 1.30)
1995	0.0 (0.0 to 0.0)	0.0 (0.0 to 0.0)	8.0 (6.9 to 9.3)	5.9 (5.1 to 6.8)	2.85 (2.44 to 3.31)	2.10 (1.80 to 2.44)
2000	0.0 (0.0 to 0.0)	0.0 (0.0 to 0.0)	6.6 (5.7 to 7.7)	4.9 (4.2 to 5.7)	2.59 (2.23 to 3.01)	1.92 (1.64 to 2.23)
2005	0.0 (0.0 to 0.0)	0.0 (0.0 to 0.0)	5.1 (4.4 to 5.9)	3.8 (3.2 to 4.4)	2.30 (1.97 to 2.68)	1.70 (1.45 to 1.97)
2010	0.0 (0.0 to 0.0)	0.0 (0.0 to 0.0)	5.7 (4.9 to 6.6)	4.2 (3.6 to 4.8)	2.82 (2.42 to 3.25)	2.08 (1.80 to 2.39)
2015	0.0 (0.0 to 0.0)	0.0 (0.0 to 0.0)	4.2 (3.6 to 4.9)	3.1 (2.7 to 3.6)	2.31 (1.99 to 2.69)	1.71 (1.47 to 1.98)
2020	0.0 (0.0 to 0.0)	0.0 (0.0 to 0.0)	2.7 (2.3 to 3.2)	2.0 (1.7 to 2.4)	1.76 (1.48 to 2.05)	1.31 (1.10 to 1.53)
2021	0.0 (0.0 to 0.0)	0.0 (0.0 to 0.0)	2.4 (1.9 to 2.9)	1.8 (1.4 to 2.2)	1.45 (1.15 to 1.76)	1.07 (0.87 to 1.31)

Oslo NOR_4910 Western Europe Stillbirth Rate / Neonatal Mortality Rate (SBR/NMR) − 20 weeks
lambda = 0.2 ; zeta = 0.99 ; scale = 5 ; amp = 0.028

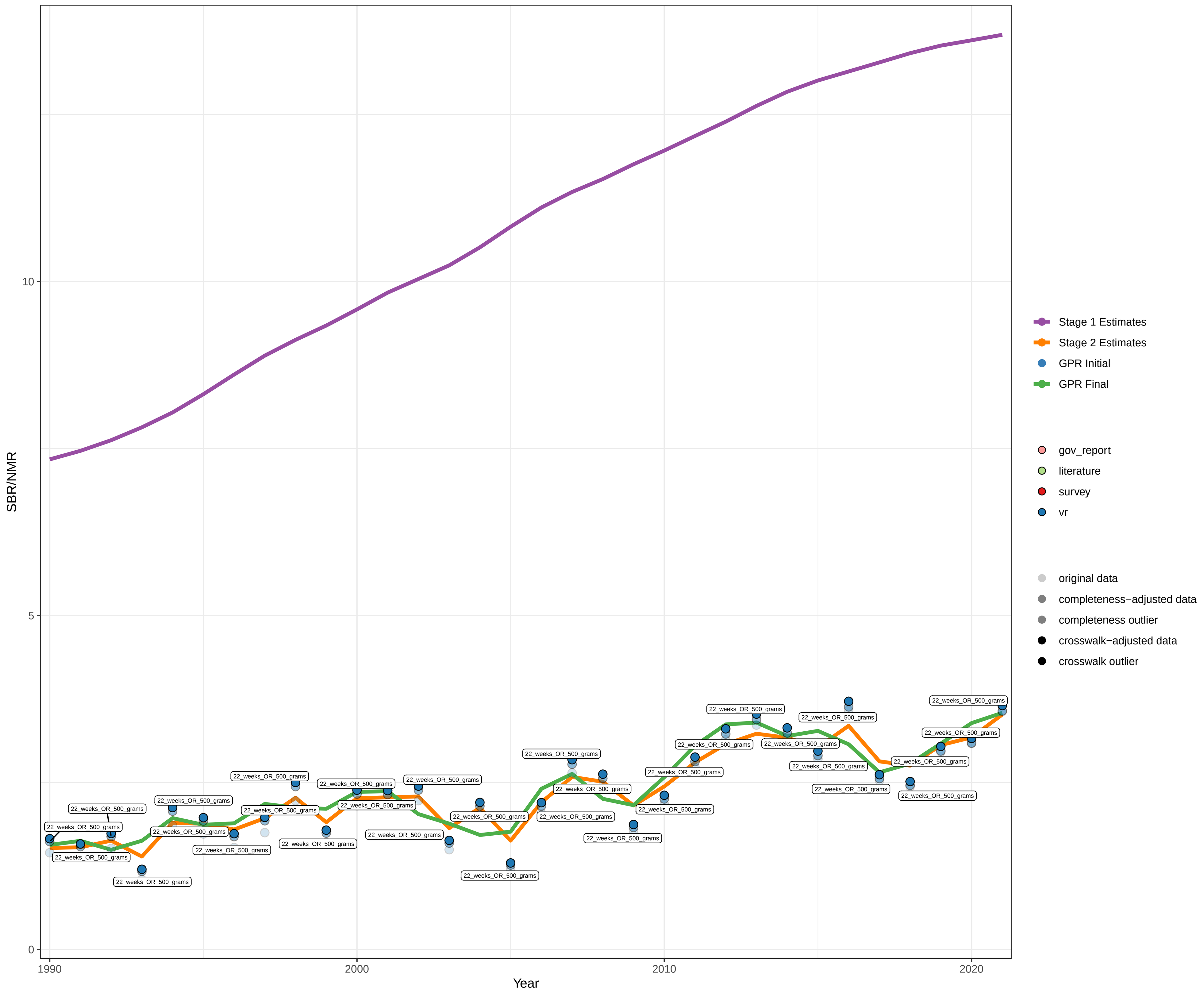

Oslo NOR_4910 Western Europe Stillbirth Rate / Neonatal Mortality Rate (SBR/NMR) − 28 weeks
lambda = 0.2 ; zeta = 0.99 ; scale = 5 ; amp = 0.028

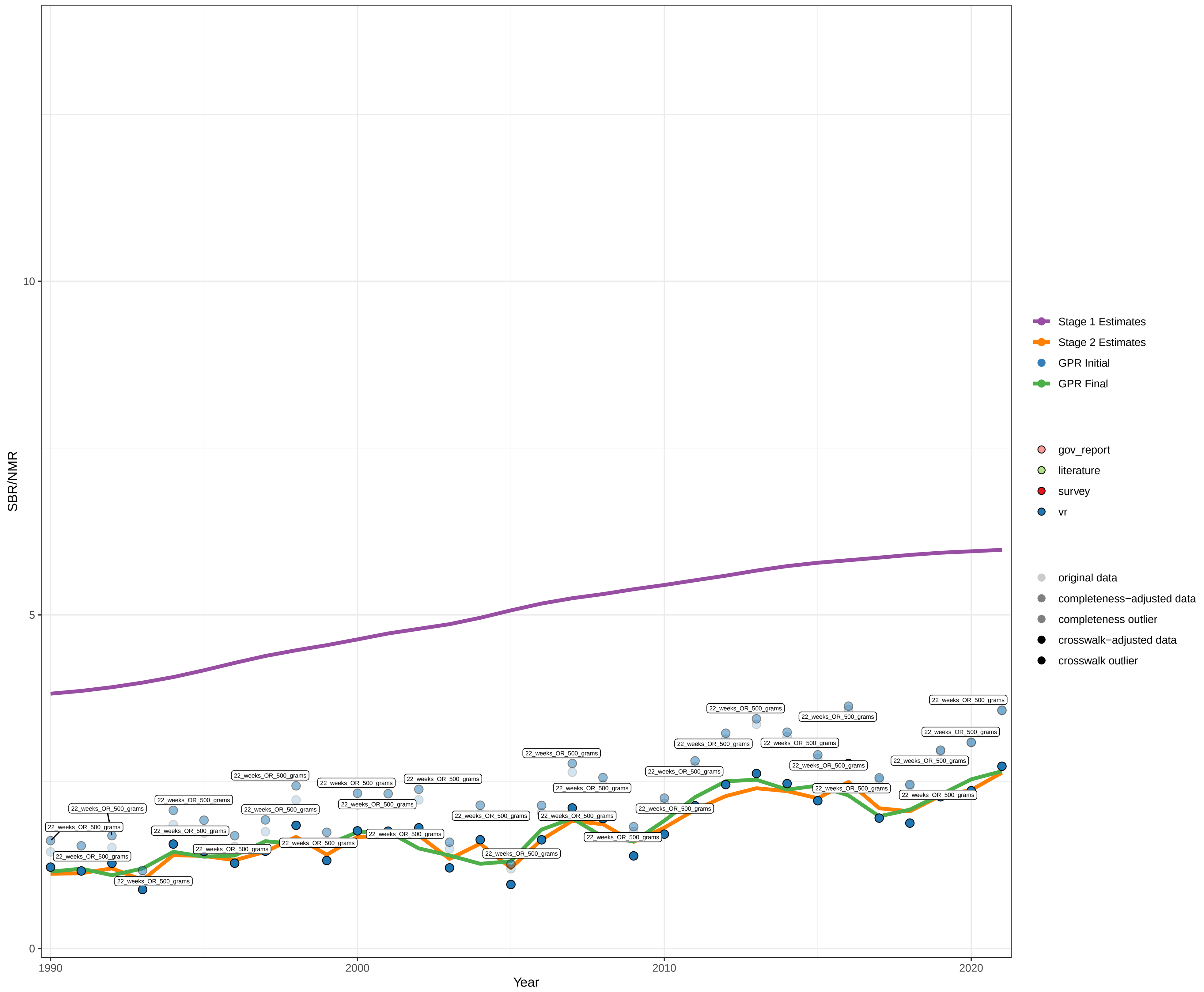

Definition	Years	Outlier	Source.Type	Title
22_weeks_OR_500_grams	1990−2021	0	VR	Norway Medical Birth Registry

Oslo NOR_4910 Western Europe Stillbirth Rate (SBR) − 20 weeks
lambda = 0.2 ; zeta = 0.99 ; scale = 5 ; amp = 0.028

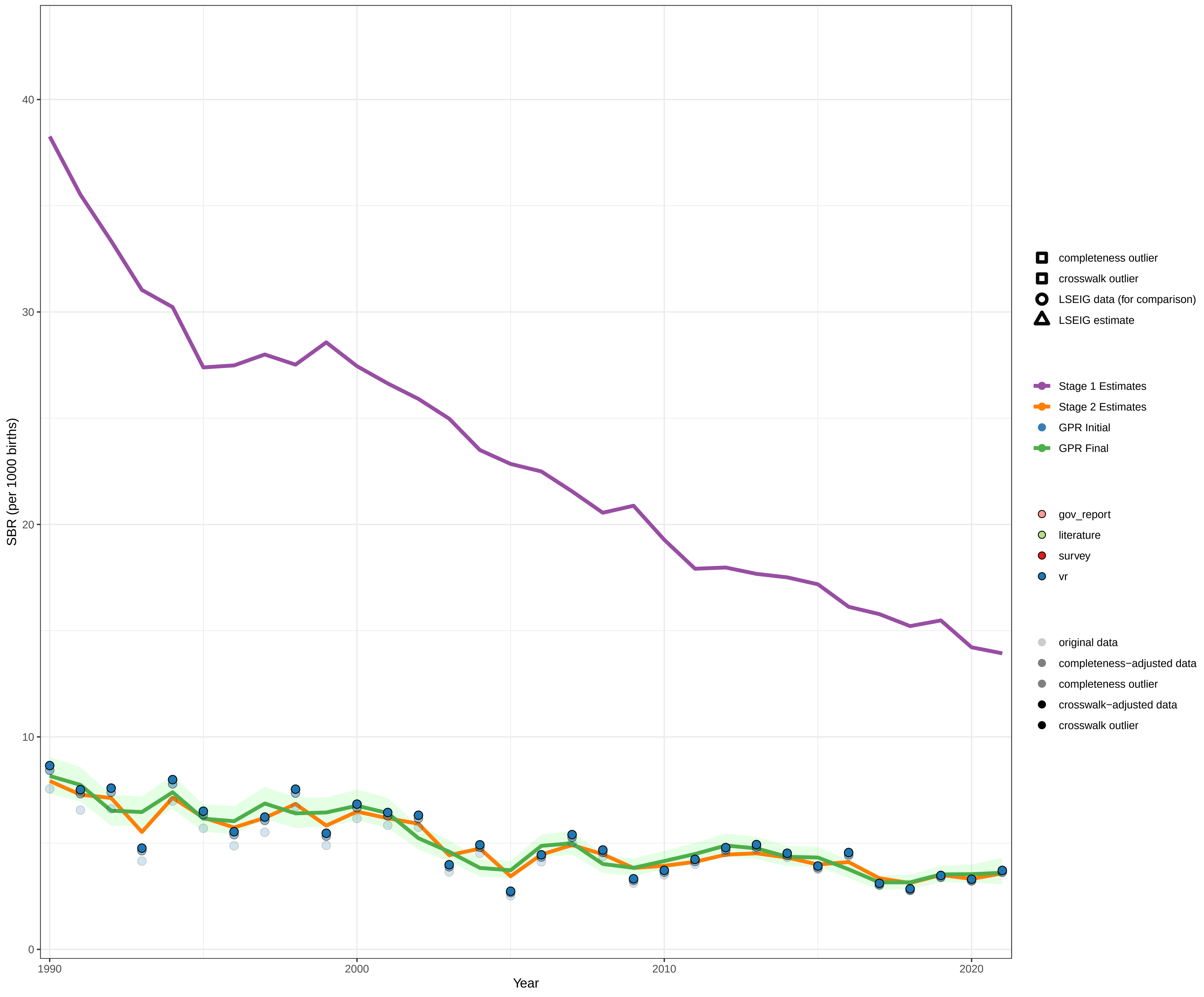

Oslo NOR_4910 Western Europe Stillbirth Rate (SBR) − 28 weeks
lambda = 0.2 ; zeta = 0.99 ; scale = 5 ; amp = 0.028

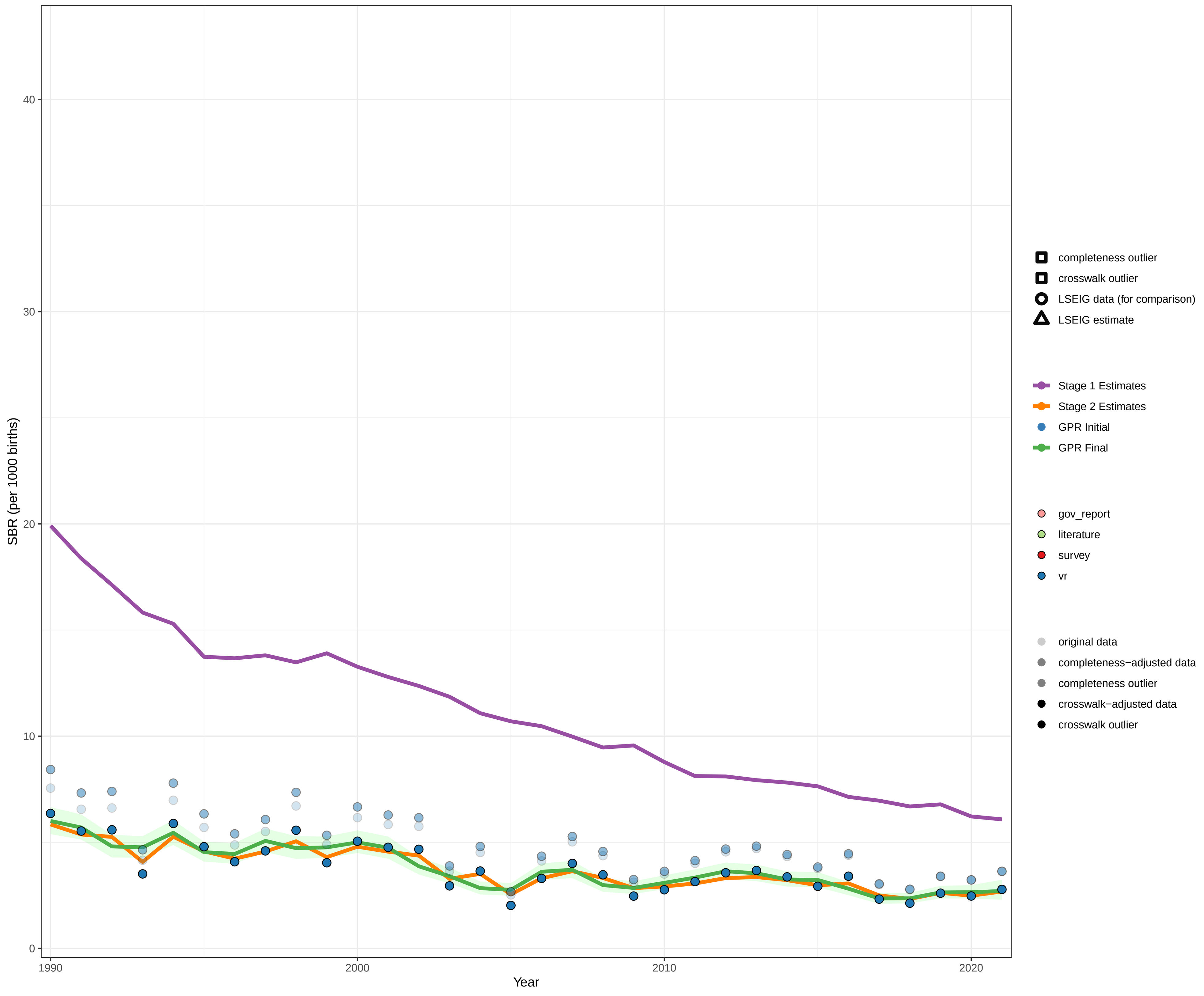

Year	Total Stillbirths (in thousands)	Total Stillbirths (in thousands)	SBR (per 1000 births)	SBR (per 1000 births)	SBR/NMR	SBR/NMR
	20 weeks	28 weeks	20 weeks	28 weeks	20 weeks	28 weeks
1990	0.1 (0.1 to 0.1)	0.0 (0.0 to 0.0)	8.2 (7.3 to 9.1)	6.0 (5.4 to 6.7)	1.57 (1.40 to 1.74)	1.15 (1.03 to 1.28)
1995	0.0 (0.0 to 0.1)	0.0 (0.0 to 0.0)	6.2 (5.5 to 6.8)	4.5 (4.1 to 5.0)	1.87 (1.68 to 2.07)	1.38 (1.24 to 1.53)
2000	0.1 (0.0 to 0.1)	0.0 (0.0 to 0.0)	6.8 (6.1 to 7.5)	5.0 (4.5 to 5.6)	2.36 (2.12 to 2.63)	1.74 (1.57 to 1.94)
2005	0.0 (0.0 to 0.0)	0.0 (0.0 to 0.0)	3.7 (3.3 to 4.2)	2.8 (2.5 to 3.1)	1.76 (1.58 to 1.97)	1.31 (1.18 to 1.46)
2010	0.0 (0.0 to 0.0)	0.0 (0.0 to 0.0)	4.2 (3.8 to 4.6)	3.1 (2.8 to 3.4)	2.58 (2.33 to 2.88)	1.92 (1.73 to 2.14)
2015	0.0 (0.0 to 0.0)	0.0 (0.0 to 0.0)	4.3 (3.9 to 4.8)	3.2 (2.9 to 3.6)	3.27 (2.92 to 3.65)	2.44 (2.18 to 2.72)
2020	0.0 (0.0 to 0.0)	0.0 (0.0 to 0.0)	3.5 (3.1 to 4.0)	2.6 (2.4 to 3.0)	3.39 (3.01 to 3.82)	2.54 (2.25 to 2.85)
2021	0.0 (0.0 to 0.0)	0.0 (0.0 to 0.0)	3.6 (3.1 to 4.3)	2.7 (2.3 to 3.2)	3.54 (3.00 to 4.24)	2.66 (2.25 to 3.18)

Rogaland NOR_4920 Western Europe Stillbirth Rate / Neonatal Mortality Rate (SBR/NMR) − 20 weeks
lambda = 0.2 ; zeta = 0.99 ; scale = 5 ; amp = 0.028

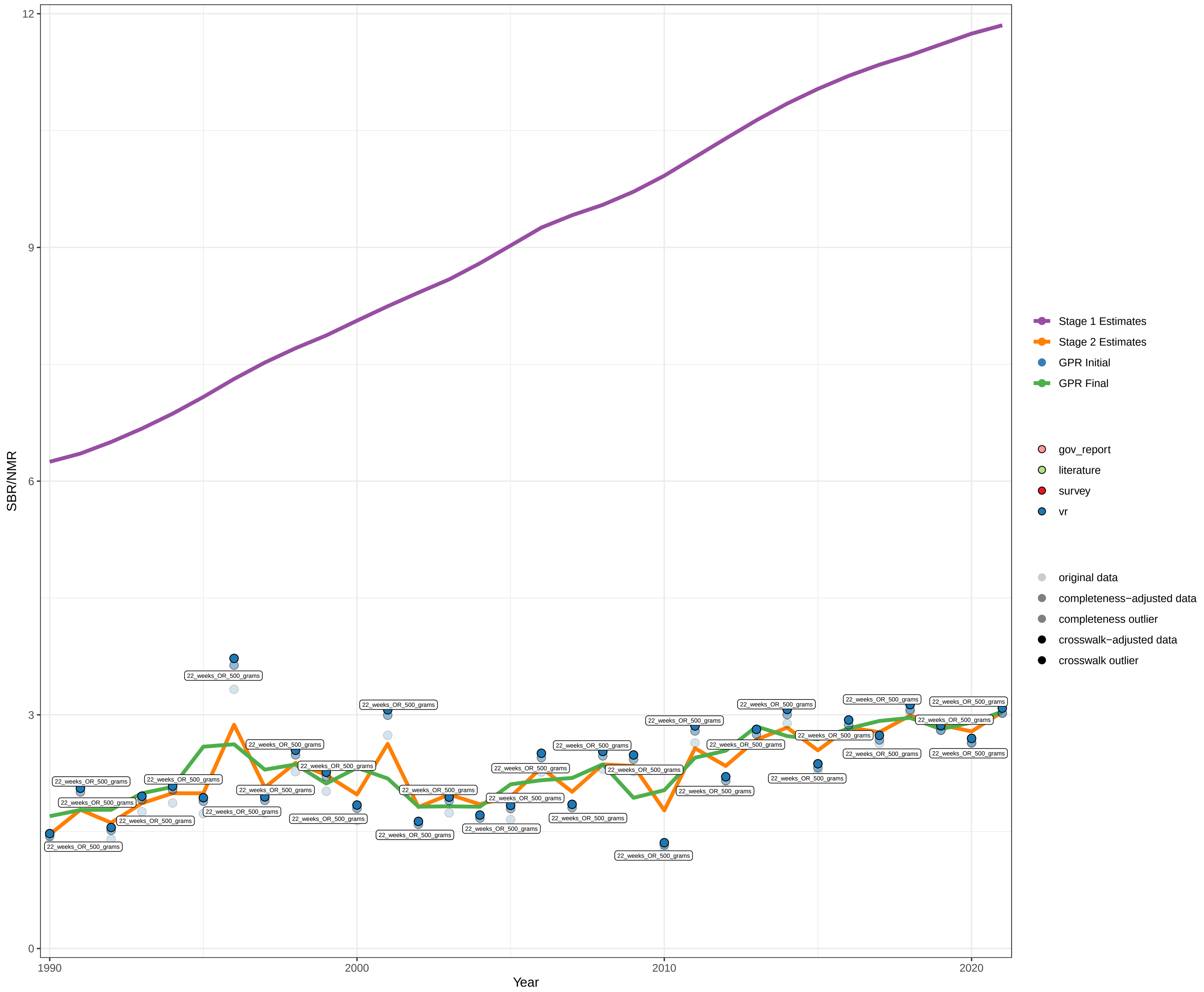

Rogaland NOR_4920 Western Europe Stillbirth Rate / Neonatal Mortality Rate (SBR/NMR) − 28 weeks
lambda = 0.2 ; zeta = 0.99 ; scale = 5 ; amp = 0.028

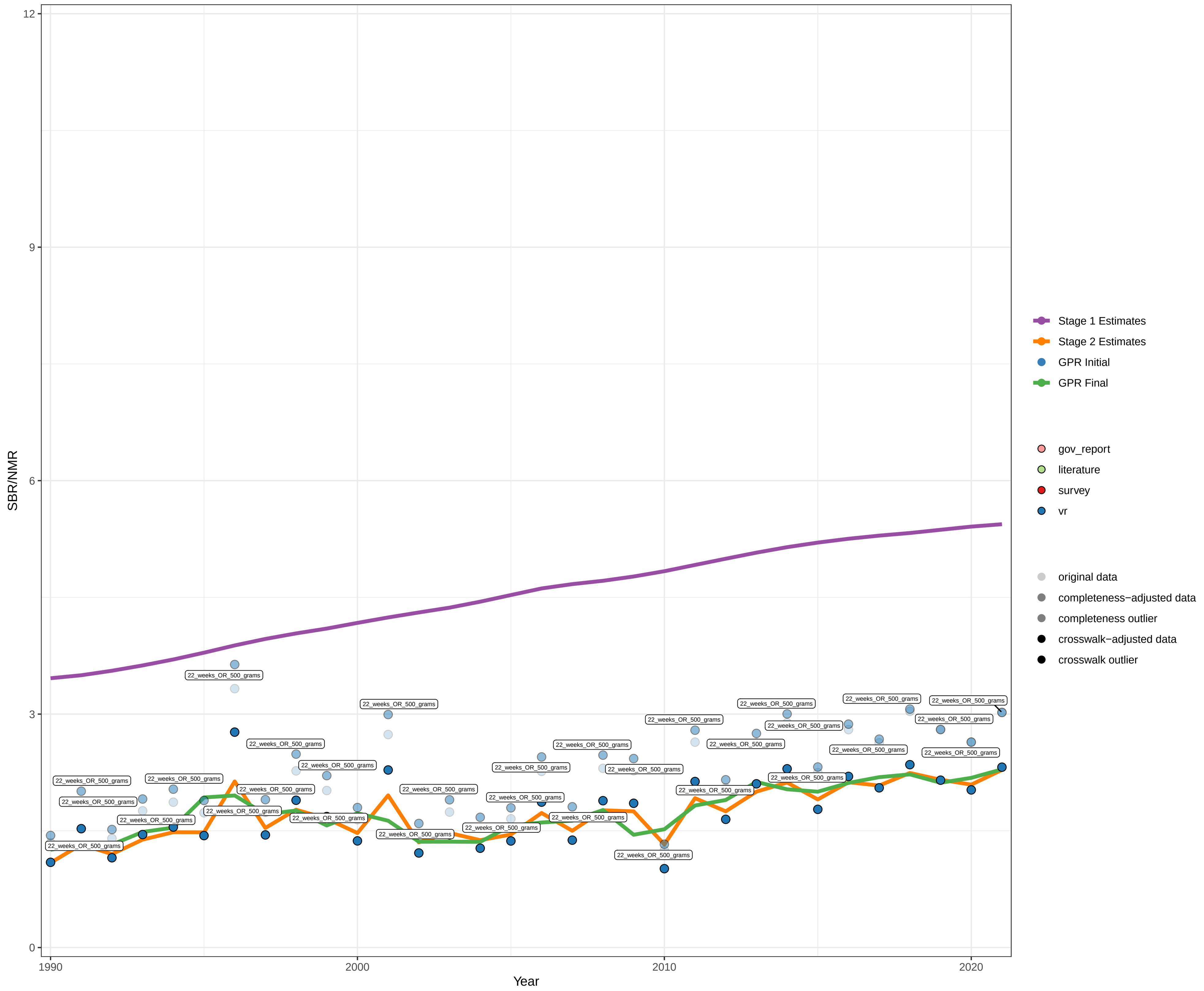

Definition	Years	Outlier	Source.Type	Title
22_weeks_OR_500_grams	1990−2021	0	VR	Norway Medical Birth Registry

Rogaland NOR_4920 Western Europe Stillbirth Rate (SBR) − 20 weeks
lambda = 0.2 ; zeta = 0.99 ; scale = 5 ; amp = 0.028

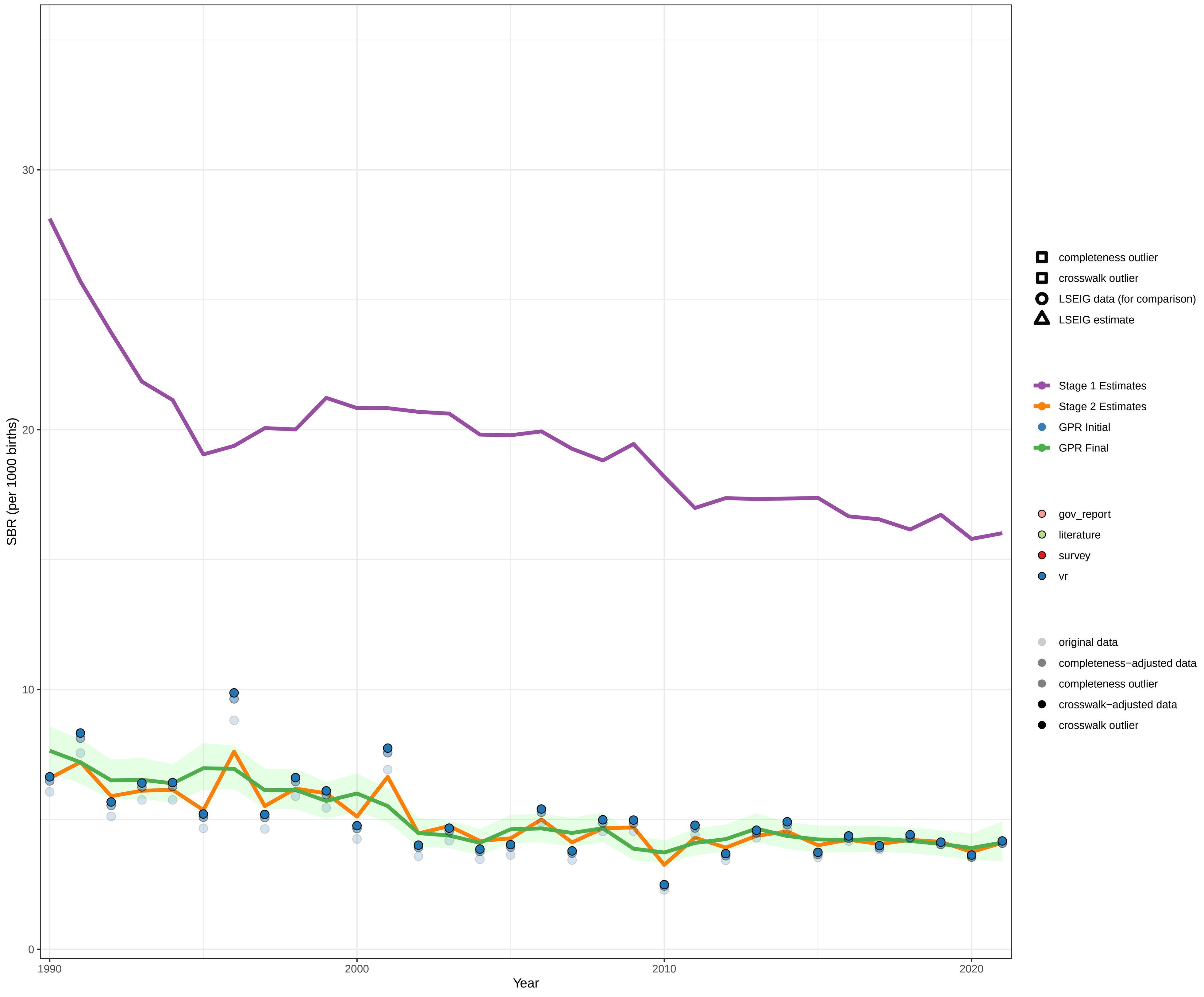

Rogaland NOR_4920 Western Europe Stillbirth Rate (SBR) − 28 weeks
lambda = 0.2 ; zeta = 0.99 ; scale = 5 ; amp = 0.028

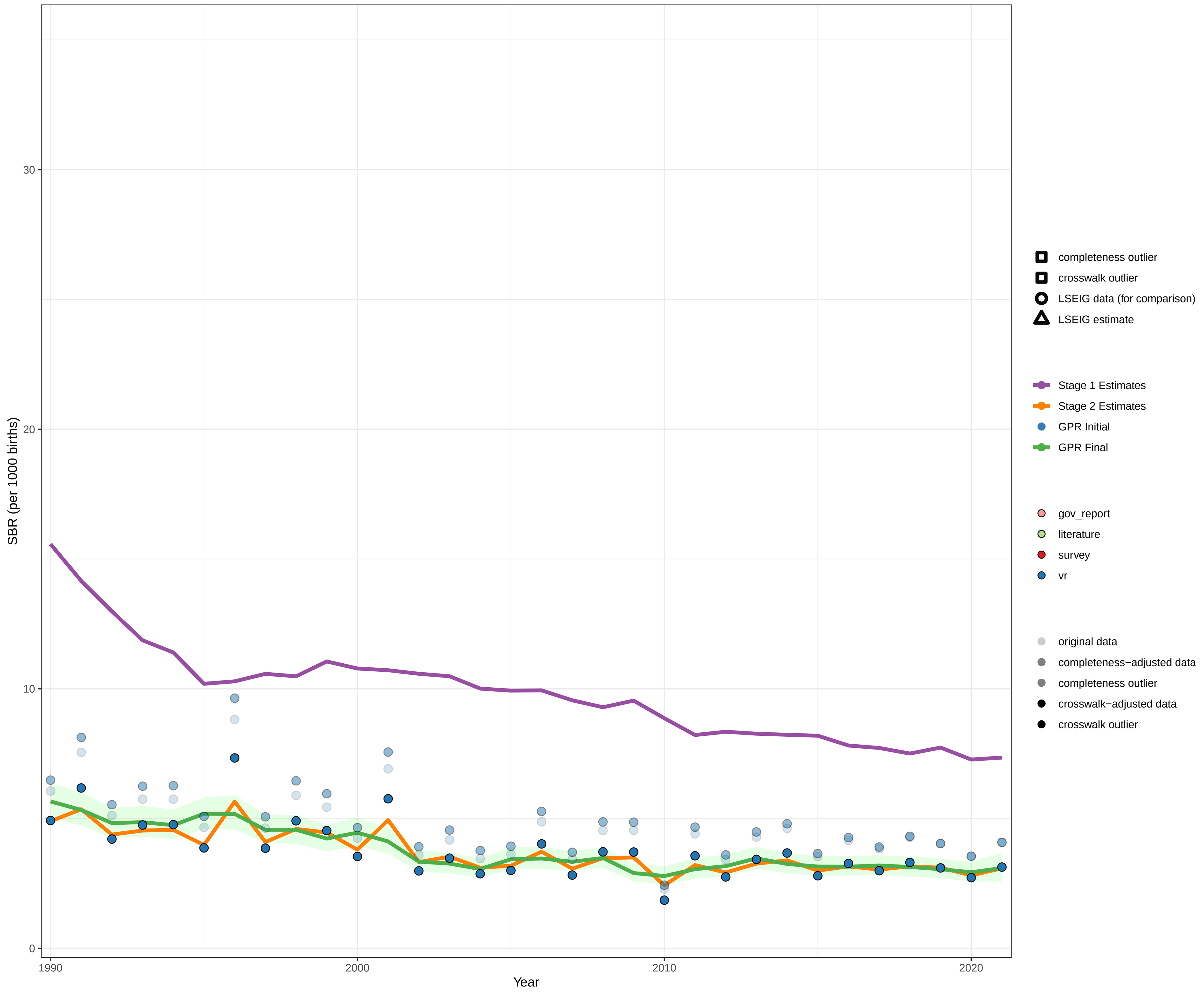

Year	Total Stillbirths (in thousands)	Total Stillbirths (in thousands)	SBR (per 1000 births)	SBR (per 1000 births)	SBR/NMR	SBR/NMR
	20 weeks	28 weeks	20 weeks	28 weeks	20 weeks	28 weeks
1990	0.0 (0.0 to 0.0)	0.0 (0.0 to 0.0)	7.6 (6.8 to 8.6)	5.7 (5.0 to 6.4)	1.70 (1.51 to 1.91)	1.26 (1.12 to 1.41)
1995	0.0 (0.0 to 0.0)	0.0 (0.0 to 0.0)	7.0 (6.1 to 7.9)	5.2 (4.6 to 5.8)	2.59 (2.28 to 2.95)	1.93 (1.71 to 2.16)
2000	0.0 (0.0 to 0.0)	0.0 (0.0 to 0.0)	6.0 (5.3 to 6.8)	4.5 (3.9 to 5.0)	2.32 (2.05 to 2.62)	1.73 (1.53 to 1.95)
2005	0.0 (0.0 to 0.0)	0.0 (0.0 to 0.0)	4.6 (4.1 to 5.2)	3.4 (3.0 to 3.9)	2.11 (1.86 to 2.36)	1.57 (1.39 to 1.78)
2010	0.0 (0.0 to 0.0)	0.0 (0.0 to 0.0)	3.7 (3.3 to 4.2)	2.8 (2.5 to 3.2)	2.03 (1.80 to 2.28)	1.52 (1.34 to 1.73)
2015	0.0 (0.0 to 0.0)	0.0 (0.0 to 0.0)	4.2 (3.7 to 4.8)	3.2 (2.8 to 3.6)	2.69 (2.37 to 3.02)	2.00 (1.77 to 2.26)
2020	0.0 (0.0 to 0.0)	0.0 (0.0 to 0.0)	3.9 (3.4 to 4.5)	2.9 (2.6 to 3.3)	2.90 (2.55 to 3.32)	2.18 (1.91 to 2.48)
2021	0.0 (0.0 to 0.0)	0.0 (0.0 to 0.0)	4.1 (3.4 to 4.9)	3.1 (2.6 to 3.7)	3.04 (2.52 to 3.64)	2.29 (1.91 to 2.72)

Troms og Finnmark NOR_60137 Western Europe Stillbirth Rate / Neonatal Mortality Rate (SBR/NMR) − 20 weeks
lambda = 0.2 ; zeta = 0.99 ; scale = 5 ; amp = 0.028

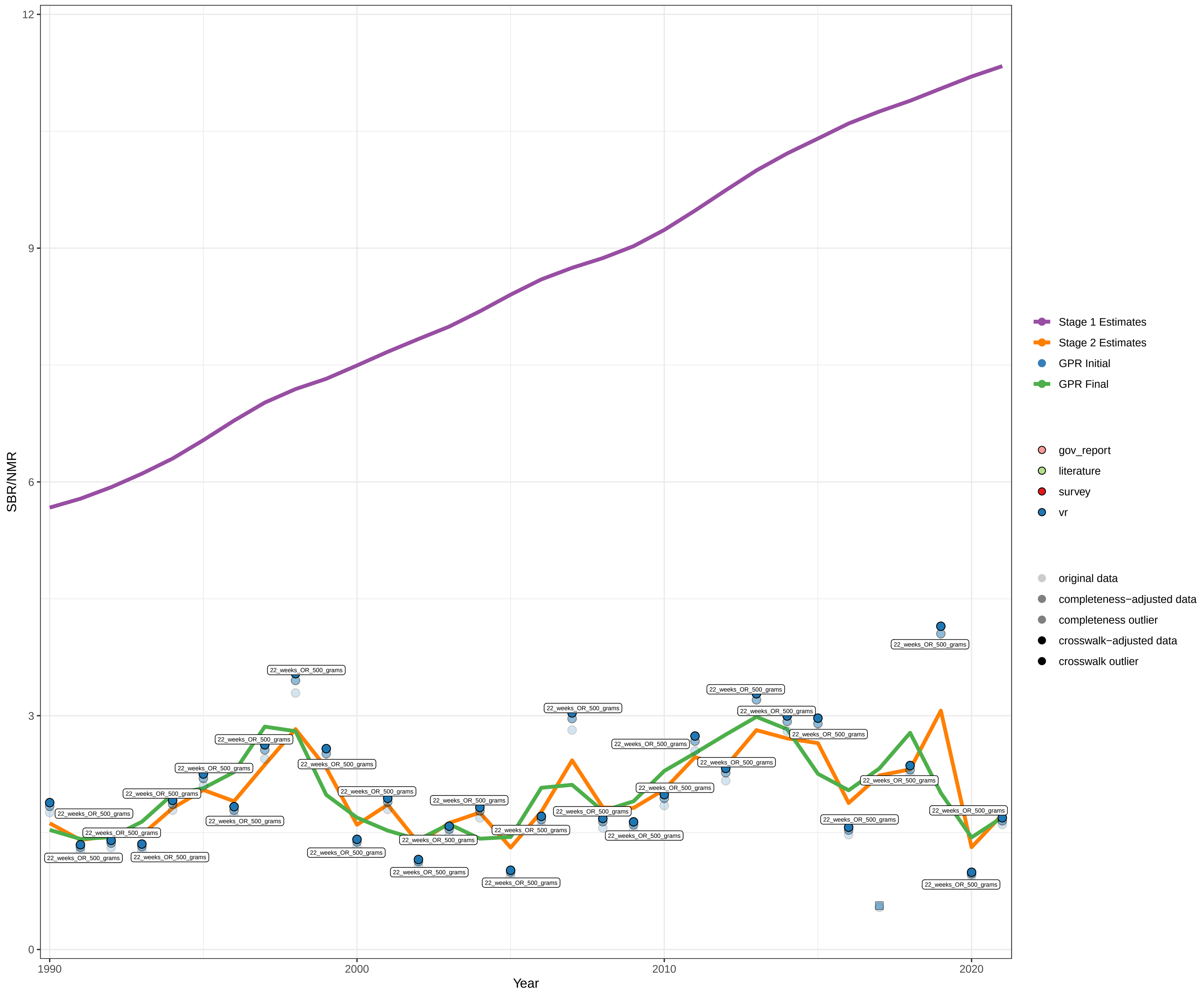

Troms og Finnmark NOR_60137 Western Europe Stillbirth Rate / Neonatal Mortality Rate (SBR/NMR) − 28 weeks
lambda = 0.2 ; zeta = 0.99 ; scale = 5 ; amp = 0.028

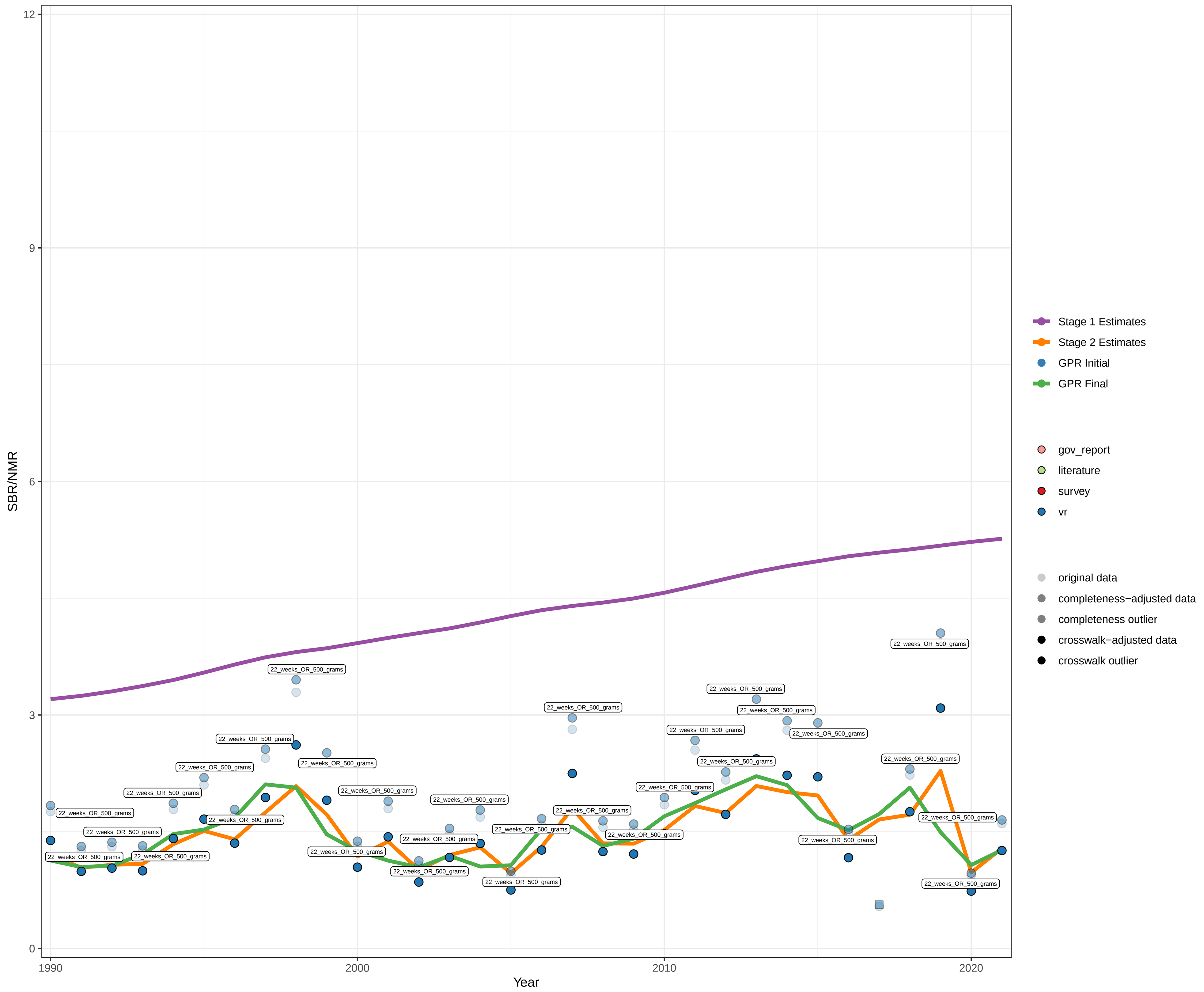

Definition	Years	Outlier	Source.Type	Title
22_weeks_OR_500_grams	1990−2016, 2018−2021	0	VR	Norway Medical Birth Registry
22_weeks_OR_500_grams	2017	1	VR	Norway Medical Birth Registry

Troms og Finnmark NOR_60137 Western Europe Stillbirth Rate (SBR) − 20 weeks
lambda = 0.2 ; zeta = 0.99 ; scale = 5 ; amp = 0.028

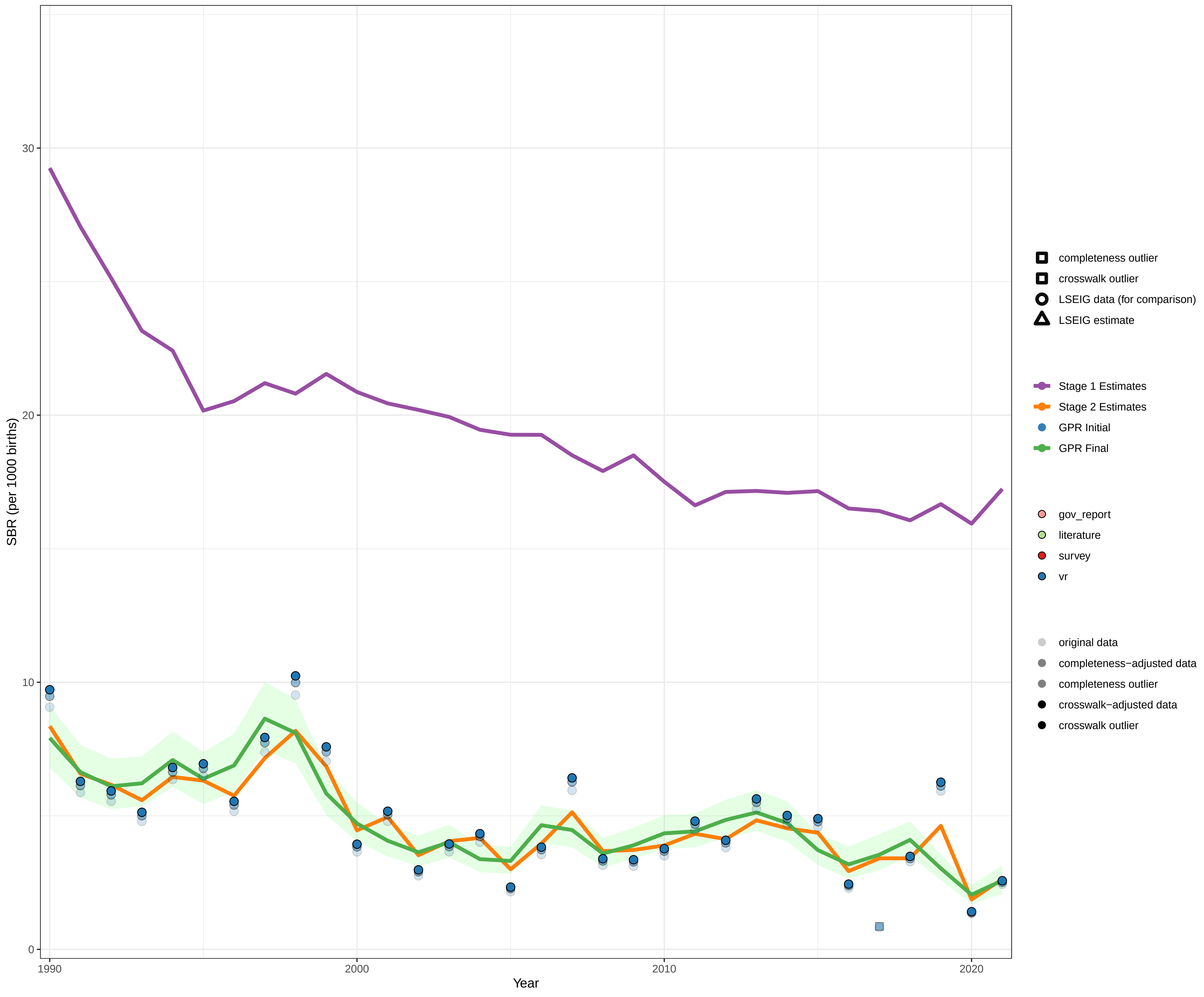

Troms og Finnmark NOR_60137 Western Europe Stillbirth Rate (SBR) − 28 weeks
lambda = 0.2 ; zeta = 0.99 ; scale = 5 ; amp = 0.028

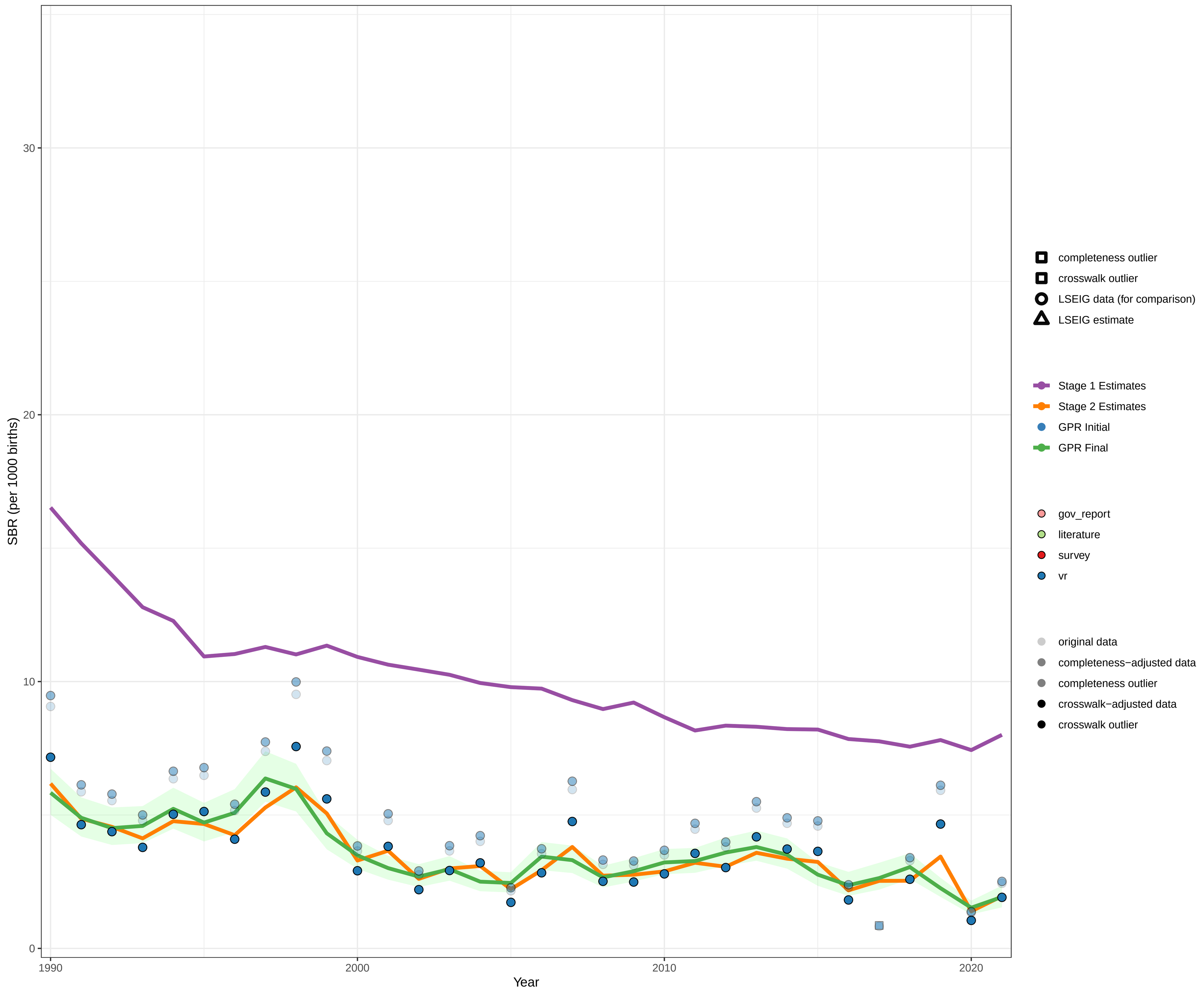

Year	Total Stillbirths (in thousands)	Total Stillbirths (in thousands)	SBR (per 1000 births)	SBR (per 1000 births)	SBR/NMR	SBR/NMR
	20 weeks	28 weeks	20 weeks	28 weeks	20 weeks	28 weeks
1990	0.0 (0.0 to 0.0)	0.0 (0.0 to 0.0)	7.9 (6.8 to 9.1)	5.8 (5.0 to 6.7)	1.53 (1.32 to 1.77)	1.13 (0.97 to 1.31)
1995	0.0 (0.0 to 0.0)	0.0 (0.0 to 0.0)	6.4 (5.4 to 7.4)	4.7 (4.0 to 5.5)	2.07 (1.76 to 2.39)	1.53 (1.30 to 1.77)
2000	0.0 (0.0 to 0.0)	0.0 (0.0 to 0.0)	4.7 (4.0 to 5.5)	3.5 (3.0 to 4.1)	1.69 (1.45 to 1.97)	1.25 (1.07 to 1.46)
2005	0.0 (0.0 to 0.0)	0.0 (0.0 to 0.0)	3.3 (2.8 to 3.8)	2.5 (2.1 to 2.9)	1.44 (1.23 to 1.68)	1.07 (0.91 to 1.24)
2010	0.0 (0.0 to 0.0)	0.0 (0.0 to 0.0)	4.3 (3.8 to 5.0)	3.2 (2.8 to 3.7)	2.29 (1.98 to 2.65)	1.70 (1.47 to 1.96)
2015	0.0 (0.0 to 0.0)	0.0 (0.0 to 0.0)	3.7 (3.2 to 4.3)	2.8 (2.3 to 3.2)	2.25 (1.91 to 2.63)	1.68 (1.42 to 1.96)
2020	0.0 (0.0 to 0.0)	0.0 (0.0 to 0.0)	2.0 (1.7 to 2.4)	1.5 (1.3 to 1.8)	1.44 (1.20 to 1.69)	1.07 (0.90 to 1.26)
2021	0.0 (0.0 to 0.0)	0.0 (0.0 to 0.0)	2.6 (2.1 to 3.2)	1.9 (1.5 to 2.4)	1.70 (1.36 to 2.07)	1.27 (1.01 to 1.55)

Trondelag NOR_53432 Western Europe Stillbirth Rate / Neonatal Mortality Rate (SBR/NMR) − 20 weeks
lambda = 0.2 ; zeta = 0.99 ; scale = 5 ; amp = 0.028

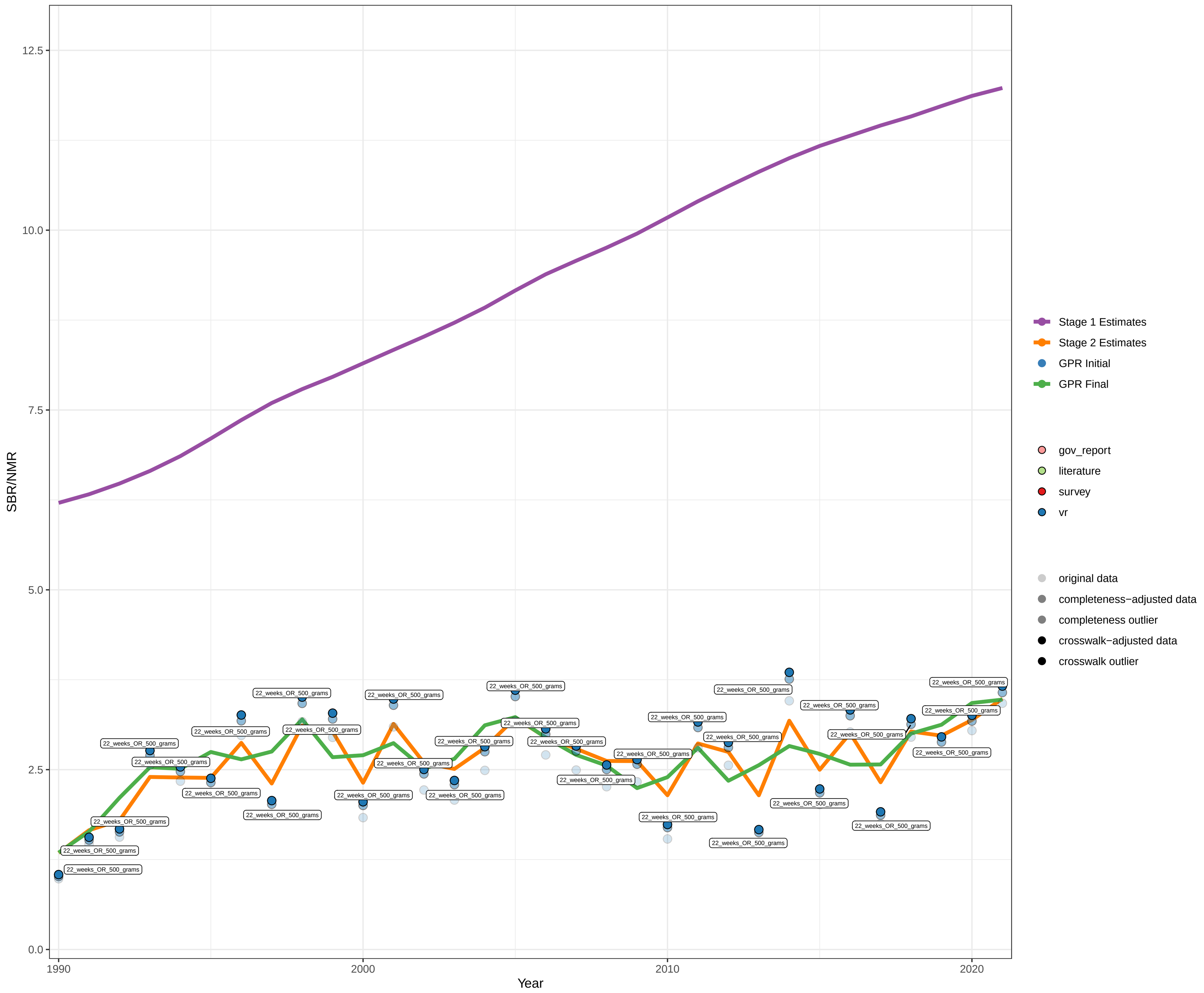

Trondelag NOR_53432 Western Europe Stillbirth Rate / Neonatal Mortality Rate (SBR/NMR) − 28 weeks
lambda = 0.2 ; zeta = 0.99 ; scale = 5 ; amp = 0.028

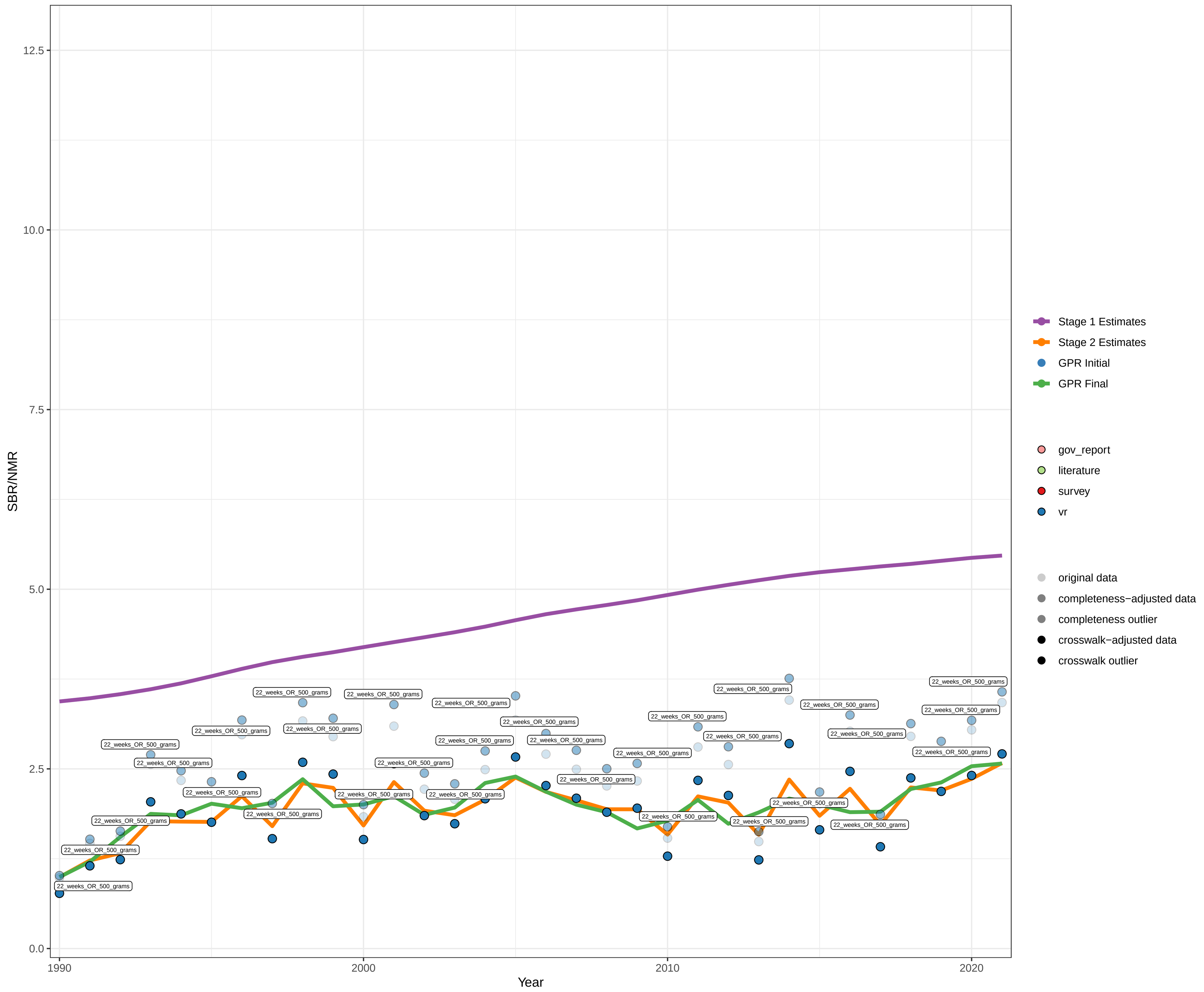

Definition	Years	Outlier	Source.Type	Title
22_weeks_OR_500_grams	1990−2021	0	VR	Norway Medical Birth Registry

Trondelag NOR_53432 Western Europe Stillbirth Rate (SBR) − 20 weeks
lambda = 0.2 ; zeta = 0.99 ; scale = 5 ; amp = 0.028

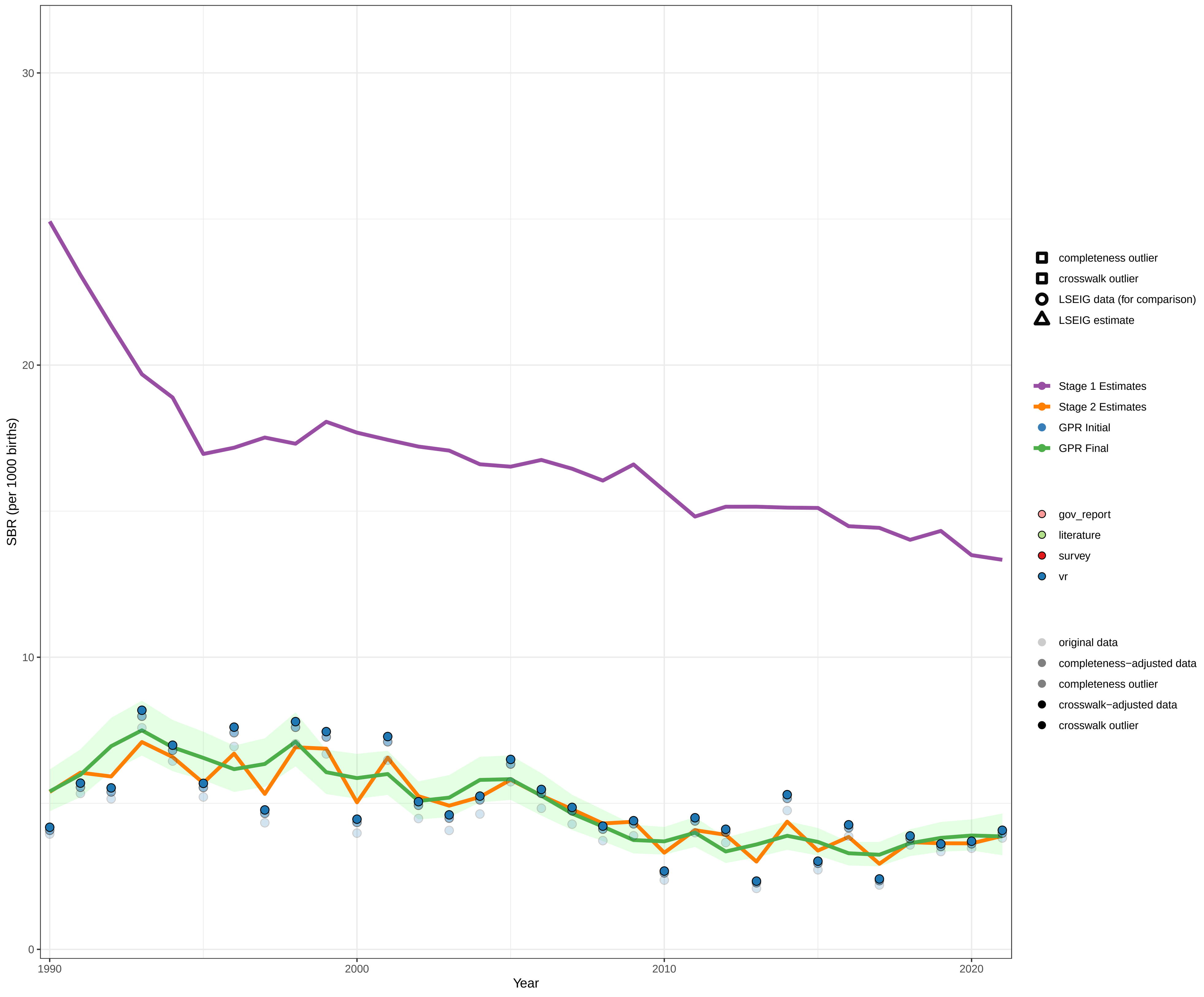

Trondelag NOR_53432 Western Europe Stillbirth Rate (SBR) − 28 weeks
lambda = 0.2 ; zeta = 0.99 ; scale = 5 ; amp = 0.028

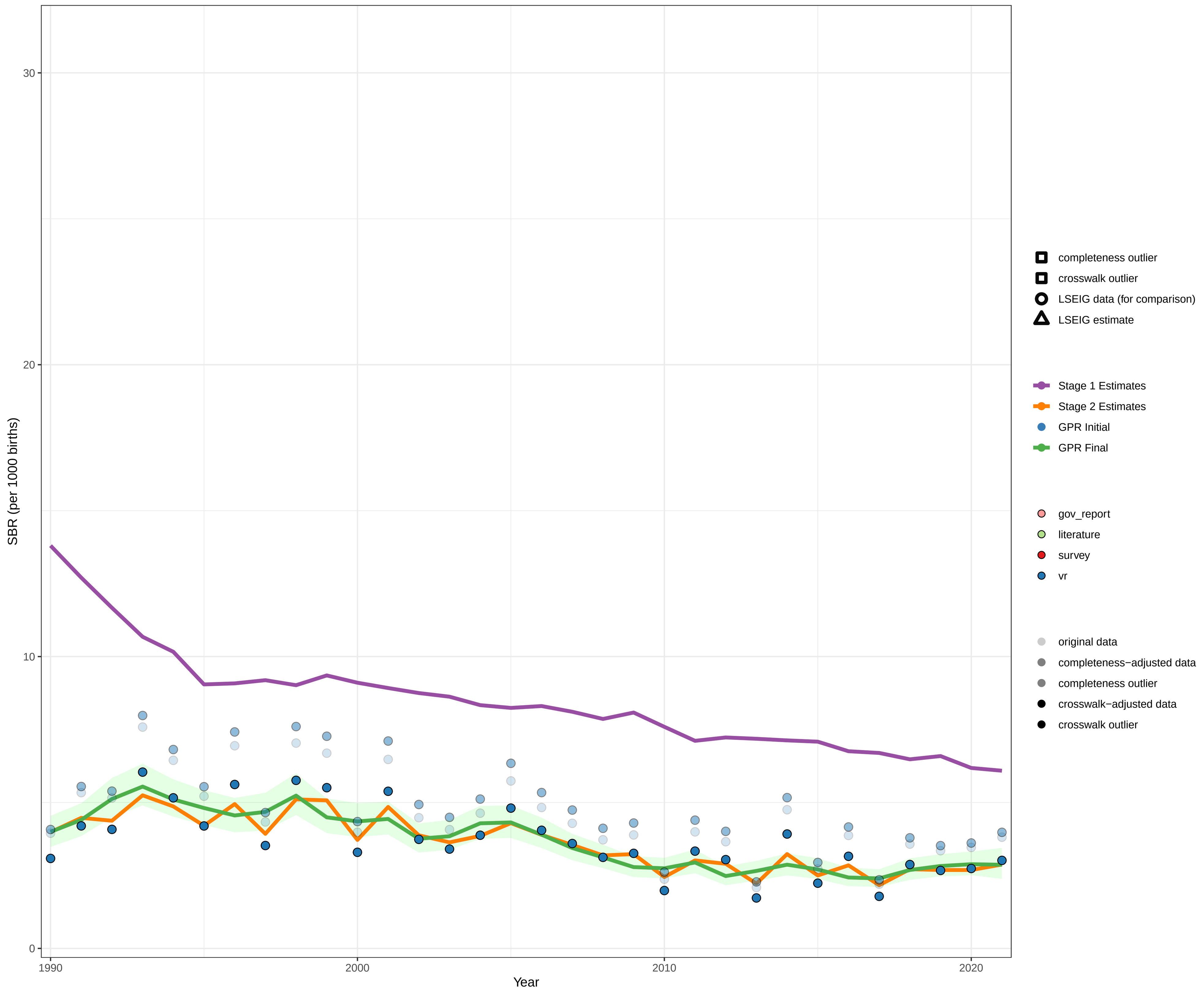

Year	Total Stillbirths (in thousands)	Total Stillbirths (in thousands)	SBR (per 1000 births)	SBR (per 1000 births)	SBR/NMR	SBR/NMR
	20 weeks	28 weeks	20 weeks	28 weeks	20 weeks	28 weeks
1990	0.0 (0.0 to 0.0)	0.0 (0.0 to 0.0)	5.4 (4.7 to 6.2)	4.0 (3.5 to 4.5)	1.35 (1.18 to 1.53)	1.00 (0.87 to 1.13)
1995	0.0 (0.0 to 0.0)	0.0 (0.0 to 0.0)	6.6 (5.8 to 7.5)	4.8 (4.2 to 5.4)	2.75 (2.43 to 3.12)	2.02 (1.77 to 2.27)
2000	0.0 (0.0 to 0.0)	0.0 (0.0 to 0.0)	5.9 (5.2 to 6.7)	4.4 (3.8 to 5.0)	2.70 (2.37 to 3.08)	2.01 (1.76 to 2.29)
2005	0.0 (0.0 to 0.0)	0.0 (0.0 to 0.0)	5.8 (5.1 to 6.6)	4.3 (3.8 to 4.9)	3.23 (2.84 to 3.68)	2.39 (2.10 to 2.71)
2010	0.0 (0.0 to 0.0)	0.0 (0.0 to 0.0)	3.7 (3.2 to 4.2)	2.7 (2.4 to 3.1)	2.40 (2.10 to 2.72)	1.78 (1.56 to 2.01)
2015	0.0 (0.0 to 0.0)	0.0 (0.0 to 0.0)	3.7 (3.2 to 4.2)	2.7 (2.4 to 3.1)	2.72 (2.37 to 3.07)	2.00 (1.76 to 2.29)
2020	0.0 (0.0 to 0.0)	0.0 (0.0 to 0.0)	3.9 (3.4 to 4.4)	2.9 (2.5 to 3.3)	3.43 (2.98 to 3.91)	2.54 (2.21 to 2.92)
2021	0.0 (0.0 to 0.0)	0.0 (0.0 to 0.0)	3.9 (3.2 to 4.6)	2.9 (2.4 to 3.4)	3.47 (2.89 to 4.17)	2.57 (2.14 to 3.09)

Vestfold og Telemark NOR_60134 Western Europe Stillbirth Rate / Neonatal Mortality Rate (SBR/NMR) − 20 weeks
lambda = 0.2 ; zeta = 0.99 ; scale = 5 ; amp = 0.028

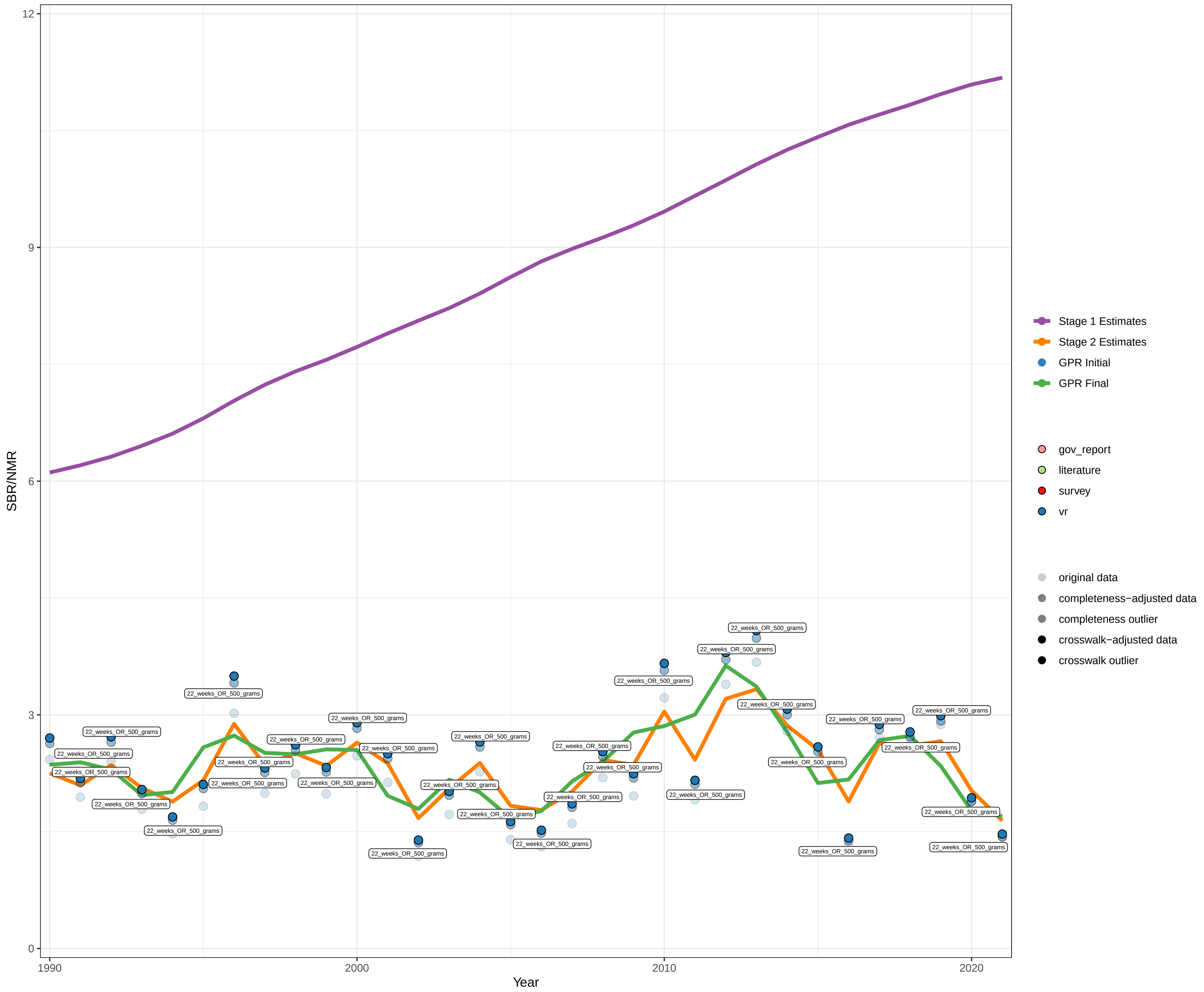

Vestfold og Telemark NOR_60134 Western Europe Stillbirth Rate / Neonatal Mortality Rate (SBR/NMR) − 28 weeks
lambda = 0.2 ; zeta = 0.99 ; scale = 5 ; amp = 0.028

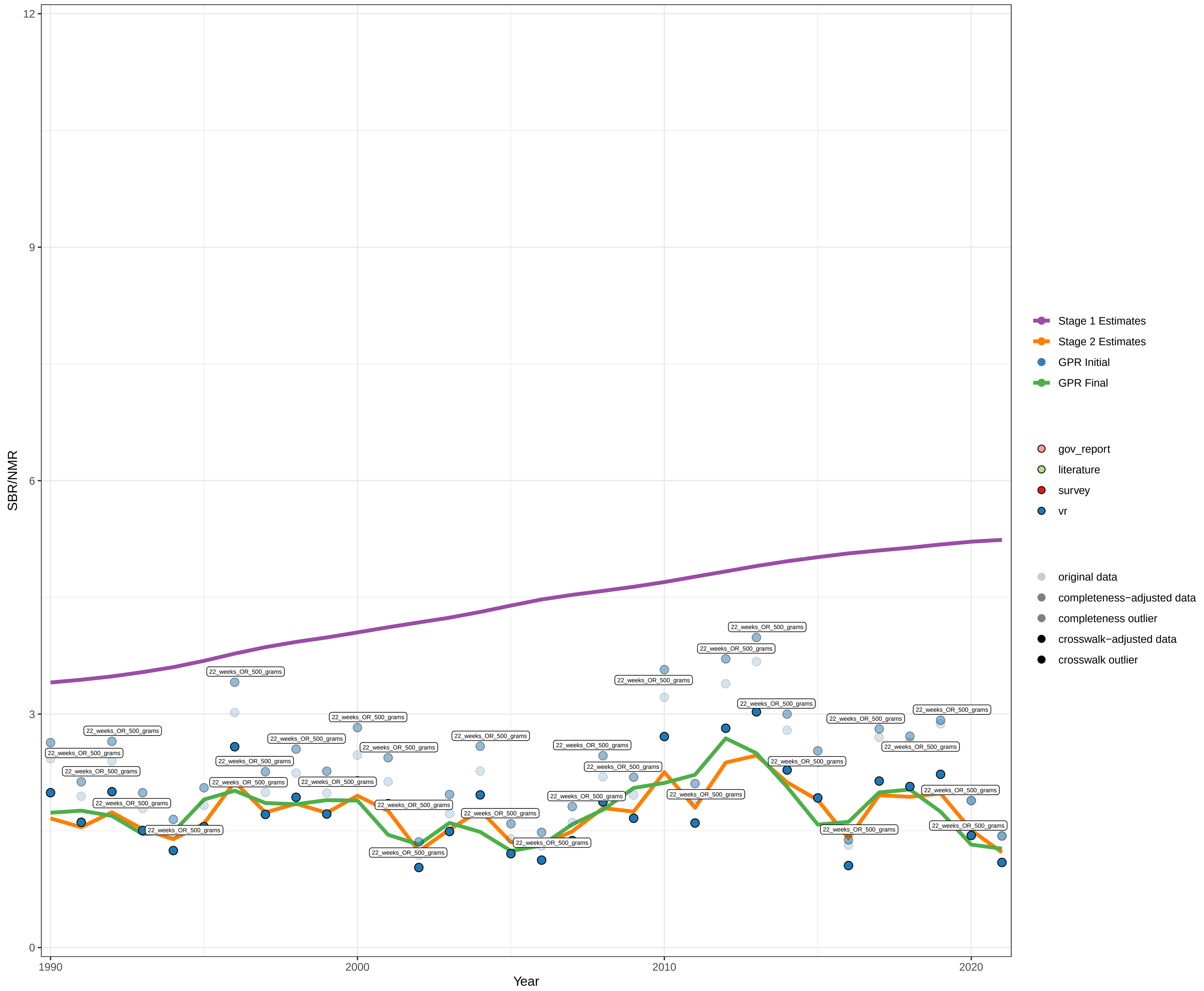

Definition	Years	Outlier	Source.Type	Title
22_weeks_OR_500_grams	1990−2021	0	VR	Norway Medical Birth Registry

Vestfold og Telemark NOR_60134 Western Europe Stillbirth Rate (SBR) − 20 weeks
lambda = 0.2 ; zeta = 0.99 ; scale = 5 ; amp = 0.028

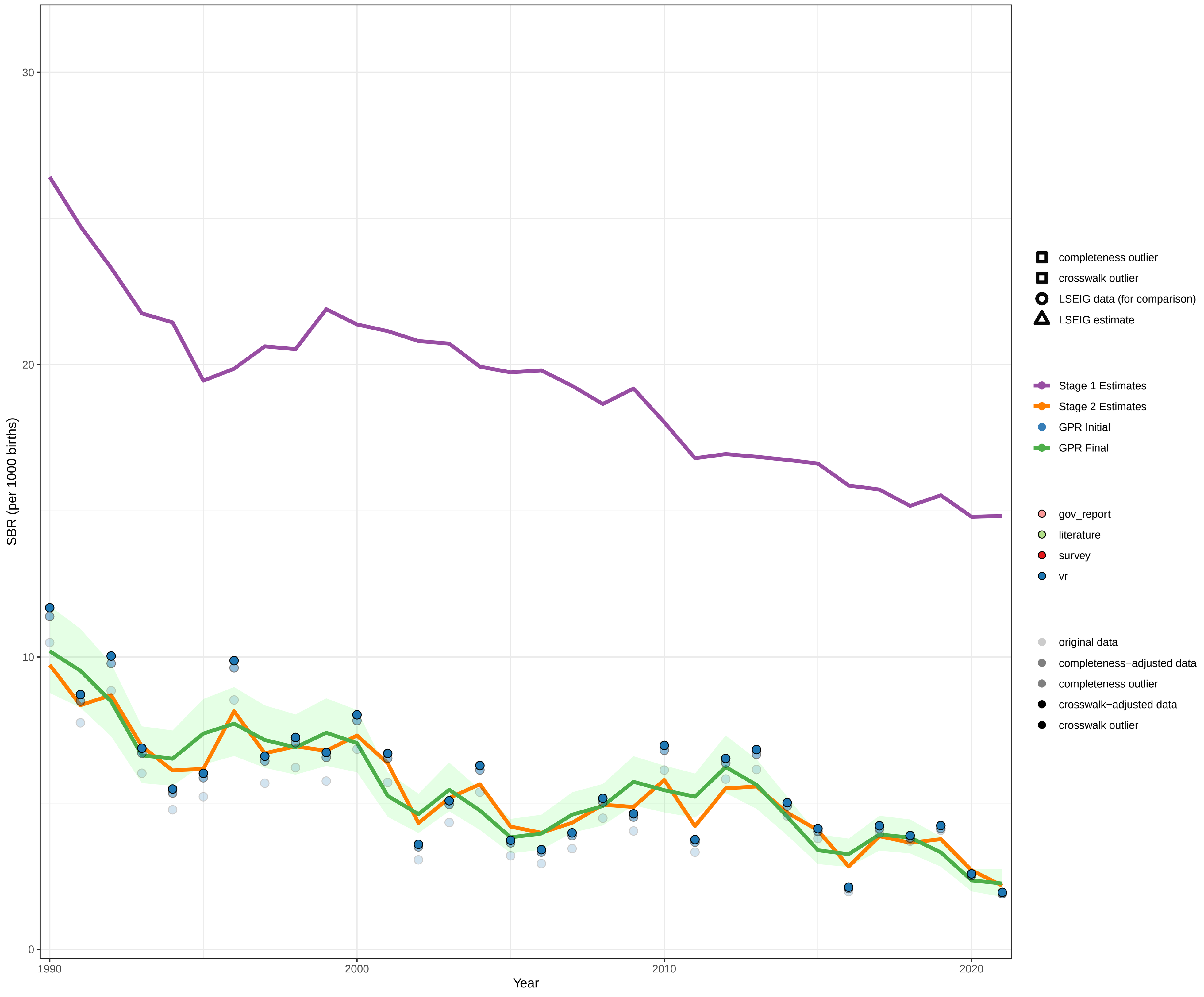

Vestfold og Telemark NOR_60134 Western Europe Stillbirth Rate (SBR) − 28 weeks
lambda = 0.2 ; zeta = 0.99 ; scale = 5 ; amp = 0.028

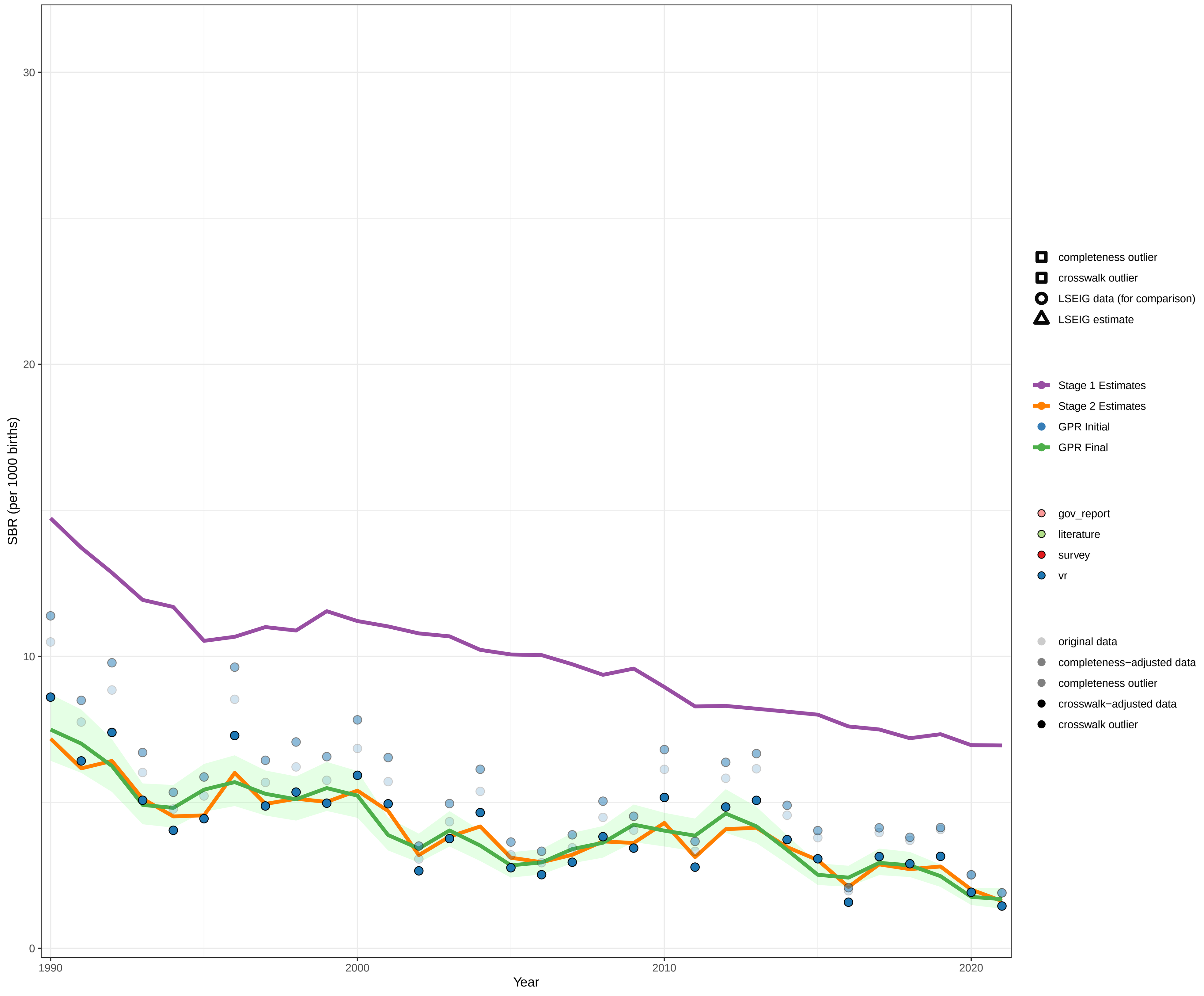

Year	Total Stillbirths (in thousands)	Total Stillbirths (in thousands)	SBR (per 1000 births)	SBR (per 1000 births)	SBR/NMR	SBR/NMR
	20 weeks	28 weeks	20 weeks	28 weeks	20 weeks	28 weeks
1990	0.0<br>(0.0 to 0.1)	0.0<br>(0.0 to 0.0)	10.2<br>(8.8 to 11.8)	7.5<br>(6.4 to 8.7)	2.36<br>(2.03 to 2.72)	1.73<br>(1.49 to 2.01)
1995	0.0<br>(0.0 to 0.0)	0.0<br>(0.0 to 0.0)	7.4<br>(6.3 to 8.6)	5.4<br>(4.7 to 6.3)	2.58<br>(2.21 to 3.00)	1.90<br>(1.64 to 2.21)
2000	0.0<br>(0.0 to 0.0)	0.0<br>(0.0 to 0.0)	7.1<br>(6.1 to 8.2)	5.2<br>(4.5 to 6.1)	2.55<br>(2.19 to 2.96)	1.89<br>(1.61 to 2.19)
2005	0.0<br>(0.0 to 0.0)	0.0<br>(0.0 to 0.0)	3.8<br>(3.3 to 4.5)	2.8<br>(2.4 to 3.3)	1.67<br>(1.43 to 1.95)	1.24<br>(1.06 to 1.44)
2010	0.0<br>(0.0 to 0.0)	0.0<br>(0.0 to 0.0)	5.4<br>(4.7 to 6.3)	4.0<br>(3.5 to 4.6)	2.86<br>(2.46 to 3.30)	2.11<br>(1.83 to 2.44)
2015	0.0<br>(0.0 to 0.0)	0.0<br>(0.0 to 0.0)	3.4<br>(2.9 to 3.9)	2.5<br>(2.2 to 2.9)	2.12<br>(1.83 to 2.47)	1.58<br>(1.36 to 1.83)
2020	0.0<br>(0.0 to 0.0)	0.0<br>(0.0 to 0.0)	2.4<br>(2.0 to 2.8)	1.8<br>(1.5 to 2.1)	1.77<br>(1.49 to 2.06)	1.32<br>(1.11 to 1.55)
2021	0.0<br>(0.0 to 0.0)	0.0<br>(0.0 to 0.0)	2.3<br>(1.8 to 2.7)	1.7<br>(1.4 to 2.1)	1.70<br>(1.35 to 2.07)	1.27<br>(1.02 to 1.55)

Vestland NOR_60132 Western Europe Stillbirth Rate / Neonatal Mortality Rate (SBR/NMR) − 20 weeks
lambda = 0.2 ; zeta = 0.99 ; scale = 5 ; amp = 0.028

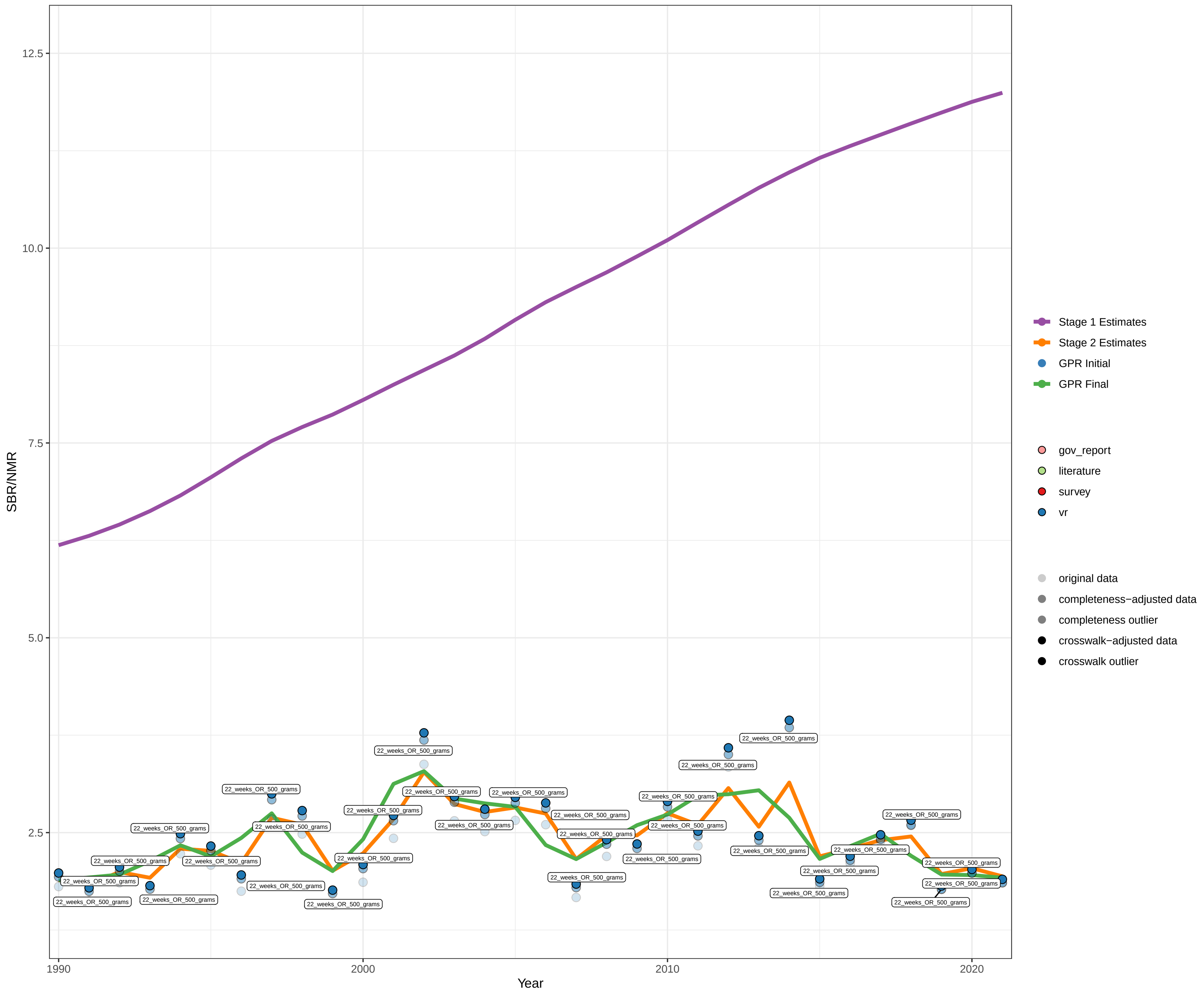

Vestland NOR_60132 Western Europe Stillbirth Rate / Neonatal Mortality Rate (SBR/NMR) − 28 weeks
lambda = 0.2 ; zeta = 0.99 ; scale = 5 ; amp = 0.028

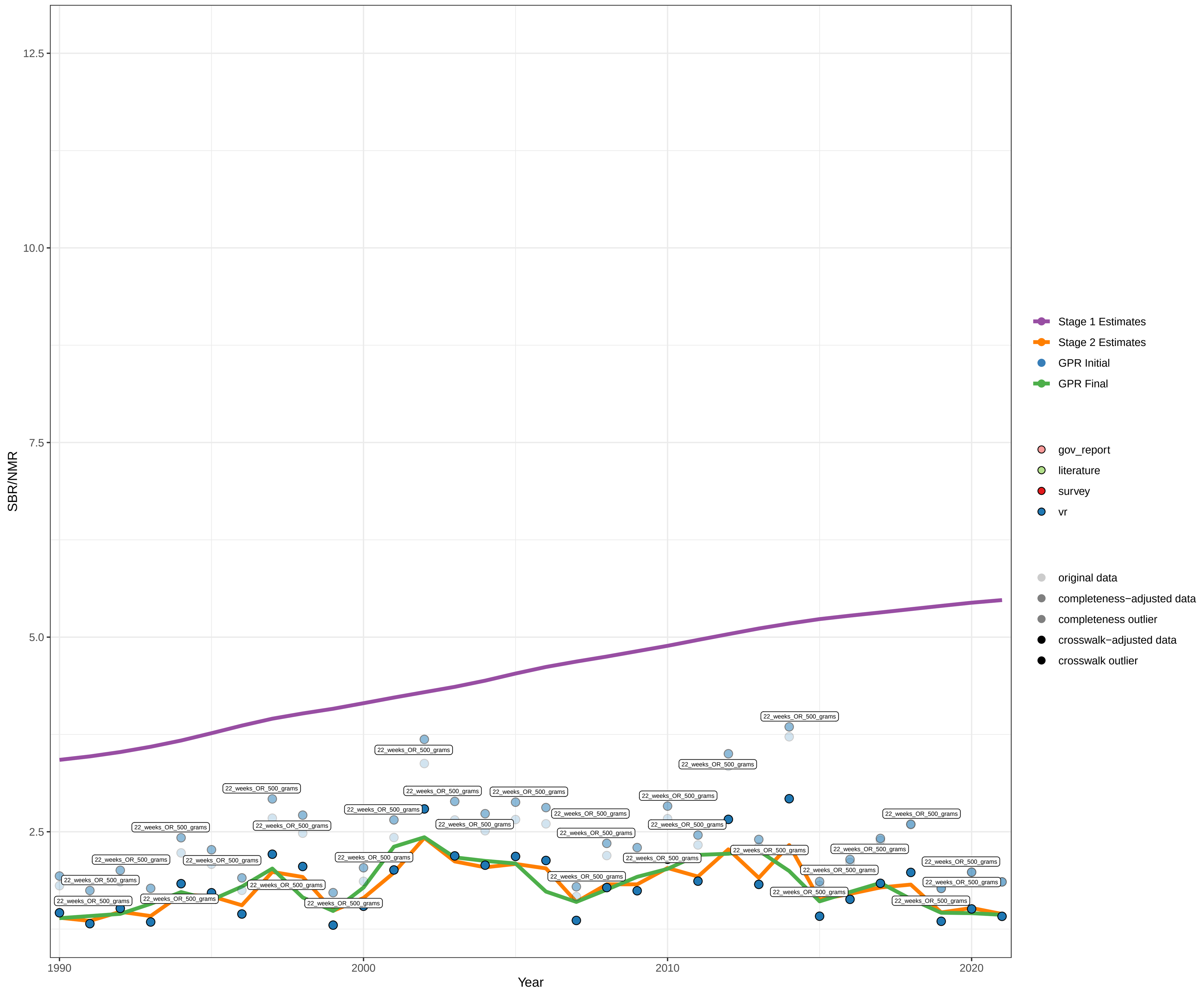

Definition	Years	Outlier	Source.Type	Title
22_weeks_OR_500_grams	1990−2021	0	VR	Norway Medical Birth Registry

Vestland NOR_60132 Western Europe Stillbirth Rate (SBR) − 20 weeks
lambda = 0.2 ; zeta = 0.99 ; scale = 5 ; amp = 0.028

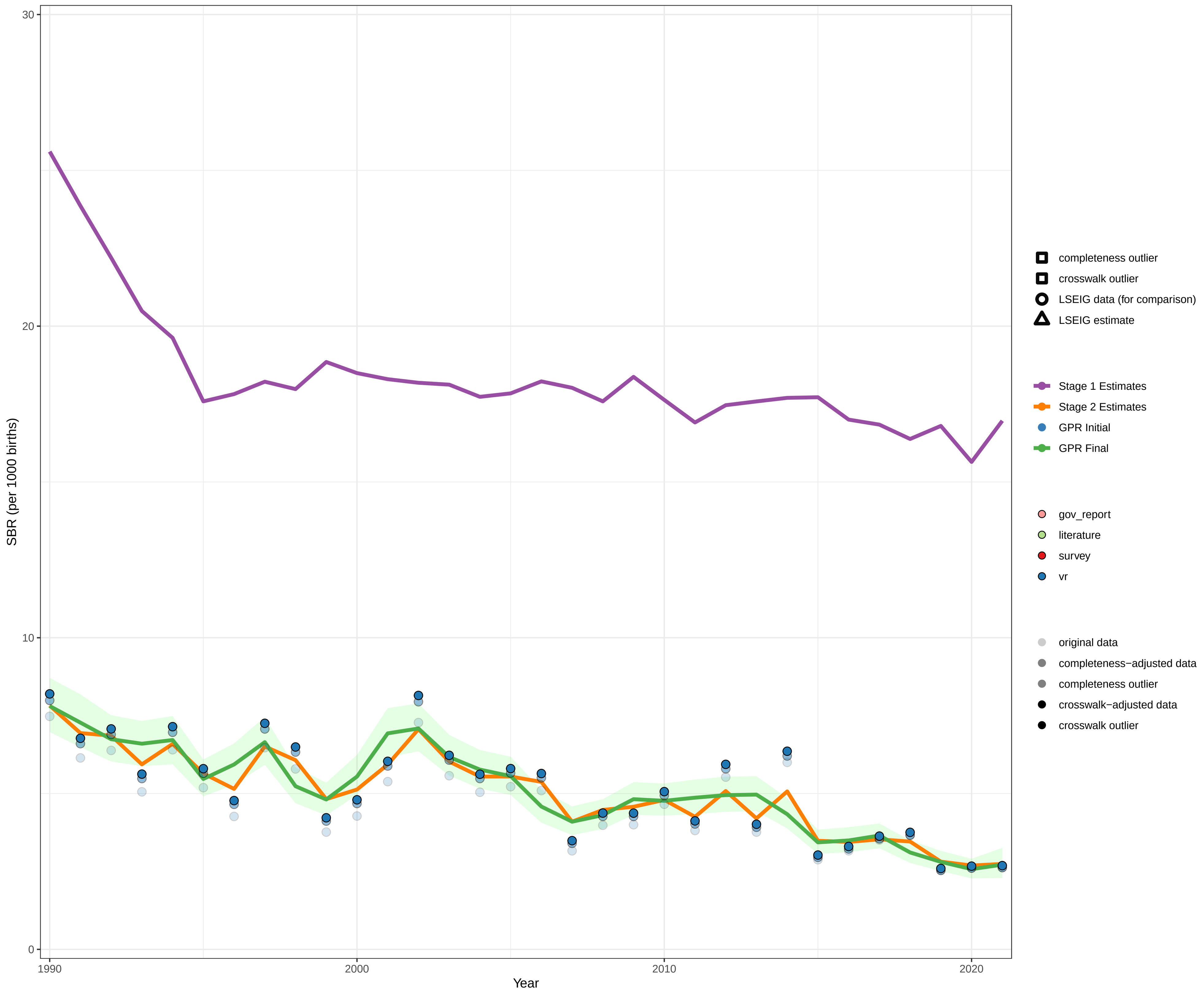

Vestland NOR_60132 Western Europe Stillbirth Rate (SBR) − 28 weeks
lambda = 0.2 ; zeta = 0.99 ; scale = 5 ; amp = 0.028

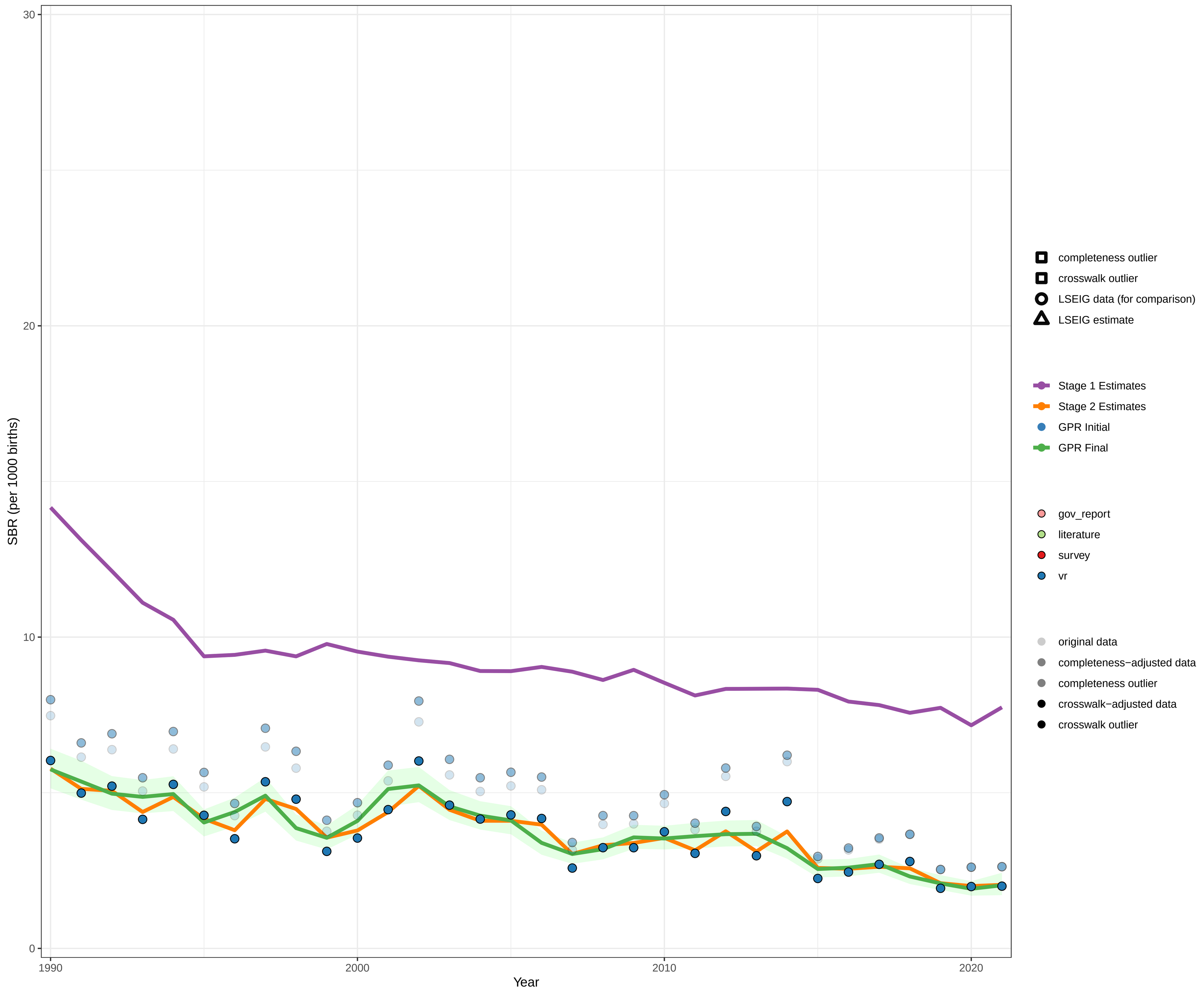

Year	Total Stillbirths (in thousands)	Total Stillbirths (in thousands)	SBR (per 1000 births)	SBR (per 1000 births)	SBR/NMR	SBR/NMR
	20 weeks	28 weeks	20 weeks	28 weeks	20 weeks	28 weeks
1990	0.1 (0.1 to 0.1)	0.0 (0.0 to 0.0)	7.8 (7.0 to 8.7)	5.8 (5.1 to 6.4)	1.89 (1.69 to 2.11)	1.39 (1.24 to 1.55)
1995	0.0 (0.0 to 0.0)	0.0 (0.0 to 0.0)	5.5 (4.9 to 6.1)	4.0 (3.6 to 4.5)	2.19 (1.97 to 2.45)	1.62 (1.44 to 1.79)
2000	0.0 (0.0 to 0.0)	0.0 (0.0 to 0.0)	5.5 (4.9 to 6.2)	4.1 (3.7 to 4.6)	2.41 (2.16 to 2.72)	1.78 (1.59 to 2.01)
2005	0.0 (0.0 to 0.0)	0.0 (0.0 to 0.0)	5.6 (5.0 to 6.2)	4.1 (3.7 to 4.6)	2.83 (2.52 to 3.15)	2.09 (1.87 to 2.33)
2010	0.0 (0.0 to 0.0)	0.0 (0.0 to 0.0)	4.8 (4.3 to 5.3)	3.5 (3.2 to 3.9)	2.73 (2.46 to 3.05)	2.02 (1.82 to 2.26)
2015	0.0 (0.0 to 0.0)	0.0 (0.0 to 0.0)	3.4 (3.1 to 3.8)	2.5 (2.3 to 2.9)	2.16 (1.93 to 2.42)	1.61 (1.43 to 1.80)
2020	0.0 (0.0 to 0.0)	0.0 (0.0 to 0.0)	2.6 (2.3 to 2.9)	1.9 (1.7 to 2.2)	1.95 (1.73 to 2.21)	1.45 (1.29 to 1.64)
2021	0.0 (0.0 to 0.0)	0.0 (0.0 to 0.0)	2.7 (2.3 to 3.3)	2.0 (1.7 to 2.4)	1.92 (1.62 to 2.31)	1.43 (1.21 to 1.72)

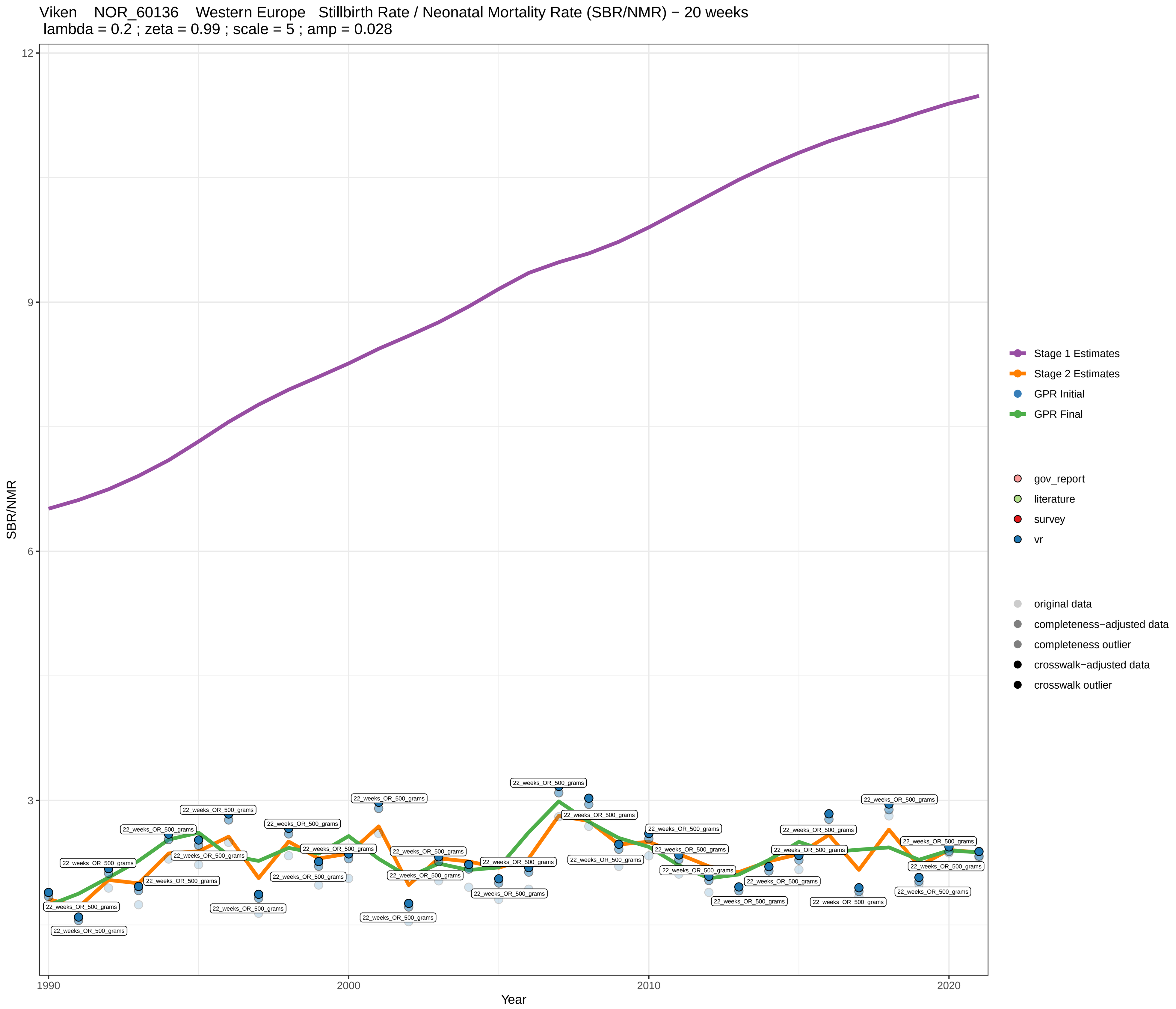

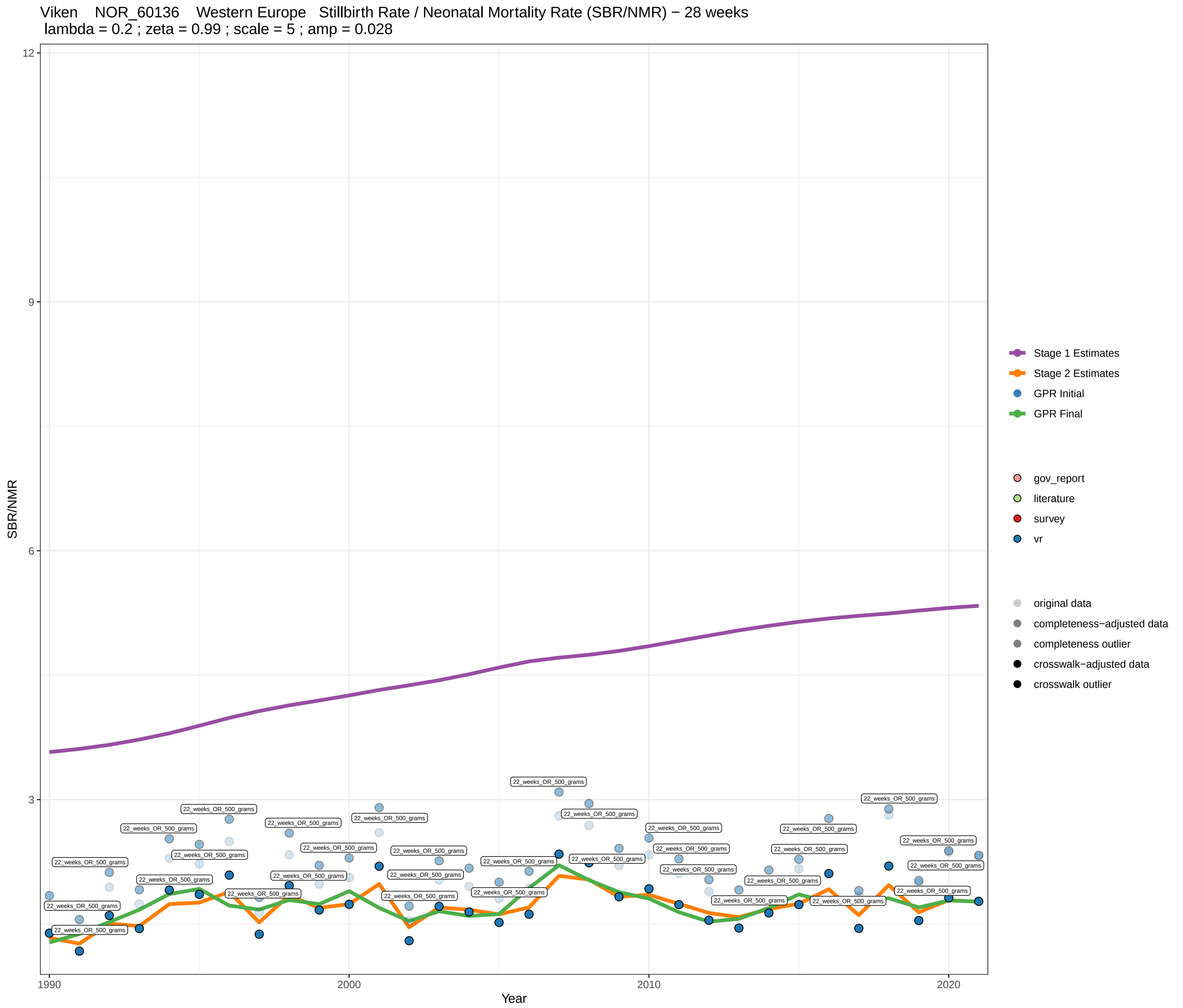

Definition	Years	Outlier	Source.Type	Title
22_weeks_OR_500_grams	1990−2021	0	VR	Norway Medical Birth Registry

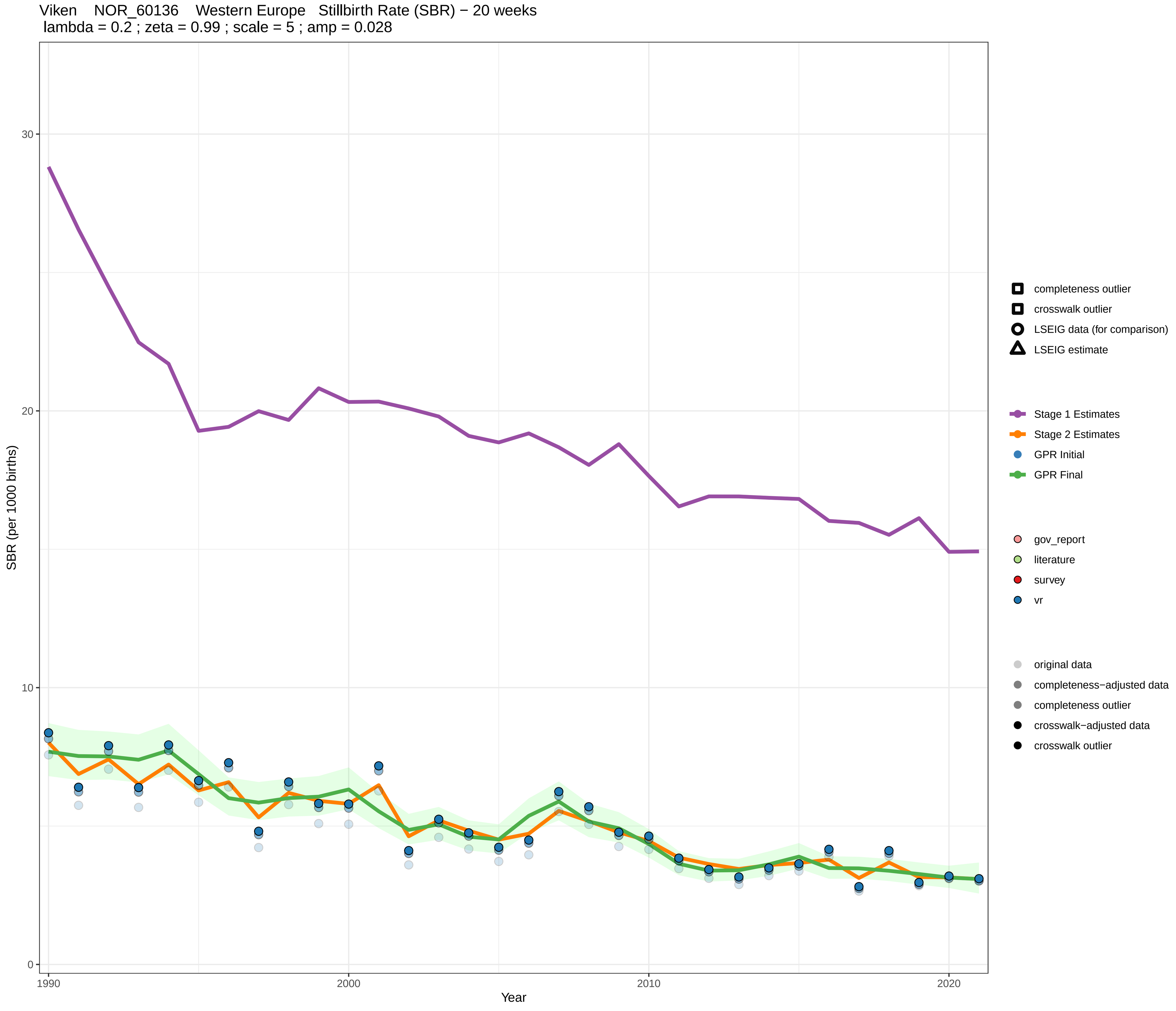

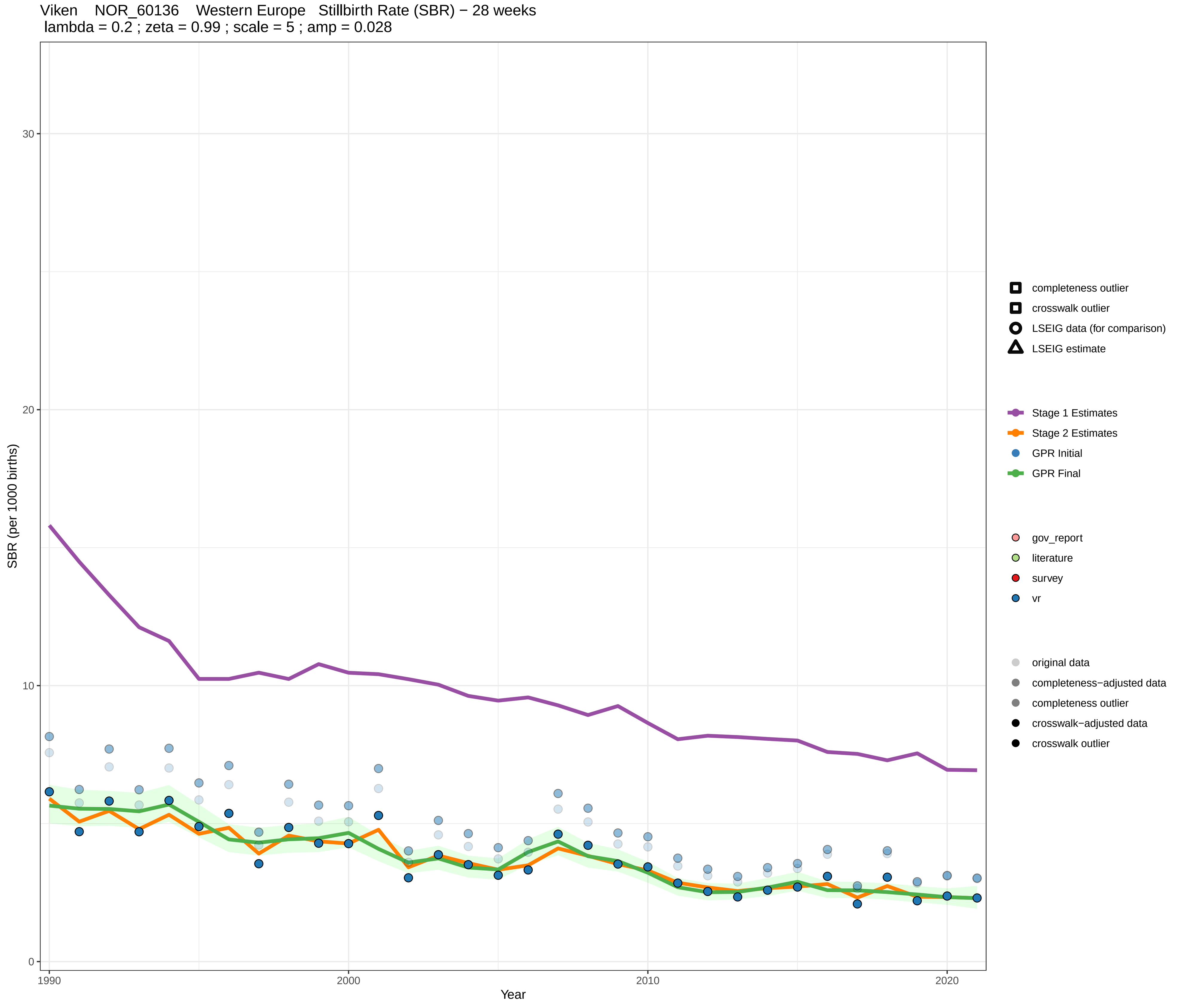

Year	Total Stillbirths (in thousands)	Total Stillbirths (in thousands)	SBR (per 1000 births)	SBR (per 1000 births)	SBR/NMR	SBR/NMR
	20 weeks	28 weeks	20 weeks	28 weeks	20 weeks	28 weeks
1990	0.1 (0.1 to 0.1)	0.1 (0.1 to 0.1)	7.7 (6.8 to 8.7)	5.7 (5.0 to 6.4)	1.74 (1.54 to 1.97)	1.28 (1.13 to 1.45)
1995	0.1 (0.1 to 0.1)	0.1 (0.1 to 0.1)	6.9 (6.1 to 7.7)	5.1 (4.5 to 5.7)	2.61 (2.32 to 2.94)	1.92 (1.71 to 2.16)
2000	0.1 (0.1 to 0.1)	0.1 (0.0 to 0.1)	6.3 (5.6 to 7.1)	4.7 (4.1 to 5.2)	2.57 (2.28 to 2.89)	1.90 (1.68 to 2.13)
2005	0.1 (0.0 to 0.1)	0.0 (0.0 to 0.0)	4.5 (4.0 to 5.1)	3.3 (3.0 to 3.7)	2.19 (1.95 to 2.46)	1.62 (1.44 to 1.82)
2010	0.1 (0.0 to 0.1)	0.0 (0.0 to 0.0)	4.4 (3.9 to 4.9)	3.2 (2.9 to 3.6)	2.44 (2.17 to 2.74)	1.81 (1.60 to 2.03)
2015	0.0 (0.0 to 0.1)	0.0 (0.0 to 0.0)	3.9 (3.5 to 4.4)	2.9 (2.6 to 3.3)	2.50 (2.22 to 2.82)	1.86 (1.65 to 2.09)
2020	0.0 (0.0 to 0.0)	0.0 (0.0 to 0.0)	3.1 (2.8 to 3.6)	2.3 (2.1 to 2.7)	2.40 (2.11 to 2.73)	1.78 (1.57 to 2.03)
2021	0.0 (0.0 to 0.0)	0.0 (0.0 to 0.0)	3.1 (2.6 to 3.7)	2.3 (1.9 to 2.7)	2.37 (1.97 to 2.83)	1.77 (1.47 to 2.11)

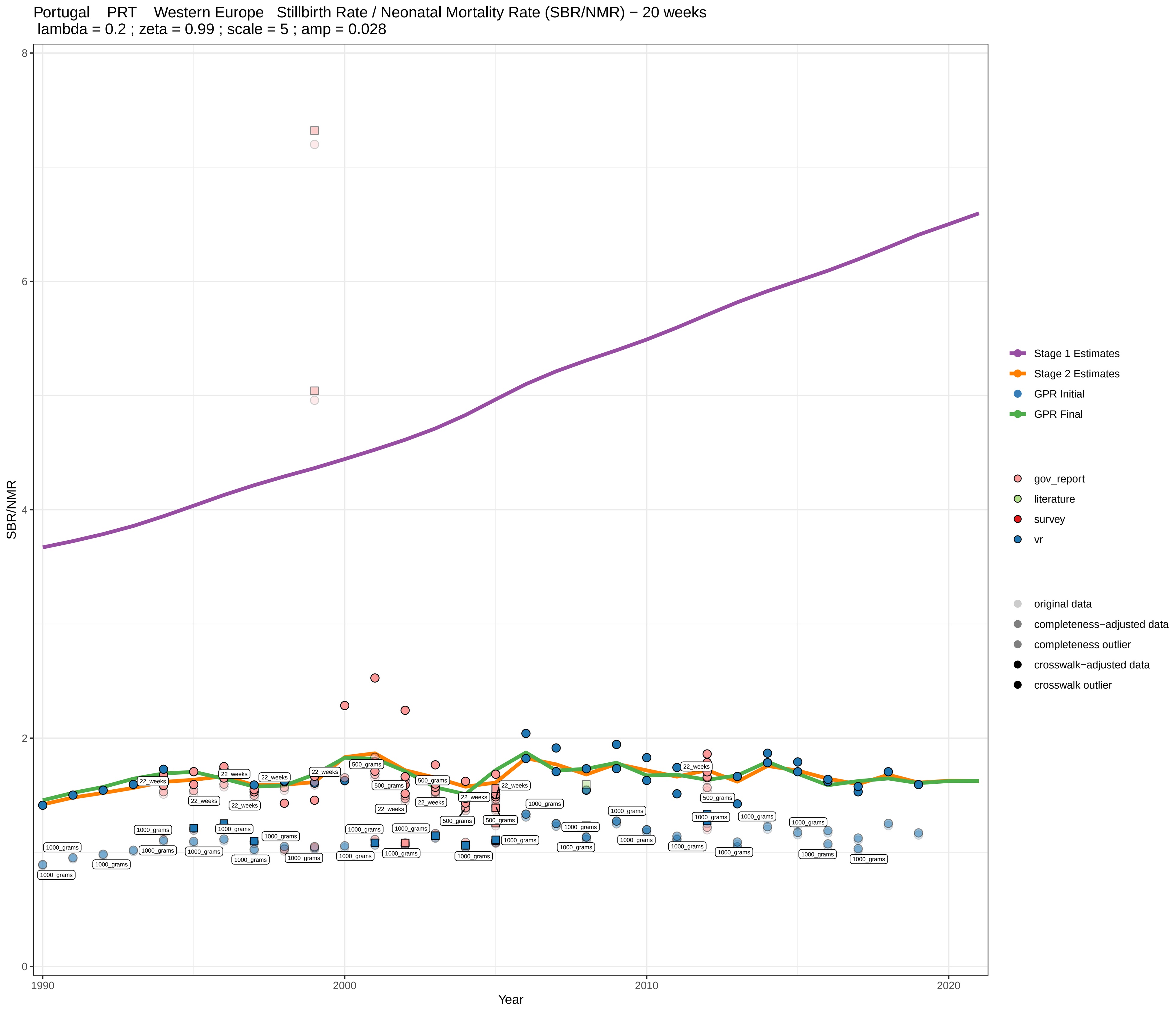

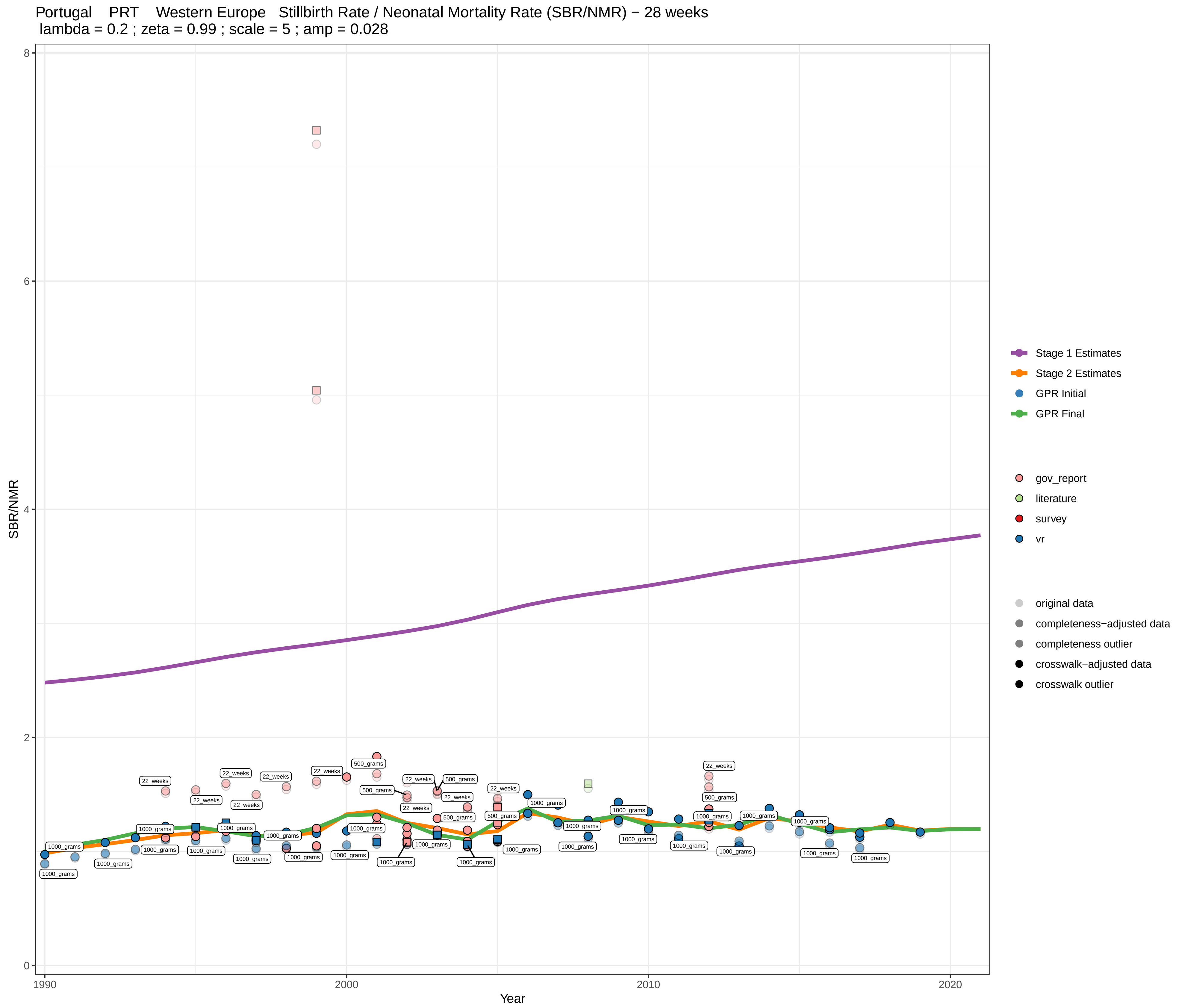

Definition	Years	Outlier	Source.Type	Title
1000_grams	1990−2017	0	VR	European Health for All Database
1000_grams, 500_grams	2001−2005, 2012	0	Statistical Report	Portugal Health Statistics
22_weeks	1994−1999	0	Statistical Report	Portugal Statistical Yearbook
22_weeks	2002−2005, 2012	0	Statistical Report	Portugal Health Statistics
28_weeks	1994−2002	0	Statistical Report	Portugal Statistical Yearbook
28_weeks	2003−2005, 2012	0	Statistical Report	Portugal Health Statistics
28_weeks	2006−2011, 2013−2019	0	VR	United Nations Demographic Yearbook
1000_grams, 500_grams	1999	1	Statistical Report	Portugal Health Statistics
24_weeks, 28_weeks	2008	1	Sci Lit	Perinatal mortality classification: an analysis of 112 cases of stillbirth
28_weeks	1995−1997, 2001−2005, 2012	2	VR	United Nations Demographic Yearbook
28_weeks	2002, 2005	2	Statistical Report	Portugal Health Statistics

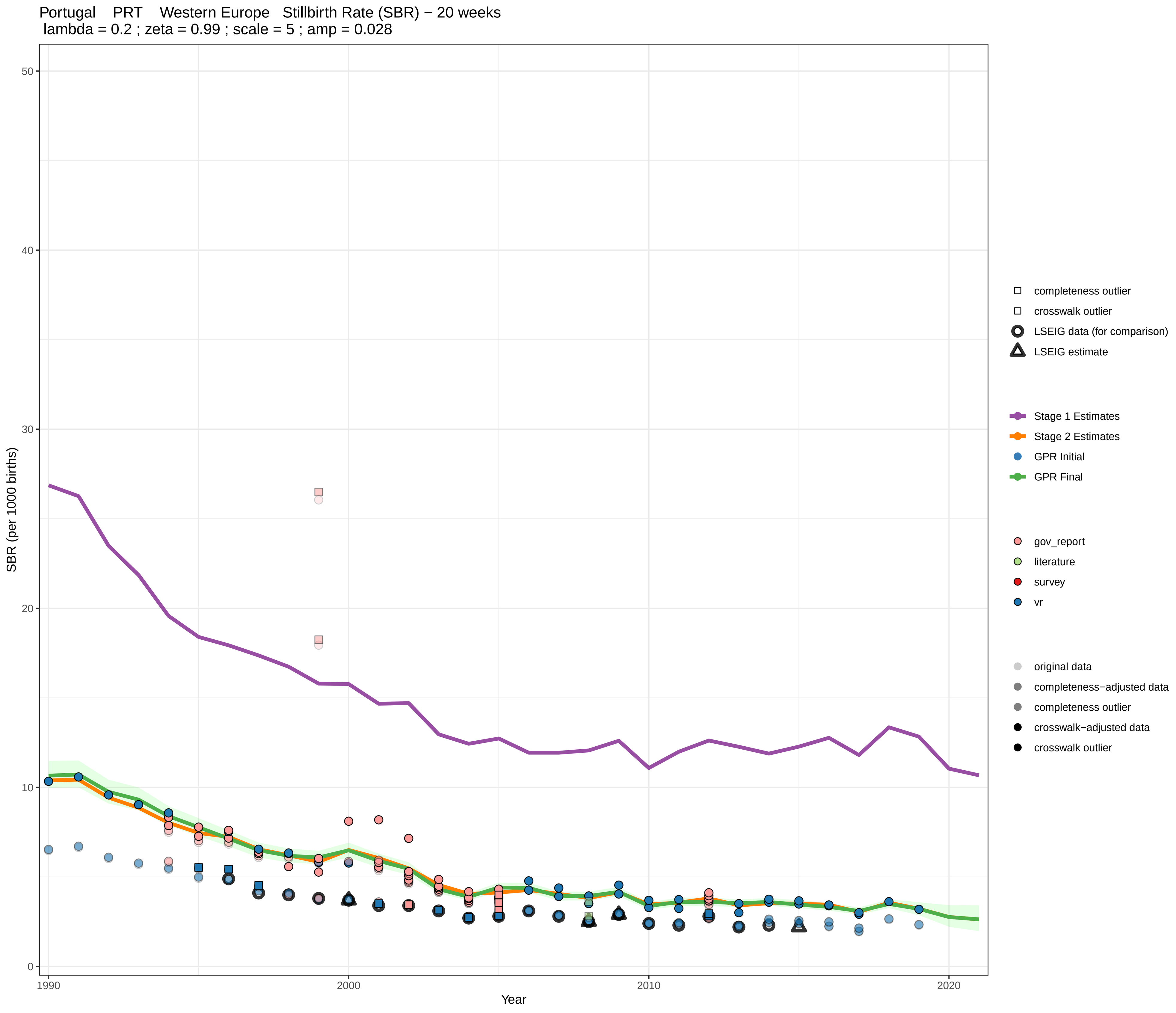

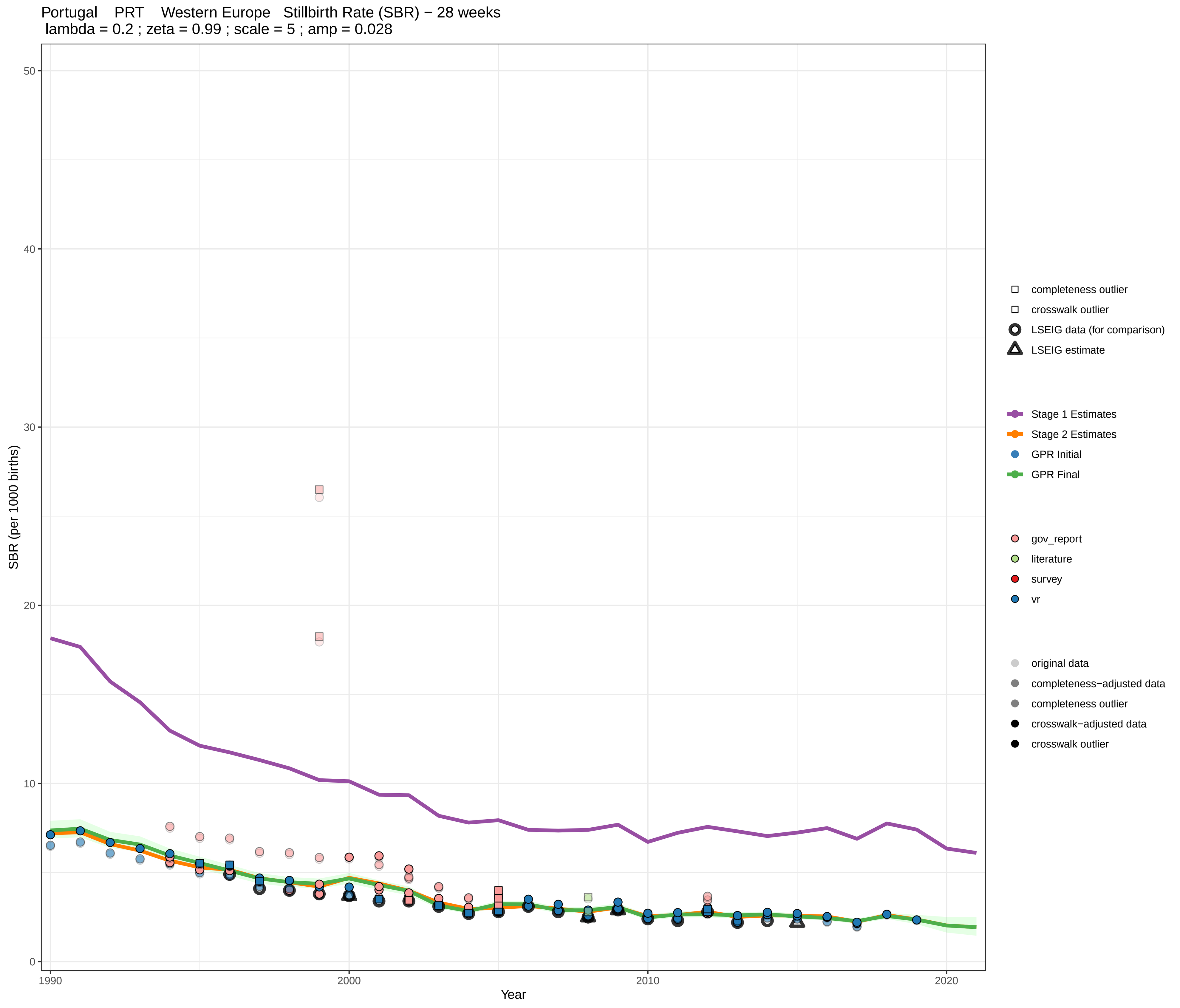

Year	Total Stillbirths (in thousands)	Total Stillbirths (in thousands)	SBR (per 1000 births)	SBR (per 1000 births)	SBR/NMR	SBR/NMR
	20 weeks	28 weeks	20 weeks	28 weeks	20 weeks	28 weeks
1990	1.3 (1.2 to 1.3)	0.9 (0.8 to 0.9)	10.7 (10.0 to 11.5)	7.4 (6.9 to 7.9)	1.46 (1.36 to 1.57)	1.01 (0.94 to 1.08)
1995	0.9 (0.8 to 0.9)	0.6 (0.6 to 0.6)	7.8 (7.3 to 8.3)	5.5 (5.2 to 5.9)	1.70 (1.60 to 1.82)	1.21 (1.14 to 1.29)
2000	0.8 (0.7 to 0.8)	0.5 (0.5 to 0.6)	6.5 (6.1 to 6.9)	4.7 (4.4 to 5.0)	1.83 (1.71 to 1.95)	1.32 (1.24 to 1.40)
2005	0.5 (0.4 to 0.5)	0.3 (0.3 to 0.4)	4.4 (4.2 to 4.7)	3.2 (3.0 to 3.4)	1.72 (1.62 to 1.82)	1.26 (1.19 to 1.33)
2010	0.3 (0.3 to 0.4)	0.2 (0.2 to 0.3)	3.4 (3.2 to 3.6)	2.5 (2.3 to 2.6)	1.67 (1.57 to 1.78)	1.23 (1.15 to 1.31)
2015	0.3 (0.3 to 0.3)	0.2 (0.2 to 0.2)	3.5 (3.3 to 3.7)	2.5 (2.4 to 2.7)	1.69 (1.59 to 1.81)	1.24 (1.17 to 1.32)
2020	0.2 (0.2 to 0.3)	0.2 (0.1 to 0.2)	2.8 (2.2 to 3.4)	2.0 (1.6 to 2.5)	1.62 (1.30 to 2.01)	1.19 (0.96 to 1.48)
2021	0.2 (0.2 to 0.3)	0.2 (0.1 to 0.2)	2.6 (2.0 to 3.4)	1.9 (1.5 to 2.5)	1.62 (1.22 to 2.11)	1.20 (0.90 to 1.55)

San Marino SMR Western Europe Stillbirth Rate / Neonatal Mortality Rate (SBR/NMR) − 20 weeks
lambda = 0.4 ; zeta = 0.9 ; scale = 10 ; amp = 0.028

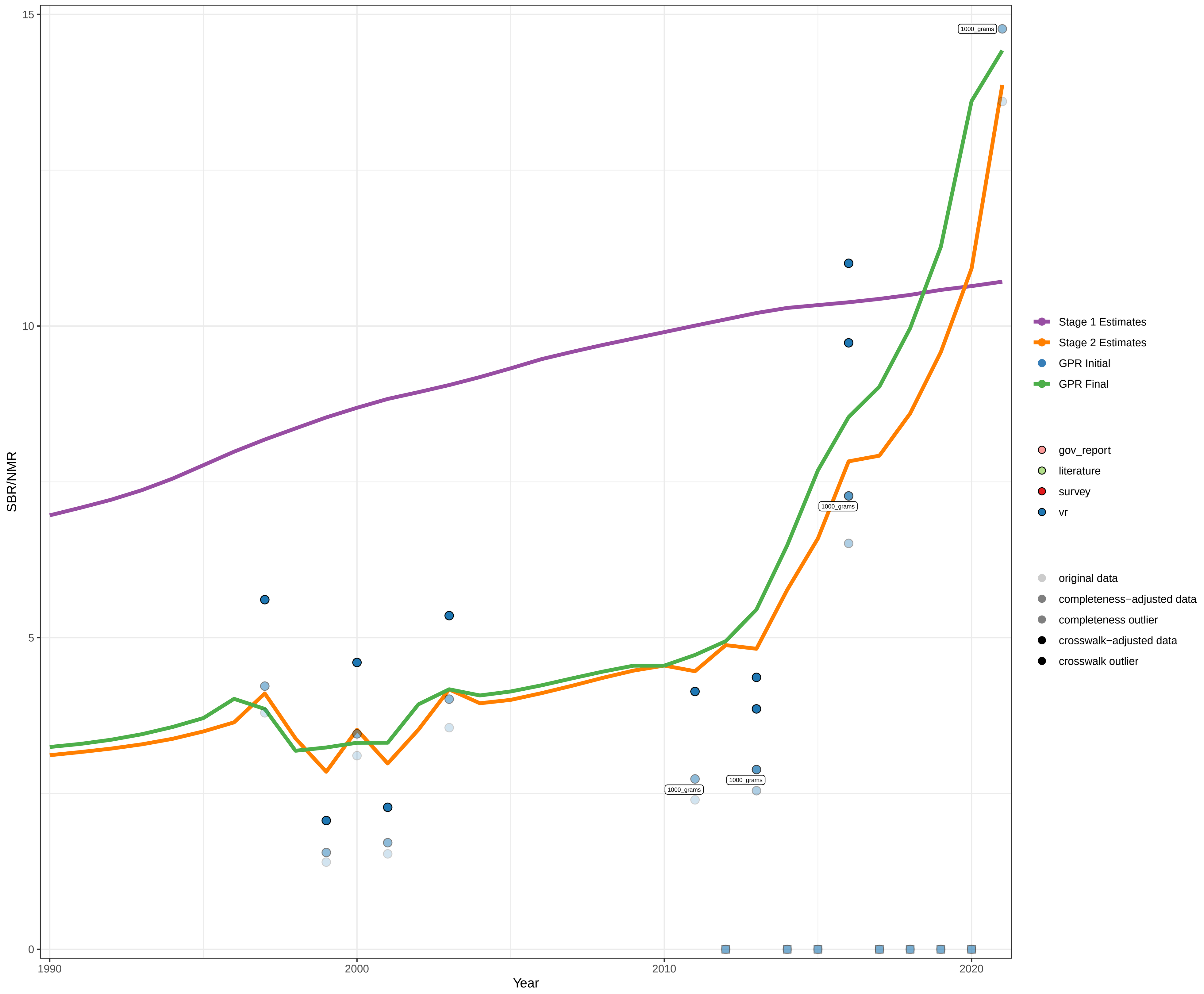

San Marino SMR Western Europe Stillbirth Rate / Neonatal Mortality Rate (SBR/NMR) − 28 weeks
lambda = 0.4 ; zeta = 0.9 ; scale = 10 ; amp = 0.028

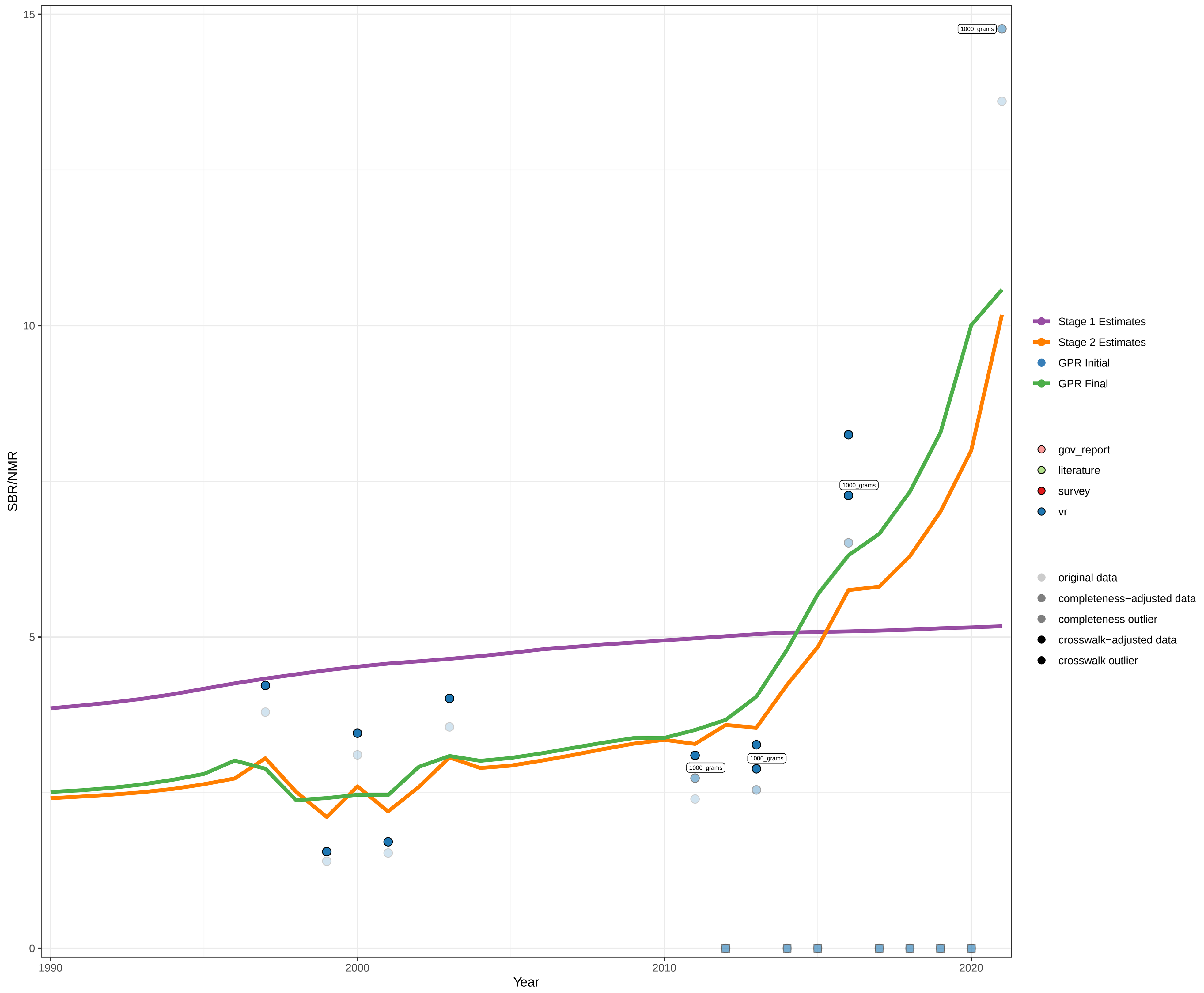

Definition	Years	Outlier	Source.Type	Title
1000_grams	2011, 2013, 2016, 2021	0	VR	European Health for All Database
28_weeks	1997, 1999−2001, 2003, 2013, 2016	0	VR	United Nations Demographic Yearbook
1000_grams	2012, 2014, 2015, 2017−2020	1	VR	European Health for All Database

San Marino SMR Western Europe Stillbirth Rate (SBR) − 20 weeks
lambda = 0.4 ; zeta = 0.9 ; scale = 10 ; amp = 0.028

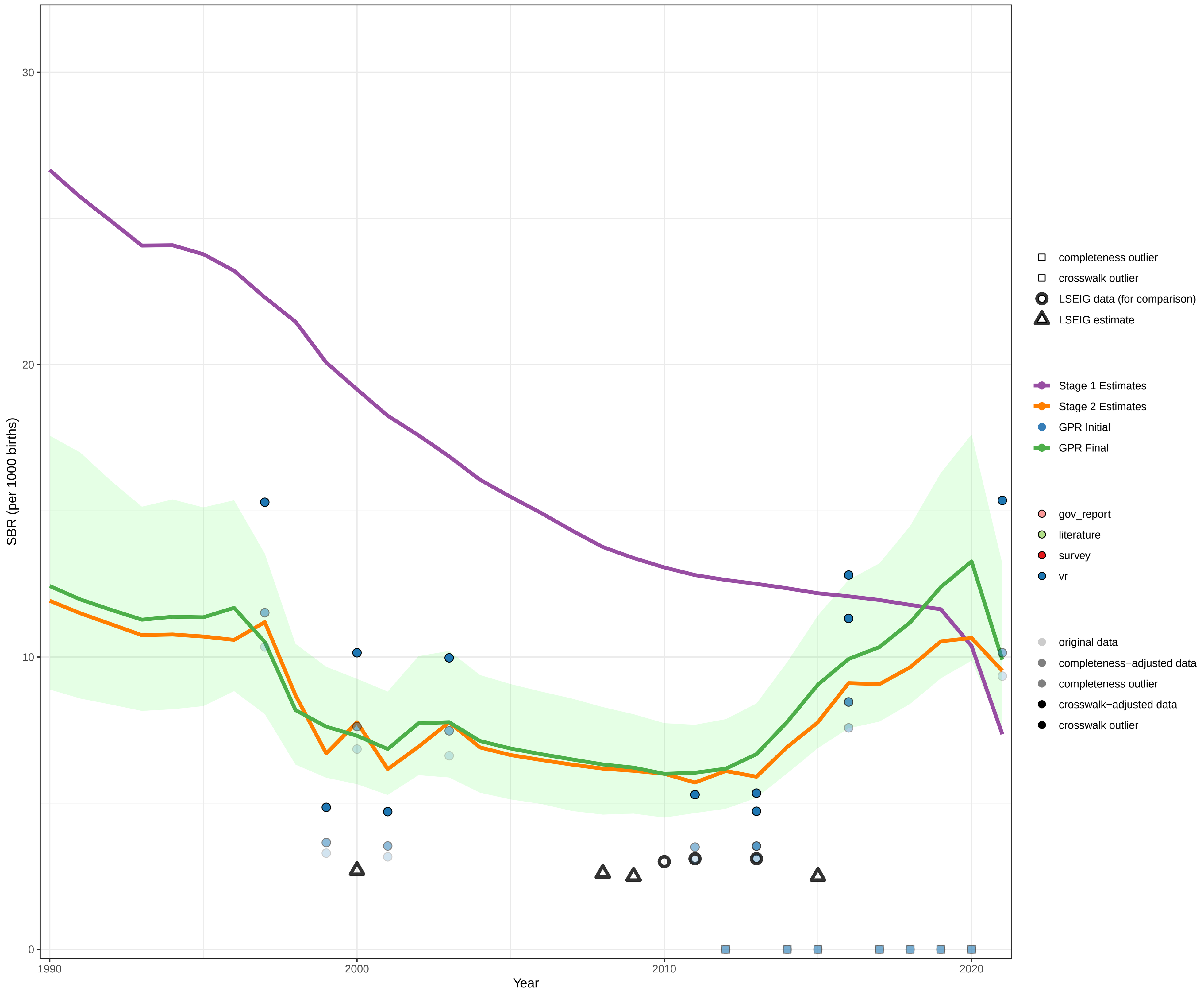

San Marino SMR Western Europe Stillbirth Rate (SBR) − 28 weeks
lambda = 0.4 ; zeta = 0.9 ; scale = 10 ; amp = 0.028

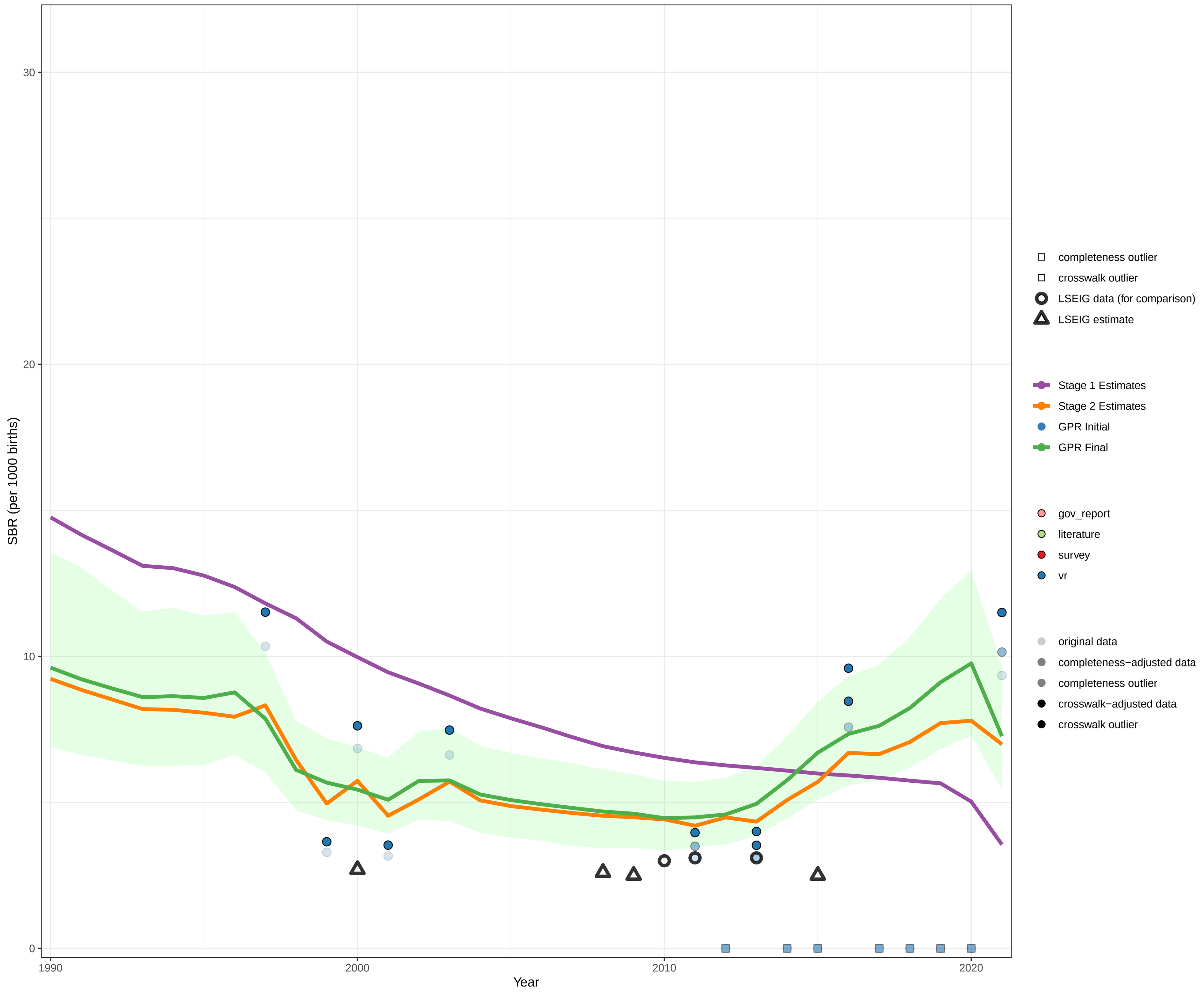

Year	Total Stillbirths (in thousands)	Total Stillbirths (in thousands)	SBR (per 1000 births)	SBR (per 1000 births)	SBR/NMR	SBR/NMR
	20 weeks	28 weeks	20 weeks	28 weeks	20 weeks	28 weeks
1990	0.0 (0.0 to 0.0)	0.0 (0.0 to 0.0)	12.4 (8.9 to 17.6)	9.6 (6.9 to 13.6)	3.25 (2.32 to 4.59)	2.51 (1.80 to 3.54)
1995	0.0 (0.0 to 0.0)	0.0 (0.0 to 0.0)	11.4 (8.3 to 15.1)	8.6 (6.3 to 11.4)	3.71 (2.72 to 4.94)	2.80 (2.05 to 3.72)
2000	0.0 (0.0 to 0.0)	0.0 (0.0 to 0.0)	7.3 (5.7 to 9.3)	5.4 (4.2 to 6.9)	3.31 (2.56 to 4.20)	2.47 (1.91 to 3.12)
2005	0.0 (0.0 to 0.0)	0.0 (0.0 to 0.0)	6.9 (5.1 to 9.1)	5.1 (3.8 to 6.7)	4.14 (3.09 to 5.46)	3.06 (2.29 to 4.03)
2010	0.0 (0.0 to 0.0)	0.0 (0.0 to 0.0)	6.0 (4.5 to 7.7)	4.5 (3.3 to 5.7)	4.55 (3.42 to 5.86)	3.38 (2.54 to 4.35)
2015	0.0 (0.0 to 0.0)	0.0 (0.0 to 0.0)	9.1 (6.9 to 11.4)	6.7 (5.1 to 8.5)	7.68 (5.84 to 9.69)	5.69 (4.32 to 7.17)
2020	0.0 (0.0 to 0.0)	0.0 (0.0 to 0.0)	13.3 (9.9 to 17.6)	9.8 (7.3 to 12.9)	13.61 (10.13 to 18.06)	10.01 (7.46 to 13.27)
2021	0.0 (0.0 to 0.0)	0.0 (0.0 to 0.0)	9.9 (7.4 to 13.2)	7.3 (5.4 to 9.7)	14.42 (10.78 to 19.21)	10.58 (7.92 to 14.07)

Spain ESP Western Europe Stillbirth Rate / Neonatal Mortality Rate (SBR/NMR) − 20 weeks
lambda = 0.2 ; zeta = 0.99 ; scale = 5 ; amp = 0.028

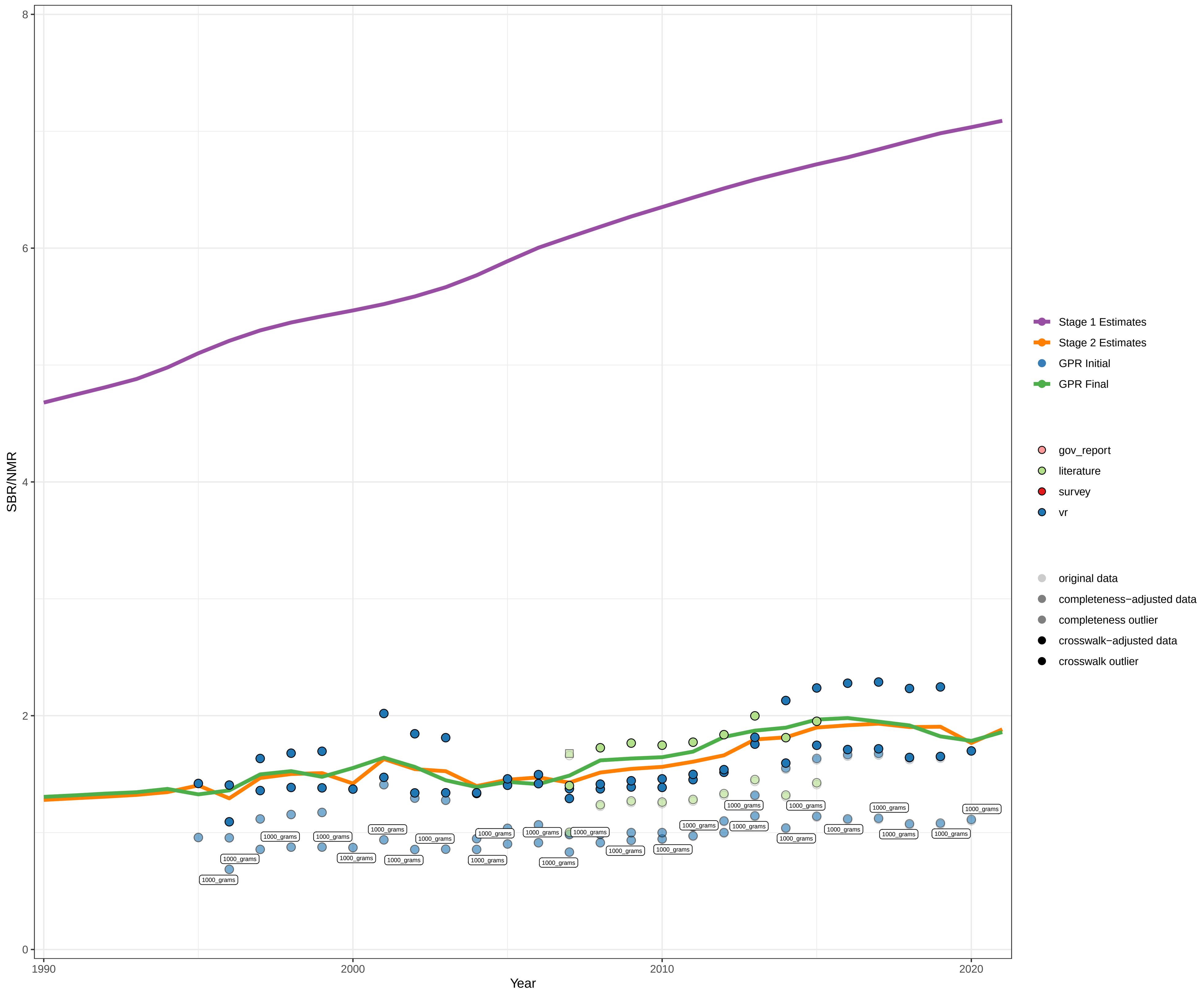

Spain ESP Western Europe Stillbirth Rate / Neonatal Mortality Rate (SBR/NMR) − 28 weeks
lambda = 0.2 ; zeta = 0.99 ; scale = 5 ; amp = 0.028

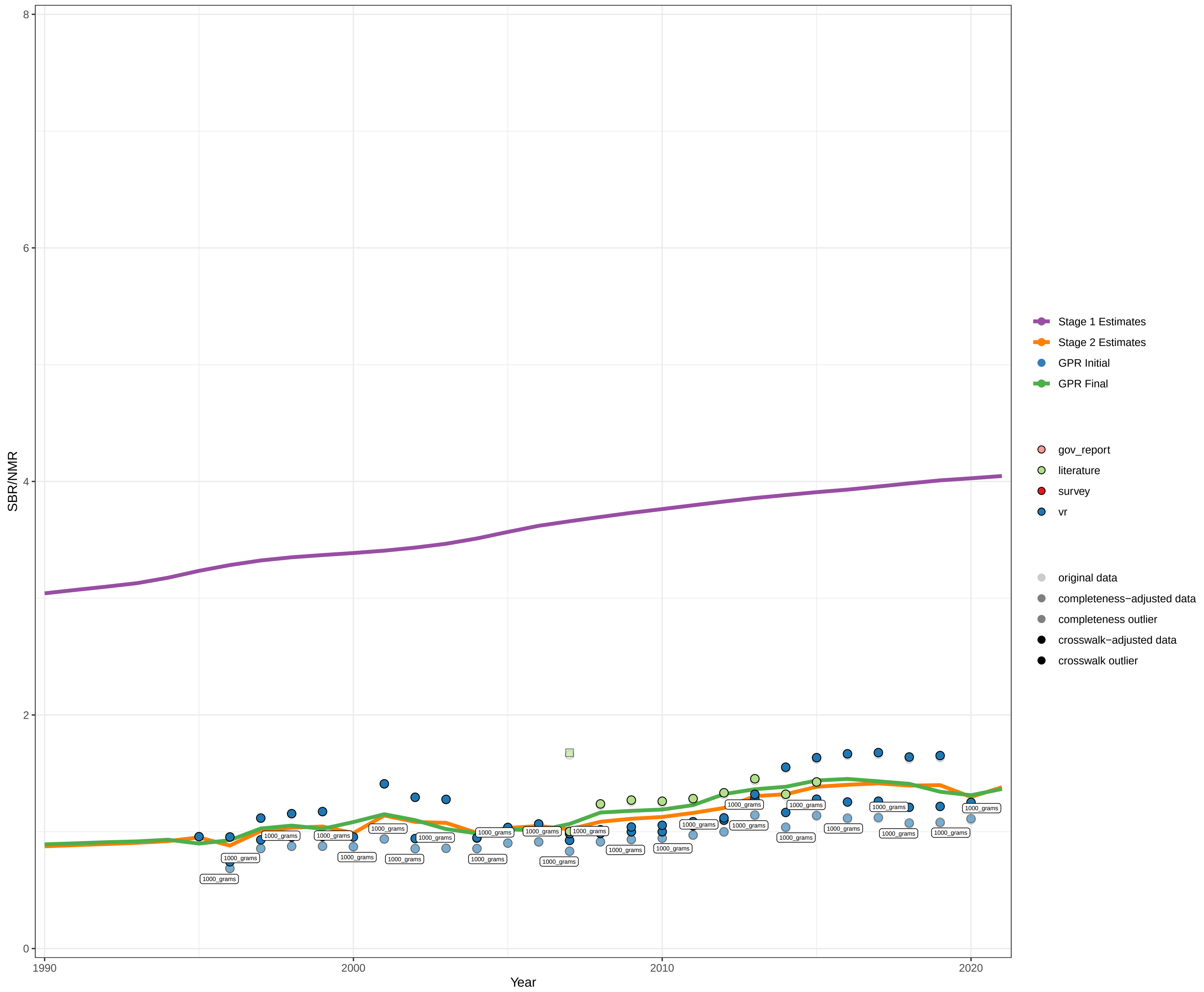

Definition	Years	Outlier	Source.Type	Title
1000_grams	1996−2020	0	VR	European Health for All Database
28_weeks	1995−1999, 2001−2019	0	VR	United Nations Demographic Yearbook
28_weeks	2007−2015	0	Sci Lit	Secular trends in stillbirth by maternal socioeconomic status in Spain 2007−15: a population−based study of 4 million births
22_weeks	2007	1	Sci Lit	Multiple birth trends in the region of Castilla y Le<f3>n (Spain) in a 13 year period

Spain ESP Western Europe Stillbirth Rate (SBR) − 20 weeks
lambda = 0.2 ; zeta = 0.99 ; scale = 5 ; amp = 0.028

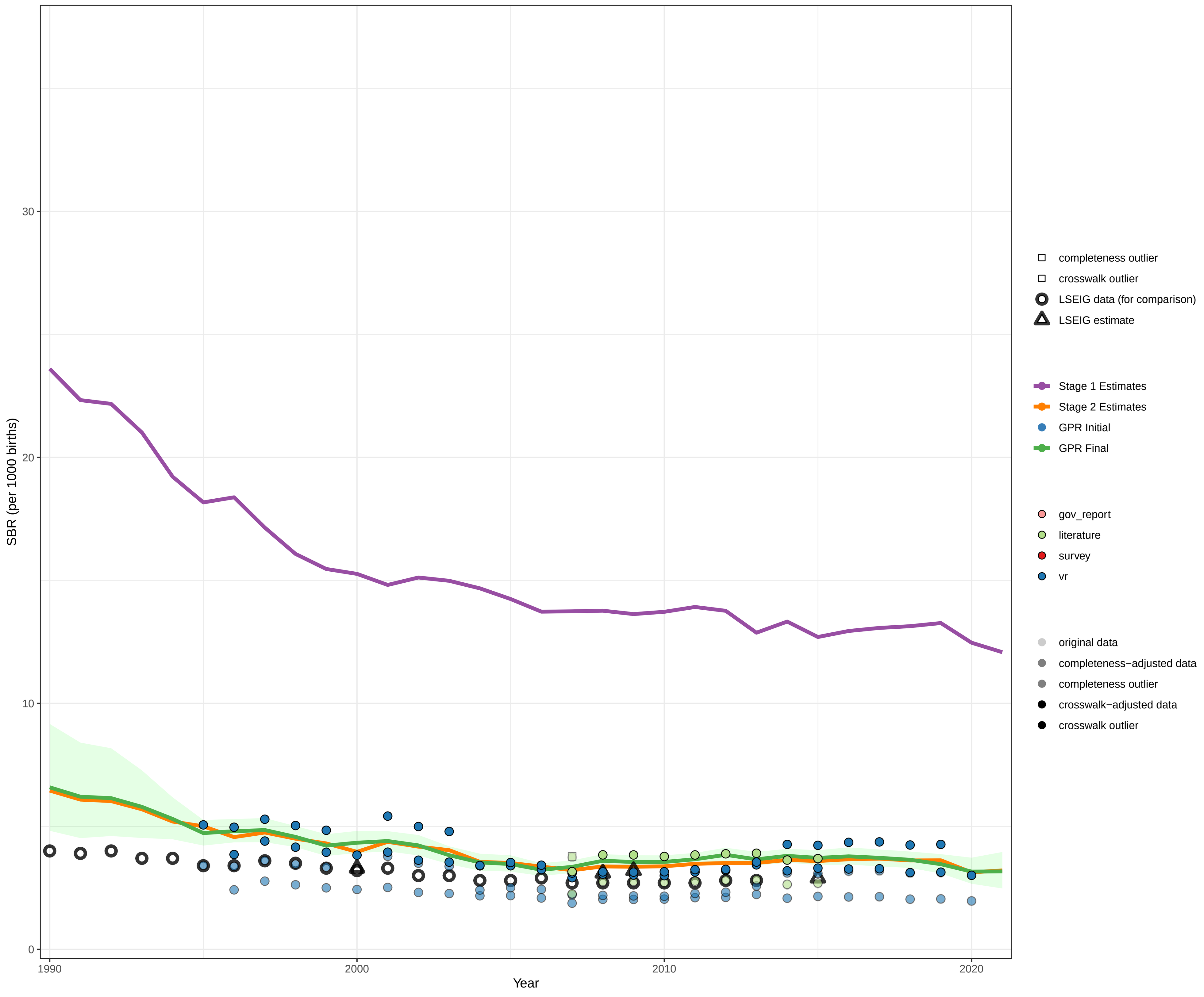

Spain ESP Western Europe Stillbirth Rate (SBR) − 28 weeks
lambda = 0.2 ; zeta = 0.99 ; scale = 5 ; amp = 0.028

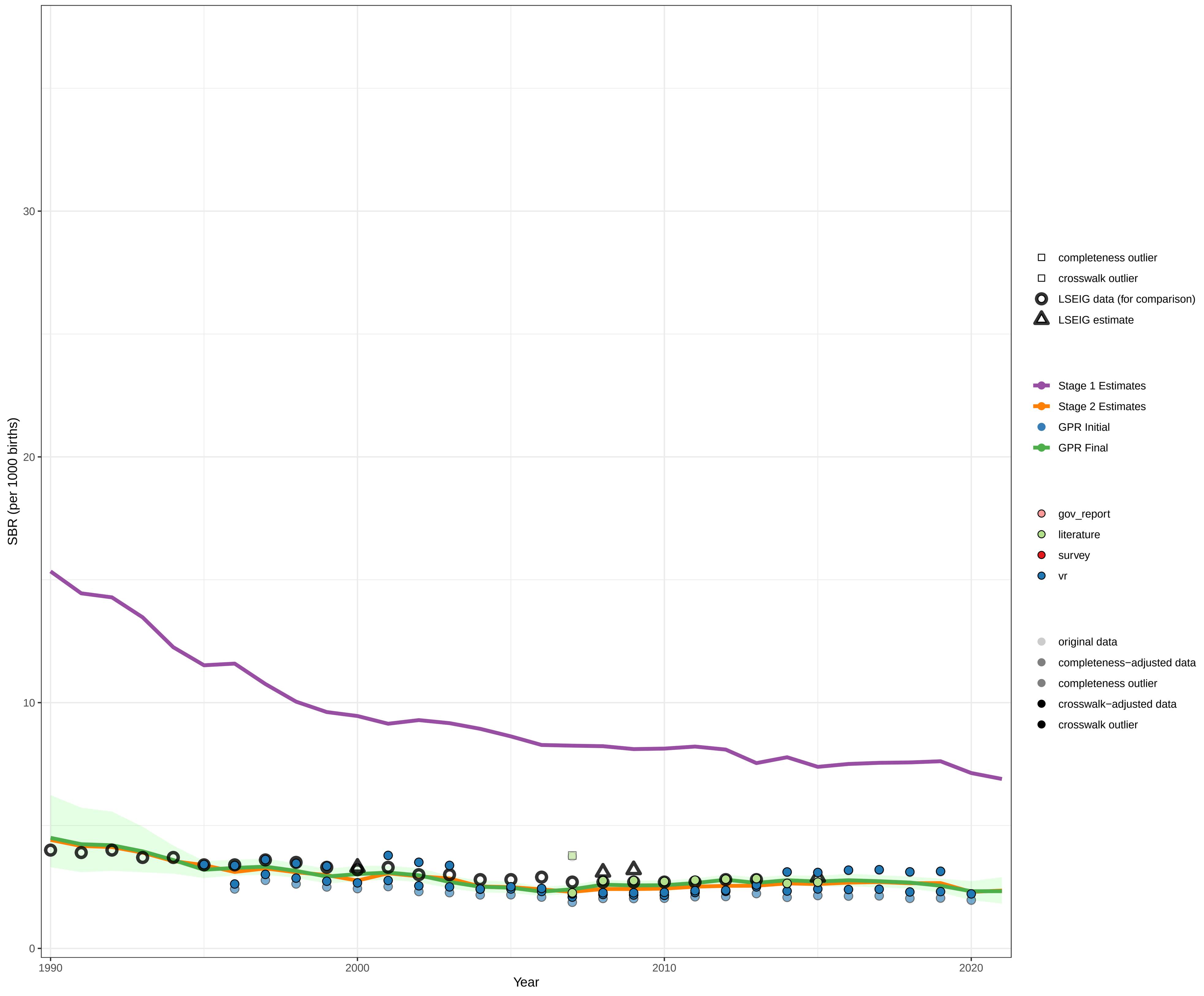

Year	Total Stillbirths (in thousands)	Total Stillbirths (in thousands)	SBR (per 1000 births)	SBR (per 1000 births)	SBR/NMR	SBR/NMR
	20 weeks	28 weeks	20 weeks	28 weeks	20 weeks	28 weeks
1990	2.6 (1.9 to 3.7)	1.8 (1.3 to 2.5)	6.6 (4.8 to 9.2)	4.5 (3.3 to 6.2)	1.31 (0.96 to 1.82)	0.89 (0.65 to 1.24)
1995	1.7 (1.5 to 1.9)	1.2 (1.0 to 1.3)	4.7 (4.2 to 5.3)	3.2 (2.9 to 3.6)	1.33 (1.18 to 1.48)	0.90 (0.80 to 1.00)
2000	1.7 (1.6 to 1.9)	1.2 (1.1 to 1.3)	4.3 (3.9 to 4.8)	3.0 (2.7 to 3.4)	1.55 (1.40 to 1.72)	1.08 (0.98 to 1.20)
2005	1.6 (1.5 to 1.8)	1.2 (1.1 to 1.3)	3.5 (3.2 to 3.8)	2.5 (2.3 to 2.7)	1.43 (1.31 to 1.58)	1.02 (0.93 to 1.12)
2010	1.7 (1.6 to 1.8)	1.2 (1.2 to 1.3)	3.6 (3.3 to 3.8)	2.6 (2.4 to 2.8)	1.64 (1.53 to 1.77)	1.19 (1.11 to 1.28)
2015	1.6 (1.4 to 1.7)	1.1 (1.1 to 1.2)	3.7 (3.4 to 4.0)	2.7 (2.5 to 2.9)	1.97 (1.82 to 2.13)	1.44 (1.33 to 1.56)
2020	1.1 (0.9 to 1.3)	0.8 (0.7 to 0.9)	3.2 (2.7 to 3.7)	2.3 (2.0 to 2.7)	1.78 (1.50 to 2.10)	1.31 (1.11 to 1.54)
2021	1.1 (0.8 to 1.3)	0.8 (0.6 to 1.0)	3.2 (2.5 to 4.0)	2.3 (1.8 to 2.9)	1.86 (1.45 to 2.32)	1.37 (1.07 to 1.70)

Sweden SWE Western Europe Stillbirth Rate / Neonatal Mortality Rate (SBR/NMR) − 20 weeks
lambda = 0.2 ; zeta = 0.99 ; scale = 5 ; amp = 0.028

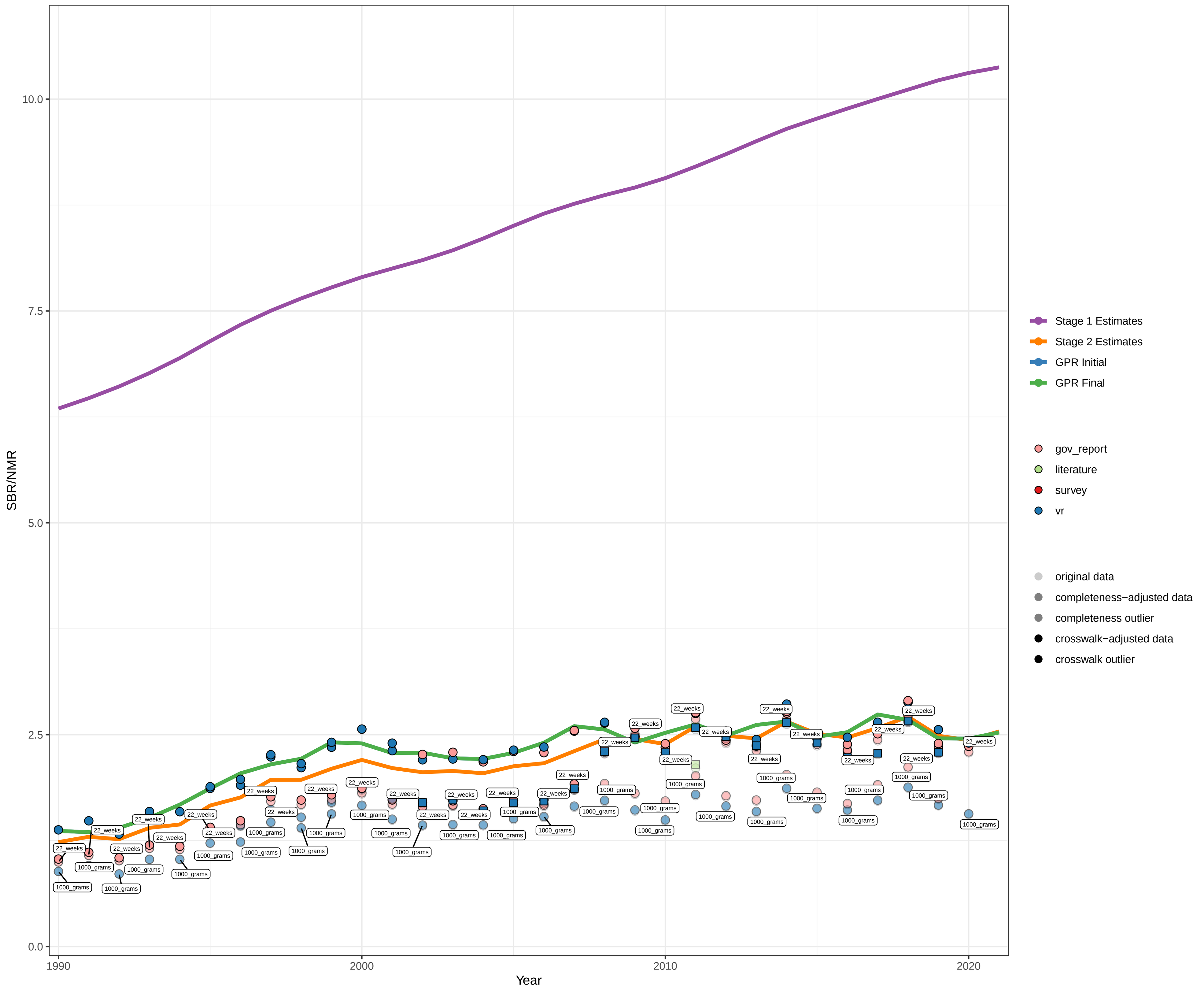

Sweden SWE Western Europe Stillbirth Rate / Neonatal Mortality Rate (SBR/NMR) − 28 weeks
lambda = 0.2 ; zeta = 0.99 ; scale = 5 ; amp = 0.028

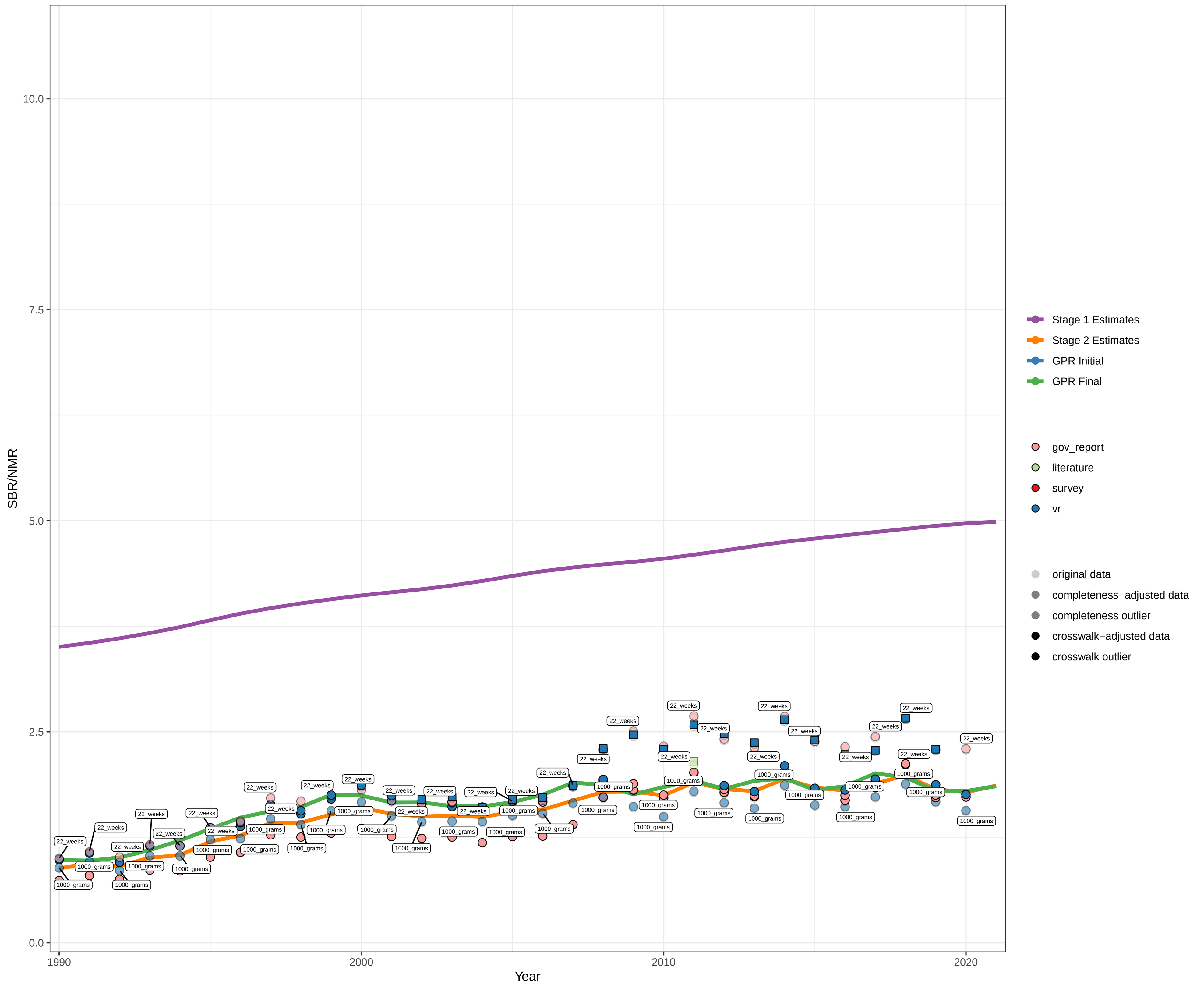

Definition	Years	Outlier	Source.Type	Title
1000_grams	1990−2020	0	VR	European Health for All Database
22_weeks	1990−2020	0	Statistical Report	Sweden Pregnancies, Deliveries, and Newborn Infants
28_weeks	1995−2001	0	VR	United Nations Demographic Yearbook
28_weeks	2002−2019	0	Statistical Report	Sweden Pregnancies, Deliveries, and Newborn Infants
22_weeks	2011	1	Sci Lit	Pregnancy weight gain by gestational age and stillbirth: a population−based cohort study
28_weeks	2002−2019	2	VR	United Nations Demographic Yearbook

Sweden SWE Western Europe Stillbirth Rate (SBR) − 20 weeks
lambda = 0.2 ; zeta = 0.99 ; scale = 5 ; amp = 0.028

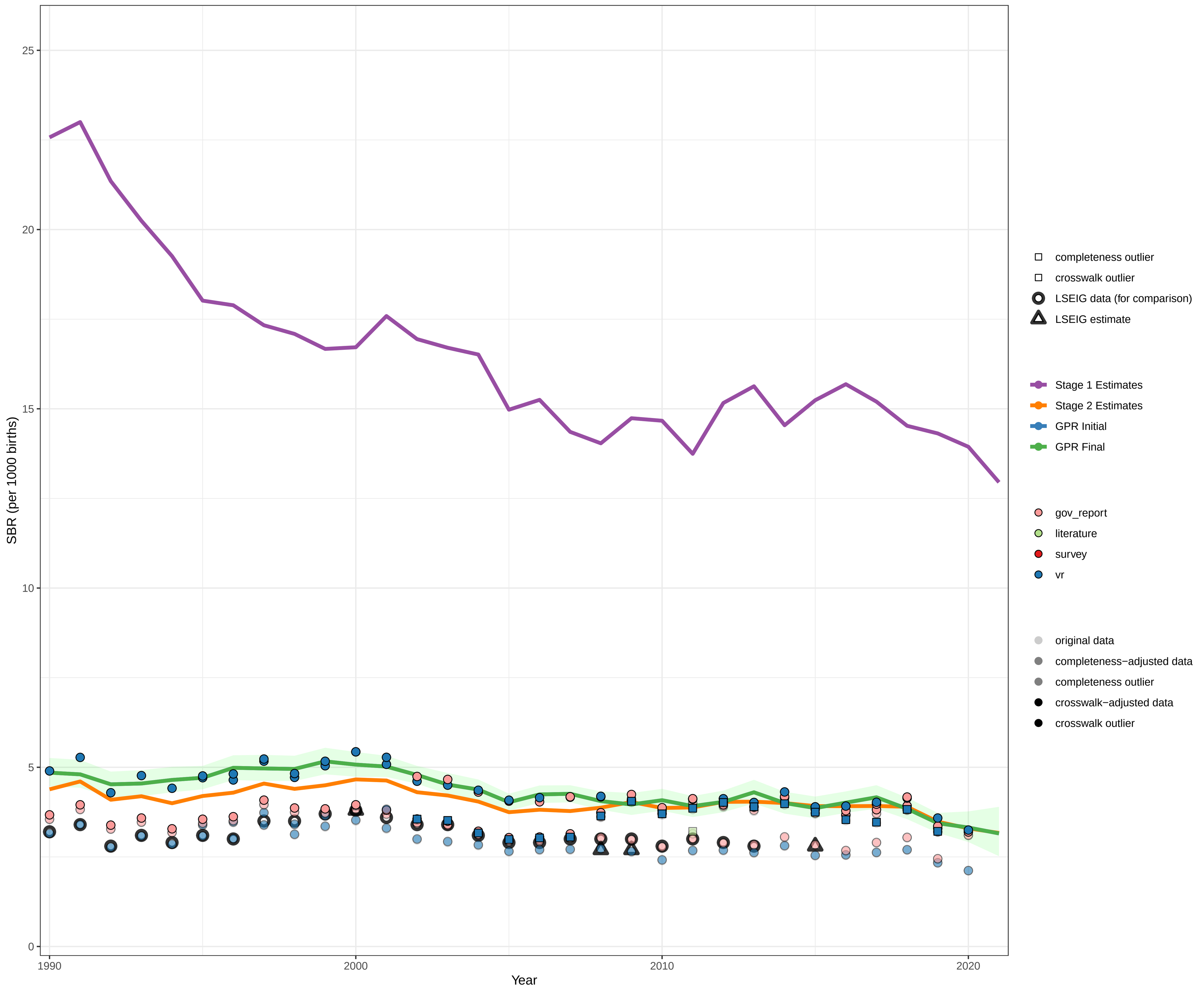

Sweden SWE Western Europe Stillbirth Rate (SBR) − 28 weeks
lambda = 0.2 ; zeta = 0.99 ; scale = 5 ; amp = 0.028

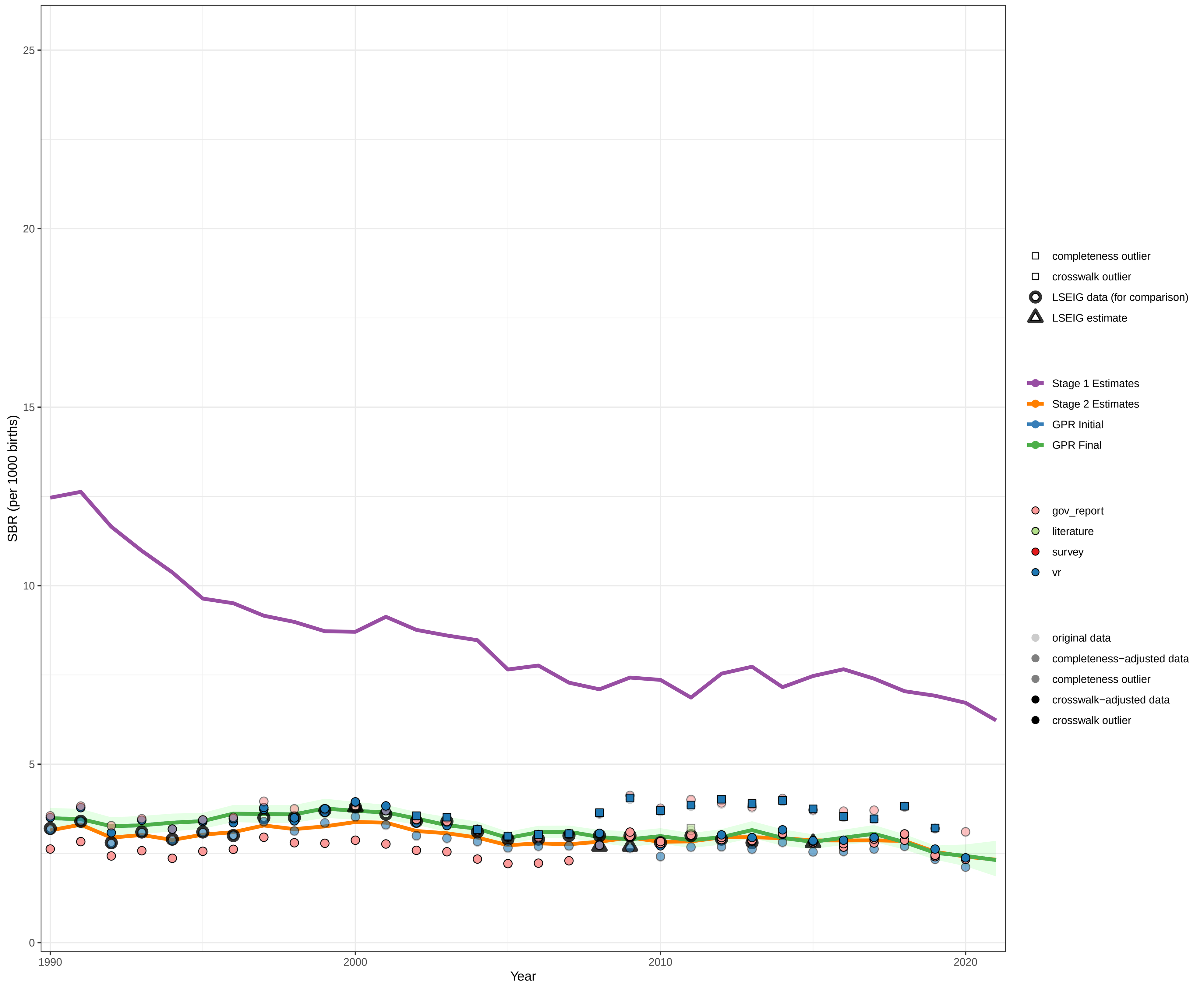

Year	Total Stillbirths (in thousands)	Total Stillbirths (in thousands)	SBR (per 1000 births)	SBR (per 1000 births)	SBR/NMR	SBR/NMR
	20 weeks	28 weeks	20 weeks	28 weeks	20 weeks	28 weeks
1990	0.6 (0.6 to 0.6)	0.4 (0.4 to 0.5)	4.8 (4.5 to 5.3)	3.5 (3.2 to 3.8)	1.36 (1.26 to 1.48)	0.98 (0.91 to 1.06)
1995	0.5 (0.4 to 0.5)	0.3 (0.3 to 0.4)	4.7 (4.4 to 5.0)	3.4 (3.2 to 3.6)	1.87 (1.74 to 2.00)	1.35 (1.26 to 1.44)
2000	0.5 (0.4 to 0.5)	0.3 (0.3 to 0.4)	5.1 (4.7 to 5.4)	3.7 (3.4 to 3.9)	2.40 (2.24 to 2.56)	1.74 (1.63 to 1.86)
2005	0.4 (0.4 to 0.4)	0.3 (0.3 to 0.3)	4.0 (3.8 to 4.3)	2.9 (2.8 to 3.1)	2.29 (2.15 to 2.42)	1.67 (1.57 to 1.76)
2010	0.5 (0.4 to 0.5)	0.3 (0.3 to 0.4)	4.1 (3.8 to 4.4)	3.0 (2.8 to 3.2)	2.52 (2.34 to 2.72)	1.85 (1.72 to 1.98)
2015	0.4 (0.4 to 0.5)	0.3 (0.3 to 0.4)	3.9 (3.6 to 4.2)	2.8 (2.6 to 3.0)	2.48 (2.29 to 2.68)	1.81 (1.68 to 1.95)
2020	0.4 (0.3 to 0.4)	0.3 (0.2 to 0.3)	3.3 (2.9 to 3.8)	2.4 (2.1 to 2.8)	2.45 (2.15 to 2.79)	1.80 (1.58 to 2.04)
2021	0.4 (0.3 to 0.4)	0.3 (0.2 to 0.3)	3.2 (2.5 to 3.9)	2.3 (1.9 to 2.9)	2.53 (2.02 to 3.12)	1.86 (1.49 to 2.29)

Stockholm SWE_4944 Western Europe Stillbirth Rate / Neonatal Mortality Rate (SBR/NMR) − 20 weeks
lambda = 1 ; zeta = 0.6 ; scale = 15 ; amp = 0.028

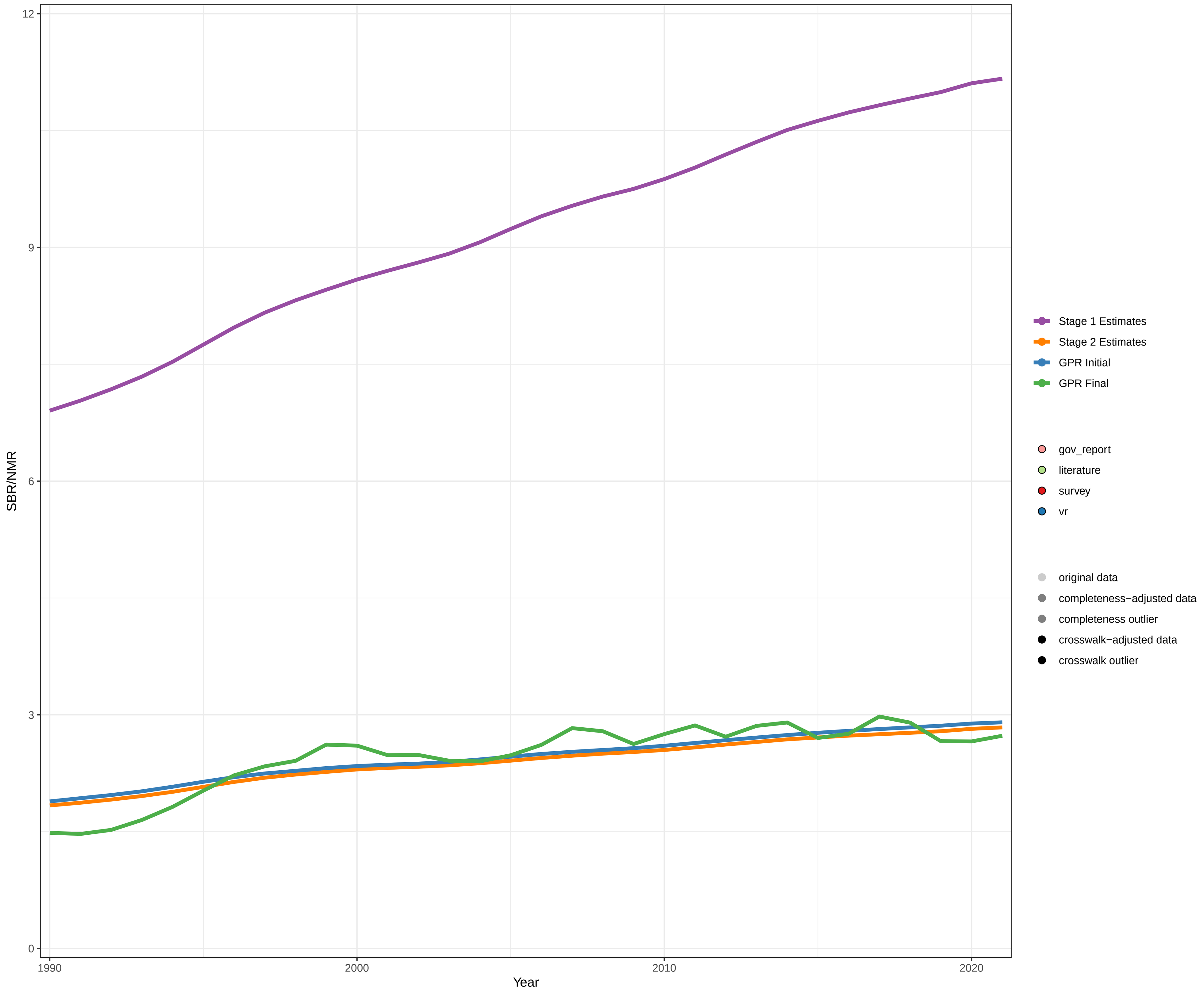

Stockholm SWE_4944 Western Europe Stillbirth Rate / Neonatal Mortality Rate (SBR/NMR) − 28 weeks
lambda = 1 ; zeta = 0.6 ; scale = 15 ; amp = 0.028

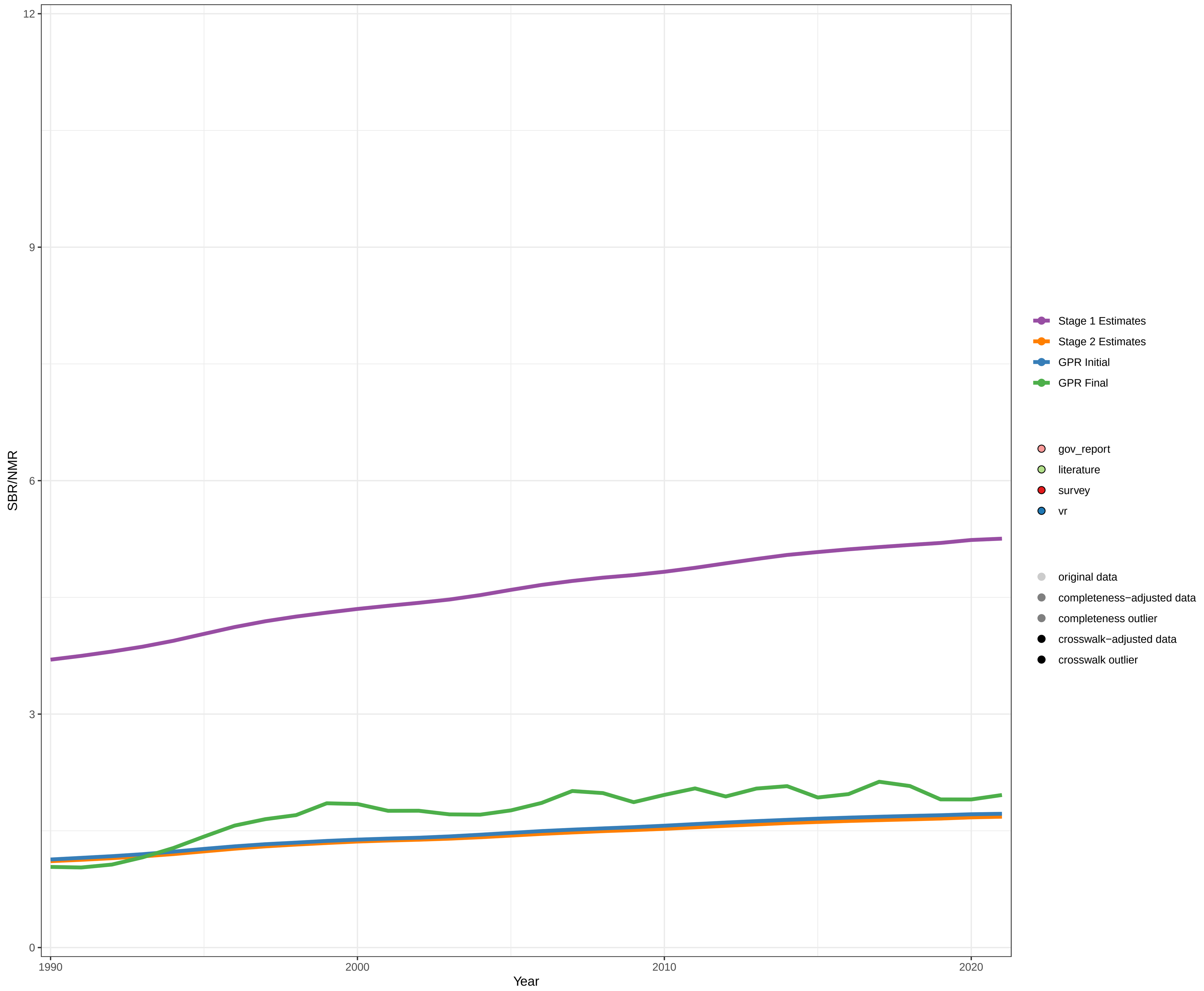

Definition	Years	Outlier	Source.Type	Title

Stockholm SWE_4944 Western Europe Stillbirth Rate (SBR) − 20 weeks
lambda = 1 ; zeta = 0.6 ; scale = 15 ; amp = 0.028

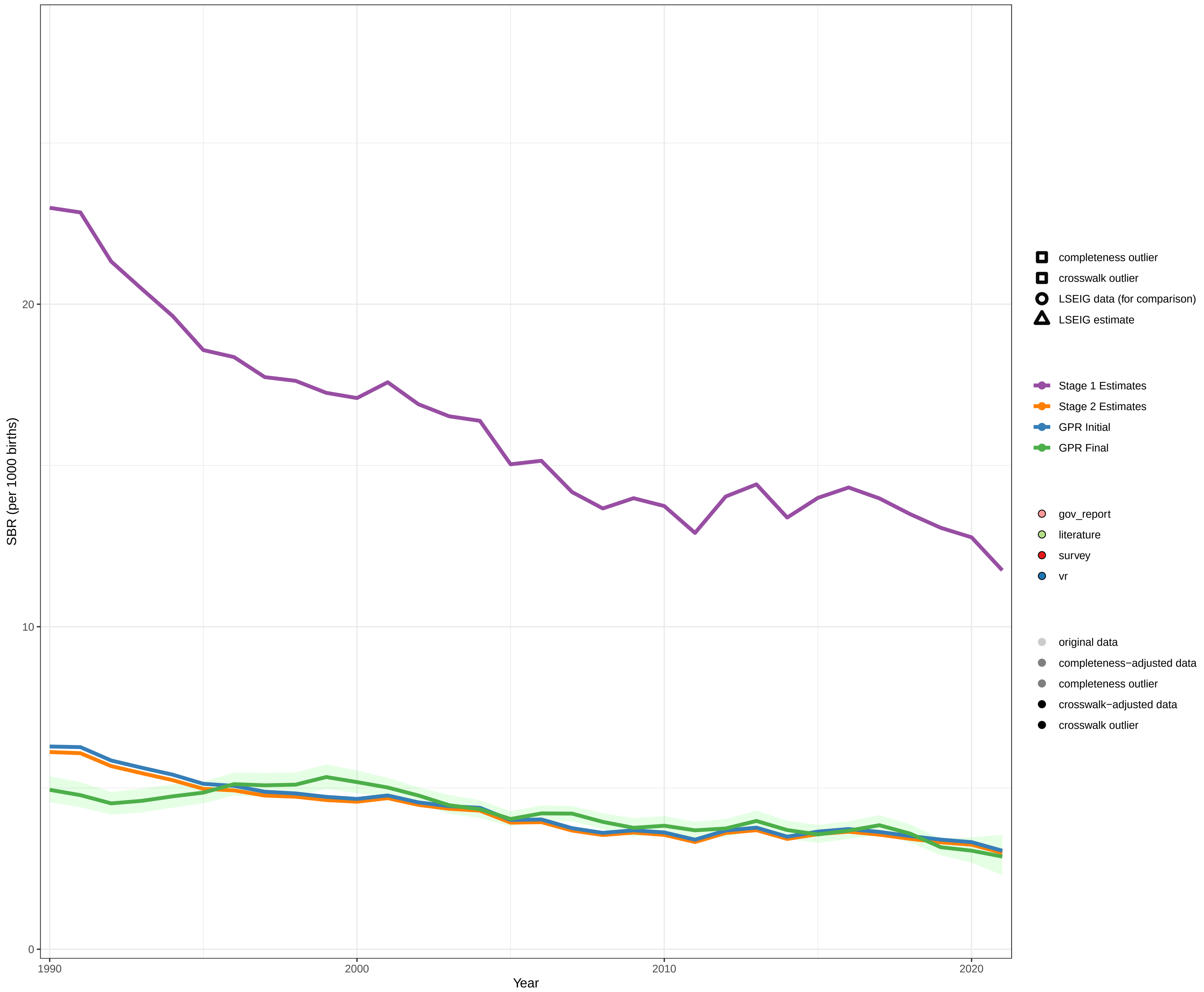

Stockholm SWE_4944 Western Europe Stillbirth Rate (SBR) − 28 weeks
lambda = 1 ; zeta = 0.6 ; scale = 15 ; amp = 0.028

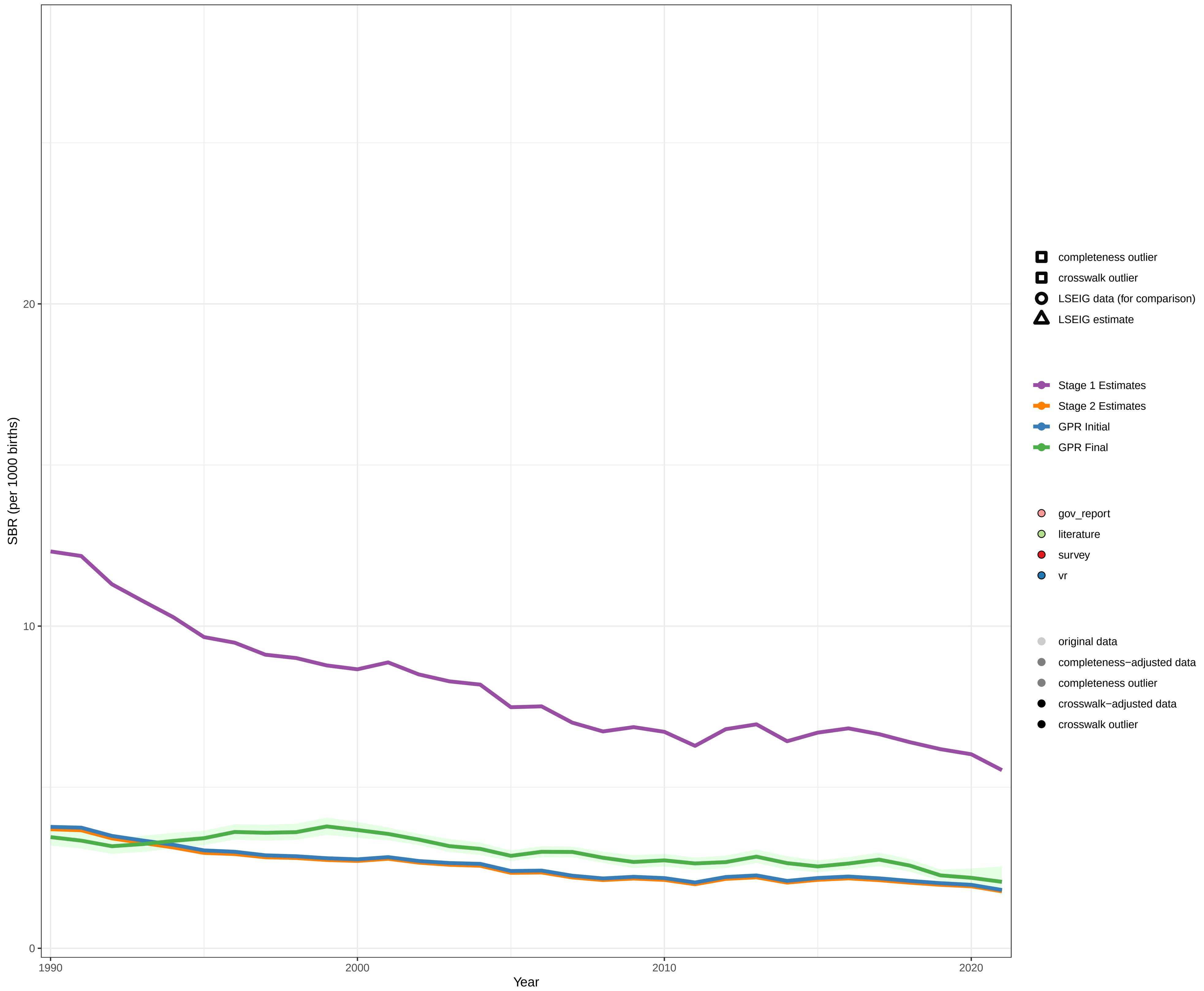

Year	Total Stillbirths (in thousands)	Total Stillbirths (in thousands)	SBR (per 1000 births)	SBR (per 1000 births)	SBR/NMR	SBR/NMR
	20 weeks	28 weeks	20 weeks	28 weeks	20 weeks	28 weeks
1990	0.1 (0.1 to 0.1)	0.1 (0.1 to 0.1)	4.9 (4.6 to 5.4)	3.5 (3.2 to 3.7)	1.48 (1.37 to 1.61)	1.04 (0.96 to 1.12)
1995	0.1 (0.1 to 0.1)	0.1 (0.1 to 0.1)	4.9 (4.5 to 5.2)	3.4 (3.2 to 3.6)	2.03 (1.89 to 2.17)	1.43 (1.34 to 1.52)
2000	0.1 (0.1 to 0.1)	0.1 (0.1 to 0.1)	5.2 (4.8 to 5.5)	3.7 (3.4 to 3.9)	2.60 (2.43 to 2.79)	1.84 (1.72 to 1.97)
2005	0.1 (0.1 to 0.1)	0.1 (0.1 to 0.1)	4.0 (3.8 to 4.3)	2.9 (2.7 to 3.0)	2.48 (2.34 to 2.63)	1.76 (1.66 to 1.87)
2010	0.1 (0.1 to 0.1)	0.1 (0.1 to 0.1)	3.8 (3.6 to 4.1)	2.7 (2.5 to 2.9)	2.75 (2.56 to 2.97)	1.96 (1.83 to 2.11)
2015	0.1 (0.1 to 0.1)	0.1 (0.1 to 0.1)	3.6 (3.3 to 3.9)	2.5 (2.4 to 2.7)	2.70 (2.50 to 2.93)	1.93 (1.79 to 2.07)
2020	0.1 (0.1 to 0.1)	0.1 (0.1 to 0.1)	3.1 (2.7 to 3.5)	2.2 (1.9 to 2.5)	2.66 (2.33 to 3.02)	1.90 (1.68 to 2.15)
2021	0.1 (0.1 to 0.1)	0.1 (0.0 to 0.1)	2.9 (2.3 to 3.5)	2.1 (1.7 to 2.5)	2.73 (2.18 to 3.37)	1.96 (1.57 to 2.41)

Sweden except Stockholm SWE_4940 Western Europe Stillbirth Rate / Neonatal Mortality Rate (SBR/NMR) − 20 weeks
lambda = 1 ; zeta = 0.6 ; scale = 15 ; amp = 0.028

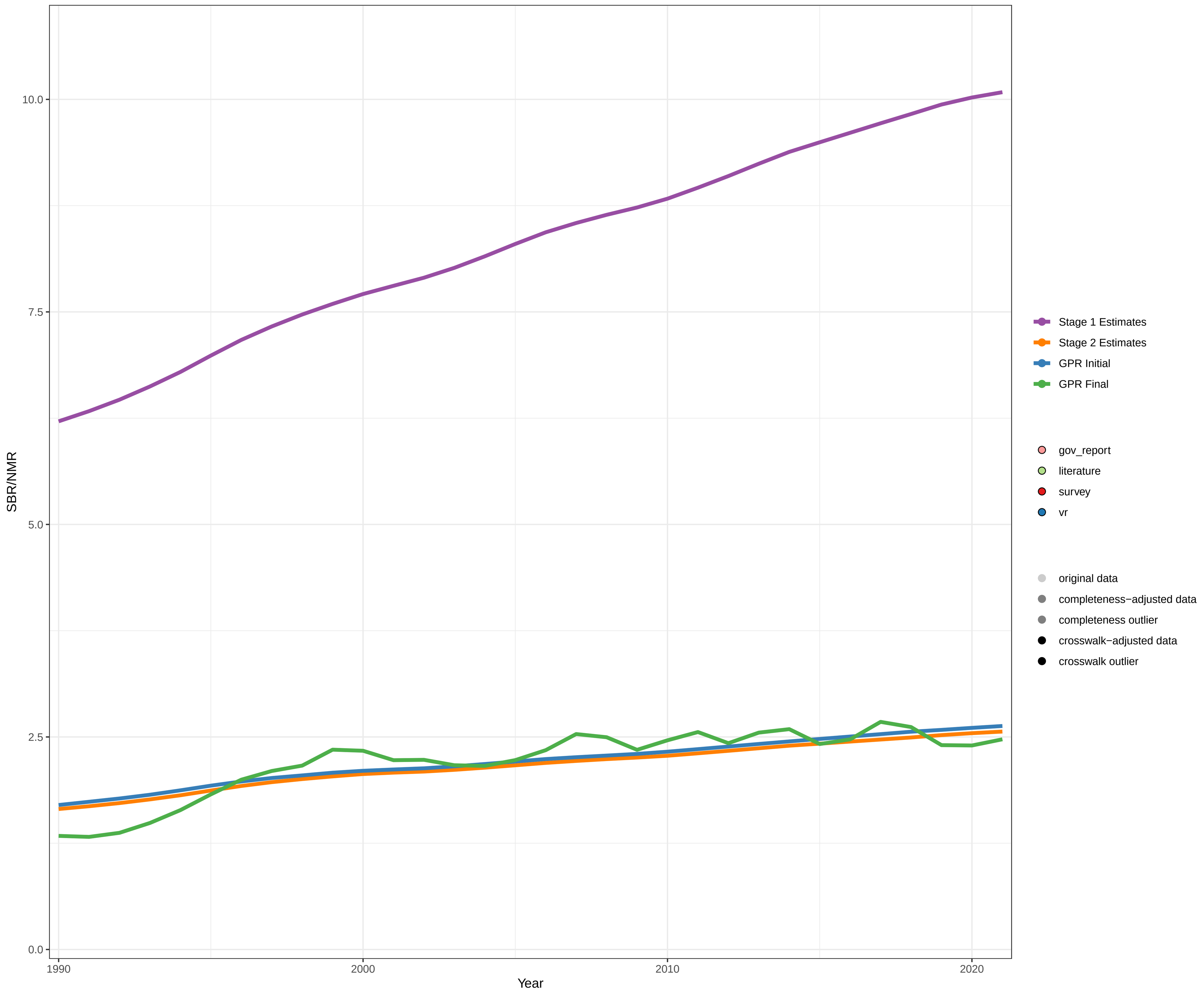

Sweden except Stockholm SWE_4940 Western Europe Stillbirth Rate / Neonatal Mortality Rate (SBR/NMR) − 28 weeks
lambda = 1 ; zeta = 0.6 ; scale = 15 ; amp = 0.028

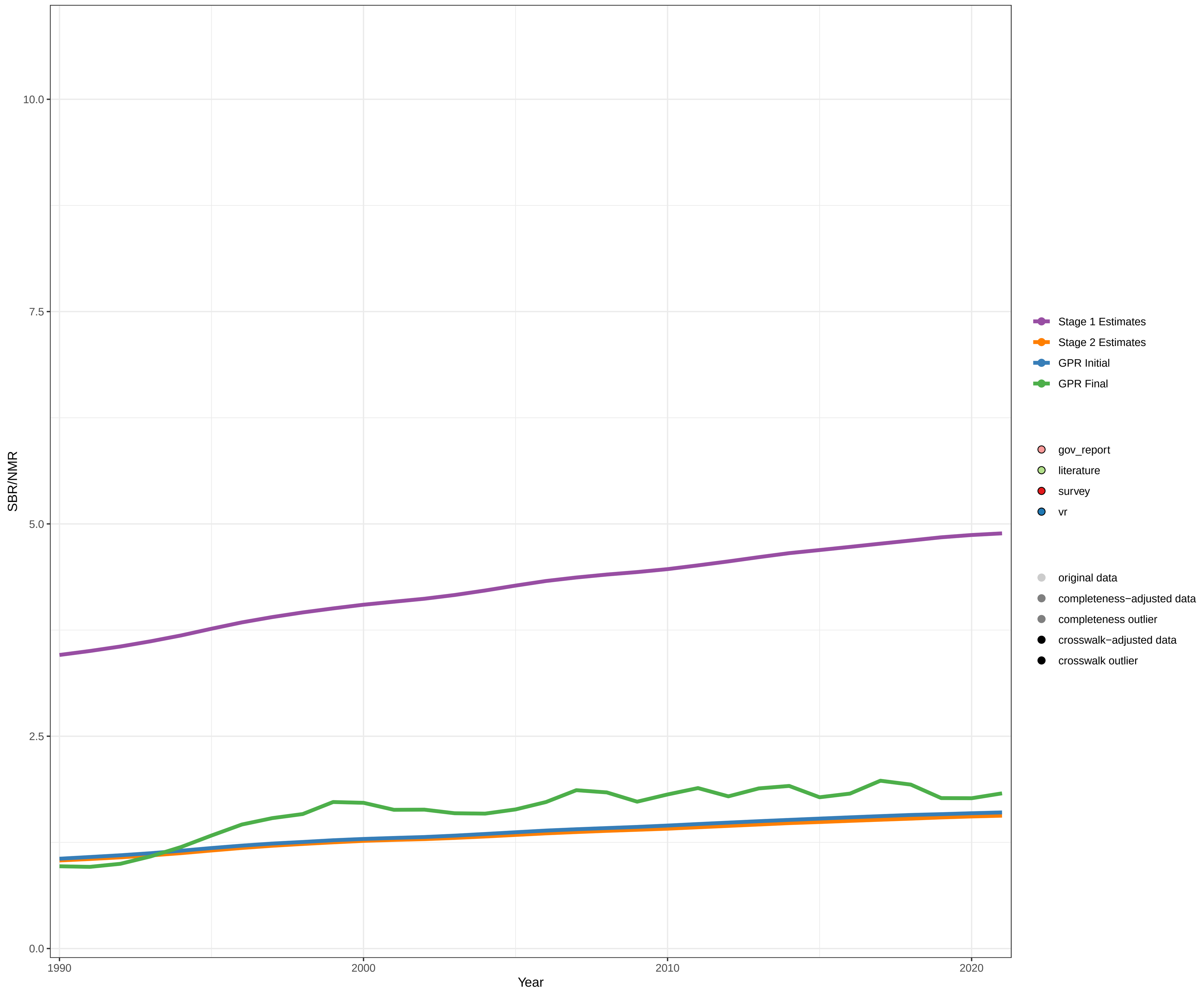

Definition	Years	Outlier	Source.Type	Title

Sweden except Stockholm SWE_4940 Western Europe Stillbirth Rate (SBR) − 20 weeks
lambda = 1 ; zeta = 0.6 ; scale = 15 ; amp = 0.028

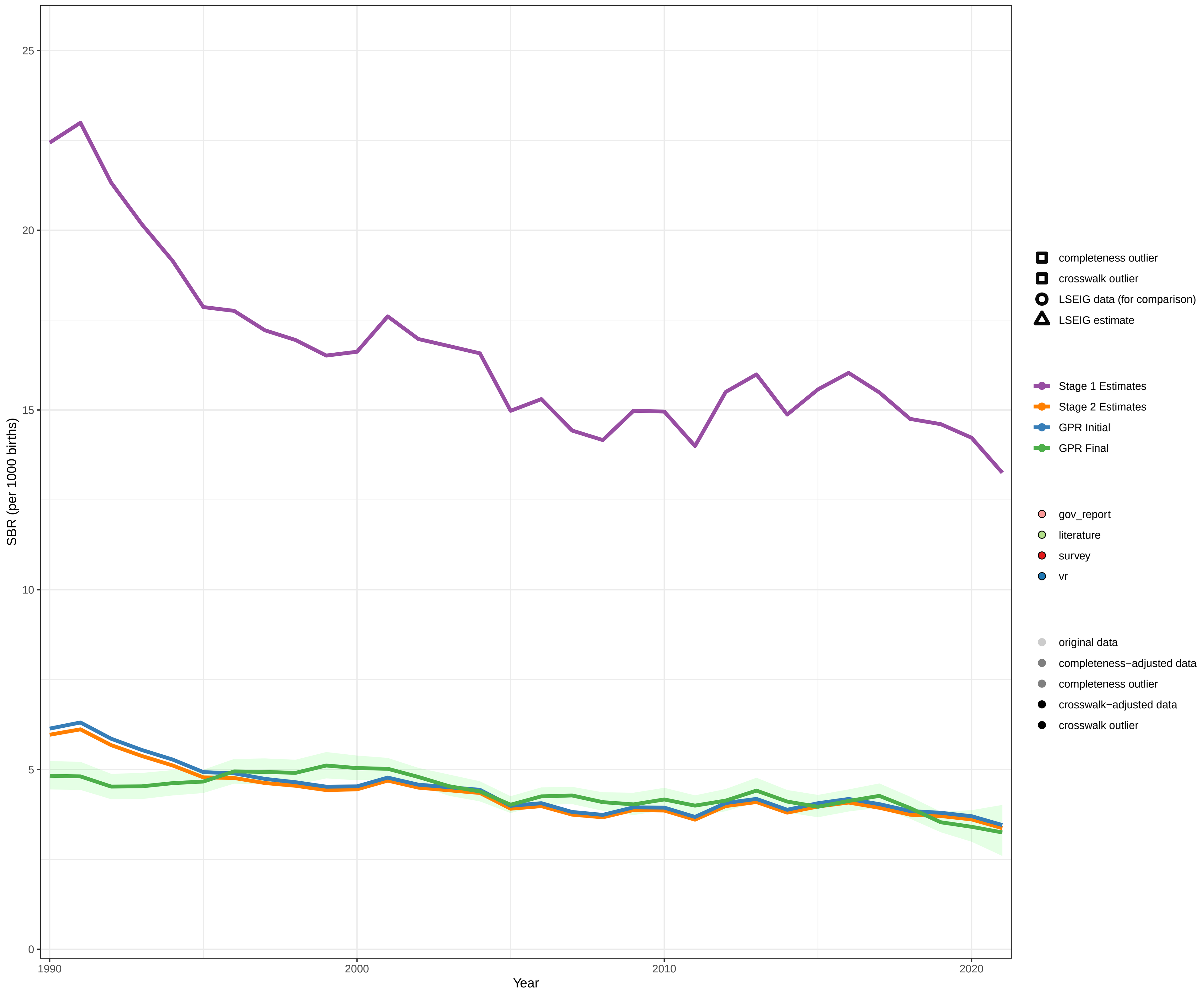

Sweden except Stockholm SWE_4940 Western Europe Stillbirth Rate (SBR) − 28 weeks
lambda = 1 ; zeta = 0.6 ; scale = 15 ; amp = 0.028

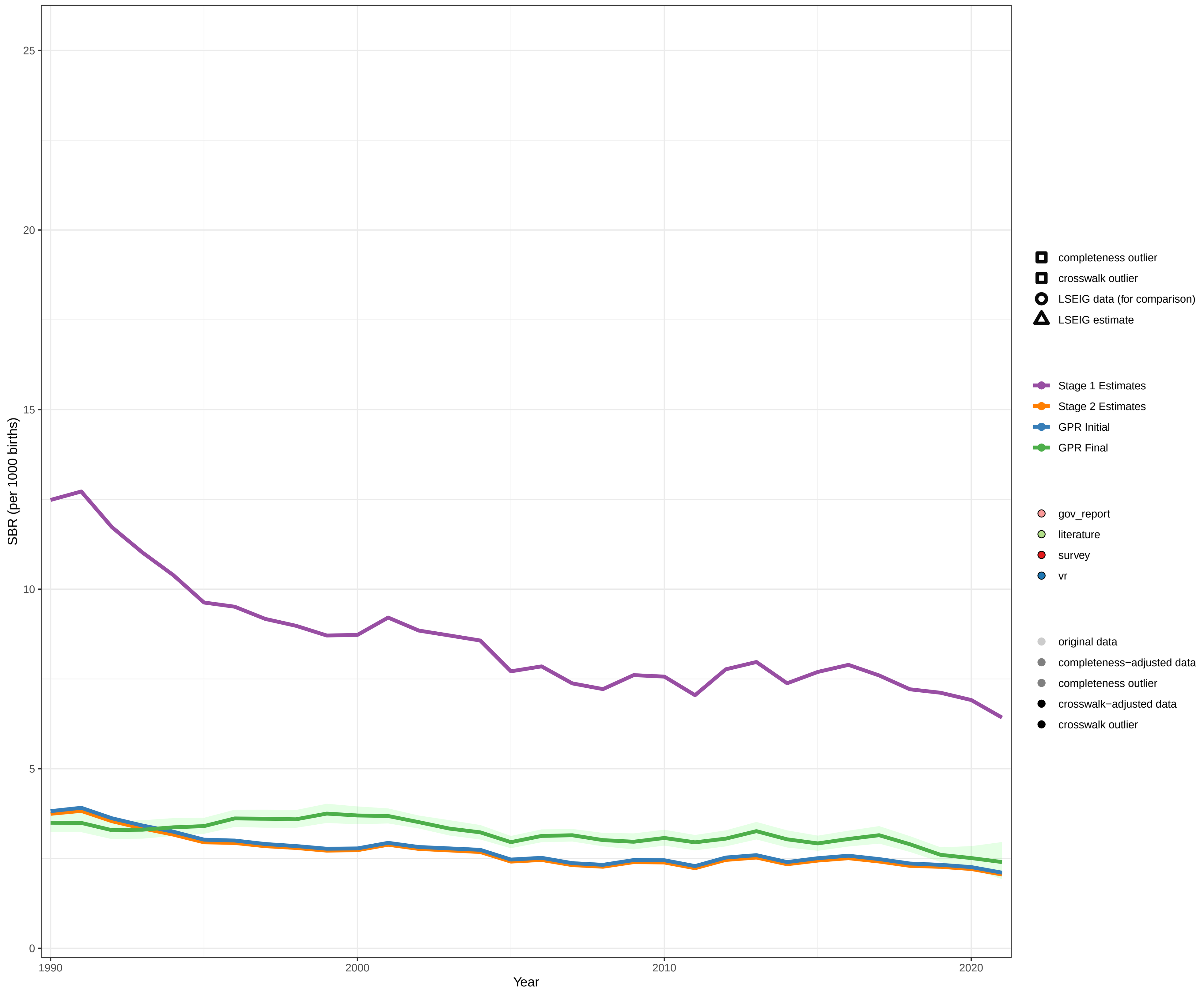

Year	Total Stillbirths (in thousands)	Total Stillbirths (in thousands)	SBR (per 1000 births)	SBR (per 1000 births)	SBR/NMR	SBR/NMR
	20 weeks	28 weeks	20 weeks	28 weeks	20 weeks	28 weeks
1990	0.5 (0.4 to 0.5)	0.3 (0.3 to 0.4)	4.8 (4.4 to 5.2)	3.5 (3.2 to 3.8)	1.34 (1.23 to 1.45)	0.97 (0.89 to 1.05)
1995	0.4 (0.3 to 0.4)	0.3 (0.2 to 0.3)	4.7 (4.3 to 5.0)	3.4 (3.2 to 3.6)	1.82 (1.70 to 1.95)	1.33 (1.25 to 1.42)
2000	0.3 (0.3 to 0.4)	0.3 (0.2 to 0.3)	5.0 (4.7 to 5.4)	3.7 (3.5 to 4.0)	2.34 (2.18 to 2.50)	1.72 (1.60 to 1.83)
2005	0.3 (0.3 to 0.3)	0.2 (0.2 to 0.2)	4.0 (3.8 to 4.3)	3.0 (2.8 to 3.1)	2.23 (2.10 to 2.36)	1.64 (1.54 to 1.73)
2010	0.4 (0.3 to 0.4)	0.3 (0.2 to 0.3)	4.2 (3.9 to 4.5)	3.1 (2.9 to 3.3)	2.46 (2.28 to 2.65)	1.81 (1.69 to 1.95)
2015	0.3 (0.3 to 0.4)	0.3 (0.2 to 0.3)	4.0 (3.7 to 4.3)	2.9 (2.7 to 3.1)	2.42 (2.24 to 2.62)	1.78 (1.66 to 1.91)
2020	0.3 (0.3 to 0.3)	0.2 (0.2 to 0.2)	3.4 (3.0 to 3.9)	2.5 (2.2 to 2.8)	2.40 (2.11 to 2.73)	1.77 (1.56 to 2.00)
2021	0.3 (0.2 to 0.3)	0.2 (0.2 to 0.3)	3.2 (2.6 to 4.0)	2.4 (1.9 to 3.0)	2.47 (1.98 to 3.05)	1.83 (1.46 to 2.25)

Switzerland CHE Western Europe Stillbirth Rate / Neonatal Mortality Rate (SBR/NMR) − 20 weeks
lambda = 0.2 ; zeta = 0.99 ; scale = 5 ; amp = 0.028

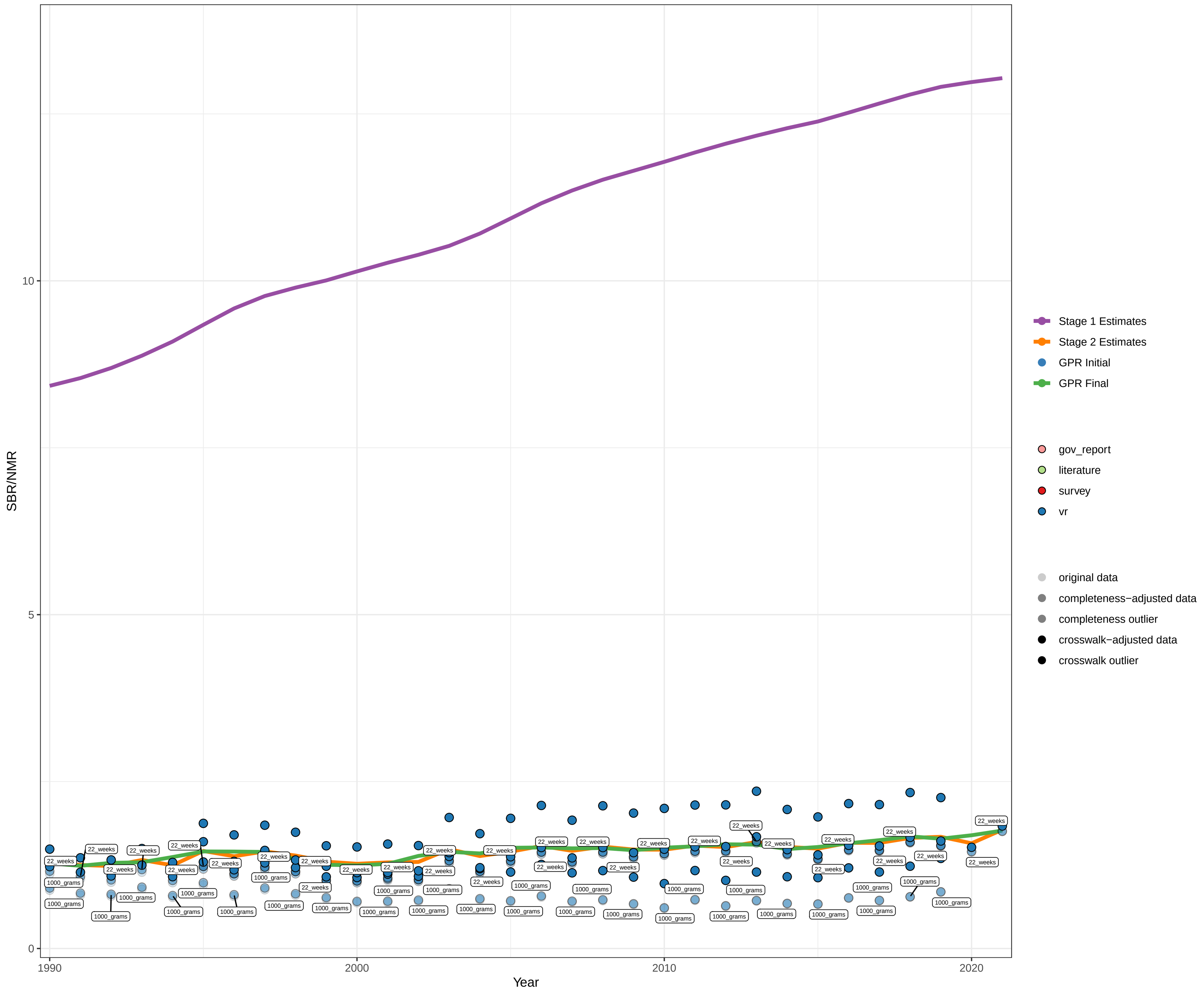

Switzerland CHE Western Europe Stillbirth Rate / Neonatal Mortality Rate (SBR/NMR) − 28 weeks
lambda = 0.2 ; zeta = 0.99 ; scale = 5 ; amp = 0.028

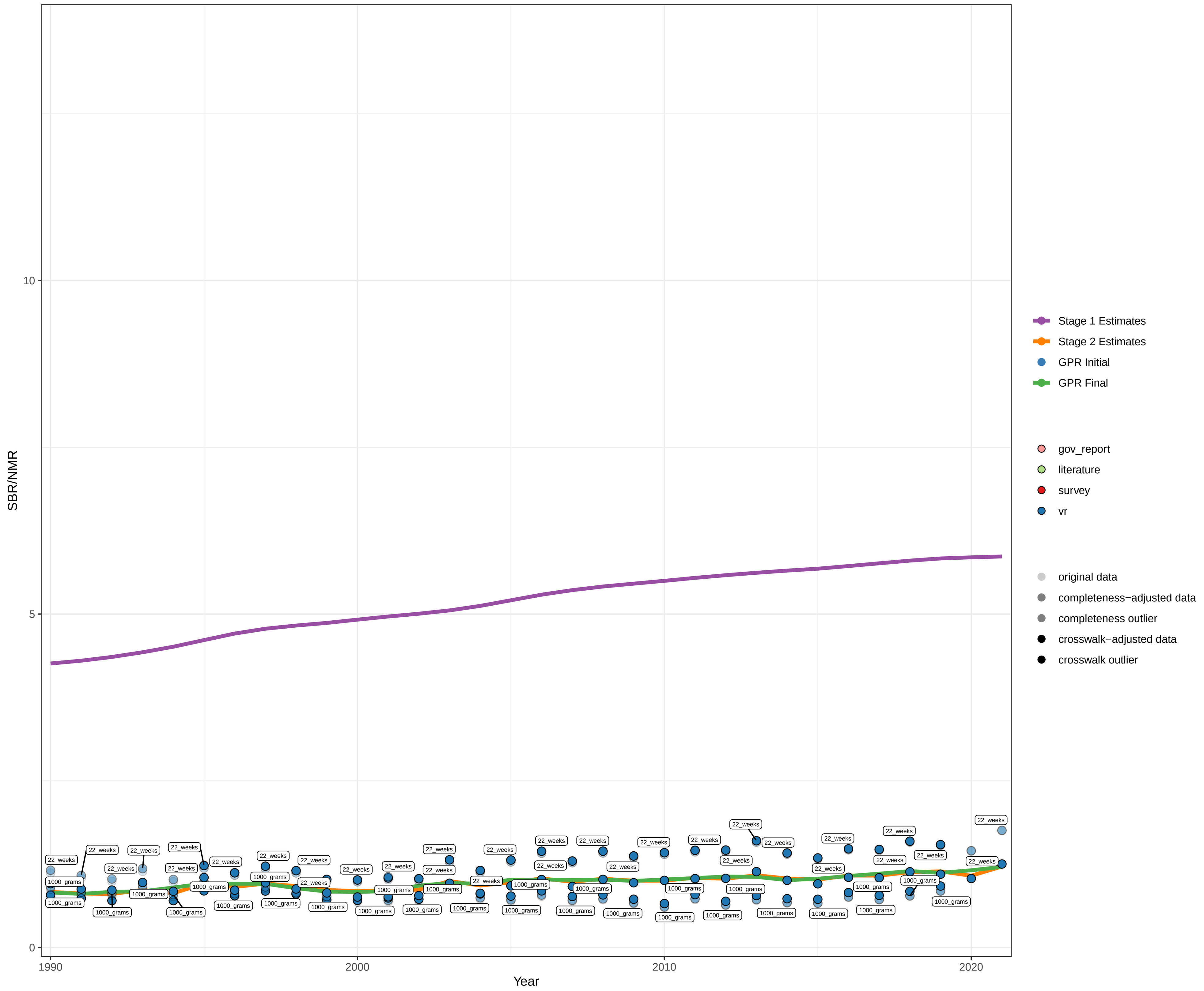

Definition	Years	Outlier	Source.Type	Title
1000_grams	1990−2019	0	VR	European Health for All Database
22_weeks	1990−2021	0	VR	Switzerland Vital Statistics − Live Births and Stillbirths since 1803
28_weeks	1995−2019	0	VR	United Nations Demographic Yearbook

Switzerland CHE Western Europe Stillbirth Rate (SBR) − 20 weeks
lambda = 0.2 ; zeta = 0.99 ; scale = 5 ; amp = 0.028

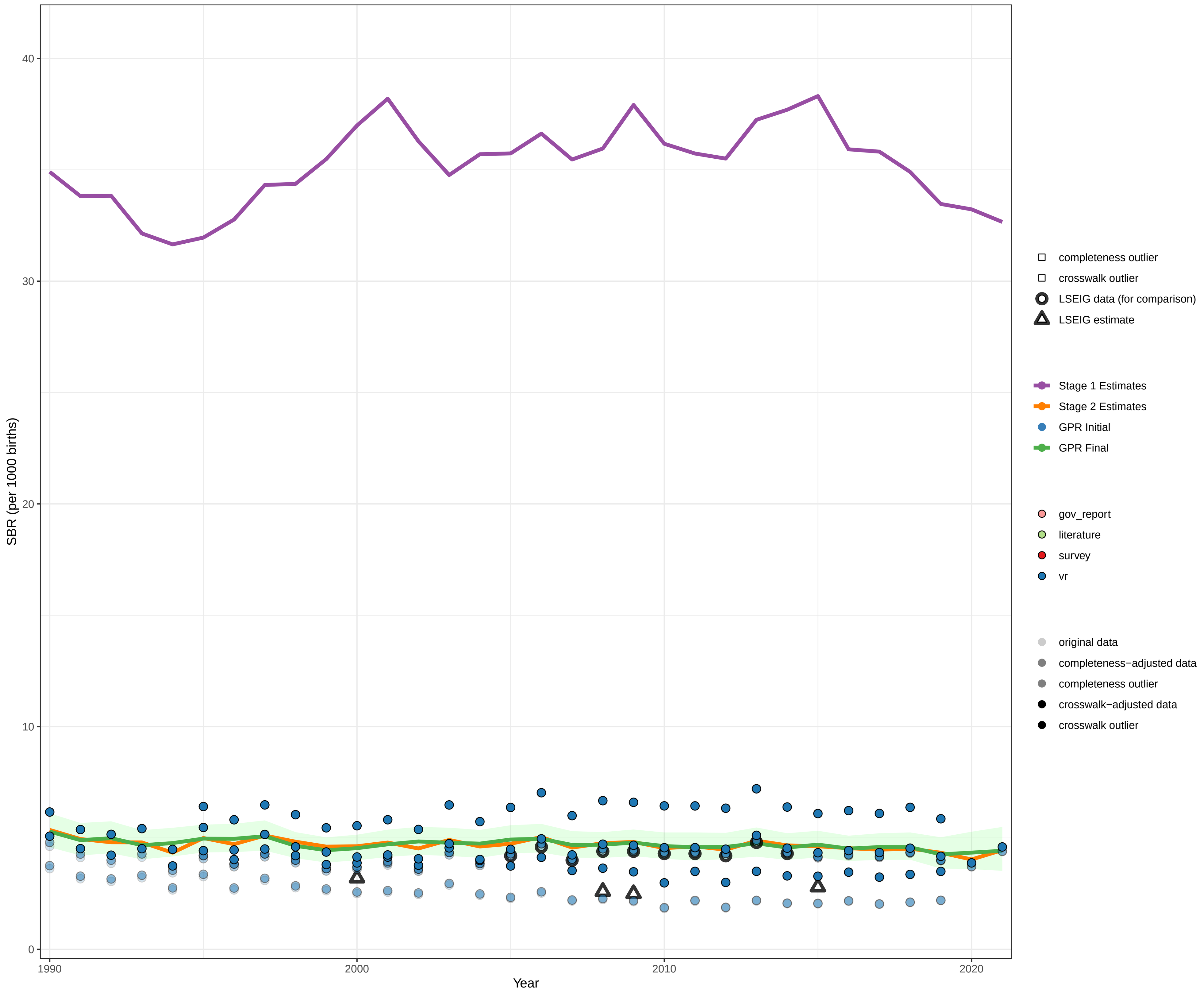

Switzerland CHE Western Europe Stillbirth Rate (SBR) − 28 weeks
lambda = 0.2 ; zeta = 0.99 ; scale = 5 ; amp = 0.028

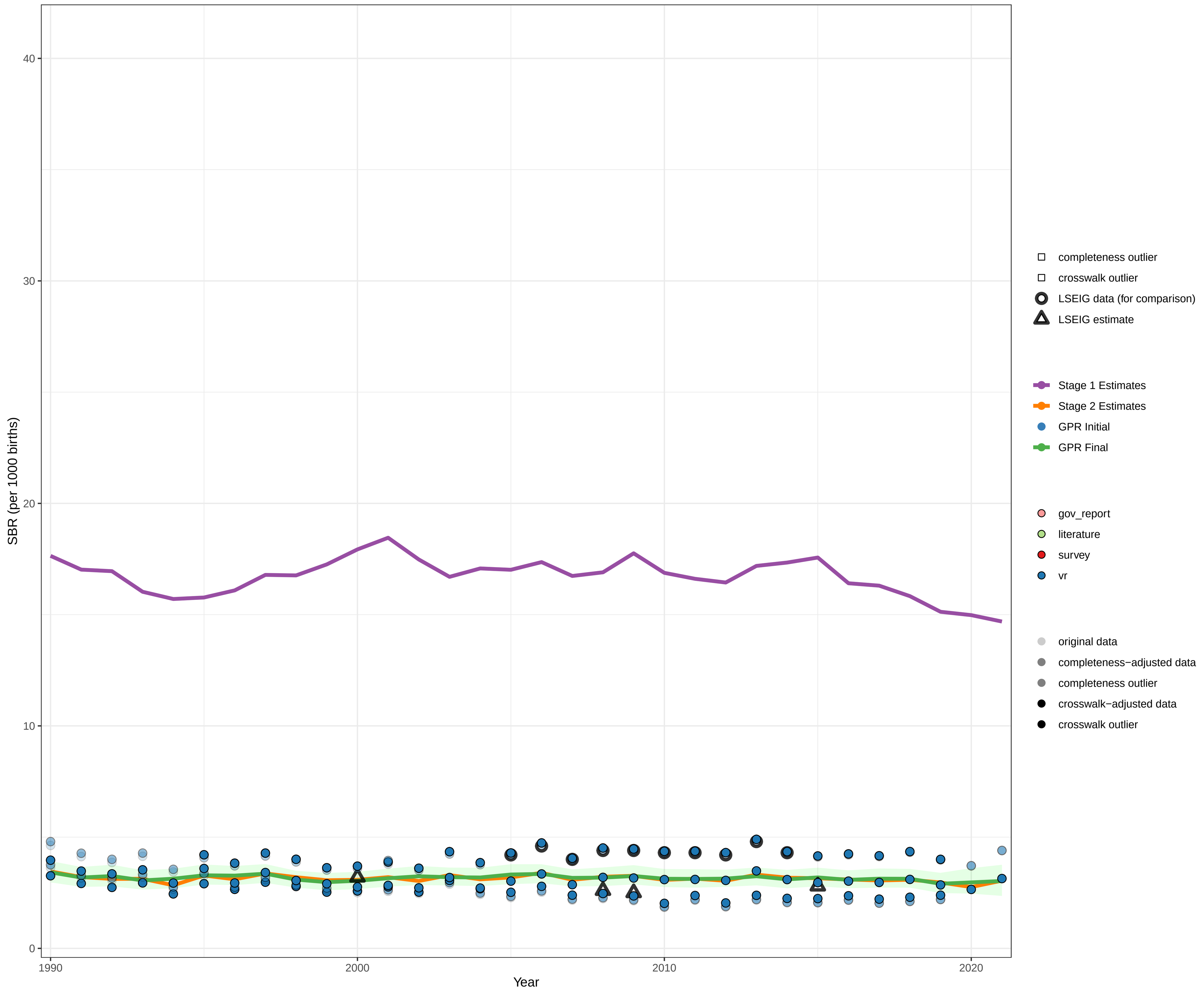

Year	Total Stillbirths (in thousands)	Total Stillbirths (in thousands)	SBR (per 1000 births)	SBR (per 1000 births)	SBR/NMR	SBR/NMR
	20 weeks	28 weeks	20 weeks	28 weeks	20 weeks	28 weeks
1990	0.5 (0.4 to 0.5)	0.3 (0.3 to 0.3)	5.3 (4.6 to 6.1)	3.4 (3.0 to 3.9)	1.28 (1.11 to 1.47)	0.83 (0.72 to 0.95)
1995	0.4 (0.4 to 0.5)	0.3 (0.2 to 0.3)	5.0 (4.4 to 5.6)	3.3 (2.9 to 3.8)	1.45 (1.27 to 1.64)	0.96 (0.84 to 1.10)
2000	0.3 (0.3 to 0.4)	0.2 (0.2 to 0.3)	4.5 (4.0 to 5.1)	3.0 (2.7 to 3.4)	1.25 (1.10 to 1.41)	0.83 (0.73 to 0.94)
2005	0.4 (0.3 to 0.4)	0.2 (0.2 to 0.3)	4.9 (4.3 to 5.6)	3.3 (2.9 to 3.8)	1.51 (1.32 to 1.70)	1.02 (0.88 to 1.16)
2010	0.4 (0.3 to 0.4)	0.3 (0.2 to 0.3)	4.6 (4.1 to 5.2)	3.1 (2.8 to 3.6)	1.51 (1.32 to 1.71)	1.02 (0.90 to 1.16)
2015	0.4 (0.4 to 0.5)	0.3 (0.2 to 0.3)	4.7 (4.1 to 5.3)	3.2 (2.8 to 3.6)	1.52 (1.33 to 1.72)	1.03 (0.90 to 1.17)
2020	0.4 (0.3 to 0.5)	0.3 (0.2 to 0.3)	4.3 (3.6 to 5.3)	3.0 (2.5 to 3.6)	1.70 (1.41 to 2.06)	1.16 (0.96 to 1.41)
2021	0.4 (0.3 to 0.5)	0.3 (0.2 to 0.3)	4.4 (3.5 to 5.5)	3.0 (2.4 to 3.8)	1.77 (1.41 to 2.19)	1.21 (0.94 to 1.50)

United Kingdom GBR Western Europe Stillbirth Rate / Neonatal Mortality Rate (SBR/NMR) − 20 weeks
lambda = 0.2 ; zeta = 0.99 ; scale = 5 ; amp = 0.028

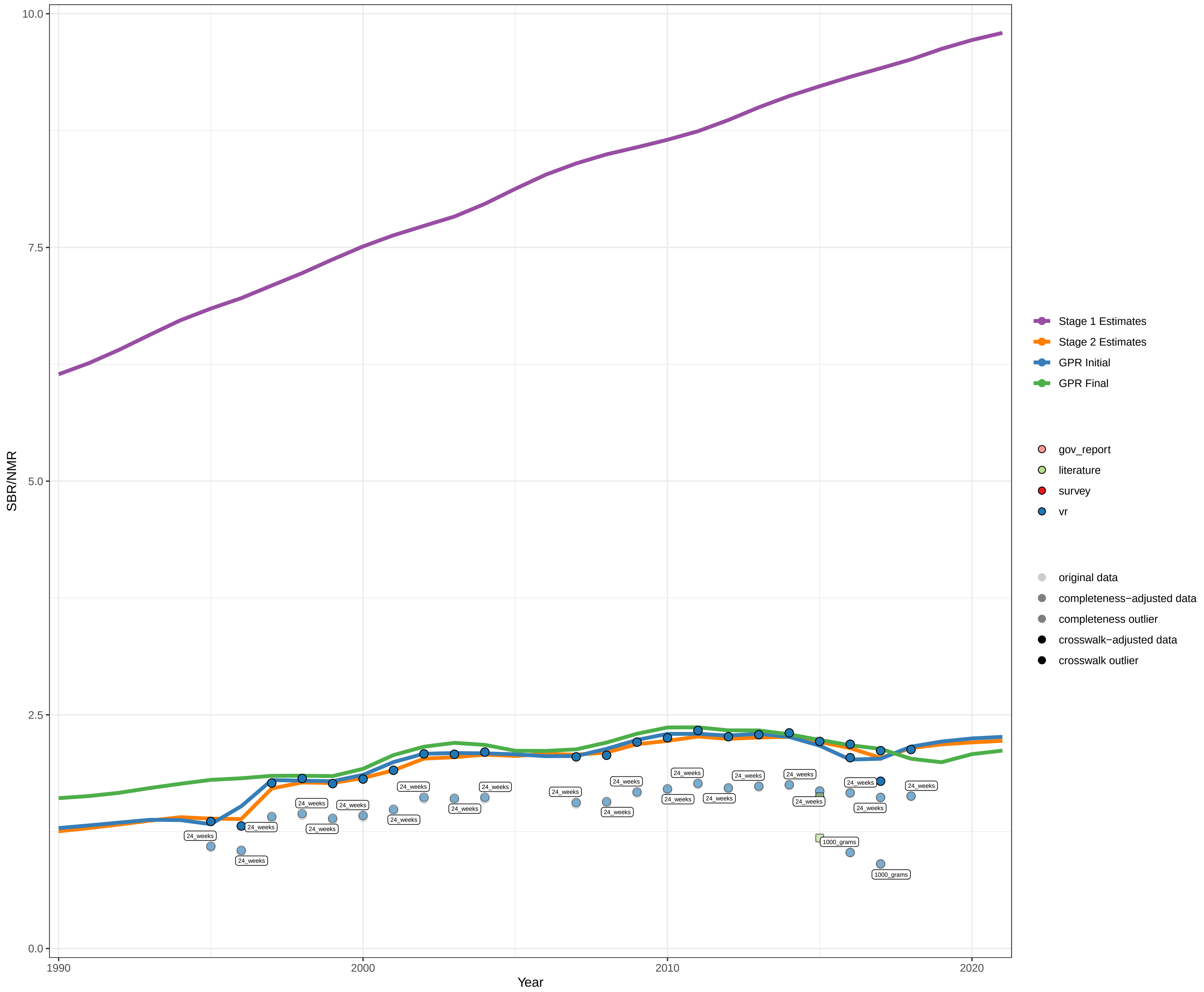

United Kingdom GBR Western Europe Stillbirth Rate / Neonatal Mortality Rate (SBR/NMR) − 28 weeks
lambda = 0.2 ; zeta = 0.99 ; scale = 5 ; amp = 0.028

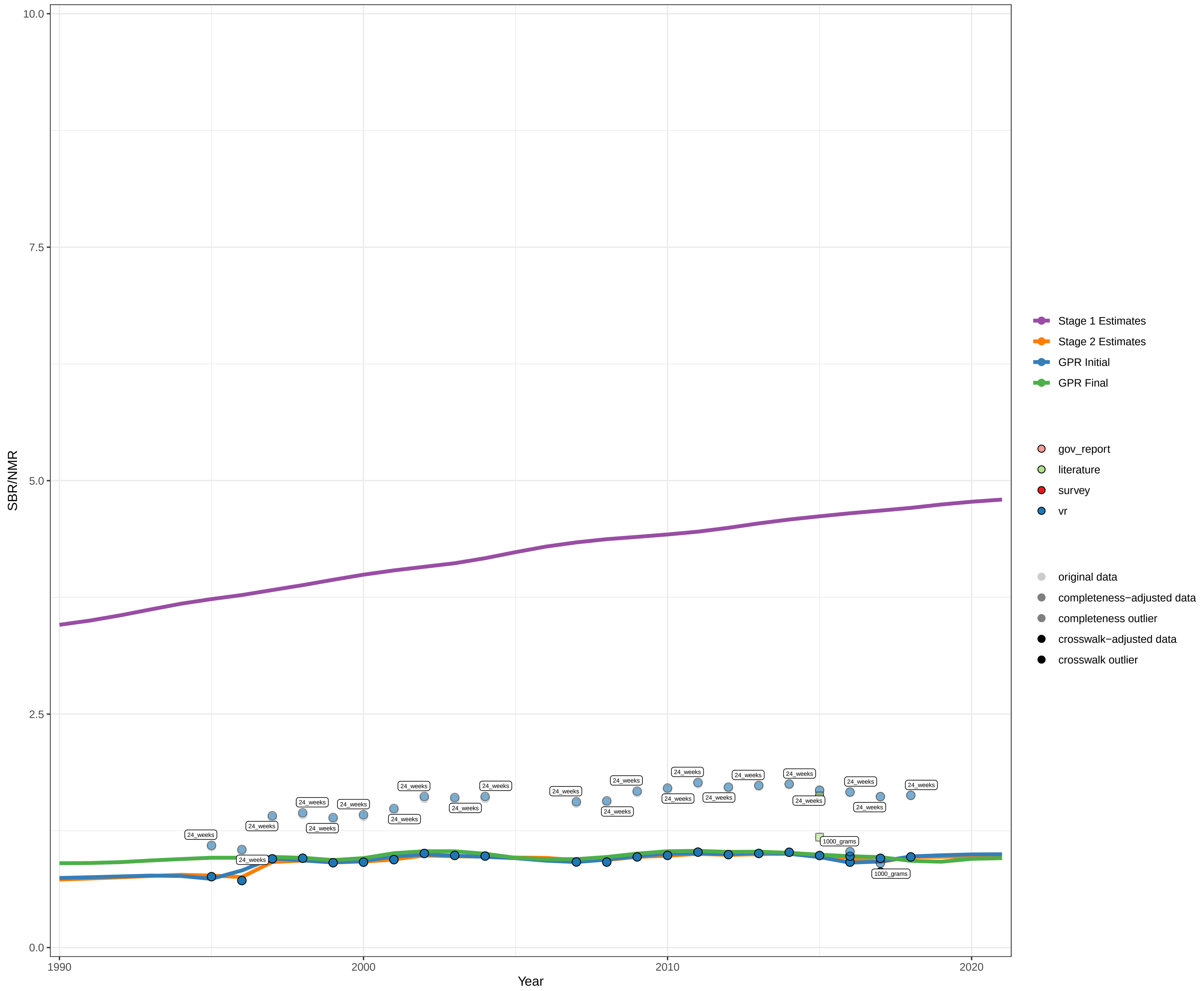

Definition	Years	Outlier	Source.Type	Title
1000_grams	2016, 2017	0	VR	European Health for All Database
24_weeks	1995−2004, 2007−2018	0	VR	United Nations Demographic Yearbook
22_weeks, 24_weeks, 28_weeks	2015	1	Sci Lit	Awareness of fetal movements and care package to reduce fetal mortality (AFFIRM): a stepped wedge, cluster−randomised trial

United Kingdom GBR Western Europe Stillbirth Rate (SBR) − 20 weeks
lambda = 0.2 ; zeta = 0.99 ; scale = 5 ; amp = 0.028

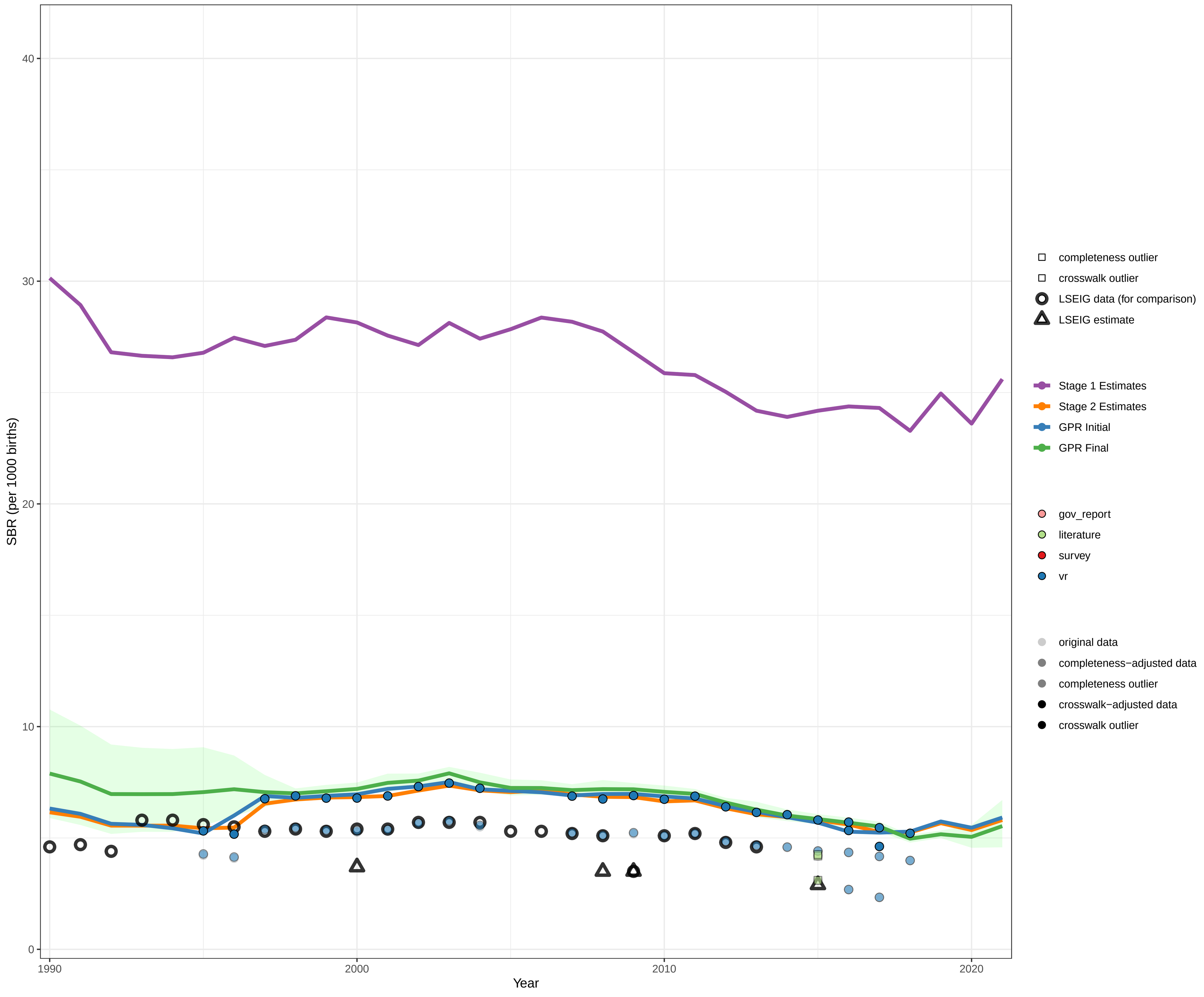

United Kingdom GBR Western Europe Stillbirth Rate (SBR) − 28 weeks
lambda = 0.2 ; zeta = 0.99 ; scale = 5 ; amp = 0.028

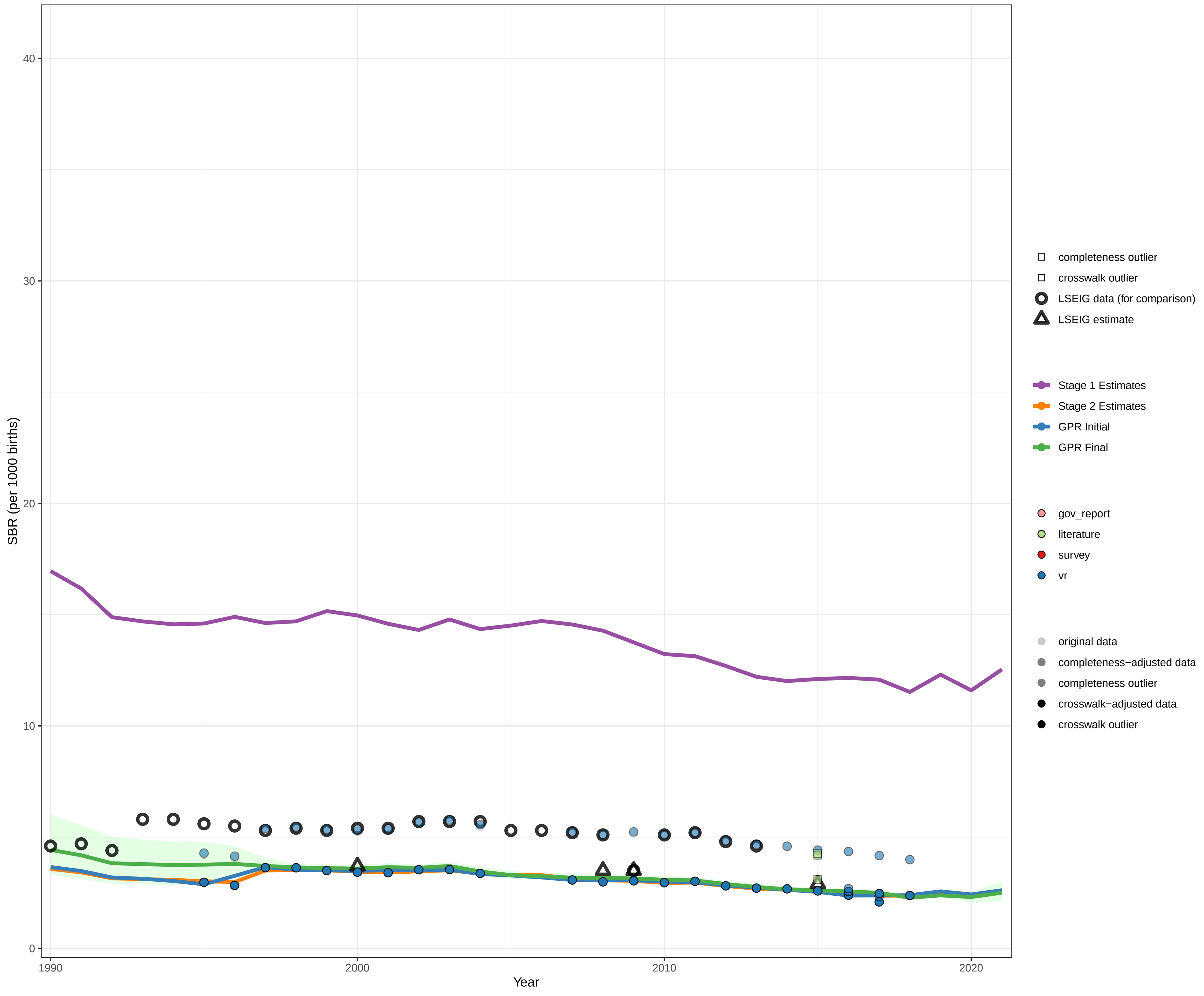

Year	Total Stillbirths (in thousands)	Total Stillbirths (in thousands)	SBR (per 1000 births)	SBR (per 1000 births)	SBR/NMR	SBR/NMR
	20 weeks	28 weeks	20 weeks	28 weeks	20 weeks	28 weeks
1990	6.3 (4.7 to 8.6)	3.5 (2.6 to 4.8)	7.9 (5.9 to 10.8)	4.4 (3.3 to 6.0)	1.61 (1.20 to 2.19)	0.90 (0.68 to 1.23)
1995	5.2 (4.1 to 6.7)	2.8 (2.2 to 3.5)	7.1 (5.6 to 9.1)	3.8 (3.0 to 4.8)	1.80 (1.42 to 2.32)	0.96 (0.76 to 1.23)
2000	4.9 (4.7 to 5.1)	2.4 (2.3 to 2.6)	7.2 (6.9 to 7.5)	3.6 (3.4 to 3.8)	1.92 (1.85 to 2.00)	0.96 (0.91 to 1.00)
2005	5.4 (5.1 to 5.7)	2.4 (2.3 to 2.6)	7.2 (6.9 to 7.6)	3.3 (3.1 to 3.5)	2.12 (2.02 to 2.23)	0.96 (0.91 to 1.01)
2010	5.7 (5.5 to 6.0)	2.5 (2.4 to 2.6)	7.1 (6.8 to 7.4)	3.1 (2.9 to 3.2)	2.36 (2.27 to 2.46)	1.03 (0.98 to 1.09)
2015	4.5 (4.4 to 4.7)	2.0 (1.9 to 2.1)	5.8 (5.6 to 6.1)	2.6 (2.5 to 2.7)	2.23 (2.15 to 2.31)	0.99 (0.95 to 1.04)
2020	3.5 (3.1 to 3.8)	1.6 (1.4 to 1.7)	5.1 (4.6 to 5.6)	2.3 (2.1 to 2.5)	2.08 (1.88 to 2.30)	0.95 (0.86 to 1.05)
2021	3.8 (3.1 to 4.6)	1.7 (1.4 to 2.1)	5.5 (4.6 to 6.7)	2.5 (2.1 to 3.0)	2.12 (1.75 to 2.57)	0.96 (0.80 to 1.15)

England GBR_4749 Western Europe Stillbirth Rate / Neonatal Mortality Rate (SBR/NMR) − 20 weeks
lambda = 0.2 ; zeta = 0.99 ; scale = 5 ; amp = 0.028

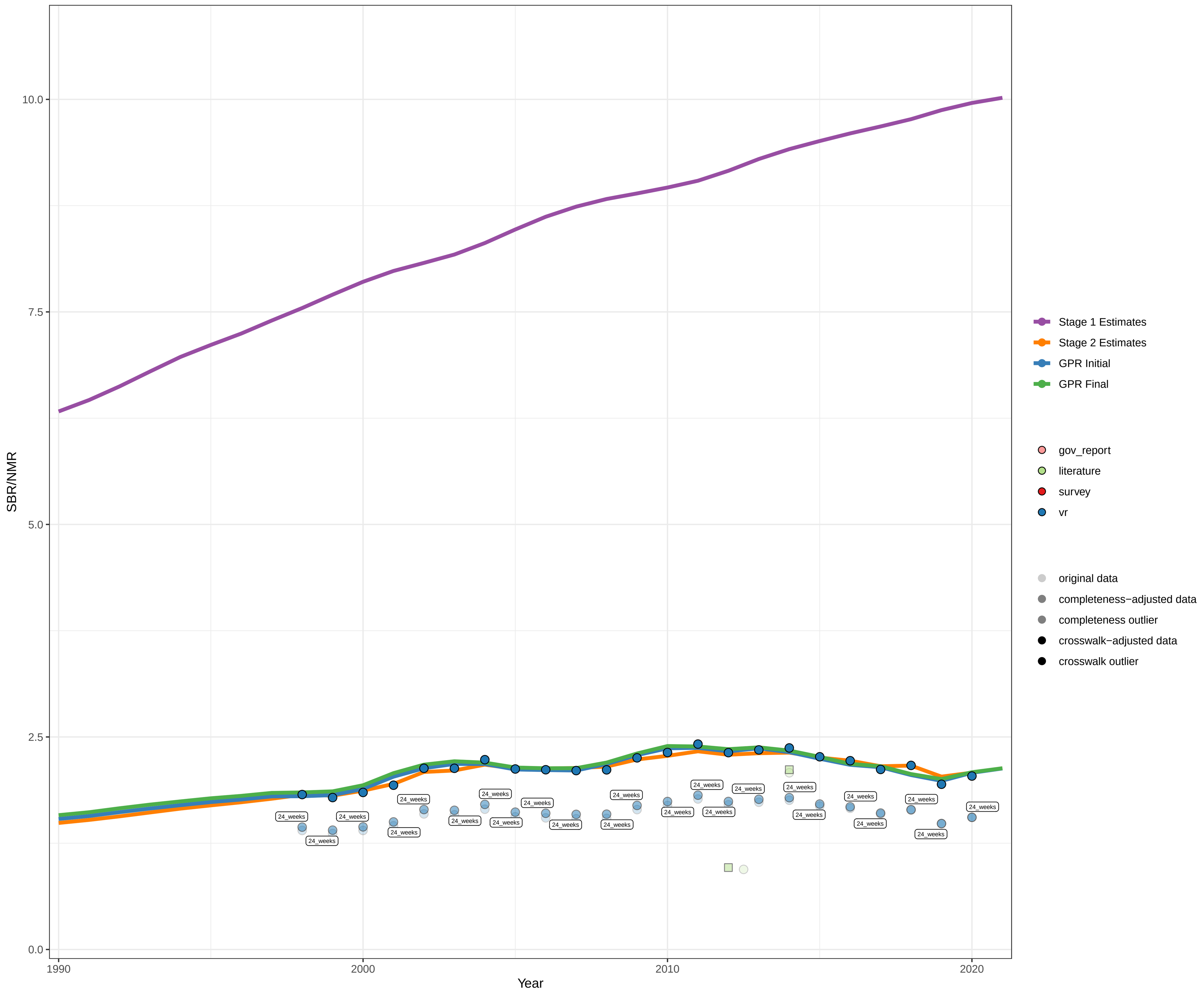

England GBR_4749 Western Europe Stillbirth Rate / Neonatal Mortality Rate (SBR/NMR) − 28 weeks
lambda = 0.2 ; zeta = 0.99 ; scale = 5 ; amp = 0.028

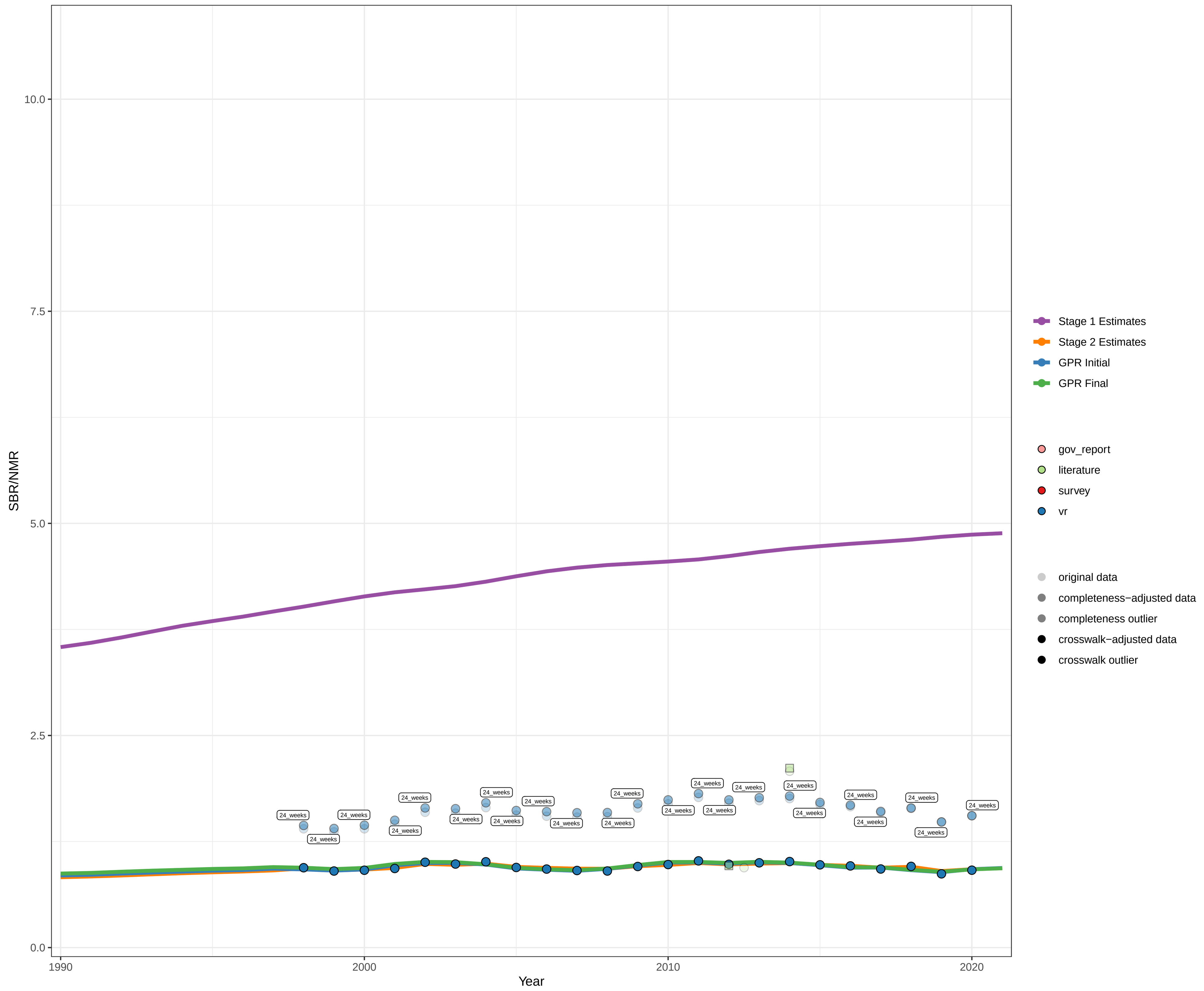

Definition	Years	Outlier	Source.Type	Title
24_weeks	1998−2007	0	VR	United Kingdom − England and Wales Birth Statistics
24_weeks	2008−2020	0	VR	United Kingdom − England and Wales Child Mortality Statistics
24_weeks	2014	1	Sci Lit	Association between maternal haemoglobin and stillbirth: acohort study among a multi−ethnic population in England
other	2012	1	Sci Lit	Umbilical and fetal middle cerebral artery Doppler at 30−34 weeks? gestation in the prediction of adverse perinatal outcome

England GBR_4749 Western Europe Stillbirth Rate (SBR) − 20 weeks
lambda = 0.2 ; zeta = 0.99 ; scale = 5 ; amp = 0.028

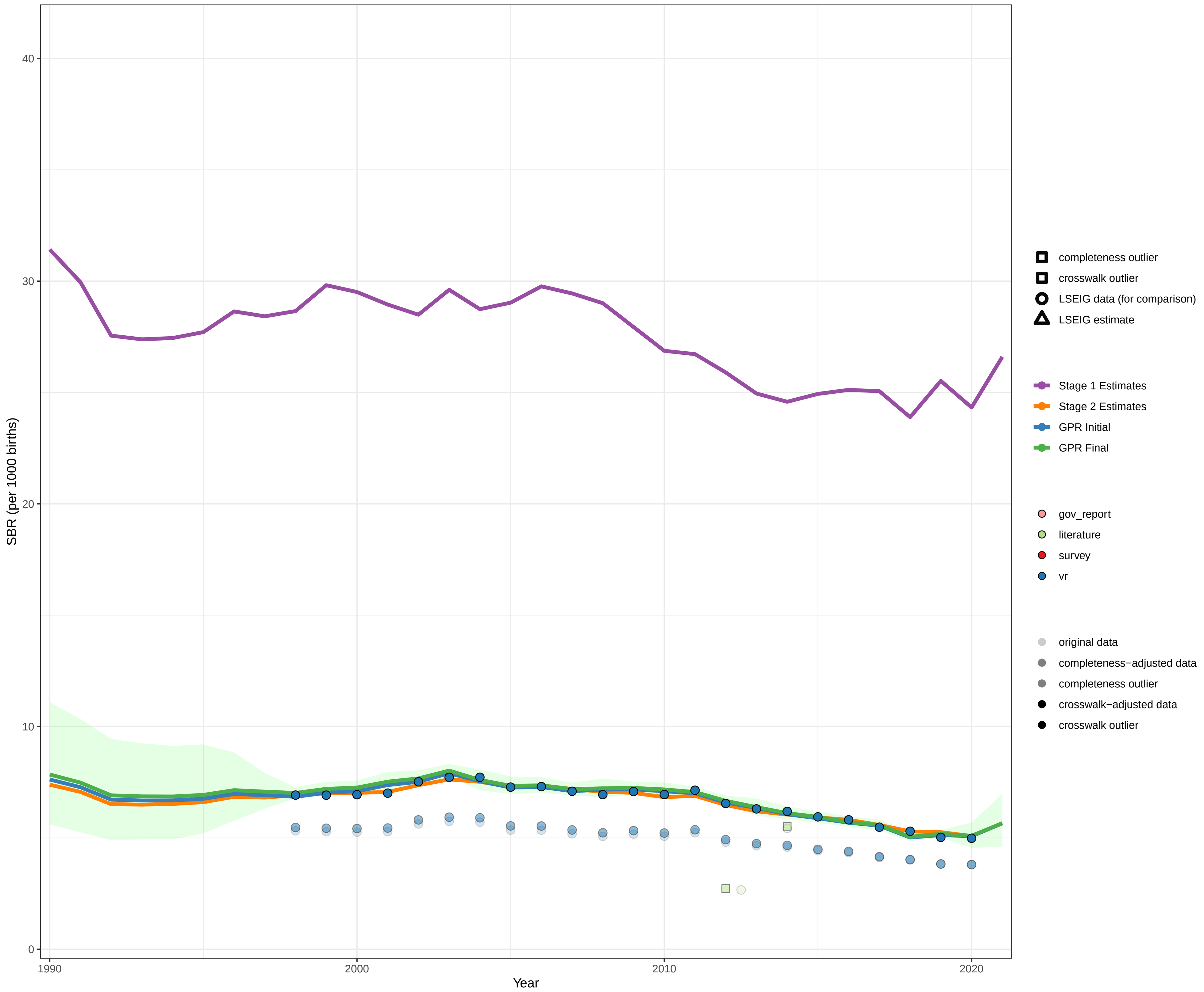

England GBR_4749 Western Europe Stillbirth Rate (SBR) − 28 weeks
lambda = 0.2 ; zeta = 0.99 ; scale = 5 ; amp = 0.028

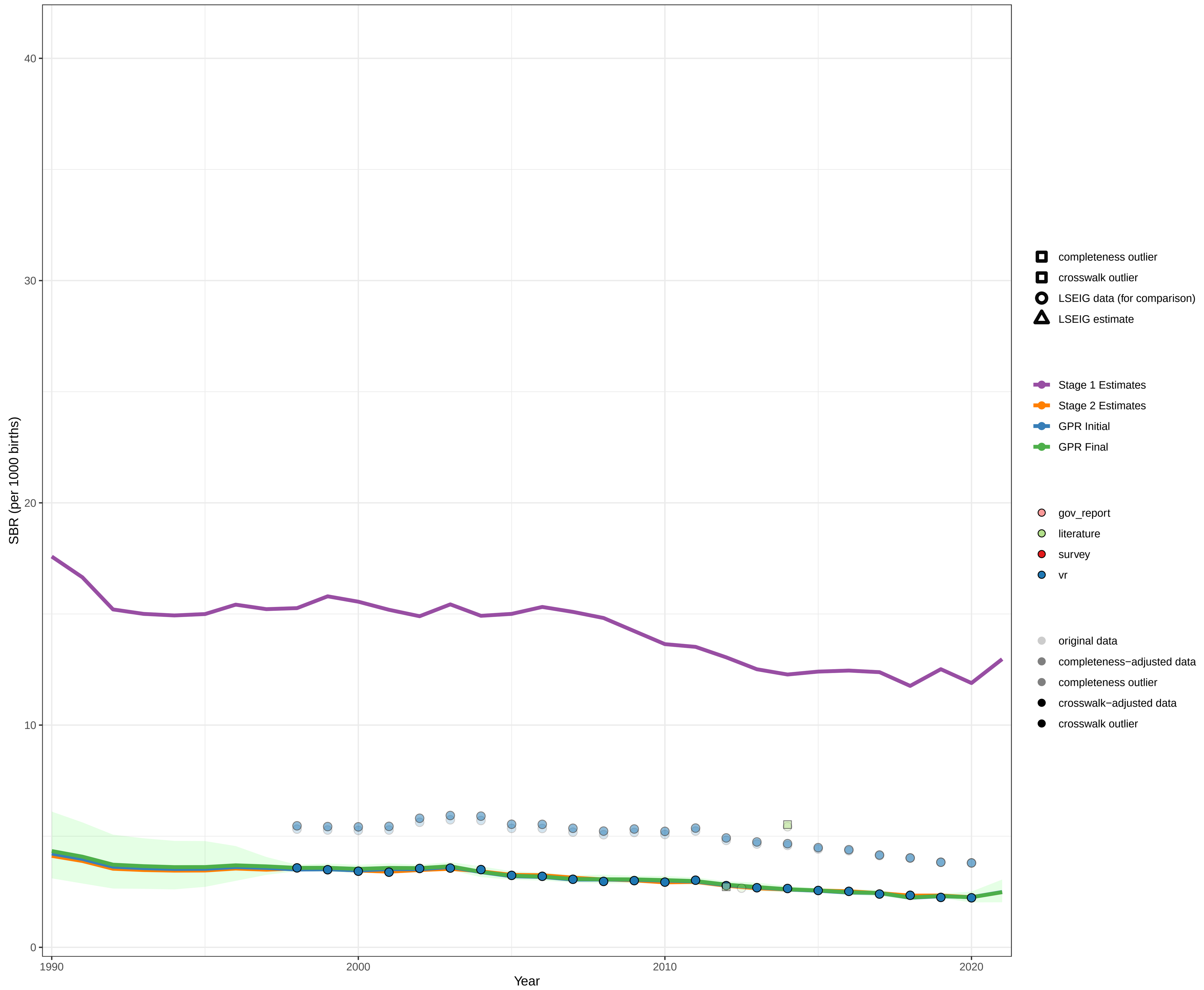

Year	Total Stillbirths (in thousands)	Total Stillbirths (in thousands)	SBR (per 1000 births)	SBR (per 1000 births)	SBR/NMR	SBR/NMR
	20 weeks	28 weeks	20 weeks	28 weeks	20 weeks	28 weeks
1990	5.2 (3.7 to 7.4)	2.9 (2.1 to 4.1)	7.8 (5.6 to 11.1)	4.3 (3.1 to 6.1)	1.58 (1.13 to 2.23)	0.87 (0.63 to 1.23)
1995	4.3 (3.2 to 5.7)	2.2 (1.7 to 3.0)	6.9 (5.2 to 9.2)	3.6 (2.7 to 4.8)	1.78 (1.33 to 2.36)	0.93 (0.70 to 1.23)
2000	4.2 (4.0 to 4.4)	2.0 (1.9 to 2.1)	7.3 (6.9 to 7.6)	3.5 (3.3 to 3.7)	1.93 (1.85 to 2.02)	0.94 (0.89 to 0.99)
2005	4.6 (4.4 to 4.9)	2.0 (1.9 to 2.1)	7.3 (7.0 to 7.8)	3.2 (3.0 to 3.4)	2.14 (2.03 to 2.26)	0.94 (0.89 to 1.00)
2010	4.9 (4.7 to 5.2)	2.1 (2.0 to 2.2)	7.2 (6.9 to 7.5)	3.0 (2.9 to 3.2)	2.39 (2.29 to 2.50)	1.01 (0.95 to 1.07)
2015	3.9 (3.8 to 4.1)	1.7 (1.6 to 1.8)	5.9 (5.7 to 6.2)	2.6 (2.5 to 2.7)	2.27 (2.17 to 2.36)	0.98 (0.94 to 1.02)
2020	3.0 (2.7 to 3.4)	1.3 (1.2 to 1.5)	5.1 (4.6 to 5.7)	2.3 (2.0 to 2.5)	2.09 (1.86 to 2.33)	0.92 (0.83 to 1.03)
2021	3.3 (2.7 to 4.1)	1.5 (1.2 to 1.8)	5.7 (4.6 to 7.0)	2.5 (2.0 to 3.0)	2.13 (1.74 to 2.63)	0.93 (0.76 to 1.15)

East Midlands GBR_4621 Western Europe Stillbirth Rate / Neonatal Mortality Rate (SBR/NMR) − 20 weeks
lambda = 0.2 ; zeta = 0.99 ; scale = 5 ; amp = 0.028

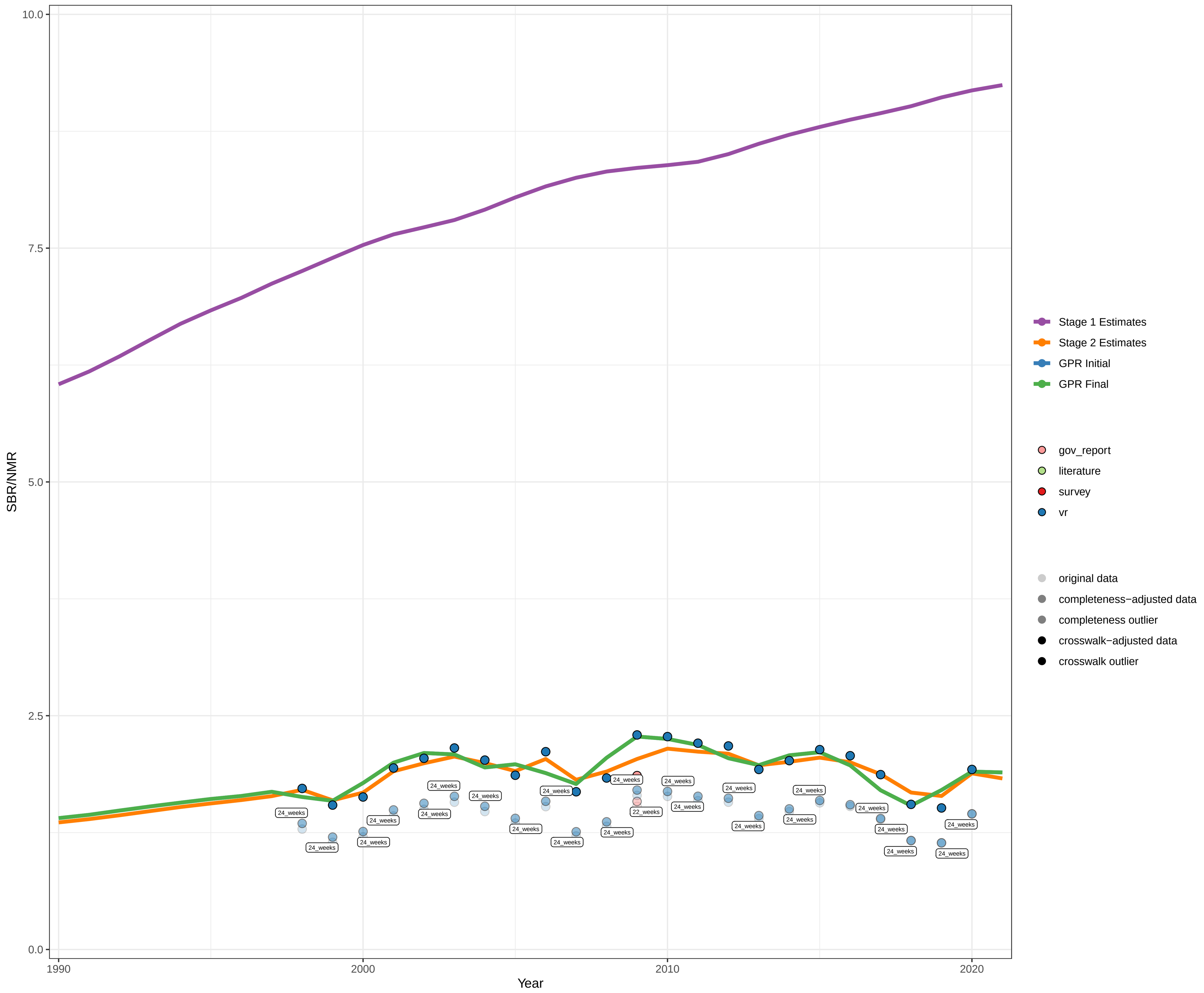

East Midlands GBR_4621 Western Europe Stillbirth Rate / Neonatal Mortality Rate (SBR/NMR) − 28 weeks
lambda = 0.2 ; zeta = 0.99 ; scale = 5 ; amp = 0.028

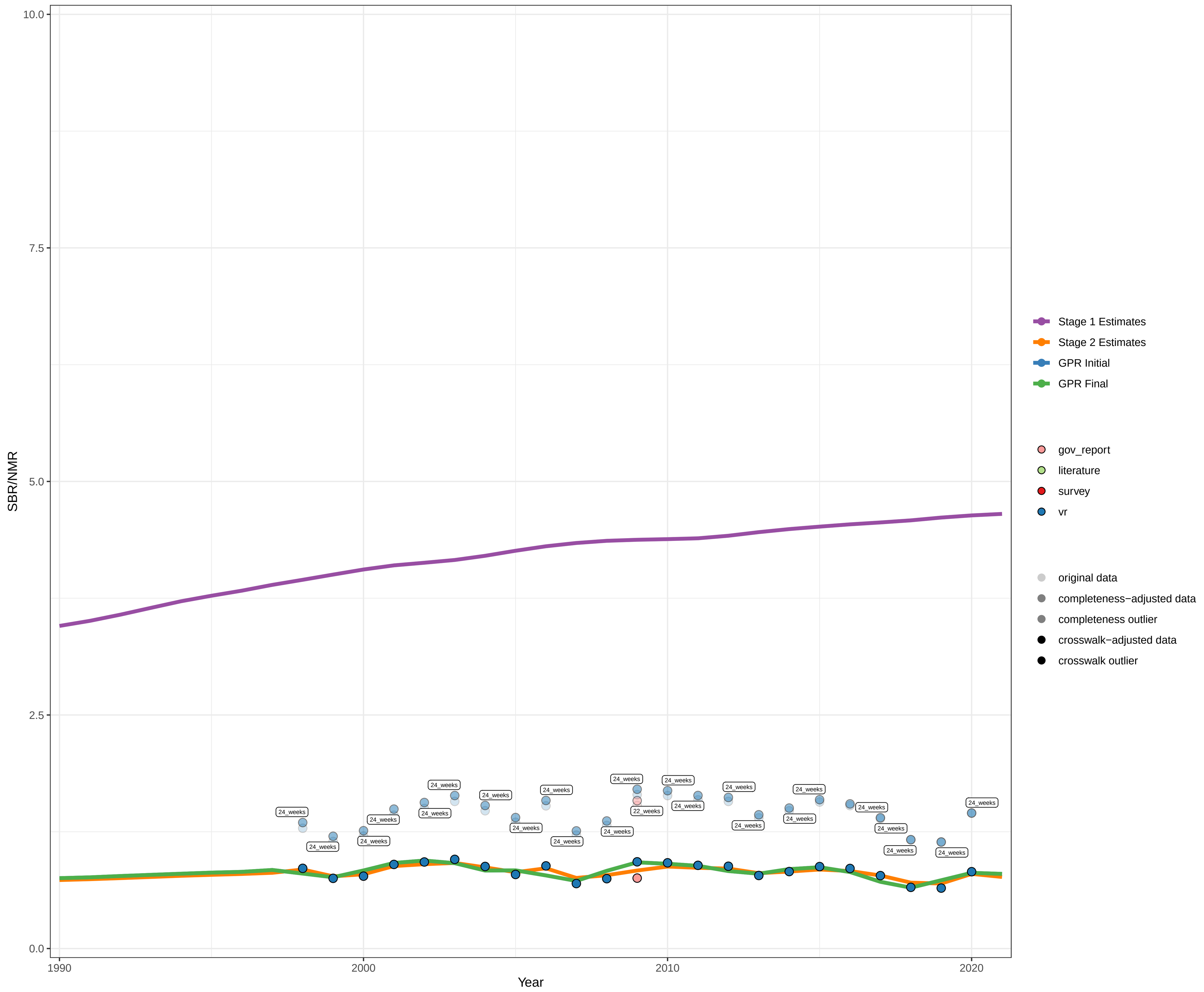

Definition	Years	Outlier	Source.Type	Title
22_weeks	2009	0	Statistical Report	United Kingdom Perinatal Mortality
24_weeks	1998−2007	0	VR	United Kingdom − England and Wales Birth Statistics
24_weeks	2008−2020	0	VR	United Kingdom − England and Wales Child Mortality Statistics

East Midlands GBR_4621 Western Europe Stillbirth Rate (SBR) − 20 weeks
lambda = 0.2 ; zeta = 0.99 ; scale = 5 ; amp = 0.028

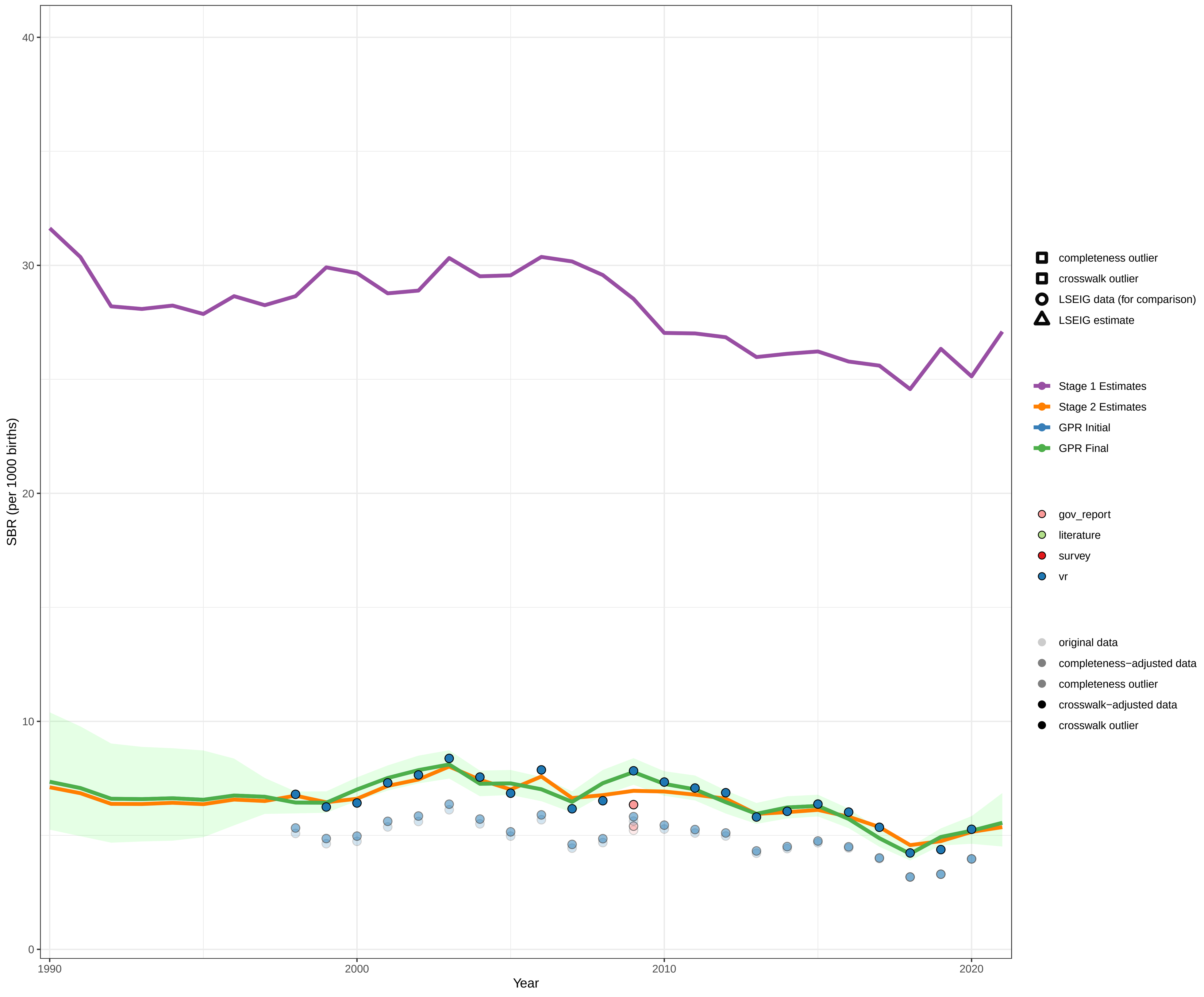

East Midlands GBR_4621 Western Europe Stillbirth Rate (SBR) − 28 weeks
lambda = 0.2 ; zeta = 0.99 ; scale = 5 ; amp = 0.028

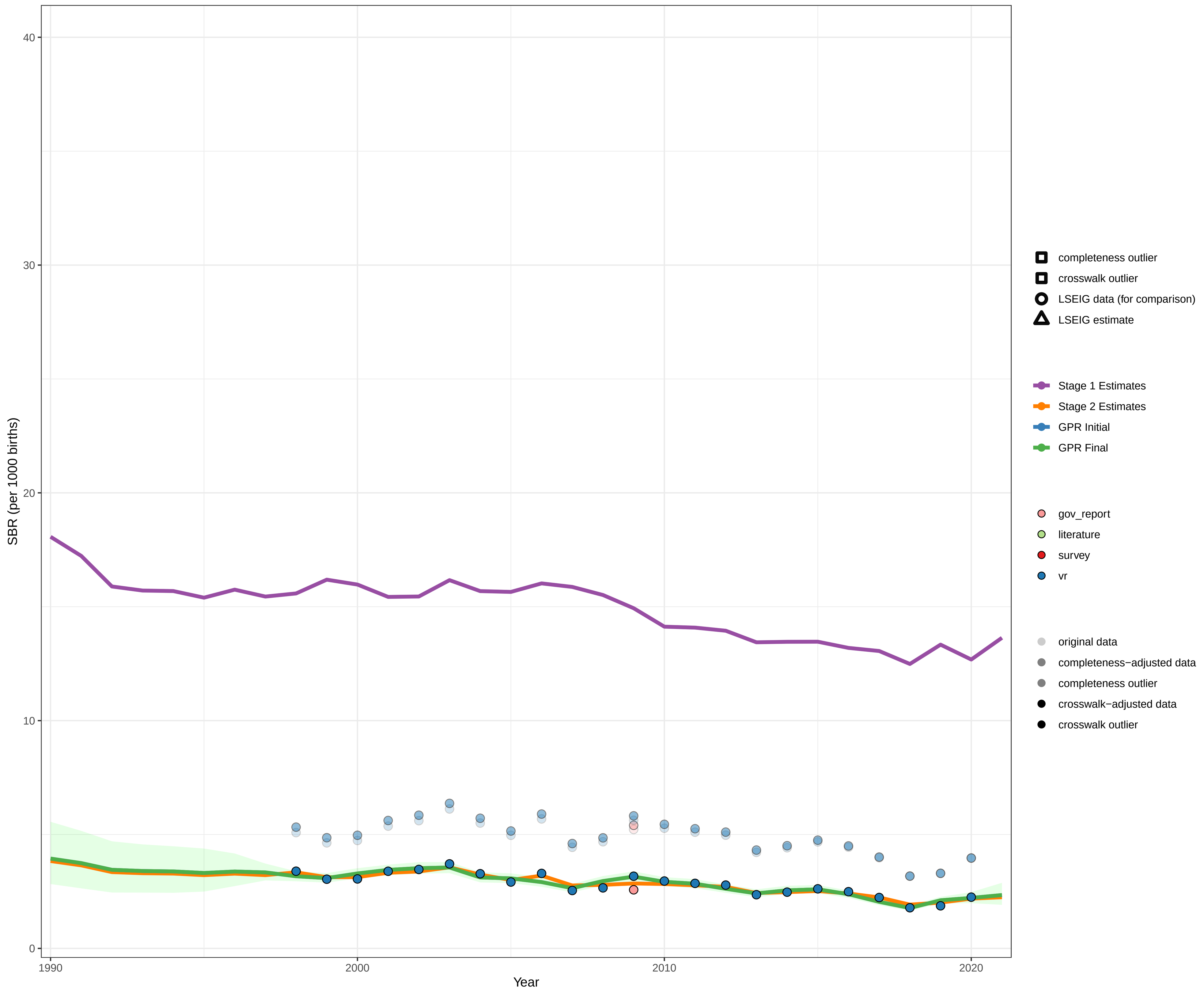

Year	Total Stillbirths (in thousands)	Total Stillbirths (in thousands)	SBR (per 1000 births)	SBR (per 1000 births)	SBR/NMR	SBR/NMR
	20 weeks	28 weeks	20 weeks	28 weeks	20 weeks	28 weeks
1990	0.4 (0.3 to 0.6)	0.2 (0.2 to 0.3)	7.4 (5.2 to 10.4)	3.9 (2.8 to 5.6)	1.40 (1.00 to 1.99)	0.75 (0.54 to 1.06)
1995	0.3 (0.2 to 0.4)	0.2 (0.1 to 0.2)	6.6 (4.9 to 8.7)	3.3 (2.5 to 4.4)	1.61 (1.21 to 2.14)	0.81 (0.61 to 1.08)
2000	0.3 (0.3 to 0.3)	0.2 (0.1 to 0.2)	7.0 (6.5 to 7.5)	3.3 (3.1 to 3.5)	1.78 (1.65 to 1.92)	0.84 (0.78 to 0.89)
2005	0.4 (0.3 to 0.4)	0.2 (0.1 to 0.2)	7.3 (6.8 to 7.9)	3.1 (2.9 to 3.3)	1.98 (1.84 to 2.14)	0.84 (0.78 to 0.90)
2010	0.4 (0.4 to 0.4)	0.2 (0.2 to 0.2)	7.3 (6.7 to 7.8)	2.9 (2.7 to 3.1)	2.25 (2.09 to 2.42)	0.91 (0.85 to 0.97)
2015	0.3 (0.3 to 0.4)	0.1 (0.1 to 0.1)	6.3 (5.8 to 6.8)	2.6 (2.4 to 2.8)	2.11 (1.96 to 2.27)	0.87 (0.81 to 0.93)
2020	0.2 (0.2 to 0.3)	0.1 (0.1 to 0.1)	5.2 (4.6 to 5.8)	2.2 (2.0 to 2.5)	1.90 (1.69 to 2.14)	0.81 (0.73 to 0.90)
2021	0.3 (0.2 to 0.3)	0.1 (0.1 to 0.1)	5.5 (4.5 to 6.9)	2.3 (1.9 to 2.9)	1.89 (1.54 to 2.34)	0.80 (0.65 to 0.98)

Derby GBR_44697 Western Europe Stillbirth Rate / Neonatal Mortality Rate (SBR/NMR) − 20 weeks
lambda = 0.8 ; zeta = 0.7 ; scale = 15 ; amp = 0.028

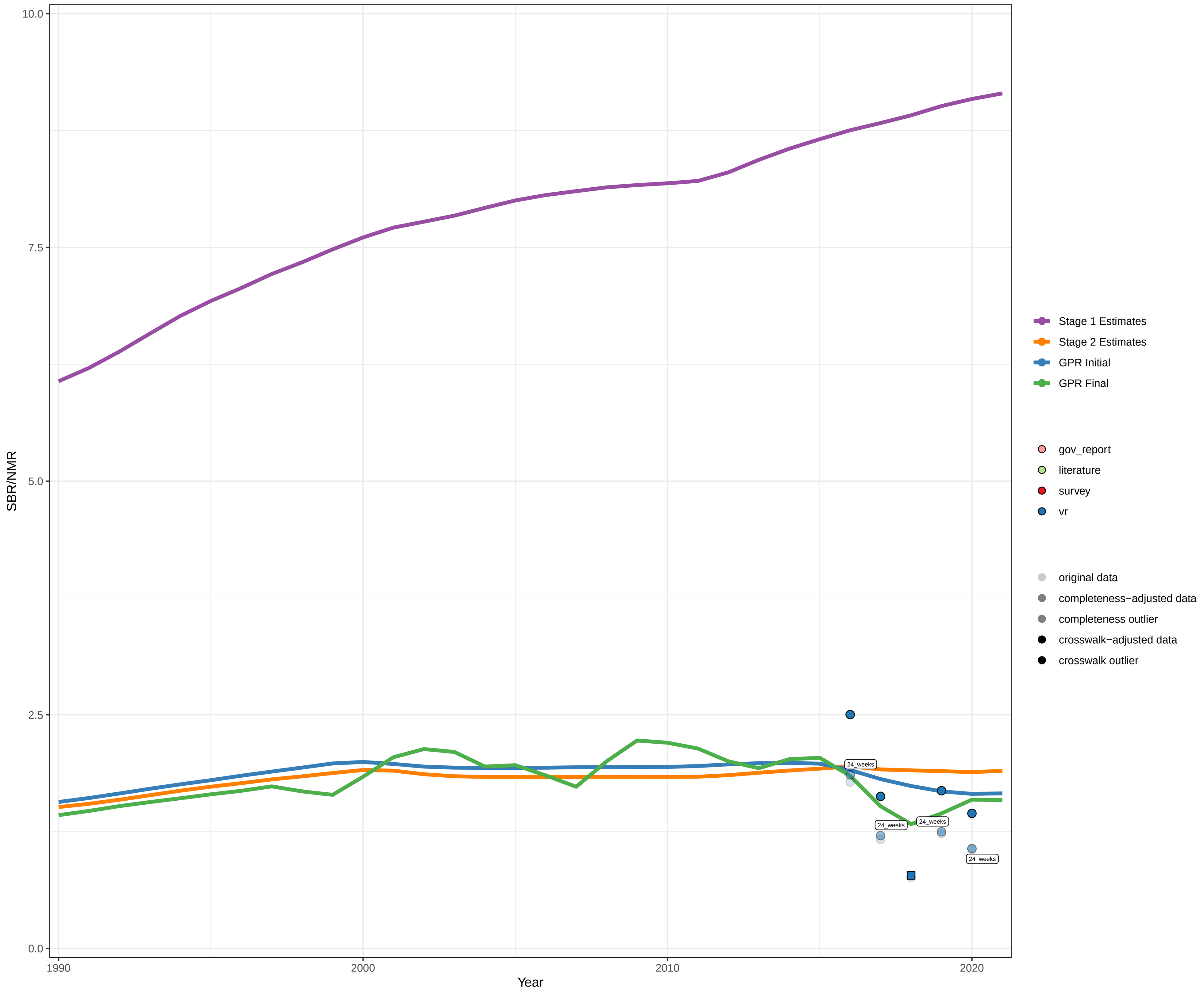

Derby GBR_44697 Western Europe Stillbirth Rate / Neonatal Mortality Rate (SBR/NMR) − 28 weeks
lambda = 0.8 ; zeta = 0.7 ; scale = 15 ; amp = 0.028

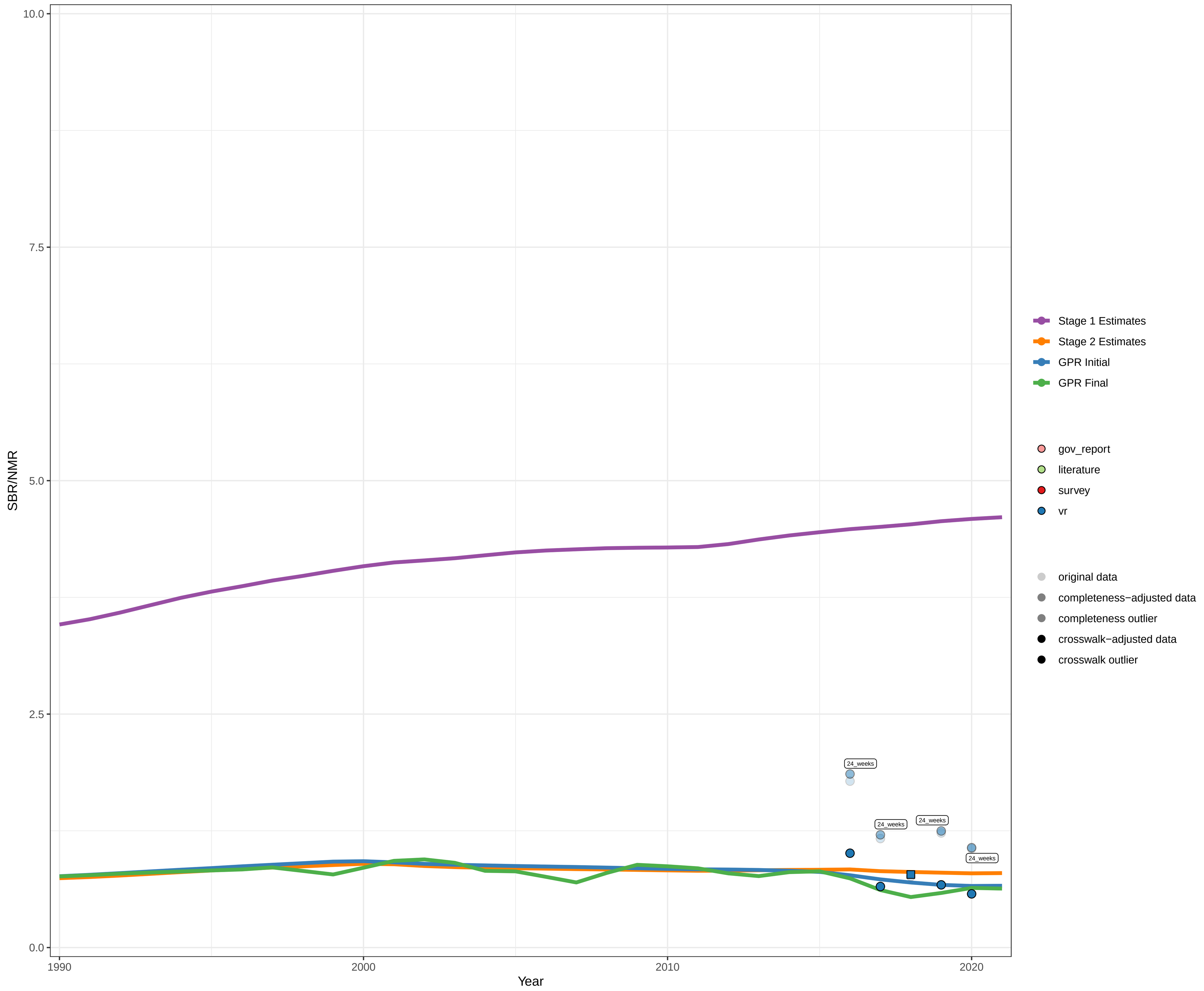

Definition	Years	Outlier	Source.Type	Title
24_weeks	2016−2020	0	VR	United Kingdom − England and Wales Child Mortality Statistics

Derby GBR_44697 Western Europe Stillbirth Rate (SBR) − 20 weeks
lambda = 0.8 ; zeta = 0.7 ; scale = 15 ; amp = 0.028

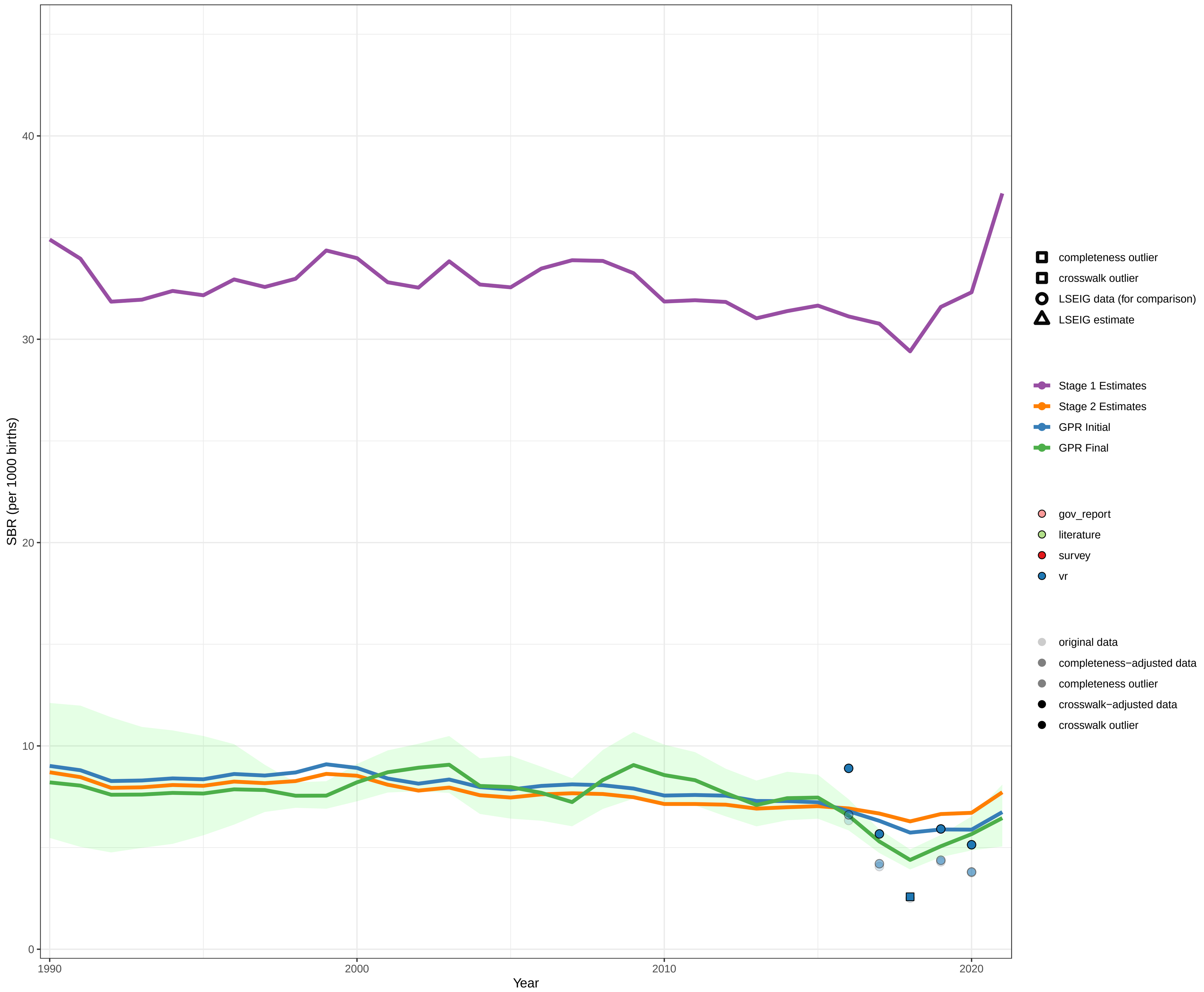

Derby GBR_44697 Western Europe Stillbirth Rate (SBR) − 28 weeks
lambda = 0.8 ; zeta = 0.7 ; scale = 15 ; amp = 0.028

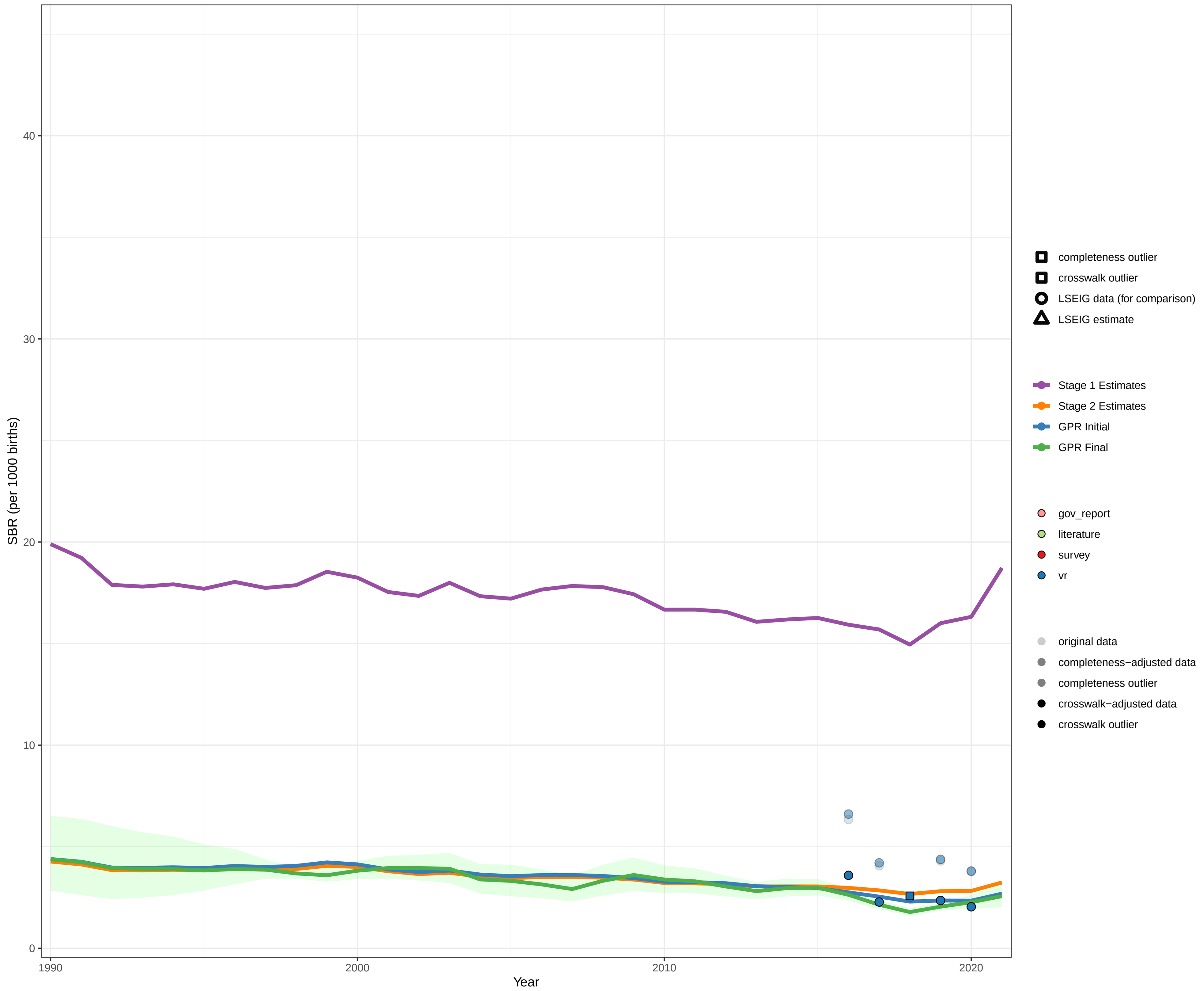

Year	Total Stillbirths (in thousands)	Total Stillbirths (in thousands)	SBR (per 1000 births)	SBR (per 1000 births)	SBR/NMR	SBR/NMR
	20 weeks	28 weeks	20 weeks	28 weeks	20 weeks	28 weeks
1990	0.0 (0.0 to 0.0)	0.0 (0.0 to 0.0)	8.2 (5.5 to 12.1)	4.4 (2.8 to 6.5)	1.43 (0.95 to 2.11)	0.76 (0.49 to 1.13)
1995	0.0 (0.0 to 0.0)	0.0 (0.0 to 0.0)	7.7 (5.6 to 10.5)	3.8 (2.8 to 5.1)	1.65 (1.21 to 2.26)	0.83 (0.61 to 1.10)
2000	0.0 (0.0 to 0.0)	0.0 (0.0 to 0.0)	8.2 (7.3 to 9.1)	3.8 (3.4 to 4.3)	1.84 (1.63 to 2.04)	0.85 (0.76 to 0.96)
2005	0.0 (0.0 to 0.0)	0.0 (0.0 to 0.0)	8.0 (6.4 to 9.5)	3.3 (2.6 to 4.1)	1.96 (1.58 to 2.34)	0.82 (0.63 to 1.01)
2010	0.0 (0.0 to 0.0)	0.0 (0.0 to 0.0)	8.6 (7.1 to 10.1)	3.4 (2.7 to 4.1)	2.20 (1.83 to 2.59)	0.87 (0.70 to 1.05)
2015	0.0 (0.0 to 0.0)	0.0 (0.0 to 0.0)	7.5 (6.4 to 8.6)	3.0 (2.6 to 3.4)	2.04 (1.76 to 2.35)	0.82 (0.71 to 0.93)
2020	0.0 (0.0 to 0.0)	0.0 (0.0 to 0.0)	5.7 (4.9 to 6.5)	2.3 (2.0 to 2.6)	1.59 (1.37 to 1.84)	0.64 (0.55 to 0.74)
2021	0.0 (0.0 to 0.0)	0.0 (0.0 to 0.0)	6.5 (5.1 to 8.1)	2.6 (2.0 to 3.2)	1.59 (1.24 to 1.99)	0.63 (0.50 to 0.79)

Derbyshire GBR_44698 Western Europe Stillbirth Rate / Neonatal Mortality Rate (SBR/NMR) − 20 weeks
lambda = 0.6 ; zeta = 0.8 ; scale = 15 ; amp = 0.028

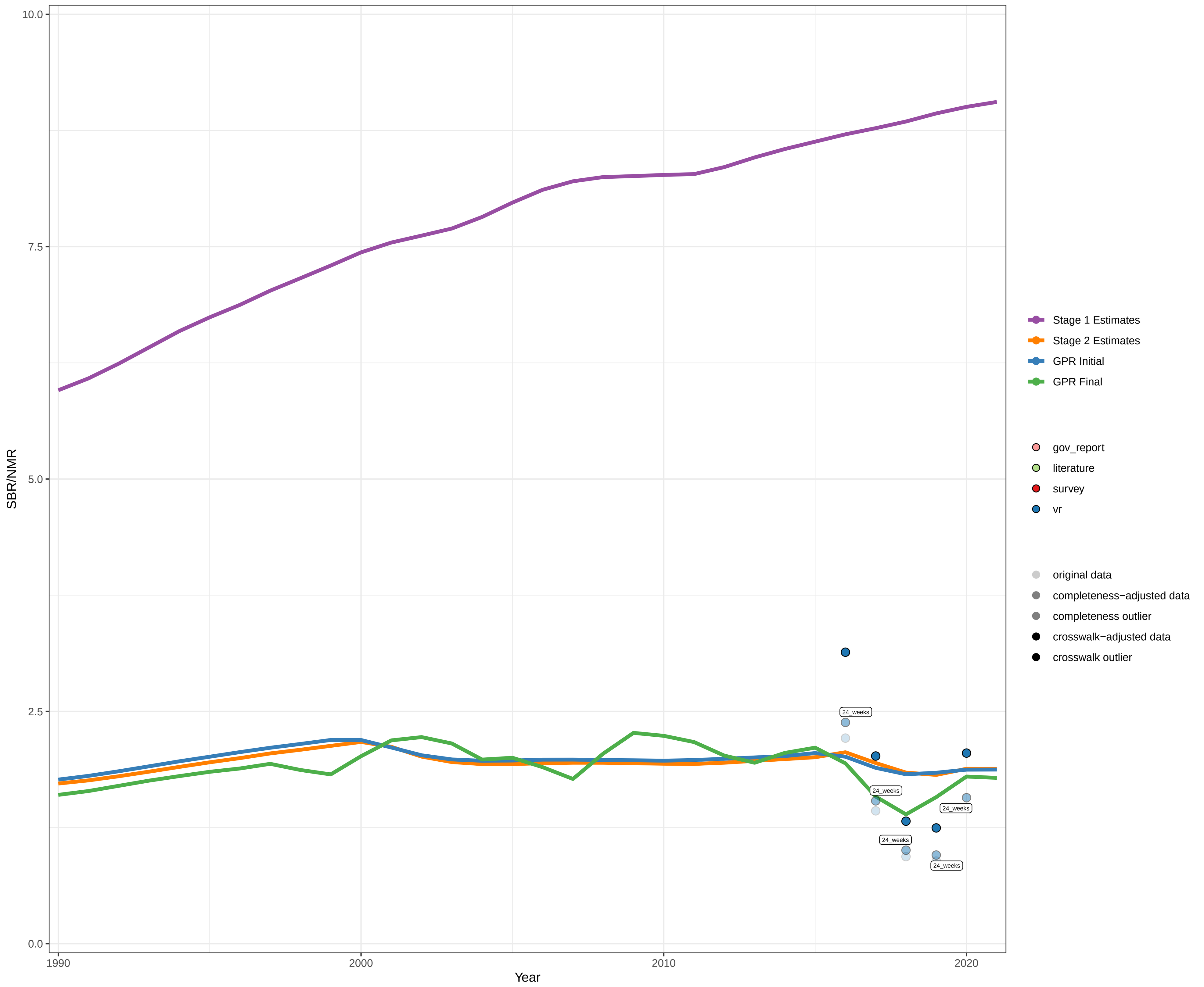

Derbyshire GBR_44698 Western Europe Stillbirth Rate / Neonatal Mortality Rate (SBR/NMR) − 28 weeks
lambda = 0.6 ; zeta = 0.8 ; scale = 15 ; amp = 0.028

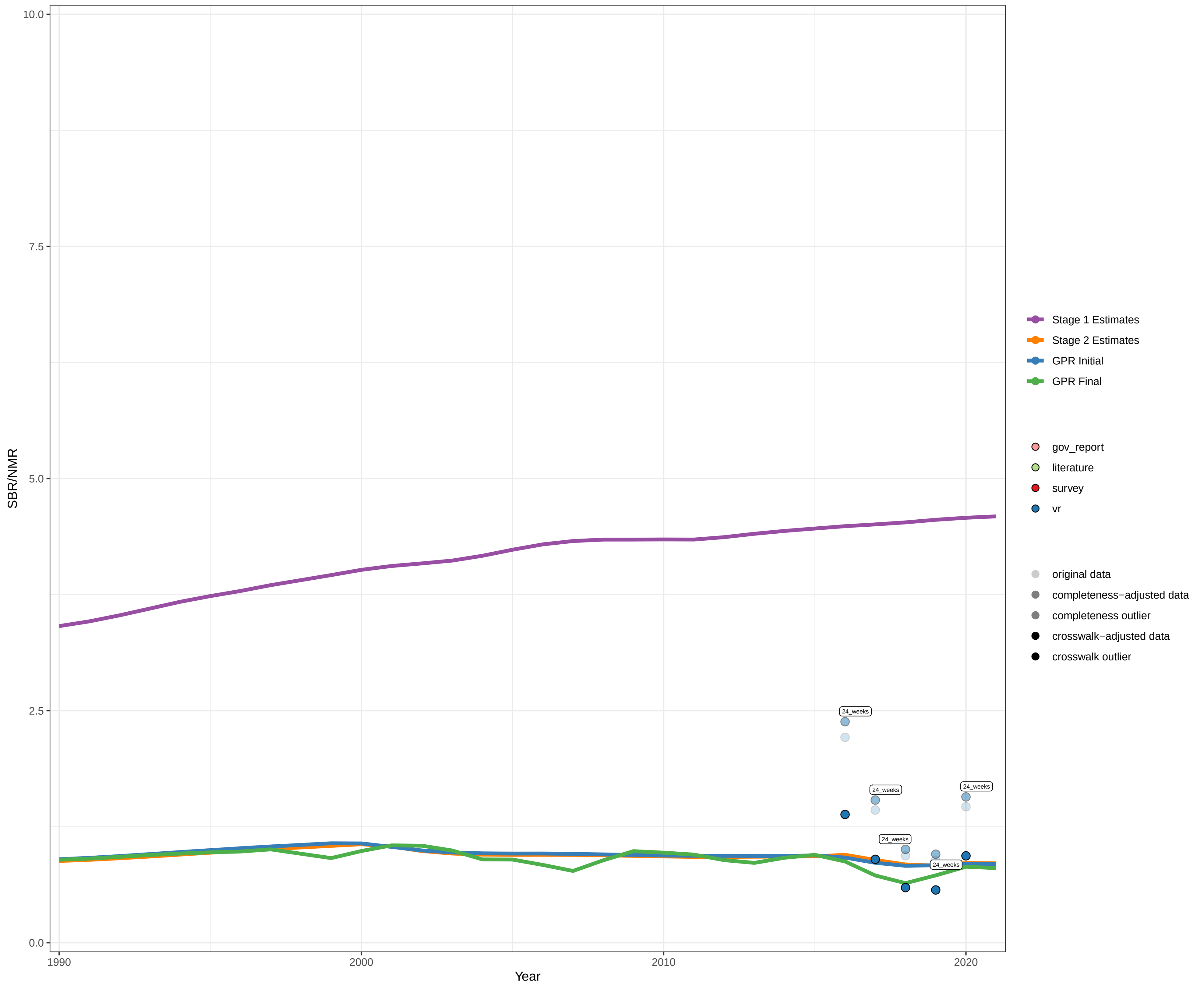

Definition	Years	Outlier	Source.Type	Title
24_weeks	2016−2020	0	VR	United Kingdom − England and Wales Child Mortality Statistics

Derbyshire GBR_44698 Western Europe Stillbirth Rate (SBR) − 20 weeks
lambda = 0.6 ; zeta = 0.8 ; scale = 15 ; amp = 0.028

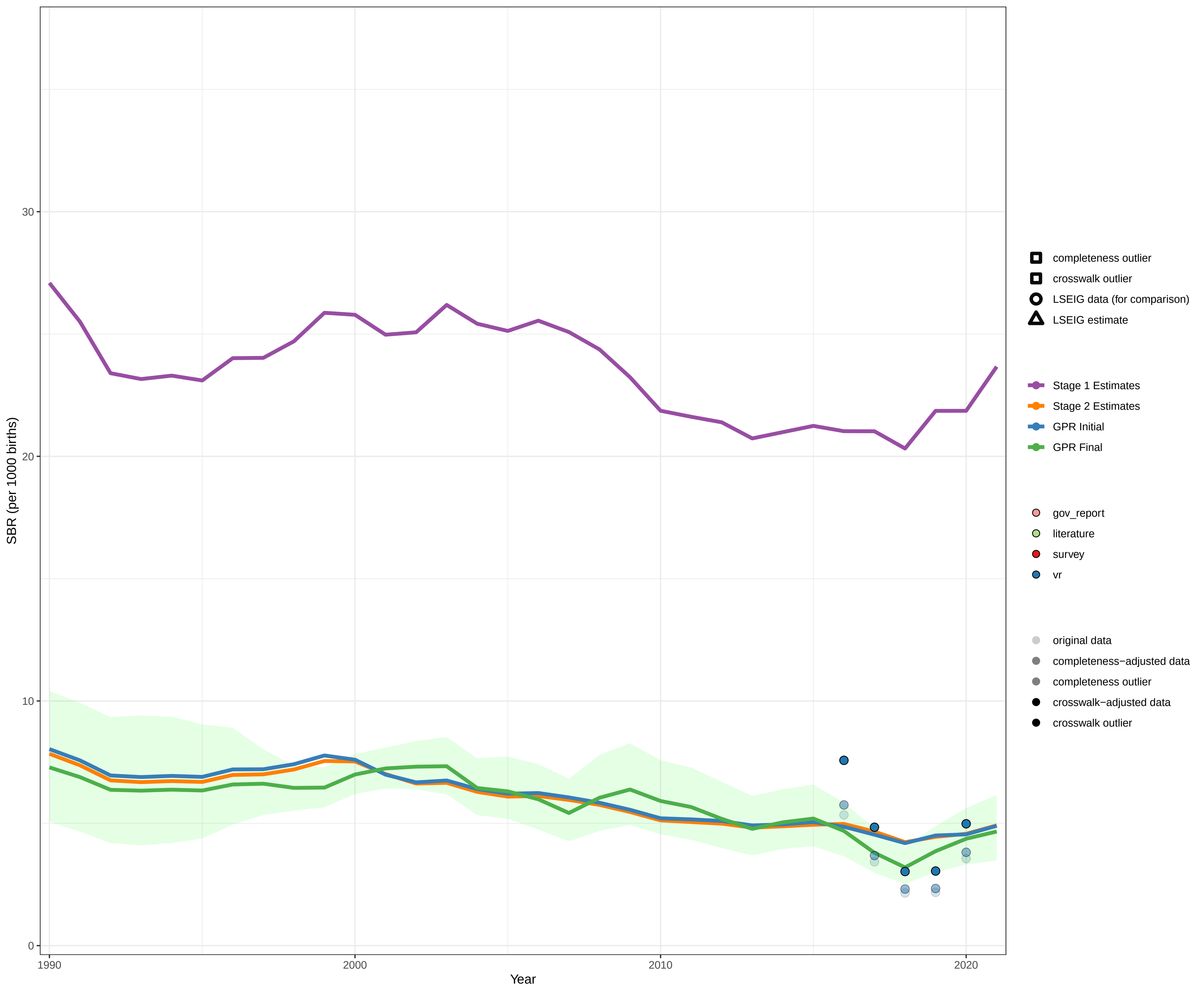

Derbyshire GBR_44698 Western Europe Stillbirth Rate (SBR) − 28 weeks
lambda = 0.6 ; zeta = 0.8 ; scale = 15 ; amp = 0.028

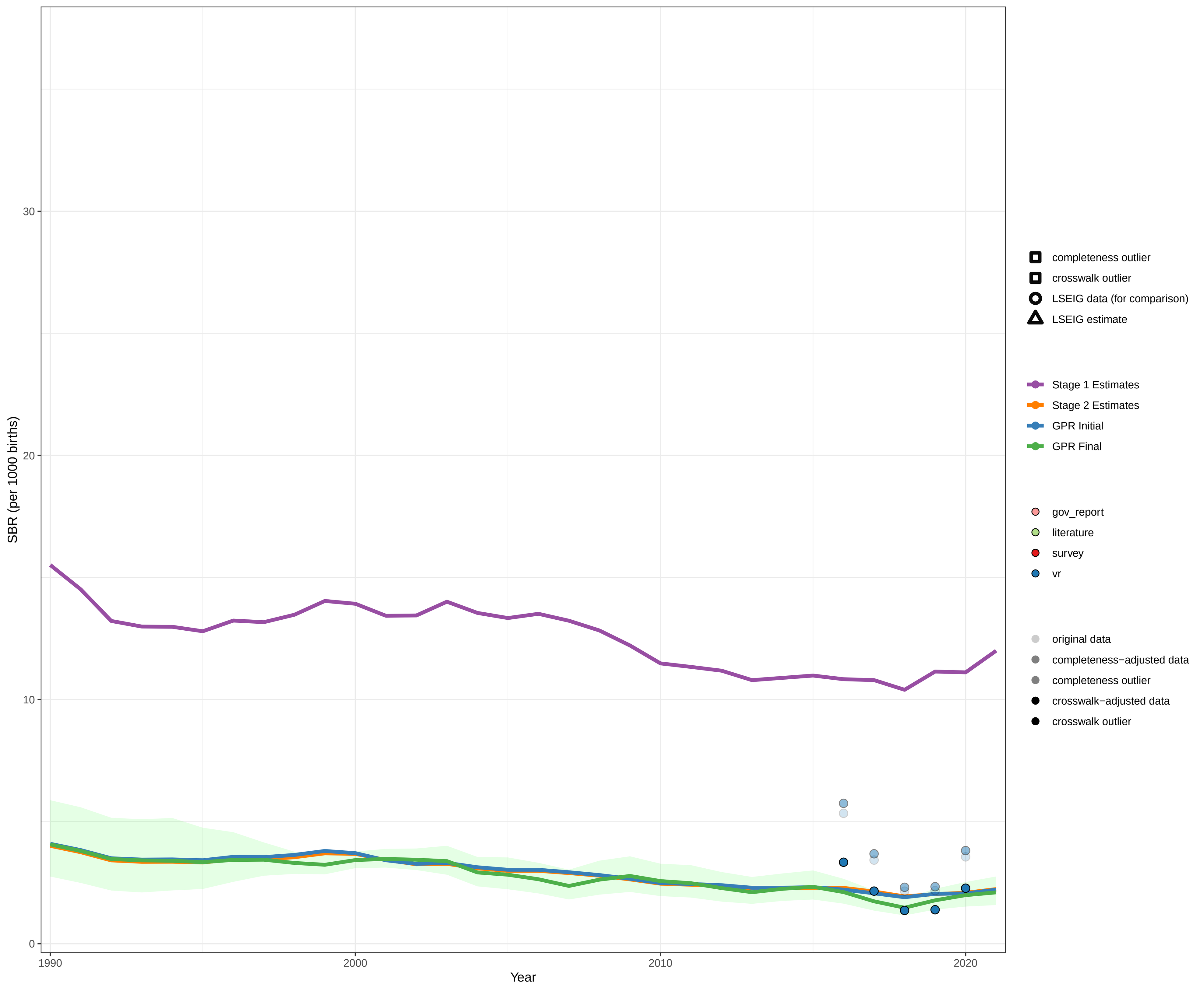

Year	Total Stillbirths (in thousands)	Total Stillbirths (in thousands)	SBR (per 1000 births)	SBR (per 1000 births)	SBR/NMR	SBR/NMR
	20 weeks	28 weeks	20 weeks	28 weeks	20 weeks	28 weeks
1990	0.1 (0.0 to 0.1)	0.0 (0.0 to 0.1)	7.3 (5.1 to 10.4)	4.1 (2.8 to 5.9)	1.60 (1.11 to 2.29)	0.89 (0.60 to 1.29)
1995	0.1 (0.0 to 0.1)	0.0 (0.0 to 0.0)	6.3 (4.4 to 9.0)	3.3 (2.2 to 4.8)	1.85 (1.28 to 2.64)	0.97 (0.65 to 1.39)
2000	0.1 (0.0 to 0.1)	0.0 (0.0 to 0.0)	7.0 (6.2 to 7.8)	3.4 (3.1 to 3.8)	2.02 (1.79 to 2.26)	0.99 (0.89 to 1.09)
2005	0.1 (0.0 to 0.1)	0.0 (0.0 to 0.0)	6.3 (5.2 to 7.7)	2.8 (2.2 to 3.5)	2.00 (1.65 to 2.45)	0.90 (0.71 to 1.12)
2010	0.1 (0.0 to 0.1)	0.0 (0.0 to 0.0)	5.9 (4.5 to 7.6)	2.6 (2.0 to 3.3)	2.24 (1.72 to 2.87)	0.97 (0.74 to 1.24)
2015	0.0 (0.0 to 0.1)	0.0 (0.0 to 0.0)	5.2 (4.1 to 6.6)	2.3 (1.8 to 3.0)	2.11 (1.65 to 2.68)	0.95 (0.74 to 1.22)
2020	0.0 (0.0 to 0.0)	0.0 (0.0 to 0.0)	4.4 (3.3 to 5.6)	2.0 (1.5 to 2.5)	1.80 (1.36 to 2.32)	0.82 (0.63 to 1.04)
2021	0.0 (0.0 to 0.0)	0.0 (0.0 to 0.0)	4.7 (3.5 to 6.2)	2.1 (1.6 to 2.8)	1.78 (1.33 to 2.36)	0.80 (0.61 to 1.05)

Leicester GBR_44701 Western Europe Stillbirth Rate / Neonatal Mortality Rate (SBR/NMR) − 20 weeks
lambda = 0.8 ; zeta = 0.7 ; scale = 15 ; amp = 0.028

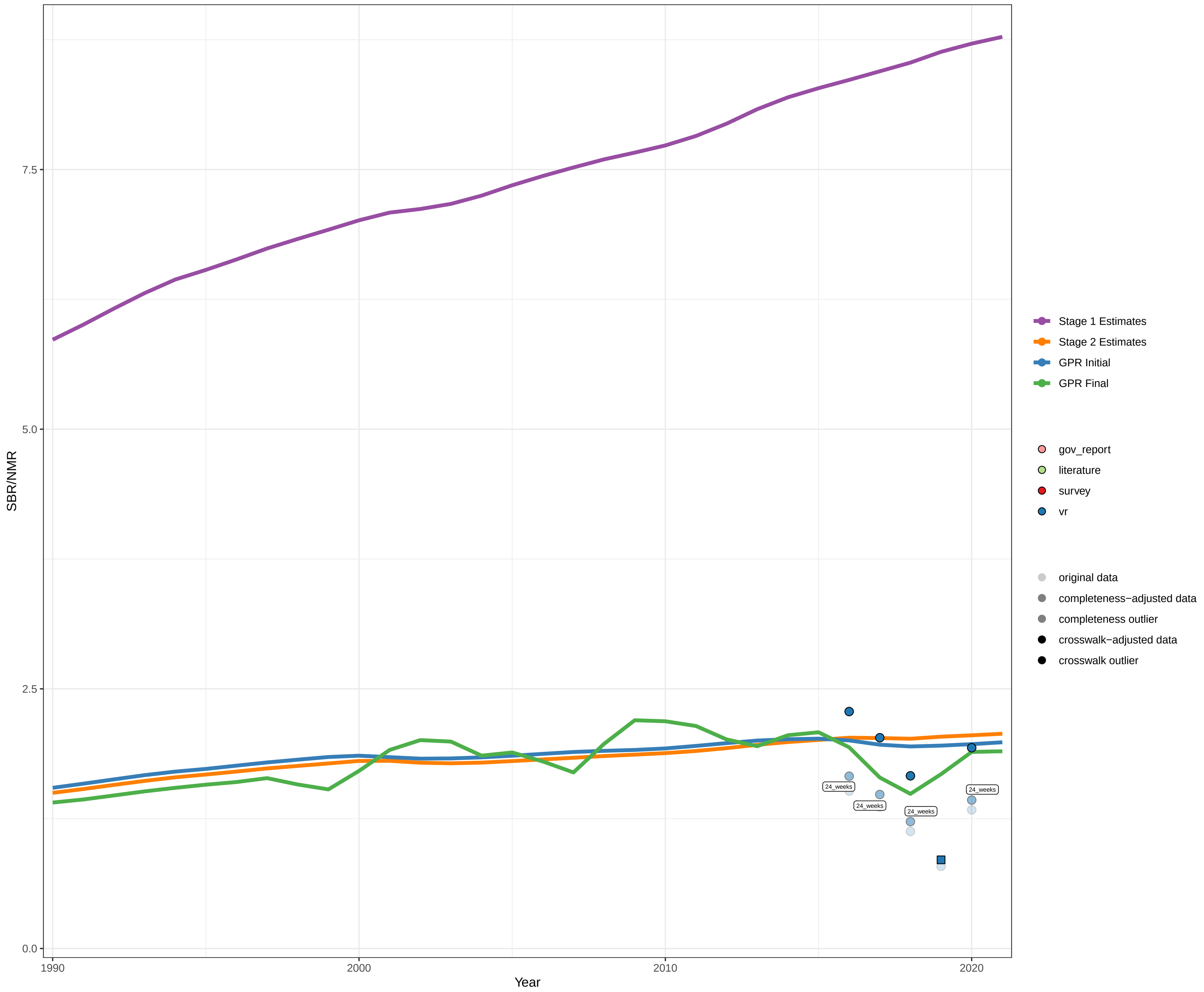

Leicester GBR_44701 Western Europe Stillbirth Rate / Neonatal Mortality Rate (SBR/NMR) − 28 weeks
lambda = 0.8 ; zeta = 0.7 ; scale = 15 ; amp = 0.028

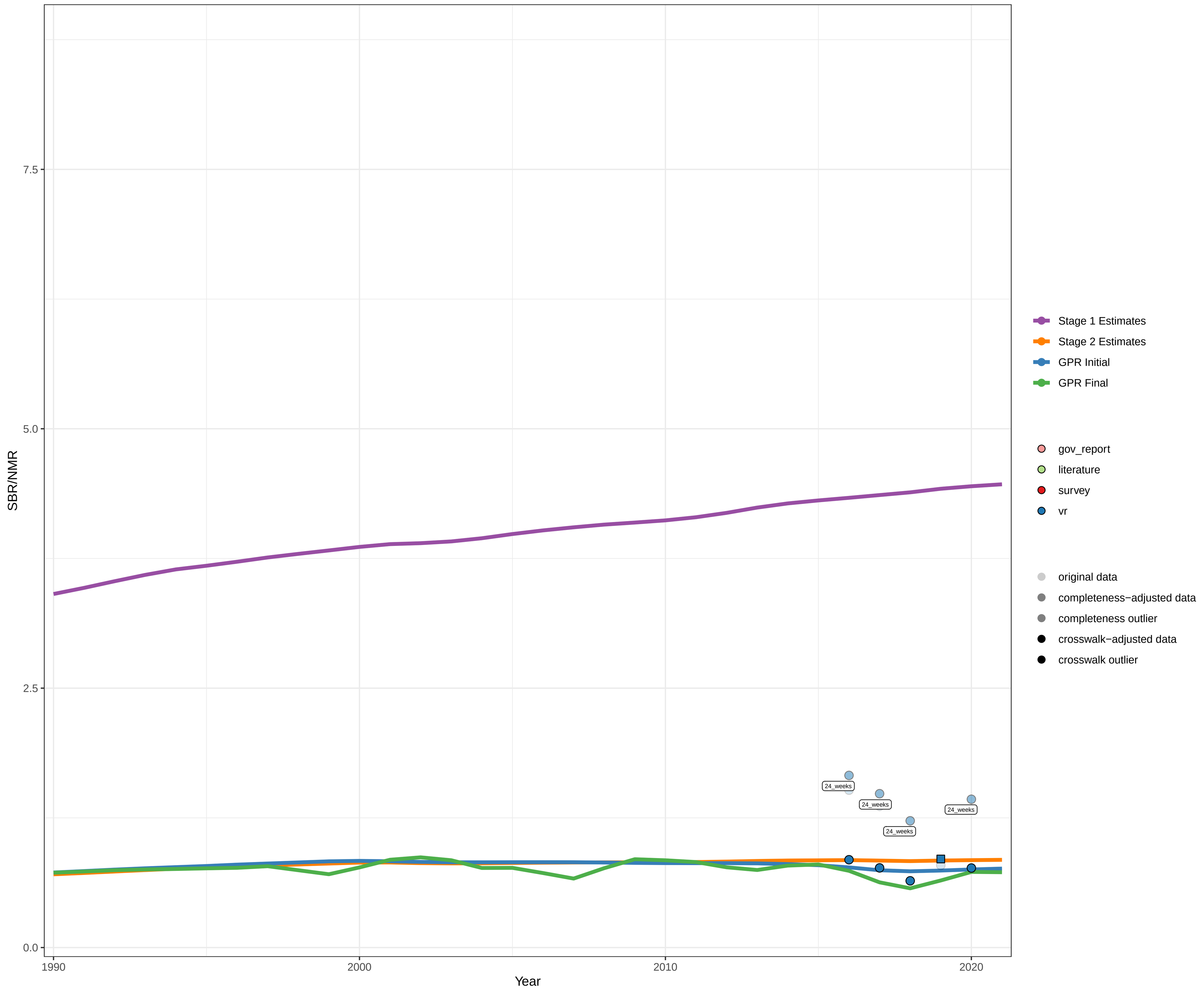

Definition	Years	Outlier	Source.Type	Title
24_weeks	2016−2020	0	VR	United Kingdom − England and Wales Child Mortality Statistics

Leicester GBR_44701 Western Europe Stillbirth Rate (SBR) − 20 weeks
lambda = 0.8 ; zeta = 0.7 ; scale = 15 ; amp = 0.028

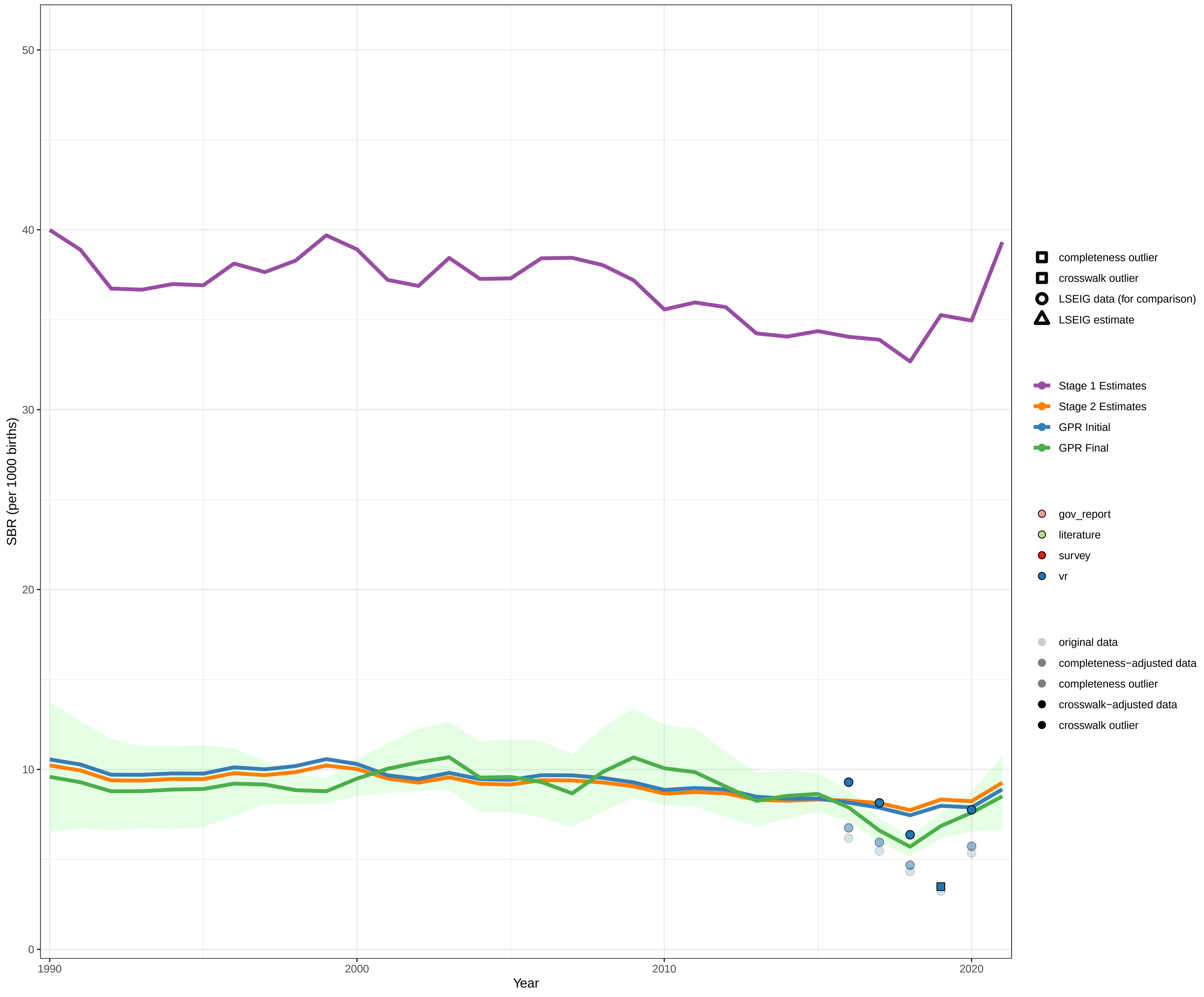

Leicester GBR_44701 Western Europe Stillbirth Rate (SBR) − 28 weeks
lambda = 0.8 ; zeta = 0.7 ; scale = 15 ; amp = 0.028

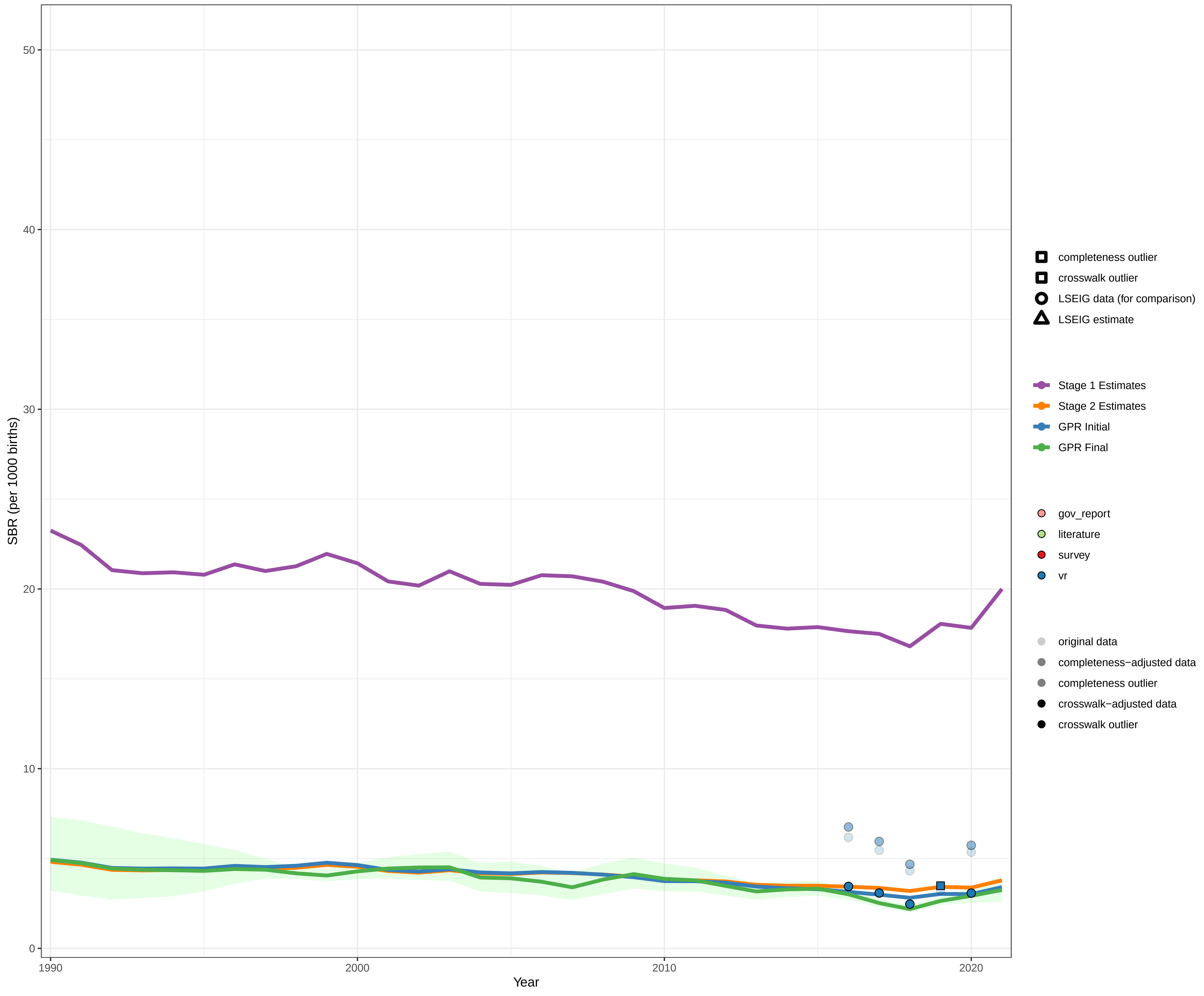

Year	Total Stillbirths (in thousands)	Total Stillbirths (in thousands)	SBR (per 1000 births)	SBR (per 1000 births)	SBR/NMR	SBR/NMR
	20 weeks	28 weeks	20 weeks	28 weeks	20 weeks	28 weeks
1990	0.0 (0.0 to 0.1)	0.0 (0.0 to 0.0)	9.6 (6.5 to 13.7)	4.9 (3.2 to 7.3)	1.41 (0.96 to 2.01)	0.72 (0.47 to 1.07)
1995	0.0 (0.0 to 0.0)	0.0 (0.0 to 0.0)	8.9 (6.8 to 11.4)	4.3 (3.2 to 5.8)	1.58 (1.20 to 2.01)	0.76 (0.56 to 1.03)
2000	0.0 (0.0 to 0.0)	0.0 (0.0 to 0.0)	9.5 (8.5 to 10.5)	4.3 (3.8 to 4.8)	1.71 (1.53 to 1.90)	0.77 (0.69 to 0.86)
2005	0.0 (0.0 to 0.1)	0.0 (0.0 to 0.0)	9.6 (7.6 to 11.7)	3.9 (3.1 to 4.8)	1.89 (1.50 to 2.30)	0.77 (0.61 to 0.95)
2010	0.1 (0.0 to 0.1)	0.0 (0.0 to 0.0)	10.1 (8.0 to 12.5)	3.9 (3.2 to 4.7)	2.19 (1.74 to 2.71)	0.84 (0.69 to 1.02)
2015	0.0 (0.0 to 0.0)	0.0 (0.0 to 0.0)	8.6 (7.6 to 9.8)	3.3 (2.9 to 3.7)	2.08 (1.84 to 2.36)	0.80 (0.71 to 0.90)
2020	0.0 (0.0 to 0.0)	0.0 (0.0 to 0.0)	7.6 (6.6 to 8.7)	2.9 (2.5 to 3.4)	1.89 (1.63 to 2.17)	0.73 (0.63 to 0.84)
2021	0.0 (0.0 to 0.0)	0.0 (0.0 to 0.0)	8.5 (6.6 to 10.8)	3.3 (2.6 to 4.0)	1.90 (1.48 to 2.40)	0.73 (0.58 to 0.90)

Leicestershire GBR_44694 Western Europe Stillbirth Rate / Neonatal Mortality Rate (SBR/NMR) − 20 weeks
lambda = 0.6 ; zeta = 0.8 ; scale = 15 ; amp = 0.028

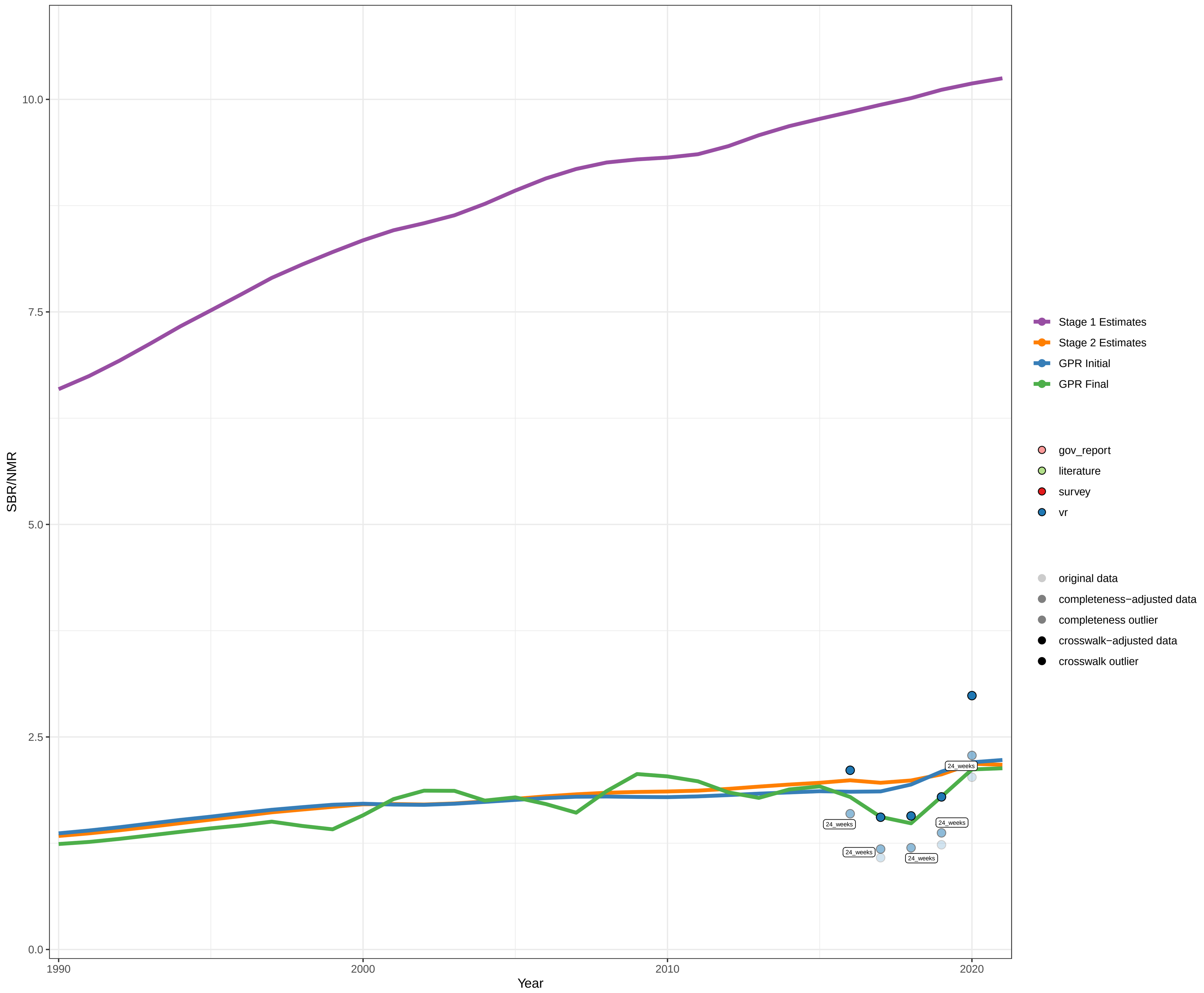

Leicestershire GBR_44694 Western Europe Stillbirth Rate / Neonatal Mortality Rate (SBR/NMR) − 28 weeks
lambda = 0.6 ; zeta = 0.8 ; scale = 15 ; amp = 0.028

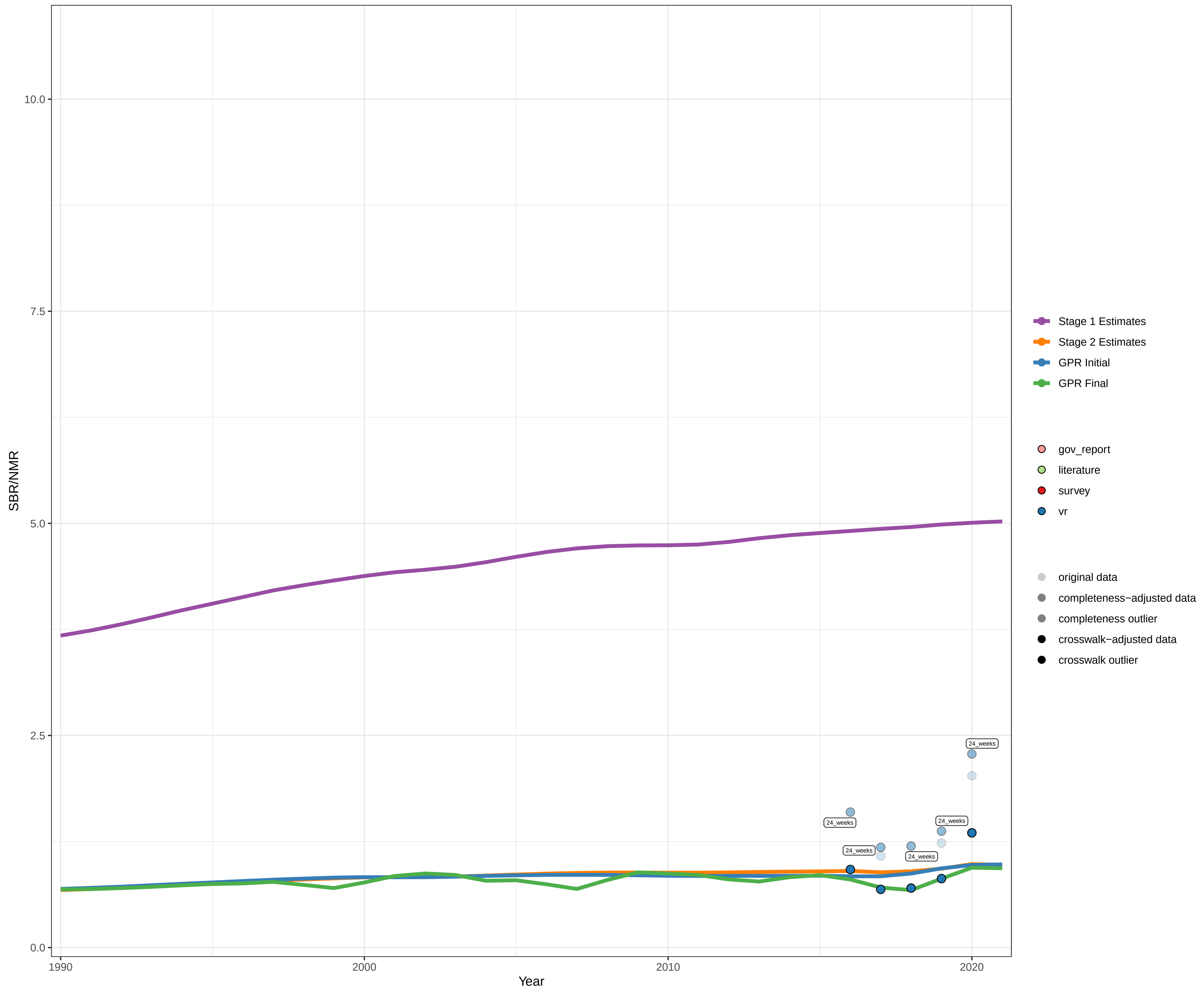

Definition	Years	Outlier	Source.Type	Title
24_weeks	2016−2020	0	VR	United Kingdom − England and Wales Child Mortality Statistics

Leicestershire GBR_44694 Western Europe Stillbirth Rate (SBR) − 20 weeks
lambda = 0.6 ; zeta = 0.8 ; scale = 15 ; amp = 0.028

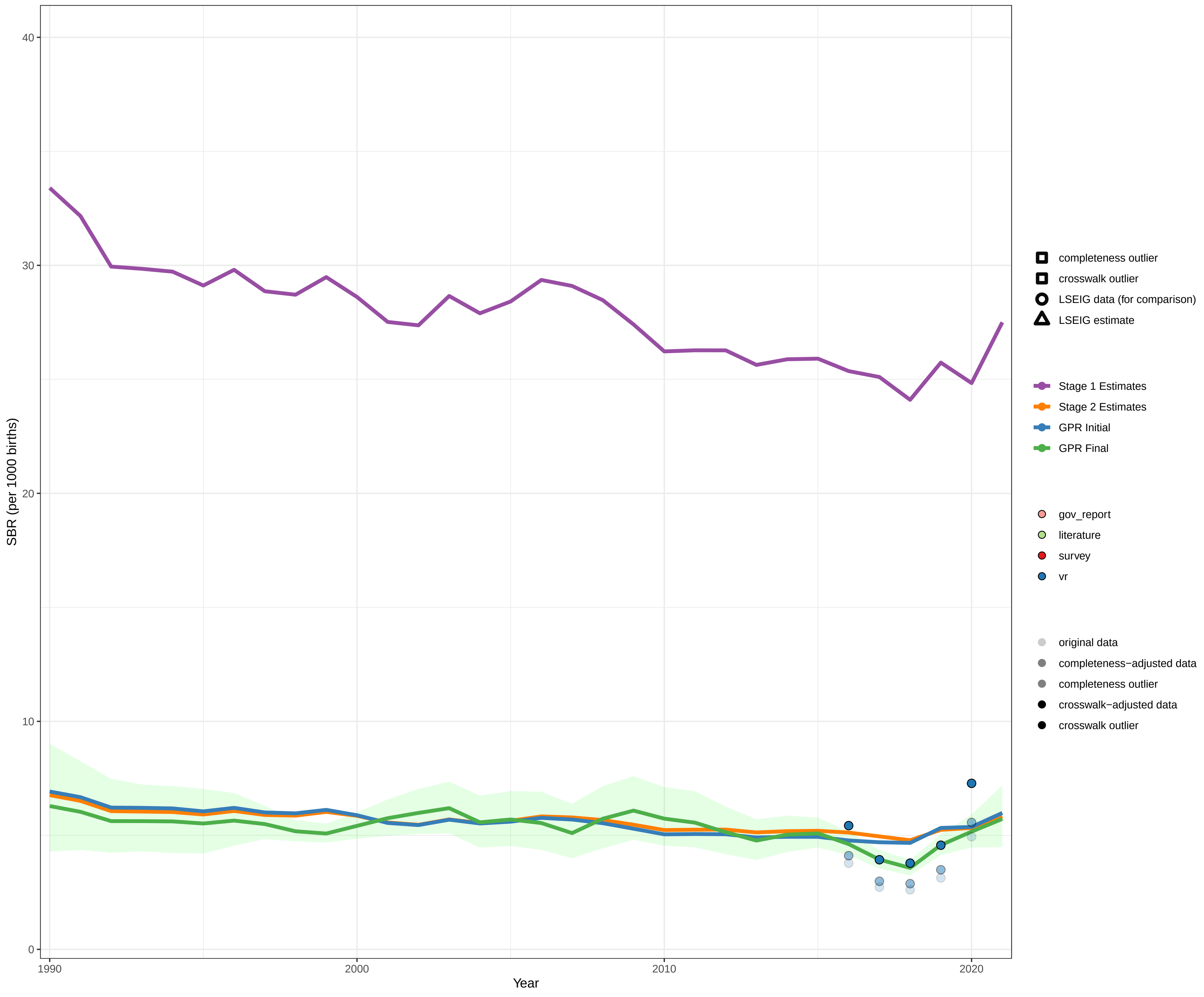

Leicestershire GBR_44694 Western Europe Stillbirth Rate (SBR) − 28 weeks
lambda = 0.6 ; zeta = 0.8 ; scale = 15 ; amp = 0.028

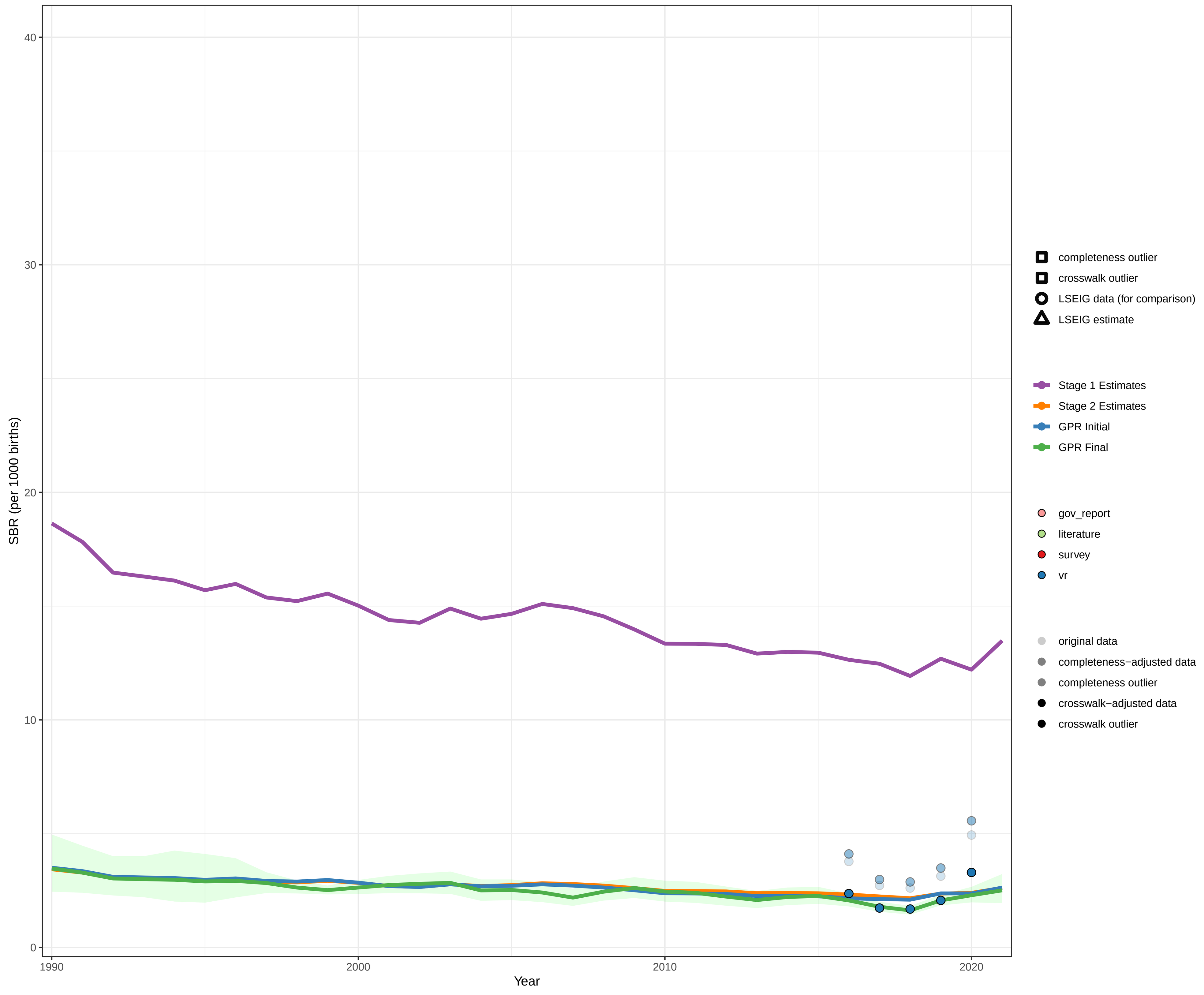

Year	Total Stillbirths (in thousands)	Total Stillbirths (in thousands)	SBR (per 1000 births)	SBR (per 1000 births)	SBR/NMR	SBR/NMR
	20 weeks	28 weeks	20 weeks	28 weeks	20 weeks	28 weeks
1990	0.0 (0.0 to 0.1)	0.0 (0.0 to 0.0)	6.3 (4.3 to 9.0)	3.5 (2.5 to 5.0)	1.24 (0.85 to 1.78)	0.68 (0.48 to 0.98)
1995	0.0 (0.0 to 0.0)	0.0 (0.0 to 0.0)	5.5 (4.2 to 7.0)	2.9 (2.0 to 4.1)	1.43 (1.09 to 1.82)	0.75 (0.51 to 1.06)
2000	0.0 (0.0 to 0.0)	0.0 (0.0 to 0.0)	5.4 (4.9 to 6.0)	2.6 (2.3 to 3.0)	1.58 (1.42 to 1.75)	0.77 (0.68 to 0.86)
2005	0.0 (0.0 to 0.0)	0.0 (0.0 to 0.0)	5.7 (4.5 to 6.9)	2.5 (2.1 to 3.0)	1.79 (1.42 to 2.18)	0.79 (0.65 to 0.94)
2010	0.0 (0.0 to 0.1)	0.0 (0.0 to 0.0)	5.7 (4.5 to 7.1)	2.5 (2.0 to 2.9)	2.04 (1.62 to 2.53)	0.87 (0.72 to 1.04)
2015	0.0 (0.0 to 0.0)	0.0 (0.0 to 0.0)	5.1 (4.5 to 5.8)	2.3 (1.9 to 2.7)	1.92 (1.68 to 2.18)	0.85 (0.72 to 1.00)
2020	0.0 (0.0 to 0.0)	0.0 (0.0 to 0.0)	5.2 (4.5 to 5.9)	2.3 (2.0 to 2.7)	2.12 (1.83 to 2.43)	0.94 (0.81 to 1.09)
2021	0.0 (0.0 to 0.0)	0.0 (0.0 to 0.0)	5.7 (4.5 to 7.2)	2.5 (1.9 to 3.2)	2.13 (1.67 to 2.68)	0.94 (0.73 to 1.20)

Lincolnshire GBR_44695 Western Europe Stillbirth Rate / Neonatal Mortality Rate (SBR/NMR) − 20 weeks
lambda = 0.6 ; zeta = 0.8 ; scale = 15 ; amp = 0.028

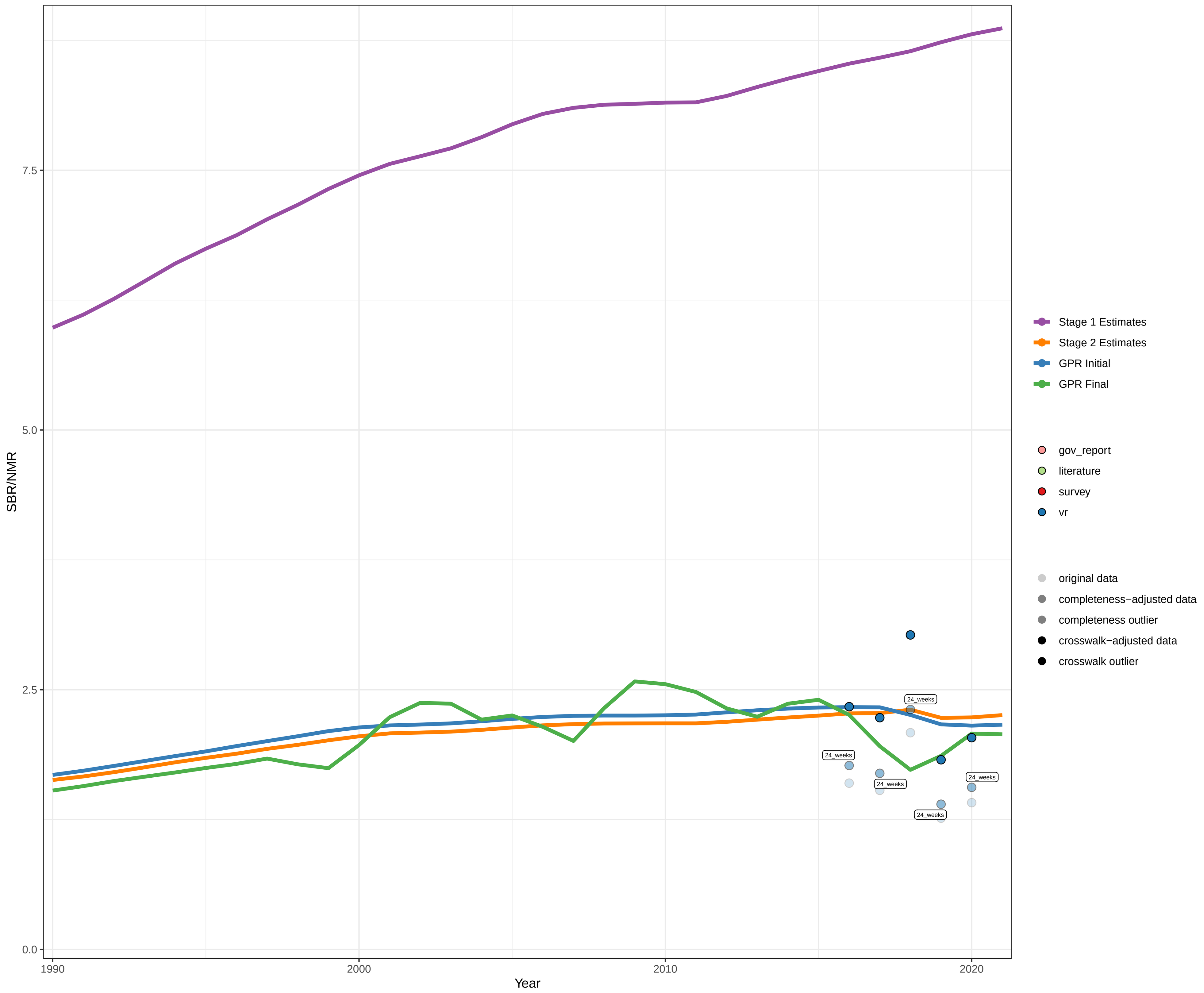

Lincolnshire GBR_44695 Western Europe Stillbirth Rate / Neonatal Mortality Rate (SBR/NMR) − 28 weeks
lambda = 0.6 ; zeta = 0.8 ; scale = 15 ; amp = 0.028

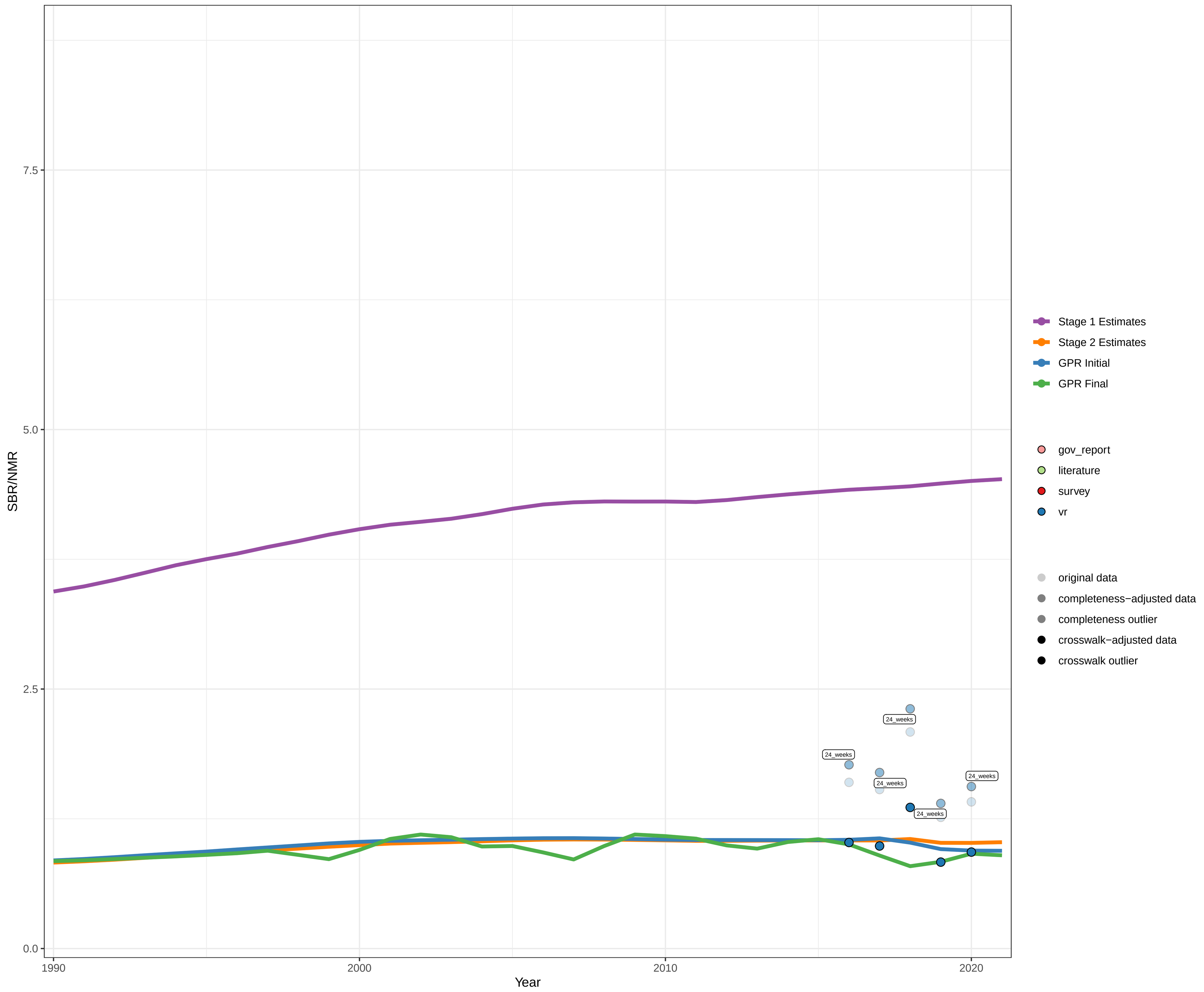

Definition	Years	Outlier	Source.Type	Title
24_weeks	2016−2020	0	VR	United Kingdom − England and Wales Child Mortality Statistics

Lincolnshire GBR_44695 Western Europe Stillbirth Rate (SBR) − 20 weeks
lambda = 0.6 ; zeta = 0.8 ; scale = 15 ; amp = 0.028

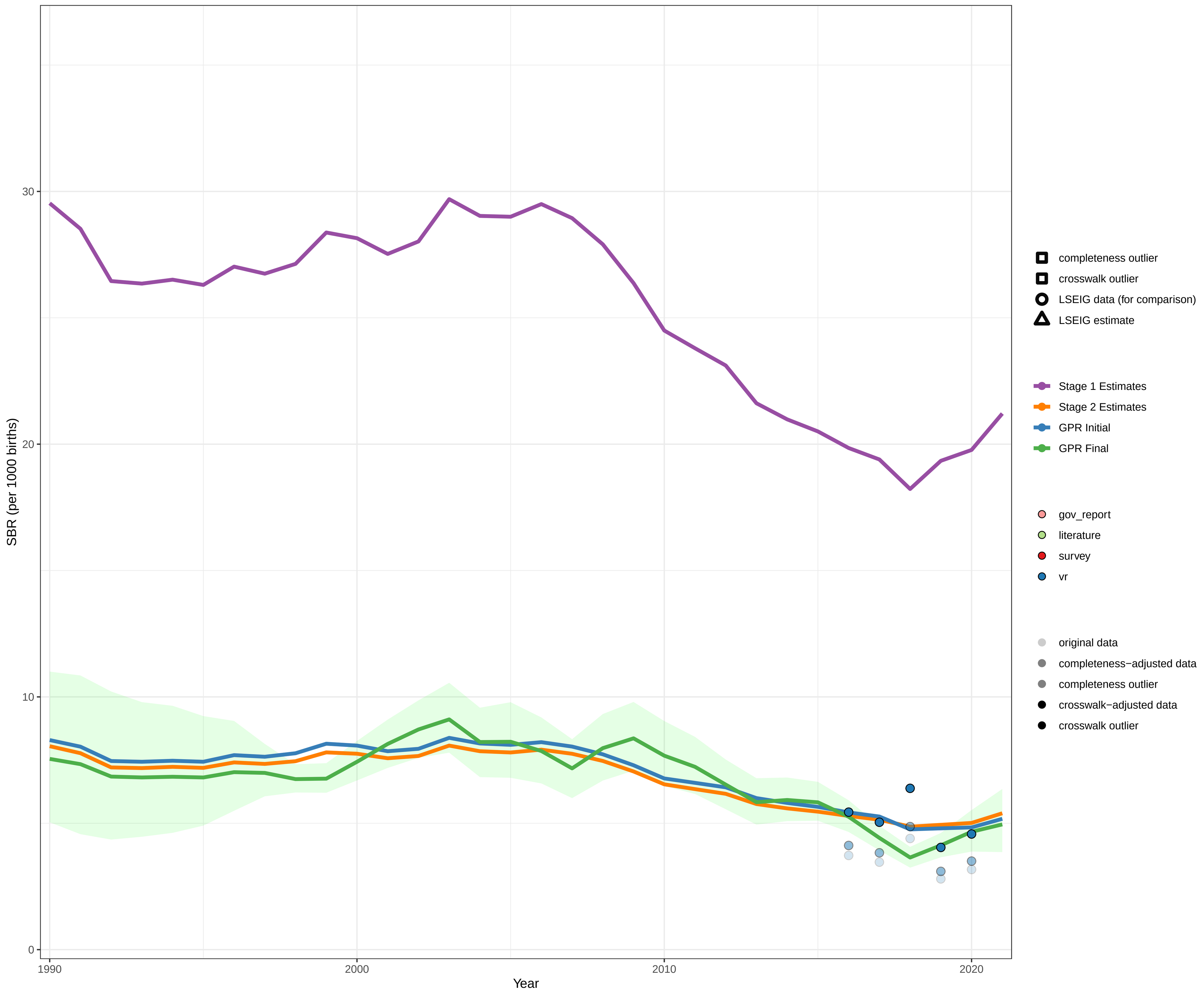

Lincolnshire GBR_44695 Western Europe Stillbirth Rate (SBR) − 28 weeks
lambda = 0.6 ; zeta = 0.8 ; scale = 15 ; amp = 0.028

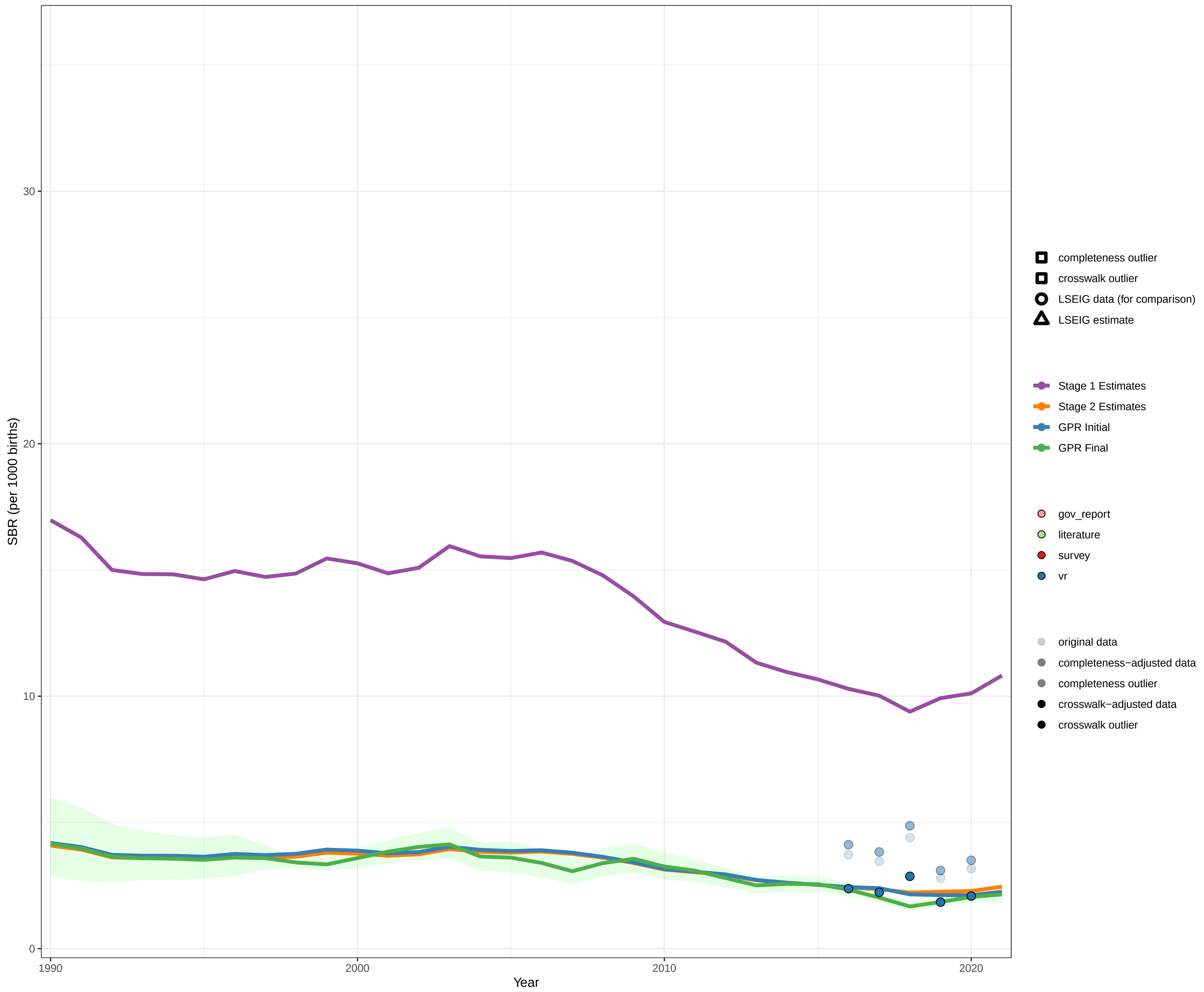

Year	Total Stillbirths (in thousands)	Total Stillbirths (in thousands)	SBR (per 1000 births)	SBR (per 1000 births)	SBR/NMR	SBR/NMR
	20 weeks	28 weeks	20 weeks	28 weeks	20 weeks	28 weeks
1990	0.1 (0.0 to 0.1)	0.0 (0.0 to 0.0)	7.5 (5.0 to 11.0)	4.2 (2.9 to 6.0)	1.53 (1.02 to 2.23)	0.84 (0.59 to 1.21)
1995	0.0 (0.0 to 0.1)	0.0 (0.0 to 0.0)	6.8 (4.9 to 9.2)	3.5 (2.8 to 4.4)	1.75 (1.26 to 2.37)	0.90 (0.71 to 1.13)
2000	0.0 (0.0 to 0.1)	0.0 (0.0 to 0.0)	7.4 (6.7 to 8.3)	3.6 (3.2 to 4.0)	1.97 (1.77 to 2.19)	0.95 (0.84 to 1.06)
2005	0.1 (0.0 to 0.1)	0.0 (0.0 to 0.0)	8.2 (6.8 to 9.8)	3.6 (3.0 to 4.2)	2.25 (1.86 to 2.68)	0.99 (0.83 to 1.16)
2010	0.1 (0.1 to 0.1)	0.0 (0.0 to 0.0)	7.7 (6.5 to 9.0)	3.3 (2.7 to 3.8)	2.55 (2.15 to 3.01)	1.08 (0.91 to 1.27)
2015	0.0 (0.0 to 0.1)	0.0 (0.0 to 0.0)	5.8 (5.1 to 6.6)	2.6 (2.3 to 2.9)	2.40 (2.10 to 2.74)	1.05 (0.93 to 1.18)
2020	0.0 (0.0 to 0.0)	0.0 (0.0 to 0.0)	4.7 (3.9 to 5.5)	2.1 (1.8 to 2.3)	2.08 (1.73 to 2.46)	0.91 (0.81 to 1.02)
2021	0.0 (0.0 to 0.0)	0.0 (0.0 to 0.0)	5.0 (3.9 to 6.4)	2.1 (1.8 to 2.6)	2.07 (1.61 to 2.66)	0.90 (0.74 to 1.09)

Northamptonshire GBR_44693 Western Europe Stillbirth Rate / Neonatal Mortality Rate (SBR/NMR) − 20 weeks
lambda = 0.8 ; zeta = 0.7 ; scale = 15 ; amp = 0.028

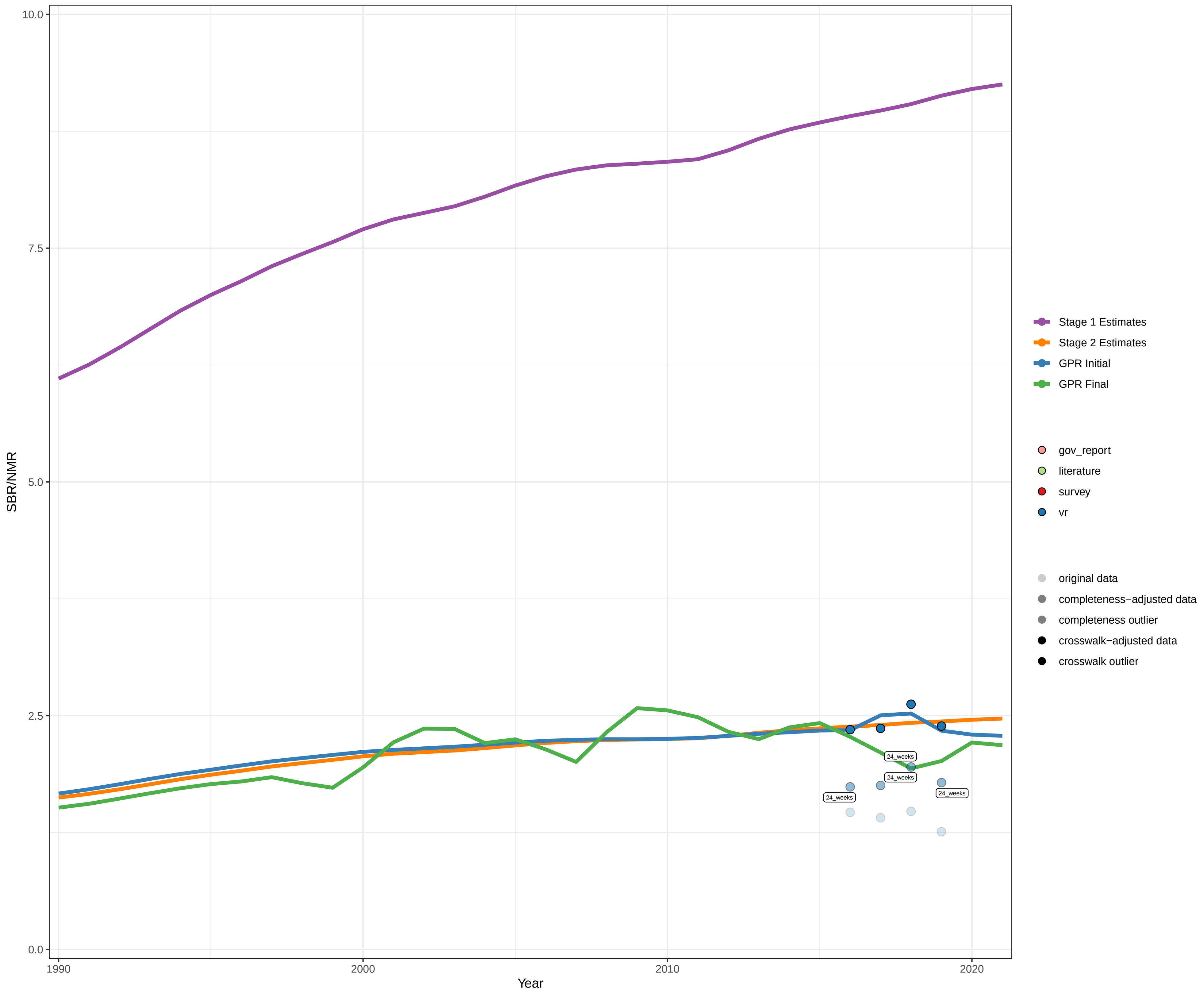

Northamptonshire GBR_44693 Western Europe Stillbirth Rate / Neonatal Mortality Rate (SBR/NMR) − 28 weeks
lambda = 0.8 ; zeta = 0.7 ; scale = 15 ; amp = 0.028

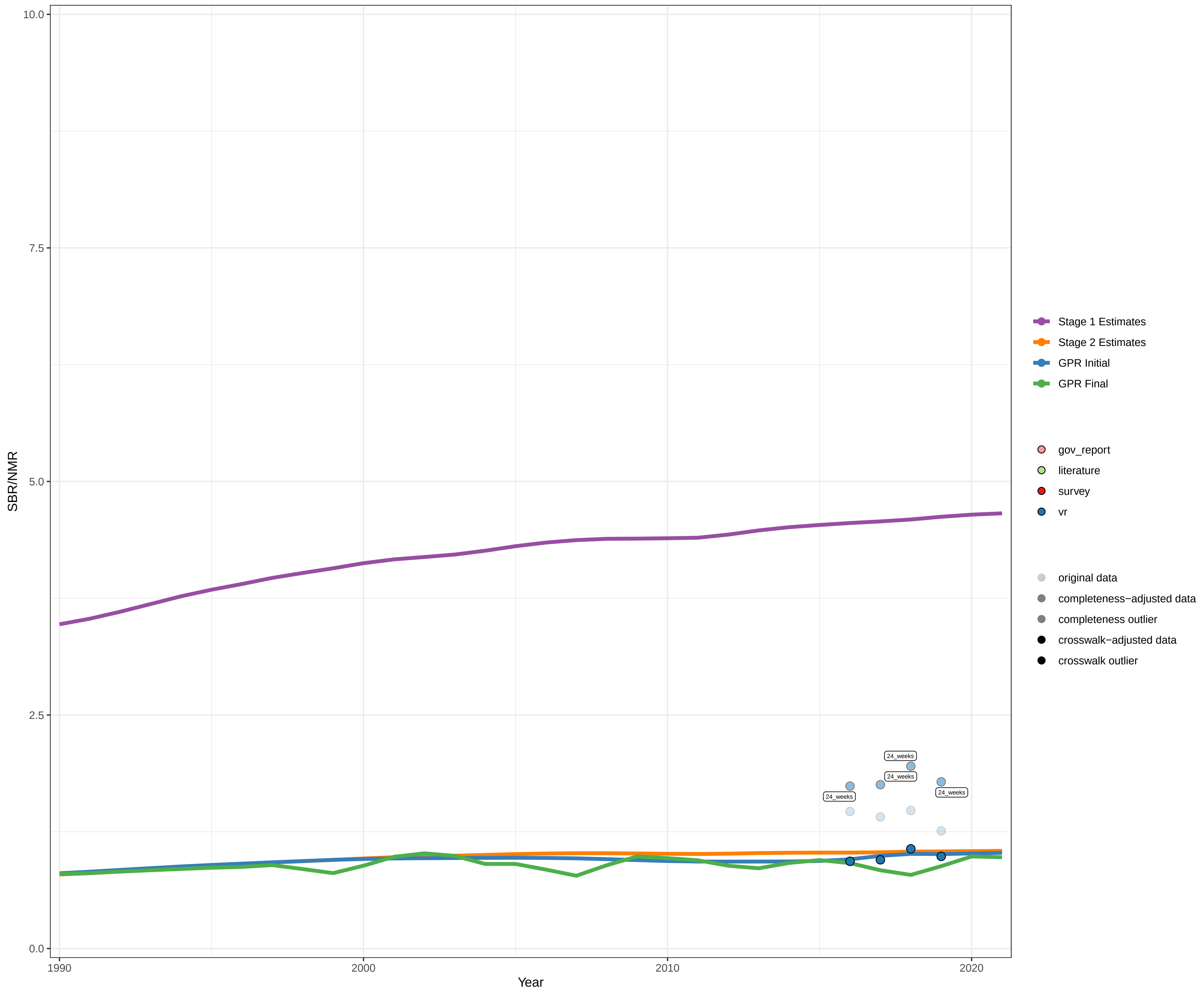

Definition	Years	Outlier	Source.Type	Title
24_weeks	2016−2019	0	VR	United Kingdom − England and Wales Child Mortality Statistics

Northamptonshire GBR_44693 Western Europe Stillbirth Rate (SBR) − 20 weeks
lambda = 0.8 ; zeta = 0.7 ; scale = 15 ; amp = 0.028

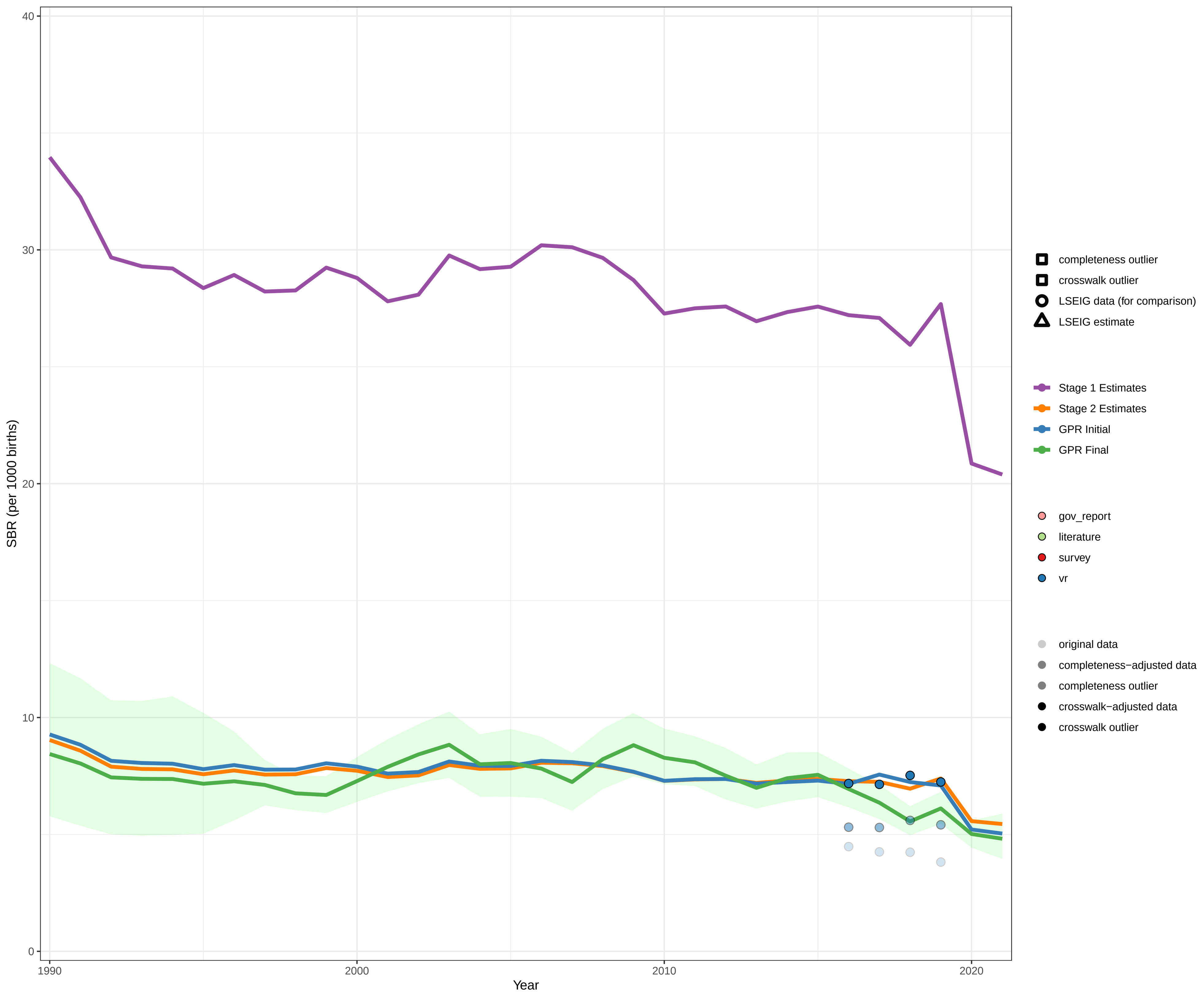

Northamptonshire GBR_44693 Western Europe Stillbirth Rate (SBR) − 28 weeks
lambda = 0.8 ; zeta = 0.7 ; scale = 15 ; amp = 0.028

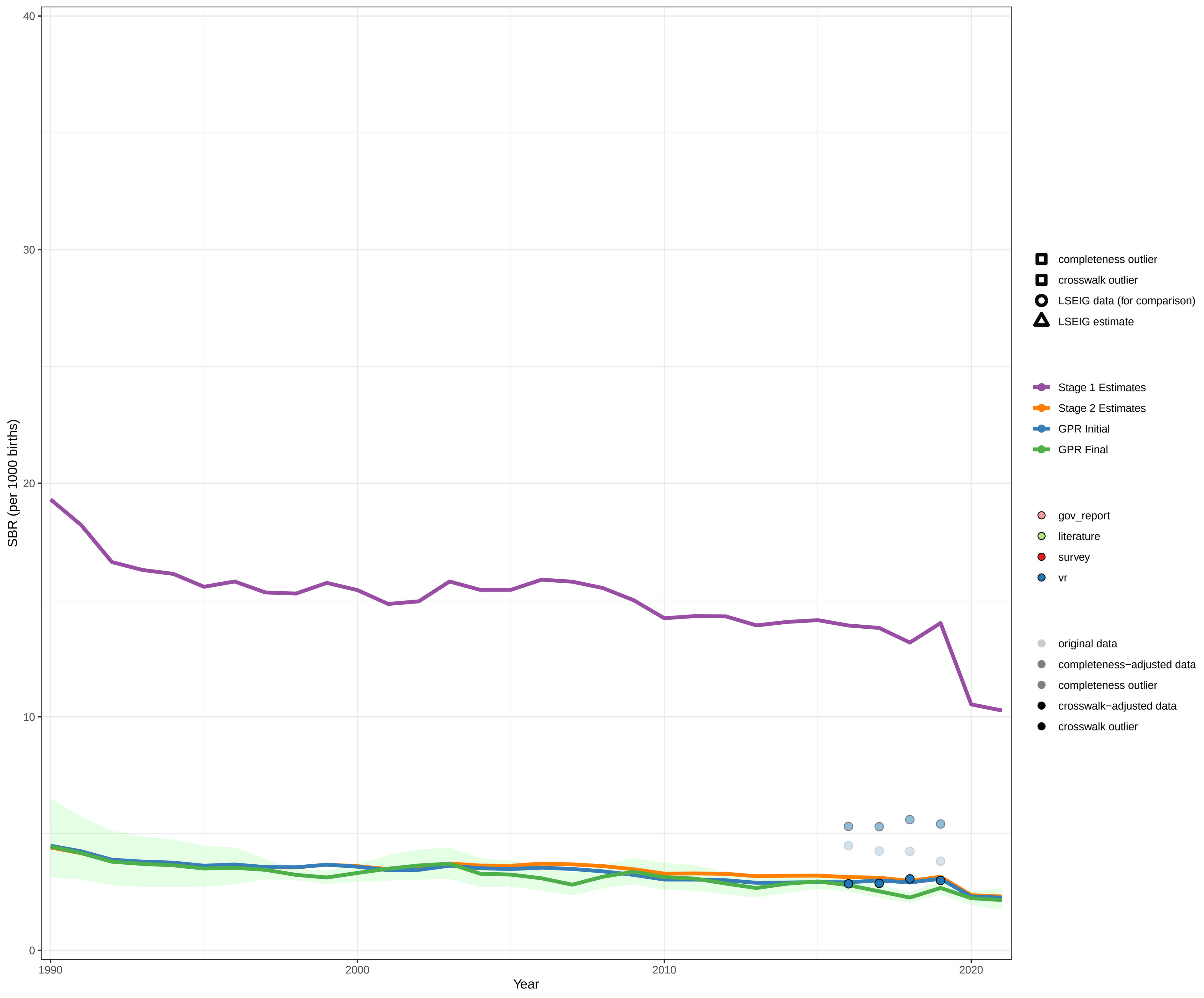

Year	Total Stillbirths (in thousands)	Total Stillbirths (in thousands)	SBR (per 1000 births)	SBR (per 1000 births)	SBR/NMR	SBR/NMR
	20 weeks	28 weeks	20 weeks	28 weeks	20 weeks	28 weeks
1990	0.1 (0.0 to 0.1)	0.0 (0.0 to 0.1)	8.4 (5.8 to 12.3)	4.4 (3.1 to 6.5)	1.52 (1.04 to 2.22)	0.80 (0.56 to 1.17)
1995	0.1 (0.0 to 0.1)	0.0 (0.0 to 0.0)	7.2 (5.0 to 10.2)	3.5 (2.7 to 4.5)	1.77 (1.24 to 2.52)	0.86 (0.67 to 1.10)
2000	0.1 (0.0 to 0.1)	0.0 (0.0 to 0.0)	7.3 (6.4 to 8.3)	3.3 (2.9 to 3.7)	1.95 (1.71 to 2.22)	0.89 (0.78 to 0.99)
2005	0.1 (0.1 to 0.1)	0.0 (0.0 to 0.0)	8.1 (6.6 to 9.5)	3.2 (2.7 to 3.8)	2.25 (1.85 to 2.65)	0.91 (0.76 to 1.07)
2010	0.1 (0.1 to 0.1)	0.0 (0.0 to 0.0)	8.3 (7.1 to 9.5)	3.1 (2.6 to 3.7)	2.56 (2.21 to 2.94)	0.97 (0.80 to 1.16)
2015	0.1 (0.1 to 0.1)	0.0 (0.0 to 0.0)	7.5 (6.6 to 8.5)	3.0 (2.6 to 3.3)	2.42 (2.11 to 2.73)	0.95 (0.85 to 1.05)
2020	0.0 (0.0 to 0.0)	0.0 (0.0 to 0.0)	5.0 (4.4 to 5.6)	2.2 (2.0 to 2.5)	2.21 (1.96 to 2.47)	0.99 (0.86 to 1.11)
2021	0.0 (0.0 to 0.0)	0.0 (0.0 to 0.0)	4.8 (4.0 to 5.9)	2.2 (1.7 to 2.7)	2.18 (1.79 to 2.67)	0.98 (0.79 to 1.21)

Nottingham GBR_44700 Western Europe Stillbirth Rate / Neonatal Mortality Rate (SBR/NMR) − 20 weeks
lambda = 0.8 ; zeta = 0.7 ; scale = 15 ; amp = 0.028

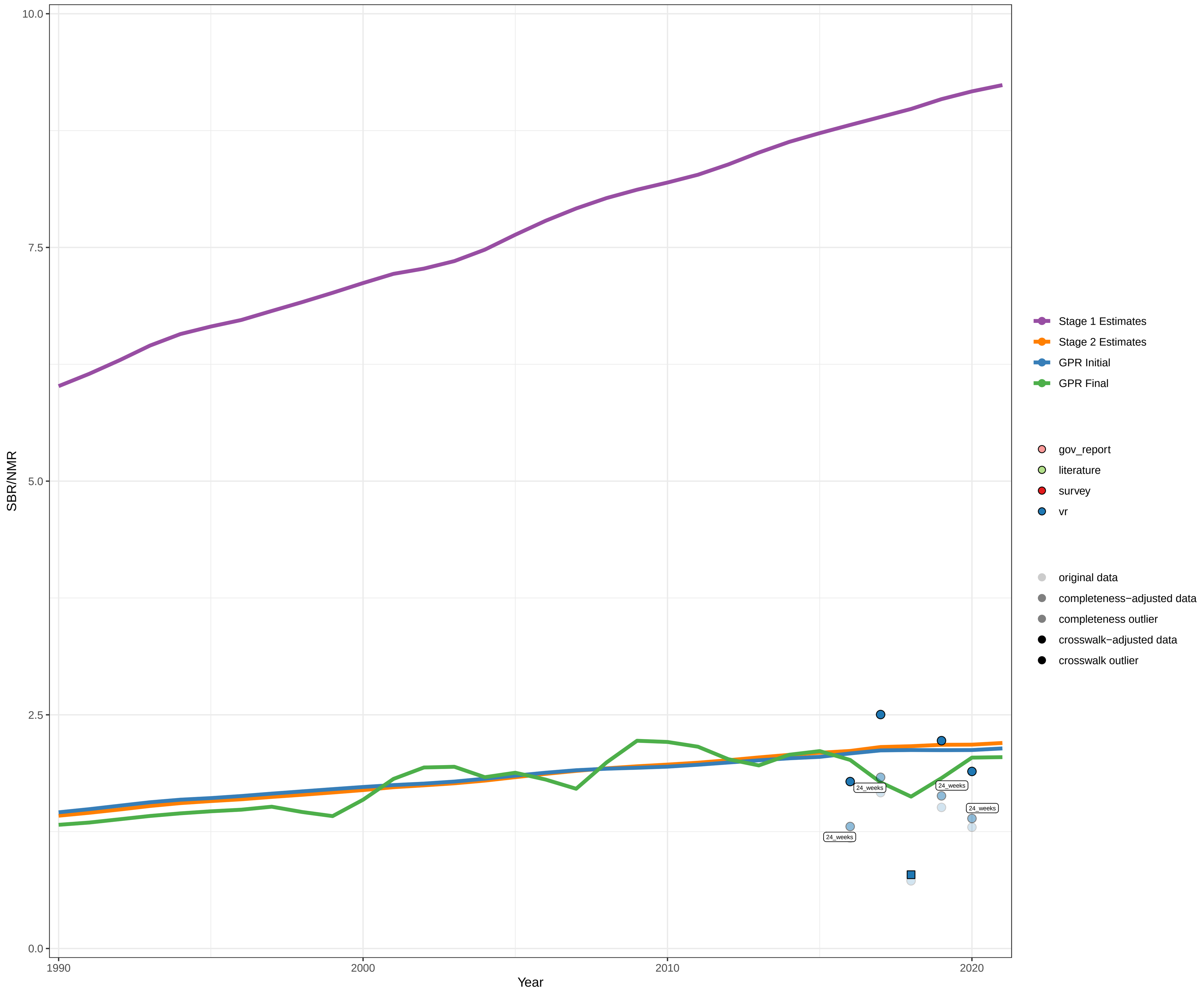

Nottingham GBR_44700 Western Europe Stillbirth Rate / Neonatal Mortality Rate (SBR/NMR) − 28 weeks
lambda = 0.8 ; zeta = 0.7 ; scale = 15 ; amp = 0.028

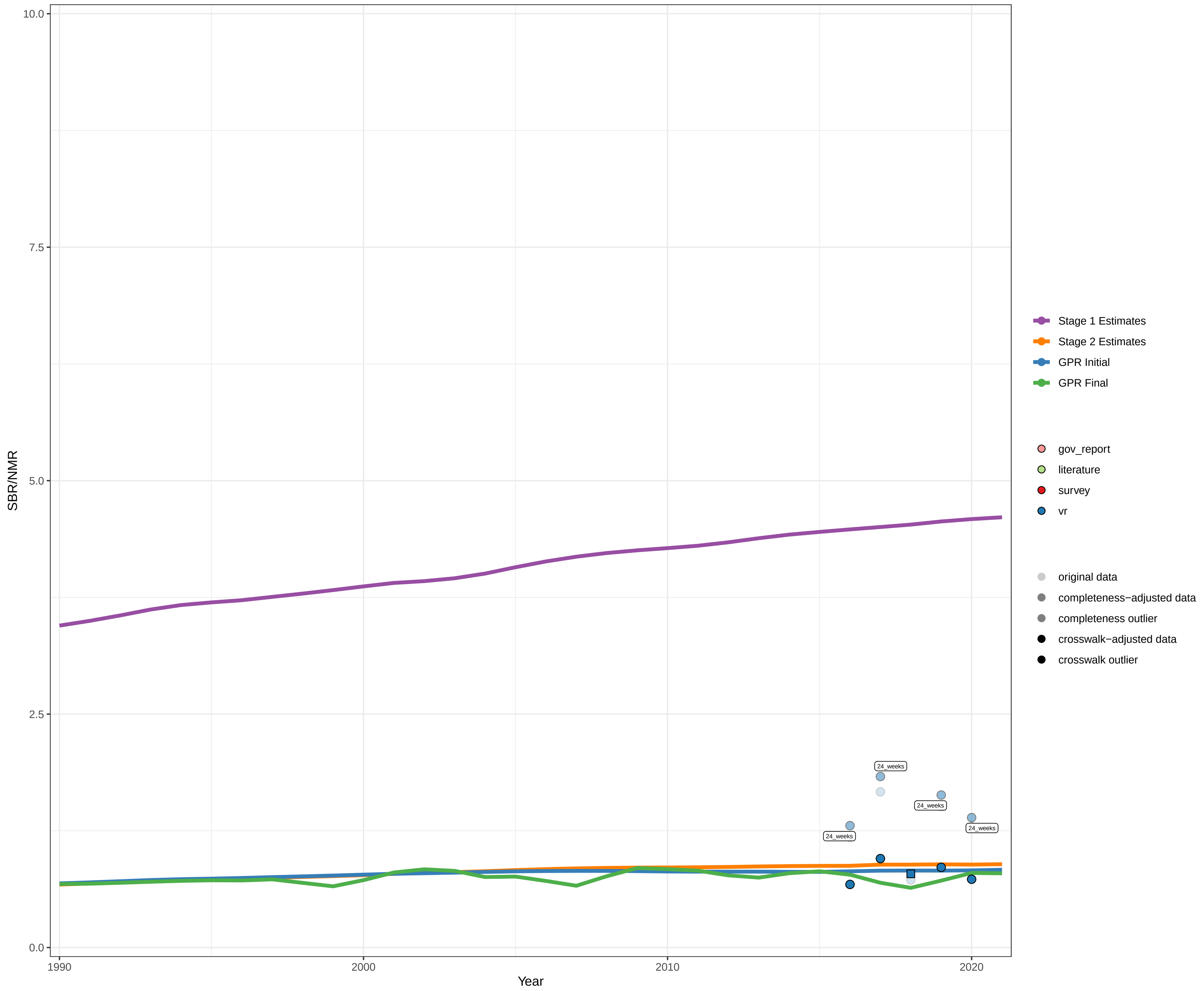

Definition	Years	Outlier	Source.Type	Title
24_weeks	2016−2020	0	VR	United Kingdom − England and Wales Child Mortality Statistics

Nottingham GBR_44700 Western Europe Stillbirth Rate (SBR) − 20 weeks
lambda = 0.8 ; zeta = 0.7 ; scale = 15 ; amp = 0.028

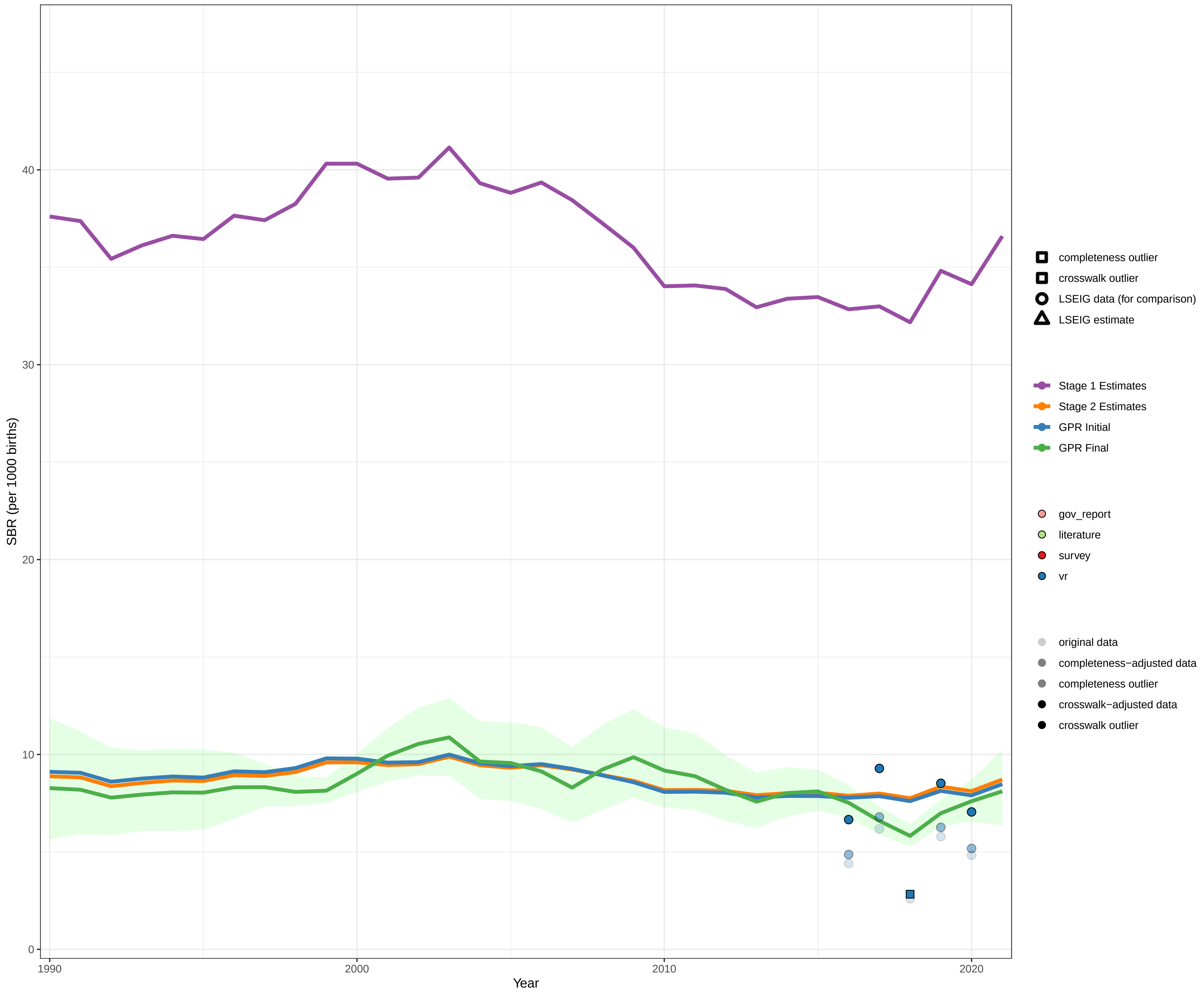

Nottingham GBR_44700 Western Europe Stillbirth Rate (SBR) − 28 weeks
lambda = 0.8 ; zeta = 0.7 ; scale = 15 ; amp = 0.028

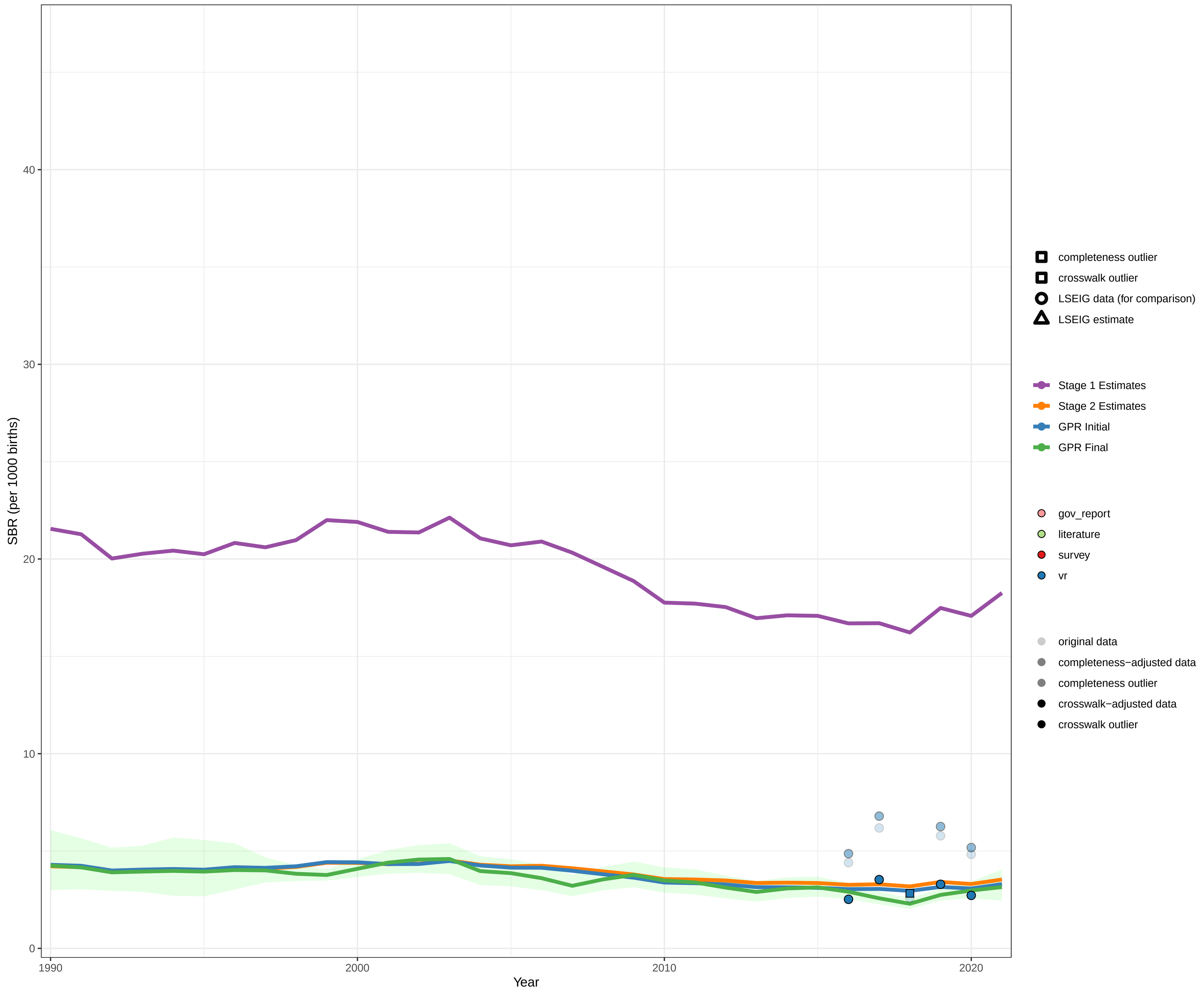

Year	Total Stillbirths (in thousands)	Total Stillbirths (in thousands)	SBR (per 1000 births)	SBR (per 1000 births)	SBR/NMR	SBR/NMR
	20 weeks	28 weeks	20 weeks	28 weeks	20 weeks	28 weeks
1990	0.0 (0.0 to 0.1)	0.0 (0.0 to 0.0)	8.3 (5.6 to 11.9)	4.3 (3.0 to 6.1)	1.32 (0.90 to 1.90)	0.68 (0.48 to 0.97)
1995	0.0 (0.0 to 0.0)	0.0 (0.0 to 0.0)	8.0 (6.1 to 10.2)	3.9 (2.7 to 5.6)	1.47 (1.12 to 1.87)	0.72 (0.49 to 1.02)
2000	0.0 (0.0 to 0.0)	0.0 (0.0 to 0.0)	9.0 (8.1 to 10.0)	4.1 (3.7 to 4.6)	1.59 (1.43 to 1.77)	0.72 (0.65 to 0.81)
2005	0.0 (0.0 to 0.0)	0.0 (0.0 to 0.0)	9.6 (7.6 to 11.7)	3.9 (3.2 to 4.6)	1.88 (1.50 to 2.29)	0.76 (0.63 to 0.90)
2010	0.0 (0.0 to 0.0)	0.0 (0.0 to 0.0)	9.2 (7.3 to 11.4)	3.5 (2.9 to 4.2)	2.21 (1.75 to 2.74)	0.84 (0.69 to 1.00)
2015	0.0 (0.0 to 0.0)	0.0 (0.0 to 0.0)	8.1 (7.1 to 9.2)	3.1 (2.7 to 3.7)	2.11 (1.85 to 2.40)	0.82 (0.69 to 0.96)
2020	0.0 (0.0 to 0.0)	0.0 (0.0 to 0.0)	7.6 (6.6 to 8.7)	3.0 (2.6 to 3.4)	2.04 (1.76 to 2.34)	0.80 (0.69 to 0.93)
2021	0.0 (0.0 to 0.0)	0.0 (0.0 to 0.0)	8.1 (6.4 to 10.2)	3.1 (2.4 to 4.0)	2.05 (1.60 to 2.59)	0.79 (0.62 to 1.02)

Nottinghamshire GBR_44699 Western Europe Stillbirth Rate / Neonatal Mortality Rate (SBR/NMR) − 20 weeks
lambda = 0.6 ; zeta = 0.8 ; scale = 15 ; amp = 0.028

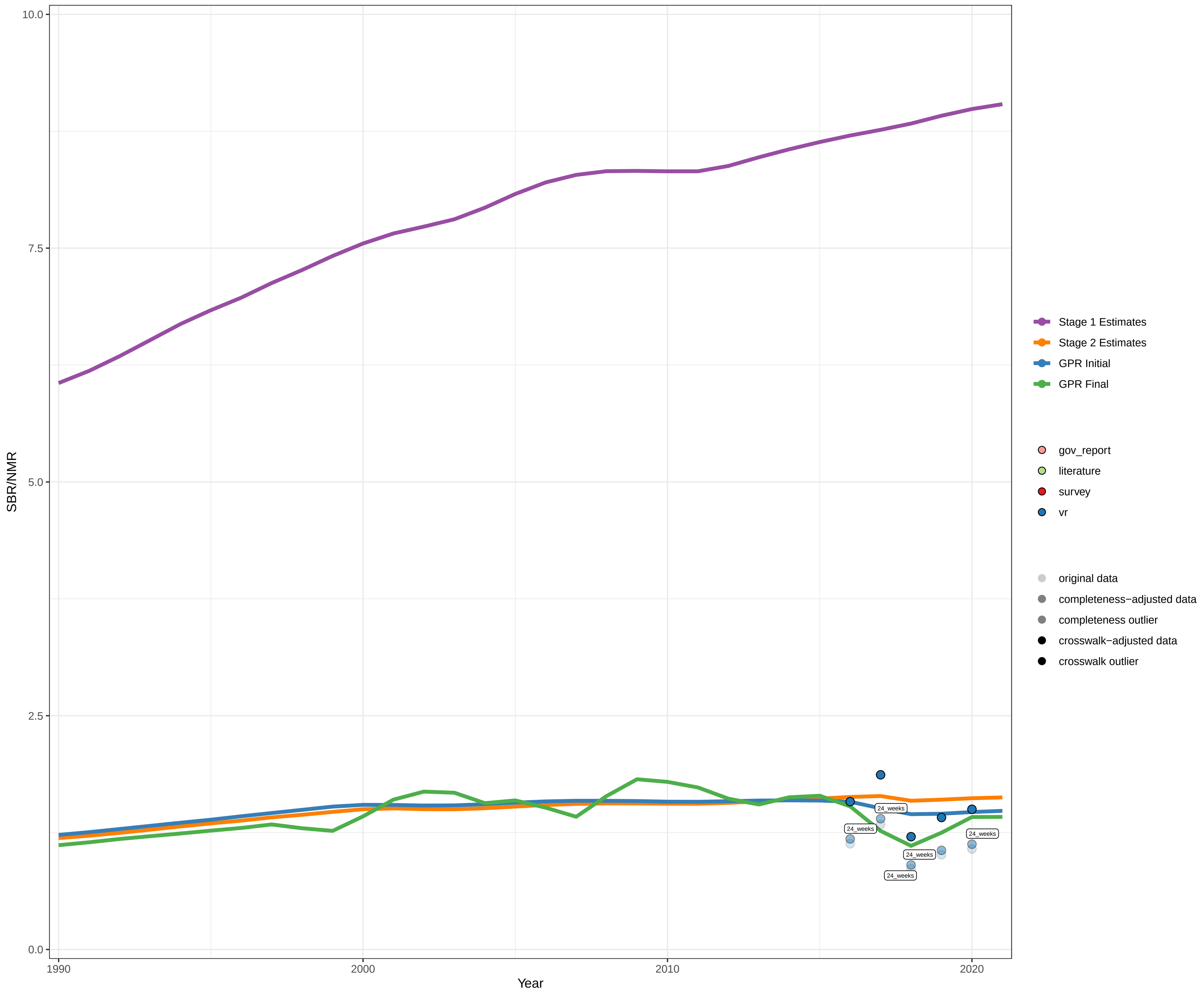

Nottinghamshire GBR_44699 Western Europe Stillbirth Rate / Neonatal Mortality Rate (SBR/NMR) − 28 weeks
lambda = 0.6 ; zeta = 0.8 ; scale = 15 ; amp = 0.028

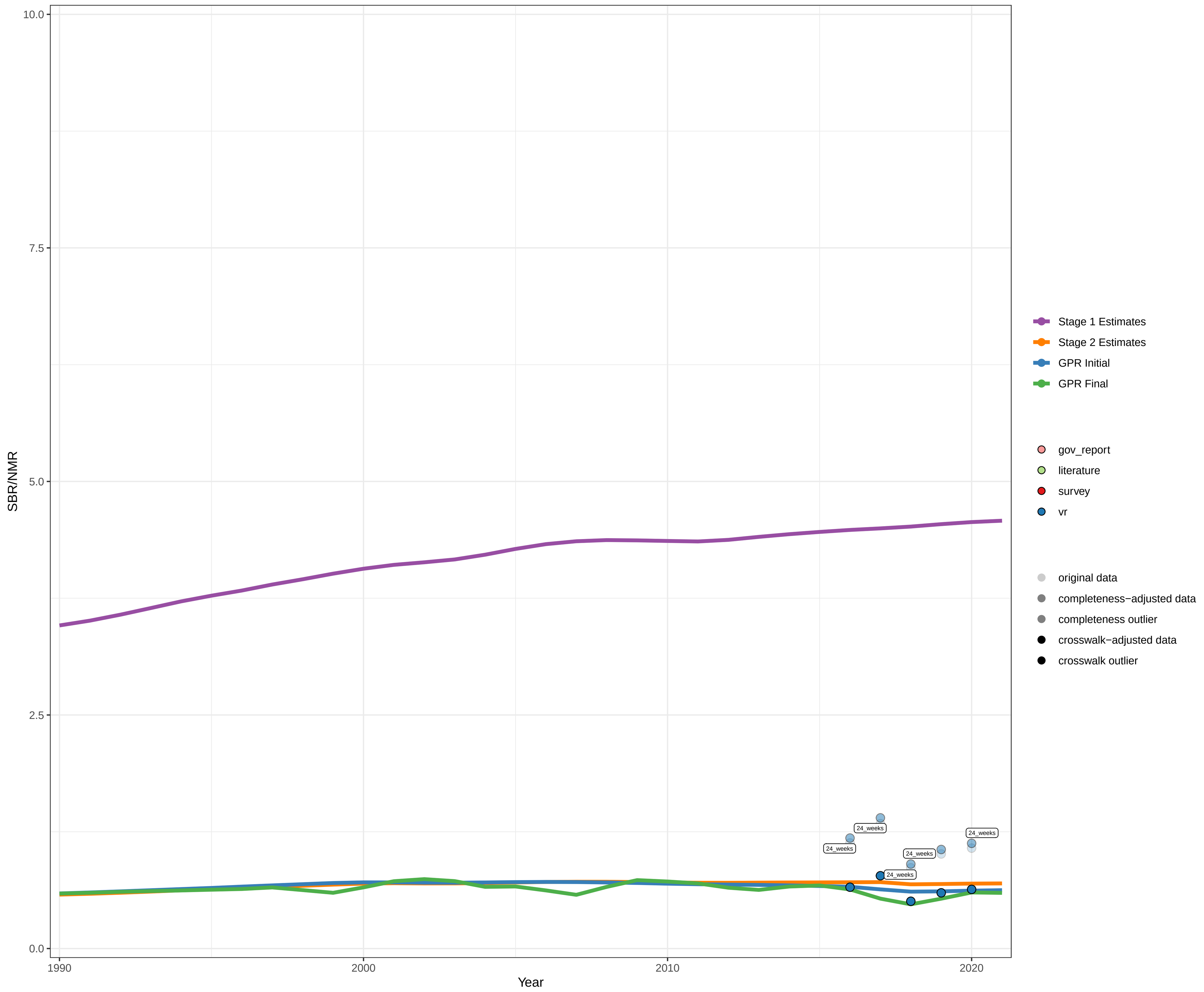

Definition	Years	Outlier	Source.Type	Title
24_weeks	2016−2020	0	VR	United Kingdom − England and Wales Child Mortality Statistics

Nottinghamshire GBR_44699 Western Europe Stillbirth Rate (SBR) − 20 weeks
lambda = 0.6 ; zeta = 0.8 ; scale = 15 ; amp = 0.028

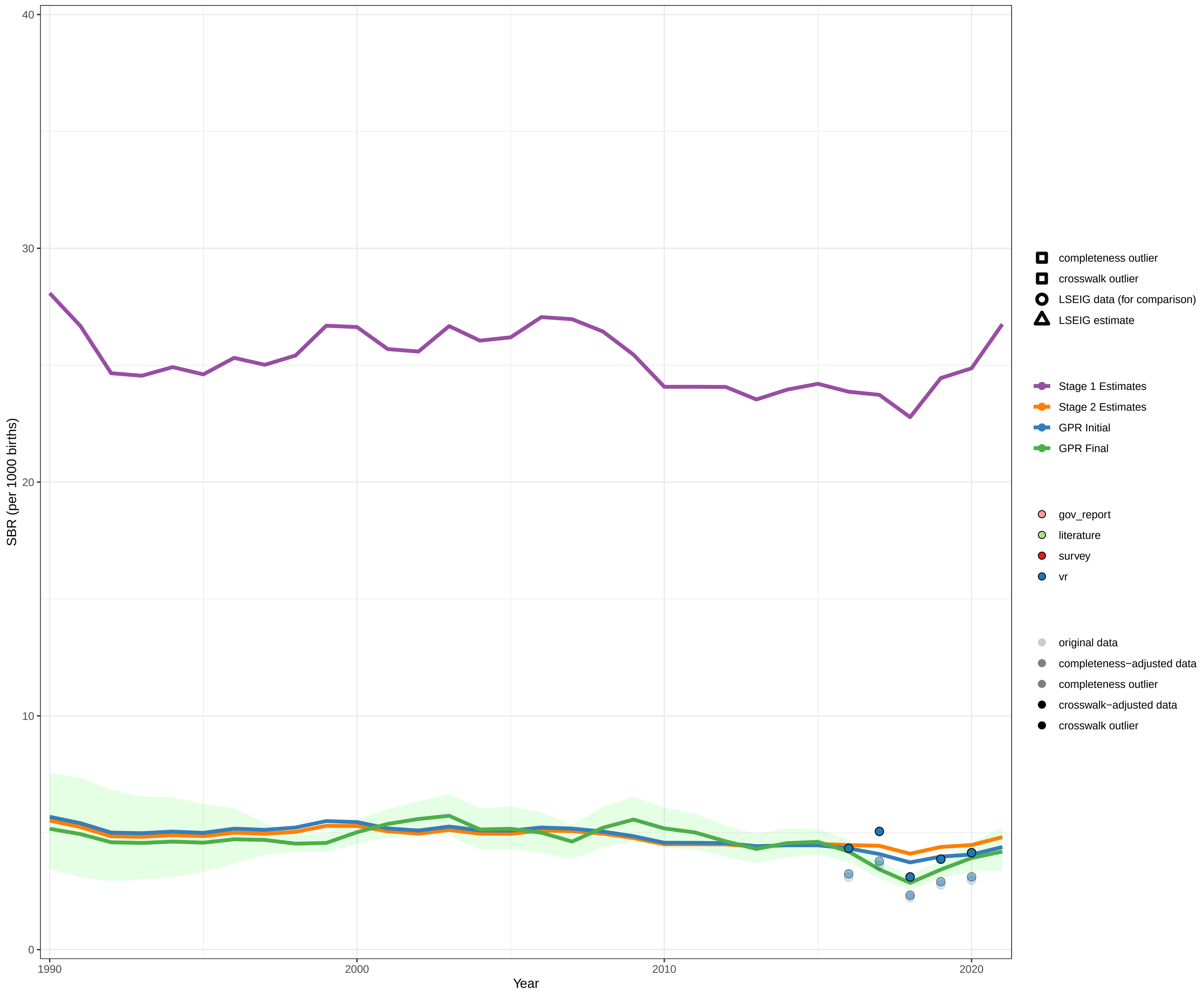

Nottinghamshire GBR_44699 Western Europe Stillbirth Rate (SBR) − 28 weeks
lambda = 0.6 ; zeta = 0.8 ; scale = 15 ; amp = 0.028

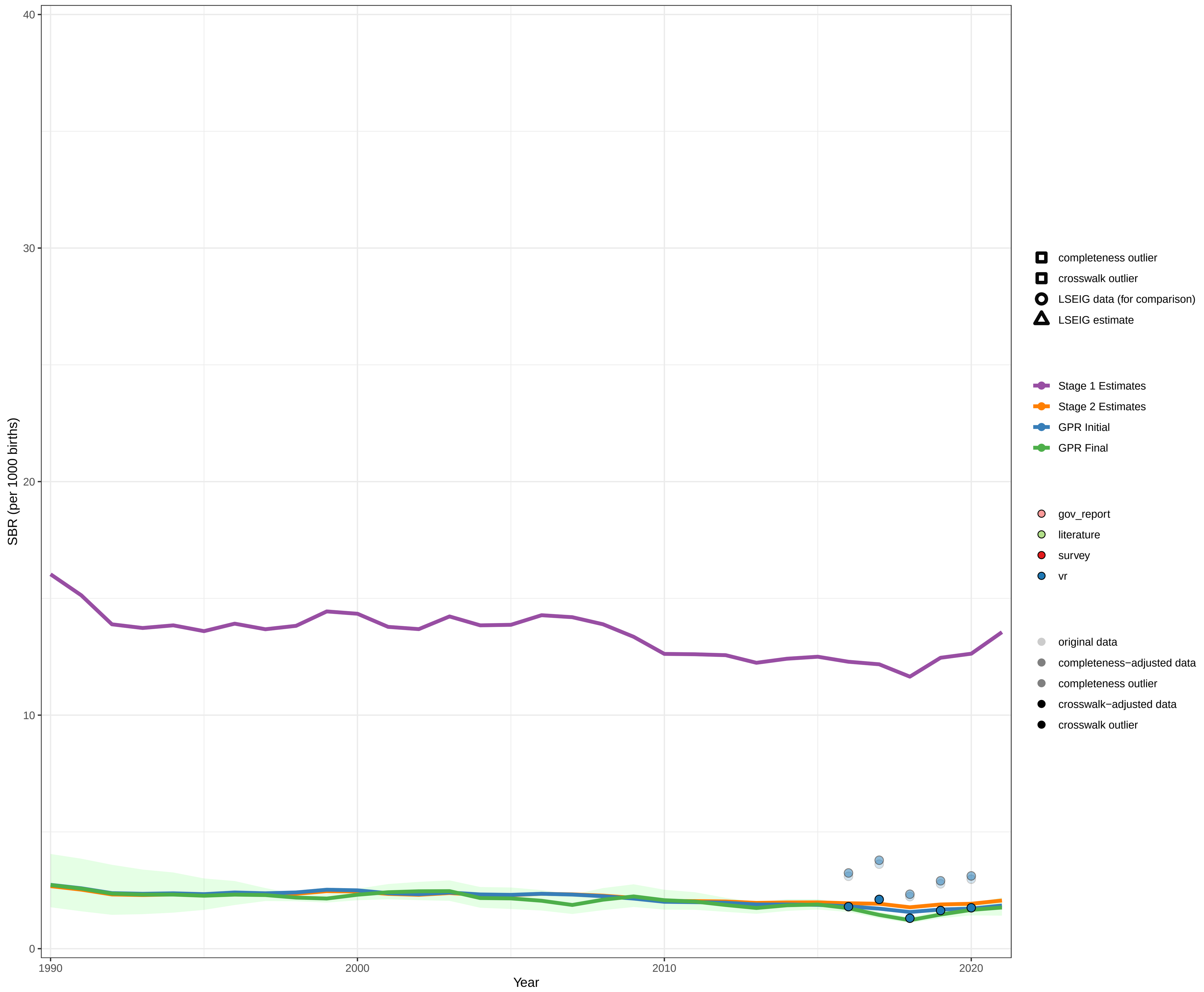

Year	Total Stillbirths (in thousands)	Total Stillbirths (in thousands)	SBR (per 1000 births)	SBR (per 1000 births)	SBR/NMR	SBR/NMR
	20 weeks	28 weeks	20 weeks	28 weeks	20 weeks	28 weeks
1990	0.0 (0.0 to 0.1)	0.0 (0.0 to 0.0)	5.2 (3.4 to 7.6)	2.7 (1.8 to 4.1)	1.12 (0.74 to 1.63)	0.59 (0.38 to 0.87)
1995	0.0 (0.0 to 0.1)	0.0 (0.0 to 0.0)	4.6 (3.3 to 6.2)	2.3 (1.7 to 3.0)	1.27 (0.92 to 1.73)	0.63 (0.46 to 0.84)
2000	0.0 (0.0 to 0.0)	0.0 (0.0 to 0.0)	5.0 (4.5 to 5.6)	2.3 (2.1 to 2.6)	1.42 (1.29 to 1.58)	0.65 (0.59 to 0.73)
2005	0.0 (0.0 to 0.1)	0.0 (0.0 to 0.0)	5.2 (4.3 to 6.1)	2.2 (1.7 to 2.6)	1.59 (1.32 to 1.89)	0.66 (0.52 to 0.81)
2010	0.0 (0.0 to 0.1)	0.0 (0.0 to 0.0)	5.2 (4.4 to 6.1)	2.1 (1.7 to 2.5)	1.79 (1.51 to 2.10)	0.72 (0.58 to 0.87)
2015	0.0 (0.0 to 0.0)	0.0 (0.0 to 0.0)	4.6 (4.1 to 5.2)	1.9 (1.7 to 2.1)	1.64 (1.45 to 1.84)	0.68 (0.60 to 0.76)
2020	0.0 (0.0 to 0.0)	0.0 (0.0 to 0.0)	3.9 (3.3 to 4.5)	1.7 (1.4 to 1.9)	1.42 (1.21 to 1.64)	0.60 (0.52 to 0.70)
2021	0.0 (0.0 to 0.0)	0.0 (0.0 to 0.0)	4.2 (3.4 to 5.2)	1.8 (1.4 to 2.2)	1.42 (1.14 to 1.77)	0.60 (0.48 to 0.74)

Rutland GBR_44696 Western Europe Stillbirth Rate / Neonatal Mortality Rate (SBR/NMR) − 20 weeks
lambda = 0.6 ; zeta = 0.8 ; scale = 15 ; amp = 0.028

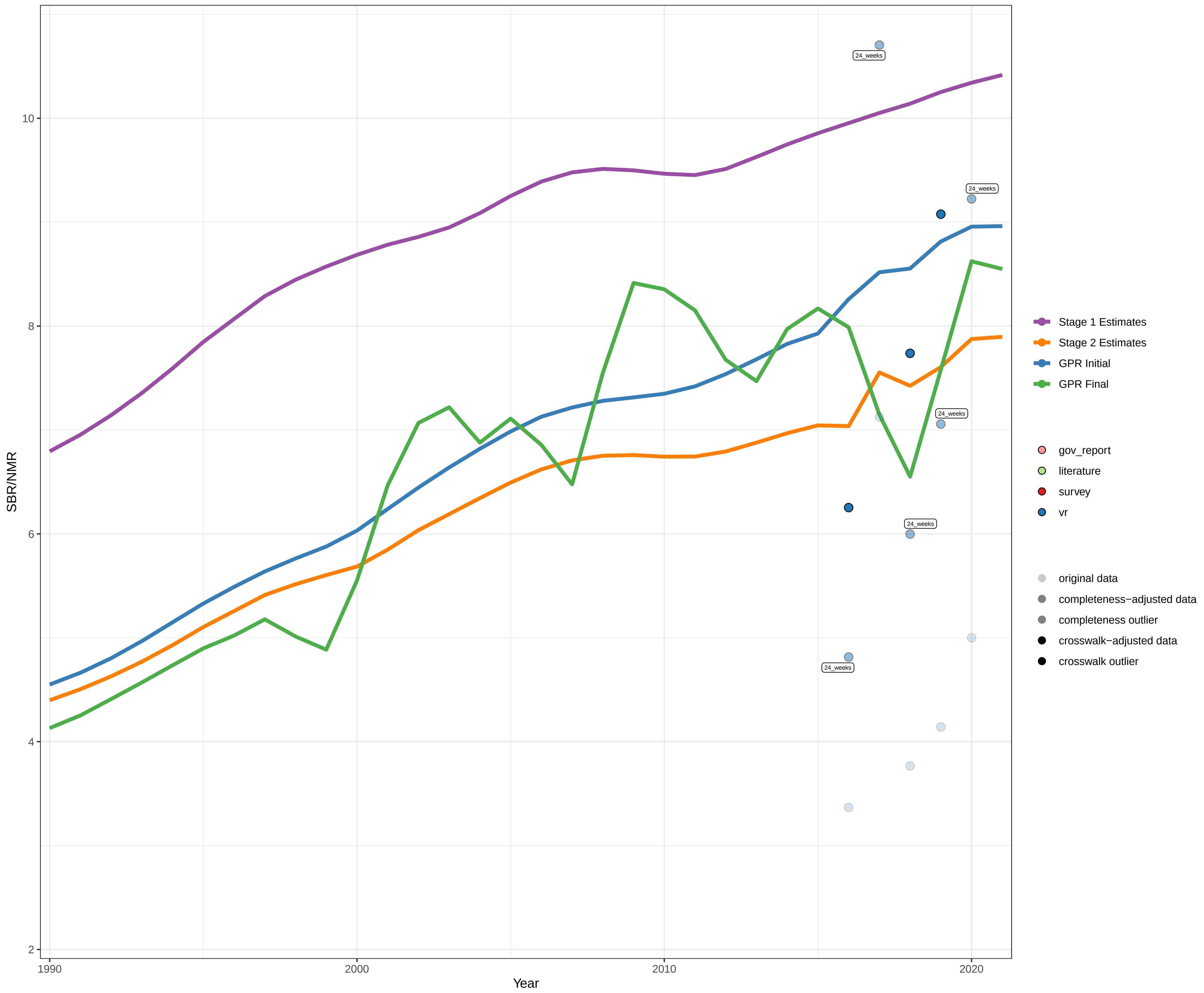

Rutland GBR_44696 Western Europe Stillbirth Rate / Neonatal Mortality Rate (SBR/NMR) − 28 weeks
lambda = 0.6 ; zeta = 0.8 ; scale = 15 ; amp = 0.028

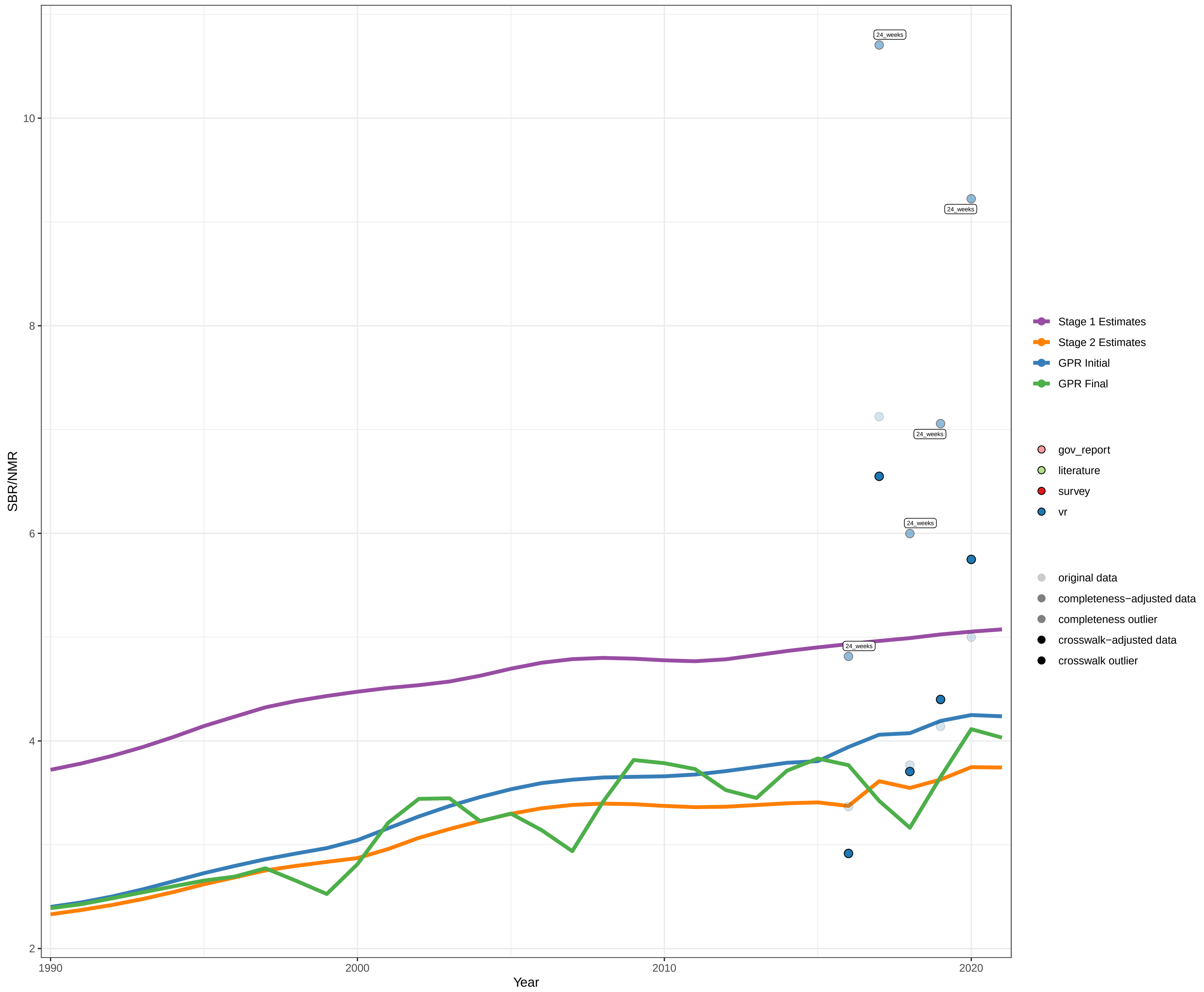

Definition	Years	Outlier	Source.Type	Title
24_weeks	2016−2020	0	VR	United Kingdom − England and Wales Child Mortality Statistics

Rutland GBR_44696 Western Europe Stillbirth Rate (SBR) − 20 weeks
lambda = 0.6 ; zeta = 0.8 ; scale = 15 ; amp = 0.028

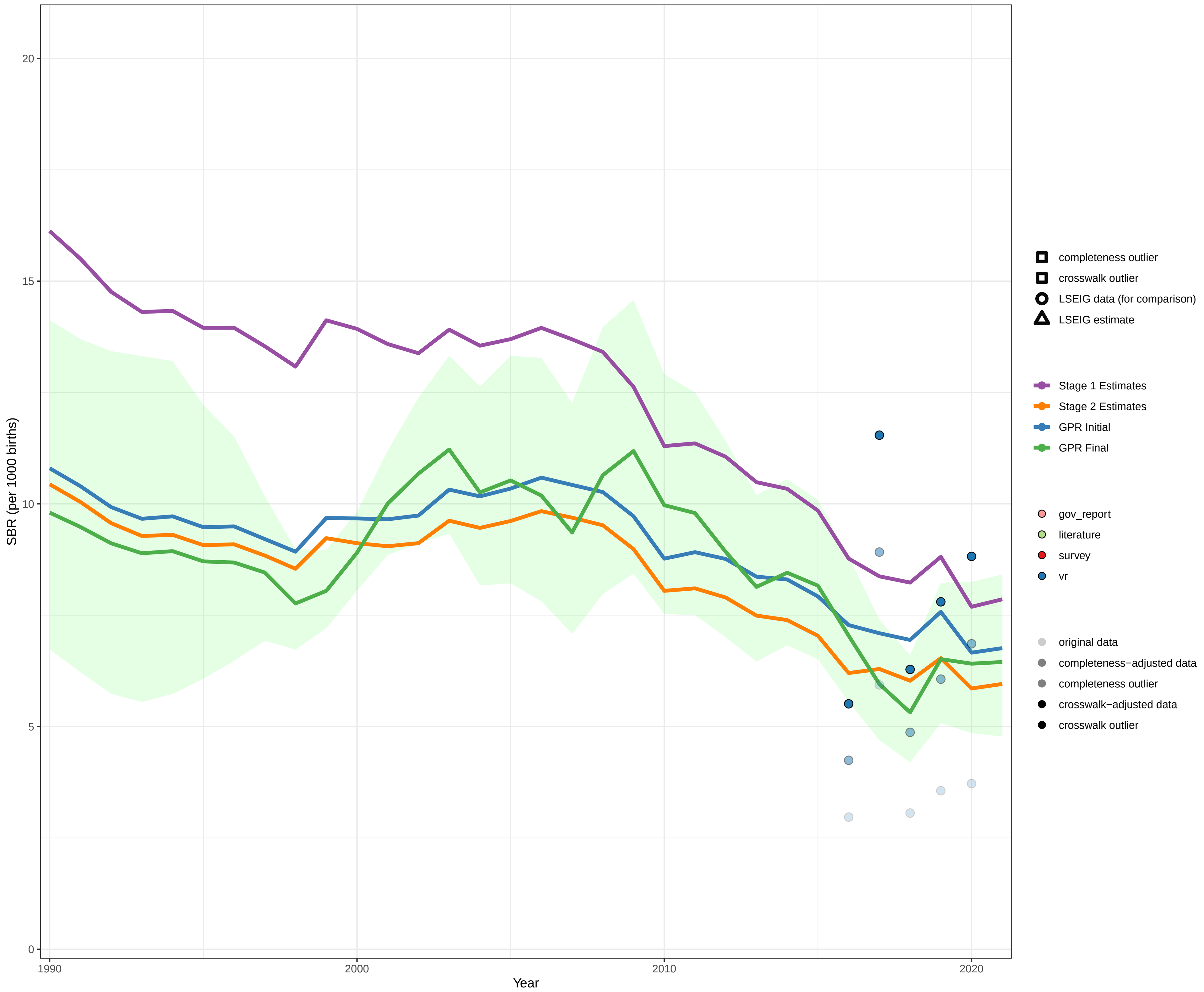

Rutland GBR_44696 Western Europe Stillbirth Rate (SBR) − 28 weeks
lambda = 0.6 ; zeta = 0.8 ; scale = 15 ; amp = 0.028

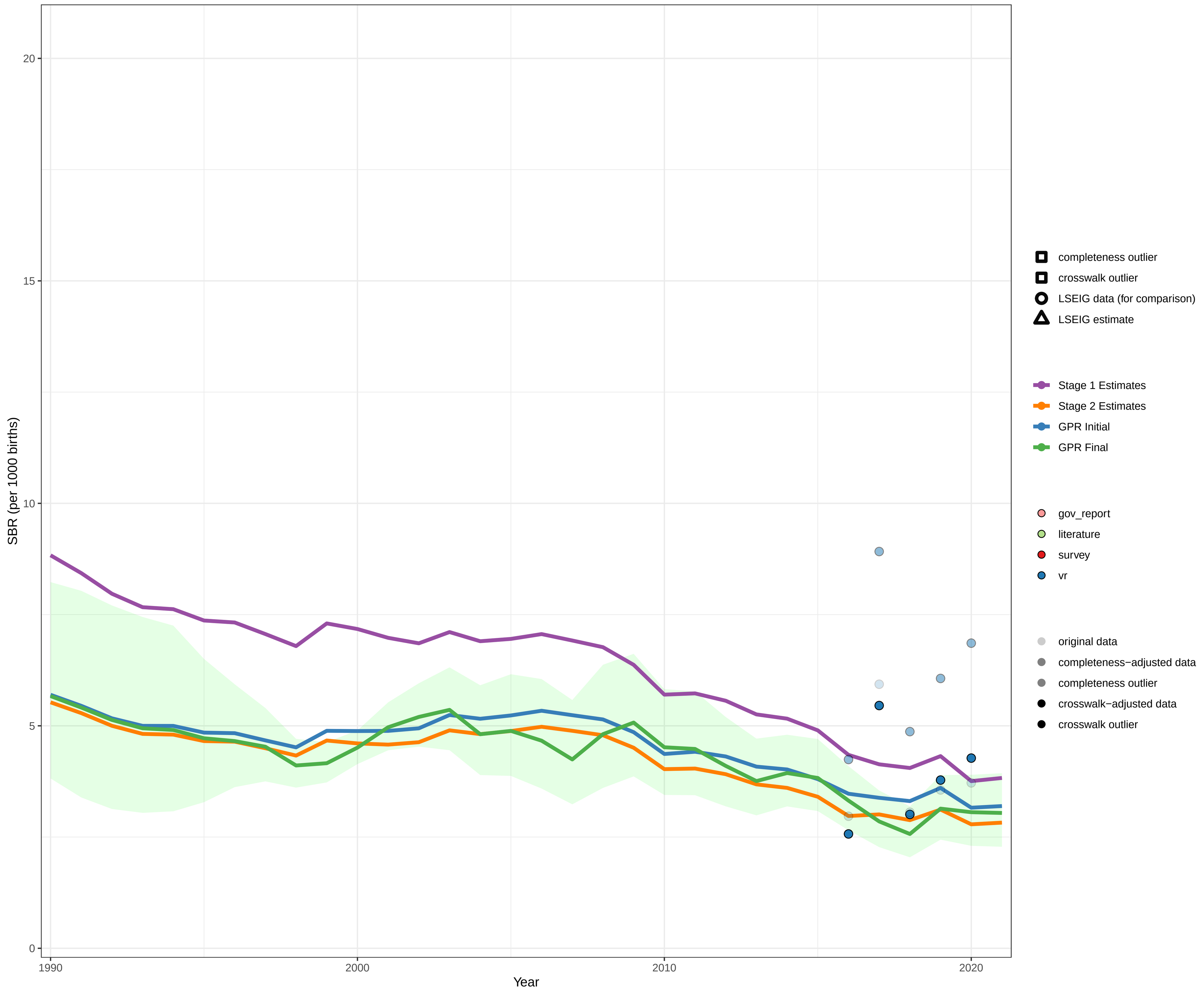

Year	Total Stillbirths (in thousands)	Total Stillbirths (in thousands)	SBR (per 1000 births)	SBR (per 1000 births)	SBR/NMR	SBR/NMR
	20 weeks	28 weeks	20 weeks	28 weeks	20 weeks	28 weeks
1990	0.0 (0.0 to 0.0)	0.0 (0.0 to 0.0)	9.8 (6.7 to 14.1)	5.7 (3.8 to 8.2)	4.13 (2.84 to 5.95)	2.39 (1.61 to 3.47)
1995	0.0 (0.0 to 0.0)	0.0 (0.0 to 0.0)	8.7 (6.1 to 12.2)	4.7 (3.3 to 6.5)	4.90 (3.42 to 6.87)	2.65 (1.85 to 3.66)
2000	0.0 (0.0 to 0.0)	0.0 (0.0 to 0.0)	8.9 (8.1 to 9.8)	4.5 (4.1 to 4.9)	5.55 (5.02 to 6.12)	2.81 (2.58 to 3.05)
2005	0.0 (0.0 to 0.0)	0.0 (0.0 to 0.0)	10.5 (8.2 to 13.3)	4.9 (3.9 to 6.2)	7.11 (5.55 to 9.00)	3.30 (2.62 to 4.16)
2010	0.0 (0.0 to 0.0)	0.0 (0.0 to 0.0)	10.0 (7.5 to 12.9)	4.5 (3.4 to 5.8)	8.35 (6.31 to 10.82)	3.79 (2.89 to 4.88)
2015	0.0 (0.0 to 0.0)	0.0 (0.0 to 0.0)	8.2 (6.5 to 10.1)	3.8 (3.1 to 4.7)	8.17 (6.51 to 10.10)	3.83 (3.09 to 4.71)
2020	0.0 (0.0 to 0.0)	0.0 (0.0 to 0.0)	6.4 (4.9 to 8.3)	3.1 (2.3 to 3.9)	8.62 (6.53 to 11.10)	4.11 (3.10 to 5.24)
2021	0.0 (0.0 to 0.0)	0.0 (0.0 to 0.0)	6.4 (4.8 to 8.4)	3.0 (2.3 to 3.9)	8.55 (6.33 to 11.16)	4.03 (3.02 to 5.22)

East of England GBR_4623 Western Europe Stillbirth Rate / Neonatal Mortality Rate (SBR/NMR) − 20 weeks
lambda = 0.2 ; zeta = 0.99 ; scale = 5 ; amp = 0.028

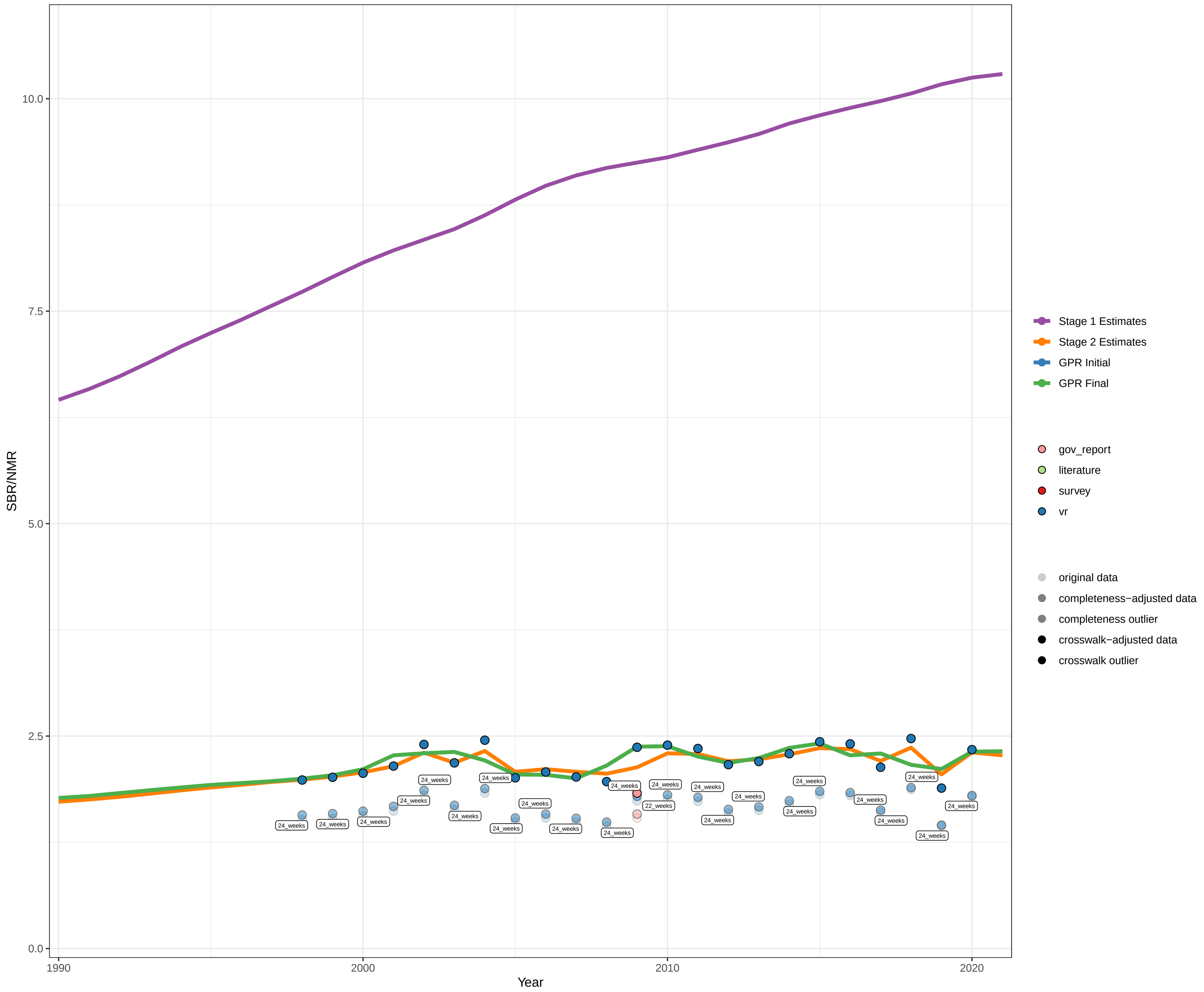

East of England GBR_4623 Western Europe Stillbirth Rate / Neonatal Mortality Rate (SBR/NMR) − 28 weeks
lambda = 0.2 ; zeta = 0.99 ; scale = 5 ; amp = 0.028

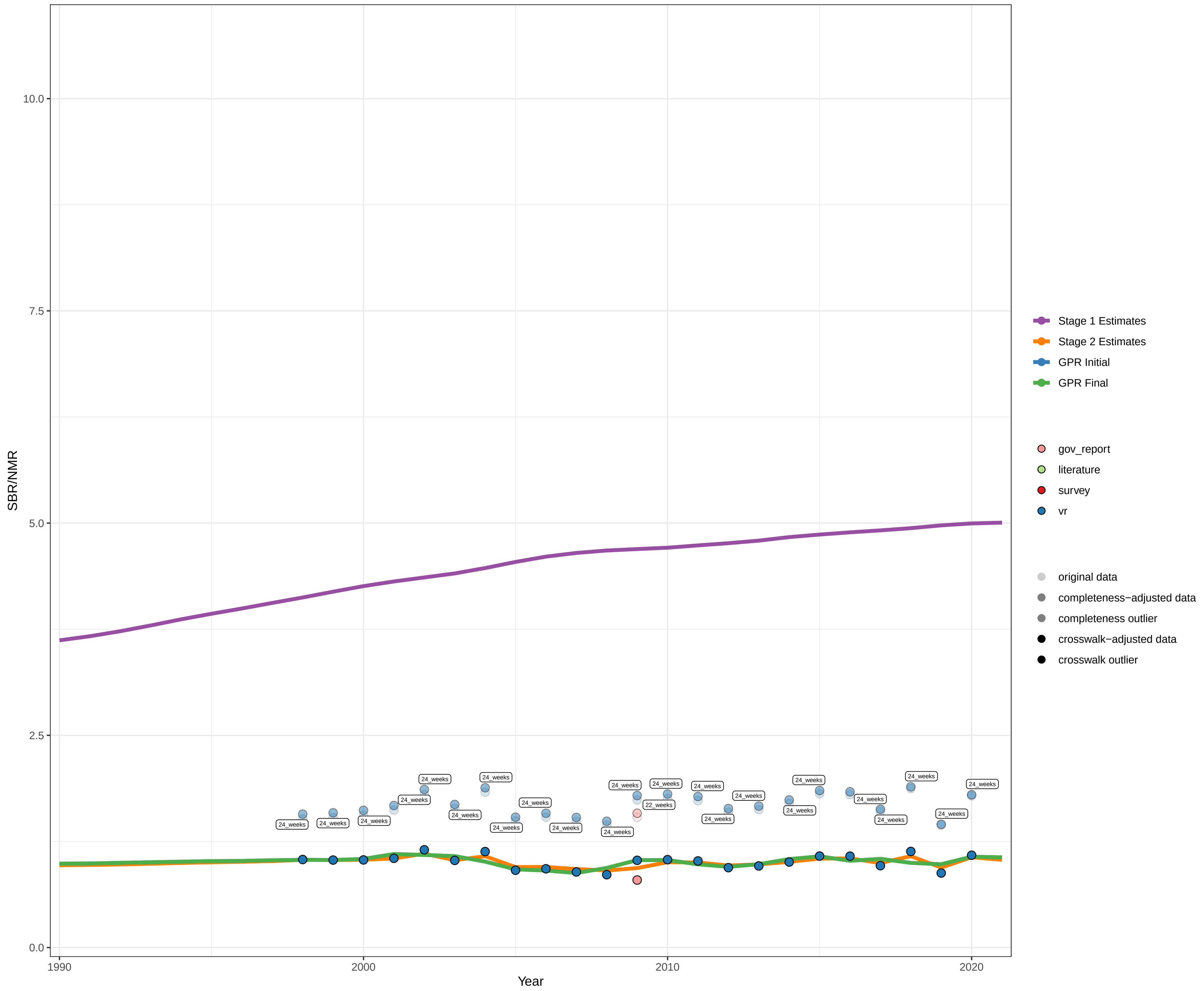

Definition	Years	Outlier	Source.Type	Title
22_weeks	2009	0	Statistical Report	United Kingdom Perinatal Mortality
24_weeks	1998−2007	0	VR	United Kingdom − England and Wales Birth Statistics
24_weeks	2008−2020	0	VR	United Kingdom − England and Wales Child Mortality Statistics

East of England GBR_4623 Western Europe Stillbirth Rate (SBR) − 20 weeks
lambda = 0.2 ; zeta = 0.99 ; scale = 5 ; amp = 0.028

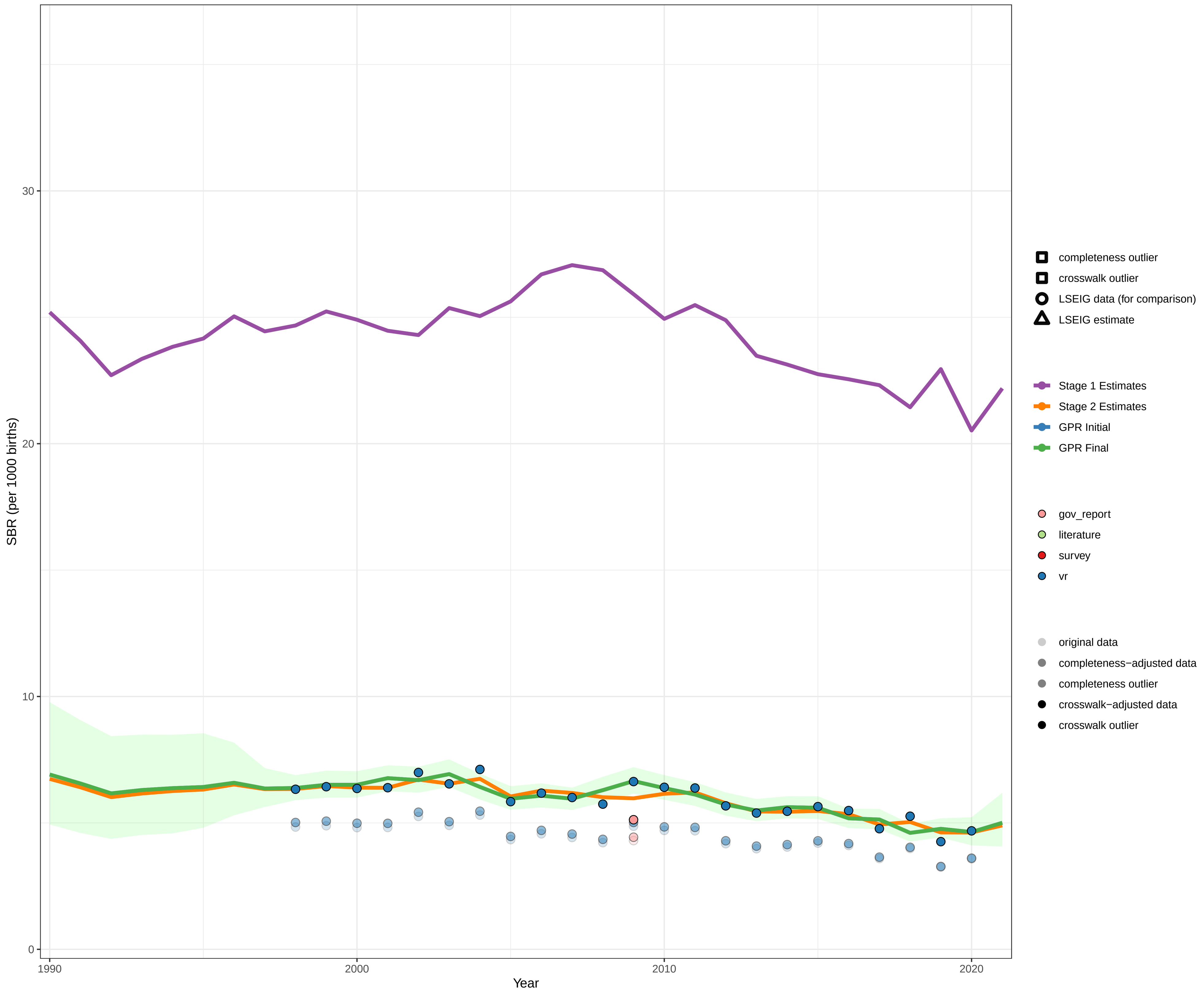

East of England GBR_4623 Western Europe Stillbirth Rate (SBR) − 28 weeks
lambda = 0.2 ; zeta = 0.99 ; scale = 5 ; amp = 0.028

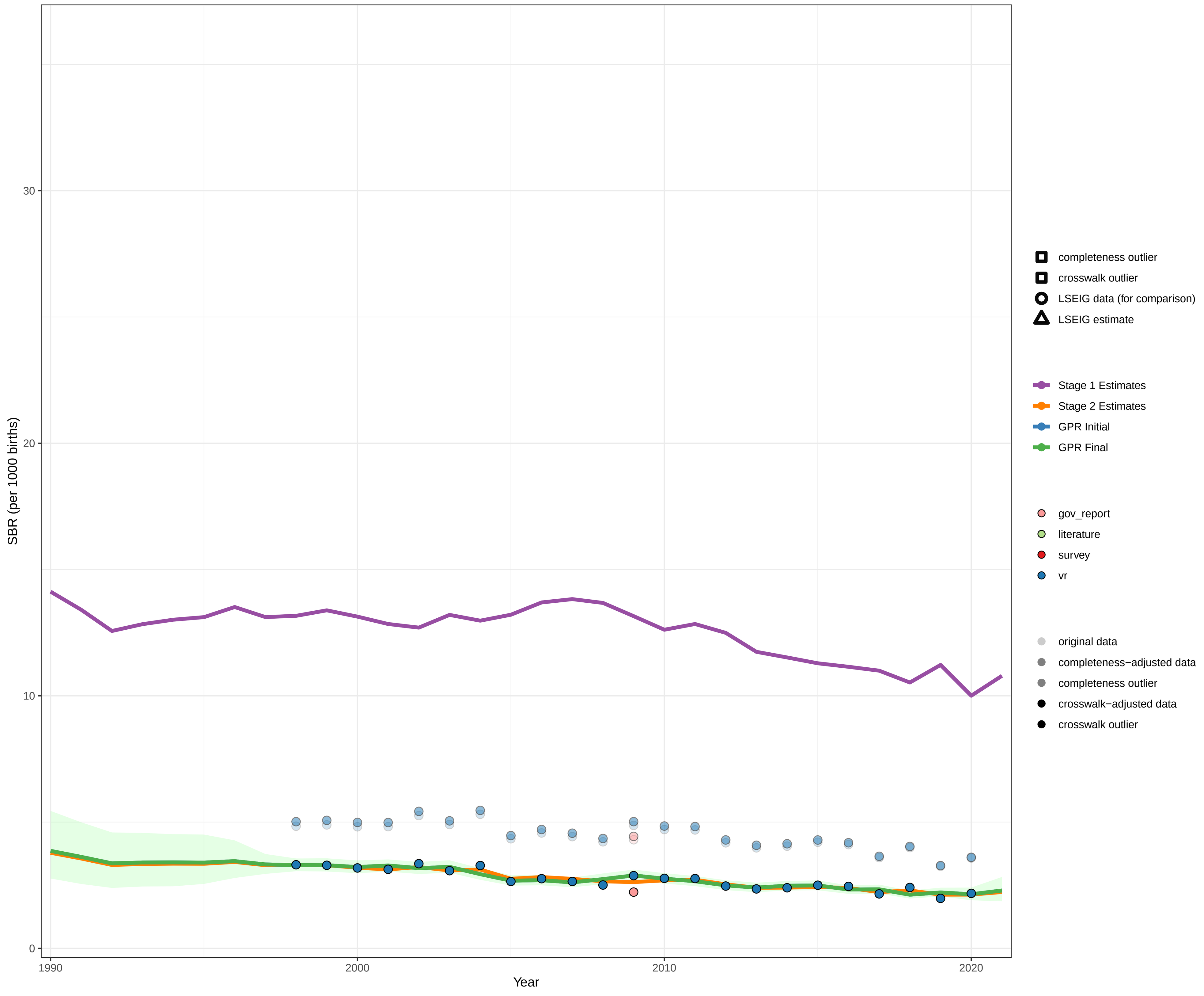

Year	Total Stillbirths (in thousands)	Total Stillbirths (in thousands)	SBR (per 1000 births)	SBR (per 1000 births)	SBR/NMR	SBR/NMR
	20 weeks	28 weeks	20 weeks	28 weeks	20 weeks	28 weeks
1990	0.5 (0.3 to 0.7)	0.3 (0.2 to 0.4)	6.9 (4.9 to 9.8)	3.9 (2.8 to 5.4)	1.77 (1.27 to 2.51)	0.99 (0.71 to 1.39)
1995	0.4 (0.3 to 0.6)	0.2 (0.2 to 0.3)	6.4 (4.8 to 8.5)	3.4 (2.6 to 4.5)	1.93 (1.44 to 2.56)	1.02 (0.77 to 1.35)
2000	0.4 (0.4 to 0.4)	0.2 (0.2 to 0.2)	6.5 (6.0 to 7.0)	3.2 (3.0 to 3.5)	2.11 (1.95 to 2.28)	1.04 (0.97 to 1.13)
2005	0.4 (0.4 to 0.4)	0.2 (0.2 to 0.2)	6.0 (5.5 to 6.5)	2.7 (2.5 to 2.9)	2.05 (1.90 to 2.22)	0.92 (0.86 to 1.00)
2010	0.5 (0.4 to 0.5)	0.2 (0.2 to 0.2)	6.4 (5.9 to 6.9)	2.8 (2.6 to 3.0)	2.38 (2.21 to 2.57)	1.03 (0.96 to 1.11)
2015	0.4 (0.4 to 0.4)	0.2 (0.2 to 0.2)	5.6 (5.2 to 6.1)	2.5 (2.3 to 2.7)	2.41 (2.22 to 2.61)	1.07 (0.99 to 1.16)
2020	0.3 (0.3 to 0.3)	0.1 (0.1 to 0.2)	4.6 (4.1 to 5.2)	2.1 (1.9 to 2.4)	2.32 (2.05 to 2.61)	1.07 (0.95 to 1.20)
2021	0.3 (0.3 to 0.4)	0.1 (0.1 to 0.2)	5.0 (4.1 to 6.2)	2.3 (1.9 to 2.8)	2.32 (1.88 to 2.87)	1.06 (0.87 to 1.31)

Bedford GBR_44716 Western Europe Stillbirth Rate / Neonatal Mortality Rate (SBR/NMR) − 20 weeks
lambda = 0.6 ; zeta = 0.8 ; scale = 15 ; amp = 0.028

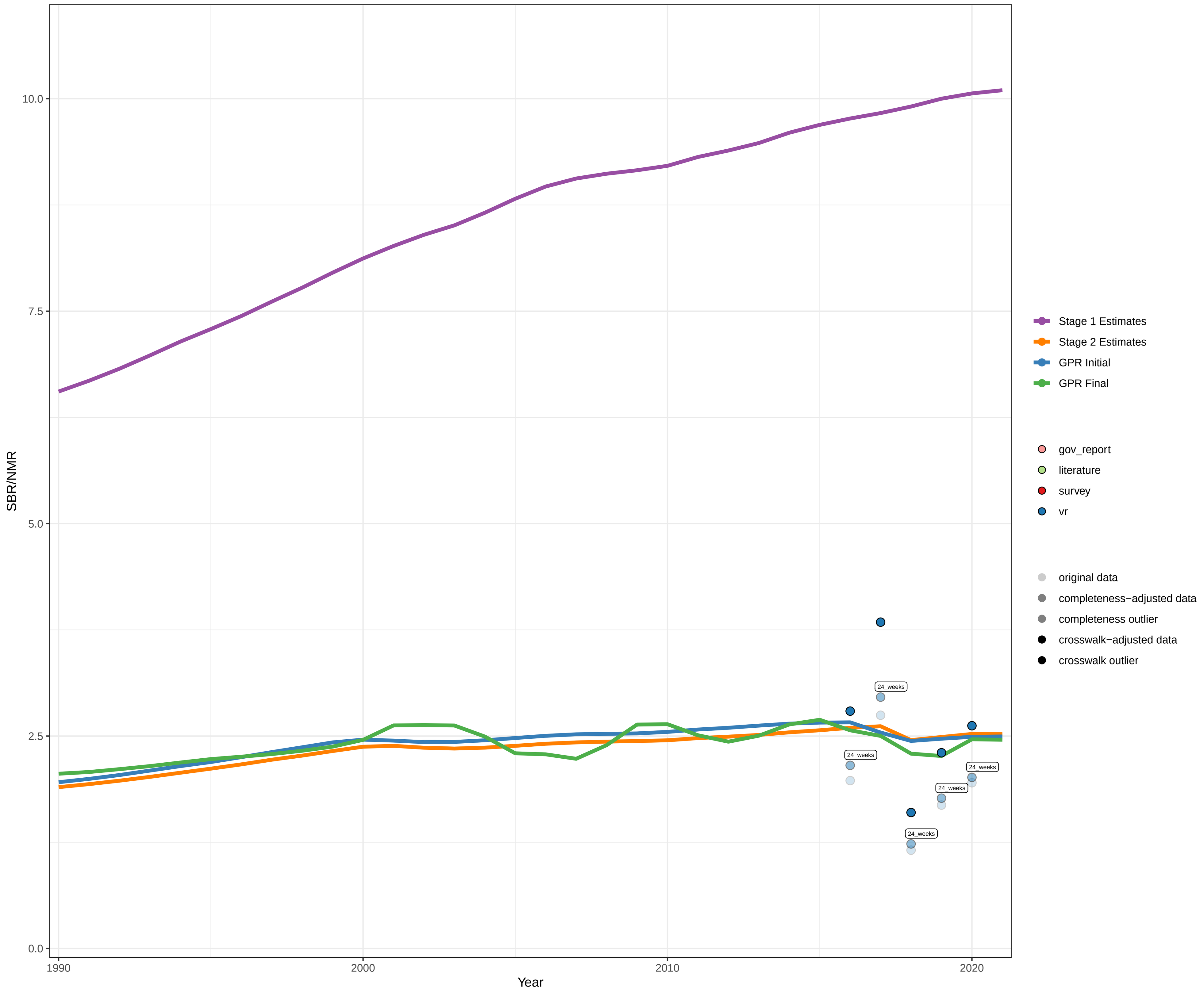

Bedford GBR_44716 Western Europe Stillbirth Rate / Neonatal Mortality Rate (SBR/NMR) − 28 weeks
lambda = 0.6 ; zeta = 0.8 ; scale = 15 ; amp = 0.028

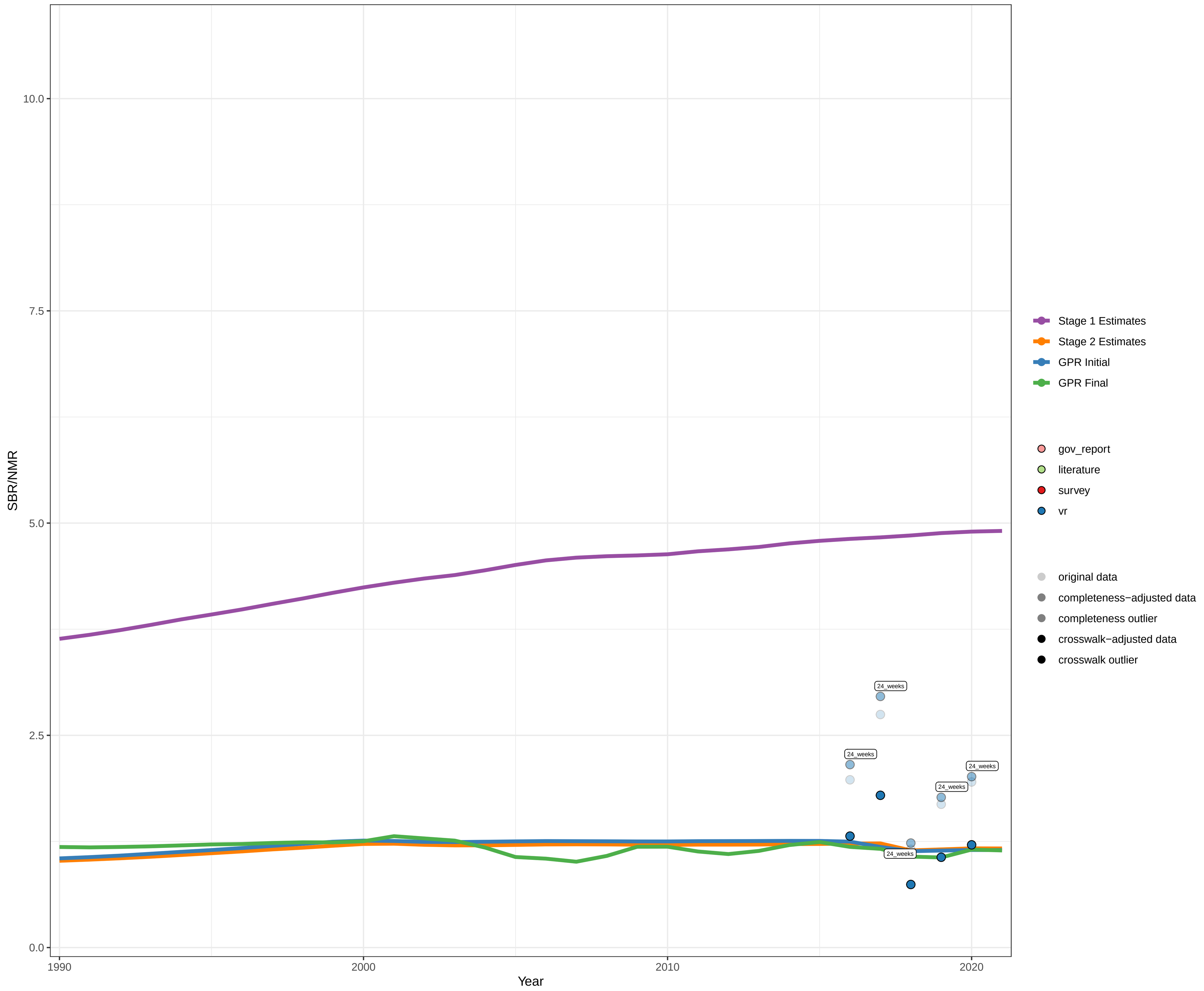

Definition	Years	Outlier	Source.Type	Title
24_weeks	2016−2020	0	VR	United Kingdom − England and Wales Child Mortality Statistics

Bedford GBR_44716 Western Europe Stillbirth Rate (SBR) − 20 weeks
lambda = 0.6 ; zeta = 0.8 ; scale = 15 ; amp = 0.028

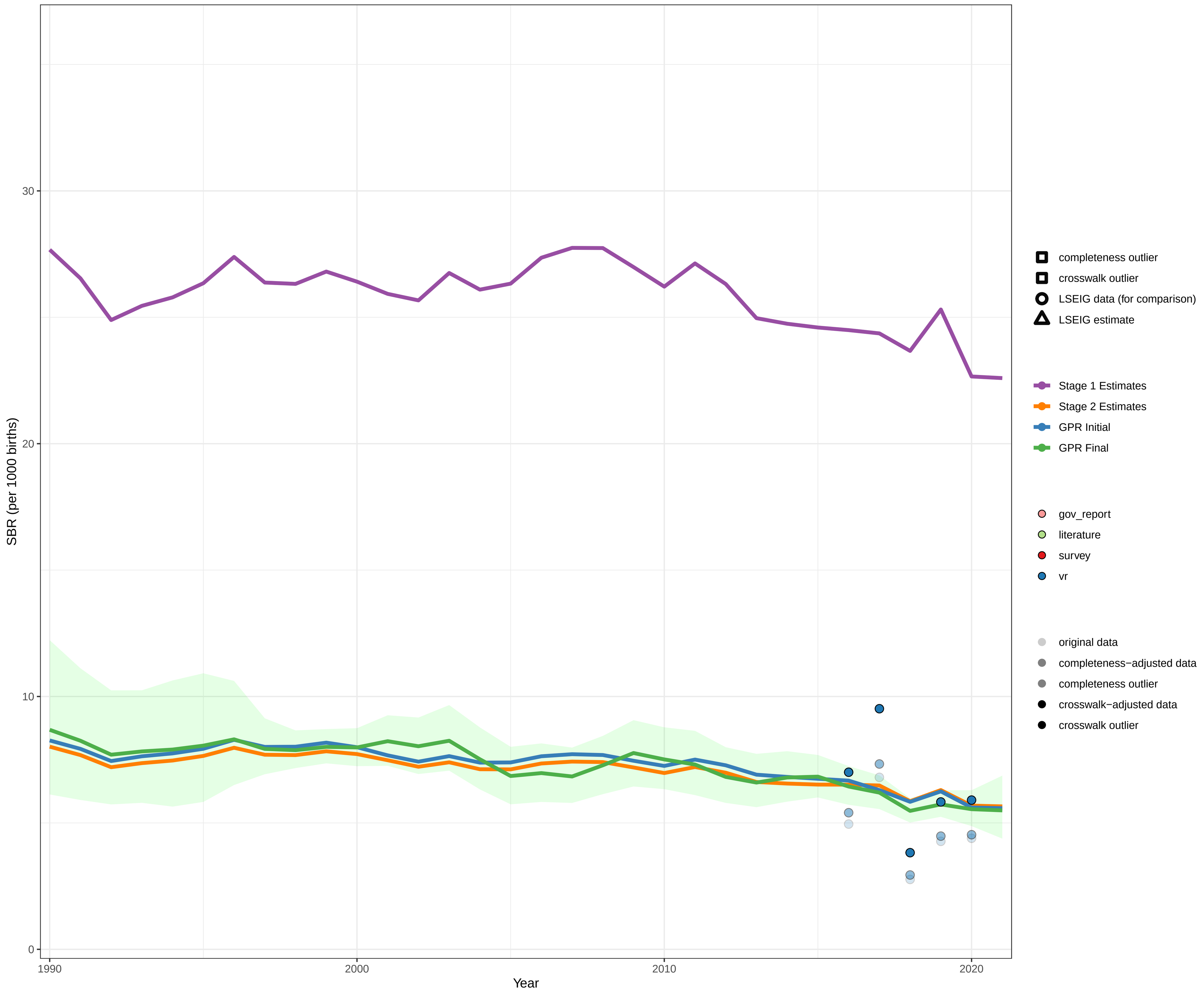

Bedford GBR_44716 Western Europe Stillbirth Rate (SBR) − 28 weeks
lambda = 0.6 ; zeta = 0.8 ; scale = 15 ; amp = 0.028

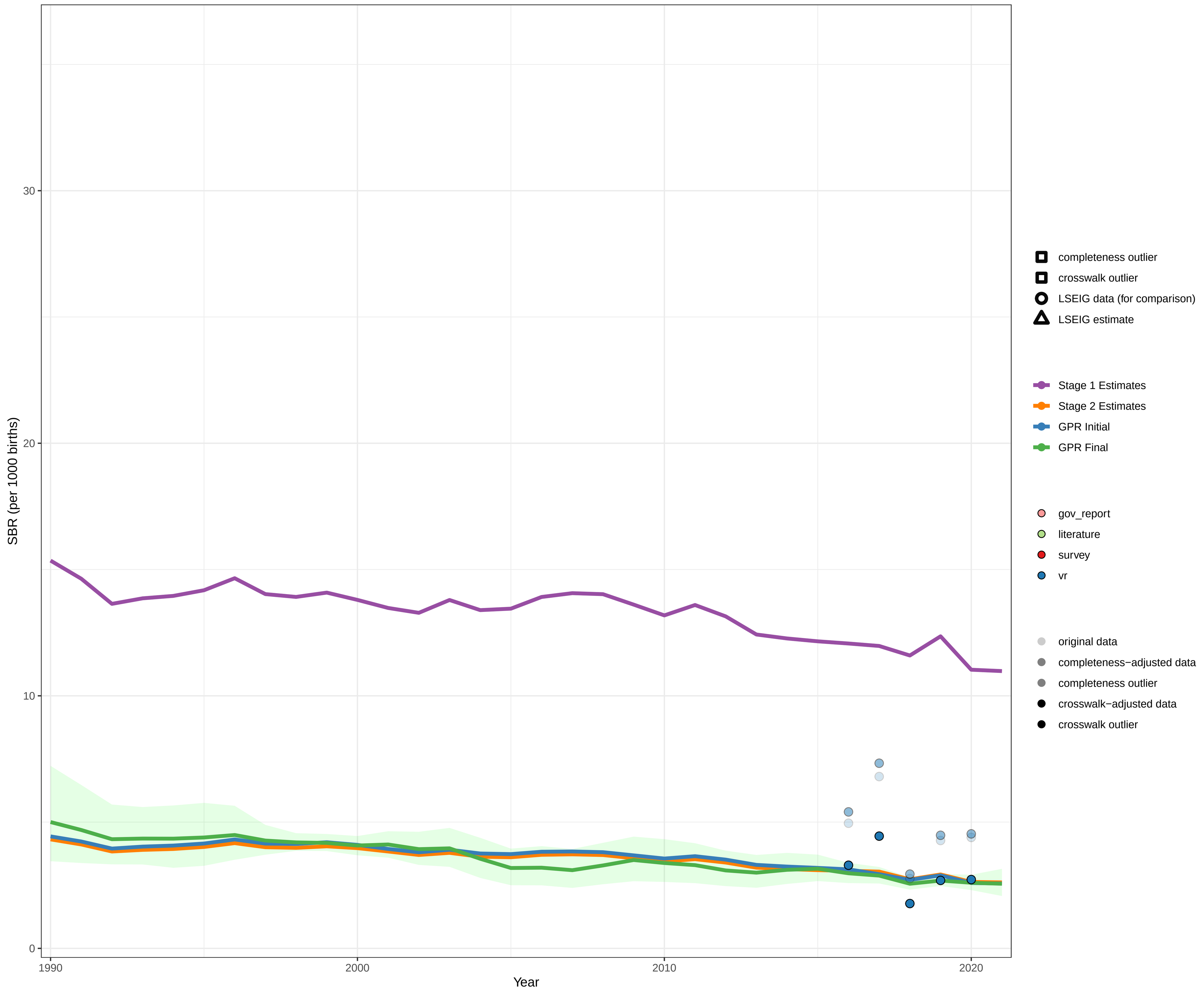

Year	Total Stillbirths (in thousands)	Total Stillbirths (in thousands)	SBR (per 1000 births)	SBR (per 1000 births)	SBR/NMR	SBR/NMR
	20 weeks	28 weeks	20 weeks	28 weeks	20 weeks	28 weeks
1990	0.0 (0.0 to 0.0)	0.0 (0.0 to 0.0)	8.7 (6.1 to 12.2)	5.0 (3.5 to 7.2)	2.06 (1.45 to 2.90)	1.18 (0.82 to 1.71)
1995	0.0 (0.0 to 0.0)	0.0 (0.0 to 0.0)	8.1 (5.8 to 10.9)	4.4 (3.3 to 5.8)	2.23 (1.61 to 3.02)	1.21 (0.90 to 1.59)
2000	0.0 (0.0 to 0.0)	0.0 (0.0 to 0.0)	8.0 (7.2 to 8.7)	4.1 (3.7 to 4.4)	2.46 (2.23 to 2.69)	1.25 (1.13 to 1.37)
2005	0.0 (0.0 to 0.0)	0.0 (0.0 to 0.0)	6.9 (5.7 to 8.0)	3.2 (2.5 to 4.0)	2.30 (1.92 to 2.68)	1.07 (0.84 to 1.32)
2010	0.0 (0.0 to 0.0)	0.0 (0.0 to 0.0)	7.5 (6.3 to 8.8)	3.4 (2.6 to 4.3)	2.64 (2.23 to 3.09)	1.19 (0.92 to 1.52)
2015	0.0 (0.0 to 0.0)	0.0 (0.0 to 0.0)	6.8 (6.0 to 7.7)	3.2 (2.7 to 3.7)	2.69 (2.37 to 3.03)	1.24 (1.05 to 1.46)
2020	0.0 (0.0 to 0.0)	0.0 (0.0 to 0.0)	5.5 (4.9 to 6.3)	2.6 (2.3 to 2.9)	2.46 (2.16 to 2.80)	1.15 (1.02 to 1.29)
2021	0.0 (0.0 to 0.0)	0.0 (0.0 to 0.0)	5.5 (4.4 to 6.9)	2.6 (2.1 to 3.1)	2.46 (1.96 to 3.07)	1.14 (0.93 to 1.41)

Cambridgeshire GBR_44721 Western Europe Stillbirth Rate / Neonatal Mortality Rate (SBR/NMR) − 20 weeks
lambda = 0.6 ; zeta = 0.8 ; scale = 15 ; amp = 0.028

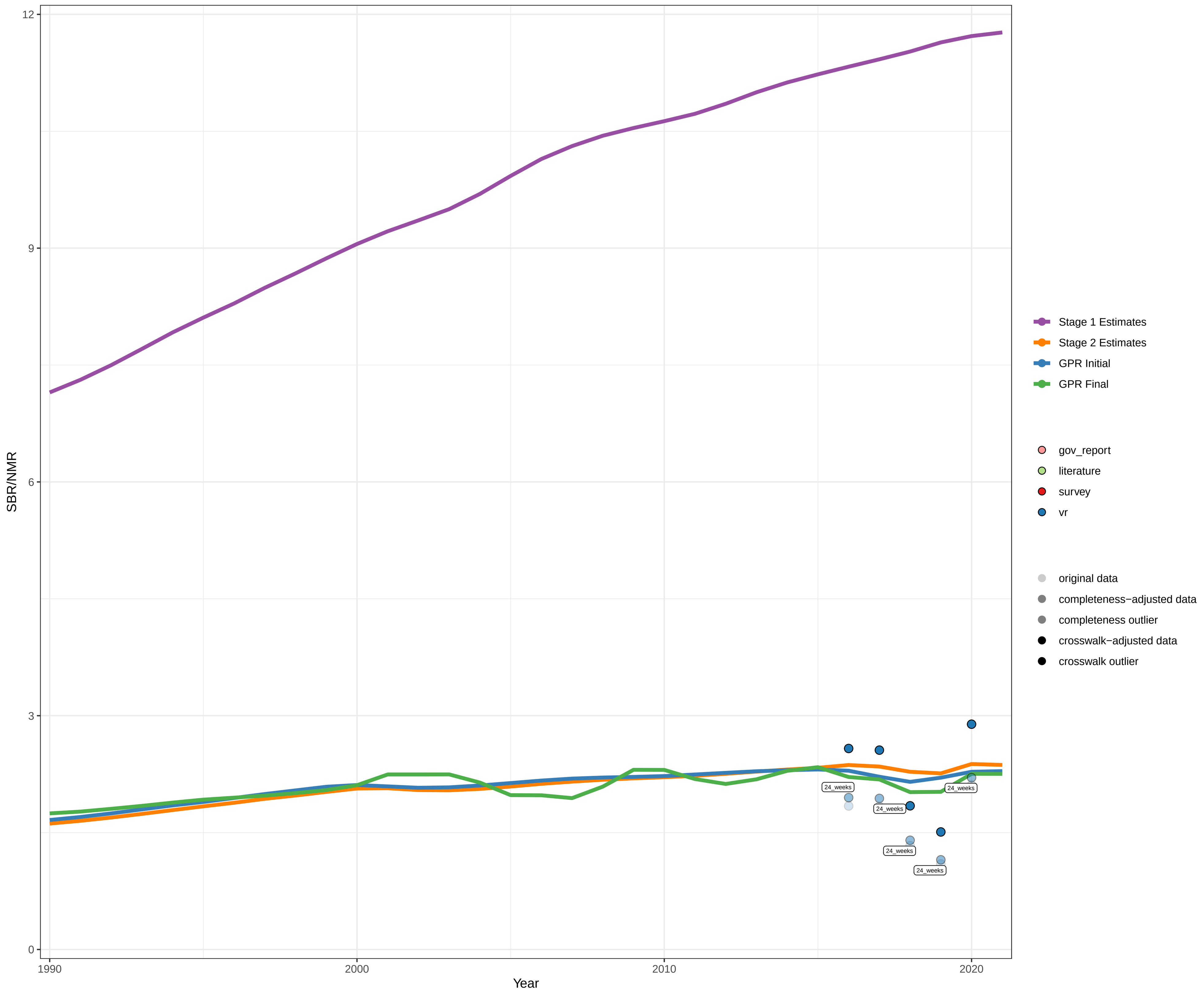

Cambridgeshire GBR_44721 Western Europe Stillbirth Rate / Neonatal Mortality Rate (SBR/NMR) − 28 weeks
lambda = 0.6 ; zeta = 0.8 ; scale = 15 ; amp = 0.028

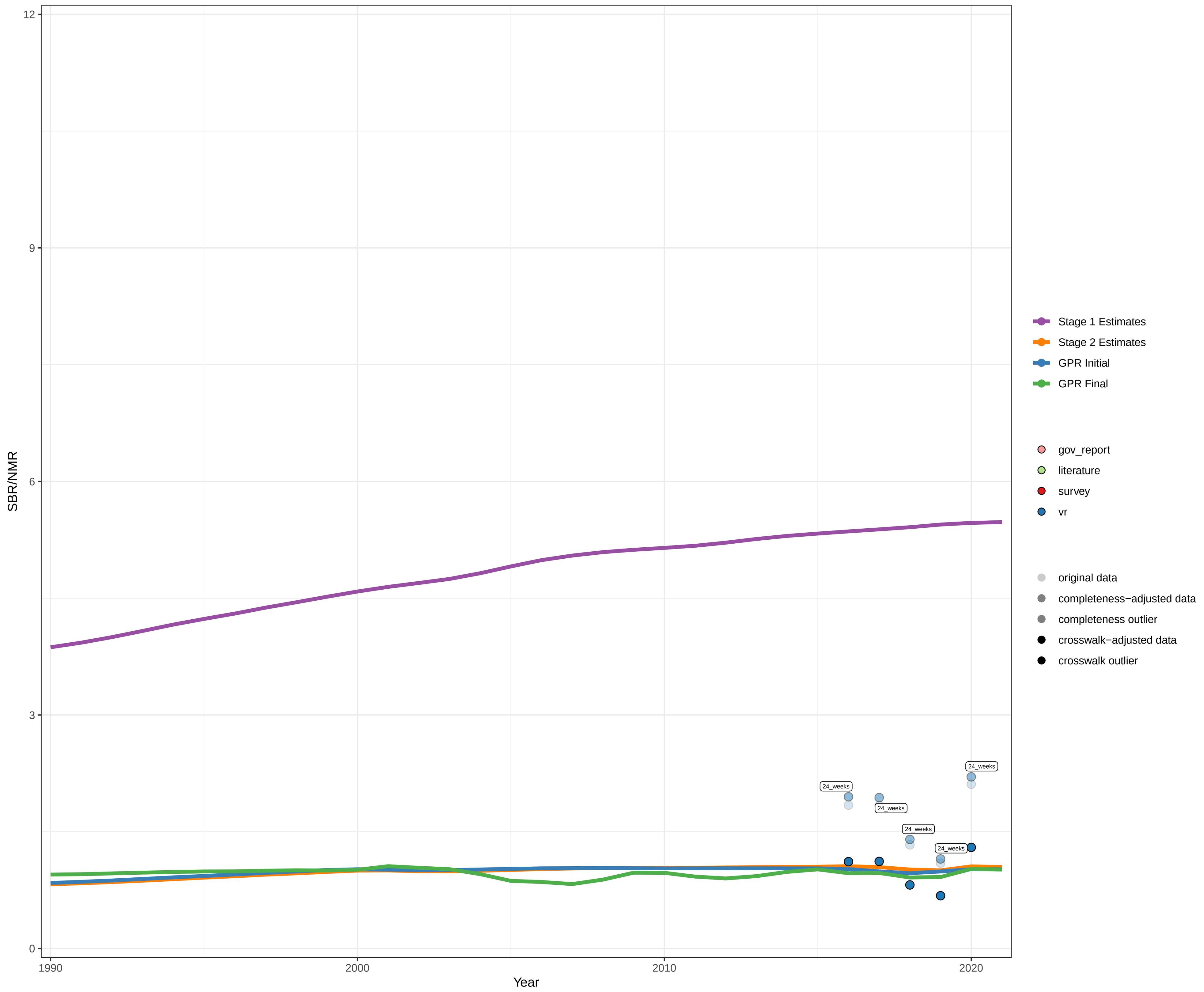

Definition	Years	Outlier	Source.Type	Title
24_weeks	2016−2020	0	VR	United Kingdom − England and Wales Child Mortality Statistics

Cambridgeshire GBR_44721 Western Europe Stillbirth Rate (SBR) − 20 weeks
lambda = 0.6 ; zeta = 0.8 ; scale = 15 ; amp = 0.028

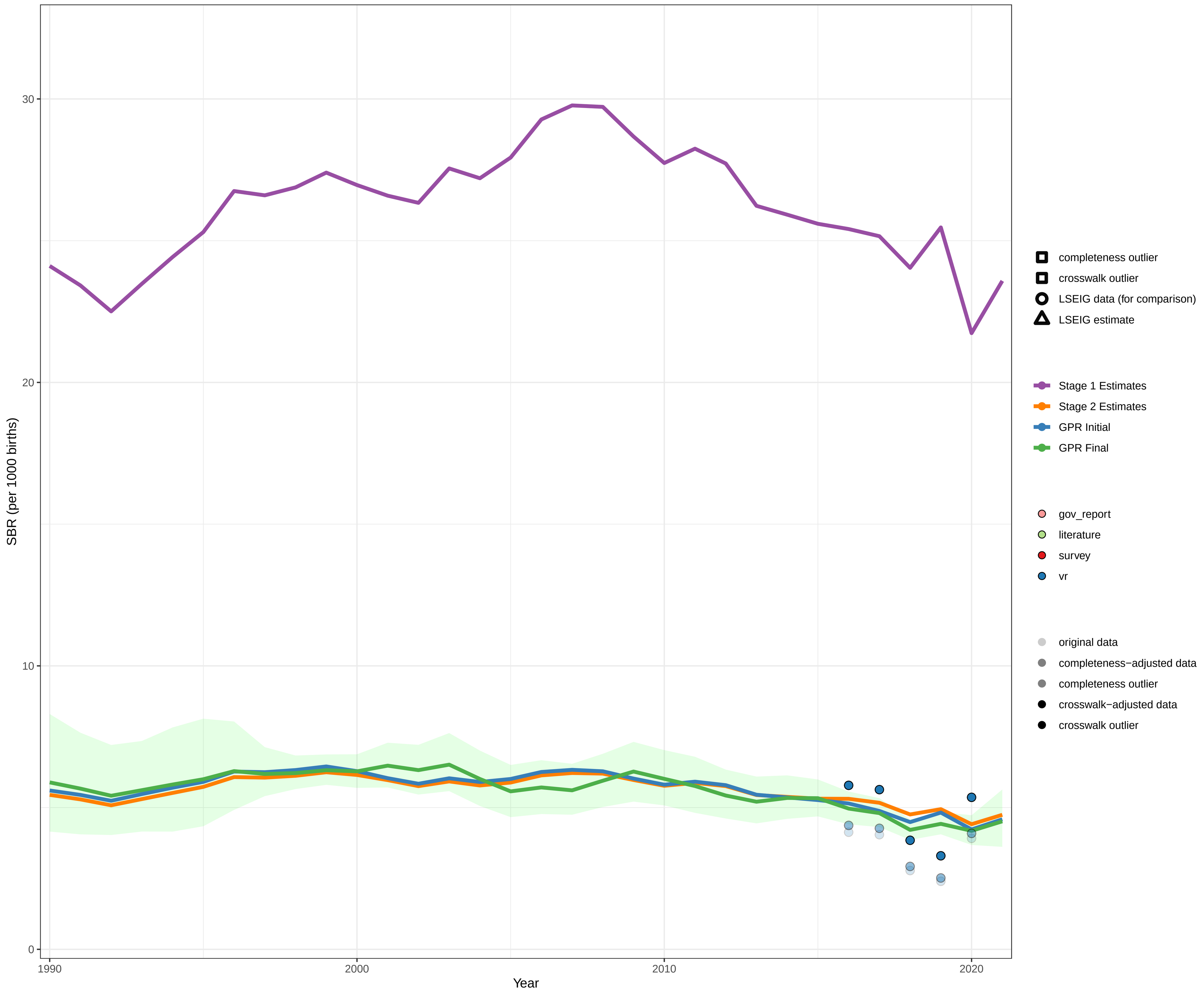

Cambridgeshire GBR_44721 Western Europe Stillbirth Rate (SBR) − 28 weeks
lambda = 0.6 ; zeta = 0.8 ; scale = 15 ; amp = 0.028

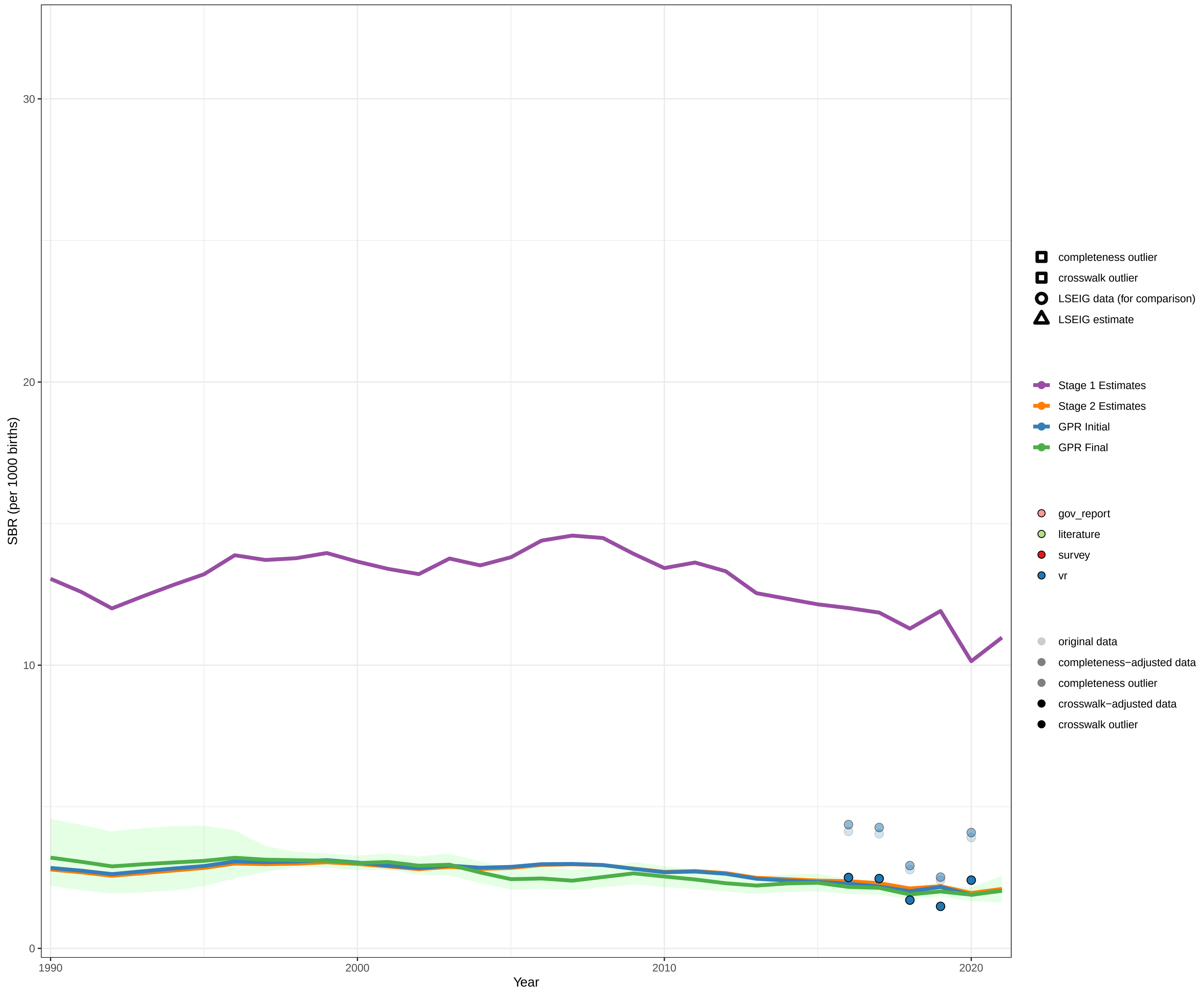

Year	Total Stillbirths (in thousands)	Total Stillbirths (in thousands)	SBR (per 1000 births)	SBR (per 1000 births)	SBR/NMR	SBR/NMR
	20 weeks	28 weeks	20 weeks	28 weeks	20 weeks	28 weeks
1990	0.0 (0.0 to 0.1)	0.0 (0.0 to 0.0)	5.9 (4.2 to 8.3)	3.2 (2.2 to 4.6)	1.75 (1.23 to 2.46)	0.95 (0.65 to 1.36)
1995	0.0 (0.0 to 0.1)	0.0 (0.0 to 0.0)	6.0 (4.3 to 8.1)	3.1 (2.2 to 4.3)	1.92 (1.39 to 2.61)	0.99 (0.71 to 1.39)
2000	0.0 (0.0 to 0.0)	0.0 (0.0 to 0.0)	6.3 (5.7 to 6.9)	3.0 (2.8 to 3.3)	2.11 (1.91 to 2.31)	1.01 (0.93 to 1.10)
2005	0.0 (0.0 to 0.0)	0.0 (0.0 to 0.0)	5.6 (4.7 to 6.5)	2.4 (2.1 to 2.8)	1.98 (1.66 to 2.31)	0.87 (0.74 to 1.01)
2010	0.0 (0.0 to 0.1)	0.0 (0.0 to 0.0)	6.0 (5.1 to 7.0)	2.5 (2.2 to 2.9)	2.30 (1.95 to 2.69)	0.97 (0.83 to 1.12)
2015	0.0 (0.0 to 0.0)	0.0 (0.0 to 0.0)	5.3 (4.7 to 6.0)	2.3 (2.0 to 2.6)	2.34 (2.06 to 2.63)	1.02 (0.88 to 1.16)
2020	0.0 (0.0 to 0.0)	0.0 (0.0 to 0.0)	4.2 (3.7 to 4.7)	1.9 (1.7 to 2.2)	2.25 (1.98 to 2.55)	1.02 (0.90 to 1.16)
2021	0.0 (0.0 to 0.0)	0.0 (0.0 to 0.0)	4.5 (3.6 to 5.6)	2.0 (1.6 to 2.6)	2.25 (1.80 to 2.81)	1.01 (0.81 to 1.28)

Central Bedfordshire GBR_44717 Western Europe Stillbirth Rate / Neonatal Mortality Rate (SBR/NMR) − 20 weeks
lambda = 0.6 ; zeta = 0.8 ; scale = 15 ; amp = 0.028

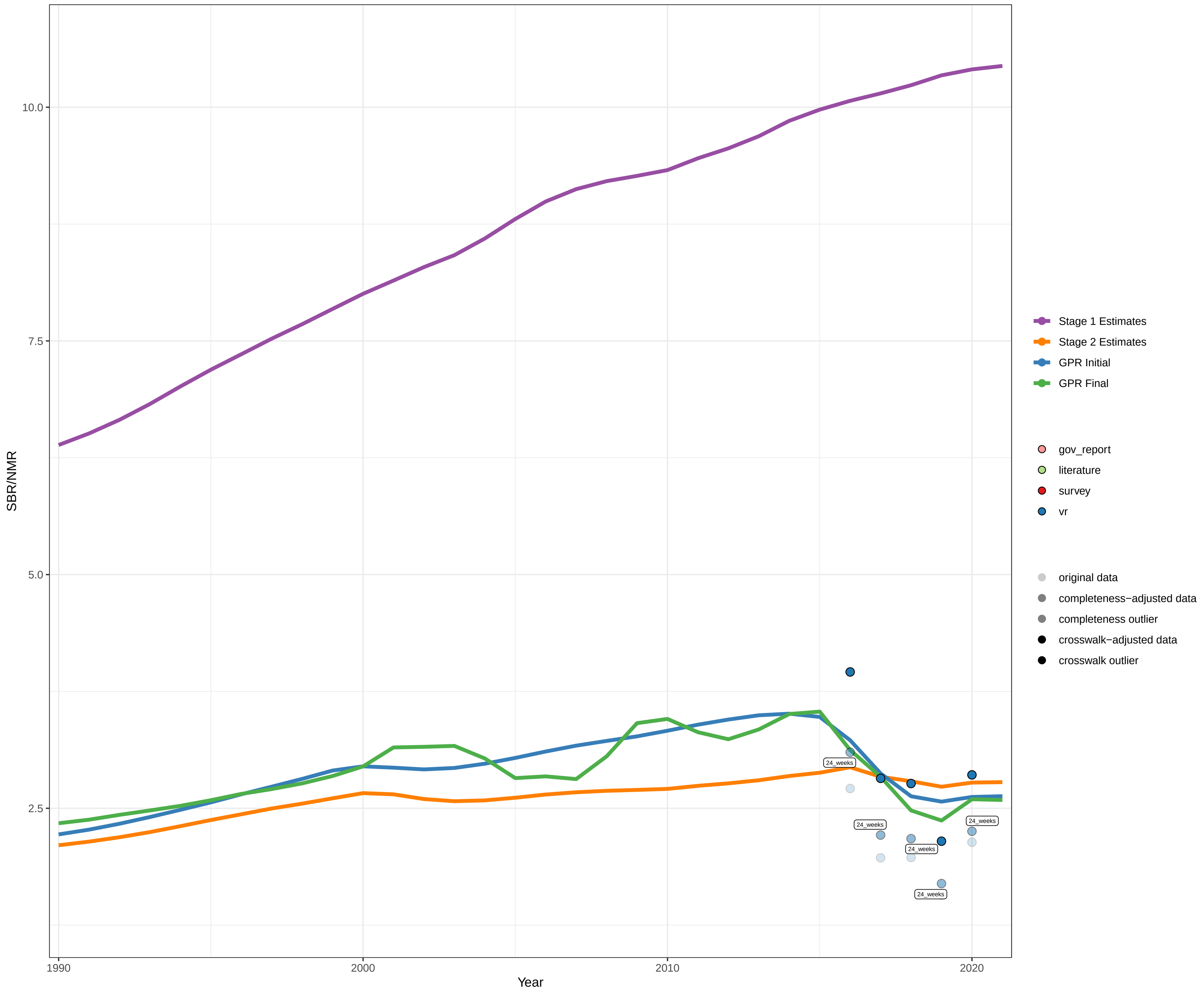

Central Bedfordshire GBR_44717 Western Europe Stillbirth Rate / Neonatal Mortality Rate (SBR/NMR) − 28 weeks
lambda = 0.6 ; zeta = 0.8 ; scale = 15 ; amp = 0.028

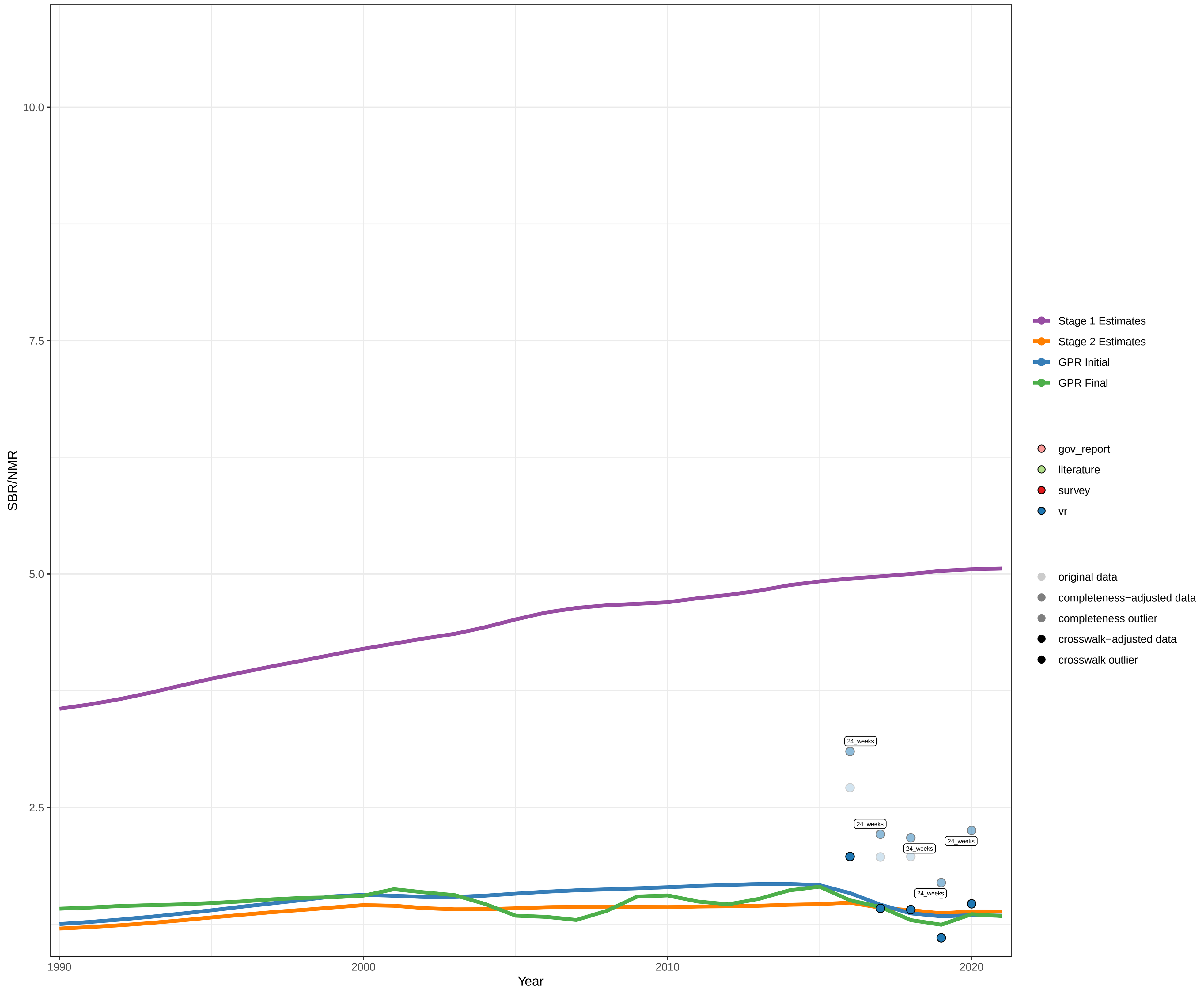

Definition	Years	Outlier	Source.Type	Title
24_weeks	2016−2020	0	VR	United Kingdom − England and Wales Child Mortality Statistics

Central Bedfordshire GBR_44717 Western Europe Stillbirth Rate (SBR) − 20 weeks
lambda = 0.6 ; zeta = 0.8 ; scale = 15 ; amp = 0.028

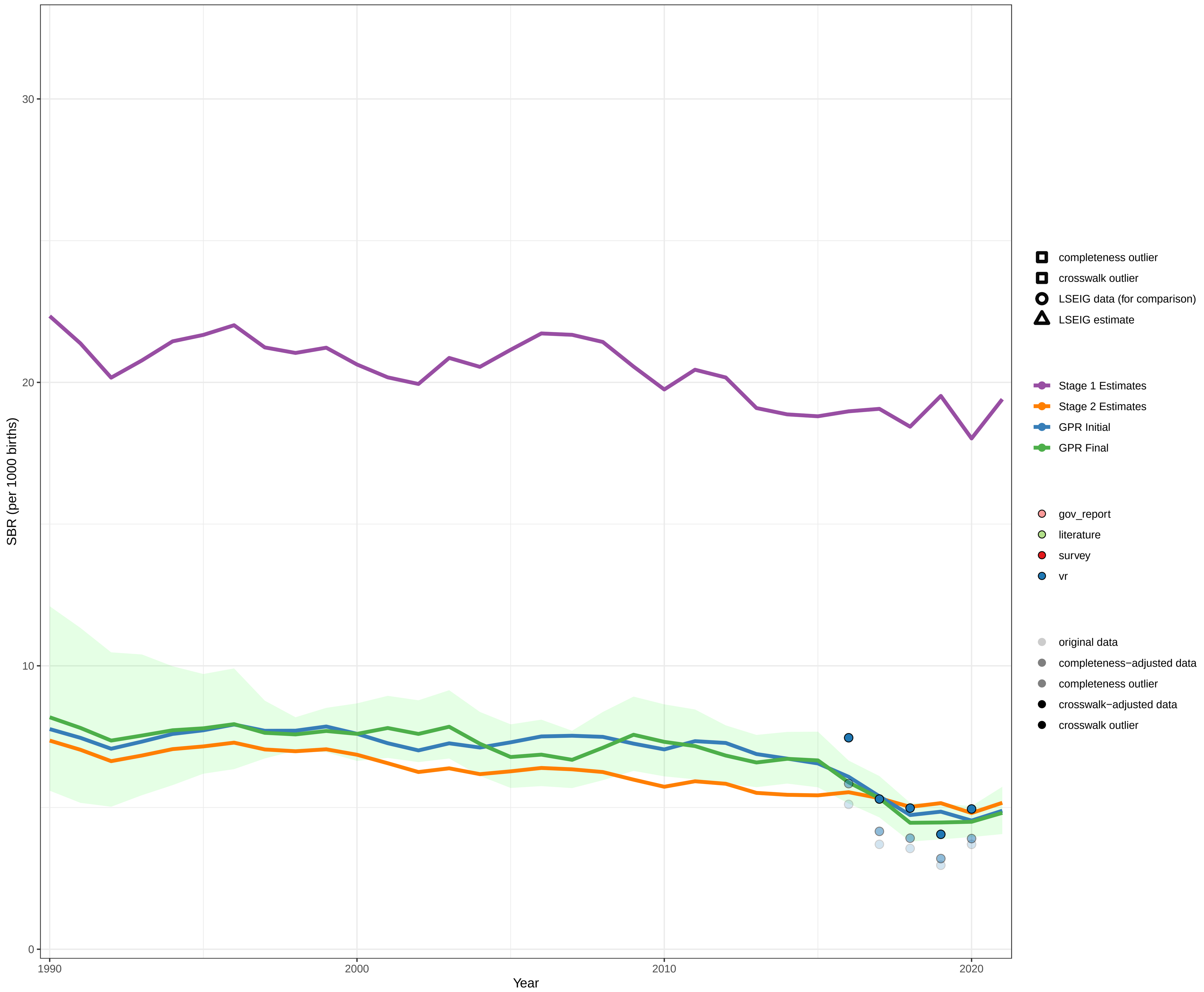

Central Bedfordshire GBR_44717 Western Europe Stillbirth Rate (SBR) − 28 weeks
lambda = 0.6 ; zeta = 0.8 ; scale = 15 ; amp = 0.028

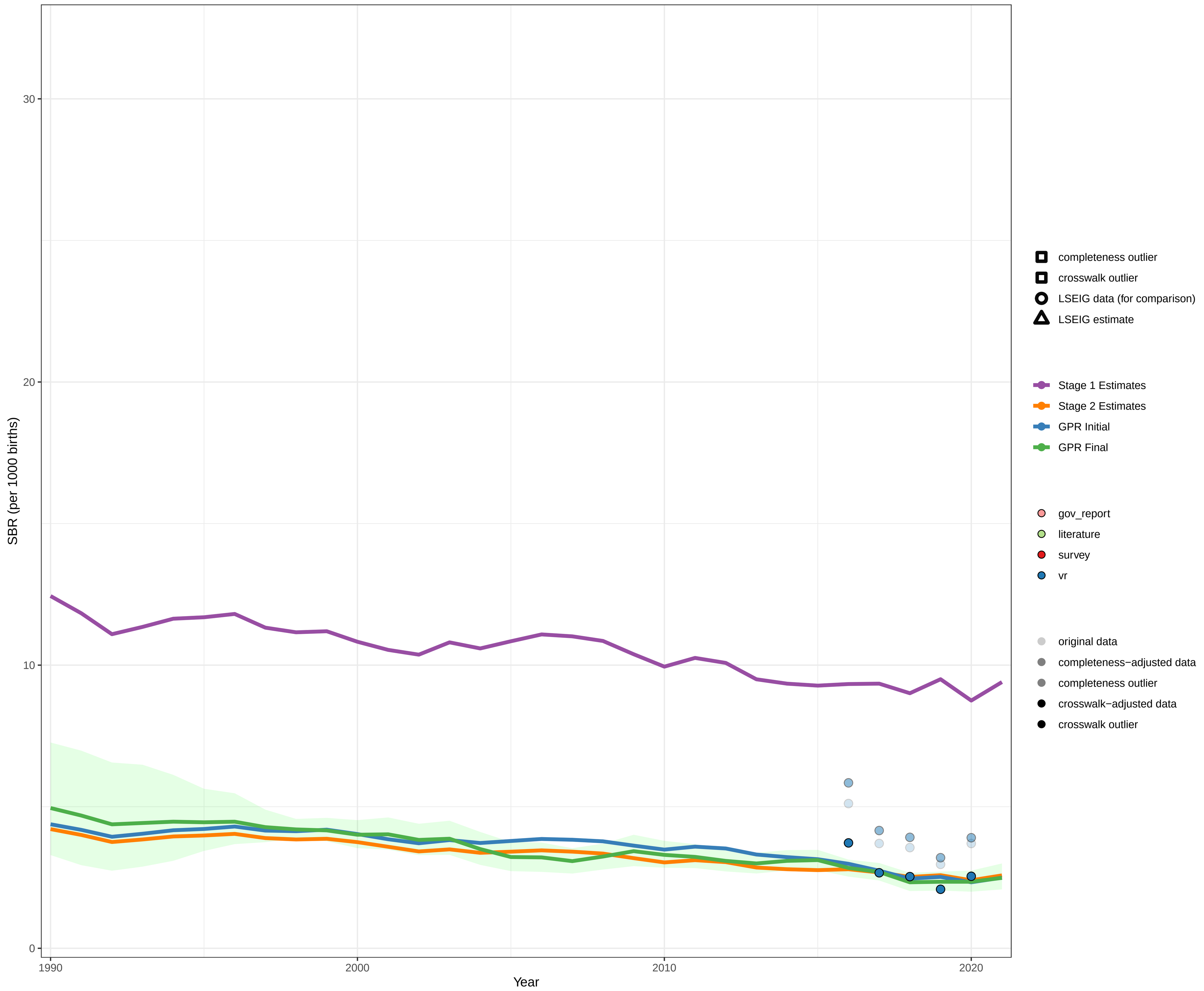

Year	Total Stillbirths (in thousands)	Total Stillbirths (in thousands)	SBR (per 1000 births)	SBR (per 1000 births)	SBR/NMR	SBR/NMR
	20 weeks	28 weeks	20 weeks	28 weeks	20 weeks	28 weeks
1990	0.0 (0.0 to 0.0)	0.0 (0.0 to 0.0)	8.2 (5.6 to 12.1)	5.0 (3.3 to 7.3)	2.34 (1.60 to 3.46)	1.42 (0.94 to 2.08)
1995	0.0 (0.0 to 0.0)	0.0 (0.0 to 0.0)	7.8 (6.2 to 9.7)	4.5 (3.4 to 5.6)	2.59 (2.05 to 3.22)	1.48 (1.14 to 1.87)
2000	0.0 (0.0 to 0.0)	0.0 (0.0 to 0.0)	7.6 (6.6 to 8.7)	4.0 (3.5 to 4.5)	2.95 (2.58 to 3.37)	1.56 (1.37 to 1.76)
2005	0.0 (0.0 to 0.0)	0.0 (0.0 to 0.0)	6.8 (5.7 to 7.9)	3.2 (2.7 to 3.8)	2.82 (2.37 to 3.30)	1.34 (1.13 to 1.56)
2010	0.0 (0.0 to 0.0)	0.0 (0.0 to 0.0)	7.3 (6.1 to 8.6)	3.3 (2.8 to 3.8)	3.46 (2.88 to 4.08)	1.56 (1.34 to 1.79)
2015	0.0 (0.0 to 0.0)	0.0 (0.0 to 0.0)	6.7 (5.7 to 7.7)	3.1 (2.8 to 3.5)	3.53 (3.03 to 4.07)	1.65 (1.46 to 1.84)
2020	0.0 (0.0 to 0.0)	0.0 (0.0 to 0.0)	4.5 (4.0 to 5.1)	2.3 (2.0 to 2.7)	2.60 (2.29 to 2.92)	1.36 (1.15 to 1.58)
2021	0.0 (0.0 to 0.0)	0.0 (0.0 to 0.0)	4.8 (4.1 to 5.7)	2.5 (2.1 to 3.0)	2.59 (2.19 to 3.08)	1.34 (1.12 to 1.61)

Essex GBR_44720 Western Europe Stillbirth Rate / Neonatal Mortality Rate (SBR/NMR) − 20 weeks
lambda = 0.6 ; zeta = 0.8 ; scale = 15 ; amp = 0.028

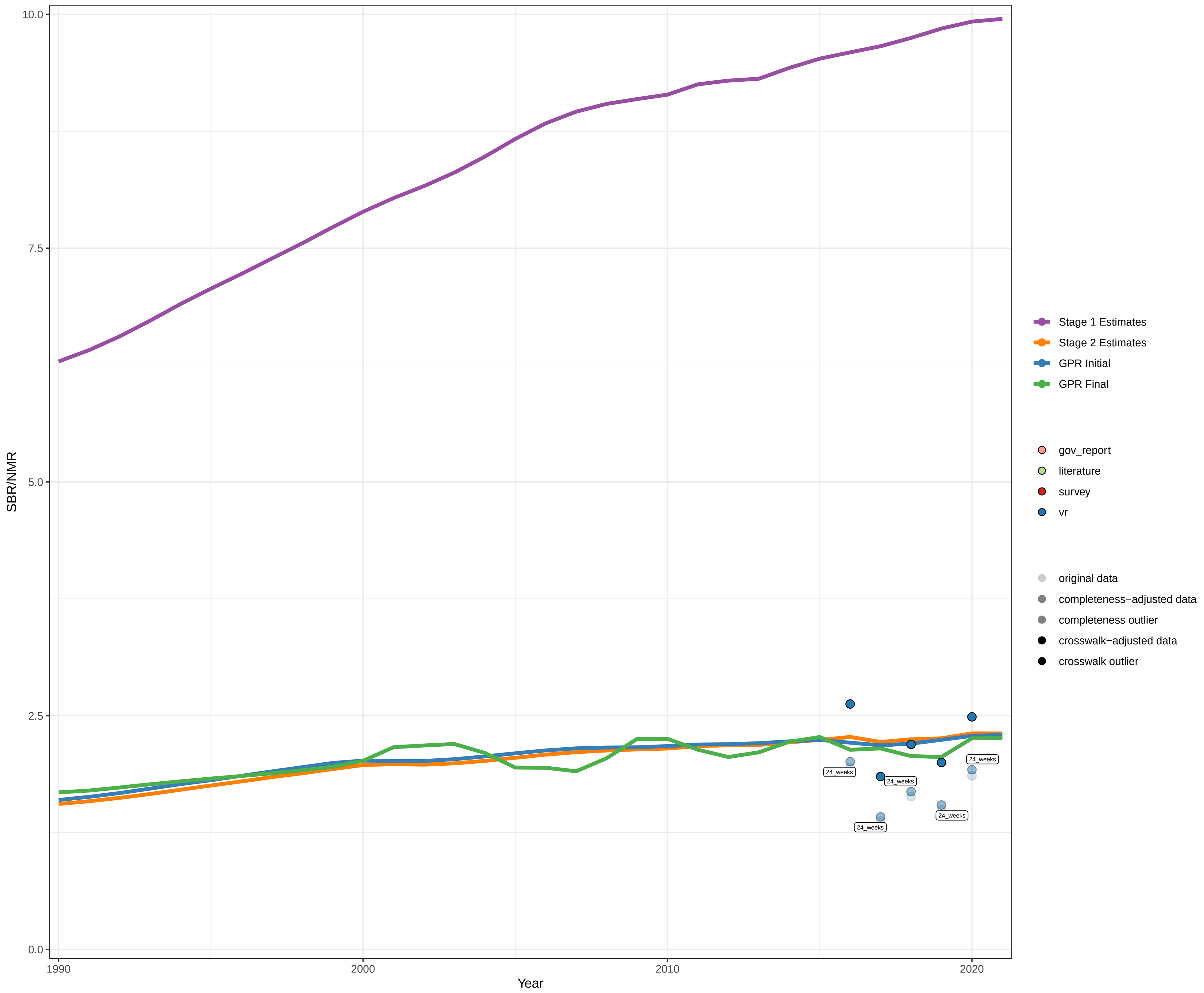

Essex GBR_44720 Western Europe Stillbirth Rate / Neonatal Mortality Rate (SBR/NMR) − 28 weeks
lambda = 0.6 ; zeta = 0.8 ; scale = 15 ; amp = 0.028

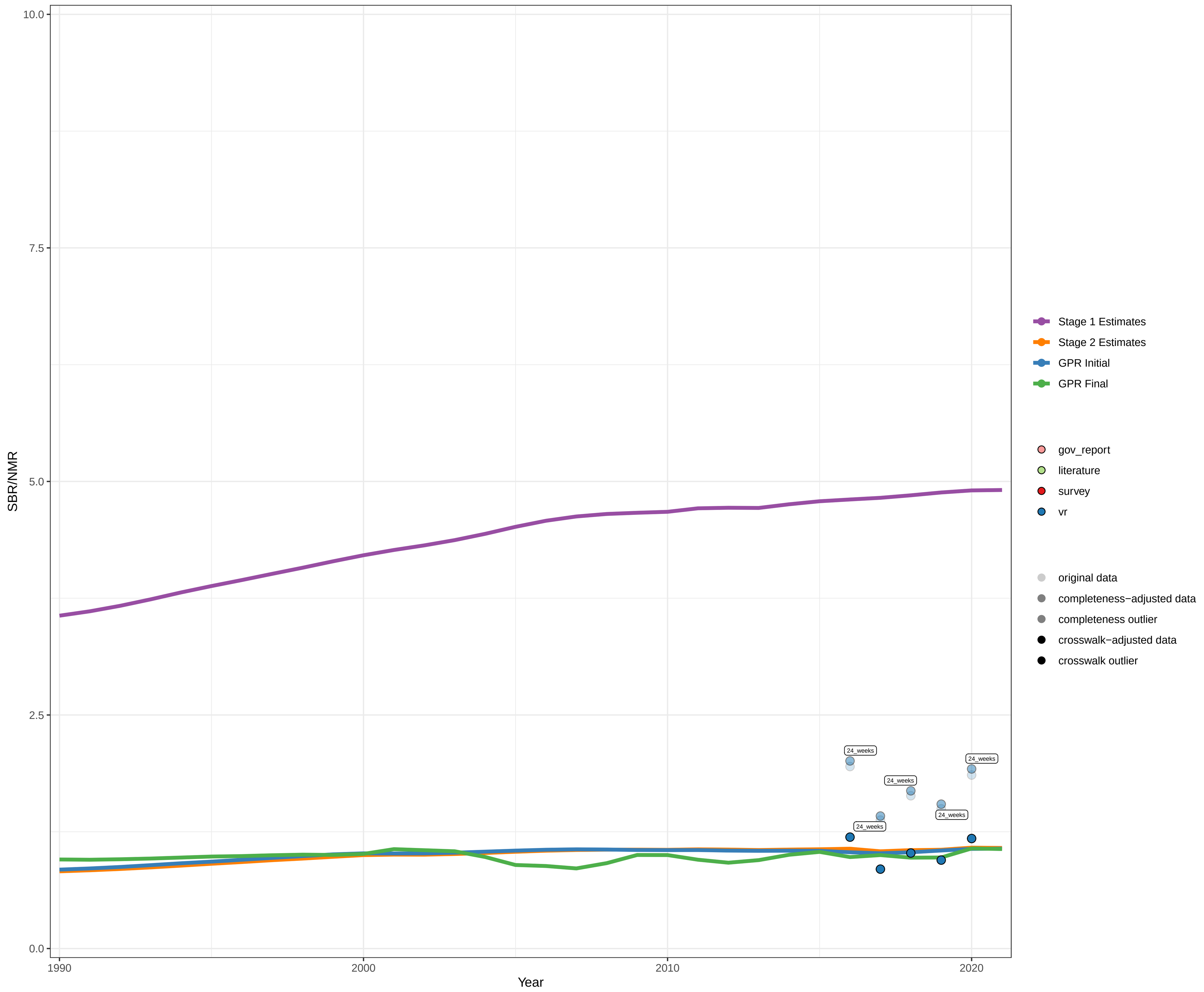

Definition	Years	Outlier	Source.Type	Title
24_weeks	2016−2020	0	VR	United Kingdom − England and Wales Child Mortality Statistics

Essex GBR_44720 Western Europe Stillbirth Rate (SBR) − 20 weeks
lambda = 0.6 ; zeta = 0.8 ; scale = 15 ; amp = 0.028

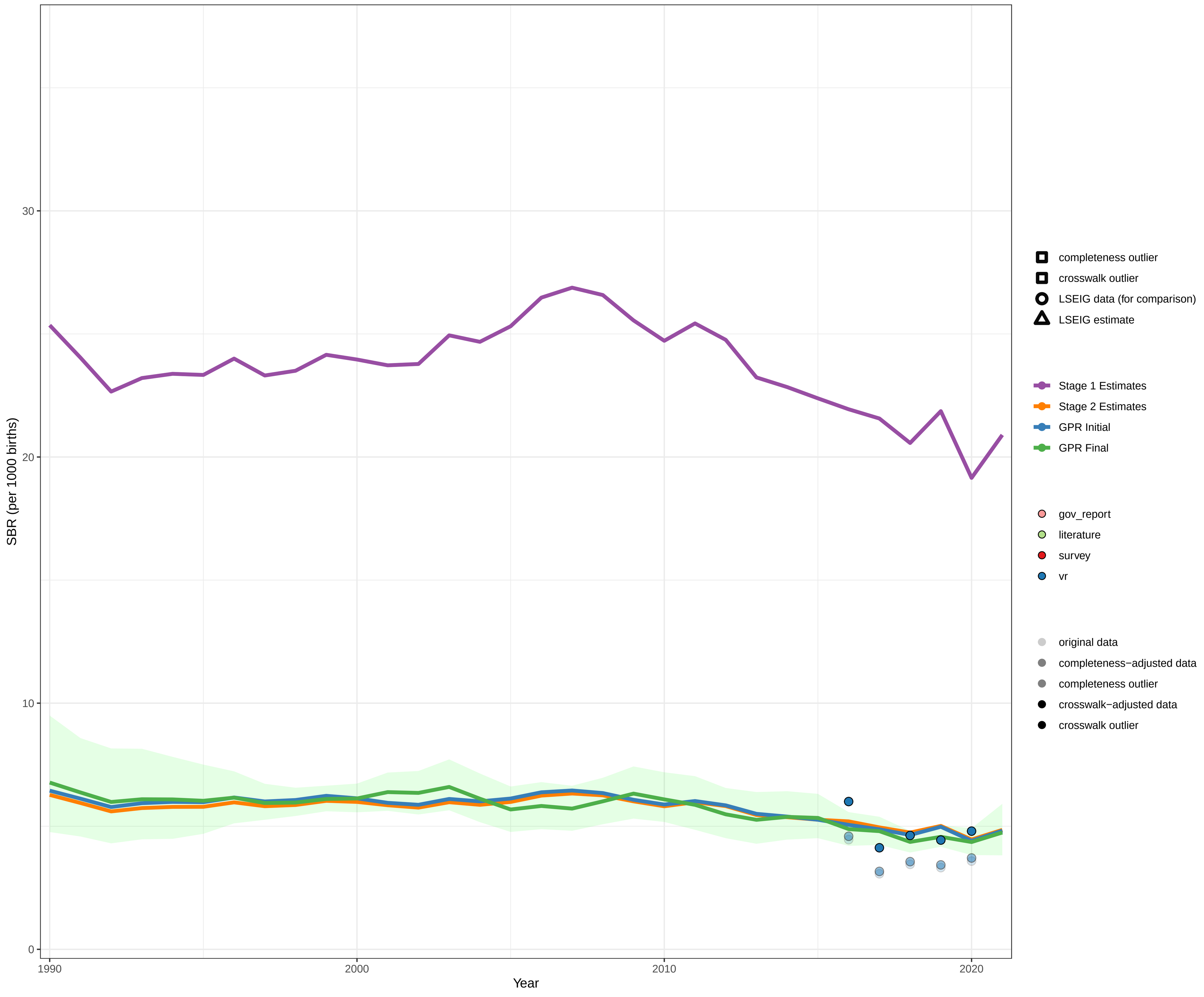

Essex GBR_44720 Western Europe Stillbirth Rate (SBR) − 28 weeks
lambda = 0.6 ; zeta = 0.8 ; scale = 15 ; amp = 0.028

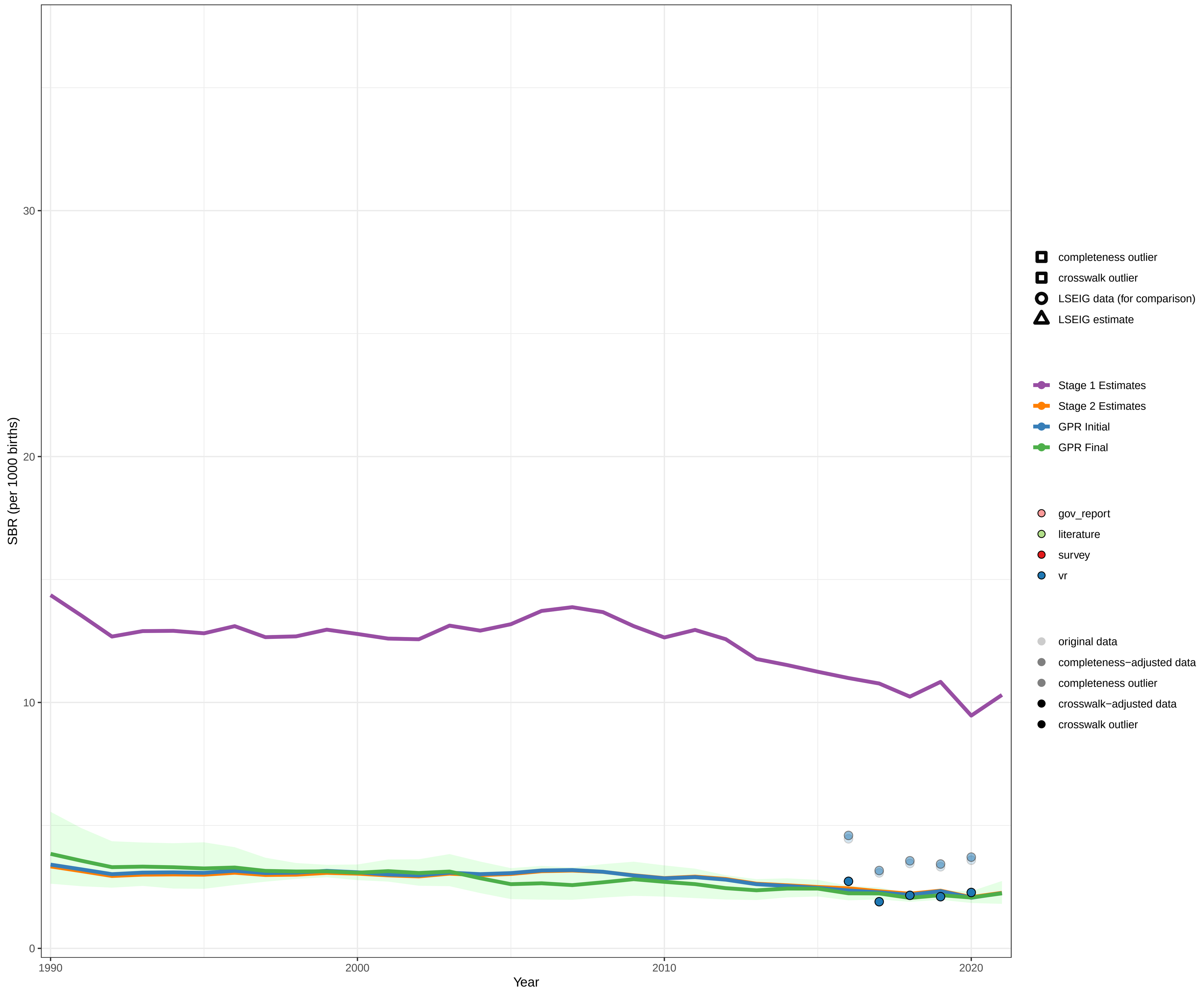

Year	Total Stillbirths (in thousands)	Total Stillbirths (in thousands)	SBR (per 1000 births)	SBR (per 1000 births)	SBR/NMR	SBR/NMR
	20 weeks	28 weeks	20 weeks	28 weeks	20 weeks	28 weeks
1990	0.1<br>(0.1 to 0.2)	0.1<br>(0.0 to 0.1)	6.8<br>(4.8 to 9.5)	3.8<br>(2.6 to 5.6)	1.68<br>(1.18 to 2.36)	0.95<br>(0.65 to 1.38)
1995	0.1<br>(0.1 to 0.1)	0.0<br>(0.0 to 0.1)	6.0<br>(4.7 to 7.5)	3.3<br>(2.4 to 4.3)	1.83<br>(1.42 to 2.27)	0.98<br>(0.73 to 1.31)
2000	0.1<br>(0.1 to 0.1)	0.0<br>(0.0 to 0.0)	6.1<br>(5.6 to 6.7)	3.1<br>(2.8 to 3.4)	2.02<br>(1.83 to 2.22)	1.01<br>(0.91 to 1.12)
2005	0.1<br>(0.1 to 0.1)	0.0<br>(0.0 to 0.0)	5.7<br>(4.8 to 6.6)	2.6<br>(2.0 to 3.3)	1.95<br>(1.63 to 2.27)	0.89<br>(0.69 to 1.12)
2010	0.1<br>(0.1 to 0.1)	0.0<br>(0.0 to 0.1)	6.1<br>(5.2 to 7.2)	2.7<br>(2.1 to 3.4)	2.25<br>(1.91 to 2.66)	1.00<br>(0.78 to 1.25)
2015	0.1<br>(0.1 to 0.1)	0.0<br>(0.0 to 0.0)	5.3<br>(4.5 to 6.3)	2.4<br>(2.1 to 2.8)	2.27<br>(1.92 to 2.69)	1.03<br>(0.90 to 1.19)
2020	0.1<br>(0.1 to 0.1)	0.0<br>(0.0 to 0.0)	4.4<br>(3.8 to 4.9)	2.1<br>(1.8 to 2.3)	2.26<br>(1.98 to 2.55)	1.07<br>(0.96 to 1.20)
2021	0.1<br>(0.1 to 0.1)	0.0<br>(0.0 to 0.0)	4.7<br>(3.8 to 5.9)	2.2<br>(1.8 to 2.7)	2.26<br>(1.82 to 2.81)	1.07<br>(0.86 to 1.31)

Hertfordshire GBR_44719 Western Europe Stillbirth Rate / Neonatal Mortality Rate (SBR/NMR) − 20 weeks
lambda = 0.6 ; zeta = 0.8 ; scale = 15 ; amp = 0.028

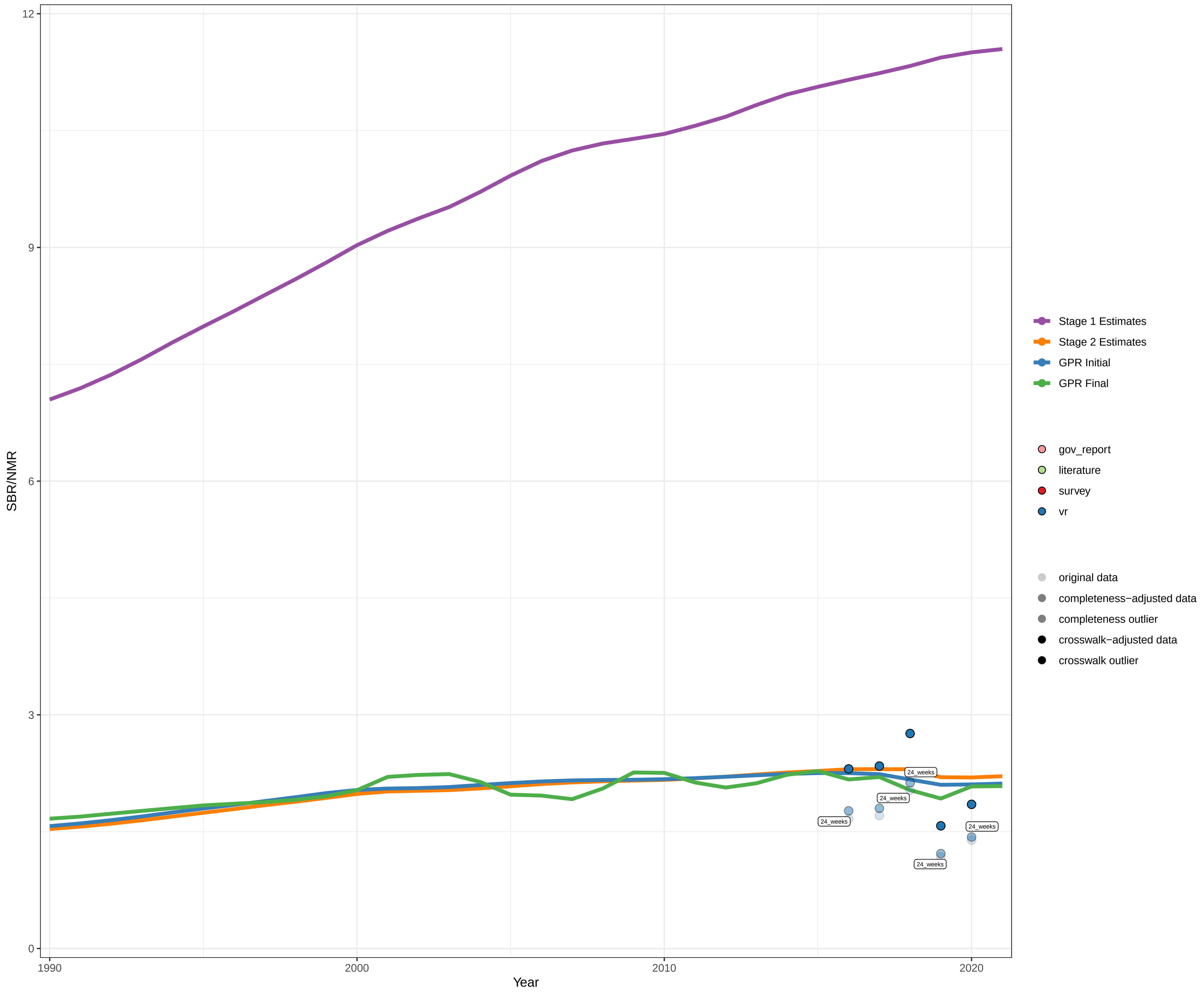

Hertfordshire GBR_44719 Western Europe Stillbirth Rate / Neonatal Mortality Rate (SBR/NMR) − 28 weeks
lambda = 0.6 ; zeta = 0.8 ; scale = 15 ; amp = 0.028

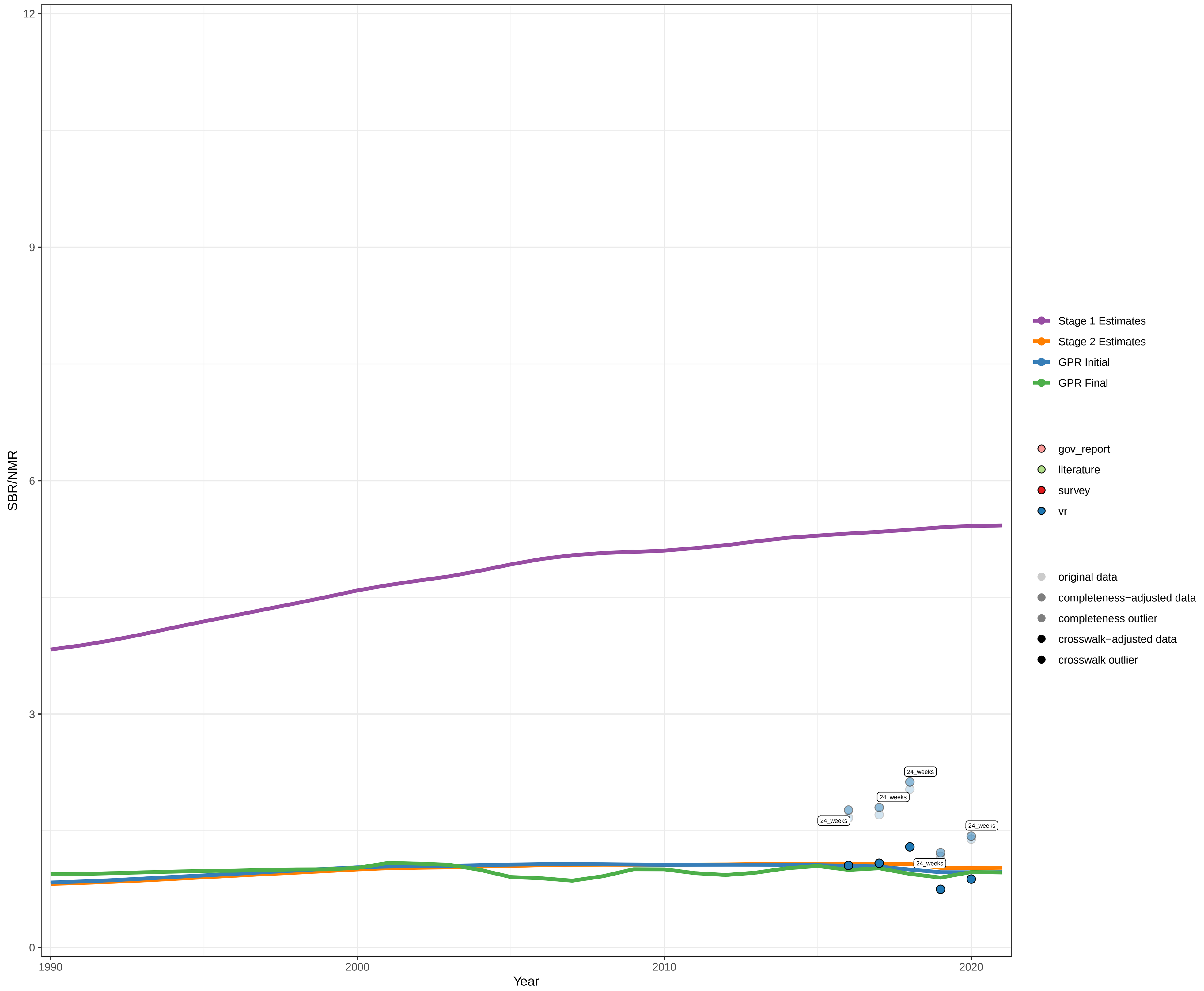

Definition	Years	Outlier	Source.Type	Title
24_weeks	2016−2020	0	VR	United Kingdom − England and Wales Child Mortality Statistics

Hertfordshire GBR_44719 Western Europe Stillbirth Rate (SBR) − 20 weeks
lambda = 0.6 ; zeta = 0.8 ; scale = 15 ; amp = 0.028

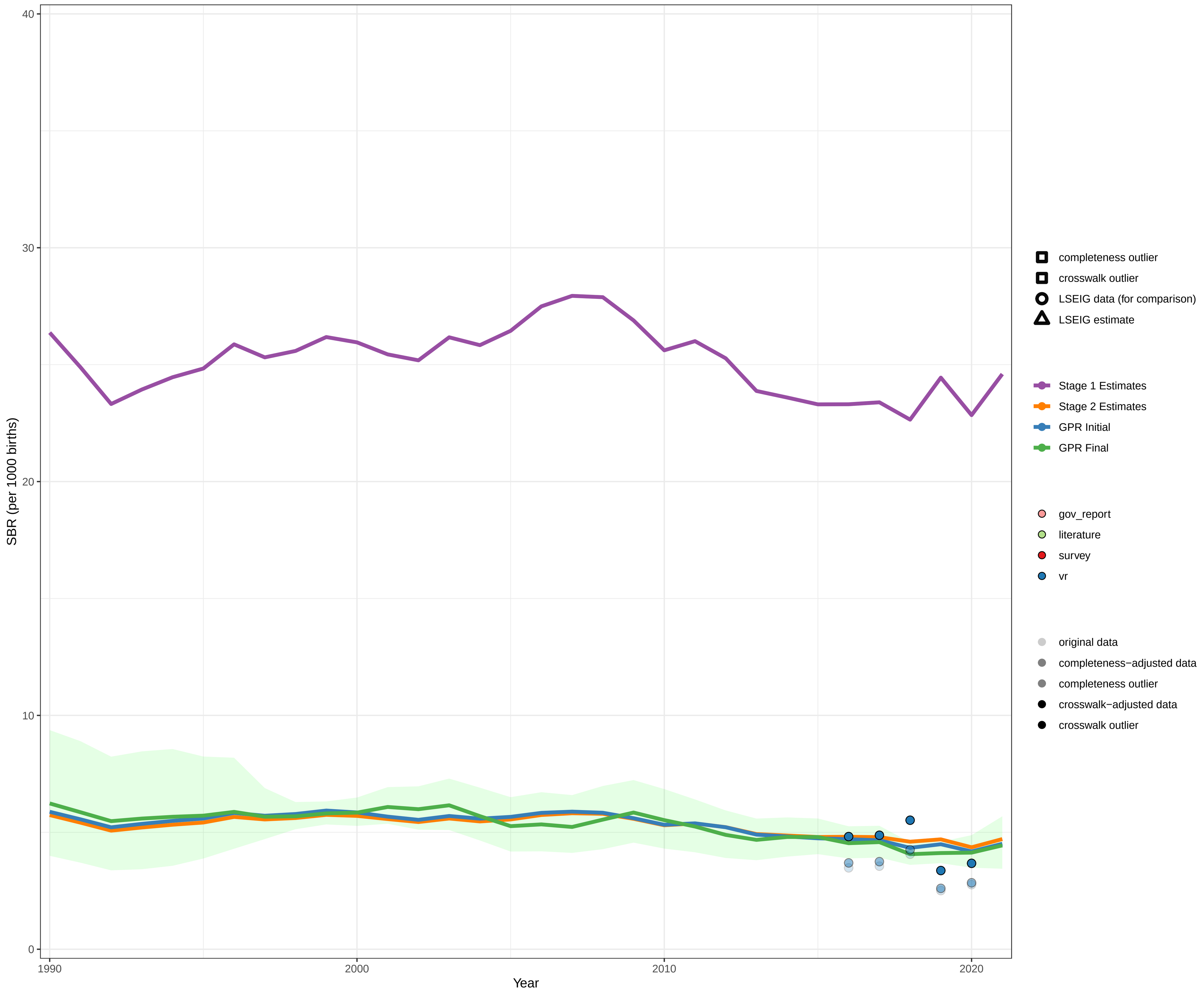

Hertfordshire GBR_44719 Western Europe Stillbirth Rate (SBR) − 28 weeks
lambda = 0.6 ; zeta = 0.8 ; scale = 15 ; amp = 0.028

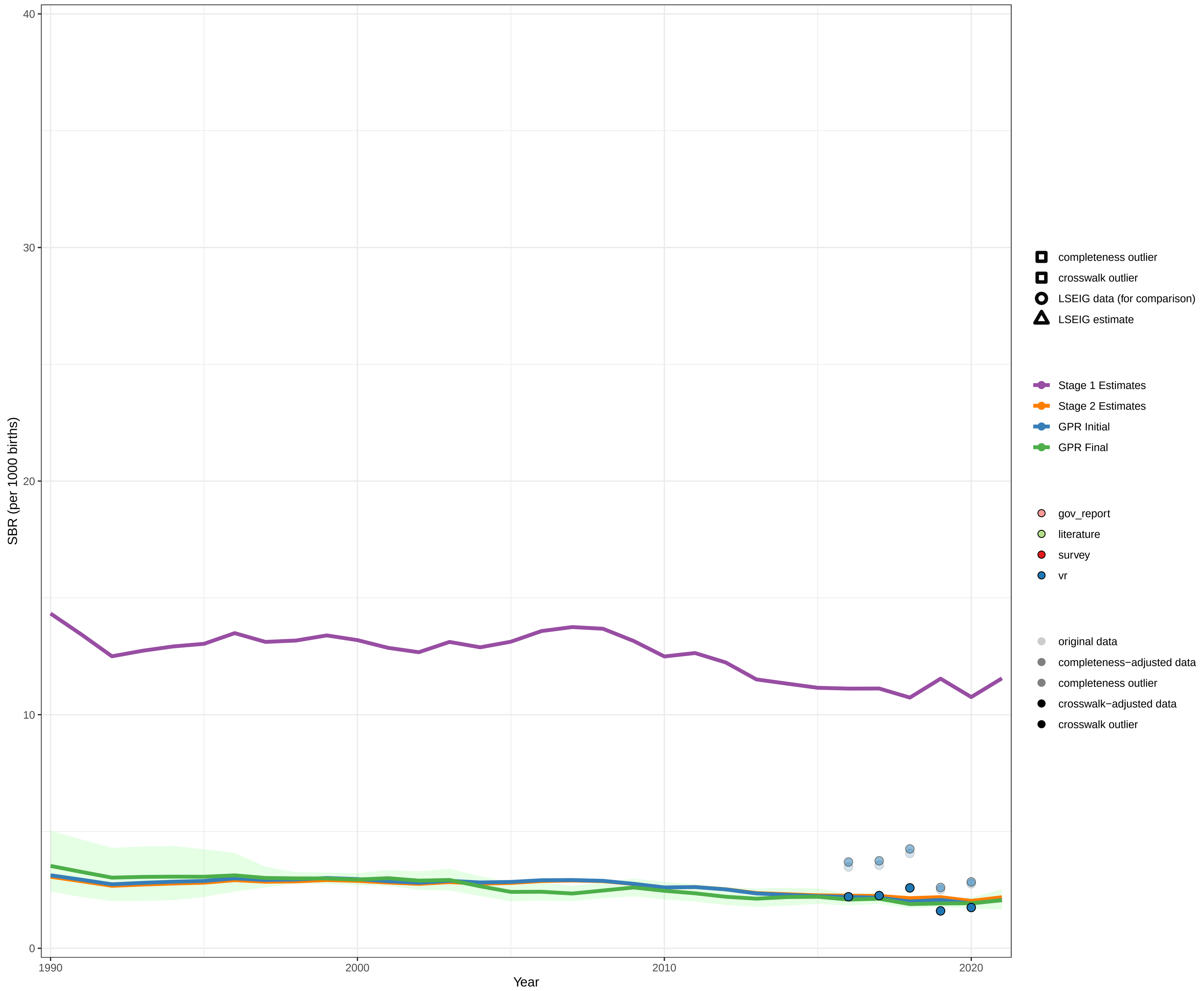

Year	Total Stillbirths (in thousands)	Total Stillbirths (in thousands)	SBR (per 1000 births)	SBR (per 1000 births)	SBR/NMR	SBR/NMR
	20 weeks	28 weeks	20 weeks	28 weeks	20 weeks	28 weeks
1990	0.1 (0.1 to 0.1)	0.0 (0.0 to 0.1)	6.2 (4.0 to 9.4)	3.5 (2.4 to 5.0)	1.67 (1.07 to 2.50)	0.94 (0.65 to 1.35)
1995	0.1 (0.1 to 0.1)	0.0 (0.0 to 0.1)	5.7 (3.9 to 8.2)	3.1 (2.2 to 4.2)	1.84 (1.25 to 2.65)	0.98 (0.71 to 1.36)
2000	0.1 (0.1 to 0.1)	0.0 (0.0 to 0.0)	5.8 (5.3 to 6.5)	2.9 (2.7 to 3.2)	2.03 (1.83 to 2.26)	1.02 (0.94 to 1.12)
2005	0.1 (0.1 to 0.1)	0.0 (0.0 to 0.0)	5.3 (4.2 to 6.5)	2.4 (2.0 to 2.8)	1.97 (1.57 to 2.44)	0.91 (0.75 to 1.06)
2010	0.1 (0.1 to 0.1)	0.0 (0.0 to 0.0)	5.5 (4.3 to 6.8)	2.5 (2.1 to 2.8)	2.25 (1.76 to 2.80)	1.00 (0.85 to 1.16)
2015	0.1 (0.1 to 0.1)	0.0 (0.0 to 0.0)	4.8 (4.1 to 5.6)	2.2 (1.9 to 2.6)	2.28 (1.93 to 2.65)	1.05 (0.90 to 1.22)
2020	0.1 (0.0 to 0.1)	0.0 (0.0 to 0.0)	4.1 (3.5 to 4.9)	1.9 (1.7 to 2.2)	2.08 (1.76 to 2.46)	0.97 (0.86 to 1.09)
2021	0.1 (0.0 to 0.1)	0.0 (0.0 to 0.0)	4.4 (3.4 to 5.7)	2.1 (1.7 to 2.5)	2.08 (1.62 to 2.67)	0.96 (0.78 to 1.19)

Luton GBR_44726 Western Europe Stillbirth Rate / Neonatal Mortality Rate (SBR/NMR) − 20 weeks
lambda = 0.8 ; zeta = 0.7 ; scale = 15 ; amp = 0.028

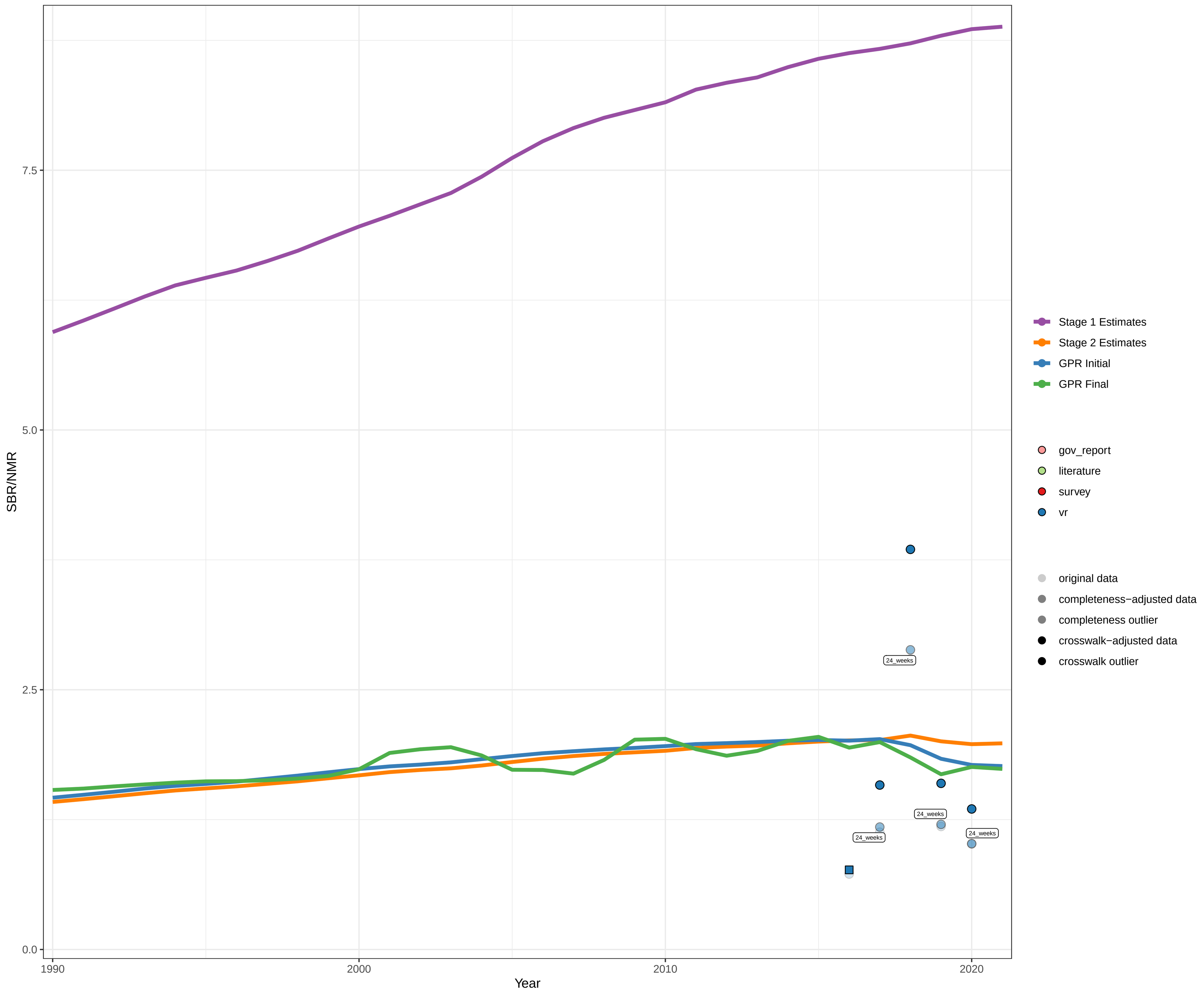

Luton GBR_44726 Western Europe Stillbirth Rate / Neonatal Mortality Rate (SBR/NMR) − 28 weeks
lambda = 0.8 ; zeta = 0.7 ; scale = 15 ; amp = 0.028

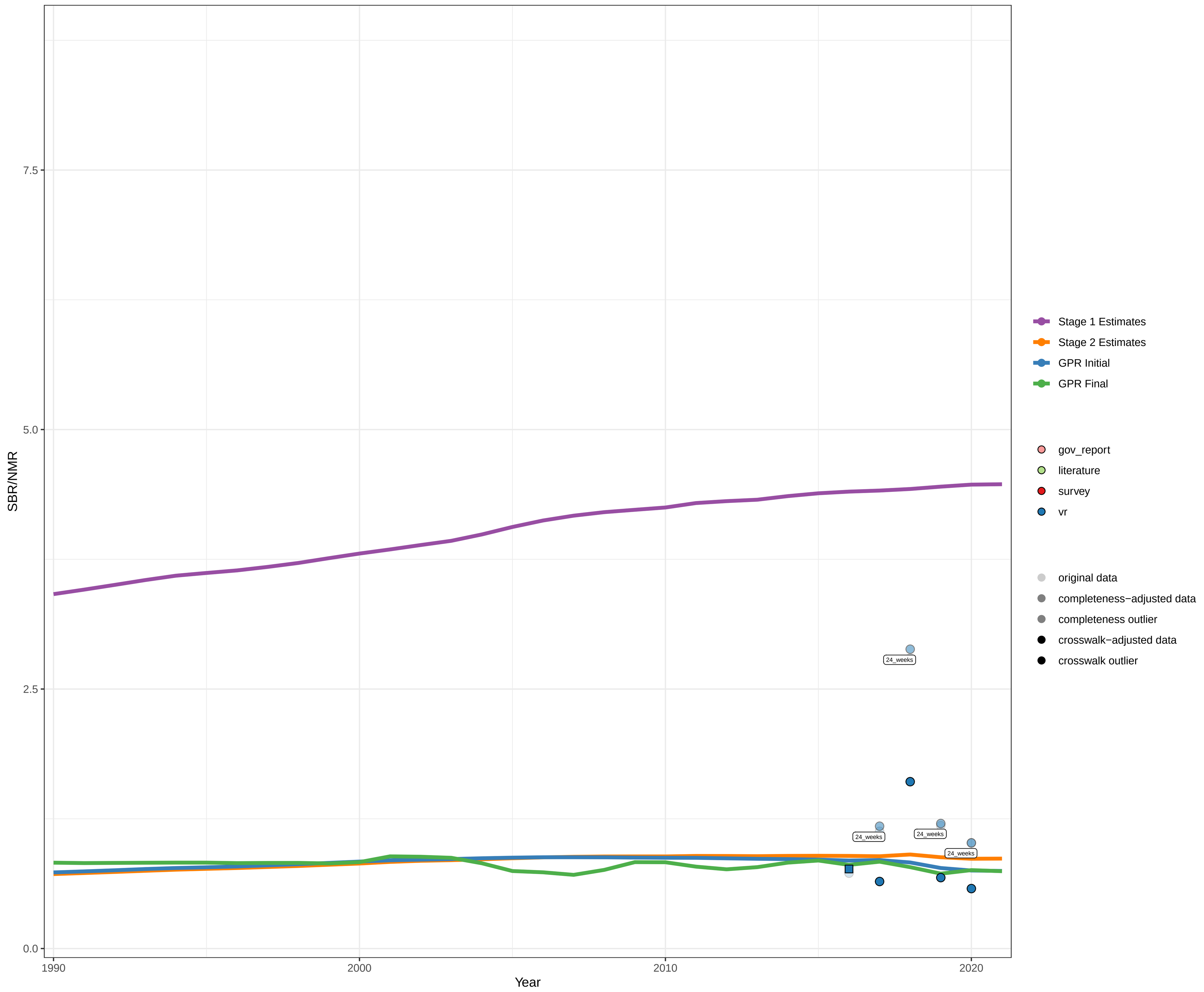

Definition	Years	Outlier	Source.Type	Title
24_weeks	2016−2020	0	VR	United Kingdom − England and Wales Child Mortality Statistics

Luton GBR_44726 Western Europe Stillbirth Rate (SBR) − 20 weeks
lambda = 0.8 ; zeta = 0.7 ; scale = 15 ; amp = 0.028

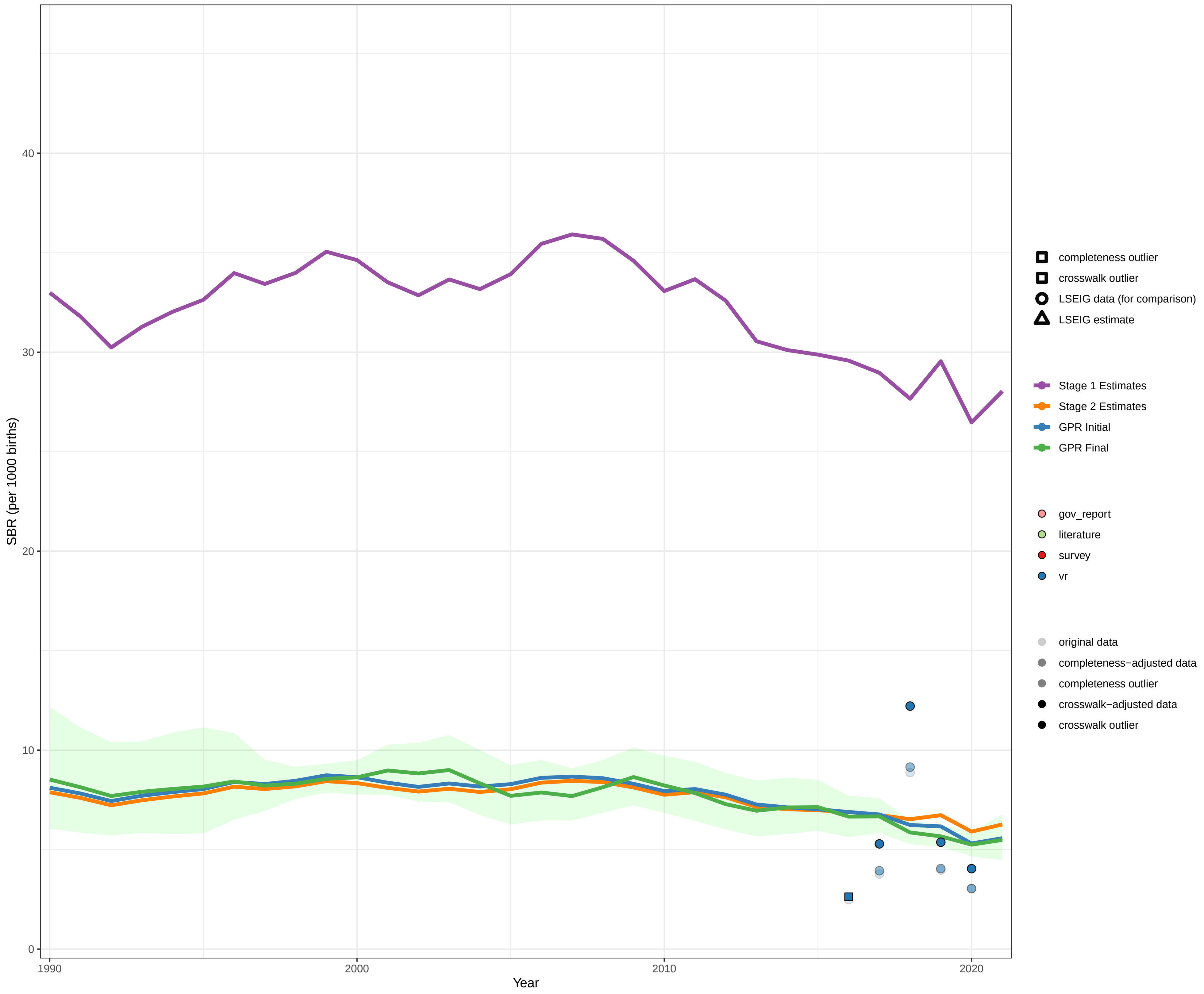

Luton GBR_44726 Western Europe Stillbirth Rate (SBR) − 28 weeks
lambda = 0.8 ; zeta = 0.7 ; scale = 15 ; amp = 0.028

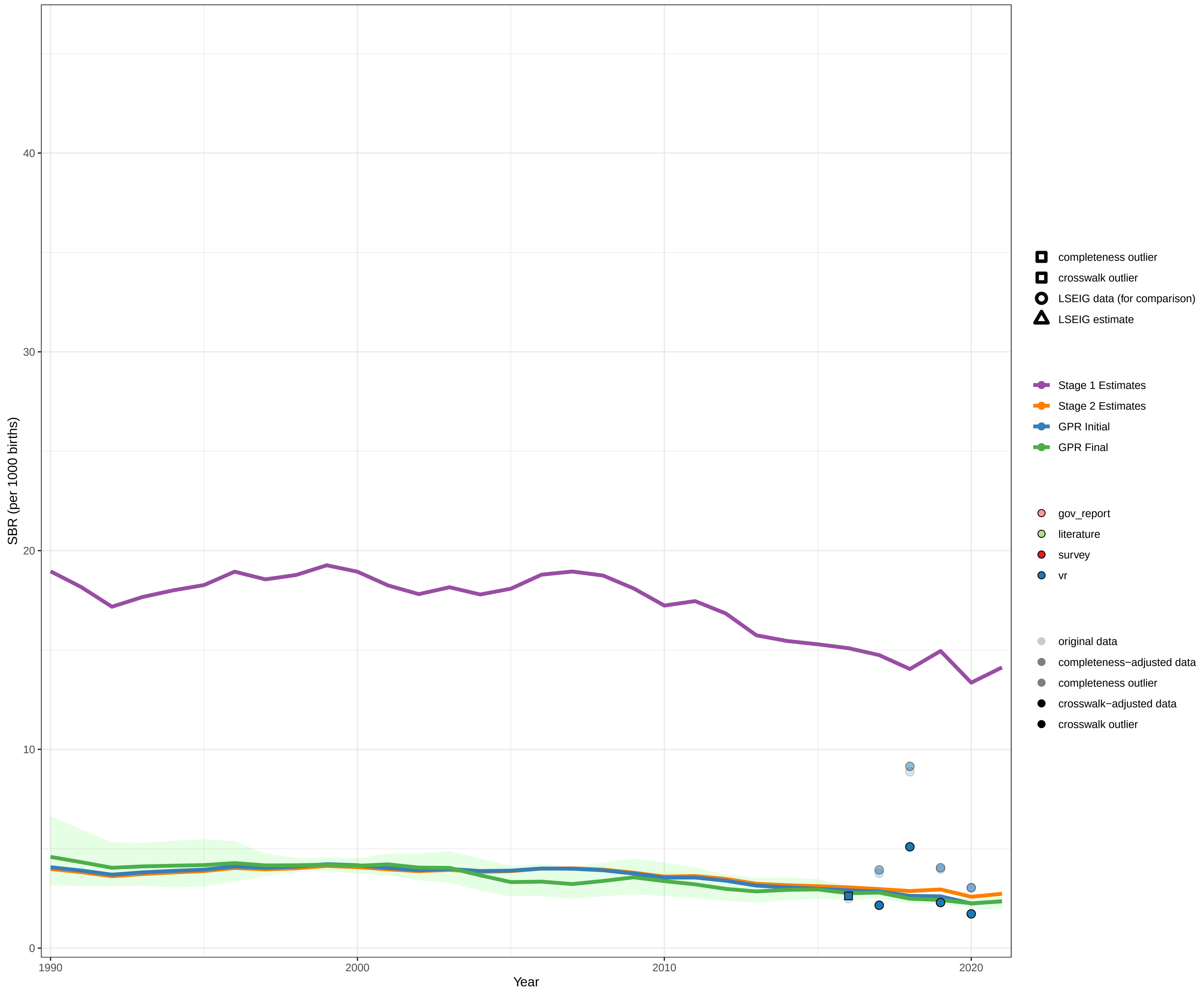

Year	Total Stillbirths (in thousands)	Total Stillbirths (in thousands)	SBR (per 1000 births)	SBR (per 1000 births)	SBR/NMR	SBR/NMR
	20 weeks	28 weeks	20 weeks	28 weeks	20 weeks	28 weeks
1990	0.0 (0.0 to 0.0)	0.0 (0.0 to 0.0)	8.5 (6.1 to 12.2)	4.6 (3.2 to 6.6)	1.54 (1.09 to 2.19)	0.83 (0.57 to 1.20)
1995	0.0 (0.0 to 0.0)	0.0 (0.0 to 0.0)	8.2 (5.8 to 11.1)	4.2 (3.1 to 5.5)	1.62 (1.15 to 2.21)	0.83 (0.62 to 1.09)
2000	0.0 (0.0 to 0.0)	0.0 (0.0 to 0.0)	8.6 (7.8 to 9.5)	4.1 (3.8 to 4.5)	1.73 (1.56 to 1.91)	0.83 (0.75 to 0.91)
2005	0.0 (0.0 to 0.0)	0.0 (0.0 to 0.0)	7.7 (6.3 to 9.2)	3.3 (2.6 to 4.1)	1.73 (1.41 to 2.08)	0.75 (0.59 to 0.93)
2010	0.0 (0.0 to 0.0)	0.0 (0.0 to 0.0)	8.2 (6.8 to 9.7)	3.4 (2.6 to 4.3)	2.03 (1.69 to 2.39)	0.83 (0.65 to 1.06)
2015	0.0 (0.0 to 0.0)	0.0 (0.0 to 0.0)	7.1 (6.0 to 8.5)	3.0 (2.5 to 3.5)	2.05 (1.71 to 2.44)	0.85 (0.72 to 1.00)
2020	0.0 (0.0 to 0.0)	0.0 (0.0 to 0.0)	5.3 (4.7 to 5.9)	2.3 (2.0 to 2.5)	1.76 (1.56 to 1.97)	0.76 (0.68 to 0.84)
2021	0.0 (0.0 to 0.0)	0.0 (0.0 to 0.0)	5.5 (4.5 to 6.8)	2.4 (1.9 to 2.8)	1.74 (1.41 to 2.14)	0.74 (0.61 to 0.90)

Norfolk GBR_44723 Western Europe Stillbirth Rate / Neonatal Mortality Rate (SBR/NMR) − 20 weeks
lambda = 0.6 ; zeta = 0.8 ; scale = 15 ; amp = 0.028

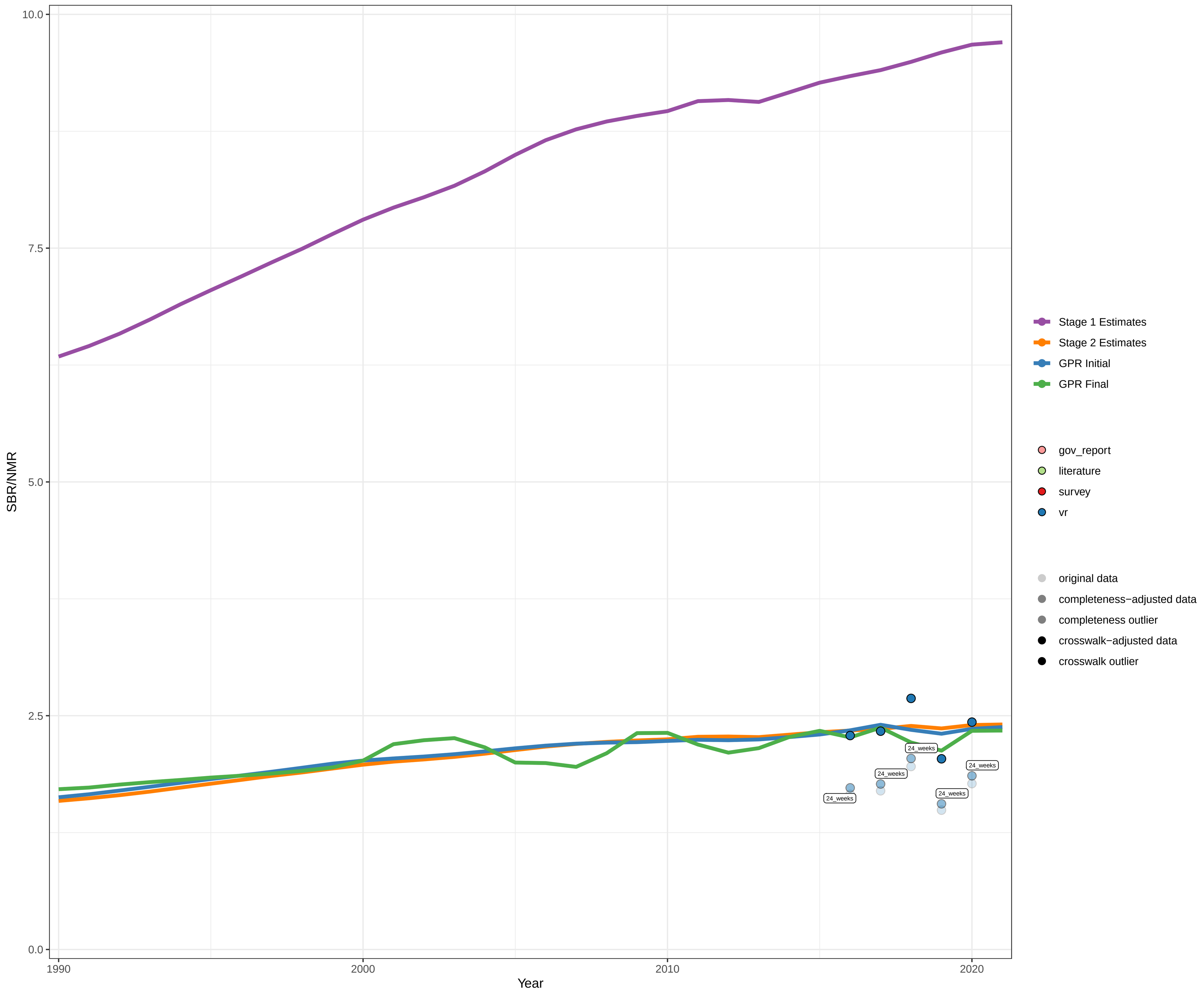

Norfolk GBR_44723 Western Europe Stillbirth Rate / Neonatal Mortality Rate (SBR/NMR) − 28 weeks
lambda = 0.6 ; zeta = 0.8 ; scale = 15 ; amp = 0.028

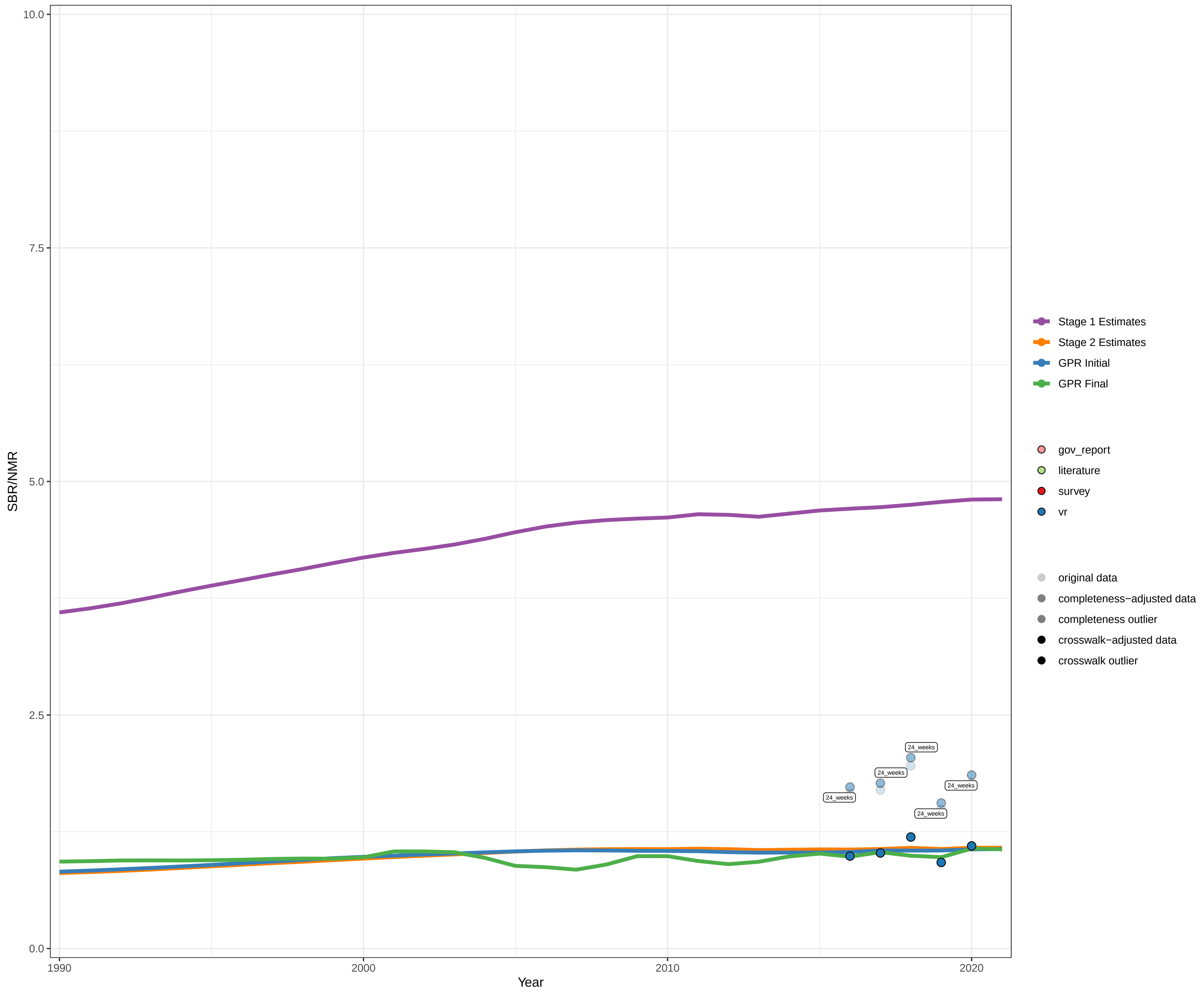

Definition	Years	Outlier	Source.Type	Title
24_weeks	2016−2020	0	VR	United Kingdom − England and Wales Child Mortality Statistics

Norfolk GBR_44723 Western Europe Stillbirth Rate (SBR) − 20 weeks
lambda = 0.6 ; zeta = 0.8 ; scale = 15 ; amp = 0.028

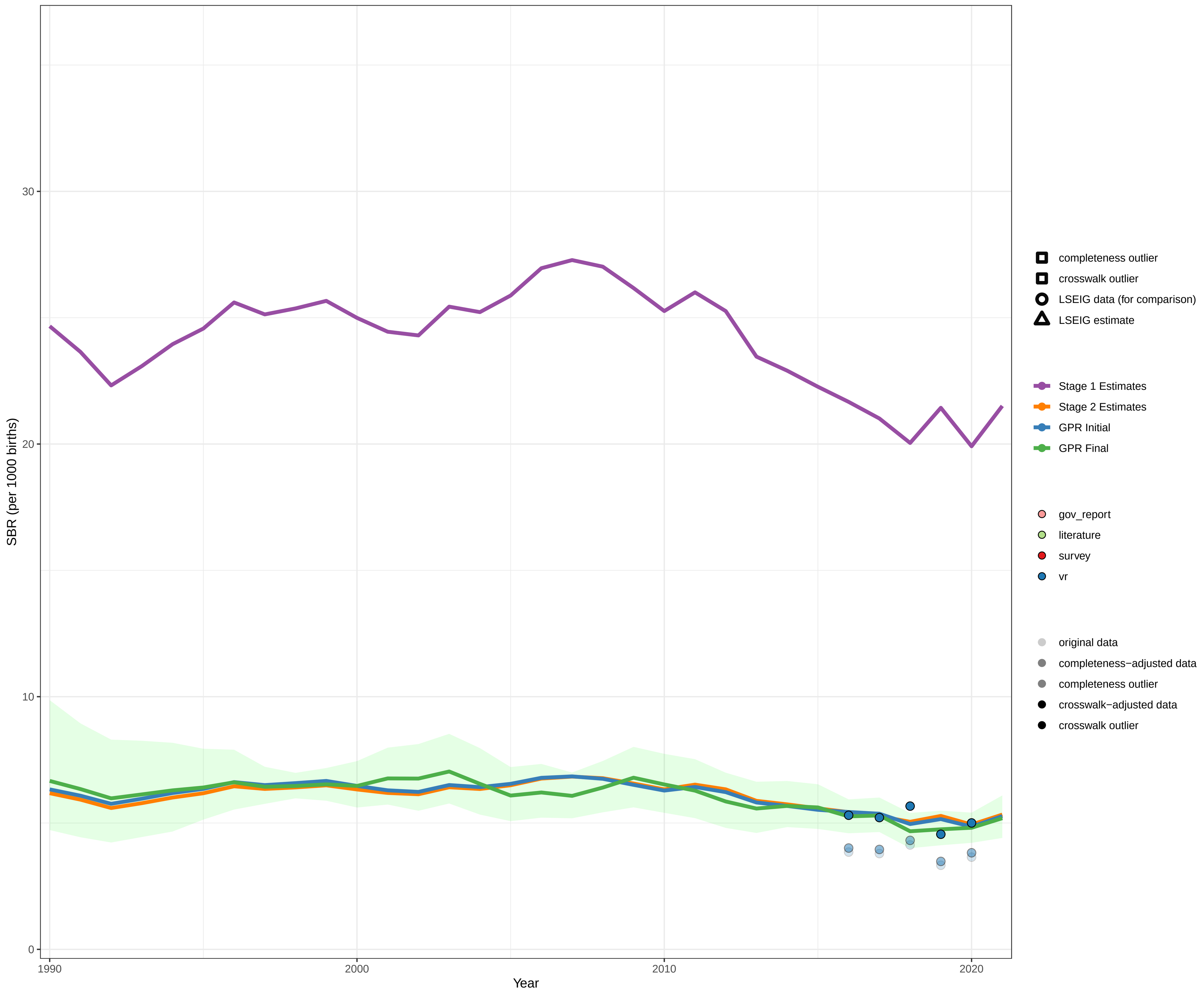

Norfolk GBR_44723 Western Europe Stillbirth Rate (SBR) − 28 weeks
lambda = 0.6 ; zeta = 0.8 ; scale = 15 ; amp = 0.028

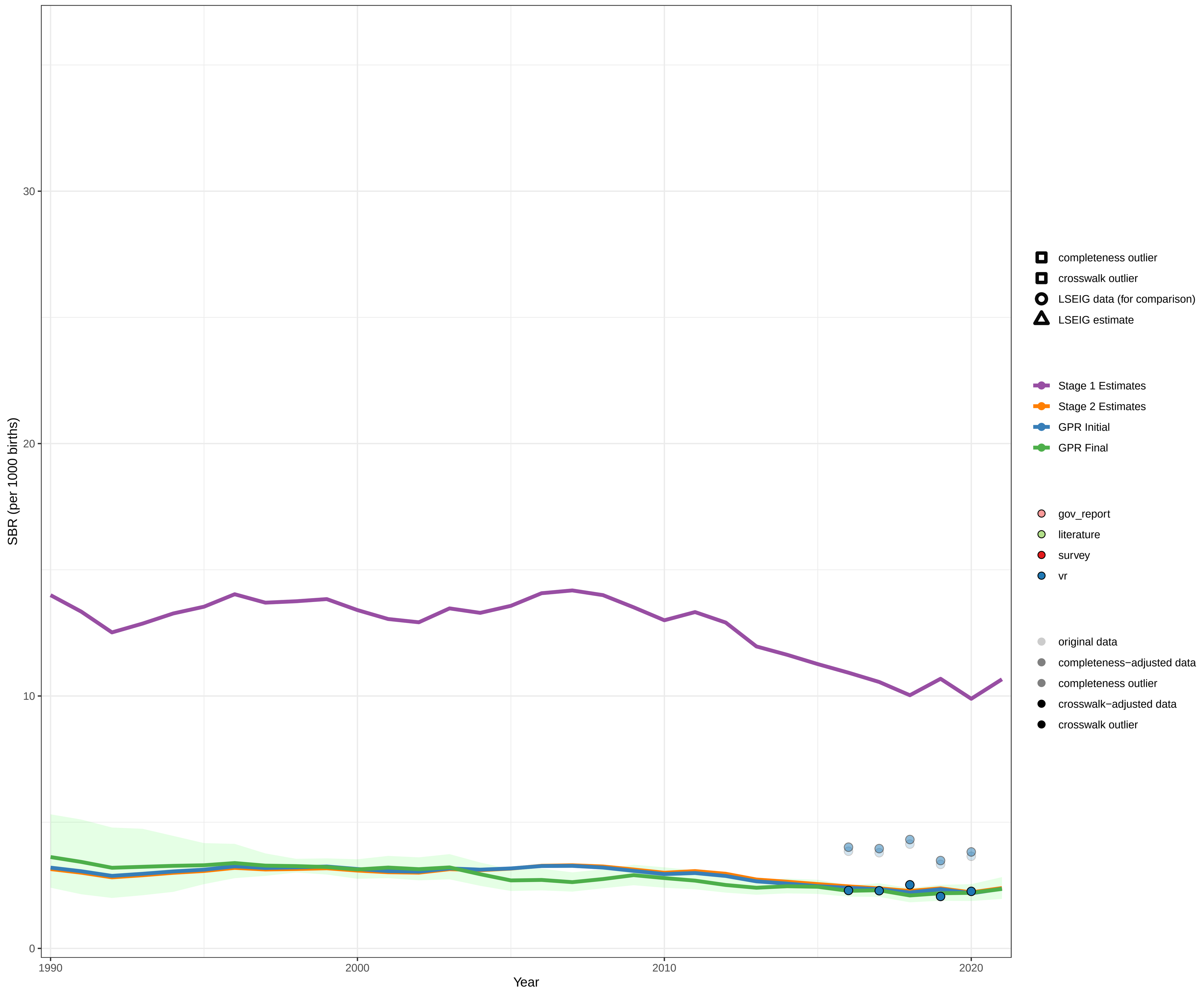

Year	Total Stillbirths (in thousands)	Total Stillbirths (in thousands)	SBR (per 1000 births)	SBR (per 1000 births)	SBR/NMR	SBR/NMR
	20 weeks	28 weeks	20 weeks	28 weeks	20 weeks	28 weeks
1990	0.1 (0.0 to 0.1)	0.0 (0.0 to 0.0)	6.7 (4.7 to 9.9)	3.6 (2.4 to 5.3)	1.71 (1.21 to 2.54)	0.93 (0.62 to 1.37)
1995	0.1 (0.0 to 0.1)	0.0 (0.0 to 0.0)	6.4 (5.1 to 7.9)	3.3 (2.5 to 4.2)	1.84 (1.47 to 2.28)	0.95 (0.73 to 1.20)
2000	0.1 (0.0 to 0.1)	0.0 (0.0 to 0.0)	6.5 (5.6 to 7.5)	3.1 (2.8 to 3.5)	2.02 (1.75 to 2.33)	0.98 (0.86 to 1.10)
2005	0.1 (0.0 to 0.1)	0.0 (0.0 to 0.0)	6.1 (5.1 to 7.2)	2.7 (2.3 to 3.1)	2.00 (1.67 to 2.37)	0.88 (0.75 to 1.03)
2010	0.1 (0.0 to 0.1)	0.0 (0.0 to 0.0)	6.5 (5.4 to 7.7)	2.8 (2.4 to 3.2)	2.32 (1.92 to 2.75)	0.99 (0.85 to 1.14)
2015	0.1 (0.0 to 0.1)	0.0 (0.0 to 0.0)	5.6 (4.8 to 6.5)	2.4 (2.2 to 2.7)	2.34 (1.98 to 2.72)	1.02 (0.90 to 1.13)
2020	0.0 (0.0 to 0.0)	0.0 (0.0 to 0.0)	4.8 (4.2 to 5.4)	2.2 (1.9 to 2.5)	2.34 (2.05 to 2.63)	1.07 (0.91 to 1.24)
2021	0.0 (0.0 to 0.0)	0.0 (0.0 to 0.0)	5.2 (4.4 to 6.1)	2.4 (2.0 to 2.8)	2.34 (1.99 to 2.75)	1.06 (0.88 to 1.27)

Peterborough GBR_44725 Western Europe Stillbirth Rate / Neonatal Mortality Rate (SBR/NMR) − 20 weeks
lambda = 0.6 ; zeta = 0.8 ; scale = 15 ; amp = 0.028

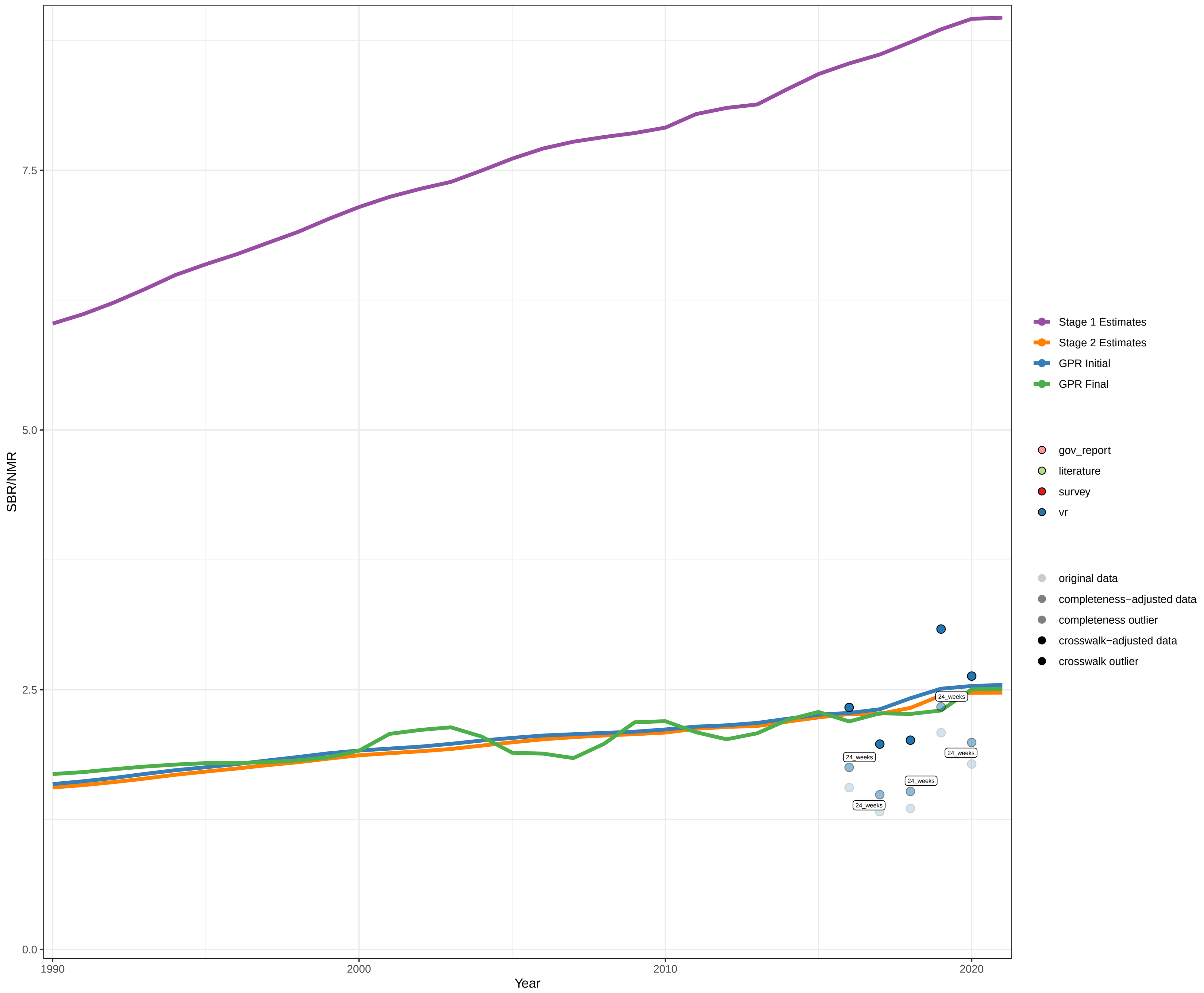

Peterborough GBR_44725 Western Europe Stillbirth Rate / Neonatal Mortality Rate (SBR/NMR) − 28 weeks
lambda = 0.6 ; zeta = 0.8 ; scale = 15 ; amp = 0.028

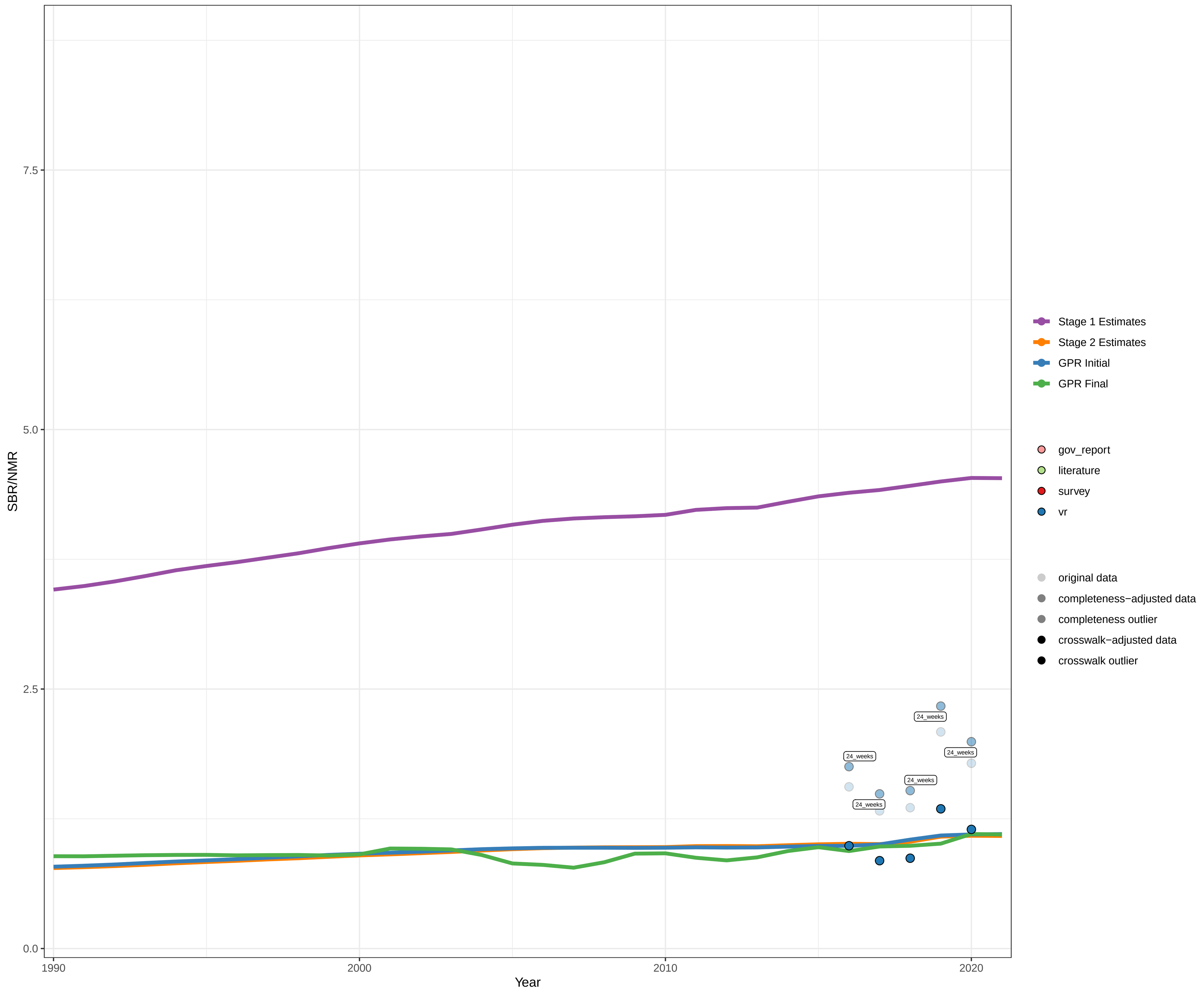

Definition	Years	Outlier	Source.Type	Title
24_weeks	2016−2020	0	VR	United Kingdom − England and Wales Child Mortality Statistics

Peterborough GBR_44725 Western Europe Stillbirth Rate (SBR) − 20 weeks
lambda = 0.6 ; zeta = 0.8 ; scale = 15 ; amp = 0.028

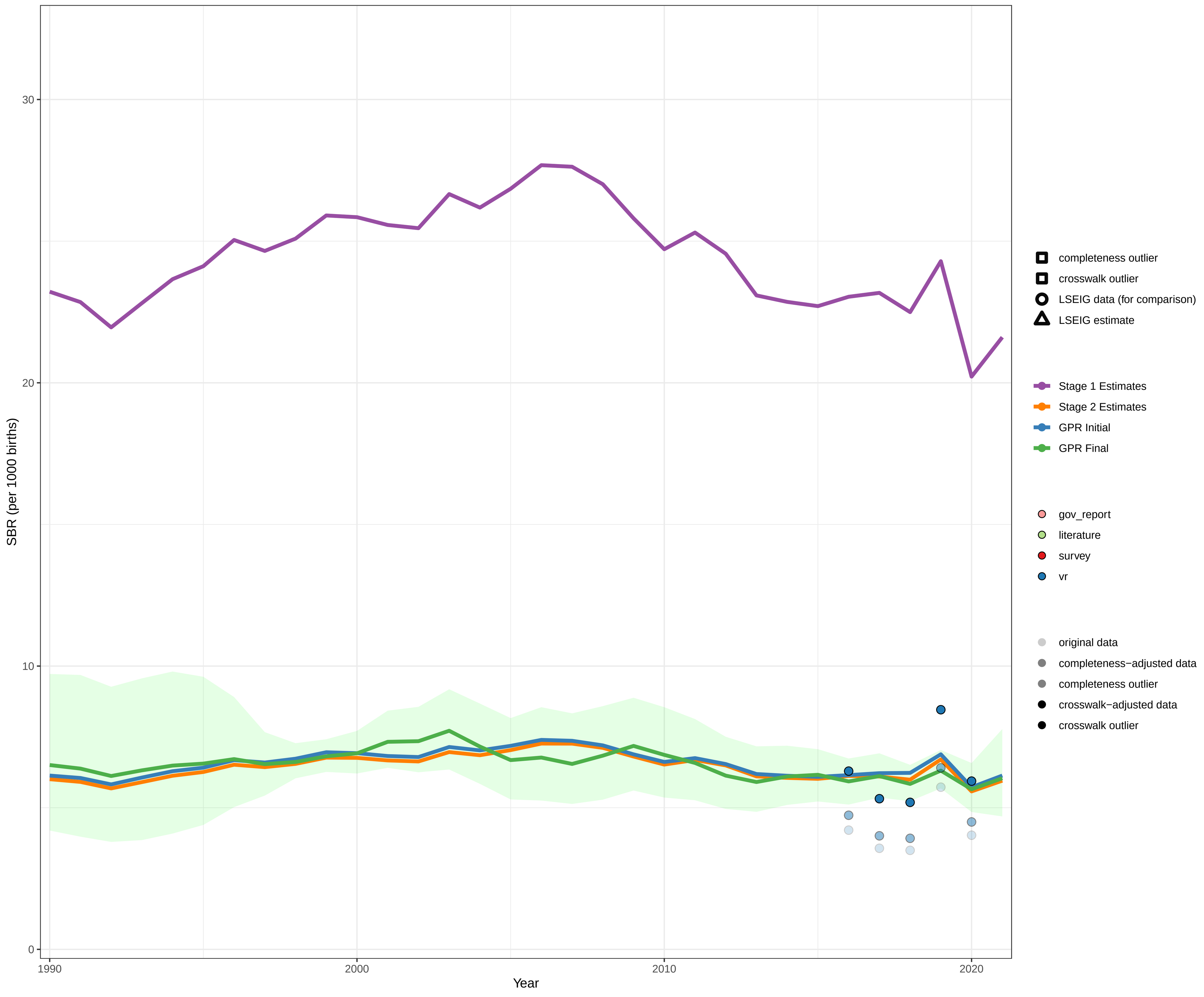

Peterborough GBR_44725 Western Europe Stillbirth Rate (SBR) − 28 weeks
lambda = 0.6 ; zeta = 0.8 ; scale = 15 ; amp = 0.028

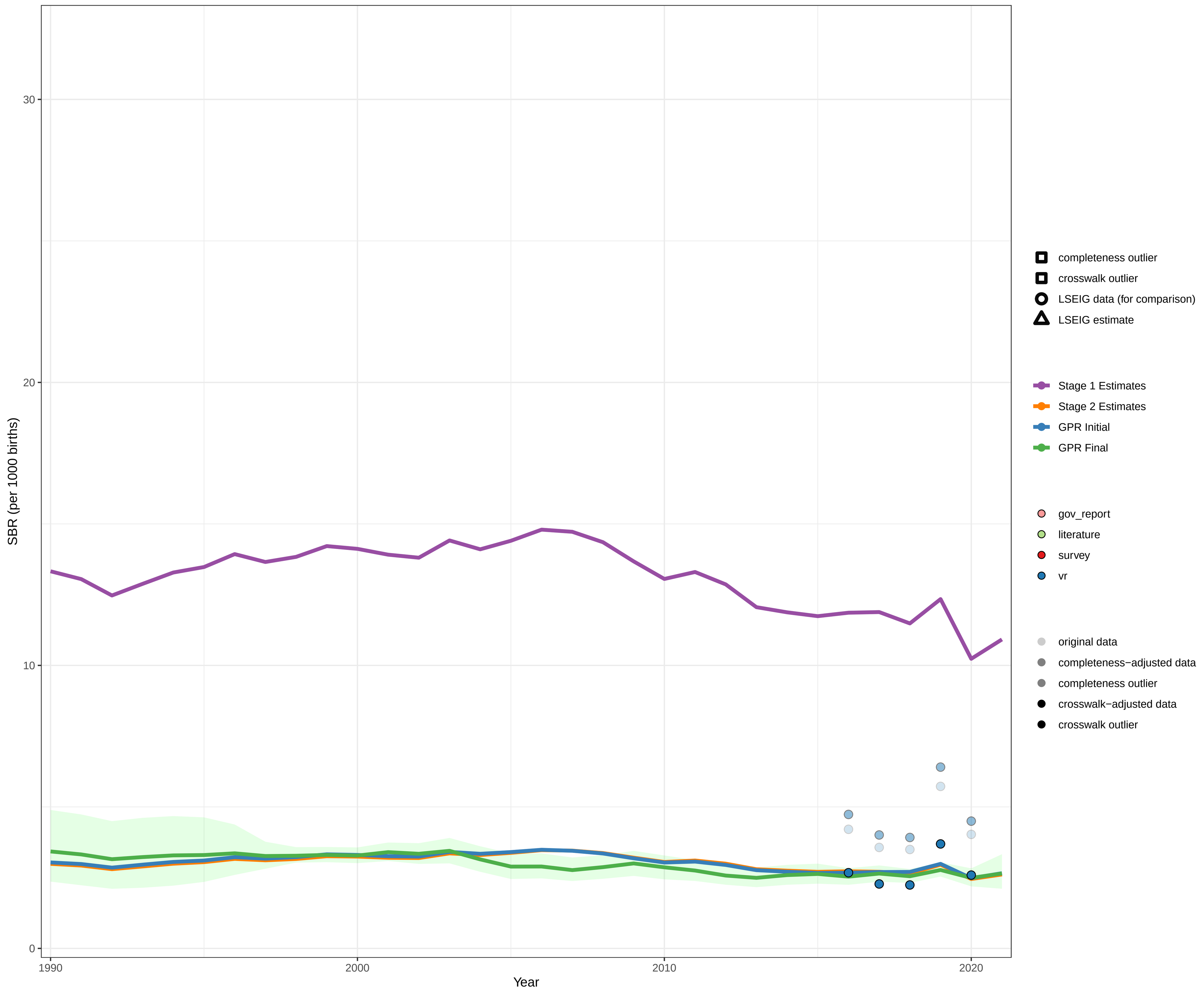

Year	Total Stillbirths (in thousands)	Total Stillbirths (in thousands)	SBR (per 1000 births)	SBR (per 1000 births)	SBR/NMR	SBR/NMR
	20 weeks	28 weeks	20 weeks	28 weeks	20 weeks	28 weeks
1990	0.0 (0.0 to 0.0)	0.0 (0.0 to 0.0)	6.5 (4.2 to 9.7)	3.4 (2.4 to 4.9)	1.69 (1.09 to 2.52)	0.89 (0.61 to 1.27)
1995	0.0 (0.0 to 0.0)	0.0 (0.0 to 0.0)	6.6 (4.4 to 9.6)	3.3 (2.3 to 4.6)	1.79 (1.20 to 2.63)	0.90 (0.64 to 1.27)
2000	0.0 (0.0 to 0.0)	0.0 (0.0 to 0.0)	6.9 (6.2 to 7.7)	3.3 (3.0 to 3.6)	1.91 (1.72 to 2.13)	0.91 (0.83 to 0.99)
2005	0.0 (0.0 to 0.0)	0.0 (0.0 to 0.0)	6.7 (5.3 to 8.2)	2.9 (2.5 to 3.4)	1.89 (1.50 to 2.31)	0.82 (0.70 to 0.95)
2010	0.0 (0.0 to 0.0)	0.0 (0.0 to 0.0)	6.9 (5.4 to 8.5)	2.9 (2.4 to 3.3)	2.20 (1.71 to 2.74)	0.92 (0.78 to 1.05)
2015	0.0 (0.0 to 0.0)	0.0 (0.0 to 0.0)	6.2 (5.2 to 7.1)	2.6 (2.3 to 3.0)	2.29 (1.94 to 2.62)	0.98 (0.85 to 1.11)
2020	0.0 (0.0 to 0.0)	0.0 (0.0 to 0.0)	5.7 (4.8 to 6.6)	2.5 (2.2 to 2.8)	2.50 (2.15 to 2.91)	1.10 (0.97 to 1.25)
2021	0.0 (0.0 to 0.0)	0.0 (0.0 to 0.0)	6.0 (4.7 to 7.8)	2.6 (2.1 to 3.3)	2.51 (1.95 to 3.23)	1.10 (0.88 to 1.38)

Southend−on−Sea GBR_44724 Western Europe Stillbirth Rate / Neonatal Mortality Rate (SBR/NMR) − 20 weeks
lambda = 0.6 ; zeta = 0.8 ; scale = 15 ; amp = 0.028

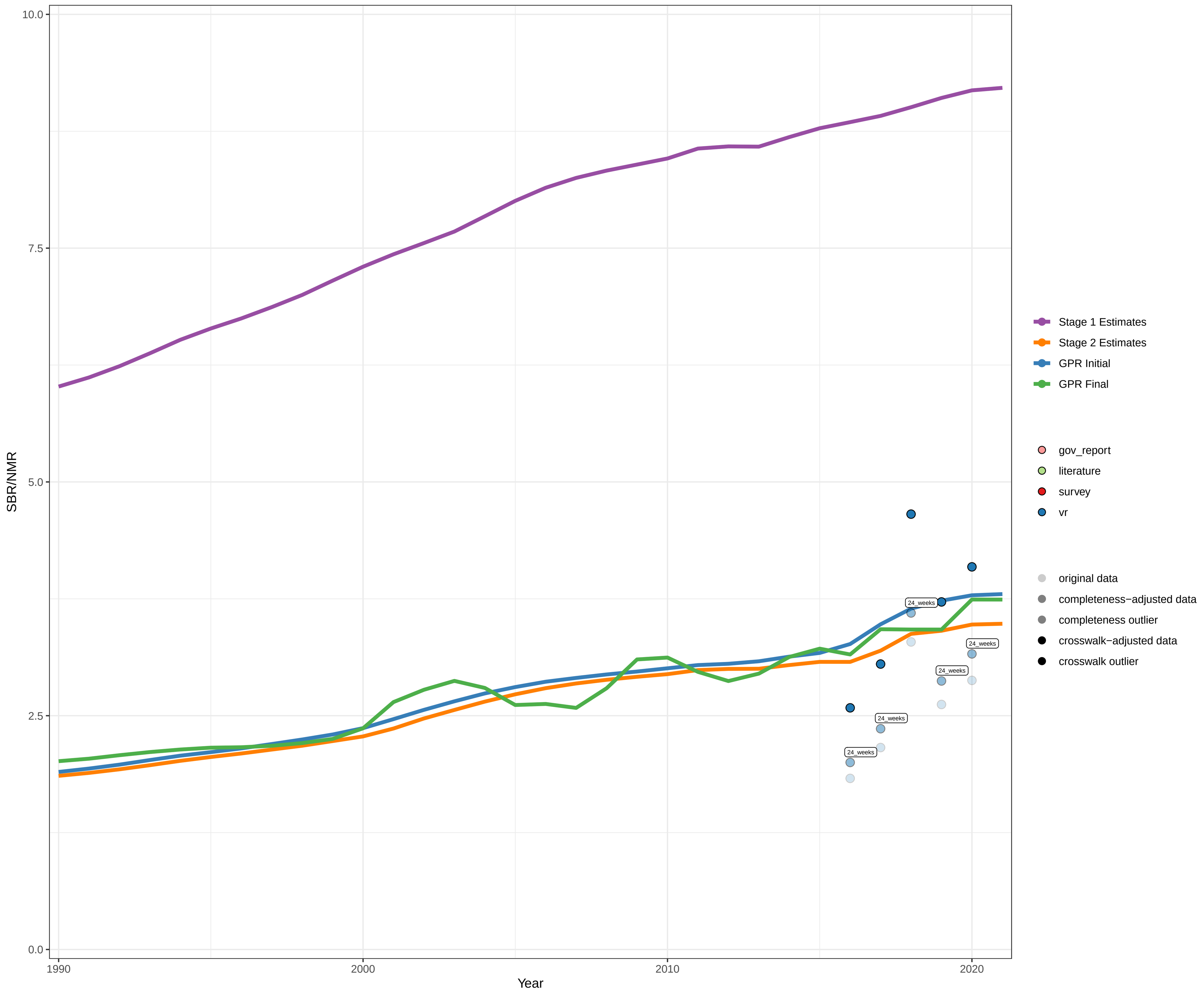

Southend−on−Sea GBR_44724 Western Europe Stillbirth Rate / Neonatal Mortality Rate (SBR/NMR) − 28 weeks
lambda = 0.6 ; zeta = 0.8 ; scale = 15 ; amp = 0.028

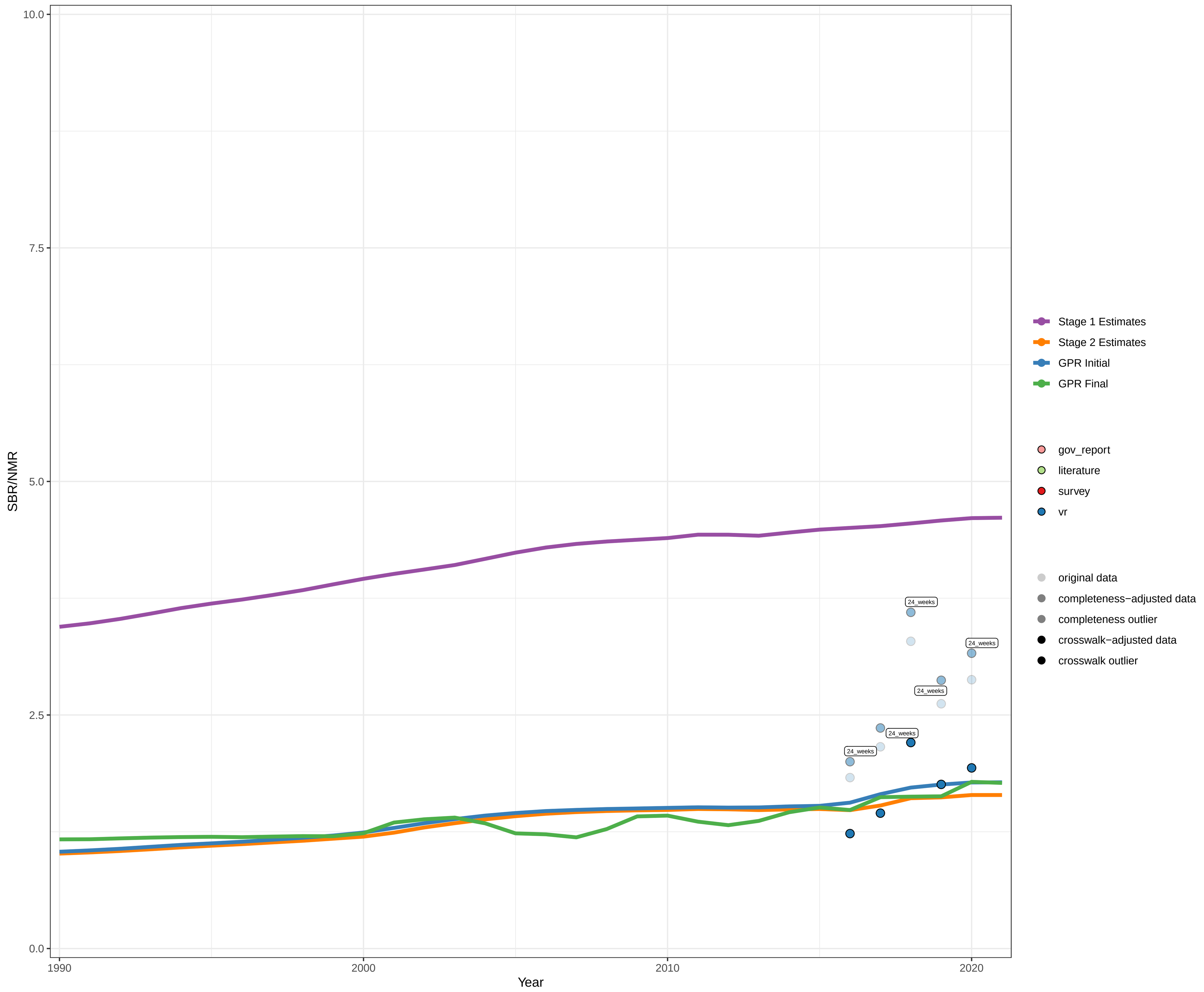

Definition	Years	Outlier	Source.Type	Title
24_weeks	2016−2020	0	VR	United Kingdom − England and Wales Child Mortality Statistics

Southend−on−Sea GBR_44724 Western Europe Stillbirth Rate (SBR) − 20 weeks
lambda = 0.6 ; zeta = 0.8 ; scale = 15 ; amp = 0.028

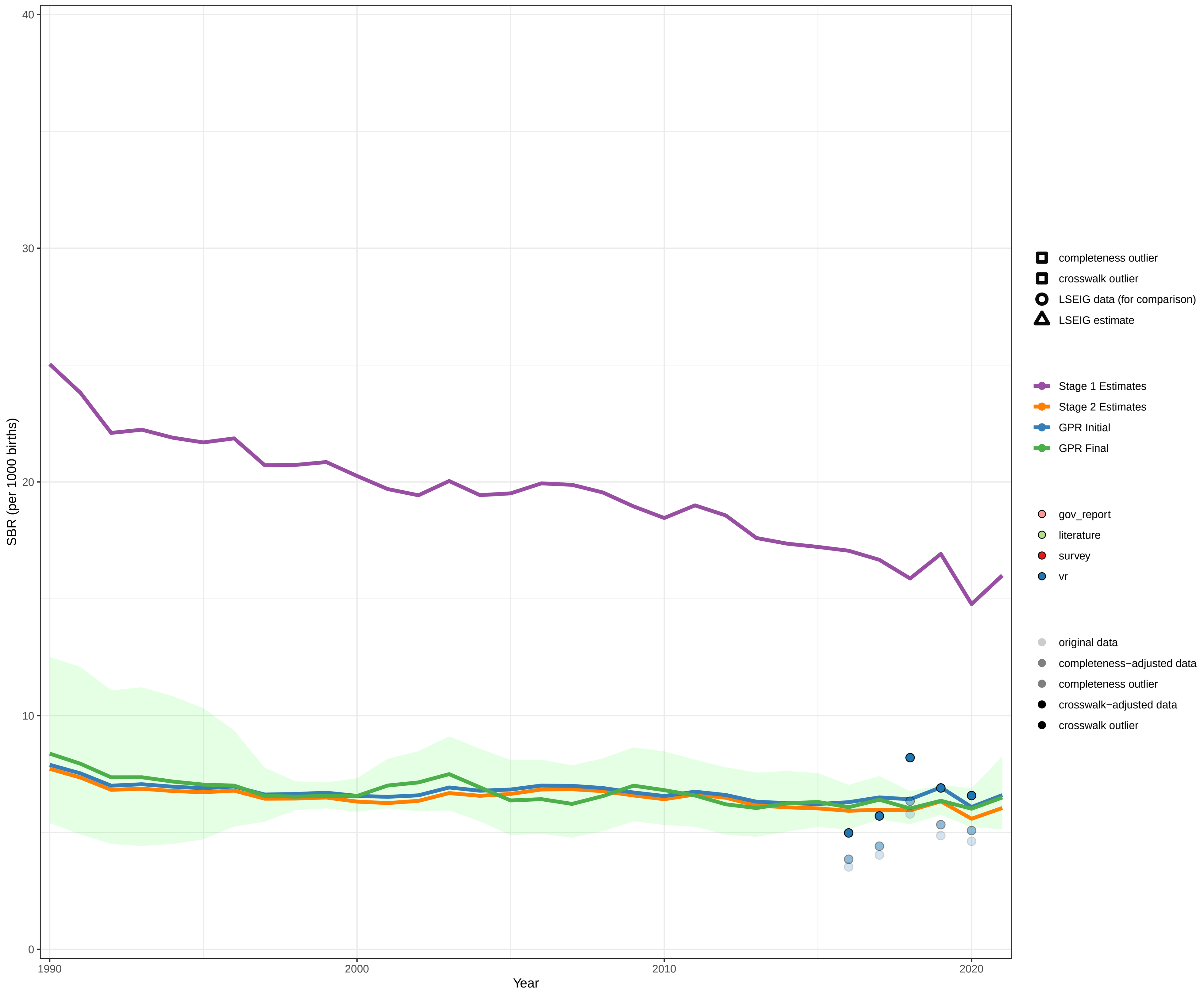

Southend−on−Sea GBR_44724 Western Europe Stillbirth Rate (SBR) − 28 weeks
lambda = 0.6 ; zeta = 0.8 ; scale = 15 ; amp = 0.028

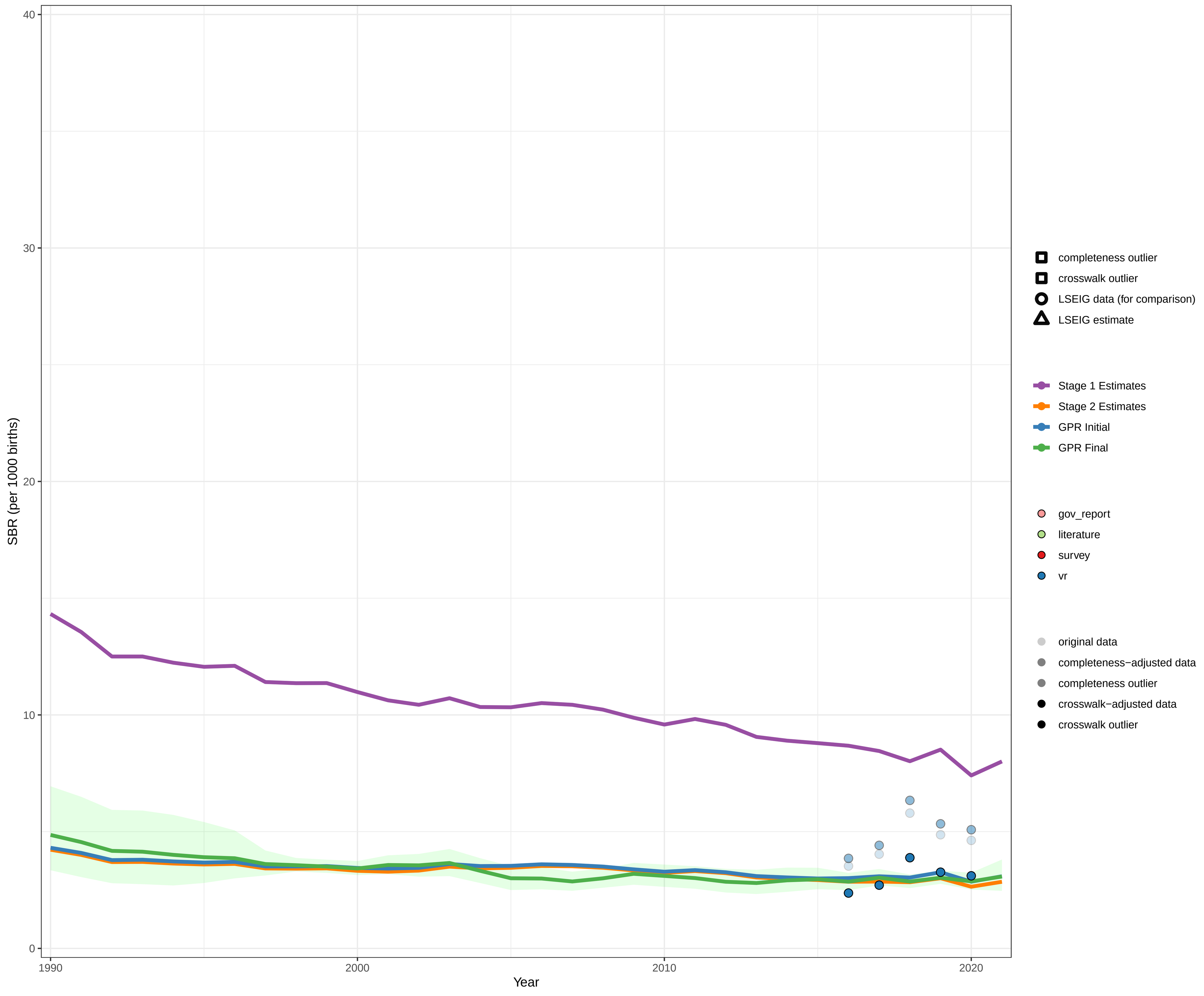

Year	Total Stillbirths (in thousands)	Total Stillbirths (in thousands)	SBR (per 1000 births)	SBR (per 1000 births)	SBR/NMR	SBR/NMR
	20 weeks	28 weeks	20 weeks	28 weeks	20 weeks	28 weeks
1990	0.0 (0.0 to 0.0)	0.0 (0.0 to 0.0)	8.4 (5.4 to 12.5)	4.9 (3.3 to 6.9)	2.01 (1.30 to 3.01)	1.17 (0.80 to 1.67)
1995	0.0 (0.0 to 0.0)	0.0 (0.0 to 0.0)	7.0 (4.7 to 10.3)	3.9 (2.8 to 5.4)	2.16 (1.44 to 3.16)	1.20 (0.86 to 1.66)
2000	0.0 (0.0 to 0.0)	0.0 (0.0 to 0.0)	6.6 (5.9 to 7.3)	3.4 (3.1 to 3.7)	2.37 (2.12 to 2.64)	1.23 (1.13 to 1.35)
2005	0.0 (0.0 to 0.0)	0.0 (0.0 to 0.0)	6.4 (4.9 to 8.1)	3.0 (2.5 to 3.5)	2.61 (2.01 to 3.33)	1.23 (1.03 to 1.45)
2010	0.0 (0.0 to 0.0)	0.0 (0.0 to 0.0)	6.8 (5.3 to 8.5)	3.1 (2.6 to 3.6)	3.12 (2.44 to 3.88)	1.42 (1.21 to 1.65)
2015	0.0 (0.0 to 0.0)	0.0 (0.0 to 0.0)	6.3 (5.2 to 7.5)	3.0 (2.5 to 3.5)	3.22 (2.67 to 3.85)	1.51 (1.29 to 1.76)
2020	0.0 (0.0 to 0.0)	0.0 (0.0 to 0.0)	6.0 (5.2 to 6.9)	2.9 (2.5 to 3.2)	3.74 (3.25 to 4.29)	1.78 (1.57 to 2.02)
2021	0.0 (0.0 to 0.0)	0.0 (0.0 to 0.0)	6.5 (5.1 to 8.3)	3.1 (2.5 to 3.8)	3.74 (2.96 to 4.75)	1.77 (1.42 to 2.19)

Suffolk GBR_44718 Western Europe Stillbirth Rate / Neonatal Mortality Rate (SBR/NMR) − 20 weeks
lambda = 0.6 ; zeta = 0.8 ; scale = 15 ; amp = 0.028

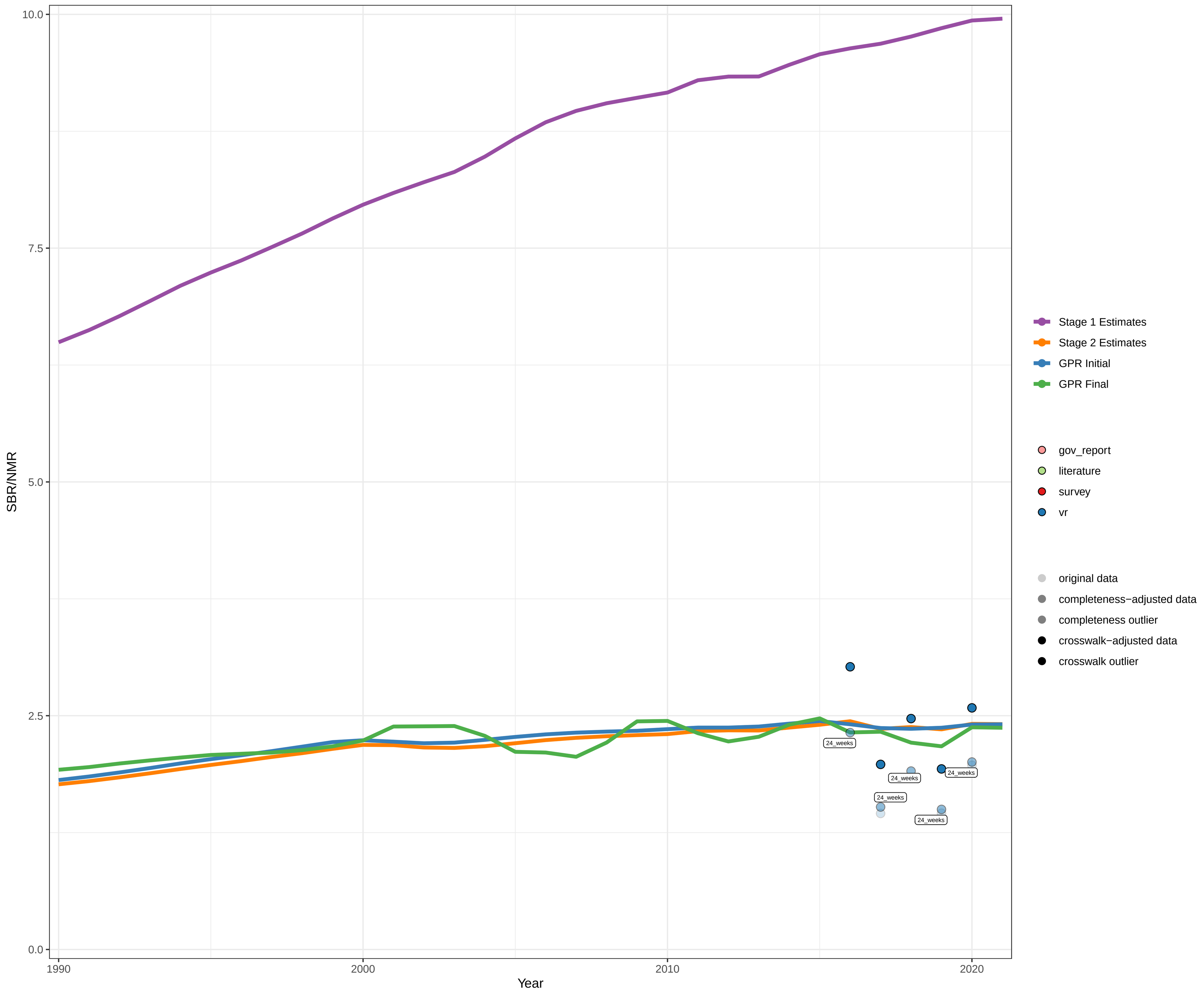

Suffolk GBR_44718 Western Europe Stillbirth Rate / Neonatal Mortality Rate (SBR/NMR) − 28 weeks
lambda = 0.6 ; zeta = 0.8 ; scale = 15 ; amp = 0.028

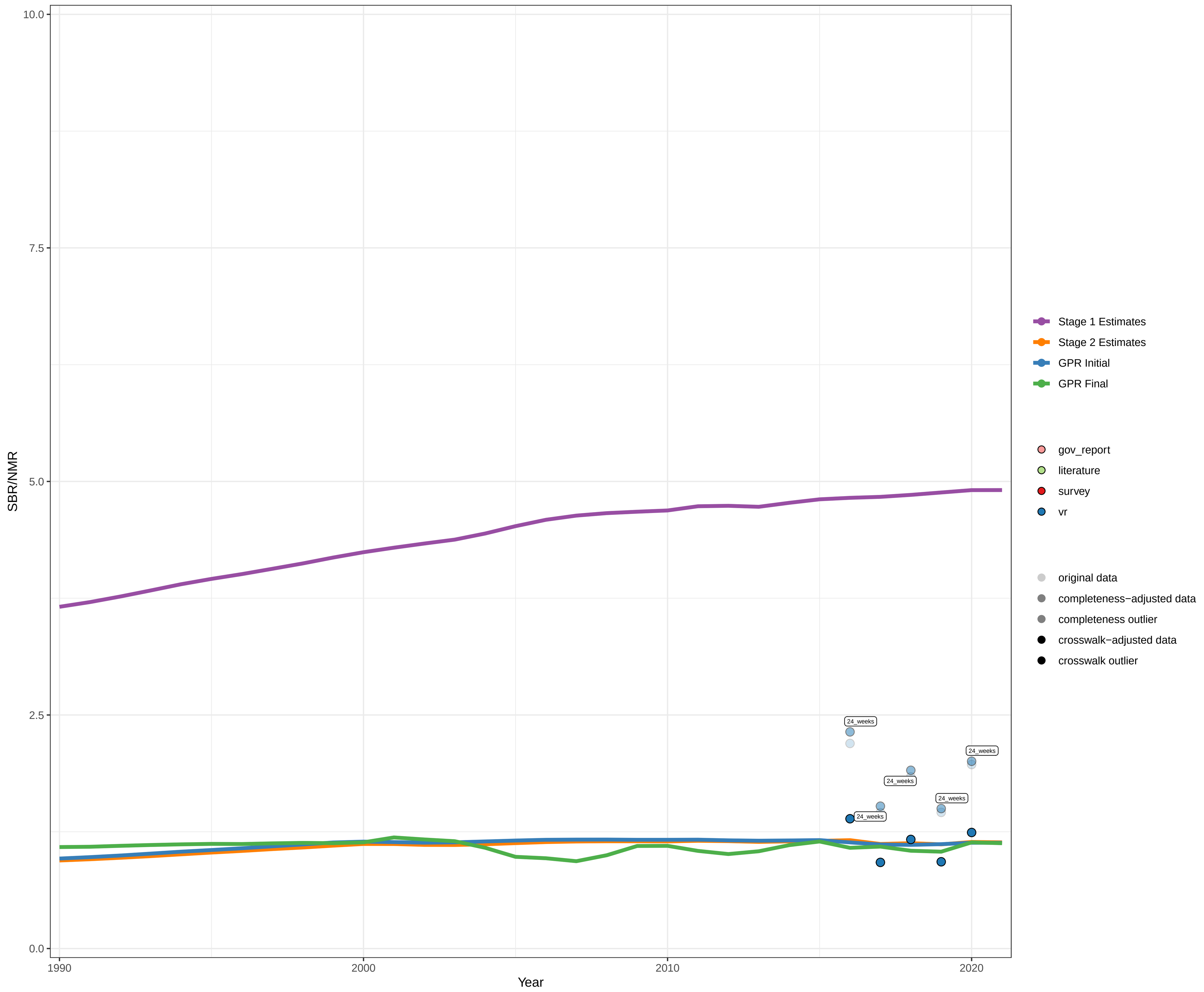

Definition	Years	Outlier	Source.Type	Title
24_weeks	2016−2020	0	VR	United Kingdom − England and Wales Child Mortality Statistics

Suffolk GBR_44718 Western Europe Stillbirth Rate (SBR) − 20 weeks
lambda = 0.6 ; zeta = 0.8 ; scale = 15 ; amp = 0.028

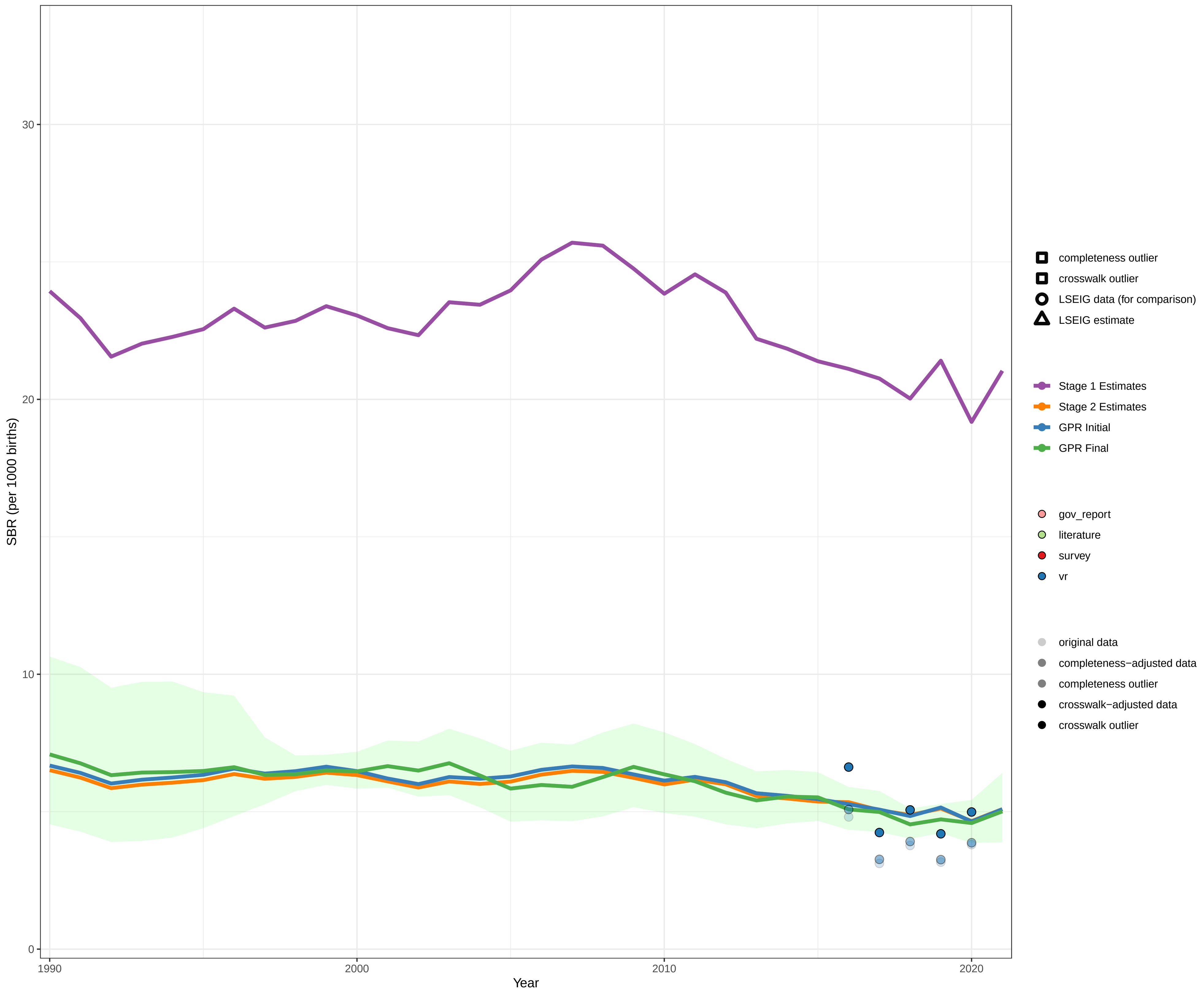

Suffolk GBR_44718 Western Europe Stillbirth Rate (SBR) − 28 weeks
lambda = 0.6 ; zeta = 0.8 ; scale = 15 ; amp = 0.028

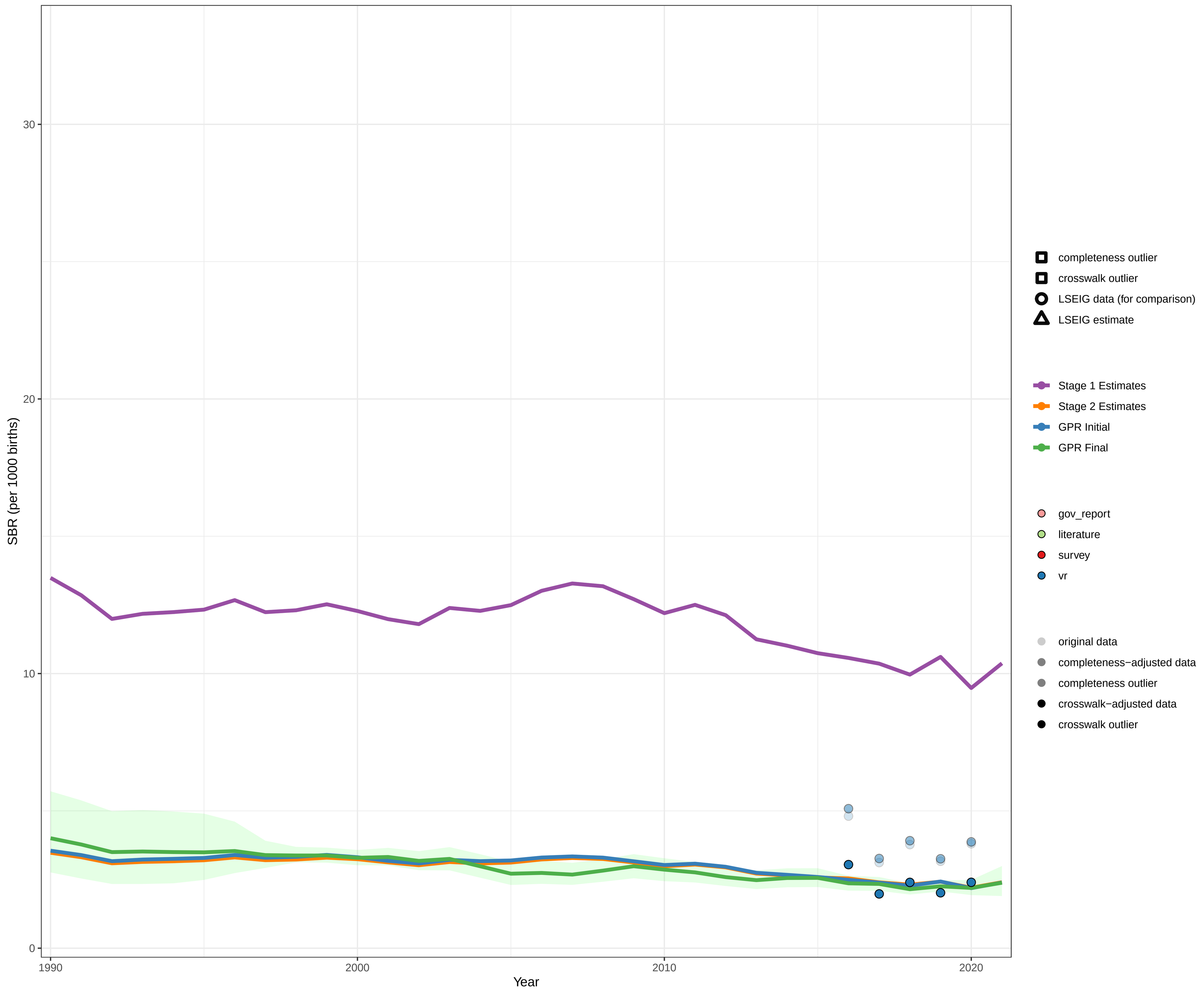

Year	Total Stillbirths (in thousands)	Total Stillbirths (in thousands)	SBR (per 1000 births)	SBR (per 1000 births)	SBR/NMR	SBR/NMR
	20 weeks	28 weeks	20 weeks	28 weeks	20 weeks	28 weeks
1990	0.1 (0.0 to 0.1)	0.0 (0.0 to 0.0)	7.1 (4.5 to 10.6)	4.0 (2.8 to 5.7)	1.92 (1.23 to 2.89)	1.09 (0.75 to 1.55)
1995	0.1 (0.0 to 0.1)	0.0 (0.0 to 0.0)	6.5 (4.4 to 9.3)	3.5 (2.5 to 4.9)	2.08 (1.41 to 3.00)	1.12 (0.80 to 1.57)
2000	0.0 (0.0 to 0.1)	0.0 (0.0 to 0.0)	6.5 (5.8 to 7.2)	3.3 (3.0 to 3.6)	2.23 (2.02 to 2.48)	1.14 (1.04 to 1.24)
2005	0.0 (0.0 to 0.1)	0.0 (0.0 to 0.0)	5.8 (4.6 to 7.2)	2.7 (2.3 to 3.2)	2.11 (1.68 to 2.61)	0.98 (0.83 to 1.14)
2010	0.1 (0.0 to 0.1)	0.0 (0.0 to 0.0)	6.4 (5.0 to 7.9)	2.9 (2.4 to 3.3)	2.44 (1.90 to 3.03)	1.10 (0.94 to 1.26)
2015	0.0 (0.0 to 0.1)	0.0 (0.0 to 0.0)	5.5 (4.7 to 6.4)	2.6 (2.2 to 2.9)	2.47 (2.09 to 2.88)	1.15 (1.00 to 1.30)
2020	0.0 (0.0 to 0.0)	0.0 (0.0 to 0.0)	4.6 (3.9 to 5.4)	2.2 (1.9 to 2.5)	2.38 (2.00 to 2.81)	1.14 (1.00 to 1.29)
2021	0.0 (0.0 to 0.0)	0.0 (0.0 to 0.0)	5.0 (3.9 to 6.4)	2.4 (1.9 to 3.0)	2.37 (1.84 to 3.04)	1.13 (0.90 to 1.42)

Thurrock GBR_44722 Western Europe Stillbirth Rate / Neonatal Mortality Rate (SBR/NMR) − 20 weeks
lambda = 0.6 ; zeta = 0.8 ; scale = 15 ; amp = 0.028

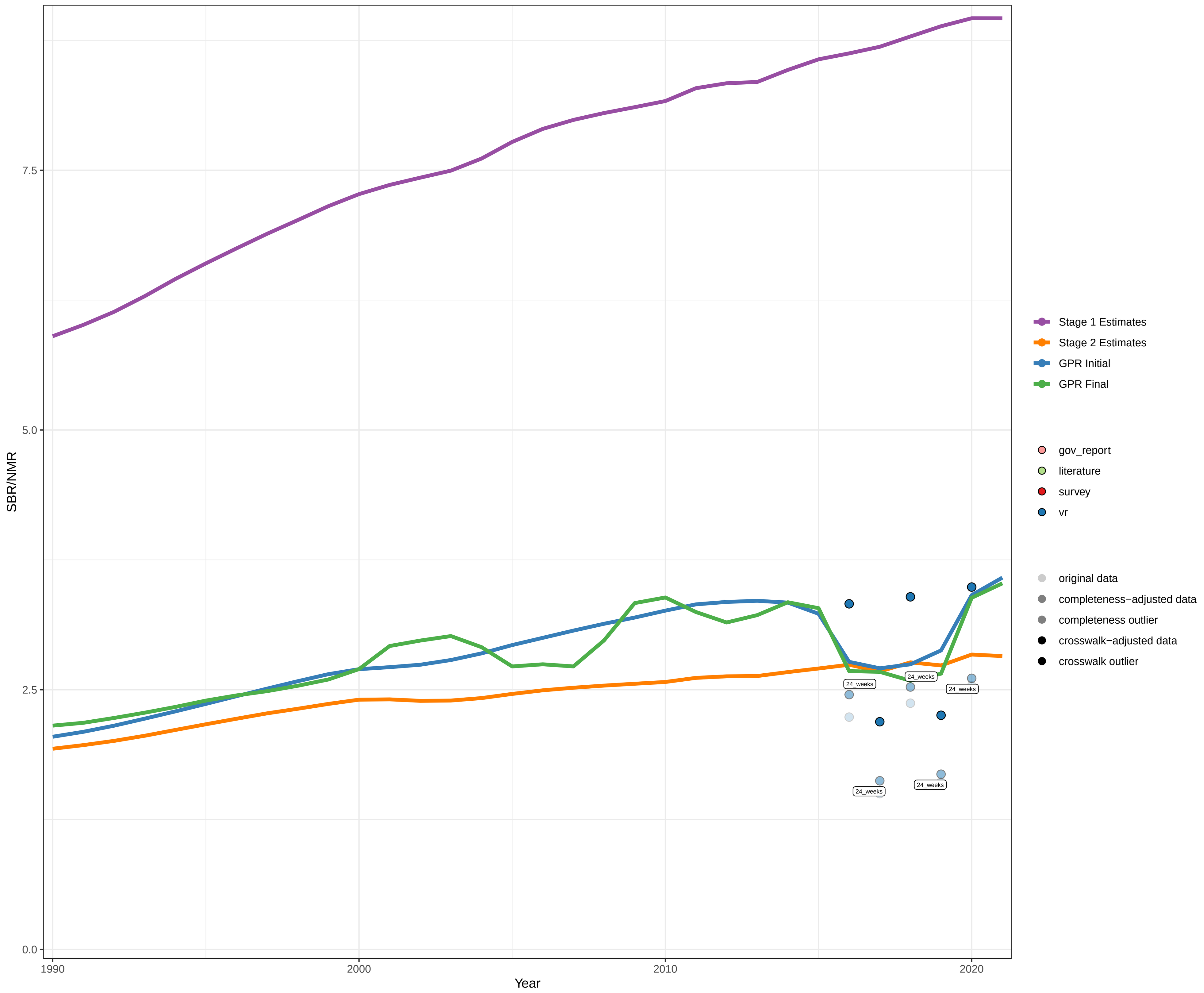

Thurrock GBR_44722 Western Europe Stillbirth Rate / Neonatal Mortality Rate (SBR/NMR) − 28 weeks
lambda = 0.6 ; zeta = 0.8 ; scale = 15 ; amp = 0.028

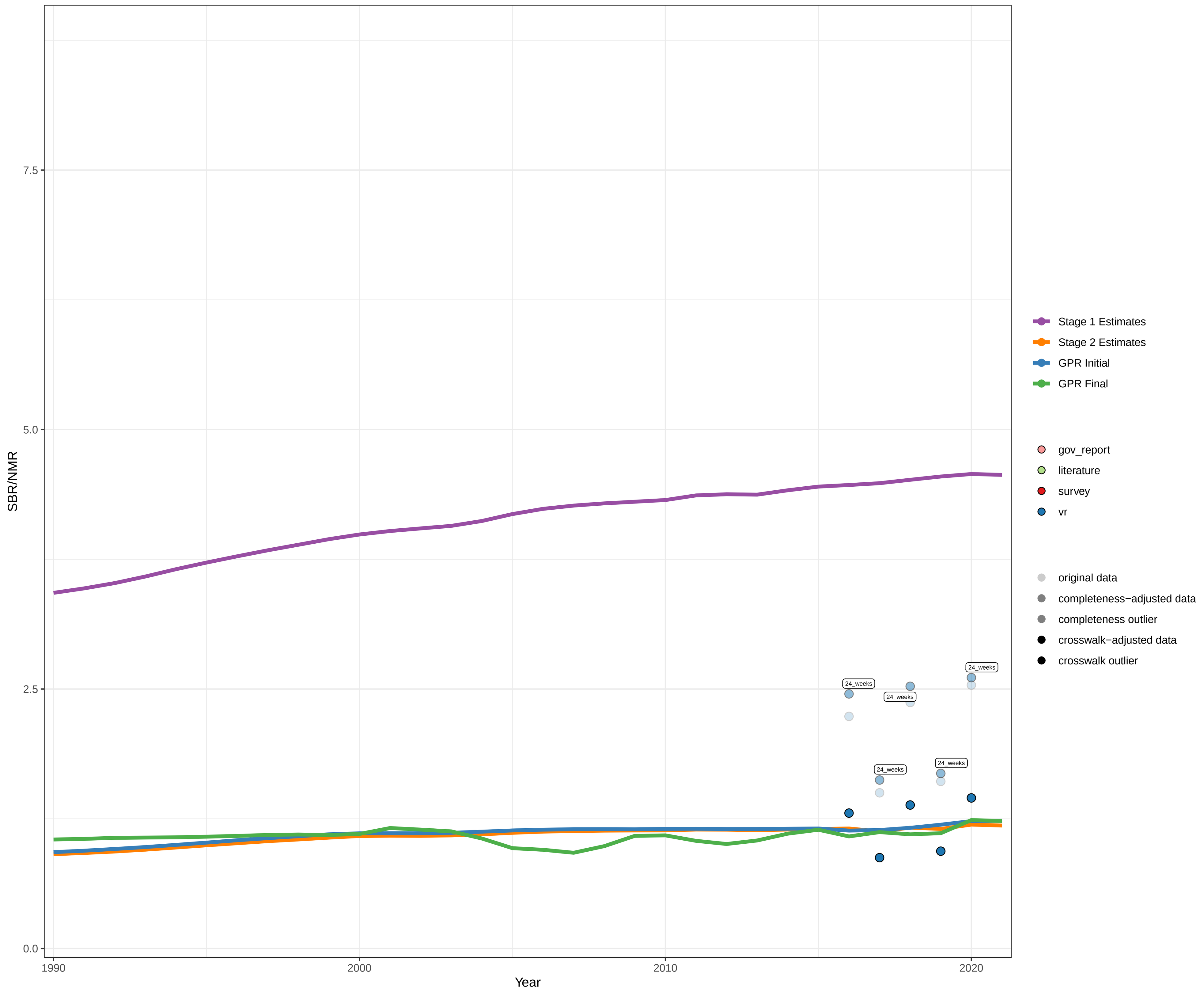

Definition	Years	Outlier	Source.Type	Title
24_weeks	2016−2020	0	VR	United Kingdom − England and Wales Child Mortality Statistics

Thurrock GBR_44722 Western Europe Stillbirth Rate (SBR) − 20 weeks
lambda = 0.6 ; zeta = 0.8 ; scale = 15 ; amp = 0.028

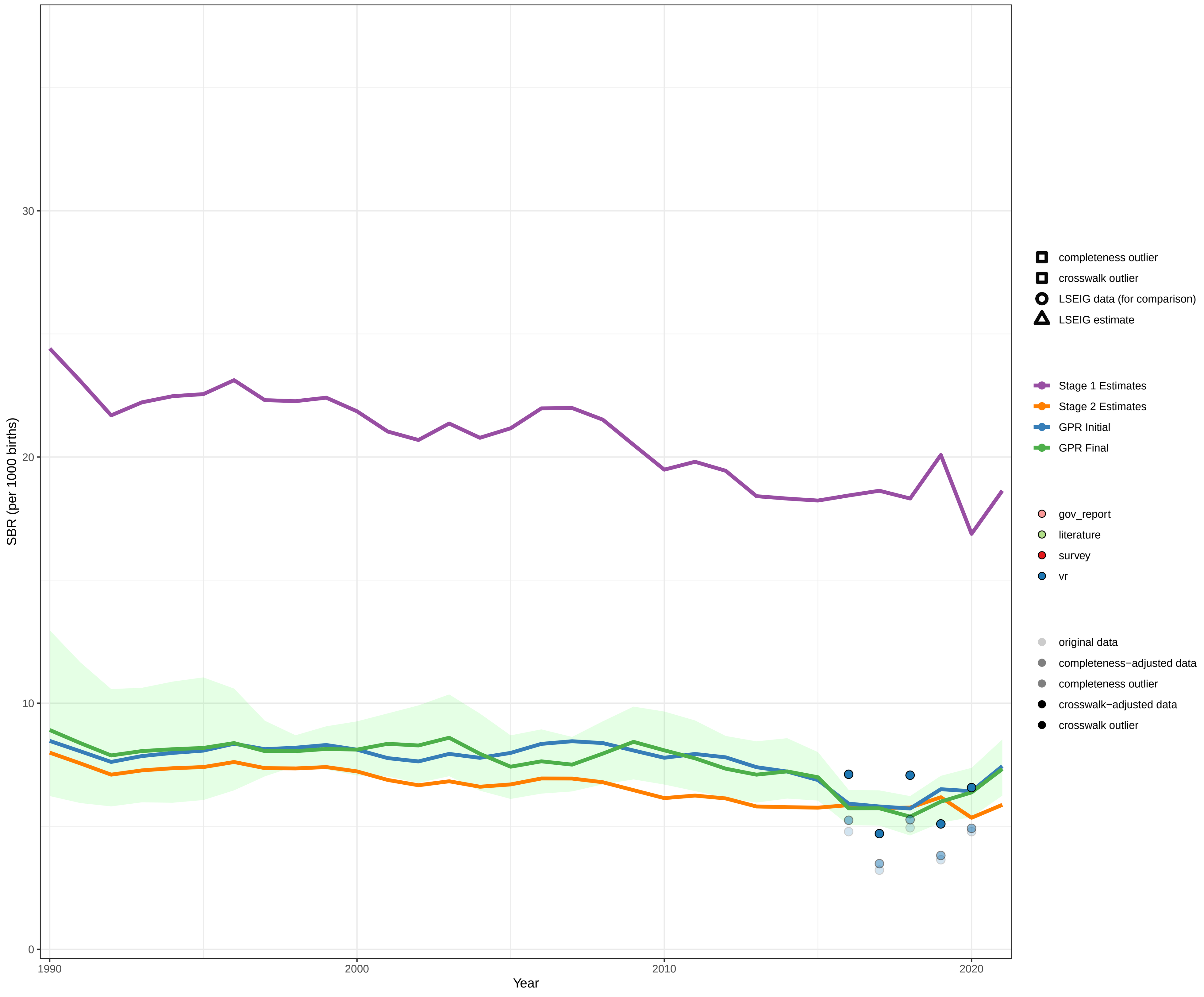

Thurrock GBR_44722 Western Europe Stillbirth Rate (SBR) − 28 weeks
lambda = 0.6 ; zeta = 0.8 ; scale = 15 ; amp = 0.028

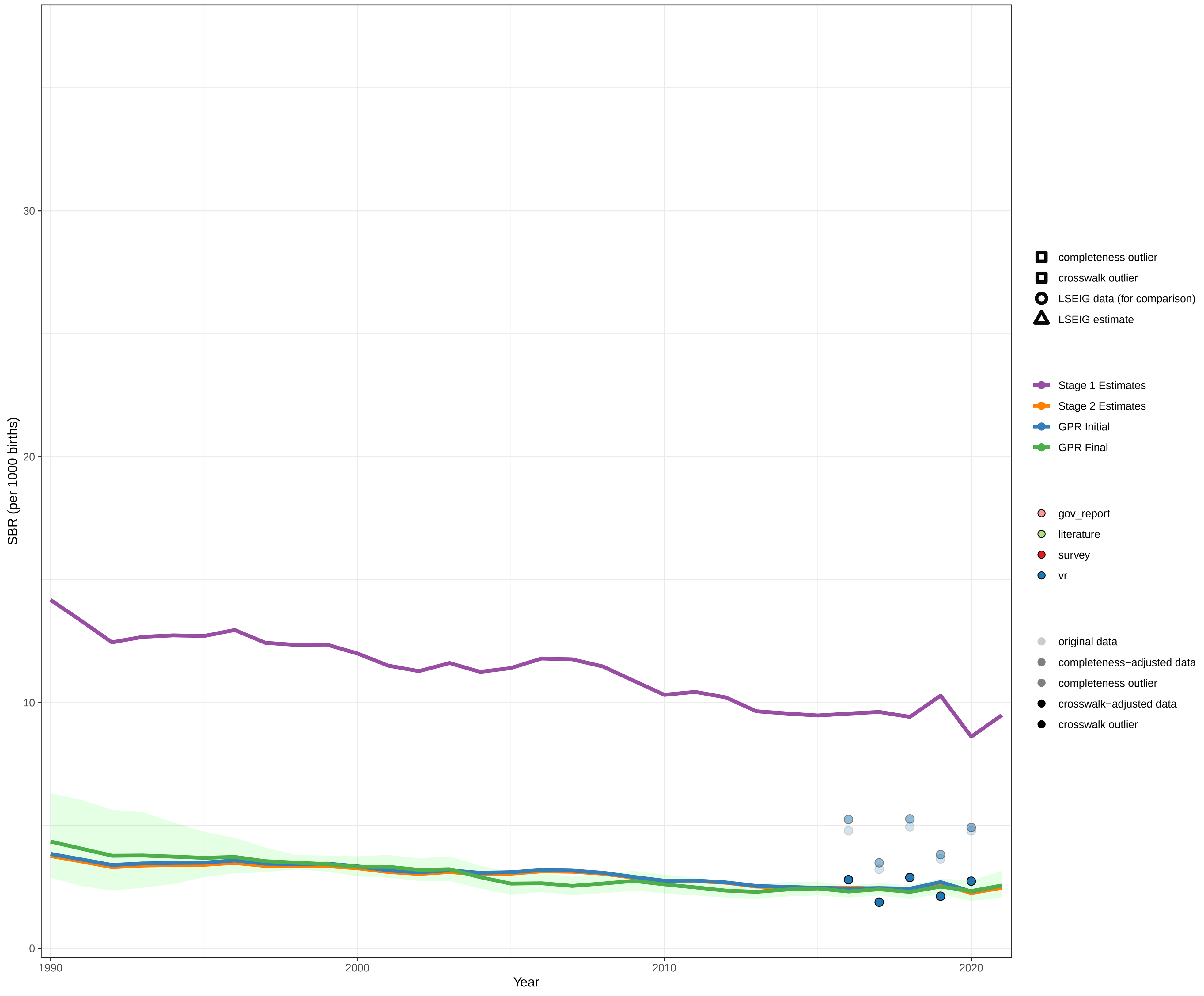

Year	Total Stillbirths (in thousands)	Total Stillbirths (in thousands)	SBR (per 1000 births)	SBR (per 1000 births)	SBR/NMR	SBR/NMR
	20 weeks	28 weeks	20 weeks	28 weeks	20 weeks	28 weeks
1990	0.0 (0.0 to 0.0)	0.0 (0.0 to 0.0)	8.9 (6.2 to 13.0)	4.3 (2.9 to 6.3)	2.15 (1.51 to 3.13)	1.05 (0.70 to 1.53)
1995	0.0 (0.0 to 0.0)	0.0 (0.0 to 0.0)	8.2 (6.1 to 11.0)	3.7 (2.9 to 4.7)	2.40 (1.78 to 3.23)	1.08 (0.85 to 1.39)
2000	0.0 (0.0 to 0.0)	0.0 (0.0 to 0.0)	8.1 (7.1 to 9.3)	3.3 (2.9 to 3.7)	2.70 (2.35 to 3.08)	1.10 (0.98 to 1.24)
2005	0.0 (0.0 to 0.0)	0.0 (0.0 to 0.0)	7.4 (6.1 to 8.7)	2.6 (2.2 to 3.1)	2.72 (2.24 to 3.19)	0.97 (0.80 to 1.14)
2010	0.0 (0.0 to 0.0)	0.0 (0.0 to 0.0)	8.1 (6.7 to 9.7)	2.6 (2.2 to 3.0)	3.39 (2.81 to 4.05)	1.09 (0.93 to 1.25)
2015	0.0 (0.0 to 0.0)	0.0 (0.0 to 0.0)	7.0 (6.0 to 8.0)	2.4 (2.2 to 2.7)	3.28 (2.84 to 3.77)	1.14 (1.01 to 1.28)
2020	0.0 (0.0 to 0.0)	0.0 (0.0 to 0.0)	6.4 (5.4 to 7.4)	2.3 (1.9 to 2.8)	3.38 (2.85 to 3.91)	1.24 (1.02 to 1.47)
2021	0.0 (0.0 to 0.0)	0.0 (0.0 to 0.0)	7.3 (6.2 to 8.5)	2.6 (2.1 to 3.1)	3.52 (3.00 to 4.10)	1.23 (1.00 to 1.51)

Greater London GBR_4624 Western Europe Stillbirth Rate / Neonatal Mortality Rate (SBR/NMR) − 20 weeks
lambda = 0.2 ; zeta = 0.99 ; scale = 5 ; amp = 0.028

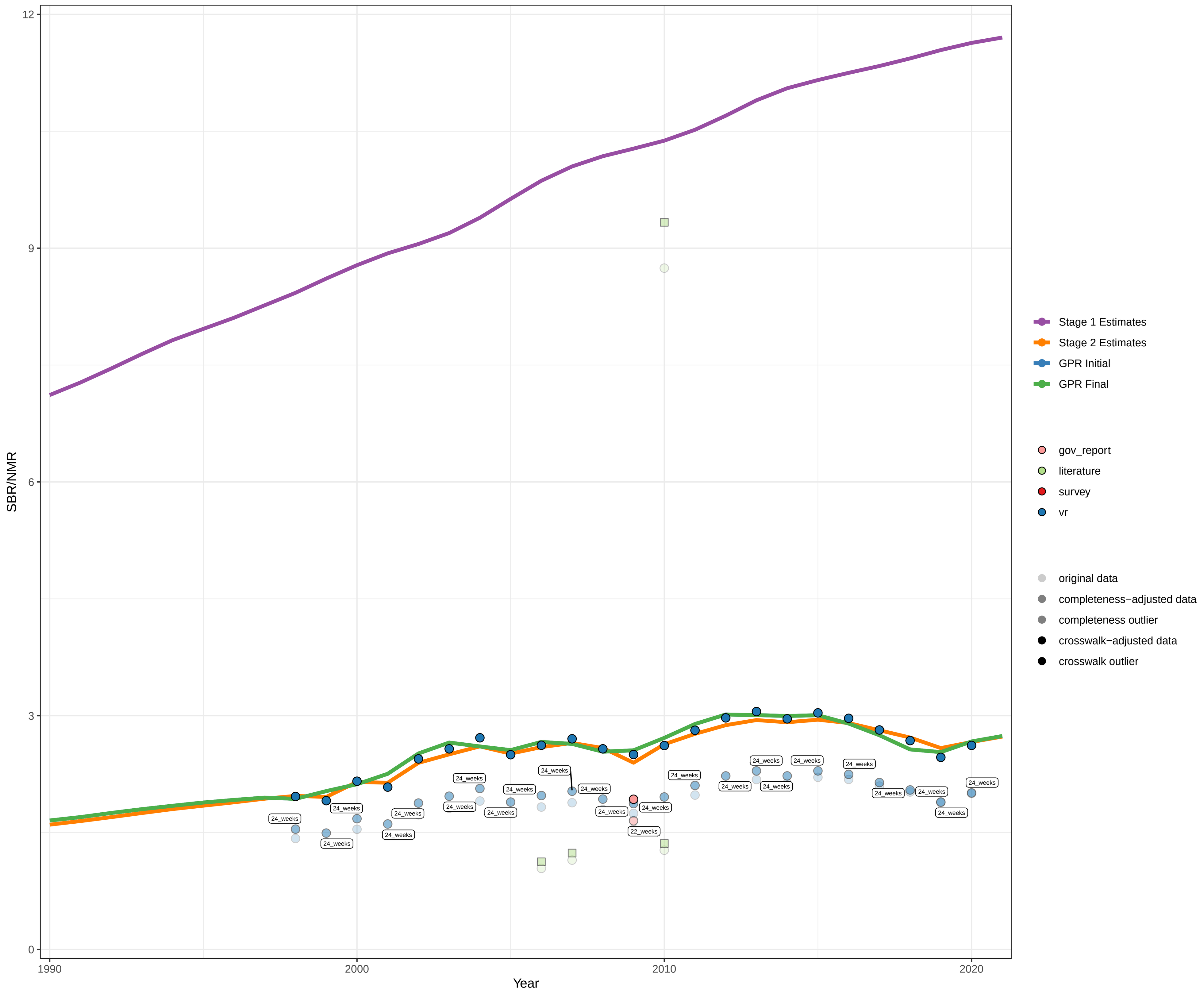

Greater London GBR_4624 Western Europe Stillbirth Rate / Neonatal Mortality Rate (SBR/NMR) − 28 weeks
lambda = 0.2 ; zeta = 0.99 ; scale = 5 ; amp = 0.028

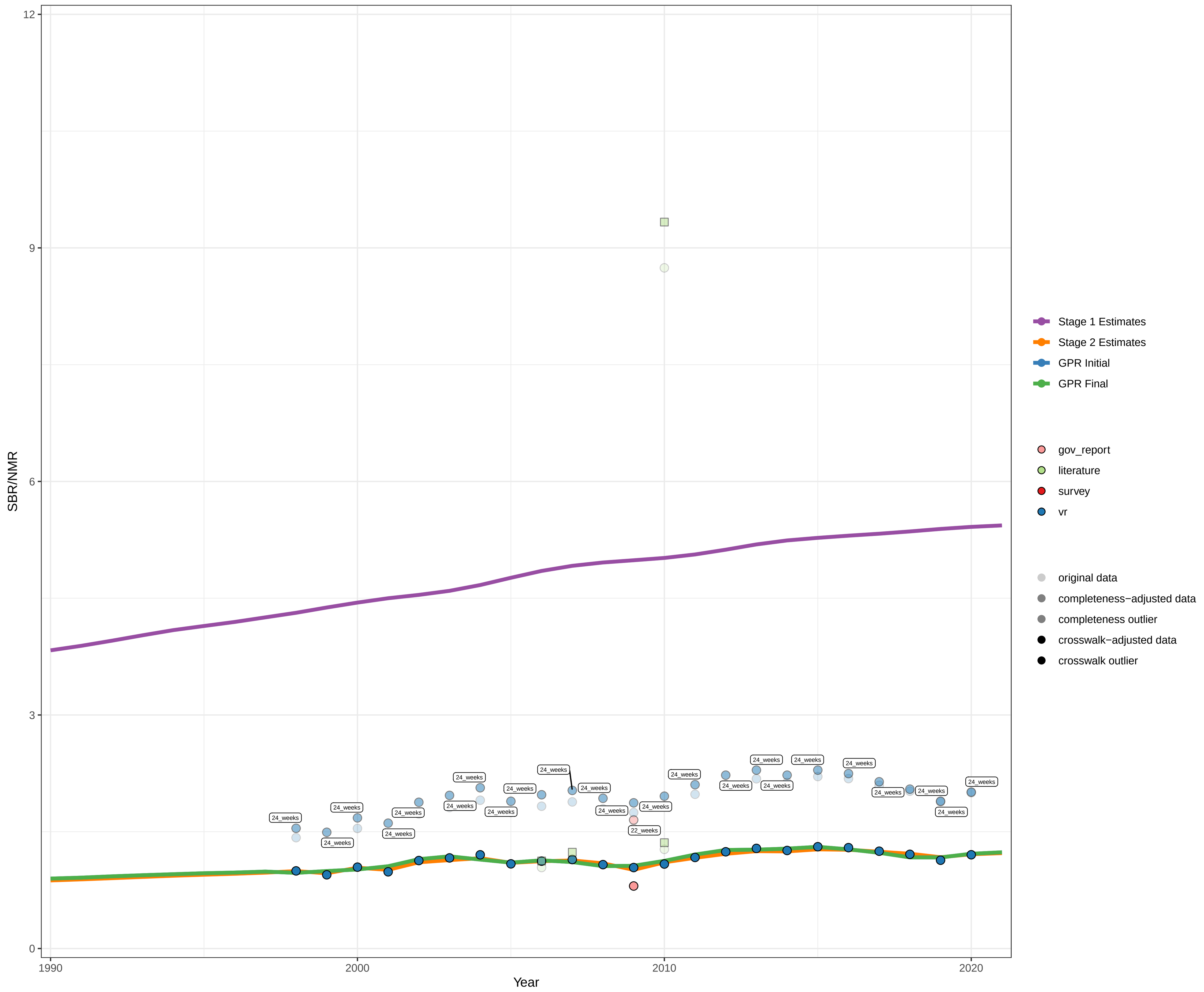

Definition	Years	Outlier	Source.Type	Title
22_weeks	2009	0	Statistical Report	United Kingdom Perinatal Mortality
24_weeks	1998−2007	0	VR	United Kingdom − England and Wales Birth Statistics
24_weeks	2008−2020	0	VR	United Kingdom − England and Wales Child Mortality Statistics
24_weeks	2006	1	Sci Lit	Value of third−trimester cerebroplacental ratio and uterine artery Doppler indices as predictors of stillbirth and perinatal loss
24_weeks	2007	1	Sci Lit	Mid−pregnancy fetal growth, uteroplacental Doppler indices and maternal demographic characteristics: role in prediction of stillbirth
24_weeks	2010	1	Sci Lit	Birth weight in live births and stillbirths
24_weeks	2010	1	Sci Lit	Prediction of stillbirth from placental growth factor at 19−24 weeks

Greater London GBR_4624 Western Europe Stillbirth Rate (SBR) − 20 weeks
lambda = 0.2 ; zeta = 0.99 ; scale = 5 ; amp = 0.028

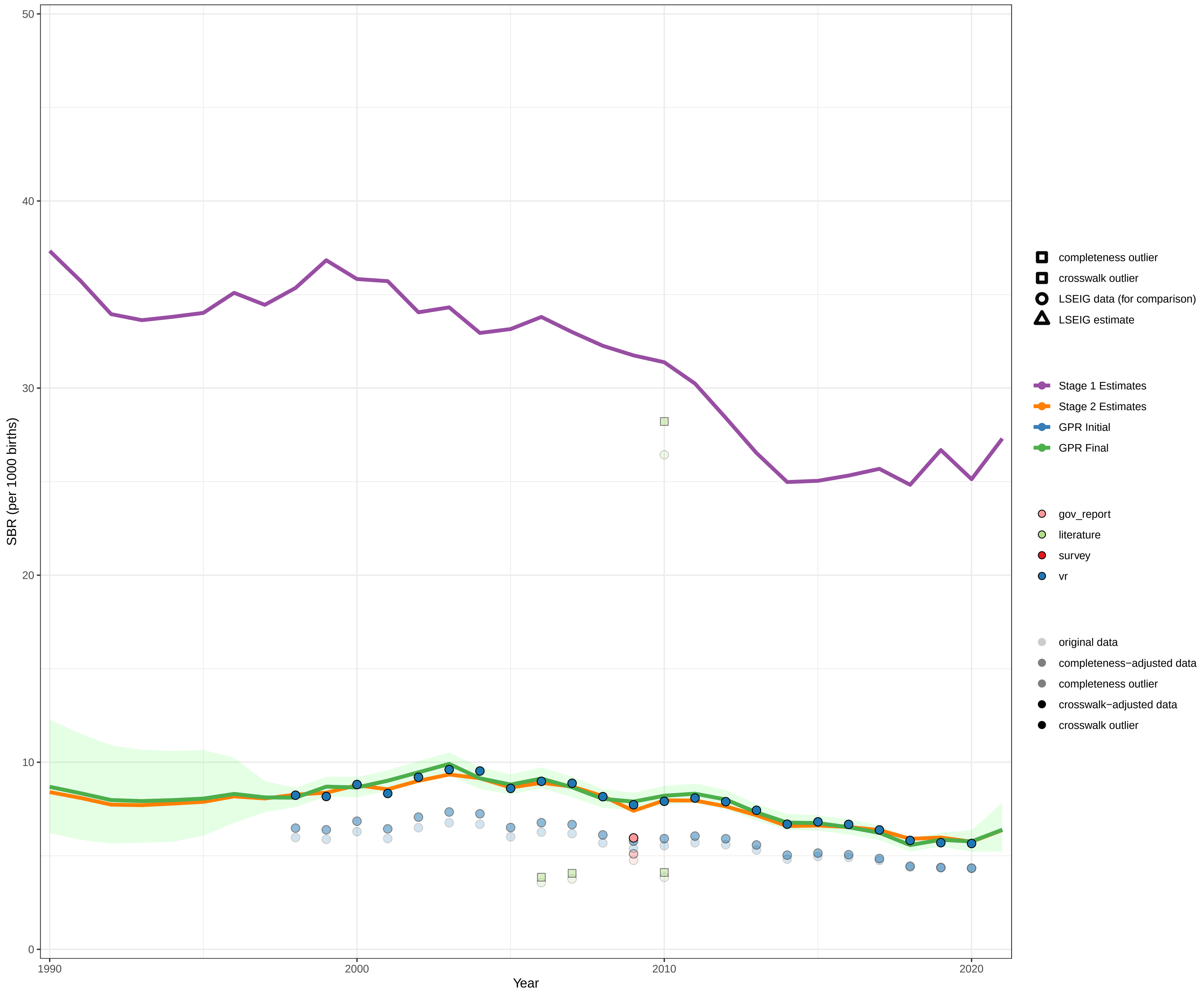

Greater London GBR_4624 Western Europe Stillbirth Rate (SBR) − 28 weeks
lambda = 0.2 ; zeta = 0.99 ; scale = 5 ; amp = 0.028

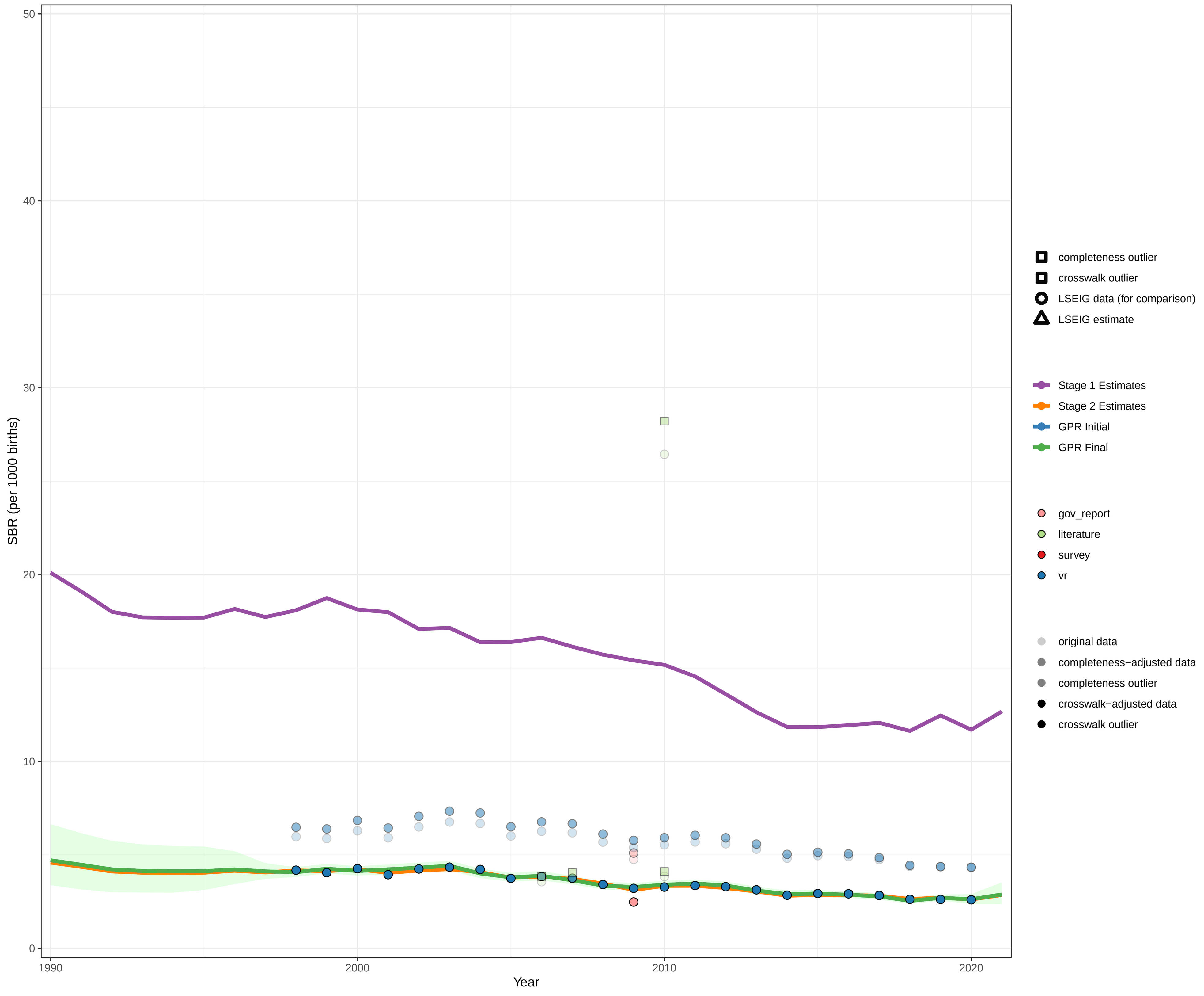

Year	Total Stillbirths (in thousands)	Total Stillbirths (in thousands)	SBR (per 1000 births)	SBR (per 1000 births)	SBR/NMR	SBR/NMR
	20 weeks	28 weeks	20 weeks	28 weeks	20 weeks	28 weeks
1990	0.9 (0.7 to 1.3)	0.5 (0.4 to 0.7)	8.7 (6.2 to 12.3)	4.7 (3.4 to 6.6)	1.66 (1.18 to 2.34)	0.90 (0.64 to 1.27)
1995	0.8 (0.6 to 1.1)	0.4 (0.3 to 0.6)	8.1 (6.1 to 10.7)	4.1 (3.1 to 5.5)	1.89 (1.42 to 2.50)	0.97 (0.73 to 1.28)
2000	0.9 (0.8 to 1.0)	0.4 (0.4 to 0.5)	8.7 (8.1 to 9.2)	4.1 (3.9 to 4.4)	2.12 (1.99 to 2.26)	1.02 (0.96 to 1.08)
2005	1.0 (1.0 to 1.1)	0.4 (0.4 to 0.5)	8.8 (8.3 to 9.4)	3.8 (3.6 to 4.0)	2.56 (2.41 to 2.72)	1.10 (1.04 to 1.18)
2010	1.1 (1.0 to 1.2)	0.4 (0.4 to 0.5)	8.2 (7.7 to 8.7)	3.4 (3.2 to 3.6)	2.72 (2.55 to 2.89)	1.13 (1.06 to 1.20)
2015	0.9 (0.8 to 0.9)	0.4 (0.4 to 0.4)	6.7 (6.3 to 7.2)	2.9 (2.8 to 3.1)	3.01 (2.83 to 3.19)	1.31 (1.23 to 1.39)
2020	0.7 (0.6 to 0.7)	0.3 (0.3 to 0.3)	5.8 (5.2 to 6.4)	2.6 (2.4 to 2.9)	2.67 (2.41 to 2.95)	1.22 (1.10 to 1.35)
2021	0.7 (0.6 to 0.9)	0.3 (0.3 to 0.4)	6.4 (5.2 to 7.8)	2.9 (2.4 to 3.5)	2.74 (2.24 to 3.36)	1.24 (1.01 to 1.51)

Barking and Dagenham GBR_44754 Western Europe Stillbirth Rate / Neonatal Mortality Rate (SBR/NMR) − 20 weeks
lambda = 0.6 ; zeta = 0.8 ; scale = 15 ; amp = 0.028

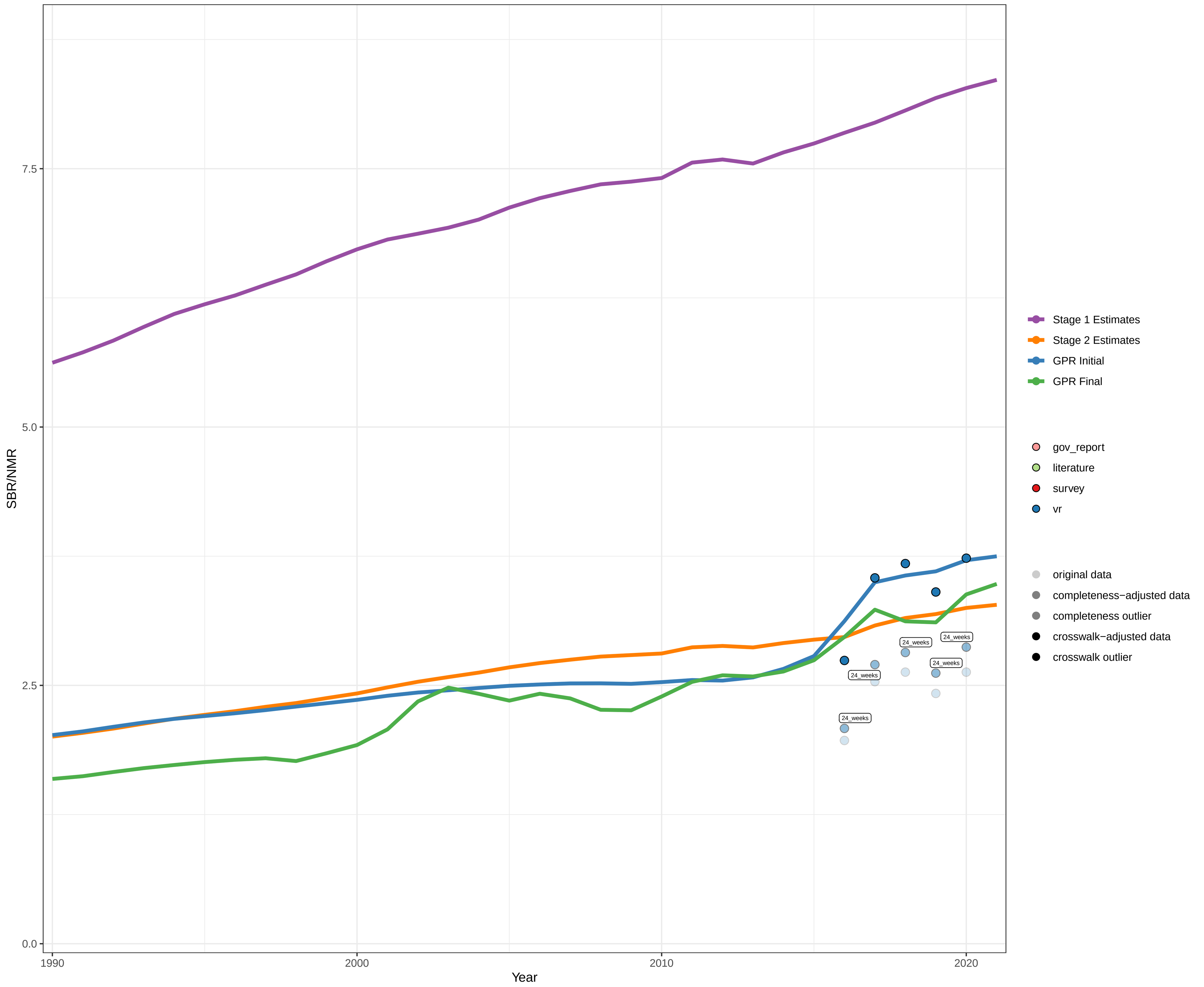

Barking and Dagenham GBR_44754 Western Europe Stillbirth Rate / Neonatal Mortality Rate (SBR/NMR) − 28 weeks
lambda = 0.6 ; zeta = 0.8 ; scale = 15 ; amp = 0.028

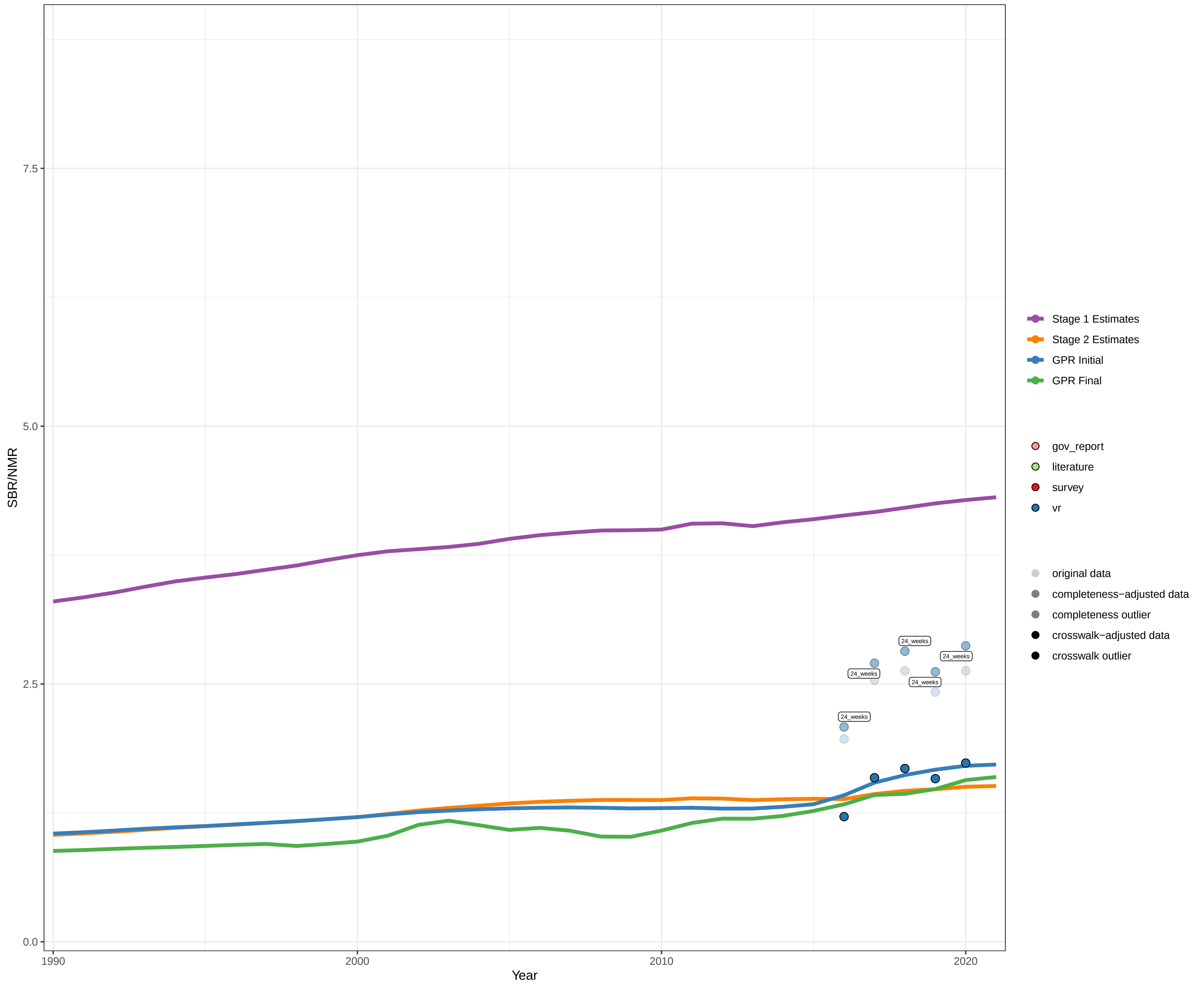

Definition	Years	Outlier	Source.Type	Title
24_weeks	2016−2020	0	VR	United Kingdom − England and Wales Child Mortality Statistics

Barking and Dagenham GBR_44754 Western Europe Stillbirth Rate (SBR) − 20 weeks
lambda = 0.6 ; zeta = 0.8 ; scale = 15 ; amp = 0.028

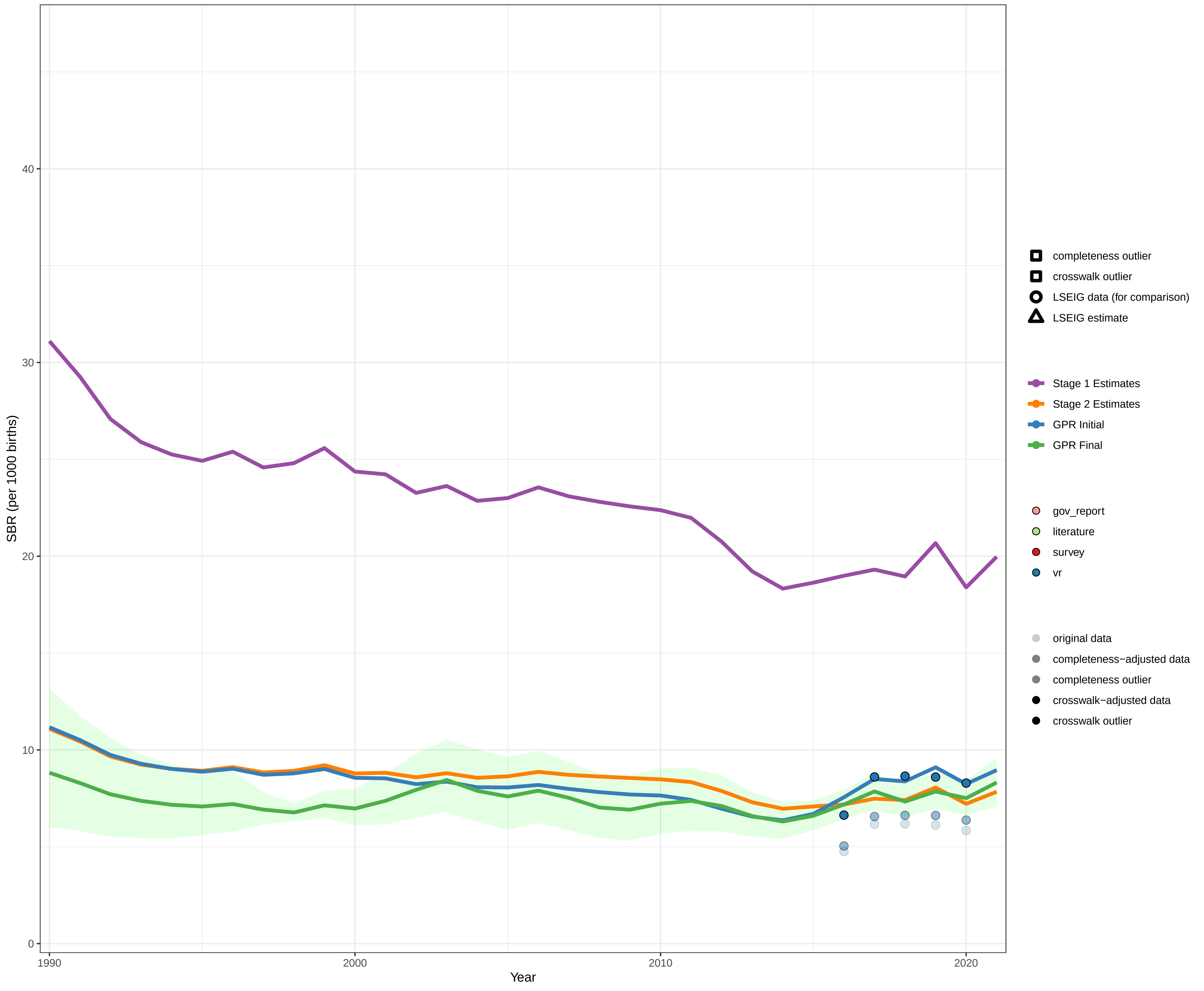

Barking and Dagenham GBR_44754 Western Europe Stillbirth Rate (SBR) − 28 weeks
lambda = 0.6 ; zeta = 0.8 ; scale = 15 ; amp = 0.028

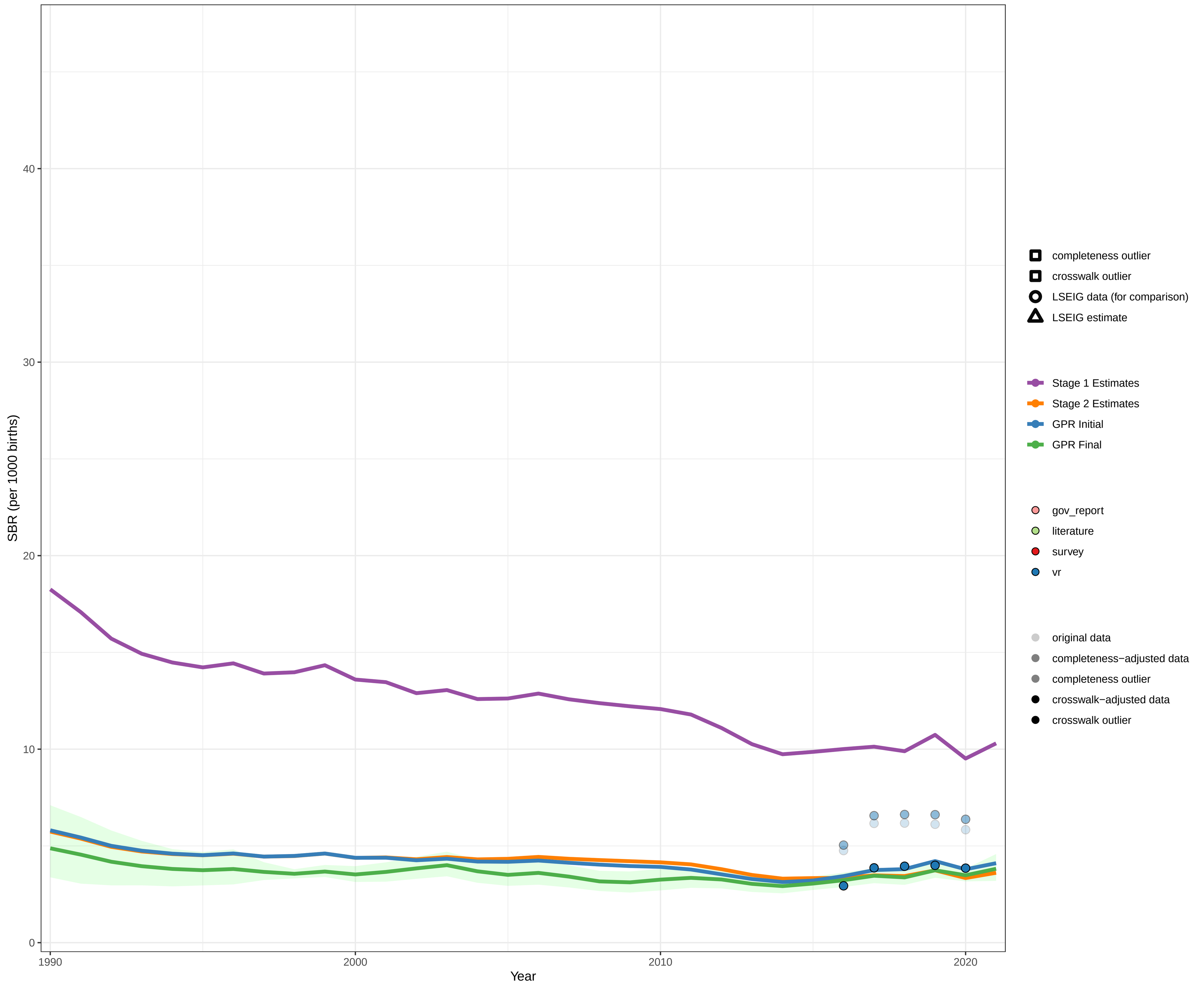

Year	Total Stillbirths (in thousands)	Total Stillbirths (in thousands)	SBR (per 1000 births)	SBR (per 1000 births)	SBR/NMR	SBR/NMR
	20 weeks	28 weeks	20 weeks	28 weeks	20 weeks	28 weeks
1990	0.0 (0.0 to 0.0)	0.0 (0.0 to 0.0)	8.8 (6.0 to 13.2)	4.9 (3.4 to 7.1)	1.59 (1.09 to 2.38)	0.88 (0.61 to 1.28)
1995	0.0 (0.0 to 0.0)	0.0 (0.0 to 0.0)	7.1 (5.6 to 8.8)	3.7 (3.0 to 4.7)	1.76 (1.40 to 2.19)	0.93 (0.73 to 1.16)
2000	0.0 (0.0 to 0.0)	0.0 (0.0 to 0.0)	7.0 (6.1 to 8.0)	3.5 (3.1 to 4.0)	1.92 (1.68 to 2.21)	0.97 (0.86 to 1.09)
2005	0.0 (0.0 to 0.0)	0.0 (0.0 to 0.0)	7.6 (5.9 to 9.6)	3.5 (2.9 to 4.1)	2.35 (1.82 to 2.98)	1.08 (0.91 to 1.27)
2010	0.0 (0.0 to 0.0)	0.0 (0.0 to 0.0)	7.2 (5.7 to 9.1)	3.3 (2.7 to 3.8)	2.39 (1.87 to 3.00)	1.08 (0.89 to 1.26)
2015	0.0 (0.0 to 0.0)	0.0 (0.0 to 0.0)	6.6 (5.9 to 7.4)	3.1 (2.7 to 3.4)	2.74 (2.43 to 3.07)	1.27 (1.13 to 1.42)
2020	0.0 (0.0 to 0.0)	0.0 (0.0 to 0.0)	7.5 (6.6 to 8.4)	3.5 (3.2 to 3.8)	3.38 (2.98 to 3.80)	1.57 (1.43 to 1.72)
2021	0.0 (0.0 to 0.0)	0.0 (0.0 to 0.0)	8.3 (7.1 to 9.6)	3.8 (3.2 to 4.6)	3.48 (2.96 to 4.01)	1.60 (1.34 to 1.92)

Barnet GBR_44729 Western Europe Stillbirth Rate / Neonatal Mortality Rate (SBR/NMR) − 20 weeks
lambda = 0.6 ; zeta = 0.8 ; scale = 15 ; amp = 0.028

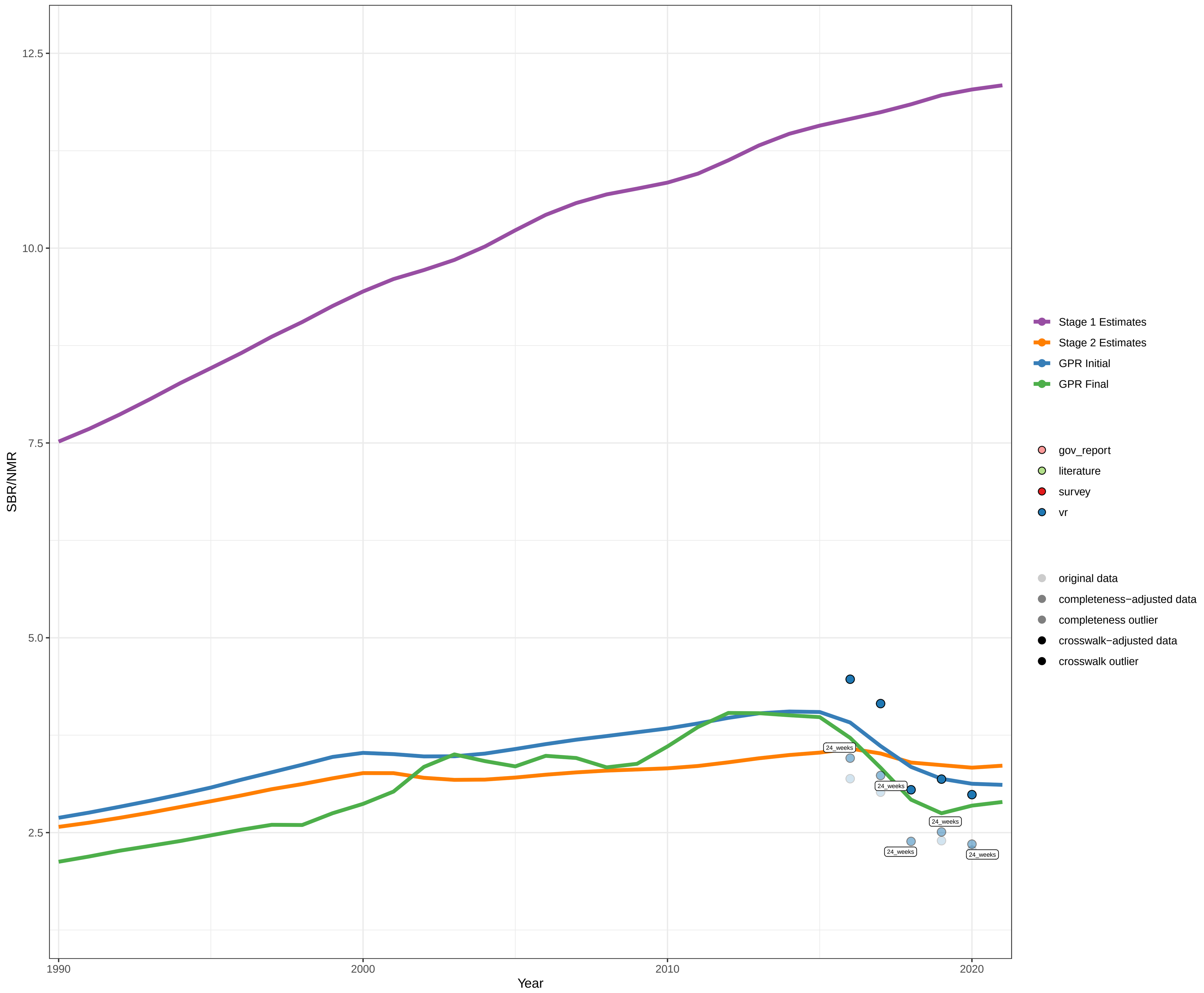

Barnet GBR_44729 Western Europe Stillbirth Rate / Neonatal Mortality Rate (SBR/NMR) − 28 weeks
lambda = 0.6 ; zeta = 0.8 ; scale = 15 ; amp = 0.028

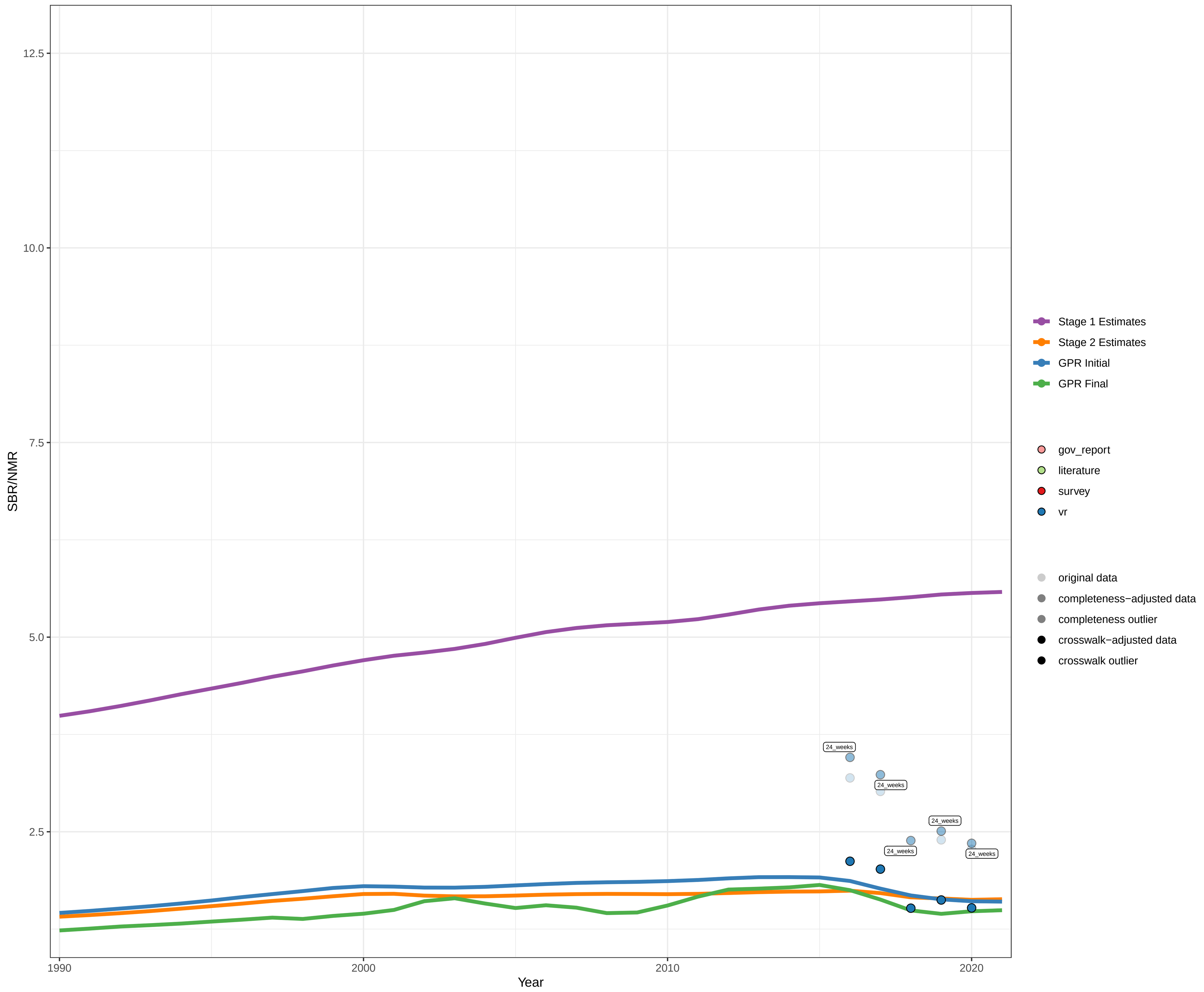

Definition	Years	Outlier	Source.Type	Title
24_weeks	2016−2020	0	VR	United Kingdom − England and Wales Child Mortality Statistics

Barnet GBR_44729 Western Europe Stillbirth Rate (SBR) − 20 weeks
lambda = 0.6 ; zeta = 0.8 ; scale = 15 ; amp = 0.028

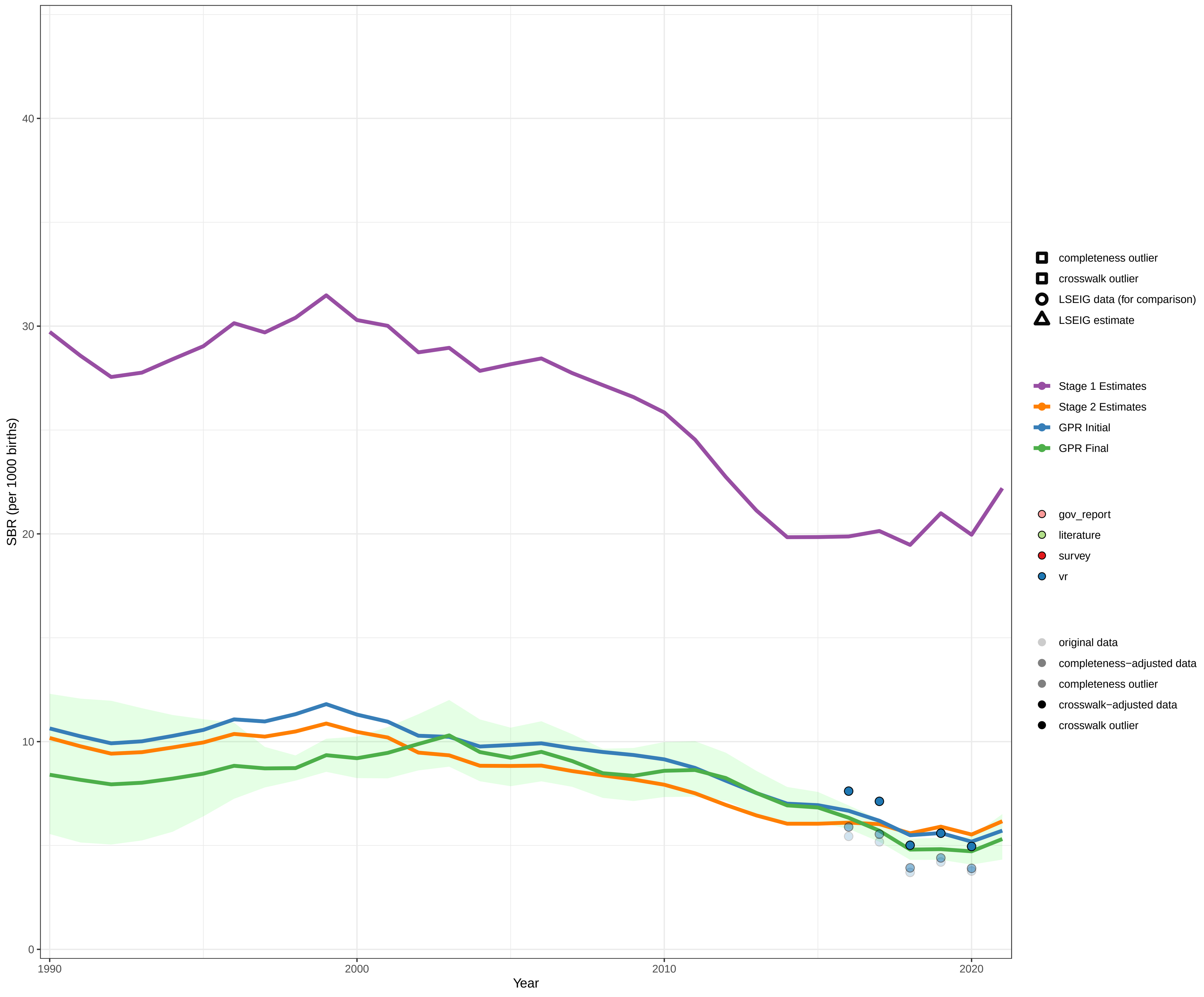

Barnet GBR_44729 Western Europe Stillbirth Rate (SBR) − 28 weeks
lambda = 0.6 ; zeta = 0.8 ; scale = 15 ; amp = 0.028

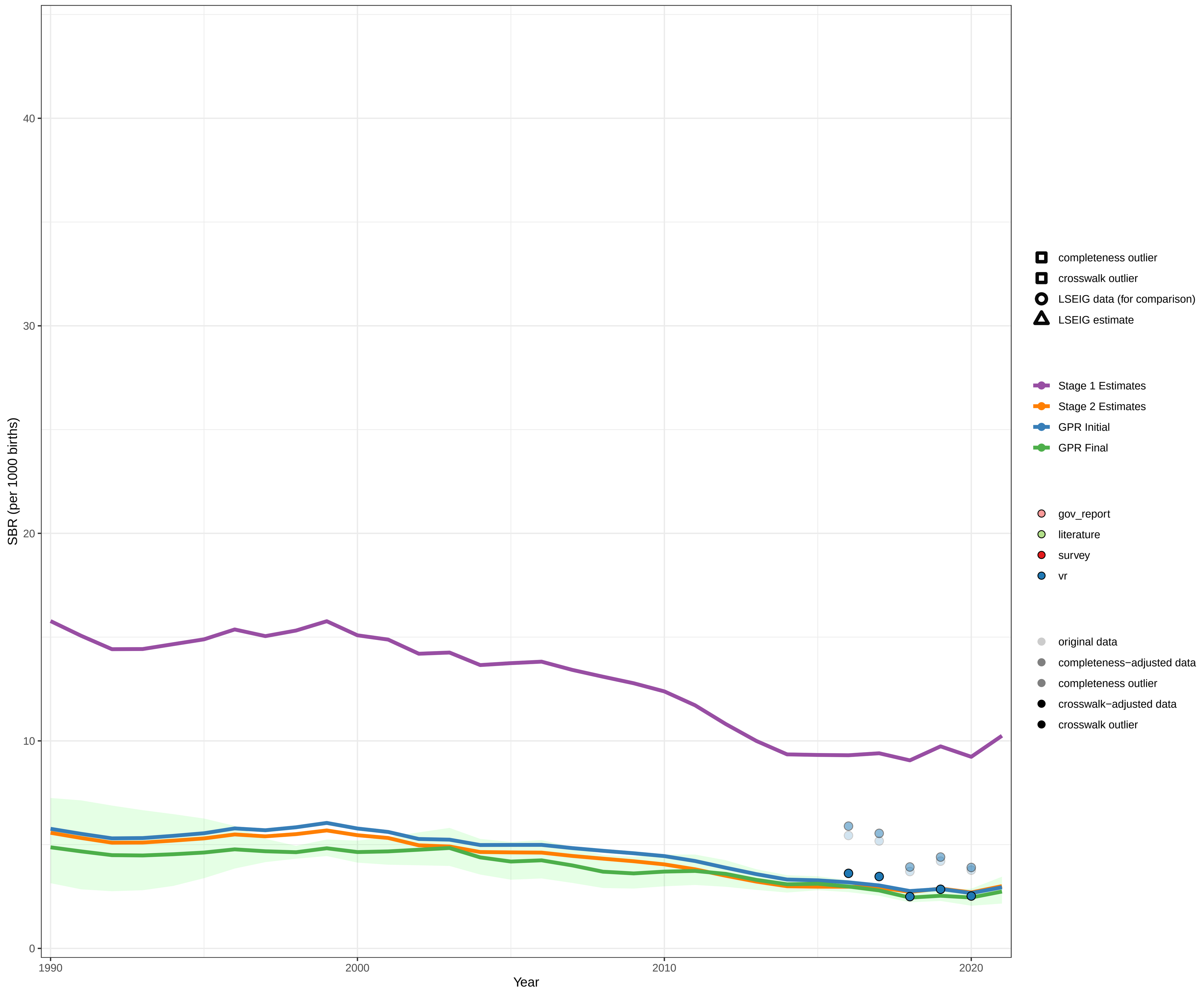

Year	Total Stillbirths (in thousands)	Total Stillbirths (in thousands)	SBR (per 1000 births)	SBR (per 1000 births)	SBR/NMR	SBR/NMR
	20 weeks	28 weeks	20 weeks	28 weeks	20 weeks	28 weeks
1990	0.0 (0.0 to 0.1)	0.0 (0.0 to 0.0)	8.4 (5.6 to 12.3)	4.9 (3.1 to 7.2)	2.13 (1.40 to 3.11)	1.23 (0.79 to 1.83)
1995	0.0 (0.0 to 0.0)	0.0 (0.0 to 0.0)	8.5 (6.4 to 11.1)	4.6 (3.4 to 6.3)	2.46 (1.87 to 3.23)	1.35 (0.99 to 1.82)
2000	0.0 (0.0 to 0.0)	0.0 (0.0 to 0.0)	9.2 (8.2 to 10.2)	4.6 (4.1 to 5.2)	2.87 (2.57 to 3.19)	1.45 (1.29 to 1.62)
2005	0.0 (0.0 to 0.1)	0.0 (0.0 to 0.0)	9.2 (7.9 to 10.7)	4.2 (3.3 to 5.1)	3.35 (2.85 to 3.88)	1.52 (1.20 to 1.86)
2010	0.0 (0.0 to 0.1)	0.0 (0.0 to 0.0)	8.6 (7.3 to 10.0)	3.7 (3.0 to 4.5)	3.61 (3.08 to 4.19)	1.55 (1.26 to 1.90)
2015	0.0 (0.0 to 0.0)	0.0 (0.0 to 0.0)	6.8 (6.1 to 7.6)	3.1 (2.8 to 3.5)	3.98 (3.57 to 4.42)	1.82 (1.63 to 2.03)
2020	0.0 (0.0 to 0.0)	0.0 (0.0 to 0.0)	4.7 (4.1 to 5.4)	2.5 (2.1 to 2.9)	2.85 (2.46 to 3.28)	1.48 (1.24 to 1.74)
2021	0.0 (0.0 to 0.0)	0.0 (0.0 to 0.0)	5.3 (4.3 to 6.5)	2.7 (2.2 to 3.5)	2.89 (2.35 to 3.55)	1.49 (1.18 to 1.88)

Bexley GBR_44732 Western Europe Stillbirth Rate / Neonatal Mortality Rate (SBR/NMR) − 20 weeks
lambda = 0.6 ; zeta = 0.8 ; scale = 15 ; amp = 0.028

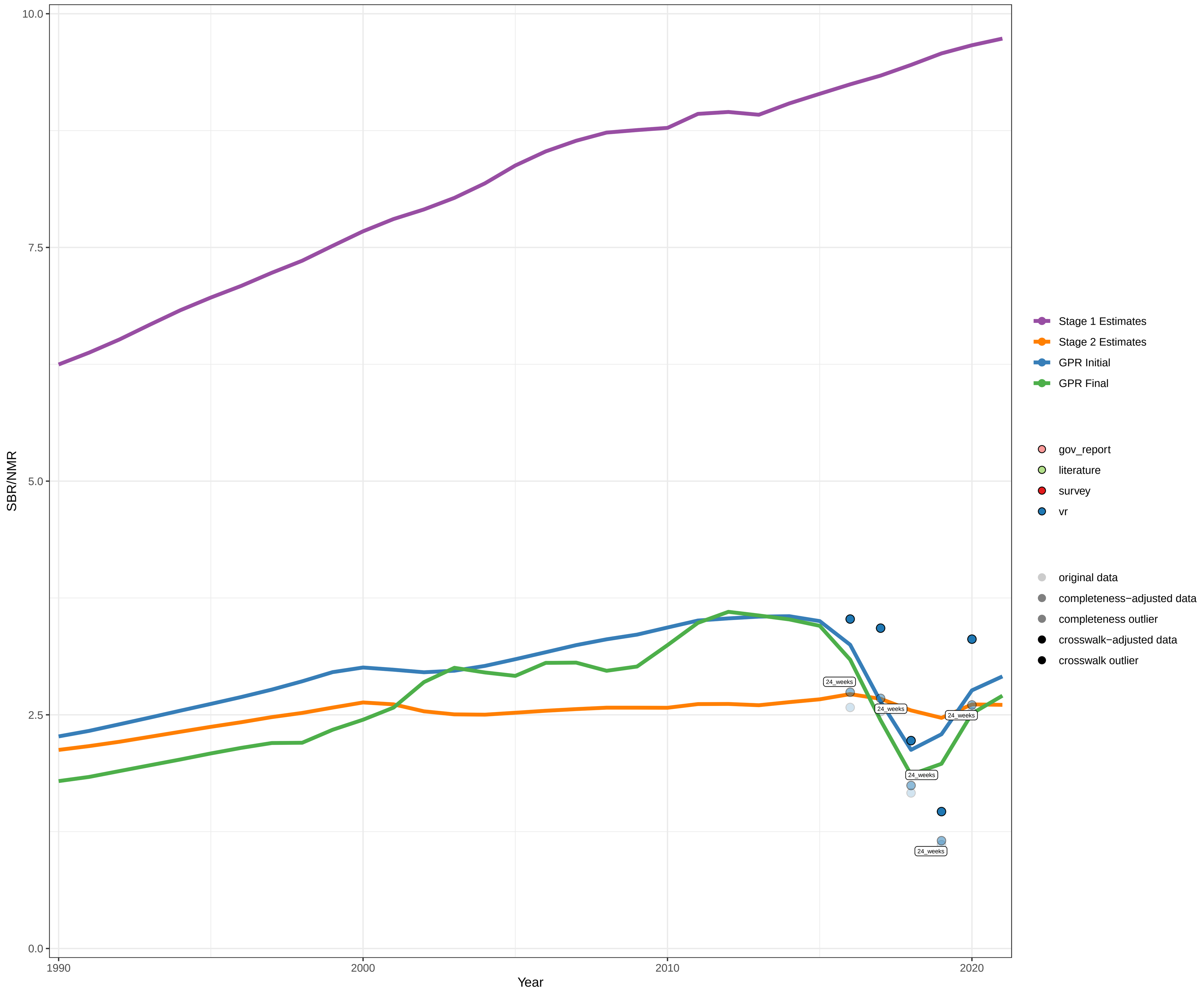

Bexley GBR_44732 Western Europe Stillbirth Rate / Neonatal Mortality Rate (SBR/NMR) − 28 weeks
lambda = 0.6 ; zeta = 0.8 ; scale = 15 ; amp = 0.028

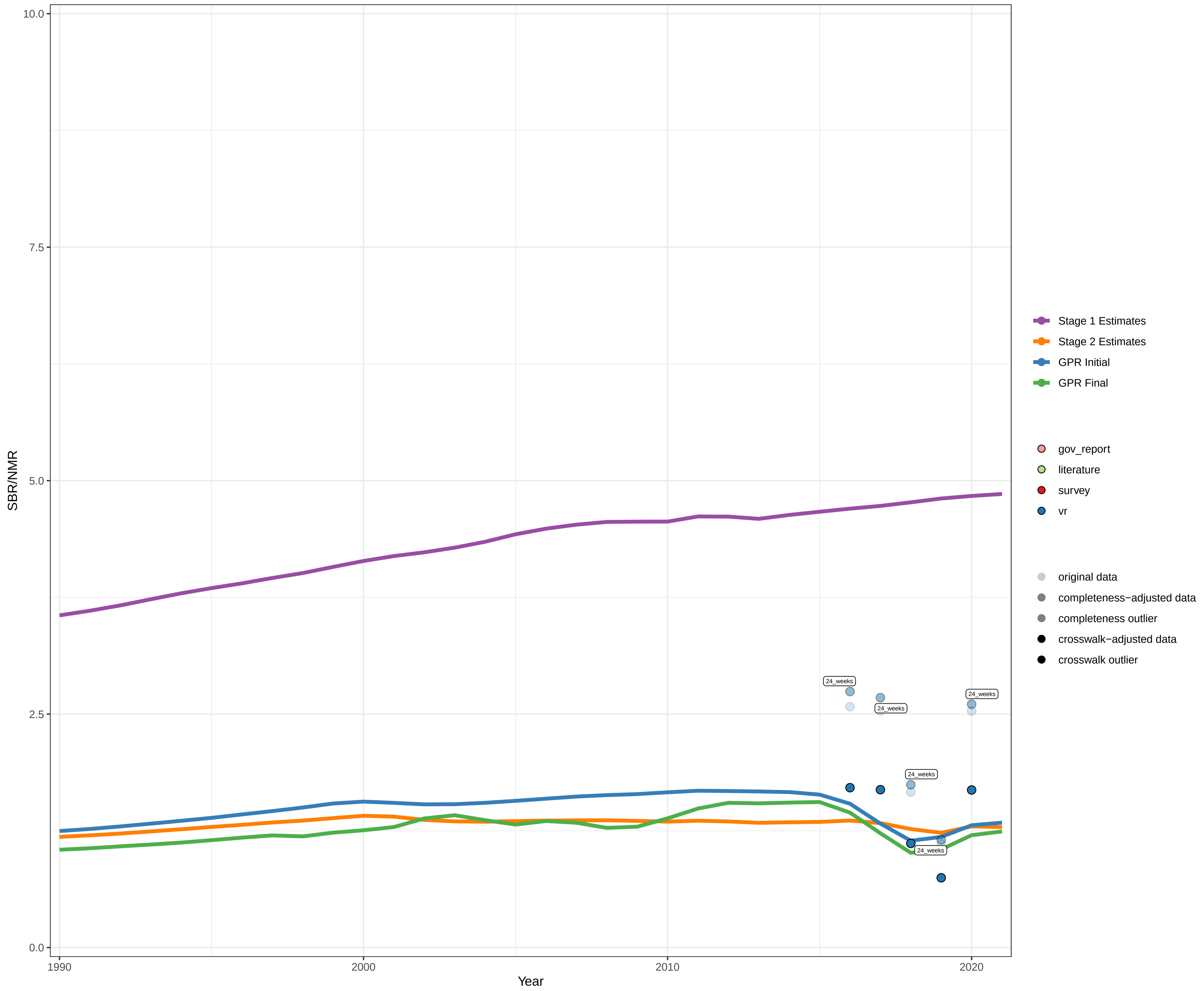

Definition	Years	Outlier	Source.Type	Title
24_weeks	2016−2020	0	VR	United Kingdom − England and Wales Child Mortality Statistics

Bexley GBR_44732 Western Europe Stillbirth Rate (SBR) − 20 weeks
lambda = 0.6 ; zeta = 0.8 ; scale = 15 ; amp = 0.028

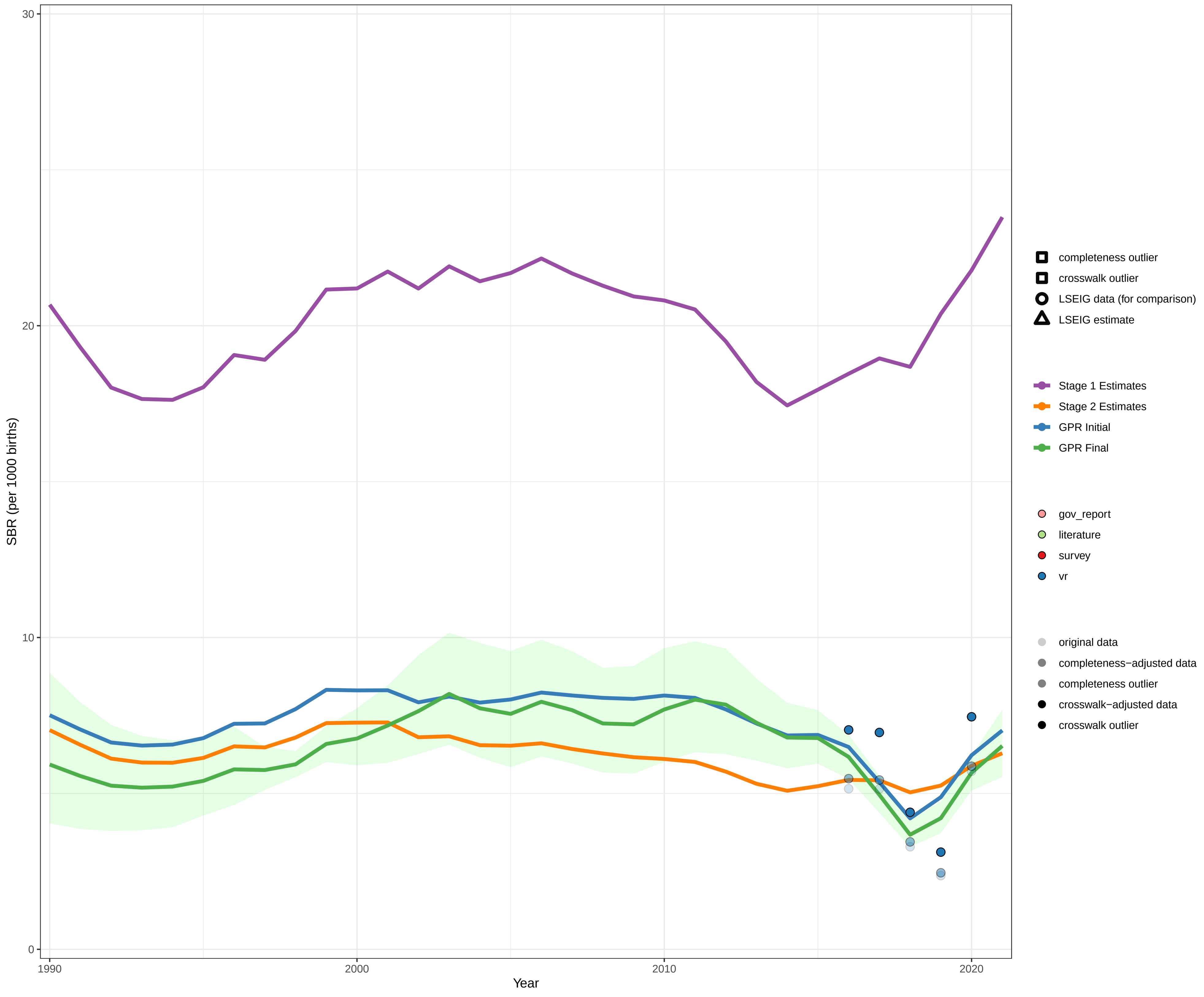

Bexley GBR_44732 Western Europe Stillbirth Rate (SBR) − 28 weeks
lambda = 0.6 ; zeta = 0.8 ; scale = 15 ; amp = 0.028

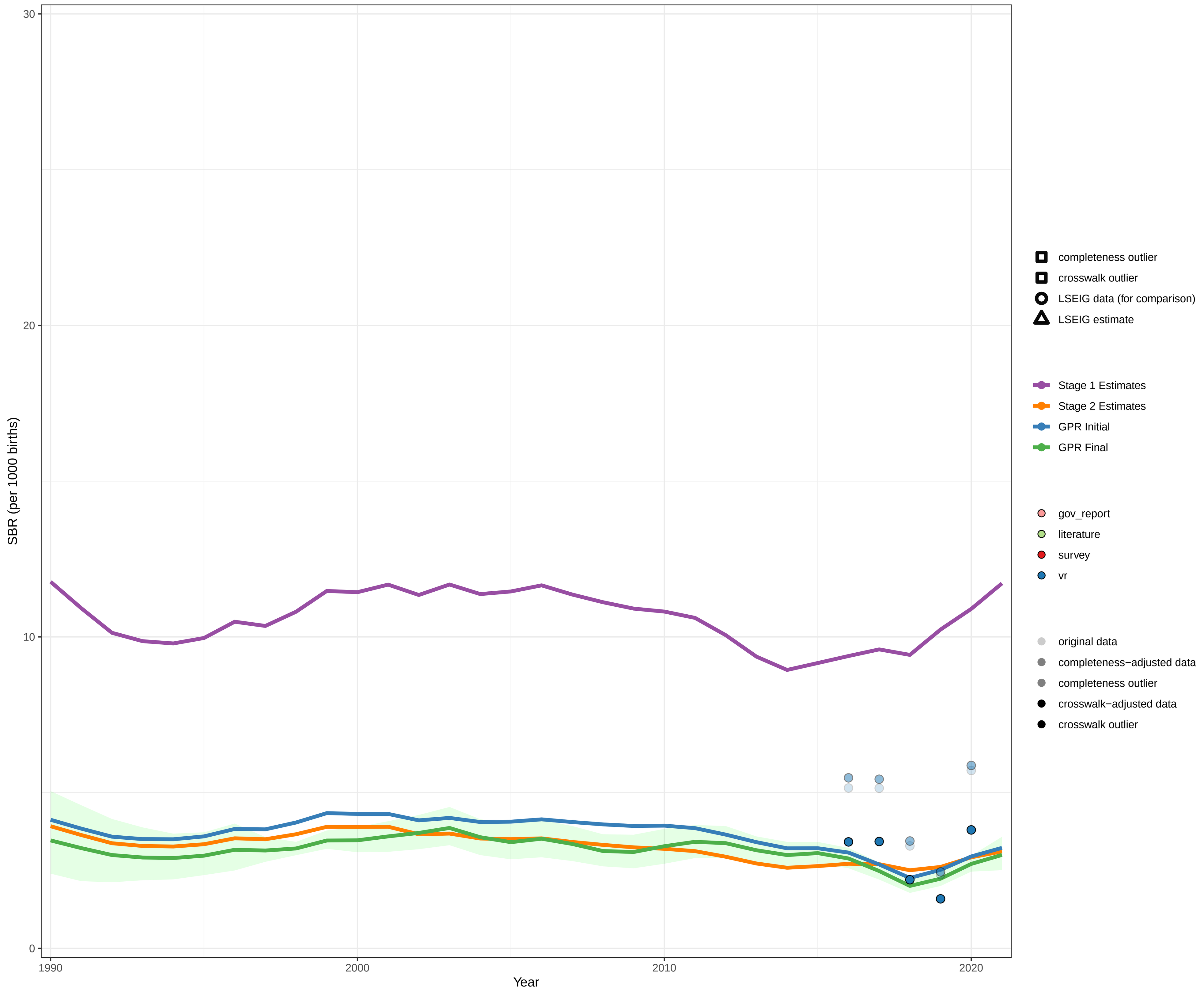

Year	Total Stillbirths (in thousands)	Total Stillbirths (in thousands)	SBR (per 1000 births)	SBR (per 1000 births)	SBR/NMR	SBR/NMR
	20 weeks	28 weeks	20 weeks	28 weeks	20 weeks	28 weeks
1990	0.0 (0.0 to 0.0)	0.0 (0.0 to 0.0)	5.9 (4.0 to 8.9)	3.5 (2.4 to 5.1)	1.79 (1.22 to 2.68)	1.05 (0.72 to 1.53)
1995	0.0 (0.0 to 0.0)	0.0 (0.0 to 0.0)	5.4 (4.3 to 6.8)	3.0 (2.4 to 3.7)	2.09 (1.66 to 2.61)	1.15 (0.91 to 1.44)
2000	0.0 (0.0 to 0.0)	0.0 (0.0 to 0.0)	6.8 (5.9 to 7.7)	3.5 (3.1 to 3.9)	2.45 (2.14 to 2.80)	1.26 (1.12 to 1.41)
2005	0.0 (0.0 to 0.0)	0.0 (0.0 to 0.0)	7.6 (5.8 to 9.6)	3.4 (2.9 to 4.0)	2.92 (2.26 to 3.70)	1.32 (1.10 to 1.54)
2010	0.0 (0.0 to 0.0)	0.0 (0.0 to 0.0)	7.7 (6.0 to 9.7)	3.3 (2.7 to 3.8)	3.25 (2.53 to 4.08)	1.38 (1.15 to 1.62)
2015	0.0 (0.0 to 0.0)	0.0 (0.0 to 0.0)	6.8 (6.0 to 7.7)	3.1 (2.7 to 3.4)	3.45 (3.03 to 3.91)	1.56 (1.39 to 1.74)
2020	0.0 (0.0 to 0.0)	0.0 (0.0 to 0.0)	5.7 (5.1 to 6.2)	2.7 (2.5 to 3.0)	2.51 (2.26 to 2.76)	1.20 (1.09 to 1.32)
2021	0.0 (0.0 to 0.0)	0.0 (0.0 to 0.0)	6.5 (5.5 to 7.7)	3.0 (2.5 to 3.6)	2.70 (2.29 to 3.19)	1.24 (1.04 to 1.48)

Brent GBR_44735 Western Europe Stillbirth Rate / Neonatal Mortality Rate (SBR/NMR) − 20 weeks
lambda = 0.6 ; zeta = 0.8 ; scale = 15 ; amp = 0.028

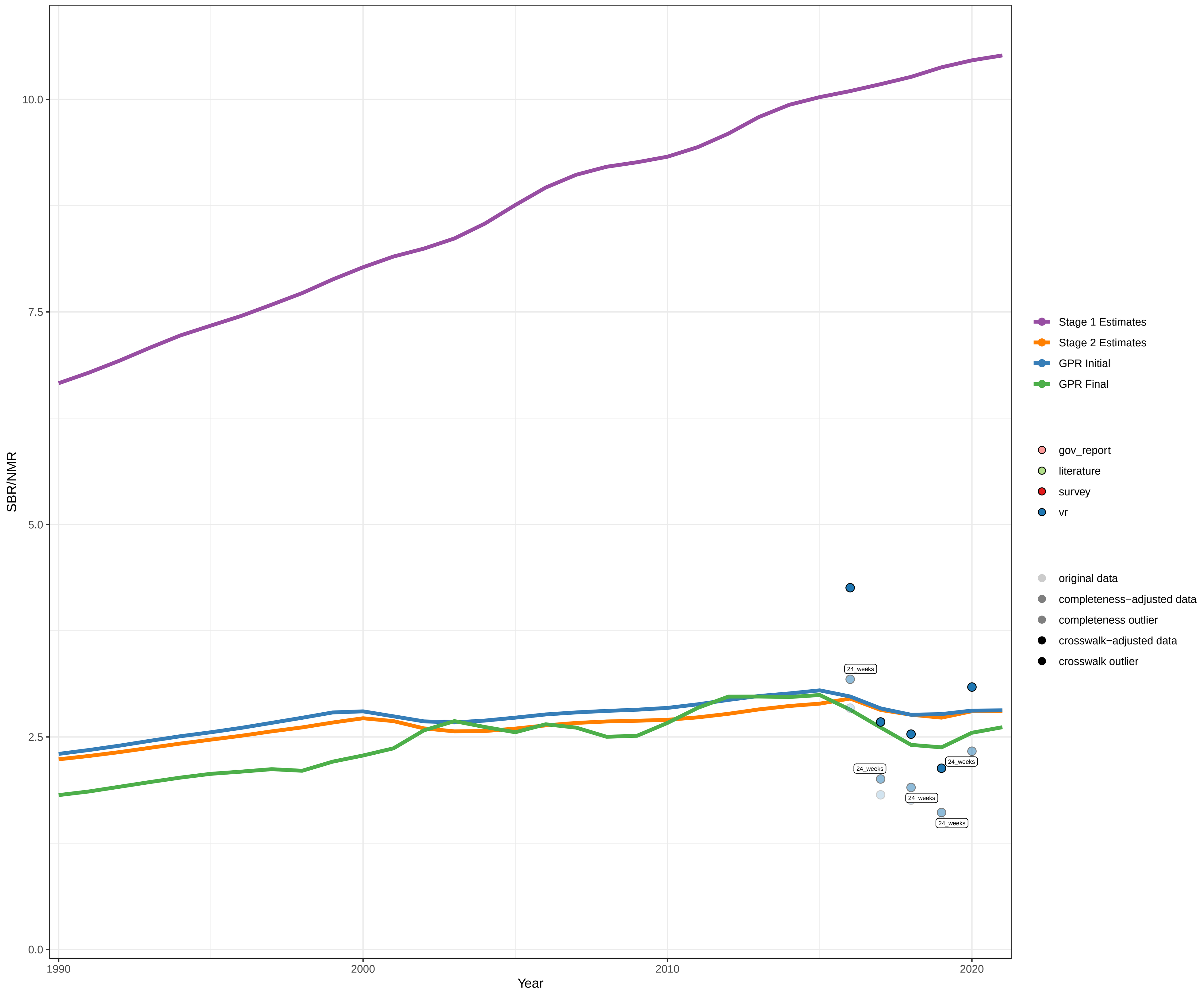

Brent GBR_44735 Western Europe Stillbirth Rate / Neonatal Mortality Rate (SBR/NMR) − 28 weeks
lambda = 0.6 ; zeta = 0.8 ; scale = 15 ; amp = 0.028

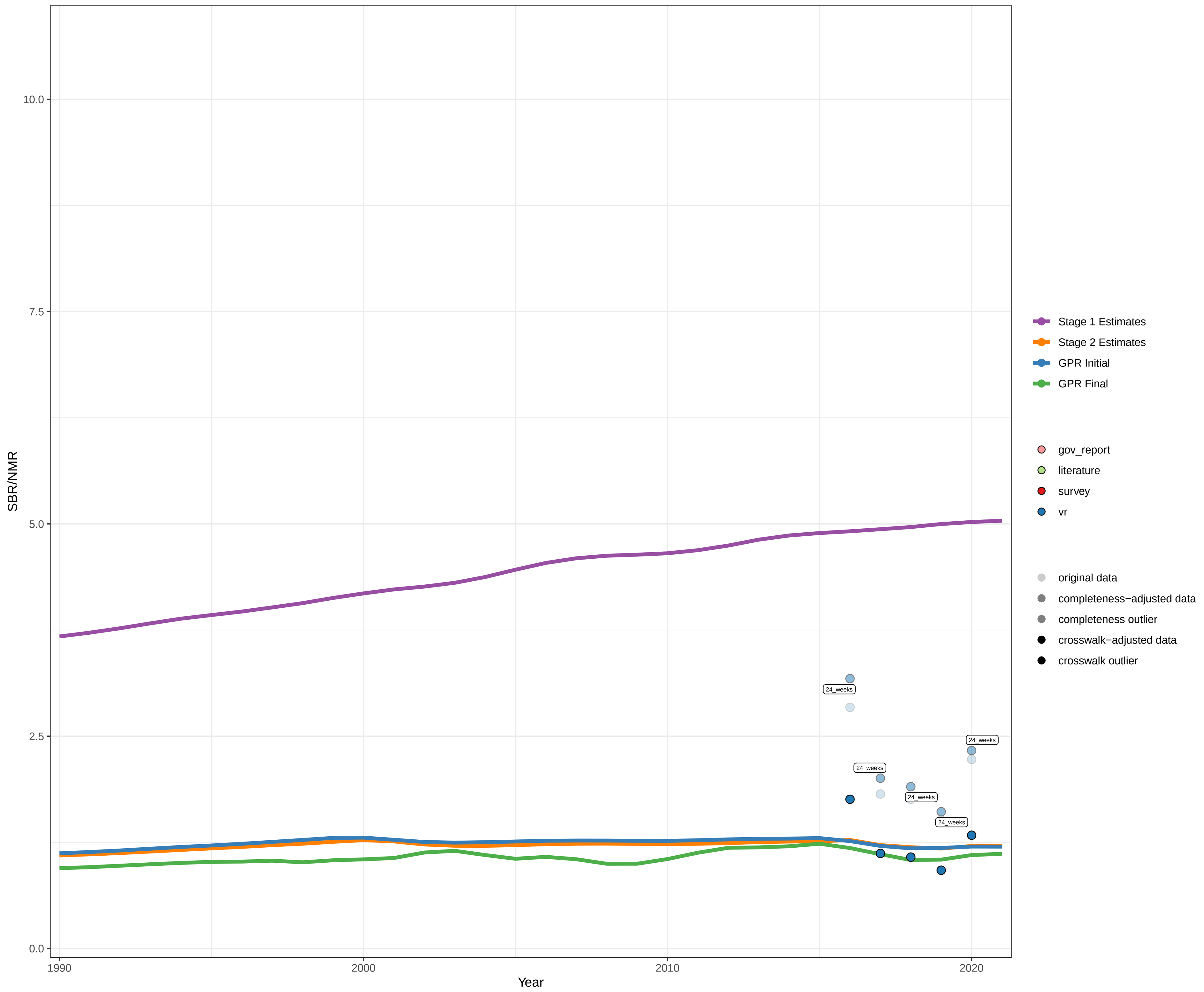

Definition	Years	Outlier	Source.Type	Title
24_weeks	2016−2020	0	VR	United Kingdom − England and Wales Child Mortality Statistics

Brent GBR_44735 Western Europe Stillbirth Rate (SBR) − 20 weeks
lambda = 0.6 ; zeta = 0.8 ; scale = 15 ; amp = 0.028

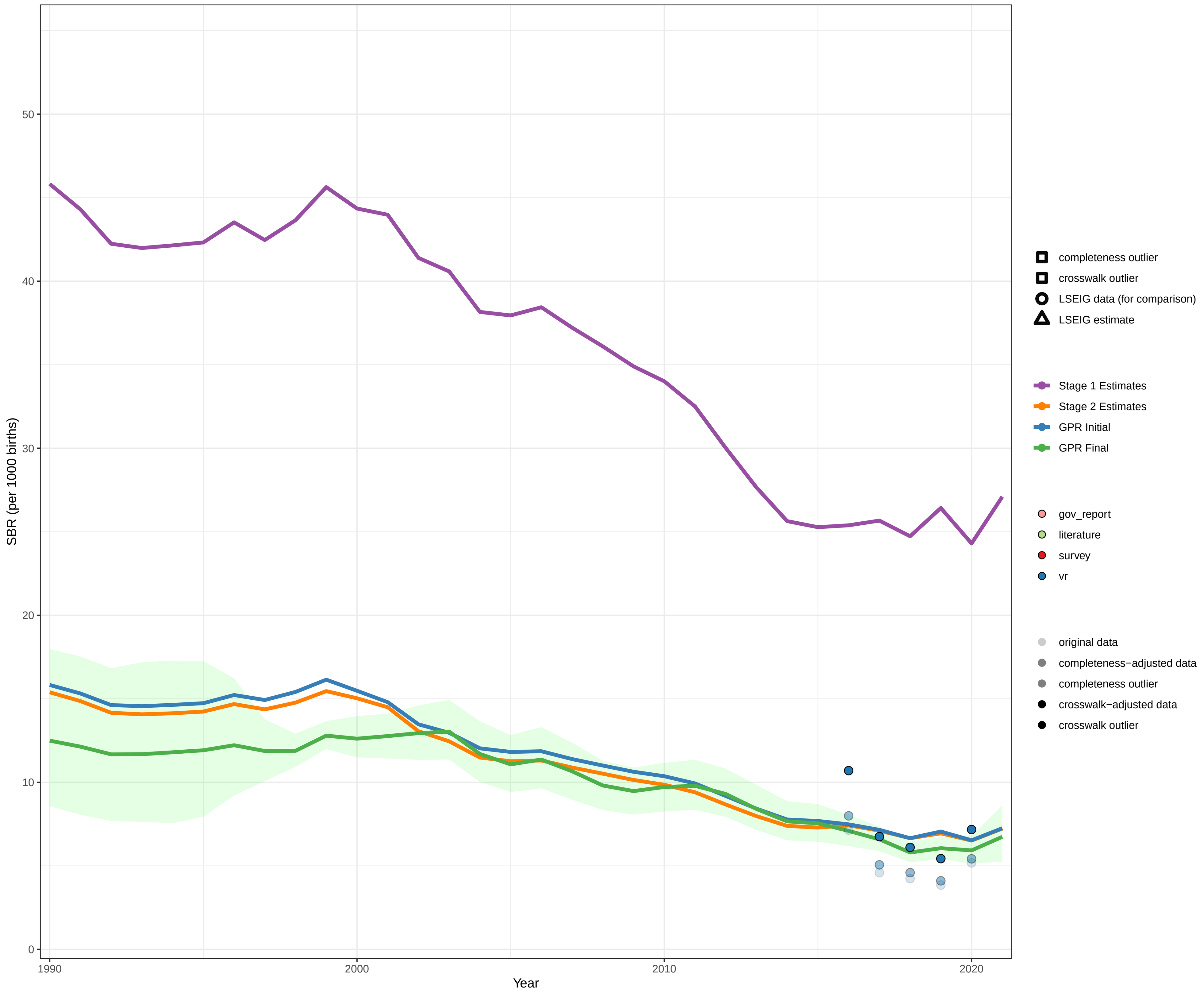

Brent GBR_44735 Western Europe Stillbirth Rate (SBR) − 28 weeks
lambda = 0.6 ; zeta = 0.8 ; scale = 15 ; amp = 0.028

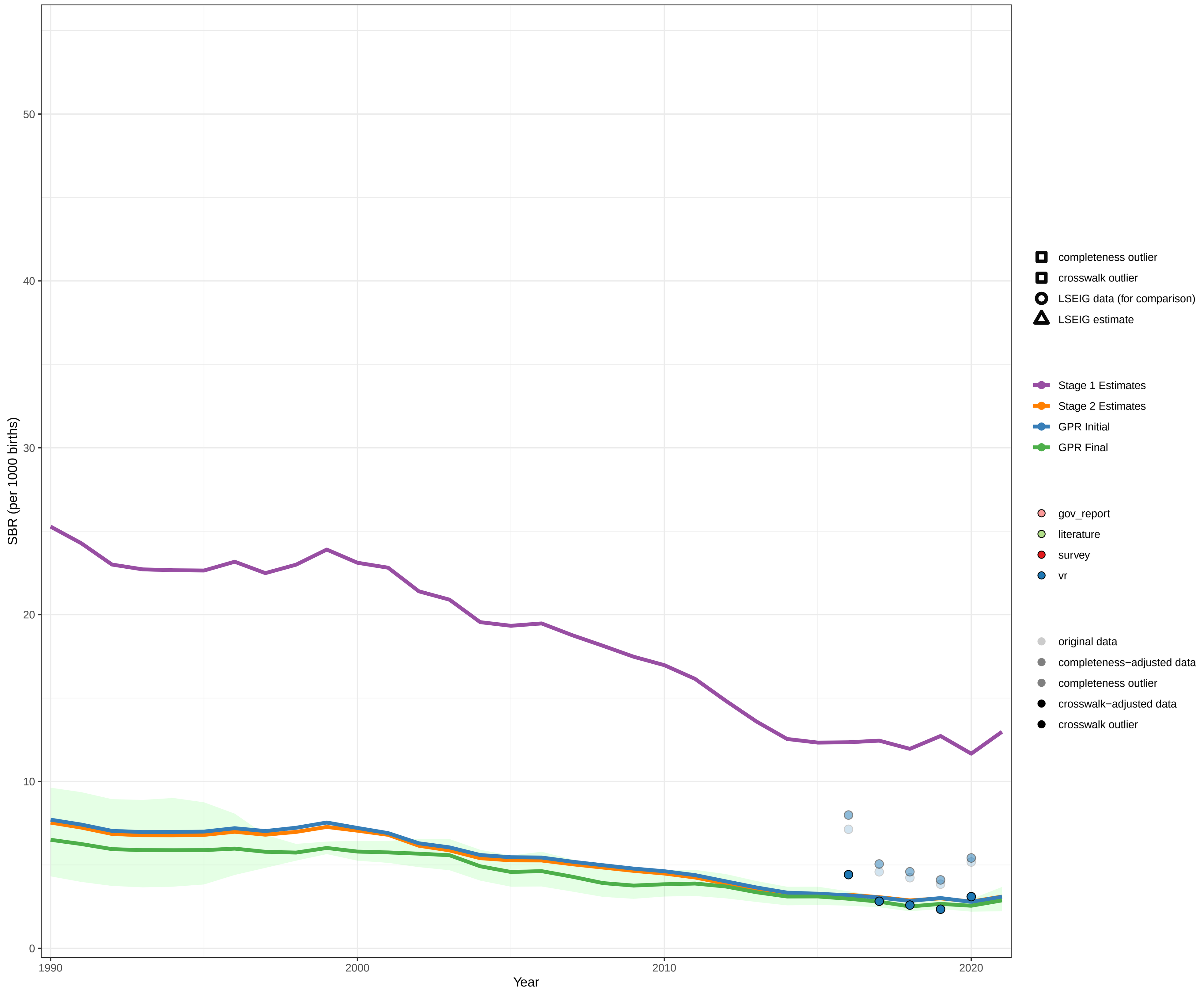

Year	Total Stillbirths (in thousands)	Total Stillbirths (in thousands)	SBR (per 1000 births)	SBR (per 1000 births)	SBR/NMR	SBR/NMR
	20 weeks	28 weeks	20 weeks	28 weeks	20 weeks	28 weeks
1990	0.0 (0.0 to 0.1)	0.0 (0.0 to 0.0)	12.5 (8.6 to 18.0)	6.5 (4.3 to 9.6)	1.82 (1.25 to 2.61)	0.95 (0.63 to 1.40)
1995	0.0 (0.0 to 0.1)	0.0 (0.0 to 0.0)	11.9 (7.9 to 17.3)	5.9 (3.8 to 8.8)	2.07 (1.38 to 2.99)	1.02 (0.66 to 1.52)
2000	0.1 (0.0 to 0.1)	0.0 (0.0 to 0.0)	12.6 (11.5 to 14.0)	5.8 (5.3 to 6.4)	2.28 (2.08 to 2.53)	1.05 (0.95 to 1.17)
2005	0.1 (0.0 to 0.1)	0.0 (0.0 to 0.0)	11.1 (9.4 to 12.8)	4.6 (3.7 to 5.6)	2.56 (2.17 to 2.96)	1.06 (0.85 to 1.28)
2010	0.1 (0.0 to 0.1)	0.0 (0.0 to 0.0)	9.7 (8.3 to 11.2)	3.8 (3.1 to 4.7)	2.67 (2.26 to 3.06)	1.05 (0.85 to 1.29)
2015	0.0 (0.0 to 0.0)	0.0 (0.0 to 0.0)	7.5 (6.4 to 8.7)	3.1 (2.6 to 3.7)	2.99 (2.56 to 3.45)	1.23 (1.03 to 1.47)
2020	0.0 (0.0 to 0.0)	0.0 (0.0 to 0.0)	5.9 (5.1 to 6.8)	2.6 (2.2 to 3.0)	2.55 (2.21 to 2.93)	1.10 (0.95 to 1.27)
2021	0.0 (0.0 to 0.0)	0.0 (0.0 to 0.0)	6.7 (5.3 to 8.6)	2.9 (2.2 to 3.7)	2.61 (2.04 to 3.34)	1.12 (0.86 to 1.43)

Bromley GBR_44731 Western Europe Stillbirth Rate / Neonatal Mortality Rate (SBR/NMR) − 20 weeks
lambda = 0.6 ; zeta = 0.8 ; scale = 15 ; amp = 0.028

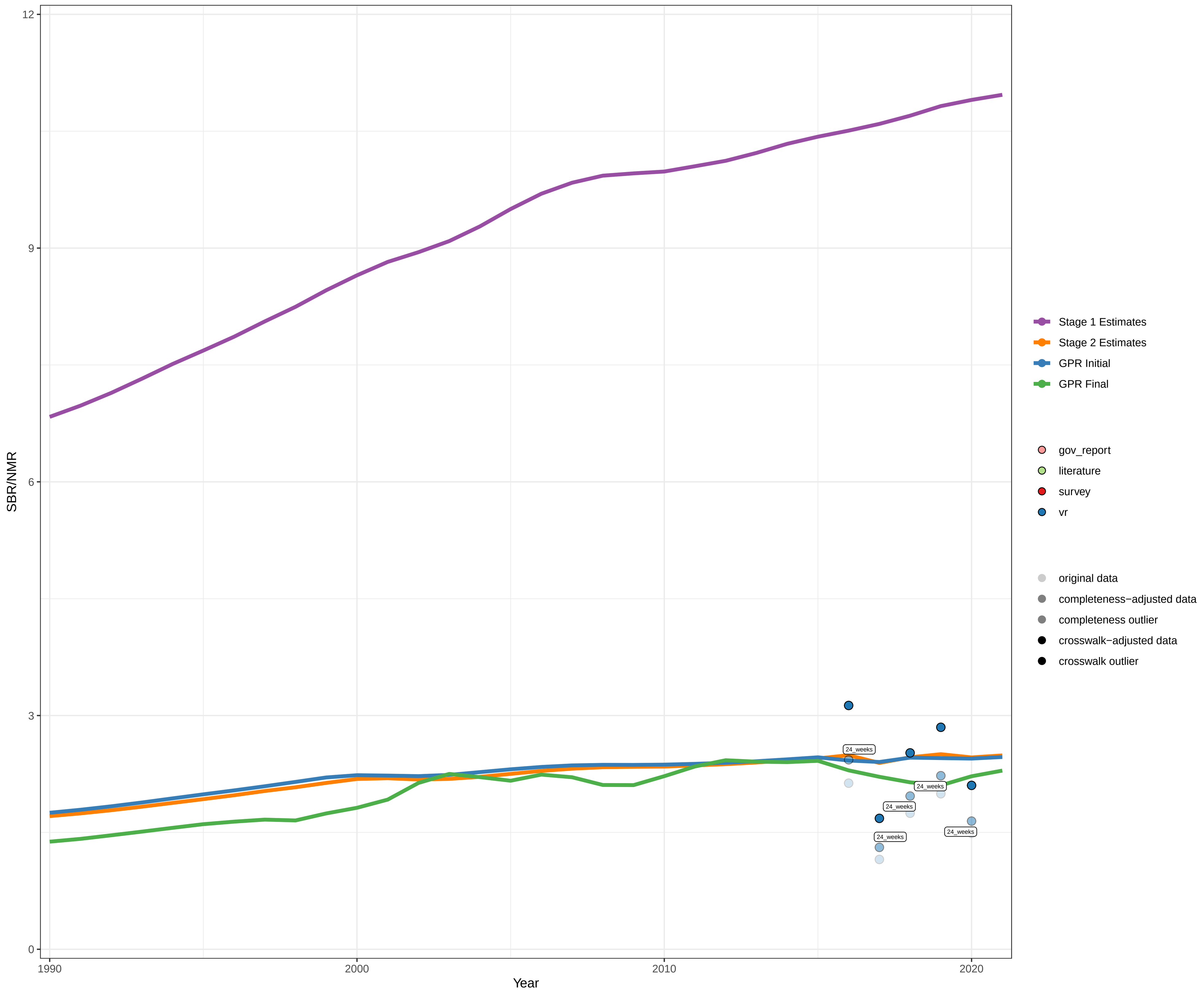

Bromley GBR_44731 Western Europe Stillbirth Rate / Neonatal Mortality Rate (SBR/NMR) − 28 weeks
lambda = 0.6 ; zeta = 0.8 ; scale = 15 ; amp = 0.028

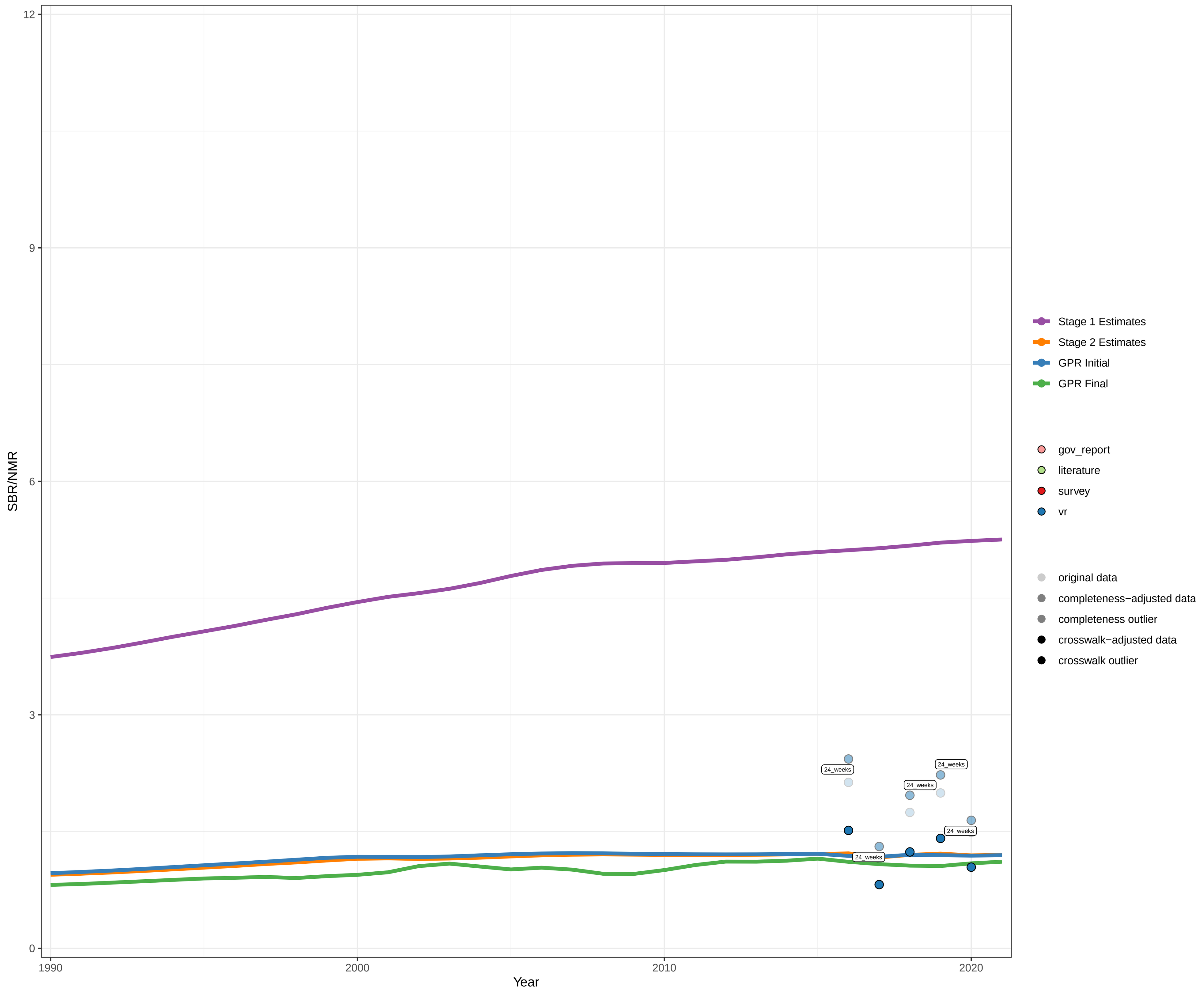

Definition	Years	Outlier	Source.Type	Title
24_weeks	2016−2020	0	VR	United Kingdom − England and Wales Child Mortality Statistics

Bromley GBR_44731 Western Europe Stillbirth Rate (SBR) − 20 weeks
lambda = 0.6 ; zeta = 0.8 ; scale = 15 ; amp = 0.028

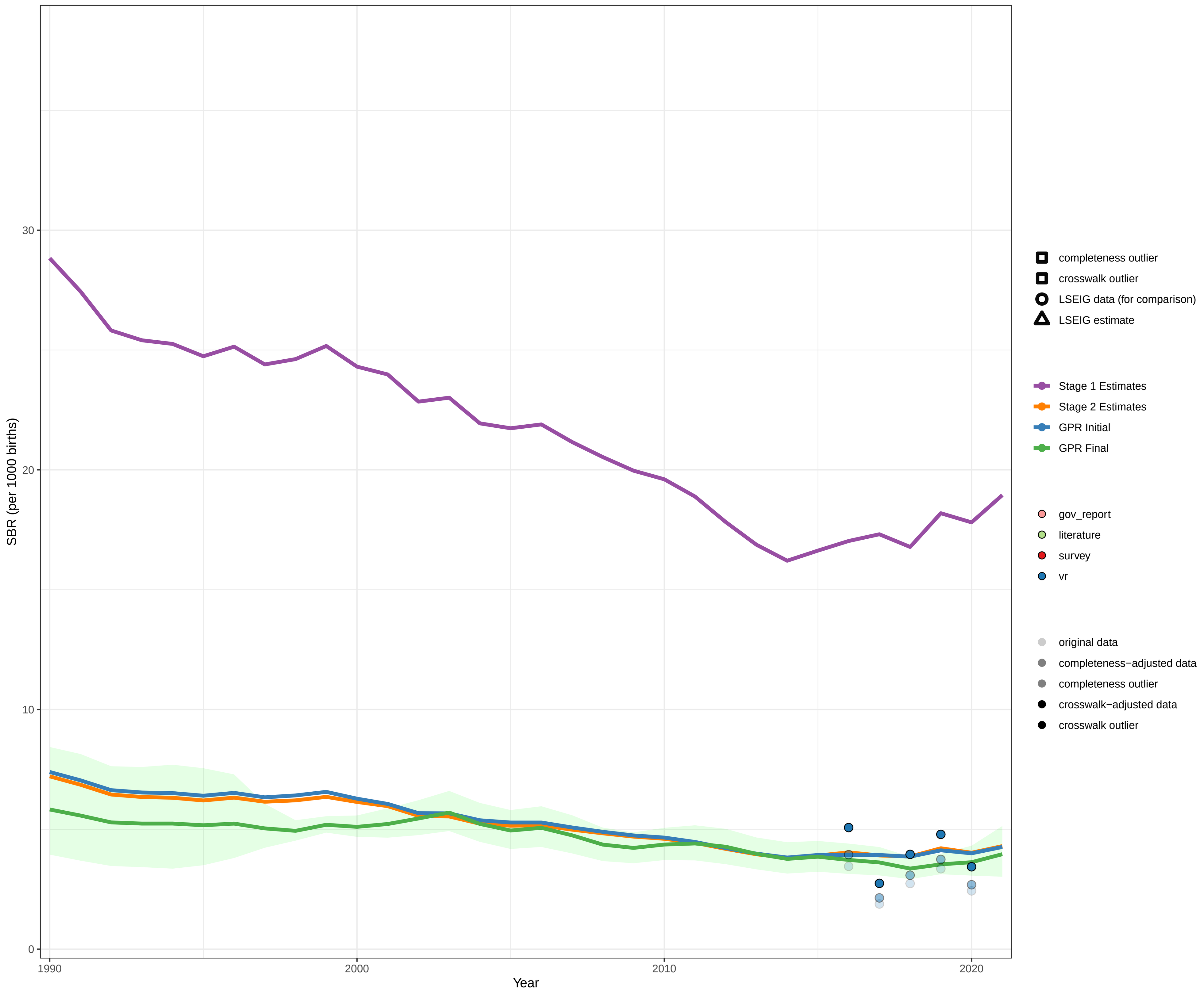

Bromley GBR_44731 Western Europe Stillbirth Rate (SBR) − 28 weeks
lambda = 0.6 ; zeta = 0.8 ; scale = 15 ; amp = 0.028

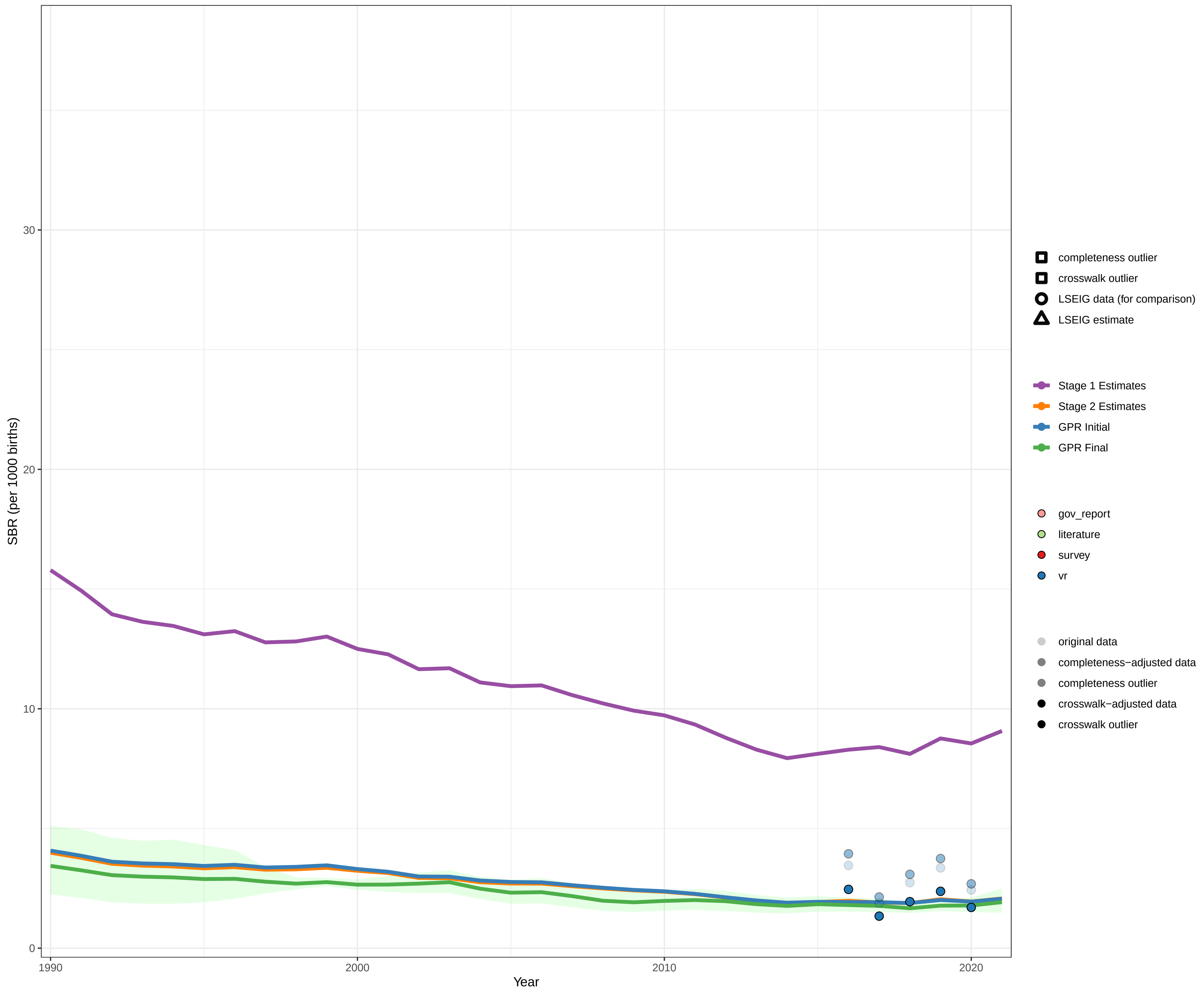

Year	Total Stillbirths (in thousands)	Total Stillbirths (in thousands)	SBR (per 1000 births)	SBR (per 1000 births)	SBR/NMR	SBR/NMR
	20 weeks	28 weeks	20 weeks	28 weeks	20 weeks	28 weeks
1990	0.0 (0.0 to 0.0)	0.0 (0.0 to 0.0)	5.8 (3.9 to 8.4)	3.4 (2.2 to 5.1)	1.38 (0.93 to 2.00)	0.81 (0.53 to 1.21)
1995	0.0 (0.0 to 0.0)	0.0 (0.0 to 0.0)	5.2 (3.5 to 7.5)	2.9 (1.9 to 4.3)	1.61 (1.09 to 2.35)	0.90 (0.59 to 1.34)
2000	0.0 (0.0 to 0.0)	0.0 (0.0 to 0.0)	5.1 (4.7 to 5.6)	2.7 (2.4 to 2.9)	1.82 (1.67 to 1.98)	0.94 (0.87 to 1.02)
2005	0.0 (0.0 to 0.0)	0.0 (0.0 to 0.0)	4.9 (4.2 to 5.8)	2.3 (1.9 to 2.8)	2.16 (1.83 to 2.54)	1.01 (0.81 to 1.24)
2010	0.0 (0.0 to 0.0)	0.0 (0.0 to 0.0)	4.4 (3.7 to 5.1)	2.0 (1.6 to 2.4)	2.22 (1.89 to 2.58)	1.01 (0.80 to 1.24)
2015	0.0 (0.0 to 0.0)	0.0 (0.0 to 0.0)	3.9 (3.2 to 4.5)	1.8 (1.5 to 2.2)	2.42 (2.03 to 2.83)	1.15 (0.96 to 1.36)
2020	0.0 (0.0 to 0.0)	0.0 (0.0 to 0.0)	3.6 (3.1 to 4.3)	1.8 (1.5 to 2.1)	2.22 (1.88 to 2.64)	1.09 (0.92 to 1.30)
2021	0.0 (0.0 to 0.0)	0.0 (0.0 to 0.0)	4.0 (3.0 to 5.1)	1.9 (1.5 to 2.5)	2.29 (1.75 to 2.97)	1.11 (0.86 to 1.44)

Camden GBR_44748 Western Europe Stillbirth Rate / Neonatal Mortality Rate (SBR/NMR) − 20 weeks
lambda = 0.6 ; zeta = 0.8 ; scale = 15 ; amp = 0.028

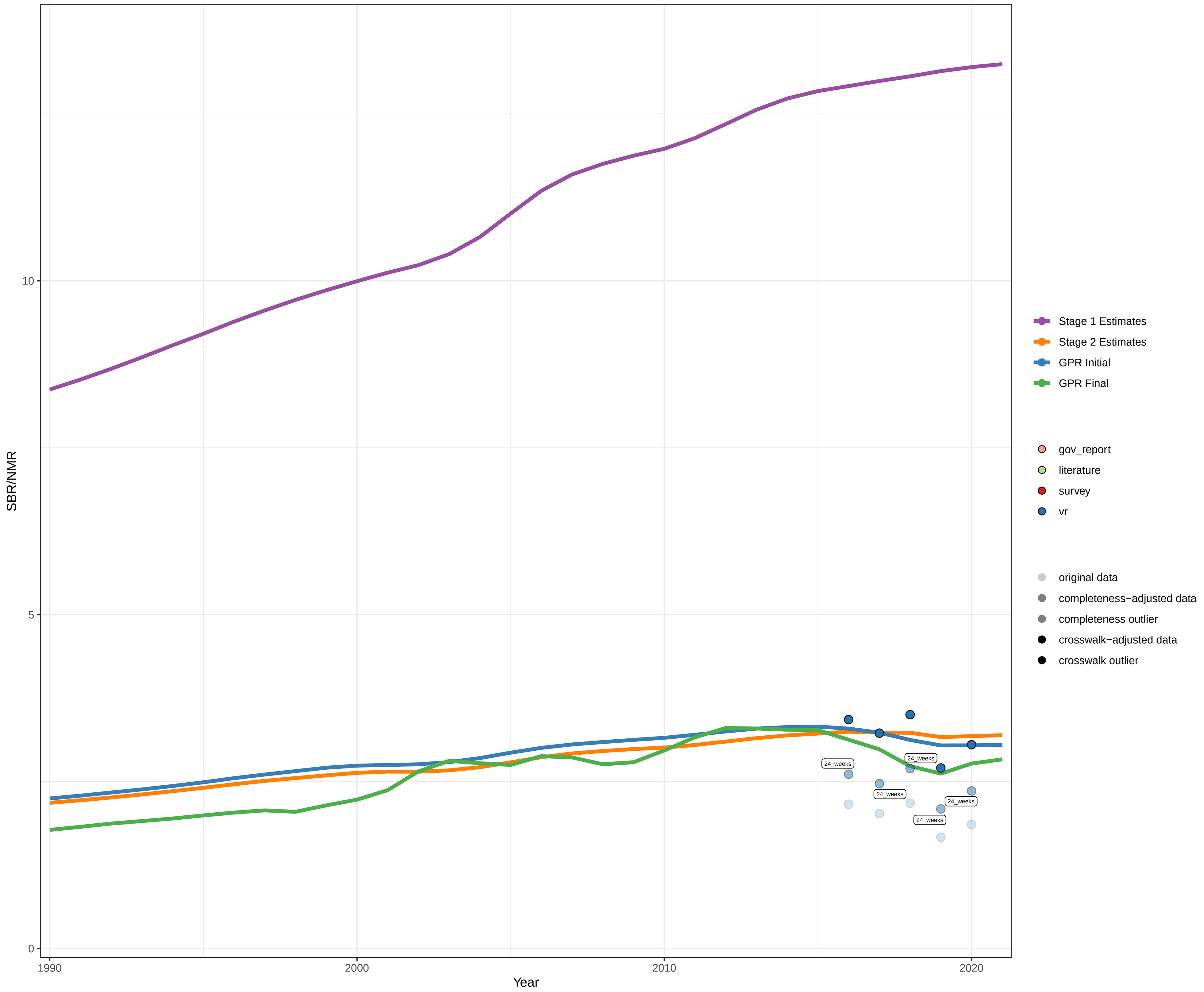

Camden GBR_44748 Western Europe Stillbirth Rate / Neonatal Mortality Rate (SBR/NMR) − 28 weeks
lambda = 0.6 ; zeta = 0.8 ; scale = 15 ; amp = 0.028

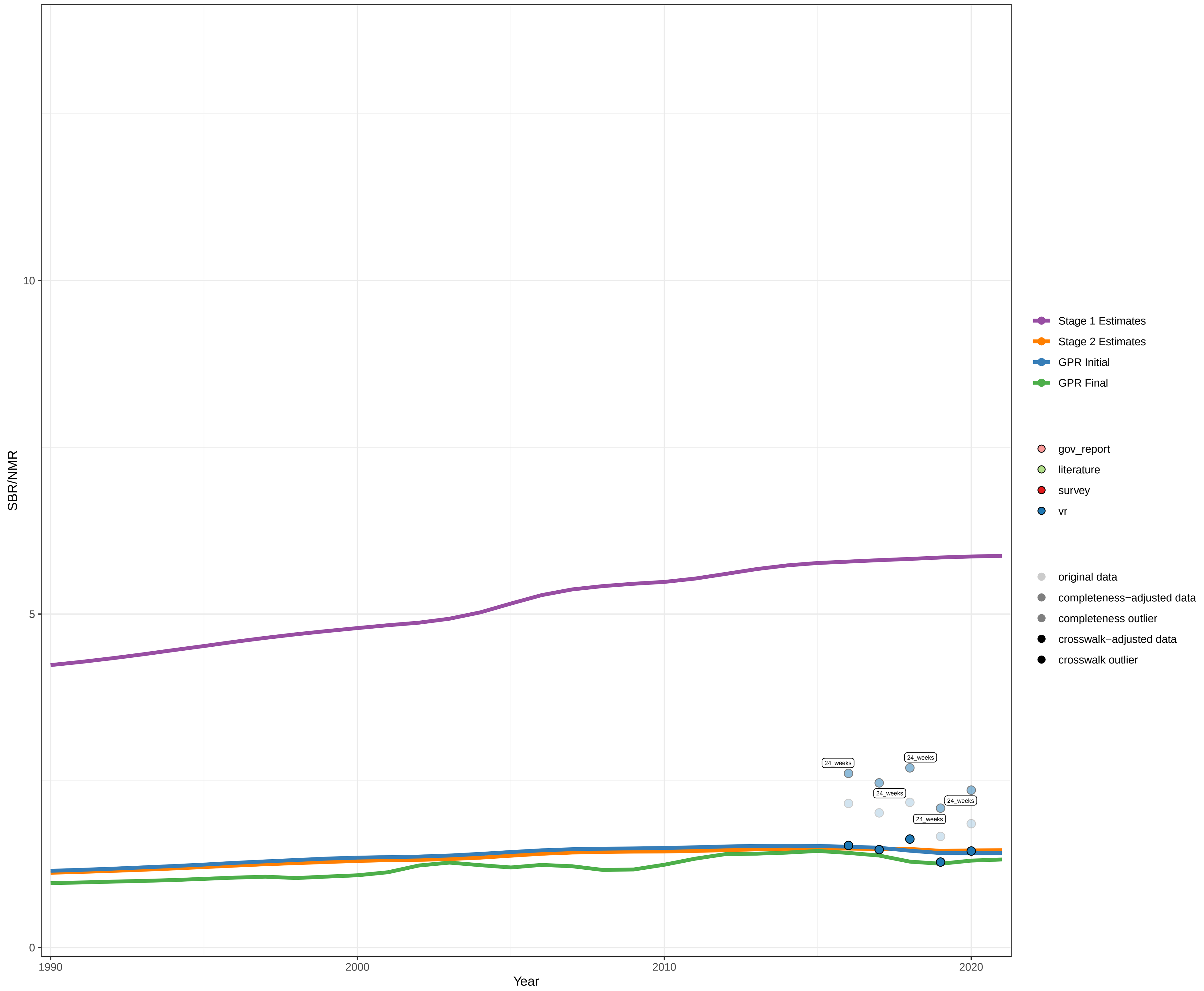

Definition	Years	Outlier	Source.Type	Title
24_weeks	2016−2020	0	VR	United Kingdom − England and Wales Child Mortality Statistics

Camden GBR_44748 Western Europe Stillbirth Rate (SBR) − 20 weeks
lambda = 0.6 ; zeta = 0.8 ; scale = 15 ; amp = 0.028

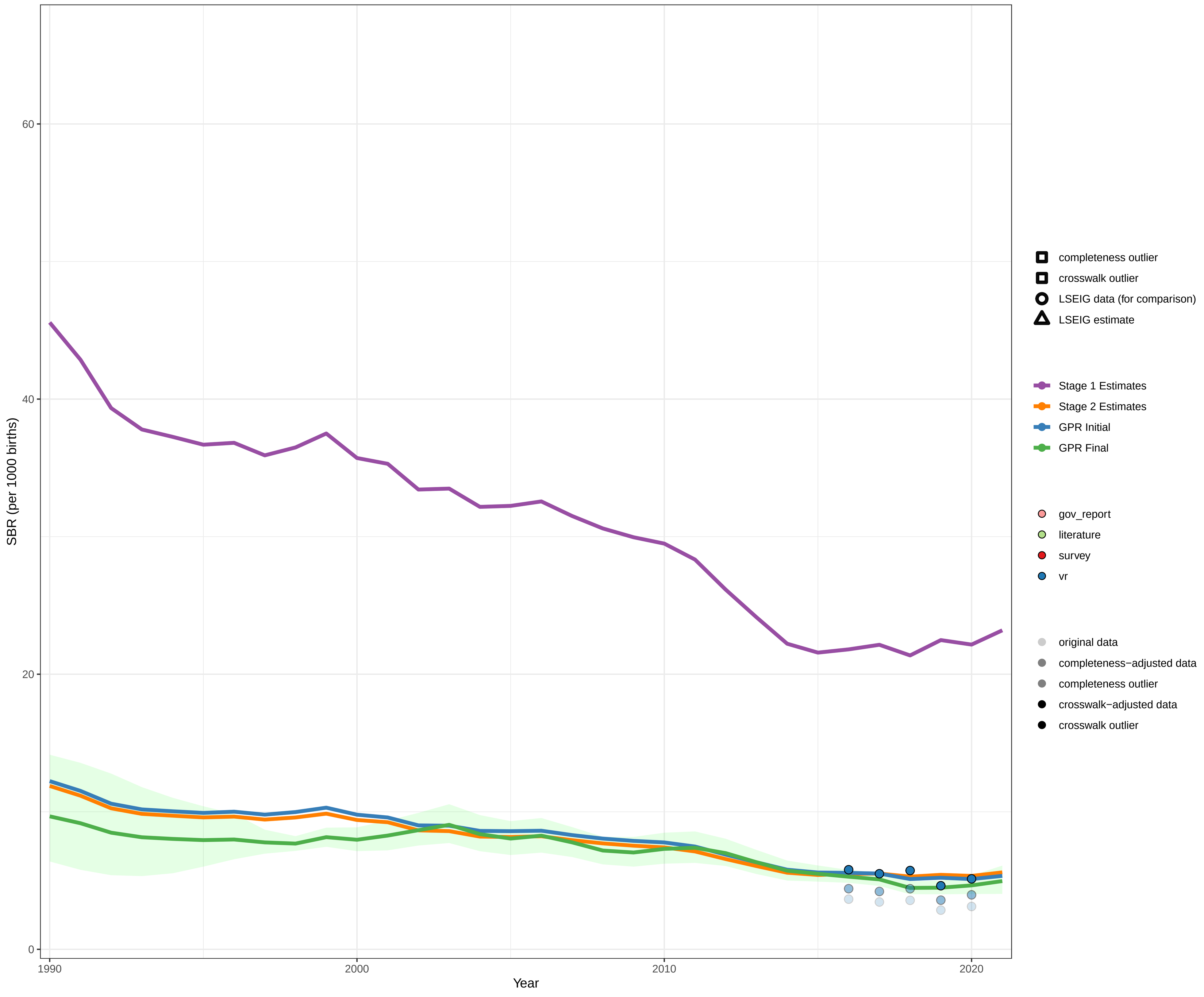

Camden GBR_44748 Western Europe Stillbirth Rate (SBR) − 28 weeks
lambda = 0.6 ; zeta = 0.8 ; scale = 15 ; amp = 0.028

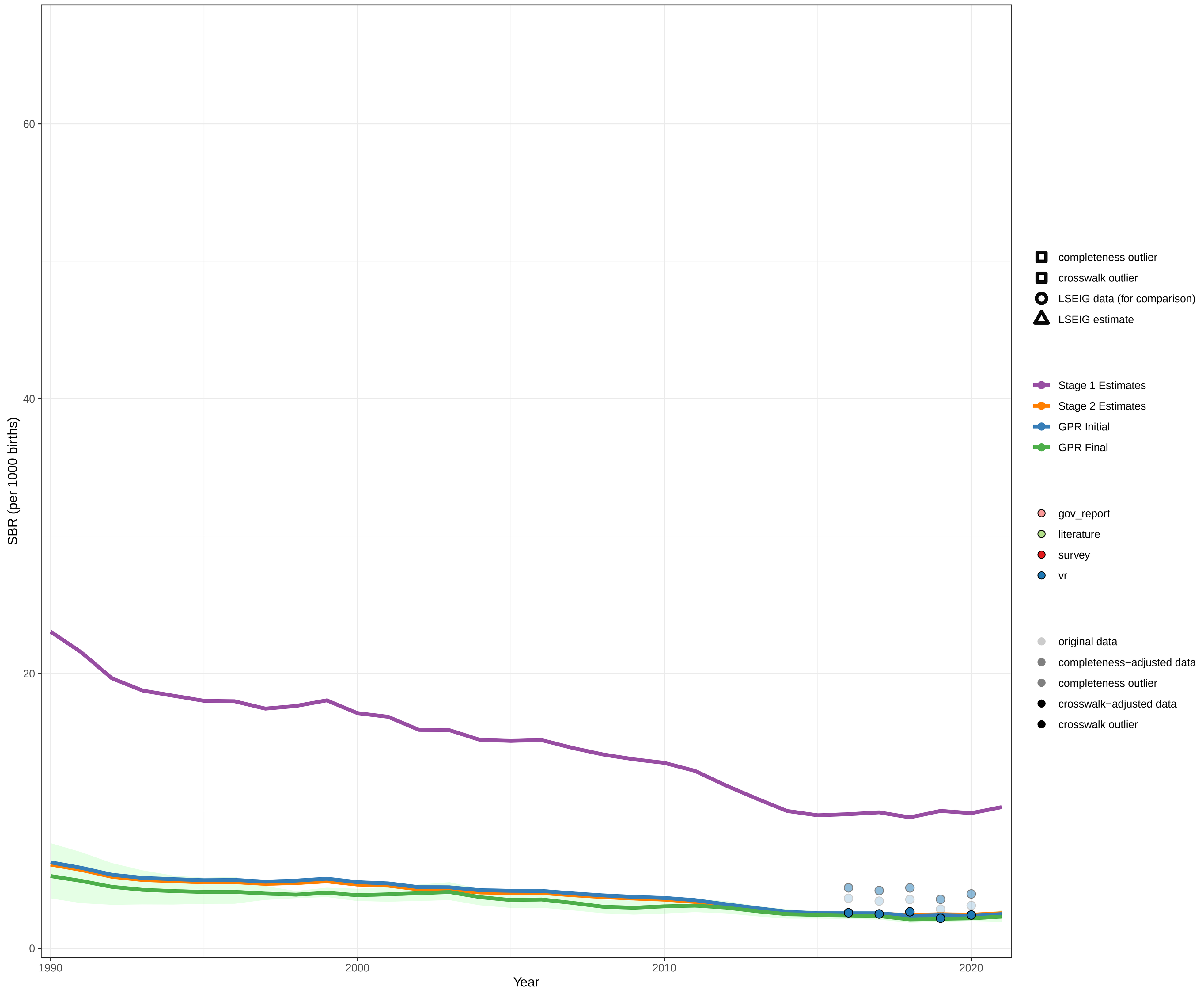

Year	Total Stillbirths (in thousands)	Total Stillbirths (in thousands)	SBR (per 1000 births)	SBR (per 1000 births)	SBR/NMR	SBR/NMR
	20 weeks	28 weeks	20 weeks	28 weeks	20 weeks	28 weeks
1990	0.0 (0.0 to 0.0)	0.0 (0.0 to 0.0)	9.7 (6.4 to 14.1)	5.3 (3.6 to 7.7)	1.78 (1.17 to 2.60)	0.97 (0.67 to 1.41)
1995	0.0 (0.0 to 0.0)	0.0 (0.0 to 0.0)	7.9 (6.0 to 10.4)	4.1 (3.2 to 5.1)	1.99 (1.51 to 2.61)	1.03 (0.81 to 1.29)
2000	0.0 (0.0 to 0.0)	0.0 (0.0 to 0.0)	8.0 (7.1 to 8.9)	3.9 (3.4 to 4.3)	2.23 (2.00 to 2.48)	1.08 (0.96 to 1.22)
2005	0.0 (0.0 to 0.0)	0.0 (0.0 to 0.0)	8.1 (6.9 to 9.3)	3.5 (2.9 to 4.1)	2.75 (2.34 to 3.18)	1.20 (1.01 to 1.41)
2010	0.0 (0.0 to 0.0)	0.0 (0.0 to 0.0)	7.3 (6.2 to 8.5)	3.1 (2.5 to 3.6)	2.97 (2.53 to 3.44)	1.24 (1.03 to 1.45)
2015	0.0 (0.0 to 0.0)	0.0 (0.0 to 0.0)	5.5 (4.9 to 6.1)	2.4 (2.2 to 2.7)	3.27 (2.93 to 3.63)	1.45 (1.29 to 1.62)
2020	0.0 (0.0 to 0.0)	0.0 (0.0 to 0.0)	4.6 (4.0 to 5.4)	2.2 (2.0 to 2.4)	2.77 (2.39 to 3.19)	1.30 (1.18 to 1.43)
2021	0.0 (0.0 to 0.0)	0.0 (0.0 to 0.0)	5.0 (4.0 to 6.1)	2.3 (1.9 to 2.8)	2.83 (2.30 to 3.48)	1.32 (1.10 to 1.58)

Croydon GBR_44742 Western Europe Stillbirth Rate / Neonatal Mortality Rate (SBR/NMR) − 20 weeks
lambda = 0.6 ; zeta = 0.8 ; scale = 15 ; amp = 0.028

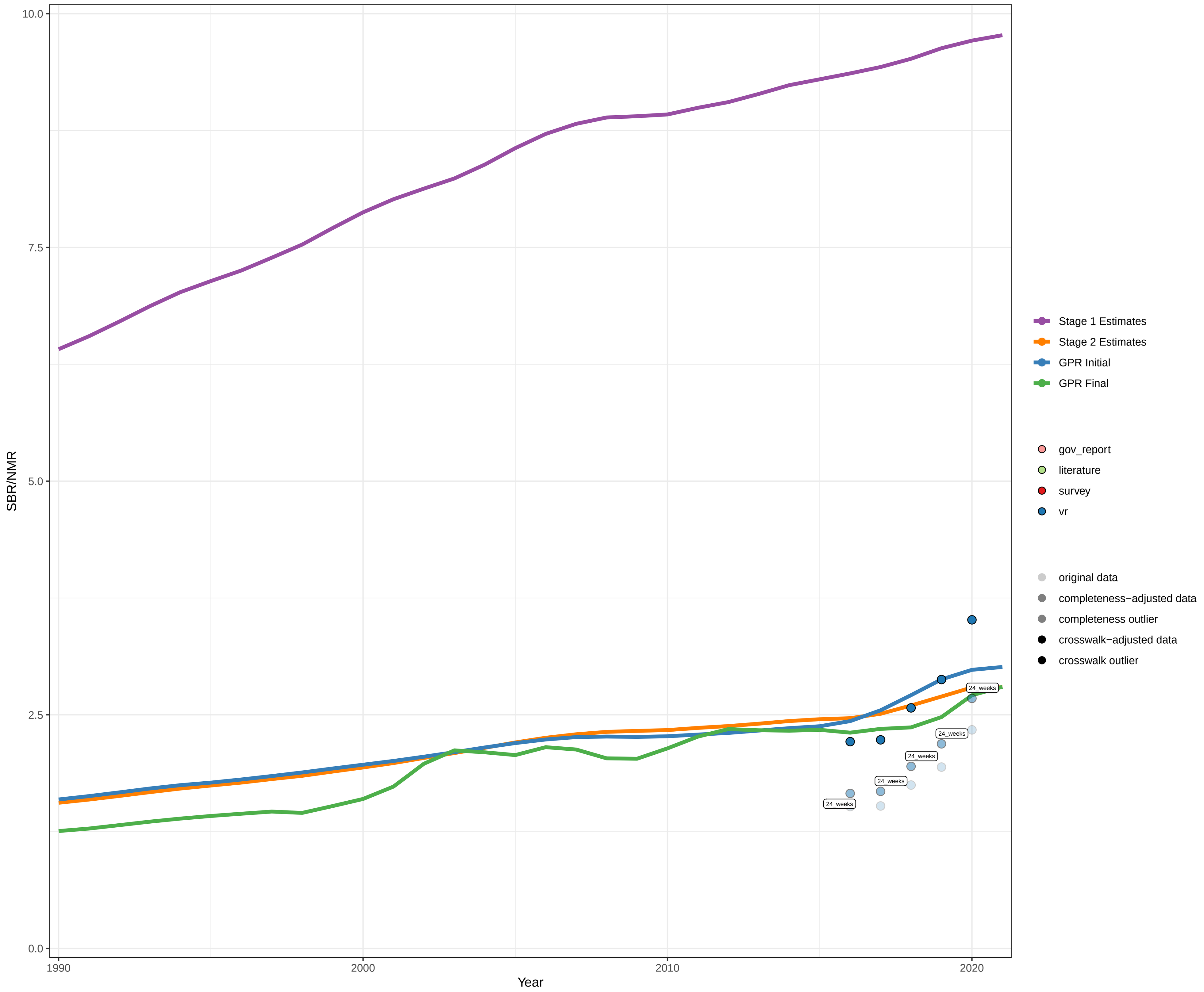

Croydon GBR_44742 Western Europe Stillbirth Rate / Neonatal Mortality Rate (SBR/NMR) − 28 weeks
lambda = 0.6 ; zeta = 0.8 ; scale = 15 ; amp = 0.028

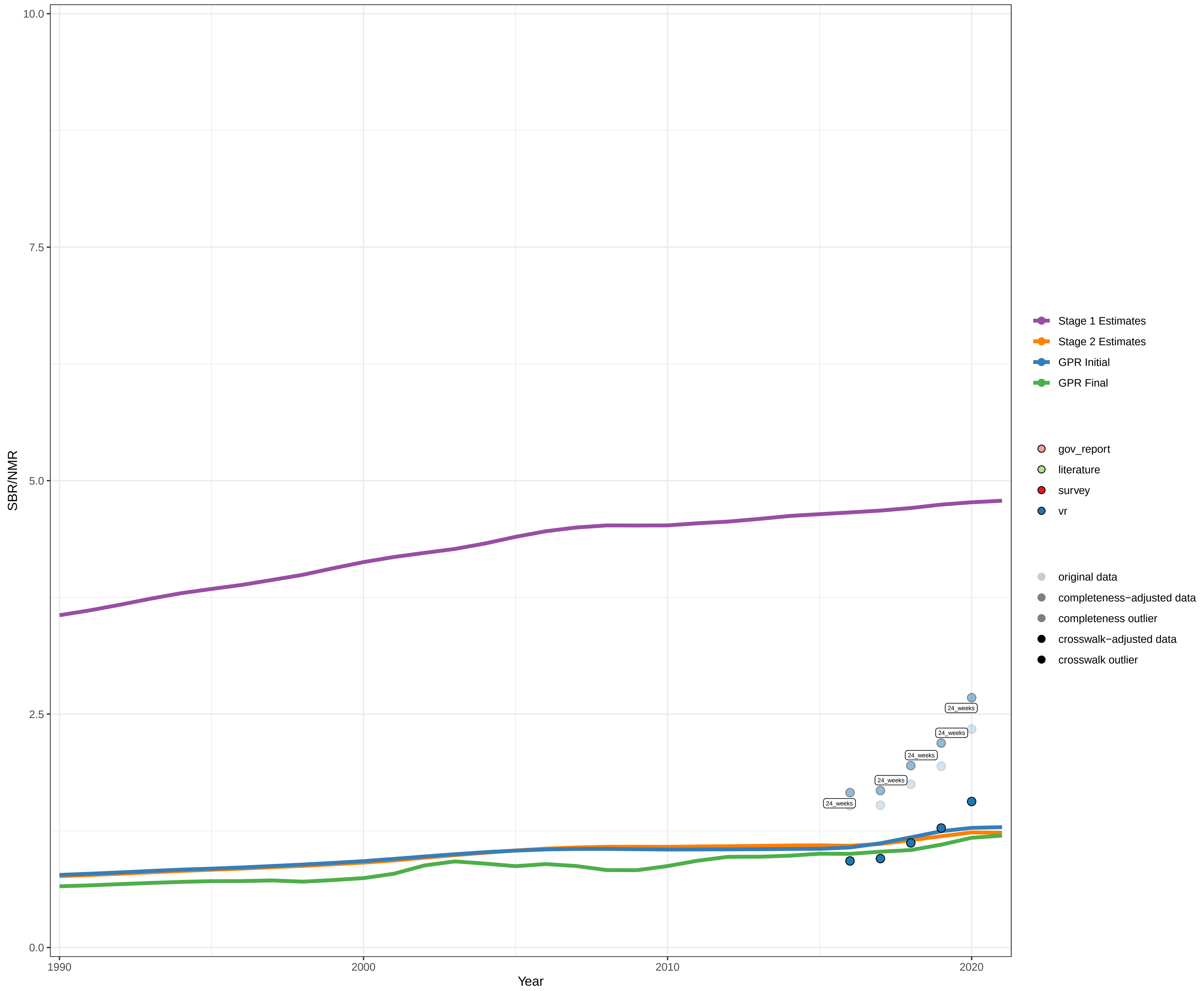

Definition	Years	Outlier	Source.Type	Title
24_weeks	2016−2020	0	VR	United Kingdom − England and Wales Child Mortality Statistics

Croydon GBR_44742 Western Europe Stillbirth Rate (SBR) − 20 weeks
lambda = 0.6 ; zeta = 0.8 ; scale = 15 ; amp = 0.028

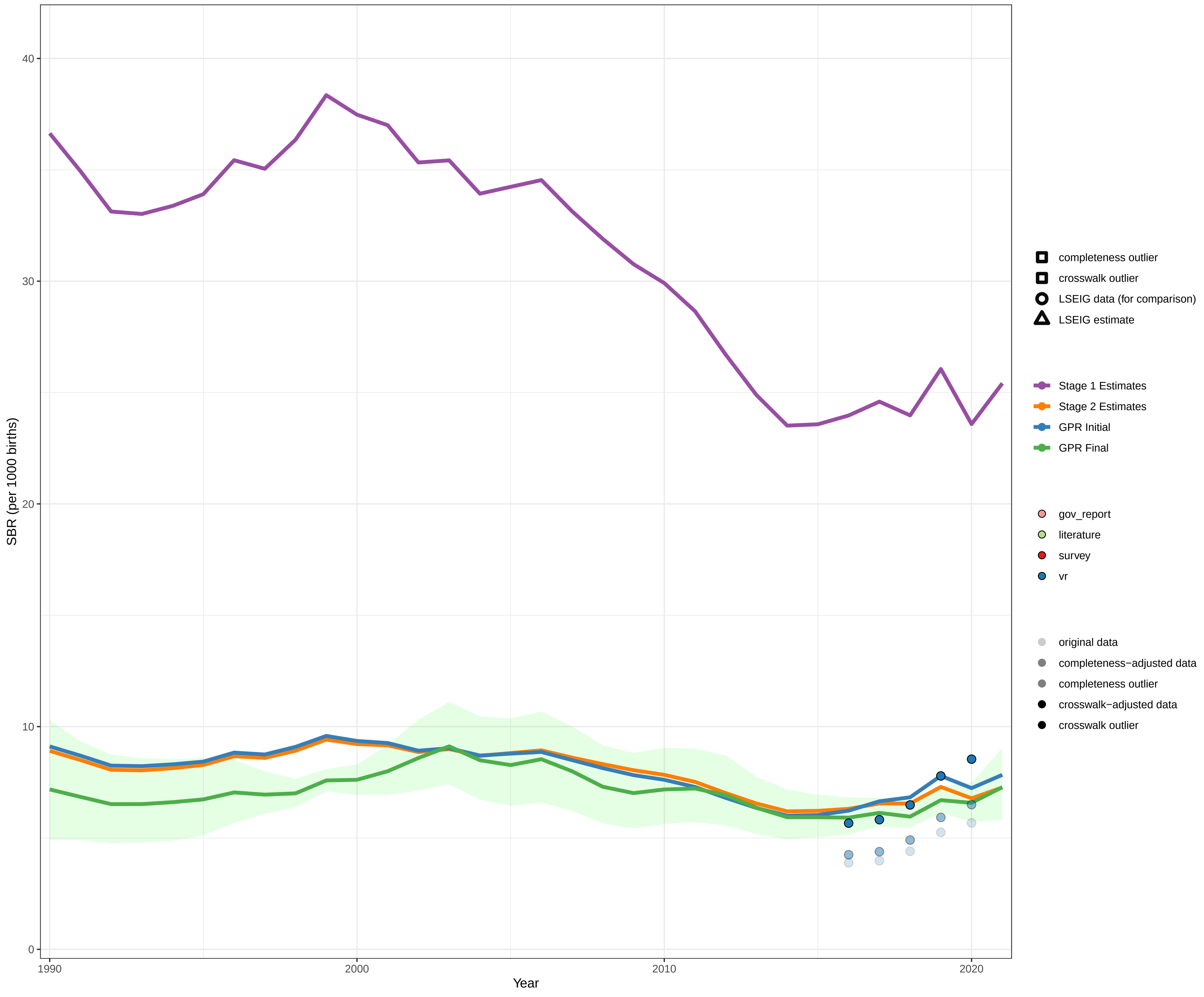

Croydon GBR_44742 Western Europe Stillbirth Rate (SBR) − 28 weeks
lambda = 0.6 ; zeta = 0.8 ; scale = 15 ; amp = 0.028

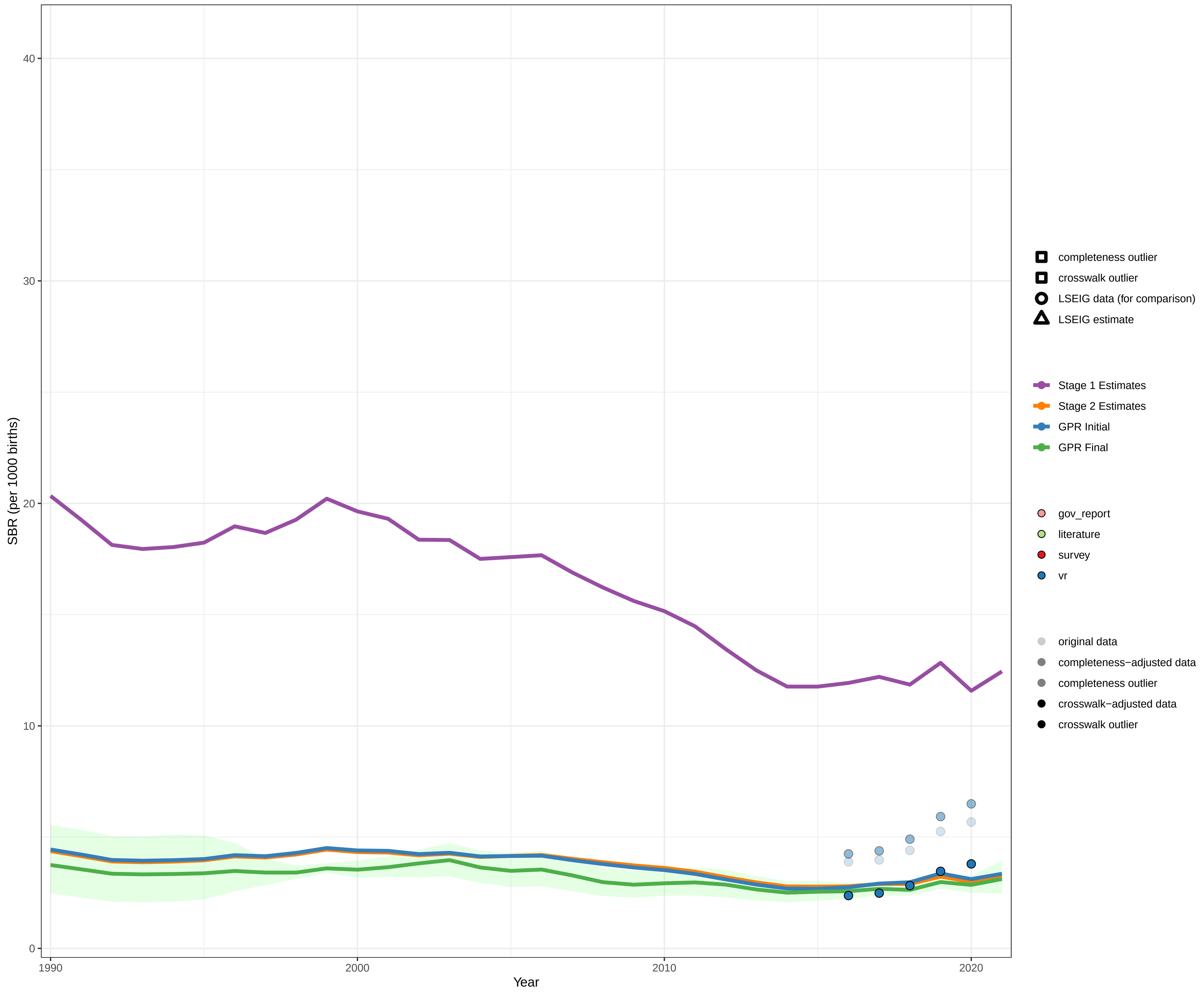

Year	Total Stillbirths (in thousands)	Total Stillbirths (in thousands)	SBR (per 1000 births)	SBR (per 1000 births)	SBR/NMR	SBR/NMR
	20 weeks	28 weeks	20 weeks	28 weeks	20 weeks	28 weeks
1990	0.0 (0.0 to 0.1)	0.0 (0.0 to 0.0)	7.2 (4.9 to 10.3)	3.7 (2.5 to 5.6)	1.26 (0.86 to 1.80)	0.66 (0.43 to 0.97)
1995	0.0 (0.0 to 0.0)	0.0 (0.0 to 0.0)	6.7 (5.1 to 8.6)	3.4 (2.2 to 5.1)	1.42 (1.08 to 1.81)	0.71 (0.46 to 1.07)
2000	0.0 (0.0 to 0.0)	0.0 (0.0 to 0.0)	7.6 (6.9 to 8.3)	3.5 (3.2 to 3.9)	1.60 (1.46 to 1.74)	0.74 (0.67 to 0.83)
2005	0.0 (0.0 to 0.1)	0.0 (0.0 to 0.0)	8.3 (6.4 to 10.4)	3.5 (2.8 to 4.3)	2.07 (1.61 to 2.59)	0.87 (0.69 to 1.08)
2010	0.0 (0.0 to 0.1)	0.0 (0.0 to 0.0)	7.2 (5.6 to 9.1)	2.9 (2.4 to 3.5)	2.14 (1.68 to 2.70)	0.87 (0.70 to 1.06)
2015	0.0 (0.0 to 0.0)	0.0 (0.0 to 0.0)	5.9 (5.0 to 6.9)	2.5 (2.1 to 3.0)	2.34 (1.99 to 2.74)	1.00 (0.85 to 1.20)
2020	0.0 (0.0 to 0.0)	0.0 (0.0 to 0.0)	6.6 (5.7 to 7.5)	2.9 (2.5 to 3.3)	2.71 (2.36 to 3.09)	1.18 (1.03 to 1.34)
2021	0.0 (0.0 to 0.0)	0.0 (0.0 to 0.0)	7.3 (5.8 to 9.0)	3.1 (2.5 to 4.0)	2.80 (2.23 to 3.48)	1.20 (0.94 to 1.52)

Ealing GBR_44744 Western Europe Stillbirth Rate / Neonatal Mortality Rate (SBR/NMR) − 20 weeks
lambda = 0.6 ; zeta = 0.8 ; scale = 15 ; amp = 0.028

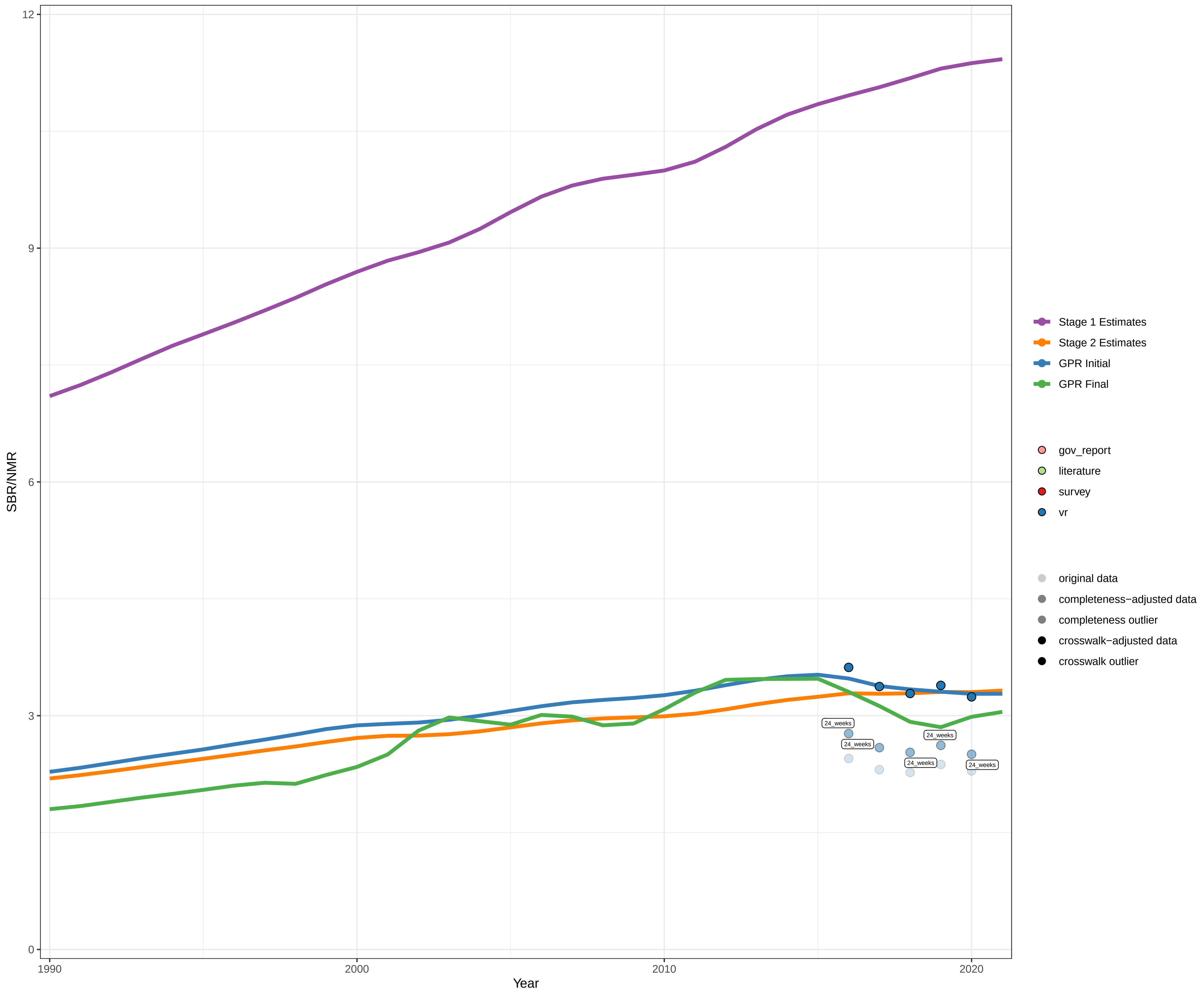

Ealing GBR_44744 Western Europe Stillbirth Rate / Neonatal Mortality Rate (SBR/NMR) − 28 weeks
lambda = 0.6 ; zeta = 0.8 ; scale = 15 ; amp = 0.028

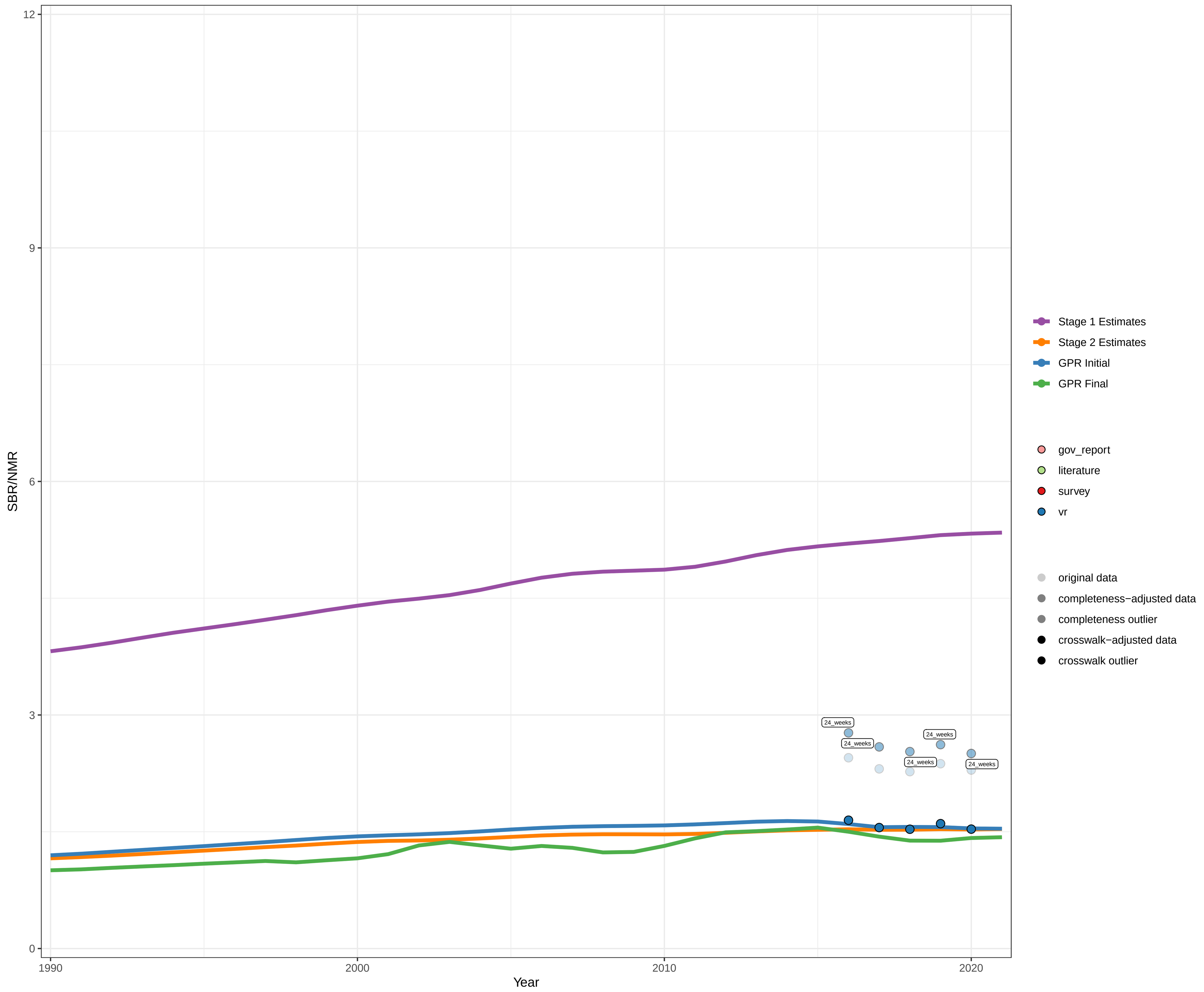

Definition	Years	Outlier	Source.Type	Title
24_weeks	2016−2020	0	VR	United Kingdom − England and Wales Child Mortality Statistics

Ealing GBR_44744 Western Europe Stillbirth Rate (SBR) − 20 weeks
lambda = 0.6 ; zeta = 0.8 ; scale = 15 ; amp = 0.028

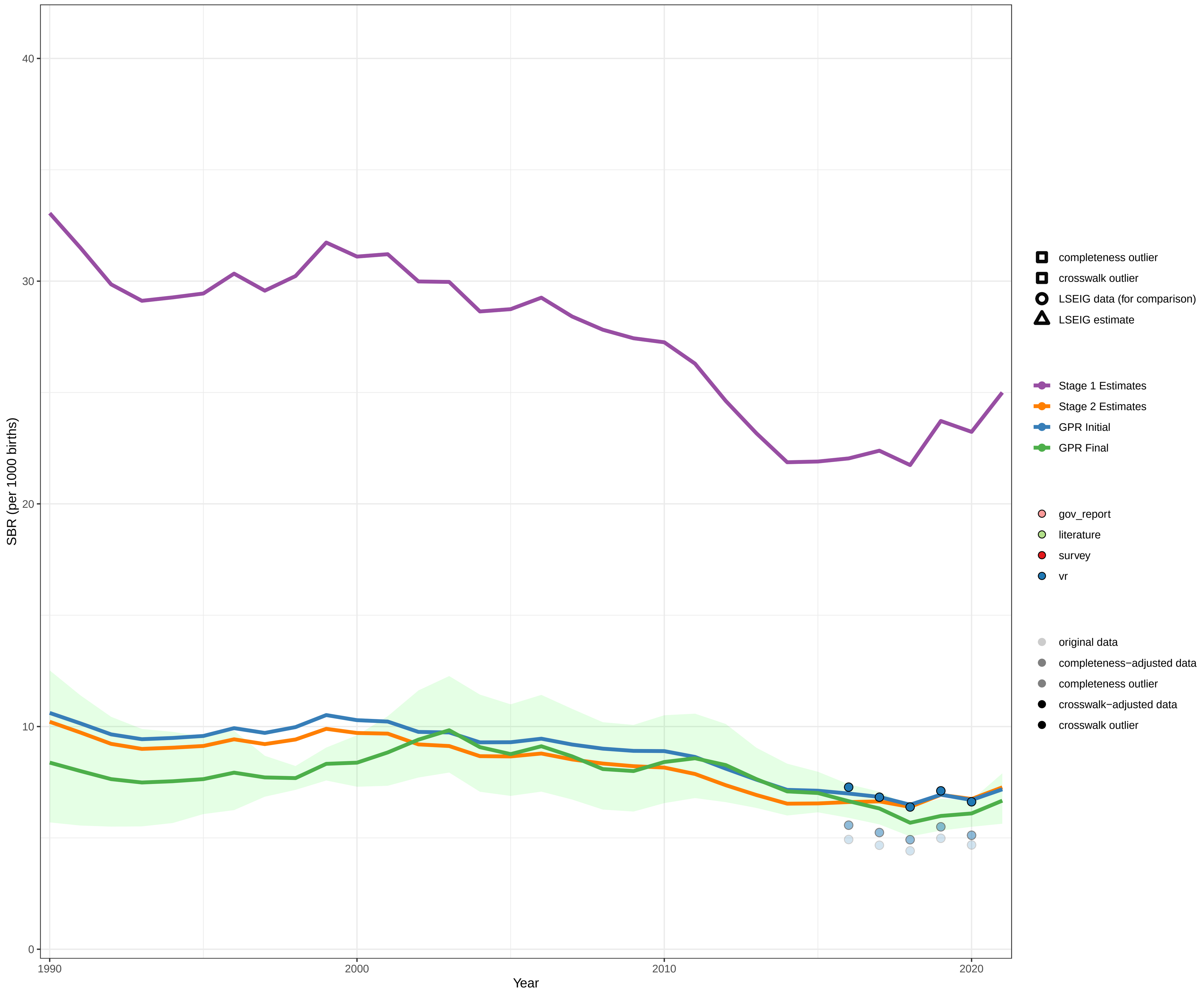

Ealing GBR_44744 Western Europe Stillbirth Rate (SBR) − 28 weeks
lambda = 0.6 ; zeta = 0.8 ; scale = 15 ; amp = 0.028

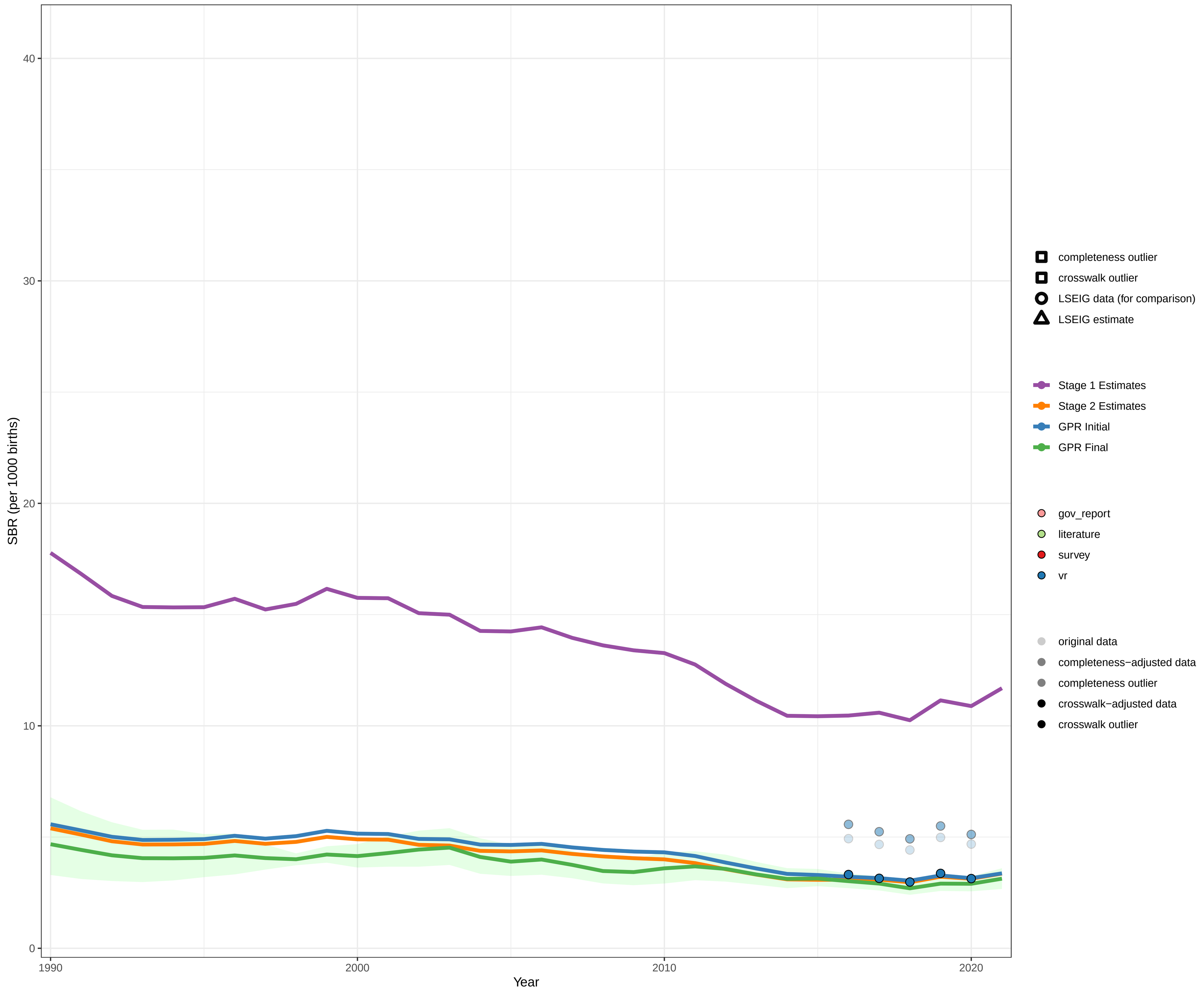

Year	Total Stillbirths (in thousands)	Total Stillbirths (in thousands)	SBR (per 1000 births)	SBR (per 1000 births)	SBR/NMR	SBR/NMR
	20 weeks	28 weeks	20 weeks	28 weeks	20 weeks	28 weeks
1990	0.0 (0.0 to 0.1)	0.0 (0.0 to 0.0)	8.4 (5.7 to 12.5)	4.7 (3.3 to 6.8)	1.80 (1.22 to 2.69)	1.01 (0.71 to 1.46)
1995	0.0 (0.0 to 0.0)	0.0 (0.0 to 0.0)	7.6 (6.1 to 9.5)	4.1 (3.2 to 5.1)	2.05 (1.63 to 2.56)	1.09 (0.86 to 1.38)
2000	0.0 (0.0 to 0.0)	0.0 (0.0 to 0.0)	8.4 (7.3 to 9.6)	4.1 (3.6 to 4.7)	2.34 (2.04 to 2.69)	1.16 (1.01 to 1.31)
2005	0.0 (0.0 to 0.1)	0.0 (0.0 to 0.0)	8.8 (6.9 to 11.0)	3.9 (3.3 to 4.7)	2.88 (2.27 to 3.62)	1.28 (1.07 to 1.53)
2010	0.0 (0.0 to 0.1)	0.0 (0.0 to 0.0)	8.4 (6.6 to 10.5)	3.6 (2.9 to 4.3)	3.08 (2.41 to 3.85)	1.32 (1.07 to 1.59)
2015	0.0 (0.0 to 0.0)	0.0 (0.0 to 0.0)	7.0 (6.1 to 8.0)	3.1 (2.8 to 3.6)	3.47 (3.05 to 3.95)	1.55 (1.38 to 1.77)
2020	0.0 (0.0 to 0.0)	0.0 (0.0 to 0.0)	6.1 (5.5 to 6.7)	2.9 (2.6 to 3.2)	2.99 (2.69 to 3.28)	1.42 (1.25 to 1.58)
2021	0.0 (0.0 to 0.0)	0.0 (0.0 to 0.0)	6.7 (5.6 to 7.9)	3.1 (2.7 to 3.6)	3.05 (2.58 to 3.61)	1.43 (1.22 to 1.64)

Enfield GBR_44741 Western Europe Stillbirth Rate / Neonatal Mortality Rate (SBR/NMR) − 20 weeks
lambda = 0.6 ; zeta = 0.8 ; scale = 15 ; amp = 0.028

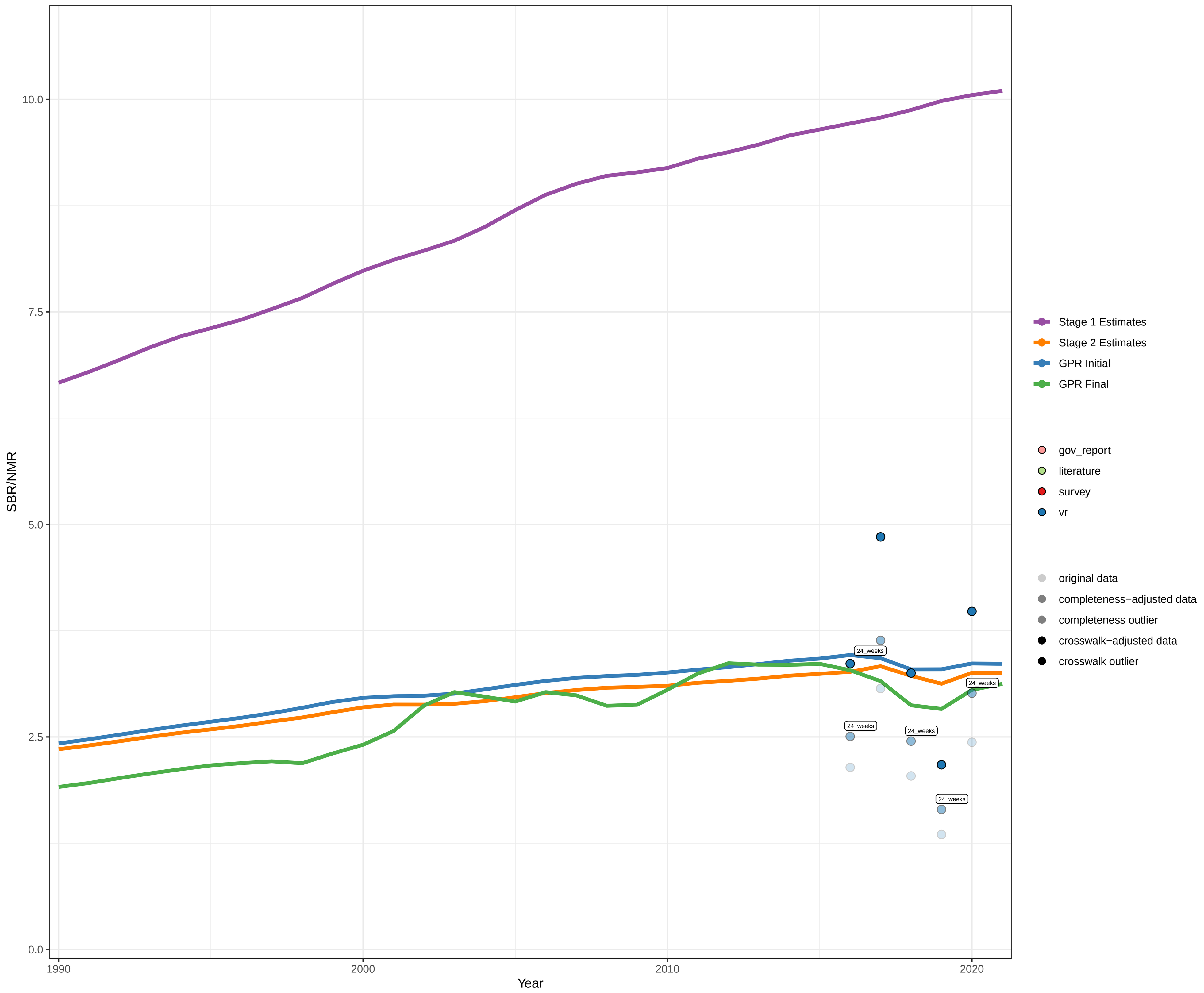

Enfield GBR_44741 Western Europe Stillbirth Rate / Neonatal Mortality Rate (SBR/NMR) − 28 weeks
lambda = 0.6 ; zeta = 0.8 ; scale = 15 ; amp = 0.028

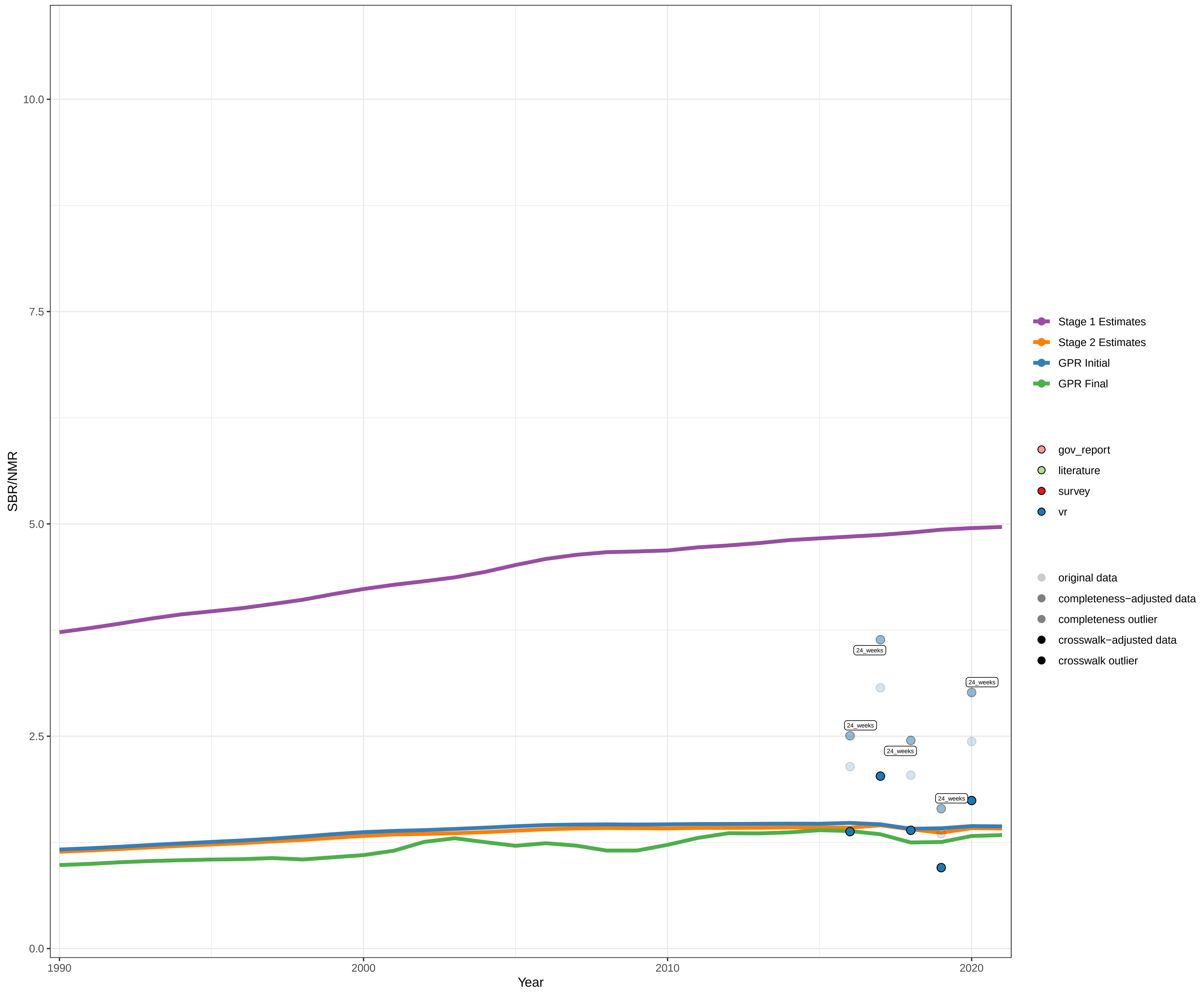

Definition	Years	Outlier	Source.Type	Title
24_weeks	2016−2020	0	VR	United Kingdom − England and Wales Child Mortality Statistics

Enfield GBR_44741 Western Europe Stillbirth Rate (SBR) − 20 weeks
lambda = 0.6 ; zeta = 0.8 ; scale = 15 ; amp = 0.028

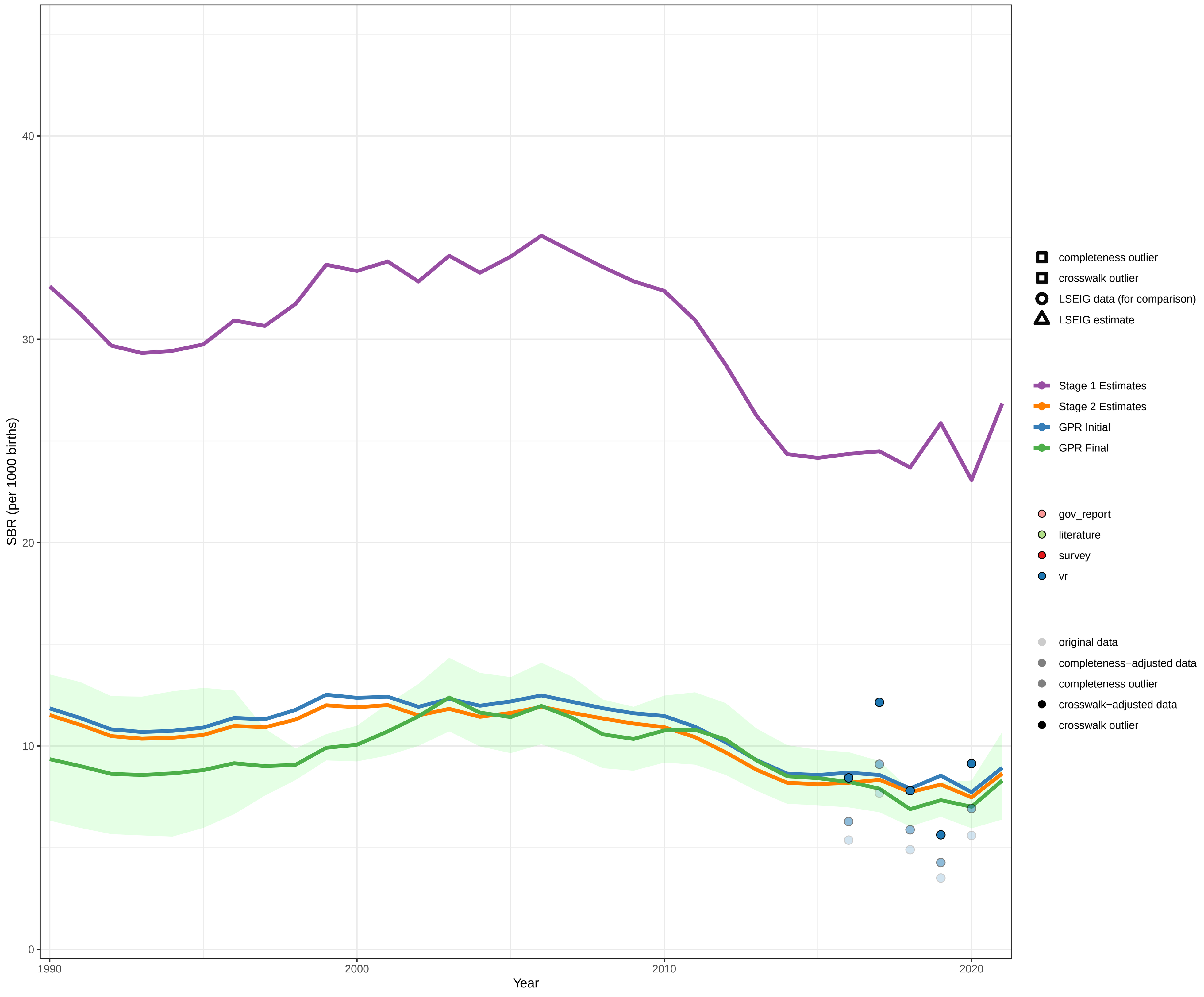

Enfield GBR_44741 Western Europe Stillbirth Rate (SBR) − 28 weeks
lambda = 0.6 ; zeta = 0.8 ; scale = 15 ; amp = 0.028

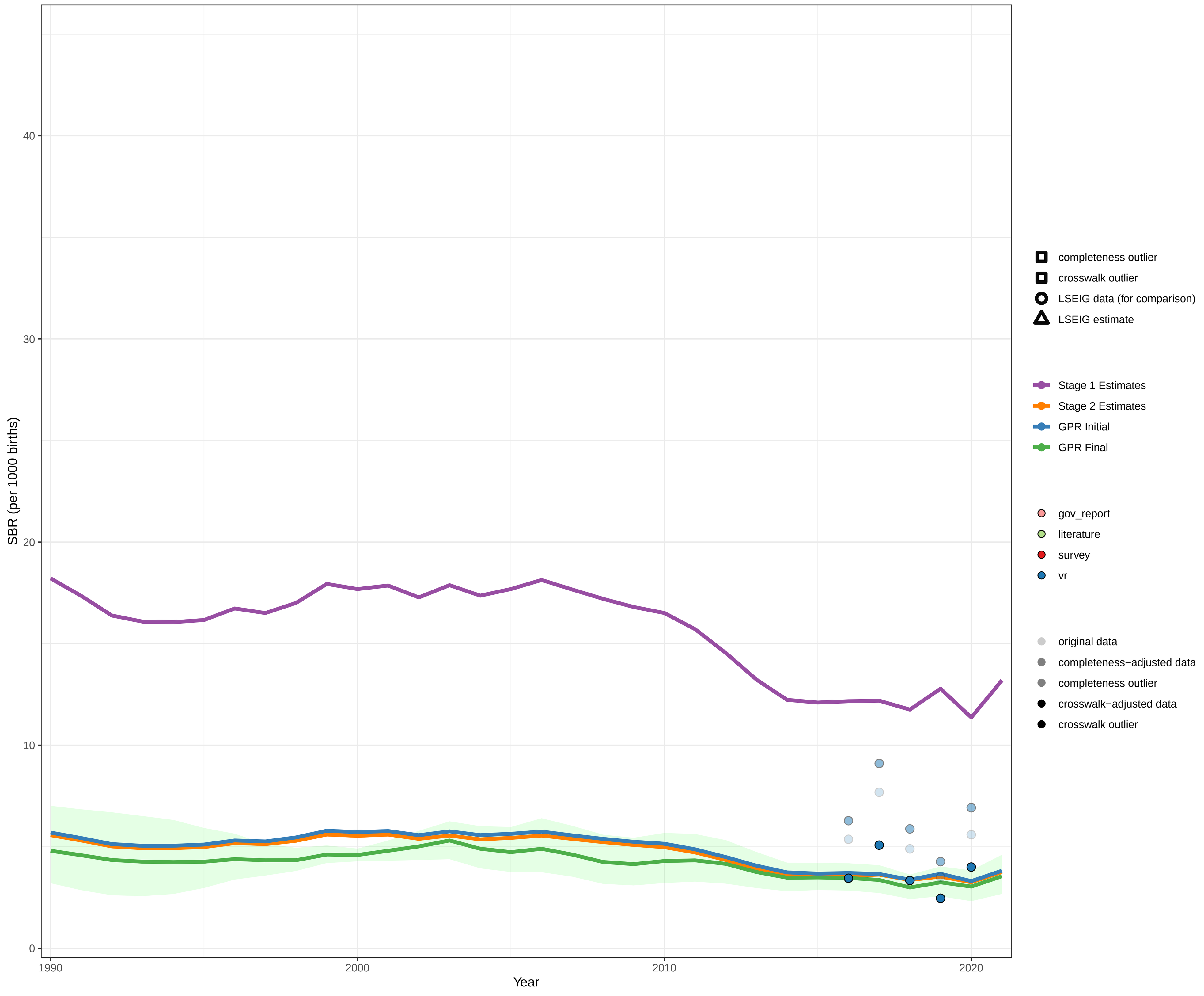

Year	Total Stillbirths (in thousands)	Total Stillbirths (in thousands)	SBR (per 1000 births)	SBR (per 1000 births)	SBR/NMR	SBR/NMR
	20 weeks	28 weeks	20 weeks	28 weeks	20 weeks	28 weeks
1990	0.0 (0.0 to 0.1)	0.0 (0.0 to 0.0)	9.3 (6.3 to 13.5)	4.8 (3.2 to 7.0)	1.91 (1.29 to 2.76)	0.98 (0.66 to 1.44)
1995	0.0 (0.0 to 0.1)	0.0 (0.0 to 0.0)	8.8 (6.0 to 12.9)	4.3 (3.0 to 5.9)	2.17 (1.47 to 3.16)	1.05 (0.73 to 1.46)
2000	0.0 (0.0 to 0.0)	0.0 (0.0 to 0.0)	10.1 (9.2 to 11.0)	4.6 (4.3 to 4.9)	2.41 (2.21 to 2.63)	1.10 (1.03 to 1.17)
2005	0.1 (0.0 to 0.1)	0.0 (0.0 to 0.0)	11.4 (9.6 to 13.4)	4.7 (3.8 to 6.0)	2.92 (2.46 to 3.42)	1.21 (0.96 to 1.53)
2010	0.1 (0.0 to 0.1)	0.0 (0.0 to 0.0)	10.8 (9.2 to 12.5)	4.3 (3.2 to 5.7)	3.06 (2.61 to 3.54)	1.22 (0.91 to 1.61)
2015	0.0 (0.0 to 0.0)	0.0 (0.0 to 0.0)	8.4 (7.1 to 9.8)	3.5 (2.9 to 4.2)	3.36 (2.83 to 3.92)	1.39 (1.15 to 1.68)
2020	0.0 (0.0 to 0.0)	0.0 (0.0 to 0.0)	7.0 (6.0 to 8.3)	3.0 (2.3 to 3.8)	3.05 (2.59 to 3.62)	1.33 (1.01 to 1.67)
2021	0.0 (0.0 to 0.0)	0.0 (0.0 to 0.0)	8.3 (6.4 to 10.7)	3.6 (2.7 to 4.6)	3.12 (2.40 to 4.02)	1.34 (1.01 to 1.73)

Greenwich GBR_44745 Western Europe Stillbirth Rate / Neonatal Mortality Rate (SBR/NMR) − 20 weeks
lambda = 0.6 ; zeta = 0.8 ; scale = 15 ; amp = 0.028

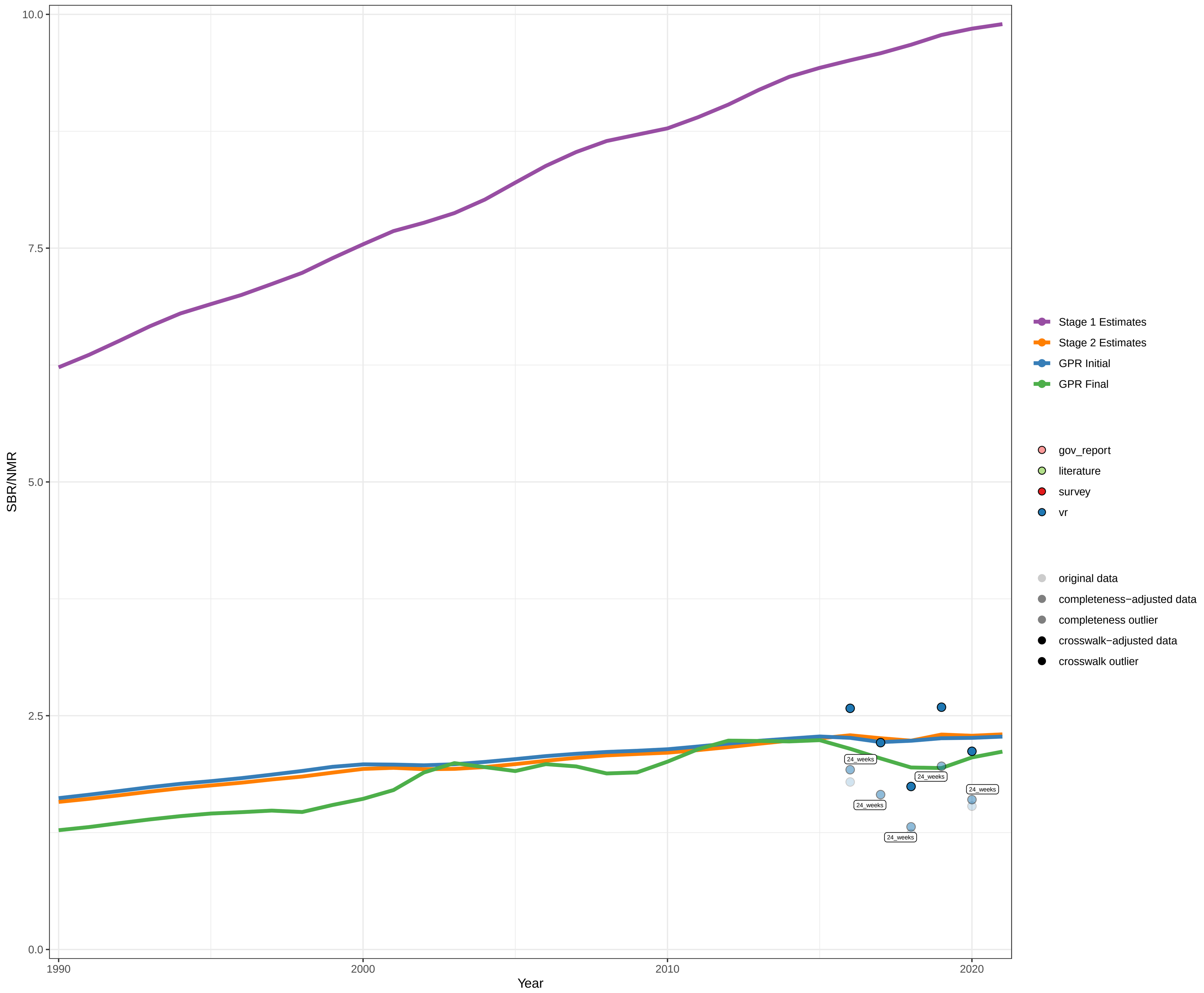

Greenwich GBR_44745 Western Europe Stillbirth Rate / Neonatal Mortality Rate (SBR/NMR) − 28 weeks
lambda = 0.6 ; zeta = 0.8 ; scale = 15 ; amp = 0.028

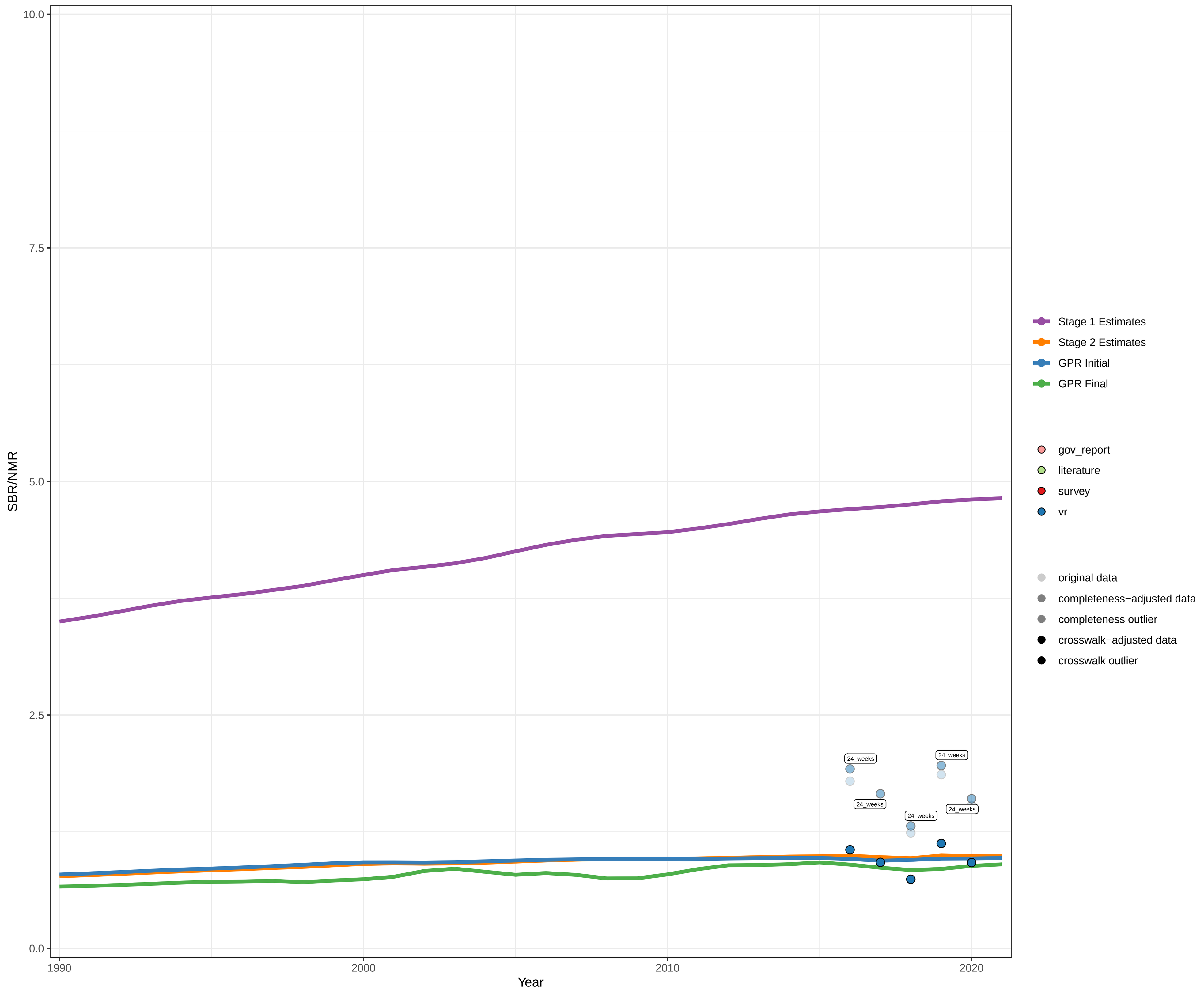

Definition	Years	Outlier	Source.Type	Title
24_weeks	2016−2020	0	VR	United Kingdom − England and Wales Child Mortality Statistics

Greenwich GBR_44745 Western Europe Stillbirth Rate (SBR) − 20 weeks
lambda = 0.6 ; zeta = 0.8 ; scale = 15 ; amp = 0.028

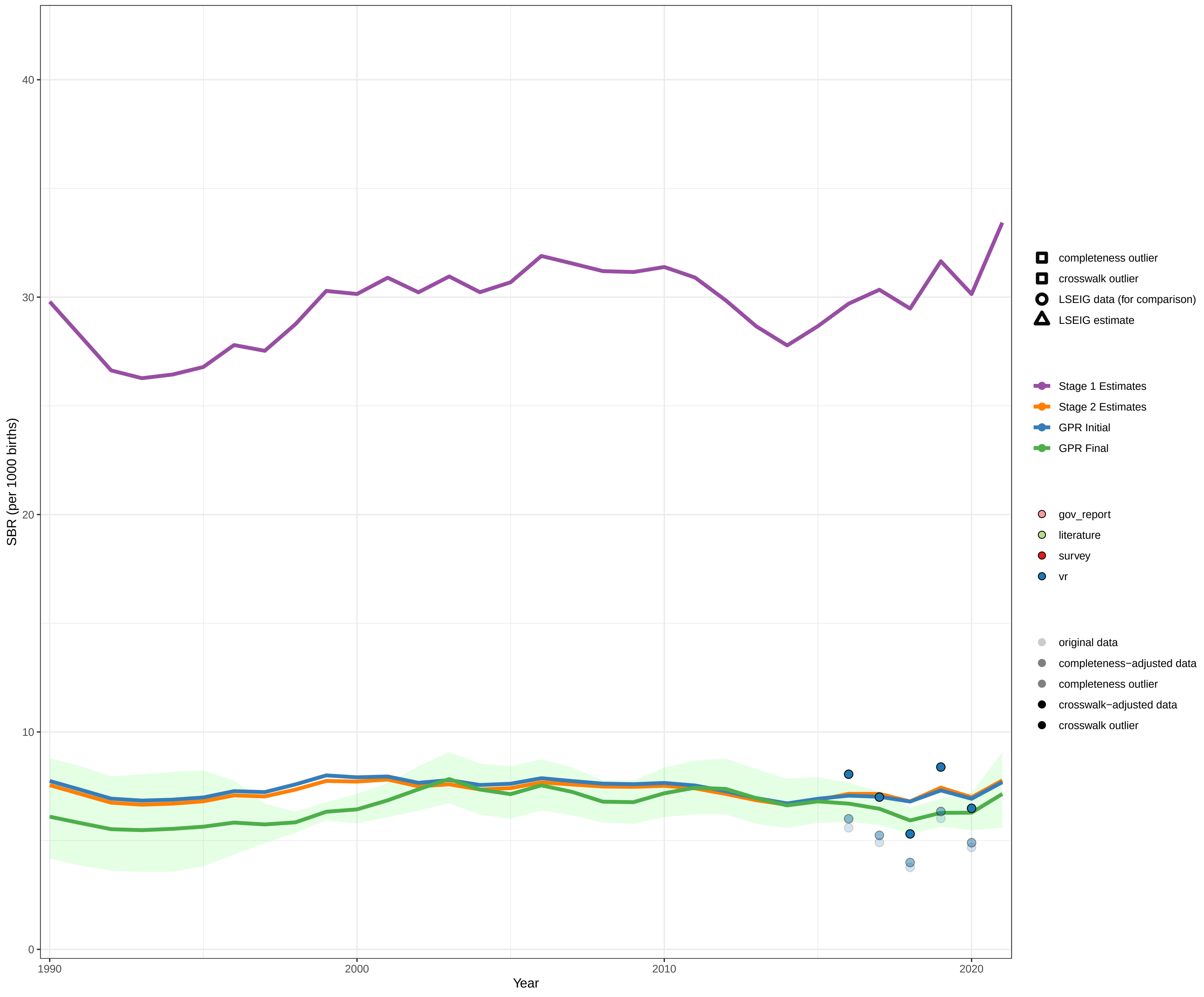

Greenwich GBR_44745 Western Europe Stillbirth Rate (SBR) − 28 weeks
lambda = 0.6 ; zeta = 0.8 ; scale = 15 ; amp = 0.028

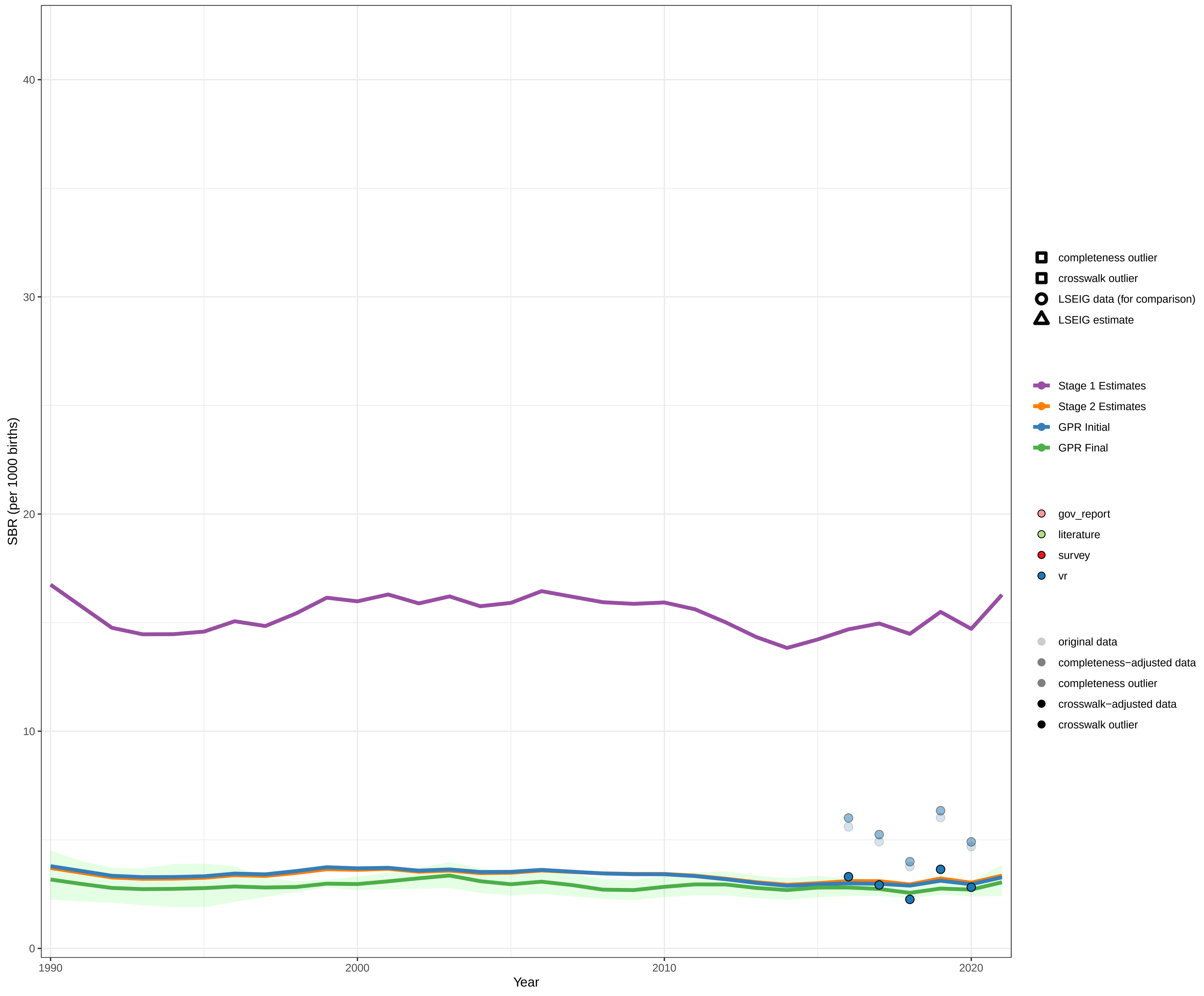

Year	Total Stillbirths (in thousands)	Total Stillbirths (in thousands)	SBR (per 1000 births)	SBR (per 1000 births)	SBR/NMR	SBR/NMR
	20 weeks	28 weeks	20 weeks	28 weeks	20 weeks	28 weeks
1990	0.0 (0.0 to 0.0)	0.0 (0.0 to 0.0)	6.1 (4.2 to 8.8)	3.2 (2.2 to 4.5)	1.28 (0.87 to 1.84)	0.66 (0.47 to 0.94)
1995	0.0 (0.0 to 0.0)	0.0 (0.0 to 0.0)	5.6 (3.8 to 8.2)	2.8 (1.9 to 3.9)	1.45 (0.98 to 2.12)	0.72 (0.49 to 1.01)
2000	0.0 (0.0 to 0.0)	0.0 (0.0 to 0.0)	6.4 (5.8 to 7.2)	3.0 (2.7 to 3.3)	1.61 (1.45 to 1.79)	0.74 (0.67 to 0.83)
2005	0.0 (0.0 to 0.0)	0.0 (0.0 to 0.0)	7.1 (6.0 to 8.4)	3.0 (2.4 to 3.5)	1.91 (1.61 to 2.25)	0.79 (0.65 to 0.93)
2010	0.0 (0.0 to 0.0)	0.0 (0.0 to 0.0)	7.2 (6.1 to 8.4)	2.8 (2.4 to 3.4)	2.01 (1.70 to 2.34)	0.79 (0.66 to 0.94)
2015	0.0 (0.0 to 0.0)	0.0 (0.0 to 0.0)	6.8 (5.8 to 7.9)	2.8 (2.3 to 3.3)	2.24 (1.91 to 2.60)	0.92 (0.77 to 1.10)
2020	0.0 (0.0 to 0.0)	0.0 (0.0 to 0.0)	6.3 (5.5 to 7.2)	2.7 (2.4 to 3.1)	2.05 (1.79 to 2.35)	0.88 (0.78 to 1.01)
2021	0.0 (0.0 to 0.0)	0.0 (0.0 to 0.0)	7.1 (5.6 to 9.1)	3.0 (2.4 to 3.9)	2.12 (1.66 to 2.69)	0.90 (0.71 to 1.14)

Hackney GBR_44756 Western Europe Stillbirth Rate / Neonatal Mortality Rate (SBR/NMR) − 20 weeks
lambda = 0.6 ; zeta = 0.8 ; scale = 15 ; amp = 0.028

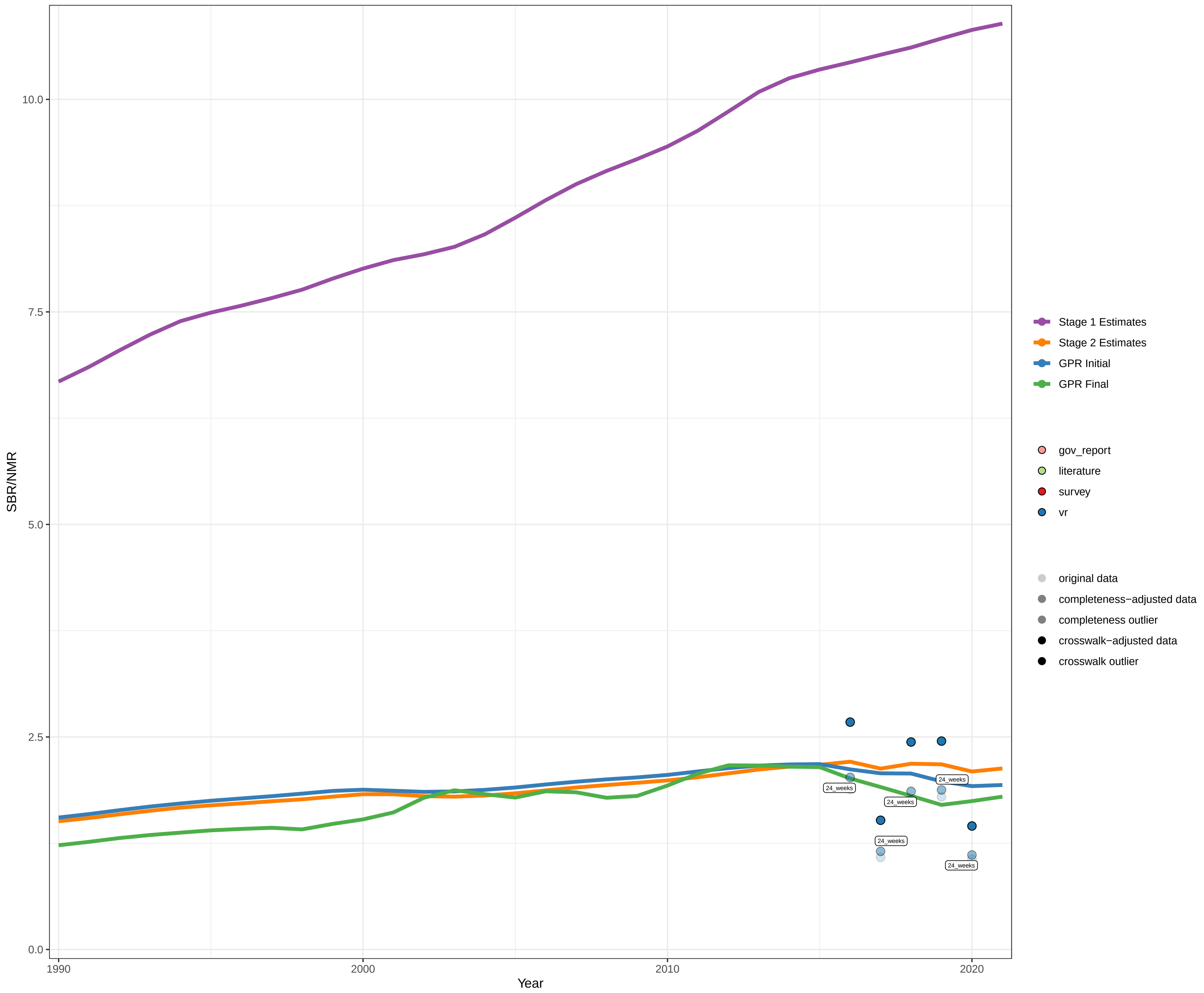

Hackney GBR_44756 Western Europe Stillbirth Rate / Neonatal Mortality Rate (SBR/NMR) − 28 weeks
lambda = 0.6 ; zeta = 0.8 ; scale = 15 ; amp = 0.028

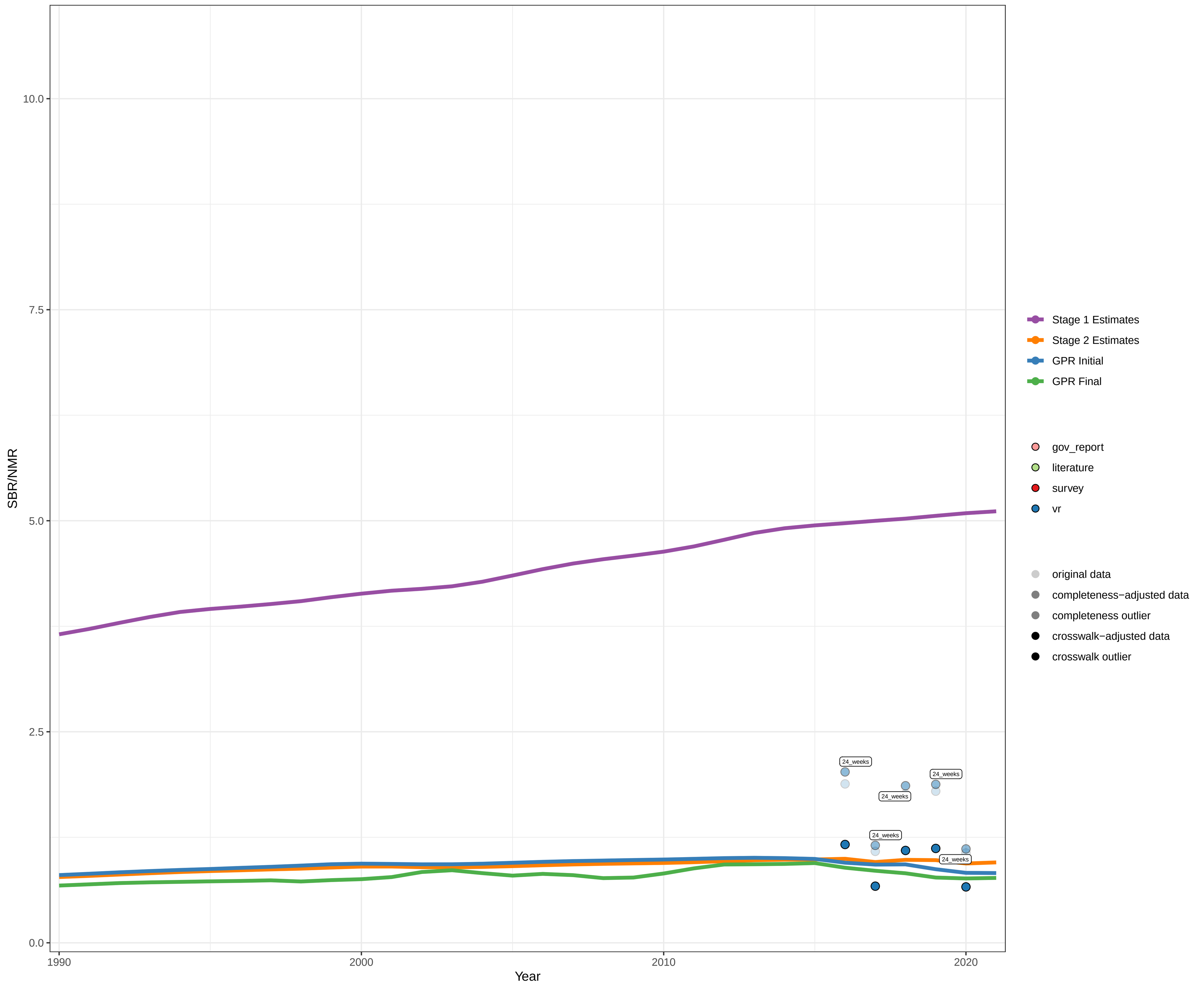

Definition	Years	Outlier	Source.Type	Title
24_weeks	2016−2020	0	VR	United Kingdom − England and Wales Child Mortality Statistics

Hackney GBR_44756 Western Europe Stillbirth Rate (SBR) − 20 weeks
lambda = 0.6 ; zeta = 0.8 ; scale = 15 ; amp = 0.028

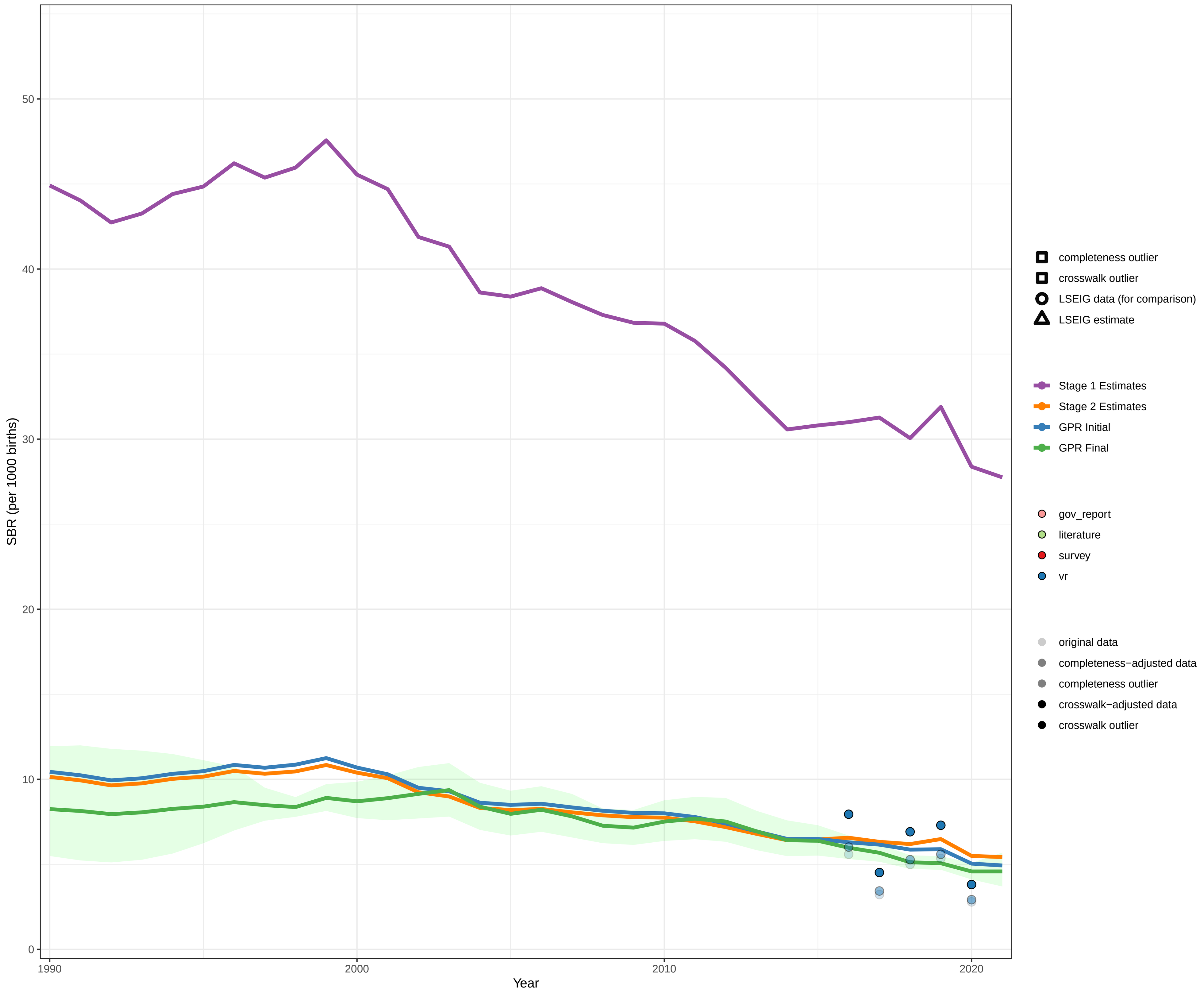

Hackney GBR_44756 Western Europe Stillbirth Rate (SBR) − 28 weeks
lambda = 0.6 ; zeta = 0.8 ; scale = 15 ; amp = 0.028

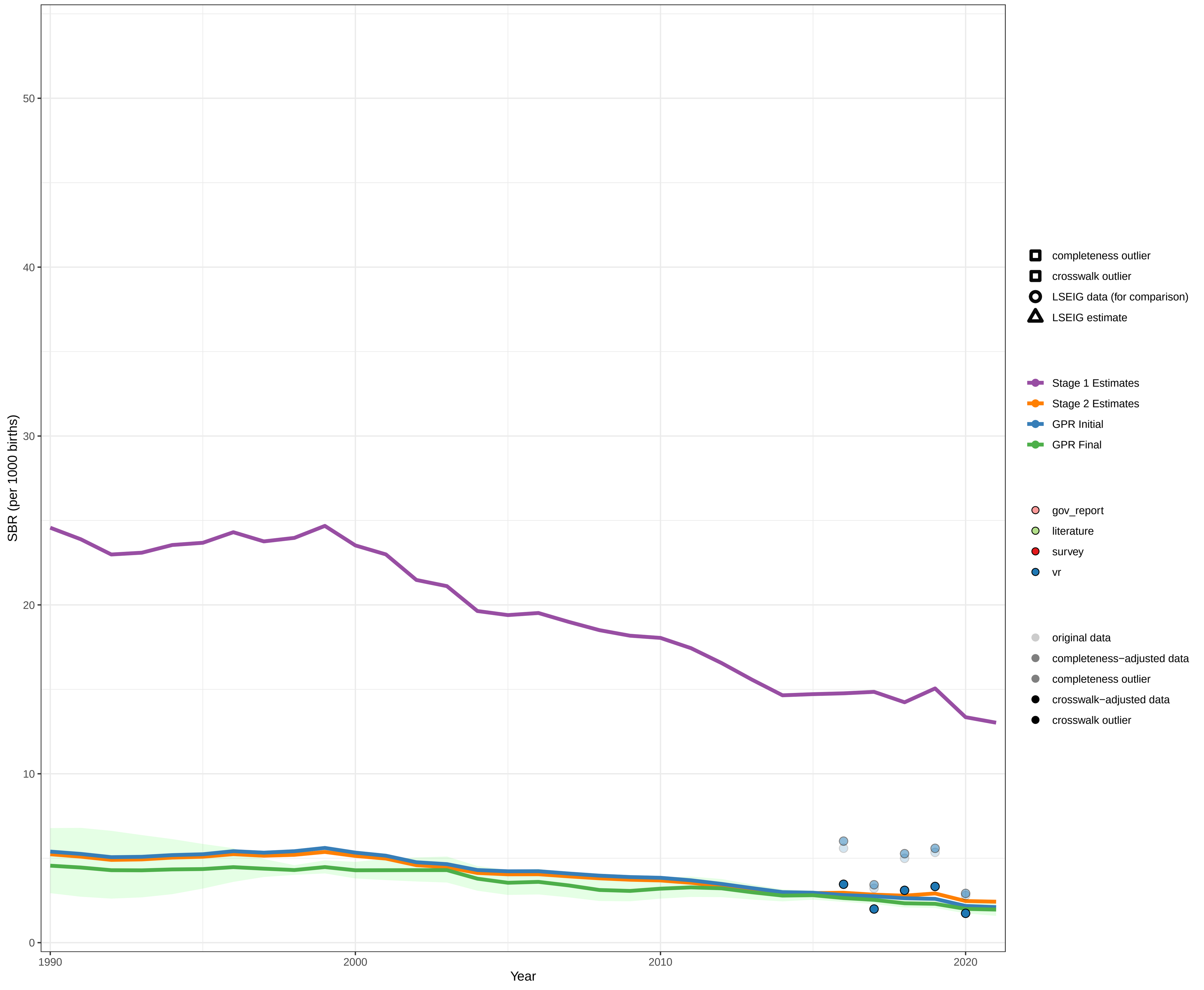

Year	Total Stillbirths (in thousands)	Total Stillbirths (in thousands)	SBR (per 1000 births)	SBR (per 1000 births)	SBR/NMR	SBR/NMR
	20 weeks	28 weeks	20 weeks	28 weeks	20 weeks	28 weeks
1990	0.0 (0.0 to 0.0)	0.0 (0.0 to 0.0)	8.2 (5.5 to 11.9)	4.6 (2.9 to 6.8)	1.23 (0.82 to 1.78)	0.68 (0.44 to 1.01)
1995	0.0 (0.0 to 0.0)	0.0 (0.0 to 0.0)	8.4 (6.2 to 11.1)	4.4 (3.2 to 5.8)	1.40 (1.04 to 1.86)	0.73 (0.53 to 0.98)
2000	0.0 (0.0 to 0.0)	0.0 (0.0 to 0.0)	8.7 (7.7 to 9.9)	4.3 (3.8 to 4.8)	1.53 (1.36 to 1.73)	0.75 (0.67 to 0.84)
2005	0.0 (0.0 to 0.0)	0.0 (0.0 to 0.0)	8.0 (6.7 to 9.3)	3.5 (2.8 to 4.3)	1.79 (1.50 to 2.09)	0.80 (0.63 to 0.96)
2010	0.0 (0.0 to 0.0)	0.0 (0.0 to 0.0)	7.5 (6.4 to 8.8)	3.2 (2.6 to 3.9)	1.93 (1.64 to 2.25)	0.82 (0.67 to 0.99)
2015	0.0 (0.0 to 0.0)	0.0 (0.0 to 0.0)	6.4 (5.5 to 7.3)	2.8 (2.5 to 3.1)	2.15 (1.85 to 2.45)	0.94 (0.86 to 1.04)
2020	0.0 (0.0 to 0.0)	0.0 (0.0 to 0.0)	4.6 (4.1 to 5.1)	2.0 (1.7 to 2.3)	1.75 (1.57 to 1.94)	0.76 (0.66 to 0.88)
2021	0.0 (0.0 to 0.0)	0.0 (0.0 to 0.0)	4.6 (3.7 to 5.7)	2.0 (1.6 to 2.4)	1.80 (1.45 to 2.23)	0.77 (0.62 to 0.94)

Hammersmith and Fulham GBR_44743 Western Europe Stillbirth Rate / Neonatal Mortality Rate (SBR/NMR) − 20 weeks
lambda = 0.6 ; zeta = 0.8 ; scale = 15 ; amp = 0.028

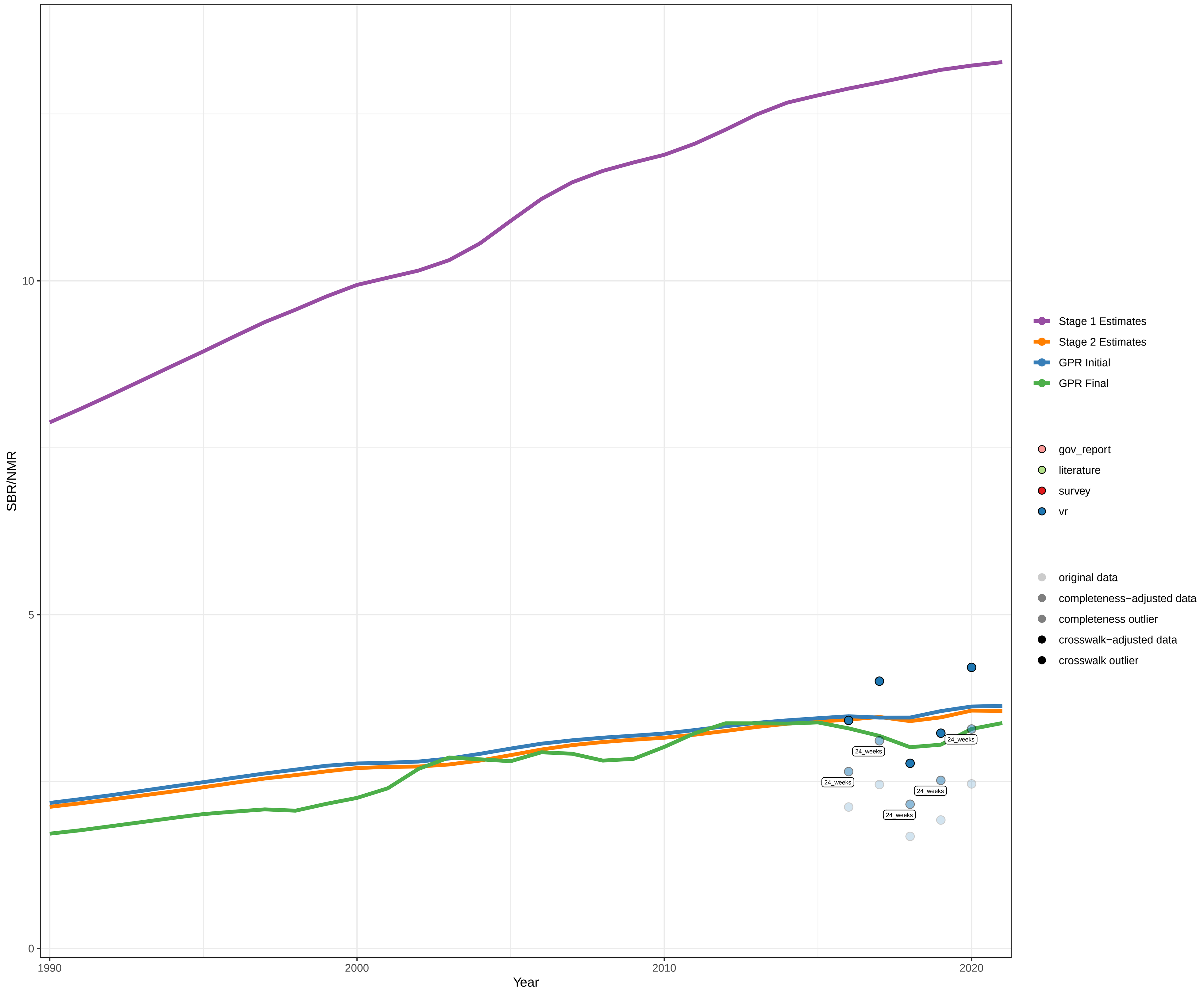

Hammersmith and Fulham GBR_44743 Western Europe Stillbirth Rate / Neonatal Mortality Rate (SBR/NMR) − 28 weeks
lambda = 0.6 ; zeta = 0.8 ; scale = 15 ; amp = 0.028

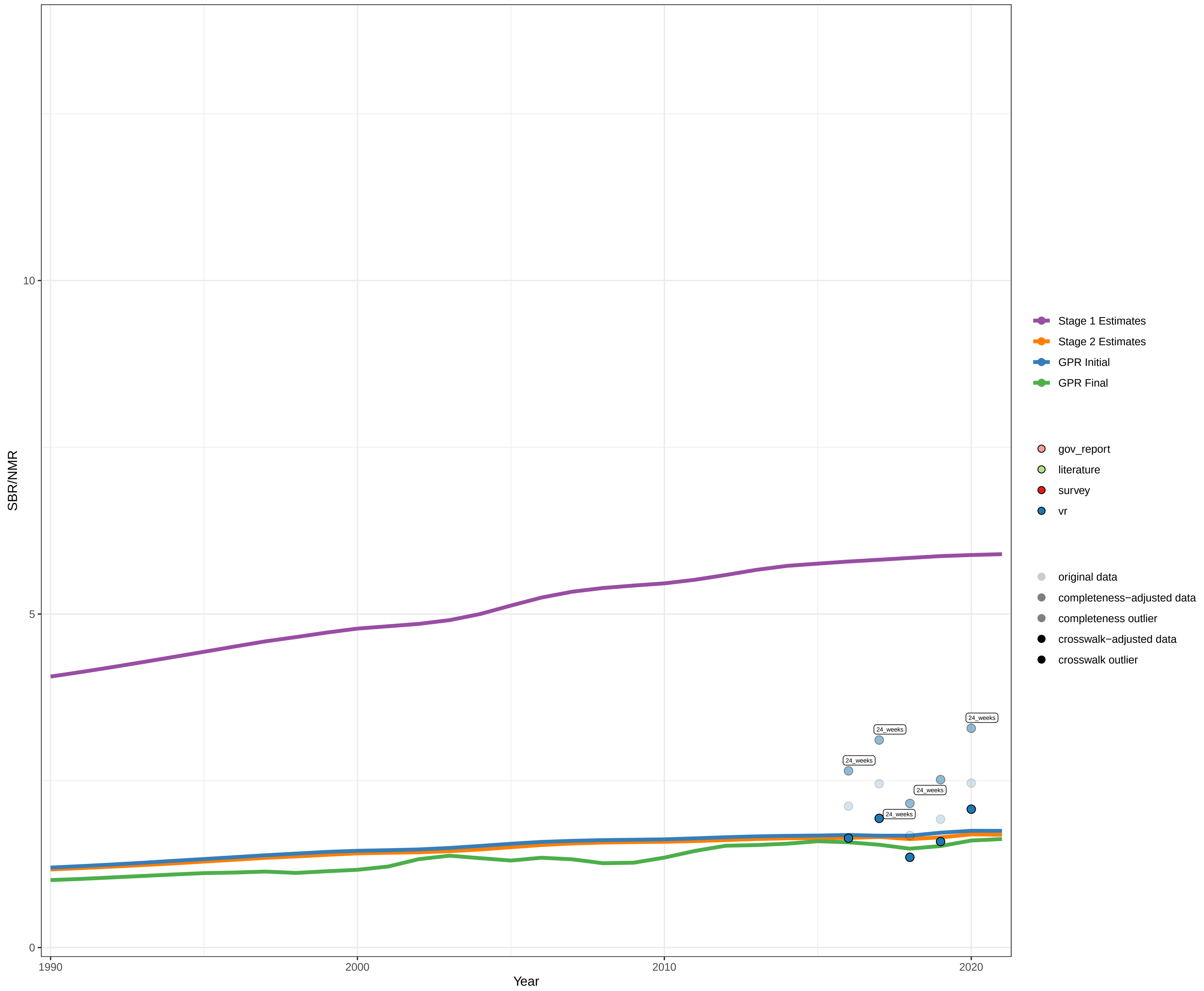

Definition	Years	Outlier	Source.Type	Title
24_weeks	2016−2020	0	VR	United Kingdom − England and Wales Child Mortality Statistics

Hammersmith and Fulham GBR_44743 Western Europe Stillbirth Rate (SBR) − 20 weeks
lambda = 0.6 ; zeta = 0.8 ; scale = 15 ; amp = 0.028

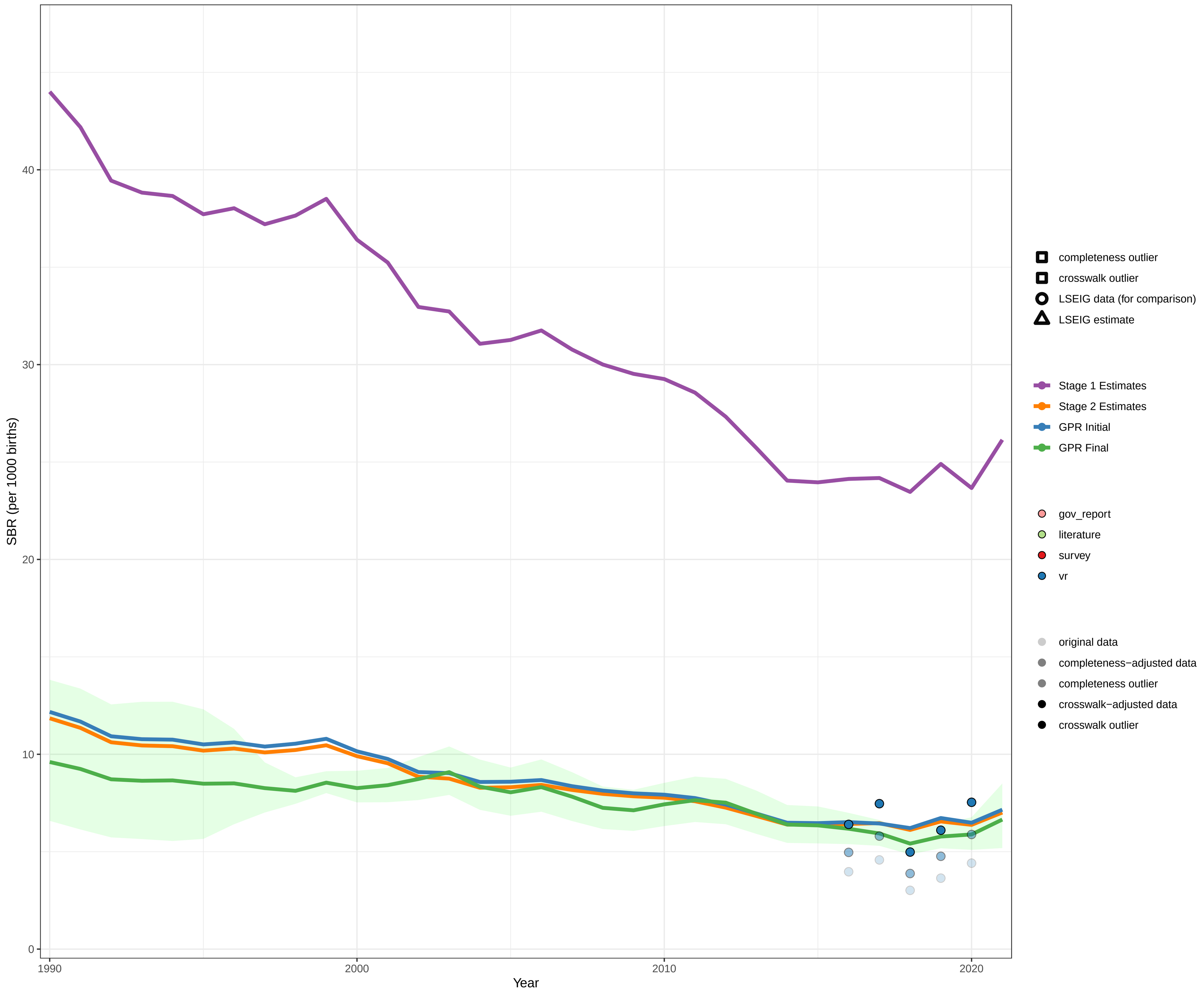

Hammersmith and Fulham GBR_44743 Western Europe Stillbirth Rate (SBR) − 28 weeks
lambda = 0.6 ; zeta = 0.8 ; scale = 15 ; amp = 0.028

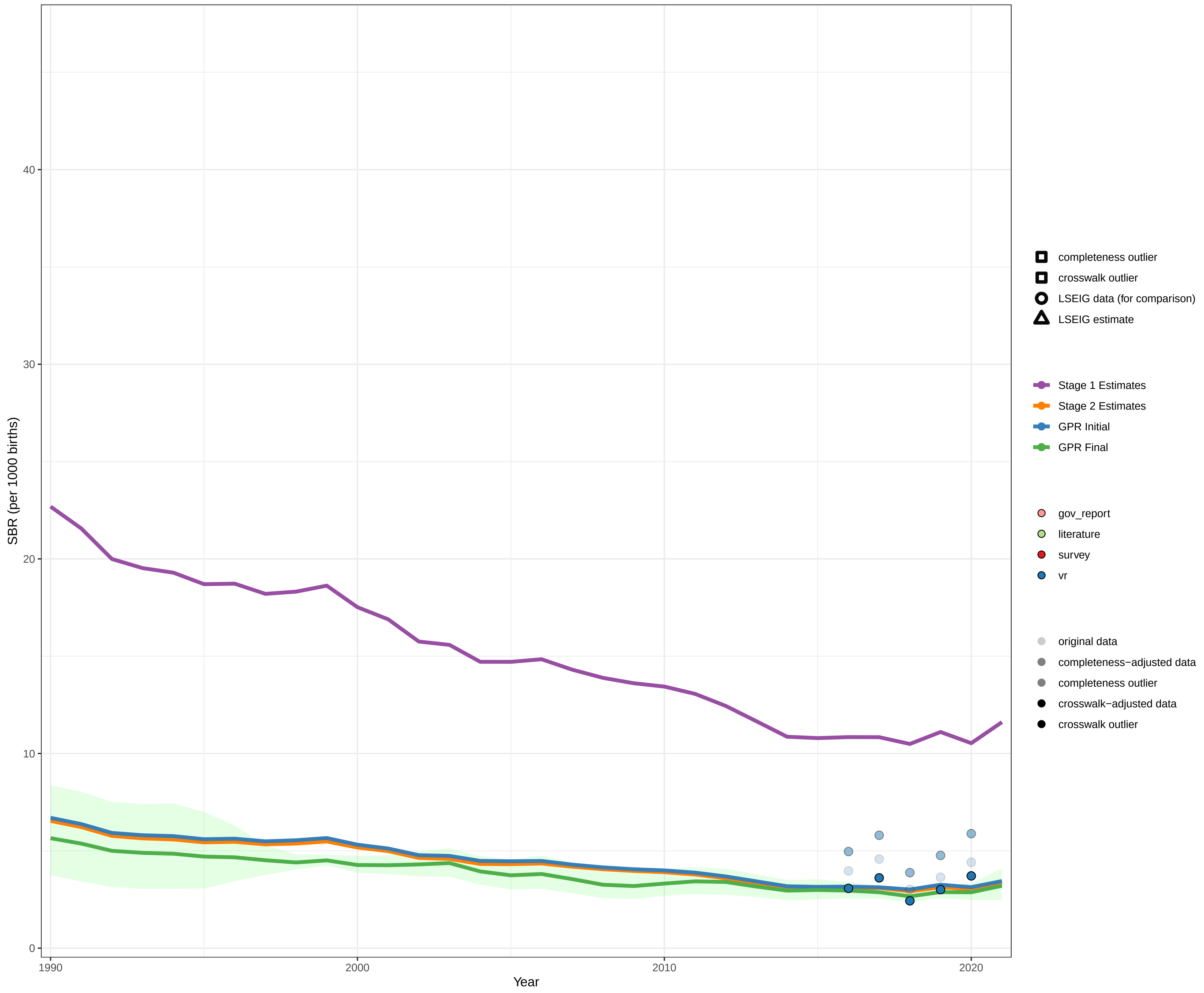

Year	Total Stillbirths (in thousands)	Total Stillbirths (in thousands)	SBR (per 1000 births)	SBR (per 1000 births)	SBR/NMR	SBR/NMR
	20 weeks	28 weeks	20 weeks	28 weeks	20 weeks	28 weeks
1990	0.0 (0.0 to 0.0)	0.0 (0.0 to 0.0)	9.6 (6.6 to 13.8)	5.6 (3.7 to 8.4)	1.72 (1.18 to 2.48)	1.01 (0.67 to 1.50)
1995	0.0 (0.0 to 0.0)	0.0 (0.0 to 0.0)	8.5 (5.7 to 12.3)	4.7 (3.1 to 7.0)	2.01 (1.34 to 2.92)	1.12 (0.73 to 1.66)
2000	0.0 (0.0 to 0.0)	0.0 (0.0 to 0.0)	8.3 (7.5 to 9.2)	4.3 (3.9 to 4.7)	2.26 (2.06 to 2.50)	1.17 (1.06 to 1.30)
2005	0.0 (0.0 to 0.0)	0.0 (0.0 to 0.0)	8.0 (6.8 to 9.3)	3.7 (3.0 to 4.5)	2.81 (2.39 to 3.25)	1.30 (1.05 to 1.58)
2010	0.0 (0.0 to 0.0)	0.0 (0.0 to 0.0)	7.4 (6.3 to 8.5)	3.3 (2.7 to 4.1)	3.02 (2.57 to 3.47)	1.35 (1.09 to 1.65)
2015	0.0 (0.0 to 0.0)	0.0 (0.0 to 0.0)	6.3 (5.4 to 7.3)	3.0 (2.5 to 3.5)	3.39 (2.89 to 3.90)	1.59 (1.33 to 1.89)
2020	0.0 (0.0 to 0.0)	0.0 (0.0 to 0.0)	5.9 (5.1 to 6.8)	2.9 (2.5 to 3.3)	3.29 (2.85 to 3.78)	1.60 (1.38 to 1.86)
2021	0.0 (0.0 to 0.0)	0.0 (0.0 to 0.0)	6.6 (5.2 to 8.5)	3.2 (2.5 to 4.1)	3.38 (2.63 to 4.32)	1.63 (1.26 to 2.08)

Haringey   GBR_44755   Western Europe   Stillbirth Rate / Neonatal Mortality Rate (SBR/NMR) − 20 weeks
lambda = 0.6 ; zeta = 0.8 ; scale = 15 ; amp = 0.028

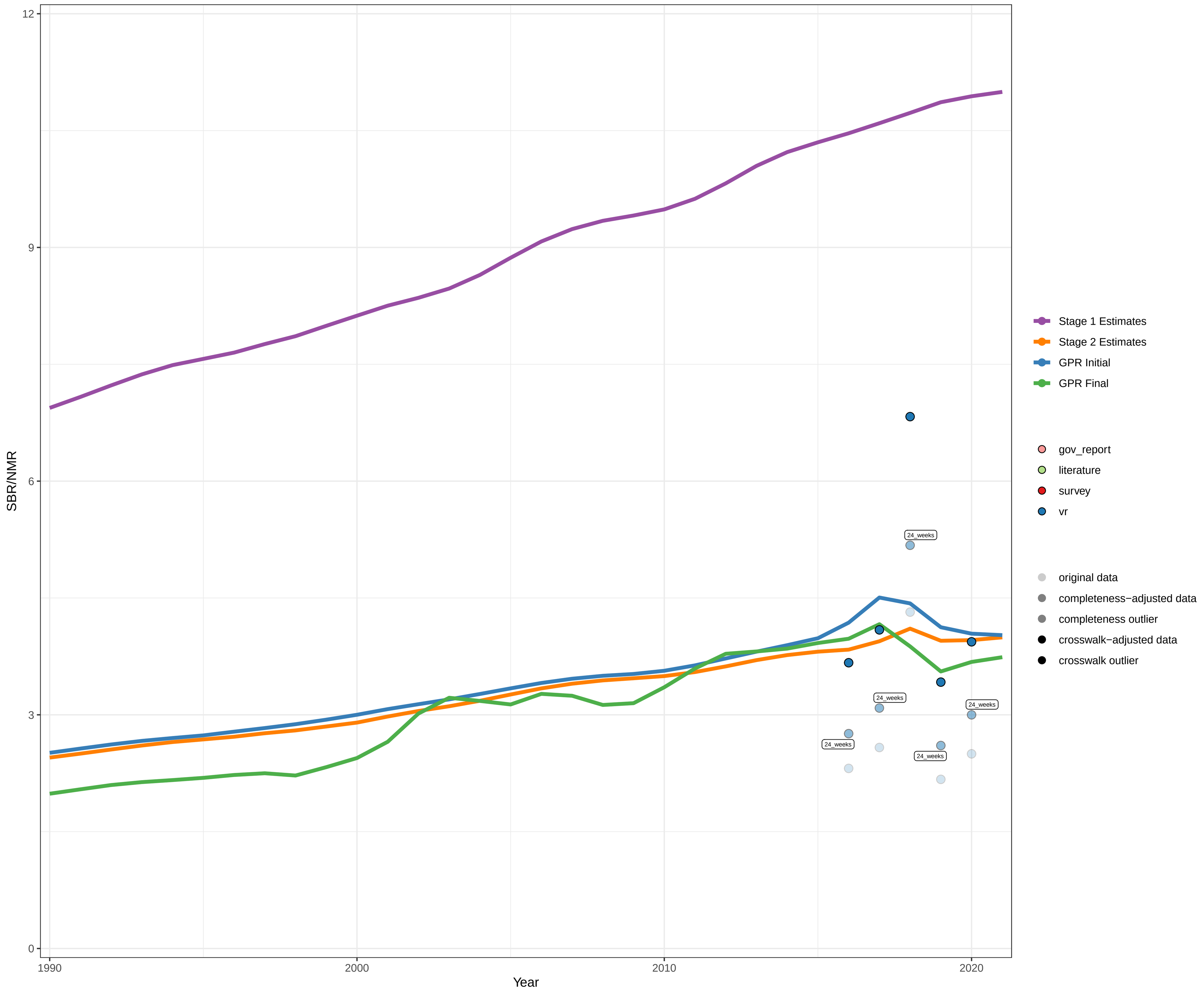

Haringey   GBR_44755   Western Europe   Stillbirth Rate / Neonatal Mortality Rate (SBR/NMR) − 28 weeks
lambda = 0.6 ; zeta = 0.8 ; scale = 15 ; amp = 0.028

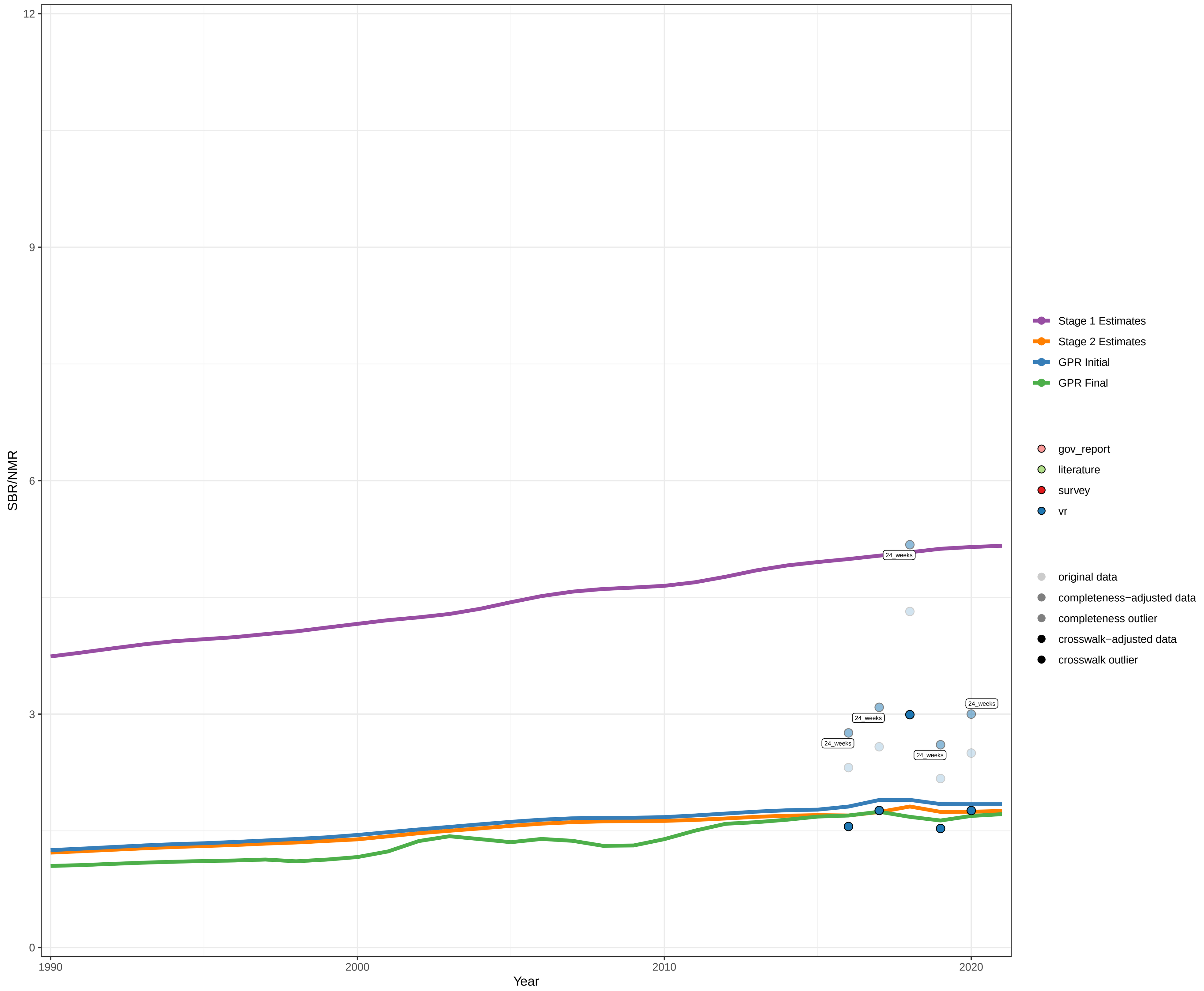

Definition	Years	Outlier	Source.Type	Title
24_weeks	2016−2020	0	VR	United Kingdom − England and Wales Child Mortality Statistics

Haringey   GBR_44755   Western Europe   Stillbirth Rate (SBR) − 20 weeks
lambda = 0.6 ; zeta = 0.8 ; scale = 15 ; amp = 0.028

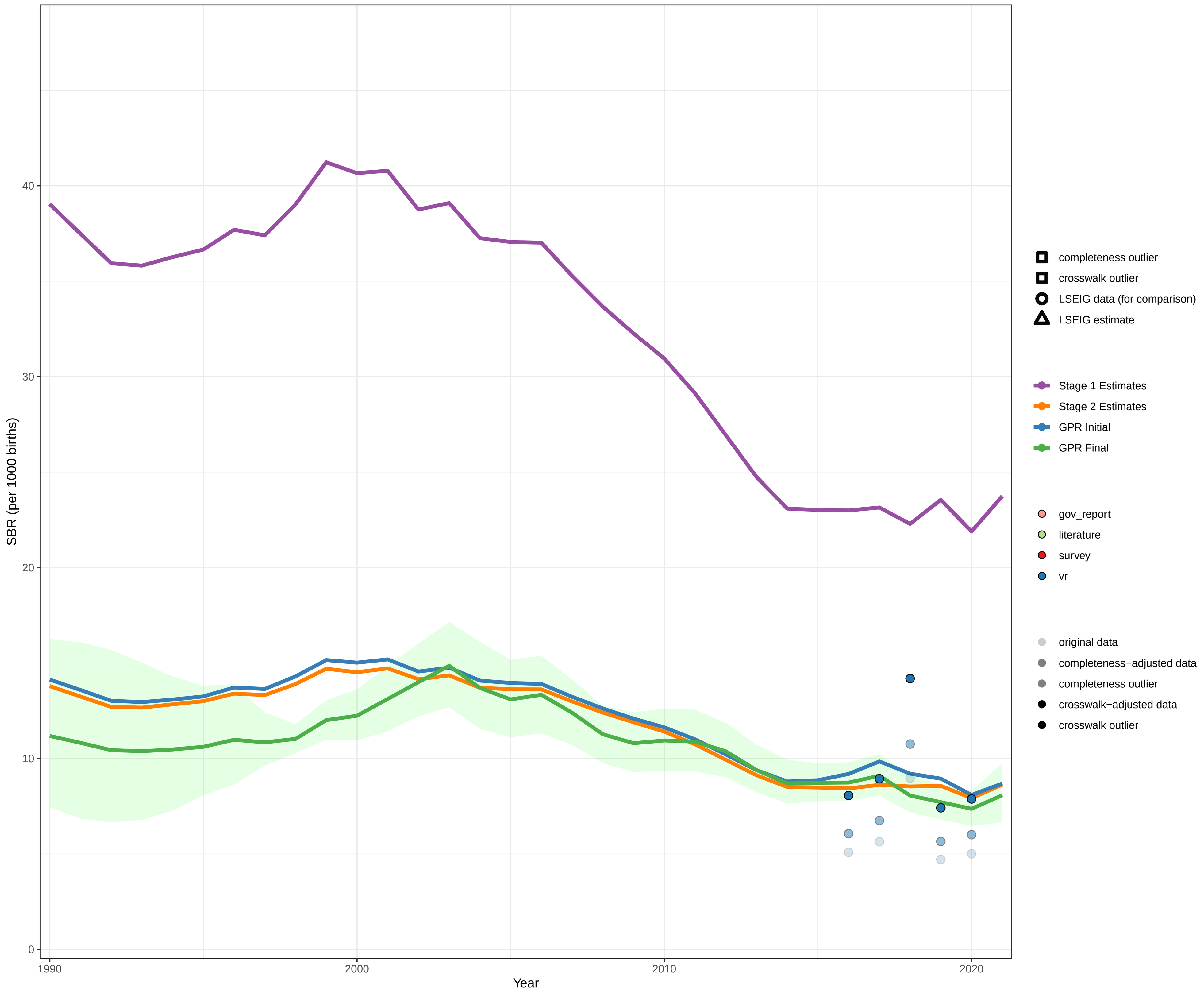

Haringey   GBR_44755   Western Europe   Stillbirth Rate (SBR) − 28 weeks
lambda = 0.6 ; zeta = 0.8 ; scale = 15 ; amp = 0.028

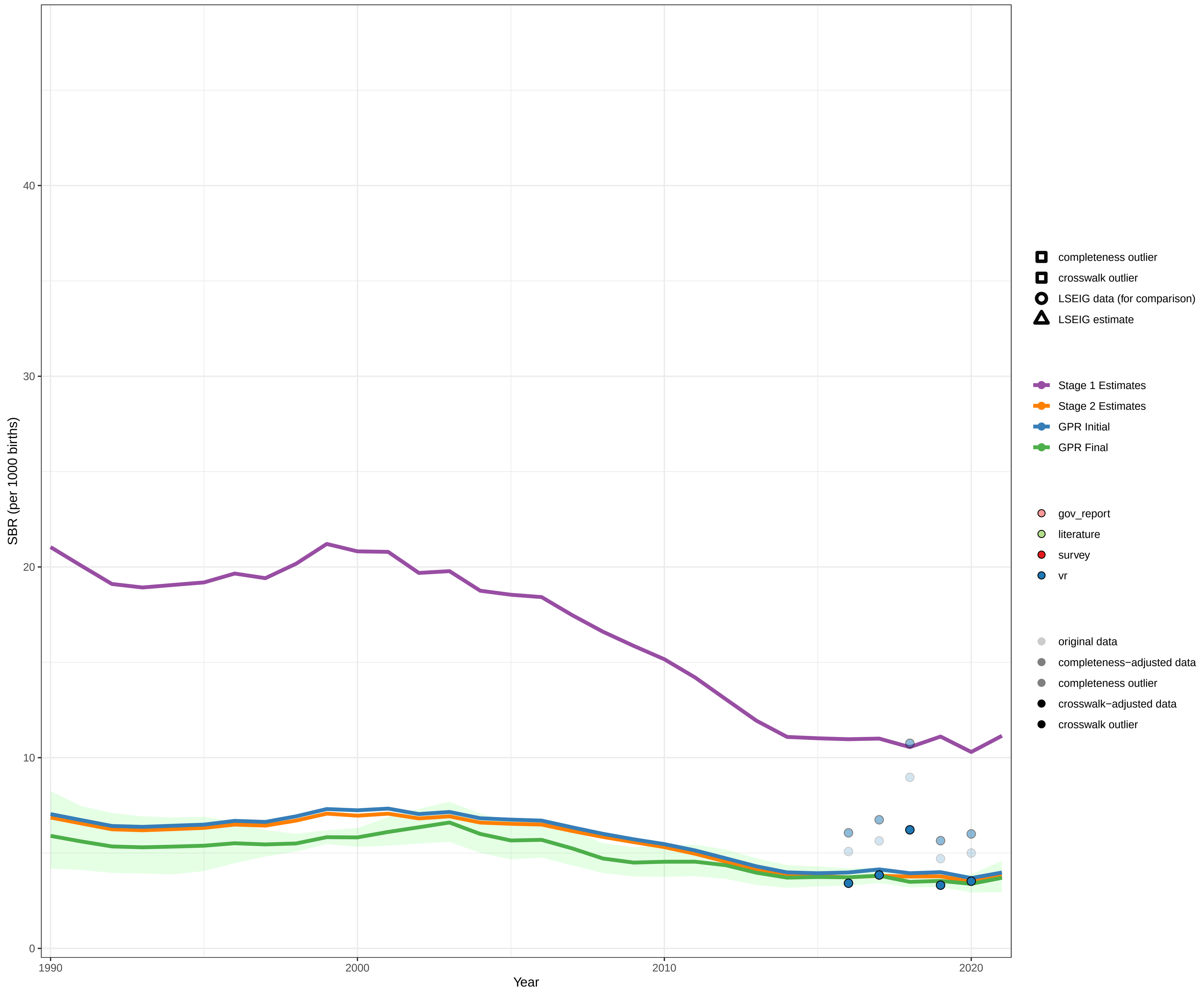

Year	Total Stillbirths (in thousands)	Total Stillbirths (in thousands)	SBR (per 1000 births)	SBR (per 1000 births)	SBR/NMR	SBR/NMR
	20 weeks	28 weeks	20 weeks	28 weeks	20 weeks	28 weeks
1990	0.0 (0.0 to 0.1)	0.0 (0.0 to 0.0)	11.2 (7.5 to 16.3)	5.9 (4.2 to 8.3)	1.99 (1.32 to 2.89)	1.05 (0.74 to 1.47)
1995	0.0 (0.0 to 0.0)	0.0 (0.0 to 0.0)	10.6 (8.1 to 13.8)	5.4 (4.1 to 6.9)	2.19 (1.67 to 2.85)	1.11 (0.84 to 1.43)
2000	0.0 (0.0 to 0.1)	0.0 (0.0 to 0.0)	12.2 (11.0 to 13.6)	5.8 (5.3 to 6.3)	2.45 (2.19 to 2.72)	1.16 (1.06 to 1.26)
2005	0.1 (0.0 to 0.1)	0.0 (0.0 to 0.0)	13.1 (11.1 to 15.2)	5.7 (4.7 to 6.7)	3.13 (2.66 to 3.63)	1.35 (1.12 to 1.59)
2010	0.0 (0.0 to 0.1)	0.0 (0.0 to 0.0)	10.9 (9.3 to 12.6)	4.5 (3.8 to 5.4)	3.35 (2.87 to 3.87)	1.39 (1.15 to 1.66)
2015	0.0 (0.0 to 0.0)	0.0 (0.0 to 0.0)	8.7 (7.7 to 9.8)	3.7 (3.2 to 4.3)	3.92 (3.48 to 4.39)	1.68 (1.46 to 1.93)
2020	0.0 (0.0 to 0.0)	0.0 (0.0 to 0.0)	7.4 (6.5 to 8.3)	3.4 (2.9 to 3.9)	3.68 (3.23 to 4.16)	1.69 (1.47 to 1.94)
2021	0.0 (0.0 to 0.0)	0.0 (0.0 to 0.0)	8.1 (6.6 to 9.7)	3.7 (2.9 to 4.6)	3.74 (3.08 to 4.51)	1.71 (1.36 to 2.13)

Harrow GBR_44740 Western Europe Stillbirth Rate / Neonatal Mortality Rate (SBR/NMR) − 20 weeks
lambda = 0.6 ; zeta = 0.8 ; scale = 15 ; amp = 0.028

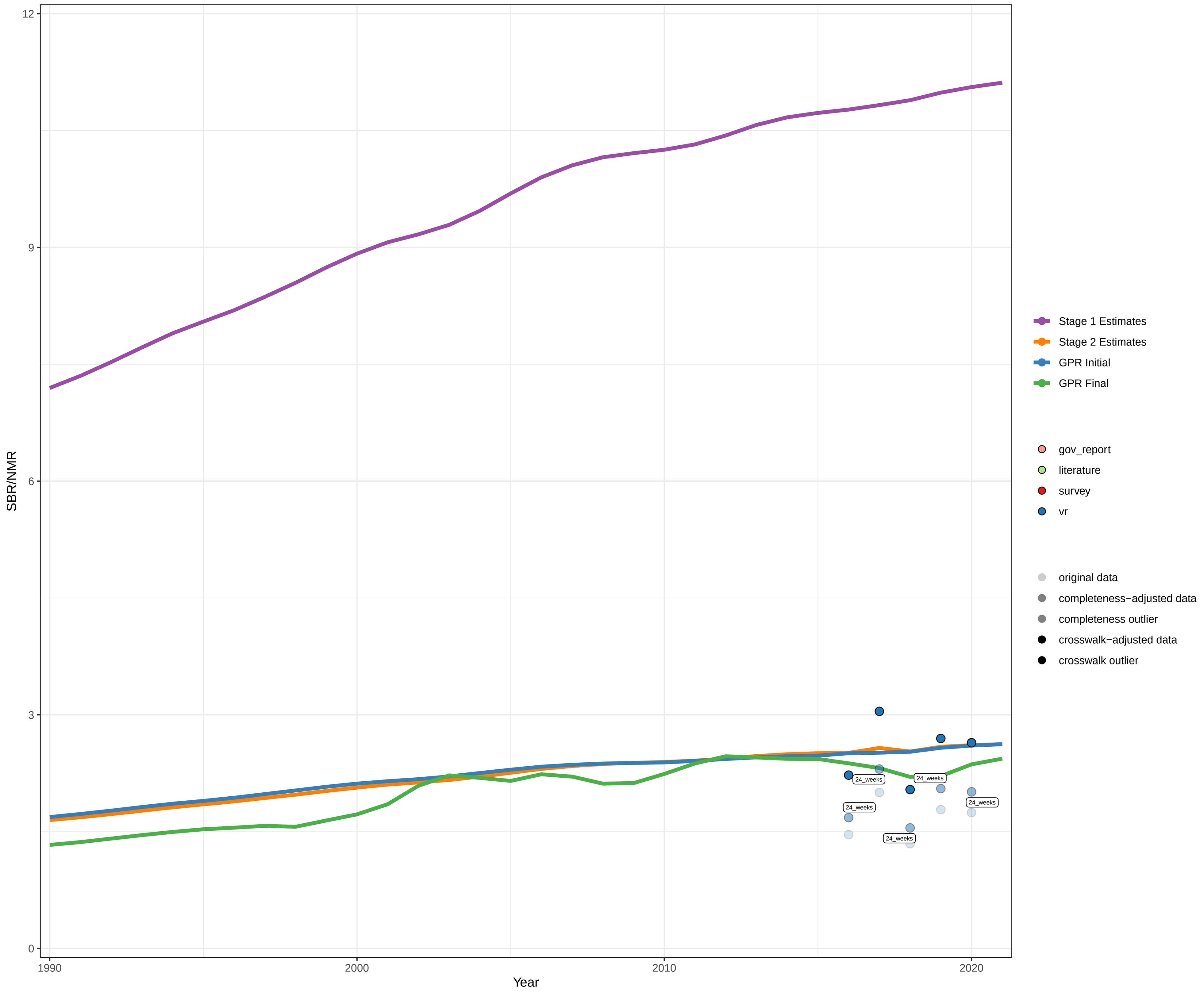

Harrow GBR_44740 Western Europe Stillbirth Rate / Neonatal Mortality Rate (SBR/NMR) − 28 weeks
lambda = 0.6 ; zeta = 0.8 ; scale = 15 ; amp = 0.028

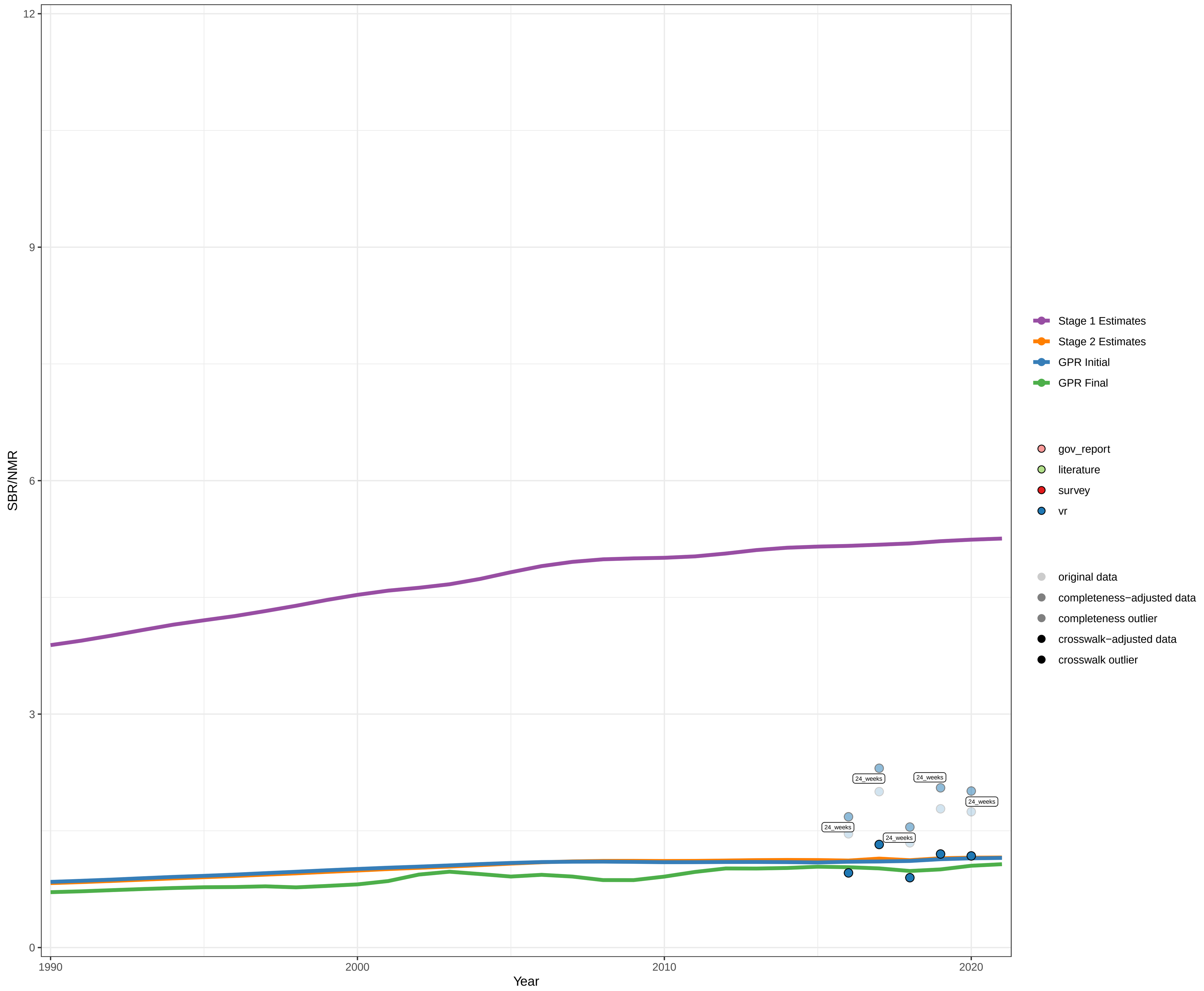

Definition	Years	Outlier	Source.Type	Title
24_weeks	2016−2020	0	VR	United Kingdom − England and Wales Child Mortality Statistics

Harrow GBR_44740 Western Europe Stillbirth Rate (SBR) − 20 weeks
lambda = 0.6 ; zeta = 0.8 ; scale = 15 ; amp = 0.028

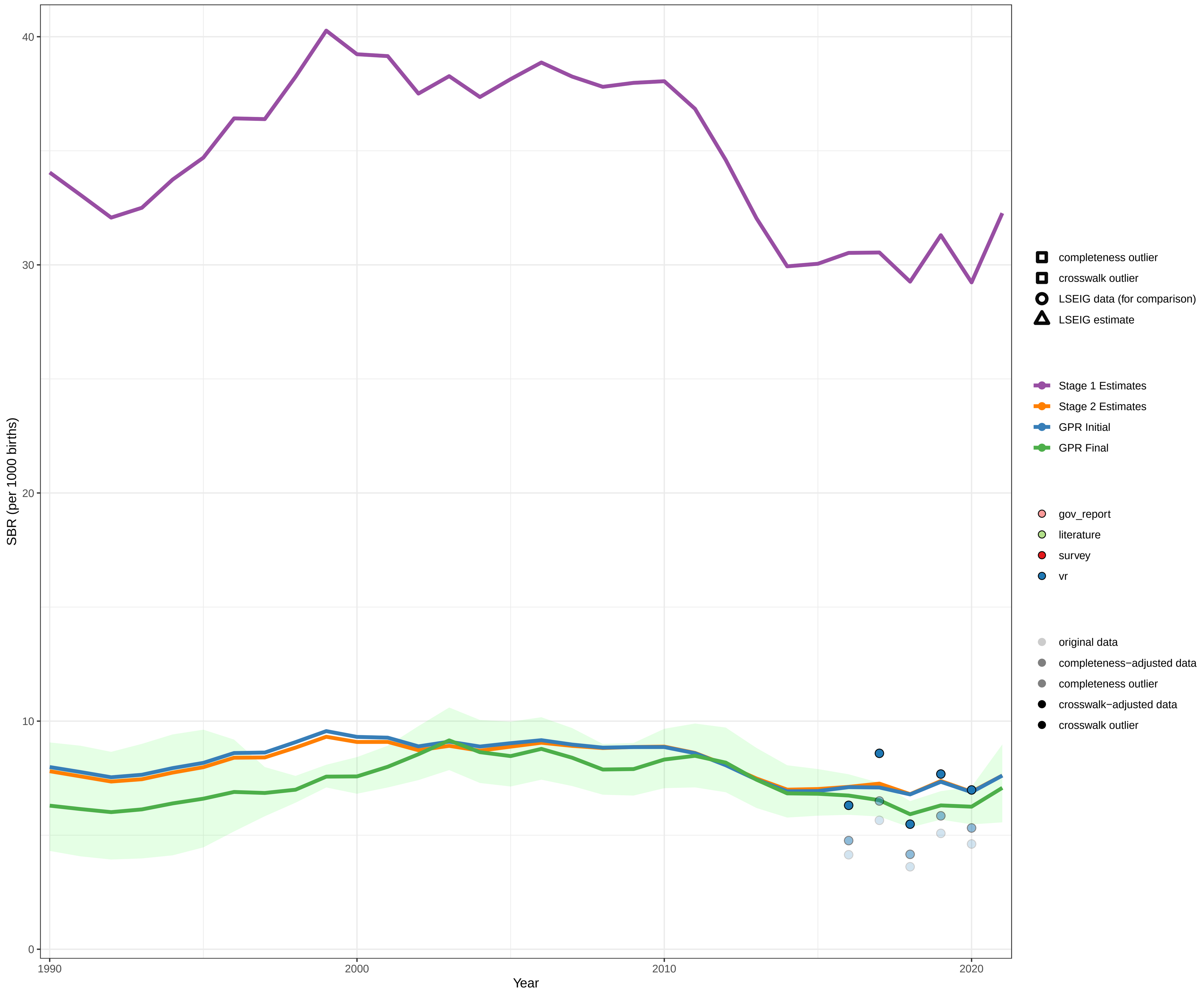

Harrow GBR_44740 Western Europe Stillbirth Rate (SBR) − 28 weeks
lambda = 0.6 ; zeta = 0.8 ; scale = 15 ; amp = 0.028

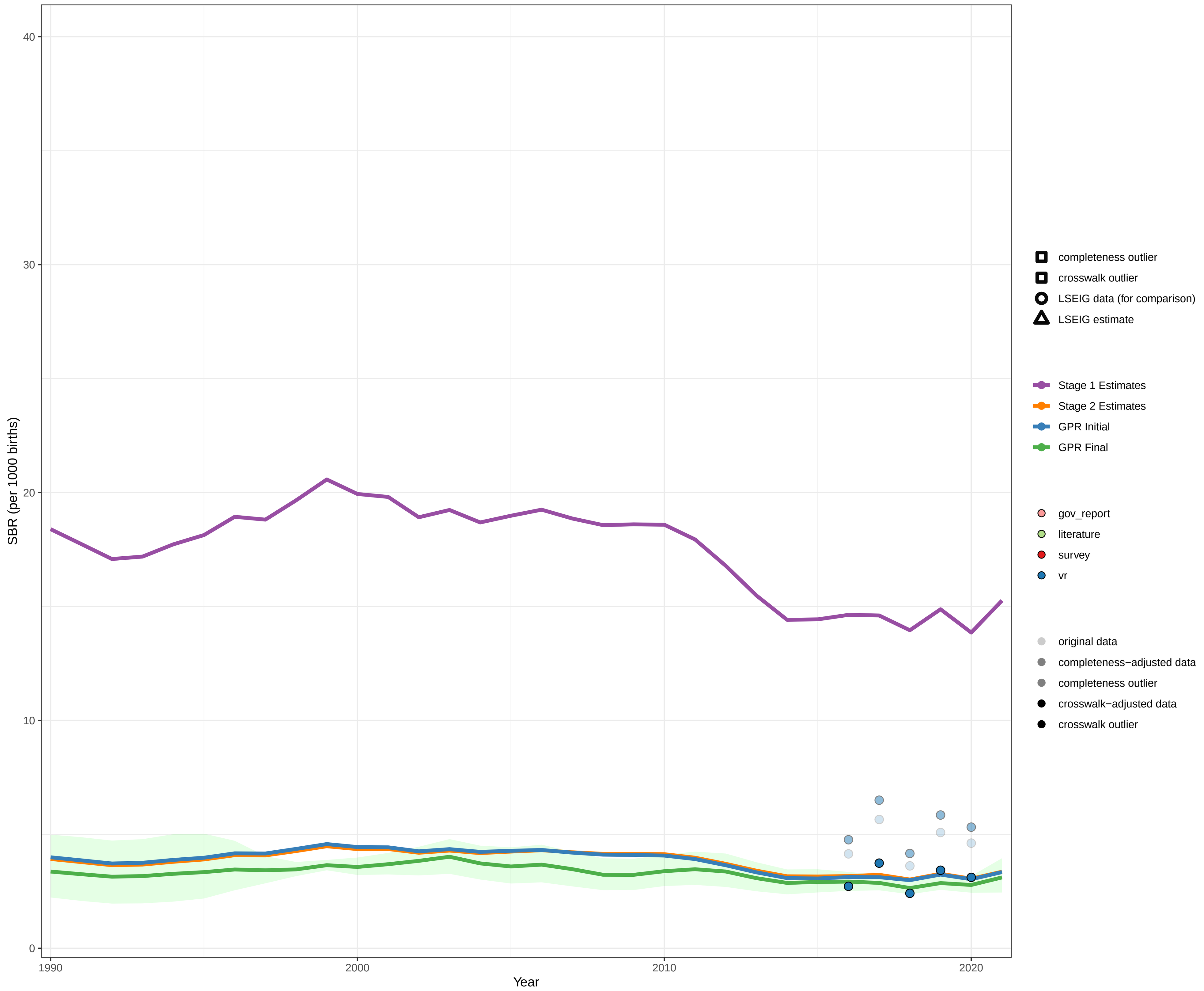

Year	Total Stillbirths (in thousands)	Total Stillbirths (in thousands)	SBR (per 1000 births)	SBR (per 1000 births)	SBR/NMR	SBR/NMR
	20 weeks	28 weeks	20 weeks	28 weeks	20 weeks	28 weeks
1990	0.0 (0.0 to 0.0)	0.0 (0.0 to 0.0)	6.3 (4.3 to 9.1)	3.4 (2.2 to 5.0)	1.33 (0.91 to 1.92)	0.71 (0.47 to 1.05)
1995	0.0 (0.0 to 0.0)	0.0 (0.0 to 0.0)	6.6 (4.5 to 9.6)	3.3 (2.2 to 5.0)	1.53 (1.04 to 2.23)	0.78 (0.51 to 1.17)
2000	0.0 (0.0 to 0.0)	0.0 (0.0 to 0.0)	7.6 (6.8 to 8.4)	3.6 (3.2 to 4.0)	1.72 (1.55 to 1.92)	0.81 (0.73 to 0.91)
2005	0.0 (0.0 to 0.0)	0.0 (0.0 to 0.0)	8.5 (7.1 to 10.0)	3.6 (2.8 to 4.4)	2.15 (1.81 to 2.54)	0.91 (0.72 to 1.13)
2010	0.0 (0.0 to 0.0)	0.0 (0.0 to 0.0)	8.3 (7.1 to 9.7)	3.4 (2.7 to 4.1)	2.24 (1.90 to 2.60)	0.91 (0.74 to 1.11)
2015	0.0 (0.0 to 0.0)	0.0 (0.0 to 0.0)	6.8 (5.9 to 7.9)	2.9 (2.4 to 3.5)	2.43 (2.09 to 2.82)	1.04 (0.87 to 1.24)
2020	0.0 (0.0 to 0.0)	0.0 (0.0 to 0.0)	6.2 (5.5 to 7.1)	2.8 (2.4 to 3.2)	2.36 (2.07 to 2.69)	1.05 (0.92 to 1.20)
2021	0.0 (0.0 to 0.0)	0.0 (0.0 to 0.0)	7.1 (5.6 to 9.0)	3.1 (2.4 to 4.0)	2.44 (1.92 to 3.09)	1.07 (0.84 to 1.36)

Havering GBR_44737 Western Europe Stillbirth Rate / Neonatal Mortality Rate (SBR/NMR) − 20 weeks
lambda = 0.6 ; zeta = 0.8 ; scale = 15 ; amp = 0.028

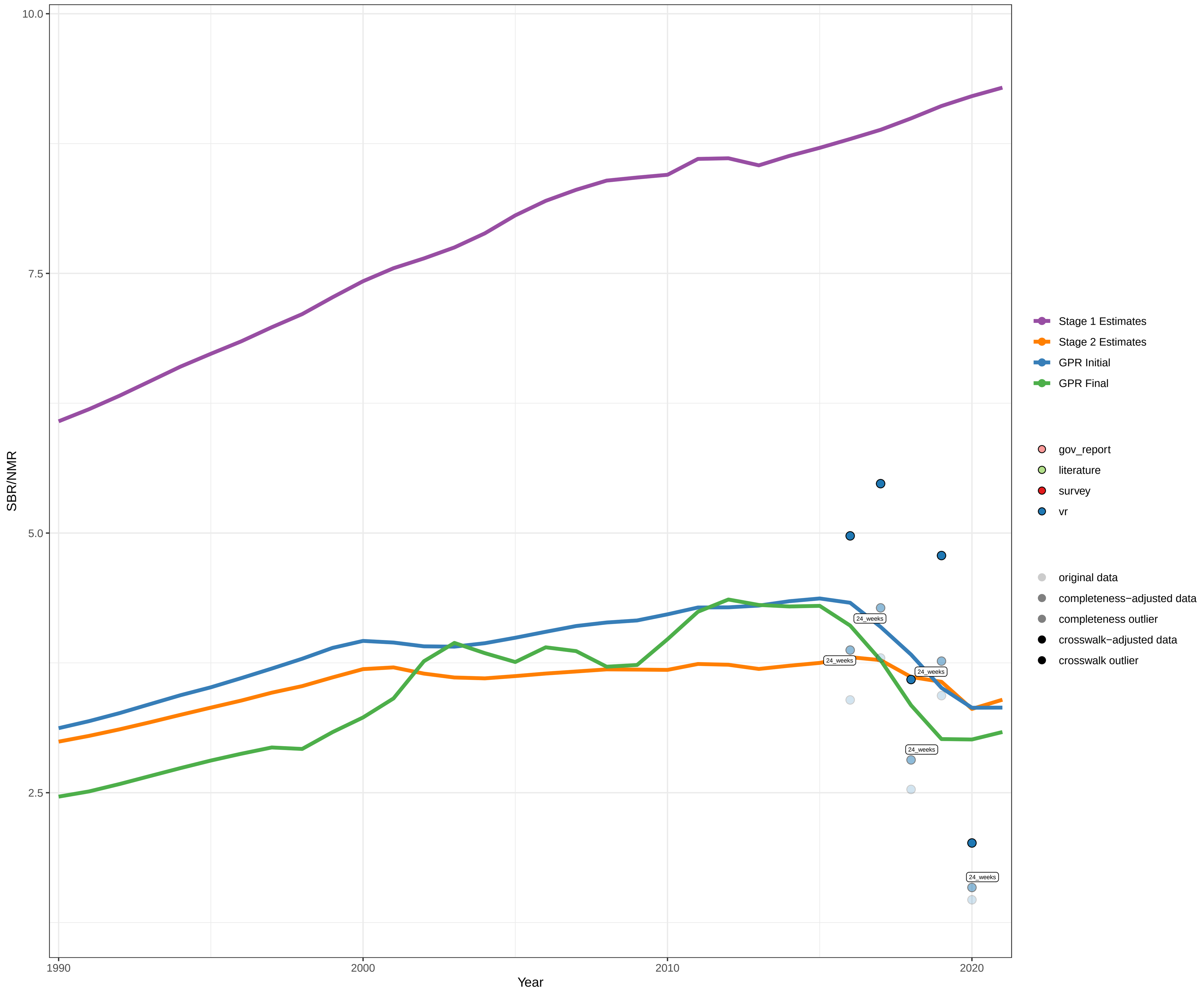

Havering GBR_44737 Western Europe Stillbirth Rate / Neonatal Mortality Rate (SBR/NMR) − 28 weeks
lambda = 0.6 ; zeta = 0.8 ; scale = 15 ; amp = 0.028

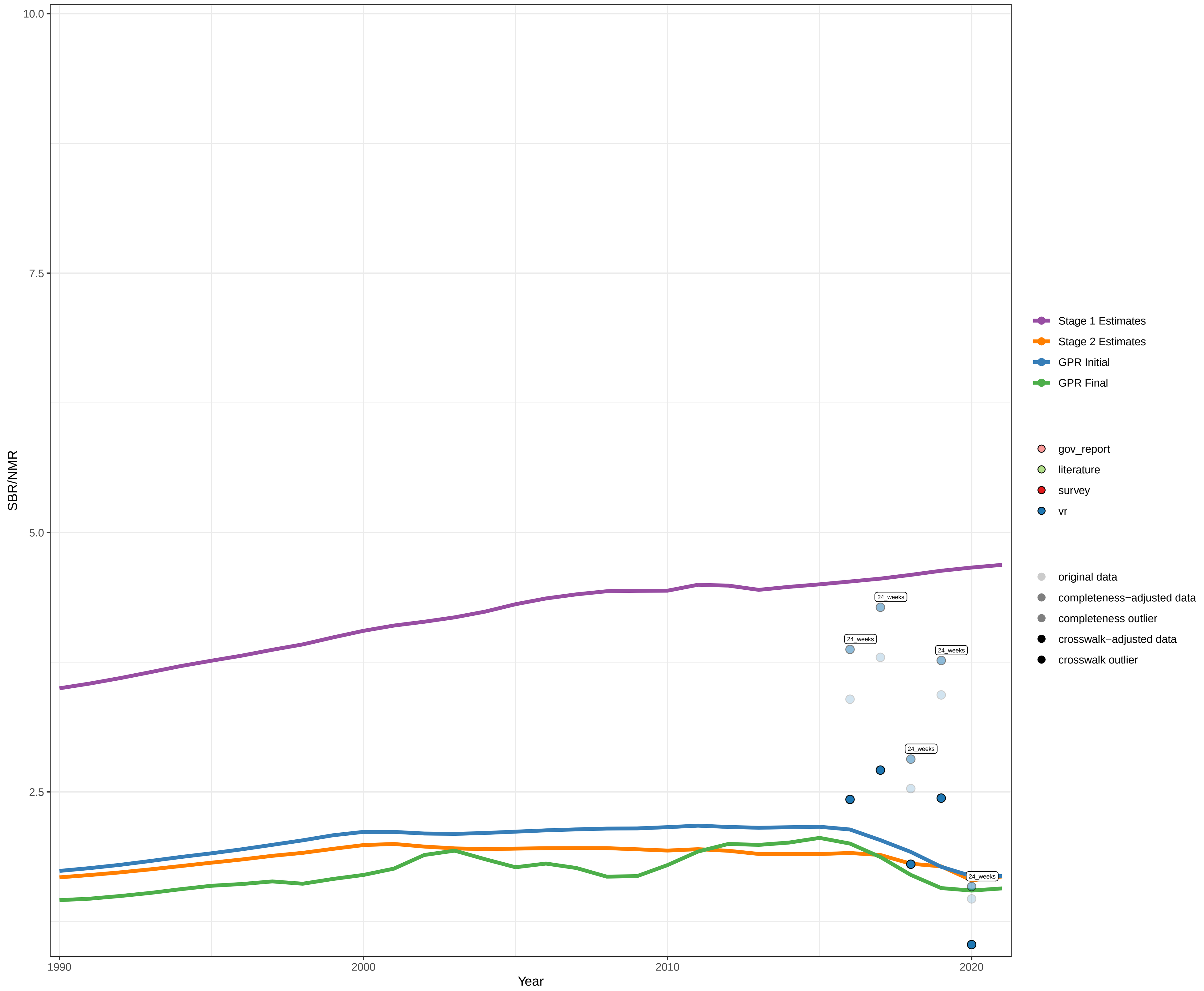

Definition	Years	Outlier	Source.Type	Title
24_weeks	2016−2020	0	VR	United Kingdom − England and Wales Child Mortality Statistics

Havering GBR_44737 Western Europe Stillbirth Rate (SBR) − 20 weeks
lambda = 0.6 ; zeta = 0.8 ; scale = 15 ; amp = 0.028

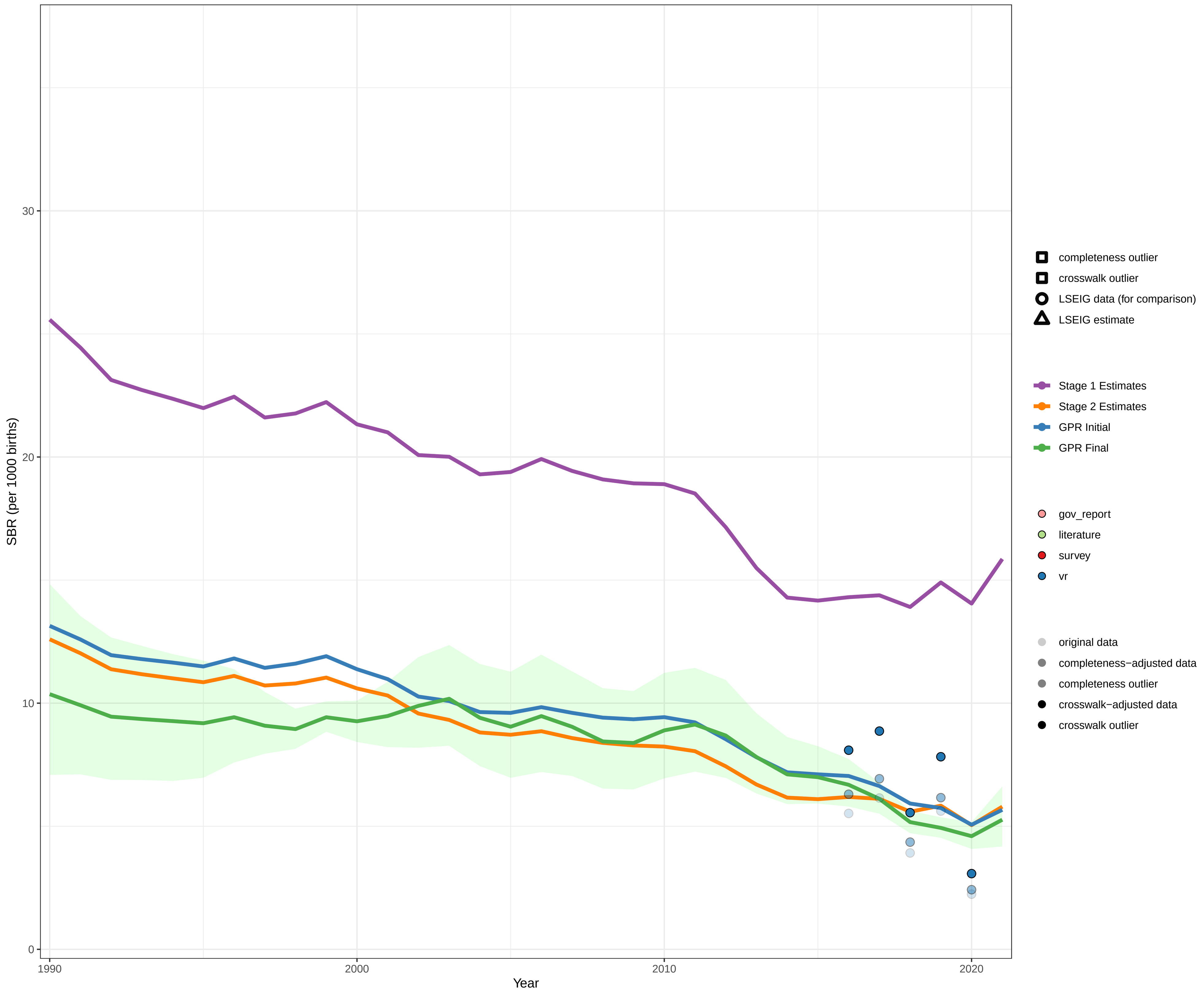

Havering GBR_44737 Western Europe Stillbirth Rate (SBR) − 28 weeks
lambda = 0.6 ; zeta = 0.8 ; scale = 15 ; amp = 0.028

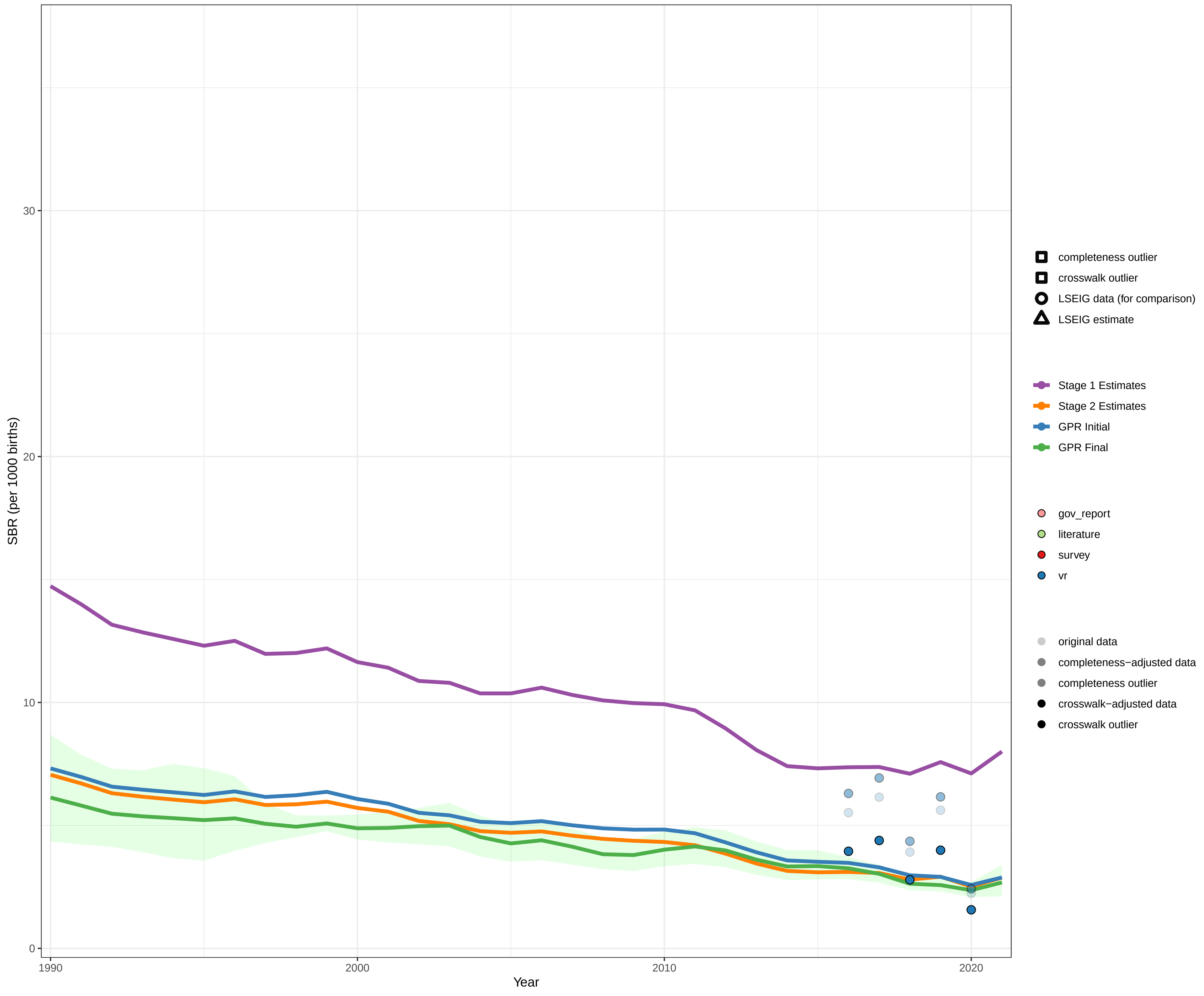

Year	Total Stillbirths (in thousands)	Total Stillbirths (in thousands)	SBR (per 1000 births)	SBR (per 1000 births)	SBR/NMR	SBR/NMR
	20 weeks	28 weeks	20 weeks	28 weeks	20 weeks	28 weeks
1990	0.0 (0.0 to 0.0)	0.0 (0.0 to 0.0)	10.4 (7.1 to 14.8)	6.1 (4.3 to 8.7)	2.46 (1.68 to 3.52)	1.46 (1.03 to 2.07)
1995	0.0 (0.0 to 0.0)	0.0 (0.0 to 0.0)	9.2 (7.0 to 11.7)	5.2 (3.6 to 7.3)	2.81 (2.13 to 3.59)	1.60 (1.09 to 2.24)
2000	0.0 (0.0 to 0.0)	0.0 (0.0 to 0.0)	9.3 (8.4 to 10.1)	4.9 (4.4 to 5.4)	3.22 (2.93 to 3.52)	1.70 (1.54 to 1.90)
2005	0.0 (0.0 to 0.0)	0.0 (0.0 to 0.0)	9.0 (7.0 to 11.3)	4.3 (3.5 to 5.1)	3.76 (2.90 to 4.69)	1.77 (1.46 to 2.10)
2010	0.0 (0.0 to 0.0)	0.0 (0.0 to 0.0)	8.9 (6.9 to 11.2)	4.0 (3.3 to 4.8)	3.98 (3.11 to 5.02)	1.79 (1.49 to 2.13)
2015	0.0 (0.0 to 0.0)	0.0 (0.0 to 0.0)	7.0 (5.9 to 8.3)	3.3 (2.8 to 4.0)	4.30 (3.64 to 5.07)	2.06 (1.72 to 2.45)
2020	0.0 (0.0 to 0.0)	0.0 (0.0 to 0.0)	4.6 (4.1 to 5.2)	2.4 (2.1 to 2.7)	3.01 (2.67 to 3.39)	1.55 (1.37 to 1.77)
2021	0.0 (0.0 to 0.0)	0.0 (0.0 to 0.0)	5.3 (4.2 to 6.6)	2.7 (2.1 to 3.4)	3.08 (2.45 to 3.88)	1.57 (1.24 to 1.99)

Hillingdon GBR_44736 Western Europe Stillbirth Rate / Neonatal Mortality Rate (SBR/NMR) − 20 weeks
lambda = 0.6 ; zeta = 0.8 ; scale = 15 ; amp = 0.028

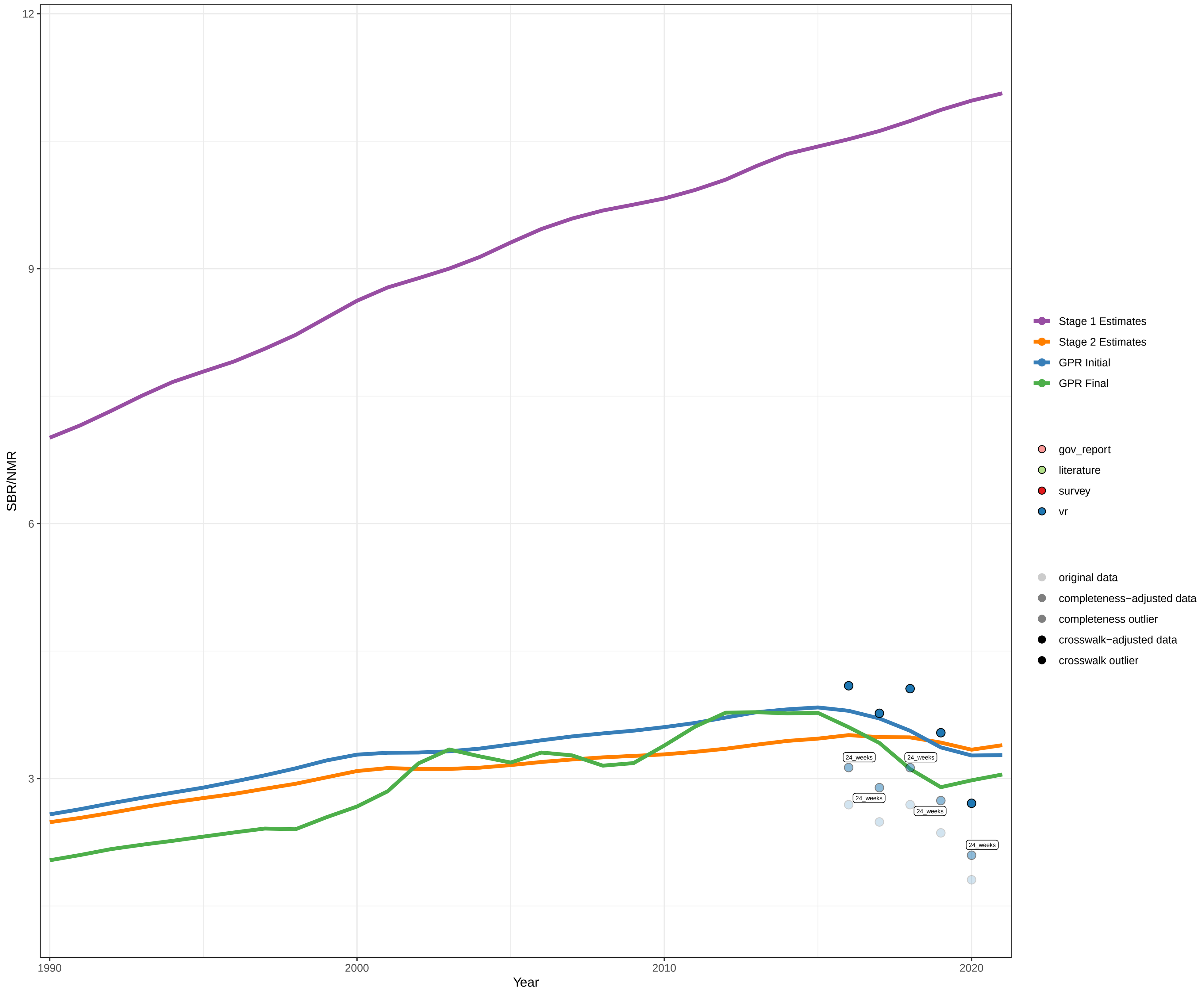

Hillingdon GBR_44736 Western Europe Stillbirth Rate / Neonatal Mortality Rate (SBR/NMR) − 28 weeks
lambda = 0.6 ; zeta = 0.8 ; scale = 15 ; amp = 0.028

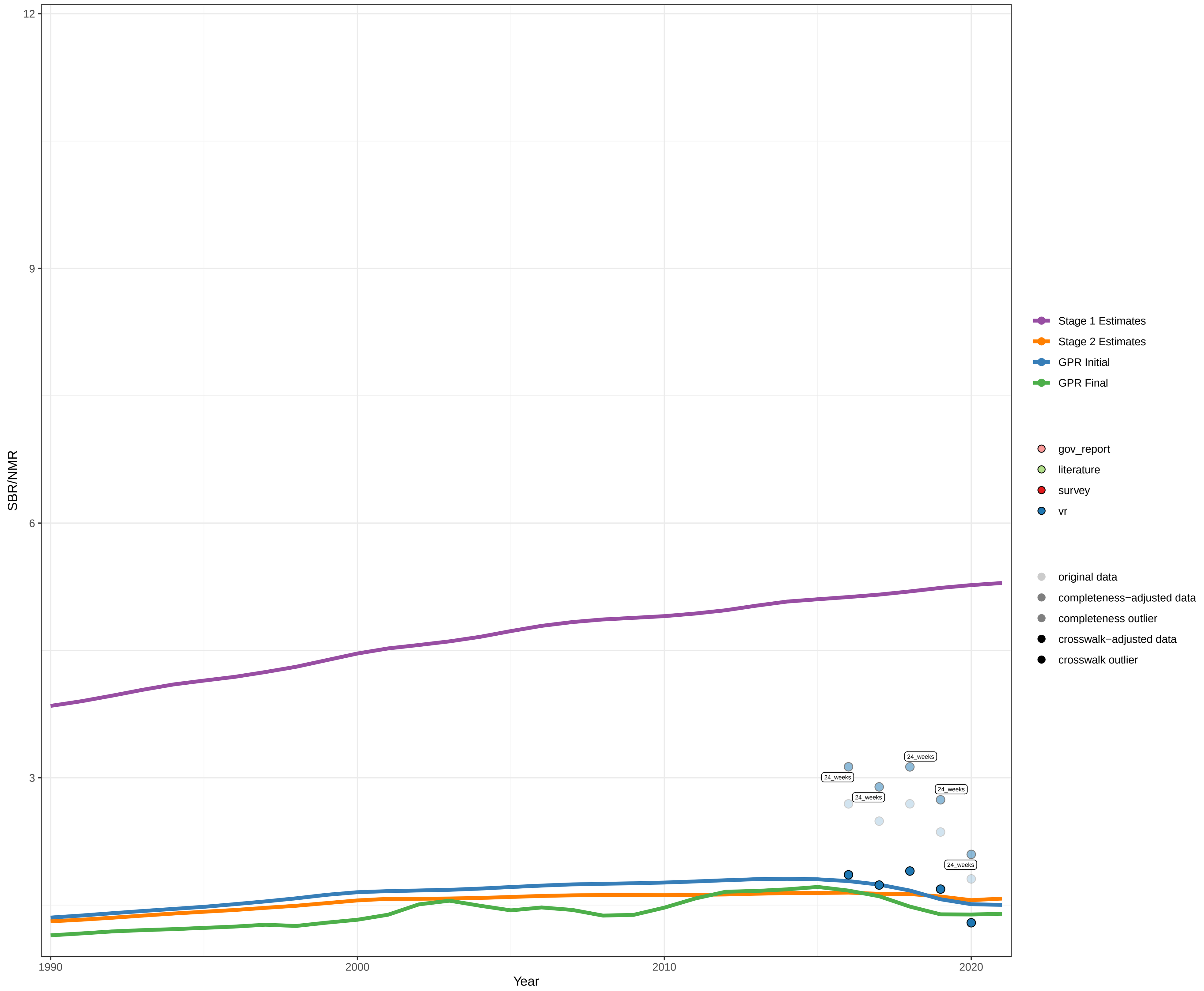

Definition	Years	Outlier	Source.Type	Title
24_weeks	2016−2020	0	VR	United Kingdom − England and Wales Child Mortality Statistics

Hillingdon GBR_44736 Western Europe Stillbirth Rate (SBR) − 20 weeks
lambda = 0.6 ; zeta = 0.8 ; scale = 15 ; amp = 0.028

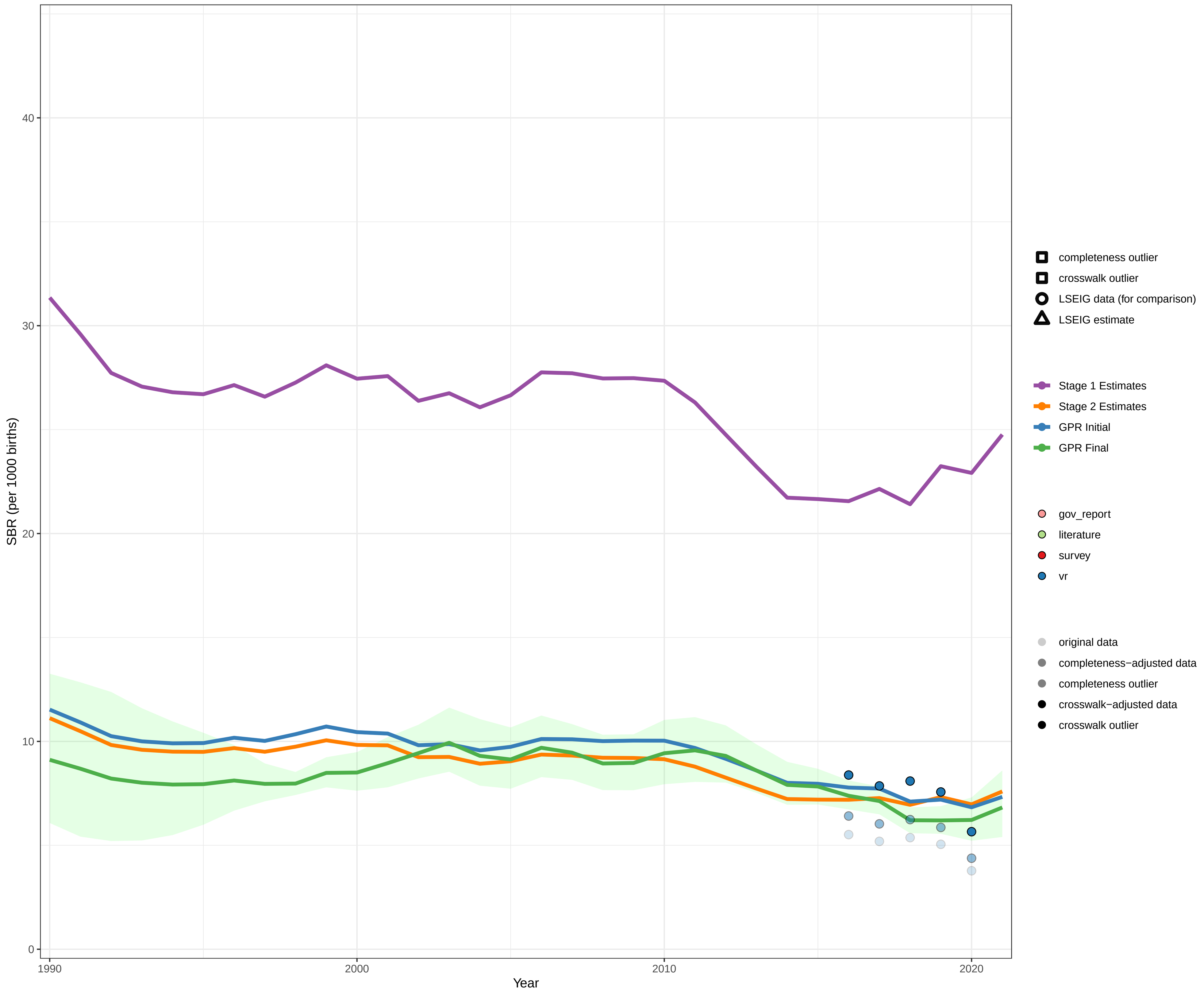

Hillingdon GBR_44736 Western Europe Stillbirth Rate (SBR) − 28 weeks
lambda = 0.6 ; zeta = 0.8 ; scale = 15 ; amp = 0.028

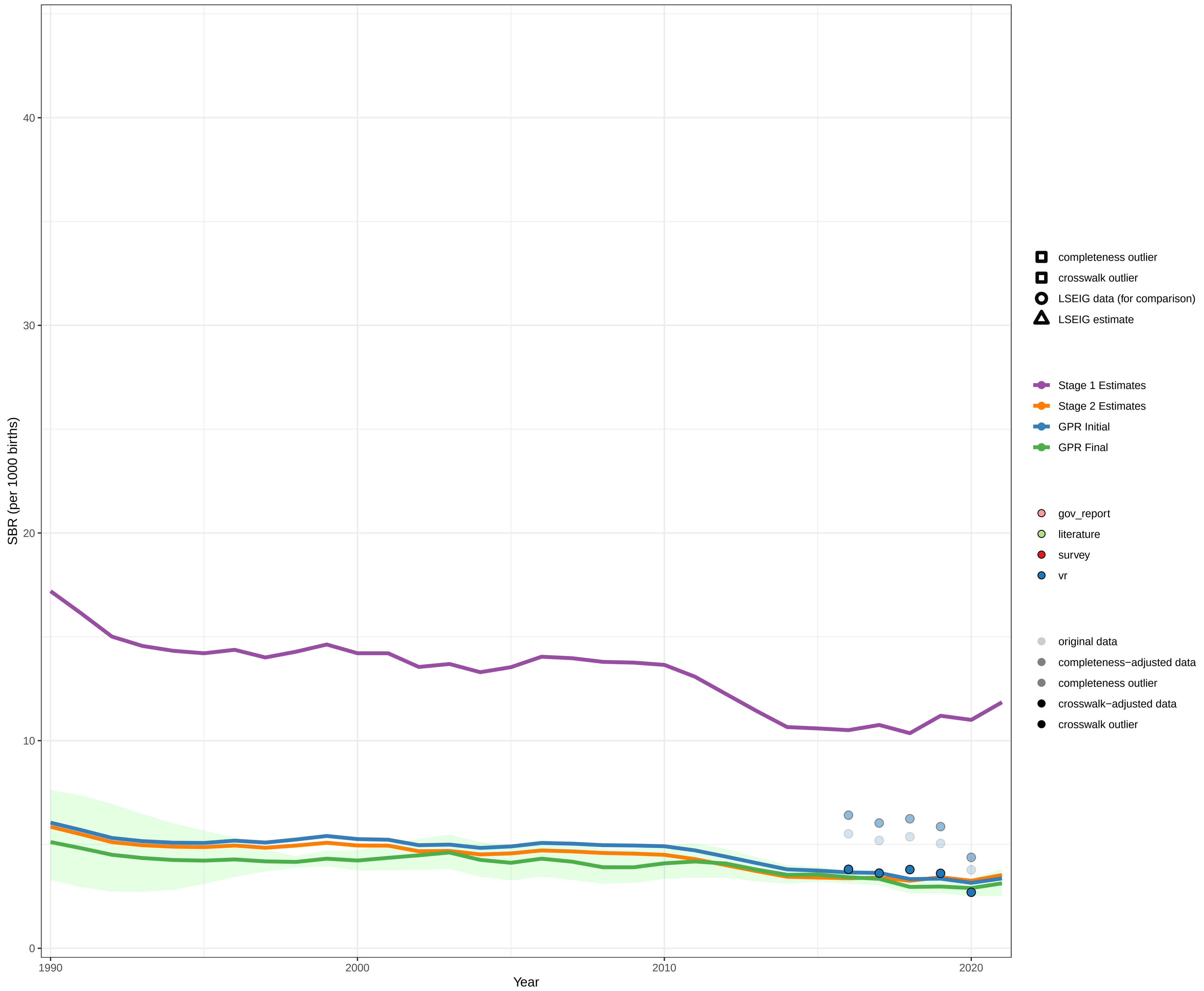

Year	Total Stillbirths (in thousands)	Total Stillbirths (in thousands)	SBR (per 1000 births)	SBR (per 1000 births)	SBR/NMR	SBR/NMR
	20 weeks	28 weeks	20 weeks	28 weeks	20 weeks	28 weeks
1990	0.0 (0.0 to 0.0)	0.0 (0.0 to 0.0)	9.1 (6.1 to 13.3)	5.1 (3.3 to 7.6)	2.04 (1.36 to 2.96)	1.14 (0.74 to 1.70)
1995	0.0 (0.0 to 0.0)	0.0 (0.0 to 0.0)	7.9 (6.0 to 10.4)	4.2 (3.1 to 5.7)	2.32 (1.75 to 3.04)	1.23 (0.91 to 1.65)
2000	0.0 (0.0 to 0.0)	0.0 (0.0 to 0.0)	8.5 (7.6 to 9.5)	4.2 (3.7 to 4.7)	2.67 (2.40 to 2.98)	1.33 (1.18 to 1.48)
2005	0.0 (0.0 to 0.0)	0.0 (0.0 to 0.0)	9.1 (7.7 to 10.7)	4.1 (3.3 to 5.0)	3.19 (2.70 to 3.73)	1.44 (1.15 to 1.74)
2010	0.0 (0.0 to 0.0)	0.0 (0.0 to 0.0)	9.4 (7.9 to 11.0)	4.1 (3.3 to 5.0)	3.39 (2.85 to 3.96)	1.47 (1.20 to 1.79)
2015	0.0 (0.0 to 0.0)	0.0 (0.0 to 0.0)	7.8 (7.0 to 8.7)	3.6 (3.2 to 3.9)	3.77 (3.36 to 4.18)	1.71 (1.54 to 1.89)
2020	0.0 (0.0 to 0.0)	0.0 (0.0 to 0.0)	6.2 (5.2 to 7.3)	2.9 (2.5 to 3.3)	2.98 (2.50 to 3.50)	1.39 (1.20 to 1.59)
2021	0.0 (0.0 to 0.0)	0.0 (0.0 to 0.0)	6.8 (5.4 to 8.6)	3.1 (2.5 to 3.8)	3.05 (2.41 to 3.84)	1.40 (1.13 to 1.72)

Hounslow GBR_44751 Western Europe Stillbirth Rate / Neonatal Mortality Rate (SBR/NMR) − 20 weeks
lambda = 0.6 ; zeta = 0.8 ; scale = 15 ; amp = 0.028

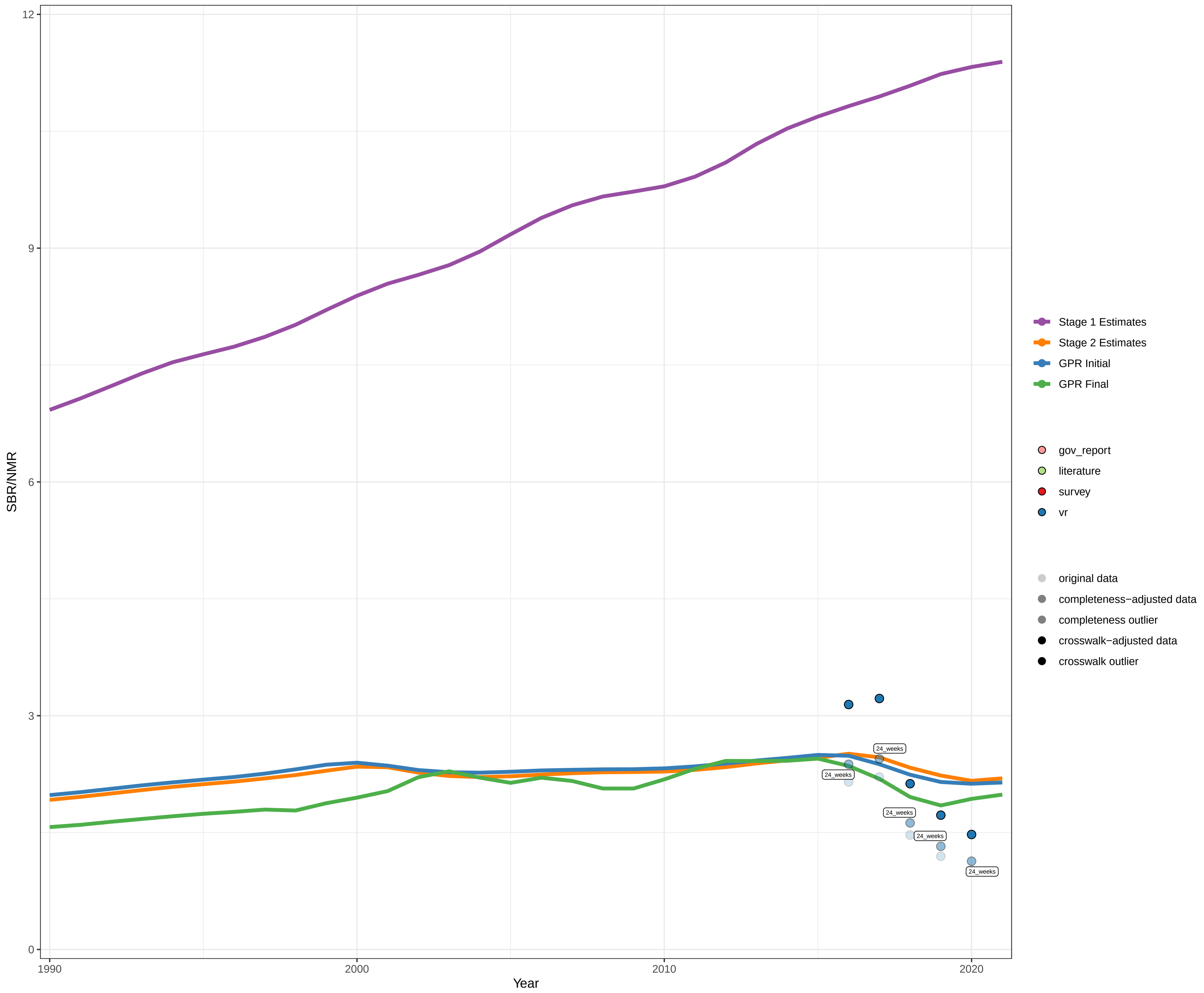

Hounslow GBR_44751 Western Europe Stillbirth Rate / Neonatal Mortality Rate (SBR/NMR) − 28 weeks
lambda = 0.6 ; zeta = 0.8 ; scale = 15 ; amp = 0.028

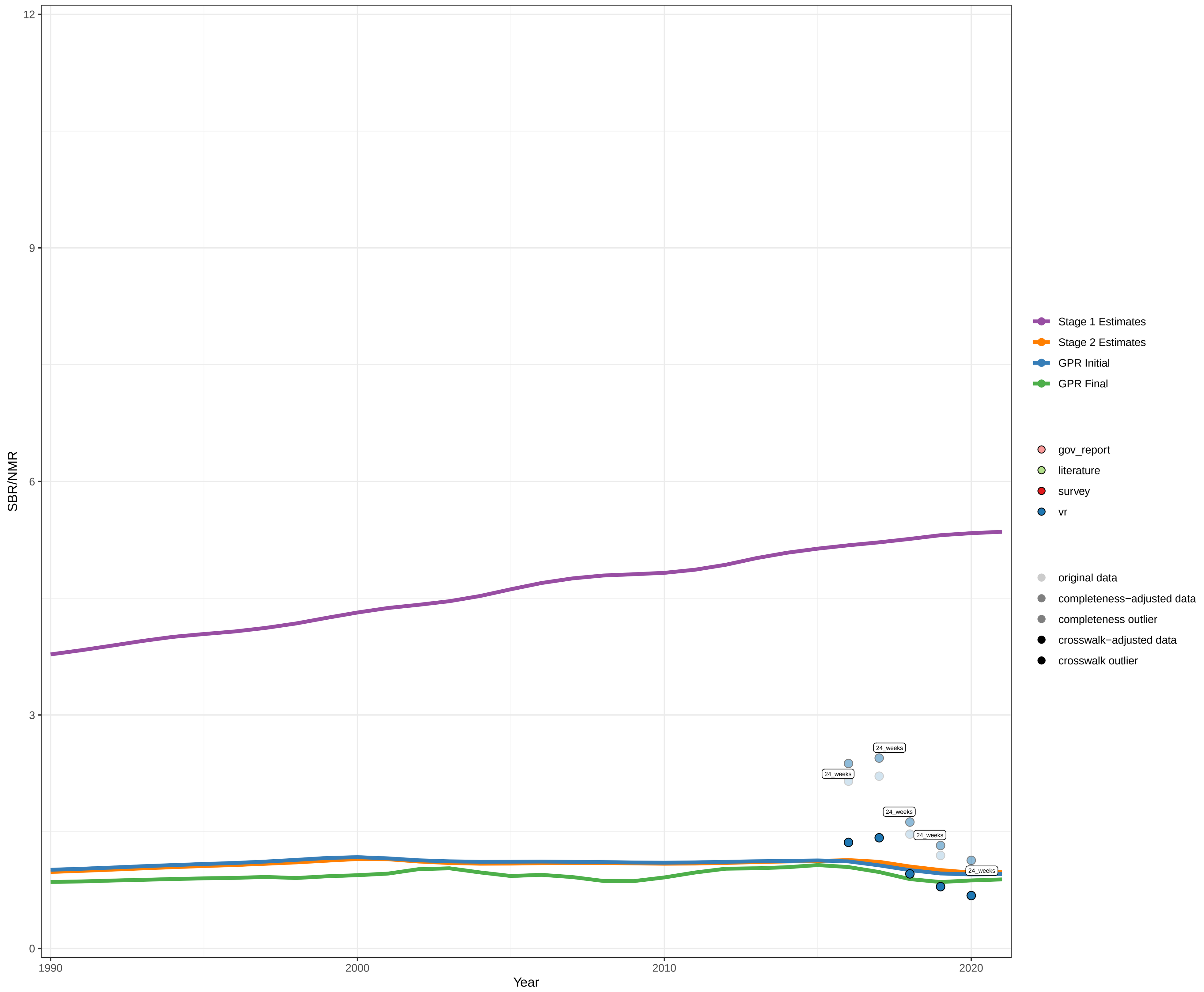

Definition	Years	Outlier	Source.Type	Title
24_weeks	2016−2020	0	VR	United Kingdom − England and Wales Child Mortality Statistics

Hounslow GBR_44751 Western Europe Stillbirth Rate (SBR) − 20 weeks
lambda = 0.6 ; zeta = 0.8 ; scale = 15 ; amp = 0.028

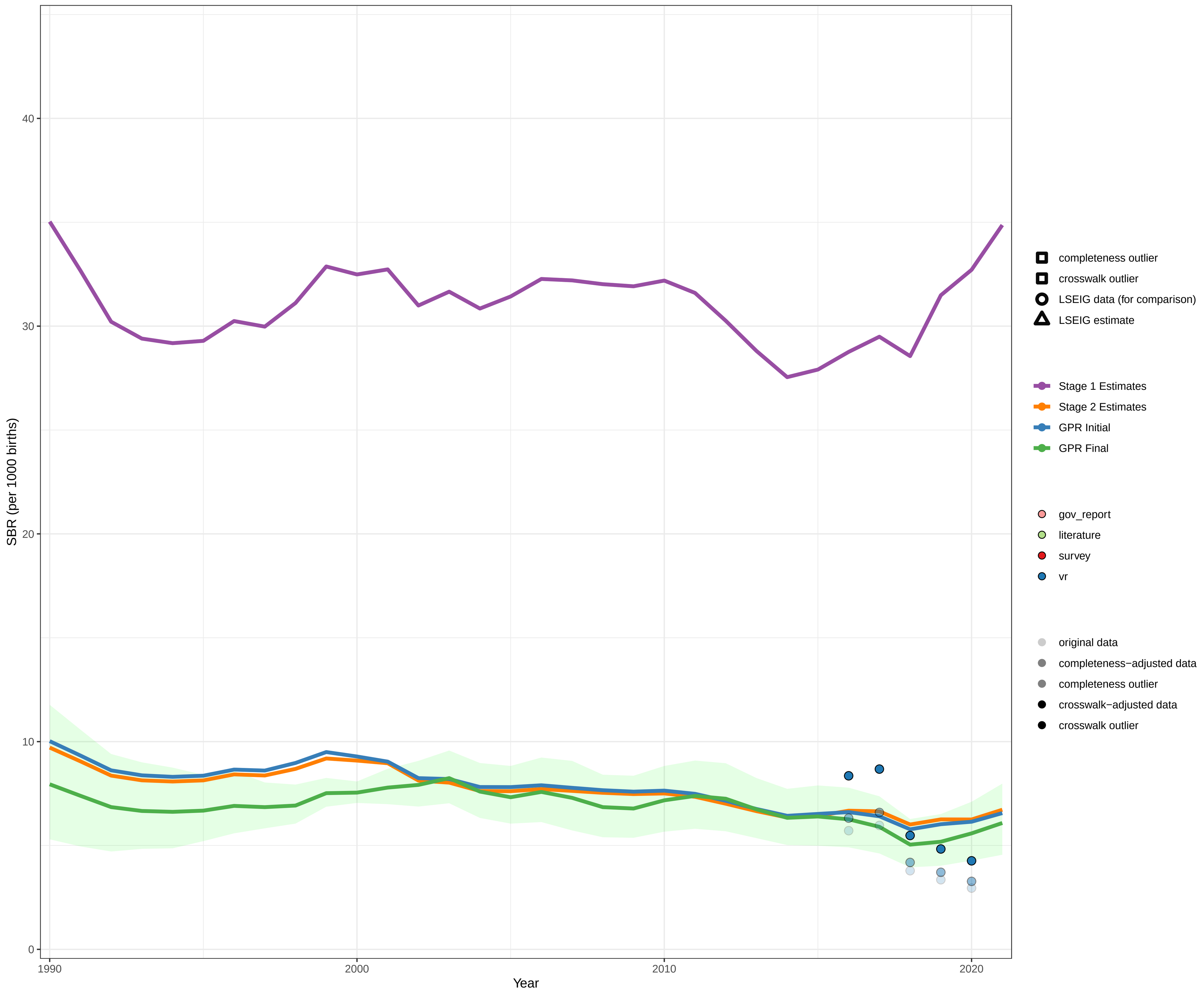

Hounslow GBR_44751 Western Europe Stillbirth Rate (SBR) − 28 weeks
lambda = 0.6 ; zeta = 0.8 ; scale = 15 ; amp = 0.028

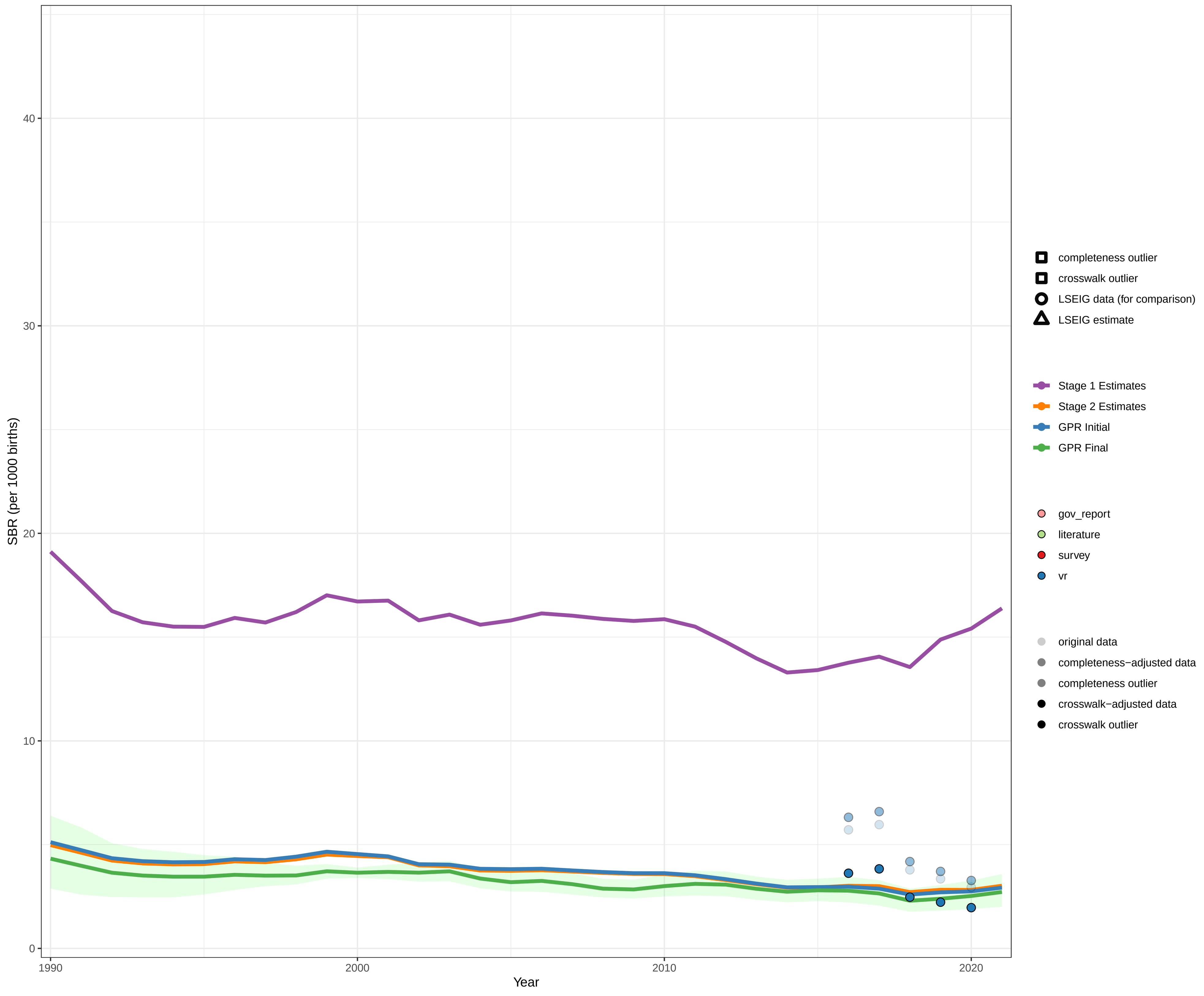

Year	Total Stillbirths (in thousands)	Total Stillbirths (in thousands)	SBR (per 1000 births)	SBR (per 1000 births)	SBR/NMR	SBR/NMR
	20 weeks	28 weeks	20 weeks	28 weeks	20 weeks	28 weeks
1990	0.0 (0.0 to 0.0)	0.0 (0.0 to 0.0)	7.9 (5.3 to 11.8)	4.3 (2.9 to 6.4)	1.57 (1.05 to 2.33)	0.86 (0.57 to 1.26)
1995	0.0 (0.0 to 0.0)	0.0 (0.0 to 0.0)	6.7 (5.2 to 8.4)	3.5 (2.6 to 4.5)	1.74 (1.36 to 2.19)	0.90 (0.68 to 1.17)
2000	0.0 (0.0 to 0.0)	0.0 (0.0 to 0.0)	7.5 (7.0 to 8.1)	3.6 (3.4 to 3.9)	1.95 (1.82 to 2.09)	0.94 (0.88 to 1.01)
2005	0.0 (0.0 to 0.0)	0.0 (0.0 to 0.0)	7.3 (6.1 to 8.8)	3.2 (2.7 to 3.7)	2.14 (1.77 to 2.58)	0.93 (0.80 to 1.08)
2010	0.0 (0.0 to 0.0)	0.0 (0.0 to 0.0)	7.2 (5.7 to 8.8)	3.0 (2.5 to 3.6)	2.18 (1.72 to 2.69)	0.91 (0.76 to 1.08)
2015	0.0 (0.0 to 0.0)	0.0 (0.0 to 0.0)	6.4 (5.0 to 7.9)	2.8 (2.3 to 3.4)	2.45 (1.91 to 3.02)	1.07 (0.87 to 1.29)
2020	0.0 (0.0 to 0.0)	0.0 (0.0 to 0.0)	5.6 (4.3 to 7.1)	2.5 (1.9 to 3.3)	1.93 (1.48 to 2.46)	0.87 (0.66 to 1.13)
2021	0.0 (0.0 to 0.0)	0.0 (0.0 to 0.0)	6.1 (4.6 to 8.0)	2.7 (2.0 to 3.6)	1.99 (1.49 to 2.61)	0.89 (0.66 to 1.17)

Islington GBR_44757 Western Europe Stillbirth Rate / Neonatal Mortality Rate (SBR/NMR) − 20 weeks
lambda = 0.6 ; zeta = 0.8 ; scale = 15 ; amp = 0.028

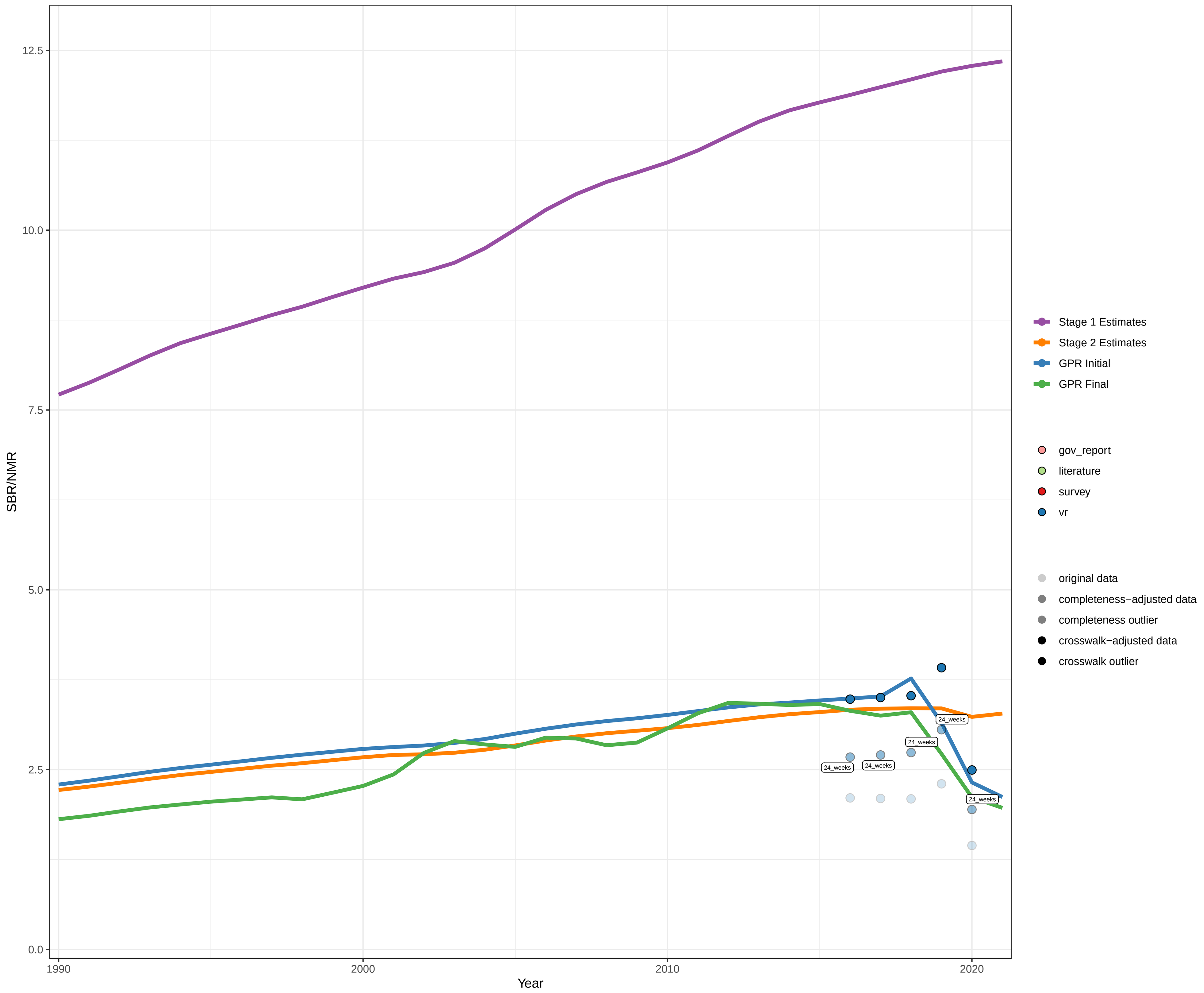

Islington GBR_44757 Western Europe Stillbirth Rate / Neonatal Mortality Rate (SBR/NMR) − 28 weeks
lambda = 0.6 ; zeta = 0.8 ; scale = 15 ; amp = 0.028

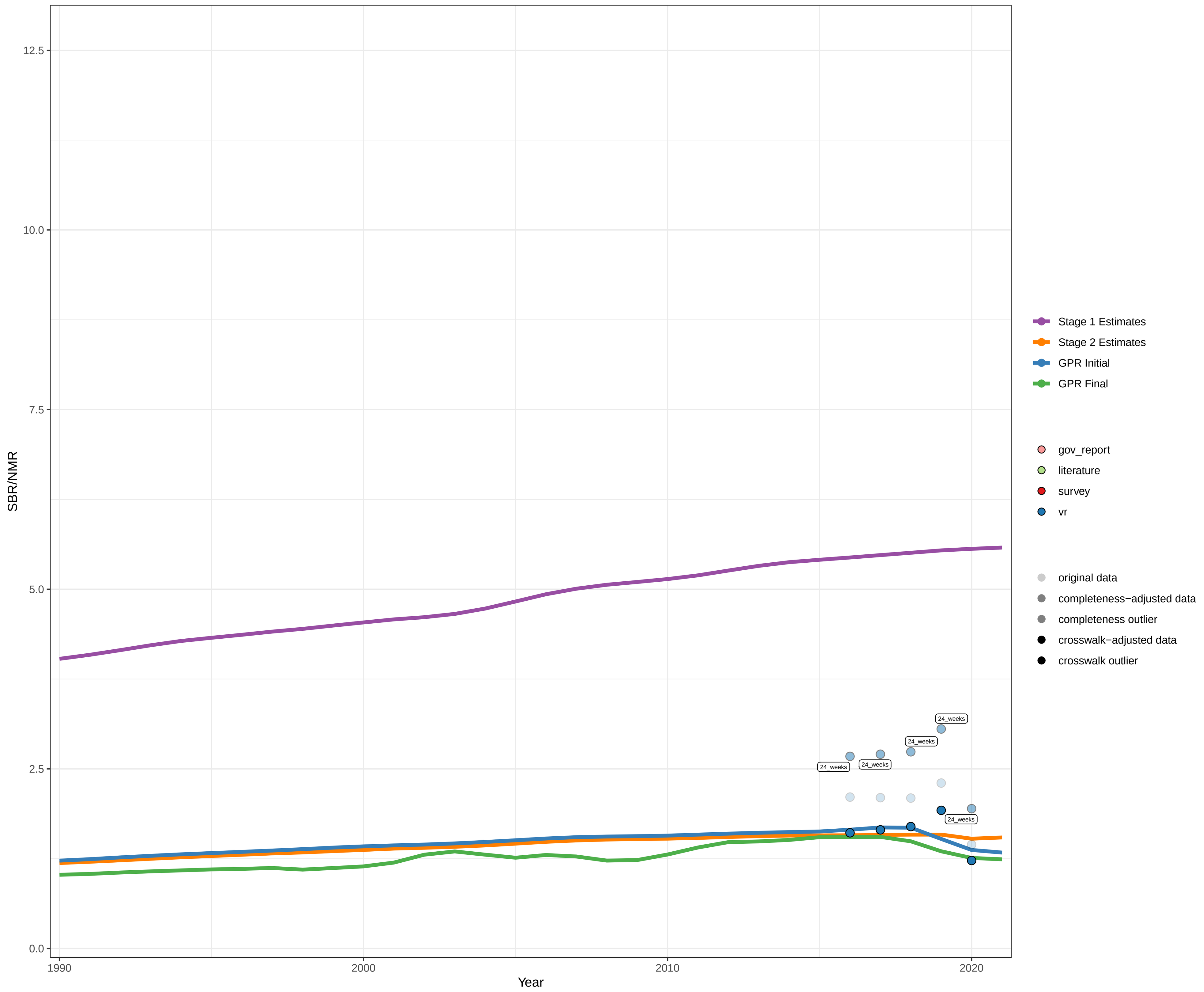

Definition	Years	Outlier	Source.Type	Title
24_weeks	2016−2020	0	VR	United Kingdom − England and Wales Child Mortality Statistics

Islington GBR_44757 Western Europe Stillbirth Rate (SBR) − 20 weeks
lambda = 0.6 ; zeta = 0.8 ; scale = 15 ; amp = 0.028

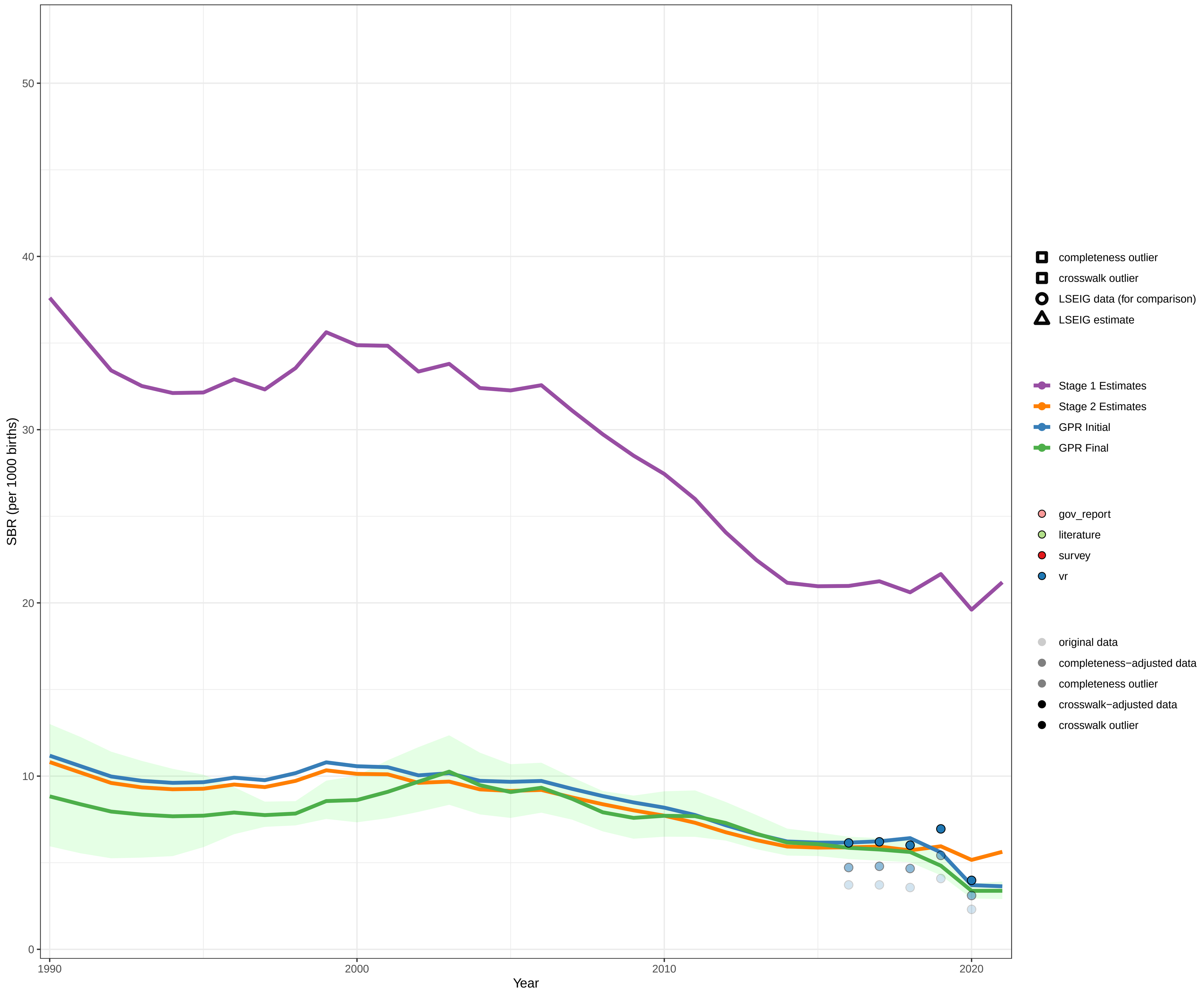

Islington GBR_44757 Western Europe Stillbirth Rate (SBR) − 28 weeks
lambda = 0.6 ; zeta = 0.8 ; scale = 15 ; amp = 0.028

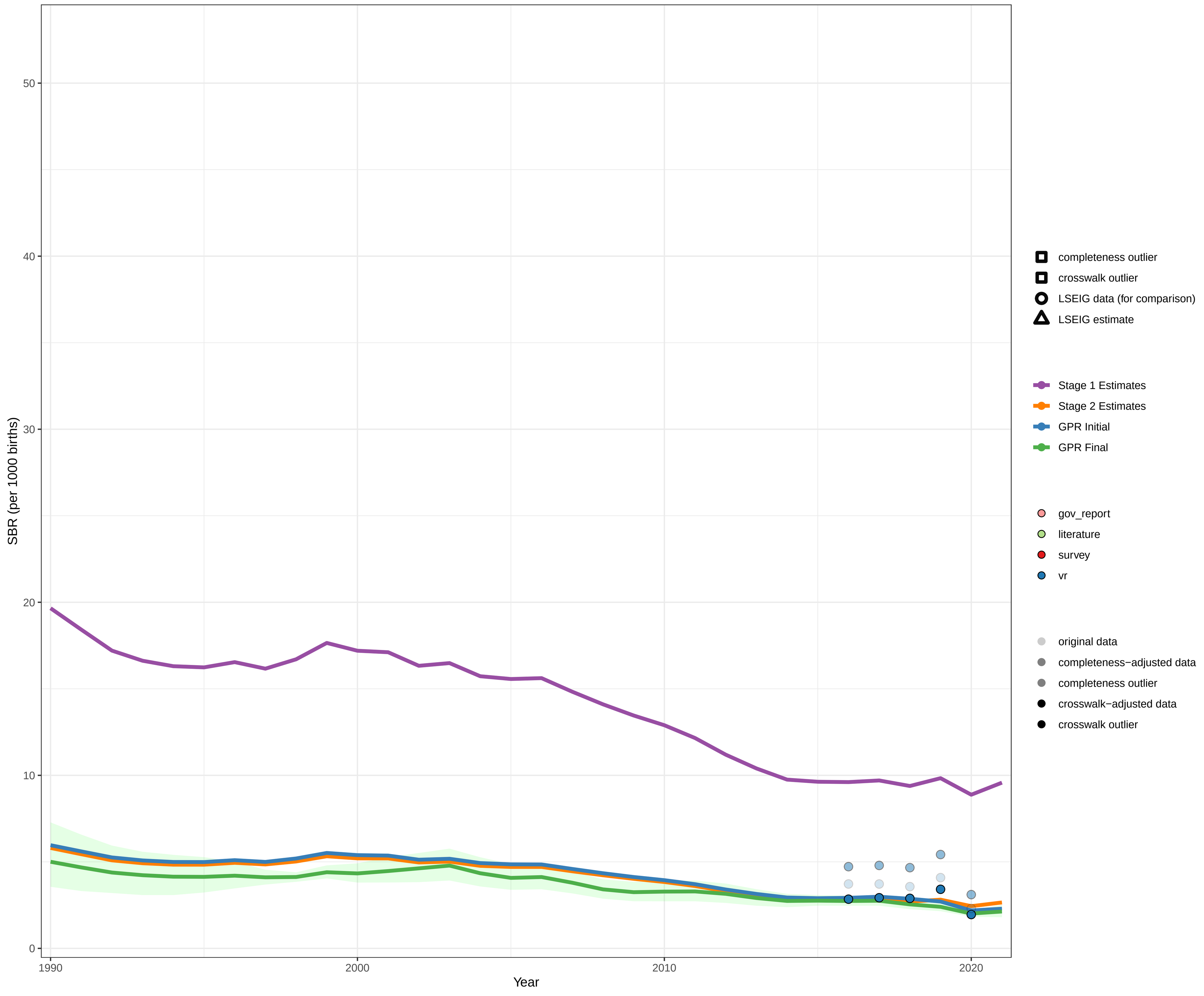

Year	Total Stillbirths (in thousands)	Total Stillbirths (in thousands)	SBR (per 1000 births)	SBR (per 1000 births)	SBR/NMR	SBR/NMR
	20 weeks	28 weeks	20 weeks	28 weeks	20 weeks	28 weeks
1990	0.0 (0.0 to 0.0)	0.0 (0.0 to 0.0)	8.8 (5.9 to 13.0)	5.0 (3.6 to 7.3)	1.81 (1.22 to 2.67)	1.03 (0.73 to 1.49)
1995	0.0 (0.0 to 0.0)	0.0 (0.0 to 0.0)	7.7 (5.9 to 10.1)	4.1 (3.2 to 5.3)	2.05 (1.57 to 2.69)	1.10 (0.86 to 1.40)
2000	0.0 (0.0 to 0.0)	0.0 (0.0 to 0.0)	8.6 (7.3 to 10.0)	4.3 (3.8 to 4.9)	2.27 (1.93 to 2.65)	1.14 (1.00 to 1.30)
2005	0.0 (0.0 to 0.0)	0.0 (0.0 to 0.0)	9.1 (7.6 to 10.7)	4.1 (3.4 to 4.9)	2.82 (2.35 to 3.32)	1.26 (1.05 to 1.51)
2010	0.0 (0.0 to 0.0)	0.0 (0.0 to 0.0)	7.7 (6.5 to 9.1)	3.3 (2.7 to 3.9)	3.07 (2.59 to 3.64)	1.31 (1.08 to 1.56)
2015	0.0 (0.0 to 0.0)	0.0 (0.0 to 0.0)	6.1 (5.4 to 6.7)	2.8 (2.5 to 3.1)	3.41 (3.02 to 3.79)	1.55 (1.39 to 1.73)
2020	0.0 (0.0 to 0.0)	0.0 (0.0 to 0.0)	3.4 (2.9 to 3.8)	2.0 (1.8 to 2.2)	2.12 (1.84 to 2.38)	1.26 (1.15 to 1.38)
2021	0.0 (0.0 to 0.0)	0.0 (0.0 to 0.0)	3.4 (2.9 to 3.9)	2.1 (1.8 to 2.5)	1.97 (1.69 to 2.28)	1.24 (1.05 to 1.47)

Kensington and Chelsea GBR_44728 Western Europe Stillbirth Rate / Neonatal Mortality Rate (SBR/NMR) − 20 weeks
lambda = 0.6 ; zeta = 0.8 ; scale = 15 ; amp = 0.028

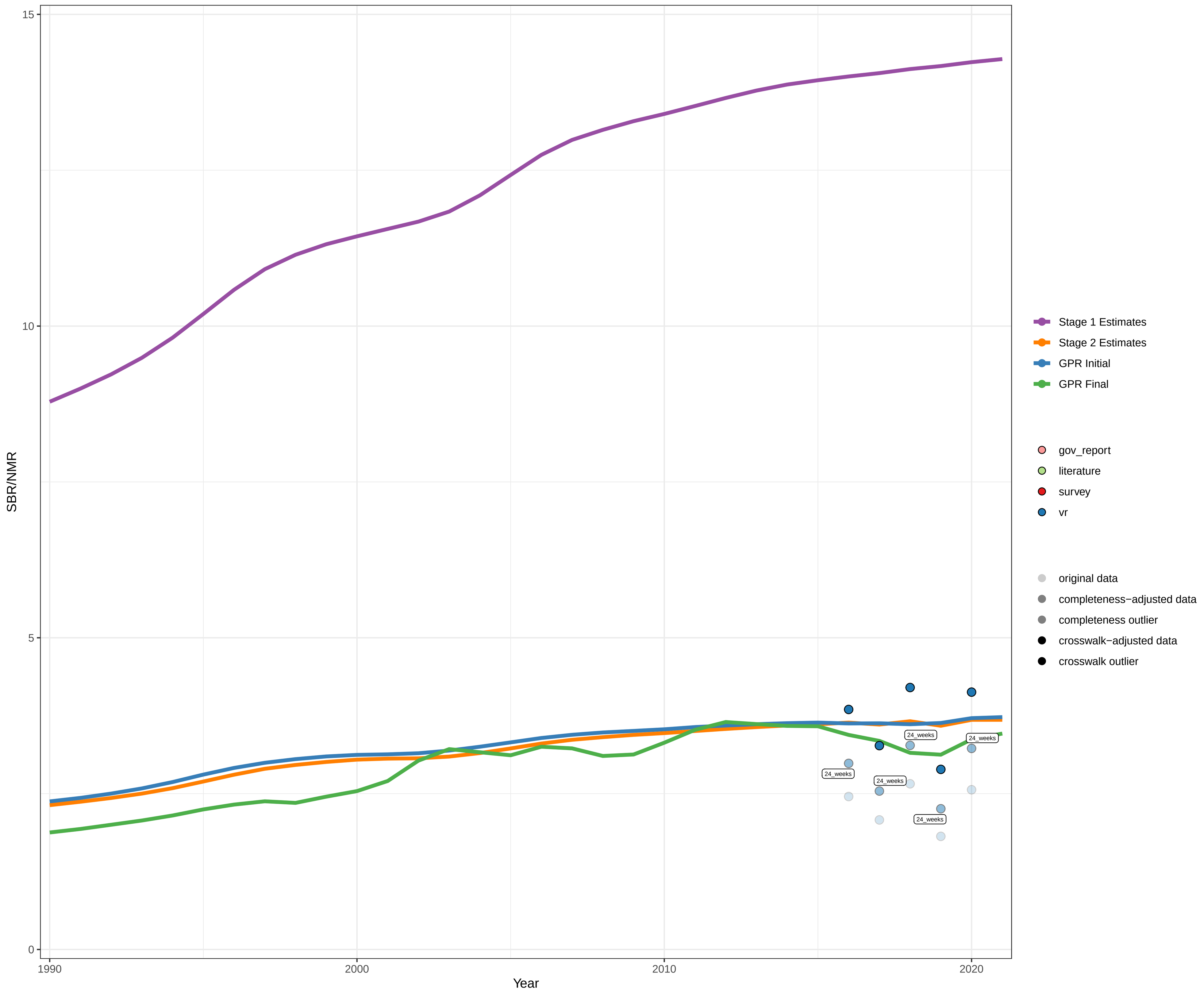

Kensington and Chelsea GBR_44728 Western Europe Stillbirth Rate / Neonatal Mortality Rate (SBR/NMR) − 28 weeks
lambda = 0.6 ; zeta = 0.8 ; scale = 15 ; amp = 0.028

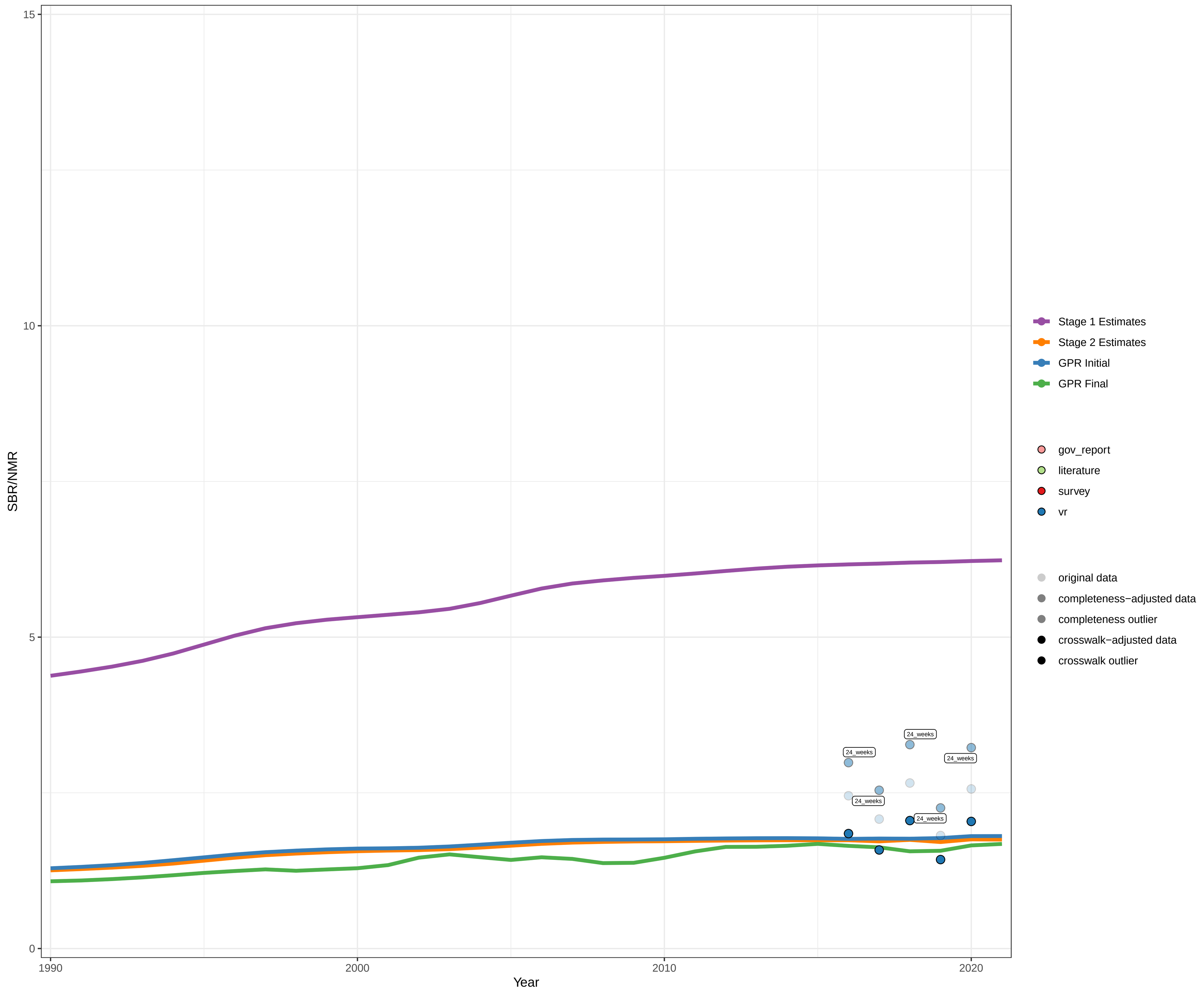

Definition	Years	Outlier	Source.Type	Title
24_weeks	2016−2020	0	VR	United Kingdom − England and Wales Child Mortality Statistics

Kensington and Chelsea GBR_44728 Western Europe Stillbirth Rate (SBR) − 20 weeks
lambda = 0.6 ; zeta = 0.8 ; scale = 15 ; amp = 0.028

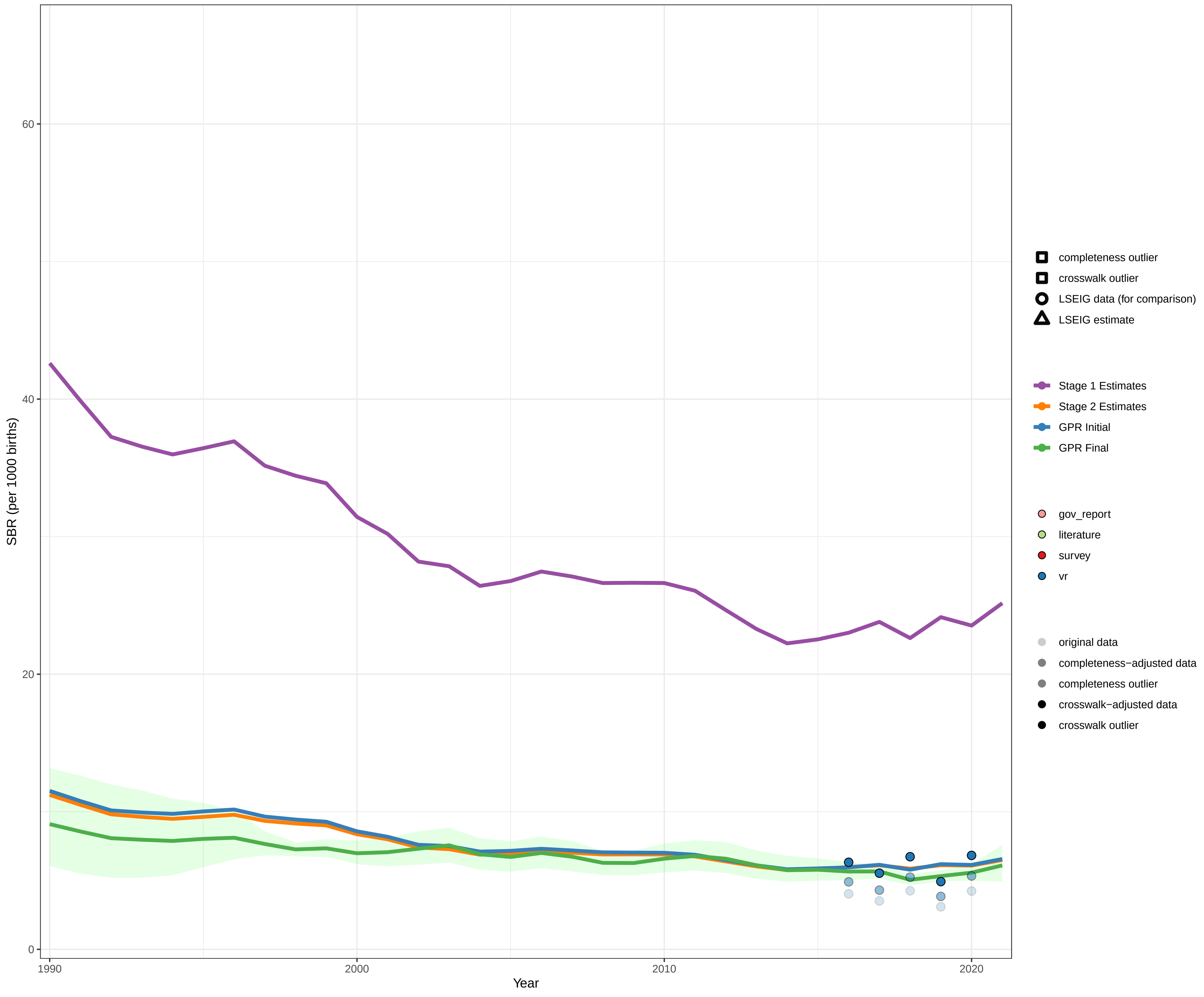

Kensington and Chelsea GBR_44728 Western Europe Stillbirth Rate (SBR) − 28 weeks
lambda = 0.6 ; zeta = 0.8 ; scale = 15 ; amp = 0.028

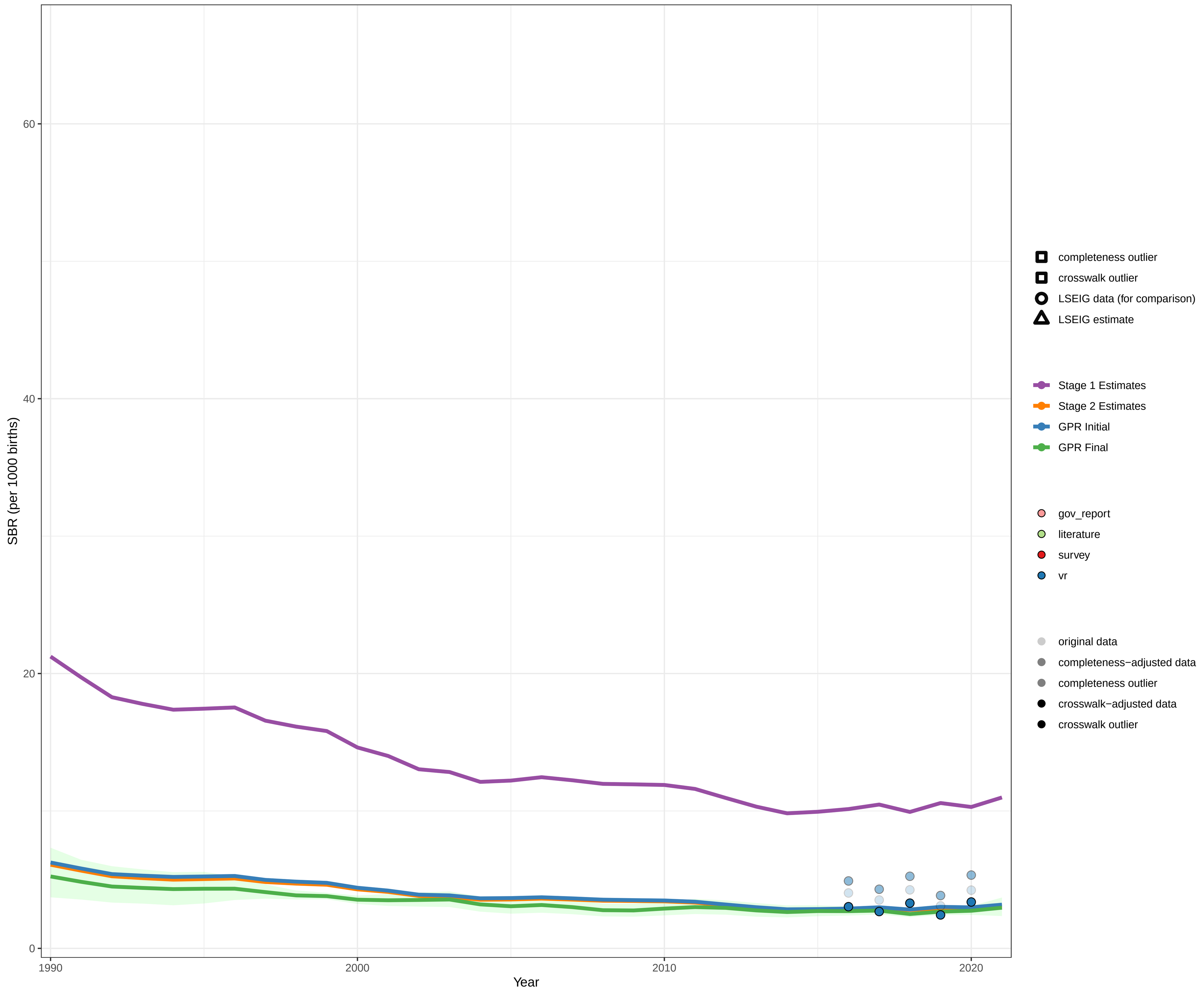

Year	Total Stillbirths (in thousands)	Total Stillbirths (in thousands)	SBR (per 1000 births)	SBR (per 1000 births)	SBR/NMR	SBR/NMR
	20 weeks	28 weeks	20 weeks	28 weeks	20 weeks	28 weeks
1990	0.0 (0.0 to 0.0)	0.0 (0.0 to 0.0)	9.1 (6.1 to 13.2)	5.2 (3.7 to 7.3)	1.88 (1.25 to 2.72)	1.08 (0.77 to 1.51)
1995	0.0 (0.0 to 0.0)	0.0 (0.0 to 0.0)	8.0 (6.0 to 10.6)	4.3 (3.3 to 5.6)	2.25 (1.67 to 2.98)	1.21 (0.91 to 1.56)
2000	0.0 (0.0 to 0.0)	0.0 (0.0 to 0.0)	7.0 (6.2 to 7.9)	3.5 (3.2 to 3.8)	2.54 (2.25 to 2.88)	1.29 (1.18 to 1.40)
2005	0.0 (0.0 to 0.0)	0.0 (0.0 to 0.0)	6.7 (5.6 to 7.9)	3.1 (2.5 to 3.6)	3.12 (2.62 to 3.65)	1.42 (1.17 to 1.68)
2010	0.0 (0.0 to 0.0)	0.0 (0.0 to 0.0)	6.6 (5.6 to 7.7)	2.9 (2.4 to 3.4)	3.32 (2.82 to 3.87)	1.46 (1.21 to 1.74)
2015	0.0 (0.0 to 0.0)	0.0 (0.0 to 0.0)	5.8 (5.0 to 6.6)	2.7 (2.3 to 3.1)	3.58 (3.09 to 4.09)	1.68 (1.45 to 1.94)
2020	0.0 (0.0 to 0.0)	0.0 (0.0 to 0.0)	5.6 (5.0 to 6.2)	2.7 (2.4 to 3.1)	3.37 (3.02 to 3.74)	1.66 (1.47 to 1.86)
2021	0.0 (0.0 to 0.0)	0.0 (0.0 to 0.0)	6.1 (4.9 to 7.6)	3.0 (2.4 to 3.7)	3.46 (2.79 to 4.30)	1.68 (1.34 to 2.10)

Kingston upon Thames GBR_44738 Western Europe Stillbirth Rate / Neonatal Mortality Rate (SBR/NMR) − 20 weeks
lambda = 0.6 ; zeta = 0.8 ; scale = 15 ; amp = 0.028

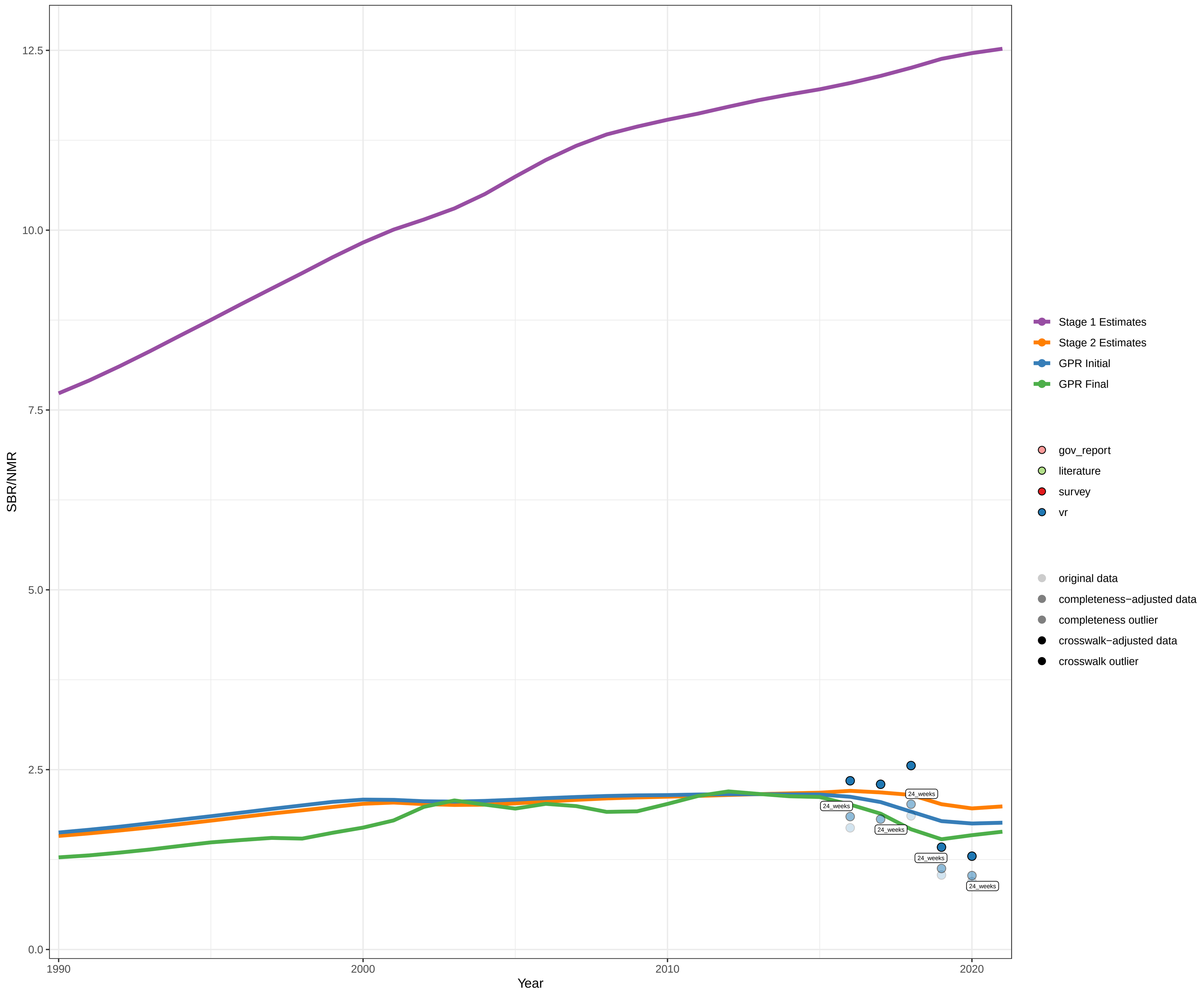

Kingston upon Thames GBR_44738 Western Europe Stillbirth Rate / Neonatal Mortality Rate (SBR/NMR) − 28 weeks
lambda = 0.6 ; zeta = 0.8 ; scale = 15 ; amp = 0.028

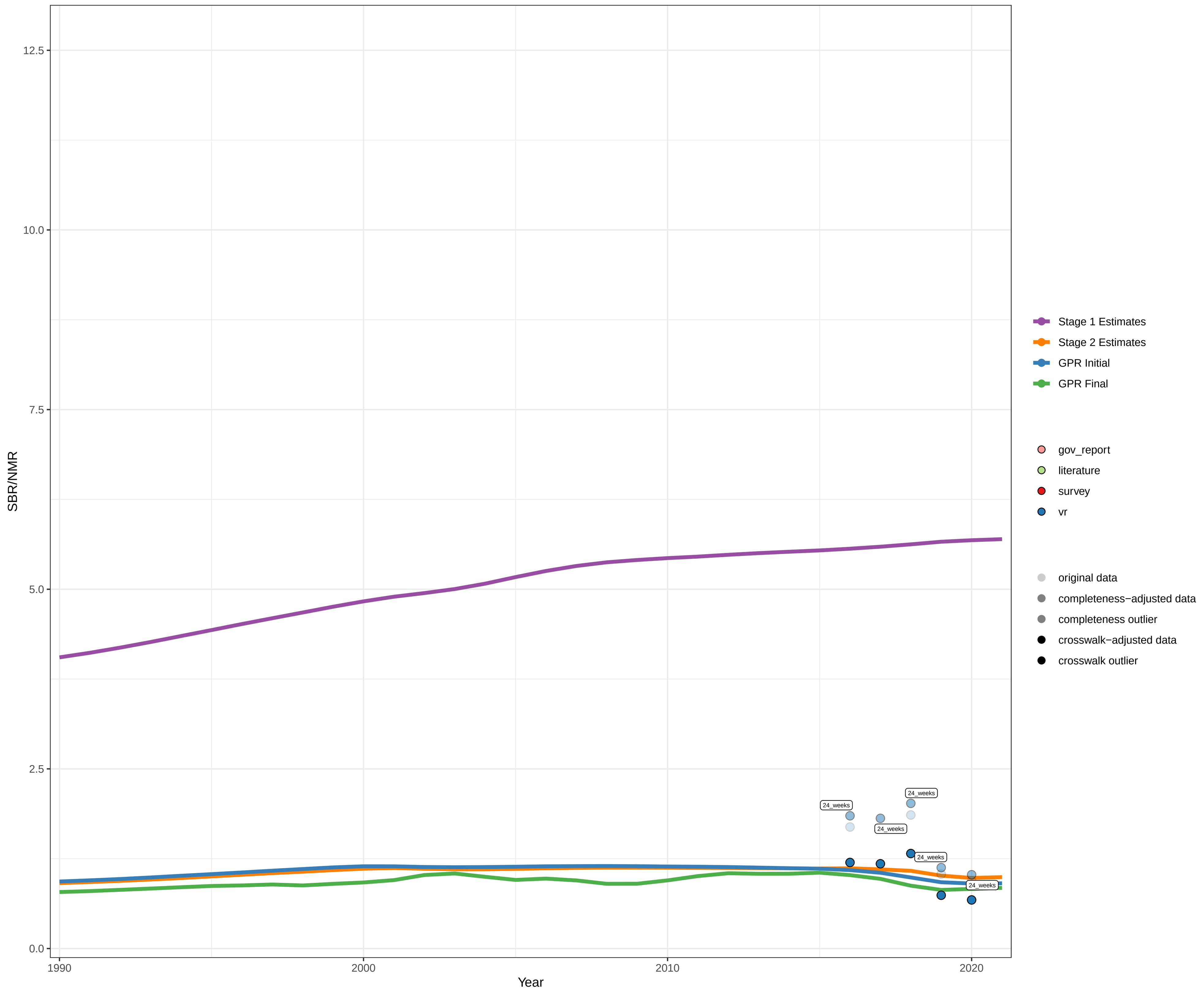

Definition	Years	Outlier	Source.Type	Title
24_weeks	2016−2020	0	VR	United Kingdom − England and Wales Child Mortality Statistics

Kingston upon Thames GBR_44738 Western Europe Stillbirth Rate (SBR) − 20 weeks
lambda = 0.6 ; zeta = 0.8 ; scale = 15 ; amp = 0.028

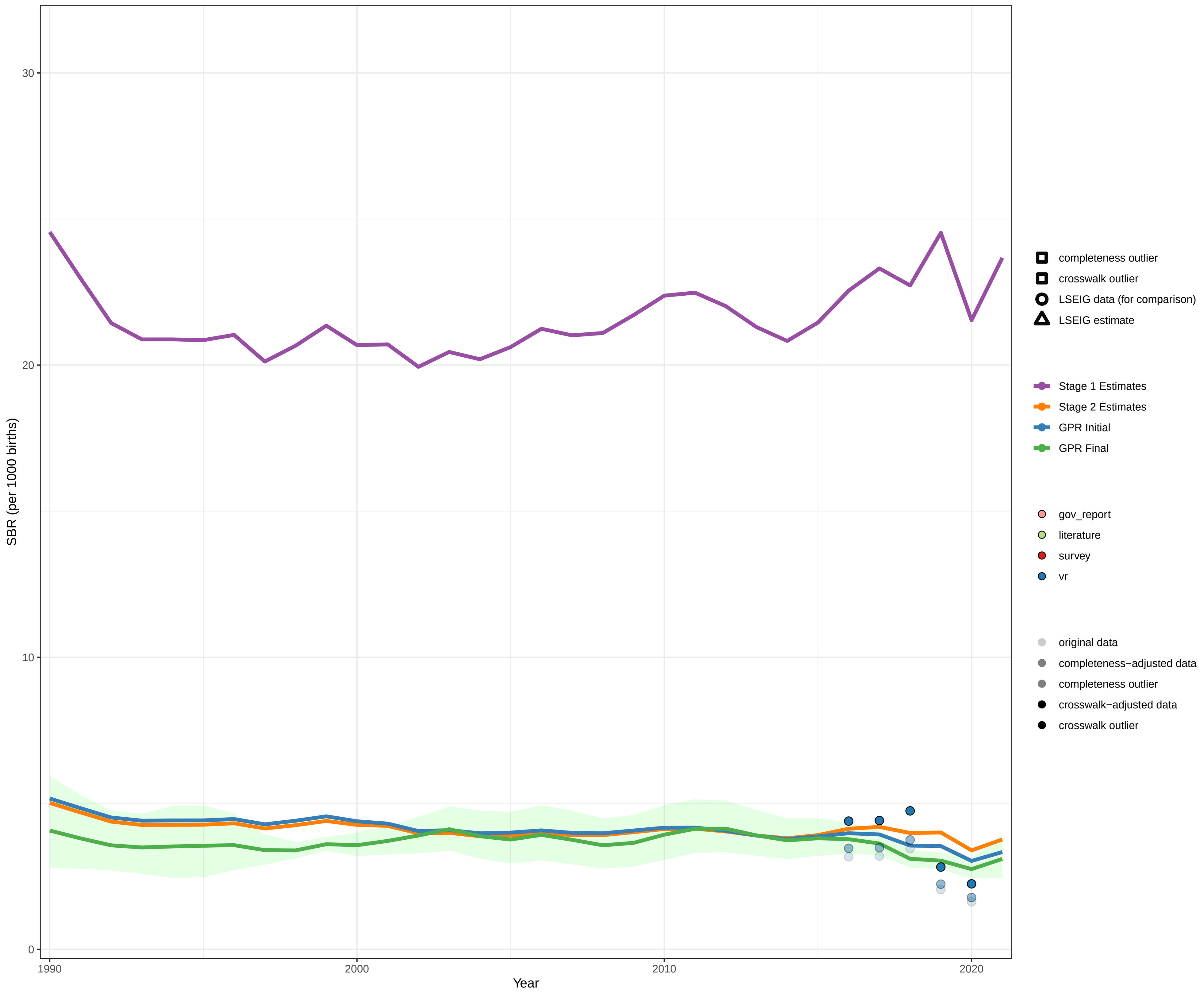

Kingston upon Thames GBR_44738 Western Europe Stillbirth Rate (SBR) − 28 weeks
lambda = 0.6 ; zeta = 0.8 ; scale = 15 ; amp = 0.028

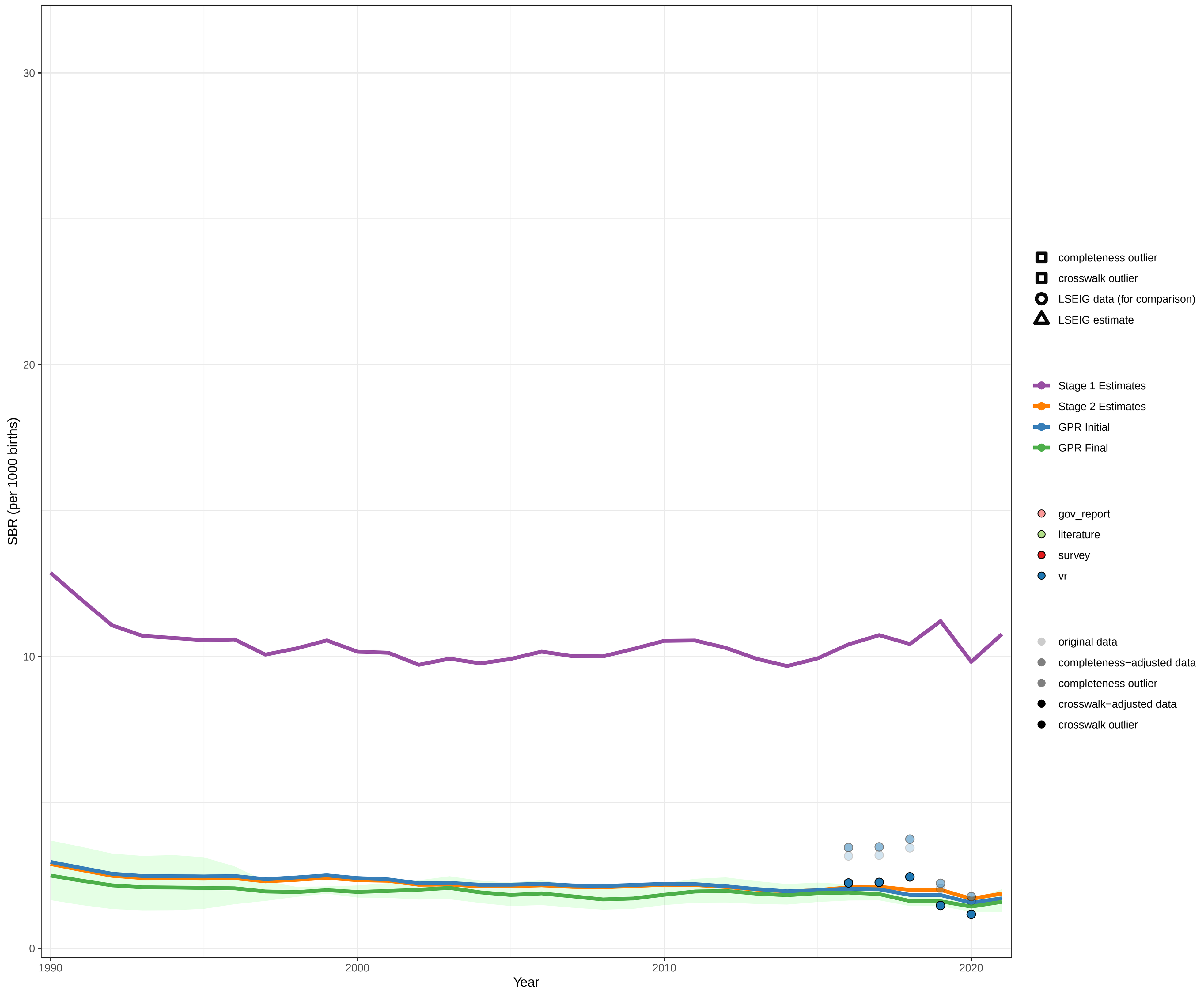

Year	Total Stillbirths (in thousands)	Total Stillbirths (in thousands)	SBR (per 1000 births)	SBR (per 1000 births)	SBR/NMR	SBR/NMR
	20 weeks	28 weeks	20 weeks	28 weeks	20 weeks	28 weeks
1990	0.0 (0.0 to 0.0)	0.0 (0.0 to 0.0)	4.1 (2.8 to 5.9)	2.5 (1.7 to 3.7)	1.28 (0.88 to 1.87)	0.79 (0.52 to 1.16)
1995	0.0 (0.0 to 0.0)	0.0 (0.0 to 0.0)	3.5 (2.5 to 4.9)	2.1 (1.4 to 3.1)	1.49 (1.03 to 2.07)	0.87 (0.57 to 1.31)
2000	0.0 (0.0 to 0.0)	0.0 (0.0 to 0.0)	3.6 (3.2 to 4.0)	1.9 (1.7 to 2.2)	1.69 (1.52 to 1.90)	0.92 (0.83 to 1.03)
2005	0.0 (0.0 to 0.0)	0.0 (0.0 to 0.0)	3.8 (2.9 to 4.7)	1.8 (1.5 to 2.3)	1.96 (1.53 to 2.45)	0.95 (0.76 to 1.18)
2010	0.0 (0.0 to 0.0)	0.0 (0.0 to 0.0)	3.9 (3.1 to 4.9)	1.8 (1.5 to 2.2)	2.02 (1.58 to 2.55)	0.95 (0.77 to 1.15)
2015	0.0 (0.0 to 0.0)	0.0 (0.0 to 0.0)	3.8 (3.2 to 4.5)	1.9 (1.6 to 2.3)	2.12 (1.78 to 2.50)	1.06 (0.89 to 1.26)
2020	0.0 (0.0 to 0.0)	0.0 (0.0 to 0.0)	2.7 (2.4 to 3.1)	1.4 (1.3 to 1.6)	1.59 (1.40 to 1.81)	0.83 (0.73 to 0.95)
2021	0.0 (0.0 to 0.0)	0.0 (0.0 to 0.0)	3.1 (2.4 to 3.9)	1.6 (1.3 to 2.0)	1.64 (1.30 to 2.08)	0.84 (0.66 to 1.08)

Lambeth GBR_44749 Western Europe Stillbirth Rate / Neonatal Mortality Rate (SBR/NMR) − 20 weeks
lambda = 0.6 ; zeta = 0.8 ; scale = 15 ; amp = 0.028

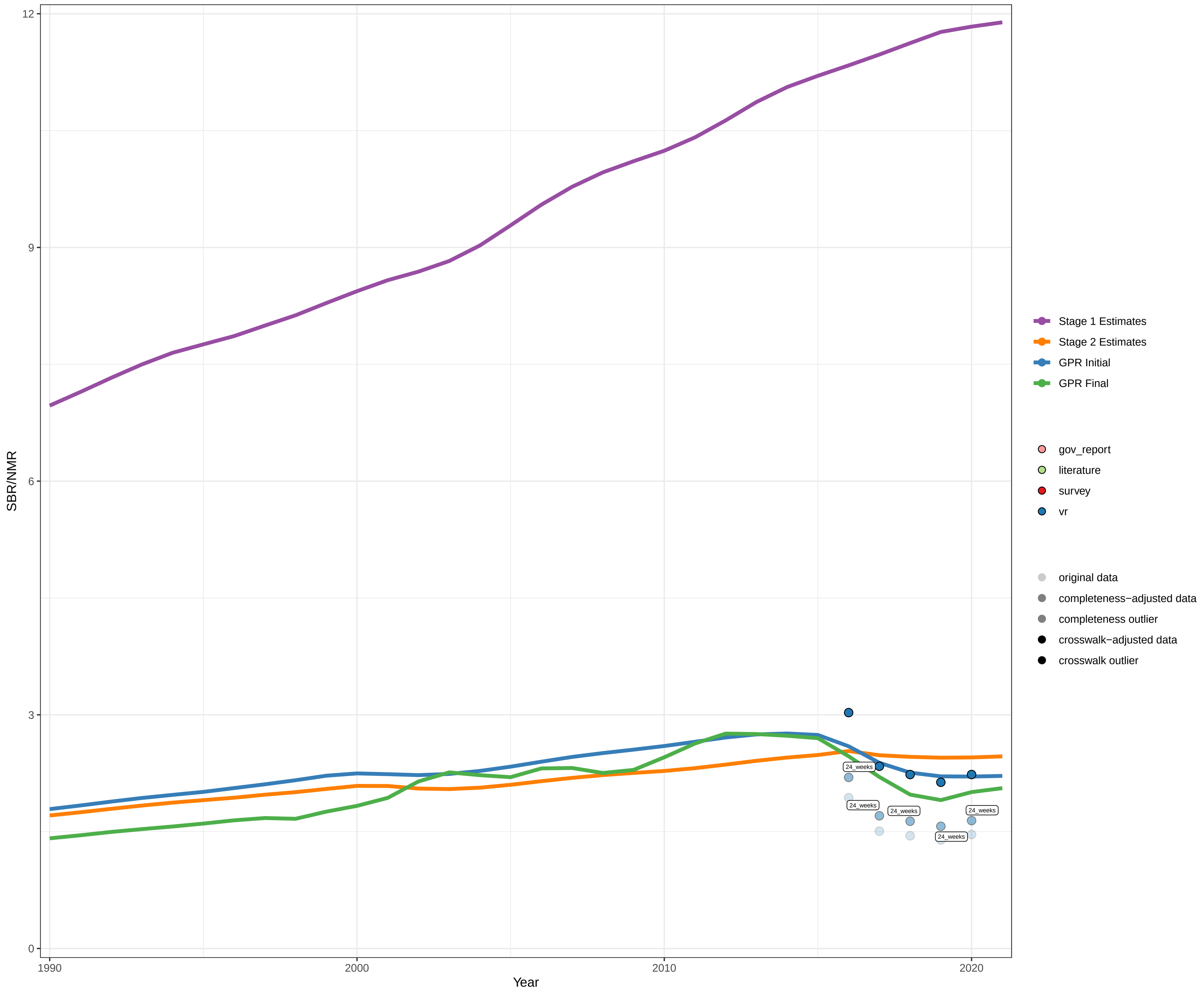

Lambeth GBR_44749 Western Europe Stillbirth Rate / Neonatal Mortality Rate (SBR/NMR) − 28 weeks
lambda = 0.6 ; zeta = 0.8 ; scale = 15 ; amp = 0.028

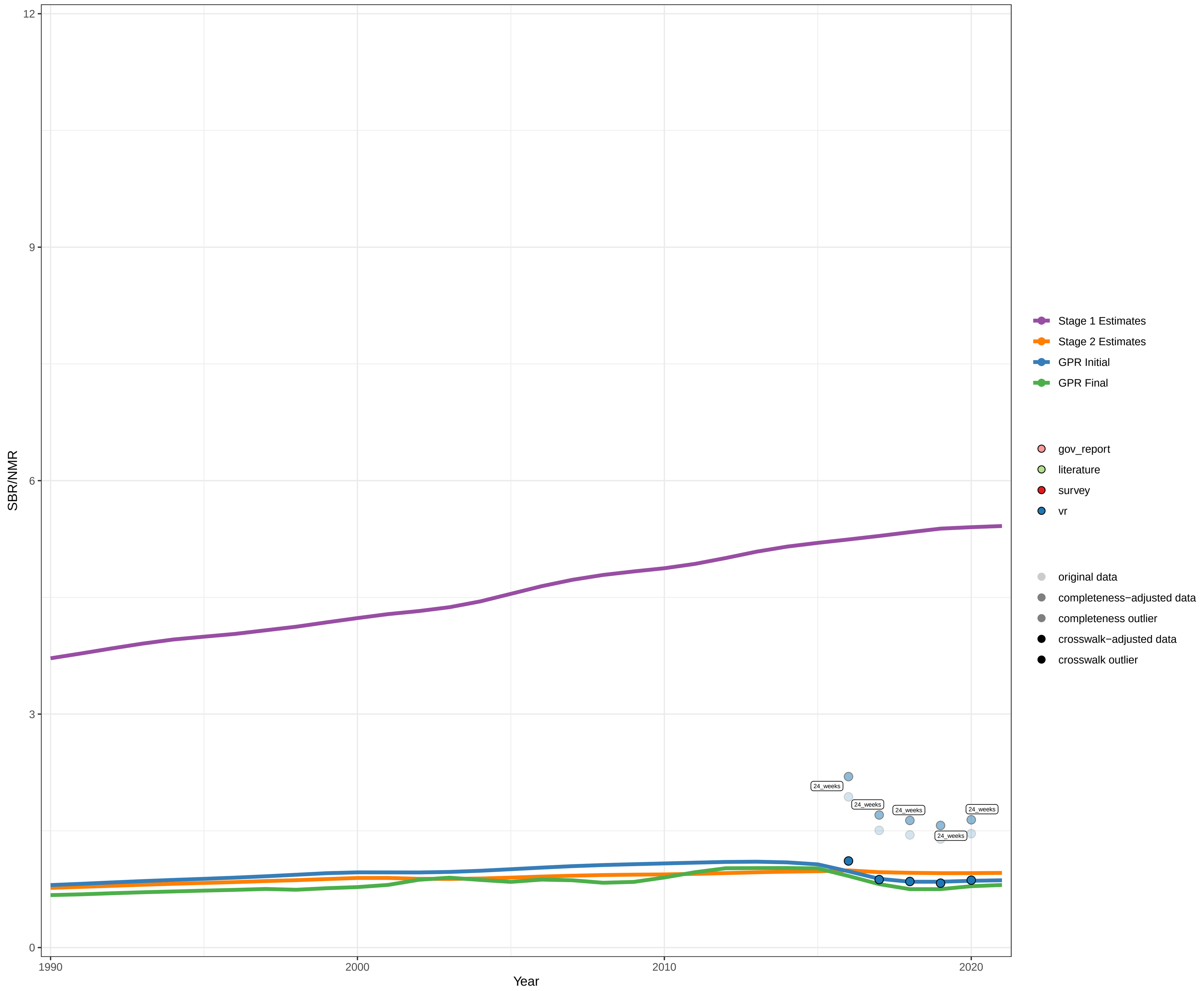

Definition	Years	Outlier	Source.Type	Title
24_weeks	2016−2020	0	VR	United Kingdom − England and Wales Child Mortality Statistics

Lambeth GBR_44749 Western Europe Stillbirth Rate (SBR) − 20 weeks
lambda = 0.6 ; zeta = 0.8 ; scale = 15 ; amp = 0.028

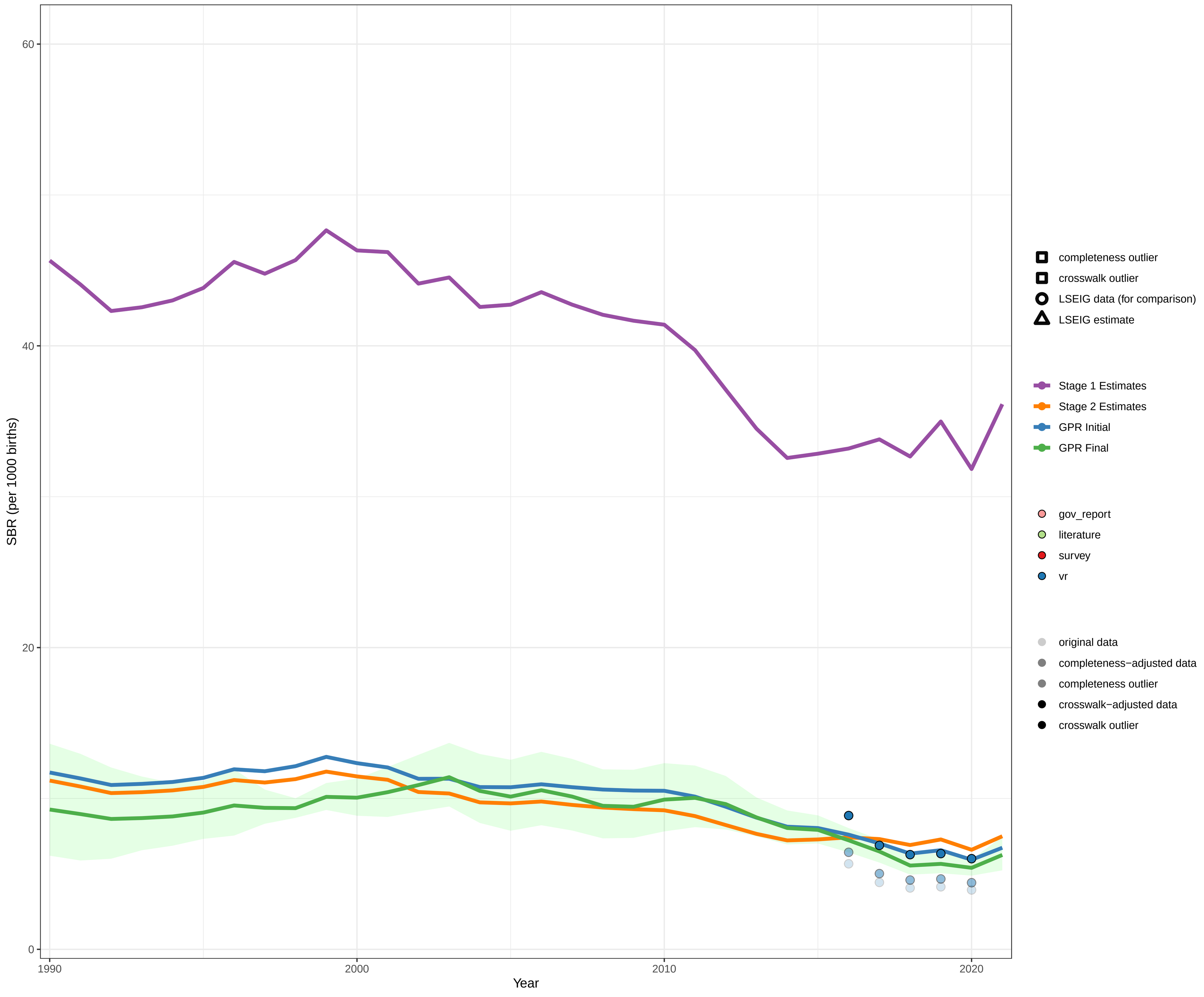

Lambeth GBR_44749 Western Europe Stillbirth Rate (SBR) − 28 weeks
lambda = 0.6 ; zeta = 0.8 ; scale = 15 ; amp = 0.028

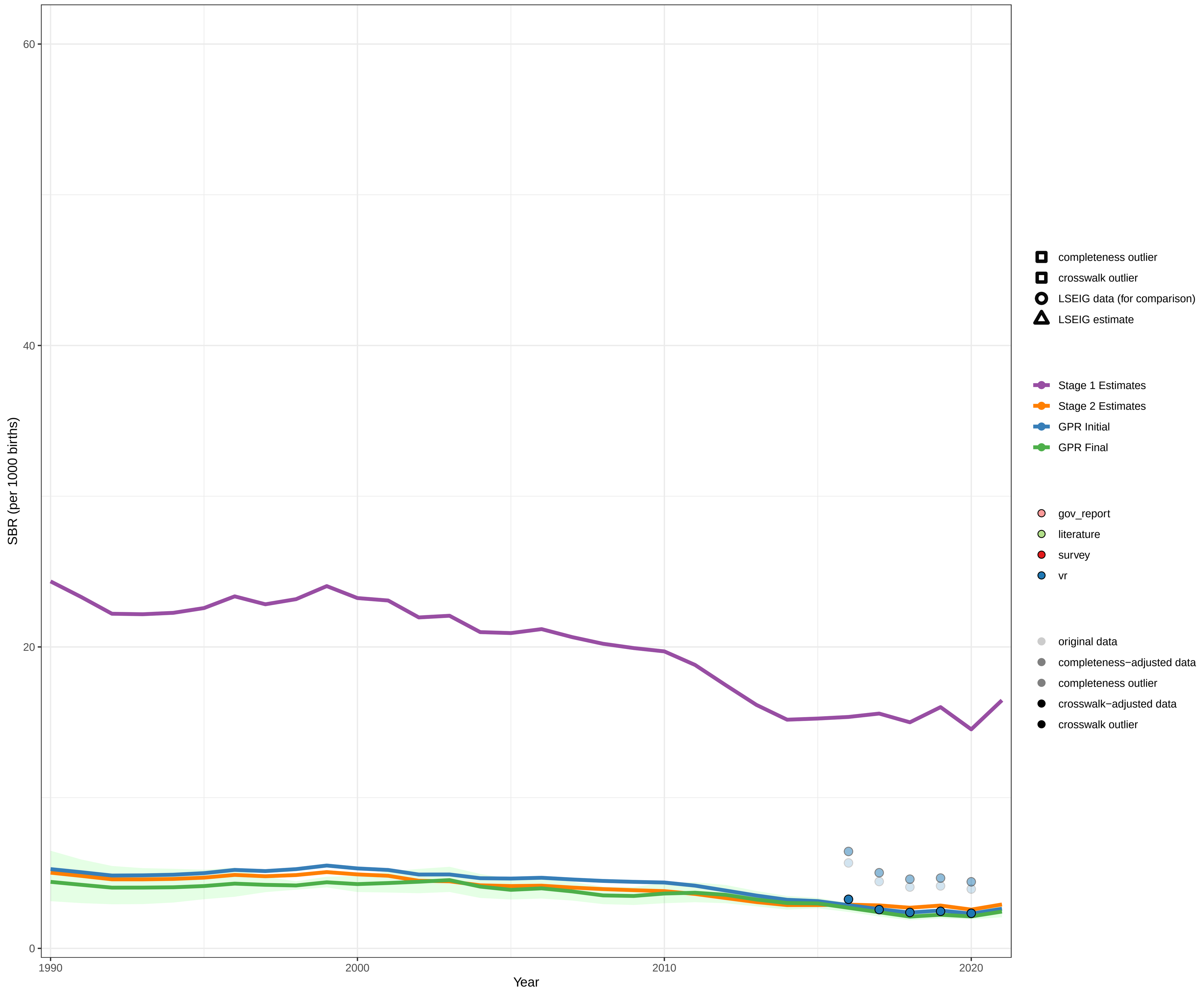

Year	Total Stillbirths (in thousands)	Total Stillbirths (in thousands)	SBR (per 1000 births)	SBR (per 1000 births)	SBR/NMR	SBR/NMR
	20 weeks	28 weeks	20 weeks	28 weeks	20 weeks	28 weeks
1990	0.0 (0.0 to 0.1)	0.0 (0.0 to 0.0)	9.3 (6.2 to 13.6)	4.4 (3.1 to 6.5)	1.41 (0.95 to 2.08)	0.67 (0.48 to 0.99)
1995	0.0 (0.0 to 0.0)	0.0 (0.0 to 0.0)	9.1 (7.3 to 11.2)	4.1 (3.3 to 5.2)	1.60 (1.30 to 1.98)	0.73 (0.58 to 0.92)
2000	0.0 (0.0 to 0.0)	0.0 (0.0 to 0.0)	10.1 (8.9 to 11.3)	4.3 (3.7 to 4.8)	1.83 (1.61 to 2.06)	0.78 (0.68 to 0.88)
2005	0.0 (0.0 to 0.1)	0.0 (0.0 to 0.0)	10.1 (7.9 to 12.6)	3.9 (3.2 to 4.6)	2.20 (1.71 to 2.73)	0.84 (0.70 to 1.00)
2010	0.0 (0.0 to 0.1)	0.0 (0.0 to 0.0)	9.9 (7.8 to 12.3)	3.6 (3.0 to 4.3)	2.45 (1.93 to 3.05)	0.90 (0.74 to 1.07)
2015	0.0 (0.0 to 0.0)	0.0 (0.0 to 0.0)	7.9 (7.0 to 8.9)	3.0 (2.7 to 3.4)	2.70 (2.39 to 3.03)	1.02 (0.91 to 1.14)
2020	0.0 (0.0 to 0.0)	0.0 (0.0 to 0.0)	5.4 (4.9 to 5.9)	2.1 (1.9 to 2.3)	2.01 (1.82 to 2.21)	0.79 (0.72 to 0.86)
2021	0.0 (0.0 to 0.0)	0.0 (0.0 to 0.0)	6.3 (5.2 to 7.5)	2.4 (2.1 to 2.9)	2.06 (1.72 to 2.46)	0.80 (0.68 to 0.96)

Lewisham GBR_44750 Western Europe Stillbirth Rate / Neonatal Mortality Rate (SBR/NMR) − 20 weeks
lambda = 0.6 ; zeta = 0.8 ; scale = 15 ; amp = 0.028

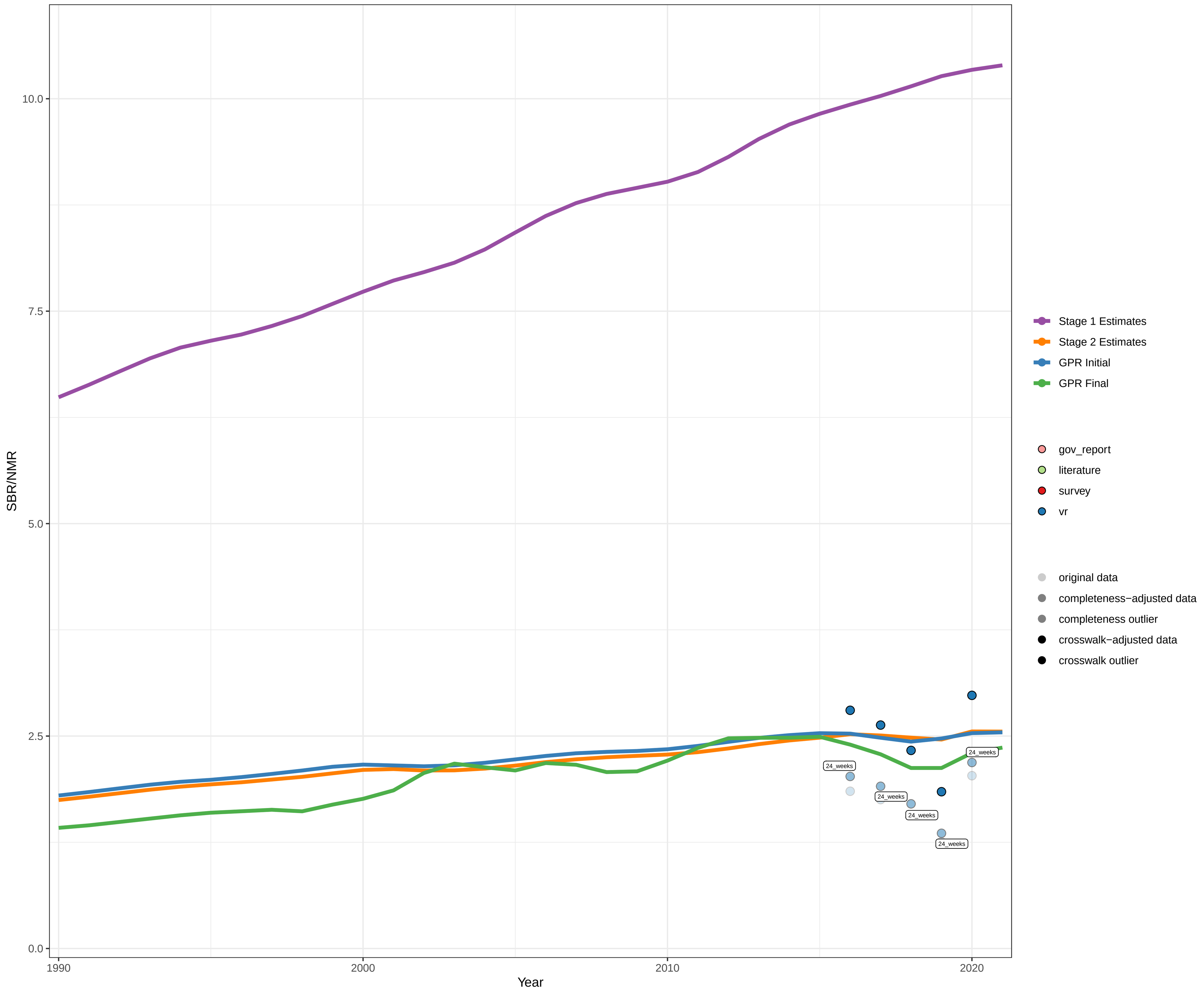

Lewisham GBR_44750 Western Europe Stillbirth Rate / Neonatal Mortality Rate (SBR/NMR) − 28 weeks
lambda = 0.6 ; zeta = 0.8 ; scale = 15 ; amp = 0.028

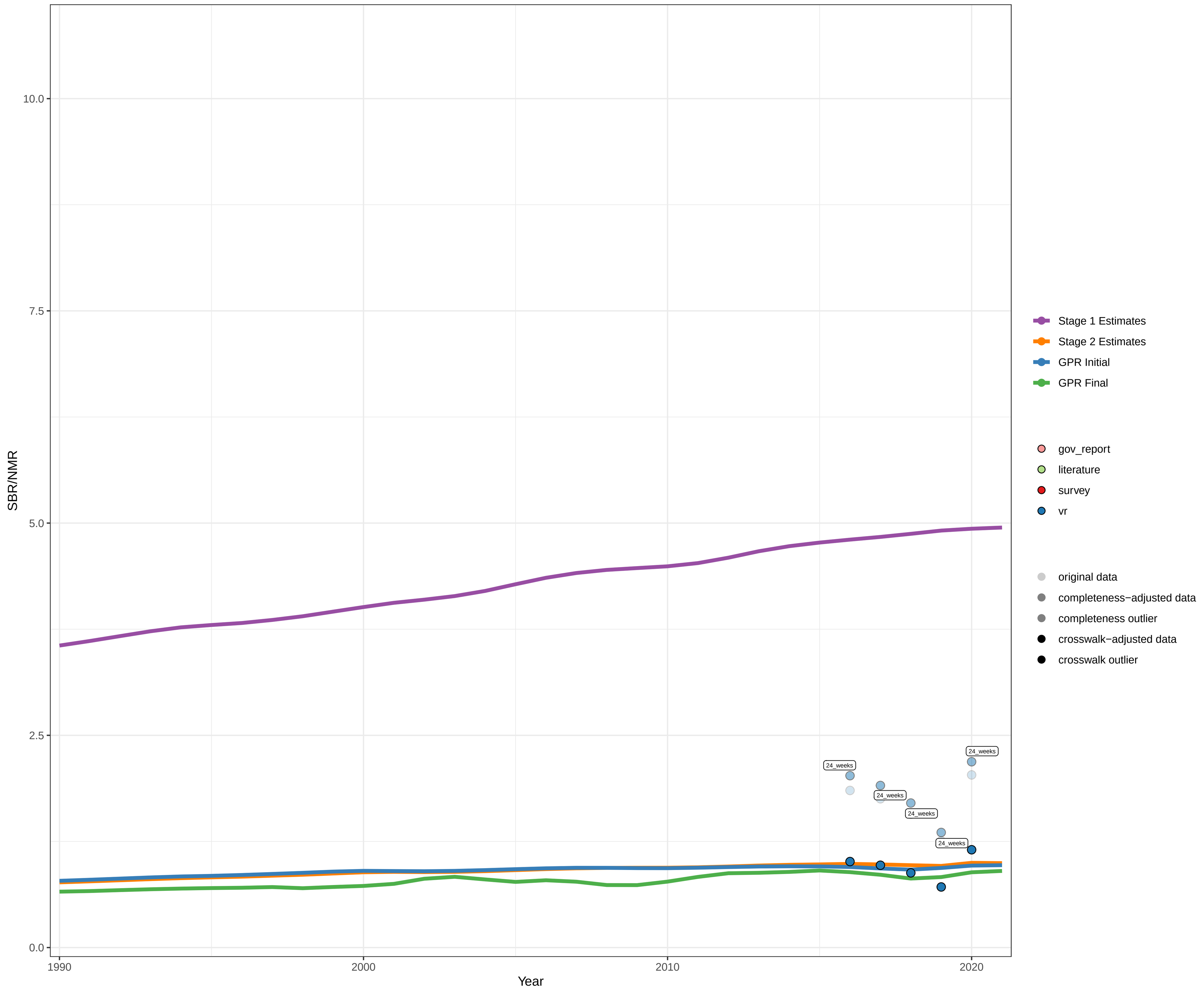

Definition	Years	Outlier	Source.Type	Title
24_weeks	2016−2020	0	VR	United Kingdom − England and Wales Child Mortality Statistics

Lewisham GBR_44750 Western Europe Stillbirth Rate (SBR) − 20 weeks
lambda = 0.6 ; zeta = 0.8 ; scale = 15 ; amp = 0.028

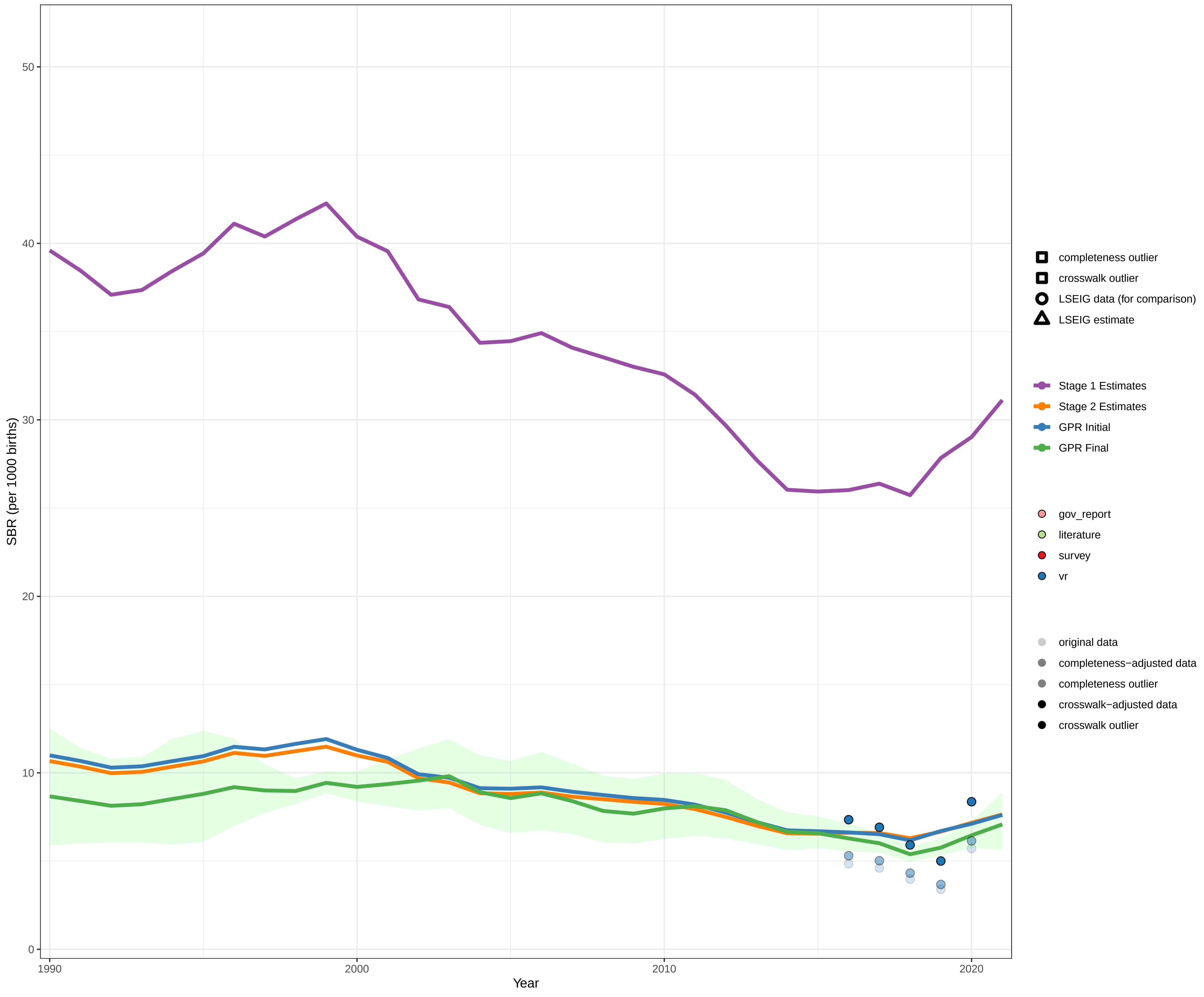

Lewisham GBR_44750 Western Europe Stillbirth Rate (SBR) − 28 weeks
lambda = 0.6 ; zeta = 0.8 ; scale = 15 ; amp = 0.028

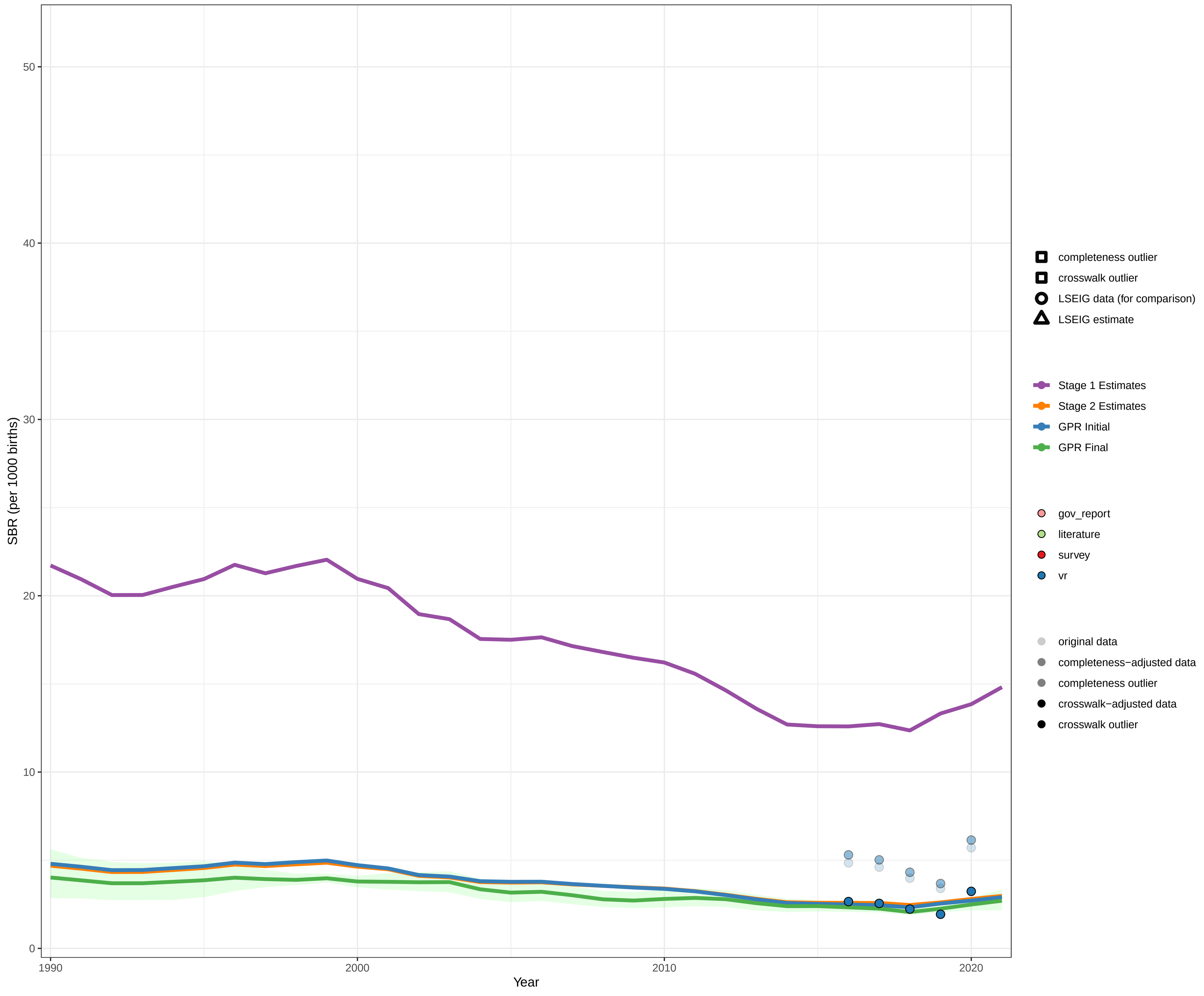

Year	Total Stillbirths (in thousands)	Total Stillbirths (in thousands)	SBR (per 1000 births)	SBR (per 1000 births)	SBR/NMR	SBR/NMR
	20 weeks	28 weeks	20 weeks	28 weeks	20 weeks	28 weeks
1990	0.0 (0.0 to 0.0)	0.0 (0.0 to 0.0)	8.7 (5.9 to 12.5)	4.0 (2.9 to 5.6)	1.42 (0.96 to 2.05)	0.66 (0.47 to 0.92)
1995	0.0 (0.0 to 0.0)	0.0 (0.0 to 0.0)	8.8 (6.1 to 12.4)	3.9 (2.9 to 5.0)	1.60 (1.10 to 2.25)	0.70 (0.53 to 0.90)
2000	0.0 (0.0 to 0.0)	0.0 (0.0 to 0.0)	9.2 (8.4 to 10.1)	3.8 (3.5 to 4.1)	1.76 (1.60 to 1.93)	0.73 (0.66 to 0.79)
2005	0.0 (0.0 to 0.0)	0.0 (0.0 to 0.0)	8.6 (6.6 to 10.7)	3.2 (2.6 to 3.7)	2.10 (1.61 to 2.61)	0.77 (0.64 to 0.91)
2010	0.0 (0.0 to 0.0)	0.0 (0.0 to 0.0)	8.0 (6.3 to 10.0)	2.8 (2.3 to 3.3)	2.21 (1.73 to 2.76)	0.78 (0.64 to 0.92)
2015	0.0 (0.0 to 0.0)	0.0 (0.0 to 0.0)	6.6 (5.7 to 7.5)	2.4 (2.1 to 2.7)	2.49 (2.17 to 2.85)	0.91 (0.79 to 1.04)
2020	0.0 (0.0 to 0.0)	0.0 (0.0 to 0.0)	6.5 (5.8 to 7.3)	2.5 (2.2 to 2.9)	2.30 (2.05 to 2.59)	0.89 (0.77 to 1.02)
2021	0.0 (0.0 to 0.0)	0.0 (0.0 to 0.0)	7.1 (5.6 to 8.9)	2.7 (2.2 to 3.3)	2.36 (1.88 to 2.97)	0.90 (0.72 to 1.12)

Merton GBR_44734 Western Europe Stillbirth Rate / Neonatal Mortality Rate (SBR/NMR) − 20 weeks
lambda = 0.6 ; zeta = 0.8 ; scale = 15 ; amp = 0.028

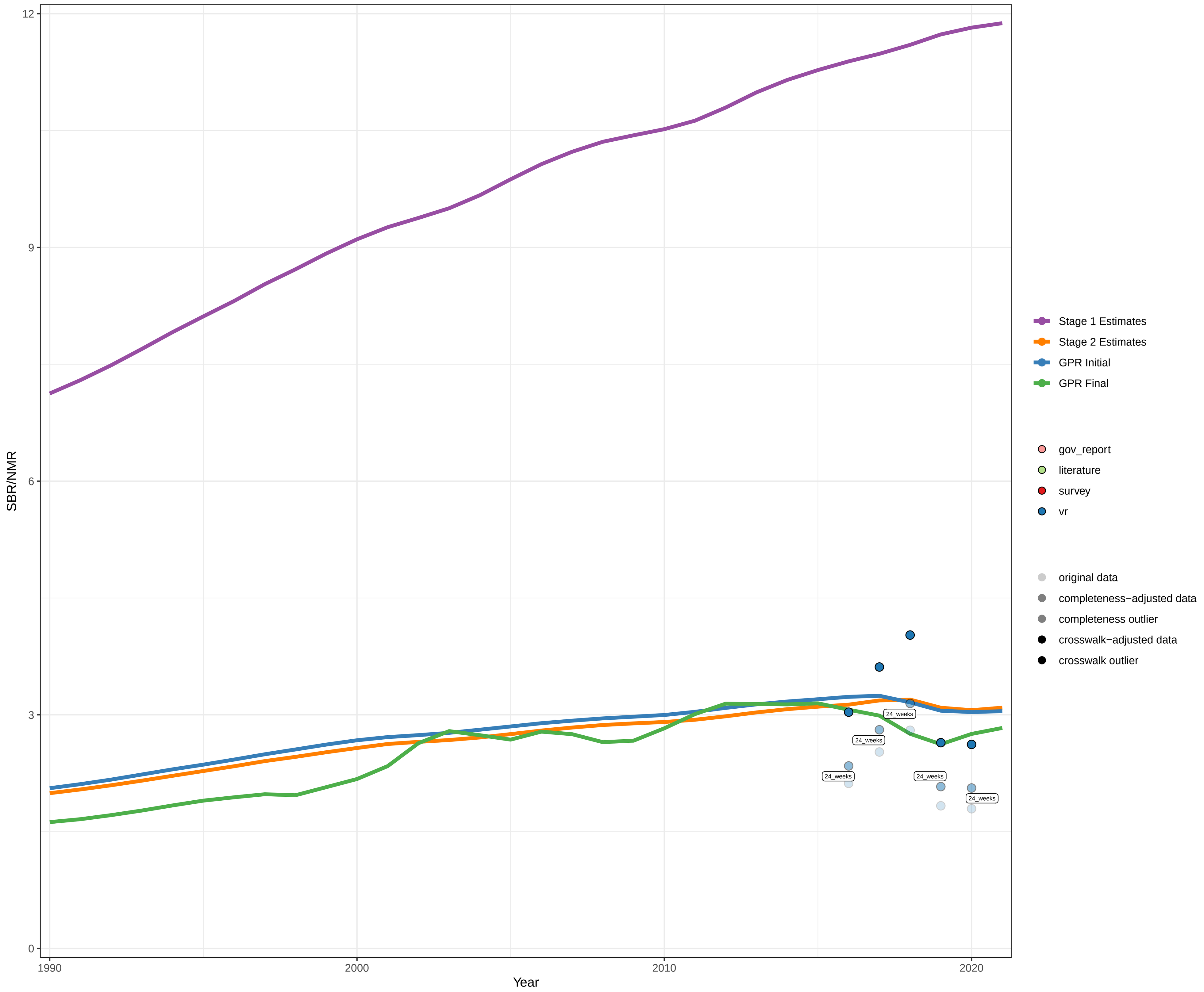

Merton GBR_44734 Western Europe Stillbirth Rate / Neonatal Mortality Rate (SBR/NMR) − 28 weeks
lambda = 0.6 ; zeta = 0.8 ; scale = 15 ; amp = 0.028

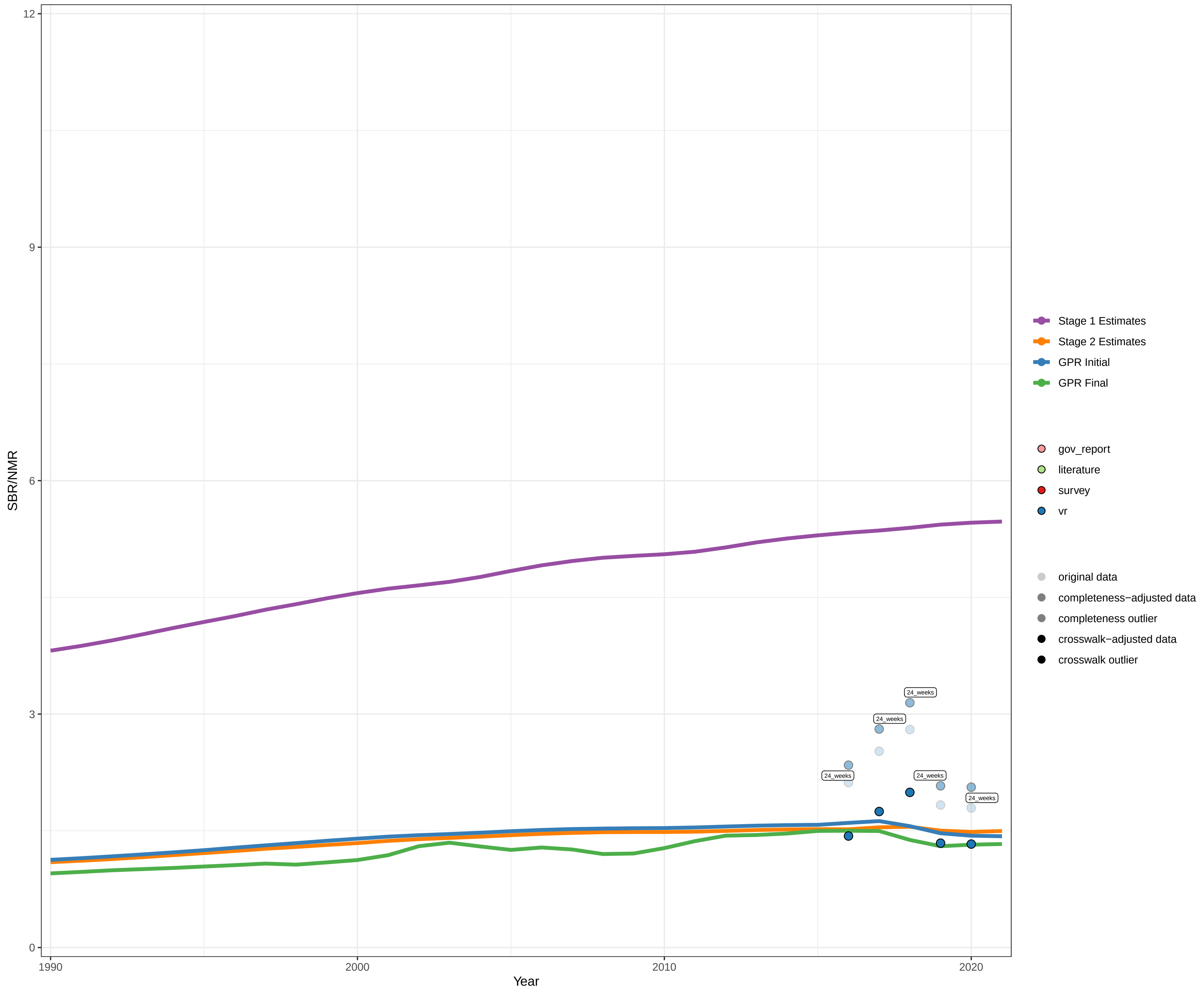

Definition	Years	Outlier	Source.Type	Title
24_weeks	2016−2020	0	VR	United Kingdom − England and Wales Child Mortality Statistics

Merton GBR_44734 Western Europe Stillbirth Rate (SBR) − 20 weeks
lambda = 0.6 ; zeta = 0.8 ; scale = 15 ; amp = 0.028

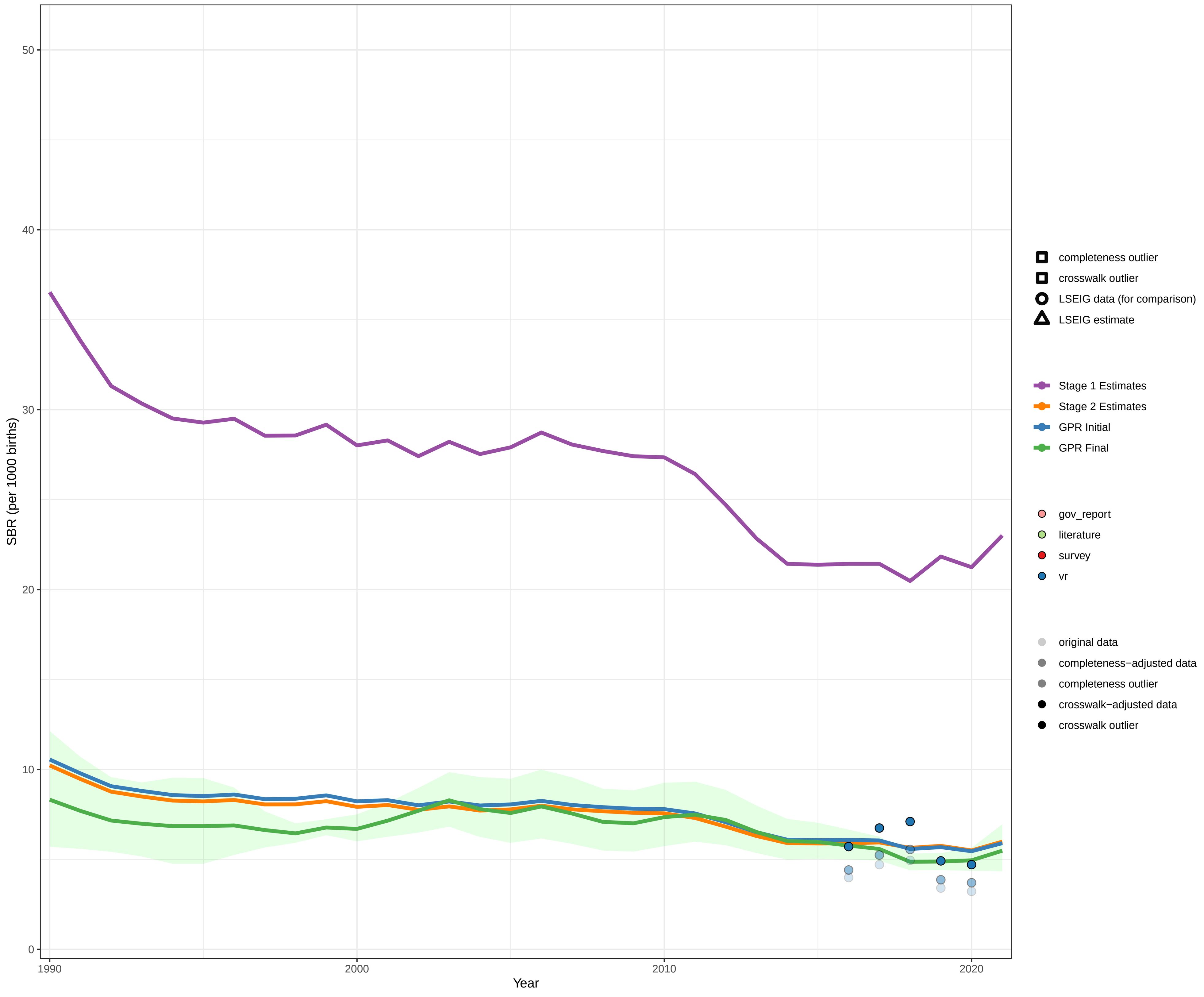

Merton GBR_44734 Western Europe Stillbirth Rate (SBR) − 28 weeks
lambda = 0.6 ; zeta = 0.8 ; scale = 15 ; amp = 0.028

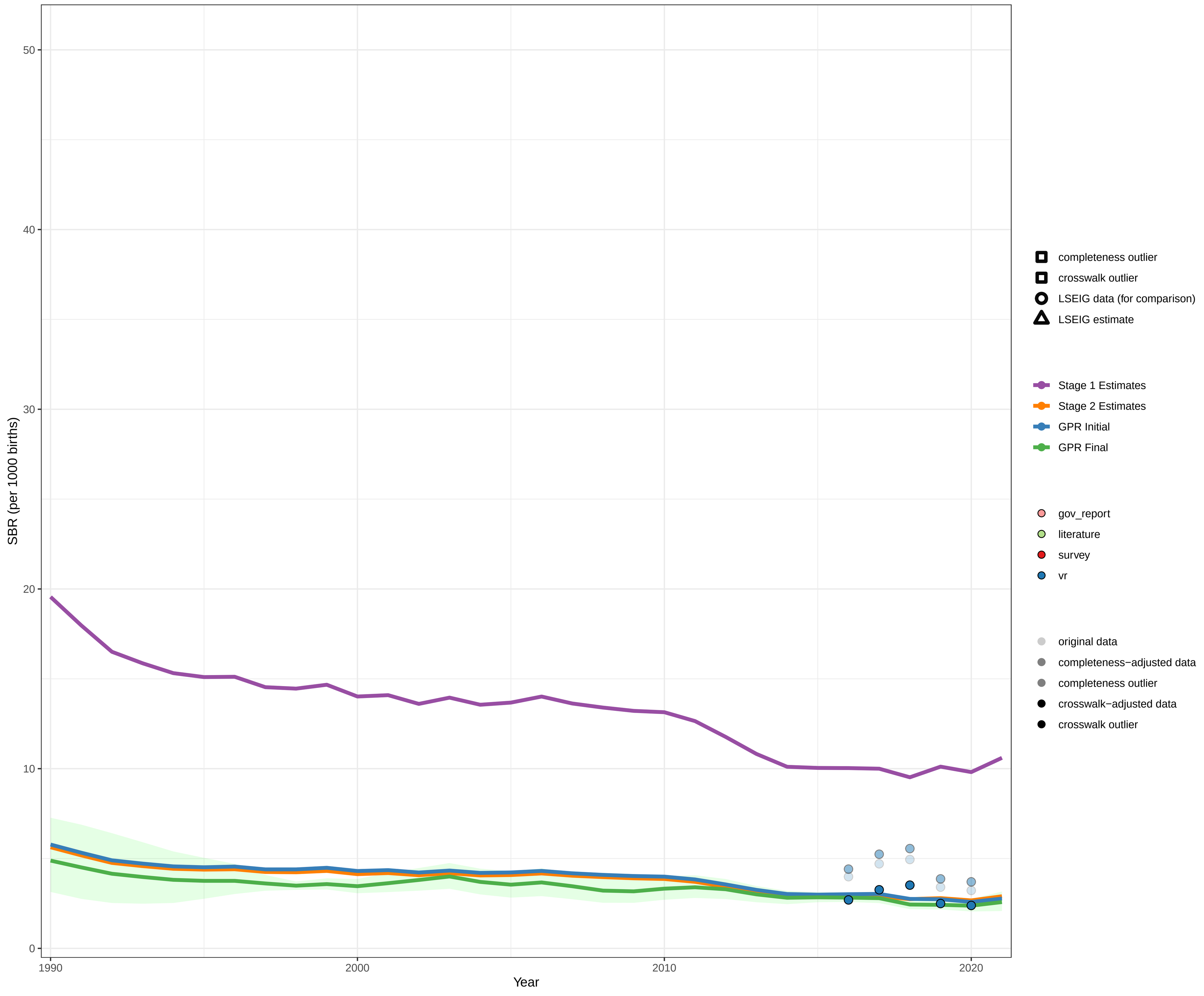

Year	Total Stillbirths (in thousands)	Total Stillbirths (in thousands)	SBR (per 1000 births)	SBR (per 1000 births)	SBR/NMR	SBR/NMR
	20 weeks	28 weeks	20 weeks	28 weeks	20 weeks	28 weeks
1990	0.0 (0.0 to 0.0)	0.0 (0.0 to 0.0)	8.3 (5.7 to 12.1)	4.9 (3.1 to 7.3)	1.62 (1.11 to 2.37)	0.95 (0.61 to 1.42)
1995	0.0 (0.0 to 0.0)	0.0 (0.0 to 0.0)	6.8 (4.8 to 9.5)	3.8 (2.8 to 5.0)	1.90 (1.32 to 2.64)	1.04 (0.77 to 1.40)
2000	0.0 (0.0 to 0.0)	0.0 (0.0 to 0.0)	6.7 (6.0 to 7.5)	3.5 (3.1 to 3.9)	2.18 (1.95 to 2.44)	1.12 (1.00 to 1.26)
2005	0.0 (0.0 to 0.0)	0.0 (0.0 to 0.0)	7.6 (5.9 to 9.5)	3.5 (2.8 to 4.3)	2.68 (2.09 to 3.36)	1.25 (1.00 to 1.52)
2010	0.0 (0.0 to 0.0)	0.0 (0.0 to 0.0)	7.3 (5.7 to 9.3)	3.3 (2.7 to 4.0)	2.83 (2.21 to 3.56)	1.28 (1.04 to 1.55)
2015	0.0 (0.0 to 0.0)	0.0 (0.0 to 0.0)	6.0 (5.0 to 7.0)	2.8 (2.6 to 3.1)	3.15 (2.65 to 3.71)	1.50 (1.35 to 1.65)
2020	0.0 (0.0 to 0.0)	0.0 (0.0 to 0.0)	4.9 (4.4 to 5.6)	2.4 (2.1 to 2.7)	2.75 (2.43 to 3.14)	1.32 (1.14 to 1.53)
2021	0.0 (0.0 to 0.0)	0.0 (0.0 to 0.0)	5.5 (4.3 to 7.0)	2.6 (2.1 to 3.2)	2.83 (2.24 to 3.59)	1.33 (1.07 to 1.63)

Newham GBR_44753 Western Europe Stillbirth Rate / Neonatal Mortality Rate (SBR/NMR) − 20 weeks
lambda = 0.6 ; zeta = 0.8 ; scale = 15 ; amp = 0.028

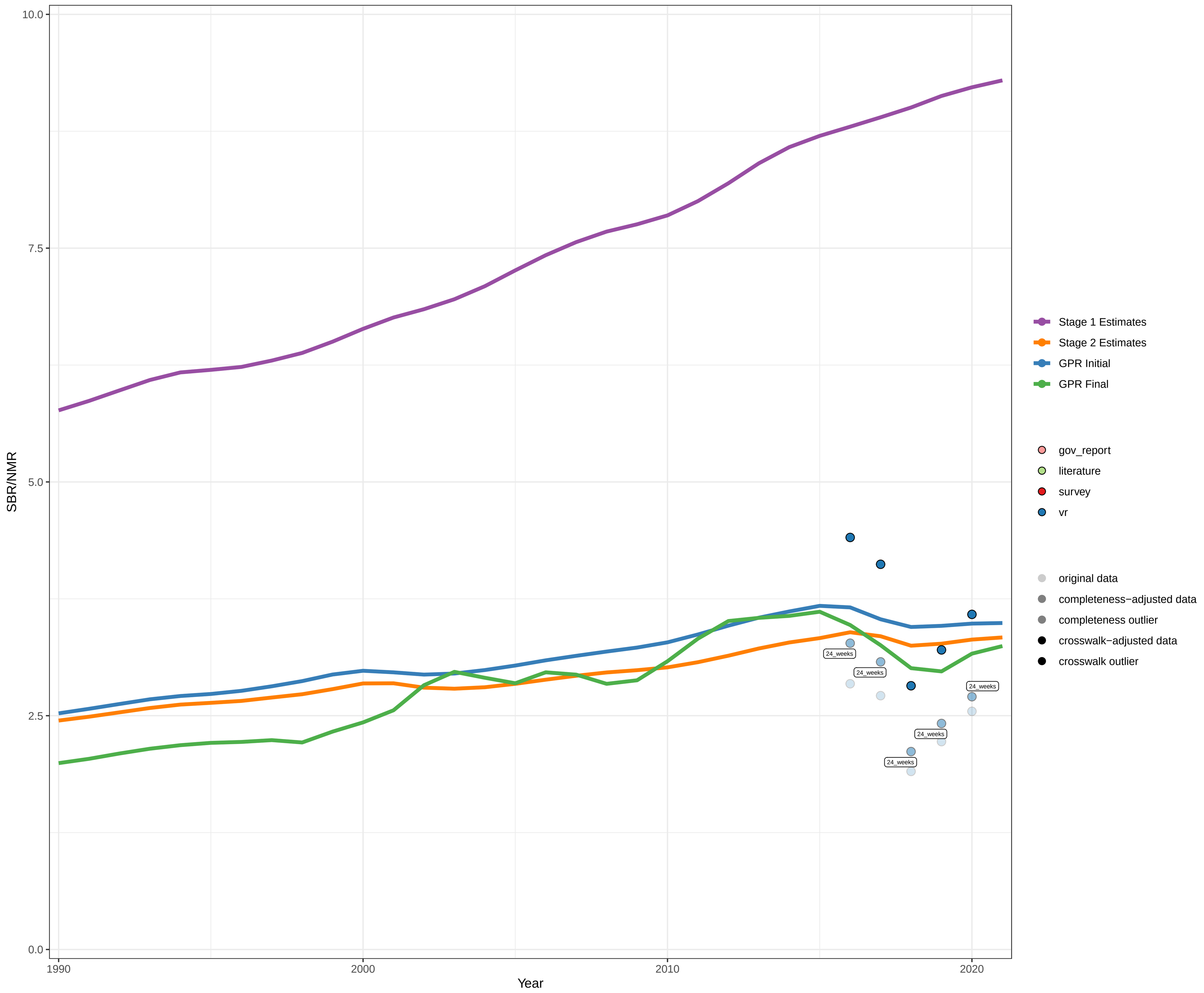

Newham GBR_44753 Western Europe Stillbirth Rate / Neonatal Mortality Rate (SBR/NMR) − 28 weeks
lambda = 0.6 ; zeta = 0.8 ; scale = 15 ; amp = 0.028

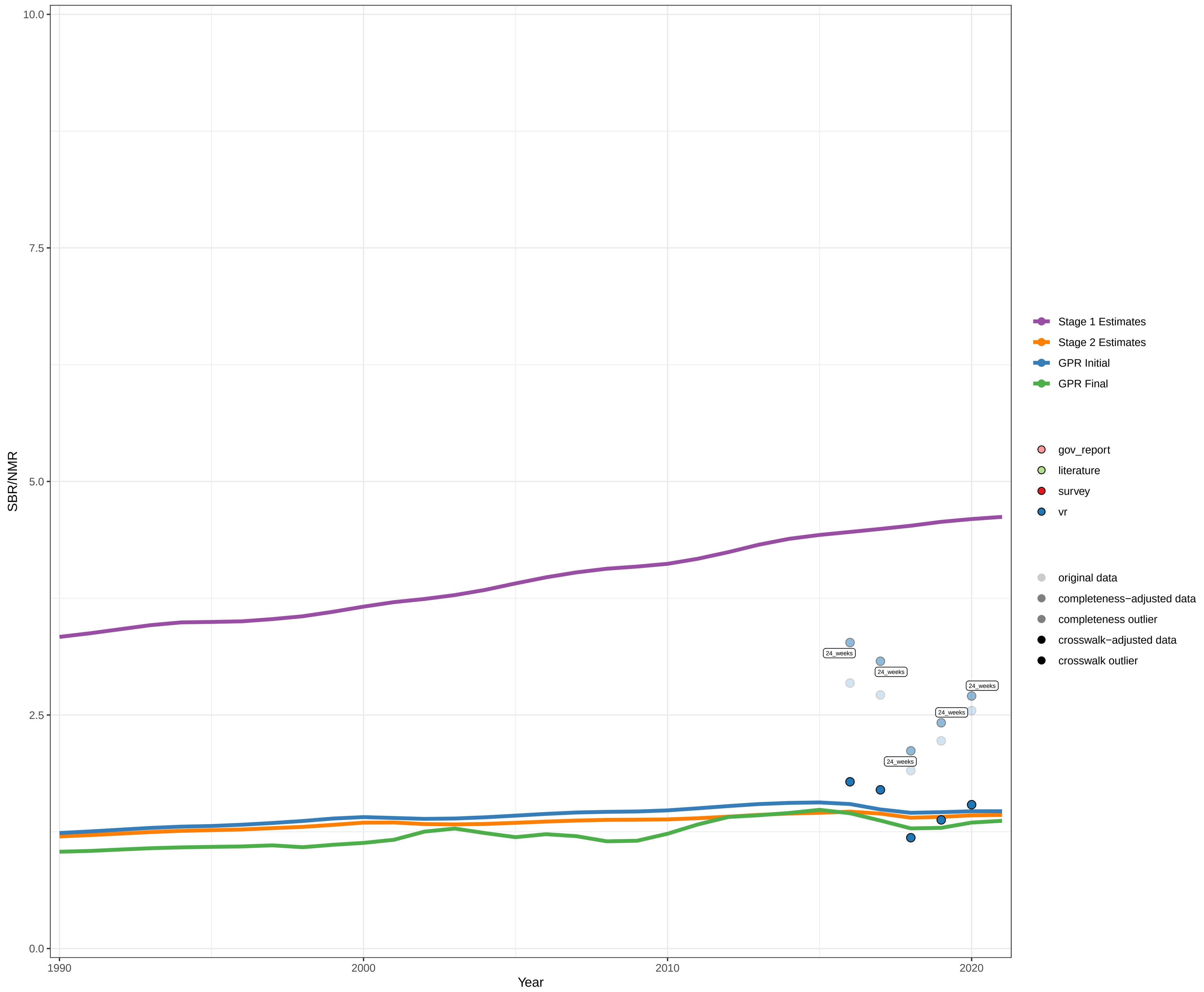

Definition	Years	Outlier	Source.Type	Title
24_weeks	2016−2020	0	VR	United Kingdom − England and Wales Child Mortality Statistics

Newham GBR_44753 Western Europe Stillbirth Rate (SBR) − 20 weeks
lambda = 0.6 ; zeta = 0.8 ; scale = 15 ; amp = 0.028

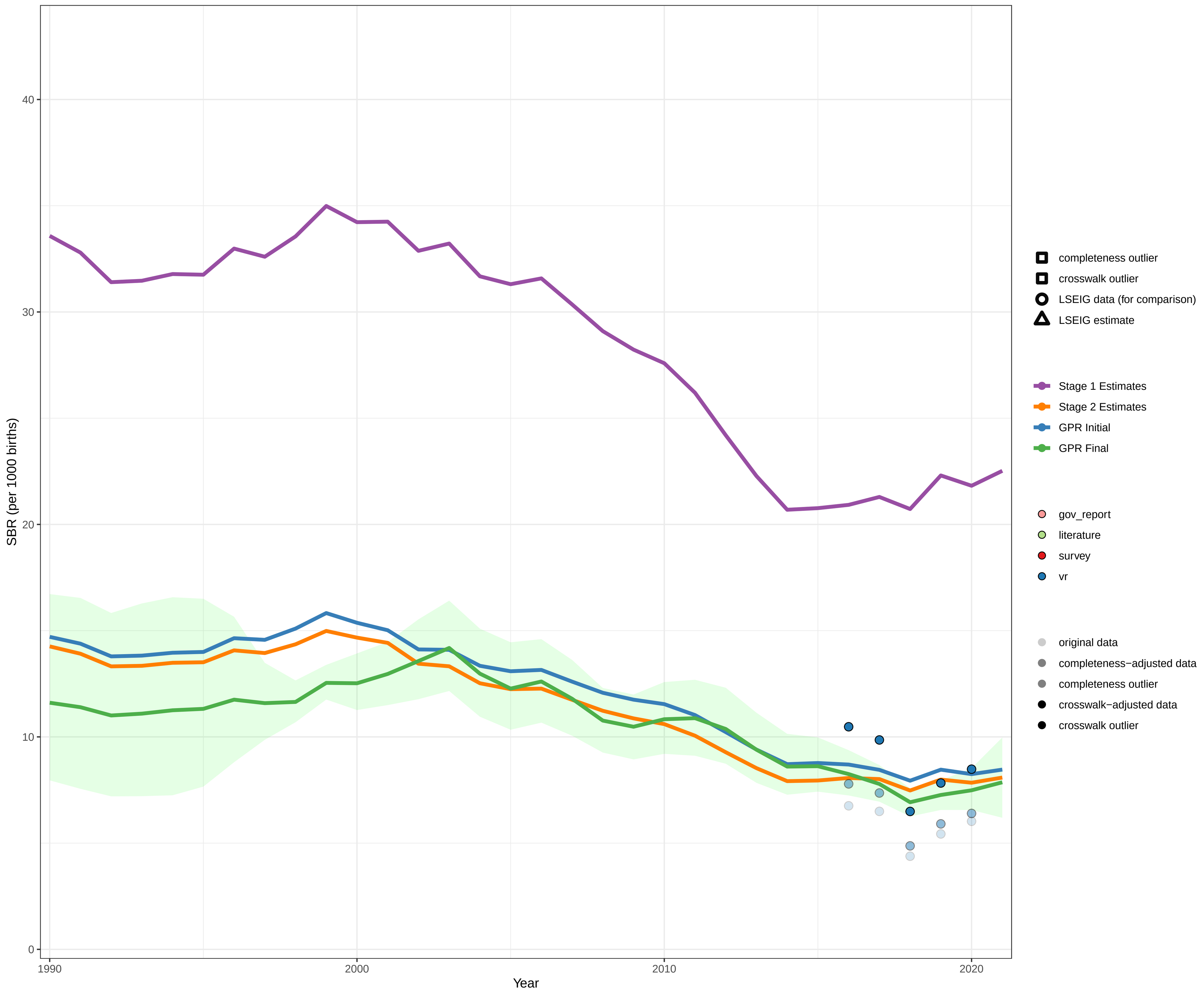

Newham GBR_44753 Western Europe Stillbirth Rate (SBR) − 28 weeks
lambda = 0.6 ; zeta = 0.8 ; scale = 15 ; amp = 0.028

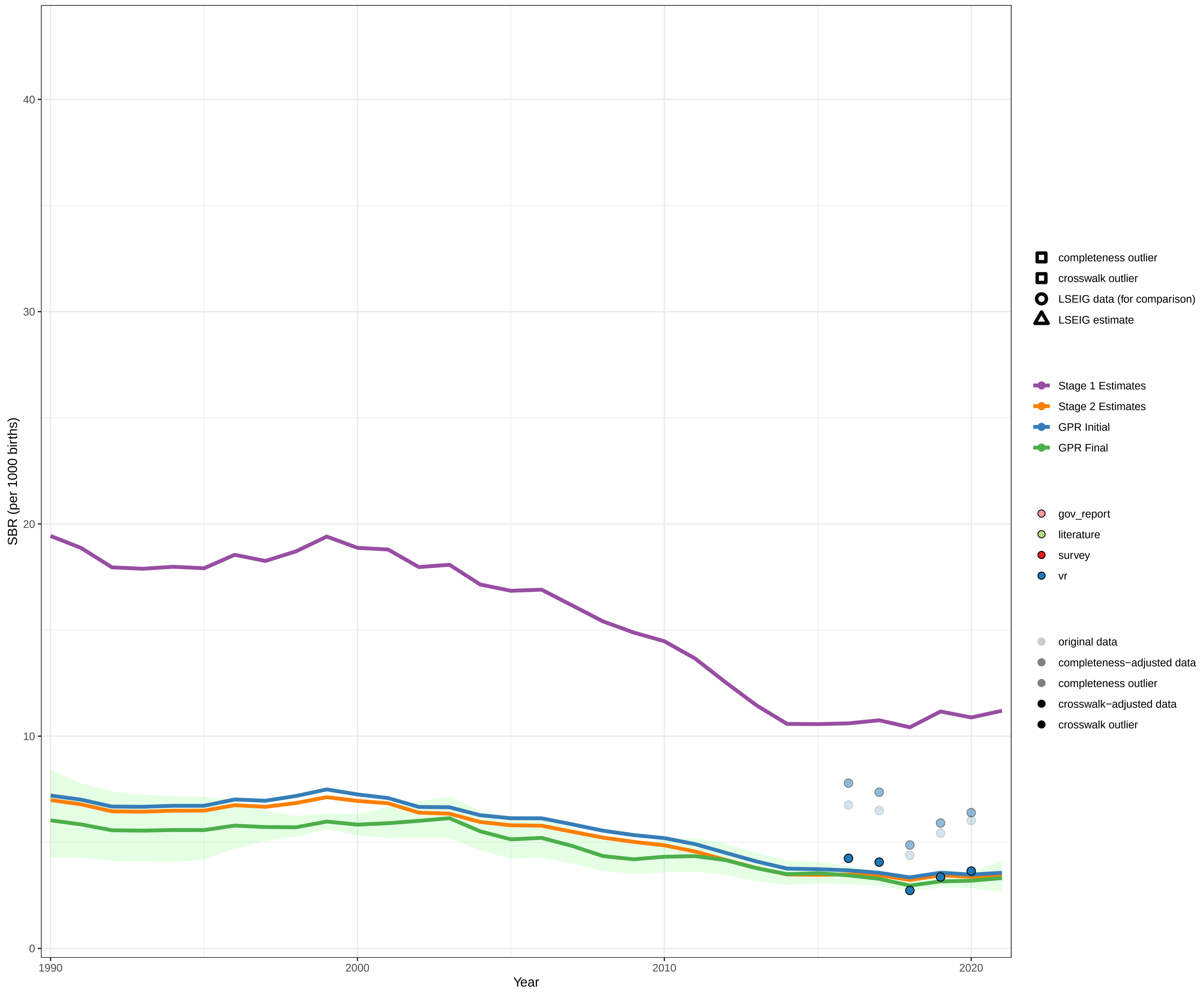

Year	Total Stillbirths (in thousands)	Total Stillbirths (in thousands)	SBR (per 1000 births)	SBR (per 1000 births)	SBR/NMR	SBR/NMR
	20 weeks	28 weeks	20 weeks	28 weeks	20 weeks	28 weeks
1990	0.1 (0.0 to 0.1)	0.0 (0.0 to 0.0)	11.6 (8.0 to 16.7)	6.0 (4.3 to 8.4)	1.99 (1.37 to 2.87)	1.04 (0.74 to 1.45)
1995	0.1 (0.0 to 0.1)	0.0 (0.0 to 0.0)	11.3 (7.7 to 16.5)	5.6 (4.2 to 7.1)	2.21 (1.50 to 3.22)	1.09 (0.82 to 1.40)
2000	0.1 (0.1 to 0.1)	0.0 (0.0 to 0.0)	12.5 (11.3 to 13.9)	5.8 (5.3 to 6.3)	2.43 (2.18 to 2.70)	1.13 (1.03 to 1.23)
2005	0.1 (0.1 to 0.1)	0.0 (0.0 to 0.0)	12.3 (10.3 to 14.5)	5.1 (4.2 to 6.1)	2.85 (2.40 to 3.35)	1.19 (0.98 to 1.41)
2010	0.1 (0.1 to 0.1)	0.0 (0.0 to 0.0)	10.8 (9.2 to 12.6)	4.3 (3.6 to 5.1)	3.08 (2.62 to 3.58)	1.23 (1.02 to 1.46)
2015	0.1 (0.0 to 0.1)	0.0 (0.0 to 0.0)	8.6 (7.4 to 10.0)	3.5 (3.1 to 4.1)	3.61 (3.11 to 4.18)	1.48 (1.28 to 1.71)
2020	0.0 (0.0 to 0.0)	0.0 (0.0 to 0.0)	7.5 (6.6 to 8.5)	3.2 (2.8 to 3.6)	3.16 (2.77 to 3.60)	1.35 (1.20 to 1.52)
2021	0.0 (0.0 to 0.1)	0.0 (0.0 to 0.0)	7.9 (6.2 to 10.0)	3.3 (2.6 to 4.1)	3.24 (2.56 to 4.12)	1.37 (1.09 to 1.71)

Redbridge GBR_44733 Western Europe Stillbirth Rate / Neonatal Mortality Rate (SBR/NMR) − 20 weeks
lambda = 0.6 ; zeta = 0.8 ; scale = 15 ; amp = 0.028

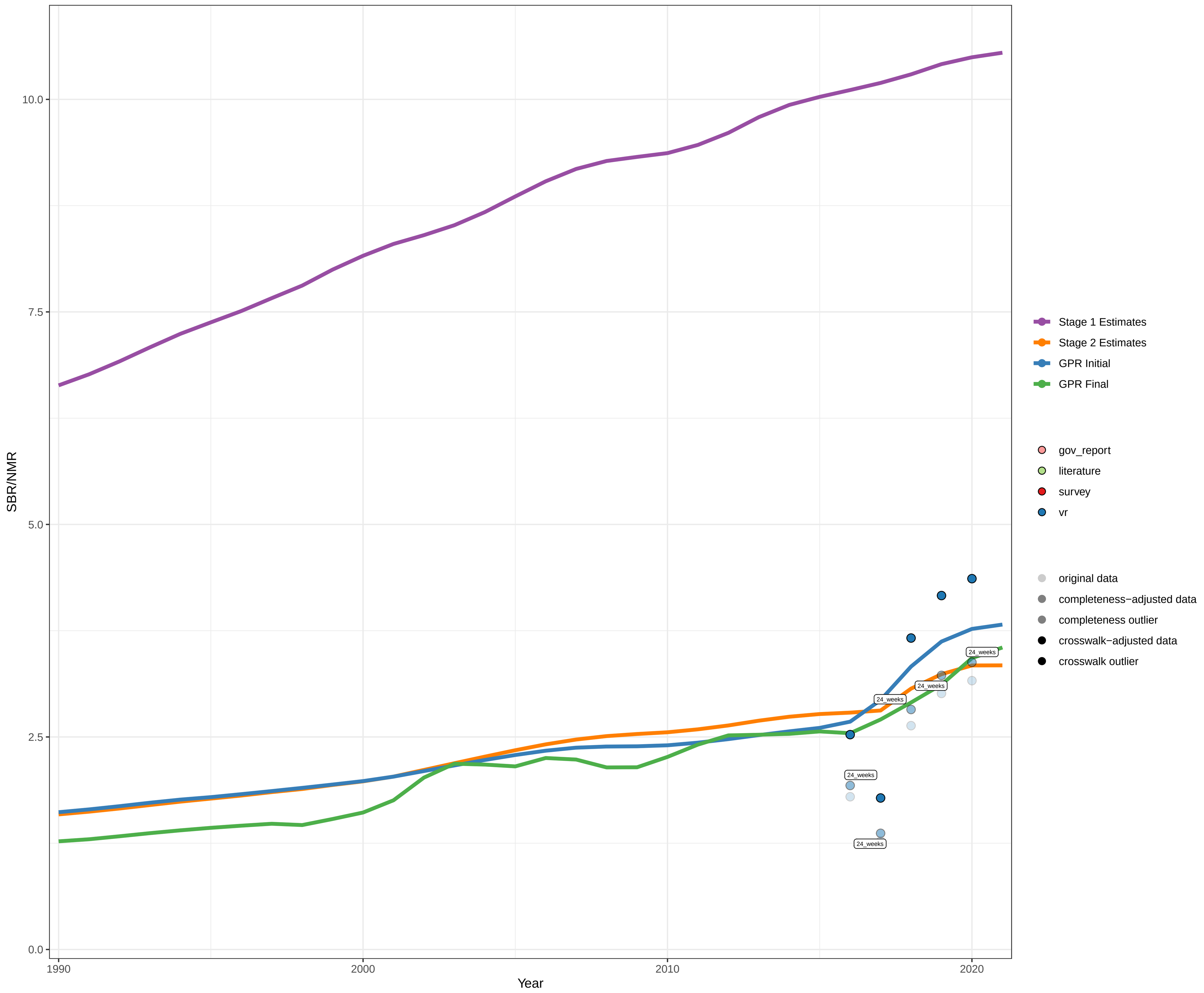

Redbridge GBR_44733 Western Europe Stillbirth Rate / Neonatal Mortality Rate (SBR/NMR) − 28 weeks
lambda = 0.6 ; zeta = 0.8 ; scale = 15 ; amp = 0.028

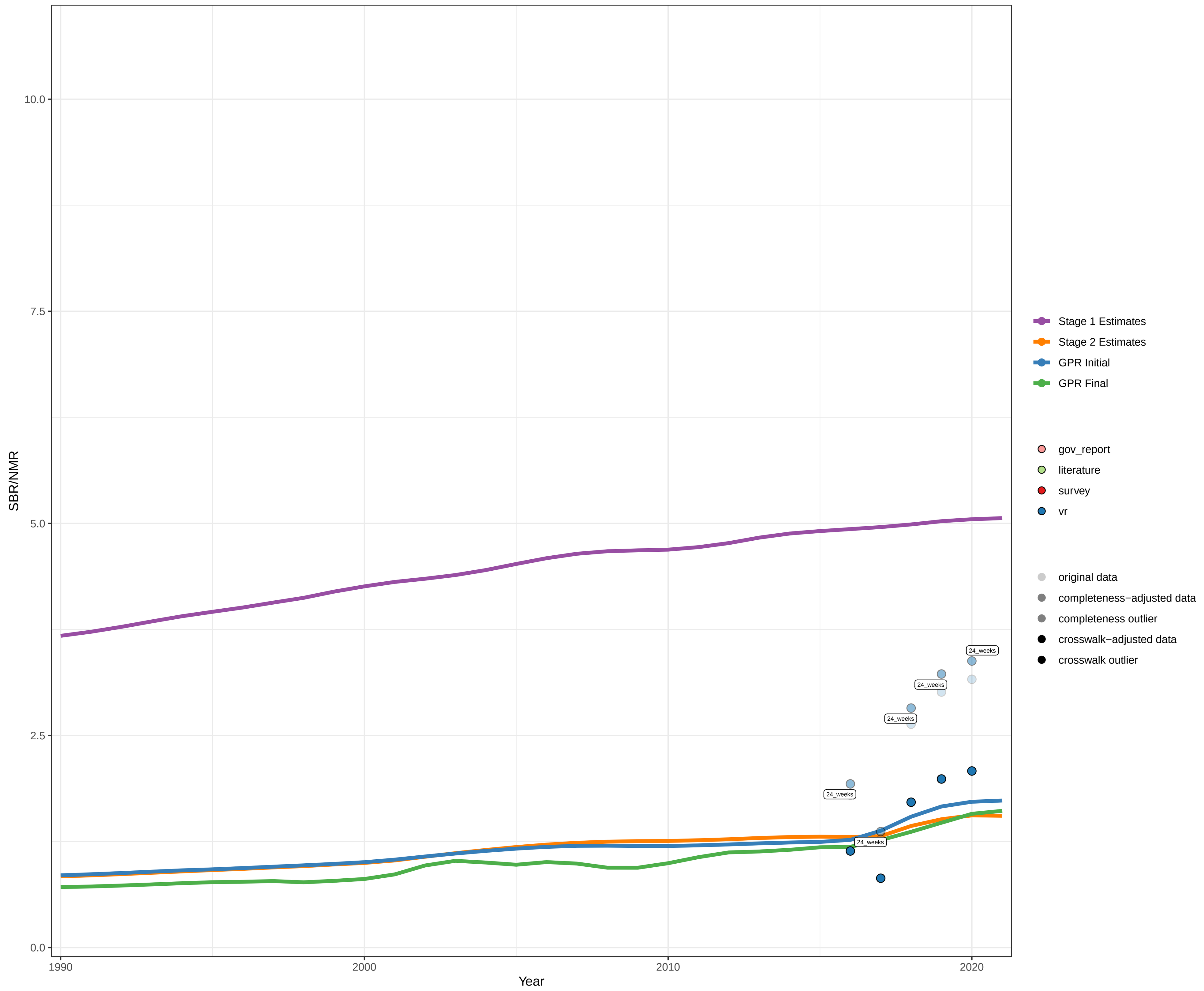

Definition	Years	Outlier	Source.Type	Title
24_weeks	2016−2020	0	VR	United Kingdom − England and Wales Child Mortality Statistics

Redbridge GBR_44733 Western Europe Stillbirth Rate (SBR) − 20 weeks
lambda = 0.6 ; zeta = 0.8 ; scale = 15 ; amp = 0.028

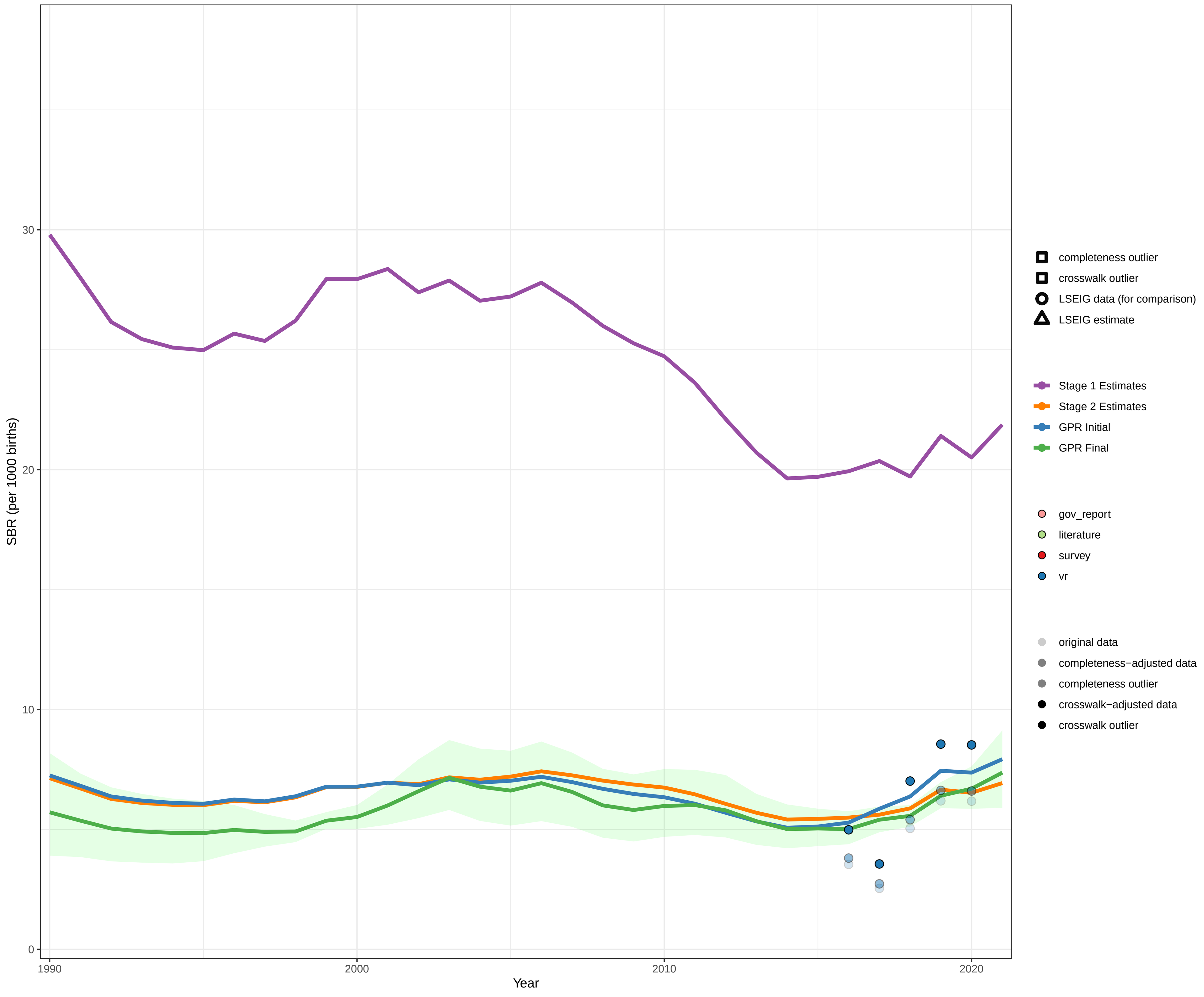

Redbridge GBR_44733 Western Europe Stillbirth Rate (SBR) − 28 weeks
lambda = 0.6 ; zeta = 0.8 ; scale = 15 ; amp = 0.028

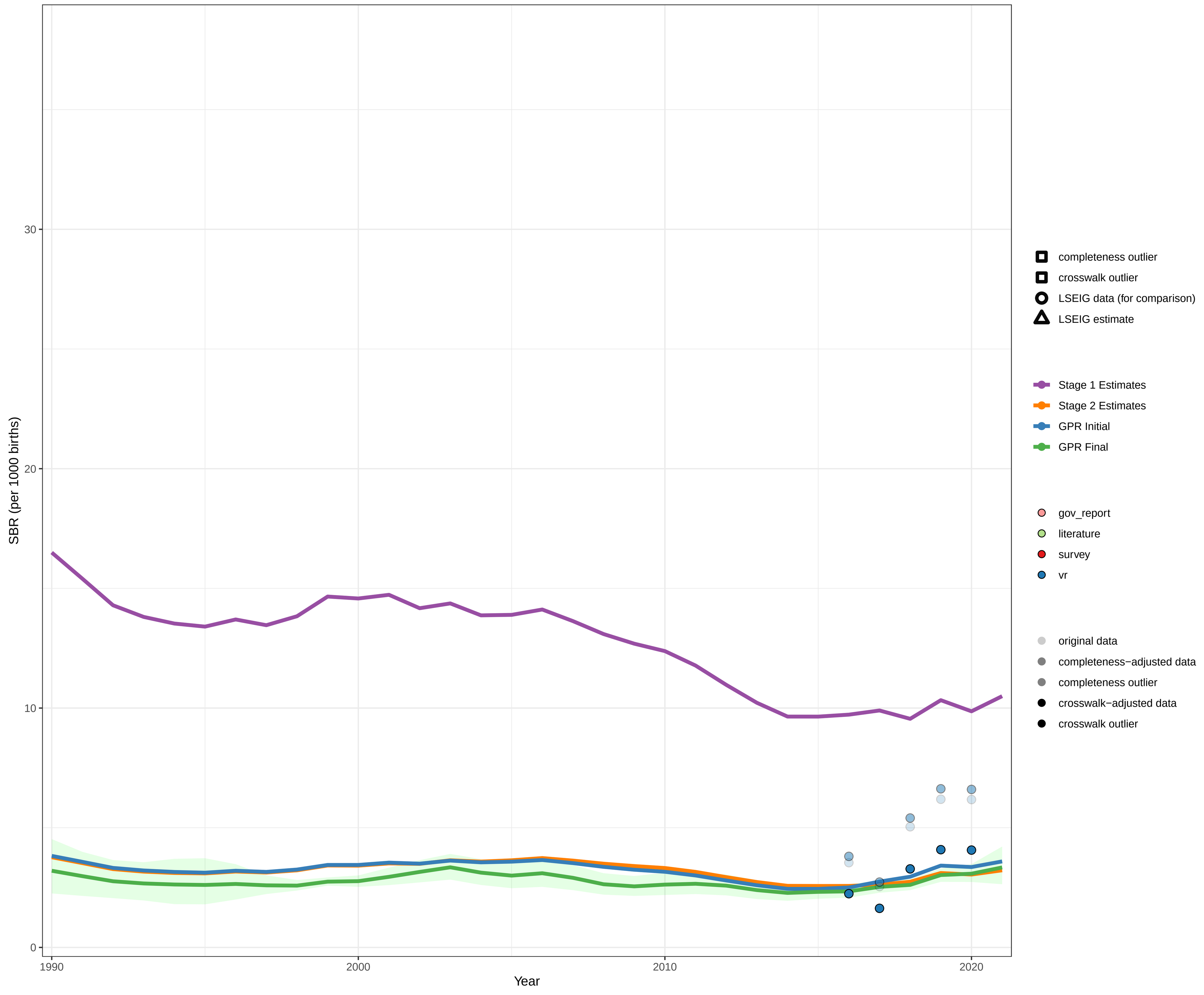

Year	Total Stillbirths (in thousands)	Total Stillbirths (in thousands)	SBR (per 1000 births)	SBR (per 1000 births)	SBR/NMR	SBR/NMR
	20 weeks	28 weeks	20 weeks	28 weeks	20 weeks	28 weeks
1990	0.0 (0.0 to 0.0)	0.0 (0.0 to 0.0)	5.7 (3.9 to 8.2)	3.2 (2.3 to 4.5)	1.27 (0.87 to 1.82)	0.71 (0.50 to 1.01)
1995	0.0 (0.0 to 0.0)	0.0 (0.0 to 0.0)	4.8 (3.7 to 6.2)	2.6 (1.8 to 3.7)	1.43 (1.09 to 1.83)	0.77 (0.53 to 1.10)
2000	0.0 (0.0 to 0.0)	0.0 (0.0 to 0.0)	5.5 (5.0 to 6.0)	2.8 (2.5 to 3.0)	1.61 (1.47 to 1.76)	0.81 (0.74 to 0.88)
2005	0.0 (0.0 to 0.0)	0.0 (0.0 to 0.0)	6.6 (5.2 to 8.3)	3.0 (2.5 to 3.5)	2.15 (1.68 to 2.69)	0.98 (0.81 to 1.15)
2010	0.0 (0.0 to 0.0)	0.0 (0.0 to 0.0)	6.0 (4.7 to 7.5)	2.6 (2.2 to 3.1)	2.27 (1.78 to 2.85)	0.99 (0.83 to 1.18)
2015	0.0 (0.0 to 0.0)	0.0 (0.0 to 0.0)	5.0 (4.3 to 5.9)	2.3 (2.0 to 2.6)	2.56 (2.19 to 2.99)	1.18 (1.03 to 1.34)
2020	0.0 (0.0 to 0.0)	0.0 (0.0 to 0.0)	6.7 (5.9 to 7.6)	3.1 (2.7 to 3.5)	3.43 (3.00 to 3.90)	1.58 (1.40 to 1.78)
2021	0.0 (0.0 to 0.0)	0.0 (0.0 to 0.0)	7.4 (5.9 to 9.1)	3.3 (2.6 to 4.2)	3.55 (2.84 to 4.40)	1.61 (1.27 to 2.03)

Richmond upon Thames GBR_44727 Western Europe Stillbirth Rate / Neonatal Mortality Rate (SBR/NMR) − 20 weeks
lambda = 0.6 ; zeta = 0.8 ; scale = 15 ; amp = 0.028

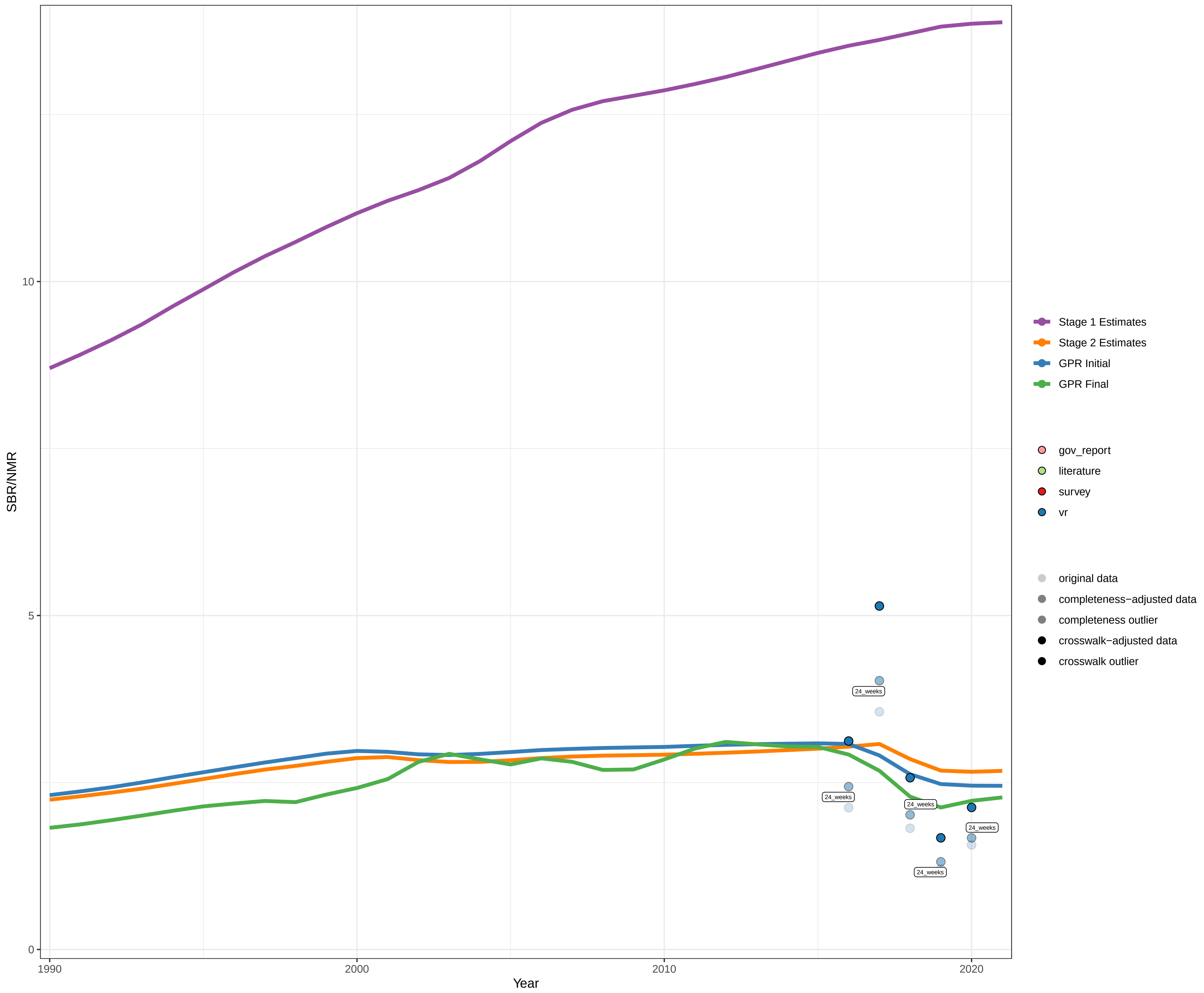

Richmond upon Thames GBR_44727 Western Europe Stillbirth Rate / Neonatal Mortality Rate (SBR/NMR) − 28 weeks
lambda = 0.6 ; zeta = 0.8 ; scale = 15 ; amp = 0.028

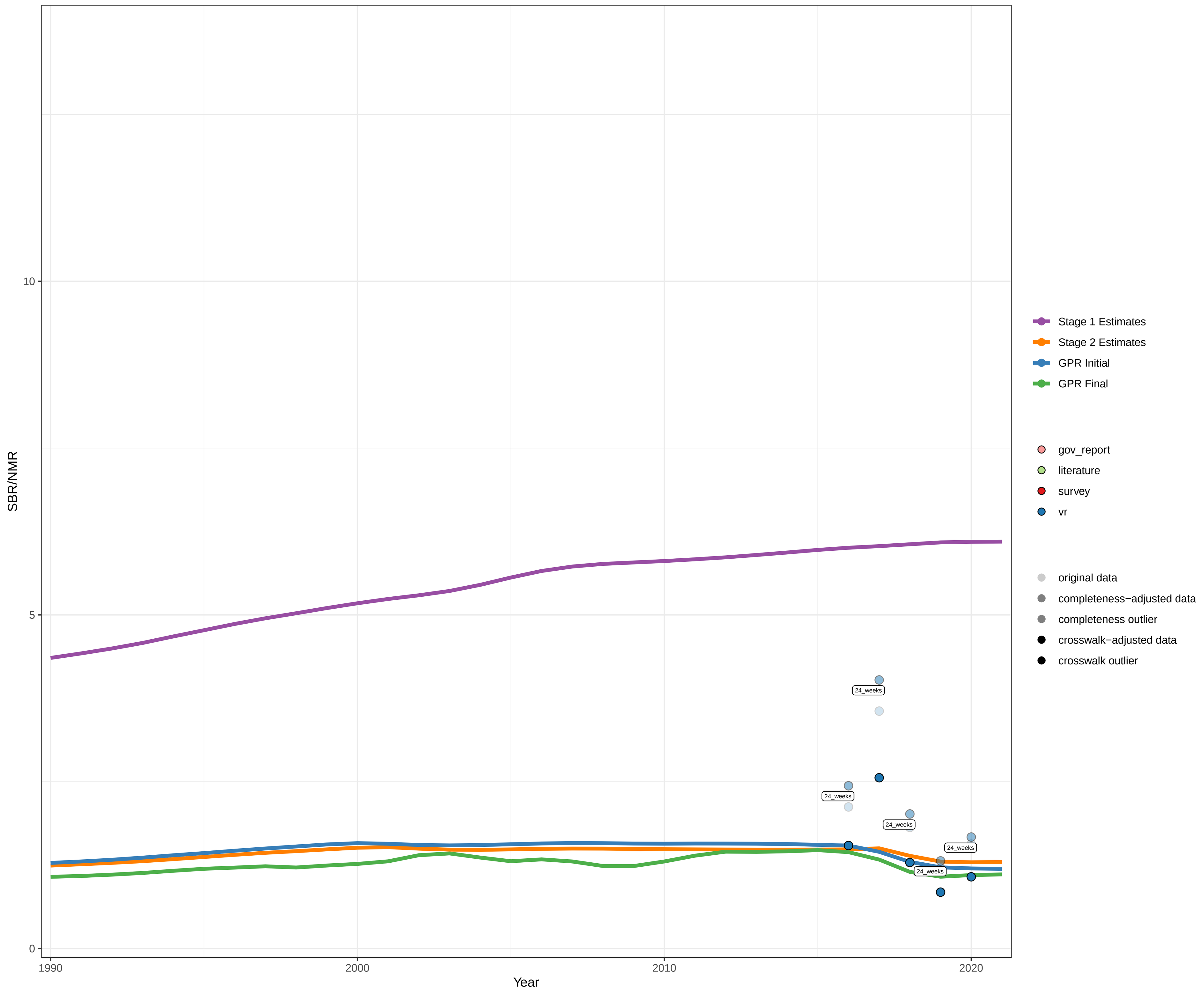

Definition	Years	Outlier	Source.Type	Title
24_weeks	2016−2020	0	VR	United Kingdom − England and Wales Child Mortality Statistics

Richmond upon Thames GBR_44727 Western Europe Stillbirth Rate (SBR) − 20 weeks
lambda = 0.6 ; zeta = 0.8 ; scale = 15 ; amp = 0.028

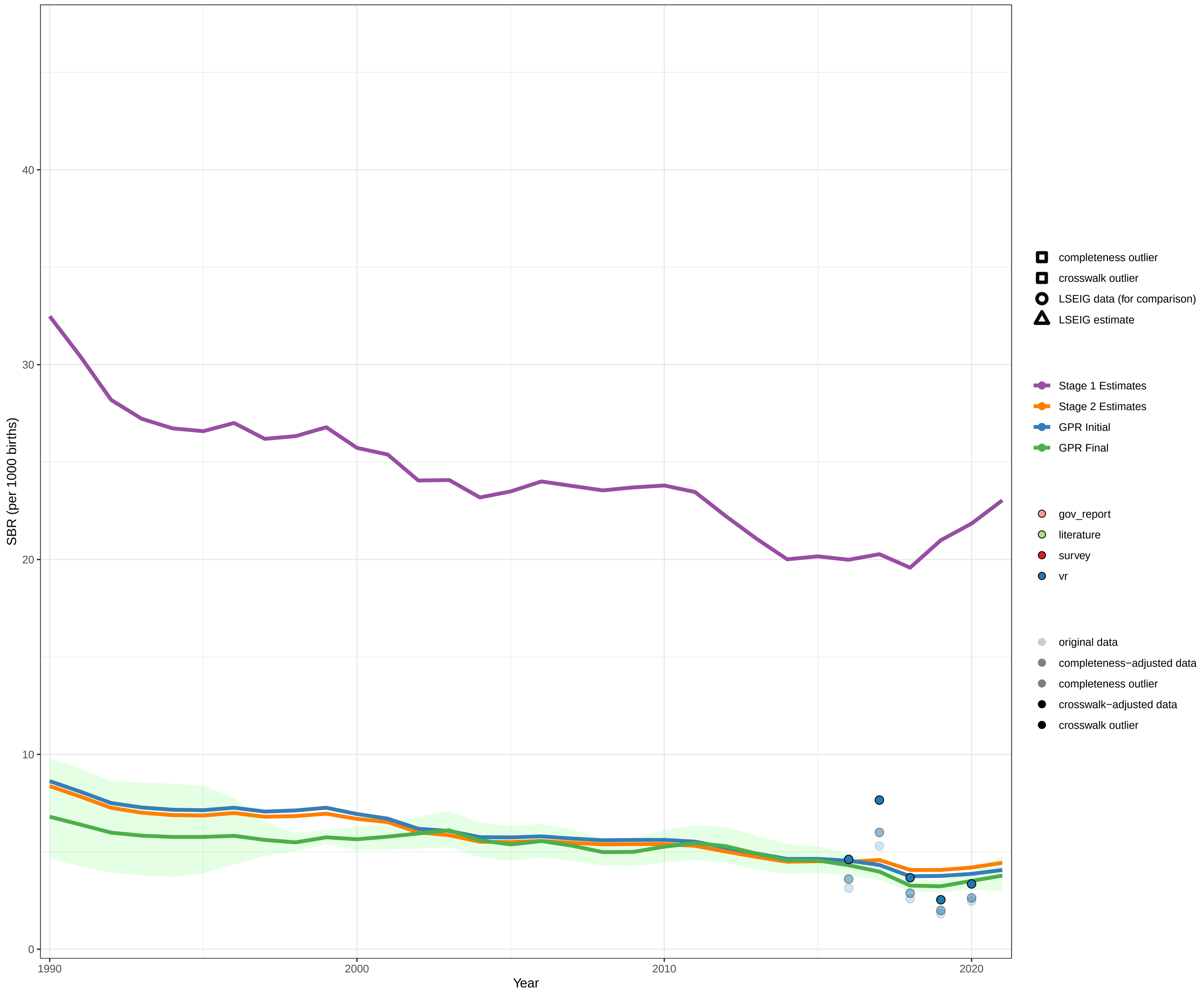

Richmond upon Thames GBR_44727 Western Europe Stillbirth Rate (SBR) − 28 weeks
lambda = 0.6 ; zeta = 0.8 ; scale = 15 ; amp = 0.028

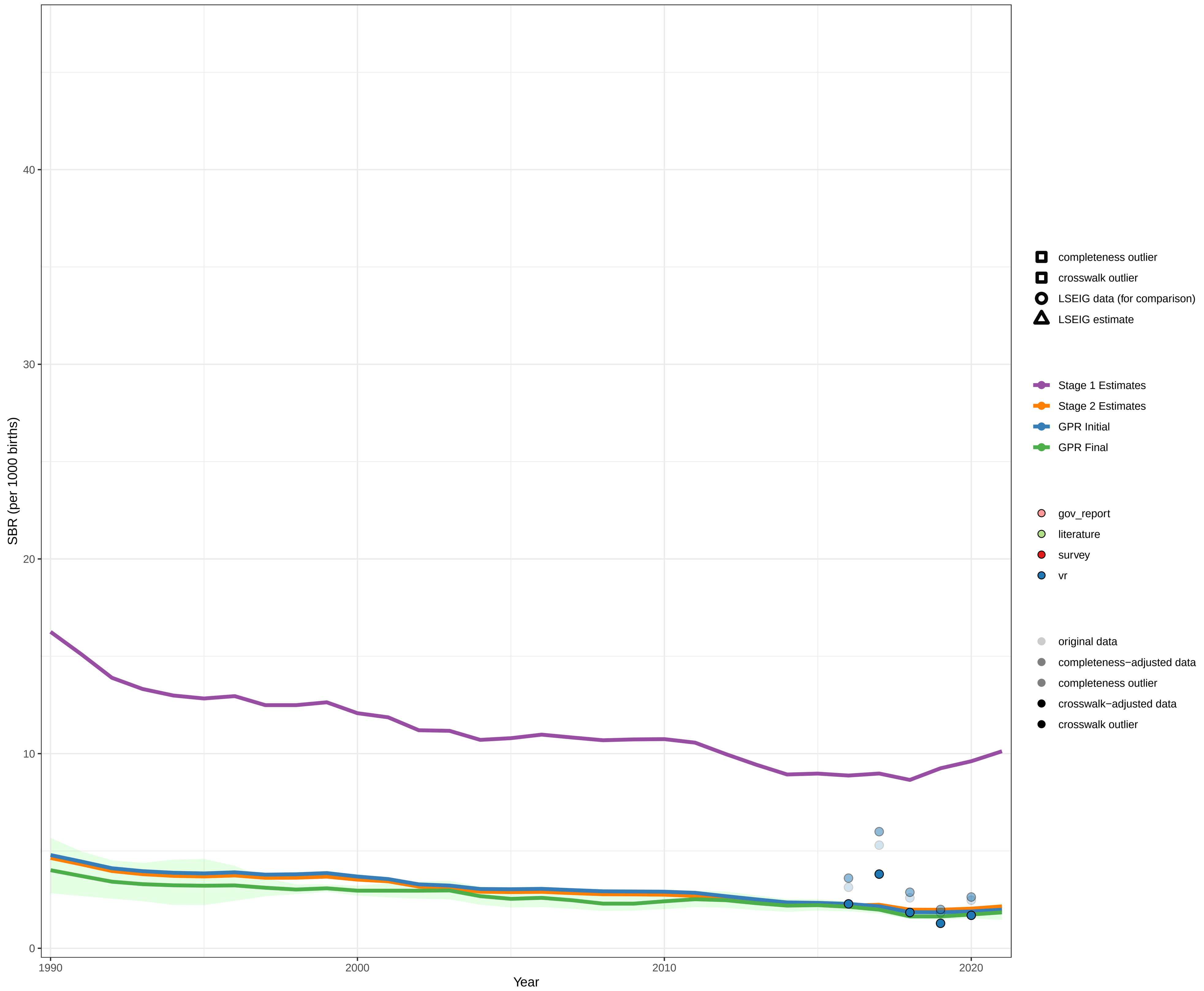

Year	Total Stillbirths (in thousands)	Total Stillbirths (in thousands)	SBR (per 1000 births)	SBR (per 1000 births)	SBR/NMR	SBR/NMR
	20 weeks	28 weeks	20 weeks	28 weeks	20 weeks	28 weeks
1990	0.0 (0.0 to 0.0)	0.0 (0.0 to 0.0)	6.8 (4.7 to 9.8)	4.0 (2.8 to 5.7)	1.82 (1.25 to 2.62)	1.07 (0.76 to 1.52)
1995	0.0 (0.0 to 0.0)	0.0 (0.0 to 0.0)	5.8 (3.9 to 8.4)	3.2 (2.2 to 4.6)	2.14 (1.45 to 3.12)	1.20 (0.82 to 1.71)
2000	0.0 (0.0 to 0.0)	0.0 (0.0 to 0.0)	5.6 (5.1 to 6.3)	3.0 (2.7 to 3.2)	2.42 (2.18 to 2.69)	1.27 (1.16 to 1.38)
2005	0.0 (0.0 to 0.0)	0.0 (0.0 to 0.0)	5.4 (4.5 to 6.3)	2.5 (2.1 to 3.0)	2.77 (2.34 to 3.26)	1.31 (1.08 to 1.54)
2010	0.0 (0.0 to 0.0)	0.0 (0.0 to 0.0)	5.3 (4.5 to 6.1)	2.4 (2.0 to 2.9)	2.84 (2.42 to 3.30)	1.30 (1.09 to 1.55)
2015	0.0 (0.0 to 0.0)	0.0 (0.0 to 0.0)	4.6 (3.9 to 5.3)	2.2 (1.9 to 2.5)	3.03 (2.61 to 3.51)	1.47 (1.29 to 1.68)
2020	0.0 (0.0 to 0.0)	0.0 (0.0 to 0.0)	3.5 (3.1 to 4.0)	1.7 (1.5 to 2.0)	2.23 (1.95 to 2.54)	1.10 (0.98 to 1.25)
2021	0.0 (0.0 to 0.0)	0.0 (0.0 to 0.0)	3.8 (3.0 to 4.8)	1.8 (1.5 to 2.3)	2.28 (1.79 to 2.89)	1.11 (0.88 to 1.40)

Southwark GBR_44752 Western Europe Stillbirth Rate / Neonatal Mortality Rate (SBR/NMR) − 20 weeks
lambda = 0.6 ; zeta = 0.8 ; scale = 15 ; amp = 0.028

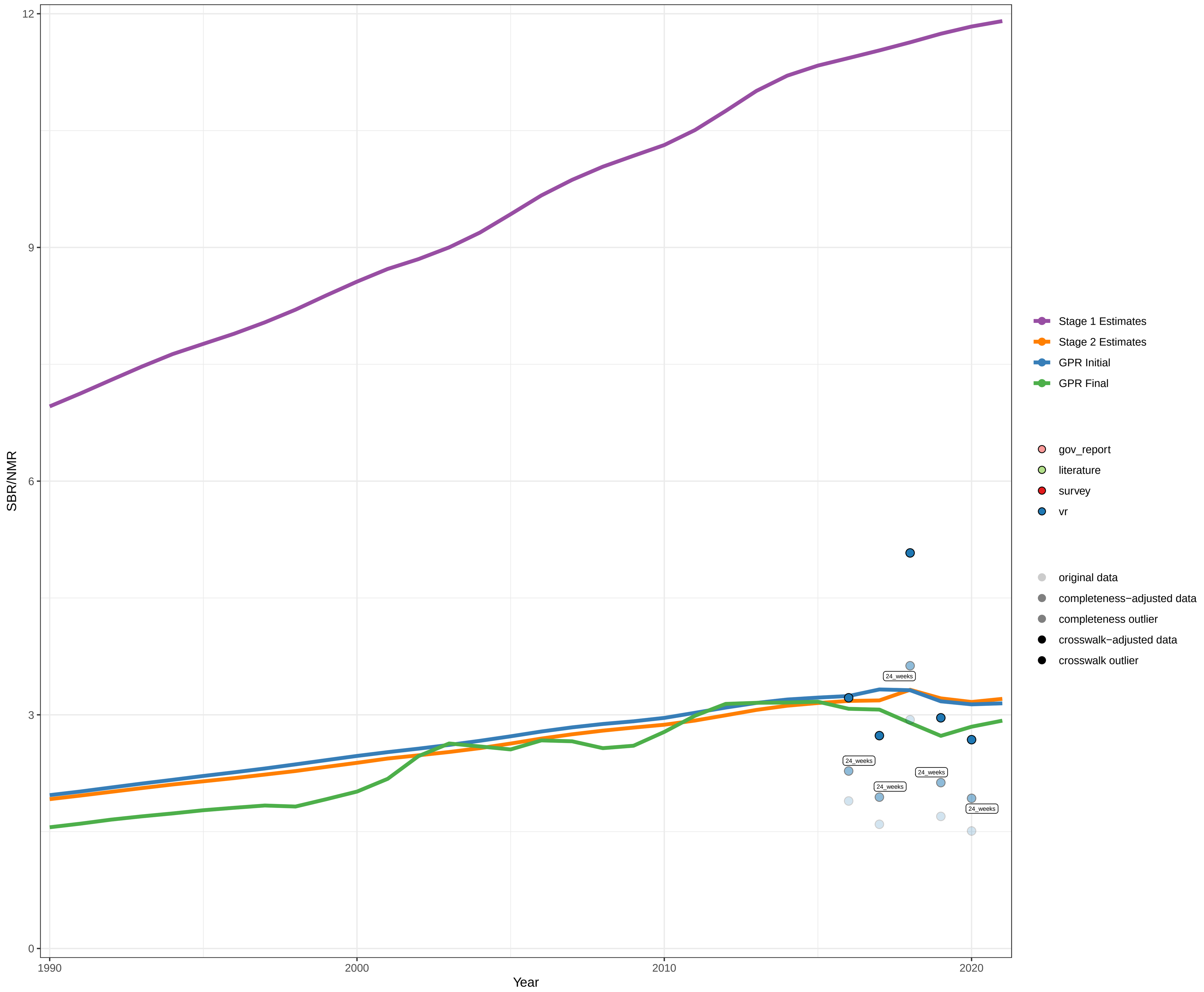

Southwark GBR_44752 Western Europe Stillbirth Rate / Neonatal Mortality Rate (SBR/NMR) − 28 weeks
lambda = 0.6 ; zeta = 0.8 ; scale = 15 ; amp = 0.028

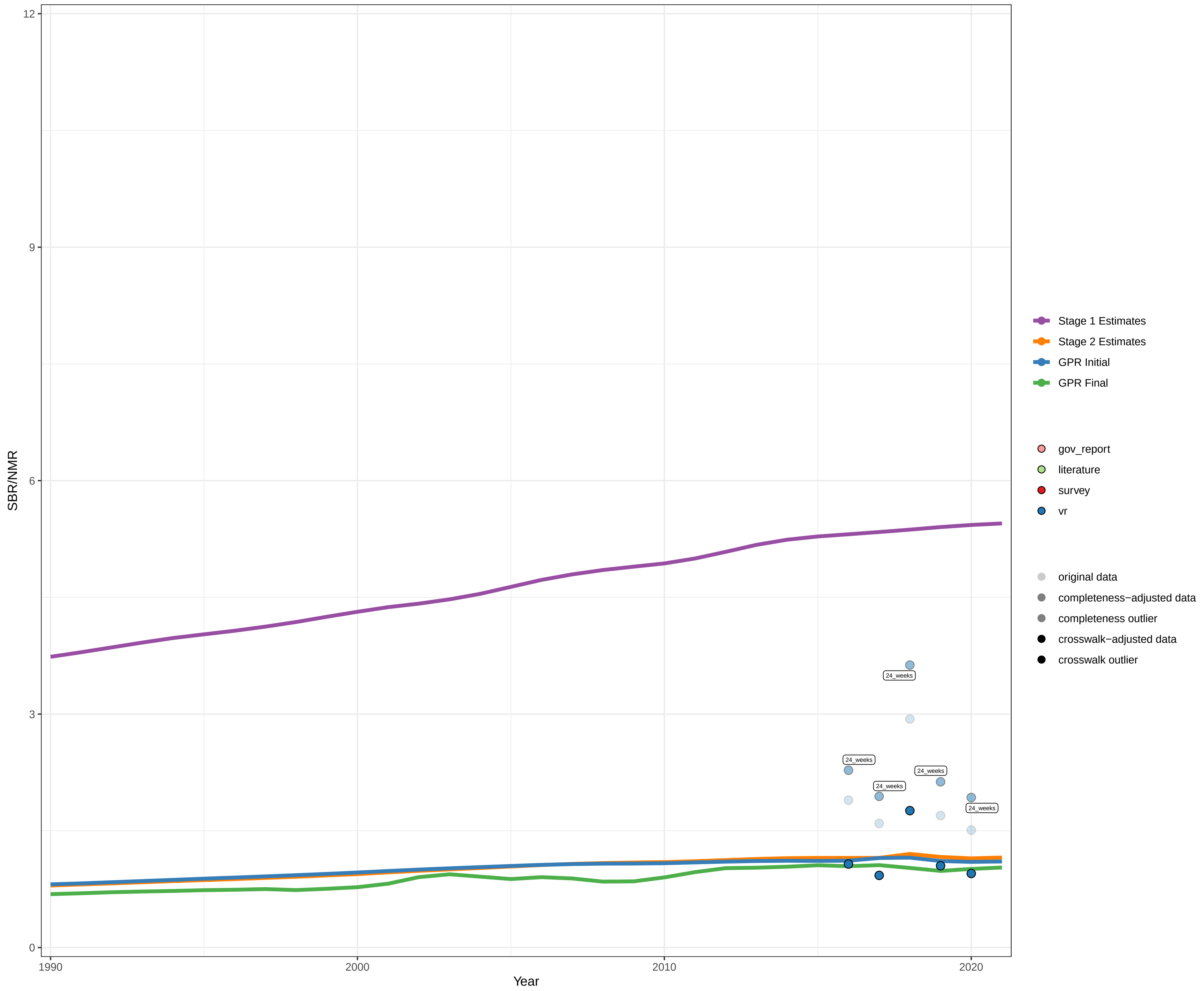

Definition	Years	Outlier	Source.Type	Title
24_weeks	2016−2020	0	VR	United Kingdom − England and Wales Child Mortality Statistics

Southwark GBR_44752 Western Europe Stillbirth Rate (SBR) − 20 weeks
lambda = 0.6 ; zeta = 0.8 ; scale = 15 ; amp = 0.028

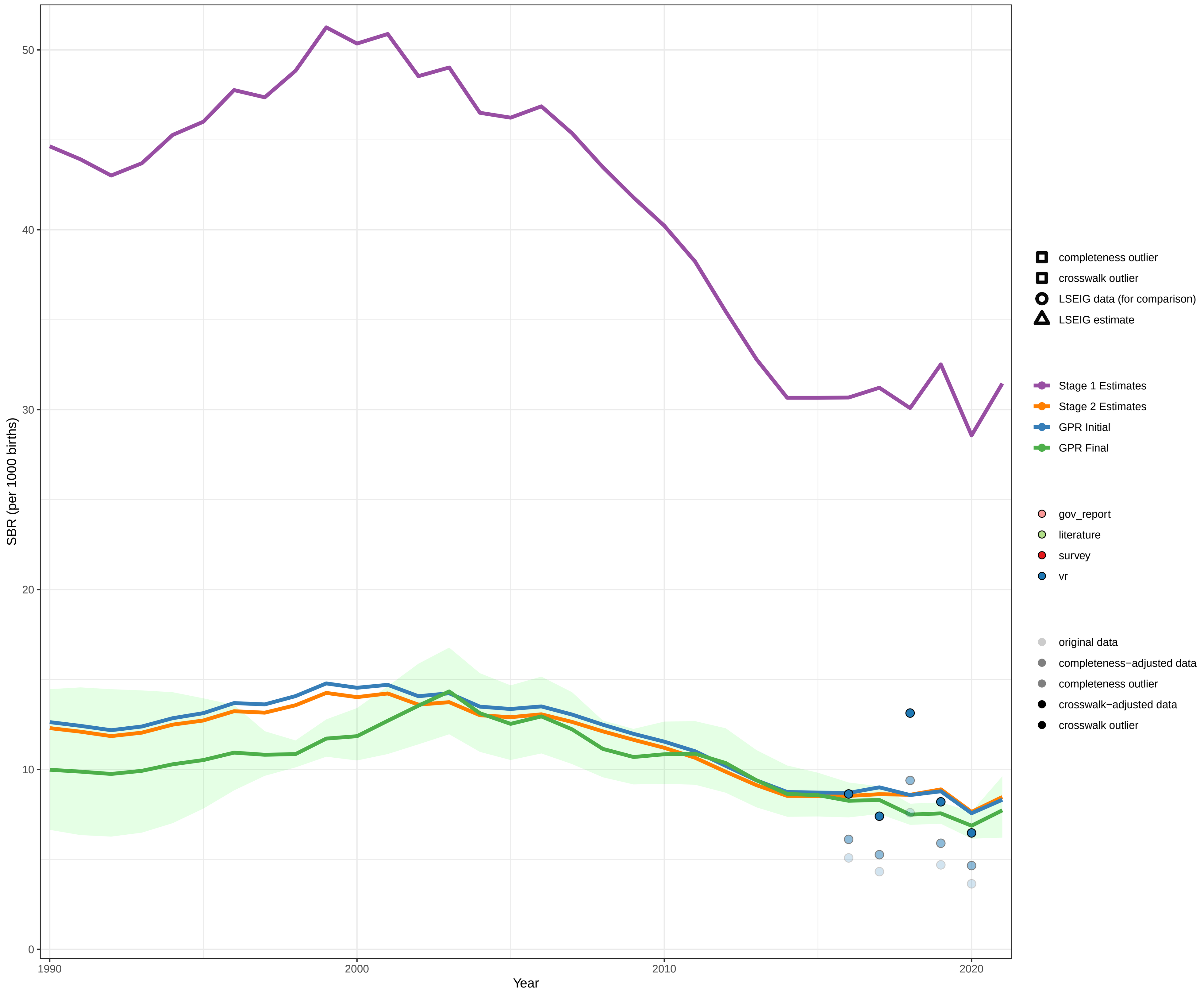

Southwark GBR_44752 Western Europe Stillbirth Rate (SBR) − 28 weeks
lambda = 0.6 ; zeta = 0.8 ; scale = 15 ; amp = 0.028

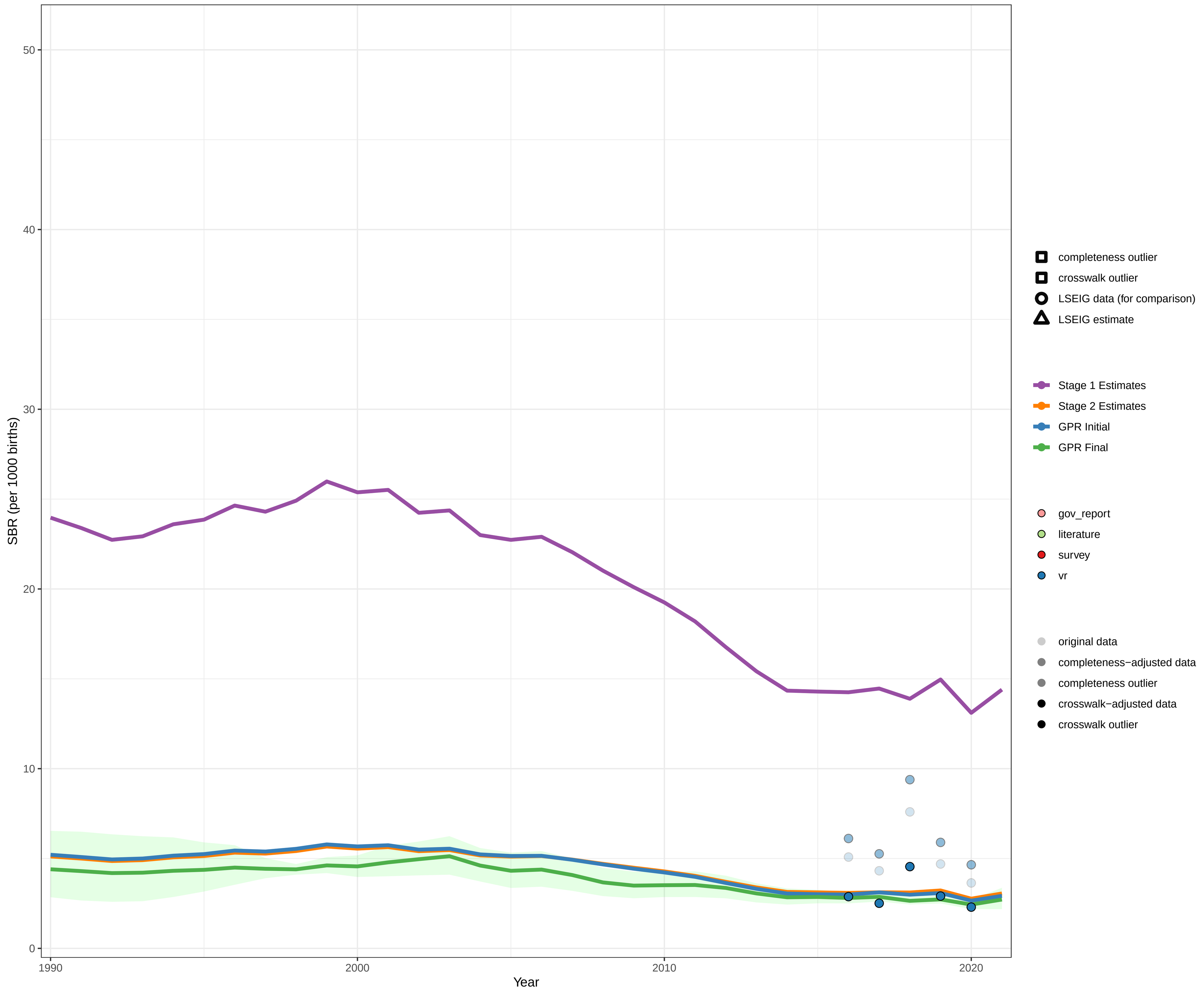

Year	Total Stillbirths (in thousands)	Total Stillbirths (in thousands)	SBR (per 1000 births)	SBR (per 1000 births)	SBR/NMR	SBR/NMR
	20 weeks	28 weeks	20 weeks	28 weeks	20 weeks	28 weeks
1990	0.0 (0.0 to 0.1)	0.0 (0.0 to 0.0)	10.0 (6.6 to 14.5)	4.4 (2.8 to 6.5)	1.56 (1.04 to 2.25)	0.69 (0.44 to 1.02)
1995	0.0 (0.0 to 0.1)	0.0 (0.0 to 0.0)	10.5 (7.8 to 14.0)	4.4 (3.2 to 5.9)	1.78 (1.32 to 2.35)	0.74 (0.53 to 1.00)
2000	0.0 (0.0 to 0.1)	0.0 (0.0 to 0.0)	11.8 (10.5 to 13.4)	4.6 (4.0 to 5.2)	2.01 (1.79 to 2.28)	0.78 (0.68 to 0.88)
2005	0.1 (0.0 to 0.1)	0.0 (0.0 to 0.0)	12.5 (10.5 to 14.7)	4.3 (3.4 to 5.3)	2.56 (2.15 to 2.99)	0.88 (0.69 to 1.09)
2010	0.1 (0.0 to 0.1)	0.0 (0.0 to 0.0)	10.8 (9.2 to 12.7)	3.5 (2.9 to 4.3)	2.78 (2.36 to 3.25)	0.90 (0.73 to 1.09)
2015	0.0 (0.0 to 0.0)	0.0 (0.0 to 0.0)	8.6 (7.4 to 9.8)	2.9 (2.5 to 3.3)	3.17 (2.73 to 3.63)	1.06 (0.93 to 1.20)
2020	0.0 (0.0 to 0.0)	0.0 (0.0 to 0.0)	6.9 (6.1 to 7.7)	2.4 (2.2 to 2.7)	2.85 (2.55 to 3.17)	1.01 (0.91 to 1.13)
2021	0.0 (0.0 to 0.0)	0.0 (0.0 to 0.0)	7.7 (6.2 to 9.6)	2.7 (2.2 to 3.4)	2.92 (2.35 to 3.64)	1.03 (0.83 to 1.28)

Sutton GBR_44739 Western Europe Stillbirth Rate / Neonatal Mortality Rate (SBR/NMR) − 20 weeks
lambda = 0.6 ; zeta = 0.8 ; scale = 15 ; amp = 0.028

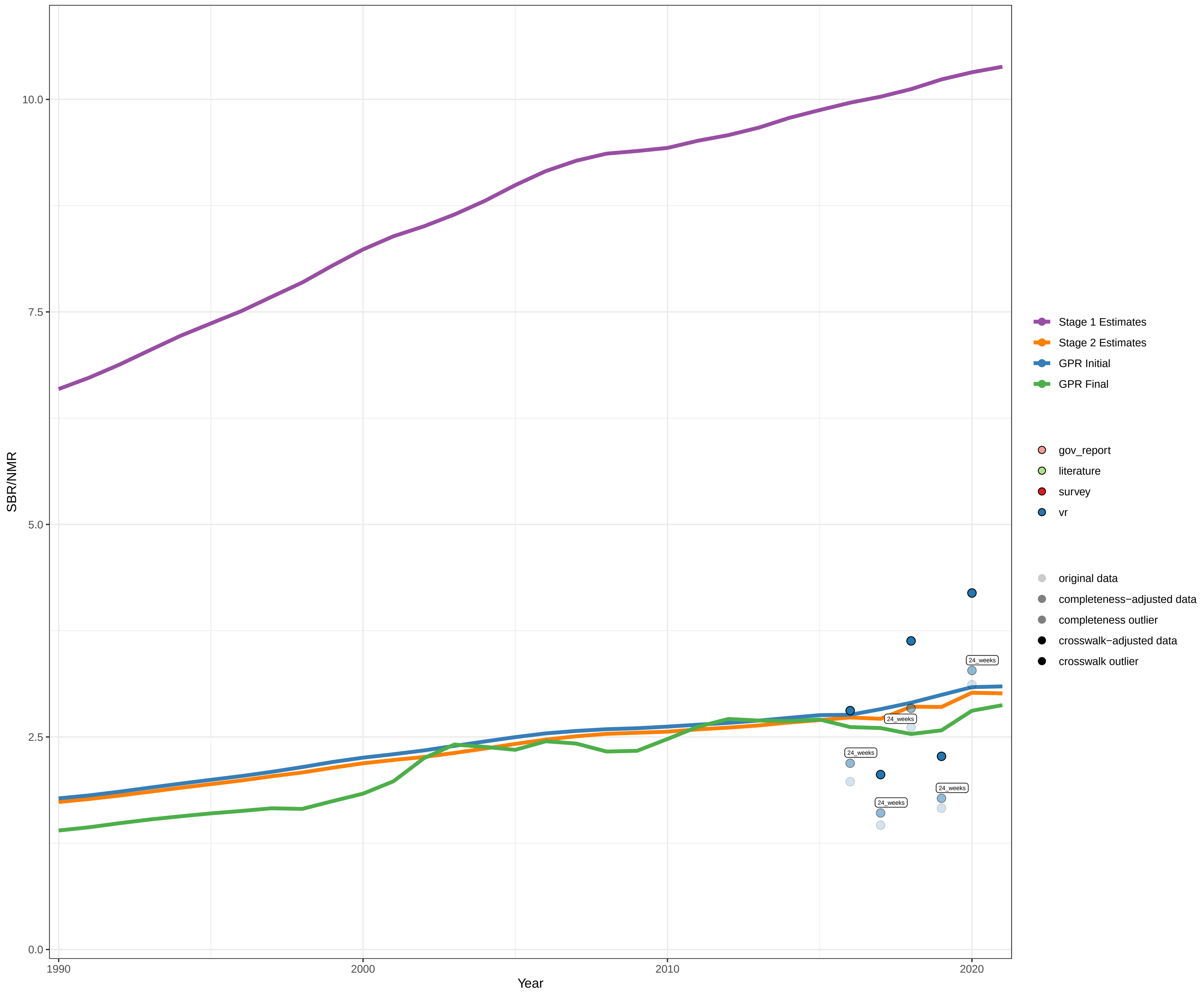

Sutton GBR_44739 Western Europe Stillbirth Rate / Neonatal Mortality Rate (SBR/NMR) − 28 weeks
lambda = 0.6 ; zeta = 0.8 ; scale = 15 ; amp = 0.028

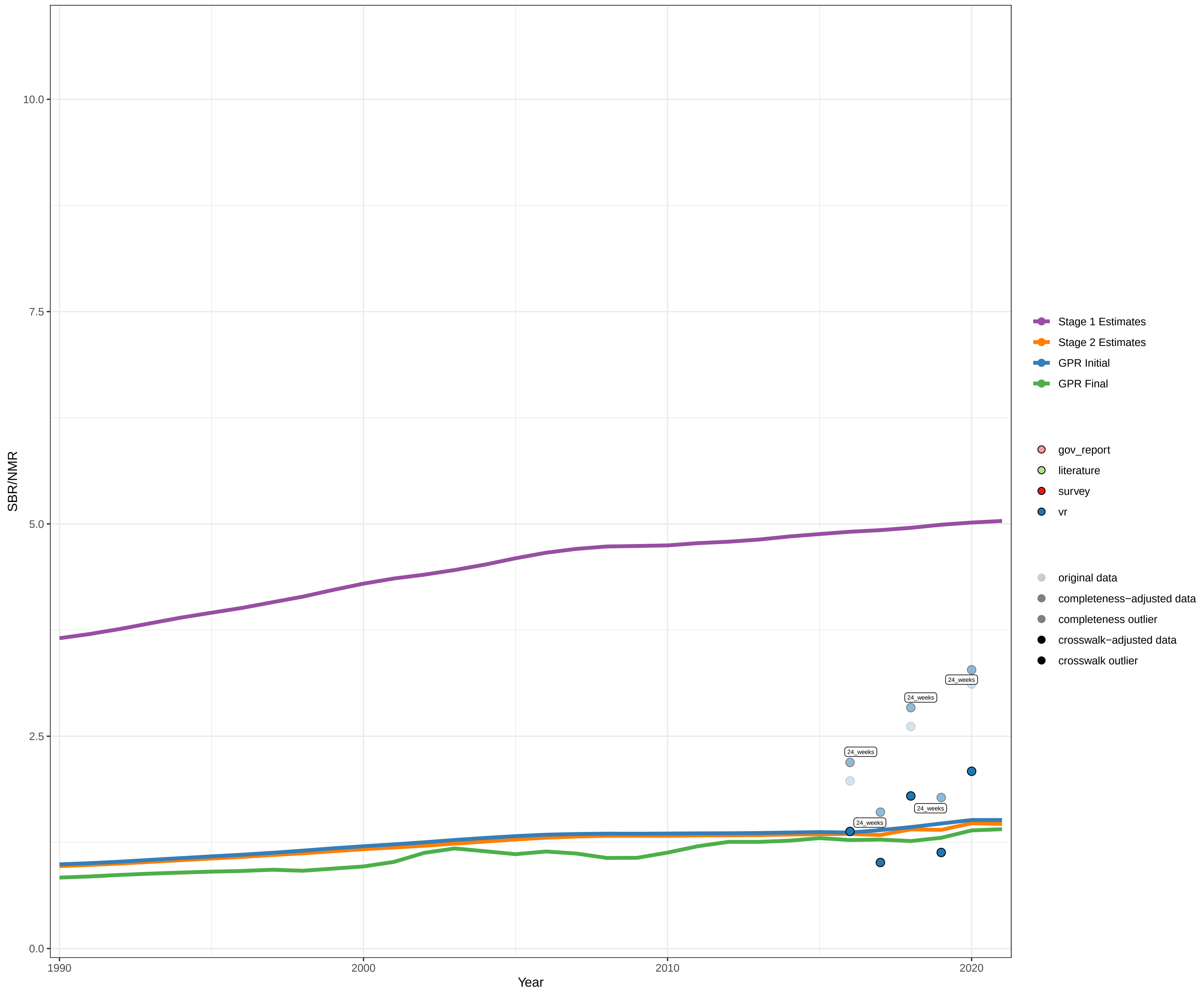

Definition	Years	Outlier	Source.Type	Title
24_weeks	2016−2020	0	VR	United Kingdom − England and Wales Child Mortality Statistics

Sutton GBR_44739 Western Europe Stillbirth Rate (SBR) − 20 weeks
lambda = 0.6 ; zeta = 0.8 ; scale = 15 ; amp = 0.028

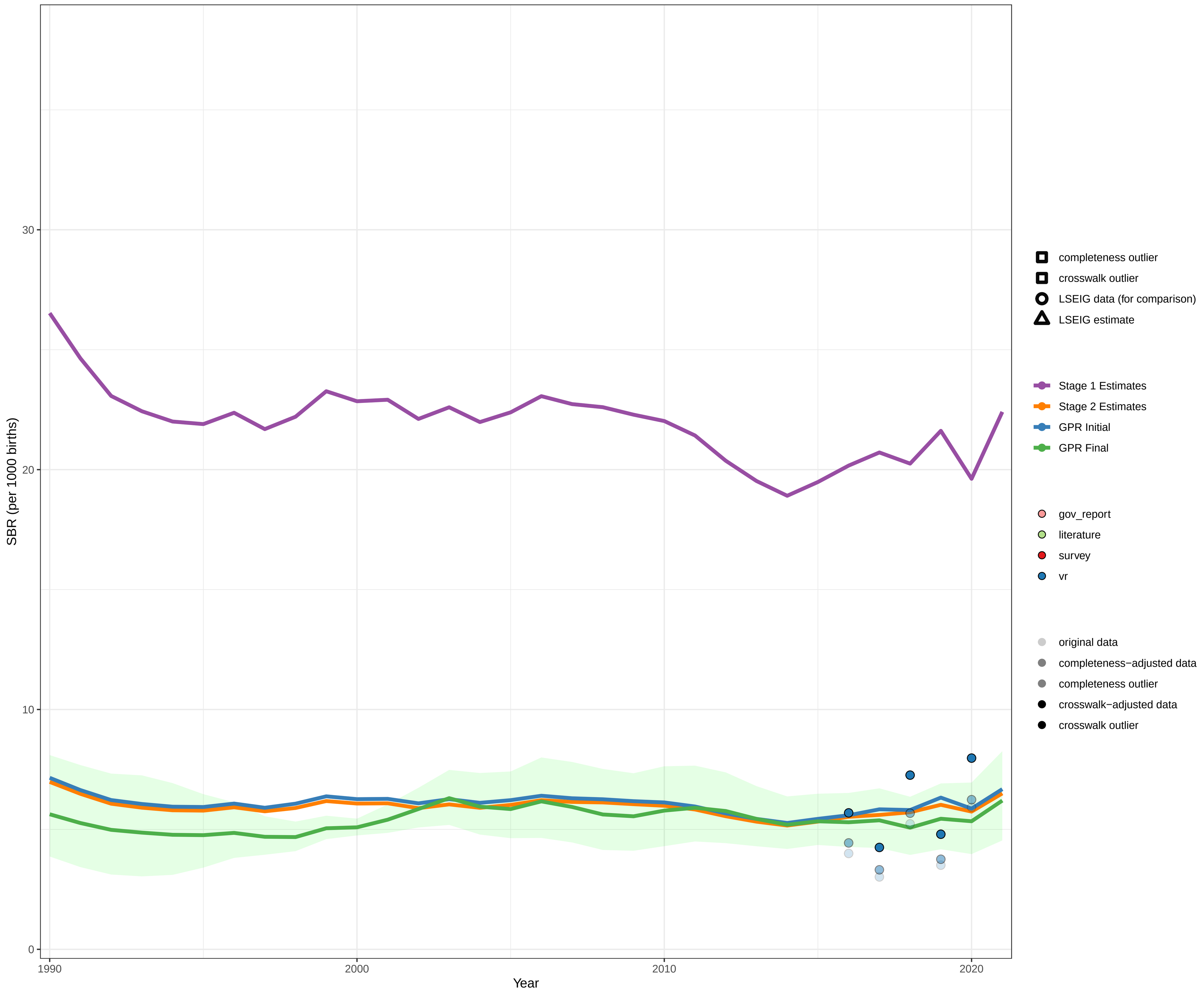

Sutton GBR_44739 Western Europe Stillbirth Rate (SBR) − 28 weeks
lambda = 0.6 ; zeta = 0.8 ; scale = 15 ; amp = 0.028

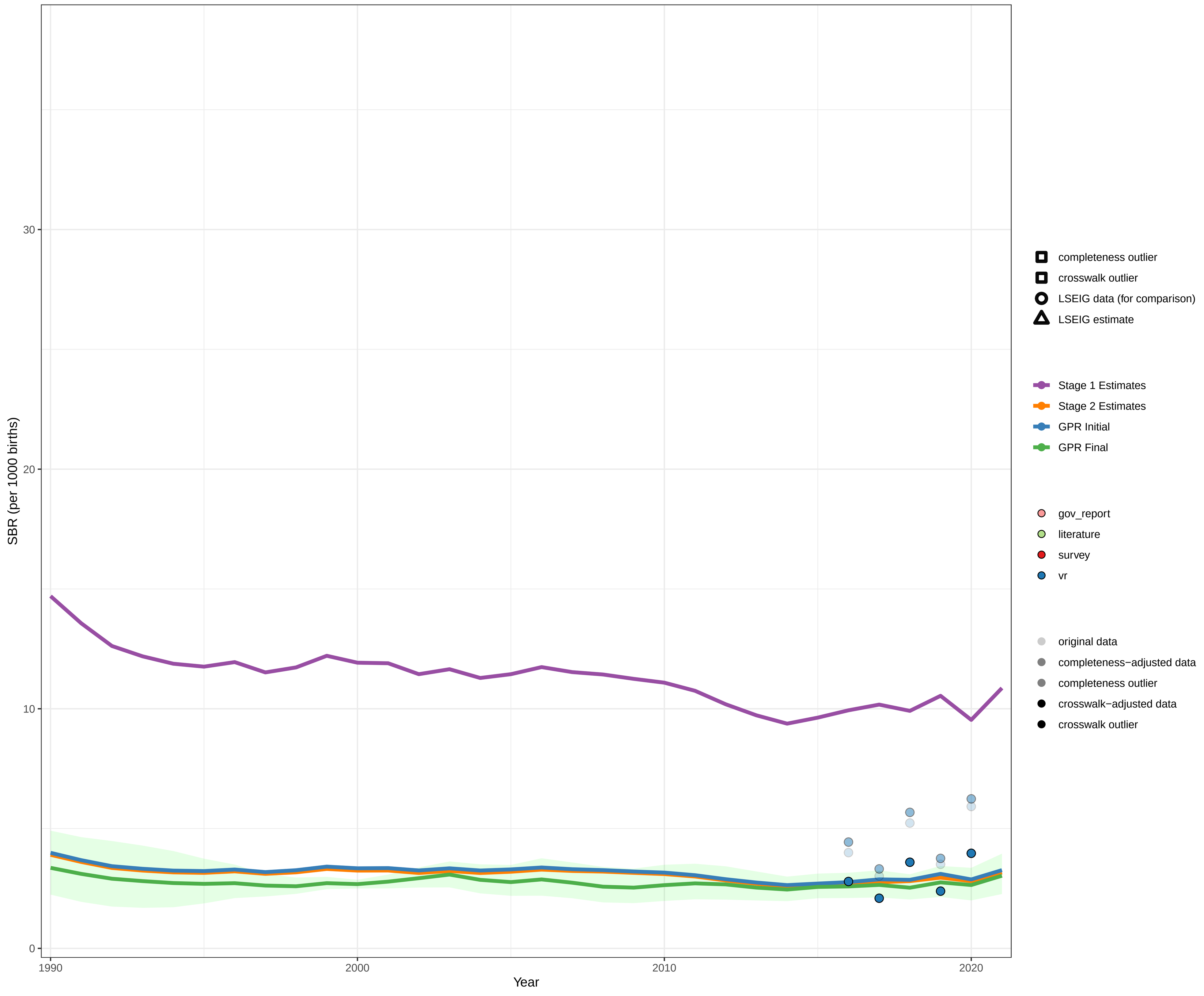

Year	Total Stillbirths (in thousands)	Total Stillbirths (in thousands)	SBR (per 1000 births)	SBR (per 1000 births)	SBR/NMR	SBR/NMR
	20 weeks	28 weeks	20 weeks	28 weeks	20 weeks	28 weeks
1990	0.0 (0.0 to 0.0)	0.0 (0.0 to 0.0)	5.6 (3.9 to 8.1)	3.4 (2.2 to 4.9)	1.40 (0.96 to 2.01)	0.84 (0.56 to 1.22)
1995	0.0 (0.0 to 0.0)	0.0 (0.0 to 0.0)	4.8 (3.4 to 6.5)	2.7 (1.9 to 3.7)	1.60 (1.15 to 2.18)	0.91 (0.63 to 1.26)
2000	0.0 (0.0 to 0.0)	0.0 (0.0 to 0.0)	5.1 (4.7 to 5.4)	2.7 (2.5 to 2.9)	1.83 (1.71 to 1.96)	0.97 (0.90 to 1.03)
2005	0.0 (0.0 to 0.0)	0.0 (0.0 to 0.0)	5.8 (4.6 to 7.4)	2.8 (2.2 to 3.5)	2.35 (1.86 to 2.98)	1.11 (0.88 to 1.40)
2010	0.0 (0.0 to 0.0)	0.0 (0.0 to 0.0)	5.8 (4.3 to 7.6)	2.6 (2.0 to 3.5)	2.48 (1.84 to 3.27)	1.13 (0.85 to 1.49)
2015	0.0 (0.0 to 0.0)	0.0 (0.0 to 0.0)	5.3 (4.4 to 6.5)	2.6 (2.1 to 3.1)	2.71 (2.21 to 3.29)	1.30 (1.06 to 1.58)
2020	0.0 (0.0 to 0.0)	0.0 (0.0 to 0.0)	5.3 (4.0 to 7.0)	2.6 (2.0 to 3.4)	2.81 (2.09 to 3.66)	1.39 (1.05 to 1.77)
2021	0.0 (0.0 to 0.0)	0.0 (0.0 to 0.0)	6.2 (4.5 to 8.3)	3.0 (2.3 to 4.0)	2.87 (2.10 to 3.83)	1.41 (1.05 to 1.83)

Tower Hamlets   GBR_44758   Western Europe   Stillbirth Rate / Neonatal Mortality Rate (SBR/NMR) − 20 weeks
lambda = 0.6 ; zeta = 0.8 ; scale = 15 ; amp = 0.028

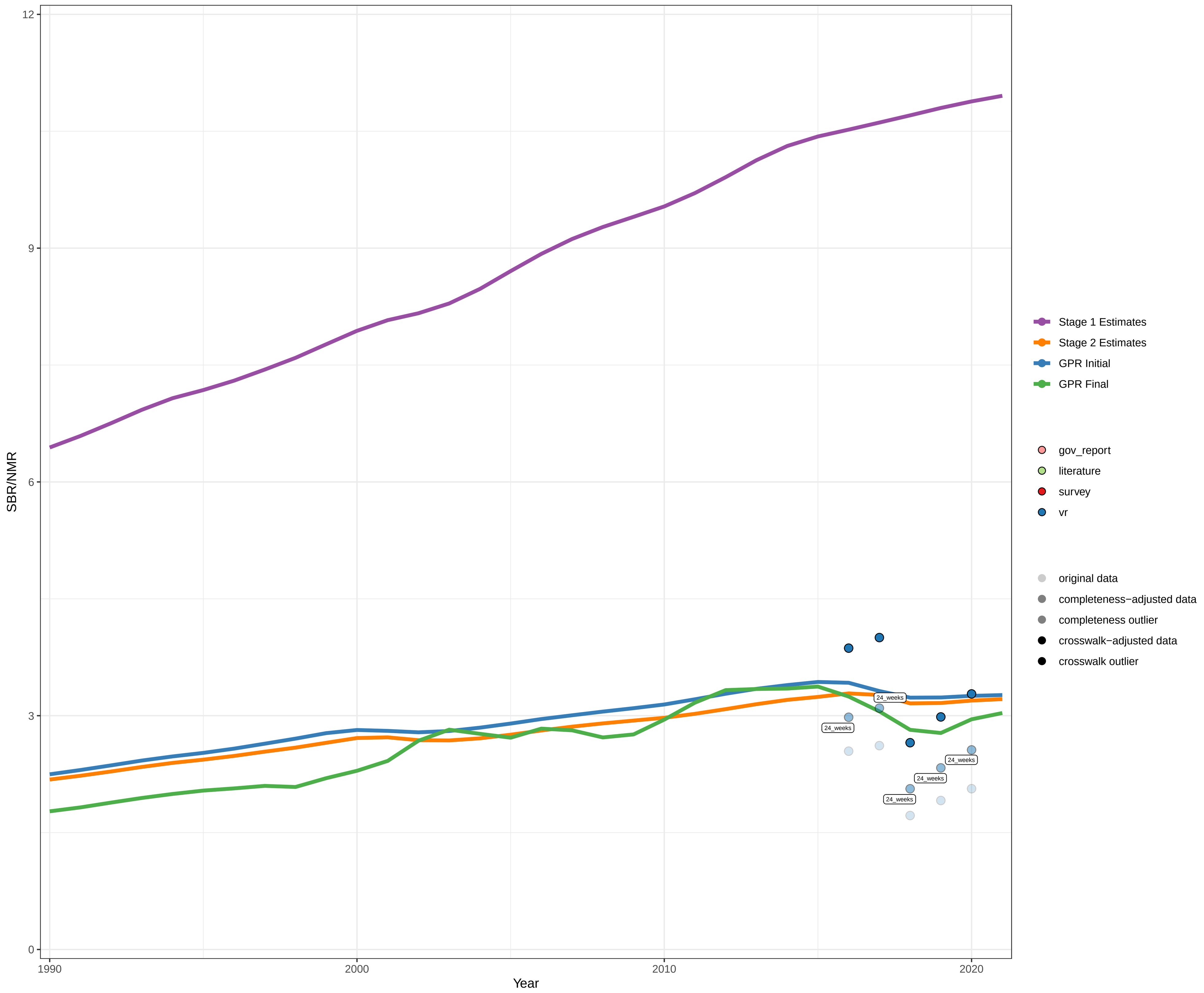

Tower Hamlets   GBR_44758   Western Europe   Stillbirth Rate / Neonatal Mortality Rate (SBR/NMR) − 28 weeks
lambda = 0.6 ; zeta = 0.8 ; scale = 15 ; amp = 0.028

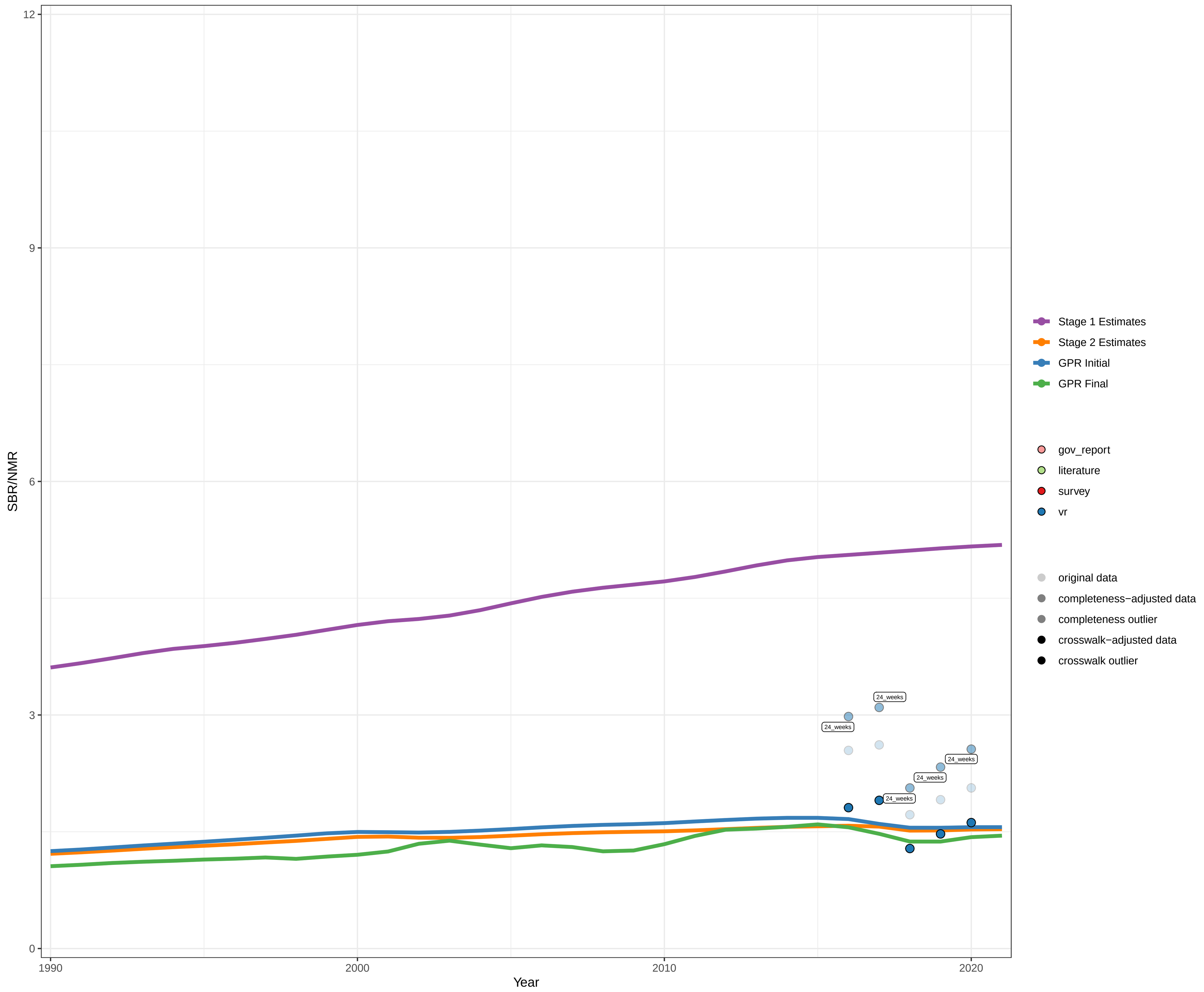

Definition	Years	Outlier	Source.Type	Title
24_weeks	2016−2020	0	VR	United Kingdom − England and Wales Child Mortality Statistics

Tower Hamlets   GBR_44758   Western Europe   Stillbirth Rate (SBR) − 20 weeks
lambda = 0.6 ; zeta = 0.8 ; scale = 15 ; amp = 0.028

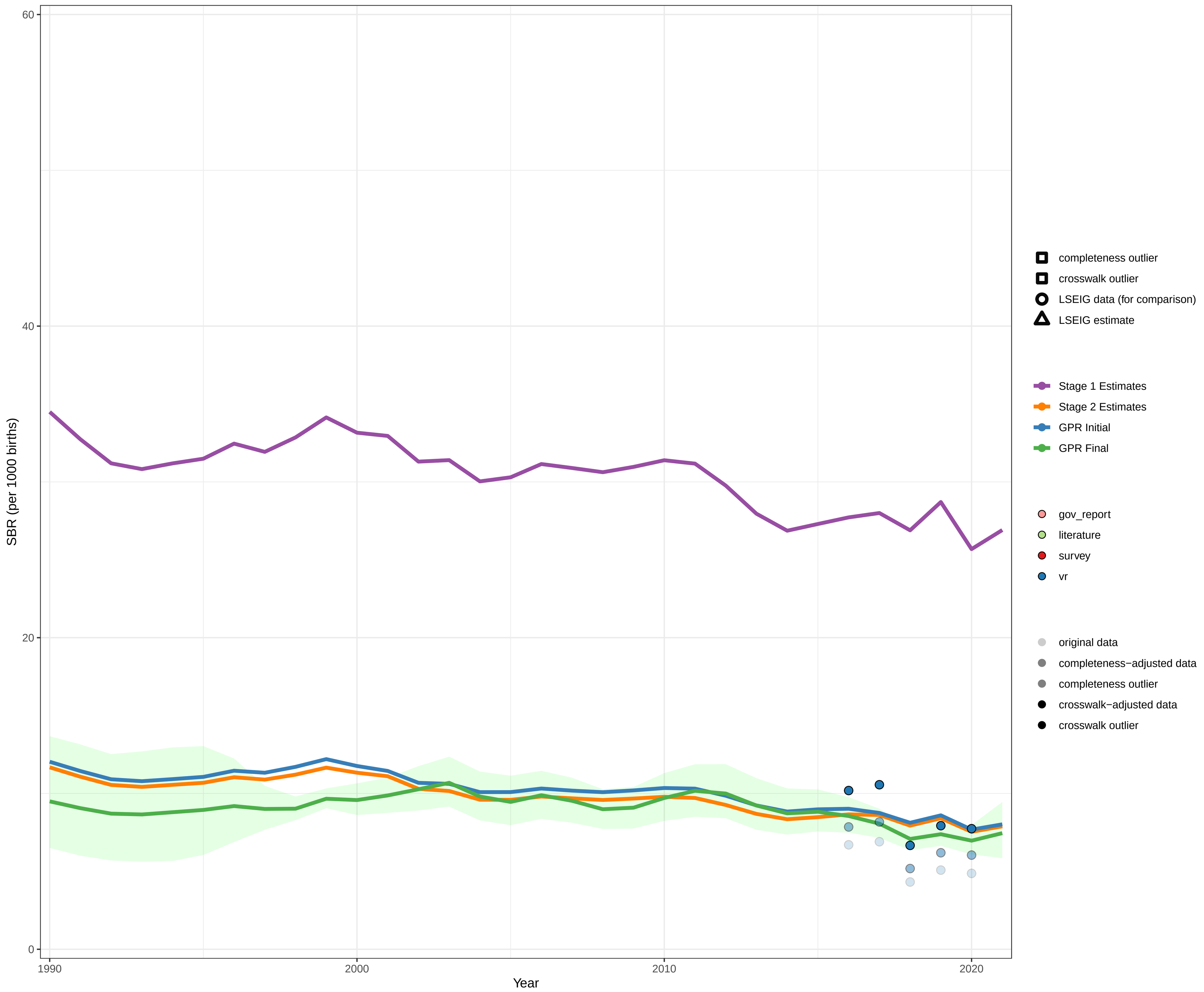

Tower Hamlets   GBR_44758   Western Europe   Stillbirth Rate (SBR) − 28 weeks
lambda = 0.6 ; zeta = 0.8 ; scale = 15 ; amp = 0.028

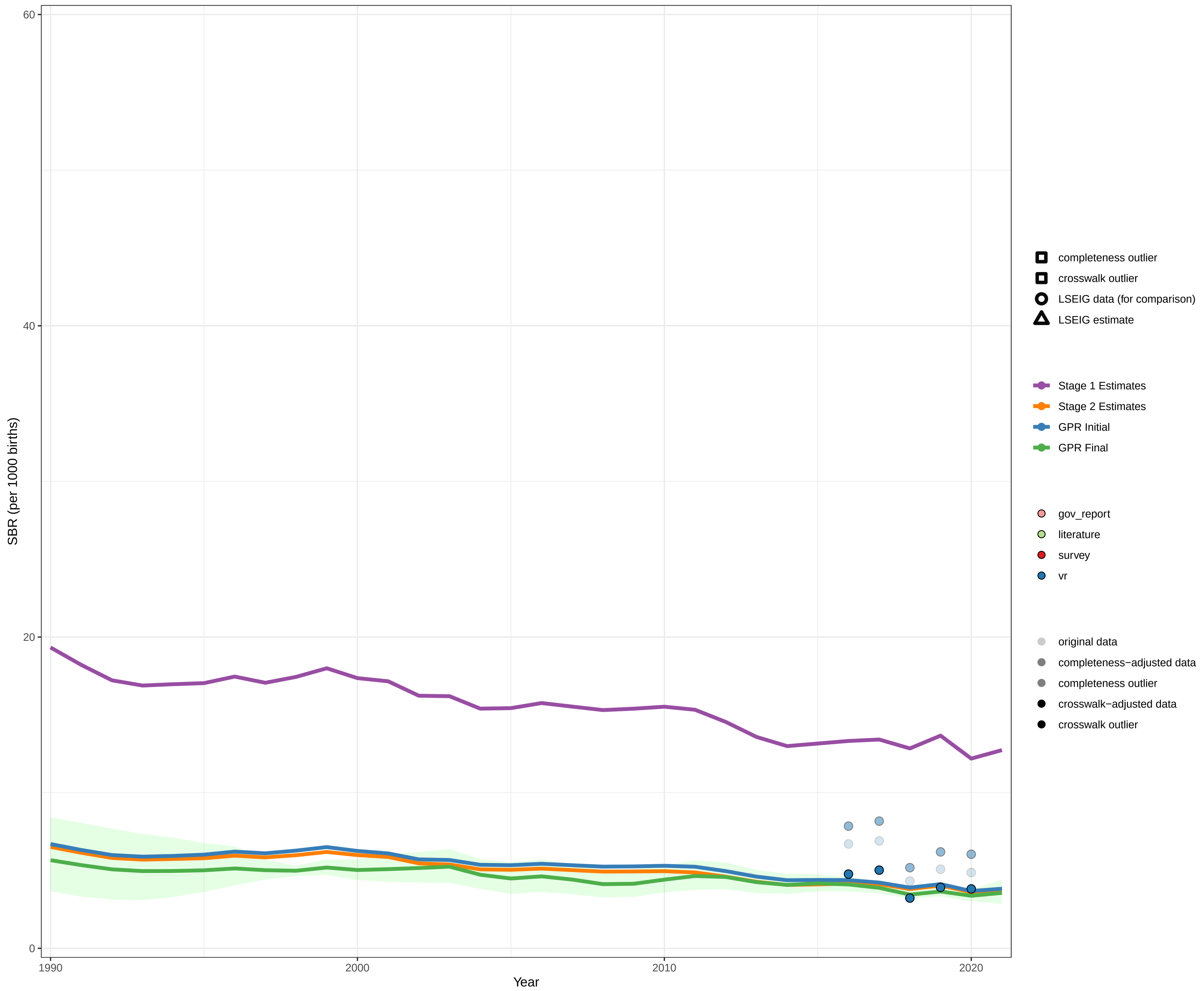

Year	Total Stillbirths (in thousands)	Total Stillbirths (in thousands)	SBR (per 1000 births)	SBR (per 1000 births)	SBR/NMR	SBR/NMR
	20 weeks	28 weeks	20 weeks	28 weeks	20 weeks	28 weeks
1990	0.0 (0.0 to 0.0)	0.0 (0.0 to 0.0)	9.5 (6.5 to 13.7)	5.7 (3.7 to 8.4)	1.77 (1.21 to 2.55)	1.06 (0.68 to 1.57)
1995	0.0 (0.0 to 0.0)	0.0 (0.0 to 0.0)	8.9 (6.1 to 13.0)	5.0 (3.6 to 6.8)	2.04 (1.38 to 2.97)	1.14 (0.83 to 1.54)
2000	0.0 (0.0 to 0.0)	0.0 (0.0 to 0.0)	9.6 (8.6 to 10.7)	5.0 (4.4 to 5.7)	2.29 (2.06 to 2.55)	1.20 (1.05 to 1.37)
2005	0.0 (0.0 to 0.0)	0.0 (0.0 to 0.0)	9.5 (8.0 to 11.1)	4.5 (3.5 to 5.5)	2.72 (2.29 to 3.20)	1.29 (1.00 to 1.59)
2010	0.0 (0.0 to 0.1)	0.0 (0.0 to 0.0)	9.7 (8.2 to 11.3)	4.4 (3.6 to 5.3)	2.95 (2.50 to 3.44)	1.34 (1.09 to 1.62)
2015	0.0 (0.0 to 0.0)	0.0 (0.0 to 0.0)	8.8 (7.6 to 10.3)	4.2 (3.7 to 4.7)	3.37 (2.89 to 3.92)	1.59 (1.40 to 1.81)
2020	0.0 (0.0 to 0.0)	0.0 (0.0 to 0.0)	7.0 (6.1 to 8.0)	3.4 (3.0 to 3.8)	2.95 (2.58 to 3.38)	1.43 (1.28 to 1.60)
2021	0.0 (0.0 to 0.0)	0.0 (0.0 to 0.0)	7.5 (5.8 to 9.5)	3.6 (2.9 to 4.4)	3.03 (2.38 to 3.85)	1.45 (1.16 to 1.80)

Waltham Forest GBR_44747 Western Europe Stillbirth Rate / Neonatal Mortality Rate (SBR/NMR) − 20 weeks
lambda = 0.6 ; zeta = 0.8 ; scale = 15 ; amp = 0.028

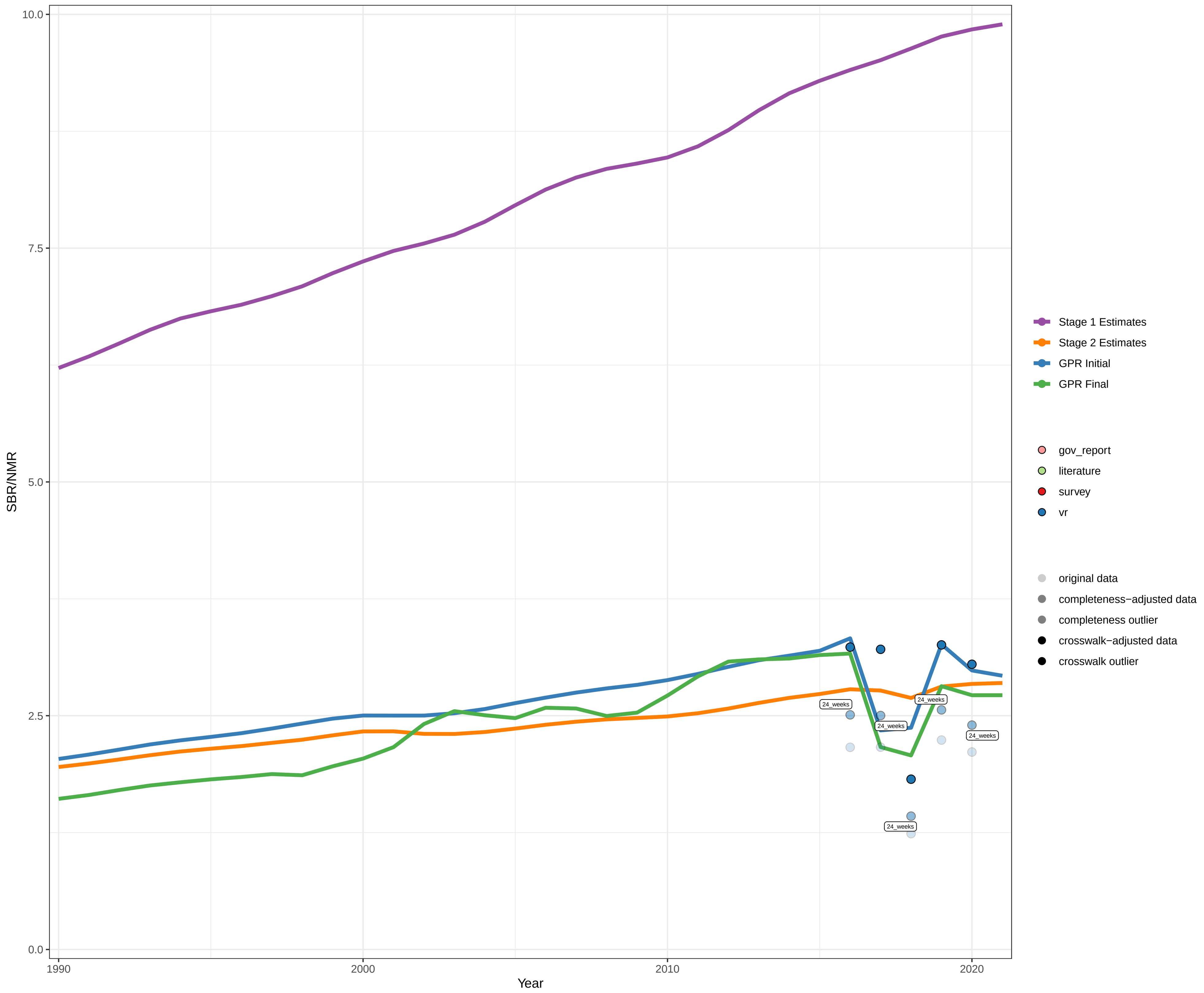

Waltham Forest GBR_44747 Western Europe Stillbirth Rate / Neonatal Mortality Rate (SBR/NMR) − 28 weeks
lambda = 0.6 ; zeta = 0.8 ; scale = 15 ; amp = 0.028

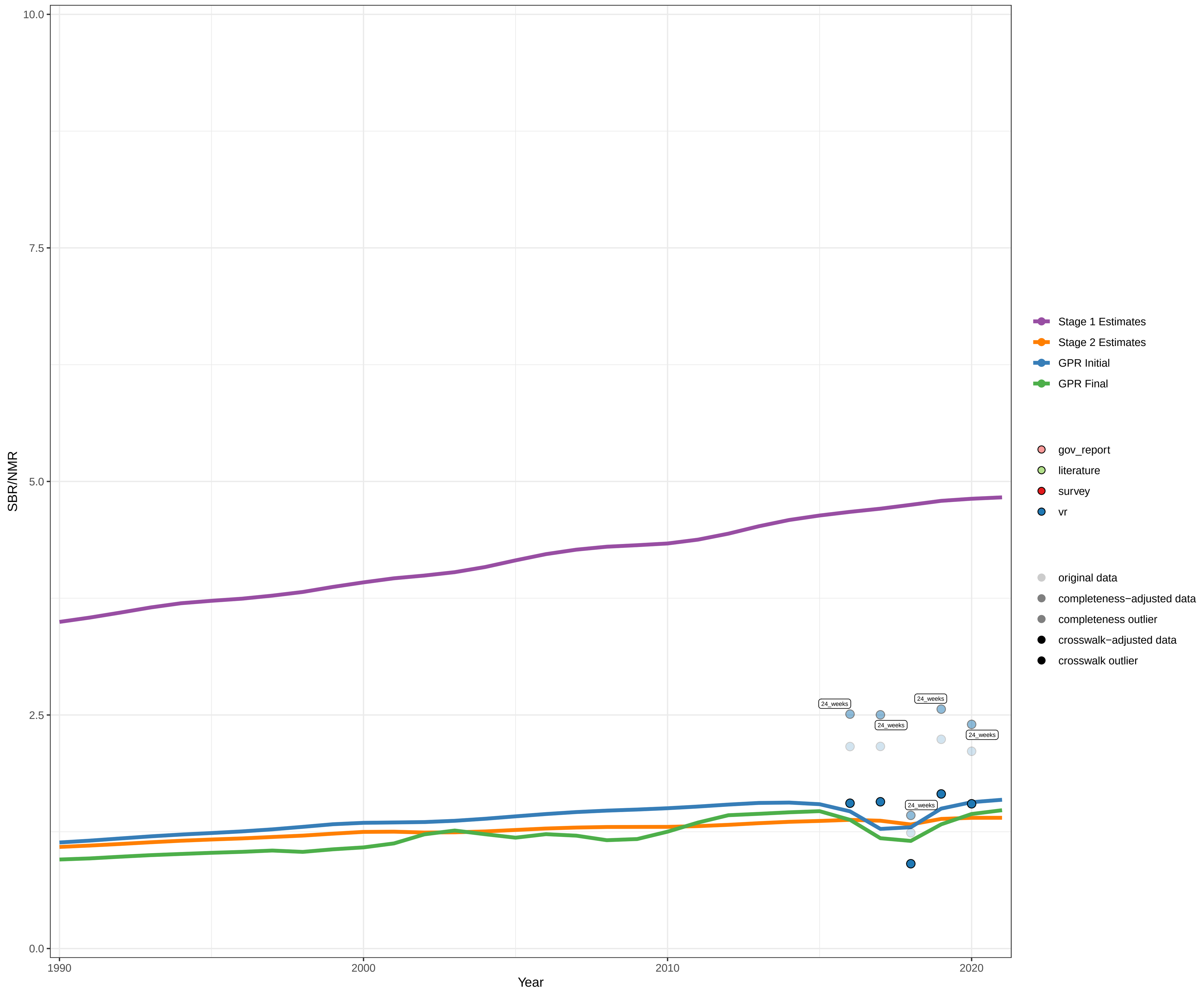

Definition	Years	Outlier	Source.Type	Title
24_weeks	2016−2020	0	VR	United Kingdom − England and Wales Child Mortality Statistics

Waltham Forest GBR_44747 Western Europe Stillbirth Rate (SBR) − 20 weeks
lambda = 0.6 ; zeta = 0.8 ; scale = 15 ; amp = 0.028

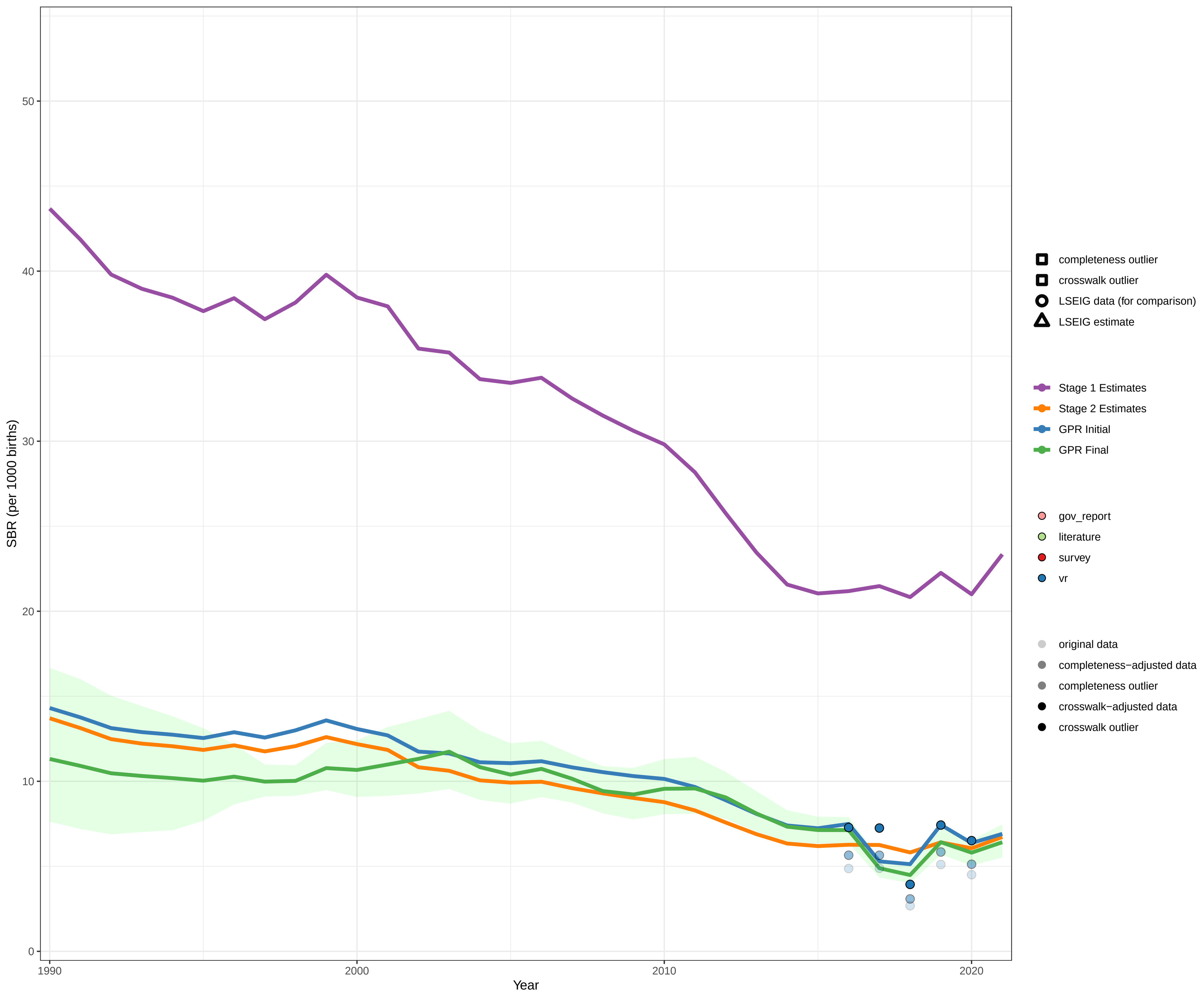

Waltham Forest GBR_44747 Western Europe Stillbirth Rate (SBR) − 28 weeks
lambda = 0.6 ; zeta = 0.8 ; scale = 15 ; amp = 0.028

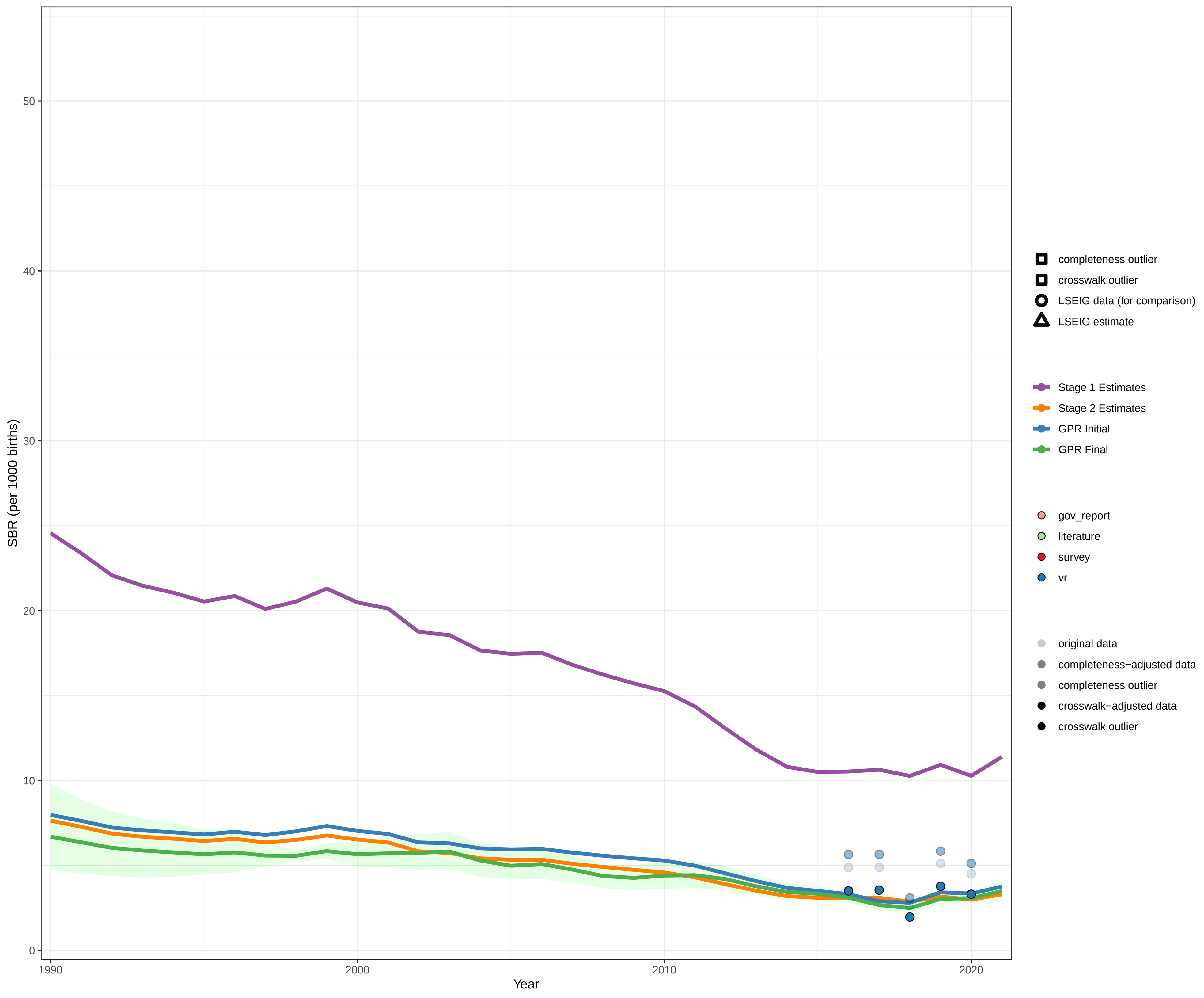

Year	Total Stillbirths (in thousands)	Total Stillbirths (in thousands)	SBR (per 1000 births)	SBR (per 1000 births)	SBR/NMR	SBR/NMR
	20 weeks	28 weeks	20 weeks	28 weeks	20 weeks	28 weeks
1990	0.0 (0.0 to 0.1)	0.0 (0.0 to 0.0)	11.3 (7.6 to 16.7)	6.7 (4.8 to 9.8)	1.61 (1.09 to 2.37)	0.95 (0.68 to 1.40)
1995	0.0 (0.0 to 0.0)	0.0 (0.0 to 0.0)	10.0 (7.7 to 13.1)	5.7 (4.5 to 7.1)	1.82 (1.39 to 2.38)	1.02 (0.81 to 1.29)
2000	0.0 (0.0 to 0.0)	0.0 (0.0 to 0.0)	10.7 (9.1 to 12.4)	5.7 (5.0 to 6.4)	2.04 (1.74 to 2.38)	1.08 (0.95 to 1.22)
2005	0.0 (0.0 to 0.1)	0.0 (0.0 to 0.0)	10.4 (8.7 to 12.2)	5.0 (4.2 to 5.9)	2.47 (2.07 to 2.91)	1.19 (0.99 to 1.41)
2010	0.0 (0.0 to 0.1)	0.0 (0.0 to 0.0)	9.6 (8.1 to 11.3)	4.4 (3.6 to 5.3)	2.72 (2.29 to 3.21)	1.25 (1.03 to 1.49)
2015	0.0 (0.0 to 0.0)	0.0 (0.0 to 0.0)	7.1 (6.3 to 7.9)	3.3 (3.0 to 3.7)	3.15 (2.80 to 3.50)	1.47 (1.31 to 1.65)
2020	0.0 (0.0 to 0.0)	0.0 (0.0 to 0.0)	5.8 (5.0 to 6.5)	3.1 (2.8 to 3.4)	2.72 (2.36 to 3.07)	1.44 (1.31 to 1.57)
2021	0.0 (0.0 to 0.0)	0.0 (0.0 to 0.0)	6.4 (5.5 to 7.5)	3.5 (3.0 to 4.2)	2.72 (2.34 to 3.16)	1.48 (1.25 to 1.77)

Wandsworth GBR_44746 Western Europe Stillbirth Rate / Neonatal Mortality Rate (SBR/NMR) − 20 weeks
lambda = 0.6 ; zeta = 0.8 ; scale = 15 ; amp = 0.028

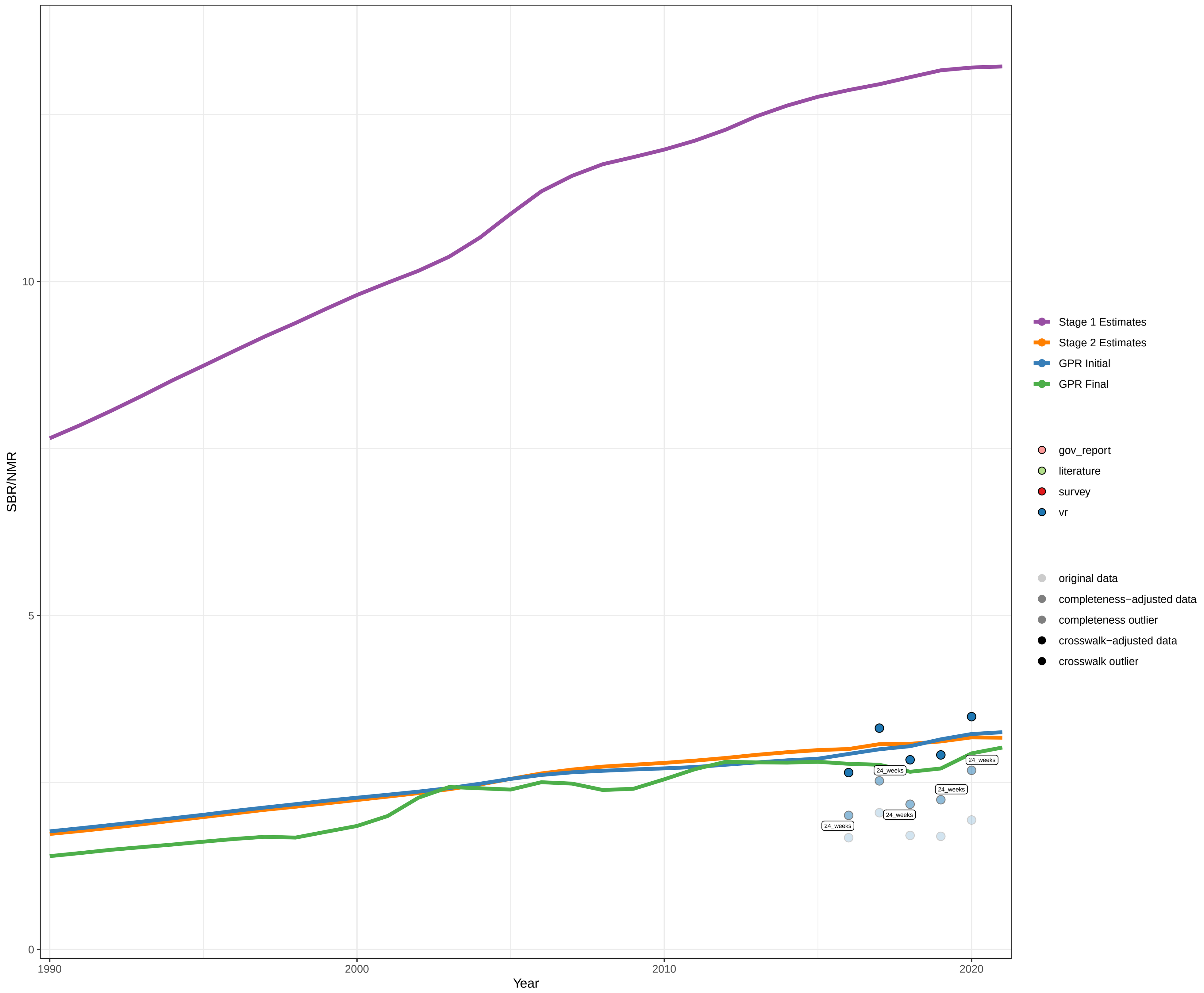

Wandsworth GBR_44746 Western Europe Stillbirth Rate / Neonatal Mortality Rate (SBR/NMR) − 28 weeks
lambda = 0.6 ; zeta = 0.8 ; scale = 15 ; amp = 0.028

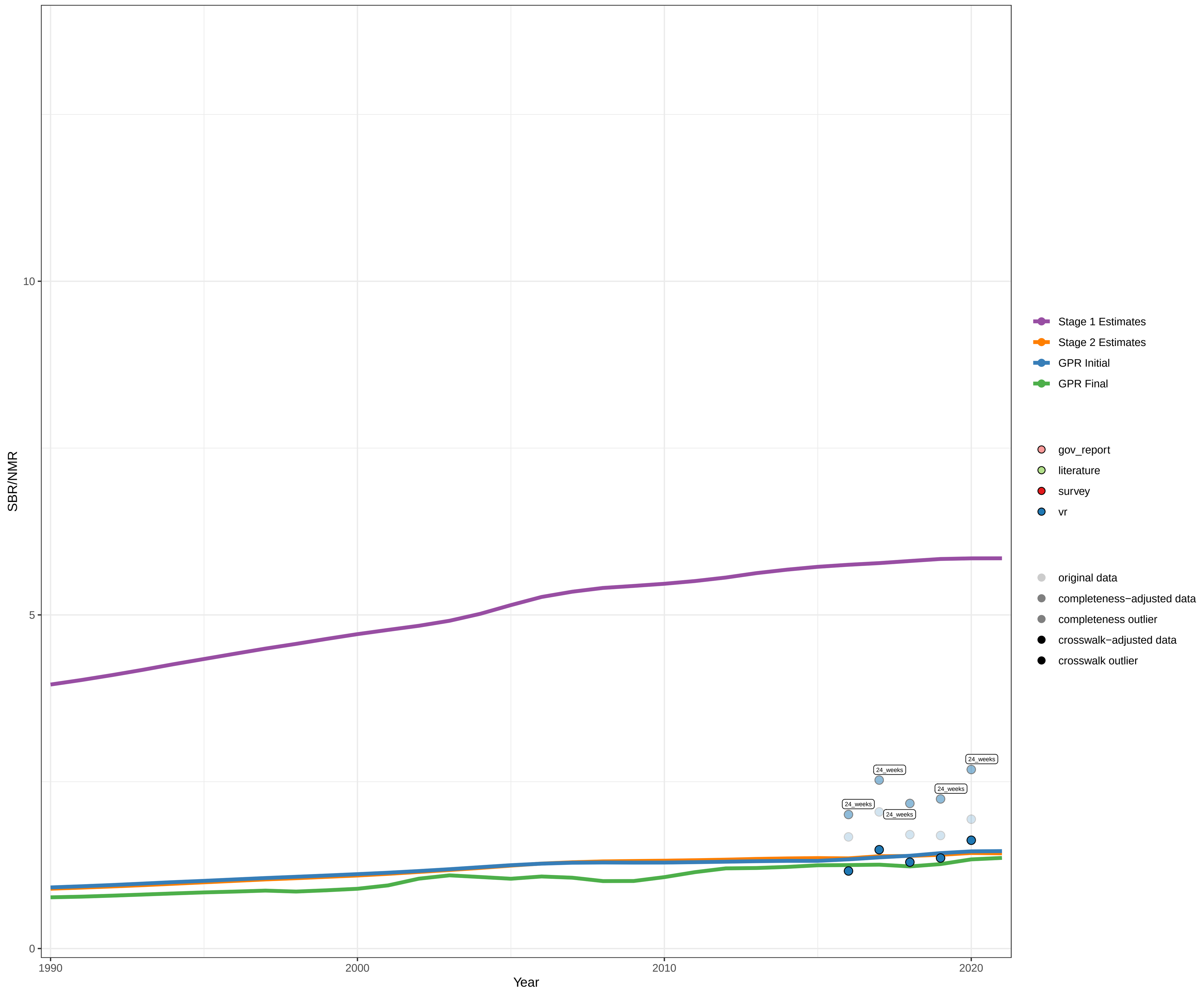

Definition	Years	Outlier	Source.Type	Title
24_weeks	2016−2020	0	VR	United Kingdom − England and Wales Child Mortality Statistics

Wandsworth GBR_44746 Western Europe Stillbirth Rate (SBR) − 20 weeks
lambda = 0.6 ; zeta = 0.8 ; scale = 15 ; amp = 0.028

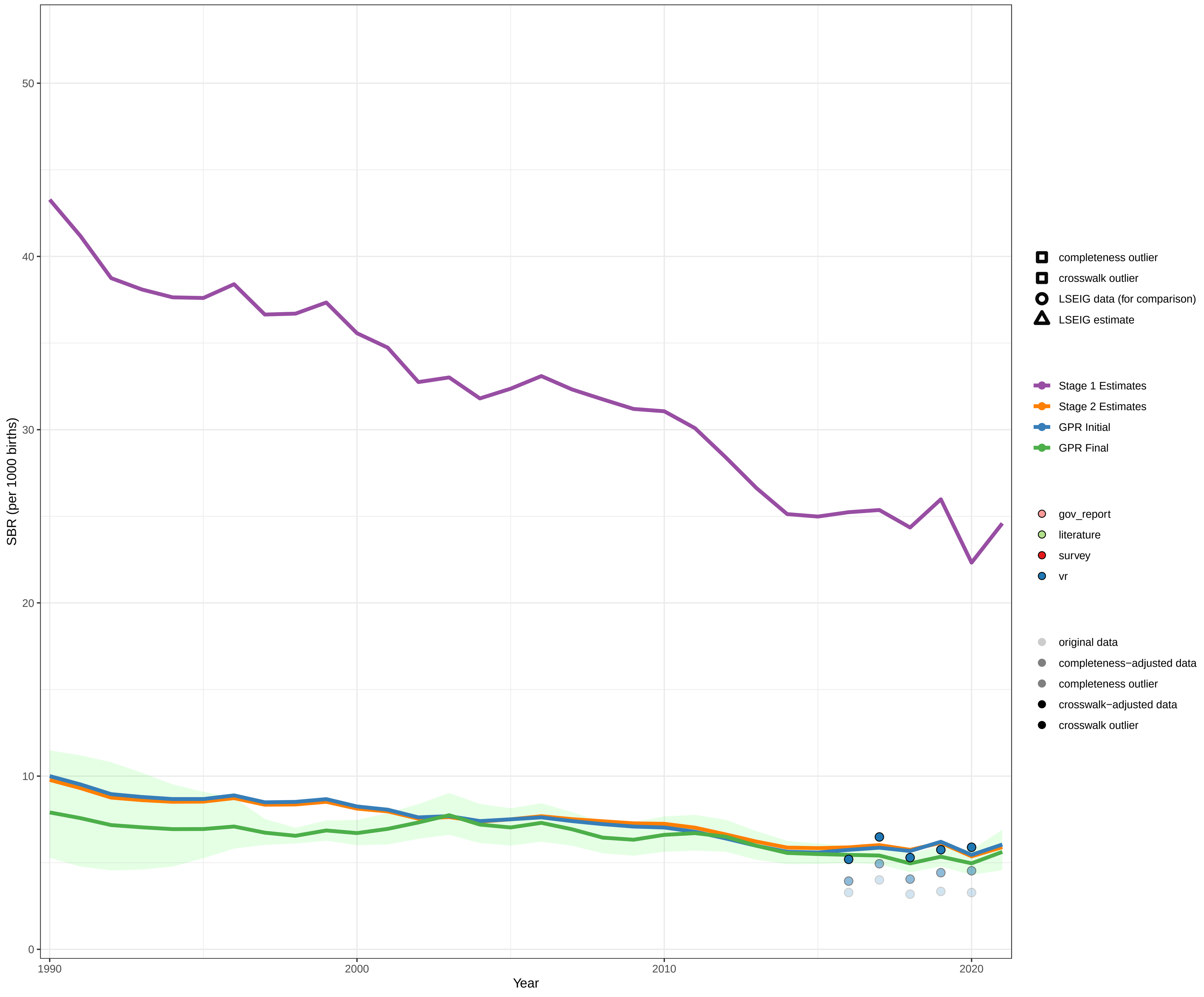

Wandsworth GBR_44746 Western Europe Stillbirth Rate (SBR) − 28 weeks
lambda = 0.6 ; zeta = 0.8 ; scale = 15 ; amp = 0.028

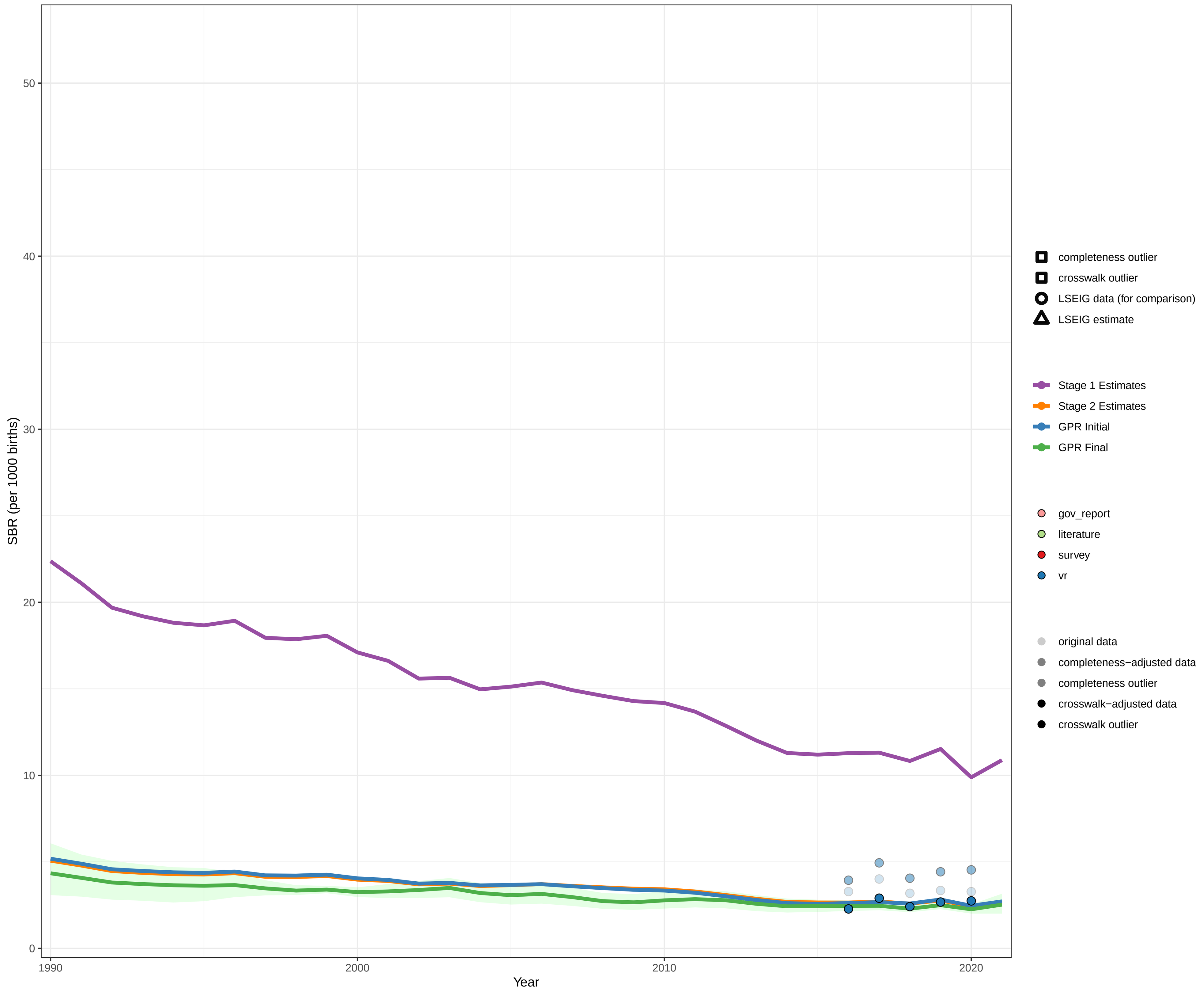

Year	Total Stillbirths (in thousands)	Total Stillbirths (in thousands)	SBR (per 1000 births)	SBR (per 1000 births)	SBR/NMR	SBR/NMR
	20 weeks	28 weeks	20 weeks	28 weeks	20 weeks	28 weeks
1990	0.0 (0.0 to 0.0)	0.0 (0.0 to 0.0)	7.9 (5.3 to 11.5)	4.3 (3.1 to 6.1)	1.40 (0.93 to 2.03)	0.77 (0.54 to 1.07)
1995	0.0 (0.0 to 0.0)	0.0 (0.0 to 0.0)	6.9 (5.3 to 9.1)	3.6 (2.7 to 4.6)	1.61 (1.22 to 2.11)	0.84 (0.63 to 1.08)
2000	0.0 (0.0 to 0.0)	0.0 (0.0 to 0.0)	6.7 (6.0 to 7.5)	3.3 (3.0 to 3.5)	1.85 (1.66 to 2.06)	0.90 (0.82 to 0.97)
2005	0.0 (0.0 to 0.0)	0.0 (0.0 to 0.0)	7.0 (6.0 to 8.1)	3.1 (2.5 to 3.6)	2.39 (2.04 to 2.77)	1.05 (0.86 to 1.24)
2010	0.0 (0.0 to 0.0)	0.0 (0.0 to 0.0)	6.6 (5.6 to 7.7)	2.8 (2.3 to 3.3)	2.55 (2.17 to 2.96)	1.07 (0.89 to 1.27)
2015	0.0 (0.0 to 0.0)	0.0 (0.0 to 0.0)	5.5 (4.9 to 6.1)	2.4 (2.1 to 2.8)	2.81 (2.52 to 3.12)	1.25 (1.08 to 1.44)
2020	0.0 (0.0 to 0.0)	0.0 (0.0 to 0.0)	5.0 (4.3 to 5.7)	2.3 (2.0 to 2.5)	2.94 (2.54 to 3.39)	1.34 (1.19 to 1.50)
2021	0.0 (0.0 to 0.0)	0.0 (0.0 to 0.0)	5.6 (4.6 to 6.9)	2.5 (2.0 to 3.2)	3.02 (2.45 to 3.71)	1.36 (1.08 to 1.70)

Westminster GBR_44730 Western Europe Stillbirth Rate / Neonatal Mortality Rate (SBR/NMR) − 20 weeks
lambda = 0.6 ; zeta = 0.8 ; scale = 15 ; amp = 0.028

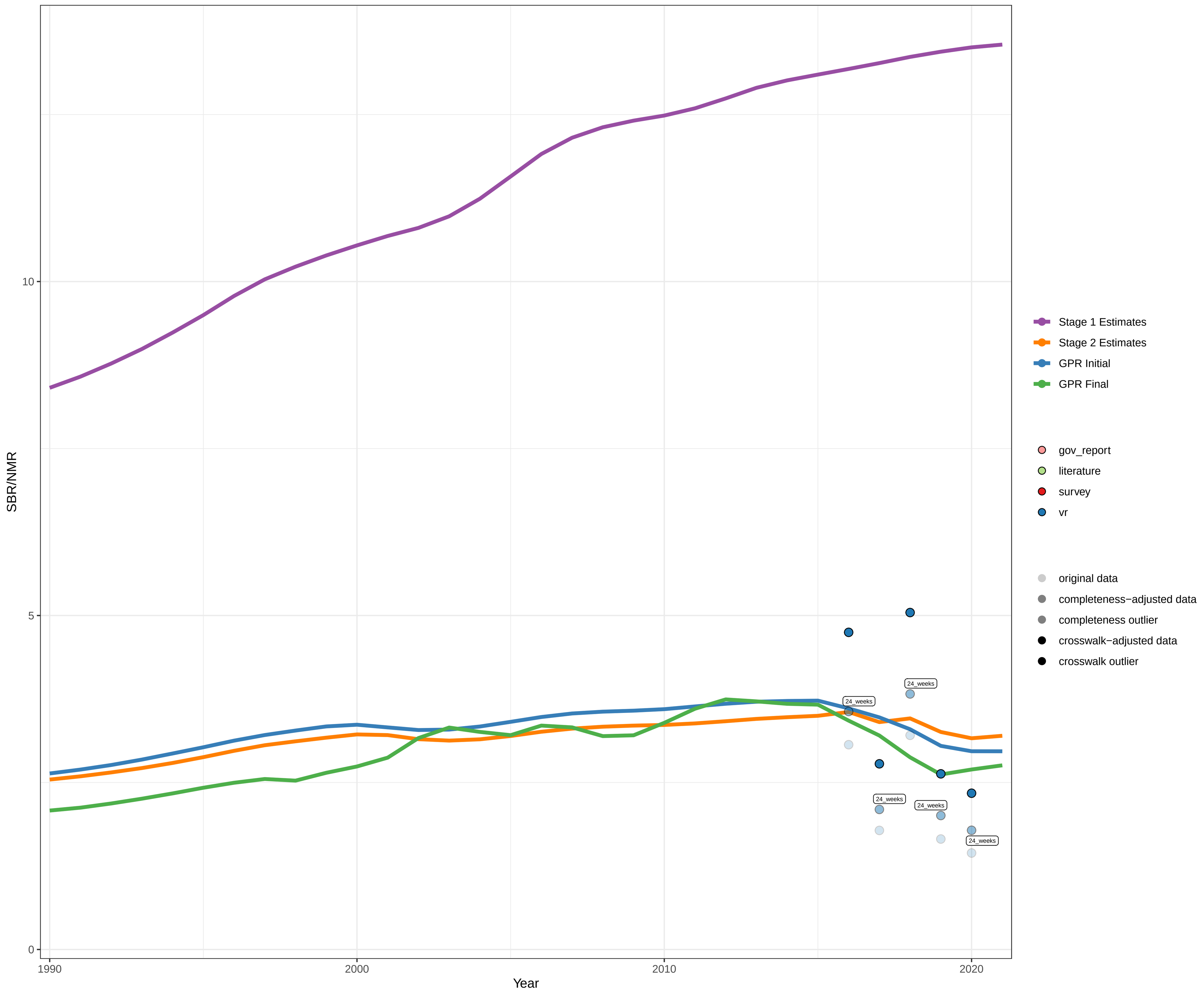

Westminster GBR_44730 Western Europe Stillbirth Rate / Neonatal Mortality Rate (SBR/NMR) − 28 weeks
lambda = 0.6 ; zeta = 0.8 ; scale = 15 ; amp = 0.028

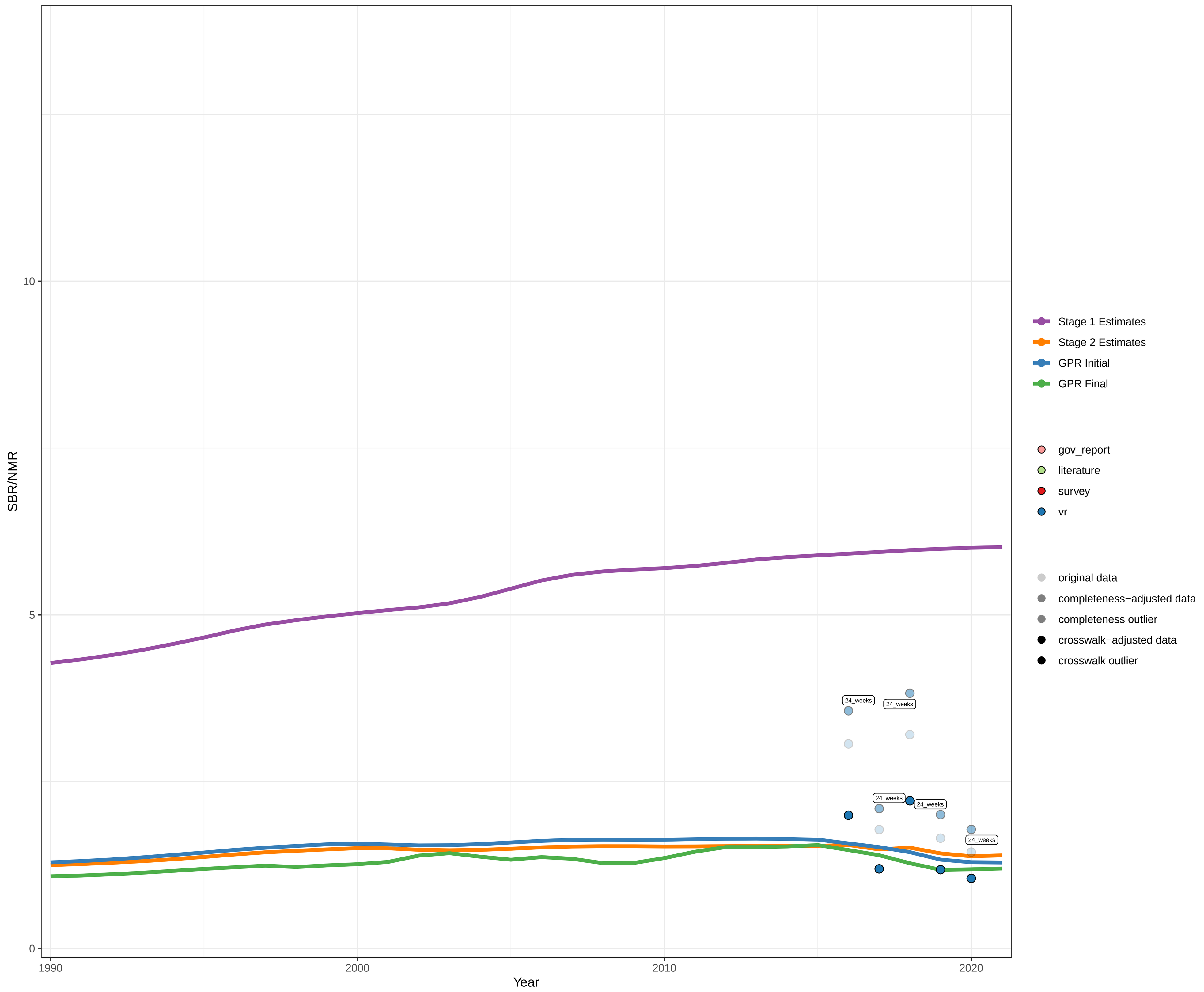

Definition	Years	Outlier	Source.Type	Title
24_weeks	2016−2020	0	VR	United Kingdom − England and Wales Child Mortality Statistics

Westminster GBR_44730 Western Europe Stillbirth Rate (SBR) − 20 weeks
lambda = 0.6 ; zeta = 0.8 ; scale = 15 ; amp = 0.028

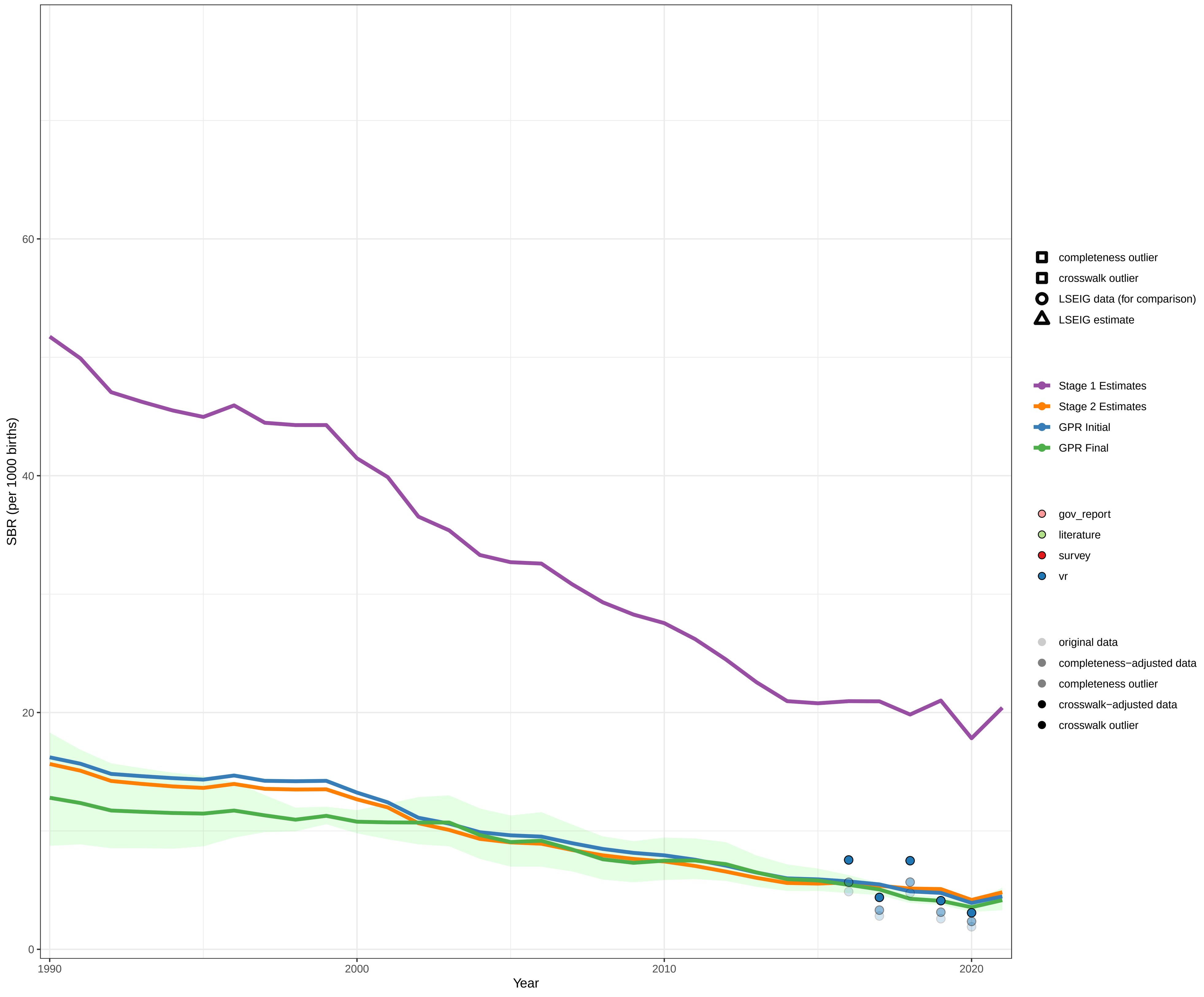

Westminster GBR_44730 Western Europe Stillbirth Rate (SBR) − 28 weeks
lambda = 0.6 ; zeta = 0.8 ; scale = 15 ; amp = 0.028

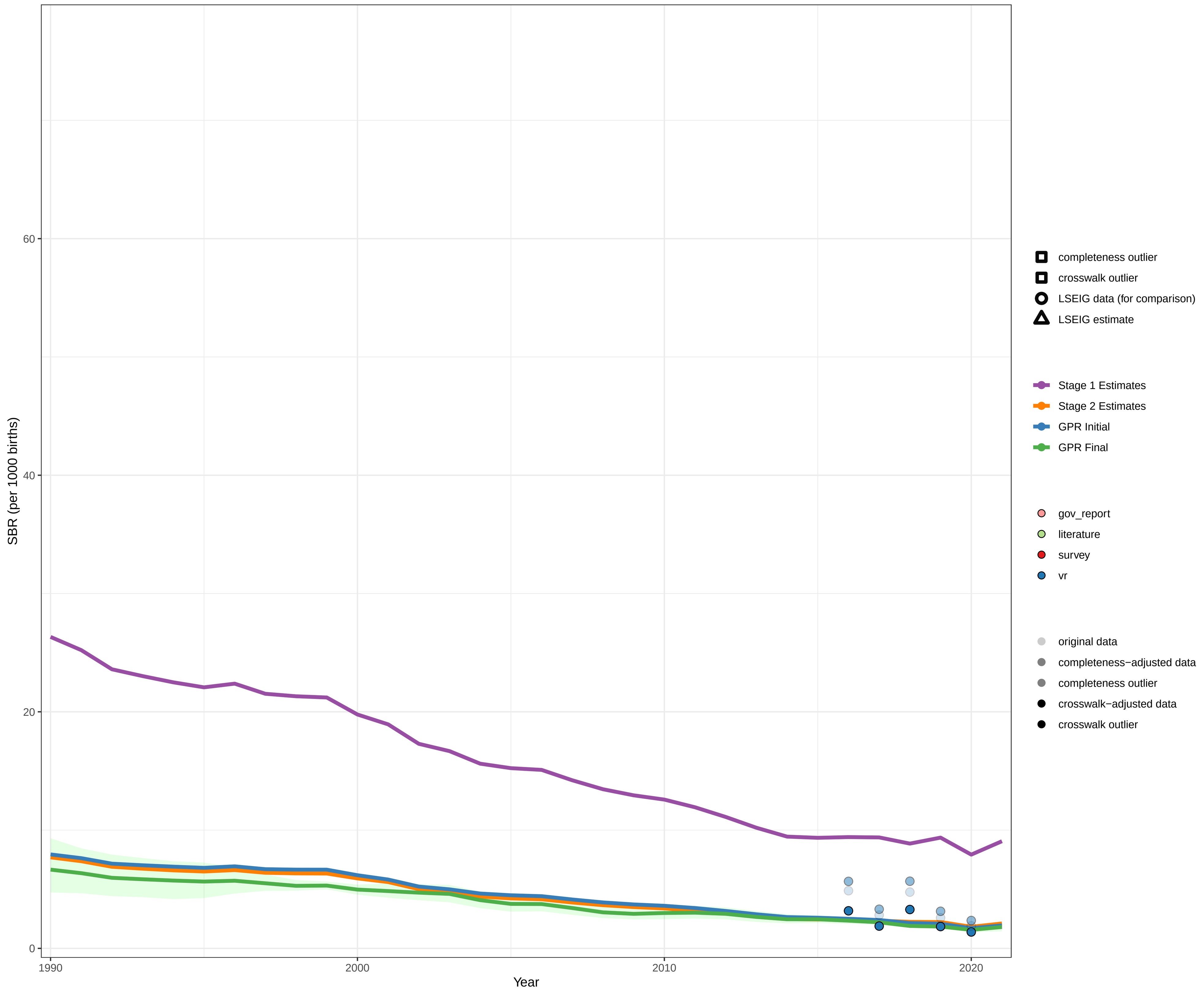

Year	Total Stillbirths (in thousands)	Total Stillbirths (in thousands)	SBR (per 1000 births)	SBR (per 1000 births)	SBR/NMR	SBR/NMR
	20 weeks	28 weeks	20 weeks	28 weeks	20 weeks	28 weeks
1990	0.0 (0.0 to 0.0)	0.0 (0.0 to 0.0)	12.8 (8.7 to 18.3)	6.7 (4.7 to 9.3)	2.08 (1.42 to 2.98)	1.08 (0.77 to 1.51)
1995	0.0 (0.0 to 0.0)	0.0 (0.0 to 0.0)	11.5 (8.7 to 14.6)	5.6 (4.3 to 7.2)	2.42 (1.84 to 3.09)	1.19 (0.90 to 1.53)
2000	0.0 (0.0 to 0.0)	0.0 (0.0 to 0.0)	10.8 (9.8 to 11.8)	5.0 (4.5 to 5.4)	2.74 (2.49 to 2.99)	1.26 (1.15 to 1.37)
2005	0.0 (0.0 to 0.0)	0.0 (0.0 to 0.0)	9.1 (7.0 to 11.3)	3.8 (3.1 to 4.4)	3.21 (2.47 to 4.00)	1.33 (1.10 to 1.57)
2010	0.0 (0.0 to 0.0)	0.0 (0.0 to 0.0)	7.5 (5.9 to 9.4)	3.0 (2.5 to 3.5)	3.39 (2.65 to 4.28)	1.36 (1.12 to 1.60)
2015	0.0 (0.0 to 0.0)	0.0 (0.0 to 0.0)	5.8 (4.9 to 6.8)	2.5 (2.1 to 2.8)	3.67 (3.11 to 4.30)	1.55 (1.35 to 1.77)
2020	0.0 (0.0 to 0.0)	0.0 (0.0 to 0.0)	3.6 (3.2 to 4.0)	1.6 (1.4 to 1.8)	2.69 (2.40 to 3.02)	1.19 (1.03 to 1.36)
2021	0.0 (0.0 to 0.0)	0.0 (0.0 to 0.0)	4.2 (3.3 to 5.2)	1.8 (1.4 to 2.2)	2.76 (2.19 to 3.46)	1.20 (0.96 to 1.48)

North East England GBR_4618 Western Europe Stillbirth Rate / Neonatal Mortality Rate (SBR/NMR) − 20 weeks
lambda = 0.2 ; zeta = 0.99 ; scale = 5 ; amp = 0.028

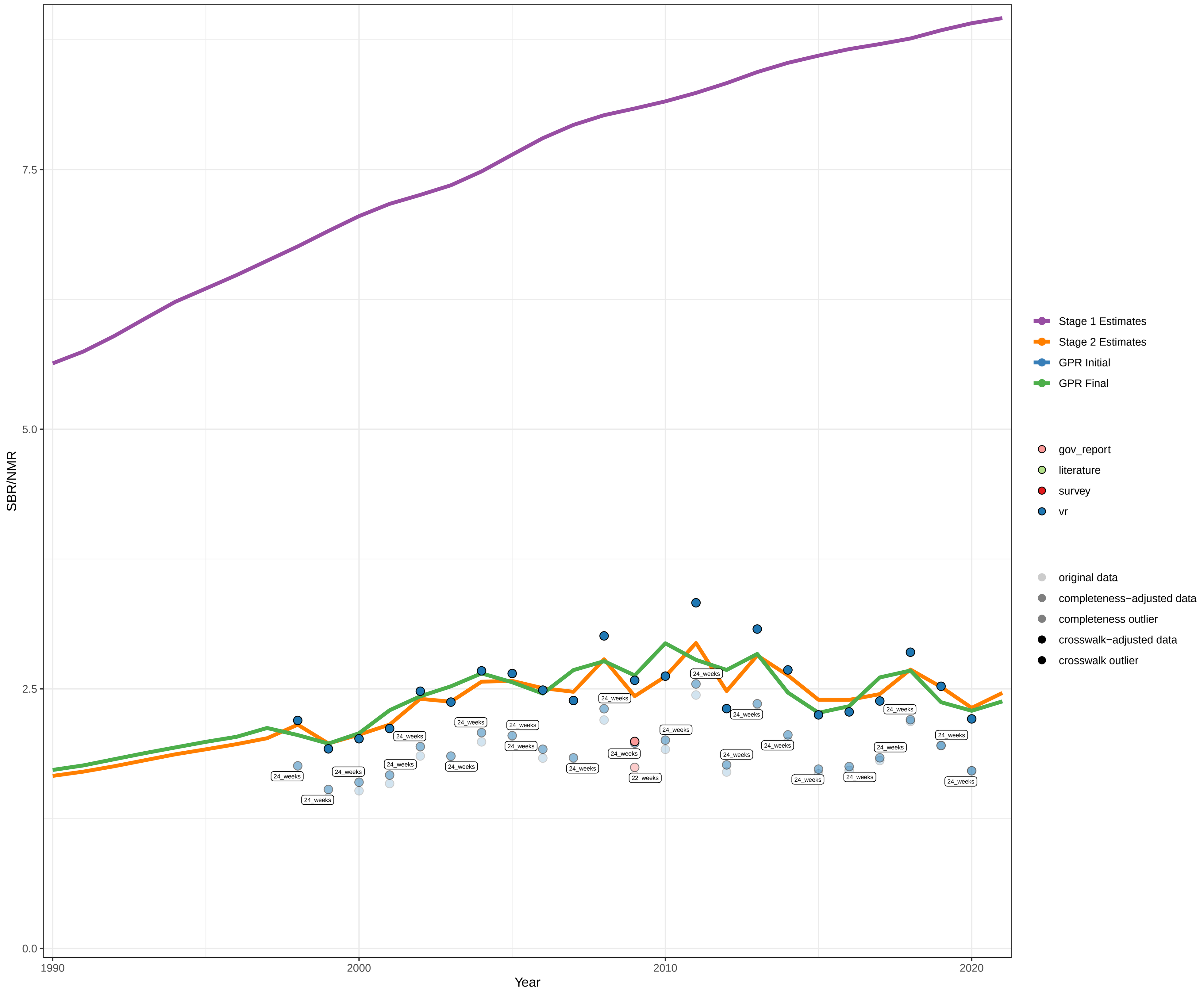

North East England GBR_4618 Western Europe Stillbirth Rate / Neonatal Mortality Rate (SBR/NMR) − 28 weeks
lambda = 0.2 ; zeta = 0.99 ; scale = 5 ; amp = 0.028

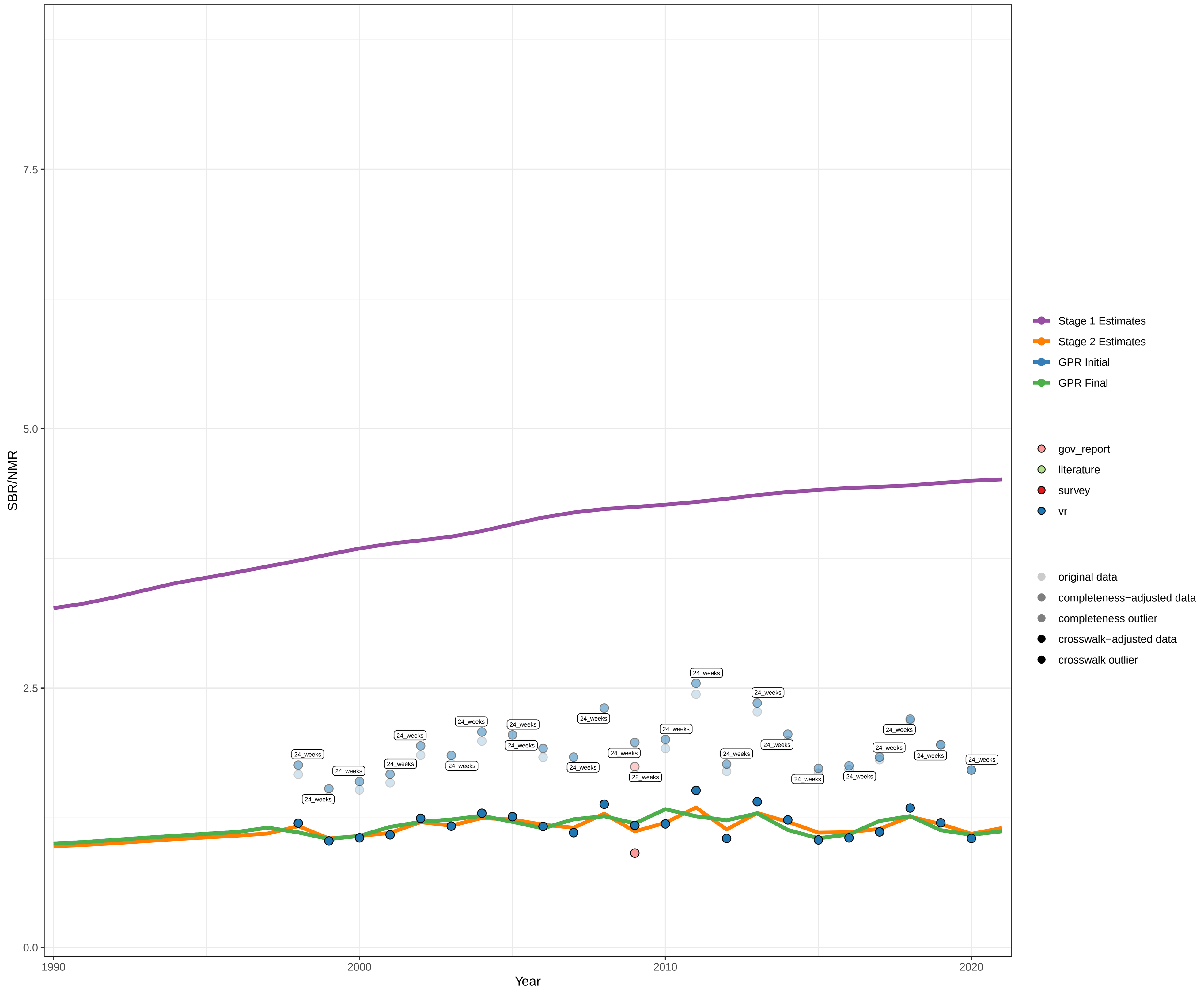

Definition	Years	Outlier	Source.Type	Title
22_weeks	2009	0	Statistical Report	United Kingdom Perinatal Mortality
24_weeks	1998−2007	0	VR	United Kingdom − England and Wales Birth Statistics
24_weeks	2008−2020	0	VR	United Kingdom − England and Wales Child Mortality Statistics

North East England GBR_4618 Western Europe Stillbirth Rate (SBR) − 20 weeks
lambda = 0.2 ; zeta = 0.99 ; scale = 5 ; amp = 0.028

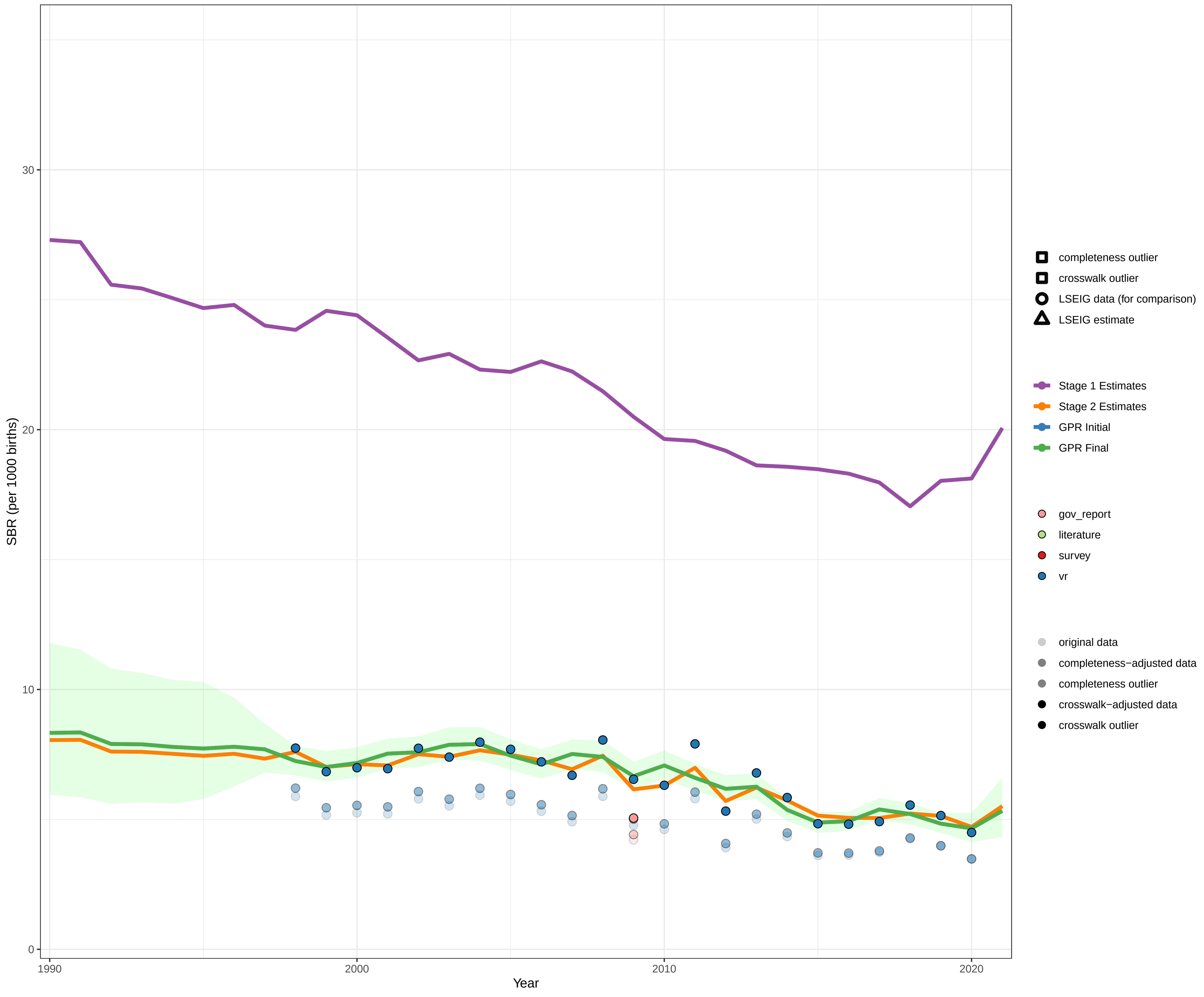

North East England GBR_4618 Western Europe Stillbirth Rate (SBR) − 28 weeks
lambda = 0.2 ; zeta = 0.99 ; scale = 5 ; amp = 0.028

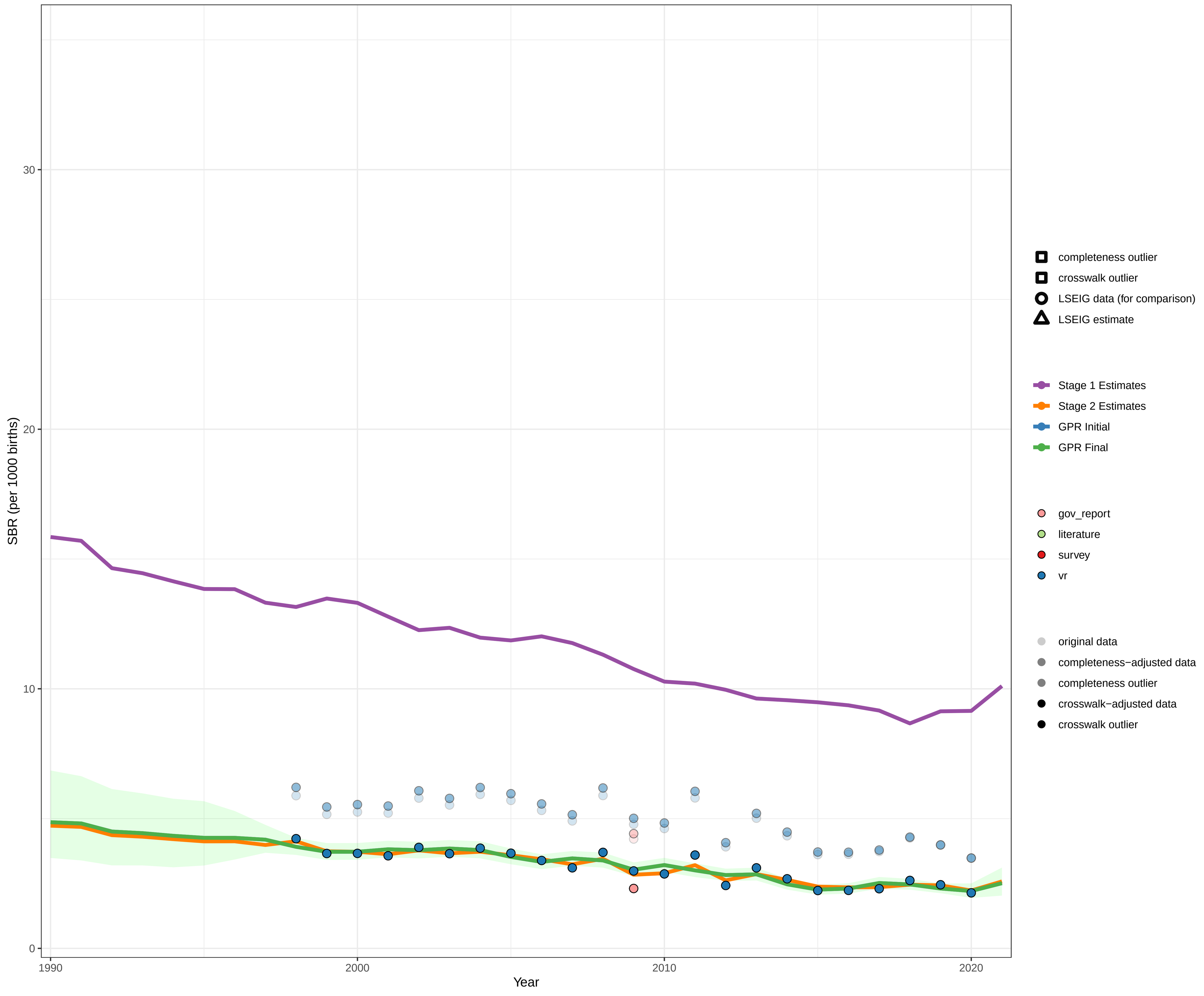

Year	Total Stillbirths (in thousands)	Total Stillbirths (in thousands)	SBR (per 1000 births)	SBR (per 1000 births)	SBR/NMR	SBR/NMR
	20 weeks	28 weeks	20 weeks	28 weeks	20 weeks	28 weeks
1990	0.3 (0.2 to 0.4)	0.2 (0.1 to 0.2)	8.3 (5.9 to 11.8)	4.9 (3.5 to 6.9)	1.72 (1.23 to 2.43)	1.00 (0.72 to 1.41)
1995	0.2 (0.2 to 0.3)	0.1 (0.1 to 0.2)	7.7 (5.8 to 10.3)	4.3 (3.2 to 5.7)	1.99 (1.49 to 2.65)	1.10 (0.82 to 1.46)
2000	0.2 (0.2 to 0.2)	0.1 (0.1 to 0.1)	7.2 (6.6 to 7.8)	3.7 (3.4 to 4.1)	2.07 (1.91 to 2.25)	1.08 (0.99 to 1.17)
2005	0.2 (0.2 to 0.2)	0.1 (0.1 to 0.1)	7.5 (6.9 to 8.1)	3.5 (3.2 to 3.8)	2.56 (2.37 to 2.78)	1.21 (1.11 to 1.32)
2010	0.2 (0.2 to 0.2)	0.1 (0.1 to 0.1)	7.1 (6.6 to 7.7)	3.2 (3.0 to 3.5)	2.94 (2.72 to 3.18)	1.33 (1.23 to 1.45)
2015	0.1 (0.1 to 0.1)	0.1 (0.1 to 0.1)	4.9 (4.5 to 5.3)	2.3 (2.1 to 2.5)	2.27 (2.09 to 2.46)	1.05 (0.96 to 1.14)
2020	0.1 (0.1 to 0.1)	0.1 (0.0 to 0.1)	4.7 (4.1 to 5.2)	2.2 (1.9 to 2.5)	2.29 (2.03 to 2.58)	1.09 (0.96 to 1.23)
2021	0.1 (0.1 to 0.2)	0.1 (0.1 to 0.1)	5.3 (4.3 to 6.6)	2.5 (2.0 to 3.1)	2.38 (1.93 to 2.95)	1.12 (0.91 to 1.39)

County Durham   GBR_44649   Western Europe   Stillbirth Rate / Neonatal Mortality Rate (SBR/NMR) − 20 weeks
lambda = 0.6 ; zeta = 0.8 ; scale = 15 ; amp = 0.028

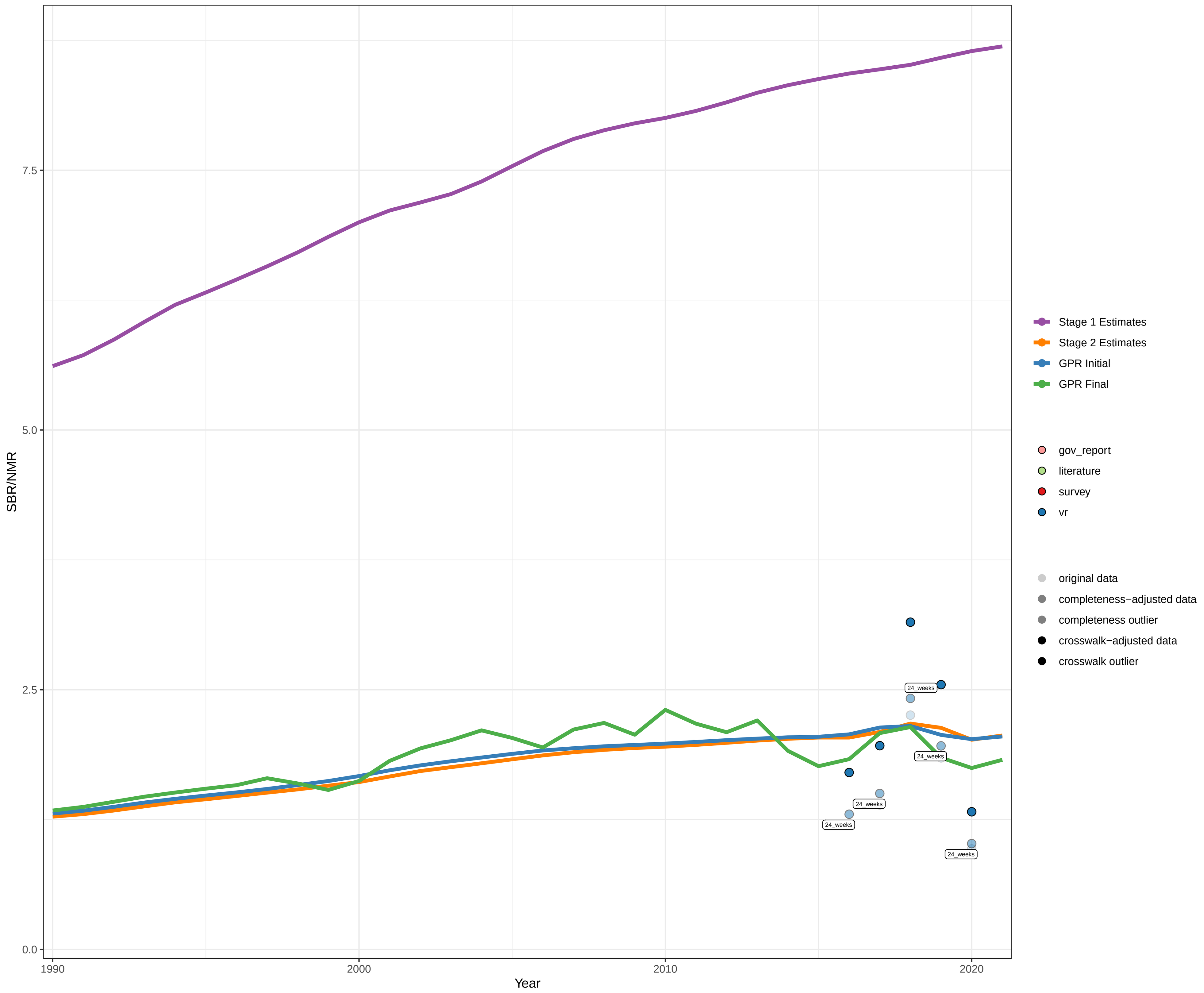

County Durham   GBR_44649   Western Europe   Stillbirth Rate / Neonatal Mortality Rate (SBR/NMR) − 28 weeks
lambda = 0.6 ; zeta = 0.8 ; scale = 15 ; amp = 0.028

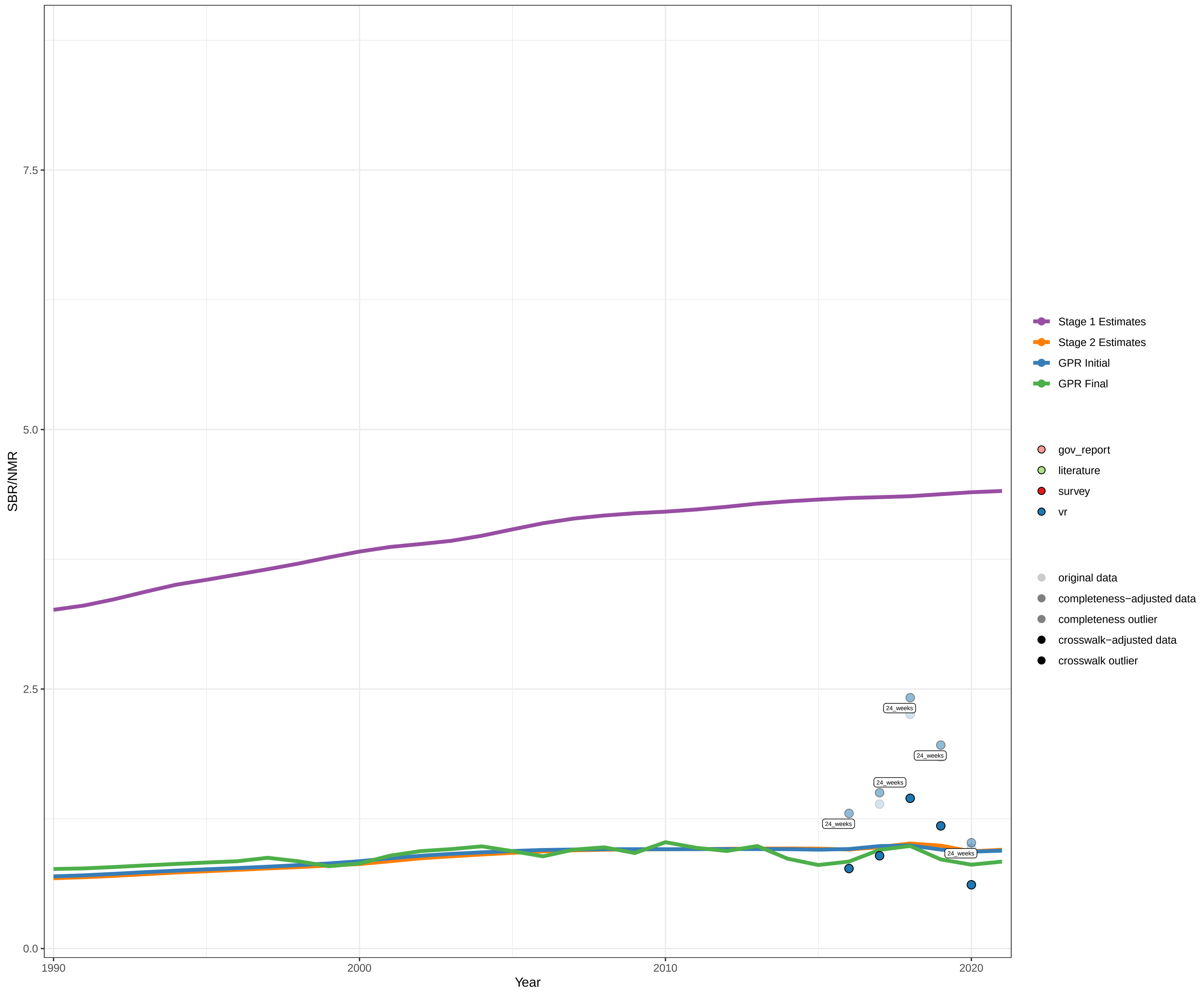

Definition	Years	Outlier	Source.Type	Title
24_weeks	2016−2020	0	VR	United Kingdom − England and Wales Child Mortality Statistics

County Durham   GBR_44649   Western Europe   Stillbirth Rate (SBR) − 20 weeks
lambda = 0.6 ; zeta = 0.8 ; scale = 15 ; amp = 0.028

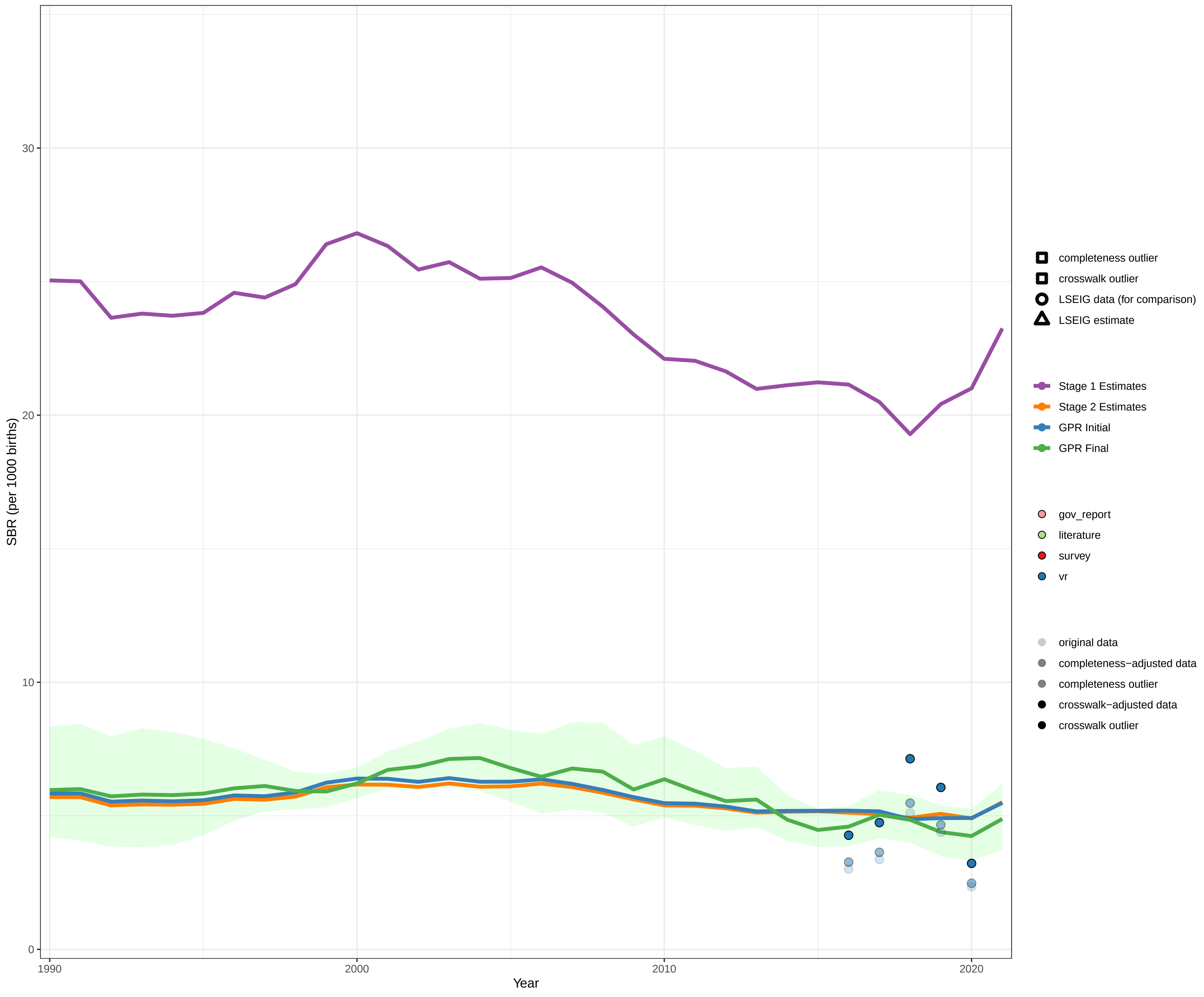

County Durham   GBR_44649   Western Europe   Stillbirth Rate (SBR) − 28 weeks
lambda = 0.6 ; zeta = 0.8 ; scale = 15 ; amp = 0.028

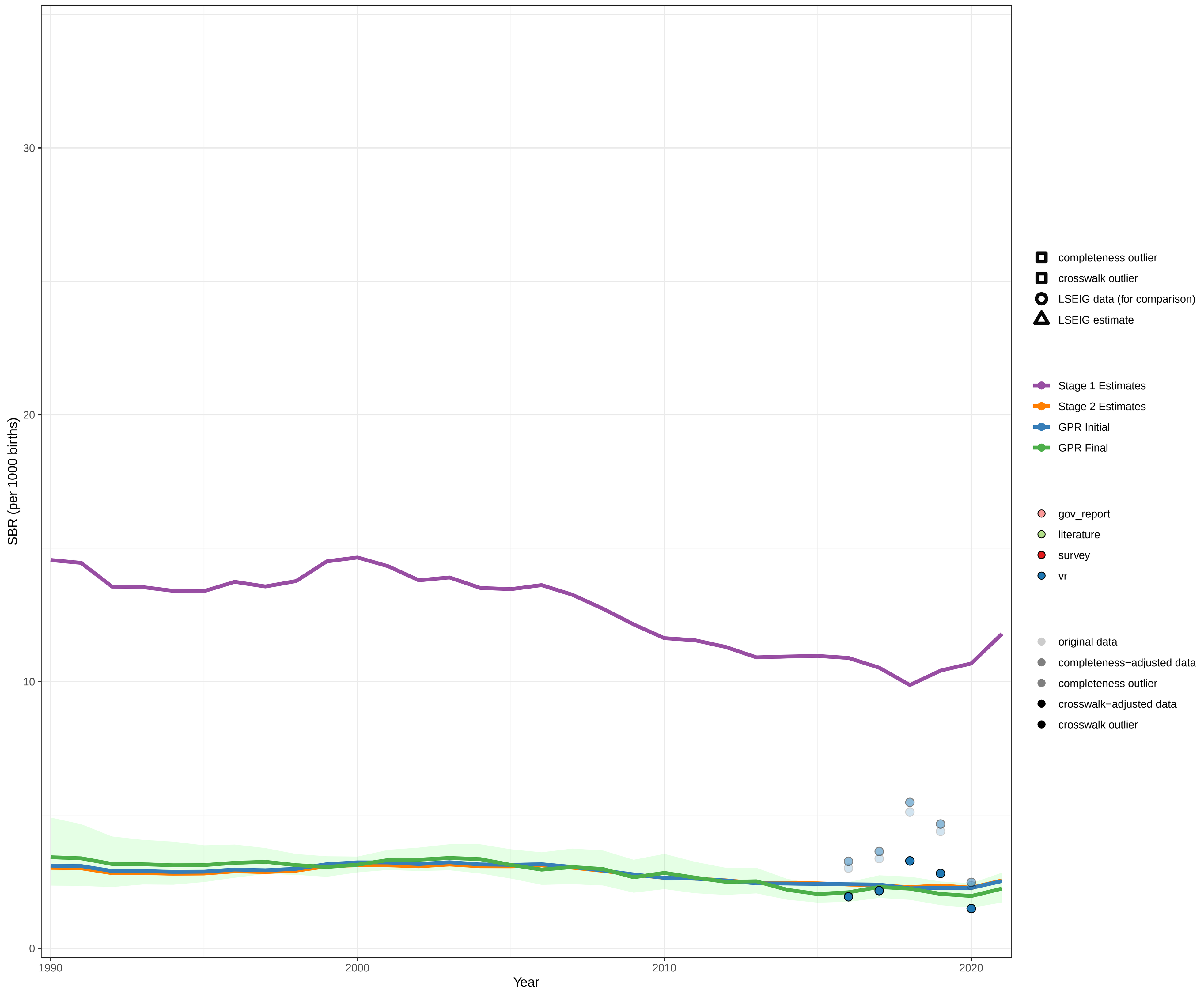

Year	Total Stillbirths (in thousands)	Total Stillbirths (in thousands)	SBR (per 1000 births)	SBR (per 1000 births)	SBR/NMR	SBR/NMR
	20 weeks	28 weeks	20 weeks	28 weeks	20 weeks	28 weeks
1990	0.0 (0.0 to 0.1)	0.0 (0.0 to 0.0)	6.0 (4.2 to 8.3)	3.4 (2.4 to 4.9)	1.34 (0.94 to 1.87)	0.77 (0.53 to 1.10)
1995	0.0 (0.0 to 0.0)	0.0 (0.0 to 0.0)	5.8 (4.3 to 7.9)	3.1 (2.5 to 3.9)	1.55 (1.13 to 2.09)	0.83 (0.66 to 1.02)
2000	0.0 (0.0 to 0.0)	0.0 (0.0 to 0.0)	6.2 (5.7 to 6.8)	3.1 (2.8 to 3.4)	1.62 (1.48 to 1.78)	0.82 (0.74 to 0.90)
2005	0.0 (0.0 to 0.0)	0.0 (0.0 to 0.0)	6.8 (5.5 to 8.2)	3.1 (2.6 to 3.7)	2.04 (1.66 to 2.47)	0.94 (0.79 to 1.11)
2010	0.0 (0.0 to 0.0)	0.0 (0.0 to 0.0)	6.4 (4.9 to 8.0)	2.8 (2.2 to 3.5)	2.31 (1.79 to 2.89)	1.02 (0.80 to 1.28)
2015	0.0 (0.0 to 0.0)	0.0 (0.0 to 0.0)	4.5 (3.8 to 5.3)	2.0 (1.7 to 2.4)	1.76 (1.51 to 2.07)	0.80 (0.68 to 0.95)
2020	0.0 (0.0 to 0.0)	0.0 (0.0 to 0.0)	4.2 (3.3 to 5.3)	2.0 (1.5 to 2.4)	1.75 (1.36 to 2.17)	0.81 (0.63 to 1.00)
2021	0.0 (0.0 to 0.0)	0.0 (0.0 to 0.0)	4.9 (3.7 to 6.2)	2.2 (1.7 to 2.8)	1.83 (1.40 to 2.31)	0.84 (0.64 to 1.06)

Darlington GBR_44643 Western Europe Stillbirth Rate / Neonatal Mortality Rate (SBR/NMR) − 20 weeks
lambda = 0.6 ; zeta = 0.8 ; scale = 15 ; amp = 0.028

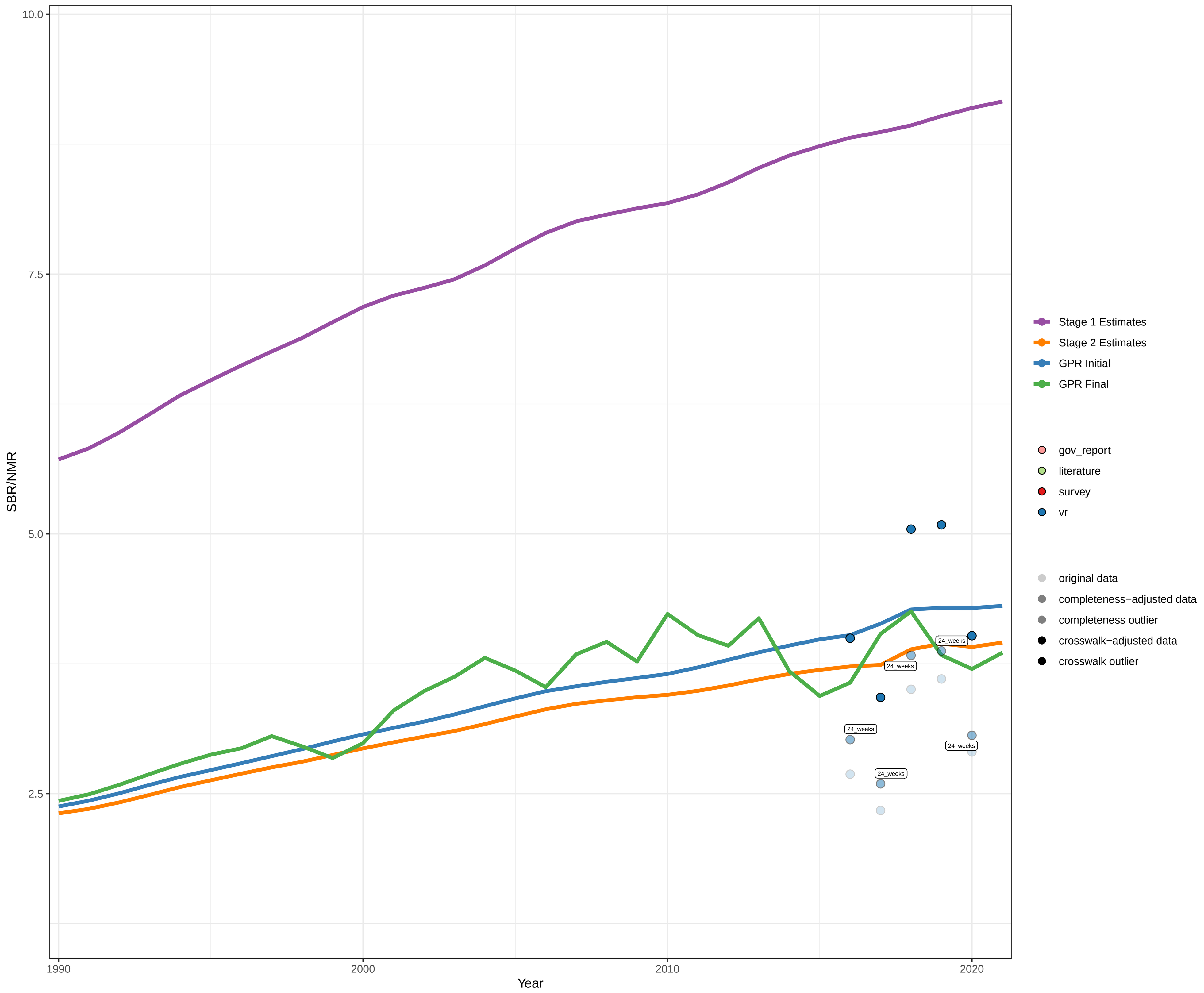

Darlington GBR_44643 Western Europe Stillbirth Rate / Neonatal Mortality Rate (SBR/NMR) − 28 weeks
lambda = 0.6 ; zeta = 0.8 ; scale = 15 ; amp = 0.028

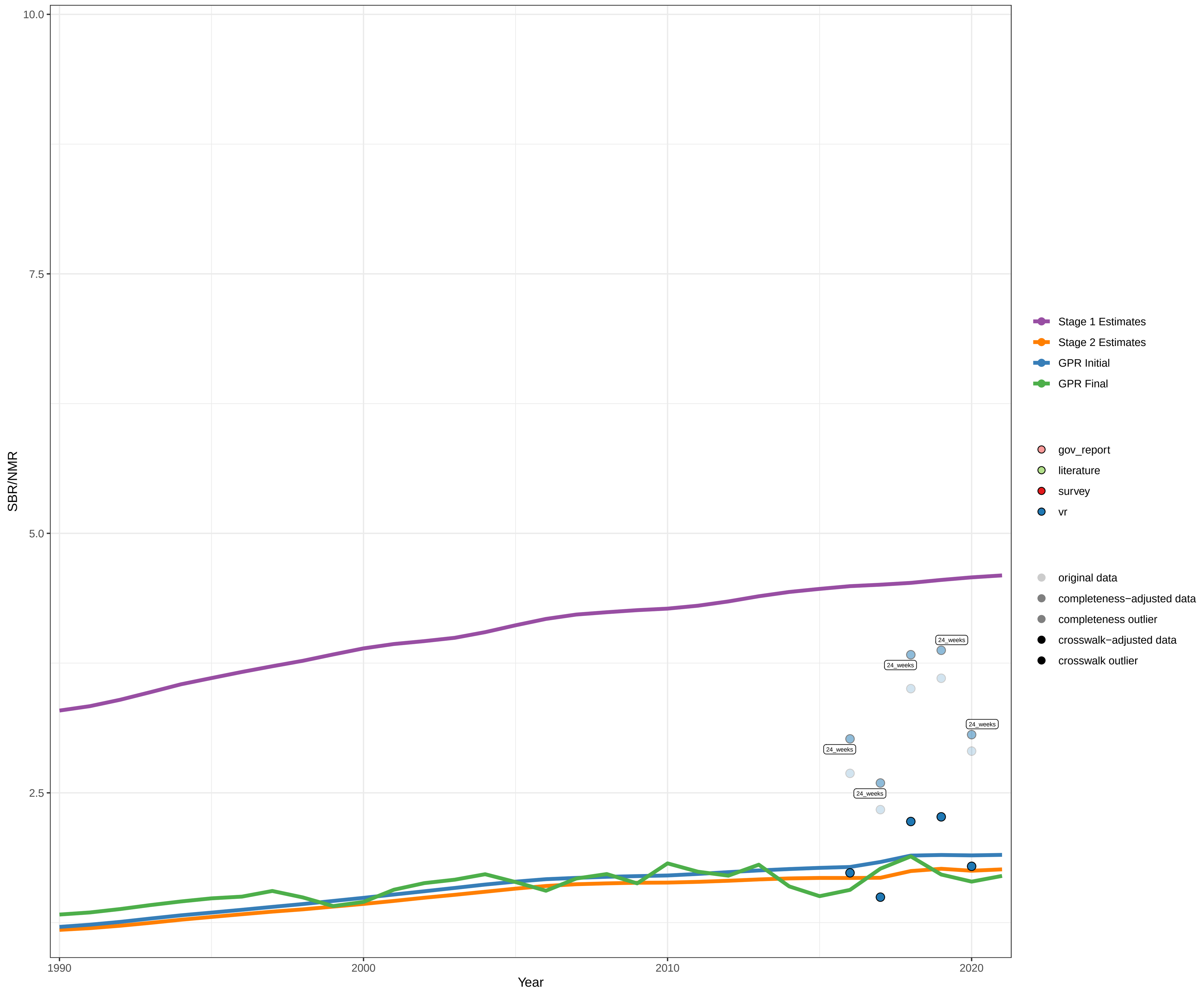

Definition	Years	Outlier	Source.Type	Title
24_weeks	2016−2020	0	VR	United Kingdom − England and Wales Child Mortality Statistics

Darlington GBR_44643 Western Europe Stillbirth Rate (SBR) − 20 weeks
lambda = 0.6 ; zeta = 0.8 ; scale = 15 ; amp = 0.028

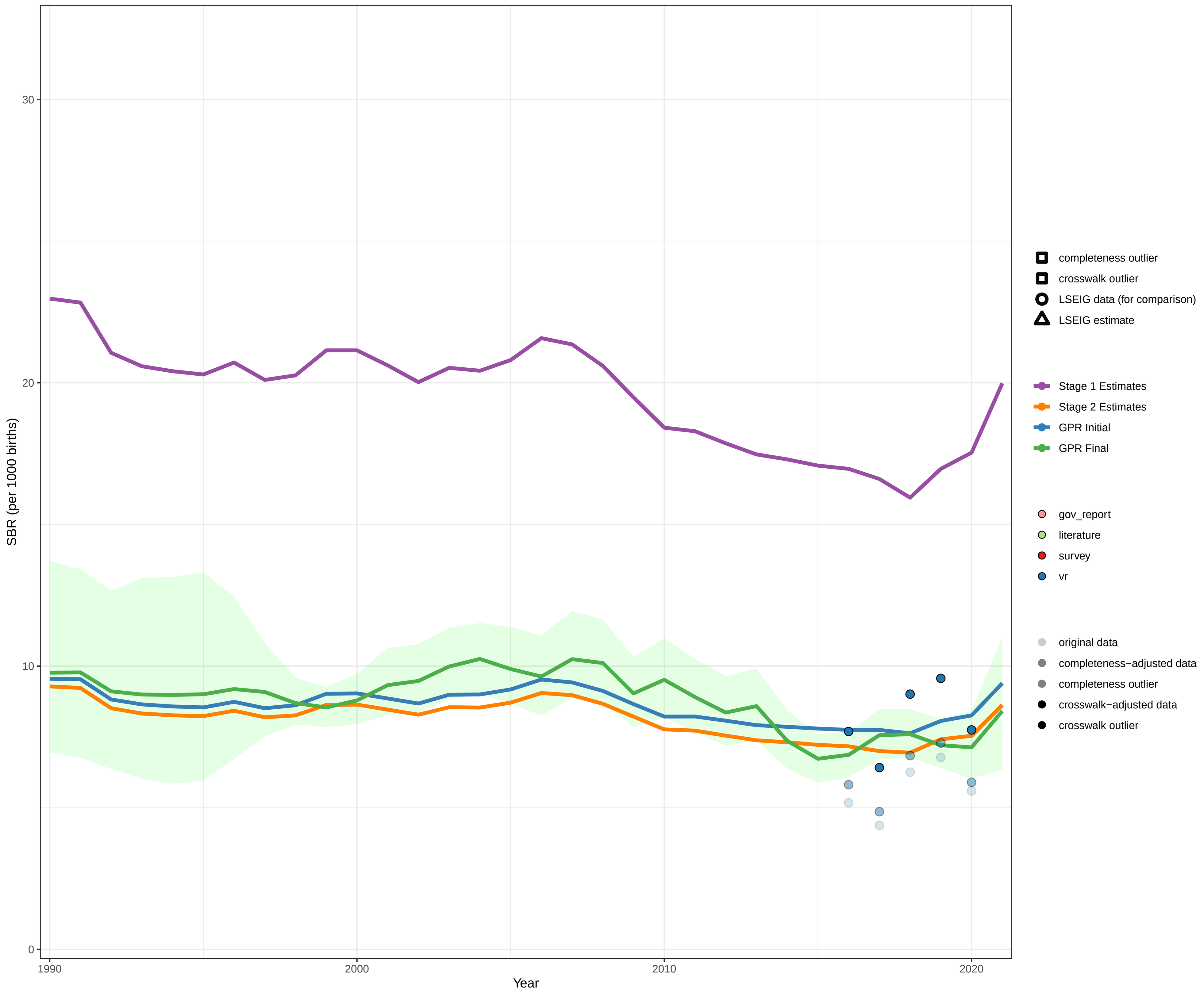

Darlington GBR_44643 Western Europe Stillbirth Rate (SBR) − 28 weeks
lambda = 0.6 ; zeta = 0.8 ; scale = 15 ; amp = 0.028

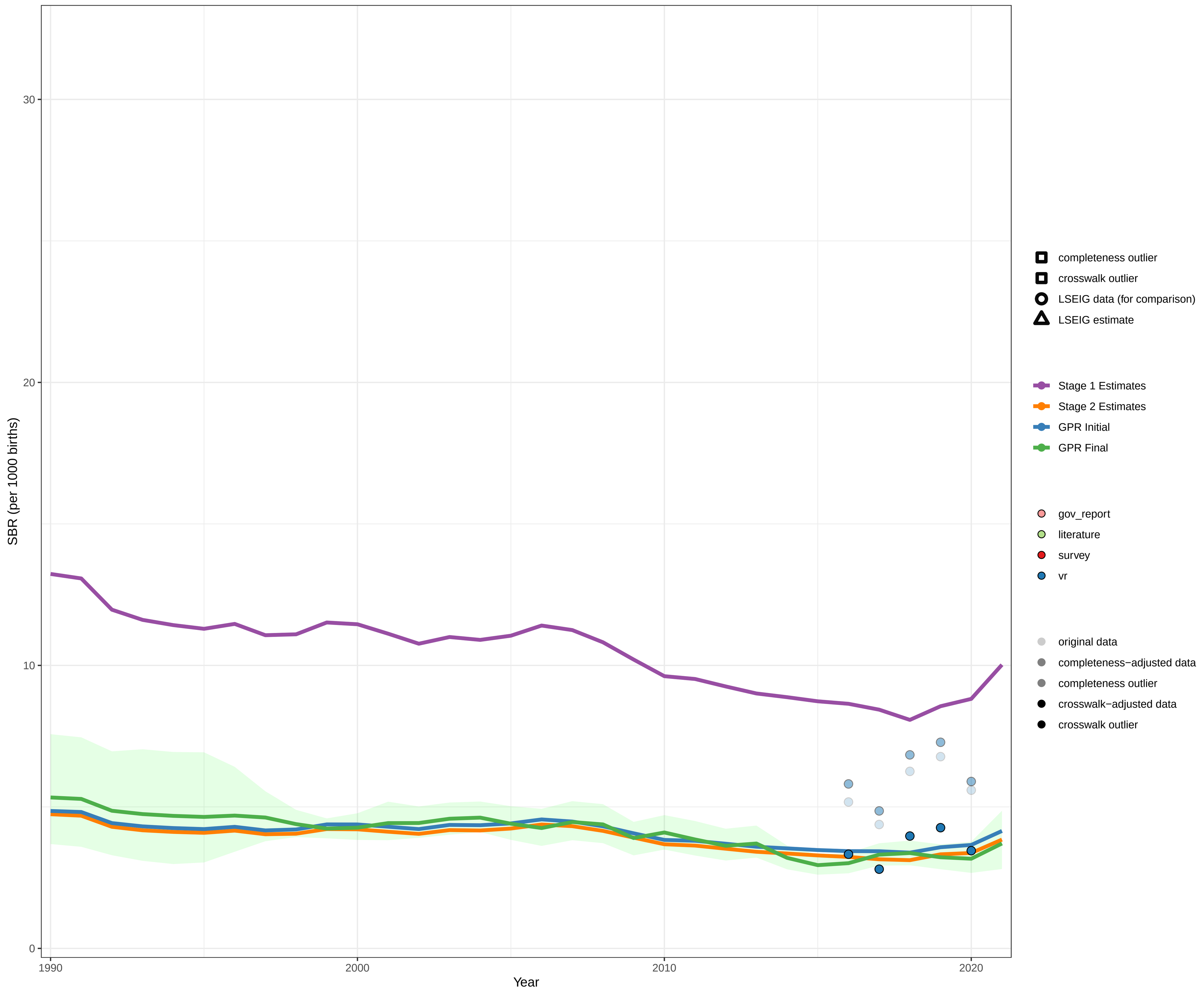

Year	Total Stillbirths (in thousands)	Total Stillbirths (in thousands)	SBR (per 1000 births)	SBR (per 1000 births)	SBR/NMR	SBR/NMR
	20 weeks	28 weeks	20 weeks	28 weeks	20 weeks	28 weeks
1990	0.0 (0.0 to 0.0)	0.0 (0.0 to 0.0)	9.8 (7.0 to 13.7)	5.3 (3.7 to 7.6)	2.43 (1.73 to 3.41)	1.33 (0.92 to 1.88)
1995	0.0 (0.0 to 0.0)	0.0 (0.0 to 0.0)	9.0 (6.0 to 13.3)	4.6 (3.0 to 6.9)	2.88 (1.90 to 4.25)	1.48 (0.97 to 2.21)
2000	0.0 (0.0 to 0.0)	0.0 (0.0 to 0.0)	8.8 (7.9 to 9.7)	4.3 (3.8 to 4.8)	2.99 (2.70 to 3.31)	1.45 (1.30 to 1.62)
2005	0.0 (0.0 to 0.0)	0.0 (0.0 to 0.0)	9.9 (8.7 to 11.4)	4.4 (3.8 to 5.0)	3.68 (3.22 to 4.24)	1.64 (1.43 to 1.87)
2010	0.0 (0.0 to 0.0)	0.0 (0.0 to 0.0)	9.5 (8.2 to 11.0)	4.1 (3.5 to 4.7)	4.23 (3.64 to 4.88)	1.82 (1.55 to 2.09)
2015	0.0 (0.0 to 0.0)	0.0 (0.0 to 0.0)	6.7 (5.9 to 7.6)	2.9 (2.6 to 3.3)	3.44 (3.01 to 3.89)	1.50 (1.33 to 1.70)
2020	0.0 (0.0 to 0.0)	0.0 (0.0 to 0.0)	7.1 (6.0 to 8.4)	3.2 (2.7 to 3.8)	3.70 (3.13 to 4.38)	1.64 (1.38 to 1.96)
2021	0.0 (0.0 to 0.0)	0.0 (0.0 to 0.0)	8.4 (6.3 to 11.0)	3.7 (2.8 to 4.9)	3.86 (2.91 to 5.06)	1.70 (1.29 to 2.23)

Gateshead GBR_44650 Western Europe Stillbirth Rate / Neonatal Mortality Rate (SBR/NMR) − 20 weeks
lambda = 0.6 ; zeta = 0.8 ; scale = 15 ; amp = 0.028

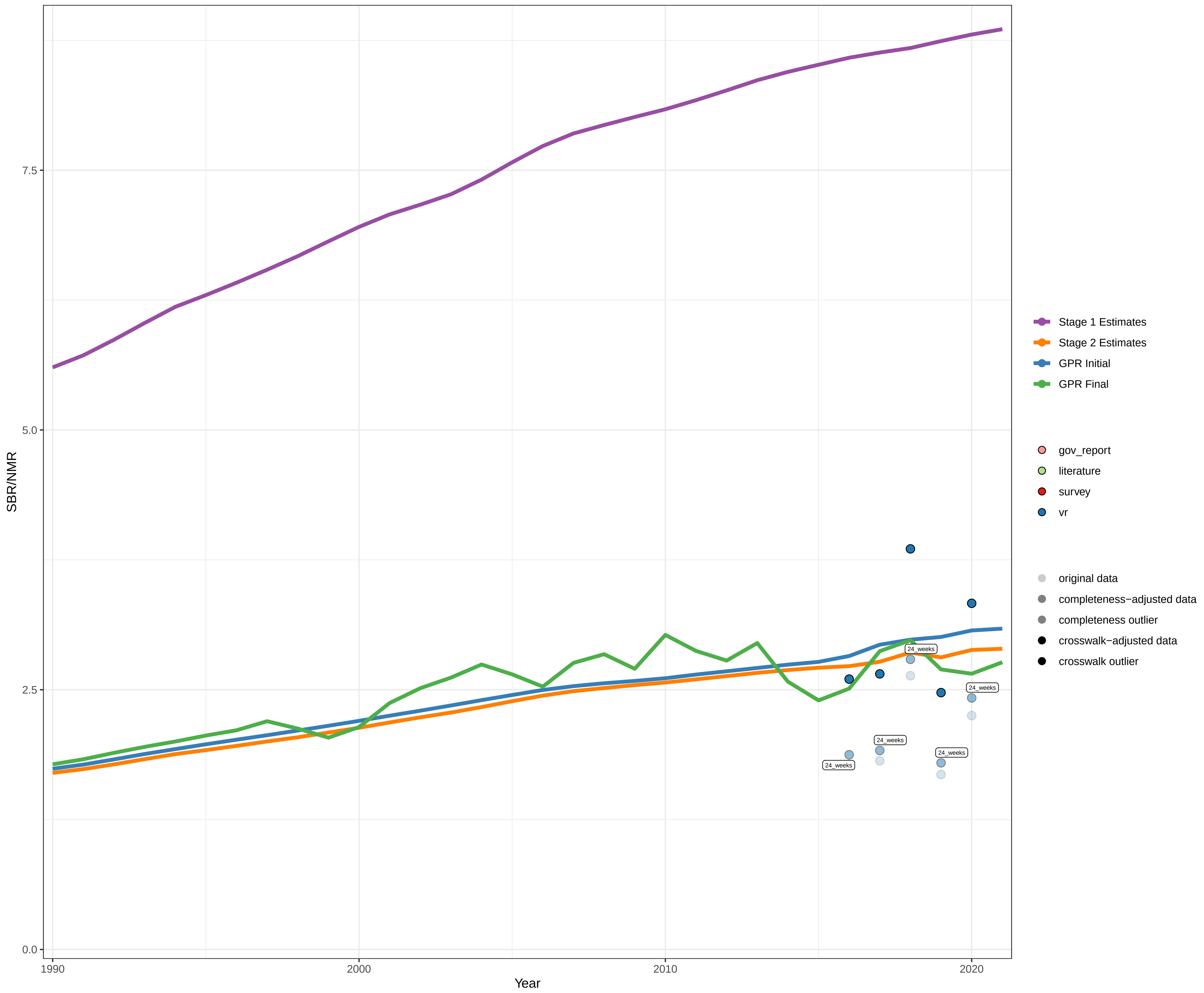

Gateshead GBR_44650 Western Europe Stillbirth Rate / Neonatal Mortality Rate (SBR/NMR) − 28 weeks
lambda = 0.6 ; zeta = 0.8 ; scale = 15 ; amp = 0.028

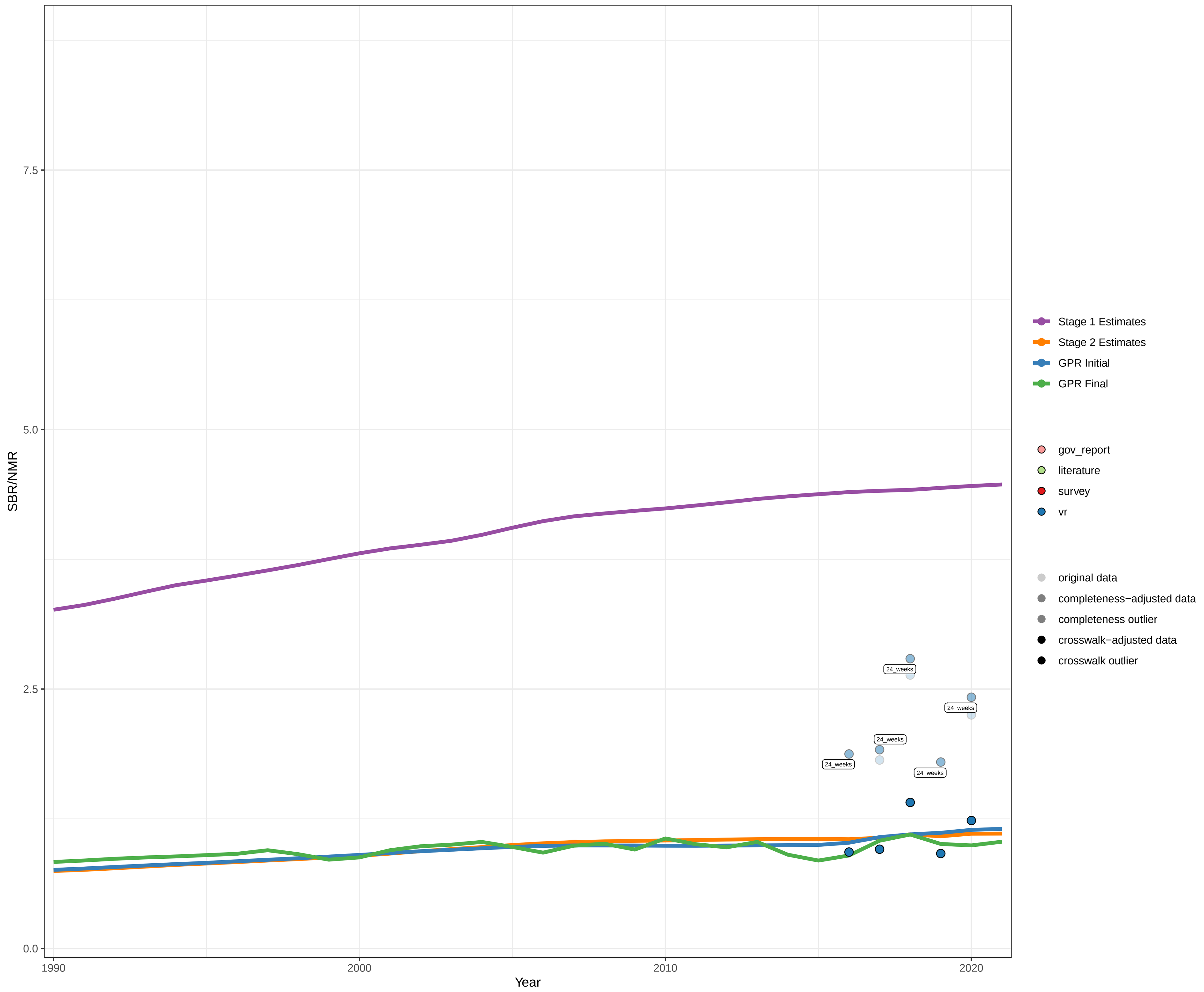

Definition	Years	Outlier	Source.Type	Title
24_weeks	2016−2020	0	VR	United Kingdom − England and Wales Child Mortality Statistics

Gateshead GBR_44650 Western Europe Stillbirth Rate (SBR) − 20 weeks
lambda = 0.6 ; zeta = 0.8 ; scale = 15 ; amp = 0.028

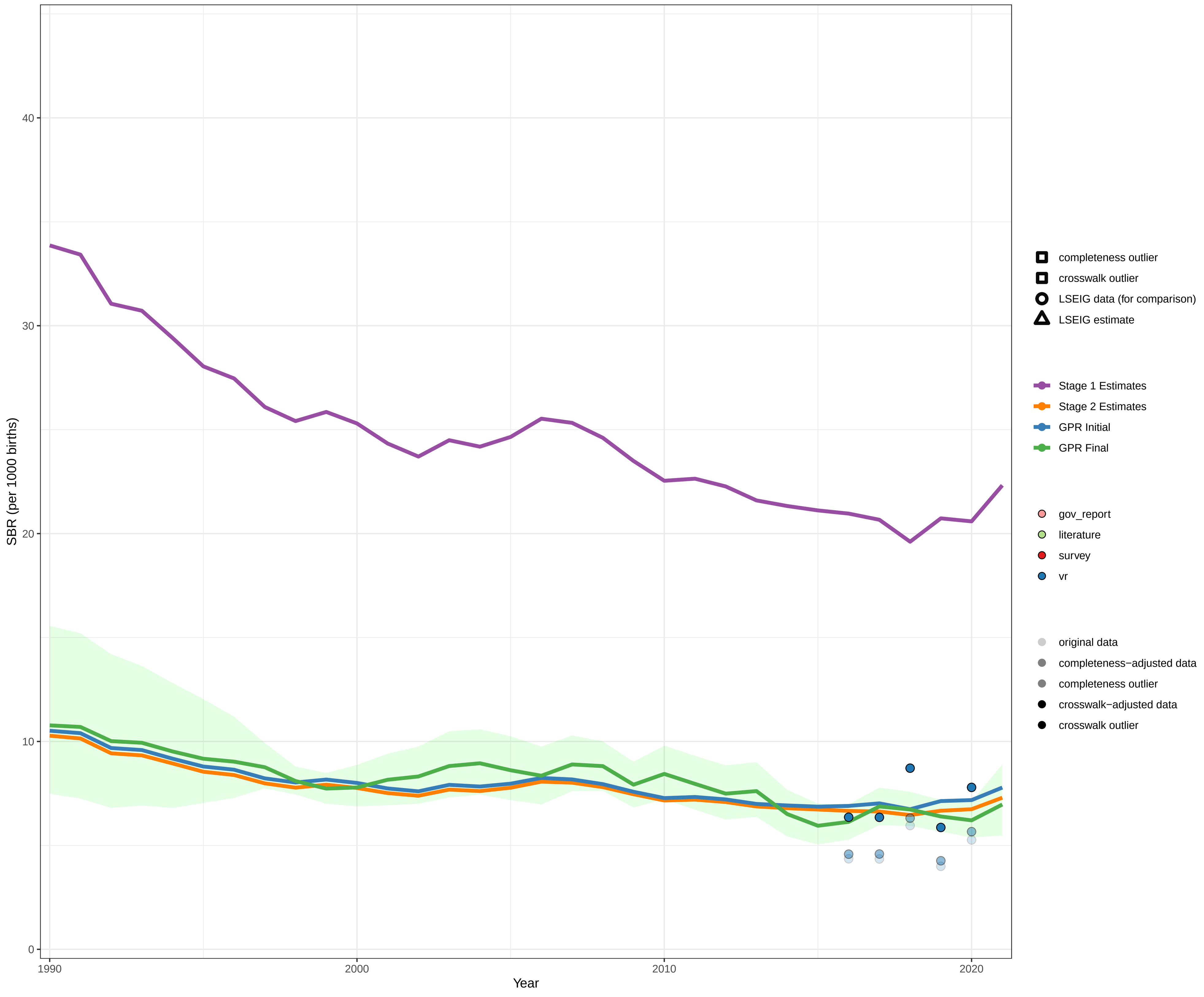

Gateshead GBR_44650 Western Europe Stillbirth Rate (SBR) − 28 weeks
lambda = 0.6 ; zeta = 0.8 ; scale = 15 ; amp = 0.028

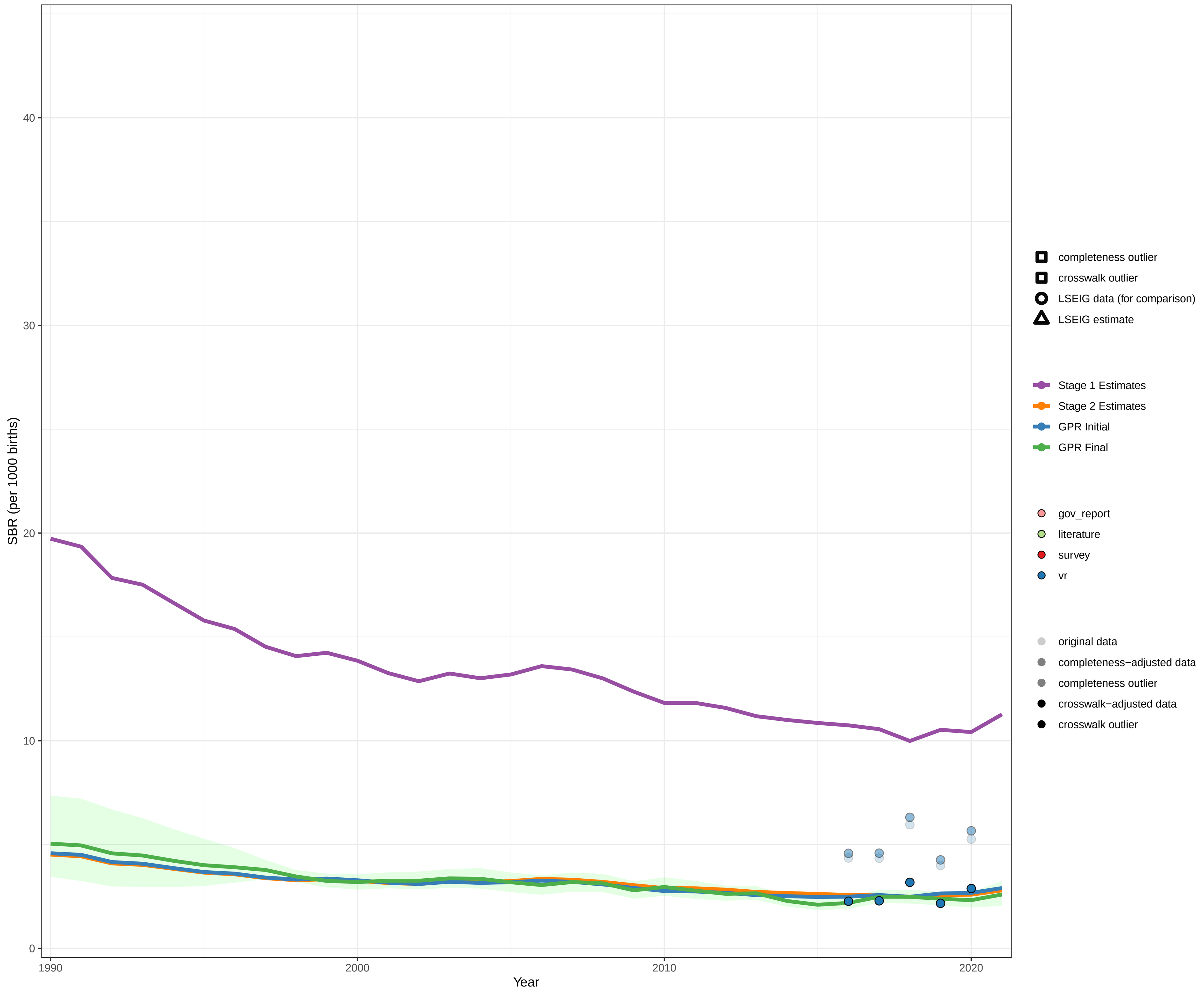

Year	Total Stillbirths (in thousands)	Total Stillbirths (in thousands)	SBR (per 1000 births)	SBR (per 1000 births)	SBR/NMR	SBR/NMR
	20 weeks	28 weeks	20 weeks	28 weeks	20 weeks	28 weeks
1990	0.0 (0.0 to 0.0)	0.0 (0.0 to 0.0)	10.8 (7.5 to 15.6)	5.0 (3.4 to 7.4)	1.78 (1.24 to 2.57)	0.83 (0.57 to 1.22)
1995	0.0 (0.0 to 0.0)	0.0 (0.0 to 0.0)	9.2 (7.0 to 12.0)	4.0 (3.0 to 5.3)	2.06 (1.58 to 2.70)	0.90 (0.67 to 1.19)
2000	0.0 (0.0 to 0.0)	0.0 (0.0 to 0.0)	7.8 (6.9 to 8.9)	3.2 (2.8 to 3.6)	2.14 (1.89 to 2.44)	0.88 (0.78 to 0.98)
2005	0.0 (0.0 to 0.0)	0.0 (0.0 to 0.0)	8.6 (7.2 to 10.3)	3.2 (2.7 to 3.7)	2.65 (2.21 to 3.15)	0.98 (0.83 to 1.12)
2010	0.0 (0.0 to 0.0)	0.0 (0.0 to 0.0)	8.4 (7.2 to 9.8)	3.0 (2.5 to 3.4)	3.03 (2.59 to 3.51)	1.06 (0.91 to 1.23)
2015	0.0 (0.0 to 0.0)	0.0 (0.0 to 0.0)	5.9 (5.0 to 7.0)	2.1 (1.8 to 2.4)	2.40 (2.03 to 2.83)	0.85 (0.74 to 0.96)
2020	0.0 (0.0 to 0.0)	0.0 (0.0 to 0.0)	6.2 (5.4 to 7.1)	2.3 (2.0 to 2.7)	2.65 (2.30 to 3.04)	0.99 (0.84 to 1.16)
2021	0.0 (0.0 to 0.0)	0.0 (0.0 to 0.0)	7.0 (5.5 to 8.9)	2.6 (2.0 to 3.2)	2.76 (2.17 to 3.53)	1.03 (0.81 to 1.29)

Hartlepool   GBR_44654   Western Europe   Stillbirth Rate / Neonatal Mortality Rate (SBR/NMR) − 20 weeks
lambda = 0.6 ; zeta = 0.8 ; scale = 15 ; amp = 0.028

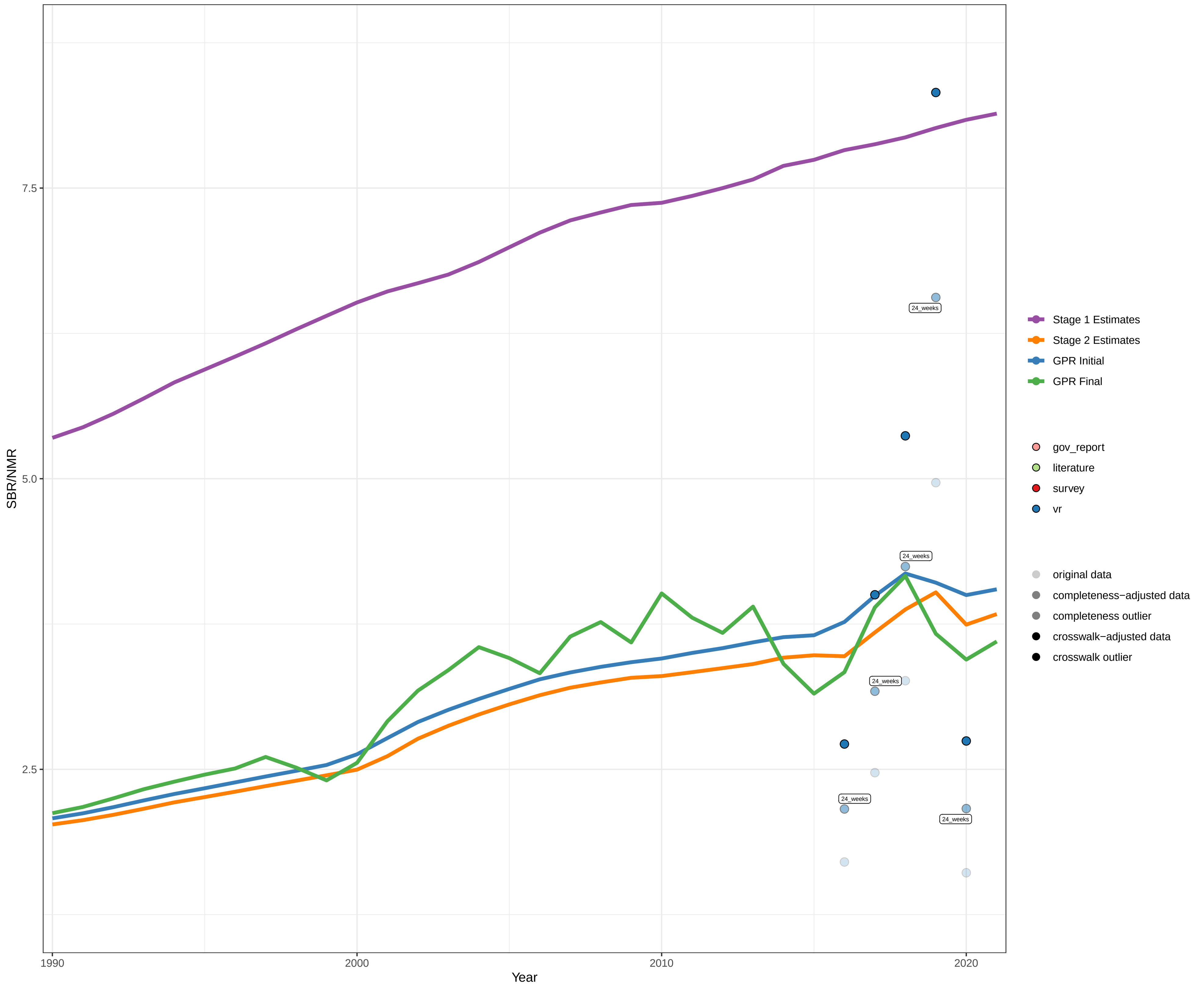

Hartlepool   GBR_44654   Western Europe   Stillbirth Rate / Neonatal Mortality Rate (SBR/NMR) − 28 weeks
lambda = 0.6 ; zeta = 0.8 ; scale = 15 ; amp = 0.028

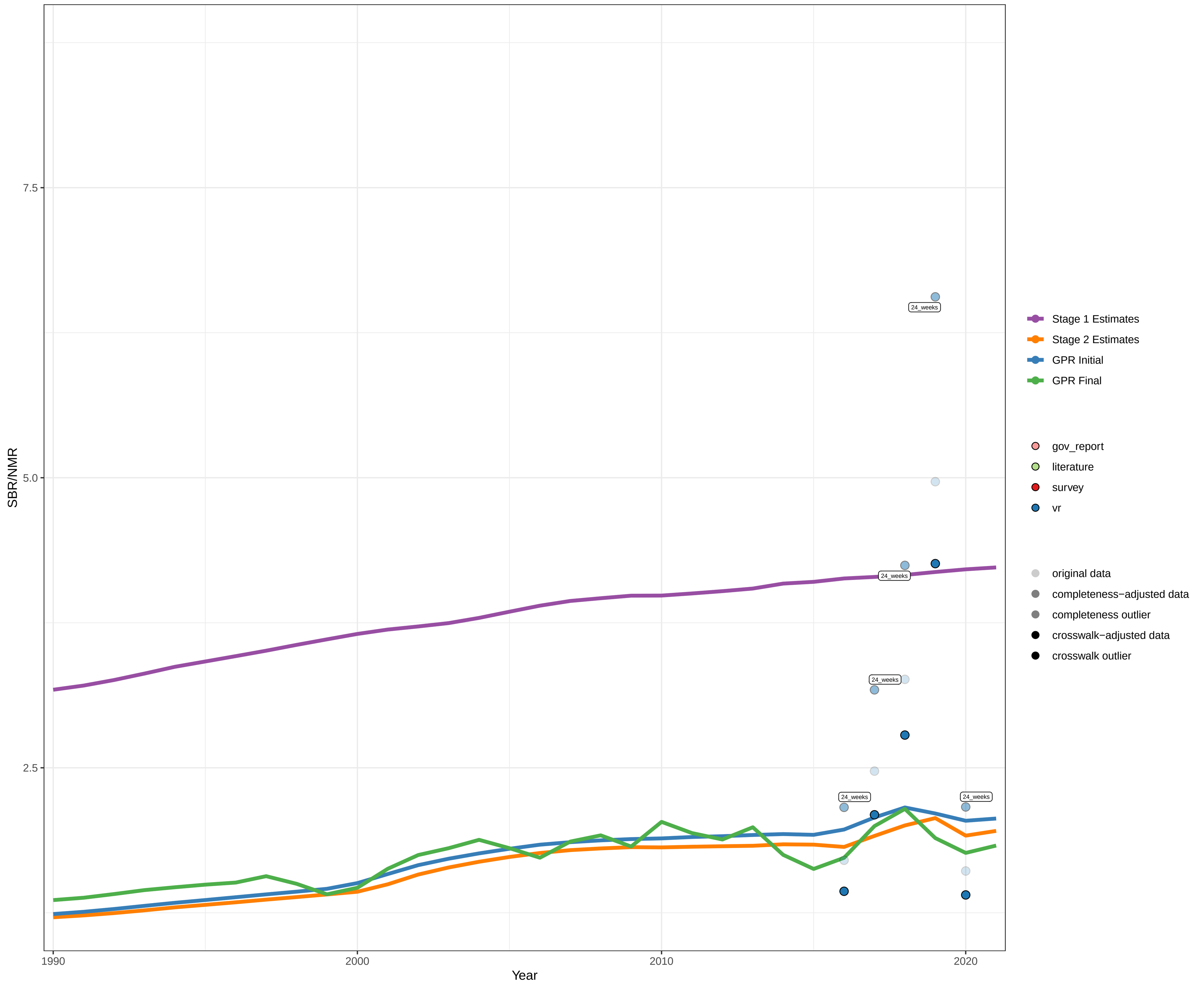

Definition	Years	Outlier	Source.Type	Title
24_weeks	2016−2020	0	VR	United Kingdom − England and Wales Child Mortality Statistics

Hartlepool   GBR_44654   Western Europe   Stillbirth Rate (SBR) − 20 weeks
lambda = 0.6 ; zeta = 0.8 ; scale = 15 ; amp = 0.028

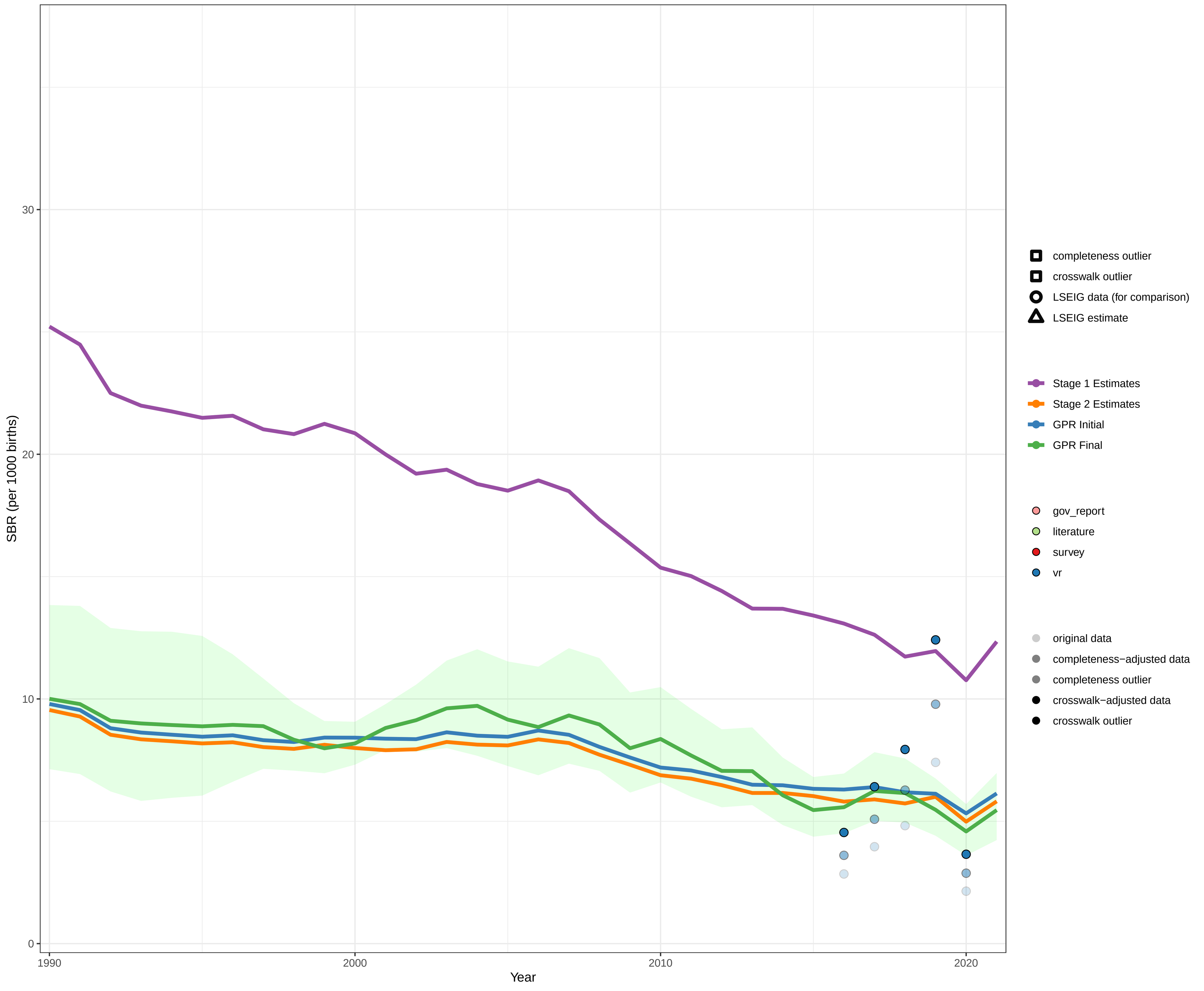

Hartlepool   GBR_44654   Western Europe   Stillbirth Rate (SBR) − 28 weeks
lambda = 0.6 ; zeta = 0.8 ; scale = 15 ; amp = 0.028

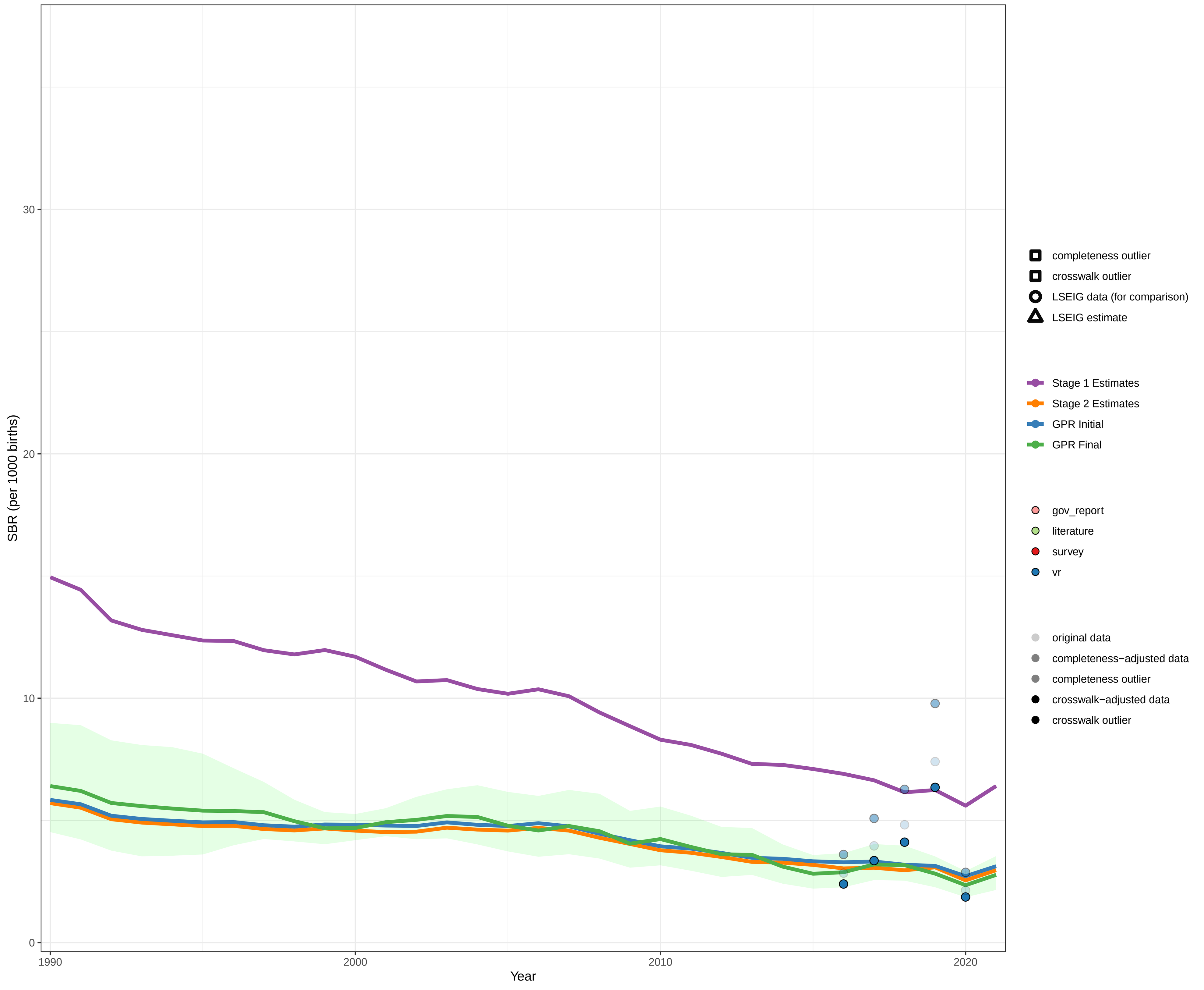

Year	Total Stillbirths (in thousands)	Total Stillbirths (in thousands)	SBR (per 1000 births)	SBR (per 1000 births)	SBR/NMR	SBR/NMR
	20 weeks	28 weeks	20 weeks	28 weeks	20 weeks	28 weeks
1990	0.0 (0.0 to 0.0)	0.0 (0.0 to 0.0)	10.0 (7.1 to 13.8)	6.4 (4.5 to 9.0)	2.12 (1.51 to 2.94)	1.36 (0.96 to 1.91)
1995	0.0 (0.0 to 0.0)	0.0 (0.0 to 0.0)	8.9 (6.0 to 12.6)	5.4 (3.6 to 7.7)	2.45 (1.67 to 3.48)	1.49 (1.00 to 2.14)
2000	0.0 (0.0 to 0.0)	0.0 (0.0 to 0.0)	8.2 (7.3 to 9.1)	4.7 (4.2 to 5.3)	2.56 (2.28 to 2.83)	1.47 (1.31 to 1.64)
2005	0.0 (0.0 to 0.0)	0.0 (0.0 to 0.0)	9.2 (7.3 to 11.5)	4.8 (3.7 to 6.2)	3.46 (2.74 to 4.35)	1.81 (1.41 to 2.33)
2010	0.0 (0.0 to 0.0)	0.0 (0.0 to 0.0)	8.4 (6.6 to 10.5)	4.2 (3.2 to 5.6)	4.01 (3.16 to 5.03)	2.03 (1.52 to 2.67)
2015	0.0 (0.0 to 0.0)	0.0 (0.0 to 0.0)	5.5 (4.4 to 6.8)	2.8 (2.2 to 3.6)	3.15 (2.52 to 3.93)	1.63 (1.28 to 2.07)
2020	0.0 (0.0 to 0.0)	0.0 (0.0 to 0.0)	4.6 (3.6 to 5.7)	2.4 (1.9 to 2.9)	3.44 (2.71 to 4.29)	1.77 (1.40 to 2.20)
2021	0.0 (0.0 to 0.0)	0.0 (0.0 to 0.0)	5.5 (4.2 to 7.0)	2.8 (2.2 to 3.5)	3.60 (2.79 to 4.60)	1.83 (1.42 to 2.34)

Middlesbrough GBR_44651 Western Europe Stillbirth Rate / Neonatal Mortality Rate (SBR/NMR) − 20 weeks
lambda = 0.6 ; zeta = 0.8 ; scale = 15 ; amp = 0.028

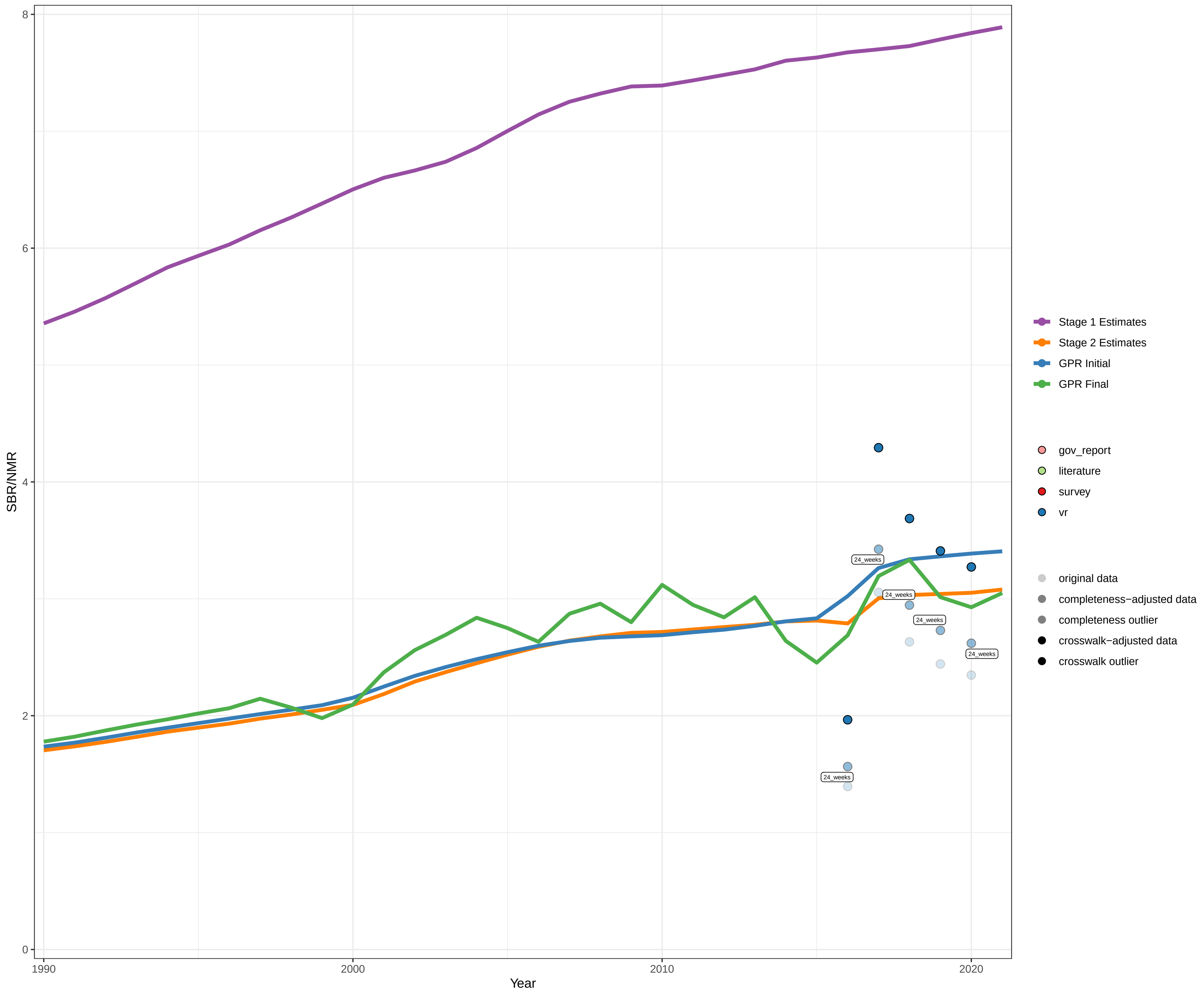

Middlesbrough GBR_44651 Western Europe Stillbirth Rate / Neonatal Mortality Rate (SBR/NMR) − 28 weeks
lambda = 0.6 ; zeta = 0.8 ; scale = 15 ; amp = 0.028

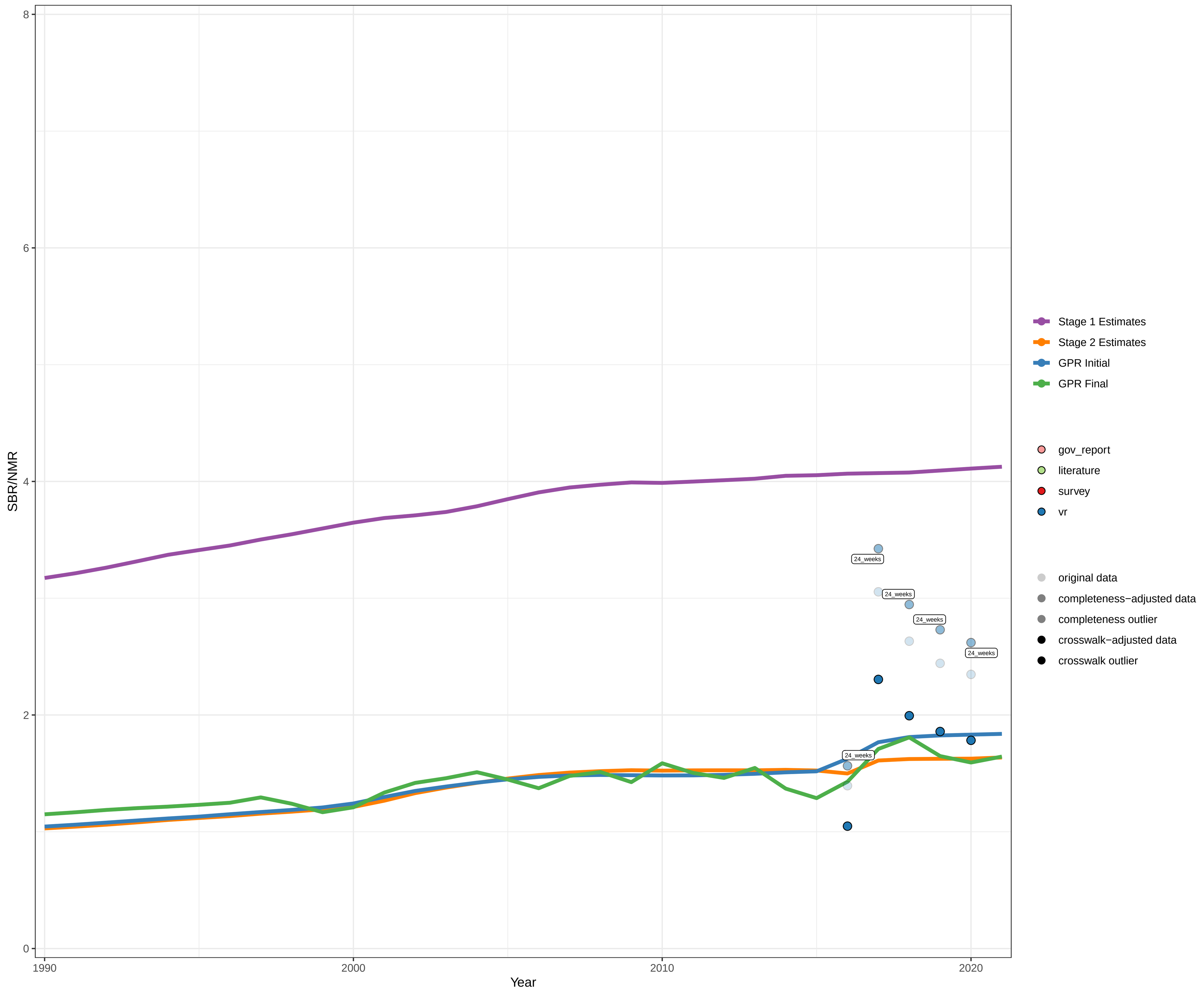

Definition	Years	Outlier	Source.Type	Title
24_weeks	2016−2020	0	VR	United Kingdom − England and Wales Child Mortality Statistics

Middlesbrough GBR_44651 Western Europe Stillbirth Rate (SBR) − 20 weeks
lambda = 0.6 ; zeta = 0.8 ; scale = 15 ; amp = 0.028

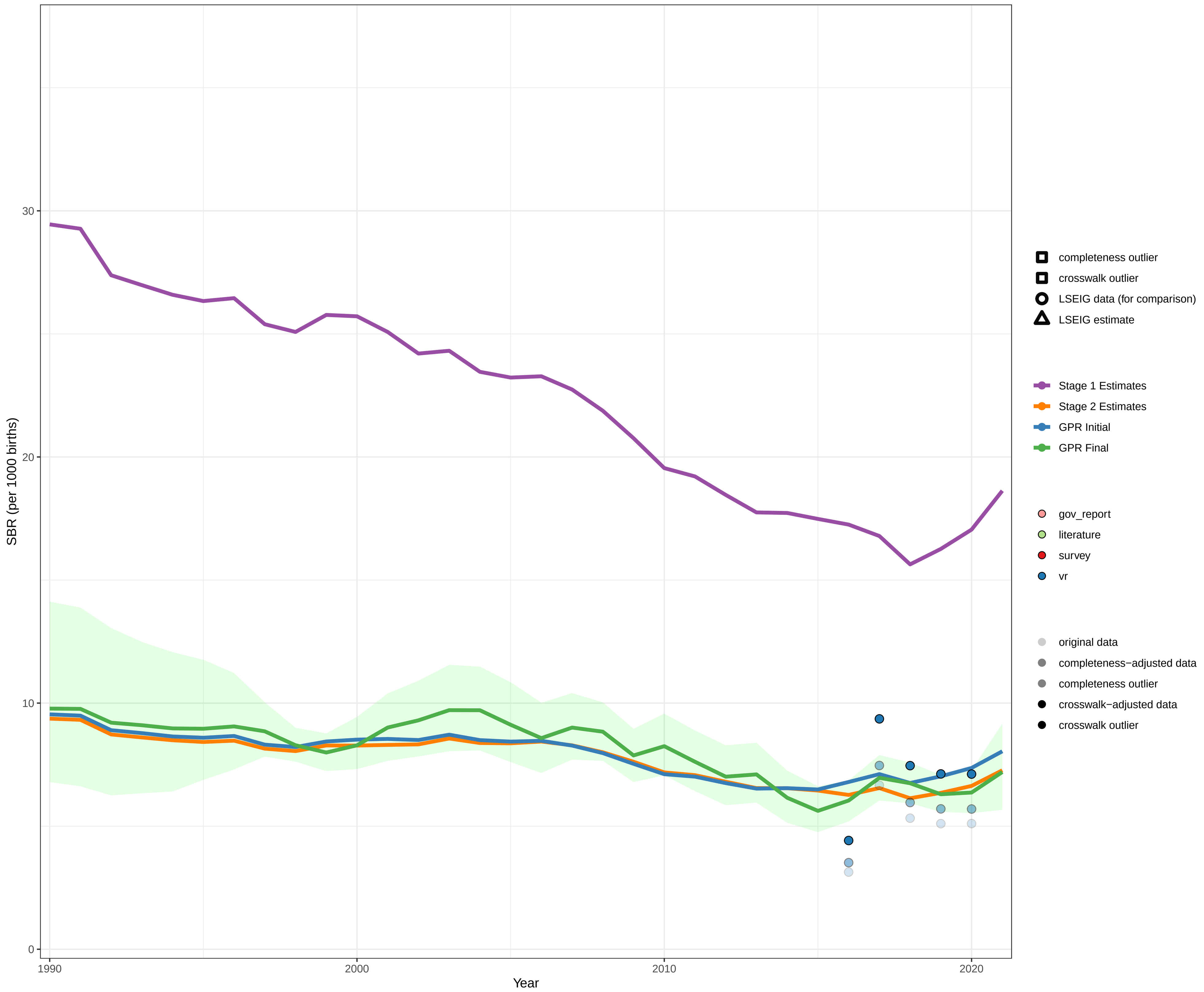

Middlesbrough GBR_44651 Western Europe Stillbirth Rate (SBR) − 28 weeks
lambda = 0.6 ; zeta = 0.8 ; scale = 15 ; amp = 0.028

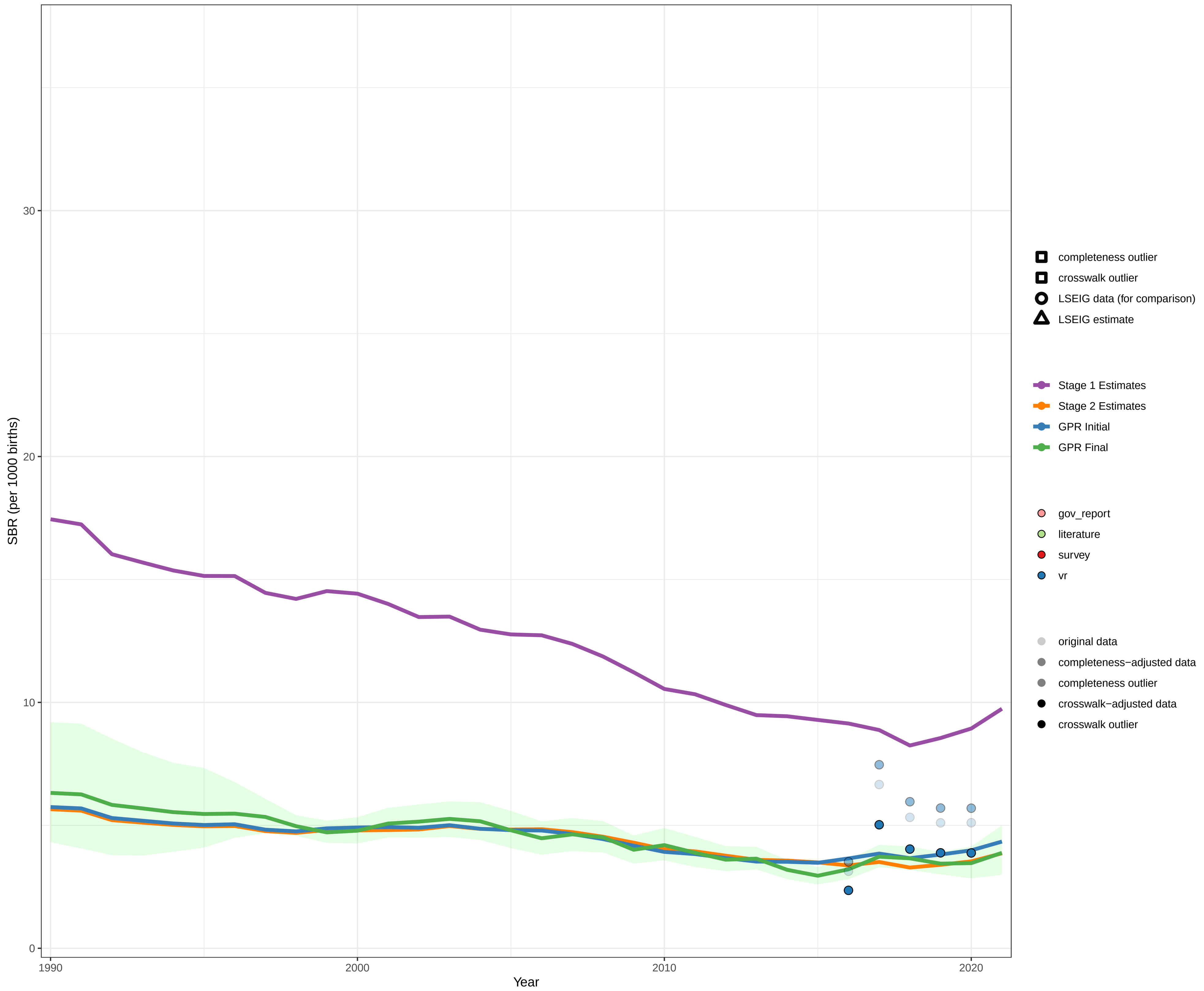

Year	Total Stillbirths (in thousands)	Total Stillbirths (in thousands)	SBR (per 1000 births)	SBR (per 1000 births)	SBR/NMR	SBR/NMR
	20 weeks	28 weeks	20 weeks	28 weeks	20 weeks	28 weeks
1990	0.0 (0.0 to 0.0)	0.0 (0.0 to 0.0)	9.8 (6.8 to 14.1)	6.3 (4.3 to 9.2)	1.78 (1.23 to 2.57)	1.15 (0.78 to 1.67)
1995	0.0 (0.0 to 0.0)	0.0 (0.0 to 0.0)	9.0 (6.9 to 11.8)	5.5 (4.1 to 7.3)	2.02 (1.55 to 2.65)	1.23 (0.92 to 1.65)
2000	0.0 (0.0 to 0.0)	0.0 (0.0 to 0.0)	8.3 (7.3 to 9.4)	4.8 (4.3 to 5.3)	2.10 (1.85 to 2.39)	1.21 (1.08 to 1.35)
2005	0.0 (0.0 to 0.0)	0.0 (0.0 to 0.0)	9.1 (7.6 to 10.8)	4.8 (4.1 to 5.6)	2.75 (2.29 to 3.27)	1.45 (1.23 to 1.69)
2010	0.0 (0.0 to 0.0)	0.0 (0.0 to 0.0)	8.2 (7.1 to 9.6)	4.2 (3.6 to 4.9)	3.12 (2.67 to 3.62)	1.59 (1.35 to 1.85)
2015	0.0 (0.0 to 0.0)	0.0 (0.0 to 0.0)	5.6 (4.8 to 6.6)	3.0 (2.6 to 3.3)	2.45 (2.08 to 2.89)	1.29 (1.13 to 1.45)
2020	0.0 (0.0 to 0.0)	0.0 (0.0 to 0.0)	6.4 (5.5 to 7.3)	3.5 (2.8 to 4.1)	2.93 (2.54 to 3.35)	1.59 (1.31 to 1.91)
2021	0.0 (0.0 to 0.0)	0.0 (0.0 to 0.0)	7.2 (5.7 to 9.2)	3.9 (3.0 to 5.0)	3.05 (2.40 to 3.88)	1.64 (1.27 to 2.12)

Newcastle upon Tyne GBR_44646 Western Europe Stillbirth Rate / Neonatal Mortality Rate (SBR/NMR) − 20 weeks
lambda = 0.6 ; zeta = 0.8 ; scale = 15 ; amp = 0.028

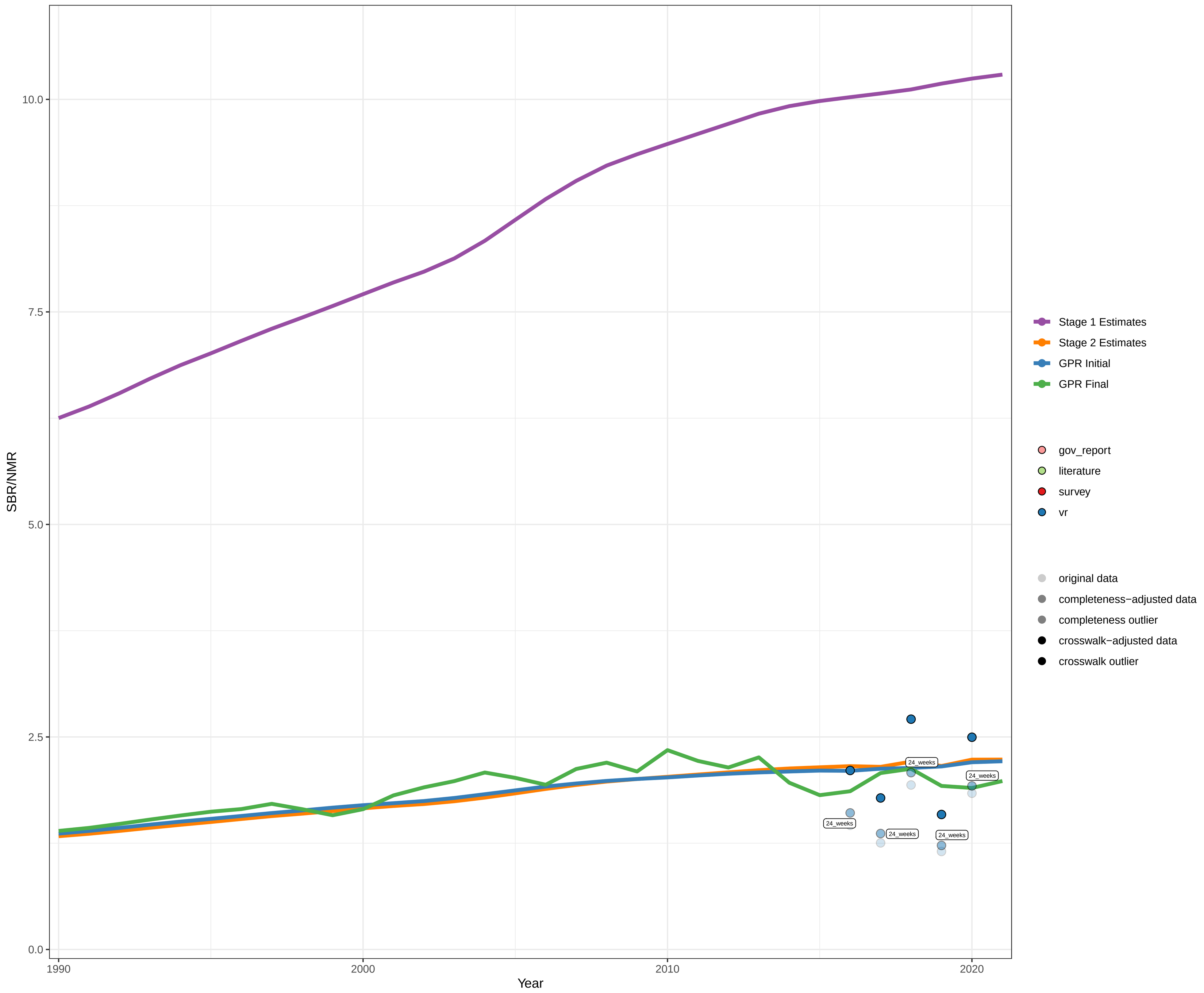

Newcastle upon Tyne GBR_44646 Western Europe Stillbirth Rate / Neonatal Mortality Rate (SBR/NMR) − 28 weeks
lambda = 0.6 ; zeta = 0.8 ; scale = 15 ; amp = 0.028

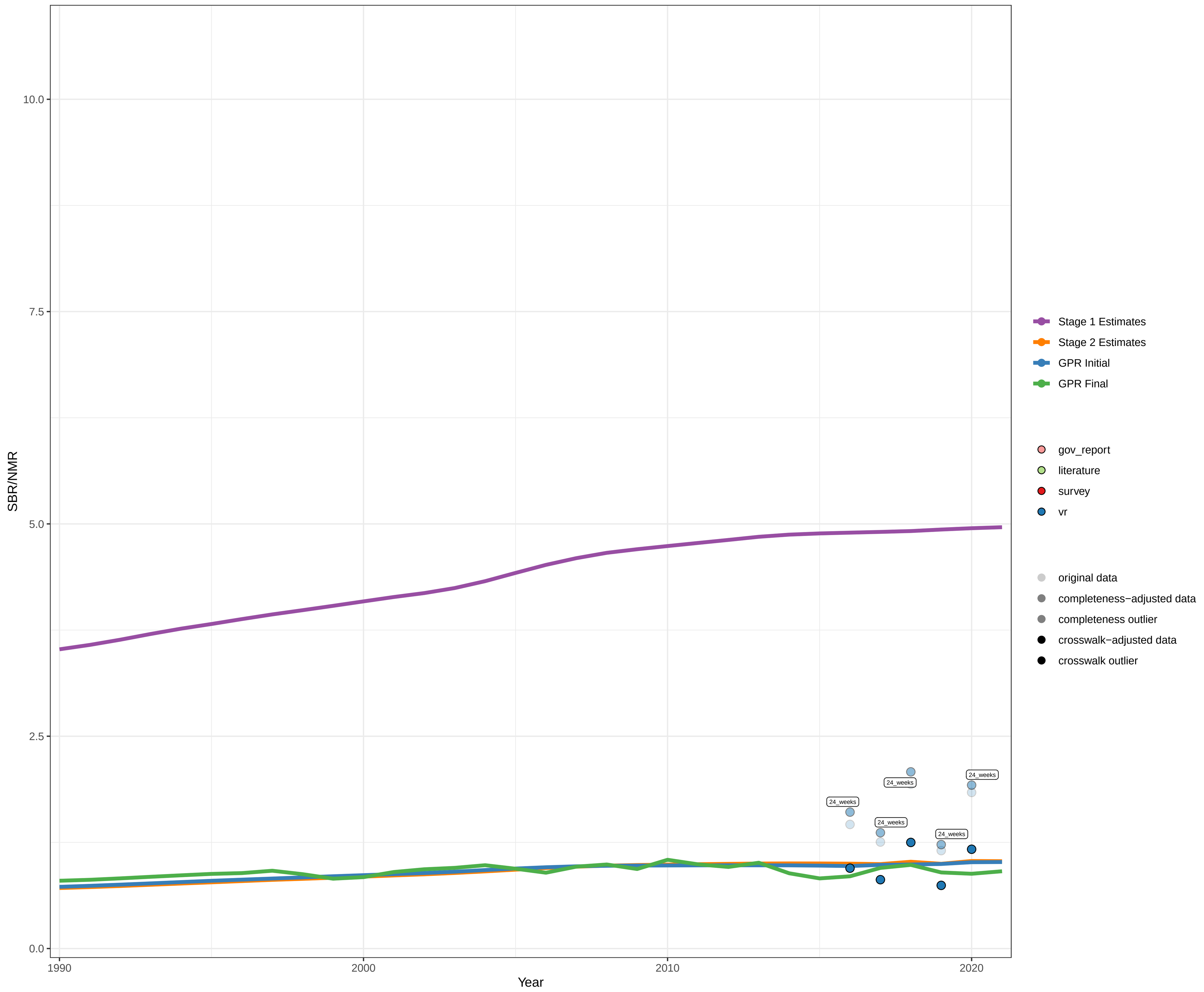

Definition	Years	Outlier	Source.Type	Title
24_weeks	2016−2020	0	VR	United Kingdom − England and Wales Child Mortality Statistics

Newcastle upon Tyne GBR_44646 Western Europe Stillbirth Rate (SBR) − 20 weeks
lambda = 0.6 ; zeta = 0.8 ; scale = 15 ; amp = 0.028

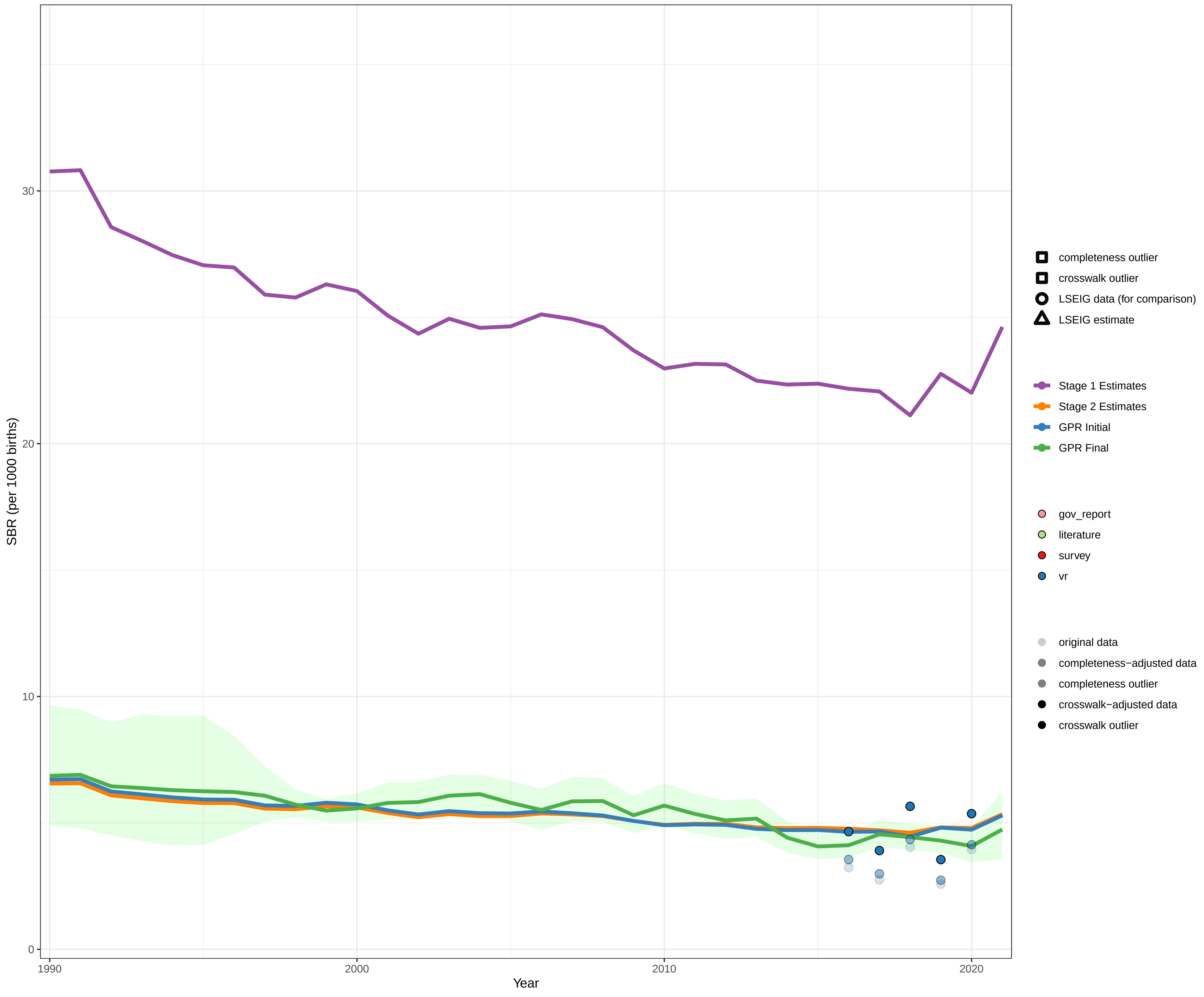

Newcastle upon Tyne GBR_44646 Western Europe Stillbirth Rate (SBR) − 28 weeks
lambda = 0.6 ; zeta = 0.8 ; scale = 15 ; amp = 0.028

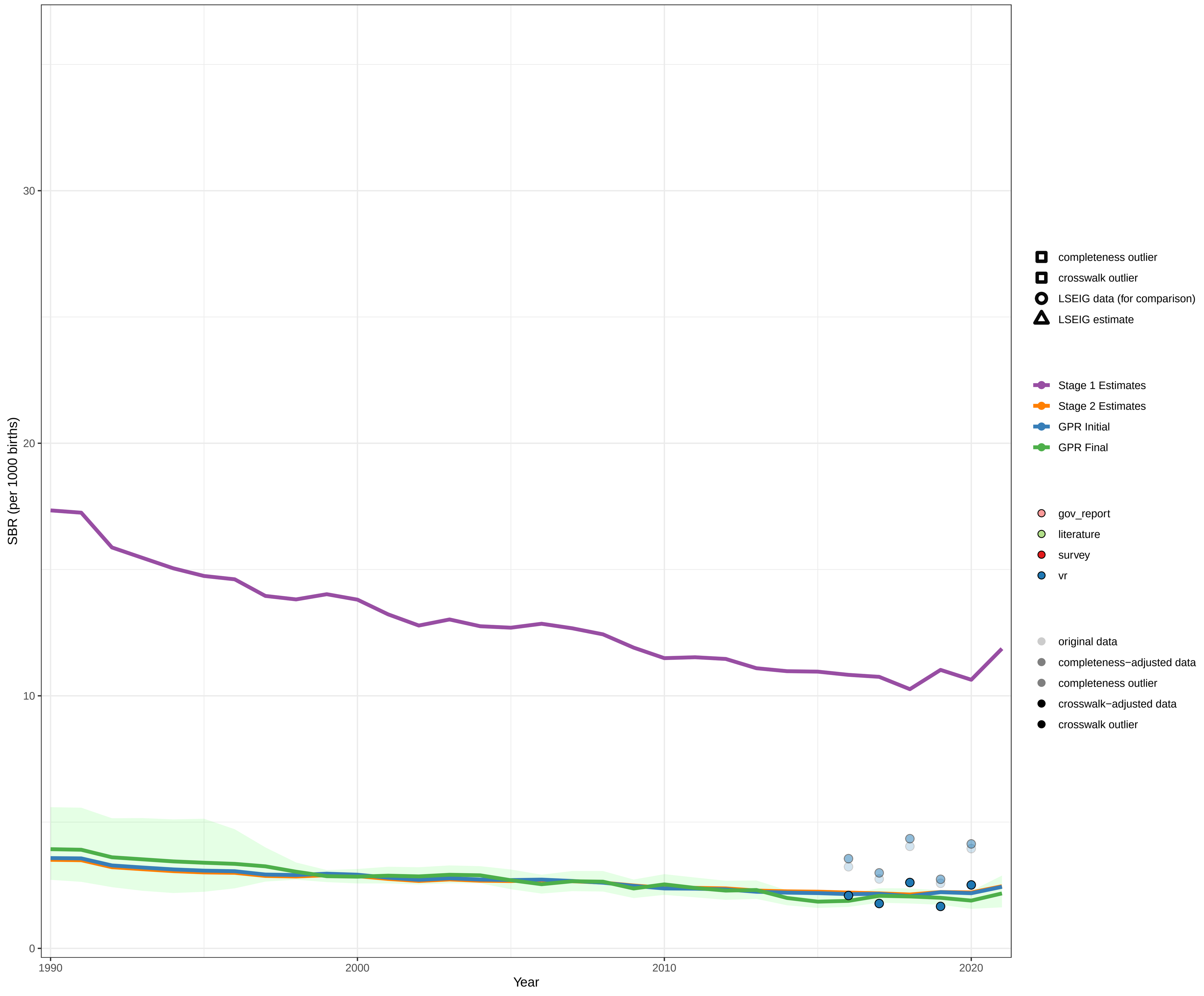

Year	Total Stillbirths (in thousands)	Total Stillbirths (in thousands)	SBR (per 1000 births)	SBR (per 1000 births)	SBR/NMR	SBR/NMR
	20 weeks	28 weeks	20 weeks	28 weeks	20 weeks	28 weeks
1990	0.0 (0.0 to 0.0)	0.0 (0.0 to 0.0)	6.9 (4.9 to 9.6)	3.9 (2.7 to 5.6)	1.39 (0.99 to 1.96)	0.80 (0.55 to 1.14)
1995	0.0 (0.0 to 0.0)	0.0 (0.0 to 0.0)	6.3 (4.1 to 9.3)	3.4 (2.2 to 5.1)	1.62 (1.07 to 2.40)	0.88 (0.58 to 1.33)
2000	0.0 (0.0 to 0.0)	0.0 (0.0 to 0.0)	5.6 (5.0 to 6.2)	2.8 (2.6 to 3.1)	1.65 (1.49 to 1.83)	0.84 (0.76 to 0.93)
2005	0.0 (0.0 to 0.0)	0.0 (0.0 to 0.0)	5.8 (5.1 to 6.7)	2.7 (2.3 to 3.1)	2.02 (1.77 to 2.32)	0.94 (0.82 to 1.09)
2010	0.0 (0.0 to 0.0)	0.0 (0.0 to 0.0)	5.7 (4.9 to 6.6)	2.5 (2.1 to 2.9)	2.34 (2.02 to 2.71)	1.05 (0.87 to 1.21)
2015	0.0 (0.0 to 0.0)	0.0 (0.0 to 0.0)	4.1 (3.6 to 4.6)	1.9 (1.6 to 2.1)	1.82 (1.59 to 2.06)	0.83 (0.71 to 0.95)
2020	0.0 (0.0 to 0.0)	0.0 (0.0 to 0.0)	4.1 (3.4 to 4.8)	1.9 (1.6 to 2.3)	1.90 (1.60 to 2.25)	0.88 (0.73 to 1.06)
2021	0.0 (0.0 to 0.0)	0.0 (0.0 to 0.0)	4.7 (3.6 to 6.2)	2.2 (1.6 to 2.9)	1.98 (1.49 to 2.61)	0.91 (0.68 to 1.21)

North Tyneside   GBR_44647   Western Europe   Stillbirth Rate / Neonatal Mortality Rate (SBR/NMR) − 20 weeks
lambda = 0.6 ; zeta = 0.8 ; scale = 15 ; amp = 0.028

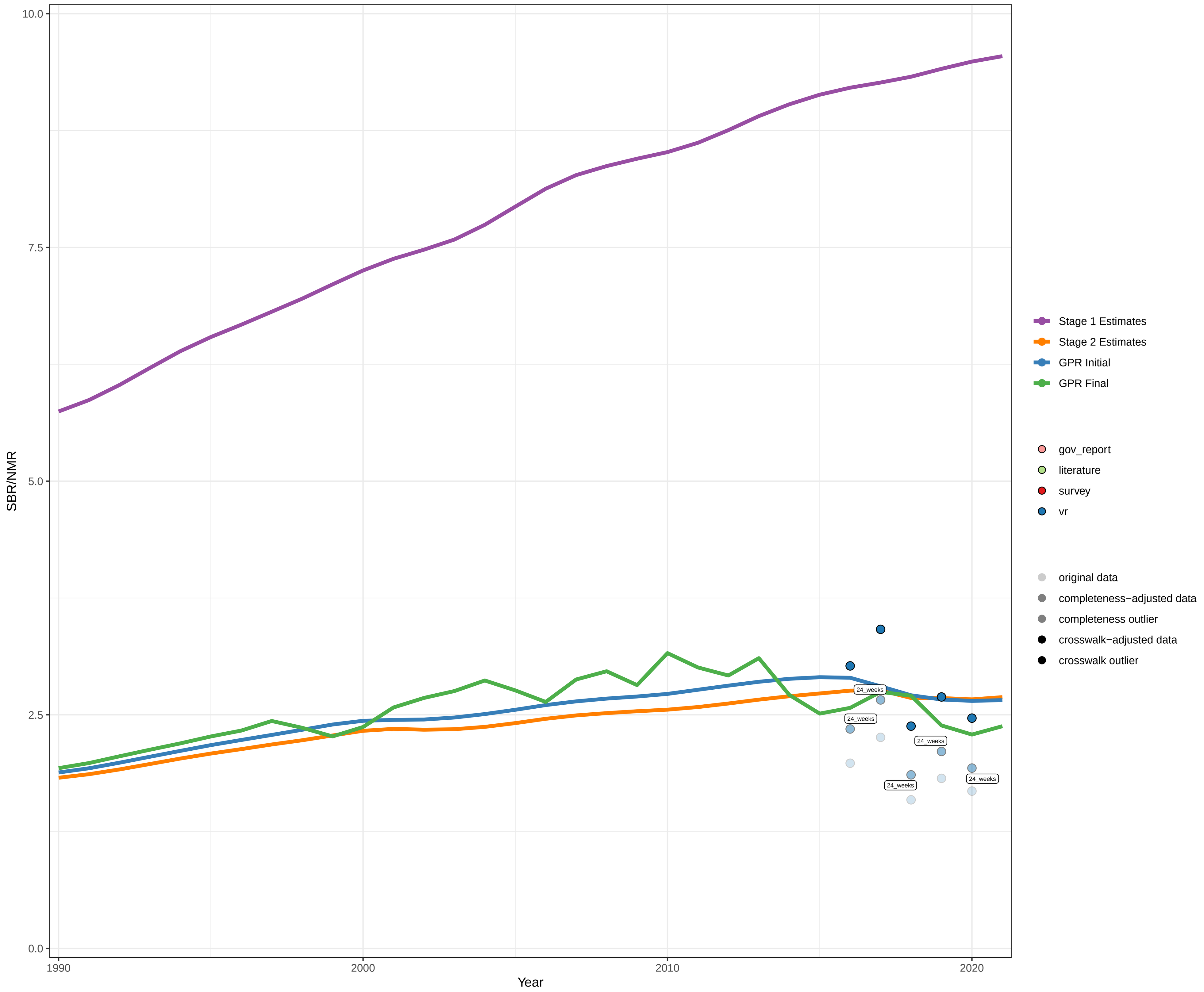

North Tyneside   GBR_44647   Western Europe   Stillbirth Rate / Neonatal Mortality Rate (SBR/NMR) − 28 weeks
lambda = 0.6 ; zeta = 0.8 ; scale = 15 ; amp = 0.028

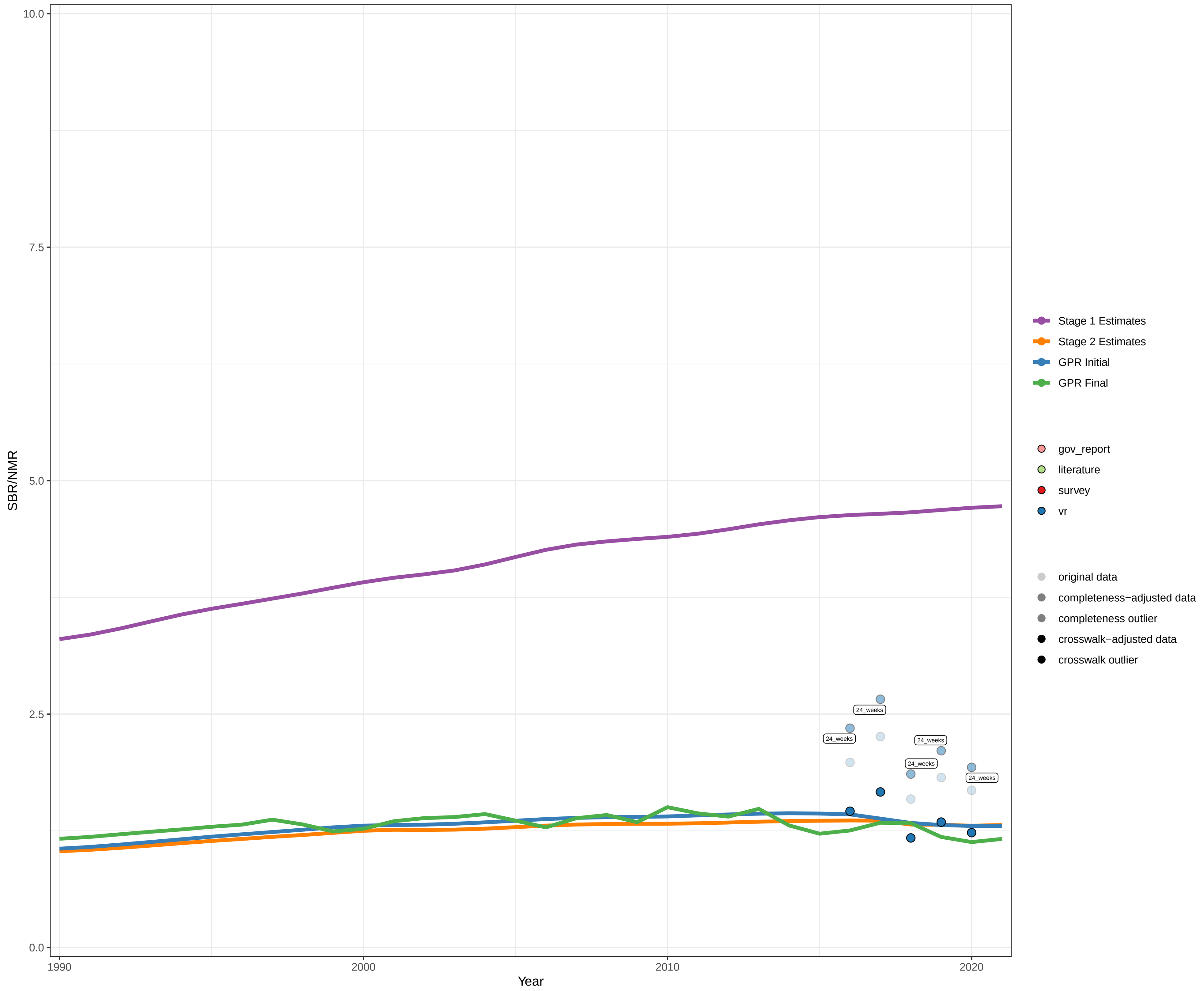

Definition	Years	Outlier	Source.Type	Title
24_weeks	2016−2020	0	VR	United Kingdom − England and Wales Child Mortality Statistics

North Tyneside   GBR_44647   Western Europe   Stillbirth Rate (SBR) − 20 weeks
lambda = 0.6 ; zeta = 0.8 ; scale = 15 ; amp = 0.028

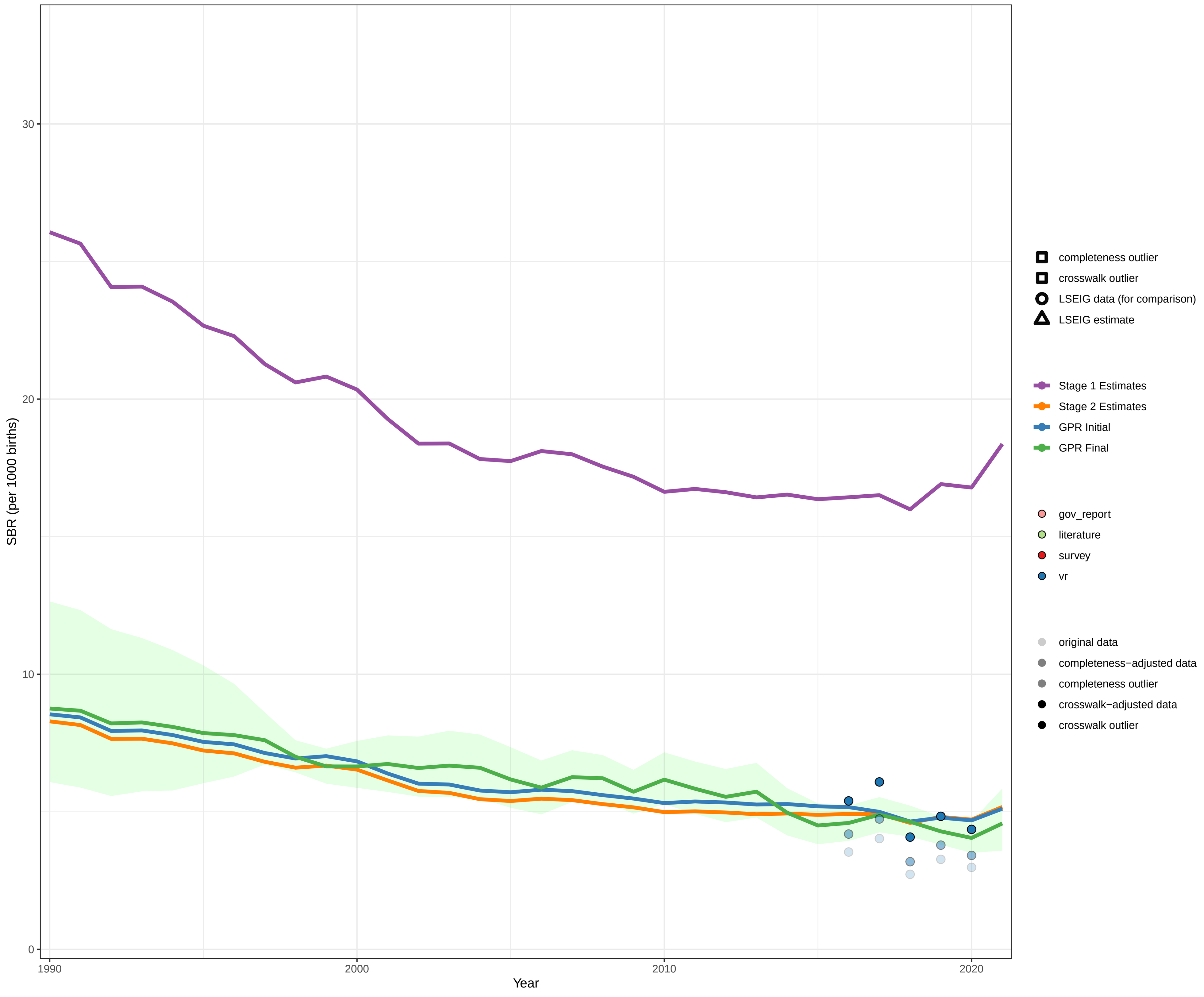

North Tyneside   GBR_44647   Western Europe   Stillbirth Rate (SBR) − 28 weeks
lambda = 0.6 ; zeta = 0.8 ; scale = 15 ; amp = 0.028

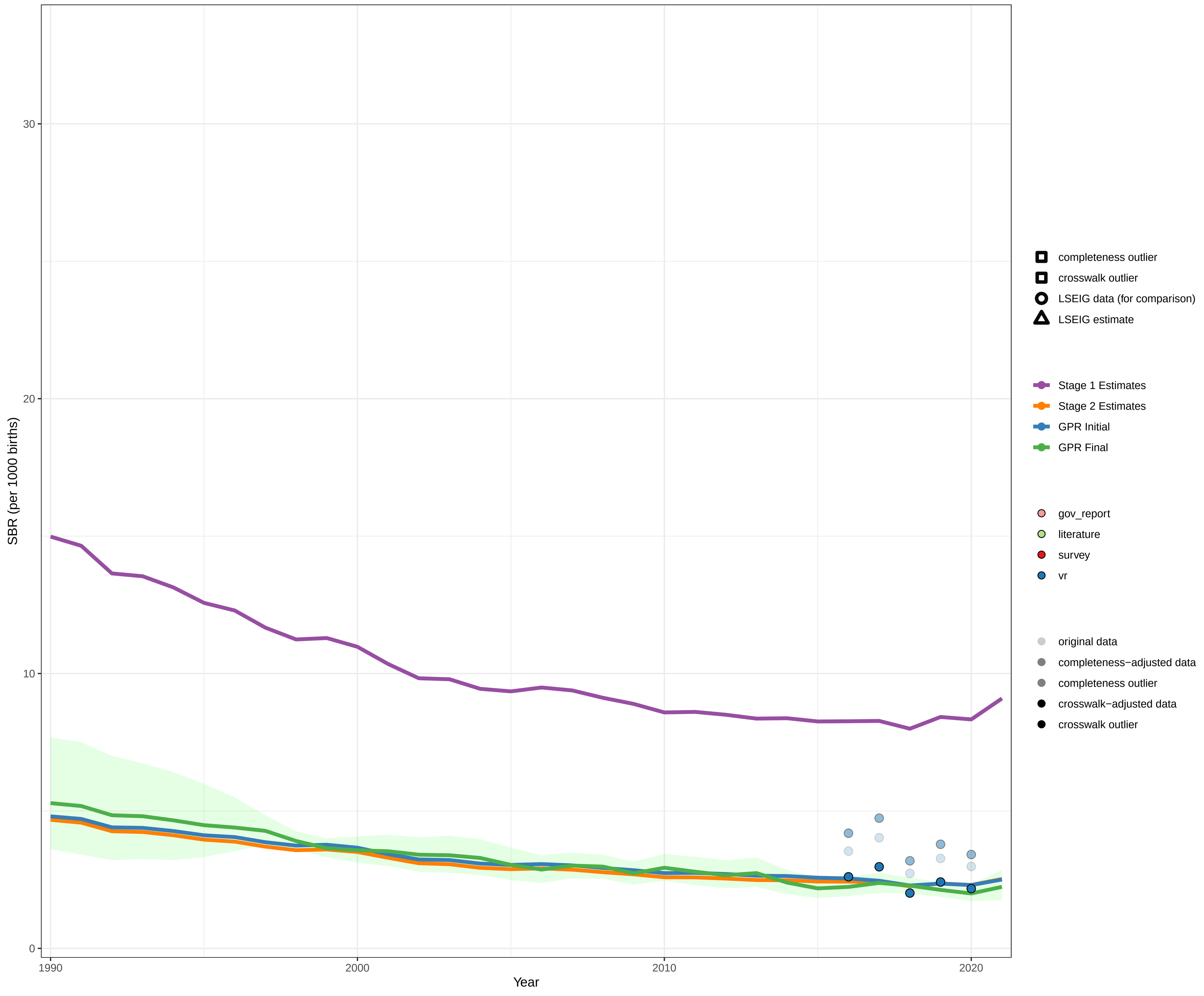

Year	Total Stillbirths (in thousands)	Total Stillbirths (in thousands)	SBR (per 1000 births)	SBR (per 1000 births)	SBR/NMR	SBR/NMR
	20 weeks	28 weeks	20 weeks	28 weeks	20 weeks	28 weeks
1990	0.0 (0.0 to 0.0)	0.0 (0.0 to 0.0)	8.8 (6.1 to 12.6)	5.3 (3.6 to 7.7)	1.93 (1.34 to 2.79)	1.16 (0.80 to 1.69)
1995	0.0 (0.0 to 0.0)	0.0 (0.0 to 0.0)	7.9 (6.0 to 10.3)	4.5 (3.3 to 6.0)	2.27 (1.74 to 2.98)	1.29 (0.96 to 1.73)
2000	0.0 (0.0 to 0.0)	0.0 (0.0 to 0.0)	6.6 (5.9 to 7.6)	3.6 (3.1 to 4.1)	2.37 (2.09 to 2.70)	1.27 (1.11 to 1.45)
2005	0.0 (0.0 to 0.0)	0.0 (0.0 to 0.0)	6.2 (5.1 to 7.3)	3.0 (2.5 to 3.7)	2.76 (2.30 to 3.29)	1.36 (1.11 to 1.64)
2010	0.0 (0.0 to 0.0)	0.0 (0.0 to 0.0)	6.2 (5.3 to 7.2)	2.9 (2.5 to 3.4)	3.16 (2.70 to 3.67)	1.50 (1.26 to 1.76)
2015	0.0 (0.0 to 0.0)	0.0 (0.0 to 0.0)	4.5 (3.8 to 5.3)	2.2 (1.8 to 2.6)	2.51 (2.13 to 2.96)	1.22 (1.02 to 1.44)
2020	0.0 (0.0 to 0.0)	0.0 (0.0 to 0.0)	4.0 (3.5 to 4.6)	2.0 (1.7 to 2.3)	2.29 (1.98 to 2.63)	1.13 (0.97 to 1.31)
2021	0.0 (0.0 to 0.0)	0.0 (0.0 to 0.0)	4.6 (3.6 to 5.8)	2.2 (1.8 to 2.9)	2.38 (1.86 to 3.04)	1.16 (0.91 to 1.49)

Northumberland   GBR_44644   Western Europe   Stillbirth Rate / Neonatal Mortality Rate (SBR/NMR) − 20 weeks
lambda = 0.6 ; zeta = 0.8 ; scale = 15 ; amp = 0.028

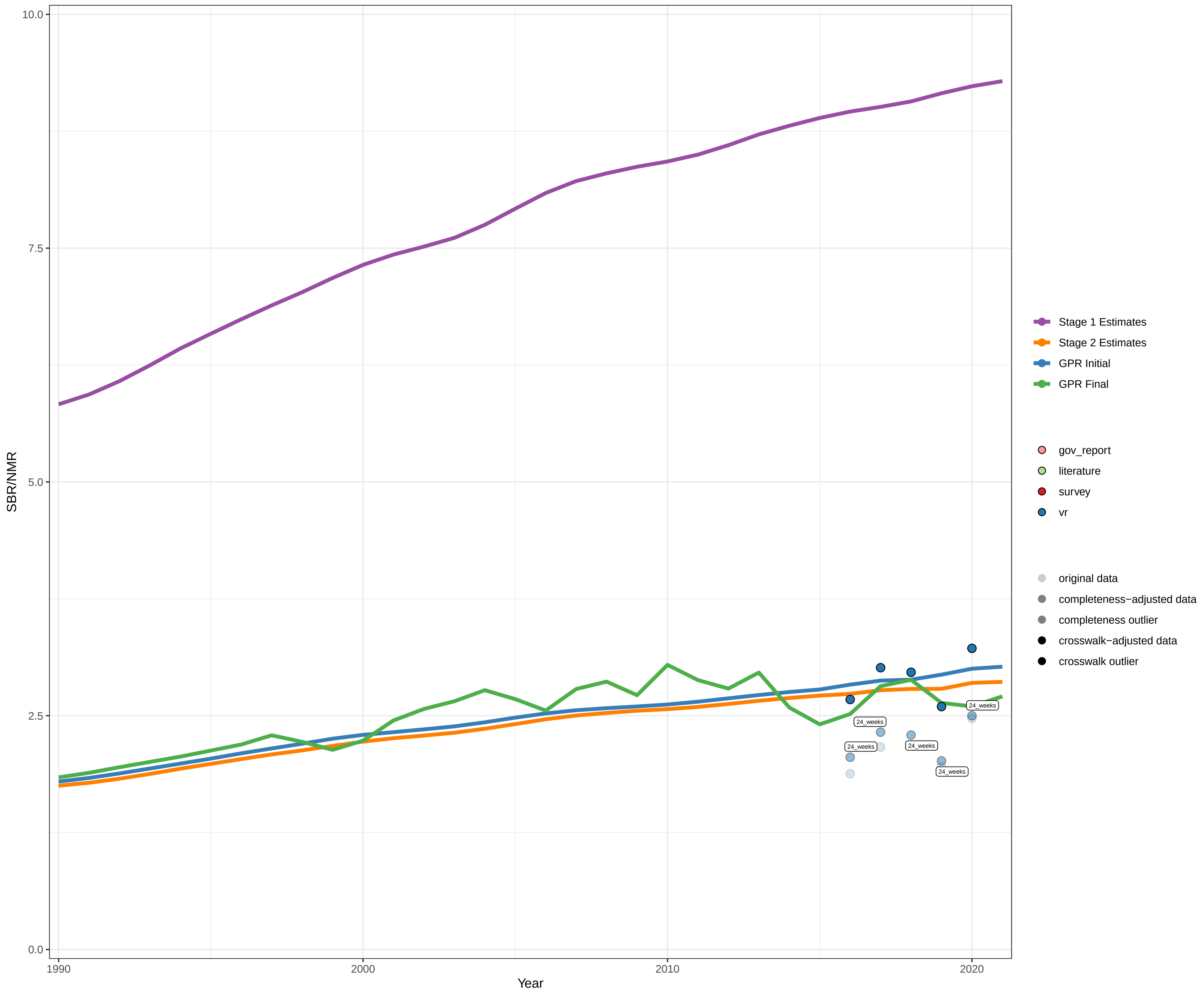

Northumberland   GBR_44644   Western Europe   Stillbirth Rate / Neonatal Mortality Rate (SBR/NMR) − 28 weeks
lambda = 0.6 ; zeta = 0.8 ; scale = 15 ; amp = 0.028

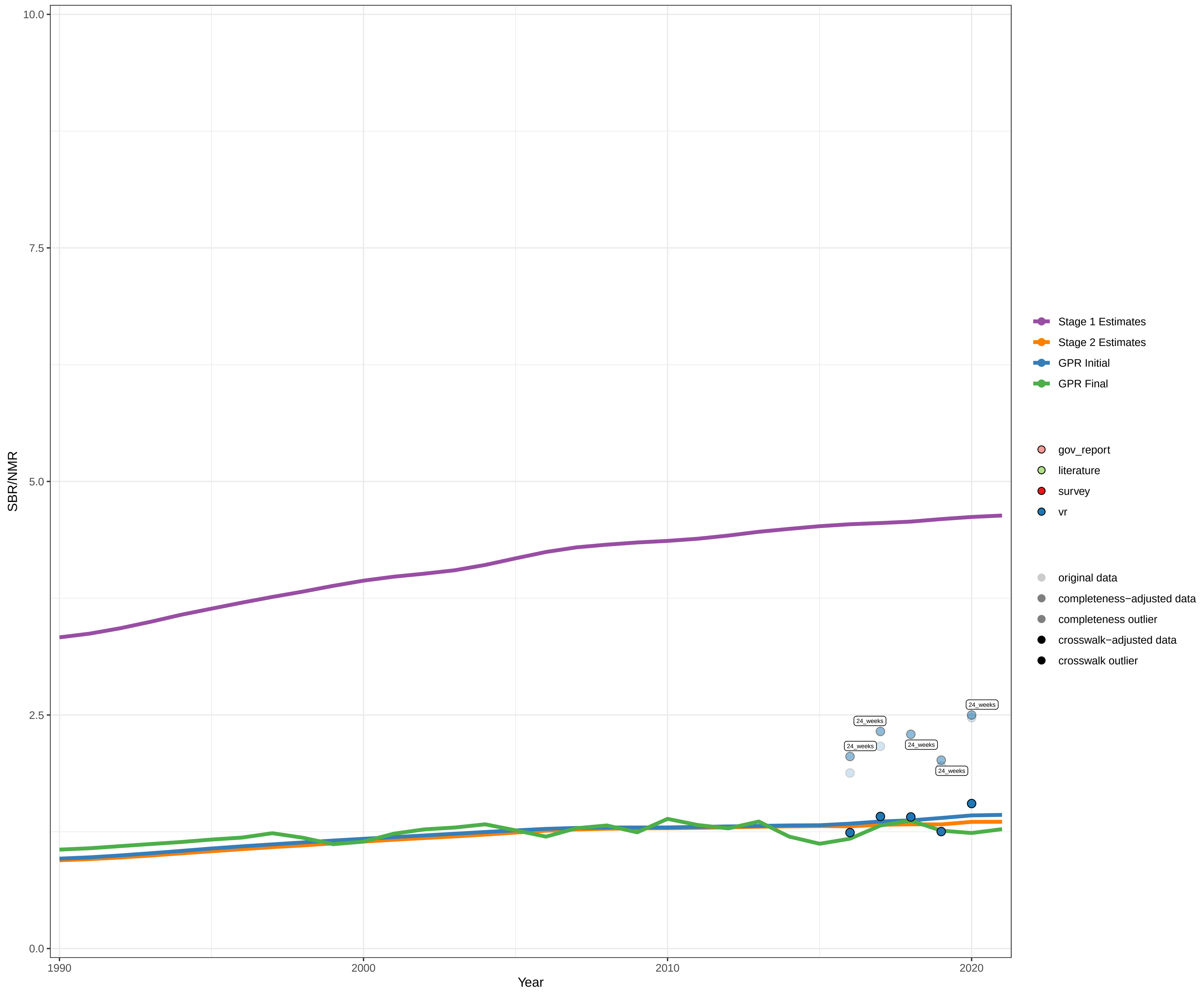

Definition	Years	Outlier	Source.Type	Title
24_weeks	2016−2020	0	VR	United Kingdom − England and Wales Child Mortality Statistics

Northumberland   GBR_44644   Western Europe   Stillbirth Rate (SBR) − 20 weeks
lambda = 0.6 ; zeta = 0.8 ; scale = 15 ; amp = 0.028

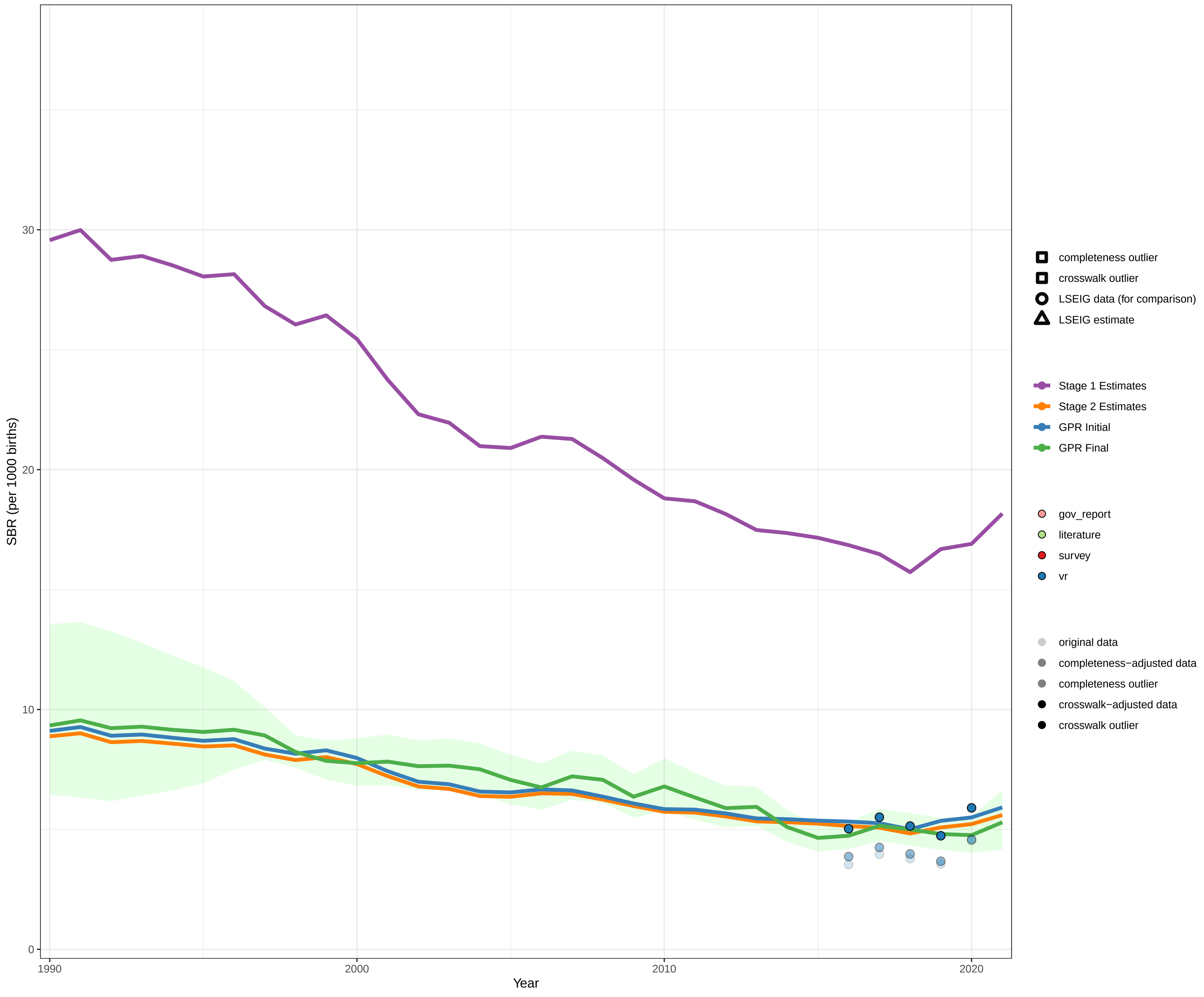

Northumberland   GBR_44644   Western Europe   Stillbirth Rate (SBR) − 28 weeks
lambda = 0.6 ; zeta = 0.8 ; scale = 15 ; amp = 0.028

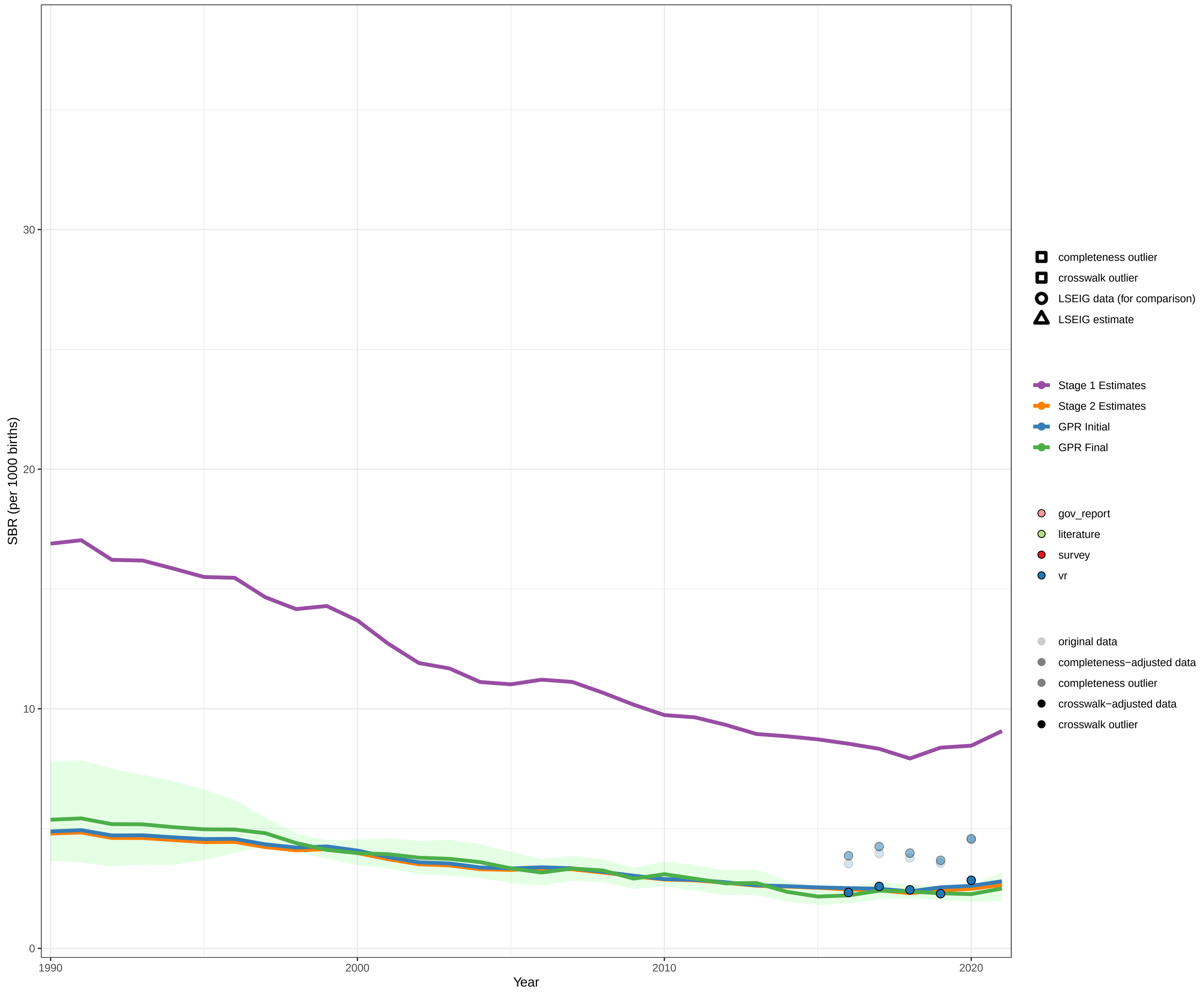

Year	Total Stillbirths (in thousands)	Total Stillbirths (in thousands)	SBR (per 1000 births)	SBR (per 1000 births)	SBR/NMR	SBR/NMR
	20 weeks	28 weeks	20 weeks	28 weeks	20 weeks	28 weeks
1990	0.0 (0.0 to 0.0)	0.0 (0.0 to 0.0)	9.3 (6.5 to 13.6)	5.4 (3.7 to 7.8)	1.84 (1.27 to 2.67)	1.06 (0.72 to 1.54)
1995	0.0 (0.0 to 0.0)	0.0 (0.0 to 0.0)	9.1 (6.9 to 11.8)	5.0 (3.7 to 6.6)	2.13 (1.63 to 2.76)	1.17 (0.87 to 1.56)
2000	0.0 (0.0 to 0.0)	0.0 (0.0 to 0.0)	7.8 (6.8 to 8.8)	4.0 (3.5 to 4.5)	2.23 (1.96 to 2.53)	1.14 (1.00 to 1.31)
2005	0.0 (0.0 to 0.0)	0.0 (0.0 to 0.0)	7.1 (6.0 to 8.1)	3.3 (2.7 to 4.0)	2.68 (2.29 to 3.07)	1.27 (1.03 to 1.53)
2010	0.0 (0.0 to 0.0)	0.0 (0.0 to 0.0)	6.8 (5.8 to 8.0)	3.1 (2.6 to 3.6)	3.04 (2.59 to 3.57)	1.39 (1.17 to 1.63)
2015	0.0 (0.0 to 0.0)	0.0 (0.0 to 0.0)	4.6 (4.1 to 5.2)	2.2 (1.8 to 2.5)	2.41 (2.11 to 2.71)	1.12 (0.94 to 1.32)
2020	0.0 (0.0 to 0.0)	0.0 (0.0 to 0.0)	4.8 (4.0 to 5.5)	2.3 (1.9 to 2.6)	2.60 (2.19 to 3.03)	1.24 (1.06 to 1.43)
2021	0.0 (0.0 to 0.0)	0.0 (0.0 to 0.0)	5.3 (4.2 to 6.6)	2.5 (2.0 to 3.2)	2.71 (2.13 to 3.40)	1.28 (1.00 to 1.63)

Redcar and Cleveland GBR_44648 Western Europe Stillbirth Rate / Neonatal Mortality Rate (SBR/NMR) − 20 weeks
lambda = 0.6 ; zeta = 0.8 ; scale = 15 ; amp = 0.028

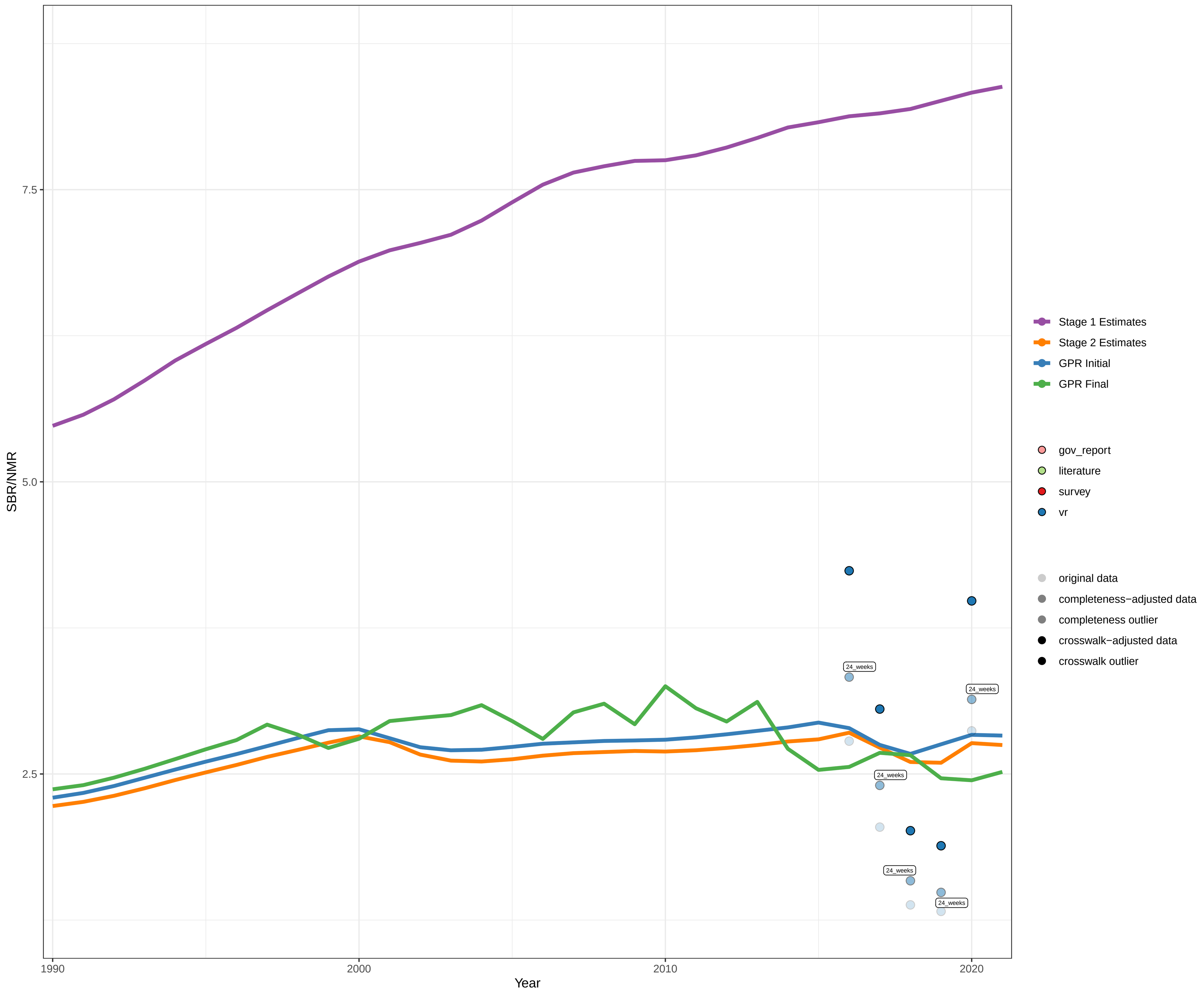

Redcar and Cleveland GBR_44648 Western Europe Stillbirth Rate / Neonatal Mortality Rate (SBR/NMR) − 28 weeks
lambda = 0.6 ; zeta = 0.8 ; scale = 15 ; amp = 0.028

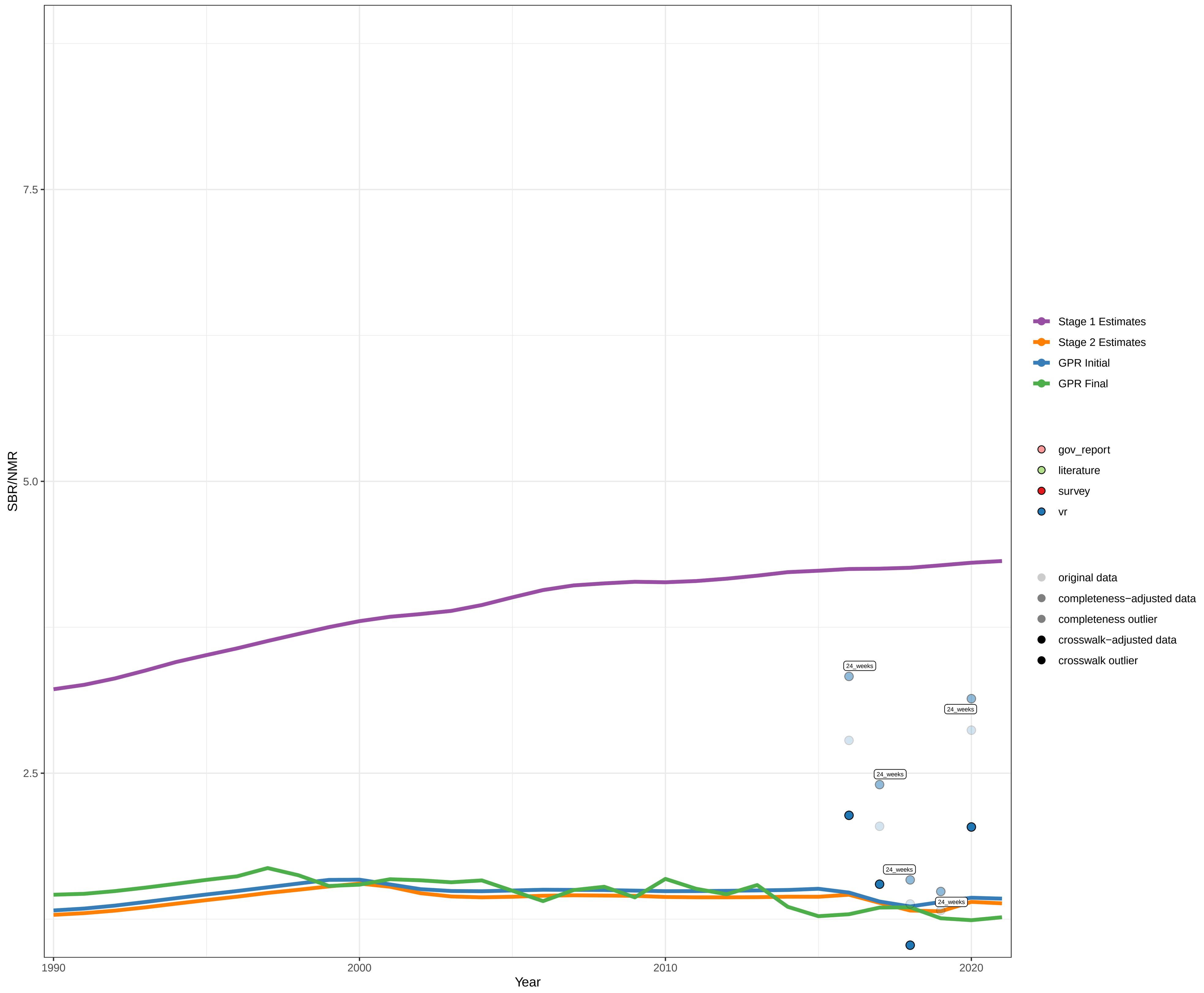

Definition	Years	Outlier	Source.Type	Title
24_weeks	2016−2020	0	VR	United Kingdom − England and Wales Child Mortality Statistics

Redcar and Cleveland GBR_44648 Western Europe Stillbirth Rate (SBR) − 20 weeks
lambda = 0.6 ; zeta = 0.8 ; scale = 15 ; amp = 0.028

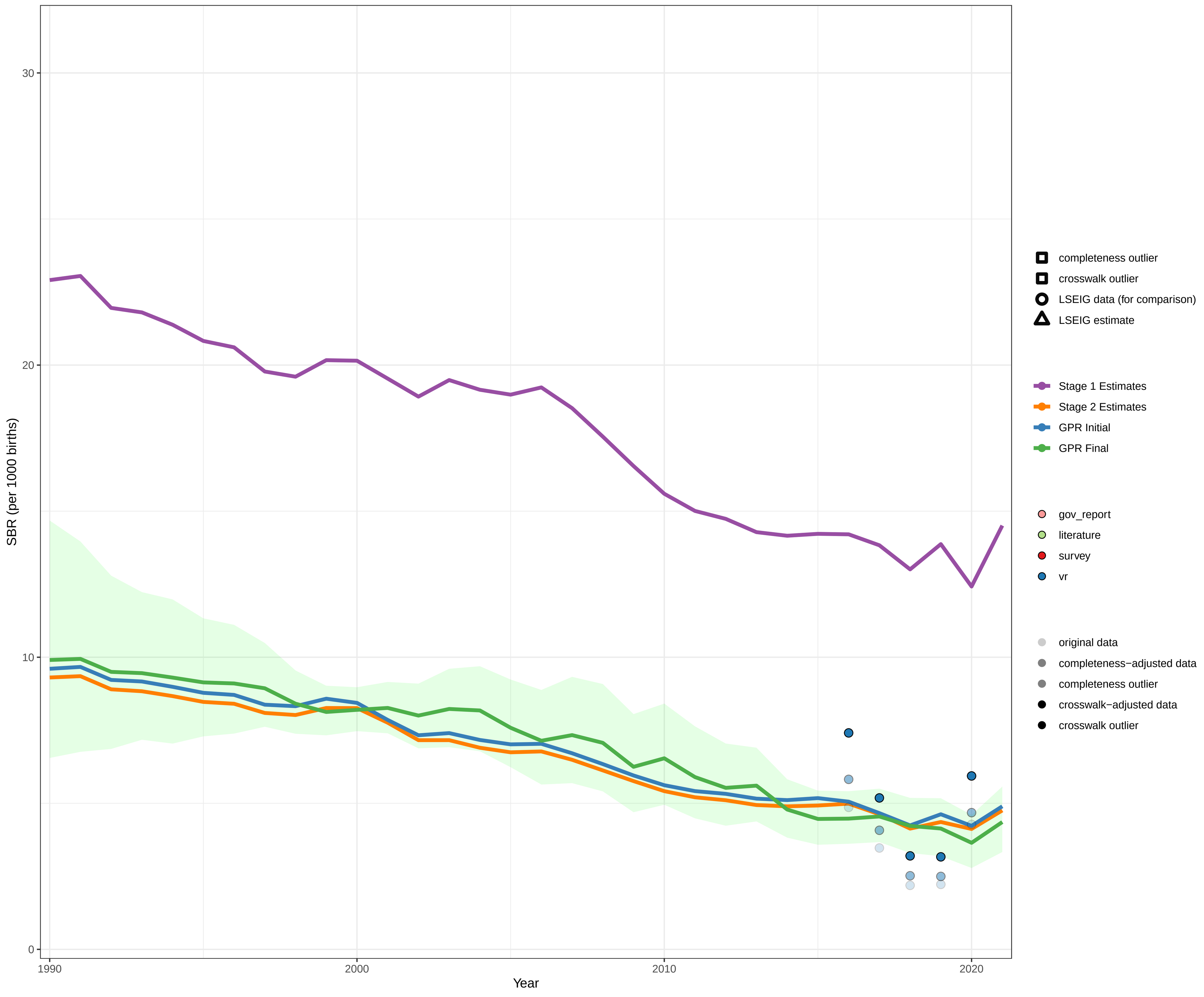

Redcar and Cleveland GBR_44648 Western Europe Stillbirth Rate (SBR) − 28 weeks
lambda = 0.6 ; zeta = 0.8 ; scale = 15 ; amp = 0.028

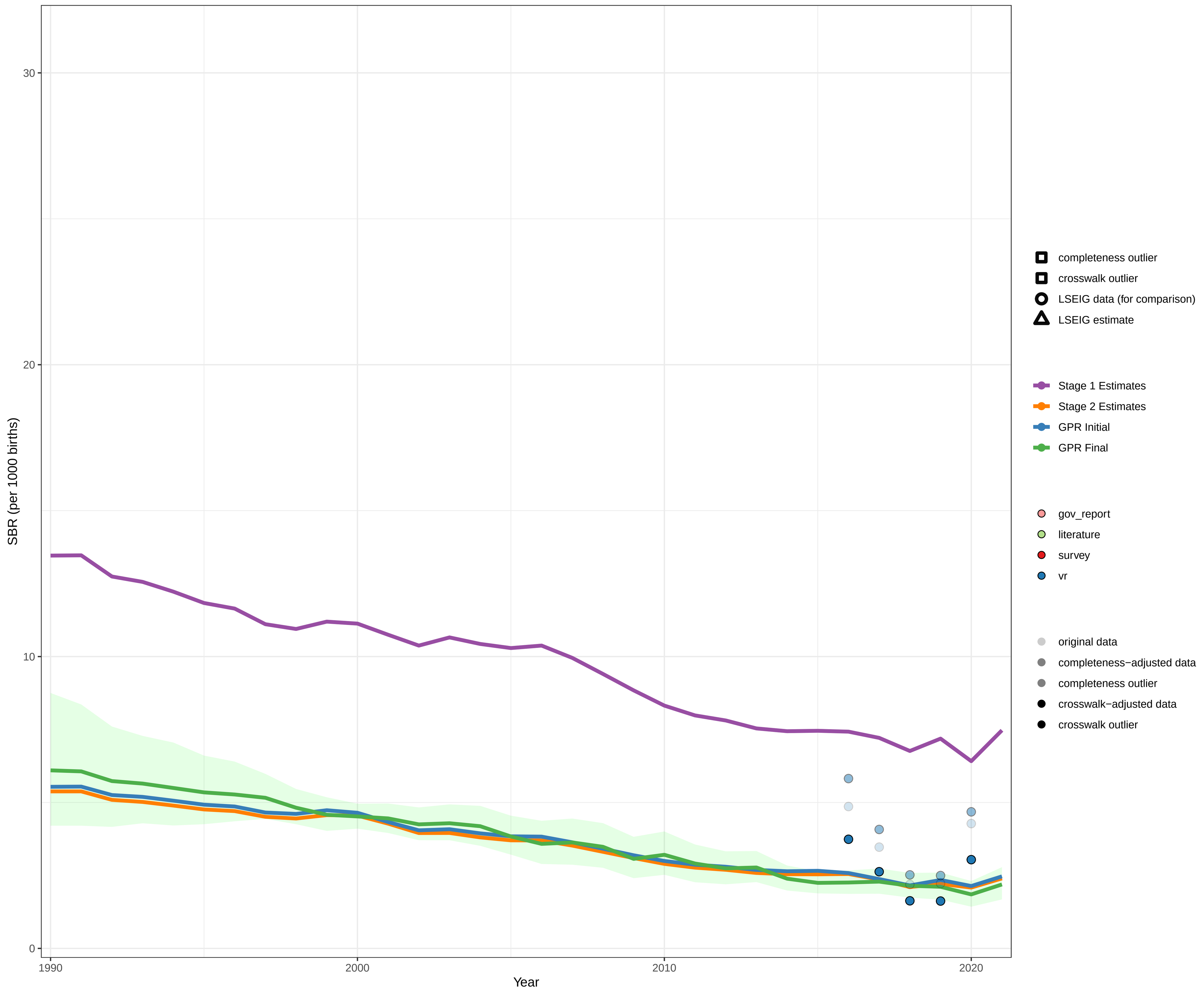

Year	Total Stillbirths (in thousands)	Total Stillbirths (in thousands)	SBR (per 1000 births)	SBR (per 1000 births)	SBR/NMR	SBR/NMR
	20 weeks	28 weeks	20 weeks	28 weeks	20 weeks	28 weeks
1990	0.0 (0.0 to 0.0)	0.0 (0.0 to 0.0)	9.9 (6.6 to 14.7)	6.1 (4.2 to 8.8)	2.37 (1.57 to 3.51)	1.46 (1.01 to 2.09)
1995	0.0 (0.0 to 0.0)	0.0 (0.0 to 0.0)	9.1 (7.3 to 11.3)	5.3 (4.3 to 6.6)	2.71 (2.16 to 3.36)	1.59 (1.26 to 1.96)
2000	0.0 (0.0 to 0.0)	0.0 (0.0 to 0.0)	8.2 (7.5 to 9.0)	4.5 (4.1 to 5.0)	2.80 (2.55 to 3.07)	1.55 (1.40 to 1.69)
2005	0.0 (0.0 to 0.0)	0.0 (0.0 to 0.0)	7.6 (6.2 to 9.2)	3.8 (3.2 to 4.5)	2.95 (2.43 to 3.60)	1.49 (1.25 to 1.77)
2010	0.0 (0.0 to 0.0)	0.0 (0.0 to 0.0)	6.5 (4.9 to 8.4)	3.2 (2.5 to 4.0)	3.25 (2.46 to 4.18)	1.59 (1.25 to 1.99)
2015	0.0 (0.0 to 0.0)	0.0 (0.0 to 0.0)	4.5 (3.6 to 5.4)	2.2 (1.9 to 2.6)	2.54 (2.03 to 3.09)	1.28 (1.07 to 1.50)
2020	0.0 (0.0 to 0.0)	0.0 (0.0 to 0.0)	3.6 (2.8 to 4.6)	1.9 (1.4 to 2.3)	2.45 (1.87 to 3.09)	1.24 (0.96 to 1.55)
2021	0.0 (0.0 to 0.0)	0.0 (0.0 to 0.0)	4.4 (3.3 to 5.6)	2.2 (1.7 to 2.8)	2.52 (1.92 to 3.22)	1.27 (0.97 to 1.61)

South Tyneside GBR_44652 Western Europe Stillbirth Rate / Neonatal Mortality Rate (SBR/NMR) − 20 weeks
lambda = 0.6 ; zeta = 0.8 ; scale = 15 ; amp = 0.028

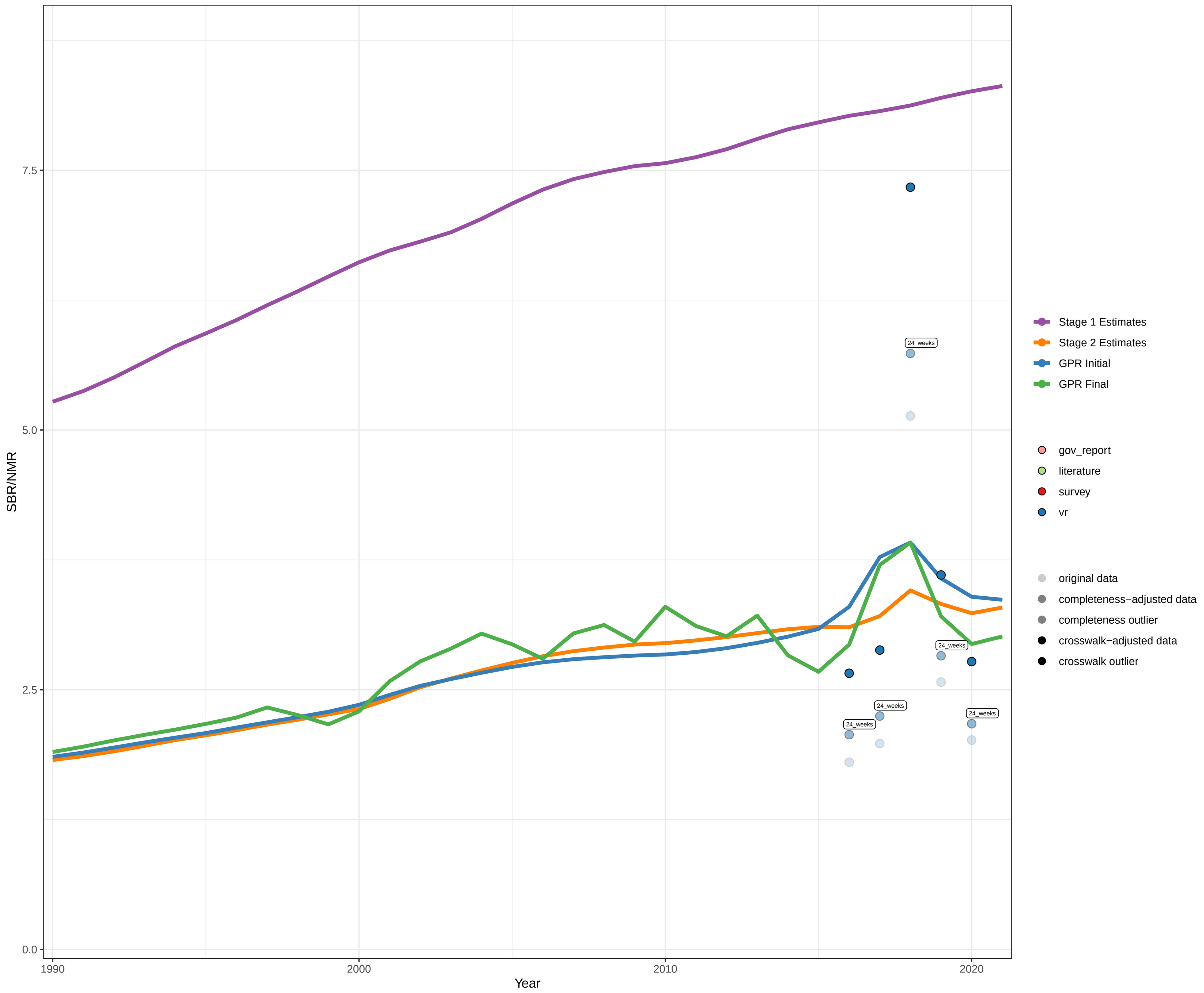

South Tyneside GBR_44652 Western Europe Stillbirth Rate / Neonatal Mortality Rate (SBR/NMR) − 28 weeks
lambda = 0.6 ; zeta = 0.8 ; scale = 15 ; amp = 0.028

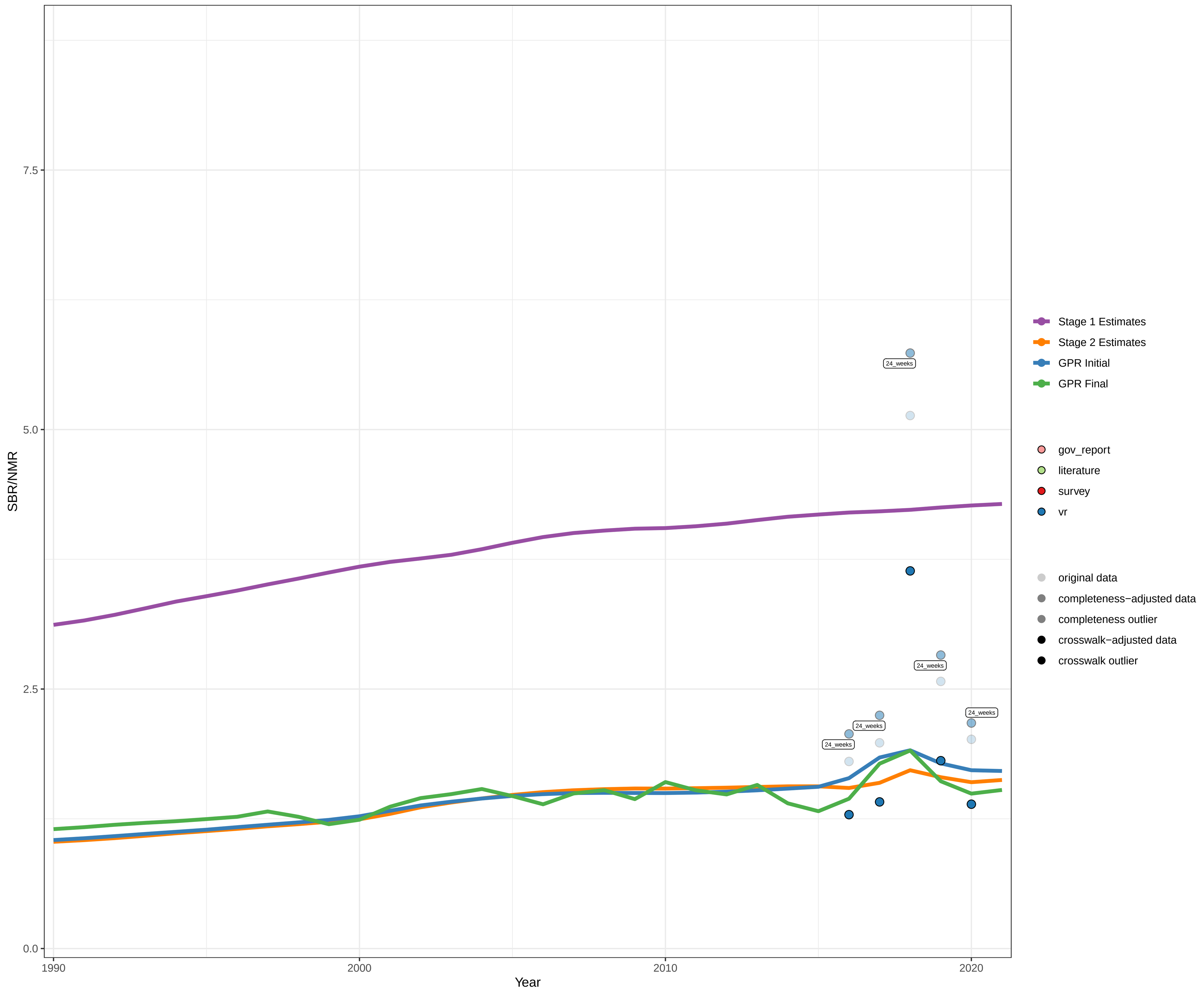

Definition	Years	Outlier	Source.Type	Title
24_weeks	2016−2020	0	VR	United Kingdom − England and Wales Child Mortality Statistics

South Tyneside GBR_44652 Western Europe Stillbirth Rate (SBR) − 20 weeks
lambda = 0.6 ; zeta = 0.8 ; scale = 15 ; amp = 0.028

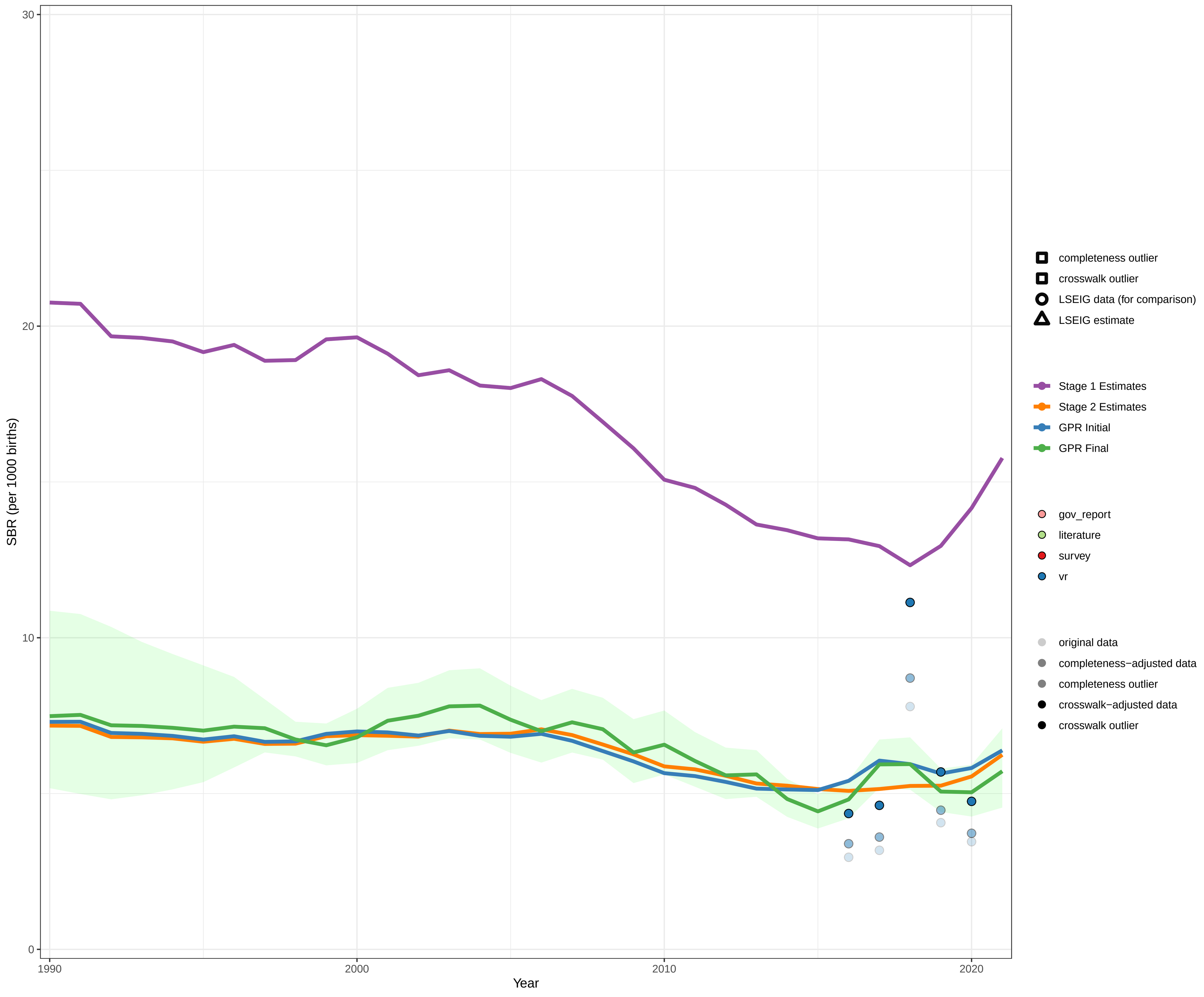

South Tyneside GBR_44652 Western Europe Stillbirth Rate (SBR) − 28 weeks
lambda = 0.6 ; zeta = 0.8 ; scale = 15 ; amp = 0.028

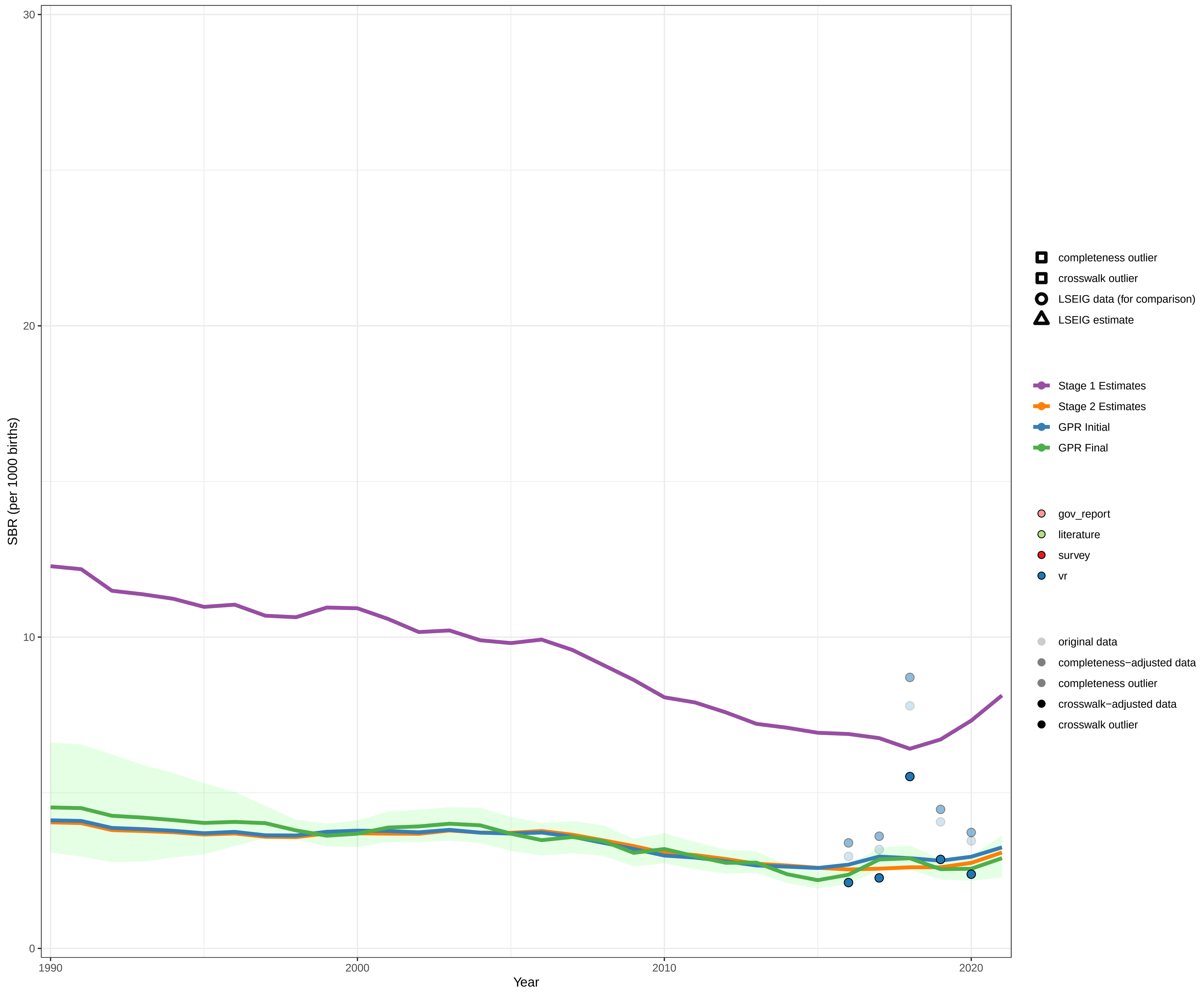

Year	Total Stillbirths (in thousands)	Total Stillbirths (in thousands)	SBR (per 1000 births)	SBR (per 1000 births)	SBR/NMR	SBR/NMR
	20 weeks	28 weeks	20 weeks	28 weeks	20 weeks	28 weeks
1990	0.0 (0.0 to 0.0)	0.0 (0.0 to 0.0)	7.5 (5.2 to 10.9)	4.5 (3.1 to 6.6)	1.90 (1.31 to 2.76)	1.15 (0.78 to 1.68)
1995	0.0 (0.0 to 0.0)	0.0 (0.0 to 0.0)	7.0 (5.4 to 9.1)	4.0 (3.0 to 5.3)	2.17 (1.66 to 2.82)	1.25 (0.93 to 1.64)
2000	0.0 (0.0 to 0.0)	0.0 (0.0 to 0.0)	6.8 (6.0 to 7.7)	3.7 (3.3 to 4.1)	2.29 (2.01 to 2.60)	1.24 (1.10 to 1.38)
2005	0.0 (0.0 to 0.0)	0.0 (0.0 to 0.0)	7.4 (6.3 to 8.5)	3.7 (3.1 to 4.2)	2.94 (2.51 to 3.37)	1.47 (1.25 to 1.68)
2010	0.0 (0.0 to 0.0)	0.0 (0.0 to 0.0)	6.6 (5.6 to 7.7)	3.2 (2.7 to 3.7)	3.30 (2.82 to 3.85)	1.60 (1.37 to 1.86)
2015	0.0 (0.0 to 0.0)	0.0 (0.0 to 0.0)	4.4 (3.9 to 5.0)	2.2 (1.9 to 2.5)	2.67 (2.34 to 3.00)	1.32 (1.16 to 1.50)
2020	0.0 (0.0 to 0.0)	0.0 (0.0 to 0.0)	5.0 (4.3 to 5.9)	2.6 (2.2 to 3.0)	2.94 (2.49 to 3.46)	1.49 (1.27 to 1.74)
2021	0.0 (0.0 to 0.0)	0.0 (0.0 to 0.0)	5.7 (4.5 to 7.1)	2.9 (2.3 to 3.6)	3.01 (2.40 to 3.74)	1.53 (1.20 to 1.91)

Stockton−on−Tees GBR_44645 Western Europe Stillbirth Rate / Neonatal Mortality Rate (SBR/NMR) − 20 weeks
lambda = 0.8 ; zeta = 0.7 ; scale = 15 ; amp = 0.028

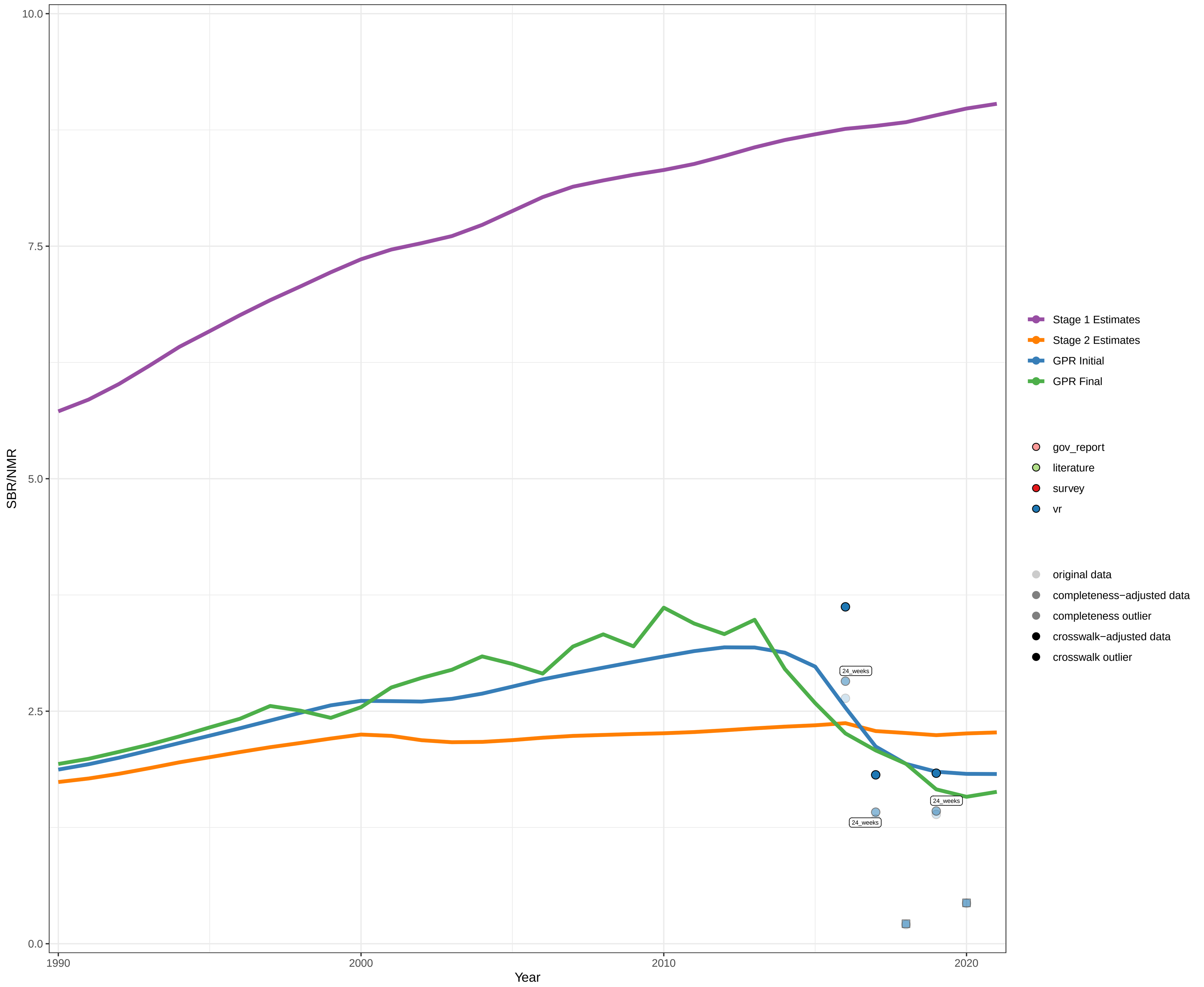

Stockton−on−Tees GBR_44645 Western Europe Stillbirth Rate / Neonatal Mortality Rate (SBR/NMR) − 28 weeks
lambda = 0.8 ; zeta = 0.7 ; scale = 15 ; amp = 0.028

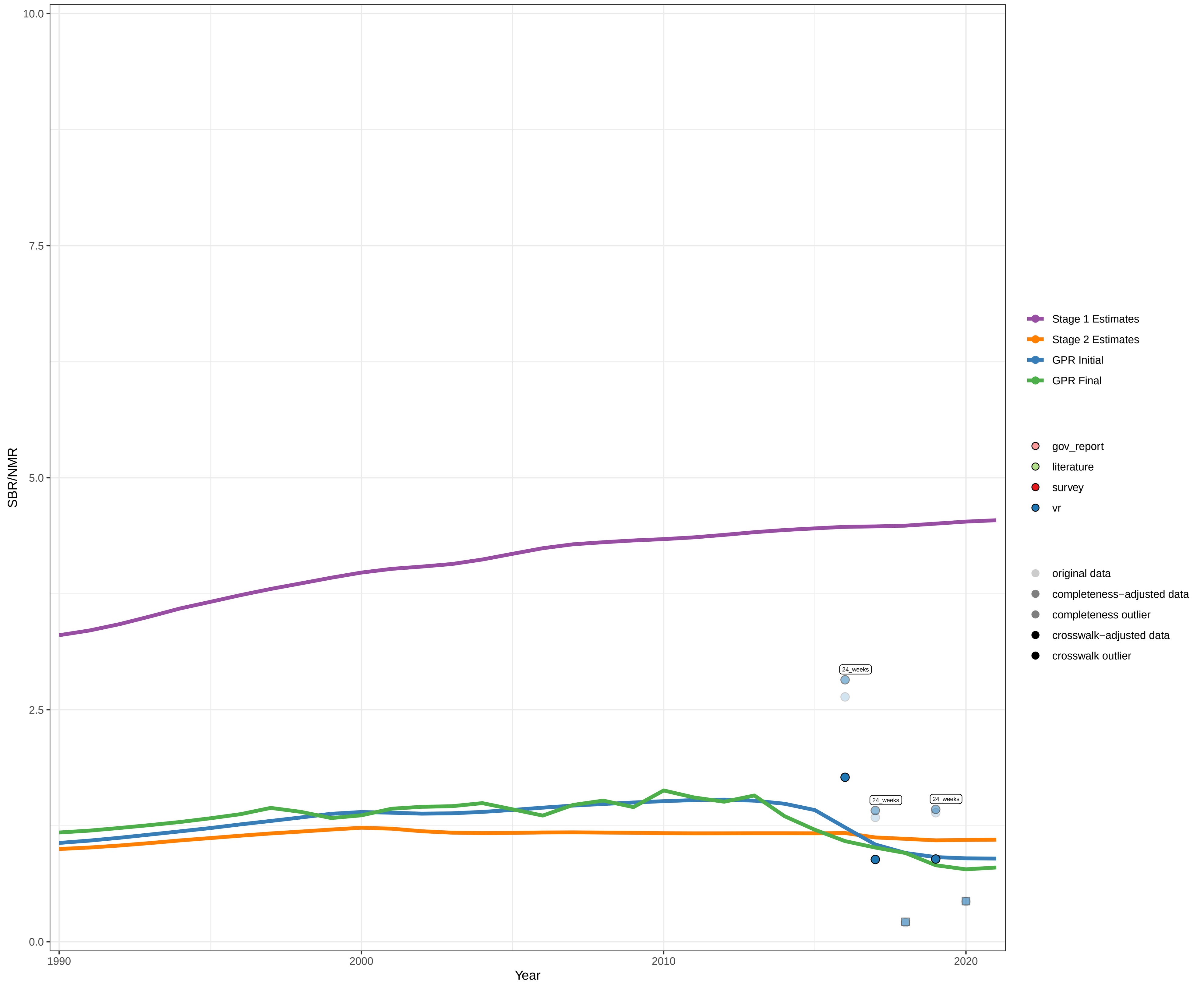

Definition	Years	Outlier	Source.Type	Title
24_weeks	2016, 2017, 2019	0	VR	United Kingdom − England and Wales Child Mortality Statistics
24_weeks	2018, 2020	1	VR	United Kingdom − England and Wales Child Mortality Statistics

Stockton−on−Tees GBR_44645 Western Europe Stillbirth Rate (SBR) − 20 weeks
lambda = 0.8 ; zeta = 0.7 ; scale = 15 ; amp = 0.028

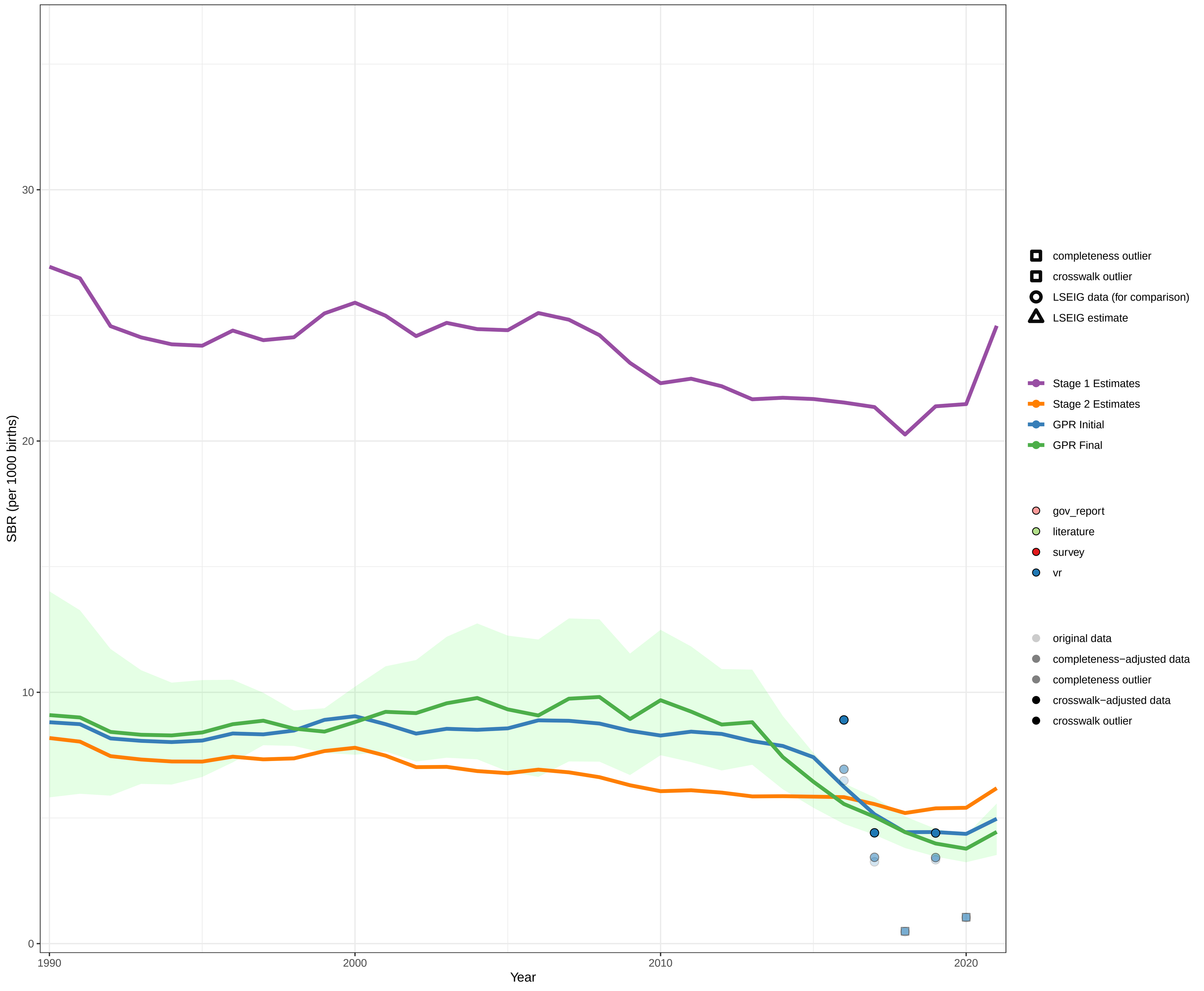

Stockton−on−Tees GBR_44645 Western Europe Stillbirth Rate (SBR) − 28 weeks
lambda = 0.8 ; zeta = 0.7 ; scale = 15 ; amp = 0.028

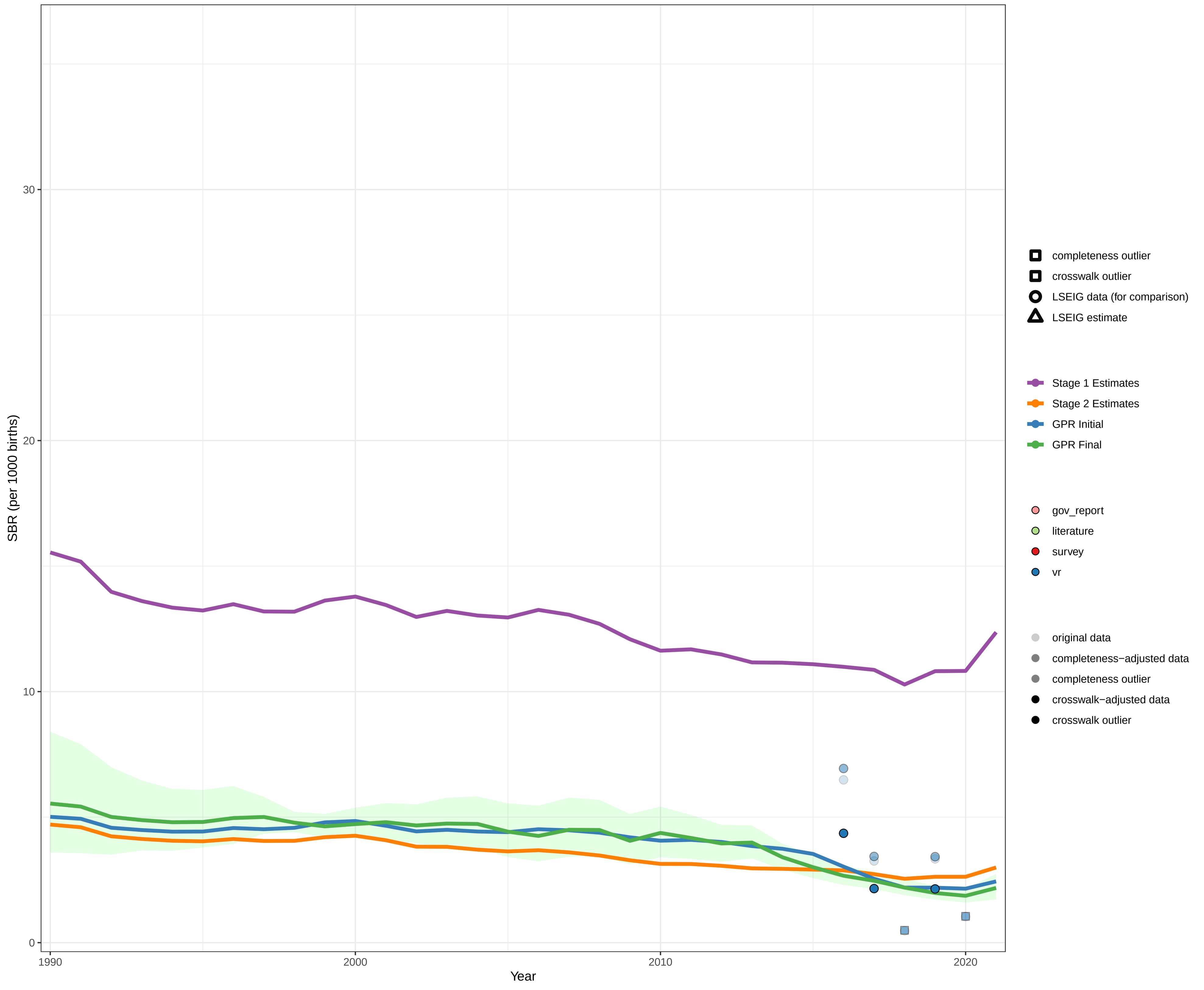

Year	Total Stillbirths (in thousands)	Total Stillbirths (in thousands)	SBR (per 1000 births)	SBR (per 1000 births)	SBR/NMR	SBR/NMR
	20 weeks	28 weeks	20 weeks	28 weeks	20 weeks	28 weeks
1990	0.0 (0.0 to 0.0)	0.0 (0.0 to 0.0)	9.1 (5.8 to 14.0)	5.5 (3.6 to 8.4)	1.93 (1.24 to 2.98)	1.18 (0.76 to 1.79)
1995	0.0 (0.0 to 0.0)	0.0 (0.0 to 0.0)	8.4 (6.6 to 10.5)	4.8 (3.8 to 6.1)	2.33 (1.84 to 2.90)	1.33 (1.05 to 1.68)
2000	0.0 (0.0 to 0.0)	0.0 (0.0 to 0.0)	8.8 (7.5 to 10.2)	4.7 (4.1 to 5.4)	2.54 (2.17 to 2.95)	1.36 (1.20 to 1.55)
2005	0.0 (0.0 to 0.0)	0.0 (0.0 to 0.0)	9.3 (6.9 to 12.3)	4.4 (3.4 to 5.5)	3.01 (2.21 to 3.96)	1.43 (1.10 to 1.79)
2010	0.0 (0.0 to 0.0)	0.0 (0.0 to 0.0)	9.7 (7.5 to 12.5)	4.4 (3.4 to 5.4)	3.61 (2.80 to 4.66)	1.63 (1.27 to 2.02)
2015	0.0 (0.0 to 0.0)	0.0 (0.0 to 0.0)	6.4 (5.4 to 7.6)	3.0 (2.6 to 3.5)	2.59 (2.17 to 3.05)	1.21 (1.03 to 1.41)
2020	0.0 (0.0 to 0.0)	0.0 (0.0 to 0.0)	3.8 (3.2 to 4.3)	1.9 (1.6 to 2.2)	1.58 (1.35 to 1.81)	0.78 (0.67 to 0.90)
2021	0.0 (0.0 to 0.0)	0.0 (0.0 to 0.0)	4.4 (3.5 to 5.6)	2.2 (1.7 to 2.7)	1.63 (1.30 to 2.05)	0.80 (0.63 to 1.01)

Sunderland GBR_44653 Western Europe Stillbirth Rate / Neonatal Mortality Rate (SBR/NMR) − 20 weeks
lambda = 0.6 ; zeta = 0.8 ; scale = 15 ; amp = 0.028

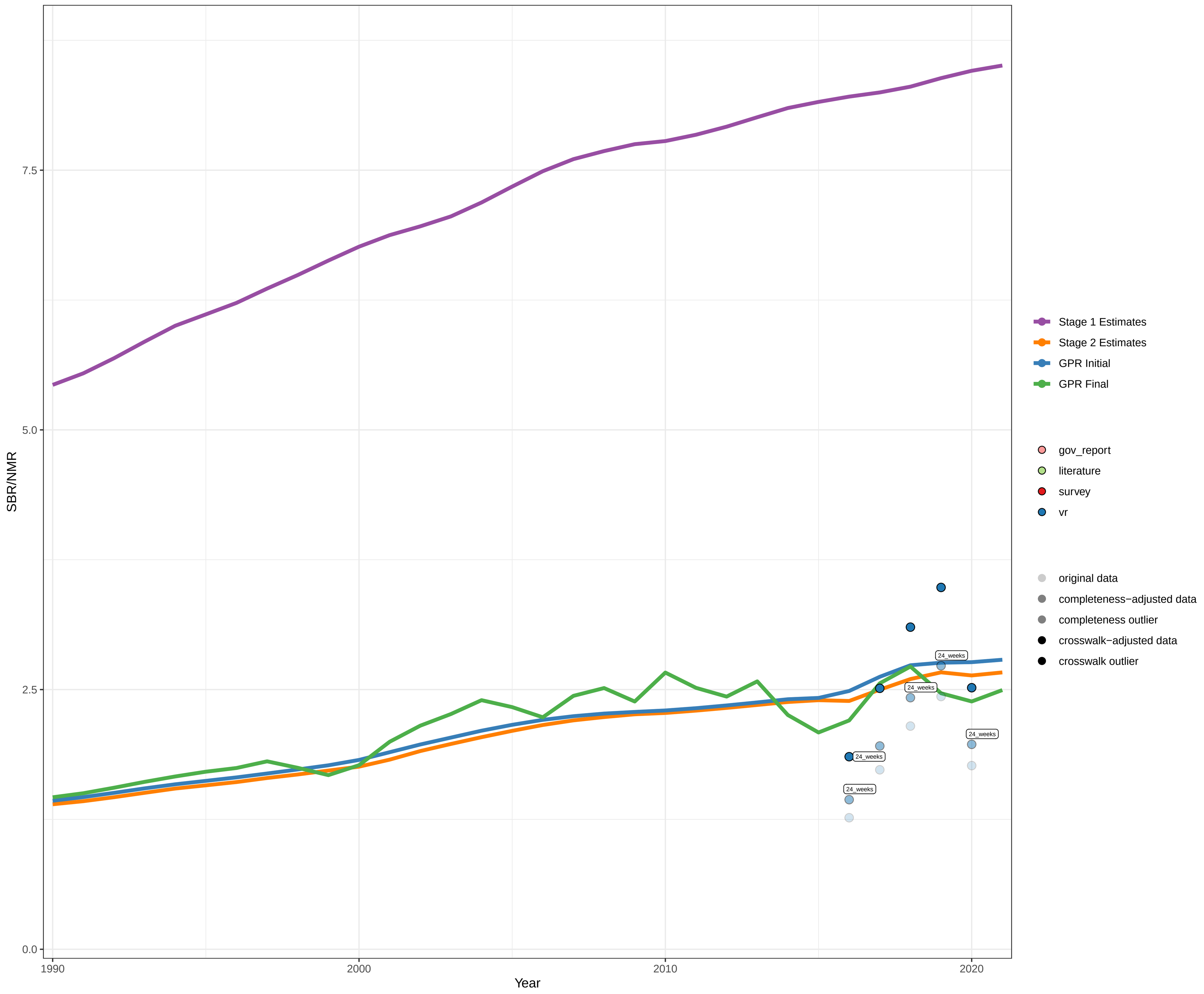

Sunderland GBR_44653 Western Europe Stillbirth Rate / Neonatal Mortality Rate (SBR/NMR) − 28 weeks
lambda = 0.6 ; zeta = 0.8 ; scale = 15 ; amp = 0.028

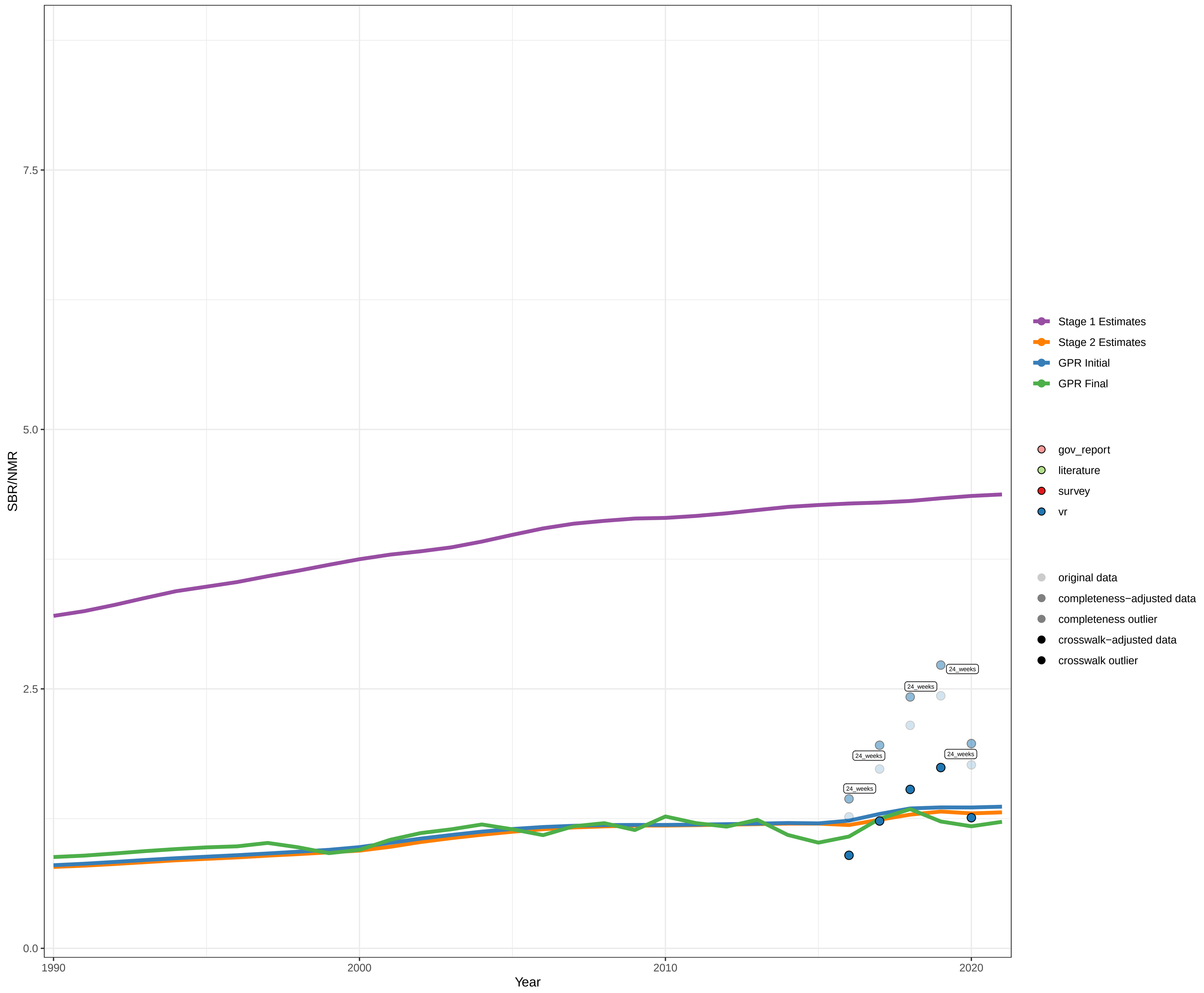

Definition	Years	Outlier	Source.Type	Title
24_weeks	2016−2020	0	VR	United Kingdom − England and Wales Child Mortality Statistics

Sunderland GBR_44653 Western Europe Stillbirth Rate (SBR) − 20 weeks
lambda = 0.6 ; zeta = 0.8 ; scale = 15 ; amp = 0.028

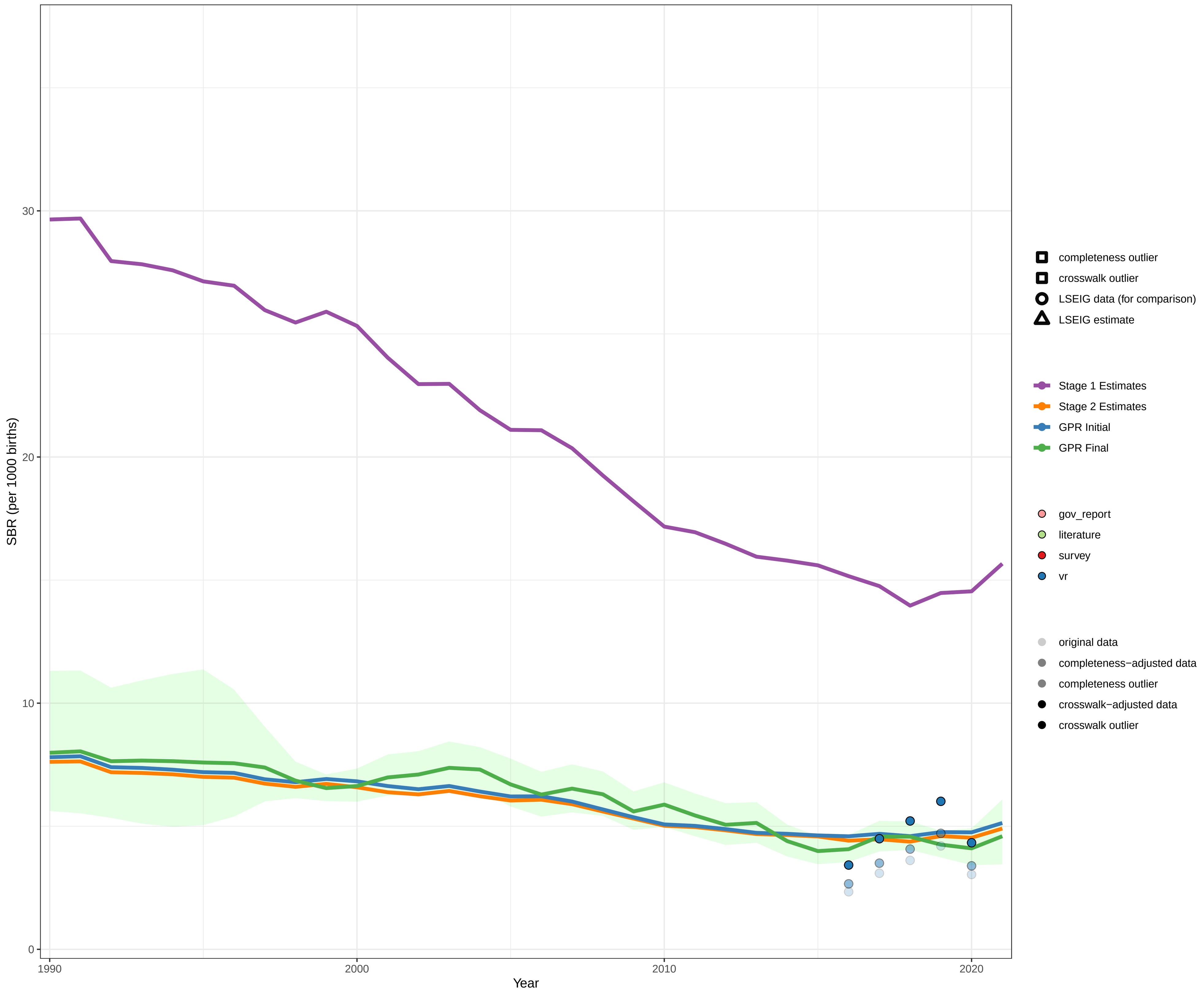

Sunderland GBR_44653 Western Europe Stillbirth Rate (SBR) − 28 weeks
lambda = 0.6 ; zeta = 0.8 ; scale = 15 ; amp = 0.028

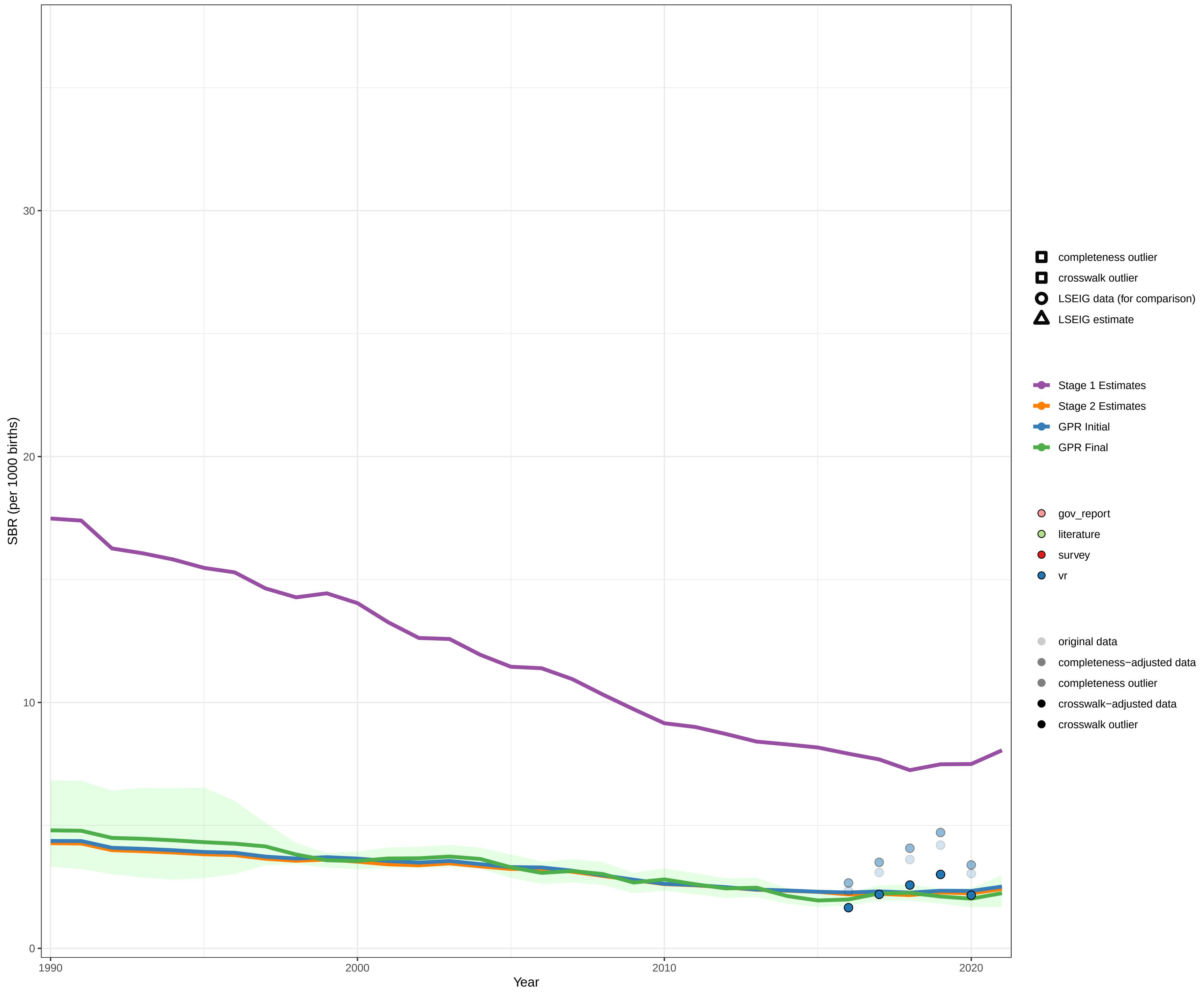

Year	Total Stillbirths (in thousands)	Total Stillbirths (in thousands)	SBR (per 1000 births)	SBR (per 1000 births)	SBR/NMR	SBR/NMR
	20 weeks	28 weeks	20 weeks	28 weeks	20 weeks	28 weeks
1990	0.0 (0.0 to 0.0)	0.0 (0.0 to 0.0)	8.0 (5.6 to 11.3)	4.8 (3.3 to 6.8)	1.46 (1.03 to 2.07)	0.88 (0.61 to 1.25)
1995	0.0 (0.0 to 0.0)	0.0 (0.0 to 0.0)	7.6 (5.0 to 11.4)	4.3 (2.9 to 6.5)	1.71 (1.14 to 2.56)	0.97 (0.64 to 1.47)
2000	0.0 (0.0 to 0.0)	0.0 (0.0 to 0.0)	6.6 (6.0 to 7.3)	3.6 (3.2 to 3.9)	1.77 (1.60 to 1.96)	0.95 (0.86 to 1.05)
2005	0.0 (0.0 to 0.0)	0.0 (0.0 to 0.0)	6.7 (5.8 to 7.7)	3.3 (2.9 to 3.8)	2.33 (2.02 to 2.70)	1.15 (1.00 to 1.33)
2010	0.0 (0.0 to 0.0)	0.0 (0.0 to 0.0)	5.9 (5.0 to 6.8)	2.8 (2.3 to 3.3)	2.66 (2.25 to 3.07)	1.27 (1.06 to 1.47)
2015	0.0 (0.0 to 0.0)	0.0 (0.0 to 0.0)	4.0 (3.5 to 4.6)	1.9 (1.7 to 2.2)	2.09 (1.81 to 2.41)	1.02 (0.88 to 1.17)
2020	0.0 (0.0 to 0.0)	0.0 (0.0 to 0.0)	4.1 (3.4 to 4.9)	2.0 (1.7 to 2.4)	2.39 (1.99 to 2.86)	1.18 (0.97 to 1.42)
2021	0.0 (0.0 to 0.0)	0.0 (0.0 to 0.0)	4.6 (3.4 to 6.1)	2.2 (1.7 to 3.0)	2.50 (1.87 to 3.31)	1.22 (0.91 to 1.62)

North West England GBR_4619 Western Europe Stillbirth Rate / Neonatal Mortality Rate (SBR/NMR) − 20 weeks
lambda = 0.2 ; zeta = 0.99 ; scale = 5 ; amp = 0.028

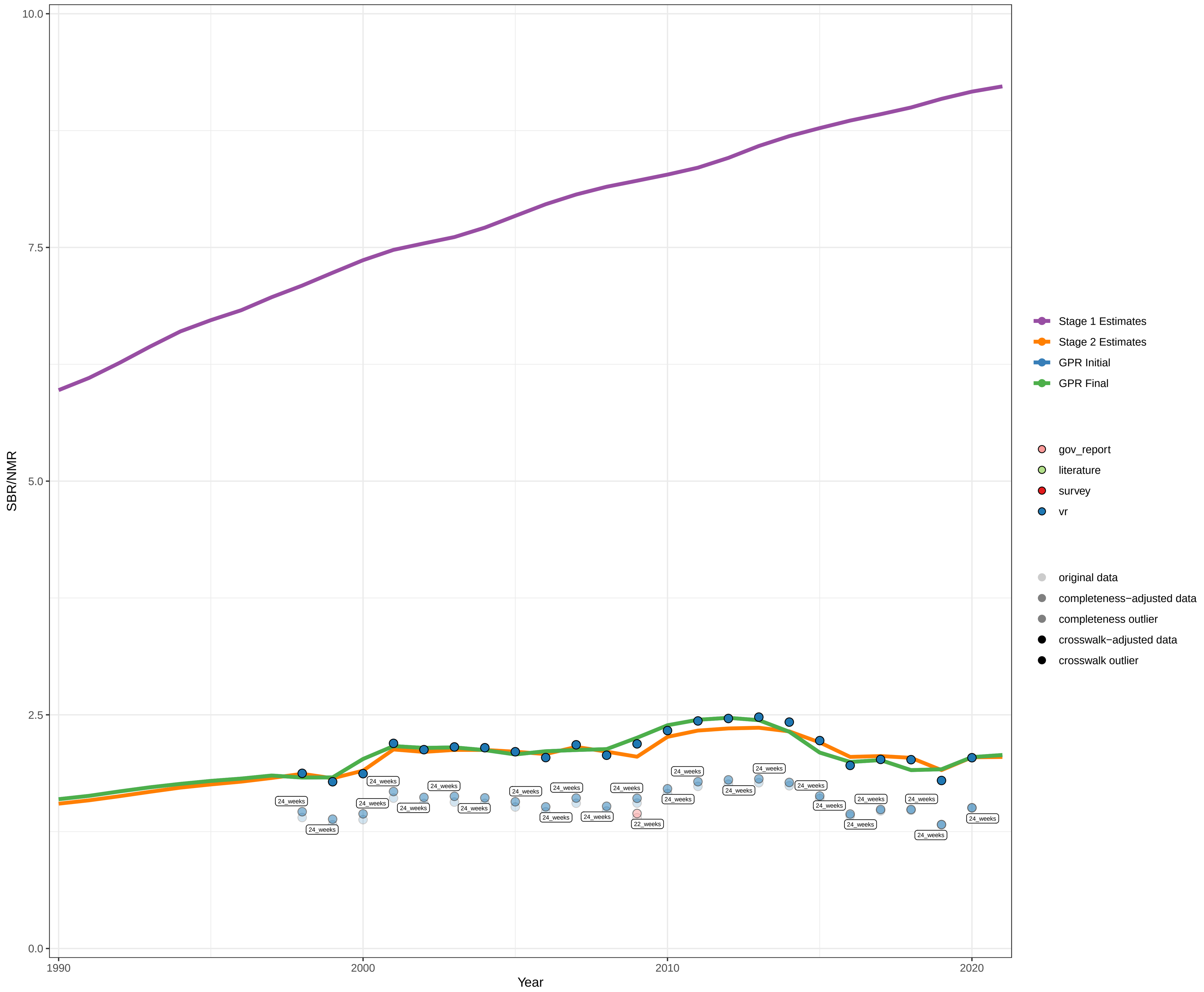

North West England GBR_4619 Western Europe Stillbirth Rate / Neonatal Mortality Rate (SBR/NMR) − 28 weeks
lambda = 0.2 ; zeta = 0.99 ; scale = 5 ; amp = 0.028

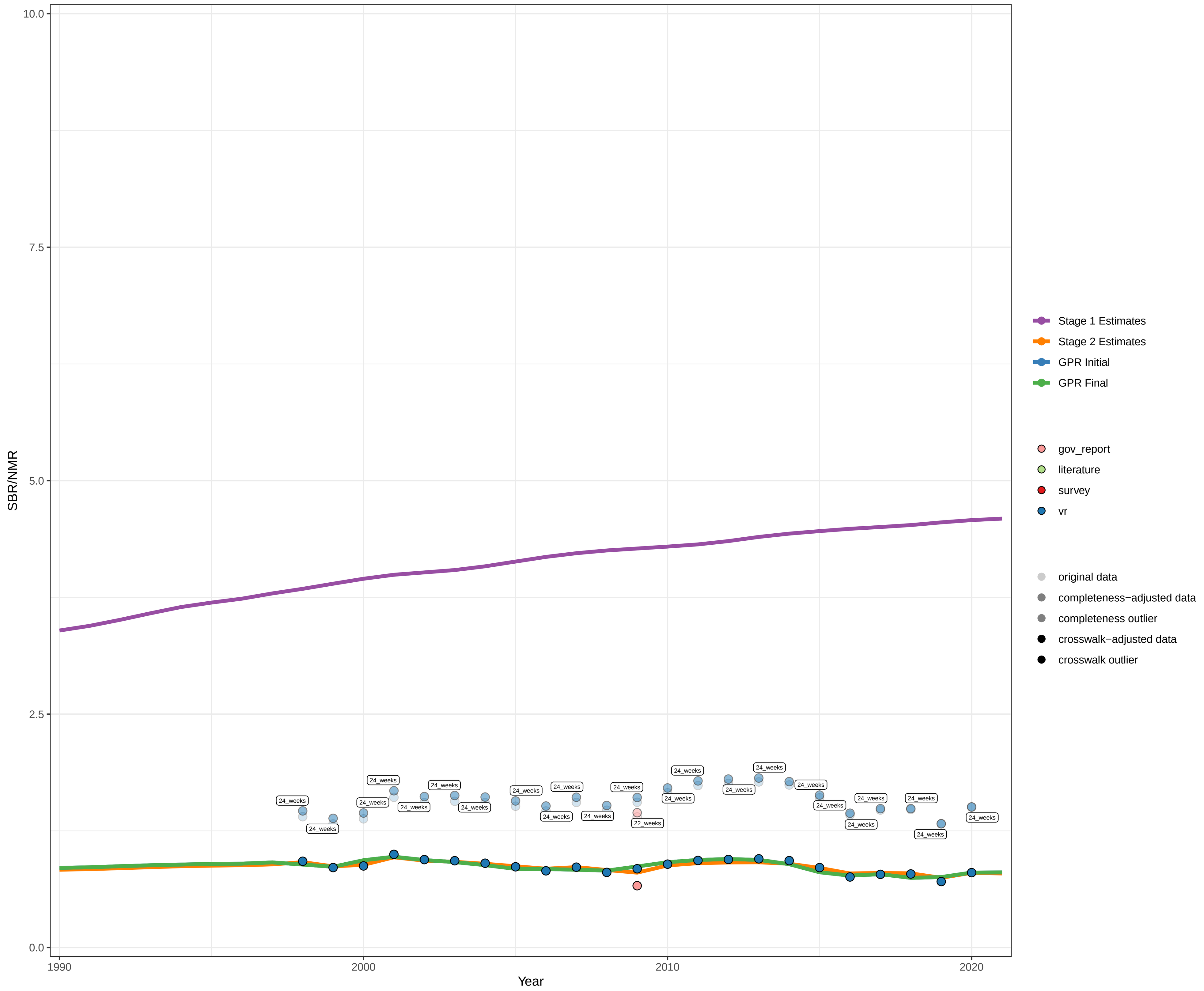

Definition	Years	Outlier	Source.Type	Title
22_weeks	2009	0	Statistical Report	United Kingdom Perinatal Mortality
24_weeks	1998−2007	0	VR	United Kingdom − England and Wales Birth Statistics
24_weeks	2008−2020	0	VR	United Kingdom − England and Wales Child Mortality Statistics

North West England GBR_4619 Western Europe Stillbirth Rate (SBR) − 20 weeks
lambda = 0.2 ; zeta = 0.99 ; scale = 5 ; amp = 0.028

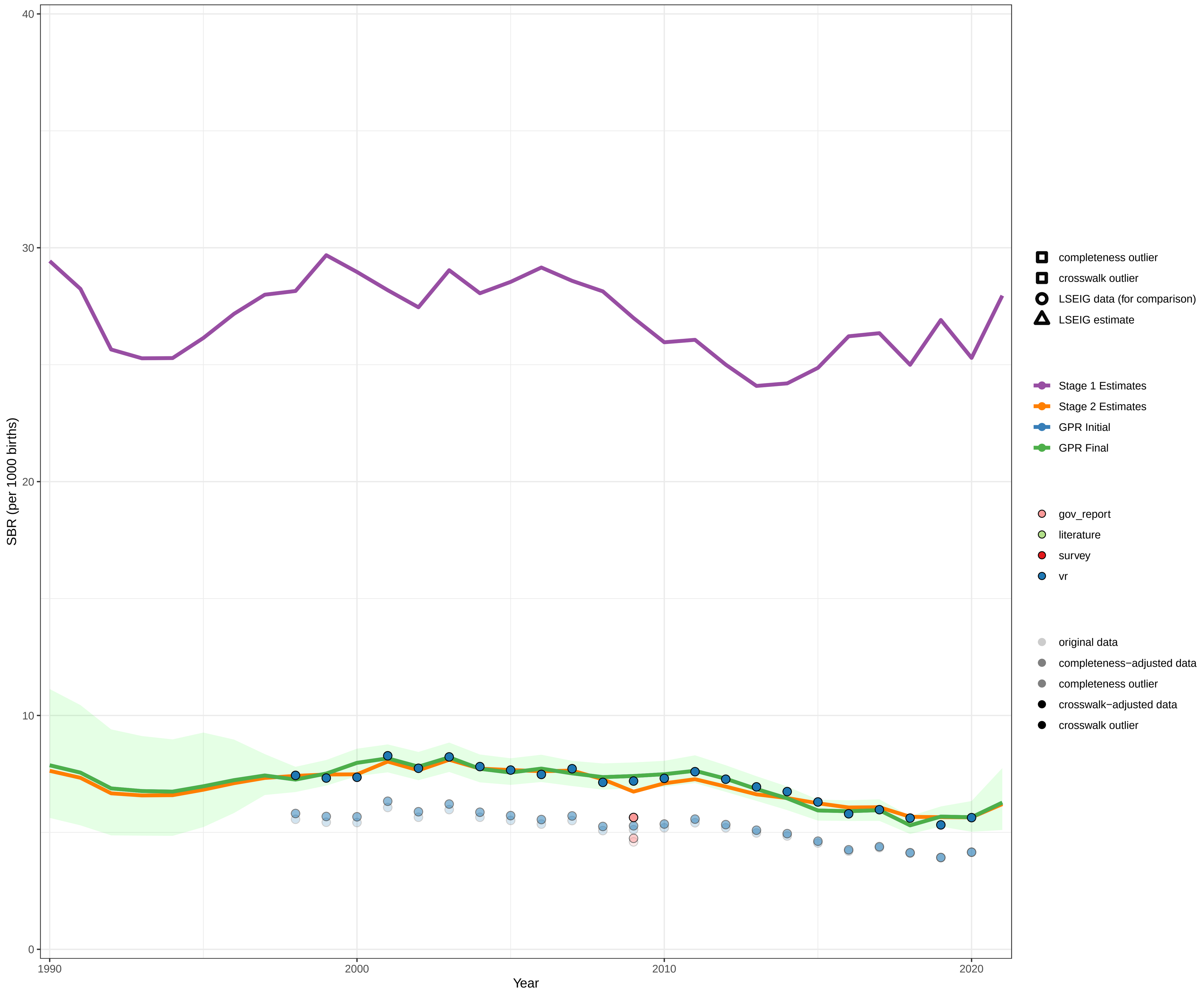

North West England GBR_4619 Western Europe Stillbirth Rate (SBR) − 28 weeks
lambda = 0.2 ; zeta = 0.99 ; scale = 5 ; amp = 0.028

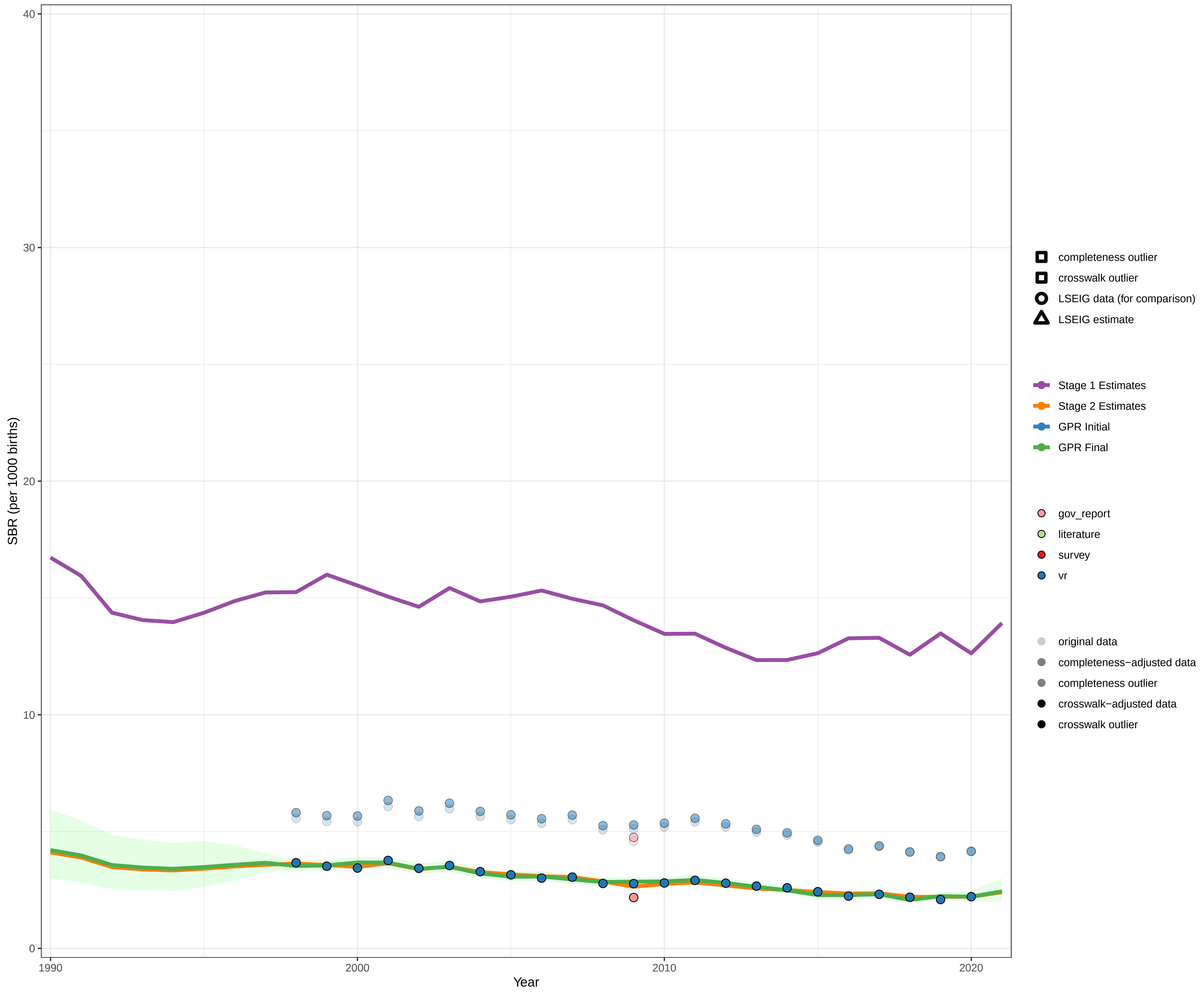

Year	Total Stillbirths (in thousands)	Total Stillbirths (in thousands)	SBR (per 1000 births)	SBR (per 1000 births)	SBR/NMR	SBR/NMR
	20 weeks	28 weeks	20 weeks	28 weeks	20 weeks	28 weeks
1990	0.8 (0.6 to 1.1)	0.4 (0.3 to 0.6)	7.9 (5.6 to 11.1)	4.2 (3.0 to 5.9)	1.60 (1.14 to 2.26)	0.85 (0.61 to 1.20)
1995	0.6 (0.4 to 0.8)	0.3 (0.2 to 0.4)	7.0 (5.2 to 9.3)	3.5 (2.6 to 4.6)	1.79 (1.34 to 2.38)	0.90 (0.67 to 1.18)
2000	0.6 (0.6 to 0.7)	0.3 (0.3 to 0.3)	8.0 (7.4 to 8.6)	3.7 (3.4 to 3.9)	2.03 (1.88 to 2.18)	0.93 (0.88 to 1.00)
2005	0.6 (0.6 to 0.7)	0.3 (0.2 to 0.3)	7.6 (7.0 to 8.2)	3.1 (2.9 to 3.3)	2.08 (1.93 to 2.24)	0.84 (0.79 to 0.90)
2010	0.7 (0.6 to 0.7)	0.3 (0.2 to 0.3)	7.5 (7.0 to 8.1)	2.9 (2.7 to 3.1)	2.39 (2.22 to 2.57)	0.91 (0.86 to 0.98)
2015	0.5 (0.5 to 0.6)	0.2 (0.2 to 0.2)	5.9 (5.5 to 6.4)	2.3 (2.1 to 2.4)	2.10 (1.94 to 2.26)	0.81 (0.75 to 0.86)
2020	0.4 (0.4 to 0.5)	0.2 (0.2 to 0.2)	5.6 (5.0 to 6.3)	2.2 (2.0 to 2.5)	2.05 (1.82 to 2.30)	0.80 (0.72 to 0.89)
2021	0.5 (0.4 to 0.6)	0.2 (0.2 to 0.2)	6.3 (5.1 to 7.7)	2.4 (2.0 to 3.0)	2.07 (1.68 to 2.56)	0.81 (0.66 to 0.99)

Blackburn with Darwen   GBR_44674   Western Europe   Stillbirth Rate / Neonatal Mortality Rate (SBR/NMR) − 20 weeks
lambda = 0.6 ; zeta = 0.8 ; scale = 15 ; amp = 0.028

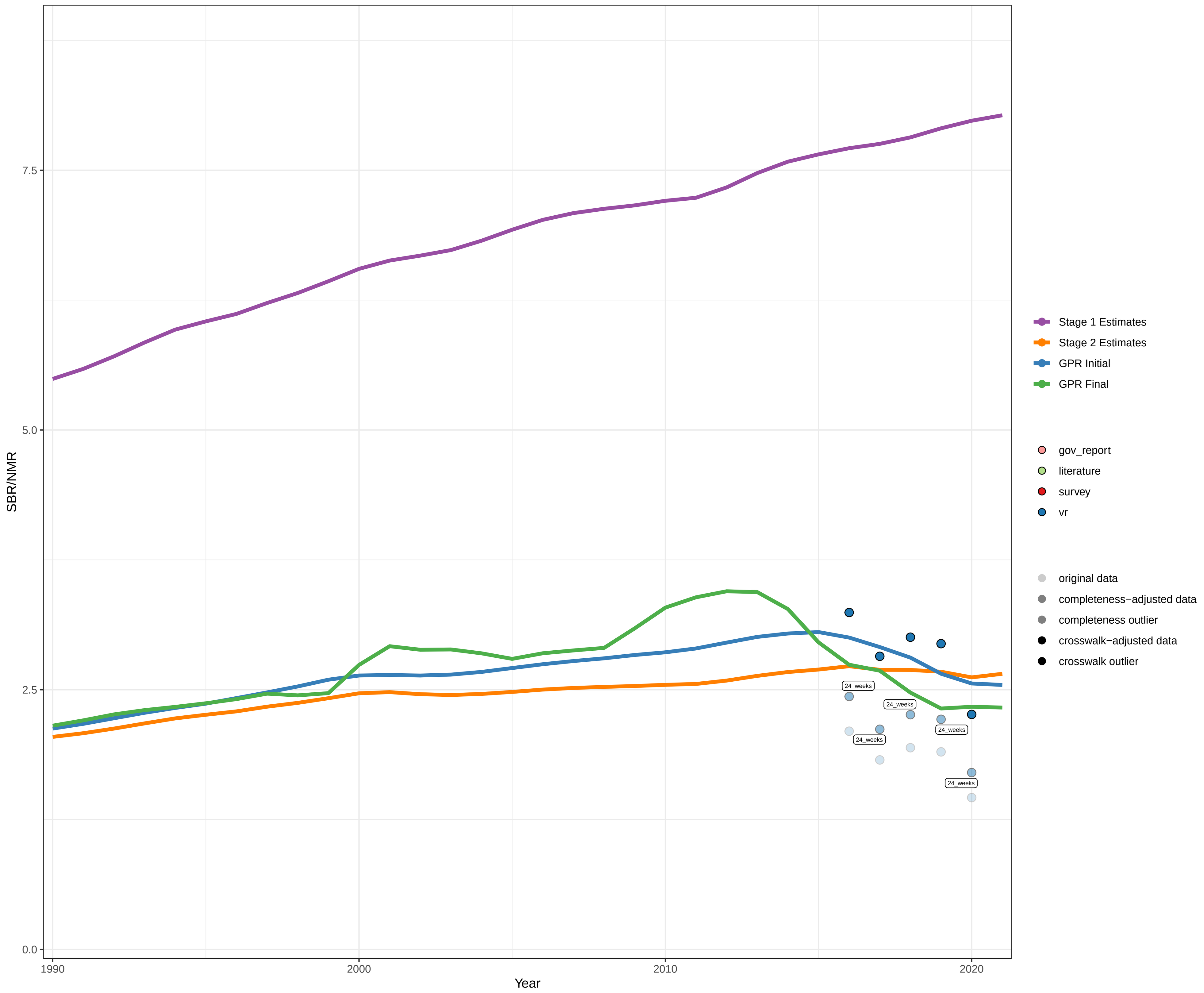

Blackburn with Darwen   GBR_44674   Western Europe   Stillbirth Rate / Neonatal Mortality Rate (SBR/NMR) − 28 weeks
lambda = 0.6 ; zeta = 0.8 ; scale = 15 ; amp = 0.028

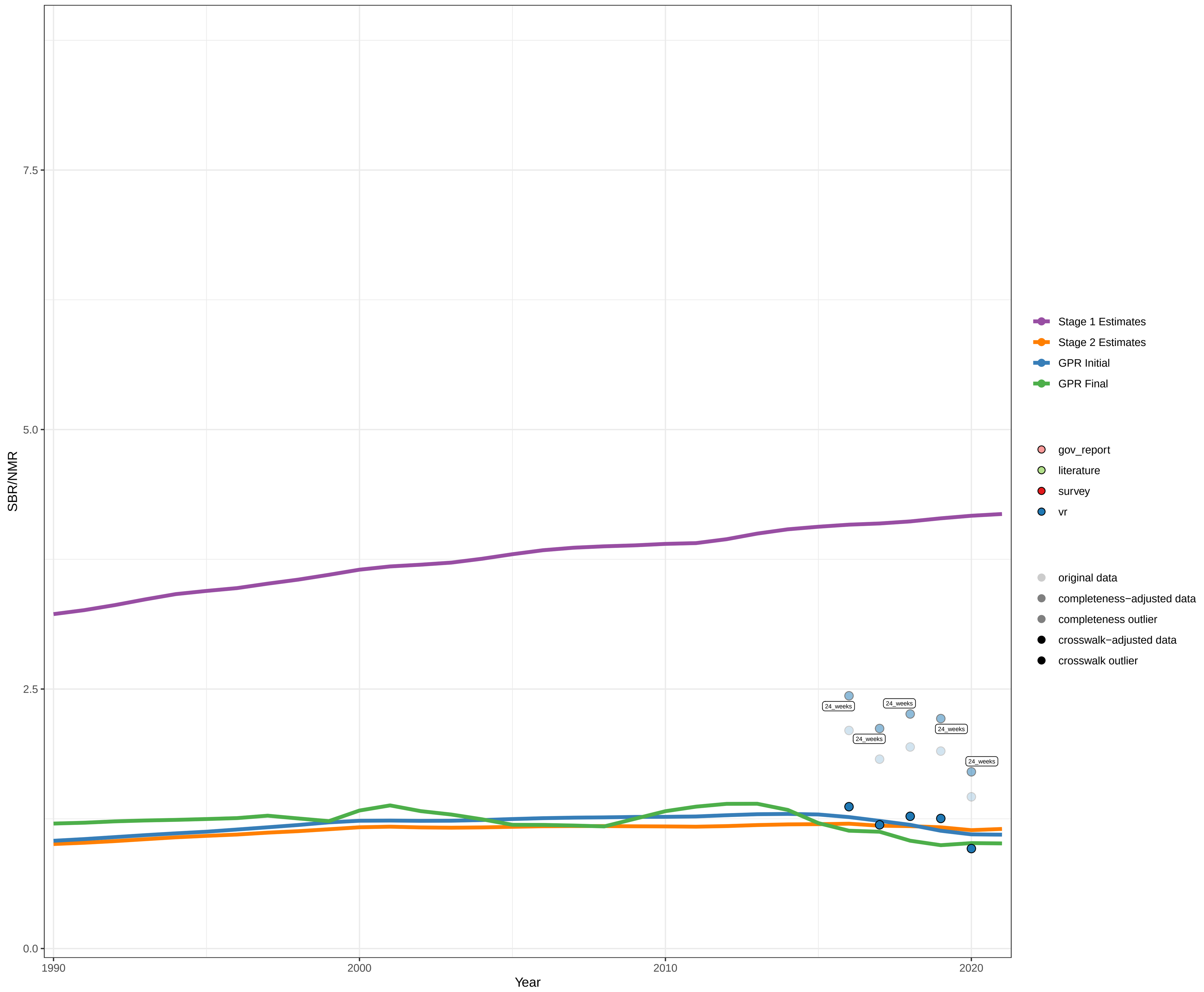

Definition	Years	Outlier	Source.Type	Title
24_weeks	2016−2020	0	VR	United Kingdom − England and Wales Child Mortality Statistics

Blackburn with Darwen   GBR_44674   Western Europe   Stillbirth Rate (SBR) − 20 weeks
lambda = 0.6 ; zeta = 0.8 ; scale = 15 ; amp = 0.028

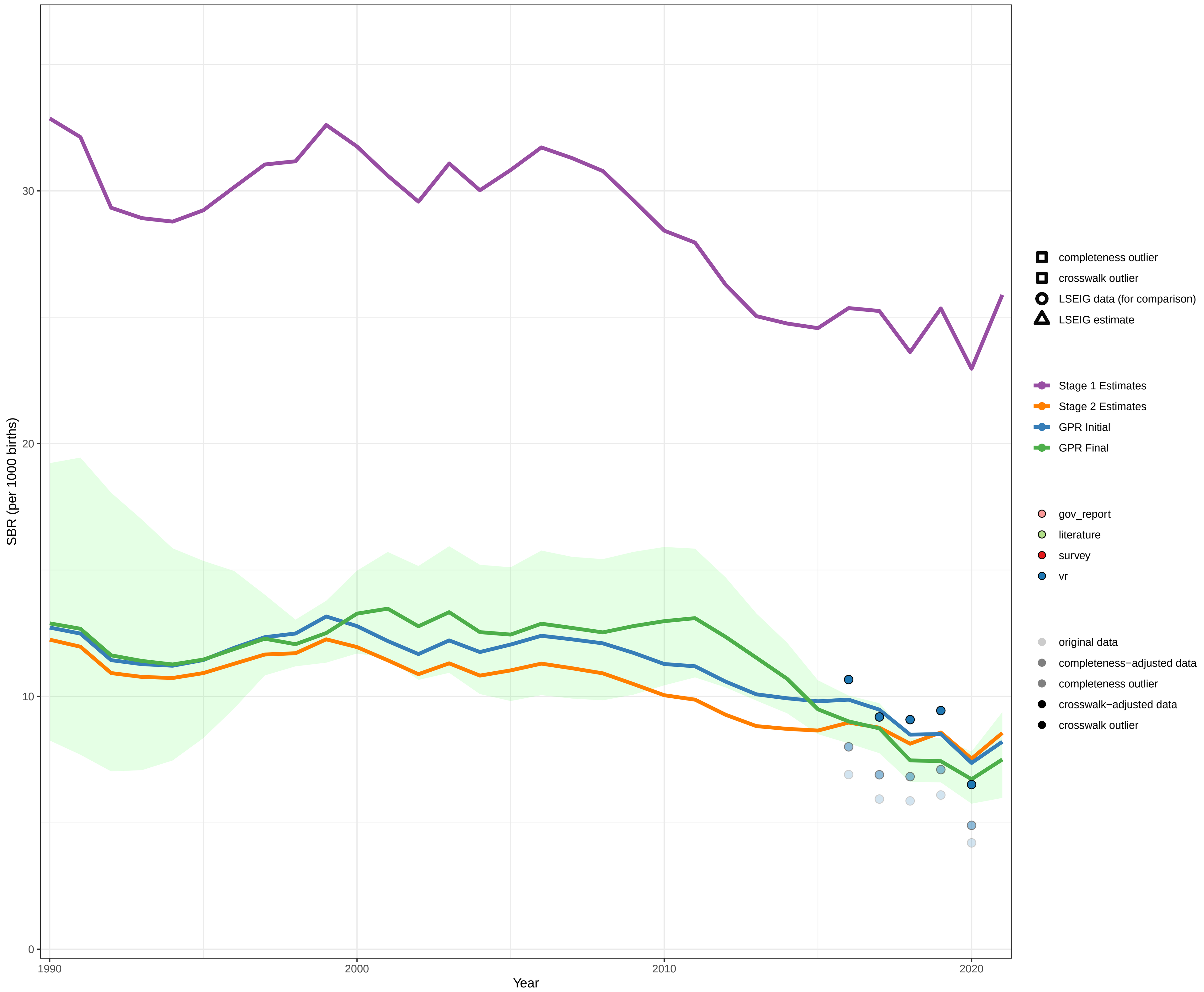

Blackburn with Darwen   GBR_44674   Western Europe   Stillbirth Rate (SBR) − 28 weeks
lambda = 0.6 ; zeta = 0.8 ; scale = 15 ; amp = 0.028

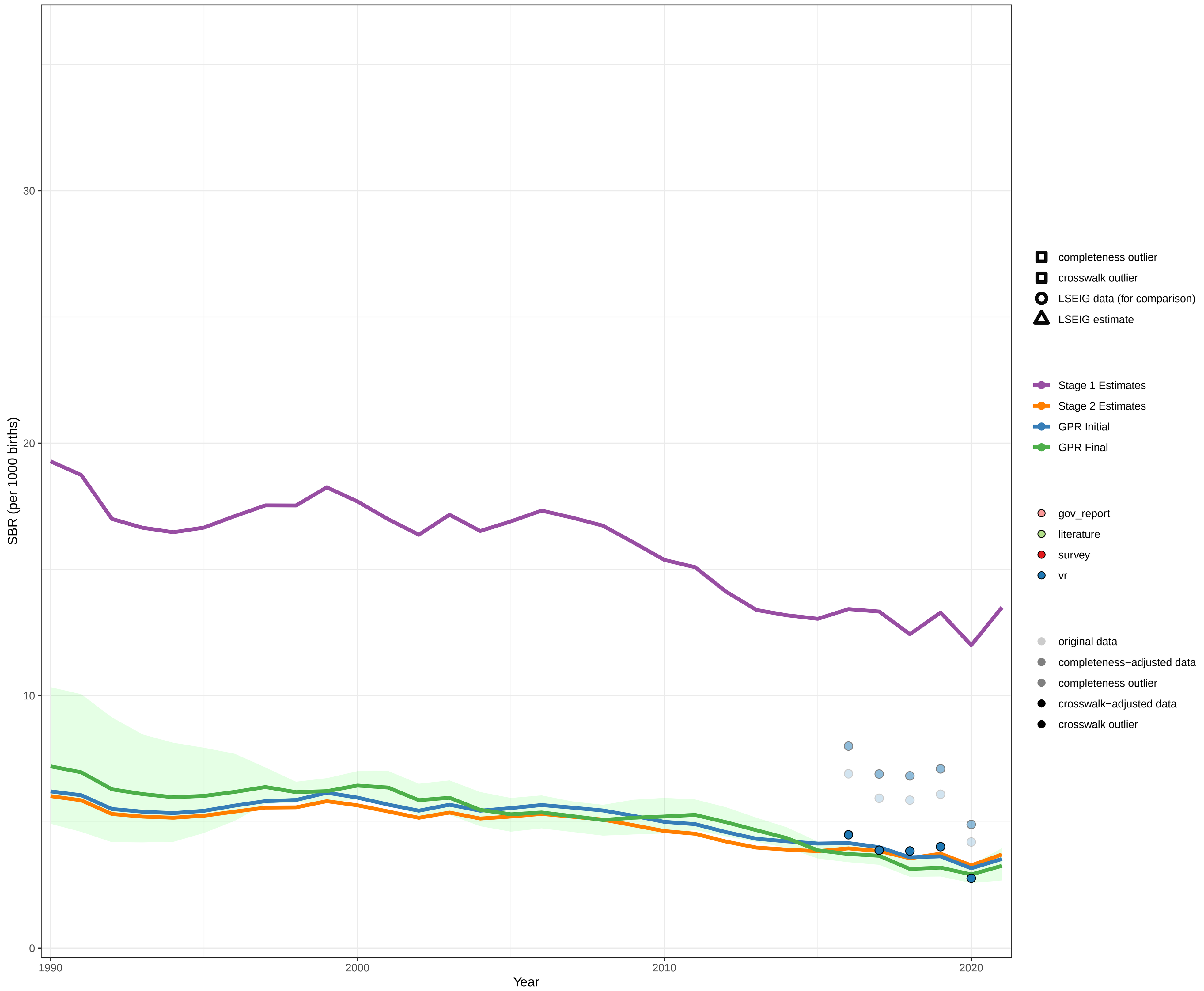

Year	Total Stillbirths (in thousands)	Total Stillbirths (in thousands)	SBR (per 1000 births)	SBR (per 1000 births)	SBR/NMR	SBR/NMR
	20 weeks	28 weeks	20 weeks	28 weeks	20 weeks	28 weeks
1990	0.0 (0.0 to 0.0)	0.0 (0.0 to 0.0)	12.9 (8.3 to 19.2)	7.2 (4.9 to 10.3)	2.15 (1.38 to 3.21)	1.20 (0.82 to 1.73)
1995	0.0 (0.0 to 0.0)	0.0 (0.0 to 0.0)	11.5 (8.4 to 15.4)	6.0 (4.6 to 7.9)	2.37 (1.73 to 3.18)	1.25 (0.94 to 1.64)
2000	0.0 (0.0 to 0.0)	0.0 (0.0 to 0.0)	13.3 (11.7 to 15.0)	6.4 (5.9 to 7.0)	2.74 (2.41 to 3.09)	1.33 (1.22 to 1.45)
2005	0.0 (0.0 to 0.0)	0.0 (0.0 to 0.0)	12.4 (9.8 to 15.1)	5.3 (4.6 to 6.0)	2.80 (2.21 to 3.40)	1.19 (1.04 to 1.34)
2010	0.0 (0.0 to 0.0)	0.0 (0.0 to 0.0)	13.0 (10.4 to 15.9)	5.2 (4.6 to 6.0)	3.29 (2.65 to 4.03)	1.32 (1.15 to 1.51)
2015	0.0 (0.0 to 0.0)	0.0 (0.0 to 0.0)	9.5 (8.5 to 10.6)	3.9 (3.6 to 4.2)	2.96 (2.65 to 3.31)	1.21 (1.11 to 1.32)
2020	0.0 (0.0 to 0.0)	0.0 (0.0 to 0.0)	6.7 (5.8 to 7.8)	2.9 (2.6 to 3.3)	2.34 (2.00 to 2.72)	1.02 (0.90 to 1.15)
2021	0.0 (0.0 to 0.0)	0.0 (0.0 to 0.0)	7.5 (6.0 to 9.4)	3.3 (2.7 to 4.0)	2.33 (1.86 to 2.91)	1.01 (0.83 to 1.23)

Blackpool   GBR_44676   Western Europe   Stillbirth Rate / Neonatal Mortality Rate (SBR/NMR) − 20 weeks
lambda = 0.6 ; zeta = 0.8 ; scale = 15 ; amp = 0.028

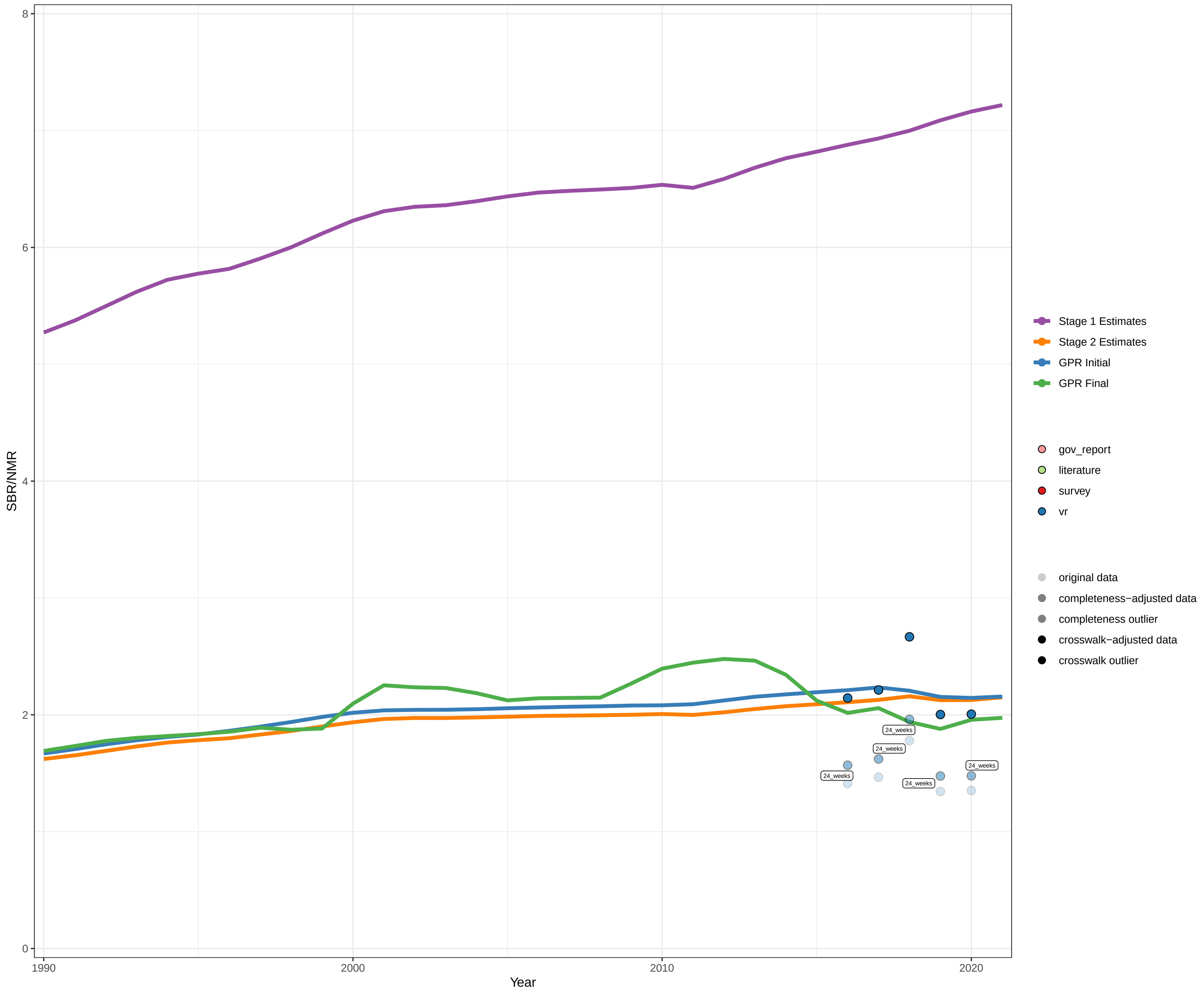

Blackpool   GBR_44676   Western Europe   Stillbirth Rate / Neonatal Mortality Rate (SBR/NMR) − 28 weeks
lambda = 0.6 ; zeta = 0.8 ; scale = 15 ; amp = 0.028

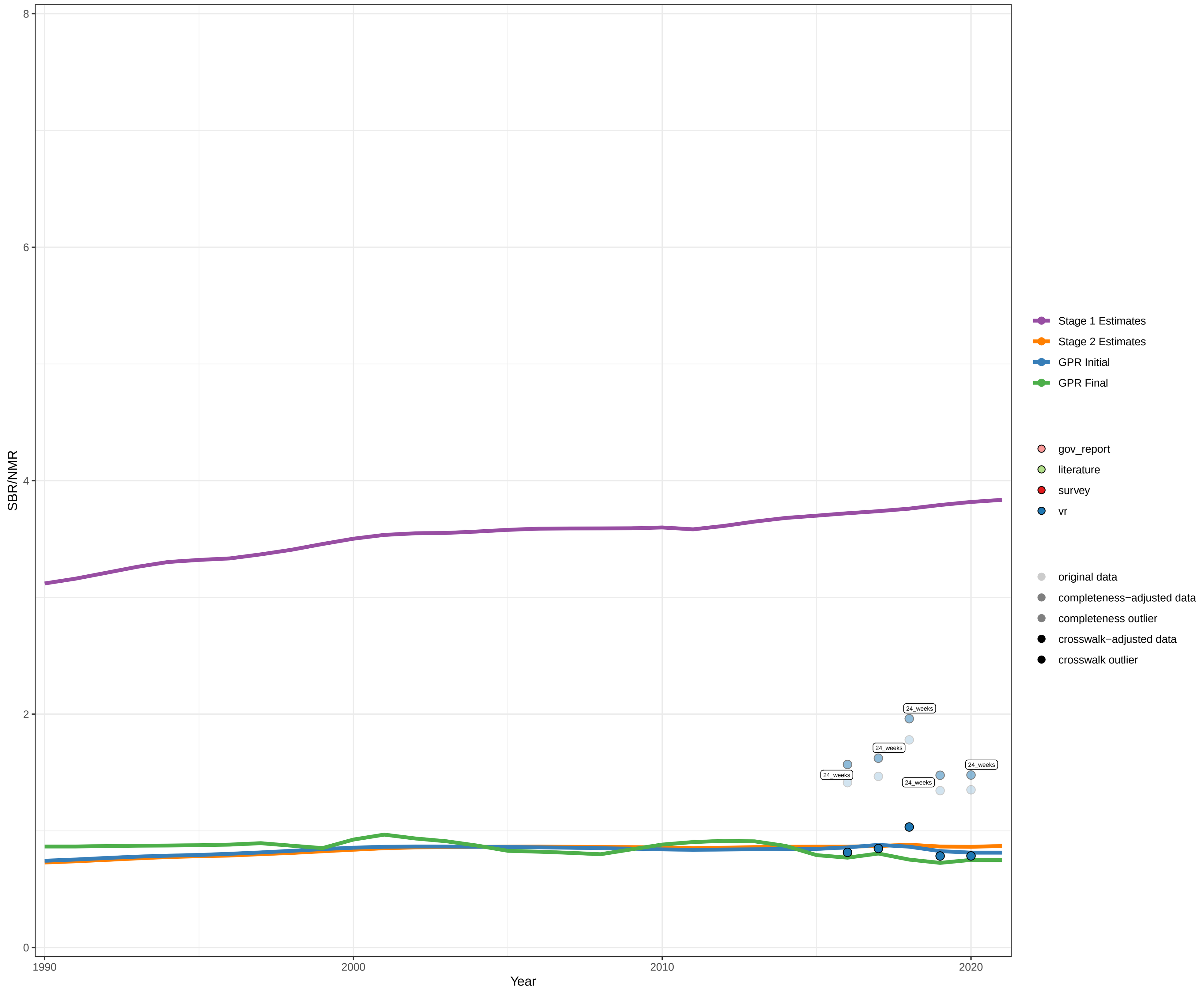

Definition	Years	Outlier	Source.Type	Title
24_weeks	2016−2020	0	VR	United Kingdom − England and Wales Child Mortality Statistics

Blackpool   GBR_44676   Western Europe   Stillbirth Rate (SBR) − 20 weeks
lambda = 0.6 ; zeta = 0.8 ; scale = 15 ; amp = 0.028

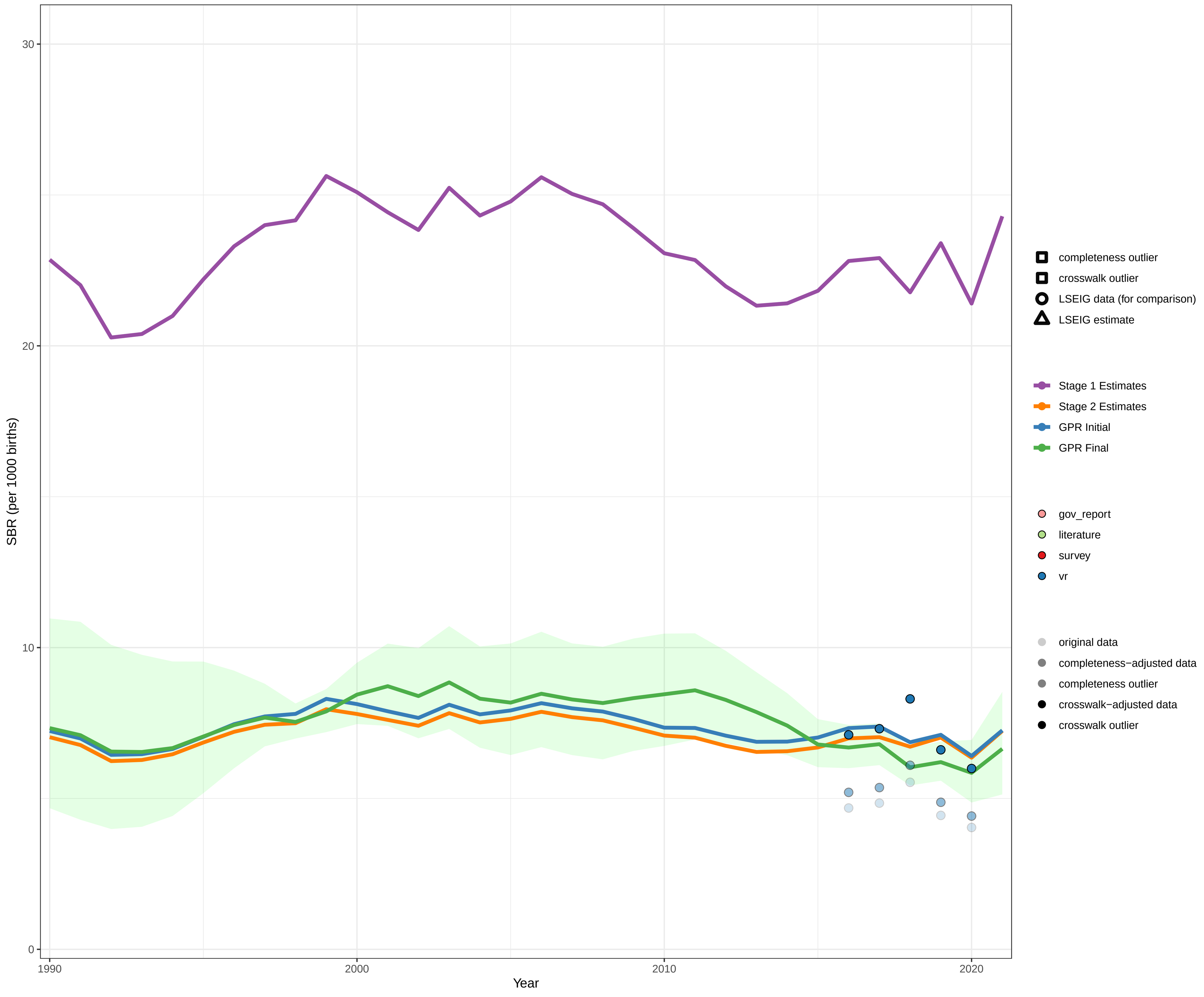

Blackpool   GBR_44676   Western Europe   Stillbirth Rate (SBR) − 28 weeks
lambda = 0.6 ; zeta = 0.8 ; scale = 15 ; amp = 0.028

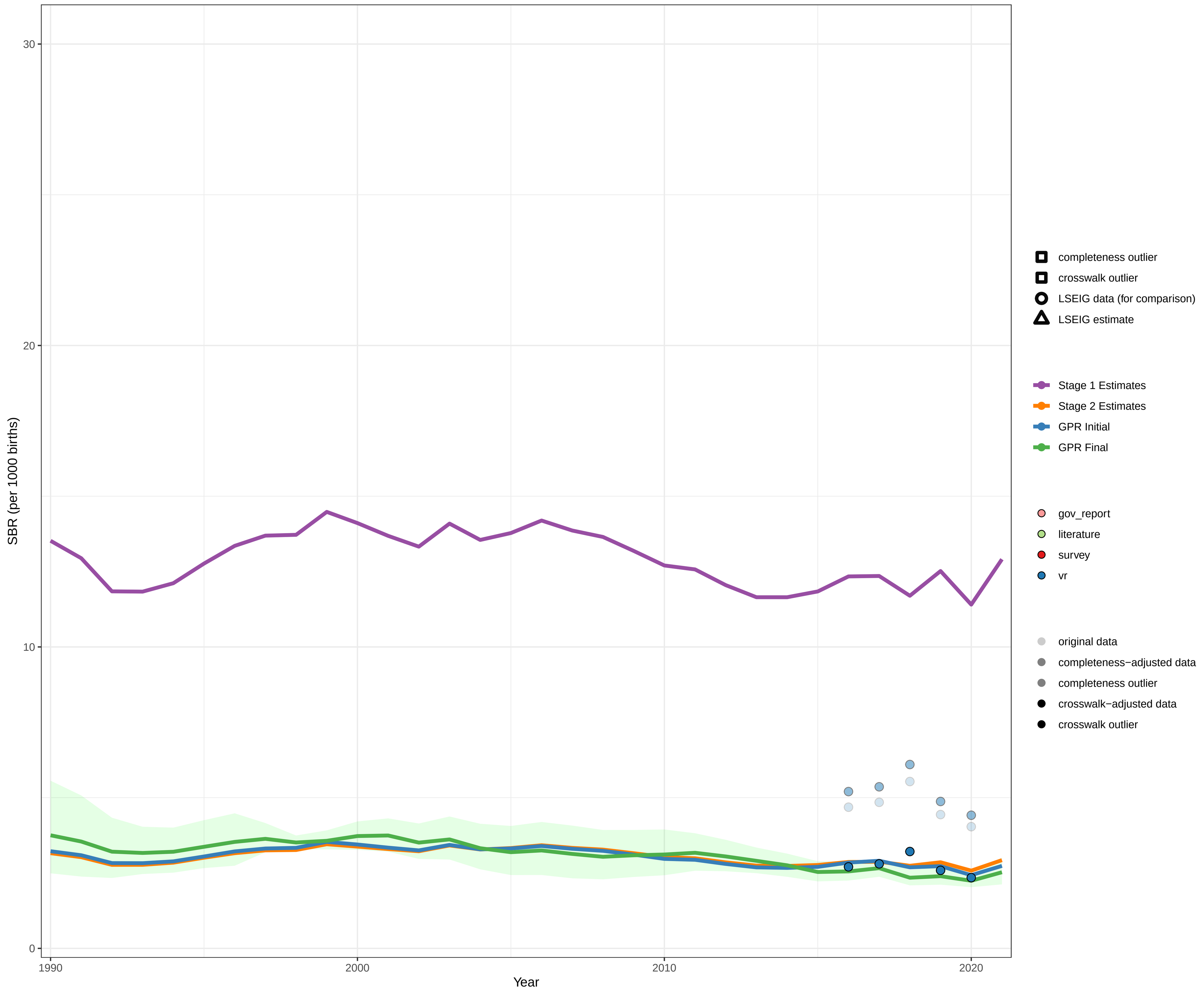

Year	Total Stillbirths (in thousands)	Total Stillbirths (in thousands)	SBR (per 1000 births)	SBR (per 1000 births)	SBR/NMR	SBR/NMR
	20 weeks	28 weeks	20 weeks	28 weeks	20 weeks	28 weeks
1990	0.0 (0.0 to 0.0)	0.0 (0.0 to 0.0)	7.3 (4.7 to 11.0)	3.8 (2.5 to 5.6)	1.69 (1.08 to 2.53)	0.87 (0.57 to 1.28)
1995	0.0 (0.0 to 0.0)	0.0 (0.0 to 0.0)	7.1 (5.2 to 9.5)	3.4 (2.7 to 4.3)	1.83 (1.35 to 2.48)	0.88 (0.69 to 1.11)
2000	0.0 (0.0 to 0.0)	0.0 (0.0 to 0.0)	8.4 (7.5 to 9.5)	3.7 (3.3 to 4.2)	2.10 (1.85 to 2.36)	0.93 (0.81 to 1.05)
2005	0.0 (0.0 to 0.0)	0.0 (0.0 to 0.0)	8.2 (6.4 to 10.1)	3.2 (2.4 to 4.1)	2.12 (1.67 to 2.63)	0.83 (0.63 to 1.05)
2010	0.0 (0.0 to 0.0)	0.0 (0.0 to 0.0)	8.5 (6.7 to 10.5)	3.1 (2.4 to 3.9)	2.39 (1.91 to 2.96)	0.88 (0.69 to 1.12)
2015	0.0 (0.0 to 0.0)	0.0 (0.0 to 0.0)	6.8 (6.0 to 7.6)	2.5 (2.2 to 2.9)	2.12 (1.89 to 2.38)	0.79 (0.69 to 0.90)
2020	0.0 (0.0 to 0.0)	0.0 (0.0 to 0.0)	5.8 (4.9 to 6.9)	2.2 (2.0 to 2.4)	1.96 (1.63 to 2.32)	0.75 (0.68 to 0.82)
2021	0.0 (0.0 to 0.0)	0.0 (0.0 to 0.0)	6.6 (5.1 to 8.5)	2.5 (2.1 to 3.0)	1.97 (1.53 to 2.54)	0.75 (0.63 to 0.89)

Bolton GBR_44662 Western Europe Stillbirth Rate / Neonatal Mortality Rate (SBR/NMR) − 20 weeks
lambda = 0.6 ; zeta = 0.8 ; scale = 15 ; amp = 0.028

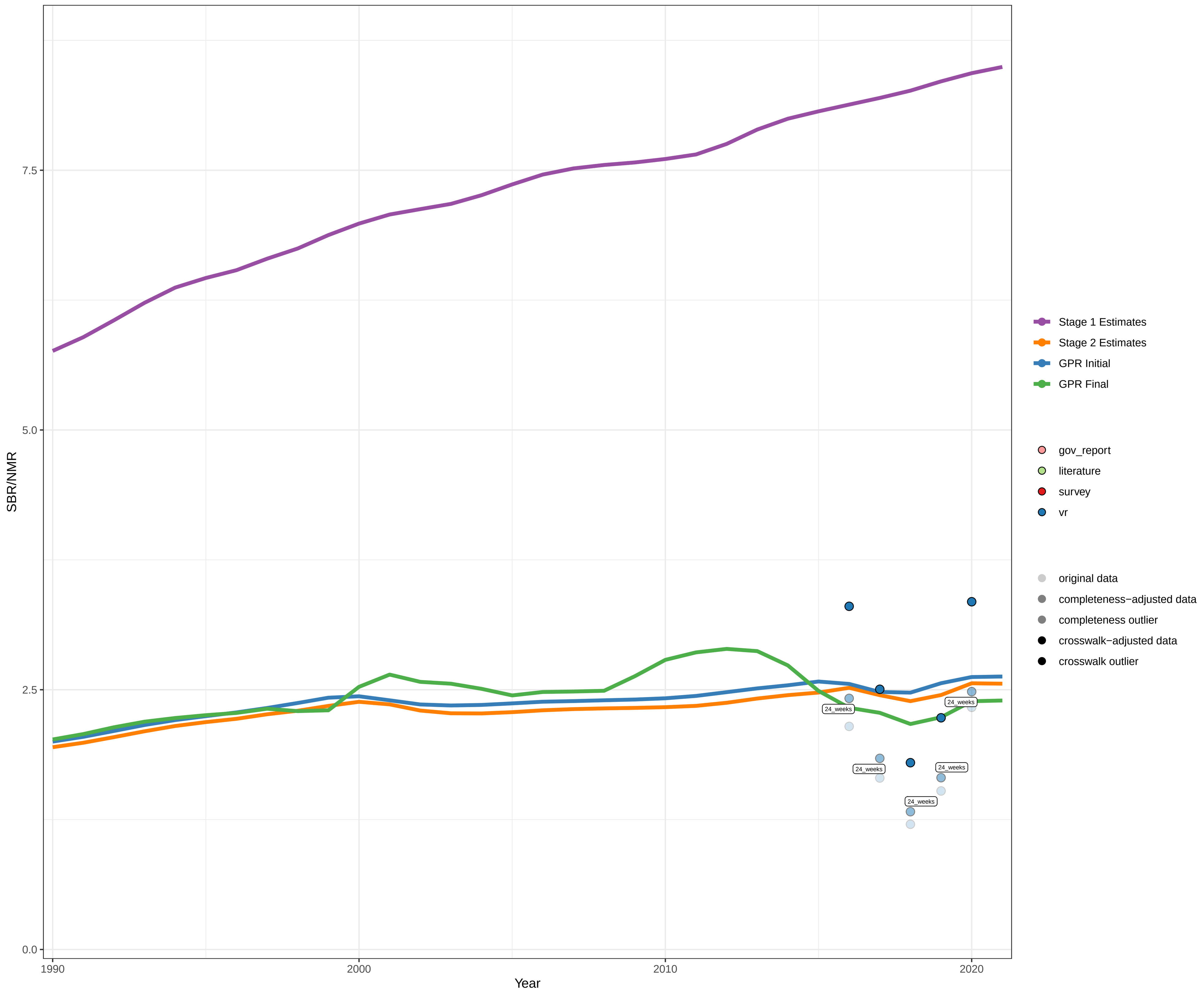

Bolton GBR_44662 Western Europe Stillbirth Rate / Neonatal Mortality Rate (SBR/NMR) − 28 weeks
lambda = 0.6 ; zeta = 0.8 ; scale = 15 ; amp = 0.028

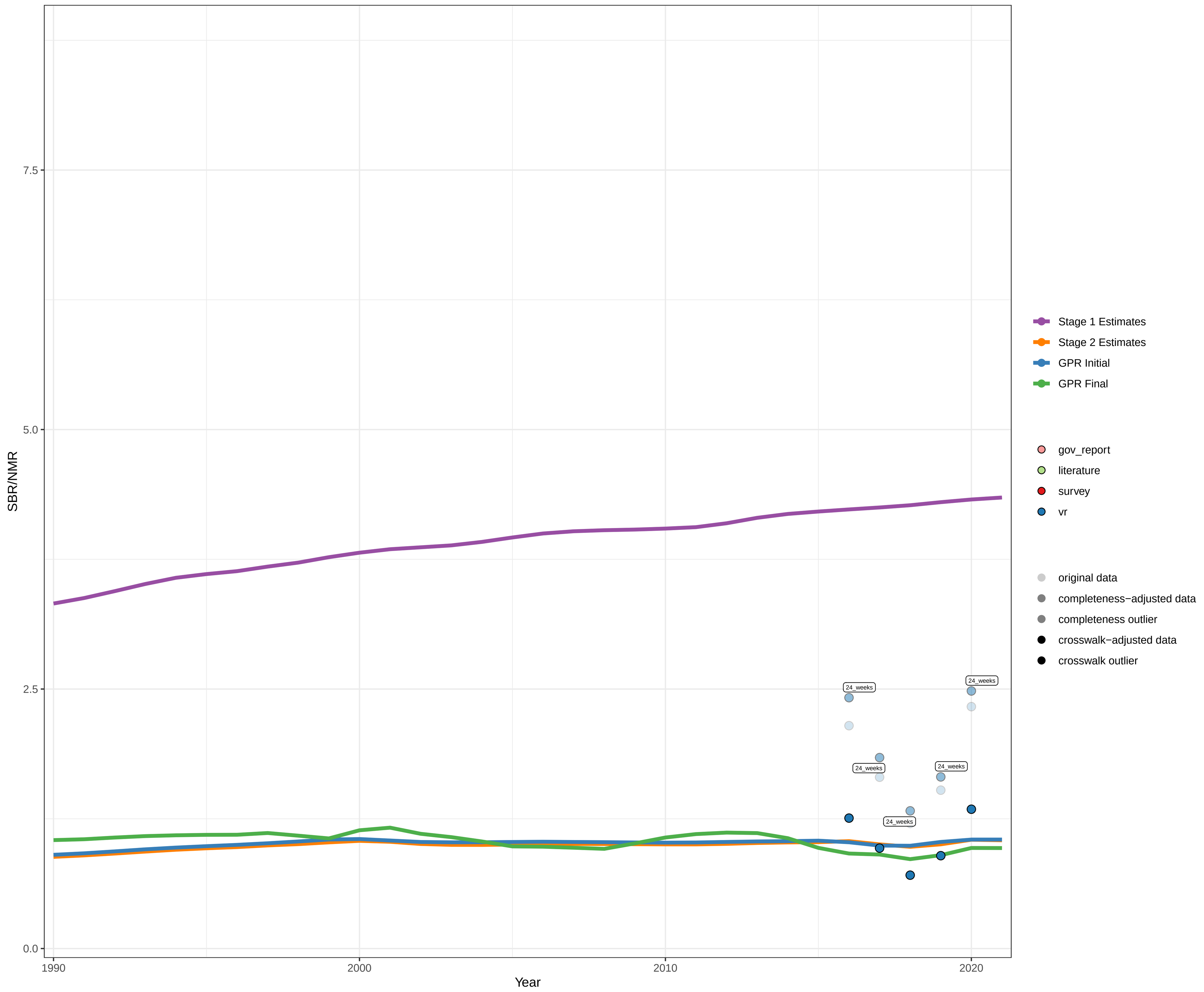

Definition	Years	Outlier	Source.Type	Title
24_weeks	2016−2020	0	VR	United Kingdom − England and Wales Child Mortality Statistics

Bolton GBR_44662 Western Europe Stillbirth Rate (SBR) − 20 weeks
lambda = 0.6 ; zeta = 0.8 ; scale = 15 ; amp = 0.028

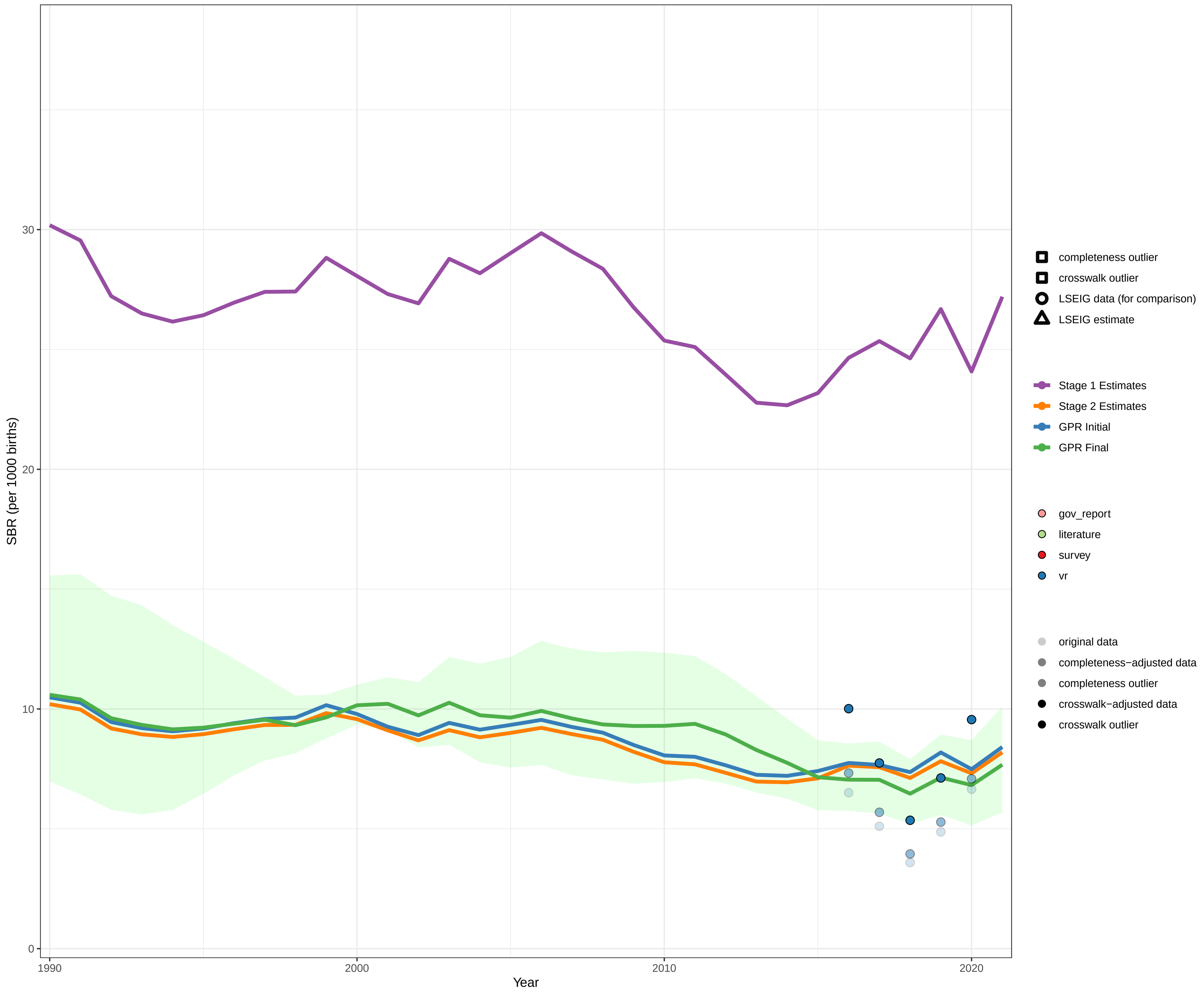

Bolton GBR_44662 Western Europe Stillbirth Rate (SBR) − 28 weeks
lambda = 0.6 ; zeta = 0.8 ; scale = 15 ; amp = 0.028

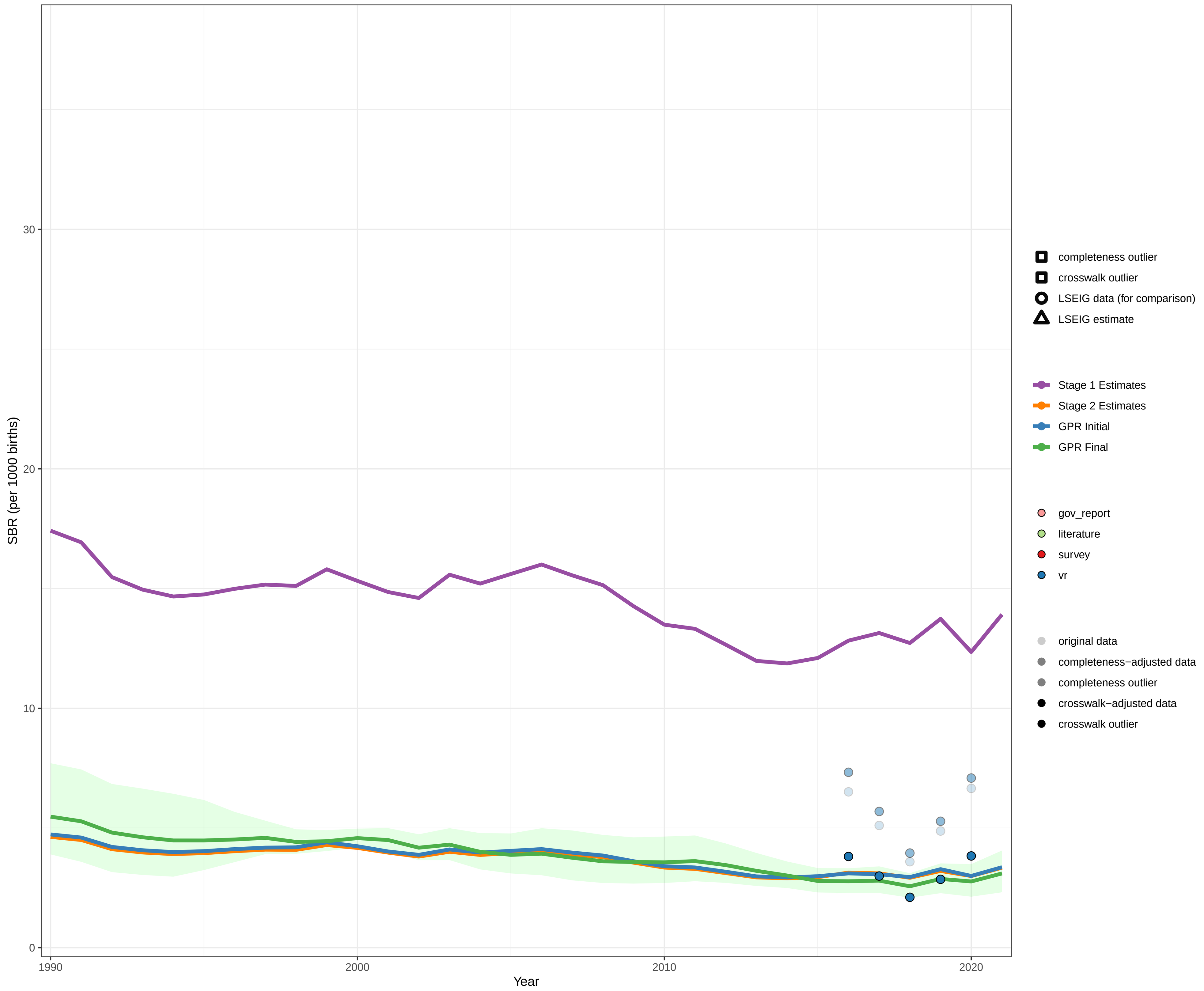

Year	Total Stillbirths (in thousands)	Total Stillbirths (in thousands)	SBR (per 1000 births)	SBR (per 1000 births)	SBR/NMR	SBR/NMR
	20 weeks	28 weeks	20 weeks	28 weeks	20 weeks	28 weeks
1990	0.0 (0.0 to 0.1)	0.0 (0.0 to 0.0)	10.6 (7.0 to 15.6)	5.5 (3.9 to 7.7)	2.02 (1.33 to 2.97)	1.04 (0.74 to 1.47)
1995	0.0 (0.0 to 0.0)	0.0 (0.0 to 0.0)	9.2 (6.5 to 12.8)	4.5 (3.2 to 6.2)	2.26 (1.58 to 3.13)	1.10 (0.79 to 1.51)
2000	0.0 (0.0 to 0.0)	0.0 (0.0 to 0.0)	10.2 (9.4 to 11.0)	4.6 (4.2 to 5.0)	2.53 (2.33 to 2.74)	1.14 (1.04 to 1.24)
2005	0.0 (0.0 to 0.0)	0.0 (0.0 to 0.0)	9.6 (7.6 to 12.2)	3.9 (3.1 to 4.8)	2.45 (1.92 to 3.09)	0.98 (0.79 to 1.21)
2010	0.0 (0.0 to 0.0)	0.0 (0.0 to 0.0)	9.3 (7.0 to 12.4)	3.6 (2.7 to 4.6)	2.79 (2.08 to 3.70)	1.07 (0.81 to 1.39)
2015	0.0 (0.0 to 0.0)	0.0 (0.0 to 0.0)	7.2 (5.8 to 8.7)	2.8 (2.3 to 3.3)	2.49 (2.01 to 3.03)	0.97 (0.80 to 1.16)
2020	0.0 (0.0 to 0.0)	0.0 (0.0 to 0.0)	6.8 (5.1 to 8.7)	2.8 (2.1 to 3.5)	2.39 (1.80 to 3.05)	0.97 (0.75 to 1.22)
2021	0.0 (0.0 to 0.0)	0.0 (0.0 to 0.0)	7.7 (5.7 to 10.1)	3.1 (2.3 to 4.1)	2.40 (1.77 to 3.15)	0.97 (0.72 to 1.27)

Bury GBR_44664 Western Europe Stillbirth Rate / Neonatal Mortality Rate (SBR/NMR) − 20 weeks
lambda = 0.6 ; zeta = 0.8 ; scale = 15 ; amp = 0.028

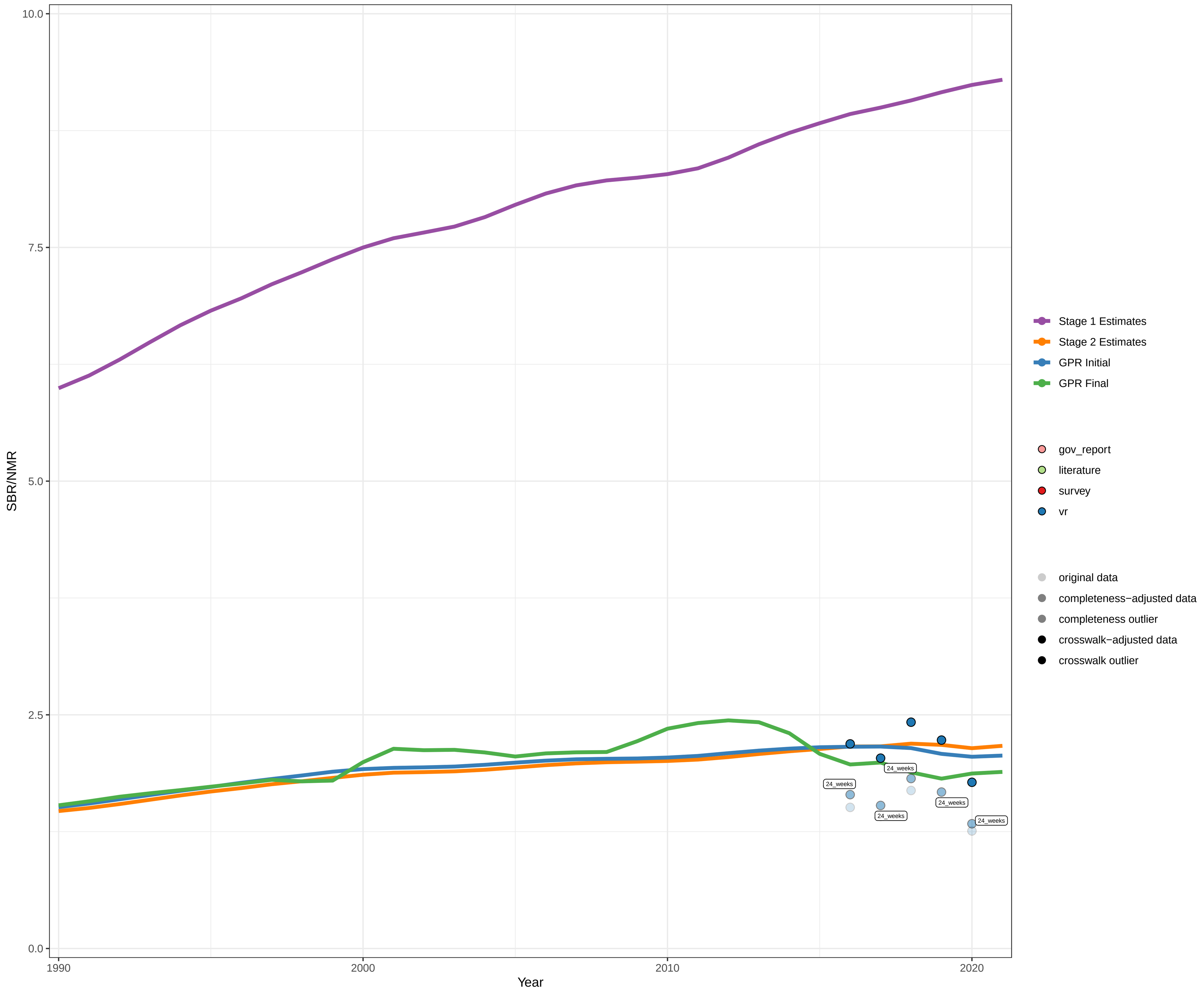

Bury GBR_44664 Western Europe Stillbirth Rate / Neonatal Mortality Rate (SBR/NMR) − 28 weeks
lambda = 0.6 ; zeta = 0.8 ; scale = 15 ; amp = 0.028

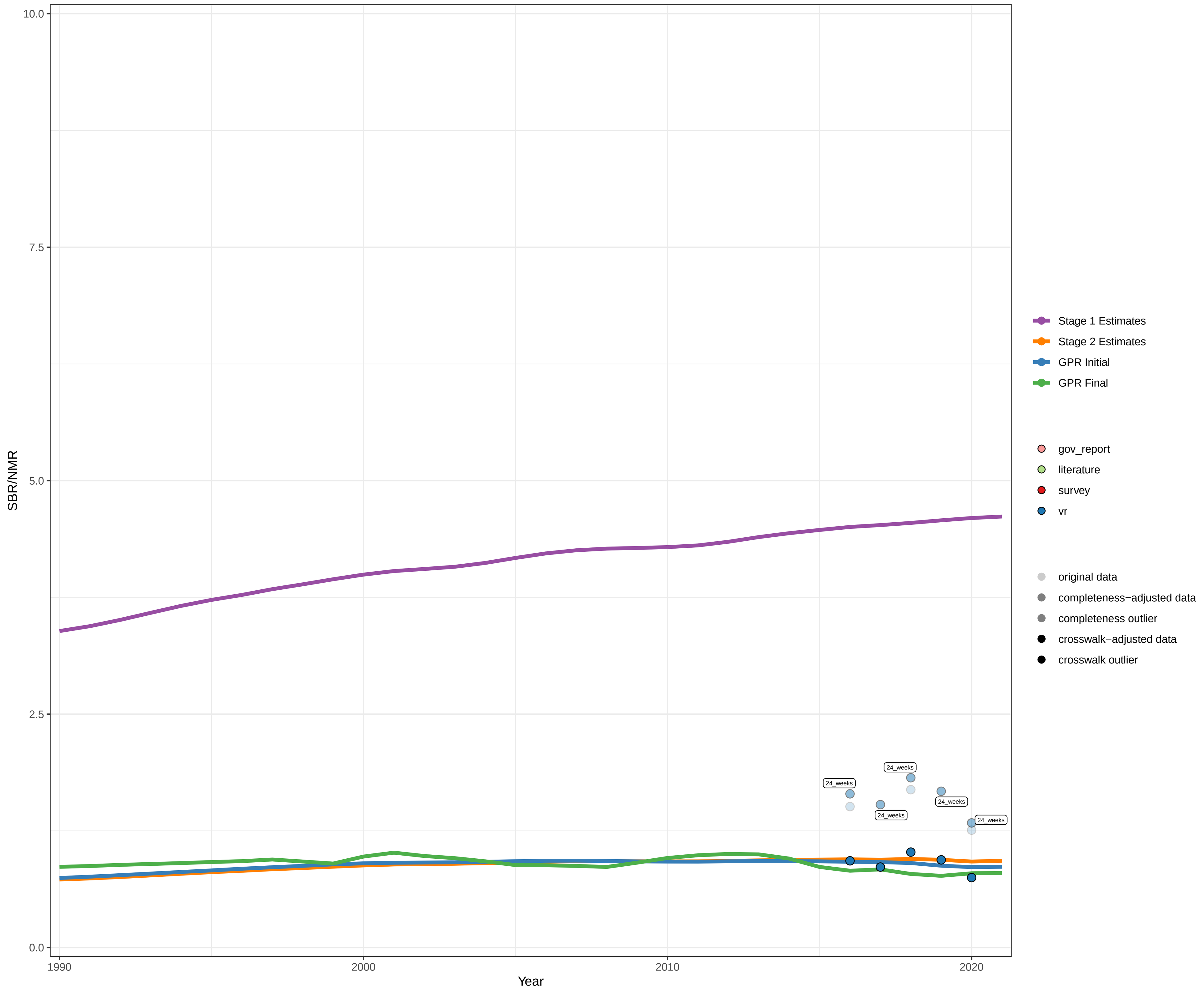

Definition	Years	Outlier	Source.Type	Title
24_weeks	2016−2020	0	VR	United Kingdom − England and Wales Child Mortality Statistics

Bury GBR_44664 Western Europe Stillbirth Rate (SBR) − 20 weeks
lambda = 0.6 ; zeta = 0.8 ; scale = 15 ; amp = 0.028

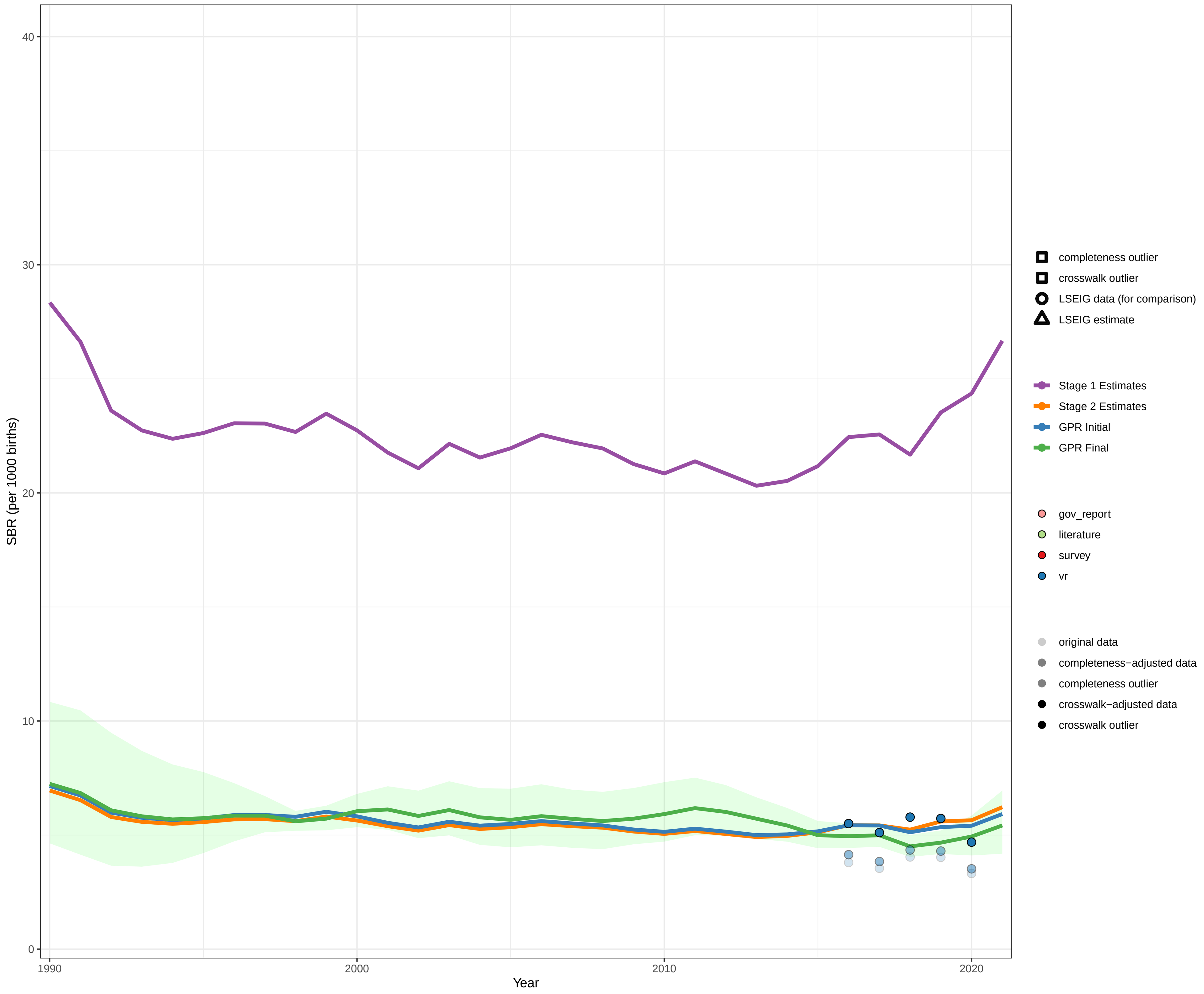

Bury GBR_44664 Western Europe Stillbirth Rate (SBR) − 28 weeks
lambda = 0.6 ; zeta = 0.8 ; scale = 15 ; amp = 0.028

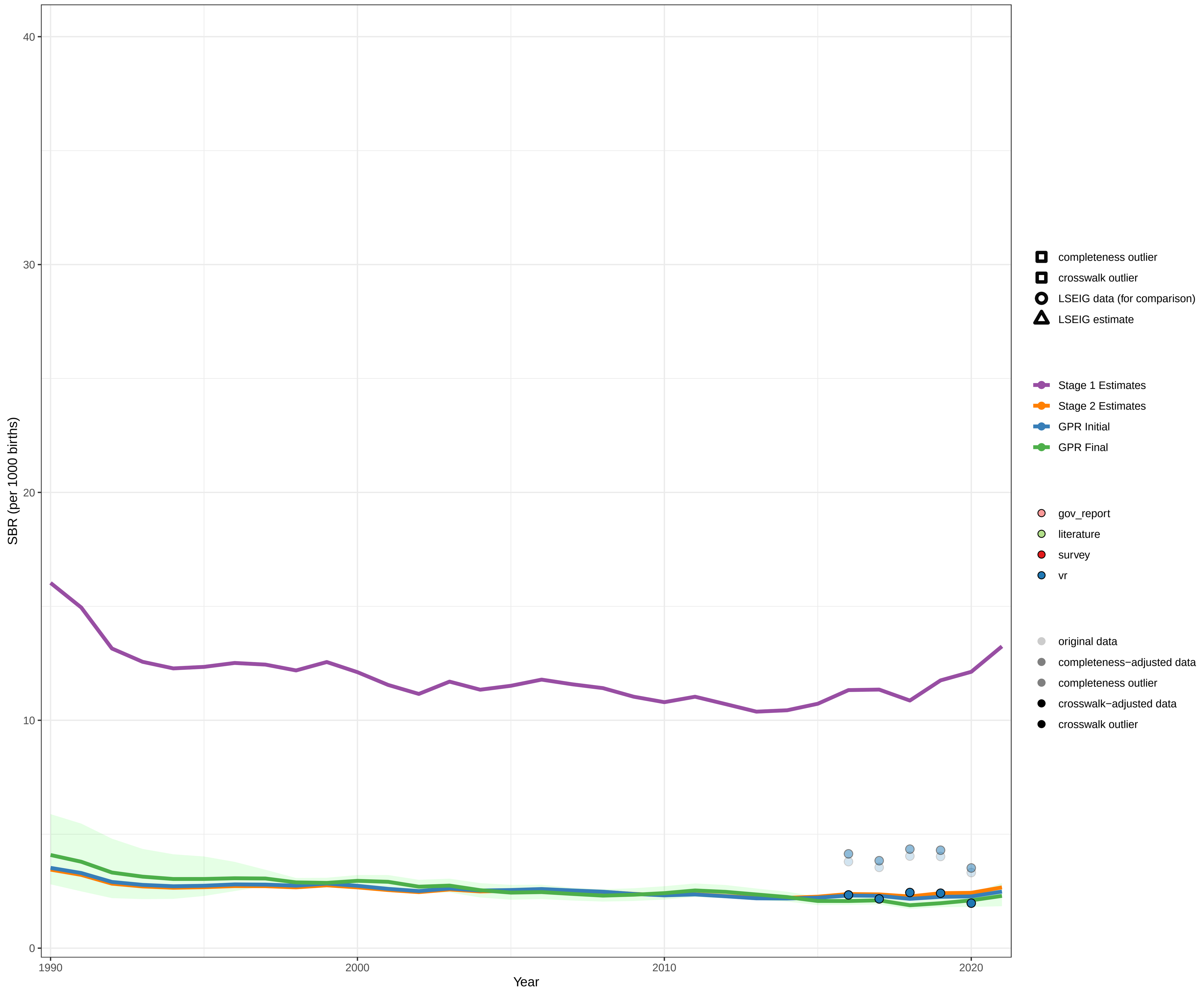

Year	Total Stillbirths (in thousands)	Total Stillbirths (in thousands)	SBR (per 1000 births)	SBR (per 1000 births)	SBR/NMR	SBR/NMR
	20 weeks	28 weeks	20 weeks	28 weeks	20 weeks	28 weeks
1990	0.0 (0.0 to 0.0)	0.0 (0.0 to 0.0)	7.2 (4.6 to 10.8)	4.1 (2.8 to 5.9)	1.53 (0.98 to 2.29)	0.86 (0.59 to 1.24)
1995	0.0 (0.0 to 0.0)	0.0 (0.0 to 0.0)	5.7 (4.2 to 7.8)	3.0 (2.3 to 4.0)	1.73 (1.27 to 2.34)	0.92 (0.69 to 1.21)
2000	0.0 (0.0 to 0.0)	0.0 (0.0 to 0.0)	6.0 (5.3 to 6.8)	3.0 (2.7 to 3.2)	1.99 (1.76 to 2.24)	0.97 (0.89 to 1.06)
2005	0.0 (0.0 to 0.0)	0.0 (0.0 to 0.0)	5.7 (4.5 to 7.0)	2.4 (2.1 to 2.8)	2.05 (1.62 to 2.55)	0.88 (0.77 to 1.01)
2010	0.0 (0.0 to 0.0)	0.0 (0.0 to 0.0)	5.9 (4.7 to 7.3)	2.4 (2.1 to 2.7)	2.35 (1.87 to 2.91)	0.96 (0.85 to 1.08)
2015	0.0 (0.0 to 0.0)	0.0 (0.0 to 0.0)	5.0 (4.4 to 5.6)	2.1 (1.9 to 2.3)	2.08 (1.84 to 2.34)	0.86 (0.79 to 0.94)
2020	0.0 (0.0 to 0.0)	0.0 (0.0 to 0.0)	4.9 (4.1 to 5.9)	2.1 (1.8 to 2.4)	1.87 (1.56 to 2.22)	0.80 (0.69 to 0.91)
2021	0.0 (0.0 to 0.0)	0.0 (0.0 to 0.0)	5.4 (4.2 to 7.0)	2.3 (1.8 to 2.9)	1.89 (1.46 to 2.42)	0.80 (0.64 to 1.00)

Cheshire East GBR_44655 Western Europe Stillbirth Rate / Neonatal Mortality Rate (SBR/NMR) − 20 weeks
lambda = 0.6 ; zeta = 0.8 ; scale = 15 ; amp = 0.028

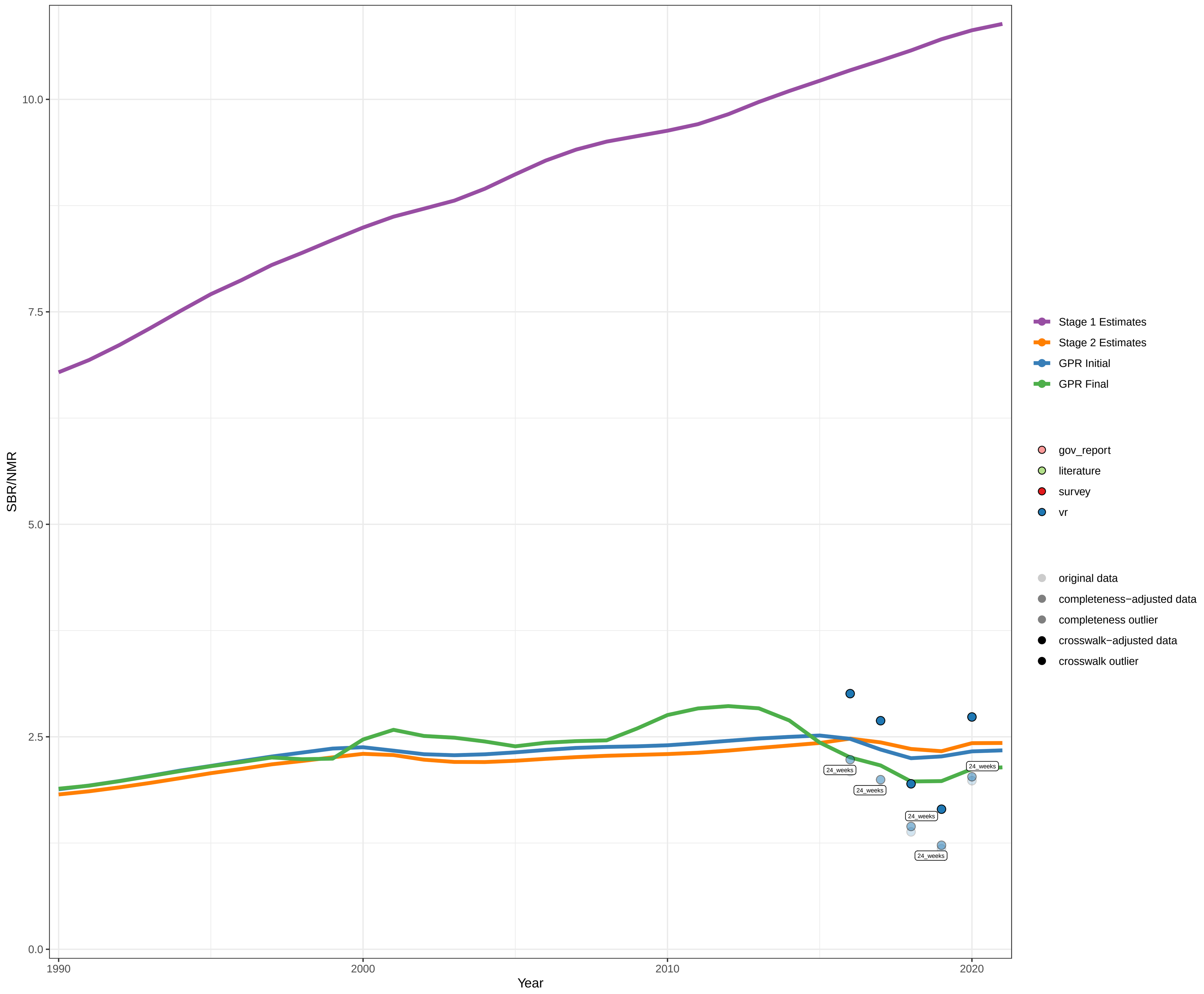

Cheshire East GBR_44655 Western Europe Stillbirth Rate / Neonatal Mortality Rate (SBR/NMR) − 28 weeks
lambda = 0.6 ; zeta = 0.8 ; scale = 15 ; amp = 0.028

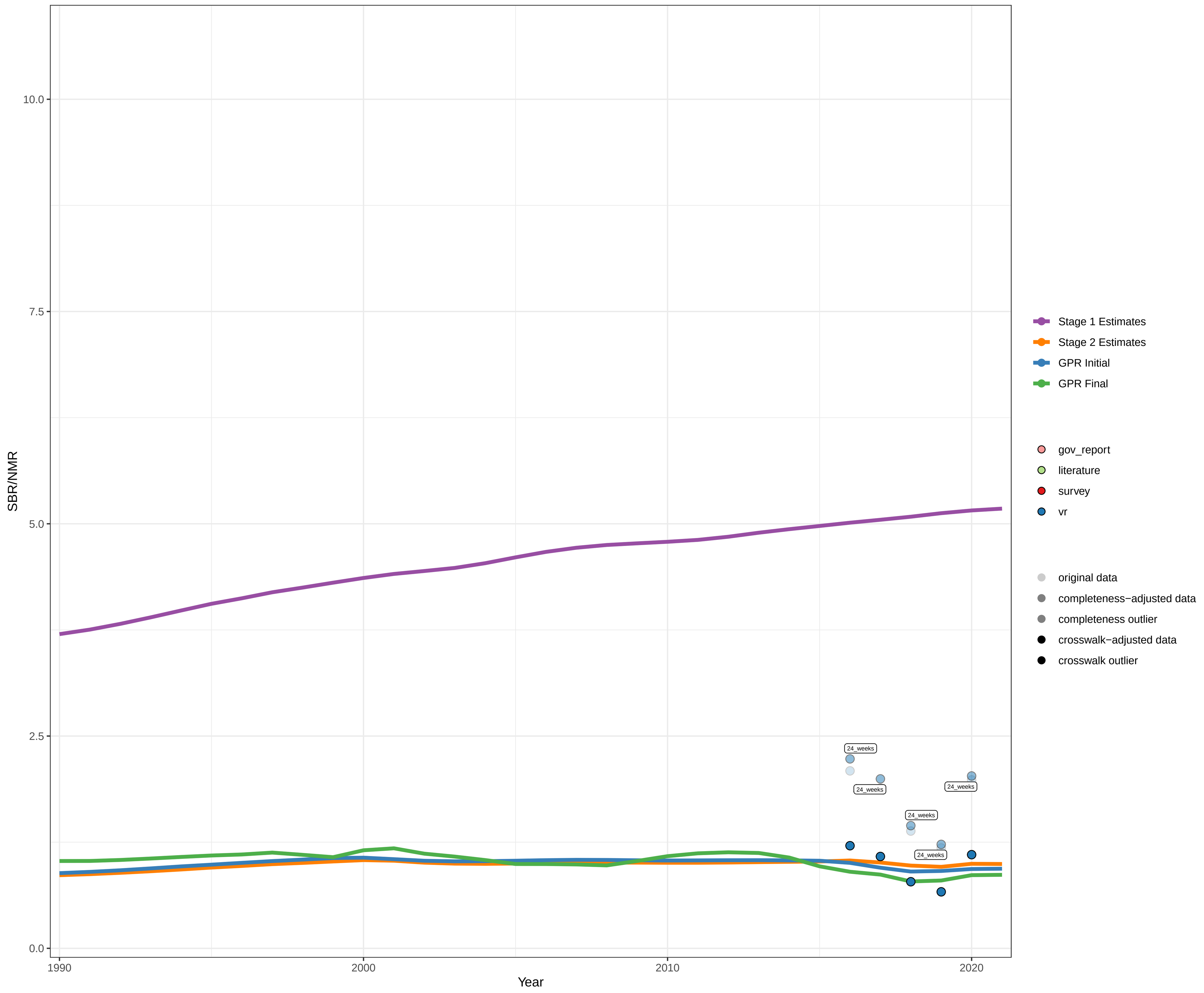

Definition	Years	Outlier	Source.Type	Title
24_weeks	2016−2020	0	VR	United Kingdom − England and Wales Child Mortality Statistics

Cheshire East GBR_44655 Western Europe Stillbirth Rate (SBR) − 20 weeks
lambda = 0.6 ; zeta = 0.8 ; scale = 15 ; amp = 0.028

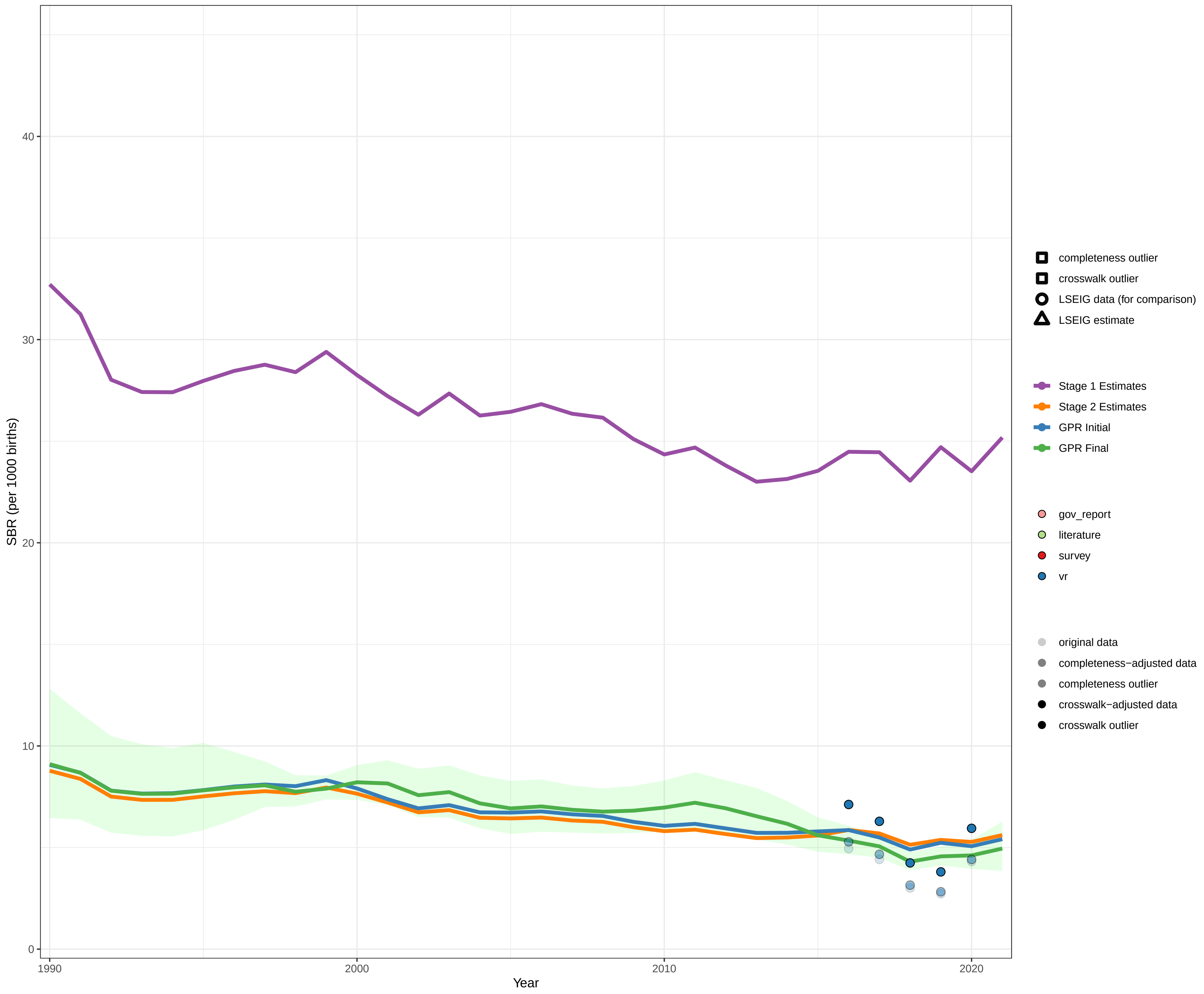

Cheshire East GBR_44655 Western Europe Stillbirth Rate (SBR) − 28 weeks
lambda = 0.6 ; zeta = 0.8 ; scale = 15 ; amp = 0.028

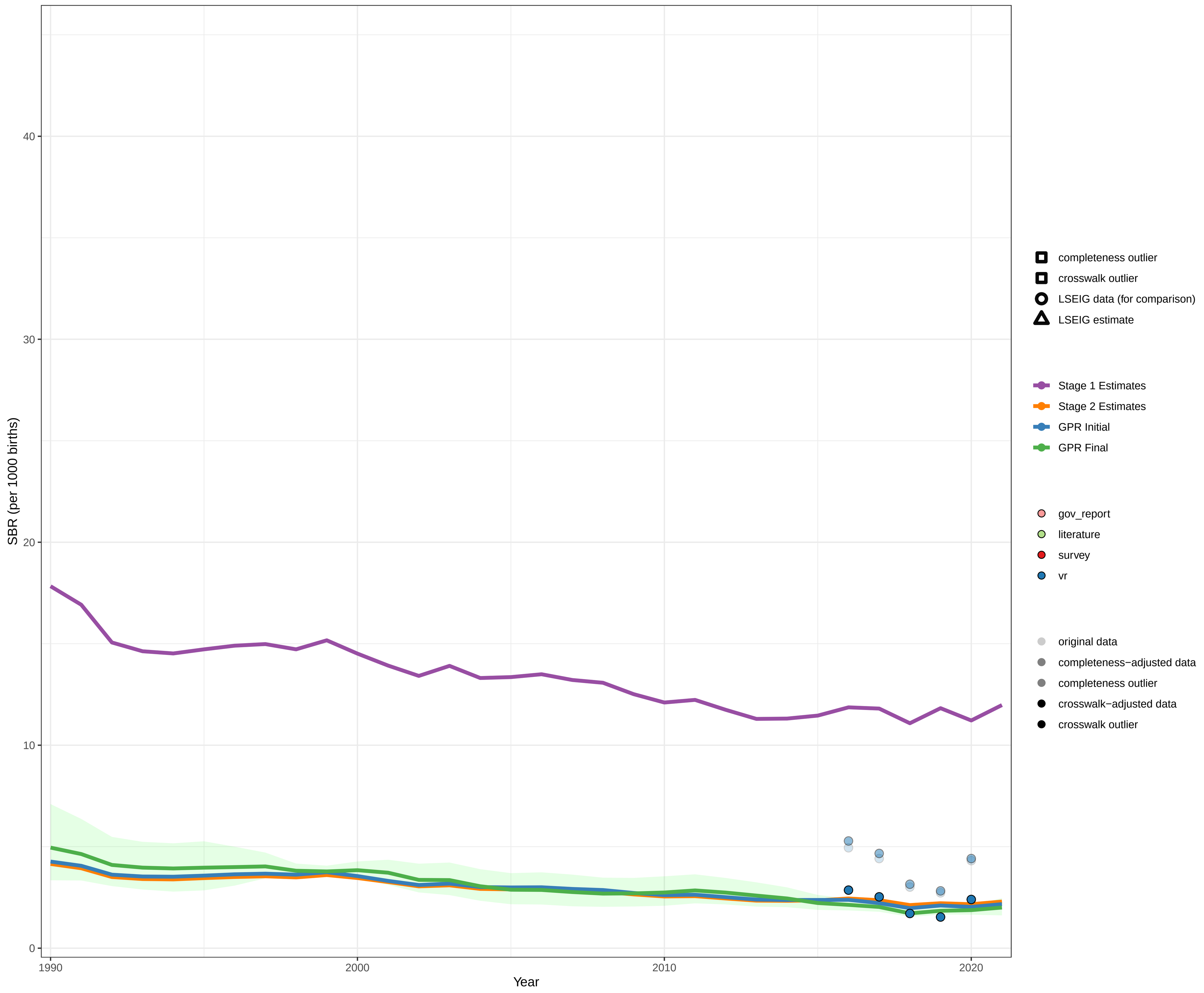

Year	Total Stillbirths (in thousands)	Total Stillbirths (in thousands)	SBR (per 1000 births)	SBR (per 1000 births)	SBR/NMR	SBR/NMR
	20 weeks	28 weeks	20 weeks	28 weeks	20 weeks	28 weeks
1990	0.0 (0.0 to 0.1)	0.0 (0.0 to 0.0)	9.1 (6.5 to 12.8)	5.0 (3.3 to 7.1)	1.89 (1.34 to 2.66)	1.03 (0.69 to 1.47)
1995	0.0 (0.0 to 0.0)	0.0 (0.0 to 0.0)	7.8 (5.9 to 10.2)	4.0 (2.8 to 5.3)	2.15 (1.61 to 2.80)	1.09 (0.78 to 1.45)
2000	0.0 (0.0 to 0.0)	0.0 (0.0 to 0.0)	8.2 (7.3 to 9.1)	3.8 (3.4 to 4.3)	2.47 (2.21 to 2.72)	1.15 (1.03 to 1.28)
2005	0.0 (0.0 to 0.0)	0.0 (0.0 to 0.0)	6.9 (5.7 to 8.3)	2.9 (2.2 to 3.7)	2.39 (1.95 to 2.86)	0.99 (0.75 to 1.27)
2010	0.0 (0.0 to 0.0)	0.0 (0.0 to 0.0)	7.0 (5.8 to 8.3)	2.7 (2.1 to 3.5)	2.76 (2.29 to 3.28)	1.09 (0.83 to 1.40)
2015	0.0 (0.0 to 0.0)	0.0 (0.0 to 0.0)	5.6 (4.8 to 6.5)	2.2 (1.9 to 2.6)	2.43 (2.08 to 2.82)	0.97 (0.82 to 1.14)
2020	0.0 (0.0 to 0.0)	0.0 (0.0 to 0.0)	4.6 (3.9 to 5.4)	1.9 (1.6 to 2.1)	2.12 (1.81 to 2.48)	0.86 (0.76 to 0.97)
2021	0.0 (0.0 to 0.0)	0.0 (0.0 to 0.0)	5.0 (3.8 to 6.3)	2.0 (1.6 to 2.5)	2.14 (1.66 to 2.72)	0.87 (0.70 to 1.07)

Cheshire West and Chester GBR_44658 Western Europe Stillbirth Rate / Neonatal Mortality Rate (SBR/NMR) − 20 weeks
lambda = 0.8 ; zeta = 0.7 ; scale = 15 ; amp = 0.028

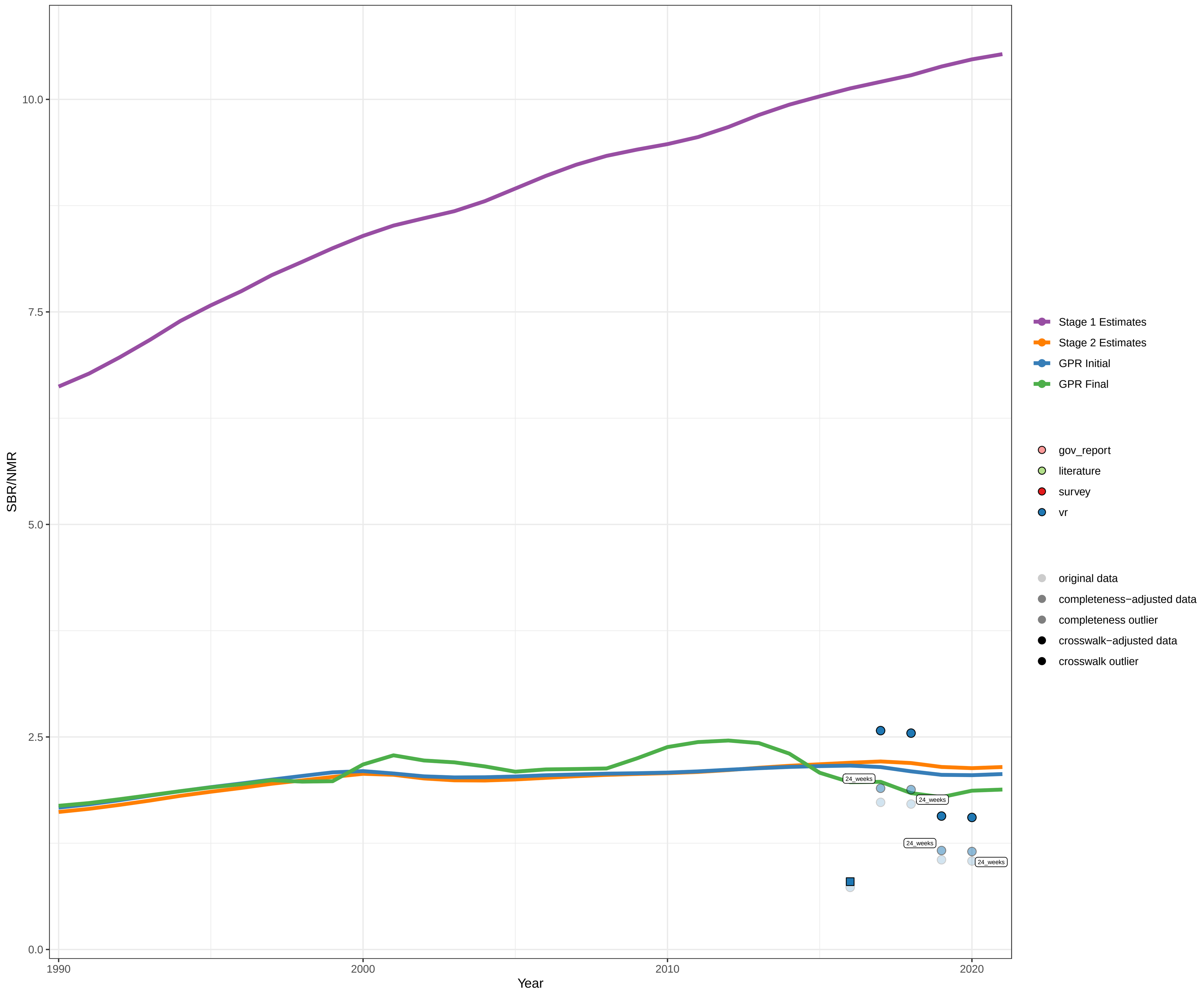

Cheshire West and Chester GBR_44658 Western Europe Stillbirth Rate / Neonatal Mortality Rate (SBR/NMR) − 28 weeks
lambda = 0.8 ; zeta = 0.7 ; scale = 15 ; amp = 0.028

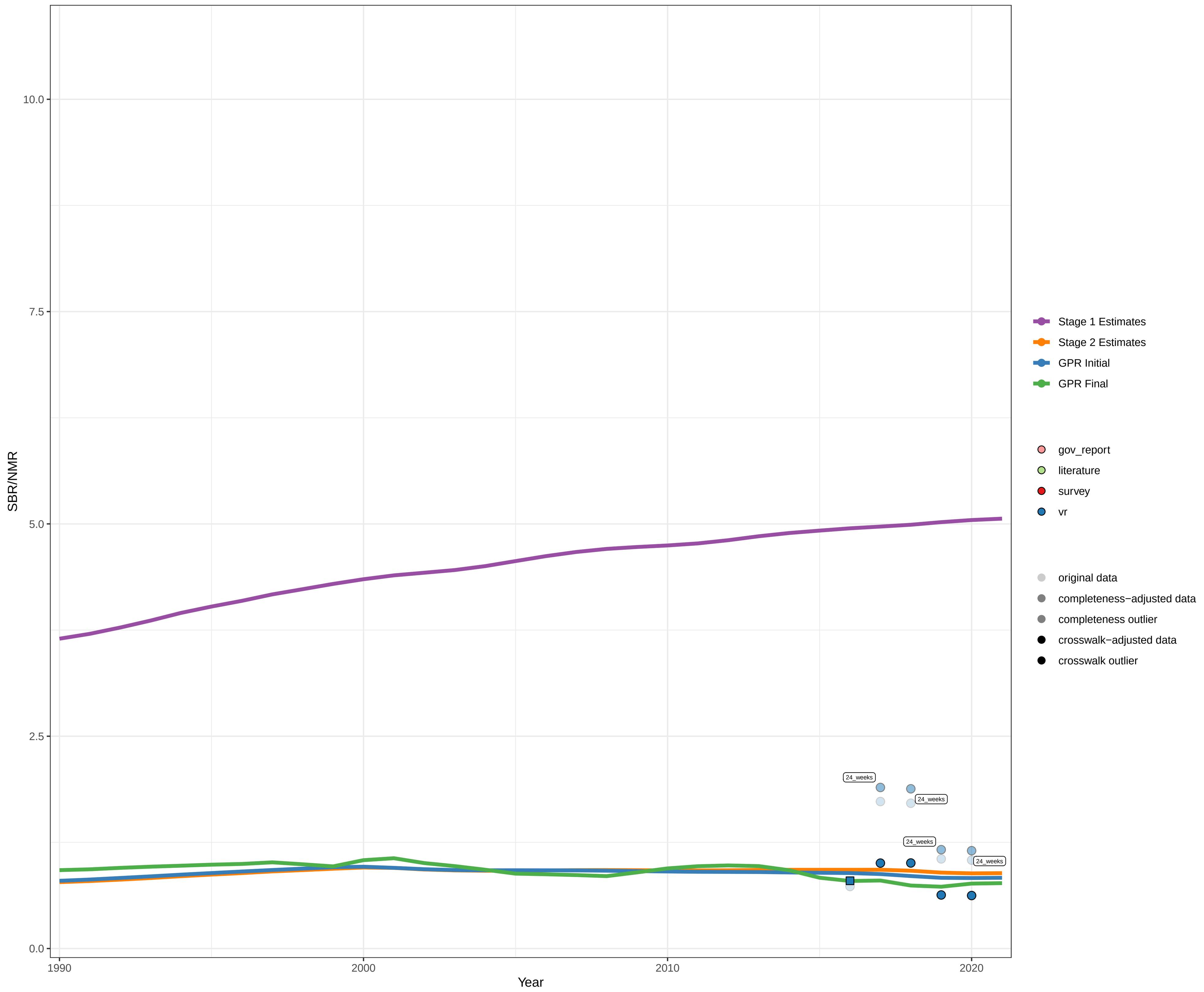

Definition	Years	Outlier	Source.Type	Title
24_weeks	2016−2020	0	VR	United Kingdom − England and Wales Child Mortality Statistics

Cheshire West and Chester GBR_44658 Western Europe Stillbirth Rate (SBR) − 20 weeks
lambda = 0.8 ; zeta = 0.7 ; scale = 15 ; amp = 0.028

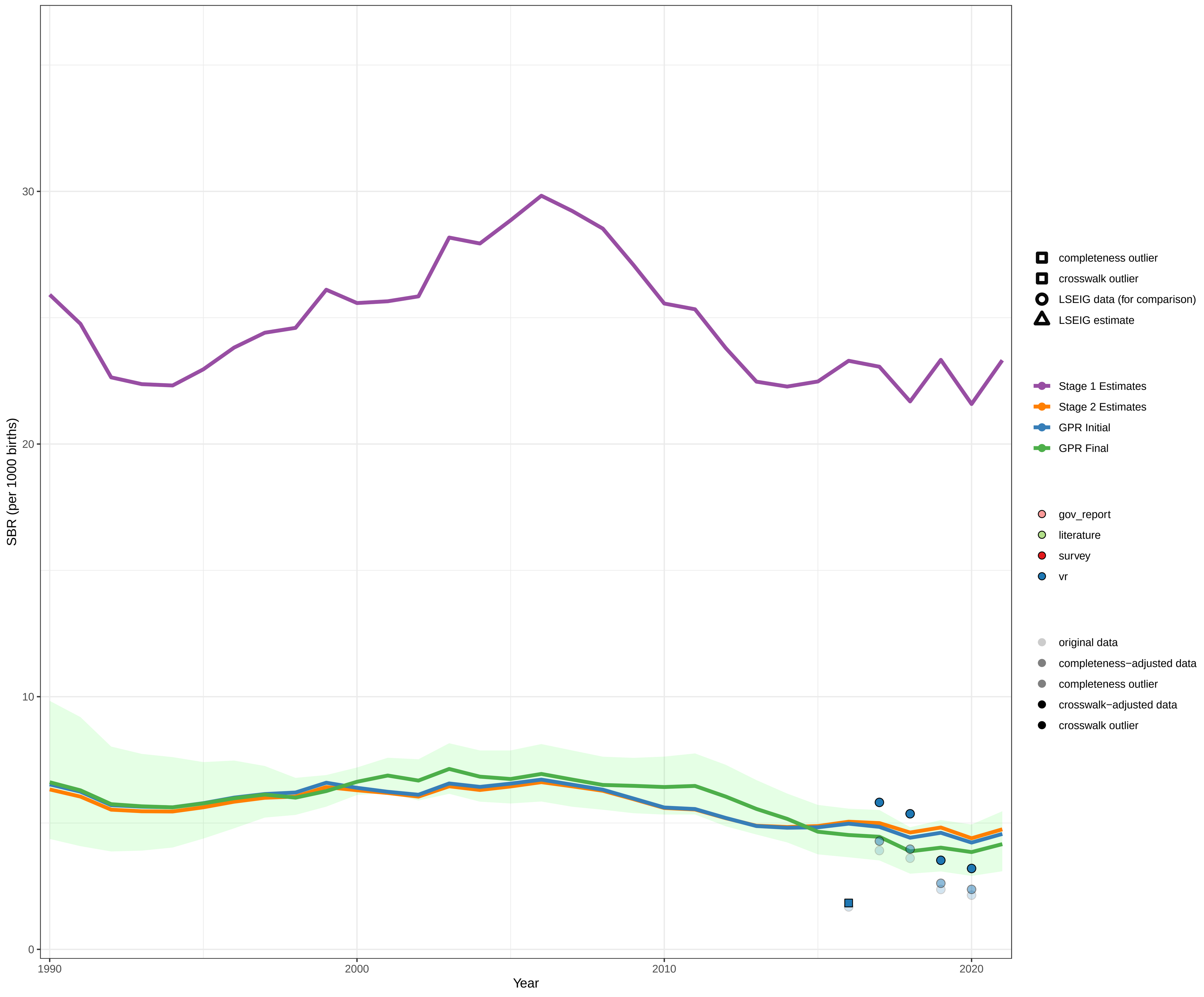

Cheshire West and Chester GBR_44658 Western Europe Stillbirth Rate (SBR) − 28 weeks
lambda = 0.8 ; zeta = 0.7 ; scale = 15 ; amp = 0.028

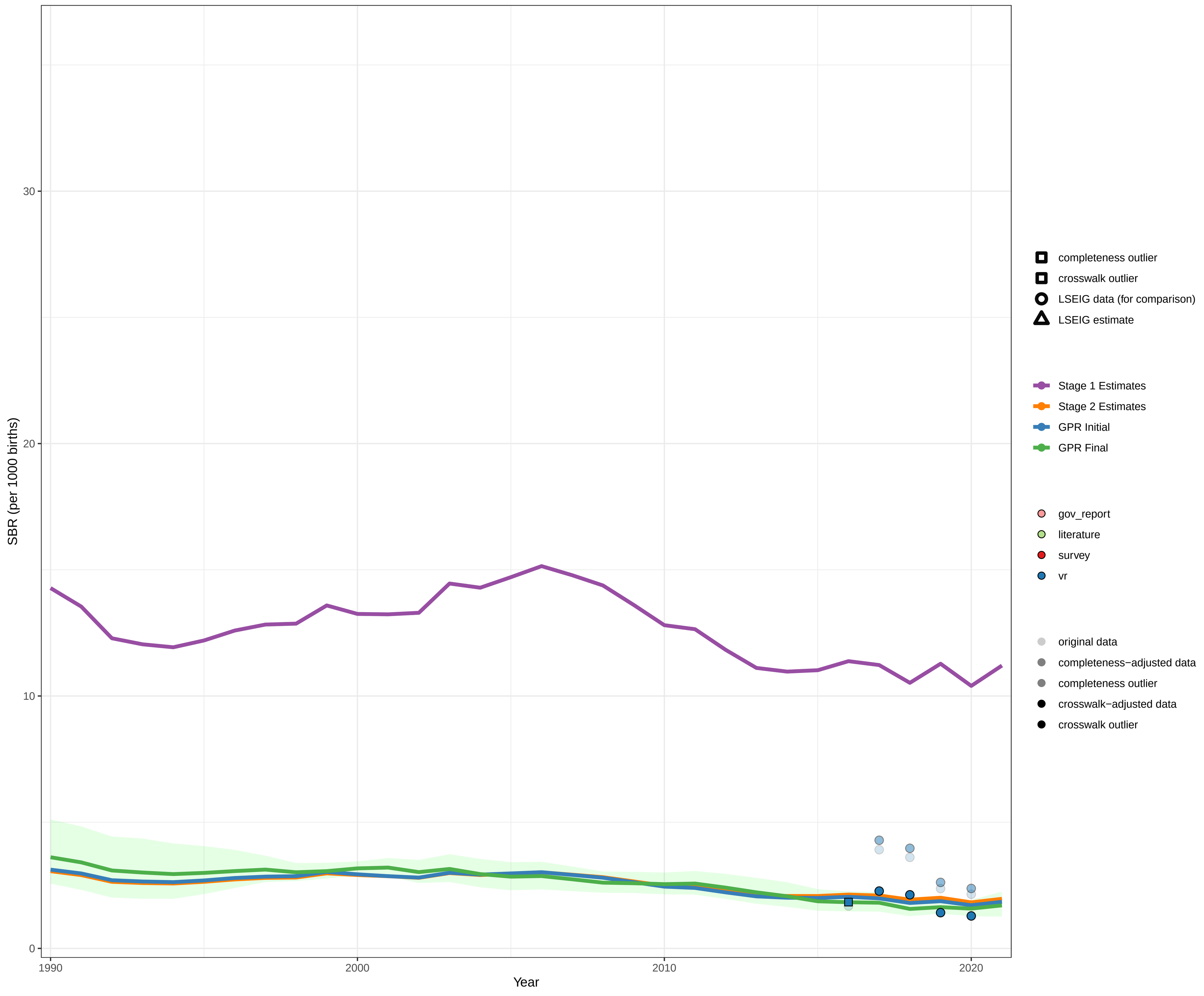

Year	Total Stillbirths (in thousands)	Total Stillbirths (in thousands)	SBR (per 1000 births)	SBR (per 1000 births)	SBR/NMR	SBR/NMR
	20 weeks	28 weeks	20 weeks	28 weeks	20 weeks	28 weeks
1990	0.0 (0.0 to 0.0)	0.0 (0.0 to 0.0)	6.6 (4.4 to 9.8)	3.6 (2.6 to 5.1)	1.69 (1.12 to 2.51)	0.92 (0.66 to 1.31)
1995	0.0 (0.0 to 0.0)	0.0 (0.0 to 0.0)	5.8 (4.4 to 7.4)	3.0 (2.2 to 4.1)	1.91 (1.45 to 2.45)	0.99 (0.71 to 1.34)
2000	0.0 (0.0 to 0.0)	0.0 (0.0 to 0.0)	6.6 (6.1 to 7.2)	3.2 (2.9 to 3.4)	2.18 (2.01 to 2.36)	1.04 (0.95 to 1.13)
2005	0.0 (0.0 to 0.0)	0.0 (0.0 to 0.0)	6.7 (5.8 to 7.9)	2.8 (2.3 to 3.4)	2.09 (1.79 to 2.44)	0.88 (0.71 to 1.06)
2010	0.0 (0.0 to 0.0)	0.0 (0.0 to 0.0)	6.4 (5.3 to 7.6)	2.5 (2.1 to 3.0)	2.38 (1.98 to 2.83)	0.94 (0.79 to 1.11)
2015	0.0 (0.0 to 0.0)	0.0 (0.0 to 0.0)	4.7 (3.8 to 5.7)	1.9 (1.5 to 2.3)	2.08 (1.68 to 2.55)	0.83 (0.67 to 1.05)
2020	0.0 (0.0 to 0.0)	0.0 (0.0 to 0.0)	3.9 (2.9 to 4.9)	1.6 (1.3 to 1.9)	1.87 (1.41 to 2.40)	0.76 (0.62 to 0.93)
2021	0.0 (0.0 to 0.0)	0.0 (0.0 to 0.0)	4.2 (3.1 to 5.5)	1.7 (1.3 to 2.3)	1.88 (1.40 to 2.47)	0.77 (0.57 to 1.02)

Cumbria GBR_44661 Western Europe Stillbirth Rate / Neonatal Mortality Rate (SBR/NMR) − 20 weeks
lambda = 0.6 ; zeta = 0.8 ; scale = 15 ; amp = 0.028

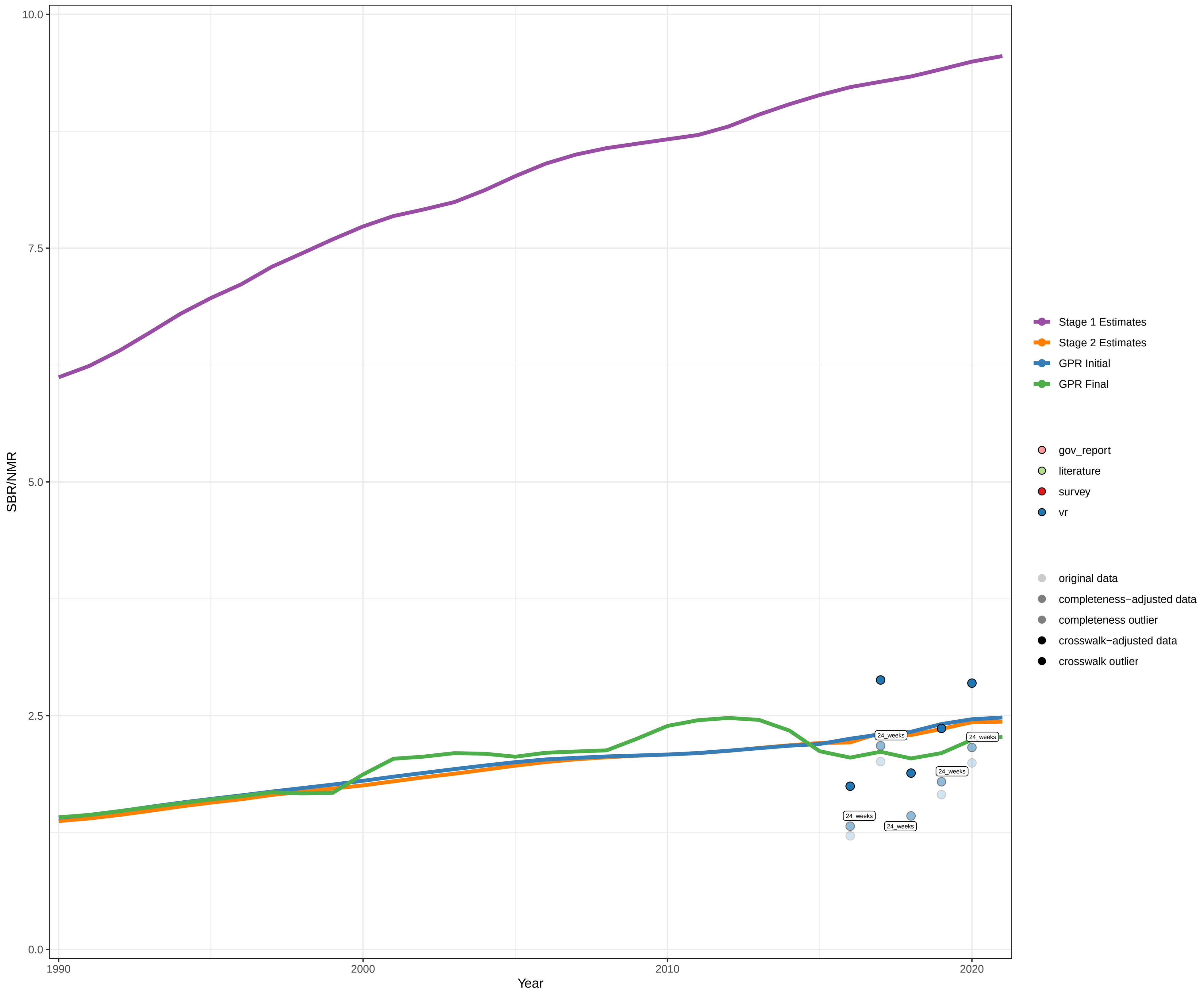

Cumbria GBR_44661 Western Europe Stillbirth Rate / Neonatal Mortality Rate (SBR/NMR) − 28 weeks
lambda = 0.6 ; zeta = 0.8 ; scale = 15 ; amp = 0.028

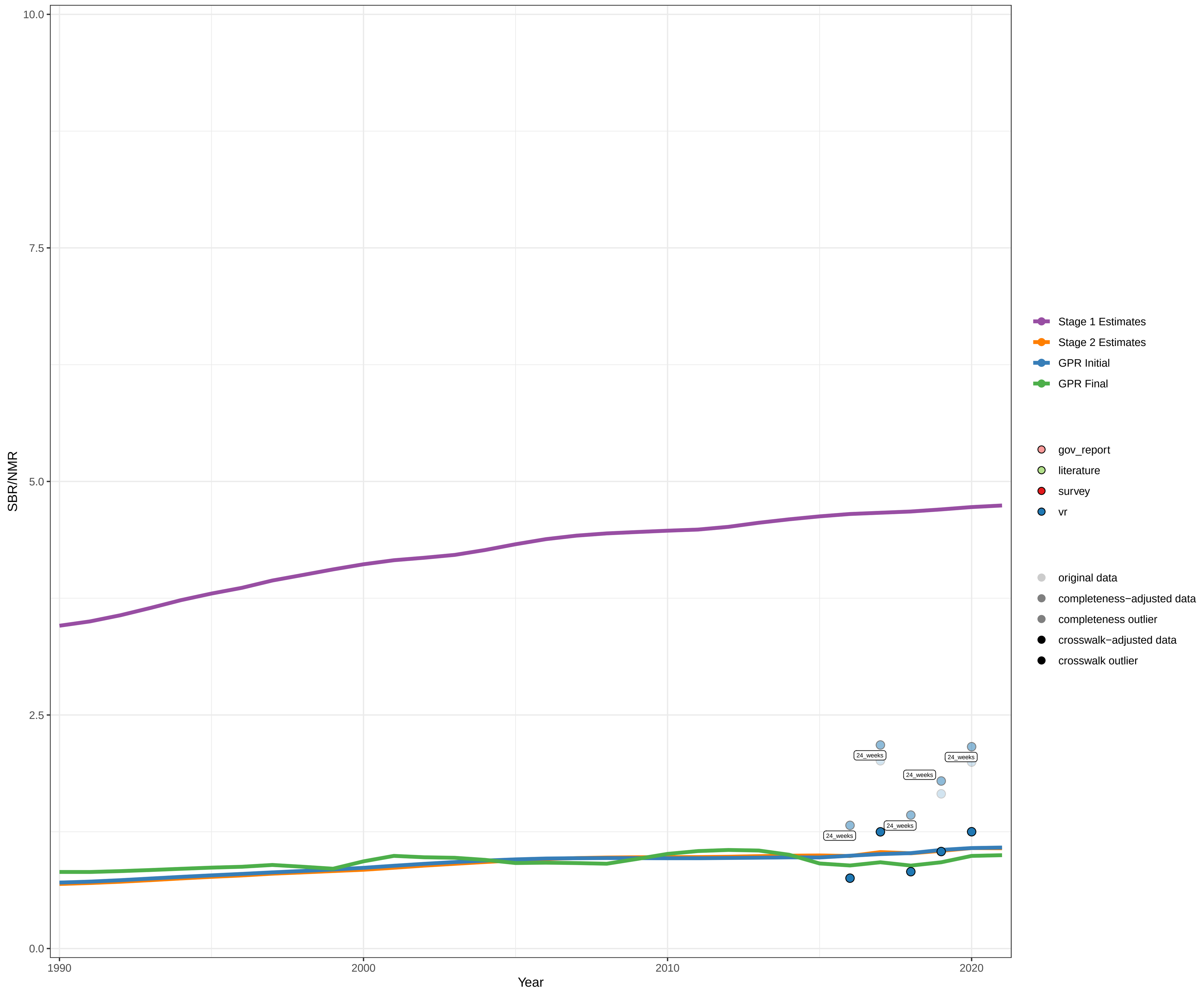

Definition	Years	Outlier	Source.Type	Title
24_weeks	2016−2020	0	VR	United Kingdom − England and Wales Child Mortality Statistics

Cumbria GBR_44661 Western Europe Stillbirth Rate (SBR) − 20 weeks
lambda = 0.6 ; zeta = 0.8 ; scale = 15 ; amp = 0.028

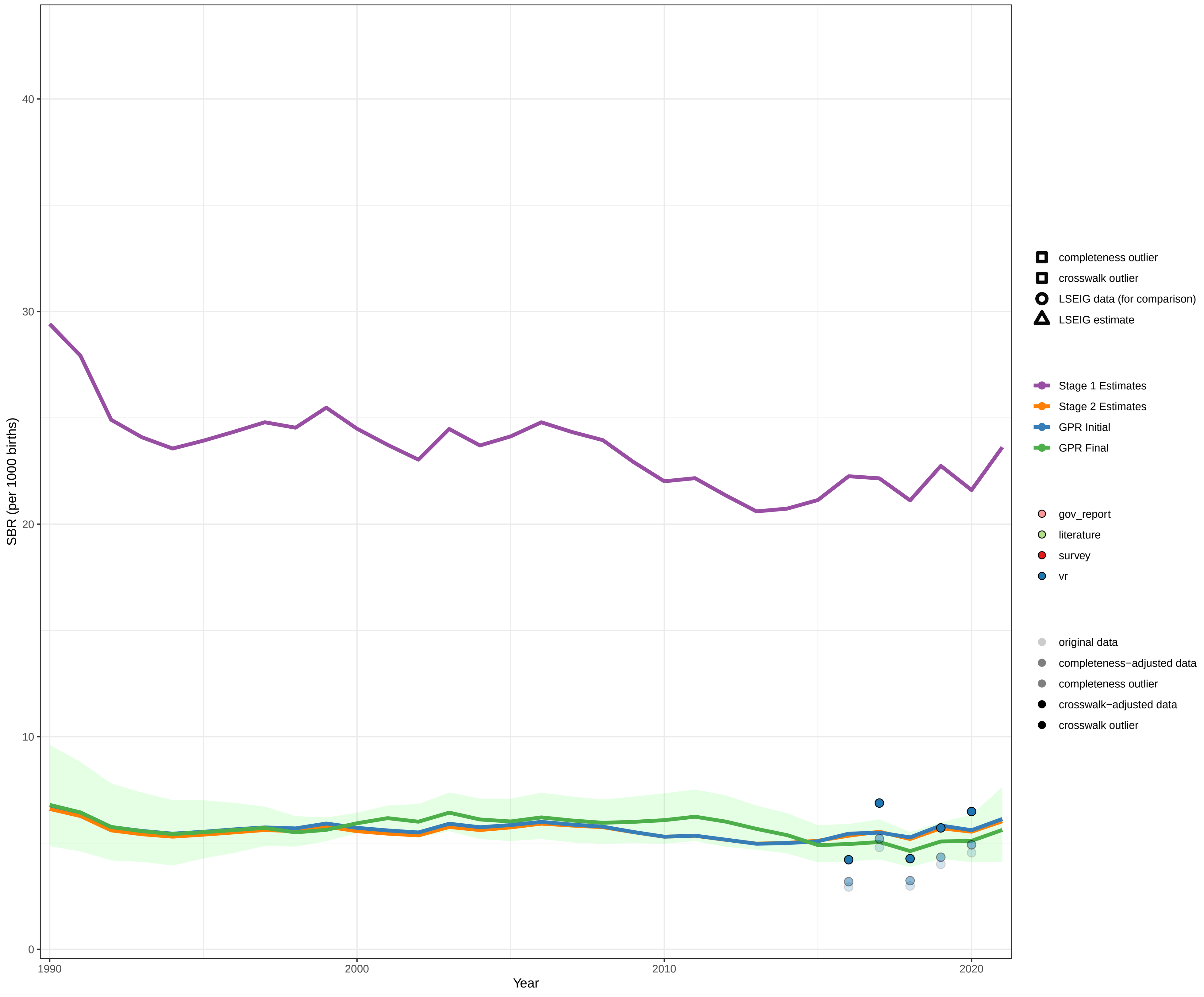

Cumbria GBR_44661 Western Europe Stillbirth Rate (SBR) − 28 weeks
lambda = 0.6 ; zeta = 0.8 ; scale = 15 ; amp = 0.028

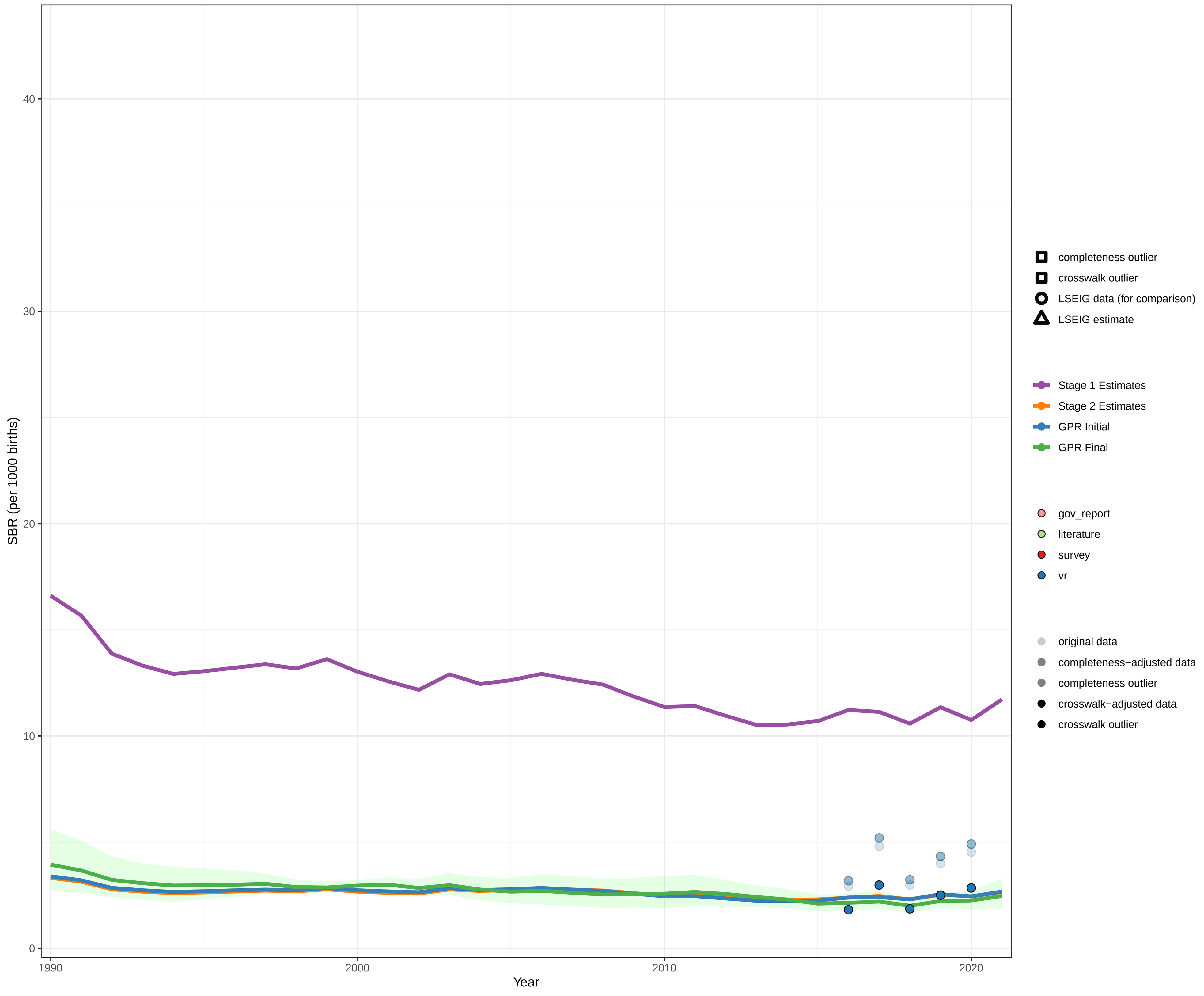

Year	Total Stillbirths (in thousands)	Total Stillbirths (in thousands)	SBR (per 1000 births)	SBR (per 1000 births)	SBR/NMR	SBR/NMR
	20 weeks	28 weeks	20 weeks	28 weeks	20 weeks	28 weeks
1990	0.0 (0.0 to 0.1)	0.0 (0.0 to 0.0)	6.8 (4.9 to 9.6)	3.9 (2.7 to 5.6)	1.41 (1.01 to 2.00)	0.82 (0.56 to 1.17)
1995	0.0 (0.0 to 0.0)	0.0 (0.0 to 0.0)	5.5 (4.3 to 7.0)	3.0 (2.3 to 3.8)	1.60 (1.24 to 2.04)	0.87 (0.67 to 1.10)
2000	0.0 (0.0 to 0.0)	0.0 (0.0 to 0.0)	5.9 (5.5 to 6.4)	3.0 (2.7 to 3.2)	1.87 (1.73 to 2.03)	0.93 (0.85 to 1.01)
2005	0.0 (0.0 to 0.0)	0.0 (0.0 to 0.0)	6.0 (5.1 to 7.1)	2.7 (2.1 to 3.3)	2.06 (1.75 to 2.43)	0.92 (0.73 to 1.14)
2010	0.0 (0.0 to 0.0)	0.0 (0.0 to 0.0)	6.1 (5.0 to 7.3)	2.6 (1.9 to 3.4)	2.39 (1.95 to 2.89)	1.01 (0.75 to 1.34)
2015	0.0 (0.0 to 0.0)	0.0 (0.0 to 0.0)	4.9 (4.1 to 5.8)	2.1 (1.7 to 2.5)	2.12 (1.77 to 2.53)	0.91 (0.75 to 1.09)
2020	0.0 (0.0 to 0.0)	0.0 (0.0 to 0.0)	5.1 (4.1 to 6.3)	2.3 (1.9 to 2.7)	2.24 (1.80 to 2.78)	0.99 (0.82 to 1.19)
2021	0.0 (0.0 to 0.0)	0.0 (0.0 to 0.0)	5.6 (4.1 to 7.6)	2.5 (1.9 to 3.3)	2.27 (1.66 to 3.09)	1.00 (0.76 to 1.33)

Halton GBR_44670 Western Europe Stillbirth Rate / Neonatal Mortality Rate (SBR/NMR) − 20 weeks
lambda = 0.6 ; zeta = 0.8 ; scale = 15 ; amp = 0.028

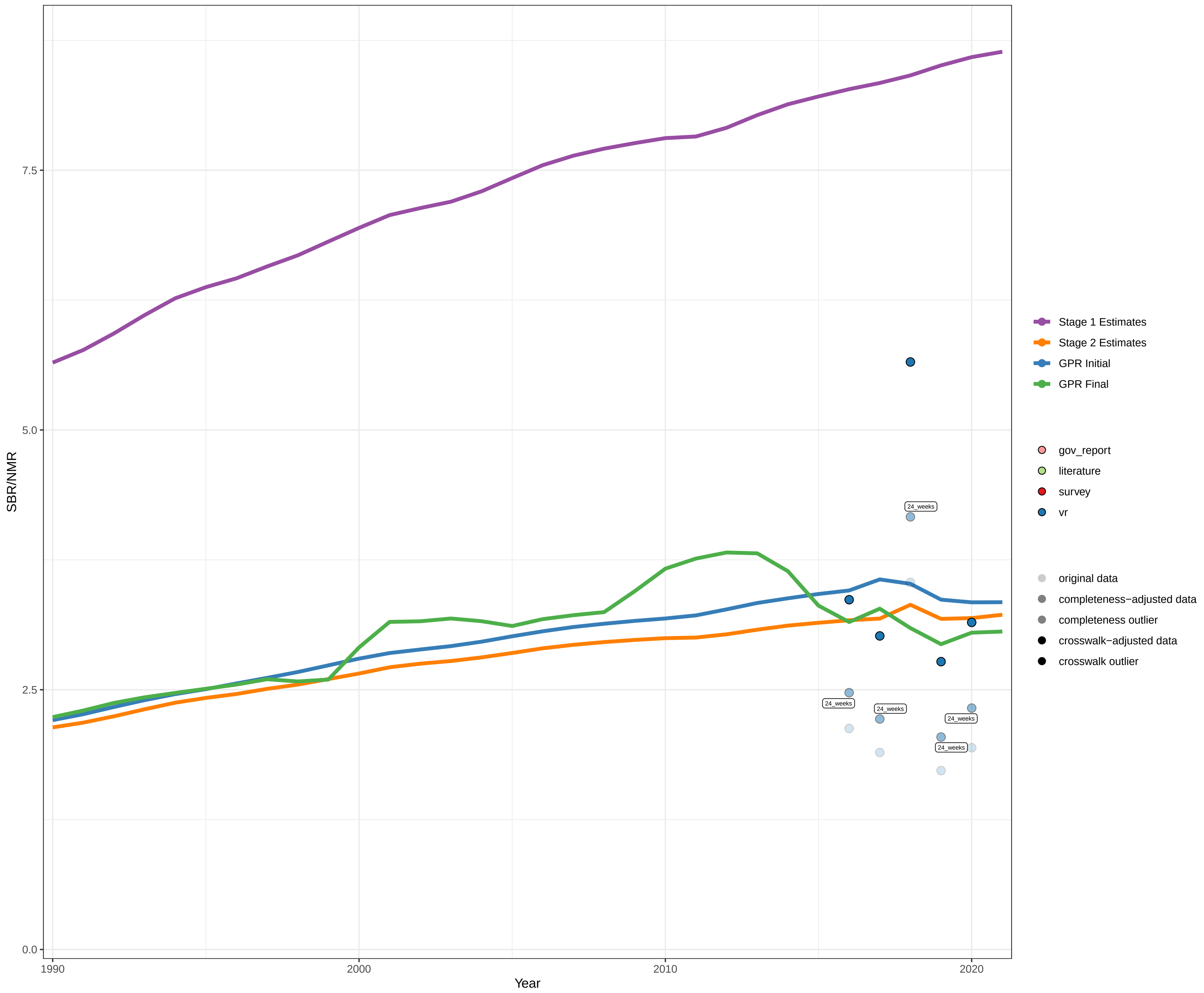

Halton GBR_44670 Western Europe Stillbirth Rate / Neonatal Mortality Rate (SBR/NMR) − 28 weeks
lambda = 0.6 ; zeta = 0.8 ; scale = 15 ; amp = 0.028

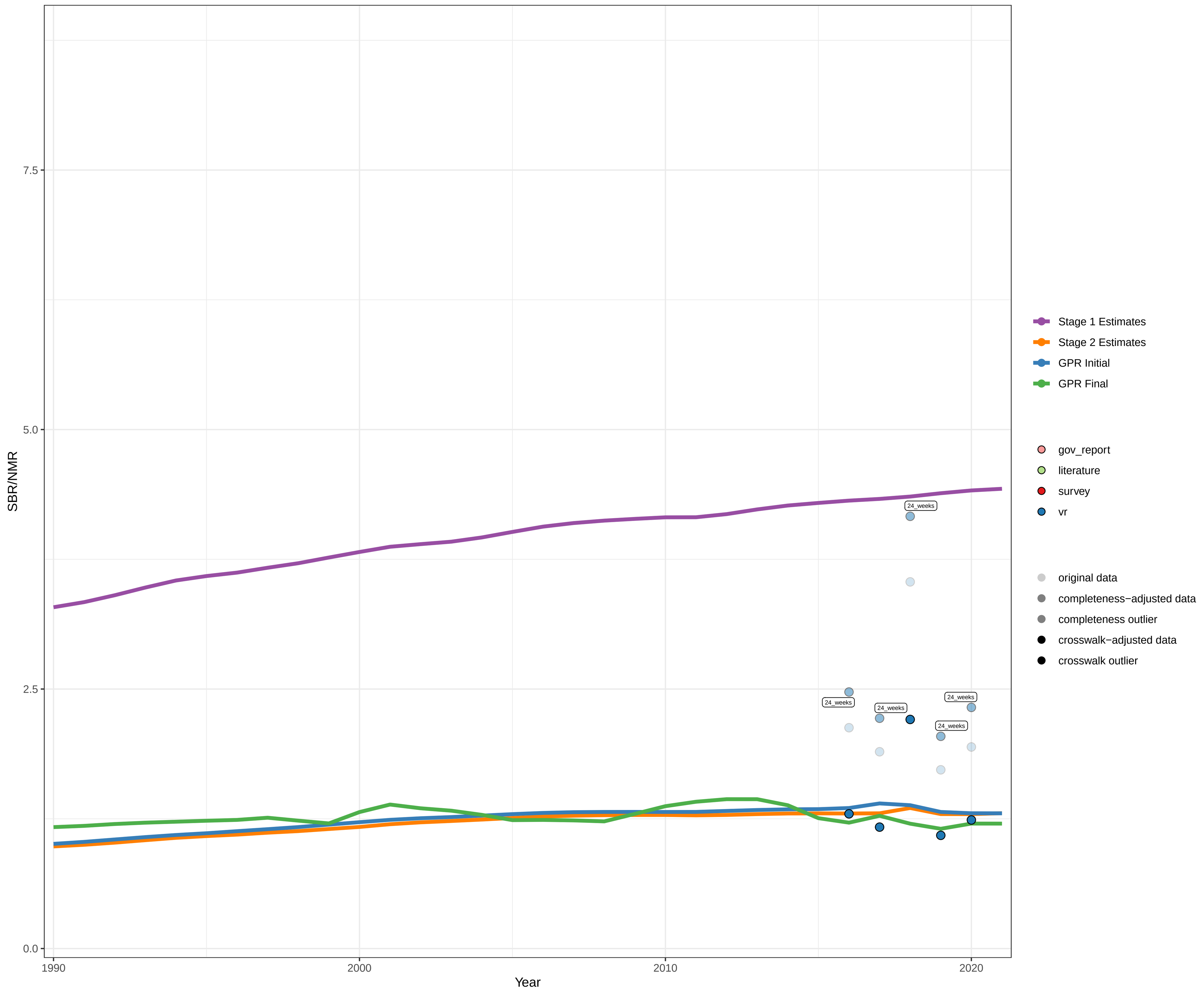

Definition	Years	Outlier	Source.Type	Title
24_weeks	2016−2020	0	VR	United Kingdom − England and Wales Child Mortality Statistics

Halton GBR_44670 Western Europe Stillbirth Rate (SBR) − 20 weeks
lambda = 0.6 ; zeta = 0.8 ; scale = 15 ; amp = 0.028

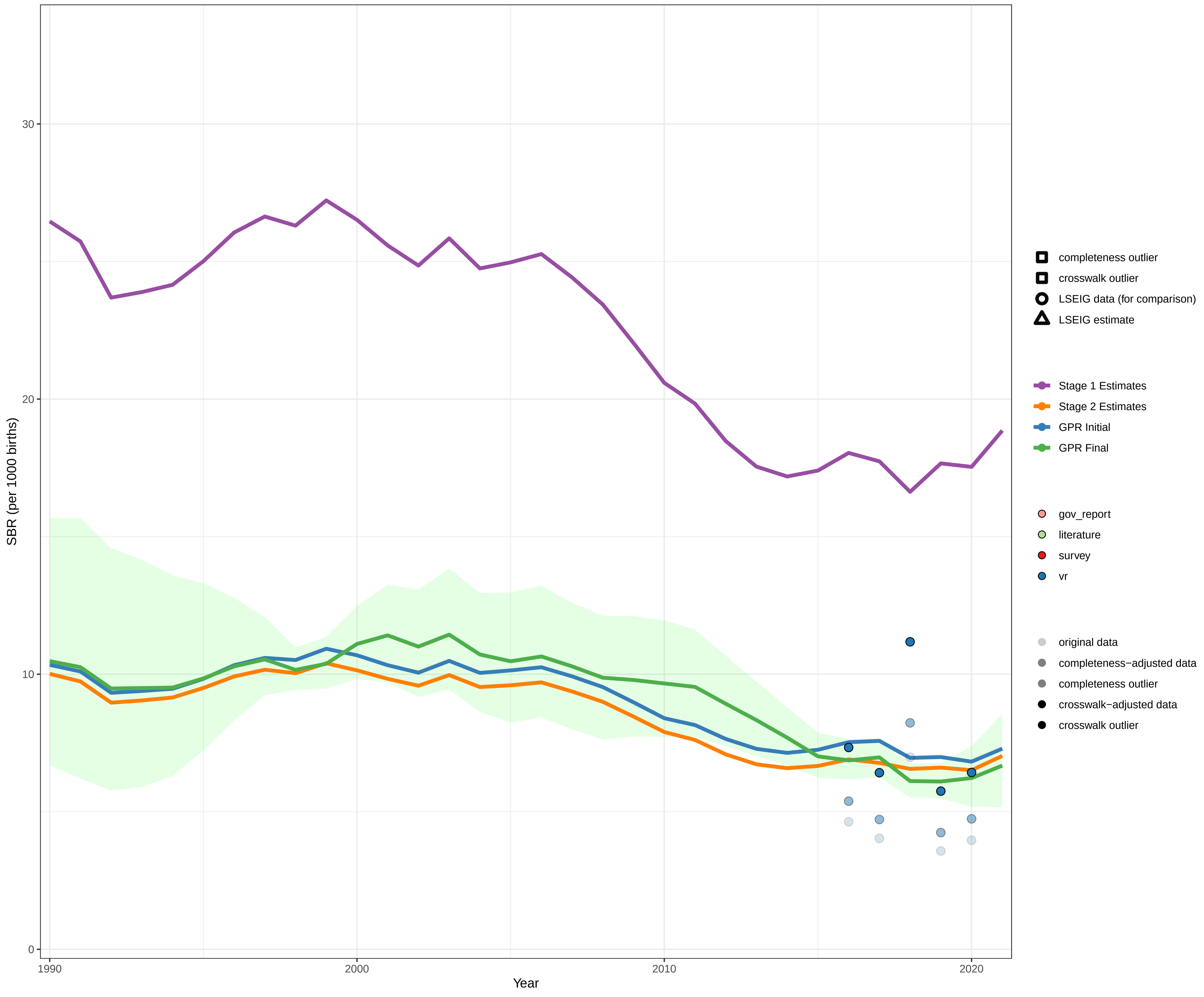

Halton GBR_44670 Western Europe Stillbirth Rate (SBR) − 28 weeks
lambda = 0.6 ; zeta = 0.8 ; scale = 15 ; amp = 0.028

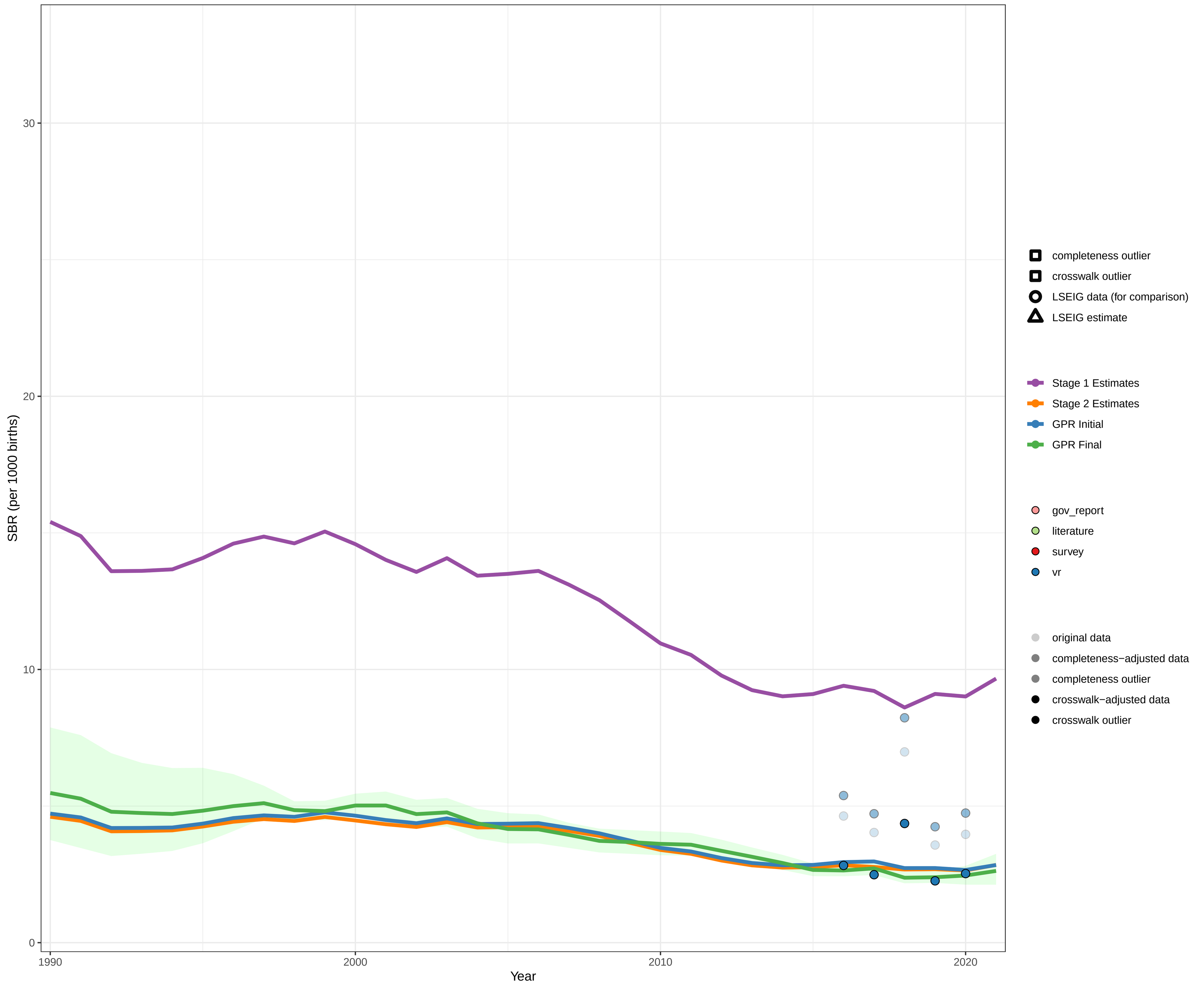

Year	Total Stillbirths (in thousands)	Total Stillbirths (in thousands)	SBR (per 1000 births)	SBR (per 1000 births)	SBR/NMR	SBR/NMR
	20 weeks	28 weeks	20 weeks	28 weeks	20 weeks	28 weeks
1990	0.0 (0.0 to 0.0)	0.0 (0.0 to 0.0)	10.5 (6.7 to 15.7)	5.5 (3.8 to 7.9)	2.24 (1.43 to 3.34)	1.17 (0.80 to 1.68)
1995	0.0 (0.0 to 0.0)	0.0 (0.0 to 0.0)	9.8 (7.2 to 13.3)	4.8 (3.6 to 6.4)	2.51 (1.84 to 3.39)	1.23 (0.93 to 1.63)
2000	0.0 (0.0 to 0.0)	0.0 (0.0 to 0.0)	11.1 (9.8 to 12.5)	5.0 (4.6 to 5.5)	2.91 (2.57 to 3.27)	1.31 (1.21 to 1.43)
2005	0.0 (0.0 to 0.0)	0.0 (0.0 to 0.0)	10.5 (8.2 to 13.0)	4.2 (3.6 to 4.7)	3.11 (2.45 to 3.86)	1.24 (1.08 to 1.41)
2010	0.0 (0.0 to 0.0)	0.0 (0.0 to 0.0)	9.7 (7.7 to 12.0)	3.6 (3.2 to 4.1)	3.67 (2.93 to 4.54)	1.37 (1.21 to 1.54)
2015	0.0 (0.0 to 0.0)	0.0 (0.0 to 0.0)	7.0 (6.2 to 7.9)	2.7 (2.4 to 2.9)	3.31 (2.94 to 3.72)	1.26 (1.15 to 1.37)
2020	0.0 (0.0 to 0.0)	0.0 (0.0 to 0.0)	6.2 (5.2 to 7.4)	2.5 (2.1 to 2.8)	3.05 (2.54 to 3.62)	1.20 (1.04 to 1.38)
2021	0.0 (0.0 to 0.0)	0.0 (0.0 to 0.0)	6.7 (5.2 to 8.6)	2.6 (2.1 to 3.3)	3.06 (2.37 to 3.92)	1.20 (0.97 to 1.49)

Knowsley GBR_44675 Western Europe Stillbirth Rate / Neonatal Mortality Rate (SBR/NMR) − 20 weeks
lambda = 0.6 ; zeta = 0.8 ; scale = 15 ; amp = 0.028

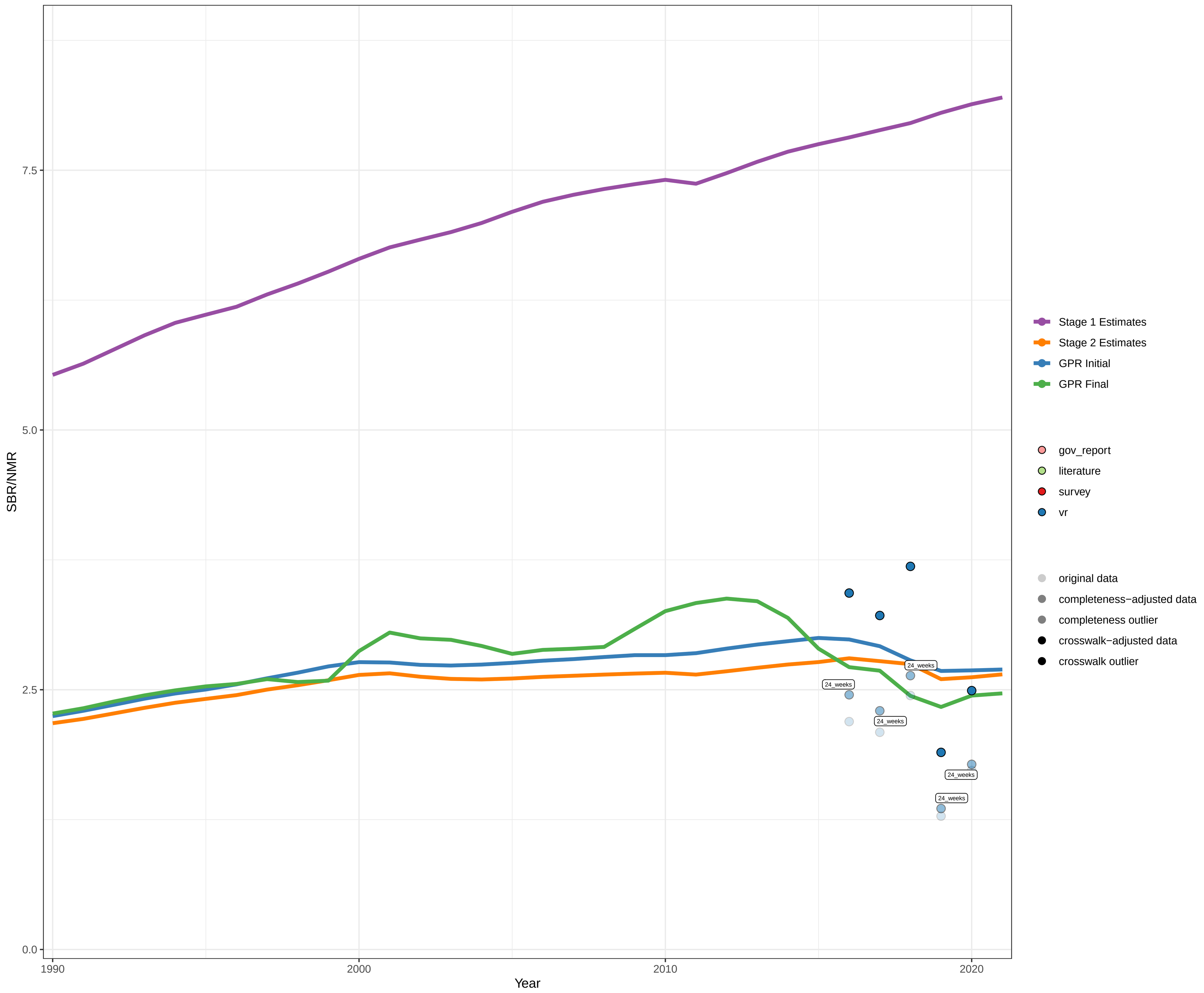

Knowsley GBR_44675 Western Europe Stillbirth Rate / Neonatal Mortality Rate (SBR/NMR) − 28 weeks
lambda = 0.6 ; zeta = 0.8 ; scale = 15 ; amp = 0.028

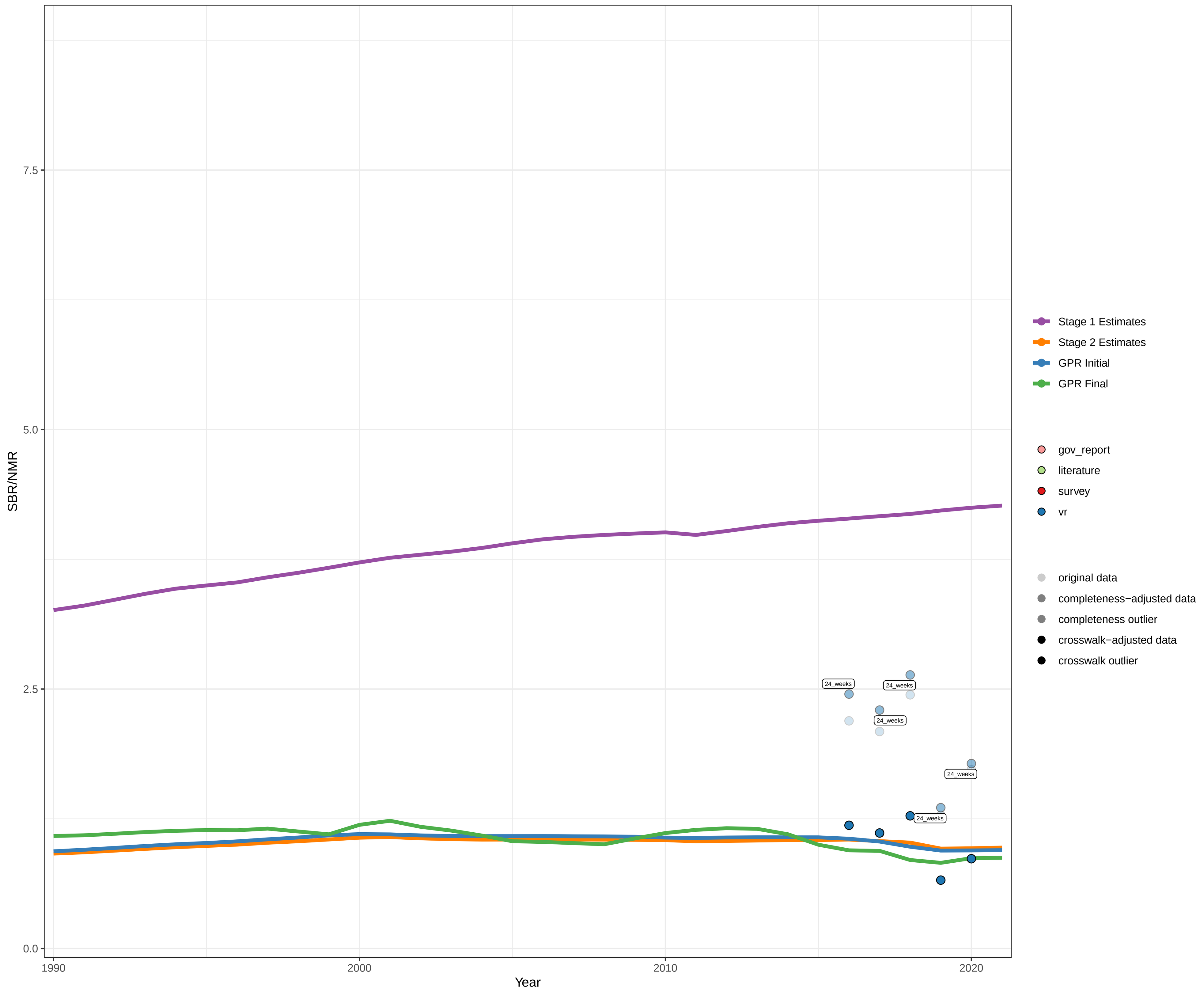

Definition	Years	Outlier	Source.Type	Title
24_weeks	2016−2020	0	VR	United Kingdom − England and Wales Child Mortality Statistics

Knowsley GBR_44675 Western Europe Stillbirth Rate (SBR) − 20 weeks
lambda = 0.6 ; zeta = 0.8 ; scale = 15 ; amp = 0.028

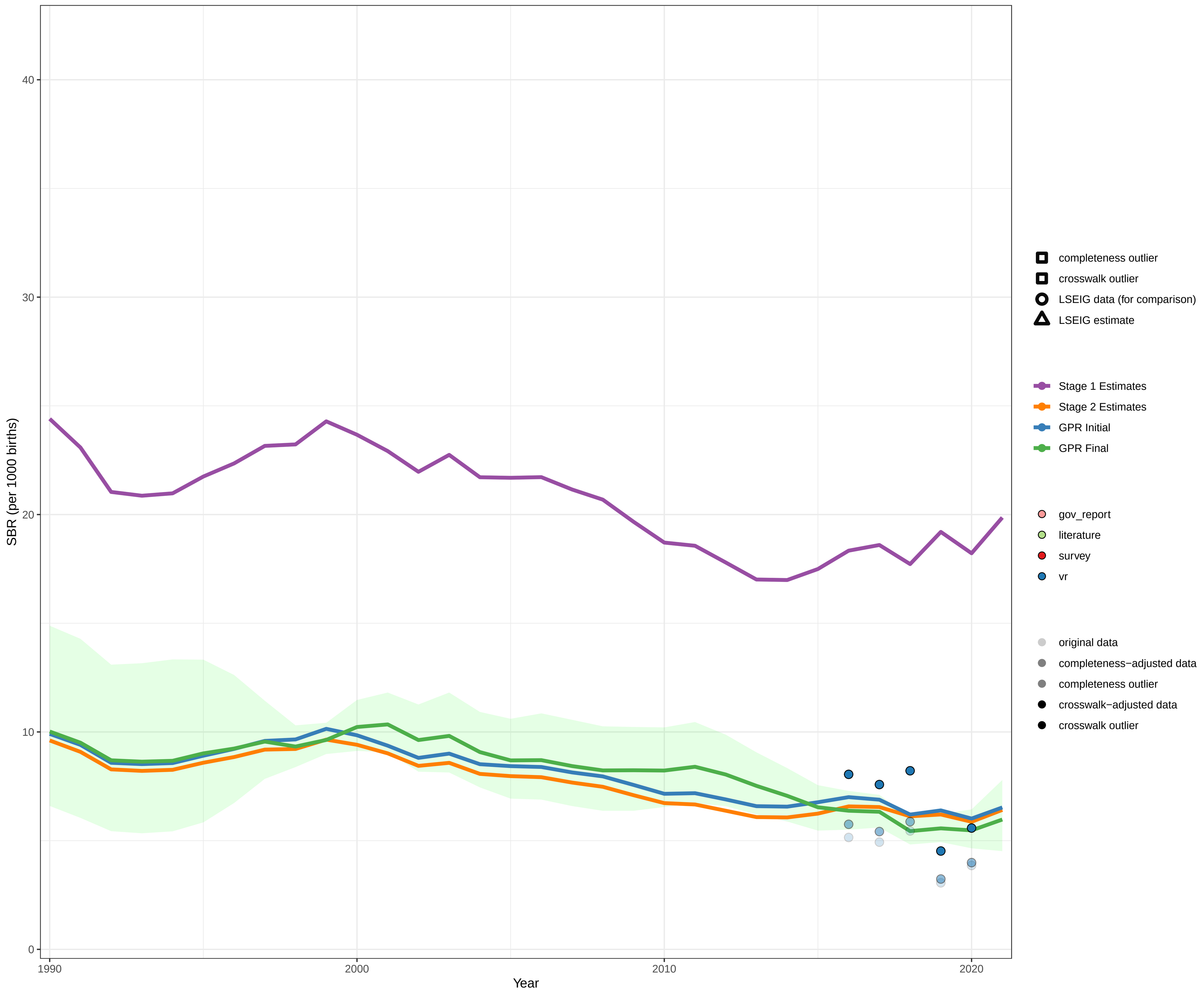

Knowsley GBR_44675 Western Europe Stillbirth Rate (SBR) − 28 weeks
lambda = 0.6 ; zeta = 0.8 ; scale = 15 ; amp = 0.028

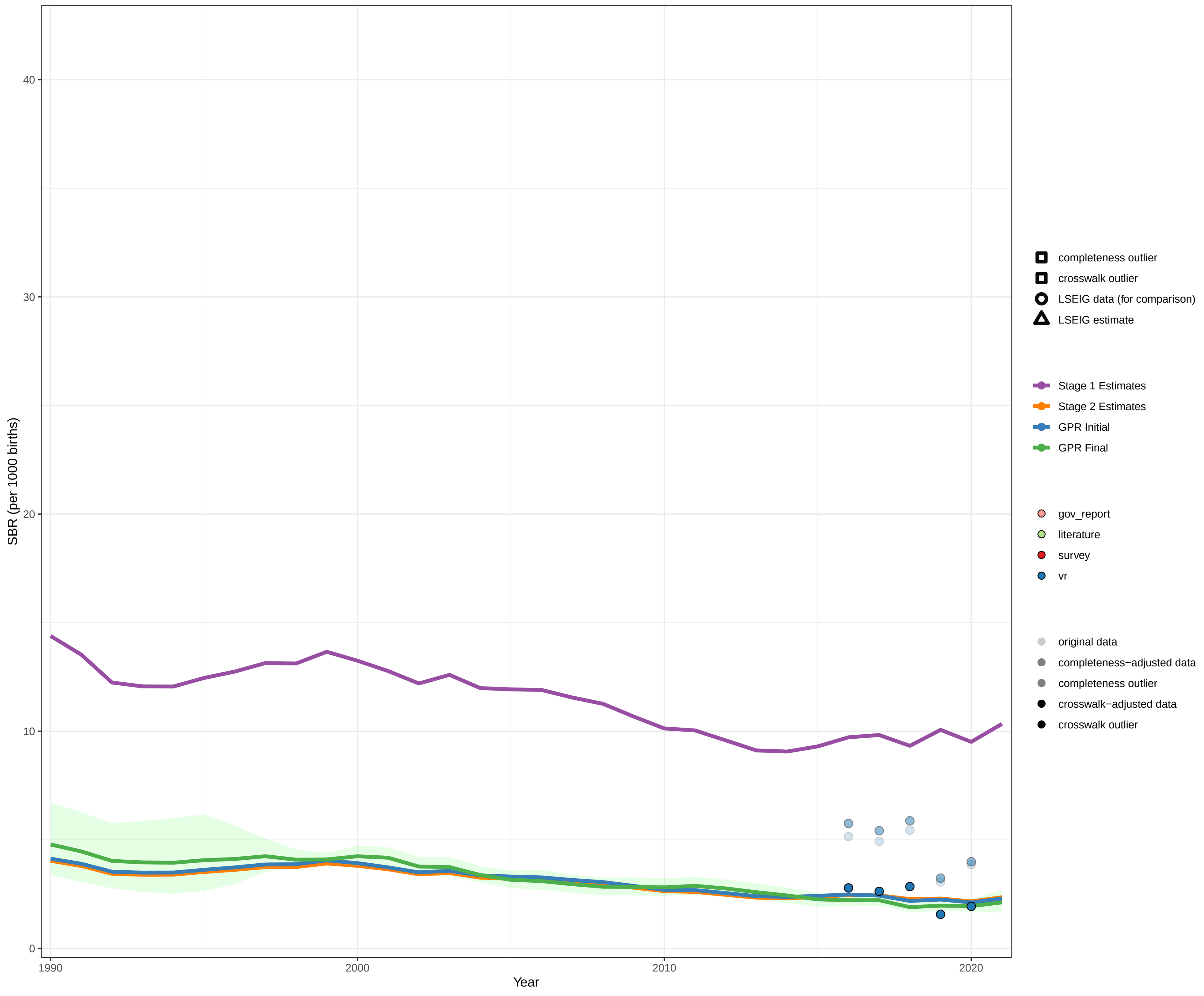

Year	Total Stillbirths (in thousands)	Total Stillbirths (in thousands)	SBR (per 1000 births)	SBR (per 1000 births)	SBR/NMR	SBR/NMR
	20 weeks	28 weeks	20 weeks	28 weeks	20 weeks	28 weeks
1990	0.0 (0.0 to 0.0)	0.0 (0.0 to 0.0)	10.0 (6.6 to 14.9)	4.8 (3.4 to 6.7)	2.27 (1.50 to 3.37)	1.08 (0.77 to 1.53)
1995	0.0 (0.0 to 0.0)	0.0 (0.0 to 0.0)	9.0 (5.8 to 13.3)	4.1 (2.6 to 6.2)	2.53 (1.64 to 3.74)	1.14 (0.74 to 1.73)
2000	0.0 (0.0 to 0.0)	0.0 (0.0 to 0.0)	10.2 (9.1 to 11.5)	4.2 (3.8 to 4.7)	2.87 (2.56 to 3.22)	1.19 (1.07 to 1.33)
2005	0.0 (0.0 to 0.0)	0.0 (0.0 to 0.0)	8.7 (6.9 to 10.6)	3.2 (2.8 to 3.6)	2.85 (2.27 to 3.47)	1.03 (0.91 to 1.17)
2010	0.0 (0.0 to 0.0)	0.0 (0.0 to 0.0)	8.2 (6.5 to 10.2)	2.8 (2.4 to 3.2)	3.26 (2.59 to 4.04)	1.11 (0.96 to 1.28)
2015	0.0 (0.0 to 0.0)	0.0 (0.0 to 0.0)	6.5 (5.5 to 7.6)	2.3 (1.9 to 2.6)	2.90 (2.42 to 3.35)	1.00 (0.85 to 1.13)
2020	0.0 (0.0 to 0.0)	0.0 (0.0 to 0.0)	5.5 (4.6 to 6.4)	1.9 (1.7 to 2.3)	2.44 (2.08 to 2.87)	0.87 (0.75 to 1.01)
2021	0.0 (0.0 to 0.0)	0.0 (0.0 to 0.0)	6.0 (4.5 to 7.8)	2.1 (1.6 to 2.7)	2.47 (1.86 to 3.22)	0.87 (0.68 to 1.12)

Lancashire GBR_44660 Western Europe Stillbirth Rate / Neonatal Mortality Rate (SBR/NMR) − 20 weeks
lambda = 0.6 ; zeta = 0.8 ; scale = 15 ; amp = 0.028

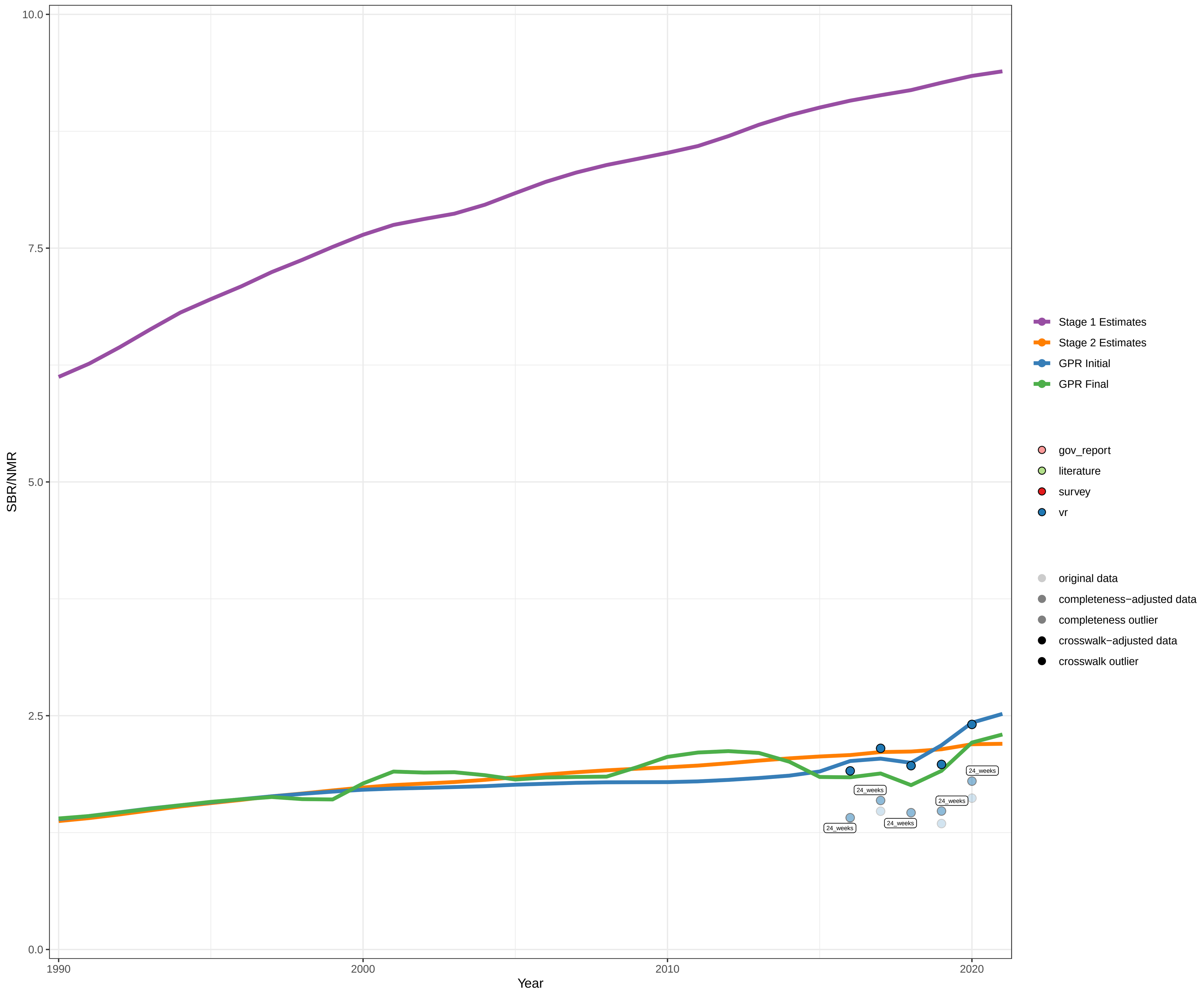

Lancashire GBR_44660 Western Europe Stillbirth Rate / Neonatal Mortality Rate (SBR/NMR) − 28 weeks
lambda = 0.6 ; zeta = 0.8 ; scale = 15 ; amp = 0.028

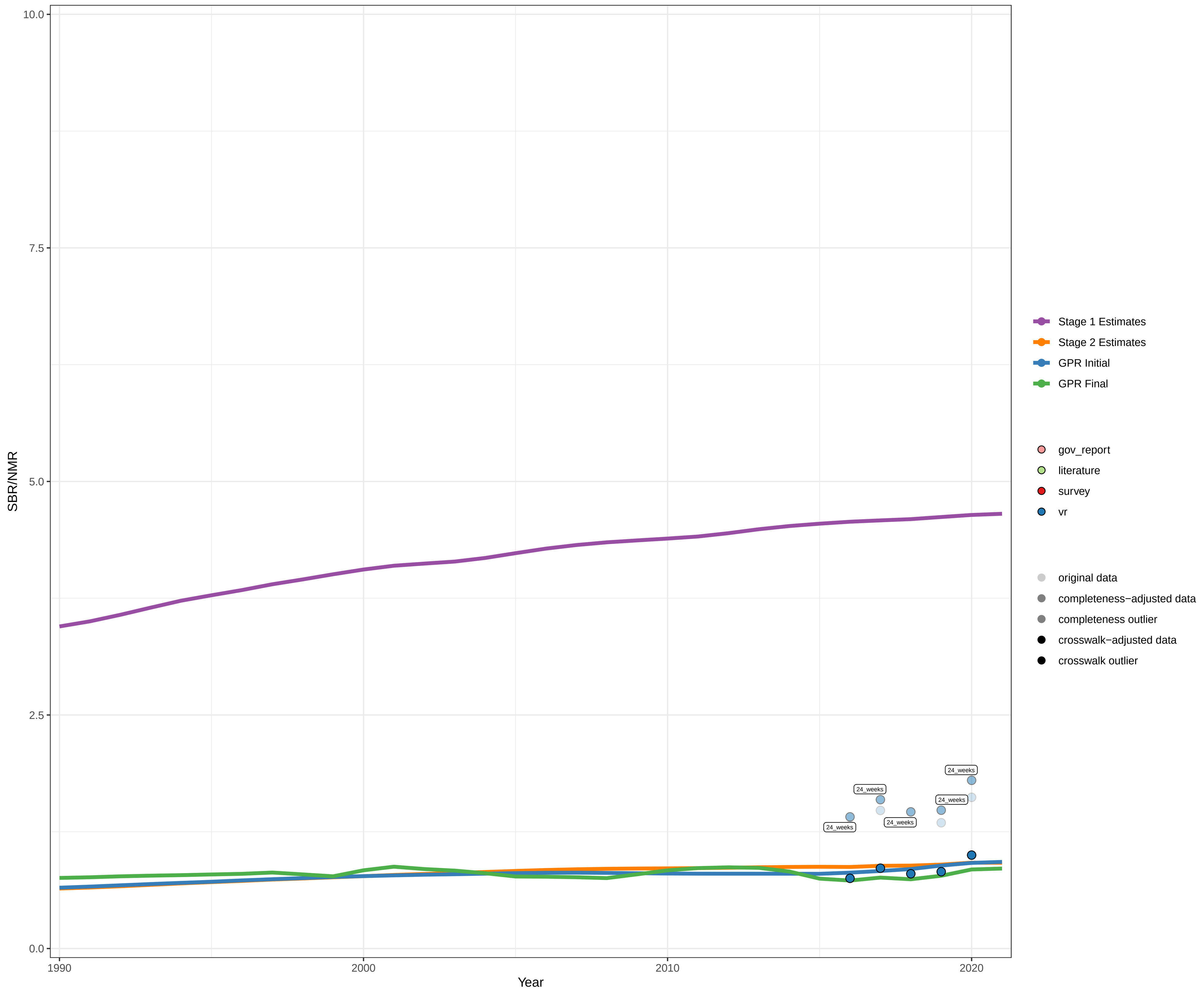

Definition	Years	Outlier	Source.Type	Title
24_weeks	2016−2020	0	VR	United Kingdom − England and Wales Child Mortality Statistics

Lancashire GBR_44660 Western Europe Stillbirth Rate (SBR) − 20 weeks
lambda = 0.6 ; zeta = 0.8 ; scale = 15 ; amp = 0.028

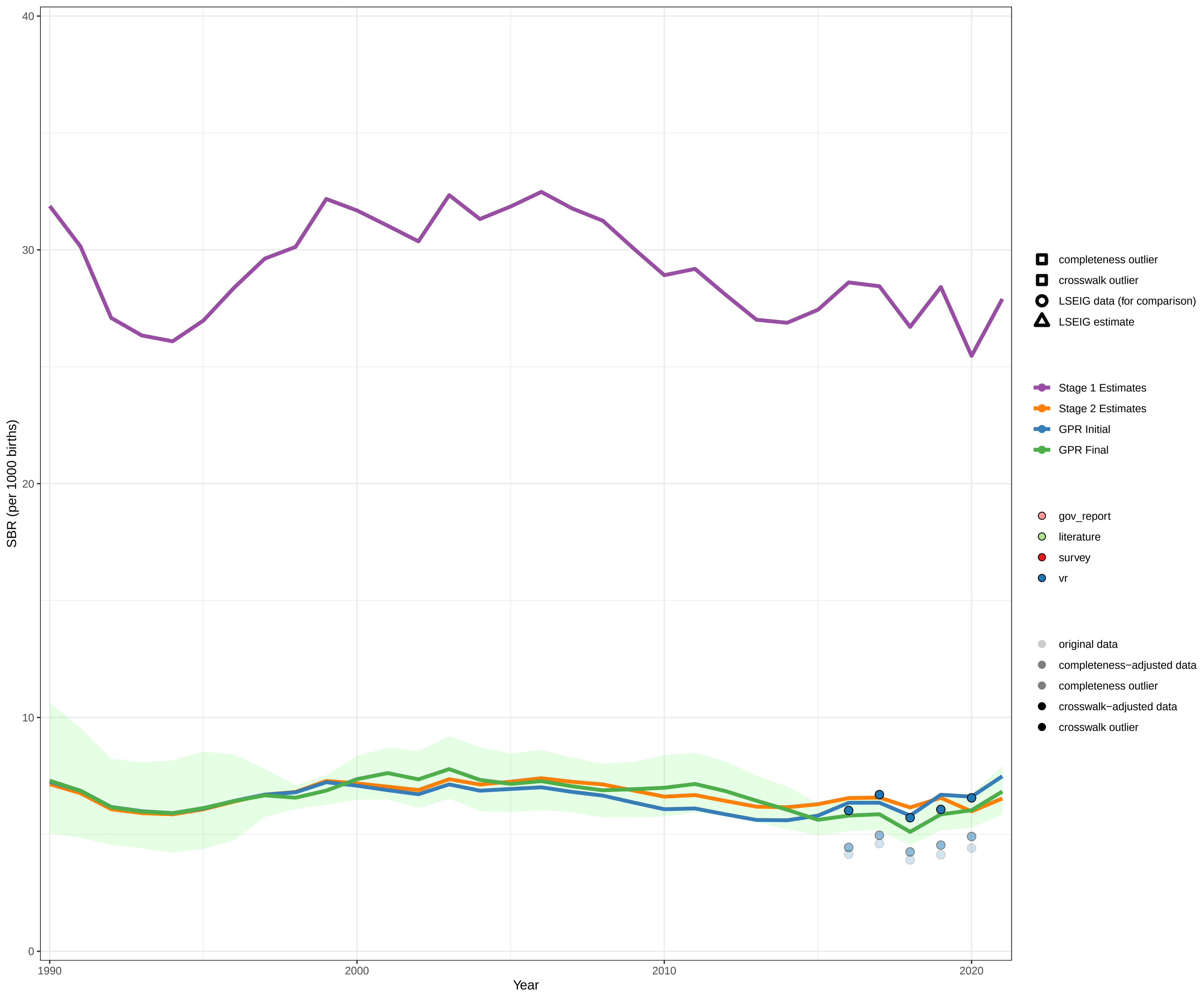

Lancashire GBR_44660 Western Europe Stillbirth Rate (SBR) − 28 weeks
lambda = 0.6 ; zeta = 0.8 ; scale = 15 ; amp = 0.028

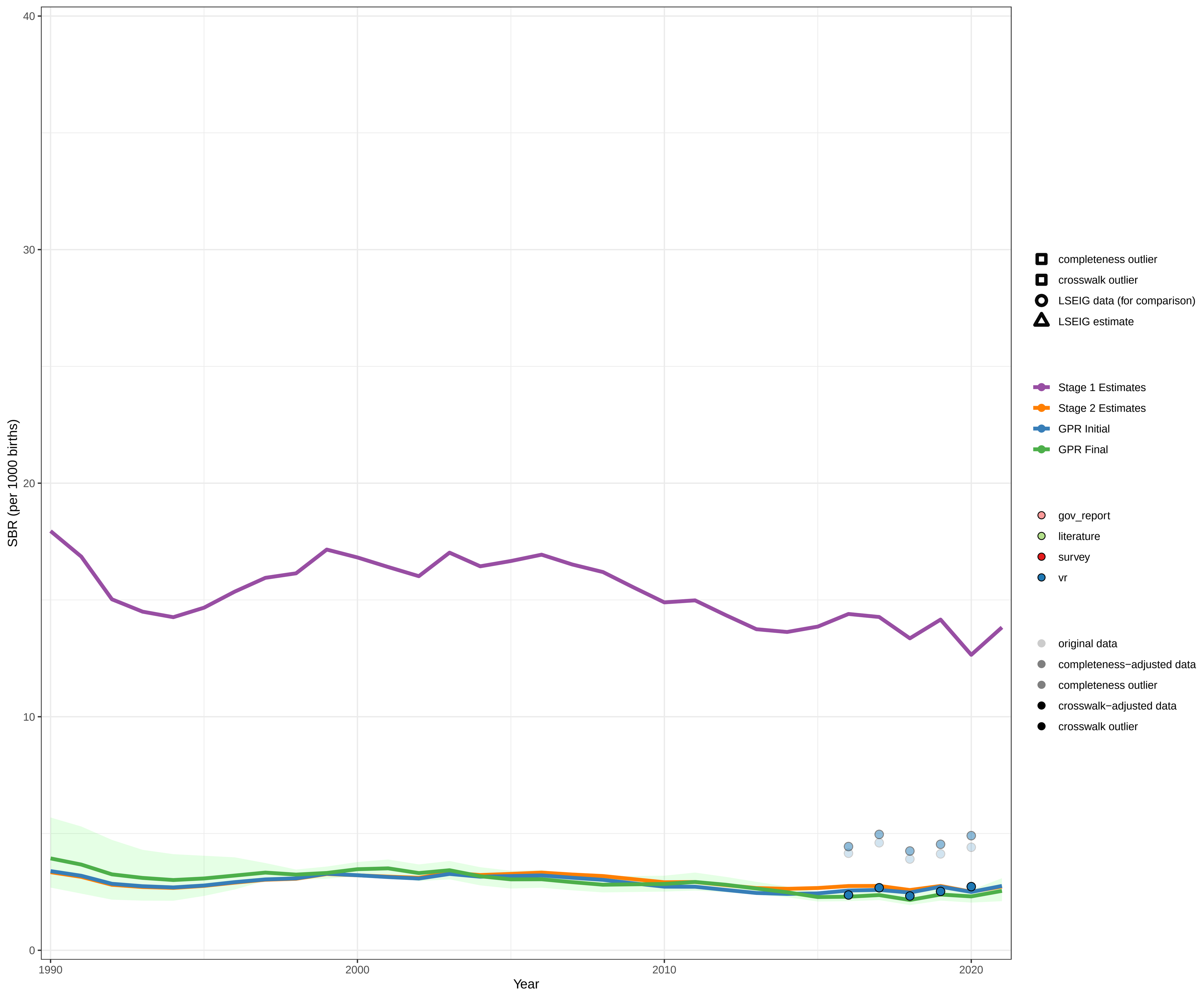

Year	Total Stillbirths (in thousands)	Total Stillbirths (in thousands)	SBR (per 1000 births)	SBR (per 1000 births)	SBR/NMR	SBR/NMR
	20 weeks	28 weeks	20 weeks	28 weeks	20 weeks	28 weeks
1990	0.1 (0.1 to 0.2)	0.1 (0.0 to 0.1)	7.3 (5.1 to 10.6)	3.9 (2.7 to 5.7)	1.40 (0.97 to 2.04)	0.76 (0.52 to 1.09)
1995	0.1 (0.1 to 0.1)	0.0 (0.0 to 0.1)	6.1 (4.4 to 8.5)	3.1 (2.3 to 4.0)	1.58 (1.13 to 2.20)	0.79 (0.60 to 1.04)
2000	0.1 (0.1 to 0.1)	0.0 (0.0 to 0.0)	7.4 (6.5 to 8.4)	3.5 (3.2 to 3.8)	1.78 (1.56 to 2.02)	0.84 (0.77 to 0.91)
2005	0.1 (0.1 to 0.1)	0.0 (0.0 to 0.0)	7.2 (6.0 to 8.5)	3.0 (2.6 to 3.4)	1.82 (1.51 to 2.15)	0.77 (0.67 to 0.86)
2010	0.1 (0.1 to 0.1)	0.0 (0.0 to 0.0)	7.0 (5.8 to 8.4)	2.8 (2.5 to 3.2)	2.06 (1.70 to 2.47)	0.84 (0.74 to 0.94)
2015	0.1 (0.1 to 0.1)	0.0 (0.0 to 0.0)	5.6 (5.0 to 6.3)	2.3 (2.1 to 2.5)	1.84 (1.63 to 2.08)	0.75 (0.69 to 0.81)
2020	0.1 (0.1 to 0.1)	0.0 (0.0 to 0.0)	6.0 (5.3 to 6.7)	2.3 (2.0 to 2.6)	2.21 (1.94 to 2.48)	0.85 (0.75 to 0.95)
2021	0.1 (0.1 to 0.1)	0.0 (0.0 to 0.0)	6.8 (5.9 to 7.9)	2.5 (2.1 to 3.1)	2.30 (1.97 to 2.67)	0.86 (0.71 to 1.04)

Liverpool GBR_44671 Western Europe Stillbirth Rate / Neonatal Mortality Rate (SBR/NMR) − 20 weeks
lambda = 0.8 ; zeta = 0.7 ; scale = 15 ; amp = 0.028

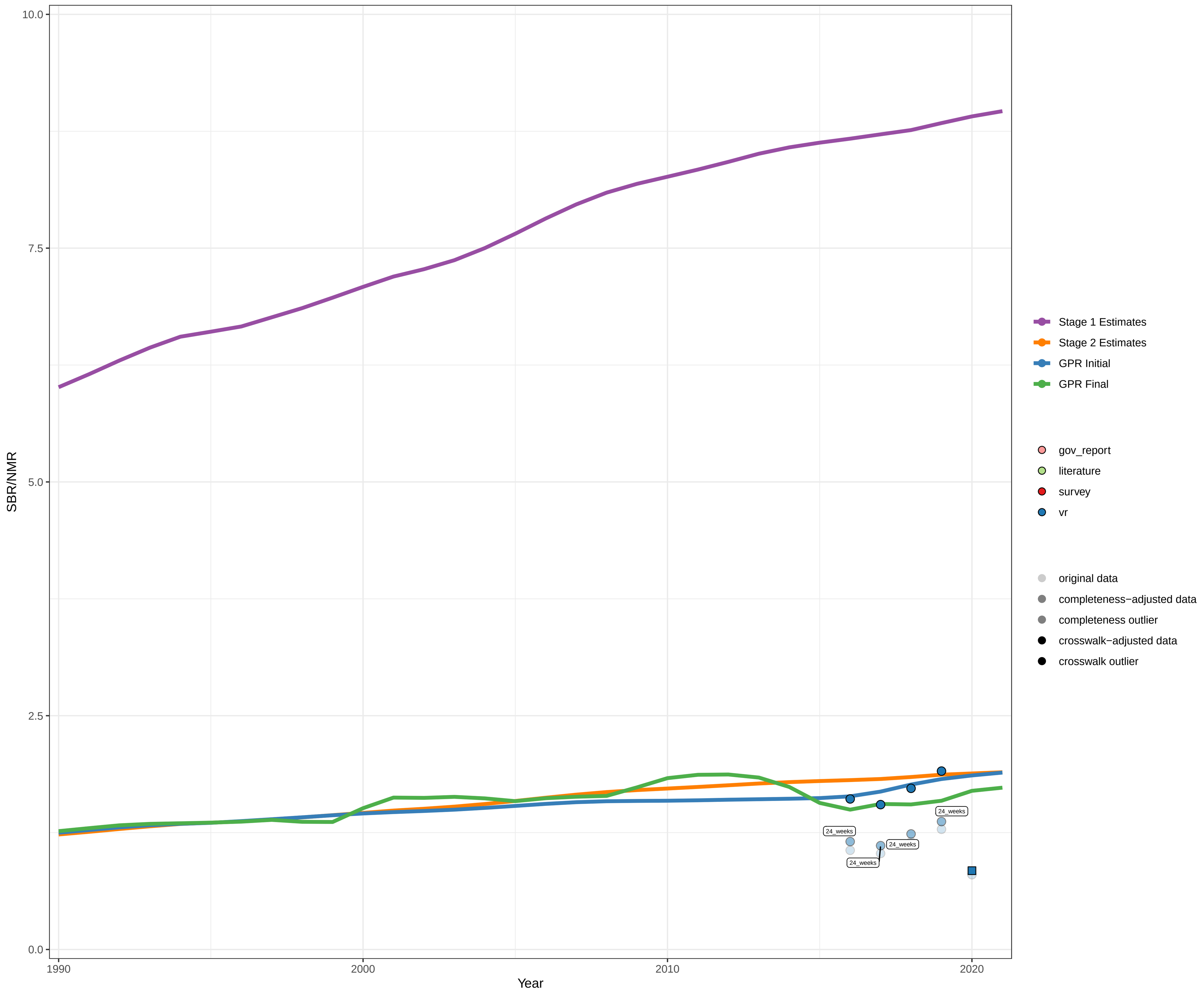

Liverpool GBR_44671 Western Europe Stillbirth Rate / Neonatal Mortality Rate (SBR/NMR) − 28 weeks
lambda = 0.8 ; zeta = 0.7 ; scale = 15 ; amp = 0.028

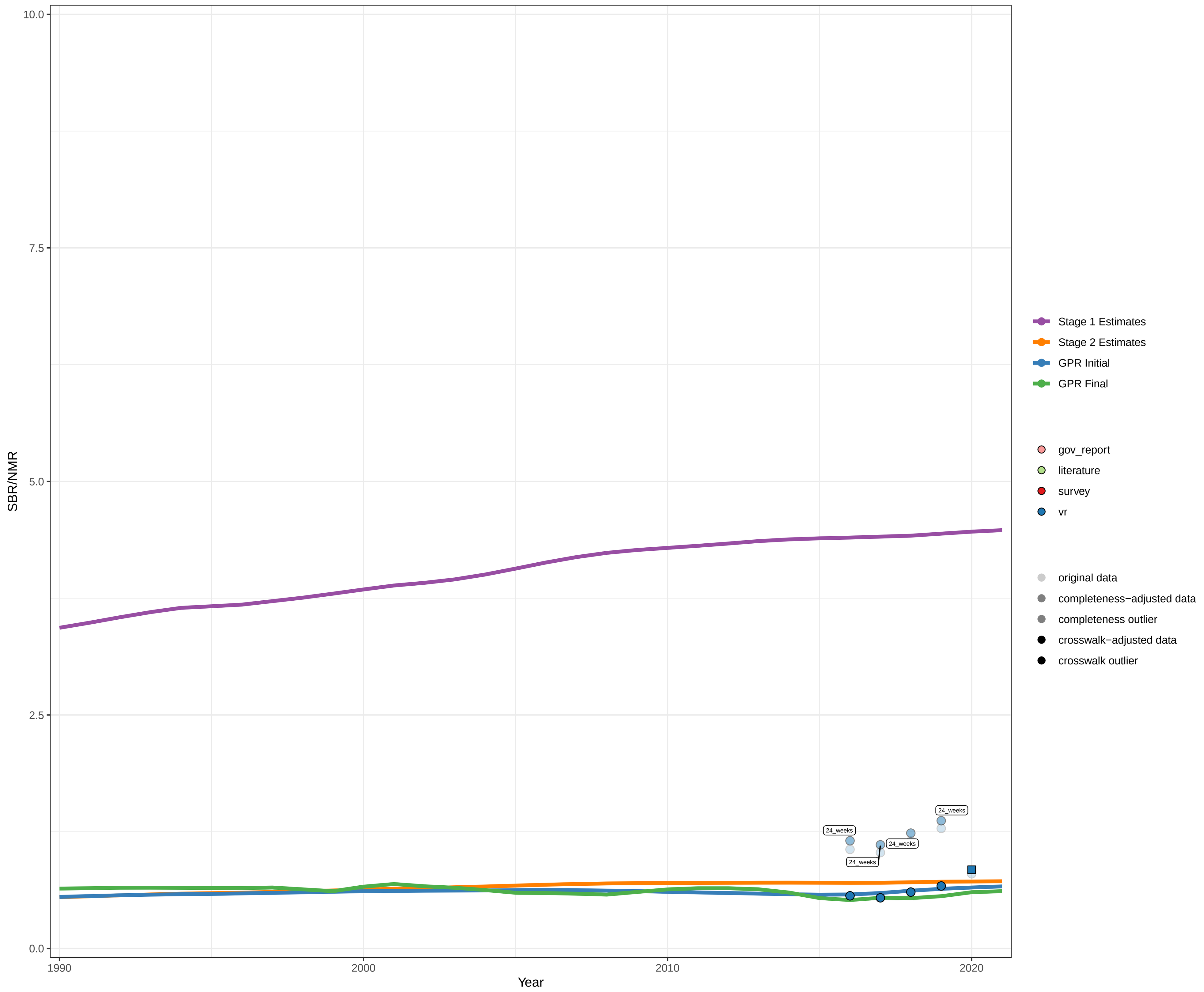

Definition	Years	Outlier	Source.Type	Title
24_weeks	2016−2020	0	VR	United Kingdom − England and Wales Child Mortality Statistics

Liverpool GBR_44671 Western Europe Stillbirth Rate (SBR) − 20 weeks
lambda = 0.8 ; zeta = 0.7 ; scale = 15 ; amp = 0.028

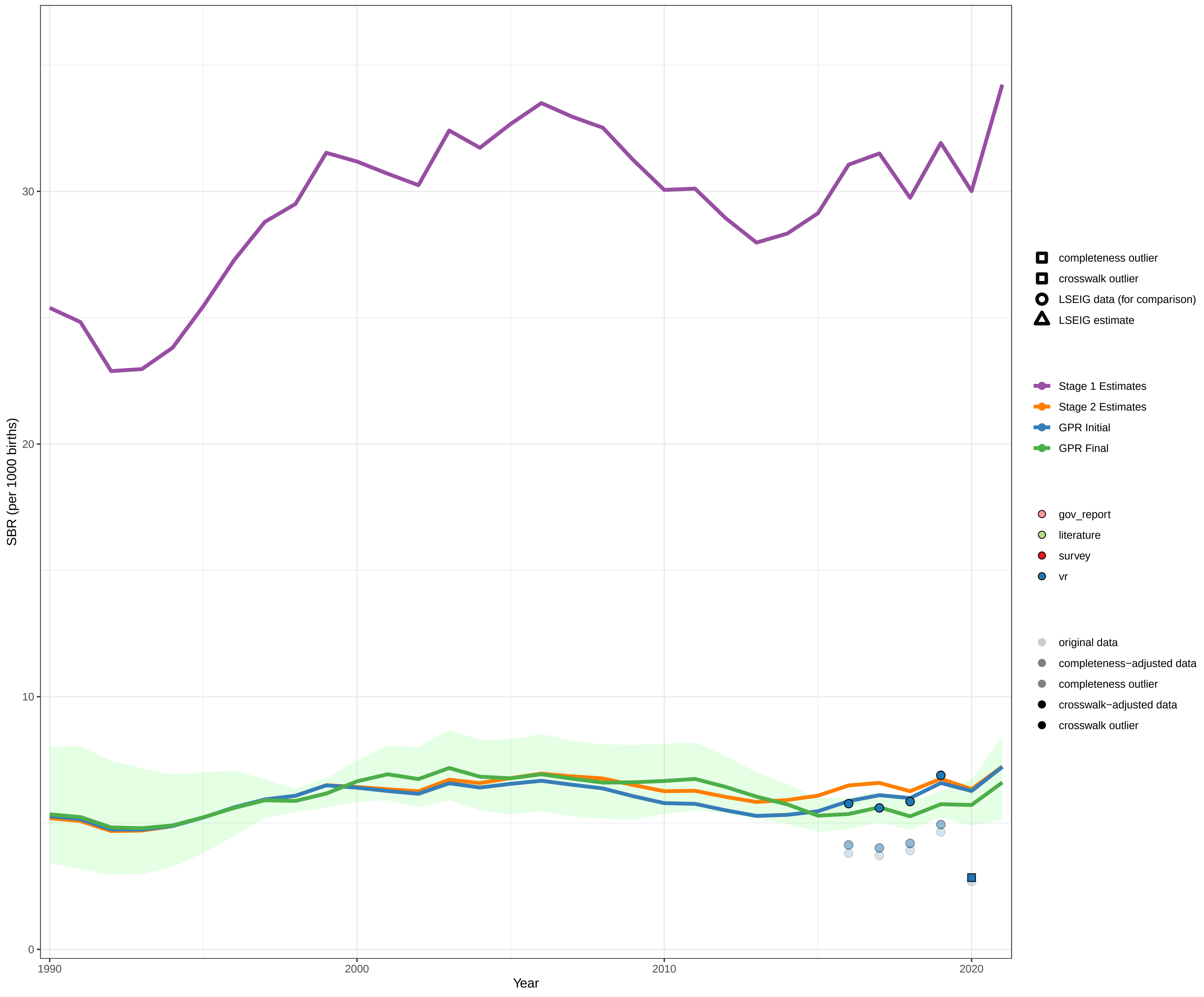

Liverpool GBR_44671 Western Europe Stillbirth Rate (SBR) − 28 weeks
lambda = 0.8 ; zeta = 0.7 ; scale = 15 ; amp = 0.028

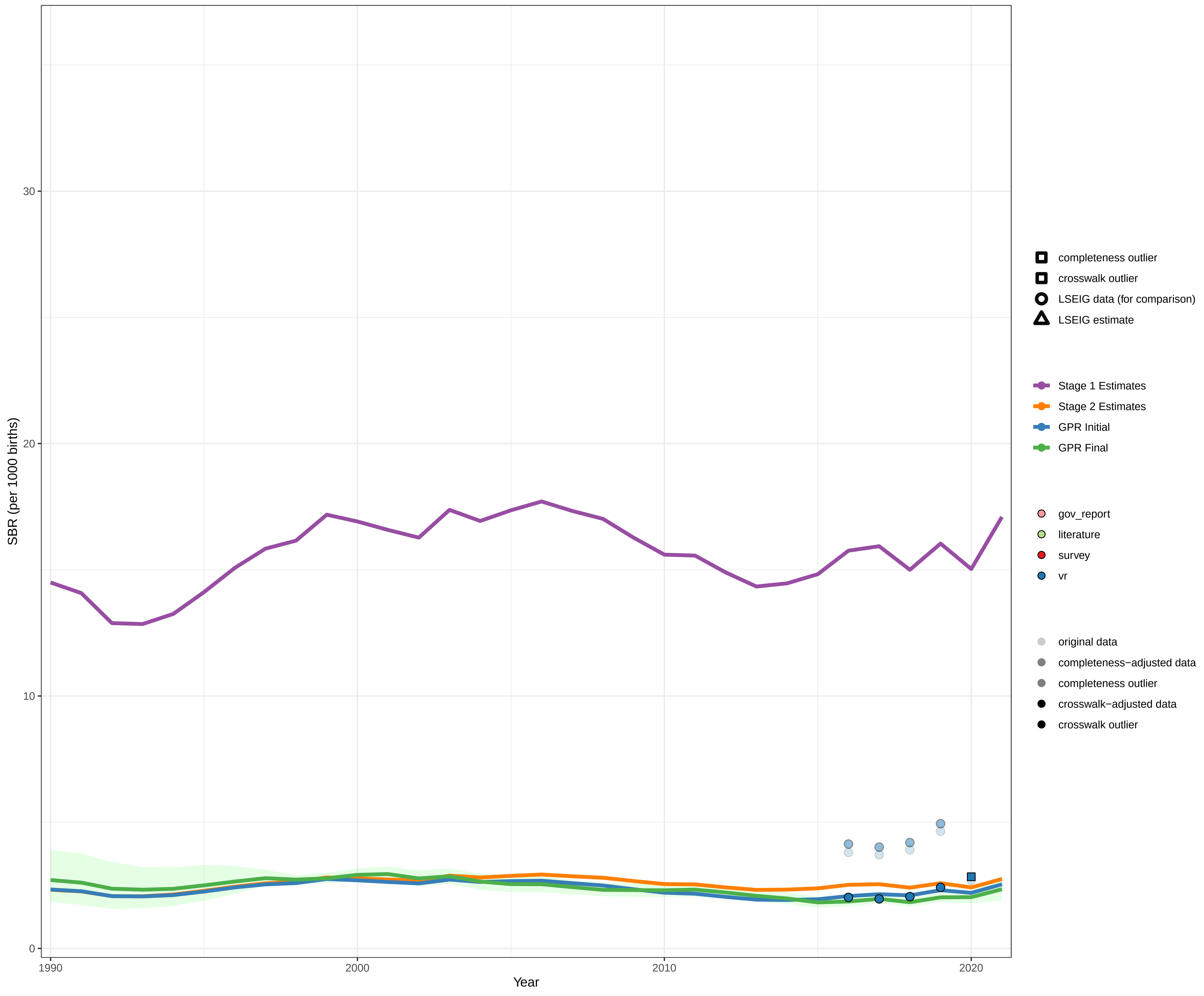

Year	Total Stillbirths (in thousands)	Total Stillbirths (in thousands)	SBR (per 1000 births)	SBR (per 1000 births)	SBR/NMR	SBR/NMR
	20 weeks	28 weeks	20 weeks	28 weeks	20 weeks	28 weeks
1990	0.0 (0.0 to 0.1)	0.0 (0.0 to 0.0)	5.3 (3.4 to 8.0)	2.7 (1.9 to 3.9)	1.27 (0.81 to 1.90)	0.64 (0.44 to 0.93)
1995	0.0 (0.0 to 0.0)	0.0 (0.0 to 0.0)	5.2 (3.8 to 7.0)	2.5 (1.9 to 3.3)	1.36 (0.99 to 1.82)	0.65 (0.49 to 0.86)
2000	0.0 (0.0 to 0.0)	0.0 (0.0 to 0.0)	6.6 (5.8 to 7.5)	2.9 (2.7 to 3.2)	1.51 (1.33 to 1.70)	0.66 (0.61 to 0.72)
2005	0.0 (0.0 to 0.0)	0.0 (0.0 to 0.0)	6.8 (5.3 to 8.3)	2.5 (2.2 to 2.9)	1.59 (1.25 to 1.95)	0.60 (0.52 to 0.68)
2010	0.0 (0.0 to 0.0)	0.0 (0.0 to 0.0)	6.7 (5.4 to 8.1)	2.3 (2.0 to 2.6)	1.83 (1.47 to 2.24)	0.63 (0.56 to 0.71)
2015	0.0 (0.0 to 0.0)	0.0 (0.0 to 0.0)	5.3 (4.7 to 6.0)	1.8 (1.6 to 2.0)	1.57 (1.38 to 1.78)	0.54 (0.48 to 0.61)
2020	0.0 (0.0 to 0.0)	0.0 (0.0 to 0.0)	5.7 (4.9 to 6.7)	2.0 (1.8 to 2.3)	1.70 (1.45 to 1.99)	0.60 (0.53 to 0.68)
2021	0.0 (0.0 to 0.0)	0.0 (0.0 to 0.0)	6.6 (5.2 to 8.4)	2.3 (1.9 to 2.9)	1.73 (1.35 to 2.21)	0.61 (0.50 to 0.76)

Manchester GBR_44677 Western Europe Stillbirth Rate / Neonatal Mortality Rate (SBR/NMR) − 20 weeks
lambda = 0.6 ; zeta = 0.8 ; scale = 15 ; amp = 0.028

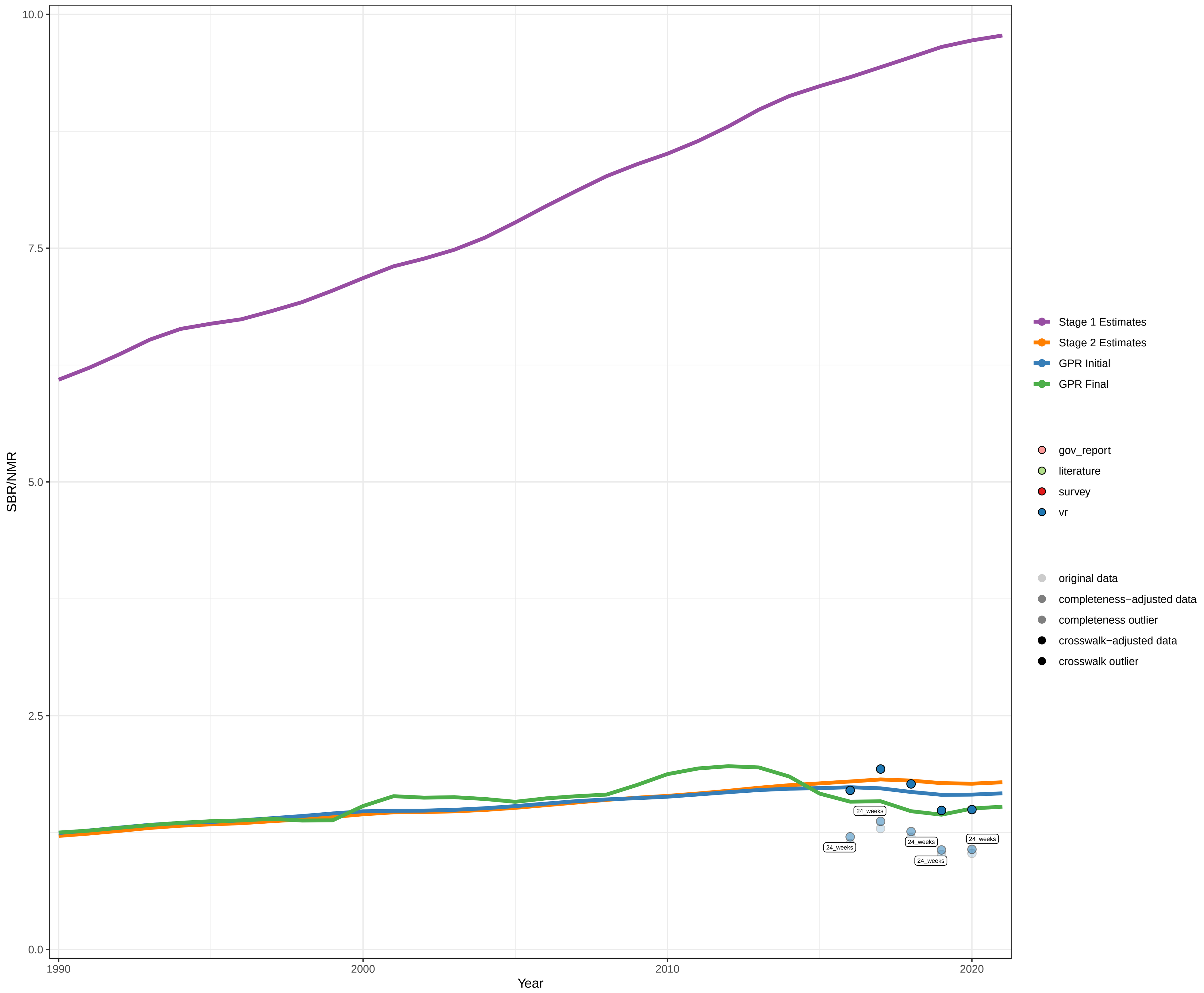

Manchester GBR_44677 Western Europe Stillbirth Rate / Neonatal Mortality Rate (SBR/NMR) − 28 weeks
lambda = 0.6 ; zeta = 0.8 ; scale = 15 ; amp = 0.028

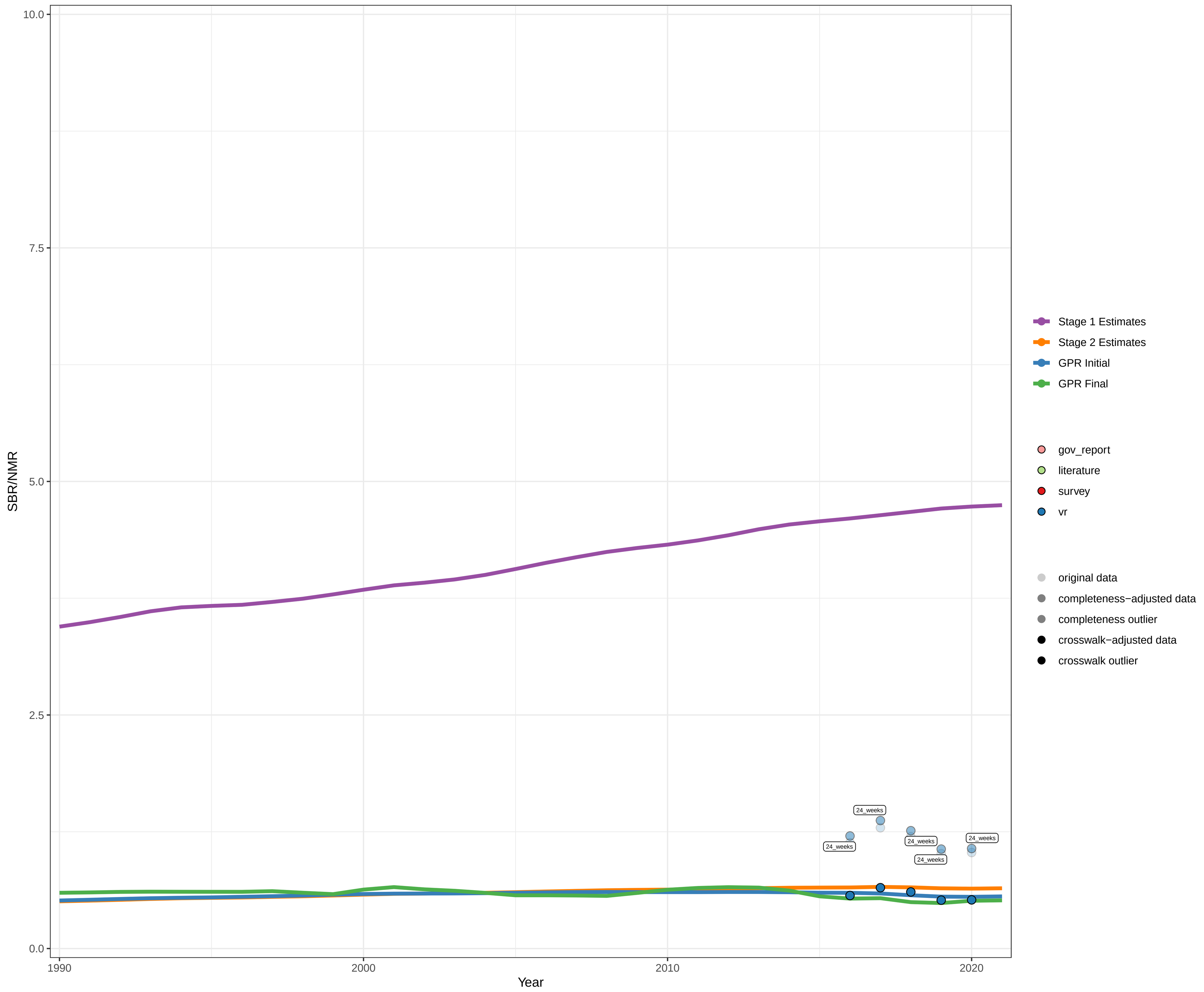

Definition	Years	Outlier	Source.Type	Title
24_weeks	2016−2020	0	VR	United Kingdom − England and Wales Child Mortality Statistics

Manchester GBR_44677 Western Europe Stillbirth Rate (SBR) − 20 weeks
lambda = 0.6 ; zeta = 0.8 ; scale = 15 ; amp = 0.028

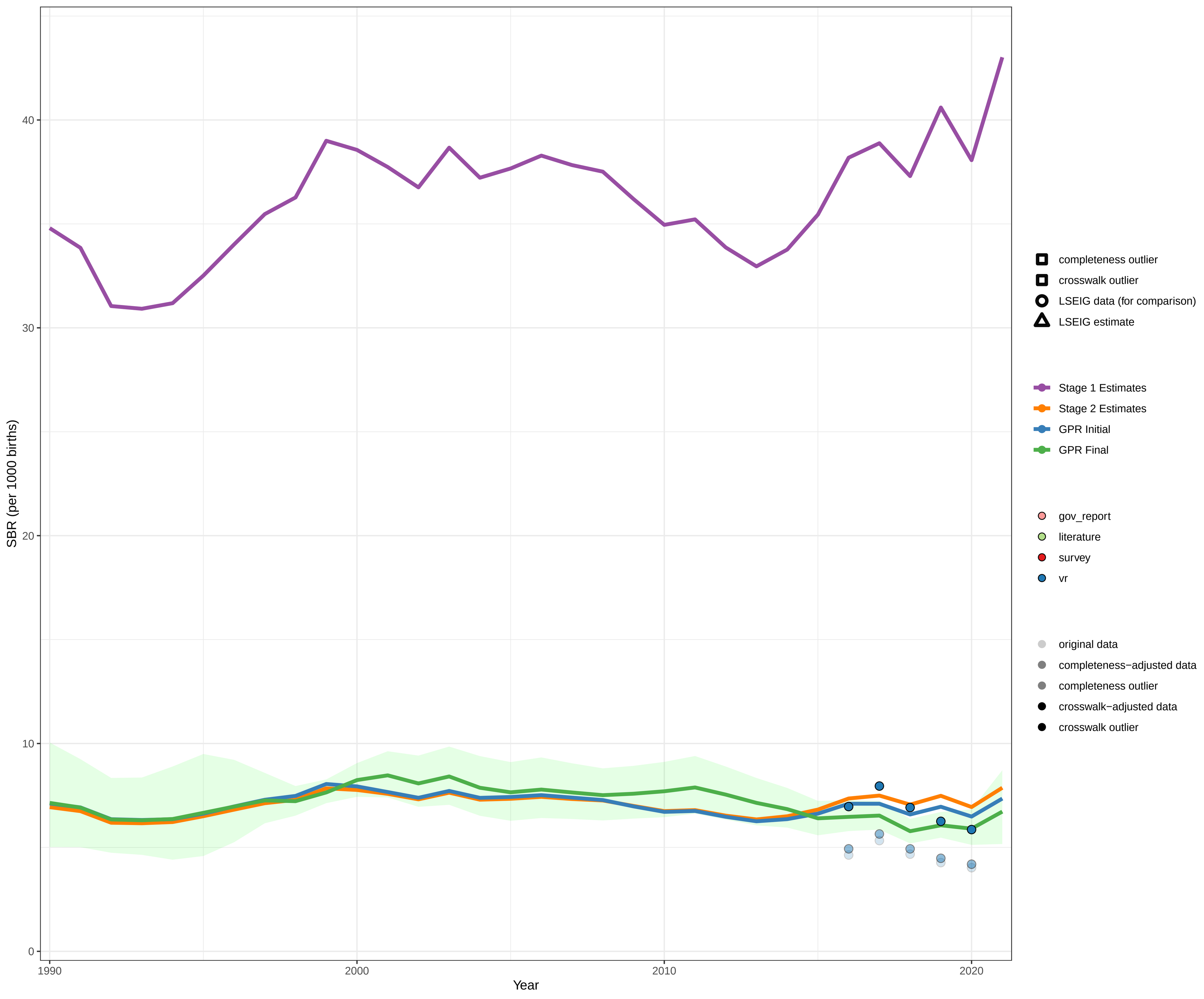

Manchester GBR_44677 Western Europe Stillbirth Rate (SBR) − 28 weeks
lambda = 0.6 ; zeta = 0.8 ; scale = 15 ; amp = 0.028

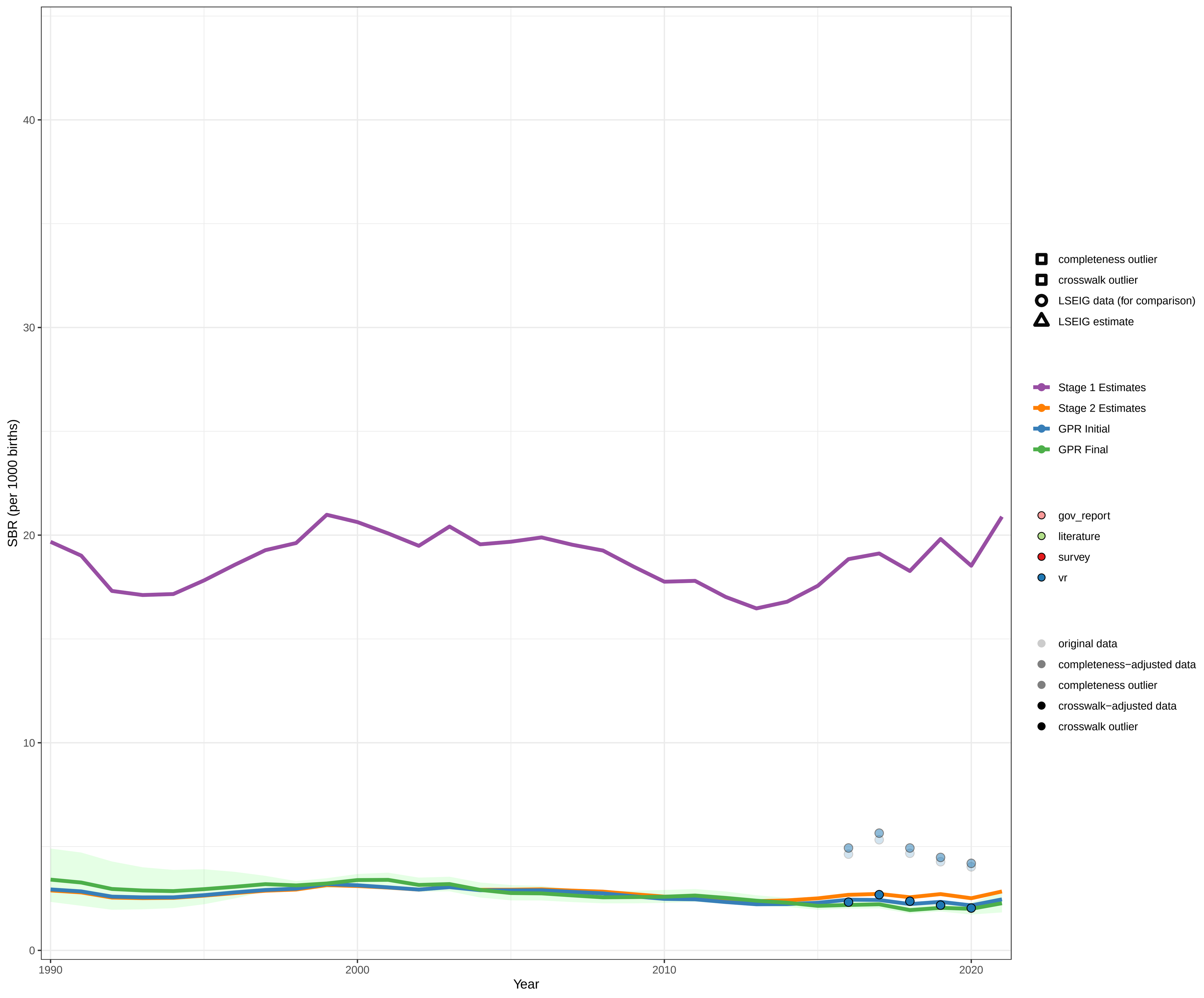

Year	Total Stillbirths (in thousands)	Total Stillbirths (in thousands)	SBR (per 1000 births)	SBR (per 1000 births)	SBR/NMR	SBR/NMR
	20 weeks	28 weeks	20 weeks	28 weeks	20 weeks	28 weeks
1990	0.1 (0.0 to 0.1)	0.0 (0.0 to 0.0)	7.1 (5.0 to 10.0)	3.4 (2.3 to 4.9)	1.25 (0.88 to 1.76)	0.60 (0.41 to 0.86)
1995	0.0 (0.0 to 0.1)	0.0 (0.0 to 0.0)	6.7 (4.6 to 9.5)	2.9 (2.2 to 3.9)	1.37 (0.94 to 1.95)	0.61 (0.46 to 0.80)
2000	0.0 (0.0 to 0.1)	0.0 (0.0 to 0.0)	8.2 (7.4 to 9.1)	3.4 (3.1 to 3.7)	1.53 (1.38 to 1.69)	0.63 (0.58 to 0.69)
2005	0.1 (0.0 to 0.1)	0.0 (0.0 to 0.0)	7.7 (6.3 to 9.1)	2.8 (2.4 to 3.1)	1.58 (1.30 to 1.88)	0.57 (0.50 to 0.65)
2010	0.1 (0.0 to 0.1)	0.0 (0.0 to 0.0)	7.7 (6.5 to 9.1)	2.6 (2.3 to 2.9)	1.88 (1.57 to 2.22)	0.63 (0.56 to 0.71)
2015	0.0 (0.0 to 0.1)	0.0 (0.0 to 0.0)	6.4 (5.6 to 7.2)	2.1 (2.0 to 2.3)	1.67 (1.45 to 1.89)	0.56 (0.51 to 0.61)
2020	0.0 (0.0 to 0.0)	0.0 (0.0 to 0.0)	5.9 (5.1 to 6.8)	2.0 (1.7 to 2.3)	1.51 (1.31 to 1.75)	0.51 (0.44 to 0.59)
2021	0.0 (0.0 to 0.1)	0.0 (0.0 to 0.0)	6.7 (5.2 to 8.7)	2.3 (1.8 to 2.8)	1.53 (1.17 to 1.98)	0.52 (0.42 to 0.64)

Oldham GBR_44667 Western Europe Stillbirth Rate / Neonatal Mortality Rate (SBR/NMR) − 20 weeks
lambda = 0.6 ; zeta = 0.8 ; scale = 15 ; amp = 0.028

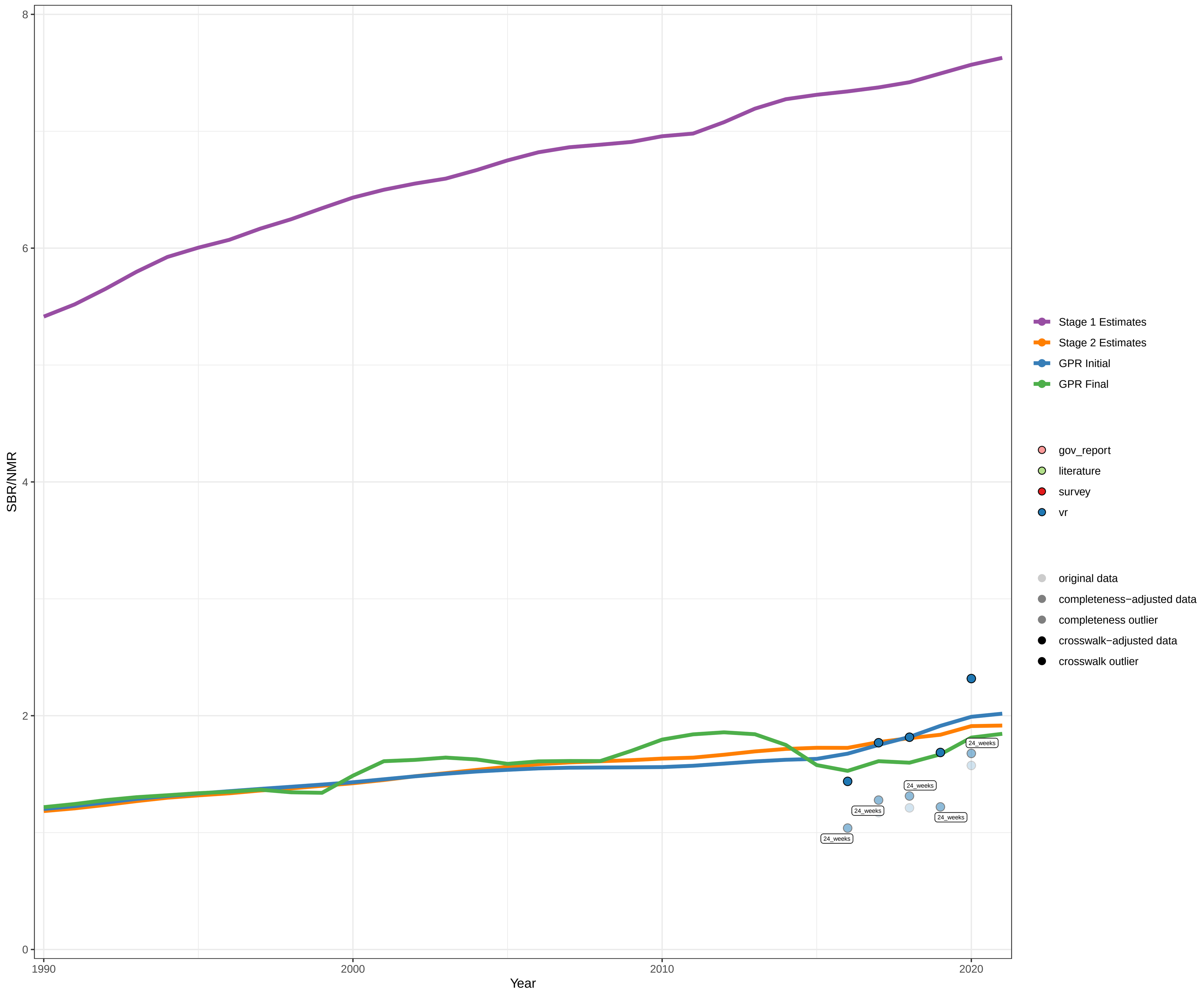

Oldham GBR_44667 Western Europe Stillbirth Rate / Neonatal Mortality Rate (SBR/NMR) − 28 weeks
lambda = 0.6 ; zeta = 0.8 ; scale = 15 ; amp = 0.028

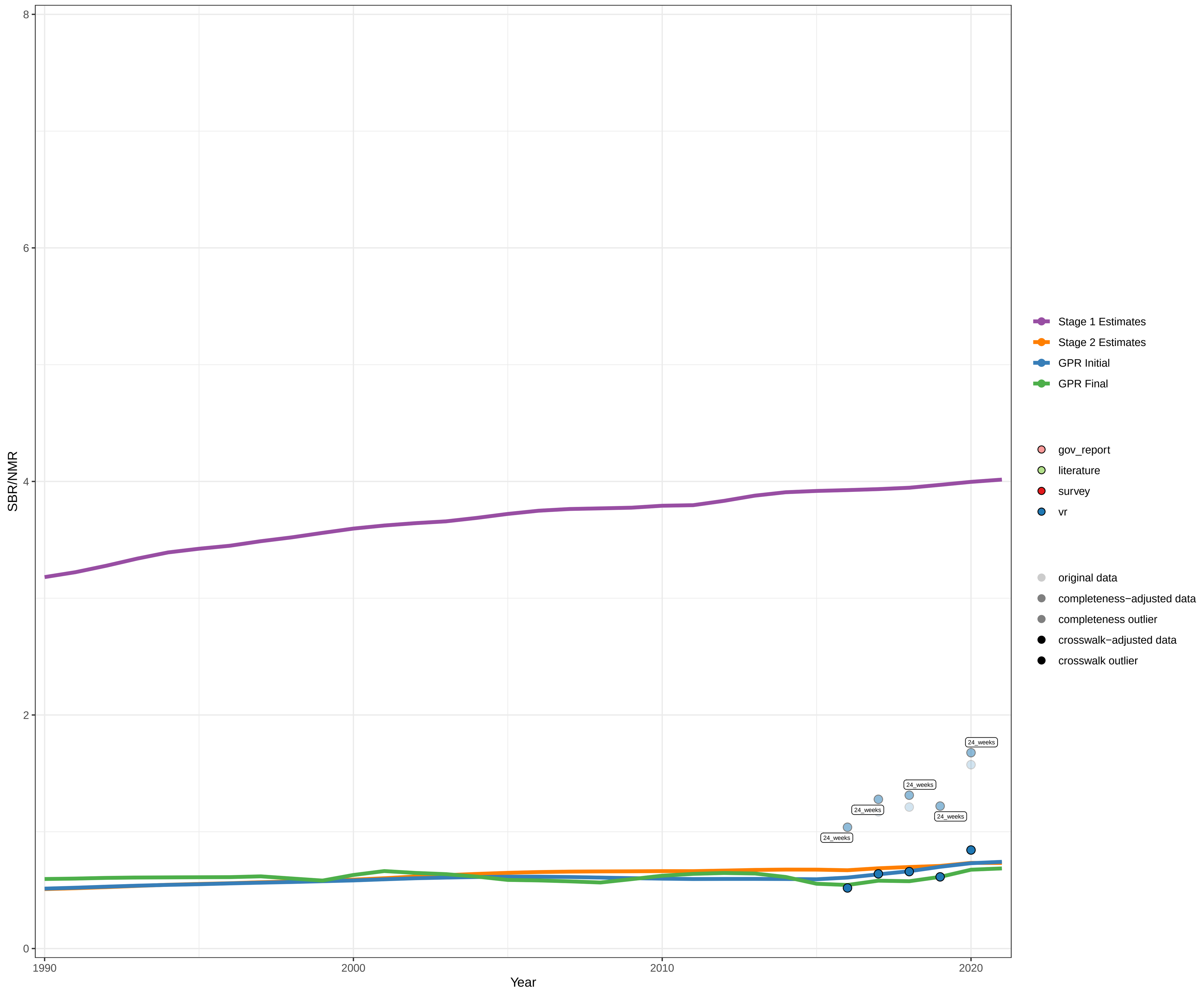

Definition	Years	Outlier	Source.Type	Title
24_weeks	2016−2020	0	VR	United Kingdom − England and Wales Child Mortality Statistics

Oldham GBR_44667 Western Europe Stillbirth Rate (SBR) − 20 weeks
lambda = 0.6 ; zeta = 0.8 ; scale = 15 ; amp = 0.028

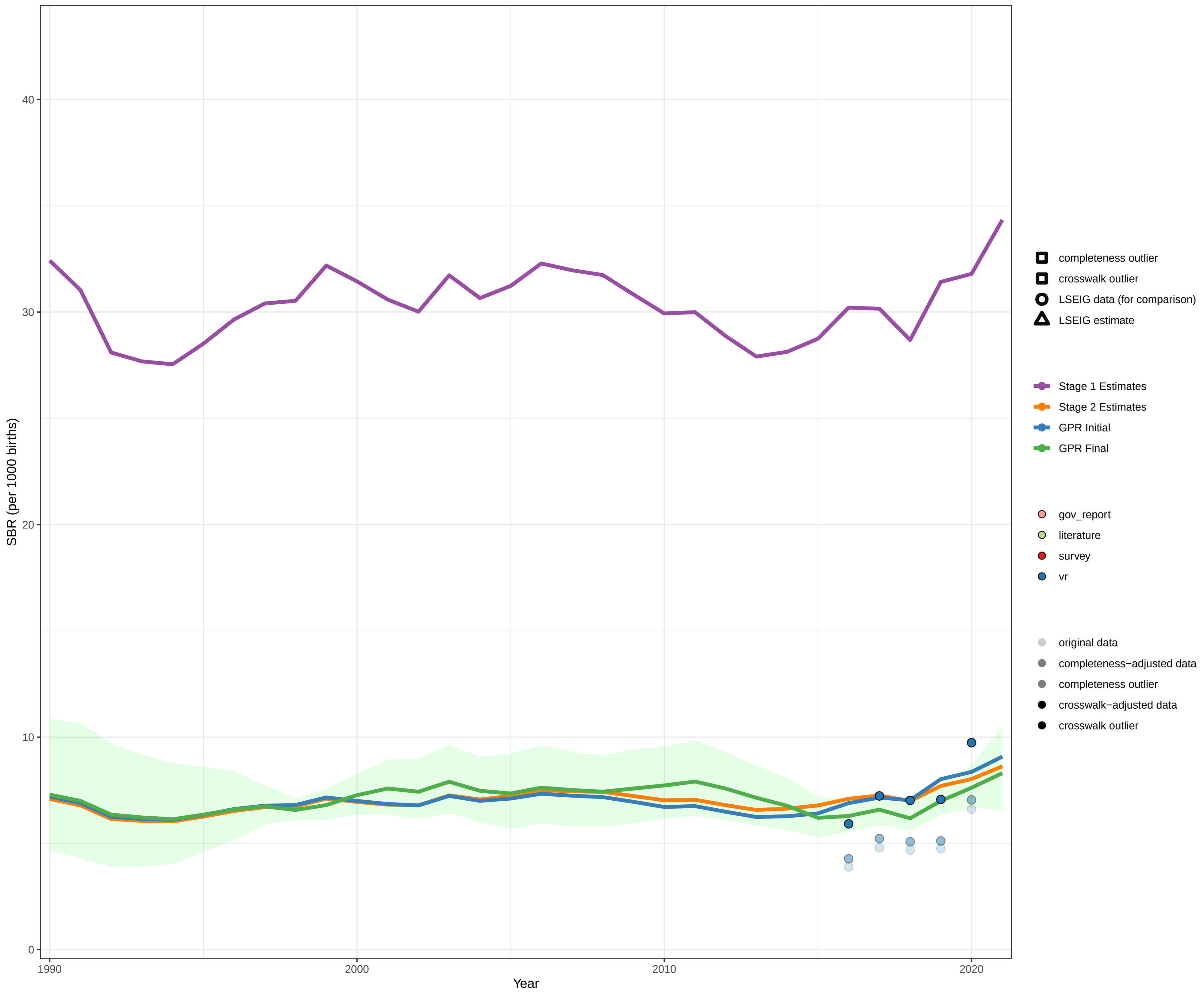

Oldham GBR_44667 Western Europe Stillbirth Rate (SBR) − 28 weeks
lambda = 0.6 ; zeta = 0.8 ; scale = 15 ; amp = 0.028

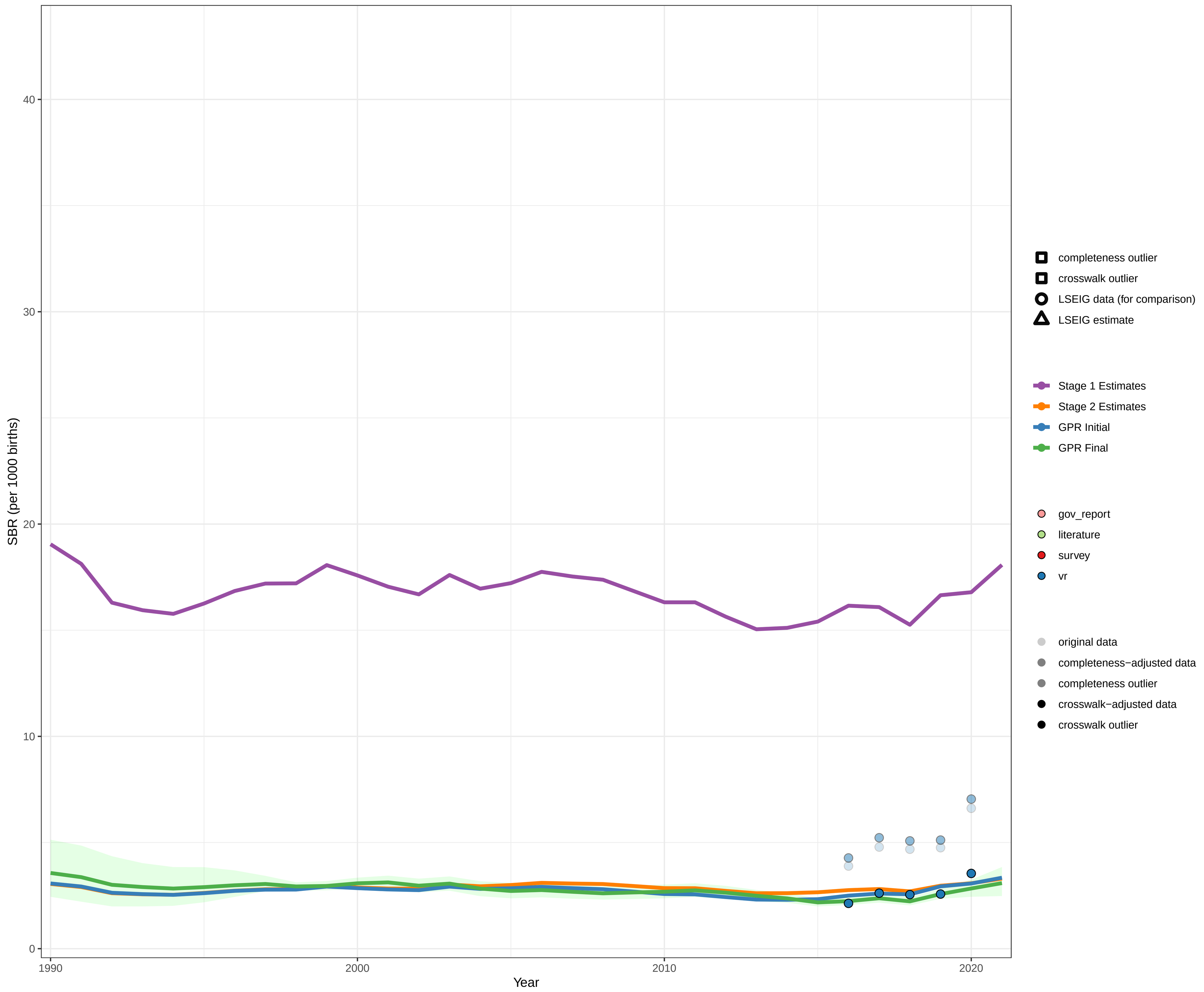

Year	Total Stillbirths (in thousands)	Total Stillbirths (in thousands)	SBR (per 1000 births)	SBR (per 1000 births)	SBR/NMR	SBR/NMR
	20 weeks	28 weeks	20 weeks	28 weeks	20 weeks	28 weeks
1990	0.0 (0.0 to 0.0)	0.0 (0.0 to 0.0)	7.3 (4.7 to 10.9)	3.6 (2.4 to 5.1)	1.22 (0.78 to 1.81)	0.60 (0.41 to 0.86)
1995	0.0 (0.0 to 0.0)	0.0 (0.0 to 0.0)	6.3 (4.6 to 8.6)	2.9 (2.2 to 3.8)	1.34 (0.96 to 1.81)	0.61 (0.46 to 0.81)
2000	0.0 (0.0 to 0.0)	0.0 (0.0 to 0.0)	7.3 (6.4 to 8.3)	3.1 (2.8 to 3.3)	1.49 (1.30 to 1.69)	0.63 (0.58 to 0.68)
2005	0.0 (0.0 to 0.0)	0.0 (0.0 to 0.0)	7.3 (5.7 to 9.2)	2.7 (2.4 to 3.1)	1.59 (1.23 to 1.99)	0.59 (0.51 to 0.67)
2010	0.0 (0.0 to 0.0)	0.0 (0.0 to 0.0)	7.7 (6.2 to 9.5)	2.7 (2.4 to 3.0)	1.80 (1.43 to 2.22)	0.62 (0.55 to 0.70)
2015	0.0 (0.0 to 0.0)	0.0 (0.0 to 0.0)	6.2 (5.3 to 7.2)	2.2 (2.0 to 2.4)	1.58 (1.35 to 1.83)	0.55 (0.51 to 0.60)
2020	0.0 (0.0 to 0.0)	0.0 (0.0 to 0.0)	7.6 (6.7 to 8.7)	2.8 (2.4 to 3.3)	1.81 (1.59 to 2.06)	0.68 (0.58 to 0.77)
2021	0.0 (0.0 to 0.0)	0.0 (0.0 to 0.0)	8.3 (6.5 to 10.5)	3.1 (2.5 to 3.8)	1.85 (1.45 to 2.34)	0.69 (0.55 to 0.85)

Rochdale GBR_44668 Western Europe Stillbirth Rate / Neonatal Mortality Rate (SBR/NMR) − 20 weeks
lambda = 0.6 ; zeta = 0.8 ; scale = 15 ; amp = 0.028

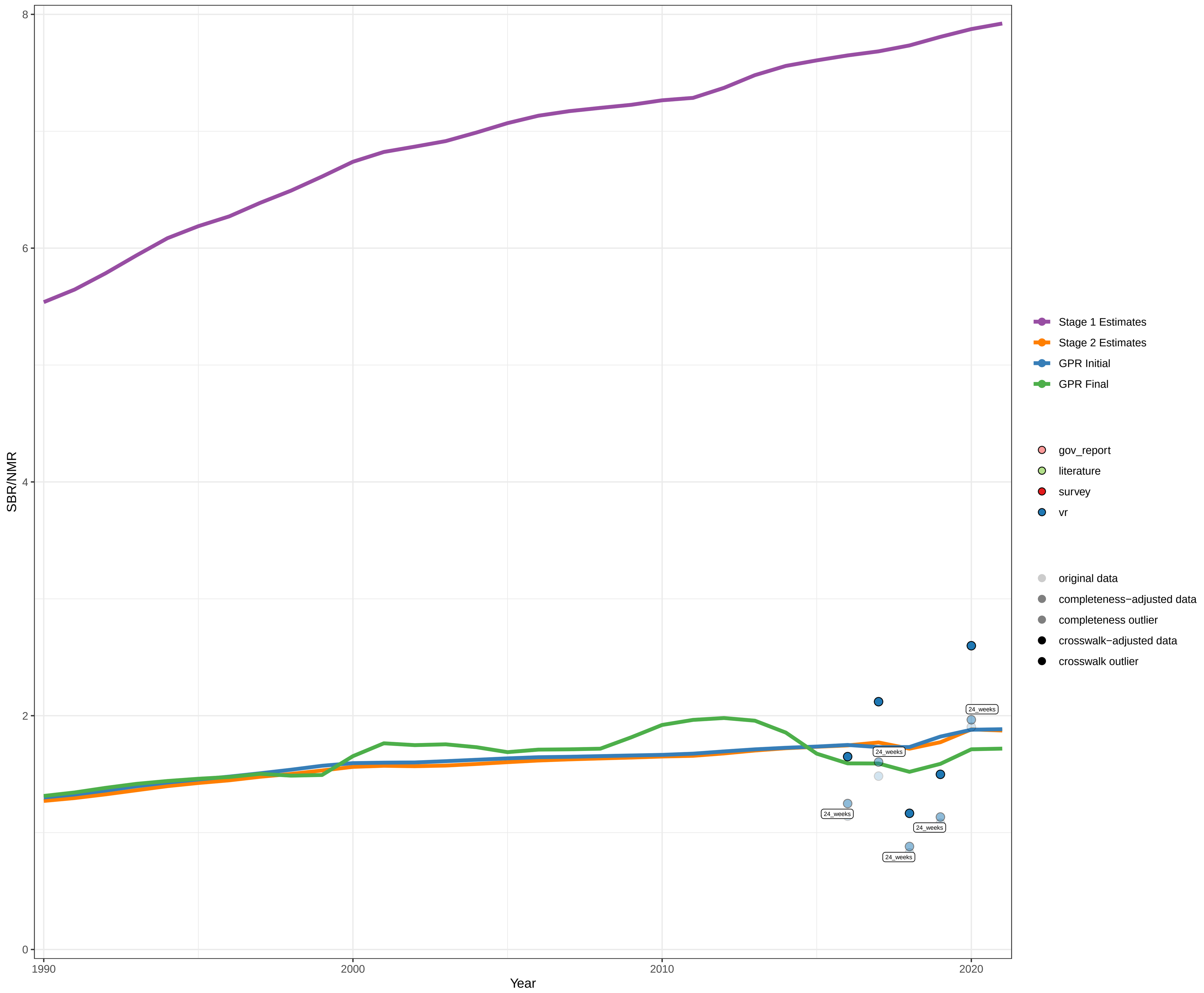

Rochdale GBR_44668 Western Europe Stillbirth Rate / Neonatal Mortality Rate (SBR/NMR) − 28 weeks
lambda = 0.6 ; zeta = 0.8 ; scale = 15 ; amp = 0.028

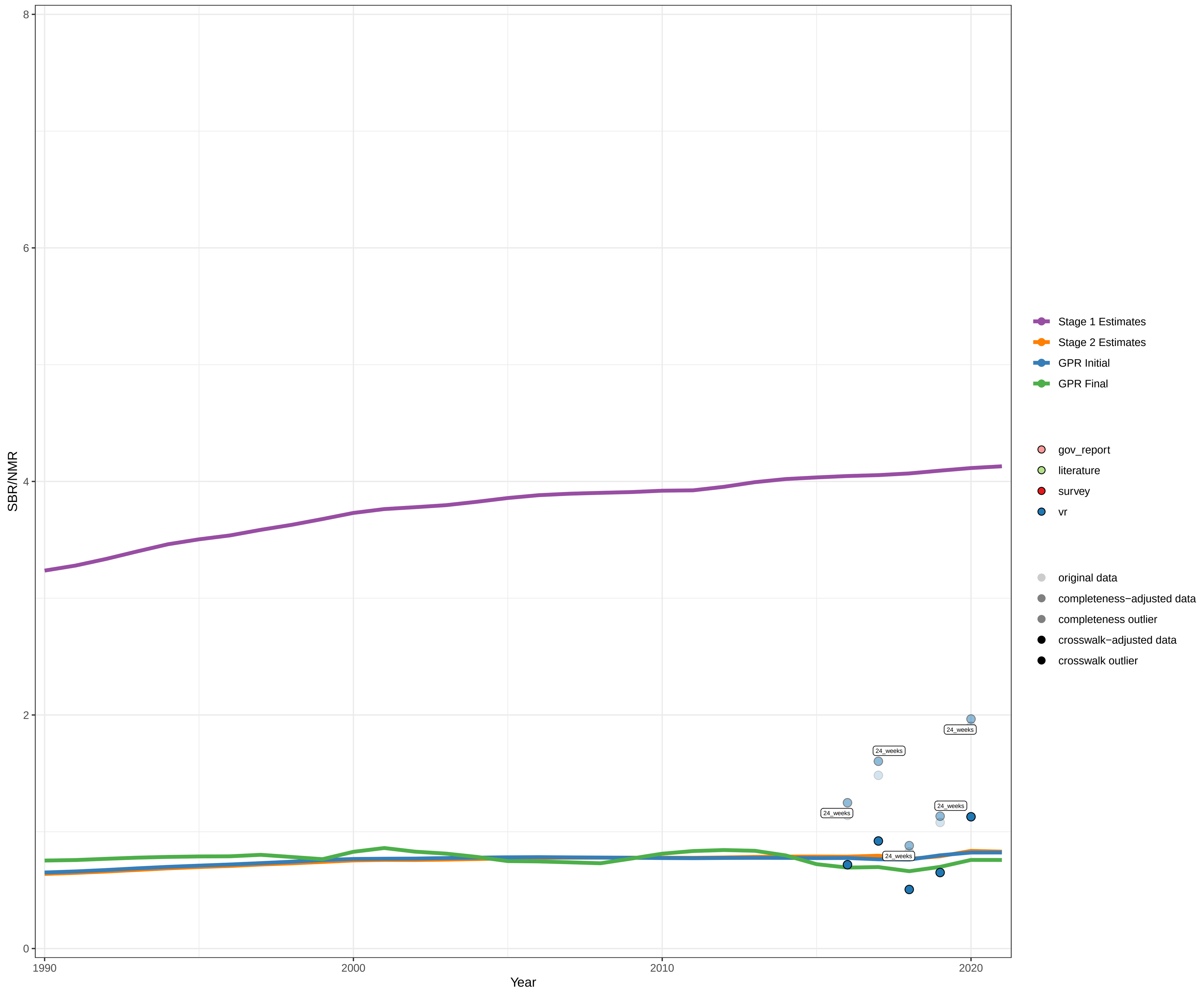

Definition	Years	Outlier	Source.Type	Title
24_weeks	2016−2020	0	VR	United Kingdom − England and Wales Child Mortality Statistics

Rochdale GBR_44668 Western Europe Stillbirth Rate (SBR) − 20 weeks
lambda = 0.6 ; zeta = 0.8 ; scale = 15 ; amp = 0.028

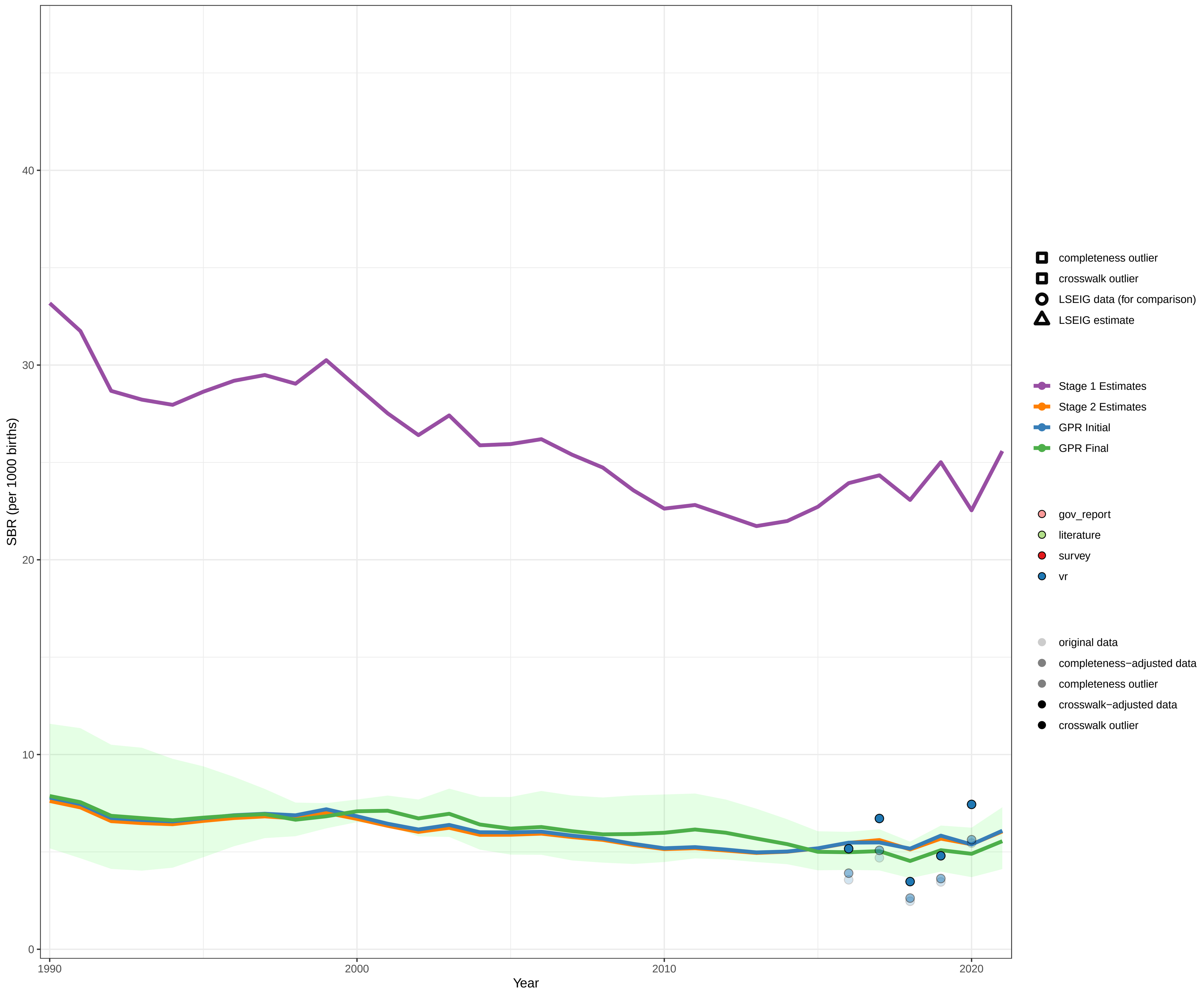

Rochdale GBR_44668 Western Europe Stillbirth Rate (SBR) − 28 weeks
lambda = 0.6 ; zeta = 0.8 ; scale = 15 ; amp = 0.028

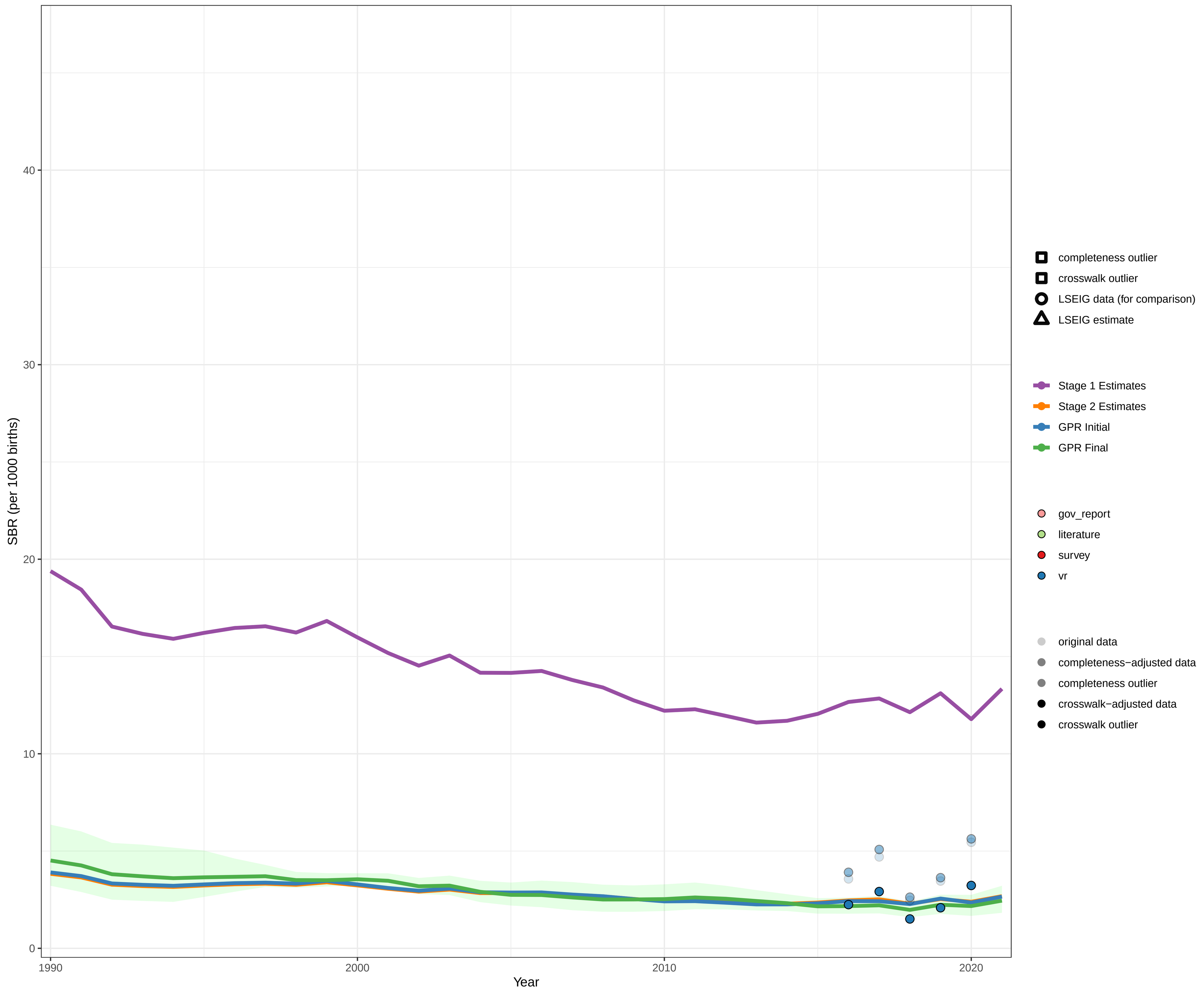

Year	Total Stillbirths (in thousands)	Total Stillbirths (in thousands)	SBR (per 1000 births)	SBR (per 1000 births)	SBR/NMR	SBR/NMR
	20 weeks	28 weeks	20 weeks	28 weeks	20 weeks	28 weeks
1990	0.0 (0.0 to 0.0)	0.0 (0.0 to 0.0)	7.9 (5.2 to 11.6)	4.5 (3.2 to 6.4)	1.31 (0.86 to 1.93)	0.75 (0.54 to 1.06)
1995	0.0 (0.0 to 0.0)	0.0 (0.0 to 0.0)	6.8 (4.7 to 9.4)	3.6 (2.6 to 5.0)	1.46 (1.02 to 2.03)	0.79 (0.57 to 1.09)
2000	0.0 (0.0 to 0.0)	0.0 (0.0 to 0.0)	7.1 (6.5 to 7.7)	3.5 (3.2 to 3.9)	1.65 (1.52 to 1.79)	0.83 (0.76 to 0.90)
2005	0.0 (0.0 to 0.0)	0.0 (0.0 to 0.0)	6.2 (4.9 to 7.8)	2.7 (2.2 to 3.4)	1.69 (1.32 to 2.13)	0.75 (0.60 to 0.92)
2010	0.0 (0.0 to 0.0)	0.0 (0.0 to 0.0)	6.0 (4.5 to 7.9)	2.5 (1.9 to 3.3)	1.92 (1.44 to 2.55)	0.81 (0.62 to 1.06)
2015	0.0 (0.0 to 0.0)	0.0 (0.0 to 0.0)	5.0 (4.1 to 6.1)	2.2 (1.8 to 2.6)	1.68 (1.36 to 2.03)	0.72 (0.60 to 0.86)
2020	0.0 (0.0 to 0.0)	0.0 (0.0 to 0.0)	4.9 (3.7 to 6.2)	2.2 (1.7 to 2.8)	1.71 (1.29 to 2.18)	0.76 (0.58 to 0.96)
2021	0.0 (0.0 to 0.0)	0.0 (0.0 to 0.0)	5.5 (4.1 to 7.3)	2.4 (1.8 to 3.2)	1.72 (1.27 to 2.26)	0.76 (0.57 to 1.00)

Salford GBR_44673 Western Europe Stillbirth Rate / Neonatal Mortality Rate (SBR/NMR) − 20 weeks
lambda = 0.8 ; zeta = 0.7 ; scale = 15 ; amp = 0.028

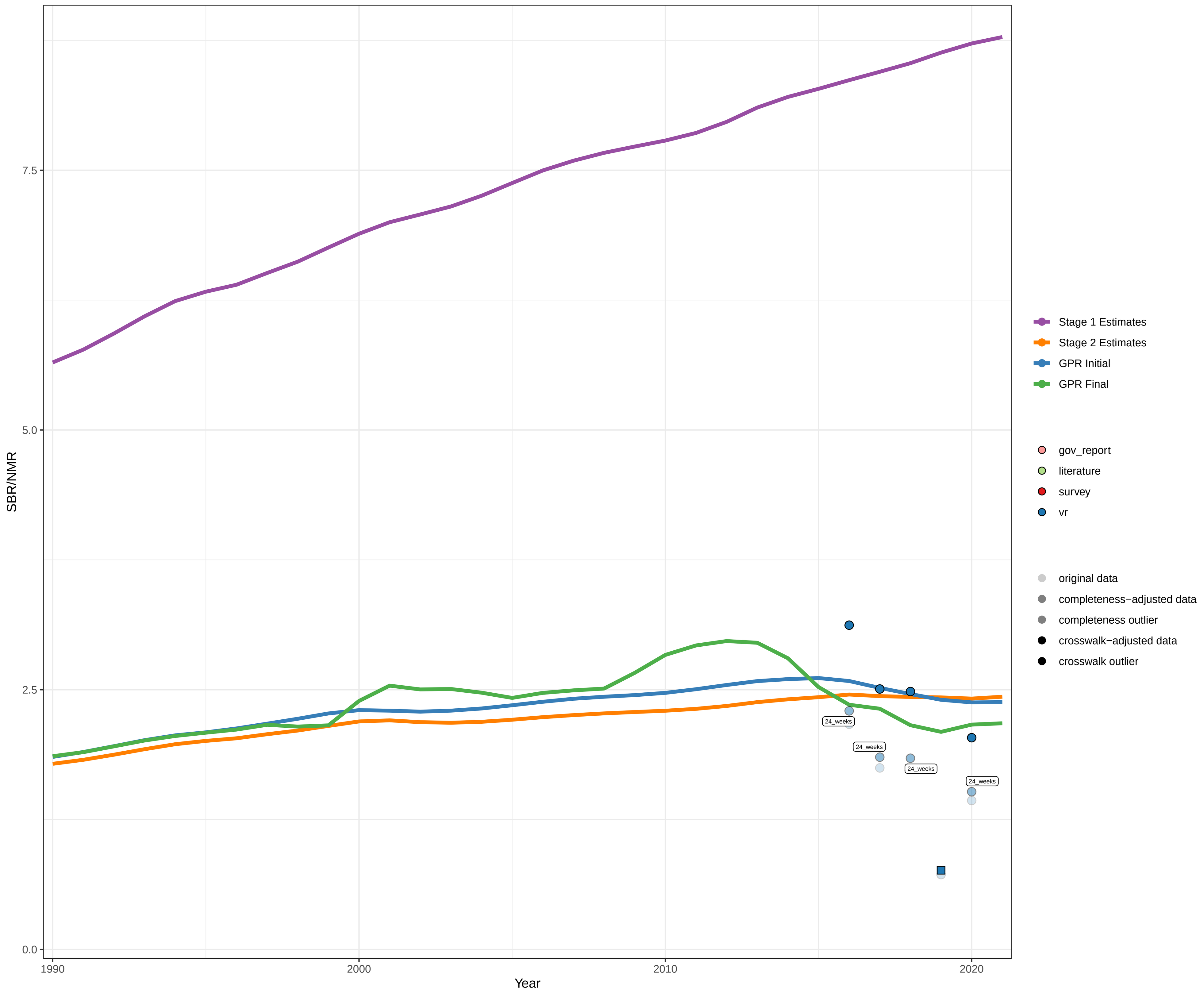

Salford GBR_44673 Western Europe Stillbirth Rate / Neonatal Mortality Rate (SBR/NMR) − 28 weeks
lambda = 0.8 ; zeta = 0.7 ; scale = 15 ; amp = 0.028

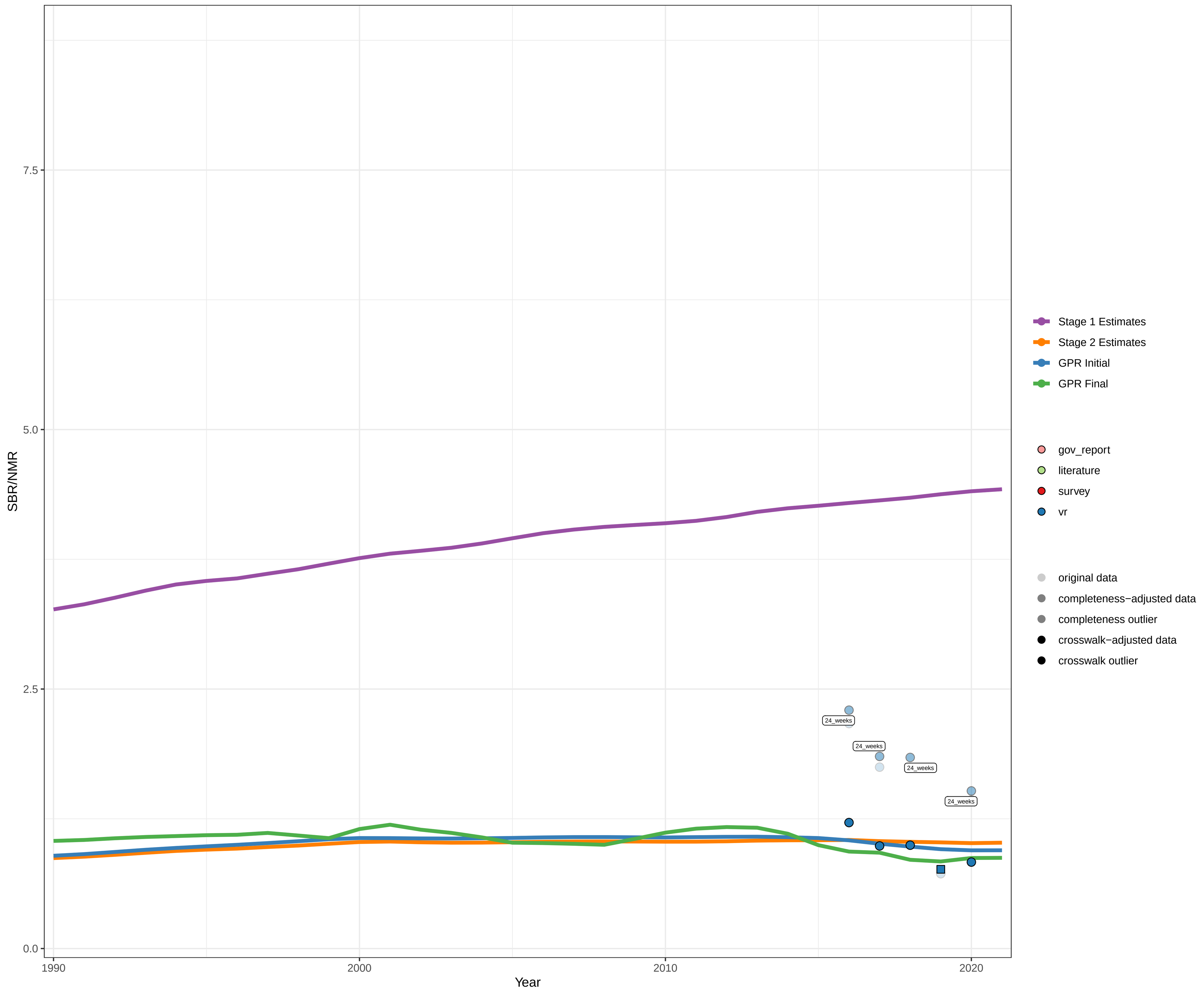

Definition	Years	Outlier	Source.Type	Title
24_weeks	2016−2020	0	VR	United Kingdom − England and Wales Child Mortality Statistics

Salford GBR_44673 Western Europe Stillbirth Rate (SBR) − 20 weeks
lambda = 0.8 ; zeta = 0.7 ; scale = 15 ; amp = 0.028

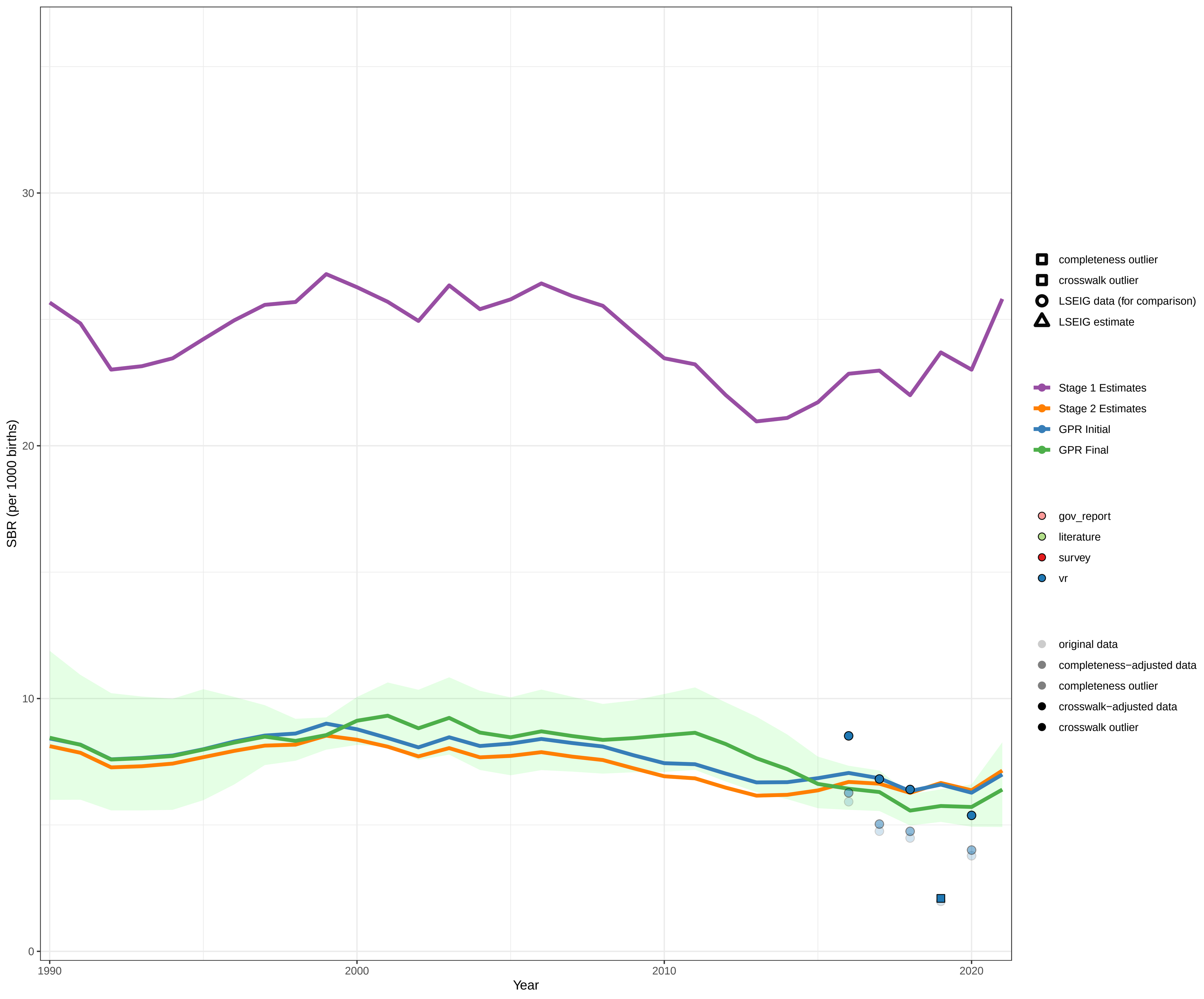

Salford GBR_44673 Western Europe Stillbirth Rate (SBR) − 28 weeks
lambda = 0.8 ; zeta = 0.7 ; scale = 15 ; amp = 0.028

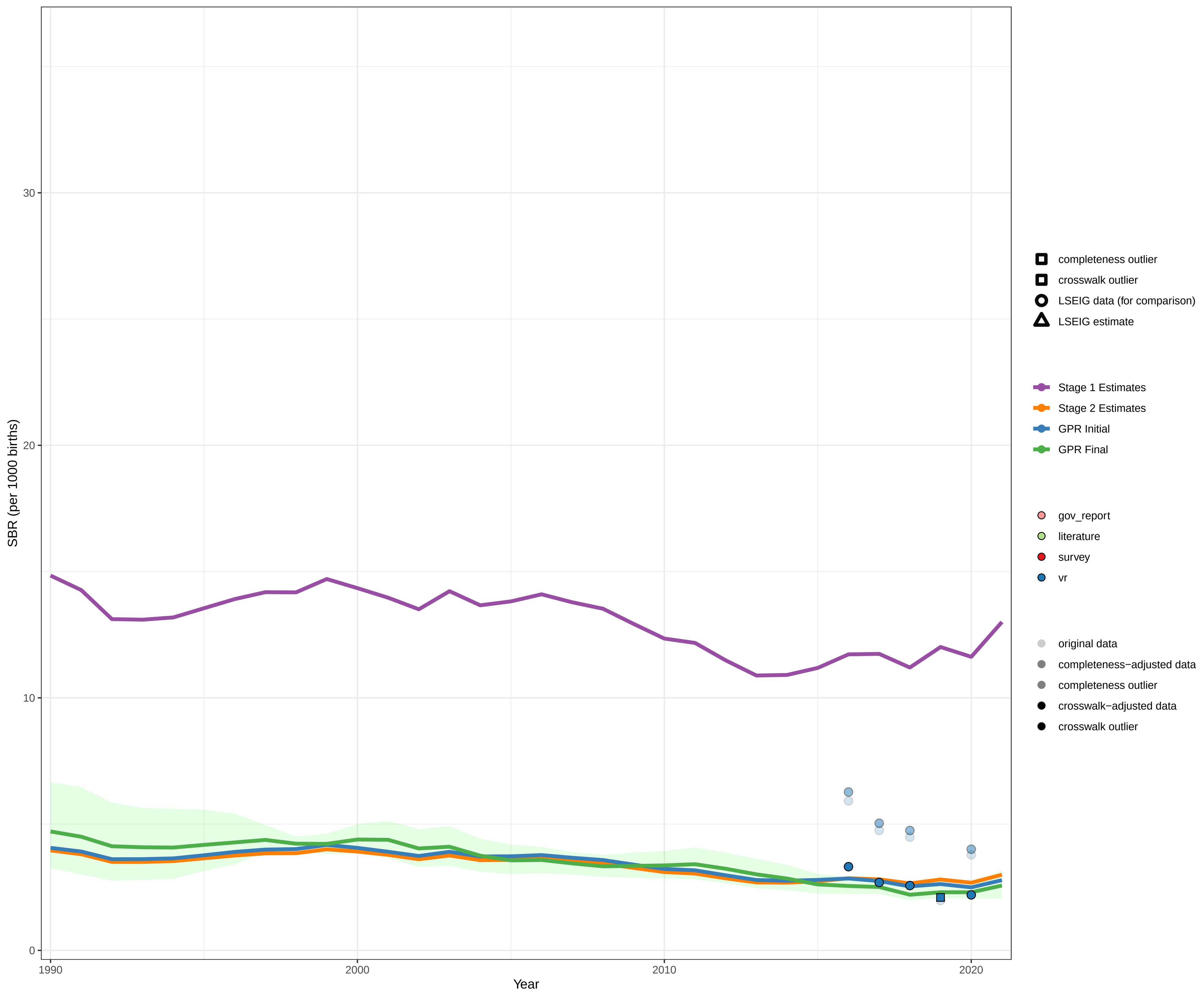

Year	Total Stillbirths (in thousands)	Total Stillbirths (in thousands)	SBR (per 1000 births)	SBR (per 1000 births)	SBR/NMR	SBR/NMR
	20 weeks	28 weeks	20 weeks	28 weeks	20 weeks	28 weeks
1990	0.0 (0.0 to 0.0)	0.0 (0.0 to 0.0)	8.5 (6.0 to 11.9)	4.7 (3.3 to 6.7)	1.86 (1.32 to 2.62)	1.04 (0.72 to 1.47)
1995	0.0 (0.0 to 0.0)	0.0 (0.0 to 0.0)	8.0 (6.0 to 10.4)	4.2 (3.1 to 5.6)	2.09 (1.56 to 2.71)	1.09 (0.82 to 1.46)
2000	0.0 (0.0 to 0.0)	0.0 (0.0 to 0.0)	9.1 (8.2 to 10.1)	4.4 (3.8 to 5.0)	2.39 (2.14 to 2.64)	1.15 (1.01 to 1.31)
2005	0.0 (0.0 to 0.0)	0.0 (0.0 to 0.0)	8.5 (7.0 to 10.0)	3.6 (3.0 to 4.2)	2.42 (1.99 to 2.87)	1.02 (0.86 to 1.20)
2010	0.0 (0.0 to 0.0)	0.0 (0.0 to 0.0)	8.5 (7.1 to 10.2)	3.4 (2.9 to 3.9)	2.84 (2.36 to 3.38)	1.12 (0.95 to 1.31)
2015	0.0 (0.0 to 0.0)	0.0 (0.0 to 0.0)	6.6 (5.7 to 7.7)	2.6 (2.2 to 3.0)	2.53 (2.16 to 2.94)	1.00 (0.86 to 1.16)
2020	0.0 (0.0 to 0.0)	0.0 (0.0 to 0.0)	5.7 (4.9 to 6.6)	2.3 (2.0 to 2.6)	2.16 (1.87 to 2.50)	0.87 (0.77 to 0.98)
2021	0.0 (0.0 to 0.0)	0.0 (0.0 to 0.0)	6.4 (4.9 to 8.3)	2.6 (2.1 to 3.2)	2.18 (1.67 to 2.81)	0.87 (0.70 to 1.08)

Sefton GBR_44659 Western Europe Stillbirth Rate / Neonatal Mortality Rate (SBR/NMR) − 20 weeks
lambda = 0.6 ; zeta = 0.8 ; scale = 15 ; amp = 0.028

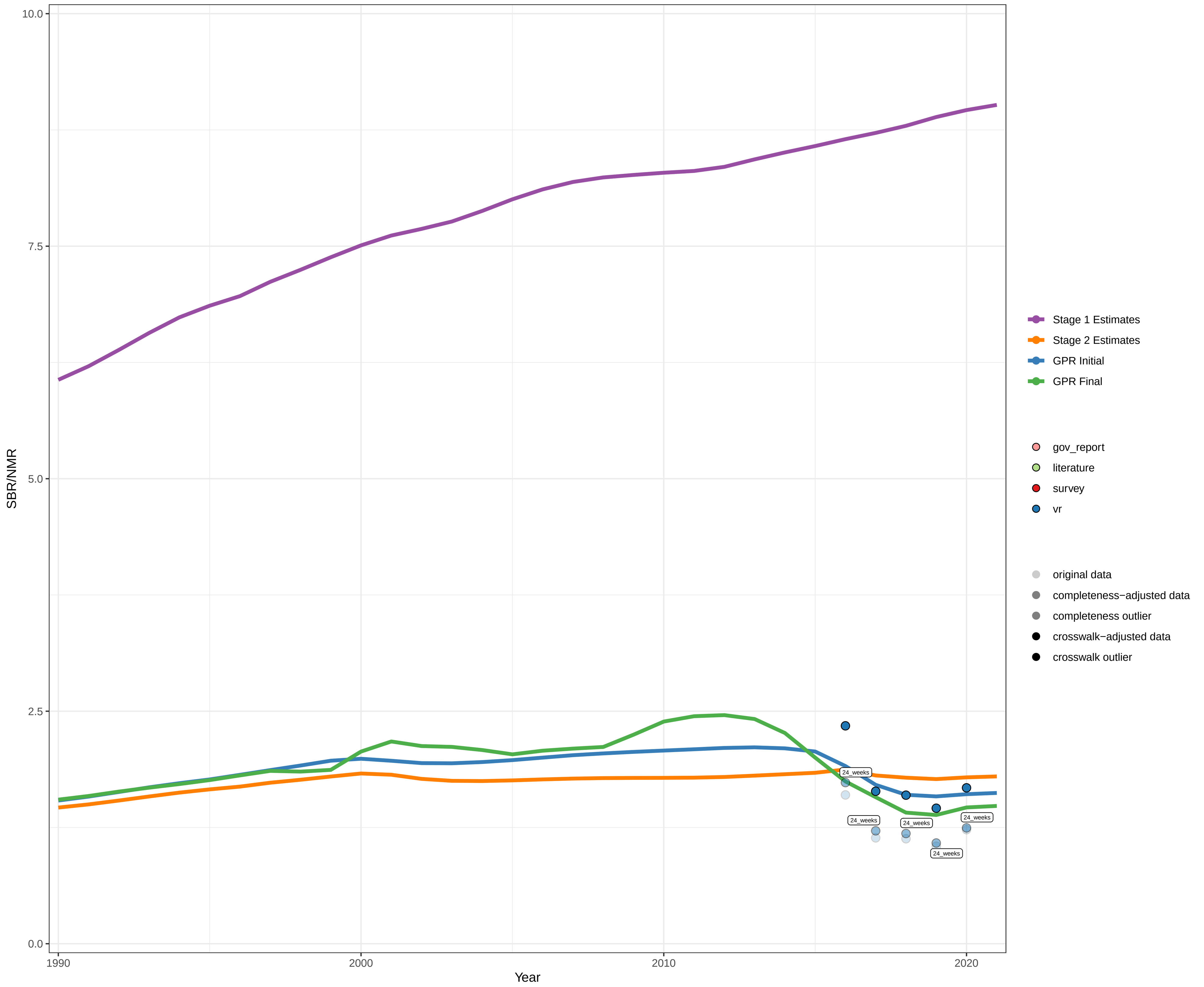

Sefton GBR_44659 Western Europe Stillbirth Rate / Neonatal Mortality Rate (SBR/NMR) − 28 weeks
lambda = 0.6 ; zeta = 0.8 ; scale = 15 ; amp = 0.028

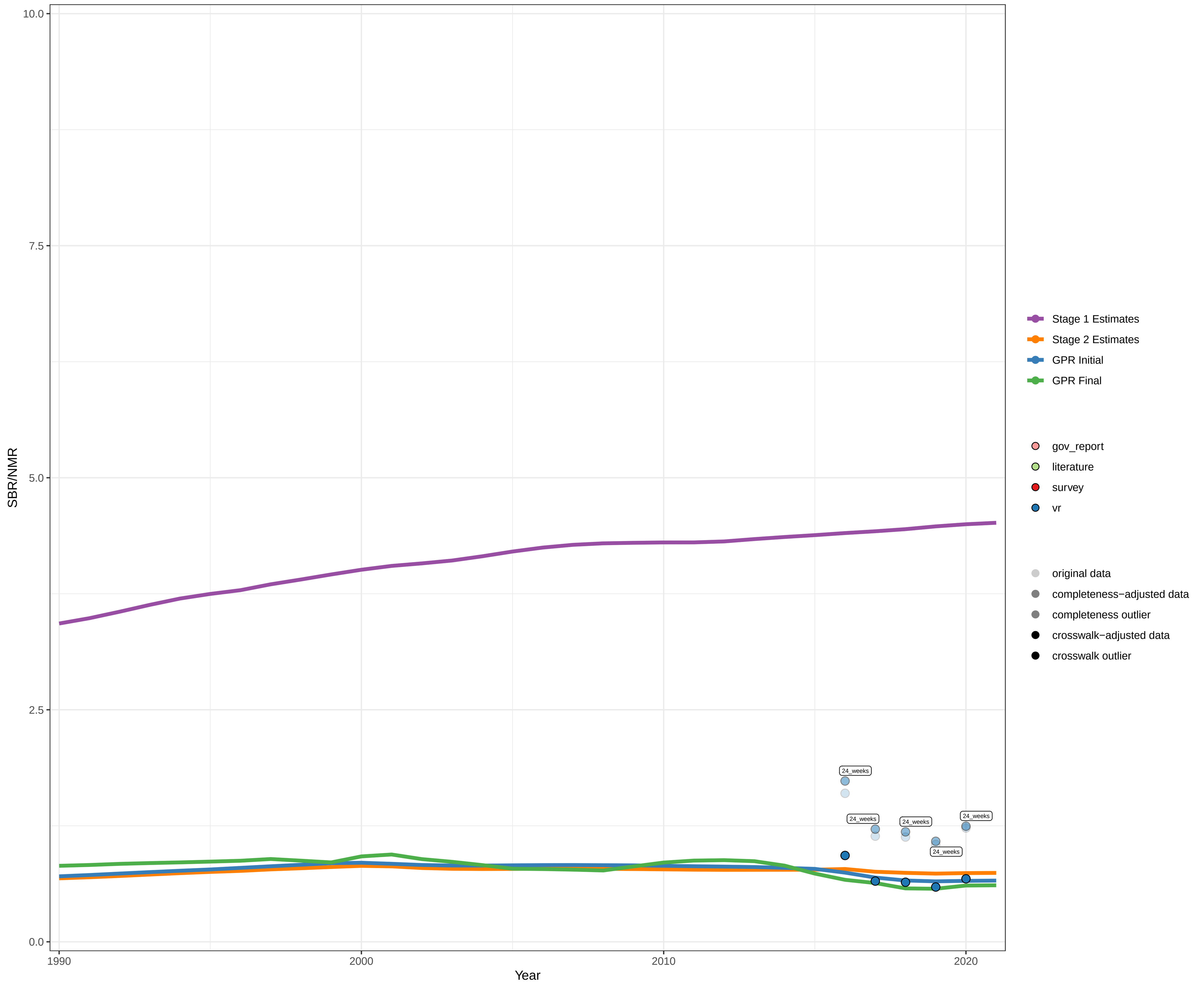

Definition	Years	Outlier	Source.Type	Title
24_weeks	2016−2020	0	VR	United Kingdom − England and Wales Child Mortality Statistics

Sefton GBR_44659 Western Europe Stillbirth Rate (SBR) − 20 weeks
lambda = 0.6 ; zeta = 0.8 ; scale = 15 ; amp = 0.028

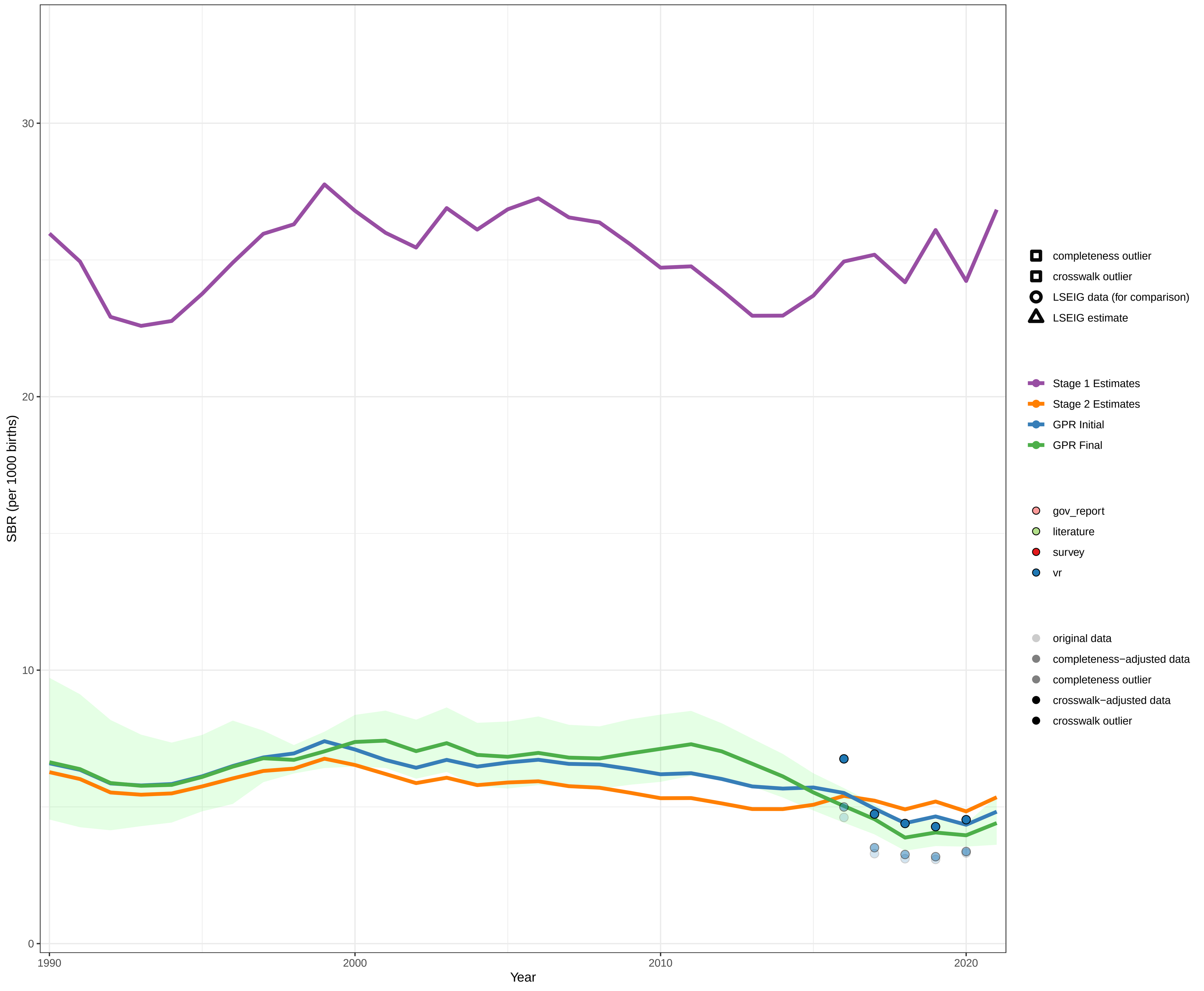

Sefton GBR_44659 Western Europe Stillbirth Rate (SBR) − 28 weeks
lambda = 0.6 ; zeta = 0.8 ; scale = 15 ; amp = 0.028

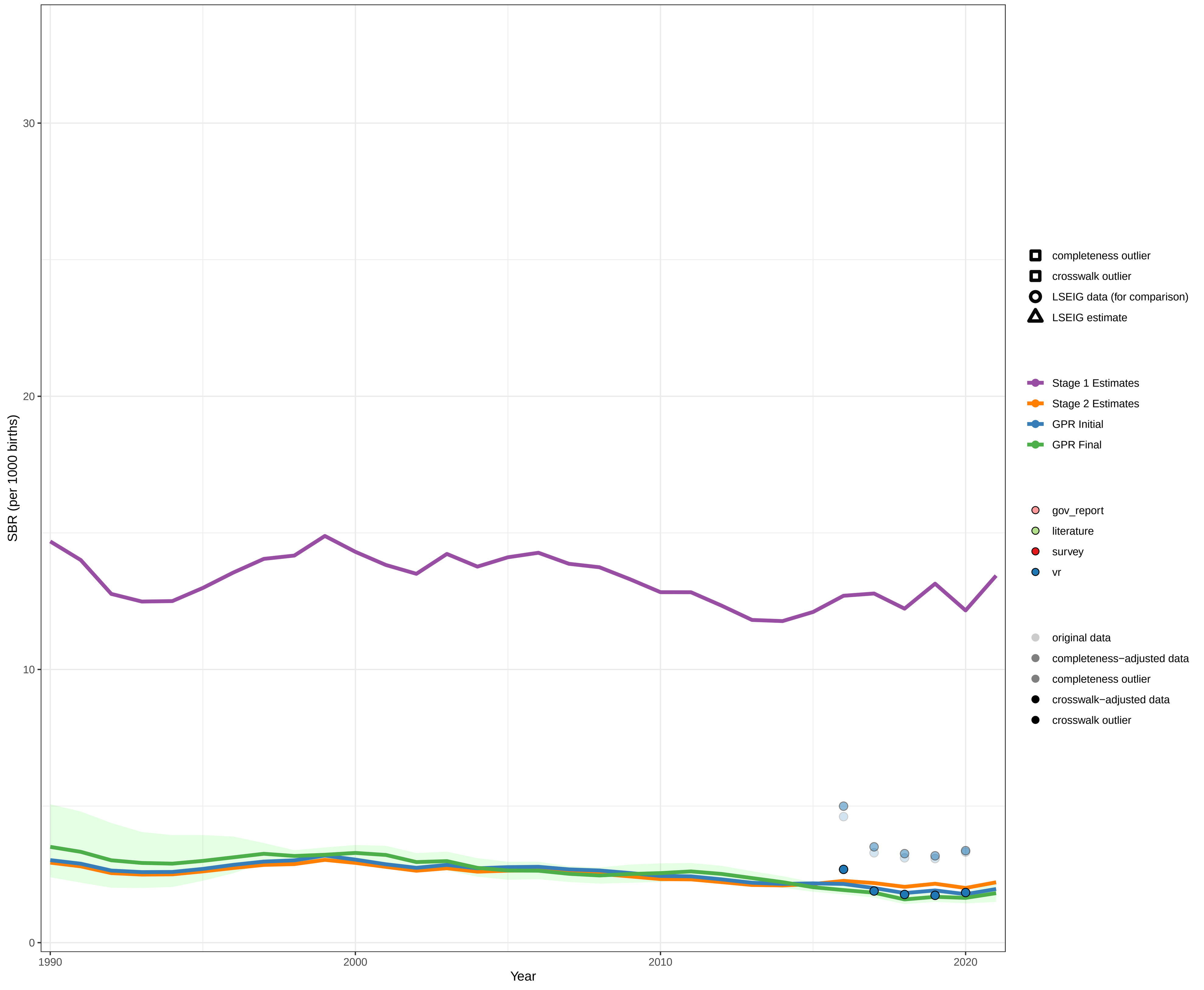

Year	Total Stillbirths (in thousands)	Total Stillbirths (in thousands)	SBR (per 1000 births)	SBR (per 1000 births)	SBR/NMR	SBR/NMR
	20 weeks	28 weeks	20 weeks	28 weeks	20 weeks	28 weeks
1990	0.0 (0.0 to 0.0)	0.0 (0.0 to 0.0)	6.6 (4.5 to 9.7)	3.5 (2.4 to 5.1)	1.55 (1.06 to 2.27)	0.82 (0.56 to 1.18)
1995	0.0 (0.0 to 0.0)	0.0 (0.0 to 0.0)	6.1 (4.8 to 7.6)	3.0 (2.3 to 3.9)	1.76 (1.40 to 2.20)	0.86 (0.65 to 1.14)
2000	0.0 (0.0 to 0.0)	0.0 (0.0 to 0.0)	7.4 (6.5 to 8.4)	3.3 (3.0 to 3.6)	2.07 (1.81 to 2.34)	0.92 (0.84 to 1.00)
2005	0.0 (0.0 to 0.0)	0.0 (0.0 to 0.0)	6.8 (5.7 to 8.1)	2.6 (2.3 to 3.0)	2.04 (1.69 to 2.42)	0.79 (0.69 to 0.88)
2010	0.0 (0.0 to 0.0)	0.0 (0.0 to 0.0)	7.1 (5.9 to 8.4)	2.5 (2.2 to 2.9)	2.39 (1.98 to 2.81)	0.85 (0.75 to 0.97)
2015	0.0 (0.0 to 0.0)	0.0 (0.0 to 0.0)	5.5 (4.9 to 6.2)	2.0 (1.9 to 2.2)	2.00 (1.76 to 2.25)	0.73 (0.67 to 0.80)
2020	0.0 (0.0 to 0.0)	0.0 (0.0 to 0.0)	4.0 (3.5 to 4.4)	1.6 (1.4 to 1.9)	1.47 (1.31 to 1.63)	0.61 (0.53 to 0.69)
2021	0.0 (0.0 to 0.0)	0.0 (0.0 to 0.0)	4.4 (3.6 to 5.4)	1.8 (1.5 to 2.2)	1.48 (1.21 to 1.81)	0.61 (0.50 to 0.74)

St Helens GBR_44665 Western Europe Stillbirth Rate / Neonatal Mortality Rate (SBR/NMR) − 20 weeks
lambda = 0.6 ; zeta = 0.8 ; scale = 15 ; amp = 0.028

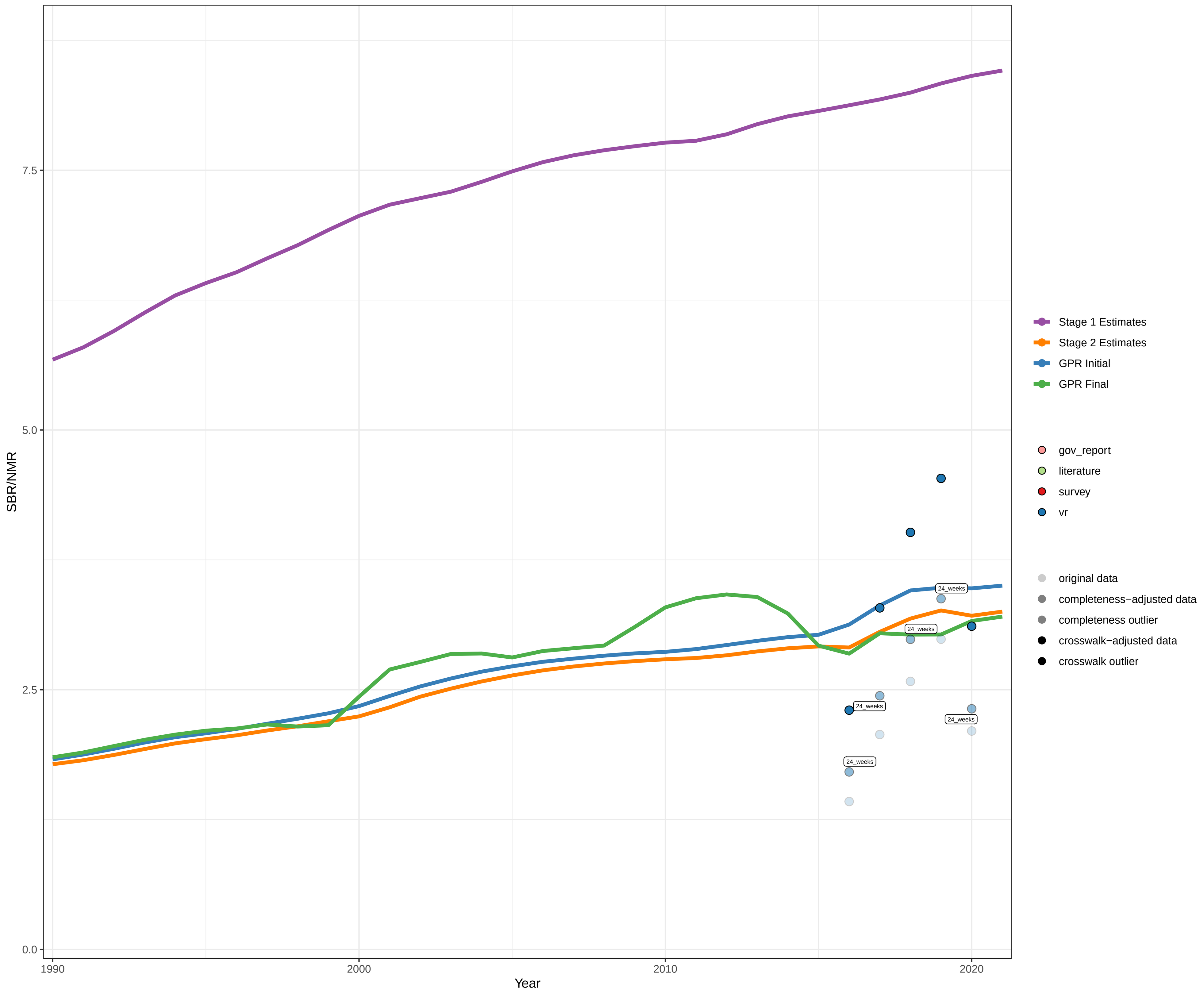

St Helens GBR_44665 Western Europe Stillbirth Rate / Neonatal Mortality Rate (SBR/NMR) − 28 weeks
lambda = 0.6 ; zeta = 0.8 ; scale = 15 ; amp = 0.028

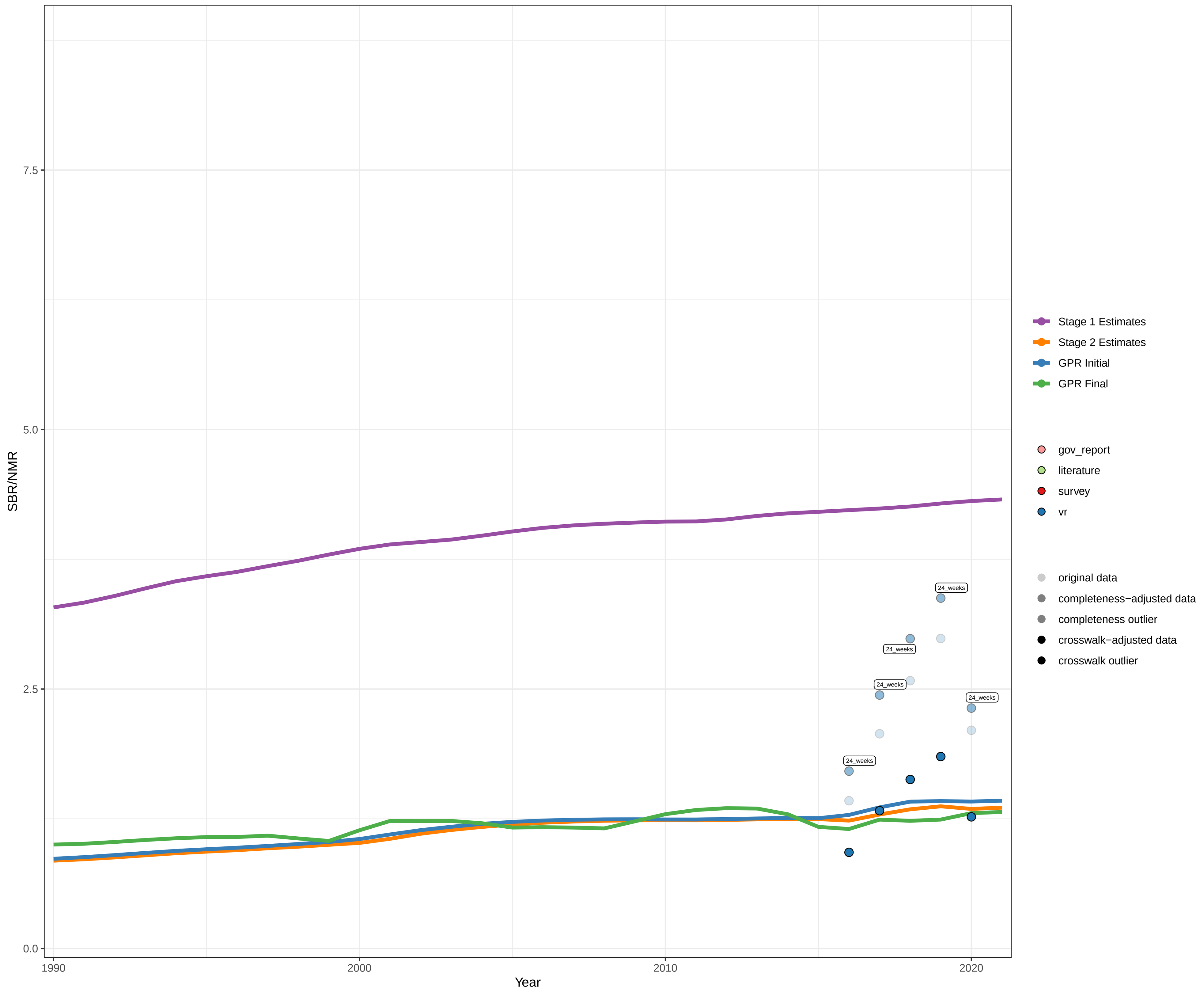

Definition	Years	Outlier	Source.Type	Title
24_weeks	2016−2020	0	VR	United Kingdom − England and Wales Child Mortality Statistics

St Helens GBR_44665 Western Europe Stillbirth Rate (SBR) − 20 weeks
lambda = 0.6 ; zeta = 0.8 ; scale = 15 ; amp = 0.028

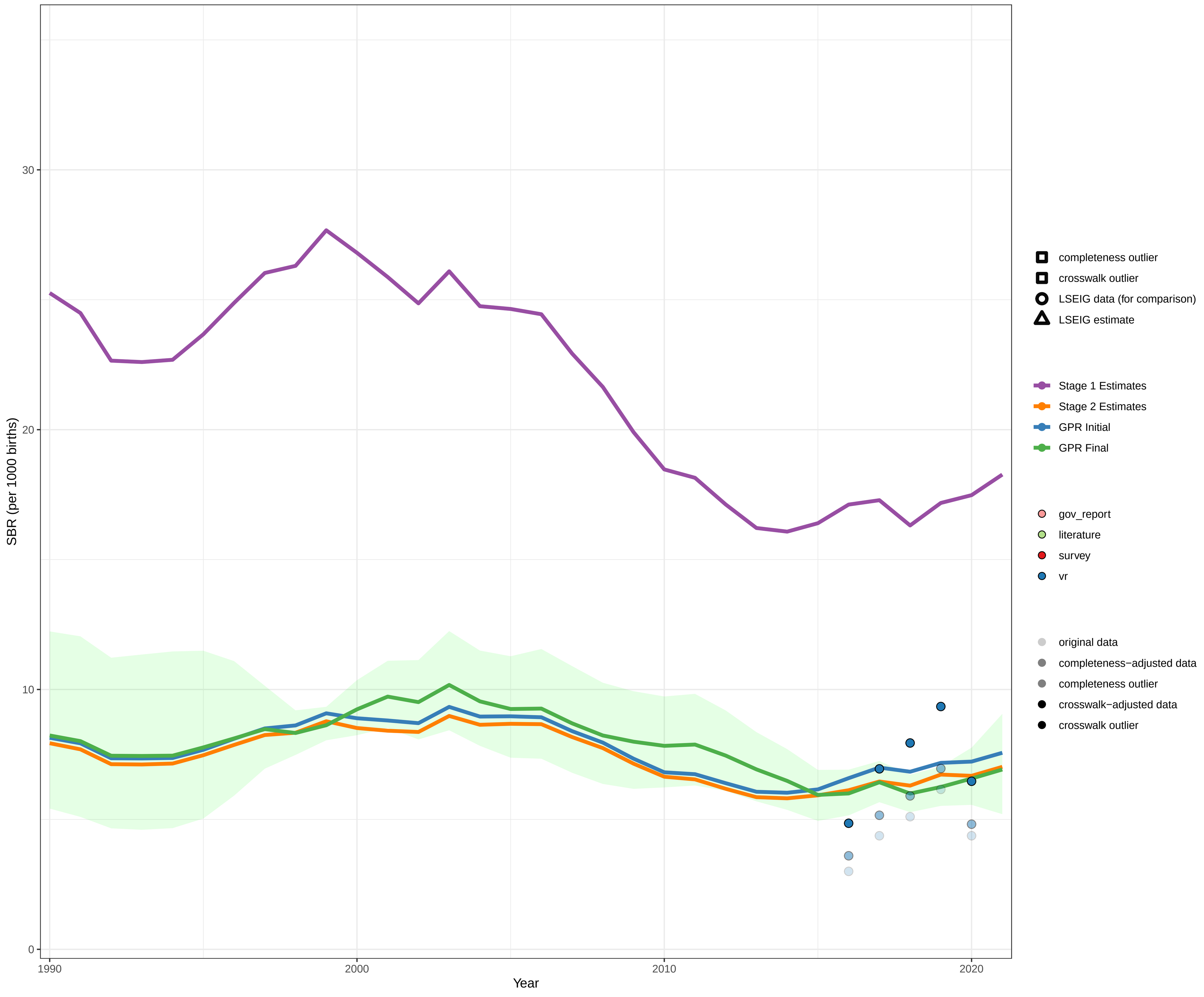

St Helens GBR_44665 Western Europe Stillbirth Rate (SBR) − 28 weeks
lambda = 0.6 ; zeta = 0.8 ; scale = 15 ; amp = 0.028

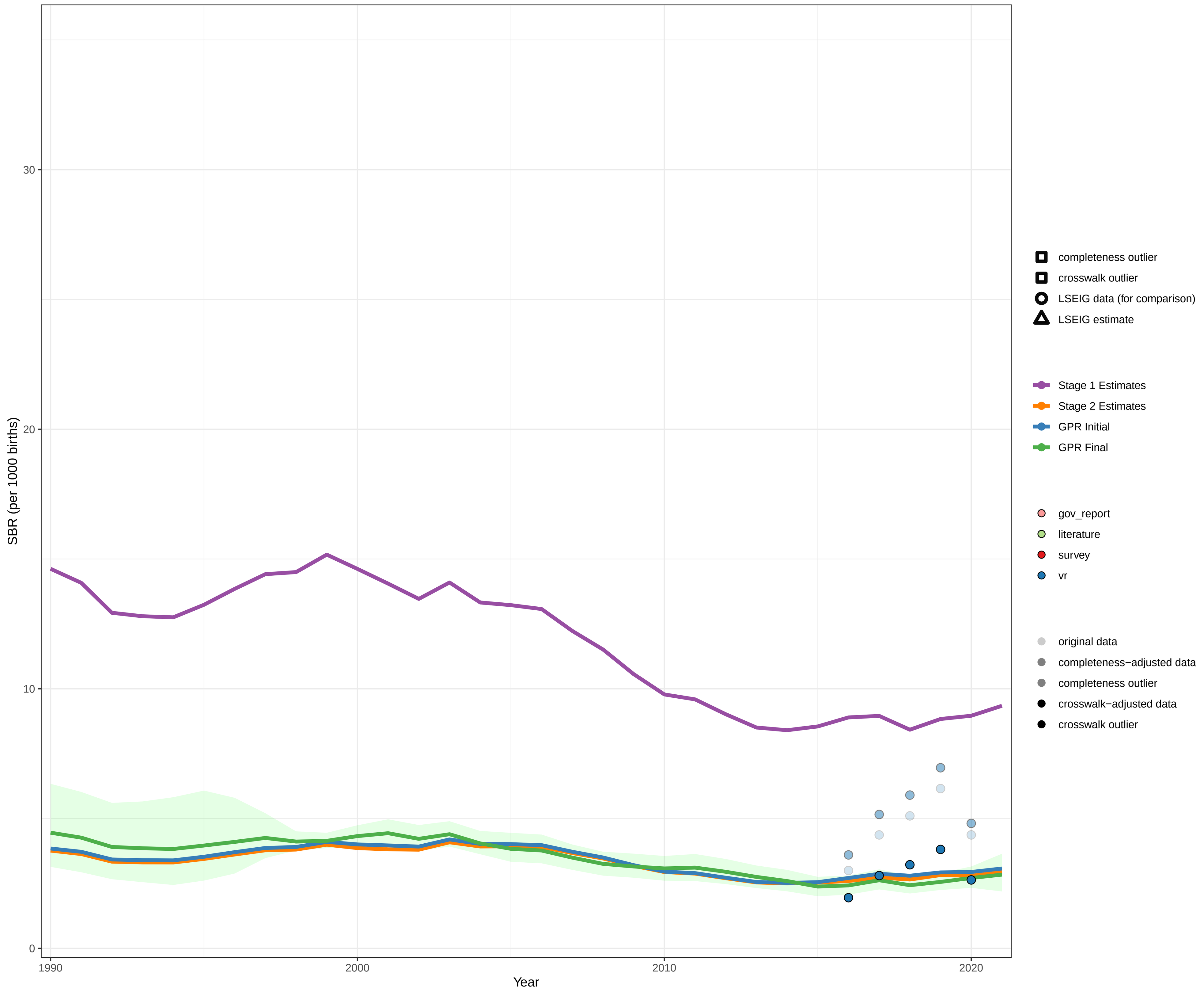

Year	Total Stillbirths (in thousands)	Total Stillbirths (in thousands)	SBR (per 1000 births)	SBR (per 1000 births)	SBR/NMR	SBR/NMR
	20 weeks	28 weeks	20 weeks	28 weeks	20 weeks	28 weeks
1990	0.0 (0.0 to 0.0)	0.0 (0.0 to 0.0)	8.2 (5.4 to 12.2)	4.5 (3.1 to 6.3)	1.85 (1.22 to 2.75)	1.00 (0.71 to 1.42)
1995	0.0 (0.0 to 0.0)	0.0 (0.0 to 0.0)	7.8 (5.0 to 11.5)	4.0 (2.6 to 6.1)	2.11 (1.36 to 3.11)	1.07 (0.71 to 1.65)
2000	0.0 (0.0 to 0.0)	0.0 (0.0 to 0.0)	9.2 (8.2 to 10.4)	4.3 (4.0 to 4.7)	2.43 (2.17 to 2.73)	1.14 (1.04 to 1.25)
2005	0.0 (0.0 to 0.0)	0.0 (0.0 to 0.0)	9.2 (7.4 to 11.3)	3.8 (3.3 to 4.5)	2.81 (2.24 to 3.43)	1.17 (1.01 to 1.35)
2010	0.0 (0.0 to 0.0)	0.0 (0.0 to 0.0)	7.8 (6.2 to 9.7)	3.1 (2.6 to 3.6)	3.29 (2.62 to 4.09)	1.29 (1.10 to 1.50)
2015	0.0 (0.0 to 0.0)	0.0 (0.0 to 0.0)	5.9 (4.9 to 6.9)	2.4 (2.0 to 2.8)	2.92 (2.43 to 3.40)	1.17 (0.99 to 1.36)
2020	0.0 (0.0 to 0.0)	0.0 (0.0 to 0.0)	6.6 (5.6 to 7.8)	2.7 (2.3 to 3.2)	3.16 (2.67 to 3.73)	1.30 (1.12 to 1.52)
2021	0.0 (0.0 to 0.0)	0.0 (0.0 to 0.0)	6.9 (5.2 to 9.1)	2.8 (2.2 to 3.6)	3.20 (2.41 to 4.20)	1.32 (1.02 to 1.69)

Stockport GBR_44656 Western Europe Stillbirth Rate / Neonatal Mortality Rate (SBR/NMR) − 20 weeks
lambda = 0.8 ; zeta = 0.7 ; scale = 15 ; amp = 0.028

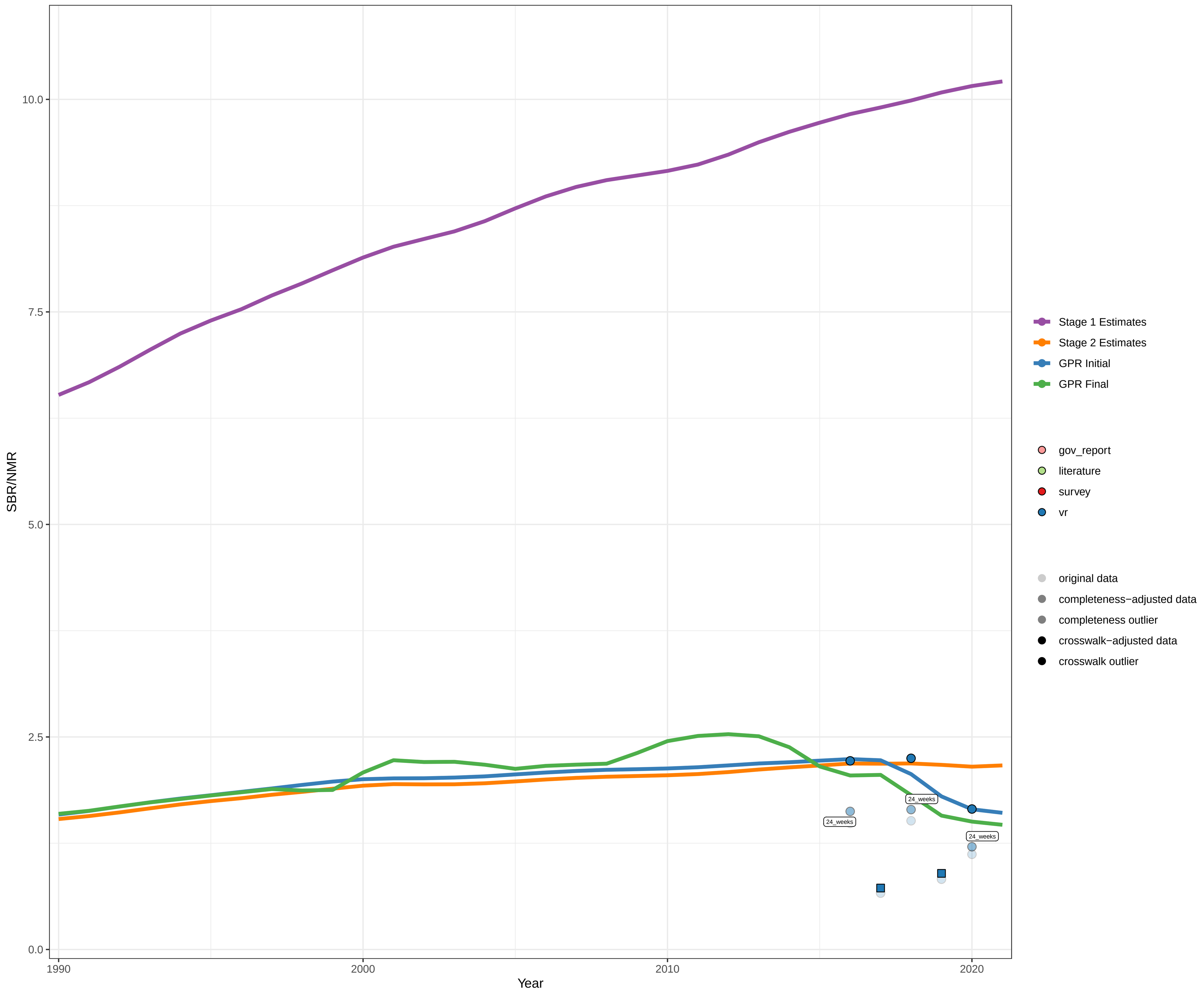

Stockport GBR_44656 Western Europe Stillbirth Rate / Neonatal Mortality Rate (SBR/NMR) − 28 weeks
lambda = 0.8 ; zeta = 0.7 ; scale = 15 ; amp = 0.028

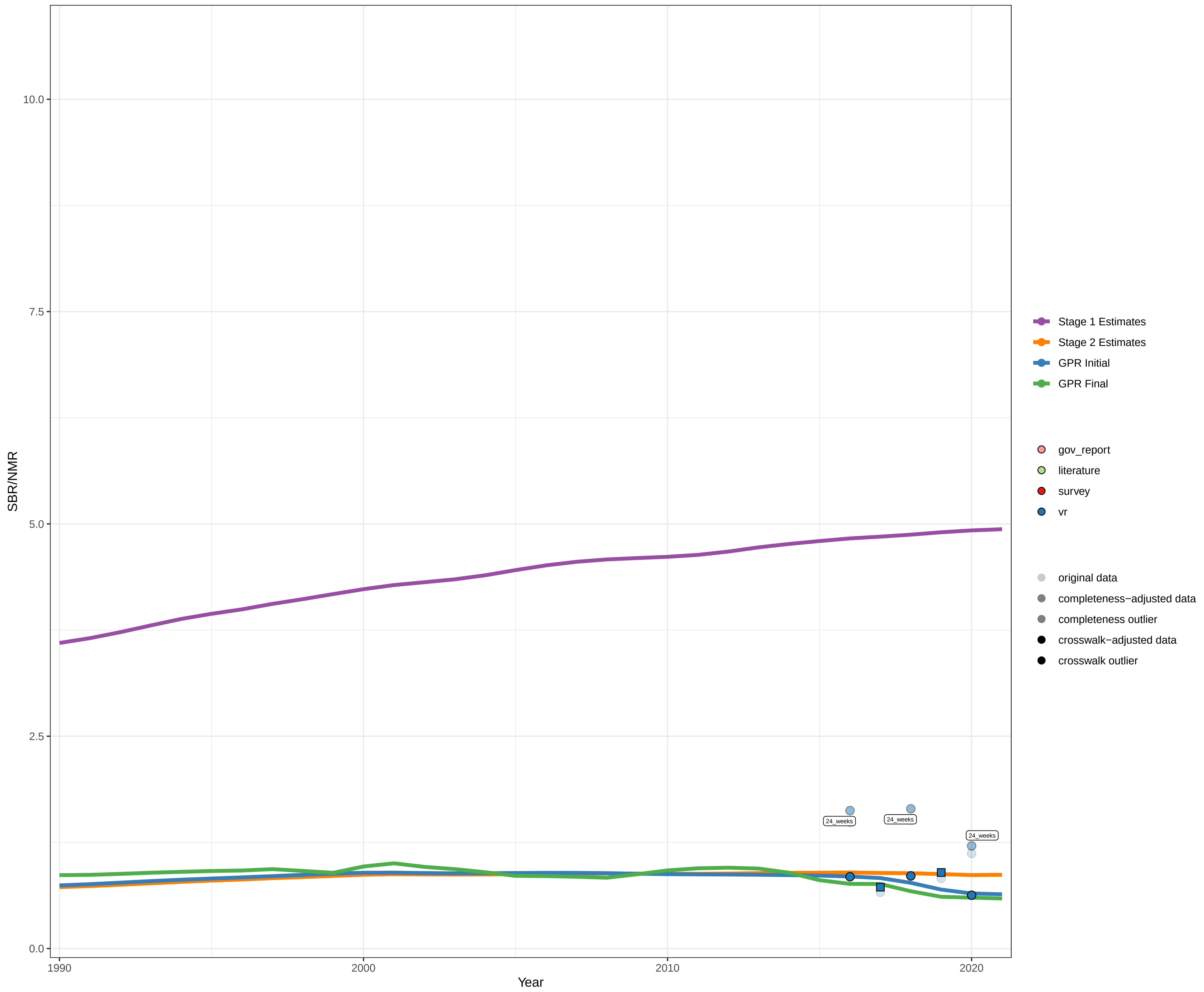

Definition	Years	Outlier	Source.Type	Title
24_weeks	2016−2020	0	VR	United Kingdom − England and Wales Child Mortality Statistics

Stockport GBR_44656 Western Europe Stillbirth Rate (SBR) − 20 weeks
lambda = 0.8 ; zeta = 0.7 ; scale = 15 ; amp = 0.028

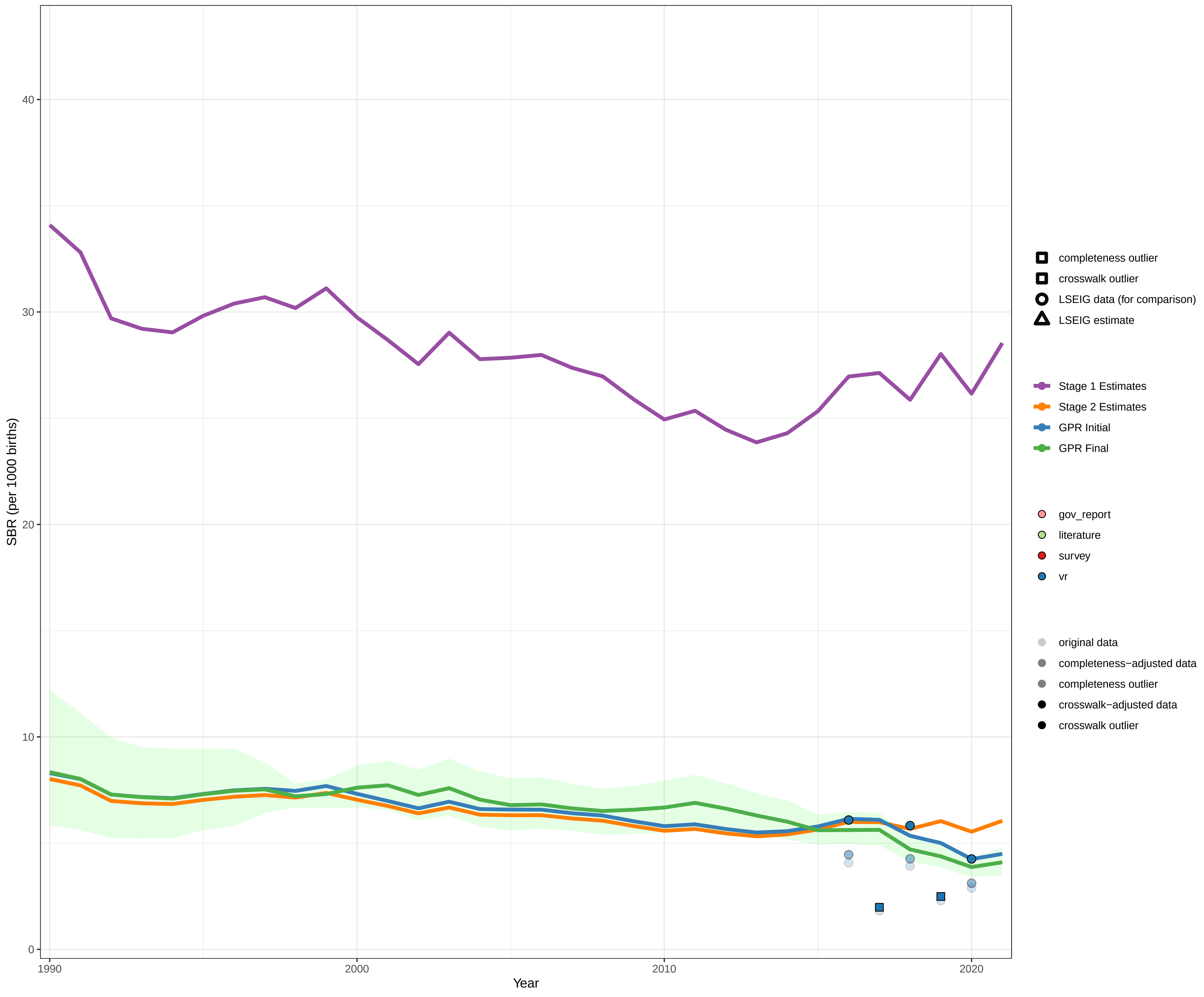

Stockport GBR_44656 Western Europe Stillbirth Rate (SBR) − 28 weeks
lambda = 0.8 ; zeta = 0.7 ; scale = 15 ; amp = 0.028

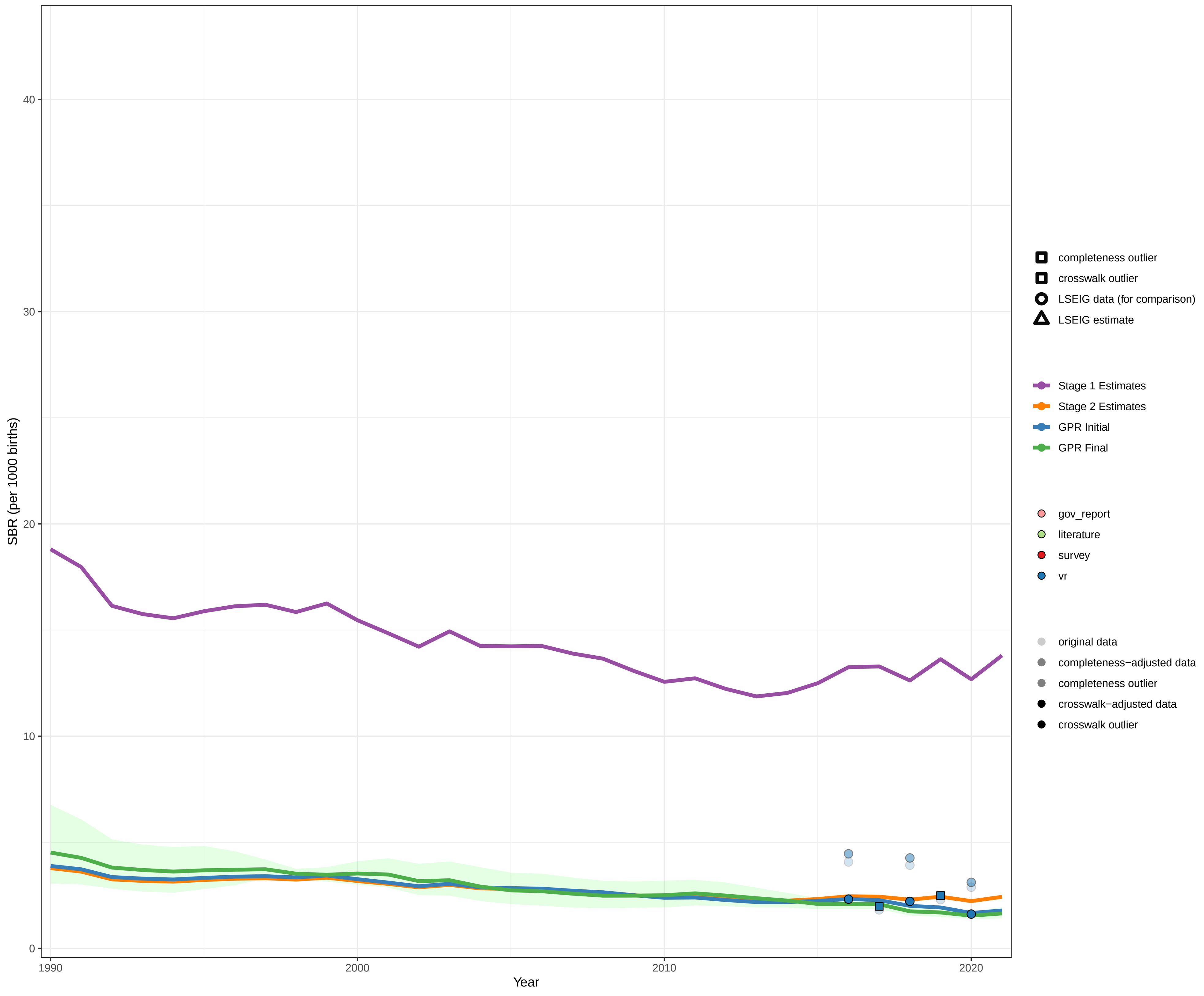

Year	Total Stillbirths (in thousands)	Total Stillbirths (in thousands)	SBR (per 1000 births)	SBR (per 1000 births)	SBR/NMR	SBR/NMR
	20 weeks	28 weeks	20 weeks	28 weeks	20 weeks	28 weeks
1990	0.0 (0.0 to 0.0)	0.0 (0.0 to 0.0)	8.3 (5.8 to 12.2)	4.5 (3.1 to 6.8)	1.60 (1.12 to 2.33)	0.86 (0.59 to 1.29)
1995	0.0 (0.0 to 0.0)	0.0 (0.0 to 0.0)	7.3 (5.6 to 9.4)	3.7 (2.8 to 4.8)	1.81 (1.39 to 2.34)	0.91 (0.69 to 1.20)
2000	0.0 (0.0 to 0.0)	0.0 (0.0 to 0.0)	7.6 (6.7 to 8.7)	3.5 (3.0 to 4.1)	2.08 (1.83 to 2.37)	0.97 (0.82 to 1.12)
2005	0.0 (0.0 to 0.0)	0.0 (0.0 to 0.0)	6.8 (5.6 to 8.1)	2.7 (2.1 to 3.6)	2.13 (1.75 to 2.52)	0.86 (0.65 to 1.12)
2010	0.0 (0.0 to 0.0)	0.0 (0.0 to 0.0)	6.7 (5.5 to 8.0)	2.5 (1.9 to 3.2)	2.45 (2.03 to 2.92)	0.92 (0.71 to 1.17)
2015	0.0 (0.0 to 0.0)	0.0 (0.0 to 0.0)	5.6 (4.9 to 6.3)	2.1 (1.8 to 2.4)	2.15 (1.89 to 2.44)	0.80 (0.71 to 0.91)
2020	0.0 (0.0 to 0.0)	0.0 (0.0 to 0.0)	3.9 (3.4 to 4.3)	1.5 (1.4 to 1.7)	1.50 (1.32 to 1.68)	0.60 (0.54 to 0.66)
2021	0.0 (0.0 to 0.0)	0.0 (0.0 to 0.0)	4.1 (3.5 to 4.8)	1.6 (1.4 to 1.9)	1.47 (1.25 to 1.71)	0.59 (0.50 to 0.70)

Tameside GBR_44672 Western Europe Stillbirth Rate / Neonatal Mortality Rate (SBR/NMR) − 20 weeks
lambda = 0.6 ; zeta = 0.8 ; scale = 15 ; amp = 0.028

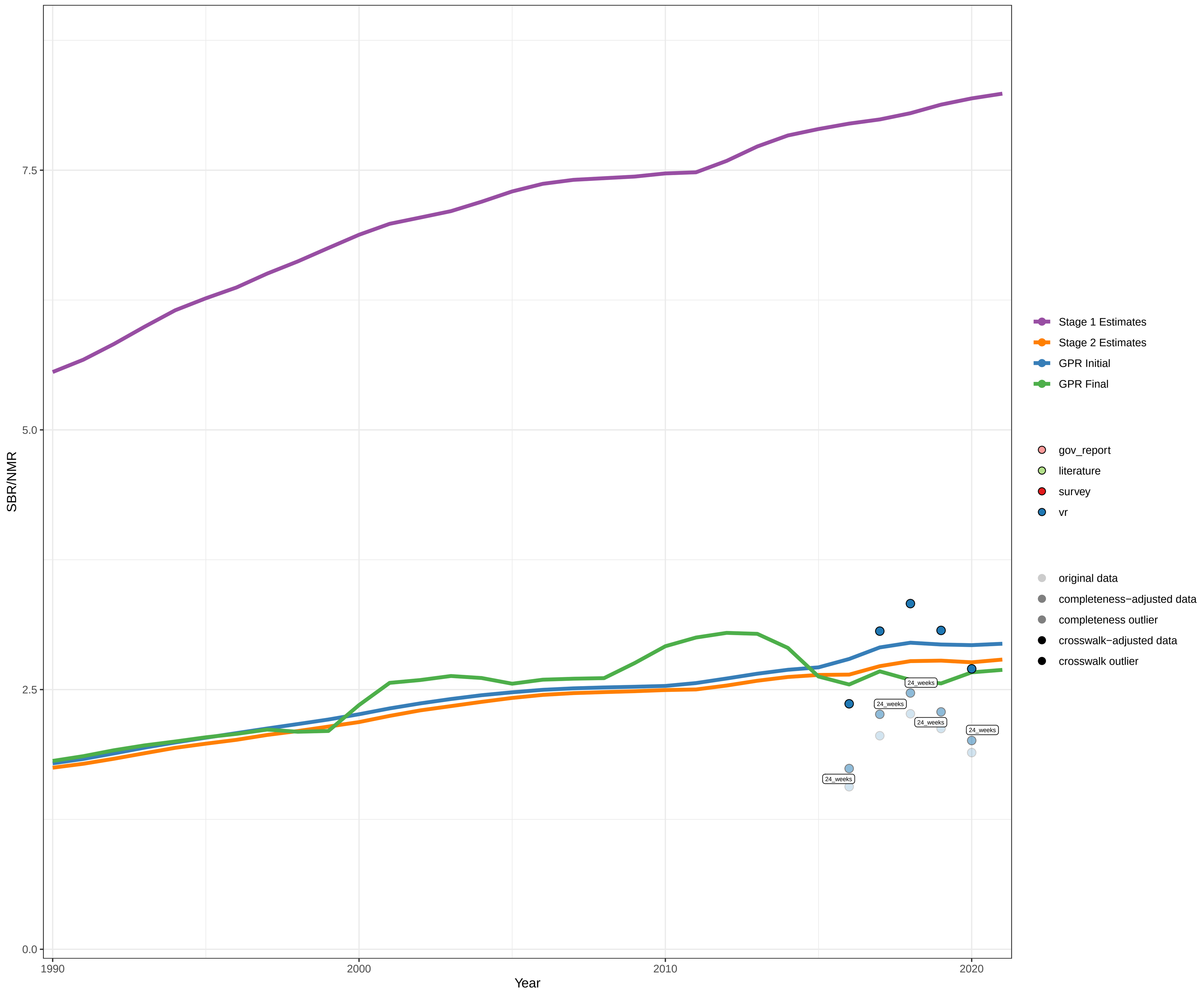

Tameside GBR_44672 Western Europe Stillbirth Rate / Neonatal Mortality Rate (SBR/NMR) − 28 weeks
lambda = 0.6 ; zeta = 0.8 ; scale = 15 ; amp = 0.028

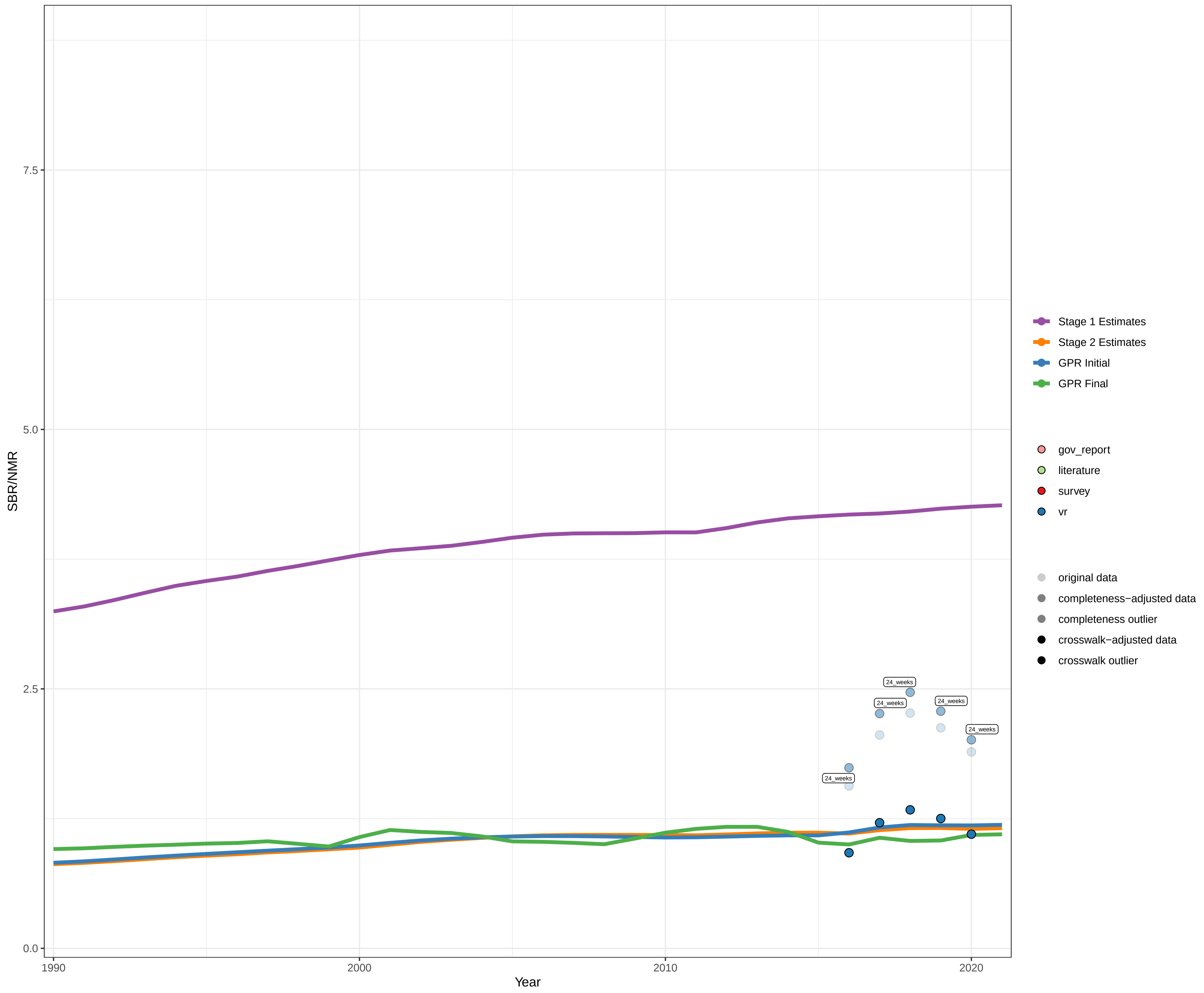

Definition	Years	Outlier	Source.Type	Title
24_weeks	2016−2020	0	VR	United Kingdom − England and Wales Child Mortality Statistics

Tameside GBR_44672 Western Europe Stillbirth Rate (SBR) − 20 weeks
lambda = 0.6 ; zeta = 0.8 ; scale = 15 ; amp = 0.028

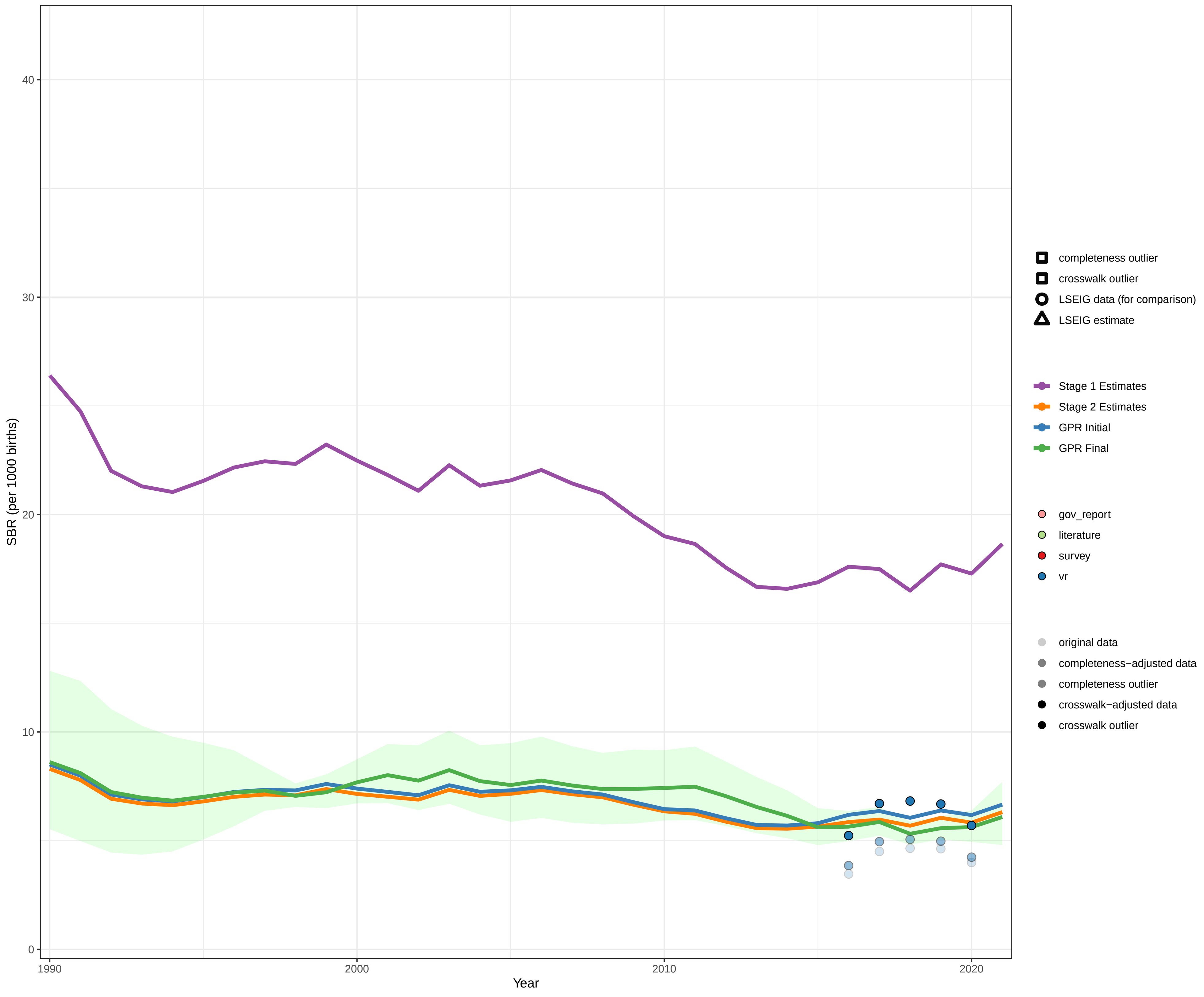

Tameside GBR_44672 Western Europe Stillbirth Rate (SBR) − 28 weeks
lambda = 0.6 ; zeta = 0.8 ; scale = 15 ; amp = 0.028

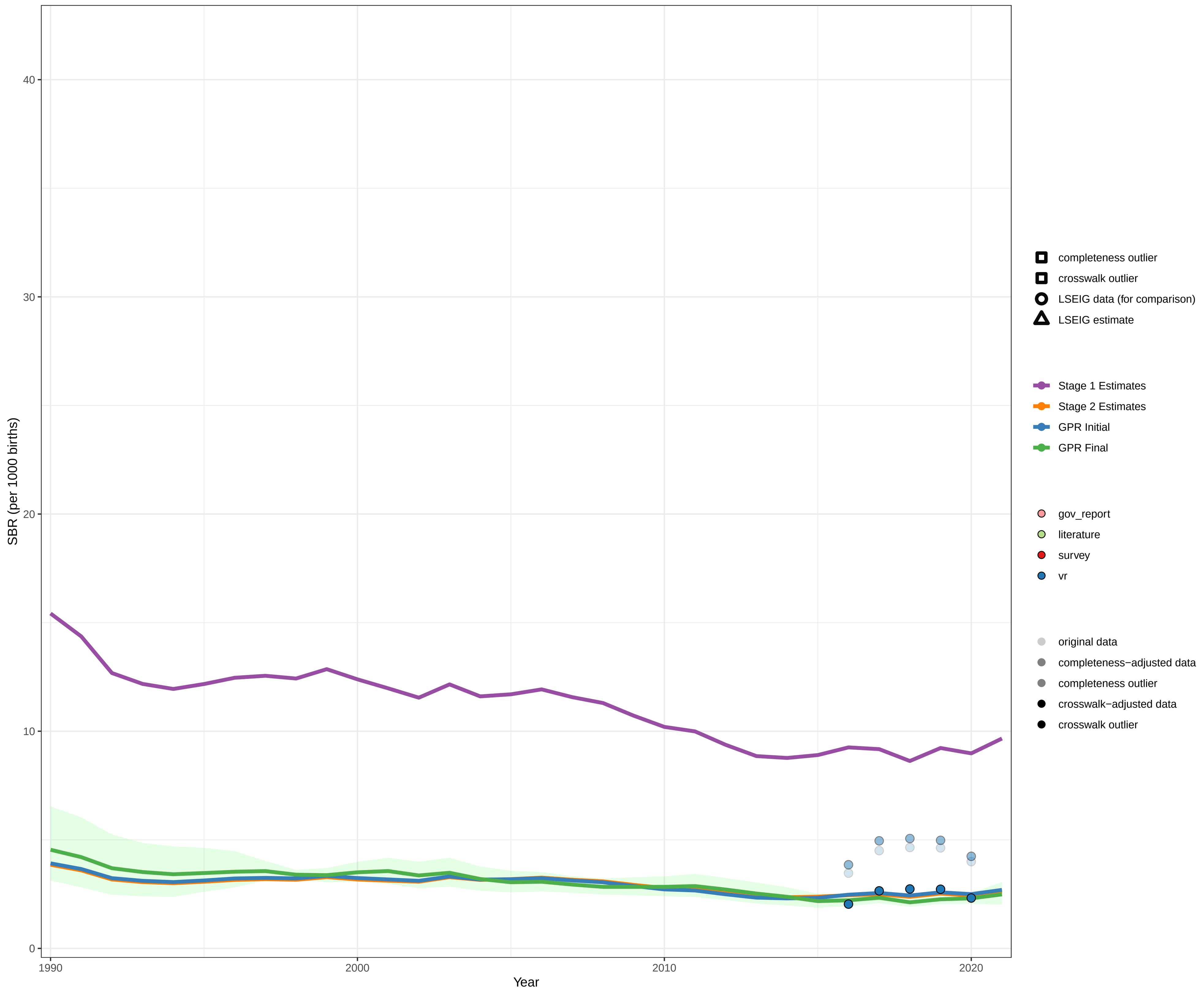

Year	Total Stillbirths (in thousands)	Total Stillbirths (in thousands)	SBR (per 1000 births)	SBR (per 1000 births)	SBR/NMR	SBR/NMR
	20 weeks	28 weeks	20 weeks	28 weeks	20 weeks	28 weeks
1990	0.0 (0.0 to 0.0)	0.0 (0.0 to 0.0)	8.6 (5.5 to 12.8)	4.5 (3.1 to 6.5)	1.81 (1.16 to 2.70)	0.96 (0.66 to 1.38)
1995	0.0 (0.0 to 0.0)	0.0 (0.0 to 0.0)	7.0 (5.1 to 9.5)	3.5 (2.6 to 4.6)	2.04 (1.47 to 2.76)	1.01 (0.76 to 1.35)
2000	0.0 (0.0 to 0.0)	0.0 (0.0 to 0.0)	7.7 (6.7 to 8.7)	3.5 (3.1 to 4.0)	2.35 (2.06 to 2.68)	1.07 (0.94 to 1.22)
2005	0.0 (0.0 to 0.0)	0.0 (0.0 to 0.0)	7.6 (5.9 to 9.5)	3.0 (2.6 to 3.6)	2.56 (1.98 to 3.21)	1.03 (0.87 to 1.21)
2010	0.0 (0.0 to 0.0)	0.0 (0.0 to 0.0)	7.4 (5.9 to 9.2)	2.8 (2.4 to 3.3)	2.92 (2.33 to 3.60)	1.11 (0.94 to 1.30)
2015	0.0 (0.0 to 0.0)	0.0 (0.0 to 0.0)	5.6 (4.8 to 6.5)	2.2 (1.9 to 2.5)	2.63 (2.24 to 3.04)	1.02 (0.88 to 1.18)
2020	0.0 (0.0 to 0.0)	0.0 (0.0 to 0.0)	5.6 (4.9 to 6.4)	2.3 (2.1 to 2.6)	2.67 (2.34 to 3.03)	1.09 (0.98 to 1.21)
2021	0.0 (0.0 to 0.0)	0.0 (0.0 to 0.0)	6.1 (4.8 to 7.7)	2.5 (2.0 to 3.1)	2.69 (2.12 to 3.41)	1.10 (0.89 to 1.35)

Trafford GBR_44657 Western Europe Stillbirth Rate / Neonatal Mortality Rate (SBR/NMR) − 20 weeks
lambda = 0.6 ; zeta = 0.8 ; scale = 15 ; amp = 0.028

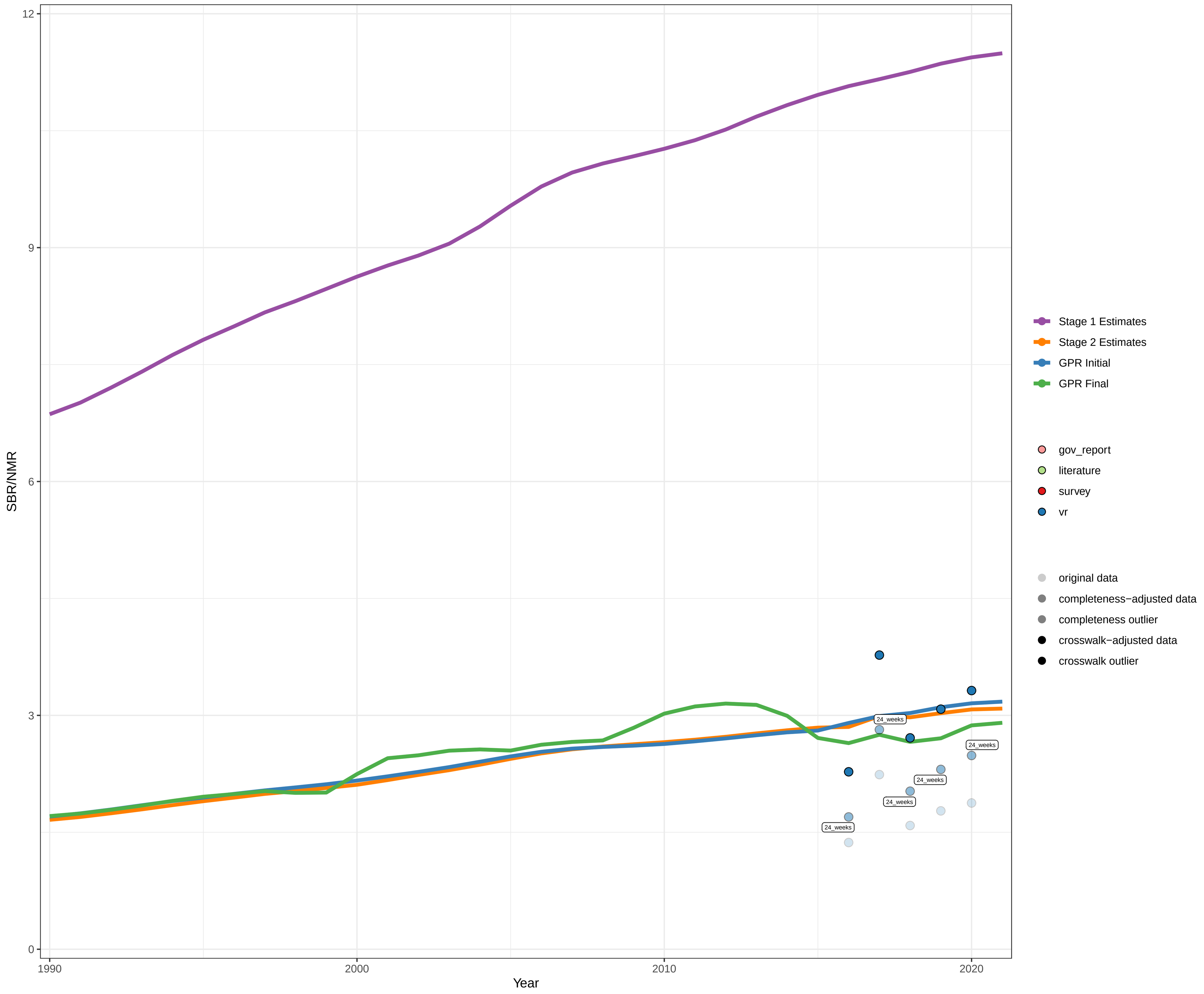

Trafford GBR_44657 Western Europe Stillbirth Rate / Neonatal Mortality Rate (SBR/NMR) − 28 weeks
lambda = 0.6 ; zeta = 0.8 ; scale = 15 ; amp = 0.028

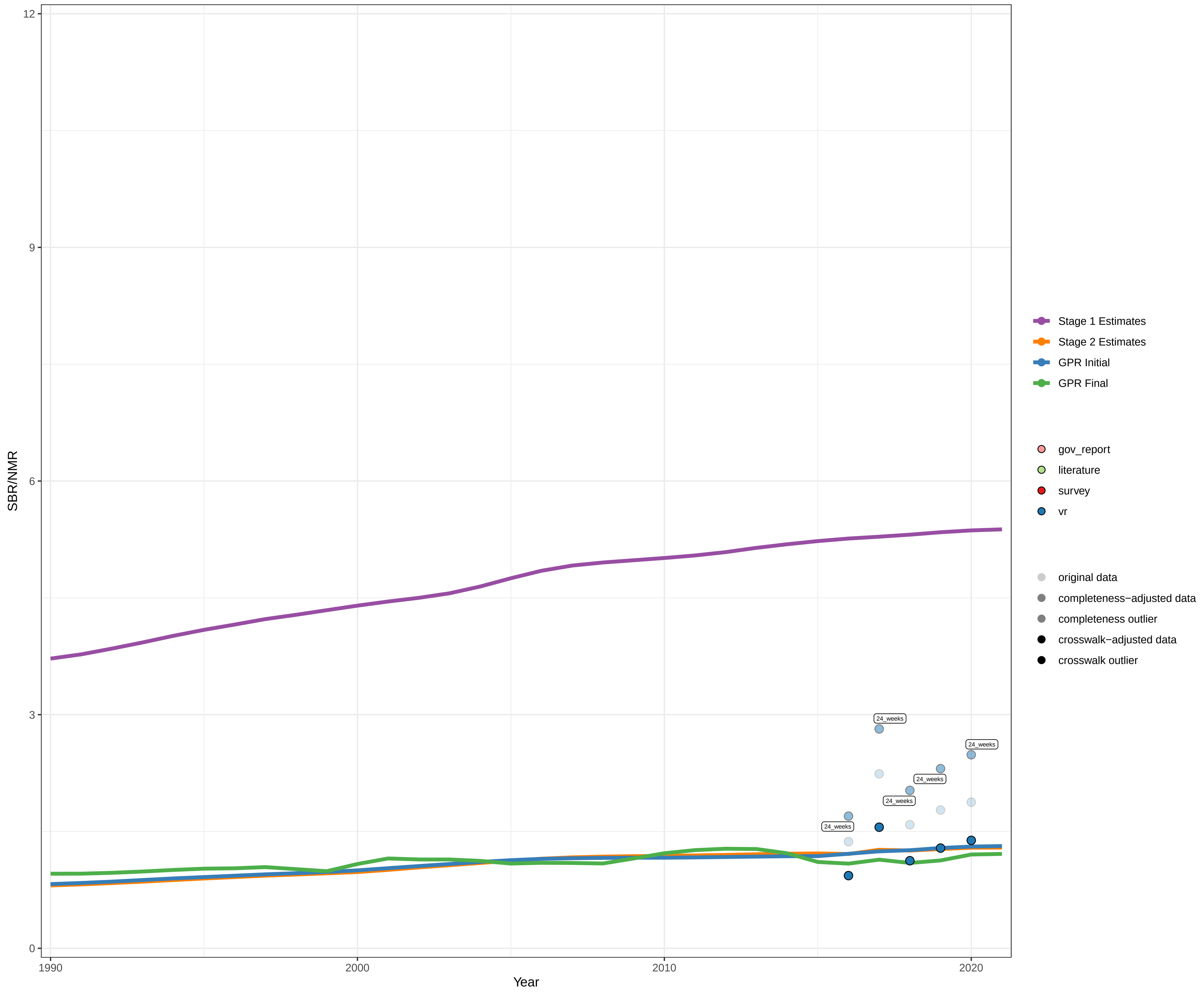

Definition	Years	Outlier	Source.Type	Title
24_weeks	2016−2020	0	VR	United Kingdom − England and Wales Child Mortality Statistics

Trafford GBR_44657 Western Europe Stillbirth Rate (SBR) − 20 weeks
lambda = 0.6 ; zeta = 0.8 ; scale = 15 ; amp = 0.028

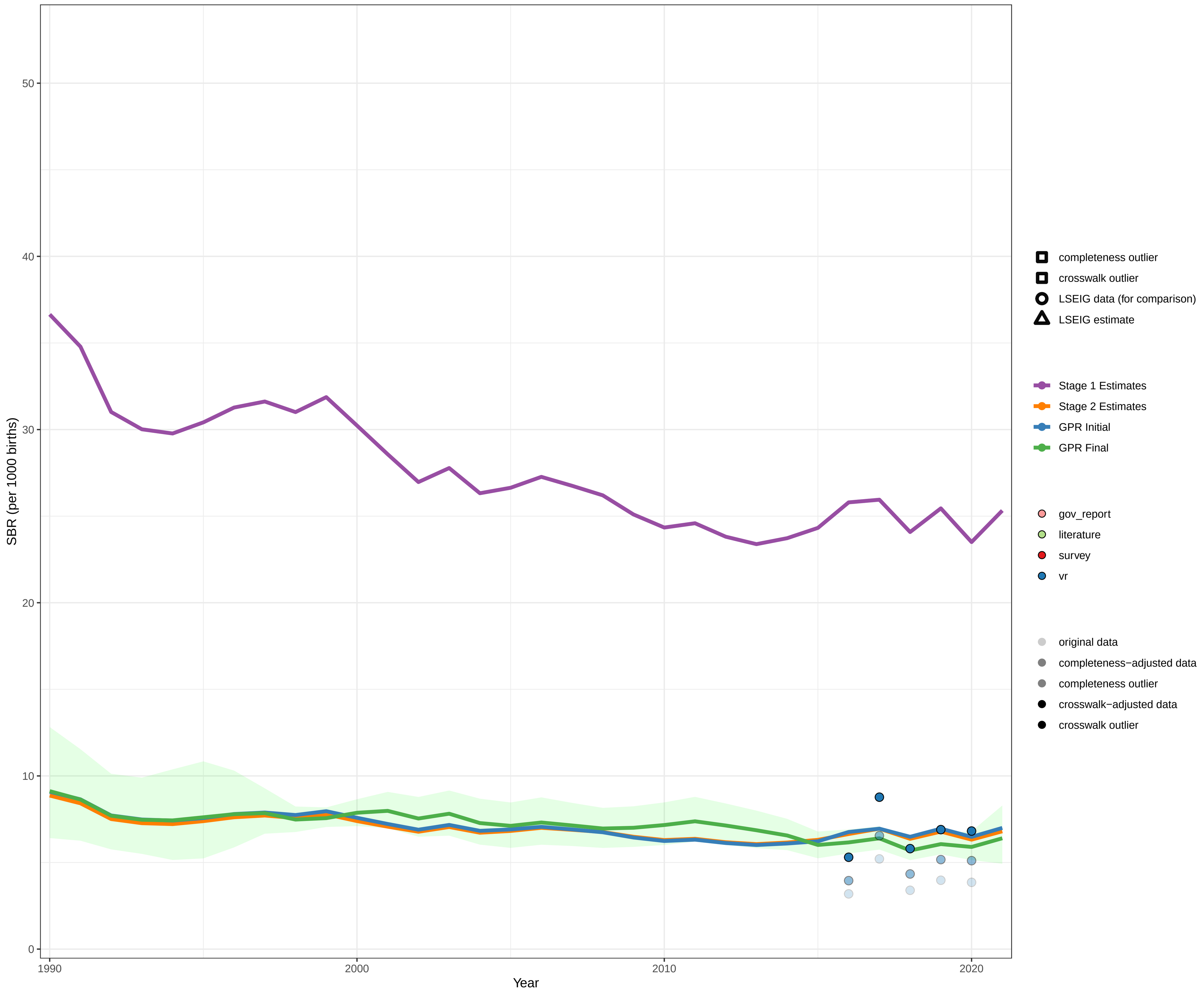

Trafford GBR_44657 Western Europe Stillbirth Rate (SBR) − 28 weeks
lambda = 0.6 ; zeta = 0.8 ; scale = 15 ; amp = 0.028

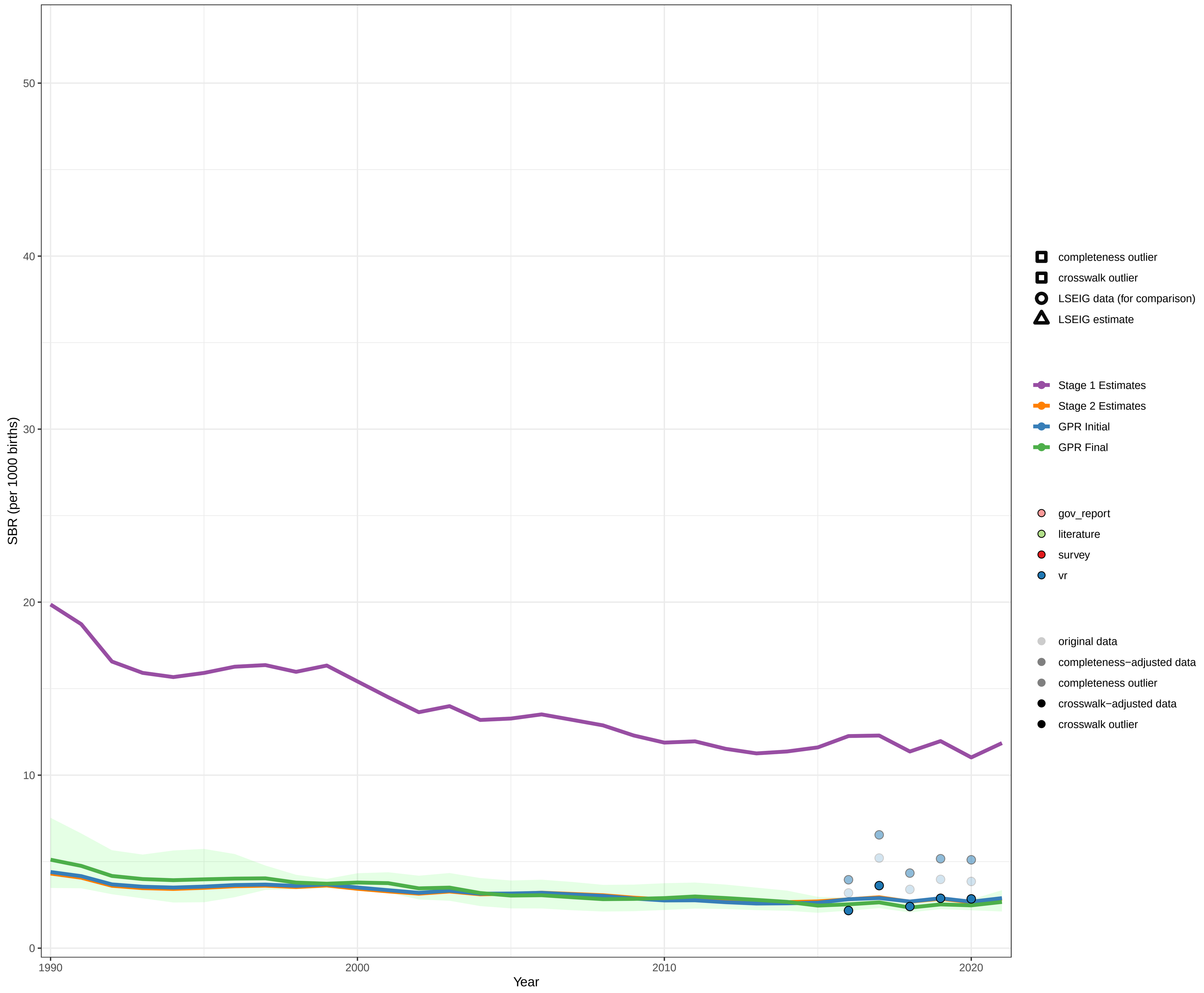

Year	Total Stillbirths (in thousands)	Total Stillbirths (in thousands)	SBR (per 1000 births)	SBR (per 1000 births)	SBR/NMR	SBR/NMR
	20 weeks	28 weeks	20 weeks	28 weeks	20 weeks	28 weeks
1990	0.0 (0.0 to 0.0)	0.0 (0.0 to 0.0)	9.1 (6.4 to 12.8)	5.1 (3.5 to 7.5)	1.71 (1.20 to 2.40)	0.96 (0.65 to 1.41)
1995	0.0 (0.0 to 0.0)	0.0 (0.0 to 0.0)	7.6 (5.2 to 10.8)	4.0 (2.7 to 5.7)	1.96 (1.35 to 2.79)	1.02 (0.68 to 1.47)
2000	0.0 (0.0 to 0.0)	0.0 (0.0 to 0.0)	7.9 (7.1 to 8.7)	3.8 (3.4 to 4.3)	2.25 (2.03 to 2.47)	1.08 (0.96 to 1.24)
2005	0.0 (0.0 to 0.0)	0.0 (0.0 to 0.0)	7.1 (5.8 to 8.5)	3.0 (2.3 to 3.9)	2.55 (2.09 to 3.03)	1.09 (0.82 to 1.40)
2010	0.0 (0.0 to 0.0)	0.0 (0.0 to 0.0)	7.2 (6.0 to 8.5)	2.9 (2.2 to 3.8)	3.02 (2.54 to 3.57)	1.22 (0.93 to 1.58)
2015	0.0 (0.0 to 0.0)	0.0 (0.0 to 0.0)	6.0 (5.2 to 6.8)	2.5 (2.0 to 3.0)	2.71 (2.36 to 3.06)	1.11 (0.92 to 1.34)
2020	0.0 (0.0 to 0.0)	0.0 (0.0 to 0.0)	5.9 (5.1 to 6.8)	2.5 (2.2 to 2.8)	2.87 (2.50 to 3.31)	1.20 (1.07 to 1.37)
2021	0.0 (0.0 to 0.0)	0.0 (0.0 to 0.0)	6.4 (4.9 to 8.3)	2.7 (2.1 to 3.4)	2.90 (2.24 to 3.76)	1.21 (0.96 to 1.52)

Warrington GBR_44666 Western Europe Stillbirth Rate / Neonatal Mortality Rate (SBR/NMR) − 20 weeks
lambda = 0.8 ; zeta = 0.7 ; scale = 15 ; amp = 0.028

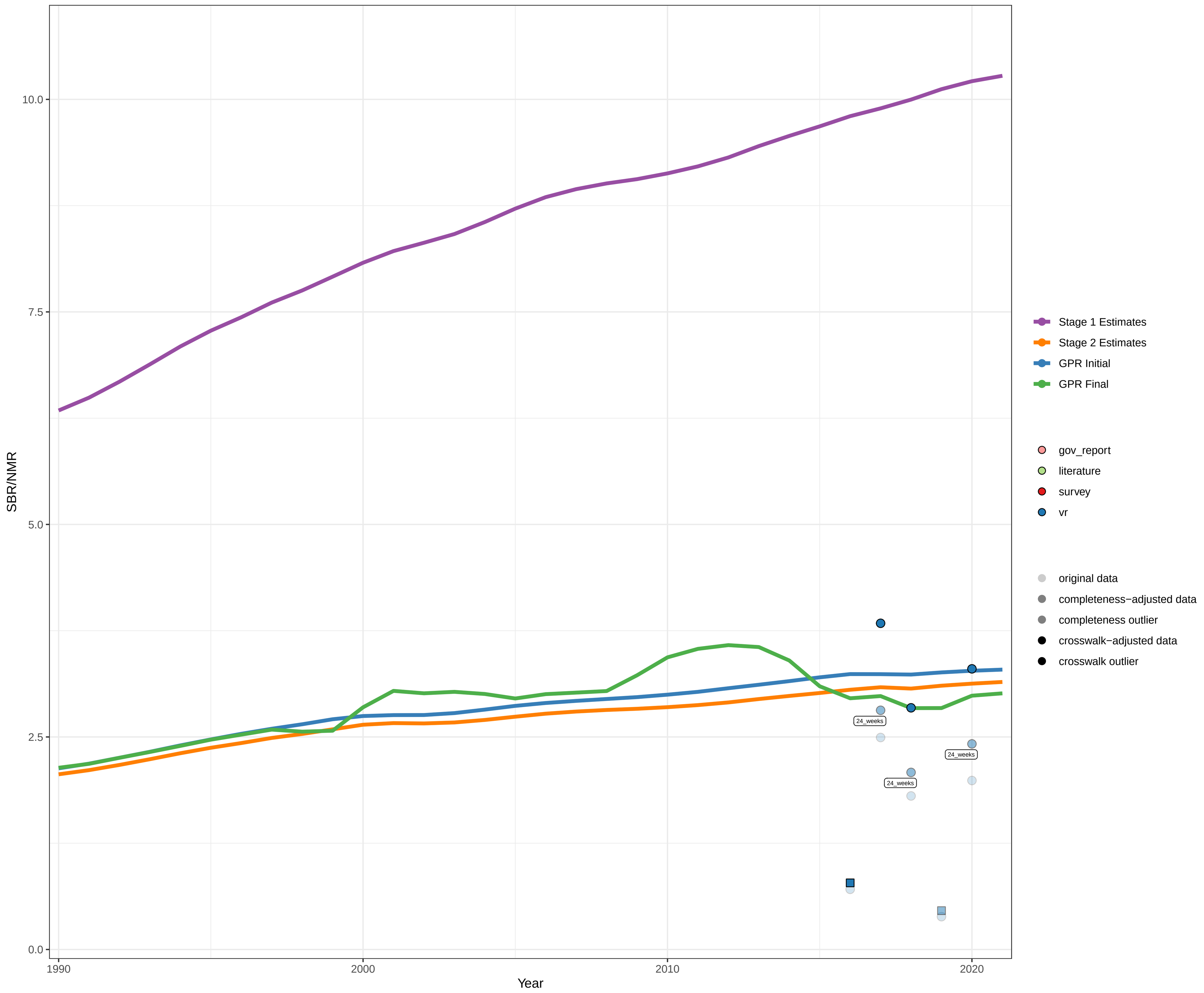

Warrington GBR_44666 Western Europe Stillbirth Rate / Neonatal Mortality Rate (SBR/NMR) − 28 weeks
lambda = 0.8 ; zeta = 0.7 ; scale = 15 ; amp = 0.028

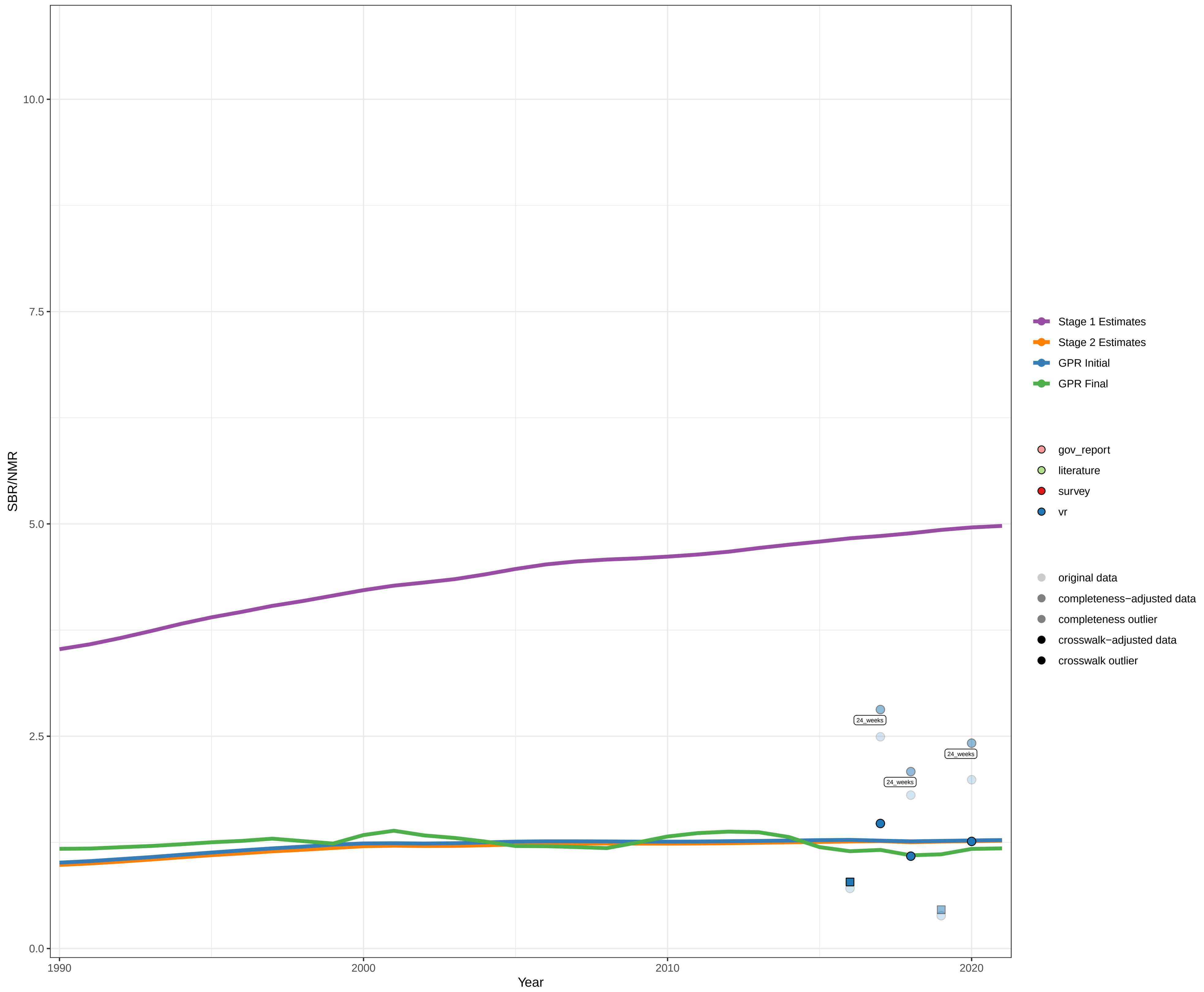

Definition	Years	Outlier	Source.Type	Title
24_weeks	2016−2018, 2020	0	VR	United Kingdom − England and Wales Child Mortality Statistics
24_weeks	2019	1	VR	United Kingdom − England and Wales Child Mortality Statistics

Warrington GBR_44666 Western Europe Stillbirth Rate (SBR) − 20 weeks
lambda = 0.8 ; zeta = 0.7 ; scale = 15 ; amp = 0.028

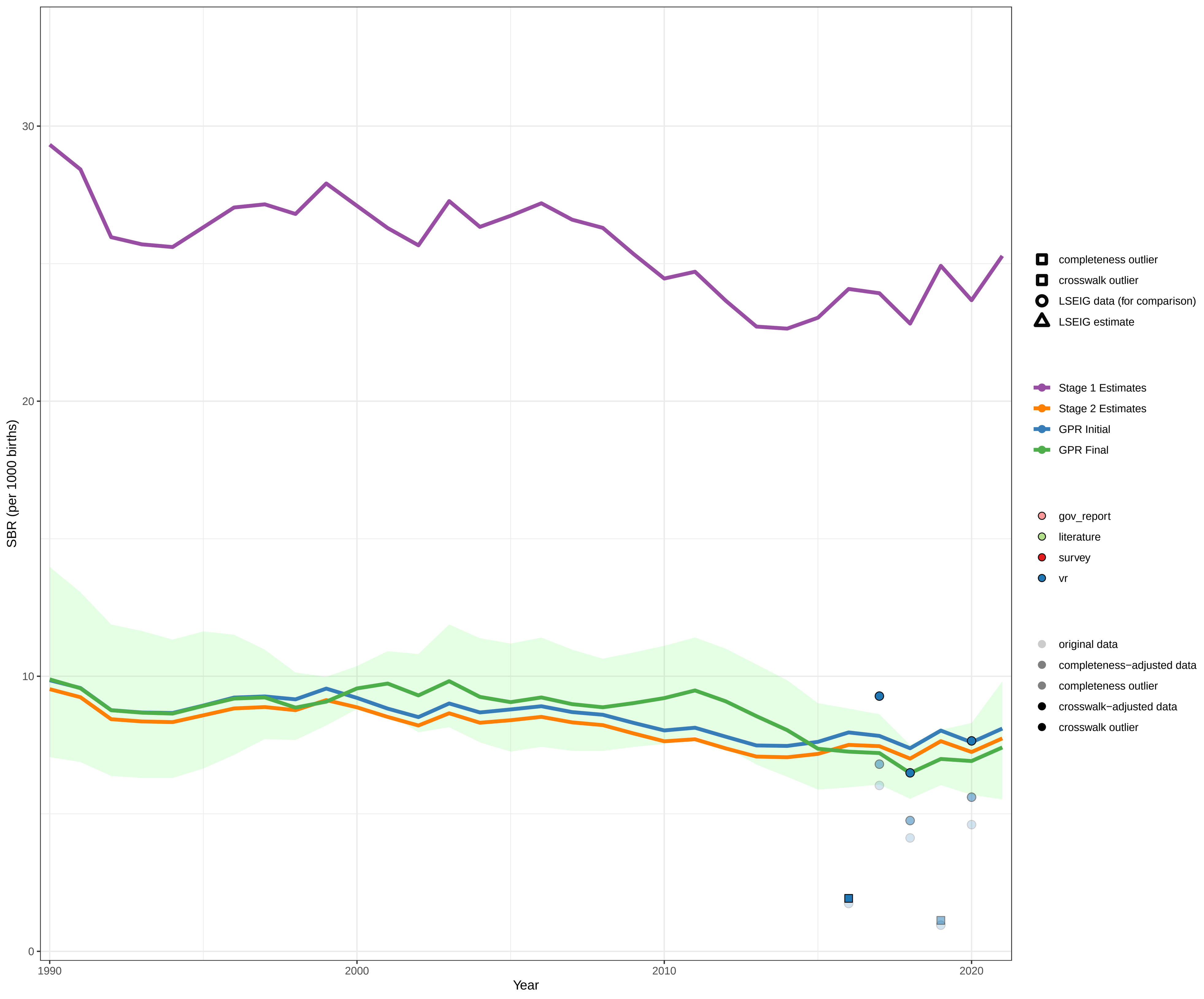

Warrington GBR_44666 Western Europe Stillbirth Rate (SBR) − 28 weeks
lambda = 0.8 ; zeta = 0.7 ; scale = 15 ; amp = 0.028

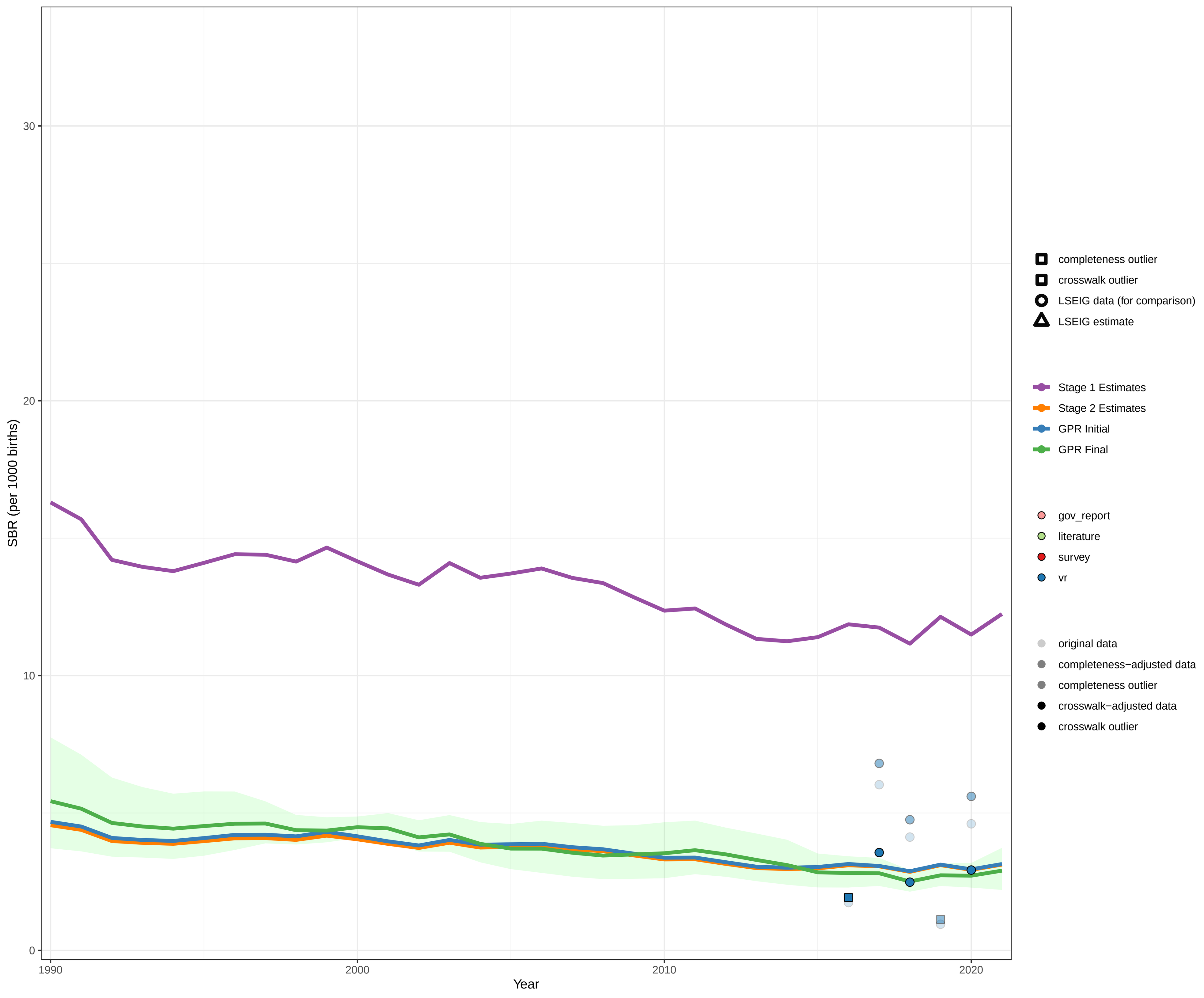

Year	Total Stillbirths (in thousands)	Total Stillbirths (in thousands)	SBR (per 1000 births)	SBR (per 1000 births)	SBR/NMR	SBR/NMR
	20 weeks	28 weeks	20 weeks	28 weeks	20 weeks	28 weeks
1990	0.0 (0.0 to 0.0)	0.0 (0.0 to 0.0)	9.9 (7.1 to 14.0)	5.4 (3.7 to 7.8)	2.14 (1.53 to 3.02)	1.17 (0.80 to 1.68)
1995	0.0 (0.0 to 0.0)	0.0 (0.0 to 0.0)	8.9 (6.6 to 11.6)	4.5 (3.4 to 5.8)	2.47 (1.84 to 3.22)	1.25 (0.95 to 1.60)
2000	0.0 (0.0 to 0.0)	0.0 (0.0 to 0.0)	9.6 (8.8 to 10.4)	4.5 (4.1 to 4.9)	2.85 (2.63 to 3.09)	1.34 (1.22 to 1.45)
2005	0.0 (0.0 to 0.0)	0.0 (0.0 to 0.0)	9.1 (7.3 to 11.2)	3.7 (3.0 to 4.6)	2.95 (2.37 to 3.65)	1.21 (0.96 to 1.50)
2010	0.0 (0.0 to 0.0)	0.0 (0.0 to 0.0)	9.2 (7.5 to 11.1)	3.5 (2.6 to 4.7)	3.44 (2.81 to 4.15)	1.32 (0.98 to 1.74)
2015	0.0 (0.0 to 0.0)	0.0 (0.0 to 0.0)	7.4 (5.9 to 9.0)	2.8 (2.3 to 3.5)	3.10 (2.47 to 3.79)	1.19 (0.96 to 1.48)
2020	0.0 (0.0 to 0.0)	0.0 (0.0 to 0.0)	6.9 (5.7 to 8.3)	2.7 (2.3 to 3.2)	2.99 (2.46 to 3.58)	1.17 (0.99 to 1.37)
2021	0.0 (0.0 to 0.0)	0.0 (0.0 to 0.0)	7.4 (5.5 to 9.8)	2.9 (2.2 to 3.7)	3.01 (2.24 to 3.99)	1.18 (0.89 to 1.52)

Wigan GBR_44669 Western Europe Stillbirth Rate / Neonatal Mortality Rate (SBR/NMR) − 20 weeks
lambda = 0.6 ; zeta = 0.8 ; scale = 15 ; amp = 0.028

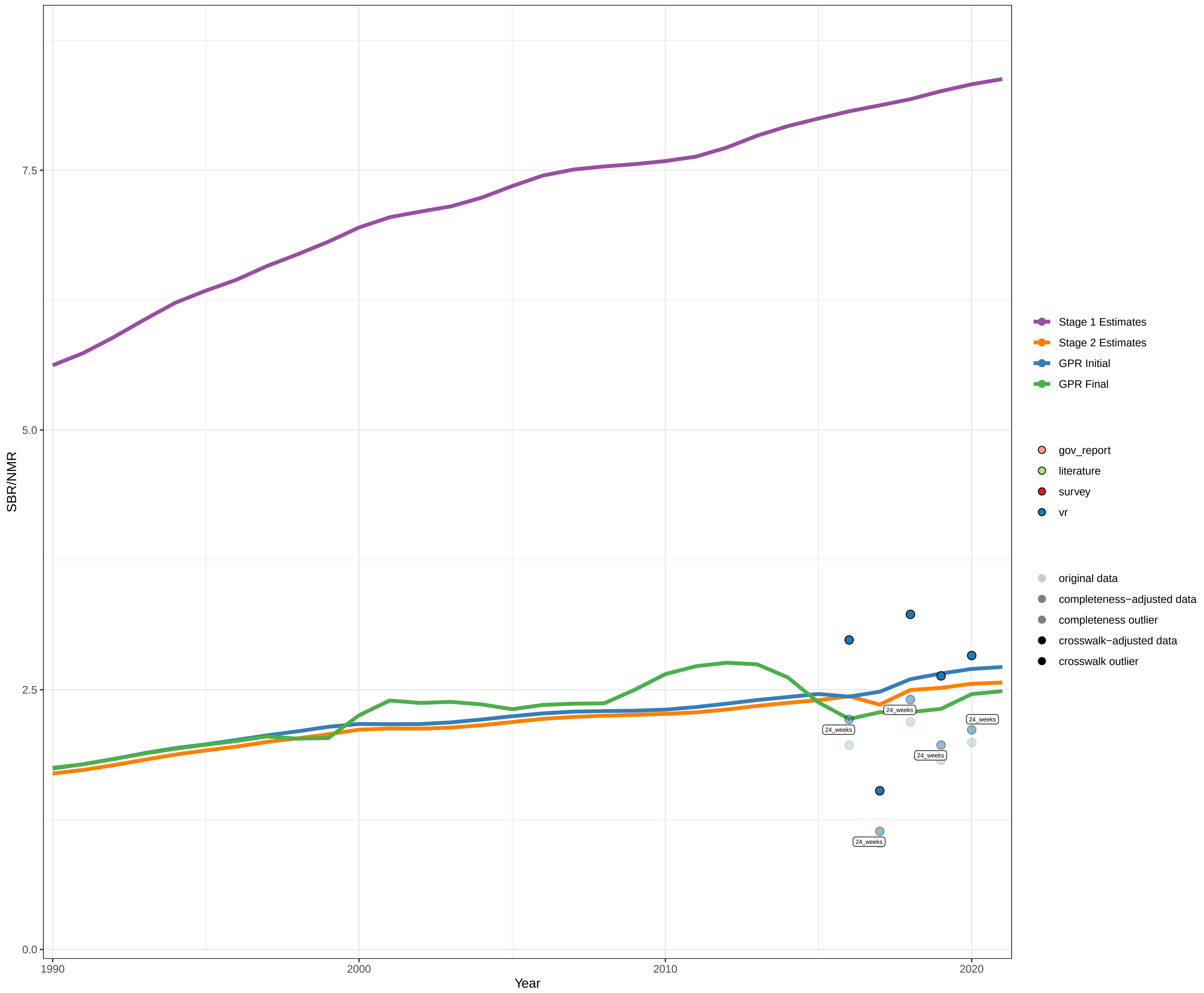

Wigan GBR_44669 Western Europe Stillbirth Rate / Neonatal Mortality Rate (SBR/NMR) − 28 weeks
lambda = 0.6 ; zeta = 0.8 ; scale = 15 ; amp = 0.028

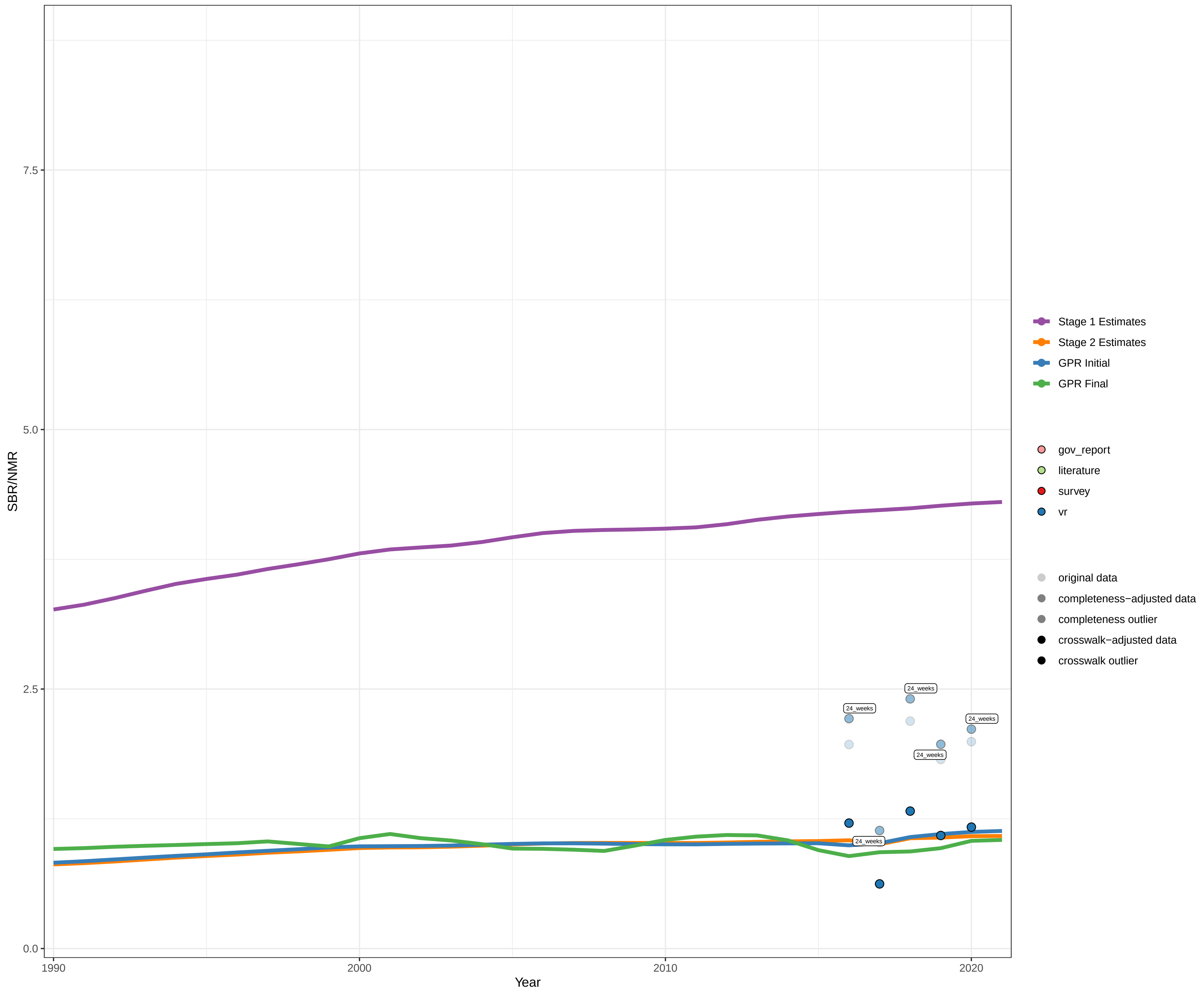

Definition	Years	Outlier	Source.Type	Title
24_weeks	2016−2020	0	VR	United Kingdom − England and Wales Child Mortality Statistics

Wigan GBR_44669 Western Europe Stillbirth Rate (SBR) − 20 weeks
lambda = 0.6 ; zeta = 0.8 ; scale = 15 ; amp = 0.028

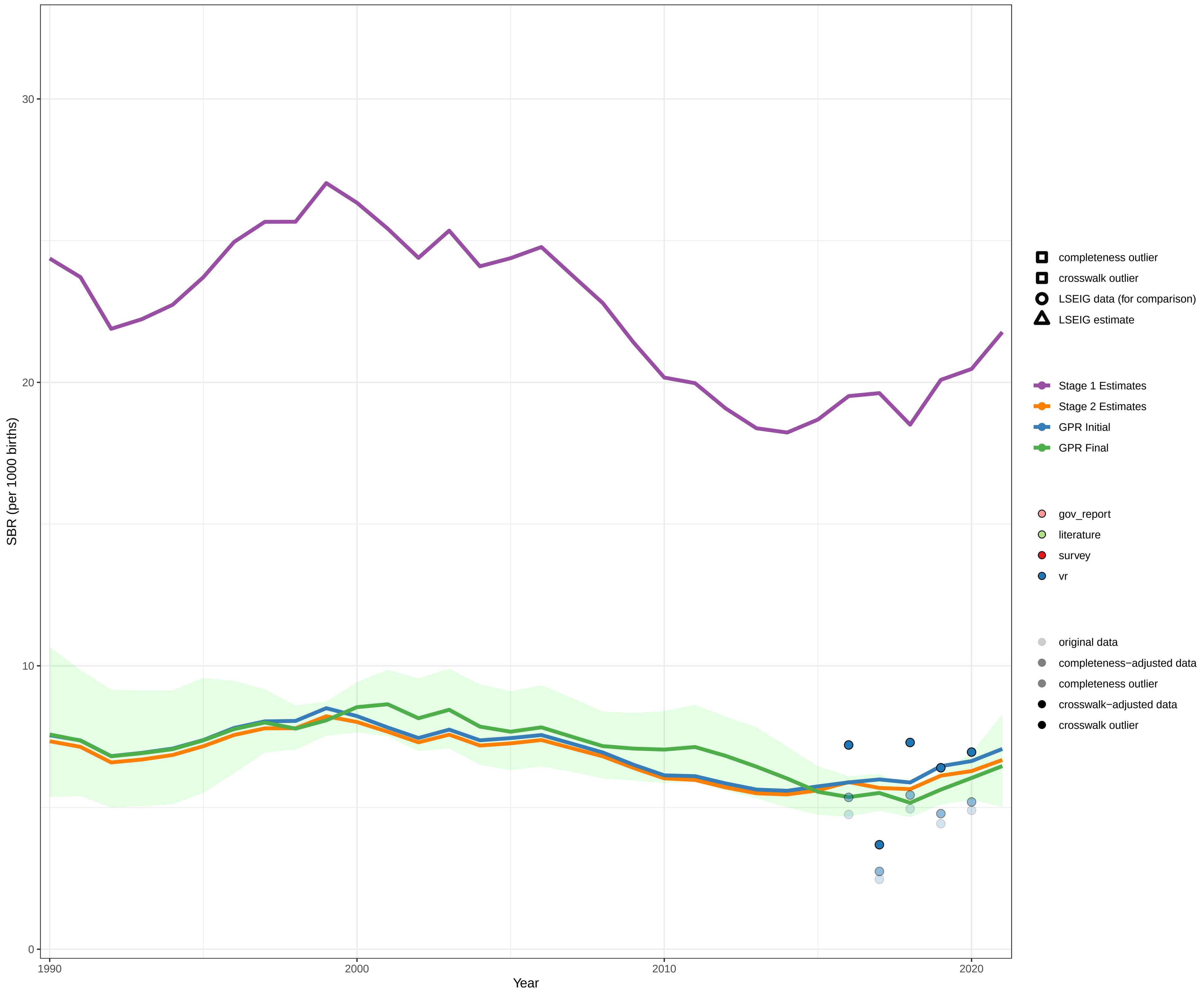

Wigan GBR_44669 Western Europe Stillbirth Rate (SBR) − 28 weeks
lambda = 0.6 ; zeta = 0.8 ; scale = 15 ; amp = 0.028

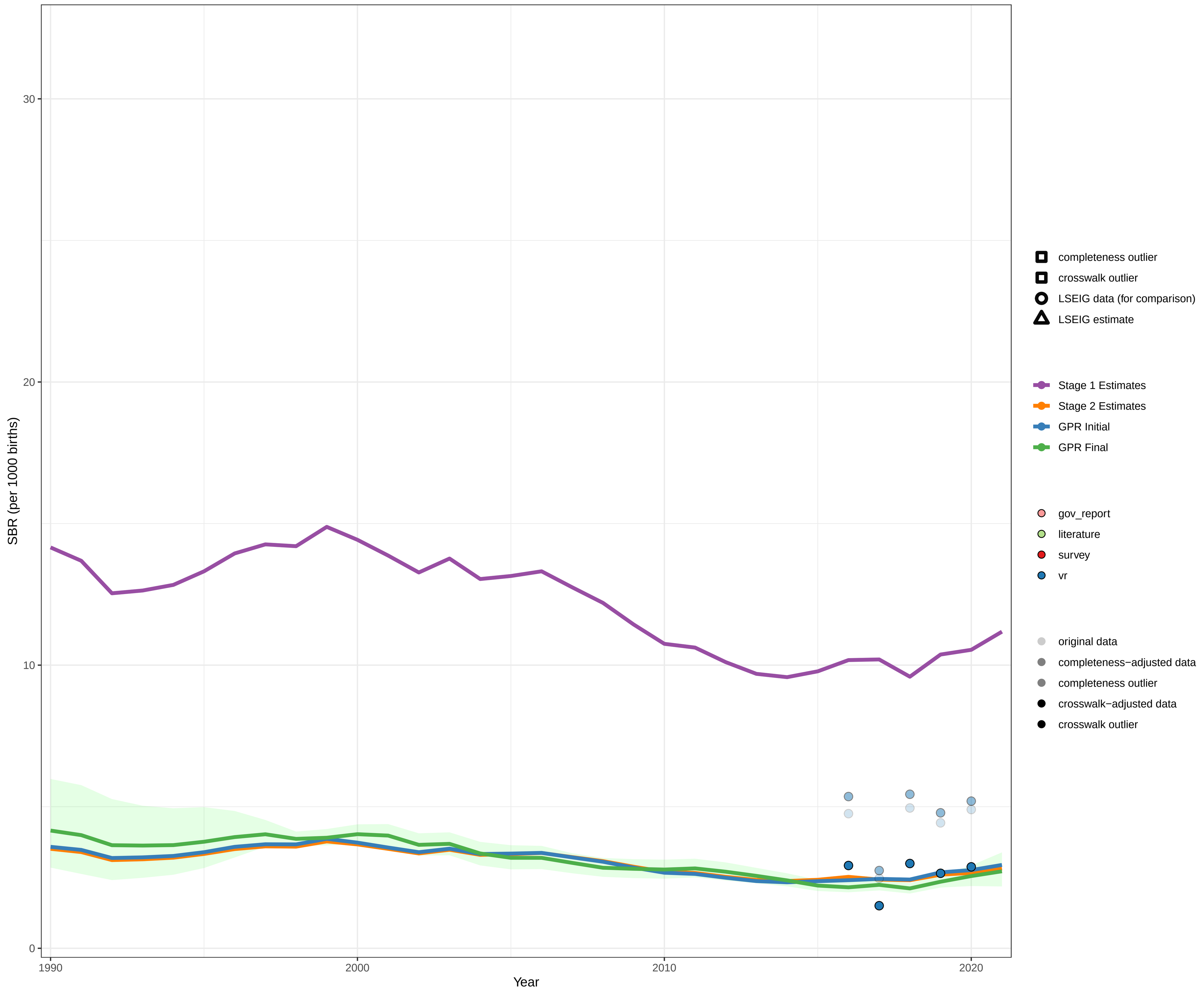

Year	Total Stillbirths (in thousands)	Total Stillbirths (in thousands)	SBR (per 1000 births)	SBR (per 1000 births)	SBR/NMR	SBR/NMR
	20 weeks	28 weeks	20 weeks	28 weeks	20 weeks	28 weeks
1990	0.0 (0.0 to 0.0)	0.0 (0.0 to 0.0)	7.6 (5.4 to 10.7)	4.2 (2.8 to 6.0)	1.75 (1.24 to 2.46)	0.96 (0.66 to 1.38)
1995	0.0 (0.0 to 0.0)	0.0 (0.0 to 0.0)	7.4 (5.5 to 9.6)	3.8 (2.8 to 5.0)	1.97 (1.48 to 2.56)	1.01 (0.76 to 1.33)
2000	0.0 (0.0 to 0.0)	0.0 (0.0 to 0.0)	8.5 (7.6 to 9.4)	4.0 (3.7 to 4.4)	2.25 (2.02 to 2.49)	1.06 (0.98 to 1.16)
2005	0.0 (0.0 to 0.0)	0.0 (0.0 to 0.0)	7.7 (6.3 to 9.1)	3.2 (2.8 to 3.6)	2.31 (1.90 to 2.74)	0.96 (0.84 to 1.10)
2010	0.0 (0.0 to 0.0)	0.0 (0.0 to 0.0)	7.0 (5.9 to 8.4)	2.8 (2.5 to 3.1)	2.65 (2.20 to 3.16)	1.05 (0.92 to 1.18)
2015	0.0 (0.0 to 0.0)	0.0 (0.0 to 0.0)	5.6 (4.7 to 6.5)	2.2 (2.0 to 2.4)	2.38 (2.03 to 2.76)	0.95 (0.87 to 1.03)
2020	0.0 (0.0 to 0.0)	0.0 (0.0 to 0.0)	6.0 (5.3 to 6.9)	2.6 (2.2 to 2.9)	2.46 (2.15 to 2.82)	1.04 (0.89 to 1.19)
2021	0.0 (0.0 to 0.0)	0.0 (0.0 to 0.0)	6.5 (5.0 to 8.3)	2.7 (2.2 to 3.4)	2.49 (1.93 to 3.19)	1.05 (0.84 to 1.30)

Wirral GBR_44663 Western Europe Stillbirth Rate / Neonatal Mortality Rate (SBR/NMR) − 20 weeks
lambda = 0.8 ; zeta = 0.7 ; scale = 15 ; amp = 0.028

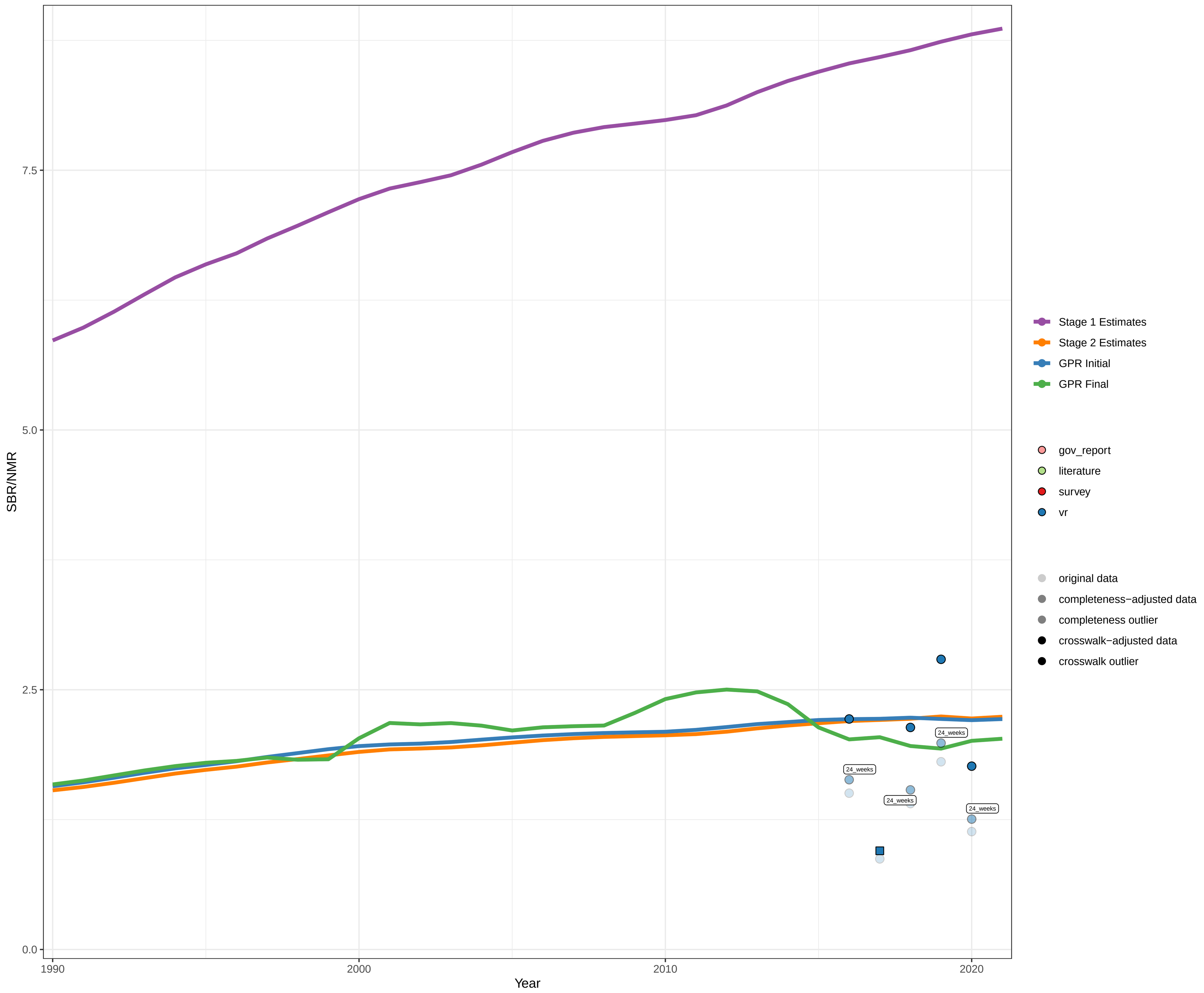

Wirral GBR_44663 Western Europe Stillbirth Rate / Neonatal Mortality Rate (SBR/NMR) − 28 weeks
lambda = 0.8 ; zeta = 0.7 ; scale = 15 ; amp = 0.028

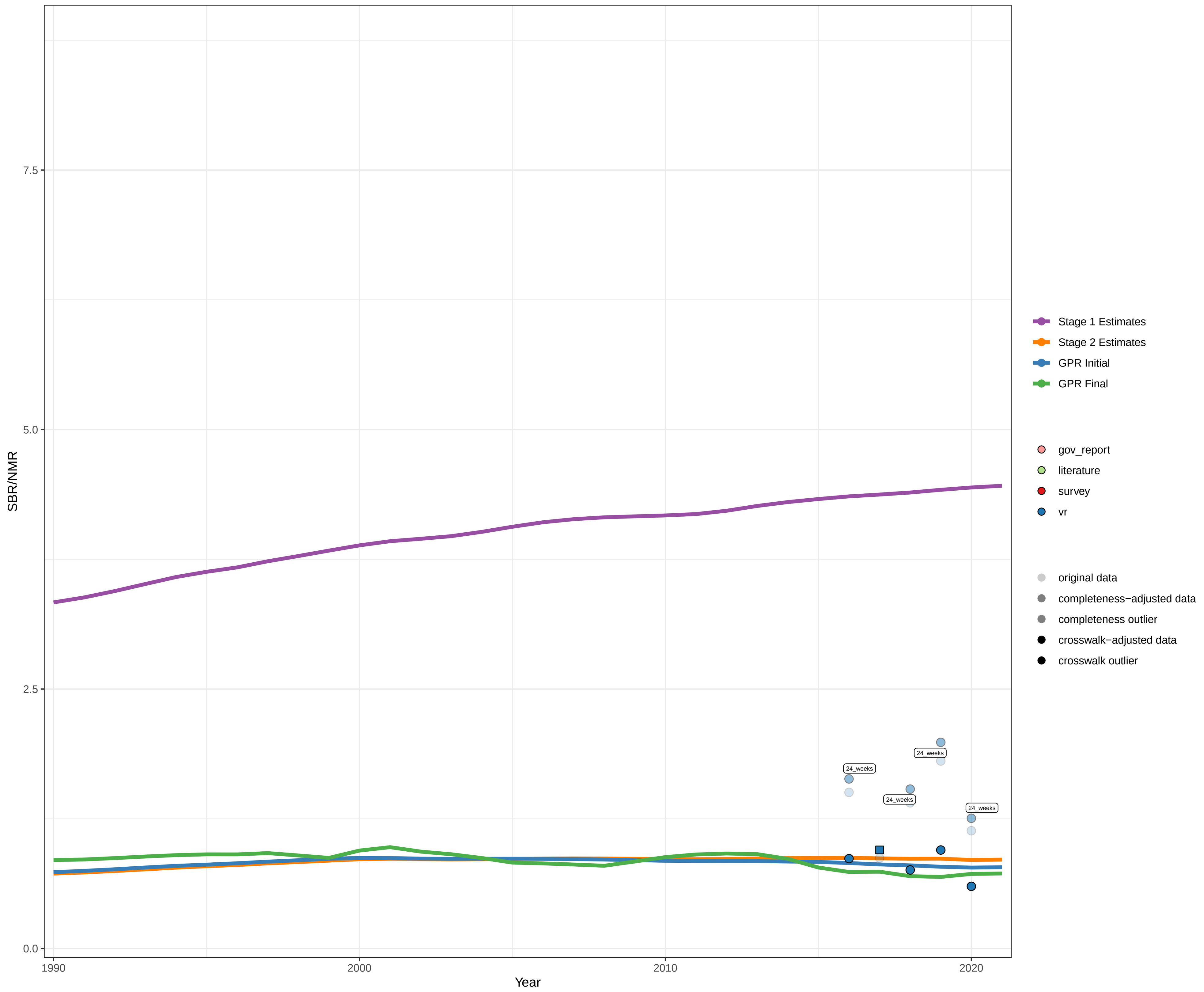

Definition	Years	Outlier	Source.Type	Title
24_weeks	2016−2020	0	VR	United Kingdom − England and Wales Child Mortality Statistics

Wirral GBR_44663 Western Europe Stillbirth Rate (SBR) − 20 weeks
lambda = 0.8 ; zeta = 0.7 ; scale = 15 ; amp = 0.028

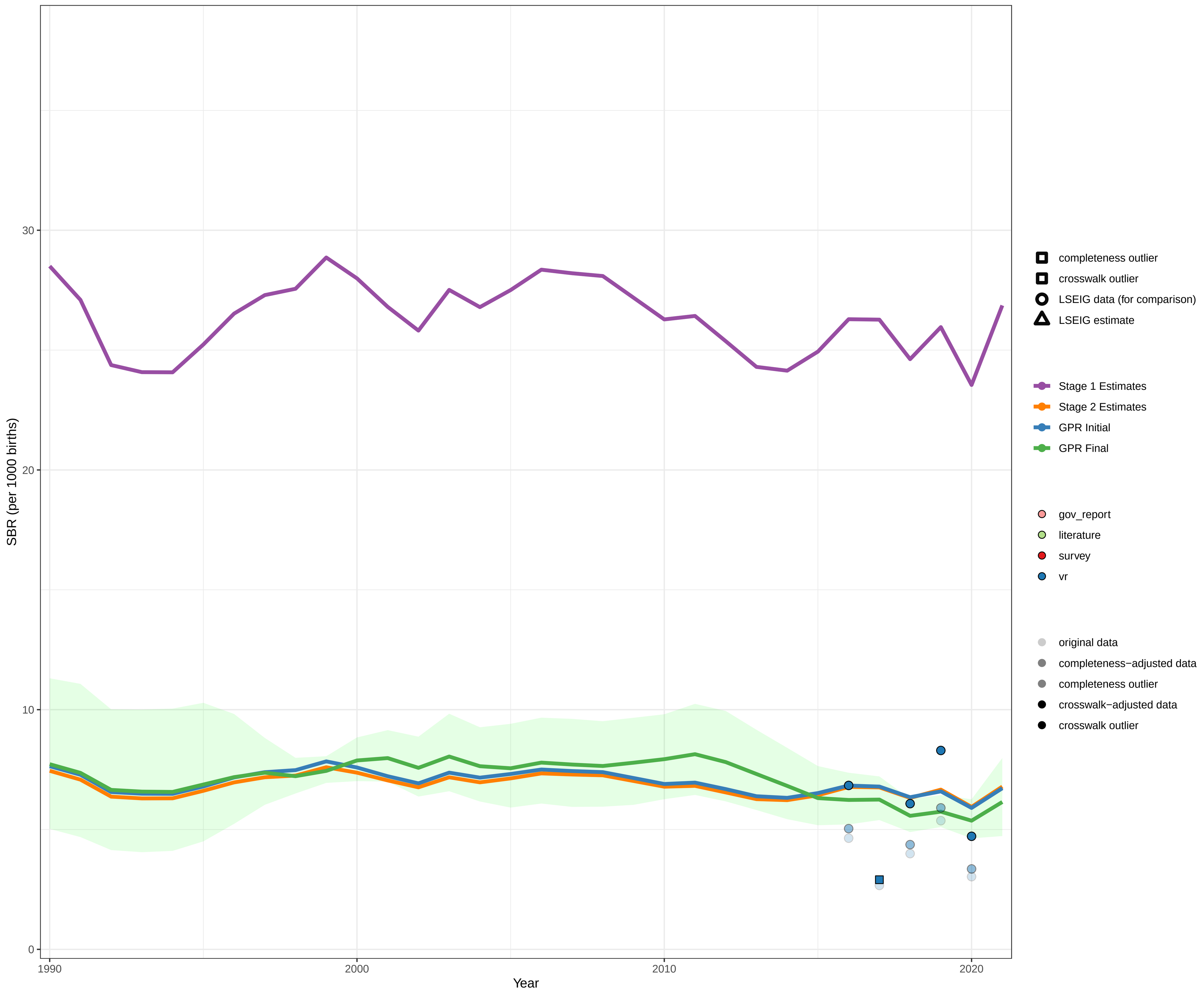

Wirral GBR_44663 Western Europe Stillbirth Rate (SBR) − 28 weeks
lambda = 0.8 ; zeta = 0.7 ; scale = 15 ; amp = 0.028

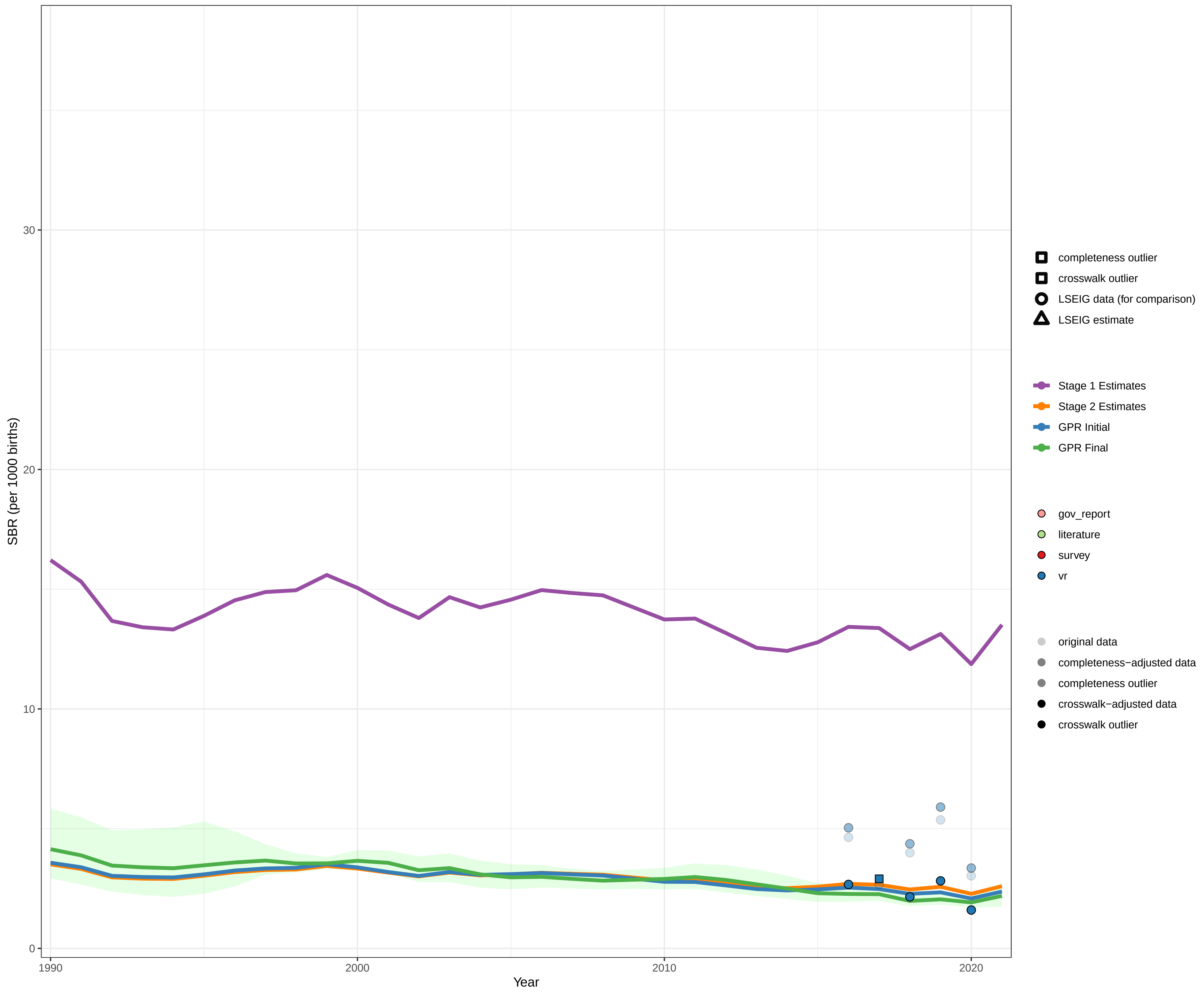

Year	Total Stillbirths (in thousands)	Total Stillbirths (in thousands)	SBR (per 1000 births)	SBR (per 1000 births)	SBR/NMR	SBR/NMR
	20 weeks	28 weeks	20 weeks	28 weeks	20 weeks	28 weeks
1990	0.0 (0.0 to 0.1)	0.0 (0.0 to 0.0)	7.7 (5.0 to 11.3)	4.1 (2.9 to 5.8)	1.59 (1.03 to 2.33)	0.85 (0.60 to 1.20)
1995	0.0 (0.0 to 0.0)	0.0 (0.0 to 0.0)	6.9 (4.5 to 10.3)	3.5 (2.3 to 5.3)	1.80 (1.18 to 2.69)	0.91 (0.60 to 1.39)
2000	0.0 (0.0 to 0.0)	0.0 (0.0 to 0.0)	7.9 (7.0 to 8.8)	3.7 (3.3 to 4.1)	2.03 (1.81 to 2.28)	0.94 (0.84 to 1.06)
2005	0.0 (0.0 to 0.0)	0.0 (0.0 to 0.0)	7.6 (5.9 to 9.4)	3.0 (2.5 to 3.5)	2.11 (1.65 to 2.63)	0.83 (0.69 to 0.98)
2010	0.0 (0.0 to 0.0)	0.0 (0.0 to 0.0)	7.9 (6.3 to 9.8)	2.9 (2.5 to 3.4)	2.41 (1.90 to 2.98)	0.88 (0.75 to 1.02)
2015	0.0 (0.0 to 0.0)	0.0 (0.0 to 0.0)	6.3 (5.2 to 7.6)	2.3 (1.9 to 2.7)	2.14 (1.75 to 2.59)	0.78 (0.66 to 0.93)
2020	0.0 (0.0 to 0.0)	0.0 (0.0 to 0.0)	5.4 (4.6 to 6.3)	1.9 (1.7 to 2.2)	2.01 (1.73 to 2.34)	0.72 (0.64 to 0.82)
2021	0.0 (0.0 to 0.0)	0.0 (0.0 to 0.0)	6.1 (4.7 to 8.0)	2.2 (1.7 to 2.7)	2.03 (1.56 to 2.63)	0.72 (0.57 to 0.91)

South East England GBR_4625 Western Europe Stillbirth Rate / Neonatal Mortality Rate (SBR/NMR) − 20 weeks
lambda = 0.2 ; zeta = 0.99 ; scale = 5 ; amp = 0.028

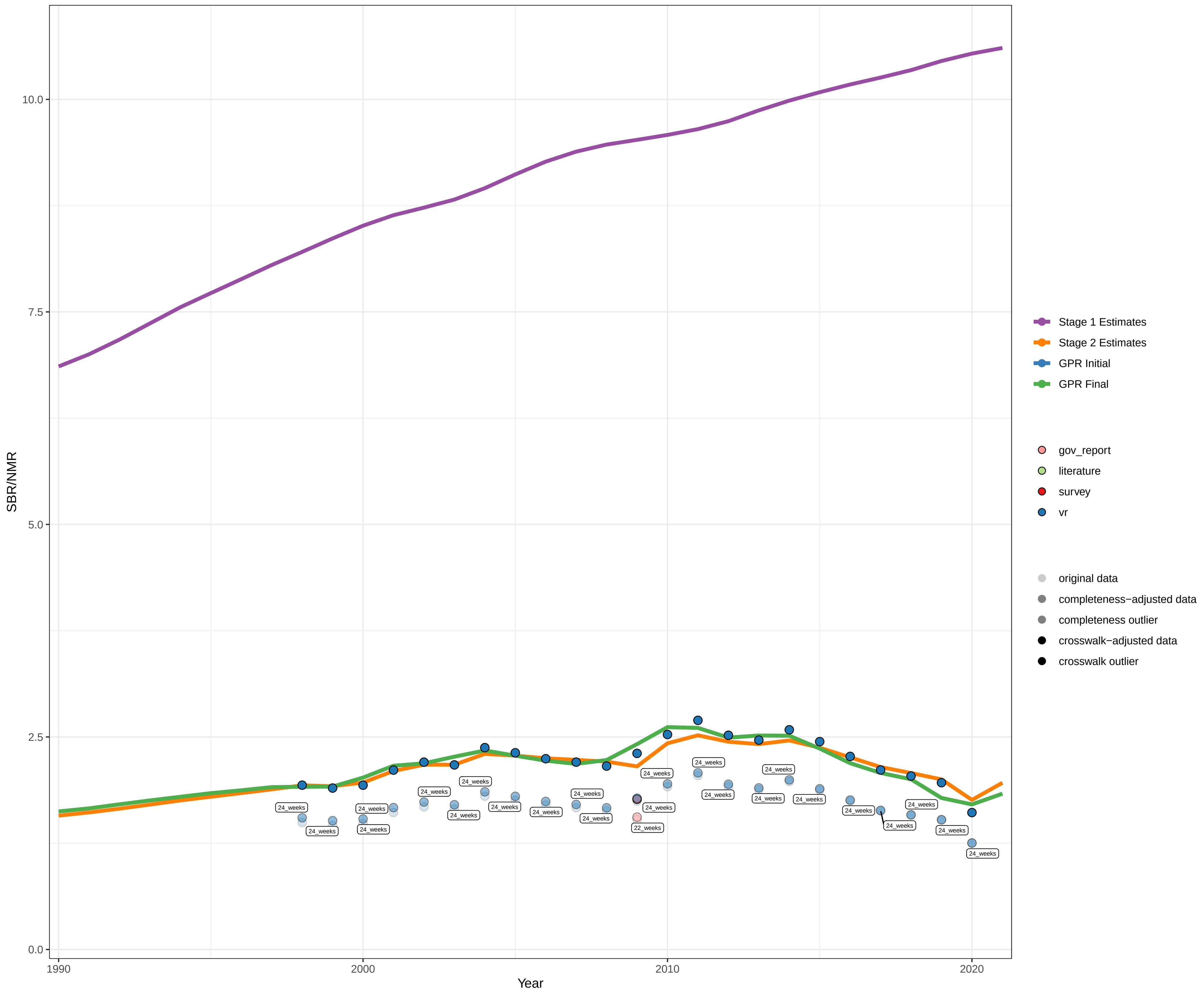

South East England GBR_4625 Western Europe Stillbirth Rate / Neonatal Mortality Rate (SBR/NMR) − 28 weeks
lambda = 0.2 ; zeta = 0.99 ; scale = 5 ; amp = 0.028

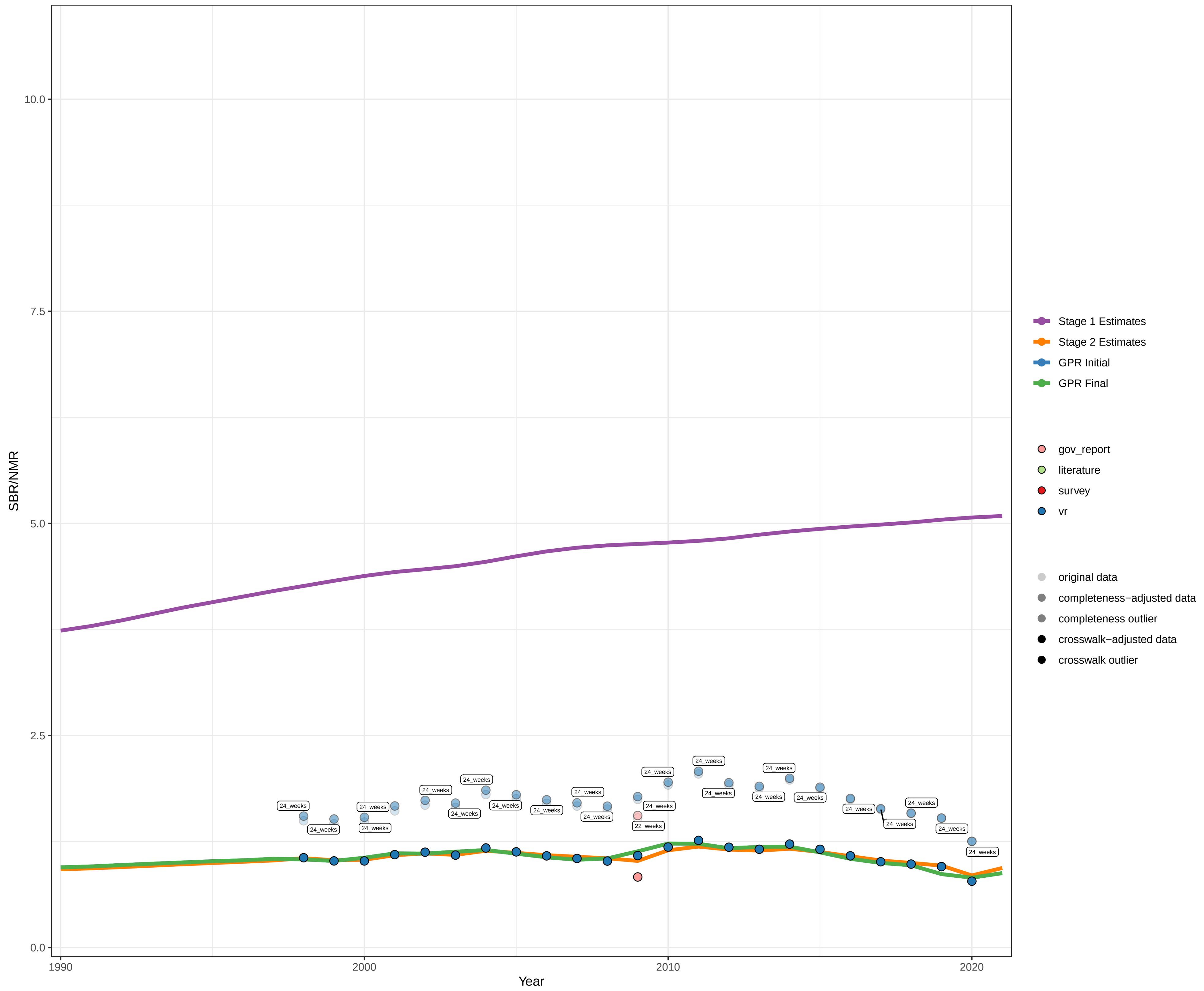

Definition	Years	Outlier	Source.Type	Title
22_weeks	2009	0	Statistical Report	United Kingdom Perinatal Mortality
24_weeks	1998−2007	0	VR	United Kingdom − England and Wales Birth Statistics
24_weeks	2008−2020	0	VR	United Kingdom − England and Wales Child Mortality Statistics

South East England GBR_4625 Western Europe Stillbirth Rate (SBR) − 20 weeks
lambda = 0.2 ; zeta = 0.99 ; scale = 5 ; amp = 0.028

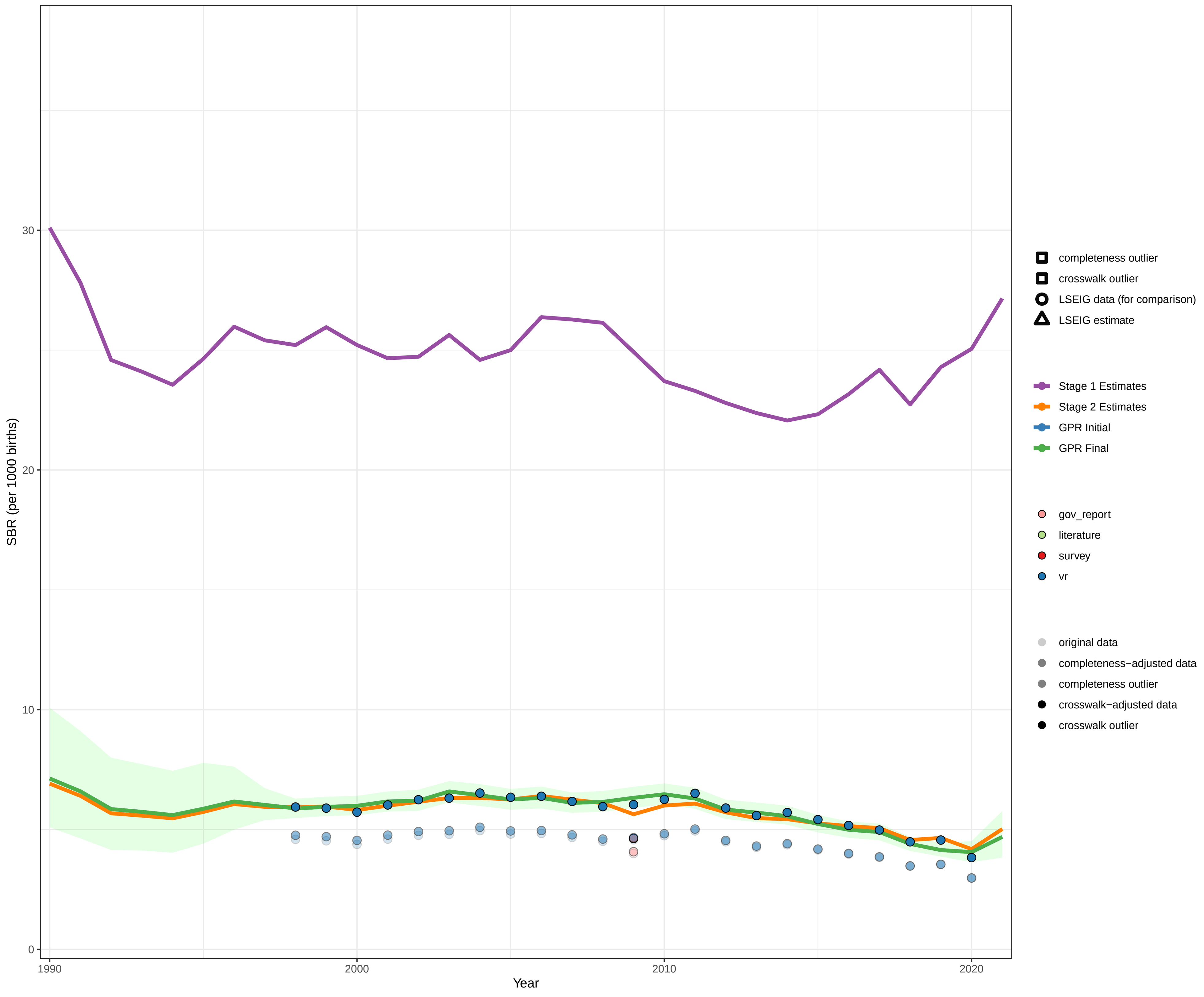

South East England GBR_4625 Western Europe Stillbirth Rate (SBR) − 28 weeks
lambda = 0.2 ; zeta = 0.99 ; scale = 5 ; amp = 0.028

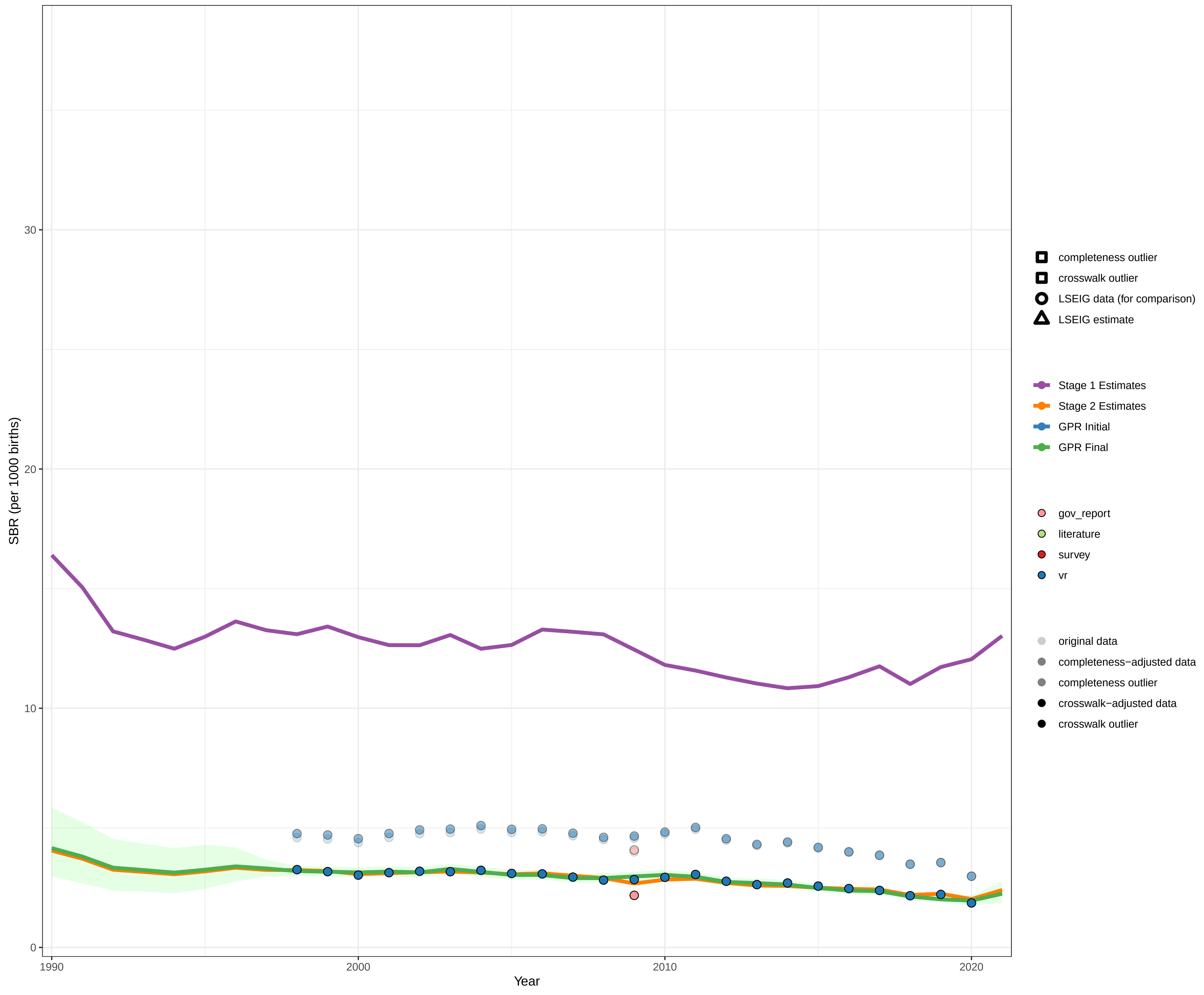

Year	Total Stillbirths (in thousands)	Total Stillbirths (in thousands)	SBR (per 1000 births)	SBR (per 1000 births)	SBR/NMR	SBR/NMR
	20 weeks	28 weeks	20 weeks	28 weeks	20 weeks	28 weeks
1990	0.7 (0.5 to 1.0)	0.4 (0.3 to 0.6)	7.1 (5.1 to 10.1)	4.2 (3.0 to 5.9)	1.62 (1.16 to 2.30)	0.95 (0.68 to 1.33)
1995	0.6 (0.4 to 0.8)	0.3 (0.2 to 0.4)	5.9 (4.4 to 7.8)	3.2 (2.4 to 4.3)	1.84 (1.38 to 2.44)	1.02 (0.77 to 1.35)
2000	0.5 (0.5 to 0.6)	0.3 (0.3 to 0.3)	6.0 (5.6 to 6.4)	3.1 (2.9 to 3.3)	2.02 (1.89 to 2.16)	1.06 (0.99 to 1.13)
2005	0.6 (0.6 to 0.6)	0.3 (0.3 to 0.3)	6.3 (5.8 to 6.7)	3.0 (2.8 to 3.2)	2.28 (2.13 to 2.44)	1.11 (1.03 to 1.18)
2010	0.7 (0.6 to 0.7)	0.3 (0.3 to 0.3)	6.5 (6.0 to 6.9)	3.0 (2.8 to 3.2)	2.62 (2.44 to 2.80)	1.23 (1.15 to 1.31)
2015	0.5 (0.5 to 0.6)	0.3 (0.2 to 0.3)	5.2 (4.9 to 5.6)	2.5 (2.3 to 2.7)	2.36 (2.20 to 2.53)	1.12 (1.05 to 1.20)
2020	0.4 (0.3 to 0.4)	0.2 (0.2 to 0.2)	4.1 (3.6 to 4.5)	2.0 (1.8 to 2.2)	1.71 (1.53 to 1.90)	0.82 (0.74 to 0.91)
2021	0.4 (0.3 to 0.5)	0.2 (0.2 to 0.3)	4.7 (3.8 to 5.8)	2.2 (1.8 to 2.8)	1.83 (1.49 to 2.26)	0.88 (0.72 to 1.08)

Bracknell Forest GBR_44765 Western Europe Stillbirth Rate / Neonatal Mortality Rate (SBR/NMR) − 20 weeks
lambda = 0.8 ; zeta = 0.7 ; scale = 15 ; amp = 0.028

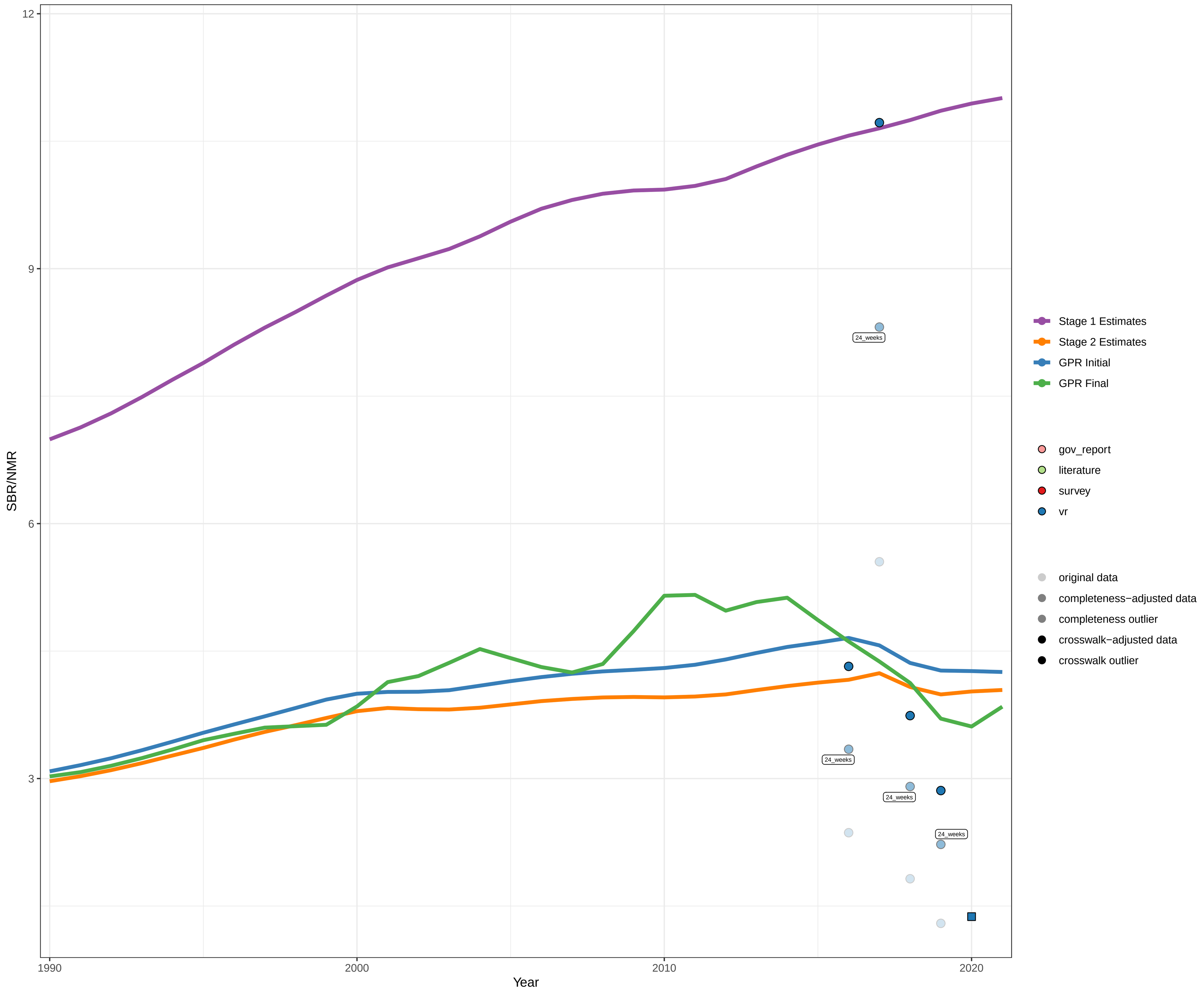

Bracknell Forest GBR_44765 Western Europe Stillbirth Rate / Neonatal Mortality Rate (SBR/NMR) − 28 weeks
lambda = 0.8 ; zeta = 0.7 ; scale = 15 ; amp = 0.028

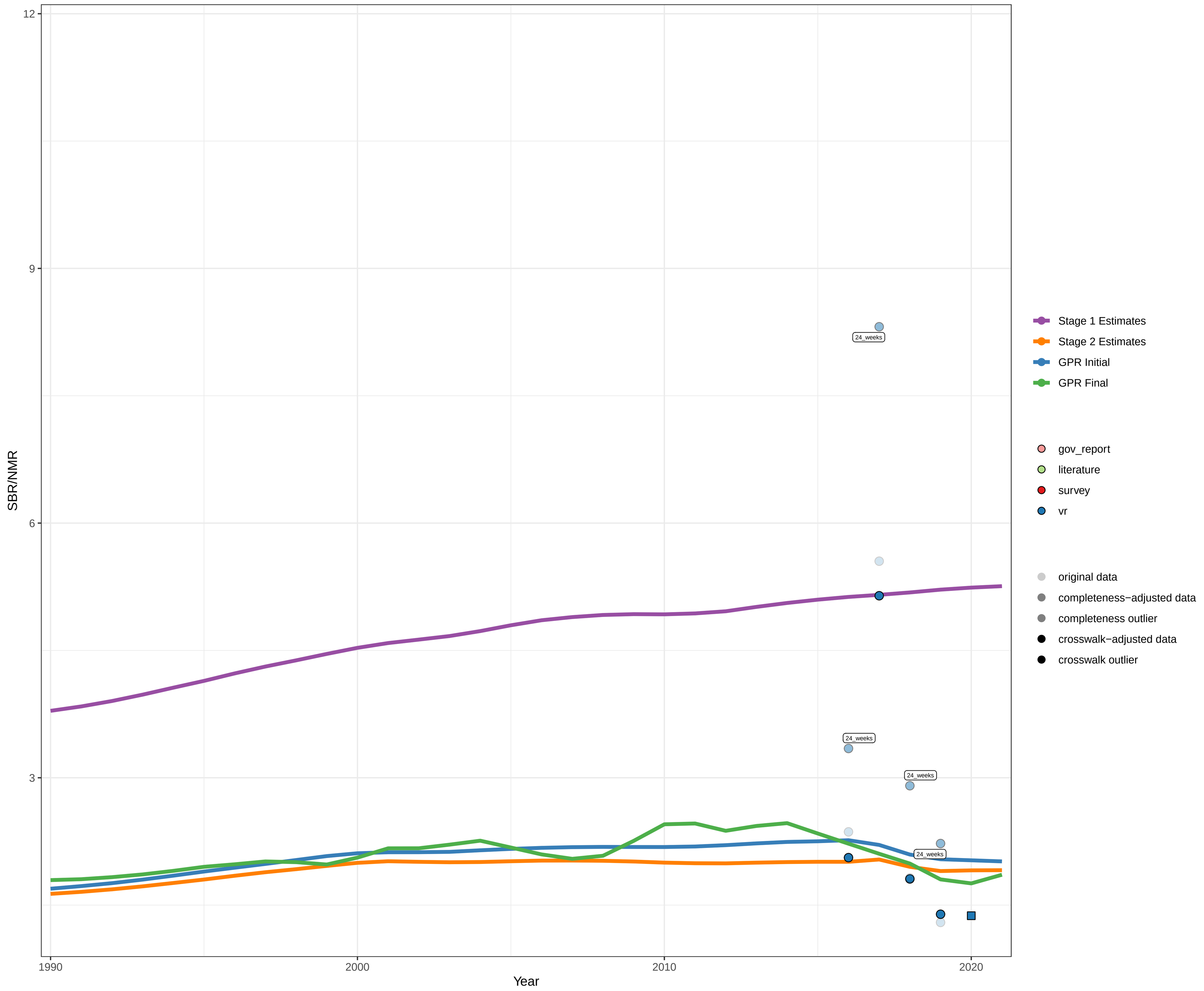

Definition	Years	Outlier	Source.Type	Title
24_weeks	2016−2020	0	VR	United Kingdom − England and Wales Child Mortality Statistics

Bracknell Forest GBR_44765 Western Europe Stillbirth Rate (SBR) − 20 weeks
lambda = 0.8 ; zeta = 0.7 ; scale = 15 ; amp = 0.028

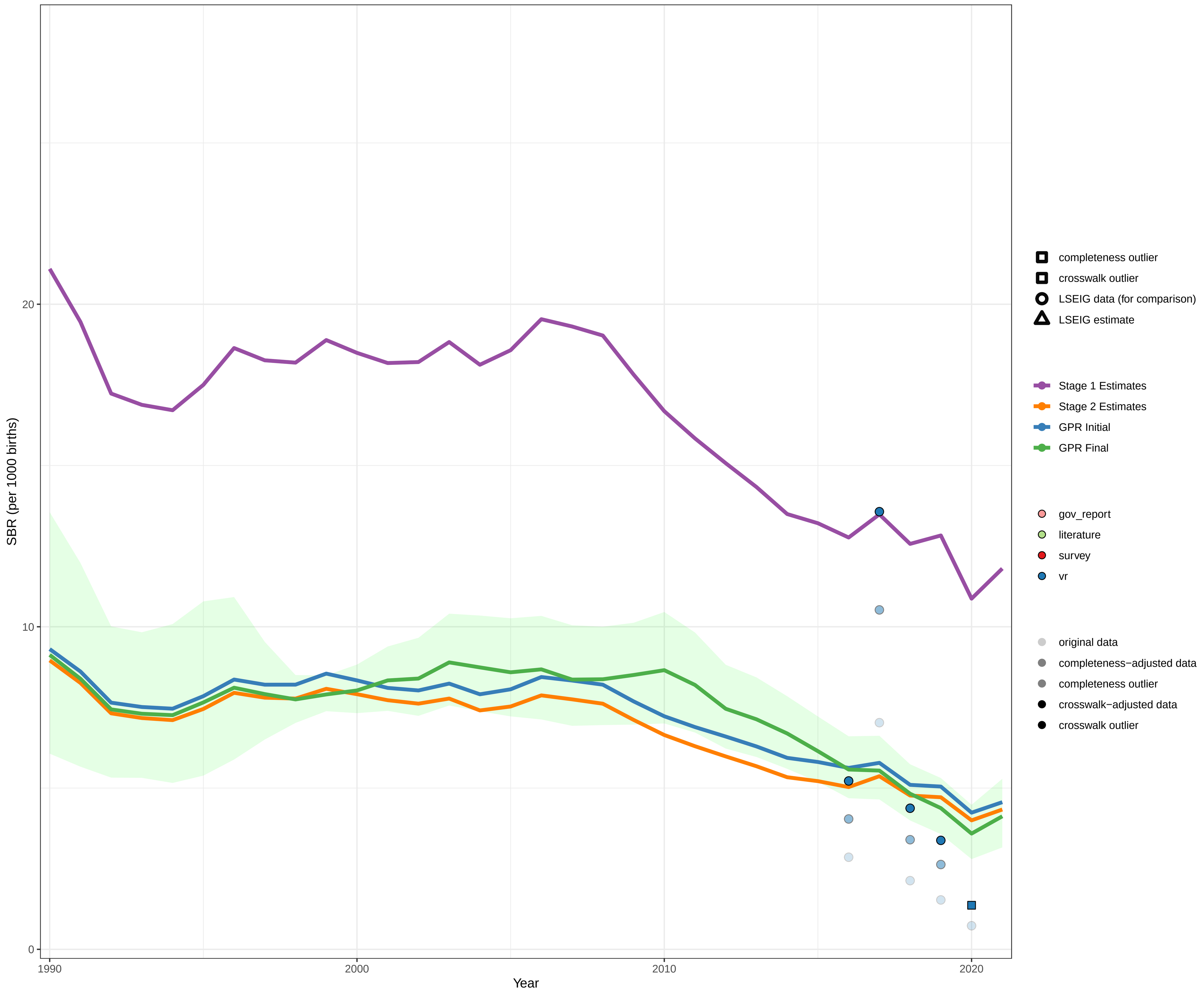

Bracknell Forest GBR_44765 Western Europe Stillbirth Rate (SBR) − 28 weeks
lambda = 0.8 ; zeta = 0.7 ; scale = 15 ; amp = 0.028

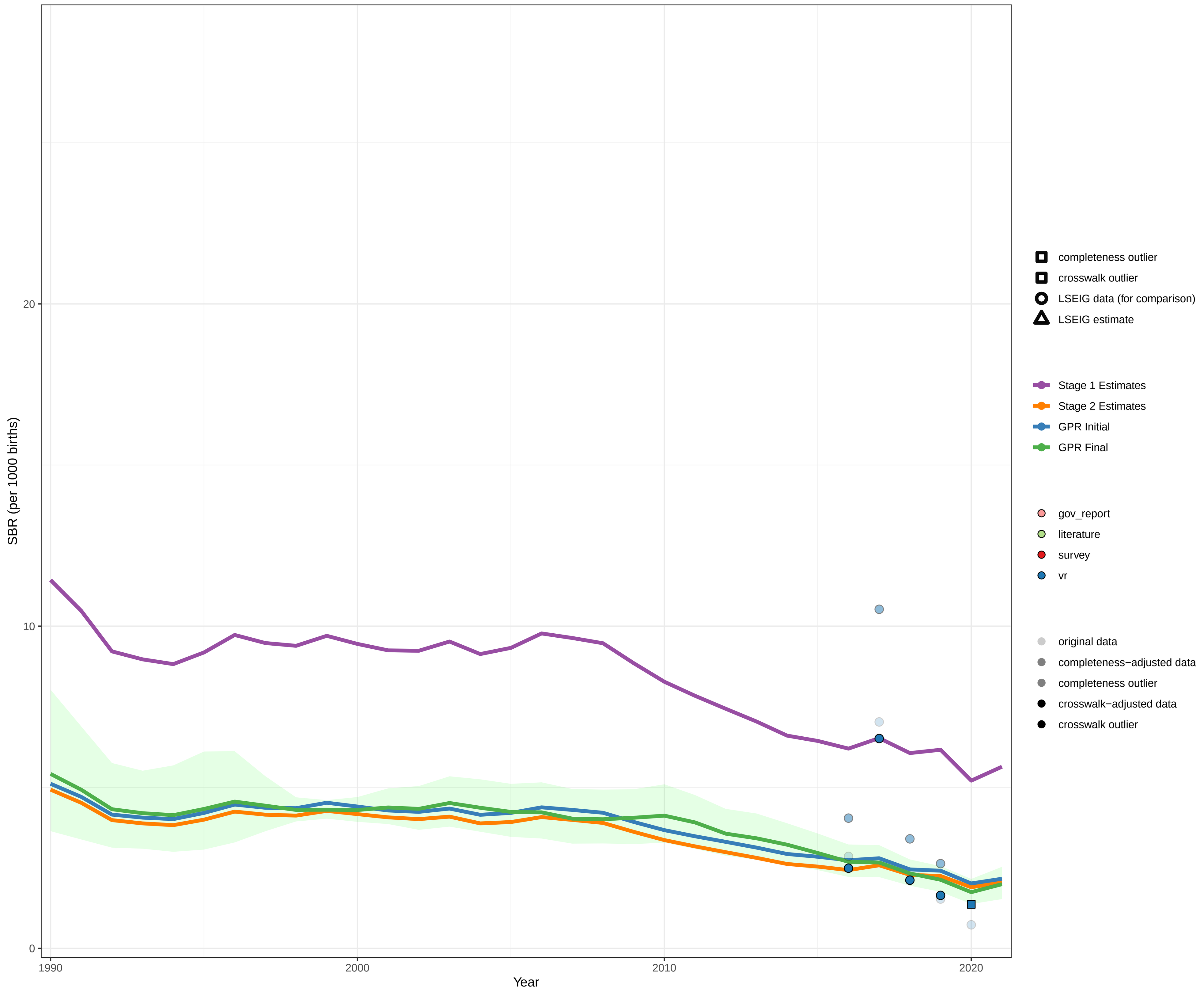

Year	Total Stillbirths (in thousands)	Total Stillbirths (in thousands)	SBR (per 1000 births)	SBR (per 1000 births)	SBR/NMR	SBR/NMR
	20 weeks	28 weeks	20 weeks	28 weeks	20 weeks	28 weeks
1990	0.0 (0.0 to 0.0)	0.0 (0.0 to 0.0)	9.1 (6.1 to 13.6)	5.4 (3.6 to 8.0)	3.03 (2.01 to 4.49)	1.79 (1.21 to 2.66)
1995	0.0 (0.0 to 0.0)	0.0 (0.0 to 0.0)	7.7 (5.4 to 10.8)	4.3 (3.1 to 6.1)	3.45 (2.43 to 4.86)	1.95 (1.38 to 2.76)
2000	0.0 (0.0 to 0.0)	0.0 (0.0 to 0.0)	8.0 (7.3 to 8.8)	4.3 (3.9 to 4.7)	3.85 (3.51 to 4.23)	2.06 (1.88 to 2.25)
2005	0.0 (0.0 to 0.0)	0.0 (0.0 to 0.0)	8.6 (7.2 to 10.3)	4.2 (3.5 to 5.1)	4.42 (3.71 to 5.28)	2.18 (1.78 to 2.63)
2010	0.0 (0.0 to 0.0)	0.0 (0.0 to 0.0)	8.7 (7.0 to 10.5)	4.1 (3.3 to 5.1)	5.15 (4.17 to 6.22)	2.45 (1.95 to 3.03)
2015	0.0 (0.0 to 0.0)	0.0 (0.0 to 0.0)	6.1 (5.2 to 7.2)	3.0 (2.4 to 3.6)	4.86 (4.11 to 5.72)	2.34 (1.92 to 2.82)
2020	0.0 (0.0 to 0.0)	0.0 (0.0 to 0.0)	3.6 (2.8 to 4.5)	1.7 (1.4 to 2.2)	3.61 (2.82 to 4.51)	1.76 (1.39 to 2.17)
2021	0.0 (0.0 to 0.0)	0.0 (0.0 to 0.0)	4.1 (3.2 to 5.3)	2.0 (1.5 to 2.5)	3.85 (2.95 to 4.93)	1.86 (1.43 to 2.36)

Brighton and Hove GBR_44770 Western Europe Stillbirth Rate / Neonatal Mortality Rate (SBR/NMR) − 20 weeks
lambda = 0.6 ; zeta = 0.8 ; scale = 15 ; amp = 0.028

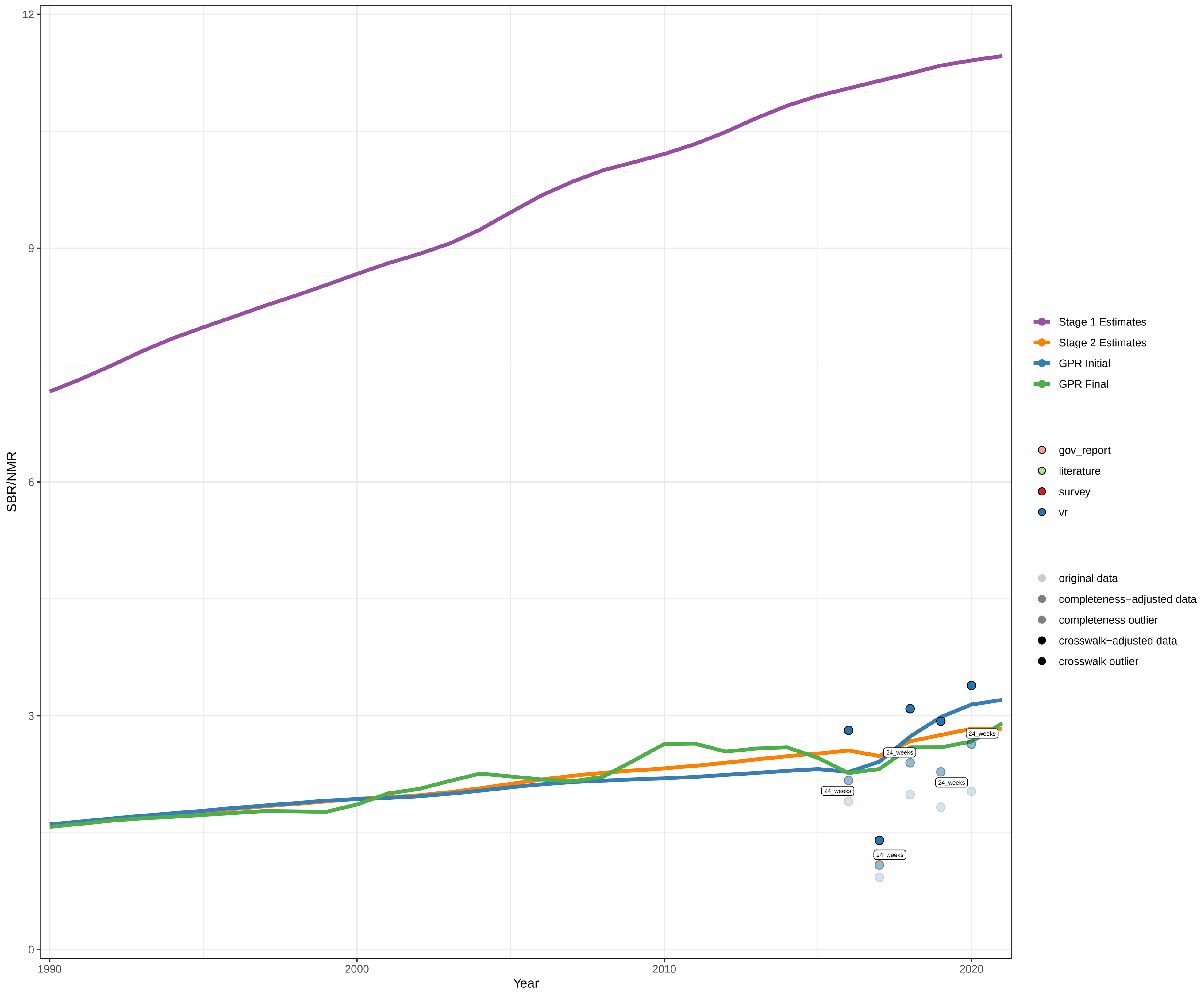

Brighton and Hove GBR_44770 Western Europe Stillbirth Rate / Neonatal Mortality Rate (SBR/NMR) − 28 weeks
lambda = 0.6 ; zeta = 0.8 ; scale = 15 ; amp = 0.028

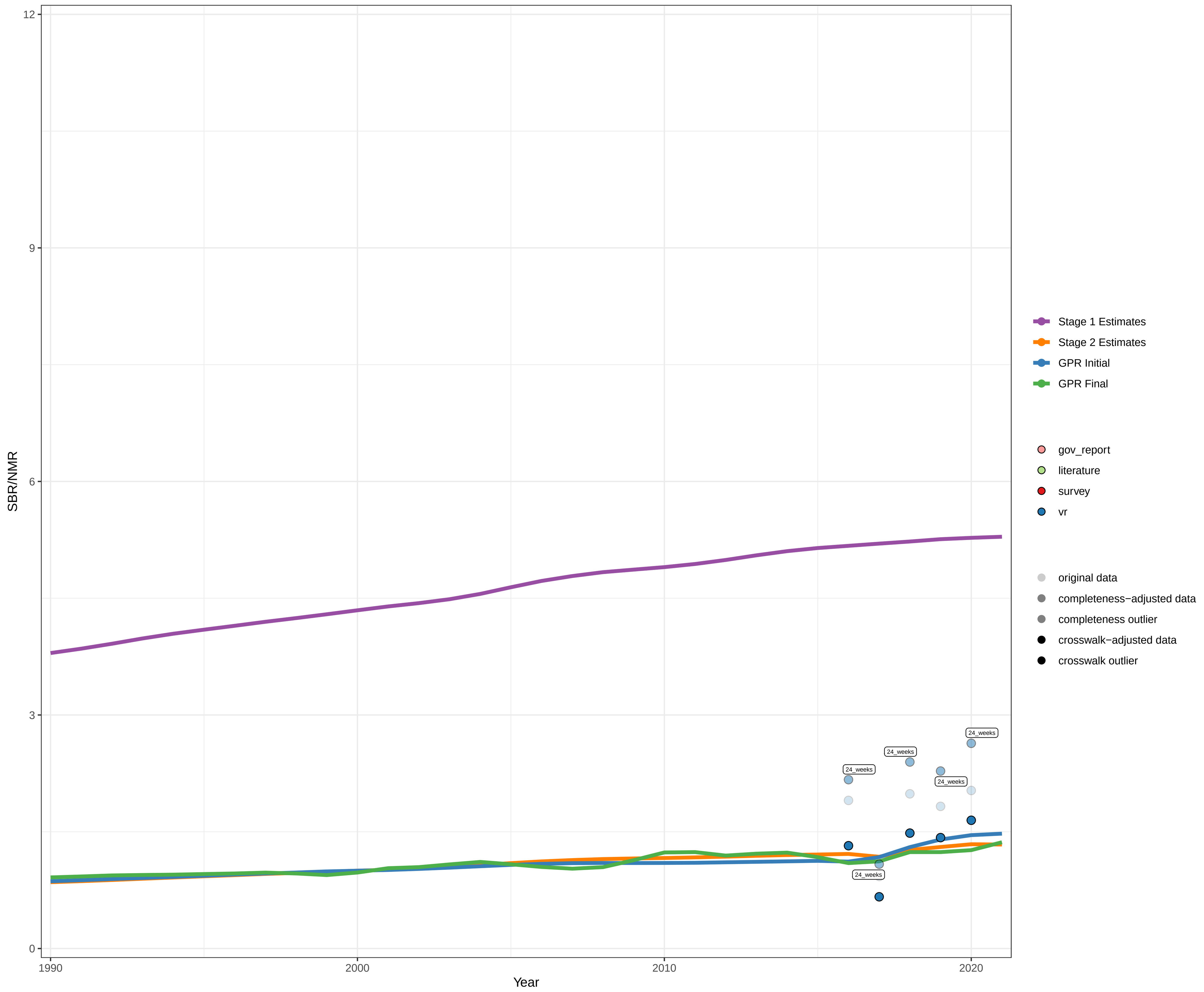

Definition	Years	Outlier	Source.Type	Title
24_weeks	2016−2020	0	VR	United Kingdom − England and Wales Child Mortality Statistics

Brighton and Hove GBR_44770 Western Europe Stillbirth Rate (SBR) − 20 weeks
lambda = 0.6 ; zeta = 0.8 ; scale = 15 ; amp = 0.028

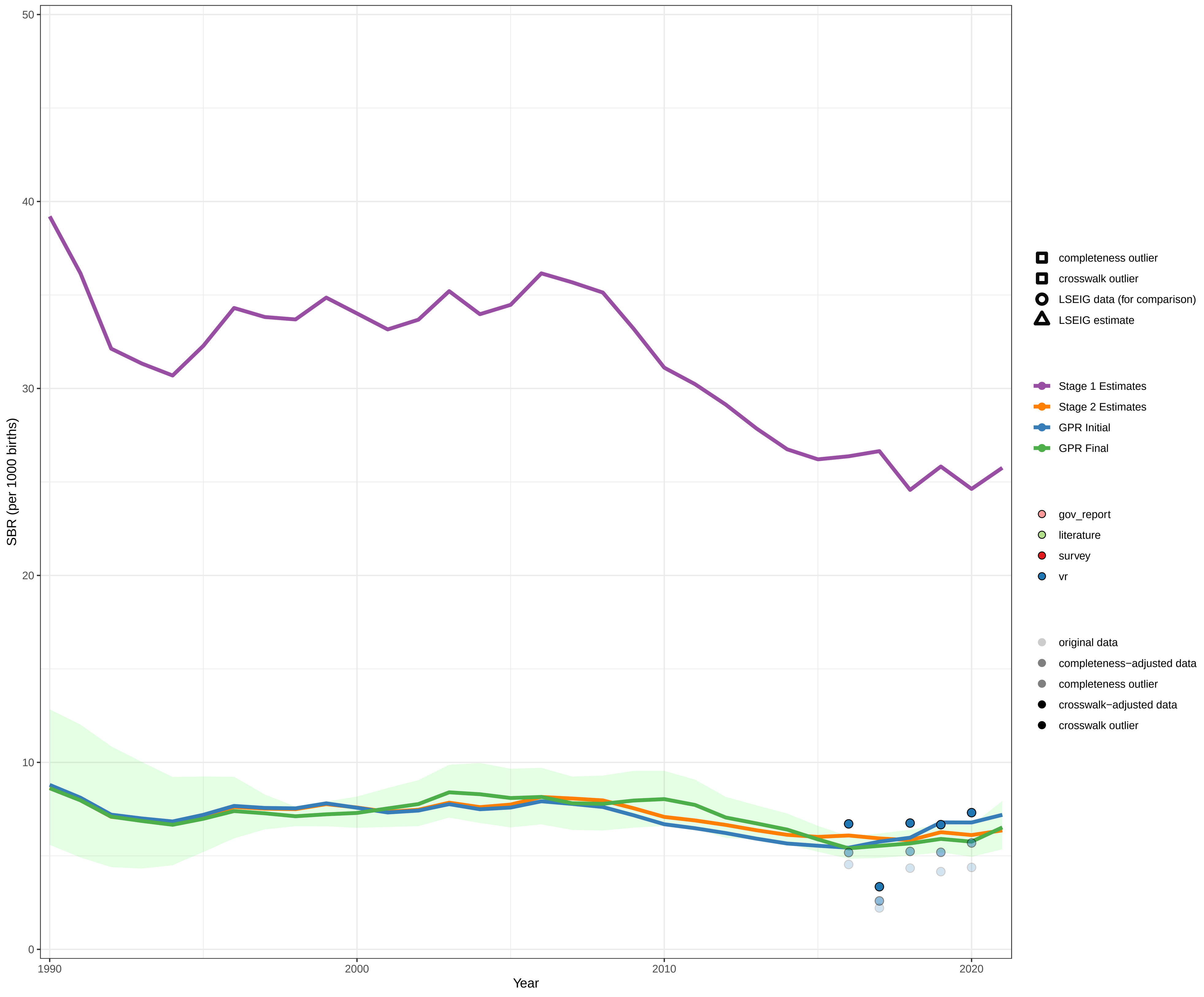

Brighton and Hove GBR_44770 Western Europe Stillbirth Rate (SBR) − 28 weeks
lambda = 0.6 ; zeta = 0.8 ; scale = 15 ; amp = 0.028

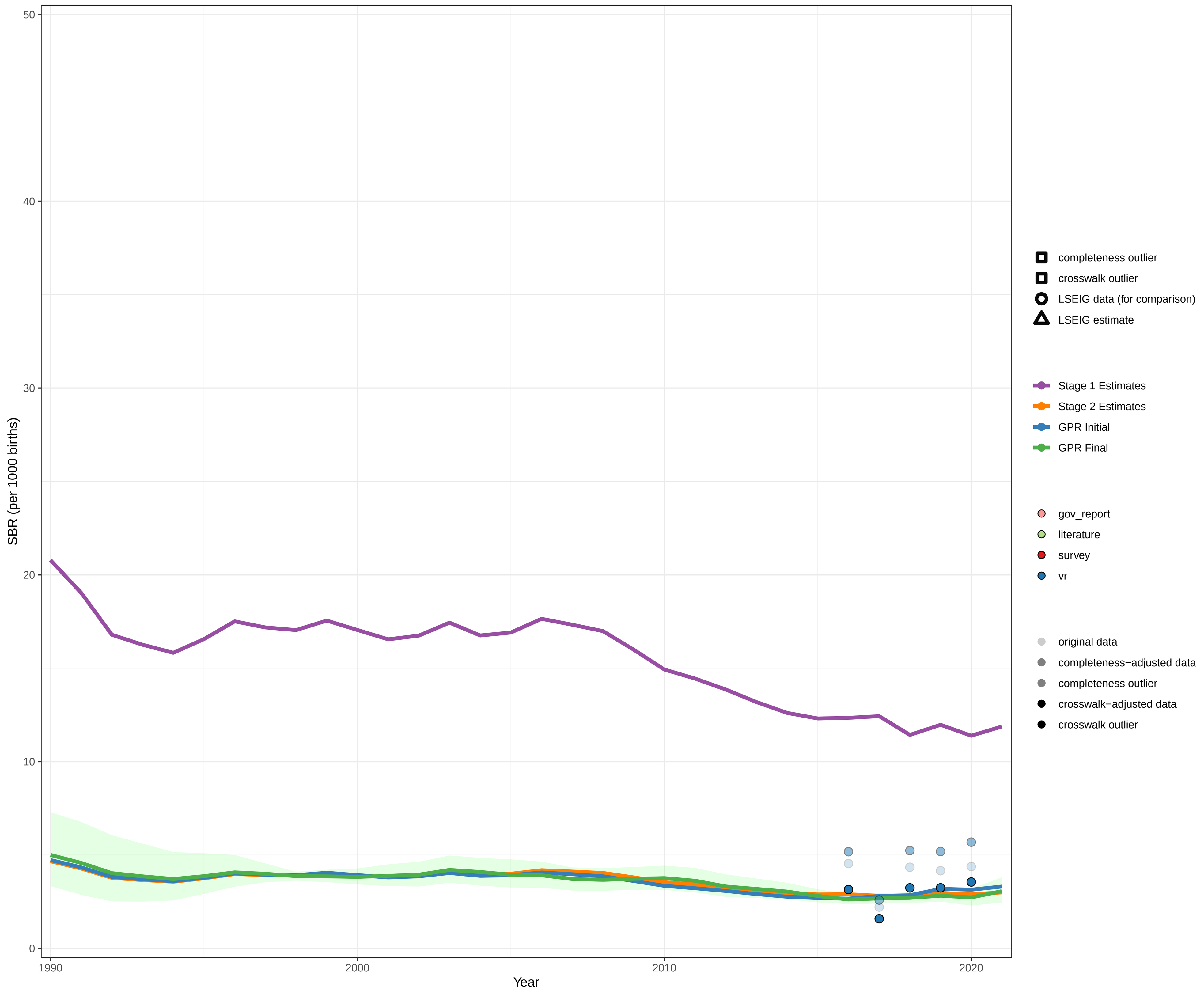

Year	Total Stillbirths (in thousands)	Total Stillbirths (in thousands)	SBR (per 1000 births)	SBR (per 1000 births)	SBR/NMR	SBR/NMR
	20 weeks	28 weeks	20 weeks	28 weeks	20 weeks	28 weeks
1990	0.0 (0.0 to 0.0)	0.0 (0.0 to 0.0)	8.6 (5.6 to 12.8)	5.0 (3.3 to 7.3)	1.57 (1.02 to 2.35)	0.91 (0.61 to 1.33)
1995	0.0 (0.0 to 0.0)	0.0 (0.0 to 0.0)	7.0 (5.2 to 9.2)	3.9 (2.9 to 5.1)	1.73 (1.29 to 2.29)	0.96 (0.72 to 1.26)
2000	0.0 (0.0 to 0.0)	0.0 (0.0 to 0.0)	7.3 (6.5 to 8.2)	3.8 (3.4 to 4.3)	1.86 (1.66 to 2.08)	0.98 (0.87 to 1.09)
2005	0.0 (0.0 to 0.0)	0.0 (0.0 to 0.0)	8.1 (6.5 to 9.7)	3.9 (3.3 to 4.8)	2.22 (1.79 to 2.65)	1.08 (0.89 to 1.31)
2010	0.0 (0.0 to 0.0)	0.0 (0.0 to 0.0)	8.0 (6.6 to 9.5)	3.8 (3.1 to 4.4)	2.64 (2.17 to 3.13)	1.23 (1.03 to 1.45)
2015	0.0 (0.0 to 0.0)	0.0 (0.0 to 0.0)	5.9 (5.2 to 6.6)	2.8 (2.5 to 3.2)	2.46 (2.18 to 2.76)	1.17 (1.03 to 1.33)
2020	0.0 (0.0 to 0.0)	0.0 (0.0 to 0.0)	5.8 (4.9 to 6.7)	2.7 (2.3 to 3.2)	2.67 (2.29 to 3.08)	1.26 (1.06 to 1.48)
2021	0.0 (0.0 to 0.0)	0.0 (0.0 to 0.0)	6.5 (5.4 to 7.9)	3.1 (2.5 to 3.8)	2.90 (2.38 to 3.53)	1.37 (1.09 to 1.70)

Buckinghamshire GBR_44760 Western Europe Stillbirth Rate / Neonatal Mortality Rate (SBR/NMR) − 20 weeks
lambda = 0.6 ; zeta = 0.8 ; scale = 15 ; amp = 0.028

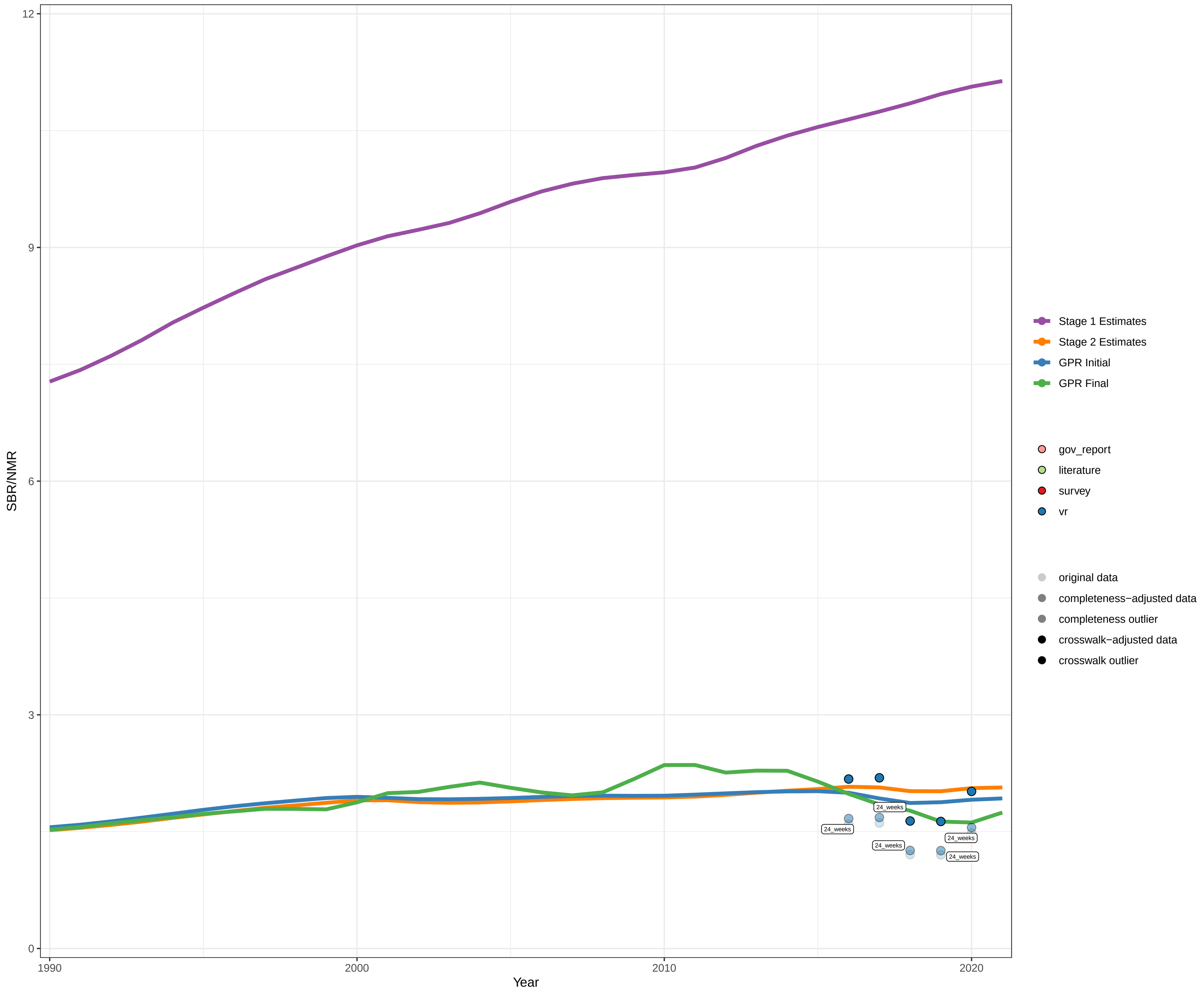

Buckinghamshire GBR_44760 Western Europe Stillbirth Rate / Neonatal Mortality Rate (SBR/NMR) − 28 weeks
lambda = 0.6 ; zeta = 0.8 ; scale = 15 ; amp = 0.028

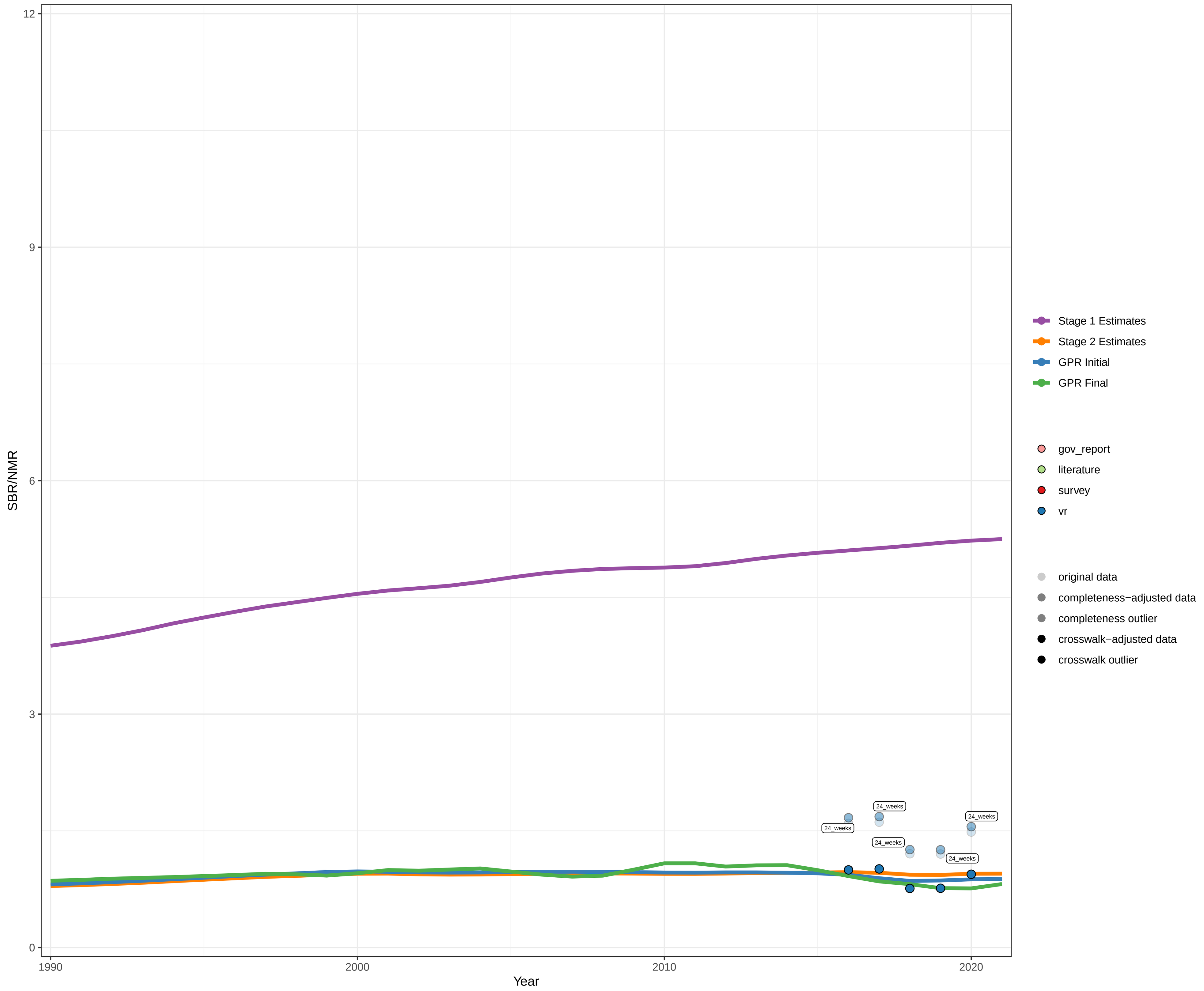

Definition	Years	Outlier	Source.Type	Title
24_weeks	2016−2020	0	VR	United Kingdom − England and Wales Child Mortality Statistics

Buckinghamshire GBR_44760 Western Europe Stillbirth Rate (SBR) − 20 weeks
lambda = 0.6 ; zeta = 0.8 ; scale = 15 ; amp = 0.028

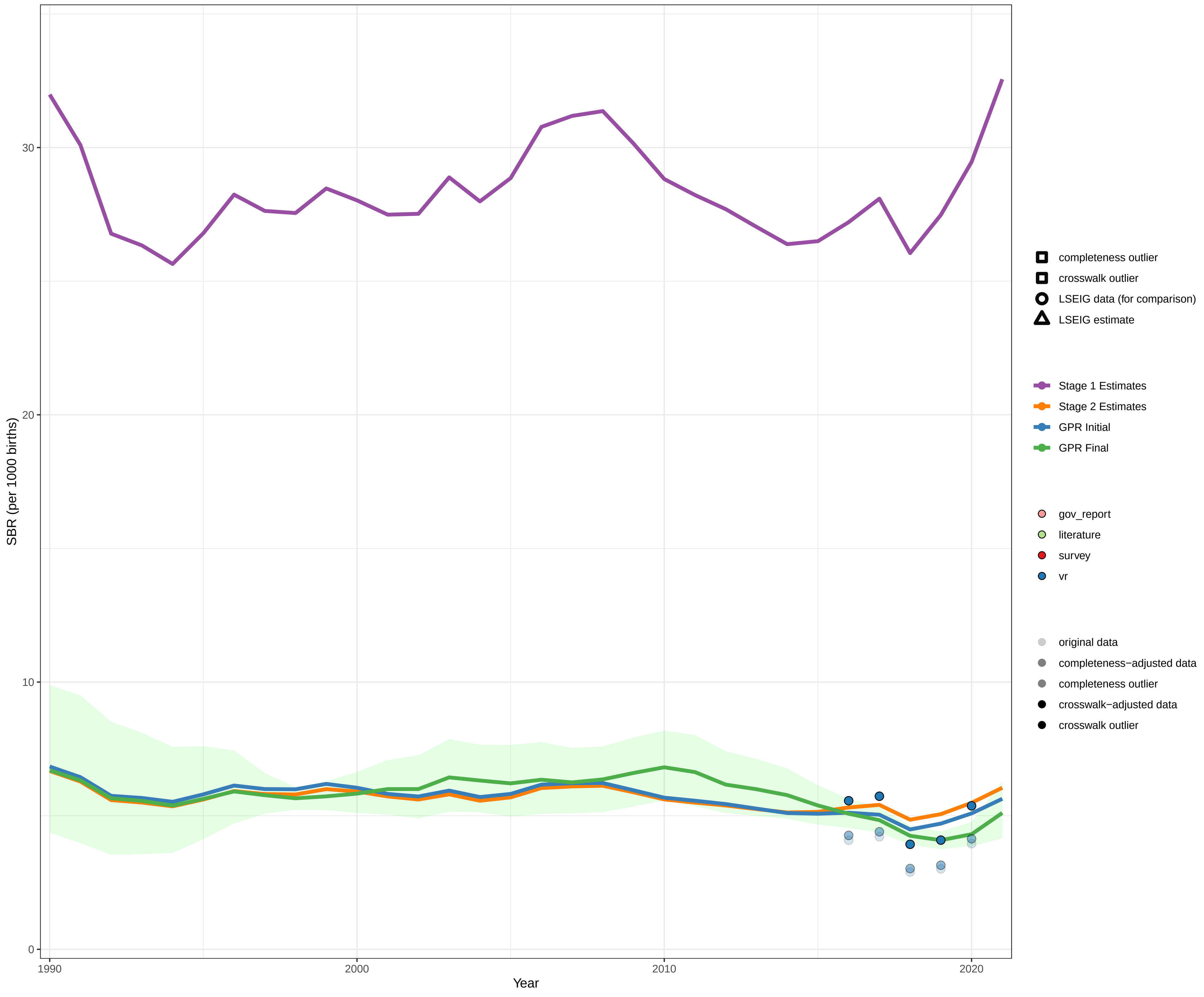

Buckinghamshire GBR_44760 Western Europe Stillbirth Rate (SBR) − 28 weeks
lambda = 0.6 ; zeta = 0.8 ; scale = 15 ; amp = 0.028

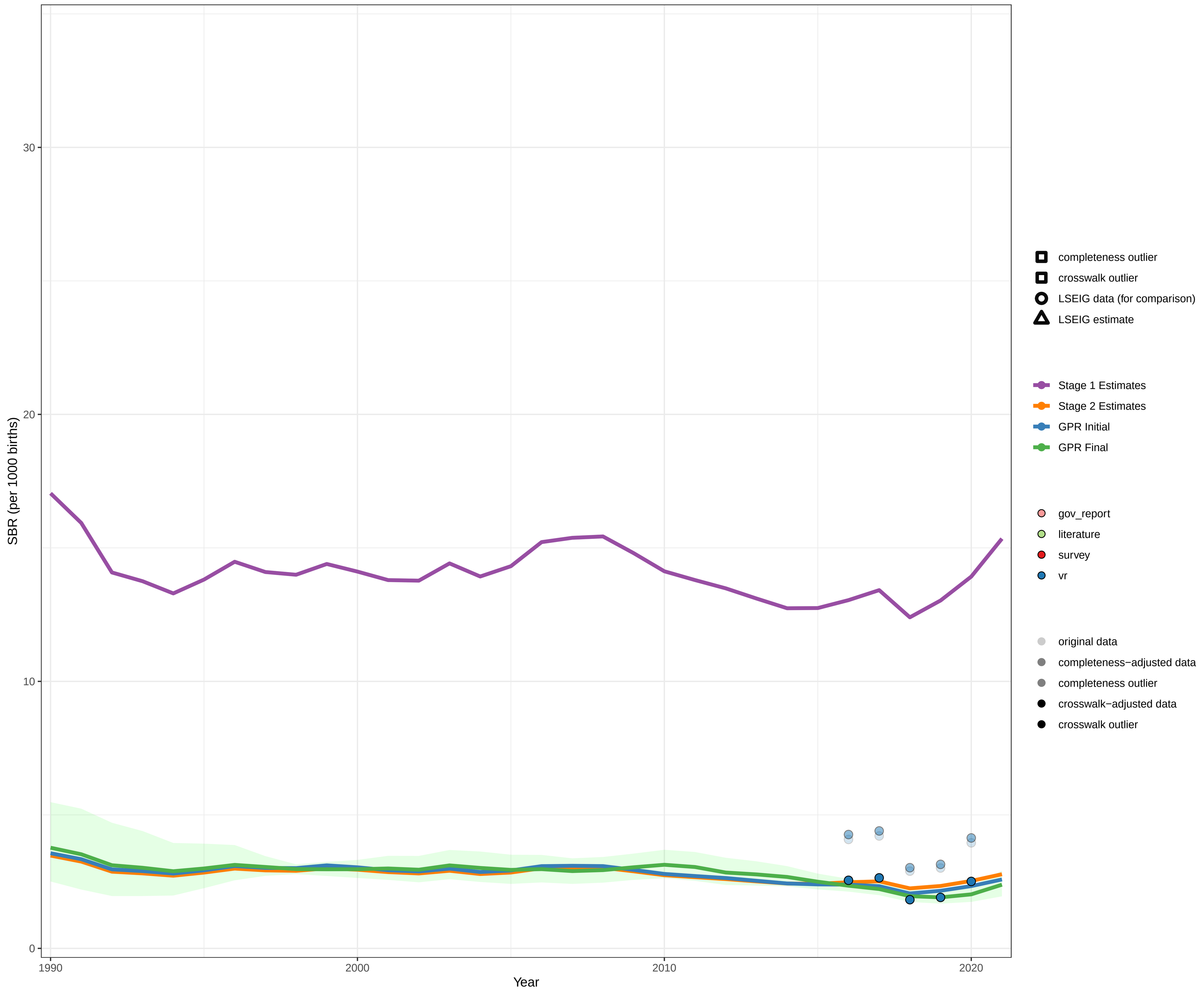

Year	Total Stillbirths (in thousands)	Total Stillbirths (in thousands)	SBR (per 1000 births)	SBR (per 1000 births)	SBR/NMR	SBR/NMR
	20 weeks	28 weeks	20 weeks	28 weeks	20 weeks	28 weeks
1990	0.0 (0.0 to 0.1)	0.0 (0.0 to 0.0)	6.7 (4.4 to 9.9)	3.8 (2.5 to 5.5)	1.52 (0.99 to 2.25)	0.86 (0.57 to 1.25)
1995	0.0 (0.0 to 0.0)	0.0 (0.0 to 0.0)	5.6 (4.1 to 7.6)	3.0 (2.3 to 3.9)	1.73 (1.27 to 2.33)	0.92 (0.69 to 1.20)
2000	0.0 (0.0 to 0.0)	0.0 (0.0 to 0.0)	5.8 (5.1 to 6.6)	3.0 (2.6 to 3.3)	1.88 (1.64 to 2.14)	0.95 (0.85 to 1.07)
2005	0.0 (0.0 to 0.0)	0.0 (0.0 to 0.0)	6.2 (5.0 to 7.6)	2.9 (2.4 to 3.5)	2.06 (1.65 to 2.54)	0.98 (0.80 to 1.17)
2010	0.0 (0.0 to 0.1)	0.0 (0.0 to 0.0)	6.8 (5.6 to 8.2)	3.1 (2.6 to 3.7)	2.35 (1.93 to 2.83)	1.08 (0.91 to 1.28)
2015	0.0 (0.0 to 0.0)	0.0 (0.0 to 0.0)	5.4 (4.7 to 6.1)	2.5 (2.2 to 2.8)	2.14 (1.86 to 2.44)	0.99 (0.88 to 1.11)
2020	0.0 (0.0 to 0.0)	0.0 (0.0 to 0.0)	4.3 (3.9 to 4.8)	2.0 (1.7 to 2.3)	1.62 (1.45 to 1.79)	0.76 (0.65 to 0.87)
2021	0.0 (0.0 to 0.0)	0.0 (0.0 to 0.0)	5.1 (4.1 to 6.3)	2.4 (1.9 to 2.9)	1.75 (1.42 to 2.15)	0.82 (0.67 to 1.00)

East Sussex GBR_44772 Western Europe Stillbirth Rate / Neonatal Mortality Rate (SBR/NMR) − 20 weeks
lambda = 0.6 ; zeta = 0.8 ; scale = 15 ; amp = 0.028

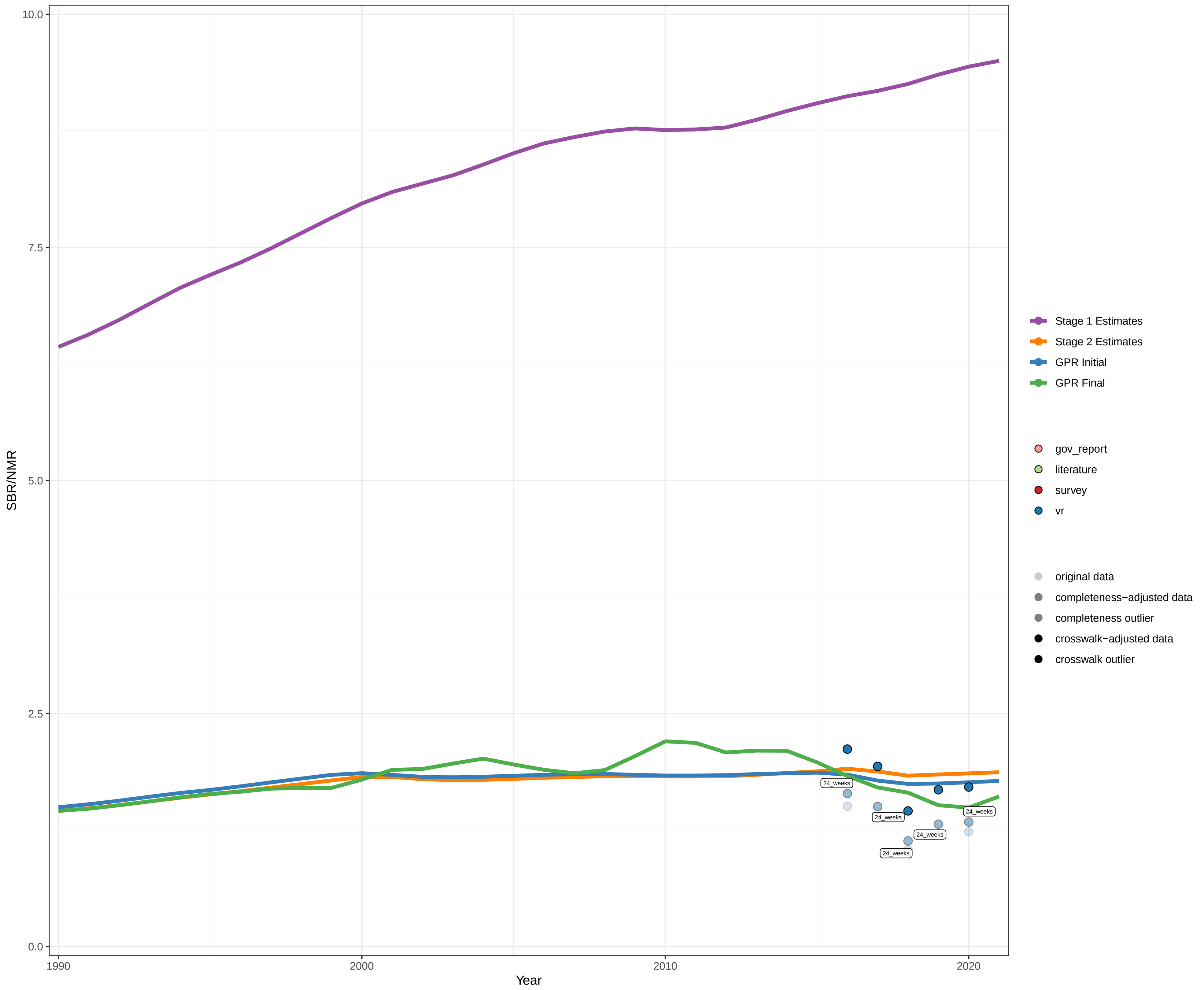

East Sussex GBR_44772 Western Europe Stillbirth Rate / Neonatal Mortality Rate (SBR/NMR) − 28 weeks
lambda = 0.6 ; zeta = 0.8 ; scale = 15 ; amp = 0.028

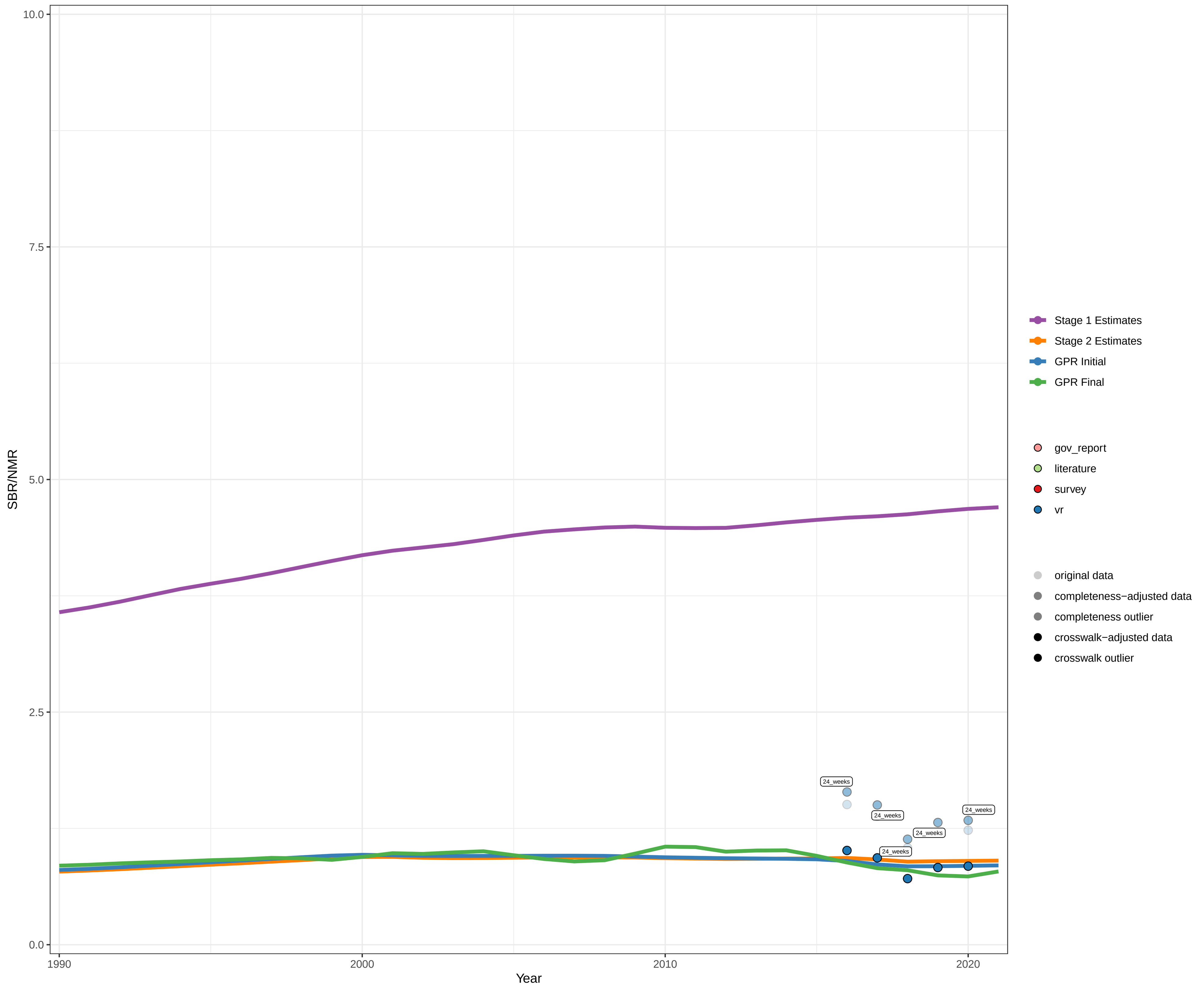

Definition	Years	Outlier	Source.Type	Title
24_weeks	2016−2020	0	VR	United Kingdom − England and Wales Child Mortality Statistics

East Sussex GBR_44772 Western Europe Stillbirth Rate (SBR) − 20 weeks
lambda = 0.6 ; zeta = 0.8 ; scale = 15 ; amp = 0.028

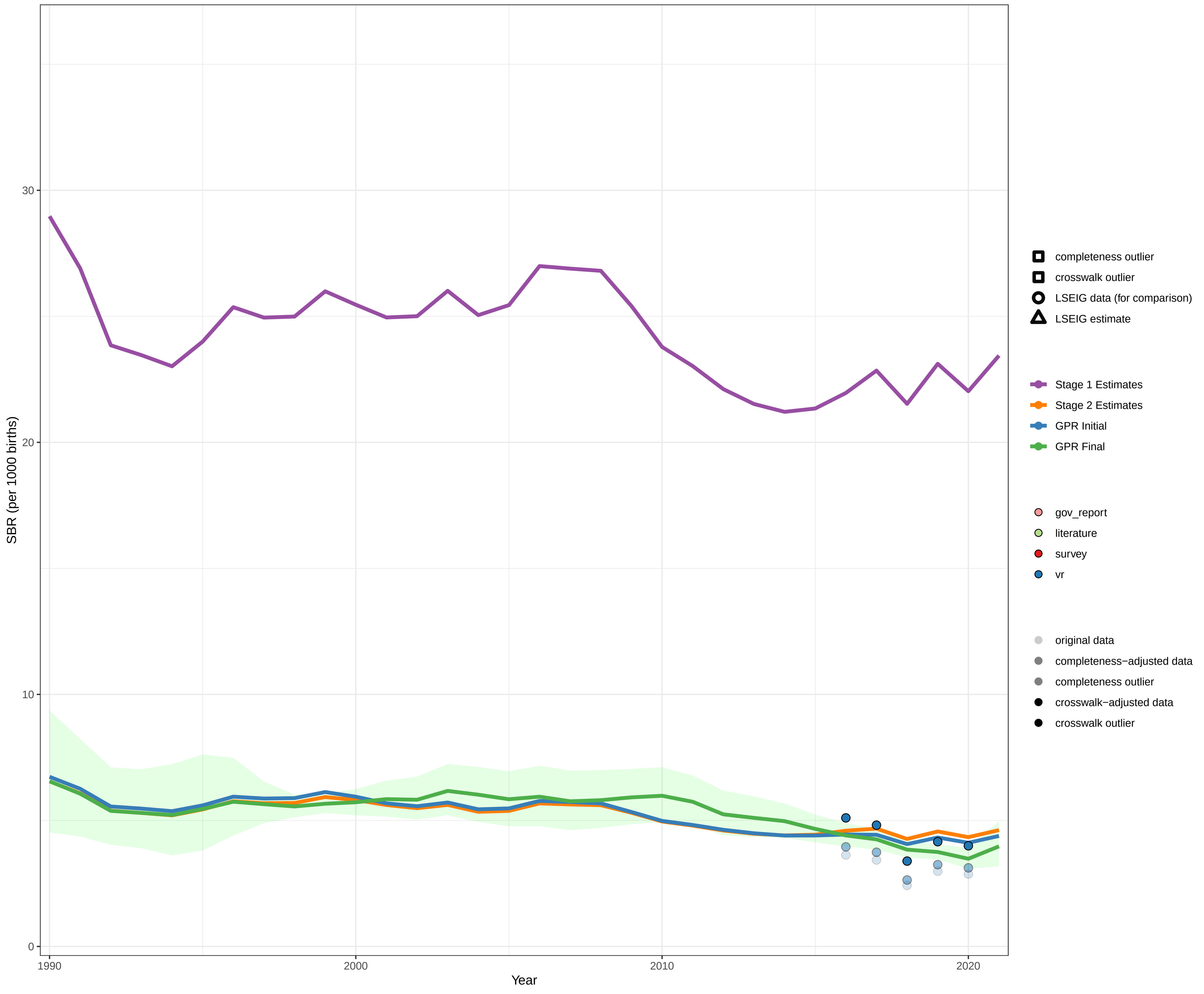

East Sussex GBR_44772 Western Europe Stillbirth Rate (SBR) − 28 weeks
lambda = 0.6 ; zeta = 0.8 ; scale = 15 ; amp = 0.028

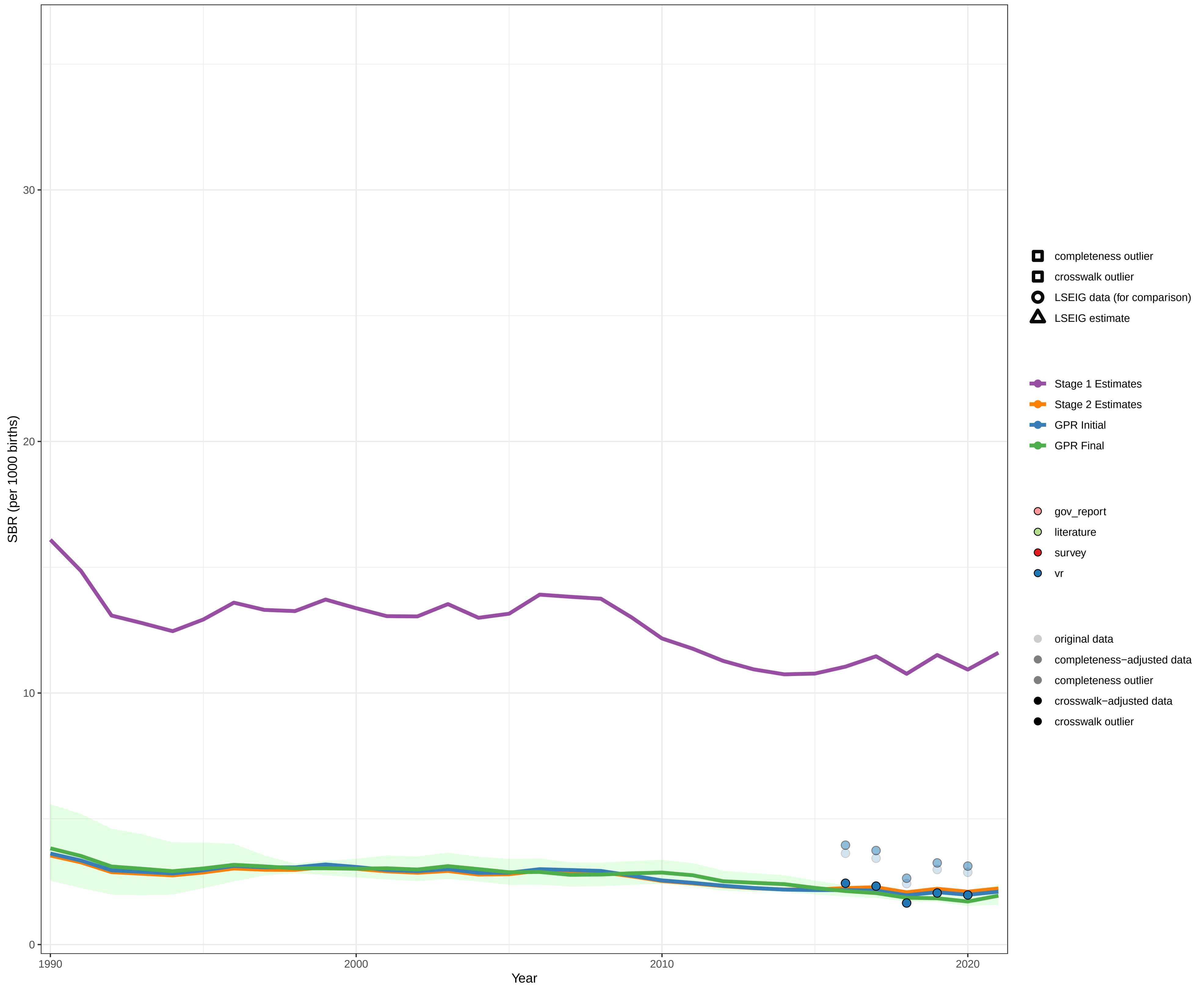

Year	Total Stillbirths (in thousands)	Total Stillbirths (in thousands)	SBR (per 1000 births)	SBR (per 1000 births)	SBR/NMR	SBR/NMR
	20 weeks	28 weeks	20 weeks	28 weeks	20 weeks	28 weeks
1990	0.0 (0.0 to 0.1)	0.0 (0.0 to 0.0)	6.6 (4.5 to 9.4)	3.8 (2.5 to 5.6)	1.46 (1.00 to 2.08)	0.85 (0.56 to 1.24)
1995	0.0 (0.0 to 0.0)	0.0 (0.0 to 0.0)	5.5 (3.8 to 7.6)	3.0 (2.2 to 4.1)	1.64 (1.14 to 2.29)	0.91 (0.67 to 1.22)
2000	0.0 (0.0 to 0.0)	0.0 (0.0 to 0.0)	5.7 (5.2 to 6.2)	3.0 (2.7 to 3.4)	1.79 (1.63 to 1.96)	0.94 (0.83 to 1.07)
2005	0.0 (0.0 to 0.0)	0.0 (0.0 to 0.0)	5.8 (4.8 to 7.0)	2.9 (2.4 to 3.4)	1.95 (1.60 to 2.32)	0.96 (0.79 to 1.14)
2010	0.0 (0.0 to 0.0)	0.0 (0.0 to 0.0)	6.0 (4.9 to 7.1)	2.9 (2.4 to 3.4)	2.20 (1.82 to 2.62)	1.05 (0.89 to 1.24)
2015	0.0 (0.0 to 0.0)	0.0 (0.0 to 0.0)	4.7 (4.1 to 5.2)	2.3 (2.0 to 2.5)	1.98 (1.75 to 2.22)	0.96 (0.84 to 1.08)
2020	0.0 (0.0 to 0.0)	0.0 (0.0 to 0.0)	3.5 (3.1 to 3.9)	1.7 (1.5 to 1.9)	1.49 (1.33 to 1.68)	0.73 (0.66 to 0.81)
2021	0.0 (0.0 to 0.0)	0.0 (0.0 to 0.0)	4.0 (3.2 to 5.0)	1.9 (1.6 to 2.4)	1.61 (1.29 to 2.02)	0.79 (0.64 to 0.97)

Hampshire GBR_44764 Western Europe Stillbirth Rate / Neonatal Mortality Rate (SBR/NMR) − 20 weeks
lambda = 0.6 ; zeta = 0.8 ; scale = 15 ; amp = 0.028

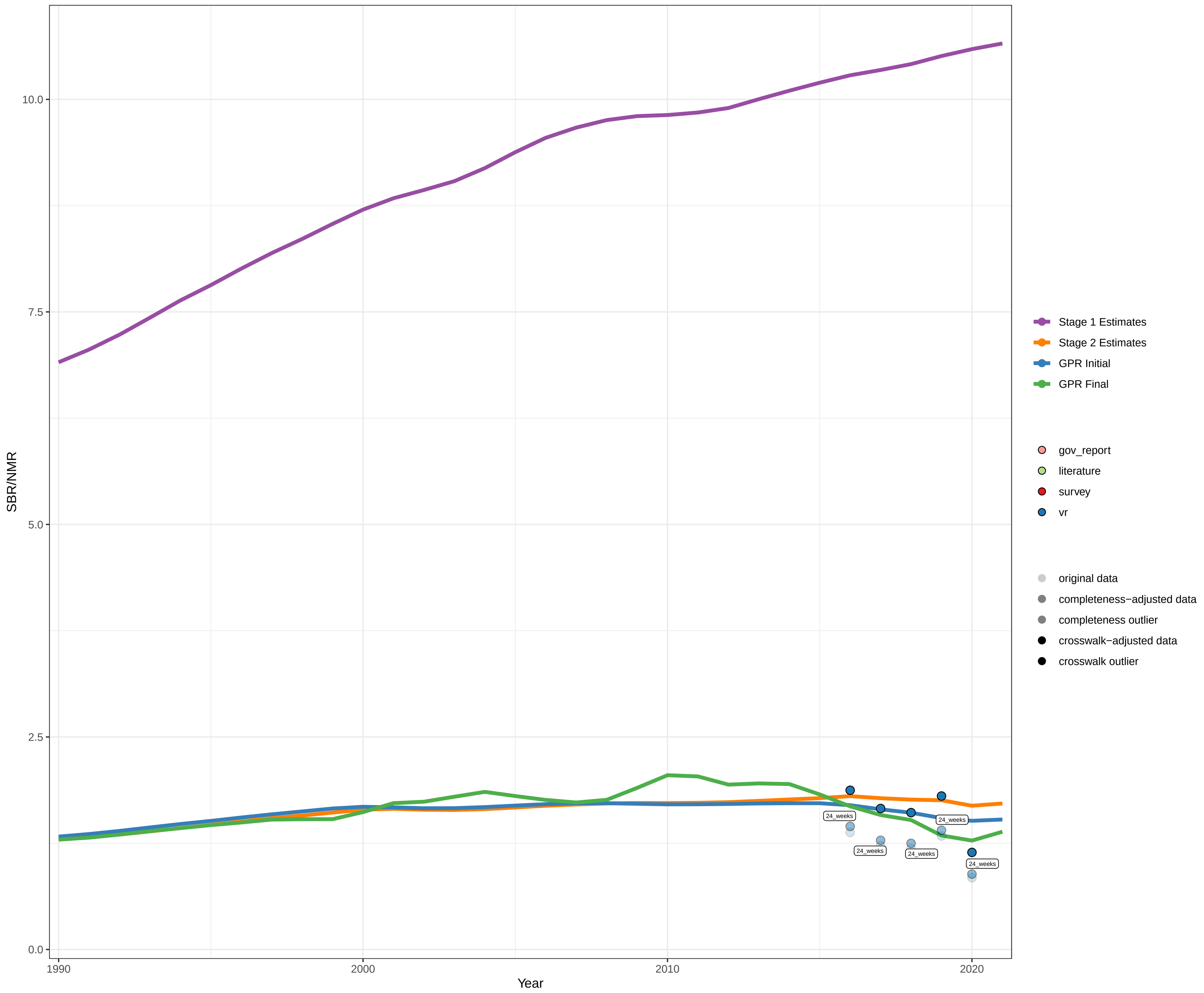

Hampshire GBR_44764 Western Europe Stillbirth Rate / Neonatal Mortality Rate (SBR/NMR) − 28 weeks
lambda = 0.6 ; zeta = 0.8 ; scale = 15 ; amp = 0.028

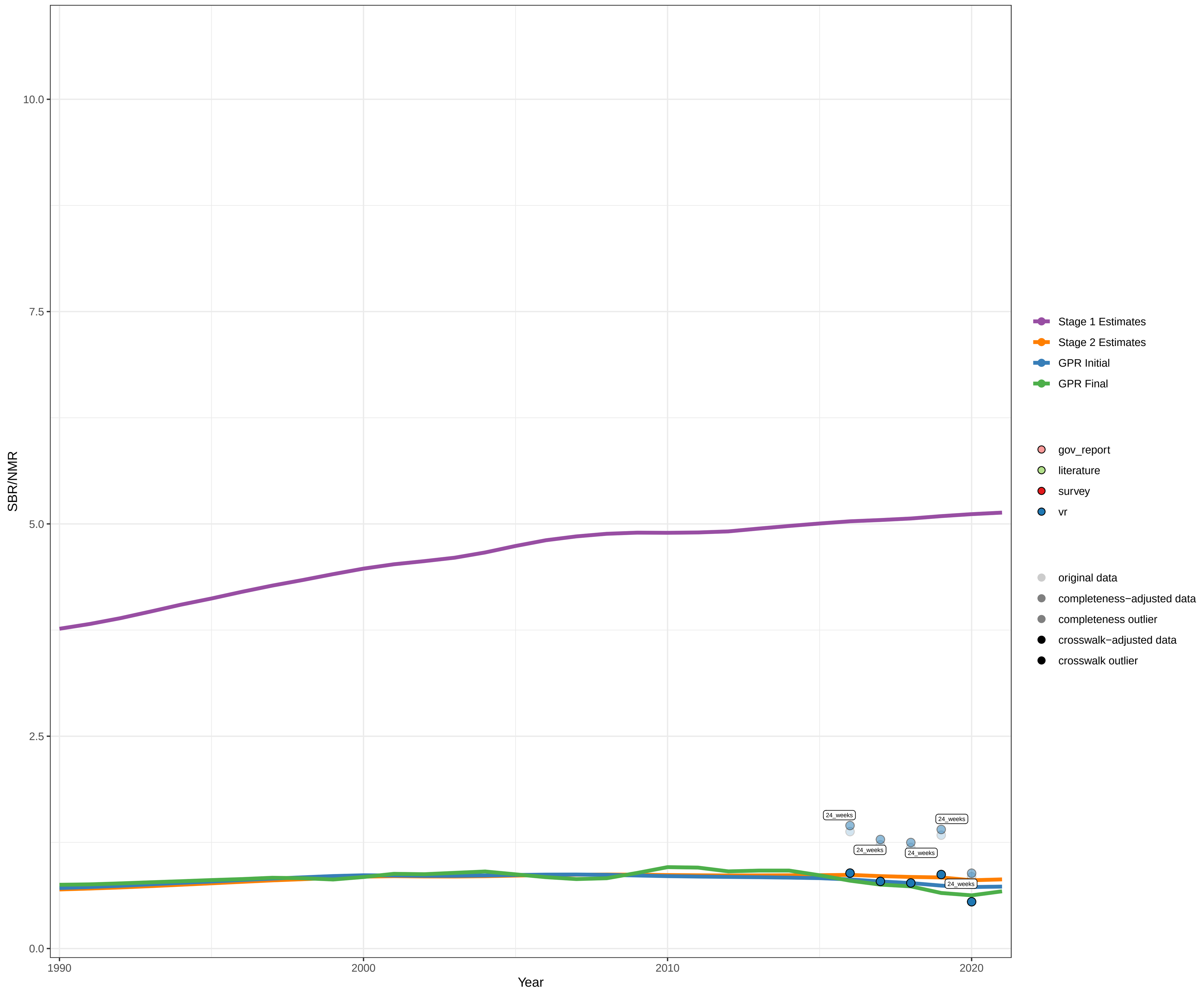

Definition	Years	Outlier	Source.Type	Title
24_weeks	2016−2020	0	VR	United Kingdom − England and Wales Child Mortality Statistics

Hampshire GBR_44764 Western Europe Stillbirth Rate (SBR) − 20 weeks
lambda = 0.6 ; zeta = 0.8 ; scale = 15 ; amp = 0.028

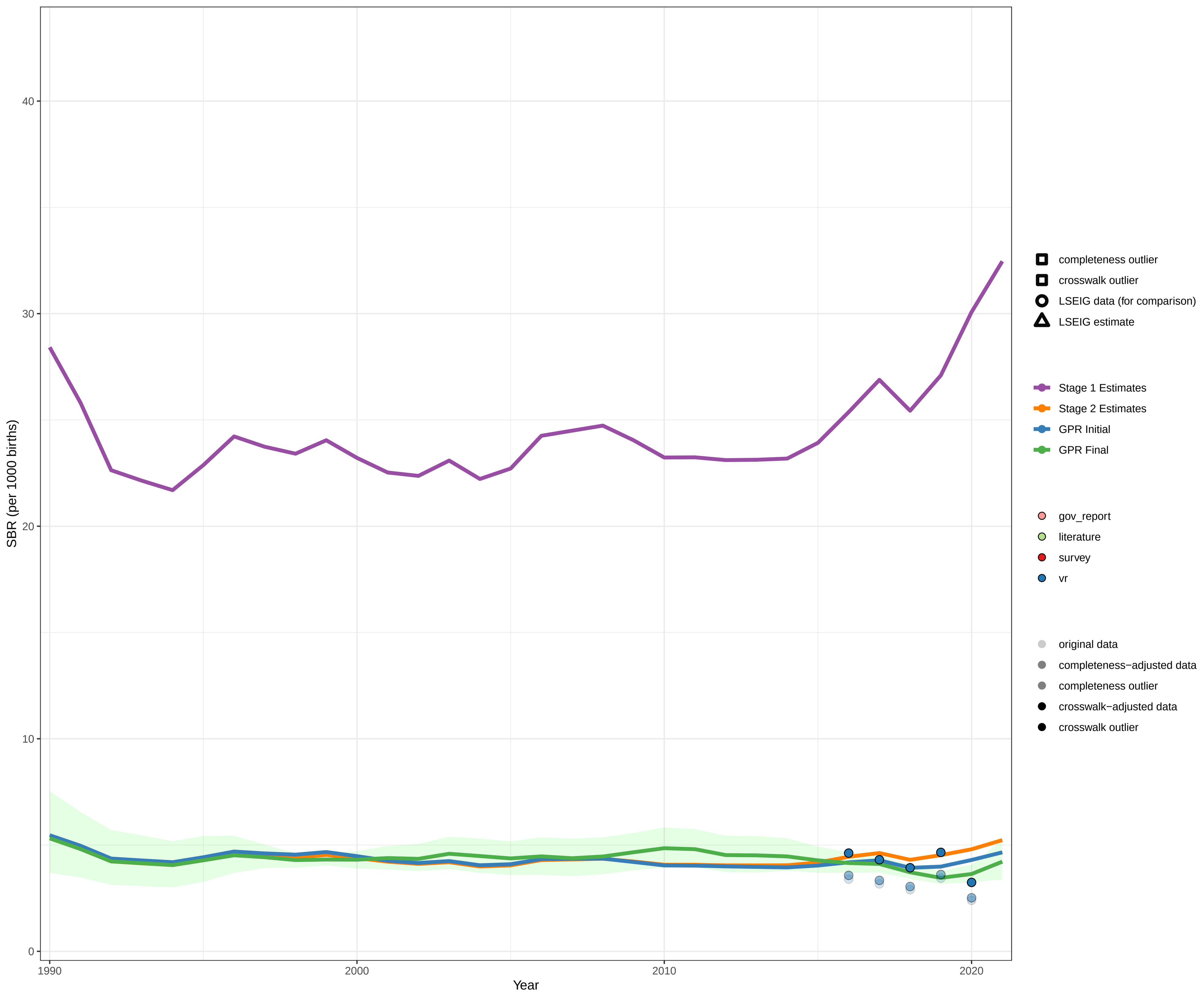

Hampshire GBR_44764 Western Europe Stillbirth Rate (SBR) − 28 weeks
lambda = 0.6 ; zeta = 0.8 ; scale = 15 ; amp = 0.028

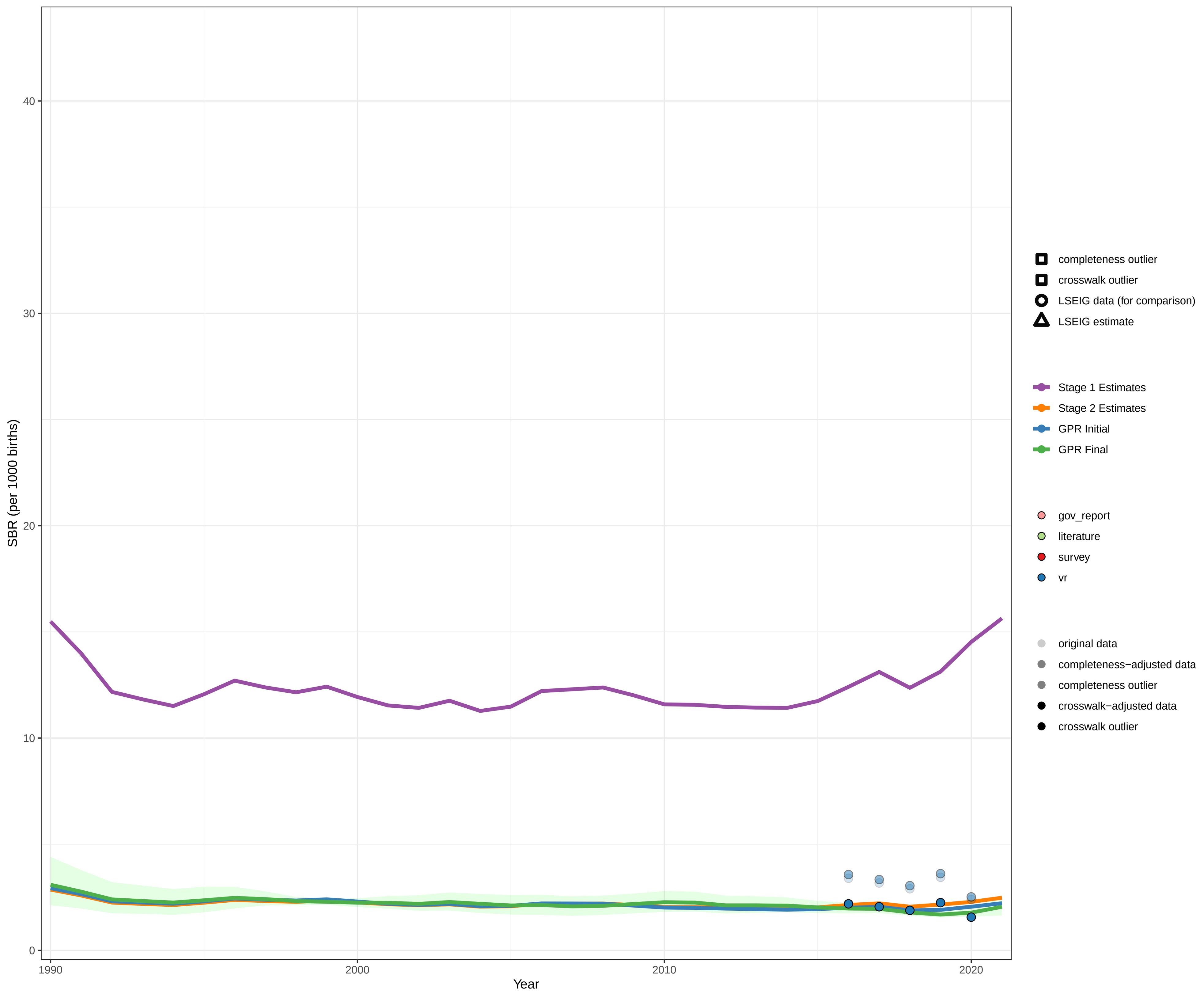

Year	Total Stillbirths (in thousands)	Total Stillbirths (in thousands)	SBR (per 1000 births)	SBR (per 1000 births)	SBR/NMR	SBR/NMR
	20 weeks	28 weeks	20 weeks	28 weeks	20 weeks	28 weeks
1990	0.1 (0.1 to 0.1)	0.0 (0.0 to 0.1)	5.3 (3.7 to 7.5)	3.1 (2.1 to 4.4)	1.29 (0.90 to 1.83)	0.75 (0.52 to 1.07)
1995	0.1 (0.0 to 0.1)	0.0 (0.0 to 0.0)	4.3 (3.3 to 5.4)	2.4 (1.8 to 3.0)	1.46 (1.12 to 1.85)	0.81 (0.61 to 1.03)
2000	0.1 (0.1 to 0.1)	0.0 (0.0 to 0.0)	4.3 (3.9 to 4.7)	2.2 (2.0 to 2.5)	1.62 (1.46 to 1.77)	0.84 (0.76 to 0.92)
2005	0.1 (0.1 to 0.1)	0.0 (0.0 to 0.0)	4.4 (3.6 to 5.2)	2.1 (1.7 to 2.6)	1.80 (1.48 to 2.14)	0.87 (0.70 to 1.08)
2010	0.1 (0.1 to 0.1)	0.0 (0.0 to 0.0)	4.9 (3.9 to 5.8)	2.3 (1.8 to 2.8)	2.05 (1.67 to 2.46)	0.96 (0.76 to 1.18)
2015	0.1 (0.1 to 0.1)	0.0 (0.0 to 0.0)	4.3 (3.7 to 4.9)	2.0 (1.8 to 2.3)	1.82 (1.57 to 2.10)	0.86 (0.75 to 0.99)
2020	0.0 (0.0 to 0.1)	0.0 (0.0 to 0.0)	3.6 (3.2 to 4.1)	1.8 (1.6 to 2.0)	1.28 (1.14 to 1.43)	0.63 (0.56 to 0.70)
2021	0.1 (0.0 to 0.1)	0.0 (0.0 to 0.0)	4.2 (3.4 to 5.2)	2.1 (1.6 to 2.5)	1.39 (1.11 to 1.72)	0.67 (0.54 to 0.83)

Isle of Wight   GBR_44774   Western Europe   Stillbirth Rate / Neonatal Mortality Rate (SBR/NMR) − 20 weeks
lambda = 0.8 ; zeta = 0.7 ; scale = 15 ; amp = 0.028

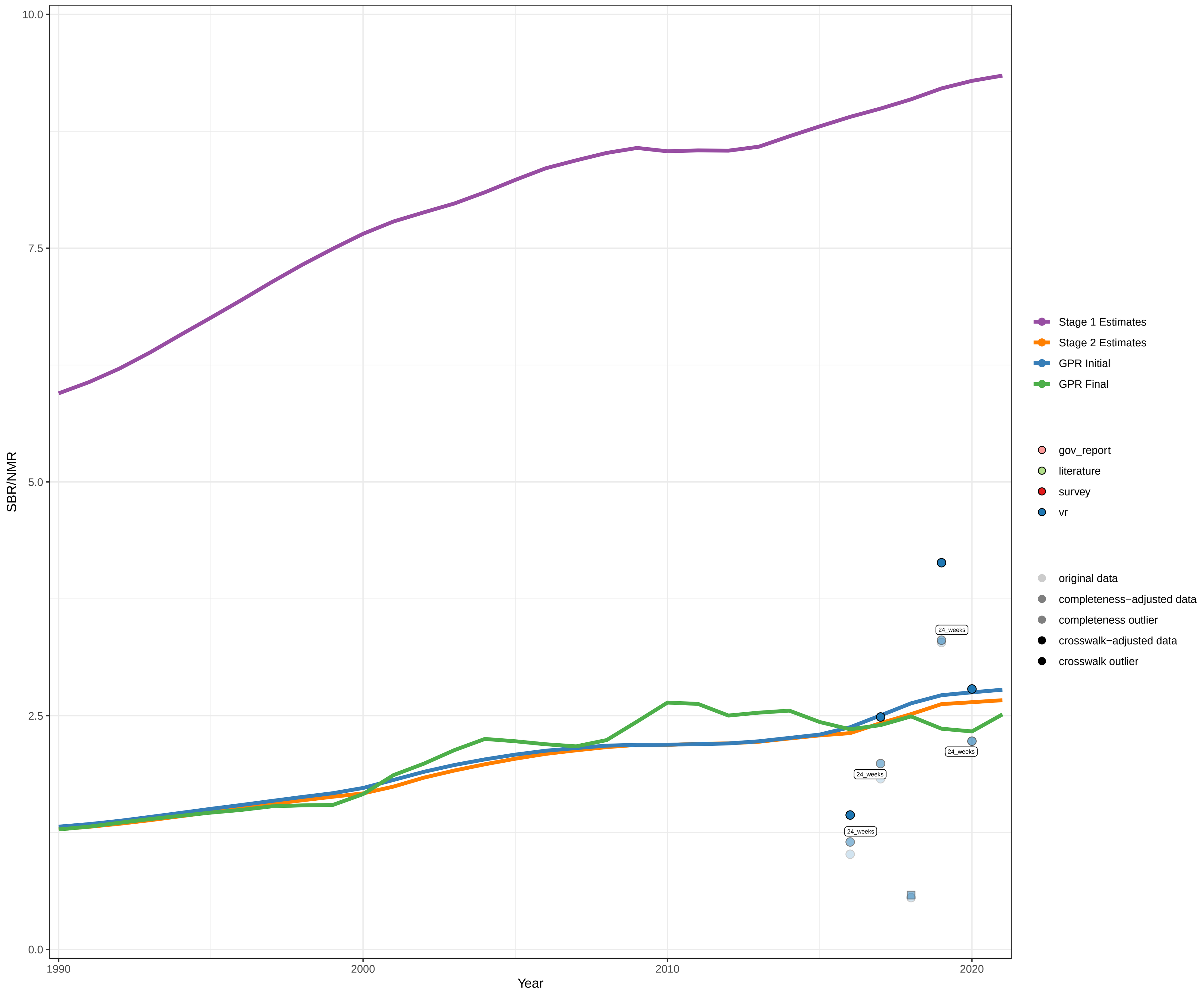

Isle of Wight   GBR_44774   Western Europe   Stillbirth Rate / Neonatal Mortality Rate (SBR/NMR) − 28 weeks
lambda = 0.8 ; zeta = 0.7 ; scale = 15 ; amp = 0.028

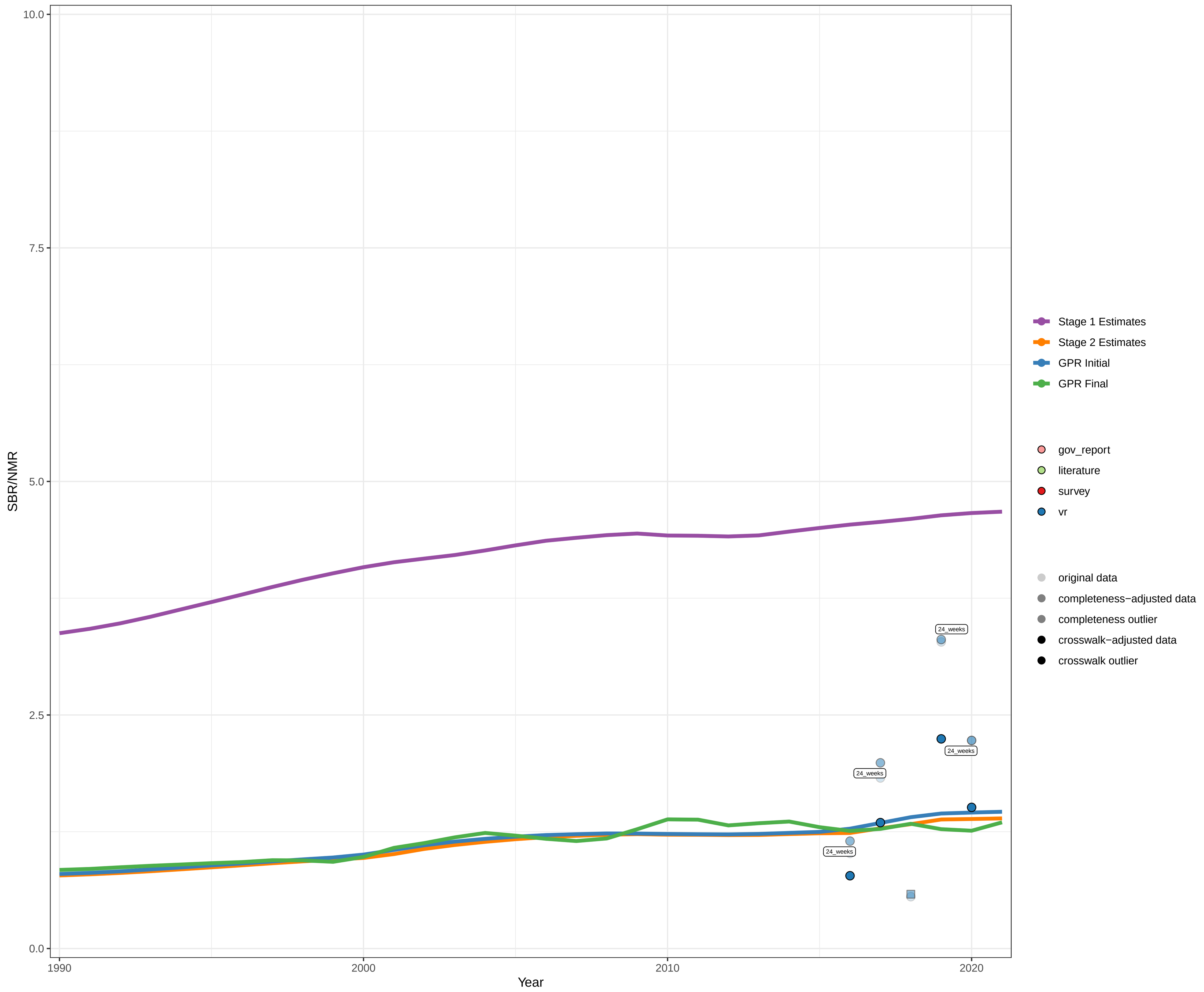

Definition	Years	Outlier	Source.Type	Title
24_weeks	2016, 2017, 2019, 2020	0	VR	United Kingdom − England and Wales Child Mortality Statistics
24_weeks	2018	1	VR	United Kingdom − England and Wales Child Mortality Statistics

Isle of Wight   GBR_44774   Western Europe   Stillbirth Rate (SBR) − 20 weeks
lambda = 0.8 ; zeta = 0.7 ; scale = 15 ; amp = 0.028

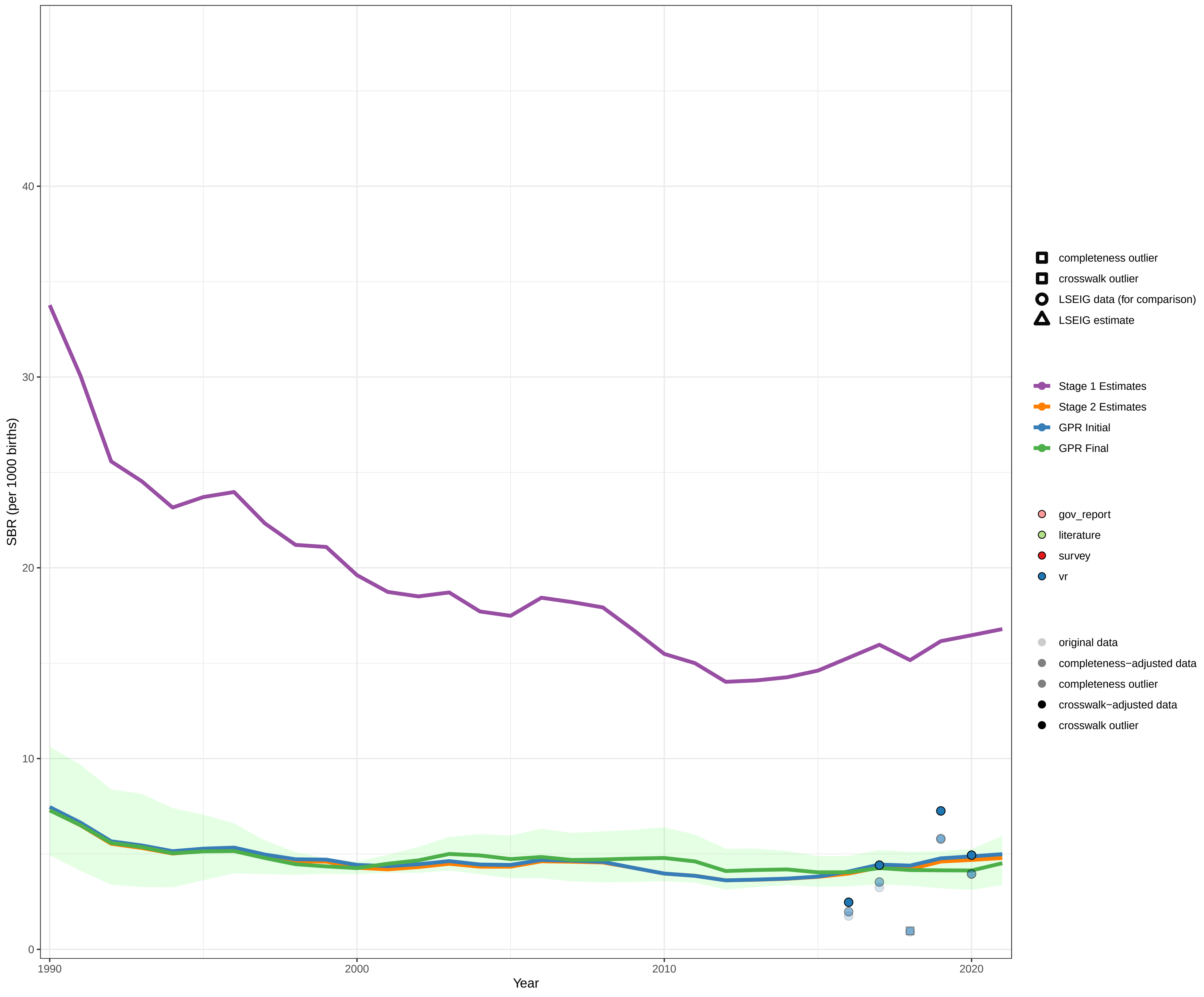

Isle of Wight   GBR_44774   Western Europe   Stillbirth Rate (SBR) − 28 weeks
lambda = 0.8 ; zeta = 0.7 ; scale = 15 ; amp = 0.028

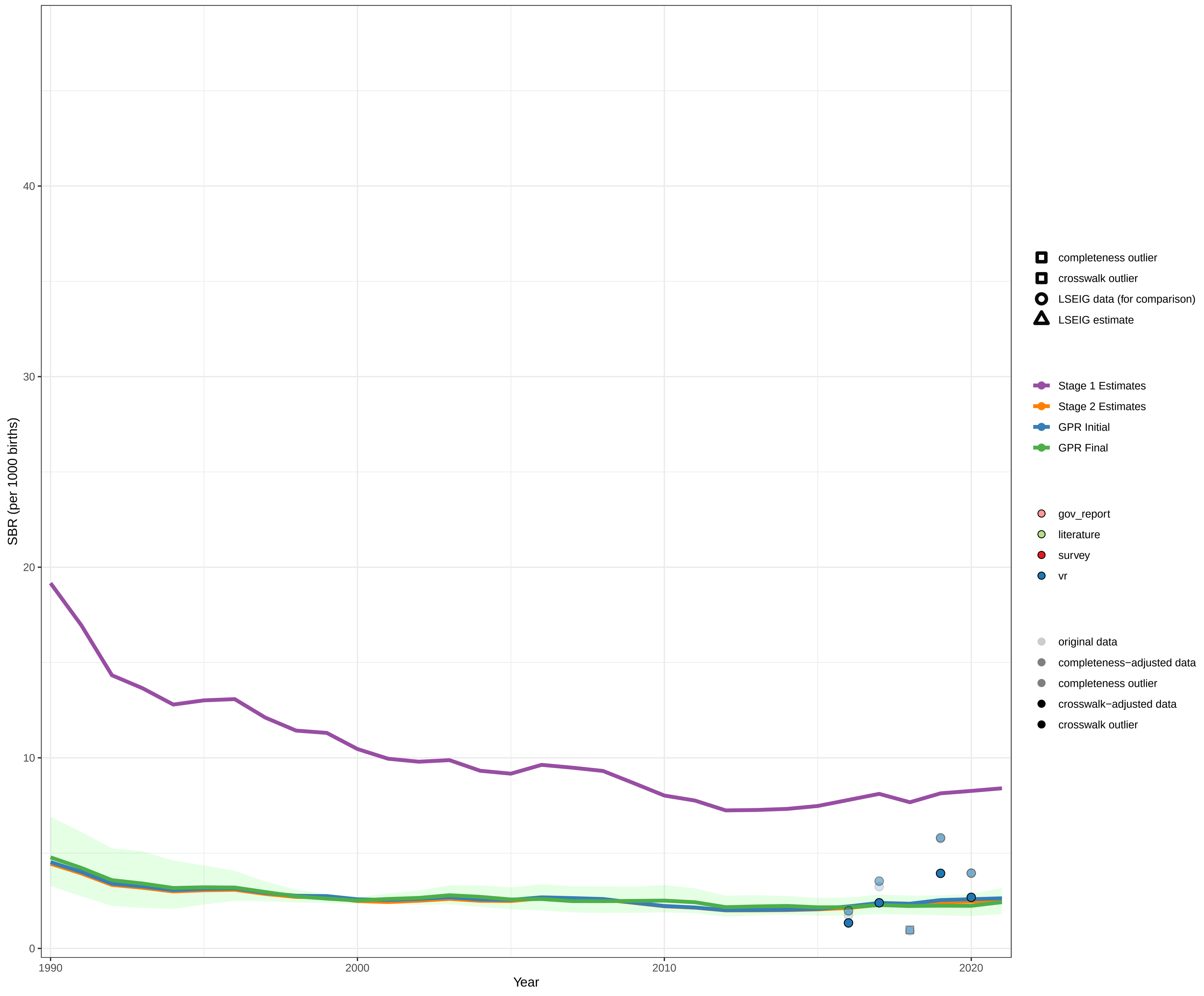

Year	Total Stillbirths (in thousands)	Total Stillbirths (in thousands)	SBR (per 1000 births)	SBR (per 1000 births)	SBR/NMR	SBR/NMR
	20 weeks	28 weeks	20 weeks	28 weeks	20 weeks	28 weeks
1990	0.0 (0.0 to 0.0)	0.0 (0.0 to 0.0)	7.3 (4.9 to 10.6)	4.8 (3.3 to 6.9)	1.28 (0.87 to 1.87)	0.84 (0.58 to 1.22)
1995	0.0 (0.0 to 0.0)	0.0 (0.0 to 0.0)	5.1 (3.6 to 7.1)	3.2 (2.3 to 4.4)	1.46 (1.03 to 2.01)	0.91 (0.66 to 1.24)
2000	0.0 (0.0 to 0.0)	0.0 (0.0 to 0.0)	4.3 (3.9 to 4.6)	2.5 (2.3 to 2.7)	1.66 (1.53 to 1.80)	0.98 (0.92 to 1.05)
2005	0.0 (0.0 to 0.0)	0.0 (0.0 to 0.0)	4.7 (3.7 to 6.0)	2.6 (2.0 to 3.2)	2.23 (1.75 to 2.81)	1.21 (0.96 to 1.51)
2010	0.0 (0.0 to 0.0)	0.0 (0.0 to 0.0)	4.8 (3.6 to 6.4)	2.5 (1.9 to 3.3)	2.64 (1.97 to 3.53)	1.38 (1.04 to 1.83)
2015	0.0 (0.0 to 0.0)	0.0 (0.0 to 0.0)	4.0 (3.3 to 4.9)	2.2 (1.7 to 2.6)	2.43 (1.97 to 2.94)	1.30 (1.03 to 1.60)
2020	0.0 (0.0 to 0.0)	0.0 (0.0 to 0.0)	4.1 (3.1 to 5.3)	2.2 (1.7 to 2.9)	2.33 (1.76 to 2.97)	1.26 (0.95 to 1.62)
2021	0.0 (0.0 to 0.0)	0.0 (0.0 to 0.0)	4.5 (3.4 to 5.9)	2.4 (1.8 to 3.2)	2.51 (1.87 to 3.31)	1.35 (1.01 to 1.76)

Kent GBR_44769 Western Europe Stillbirth Rate / Neonatal Mortality Rate (SBR/NMR) − 20 weeks
lambda = 0.6 ; zeta = 0.8 ; scale = 15 ; amp = 0.028

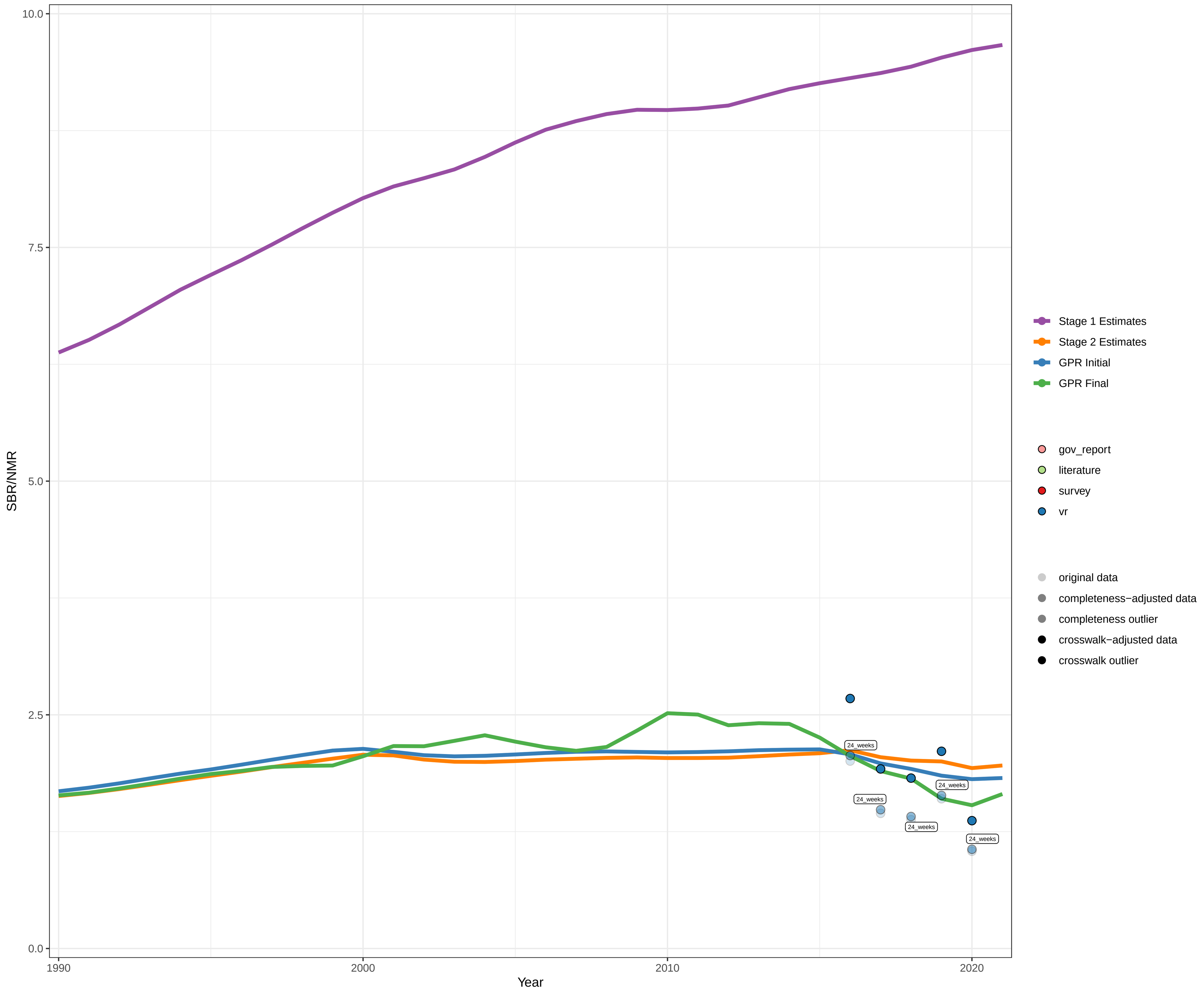

Kent GBR_44769 Western Europe Stillbirth Rate / Neonatal Mortality Rate (SBR/NMR) − 28 weeks
lambda = 0.6 ; zeta = 0.8 ; scale = 15 ; amp = 0.028

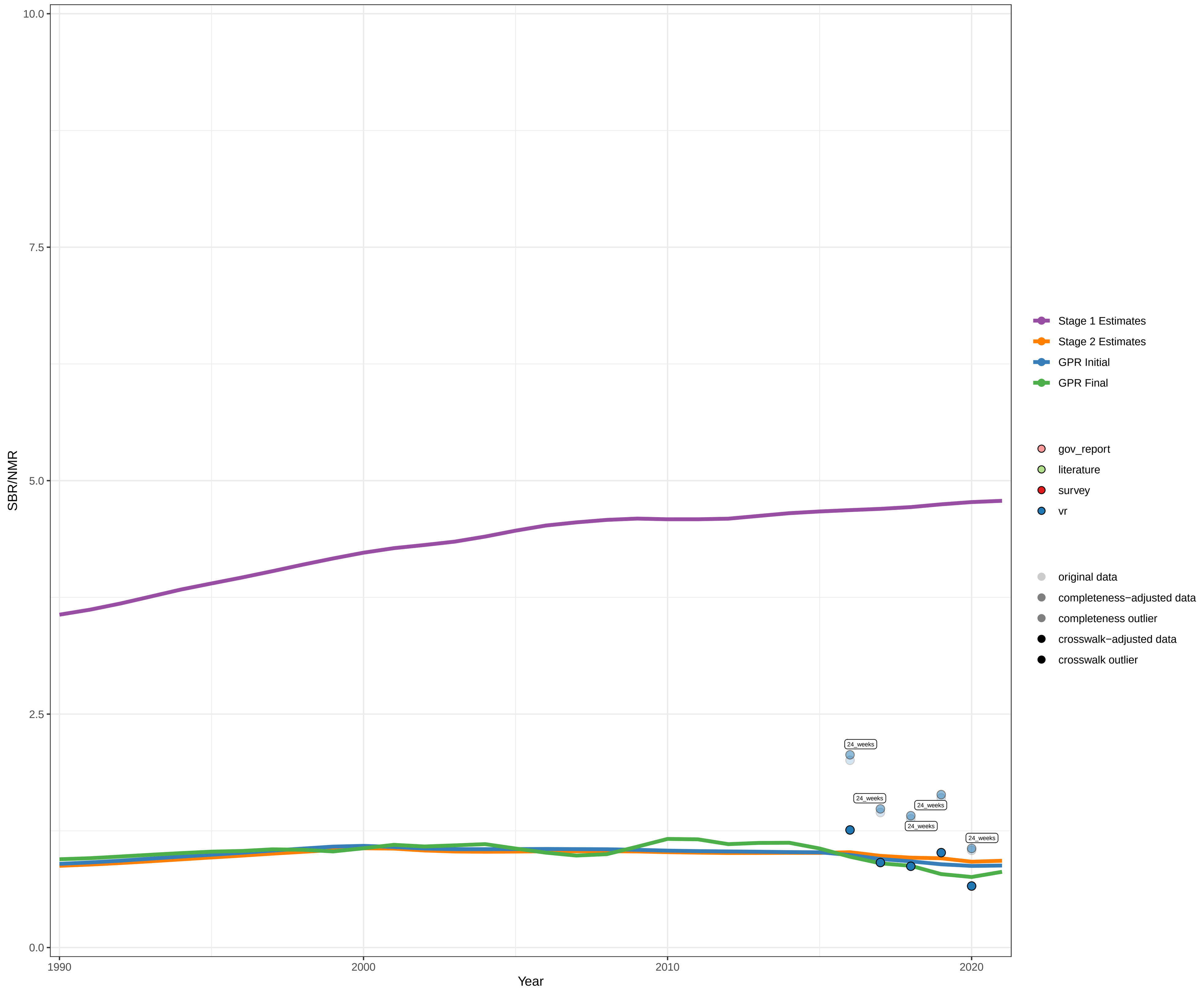

Definition	Years	Outlier	Source.Type	Title
24_weeks	2016−2020	0	VR	United Kingdom − England and Wales Child Mortality Statistics

Kent GBR_44769 Western Europe Stillbirth Rate (SBR) − 20 weeks
lambda = 0.6 ; zeta = 0.8 ; scale = 15 ; amp = 0.028

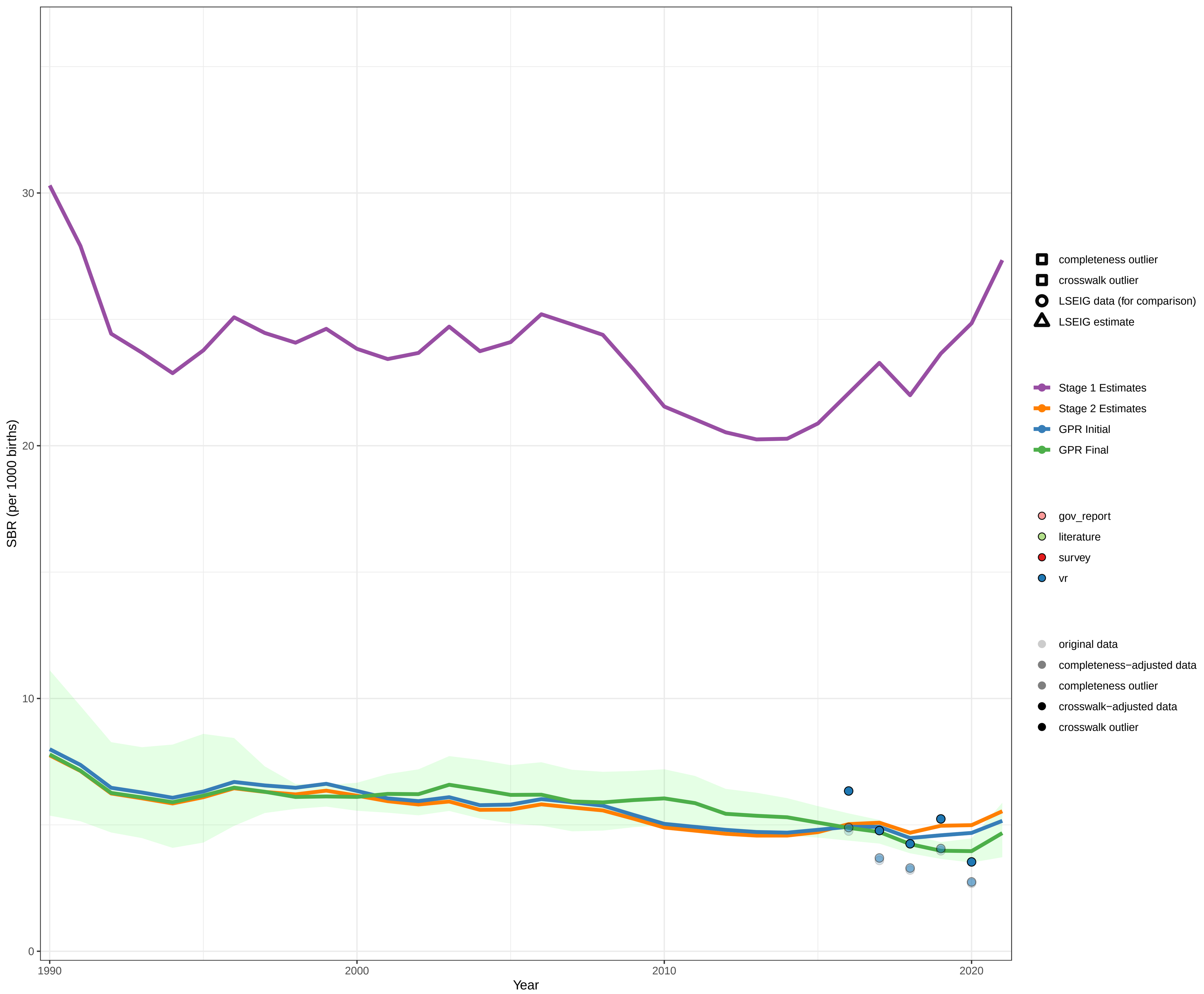

Kent GBR_44769 Western Europe Stillbirth Rate (SBR) − 28 weeks
lambda = 0.6 ; zeta = 0.8 ; scale = 15 ; amp = 0.028

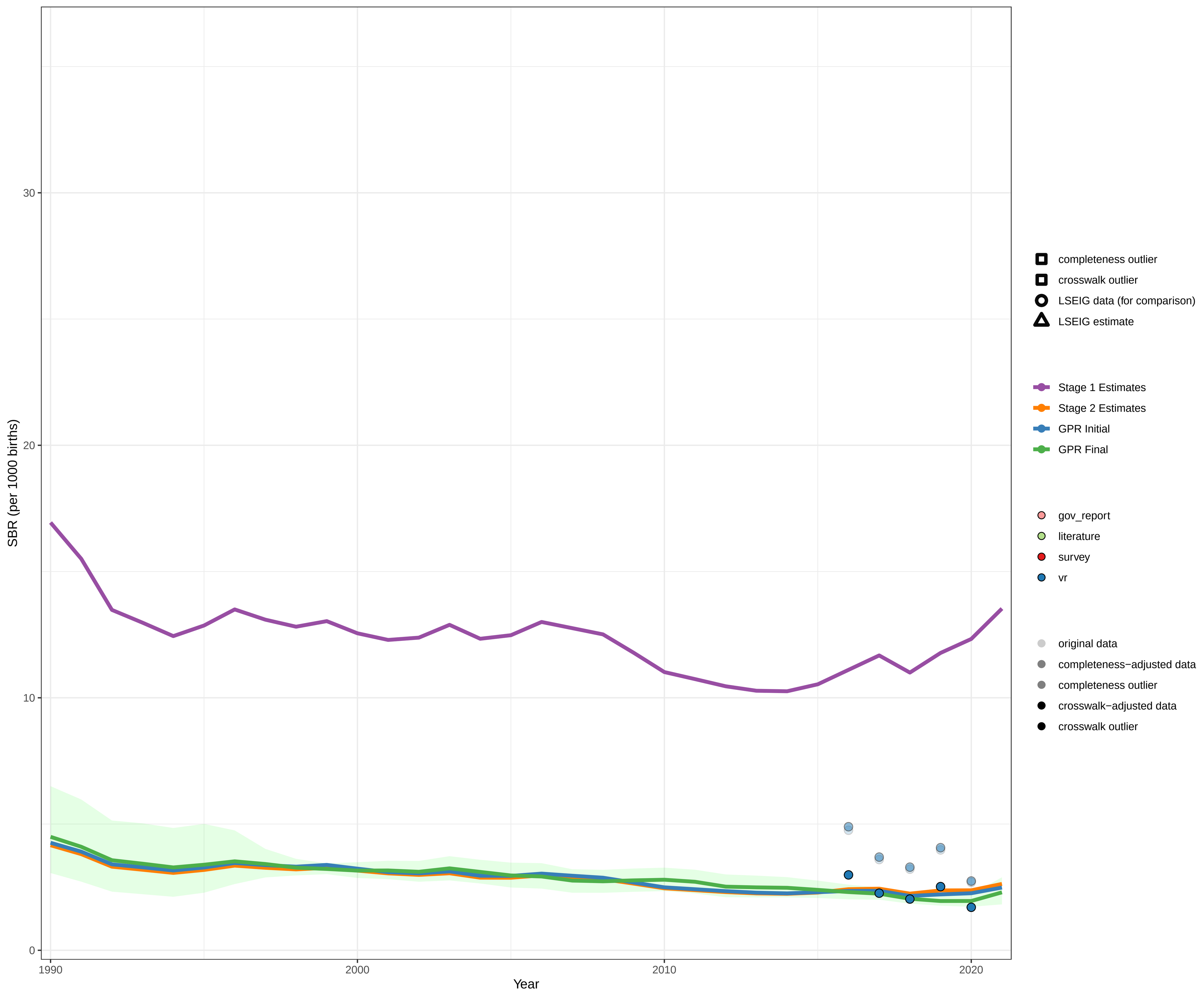

Year	Total Stillbirths (in thousands)	Total Stillbirths (in thousands)	SBR (per 1000 births)	SBR (per 1000 births)	SBR/NMR	SBR/NMR
	20 weeks	28 weeks	20 weeks	28 weeks	20 weeks	28 weeks
1990	0.1<br>(0.1 to 0.2)	0.1<br>(0.1 to 0.1)	7.8<br>(5.4 to 11.1)	4.5<br>(3.1 to 6.5)	1.64<br>(1.13 to 2.34)	0.95<br>(0.64 to 1.37)
1995	0.1<br>(0.1 to 0.1)	0.1<br>(0.0 to 0.1)	6.2<br>(4.3 to 8.6)	3.4<br>(2.3 to 5.0)	1.87<br>(1.30 to 2.61)	1.03<br>(0.69 to 1.52)
2000	0.1<br>(0.1 to 0.1)	0.0<br>(0.0 to 0.1)	6.1<br>(5.6 to 6.7)	3.2<br>(2.9 to 3.5)	2.06<br>(1.87 to 2.25)	1.06<br>(0.96 to 1.18)
2005	0.1<br>(0.1 to 0.1)	0.0<br>(0.0 to 0.1)	6.2<br>(5.0 to 7.4)	3.0<br>(2.5 to 3.5)	2.21<br>(1.81 to 2.63)	1.06<br>(0.89 to 1.24)
2010	0.1<br>(0.1 to 0.1)	0.0<br>(0.0 to 0.1)	6.0<br>(5.0 to 7.2)	2.8<br>(2.3 to 3.3)	2.52<br>(2.07 to 3.00)	1.16<br>(0.98 to 1.36)
2015	0.1<br>(0.1 to 0.1)	0.0<br>(0.0 to 0.0)	5.1<br>(4.5 to 5.7)	2.4<br>(2.1 to 2.8)	2.26<br>(1.99 to 2.55)	1.06<br>(0.92 to 1.22)
2020	0.1<br>(0.1 to 0.1)	0.0<br>(0.0 to 0.0)	4.0<br>(3.5 to 4.5)	2.0<br>(1.7 to 2.2)	1.53<br>(1.36 to 1.73)	0.76<br>(0.67 to 0.86)
2021	0.1<br>(0.1 to 0.1)	0.0<br>(0.0 to 0.0)	4.7<br>(3.7 to 5.9)	2.3<br>(1.8 to 2.9)	1.65<br>(1.32 to 2.08)	0.81<br>(0.64 to 1.02)

Medway GBR_44771 Western Europe Stillbirth Rate / Neonatal Mortality Rate (SBR/NMR) − 20 weeks
lambda = 0.6 ; zeta = 0.8 ; scale = 15 ; amp = 0.028

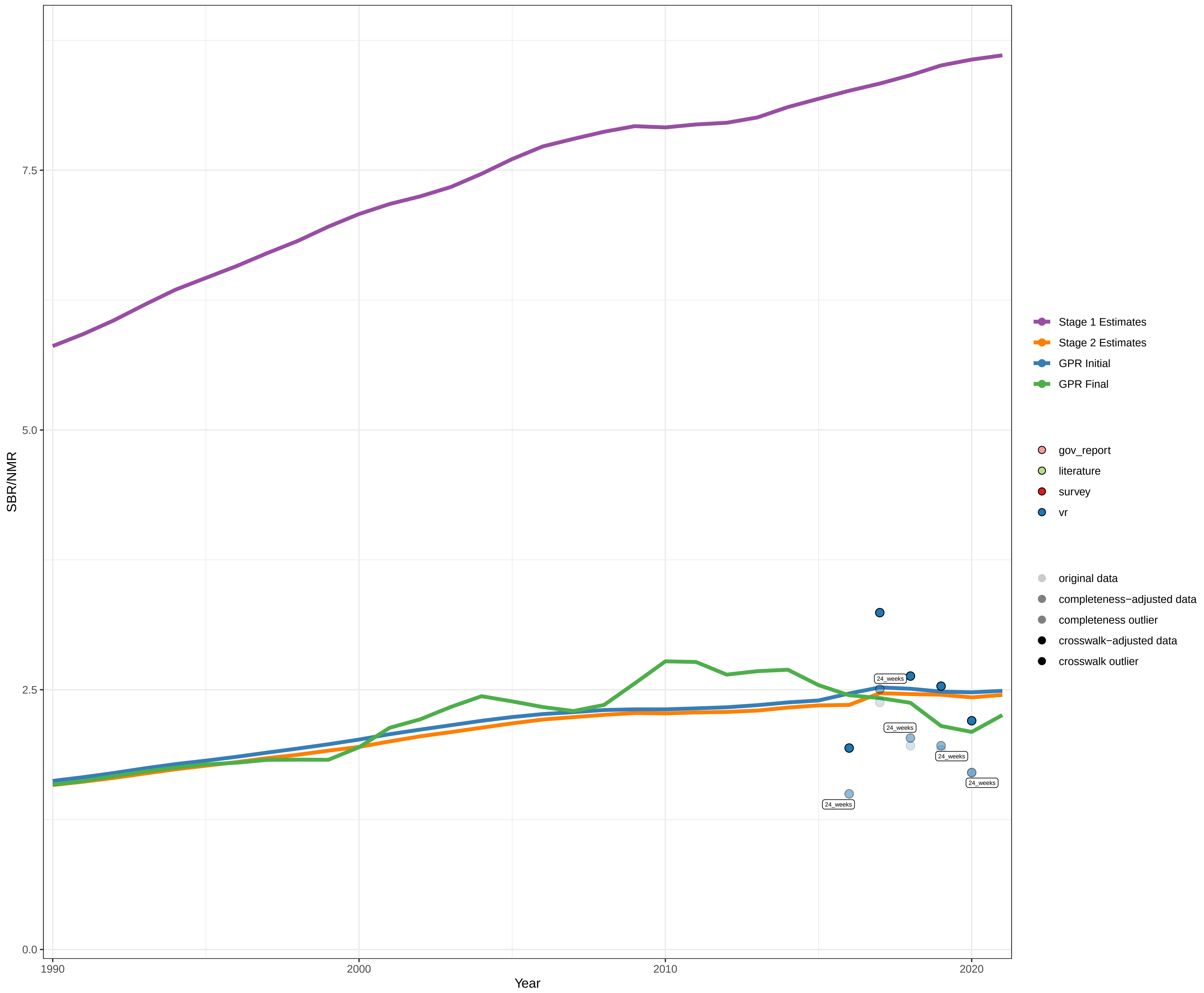

Medway GBR_44771 Western Europe Stillbirth Rate / Neonatal Mortality Rate (SBR/NMR) − 28 weeks
lambda = 0.6 ; zeta = 0.8 ; scale = 15 ; amp = 0.028

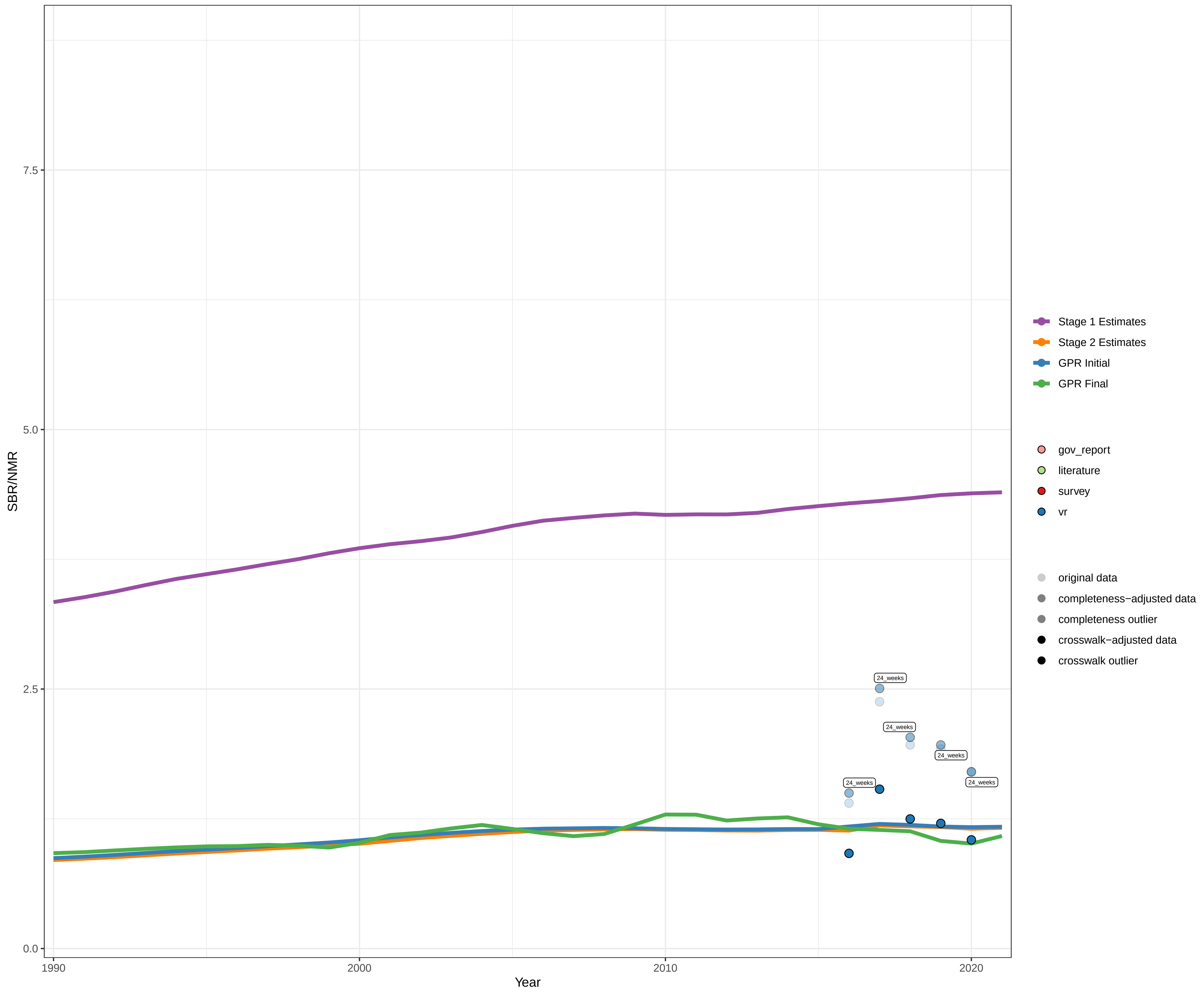

Definition	Years	Outlier	Source.Type	Title
24_weeks	2016−2020	0	VR	United Kingdom − England and Wales Child Mortality Statistics

Medway GBR_44771 Western Europe Stillbirth Rate (SBR) − 20 weeks
lambda = 0.6 ; zeta = 0.8 ; scale = 15 ; amp = 0.028

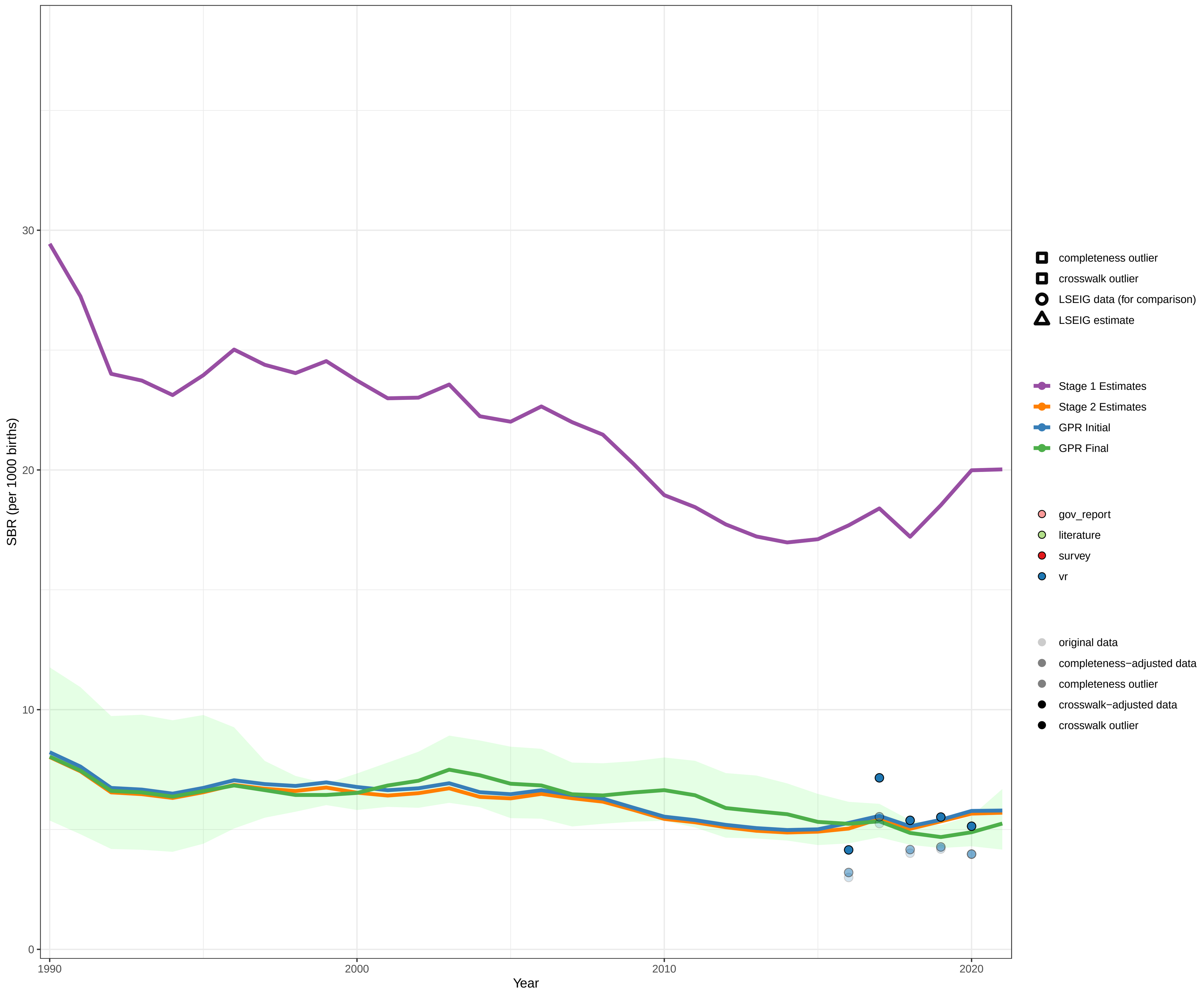

Medway GBR_44771 Western Europe Stillbirth Rate (SBR) − 28 weeks
lambda = 0.6 ; zeta = 0.8 ; scale = 15 ; amp = 0.028

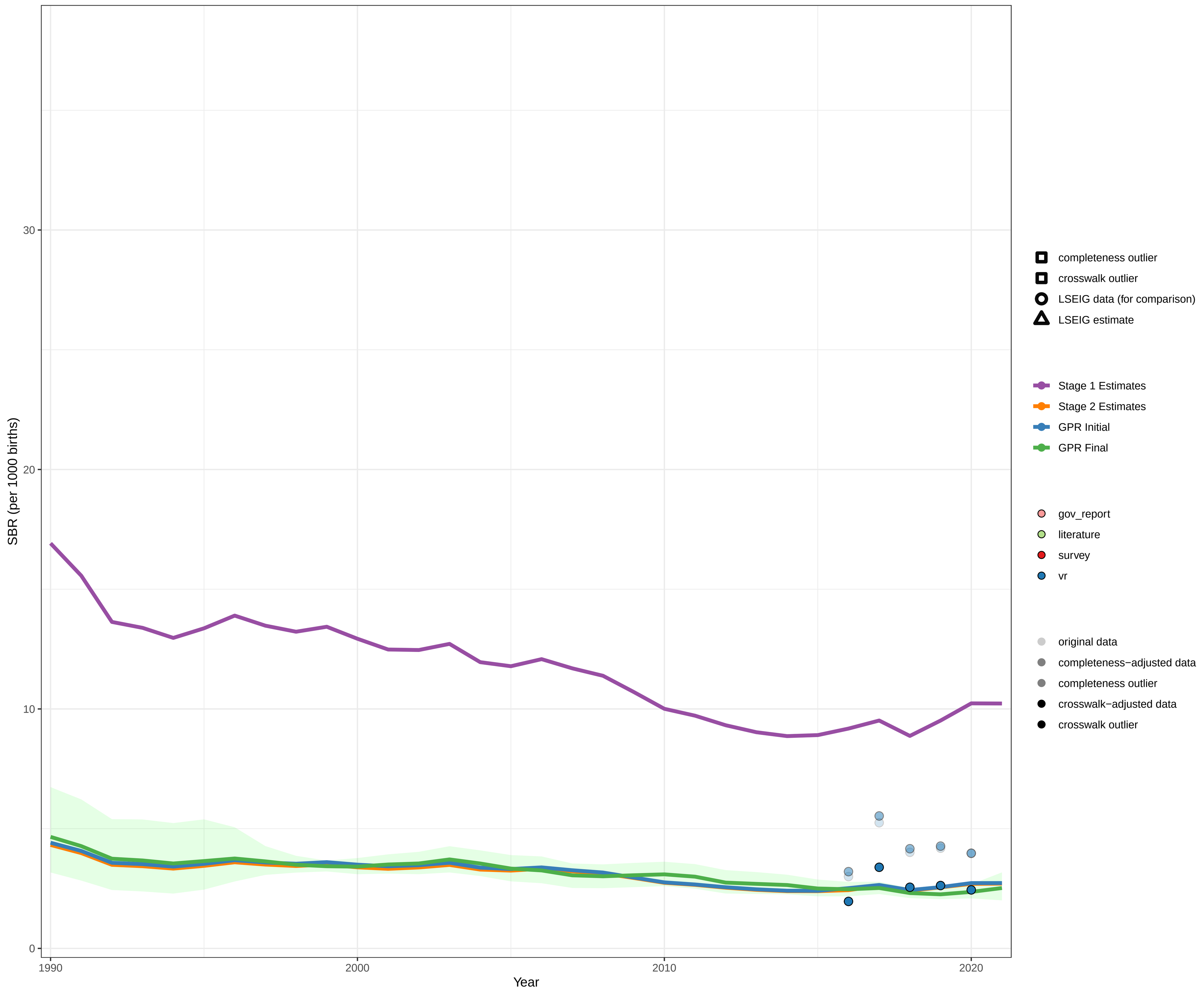

Year	Total Stillbirths (in thousands)	Total Stillbirths (in thousands)	SBR (per 1000 births)	SBR (per 1000 births)	SBR/NMR	SBR/NMR
	20 weeks	28 weeks	20 weeks	28 weeks	20 weeks	28 weeks
1990	0.0 (0.0 to 0.0)	0.0 (0.0 to 0.0)	8.0 (5.4 to 11.8)	4.7 (3.2 to 6.7)	1.59 (1.06 to 2.32)	0.92 (0.63 to 1.33)
1995	0.0 (0.0 to 0.0)	0.0 (0.0 to 0.0)	6.6 (4.4 to 9.8)	3.6 (2.5 to 5.4)	1.78 (1.19 to 2.64)	0.98 (0.66 to 1.45)
2000	0.0 (0.0 to 0.0)	0.0 (0.0 to 0.0)	6.5 (5.8 to 7.3)	3.4 (3.1 to 3.8)	1.95 (1.73 to 2.19)	1.02 (0.92 to 1.13)
2005	0.0 (0.0 to 0.0)	0.0 (0.0 to 0.0)	6.9 (5.5 to 8.5)	3.3 (2.8 to 3.9)	2.39 (1.89 to 2.92)	1.15 (0.97 to 1.35)
2010	0.0 (0.0 to 0.0)	0.0 (0.0 to 0.0)	6.6 (5.4 to 8.0)	3.1 (2.6 to 3.6)	2.77 (2.24 to 3.34)	1.29 (1.09 to 1.51)
2015	0.0 (0.0 to 0.0)	0.0 (0.0 to 0.0)	5.3 (4.4 to 6.5)	2.5 (2.2 to 2.9)	2.54 (2.08 to 3.10)	1.20 (1.04 to 1.38)
2020	0.0 (0.0 to 0.0)	0.0 (0.0 to 0.0)	4.9 (4.3 to 5.6)	2.4 (2.1 to 2.7)	2.09 (1.84 to 2.39)	1.01 (0.90 to 1.15)
2021	0.0 (0.0 to 0.0)	0.0 (0.0 to 0.0)	5.2 (4.2 to 6.7)	2.5 (2.0 to 3.2)	2.26 (1.79 to 2.87)	1.08 (0.86 to 1.36)

Milton Keynes GBR_44775 Western Europe Stillbirth Rate / Neonatal Mortality Rate (SBR/NMR) − 20 weeks
lambda = 0.6 ; zeta = 0.8 ; scale = 15 ; amp = 0.028

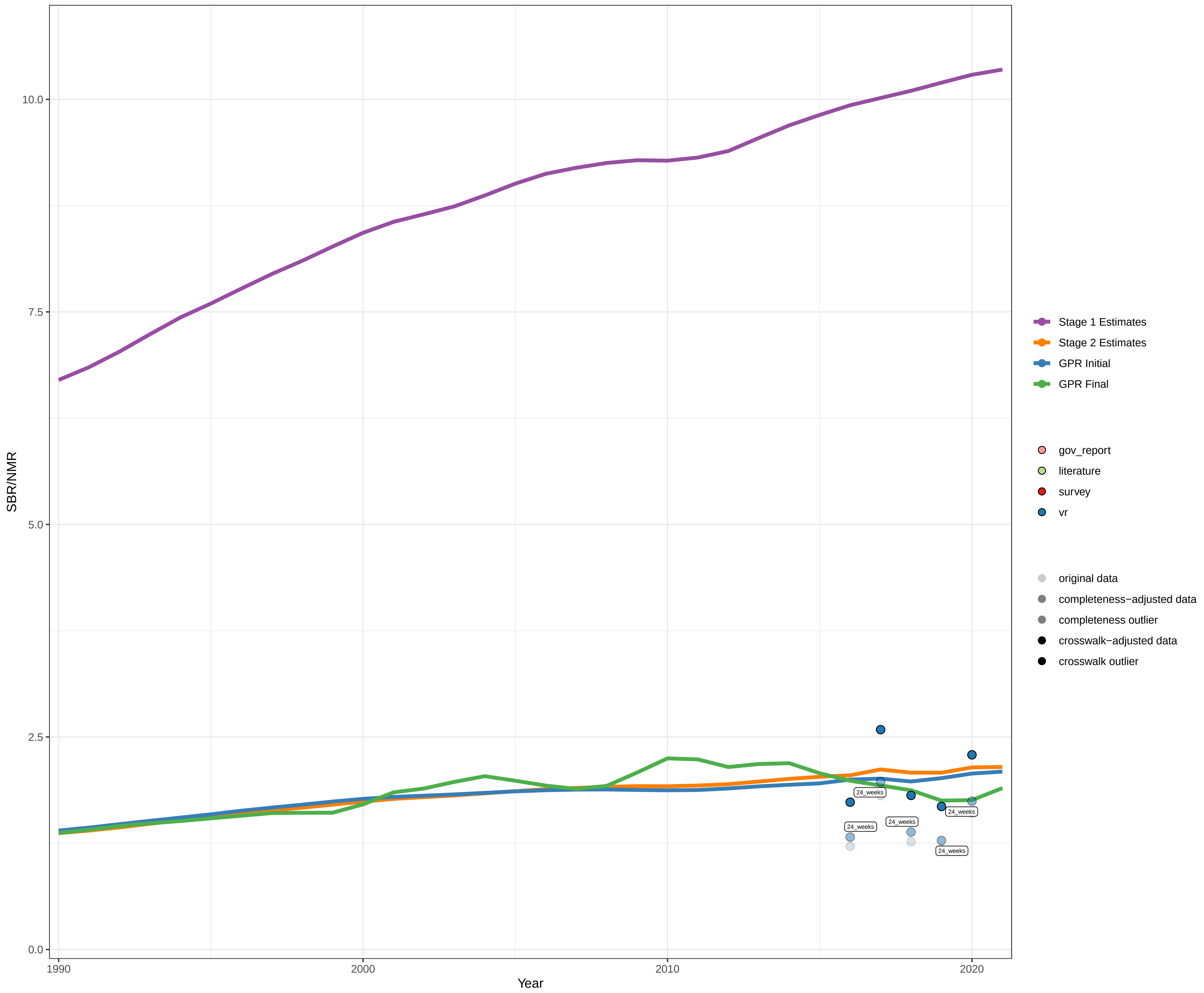

Milton Keynes GBR_44775 Western Europe Stillbirth Rate / Neonatal Mortality Rate (SBR/NMR) − 28 weeks
lambda = 0.6 ; zeta = 0.8 ; scale = 15 ; amp = 0.028

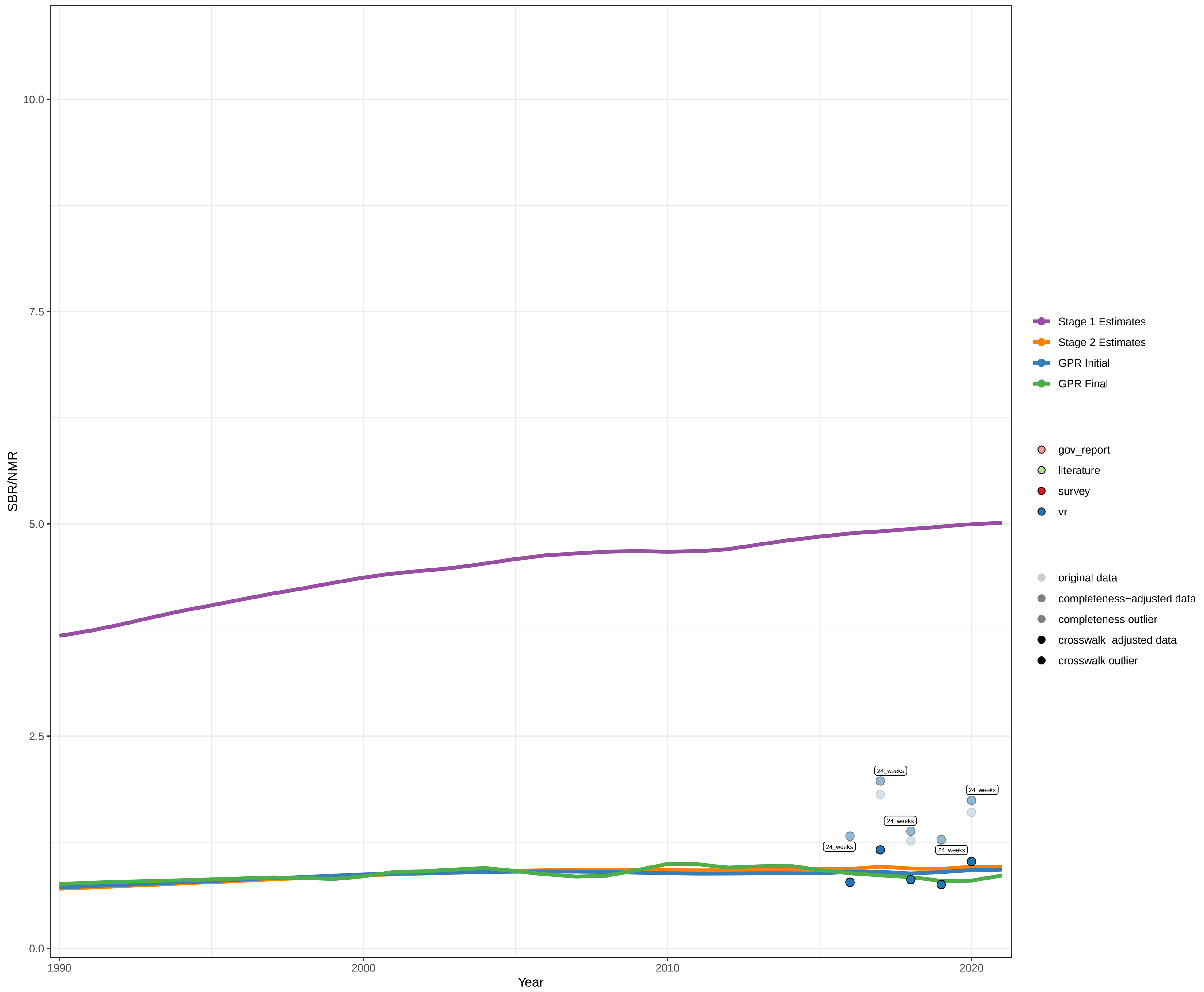

Definition	Years	Outlier	Source.Type	Title
24_weeks	2016−2020	0	VR	United Kingdom − England and Wales Child Mortality Statistics

Milton Keynes GBR_44775 Western Europe Stillbirth Rate (SBR) − 20 weeks
lambda = 0.6 ; zeta = 0.8 ; scale = 15 ; amp = 0.028

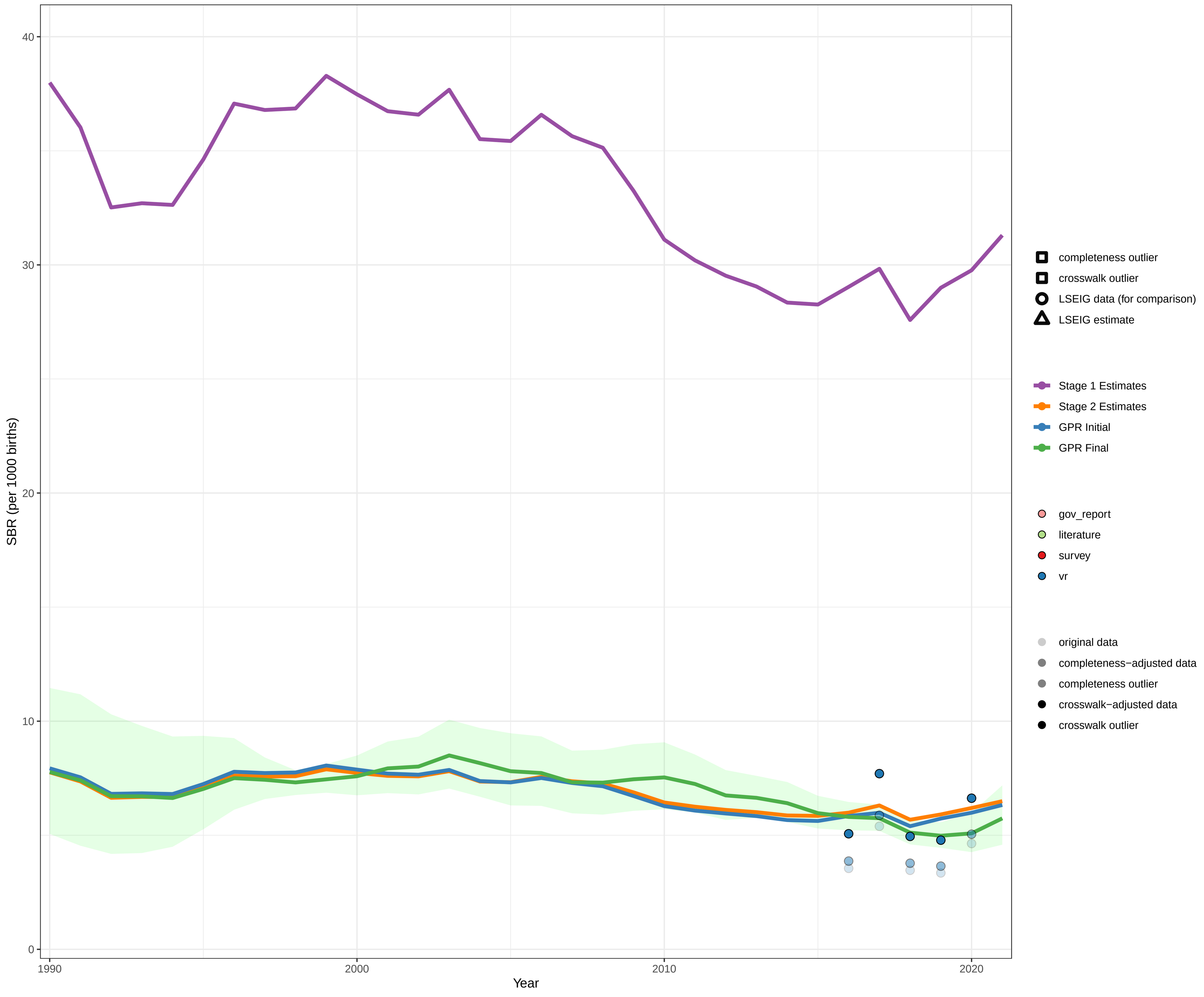

Milton Keynes GBR_44775 Western Europe Stillbirth Rate (SBR) − 28 weeks
lambda = 0.6 ; zeta = 0.8 ; scale = 15 ; amp = 0.028

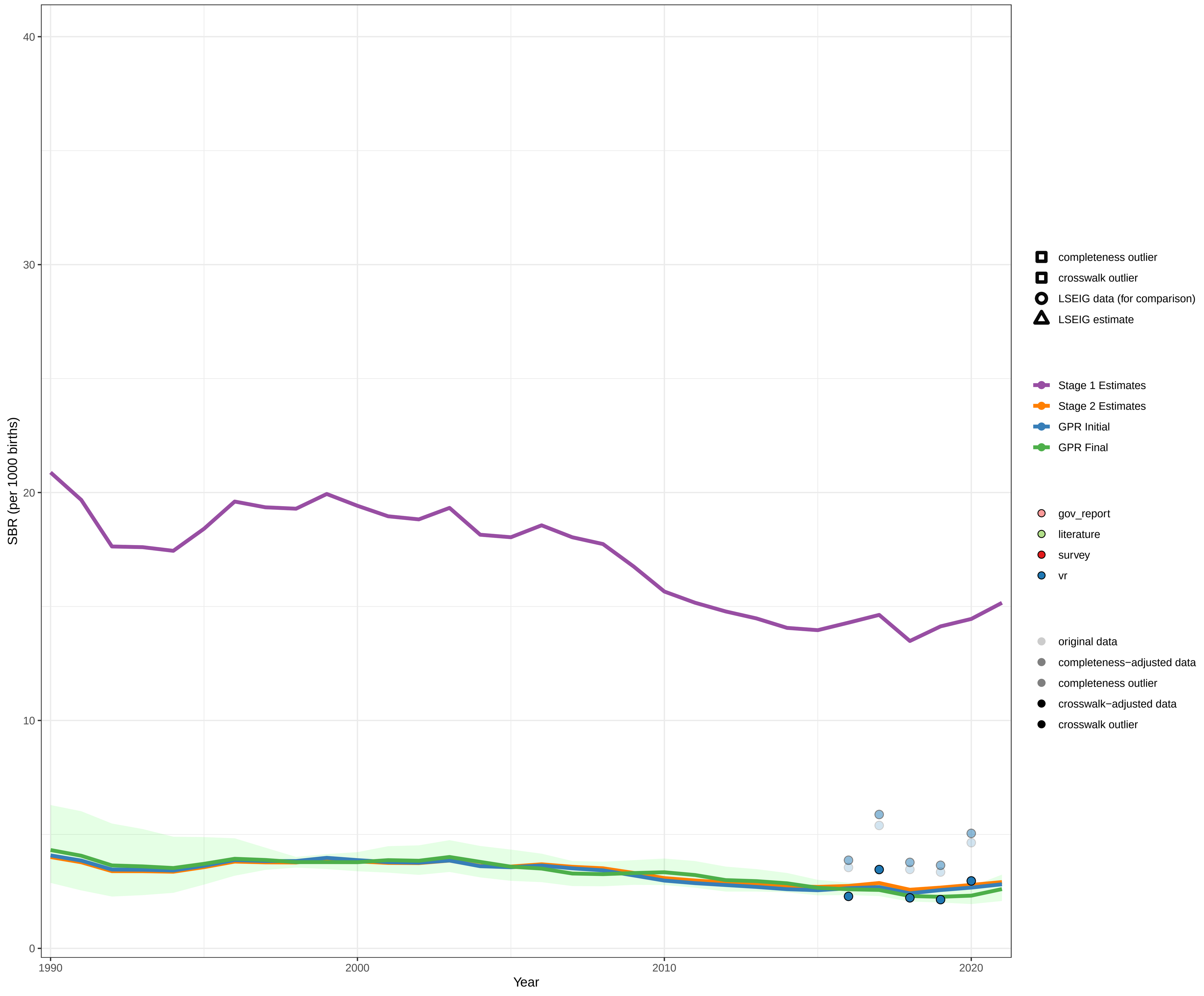

Year	Total Stillbirths (in thousands)	Total Stillbirths (in thousands)	SBR (per 1000 births)	SBR (per 1000 births)	SBR/NMR	SBR/NMR
	20 weeks	28 weeks	20 weeks	28 weeks	20 weeks	28 weeks
1990	0.0 (0.0 to 0.0)	0.0 (0.0 to 0.0)	7.8 (5.1 to 11.5)	4.3 (2.9 to 6.3)	1.37 (0.89 to 2.02)	0.76 (0.51 to 1.11)
1995	0.0 (0.0 to 0.0)	0.0 (0.0 to 0.0)	7.0 (5.3 to 9.4)	3.7 (2.8 to 4.9)	1.54 (1.16 to 2.05)	0.81 (0.61 to 1.07)
2000	0.0 (0.0 to 0.0)	0.0 (0.0 to 0.0)	7.6 (6.8 to 8.5)	3.8 (3.4 to 4.2)	1.71 (1.52 to 1.91)	0.85 (0.76 to 0.95)
2005	0.0 (0.0 to 0.0)	0.0 (0.0 to 0.0)	7.8 (6.3 to 9.5)	3.6 (3.0 to 4.3)	1.99 (1.60 to 2.41)	0.91 (0.75 to 1.10)
2010	0.0 (0.0 to 0.0)	0.0 (0.0 to 0.0)	7.5 (6.1 to 9.1)	3.3 (2.8 to 3.9)	2.25 (1.83 to 2.71)	1.00 (0.83 to 1.18)
2015	0.0 (0.0 to 0.0)	0.0 (0.0 to 0.0)	6.0 (5.3 to 6.7)	2.7 (2.3 to 3.0)	2.07 (1.84 to 2.34)	0.92 (0.81 to 1.04)
2020	0.0 (0.0 to 0.0)	0.0 (0.0 to 0.0)	5.1 (4.3 to 6.0)	2.3 (1.9 to 2.7)	1.76 (1.47 to 2.07)	0.80 (0.67 to 0.94)
2021	0.0 (0.0 to 0.0)	0.0 (0.0 to 0.0)	5.7 (4.6 to 7.2)	2.6 (2.1 to 3.2)	1.90 (1.52 to 2.38)	0.86 (0.69 to 1.07)

Oxfordshire GBR_44767 Western Europe Stillbirth Rate / Neonatal Mortality Rate (SBR/NMR) − 20 weeks
lambda = 0.6 ; zeta = 0.8 ; scale = 15 ; amp = 0.028

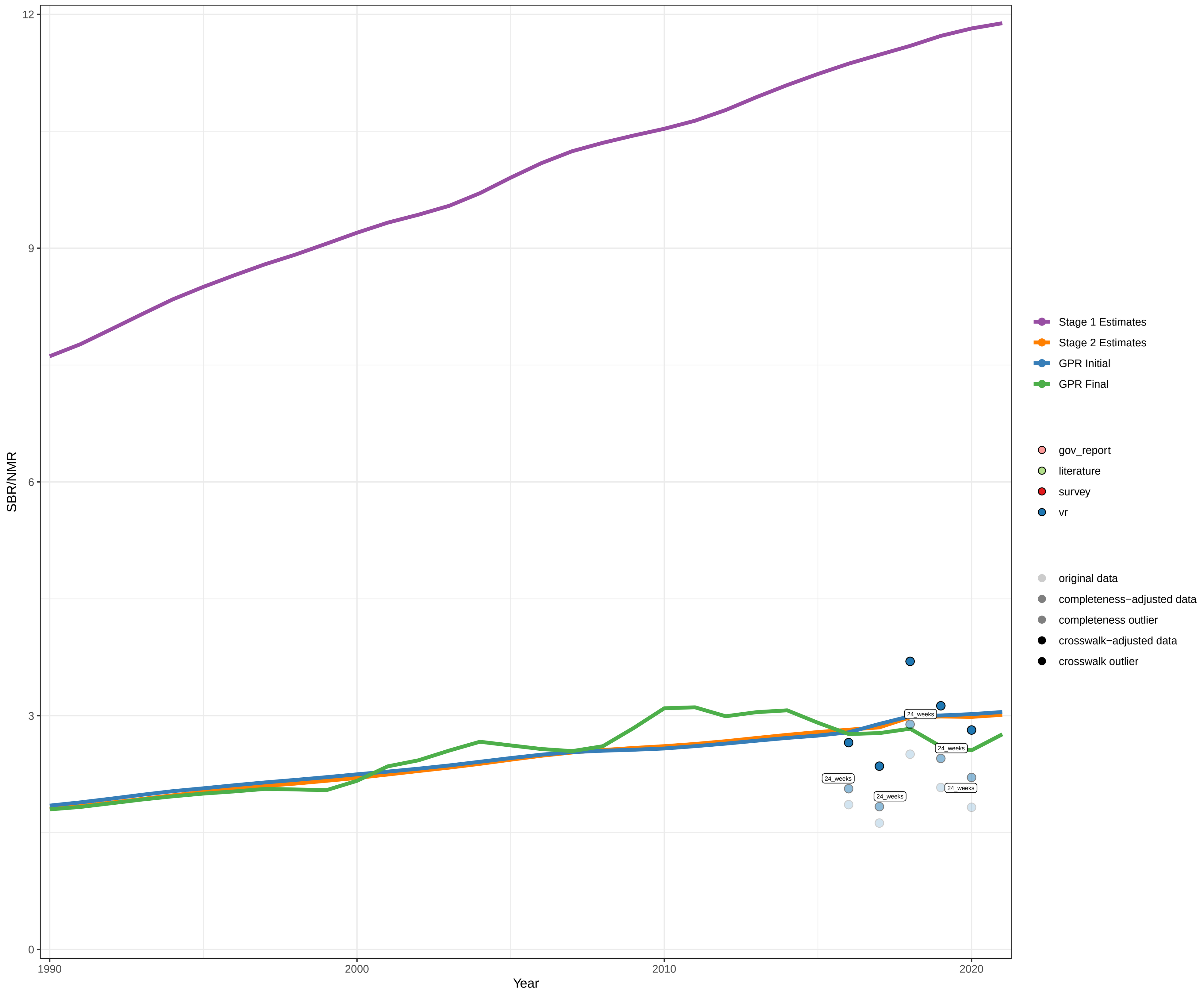

Oxfordshire GBR_44767 Western Europe Stillbirth Rate / Neonatal Mortality Rate (SBR/NMR) − 28 weeks
lambda = 0.6 ; zeta = 0.8 ; scale = 15 ; amp = 0.028

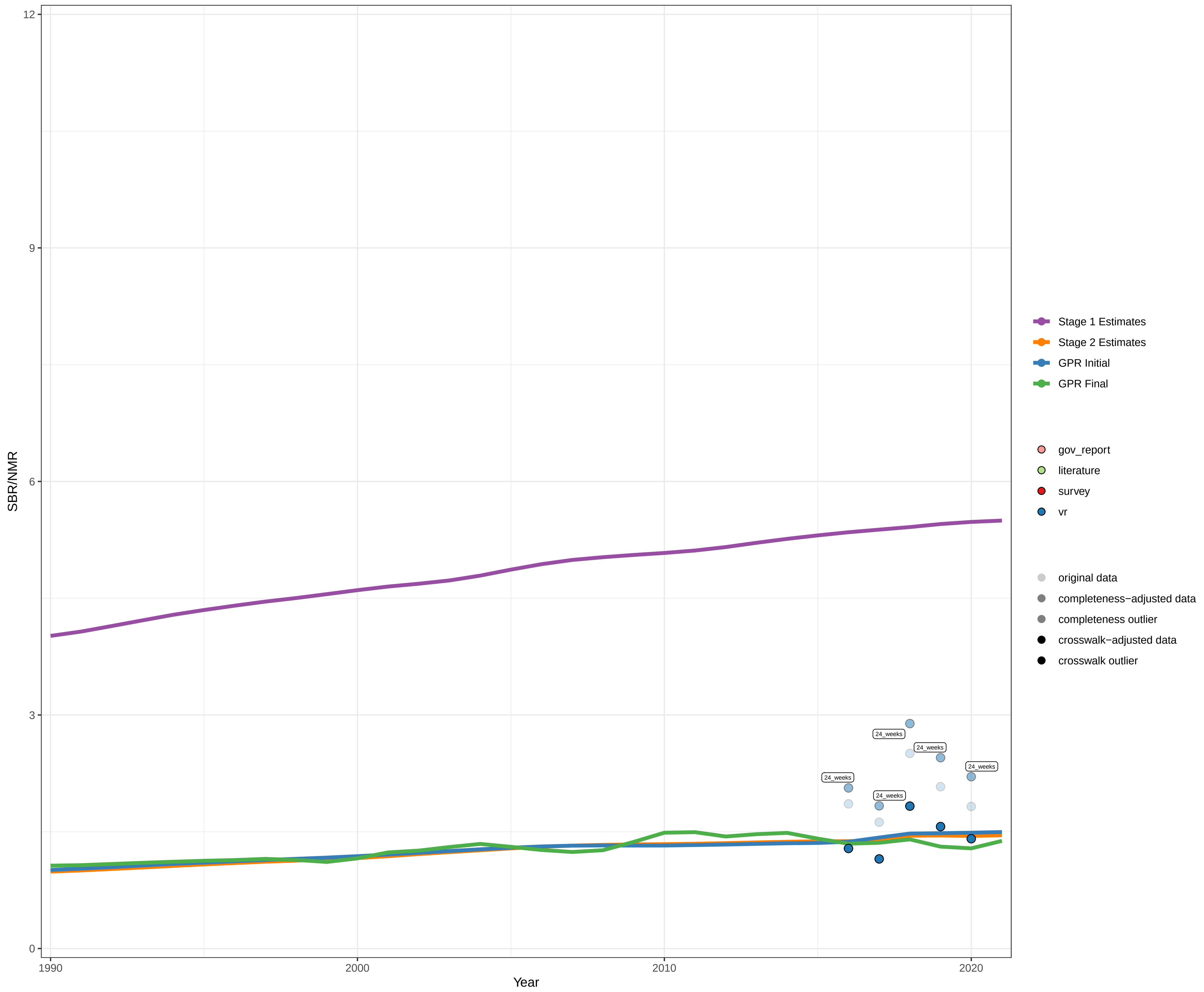

Definition	Years	Outlier	Source.Type	Title
24_weeks	2016−2020	0	VR	United Kingdom − England and Wales Child Mortality Statistics

Oxfordshire GBR_44767 Western Europe Stillbirth Rate (SBR) − 20 weeks
lambda = 0.6 ; zeta = 0.8 ; scale = 15 ; amp = 0.028

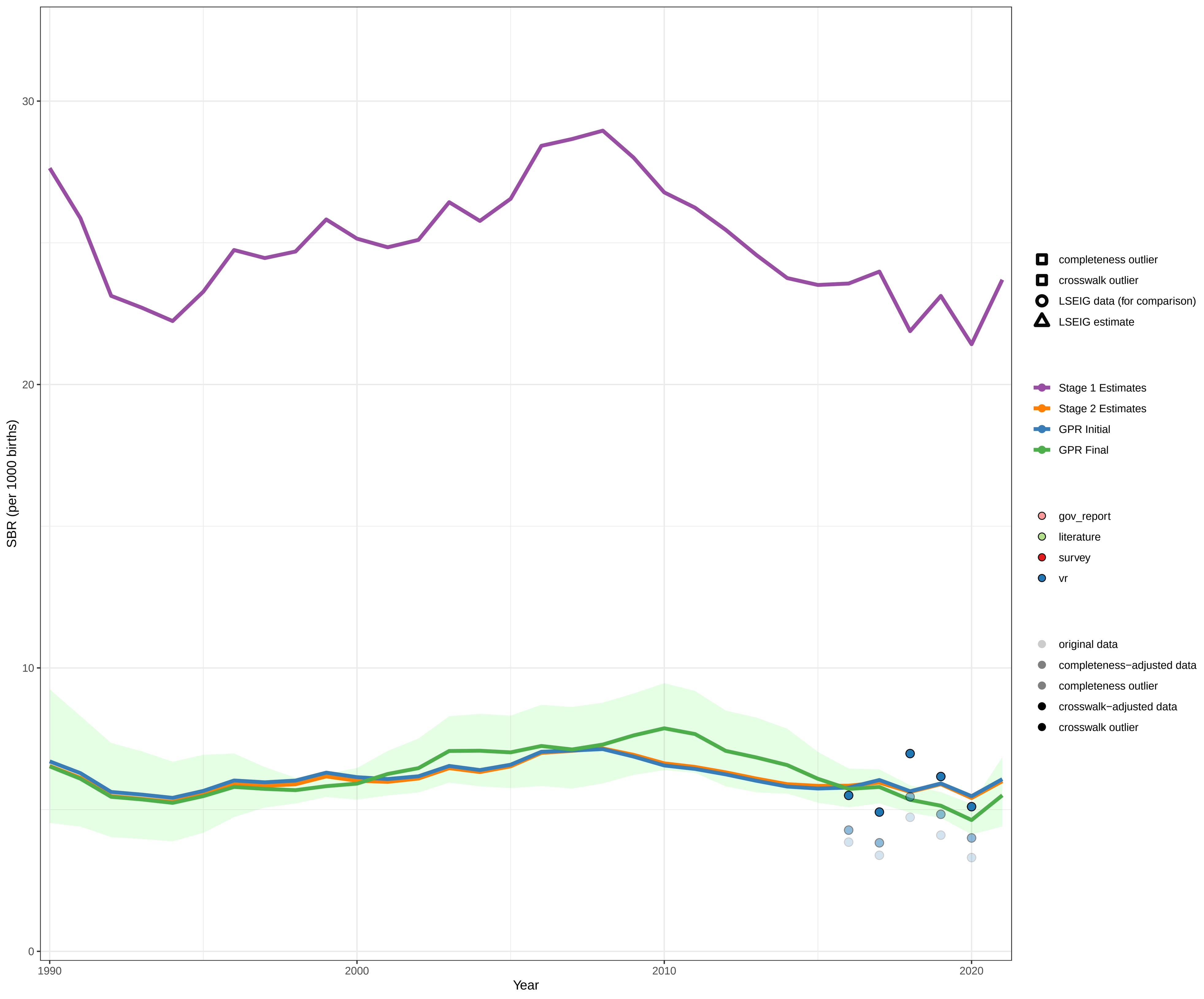

Oxfordshire GBR_44767 Western Europe Stillbirth Rate (SBR) − 28 weeks
lambda = 0.6 ; zeta = 0.8 ; scale = 15 ; amp = 0.028

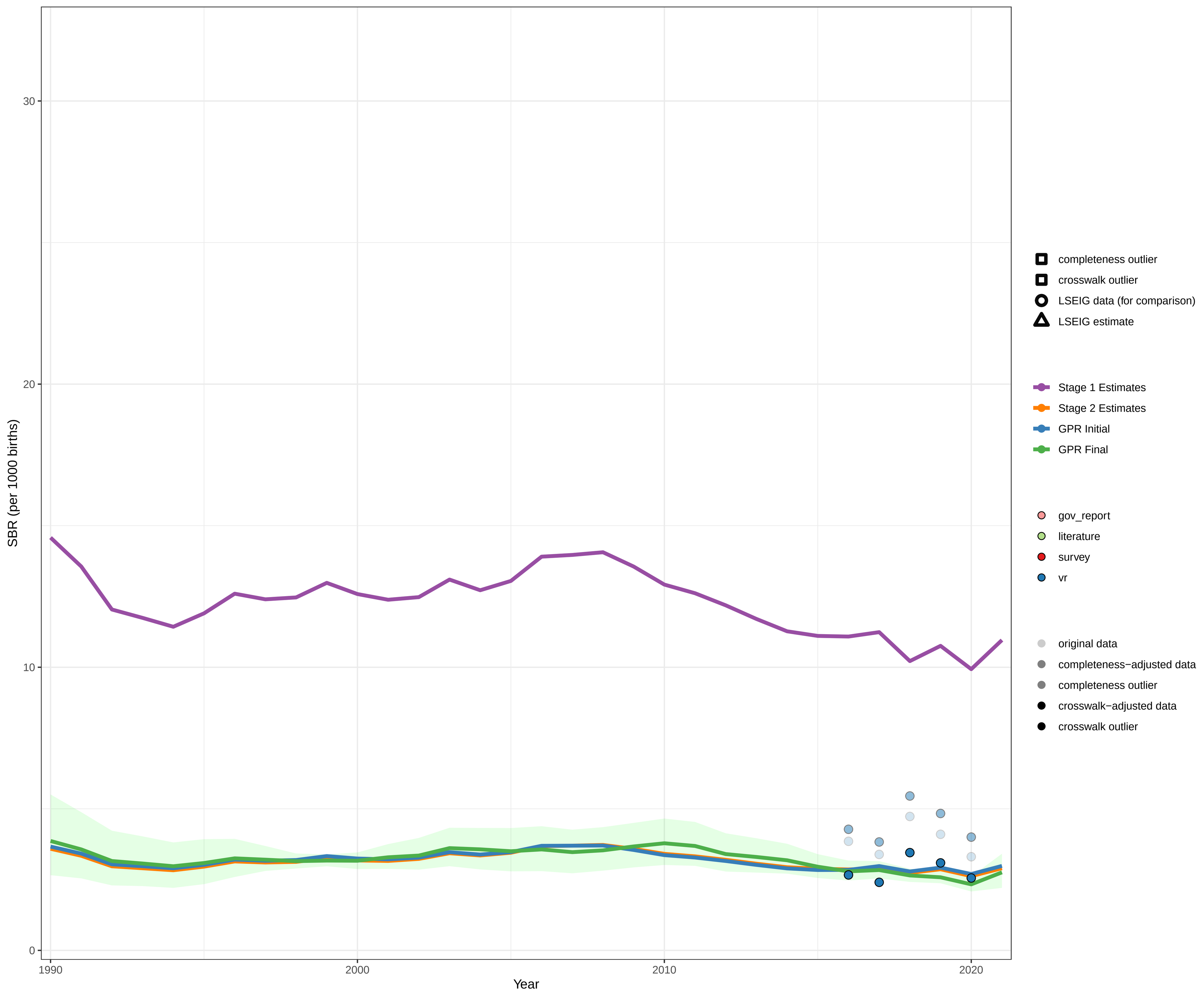

Year	Total Stillbirths (in thousands)	Total Stillbirths (in thousands)	SBR (per 1000 births)	SBR (per 1000 births)	SBR/NMR	SBR/NMR
	20 weeks	28 weeks	20 weeks	28 weeks	20 weeks	28 weeks
1990	0.1 (0.0 to 0.1)	0.0 (0.0 to 0.0)	6.5 (4.5 to 9.2)	3.9 (2.7 to 5.5)	1.80 (1.25 to 2.55)	1.06 (0.73 to 1.52)
1995	0.0 (0.0 to 0.1)	0.0 (0.0 to 0.0)	5.5 (4.2 to 6.9)	3.1 (2.3 to 3.9)	2.00 (1.53 to 2.53)	1.13 (0.85 to 1.43)
2000	0.0 (0.0 to 0.0)	0.0 (0.0 to 0.0)	5.9 (5.4 to 6.5)	3.2 (2.9 to 3.5)	2.16 (1.96 to 2.37)	1.16 (1.05 to 1.27)
2005	0.1 (0.0 to 0.1)	0.0 (0.0 to 0.0)	7.0 (5.8 to 8.3)	3.5 (2.8 to 4.3)	2.62 (2.15 to 3.10)	1.31 (1.04 to 1.61)
2010	0.1 (0.1 to 0.1)	0.0 (0.0 to 0.0)	7.9 (6.4 to 9.5)	3.8 (3.0 to 4.7)	3.10 (2.51 to 3.72)	1.49 (1.19 to 1.83)
2015	0.0 (0.0 to 0.1)	0.0 (0.0 to 0.0)	6.1 (5.2 to 7.0)	3.0 (2.6 to 3.4)	2.91 (2.51 to 3.36)	1.41 (1.22 to 1.62)
2020	0.0 (0.0 to 0.0)	0.0 (0.0 to 0.0)	4.6 (4.1 to 5.2)	2.3 (2.1 to 2.6)	2.56 (2.28 to 2.87)	1.29 (1.15 to 1.44)
2021	0.0 (0.0 to 0.0)	0.0 (0.0 to 0.0)	5.5 (4.4 to 6.9)	2.8 (2.2 to 3.4)	2.76 (2.21 to 3.44)	1.38 (1.11 to 1.71)

Portsmouth GBR_44773 Western Europe Stillbirth Rate / Neonatal Mortality Rate (SBR/NMR) − 20 weeks
lambda = 0.6 ; zeta = 0.8 ; scale = 15 ; amp = 0.028

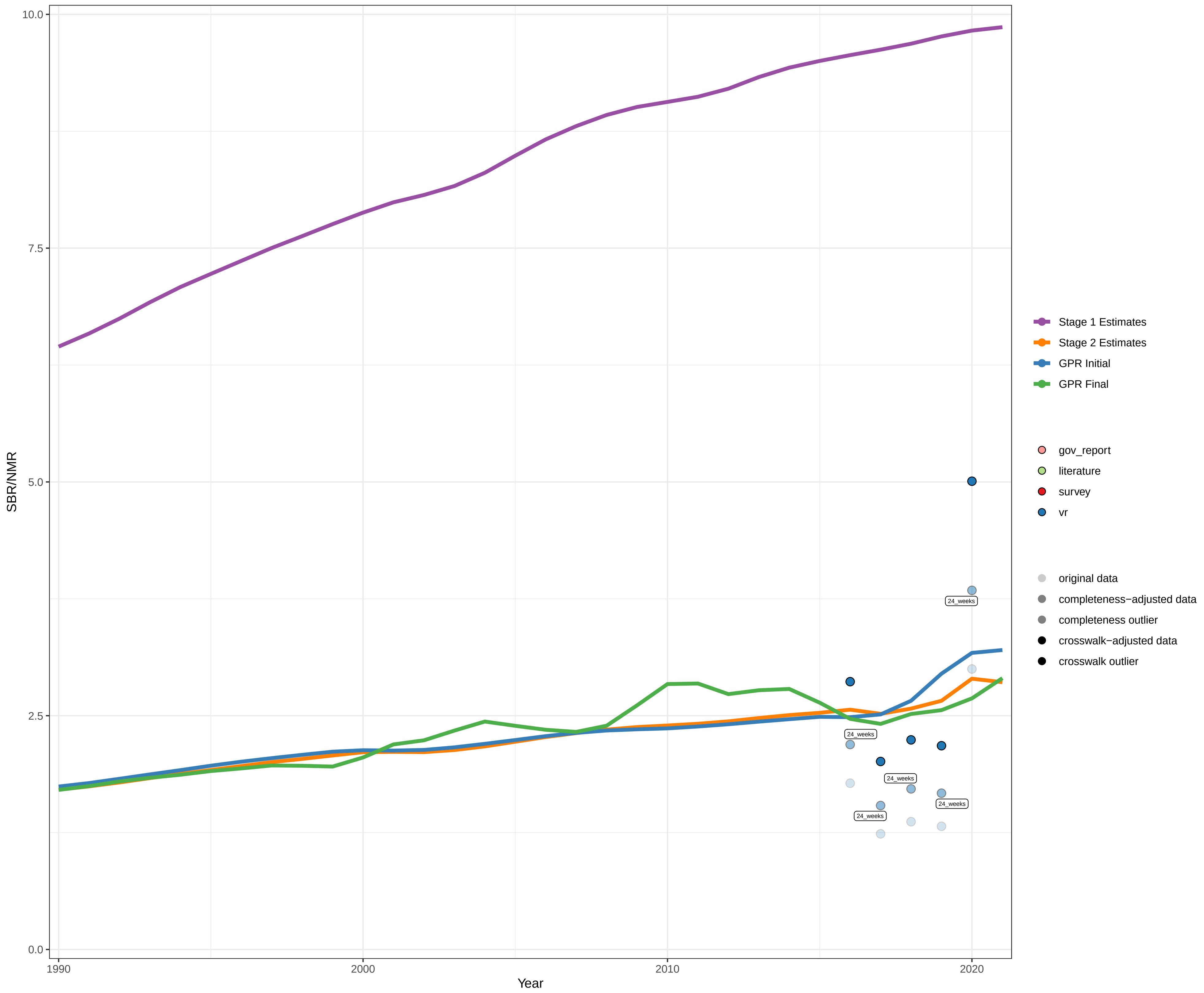

Portsmouth GBR_44773 Western Europe Stillbirth Rate / Neonatal Mortality Rate (SBR/NMR) − 28 weeks
lambda = 0.6 ; zeta = 0.8 ; scale = 15 ; amp = 0.028

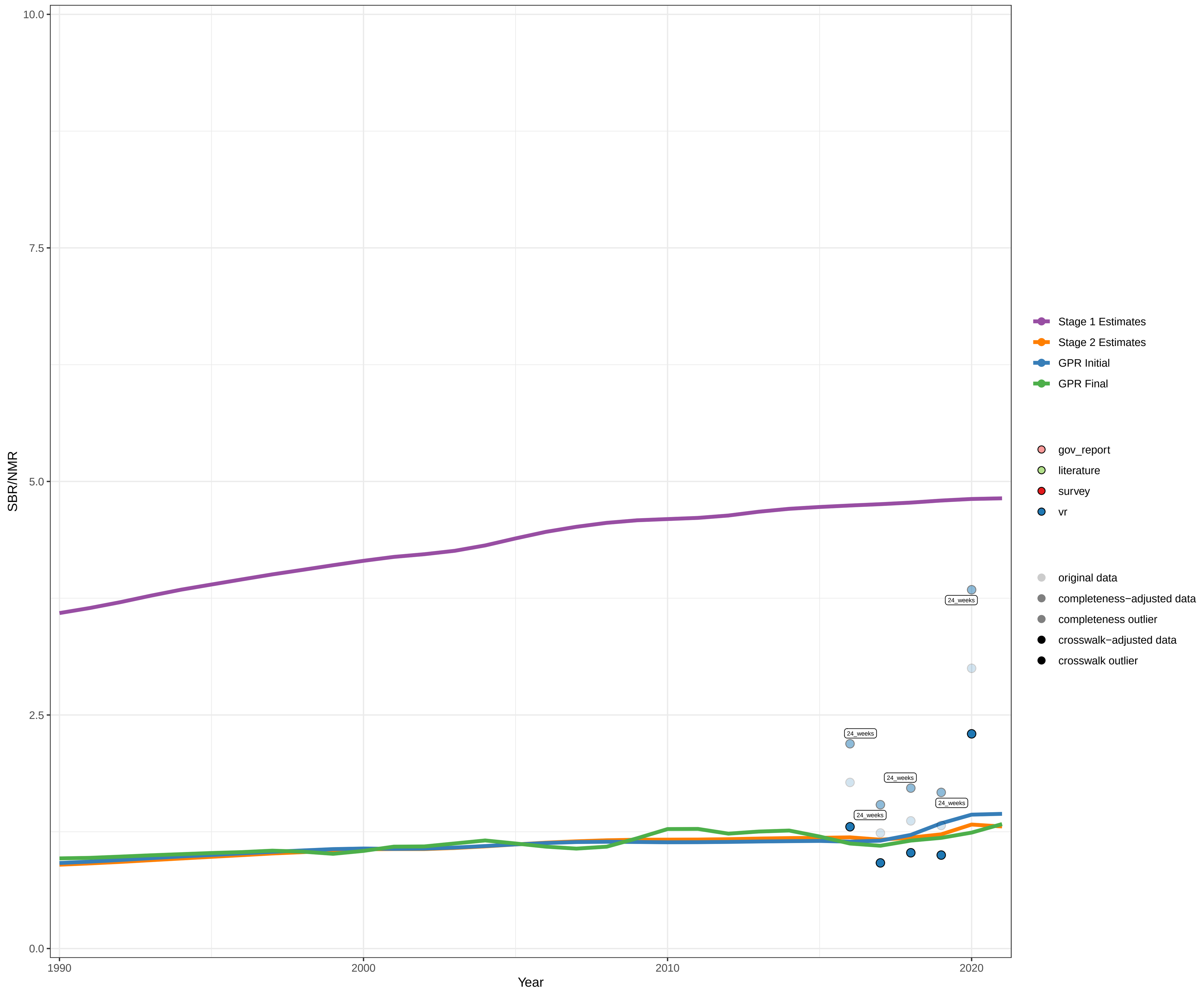

Definition	Years	Outlier	Source.Type	Title
24_weeks	2016−2020	0	VR	United Kingdom − England and Wales Child Mortality Statistics

Portsmouth GBR_44773 Western Europe Stillbirth Rate (SBR) − 20 weeks
lambda = 0.6 ; zeta = 0.8 ; scale = 15 ; amp = 0.028

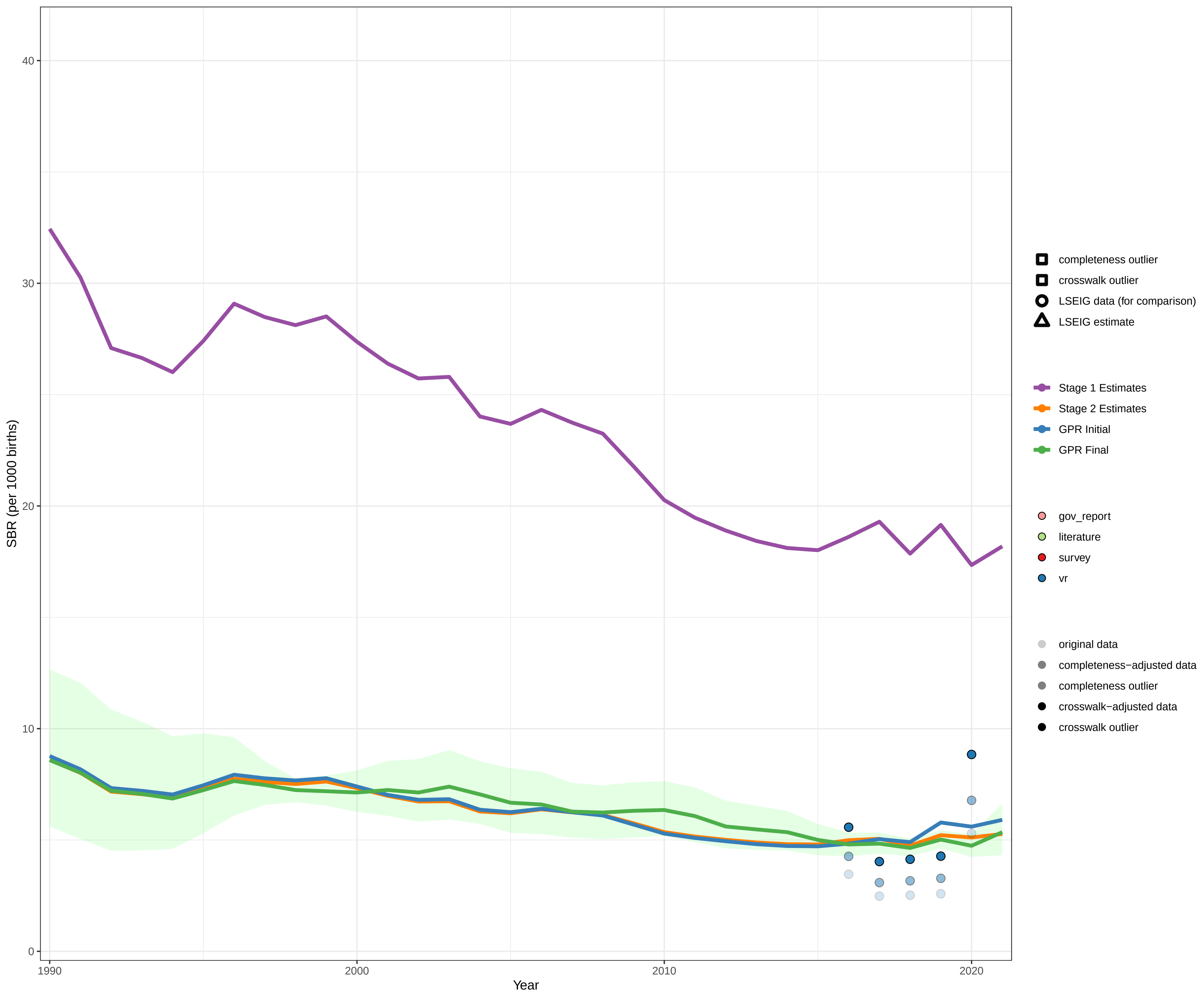

Portsmouth GBR_44773 Western Europe Stillbirth Rate (SBR) − 28 weeks
lambda = 0.6 ; zeta = 0.8 ; scale = 15 ; amp = 0.028

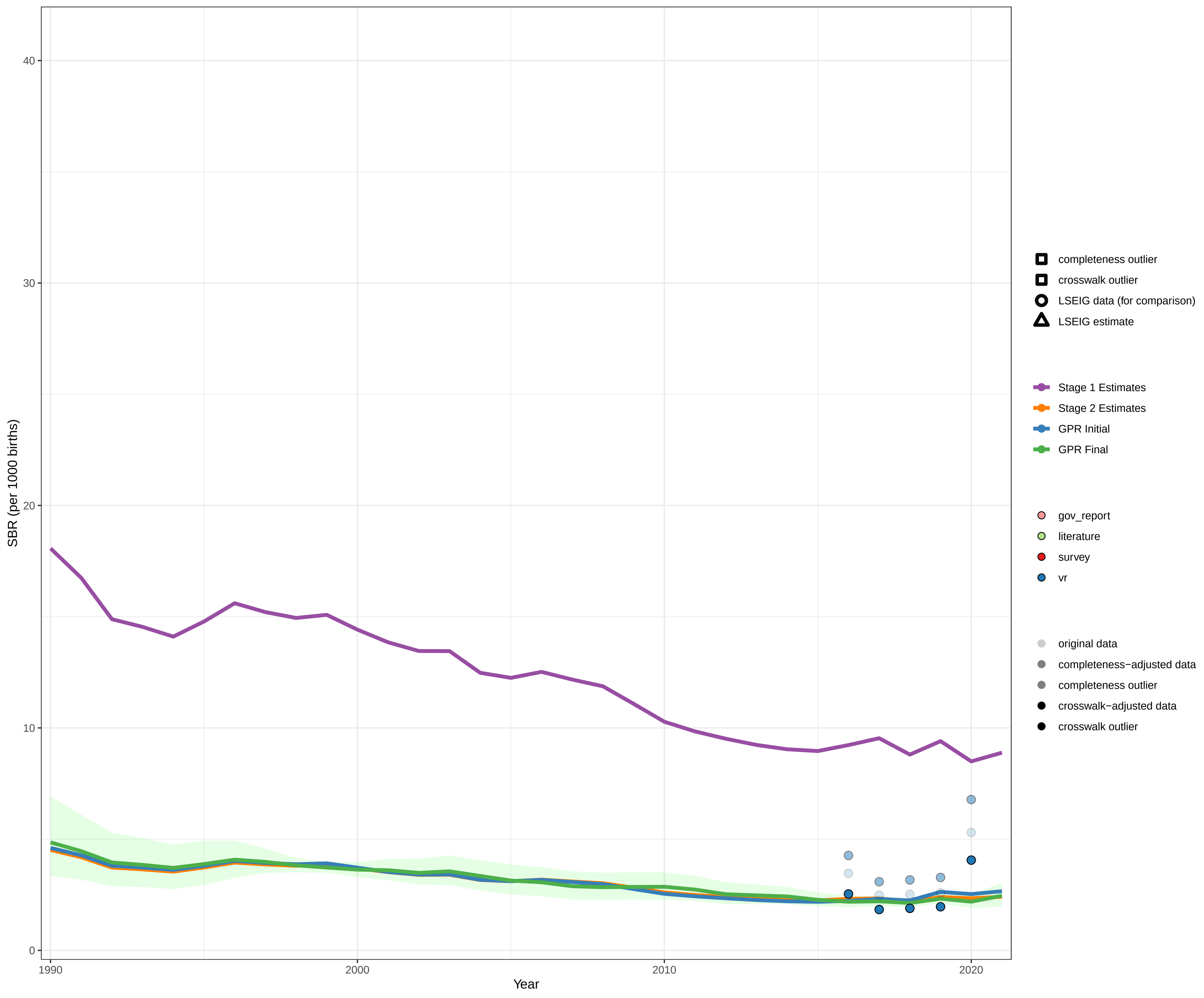

Year	Total Stillbirths (in thousands)	Total Stillbirths (in thousands)	SBR (per 1000 births)	SBR (per 1000 births)	SBR/NMR	SBR/NMR
	20 weeks	28 weeks	20 weeks	28 weeks	20 weeks	28 weeks
1990	0.0 (0.0 to 0.0)	0.0 (0.0 to 0.0)	8.6 (5.6 to 12.7)	4.9 (3.3 to 6.9)	1.71 (1.11 to 2.52)	0.97 (0.66 to 1.38)
1995	0.0 (0.0 to 0.0)	0.0 (0.0 to 0.0)	7.2 (5.3 to 9.8)	3.9 (2.9 to 4.9)	1.91 (1.40 to 2.58)	1.02 (0.78 to 1.30)
2000	0.0 (0.0 to 0.0)	0.0 (0.0 to 0.0)	7.1 (6.3 to 8.1)	3.6 (3.3 to 4.0)	2.05 (1.80 to 2.34)	1.05 (0.95 to 1.14)
2005	0.0 (0.0 to 0.0)	0.0 (0.0 to 0.0)	6.7 (5.3 to 8.2)	3.1 (2.5 to 3.9)	2.39 (1.91 to 2.95)	1.12 (0.90 to 1.39)
2010	0.0 (0.0 to 0.0)	0.0 (0.0 to 0.0)	6.3 (5.2 to 7.6)	2.9 (2.3 to 3.5)	2.84 (2.32 to 3.42)	1.28 (1.02 to 1.57)
2015	0.0 (0.0 to 0.0)	0.0 (0.0 to 0.0)	5.0 (4.3 to 5.7)	2.3 (2.0 to 2.6)	2.64 (2.28 to 3.02)	1.20 (1.04 to 1.38)
2020	0.0 (0.0 to 0.0)	0.0 (0.0 to 0.0)	4.7 (4.2 to 5.3)	2.2 (1.9 to 2.5)	2.69 (2.40 to 2.98)	1.24 (1.08 to 1.43)
2021	0.0 (0.0 to 0.0)	0.0 (0.0 to 0.0)	5.4 (4.3 to 6.6)	2.5 (2.0 to 3.0)	2.90 (2.34 to 3.60)	1.33 (1.07 to 1.65)

Reading GBR_44768 Western Europe Stillbirth Rate / Neonatal Mortality Rate (SBR/NMR) − 20 weeks
lambda = 0.6 ; zeta = 0.8 ; scale = 15 ; amp = 0.028

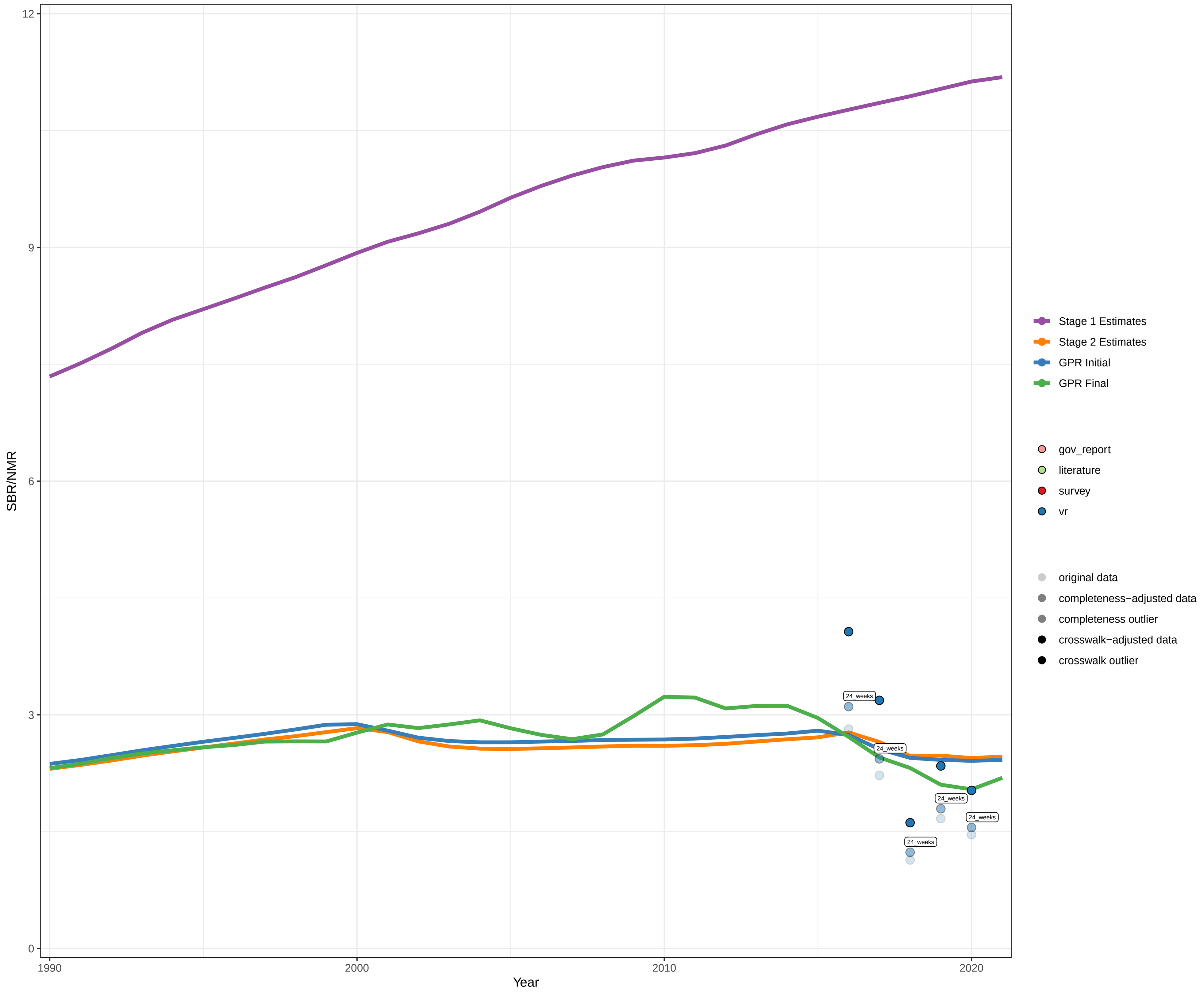

Reading GBR_44768 Western Europe Stillbirth Rate / Neonatal Mortality Rate (SBR/NMR) − 28 weeks
lambda = 0.6 ; zeta = 0.8 ; scale = 15 ; amp = 0.028

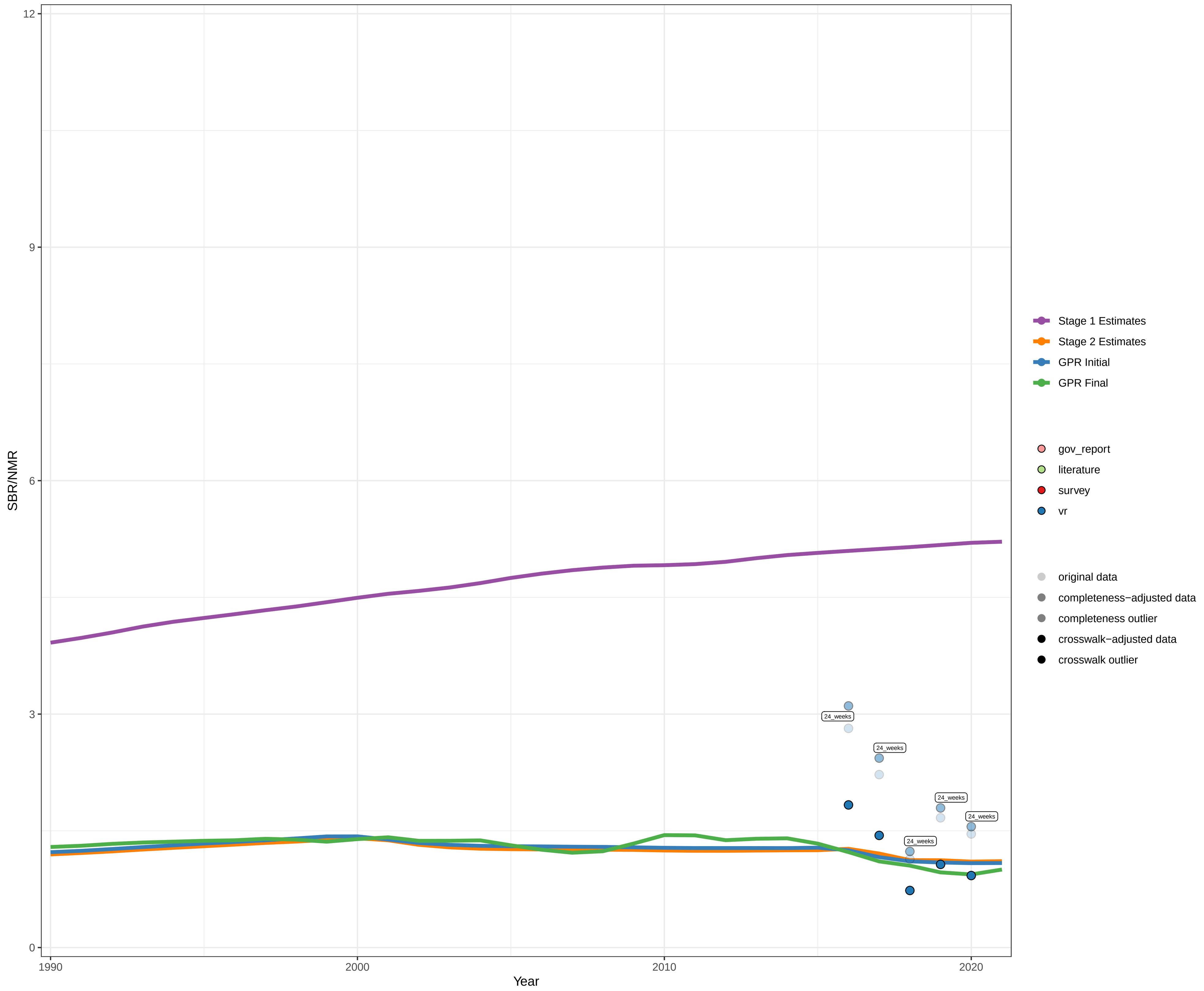

Definition	Years	Outlier	Source.Type	Title
24_weeks	2016−2020	0	VR	United Kingdom − England and Wales Child Mortality Statistics

Reading GBR_44768 Western Europe Stillbirth Rate (SBR) − 20 weeks
lambda = 0.6 ; zeta = 0.8 ; scale = 15 ; amp = 0.028

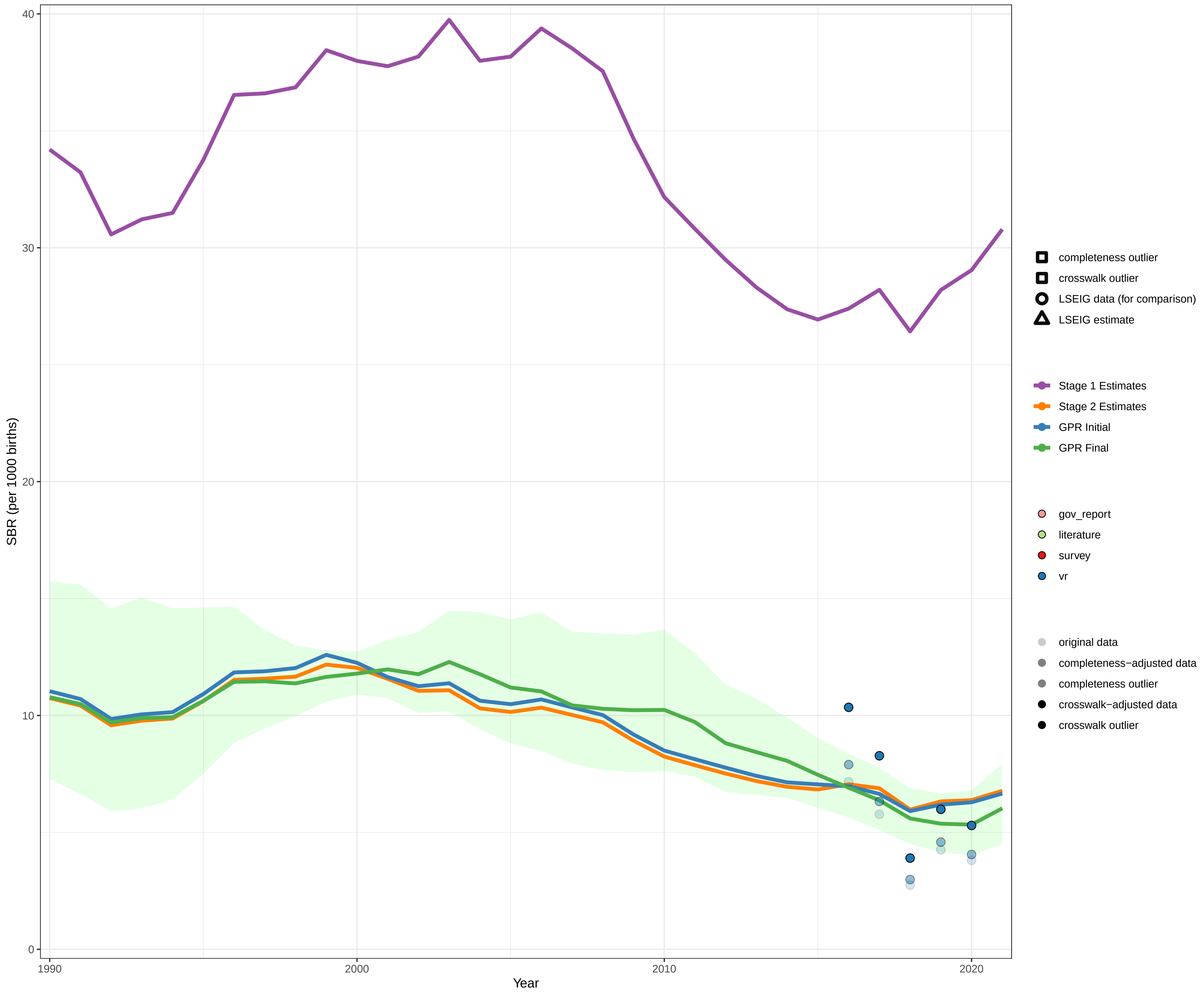

Reading GBR_44768 Western Europe Stillbirth Rate (SBR) − 28 weeks
lambda = 0.6 ; zeta = 0.8 ; scale = 15 ; amp = 0.028

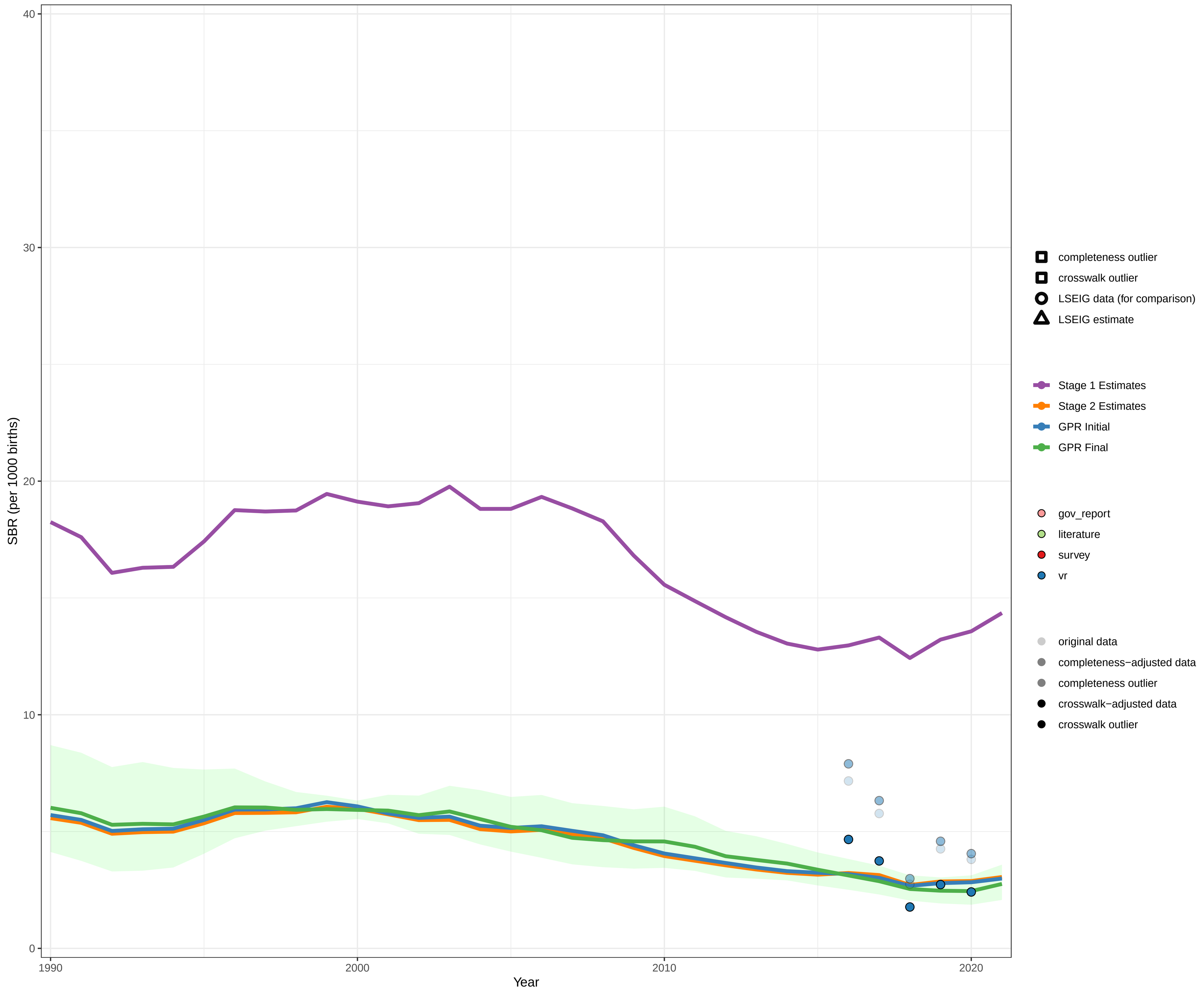

Year	Total Stillbirths (in thousands)	Total Stillbirths (in thousands)	SBR (per 1000 births)	SBR (per 1000 births)	SBR/NMR	SBR/NMR
	20 weeks	28 weeks	20 weeks	28 weeks	20 weeks	28 weeks
1990	0.0 (0.0 to 0.0)	0.0 (0.0 to 0.0)	10.8 (7.3 to 15.7)	6.0 (4.1 to 8.7)	2.32 (1.56 to 3.38)	1.29 (0.89 to 1.87)
1995	0.0 (0.0 to 0.0)	0.0 (0.0 to 0.0)	10.6 (7.5 to 14.6)	5.6 (4.1 to 7.7)	2.58 (1.82 to 3.55)	1.37 (0.99 to 1.86)
2000	0.0 (0.0 to 0.0)	0.0 (0.0 to 0.0)	11.8 (10.9 to 12.7)	5.9 (5.5 to 6.3)	2.77 (2.56 to 2.99)	1.39 (1.30 to 1.49)
2005	0.0 (0.0 to 0.0)	0.0 (0.0 to 0.0)	11.2 (8.8 to 14.1)	5.2 (4.1 to 6.5)	2.83 (2.22 to 3.57)	1.31 (1.05 to 1.64)
2010	0.0 (0.0 to 0.0)	0.0 (0.0 to 0.0)	10.2 (7.6 to 13.7)	4.6 (3.4 to 6.1)	3.23 (2.41 to 4.31)	1.44 (1.09 to 1.92)
2015	0.0 (0.0 to 0.0)	0.0 (0.0 to 0.0)	7.5 (6.0 to 9.0)	3.4 (2.7 to 4.1)	2.96 (2.40 to 3.58)	1.34 (1.07 to 1.63)
2020	0.0 (0.0 to 0.0)	0.0 (0.0 to 0.0)	5.3 (4.0 to 6.8)	2.5 (1.9 to 3.1)	2.04 (1.54 to 2.61)	0.94 (0.72 to 1.20)
2021	0.0 (0.0 to 0.0)	0.0 (0.0 to 0.0)	6.0 (4.5 to 7.9)	2.8 (2.1 to 3.6)	2.19 (1.63 to 2.88)	1.00 (0.75 to 1.30)

Slough GBR_44777 Western Europe Stillbirth Rate / Neonatal Mortality Rate (SBR/NMR) − 20 weeks
lambda = 0.6 ; zeta = 0.8 ; scale = 15 ; amp = 0.028

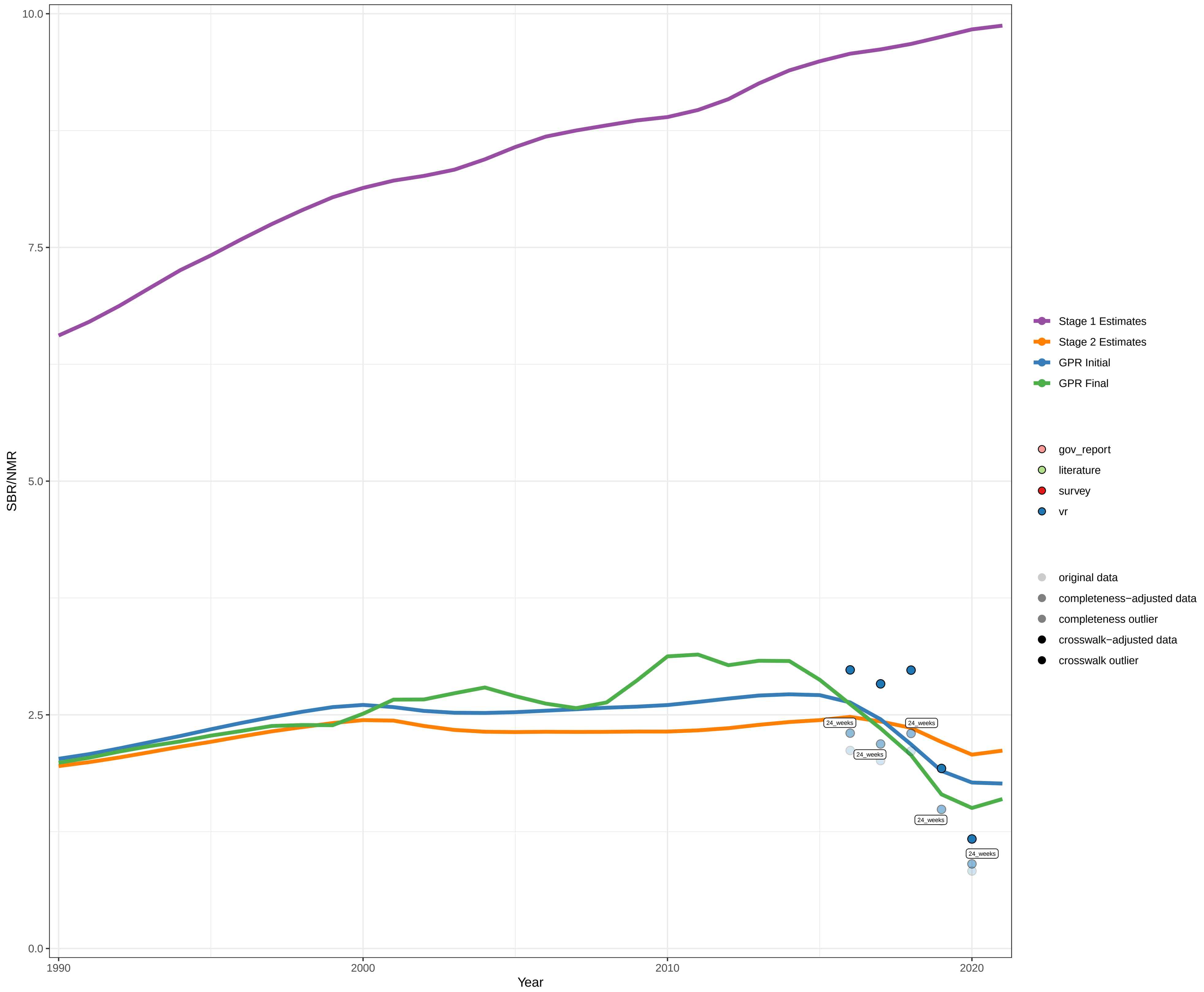

Slough GBR_44777 Western Europe Stillbirth Rate / Neonatal Mortality Rate (SBR/NMR) − 28 weeks
lambda = 0.6 ; zeta = 0.8 ; scale = 15 ; amp = 0.028

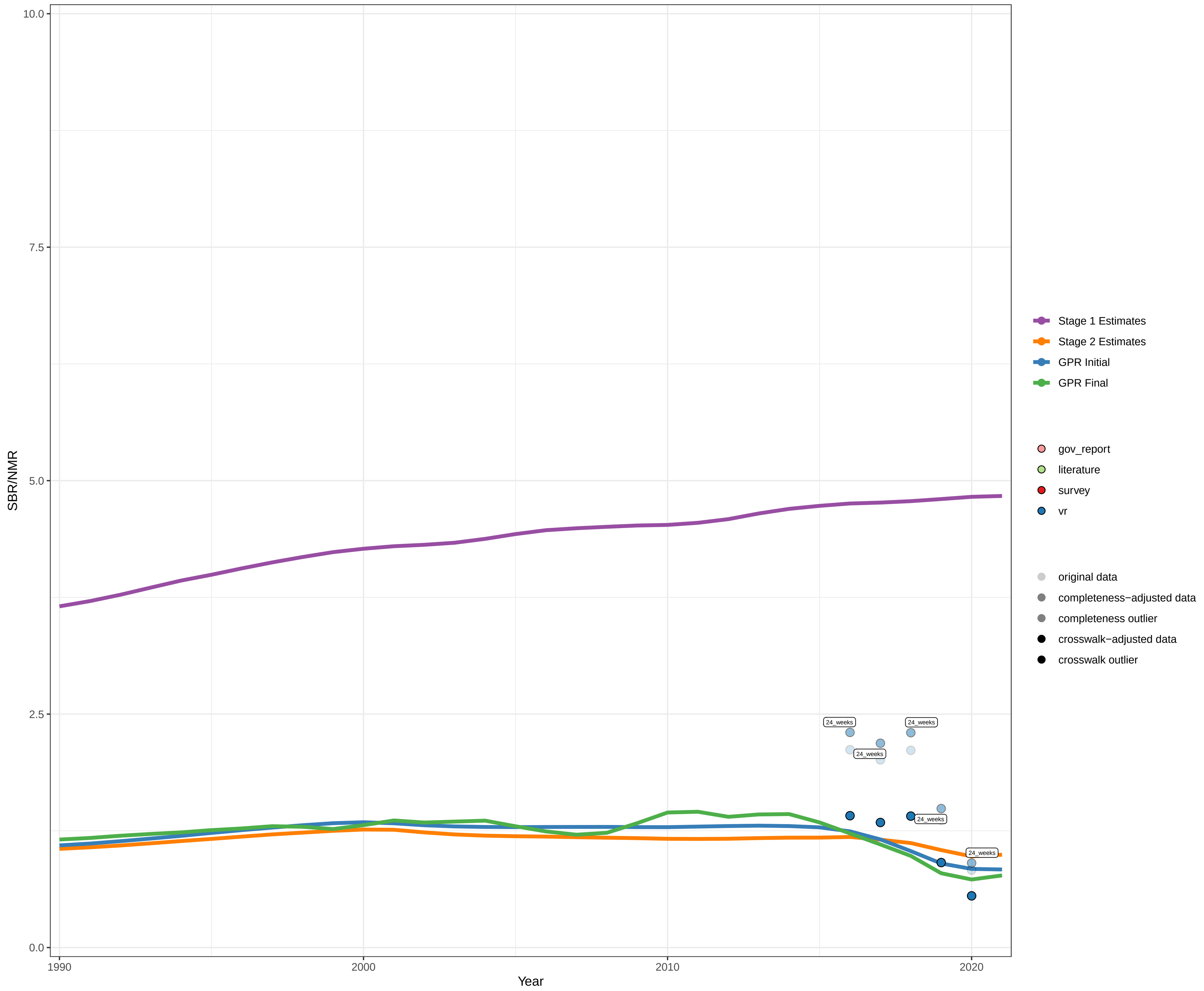

Definition	Years	Outlier	Source.Type	Title
24_weeks	2016−2020	0	VR	United Kingdom − England and Wales Child Mortality Statistics

Slough GBR_44777 Western Europe Stillbirth Rate (SBR) − 20 weeks
lambda = 0.6 ; zeta = 0.8 ; scale = 15 ; amp = 0.028

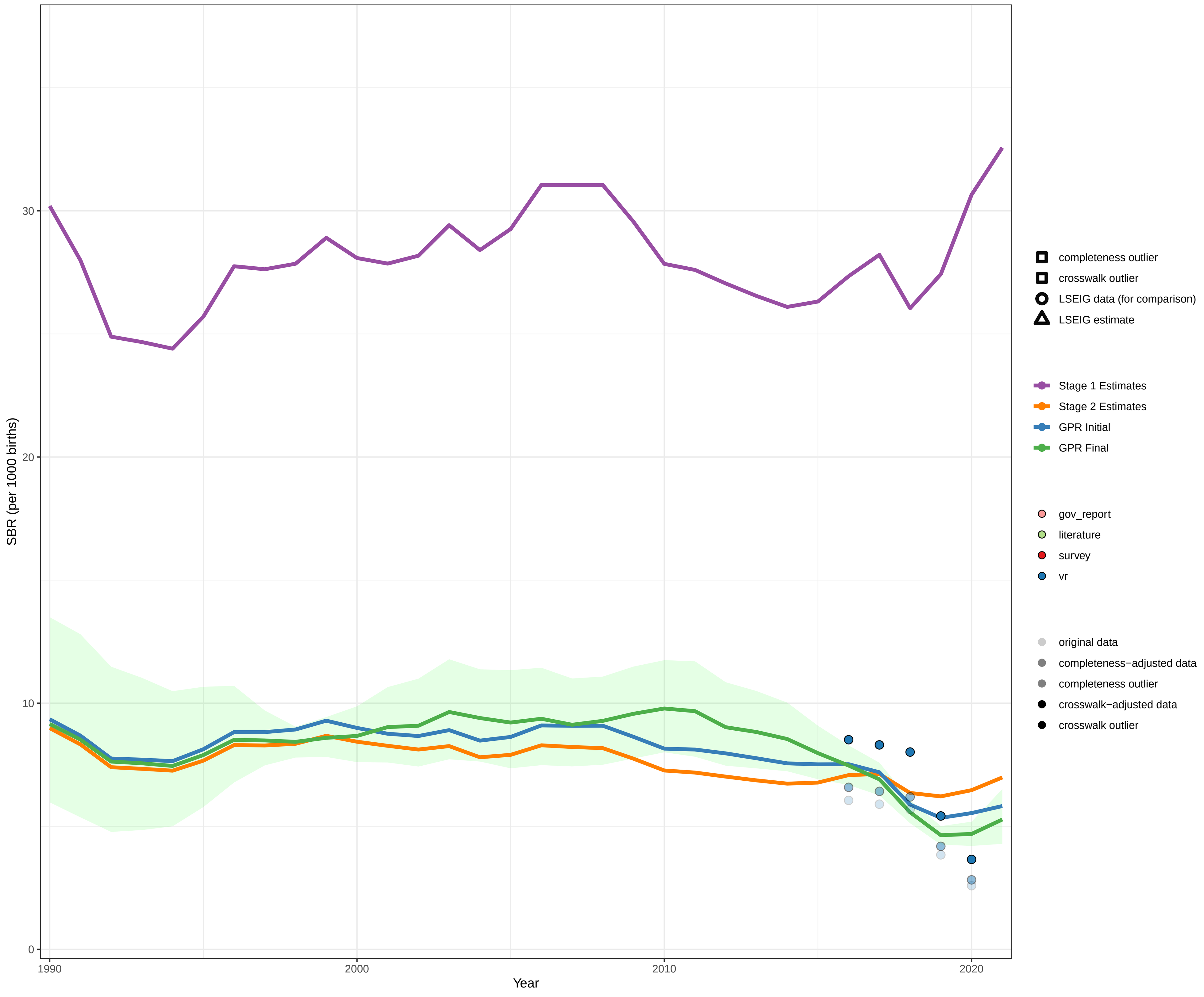

Slough GBR_44777 Western Europe Stillbirth Rate (SBR) − 28 weeks
lambda = 0.6 ; zeta = 0.8 ; scale = 15 ; amp = 0.028

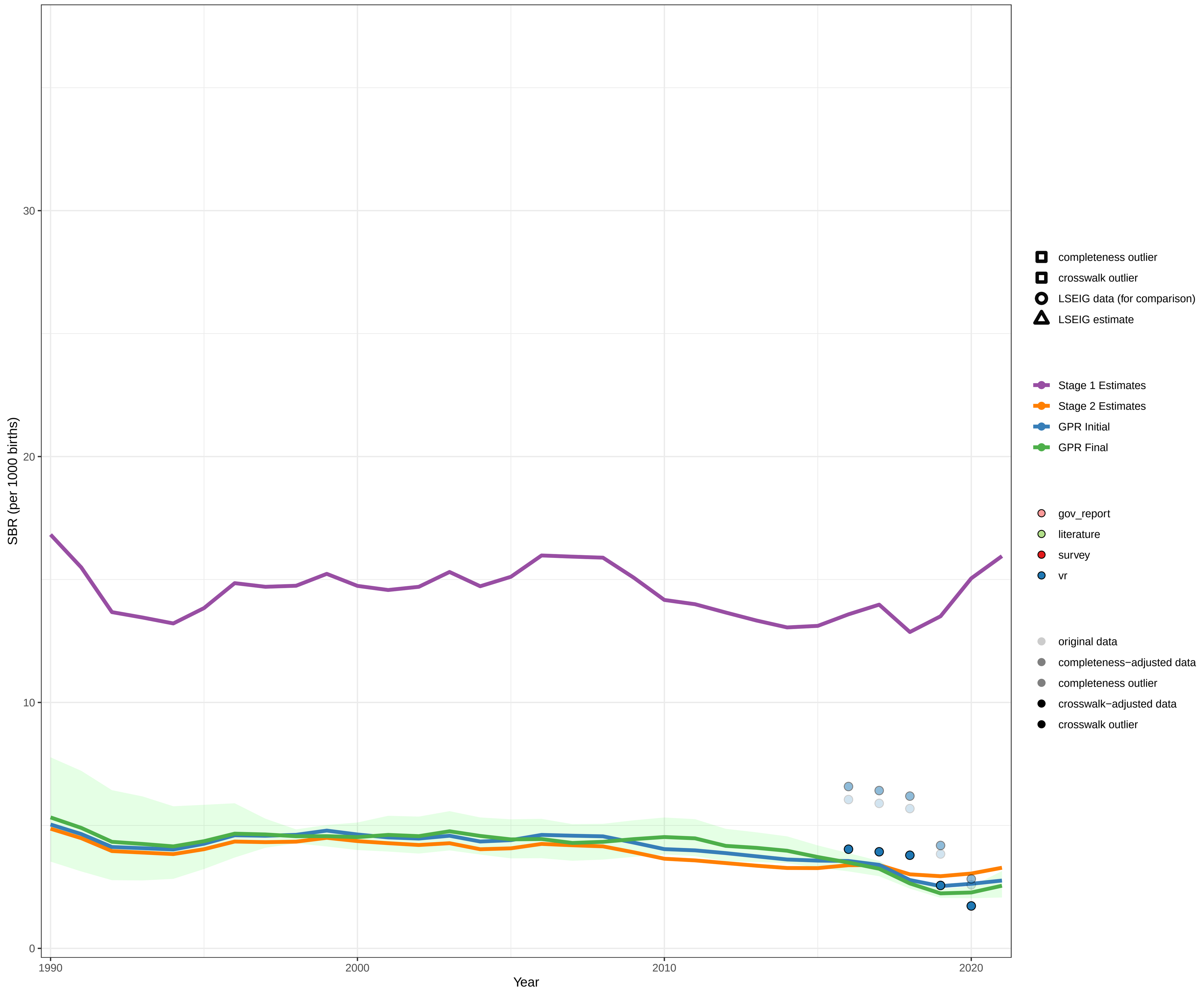

Year	Total Stillbirths (in thousands)	Total Stillbirths (in thousands)	SBR (per 1000 births)	SBR (per 1000 births)	SBR/NMR	SBR/NMR
	20 weeks	28 weeks	20 weeks	28 weeks	20 weeks	28 weeks
1990	0.0 (0.0 to 0.0)	0.0 (0.0 to 0.0)	9.2 (6.0 to 13.5)	5.3 (3.5 to 7.8)	1.99 (1.30 to 2.93)	1.16 (0.77 to 1.69)
1995	0.0 (0.0 to 0.0)	0.0 (0.0 to 0.0)	7.9 (5.8 to 10.7)	4.4 (3.2 to 5.8)	2.28 (1.67 to 3.08)	1.26 (0.93 to 1.68)
2000	0.0 (0.0 to 0.0)	0.0 (0.0 to 0.0)	8.7 (7.6 to 9.9)	4.5 (4.0 to 5.1)	2.51 (2.20 to 2.86)	1.31 (1.16 to 1.48)
2005	0.0 (0.0 to 0.0)	0.0 (0.0 to 0.0)	9.2 (7.4 to 11.3)	4.4 (3.7 to 5.2)	2.70 (2.15 to 3.32)	1.30 (1.07 to 1.54)
2010	0.0 (0.0 to 0.0)	0.0 (0.0 to 0.0)	9.8 (8.0 to 11.7)	4.5 (3.8 to 5.3)	3.13 (2.56 to 3.75)	1.45 (1.22 to 1.70)
2015	0.0 (0.0 to 0.0)	0.0 (0.0 to 0.0)	8.0 (6.9 to 9.1)	3.7 (3.3 to 4.2)	2.87 (2.49 to 3.28)	1.34 (1.18 to 1.51)
2020	0.0 (0.0 to 0.0)	0.0 (0.0 to 0.0)	4.7 (4.2 to 5.2)	2.3 (2.0 to 2.5)	1.50 (1.35 to 1.66)	0.73 (0.65 to 0.81)
2021	0.0 (0.0 to 0.0)	0.0 (0.0 to 0.0)	5.3 (4.3 to 6.5)	2.5 (2.1 to 3.1)	1.60 (1.30 to 1.97)	0.77 (0.63 to 0.95)

Southampton GBR_44776 Western Europe Stillbirth Rate / Neonatal Mortality Rate (SBR/NMR) − 20 weeks
lambda = 0.6 ; zeta = 0.8 ; scale = 15 ; amp = 0.028

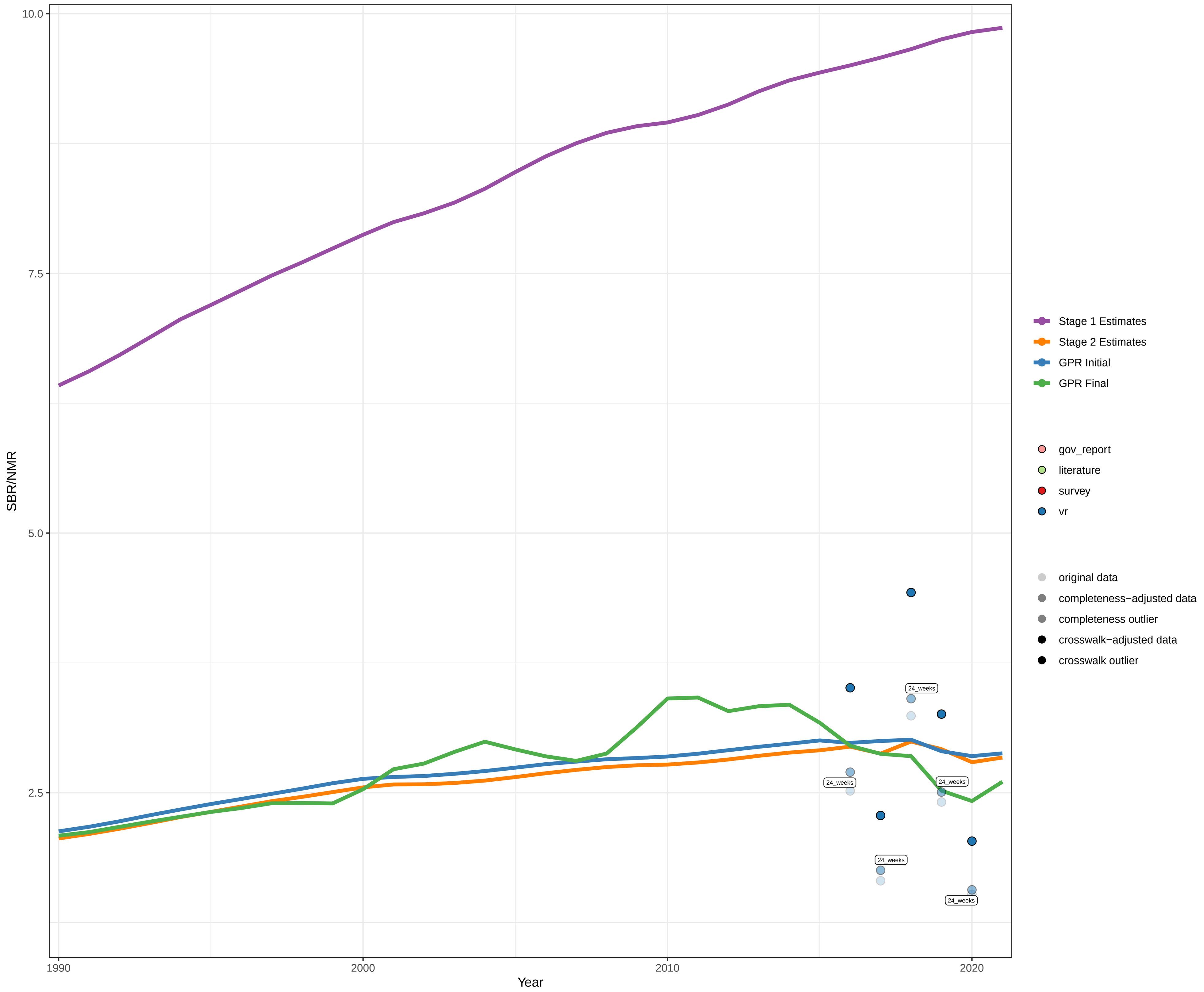

Southampton GBR_44776 Western Europe Stillbirth Rate / Neonatal Mortality Rate (SBR/NMR) − 28 weeks
lambda = 0.6 ; zeta = 0.8 ; scale = 15 ; amp = 0.028

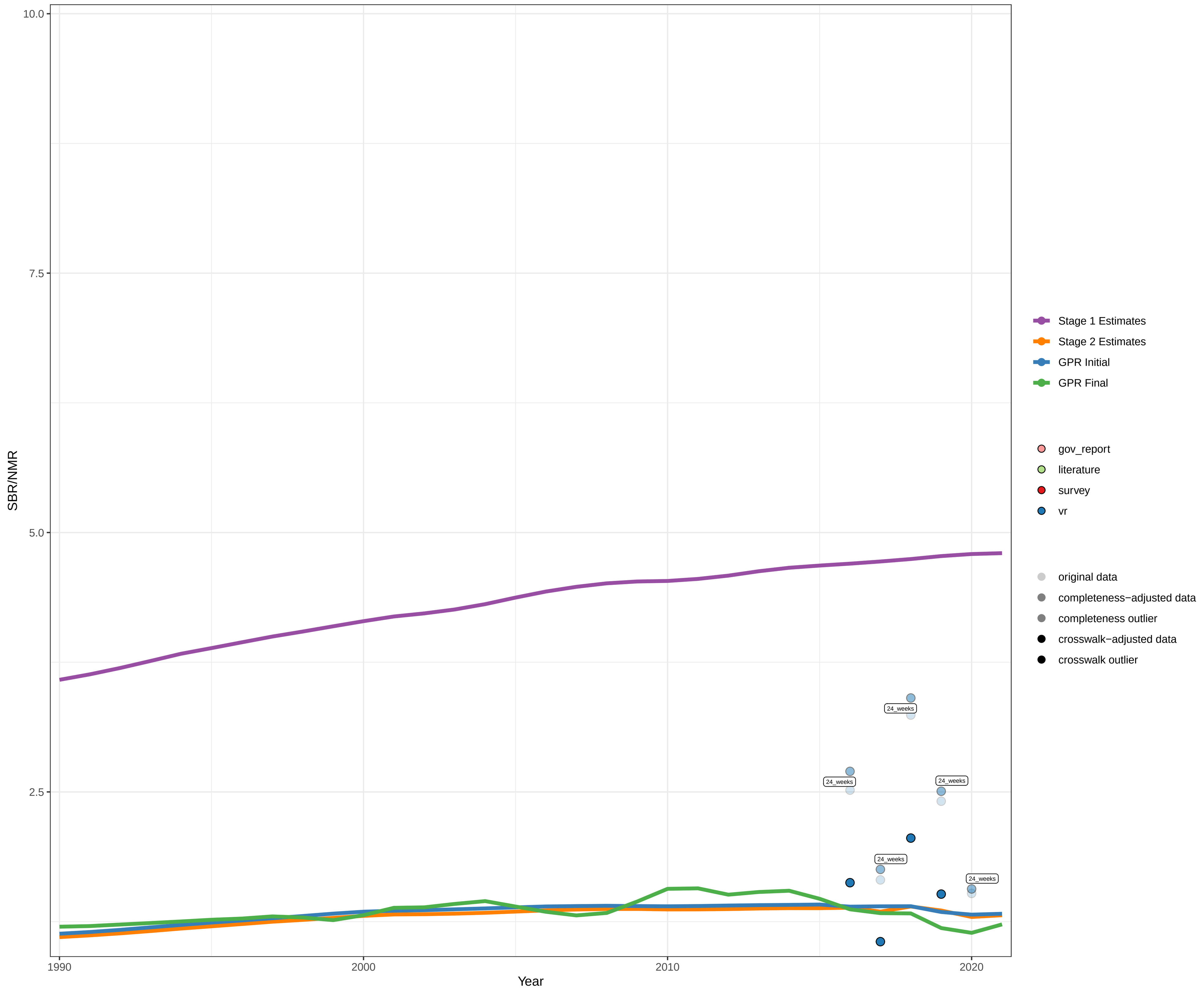

Definition	Years	Outlier	Source.Type	Title
24_weeks	2016−2020	0	VR	United Kingdom − England and Wales Child Mortality Statistics

Southampton GBR_44776 Western Europe Stillbirth Rate (SBR) − 20 weeks
lambda = 0.6 ; zeta = 0.8 ; scale = 15 ; amp = 0.028

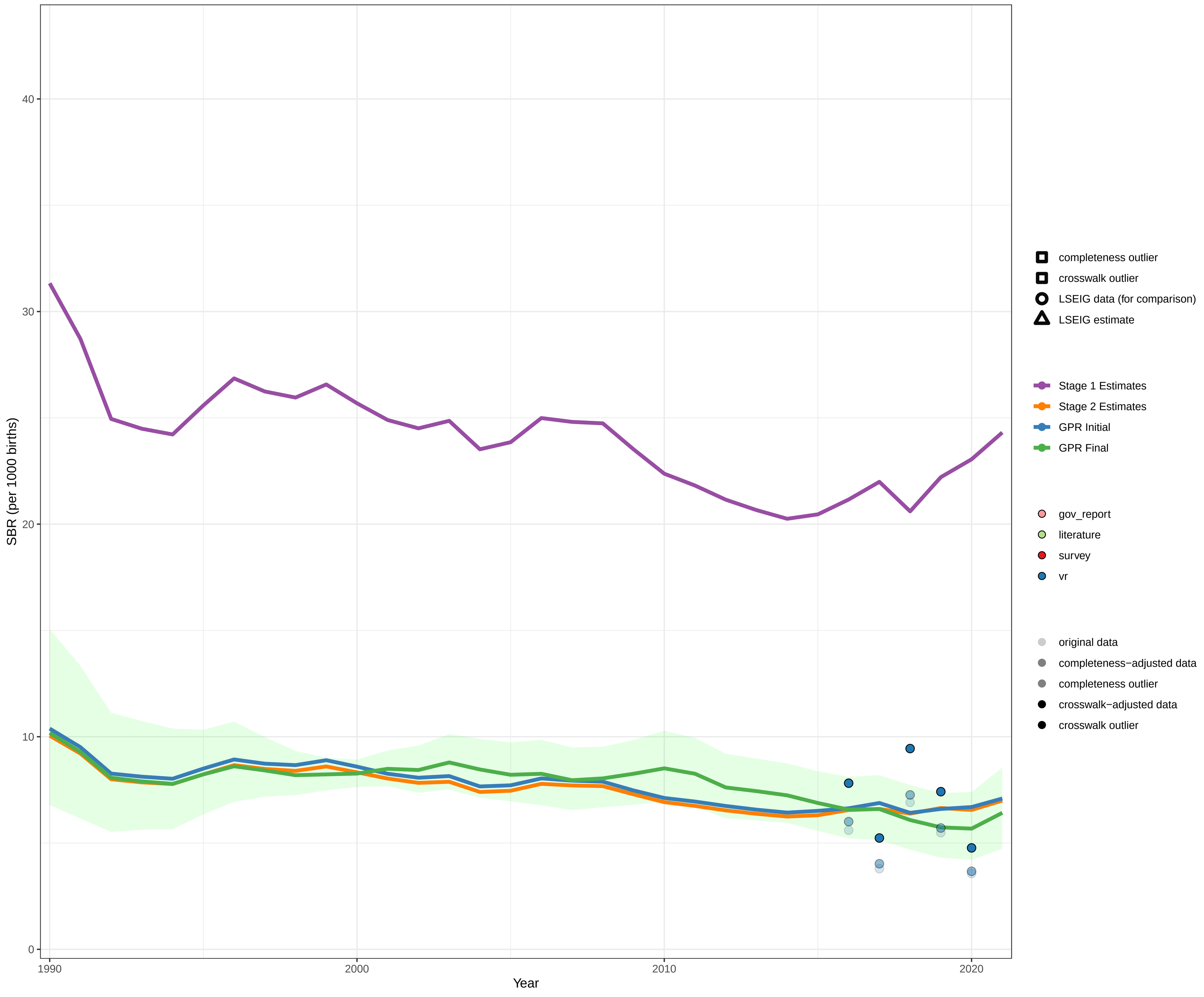

Southampton GBR_44776 Western Europe Stillbirth Rate (SBR) − 28 weeks
lambda = 0.6 ; zeta = 0.8 ; scale = 15 ; amp = 0.028

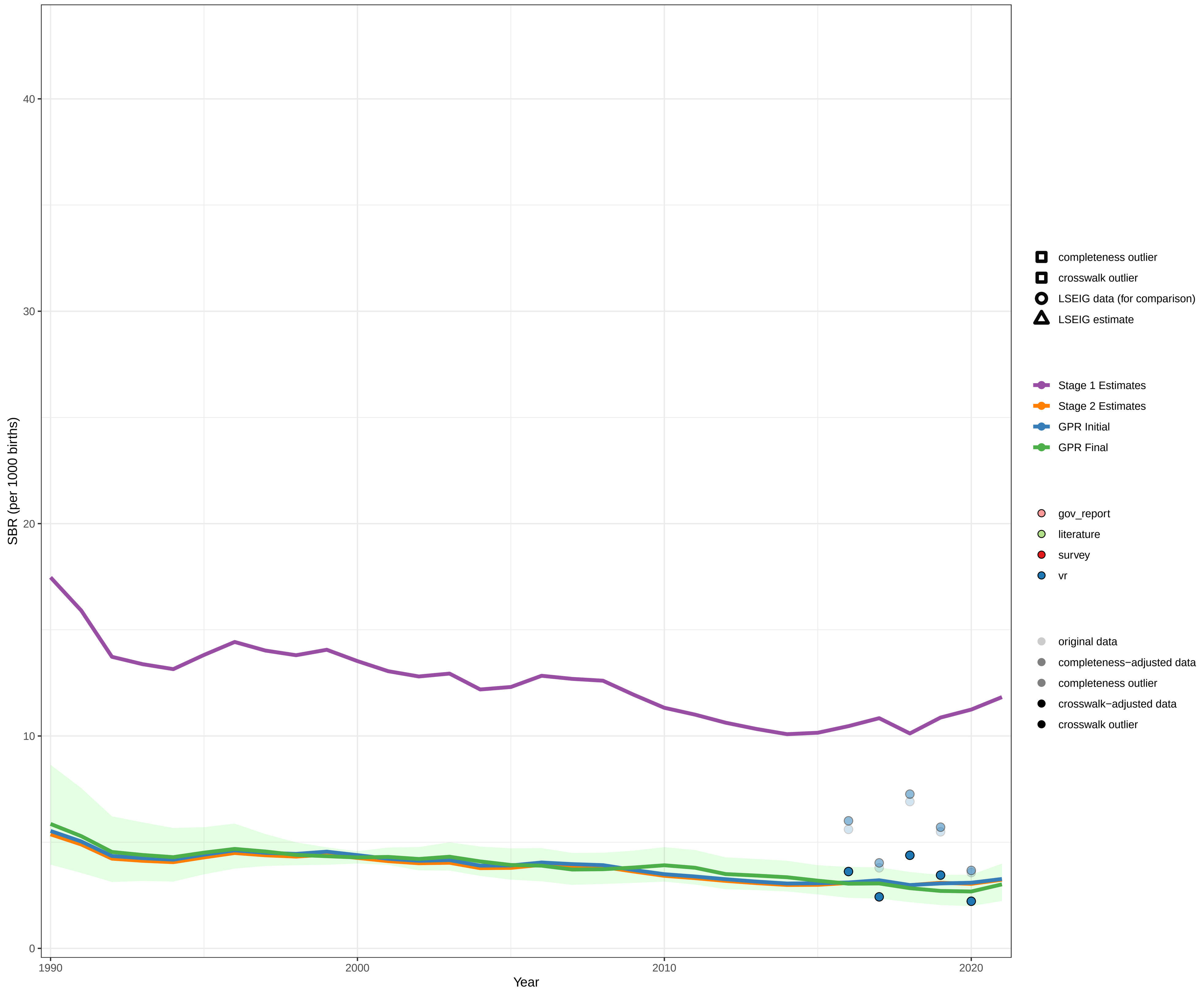

Year	Total Stillbirths (in thousands)	Total Stillbirths (in thousands)	SBR (per 1000 births)	SBR (per 1000 births)	SBR/NMR	SBR/NMR
	20 weeks	28 weeks	20 weeks	28 weeks	20 weeks	28 weeks
1990	0.0 (0.0 to 0.0)	0.0 (0.0 to 0.0)	10.2 (6.8 to 15.1)	5.9 (3.9 to 8.6)	2.09 (1.39 to 3.09)	1.20 (0.81 to 1.77)
1995	0.0 (0.0 to 0.0)	0.0 (0.0 to 0.0)	8.2 (6.3 to 10.3)	4.5 (3.5 to 5.7)	2.31 (1.78 to 2.90)	1.27 (0.98 to 1.61)
2000	0.0 (0.0 to 0.0)	0.0 (0.0 to 0.0)	8.3 (7.6 to 8.9)	4.3 (4.0 to 4.6)	2.53 (2.34 to 2.74)	1.31 (1.23 to 1.40)
2005	0.0 (0.0 to 0.0)	0.0 (0.0 to 0.0)	8.2 (7.0 to 9.7)	3.9 (3.2 to 4.7)	2.92 (2.47 to 3.46)	1.40 (1.15 to 1.67)
2010	0.0 (0.0 to 0.0)	0.0 (0.0 to 0.0)	8.5 (6.9 to 10.3)	3.9 (3.1 to 4.8)	3.41 (2.78 to 4.12)	1.57 (1.26 to 1.91)
2015	0.0 (0.0 to 0.0)	0.0 (0.0 to 0.0)	6.9 (5.6 to 8.4)	3.2 (2.5 to 3.9)	3.17 (2.57 to 3.86)	1.47 (1.17 to 1.81)
2020	0.0 (0.0 to 0.0)	0.0 (0.0 to 0.0)	5.7 (4.2 to 7.4)	2.7 (2.0 to 3.5)	2.42 (1.79 to 3.16)	1.14 (0.85 to 1.48)
2021	0.0 (0.0 to 0.0)	0.0 (0.0 to 0.0)	6.4 (4.7 to 8.6)	3.0 (2.2 to 4.0)	2.61 (1.92 to 3.47)	1.22 (0.91 to 1.62)

Surrey GBR_44761 Western Europe Stillbirth Rate / Neonatal Mortality Rate (SBR/NMR) − 20 weeks
lambda = 0.6 ; zeta = 0.8 ; scale = 15 ; amp = 0.028

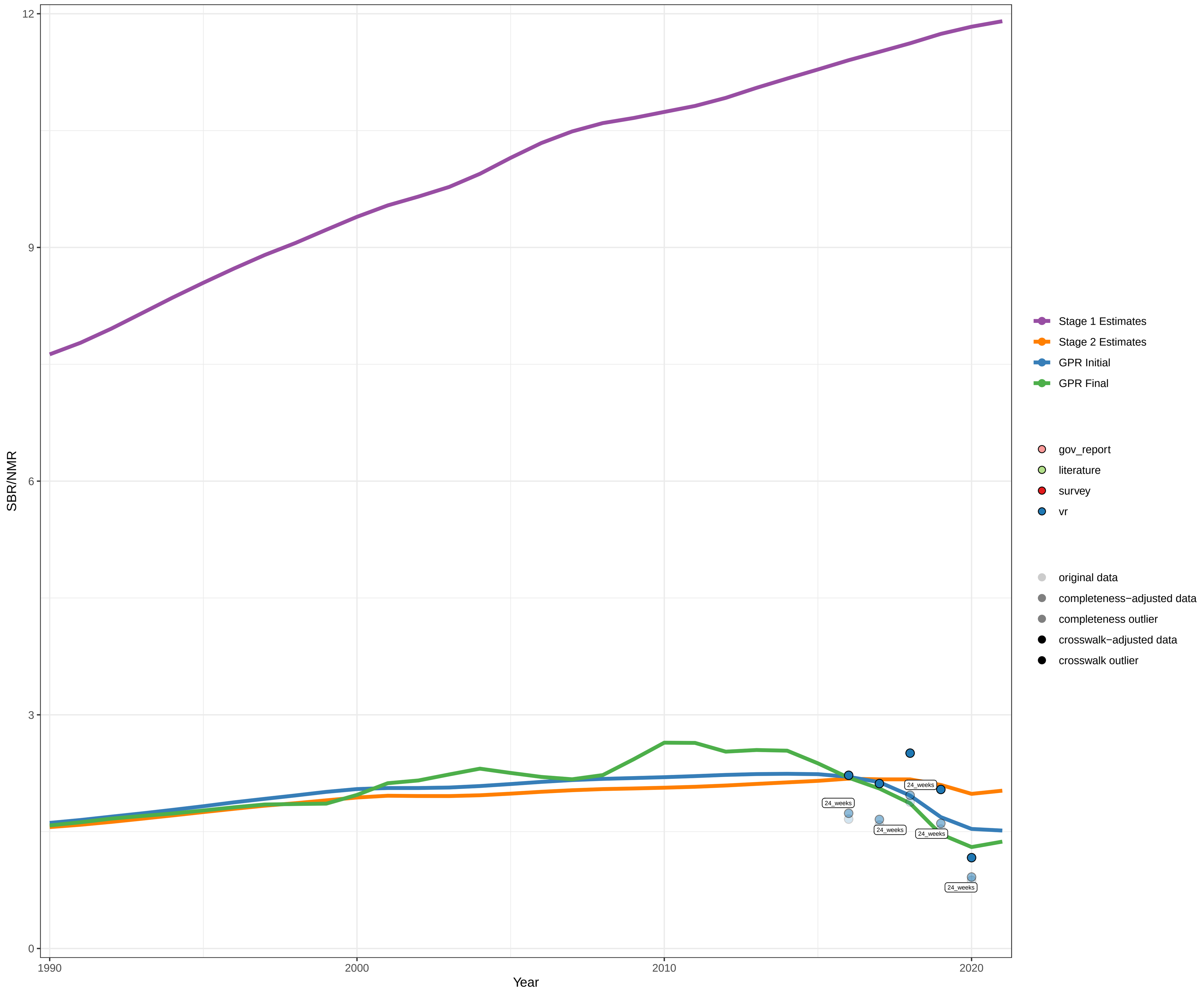

Surrey GBR_44761 Western Europe Stillbirth Rate / Neonatal Mortality Rate (SBR/NMR) − 28 weeks
lambda = 0.6 ; zeta = 0.8 ; scale = 15 ; amp = 0.028

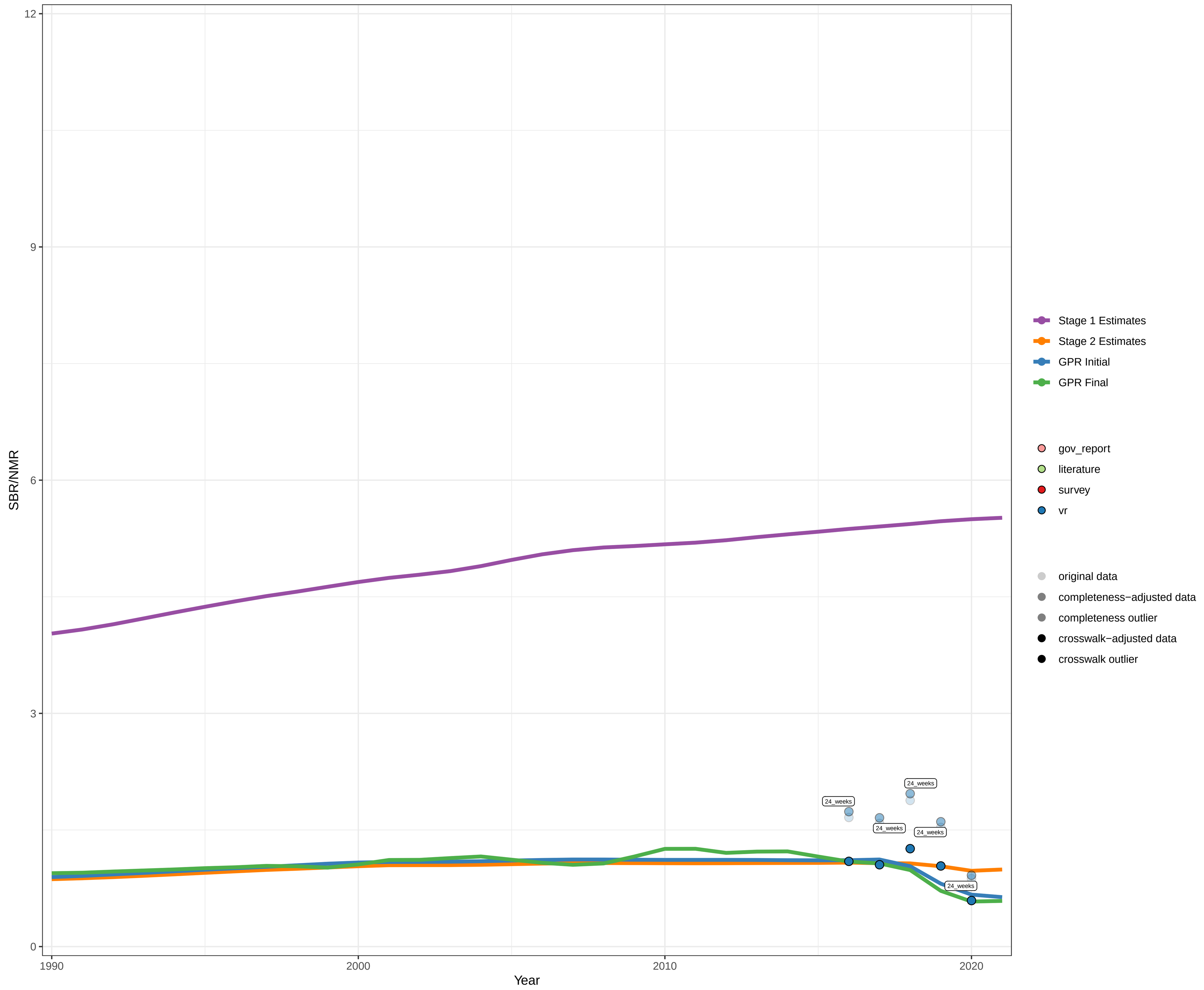

Definition	Years	Outlier	Source.Type	Title
24_weeks	2016−2020	0	VR	United Kingdom − England and Wales Child Mortality Statistics

Surrey GBR_44761 Western Europe Stillbirth Rate (SBR) − 20 weeks
lambda = 0.6 ; zeta = 0.8 ; scale = 15 ; amp = 0.028

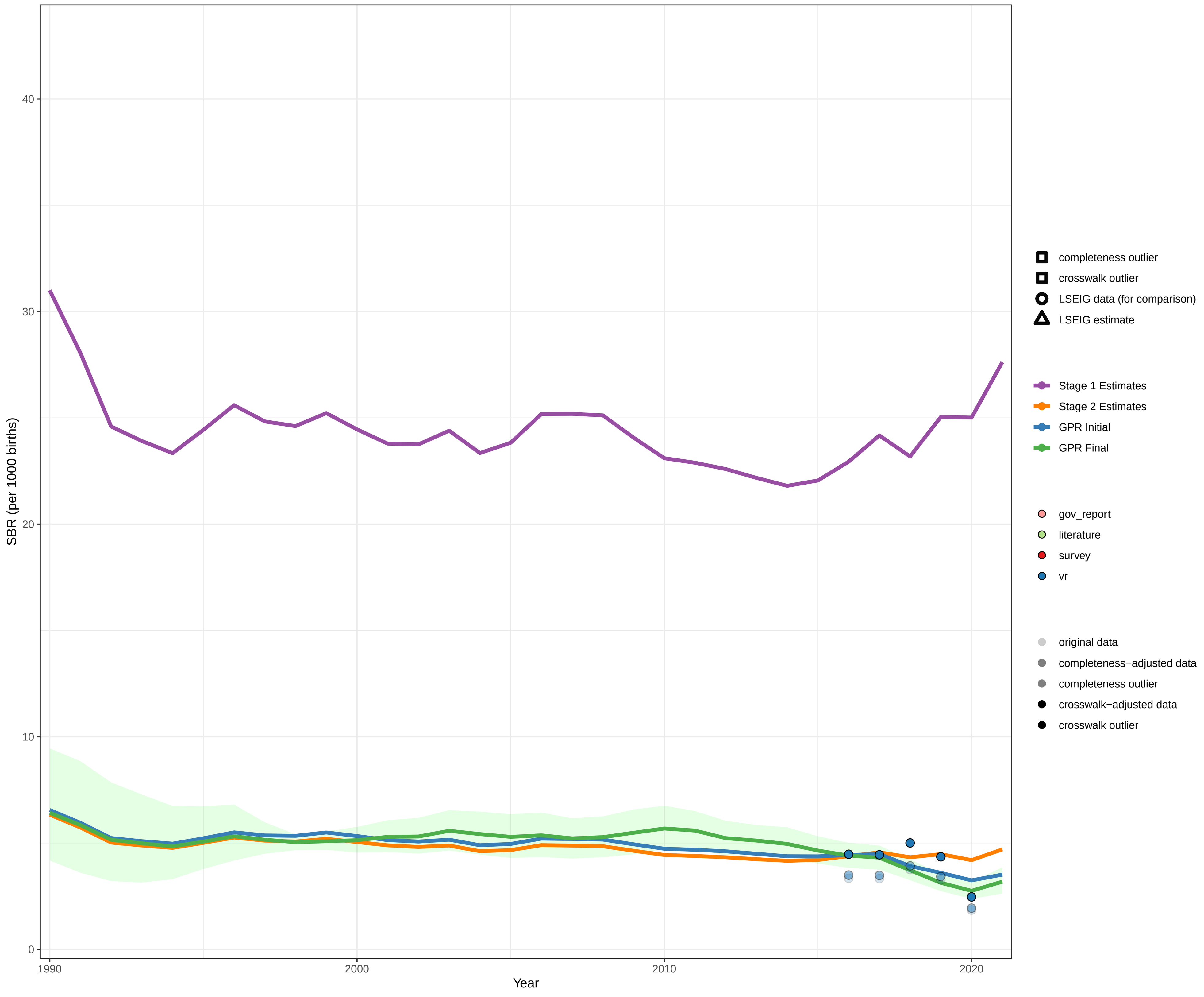

Surrey GBR_44761 Western Europe Stillbirth Rate (SBR) − 28 weeks
lambda = 0.6 ; zeta = 0.8 ; scale = 15 ; amp = 0.028

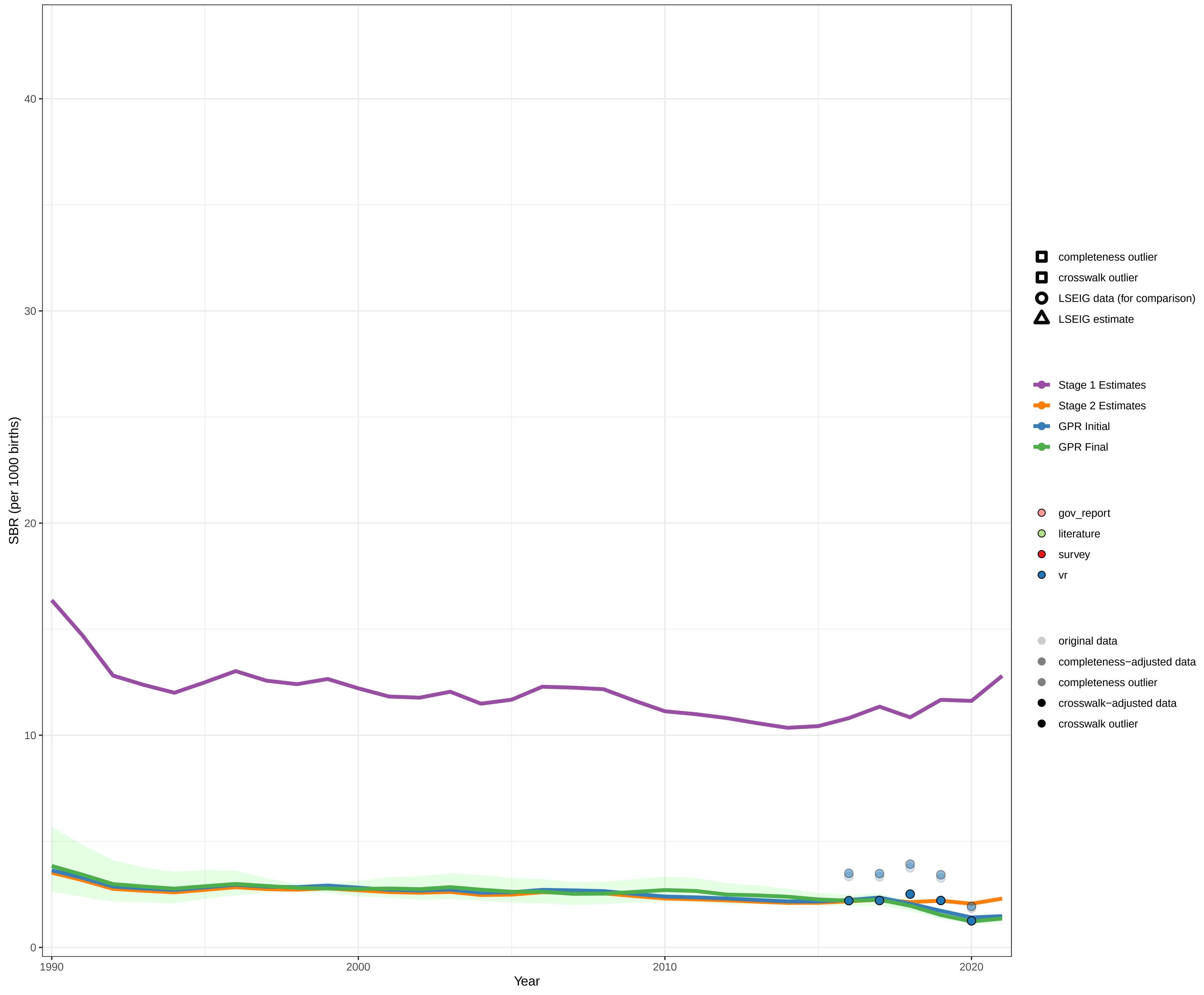

Year	Total Stillbirths (in thousands)	Total Stillbirths (in thousands)	SBR (per 1000 births)	SBR (per 1000 births)	SBR/NMR	SBR/NMR
	20 weeks	28 weeks	20 weeks	28 weeks	20 weeks	28 weeks
1990	0.1 (0.1 to 0.1)	0.0 (0.0 to 0.1)	6.4 (4.2 to 9.5)	3.8 (2.6 to 5.7)	1.58 (1.03 to 2.33)	0.94 (0.65 to 1.40)
1995	0.1 (0.0 to 0.1)	0.0 (0.0 to 0.0)	5.1 (3.8 to 6.7)	2.9 (2.3 to 3.6)	1.77 (1.32 to 2.35)	1.01 (0.80 to 1.28)
2000	0.1 (0.1 to 0.1)	0.0 (0.0 to 0.0)	5.1 (4.6 to 5.8)	2.8 (2.4 to 3.1)	1.97 (1.75 to 2.21)	1.06 (0.92 to 1.21)
2005	0.1 (0.1 to 0.1)	0.0 (0.0 to 0.0)	5.3 (4.3 to 6.4)	2.6 (2.1 to 3.3)	2.25 (1.83 to 2.71)	1.12 (0.90 to 1.39)
2010	0.1 (0.1 to 0.1)	0.0 (0.0 to 0.0)	5.7 (4.7 to 6.8)	2.7 (2.2 to 3.3)	2.64 (2.20 to 3.14)	1.26 (1.00 to 1.55)
2015	0.1 (0.1 to 0.1)	0.0 (0.0 to 0.0)	4.6 (4.0 to 5.3)	2.3 (2.0 to 2.6)	2.38 (2.06 to 2.72)	1.16 (1.01 to 1.31)
2020	0.0 (0.0 to 0.0)	0.0 (0.0 to 0.0)	2.8 (2.4 to 3.2)	1.2 (1.1 to 1.3)	1.30 (1.12 to 1.50)	0.58 (0.52 to 0.64)
2021	0.0 (0.0 to 0.0)	0.0 (0.0 to 0.0)	3.2 (2.6 to 3.9)	1.4 (1.2 to 1.6)	1.37 (1.13 to 1.66)	0.59 (0.50 to 0.69)

West Berkshire GBR_44763 Western Europe Stillbirth Rate / Neonatal Mortality Rate (SBR/NMR) − 20 weeks
lambda = 0.6 ; zeta = 0.8 ; scale = 15 ; amp = 0.028

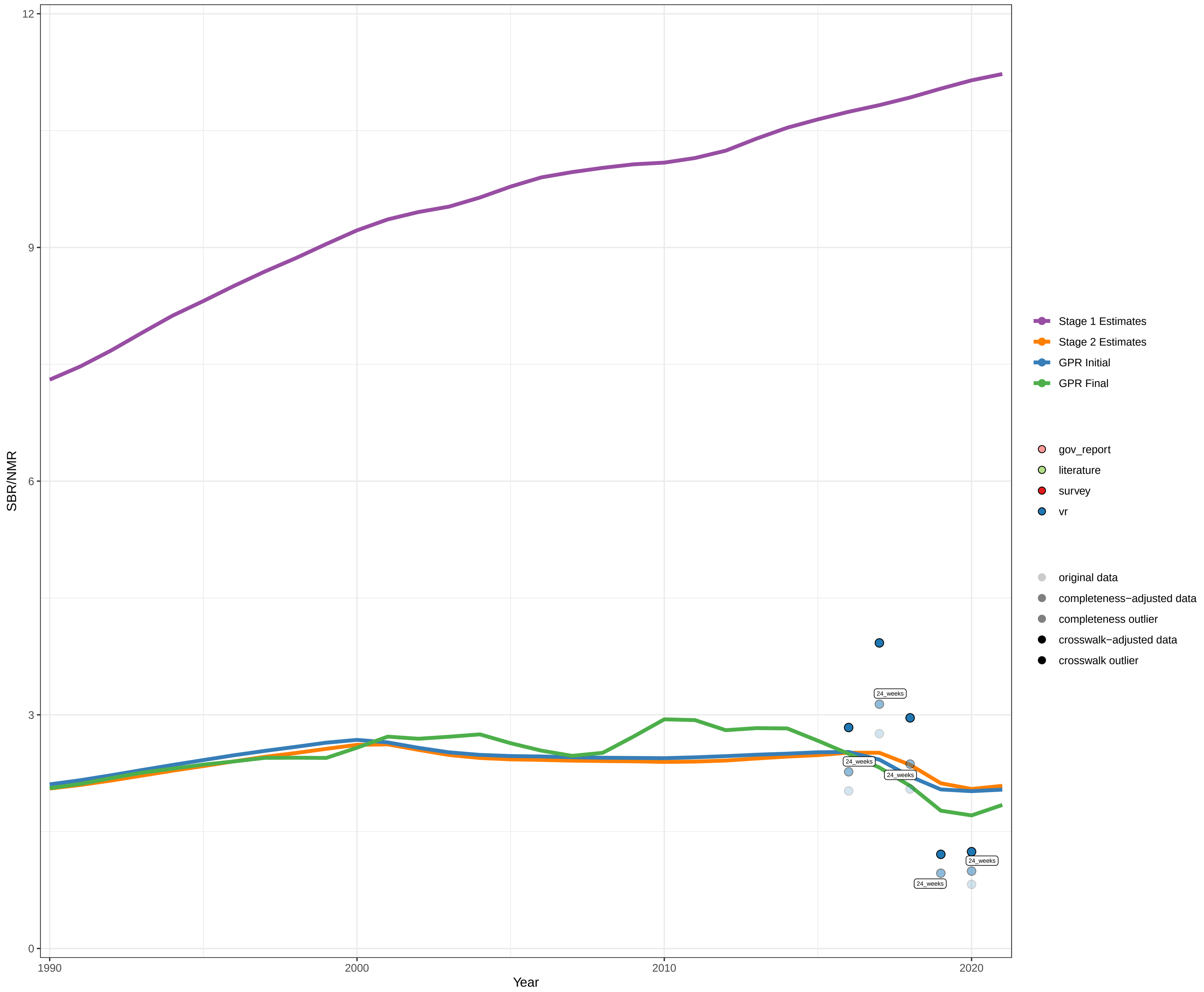

West Berkshire GBR_44763 Western Europe Stillbirth Rate / Neonatal Mortality Rate (SBR/NMR) − 28 weeks
lambda = 0.6 ; zeta = 0.8 ; scale = 15 ; amp = 0.028

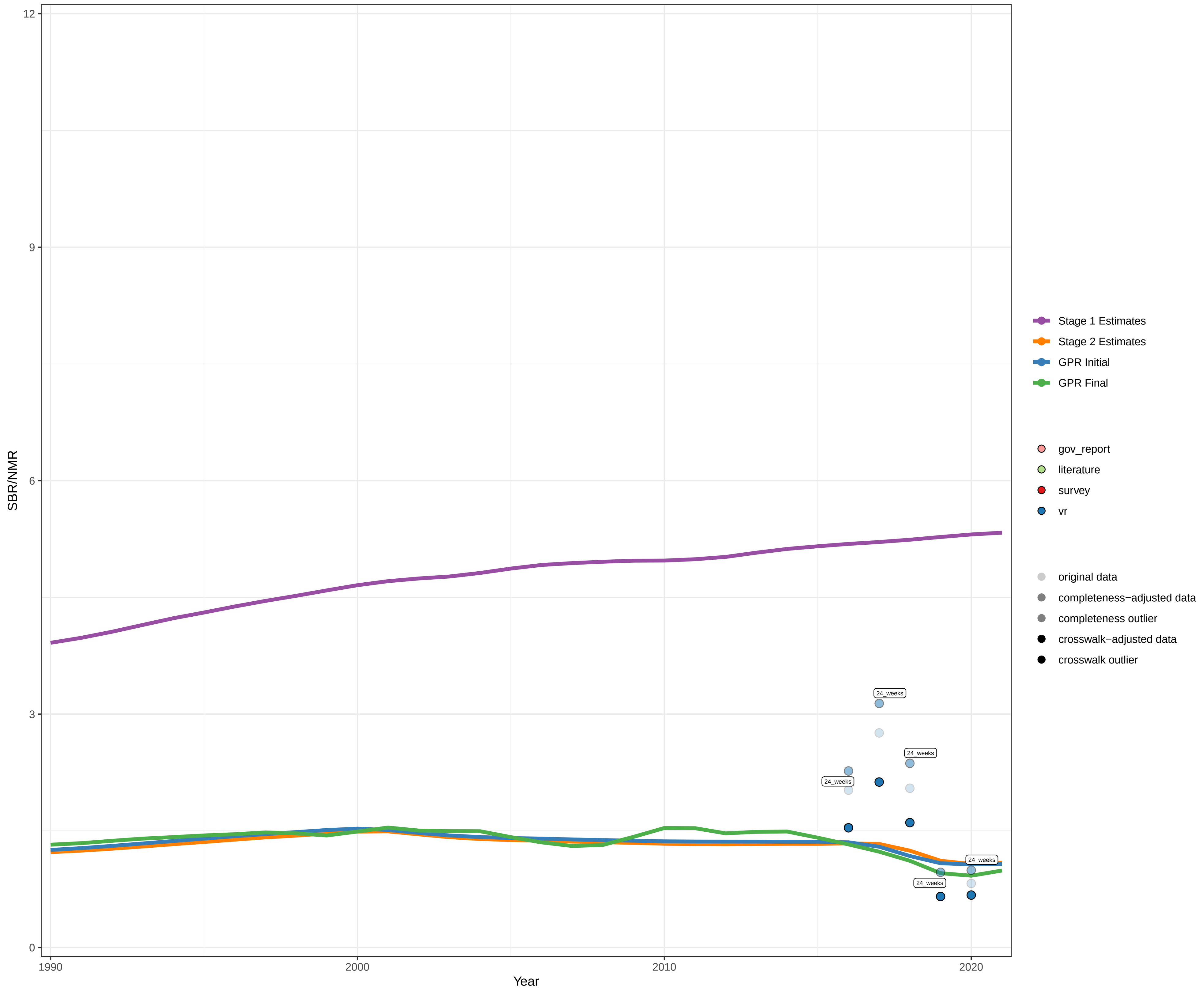

Definition	Years	Outlier	Source.Type	Title
24_weeks	2016−2020	0	VR	United Kingdom − England and Wales Child Mortality Statistics

West Berkshire GBR_44763 Western Europe Stillbirth Rate (SBR) − 20 weeks
lambda = 0.6 ; zeta = 0.8 ; scale = 15 ; amp = 0.028

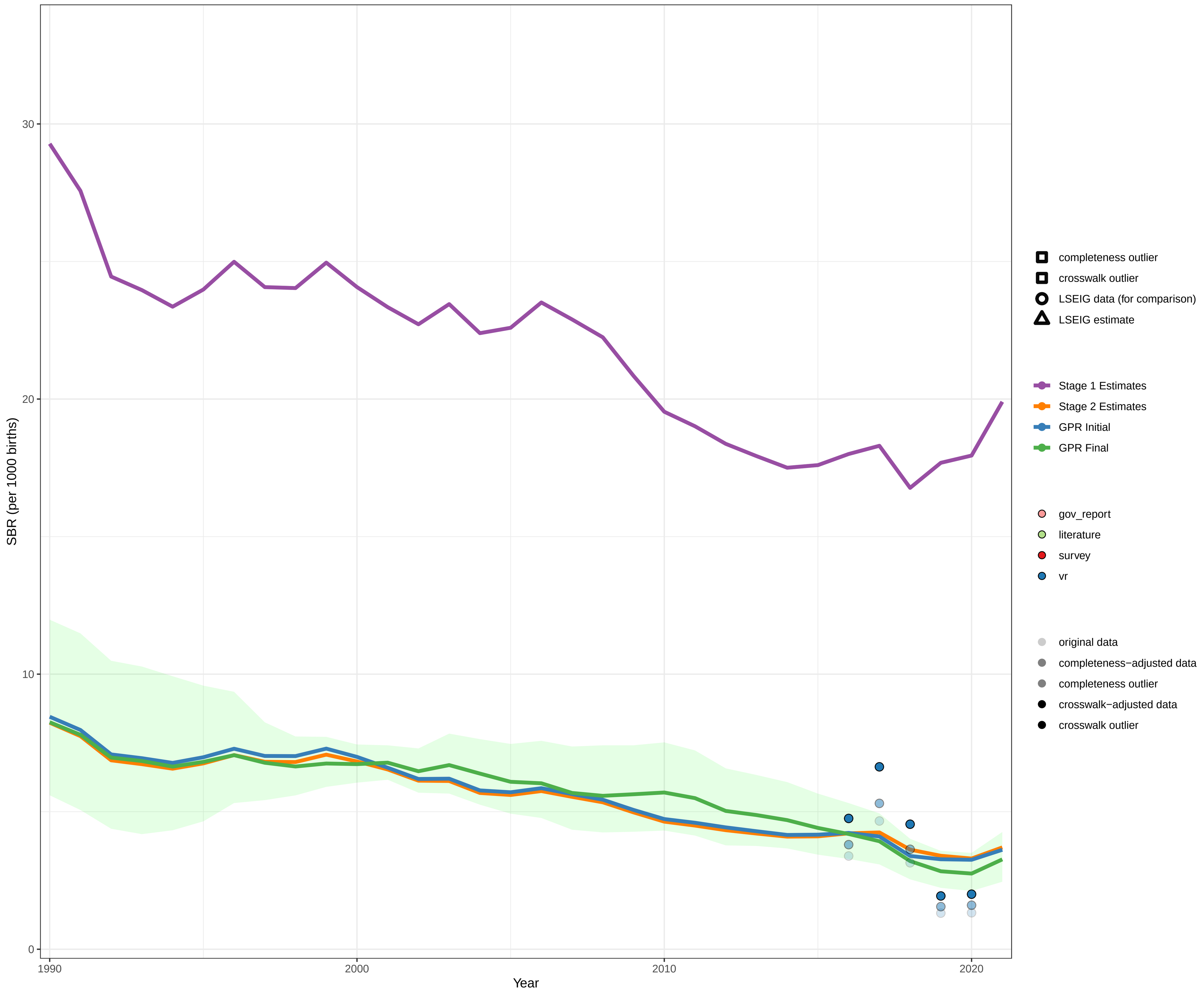

West Berkshire GBR_44763 Western Europe Stillbirth Rate (SBR) − 28 weeks
lambda = 0.6 ; zeta = 0.8 ; scale = 15 ; amp = 0.028

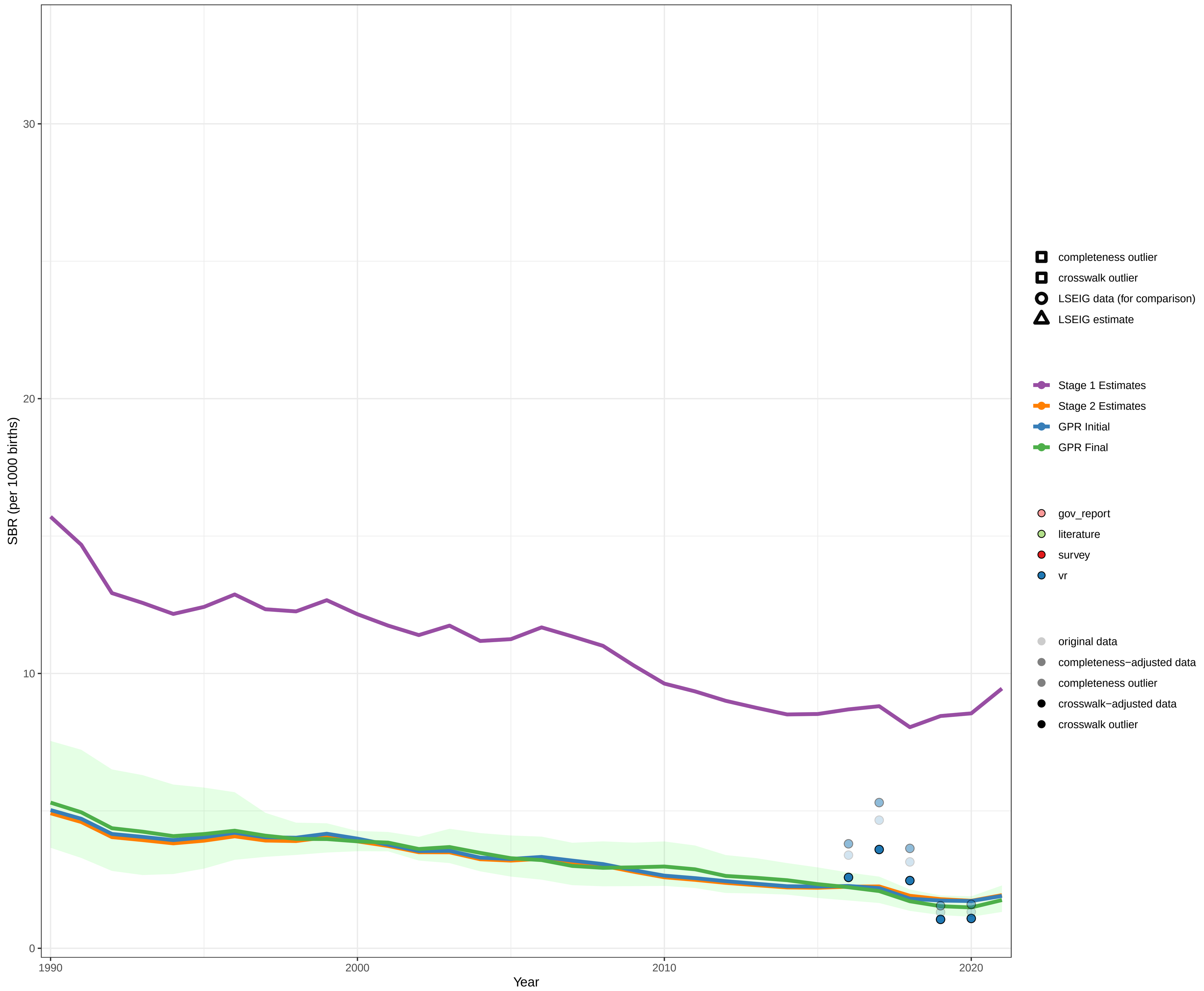

Year	Total Stillbirths (in thousands)	Total Stillbirths (in thousands)	SBR (per 1000 births)	SBR (per 1000 births)	SBR/NMR	SBR/NMR
	20 weeks	28 weeks	20 weeks	28 weeks	20 weeks	28 weeks
1990	0.0 (0.0 to 0.0)	0.0 (0.0 to 0.0)	8.3 (5.6 to 12.0)	5.3 (3.7 to 7.5)	2.06 (1.40 to 2.99)	1.32 (0.91 to 1.88)
1995	0.0 (0.0 to 0.0)	0.0 (0.0 to 0.0)	6.8 (4.6 to 9.6)	4.2 (2.9 to 5.8)	2.36 (1.61 to 3.32)	1.44 (1.00 to 2.03)
2000	0.0 (0.0 to 0.0)	0.0 (0.0 to 0.0)	6.7 (6.1 to 7.4)	3.9 (3.5 to 4.3)	2.58 (2.32 to 2.85)	1.49 (1.35 to 1.63)
2005	0.0 (0.0 to 0.0)	0.0 (0.0 to 0.0)	6.1 (4.9 to 7.5)	3.3 (2.6 to 4.1)	2.64 (2.13 to 3.23)	1.42 (1.13 to 1.78)
2010	0.0 (0.0 to 0.0)	0.0 (0.0 to 0.0)	5.7 (4.3 to 7.5)	3.0 (2.3 to 3.9)	2.94 (2.23 to 3.88)	1.54 (1.17 to 2.01)
2015	0.0 (0.0 to 0.0)	0.0 (0.0 to 0.0)	4.4 (3.4 to 5.7)	2.3 (1.8 to 2.9)	2.67 (2.08 to 3.42)	1.41 (1.11 to 1.78)
2020	0.0 (0.0 to 0.0)	0.0 (0.0 to 0.0)	2.8 (2.1 to 3.5)	1.5 (1.1 to 1.9)	1.71 (1.32 to 2.18)	0.92 (0.71 to 1.17)
2021	0.0 (0.0 to 0.0)	0.0 (0.0 to 0.0)	3.3 (2.5 to 4.3)	1.8 (1.3 to 2.3)	1.84 (1.38 to 2.40)	0.99 (0.75 to 1.29)

West Sussex GBR_44766 Western Europe Stillbirth Rate / Neonatal Mortality Rate (SBR/NMR) − 20 weeks
lambda = 0.6 ; zeta = 0.8 ; scale = 15 ; amp = 0.028

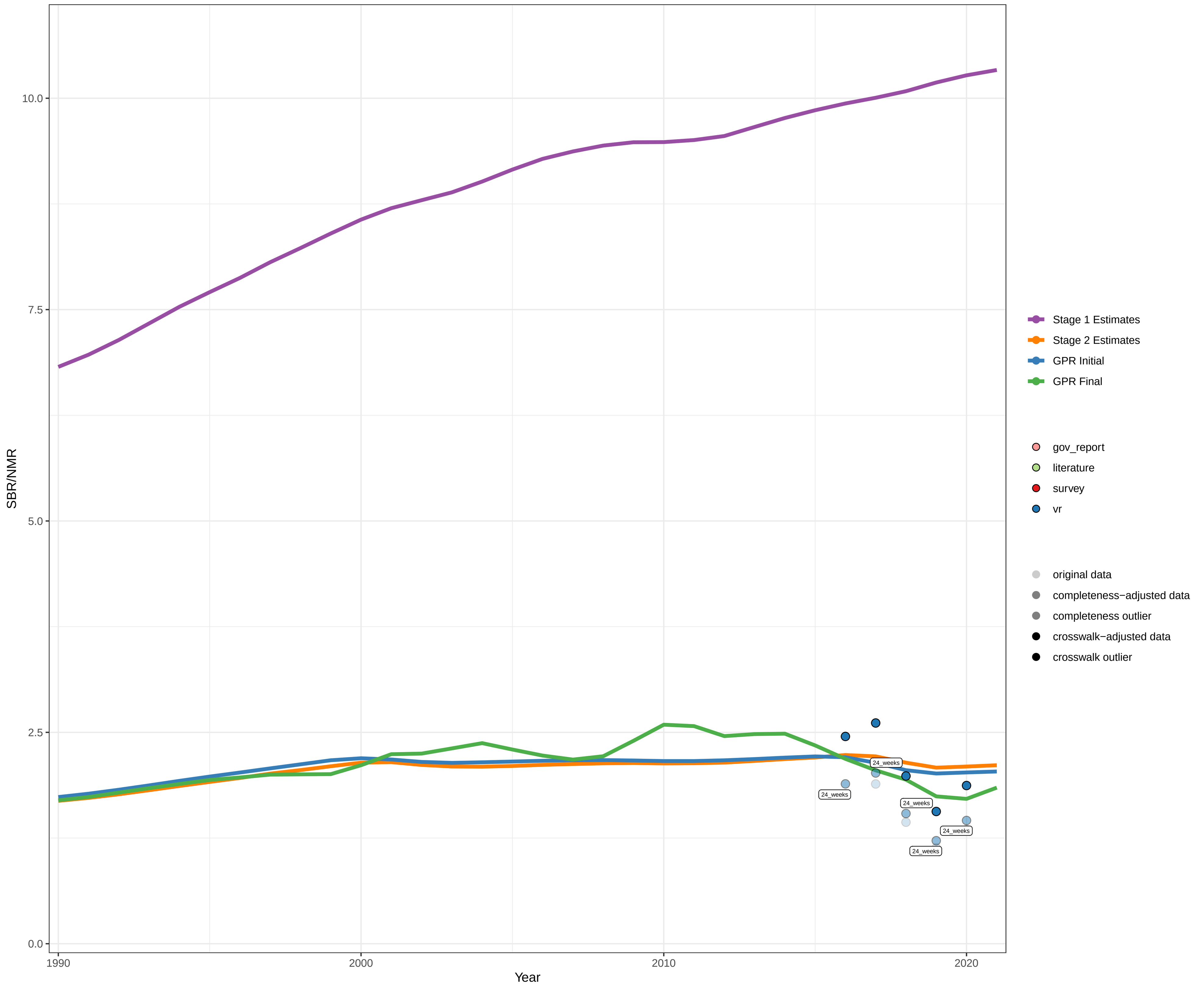

West Sussex GBR_44766 Western Europe Stillbirth Rate / Neonatal Mortality Rate (SBR/NMR) − 28 weeks
lambda = 0.6 ; zeta = 0.8 ; scale = 15 ; amp = 0.028

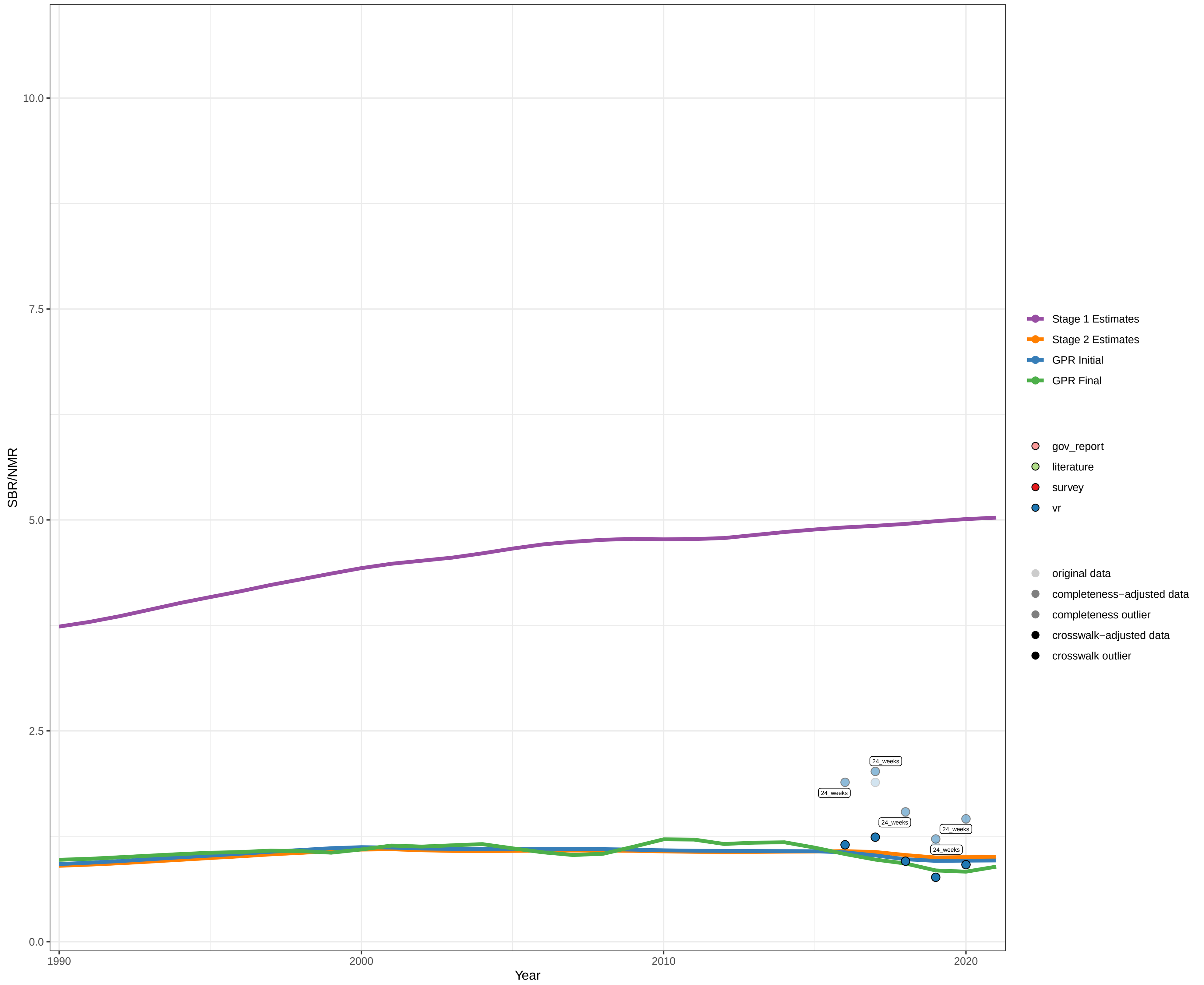

Definition	Years	Outlier	Source.Type	Title
24_weeks	2016−2020	0	VR	United Kingdom − England and Wales Child Mortality Statistics

West Sussex GBR_44766 Western Europe Stillbirth Rate (SBR) − 20 weeks
lambda = 0.6 ; zeta = 0.8 ; scale = 15 ; amp = 0.028

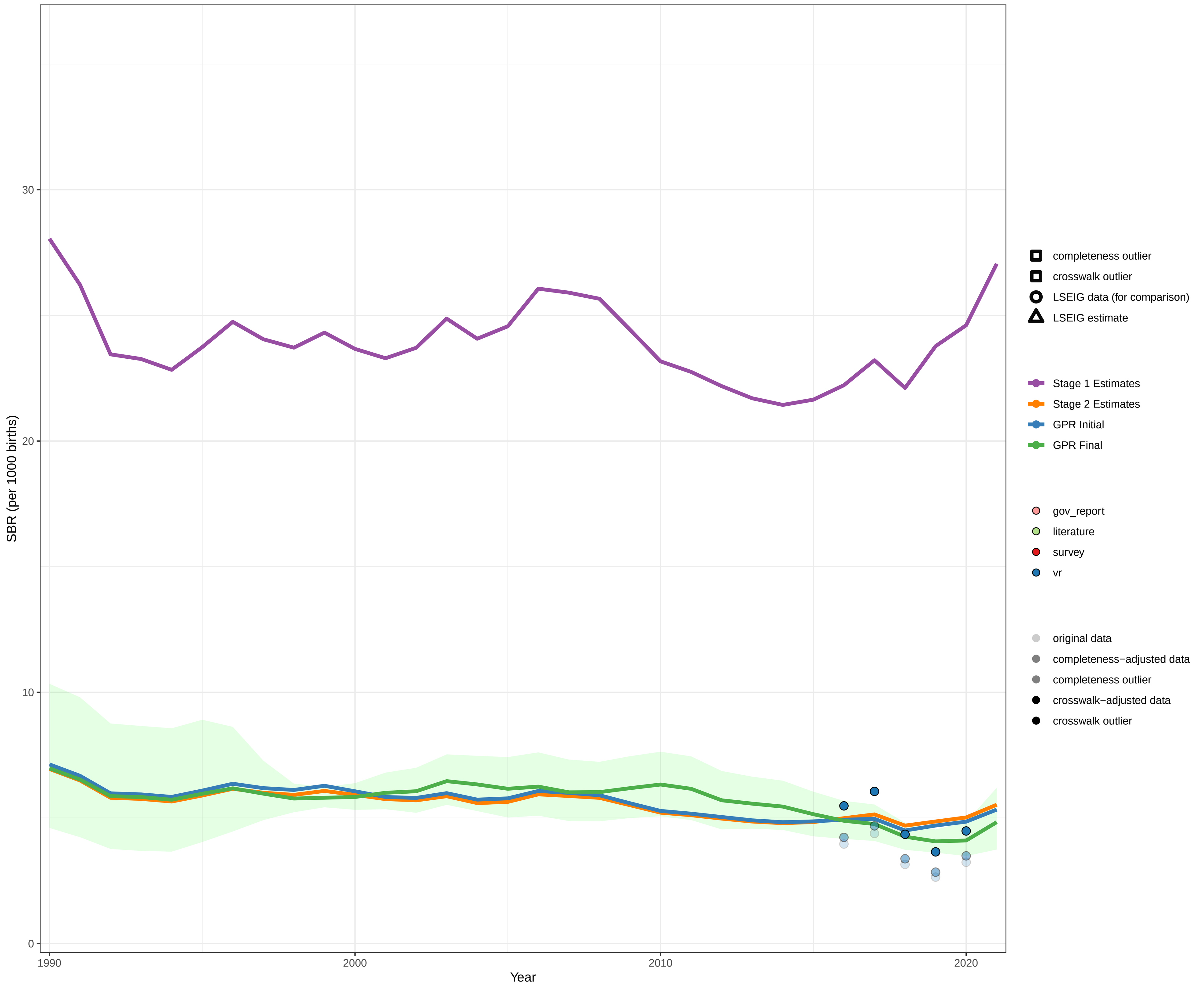

West Sussex GBR_44766 Western Europe Stillbirth Rate (SBR) − 28 weeks
lambda = 0.6 ; zeta = 0.8 ; scale = 15 ; amp = 0.028

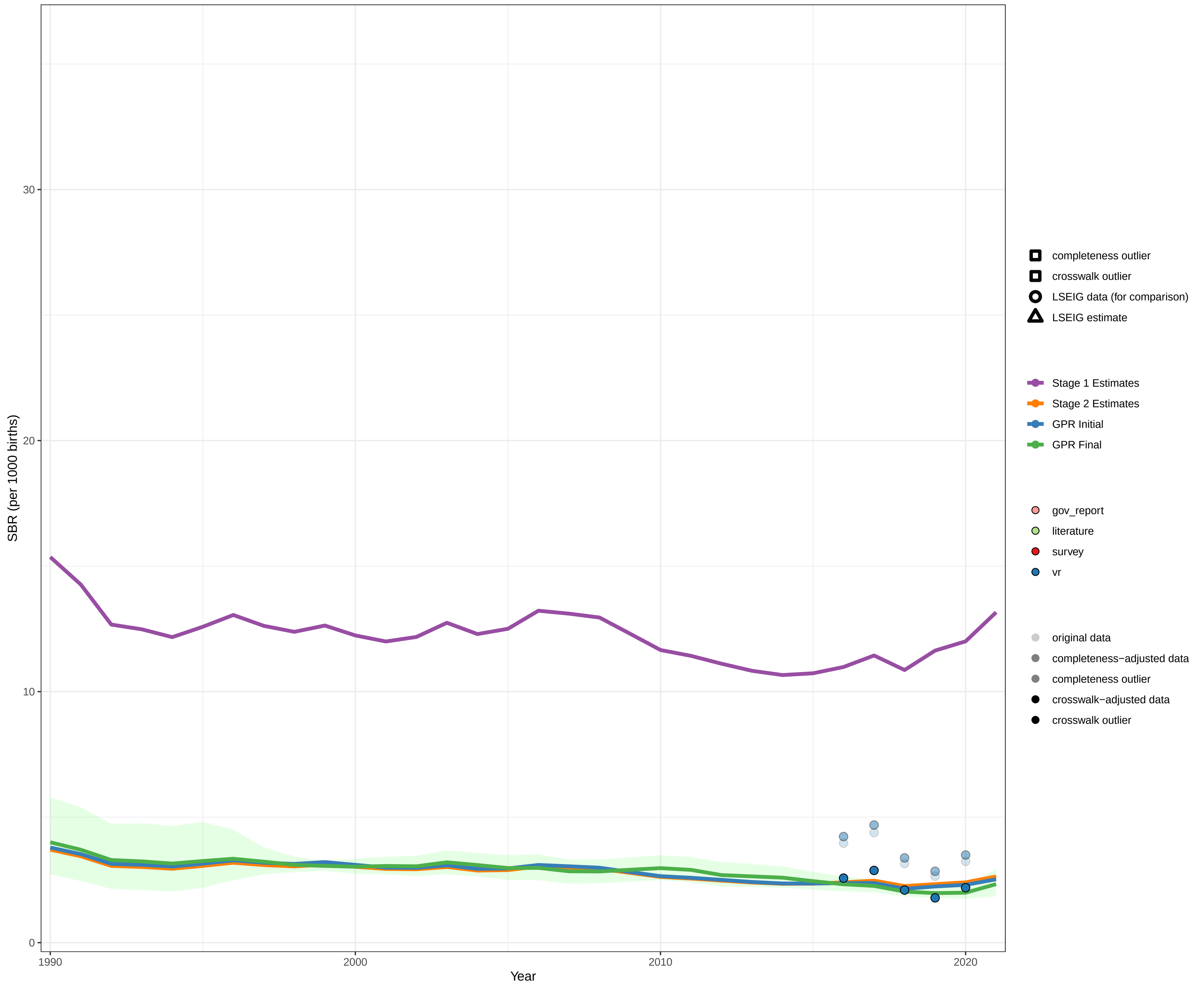

Year	Total Stillbirths (in thousands)	Total Stillbirths (in thousands)	SBR (per 1000 births)	SBR (per 1000 births)	SBR/NMR	SBR/NMR
	20 weeks	28 weeks	20 weeks	28 weeks	20 weeks	28 weeks
1990	0.1 (0.0 to 0.1)	0.0 (0.0 to 0.0)	7.0 (4.6 to 10.3)	4.0 (2.7 to 5.8)	1.70 (1.12 to 2.51)	0.97 (0.66 to 1.41)
1995	0.0 (0.0 to 0.1)	0.0 (0.0 to 0.0)	6.0 (4.0 to 8.9)	3.3 (2.2 to 4.8)	1.94 (1.31 to 2.89)	1.06 (0.71 to 1.56)
2000	0.0 (0.0 to 0.1)	0.0 (0.0 to 0.0)	5.8 (5.3 to 6.4)	3.0 (2.7 to 3.3)	2.11 (1.93 to 2.31)	1.09 (0.99 to 1.21)
2005	0.1 (0.0 to 0.1)	0.0 (0.0 to 0.0)	6.2 (5.0 to 7.4)	3.0 (2.5 to 3.5)	2.30 (1.87 to 2.77)	1.11 (0.93 to 1.30)
2010	0.1 (0.0 to 0.1)	0.0 (0.0 to 0.0)	6.3 (5.1 to 7.6)	3.0 (2.5 to 3.5)	2.59 (2.07 to 3.12)	1.21 (1.02 to 1.42)
2015	0.0 (0.0 to 0.1)	0.0 (0.0 to 0.0)	5.2 (4.3 to 6.0)	2.5 (2.1 to 2.8)	2.35 (1.94 to 2.75)	1.12 (0.96 to 1.29)
2020	0.0 (0.0 to 0.0)	0.0 (0.0 to 0.0)	4.1 (3.5 to 4.8)	2.0 (1.8 to 2.3)	1.71 (1.45 to 2.02)	0.83 (0.73 to 0.95)
2021	0.0 (0.0 to 0.0)	0.0 (0.0 to 0.0)	4.8 (3.7 to 6.2)	2.3 (1.8 to 2.9)	1.85 (1.43 to 2.37)	0.89 (0.71 to 1.12)

Windsor and Maidenhead GBR_44762 Western Europe Stillbirth Rate / Neonatal Mortality Rate (SBR/NMR) − 20 weeks
lambda = 0.6 ; zeta = 0.8 ; scale = 15 ; amp = 0.028

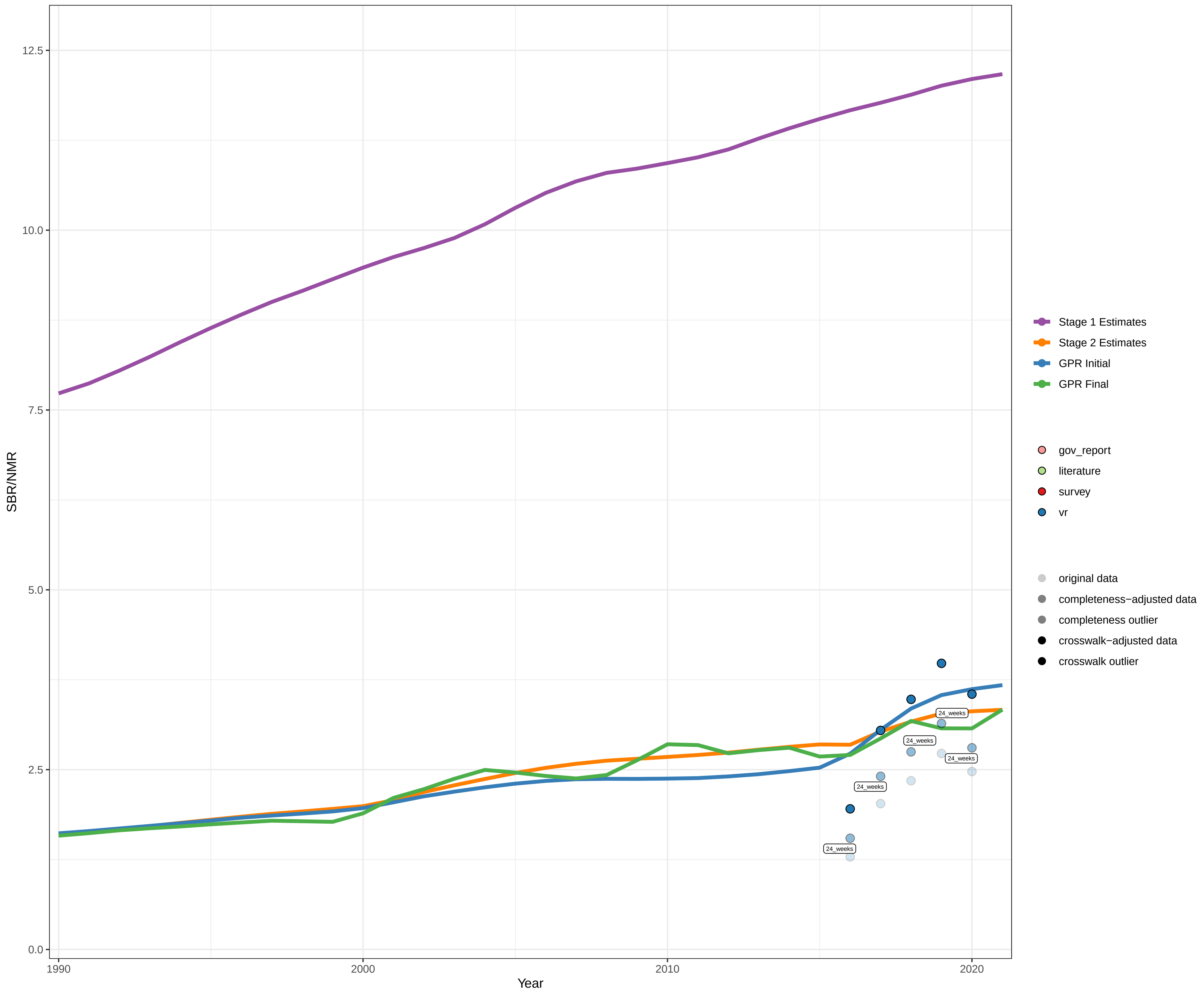

Windsor and Maidenhead GBR_44762 Western Europe Stillbirth Rate / Neonatal Mortality Rate (SBR/NMR) − 28 weeks
lambda = 0.6 ; zeta = 0.8 ; scale = 15 ; amp = 0.028

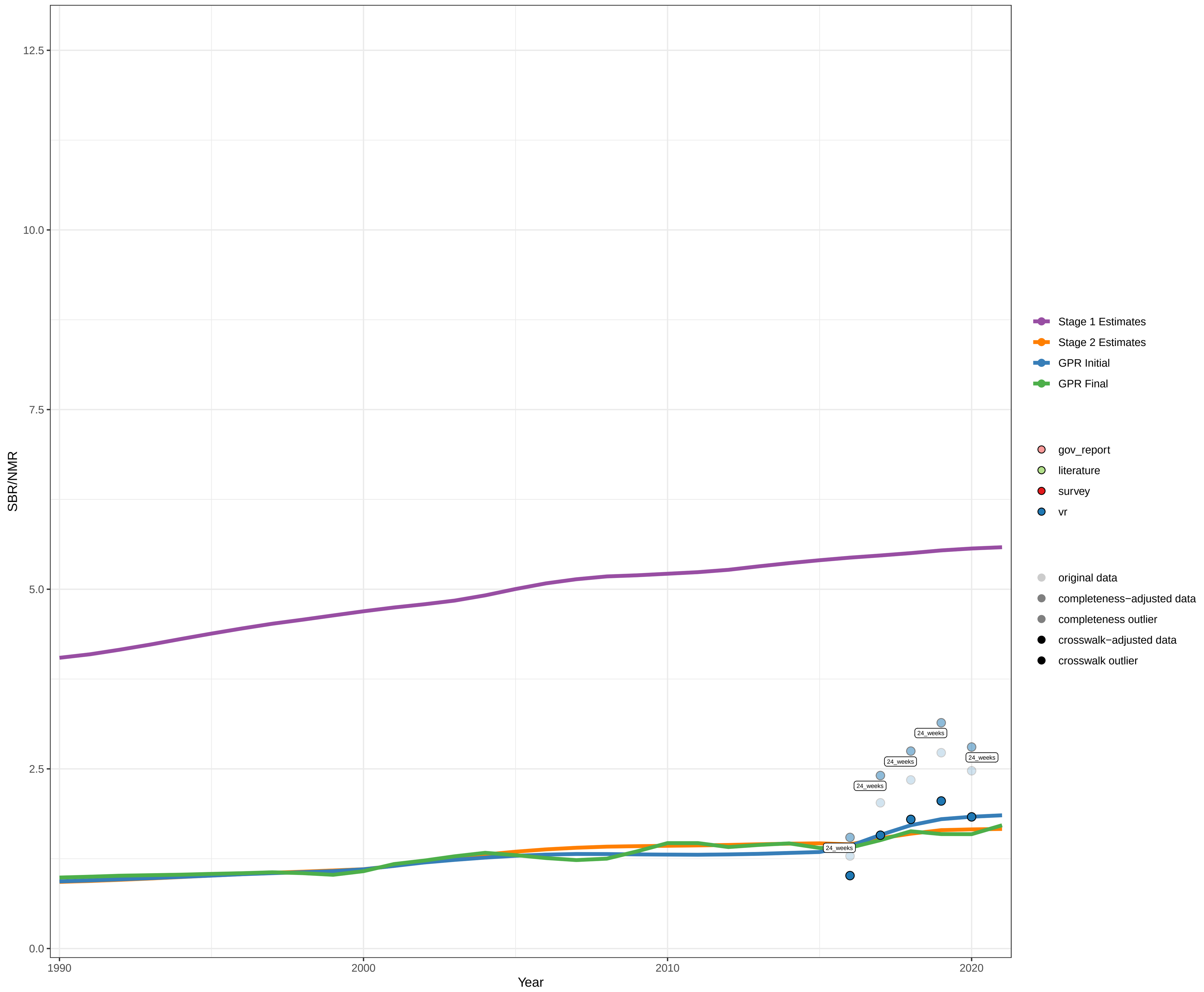

Definition	Years	Outlier	Source.Type	Title
24_weeks	2016−2020	0	VR	United Kingdom − England and Wales Child Mortality Statistics

Windsor and Maidenhead GBR_44762 Western Europe Stillbirth Rate (SBR) − 20 weeks
lambda = 0.6 ; zeta = 0.8 ; scale = 15 ; amp = 0.028

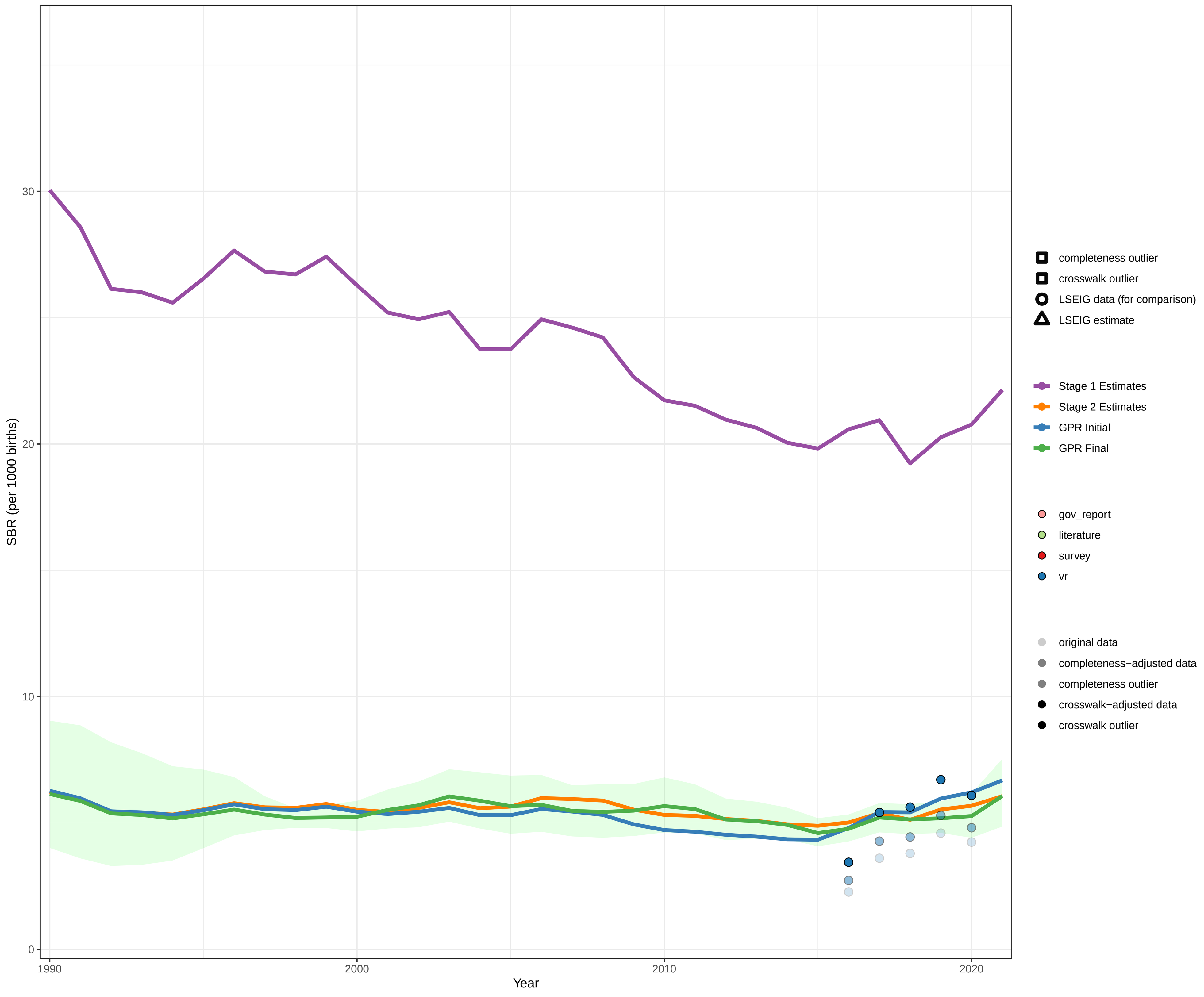

Windsor and Maidenhead GBR_44762 Western Europe Stillbirth Rate (SBR) − 28 weeks
lambda = 0.6 ; zeta = 0.8 ; scale = 15 ; amp = 0.028

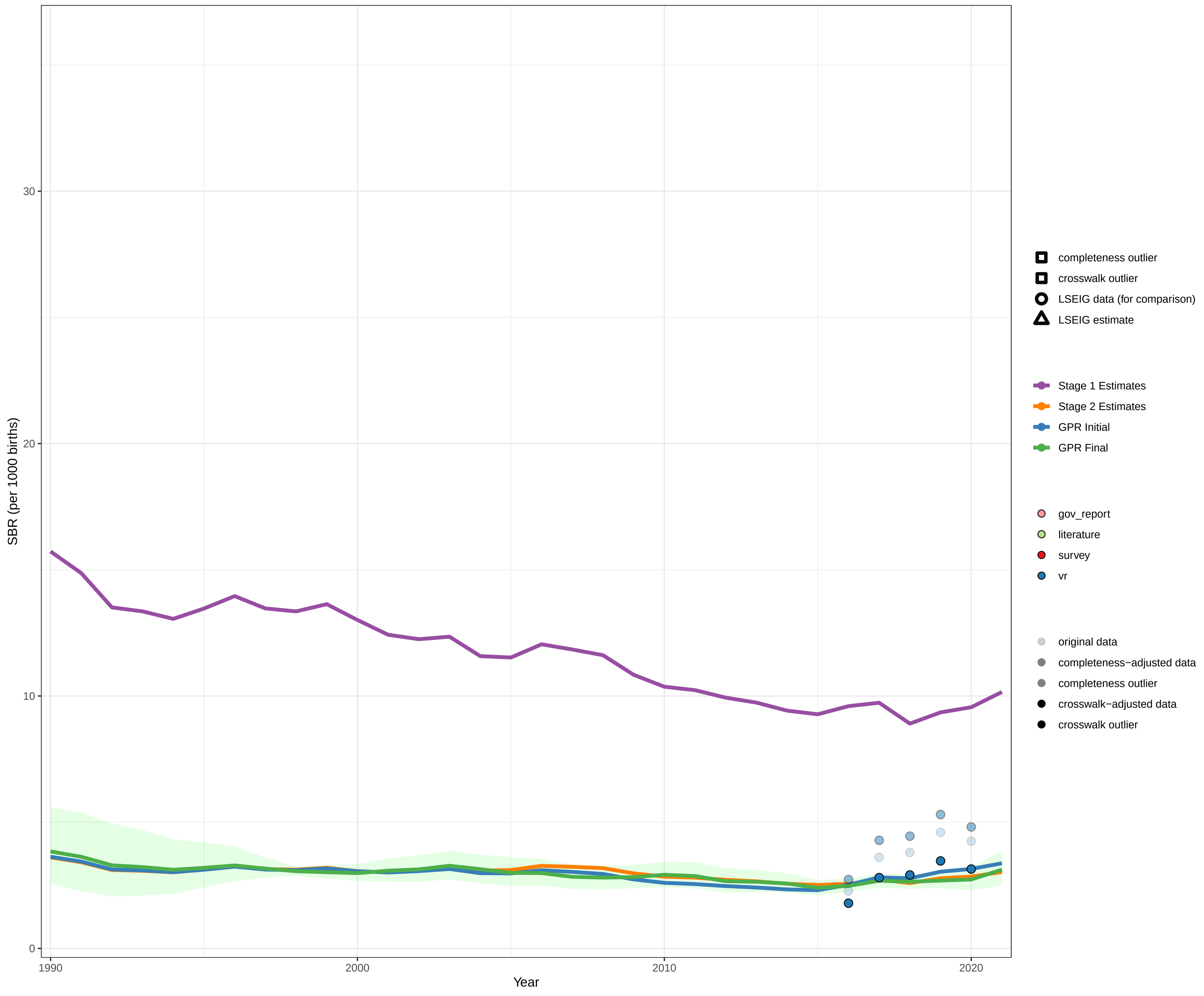

Year	Total Stillbirths (in thousands)	Total Stillbirths (in thousands)	SBR (per 1000 births)	SBR (per 1000 births)	SBR/NMR	SBR/NMR
	20 weeks	28 weeks	20 weeks	28 weeks	20 weeks	28 weeks
1990	0.0 (0.0 to 0.0)	0.0 (0.0 to 0.0)	6.2 (4.0 to 9.1)	3.8 (2.6 to 5.6)	1.58 (1.03 to 2.33)	0.99 (0.66 to 1.44)
1995	0.0 (0.0 to 0.0)	0.0 (0.0 to 0.0)	5.3 (4.0 to 7.1)	3.2 (2.4 to 4.2)	1.74 (1.30 to 2.32)	1.04 (0.78 to 1.37)
2000	0.0 (0.0 to 0.0)	0.0 (0.0 to 0.0)	5.2 (4.7 to 5.9)	3.0 (2.7 to 3.3)	1.89 (1.68 to 2.12)	1.08 (0.96 to 1.20)
2005	0.0 (0.0 to 0.0)	0.0 (0.0 to 0.0)	5.7 (4.6 to 6.9)	3.0 (2.5 to 3.6)	2.46 (1.99 to 2.99)	1.30 (1.07 to 1.57)
2010	0.0 (0.0 to 0.0)	0.0 (0.0 to 0.0)	5.7 (4.6 to 6.8)	2.9 (2.4 to 3.4)	2.85 (2.33 to 3.42)	1.47 (1.22 to 1.73)
2015	0.0 (0.0 to 0.0)	0.0 (0.0 to 0.0)	4.6 (4.1 to 5.2)	2.4 (2.1 to 2.7)	2.68 (2.38 to 3.03)	1.40 (1.22 to 1.58)
2020	0.0 (0.0 to 0.0)	0.0 (0.0 to 0.0)	5.3 (4.4 to 6.2)	2.7 (2.3 to 3.2)	3.07 (2.57 to 3.60)	1.59 (1.34 to 1.87)
2021	0.0 (0.0 to 0.0)	0.0 (0.0 to 0.0)	6.1 (4.9 to 7.5)	3.1 (2.5 to 3.9)	3.33 (2.67 to 4.15)	1.72 (1.37 to 2.13)

Wokingham GBR_44759 Western Europe Stillbirth Rate / Neonatal Mortality Rate (SBR/NMR) − 20 weeks
lambda = 0.6 ; zeta = 0.8 ; scale = 15 ; amp = 0.028

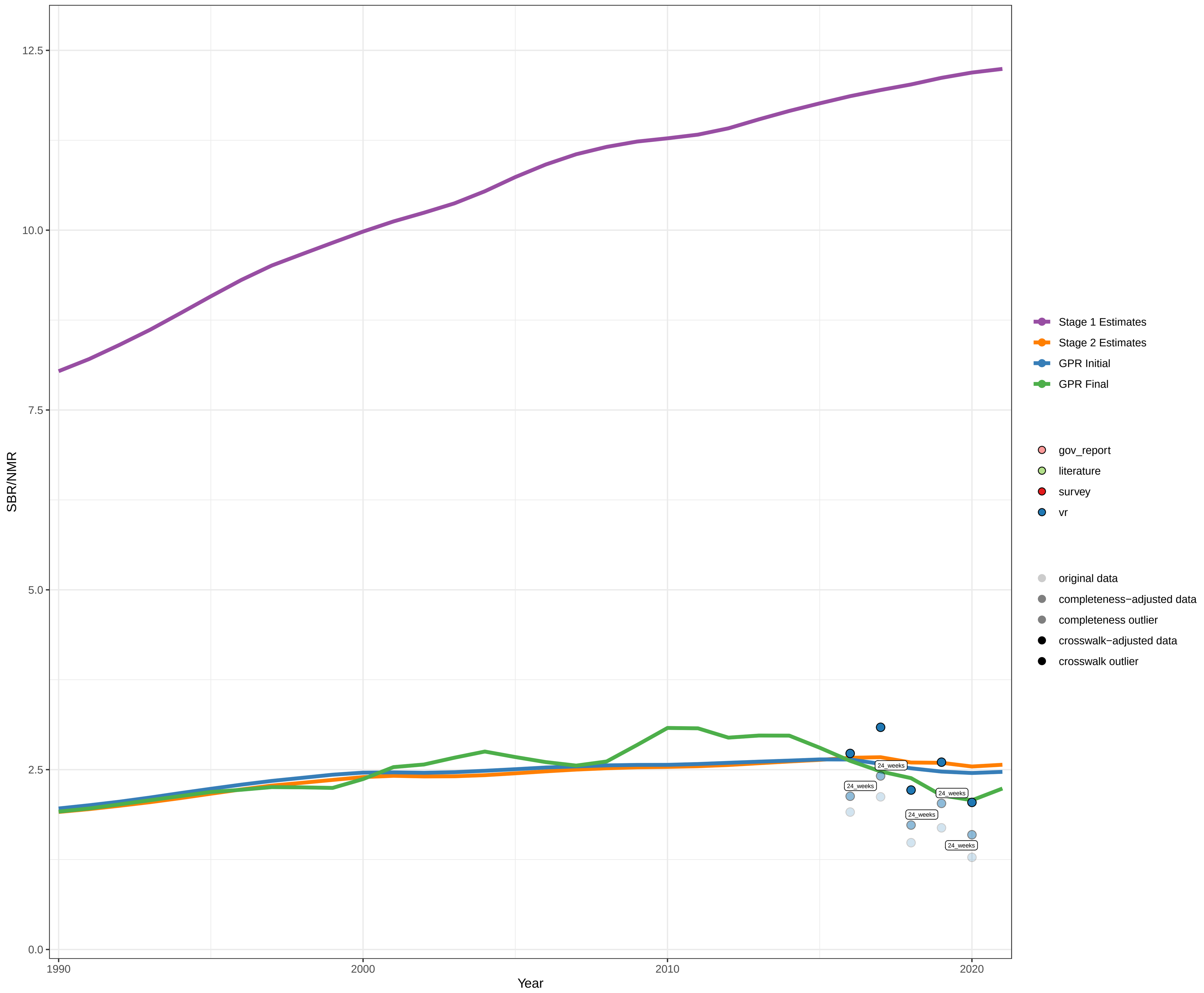

Wokingham GBR_44759 Western Europe Stillbirth Rate / Neonatal Mortality Rate (SBR/NMR) − 28 weeks
lambda = 0.6 ; zeta = 0.8 ; scale = 15 ; amp = 0.028

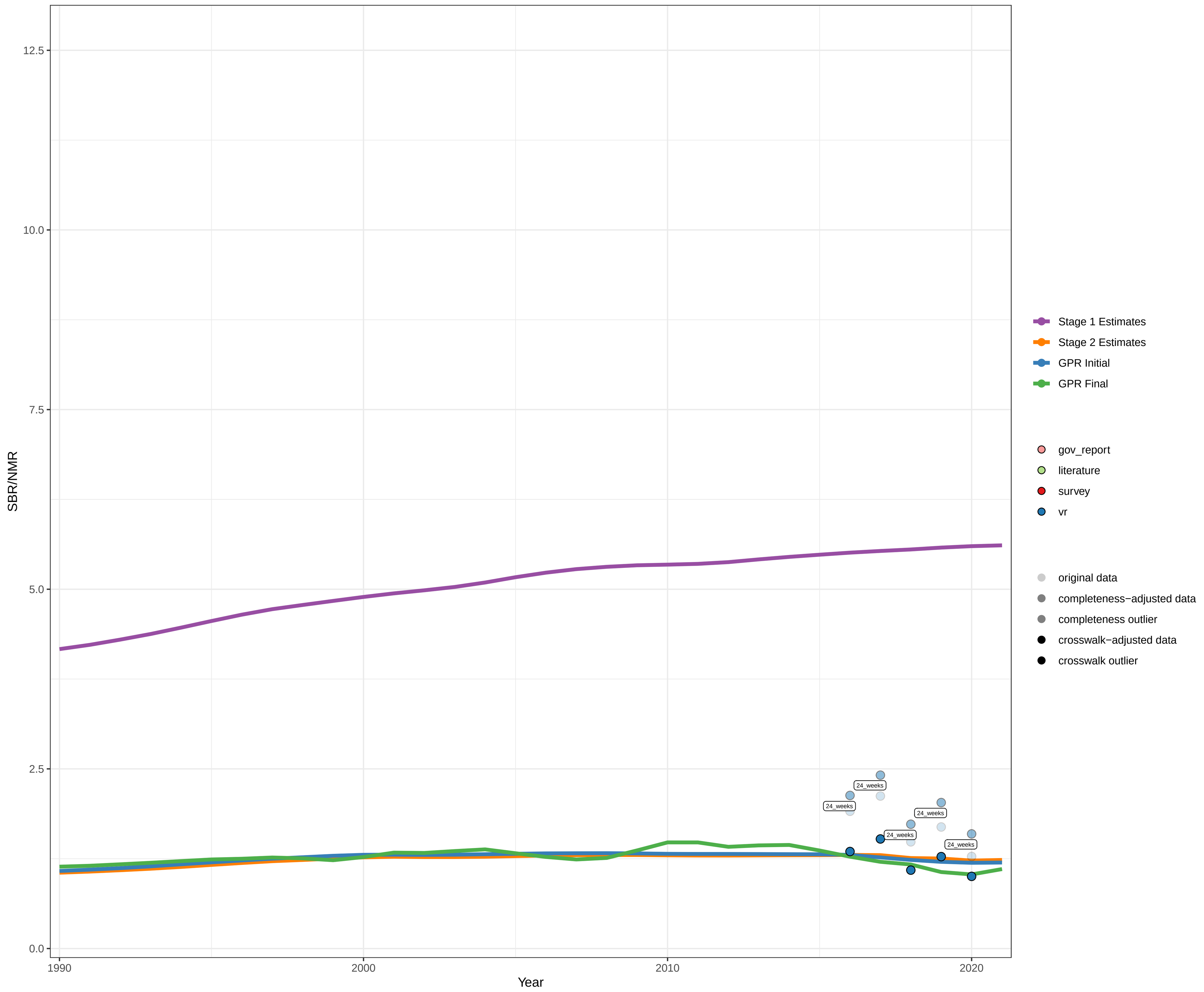

Definition	Years	Outlier	Source.Type	Title
24_weeks	2016−2020	0	VR	United Kingdom − England and Wales Child Mortality Statistics

Wokingham GBR_44759 Western Europe Stillbirth Rate (SBR) − 20 weeks
lambda = 0.6 ; zeta = 0.8 ; scale = 15 ; amp = 0.028

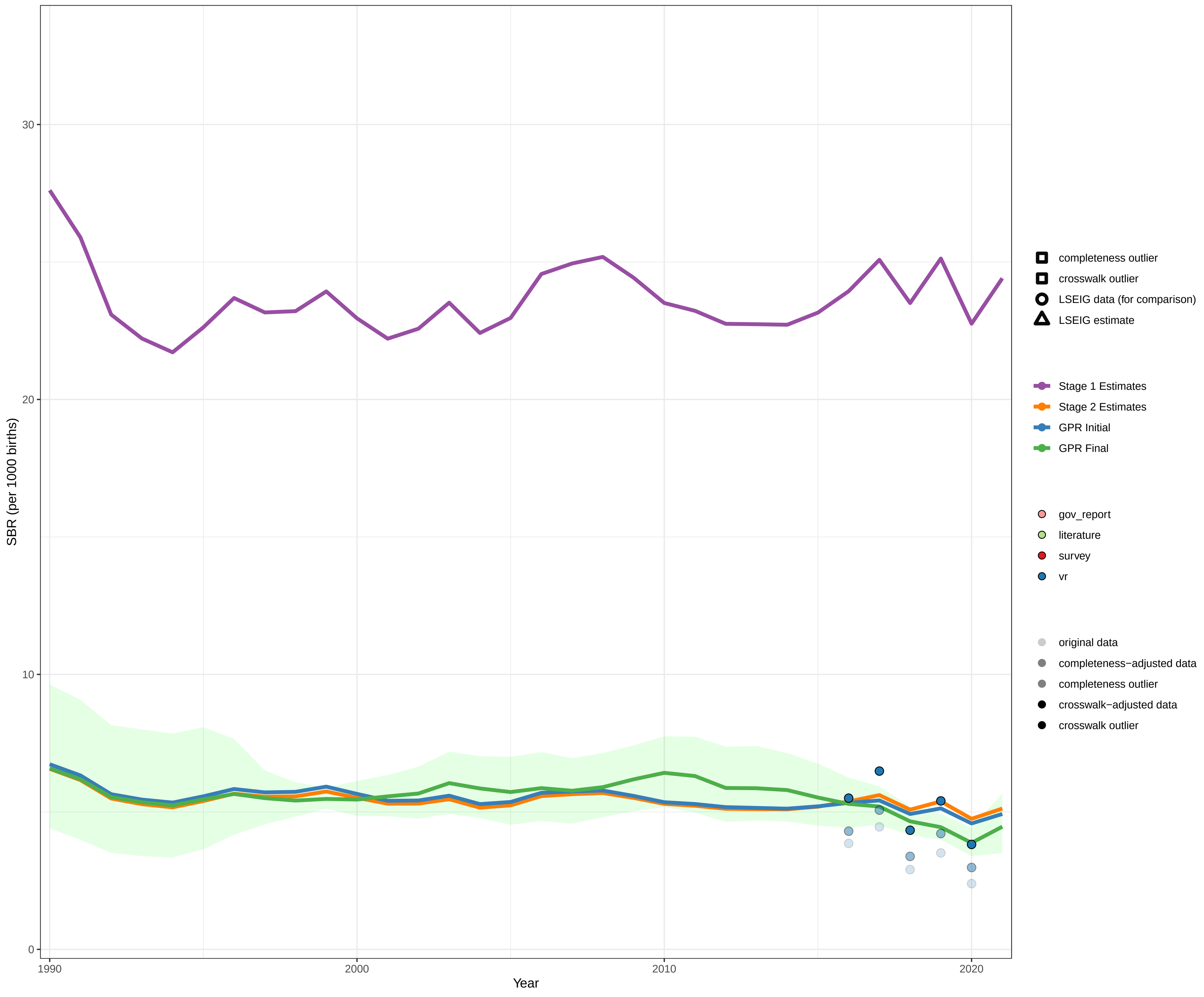

Wokingham GBR_44759 Western Europe Stillbirth Rate (SBR) − 28 weeks
lambda = 0.6 ; zeta = 0.8 ; scale = 15 ; amp = 0.028

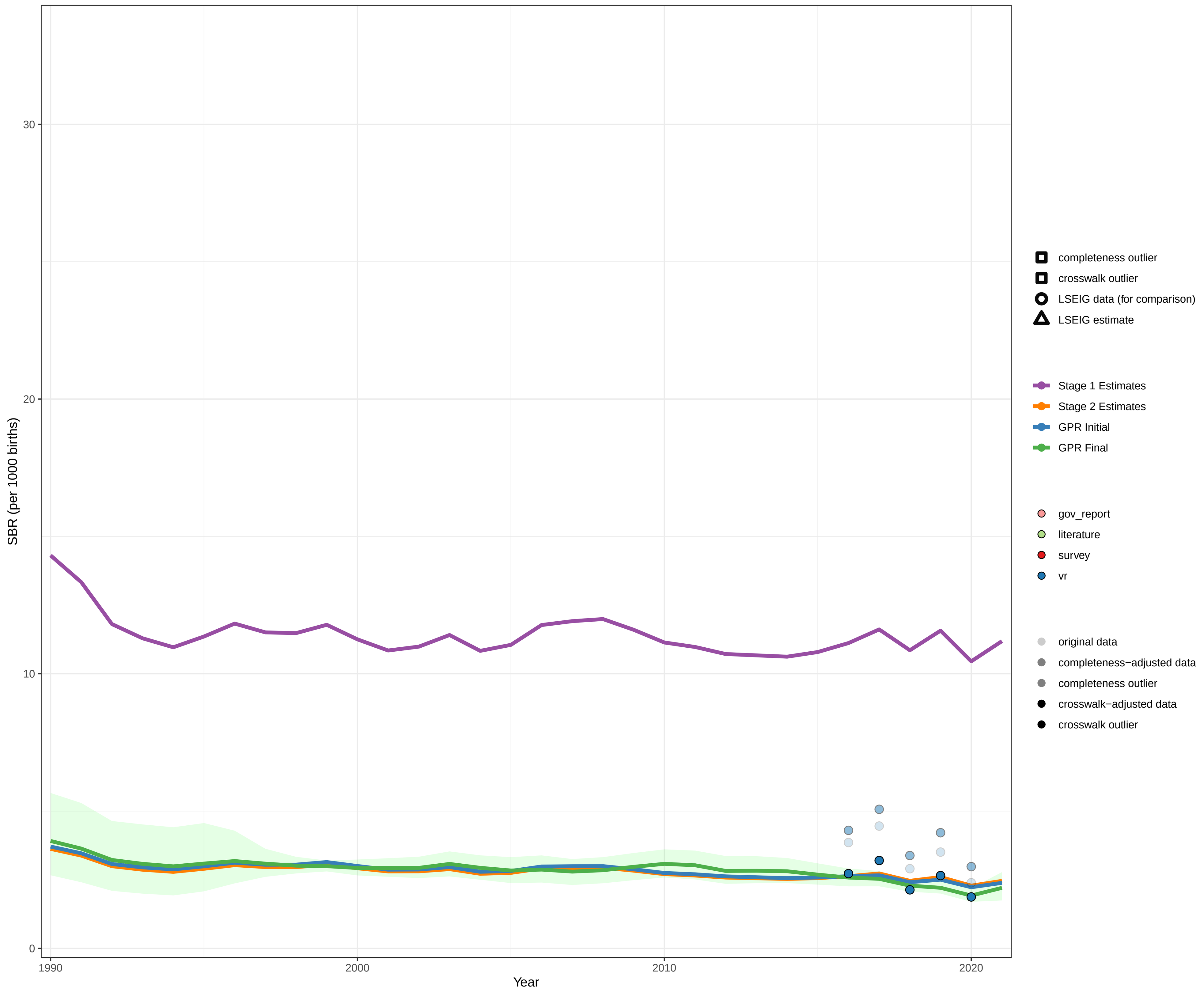

Year	Total Stillbirths (in thousands)	Total Stillbirths (in thousands)	SBR (per 1000 births)	SBR (per 1000 births)	SBR/NMR	SBR/NMR
	20 weeks	28 weeks	20 weeks	28 weeks	20 weeks	28 weeks
1990	0.0 (0.0 to 0.0)	0.0 (0.0 to 0.0)	6.6 (4.4 to 9.6)	3.9 (2.7 to 5.7)	1.92 (1.28 to 2.81)	1.14 (0.78 to 1.65)
1995	0.0 (0.0 to 0.0)	0.0 (0.0 to 0.0)	5.5 (3.6 to 8.1)	3.1 (2.1 to 4.6)	2.19 (1.46 to 3.24)	1.24 (0.83 to 1.83)
2000	0.0 (0.0 to 0.0)	0.0 (0.0 to 0.0)	5.4 (4.9 to 6.1)	2.9 (2.7 to 3.2)	2.37 (2.11 to 2.66)	1.27 (1.16 to 1.41)
2005	0.0 (0.0 to 0.0)	0.0 (0.0 to 0.0)	5.7 (4.5 to 7.0)	2.8 (2.4 to 3.3)	2.68 (2.12 to 3.27)	1.33 (1.11 to 1.55)
2010	0.0 (0.0 to 0.0)	0.0 (0.0 to 0.0)	6.4 (5.2 to 7.7)	3.1 (2.6 to 3.6)	3.08 (2.48 to 3.72)	1.48 (1.24 to 1.73)
2015	0.0 (0.0 to 0.0)	0.0 (0.0 to 0.0)	5.5 (4.5 to 6.8)	2.7 (2.3 to 3.1)	2.80 (2.28 to 3.43)	1.36 (1.18 to 1.57)
2020	0.0 (0.0 to 0.0)	0.0 (0.0 to 0.0)	3.9 (3.4 to 4.4)	1.9 (1.7 to 2.2)	2.07 (1.82 to 2.37)	1.03 (0.91 to 1.17)
2021	0.0 (0.0 to 0.0)	0.0 (0.0 to 0.0)	4.5 (3.5 to 5.7)	2.2 (1.7 to 2.8)	2.24 (1.76 to 2.85)	1.11 (0.88 to 1.39)

South West England GBR_4626 Western Europe Stillbirth Rate / Neonatal Mortality Rate (SBR/NMR) − 20 weeks
lambda = 0.2 ; zeta = 0.99 ; scale = 5 ; amp = 0.028

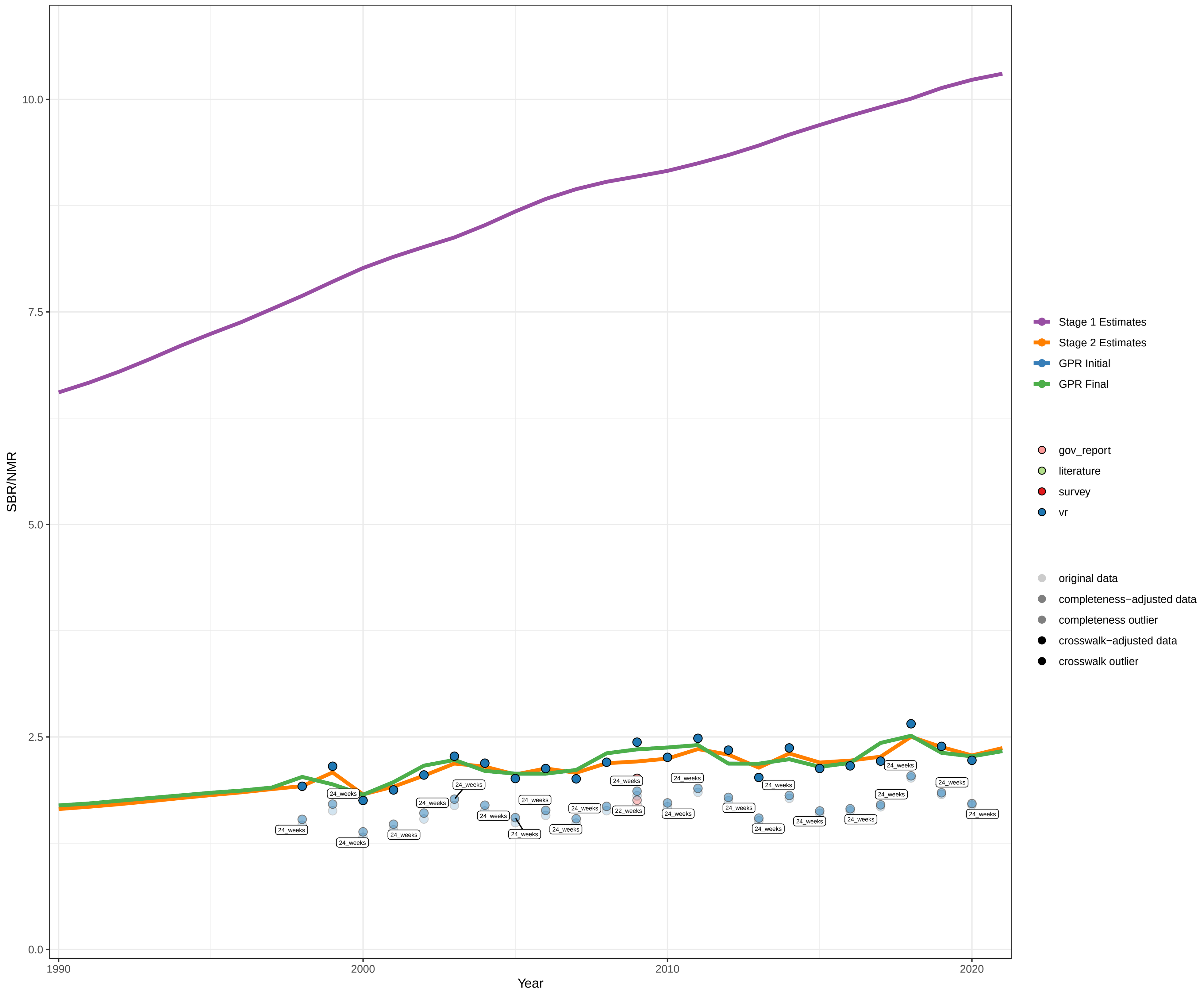

South West England GBR_4626 Western Europe Stillbirth Rate / Neonatal Mortality Rate (SBR/NMR) − 28 weeks
lambda = 0.2 ; zeta = 0.99 ; scale = 5 ; amp = 0.028

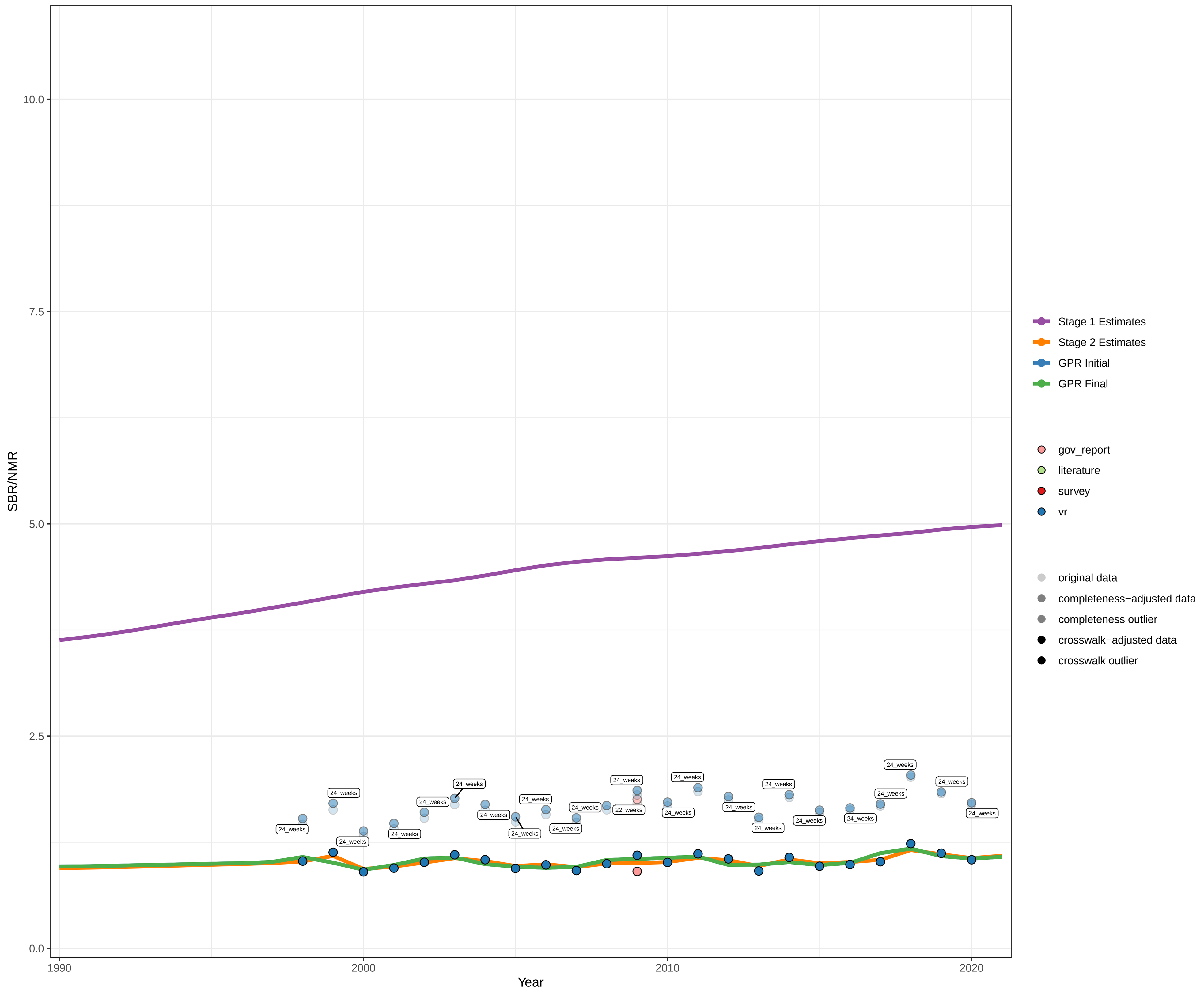

Definition	Years	Outlier	Source.Type	Title
22_weeks	2009	0	Statistical Report	United Kingdom Perinatal Mortality
24_weeks	1998−2007	0	VR	United Kingdom − England and Wales Birth Statistics
24_weeks	2008−2020	0	VR	United Kingdom − England and Wales Child Mortality Statistics

South West England GBR_4626 Western Europe Stillbirth Rate (SBR) − 20 weeks
lambda = 0.2 ; zeta = 0.99 ; scale = 5 ; amp = 0.028

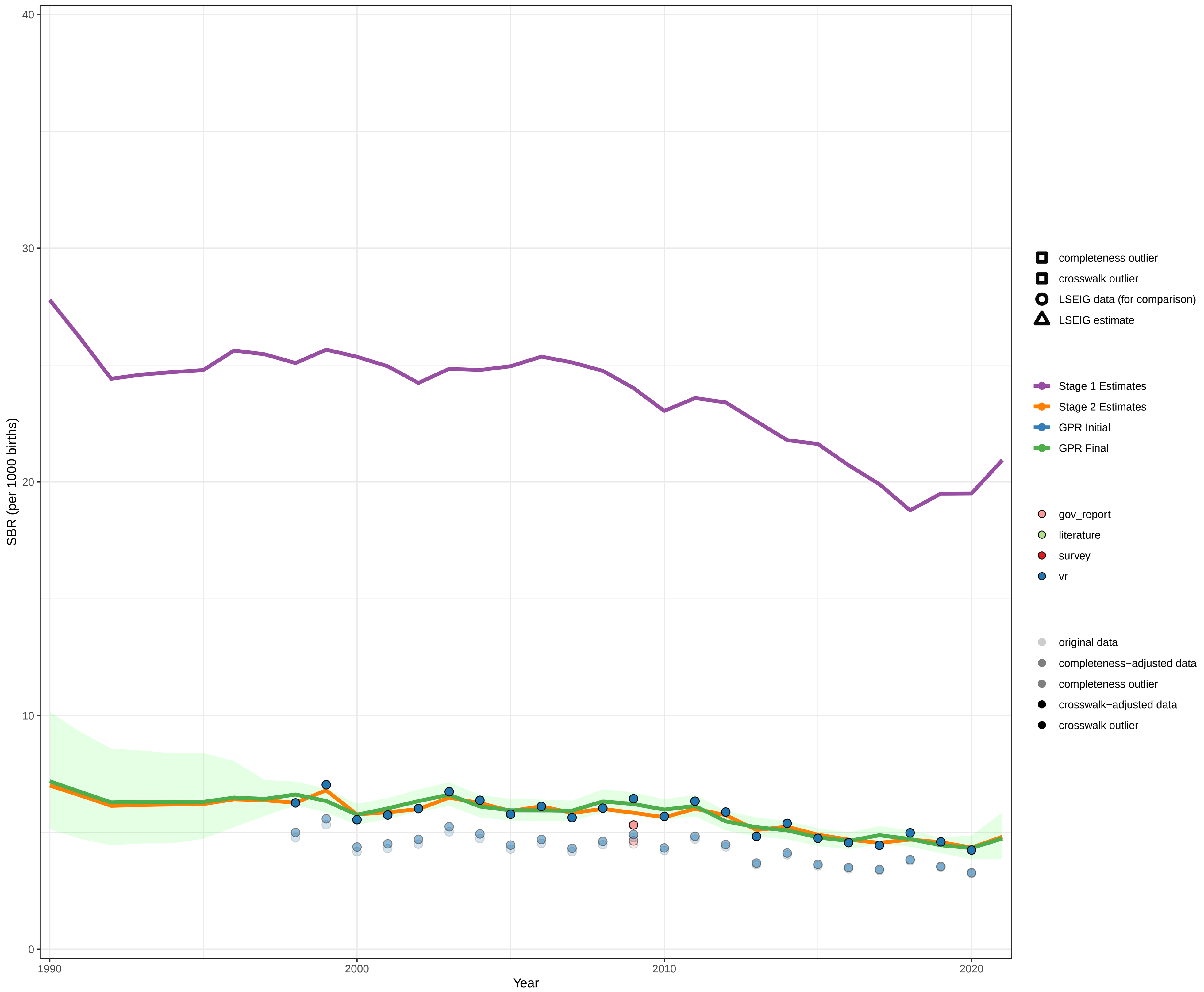

South West England GBR_4626 Western Europe Stillbirth Rate (SBR) − 28 weeks
lambda = 0.2 ; zeta = 0.99 ; scale = 5 ; amp = 0.028

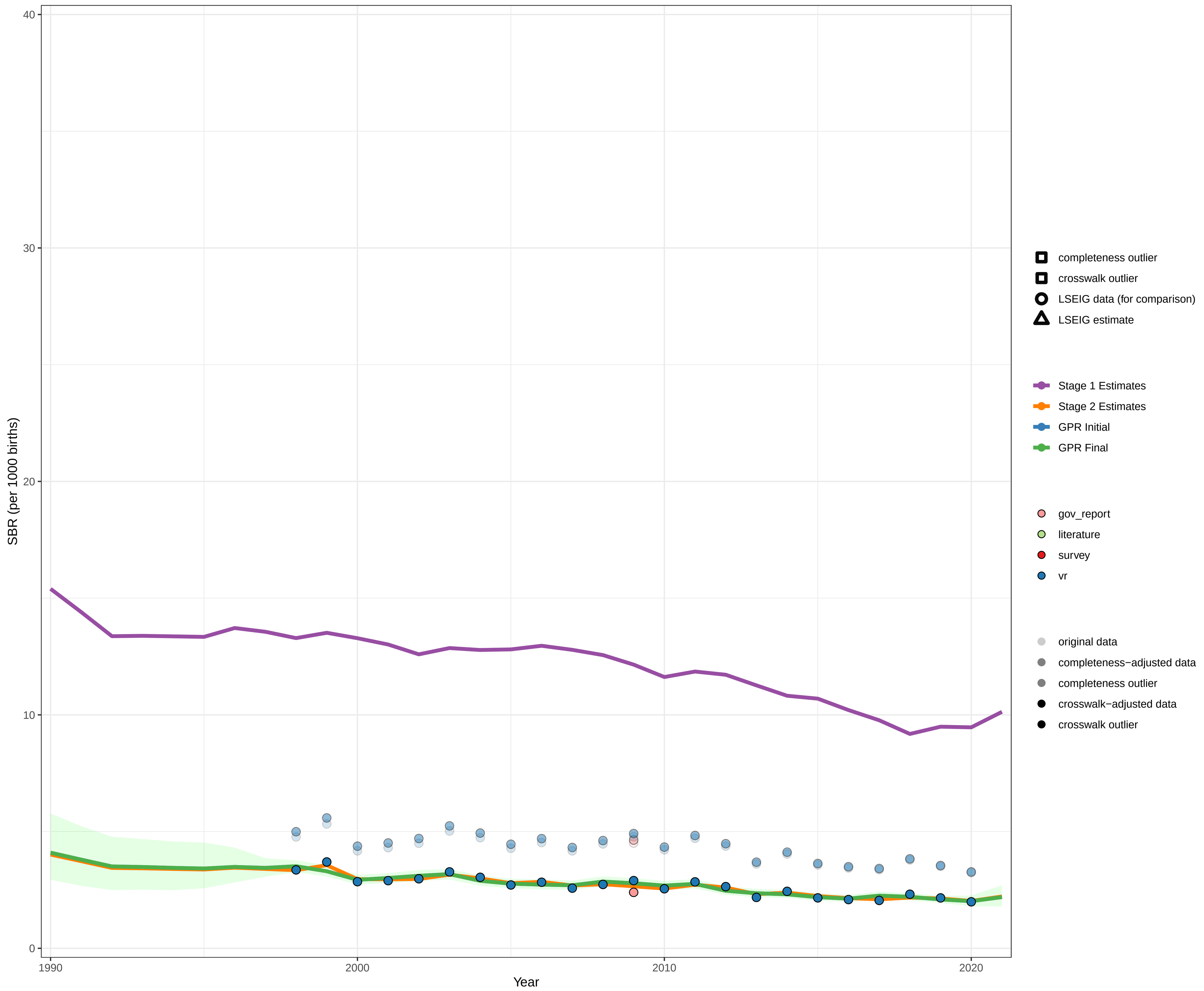

Year	Total Stillbirths (in thousands)	Total Stillbirths (in thousands)	SBR (per 1000 births)	SBR (per 1000 births)	SBR/NMR	SBR/NMR
	20 weeks	28 weeks	20 weeks	28 weeks	20 weeks	28 weeks
1990	0.4 (0.3 to 0.6)	0.2 (0.2 to 0.3)	7.2 (5.1 to 10.2)	4.1 (2.9 to 5.8)	1.69 (1.21 to 2.40)	0.97 (0.69 to 1.36)
1995	0.3 (0.3 to 0.5)	0.2 (0.1 to 0.3)	6.3 (4.7 to 8.4)	3.4 (2.6 to 4.5)	1.84 (1.38 to 2.45)	1.00 (0.75 to 1.32)
2000	0.3 (0.3 to 0.3)	0.1 (0.1 to 0.2)	5.8 (5.3 to 6.2)	2.9 (2.7 to 3.1)	1.82 (1.68 to 1.97)	0.93 (0.86 to 1.00)
2005	0.3 (0.3 to 0.3)	0.1 (0.1 to 0.2)	5.9 (5.5 to 6.4)	2.8 (2.6 to 3.0)	2.07 (1.92 to 2.24)	0.96 (0.90 to 1.04)
2010	0.4 (0.3 to 0.4)	0.2 (0.2 to 0.2)	6.0 (5.5 to 6.4)	2.7 (2.5 to 2.9)	2.38 (2.20 to 2.55)	1.07 (0.99 to 1.15)
2015	0.3 (0.3 to 0.3)	0.1 (0.1 to 0.1)	4.8 (4.4 to 5.2)	2.2 (2.0 to 2.4)	2.15 (1.98 to 2.32)	0.98 (0.91 to 1.06)
2020	0.2 (0.2 to 0.2)	0.1 (0.1 to 0.1)	4.3 (3.8 to 4.9)	2.0 (1.8 to 2.3)	2.27 (2.02 to 2.55)	1.06 (0.94 to 1.19)
2021	0.2 (0.2 to 0.3)	0.1 (0.1 to 0.1)	4.7 (3.9 to 5.9)	2.2 (1.8 to 2.7)	2.33 (1.90 to 2.88)	1.08 (0.88 to 1.33)

Bath and North East Somerset GBR_44784 Western Europe Stillbirth Rate / Neonatal Mortality Rate (SBR/NMR) − 20 weeks
lambda = 0.8 ; zeta = 0.7 ; scale = 15 ; amp = 0.028

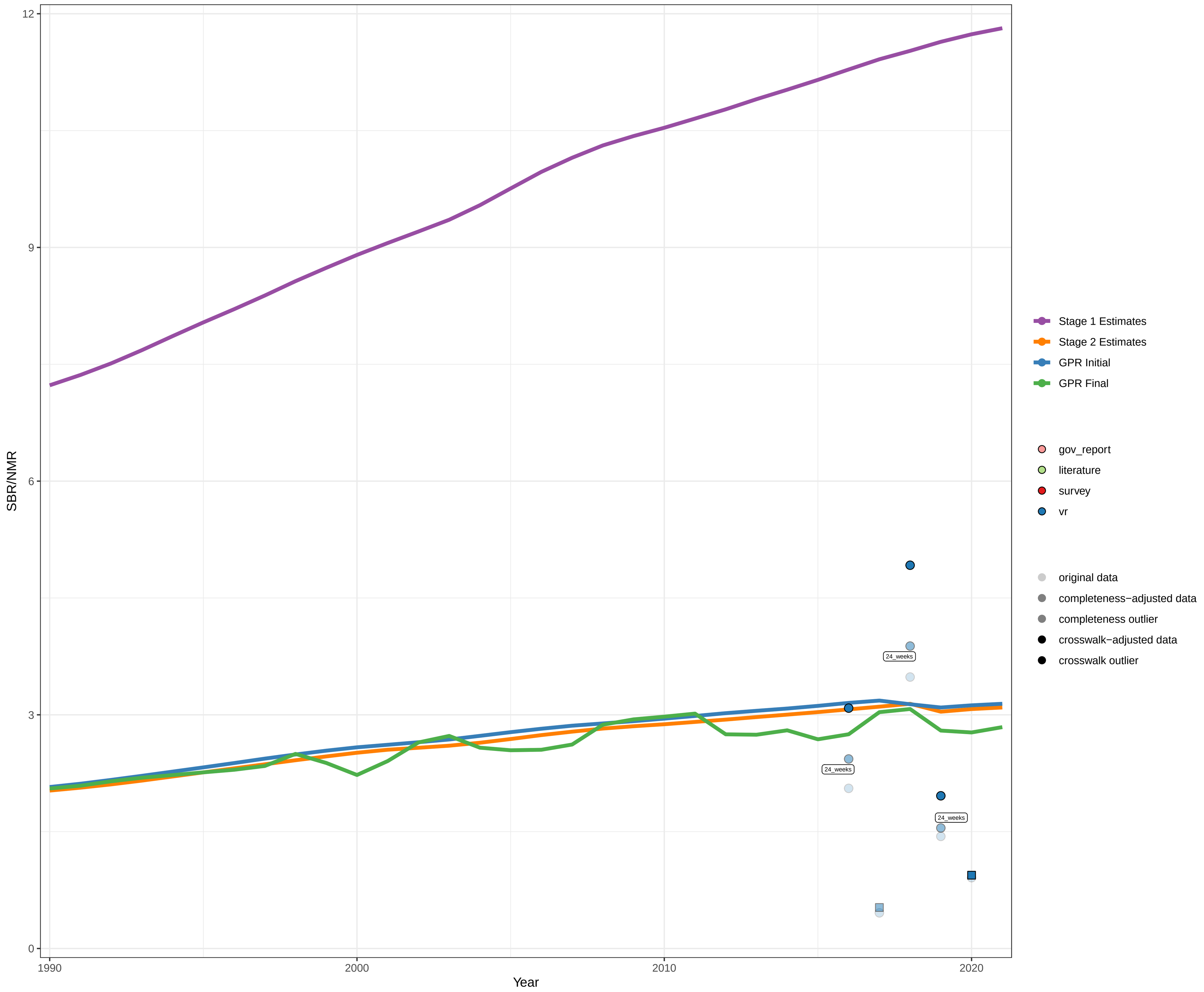

Bath and North East Somerset GBR_44784 Western Europe Stillbirth Rate / Neonatal Mortality Rate (SBR/NMR) − 28 weeks
lambda = 0.8 ; zeta = 0.7 ; scale = 15 ; amp = 0.028

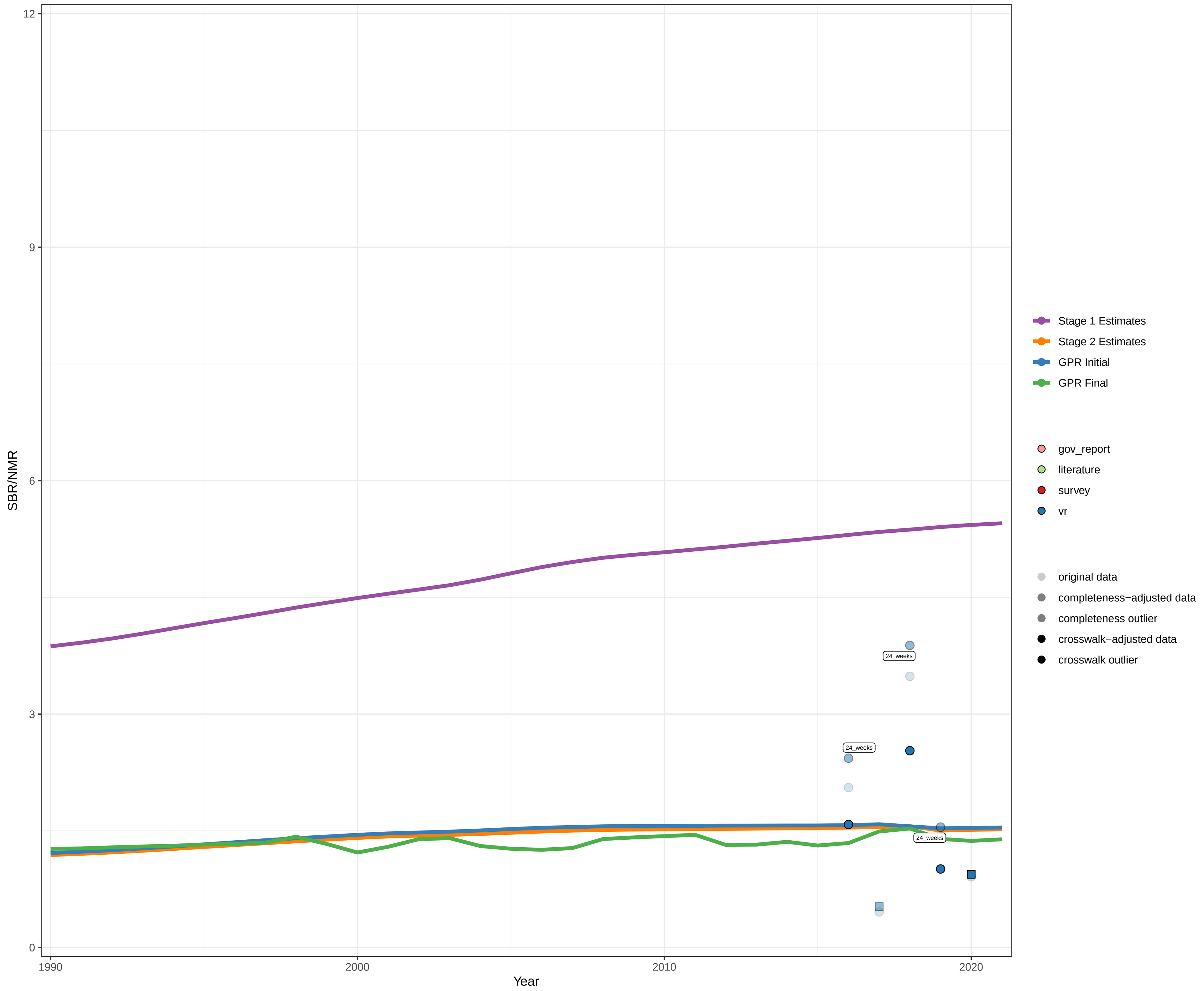

Definition	Years	Outlier	Source.Type	Title
24_weeks	2016, 2018−2020	0	VR	United Kingdom − England and Wales Child Mortality Statistics
24_weeks	2017	1	VR	United Kingdom − England and Wales Child Mortality Statistics

Bath and North East Somerset GBR_44784 Western Europe Stillbirth Rate (SBR) − 20 weeks
lambda = 0.8 ; zeta = 0.7 ; scale = 15 ; amp = 0.028

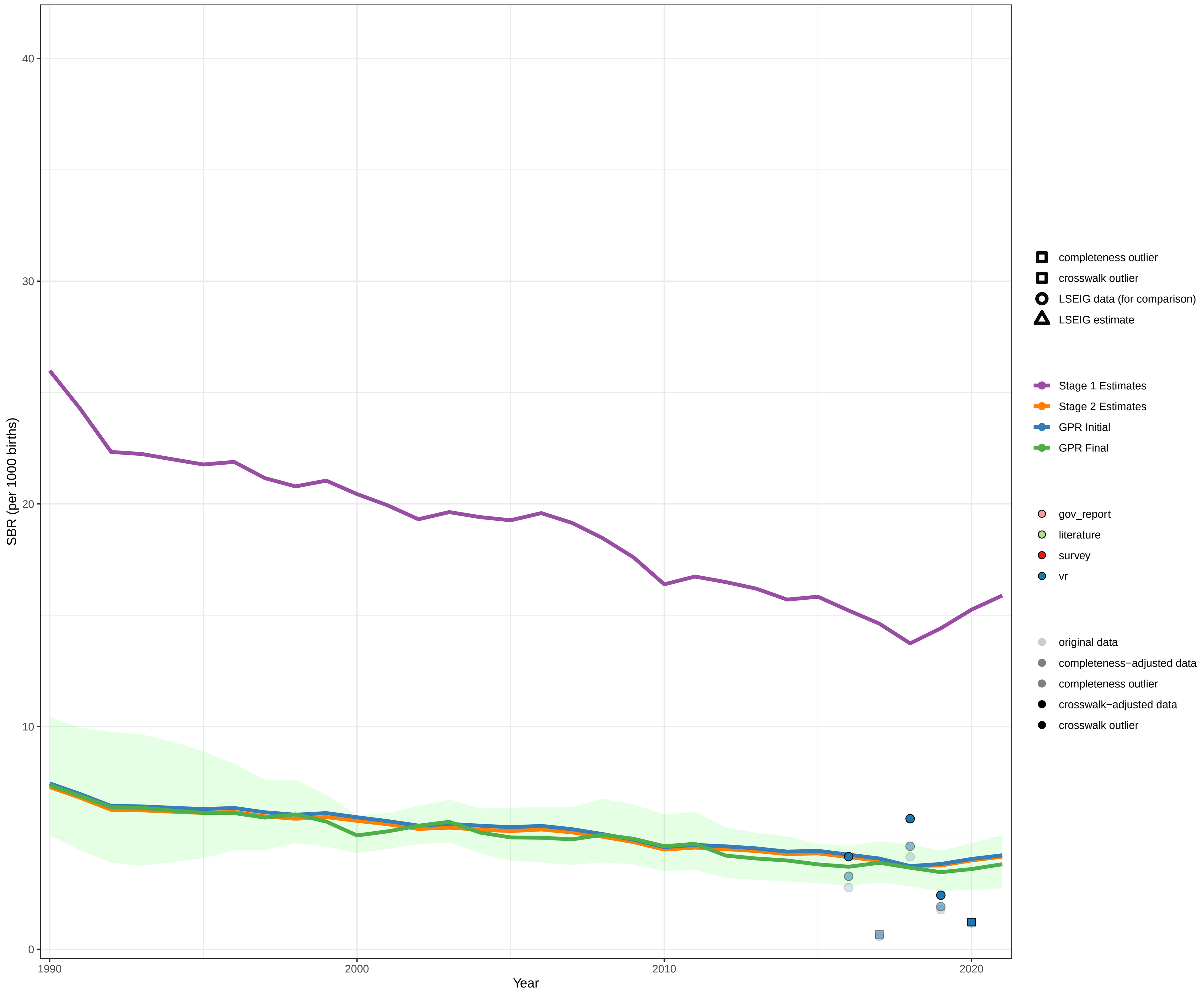

Bath and North East Somerset GBR_44784 Western Europe Stillbirth Rate (SBR) − 28 weeks
lambda = 0.8 ; zeta = 0.7 ; scale = 15 ; amp = 0.028

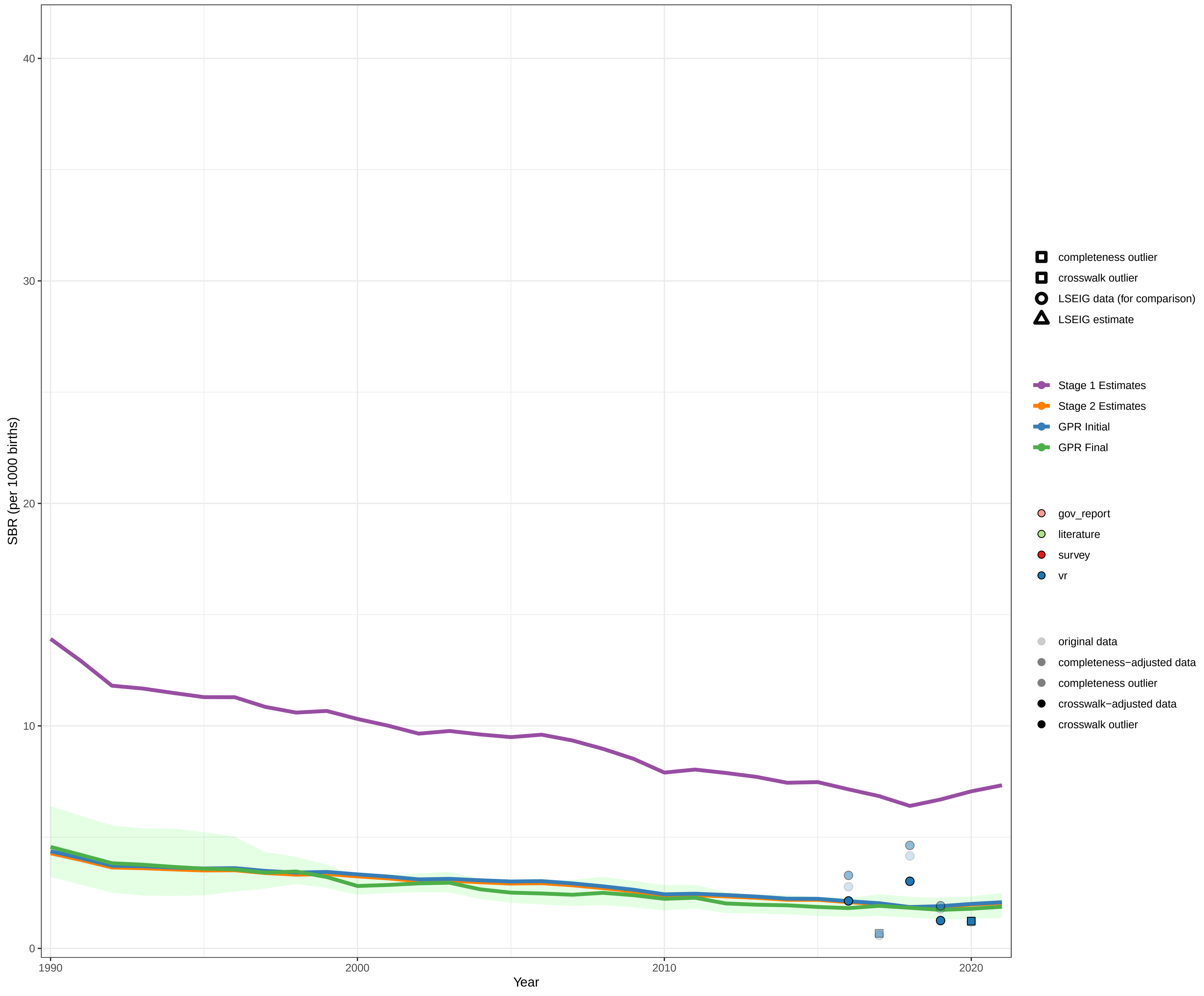

Year	Total Stillbirths (in thousands)	Total Stillbirths (in thousands)	SBR (per 1000 births)	SBR (per 1000 births)	SBR/NMR	SBR/NMR
	20 weeks	28 weeks	20 weeks	28 weeks	20 weeks	28 weeks
1990	0.0 (0.0 to 0.0)	0.0 (0.0 to 0.0)	7.4 (5.1 to 10.4)	4.6 (3.2 to 6.4)	2.05 (1.43 to 2.91)	1.27 (0.89 to 1.78)
1995	0.0 (0.0 to 0.0)	0.0 (0.0 to 0.0)	6.1 (4.1 to 8.9)	3.6 (2.4 to 5.2)	2.26 (1.51 to 3.28)	1.32 (0.88 to 1.93)
2000	0.0 (0.0 to 0.0)	0.0 (0.0 to 0.0)	5.1 (4.3 to 6.0)	2.8 (2.4 to 3.2)	2.23 (1.88 to 2.62)	1.22 (1.05 to 1.41)
2005	0.0 (0.0 to 0.0)	0.0 (0.0 to 0.0)	5.0 (4.0 to 6.3)	2.5 (2.0 to 3.1)	2.55 (2.01 to 3.21)	1.27 (1.04 to 1.55)
2010	0.0 (0.0 to 0.0)	0.0 (0.0 to 0.0)	4.6 (3.5 to 6.0)	2.2 (1.7 to 2.8)	2.98 (2.26 to 3.89)	1.43 (1.10 to 1.83)
2015	0.0 (0.0 to 0.0)	0.0 (0.0 to 0.0)	3.8 (3.0 to 4.7)	1.9 (1.5 to 2.3)	2.69 (2.09 to 3.34)	1.31 (1.02 to 1.63)
2020	0.0 (0.0 to 0.0)	0.0 (0.0 to 0.0)	3.6 (2.7 to 4.8)	1.8 (1.3 to 2.3)	2.77 (2.05 to 3.67)	1.37 (1.02 to 1.80)
2021	0.0 (0.0 to 0.0)	0.0 (0.0 to 0.0)	3.8 (2.7 to 5.1)	1.9 (1.4 to 2.5)	2.84 (2.04 to 3.82)	1.39 (1.01 to 1.85)

Bournemouth GBR_44790 Western Europe Stillbirth Rate / Neonatal Mortality Rate (SBR/NMR) − 20 weeks
lambda = 0.8 ; zeta = 0.7 ; scale = 15 ; amp = 0.028

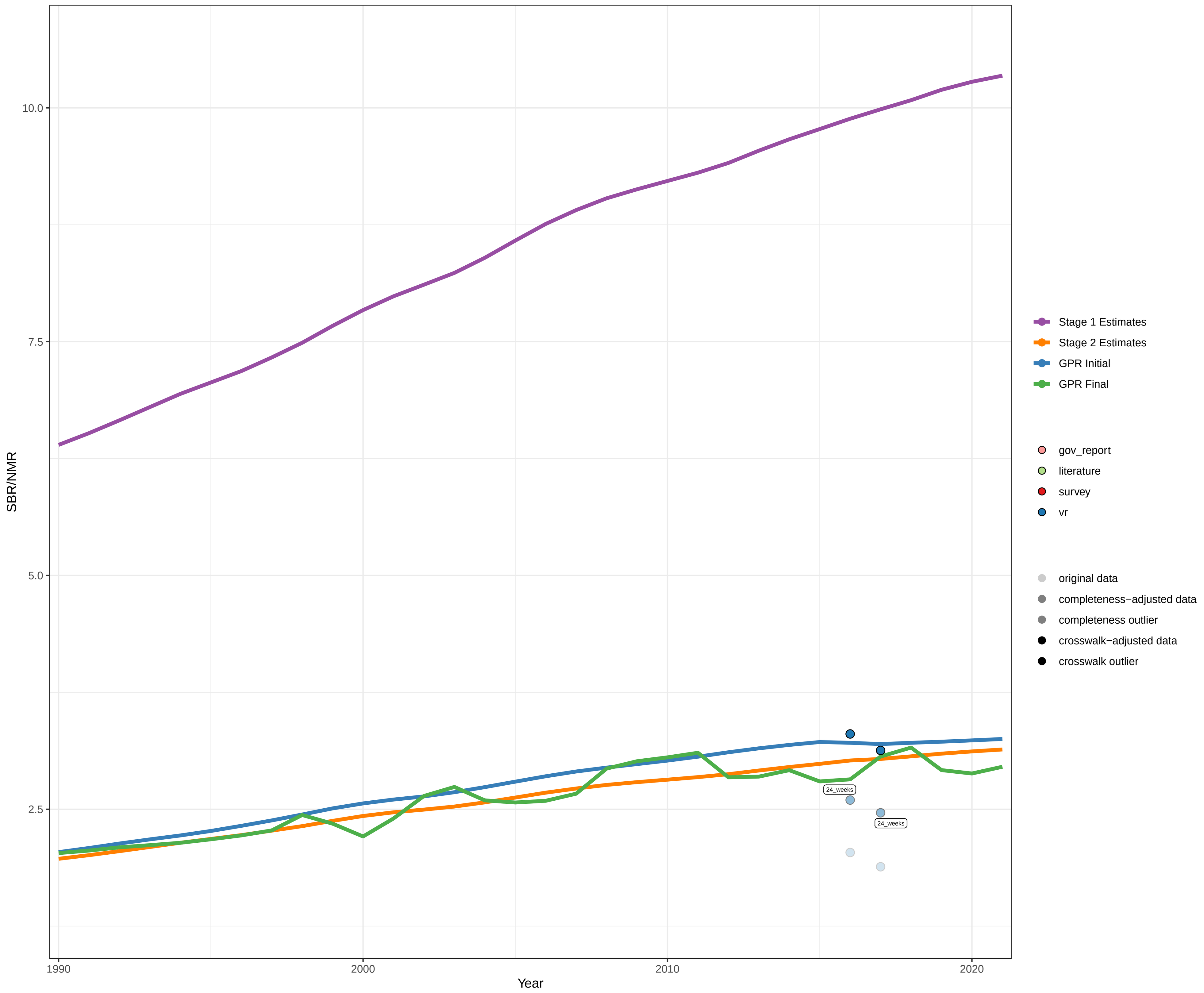

Bournemouth GBR_44790 Western Europe Stillbirth Rate / Neonatal Mortality Rate (SBR/NMR) − 28 weeks
lambda = 0.8 ; zeta = 0.7 ; scale = 15 ; amp = 0.028

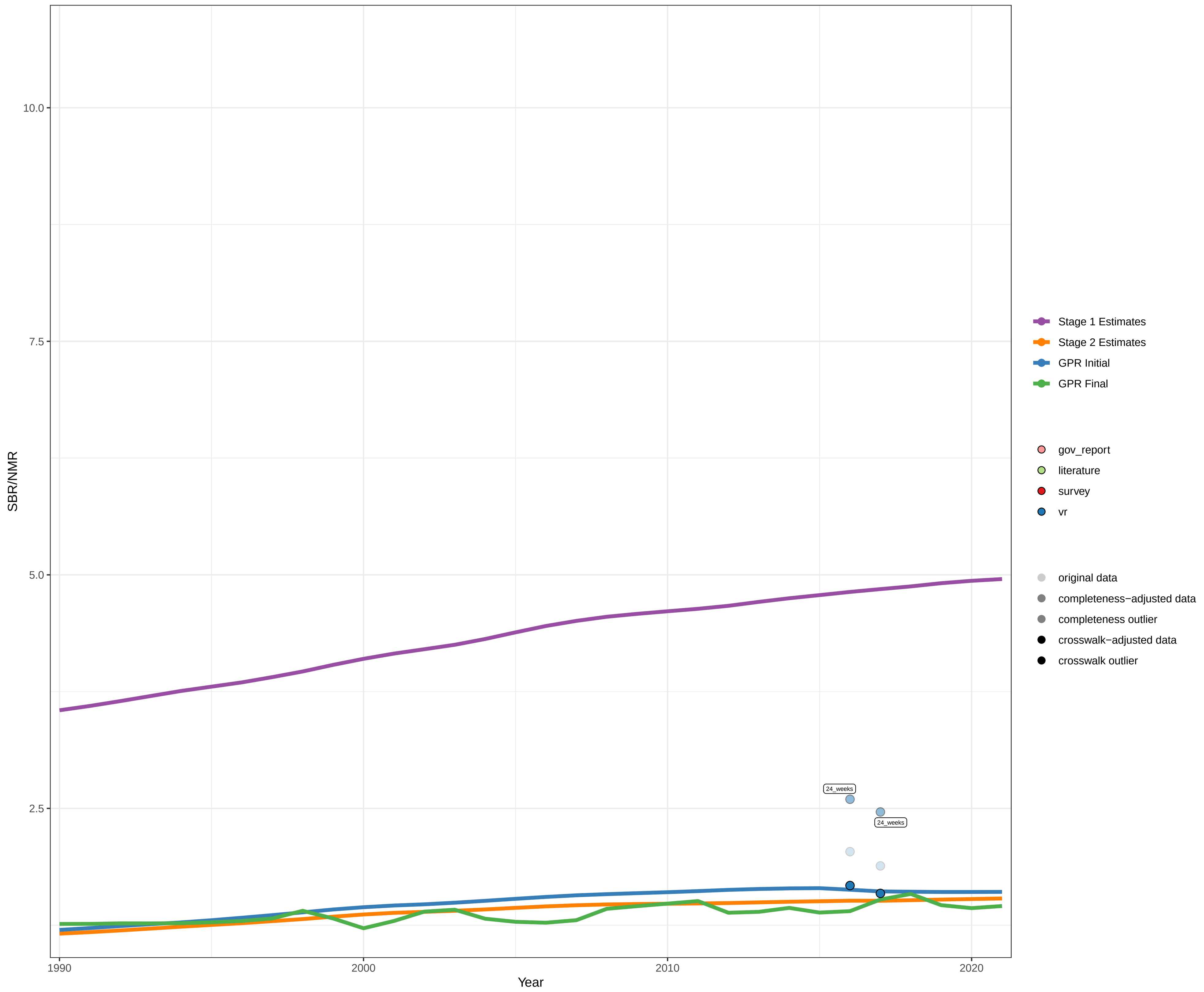

Definition	Years	Outlier	Source.Type	Title
24_weeks	2016, 2017	0	VR	United Kingdom − England and Wales Child Mortality Statistics

Bournemouth GBR_44790 Western Europe Stillbirth Rate (SBR) − 20 weeks
lambda = 0.8 ; zeta = 0.7 ; scale = 15 ; amp = 0.028

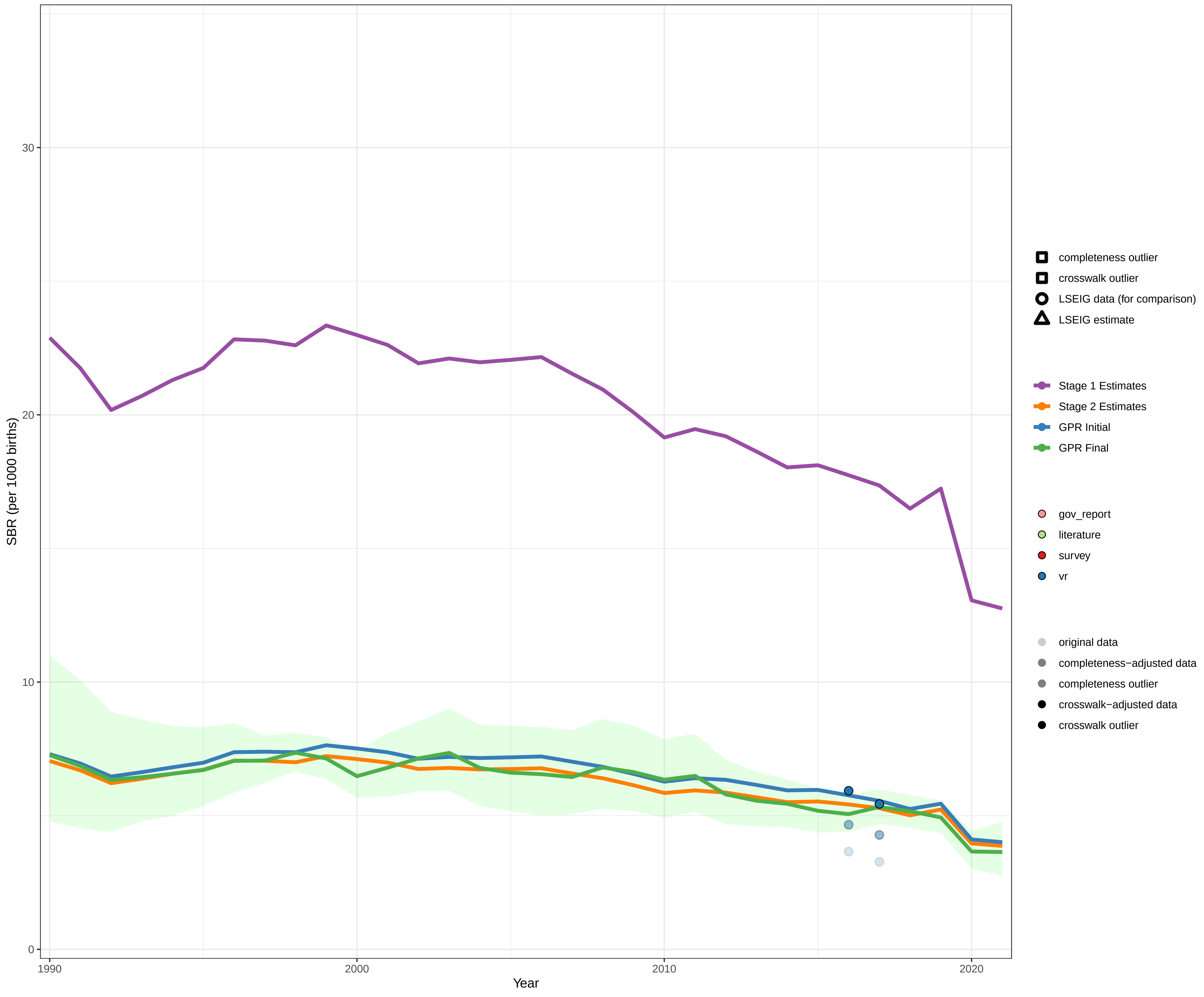

Bournemouth GBR_44790 Western Europe Stillbirth Rate (SBR) − 28 weeks
lambda = 0.8 ; zeta = 0.7 ; scale = 15 ; amp = 0.028

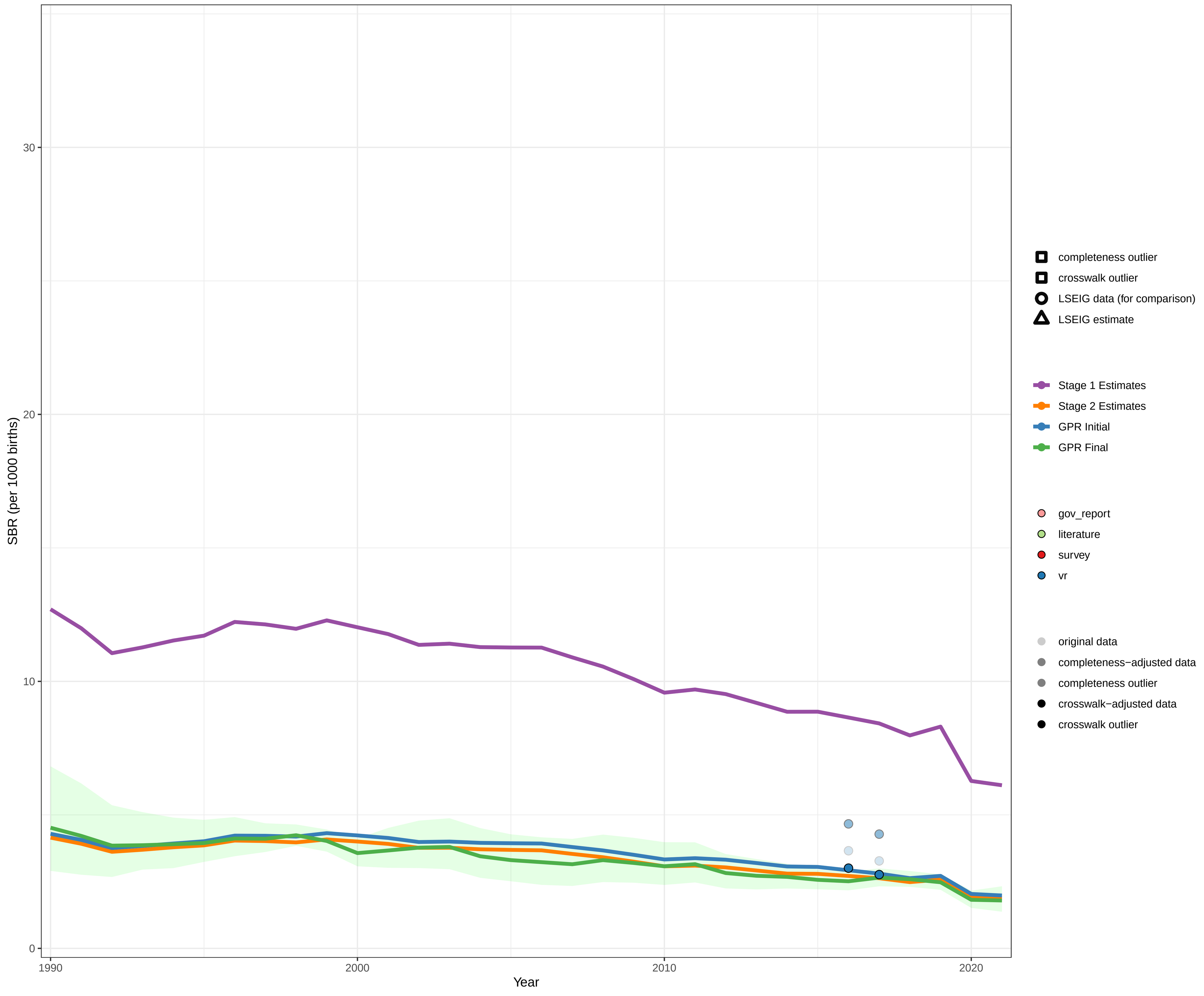

Year	Total Stillbirths (in thousands)	Total Stillbirths (in thousands)	SBR (per 1000 births)	SBR (per 1000 births)	SBR/NMR	SBR/NMR
	20 weeks	28 weeks	20 weeks	28 weeks	20 weeks	28 weeks
1990	0.0 (0.0 to 0.0)	0.0 (0.0 to 0.0)	7.3 (4.8 to 11.0)	4.5 (2.9 to 6.8)	2.03 (1.34 to 3.07)	1.26 (0.81 to 1.91)
1995	0.0 (0.0 to 0.0)	0.0 (0.0 to 0.0)	6.7 (5.4 to 8.3)	3.9 (3.2 to 4.8)	2.18 (1.74 to 2.70)	1.28 (1.05 to 1.56)
2000	0.0 (0.0 to 0.0)	0.0 (0.0 to 0.0)	6.5 (5.7 to 7.4)	3.6 (3.1 to 4.1)	2.21 (1.94 to 2.53)	1.22 (1.05 to 1.40)
2005	0.0 (0.0 to 0.0)	0.0 (0.0 to 0.0)	6.6 (5.2 to 8.4)	3.3 (2.5 to 4.3)	2.57 (2.01 to 3.25)	1.29 (0.98 to 1.66)
2010	0.0 (0.0 to 0.0)	0.0 (0.0 to 0.0)	6.3 (4.9 to 7.9)	3.1 (2.4 to 4.0)	3.05 (2.37 to 3.79)	1.48 (1.14 to 1.91)
2015	0.0 (0.0 to 0.0)	0.0 (0.0 to 0.0)	5.2 (4.4 to 6.0)	2.6 (2.2 to 3.0)	2.80 (2.36 to 3.25)	1.39 (1.20 to 1.59)
2020	0.0 (0.0 to 0.0)	0.0 (0.0 to 0.0)	3.7 (3.0 to 4.4)	1.8 (1.5 to 2.2)	2.88 (2.38 to 3.48)	1.43 (1.19 to 1.70)
2021	0.0 (0.0 to 0.0)	0.0 (0.0 to 0.0)	3.6 (2.7 to 4.8)	1.8 (1.4 to 2.3)	2.95 (2.23 to 3.87)	1.45 (1.11 to 1.89)

Bristol, City of GBR_44789 Western Europe Stillbirth Rate / Neonatal Mortality Rate (SBR/NMR) − 20 weeks
lambda = 0.6 ; zeta = 0.8 ; scale = 15 ; amp = 0.028

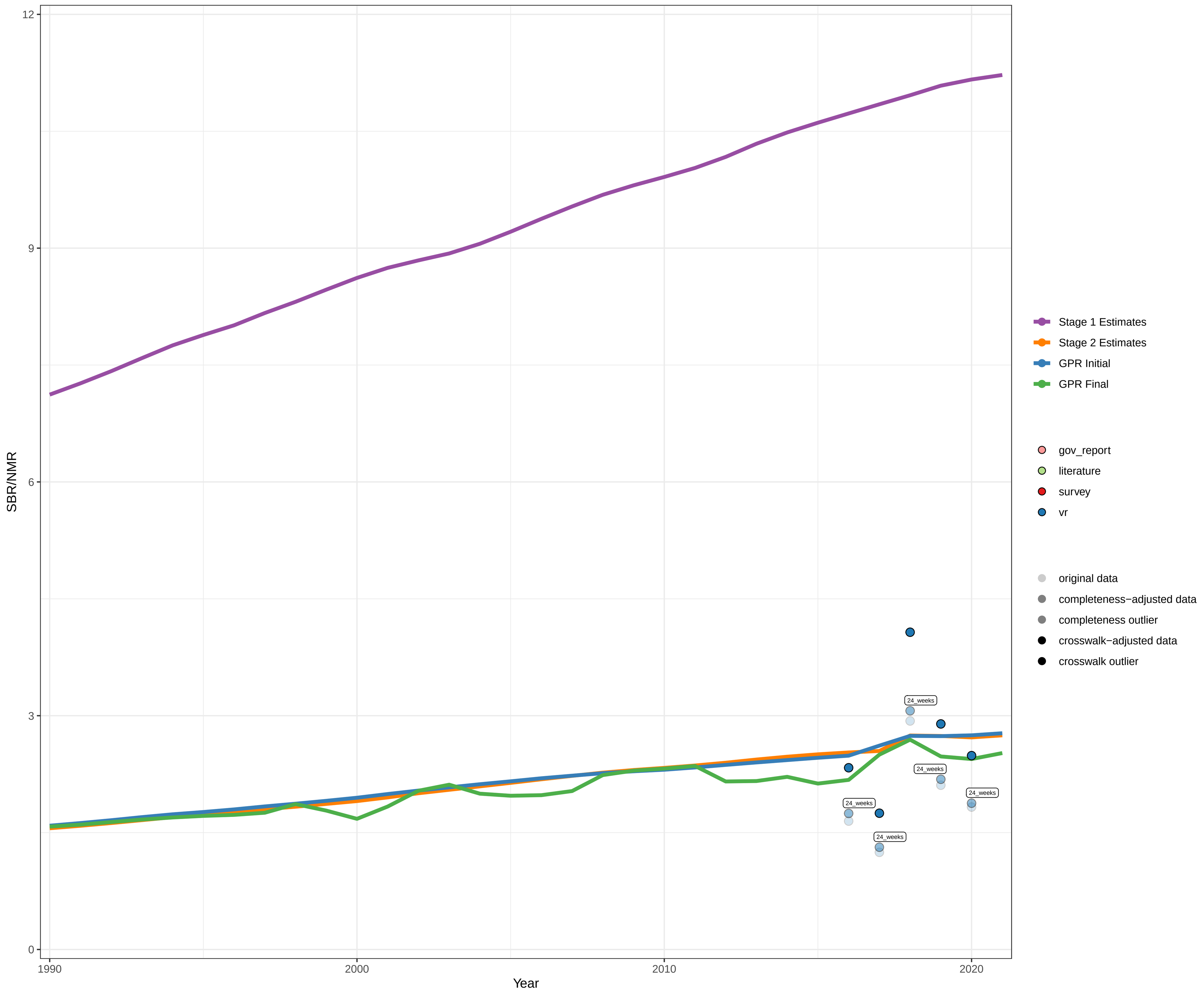

Bristol, City of GBR_44789 Western Europe Stillbirth Rate / Neonatal Mortality Rate (SBR/NMR) − 28 weeks
lambda = 0.6 ; zeta = 0.8 ; scale = 15 ; amp = 0.028

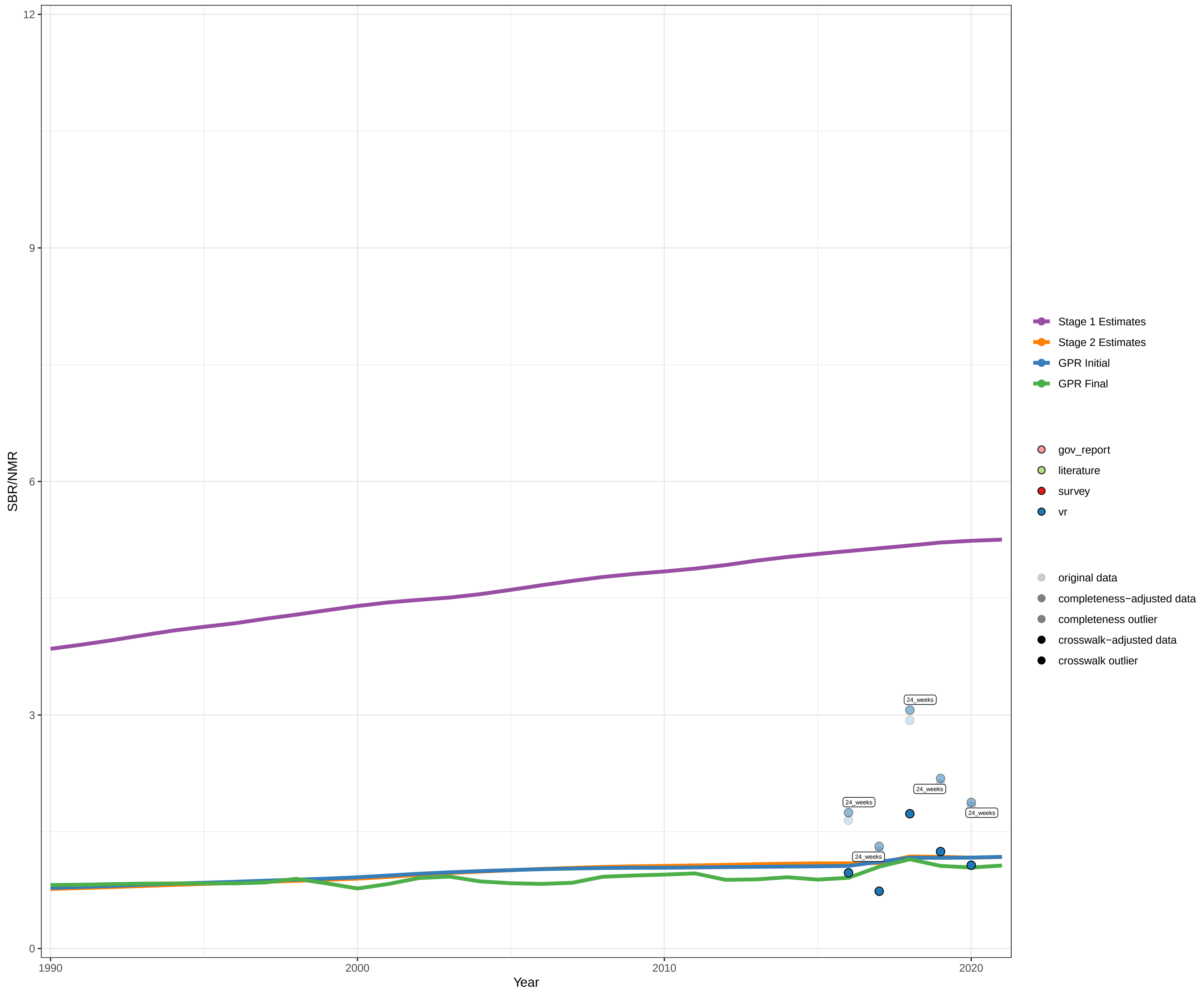

Definition	Years	Outlier	Source.Type	Title
24_weeks	2016−2020	0	VR	United Kingdom − England and Wales Child Mortality Statistics

Bristol, City of GBR_44789 Western Europe Stillbirth Rate (SBR) − 20 weeks
lambda = 0.6 ; zeta = 0.8 ; scale = 15 ; amp = 0.028

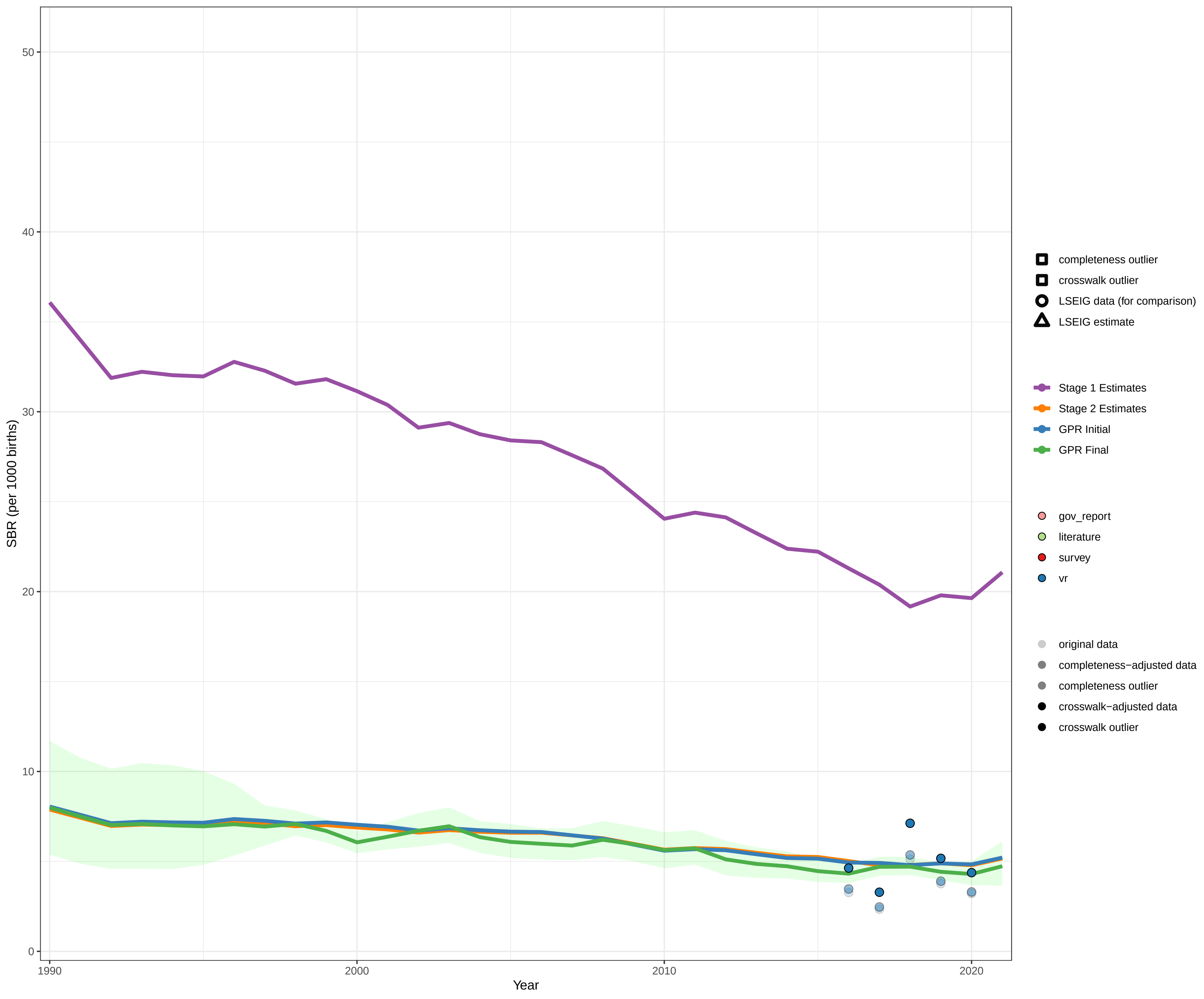

Bristol, City of GBR_44789 Western Europe Stillbirth Rate (SBR) − 28 weeks
lambda = 0.6 ; zeta = 0.8 ; scale = 15 ; amp = 0.028

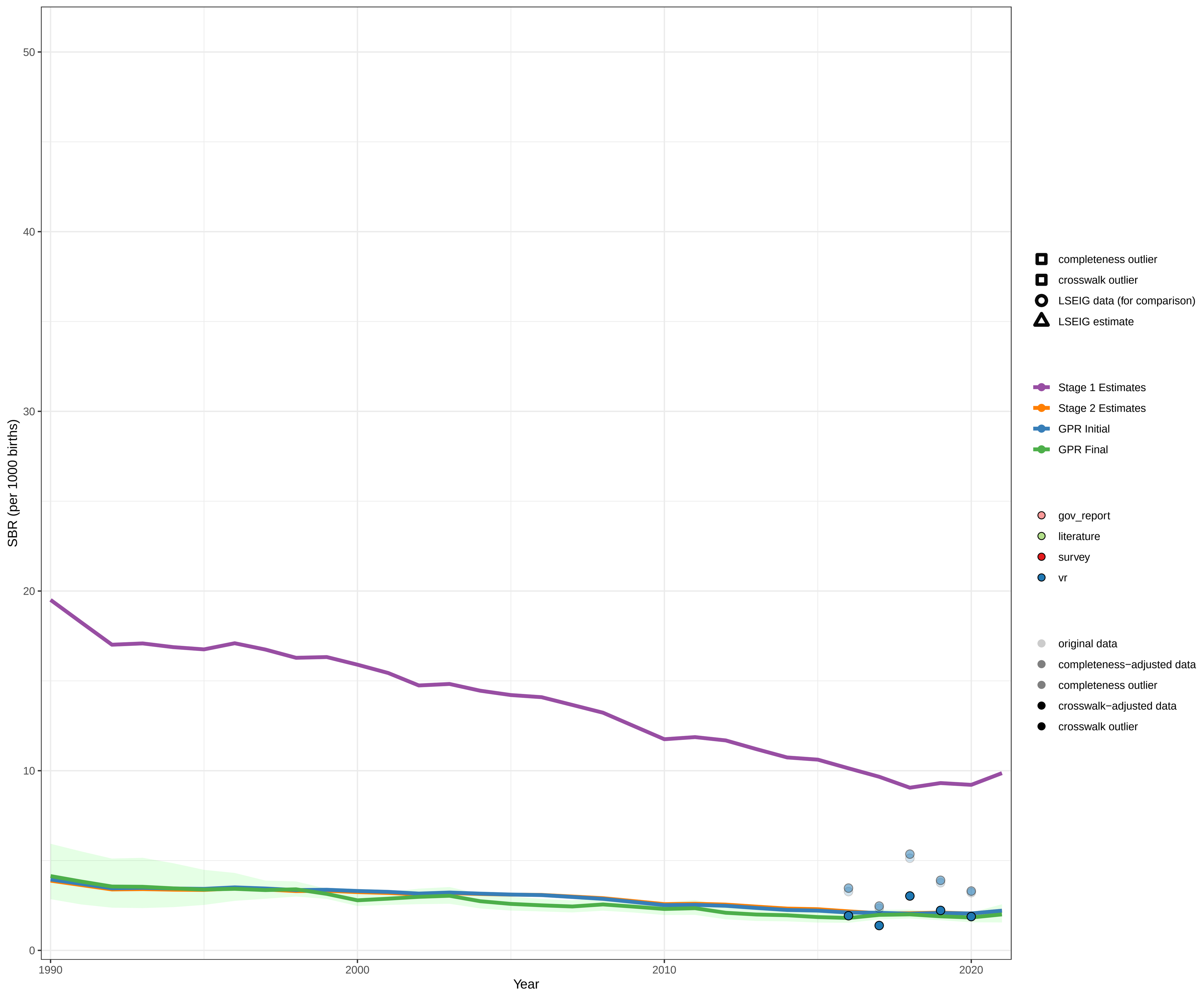

Year	Total Stillbirths (in thousands)	Total Stillbirths (in thousands)	SBR (per 1000 births)	SBR (per 1000 births)	SBR/NMR	SBR/NMR
	20 weeks	28 weeks	20 weeks	28 weeks	20 weeks	28 weeks
1990	0.0 (0.0 to 0.1)	0.0 (0.0 to 0.0)	8.0 (5.4 to 11.7)	4.1 (2.9 to 5.9)	1.58 (1.06 to 2.31)	0.82 (0.56 to 1.17)
1995	0.0 (0.0 to 0.1)	0.0 (0.0 to 0.0)	6.9 (4.8 to 10.0)	3.4 (2.5 to 4.5)	1.71 (1.18 to 2.47)	0.84 (0.62 to 1.11)
2000	0.0 (0.0 to 0.0)	0.0 (0.0 to 0.0)	6.1 (5.5 to 6.7)	2.8 (2.5 to 3.1)	1.68 (1.51 to 1.85)	0.77 (0.68 to 0.87)
2005	0.0 (0.0 to 0.0)	0.0 (0.0 to 0.0)	6.1 (5.2 to 7.1)	2.6 (2.2 to 3.0)	1.97 (1.69 to 2.29)	0.84 (0.72 to 0.97)
2010	0.0 (0.0 to 0.0)	0.0 (0.0 to 0.0)	5.6 (4.6 to 6.6)	2.3 (2.0 to 2.7)	2.32 (1.90 to 2.73)	0.95 (0.81 to 1.10)
2015	0.0 (0.0 to 0.0)	0.0 (0.0 to 0.0)	4.5 (3.9 to 5.1)	1.9 (1.5 to 2.2)	2.13 (1.84 to 2.44)	0.89 (0.74 to 1.06)
2020	0.0 (0.0 to 0.0)	0.0 (0.0 to 0.0)	4.3 (3.7 to 5.0)	1.8 (1.6 to 2.1)	2.44 (2.09 to 2.85)	1.04 (0.89 to 1.20)
2021	0.0 (0.0 to 0.0)	0.0 (0.0 to 0.0)	4.7 (3.6 to 6.1)	2.0 (1.6 to 2.6)	2.52 (1.94 to 3.26)	1.07 (0.83 to 1.37)

Cornwall GBR_44791 Western Europe Stillbirth Rate / Neonatal Mortality Rate (SBR/NMR) − 20 weeks
lambda = 1 ; zeta = 0.6 ; scale = 15 ; amp = 0.028

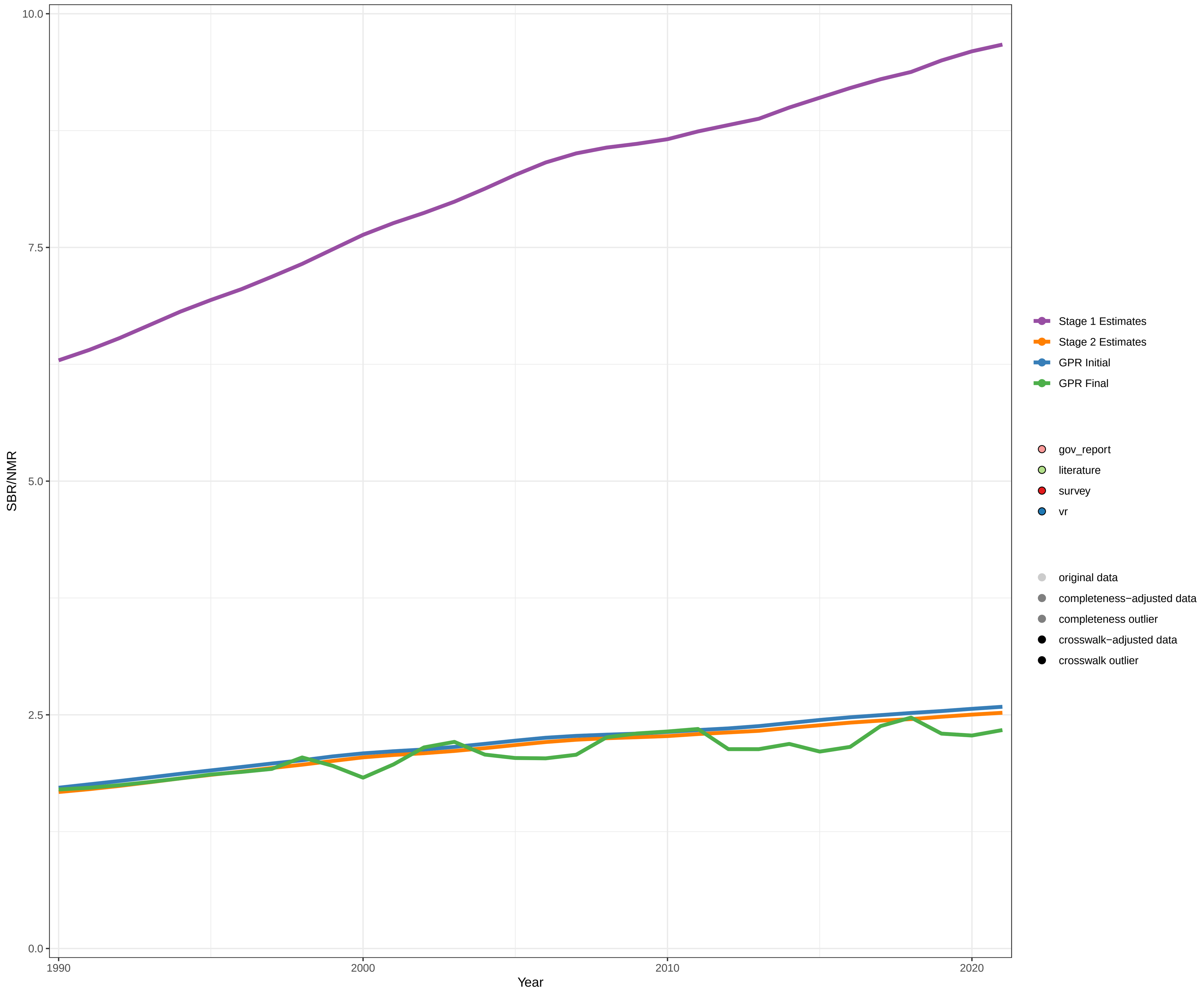

Cornwall GBR_44791 Western Europe Stillbirth Rate / Neonatal Mortality Rate (SBR/NMR) − 28 weeks
lambda = 1 ; zeta = 0.6 ; scale = 15 ; amp = 0.028

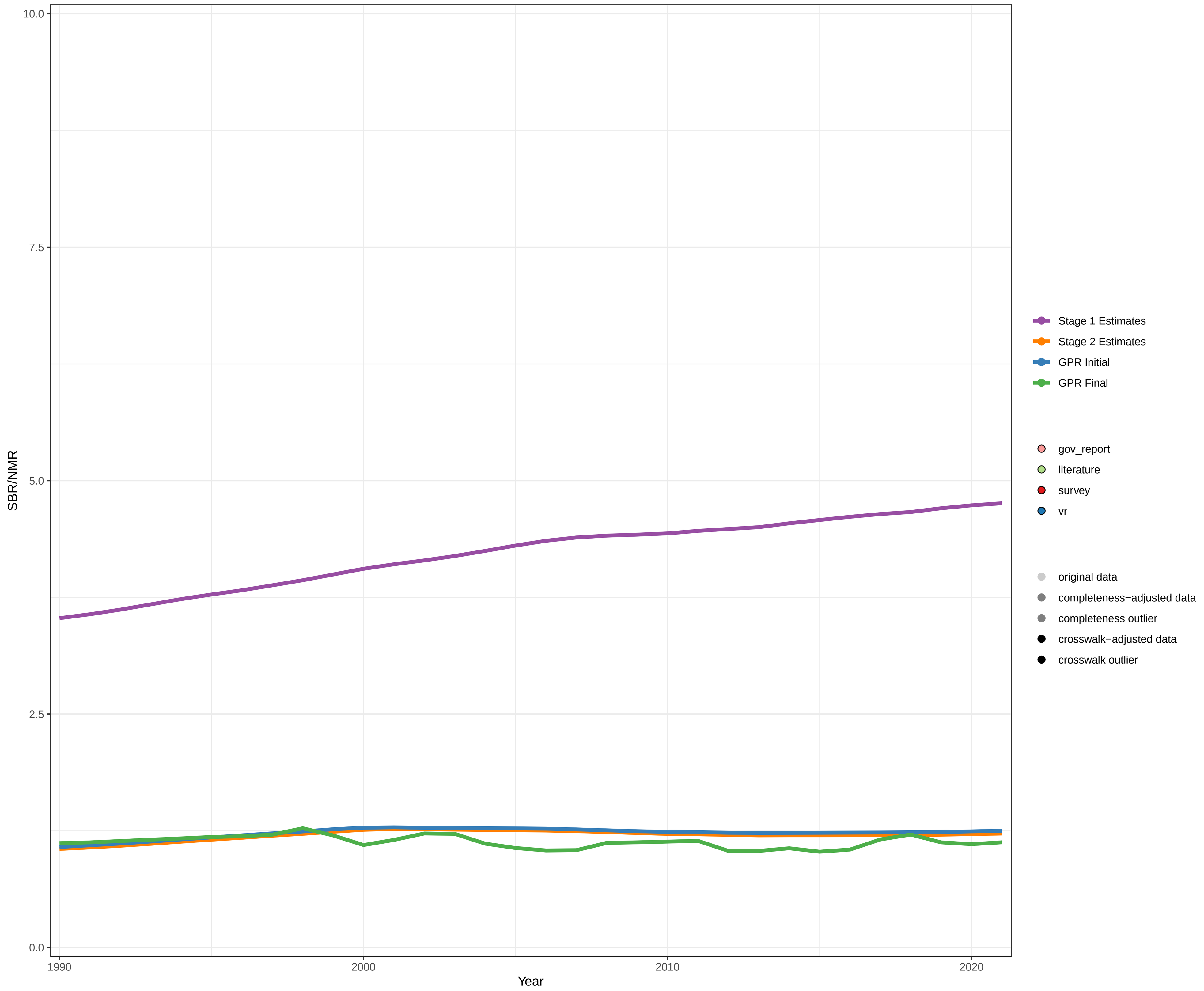

Definition	Years	Outlier	Source.Type	Title

Cornwall GBR_44791 Western Europe Stillbirth Rate (SBR) − 20 weeks
lambda = 1 ; zeta = 0.6 ; scale = 15 ; amp = 0.028

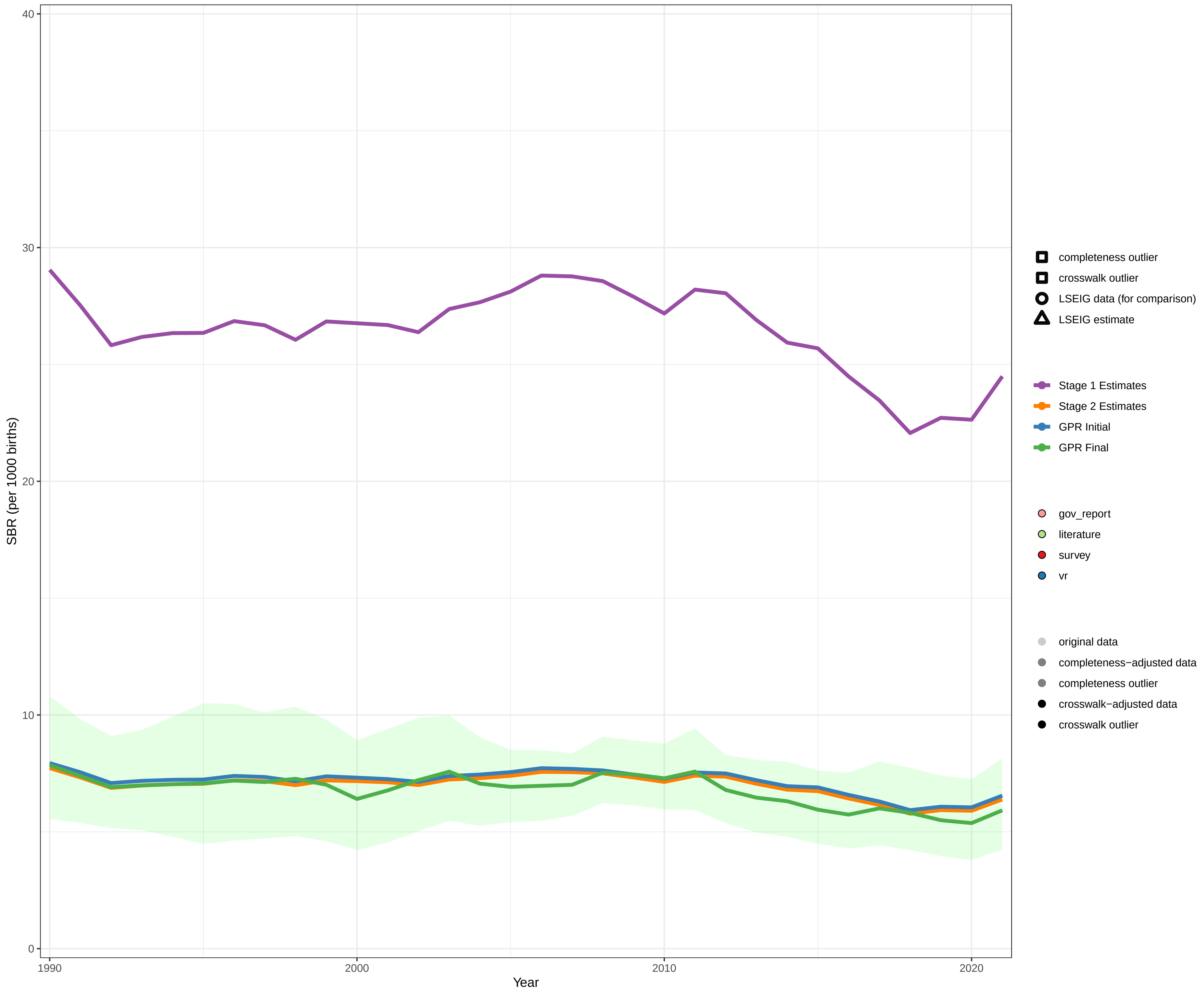

Cornwall GBR_44791 Western Europe Stillbirth Rate (SBR) − 28 weeks
lambda = 1 ; zeta = 0.6 ; scale = 15 ; amp = 0.028

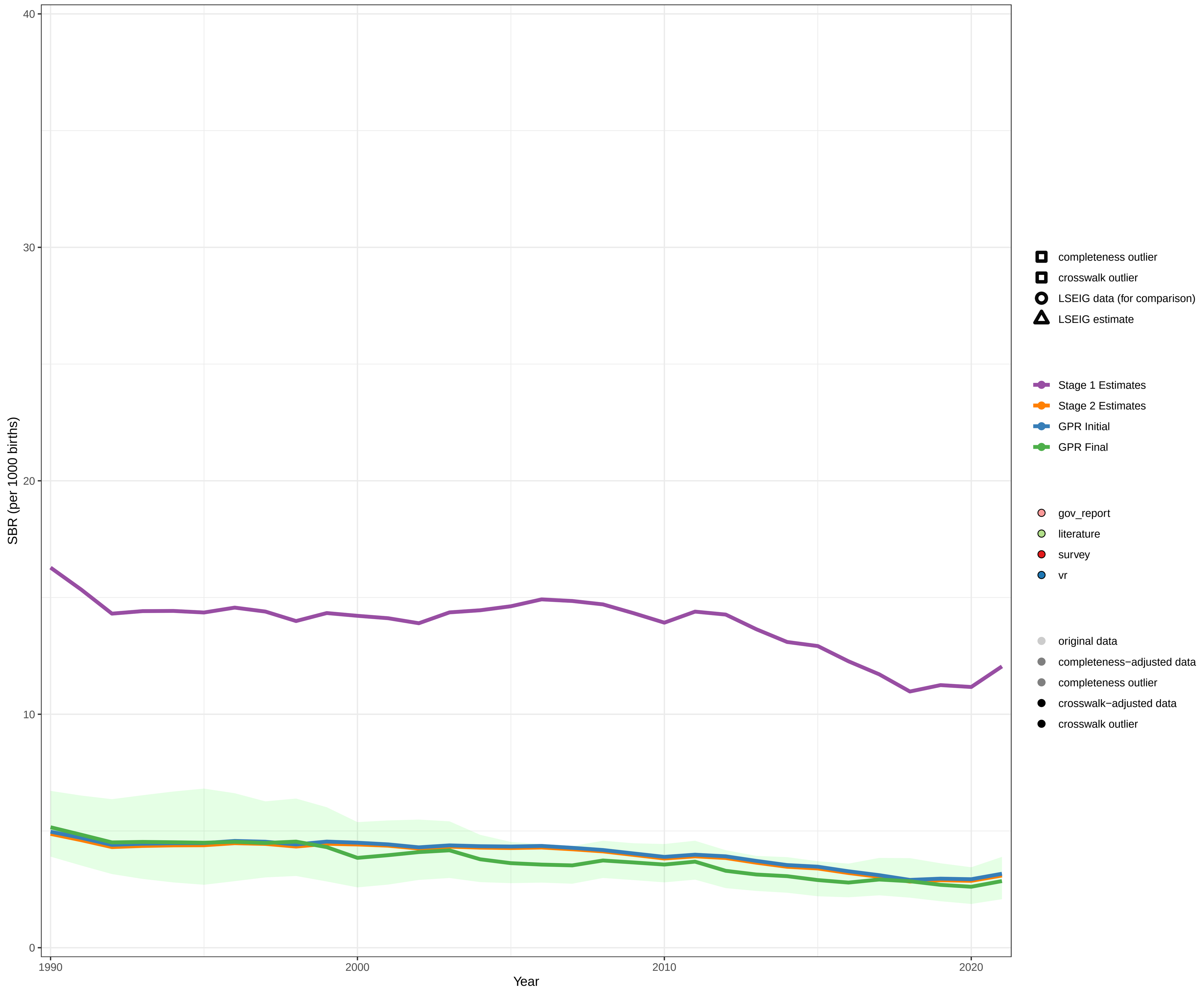

Year	Total Stillbirths (in thousands)	Total Stillbirths (in thousands)	SBR (per 1000 births)	SBR (per 1000 births)	SBR/NMR	SBR/NMR
	20 weeks	28 weeks	20 weeks	28 weeks	20 weeks	28 weeks
1990	0.0 (0.0 to 0.1)	0.0 (0.0 to 0.0)	7.9 (5.5 to 10.8)	5.2 (3.9 to 6.7)	1.70 (1.20 to 2.34)	1.12 (0.85 to 1.46)
1995	0.0 (0.0 to 0.1)	0.0 (0.0 to 0.0)	7.1 (4.5 to 10.5)	4.5 (2.7 to 6.8)	1.86 (1.18 to 2.77)	1.18 (0.71 to 1.79)
2000	0.0 (0.0 to 0.0)	0.0 (0.0 to 0.0)	6.4 (4.2 to 8.9)	3.8 (2.6 to 5.4)	1.83 (1.20 to 2.55)	1.10 (0.74 to 1.53)
2005	0.0 (0.0 to 0.0)	0.0 (0.0 to 0.0)	6.9 (5.4 to 8.5)	3.6 (2.8 to 4.5)	2.04 (1.59 to 2.51)	1.07 (0.81 to 1.33)
2010	0.0 (0.0 to 0.0)	0.0 (0.0 to 0.0)	7.3 (6.0 to 8.8)	3.6 (2.8 to 4.4)	2.32 (1.90 to 2.80)	1.13 (0.89 to 1.41)
2015	0.0 (0.0 to 0.0)	0.0 (0.0 to 0.0)	5.9 (4.5 to 7.6)	2.9 (2.2 to 3.7)	2.11 (1.59 to 2.70)	1.03 (0.78 to 1.31)
2020	0.0 (0.0 to 0.0)	0.0 (0.0 to 0.0)	5.4 (3.8 to 7.3)	2.6 (1.9 to 3.4)	2.28 (1.61 to 3.08)	1.11 (0.79 to 1.46)
2021	0.0 (0.0 to 0.0)	0.0 (0.0 to 0.0)	5.9 (4.2 to 8.1)	2.9 (2.1 to 3.9)	2.34 (1.67 to 3.21)	1.13 (0.82 to 1.54)

Devon GBR_44782 Western Europe Stillbirth Rate / Neonatal Mortality Rate (SBR/NMR) − 20 weeks
lambda = 0.6 ; zeta = 0.8 ; scale = 15 ; amp = 0.028

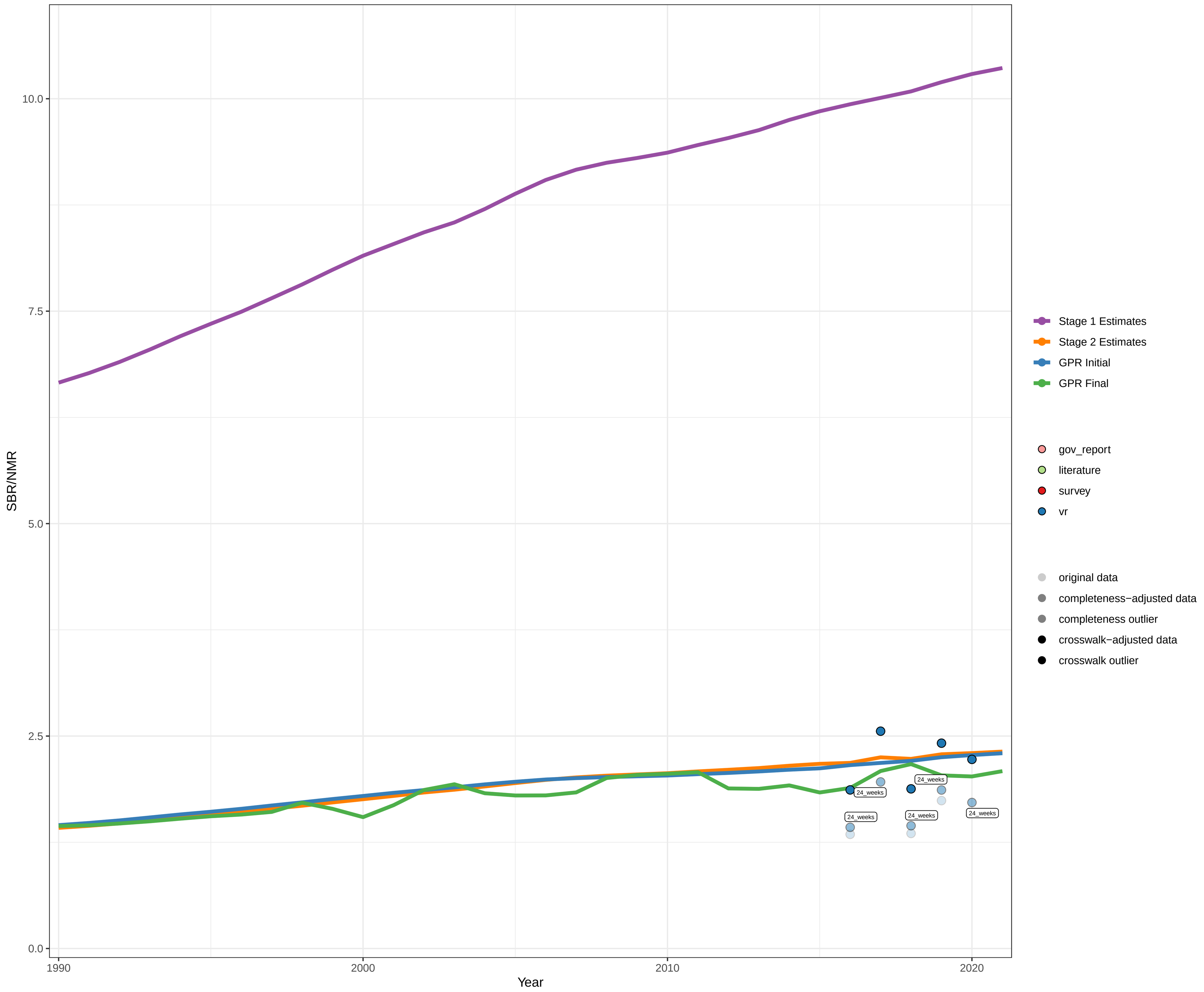

Devon GBR_44782 Western Europe Stillbirth Rate / Neonatal Mortality Rate (SBR/NMR) − 28 weeks
lambda = 0.6 ; zeta = 0.8 ; scale = 15 ; amp = 0.028

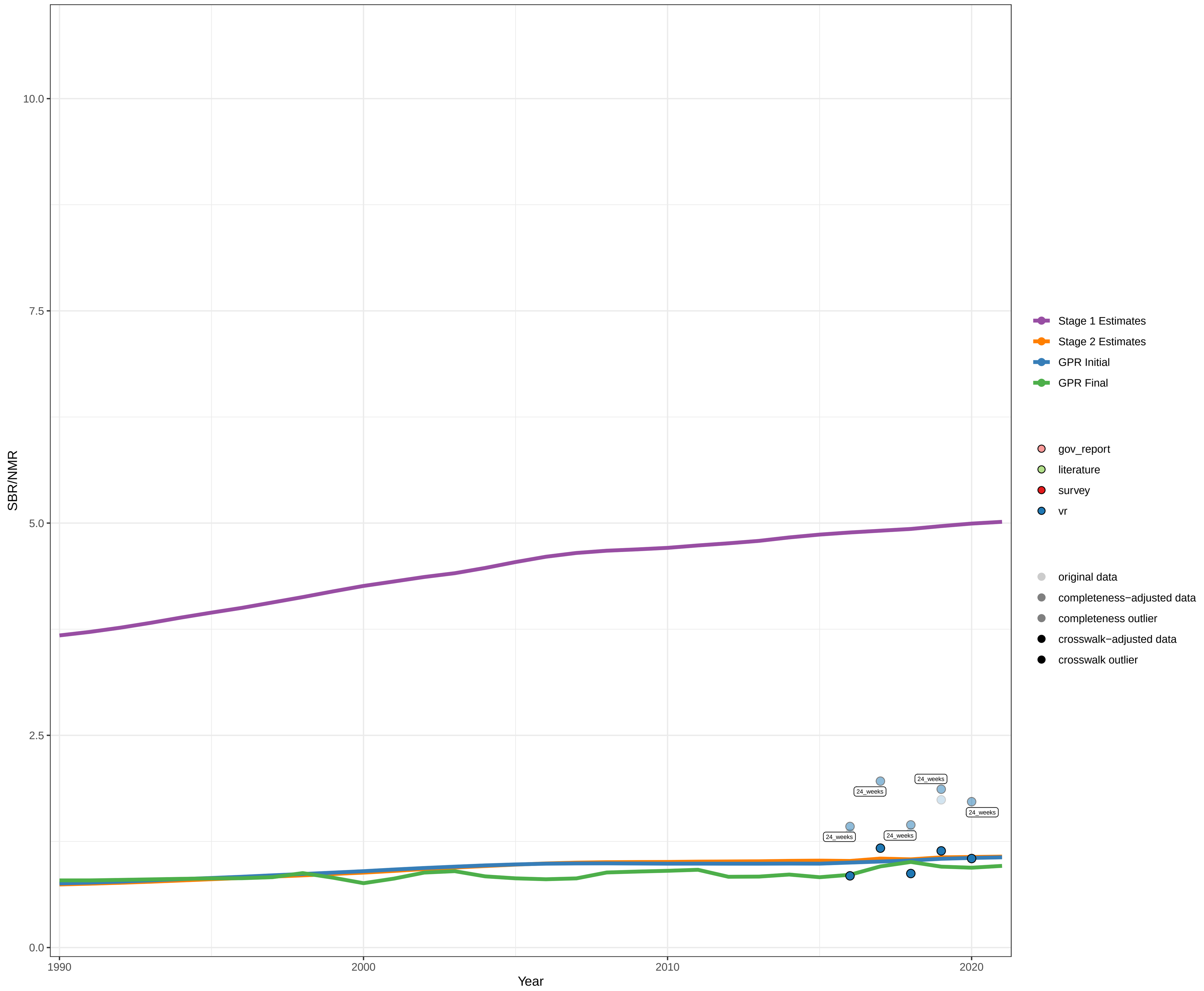

Definition	Years	Outlier	Source.Type	Title
24_weeks	2016−2020	0	VR	United Kingdom − England and Wales Child Mortality Statistics

Devon GBR_44782 Western Europe Stillbirth Rate (SBR) − 20 weeks
lambda = 0.6 ; zeta = 0.8 ; scale = 15 ; amp = 0.028

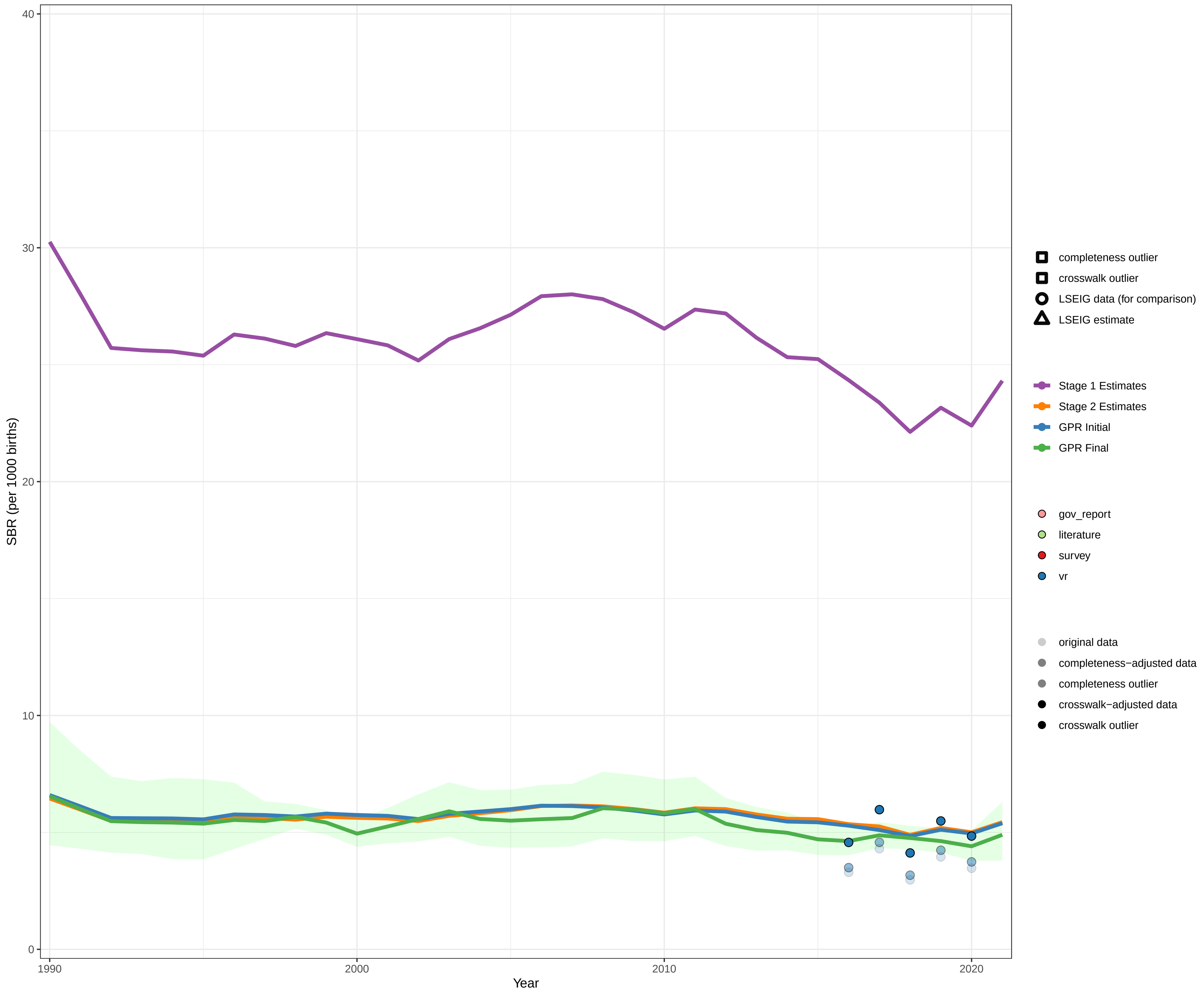

Devon GBR_44782 Western Europe Stillbirth Rate (SBR) − 28 weeks
lambda = 0.6 ; zeta = 0.8 ; scale = 15 ; amp = 0.028

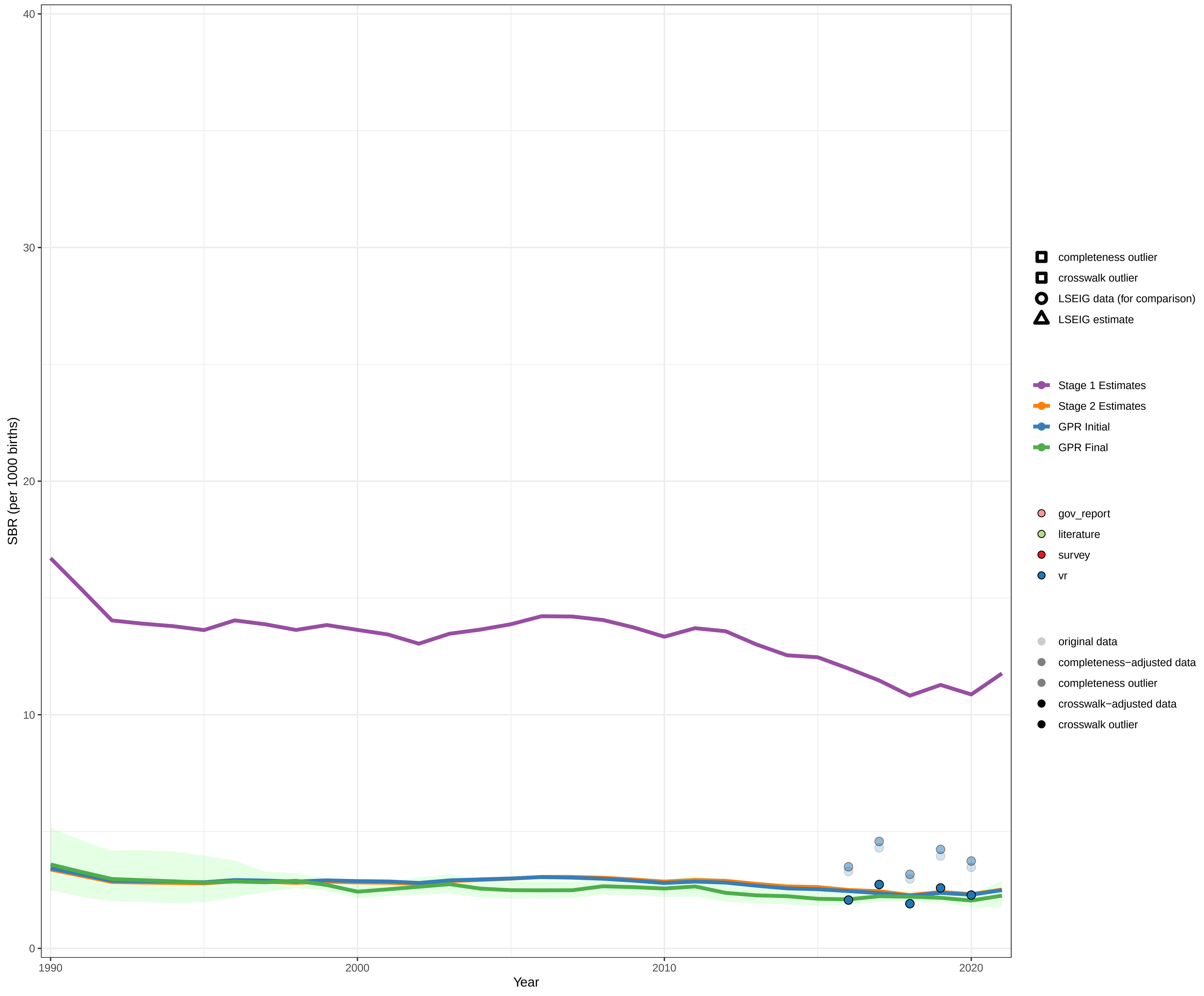

Year	Total Stillbirths (in thousands)	Total Stillbirths (in thousands)	SBR (per 1000 births)	SBR (per 1000 births)	SBR/NMR	SBR/NMR
	20 weeks	28 weeks	20 weeks	28 weeks	20 weeks	28 weeks
1990	0.0 (0.0 to 0.1)	0.0 (0.0 to 0.0)	6.5 (4.5 to 9.7)	3.6 (2.5 to 5.2)	1.44 (0.98 to 2.14)	0.79 (0.55 to 1.13)
1995	0.0 (0.0 to 0.1)	0.0 (0.0 to 0.0)	5.4 (3.8 to 7.3)	2.8 (2.0 to 4.0)	1.56 (1.11 to 2.11)	0.81 (0.57 to 1.15)
2000	0.0 (0.0 to 0.0)	0.0 (0.0 to 0.0)	4.9 (4.4 to 5.5)	2.4 (2.1 to 2.7)	1.55 (1.37 to 1.72)	0.76 (0.67 to 0.85)
2005	0.0 (0.0 to 0.0)	0.0 (0.0 to 0.0)	5.5 (4.3 to 6.8)	2.5 (2.1 to 2.9)	1.80 (1.42 to 2.24)	0.82 (0.70 to 0.94)
2010	0.0 (0.0 to 0.1)	0.0 (0.0 to 0.0)	5.8 (4.6 to 7.3)	2.6 (2.2 to 2.9)	2.06 (1.63 to 2.56)	0.90 (0.77 to 1.04)
2015	0.0 (0.0 to 0.0)	0.0 (0.0 to 0.0)	4.7 (4.0 to 5.4)	2.1 (1.8 to 2.5)	1.84 (1.57 to 2.11)	0.83 (0.71 to 0.96)
2020	0.0 (0.0 to 0.0)	0.0 (0.0 to 0.0)	4.4 (3.8 to 5.1)	2.0 (1.8 to 2.3)	2.02 (1.74 to 2.34)	0.94 (0.82 to 1.08)
2021	0.0 (0.0 to 0.0)	0.0 (0.0 to 0.0)	4.9 (3.8 to 6.3)	2.3 (1.8 to 2.9)	2.09 (1.62 to 2.68)	0.96 (0.75 to 1.23)

Dorset GBR_44779 Western Europe Stillbirth Rate / Neonatal Mortality Rate (SBR/NMR) − 20 weeks
lambda = 0.6 ; zeta = 0.8 ; scale = 15 ; amp = 0.028

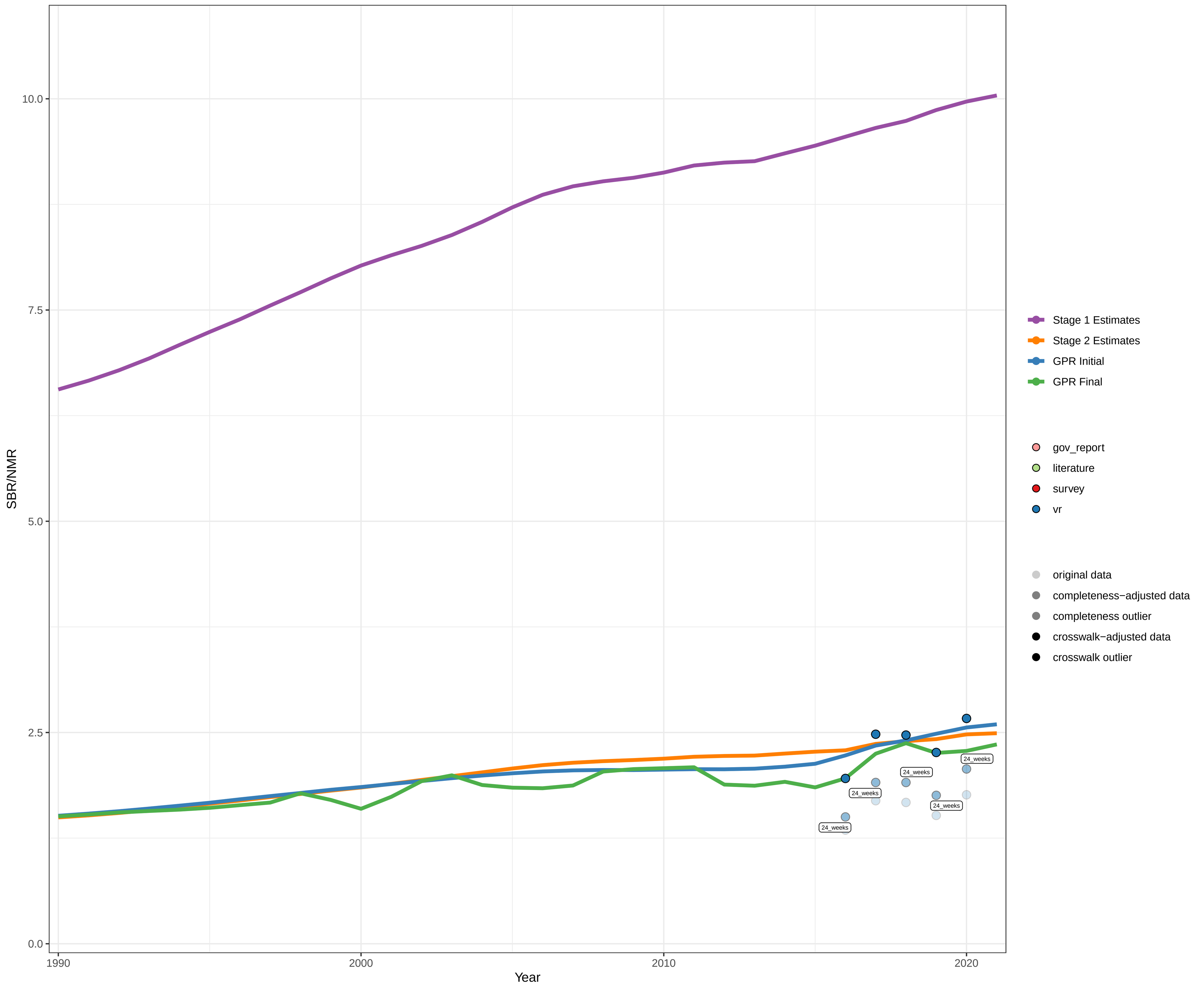

Dorset GBR_44779 Western Europe Stillbirth Rate / Neonatal Mortality Rate (SBR/NMR) − 28 weeks
lambda = 0.6 ; zeta = 0.8 ; scale = 15 ; amp = 0.028

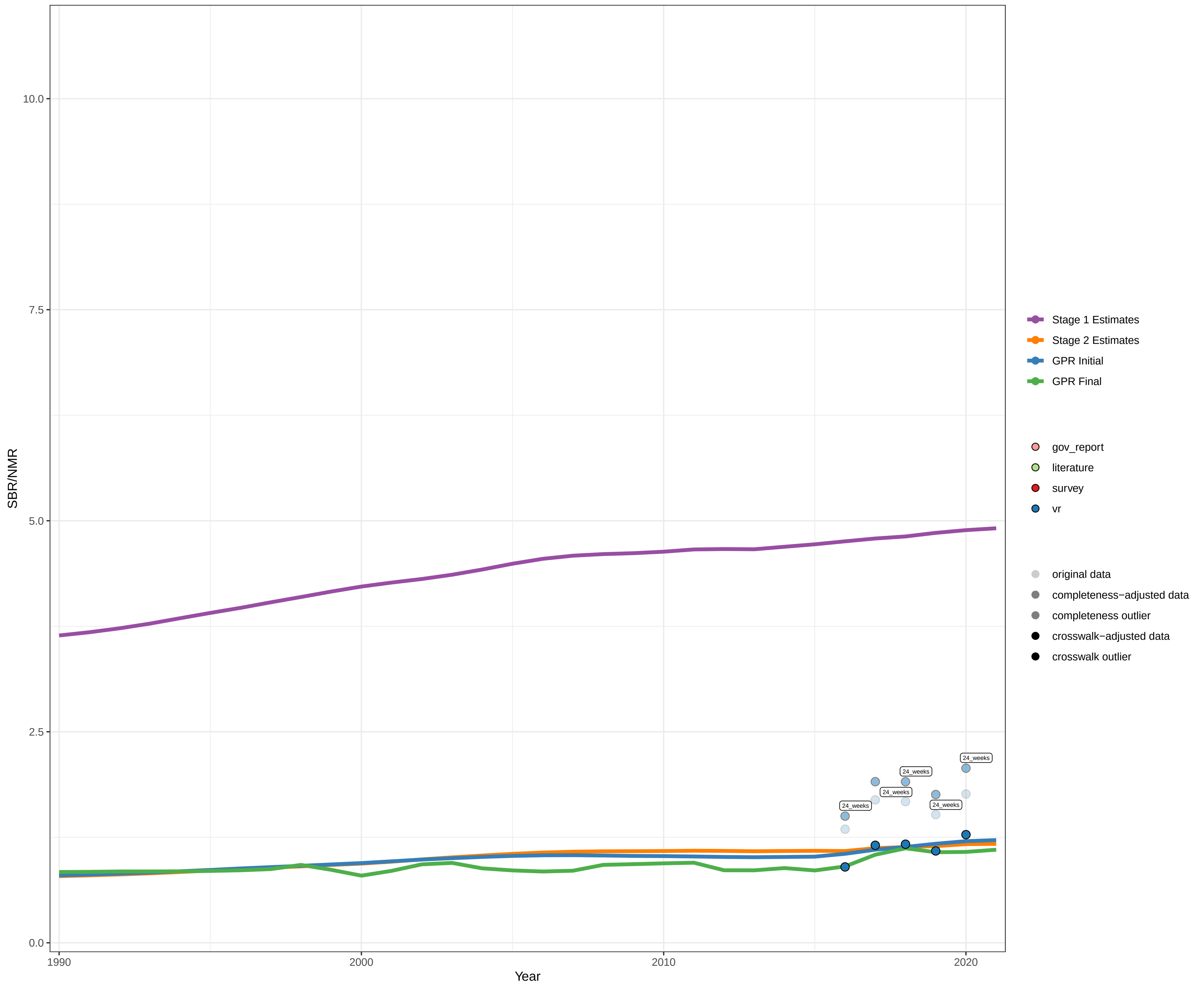

Definition	Years	Outlier	Source.Type	Title
24_weeks	2016−2020	0	VR	United Kingdom − England and Wales Child Mortality Statistics

Dorset GBR_44779 Western Europe Stillbirth Rate (SBR) − 20 weeks
lambda = 0.6 ; zeta = 0.8 ; scale = 15 ; amp = 0.028

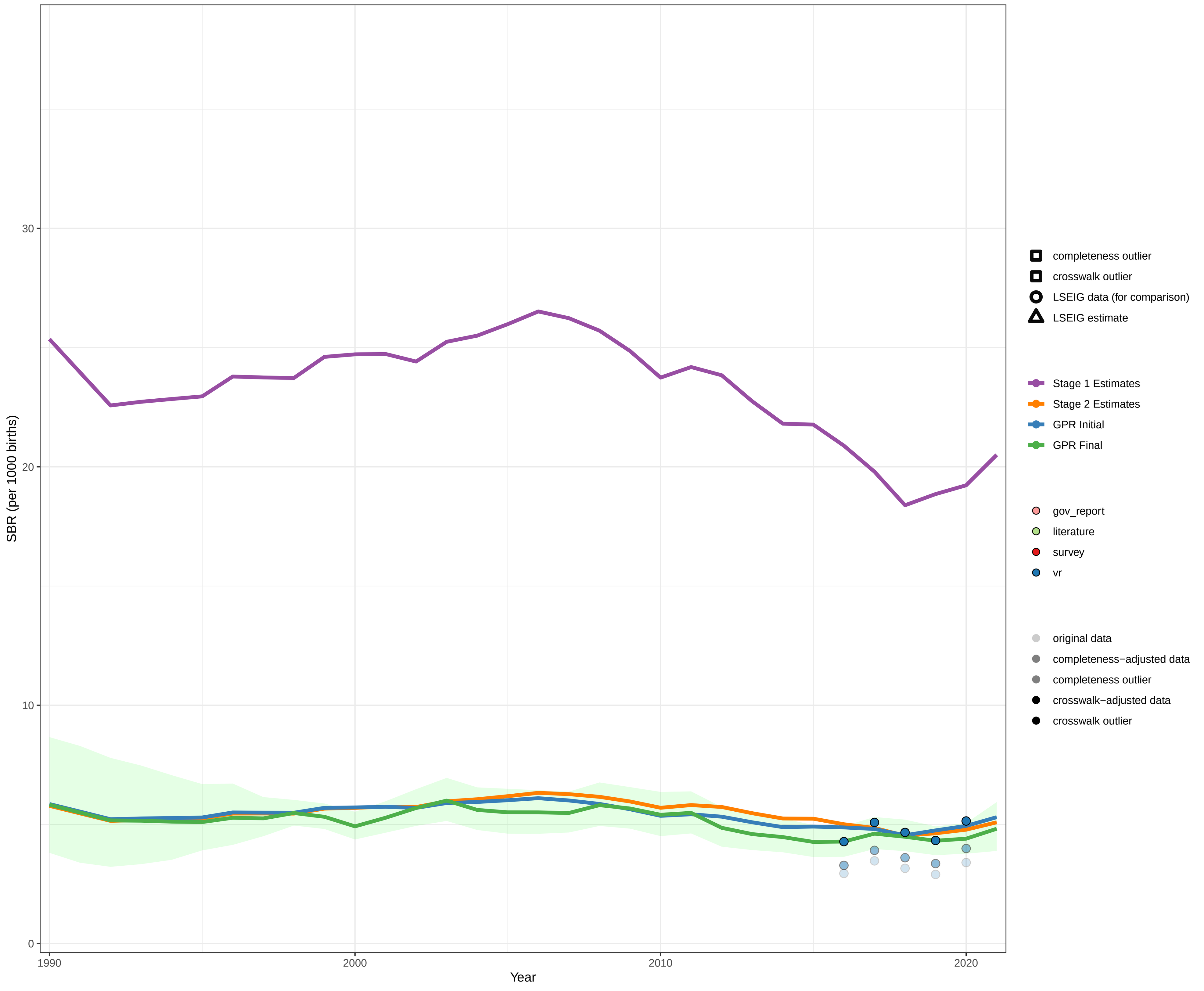

Dorset GBR_44779 Western Europe Stillbirth Rate (SBR) − 28 weeks
lambda = 0.6 ; zeta = 0.8 ; scale = 15 ; amp = 0.028

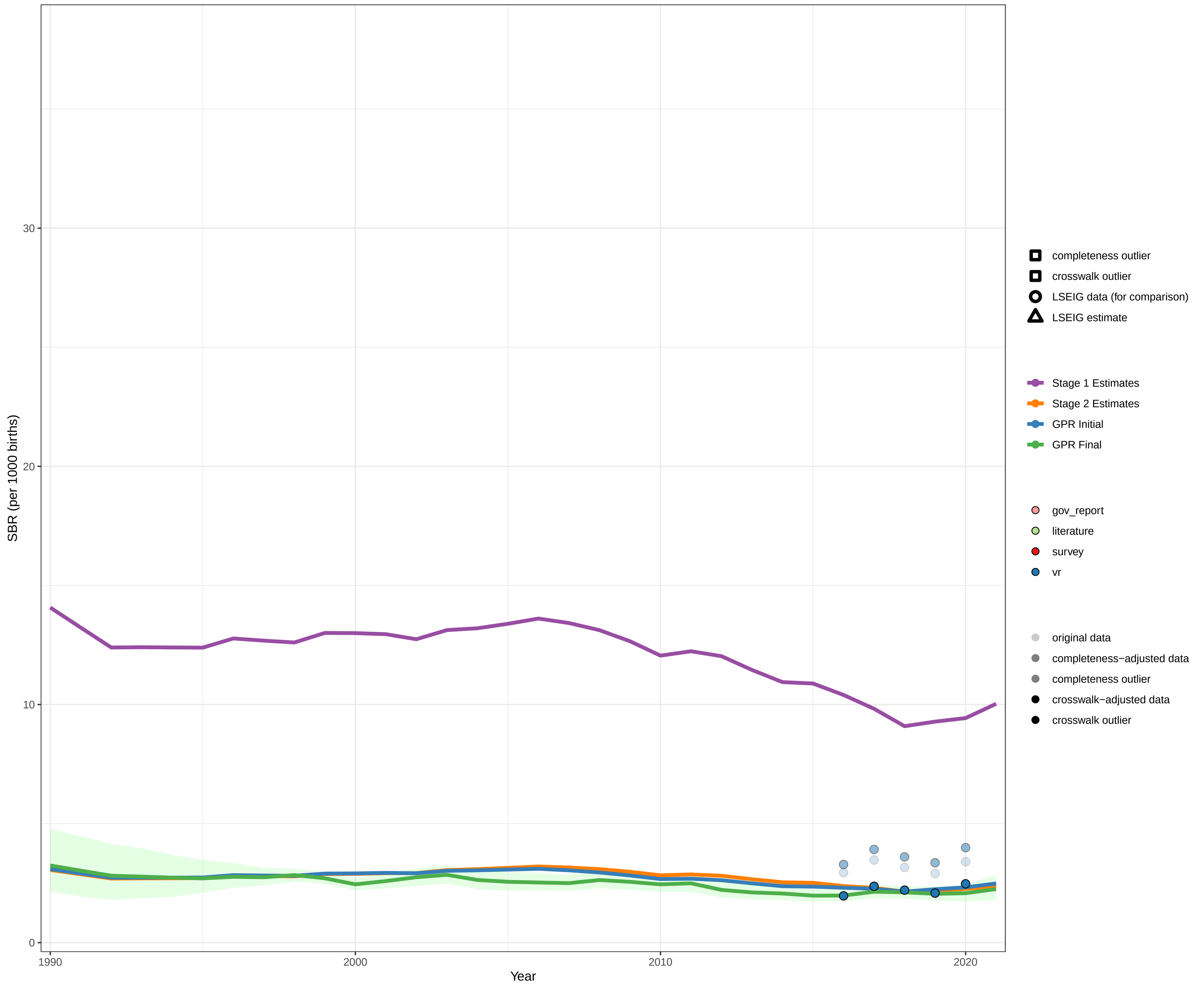

Year	Total Stillbirths (in thousands)	Total Stillbirths (in thousands)	SBR (per 1000 births)	SBR (per 1000 births)	SBR/NMR	SBR/NMR
	20 weeks	28 weeks	20 weeks	28 weeks	20 weeks	28 weeks
1990	0.0 (0.0 to 0.0)	0.0 (0.0 to 0.0)	5.8 (3.8 to 8.7)	3.2 (2.2 to 4.8)	1.51 (0.99 to 2.24)	0.84 (0.56 to 1.23)
1995	0.0 (0.0 to 0.0)	0.0 (0.0 to 0.0)	5.1 (3.9 to 6.7)	2.7 (2.1 to 3.5)	1.61 (1.23 to 2.11)	0.85 (0.66 to 1.10)
2000	0.0 (0.0 to 0.0)	0.0 (0.0 to 0.0)	4.9 (4.4 to 5.5)	2.4 (2.2 to 2.7)	1.60 (1.42 to 1.80)	0.80 (0.72 to 0.88)
2005	0.0 (0.0 to 0.0)	0.0 (0.0 to 0.0)	5.5 (4.6 to 6.5)	2.6 (2.2 to 3.0)	1.85 (1.55 to 2.18)	0.86 (0.73 to 1.00)
2010	0.0 (0.0 to 0.0)	0.0 (0.0 to 0.0)	5.4 (4.5 to 6.4)	2.4 (2.1 to 2.8)	2.08 (1.73 to 2.45)	0.94 (0.82 to 1.09)
2015	0.0 (0.0 to 0.0)	0.0 (0.0 to 0.0)	4.3 (3.6 to 5.0)	2.0 (1.7 to 2.2)	1.85 (1.58 to 2.16)	0.86 (0.75 to 0.98)
2020	0.0 (0.0 to 0.0)	0.0 (0.0 to 0.0)	4.4 (3.8 to 5.1)	2.1 (1.7 to 2.5)	2.28 (1.96 to 2.62)	1.08 (0.90 to 1.27)
2021	0.0 (0.0 to 0.0)	0.0 (0.0 to 0.0)	4.8 (3.9 to 5.9)	2.3 (1.8 to 2.8)	2.36 (1.90 to 2.91)	1.10 (0.87 to 1.39)

Gloucestershire GBR_44785 Western Europe Stillbirth Rate / Neonatal Mortality Rate (SBR/NMR) − 20 weeks
lambda = 0.6 ; zeta = 0.8 ; scale = 15 ; amp = 0.028

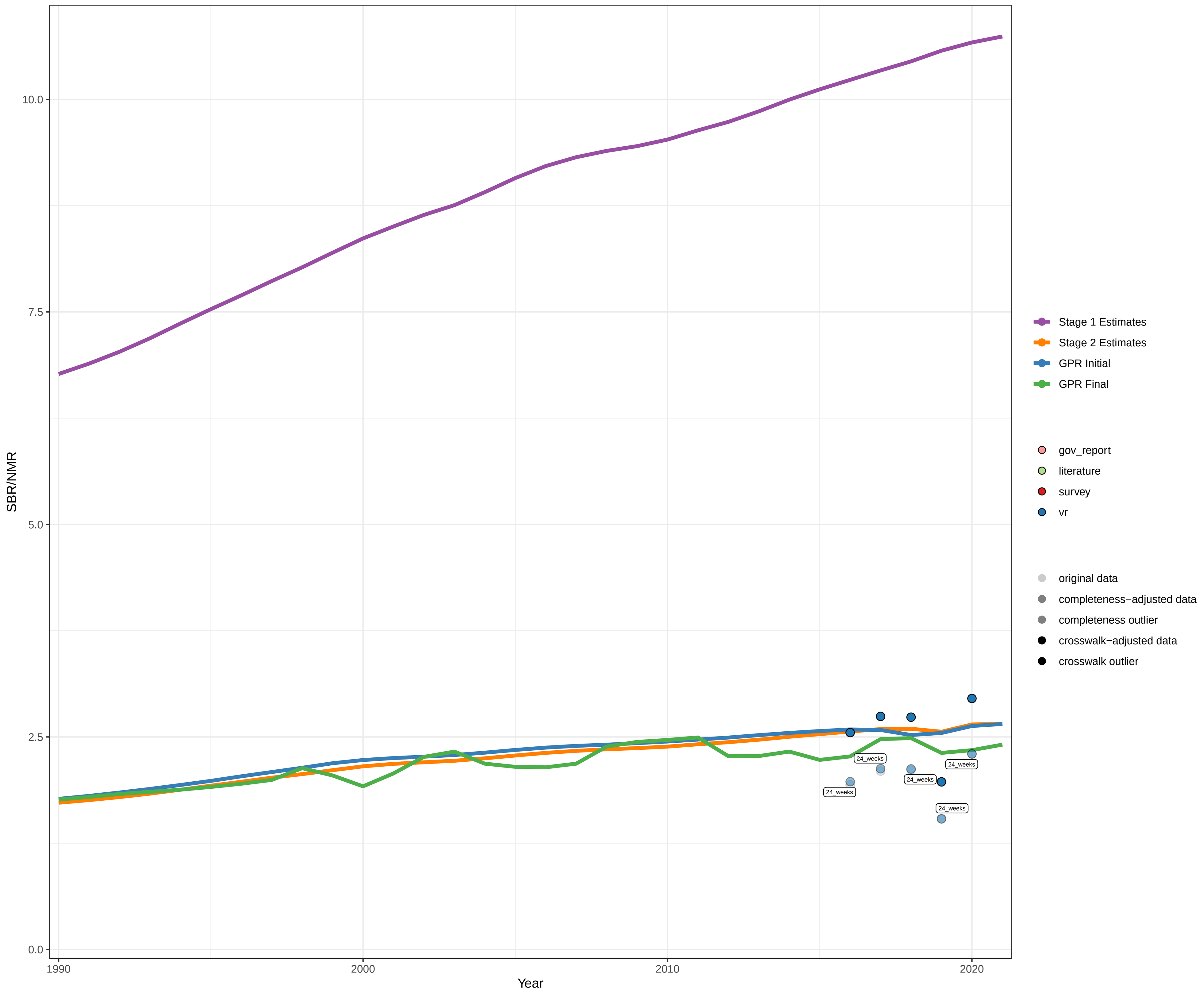

Gloucestershire GBR_44785 Western Europe Stillbirth Rate / Neonatal Mortality Rate (SBR/NMR) − 28 weeks
lambda = 0.6 ; zeta = 0.8 ; scale = 15 ; amp = 0.028

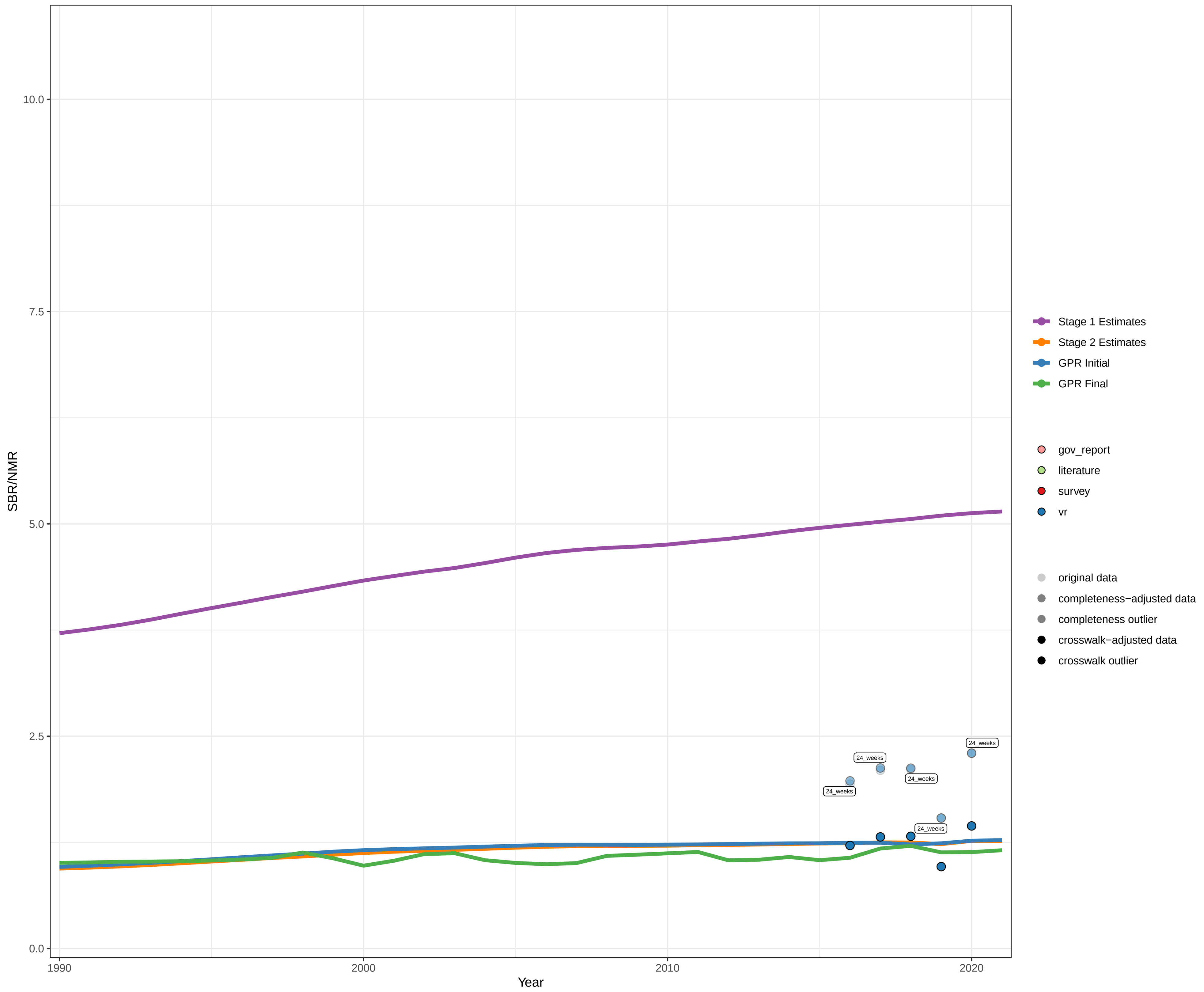

Definition	Years	Outlier	Source.Type	Title
24_weeks	2016−2020	0	VR	United Kingdom − England and Wales Child Mortality Statistics

Gloucestershire GBR_44785 Western Europe Stillbirth Rate (SBR) − 20 weeks
lambda = 0.6 ; zeta = 0.8 ; scale = 15 ; amp = 0.028

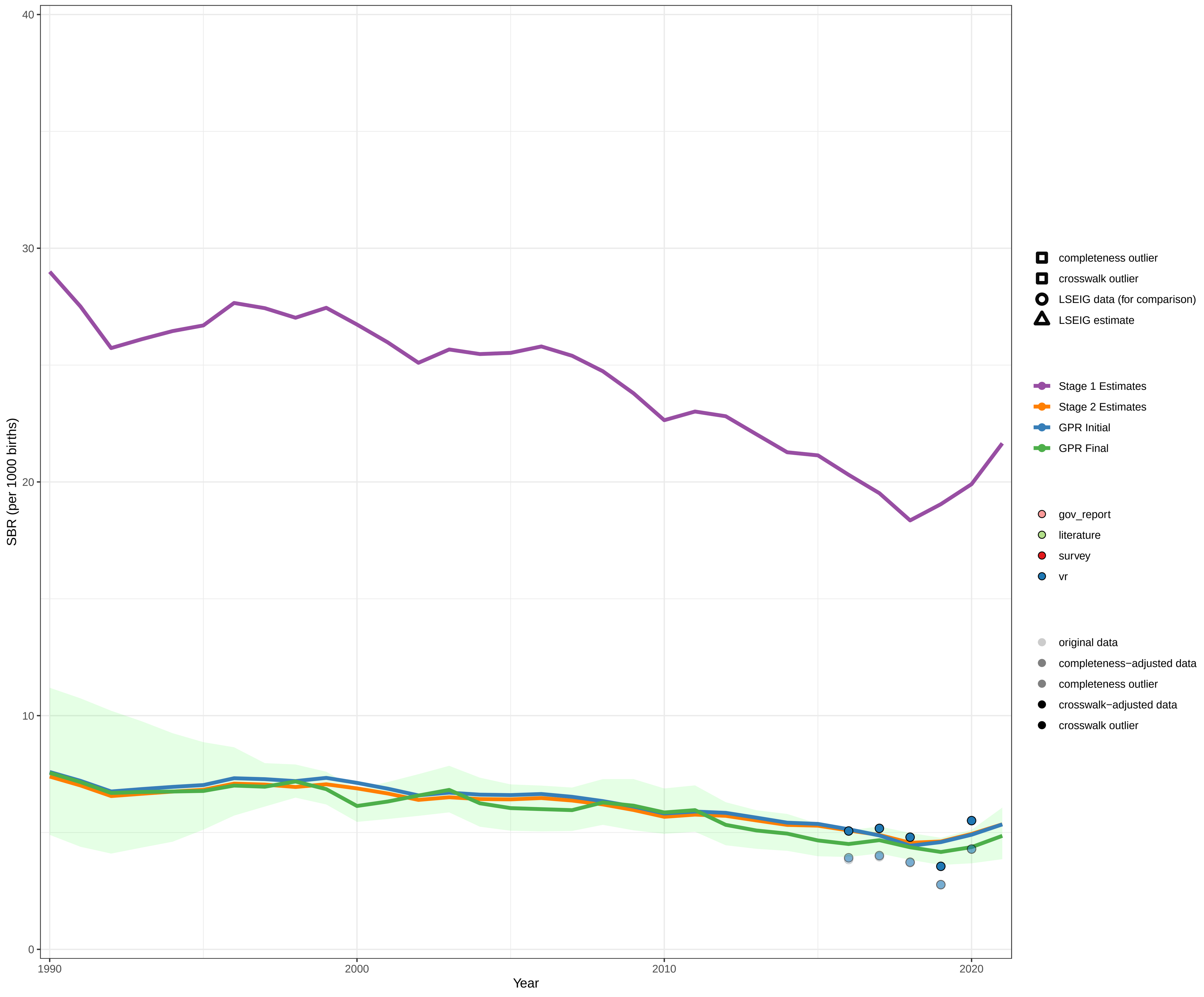

Gloucestershire GBR_44785 Western Europe Stillbirth Rate (SBR) − 28 weeks
lambda = 0.6 ; zeta = 0.8 ; scale = 15 ; amp = 0.028

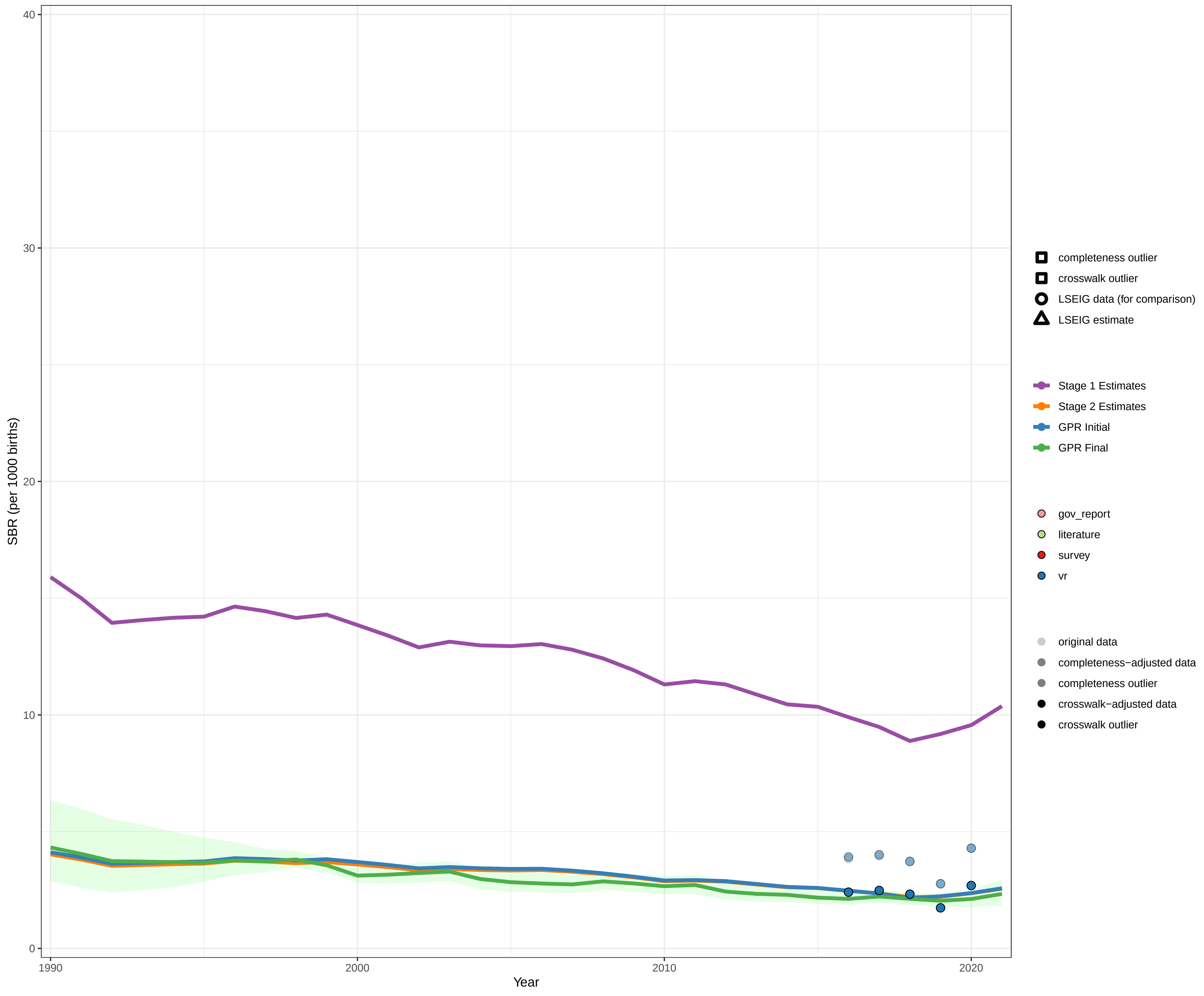

Year	Total Stillbirths (in thousands)	Total Stillbirths (in thousands)	SBR (per 1000 births)	SBR (per 1000 births)	SBR/NMR	SBR/NMR
	20 weeks	28 weeks	20 weeks	28 weeks	20 weeks	28 weeks
1990	0.1 (0.0 to 0.1)	0.0 (0.0 to 0.0)	7.6 (4.9 to 11.2)	4.3 (2.9 to 6.4)	1.76 (1.14 to 2.61)	1.01 (0.67 to 1.48)
1995	0.0 (0.0 to 0.1)	0.0 (0.0 to 0.0)	6.8 (5.1 to 8.9)	3.7 (2.9 to 4.7)	1.91 (1.44 to 2.50)	1.04 (0.80 to 1.34)
2000	0.0 (0.0 to 0.0)	0.0 (0.0 to 0.0)	6.1 (5.5 to 6.9)	3.1 (2.8 to 3.5)	1.92 (1.71 to 2.15)	0.98 (0.88 to 1.08)
2005	0.0 (0.0 to 0.0)	0.0 (0.0 to 0.0)	6.0 (5.1 to 7.1)	2.8 (2.4 to 3.3)	2.15 (1.80 to 2.51)	1.01 (0.86 to 1.17)
2010	0.0 (0.0 to 0.0)	0.0 (0.0 to 0.0)	5.9 (4.9 to 6.9)	2.7 (2.3 to 3.1)	2.46 (2.08 to 2.90)	1.12 (0.97 to 1.30)
2015	0.0 (0.0 to 0.0)	0.0 (0.0 to 0.0)	4.7 (4.0 to 5.4)	2.2 (1.9 to 2.5)	2.23 (1.91 to 2.57)	1.04 (0.91 to 1.19)
2020	0.0 (0.0 to 0.0)	0.0 (0.0 to 0.0)	4.4 (3.7 to 5.1)	2.1 (1.8 to 2.5)	2.35 (1.97 to 2.74)	1.14 (0.95 to 1.35)
2021	0.0 (0.0 to 0.0)	0.0 (0.0 to 0.0)	4.9 (3.9 to 6.1)	2.3 (1.8 to 3.0)	2.41 (1.91 to 3.01)	1.16 (0.91 to 1.47)

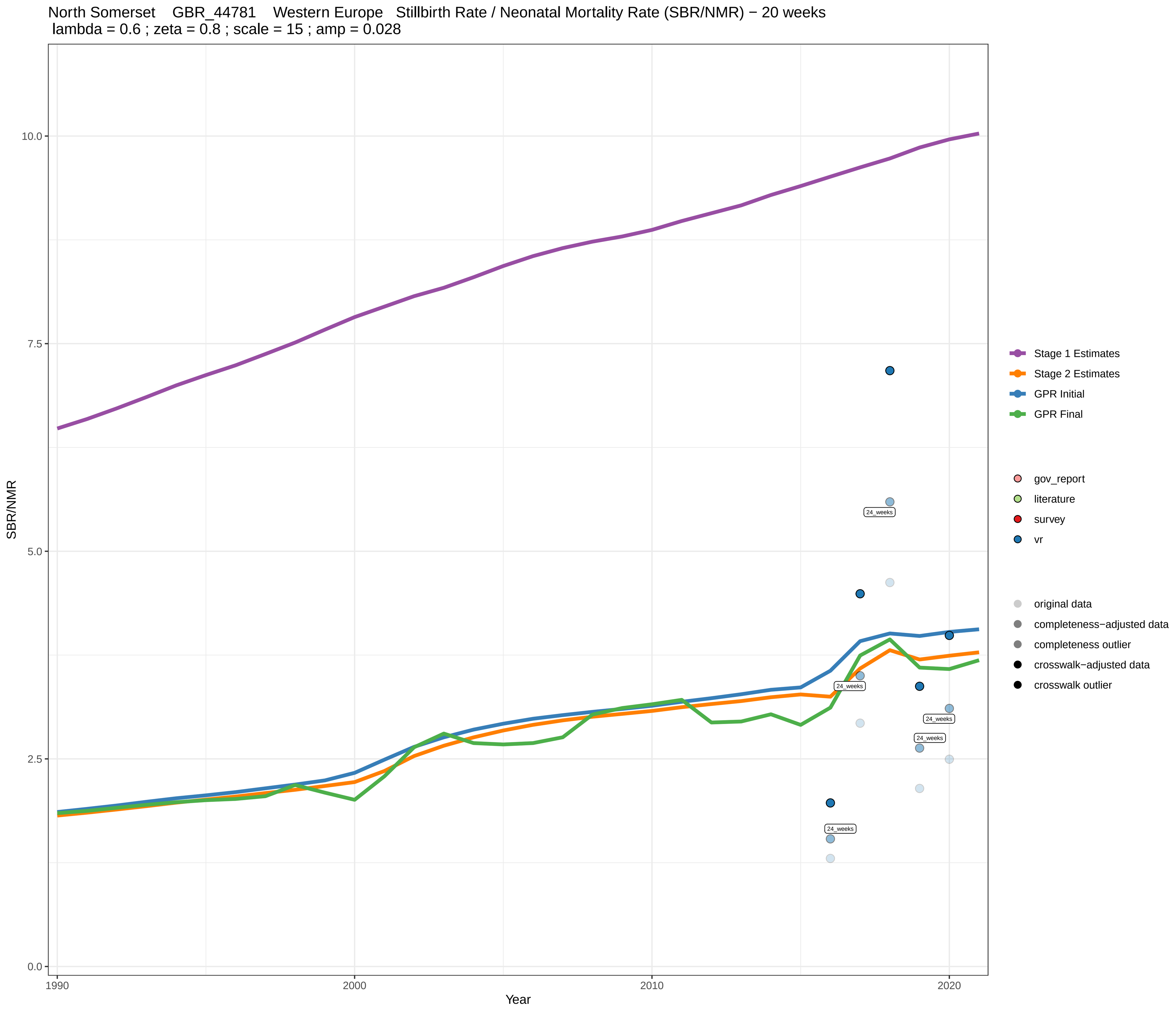

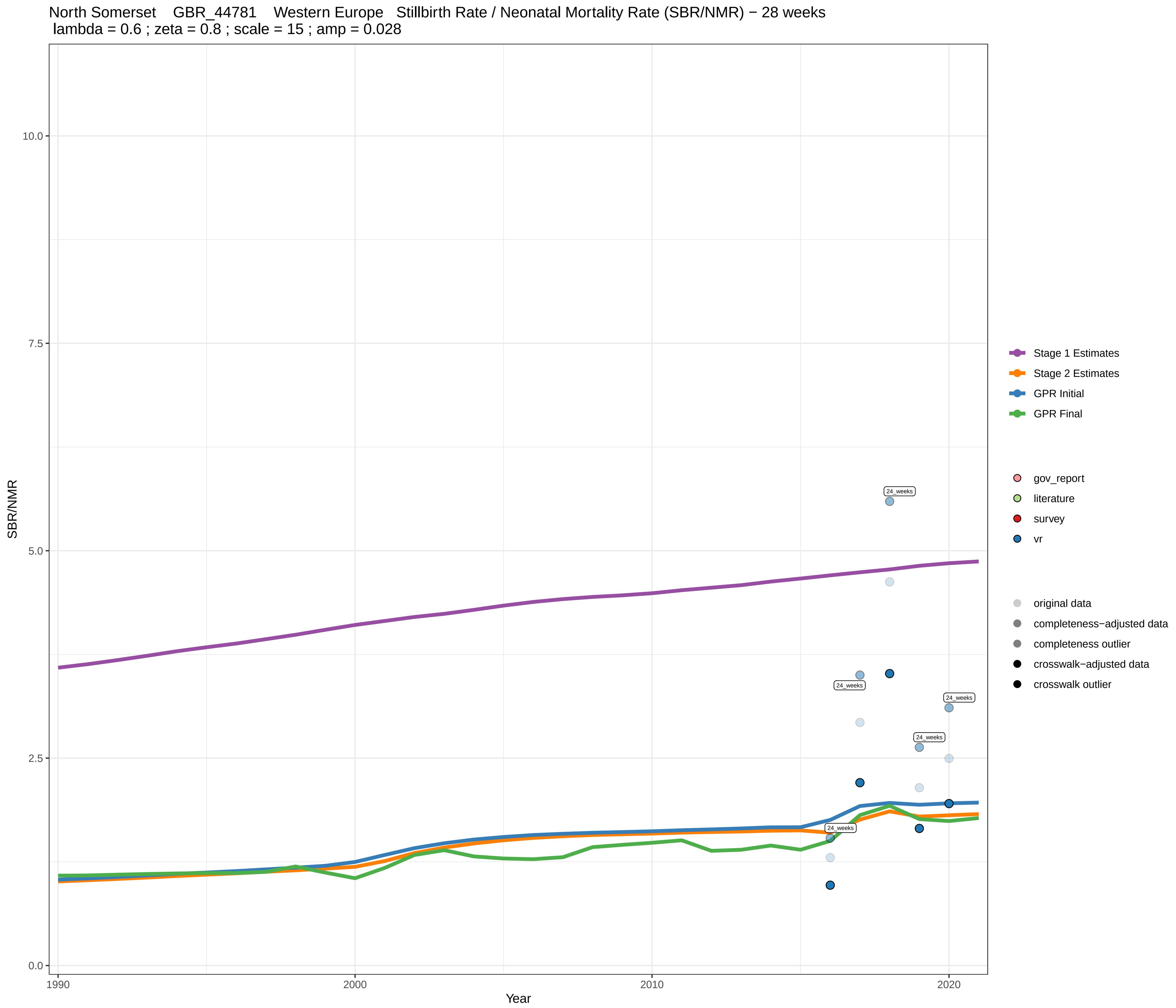

Definition	Years	Outlier	Source.Type	Title
24_weeks	2016−2020	0	VR	United Kingdom − England and Wales Child Mortality Statistics

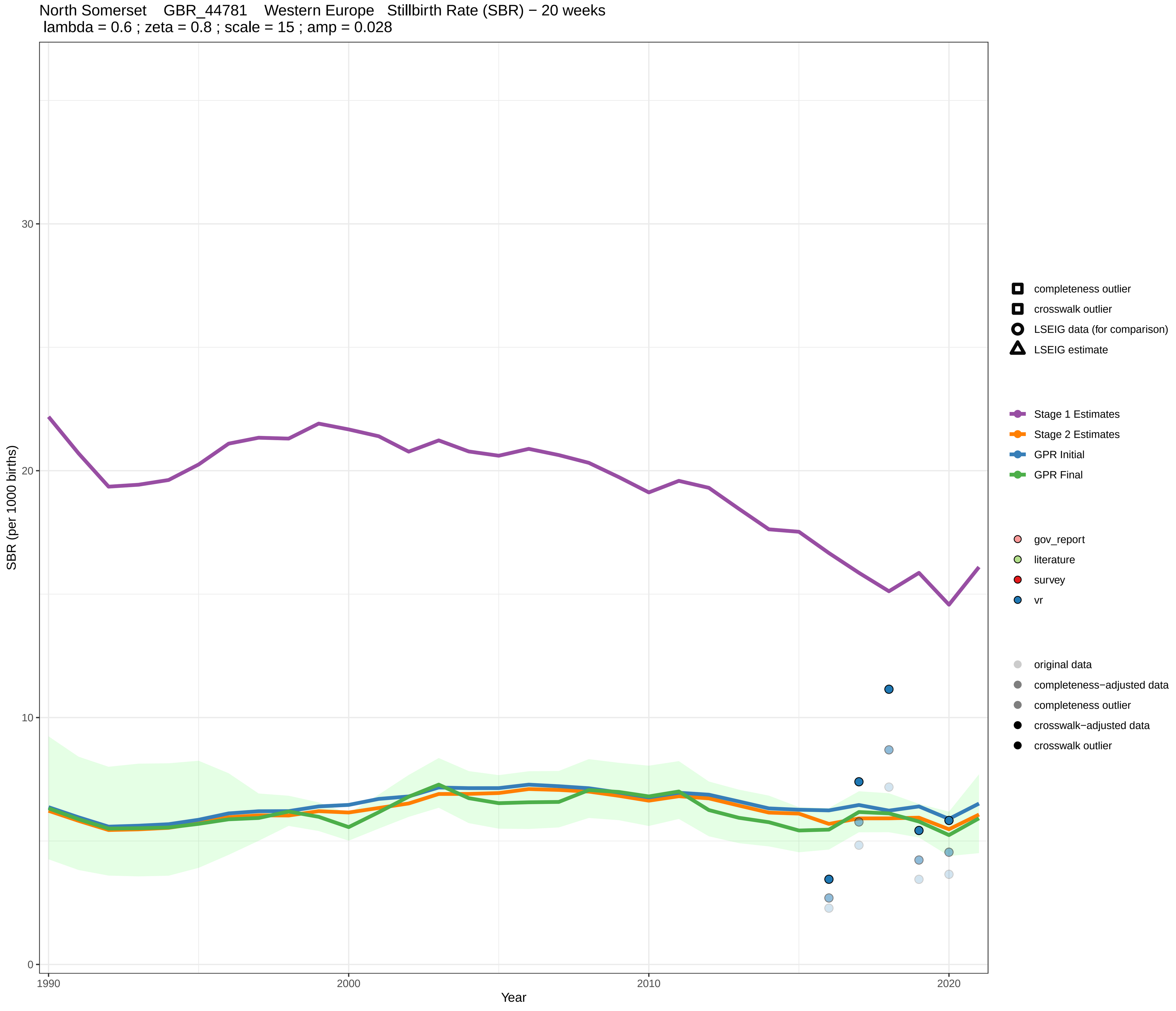

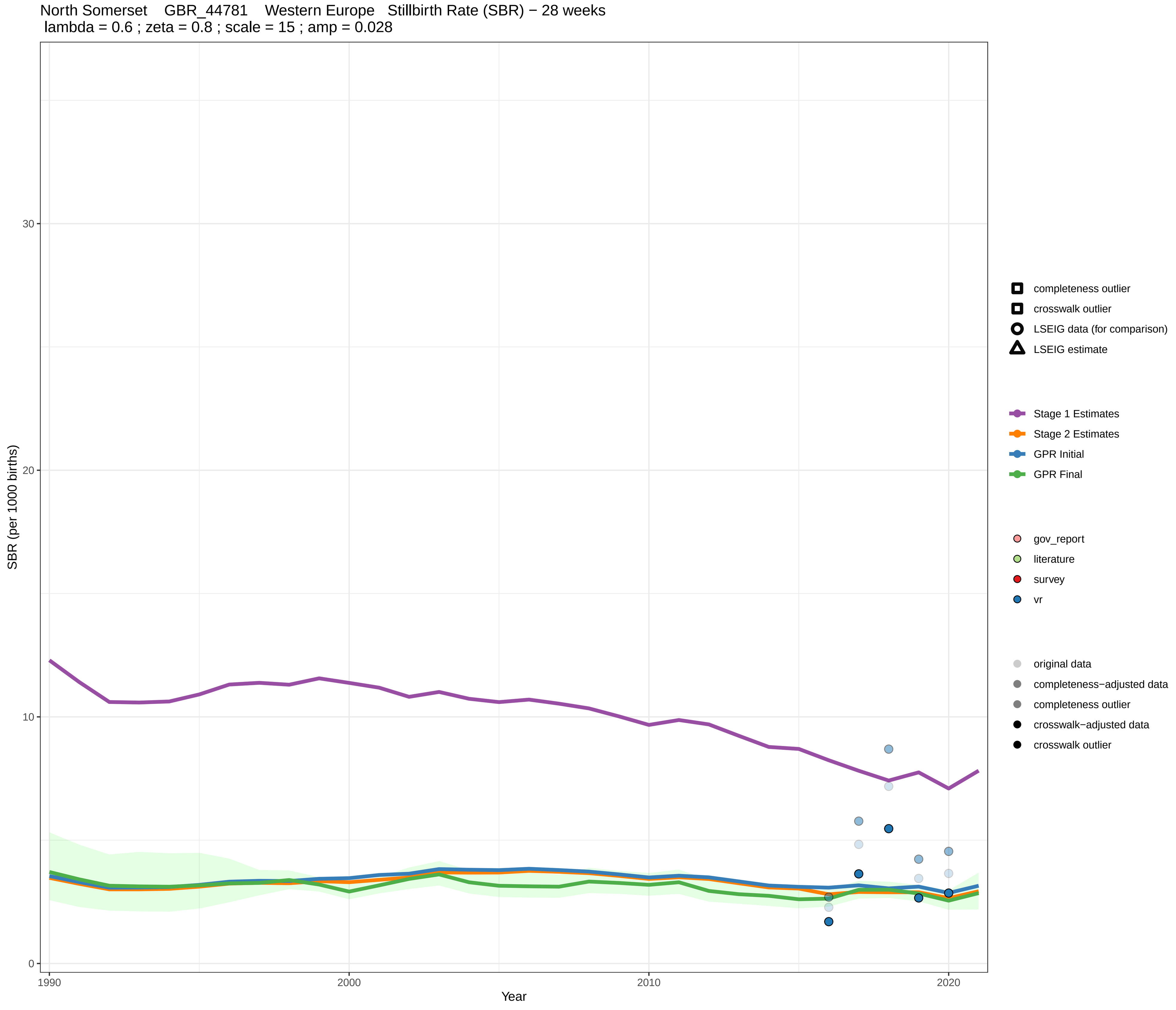

Year	Total Stillbirths (in thousands)	Total Stillbirths (in thousands)	SBR (per 1000 births)	SBR (per 1000 births)	SBR/NMR	SBR/NMR
	20 weeks	28 weeks	20 weeks	28 weeks	20 weeks	28 weeks
1990	0.0 (0.0 to 0.0)	0.0 (0.0 to 0.0)	6.3 (4.3 to 9.2)	3.7 (2.6 to 5.3)	1.85 (1.24 to 2.70)	1.08 (0.75 to 1.55)
1995	0.0 (0.0 to 0.0)	0.0 (0.0 to 0.0)	5.7 (3.9 to 8.3)	3.2 (2.2 to 4.5)	2.00 (1.38 to 2.90)	1.11 (0.78 to 1.58)
2000	0.0 (0.0 to 0.0)	0.0 (0.0 to 0.0)	5.6 (5.0 to 6.1)	2.9 (2.6 to 3.3)	2.01 (1.81 to 2.21)	1.05 (0.94 to 1.18)
2005	0.0 (0.0 to 0.0)	0.0 (0.0 to 0.0)	6.5 (5.5 to 7.7)	3.2 (2.7 to 3.6)	2.67 (2.25 to 3.14)	1.29 (1.10 to 1.49)
2010	0.0 (0.0 to 0.0)	0.0 (0.0 to 0.0)	6.8 (5.6 to 8.1)	3.2 (2.7 to 3.7)	3.16 (2.60 to 3.74)	1.48 (1.28 to 1.70)
2015	0.0 (0.0 to 0.0)	0.0 (0.0 to 0.0)	5.4 (4.5 to 6.4)	2.6 (2.2 to 3.0)	2.91 (2.44 to 3.42)	1.40 (1.20 to 1.59)
2020	0.0 (0.0 to 0.0)	0.0 (0.0 to 0.0)	5.2 (4.4 to 6.2)	2.5 (2.2 to 3.0)	3.58 (3.00 to 4.23)	1.74 (1.49 to 2.03)
2021	0.0 (0.0 to 0.0)	0.0 (0.0 to 0.0)	5.9 (4.5 to 7.7)	2.9 (2.2 to 3.7)	3.69 (2.81 to 4.80)	1.78 (1.36 to 2.30)

Plymouth GBR_44792 Western Europe Stillbirth Rate / Neonatal Mortality Rate (SBR/NMR) − 20 weeks
lambda = 0.8 ; zeta = 0.7 ; scale = 15 ; amp = 0.028

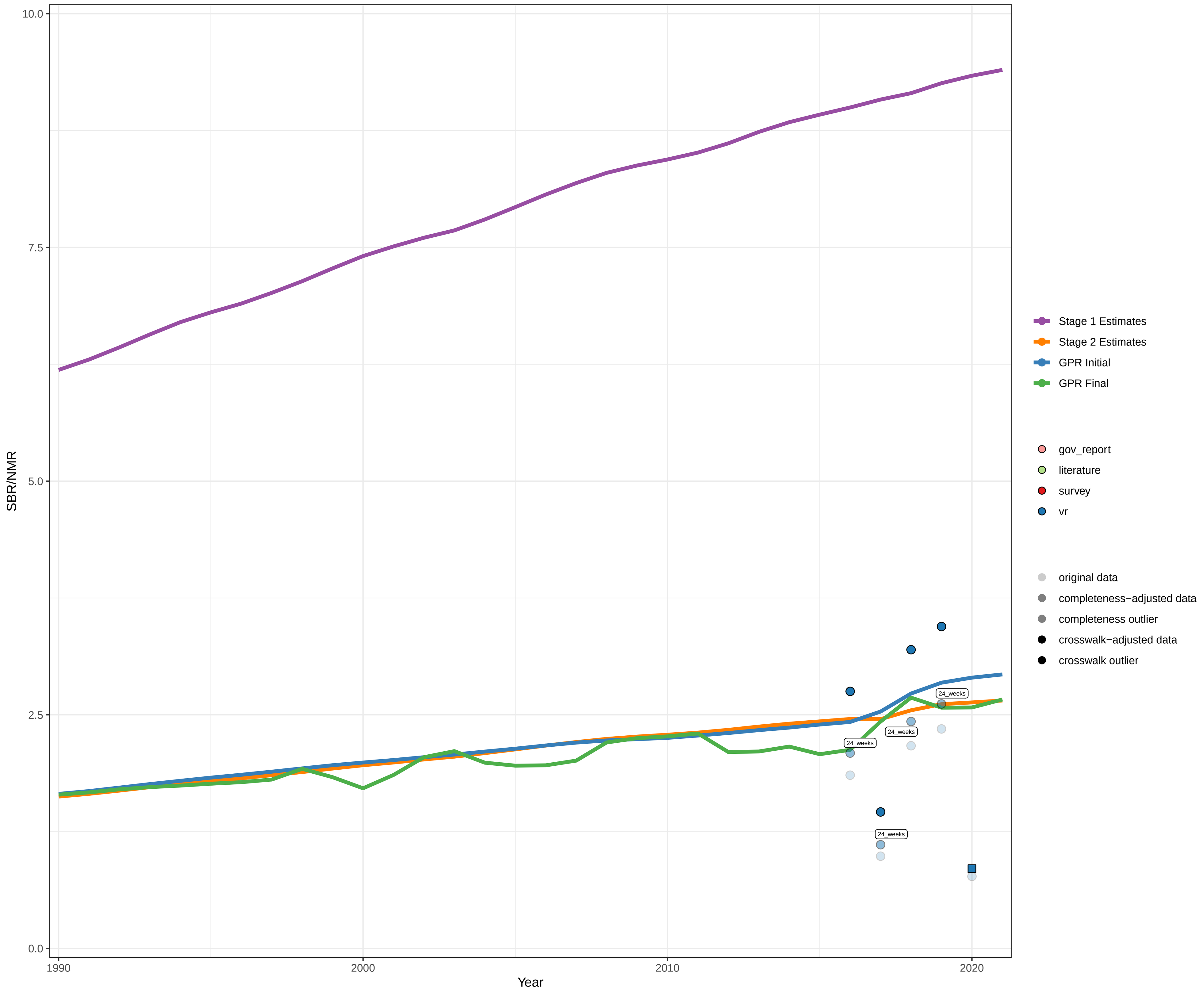

Plymouth GBR_44792 Western Europe Stillbirth Rate / Neonatal Mortality Rate (SBR/NMR) − 28 weeks
lambda = 0.8 ; zeta = 0.7 ; scale = 15 ; amp = 0.028

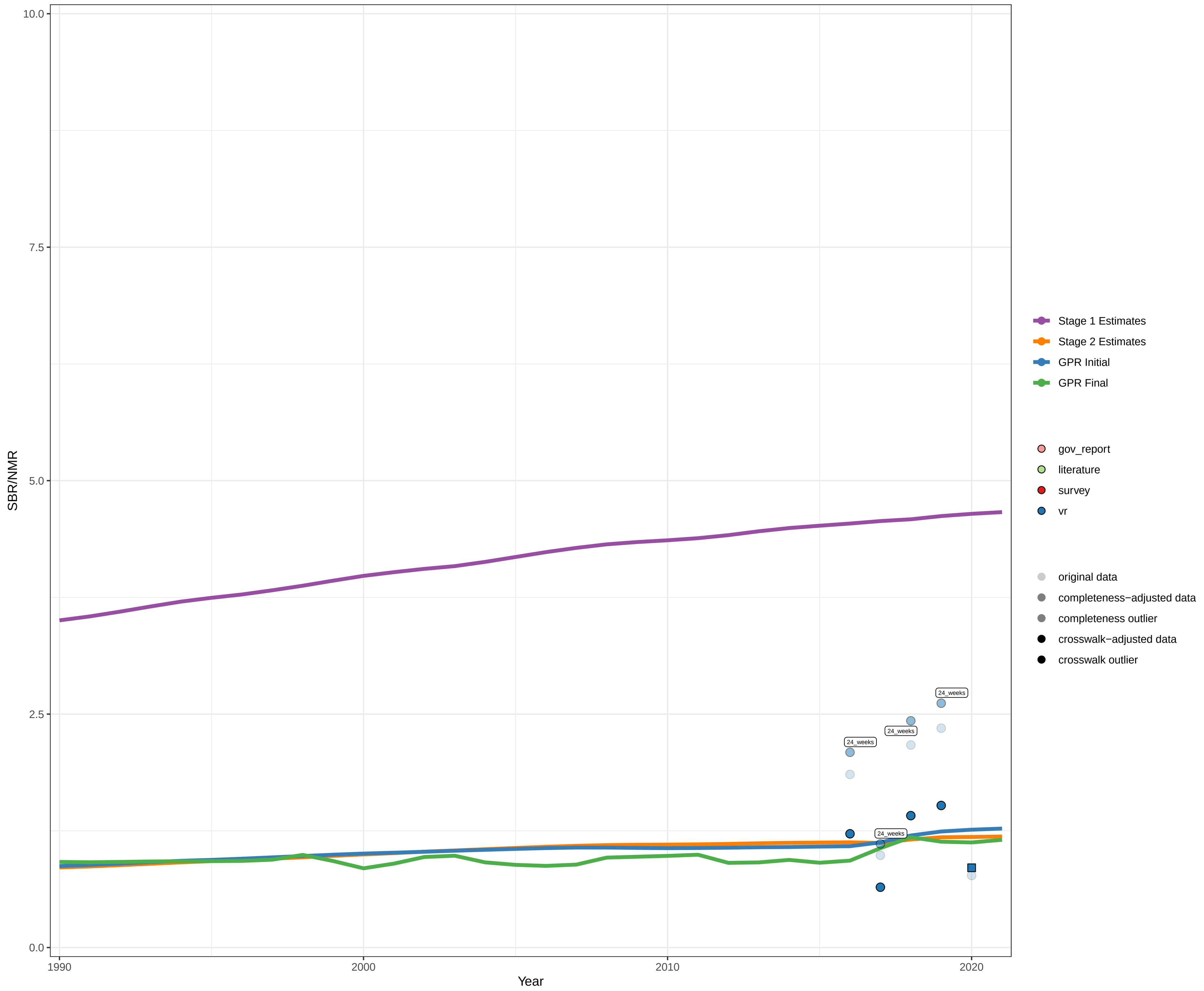

Definition	Years	Outlier	Source.Type	Title
24_weeks	2016−2020	0	VR	United Kingdom − England and Wales Child Mortality Statistics

Plymouth GBR_44792 Western Europe Stillbirth Rate (SBR) − 20 weeks
lambda = 0.8 ; zeta = 0.7 ; scale = 15 ; amp = 0.028

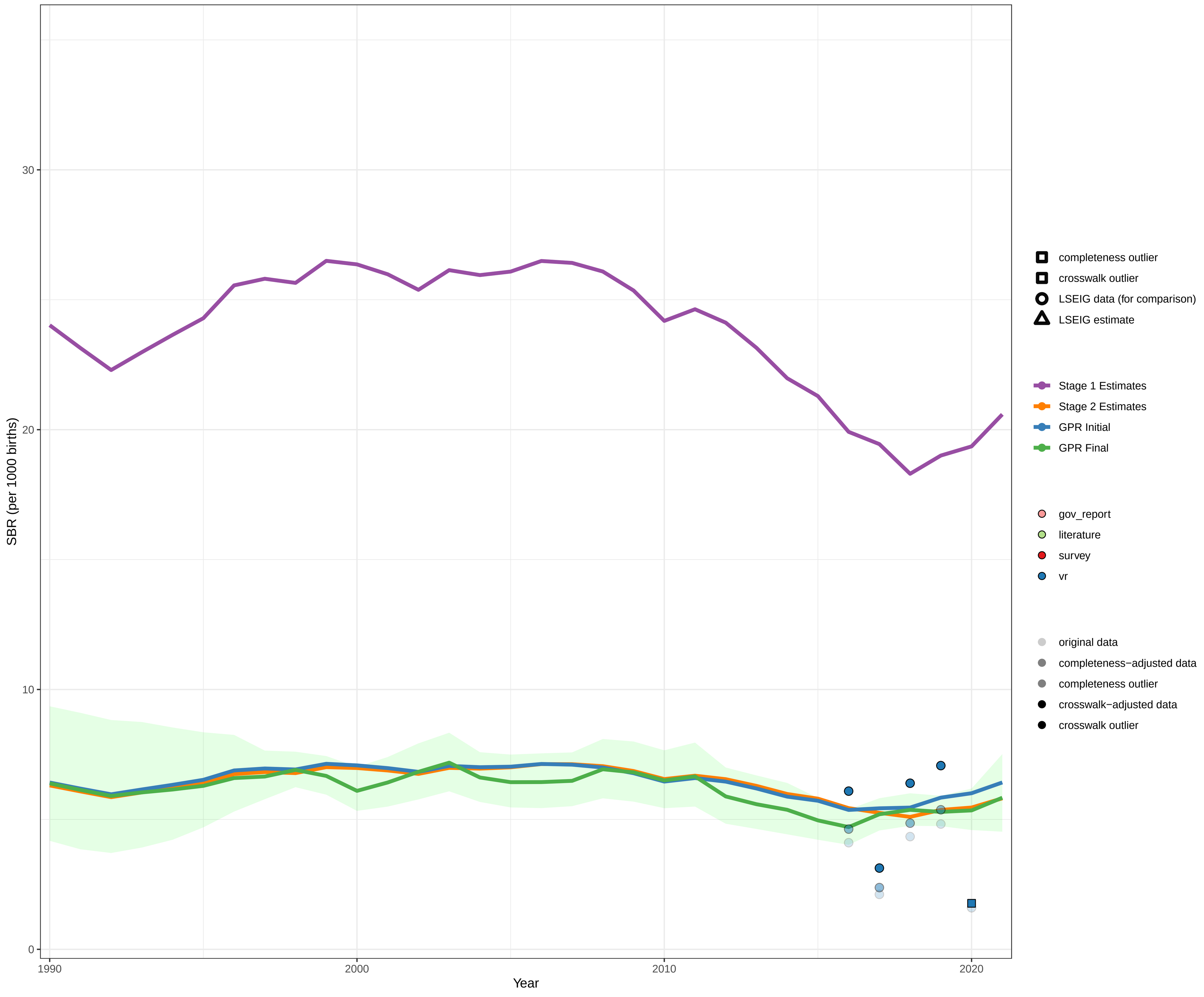

Plymouth GBR_44792 Western Europe Stillbirth Rate (SBR) − 28 weeks
lambda = 0.8 ; zeta = 0.7 ; scale = 15 ; amp = 0.028

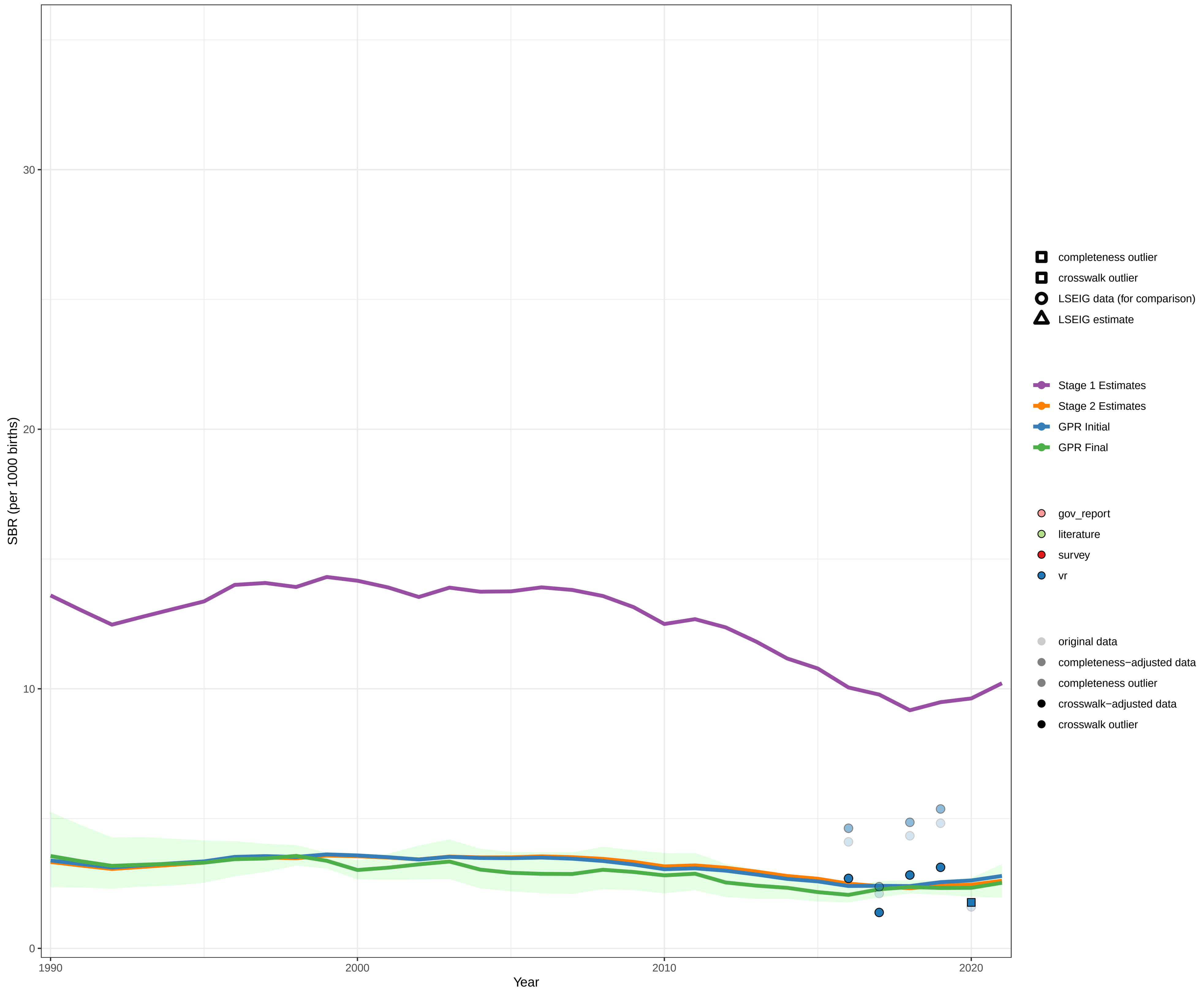

Year	Total Stillbirths (in thousands)	Total Stillbirths (in thousands)	SBR (per 1000 births)	SBR (per 1000 births)	SBR/NMR	SBR/NMR
	20 weeks	28 weeks	20 weeks	28 weeks	20 weeks	28 weeks
1990	0.0 (0.0 to 0.0)	0.0 (0.0 to 0.0)	6.4 (4.2 to 9.4)	3.6 (2.4 to 5.2)	1.65 (1.08 to 2.41)	0.92 (0.61 to 1.35)
1995	0.0 (0.0 to 0.0)	0.0 (0.0 to 0.0)	6.3 (4.7 to 8.4)	3.3 (2.5 to 4.2)	1.76 (1.32 to 2.34)	0.93 (0.71 to 1.17)
2000	0.0 (0.0 to 0.0)	0.0 (0.0 to 0.0)	6.1 (5.3 to 7.0)	3.0 (2.7 to 3.4)	1.71 (1.50 to 1.97)	0.85 (0.75 to 0.95)
2005	0.0 (0.0 to 0.0)	0.0 (0.0 to 0.0)	6.4 (5.5 to 7.5)	2.9 (2.2 to 3.7)	1.96 (1.66 to 2.28)	0.89 (0.67 to 1.13)
2010	0.0 (0.0 to 0.0)	0.0 (0.0 to 0.0)	6.5 (5.4 to 7.7)	2.8 (2.1 to 3.7)	2.27 (1.90 to 2.67)	0.98 (0.74 to 1.28)
2015	0.0 (0.0 to 0.0)	0.0 (0.0 to 0.0)	5.0 (4.2 to 5.8)	2.2 (1.8 to 2.6)	2.08 (1.77 to 2.44)	0.91 (0.76 to 1.07)
2020	0.0 (0.0 to 0.0)	0.0 (0.0 to 0.0)	5.3 (4.6 to 6.2)	2.3 (2.0 to 2.7)	2.58 (2.21 to 2.99)	1.13 (0.96 to 1.31)
2021	0.0 (0.0 to 0.0)	0.0 (0.0 to 0.0)	5.8 (4.5 to 7.5)	2.5 (2.0 to 3.2)	2.66 (2.07 to 3.43)	1.15 (0.89 to 1.48)

Poole GBR_44783 Western Europe Stillbirth Rate / Neonatal Mortality Rate (SBR/NMR) − 20 weeks
lambda = 0.8 ; zeta = 0.7 ; scale = 15 ; amp = 0.028

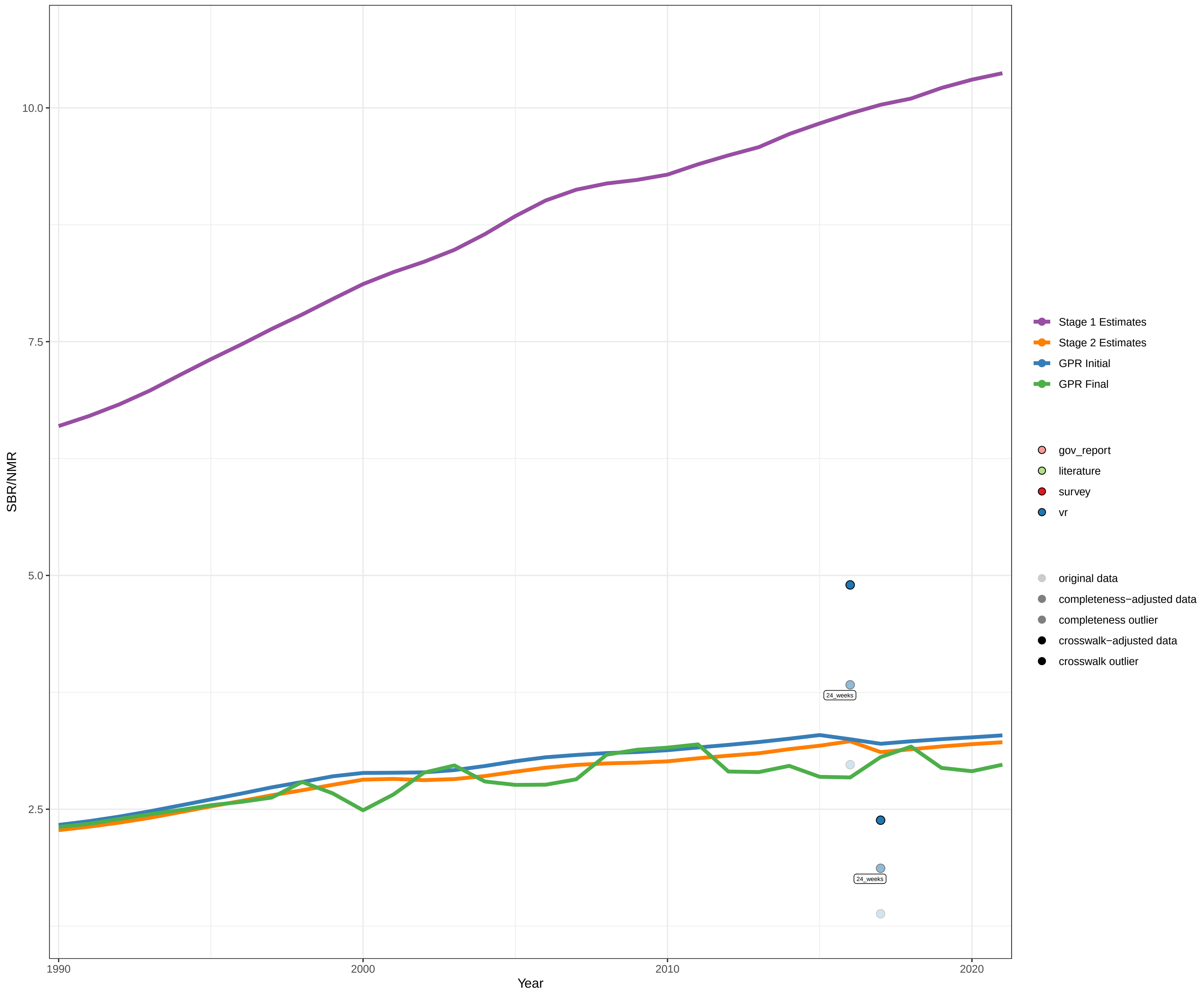

Poole GBR_44783 Western Europe Stillbirth Rate / Neonatal Mortality Rate (SBR/NMR) − 28 weeks
lambda = 0.8 ; zeta = 0.7 ; scale = 15 ; amp = 0.028

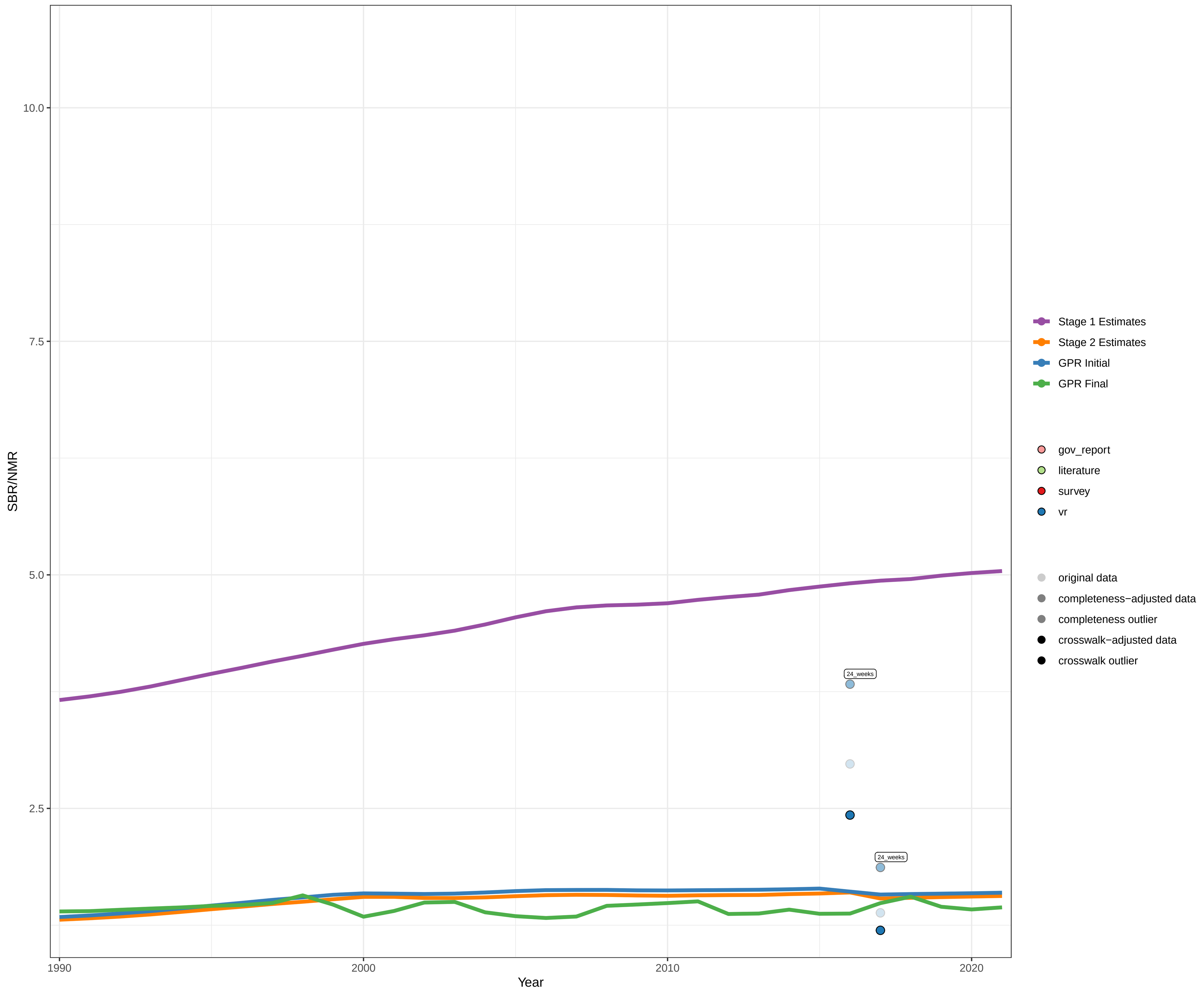

Definition	Years	Outlier	Source.Type	Title
24_weeks	2016, 2017	0	VR	United Kingdom − England and Wales Child Mortality Statistics

Poole GBR_44783 Western Europe Stillbirth Rate (SBR) − 20 weeks
lambda = 0.8 ; zeta = 0.7 ; scale = 15 ; amp = 0.028

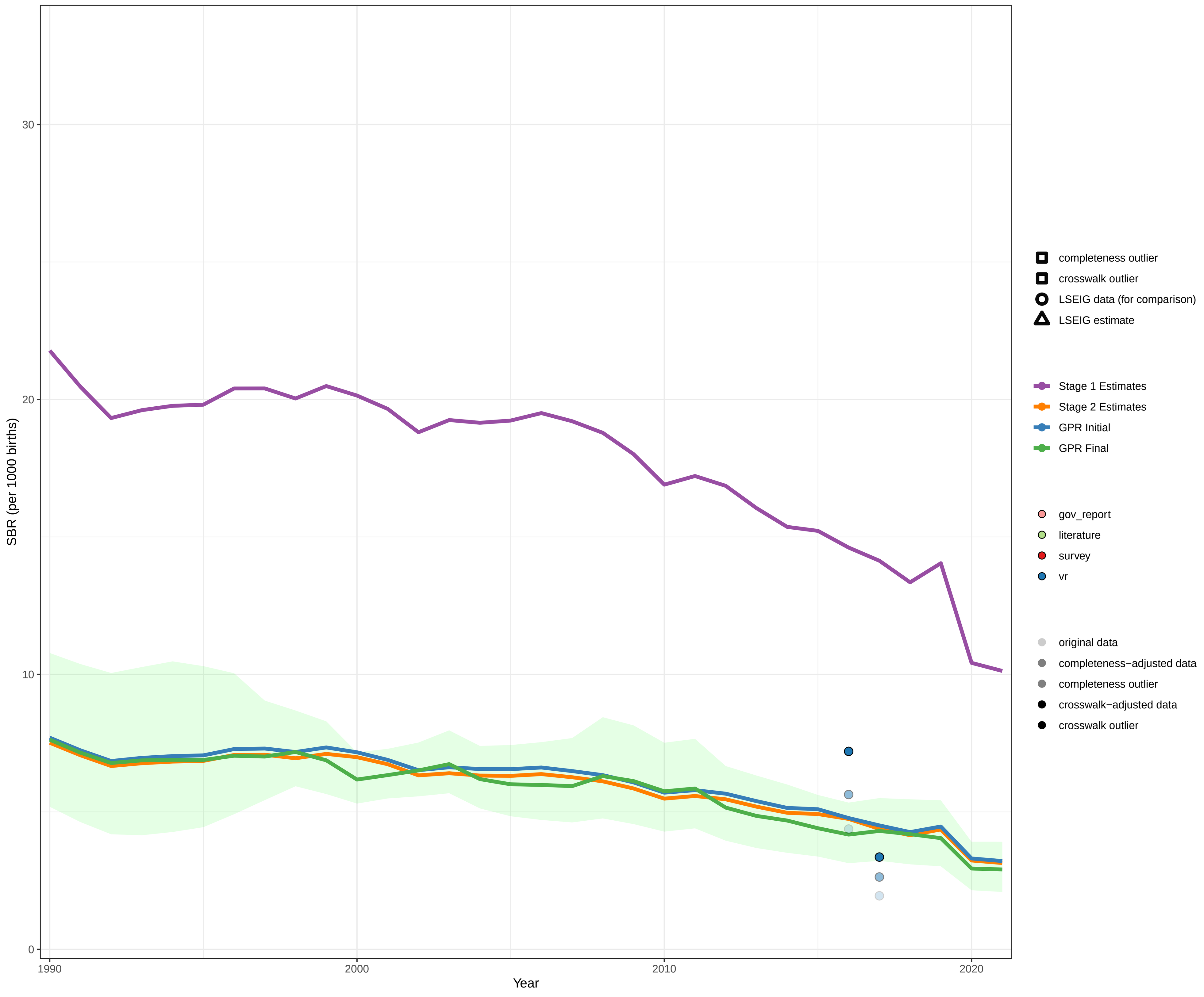

Poole GBR_44783 Western Europe Stillbirth Rate (SBR) − 28 weeks
lambda = 0.8 ; zeta = 0.7 ; scale = 15 ; amp = 0.028

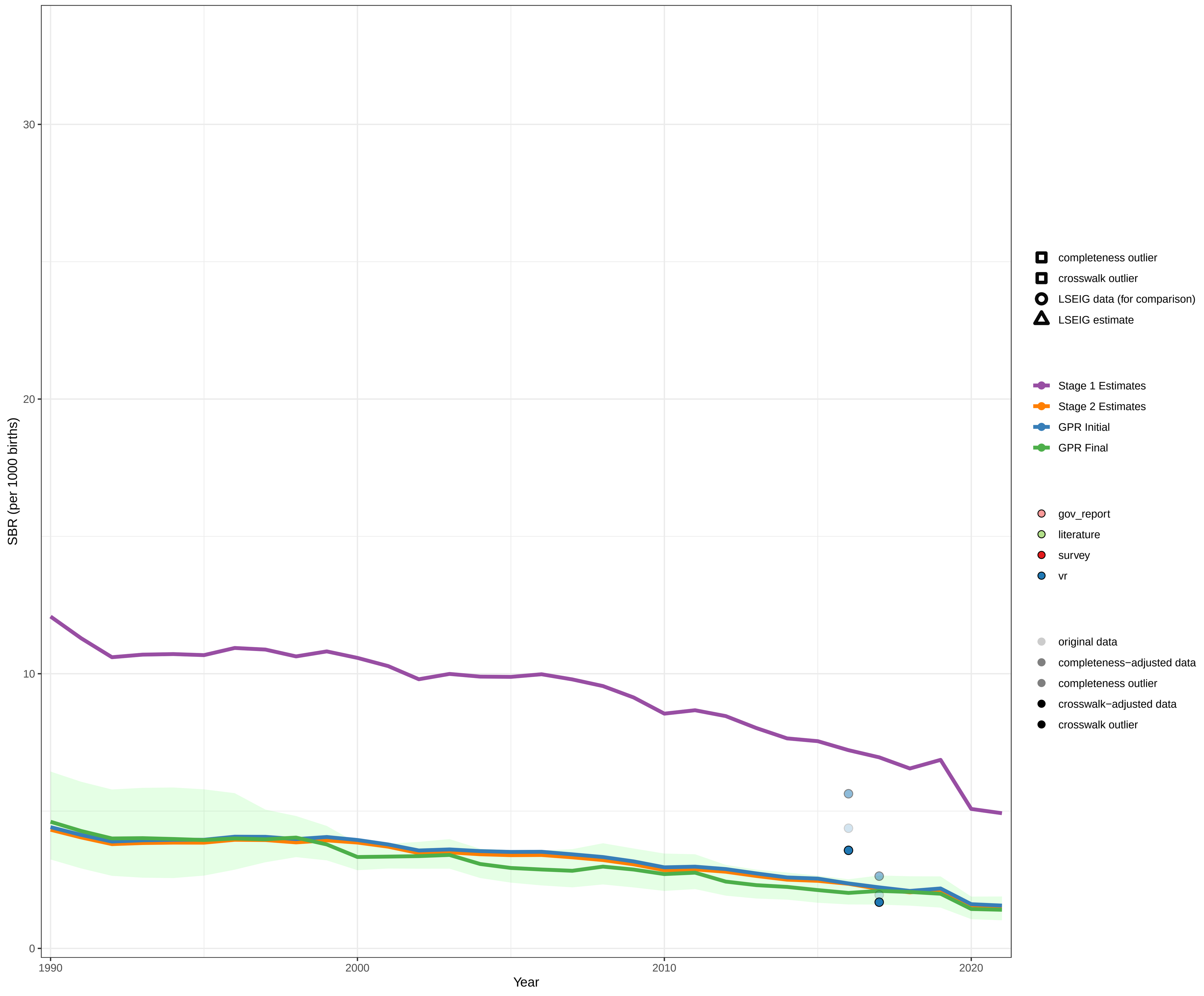

Year	Total Stillbirths (in thousands)	Total Stillbirths (in thousands)	SBR (per 1000 births)	SBR (per 1000 births)	SBR/NMR	SBR/NMR
	20 weeks	28 weeks	20 weeks	28 weeks	20 weeks	28 weeks
1990	0.0 (0.0 to 0.0)	0.0 (0.0 to 0.0)	7.6 (5.2 to 10.8)	4.6 (3.2 to 6.4)	2.31 (1.57 to 3.27)	1.40 (0.98 to 1.95)
1995	0.0 (0.0 to 0.0)	0.0 (0.0 to 0.0)	6.9 (4.4 to 10.3)	3.9 (2.7 to 5.8)	2.54 (1.64 to 3.80)	1.46 (0.98 to 2.14)
2000	0.0 (0.0 to 0.0)	0.0 (0.0 to 0.0)	6.2 (5.3 to 7.2)	3.3 (2.8 to 3.8)	2.49 (2.14 to 2.89)	1.34 (1.15 to 1.54)
2005	0.0 (0.0 to 0.0)	0.0 (0.0 to 0.0)	6.0 (4.8 to 7.4)	2.9 (2.4 to 3.6)	2.76 (2.23 to 3.42)	1.35 (1.10 to 1.65)
2010	0.0 (0.0 to 0.0)	0.0 (0.0 to 0.0)	5.7 (4.3 to 7.5)	2.7 (2.1 to 3.5)	3.16 (2.35 to 4.13)	1.49 (1.15 to 1.90)
2015	0.0 (0.0 to 0.0)	0.0 (0.0 to 0.0)	4.4 (3.4 to 5.6)	2.1 (1.7 to 2.6)	2.85 (2.18 to 3.63)	1.37 (1.07 to 1.71)
2020	0.0 (0.0 to 0.0)	0.0 (0.0 to 0.0)	2.9 (2.1 to 3.9)	1.4 (1.1 to 1.9)	2.91 (2.13 to 3.87)	1.42 (1.05 to 1.87)
2021	0.0 (0.0 to 0.0)	0.0 (0.0 to 0.0)	2.9 (2.1 to 3.9)	1.4 (1.0 to 1.9)	2.98 (2.14 to 4.01)	1.44 (1.05 to 1.94)

Somerset GBR_44786 Western Europe Stillbirth Rate / Neonatal Mortality Rate (SBR/NMR) − 20 weeks
lambda = 0.6 ; zeta = 0.8 ; scale = 15 ; amp = 0.028

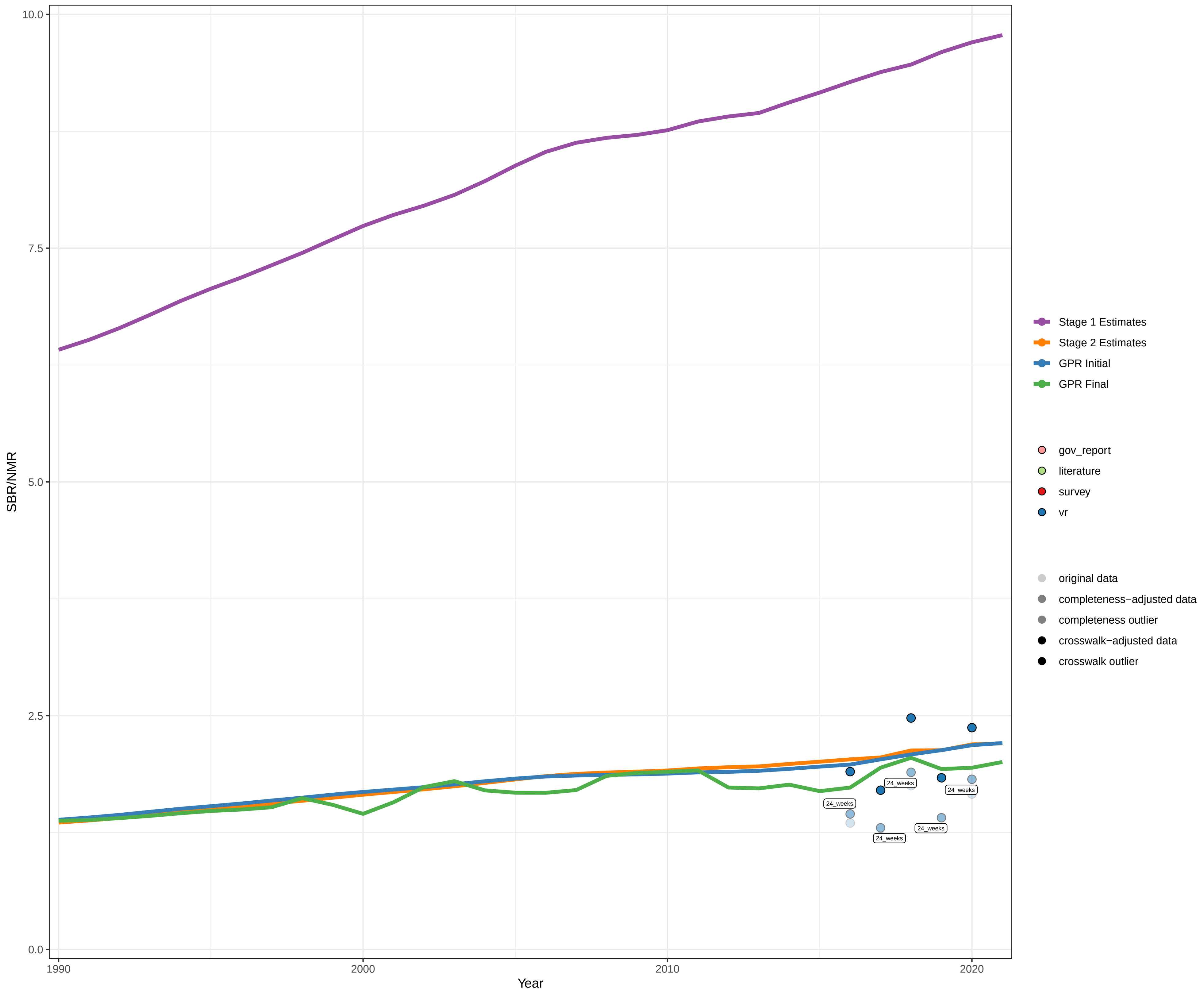

Somerset GBR_44786 Western Europe Stillbirth Rate / Neonatal Mortality Rate (SBR/NMR) − 28 weeks
lambda = 0.6 ; zeta = 0.8 ; scale = 15 ; amp = 0.028

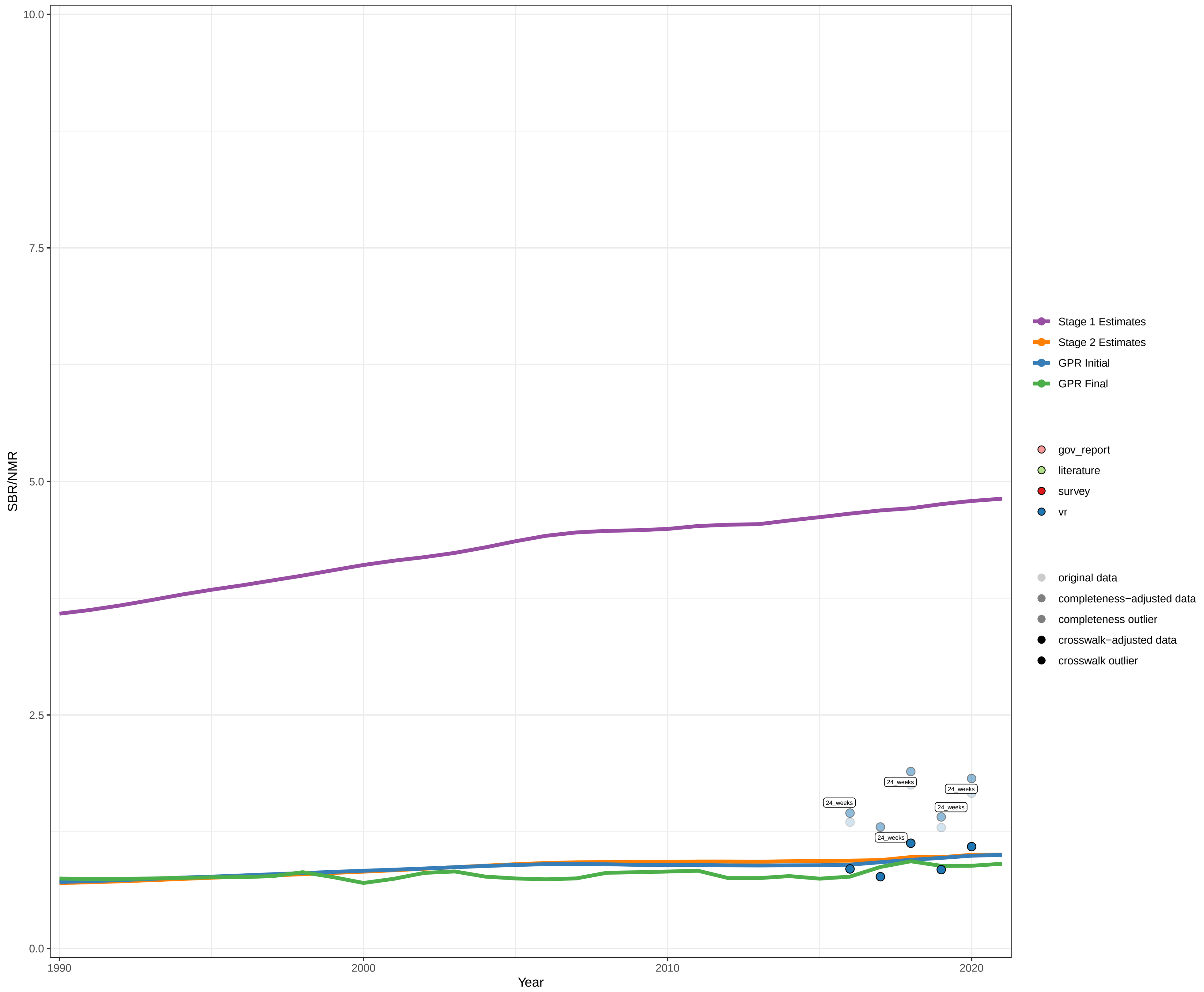

Definition	Years	Outlier	Source.Type	Title
24_weeks	2016−2020	0	VR	United Kingdom − England and Wales Child Mortality Statistics

Somerset GBR_44786 Western Europe Stillbirth Rate (SBR) − 20 weeks
lambda = 0.6 ; zeta = 0.8 ; scale = 15 ; amp = 0.028

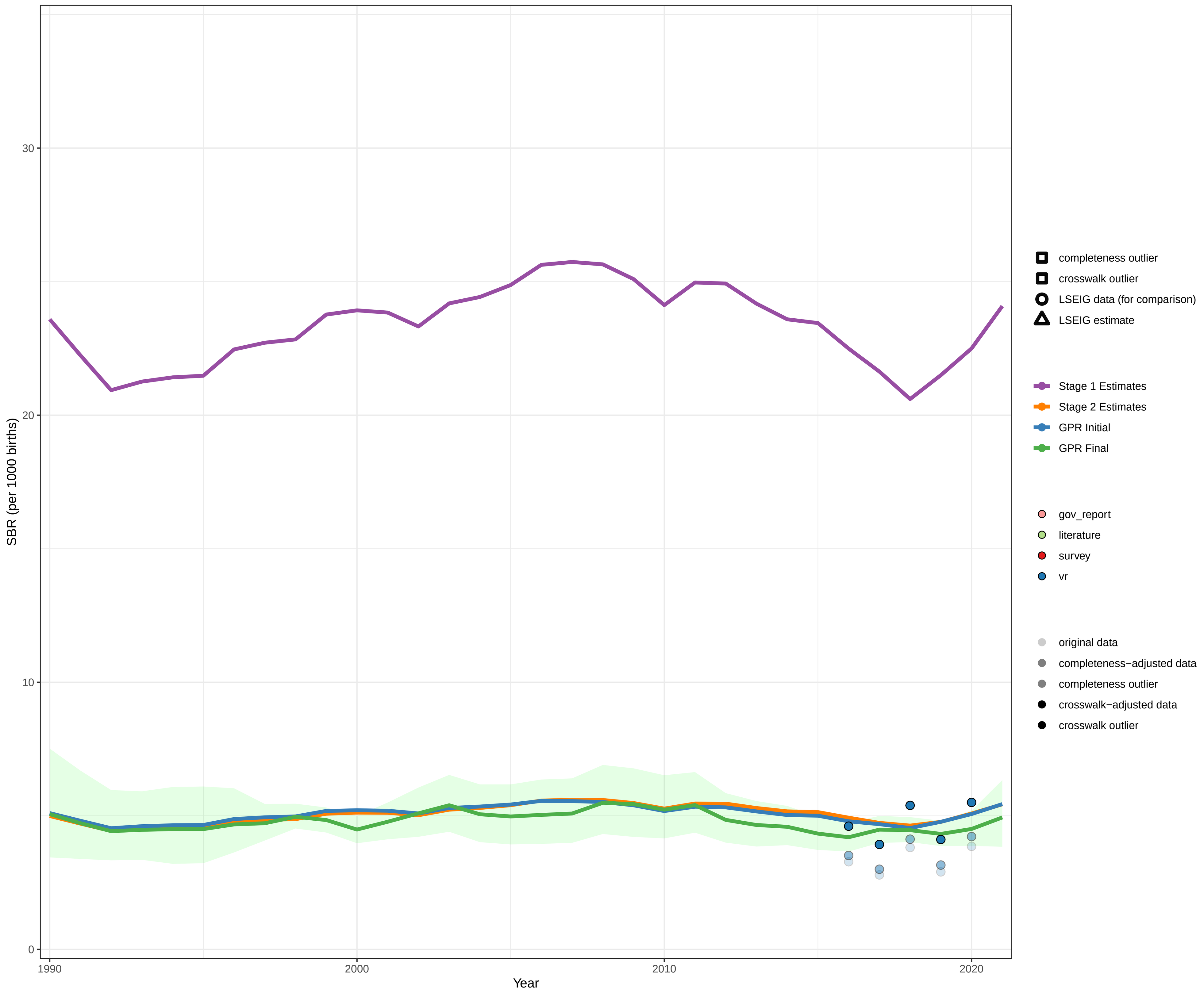

Somerset GBR_44786 Western Europe Stillbirth Rate (SBR) − 28 weeks
lambda = 0.6 ; zeta = 0.8 ; scale = 15 ; amp = 0.028

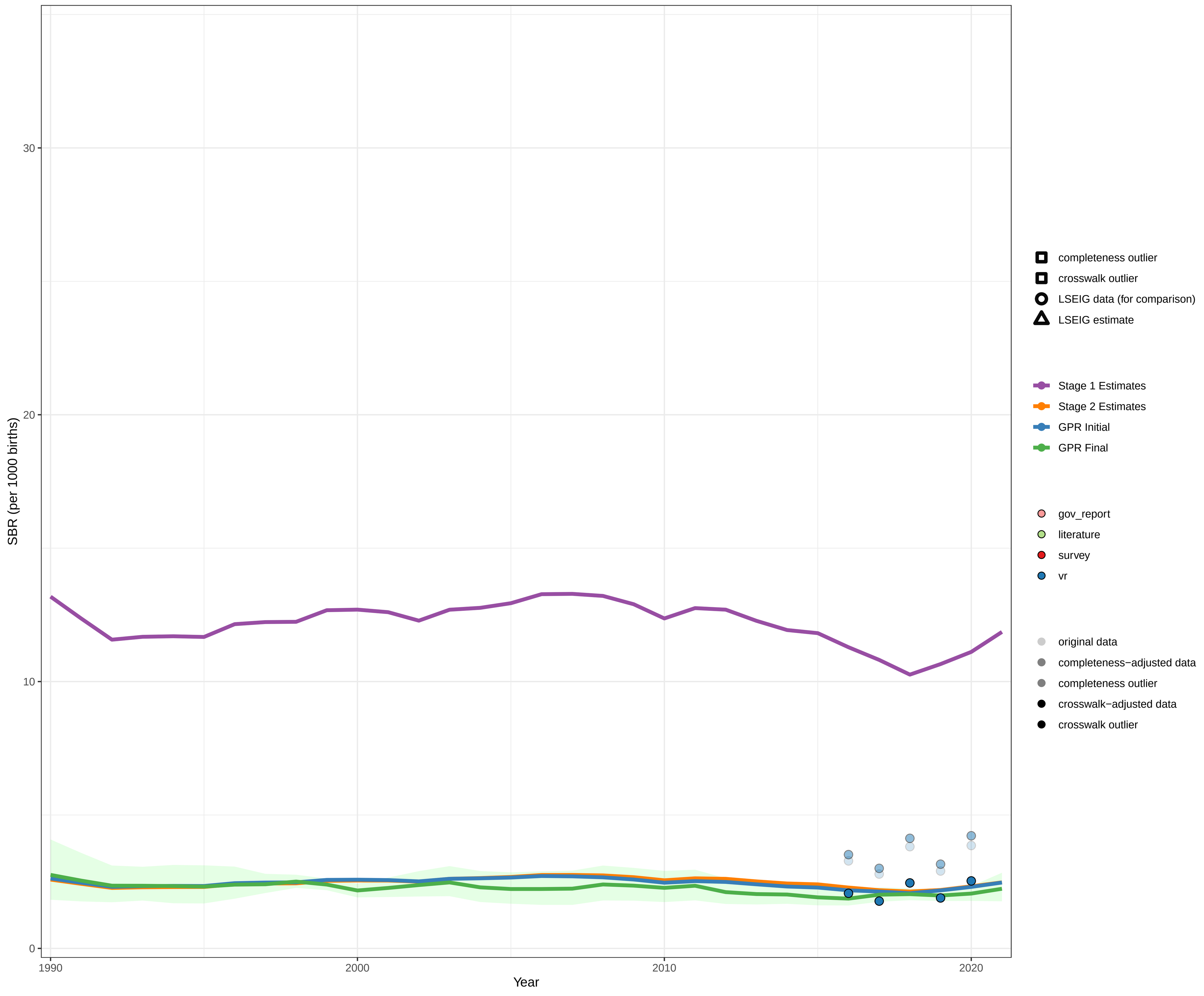

Year	Total Stillbirths (in thousands)	Total Stillbirths (in thousands)	SBR (per 1000 births)	SBR (per 1000 births)	SBR/NMR	SBR/NMR
	20 weeks	28 weeks	20 weeks	28 weeks	20 weeks	28 weeks
1990	0.0 (0.0 to 0.0)	0.0 (0.0 to 0.0)	5.1 (3.4 to 7.5)	2.8 (1.8 to 4.1)	1.38 (0.94 to 2.04)	0.75 (0.50 to 1.11)
1995	0.0 (0.0 to 0.0)	0.0 (0.0 to 0.0)	4.5 (3.2 to 6.1)	2.3 (1.7 to 3.1)	1.48 (1.06 to 2.01)	0.76 (0.55 to 1.02)
2000	0.0 (0.0 to 0.0)	0.0 (0.0 to 0.0)	4.5 (4.0 to 5.0)	2.2 (1.9 to 2.4)	1.45 (1.28 to 1.62)	0.70 (0.62 to 0.79)
2005	0.0 (0.0 to 0.0)	0.0 (0.0 to 0.0)	5.0 (3.9 to 6.2)	2.2 (1.7 to 2.9)	1.68 (1.32 to 2.08)	0.75 (0.56 to 0.96)
2010	0.0 (0.0 to 0.0)	0.0 (0.0 to 0.0)	5.2 (4.2 to 6.5)	2.3 (1.7 to 2.9)	1.90 (1.51 to 2.37)	0.82 (0.63 to 1.06)
2015	0.0 (0.0 to 0.0)	0.0 (0.0 to 0.0)	4.3 (3.7 to 5.0)	1.9 (1.6 to 2.2)	1.69 (1.45 to 1.94)	0.75 (0.63 to 0.87)
2020	0.0 (0.0 to 0.0)	0.0 (0.0 to 0.0)	4.5 (3.9 to 5.2)	2.1 (1.8 to 2.3)	1.94 (1.67 to 2.24)	0.89 (0.77 to 1.01)
2021	0.0 (0.0 to 0.0)	0.0 (0.0 to 0.0)	4.9 (3.8 to 6.3)	2.2 (1.8 to 2.8)	2.01 (1.56 to 2.57)	0.91 (0.72 to 1.15)

South Gloucestershire GBR_44778 Western Europe Stillbirth Rate / Neonatal Mortality Rate (SBR/NMR) − 20 weeks
lambda = 0.6 ; zeta = 0.8 ; scale = 15 ; amp = 0.028

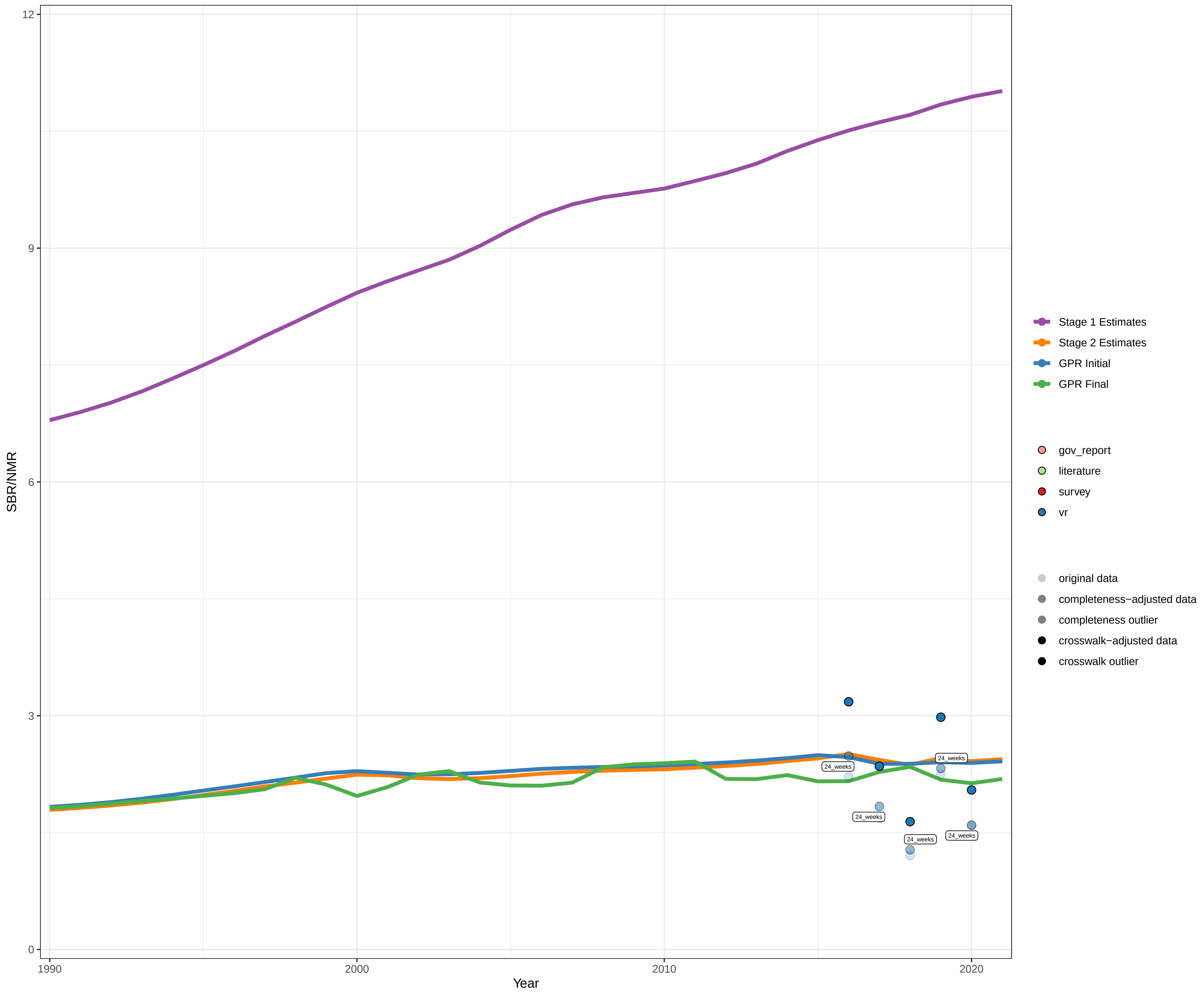

South Gloucestershire GBR_44778 Western Europe Stillbirth Rate / Neonatal Mortality Rate (SBR/NMR) − 28 weeks
lambda = 0.6 ; zeta = 0.8 ; scale = 15 ; amp = 0.028

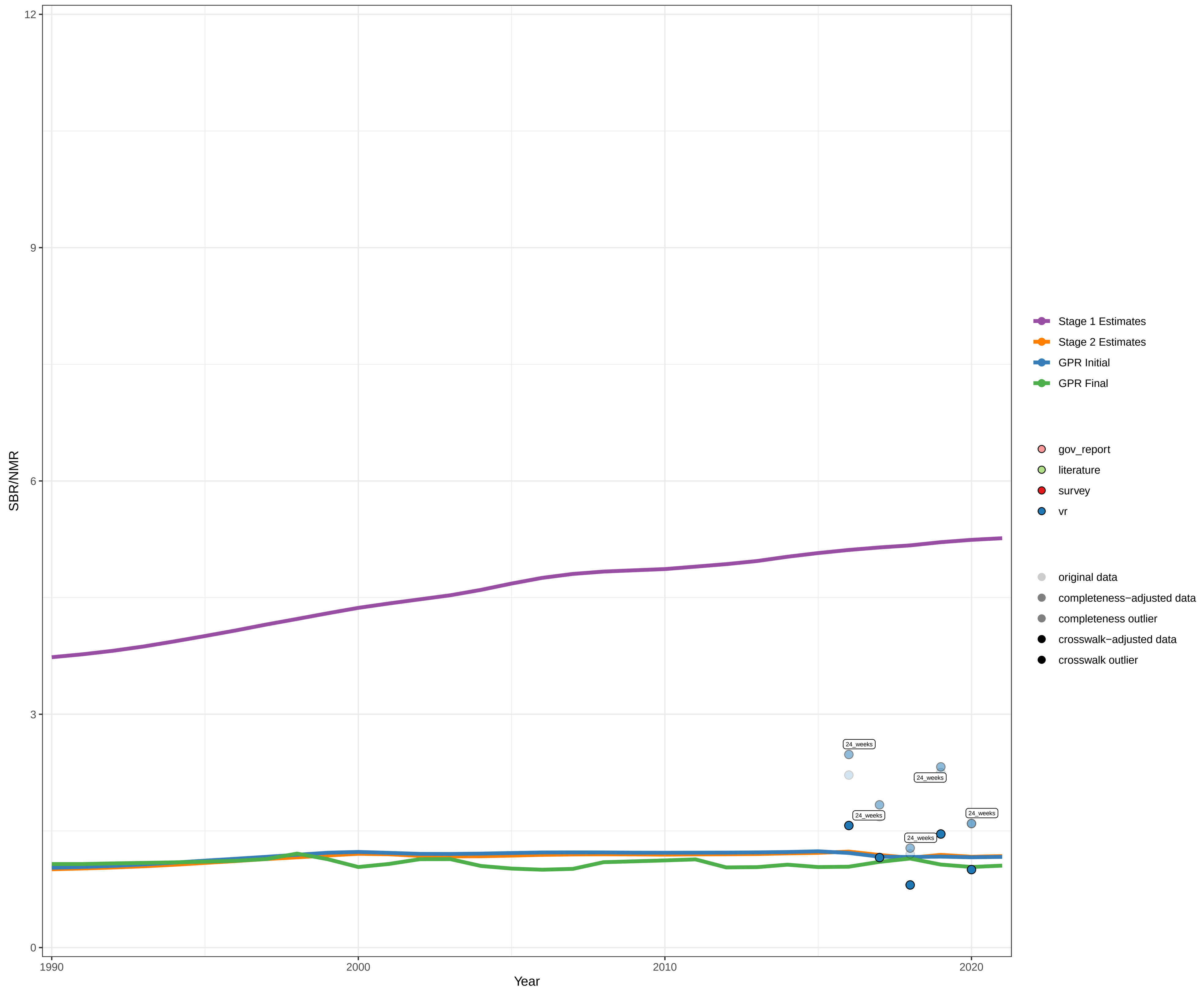

Definition	Years	Outlier	Source.Type	Title
24_weeks	2016−2020	0	VR	United Kingdom − England and Wales Child Mortality Statistics

South Gloucestershire GBR_44778 Western Europe Stillbirth Rate (SBR) − 20 weeks
lambda = 0.6 ; zeta = 0.8 ; scale = 15 ; amp = 0.028

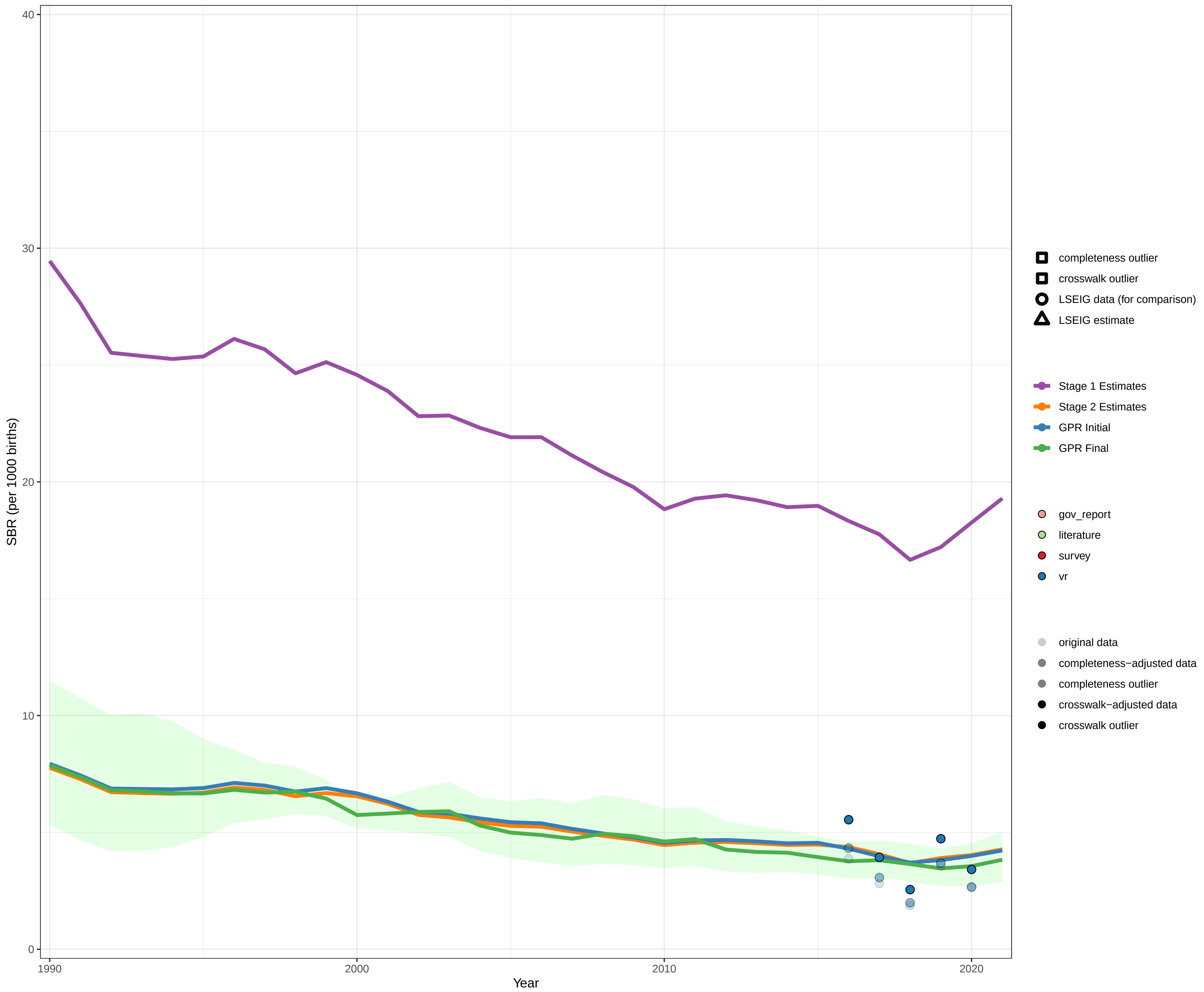

South Gloucestershire GBR_44778 Western Europe Stillbirth Rate (SBR) − 28 weeks
lambda = 0.6 ; zeta = 0.8 ; scale = 15 ; amp = 0.028

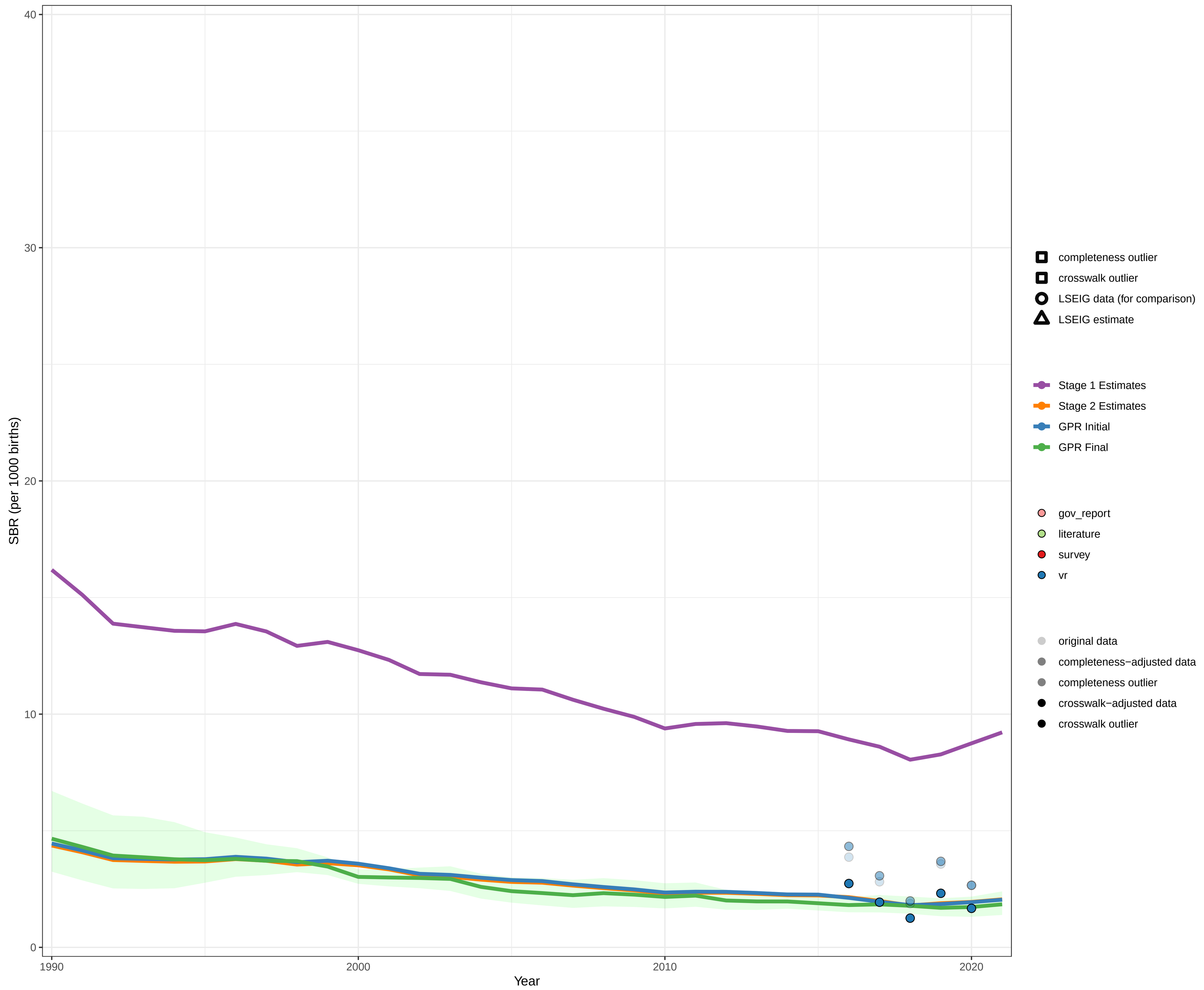

Year	Total Stillbirths (in thousands)	Total Stillbirths (in thousands)	SBR (per 1000 births)	SBR (per 1000 births)	SBR/NMR	SBR/NMR
	20 weeks	28 weeks	20 weeks	28 weeks	20 weeks	28 weeks
1990	0.0 (0.0 to 0.0)	0.0 (0.0 to 0.0)	7.9 (5.3 to 11.5)	4.7 (3.2 to 6.7)	1.82 (1.22 to 2.65)	1.07 (0.75 to 1.55)
1995	0.0 (0.0 to 0.0)	0.0 (0.0 to 0.0)	6.7 (4.8 to 9.0)	3.7 (2.8 to 4.9)	1.97 (1.42 to 2.66)	1.11 (0.82 to 1.46)
2000	0.0 (0.0 to 0.0)	0.0 (0.0 to 0.0)	5.7 (5.2 to 6.4)	3.0 (2.7 to 3.3)	1.97 (1.77 to 2.18)	1.04 (0.93 to 1.15)
2005	0.0 (0.0 to 0.0)	0.0 (0.0 to 0.0)	5.0 (3.9 to 6.3)	2.4 (1.9 to 3.0)	2.10 (1.65 to 2.67)	1.02 (0.81 to 1.26)
2010	0.0 (0.0 to 0.0)	0.0 (0.0 to 0.0)	4.6 (3.5 to 6.0)	2.2 (1.7 to 2.7)	2.39 (1.79 to 3.13)	1.12 (0.87 to 1.43)
2015	0.0 (0.0 to 0.0)	0.0 (0.0 to 0.0)	3.9 (3.2 to 4.8)	1.9 (1.6 to 2.2)	2.16 (1.75 to 2.64)	1.04 (0.86 to 1.22)
2020	0.0 (0.0 to 0.0)	0.0 (0.0 to 0.0)	3.6 (2.7 to 4.5)	1.7 (1.3 to 2.2)	2.13 (1.61 to 2.72)	1.04 (0.78 to 1.30)
2021	0.0 (0.0 to 0.0)	0.0 (0.0 to 0.0)	3.8 (2.9 to 5.0)	1.8 (1.4 to 2.4)	2.19 (1.64 to 2.87)	1.05 (0.79 to 1.37)

Swindon GBR_44787 Western Europe Stillbirth Rate / Neonatal Mortality Rate (SBR/NMR) − 20 weeks
lambda = 0.6 ; zeta = 0.8 ; scale = 15 ; amp = 0.028

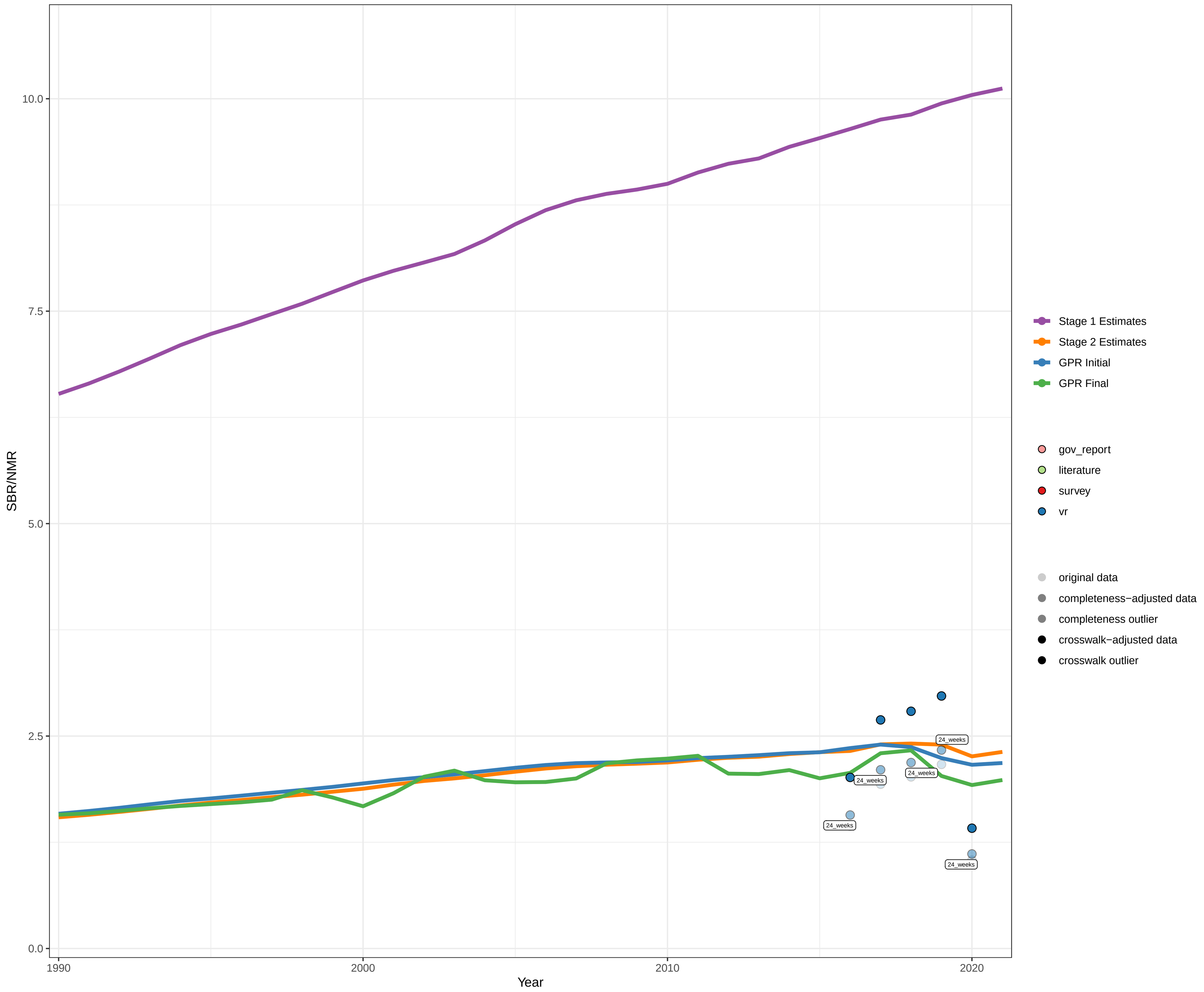

Swindon GBR_44787 Western Europe Stillbirth Rate / Neonatal Mortality Rate (SBR/NMR) − 28 weeks
lambda = 0.6 ; zeta = 0.8 ; scale = 15 ; amp = 0.028

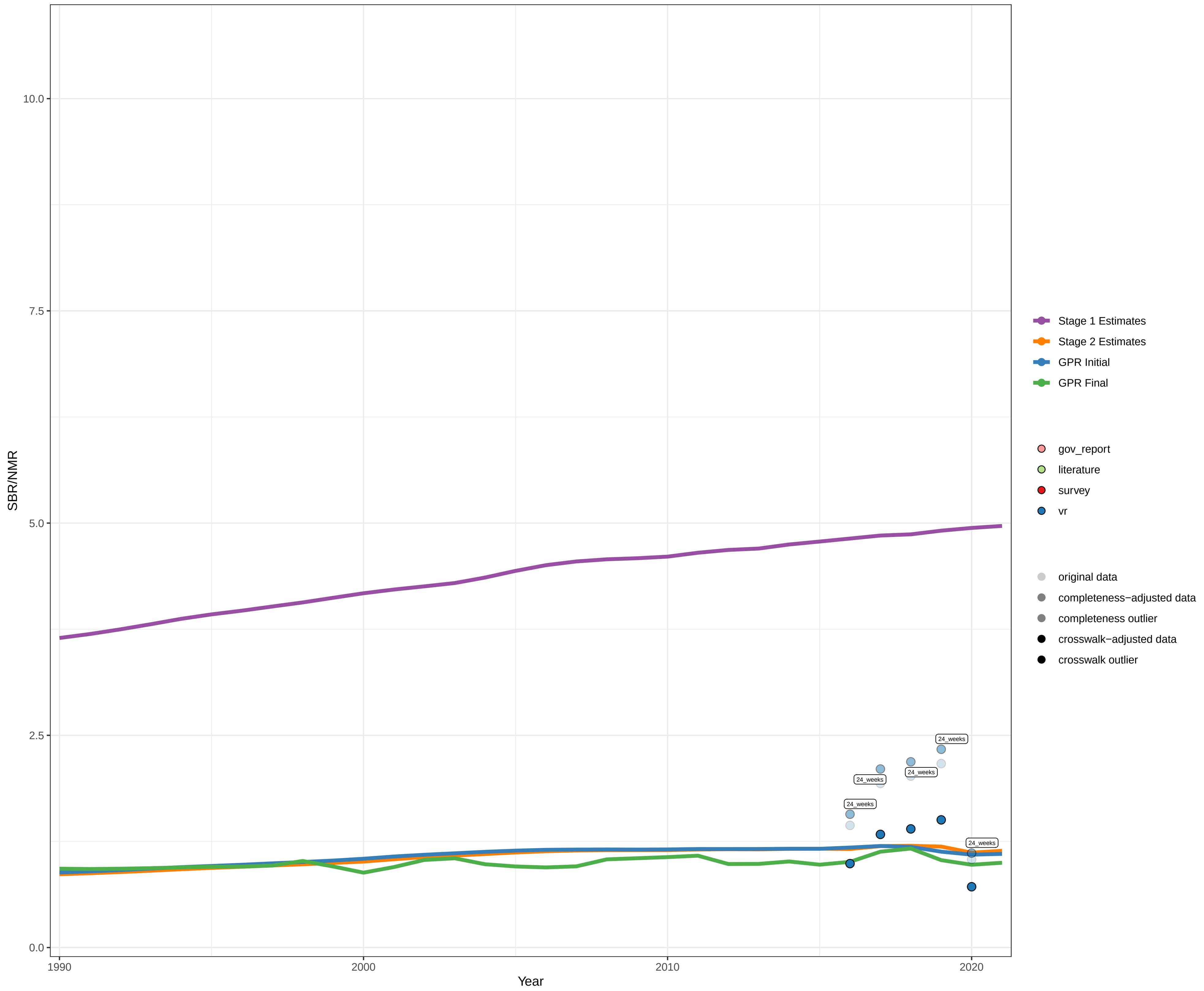

Definition	Years	Outlier	Source.Type	Title
24_weeks	2016−2020	0	VR	United Kingdom − England and Wales Child Mortality Statistics

Swindon GBR_44787 Western Europe Stillbirth Rate (SBR) − 20 weeks
lambda = 0.6 ; zeta = 0.8 ; scale = 15 ; amp = 0.028

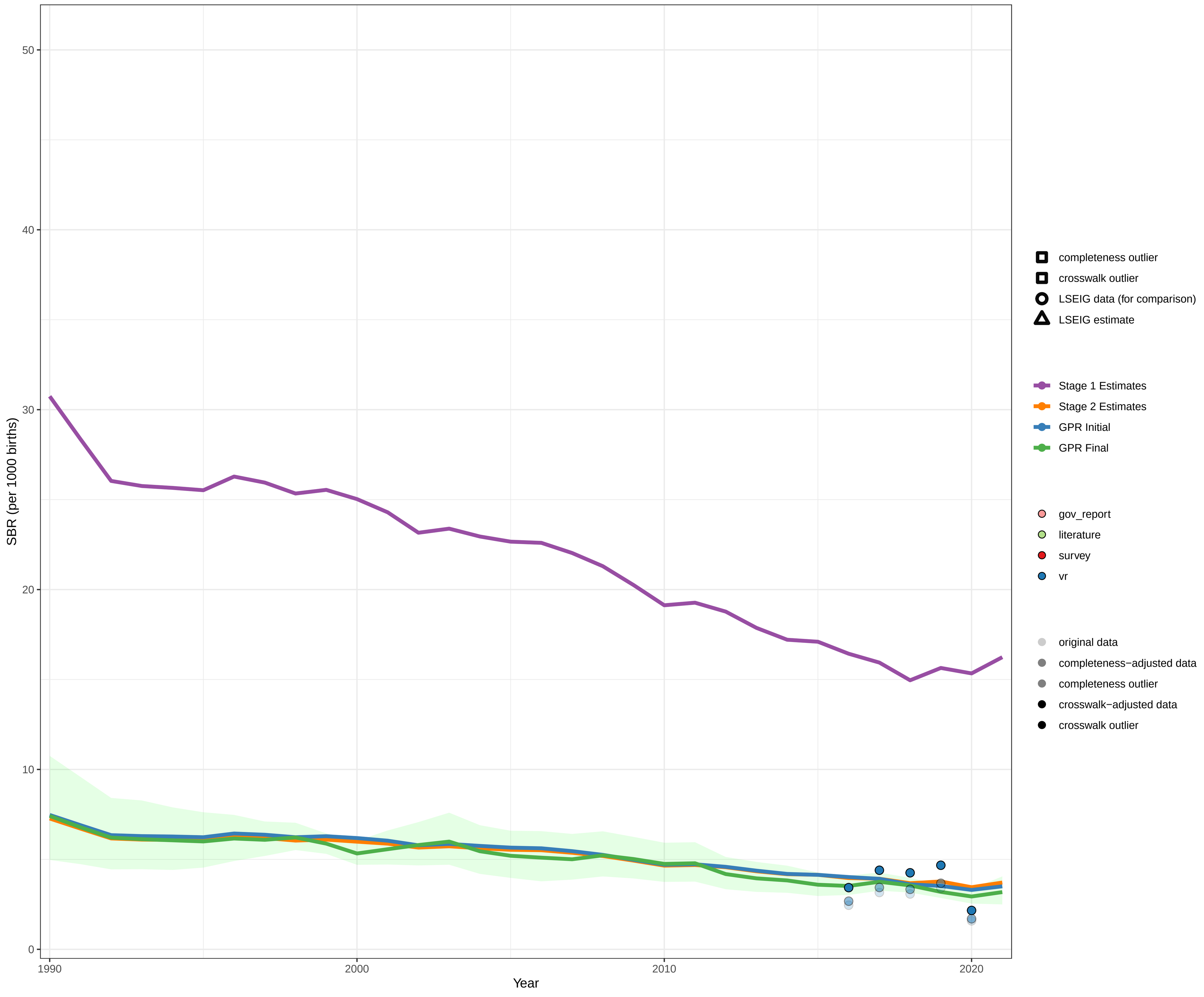

Swindon GBR_44787 Western Europe Stillbirth Rate (SBR) − 28 weeks
lambda = 0.6 ; zeta = 0.8 ; scale = 15 ; amp = 0.028

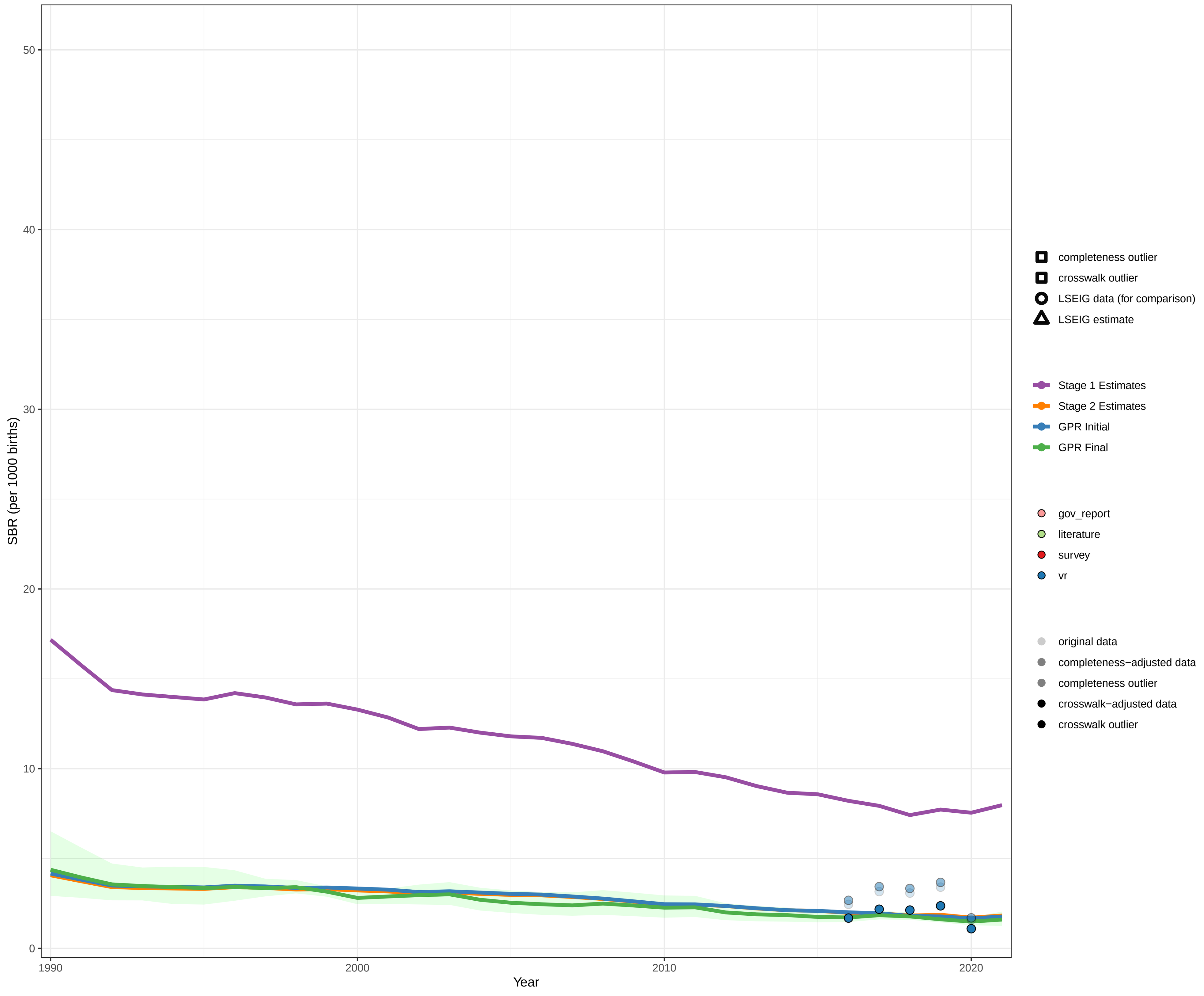

Year	Total Stillbirths (in thousands)	Total Stillbirths (in thousands)	SBR (per 1000 births)	SBR (per 1000 births)	SBR/NMR	SBR/NMR
	20 weeks	28 weeks	20 weeks	28 weeks	20 weeks	28 weeks
1990	0.0 (0.0 to 0.0)	0.0 (0.0 to 0.0)	7.4 (5.0 to 10.8)	4.4 (2.9 to 6.5)	1.57 (1.06 to 2.28)	0.93 (0.62 to 1.38)
1995	0.0 (0.0 to 0.0)	0.0 (0.0 to 0.0)	6.0 (4.5 to 7.6)	3.4 (2.4 to 4.5)	1.70 (1.29 to 2.16)	0.95 (0.69 to 1.28)
2000	0.0 (0.0 to 0.0)	0.0 (0.0 to 0.0)	5.3 (4.7 to 6.0)	2.8 (2.5 to 3.2)	1.67 (1.48 to 1.90)	0.88 (0.77 to 1.01)
2005	0.0 (0.0 to 0.0)	0.0 (0.0 to 0.0)	5.2 (4.0 to 6.6)	2.5 (2.0 to 3.2)	1.96 (1.49 to 2.48)	0.96 (0.74 to 1.21)
2010	0.0 (0.0 to 0.0)	0.0 (0.0 to 0.0)	4.7 (3.7 to 5.9)	2.3 (1.7 to 2.9)	2.23 (1.76 to 2.79)	1.07 (0.80 to 1.39)
2015	0.0 (0.0 to 0.0)	0.0 (0.0 to 0.0)	3.6 (3.0 to 4.3)	1.8 (1.5 to 2.1)	2.00 (1.65 to 2.38)	0.98 (0.81 to 1.17)
2020	0.0 (0.0 to 0.0)	0.0 (0.0 to 0.0)	2.9 (2.5 to 3.4)	1.5 (1.3 to 1.7)	1.92 (1.67 to 2.20)	0.98 (0.84 to 1.12)
2021	0.0 (0.0 to 0.0)	0.0 (0.0 to 0.0)	3.2 (2.5 to 4.1)	1.6 (1.3 to 2.0)	1.98 (1.56 to 2.53)	1.00 (0.78 to 1.28)

Torbay GBR_44788 Western Europe Stillbirth Rate / Neonatal Mortality Rate (SBR/NMR) − 20 weeks
lambda = 0.8 ; zeta = 0.7 ; scale = 15 ; amp = 0.028

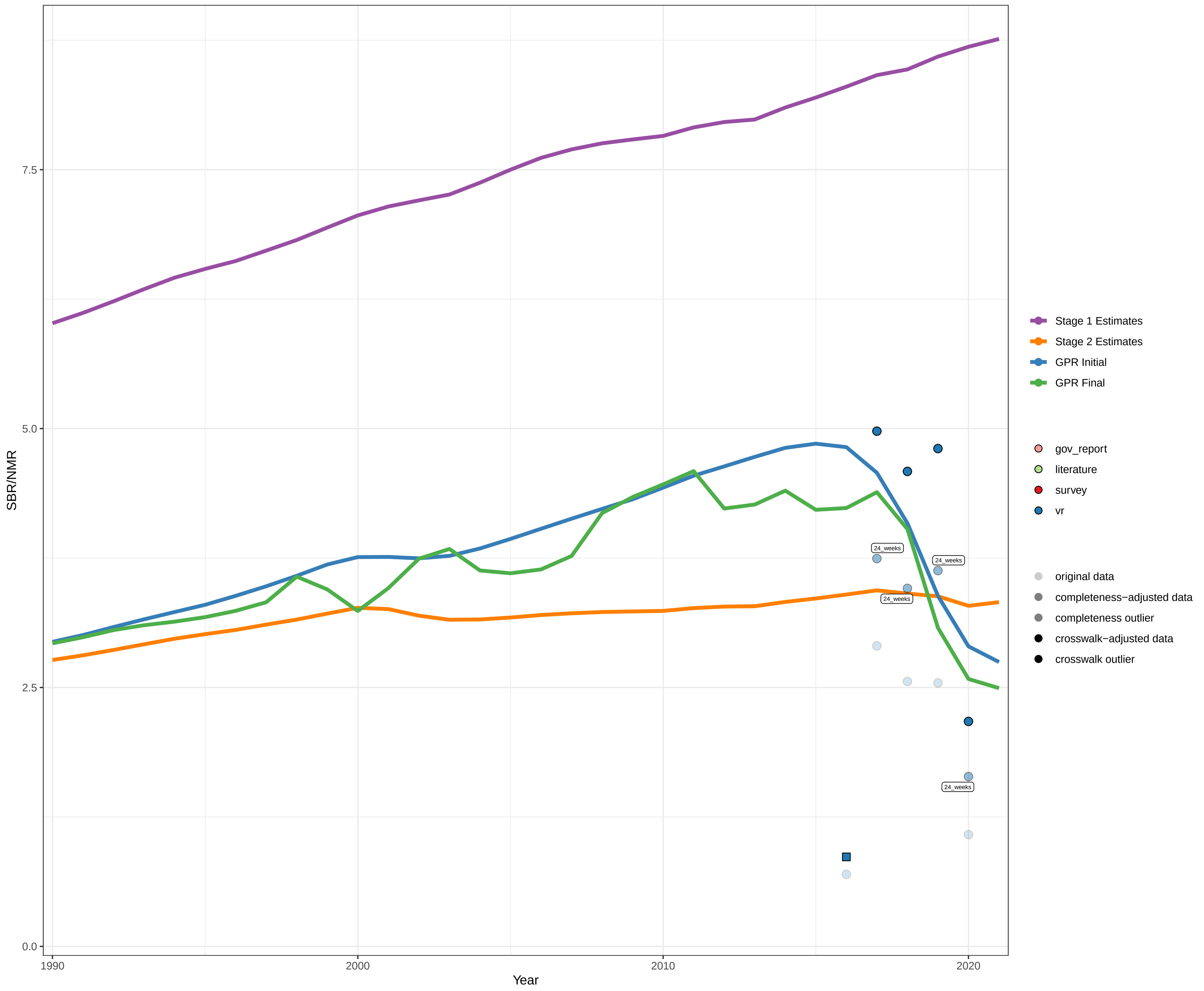

Torbay GBR_44788 Western Europe Stillbirth Rate / Neonatal Mortality Rate (SBR/NMR) − 28 weeks
lambda = 0.8 ; zeta = 0.7 ; scale = 15 ; amp = 0.028

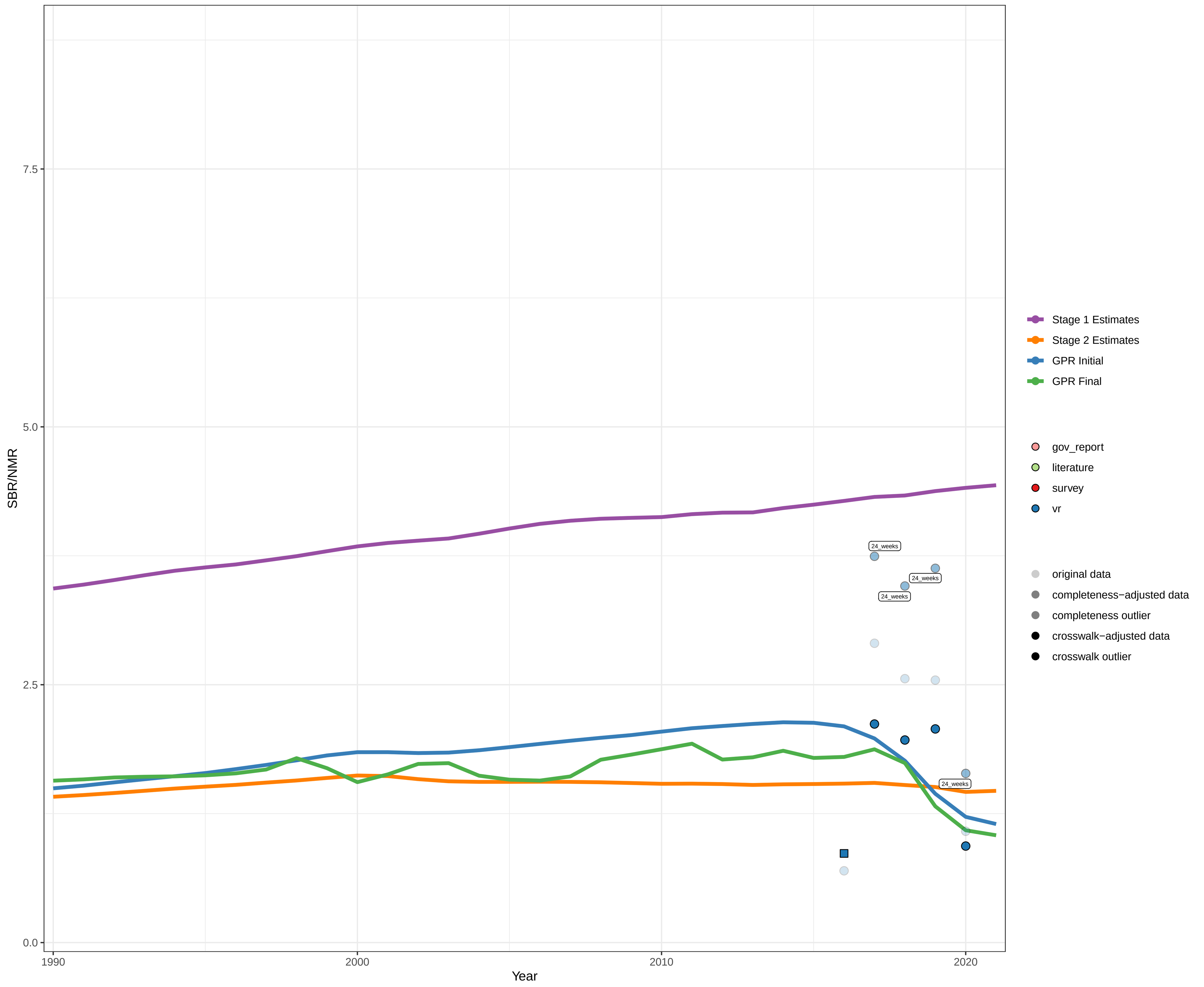

Definition	Years	Outlier	Source.Type	Title
24_weeks	2016−2020	0	VR	United Kingdom − England and Wales Child Mortality Statistics

Torbay GBR_44788 Western Europe Stillbirth Rate (SBR) − 20 weeks
lambda = 0.8 ; zeta = 0.7 ; scale = 15 ; amp = 0.028

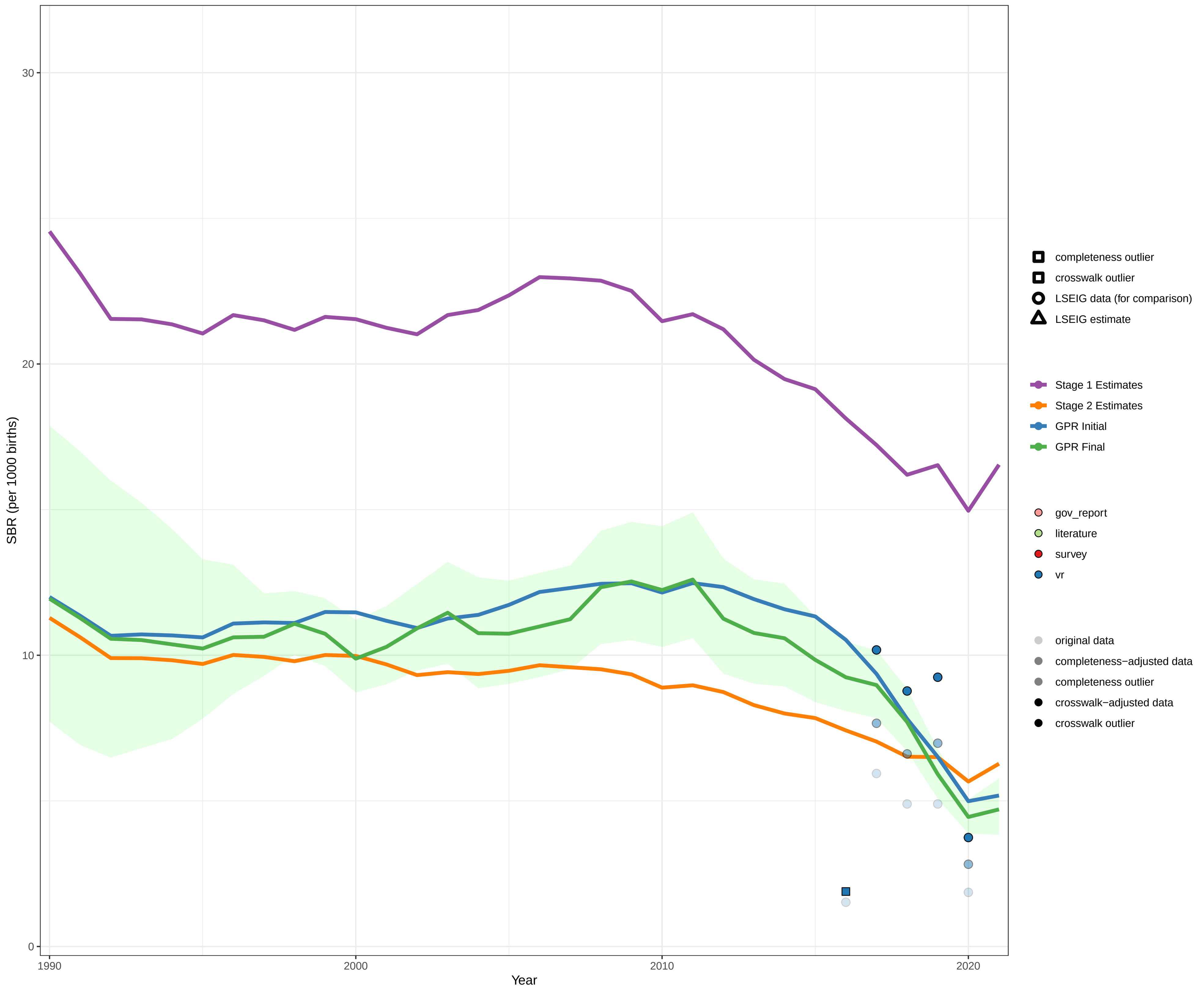

Torbay GBR_44788 Western Europe Stillbirth Rate (SBR) − 28 weeks
lambda = 0.8 ; zeta = 0.7 ; scale = 15 ; amp = 0.028

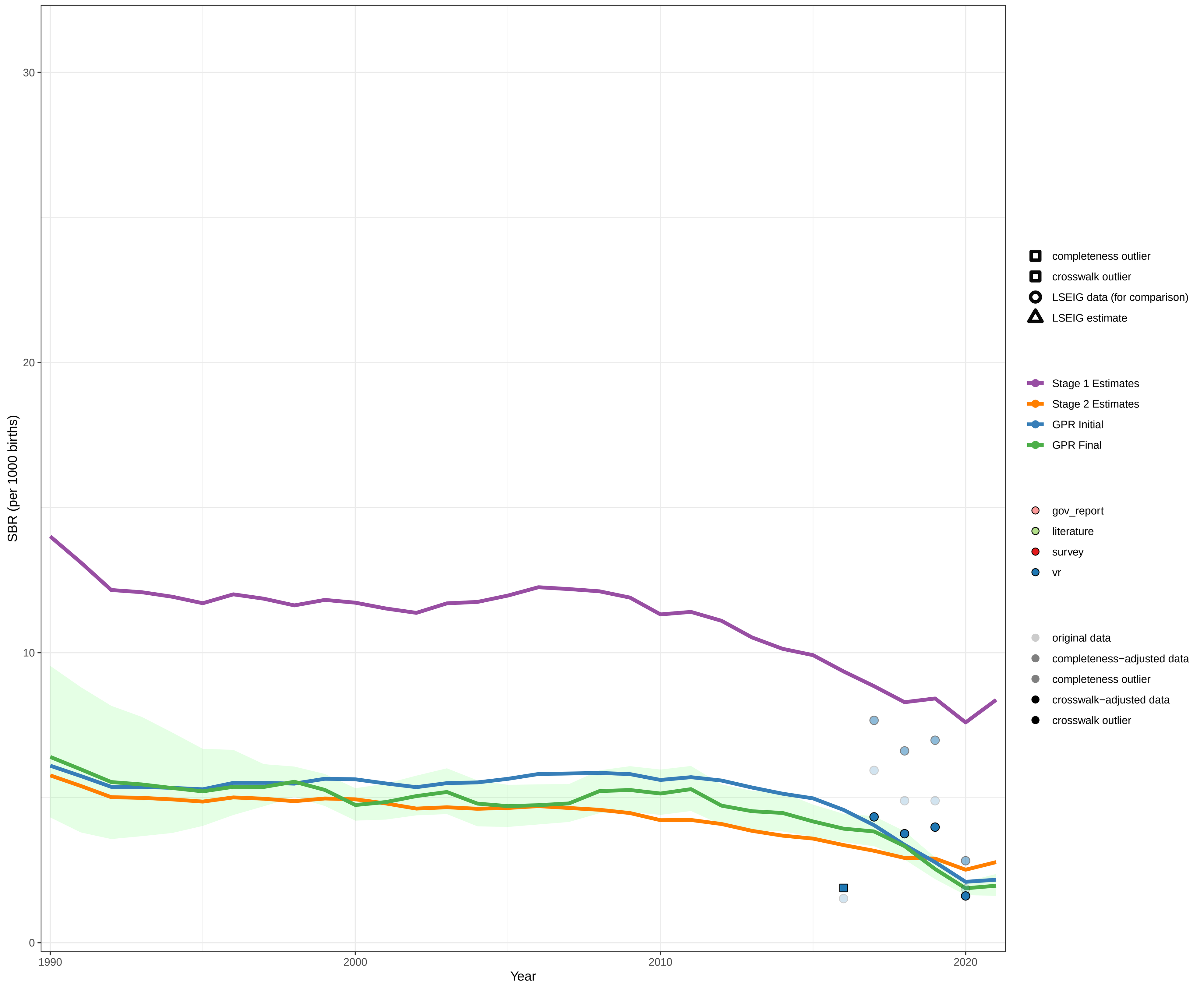

Year	Total Stillbirths (in thousands)	Total Stillbirths (in thousands)	SBR (per 1000 births)	SBR (per 1000 births)	SBR/NMR	SBR/NMR
	20 weeks	28 weeks	20 weeks	28 weeks	20 weeks	28 weeks
1990	0.0 (0.0 to 0.0)	0.0 (0.0 to 0.0)	11.9 (7.7 to 17.9)	6.4 (4.3 to 9.5)	2.93 (1.89 to 4.38)	1.57 (1.06 to 2.34)
1995	0.0 (0.0 to 0.0)	0.0 (0.0 to 0.0)	10.2 (7.8 to 13.3)	5.2 (4.0 to 6.7)	3.18 (2.43 to 4.13)	1.62 (1.25 to 2.08)
2000	0.0 (0.0 to 0.0)	0.0 (0.0 to 0.0)	9.9 (8.7 to 11.2)	4.7 (4.2 to 5.3)	3.24 (2.86 to 3.67)	1.56 (1.38 to 1.75)
2005	0.0 (0.0 to 0.0)	0.0 (0.0 to 0.0)	10.7 (9.0 to 12.6)	4.7 (4.0 to 5.4)	3.60 (3.03 to 4.21)	1.58 (1.34 to 1.83)
2010	0.0 (0.0 to 0.0)	0.0 (0.0 to 0.0)	12.2 (10.3 to 14.4)	5.1 (4.4 to 6.0)	4.46 (3.75 to 5.26)	1.87 (1.61 to 2.18)
2015	0.0 (0.0 to 0.0)	0.0 (0.0 to 0.0)	9.8 (8.4 to 11.3)	4.2 (3.6 to 4.8)	4.22 (3.60 to 4.86)	1.79 (1.56 to 2.04)
2020	0.0 (0.0 to 0.0)	0.0 (0.0 to 0.0)	4.4 (3.9 to 5.1)	1.9 (1.6 to 2.1)	2.58 (2.25 to 2.93)	1.09 (0.95 to 1.22)
2021	0.0 (0.0 to 0.0)	0.0 (0.0 to 0.0)	4.7 (3.8 to 5.8)	2.0 (1.6 to 2.4)	2.49 (2.03 to 3.05)	1.04 (0.86 to 1.25)

Wiltshire GBR_44780 Western Europe Stillbirth Rate / Neonatal Mortality Rate (SBR/NMR) − 20 weeks
lambda = 0.6 ; zeta = 0.8 ; scale = 15 ; amp = 0.028

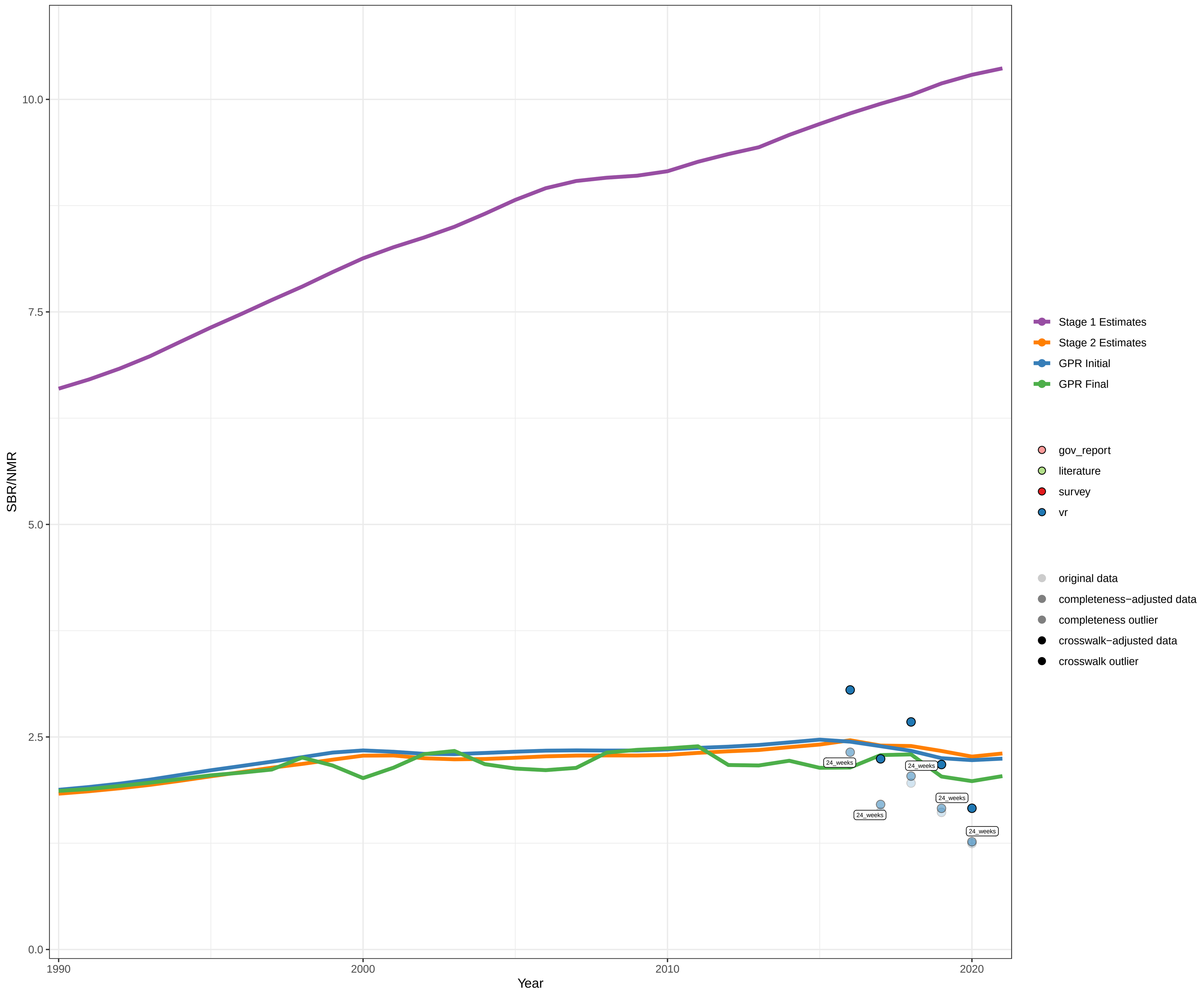

Wiltshire GBR_44780 Western Europe Stillbirth Rate / Neonatal Mortality Rate (SBR/NMR) − 28 weeks
lambda = 0.6 ; zeta = 0.8 ; scale = 15 ; amp = 0.028

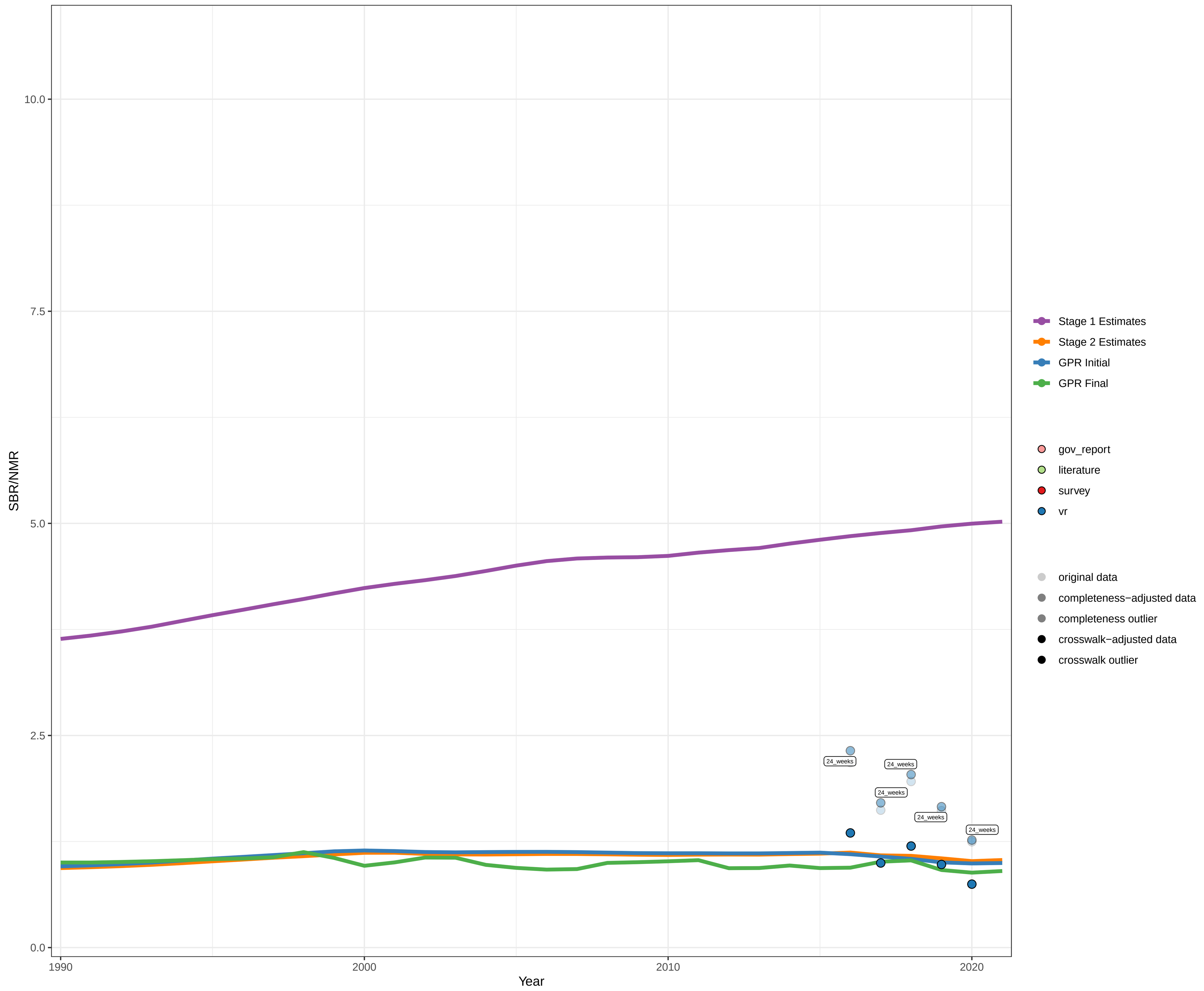

Definition	Years	Outlier	Source.Type	Title
24_weeks	2016−2020	0	VR	United Kingdom − England and Wales Child Mortality Statistics

Wiltshire GBR_44780 Western Europe Stillbirth Rate (SBR) − 20 weeks
lambda = 0.6 ; zeta = 0.8 ; scale = 15 ; amp = 0.028

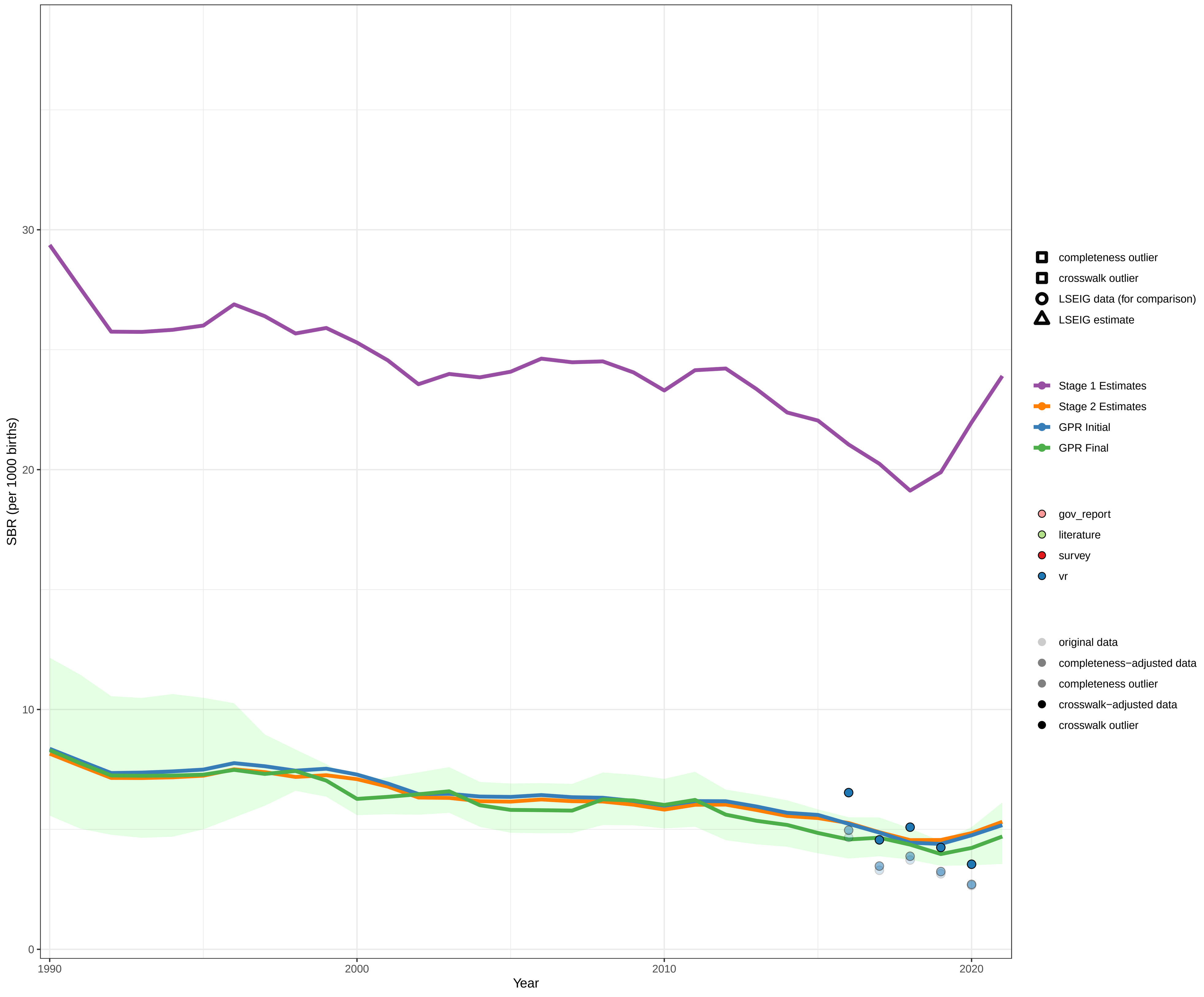

Wiltshire GBR_44780 Western Europe Stillbirth Rate (SBR) − 28 weeks
lambda = 0.6 ; zeta = 0.8 ; scale = 15 ; amp = 0.028

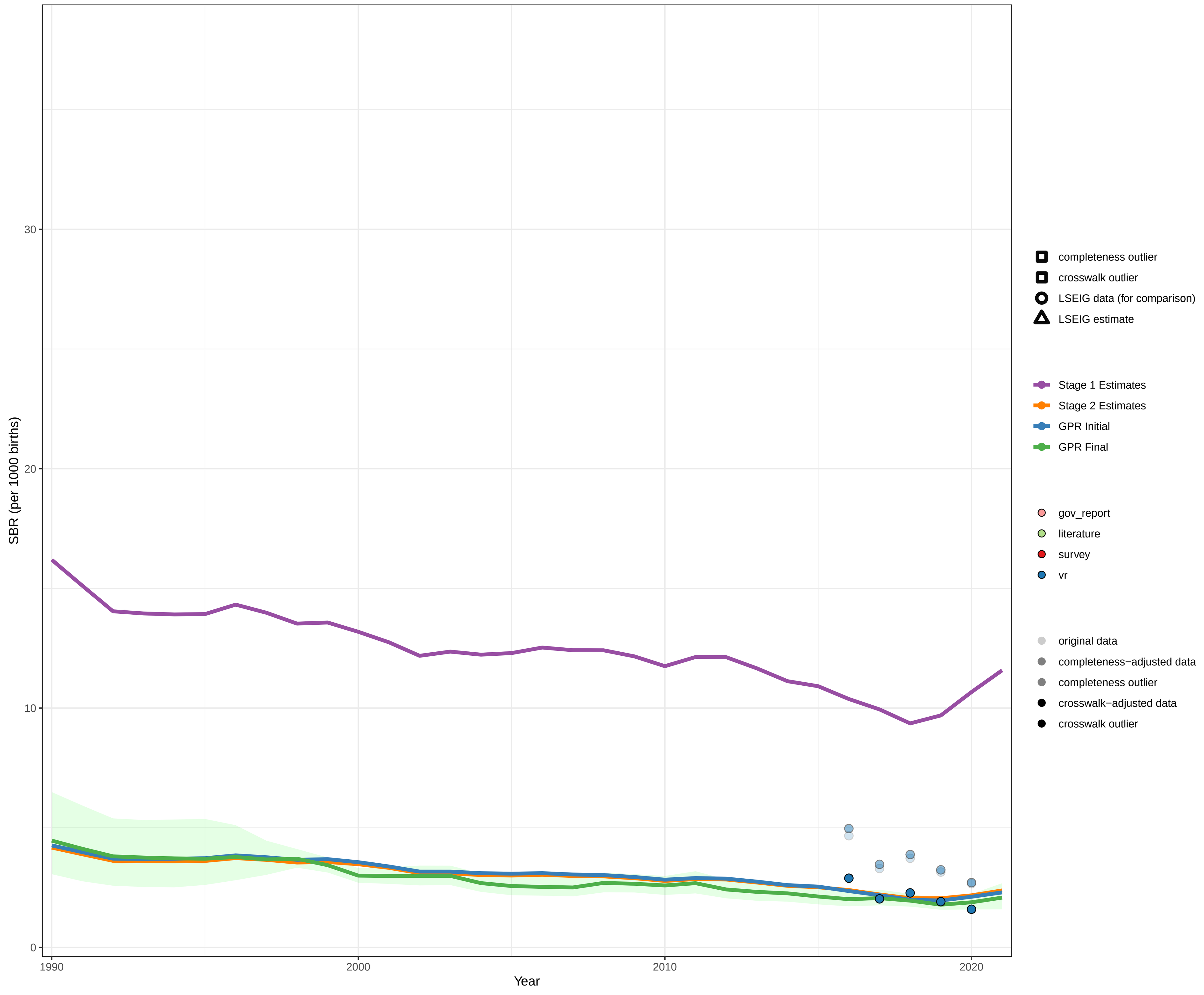

Year	Total Stillbirths (in thousands)	Total Stillbirths (in thousands)	SBR (per 1000 births)	SBR (per 1000 births)	SBR/NMR	SBR/NMR
	20 weeks	28 weeks	20 weeks	28 weeks	20 weeks	28 weeks
1990	0.0 (0.0 to 0.1)	0.0 (0.0 to 0.0)	8.3 (5.6 to 12.2)	4.5 (3.1 to 6.5)	1.87 (1.25 to 2.73)	1.00 (0.69 to 1.46)
1995	0.0 (0.0 to 0.1)	0.0 (0.0 to 0.0)	7.3 (5.0 to 10.5)	3.7 (2.6 to 5.4)	2.05 (1.41 to 2.95)	1.04 (0.73 to 1.51)
2000	0.0 (0.0 to 0.0)	0.0 (0.0 to 0.0)	6.3 (5.6 to 7.0)	3.0 (2.7 to 3.3)	2.02 (1.80 to 2.25)	0.96 (0.87 to 1.06)
2005	0.0 (0.0 to 0.0)	0.0 (0.0 to 0.0)	5.8 (4.9 to 6.9)	2.6 (2.2 to 3.0)	2.13 (1.78 to 2.53)	0.94 (0.80 to 1.10)
2010	0.0 (0.0 to 0.0)	0.0 (0.0 to 0.0)	6.0 (5.0 to 7.1)	2.6 (2.2 to 3.0)	2.37 (1.98 to 2.79)	1.02 (0.86 to 1.17)
2015	0.0 (0.0 to 0.0)	0.0 (0.0 to 0.0)	4.9 (4.0 to 5.8)	2.1 (1.8 to 2.5)	2.14 (1.76 to 2.57)	0.94 (0.79 to 1.09)
2020	0.0 (0.0 to 0.0)	0.0 (0.0 to 0.0)	4.2 (3.5 to 5.1)	1.9 (1.6 to 2.2)	1.98 (1.64 to 2.39)	0.88 (0.74 to 1.05)
2021	0.0 (0.0 to 0.0)	0.0 (0.0 to 0.0)	4.7 (3.6 to 6.1)	2.1 (1.6 to 2.7)	2.04 (1.54 to 2.66)	0.90 (0.69 to 1.16)

West Midlands GBR_4622 Western Europe Stillbirth Rate / Neonatal Mortality Rate (SBR/NMR) − 20 weeks
lambda = 0.2 ; zeta = 0.99 ; scale = 5 ; amp = 0.028

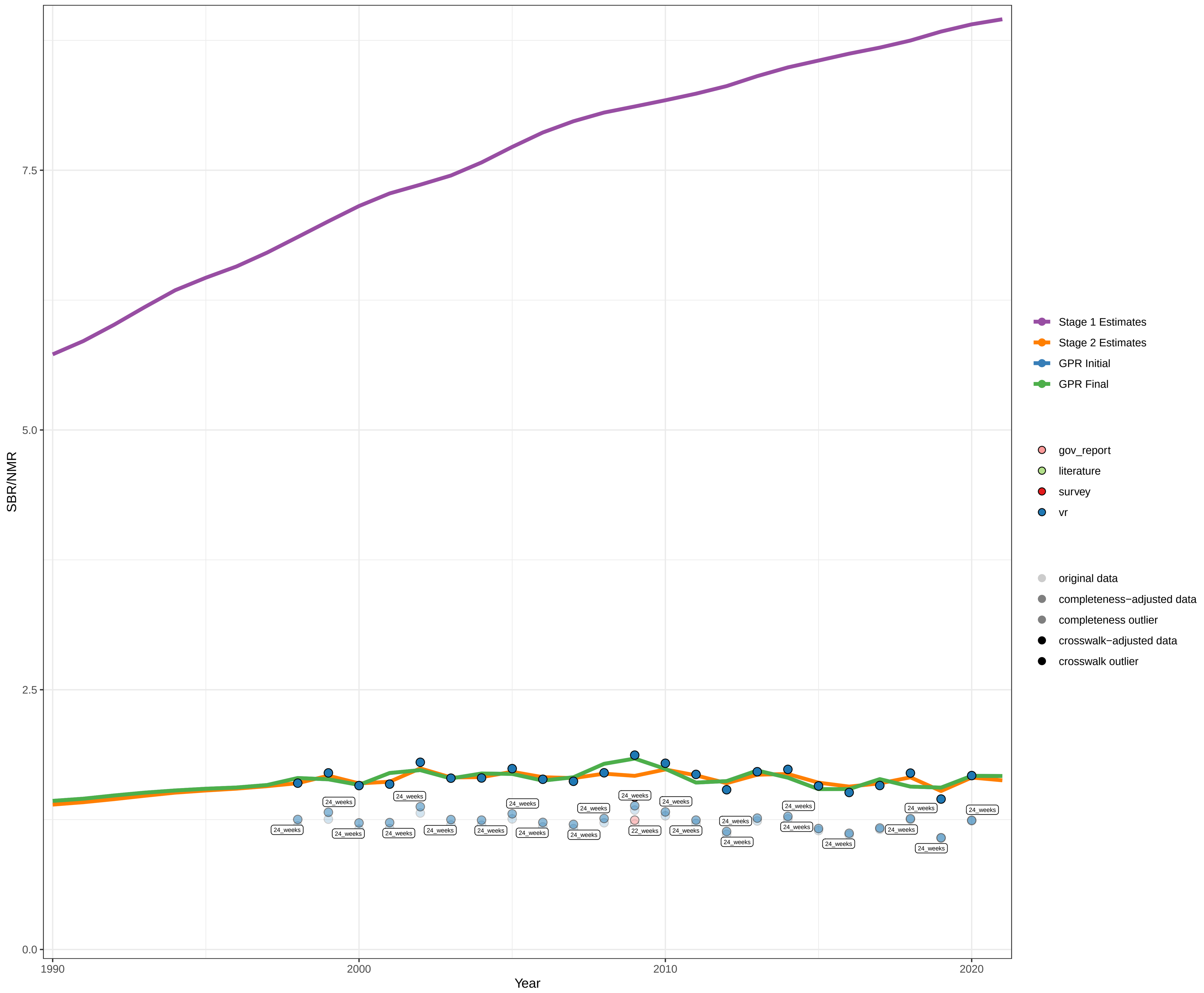

West Midlands GBR_4622 Western Europe Stillbirth Rate / Neonatal Mortality Rate (SBR/NMR) − 28 weeks
lambda = 0.2 ; zeta = 0.99 ; scale = 5 ; amp = 0.028

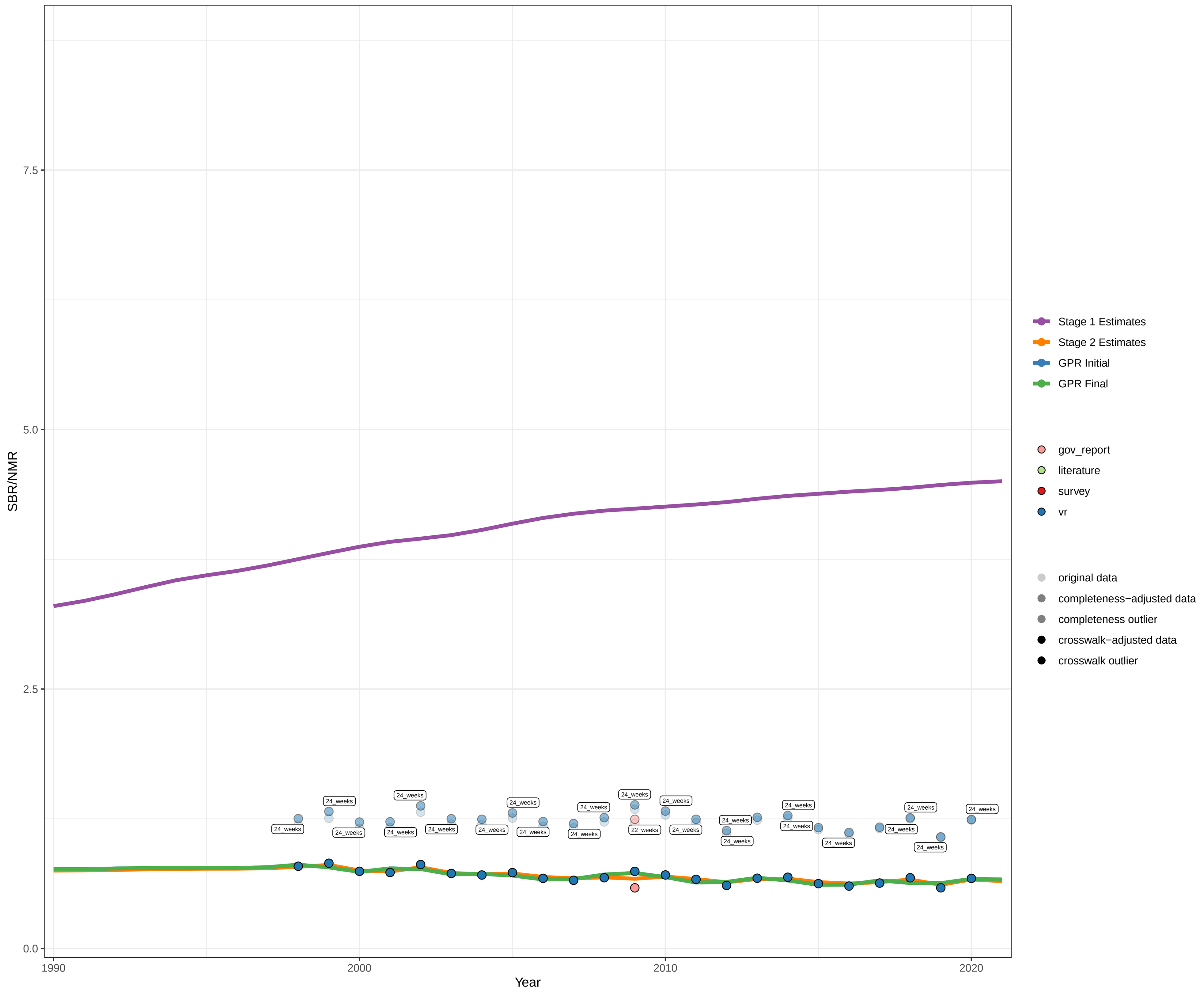

Definition	Years	Outlier	Source.Type	Title
22_weeks	2009	0	Statistical Report	United Kingdom Perinatal Mortality
24_weeks	1998−2007	0	VR	United Kingdom − England and Wales Birth Statistics
24_weeks	2008−2020	0	VR	United Kingdom − England and Wales Child Mortality Statistics

West Midlands GBR_4622 Western Europe Stillbirth Rate (SBR) − 20 weeks
lambda = 0.2 ; zeta = 0.99 ; scale = 5 ; amp = 0.028

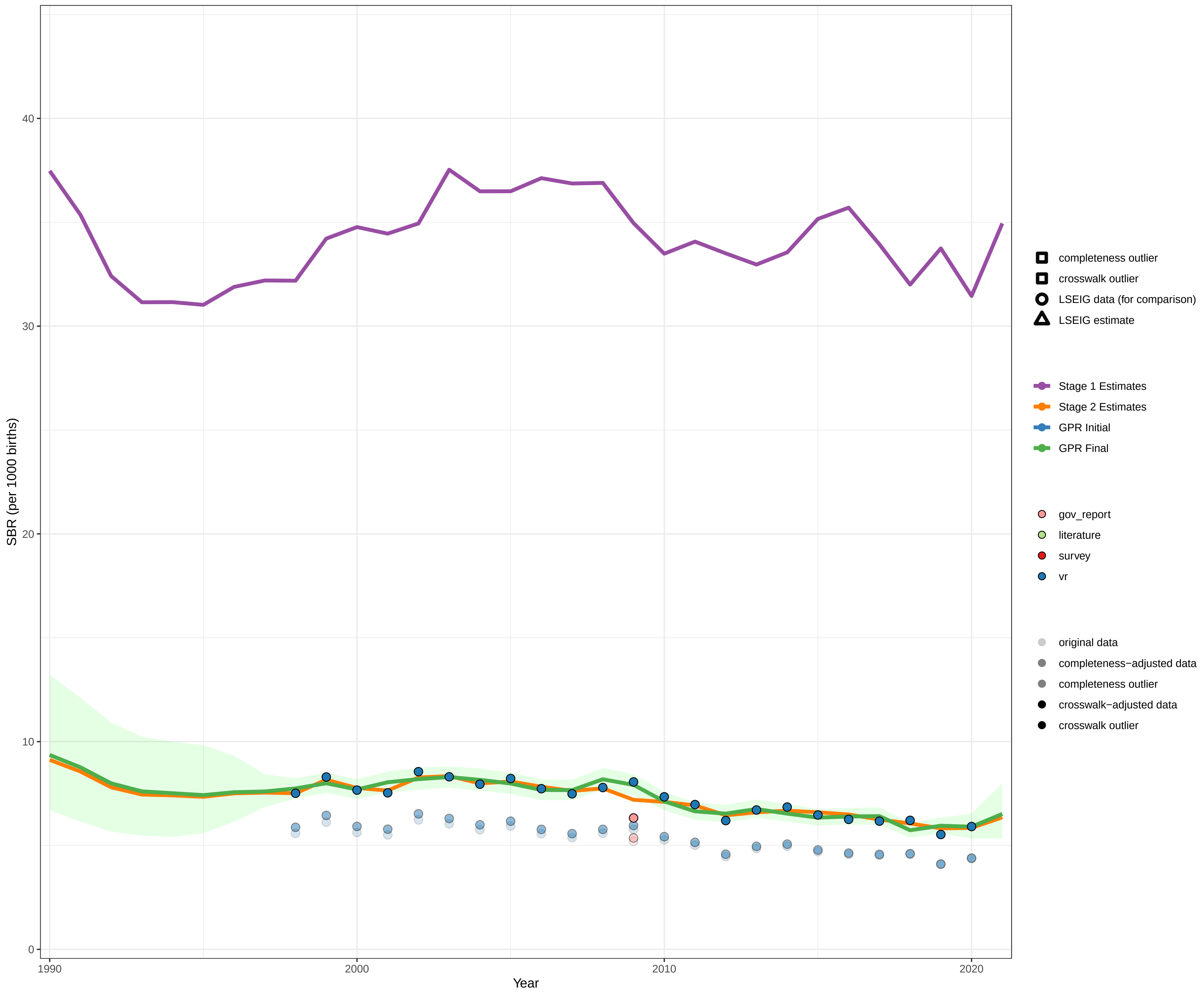

West Midlands GBR_4622 Western Europe Stillbirth Rate (SBR) − 28 weeks
lambda = 0.2 ; zeta = 0.99 ; scale = 5 ; amp = 0.028

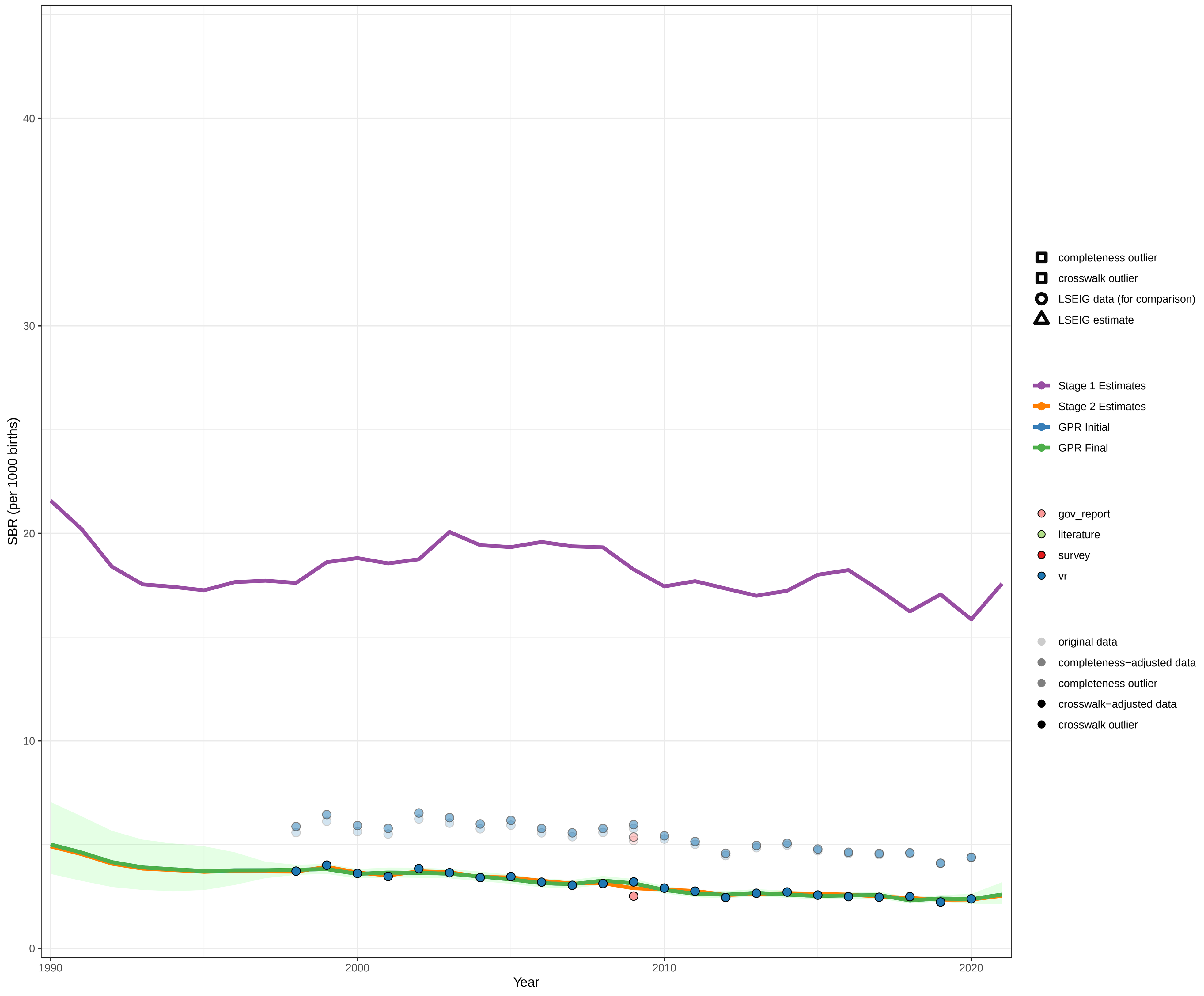

Year	Total Stillbirths (in thousands)	Total Stillbirths (in thousands)	SBR (per 1000 births)	SBR (per 1000 births)	SBR/NMR	SBR/NMR
	20 weeks	28 weeks	20 weeks	28 weeks	20 weeks	28 weeks
1990	0.7 (0.5 to 1.0)	0.4 (0.3 to 0.5)	9.4 (6.7 to 13.2)	5.0 (3.6 to 7.1)	1.43 (1.02 to 2.02)	0.77 (0.55 to 1.08)
1995	0.5 (0.4 to 0.7)	0.3 (0.2 to 0.3)	7.4 (5.6 to 9.8)	3.7 (2.8 to 4.9)	1.55 (1.16 to 2.05)	0.78 (0.59 to 1.03)
2000	0.5 (0.4 to 0.5)	0.2 (0.2 to 0.2)	7.7 (7.2 to 8.2)	3.6 (3.4 to 3.8)	1.58 (1.49 to 1.69)	0.74 (0.69 to 0.79)
2005	0.5 (0.5 to 0.6)	0.2 (0.2 to 0.2)	8.0 (7.5 to 8.5)	3.3 (3.1 to 3.5)	1.69 (1.58 to 1.80)	0.70 (0.66 to 0.75)
2010	0.5 (0.5 to 0.6)	0.2 (0.2 to 0.2)	7.1 (6.7 to 7.6)	2.8 (2.6 to 3.0)	1.74 (1.63 to 1.85)	0.69 (0.65 to 0.73)
2015	0.4 (0.4 to 0.5)	0.2 (0.2 to 0.2)	6.3 (5.9 to 6.8)	2.5 (2.4 to 2.7)	1.54 (1.45 to 1.64)	0.61 (0.58 to 0.65)
2020	0.4 (0.3 to 0.4)	0.2 (0.1 to 0.2)	5.9 (5.3 to 6.5)	2.4 (2.1 to 2.6)	1.67 (1.51 to 1.85)	0.67 (0.60 to 0.74)
2021	0.4 (0.3 to 0.5)	0.2 (0.1 to 0.2)	6.5 (5.3 to 8.0)	2.6 (2.1 to 3.2)	1.67 (1.37 to 2.05)	0.67 (0.54 to 0.82)

Birmingham   GBR_44714   Western Europe   Stillbirth Rate / Neonatal Mortality Rate (SBR/NMR) − 20 weeks
lambda = 0.6 ; zeta = 0.8 ; scale = 15 ; amp = 0.028

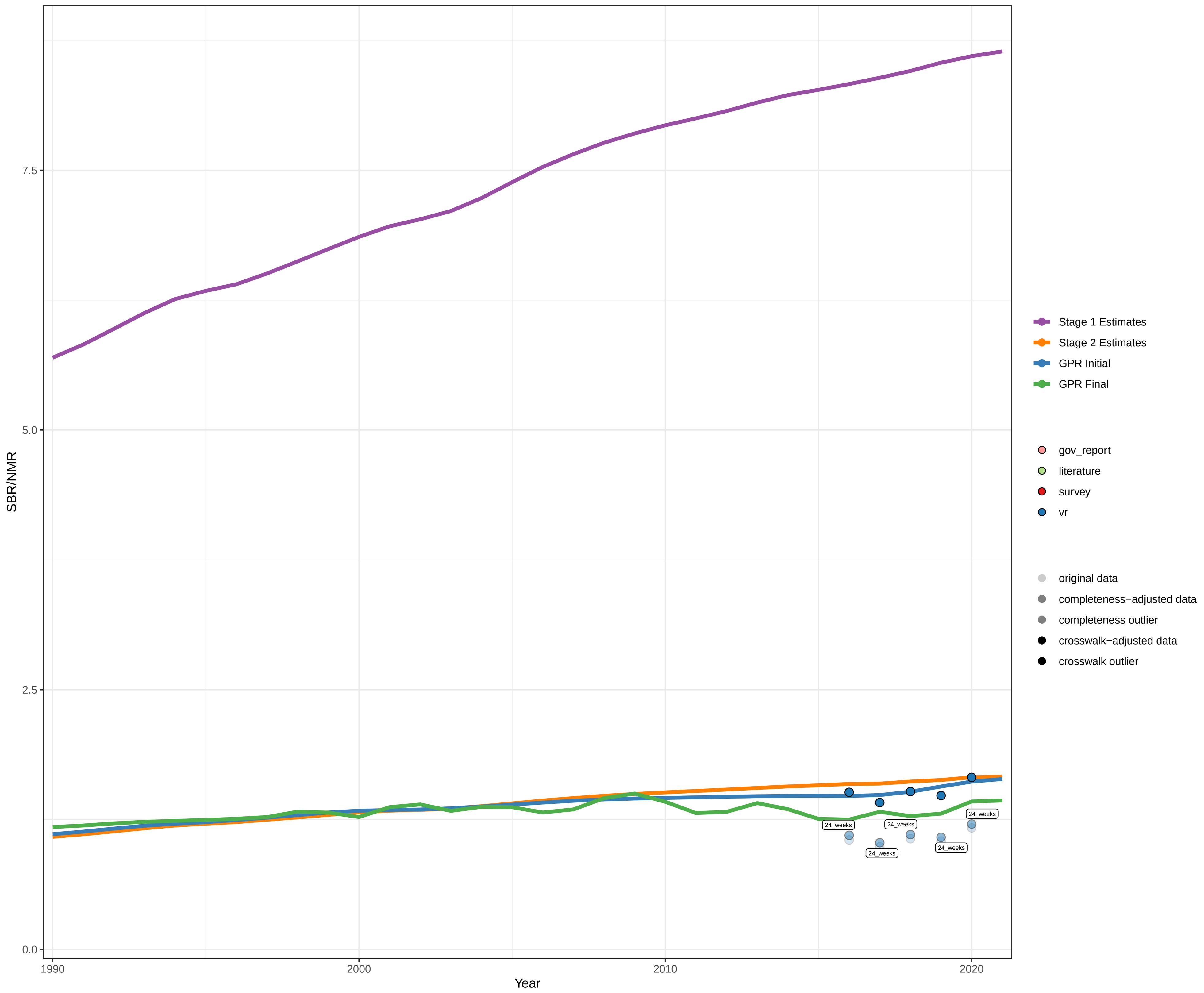

Birmingham   GBR_44714   Western Europe   Stillbirth Rate / Neonatal Mortality Rate (SBR/NMR) − 28 weeks
lambda = 0.6 ; zeta = 0.8 ; scale = 15 ; amp = 0.028

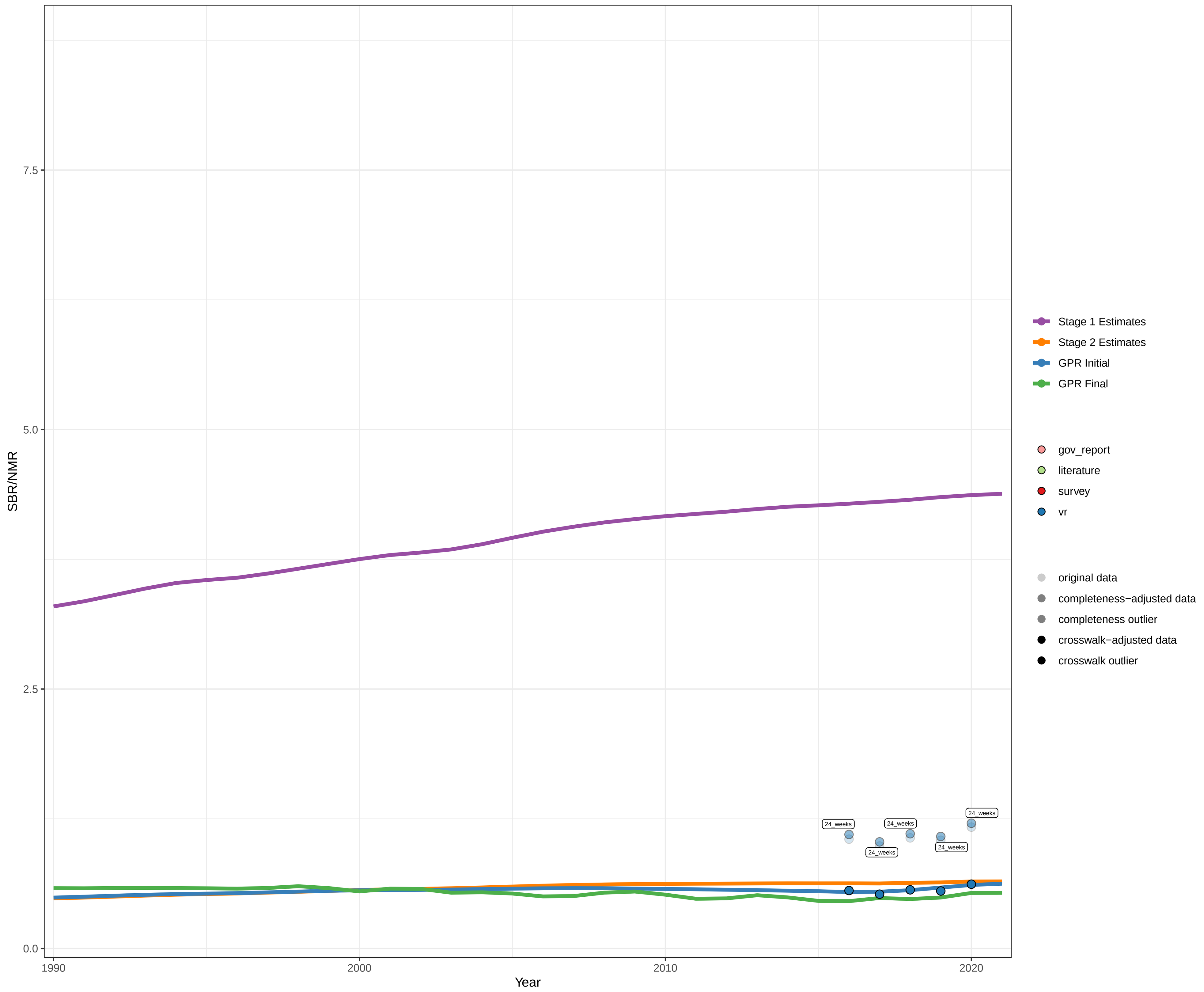

Definition	Years	Outlier	Source.Type	Title
24_weeks	2016−2020	0	VR	United Kingdom − England and Wales Child Mortality Statistics

Birmingham   GBR_44714   Western Europe   Stillbirth Rate (SBR) − 20 weeks
lambda = 0.6 ; zeta = 0.8 ; scale = 15 ; amp = 0.028

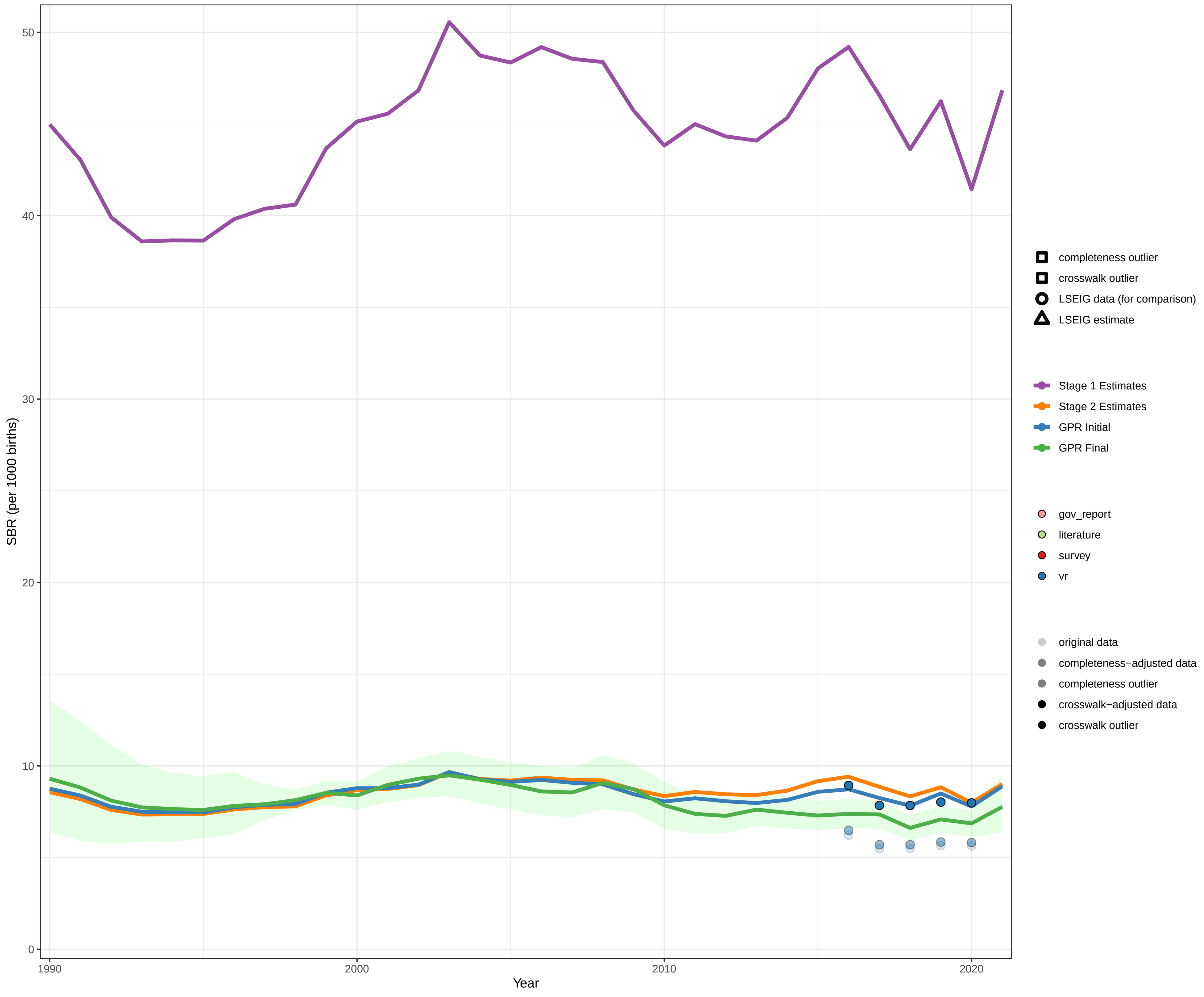

Birmingham   GBR_44714   Western Europe   Stillbirth Rate (SBR) − 28 weeks
lambda = 0.6 ; zeta = 0.8 ; scale = 15 ; amp = 0.028

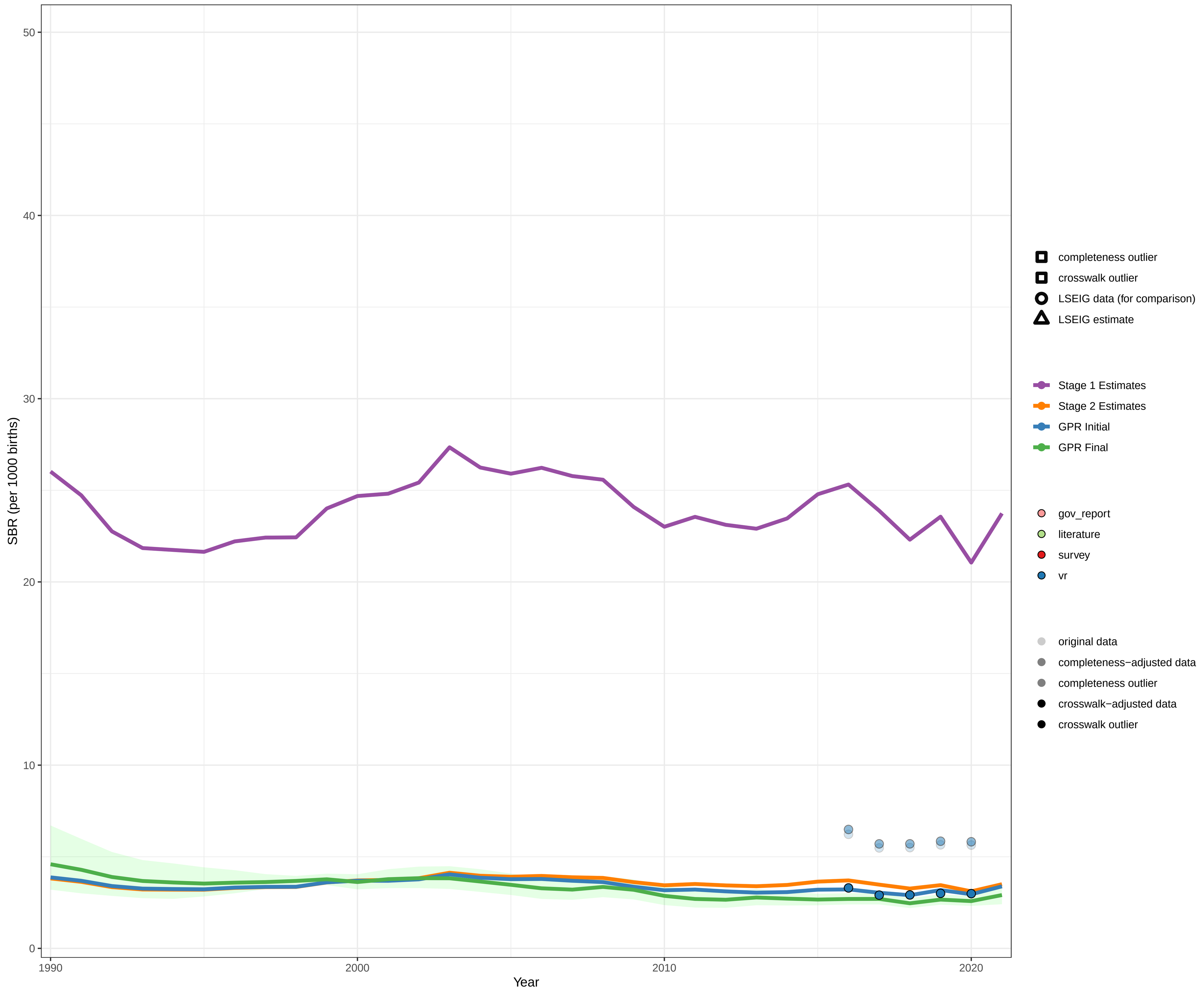

Year	Total Stillbirths (in thousands)	Total Stillbirths (in thousands)	SBR (per 1000 births)	SBR (per 1000 births)	SBR/NMR	SBR/NMR
	20 weeks	28 weeks	20 weeks	28 weeks	20 weeks	28 weeks
1990	0.2<br>(0.1 to 0.2)	0.1<br>(0.1 to 0.1)	9.3<br>(6.4 to 13.6)	4.6<br>(3.2 to 6.7)	1.18<br>(0.81 to 1.72)	0.58<br>(0.41 to 0.85)
1995	0.1<br>(0.1 to 0.1)	0.1<br>(0.0 to 0.1)	7.6<br>(6.1 to 9.4)	3.5<br>(2.8 to 4.4)	1.25<br>(0.99 to 1.55)	0.58<br>(0.46 to 0.73)
2000	0.1<br>(0.1 to 0.1)	0.1<br>(0.0 to 0.1)	8.4<br>(7.6 to 9.2)	3.6<br>(3.2 to 4.0)	1.28<br>(1.16 to 1.39)	0.55<br>(0.49 to 0.61)
2005	0.1<br>(0.1 to 0.2)	0.1<br>(0.0 to 0.1)	9.0<br>(7.6 to 10.2)	3.5<br>(2.9 to 4.1)	1.37<br>(1.17 to 1.56)	0.53<br>(0.44 to 0.62)
2010	0.1<br>(0.1 to 0.2)	0.0<br>(0.0 to 0.1)	7.9<br>(6.6 to 9.1)	2.9<br>(2.4 to 3.4)	1.42<br>(1.19 to 1.65)	0.52<br>(0.43 to 0.62)
2015	0.1<br>(0.1 to 0.1)	0.0<br>(0.0 to 0.1)	7.3<br>(6.5 to 8.1)	2.7<br>(2.4 to 3.0)	1.26<br>(1.12 to 1.39)	0.46<br>(0.41 to 0.51)
2020	0.1<br>(0.1 to 0.1)	0.0<br>(0.0 to 0.0)	6.9<br>(6.1 to 7.6)	2.6<br>(2.3 to 2.9)	1.42<br>(1.27 to 1.58)	0.54<br>(0.48 to 0.59)
2021	0.1<br>(0.1 to 0.1)	0.0<br>(0.0 to 0.1)	7.8<br>(6.4 to 9.5)	2.9<br>(2.4 to 3.5)	1.43<br>(1.17 to 1.76)	0.54<br>(0.44 to 0.65)

Coventry GBR_44709 Western Europe Stillbirth Rate / Neonatal Mortality Rate (SBR/NMR) − 20 weeks
lambda = 0.6 ; zeta = 0.8 ; scale = 15 ; amp = 0.028

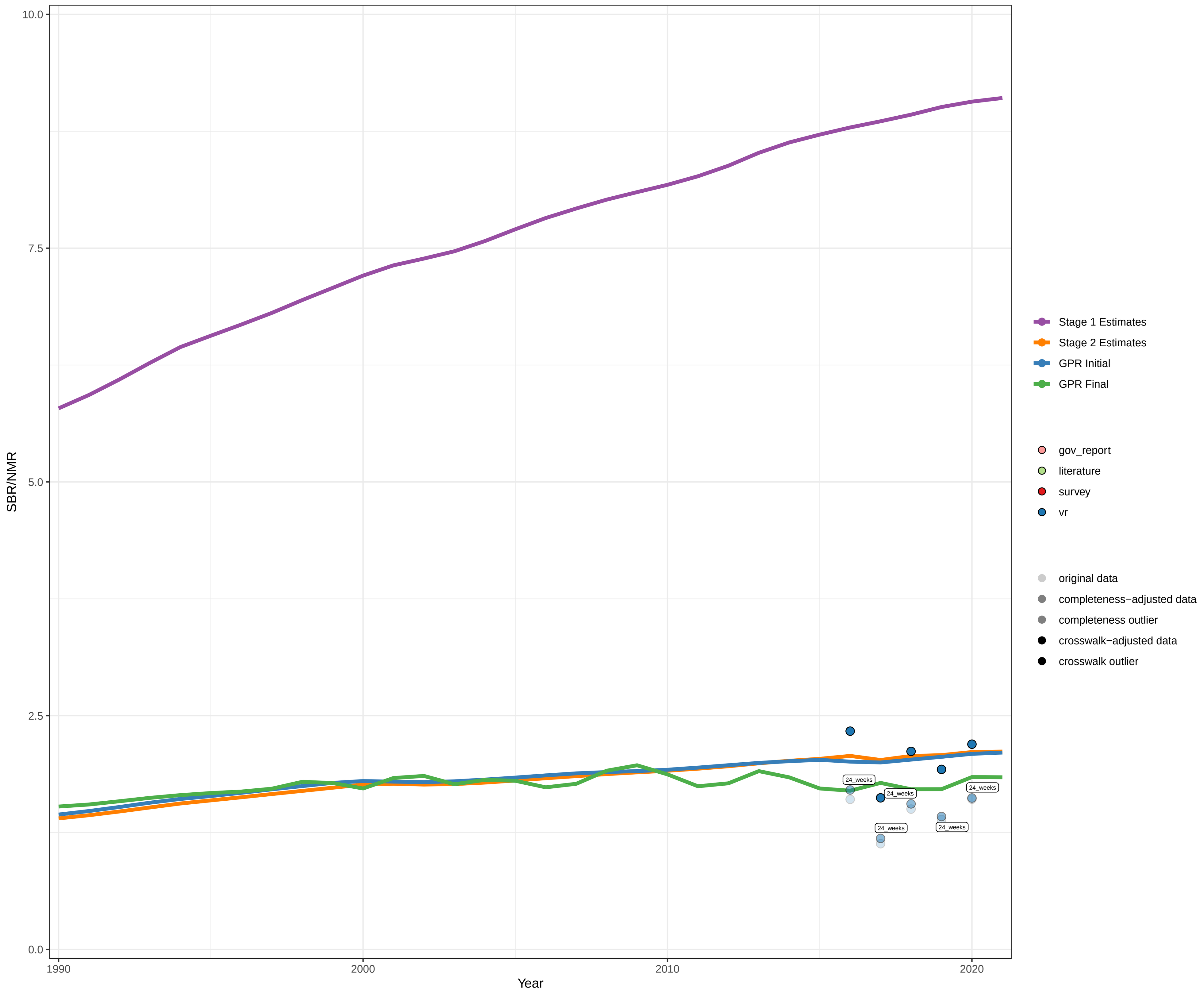

Coventry GBR_44709 Western Europe Stillbirth Rate / Neonatal Mortality Rate (SBR/NMR) − 28 weeks
lambda = 0.6 ; zeta = 0.8 ; scale = 15 ; amp = 0.028

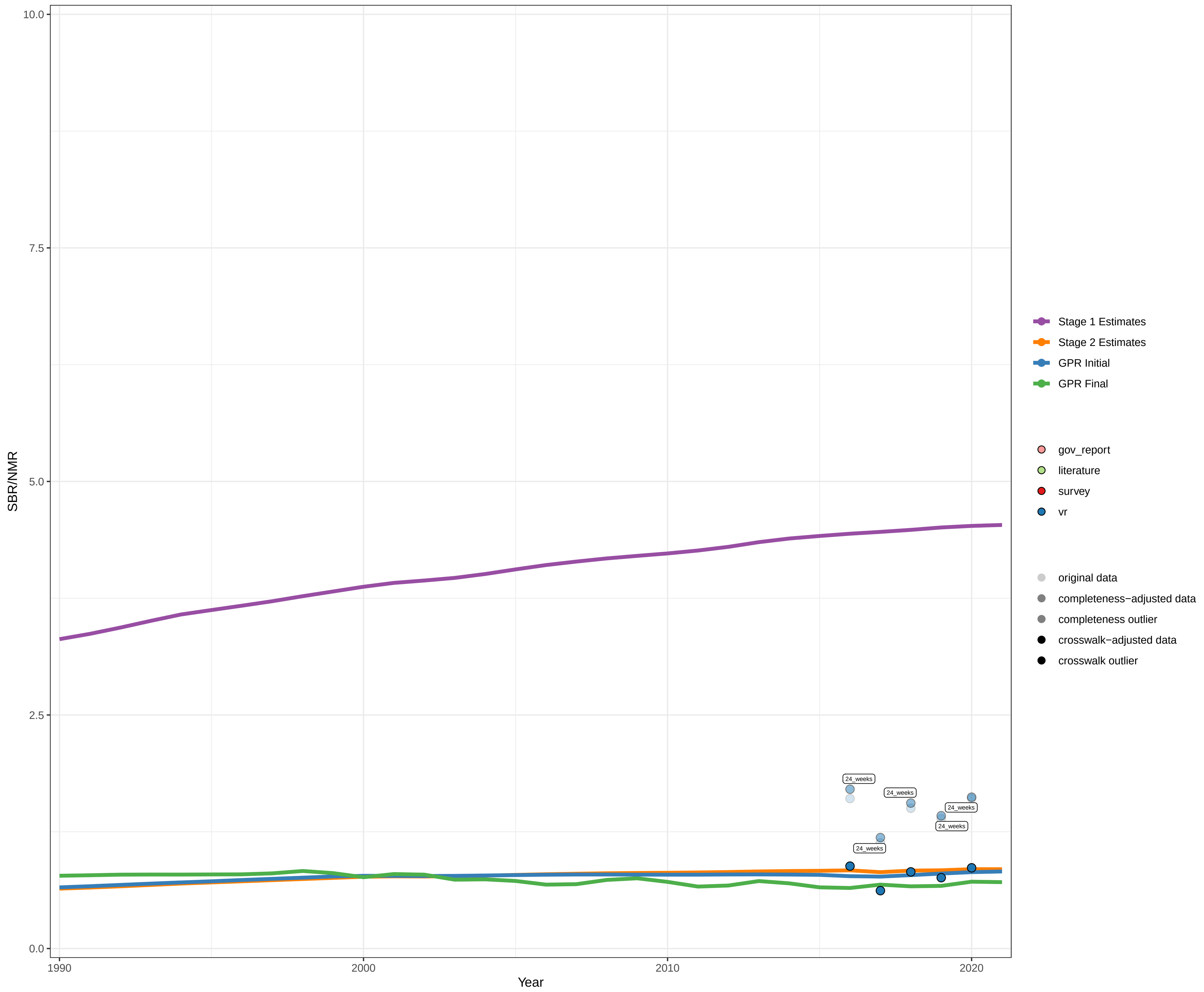

Definition	Years	Outlier	Source.Type	Title
24_weeks	2016−2020	0	VR	United Kingdom − England and Wales Child Mortality Statistics

Coventry GBR_44709 Western Europe Stillbirth Rate (SBR) − 20 weeks
lambda = 0.6 ; zeta = 0.8 ; scale = 15 ; amp = 0.028

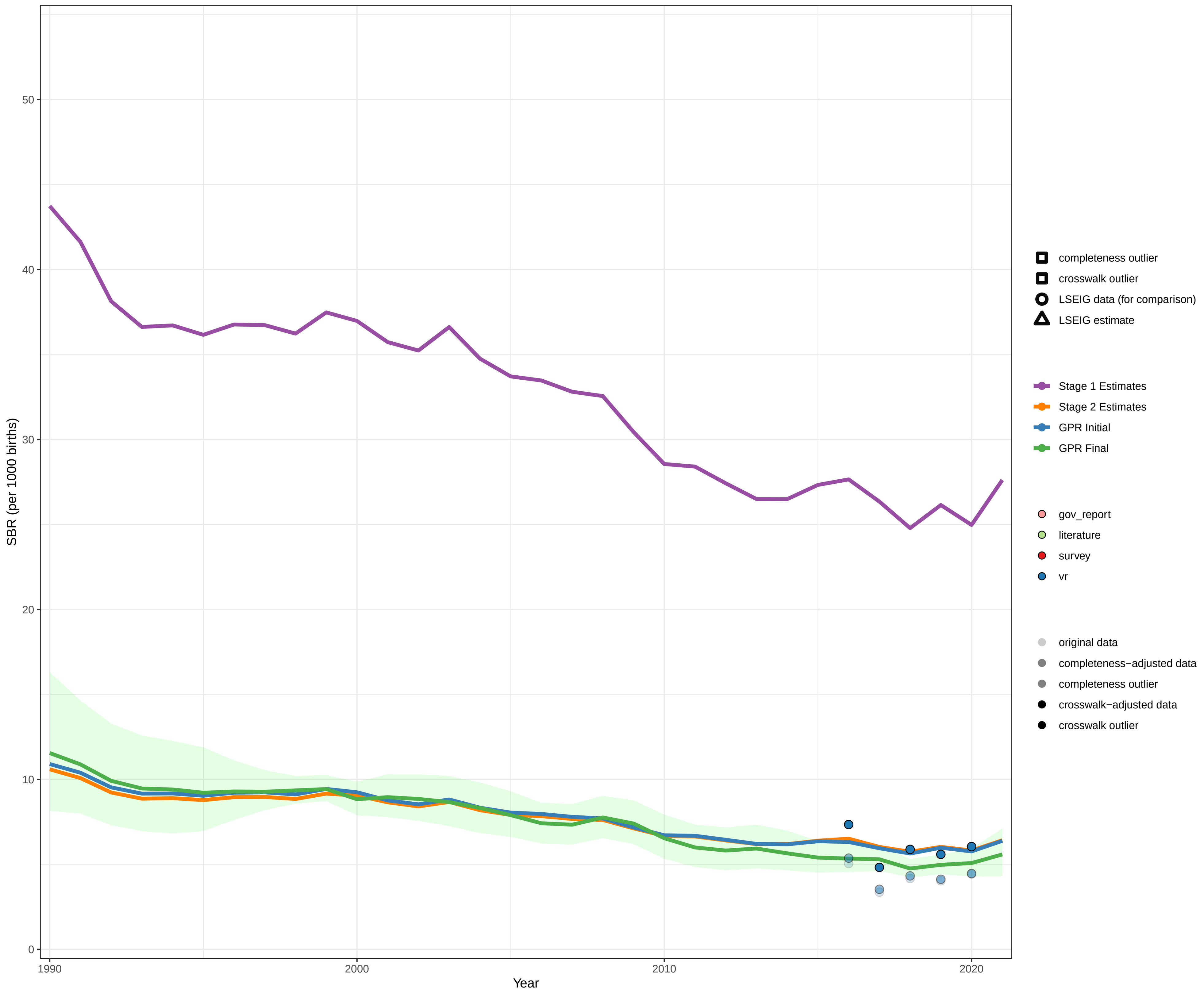

Coventry GBR_44709 Western Europe Stillbirth Rate (SBR) − 28 weeks
lambda = 0.6 ; zeta = 0.8 ; scale = 15 ; amp = 0.028

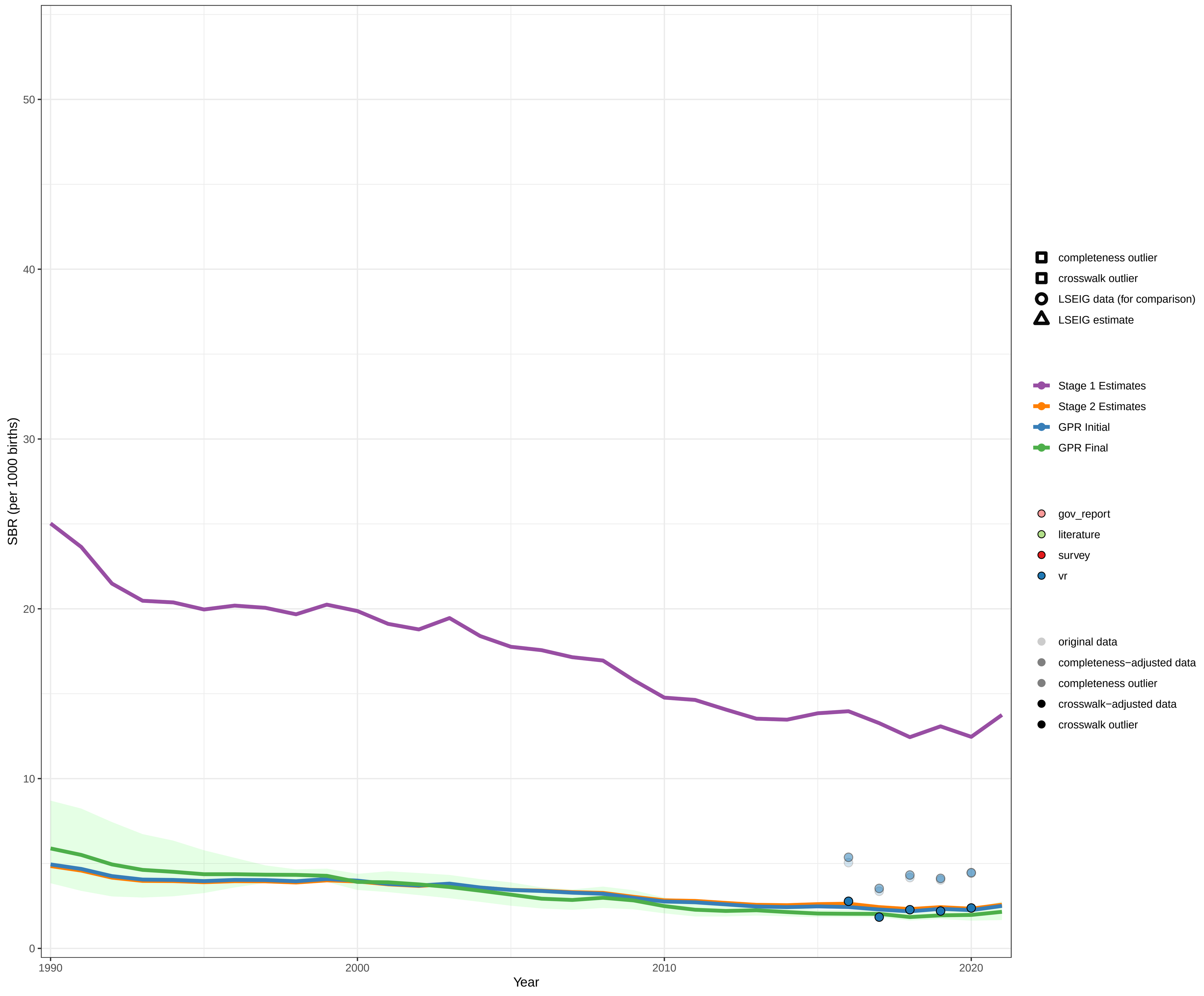

Year	Total Stillbirths (in thousands)	Total Stillbirths (in thousands)	SBR (per 1000 births)	SBR (per 1000 births)	SBR/NMR	SBR/NMR
	20 weeks	28 weeks	20 weeks	28 weeks	20 weeks	28 weeks
1990	0.1 (0.0 to 0.1)	0.0 (0.0 to 0.0)	11.6 (8.1 to 16.3)	5.9 (3.8 to 8.7)	1.53 (1.08 to 2.16)	0.78 (0.51 to 1.15)
1995	0.0 (0.0 to 0.0)	0.0 (0.0 to 0.0)	9.2 (7.0 to 11.9)	4.4 (3.3 to 5.8)	1.67 (1.26 to 2.16)	0.79 (0.59 to 1.05)
2000	0.0 (0.0 to 0.0)	0.0 (0.0 to 0.0)	8.8 (7.9 to 9.9)	3.9 (3.4 to 4.4)	1.72 (1.54 to 1.92)	0.76 (0.67 to 0.86)
2005	0.0 (0.0 to 0.0)	0.0 (0.0 to 0.0)	7.9 (6.6 to 9.3)	3.2 (2.5 to 3.9)	1.80 (1.51 to 2.13)	0.72 (0.57 to 0.89)
2010	0.0 (0.0 to 0.0)	0.0 (0.0 to 0.0)	6.5 (5.3 to 7.9)	2.5 (2.1 to 3.0)	1.87 (1.53 to 2.27)	0.71 (0.59 to 0.86)
2015	0.0 (0.0 to 0.0)	0.0 (0.0 to 0.0)	5.4 (4.5 to 6.4)	2.1 (1.8 to 2.3)	1.72 (1.44 to 2.04)	0.65 (0.59 to 0.73)
2020	0.0 (0.0 to 0.0)	0.0 (0.0 to 0.0)	5.1 (4.3 to 5.9)	2.0 (1.6 to 2.3)	1.84 (1.56 to 2.15)	0.72 (0.59 to 0.85)
2021	0.0 (0.0 to 0.0)	0.0 (0.0 to 0.0)	5.6 (4.3 to 7.1)	2.2 (1.7 to 2.8)	1.84 (1.42 to 2.34)	0.71 (0.55 to 0.91)

Dudley GBR_44708 Western Europe Stillbirth Rate / Neonatal Mortality Rate (SBR/NMR) − 20 weeks
lambda = 0.6 ; zeta = 0.8 ; scale = 15 ; amp = 0.028

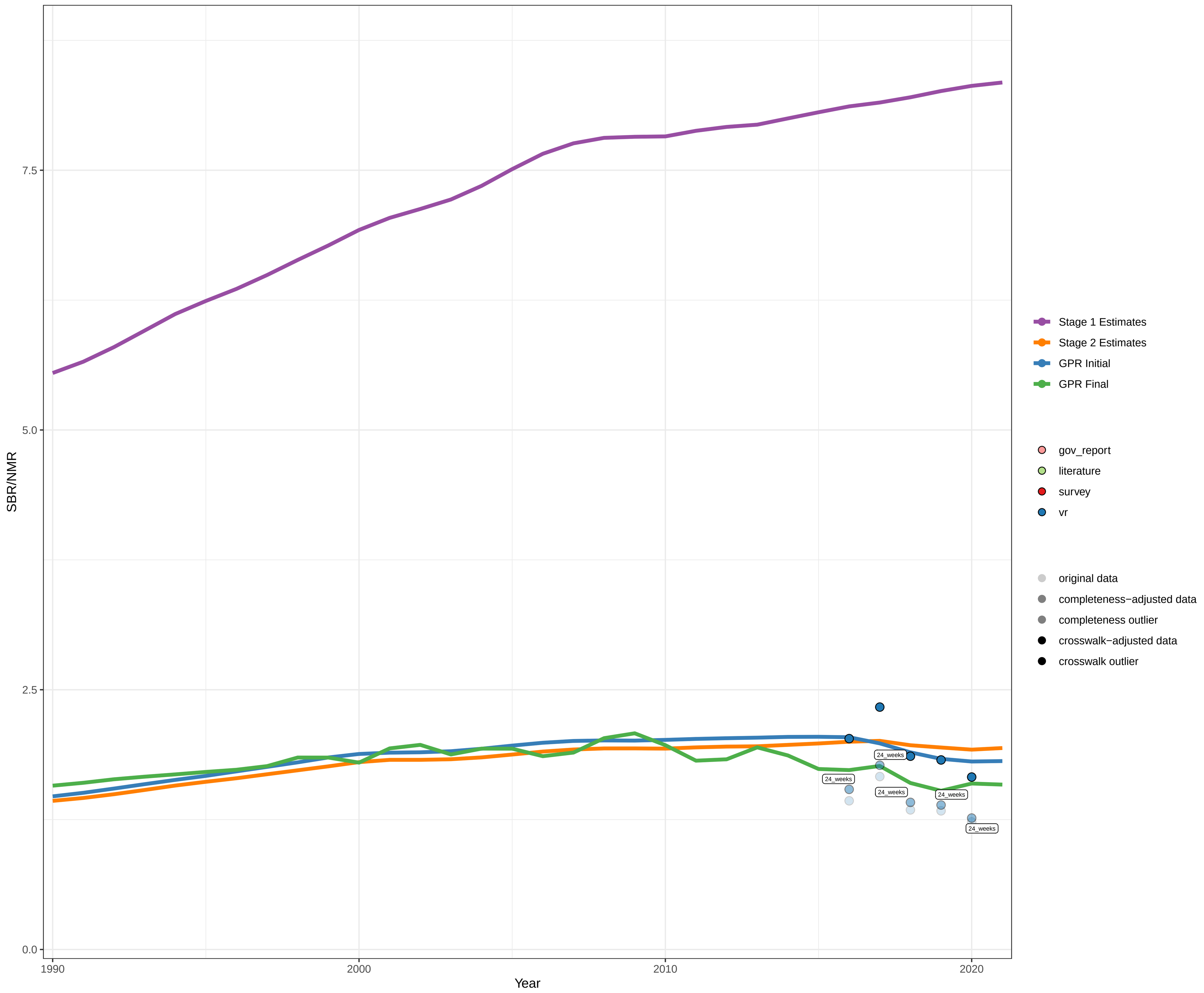

Dudley GBR_44708 Western Europe Stillbirth Rate / Neonatal Mortality Rate (SBR/NMR) − 28 weeks
lambda = 0.6 ; zeta = 0.8 ; scale = 15 ; amp = 0.028

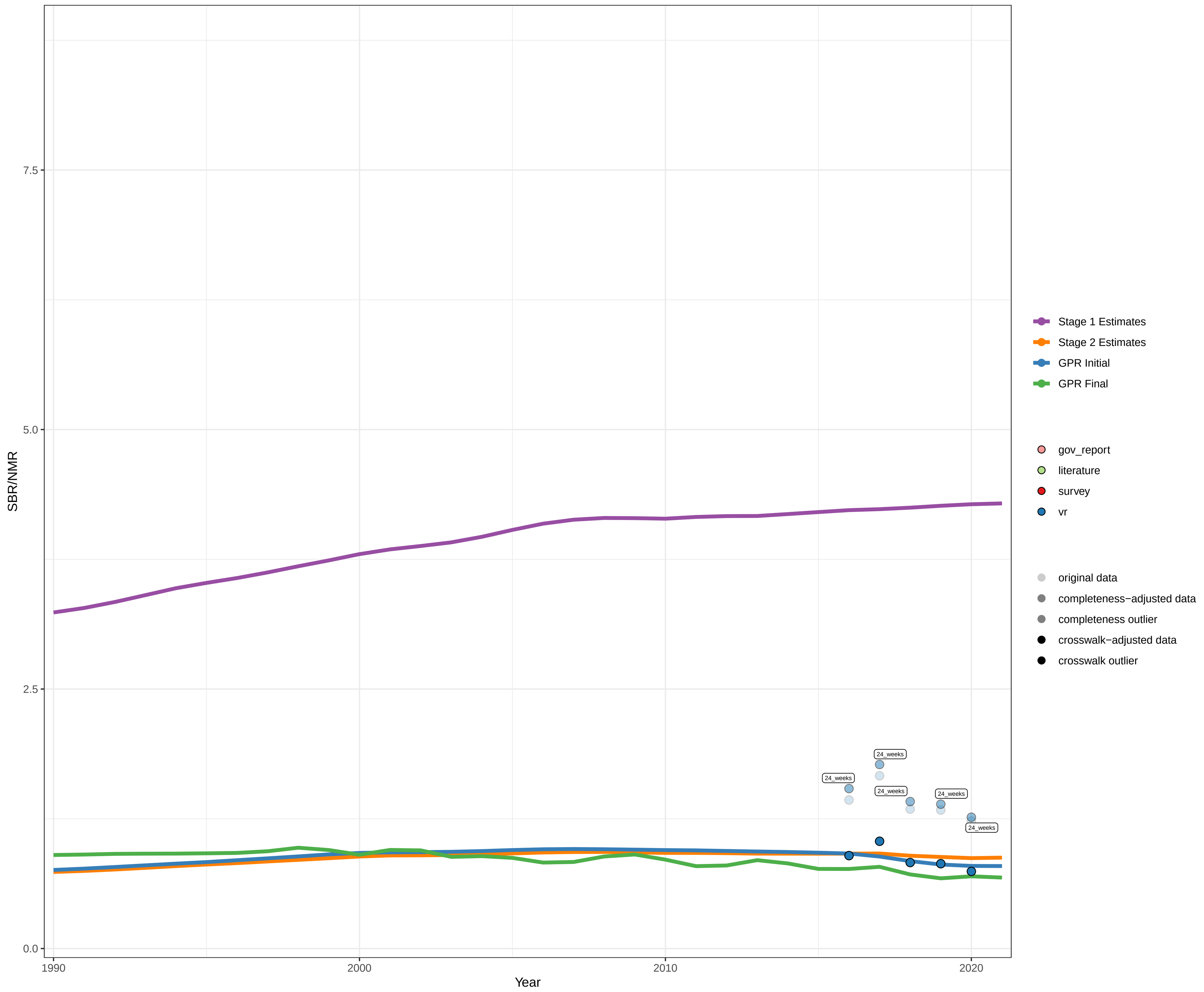

Definition	Years	Outlier	Source.Type	Title
24_weeks	2016−2020	0	VR	United Kingdom − England and Wales Child Mortality Statistics

Dudley GBR_44708 Western Europe Stillbirth Rate (SBR) − 20 weeks
lambda = 0.6 ; zeta = 0.8 ; scale = 15 ; amp = 0.028

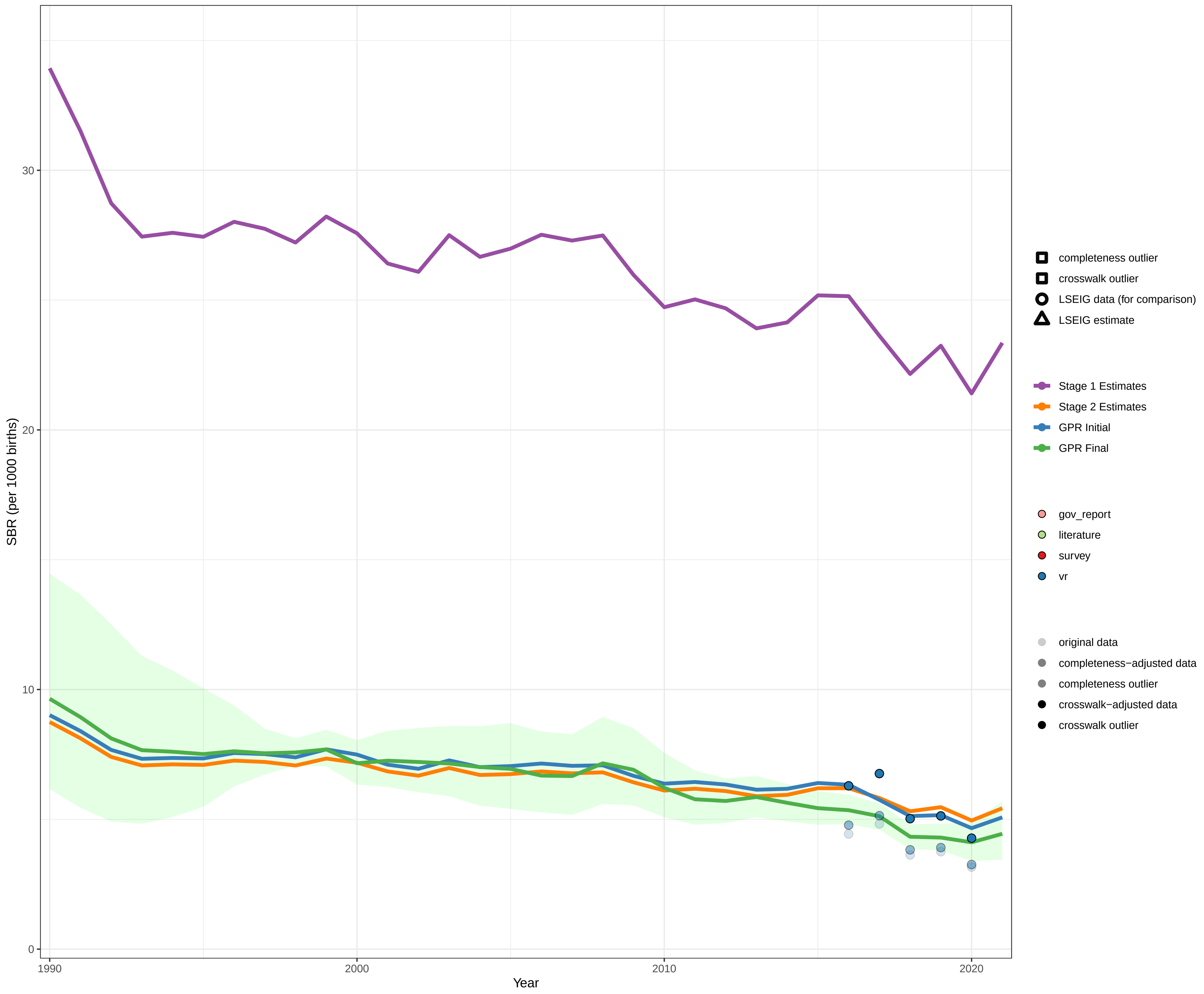

Dudley GBR_44708 Western Europe Stillbirth Rate (SBR) − 28 weeks
lambda = 0.6 ; zeta = 0.8 ; scale = 15 ; amp = 0.028

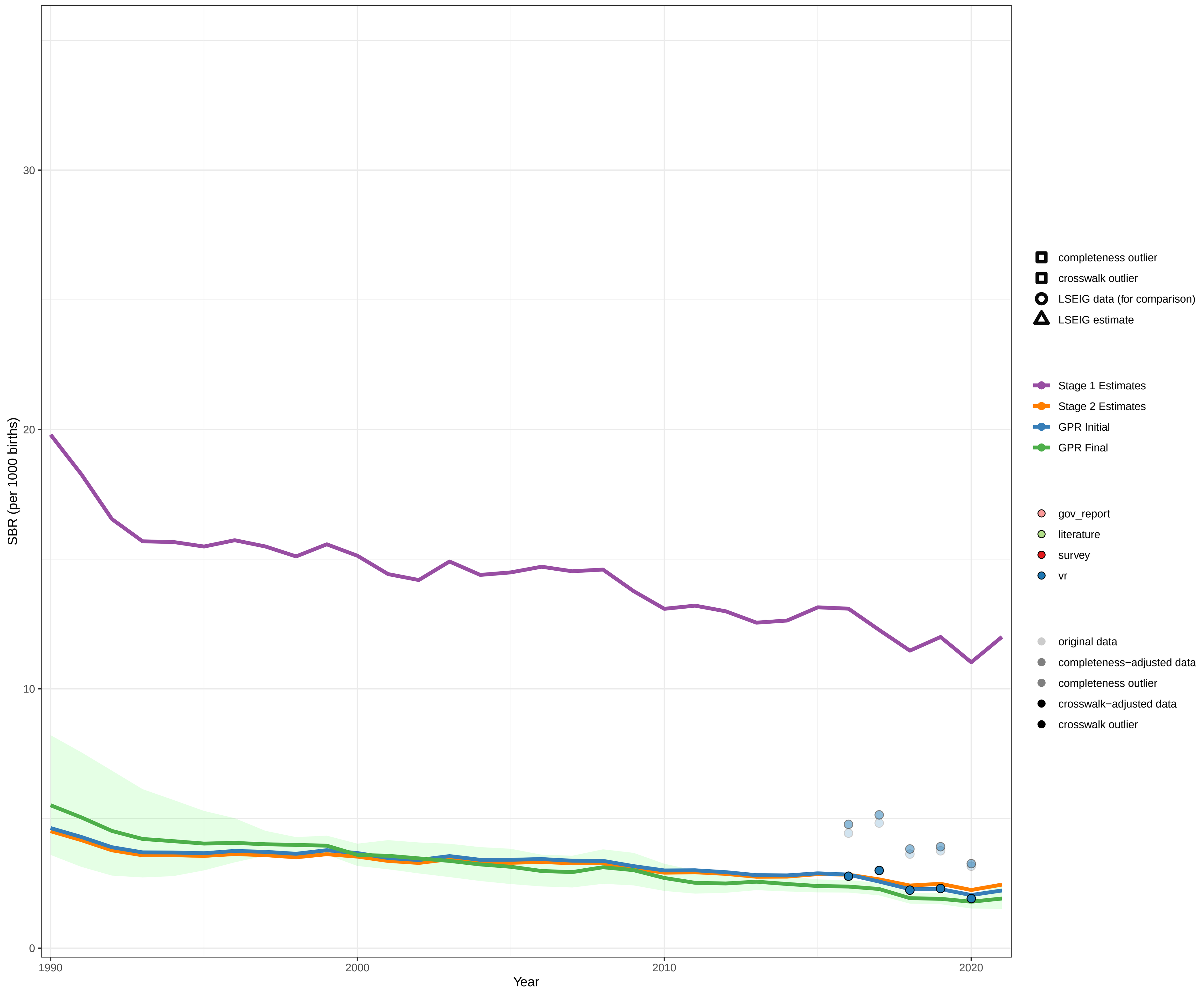

Year	Total Stillbirths (in thousands)	Total Stillbirths (in thousands)	SBR (per 1000 births)	SBR (per 1000 births)	SBR/NMR	SBR/NMR
	20 weeks	28 weeks	20 weeks	28 weeks	20 weeks	28 weeks
1990	0.0 (0.0 to 0.1)	0.0 (0.0 to 0.0)	9.6 (6.2 to 14.5)	5.5 (3.6 to 8.2)	1.58 (1.01 to 2.36)	0.90 (0.59 to 1.34)
1995	0.0 (0.0 to 0.0)	0.0 (0.0 to 0.0)	7.5 (5.5 to 10.0)	4.0 (3.0 to 5.3)	1.71 (1.25 to 2.29)	0.92 (0.68 to 1.20)
2000	0.0 (0.0 to 0.0)	0.0 (0.0 to 0.0)	7.2 (6.3 to 8.0)	3.6 (3.2 to 4.0)	1.80 (1.59 to 2.02)	0.90 (0.80 to 1.01)
2005	0.0 (0.0 to 0.0)	0.0 (0.0 to 0.0)	6.9 (5.4 to 8.7)	3.1 (2.5 to 3.8)	1.93 (1.50 to 2.42)	0.87 (0.69 to 1.07)
2010	0.0 (0.0 to 0.0)	0.0 (0.0 to 0.0)	6.2 (5.1 to 7.6)	2.7 (2.2 to 3.3)	1.97 (1.61 to 2.39)	0.86 (0.70 to 1.03)
2015	0.0 (0.0 to 0.0)	0.0 (0.0 to 0.0)	5.4 (4.8 to 6.1)	2.4 (2.1 to 2.7)	1.74 (1.53 to 1.95)	0.77 (0.69 to 0.85)
2020	0.0 (0.0 to 0.0)	0.0 (0.0 to 0.0)	4.1 (3.4 to 4.9)	1.8 (1.5 to 2.1)	1.60 (1.32 to 1.90)	0.70 (0.59 to 0.81)
2021	0.0 (0.0 to 0.0)	0.0 (0.0 to 0.0)	4.4 (3.4 to 5.7)	1.9 (1.5 to 2.4)	1.59 (1.23 to 2.04)	0.68 (0.54 to 0.86)

Herefordshire, County of GBR_44703 Western Europe Stillbirth Rate / Neonatal Mortality Rate (SBR/NMR) − 20 weeks
lambda = 0.8 ; zeta = 0.7 ; scale = 15 ; amp = 0.028

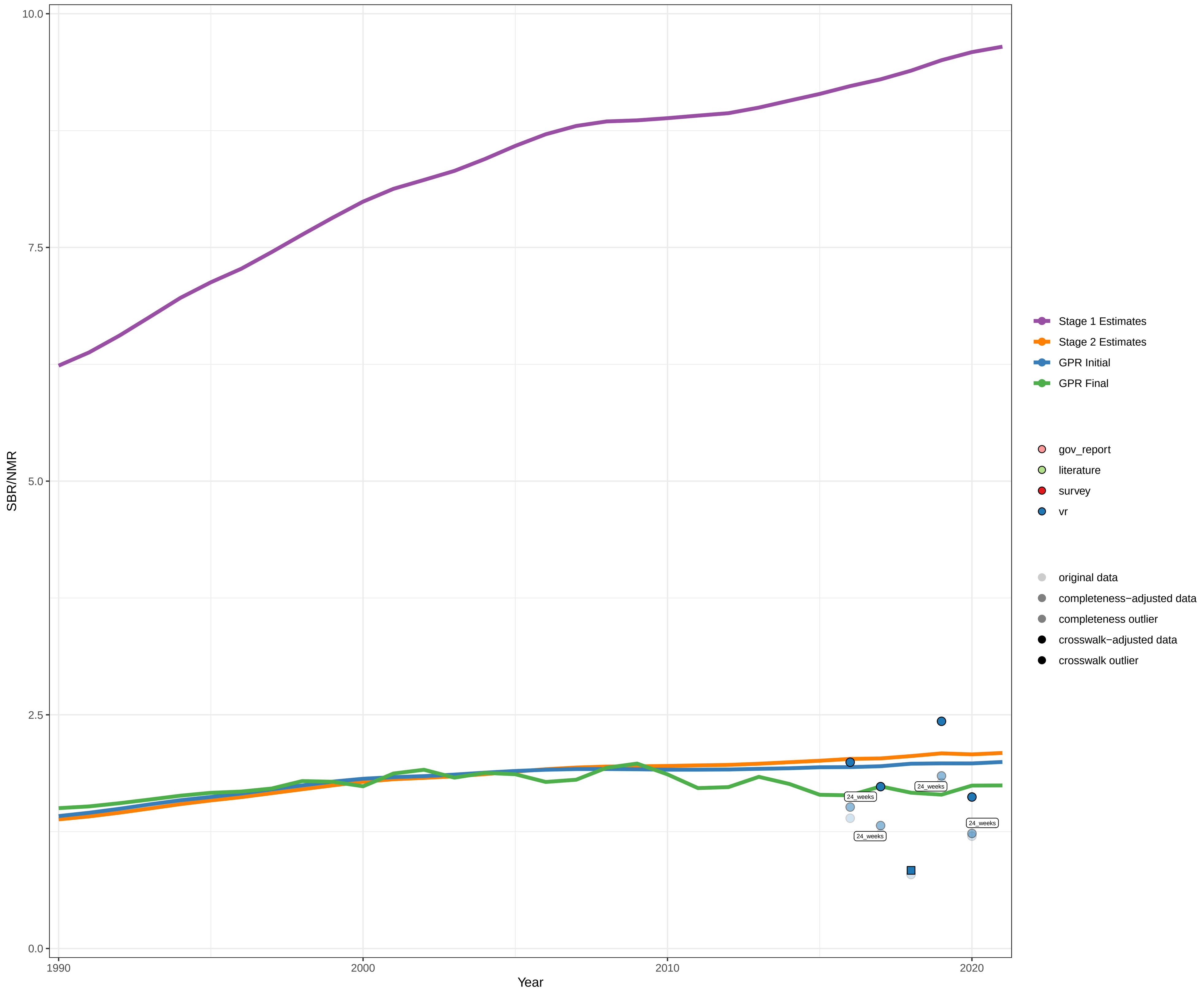

Herefordshire, County of GBR_44703 Western Europe Stillbirth Rate / Neonatal Mortality Rate (SBR/NMR) − 28 weeks
lambda = 0.8 ; zeta = 0.7 ; scale = 15 ; amp = 0.028

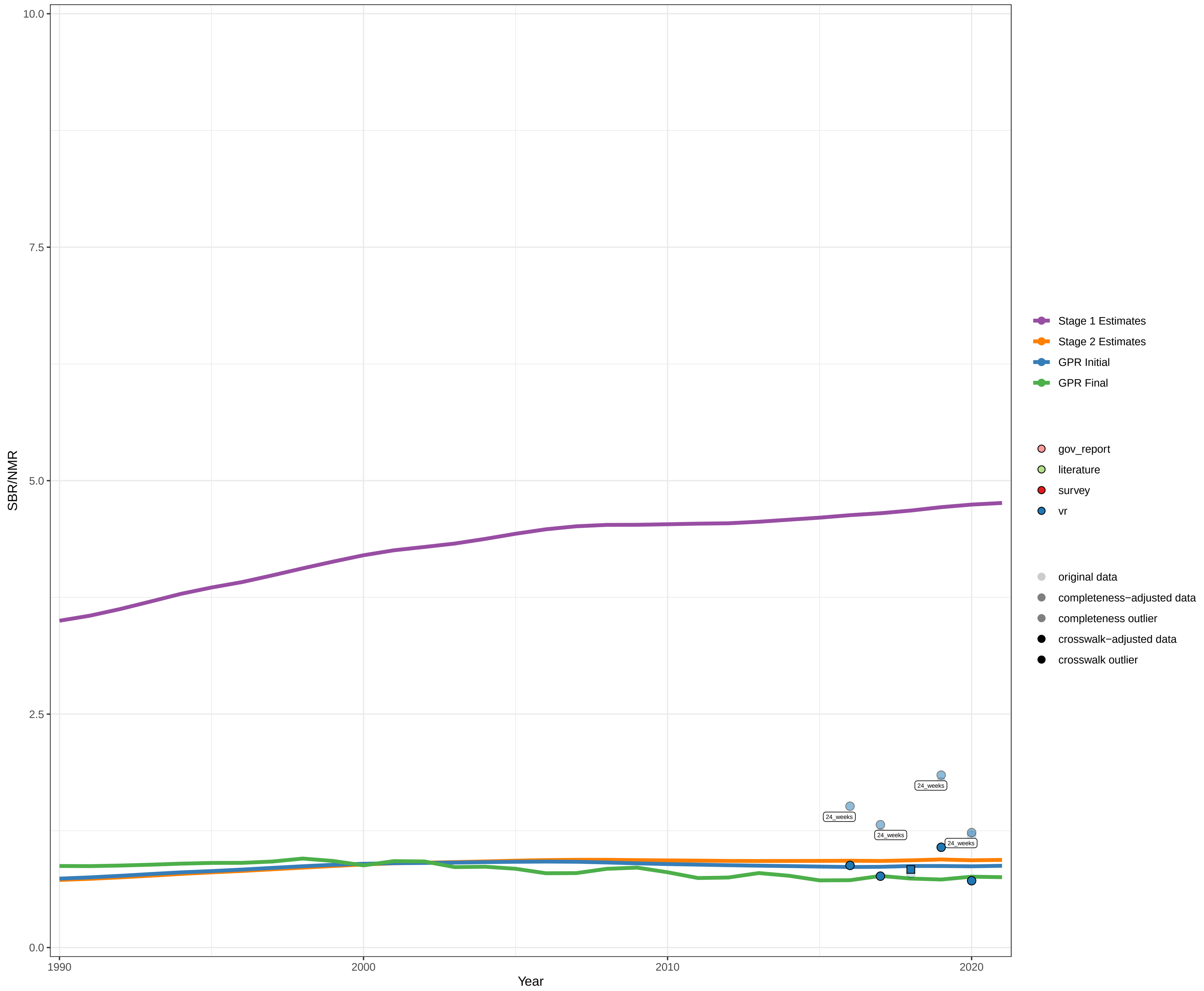

Definition	Years	Outlier	Source.Type	Title
24_weeks	2016−2020	0	VR	United Kingdom − England and Wales Child Mortality Statistics

Herefordshire, County of GBR_44703 Western Europe Stillbirth Rate (SBR) − 20 weeks
lambda = 0.8 ; zeta = 0.7 ; scale = 15 ; amp = 0.028

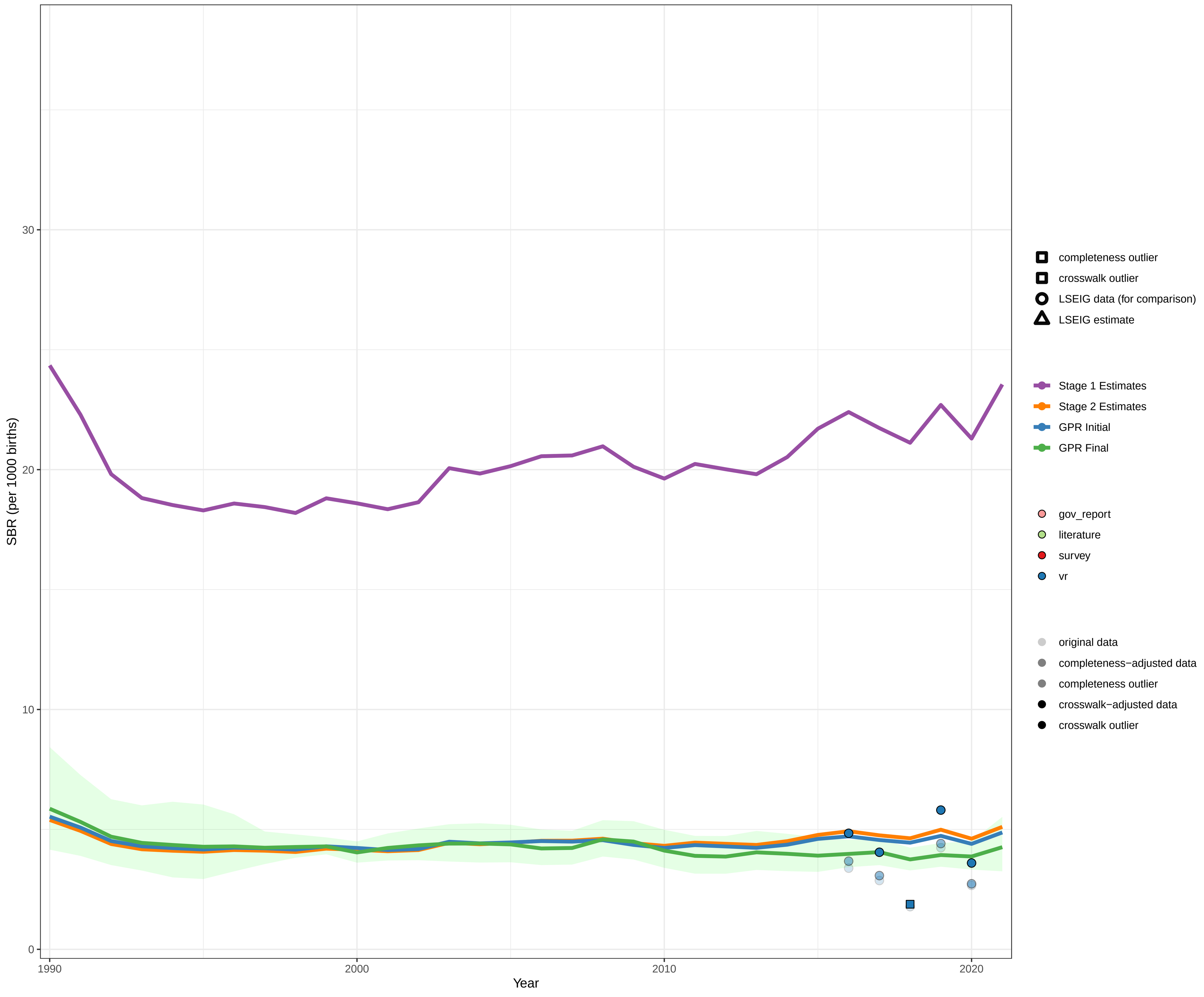

Herefordshire, County of GBR_44703 Western Europe Stillbirth Rate (SBR) − 28 weeks
lambda = 0.8 ; zeta = 0.7 ; scale = 15 ; amp = 0.028

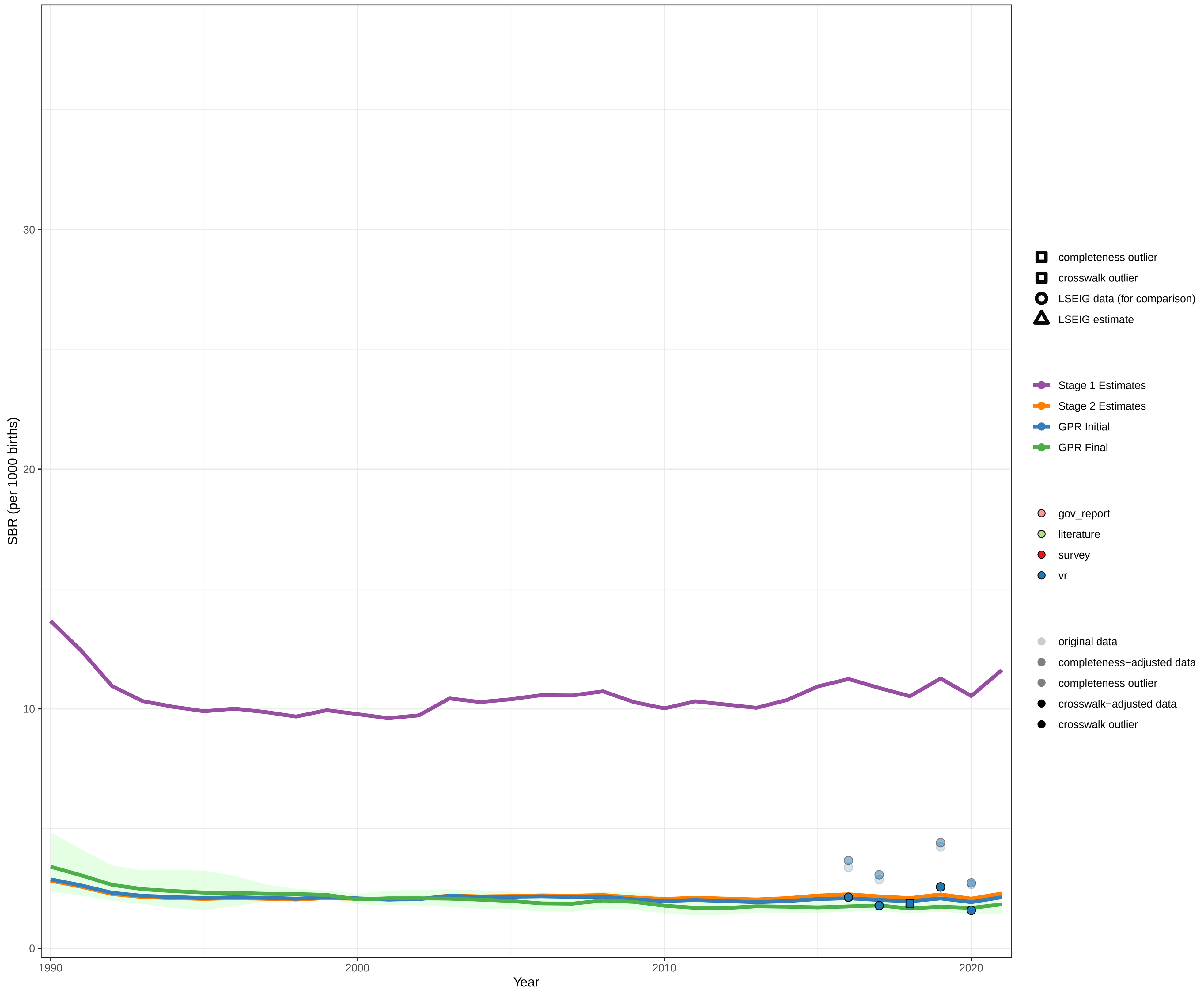

Year	Total Stillbirths (in thousands)	Total Stillbirths (in thousands)	SBR (per 1000 births)	SBR (per 1000 births)	SBR/NMR	SBR/NMR
	20 weeks	28 weeks	20 weeks	28 weeks	20 weeks	28 weeks
1990	0.0 (0.0 to 0.0)	0.0 (0.0 to 0.0)	5.9 (4.2 to 8.4)	3.4 (2.4 to 4.8)	1.50 (1.06 to 2.16)	0.87 (0.61 to 1.24)
1995	0.0 (0.0 to 0.0)	0.0 (0.0 to 0.0)	4.3 (2.9 to 6.0)	2.3 (1.6 to 3.2)	1.67 (1.14 to 2.35)	0.91 (0.63 to 1.27)
2000	0.0 (0.0 to 0.0)	0.0 (0.0 to 0.0)	4.0 (3.6 to 4.5)	2.0 (1.8 to 2.3)	1.74 (1.55 to 1.93)	0.88 (0.79 to 0.98)
2005	0.0 (0.0 to 0.0)	0.0 (0.0 to 0.0)	4.4 (3.6 to 5.2)	2.0 (1.6 to 2.4)	1.86 (1.55 to 2.21)	0.84 (0.69 to 1.01)
2010	0.0 (0.0 to 0.0)	0.0 (0.0 to 0.0)	4.1 (3.4 to 5.0)	1.8 (1.5 to 2.2)	1.86 (1.54 to 2.26)	0.81 (0.66 to 0.98)
2015	0.0 (0.0 to 0.0)	0.0 (0.0 to 0.0)	3.9 (3.2 to 4.7)	1.7 (1.5 to 1.9)	1.64 (1.36 to 1.97)	0.72 (0.62 to 0.82)
2020	0.0 (0.0 to 0.0)	0.0 (0.0 to 0.0)	3.9 (3.3 to 4.5)	1.7 (1.4 to 2.0)	1.74 (1.50 to 2.03)	0.76 (0.65 to 0.89)
2021	0.0 (0.0 to 0.0)	0.0 (0.0 to 0.0)	4.3 (3.3 to 5.5)	1.8 (1.4 to 2.4)	1.74 (1.33 to 2.26)	0.75 (0.58 to 0.97)

Sandwell GBR_44715 Western Europe Stillbirth Rate / Neonatal Mortality Rate (SBR/NMR) − 20 weeks
lambda = 0.8 ; zeta = 0.7 ; scale = 15 ; amp = 0.028

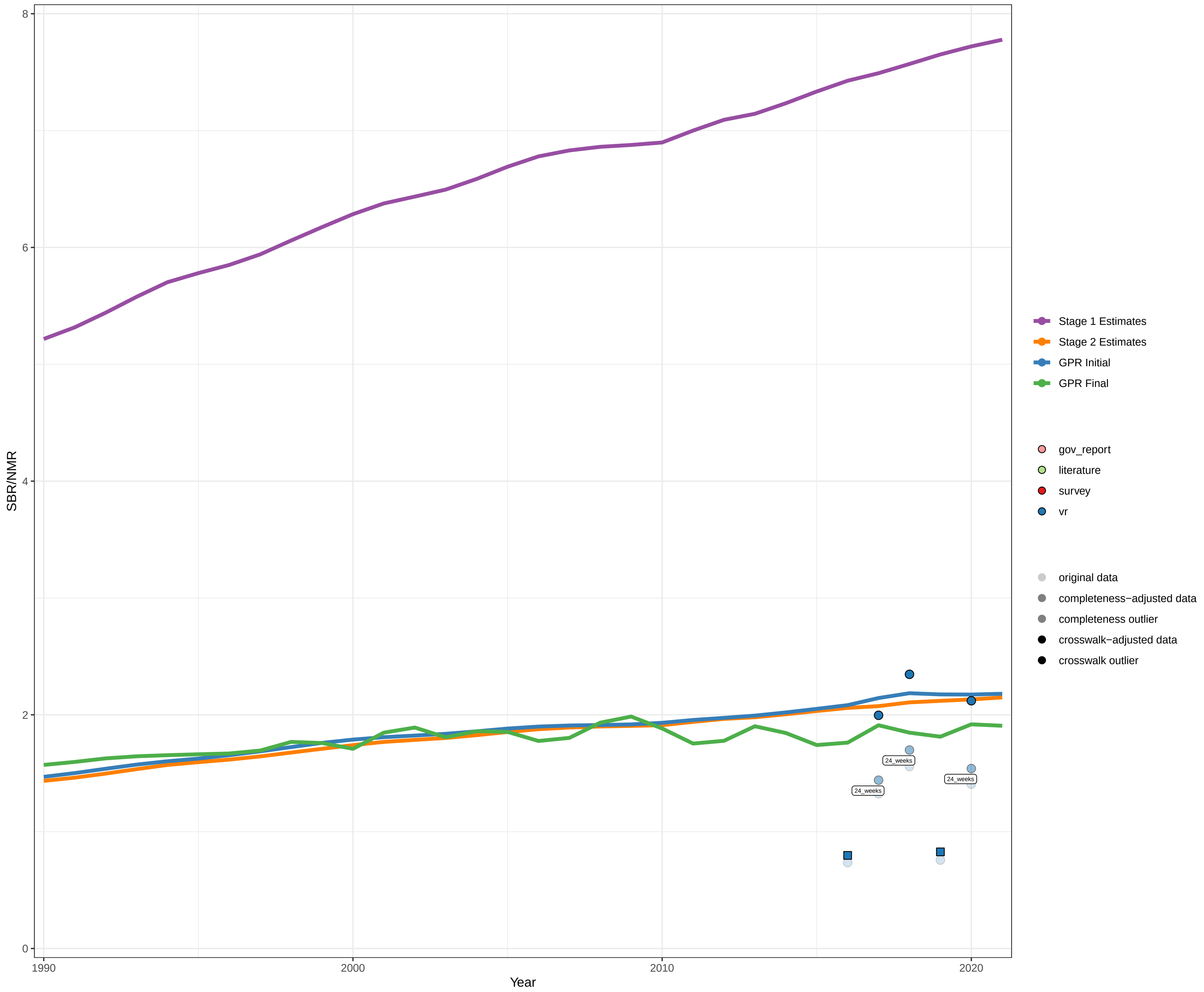

Sandwell GBR_44715 Western Europe Stillbirth Rate / Neonatal Mortality Rate (SBR/NMR) − 28 weeks
lambda = 0.8 ; zeta = 0.7 ; scale = 15 ; amp = 0.028

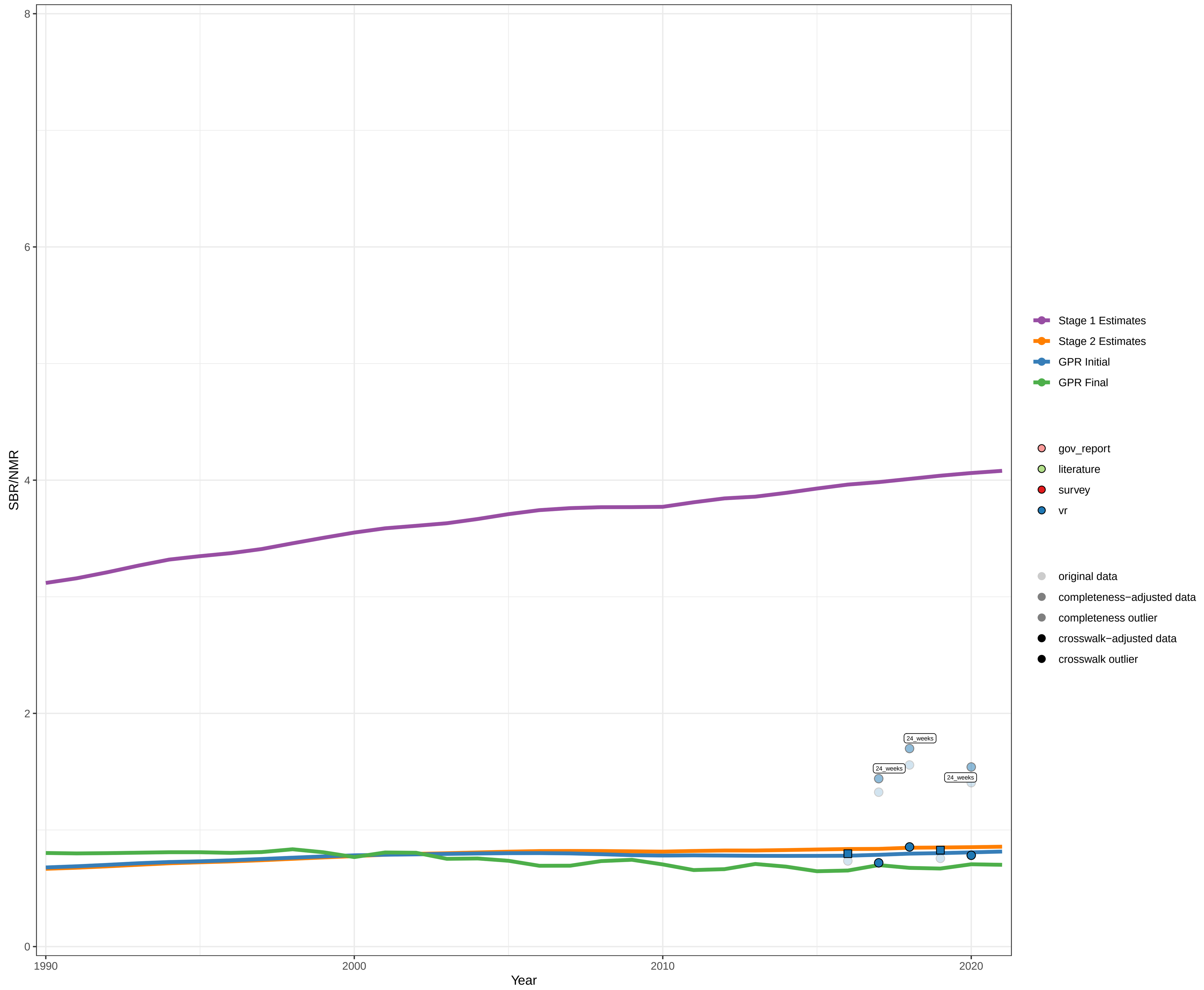

Definition	Years	Outlier	Source.Type	Title
24_weeks	2016−2020	0	VR	United Kingdom − England and Wales Child Mortality Statistics

Sandwell GBR_44715 Western Europe Stillbirth Rate (SBR) − 20 weeks
lambda = 0.8 ; zeta = 0.7 ; scale = 15 ; amp = 0.028

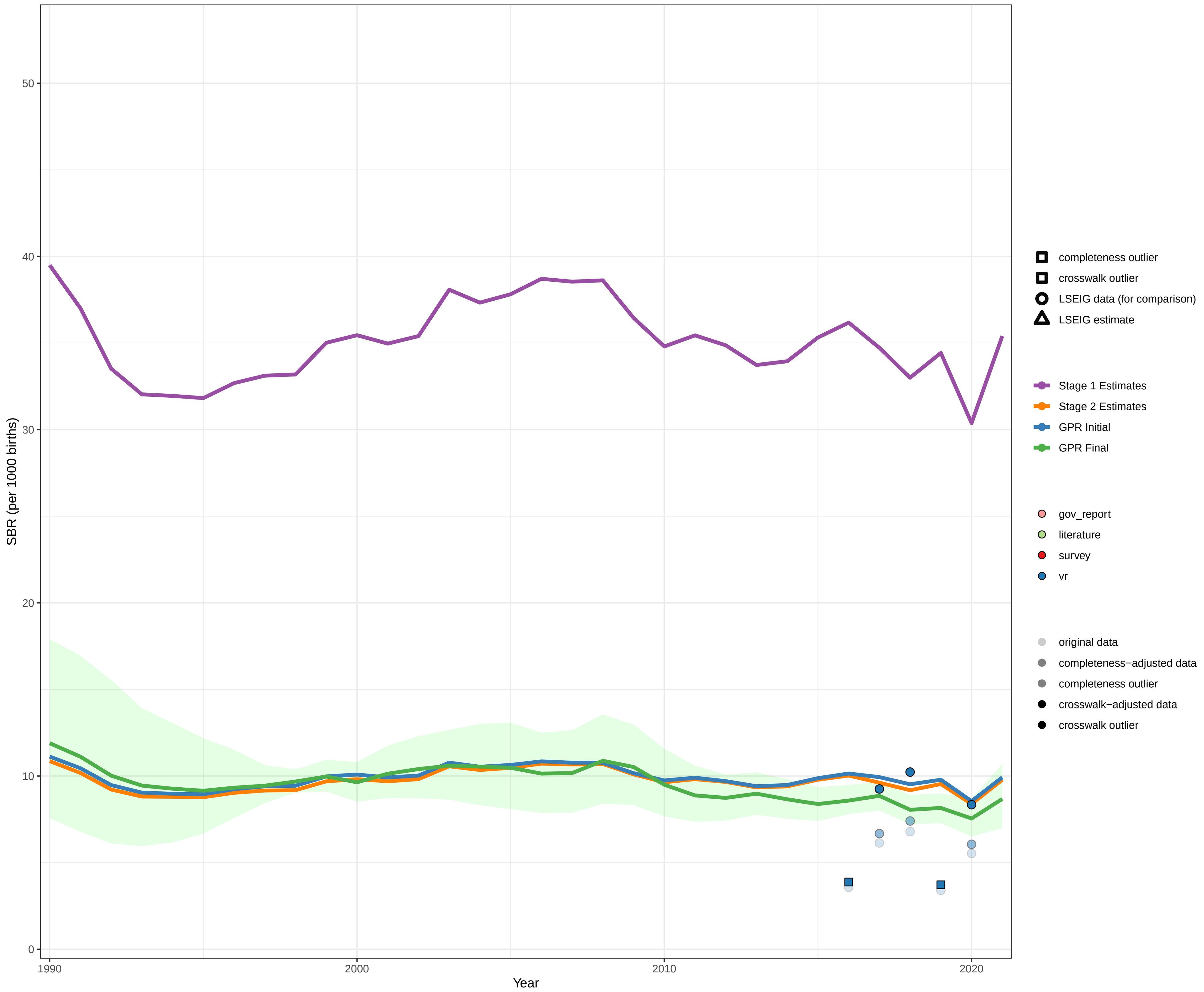

Sandwell GBR_44715 Western Europe Stillbirth Rate (SBR) − 28 weeks
lambda = 0.8 ; zeta = 0.7 ; scale = 15 ; amp = 0.028

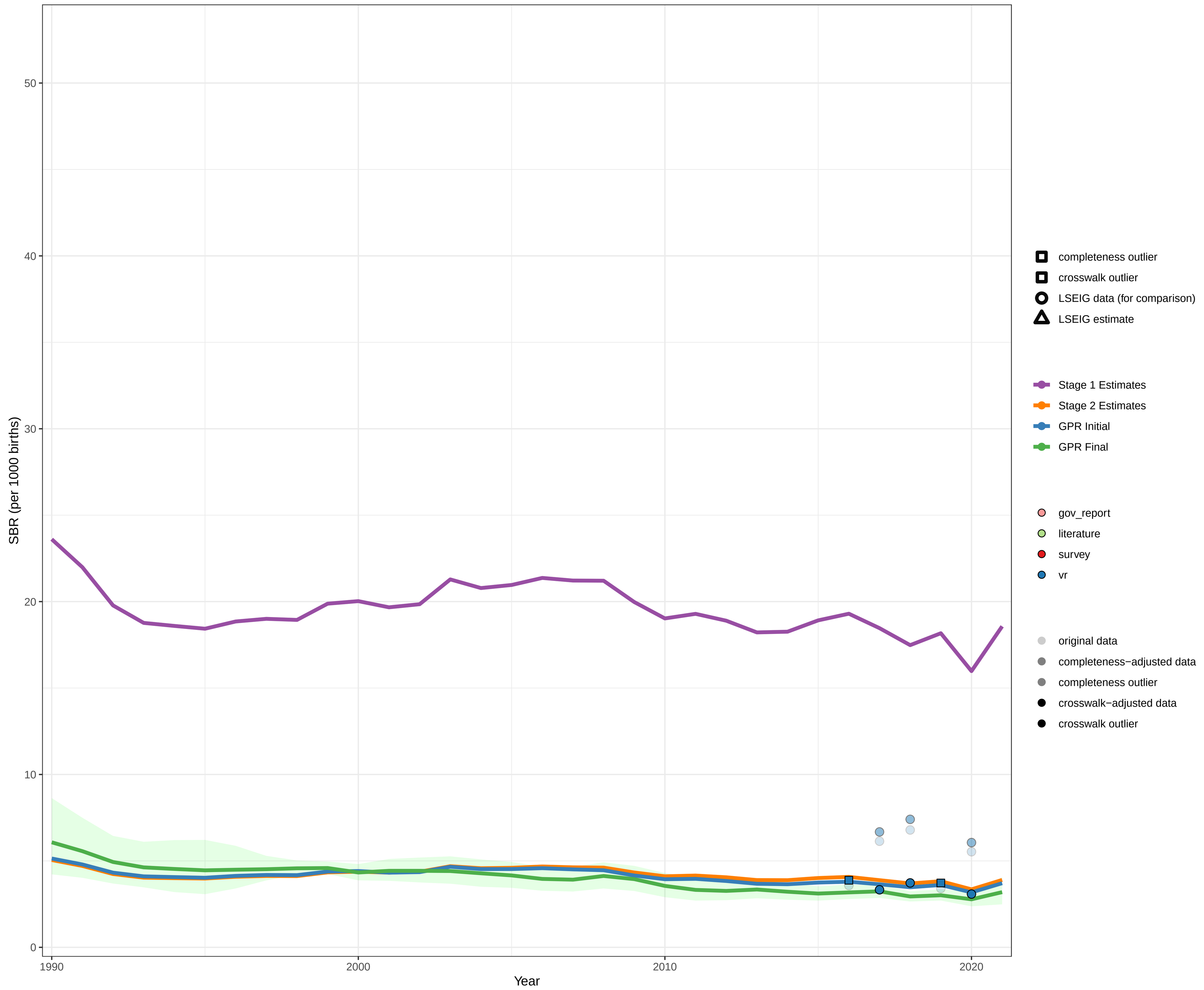

Year	Total Stillbirths (in thousands)	Total Stillbirths (in thousands)	SBR (per 1000 births)	SBR (per 1000 births)	SBR/NMR	SBR/NMR
	20 weeks	28 weeks	20 weeks	28 weeks	20 weeks	28 weeks
1990	0.1 (0.0 to 0.1)	0.0 (0.0 to 0.0)	11.9 (7.6 to 17.9)	6.1 (4.2 to 8.6)	1.57 (1.00 to 2.37)	0.80 (0.56 to 1.14)
1995	0.0 (0.0 to 0.1)	0.0 (0.0 to 0.0)	9.2 (6.7 to 12.2)	4.5 (3.1 to 6.2)	1.66 (1.21 to 2.21)	0.81 (0.56 to 1.13)
2000	0.0 (0.0 to 0.0)	0.0 (0.0 to 0.0)	9.6 (8.5 to 10.8)	4.3 (3.9 to 4.8)	1.71 (1.51 to 1.92)	0.77 (0.69 to 0.85)
2005	0.0 (0.0 to 0.1)	0.0 (0.0 to 0.0)	10.5 (8.1 to 13.1)	4.2 (3.4 to 4.9)	1.85 (1.43 to 2.31)	0.74 (0.61 to 0.87)
2010	0.0 (0.0 to 0.1)	0.0 (0.0 to 0.0)	9.5 (7.7 to 11.6)	3.6 (2.9 to 4.3)	1.88 (1.52 to 2.30)	0.70 (0.57 to 0.85)
2015	0.0 (0.0 to 0.0)	0.0 (0.0 to 0.0)	8.4 (7.4 to 9.4)	3.1 (2.7 to 3.5)	1.74 (1.54 to 1.94)	0.65 (0.56 to 0.73)
2020	0.0 (0.0 to 0.0)	0.0 (0.0 to 0.0)	7.5 (6.5 to 8.7)	2.8 (2.4 to 3.2)	1.92 (1.65 to 2.21)	0.71 (0.60 to 0.82)
2021	0.0 (0.0 to 0.0)	0.0 (0.0 to 0.0)	8.7 (7.0 to 10.7)	3.2 (2.5 to 4.0)	1.91 (1.54 to 2.35)	0.70 (0.55 to 0.89)

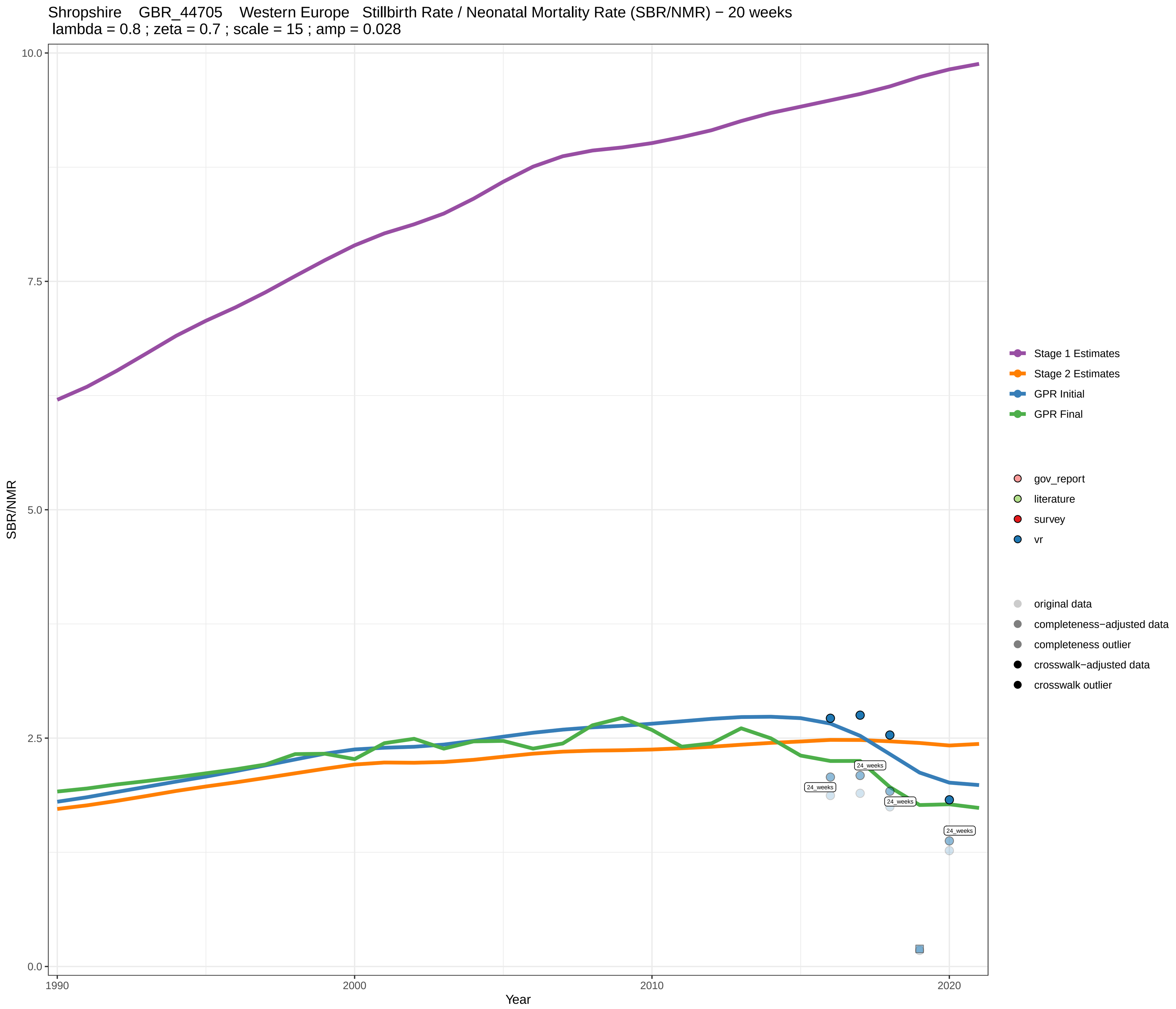

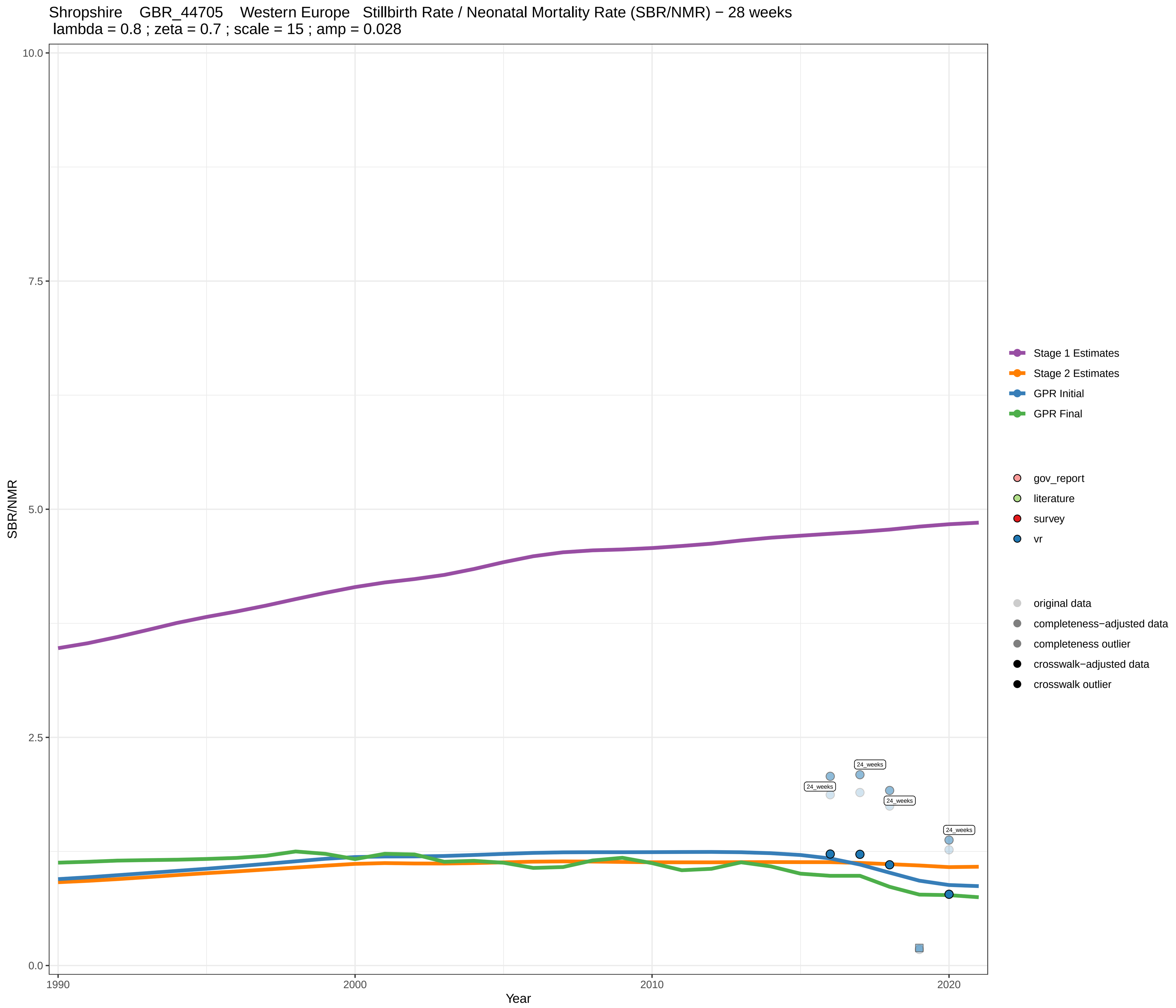

Definition	Years	Outlier	Source.Type	Title
24_weeks	2016−2018, 2020	0	VR	United Kingdom − England and Wales Child Mortality Statistics
24_weeks	2019	1	VR	United Kingdom − England and Wales Child Mortality Statistics

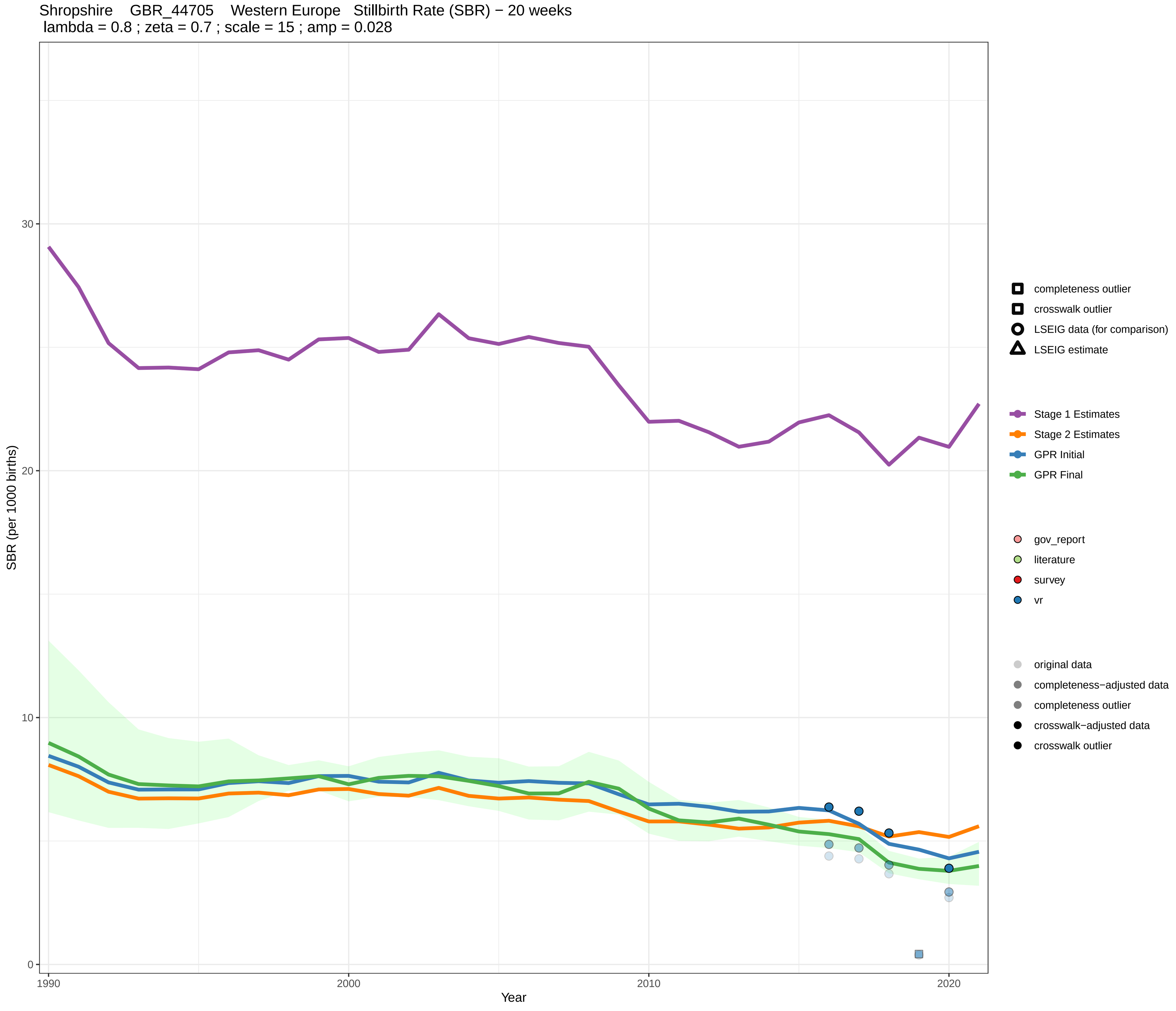

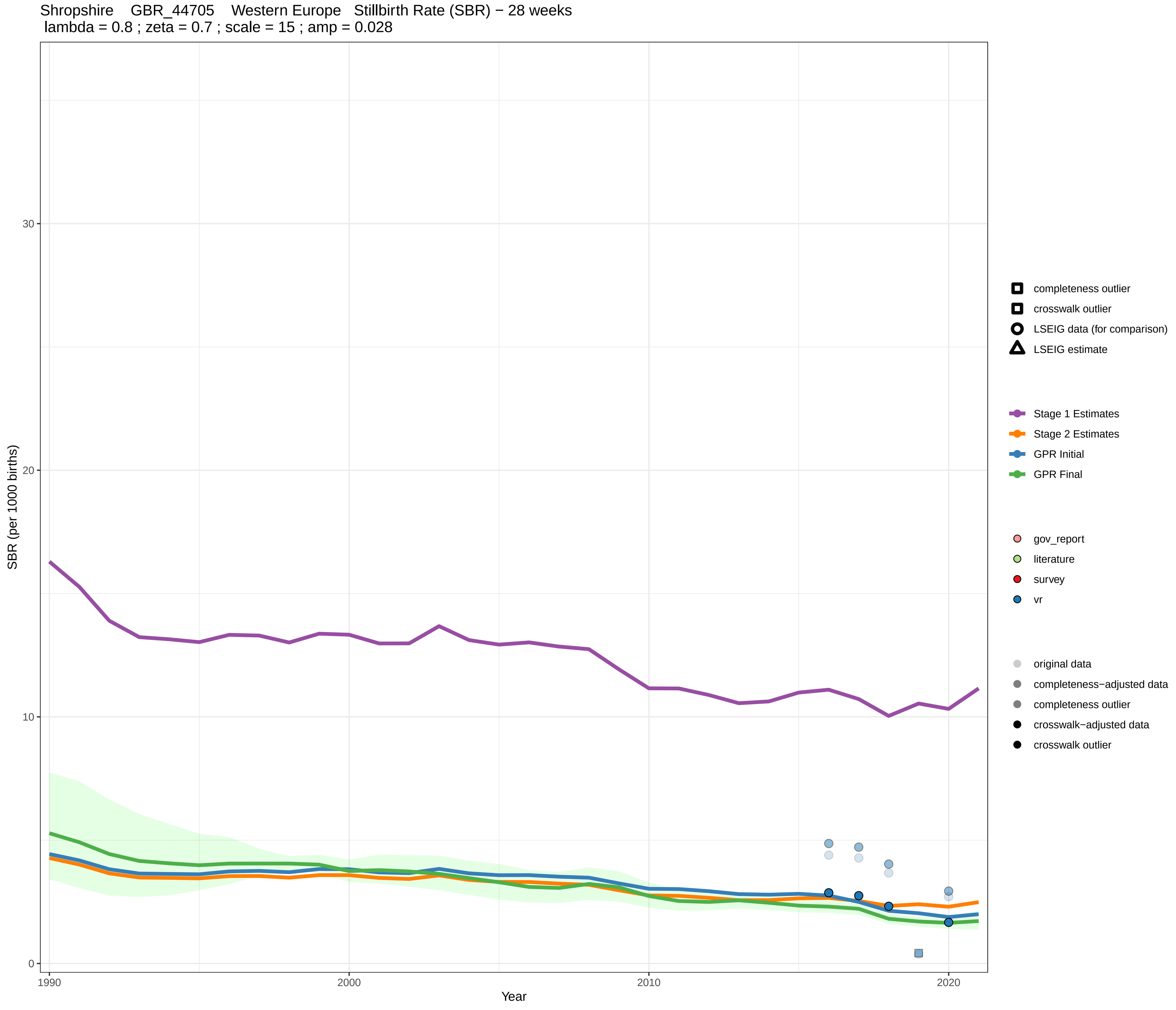

Year	Total Stillbirths (in thousands)	Total Stillbirths (in thousands)	SBR (per 1000 births)	SBR (per 1000 births)	SBR/NMR	SBR/NMR
	20 weeks	28 weeks	20 weeks	28 weeks	20 weeks	28 weeks
1990	0.0 (0.0 to 0.0)	0.0 (0.0 to 0.0)	9.0 (6.2 to 13.1)	5.3 (3.4 to 7.7)	1.92 (1.32 to 2.80)	1.13 (0.73 to 1.65)
1995	0.0 (0.0 to 0.0)	0.0 (0.0 to 0.0)	7.2 (5.7 to 9.0)	4.0 (3.0 to 5.3)	2.11 (1.67 to 2.65)	1.17 (0.87 to 1.54)
2000	0.0 (0.0 to 0.0)	0.0 (0.0 to 0.0)	7.3 (6.6 to 8.0)	3.7 (3.3 to 4.2)	2.27 (2.06 to 2.50)	1.17 (1.03 to 1.31)
2005	0.0 (0.0 to 0.0)	0.0 (0.0 to 0.0)	7.2 (6.2 to 8.4)	3.3 (2.6 to 4.0)	2.47 (2.13 to 2.86)	1.13 (0.88 to 1.38)
2010	0.0 (0.0 to 0.0)	0.0 (0.0 to 0.0)	6.3 (5.3 to 7.4)	2.7 (2.3 to 3.3)	2.59 (2.17 to 3.03)	1.12 (0.93 to 1.34)
2015	0.0 (0.0 to 0.0)	0.0 (0.0 to 0.0)	5.4 (4.8 to 6.0)	2.3 (2.1 to 2.6)	2.31 (2.06 to 2.56)	1.01 (0.89 to 1.13)
2020	0.0 (0.0 to 0.0)	0.0 (0.0 to 0.0)	3.8 (3.3 to 4.4)	1.6 (1.4 to 1.9)	1.78 (1.53 to 2.04)	0.77 (0.66 to 0.89)
2021	0.0 (0.0 to 0.0)	0.0 (0.0 to 0.0)	4.0 (3.2 to 5.0)	1.7 (1.4 to 2.1)	1.74 (1.39 to 2.16)	0.75 (0.60 to 0.93)

Solihull GBR_44704 Western Europe Stillbirth Rate / Neonatal Mortality Rate (SBR/NMR) − 20 weeks
lambda = 0.8 ; zeta = 0.7 ; scale = 15 ; amp = 0.028

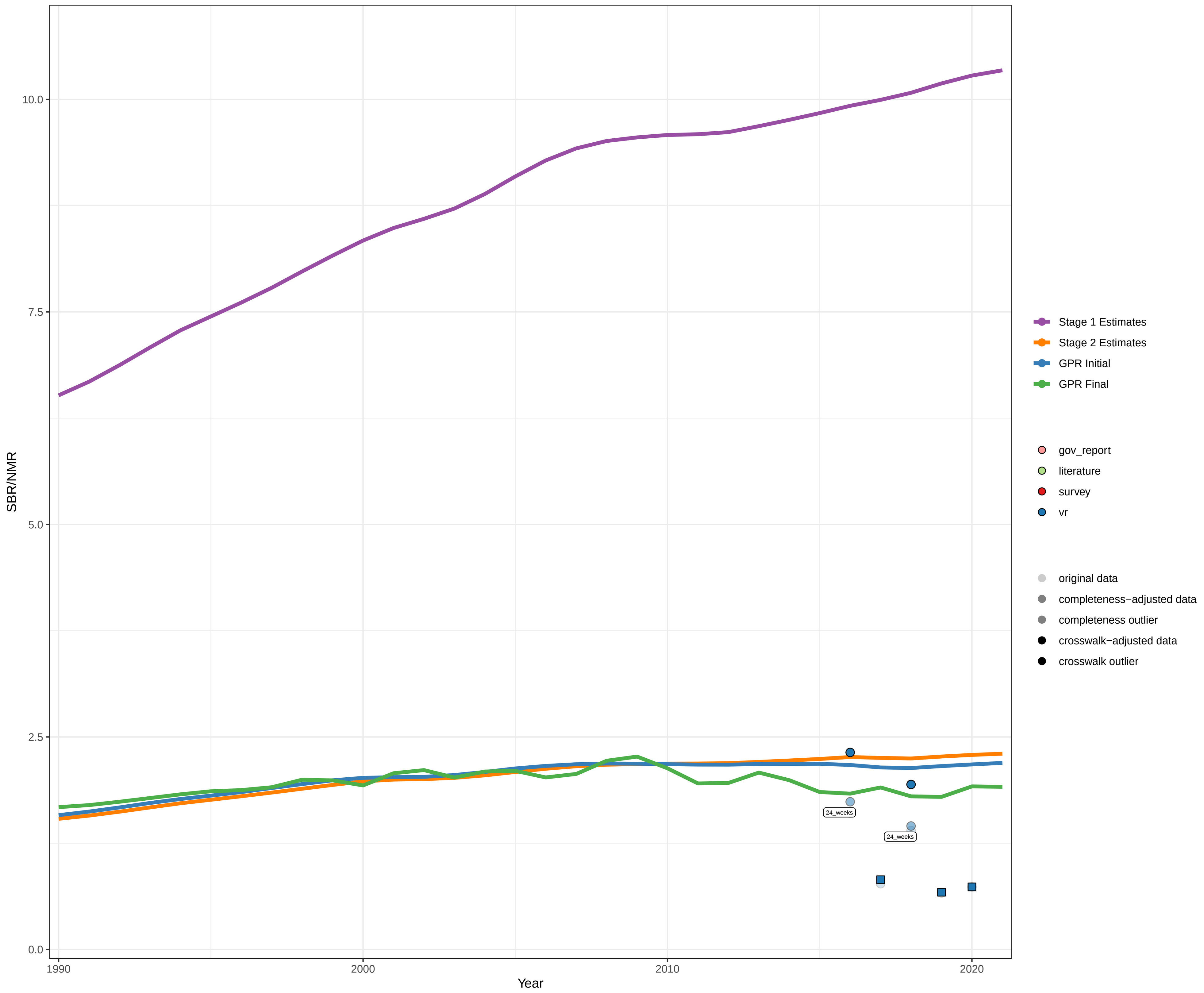

Solihull GBR_44704 Western Europe Stillbirth Rate / Neonatal Mortality Rate (SBR/NMR) − 28 weeks
lambda = 0.8 ; zeta = 0.7 ; scale = 15 ; amp = 0.028

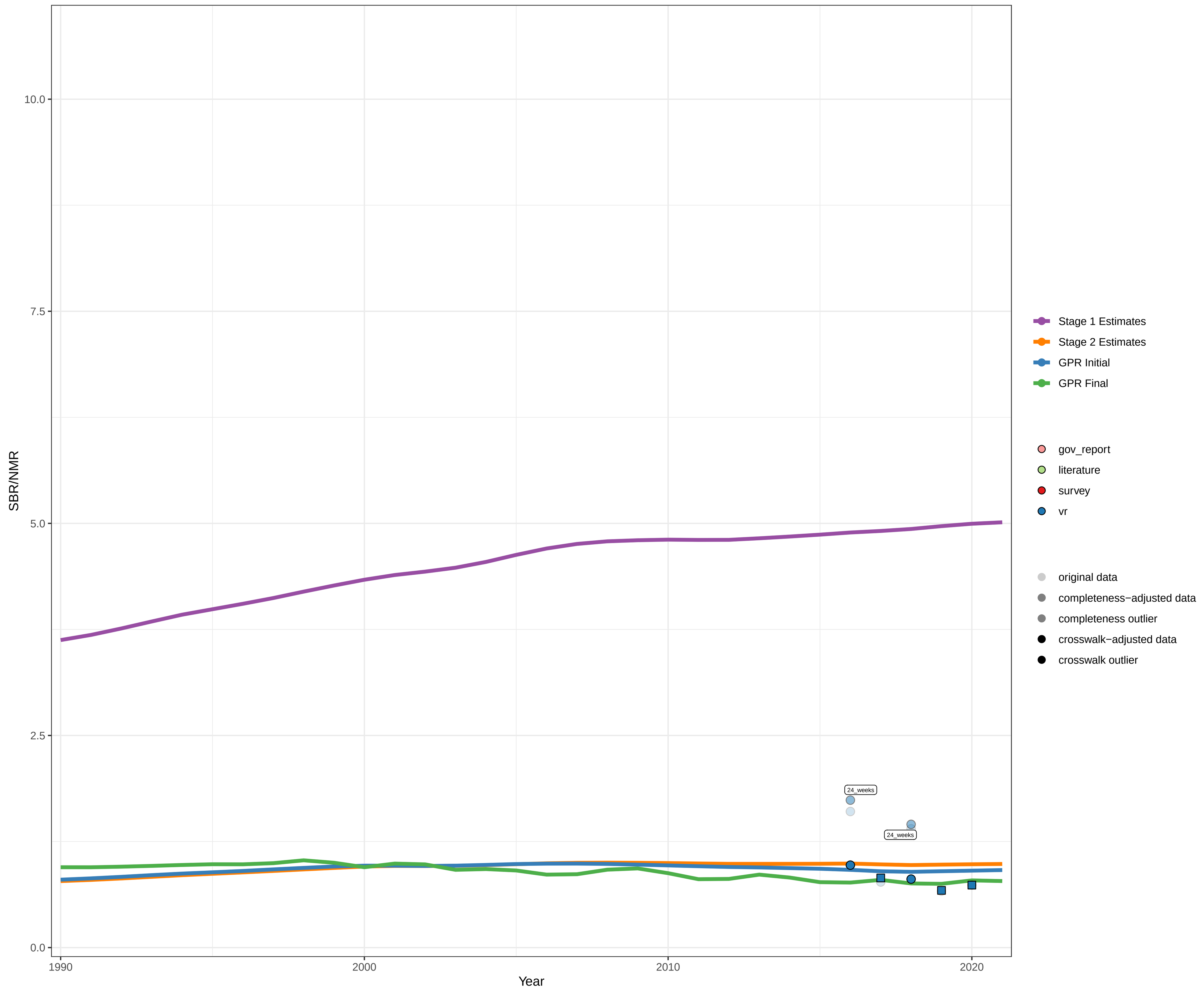

Definition	Years	Outlier	Source.Type	Title
24_weeks	2016−2020	0	VR	United Kingdom − England and Wales Child Mortality Statistics

Solihull GBR_44704 Western Europe Stillbirth Rate (SBR) − 20 weeks
lambda = 0.8 ; zeta = 0.7 ; scale = 15 ; amp = 0.028

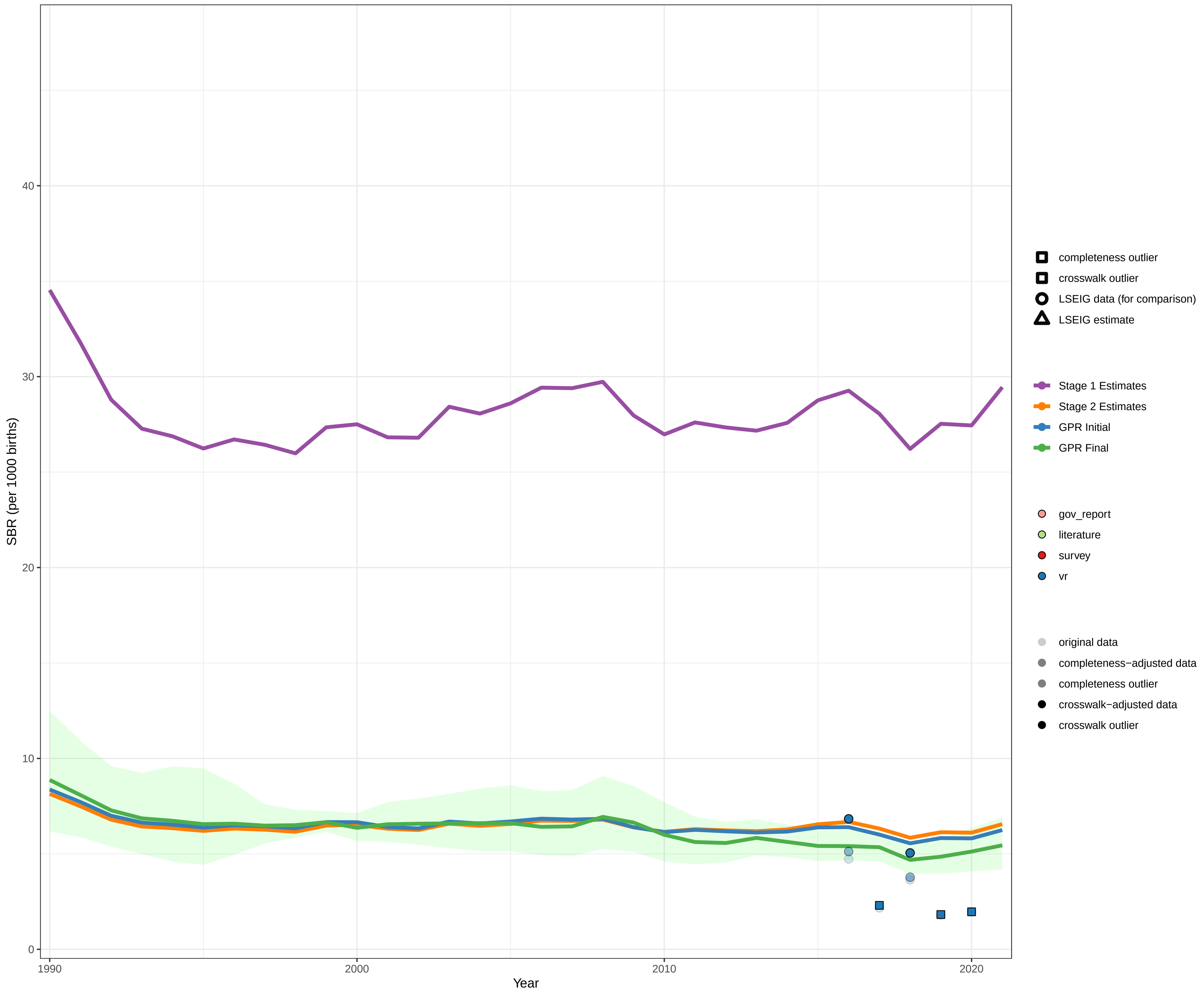

Solihull GBR_44704 Western Europe Stillbirth Rate (SBR) − 28 weeks
lambda = 0.8 ; zeta = 0.7 ; scale = 15 ; amp = 0.028

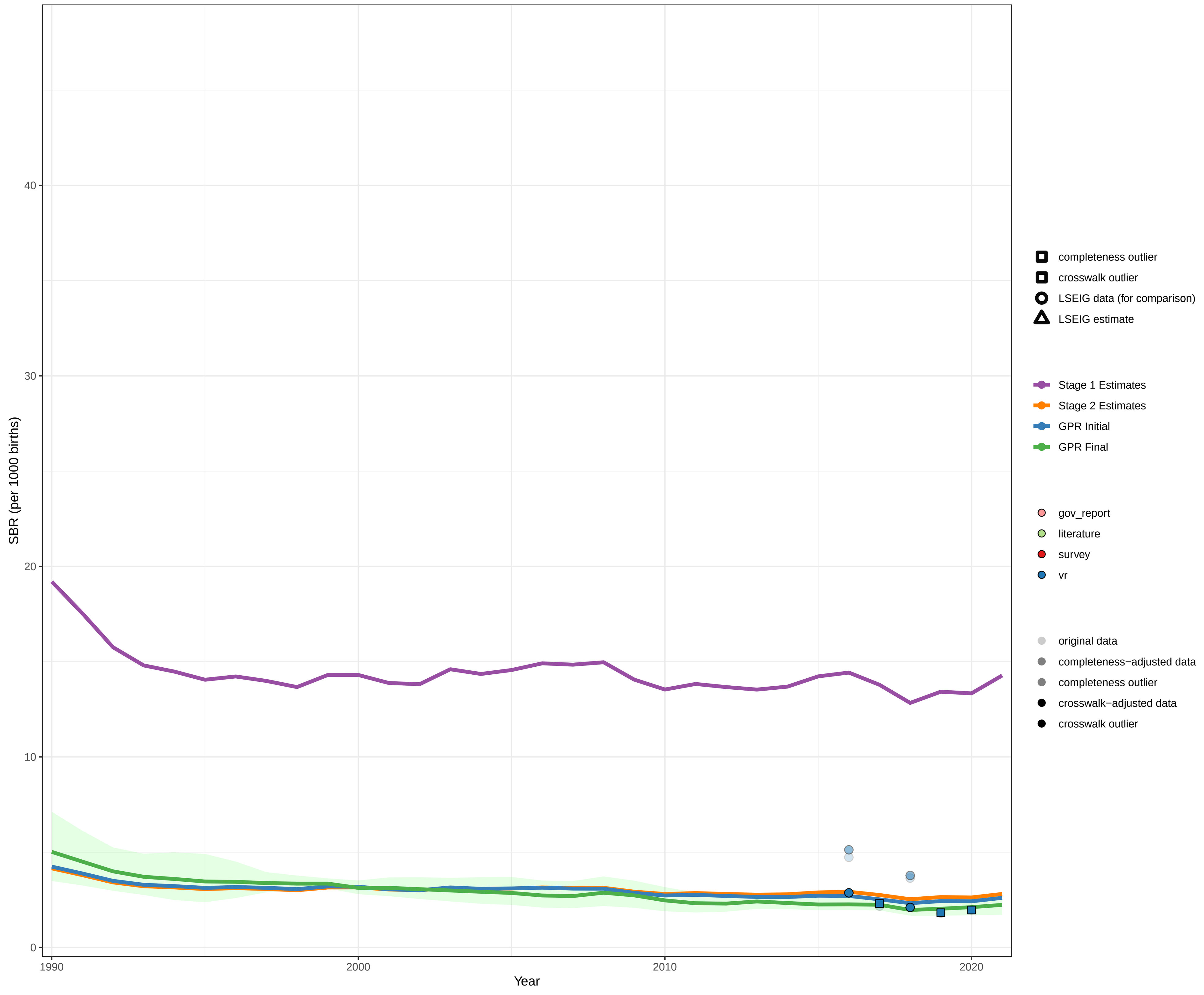

Year	Total Stillbirths (in thousands)	Total Stillbirths (in thousands)	SBR (per 1000 births)	SBR (per 1000 births)	SBR/NMR	SBR/NMR
	20 weeks	28 weeks	20 weeks	28 weeks	20 weeks	28 weeks
1990	0.0 (0.0 to 0.0)	0.0 (0.0 to 0.0)	8.9 (6.2 to 12.5)	5.0 (3.5 to 7.1)	1.67 (1.17 to 2.36)	0.95 (0.66 to 1.35)
1995	0.0 (0.0 to 0.0)	0.0 (0.0 to 0.0)	6.6 (4.4 to 9.5)	3.5 (2.4 to 4.9)	1.86 (1.26 to 2.69)	0.98 (0.67 to 1.39)
2000	0.0 (0.0 to 0.0)	0.0 (0.0 to 0.0)	6.4 (5.7 to 7.1)	3.1 (2.8 to 3.5)	1.93 (1.72 to 2.16)	0.95 (0.85 to 1.07)
2005	0.0 (0.0 to 0.0)	0.0 (0.0 to 0.0)	6.6 (5.1 to 8.6)	2.9 (2.2 to 3.7)	2.10 (1.63 to 2.73)	0.91 (0.71 to 1.18)
2010	0.0 (0.0 to 0.0)	0.0 (0.0 to 0.0)	6.0 (4.6 to 7.7)	2.5 (1.9 to 3.2)	2.13 (1.63 to 2.74)	0.88 (0.67 to 1.12)
2015	0.0 (0.0 to 0.0)	0.0 (0.0 to 0.0)	5.4 (4.6 to 6.2)	2.3 (1.9 to 2.6)	1.85 (1.58 to 2.13)	0.77 (0.67 to 0.88)
2020	0.0 (0.0 to 0.0)	0.0 (0.0 to 0.0)	5.1 (4.1 to 6.4)	2.1 (1.7 to 2.6)	1.92 (1.53 to 2.38)	0.79 (0.63 to 0.98)
2021	0.0 (0.0 to 0.0)	0.0 (0.0 to 0.0)	5.4 (4.2 to 6.9)	2.2 (1.7 to 2.8)	1.91 (1.47 to 2.44)	0.78 (0.60 to 0.99)

Staffordshire GBR_44707 Western Europe Stillbirth Rate / Neonatal Mortality Rate (SBR/NMR) − 20 weeks
lambda = 0.8 ; zeta = 0.7 ; scale = 15 ; amp = 0.028

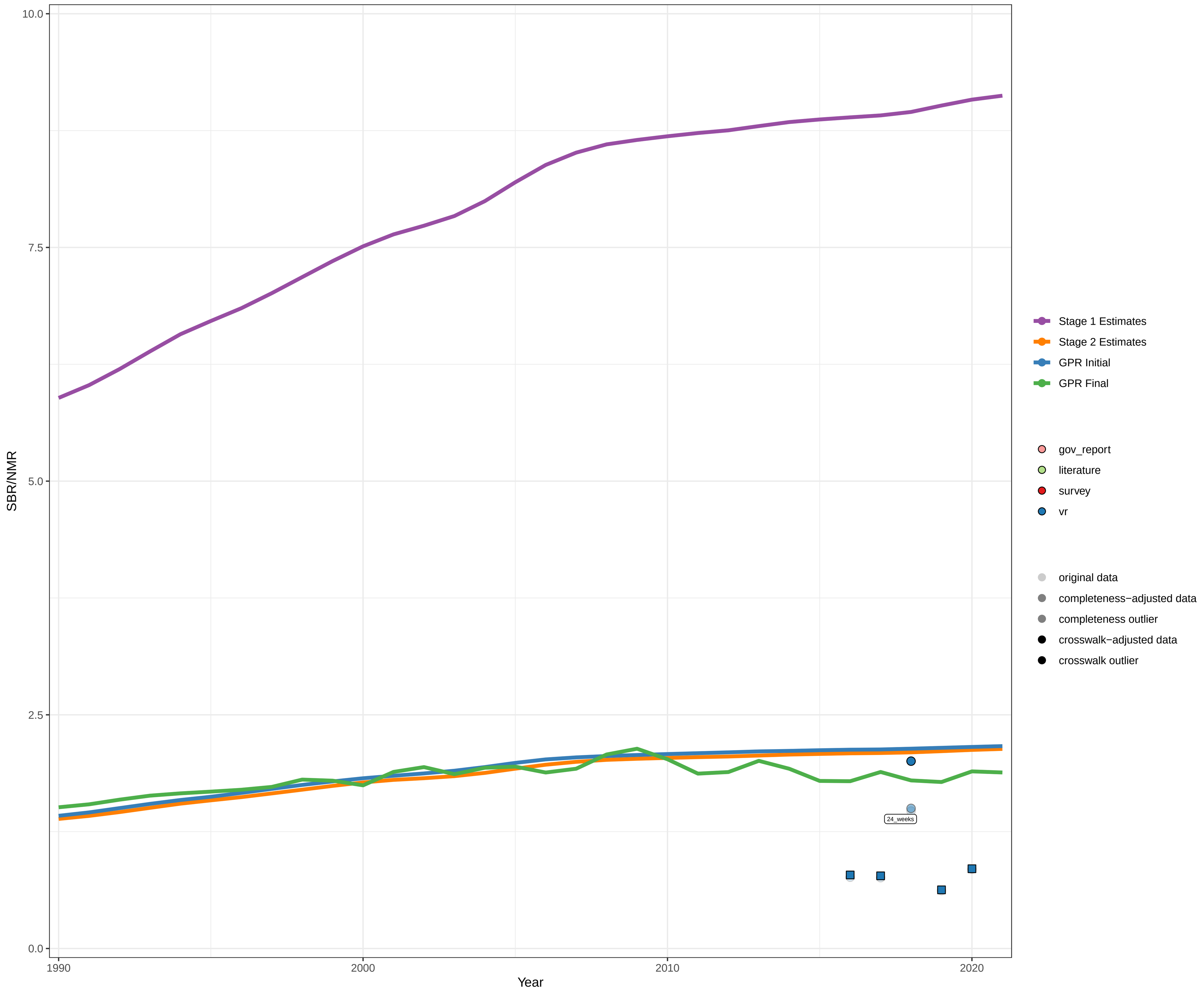

Staffordshire GBR_44707 Western Europe Stillbirth Rate / Neonatal Mortality Rate (SBR/NMR) − 28 weeks
lambda = 0.8 ; zeta = 0.7 ; scale = 15 ; amp = 0.028

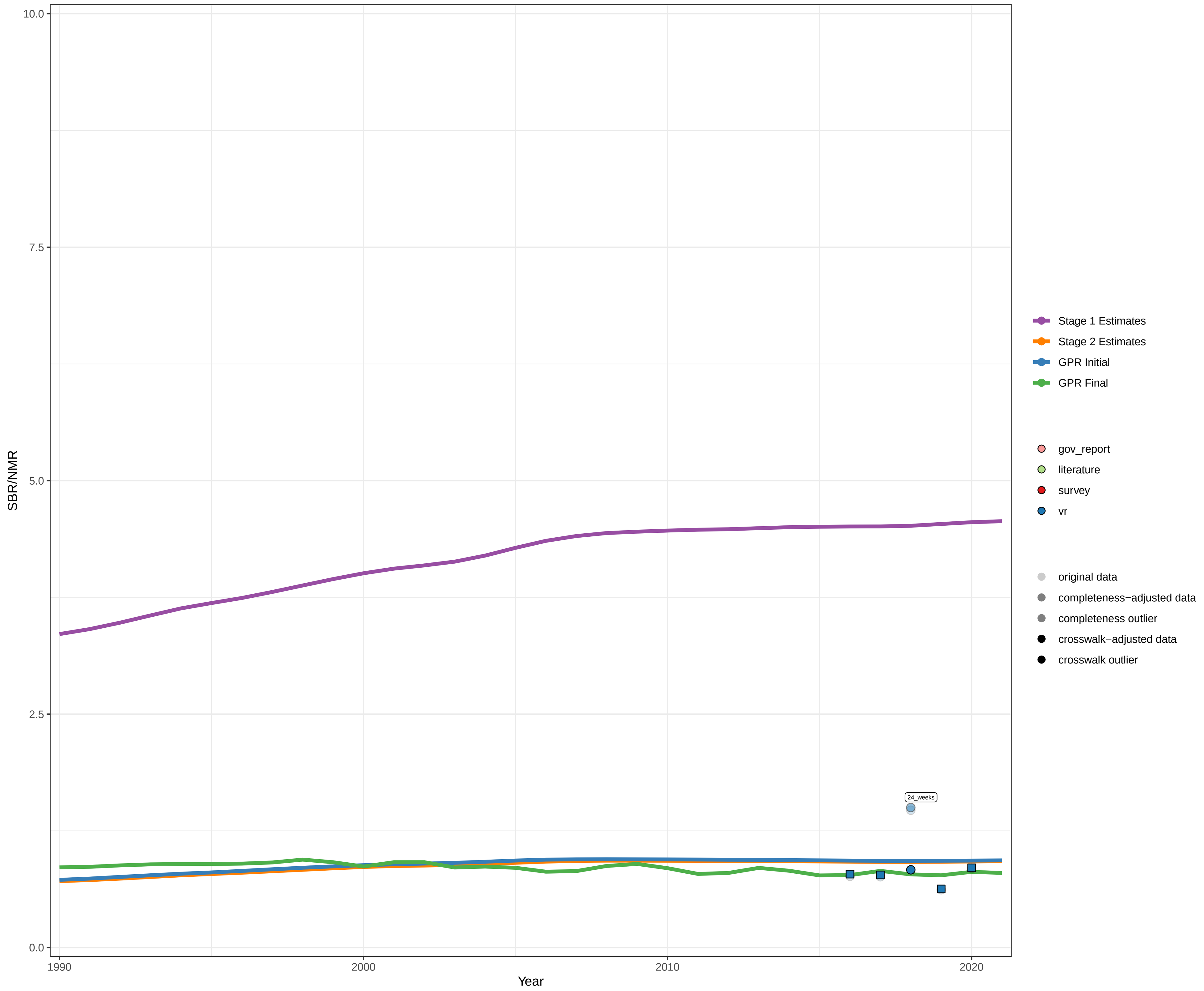

Definition	Years	Outlier	Source.Type	Title
24_weeks	2016−2020	0	VR	United Kingdom − England and Wales Child Mortality Statistics

Staffordshire GBR_44707 Western Europe Stillbirth Rate (SBR) − 20 weeks
lambda = 0.8 ; zeta = 0.7 ; scale = 15 ; amp = 0.028

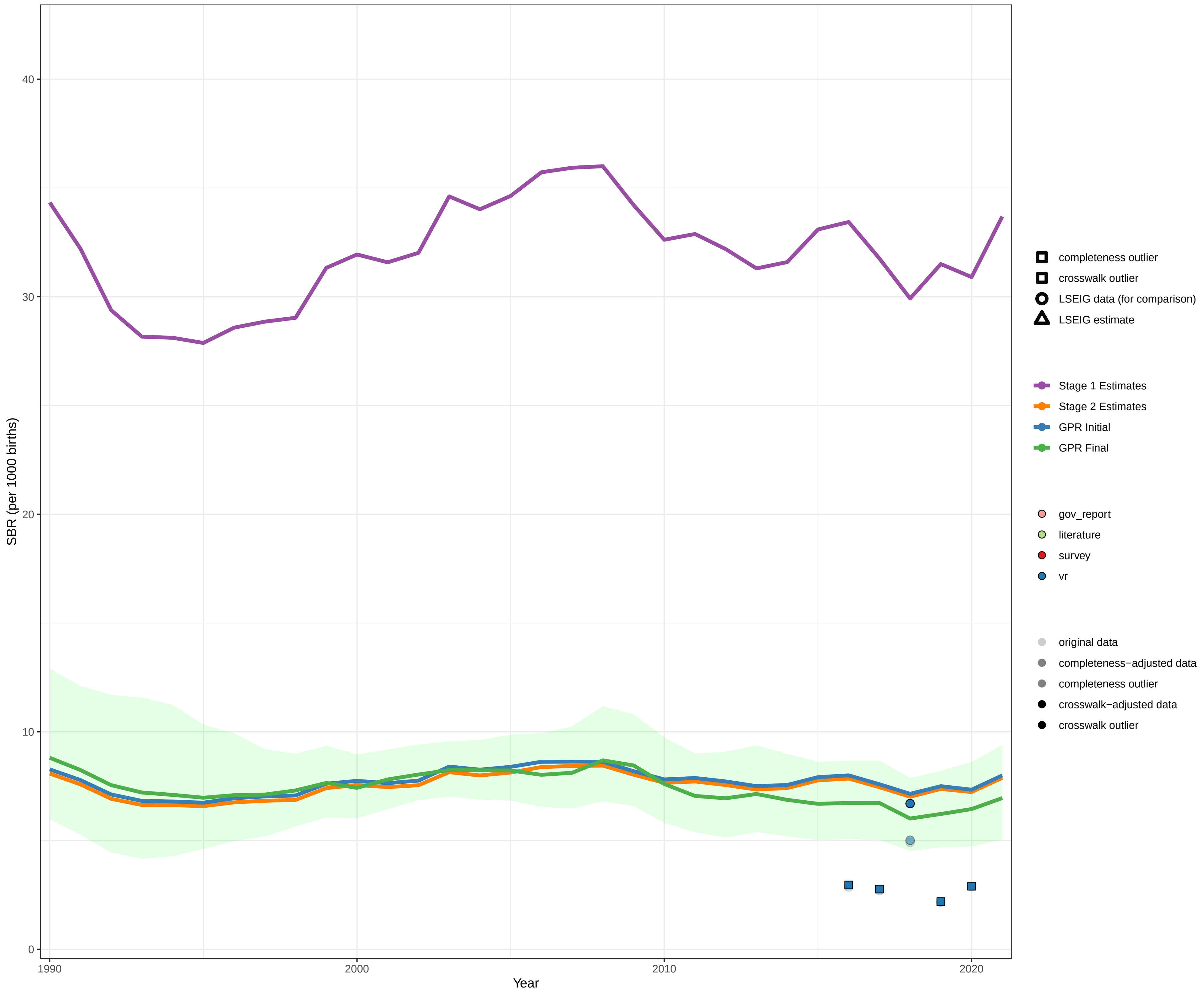

Staffordshire GBR_44707 Western Europe Stillbirth Rate (SBR) − 28 weeks
lambda = 0.8 ; zeta = 0.7 ; scale = 15 ; amp = 0.028

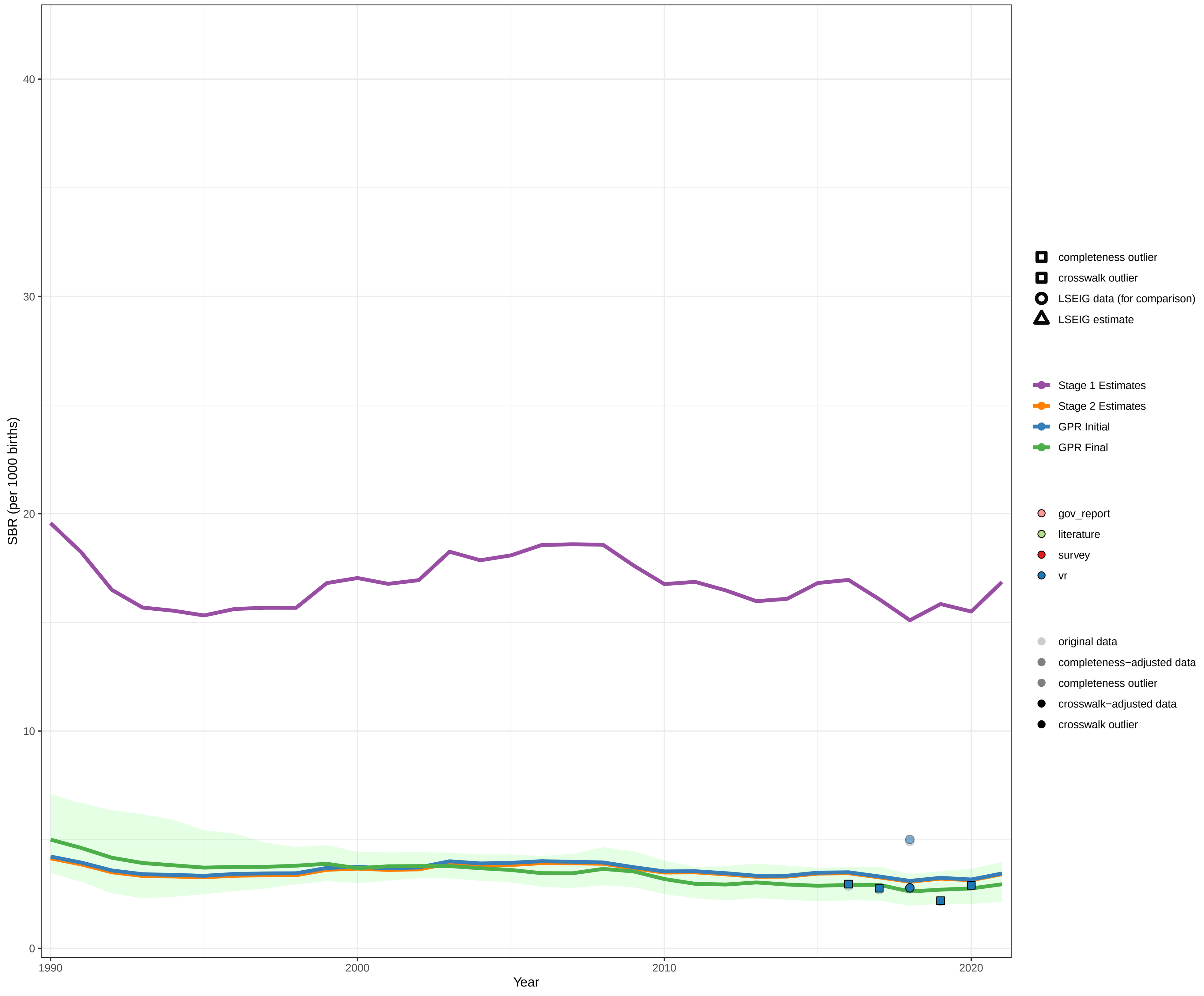

Year	Total Stillbirths (in thousands)	Total Stillbirths (in thousands)	SBR (per 1000 births)	SBR (per 1000 births)	SBR/NMR	SBR/NMR
	20 weeks	28 weeks	20 weeks	28 weeks	20 weeks	28 weeks
1990	0.1 (0.1 to 0.1)	0.1 (0.0 to 0.1)	8.8 (6.0 to 12.9)	5.0 (3.5 to 7.1)	1.51 (1.03 to 2.21)	0.86 (0.59 to 1.22)
1995	0.1 (0.0 to 0.1)	0.0 (0.0 to 0.0)	7.0 (4.6 to 10.3)	3.7 (2.5 to 5.4)	1.68 (1.11 to 2.49)	0.90 (0.60 to 1.31)
2000	0.1 (0.0 to 0.1)	0.0 (0.0 to 0.0)	7.4 (6.0 to 9.0)	3.7 (3.0 to 4.4)	1.75 (1.42 to 2.11)	0.87 (0.71 to 1.05)
2005	0.1 (0.1 to 0.1)	0.0 (0.0 to 0.0)	8.2 (6.8 to 9.9)	3.6 (3.0 to 4.3)	1.95 (1.62 to 2.34)	0.85 (0.72 to 1.02)
2010	0.1 (0.1 to 0.1)	0.0 (0.0 to 0.0)	7.6 (5.8 to 9.7)	3.2 (2.5 to 4.0)	2.02 (1.55 to 2.60)	0.85 (0.67 to 1.07)
2015	0.1 (0.0 to 0.1)	0.0 (0.0 to 0.0)	6.7 (5.0 to 8.6)	2.9 (2.2 to 3.7)	1.79 (1.35 to 2.31)	0.77 (0.58 to 0.99)
2020	0.1 (0.0 to 0.1)	0.0 (0.0 to 0.0)	6.4 (4.7 to 8.6)	2.8 (2.0 to 3.7)	1.89 (1.39 to 2.53)	0.81 (0.60 to 1.08)
2021	0.1 (0.0 to 0.1)	0.0 (0.0 to 0.0)	7.0 (5.0 to 9.4)	2.9 (2.1 to 4.0)	1.88 (1.36 to 2.55)	0.80 (0.58 to 1.08)

Stoke−on−Trent GBR_44711 Western Europe Stillbirth Rate / Neonatal Mortality Rate (SBR/NMR) − 20 weeks
lambda = 0.6 ; zeta = 0.8 ; scale = 15 ; amp = 0.028

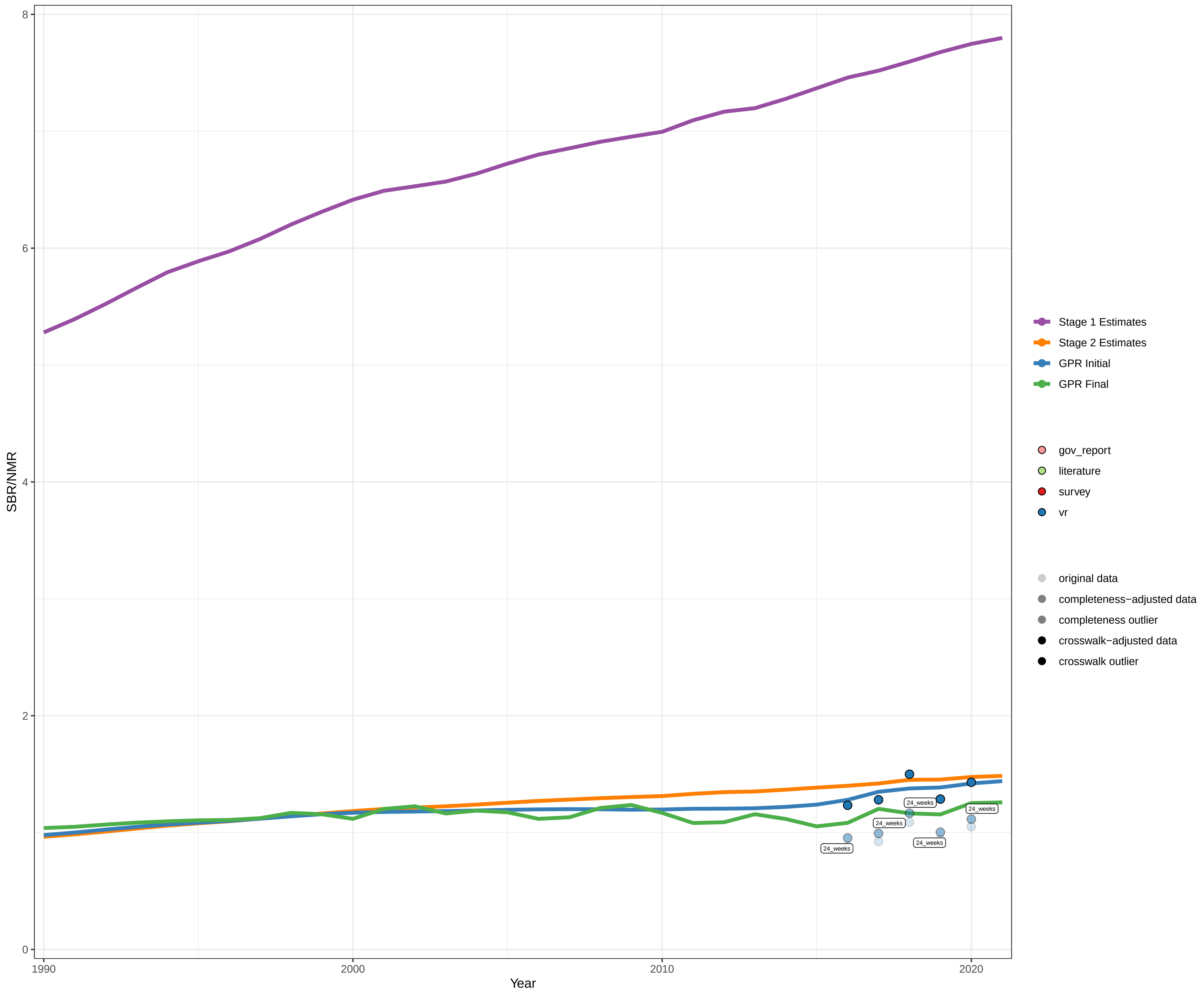

Stoke−on−Trent GBR_44711 Western Europe Stillbirth Rate / Neonatal Mortality Rate (SBR/NMR) − 28 weeks
lambda = 0.6 ; zeta = 0.8 ; scale = 15 ; amp = 0.028

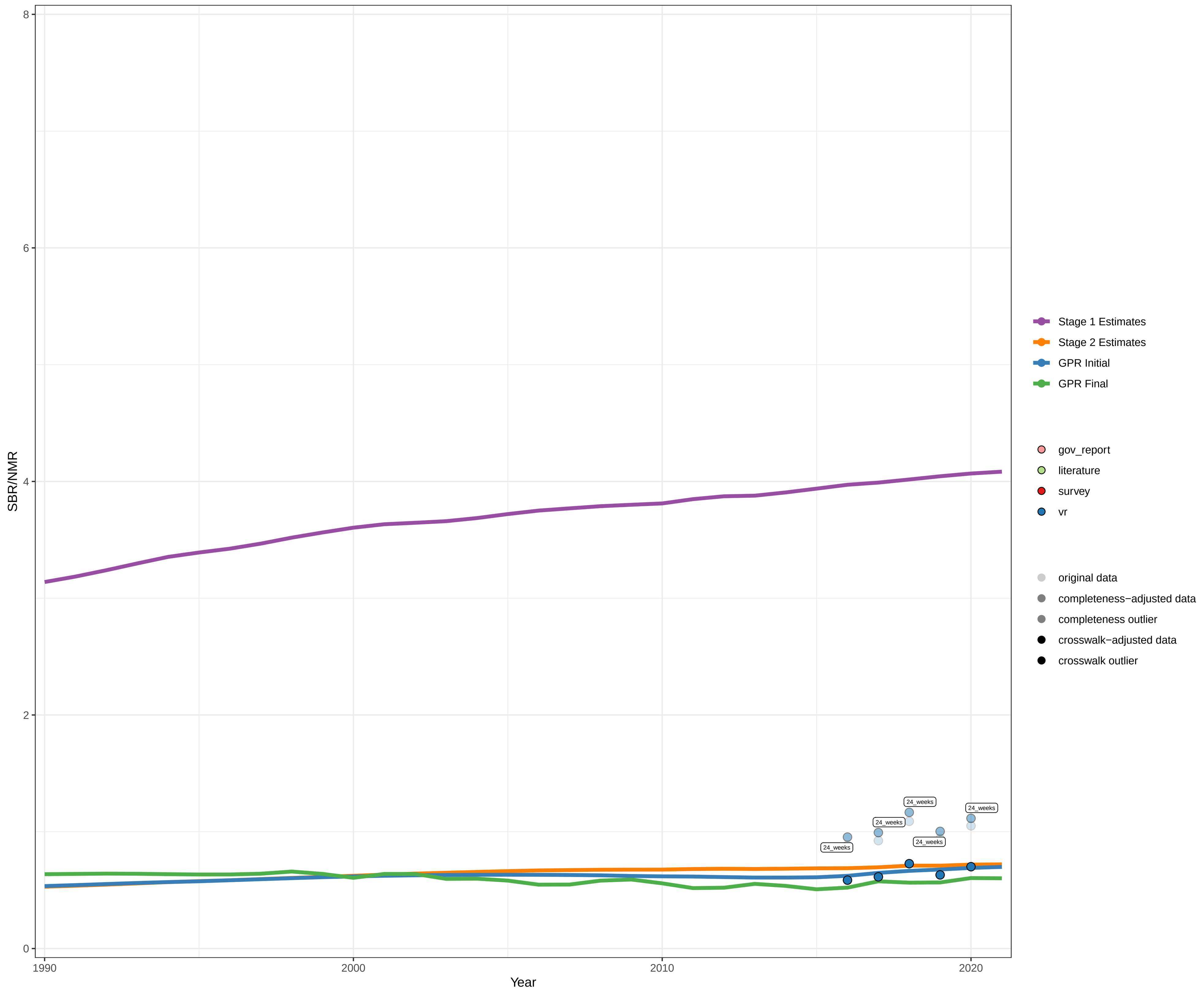

Definition	Years	Outlier	Source.Type	Title
24_weeks	2016−2020	0	VR	United Kingdom − England and Wales Child Mortality Statistics

Stoke−on−Trent GBR_44711 Western Europe Stillbirth Rate (SBR) − 20 weeks
lambda = 0.6 ; zeta = 0.8 ; scale = 15 ; amp = 0.028

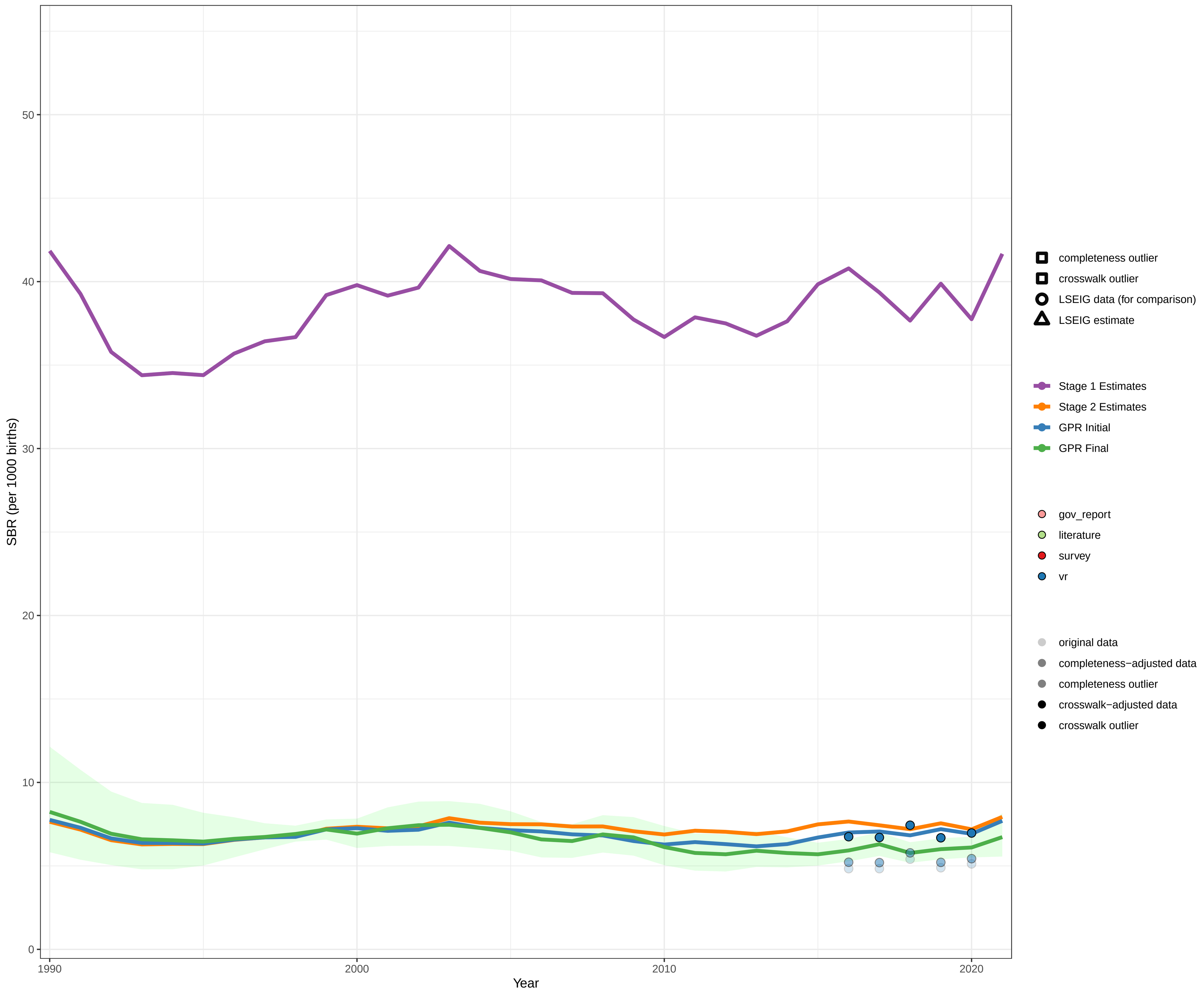

Stoke−on−Trent GBR_44711 Western Europe Stillbirth Rate (SBR) − 28 weeks
lambda = 0.6 ; zeta = 0.8 ; scale = 15 ; amp = 0.028

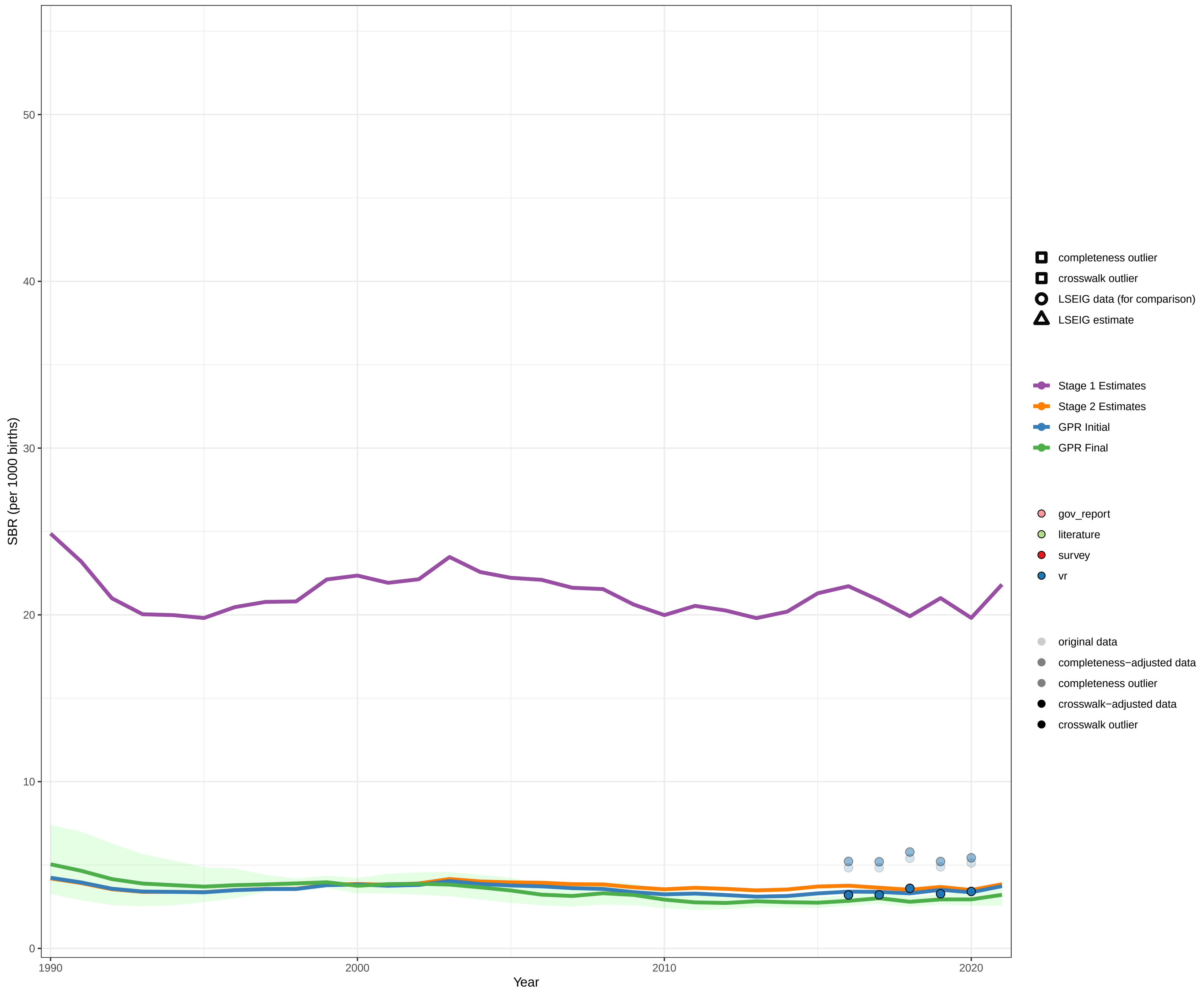

Year	Total Stillbirths (in thousands)	Total Stillbirths (in thousands)	SBR (per 1000 births)	SBR (per 1000 births)	SBR/NMR	SBR/NMR
	20 weeks	28 weeks	20 weeks	28 weeks	20 weeks	28 weeks
1990	0.0 (0.0 to 0.0)	0.0 (0.0 to 0.0)	8.2 (5.8 to 12.1)	5.0 (3.3 to 7.4)	1.04 (0.73 to 1.53)	0.64 (0.41 to 0.93)
1995	0.0 (0.0 to 0.0)	0.0 (0.0 to 0.0)	6.5 (5.0 to 8.2)	3.7 (2.8 to 4.9)	1.11 (0.86 to 1.40)	0.63 (0.48 to 0.83)
2000	0.0 (0.0 to 0.0)	0.0 (0.0 to 0.0)	6.9 (6.1 to 7.8)	3.8 (3.3 to 4.2)	1.12 (0.98 to 1.26)	0.61 (0.53 to 0.68)
2005	0.0 (0.0 to 0.0)	0.0 (0.0 to 0.0)	7.0 (5.9 to 8.3)	3.5 (2.7 to 4.2)	1.17 (0.99 to 1.38)	0.58 (0.46 to 0.71)
2010	0.0 (0.0 to 0.0)	0.0 (0.0 to 0.0)	6.1 (5.0 to 7.4)	2.9 (2.4 to 3.5)	1.17 (0.96 to 1.41)	0.56 (0.46 to 0.66)
2015	0.0 (0.0 to 0.0)	0.0 (0.0 to 0.0)	5.7 (5.0 to 6.4)	2.7 (2.4 to 3.1)	1.05 (0.93 to 1.18)	0.51 (0.45 to 0.57)
2020	0.0 (0.0 to 0.0)	0.0 (0.0 to 0.0)	6.1 (5.5 to 6.7)	2.9 (2.6 to 3.4)	1.25 (1.13 to 1.39)	0.60 (0.53 to 0.69)
2021	0.0 (0.0 to 0.0)	0.0 (0.0 to 0.0)	6.7 (5.6 to 8.2)	3.2 (2.6 to 4.0)	1.26 (1.04 to 1.53)	0.60 (0.48 to 0.74)

Telford and Wrekin GBR_44710 Western Europe Stillbirth Rate / Neonatal Mortality Rate (SBR/NMR) − 20 weeks
lambda = 0.6 ; zeta = 0.8 ; scale = 15 ; amp = 0.028

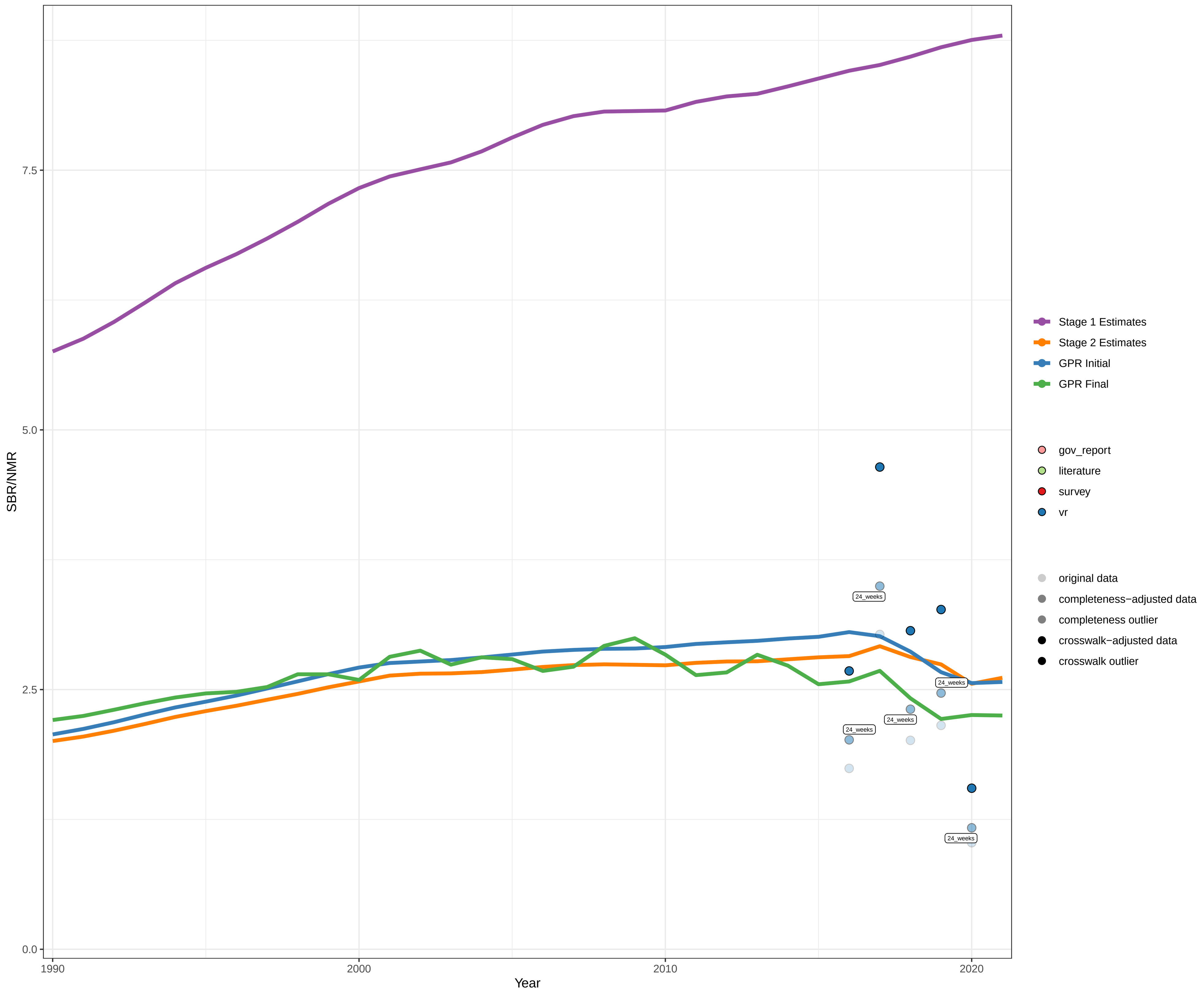

Telford and Wrekin GBR_44710 Western Europe Stillbirth Rate / Neonatal Mortality Rate (SBR/NMR) − 28 weeks
lambda = 0.6 ; zeta = 0.8 ; scale = 15 ; amp = 0.028

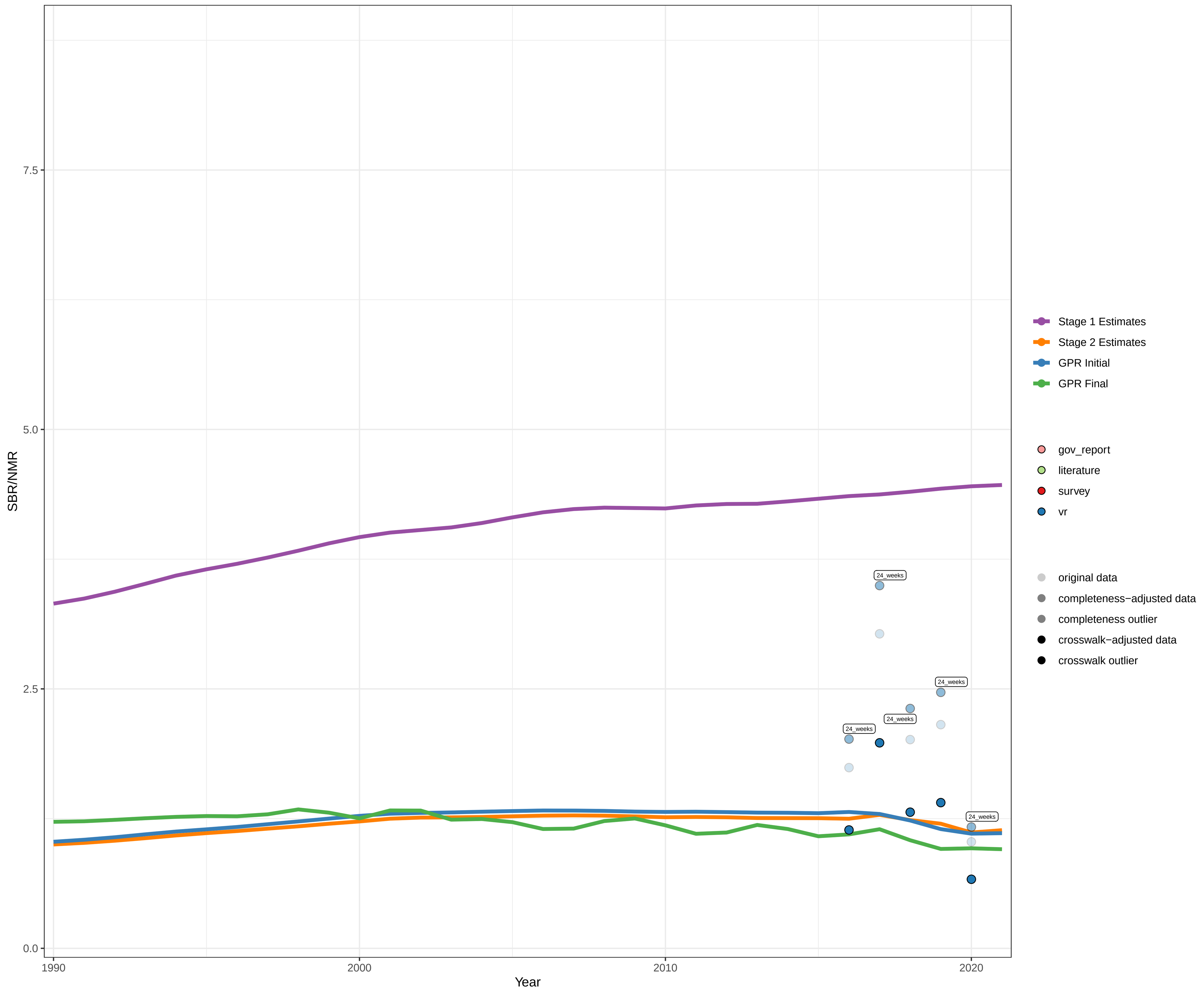

Definition	Years	Outlier	Source.Type	Title
24_weeks	2016−2020	0	VR	United Kingdom − England and Wales Child Mortality Statistics

Telford and Wrekin GBR_44710 Western Europe Stillbirth Rate (SBR) − 20 weeks
lambda = 0.6 ; zeta = 0.8 ; scale = 15 ; amp = 0.028

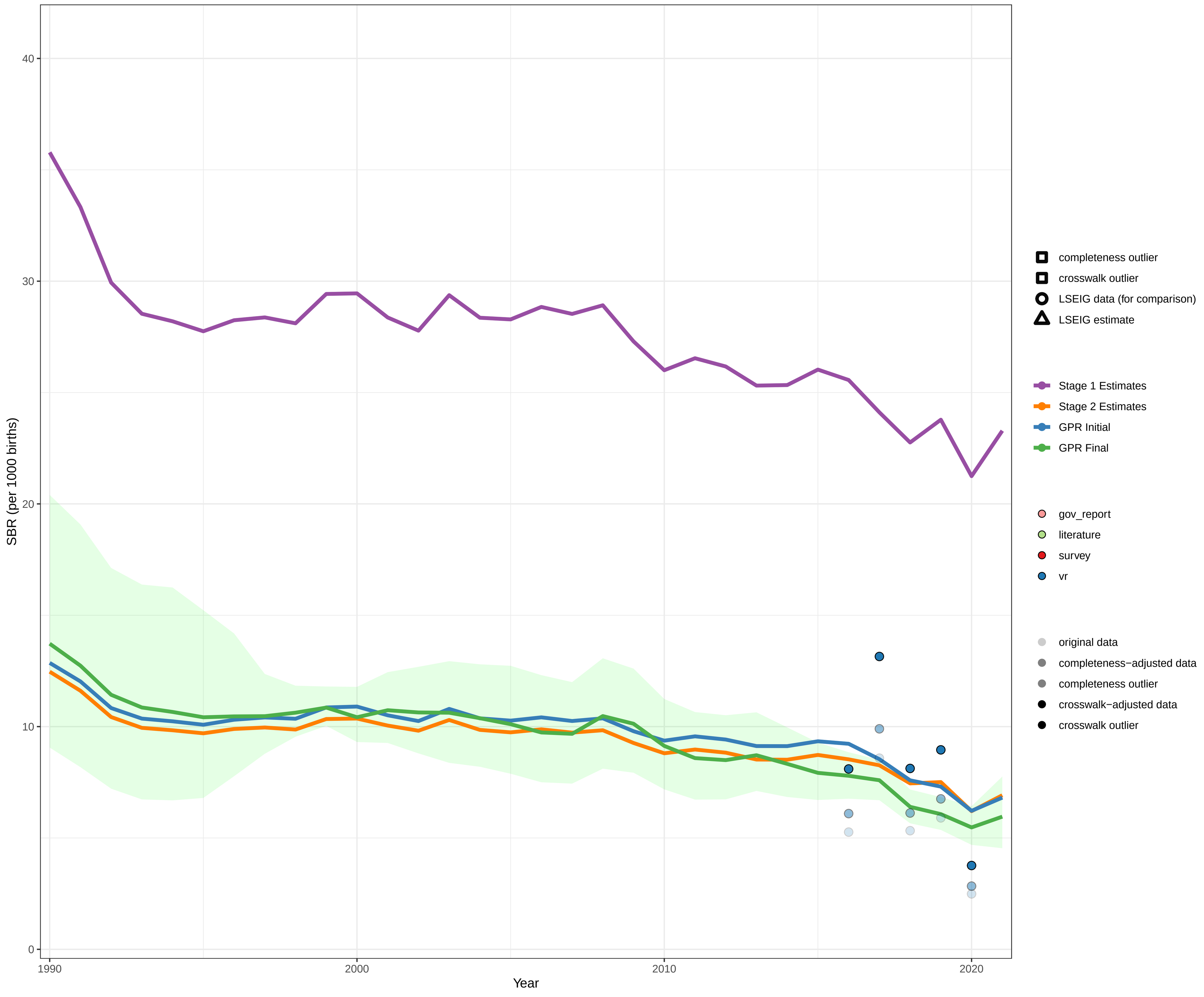

Telford and Wrekin GBR_44710 Western Europe Stillbirth Rate (SBR) − 28 weeks
lambda = 0.6 ; zeta = 0.8 ; scale = 15 ; amp = 0.028

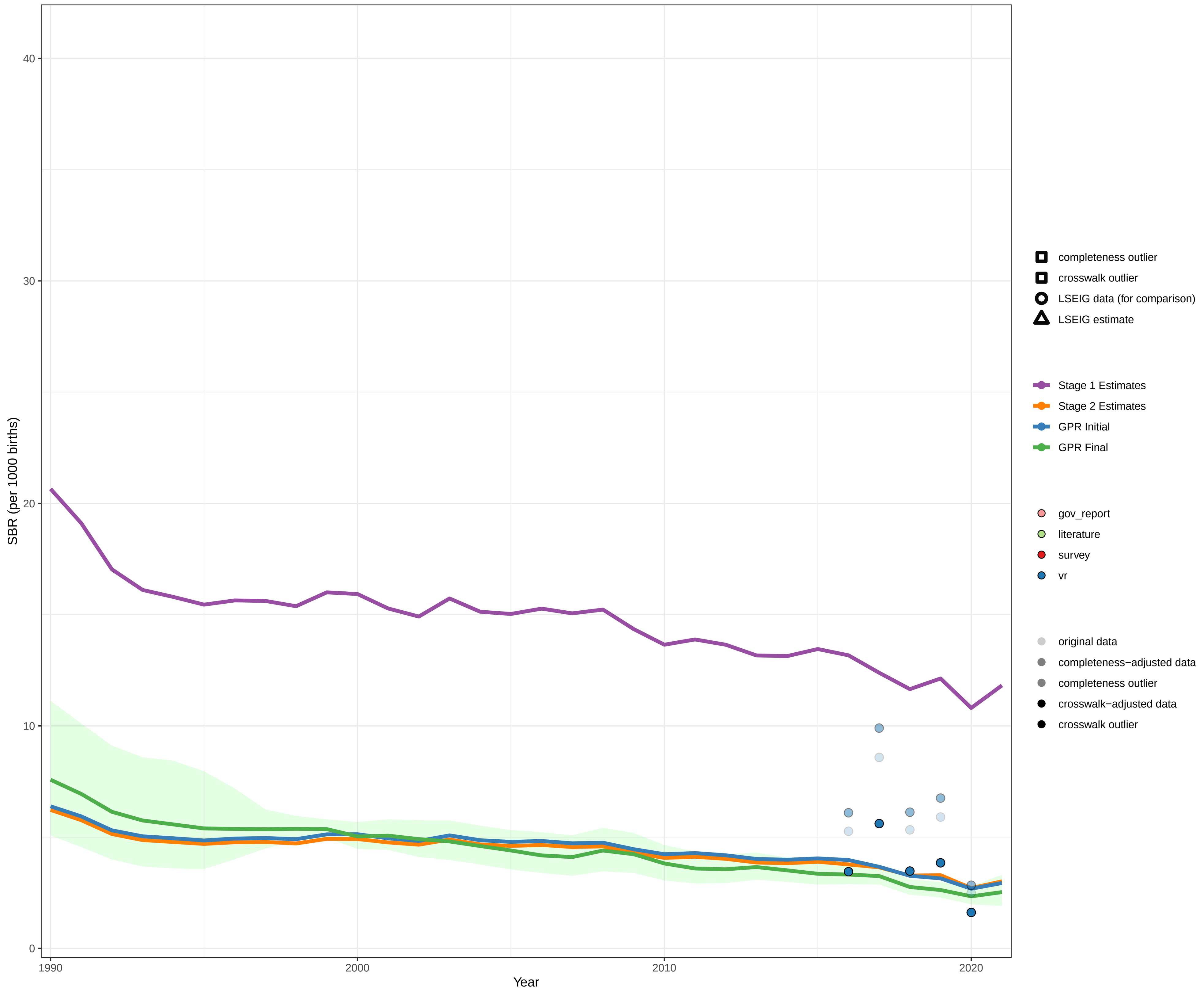

Year	Total Stillbirths (in thousands)	Total Stillbirths (in thousands)	SBR (per 1000 births)	SBR (per 1000 births)	SBR/NMR	SBR/NMR
	20 weeks	28 weeks	20 weeks	28 weeks	20 weeks	28 weeks
1990	0.0 (0.0 to 0.0)	0.0 (0.0 to 0.0)	13.7 (9.1 to 20.4)	7.6 (5.1 to 11.1)	2.21 (1.46 to 3.28)	1.22 (0.82 to 1.79)
1995	0.0 (0.0 to 0.0)	0.0 (0.0 to 0.0)	10.4 (6.8 to 15.2)	5.4 (3.6 to 8.0)	2.46 (1.61 to 3.60)	1.27 (0.84 to 1.88)
2000	0.0 (0.0 to 0.0)	0.0 (0.0 to 0.0)	10.4 (9.3 to 11.8)	5.0 (4.5 to 5.7)	2.59 (2.32 to 2.93)	1.25 (1.11 to 1.41)
2005	0.0 (0.0 to 0.0)	0.0 (0.0 to 0.0)	10.1 (7.9 to 12.7)	4.4 (3.5 to 5.3)	2.79 (2.18 to 3.52)	1.22 (0.98 to 1.47)
2010	0.0 (0.0 to 0.0)	0.0 (0.0 to 0.0)	9.1 (7.2 to 11.2)	3.8 (3.0 to 4.6)	2.84 (2.23 to 3.49)	1.19 (0.95 to 1.44)
2015	0.0 (0.0 to 0.0)	0.0 (0.0 to 0.0)	7.9 (6.7 to 9.3)	3.4 (2.9 to 3.8)	2.55 (2.16 to 2.99)	1.08 (0.92 to 1.22)
2020	0.0 (0.0 to 0.0)	0.0 (0.0 to 0.0)	5.5 (4.7 to 6.4)	2.3 (2.0 to 2.8)	2.26 (1.93 to 2.65)	0.96 (0.81 to 1.15)
2021	0.0 (0.0 to 0.0)	0.0 (0.0 to 0.0)	6.0 (4.5 to 7.8)	2.5 (1.9 to 3.3)	2.25 (1.71 to 2.93)	0.96 (0.72 to 1.25)

Walsall GBR_44712 Western Europe Stillbirth Rate / Neonatal Mortality Rate (SBR/NMR) − 20 weeks
lambda = 0.6 ; zeta = 0.8 ; scale = 15 ; amp = 0.028

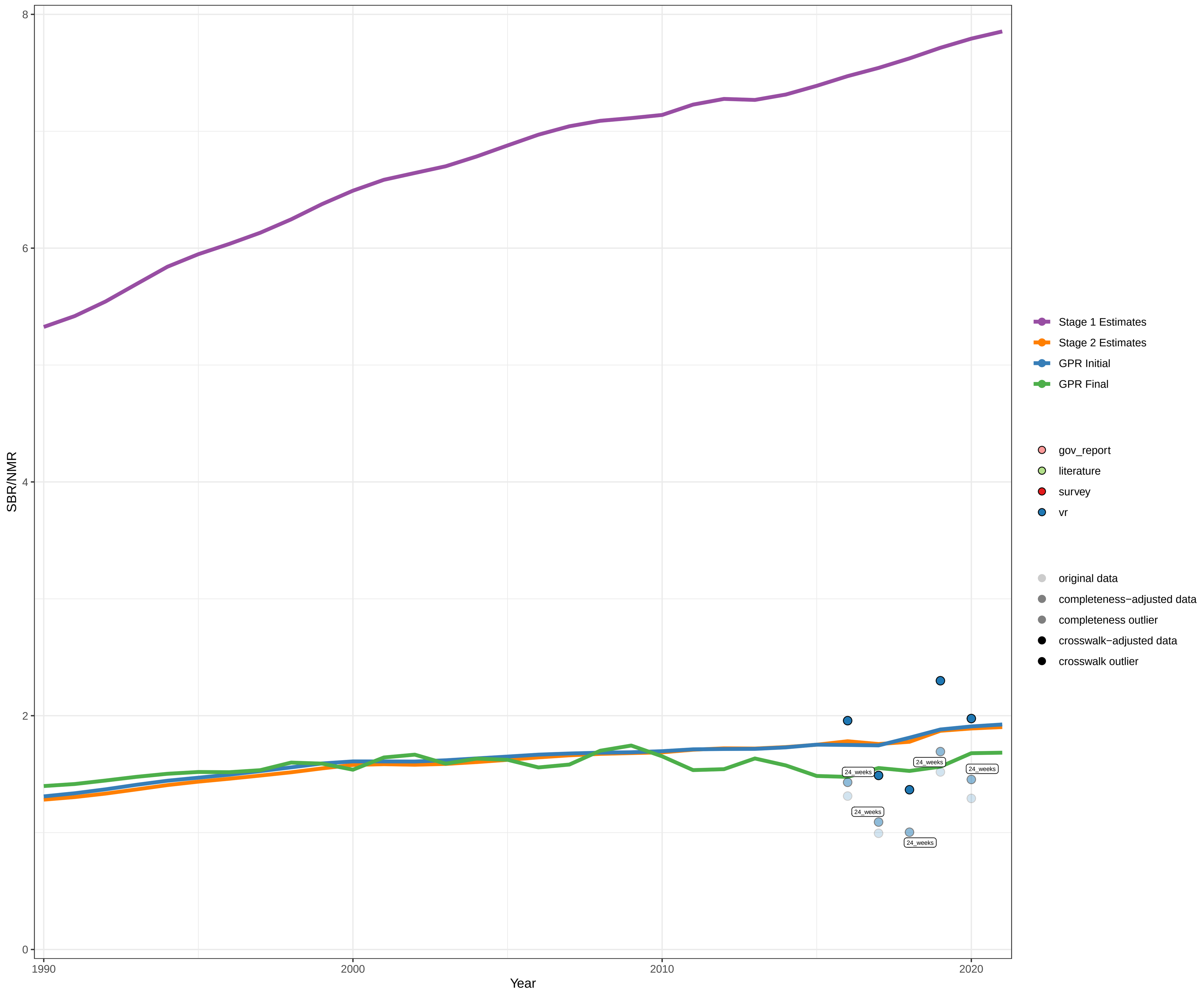

Walsall GBR_44712 Western Europe Stillbirth Rate / Neonatal Mortality Rate (SBR/NMR) − 28 weeks
lambda = 0.6 ; zeta = 0.8 ; scale = 15 ; amp = 0.028

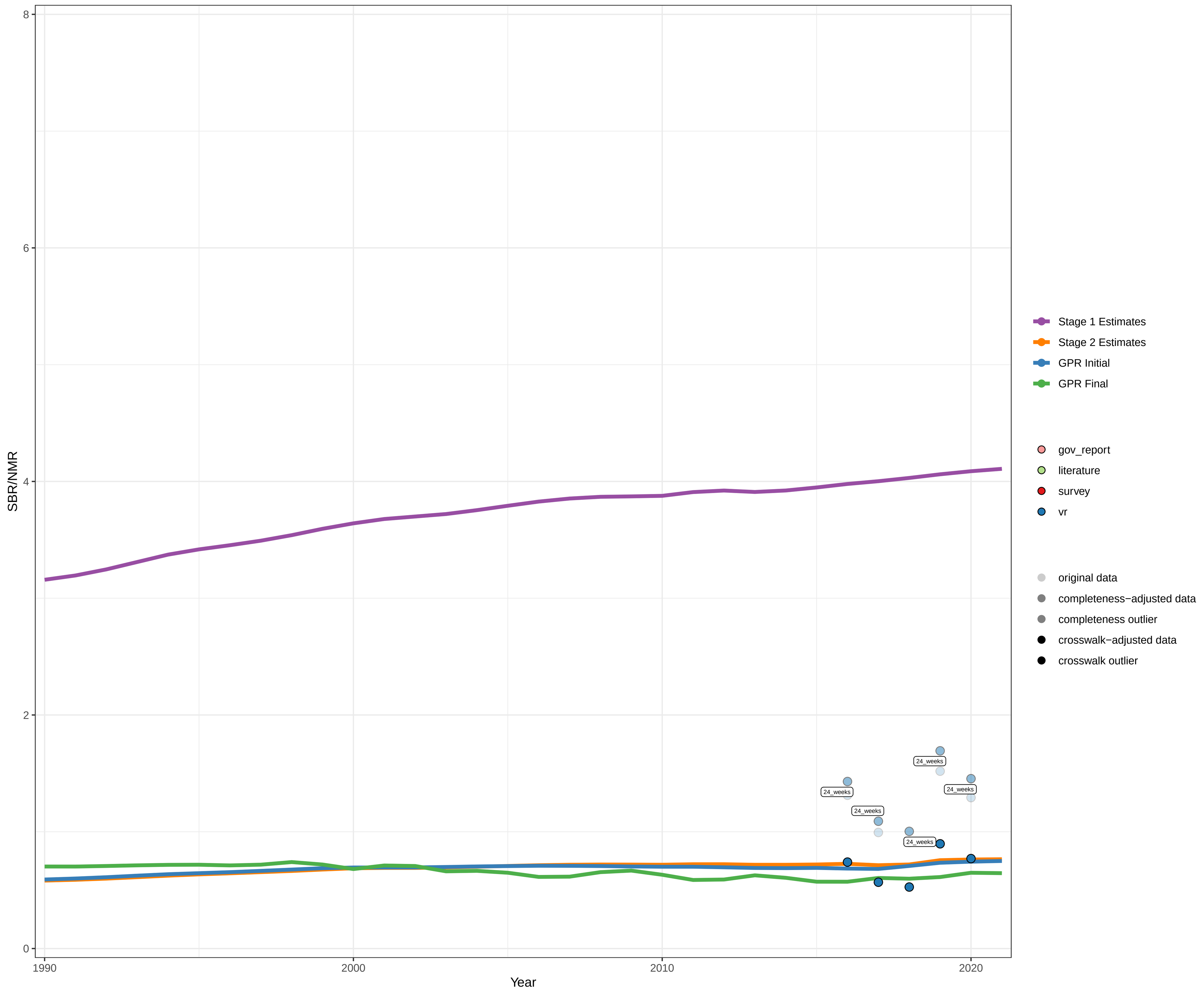

Definition	Years	Outlier	Source.Type	Title
24_weeks	2016−2020	0	VR	United Kingdom − England and Wales Child Mortality Statistics

Walsall GBR_44712 Western Europe Stillbirth Rate (SBR) − 20 weeks
lambda = 0.6 ; zeta = 0.8 ; scale = 15 ; amp = 0.028

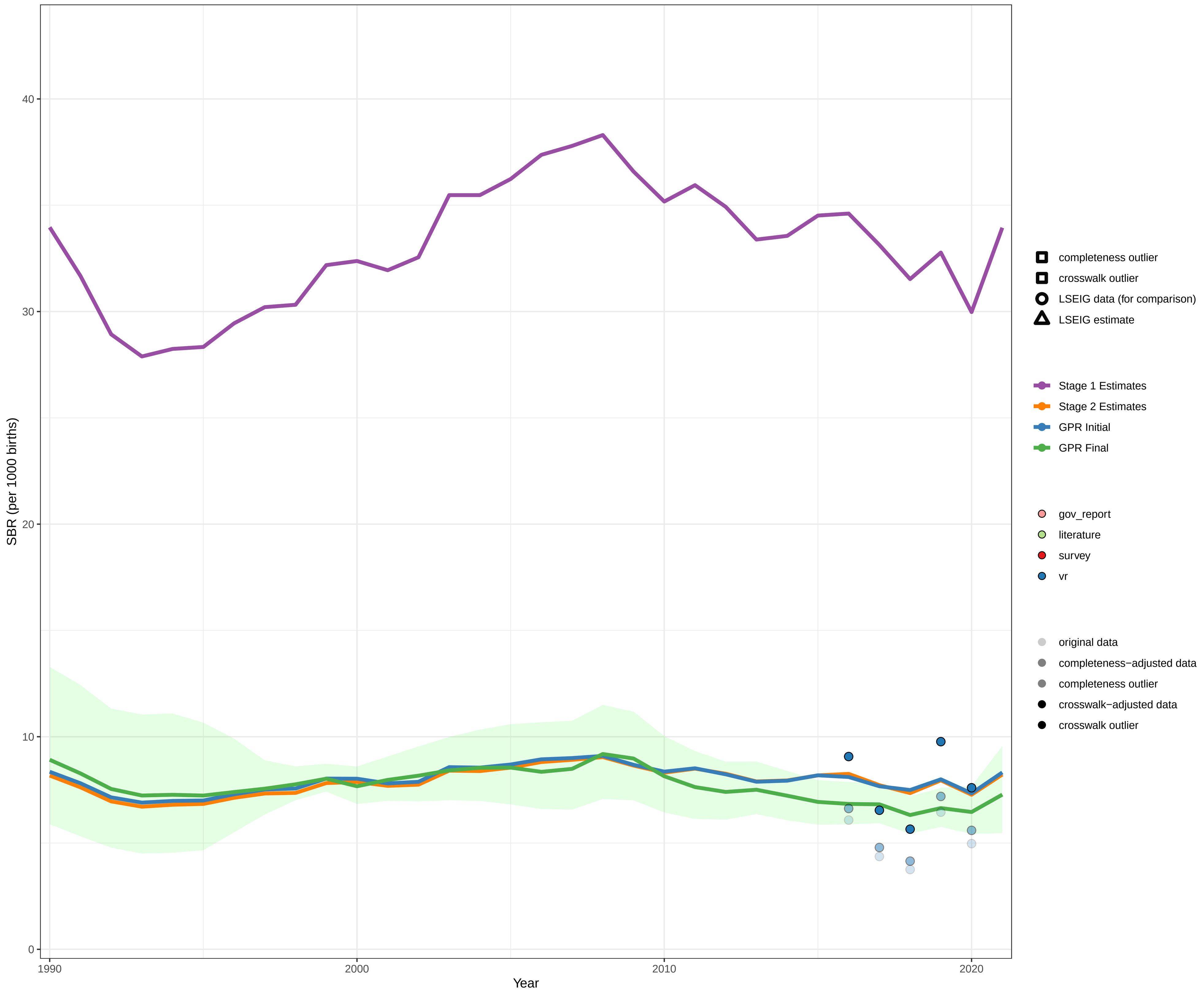

Walsall GBR_44712 Western Europe Stillbirth Rate (SBR) − 28 weeks
lambda = 0.6 ; zeta = 0.8 ; scale = 15 ; amp = 0.028

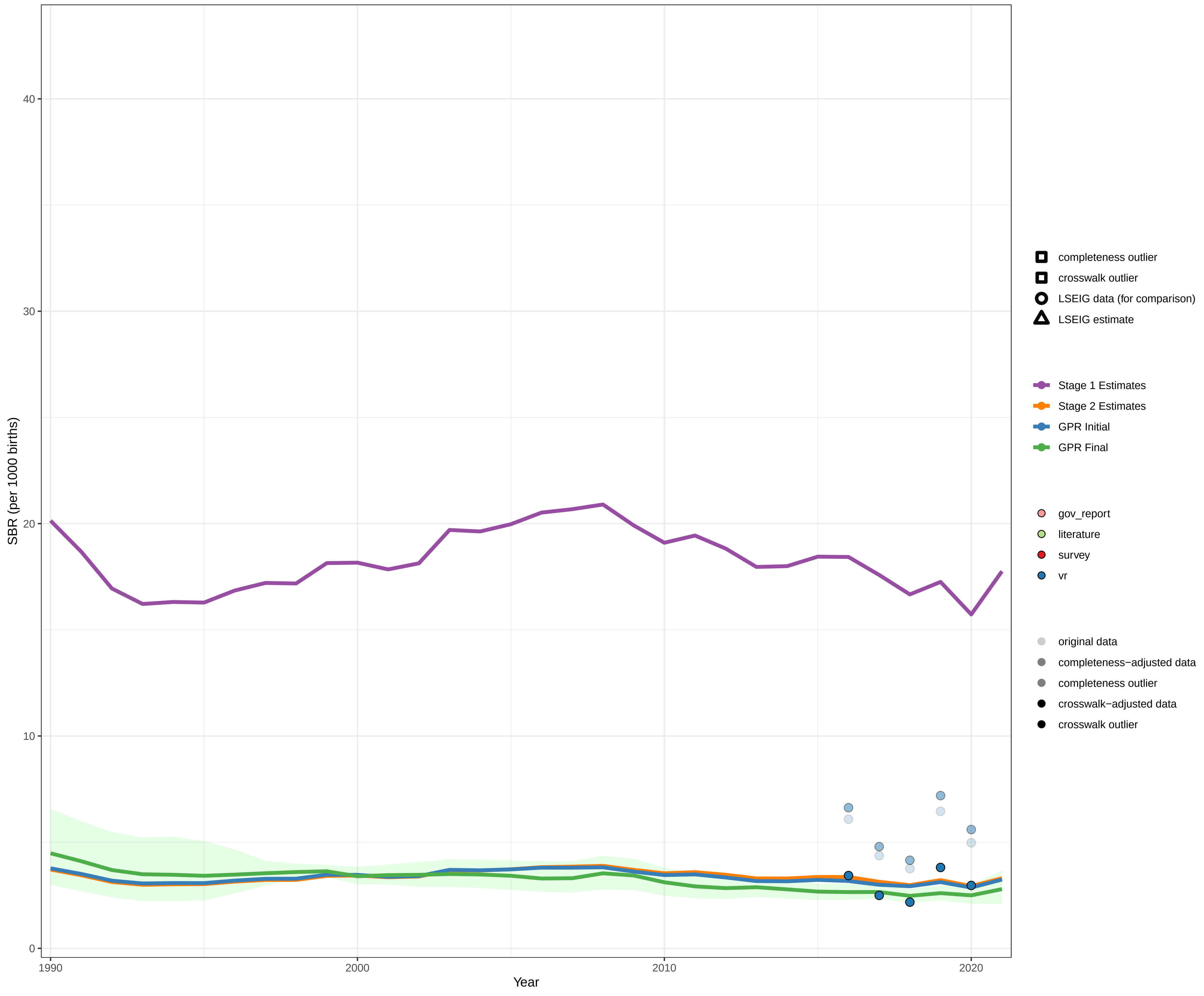

Year	Total Stillbirths (in thousands)	Total Stillbirths (in thousands)	SBR (per 1000 births)	SBR (per 1000 births)	SBR/NMR	SBR/NMR
	20 weeks	28 weeks	20 weeks	28 weeks	20 weeks	28 weeks
1990	0.0 (0.0 to 0.1)	0.0 (0.0 to 0.0)	8.9 (5.9 to 13.3)	4.5 (3.0 to 6.6)	1.40 (0.92 to 2.08)	0.70 (0.47 to 1.03)
1995	0.0 (0.0 to 0.0)	0.0 (0.0 to 0.0)	7.2 (4.7 to 10.7)	3.4 (2.3 to 5.1)	1.52 (0.98 to 2.24)	0.72 (0.47 to 1.06)
2000	0.0 (0.0 to 0.0)	0.0 (0.0 to 0.0)	7.7 (6.8 to 8.6)	3.4 (3.0 to 3.8)	1.54 (1.37 to 1.72)	0.68 (0.60 to 0.77)
2005	0.0 (0.0 to 0.0)	0.0 (0.0 to 0.0)	8.5 (6.8 to 10.6)	3.4 (2.8 to 4.1)	1.62 (1.29 to 2.01)	0.65 (0.52 to 0.79)
2010	0.0 (0.0 to 0.0)	0.0 (0.0 to 0.0)	8.1 (6.4 to 10.0)	3.1 (2.5 to 3.8)	1.65 (1.31 to 2.04)	0.63 (0.50 to 0.77)
2015	0.0 (0.0 to 0.0)	0.0 (0.0 to 0.0)	6.9 (5.9 to 8.0)	2.7 (2.3 to 3.0)	1.48 (1.25 to 1.70)	0.57 (0.49 to 0.65)
2020	0.0 (0.0 to 0.0)	0.0 (0.0 to 0.0)	6.5 (5.4 to 7.7)	2.5 (2.1 to 3.0)	1.68 (1.41 to 2.00)	0.65 (0.55 to 0.77)
2021	0.0 (0.0 to 0.0)	0.0 (0.0 to 0.0)	7.3 (5.5 to 9.6)	2.8 (2.1 to 3.7)	1.68 (1.26 to 2.21)	0.65 (0.49 to 0.85)

Warwickshire GBR_44702 Western Europe Stillbirth Rate / Neonatal Mortality Rate (SBR/NMR) − 20 weeks
lambda = 0.8 ; zeta = 0.7 ; scale = 15 ; amp = 0.028

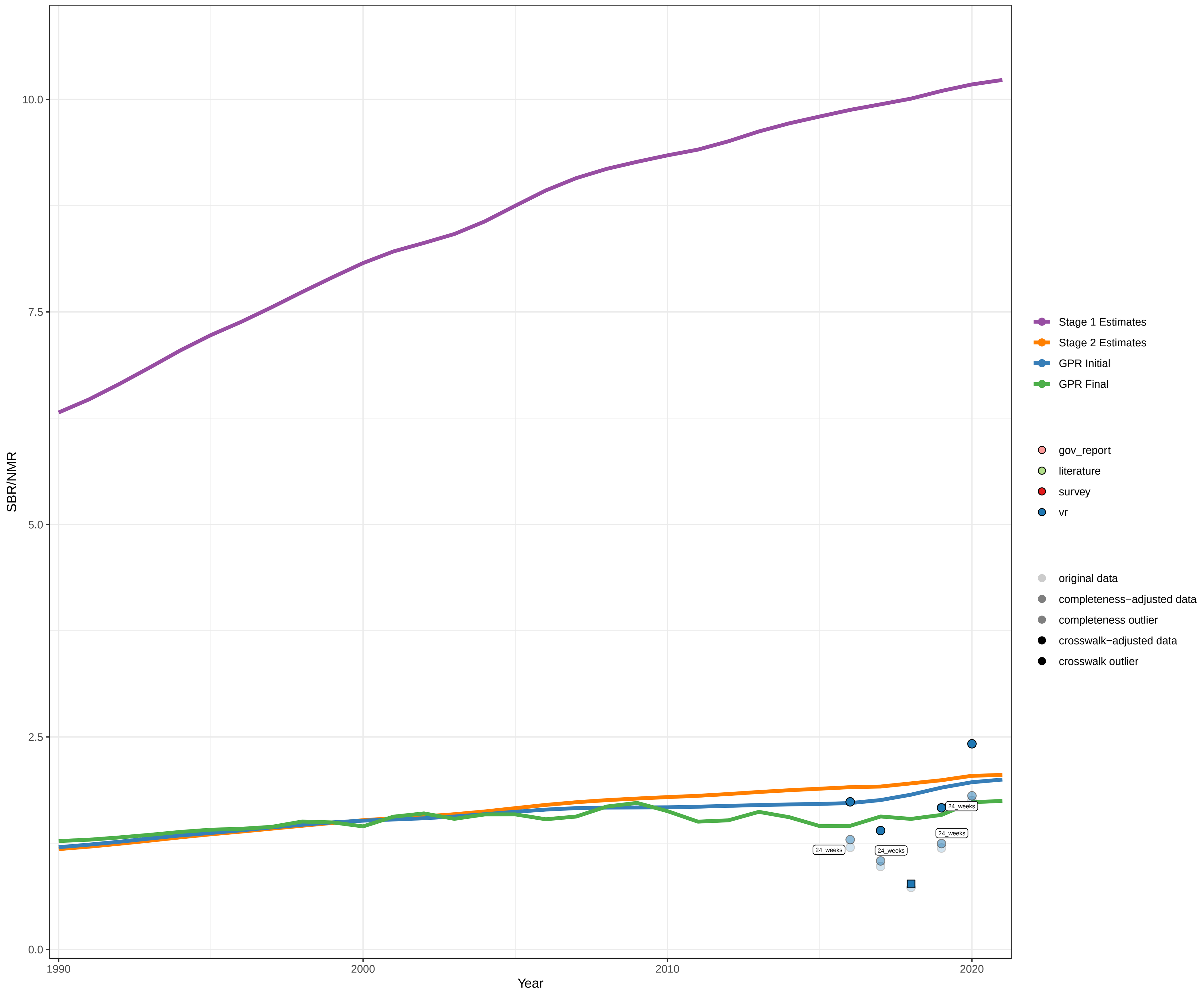

Warwickshire GBR_44702 Western Europe Stillbirth Rate / Neonatal Mortality Rate (SBR/NMR) − 28 weeks
lambda = 0.8 ; zeta = 0.7 ; scale = 15 ; amp = 0.028

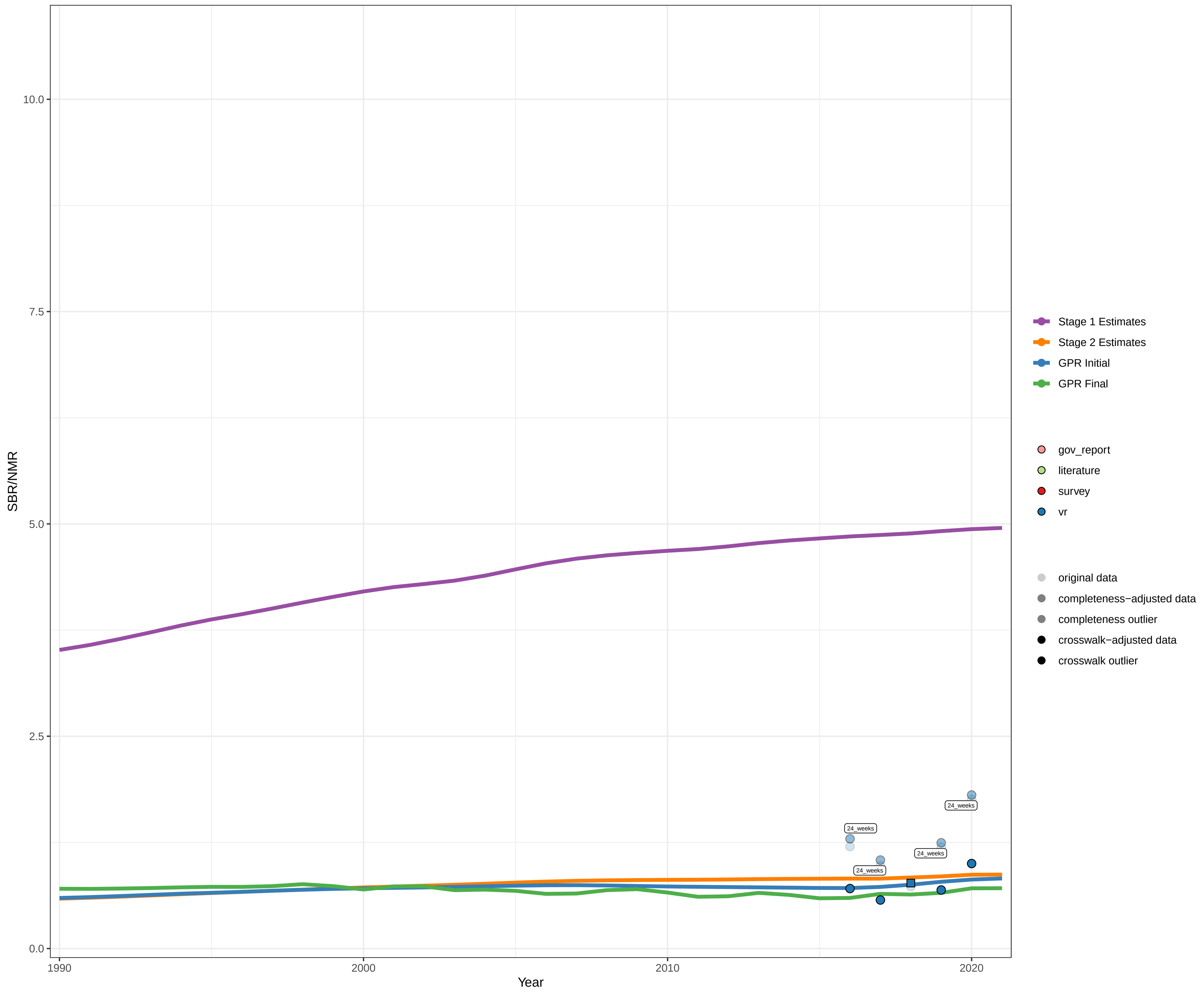

Definition	Years	Outlier	Source.Type	Title
24_weeks	2016−2020	0	VR	United Kingdom − England and Wales Child Mortality Statistics

Warwickshire GBR_44702 Western Europe Stillbirth Rate (SBR) − 20 weeks
lambda = 0.8 ; zeta = 0.7 ; scale = 15 ; amp = 0.028

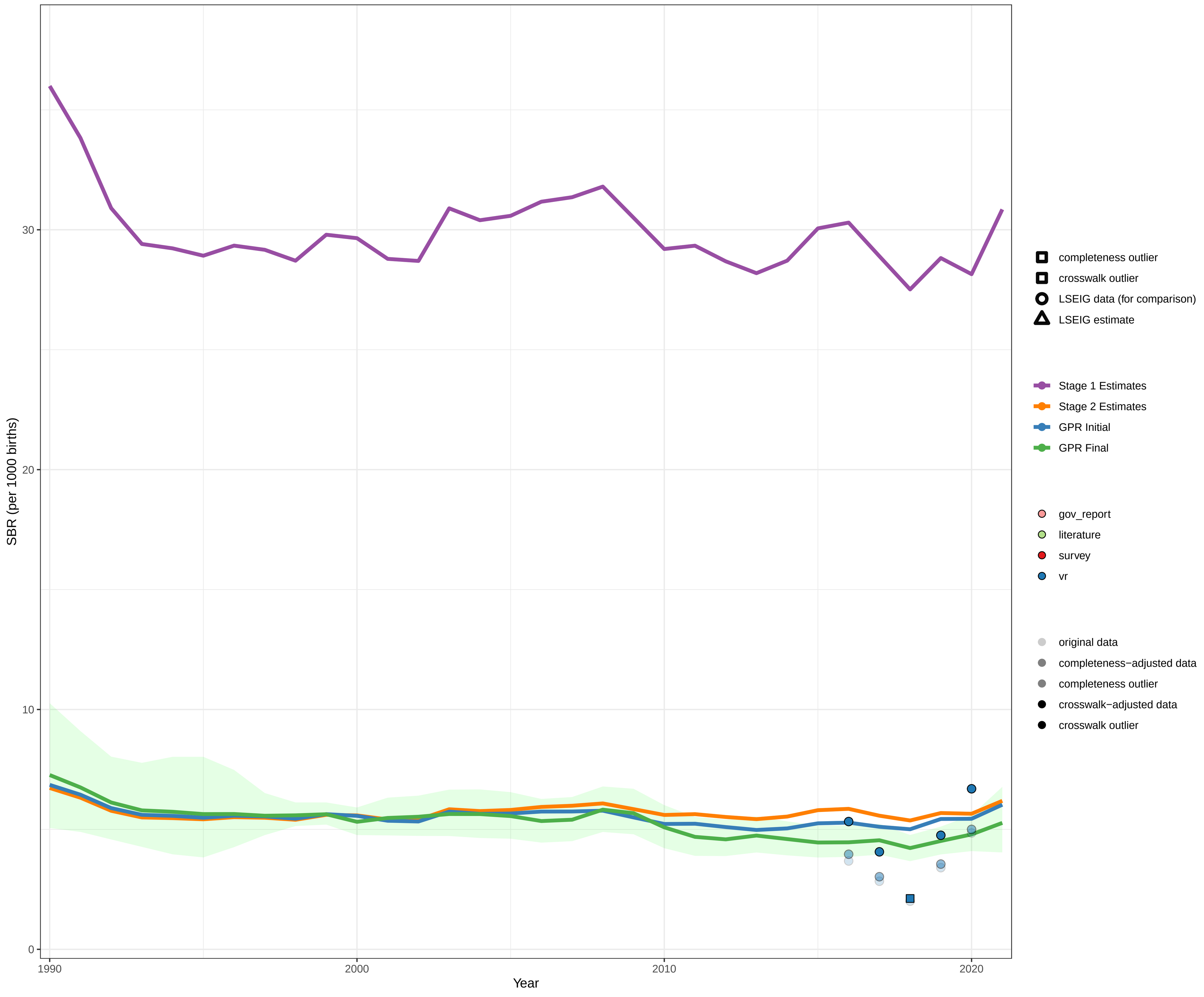

Warwickshire GBR_44702 Western Europe Stillbirth Rate (SBR) − 28 weeks
lambda = 0.8 ; zeta = 0.7 ; scale = 15 ; amp = 0.028

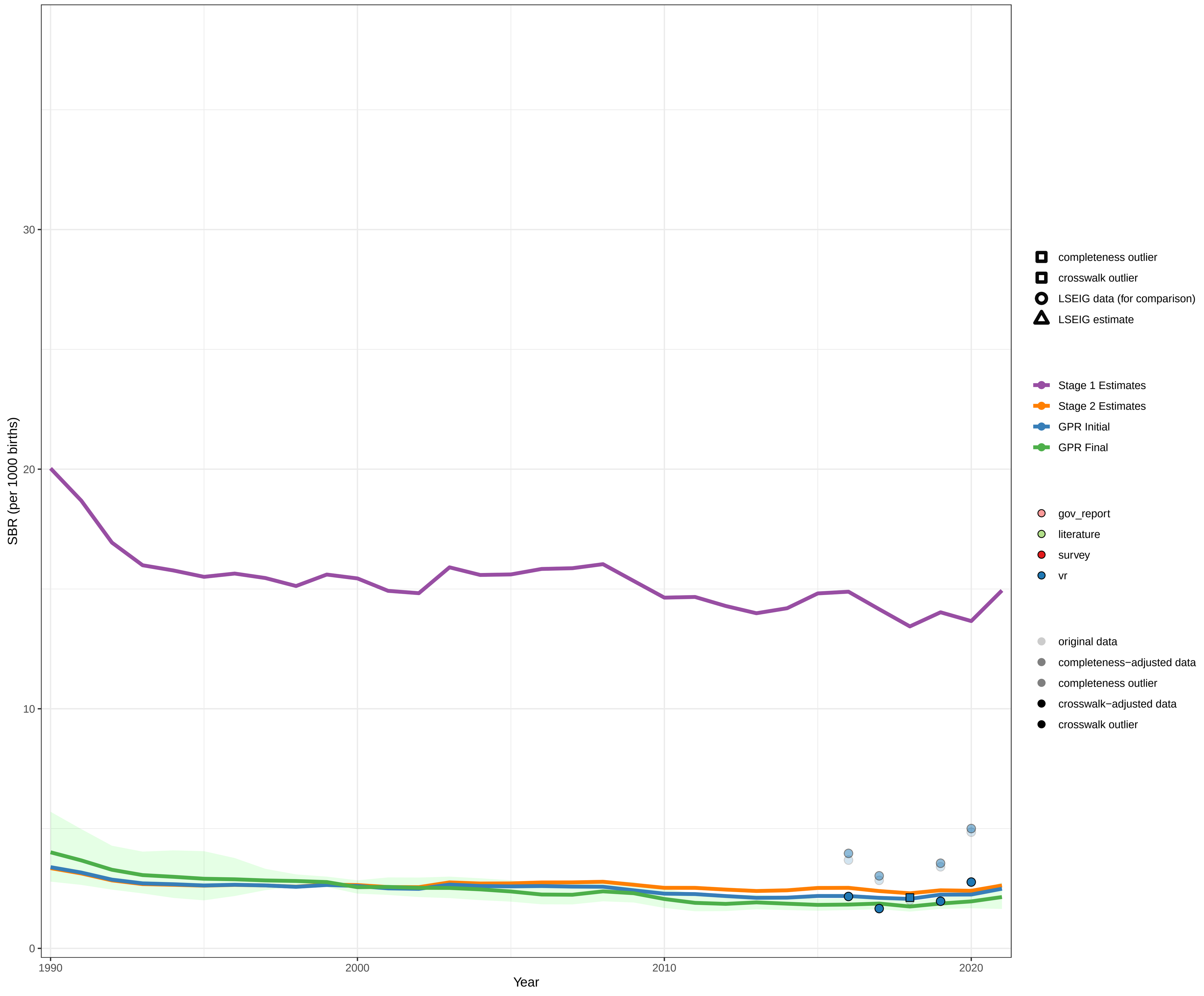

Year	Total Stillbirths (in thousands)	Total Stillbirths (in thousands)	SBR (per 1000 births)	SBR (per 1000 births)	SBR/NMR	SBR/NMR
	20 weeks	28 weeks	20 weeks	28 weeks	20 weeks	28 weeks
1990	0.0 (0.0 to 0.1)	0.0 (0.0 to 0.0)	7.3 (5.1 to 10.3)	4.0 (2.8 to 5.7)	1.28 (0.89 to 1.80)	0.70 (0.49 to 1.00)
1995	0.0 (0.0 to 0.0)	0.0 (0.0 to 0.0)	5.6 (3.8 to 8.0)	2.9 (2.0 to 4.1)	1.41 (0.96 to 2.01)	0.73 (0.50 to 1.01)
2000	0.0 (0.0 to 0.0)	0.0 (0.0 to 0.0)	5.3 (4.8 to 5.9)	2.6 (2.3 to 2.8)	1.45 (1.30 to 1.61)	0.70 (0.62 to 0.77)
2005	0.0 (0.0 to 0.0)	0.0 (0.0 to 0.0)	5.6 (4.6 to 6.6)	2.4 (2.0 to 2.8)	1.59 (1.32 to 1.87)	0.68 (0.56 to 0.81)
2010	0.0 (0.0 to 0.0)	0.0 (0.0 to 0.0)	5.1 (4.2 to 6.0)	2.1 (1.7 to 2.5)	1.63 (1.35 to 1.92)	0.66 (0.54 to 0.80)
2015	0.0 (0.0 to 0.0)	0.0 (0.0 to 0.0)	4.5 (3.8 to 5.2)	1.8 (1.6 to 2.1)	1.45 (1.25 to 1.68)	0.59 (0.51 to 0.67)
2020	0.0 (0.0 to 0.0)	0.0 (0.0 to 0.0)	4.8 (4.1 to 5.6)	2.0 (1.7 to 2.3)	1.73 (1.48 to 2.02)	0.71 (0.61 to 0.83)
2021	0.0 (0.0 to 0.0)	0.0 (0.0 to 0.0)	5.3 (4.0 to 6.8)	2.1 (1.6 to 2.8)	1.75 (1.34 to 2.25)	0.71 (0.55 to 0.91)

Wolverhampton GBR_44713 Western Europe Stillbirth Rate / Neonatal Mortality Rate (SBR/NMR) − 20 weeks
lambda = 0.8 ; zeta = 0.7 ; scale = 15 ; amp = 0.028

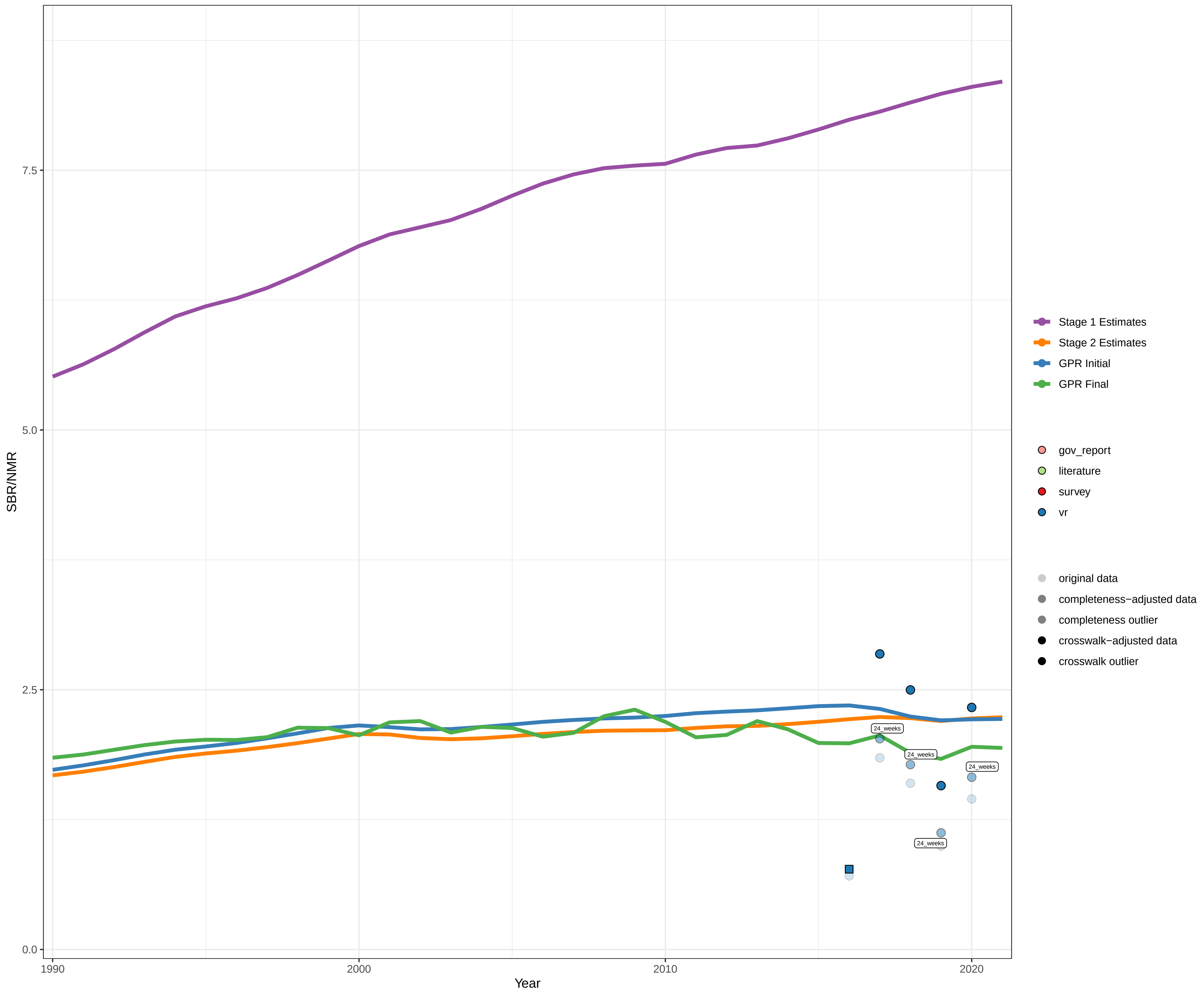

Wolverhampton GBR_44713 Western Europe Stillbirth Rate / Neonatal Mortality Rate (SBR/NMR) − 28 weeks
lambda = 0.8 ; zeta = 0.7 ; scale = 15 ; amp = 0.028

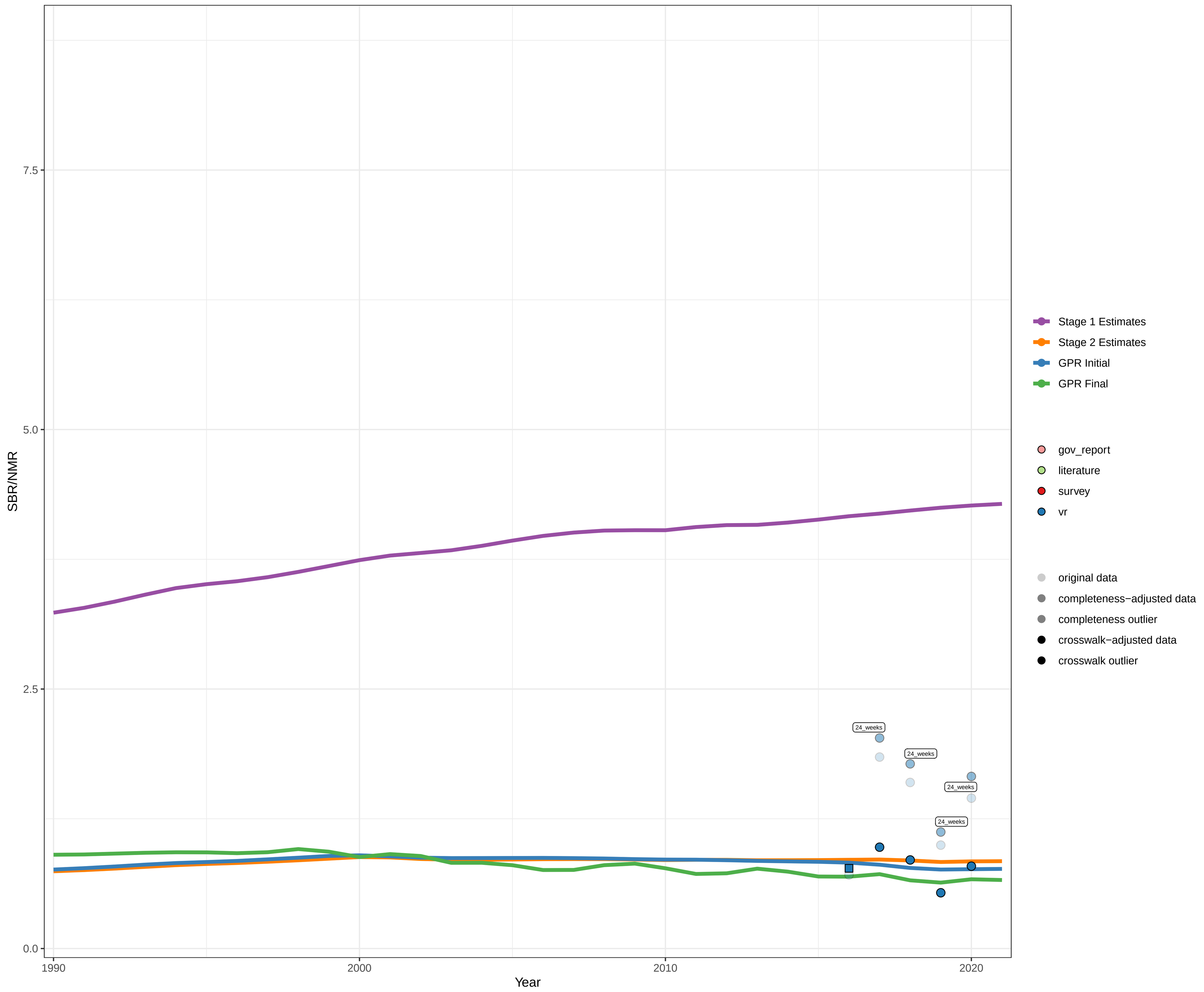

Definition	Years	Outlier	Source.Type	Title
24_weeks	2016−2020	0	VR	United Kingdom − England and Wales Child Mortality Statistics

Wolverhampton GBR_44713 Western Europe Stillbirth Rate (SBR) − 20 weeks
lambda = 0.8 ; zeta = 0.7 ; scale = 15 ; amp = 0.028

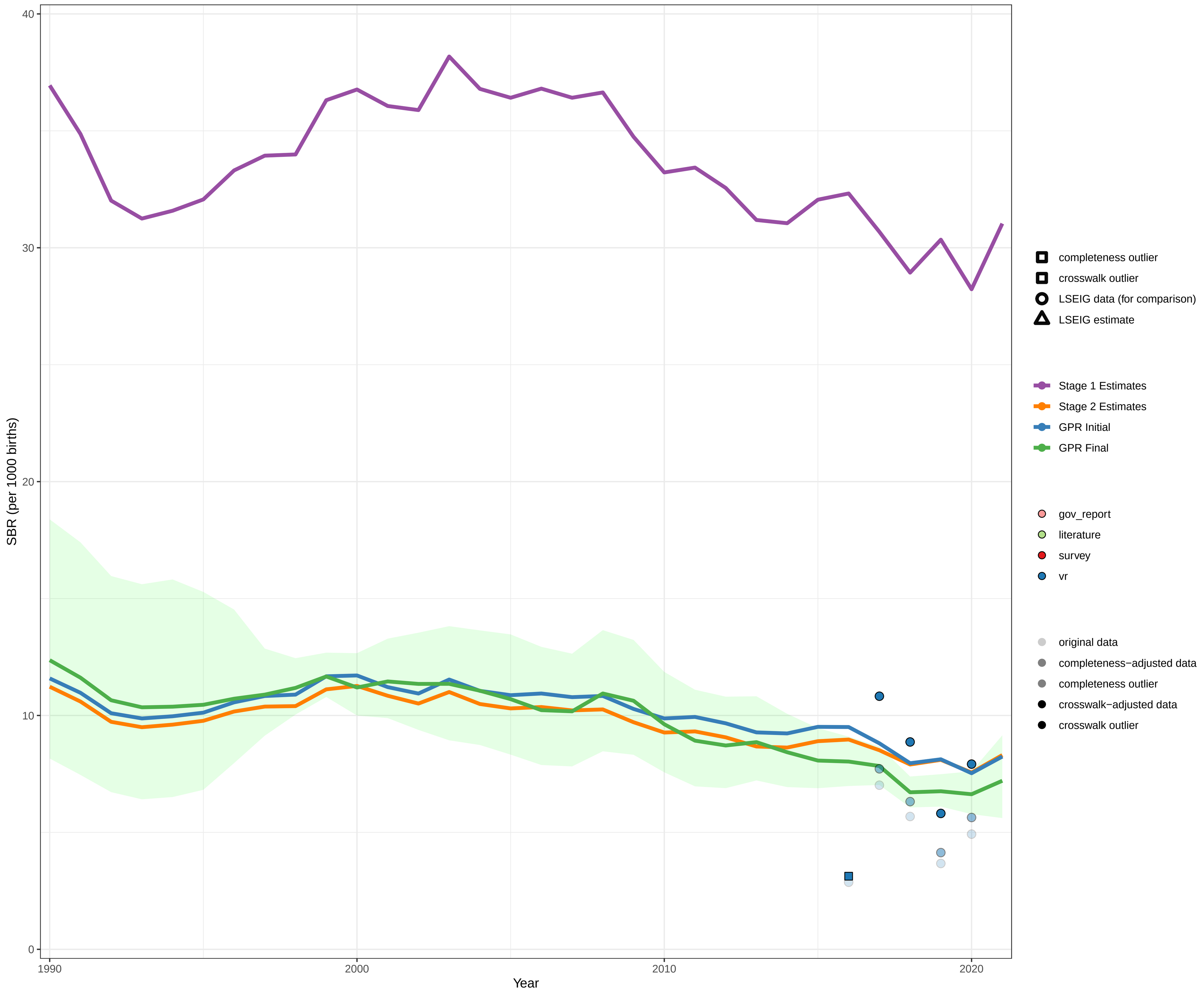

Wolverhampton GBR_44713 Western Europe Stillbirth Rate (SBR) − 28 weeks
lambda = 0.8 ; zeta = 0.7 ; scale = 15 ; amp = 0.028

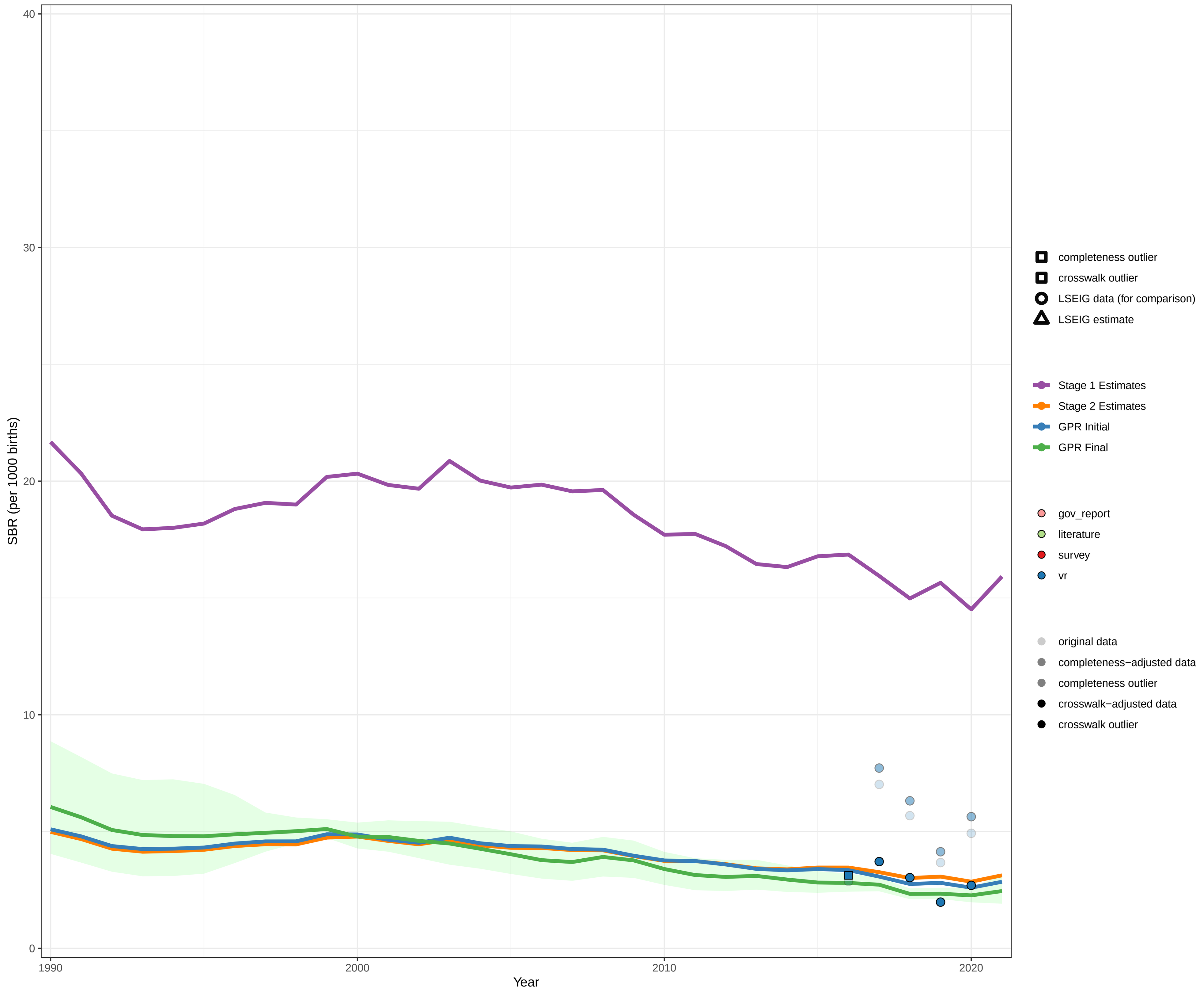

Year	Total Stillbirths (in thousands)	Total Stillbirths (in thousands)	SBR (per 1000 births)	SBR (per 1000 births)	SBR/NMR	SBR/NMR
	20 weeks	28 weeks	20 weeks	28 weeks	20 weeks	28 weeks
1990	0.0 (0.0 to 0.1)	0.0 (0.0 to 0.0)	12.4 (8.2 to 18.4)	6.1 (4.0 to 8.9)	1.85 (1.22 to 2.74)	0.90 (0.60 to 1.32)
1995	0.0 (0.0 to 0.1)	0.0 (0.0 to 0.0)	10.5 (6.8 to 15.3)	4.8 (3.2 to 7.0)	2.02 (1.32 to 2.95)	0.93 (0.62 to 1.36)
2000	0.0 (0.0 to 0.0)	0.0 (0.0 to 0.0)	11.2 (10.0 to 12.7)	4.8 (4.3 to 5.4)	2.06 (1.84 to 2.33)	0.88 (0.79 to 0.99)
2005	0.0 (0.0 to 0.0)	0.0 (0.0 to 0.0)	10.7 (8.3 to 13.5)	4.0 (3.2 to 5.0)	2.13 (1.66 to 2.68)	0.80 (0.63 to 1.00)
2010	0.0 (0.0 to 0.0)	0.0 (0.0 to 0.0)	9.6 (7.6 to 11.9)	3.4 (2.7 to 4.1)	2.19 (1.72 to 2.70)	0.77 (0.62 to 0.94)
2015	0.0 (0.0 to 0.0)	0.0 (0.0 to 0.0)	8.1 (6.9 to 9.5)	2.8 (2.4 to 3.3)	1.99 (1.70 to 2.33)	0.69 (0.59 to 0.81)
2020	0.0 (0.0 to 0.0)	0.0 (0.0 to 0.0)	6.6 (5.8 to 7.6)	2.3 (2.0 to 2.6)	1.95 (1.70 to 2.24)	0.67 (0.58 to 0.77)
2021	0.0 (0.0 to 0.0)	0.0 (0.0 to 0.0)	7.2 (5.6 to 9.2)	2.5 (1.9 to 3.1)	1.94 (1.51 to 2.46)	0.66 (0.51 to 0.84)

Worcestershire GBR_44706 Western Europe Stillbirth Rate / Neonatal Mortality Rate (SBR/NMR) − 20 weeks
lambda = 0.6 ; zeta = 0.8 ; scale = 15 ; amp = 0.028

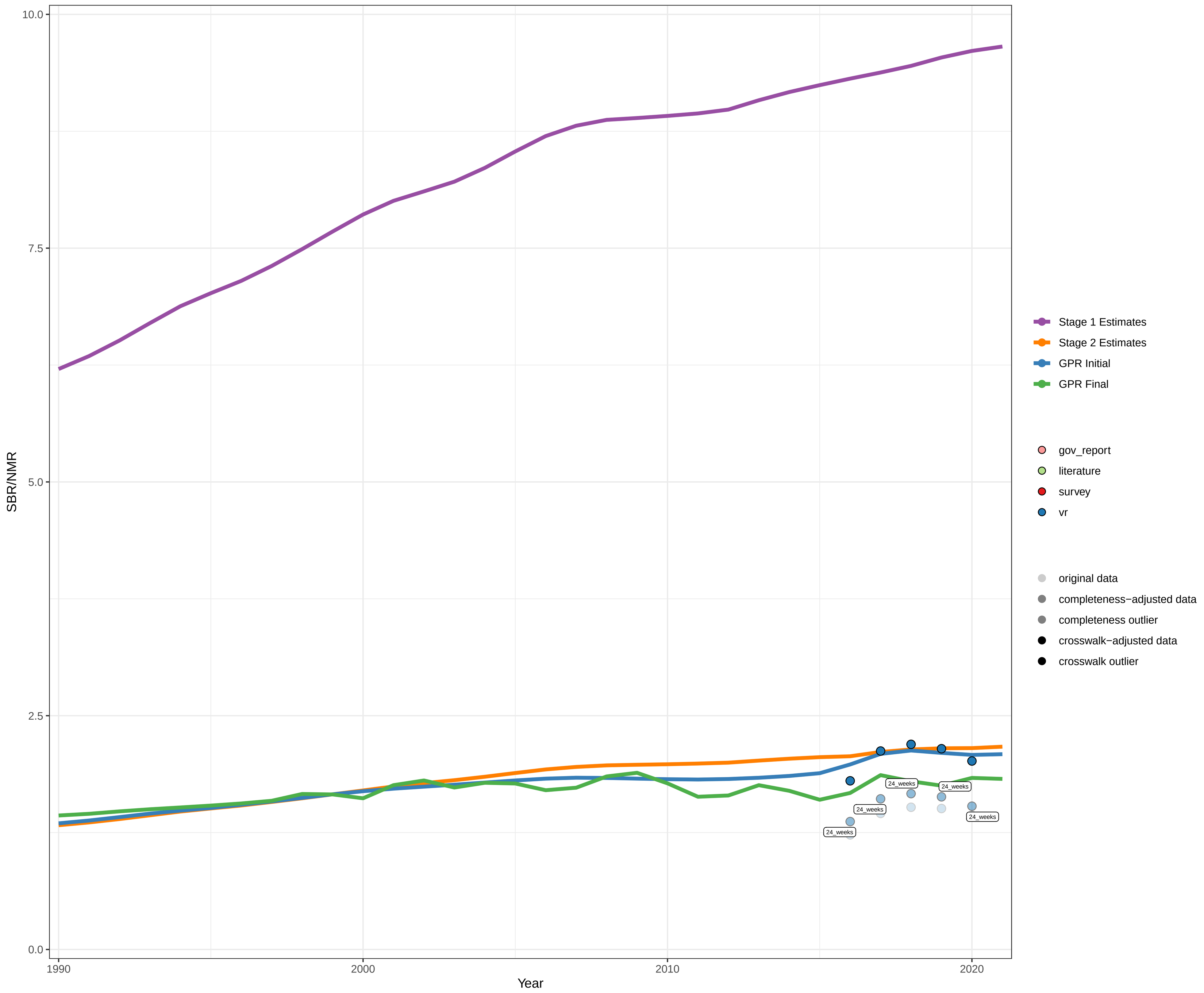

Worcestershire GBR_44706 Western Europe Stillbirth Rate / Neonatal Mortality Rate (SBR/NMR) − 28 weeks
lambda = 0.6 ; zeta = 0.8 ; scale = 15 ; amp = 0.028

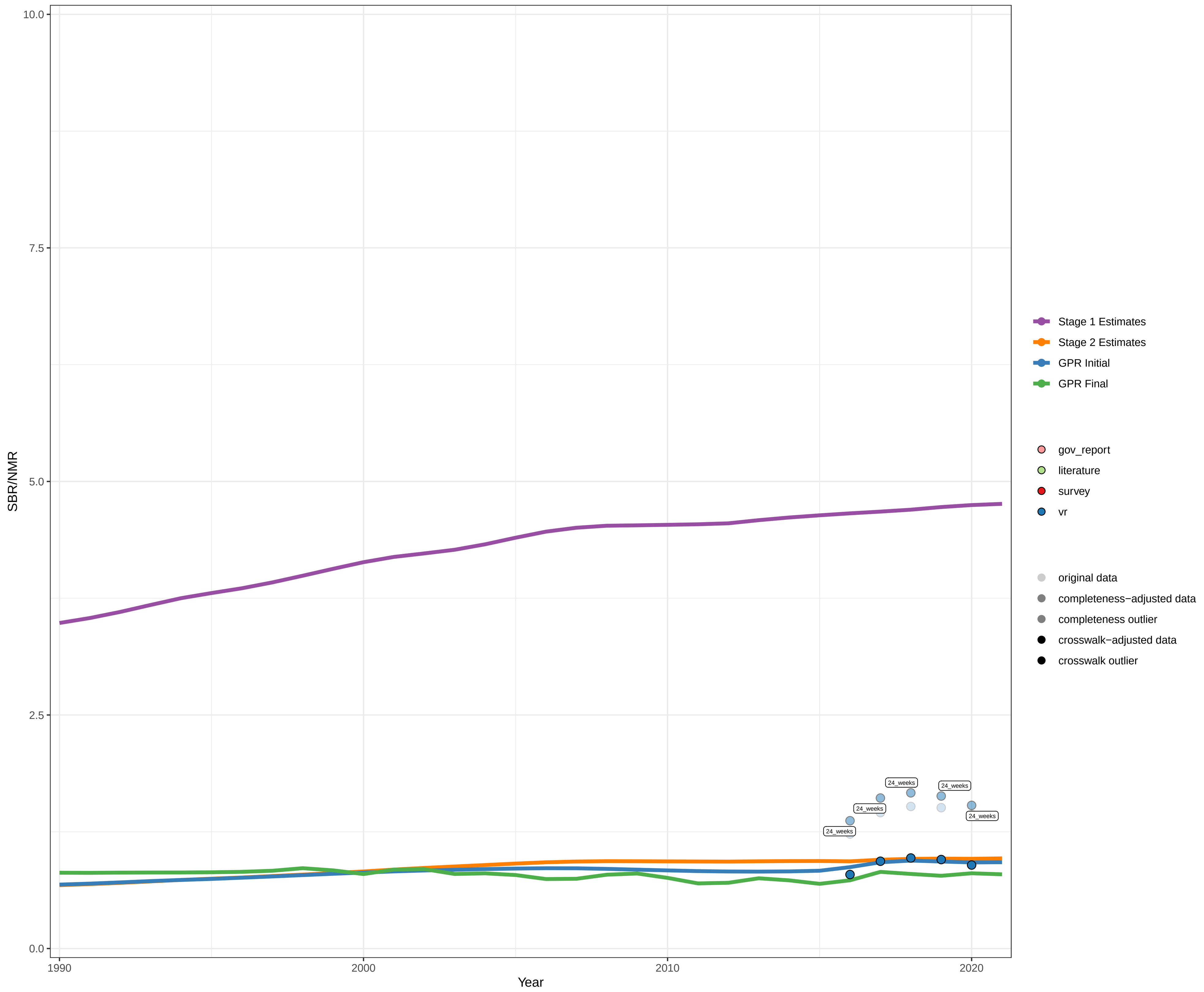

Definition	Years	Outlier	Source.Type	Title
24_weeks	2016−2020	0	VR	United Kingdom − England and Wales Child Mortality Statistics

Worcestershire GBR_44706 Western Europe Stillbirth Rate (SBR) − 20 weeks
lambda = 0.6 ; zeta = 0.8 ; scale = 15 ; amp = 0.028

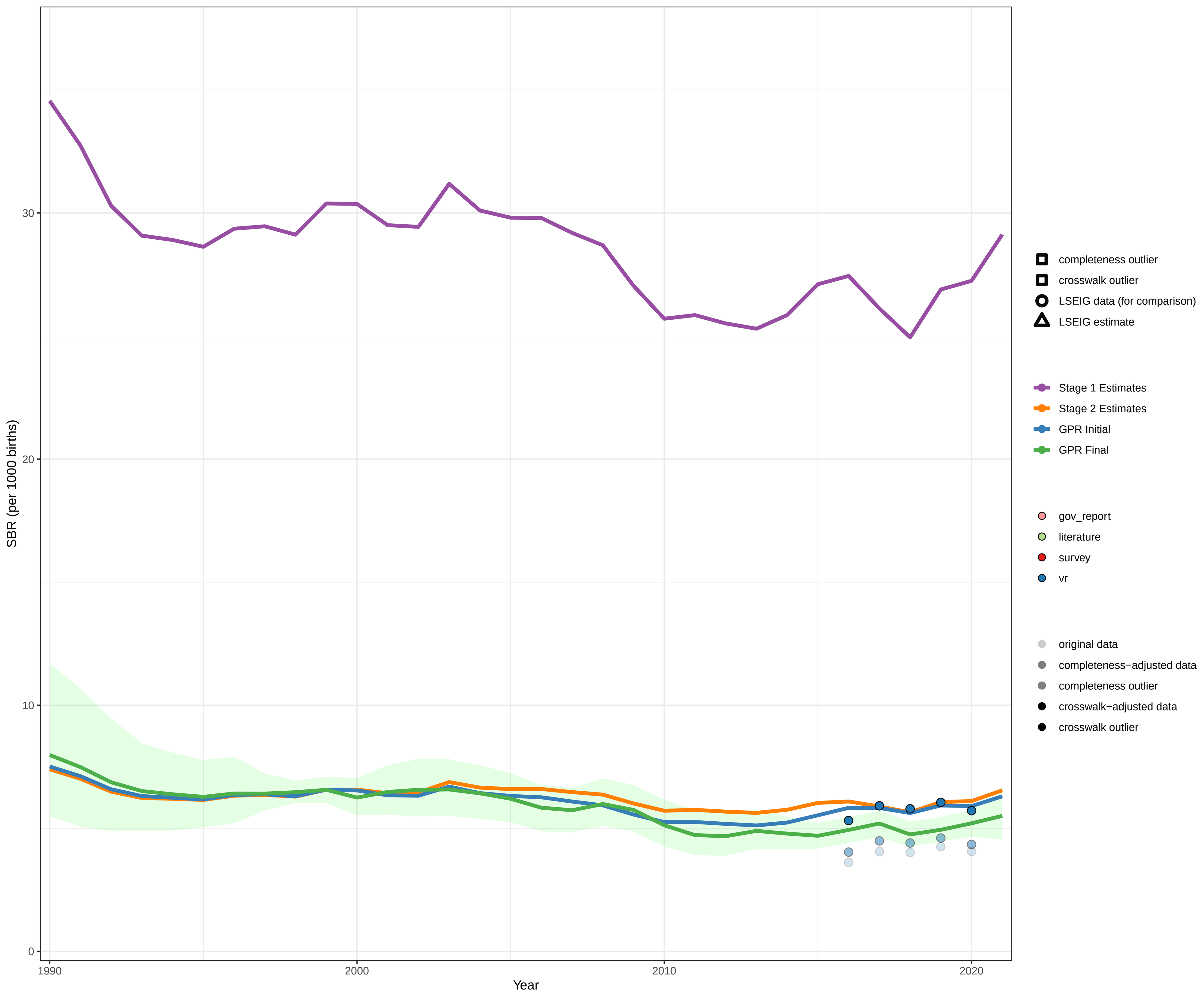

Worcestershire GBR_44706 Western Europe Stillbirth Rate (SBR) − 28 weeks
lambda = 0.6 ; zeta = 0.8 ; scale = 15 ; amp = 0.028

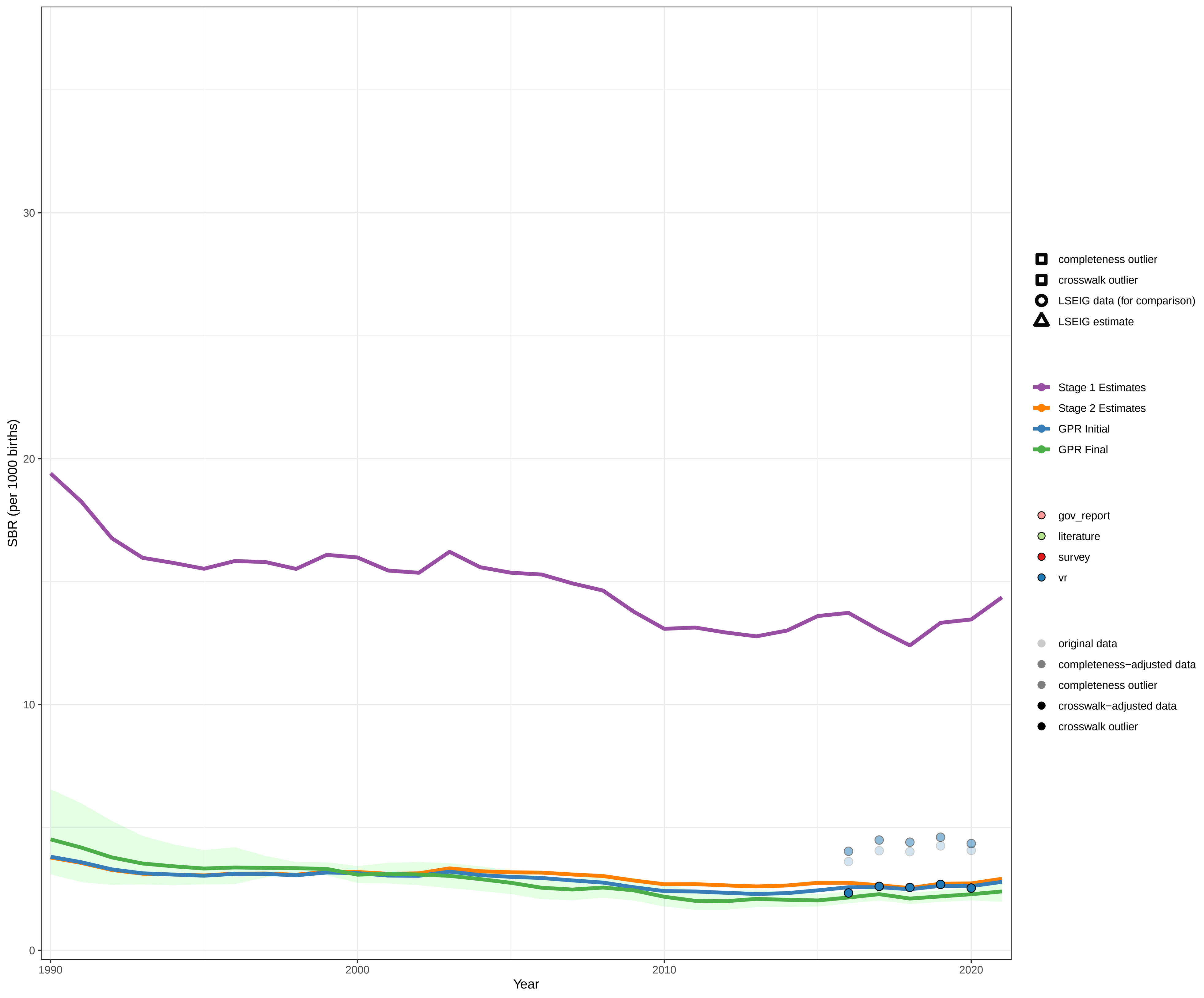

Year	Total Stillbirths (in thousands)	Total Stillbirths (in thousands)	SBR (per 1000 births)	SBR (per 1000 births)	SBR/NMR	SBR/NMR
	20 weeks	28 weeks	20 weeks	28 weeks	20 weeks	28 weeks
1990	0.1 (0.0 to 0.1)	0.0 (0.0 to 0.0)	8.0 (5.5 to 11.7)	4.5 (3.1 to 6.6)	1.43 (0.98 to 2.10)	0.81 (0.55 to 1.18)
1995	0.0 (0.0 to 0.0)	0.0 (0.0 to 0.0)	6.3 (5.0 to 7.8)	3.3 (2.7 to 4.1)	1.54 (1.24 to 1.91)	0.82 (0.66 to 1.00)
2000	0.0 (0.0 to 0.0)	0.0 (0.0 to 0.0)	6.2 (5.5 to 7.0)	3.1 (2.7 to 3.4)	1.62 (1.43 to 1.82)	0.80 (0.71 to 0.89)
2005	0.0 (0.0 to 0.0)	0.0 (0.0 to 0.0)	6.2 (5.2 to 7.2)	2.7 (2.3 to 3.3)	1.78 (1.50 to 2.07)	0.79 (0.66 to 0.93)
2010	0.0 (0.0 to 0.0)	0.0 (0.0 to 0.0)	5.1 (4.3 to 6.2)	2.2 (1.8 to 2.7)	1.78 (1.48 to 2.14)	0.76 (0.62 to 0.93)
2015	0.0 (0.0 to 0.0)	0.0 (0.0 to 0.0)	4.7 (4.2 to 5.2)	2.0 (1.8 to 2.3)	1.60 (1.42 to 1.78)	0.69 (0.61 to 0.78)
2020	0.0 (0.0 to 0.0)	0.0 (0.0 to 0.0)	5.2 (4.7 to 5.8)	2.3 (2.0 to 2.5)	1.83 (1.64 to 2.03)	0.81 (0.72 to 0.89)
2021	0.0 (0.0 to 0.0)	0.0 (0.0 to 0.0)	5.5 (4.5 to 6.7)	2.4 (2.0 to 2.9)	1.82 (1.50 to 2.22)	0.79 (0.65 to 0.97)

Yorkshire and the Humber GBR_4620 Western Europe Stillbirth Rate / Neonatal Mortality Rate (SBR/NMR) − 20 weeks
lambda = 0.2 ; zeta = 0.99 ; scale = 5 ; amp = 0.028

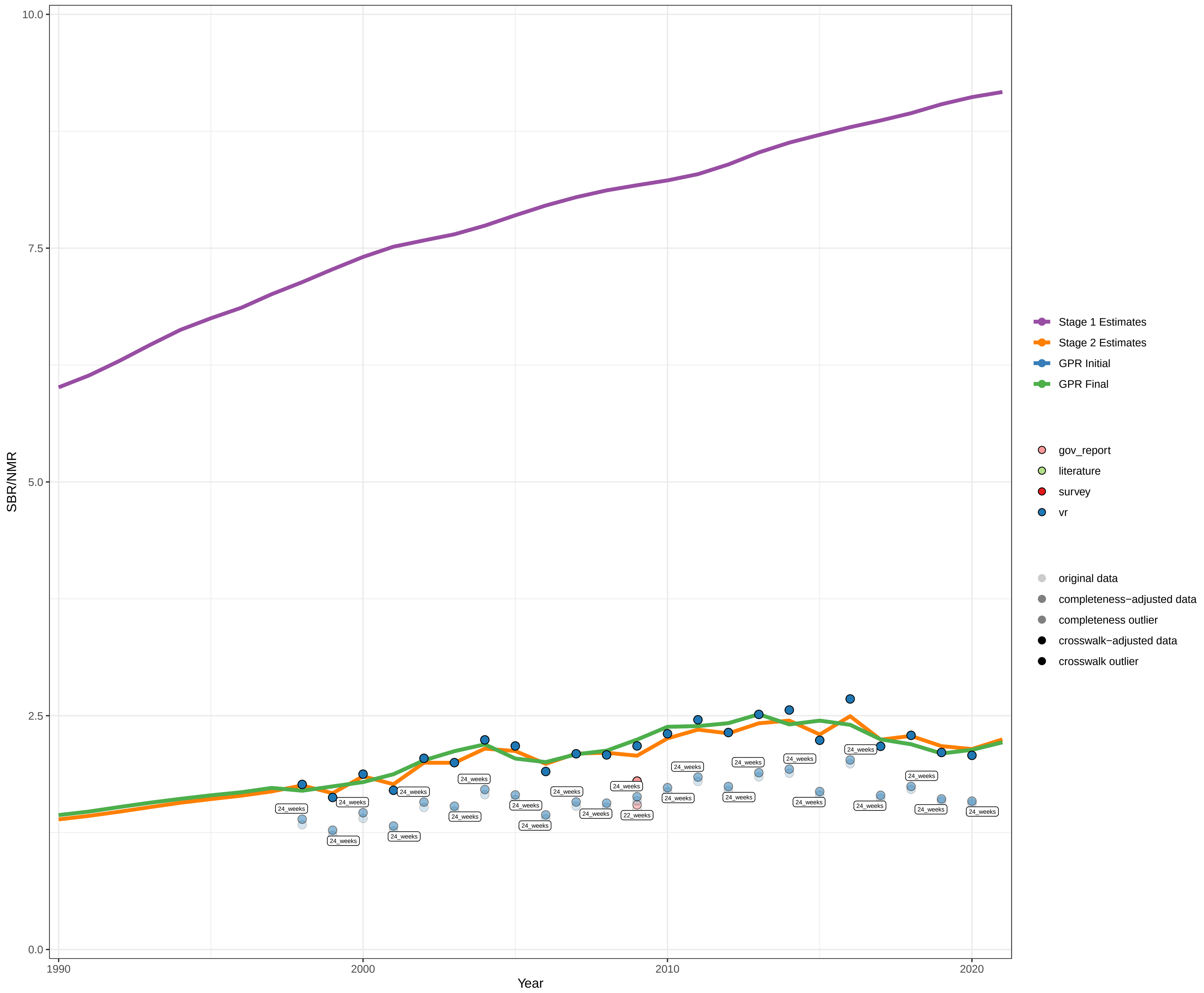

Yorkshire and the Humber GBR_4620 Western Europe Stillbirth Rate / Neonatal Mortality Rate (SBR/NMR) − 28 weeks
lambda = 0.2 ; zeta = 0.99 ; scale = 5 ; amp = 0.028

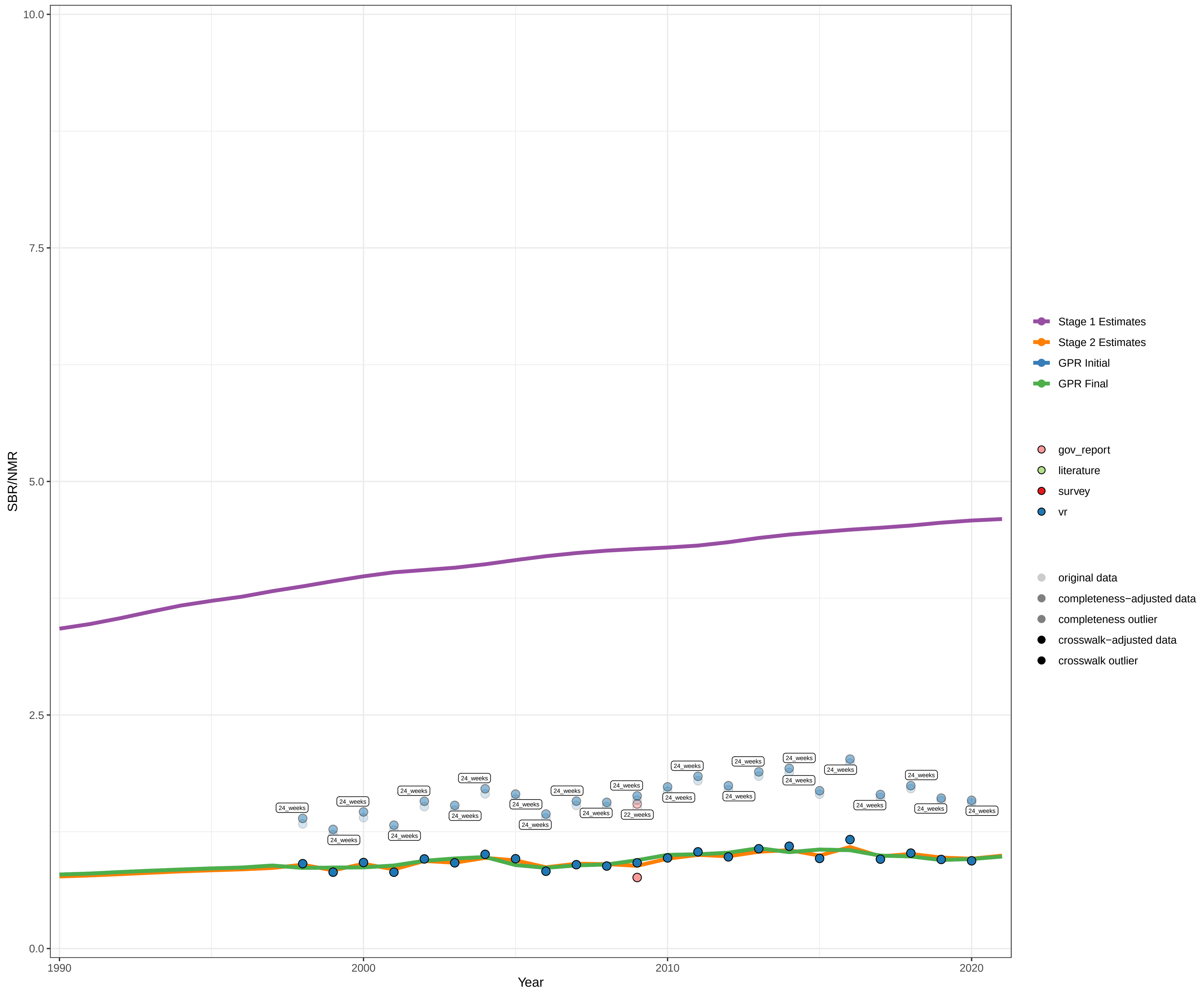

Definition	Years	Outlier	Source.Type	Title
22_weeks	2009	0	Statistical Report	United Kingdom Perinatal Mortality
24_weeks	1998−2007	0	VR	United Kingdom − England and Wales Birth Statistics
24_weeks	2008−2020	0	VR	United Kingdom − England and Wales Child Mortality Statistics

Yorkshire and the Humber GBR_4620 Western Europe Stillbirth Rate (SBR) − 20 weeks
lambda = 0.2 ; zeta = 0.99 ; scale = 5 ; amp = 0.028

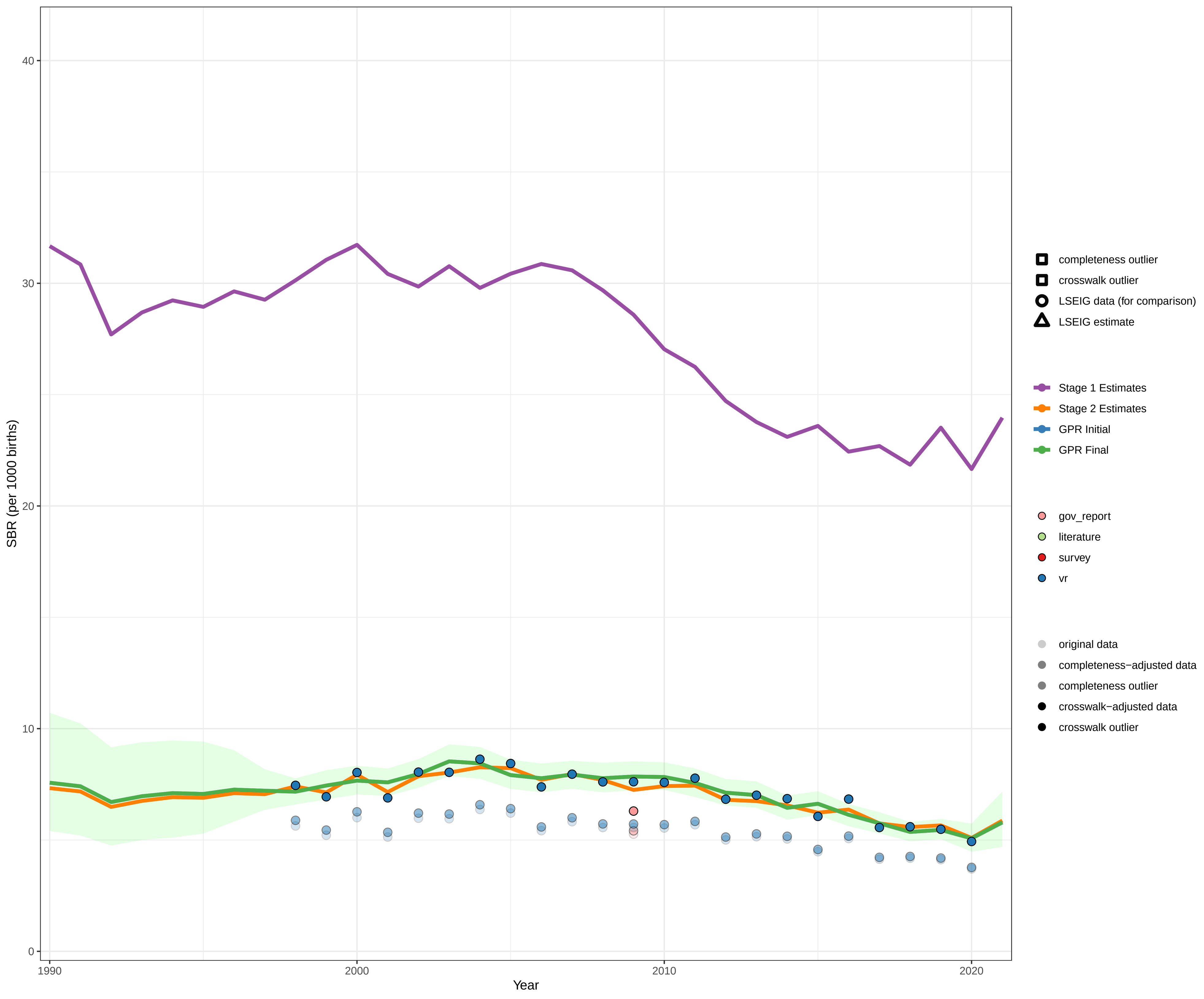

Yorkshire and the Humber GBR_4620 Western Europe Stillbirth Rate (SBR) − 28 weeks
lambda = 0.2 ; zeta = 0.99 ; scale = 5 ; amp = 0.028

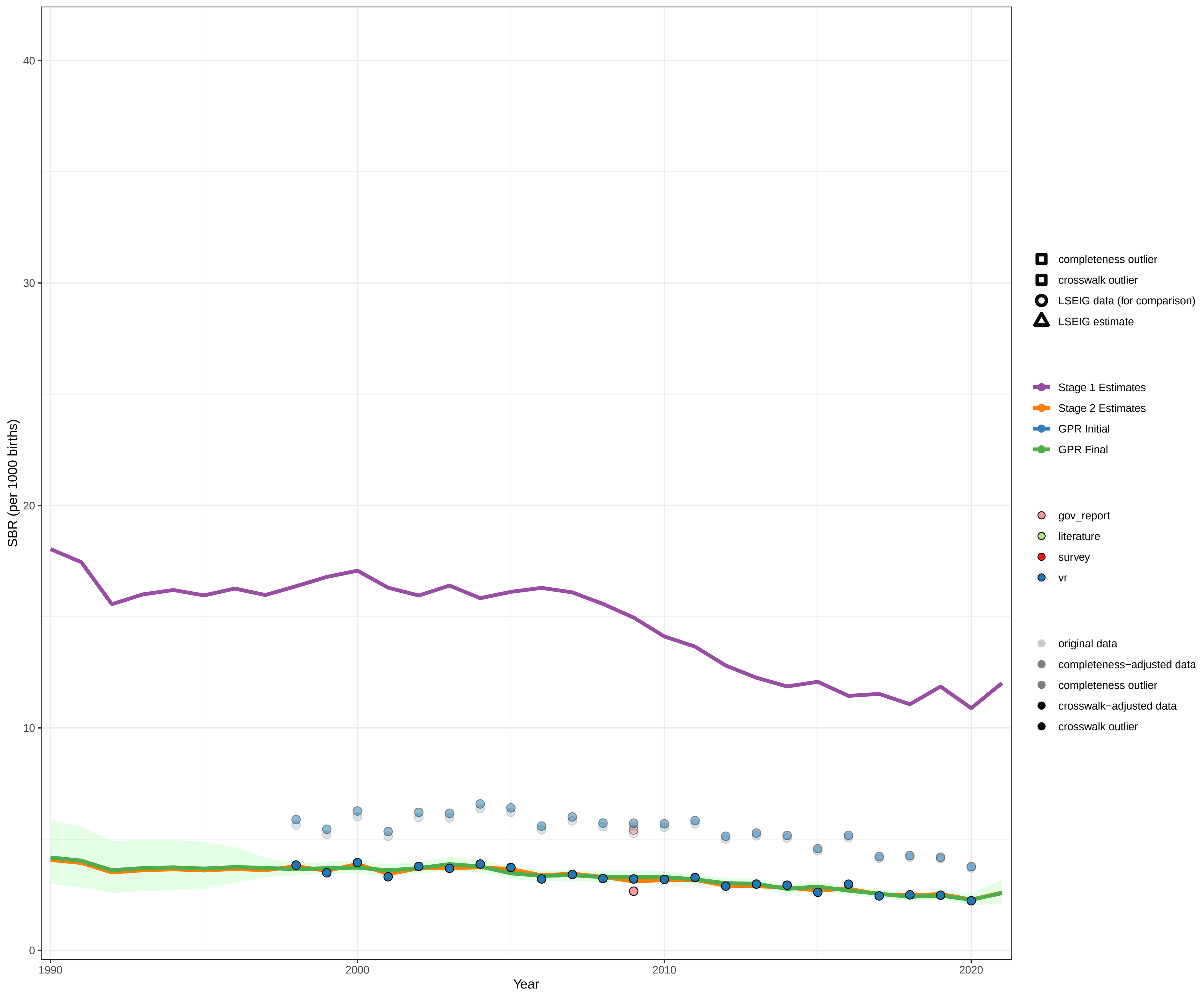

Year	Total Stillbirths (in thousands)	Total Stillbirths (in thousands)	SBR (per 1000 births)	SBR (per 1000 births)	SBR/NMR	SBR/NMR
	20 weeks	28 weeks	20 weeks	28 weeks	20 weeks	28 weeks
1990	0.5 (0.4 to 0.7)	0.3 (0.2 to 0.4)	7.6 (5.4 to 10.7)	4.2 (3.0 to 5.9)	1.44 (1.03 to 2.03)	0.79 (0.57 to 1.12)
1995	0.4 (0.3 to 0.6)	0.2 (0.2 to 0.3)	7.1 (5.3 to 9.4)	3.7 (2.8 to 4.9)	1.65 (1.23 to 2.20)	0.86 (0.64 to 1.14)
2000	0.4 (0.4 to 0.5)	0.2 (0.2 to 0.2)	7.7 (7.0 to 8.3)	3.7 (3.4 to 4.0)	1.79 (1.64 to 1.95)	0.87 (0.80 to 0.94)
2005	0.5 (0.5 to 0.5)	0.2 (0.2 to 0.2)	7.9 (7.3 to 8.6)	3.5 (3.2 to 3.7)	2.04 (1.88 to 2.22)	0.89 (0.83 to 0.97)
2010	0.5 (0.5 to 0.6)	0.2 (0.2 to 0.2)	7.8 (7.2 to 8.5)	3.3 (3.1 to 3.6)	2.38 (2.20 to 2.58)	1.00 (0.93 to 1.08)
2015	0.4 (0.4 to 0.5)	0.2 (0.2 to 0.2)	6.6 (6.1 to 7.2)	2.9 (2.6 to 3.1)	2.45 (2.26 to 2.65)	1.06 (0.98 to 1.14)
2020	0.3 (0.3 to 0.3)	0.1 (0.1 to 0.1)	5.1 (4.5 to 5.7)	2.3 (2.0 to 2.6)	2.13 (1.88 to 2.42)	0.96 (0.85 to 1.08)
2021	0.3 (0.3 to 0.4)	0.1 (0.1 to 0.2)	5.8 (4.7 to 7.2)	2.6 (2.1 to 3.2)	2.22 (1.79 to 2.75)	0.98 (0.80 to 1.22)

Barnsley GBR_44692 Western Europe Stillbirth Rate / Neonatal Mortality Rate (SBR/NMR) − 20 weeks
lambda = 0.6 ; zeta = 0.8 ; scale = 15 ; amp = 0.028

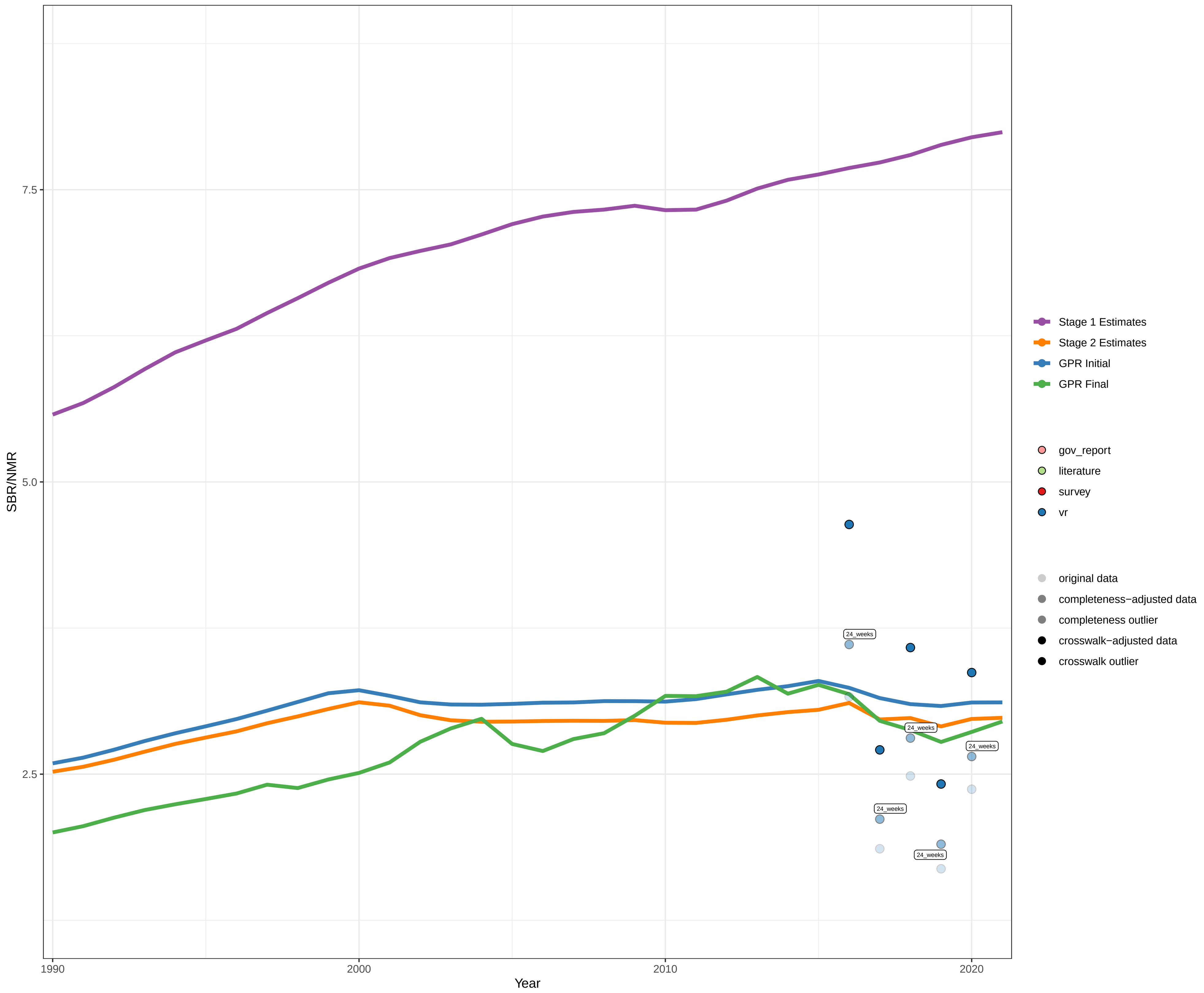

Barnsley GBR_44692 Western Europe Stillbirth Rate / Neonatal Mortality Rate (SBR/NMR) − 28 weeks
lambda = 0.6 ; zeta = 0.8 ; scale = 15 ; amp = 0.028

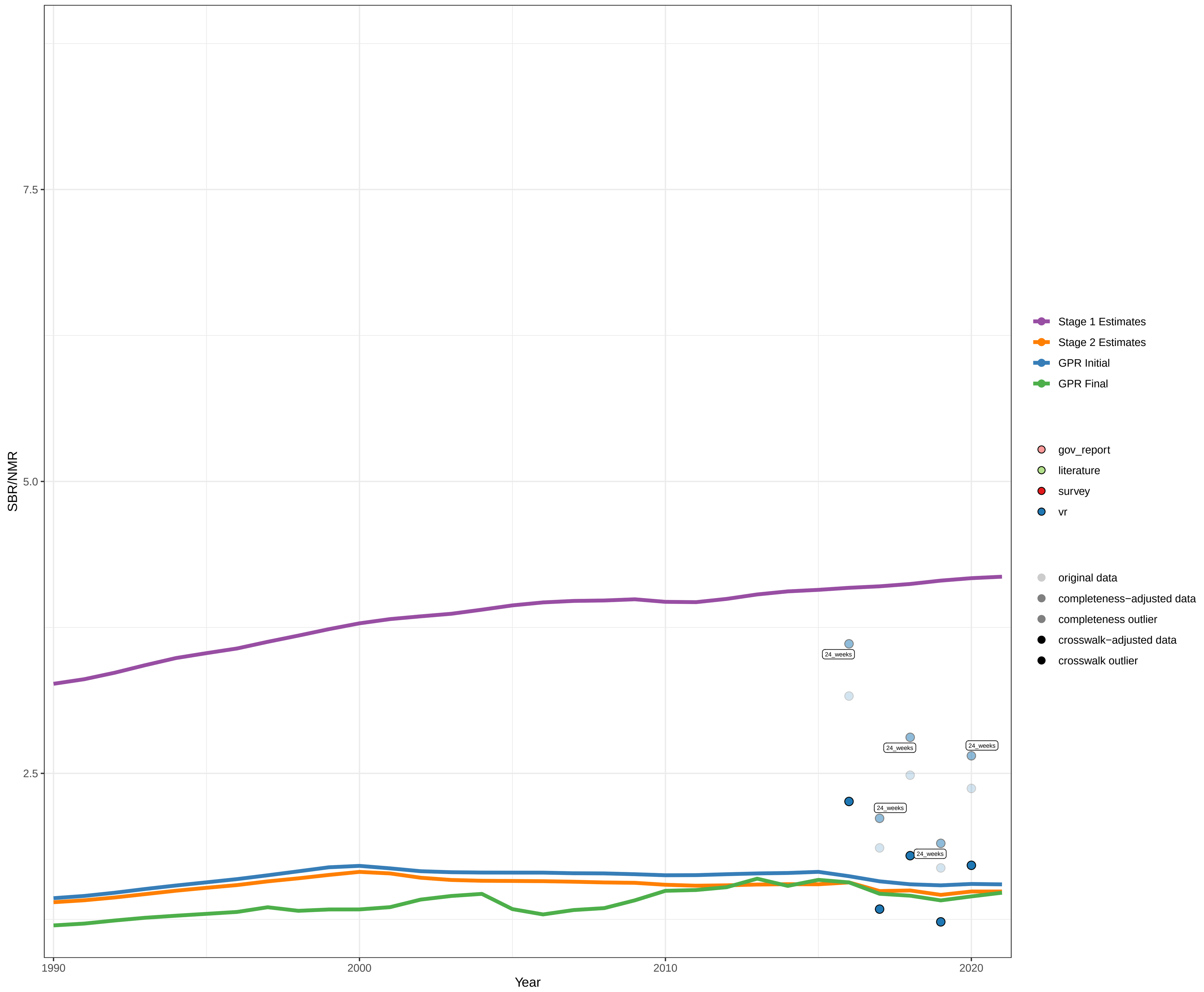

Definition	Years	Outlier	Source.Type	Title
24_weeks	2016−2020	0	VR	United Kingdom − England and Wales Child Mortality Statistics

Barnsley GBR_44692 Western Europe Stillbirth Rate (SBR) − 20 weeks
lambda = 0.6 ; zeta = 0.8 ; scale = 15 ; amp = 0.028

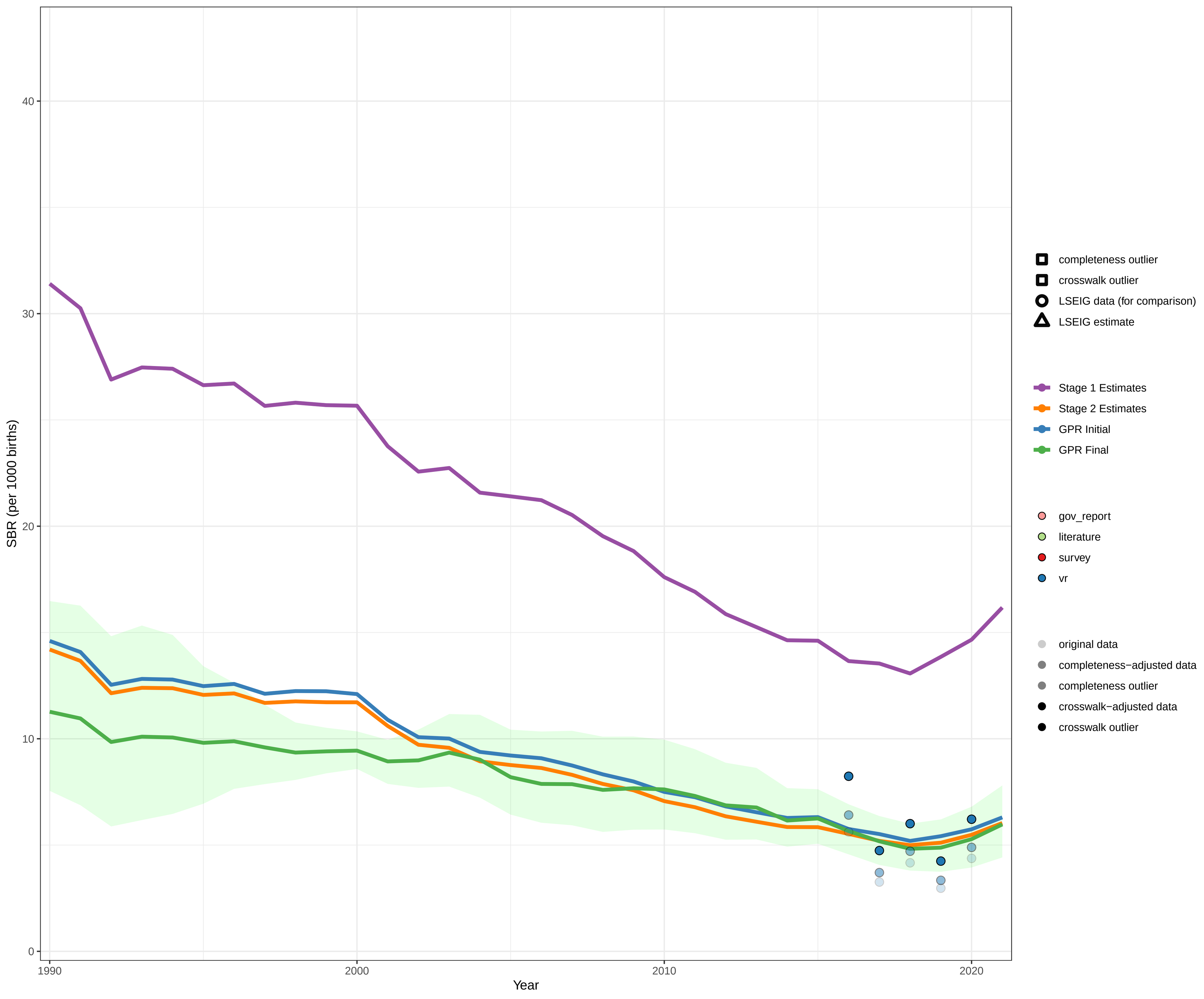

Barnsley GBR_44692 Western Europe Stillbirth Rate (SBR) − 28 weeks
lambda = 0.6 ; zeta = 0.8 ; scale = 15 ; amp = 0.028

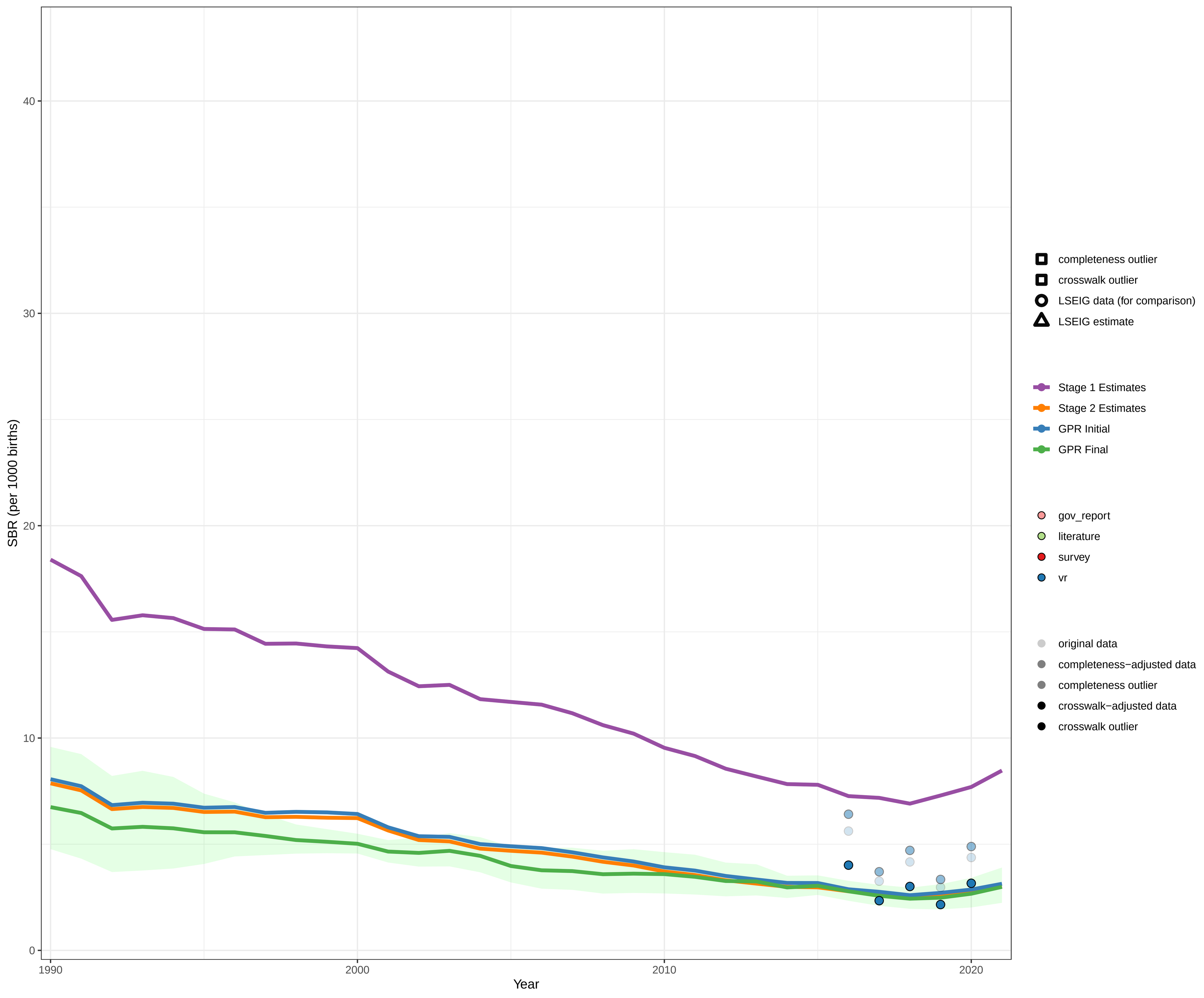

Year	Total Stillbirths (in thousands)	Total Stillbirths (in thousands)	SBR (per 1000 births)	SBR (per 1000 births)	SBR/NMR	SBR/NMR
	20 weeks	28 weeks	20 weeks	28 weeks	20 weeks	28 weeks
1990	0.0 (0.0 to 0.1)	0.0 (0.0 to 0.0)	11.3 (7.5 to 16.5)	6.7 (4.8 to 9.6)	2.00 (1.34 to 2.93)	1.20 (0.85 to 1.70)
1995	0.0 (0.0 to 0.0)	0.0 (0.0 to 0.0)	9.8 (6.9 to 13.4)	5.6 (4.1 to 7.4)	2.29 (1.62 to 3.13)	1.30 (0.95 to 1.72)
2000	0.0 (0.0 to 0.0)	0.0 (0.0 to 0.0)	9.4 (8.6 to 10.3)	5.0 (4.6 to 5.5)	2.51 (2.28 to 2.75)	1.33 (1.21 to 1.46)
2005	0.0 (0.0 to 0.0)	0.0 (0.0 to 0.0)	8.2 (6.4 to 10.4)	4.0 (3.2 to 4.9)	2.76 (2.17 to 3.51)	1.34 (1.08 to 1.65)
2010	0.0 (0.0 to 0.0)	0.0 (0.0 to 0.0)	7.6 (5.7 to 10.0)	3.6 (2.7 to 4.6)	3.17 (2.38 to 4.14)	1.49 (1.12 to 1.93)
2015	0.0 (0.0 to 0.0)	0.0 (0.0 to 0.0)	6.3 (5.1 to 7.6)	3.0 (2.6 to 3.5)	3.26 (2.64 to 3.98)	1.59 (1.36 to 1.84)
2020	0.0 (0.0 to 0.0)	0.0 (0.0 to 0.0)	5.3 (3.9 to 6.8)	2.7 (2.0 to 3.4)	2.86 (2.14 to 3.69)	1.45 (1.10 to 1.85)
2021	0.0 (0.0 to 0.0)	0.0 (0.0 to 0.0)	6.0 (4.4 to 7.8)	3.0 (2.2 to 3.9)	2.95 (2.18 to 3.86)	1.48 (1.11 to 1.93)

Bradford GBR_44684 Western Europe Stillbirth Rate / Neonatal Mortality Rate (SBR/NMR) − 20 weeks
lambda = 0.6 ; zeta = 0.8 ; scale = 15 ; amp = 0.028

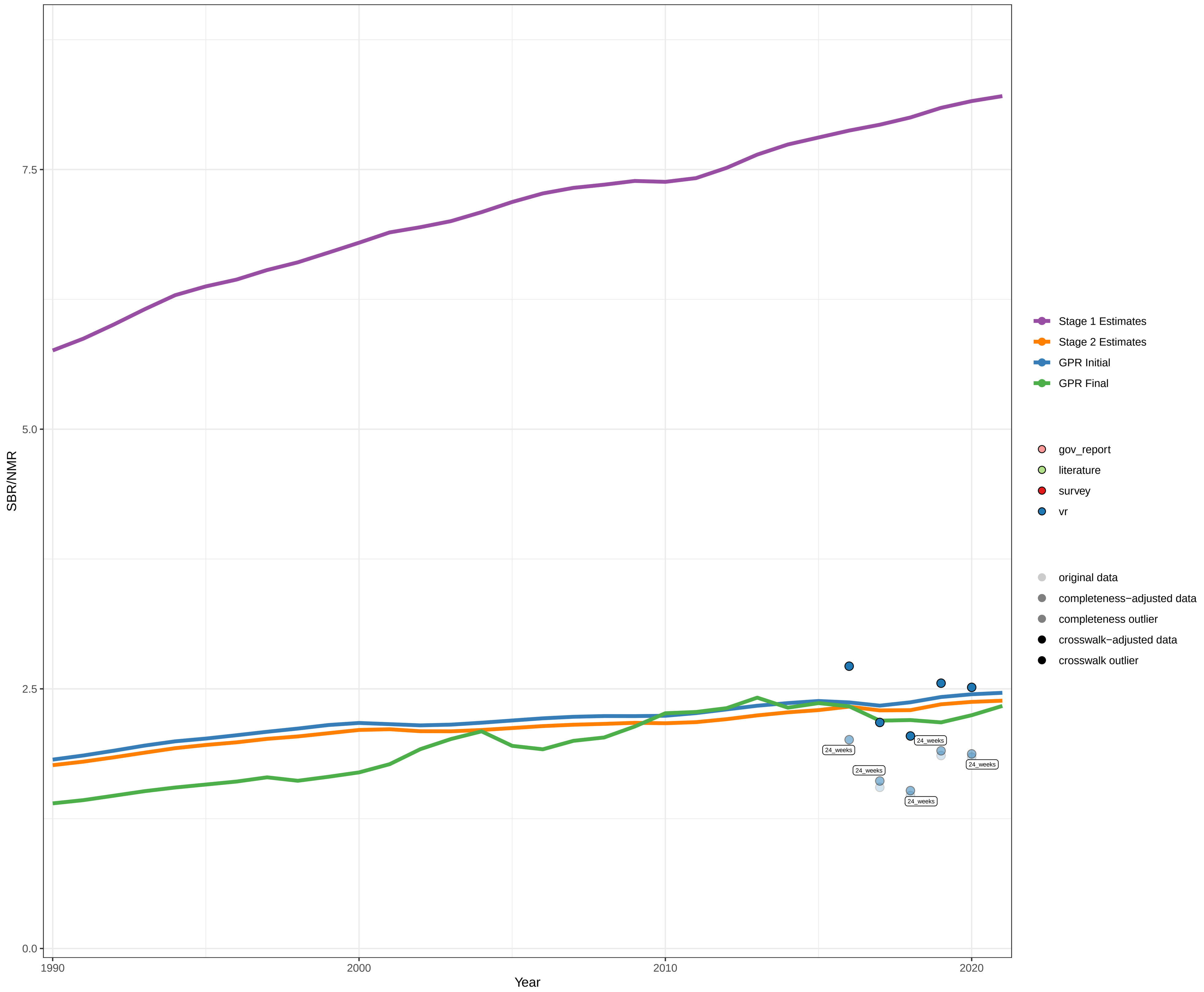

Bradford GBR_44684 Western Europe Stillbirth Rate / Neonatal Mortality Rate (SBR/NMR) − 28 weeks
lambda = 0.6 ; zeta = 0.8 ; scale = 15 ; amp = 0.028

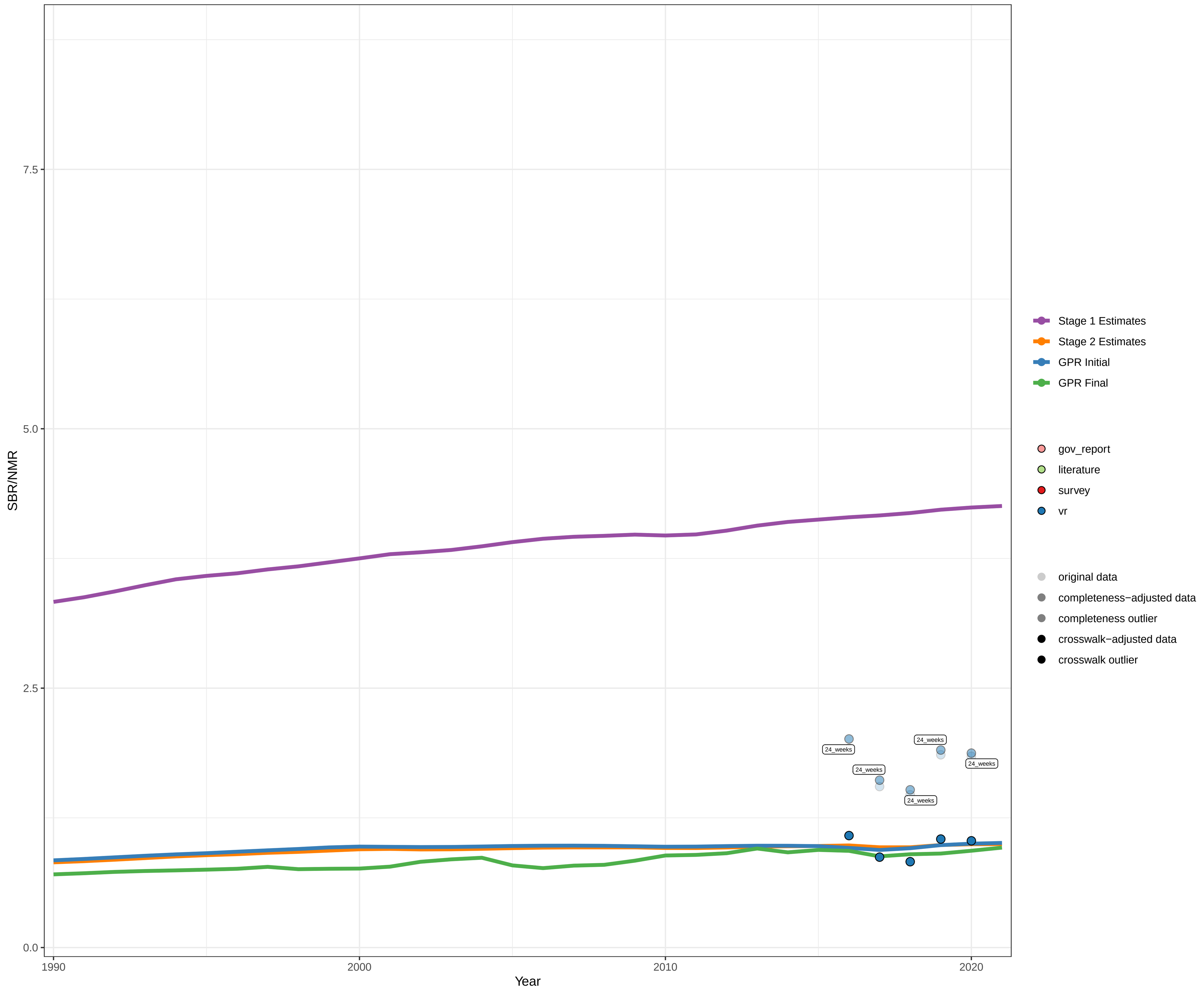

Definition	Years	Outlier	Source.Type	Title
24_weeks	2016−2020	0	VR	United Kingdom − England and Wales Child Mortality Statistics

Bradford GBR_44684 Western Europe Stillbirth Rate (SBR) − 20 weeks
lambda = 0.6 ; zeta = 0.8 ; scale = 15 ; amp = 0.028

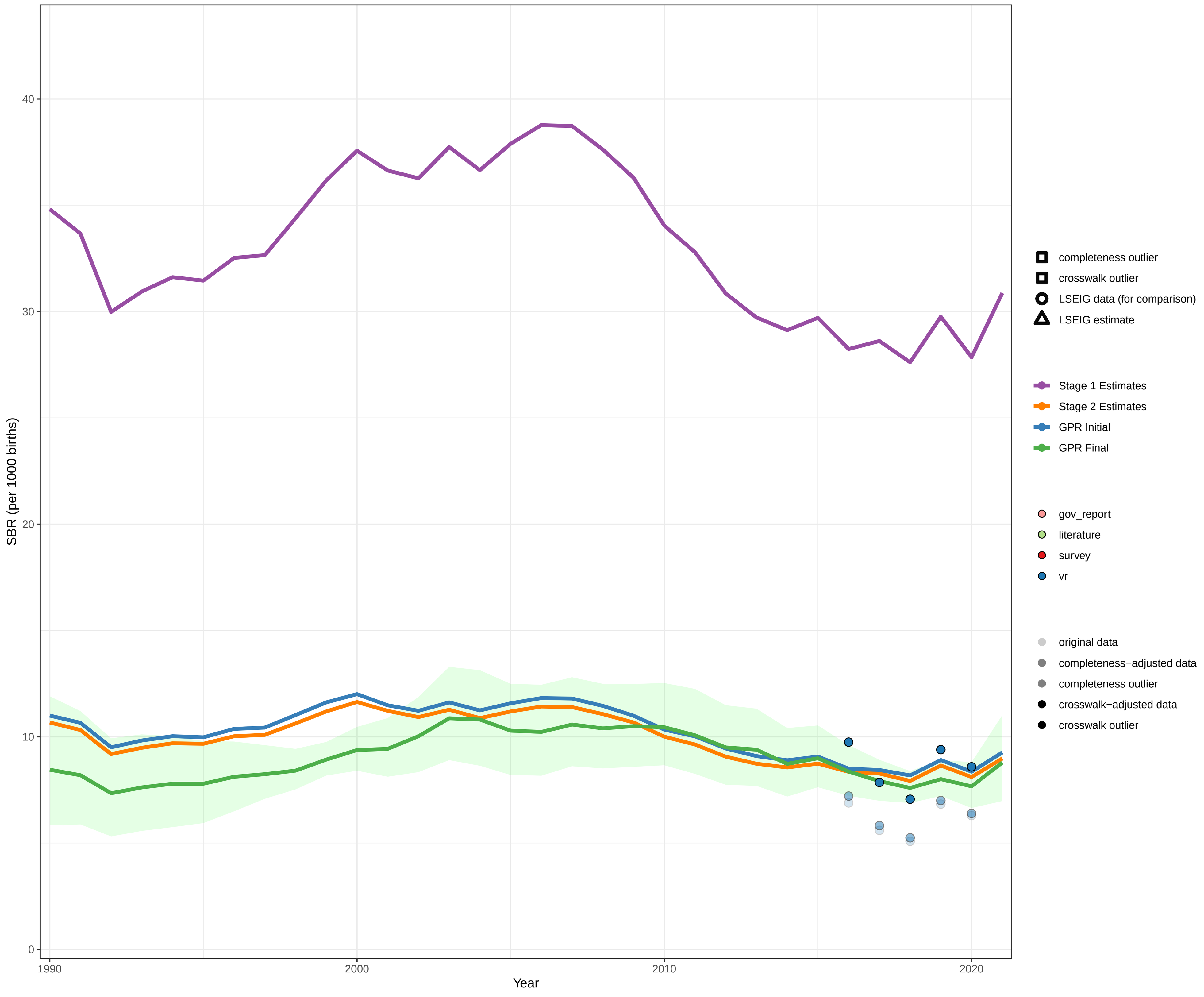

Bradford GBR_44684 Western Europe Stillbirth Rate (SBR) − 28 weeks
lambda = 0.6 ; zeta = 0.8 ; scale = 15 ; amp = 0.028

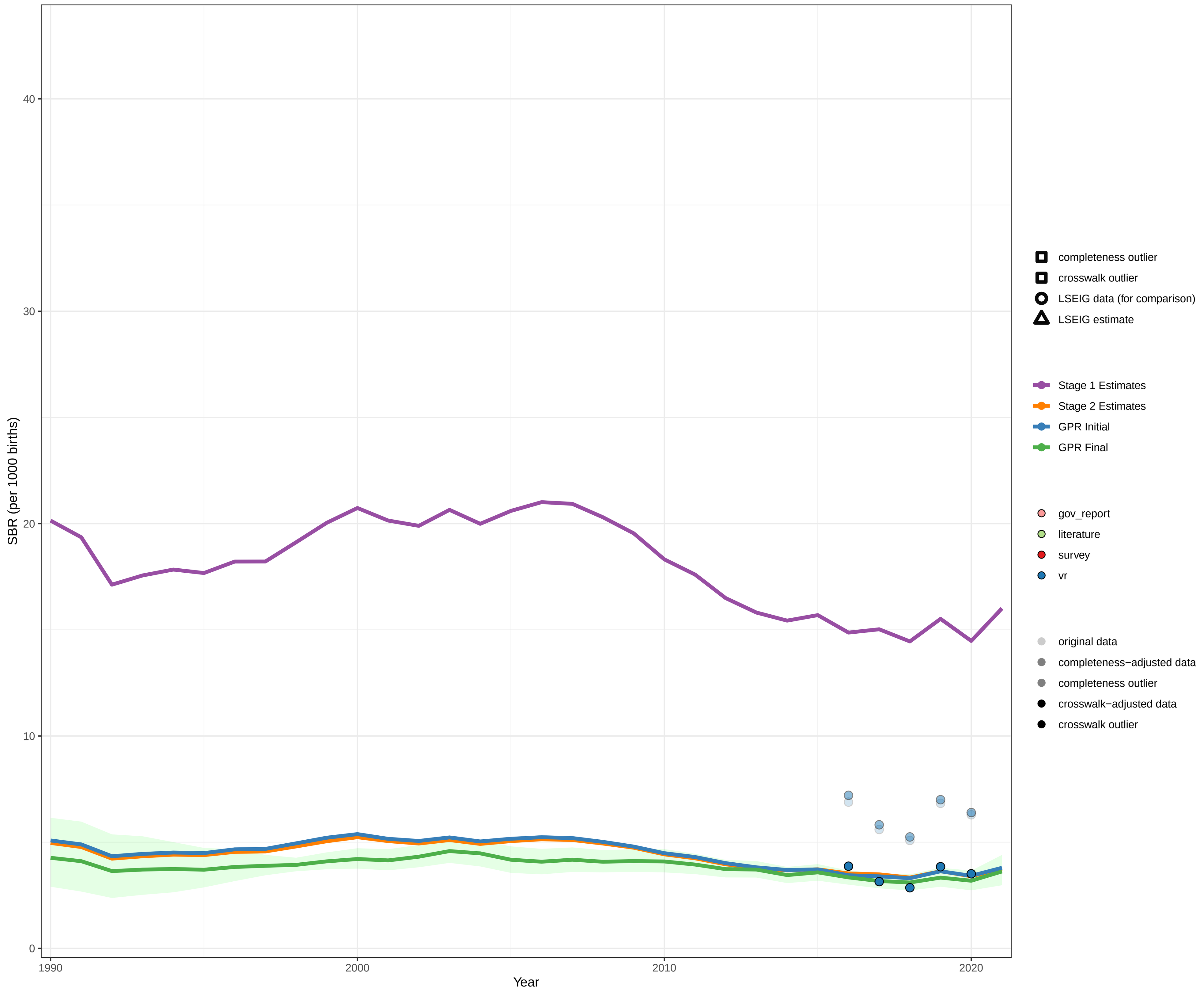

Year	Total Stillbirths (in thousands)	Total Stillbirths (in thousands)	SBR (per 1000 births)	SBR (per 1000 births)	SBR/NMR	SBR/NMR
	20 weeks	28 weeks	20 weeks	28 weeks	20 weeks	28 weeks
1990	0.1 (0.0 to 0.1)	0.0 (0.0 to 0.0)	8.4 (5.8 to 11.9)	4.3 (2.9 to 6.1)	1.40 (0.96 to 1.97)	0.71 (0.48 to 1.02)
1995	0.1 (0.0 to 0.1)	0.0 (0.0 to 0.0)	7.8 (5.9 to 9.8)	3.7 (2.9 to 4.7)	1.58 (1.20 to 1.99)	0.75 (0.58 to 0.96)
2000	0.1 (0.1 to 0.1)	0.0 (0.0 to 0.0)	9.4 (8.4 to 10.5)	4.2 (3.8 to 4.7)	1.70 (1.52 to 1.89)	0.76 (0.68 to 0.85)
2005	0.1 (0.1 to 0.1)	0.0 (0.0 to 0.0)	10.3 (8.2 to 12.5)	4.2 (3.6 to 4.8)	1.95 (1.56 to 2.37)	0.79 (0.67 to 0.91)
2010	0.1 (0.1 to 0.1)	0.0 (0.0 to 0.0)	10.4 (8.7 to 12.5)	4.1 (3.6 to 4.7)	2.27 (1.88 to 2.72)	0.89 (0.78 to 1.01)
2015	0.1 (0.1 to 0.1)	0.0 (0.0 to 0.0)	9.0 (7.6 to 10.5)	3.6 (3.2 to 4.0)	2.36 (2.00 to 2.77)	0.94 (0.84 to 1.04)
2020	0.1 (0.0 to 0.1)	0.0 (0.0 to 0.0)	7.7 (6.6 to 8.8)	3.2 (2.7 to 3.7)	2.25 (1.95 to 2.58)	0.93 (0.80 to 1.07)
2021	0.1 (0.0 to 0.1)	0.0 (0.0 to 0.0)	8.8 (7.0 to 11.0)	3.6 (3.0 to 4.4)	2.34 (1.85 to 2.93)	0.96 (0.79 to 1.17)

Calderdale GBR_44682 Western Europe Stillbirth Rate / Neonatal Mortality Rate (SBR/NMR) − 20 weeks
lambda = 0.8 ; zeta = 0.7 ; scale = 15 ; amp = 0.028

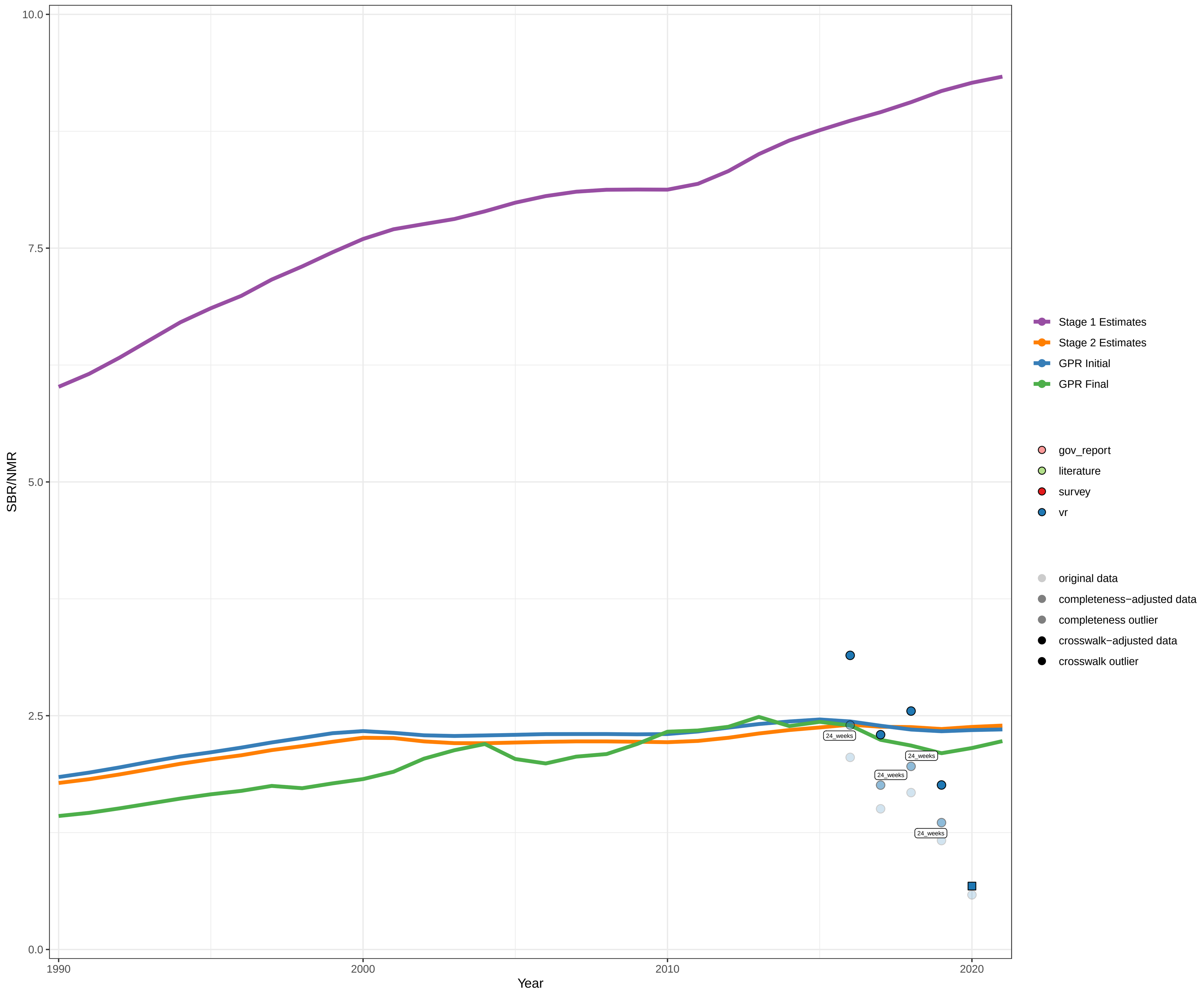

Calderdale GBR_44682 Western Europe Stillbirth Rate / Neonatal Mortality Rate (SBR/NMR) − 28 weeks
lambda = 0.8 ; zeta = 0.7 ; scale = 15 ; amp = 0.028

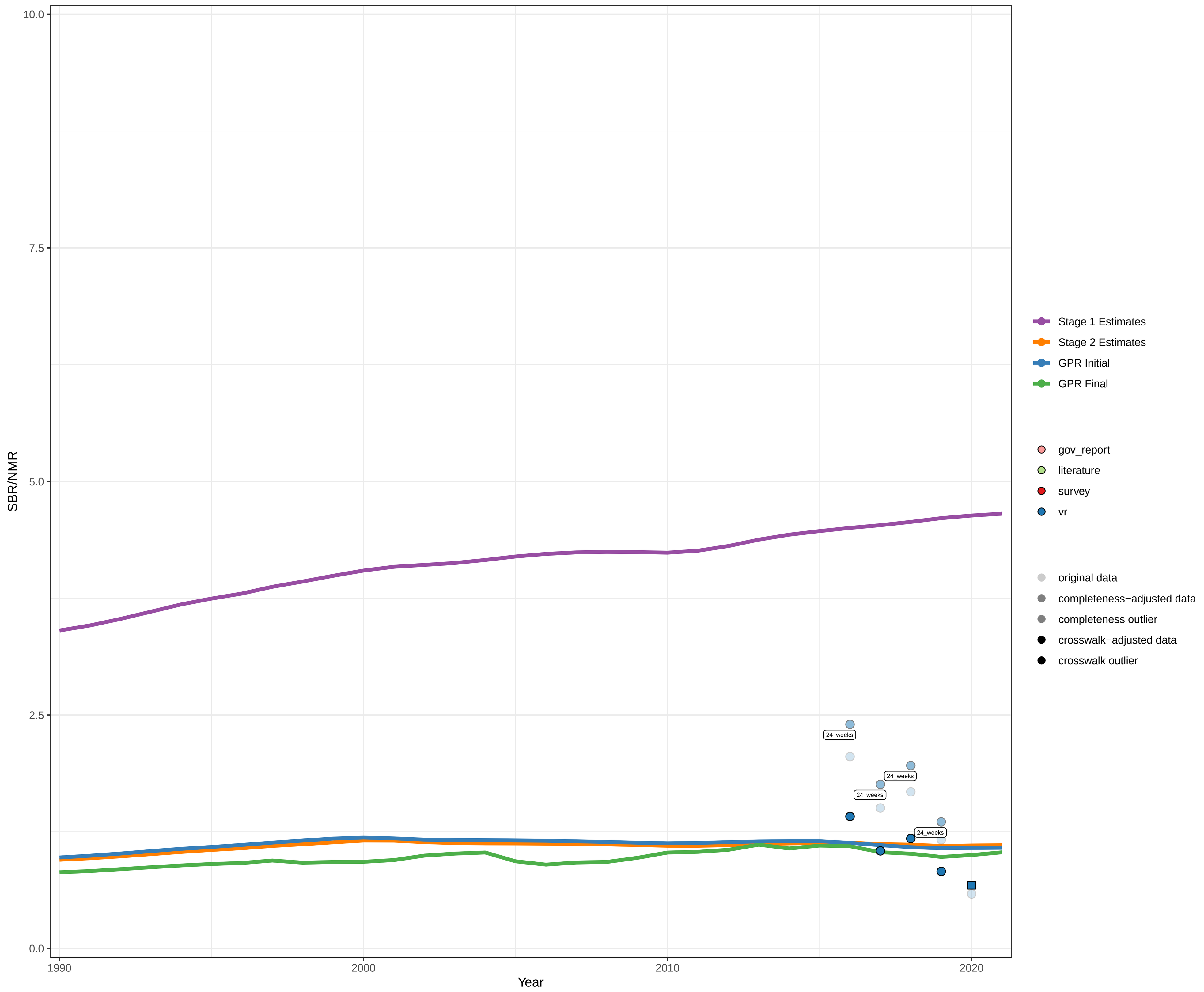

Definition	Years	Outlier	Source.Type	Title
24_weeks	2016−2020	0	VR	United Kingdom − England and Wales Child Mortality Statistics

Calderdale GBR_44682 Western Europe Stillbirth Rate (SBR) − 20 weeks
lambda = 0.8 ; zeta = 0.7 ; scale = 15 ; amp = 0.028

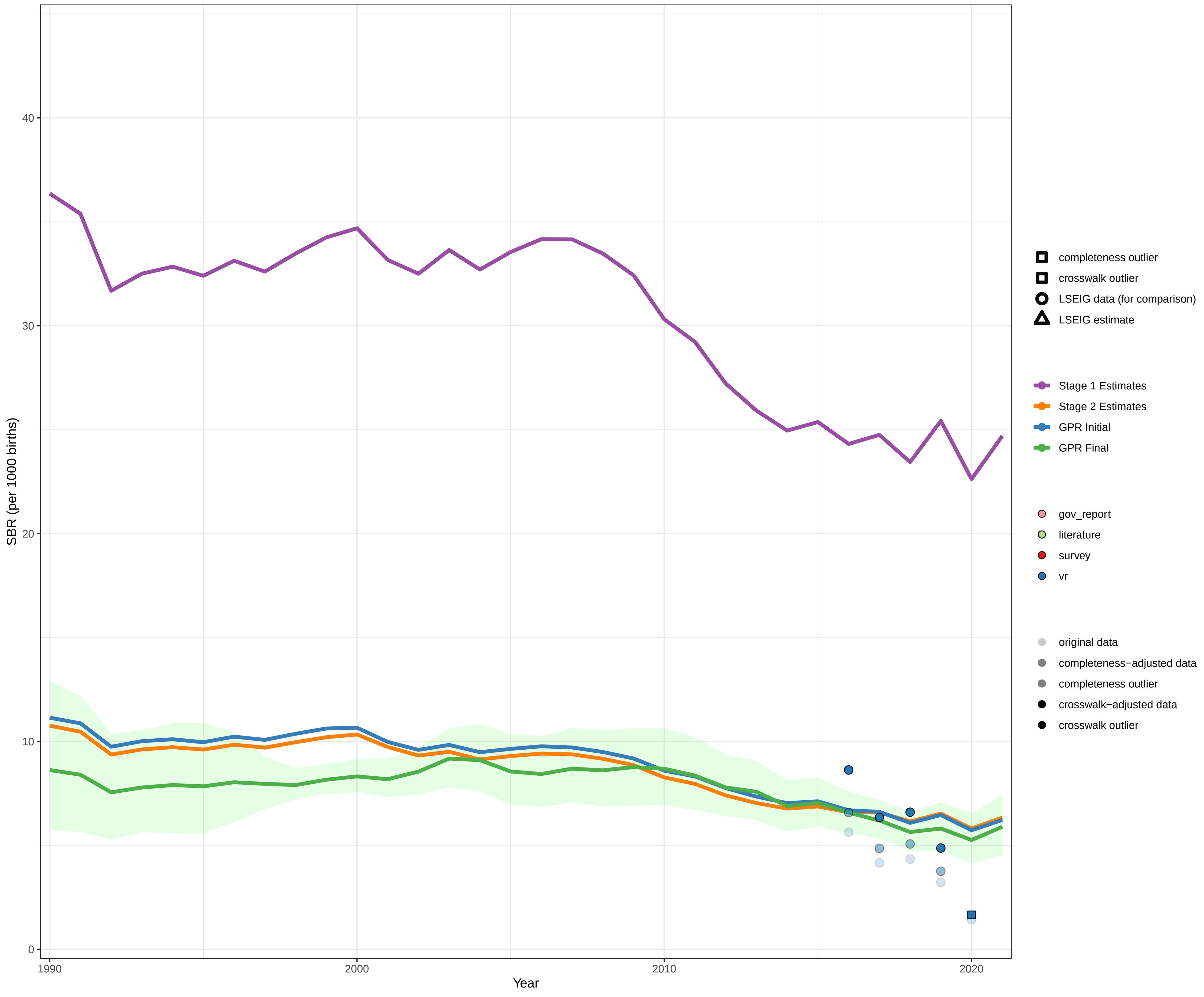

Calderdale GBR_44682 Western Europe Stillbirth Rate (SBR) − 28 weeks
lambda = 0.8 ; zeta = 0.7 ; scale = 15 ; amp = 0.028

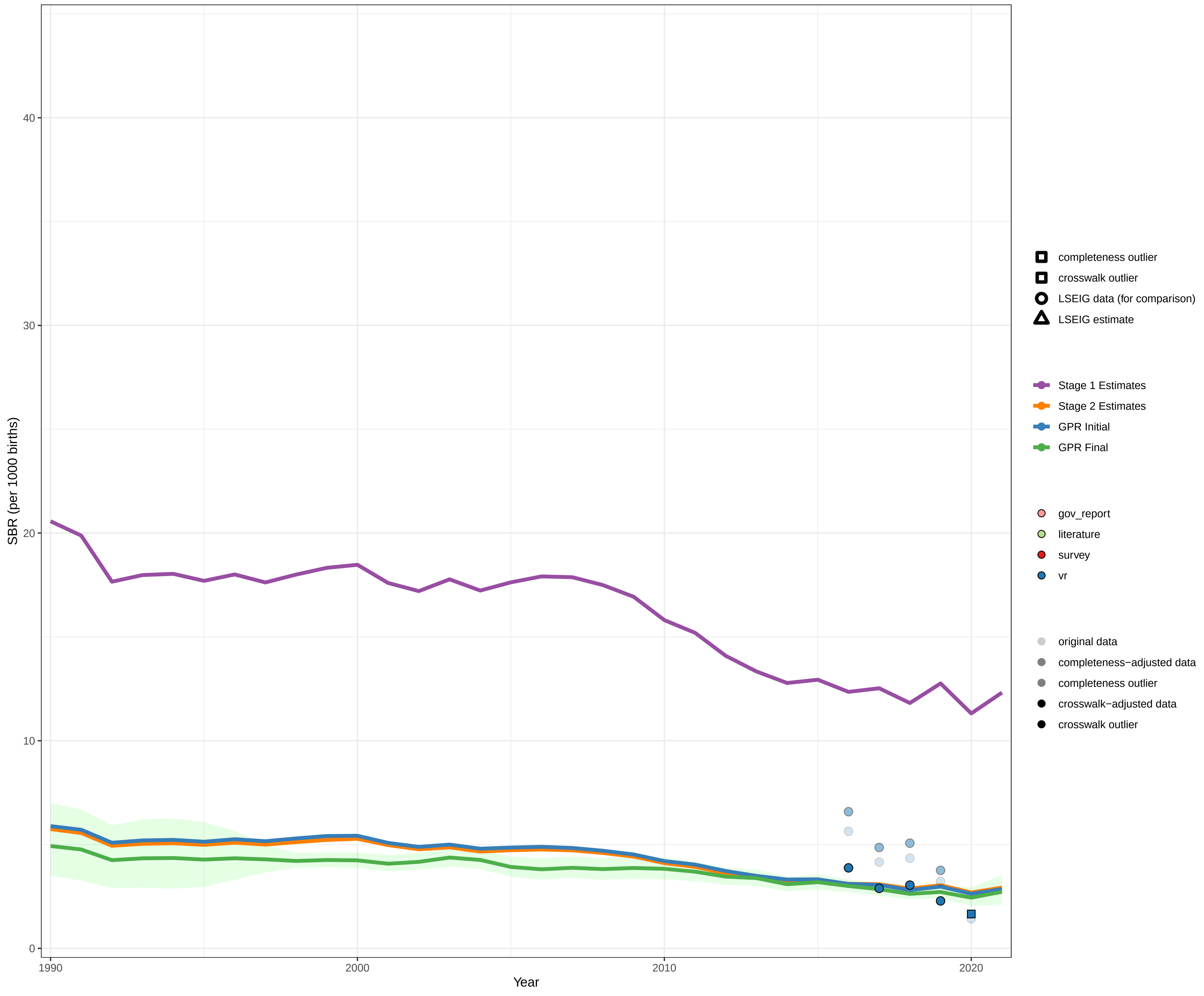

Year	Total Stillbirths (in thousands)	Total Stillbirths (in thousands)	SBR (per 1000 births)	SBR (per 1000 births)	SBR/NMR	SBR/NMR
	20 weeks	28 weeks	20 weeks	28 weeks	20 weeks	28 weeks
1990	0.0 (0.0 to 0.0)	0.0 (0.0 to 0.0)	8.6 (5.7 to 12.9)	4.9 (3.5 to 7.0)	1.43 (0.95 to 2.14)	0.82 (0.58 to 1.16)
1995	0.0 (0.0 to 0.0)	0.0 (0.0 to 0.0)	7.8 (5.6 to 10.9)	4.3 (3.0 to 6.1)	1.66 (1.18 to 2.31)	0.90 (0.63 to 1.29)
2000	0.0 (0.0 to 0.0)	0.0 (0.0 to 0.0)	8.3 (7.6 to 9.1)	4.2 (3.9 to 4.6)	1.82 (1.66 to 2.00)	0.93 (0.85 to 1.01)
2005	0.0 (0.0 to 0.0)	0.0 (0.0 to 0.0)	8.6 (7.0 to 10.3)	3.9 (3.5 to 4.4)	2.04 (1.66 to 2.46)	0.93 (0.82 to 1.05)
2010	0.0 (0.0 to 0.0)	0.0 (0.0 to 0.0)	8.7 (6.9 to 10.6)	3.8 (3.3 to 4.4)	2.33 (1.86 to 2.85)	1.03 (0.89 to 1.17)
2015	0.0 (0.0 to 0.0)	0.0 (0.0 to 0.0)	7.0 (5.9 to 8.3)	3.2 (2.8 to 3.6)	2.43 (2.03 to 2.85)	1.10 (0.98 to 1.23)
2020	0.0 (0.0 to 0.0)	0.0 (0.0 to 0.0)	5.3 (4.1 to 6.5)	2.4 (2.1 to 2.9)	2.15 (1.69 to 2.68)	1.00 (0.84 to 1.20)
2021	0.0 (0.0 to 0.0)	0.0 (0.0 to 0.0)	5.9 (4.6 to 7.4)	2.7 (2.1 to 3.5)	2.23 (1.72 to 2.80)	1.03 (0.79 to 1.33)

Doncaster GBR_44690 Western Europe Stillbirth Rate / Neonatal Mortality Rate (SBR/NMR) − 20 weeks
lambda = 0.6 ; zeta = 0.8 ; scale = 15 ; amp = 0.028

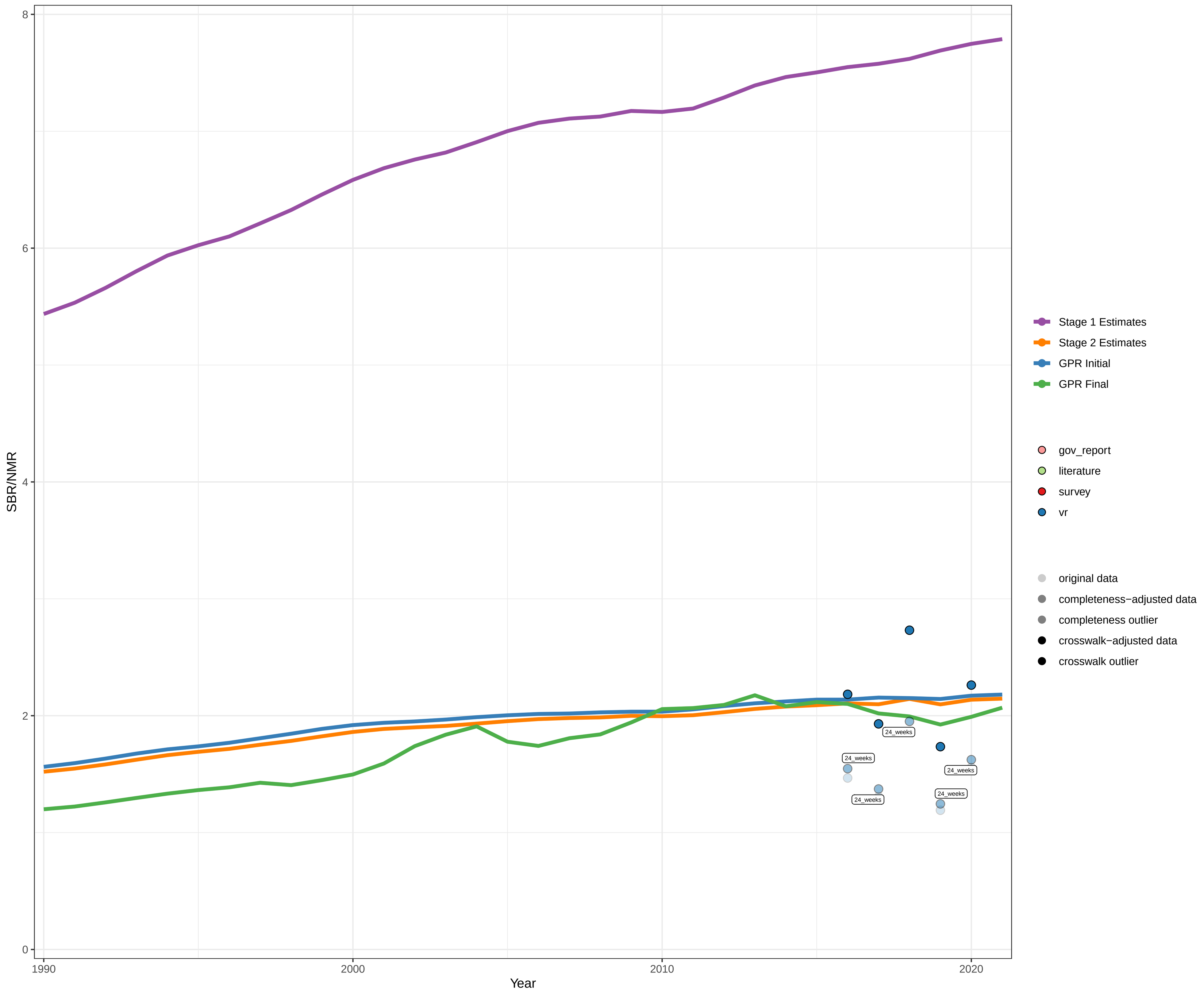

Doncaster GBR_44690 Western Europe Stillbirth Rate / Neonatal Mortality Rate (SBR/NMR) − 28 weeks
lambda = 0.6 ; zeta = 0.8 ; scale = 15 ; amp = 0.028

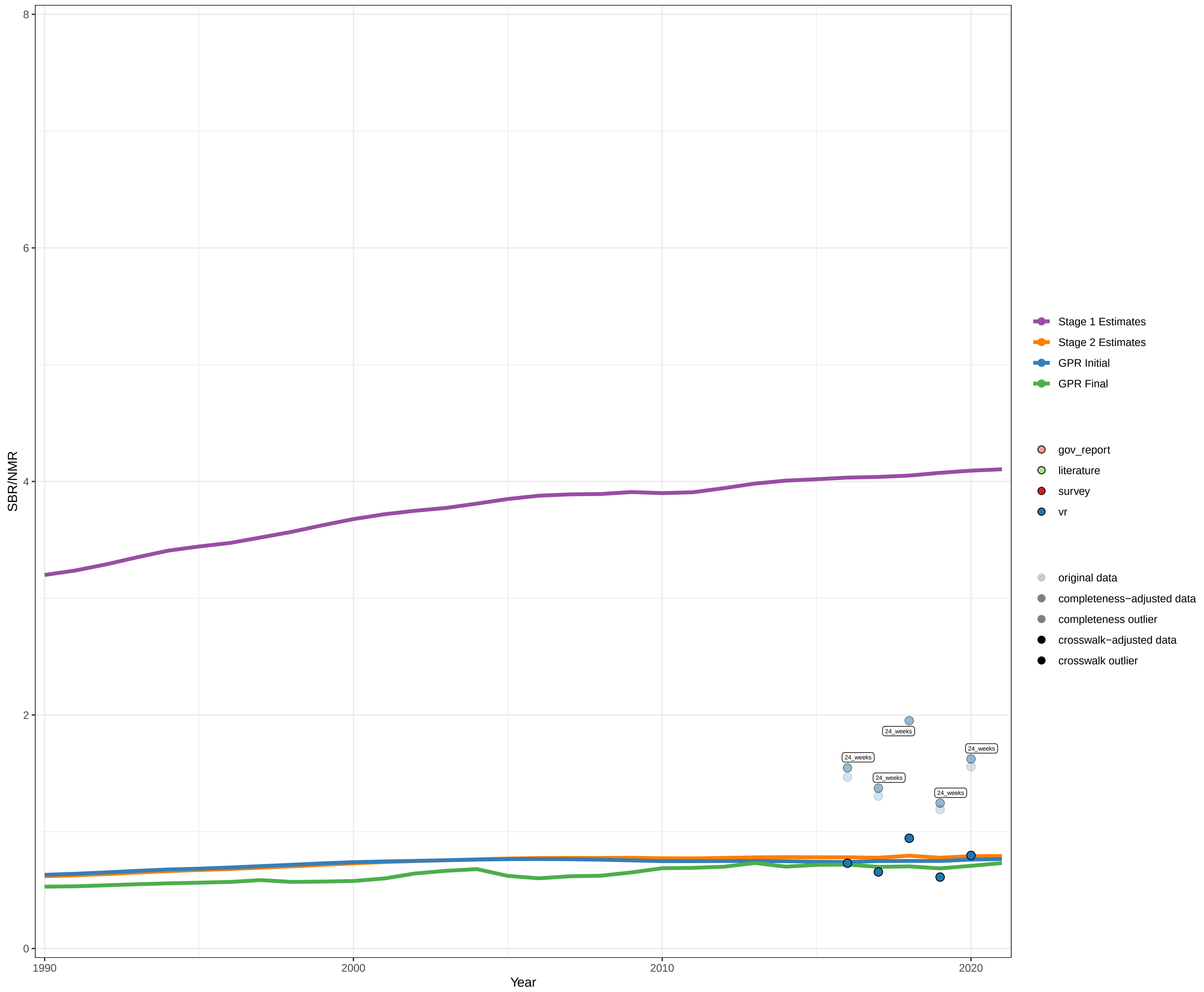

Definition	Years	Outlier	Source.Type	Title
24_weeks	2016−2020	0	VR	United Kingdom − England and Wales Child Mortality Statistics

Doncaster GBR_44690 Western Europe Stillbirth Rate (SBR) − 20 weeks
lambda = 0.6 ; zeta = 0.8 ; scale = 15 ; amp = 0.028

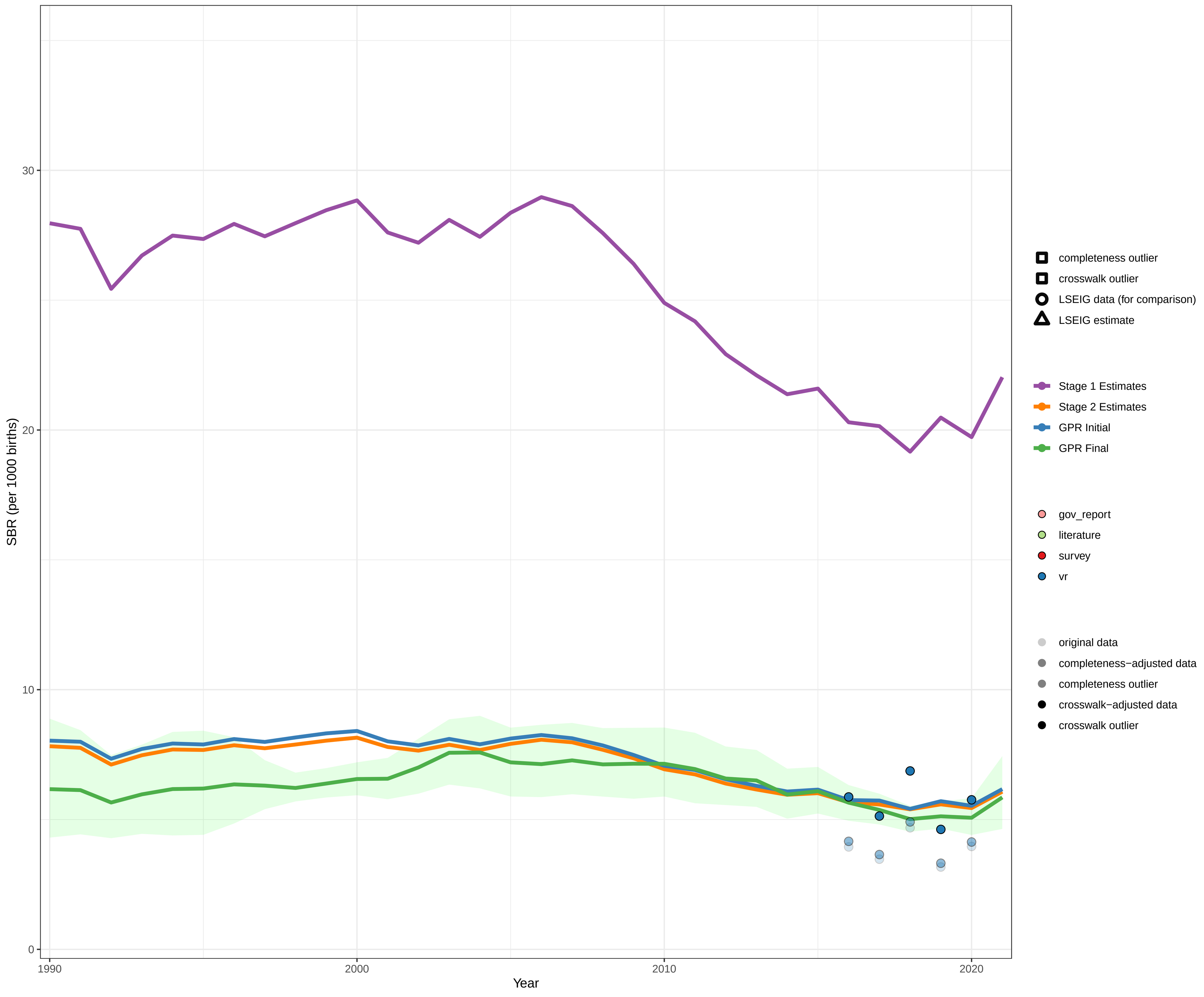

Doncaster GBR_44690 Western Europe Stillbirth Rate (SBR) − 28 weeks
lambda = 0.6 ; zeta = 0.8 ; scale = 15 ; amp = 0.028

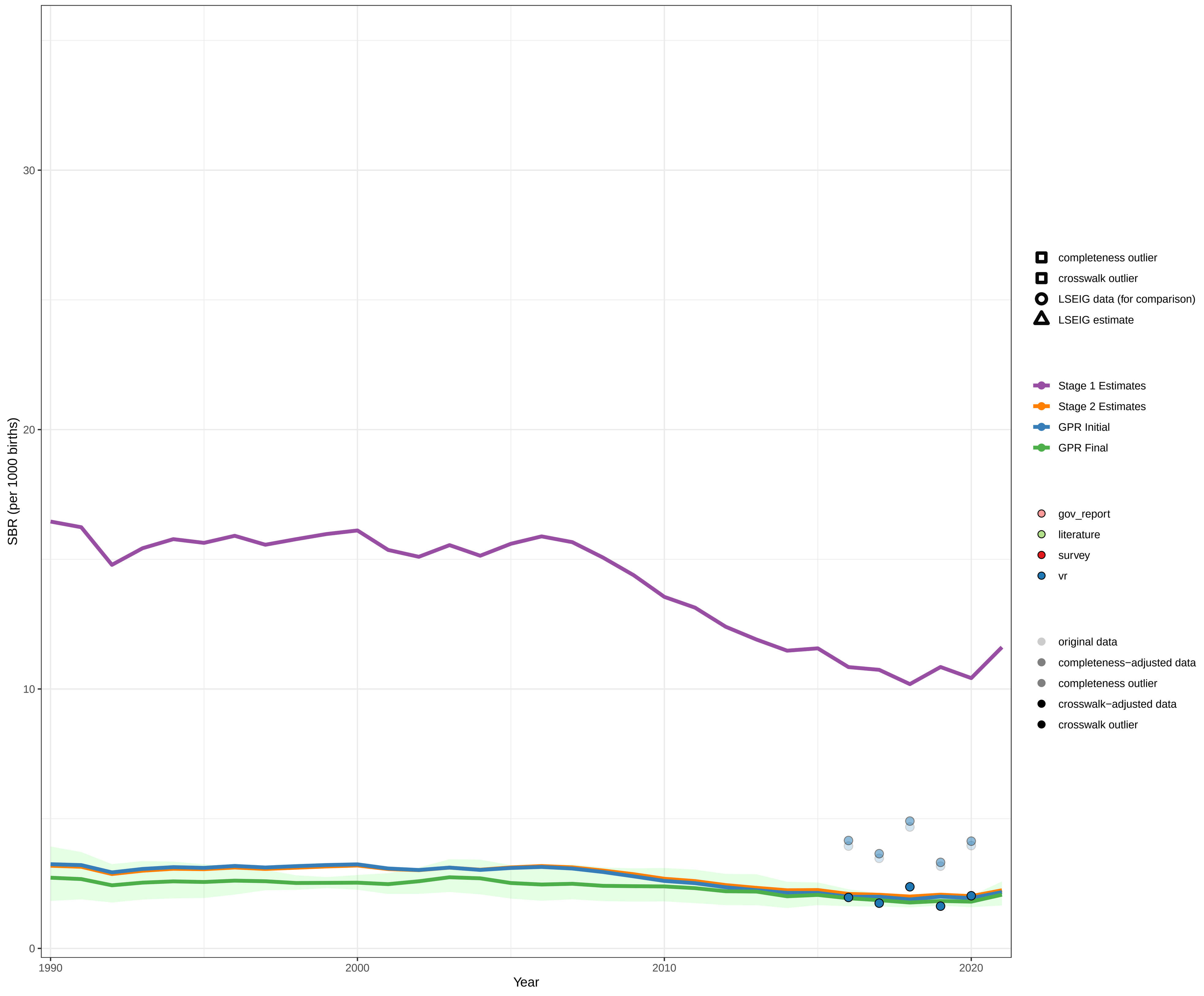

Year	Total Stillbirths (in thousands)	Total Stillbirths (in thousands)	SBR (per 1000 births)	SBR (per 1000 births)	SBR/NMR	SBR/NMR
	20 weeks	28 weeks	20 weeks	28 weeks	20 weeks	28 weeks
1990	0.0 (0.0 to 0.0)	0.0 (0.0 to 0.0)	6.2 (4.3 to 8.9)	2.7 (1.8 to 3.9)	1.20 (0.84 to 1.73)	0.53 (0.36 to 0.76)
1995	0.0 (0.0 to 0.0)	0.0 (0.0 to 0.0)	6.2 (4.4 to 8.4)	2.6 (1.9 to 3.2)	1.36 (0.97 to 1.85)	0.56 (0.43 to 0.71)
2000	0.0 (0.0 to 0.0)	0.0 (0.0 to 0.0)	6.6 (5.9 to 7.2)	2.5 (2.3 to 2.8)	1.50 (1.35 to 1.64)	0.58 (0.52 to 0.65)
2005	0.0 (0.0 to 0.0)	0.0 (0.0 to 0.0)	7.2 (5.9 to 8.5)	2.5 (1.9 to 3.2)	1.78 (1.45 to 2.11)	0.62 (0.47 to 0.79)
2010	0.0 (0.0 to 0.0)	0.0 (0.0 to 0.0)	7.1 (5.9 to 8.5)	2.4 (1.8 to 3.1)	2.06 (1.69 to 2.46)	0.69 (0.52 to 0.89)
2015	0.0 (0.0 to 0.0)	0.0 (0.0 to 0.0)	6.1 (5.2 to 7.0)	2.1 (1.7 to 2.5)	2.12 (1.82 to 2.44)	0.72 (0.58 to 0.88)
2020	0.0 (0.0 to 0.0)	0.0 (0.0 to 0.0)	5.1 (4.4 to 5.8)	1.8 (1.6 to 2.0)	1.99 (1.73 to 2.29)	0.71 (0.62 to 0.80)
2021	0.0 (0.0 to 0.0)	0.0 (0.0 to 0.0)	5.9 (4.6 to 7.4)	2.1 (1.7 to 2.6)	2.07 (1.64 to 2.63)	0.73 (0.58 to 0.91)

East Riding of Yorkshire GBR_44679 Western Europe Stillbirth Rate / Neonatal Mortality Rate (SBR/NMR) − 20 weeks
lambda = 0.6 ; zeta = 0.8 ; scale = 15 ; amp = 0.028

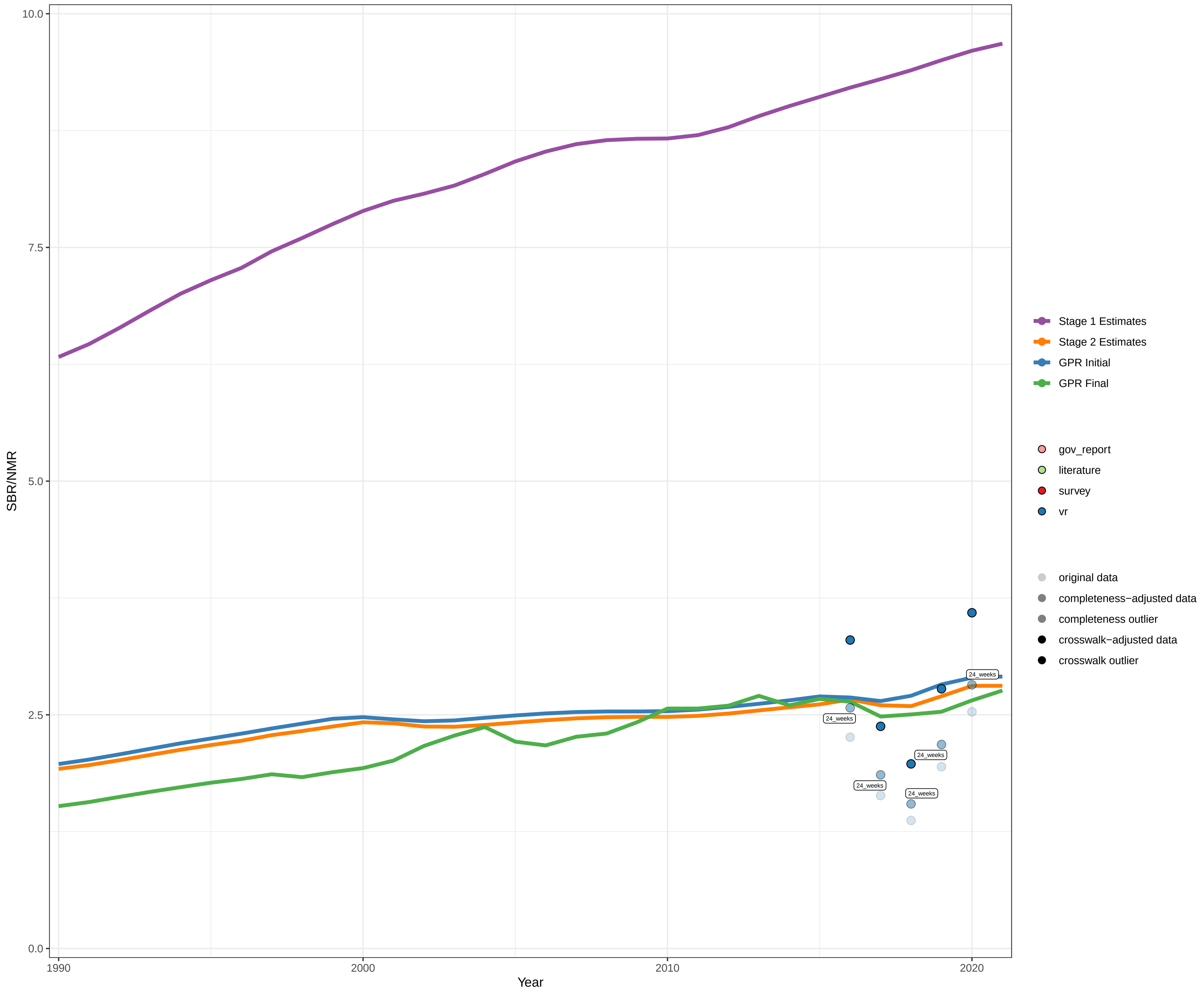

East Riding of Yorkshire GBR_44679 Western Europe Stillbirth Rate / Neonatal Mortality Rate (SBR/NMR) − 28 weeks
lambda = 0.6 ; zeta = 0.8 ; scale = 15 ; amp = 0.028

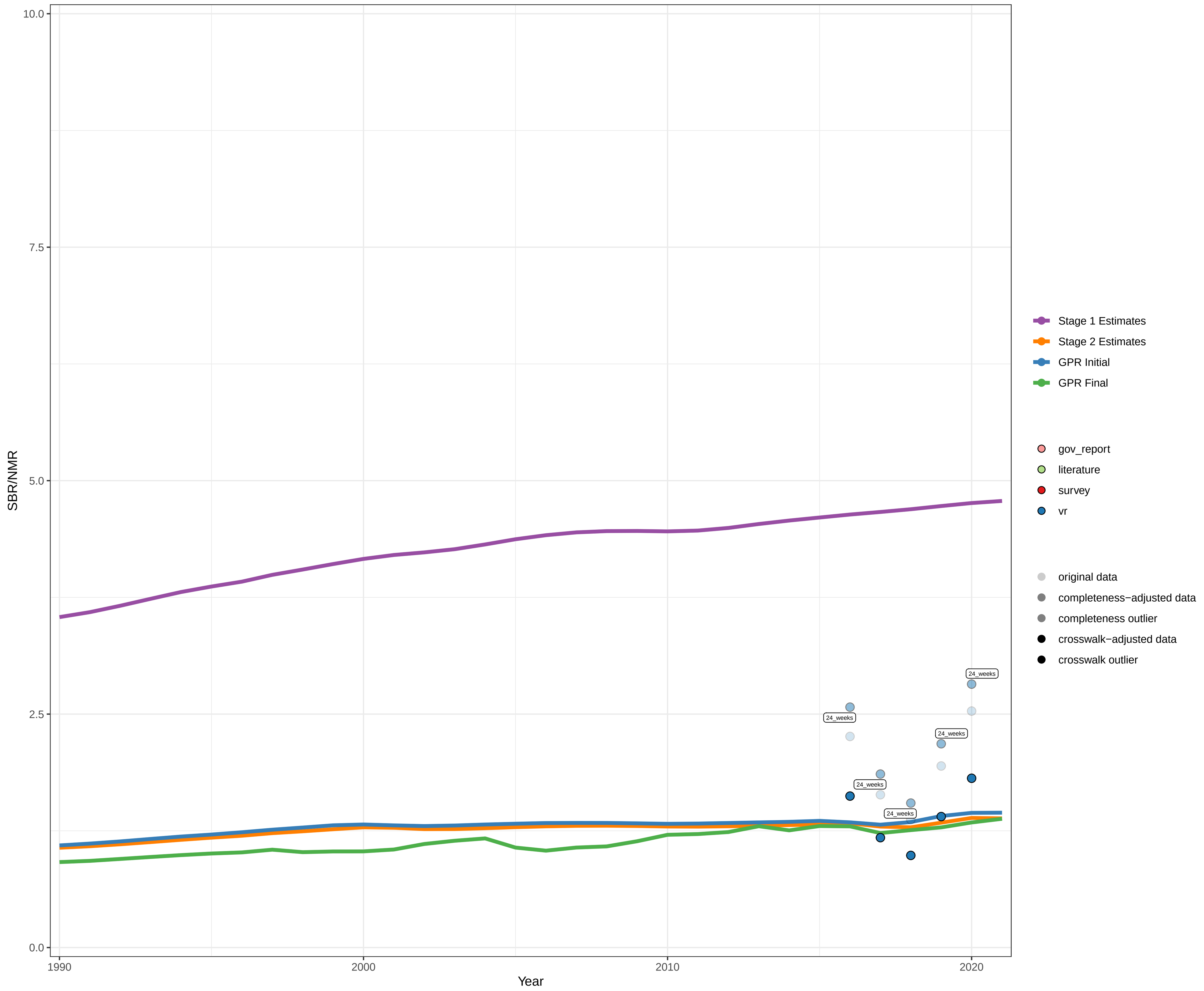

Definition	Years	Outlier	Source.Type	Title
24_weeks	2016−2020	0	VR	United Kingdom − England and Wales Child Mortality Statistics

East Riding of Yorkshire GBR_44679 Western Europe Stillbirth Rate (SBR) − 20 weeks
lambda = 0.6 ; zeta = 0.8 ; scale = 15 ; amp = 0.028

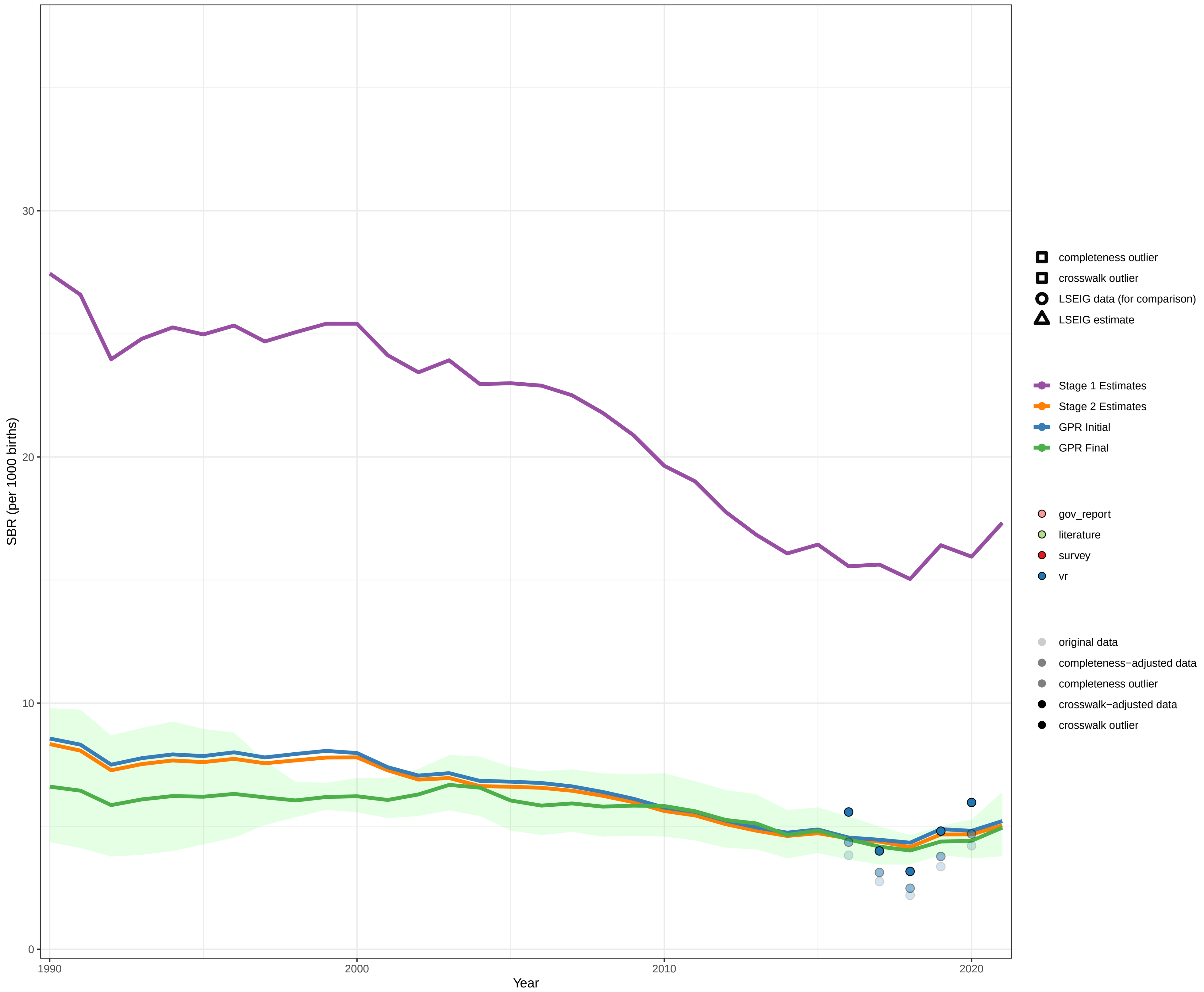

East Riding of Yorkshire GBR_44679 Western Europe Stillbirth Rate (SBR) − 28 weeks
lambda = 0.6 ; zeta = 0.8 ; scale = 15 ; amp = 0.028

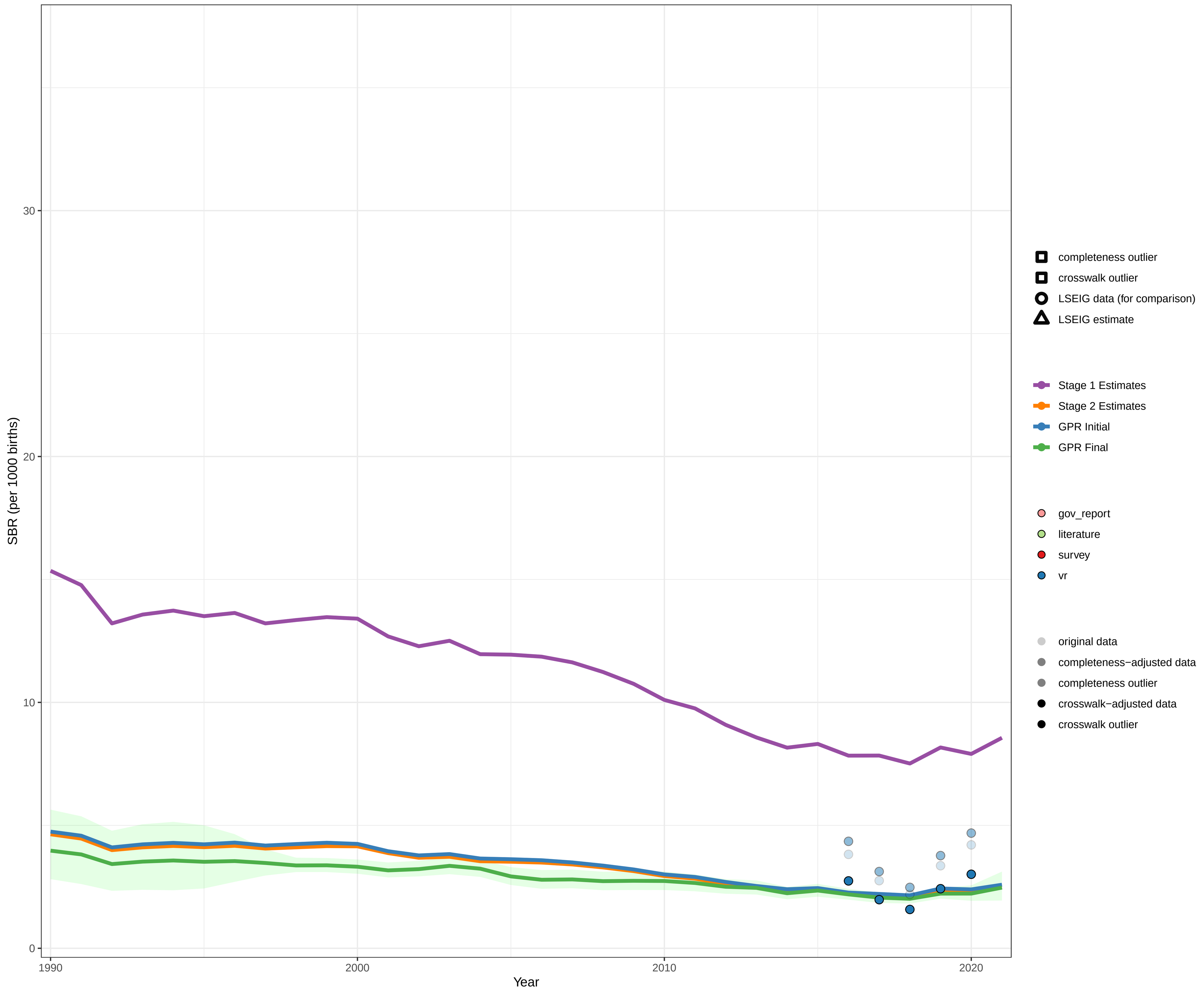

Year	Total Stillbirths (in thousands)	Total Stillbirths (in thousands)	SBR (per 1000 births)	SBR (per 1000 births)	SBR/NMR	SBR/NMR
	20 weeks	28 weeks	20 weeks	28 weeks	20 weeks	28 weeks
1990	0.0 (0.0 to 0.0)	0.0 (0.0 to 0.0)	6.6 (4.3 to 9.8)	4.0 (2.8 to 5.6)	1.52 (1.00 to 2.26)	0.92 (0.65 to 1.30)
1995	0.0 (0.0 to 0.0)	0.0 (0.0 to 0.0)	6.2 (4.3 to 9.0)	3.5 (2.4 to 5.0)	1.77 (1.22 to 2.56)	1.01 (0.70 to 1.43)
2000	0.0 (0.0 to 0.0)	0.0 (0.0 to 0.0)	6.2 (5.6 to 7.0)	3.3 (3.0 to 3.6)	1.93 (1.73 to 2.16)	1.03 (0.94 to 1.12)
2005	0.0 (0.0 to 0.0)	0.0 (0.0 to 0.0)	6.0 (4.8 to 7.4)	2.9 (2.6 to 3.3)	2.21 (1.76 to 2.71)	1.07 (0.94 to 1.21)
2010	0.0 (0.0 to 0.0)	0.0 (0.0 to 0.0)	5.8 (4.6 to 7.2)	2.7 (2.4 to 3.1)	2.57 (2.02 to 3.16)	1.21 (1.05 to 1.38)
2015	0.0 (0.0 to 0.0)	0.0 (0.0 to 0.0)	4.8 (3.9 to 5.8)	2.3 (2.1 to 2.6)	2.67 (2.16 to 3.19)	1.30 (1.16 to 1.45)
2020	0.0 (0.0 to 0.0)	0.0 (0.0 to 0.0)	4.4 (3.7 to 5.3)	2.2 (1.9 to 2.6)	2.65 (2.22 to 3.18)	1.34 (1.16 to 1.54)
2021	0.0 (0.0 to 0.0)	0.0 (0.0 to 0.0)	4.9 (3.8 to 6.4)	2.5 (1.9 to 3.1)	2.76 (2.11 to 3.57)	1.38 (1.09 to 1.74)

Kingston upon Hull, City of GBR_44691 Western Europe Stillbirth Rate / Neonatal Mortality Rate (SBR/NMR) − 20 weeks
lambda = 0.6 ; zeta = 0.8 ; scale = 15 ; amp = 0.028

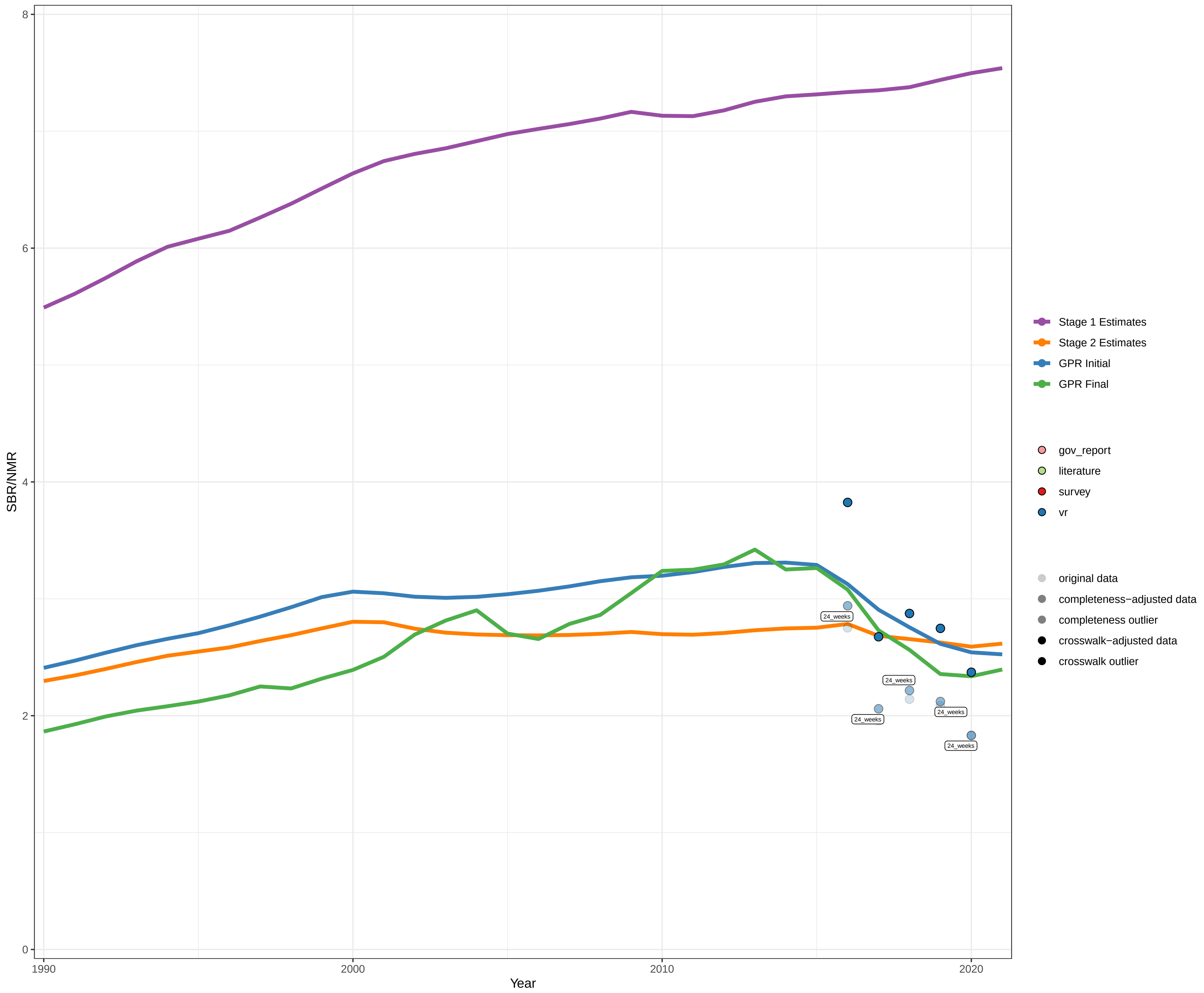

Kingston upon Hull, City of GBR_44691 Western Europe Stillbirth Rate / Neonatal Mortality Rate (SBR/NMR) − 28 weeks
lambda = 0.6 ; zeta = 0.8 ; scale = 15 ; amp = 0.028

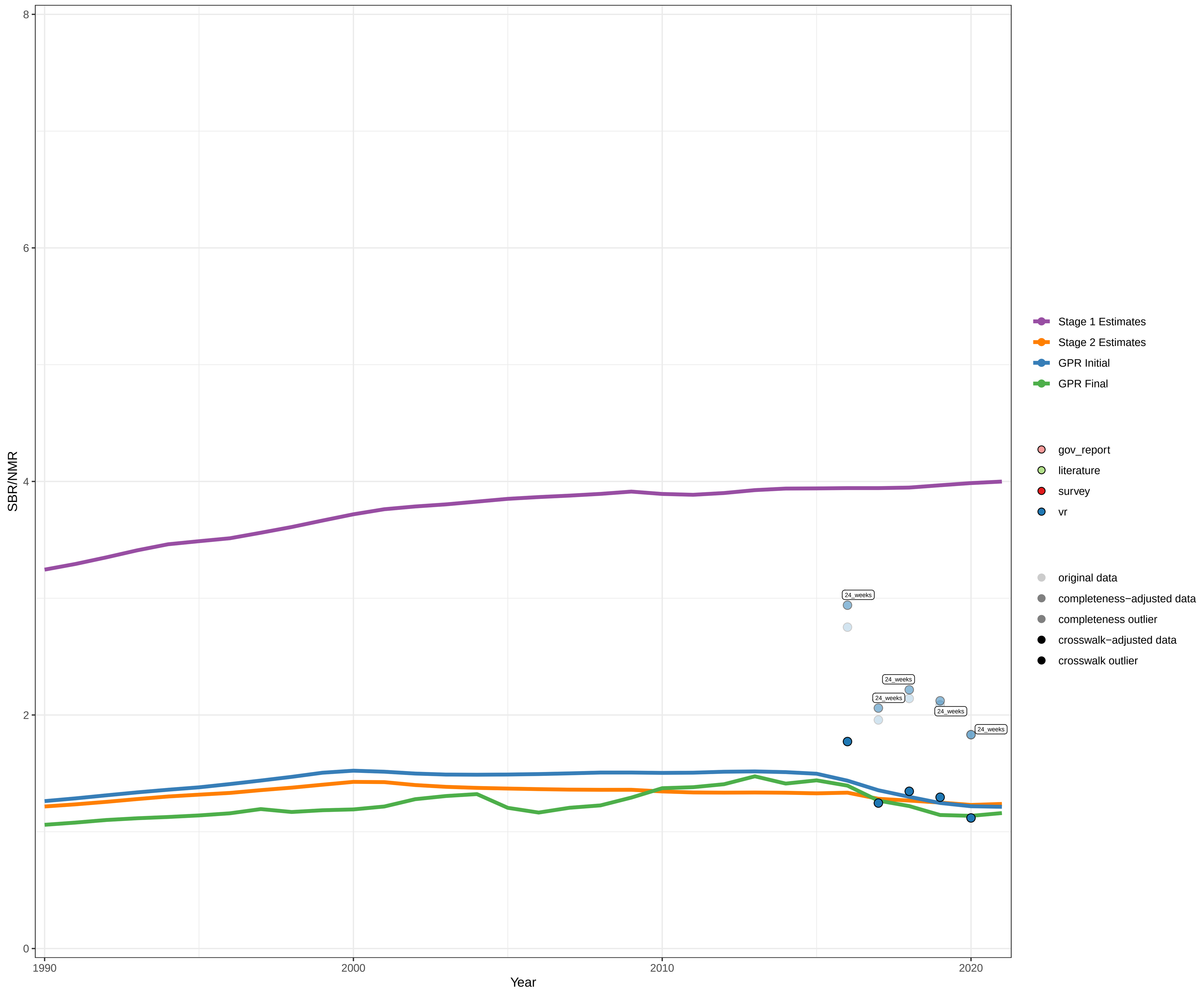

Definition	Years	Outlier	Source.Type	Title
24_weeks	2016−2020	0	VR	United Kingdom − England and Wales Child Mortality Statistics

Kingston upon Hull, City of GBR_44691 Western Europe Stillbirth Rate (SBR) − 20 weeks
lambda = 0.6 ; zeta = 0.8 ; scale = 15 ; amp = 0.028

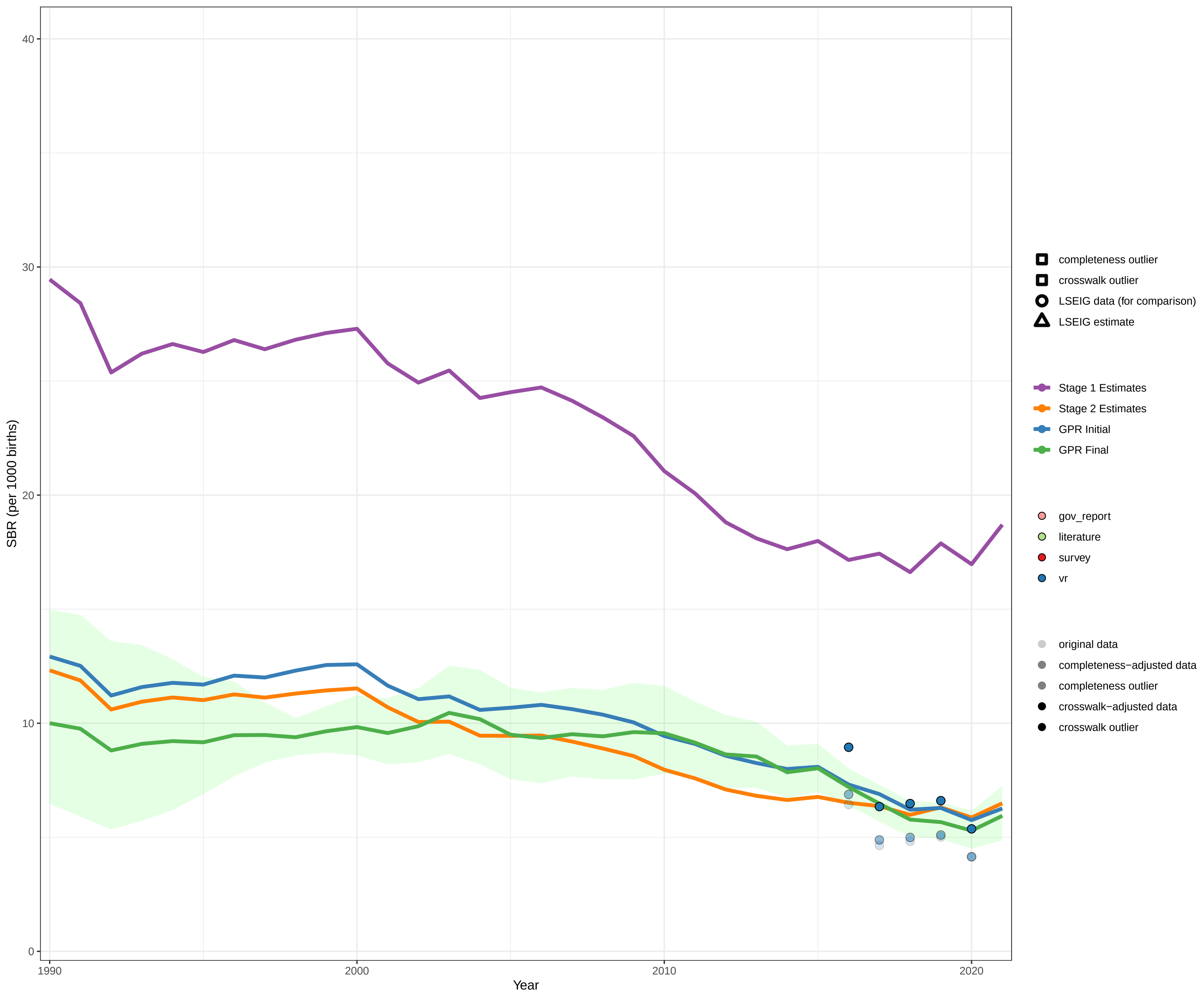

Kingston upon Hull, City of GBR_44691 Western Europe Stillbirth Rate (SBR) − 28 weeks
lambda = 0.6 ; zeta = 0.8 ; scale = 15 ; amp = 0.028

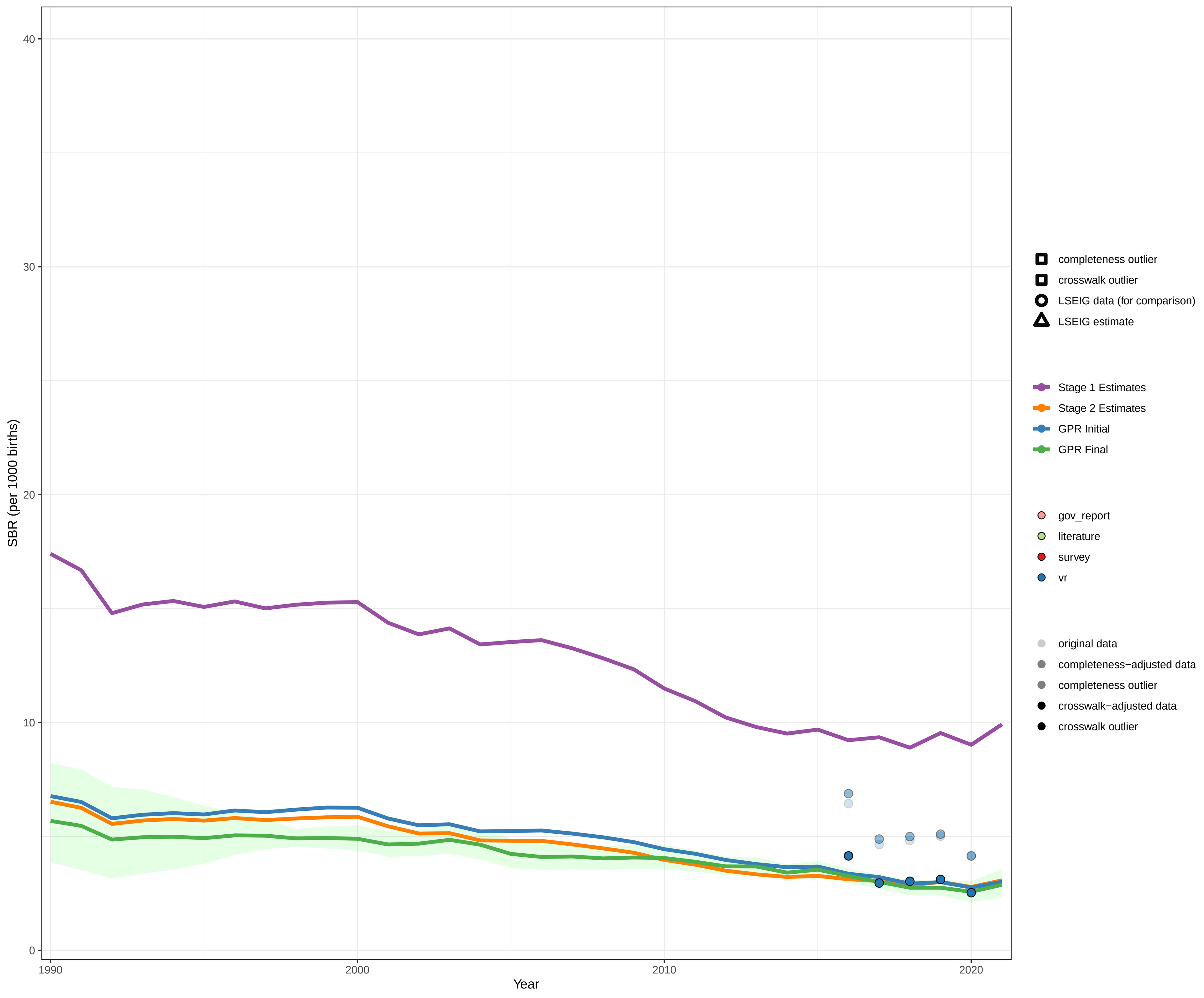

Year	Total Stillbirths (in thousands)	Total Stillbirths (in thousands)	SBR (per 1000 births)	SBR (per 1000 births)	SBR/NMR	SBR/NMR
	20 weeks	28 weeks	20 weeks	28 weeks	20 weeks	28 weeks
1990	0.0 (0.0 to 0.1)	0.0 (0.0 to 0.0)	10.0 (6.5 to 15.0)	5.7 (3.9 to 8.2)	1.86 (1.20 to 2.79)	1.06 (0.72 to 1.54)
1995	0.0 (0.0 to 0.0)	0.0 (0.0 to 0.0)	9.2 (6.9 to 12.0)	4.9 (3.8 to 6.3)	2.12 (1.59 to 2.78)	1.14 (0.88 to 1.47)
2000	0.0 (0.0 to 0.0)	0.0 (0.0 to 0.0)	9.8 (8.6 to 11.2)	4.9 (4.4 to 5.5)	2.39 (2.09 to 2.73)	1.19 (1.07 to 1.34)
2005	0.0 (0.0 to 0.0)	0.0 (0.0 to 0.0)	9.5 (7.5 to 11.6)	4.2 (3.6 to 4.9)	2.70 (2.15 to 3.29)	1.20 (1.03 to 1.39)
2010	0.0 (0.0 to 0.0)	0.0 (0.0 to 0.0)	9.6 (7.8 to 11.6)	4.1 (3.6 to 4.6)	3.24 (2.64 to 3.94)	1.37 (1.20 to 1.57)
2015	0.0 (0.0 to 0.0)	0.0 (0.0 to 0.0)	8.0 (7.0 to 9.1)	3.5 (3.2 to 3.9)	3.26 (2.84 to 3.70)	1.44 (1.28 to 1.59)
2020	0.0 (0.0 to 0.0)	0.0 (0.0 to 0.0)	5.3 (4.5 to 6.2)	2.6 (2.1 to 3.0)	2.34 (1.99 to 2.73)	1.14 (0.94 to 1.34)
2021	0.0 (0.0 to 0.0)	0.0 (0.0 to 0.0)	5.9 (4.9 to 7.3)	2.9 (2.3 to 3.6)	2.40 (1.96 to 2.92)	1.16 (0.93 to 1.44)

Kirklees GBR_44685 Western Europe Stillbirth Rate / Neonatal Mortality Rate (SBR/NMR) − 20 weeks
lambda = 0.6 ; zeta = 0.8 ; scale = 15 ; amp = 0.028

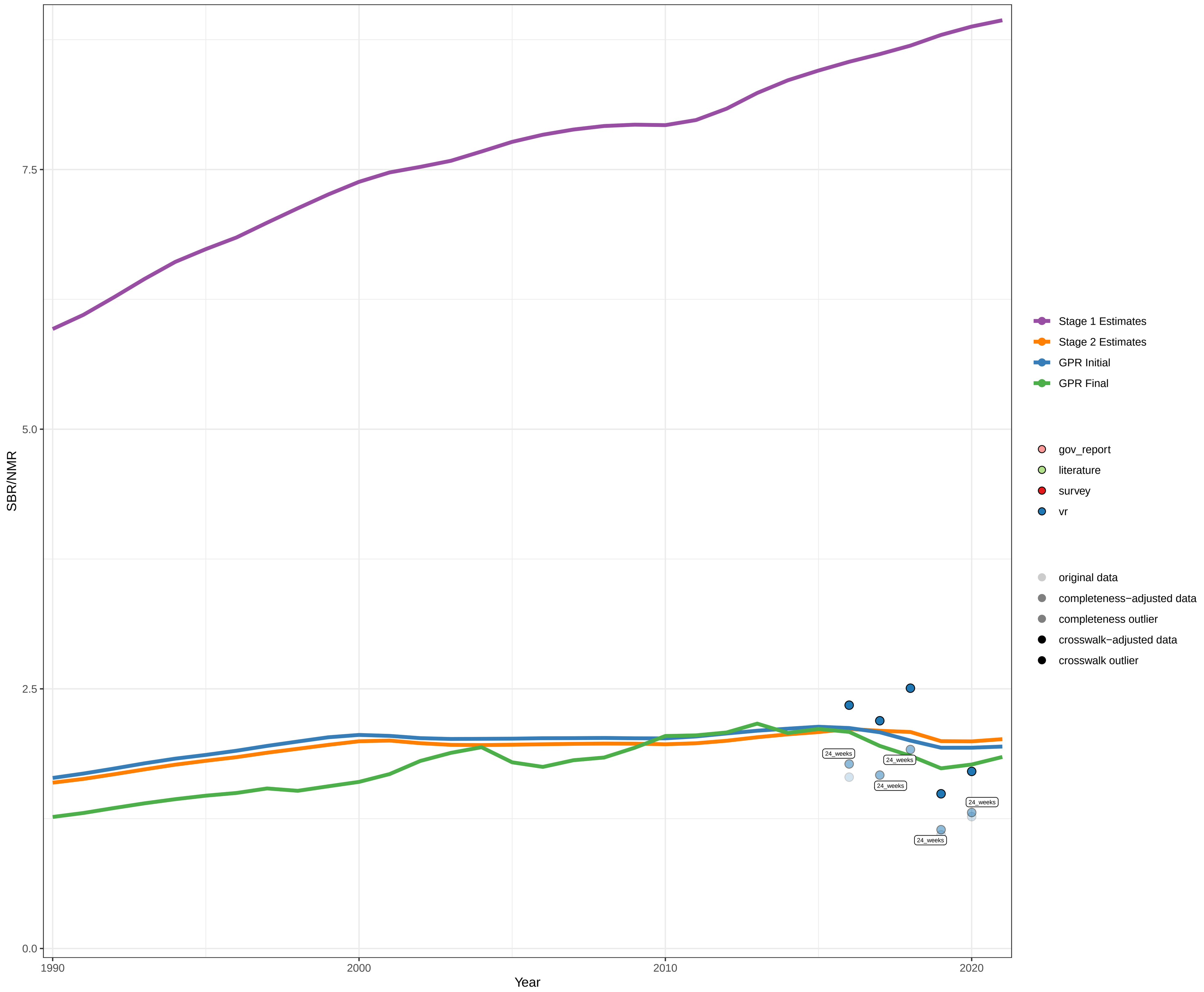

Kirklees GBR_44685 Western Europe Stillbirth Rate / Neonatal Mortality Rate (SBR/NMR) − 28 weeks
lambda = 0.6 ; zeta = 0.8 ; scale = 15 ; amp = 0.028

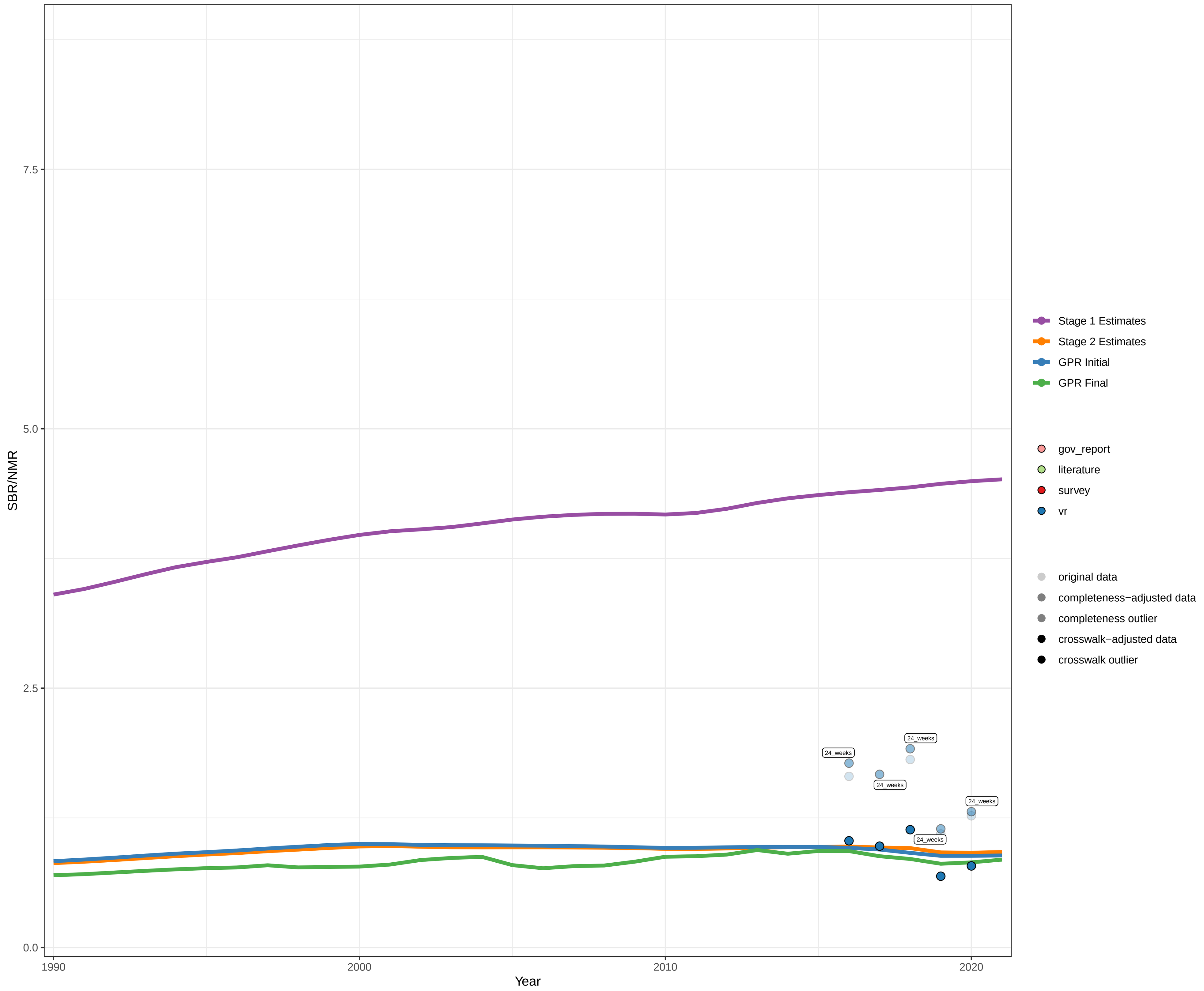

Definition	Years	Outlier	Source.Type	Title
24_weeks	2016−2020	0	VR	United Kingdom − England and Wales Child Mortality Statistics

Kirklees GBR_44685 Western Europe Stillbirth Rate (SBR) − 20 weeks
lambda = 0.6 ; zeta = 0.8 ; scale = 15 ; amp = 0.028

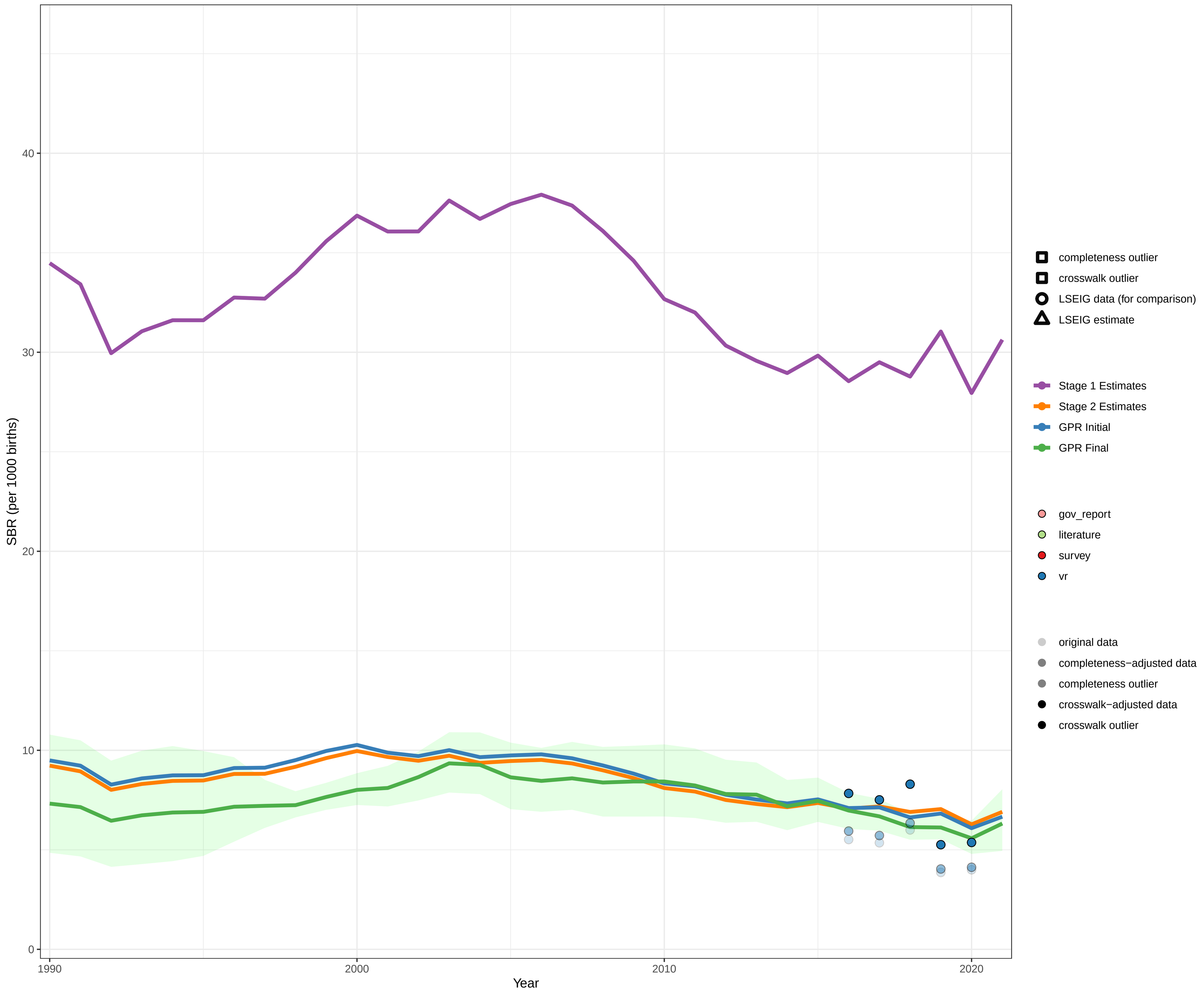

Kirklees GBR_44685 Western Europe Stillbirth Rate (SBR) − 28 weeks
lambda = 0.6 ; zeta = 0.8 ; scale = 15 ; amp = 0.028

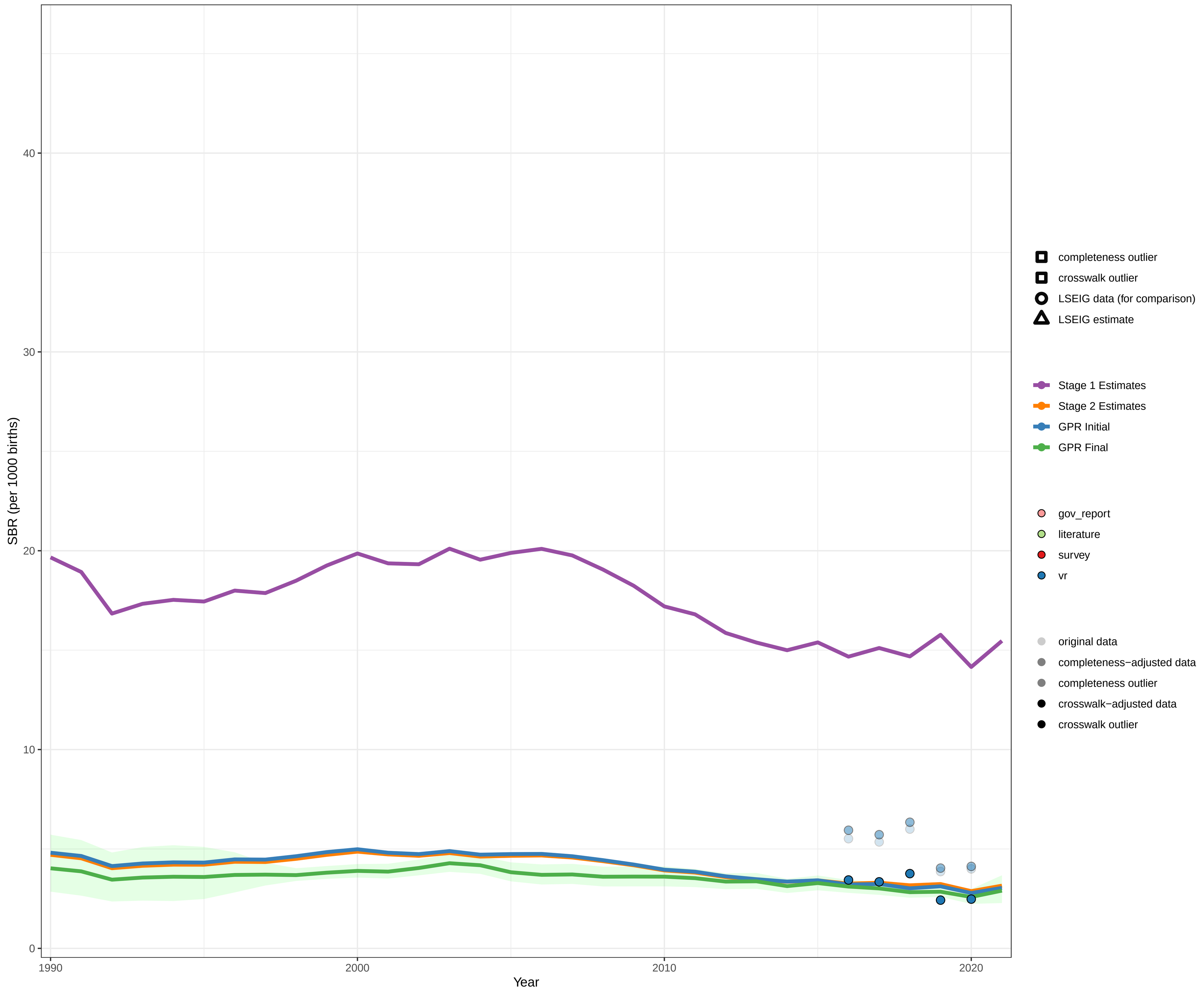

Year	Total Stillbirths (in thousands)	Total Stillbirths (in thousands)	SBR (per 1000 births)	SBR (per 1000 births)	SBR/NMR	SBR/NMR
	20 weeks	28 weeks	20 weeks	28 weeks	20 weeks	28 weeks
1990	0.0 (0.0 to 0.1)	0.0 (0.0 to 0.0)	7.3 (4.9 to 10.8)	4.0 (2.9 to 5.7)	1.27 (0.84 to 1.87)	0.70 (0.49 to 0.99)
1995	0.0 (0.0 to 0.1)	0.0 (0.0 to 0.0)	6.9 (4.7 to 10.0)	3.6 (2.5 to 5.1)	1.47 (1.00 to 2.12)	0.77 (0.53 to 1.09)
2000	0.0 (0.0 to 0.0)	0.0 (0.0 to 0.0)	8.0 (7.3 to 8.9)	3.9 (3.6 to 4.2)	1.60 (1.45 to 1.77)	0.78 (0.71 to 0.85)
2005	0.0 (0.0 to 0.1)	0.0 (0.0 to 0.0)	8.6 (7.0 to 10.4)	3.8 (3.4 to 4.3)	1.79 (1.46 to 2.15)	0.79 (0.70 to 0.90)
2010	0.0 (0.0 to 0.1)	0.0 (0.0 to 0.0)	8.4 (6.7 to 10.3)	3.6 (3.1 to 4.1)	2.05 (1.62 to 2.50)	0.88 (0.76 to 1.00)
2015	0.0 (0.0 to 0.0)	0.0 (0.0 to 0.0)	7.5 (6.4 to 8.6)	3.3 (2.9 to 3.7)	2.11 (1.81 to 2.44)	0.93 (0.83 to 1.04)
2020	0.0 (0.0 to 0.0)	0.0 (0.0 to 0.0)	5.6 (4.8 to 6.5)	2.6 (2.2 to 3.0)	1.77 (1.52 to 2.05)	0.82 (0.71 to 0.95)
2021	0.0 (0.0 to 0.0)	0.0 (0.0 to 0.0)	6.3 (4.9 to 8.0)	2.9 (2.3 to 3.7)	1.84 (1.44 to 2.35)	0.85 (0.67 to 1.07)

Leeds GBR_44686 Western Europe Stillbirth Rate / Neonatal Mortality Rate (SBR/NMR) − 20 weeks
lambda = 0.6 ; zeta = 0.8 ; scale = 15 ; amp = 0.028

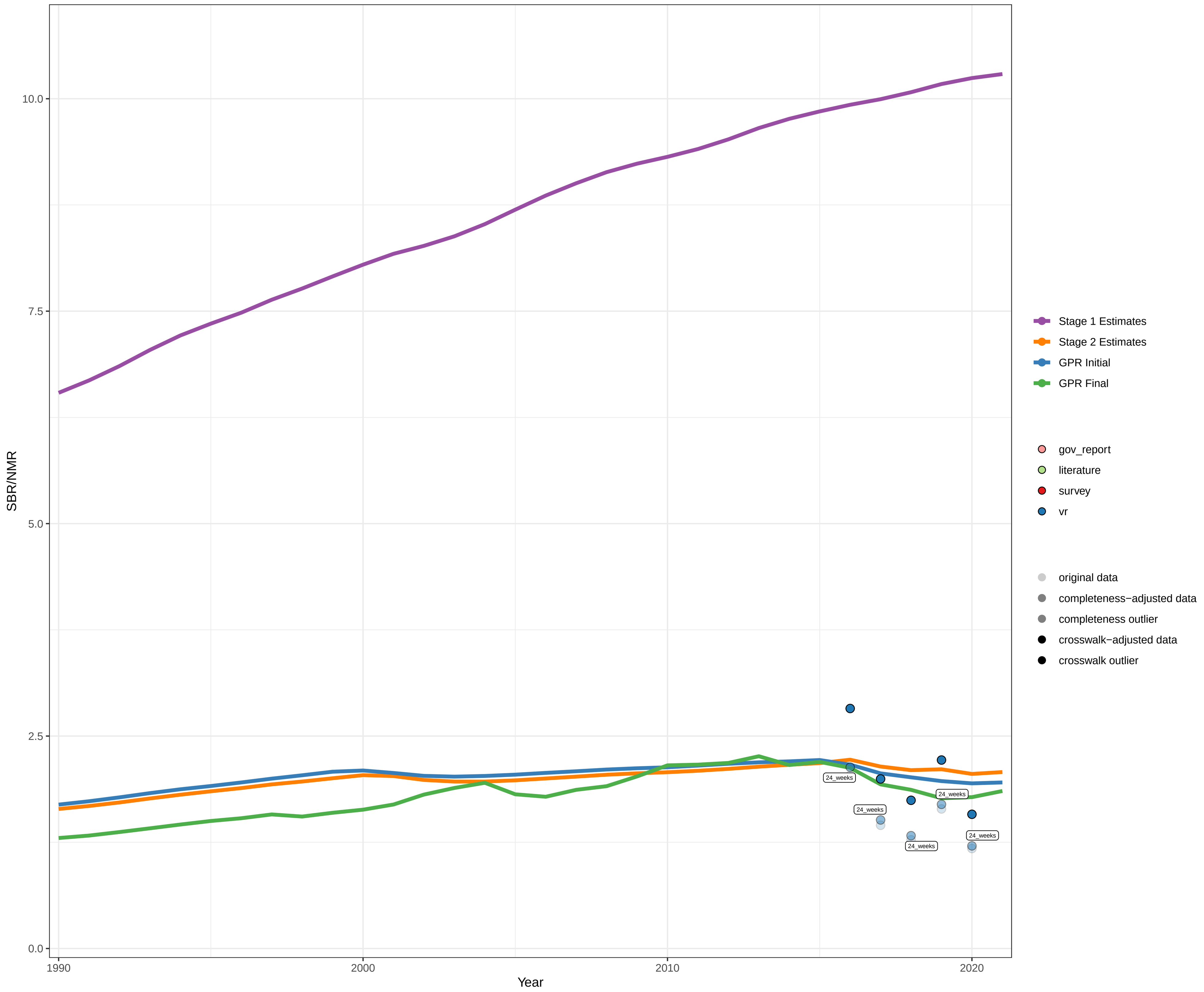

Leeds GBR_44686 Western Europe Stillbirth Rate / Neonatal Mortality Rate (SBR/NMR) − 28 weeks
lambda = 0.6 ; zeta = 0.8 ; scale = 15 ; amp = 0.028

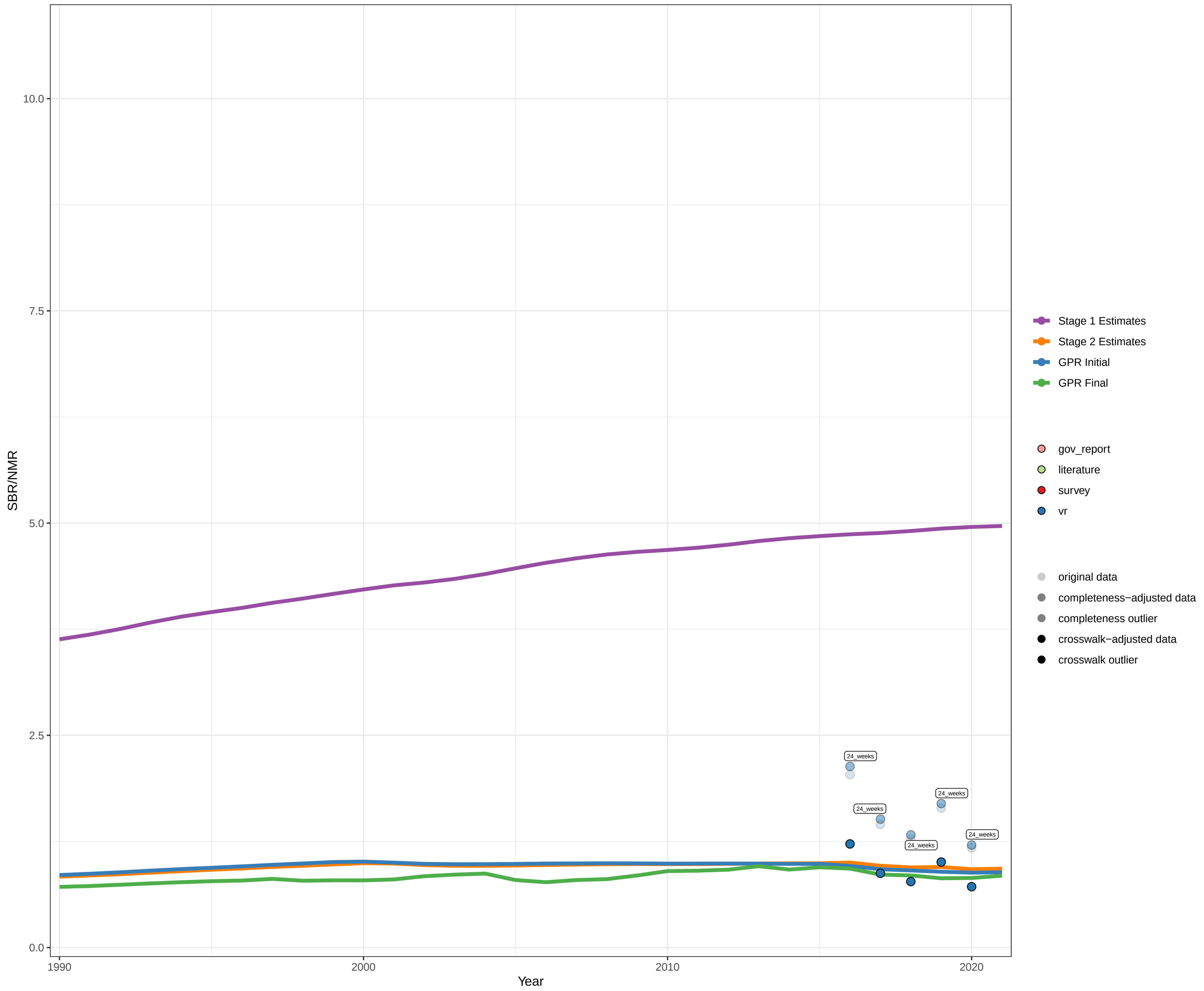

Definition	Years	Outlier	Source.Type	Title
24_weeks	2016−2020	0	VR	United Kingdom − England and Wales Child Mortality Statistics

Leeds GBR_44686 Western Europe Stillbirth Rate (SBR) − 20 weeks
lambda = 0.6 ; zeta = 0.8 ; scale = 15 ; amp = 0.028

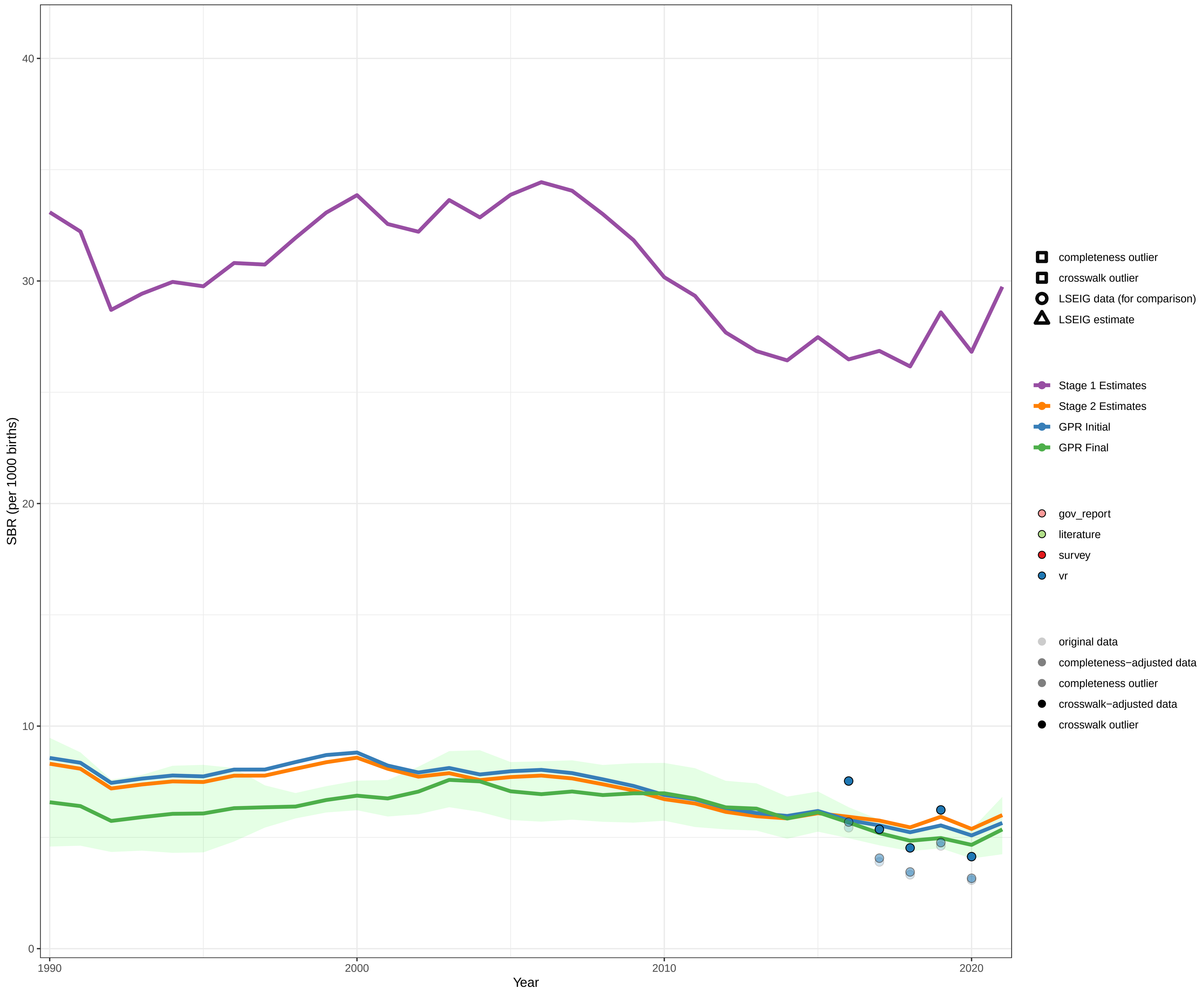

Leeds GBR_44686 Western Europe Stillbirth Rate (SBR) − 28 weeks
lambda = 0.6 ; zeta = 0.8 ; scale = 15 ; amp = 0.028

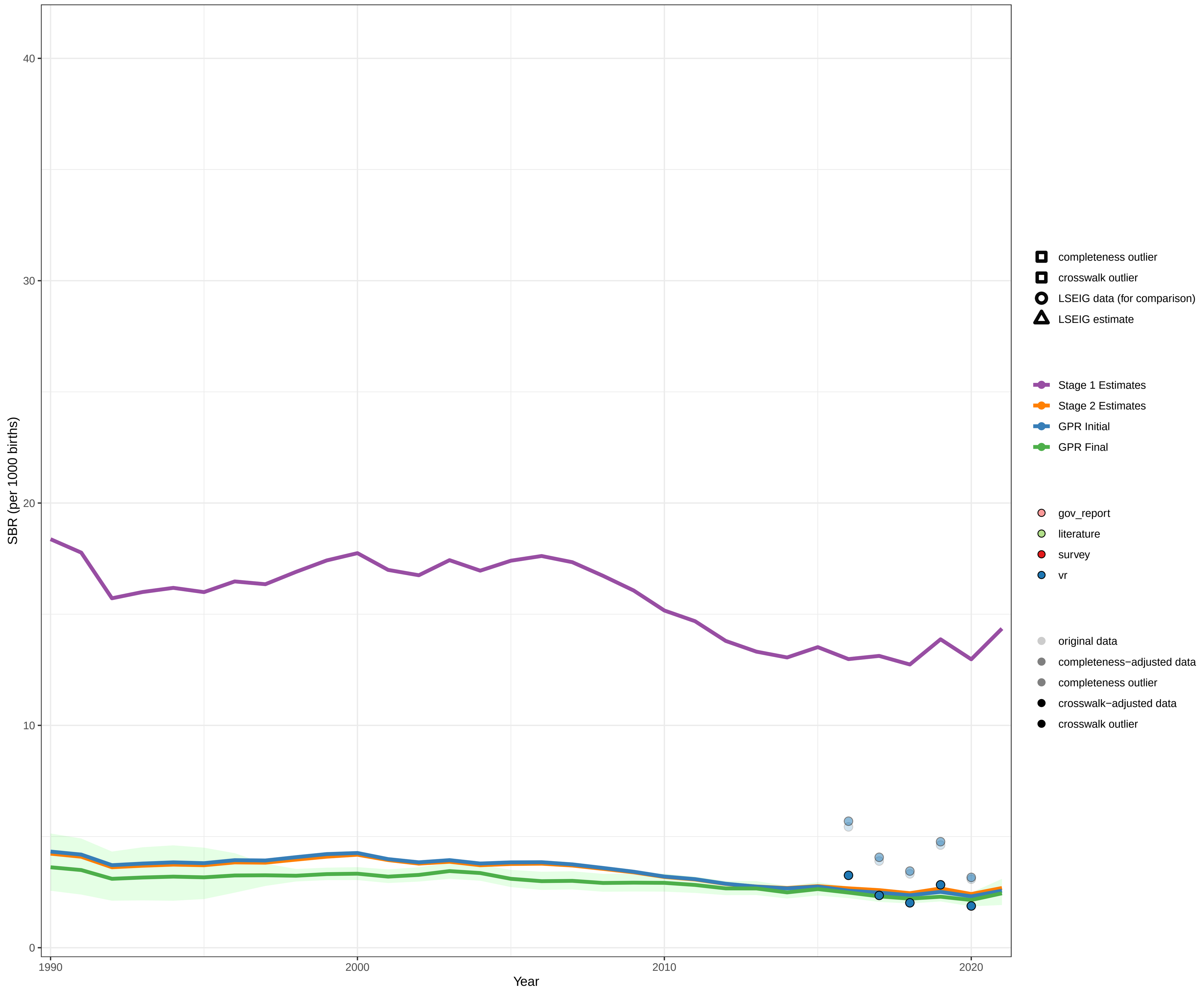

Year	Total Stillbirths (in thousands)	Total Stillbirths (in thousands)	SBR (per 1000 births)	SBR (per 1000 births)	SBR/NMR	SBR/NMR
	20 weeks	28 weeks	20 weeks	28 weeks	20 weeks	28 weeks
1990	0.1<br>(0.0 to 0.1)	0.0<br>(0.0 to 0.1)	6.6<br>(4.6 to 9.5)	3.6<br>(2.6 to 5.1)	1.30<br>(0.91 to 1.87)	0.71<br>(0.51 to 1.02)
1995	0.1<br>(0.0 to 0.1)	0.0<br>(0.0 to 0.0)	6.1<br>(4.3 to 8.3)	3.2<br>(2.2 to 4.5)	1.50<br>(1.07 to 2.04)	0.78<br>(0.54 to 1.11)
2000	0.1<br>(0.0 to 0.1)	0.0<br>(0.0 to 0.0)	6.9<br>(6.2 to 7.5)	3.3<br>(3.0 to 3.6)	1.63<br>(1.48 to 1.79)	0.79<br>(0.72 to 0.86)
2005	0.1<br>(0.1 to 0.1)	0.0<br>(0.0 to 0.0)	7.1<br>(5.8 to 8.4)	3.1<br>(2.7 to 3.5)	1.81<br>(1.48 to 2.15)	0.80<br>(0.70 to 0.90)
2010	0.1<br>(0.1 to 0.1)	0.0<br>(0.0 to 0.0)	7.0<br>(5.7 to 8.3)	2.9<br>(2.5 to 3.3)	2.16<br>(1.77 to 2.58)	0.90<br>(0.78 to 1.03)
2015	0.1<br>(0.1 to 0.1)	0.0<br>(0.0 to 0.0)	6.1<br>(5.3 to 7.1)	2.6<br>(2.3 to 2.9)	2.20<br>(1.88 to 2.53)	0.95<br>(0.84 to 1.06)
2020	0.0<br>(0.0 to 0.0)	0.0<br>(0.0 to 0.0)	4.7<br>(4.1 to 5.4)	2.1<br>(1.9 to 2.5)	1.78<br>(1.55 to 2.05)	0.82<br>(0.71 to 0.95)
2021	0.0<br>(0.0 to 0.1)	0.0<br>(0.0 to 0.0)	5.4<br>(4.2 to 6.8)	2.4<br>(1.9 to 3.1)	1.85<br>(1.47 to 2.36)	0.85<br>(0.66 to 1.07)

North East Lincolnshire   GBR_44681   Western Europe   Stillbirth Rate / Neonatal Mortality Rate (SBR/NMR) − 20 weeks
lambda = 0.6 ; zeta = 0.8 ; scale = 15 ; amp = 0.028

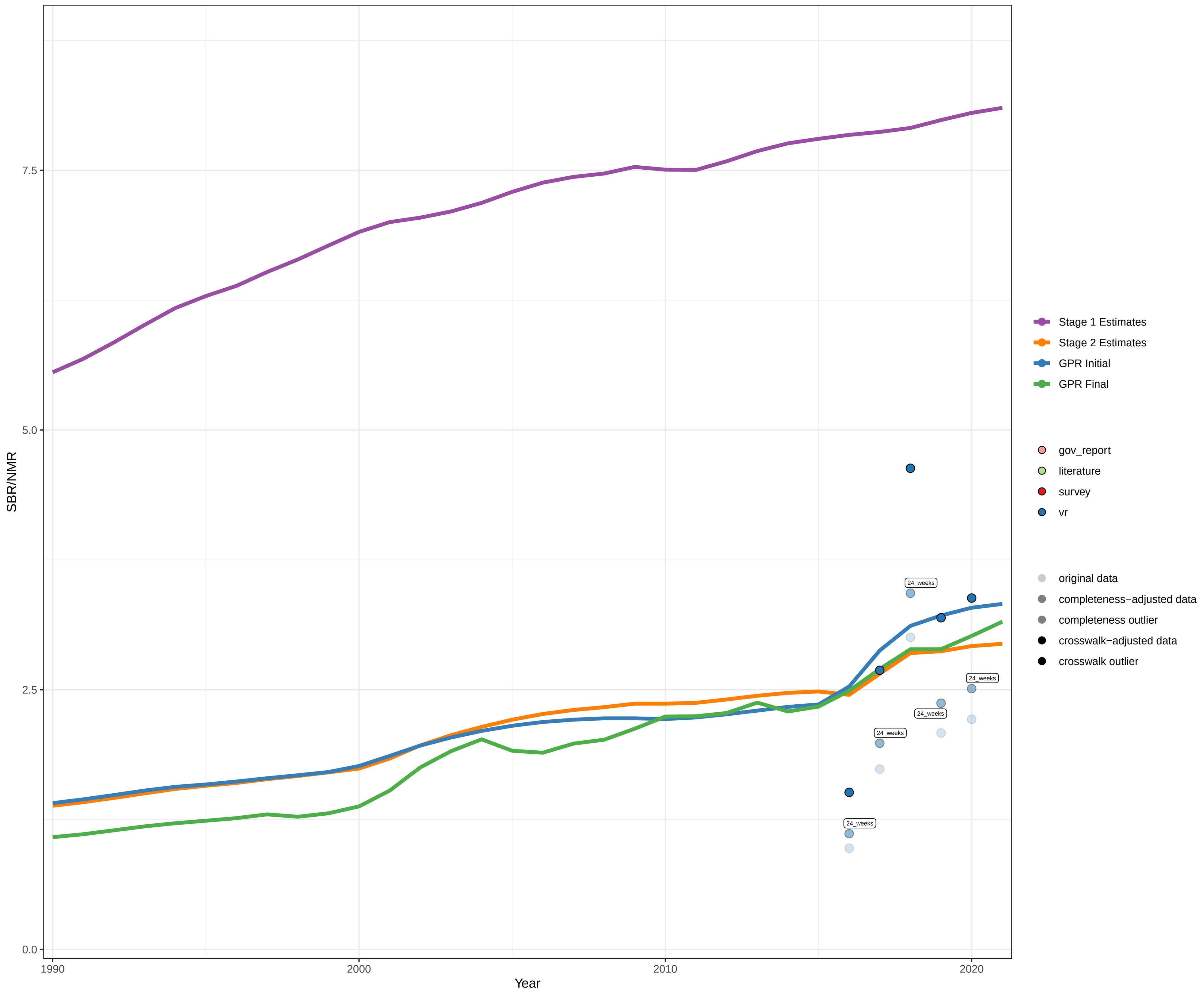

North East Lincolnshire   GBR_44681   Western Europe   Stillbirth Rate / Neonatal Mortality Rate (SBR/NMR) − 28 weeks
lambda = 0.6 ; zeta = 0.8 ; scale = 15 ; amp = 0.028

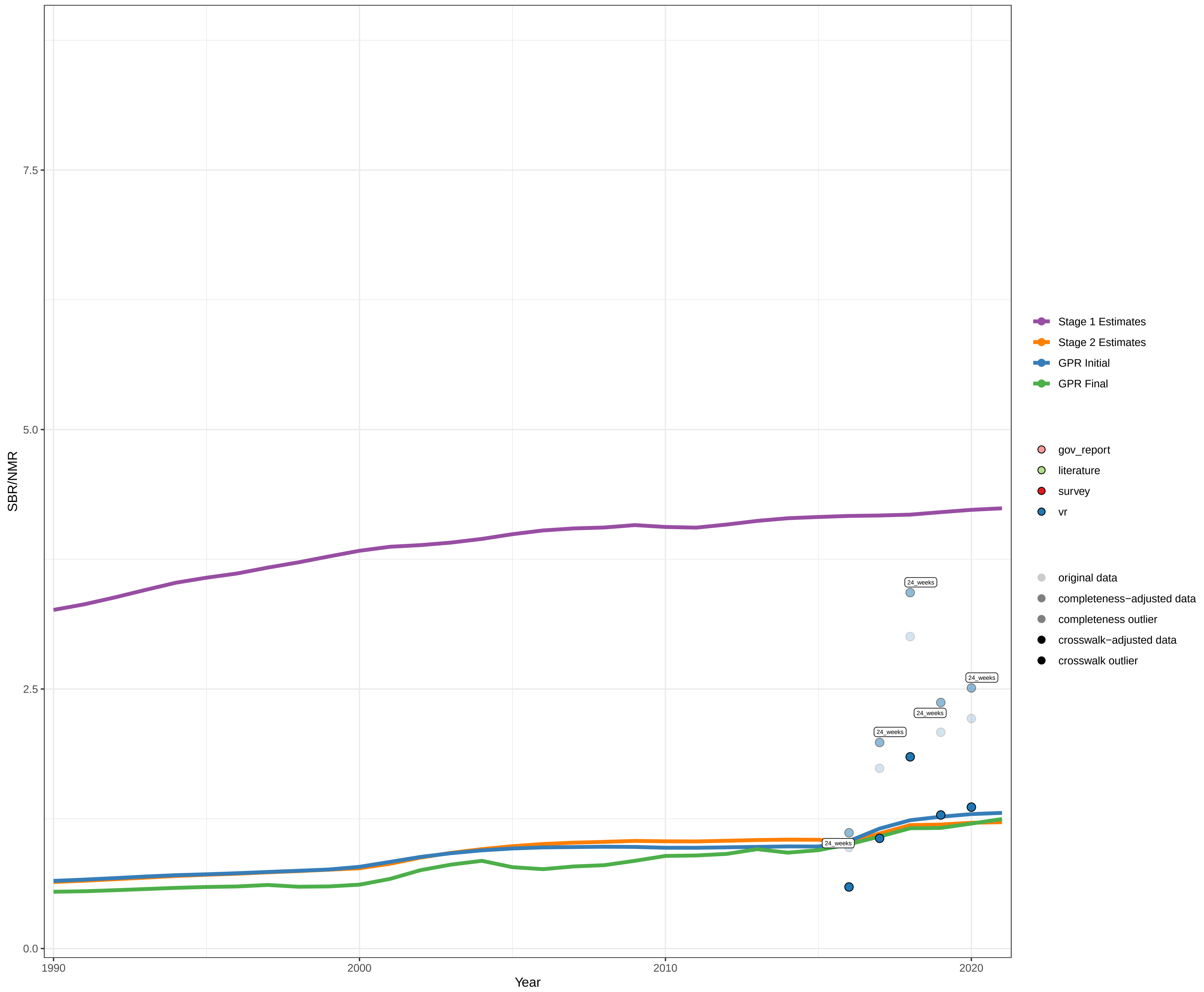

Definition	Years	Outlier	Source.Type	Title
24_weeks	2016−2020	0	VR	United Kingdom − England and Wales Child Mortality Statistics

North East Lincolnshire   GBR_44681   Western Europe   Stillbirth Rate (SBR) − 20 weeks
lambda = 0.6 ; zeta = 0.8 ; scale = 15 ; amp = 0.028

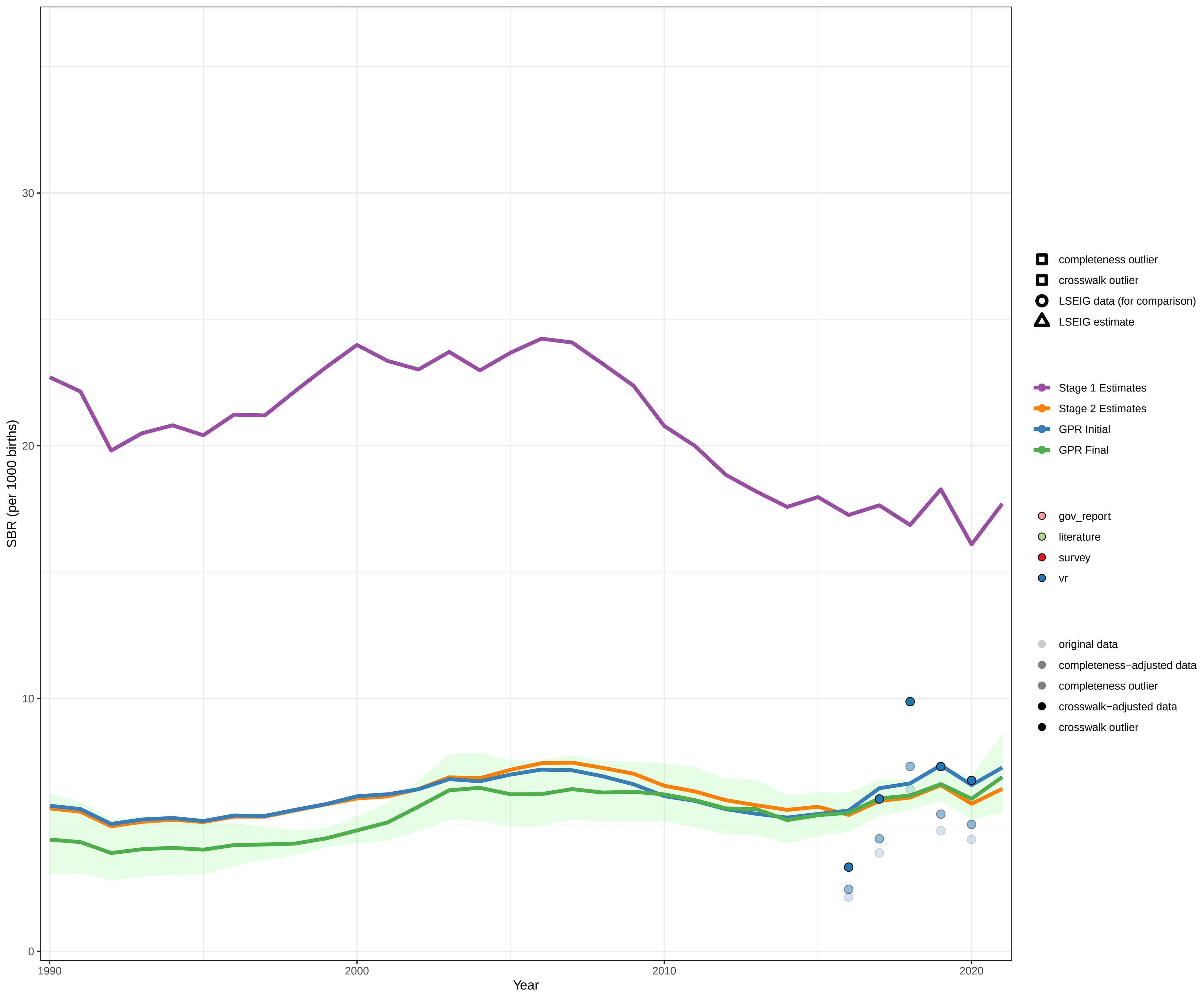

North East Lincolnshire   GBR_44681   Western Europe   Stillbirth Rate (SBR) − 28 weeks
lambda = 0.6 ; zeta = 0.8 ; scale = 15 ; amp = 0.028

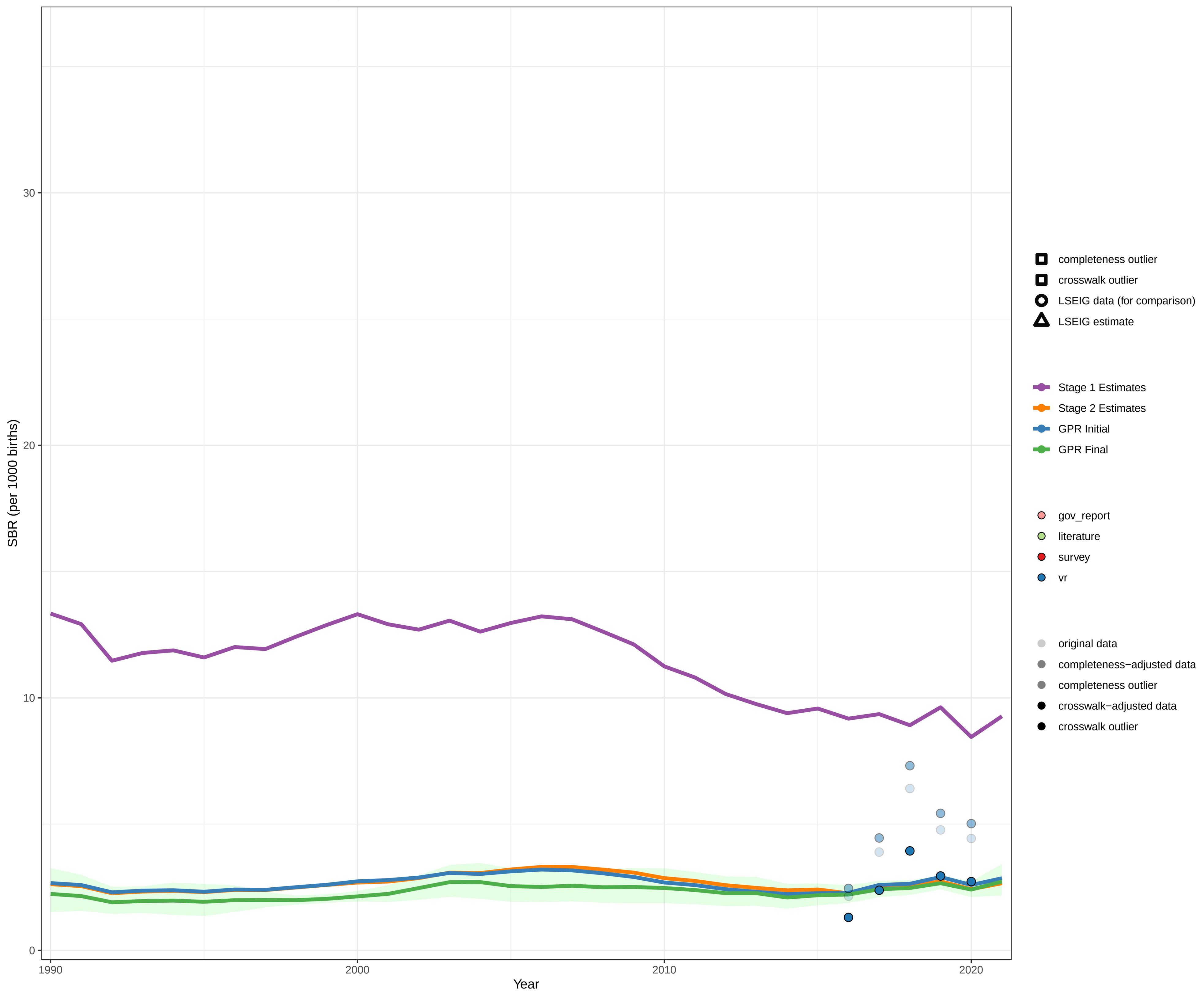

Year	Total Stillbirths (in thousands)	Total Stillbirths (in thousands)	SBR (per 1000 births)	SBR (per 1000 births)	SBR/NMR	SBR/NMR
	20 weeks	28 weeks	20 weeks	28 weeks	20 weeks	28 weeks
1990	0.0 (0.0 to 0.0)	0.0 (0.0 to 0.0)	4.4 (3.0 to 6.2)	2.2 (1.5 to 3.3)	1.08 (0.75 to 1.53)	0.55 (0.37 to 0.80)
1995	0.0 (0.0 to 0.0)	0.0 (0.0 to 0.0)	4.0 (3.1 to 5.1)	1.9 (1.4 to 2.6)	1.24 (0.94 to 1.56)	0.59 (0.42 to 0.81)
2000	0.0 (0.0 to 0.0)	0.0 (0.0 to 0.0)	4.8 (4.3 to 5.3)	2.1 (1.9 to 2.4)	1.38 (1.23 to 1.54)	0.62 (0.56 to 0.68)
2005	0.0 (0.0 to 0.0)	0.0 (0.0 to 0.0)	6.2 (4.9 to 7.5)	2.5 (1.9 to 3.3)	1.91 (1.52 to 2.32)	0.78 (0.59 to 1.00)
2010	0.0 (0.0 to 0.0)	0.0 (0.0 to 0.0)	6.2 (5.2 to 7.4)	2.5 (1.9 to 3.2)	2.24 (1.86 to 2.69)	0.89 (0.68 to 1.17)
2015	0.0 (0.0 to 0.0)	0.0 (0.0 to 0.0)	5.4 (4.6 to 6.3)	2.2 (1.8 to 2.6)	2.34 (1.98 to 2.73)	0.95 (0.78 to 1.15)
2020	0.0 (0.0 to 0.0)	0.0 (0.0 to 0.0)	6.0 (5.2 to 6.9)	2.4 (2.1 to 2.7)	3.02 (2.62 to 3.46)	1.20 (1.06 to 1.37)
2021	0.0 (0.0 to 0.0)	0.0 (0.0 to 0.0)	6.9 (5.5 to 8.6)	2.7 (2.2 to 3.4)	3.15 (2.51 to 3.95)	1.25 (1.00 to 1.56)

North Lincolnshire GBR_44683 Western Europe Stillbirth Rate / Neonatal Mortality Rate (SBR/NMR) − 20 weeks
lambda = 0.8 ; zeta = 0.7 ; scale = 15 ; amp = 0.028

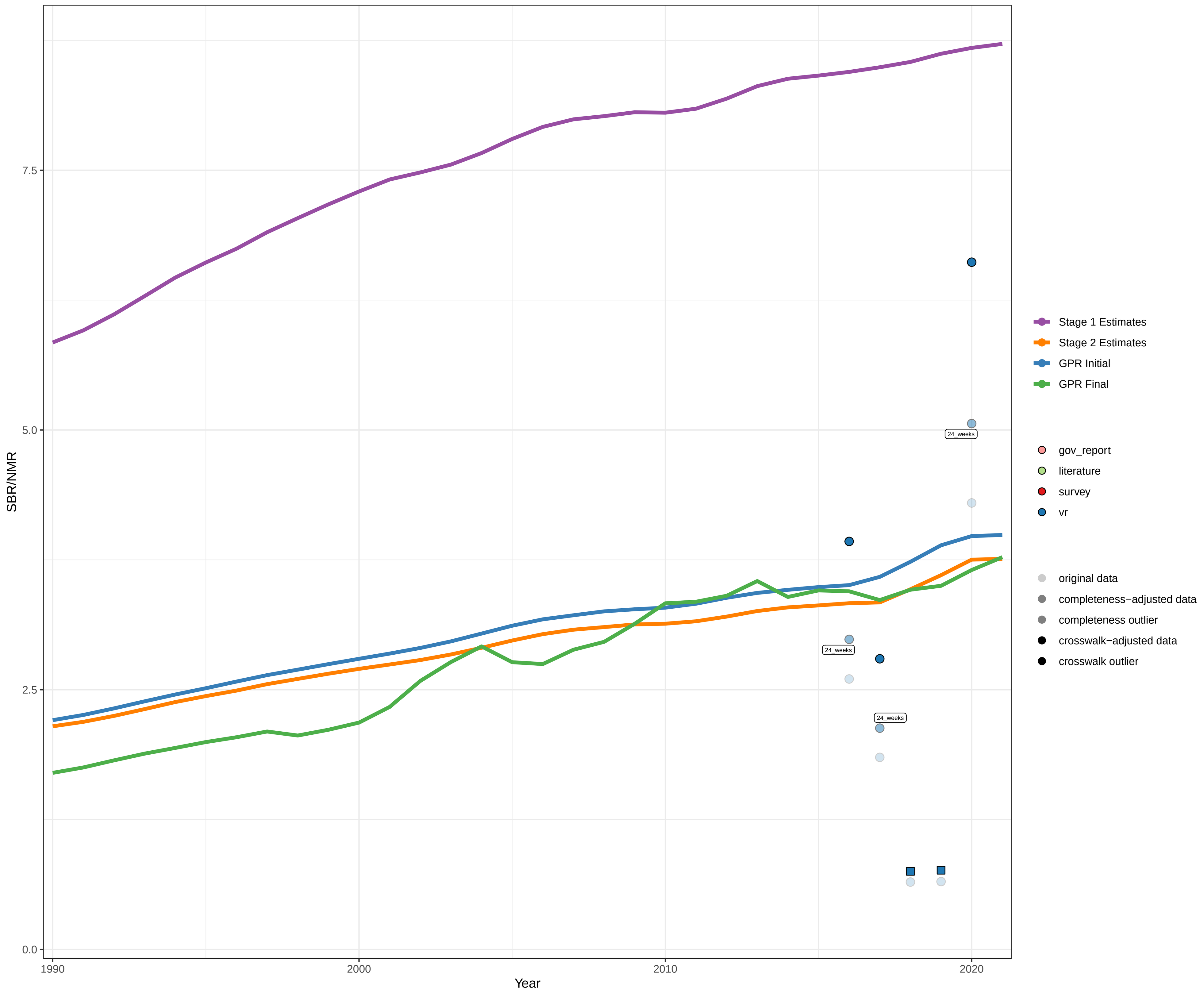

North Lincolnshire GBR_44683 Western Europe Stillbirth Rate / Neonatal Mortality Rate (SBR/NMR) − 28 weeks
lambda = 0.8 ; zeta = 0.7 ; scale = 15 ; amp = 0.028

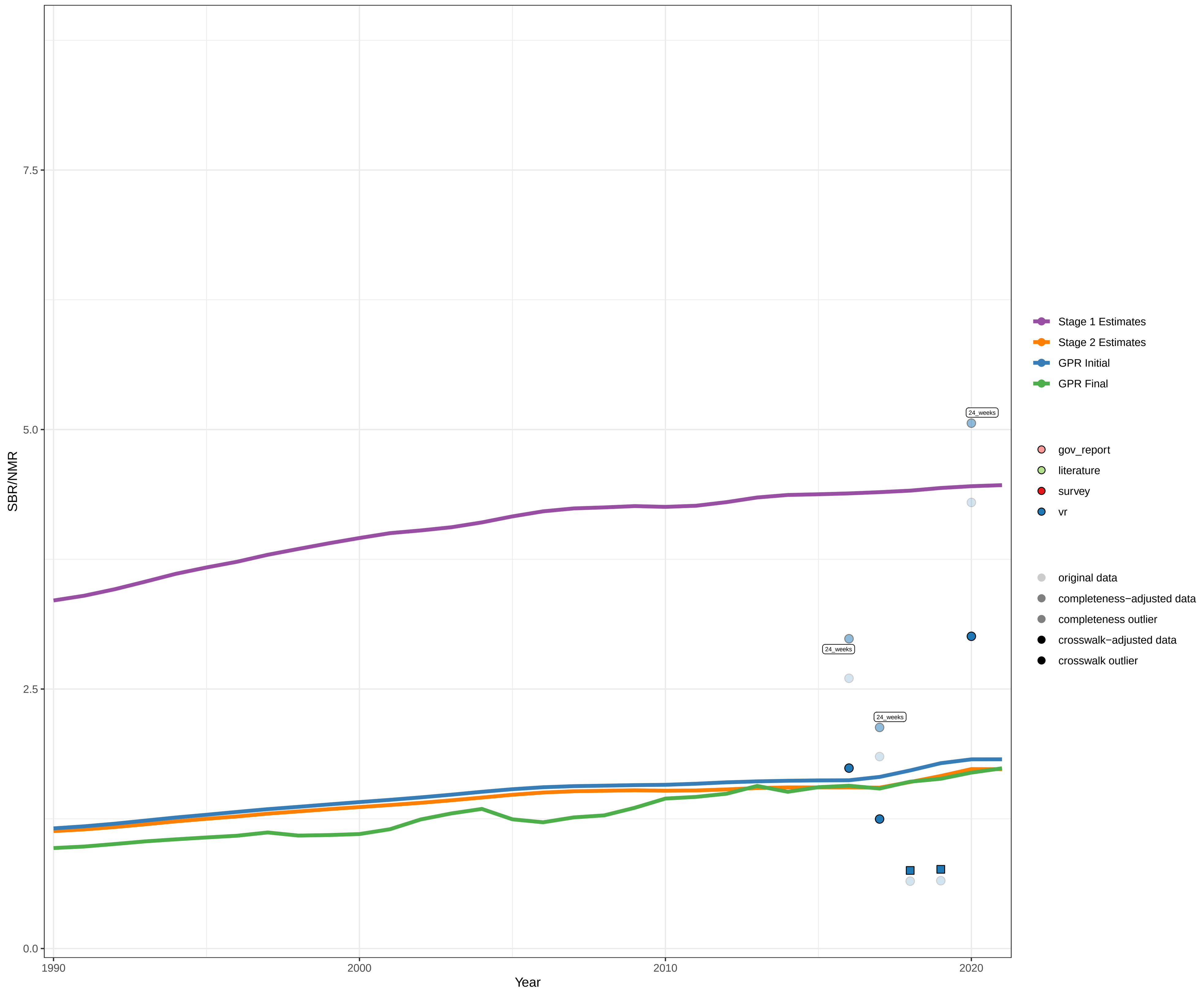

Definition	Years	Outlier	Source.Type	Title
24_weeks	2016−2020	0	VR	United Kingdom − England and Wales Child Mortality Statistics

North Lincolnshire GBR_44683 Western Europe Stillbirth Rate (SBR) − 20 weeks
lambda = 0.8 ; zeta = 0.7 ; scale = 15 ; amp = 0.028

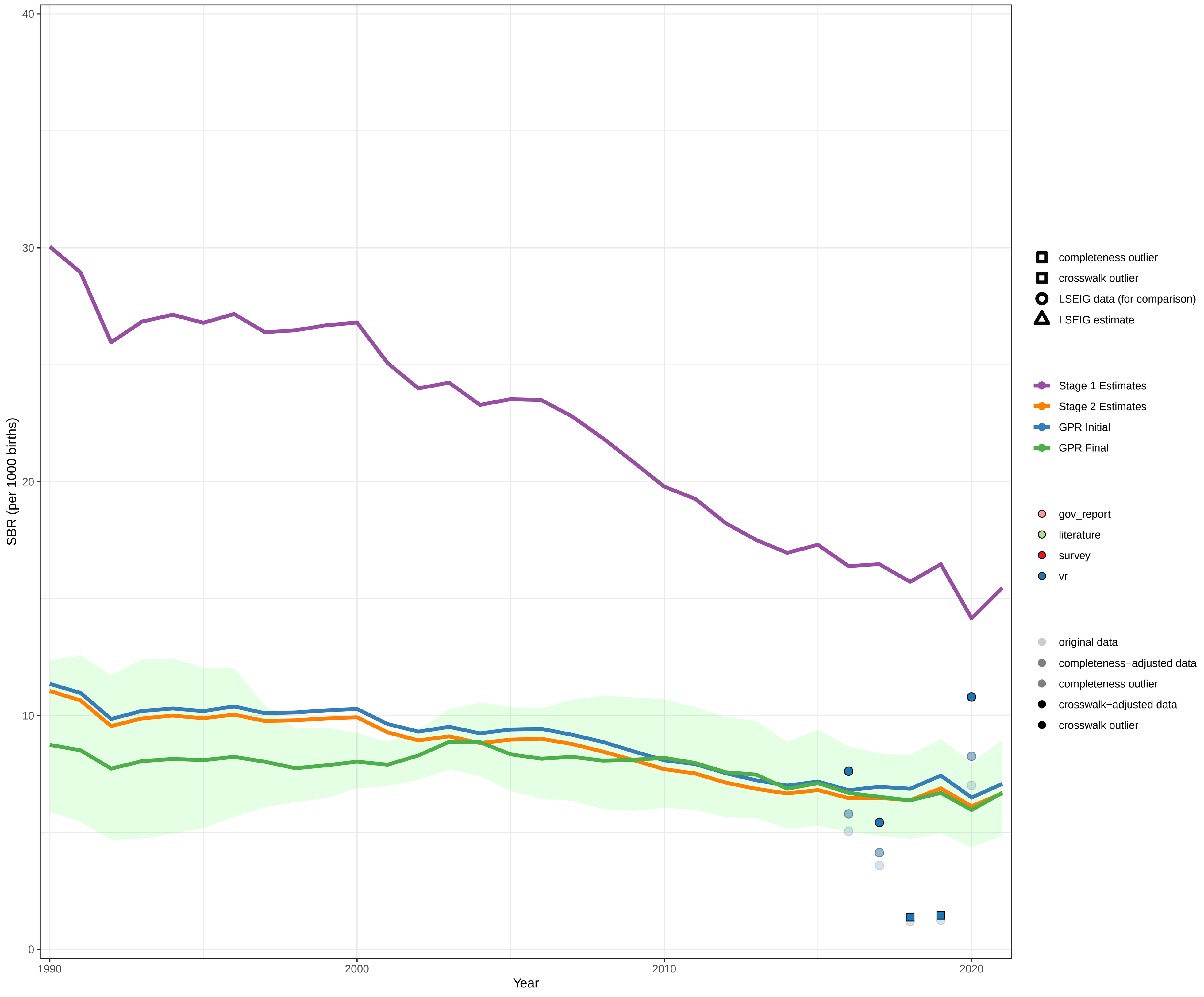

North Lincolnshire GBR_44683 Western Europe Stillbirth Rate (SBR) − 28 weeks
lambda = 0.8 ; zeta = 0.7 ; scale = 15 ; amp = 0.028

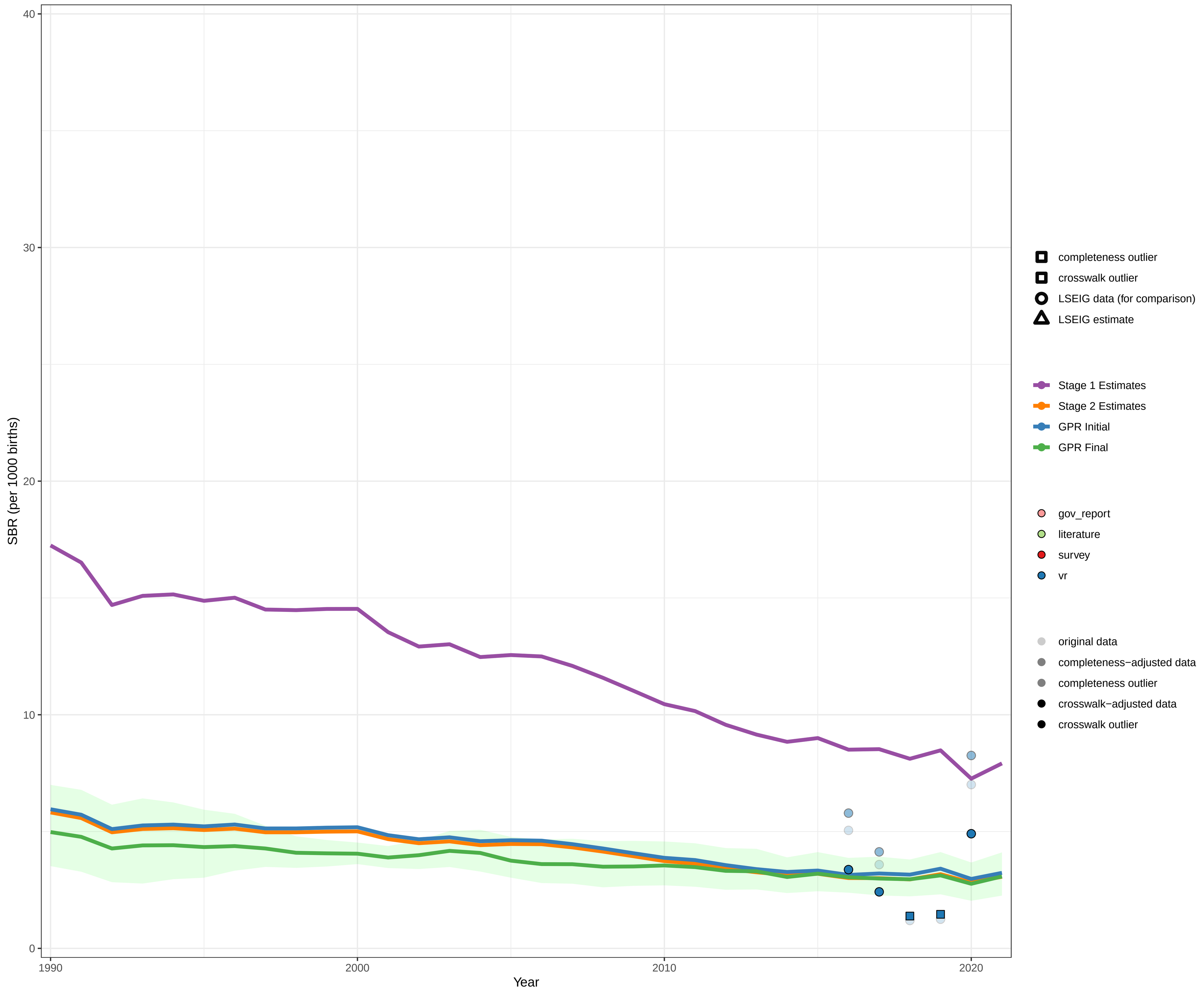

Year	Total Stillbirths (in thousands)	Total Stillbirths (in thousands)	SBR (per 1000 births)	SBR (per 1000 births)	SBR/NMR	SBR/NMR
	20 weeks	28 weeks	20 weeks	28 weeks	20 weeks	28 weeks
1990	0.0<br>(0.0 to 0.0)	0.0<br>(0.0 to 0.0)	8.7<br>(5.9 to 12.4)	5.0<br>(3.5 to 7.0)	1.70<br>(1.14 to 2.40)	0.97<br>(0.68 to 1.36)
1995	0.0<br>(0.0 to 0.0)	0.0<br>(0.0 to 0.0)	8.1<br>(5.2 to 12.0)	4.3<br>(3.0 to 5.9)	2.00<br>(1.28 to 2.97)	1.07<br>(0.75 to 1.47)
2000	0.0<br>(0.0 to 0.0)	0.0<br>(0.0 to 0.0)	8.0<br>(6.9 to 9.2)	4.1<br>(3.6 to 4.5)	2.18<br>(1.88 to 2.52)	1.10<br>(0.98 to 1.23)
2005	0.0<br>(0.0 to 0.0)	0.0<br>(0.0 to 0.0)	8.3<br>(6.8 to 10.4)	3.8<br>(3.0 to 4.8)	2.76<br>(2.24 to 3.43)	1.24<br>(1.00 to 1.58)
2010	0.0<br>(0.0 to 0.0)	0.0<br>(0.0 to 0.0)	8.2<br>(6.1 to 10.7)	3.5<br>(2.7 to 4.6)	3.33<br>(2.46 to 4.35)	1.44<br>(1.10 to 1.86)
2015	0.0<br>(0.0 to 0.0)	0.0<br>(0.0 to 0.0)	7.1<br>(5.3 to 9.4)	3.2<br>(2.4 to 4.1)	3.46<br>(2.57 to 4.57)	1.55<br>(1.19 to 2.00)
2020	0.0<br>(0.0 to 0.0)	0.0<br>(0.0 to 0.0)	6.0<br>(4.4 to 8.0)	2.8<br>(2.0 to 3.7)	3.65<br>(2.67 to 4.88)	1.69<br>(1.25 to 2.25)
2021	0.0<br>(0.0 to 0.0)	0.0<br>(0.0 to 0.0)	6.7<br>(4.8 to 9.0)	3.1<br>(2.3 to 4.1)	3.78<br>(2.73 to 5.06)	1.74<br>(1.27 to 2.32)

North Yorkshire GBR_44678 Western Europe Stillbirth Rate / Neonatal Mortality Rate (SBR/NMR) − 20 weeks
lambda = 0.6 ; zeta = 0.8 ; scale = 15 ; amp = 0.028

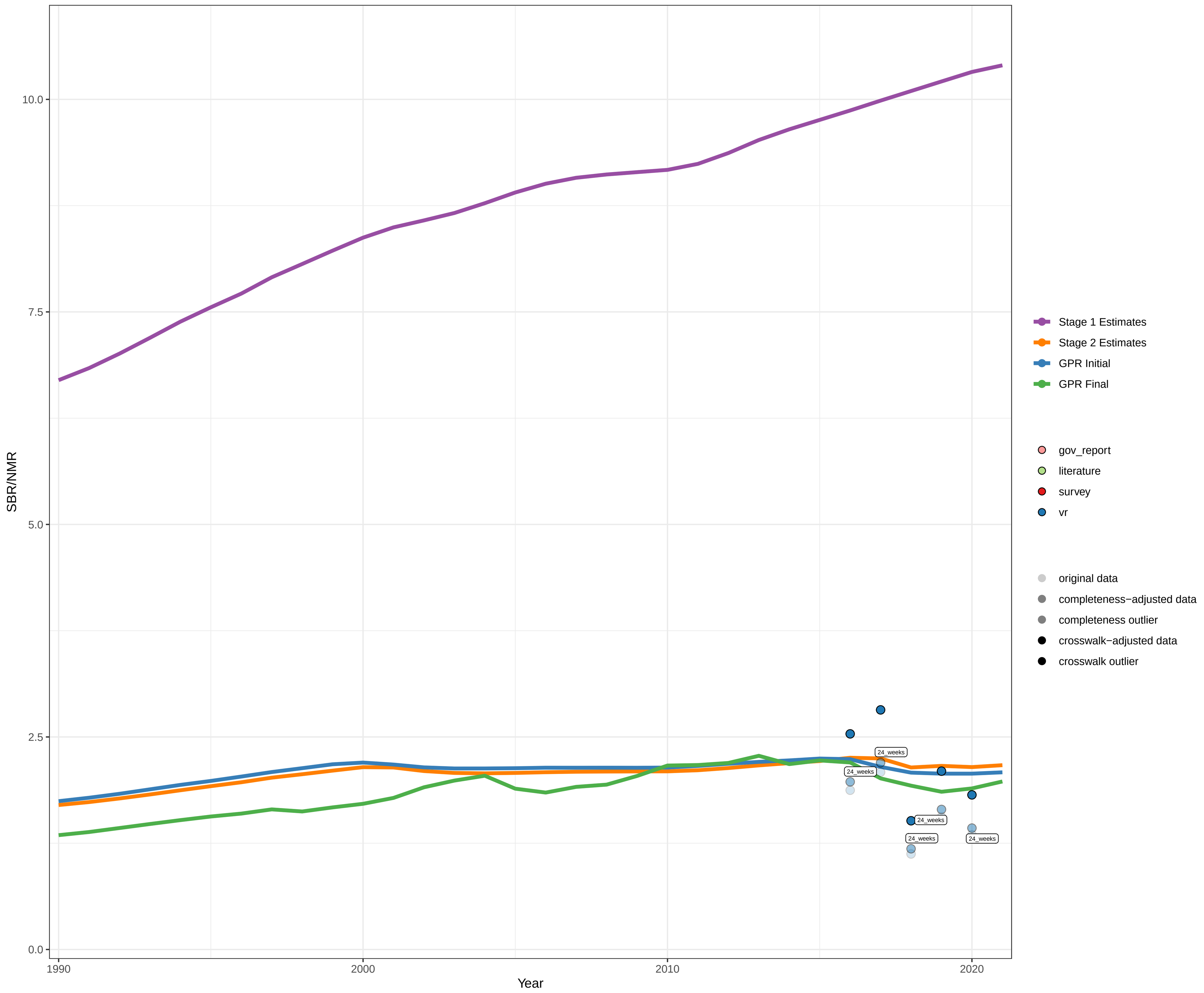

North Yorkshire GBR_44678 Western Europe Stillbirth Rate / Neonatal Mortality Rate (SBR/NMR) − 28 weeks
lambda = 0.6 ; zeta = 0.8 ; scale = 15 ; amp = 0.028

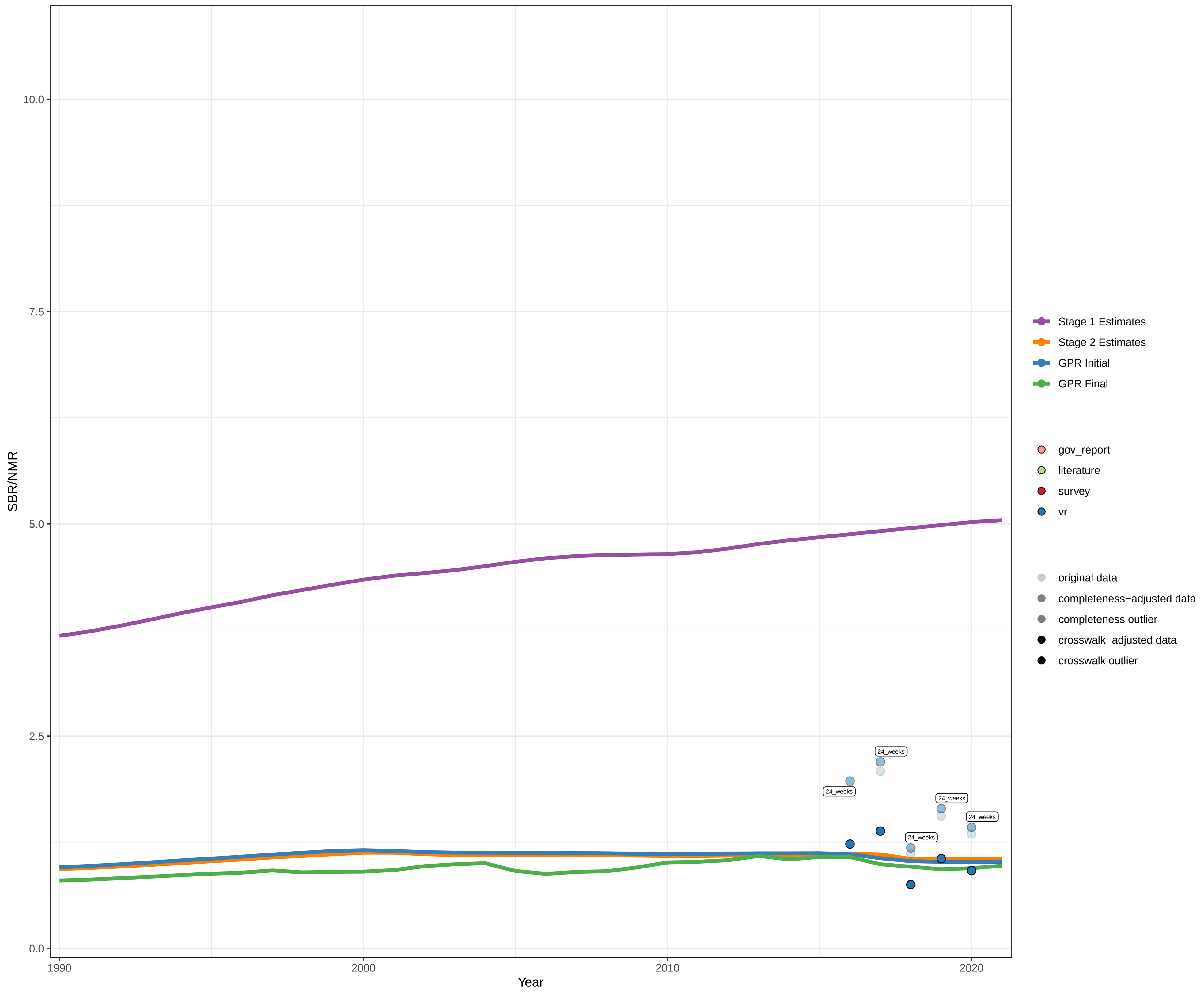

Definition	Years	Outlier	Source.Type	Title
24_weeks	2016−2020	0	VR	United Kingdom − England and Wales Child Mortality Statistics

North Yorkshire GBR_44678 Western Europe Stillbirth Rate (SBR) − 20 weeks
lambda = 0.6 ; zeta = 0.8 ; scale = 15 ; amp = 0.028

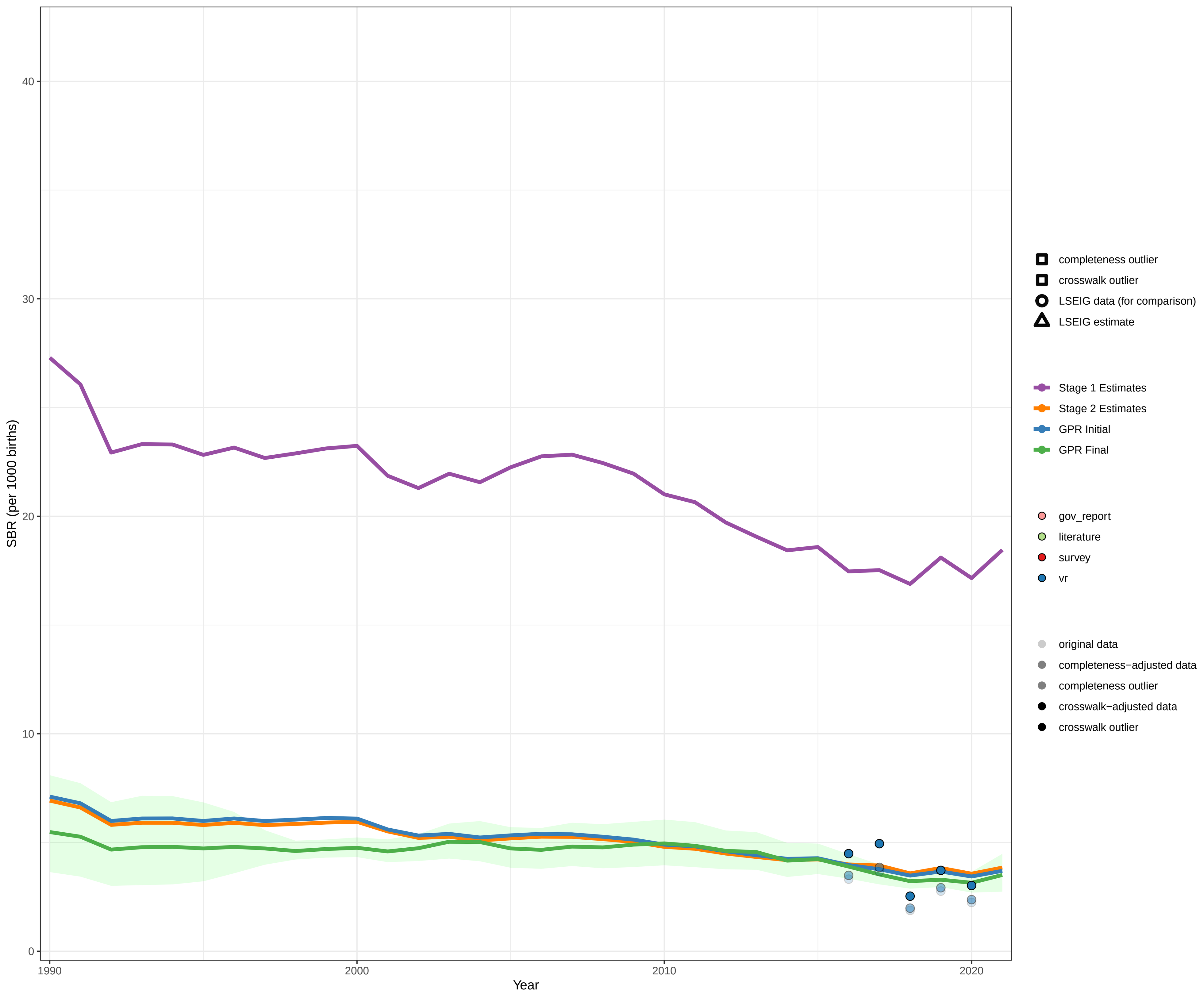

North Yorkshire GBR_44678 Western Europe Stillbirth Rate (SBR) − 28 weeks
lambda = 0.6 ; zeta = 0.8 ; scale = 15 ; amp = 0.028

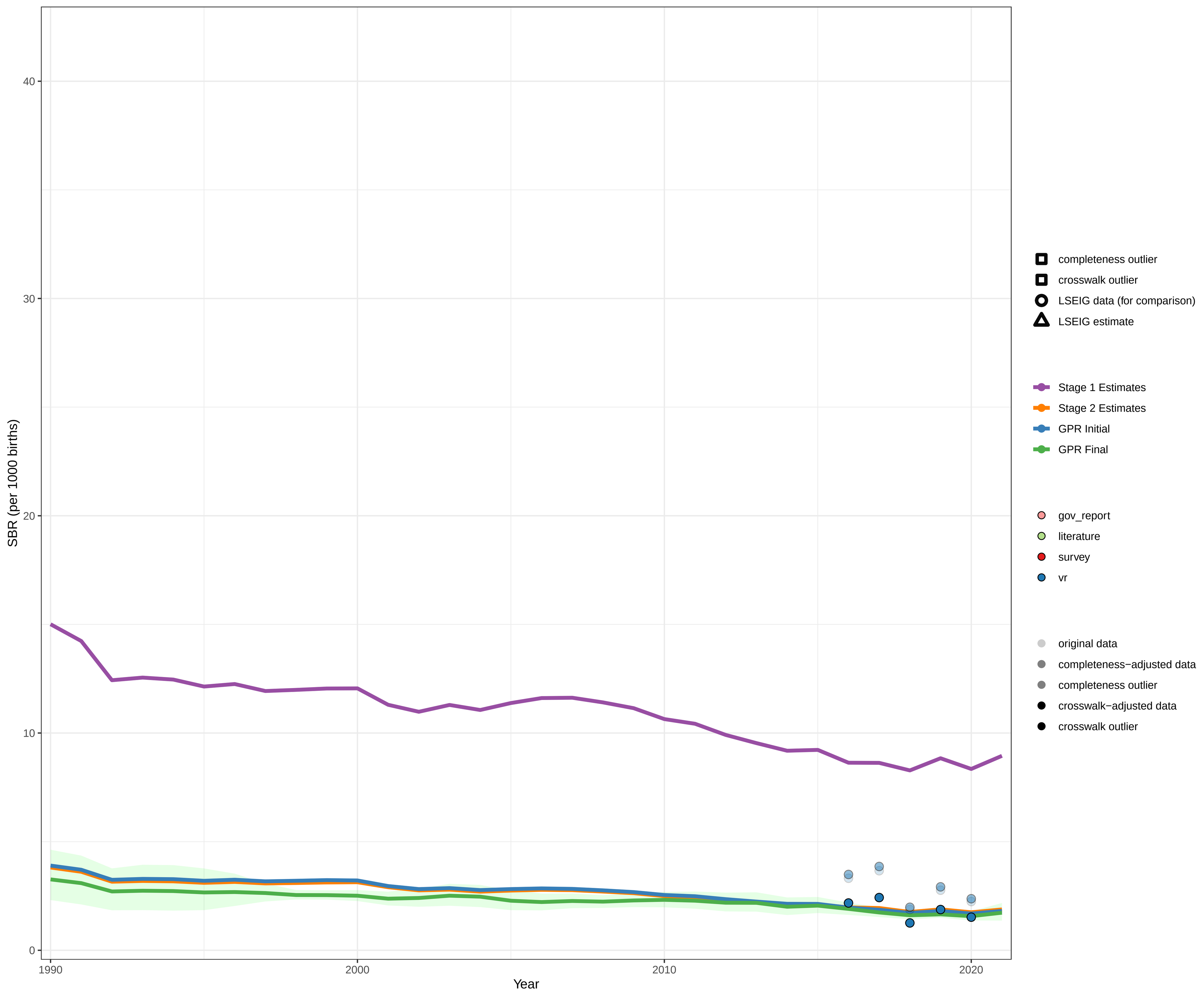

Year	Total Stillbirths (in thousands)	Total Stillbirths (in thousands)	SBR (per 1000 births)	SBR (per 1000 births)	SBR/NMR	SBR/NMR
	20 weeks	28 weeks	20 weeks	28 weeks	20 weeks	28 weeks
1990	0.0 (0.0 to 0.1)	0.0 (0.0 to 0.0)	5.5 (3.6 to 8.1)	3.3 (2.3 to 4.6)	1.34 (0.89 to 1.99)	0.80 (0.57 to 1.14)
1995	0.0 (0.0 to 0.0)	0.0 (0.0 to 0.0)	4.7 (3.2 to 6.8)	2.7 (1.8 to 3.8)	1.56 (1.06 to 2.27)	0.88 (0.61 to 1.25)
2000	0.0 (0.0 to 0.0)	0.0 (0.0 to 0.0)	4.8 (4.3 to 5.2)	2.5 (2.3 to 2.8)	1.71 (1.56 to 1.89)	0.91 (0.82 to 1.00)
2005	0.0 (0.0 to 0.0)	0.0 (0.0 to 0.0)	4.7 (3.8 to 5.7)	2.3 (1.8 to 2.8)	1.89 (1.54 to 2.28)	0.91 (0.74 to 1.10)
2010	0.0 (0.0 to 0.0)	0.0 (0.0 to 0.0)	5.0 (4.0 to 6.1)	2.3 (2.0 to 2.7)	2.16 (1.73 to 2.64)	1.01 (0.86 to 1.17)
2015	0.0 (0.0 to 0.0)	0.0 (0.0 to 0.0)	4.2 (3.5 to 5.0)	2.1 (1.7 to 2.5)	2.22 (1.86 to 2.60)	1.08 (0.90 to 1.29)
2020	0.0 (0.0 to 0.0)	0.0 (0.0 to 0.0)	3.1 (2.7 to 3.7)	1.6 (1.4 to 1.8)	1.89 (1.62 to 2.20)	0.95 (0.83 to 1.08)
2021	0.0 (0.0 to 0.0)	0.0 (0.0 to 0.0)	3.5 (2.7 to 4.5)	1.7 (1.4 to 2.2)	1.98 (1.54 to 2.52)	0.98 (0.77 to 1.23)

Rotherham GBR_44689 Western Europe Stillbirth Rate / Neonatal Mortality Rate (SBR/NMR) − 20 weeks
lambda = 0.6 ; zeta = 0.8 ; scale = 15 ; amp = 0.028

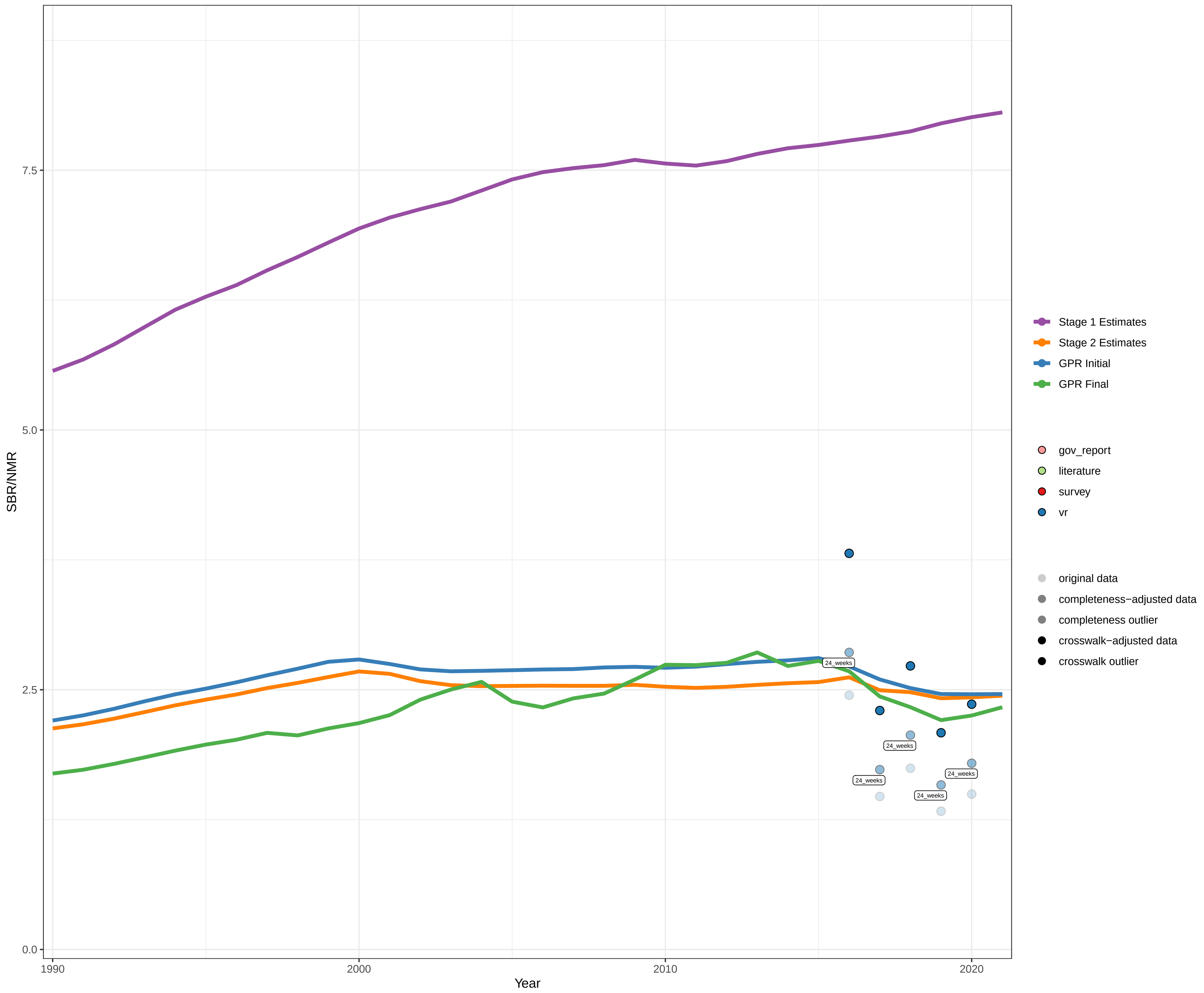

Rotherham GBR_44689 Western Europe Stillbirth Rate / Neonatal Mortality Rate (SBR/NMR) − 28 weeks
lambda = 0.6 ; zeta = 0.8 ; scale = 15 ; amp = 0.028

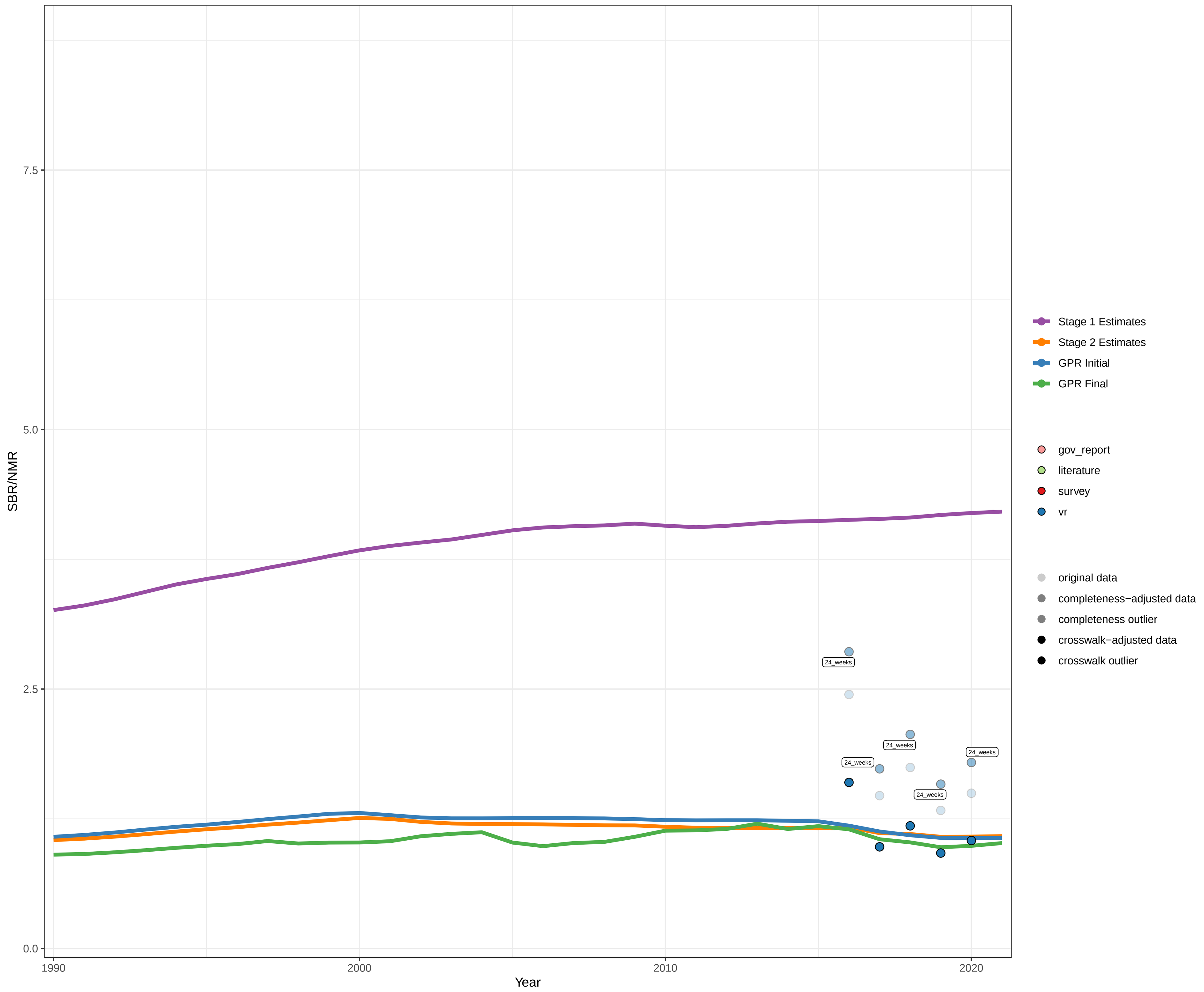

Definition	Years	Outlier	Source.Type	Title
24_weeks	2016−2020	0	VR	United Kingdom − England and Wales Child Mortality Statistics

Rotherham GBR_44689 Western Europe Stillbirth Rate (SBR) − 20 weeks
lambda = 0.6 ; zeta = 0.8 ; scale = 15 ; amp = 0.028

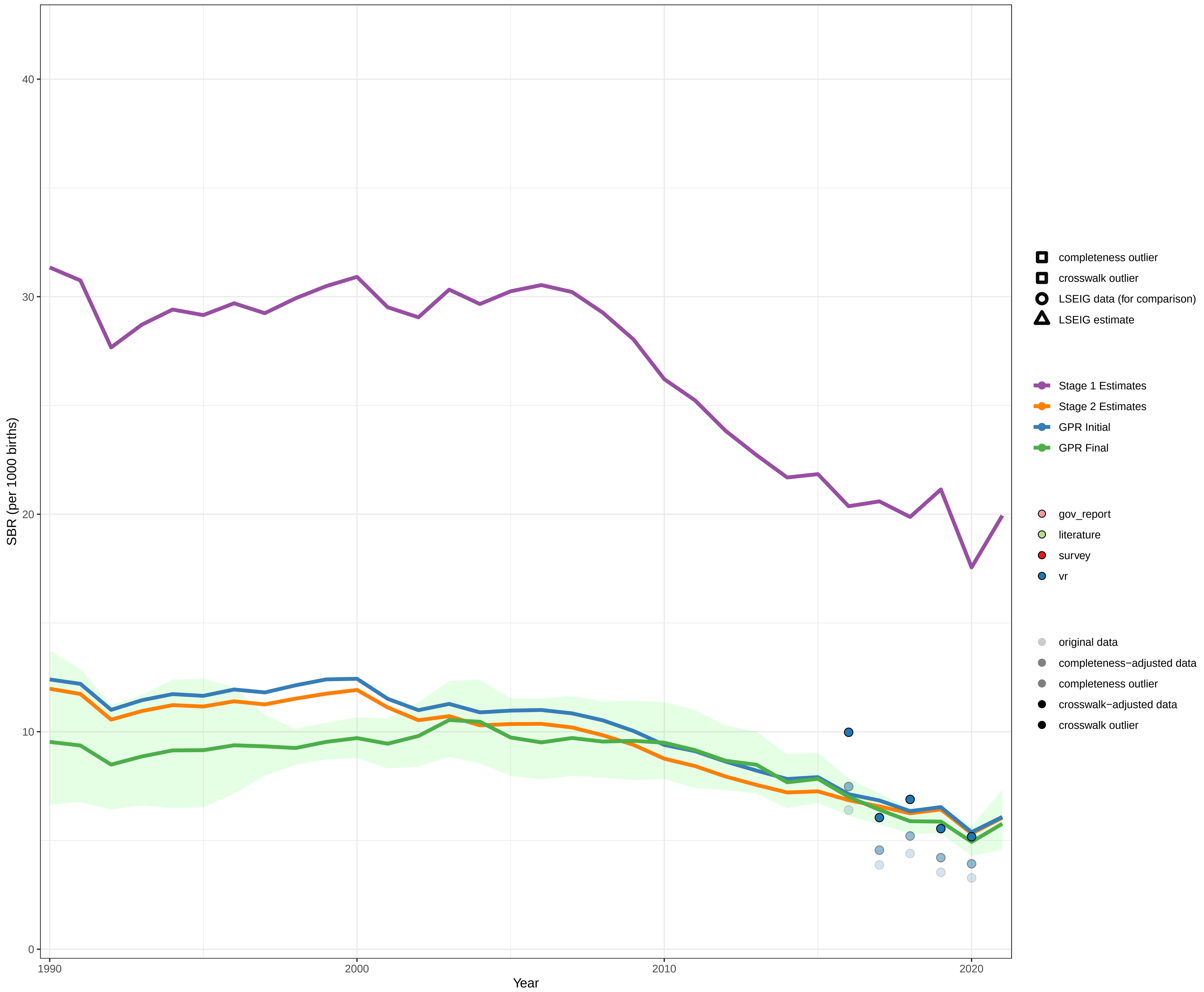

Rotherham GBR_44689 Western Europe Stillbirth Rate (SBR) − 28 weeks
lambda = 0.6 ; zeta = 0.8 ; scale = 15 ; amp = 0.028

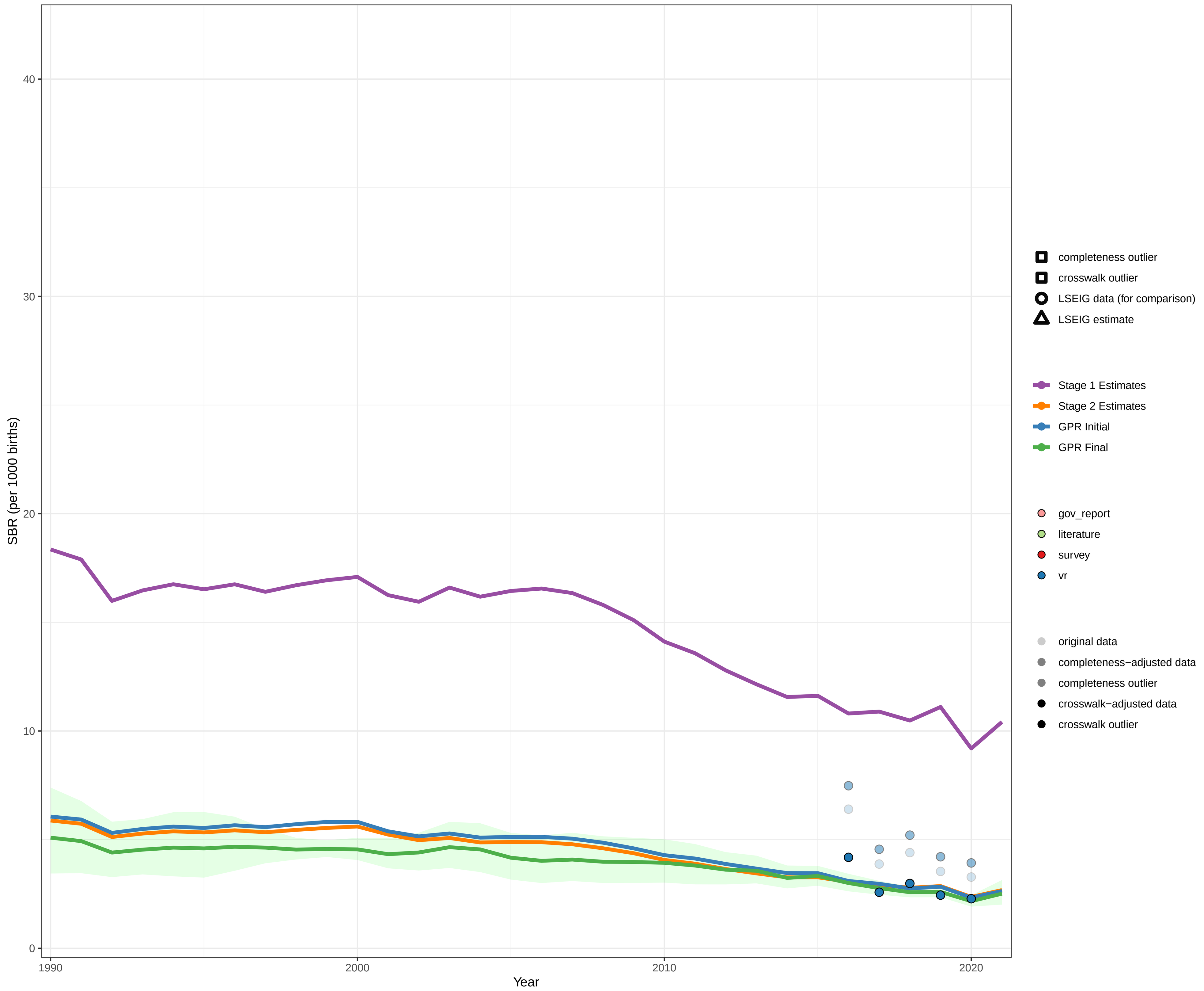

Year	Total Stillbirths (in thousands)	Total Stillbirths (in thousands)	SBR (per 1000 births)	SBR (per 1000 births)	SBR/NMR	SBR/NMR
	20 weeks	28 weeks	20 weeks	28 weeks	20 weeks	28 weeks
1990	0.0 (0.0 to 0.1)	0.0 (0.0 to 0.0)	9.5 (6.6 to 13.7)	5.1 (3.4 to 7.4)	1.69 (1.18 to 2.44)	0.90 (0.61 to 1.31)
1995	0.0 (0.0 to 0.0)	0.0 (0.0 to 0.0)	9.2 (6.5 to 12.4)	4.6 (3.3 to 6.3)	1.97 (1.40 to 2.68)	0.99 (0.70 to 1.35)
2000	0.0 (0.0 to 0.0)	0.0 (0.0 to 0.0)	9.7 (8.8 to 10.7)	4.6 (4.1 to 5.1)	2.18 (1.97 to 2.39)	1.02 (0.91 to 1.14)
2005	0.0 (0.0 to 0.0)	0.0 (0.0 to 0.0)	9.7 (8.0 to 11.5)	4.2 (3.2 to 5.3)	2.39 (1.95 to 2.83)	1.02 (0.77 to 1.30)
2010	0.0 (0.0 to 0.0)	0.0 (0.0 to 0.0)	9.5 (7.8 to 11.4)	3.9 (3.0 to 5.0)	2.74 (2.26 to 3.28)	1.14 (0.87 to 1.45)
2015	0.0 (0.0 to 0.0)	0.0 (0.0 to 0.0)	7.8 (6.7 to 9.0)	3.3 (2.9 to 3.8)	2.78 (2.38 to 3.20)	1.18 (1.02 to 1.34)
2020	0.0 (0.0 to 0.0)	0.0 (0.0 to 0.0)	4.9 (4.3 to 5.7)	2.2 (1.9 to 2.4)	2.25 (1.96 to 2.59)	0.99 (0.88 to 1.12)
2021	0.0 (0.0 to 0.0)	0.0 (0.0 to 0.0)	5.8 (4.6 to 7.3)	2.5 (2.0 to 3.1)	2.33 (1.85 to 2.97)	1.02 (0.81 to 1.27)

Sheffield GBR_44687 Western Europe Stillbirth Rate / Neonatal Mortality Rate (SBR/NMR) − 20 weeks
lambda = 0.6 ; zeta = 0.8 ; scale = 15 ; amp = 0.028

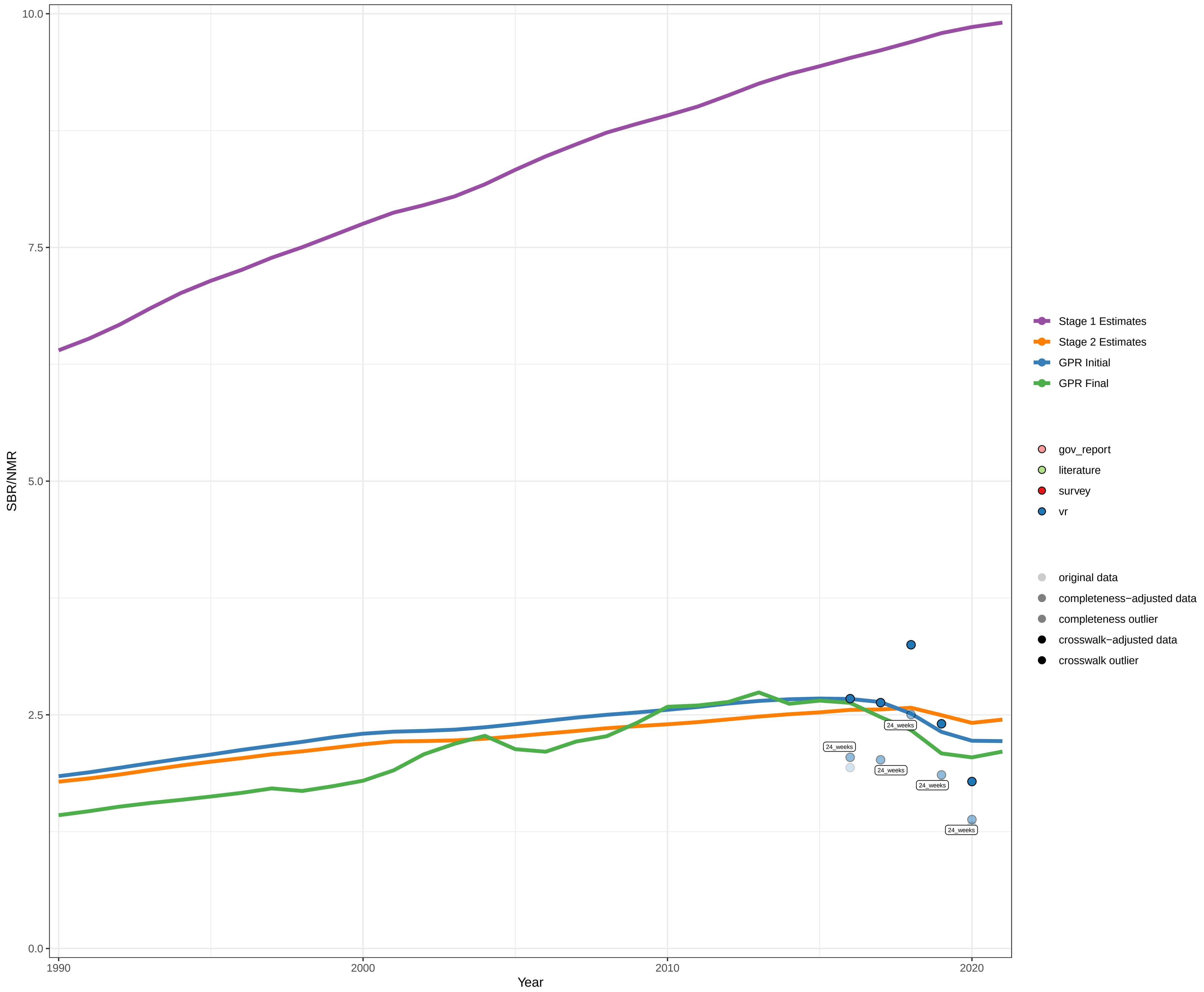

Sheffield GBR_44687 Western Europe Stillbirth Rate / Neonatal Mortality Rate (SBR/NMR) − 28 weeks
lambda = 0.6 ; zeta = 0.8 ; scale = 15 ; amp = 0.028

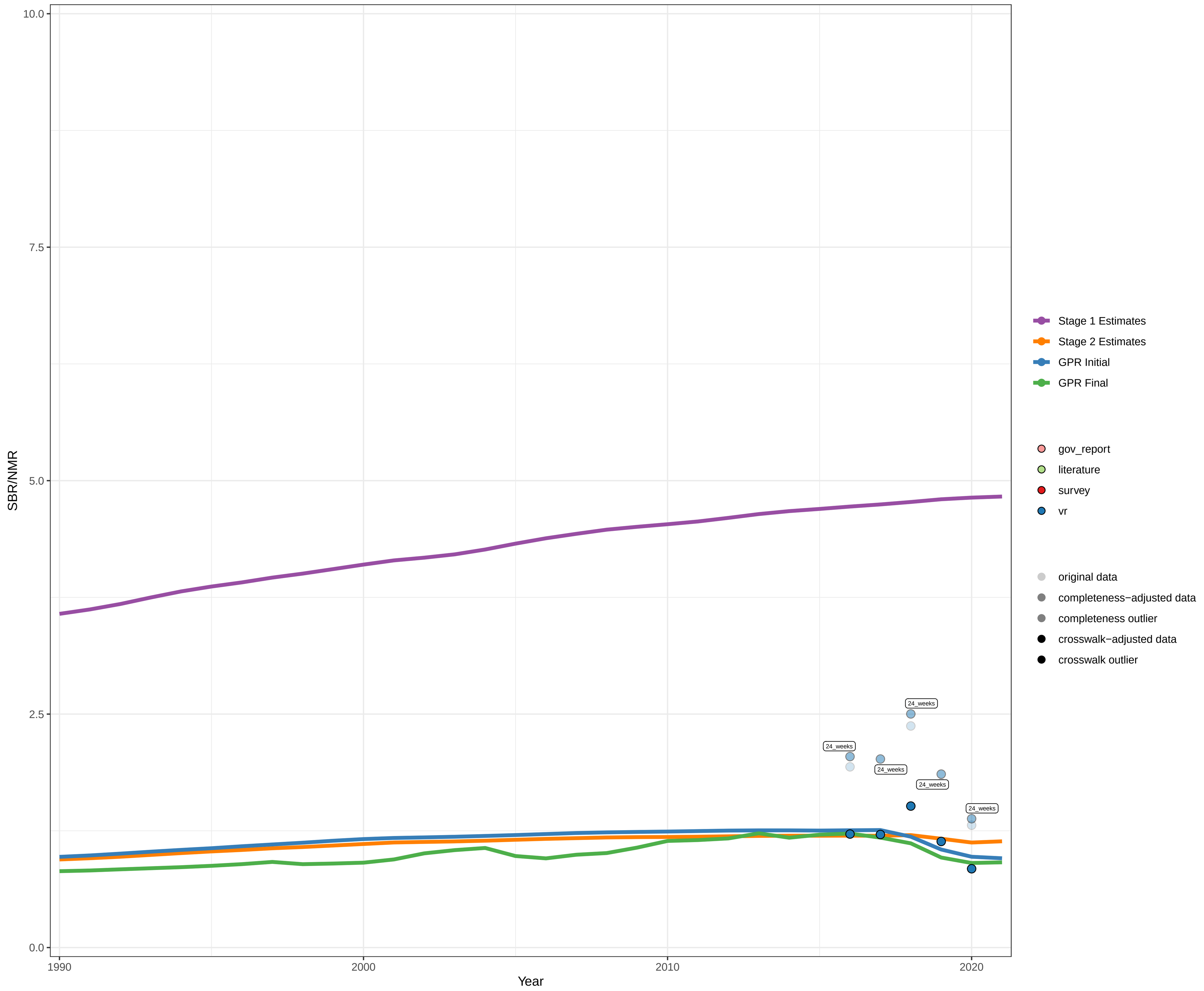

Definition	Years	Outlier	Source.Type	Title
24_weeks	2016−2020	0	VR	United Kingdom − England and Wales Child Mortality Statistics

Sheffield GBR_44687 Western Europe Stillbirth Rate (SBR) − 20 weeks
lambda = 0.6 ; zeta = 0.8 ; scale = 15 ; amp = 0.028

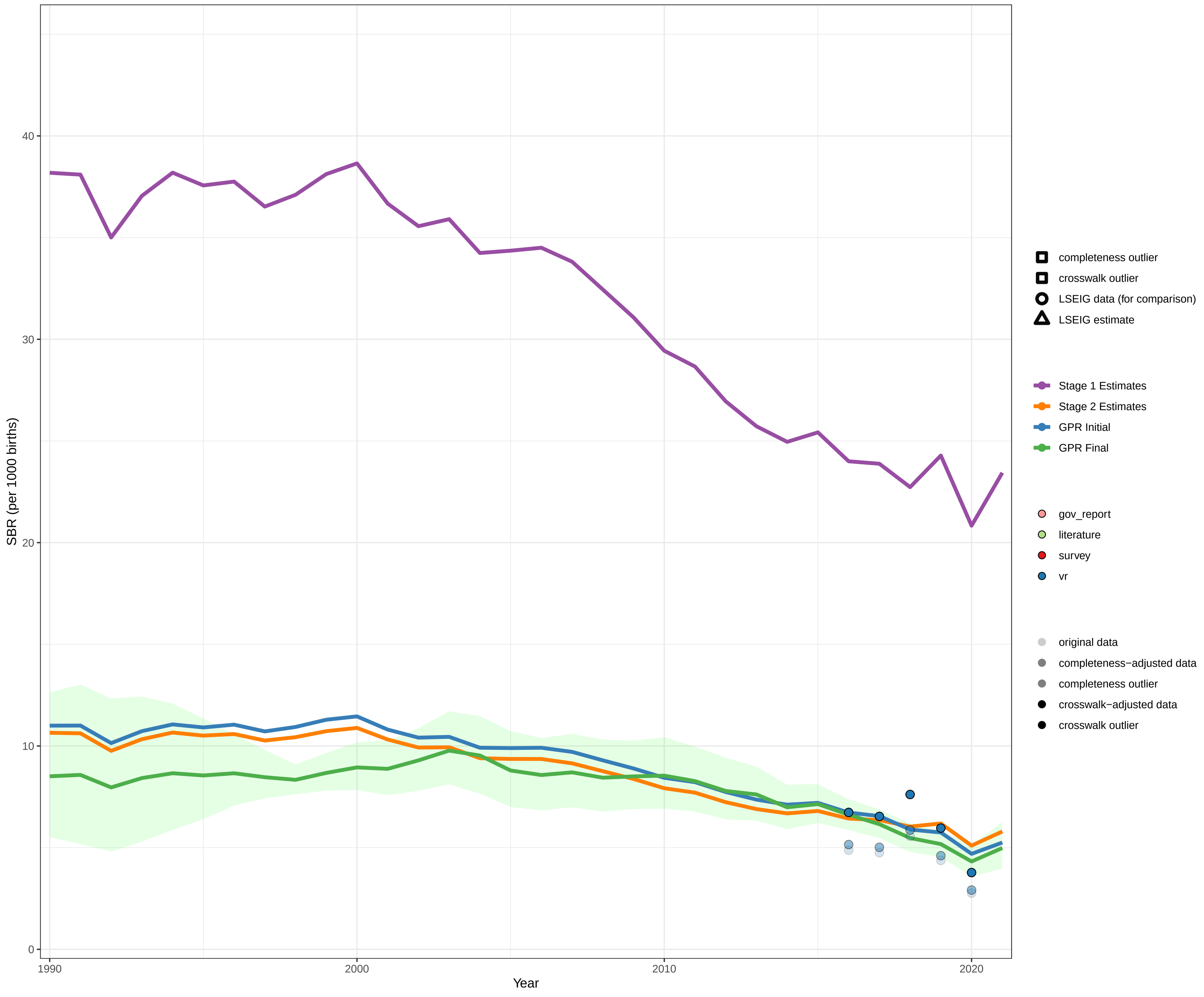

Sheffield GBR_44687 Western Europe Stillbirth Rate (SBR) − 28 weeks
lambda = 0.6 ; zeta = 0.8 ; scale = 15 ; amp = 0.028

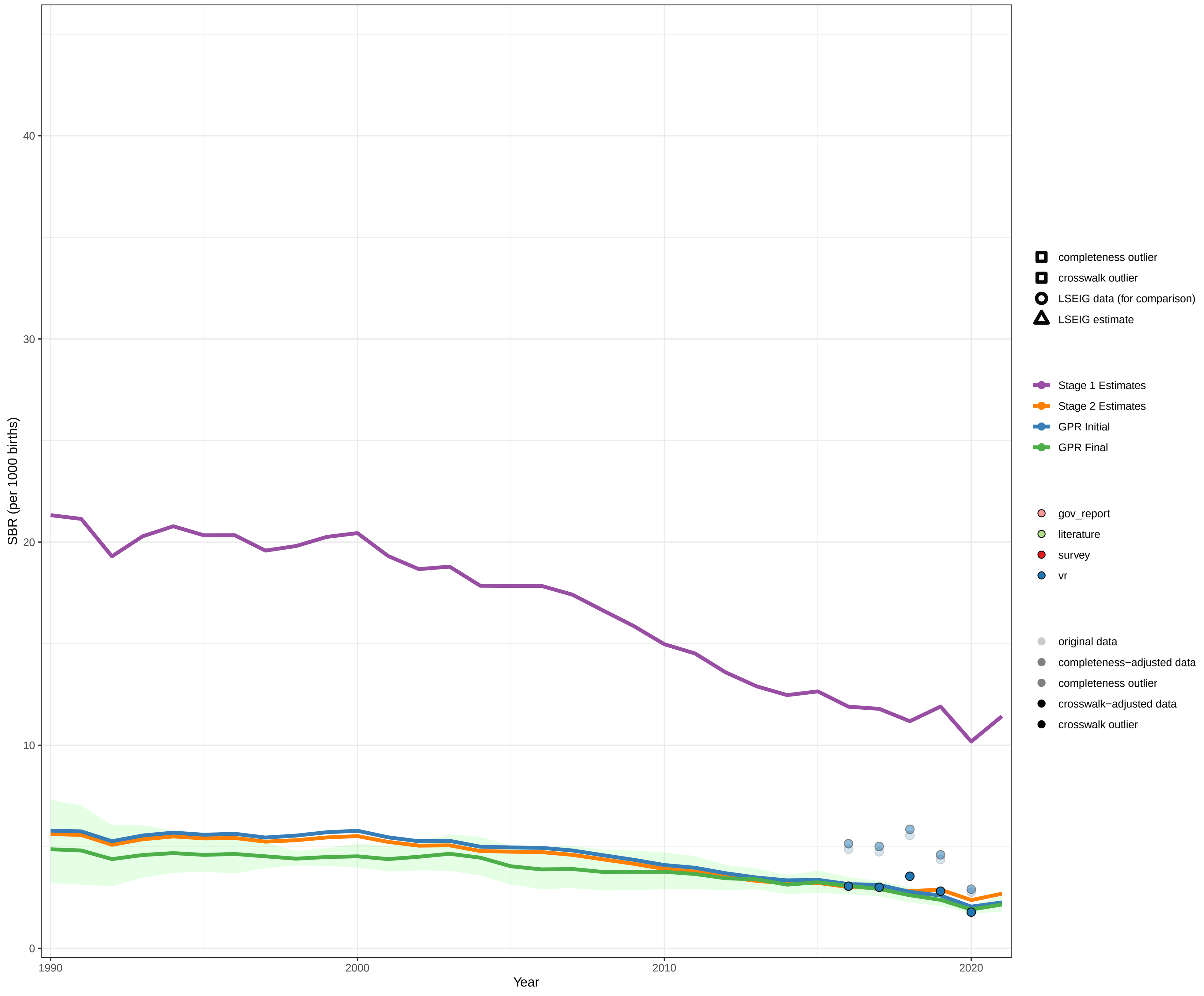

Year	Total Stillbirths (in thousands)	Total Stillbirths (in thousands)	SBR (per 1000 births)	SBR (per 1000 births)	SBR/NMR	SBR/NMR
	20 weeks	28 weeks	20 weeks	28 weeks	20 weeks	28 weeks
1990	0.1 (0.0 to 0.1)	0.0 (0.0 to 0.0)	8.5 (5.5 to 12.6)	4.9 (3.2 to 7.3)	1.43 (0.92 to 2.12)	0.82 (0.54 to 1.23)
1995	0.1 (0.0 to 0.1)	0.0 (0.0 to 0.0)	8.5 (6.4 to 11.3)	4.6 (3.8 to 5.6)	1.63 (1.22 to 2.16)	0.88 (0.72 to 1.06)
2000	0.1 (0.0 to 0.1)	0.0 (0.0 to 0.0)	8.9 (7.8 to 10.2)	4.5 (4.0 to 5.2)	1.79 (1.57 to 2.04)	0.91 (0.80 to 1.04)
2005	0.1 (0.0 to 0.1)	0.0 (0.0 to 0.0)	8.8 (7.0 to 10.7)	4.0 (3.1 to 5.1)	2.13 (1.70 to 2.60)	0.98 (0.76 to 1.23)
2010	0.1 (0.0 to 0.1)	0.0 (0.0 to 0.0)	8.5 (6.9 to 10.4)	3.8 (2.9 to 4.7)	2.59 (2.09 to 3.16)	1.14 (0.88 to 1.43)
2015	0.0 (0.0 to 0.1)	0.0 (0.0 to 0.0)	7.1 (6.2 to 8.1)	3.3 (2.7 to 3.8)	2.65 (2.31 to 3.02)	1.21 (1.02 to 1.42)
2020	0.0 (0.0 to 0.0)	0.0 (0.0 to 0.0)	4.3 (3.6 to 5.1)	1.9 (1.7 to 2.1)	2.04 (1.69 to 2.44)	0.91 (0.81 to 1.01)
2021	0.0 (0.0 to 0.0)	0.0 (0.0 to 0.0)	5.0 (4.0 to 6.3)	2.2 (1.8 to 2.6)	2.11 (1.68 to 2.64)	0.91 (0.77 to 1.09)

Wakefield GBR_44688 Western Europe Stillbirth Rate / Neonatal Mortality Rate (SBR/NMR) − 20 weeks
lambda = 0.6 ; zeta = 0.8 ; scale = 15 ; amp = 0.028

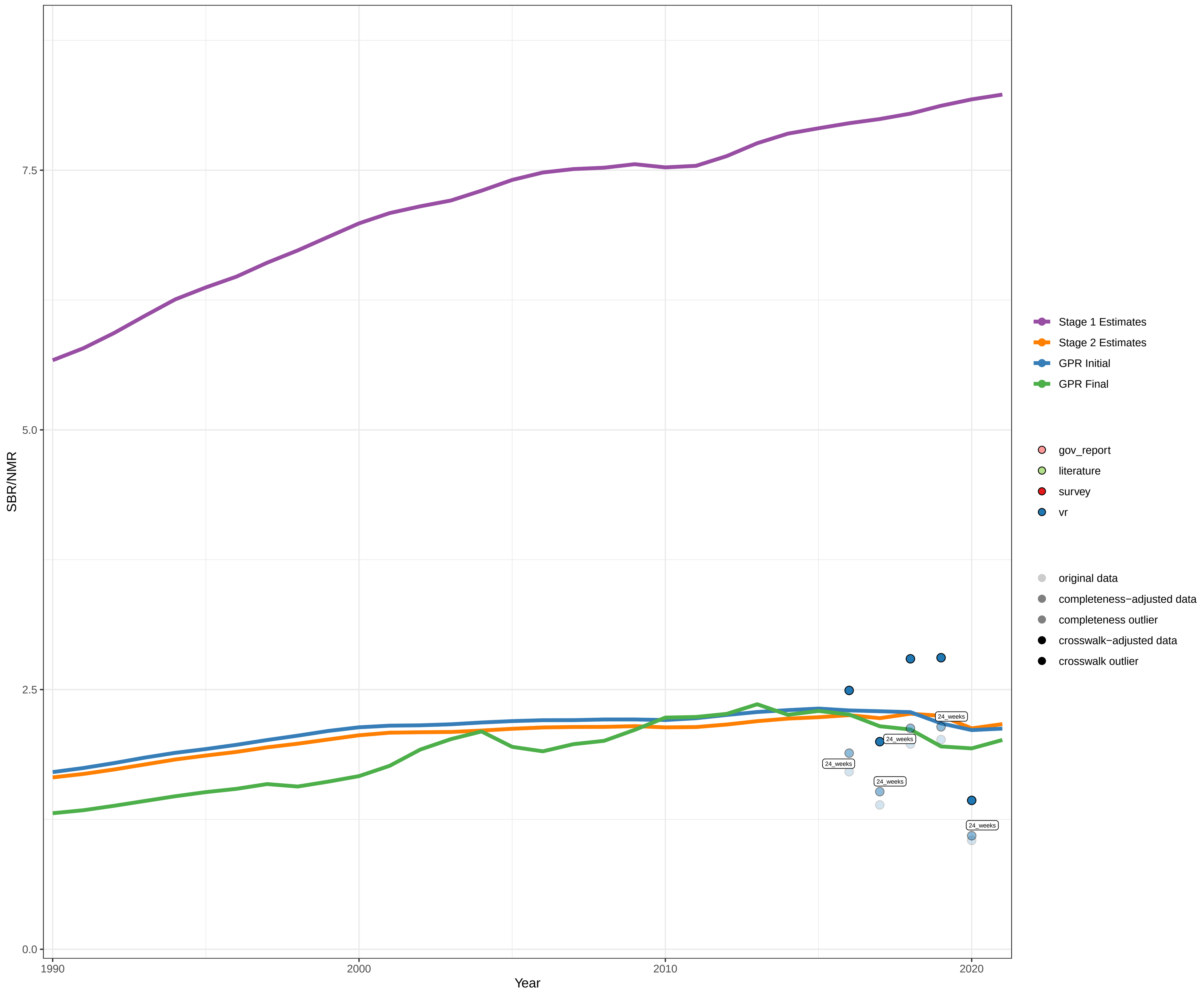

Wakefield GBR_44688 Western Europe Stillbirth Rate / Neonatal Mortality Rate (SBR/NMR) − 28 weeks
lambda = 0.6 ; zeta = 0.8 ; scale = 15 ; amp = 0.028

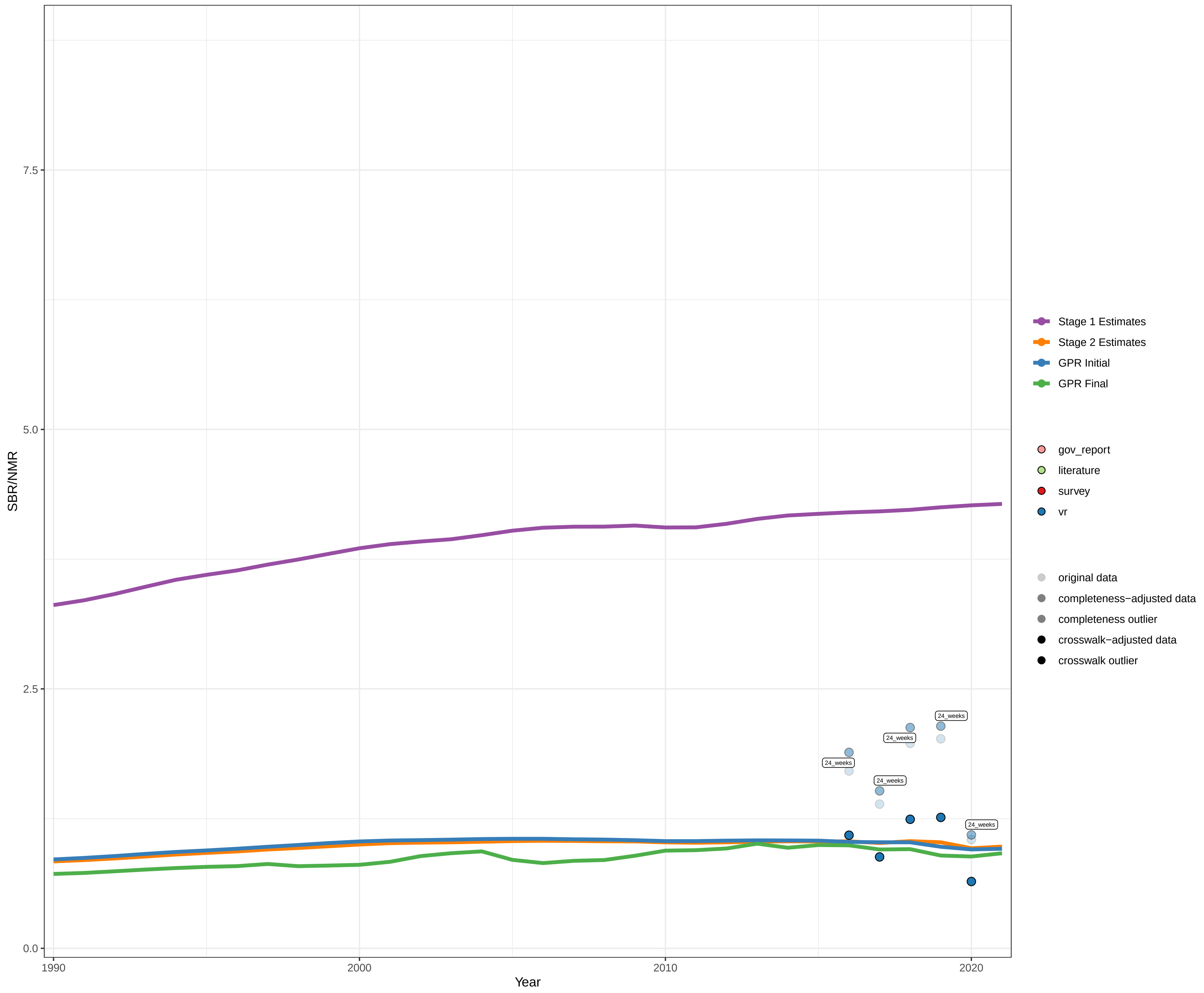

Definition	Years	Outlier	Source.Type	Title
24_weeks	2016−2020	0	VR	United Kingdom − England and Wales Child Mortality Statistics

Wakefield GBR_44688 Western Europe Stillbirth Rate (SBR) − 20 weeks
lambda = 0.6 ; zeta = 0.8 ; scale = 15 ; amp = 0.028

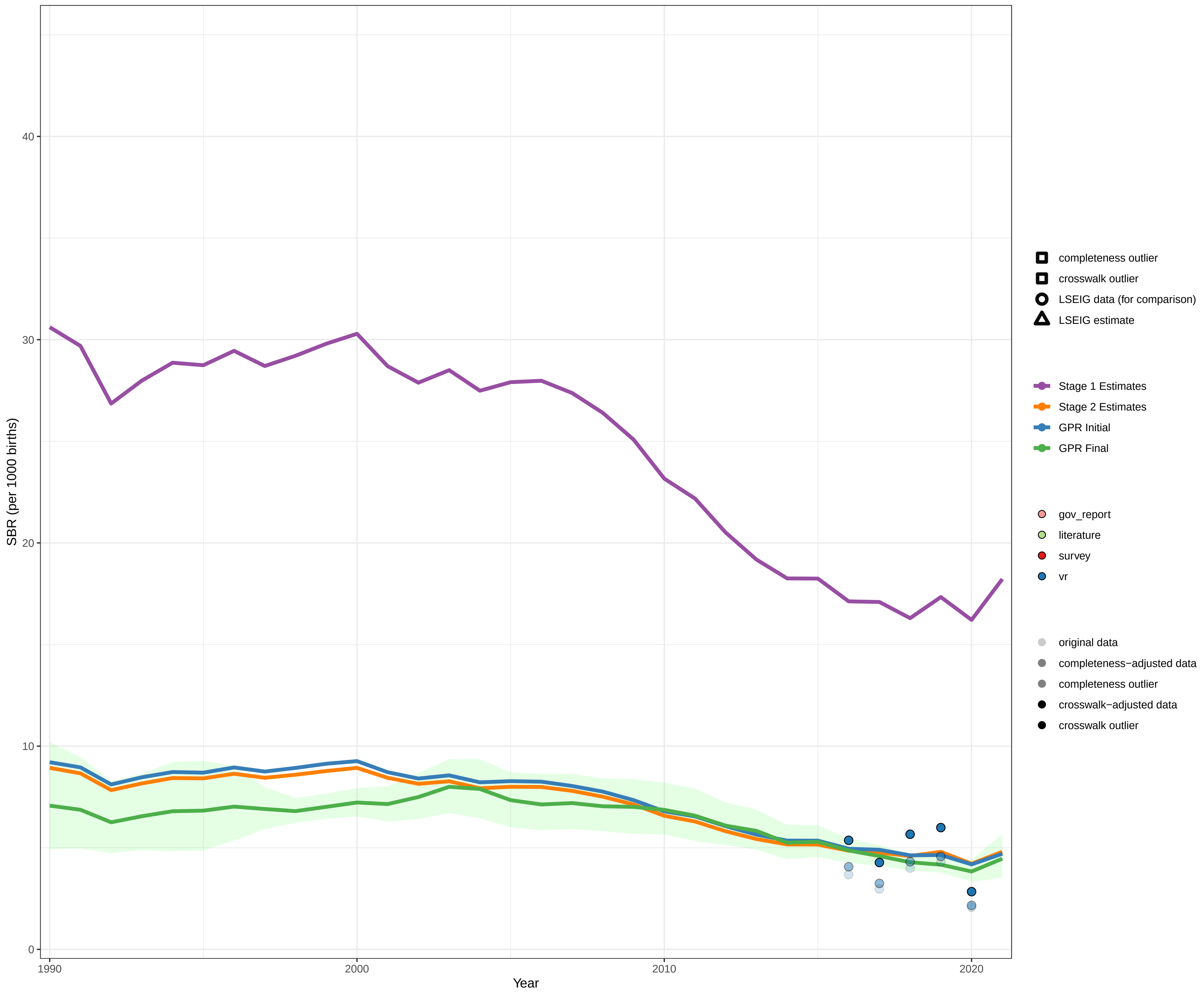

Wakefield GBR_44688 Western Europe Stillbirth Rate (SBR) − 28 weeks
lambda = 0.6 ; zeta = 0.8 ; scale = 15 ; amp = 0.028

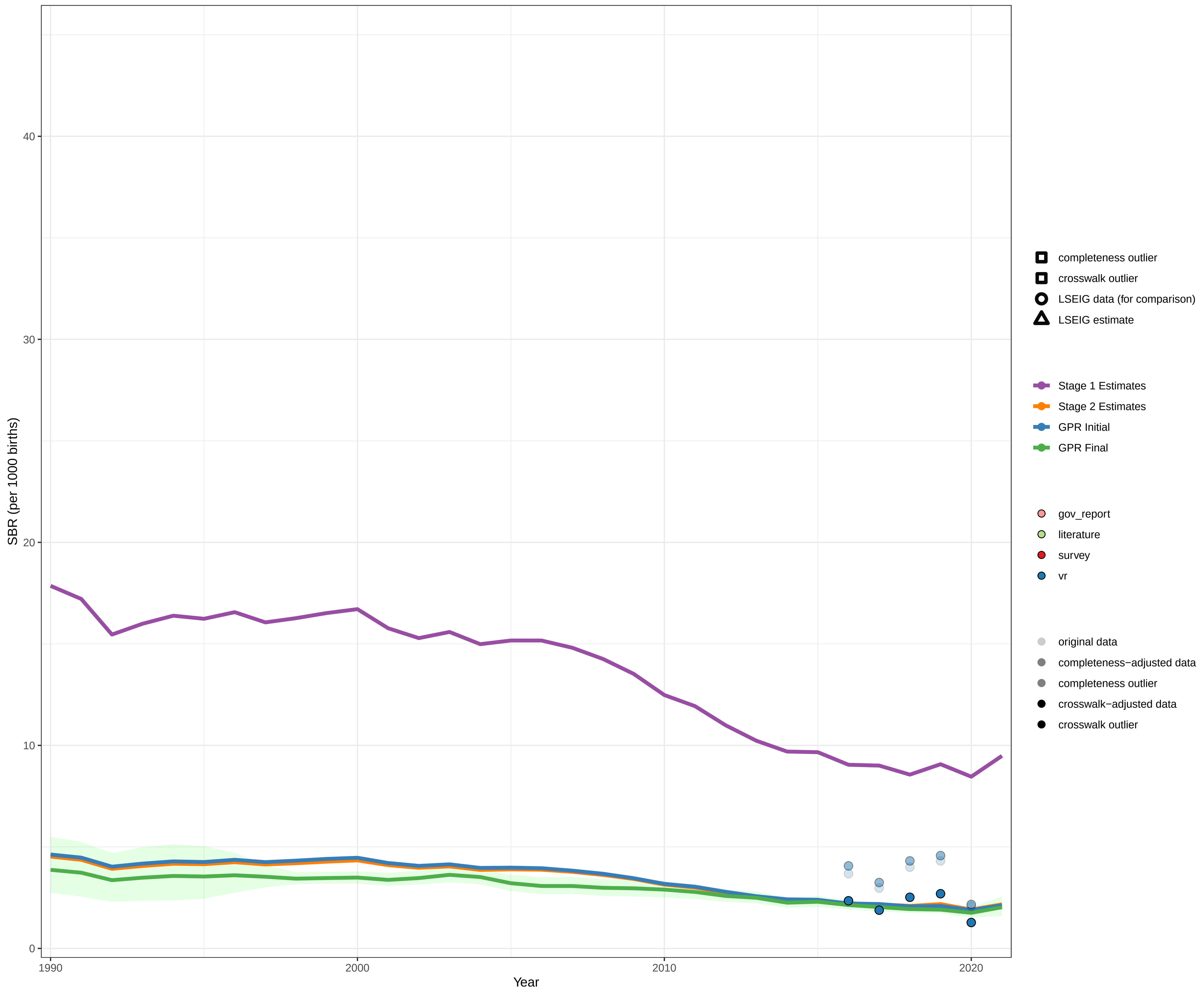

Year	Total Stillbirths (in thousands)	Total Stillbirths (in thousands)	SBR (per 1000 births)	SBR (per 1000 births)	SBR/NMR	SBR/NMR
	20 weeks	28 weeks	20 weeks	28 weeks	20 weeks	28 weeks
1990	0.0 (0.0 to 0.0)	0.0 (0.0 to 0.0)	7.1 (4.9 to 10.2)	3.9 (2.7 to 5.5)	1.31 (0.91 to 1.89)	0.72 (0.51 to 1.02)
1995	0.0 (0.0 to 0.0)	0.0 (0.0 to 0.0)	6.8 (4.9 to 9.3)	3.5 (2.4 to 5.0)	1.51 (1.08 to 2.06)	0.78 (0.54 to 1.12)
2000	0.0 (0.0 to 0.0)	0.0 (0.0 to 0.0)	7.2 (6.5 to 7.9)	3.5 (3.2 to 3.8)	1.67 (1.51 to 1.83)	0.80 (0.74 to 0.88)
2005	0.0 (0.0 to 0.0)	0.0 (0.0 to 0.0)	7.3 (6.0 to 8.7)	3.2 (2.8 to 3.6)	1.95 (1.59 to 2.31)	0.85 (0.75 to 0.96)
2010	0.0 (0.0 to 0.0)	0.0 (0.0 to 0.0)	6.9 (5.7 to 8.2)	2.9 (2.5 to 3.3)	2.23 (1.84 to 2.67)	0.94 (0.81 to 1.08)
2015	0.0 (0.0 to 0.0)	0.0 (0.0 to 0.0)	5.3 (4.5 to 6.1)	2.3 (2.0 to 2.6)	2.29 (1.97 to 2.65)	1.00 (0.89 to 1.11)
2020	0.0 (0.0 to 0.0)	0.0 (0.0 to 0.0)	3.8 (3.3 to 4.4)	1.8 (1.5 to 2.0)	1.93 (1.68 to 2.22)	0.88 (0.77 to 1.02)
2021	0.0 (0.0 to 0.0)	0.0 (0.0 to 0.0)	4.5 (3.5 to 5.7)	2.0 (1.6 to 2.6)	2.01 (1.59 to 2.57)	0.92 (0.72 to 1.16)

York GBR_44680 Western Europe Stillbirth Rate / Neonatal Mortality Rate (SBR/NMR) − 20 weeks
lambda = 0.8 ; zeta = 0.7 ; scale = 15 ; amp = 0.028

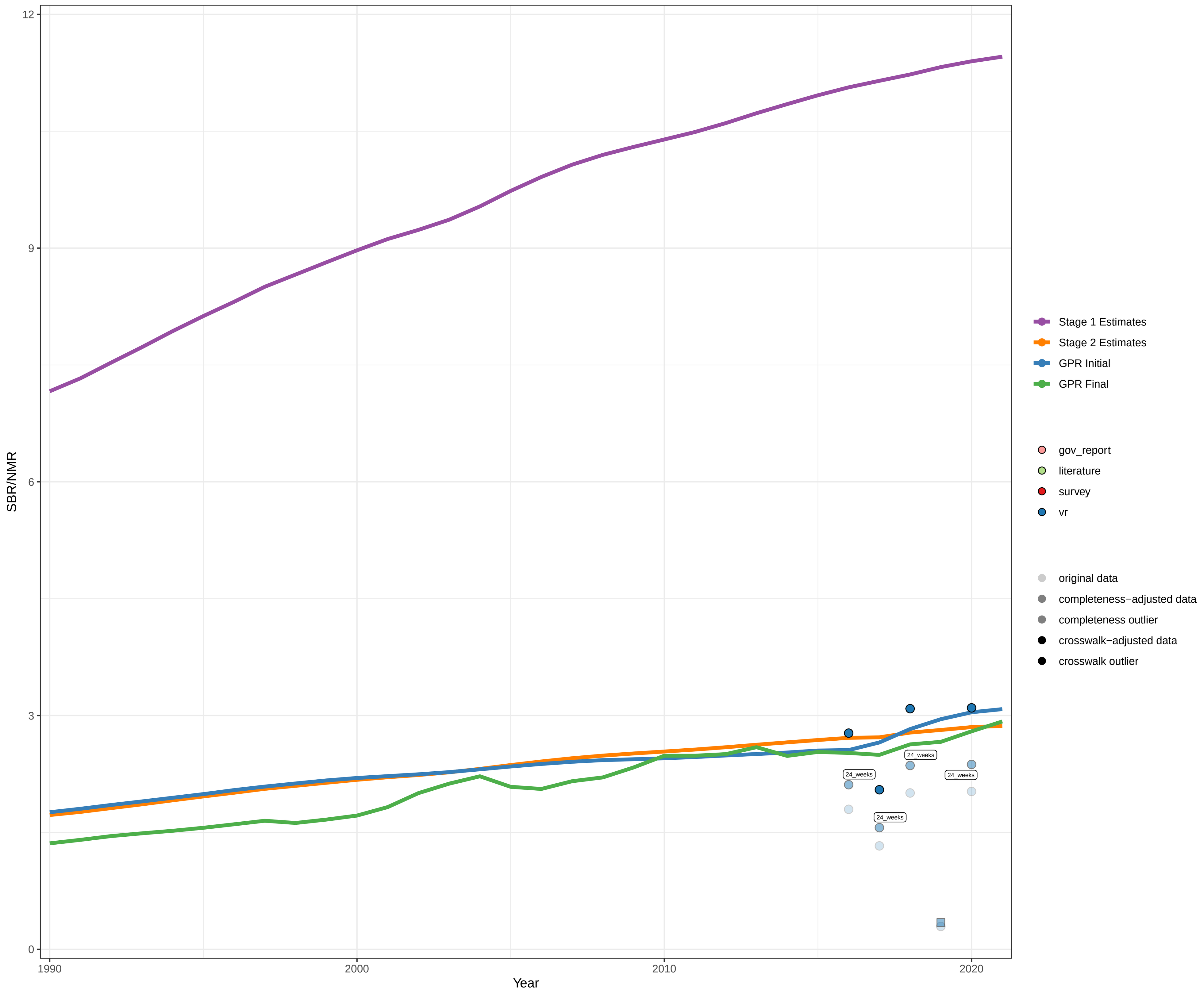

York GBR_44680 Western Europe Stillbirth Rate / Neonatal Mortality Rate (SBR/NMR) − 28 weeks
lambda = 0.8 ; zeta = 0.7 ; scale = 15 ; amp = 0.028

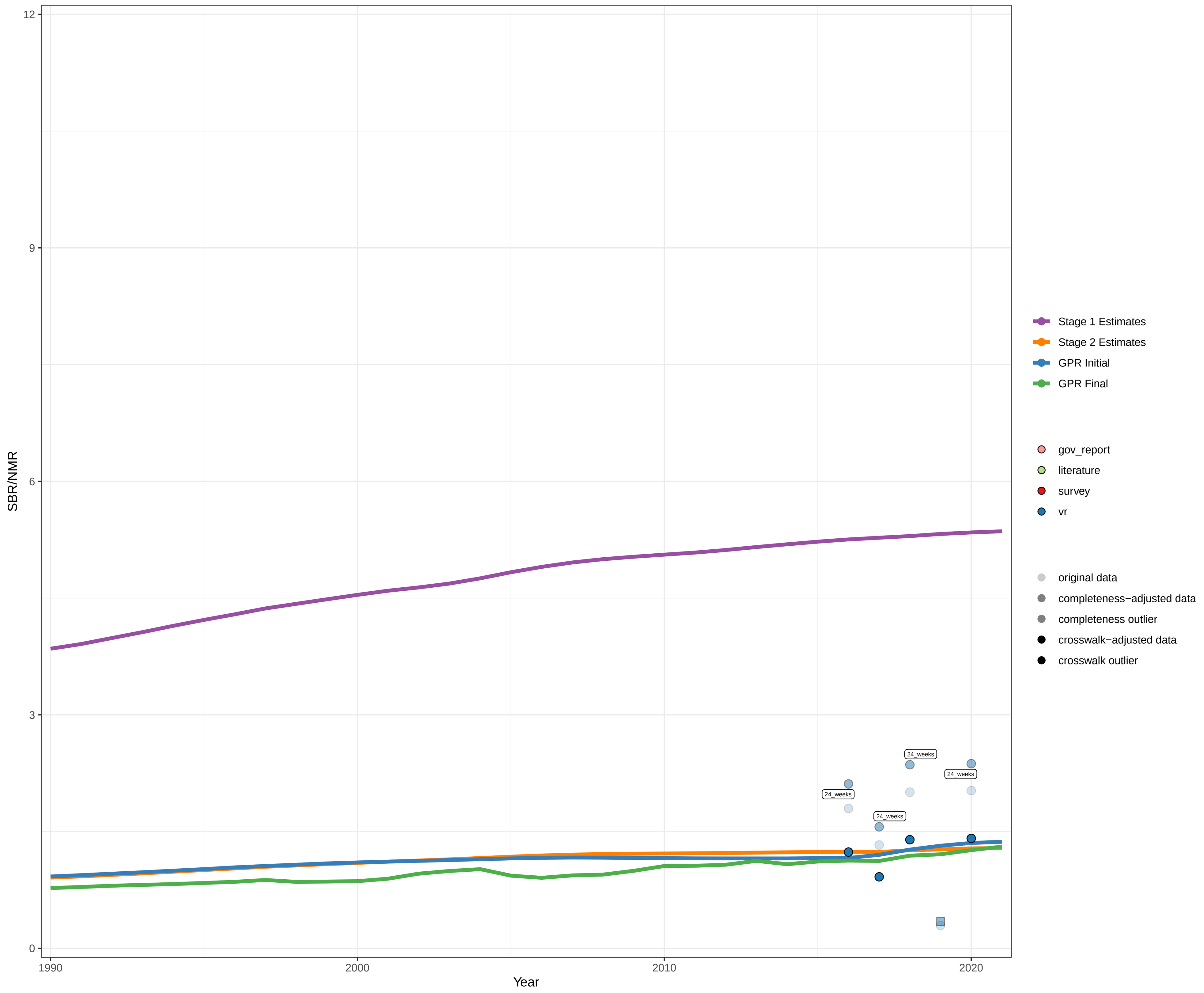

Definition	Years	Outlier	Source.Type	Title
24_weeks	2016−2018, 2020	0	VR	United Kingdom − England and Wales Child Mortality Statistics
24_weeks	2019	1	VR	United Kingdom − England and Wales Child Mortality Statistics

York GBR_44680 Western Europe Stillbirth Rate (SBR) − 20 weeks
lambda = 0.8 ; zeta = 0.7 ; scale = 15 ; amp = 0.028

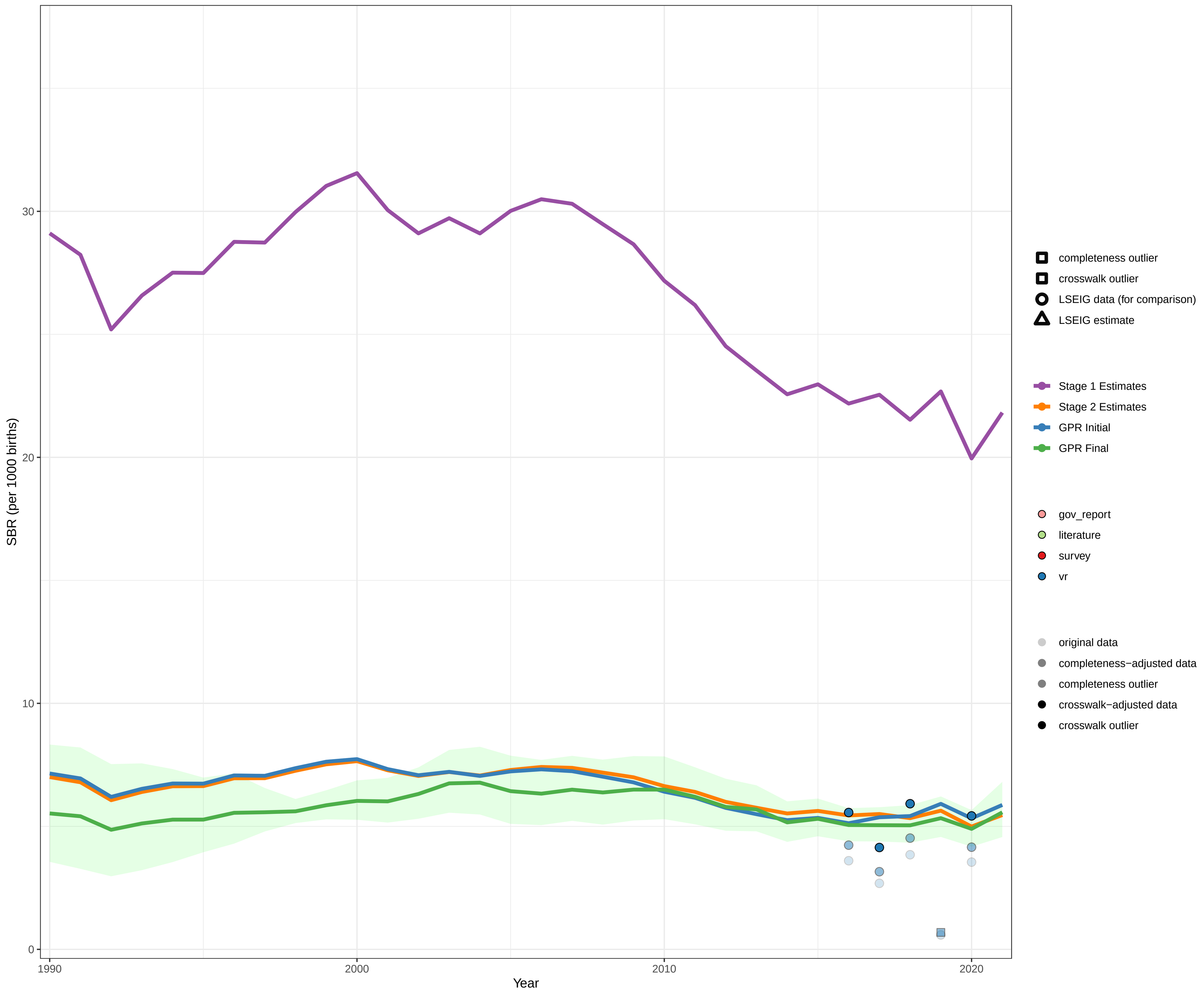

York GBR_44680 Western Europe Stillbirth Rate (SBR) − 28 weeks
lambda = 0.8 ; zeta = 0.7 ; scale = 15 ; amp = 0.028

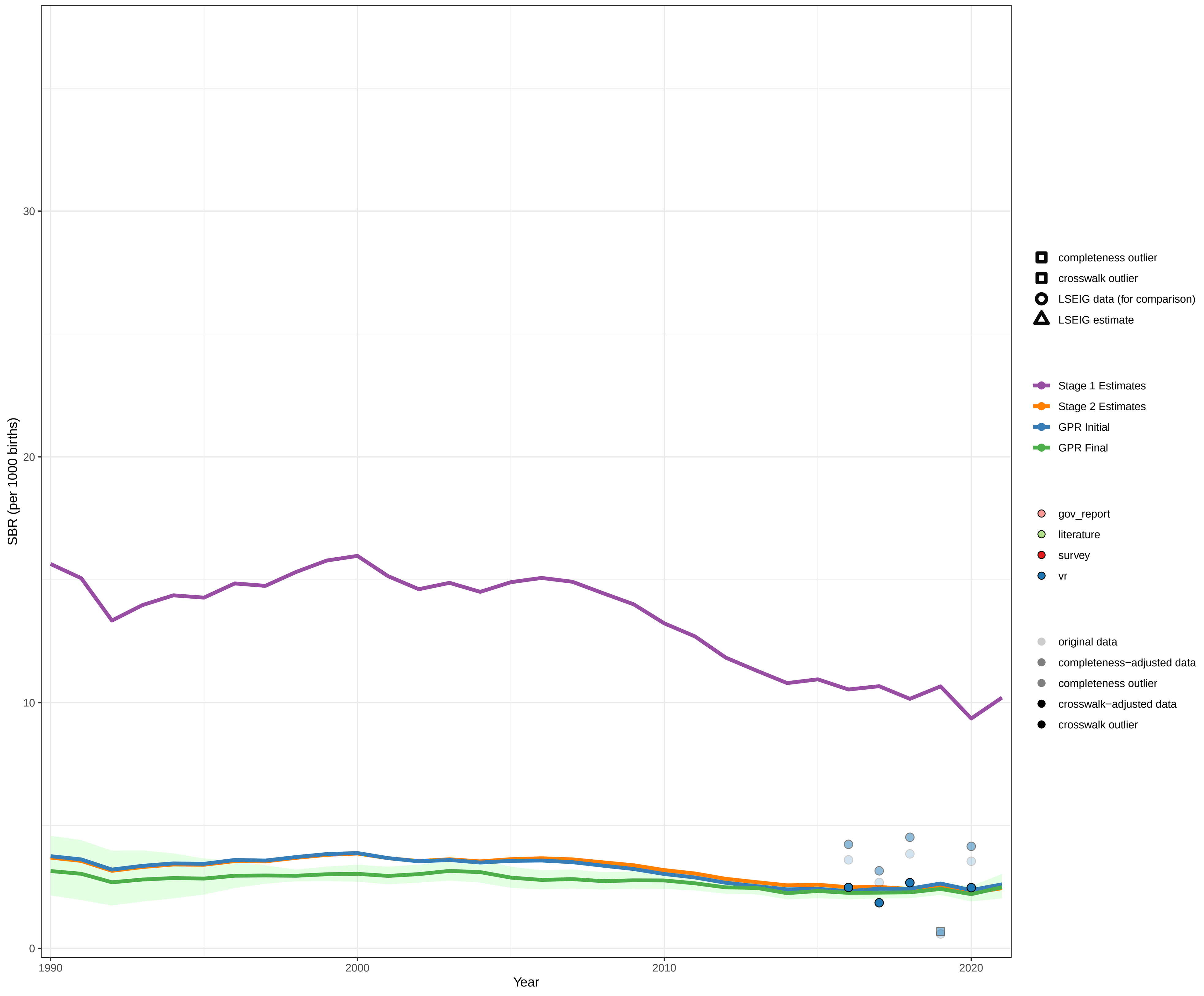

Year	Total Stillbirths (in thousands)	Total Stillbirths (in thousands)	SBR (per 1000 births)	SBR (per 1000 births)	SBR/NMR	SBR/NMR
	20 weeks	28 weeks	20 weeks	28 weeks	20 weeks	28 weeks
1990	0.0 (0.0 to 0.0)	0.0 (0.0 to 0.0)	5.5 (3.6 to 8.3)	3.1 (2.2 to 4.6)	1.36 (0.88 to 2.05)	0.77 (0.53 to 1.13)
1995	0.0 (0.0 to 0.0)	0.0 (0.0 to 0.0)	5.3 (3.9 to 7.0)	2.8 (2.2 to 3.7)	1.56 (1.17 to 2.06)	0.84 (0.65 to 1.08)
2000	0.0 (0.0 to 0.0)	0.0 (0.0 to 0.0)	6.0 (5.3 to 6.9)	3.0 (2.7 to 3.4)	1.72 (1.50 to 1.95)	0.86 (0.77 to 0.97)
2005	0.0 (0.0 to 0.0)	0.0 (0.0 to 0.0)	6.4 (5.1 to 7.9)	2.9 (2.5 to 3.3)	2.09 (1.65 to 2.55)	0.93 (0.80 to 1.08)
2010	0.0 (0.0 to 0.0)	0.0 (0.0 to 0.0)	6.5 (5.3 to 7.8)	2.8 (2.4 to 3.1)	2.48 (2.02 to 3.00)	1.06 (0.93 to 1.20)
2015	0.0 (0.0 to 0.0)	0.0 (0.0 to 0.0)	5.3 (4.6 to 6.1)	2.3 (2.0 to 2.6)	2.53 (2.19 to 2.93)	1.12 (0.98 to 1.26)
2020	0.0 (0.0 to 0.0)	0.0 (0.0 to 0.0)	4.9 (4.2 to 5.7)	2.2 (1.9 to 2.5)	2.80 (2.38 to 3.23)	1.26 (1.09 to 1.45)
2021	0.0 (0.0 to 0.0)	0.0 (0.0 to 0.0)	5.6 (4.6 to 6.8)	2.5 (2.0 to 3.0)	2.92 (2.40 to 3.58)	1.31 (1.07 to 1.59)

Northern Ireland GBR_433 Western Europe Stillbirth Rate / Neonatal Mortality Rate (SBR/NMR) − 20 weeks
lambda = 0.2 ; zeta = 0.99 ; scale = 5 ; amp = 0.028

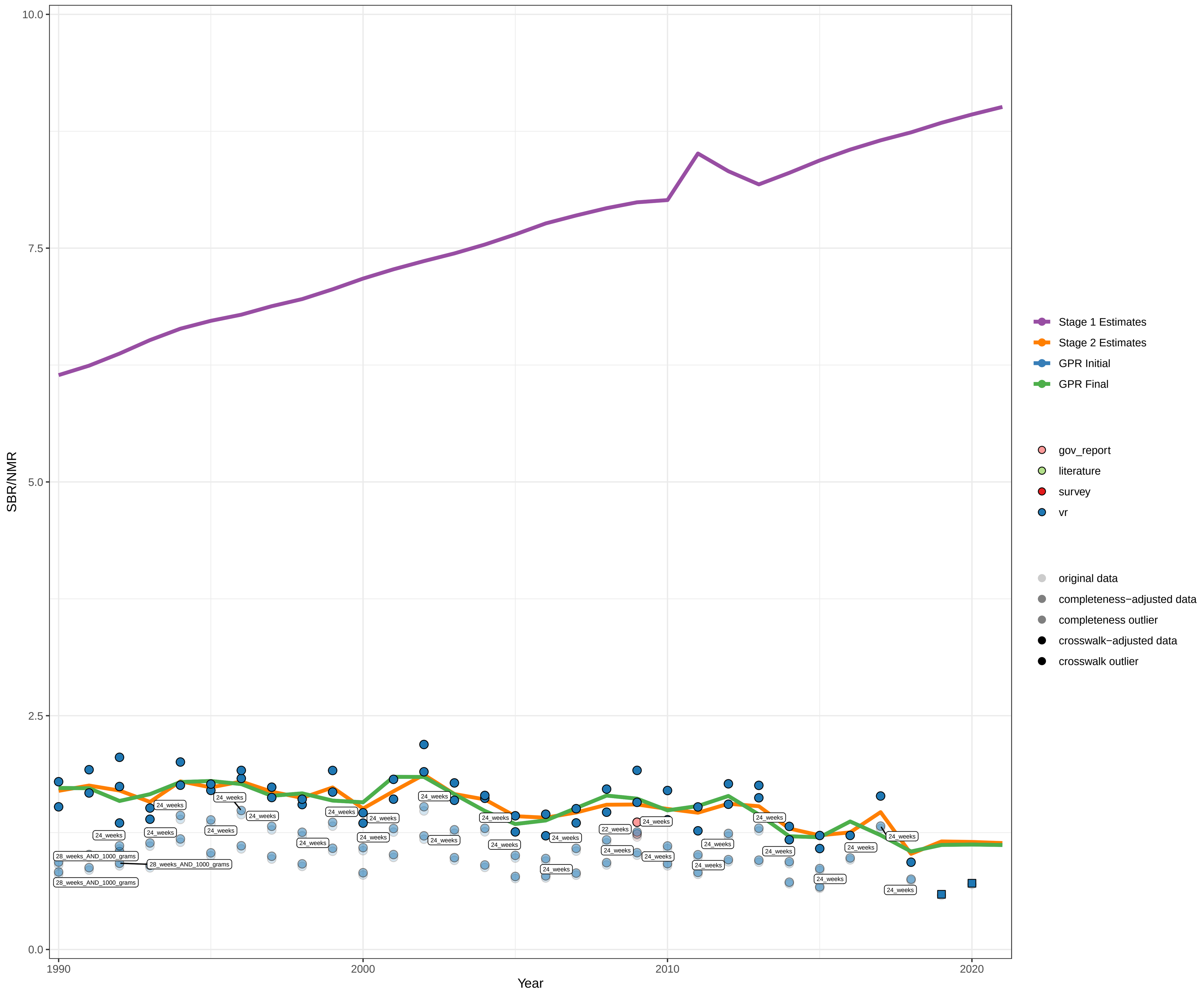

Northern Ireland GBR_433 Western Europe Stillbirth Rate / Neonatal Mortality Rate (SBR/NMR) − 28 weeks
lambda = 0.2 ; zeta = 0.99 ; scale = 5 ; amp = 0.028

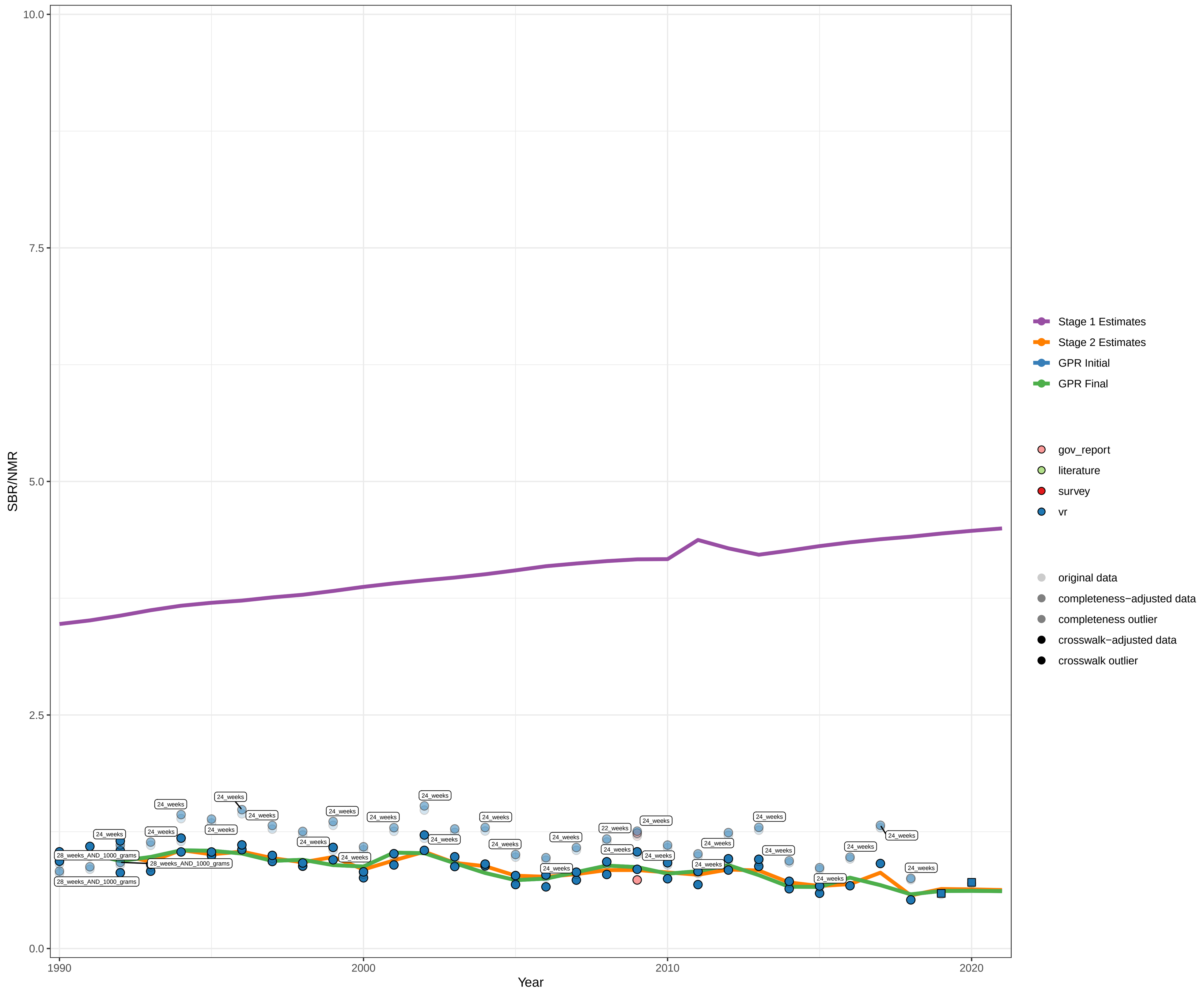

Definition	Years	Outlier	Source.Type	Title
22_weeks	2009	0	Statistical Report	United Kingdom Perinatal Mortality
24_weeks	1992−2020	0	VR	United Kingdom − Northern Ireland Registrar General Annual Report
28_weeks	1990−2015	0	VR	United Kingdom − Northern Ireland Registrar General Annual Report
28_weeks_AND_1000_grams	1990−1992	0	VR	United Kingdom − Northern Ireland Registrar General Annual Report

Northern Ireland GBR_433 Western Europe Stillbirth Rate (SBR) − 20 weeks
lambda = 0.2 ; zeta = 0.99 ; scale = 5 ; amp = 0.028

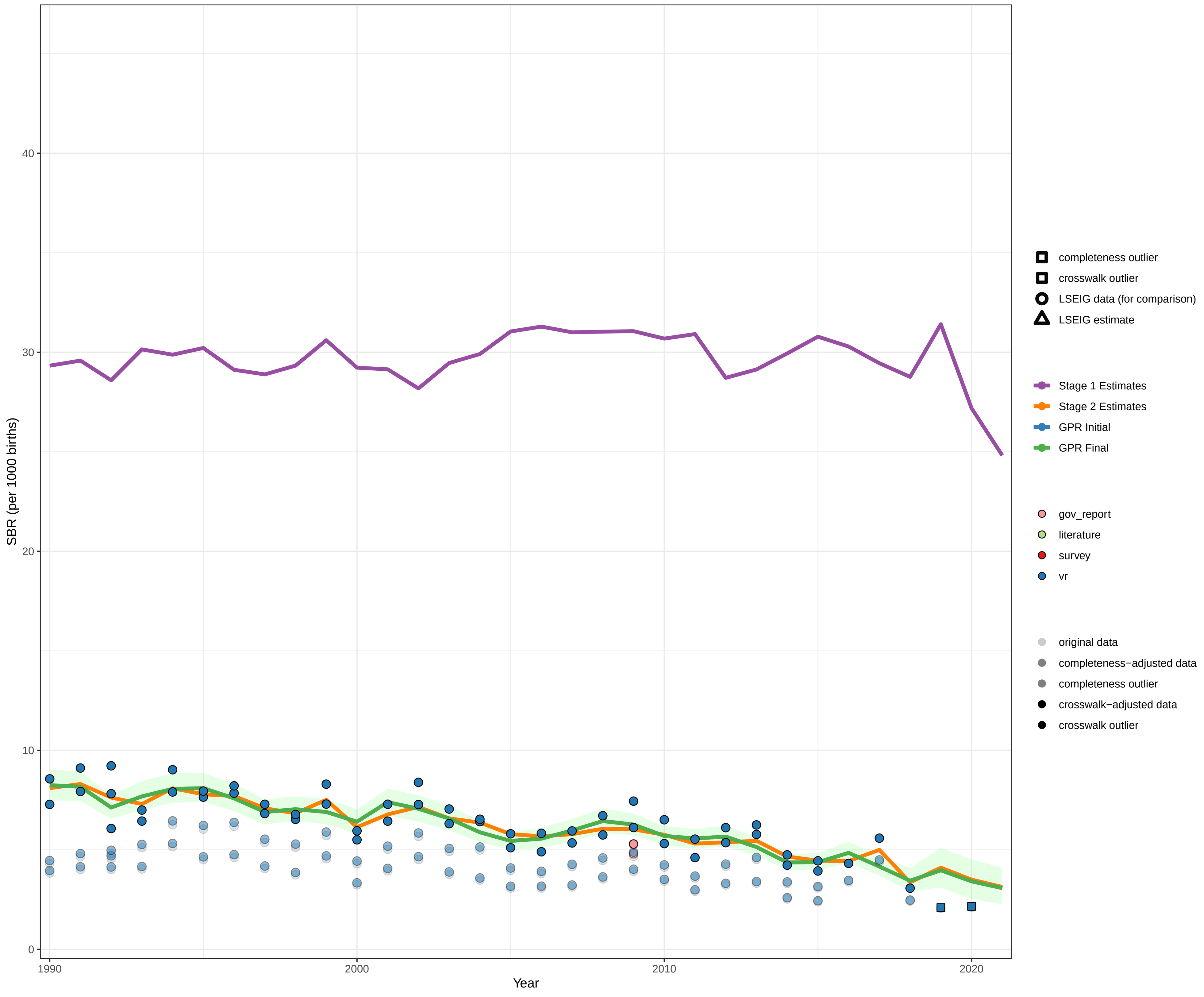

Northern Ireland GBR_433 Western Europe Stillbirth Rate (SBR) − 28 weeks
lambda = 0.2 ; zeta = 0.99 ; scale = 5 ; amp = 0.028

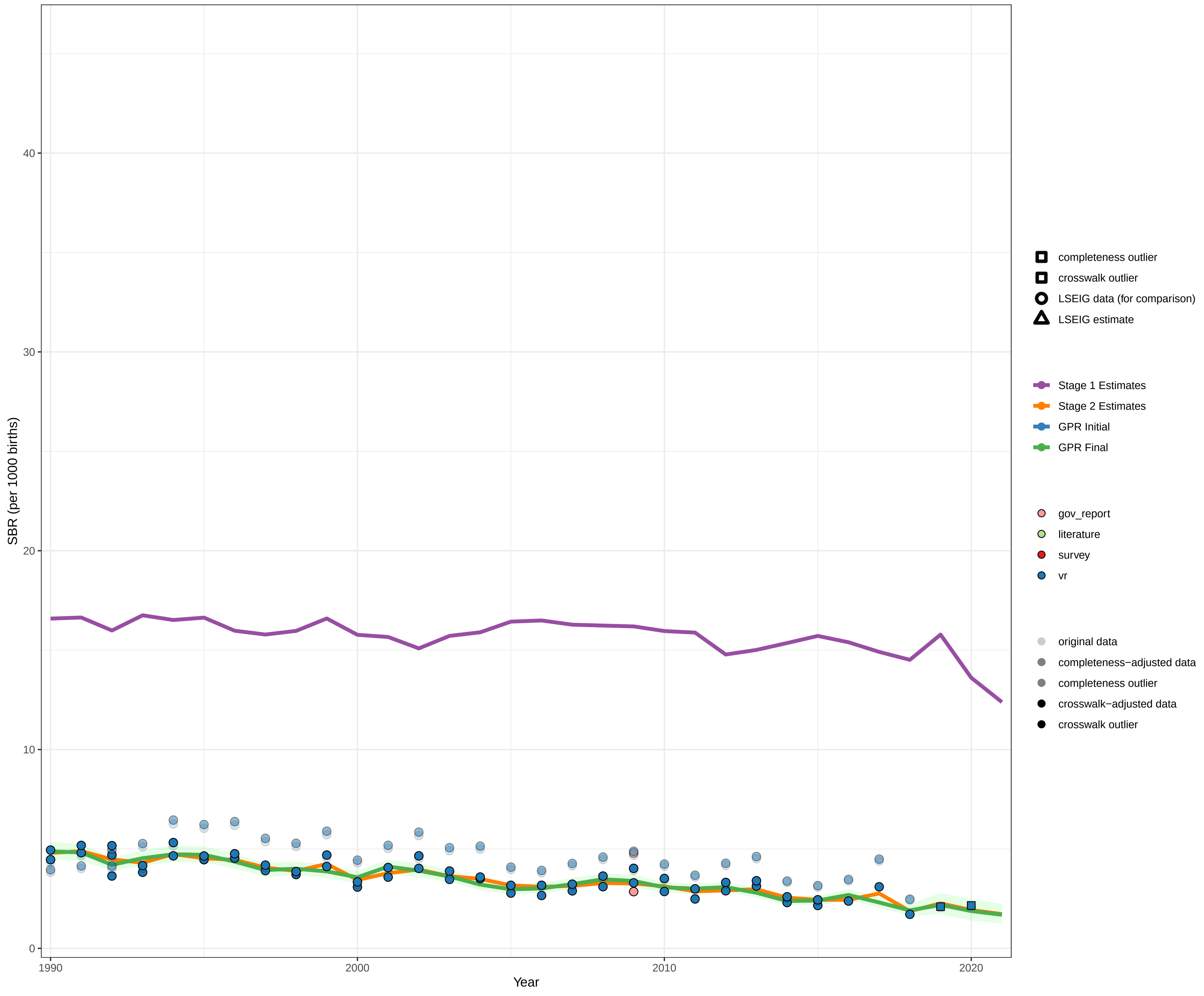

Year	Total Stillbirths (in thousands)	Total Stillbirths (in thousands)	SBR (per 1000 births)	SBR (per 1000 births)	SBR/NMR	SBR/NMR
	20 weeks	28 weeks	20 weeks	28 weeks	20 weeks	28 weeks
1990	0.2 (0.2 to 0.2)	0.1 (0.1 to 0.1)	8.2 (7.5 to 9.0)	4.9 (4.5 to 5.4)	1.73 (1.56 to 1.89)	1.02 (0.93 to 1.12)
1995	0.2 (0.2 to 0.2)	0.1 (0.1 to 0.1)	8.1 (7.4 to 8.9)	4.7 (4.3 to 5.1)	1.80 (1.65 to 1.97)	1.05 (0.96 to 1.14)
2000	0.1 (0.1 to 0.2)	0.1 (0.1 to 0.1)	6.4 (5.8 to 7.0)	3.6 (3.3 to 3.9)	1.57 (1.43 to 1.73)	0.88 (0.80 to 0.96)
2005	0.1 (0.1 to 0.1)	0.1 (0.1 to 0.1)	5.4 (5.0 to 5.9)	3.0 (2.7 to 3.2)	1.34 (1.23 to 1.45)	0.73 (0.67 to 0.80)
2010	0.1 (0.1 to 0.2)	0.1 (0.1 to 0.1)	5.7 (5.2 to 6.2)	3.1 (2.8 to 3.3)	1.49 (1.36 to 1.62)	0.80 (0.73 to 0.87)
2015	0.1 (0.1 to 0.1)	0.1 (0.1 to 0.1)	4.4 (4.0 to 4.8)	2.4 (2.2 to 2.7)	1.20 (1.09 to 1.33)	0.66 (0.60 to 0.73)
2020	0.1 (0.1 to 0.1)	0.0 (0.0 to 0.1)	3.4 (2.5 to 4.5)	1.9 (1.4 to 2.5)	1.12 (0.84 to 1.48)	0.62 (0.46 to 0.81)
2021	0.1 (0.0 to 0.1)	0.0 (0.0 to 0.0)	3.1 (2.3 to 4.1)	1.7 (1.2 to 2.2)	1.12 (0.82 to 1.49)	0.61 (0.45 to 0.82)

Scotland GBR_434 Western Europe Stillbirth Rate / Neonatal Mortality Rate (SBR/NMR) − 20 weeks
lambda = 0.2 ; zeta = 0.99 ; scale = 5 ; amp = 0.028

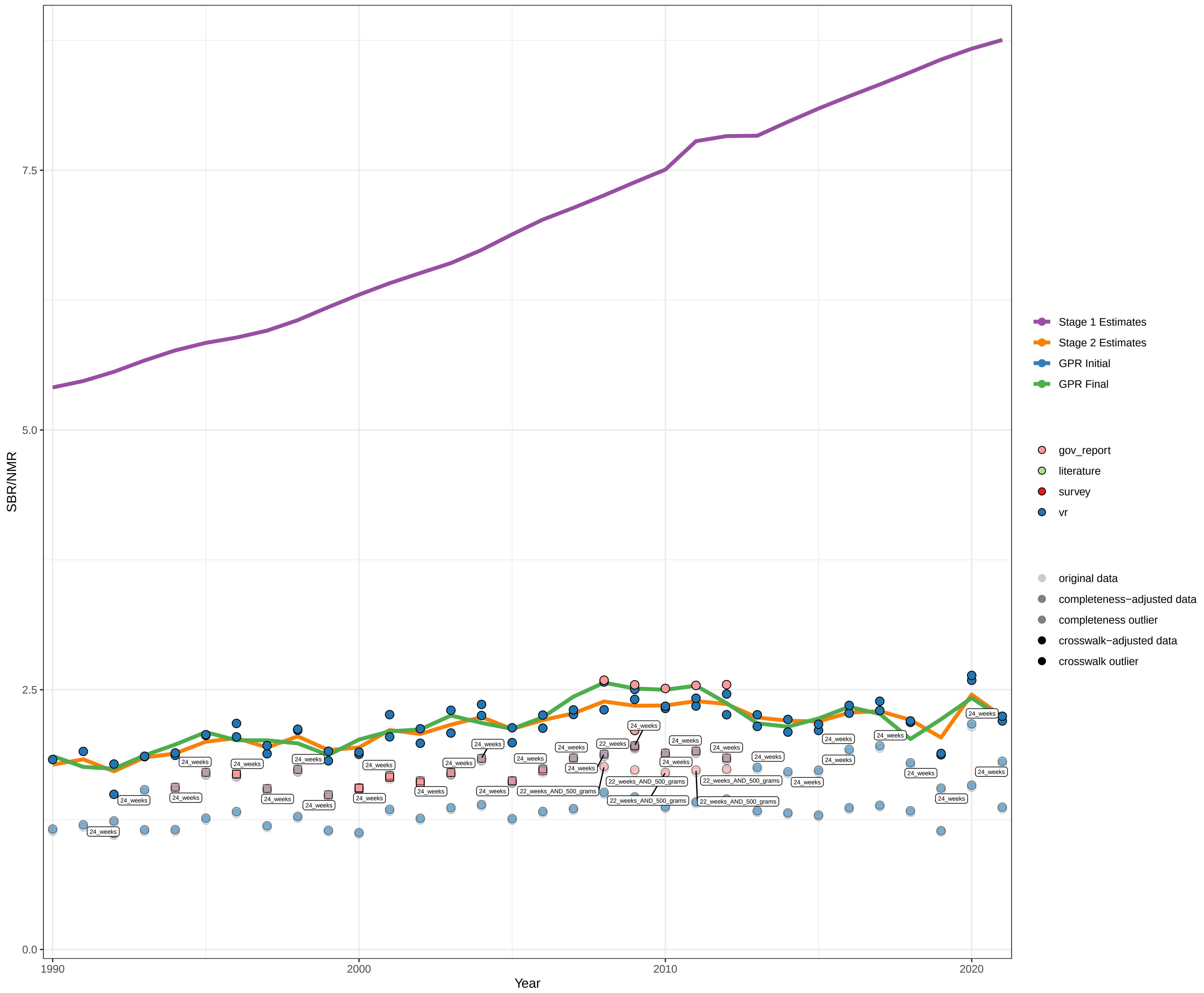

Scotland GBR_434 Western Europe Stillbirth Rate / Neonatal Mortality Rate (SBR/NMR) − 28 weeks
lambda = 0.2 ; zeta = 0.99 ; scale = 5 ; amp = 0.028

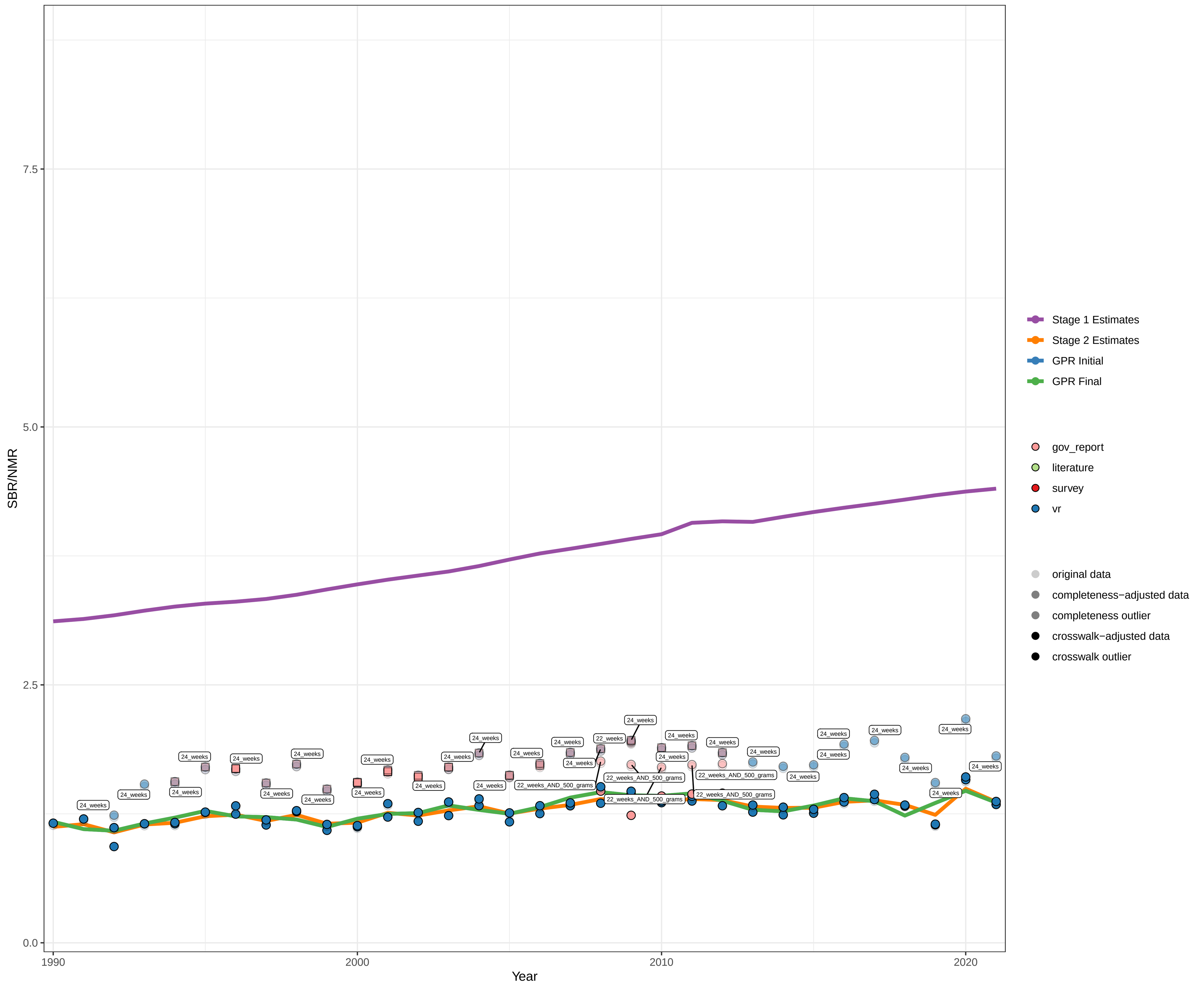

Definition	Years	Outlier	Source.Type	Title
22_weeks	2009	0	Statistical Report	United Kingdom Perinatal Mortality
22_weeks_AND_500_grams	2008−2012	0	Statistical Report	United Kingdom − Scotland Perinatal and Infant Mortality and Morbidity Report
24_weeks	1992−2021	0	VR	United Kingdom − Scotland Vital Events Reference Tables
24_weeks	1996	0	Statistical Report	United Kingdom − Scotland Stillbirth and Infant Death Report
24_weeks	2000−2002	0	Statistical Report	United Kingdom − Scotland Perinatal and Infant Mortality and Morbidity Report
28_weeks	1990−2021	0	VR	United Kingdom − Scotland Vital Events Reference Tables
24_weeks	1994, 1995, 1997−1999	1	Statistical Report	United Kingdom − Scotland Stillbirth and Infant Death Report
24_weeks	2000−2012	1	Statistical Report	United Kingdom − Scotland Perinatal and Infant Mortality and Morbidity Report

Scotland GBR_434 Western Europe Stillbirth Rate (SBR) − 20 weeks
lambda = 0.2 ; zeta = 0.99 ; scale = 5 ; amp = 0.028

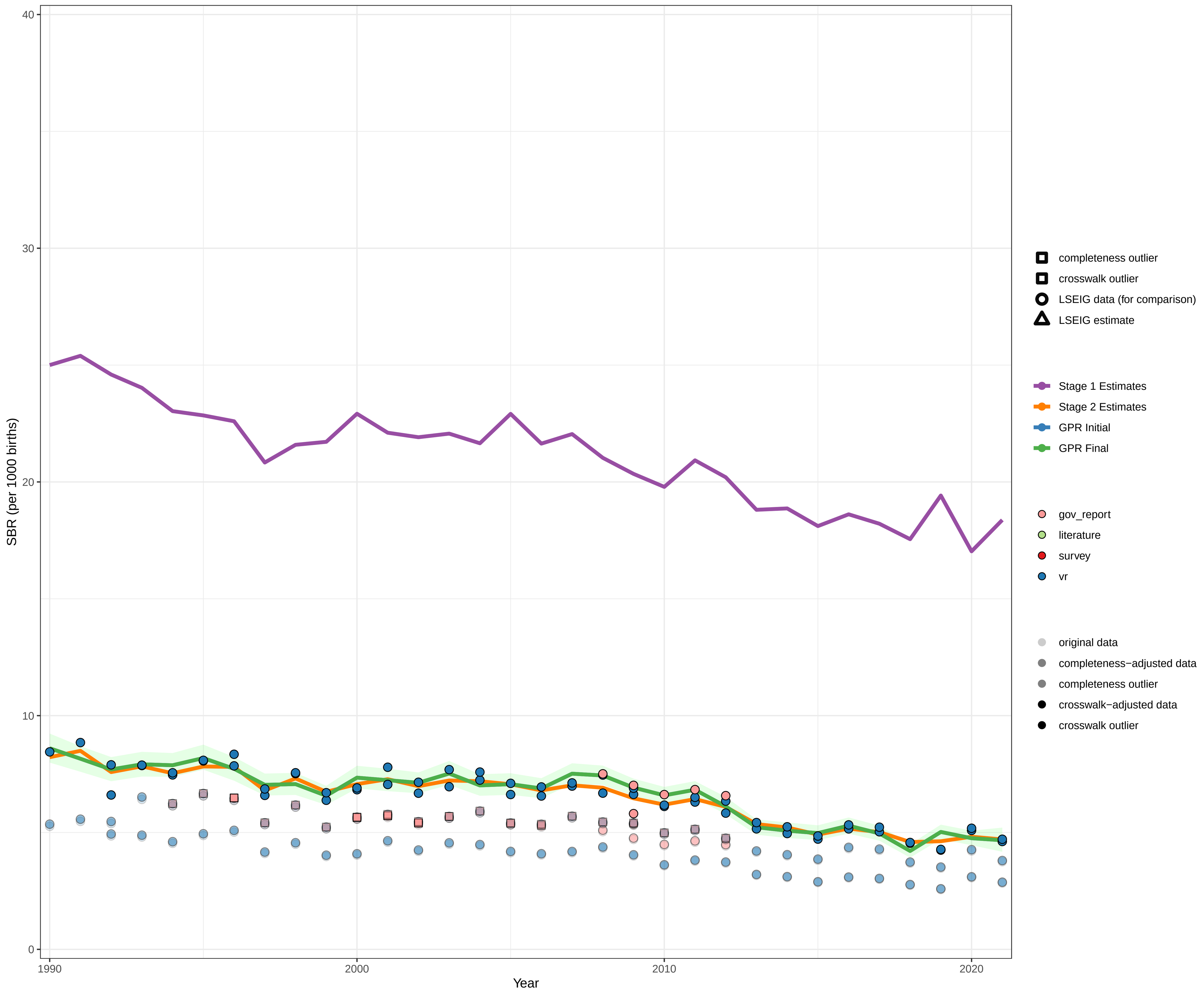

Scotland GBR_434 Western Europe Stillbirth Rate (SBR) − 28 weeks
lambda = 0.2 ; zeta = 0.99 ; scale = 5 ; amp = 0.028

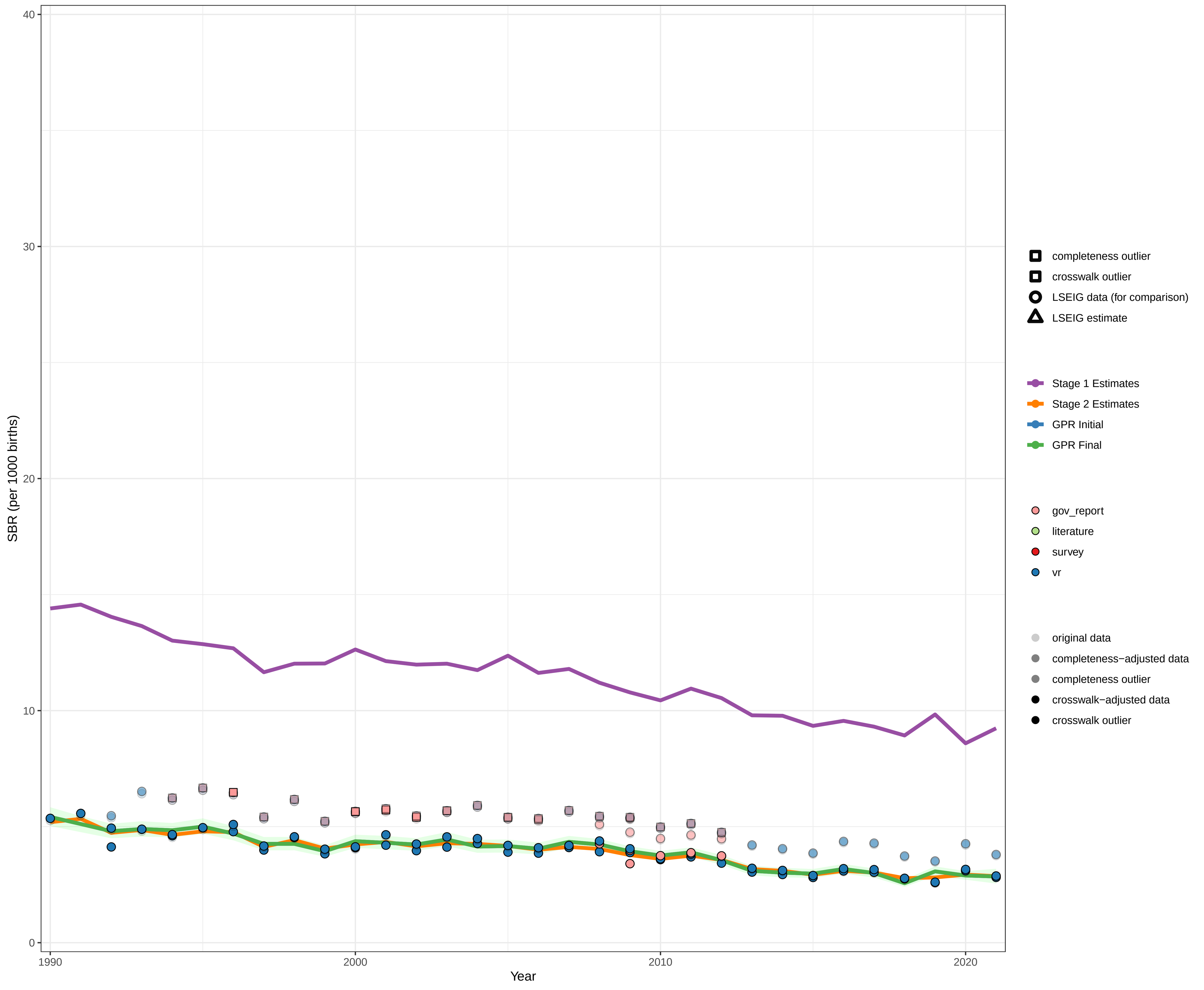

Year	Total Stillbirths (in thousands)	Total Stillbirths (in thousands)	SBR (per 1000 births)	SBR (per 1000 births)	SBR/NMR	SBR/NMR
	20 weeks	28 weeks	20 weeks	28 weeks	20 weeks	28 weeks
1990	0.6 (0.5 to 0.6)	0.4 (0.3 to 0.4)	8.6 (8.0 to 9.2)	5.4 (5.0 to 5.8)	1.86 (1.73 to 2.00)	1.17 (1.09 to 1.26)
1995	0.5 (0.5 to 0.5)	0.3 (0.3 to 0.3)	8.2 (7.7 to 8.8)	5.0 (4.7 to 5.4)	2.09 (1.96 to 2.24)	1.28 (1.20 to 1.37)
2000	0.4 (0.4 to 0.4)	0.2 (0.2 to 0.2)	7.3 (6.9 to 7.9)	4.4 (4.1 to 4.7)	2.02 (1.89 to 2.16)	1.20 (1.12 to 1.28)
2005	0.4 (0.4 to 0.4)	0.2 (0.2 to 0.2)	7.1 (6.6 to 7.5)	4.2 (3.9 to 4.4)	2.12 (1.99 to 2.26)	1.25 (1.17 to 1.34)
2010	0.4 (0.4 to 0.4)	0.2 (0.2 to 0.2)	6.6 (6.2 to 7.0)	3.8 (3.6 to 4.0)	2.50 (2.37 to 2.64)	1.42 (1.35 to 1.50)
2015	0.3 (0.3 to 0.3)	0.2 (0.2 to 0.2)	5.0 (4.7 to 5.3)	3.0 (2.8 to 3.2)	2.22 (2.09 to 2.37)	1.33 (1.25 to 1.42)
2020	0.2 (0.2 to 0.2)	0.1 (0.1 to 0.1)	4.8 (4.5 to 5.1)	2.9 (2.7 to 3.1)	2.42 (2.27 to 2.59)	1.47 (1.38 to 1.57)
2021	0.2 (0.2 to 0.3)	0.1 (0.1 to 0.2)	4.7 (4.2 to 5.2)	2.9 (2.6 to 3.2)	2.22 (1.99 to 2.48)	1.36 (1.22 to 1.52)

Wales GBR_4636 Western Europe Stillbirth Rate / Neonatal Mortality Rate (SBR/NMR) − 20 weeks
lambda = 0.2 ; zeta = 0.99 ; scale = 5 ; amp = 0.028

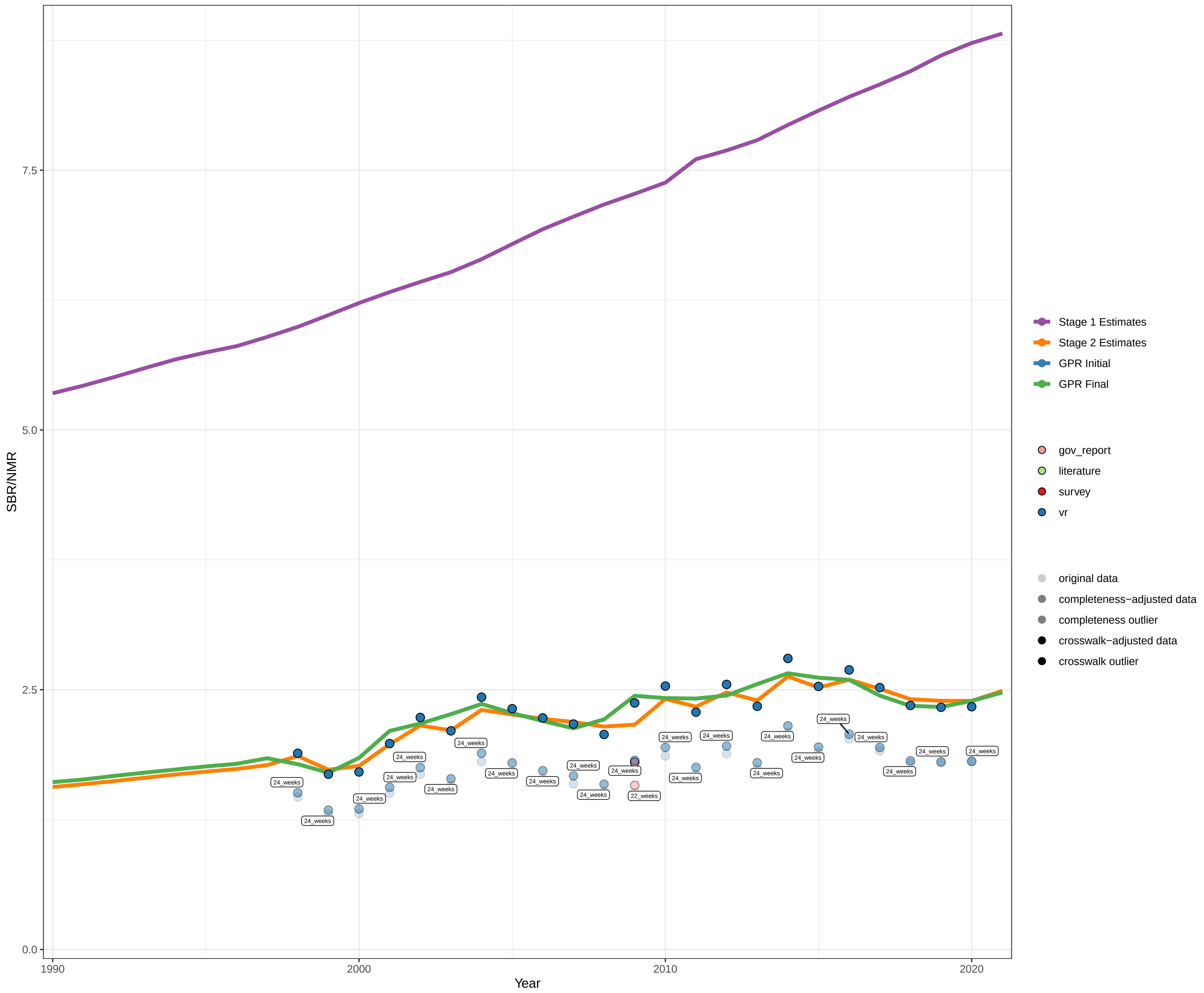

Wales GBR_4636 Western Europe Stillbirth Rate / Neonatal Mortality Rate (SBR/NMR) − 28 weeks
lambda = 0.2 ; zeta = 0.99 ; scale = 5 ; amp = 0.028

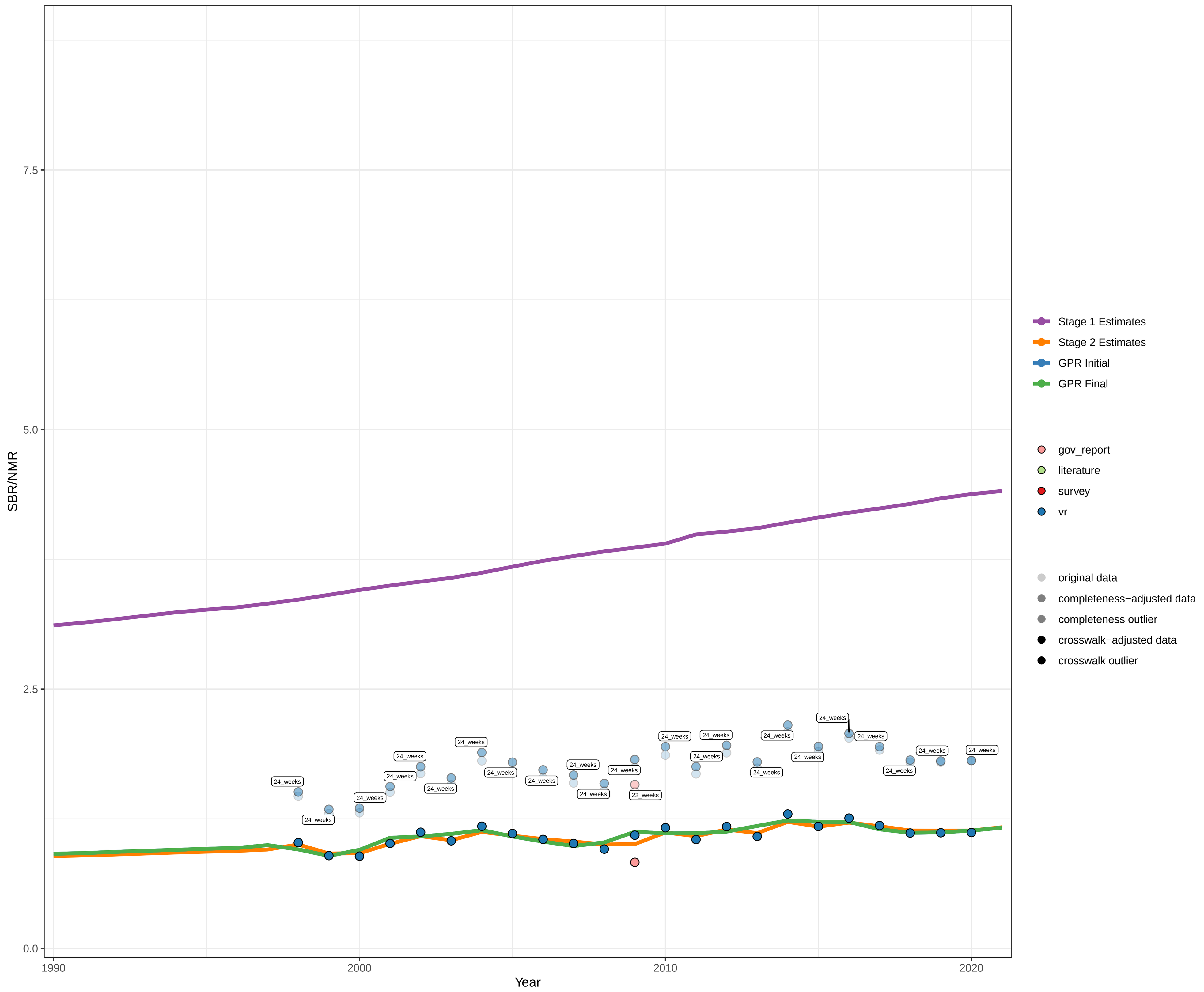

Definition	Years	Outlier	Source.Type	Title
22_weeks	2009	0	Statistical Report	United Kingdom Perinatal Mortality
24_weeks	1998−2007	0	VR	United Kingdom − England and Wales Birth Statistics
24_weeks	2008−2020	0	VR	United Kingdom − England and Wales Child Mortality Statistics

Wales GBR_4636 Western Europe Stillbirth Rate (SBR) − 20 weeks
lambda = 0.2 ; zeta = 0.99 ; scale = 5 ; amp = 0.028

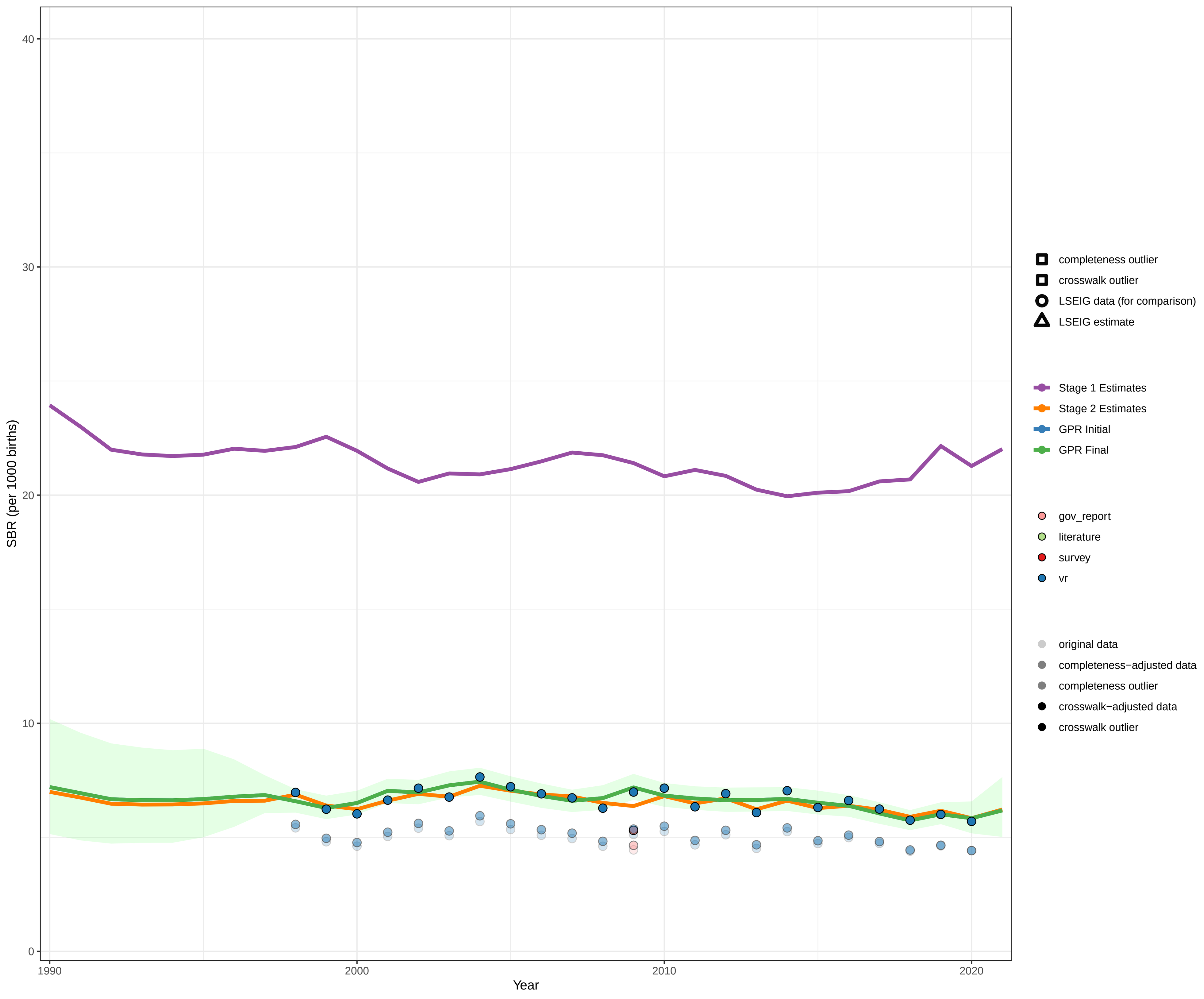

Wales GBR_4636 Western Europe Stillbirth Rate (SBR) − 28 weeks
lambda = 0.2 ; zeta = 0.99 ; scale = 5 ; amp = 0.028

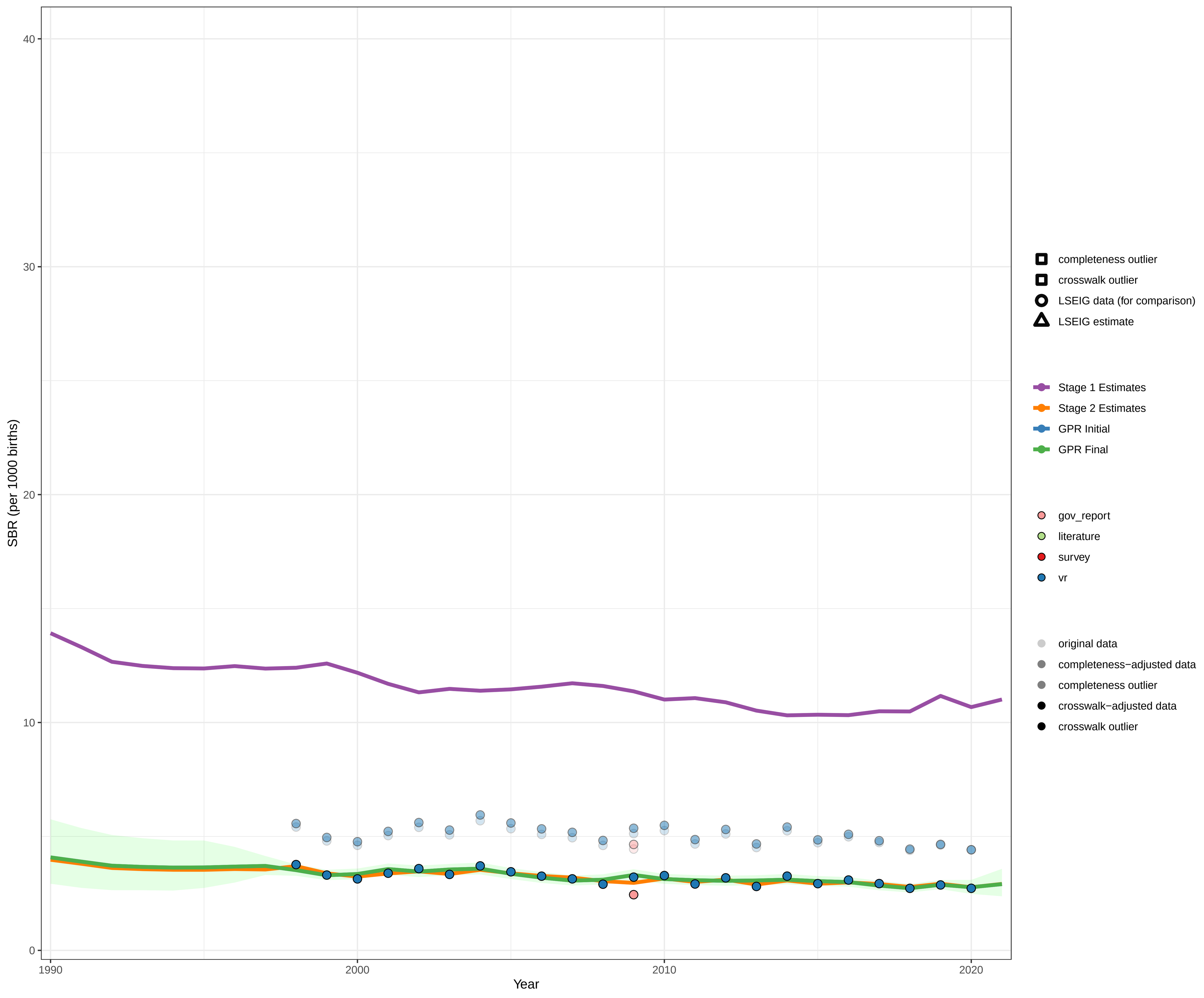

Year	Total Stillbirths (in thousands)	Total Stillbirths (in thousands)	SBR (per 1000 births)	SBR (per 1000 births)	SBR/NMR	SBR/NMR
	20 weeks	28 weeks	20 weeks	28 weeks	20 weeks	28 weeks
1990	0.3 (0.2 to 0.4)	0.2 (0.1 to 0.2)	7.2 (5.1 to 10.2)	4.1 (2.9 to 5.8)	1.61 (1.15 to 2.28)	0.91 (0.65 to 1.29)
1995	0.2 (0.2 to 0.3)	0.1 (0.1 to 0.2)	6.7 (5.0 to 8.9)	3.6 (2.7 to 4.8)	1.76 (1.32 to 2.34)	0.96 (0.72 to 1.27)
2000	0.2 (0.2 to 0.2)	0.1 (0.1 to 0.1)	6.5 (6.0 to 7.0)	3.4 (3.1 to 3.6)	1.84 (1.70 to 2.00)	0.95 (0.89 to 1.02)
2005	0.2 (0.2 to 0.3)	0.1 (0.1 to 0.1)	7.1 (6.6 to 7.7)	3.4 (3.1 to 3.6)	2.28 (2.11 to 2.46)	1.08 (1.01 to 1.16)
2010	0.2 (0.2 to 0.3)	0.1 (0.1 to 0.1)	6.8 (6.3 to 7.4)	3.1 (2.9 to 3.4)	2.42 (2.25 to 2.62)	1.11 (1.03 to 1.19)
2015	0.2 (0.2 to 0.2)	0.1 (0.1 to 0.1)	6.5 (6.0 to 7.0)	3.0 (2.8 to 3.3)	2.62 (2.41 to 2.83)	1.22 (1.14 to 1.31)
2020	0.2 (0.1 to 0.2)	0.1 (0.1 to 0.1)	5.8 (5.2 to 6.6)	2.8 (2.5 to 3.1)	2.39 (2.12 to 2.69)	1.14 (1.02 to 1.27)
2021	0.2 (0.1 to 0.2)	0.1 (0.1 to 0.1)	6.2 (5.0 to 7.6)	2.9 (2.4 to 3.6)	2.47 (2.01 to 3.06)	1.16 (0.95 to 1.43)

Latin America and Caribbean S7 NA Stillbirth Rate / Neonatal Mortality Rate (SBR/NMR) − 20 weeks
lambda = 1 ; zeta = 0.6 ; scale = 15 ; amp = 0.028

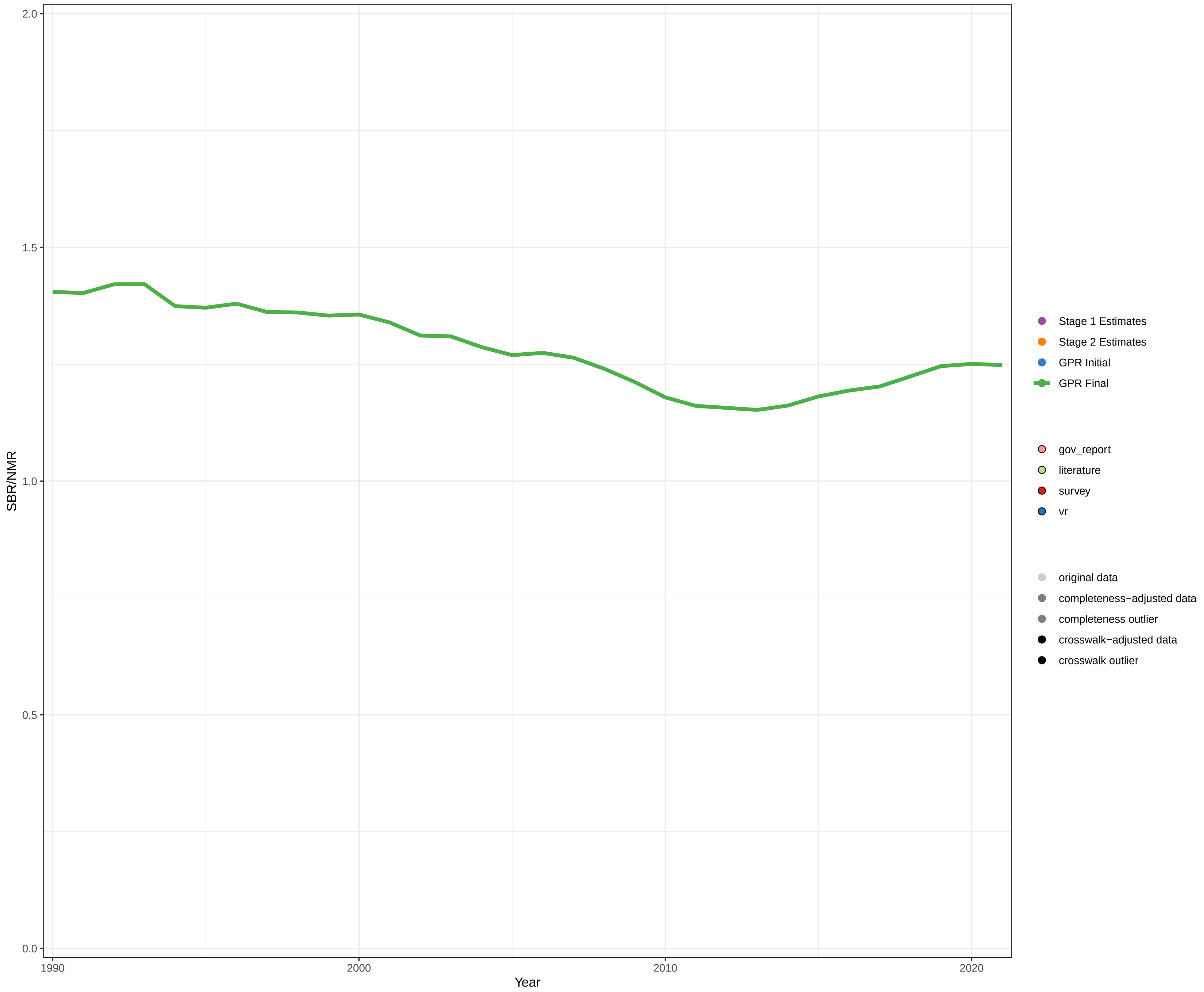

Latin America and Caribbean S7 NA Stillbirth Rate / Neonatal Mortality Rate (SBR/NMR) − 28 weeks
lambda = 1 ; zeta = 0.6 ; scale = 15 ; amp = 0.028

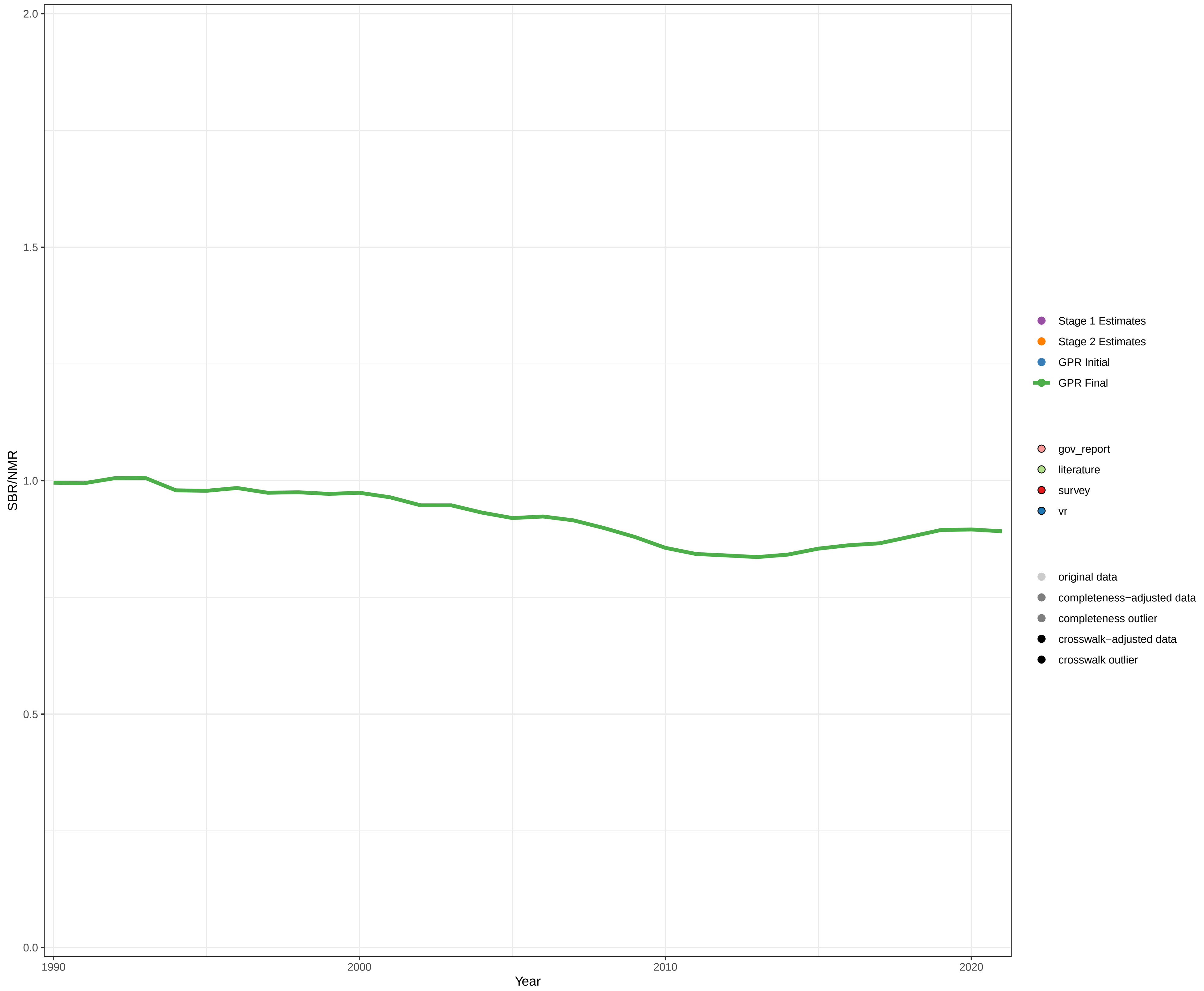

Definition	Years	Outlier	Source.Type	Title

Latin America and Caribbean S7 NA Stillbirth Rate (SBR) − 20 weeks
lambda = 1 ; zeta = 0.6 ; scale = 15 ; amp = 0.028

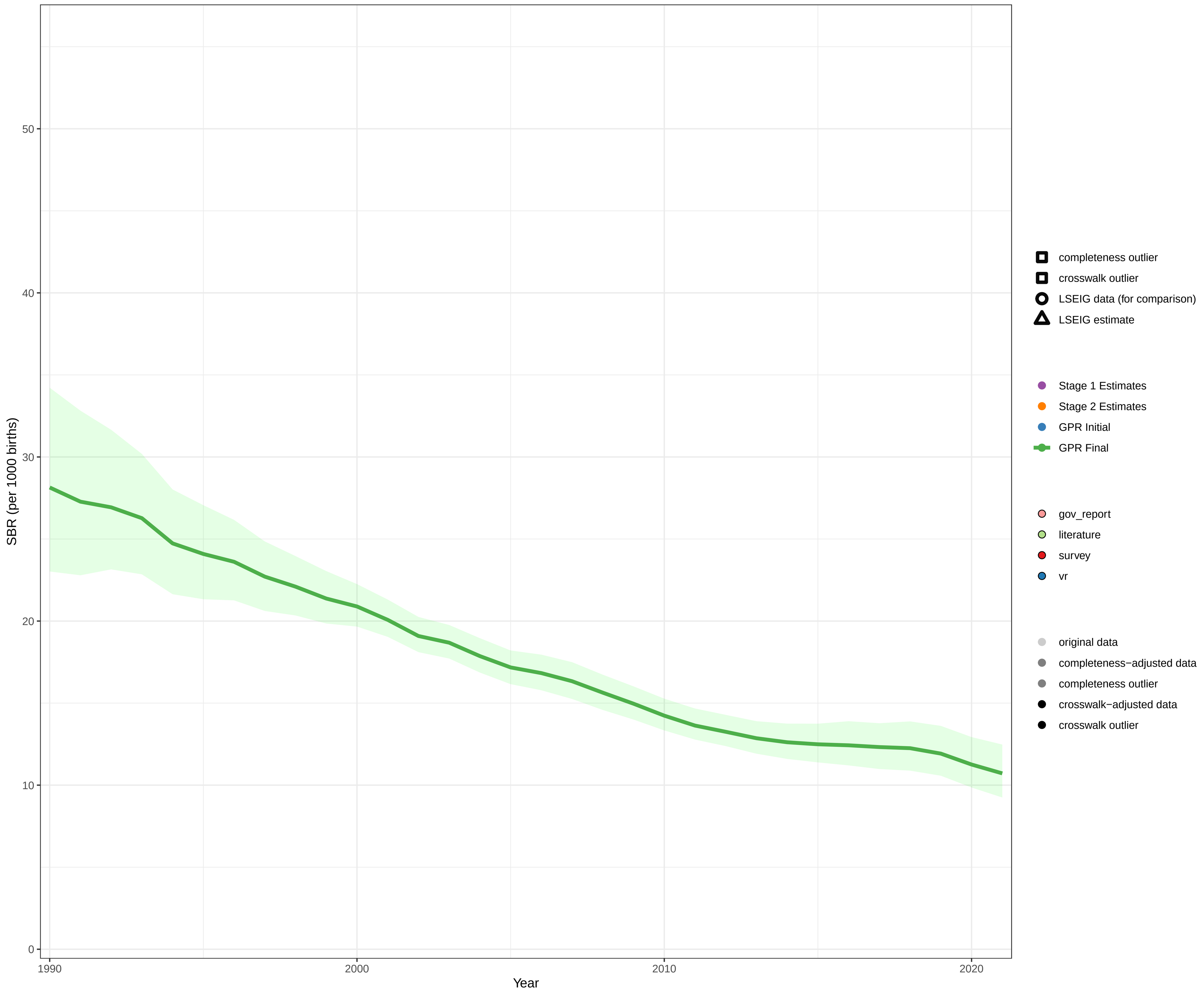

Latin America and Caribbean S7 NA Stillbirth Rate (SBR) − 28 weeks
lambda = 1 ; zeta = 0.6 ; scale = 15 ; amp = 0.028

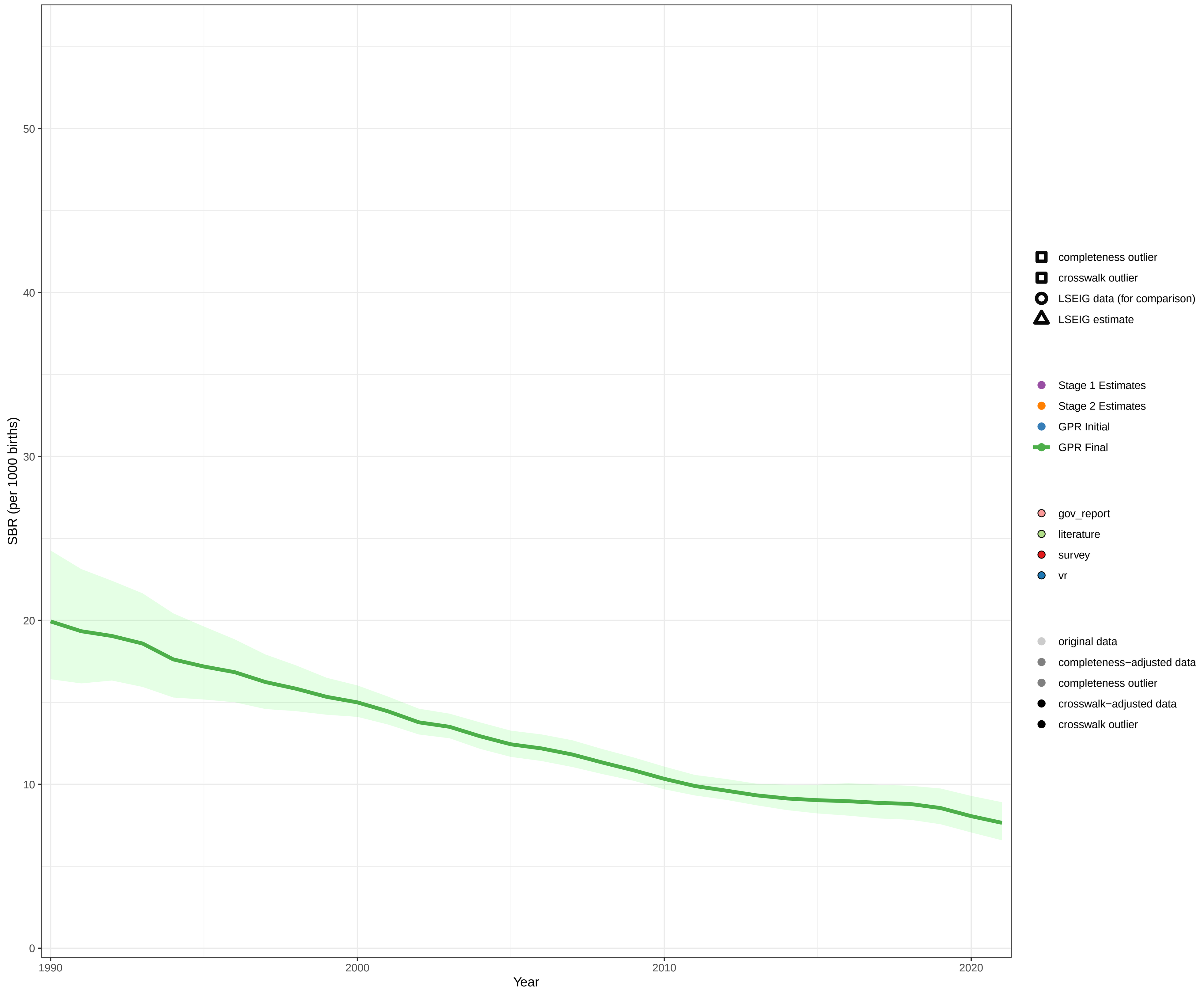

Year	Total Stillbirths (in thousands)	Total Stillbirths (in thousands)	SBR (per 1000 births)	SBR (per 1000 births)	SBR/NMR	SBR/NMR
	20 weeks	28 weeks	20 weeks	28 weeks	20 weeks	28 weeks
1990	302.0 (245.7 to 369.4)	212.2 (174.0 to 259.3)	28.1 (23.0 to 34.2)	19.9 (16.4 to 24.3)	1.40 (1.15 to 1.71)	1.00 (0.82 to 1.21)
1995	264.1 (233.1 to 297.5)	187.1 (164.8 to 214.2)	24.1 (21.3 to 27.1)	17.2 (15.2 to 19.6)	1.37 (1.21 to 1.54)	0.98 (0.86 to 1.12)
2000	227.7 (214.1 to 242.9)	162.5 (152.8 to 173.9)	20.9 (19.7 to 22.3)	15.0 (14.1 to 16.0)	1.36 (1.28 to 1.45)	0.97 (0.92 to 1.04)
2005	182.0 (171.1 to 193.2)	131.3 (123.1 to 140.2)	17.2 (16.2 to 18.2)	12.4 (11.7 to 13.3)	1.27 (1.19 to 1.35)	0.92 (0.86 to 0.98)
2010	150.9 (141.2 to 162.1)	109.1 (102.4 to 117.1)	14.2 (13.3 to 15.3)	10.3 (9.7 to 11.1)	1.18 (1.10 to 1.26)	0.86 (0.80 to 0.92)
2015	130.9 (119.2 to 144.3)	94.4 (85.9 to 104.4)	12.5 (11.4 to 13.7)	9.0 (8.2 to 10.0)	1.18 (1.08 to 1.30)	0.85 (0.78 to 0.94)
2020	108.7 (95.0 to 125.0)	77.5 (67.9 to 89.5)	11.3 (9.9 to 12.9)	8.1 (7.1 to 9.3)	1.25 (1.10 to 1.44)	0.90 (0.78 to 1.03)
2021	101.6 (87.5 to 118.5)	72.3 (62.2 to 84.3)	10.7 (9.2 to 12.5)	7.7 (6.6 to 8.9)	1.25 (1.08 to 1.45)	0.89 (0.77 to 1.04)

Andean Latin America R11 Andean Latin America Stillbirth Rate / Neonatal Mortality Rate (SBR/NMR) − 20 weeks
lambda = 1 ; zeta = 0.6 ; scale = 15 ; amp = 0.028

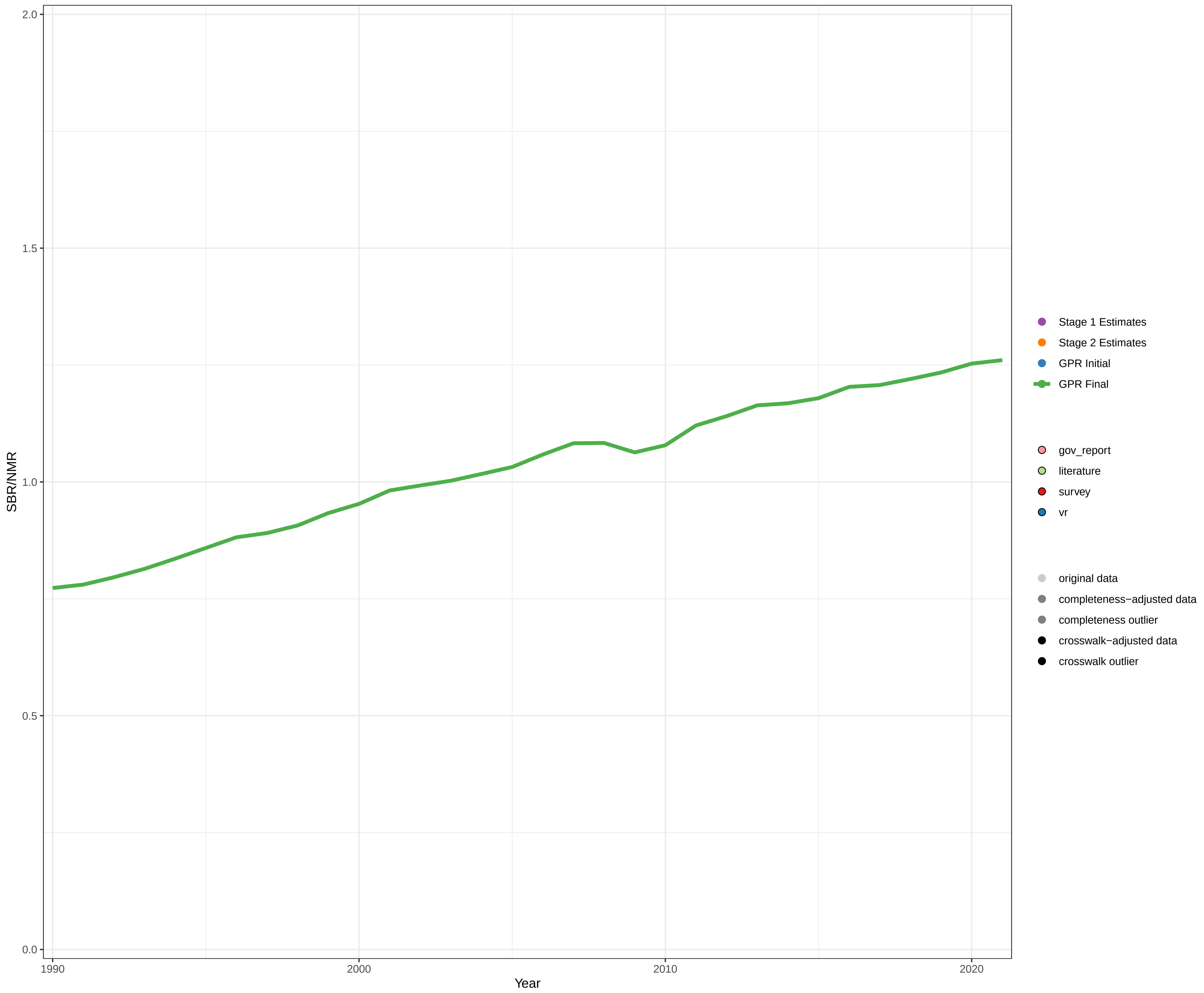

Andean Latin America R11 Andean Latin America Stillbirth Rate / Neonatal Mortality Rate (SBR/NMR) − 28 weeks
lambda = 1 ; zeta = 0.6 ; scale = 15 ; amp = 0.028

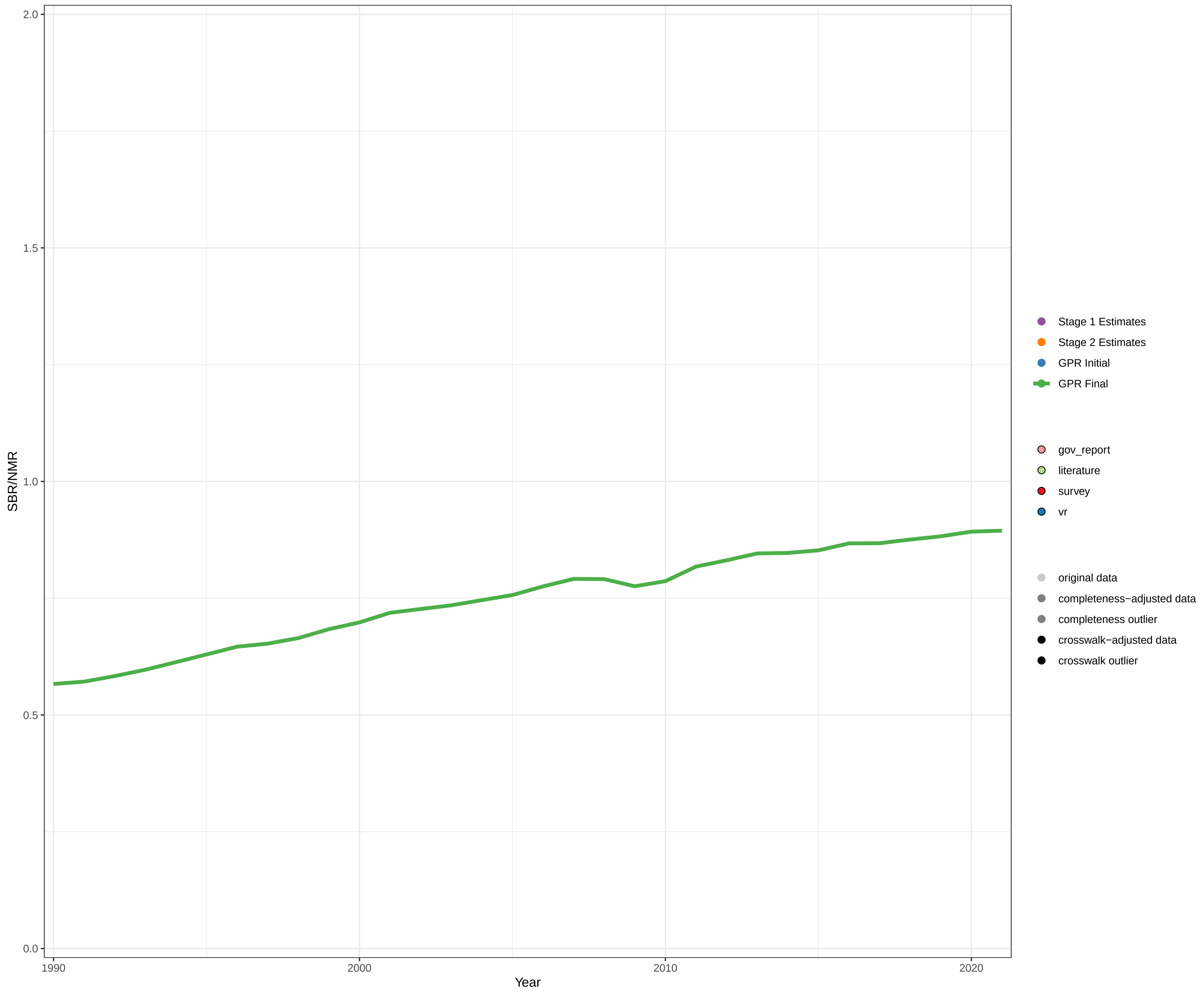

Definition	Years	Outlier	Source.Type	Title

Andean Latin America R11 Andean Latin America Stillbirth Rate (SBR) − 20 weeks
lambda = 1 ; zeta = 0.6 ; scale = 15 ; amp = 0.028

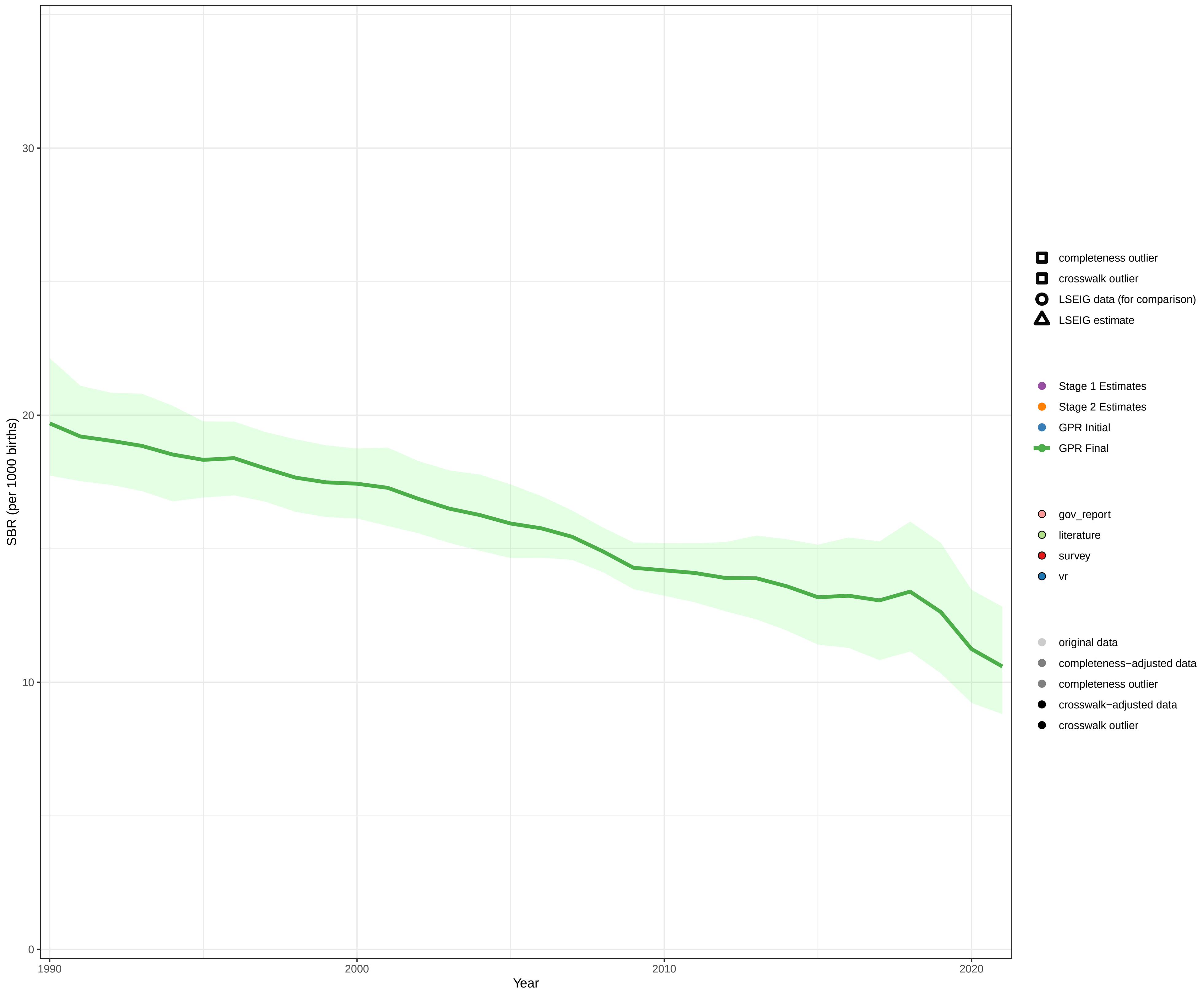

Andean Latin America R11 Andean Latin America Stillbirth Rate (SBR) − 28 weeks
lambda = 1 ; zeta = 0.6 ; scale = 15 ; amp = 0.028

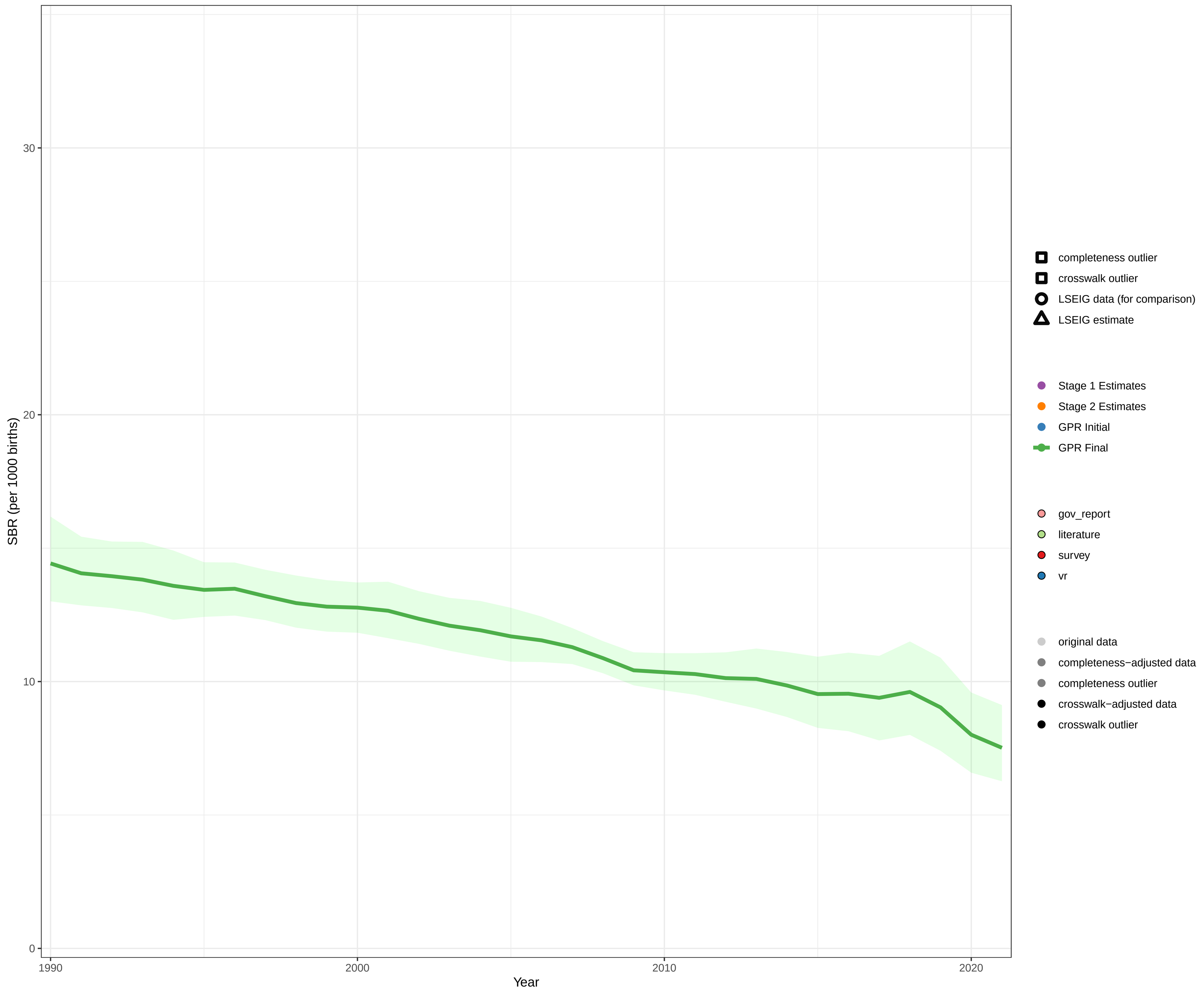

Year	Total Stillbirths (in thousands)	Total Stillbirths (in thousands)	SBR (per 1000 births)	SBR (per 1000 births)	SBR/NMR	SBR/NMR
	20 weeks	28 weeks	20 weeks	28 weeks	20 weeks	28 weeks
1990	23.8 (21.4 to 26.8)	17.3 (15.6 to 19.5)	19.7 (17.7 to 22.1)	14.4 (13.0 to 16.2)	0.77 (0.70 to 0.87)	0.57 (0.51 to 0.64)
1995	22.8 (21.0 to 24.6)	16.6 (15.4 to 17.9)	18.3 (16.9 to 19.8)	13.4 (12.4 to 14.5)	0.86 (0.79 to 0.93)	0.63 (0.58 to 0.68)
2000	21.5 (19.9 to 23.2)	15.7 (14.5 to 16.9)	17.4 (16.1 to 18.8)	12.8 (11.8 to 13.7)	0.95 (0.88 to 1.03)	0.70 (0.65 to 0.75)
2005	19.3 (17.7 to 21.1)	14.1 (13.0 to 15.4)	15.9 (14.7 to 17.4)	11.7 (10.7 to 12.8)	1.03 (0.95 to 1.13)	0.76 (0.70 to 0.83)
2010	17.6 (16.4 to 18.9)	12.8 (12.0 to 13.7)	14.2 (13.2 to 15.2)	10.3 (9.7 to 11.1)	1.08 (1.01 to 1.16)	0.79 (0.73 to 0.84)
2015	16.8 (14.5 to 19.4)	12.1 (10.5 to 13.9)	13.2 (11.4 to 15.2)	9.5 (8.3 to 10.9)	1.18 (1.02 to 1.36)	0.85 (0.74 to 0.98)
2020	14.2 (11.6 to 17.1)	10.1 (8.3 to 12.1)	11.2 (9.2 to 13.5)	8.0 (6.6 to 9.6)	1.25 (1.03 to 1.50)	0.89 (0.73 to 1.07)
2021	13.3 (11.0 to 16.2)	9.4 (7.8 to 11.4)	10.6 (8.8 to 12.8)	7.5 (6.3 to 9.1)	1.26 (1.05 to 1.53)	0.89 (0.75 to 1.09)

Bolivia (Plurinational State of)   BOL   Andean Latin America   Stillbirth Rate / Neonatal Mortality Rate (SBR/NMR) − 20 weeks
lambda = 0.8 ; zeta = 0.7 ; scale = 15 ; amp = 0.028

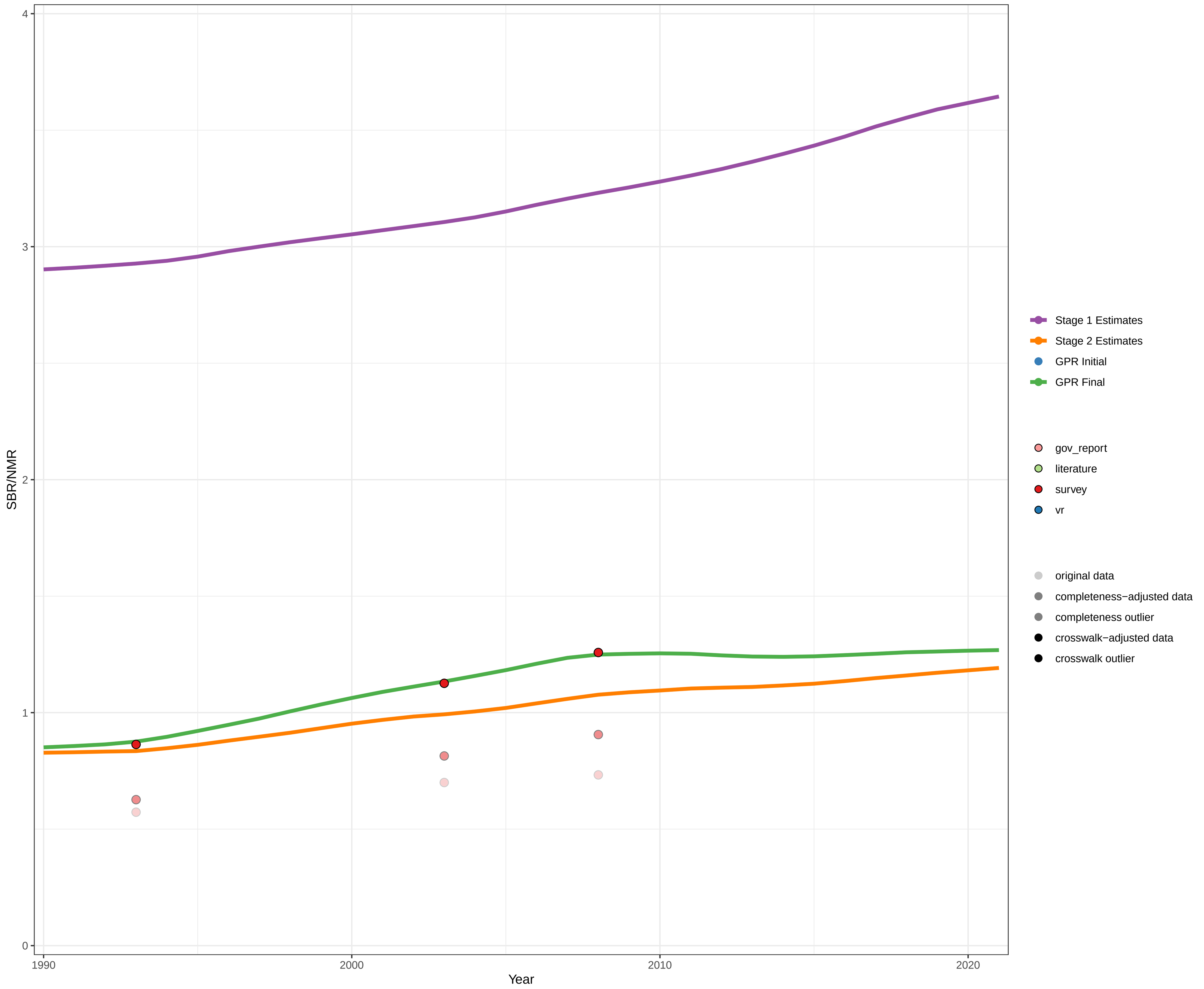

Bolivia (Plurinational State of)   BOL   Andean Latin America   Stillbirth Rate / Neonatal Mortality Rate (SBR/NMR) − 28 weeks
lambda = 0.8 ; zeta = 0.7 ; scale = 15 ; amp = 0.028

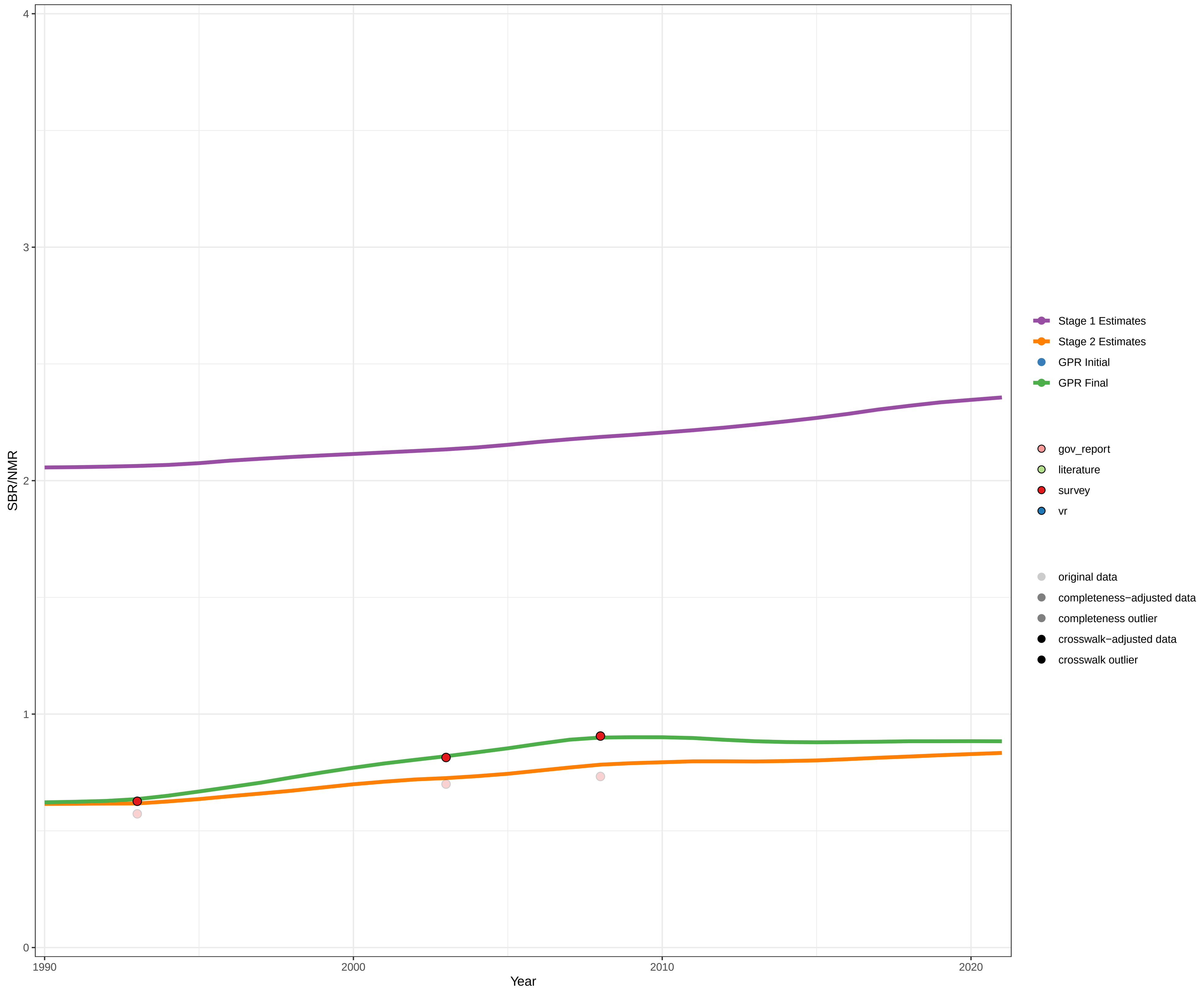

Definition	Years	Outlier	Source.Type	Title
28_weeks	1993	0	Survey	Bolivia Demographic and Health Survey 1993−1994
28_weeks	2003	0	Survey	Bolivia Demographic and Health Survey 2003−2004
28_weeks	2008	0	Survey	Bolivia Demographic and Health Survey 2008

Bolivia (Plurinational State of)   BOL   Andean Latin America   Stillbirth Rate (SBR) − 20 weeks
lambda = 0.8 ; zeta = 0.7 ; scale = 15 ; amp = 0.028

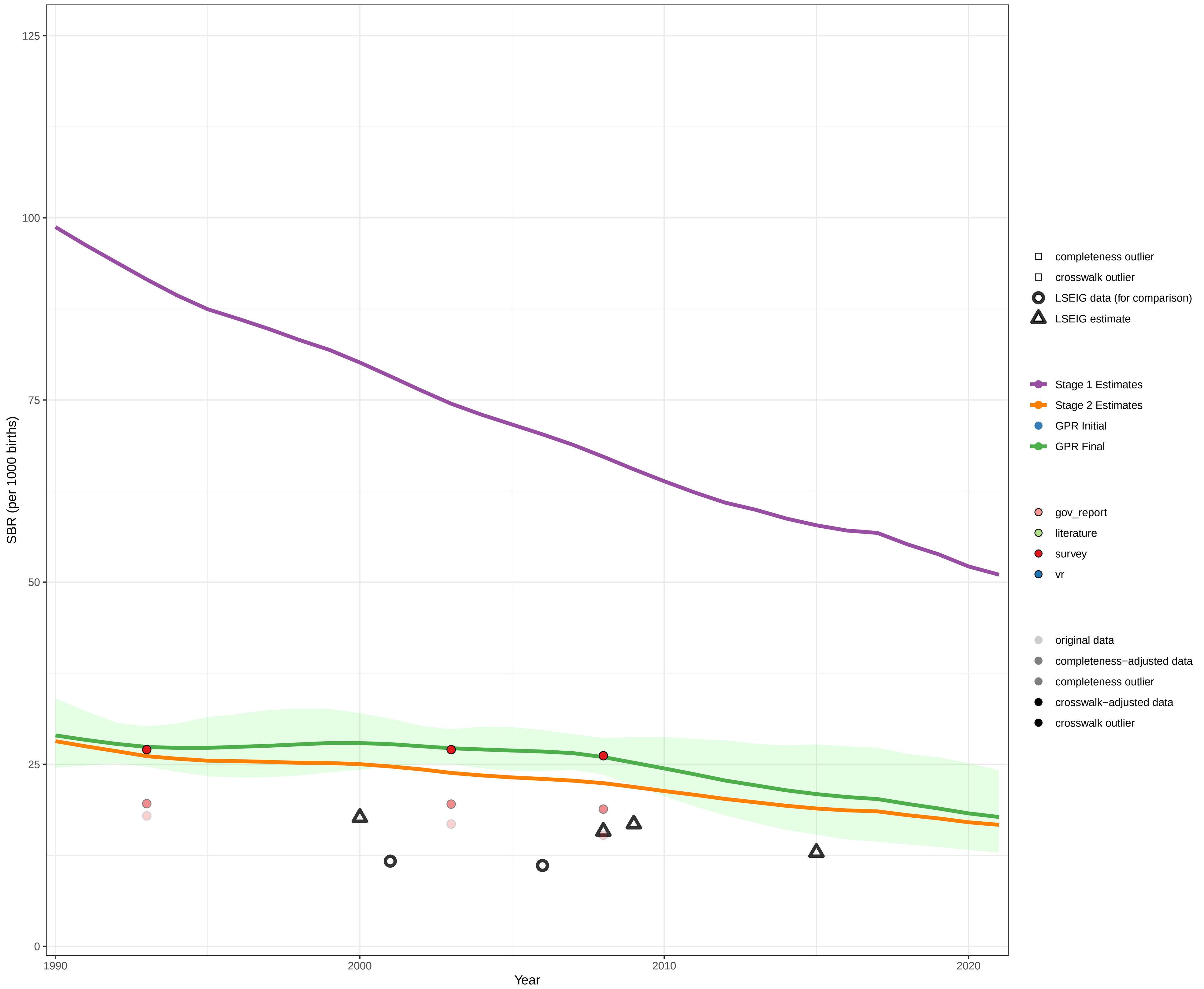

Bolivia (Plurinational State of)   BOL   Andean Latin America   Stillbirth Rate (SBR) − 28 weeks
lambda = 0.8 ; zeta = 0.7 ; scale = 15 ; amp = 0.028

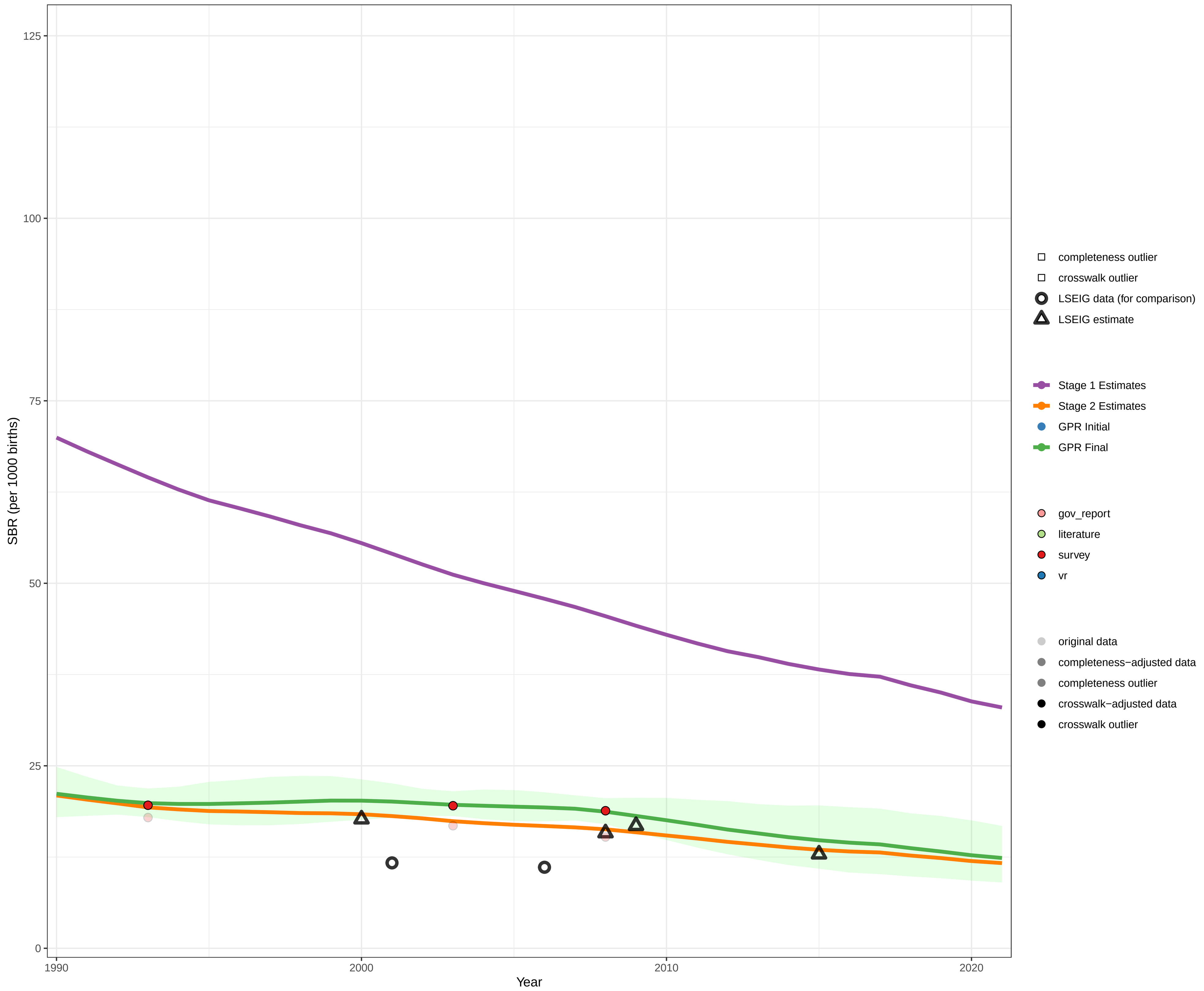

Year	Total Stillbirths (in thousands)	Total Stillbirths (in thousands)	SBR (per 1000 births)	SBR (per 1000 births)	SBR/NMR	SBR/NMR
	20 weeks	28 weeks	20 weeks	28 weeks	20 weeks	28 weeks
1990	6.8 (5.7 to 8.0)	4.9 (4.2 to 5.8)	29.0 (24.5 to 34.0)	21.2 (18.0 to 24.8)	0.85 (0.72 to 1.00)	0.62 (0.53 to 0.73)
1995	6.6 (5.6 to 7.7)	4.8 (4.1 to 5.5)	27.3 (23.3 to 31.5)	19.8 (17.0 to 22.8)	0.92 (0.79 to 1.06)	0.67 (0.57 to 0.77)
2000	6.9 (6.0 to 7.9)	5.0 (4.3 to 5.7)	27.9 (24.3 to 32.0)	20.2 (17.6 to 23.1)	1.06 (0.92 to 1.22)	0.77 (0.67 to 0.88)
2005	6.8 (6.0 to 7.6)	4.9 (4.3 to 5.4)	26.9 (24.1 to 30.1)	19.4 (17.4 to 21.7)	1.18 (1.06 to 1.32)	0.85 (0.77 to 0.95)
2010	6.4 (5.4 to 7.5)	4.5 (3.8 to 5.4)	24.4 (20.6 to 28.7)	17.5 (14.8 to 20.6)	1.25 (1.06 to 1.48)	0.90 (0.76 to 1.06)
2015	5.5 (4.0 to 7.3)	3.9 (2.8 to 5.1)	20.9 (15.3 to 27.7)	14.8 (10.9 to 19.6)	1.24 (0.91 to 1.65)	0.88 (0.65 to 1.16)
2020	4.6 (3.3 to 6.4)	3.2 (2.3 to 4.4)	18.3 (13.2 to 25.2)	12.7 (9.3 to 17.5)	1.27 (0.92 to 1.75)	0.88 (0.64 to 1.22)
2021	4.4 (3.2 to 6.1)	3.1 (2.2 to 4.2)	17.8 (12.9 to 24.2)	12.4 (9.0 to 16.8)	1.27 (0.92 to 1.73)	0.88 (0.64 to 1.20)

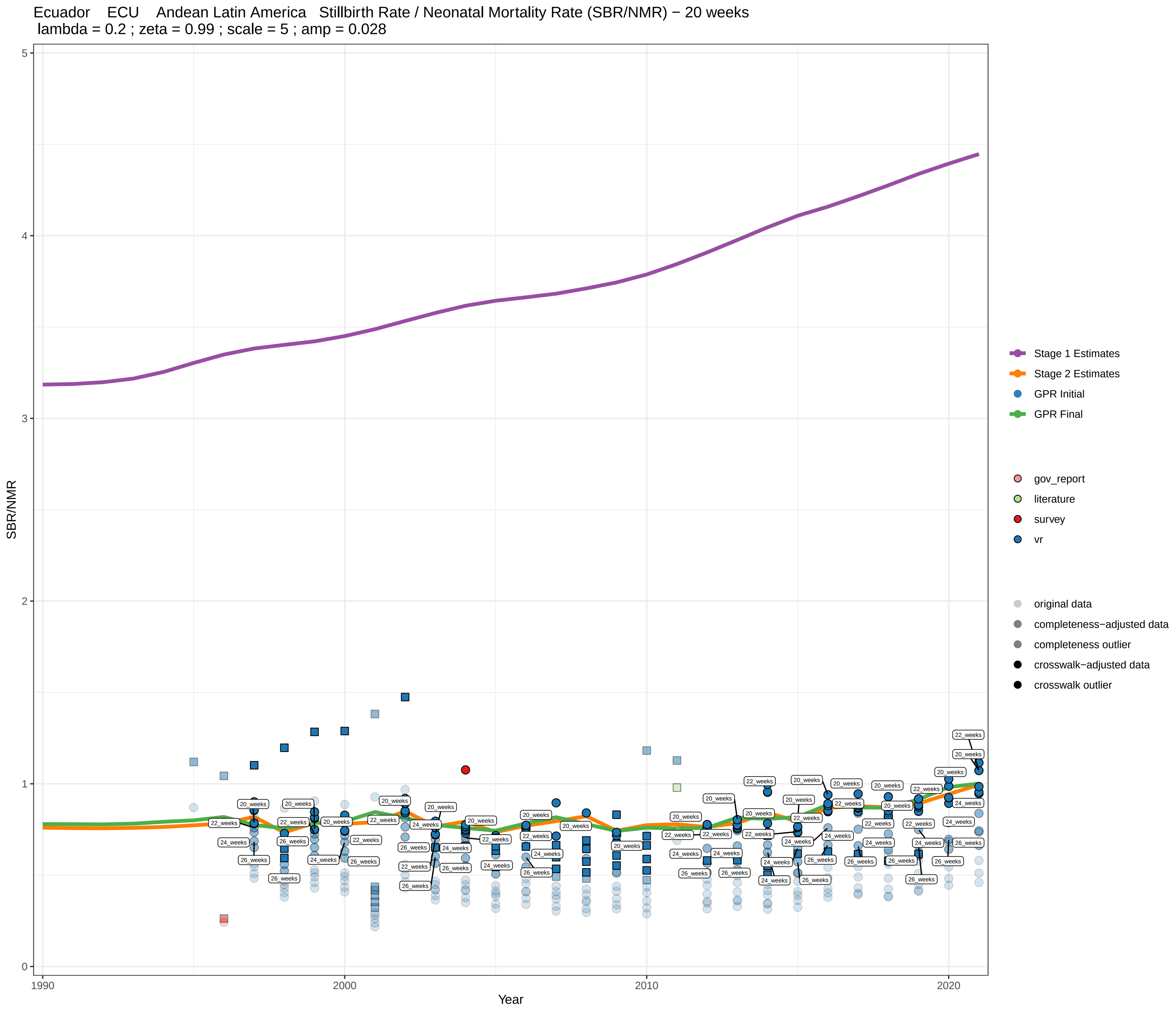

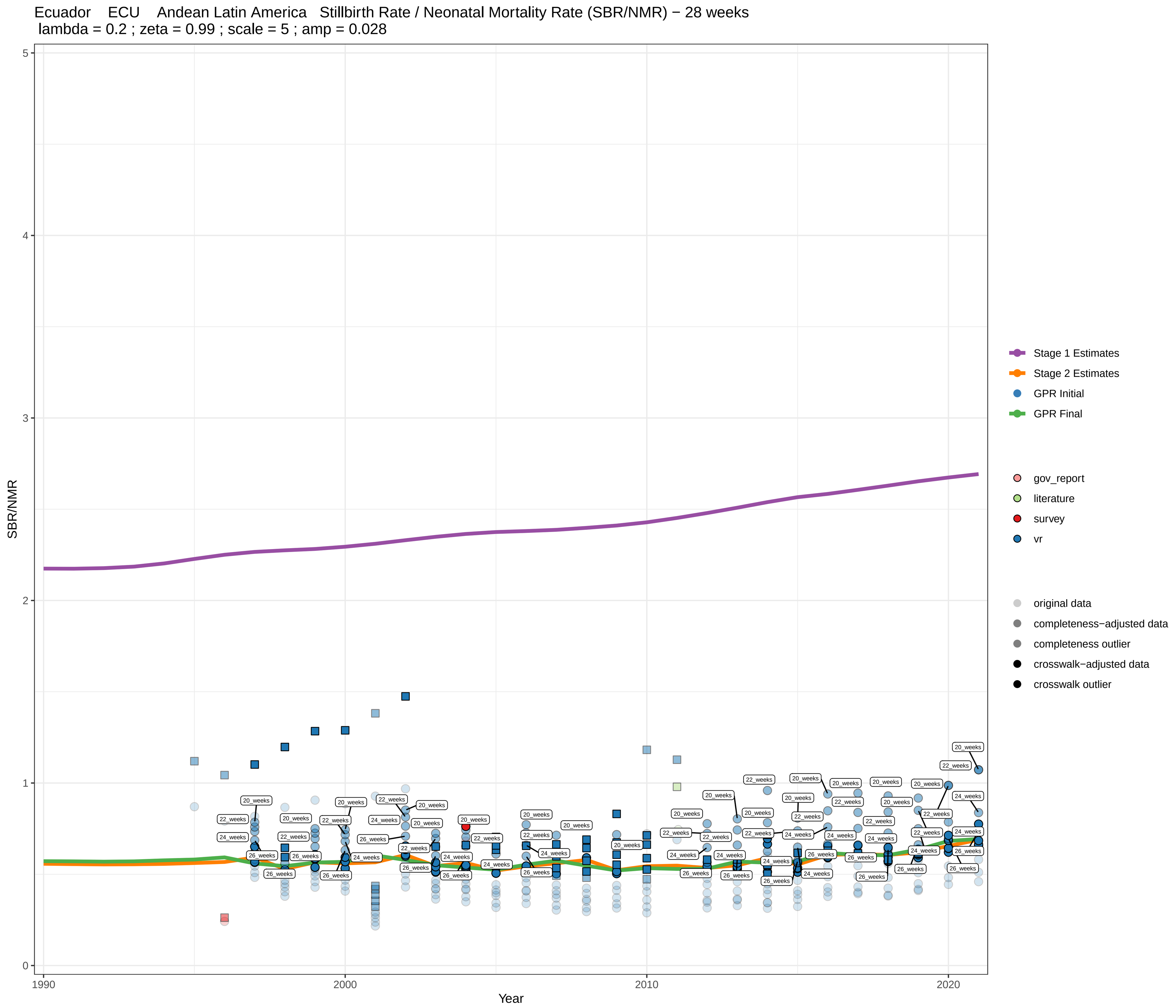

Definition	Years	Outlier	Source.Type	Title
20_weeks	1997, 1999, 2000, 2002−2004, 2006, 2007, 2009, 2010, 2012−2021	0	VR	Ecuador Fetal Deaths
22_weeks, 24_weeks, 26_weeks	1997−2000, 2002−2010, 2012−2021	0	VR	Ecuador Fetal Deaths
22_weeks, 28_weeks	2014	0	VR	Ecuador Vital Statistics Yearbook: Births and Deaths
28_weeks	1997−2000, 2002−2006, 2009, 2012, 2013, 2015−2021	0	VR	Ecuador Fetal Deaths
28_weeks	2004	0	Survey	Ecuador Reproductive Health Survey 2004
28_weeks	2007, 2008	0	VR	United Nations Demographic Yearbook
20_weeks, 22_weeks, 24_weeks, 26_weeks	2001	1	VR	Ecuador Fetal Deaths
28_weeks	1995, 1996, 2001, 2010, 2011	1	VR	United Nations Demographic Yearbook
28_weeks	1996	1	Survey	Ecuador Reproductive Health Survey 1999
28_weeks	2001, 2007, 2008, 2010	1	VR	Ecuador Fetal Deaths
28_weeks_OR_1000_grams	2011	1	Sci Lit	Maternal complications and perinatal mortality: findings of the World Health Organization Multicountry Survey on Maternal and Newborn Health
20_weeks	1998, 2005, 2008	2	VR	Ecuador Fetal Deaths
28_weeks	1997−2000, 2002−2006, 2009, 2012−2019	2	VR	United Nations Demographic Yearbook
28_weeks	2014	2	VR	Ecuador Fetal Deaths

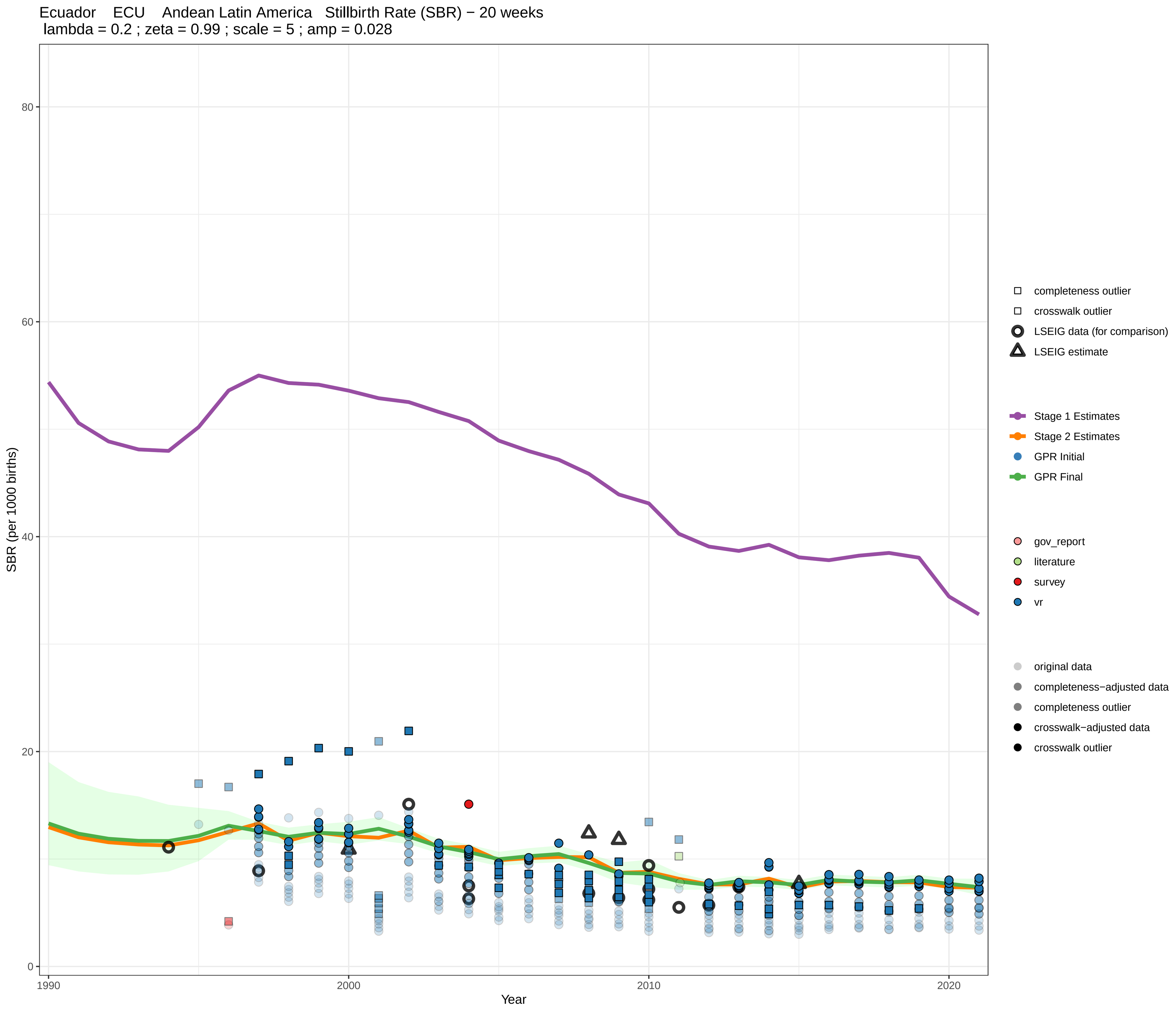

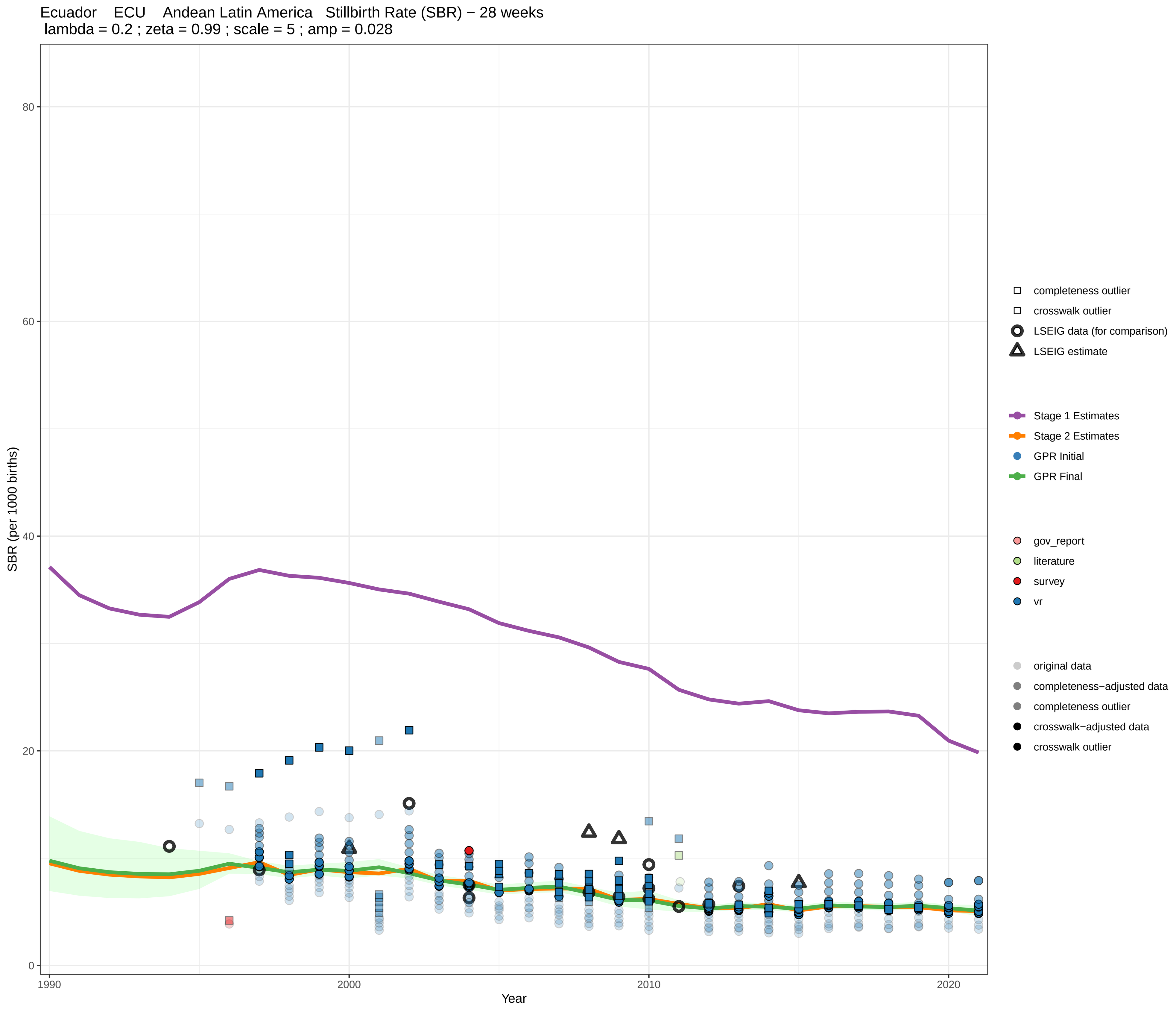

Year	Total Stillbirths (in thousands)	Total Stillbirths (in thousands)	SBR (per 1000 births)	SBR (per 1000 births)	SBR/NMR	SBR/NMR
	20 weeks	28 weeks	20 weeks	28 weeks	20 weeks	28 weeks
1990	4.0 (2.9 to 5.8)	3.0 (2.1 to 4.2)	13.3 (9.4 to 19.0)	9.8 (6.9 to 13.9)	0.78 (0.55 to 1.11)	0.57 (0.41 to 0.81)
1995	4.0 (3.2 to 4.9)	2.9 (2.3 to 3.5)	12.2 (9.8 to 14.8)	8.8 (7.1 to 10.7)	0.80 (0.65 to 0.97)	0.58 (0.47 to 0.70)
2000	4.2 (3.8 to 4.6)	3.0 (2.7 to 3.3)	12.3 (11.3 to 13.5)	8.8 (8.1 to 9.6)	0.79 (0.73 to 0.87)	0.57 (0.52 to 0.62)
2005	3.3 (3.0 to 3.5)	2.3 (2.1 to 2.5)	10.0 (9.3 to 10.7)	7.1 (6.6 to 7.5)	0.74 (0.70 to 0.79)	0.53 (0.49 to 0.56)
2010	3.0 (2.6 to 3.5)	2.1 (1.8 to 2.4)	8.6 (7.4 to 9.9)	6.1 (5.2 to 7.0)	0.76 (0.65 to 0.87)	0.53 (0.46 to 0.61)
2015	2.6 (2.4 to 2.8)	1.8 (1.7 to 1.9)	7.6 (7.1 to 8.1)	5.3 (5.0 to 5.6)	0.82 (0.77 to 0.87)	0.57 (0.54 to 0.61)
2020	2.5 (2.4 to 2.7)	1.8 (1.7 to 1.9)	7.7 (7.2 to 8.2)	5.3 (5.0 to 5.7)	0.98 (0.92 to 1.04)	0.68 (0.64 to 0.73)
2021	2.4 (2.1 to 2.6)	1.7 (1.5 to 1.8)	7.4 (6.6 to 8.2)	5.1 (4.6 to 5.7)	1.00 (0.90 to 1.11)	0.69 (0.62 to 0.77)

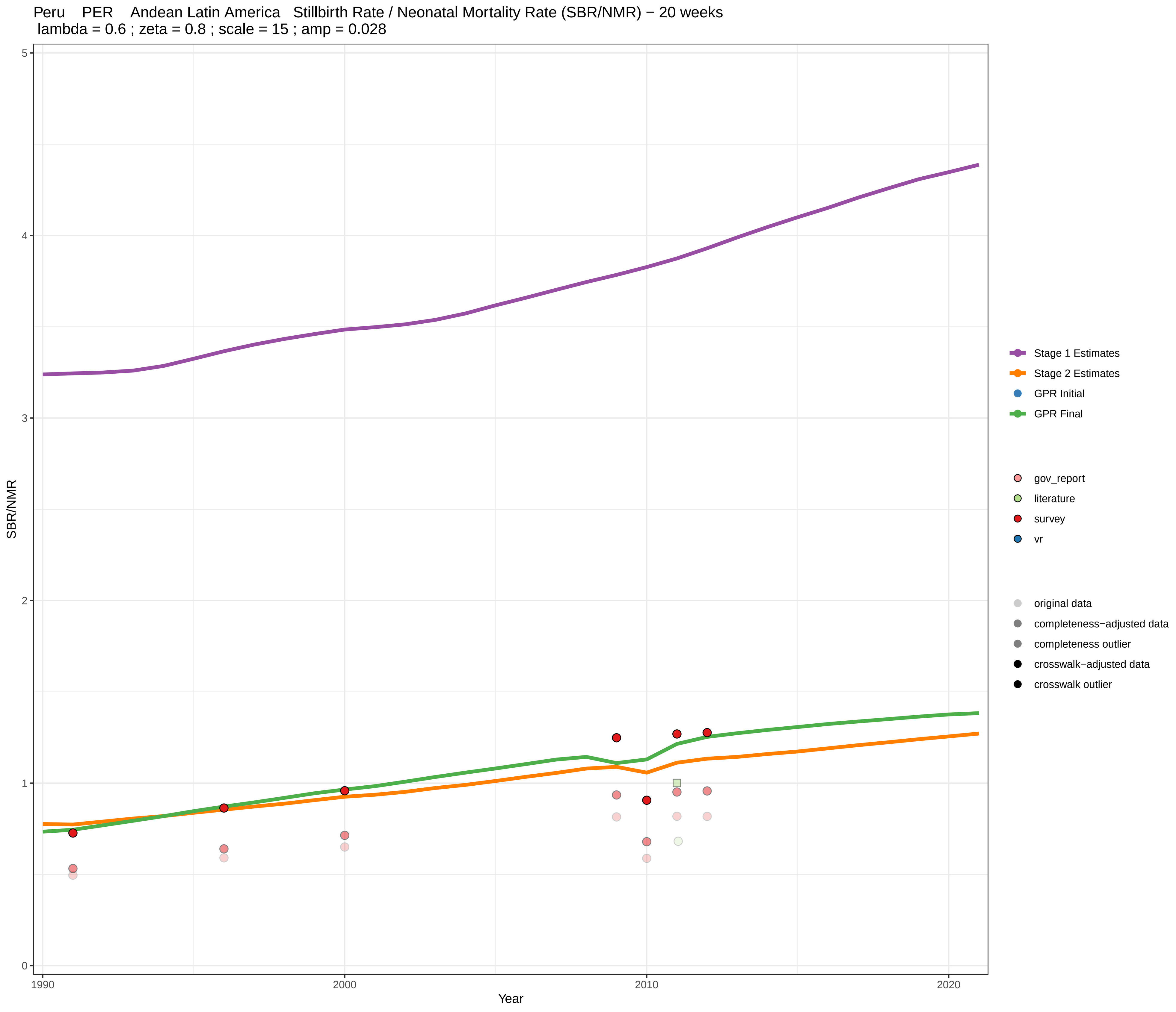

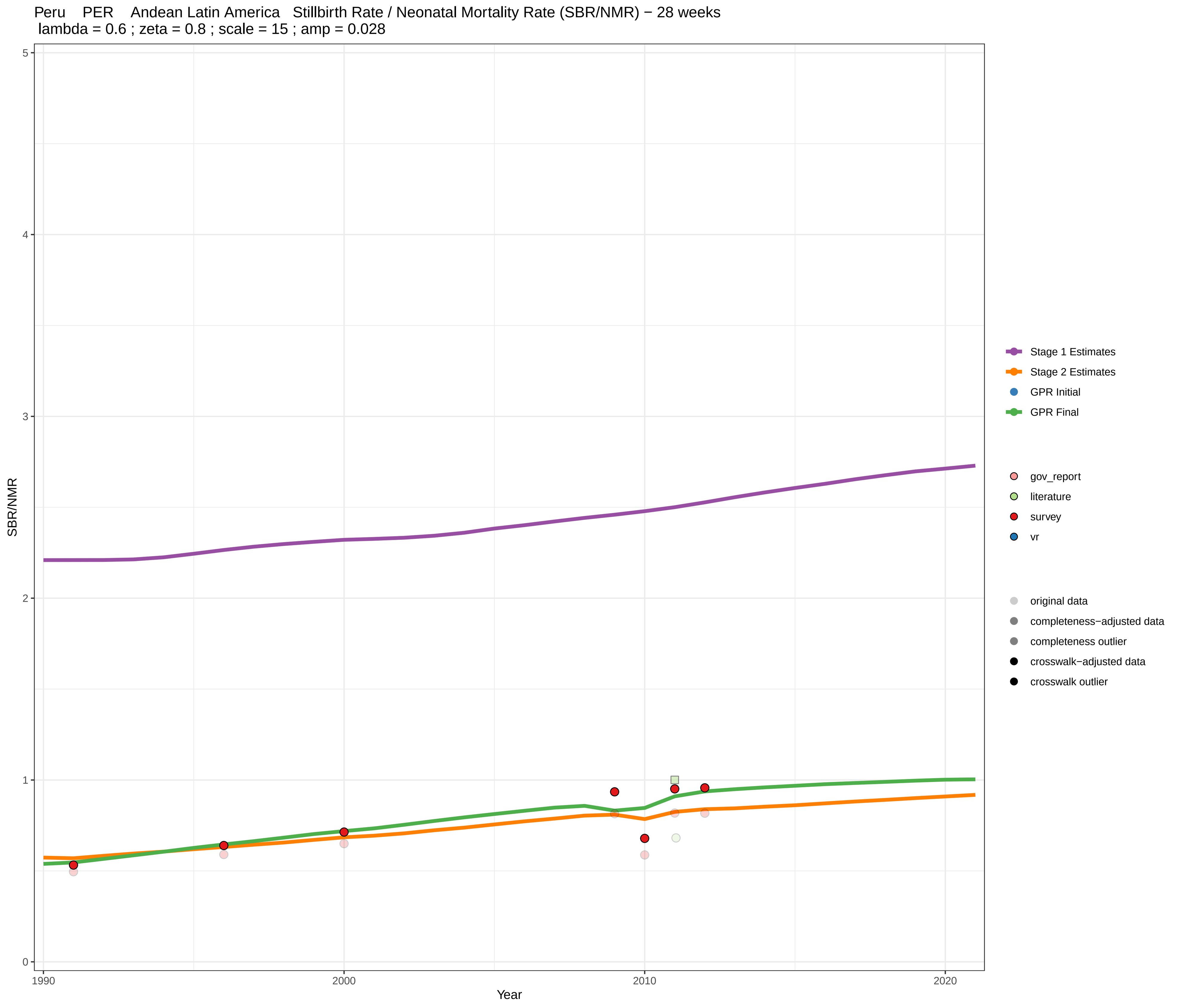

Definition	Years	Outlier	Source.Type	Title
28_weeks	1991	0	Survey	Peru Demographic and Health Survey 1991−1992
28_weeks	1996	0	Survey	Peru Demographic and Health Survey 1996
28_weeks	2000	0	Survey	Peru Demographic and Health Survey 2000
28_weeks	2009	0	Survey	Peru Continuous Demographic and Health Survey 2009
28_weeks	2010	0	Survey	Peru Continuous Demographic and Health Survey 2010
28_weeks	2011	0	Survey	Peru Continuous Demographic and Health Survey 2011
28_weeks	2012	0	Survey	Peru Continuous Demographic and Health Survey 2012
28_weeks_OR_1000_grams	2011	1	Sci Lit	Maternal complications and perinatal mortality: findings of the World Health Organization Multicountry Survey on Maternal and Newborn Health

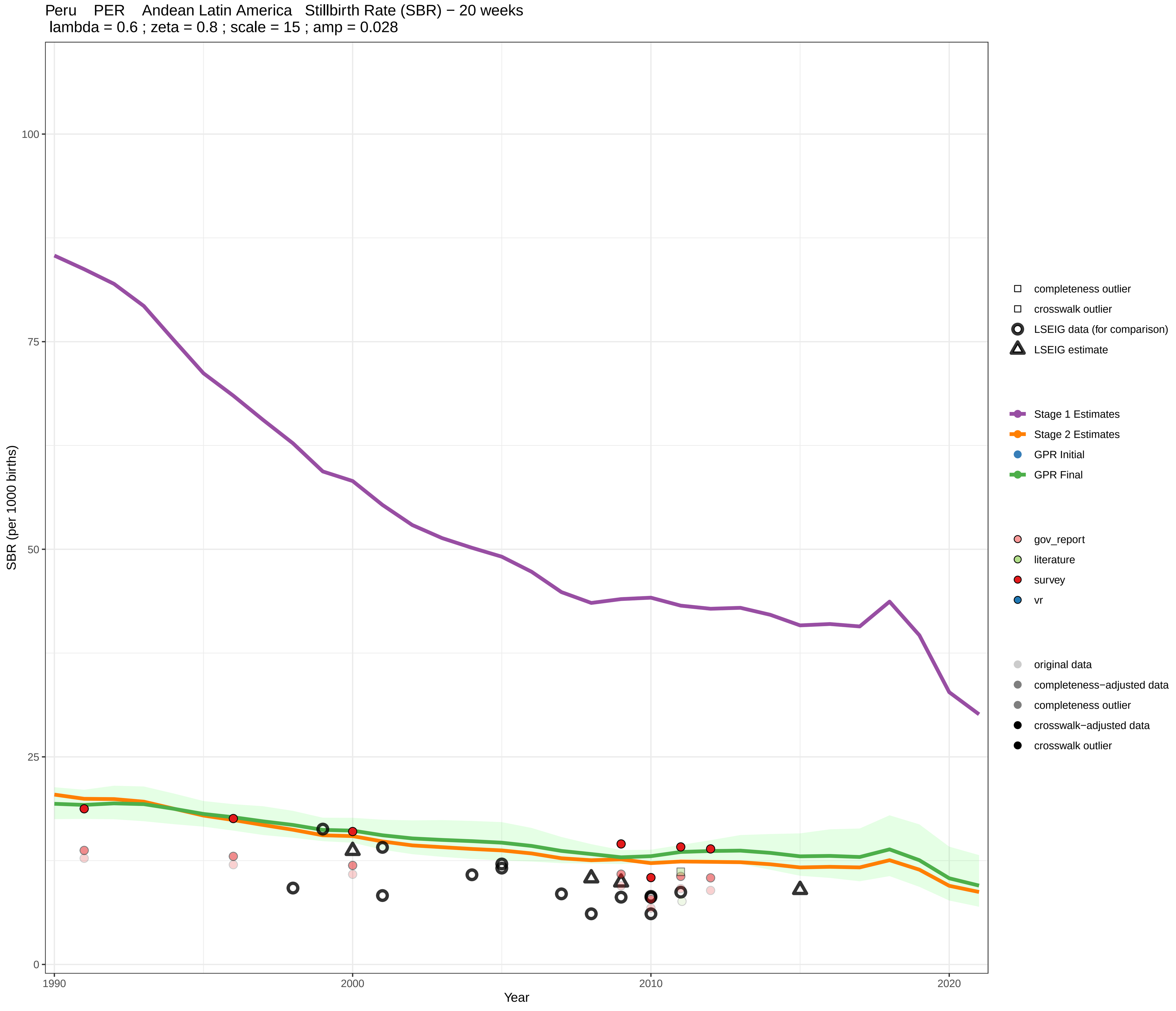

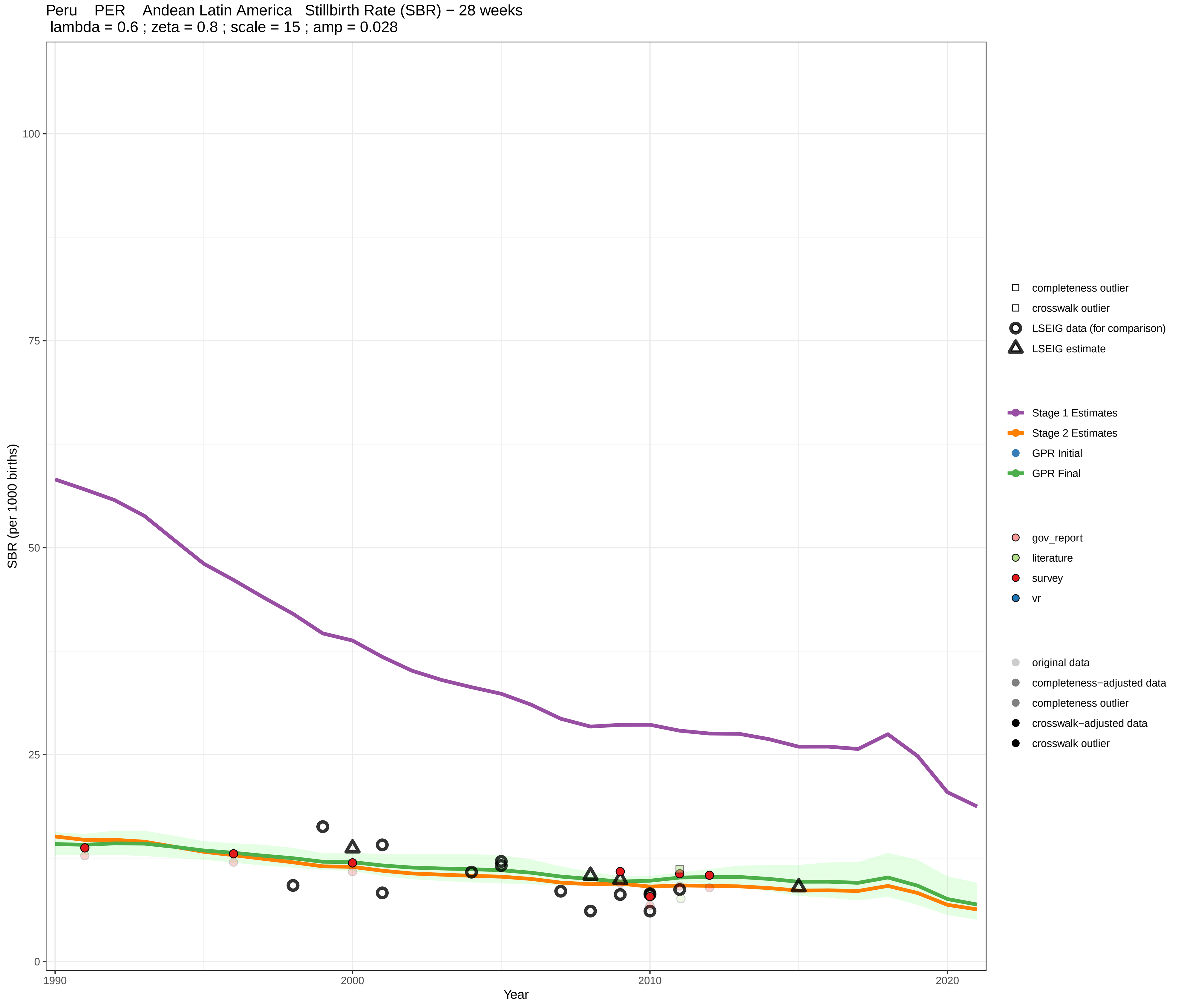

Year	Total Stillbirths (in thousands)	Total Stillbirths (in thousands)	SBR (per 1000 births)	SBR (per 1000 births)	SBR/NMR	SBR/NMR
	20 weeks	28 weeks	20 weeks	28 weeks	20 weeks	28 weeks
1990	13.0 (11.7 to 14.4)	9.5 (8.6 to 10.5)	19.3 (17.5 to 21.3)	14.2 (12.9 to 15.6)	0.73 (0.66 to 0.81)	0.54 (0.49 to 0.59)
1995	12.2 (11.2 to 13.3)	9.0 (8.2 to 9.8)	18.1 (16.6 to 19.7)	13.4 (12.3 to 14.6)	0.85 (0.77 to 0.92)	0.63 (0.57 to 0.68)
2000	10.5 (9.5 to 11.5)	7.8 (7.1 to 8.5)	16.1 (14.7 to 17.7)	12.0 (10.9 to 13.1)	0.96 (0.88 to 1.06)	0.72 (0.66 to 0.79)
2005	9.3 (7.9 to 10.9)	7.0 (6.0 to 8.1)	14.7 (12.5 to 17.1)	11.0 (9.4 to 12.9)	1.08 (0.92 to 1.26)	0.81 (0.70 to 0.95)
2010	8.2 (7.8 to 8.7)	6.1 (5.8 to 6.5)	13.0 (12.3 to 13.8)	9.8 (9.2 to 10.3)	1.13 (1.07 to 1.20)	0.85 (0.80 to 0.90)
2015	8.7 (7.1 to 10.6)	6.4 (5.3 to 7.8)	13.0 (10.7 to 15.8)	9.6 (7.9 to 11.7)	1.31 (1.07 to 1.59)	0.97 (0.80 to 1.17)
2020	7.1 (5.2 to 9.7)	5.2 (3.8 to 7.0)	10.4 (7.7 to 14.2)	7.6 (5.6 to 10.3)	1.38 (1.02 to 1.88)	1.00 (0.75 to 1.37)
2021	6.5 (4.7 to 9.0)	4.7 (3.4 to 6.5)	9.5 (7.0 to 13.2)	6.9 (5.1 to 9.5)	1.38 (1.01 to 1.92)	1.00 (0.74 to 1.39)

Caribbean R7 Caribbean Stillbirth Rate / Neonatal Mortality Rate (SBR/NMR) − 20 weeks
lambda = 1 ; zeta = 0.6 ; scale = 15 ; amp = 0.028

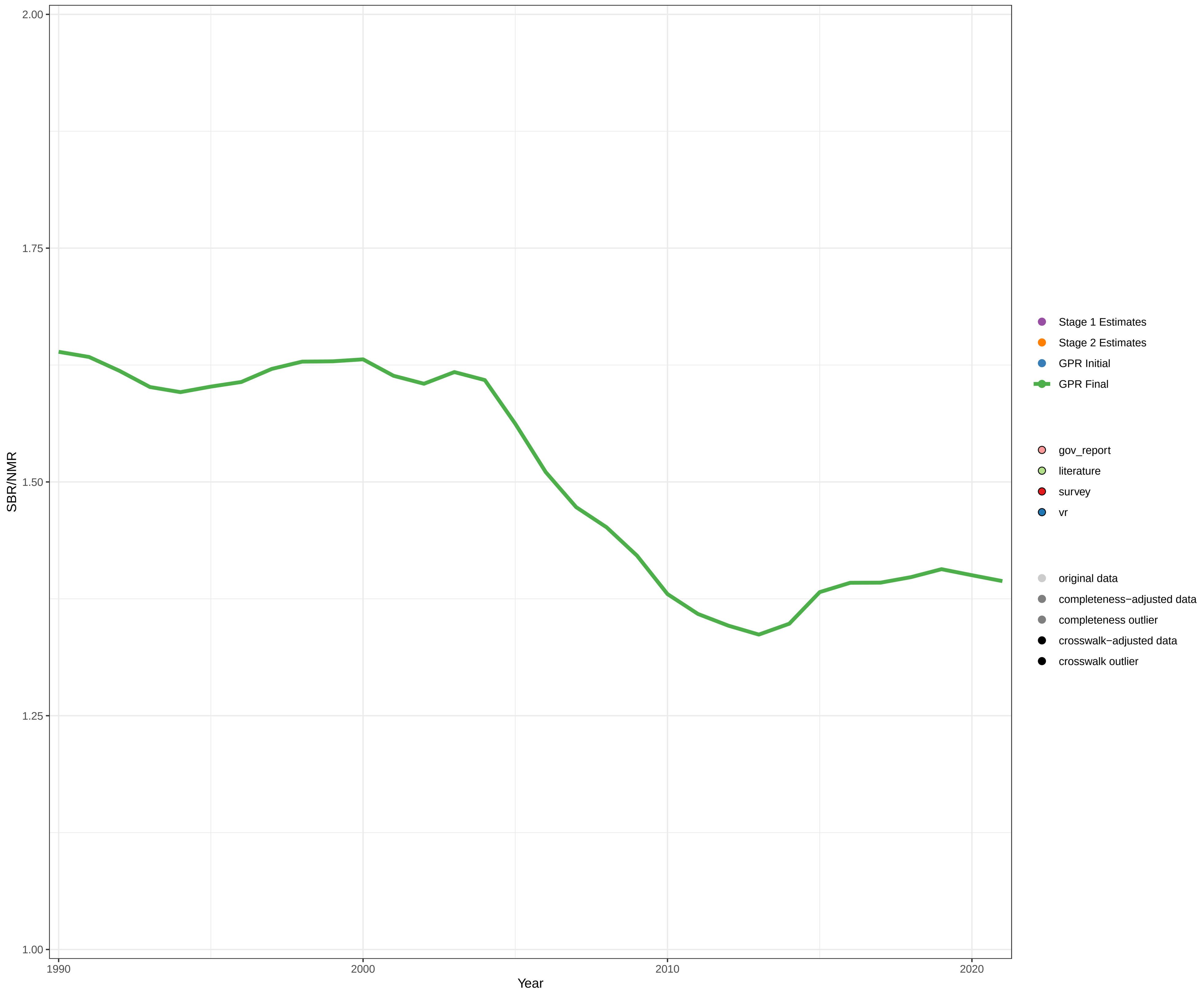

Caribbean R7 Caribbean Stillbirth Rate / Neonatal Mortality Rate (SBR/NMR) − 28 weeks
lambda = 1 ; zeta = 0.6 ; scale = 15 ; amp = 0.028

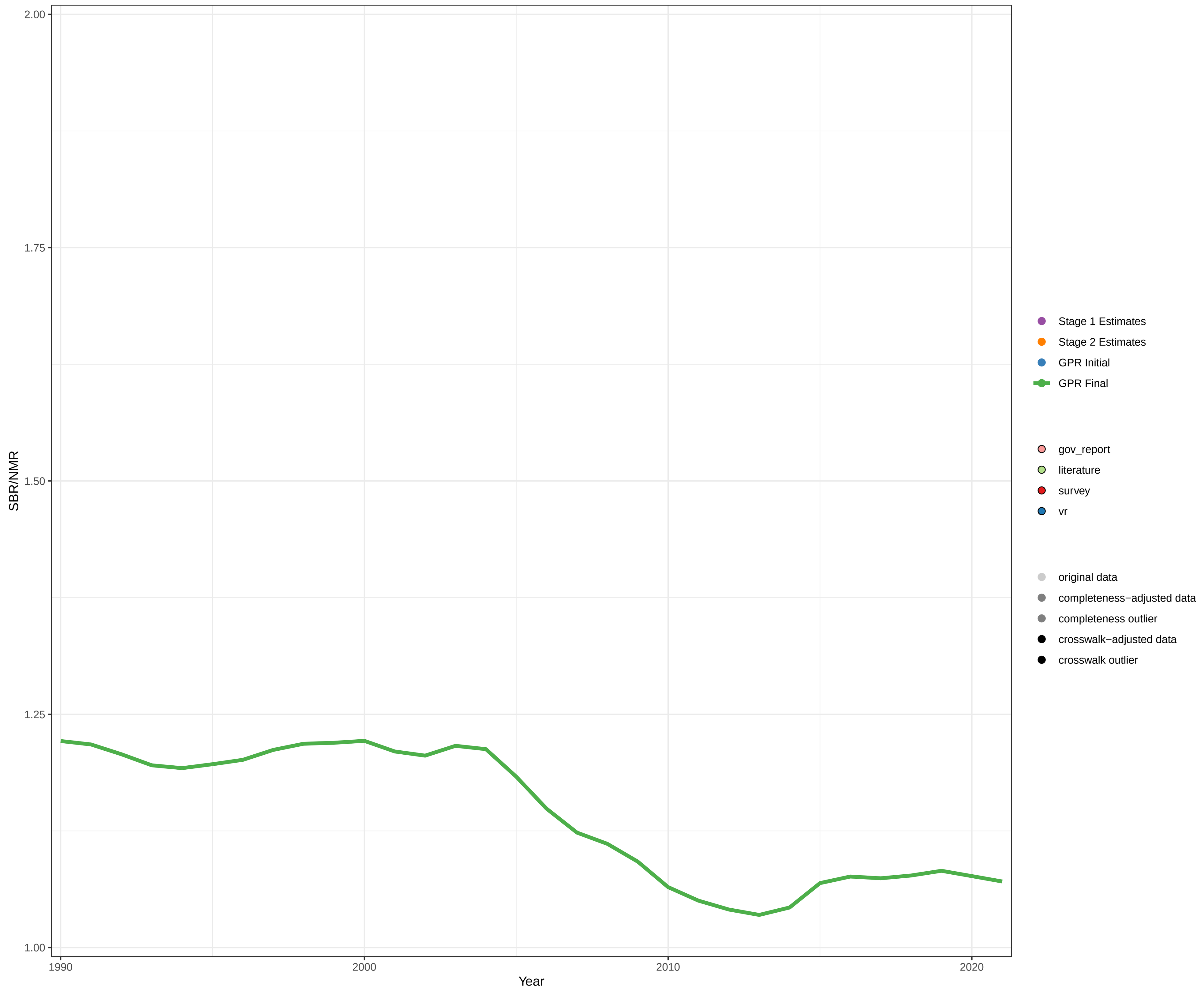

Definition	Years	Outlier	Source.Type	Title

Caribbean R7 Caribbean Stillbirth Rate (SBR) − 20 weeks
lambda = 1 ; zeta = 0.6 ; scale = 15 ; amp = 0.028

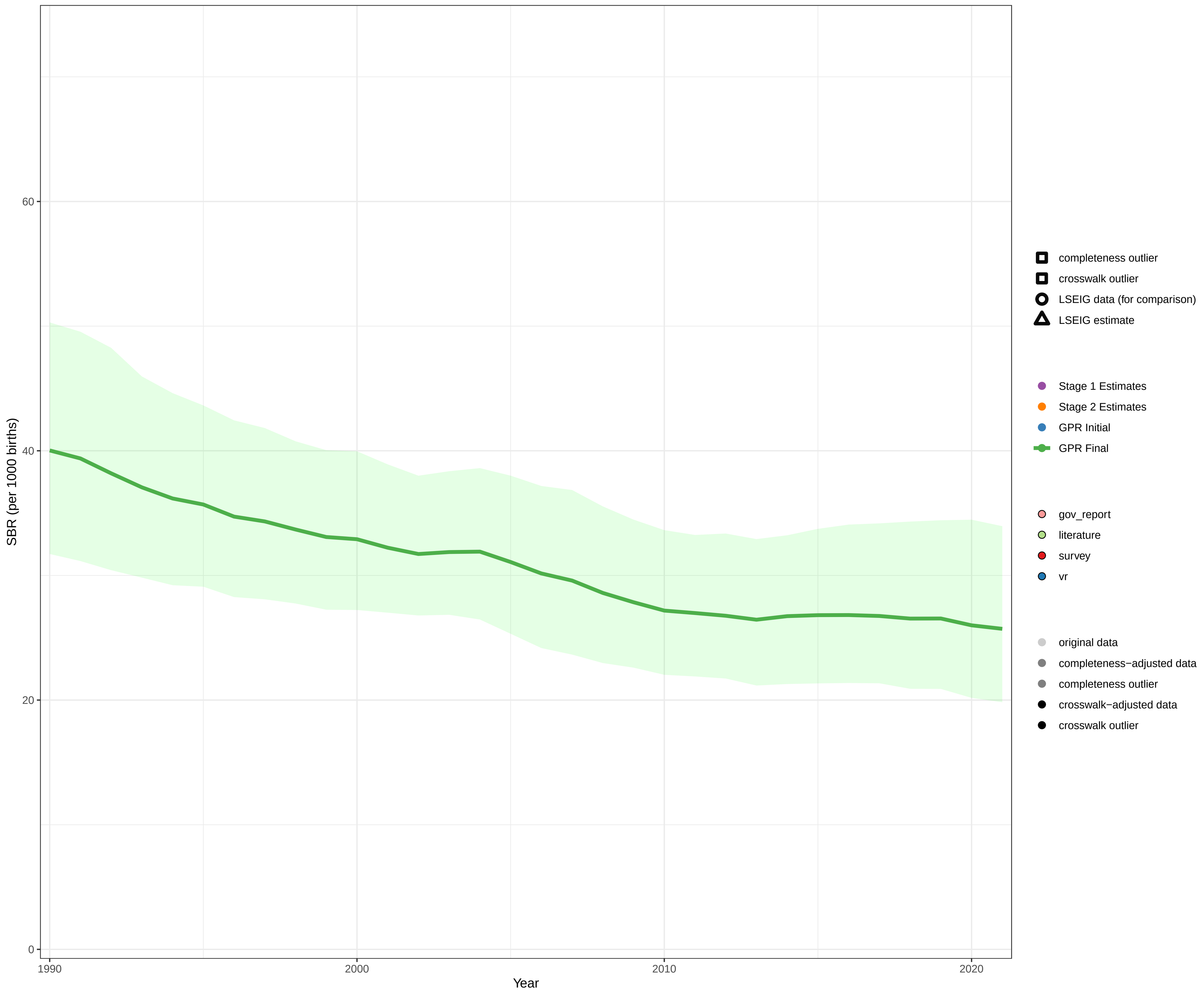

Caribbean R7 Caribbean Stillbirth Rate (SBR) − 28 weeks
lambda = 1 ; zeta = 0.6 ; scale = 15 ; amp = 0.028

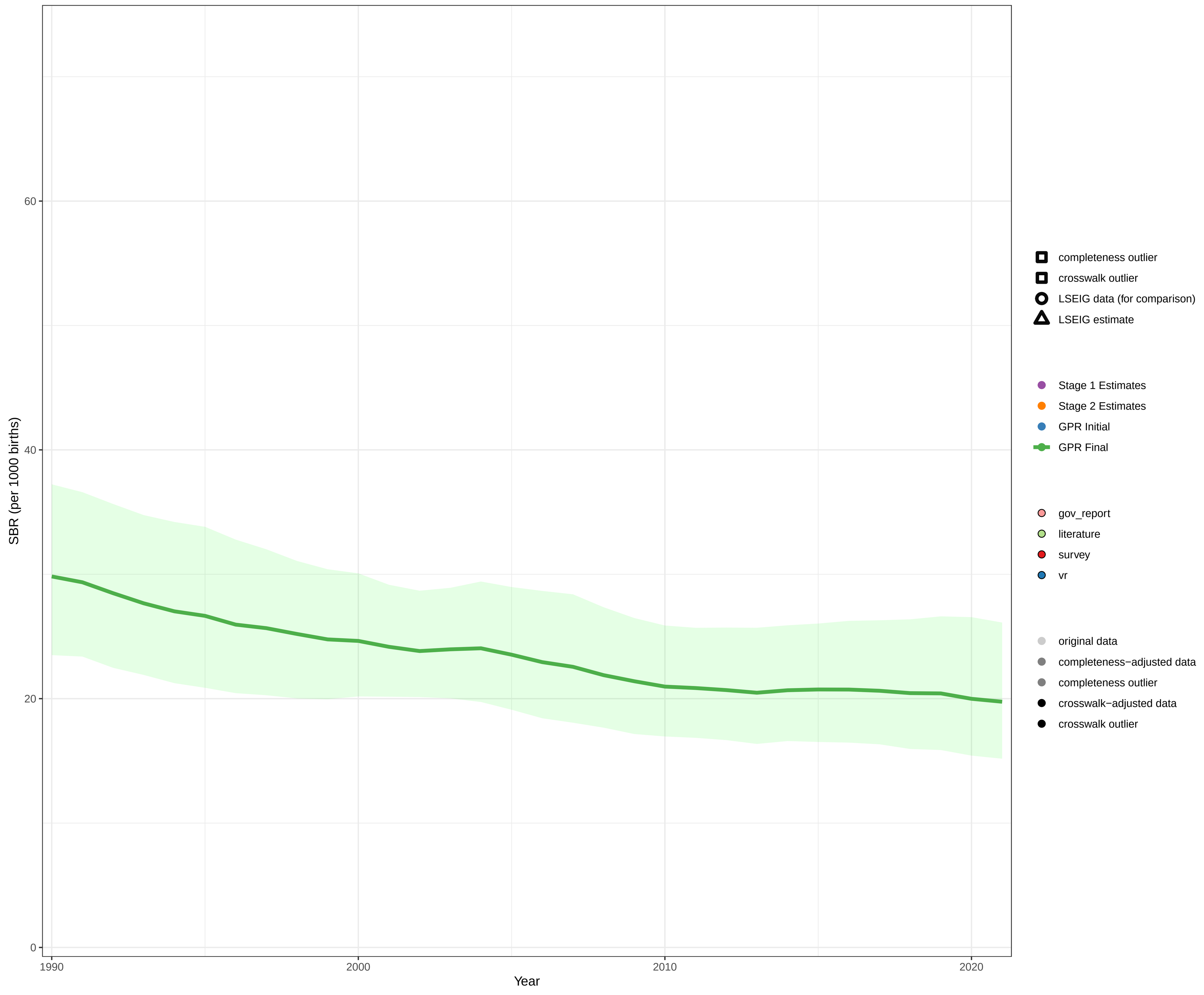

Year	Total Stillbirths (in thousands)	Total Stillbirths (in thousands)	SBR (per 1000 births)	SBR (per 1000 births)	SBR/NMR	SBR/NMR
	20 weeks	28 weeks	20 weeks	28 weeks	20 weeks	28 weeks
1990	37.8 (29.7 to 48.0)	27.9 (21.8 to 35.0)	40.0 (31.7 to 50.3)	29.8 (23.5 to 37.2)	1.64 (1.30 to 2.06)	1.22 (0.96 to 1.52)
1995	32.5 (26.3 to 40.1)	24.1 (18.7 to 30.8)	35.7 (29.1 to 43.6)	26.7 (20.9 to 33.8)	1.60 (1.31 to 1.96)	1.20 (0.94 to 1.52)
2000	29.3 (24.1 to 35.8)	21.7 (17.7 to 26.7)	32.9 (27.2 to 40.0)	24.6 (20.2 to 30.1)	1.63 (1.35 to 1.98)	1.22 (1.00 to 1.49)
2005	26.1 (21.2 to 32.2)	19.6 (15.9 to 24.3)	31.1 (25.3 to 38.0)	23.5 (19.1 to 29.0)	1.56 (1.27 to 1.91)	1.18 (0.96 to 1.46)
2010	23.2 (18.7 to 28.9)	17.8 (14.3 to 22.1)	27.2 (22.0 to 33.6)	21.0 (17.0 to 25.9)	1.38 (1.12 to 1.71)	1.06 (0.86 to 1.31)
2015	22.8 (18.0 to 28.8)	17.5 (13.9 to 22.1)	26.8 (21.3 to 33.7)	20.7 (16.5 to 26.0)	1.38 (1.10 to 1.74)	1.07 (0.85 to 1.34)
2020	21.4 (16.5 to 28.7)	16.4 (12.6 to 21.9)	26.0 (20.2 to 34.5)	20.0 (15.4 to 26.6)	1.40 (1.09 to 1.86)	1.08 (0.83 to 1.43)
2021	21.1 (16.1 to 28.0)	16.1 (12.3 to 21.4)	25.7 (19.8 to 34.0)	19.8 (15.2 to 26.1)	1.39 (1.07 to 1.84)	1.07 (0.82 to 1.42)

Antigua and Barbuda ATG Caribbean Stillbirth Rate / Neonatal Mortality Rate (SBR/NMR) − 20 weeks
lambda = 0.8 ; zeta = 0.7 ; scale = 15 ; amp = 0.028

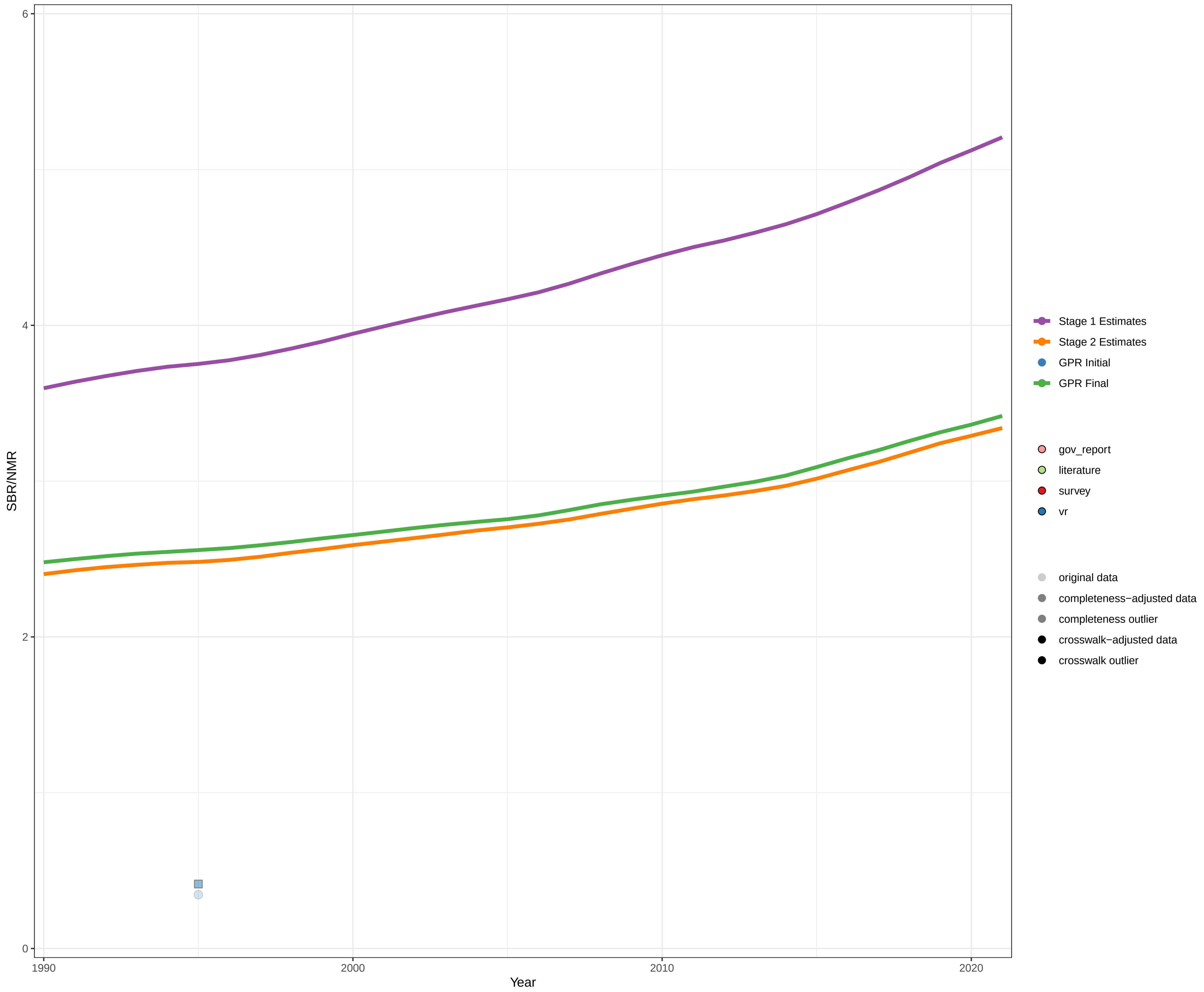

Antigua and Barbuda ATG Caribbean Stillbirth Rate / Neonatal Mortality Rate (SBR/NMR) − 28 weeks
lambda = 0.8 ; zeta = 0.7 ; scale = 15 ; amp = 0.028

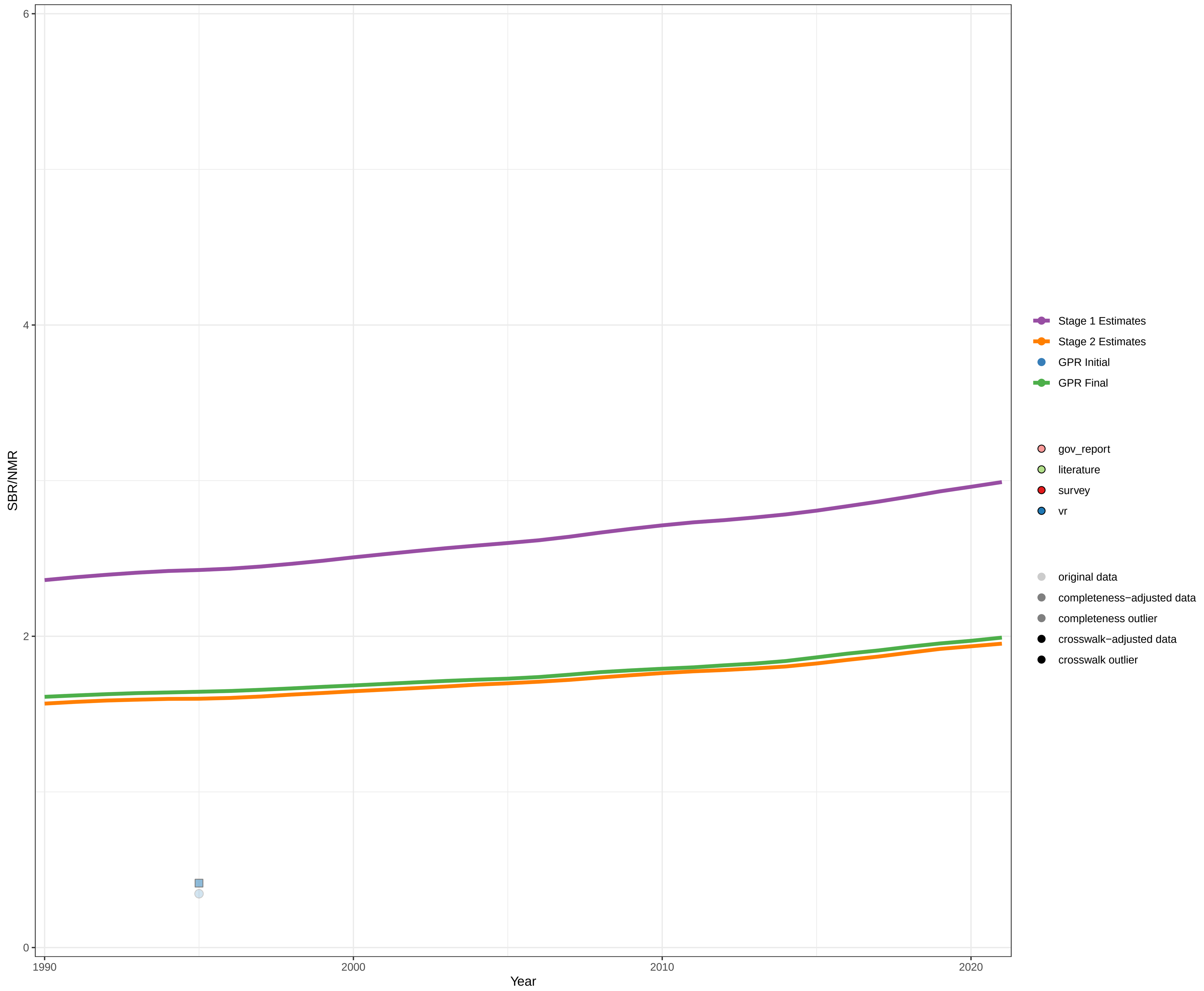

Definition	Years	Outlier	Source.Type	Title
28_weeks	1995	1	VR	United Nations Demographic Yearbook

Antigua and Barbuda ATG Caribbean Stillbirth Rate (SBR) − 20 weeks
lambda = 0.8 ; zeta = 0.7 ; scale = 15 ; amp = 0.028

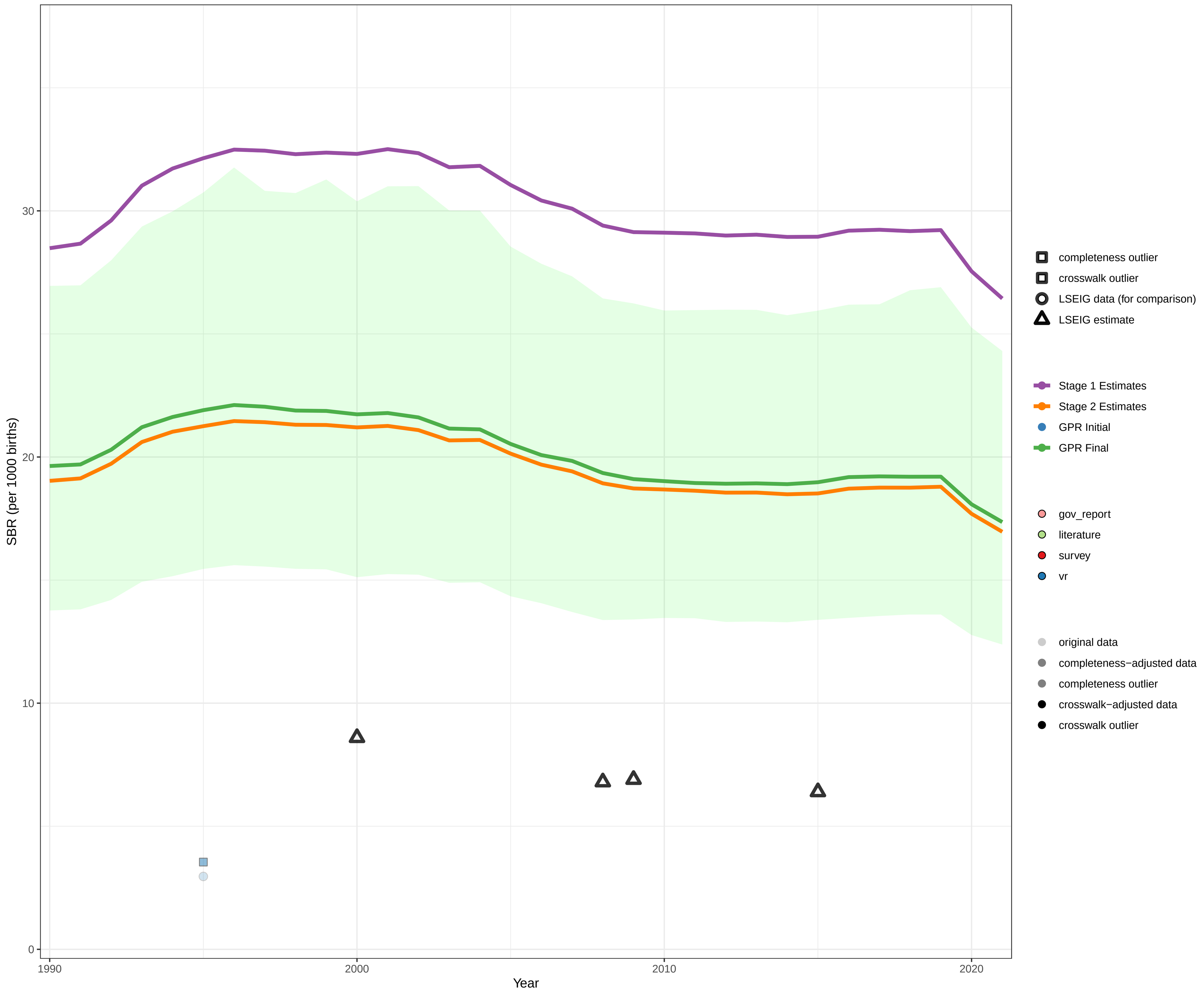

Antigua and Barbuda ATG Caribbean Stillbirth Rate (SBR) − 28 weeks
lambda = 0.8 ; zeta = 0.7 ; scale = 15 ; amp = 0.028

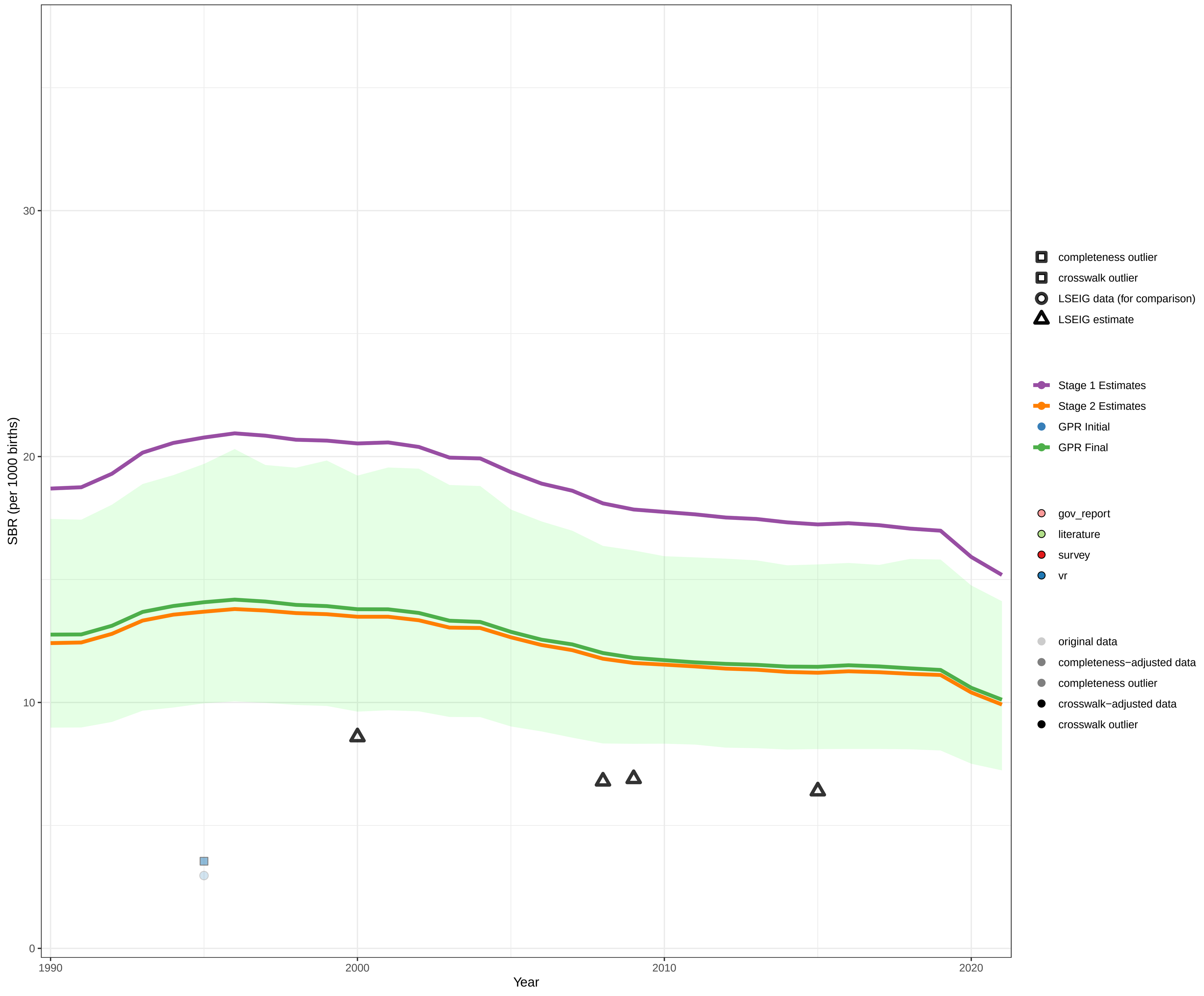

Year	Total Stillbirths (in thousands)	Total Stillbirths (in thousands)	SBR (per 1000 births)	SBR (per 1000 births)	SBR/NMR	SBR/NMR
	20 weeks	28 weeks	20 weeks	28 weeks	20 weeks	28 weeks
1990	0.0 (0.0 to 0.0)	0.0 (0.0 to 0.0)	19.6 (13.8 to 27.0)	12.8 (9.0 to 17.5)	2.48 (1.74 to 3.40)	1.61 (1.13 to 2.21)
1995	0.0 (0.0 to 0.0)	0.0 (0.0 to 0.0)	21.9 (15.5 to 30.7)	14.1 (10.0 to 19.7)	2.56 (1.80 to 3.59)	1.64 (1.16 to 2.30)
2000	0.0 (0.0 to 0.0)	0.0 (0.0 to 0.0)	21.7 (15.1 to 30.4)	13.8 (9.6 to 19.2)	2.65 (1.85 to 3.71)	1.68 (1.18 to 2.35)
2005	0.0 (0.0 to 0.0)	0.0 (0.0 to 0.0)	20.5 (14.3 to 28.5)	12.9 (9.0 to 17.8)	2.76 (1.93 to 3.83)	1.73 (1.21 to 2.40)
2010	0.0 (0.0 to 0.0)	0.0 (0.0 to 0.0)	19.0 (13.5 to 26.0)	11.7 (8.3 to 15.9)	2.91 (2.06 to 3.97)	1.79 (1.27 to 2.44)
2015	0.0 (0.0 to 0.0)	0.0 (0.0 to 0.0)	19.0 (13.4 to 25.9)	11.5 (8.1 to 15.6)	3.09 (2.18 to 4.23)	1.86 (1.32 to 2.54)
2020	0.0 (0.0 to 0.0)	0.0 (0.0 to 0.0)	18.1 (12.8 to 25.3)	10.6 (7.5 to 14.8)	3.36 (2.37 to 4.70)	1.97 (1.40 to 2.75)
2021	0.0 (0.0 to 0.0)	0.0 (0.0 to 0.0)	17.4 (12.4 to 24.3)	10.1 (7.2 to 14.1)	3.42 (2.44 to 4.79)	1.99 (1.43 to 2.78)

Bahamas   BHS   Caribbean   Stillbirth Rate / Neonatal Mortality Rate (SBR/NMR) − 20 weeks
lambda = 0.4 ; zeta = 0.9 ; scale = 10 ; amp = 0.028

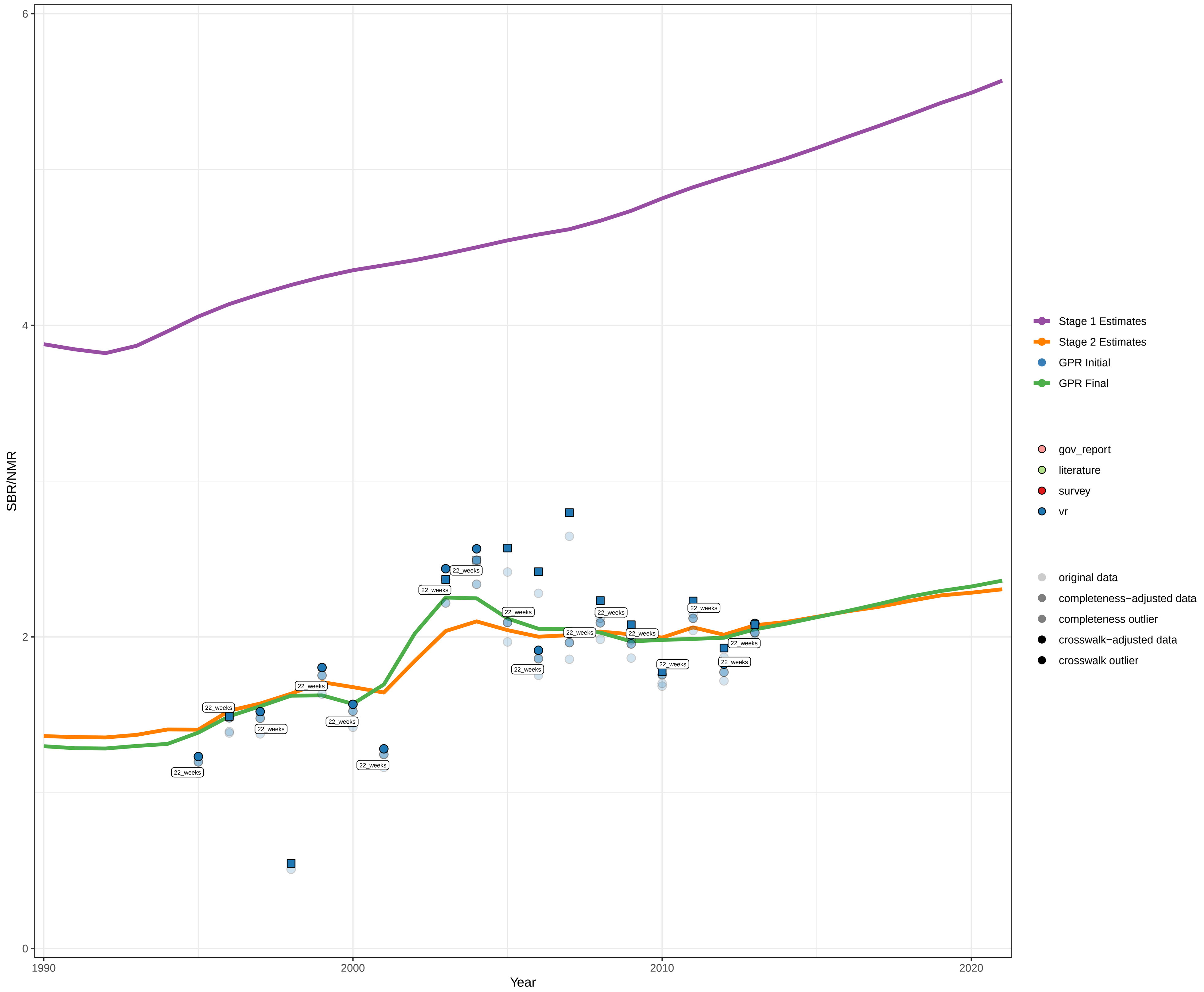

Bahamas   BHS   Caribbean   Stillbirth Rate / Neonatal Mortality Rate (SBR/NMR) − 28 weeks
lambda = 0.4 ; zeta = 0.9 ; scale = 10 ; amp = 0.028

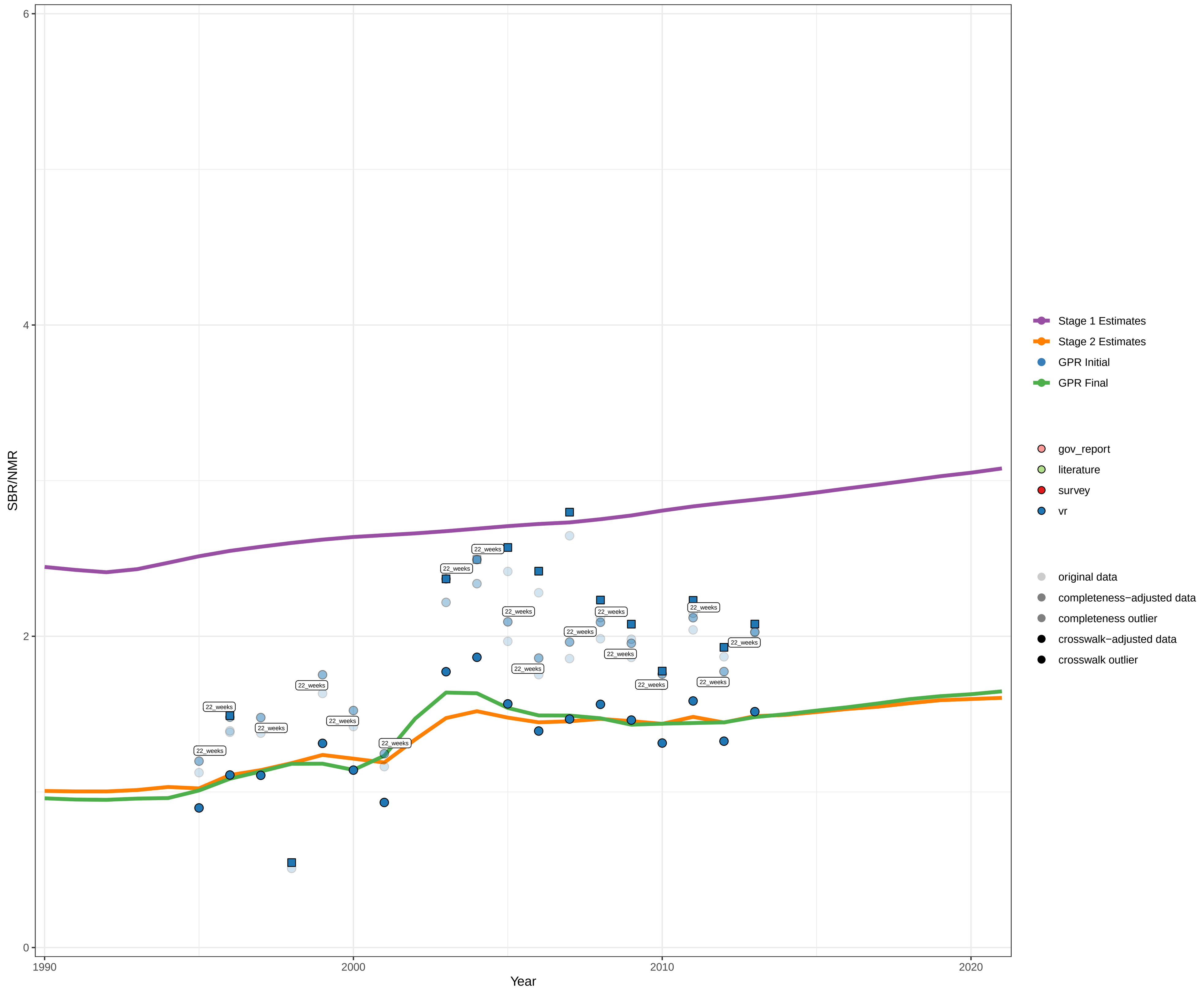

Definition	Years	Outlier	Source.Type	Title
22_weeks	1995, 1996, 2003, 2005−2013	0	VR	United Nations Demographic Yearbook
22_weeks	1996−2001, 2003−2013	0	VR	Bahamas Vital Statistics Report
22_weeks	2004	1	VR	United Nations Demographic Yearbook

Bahamas   BHS   Caribbean   Stillbirth Rate (SBR) − 20 weeks
lambda = 0.4 ; zeta = 0.9 ; scale = 10 ; amp = 0.028

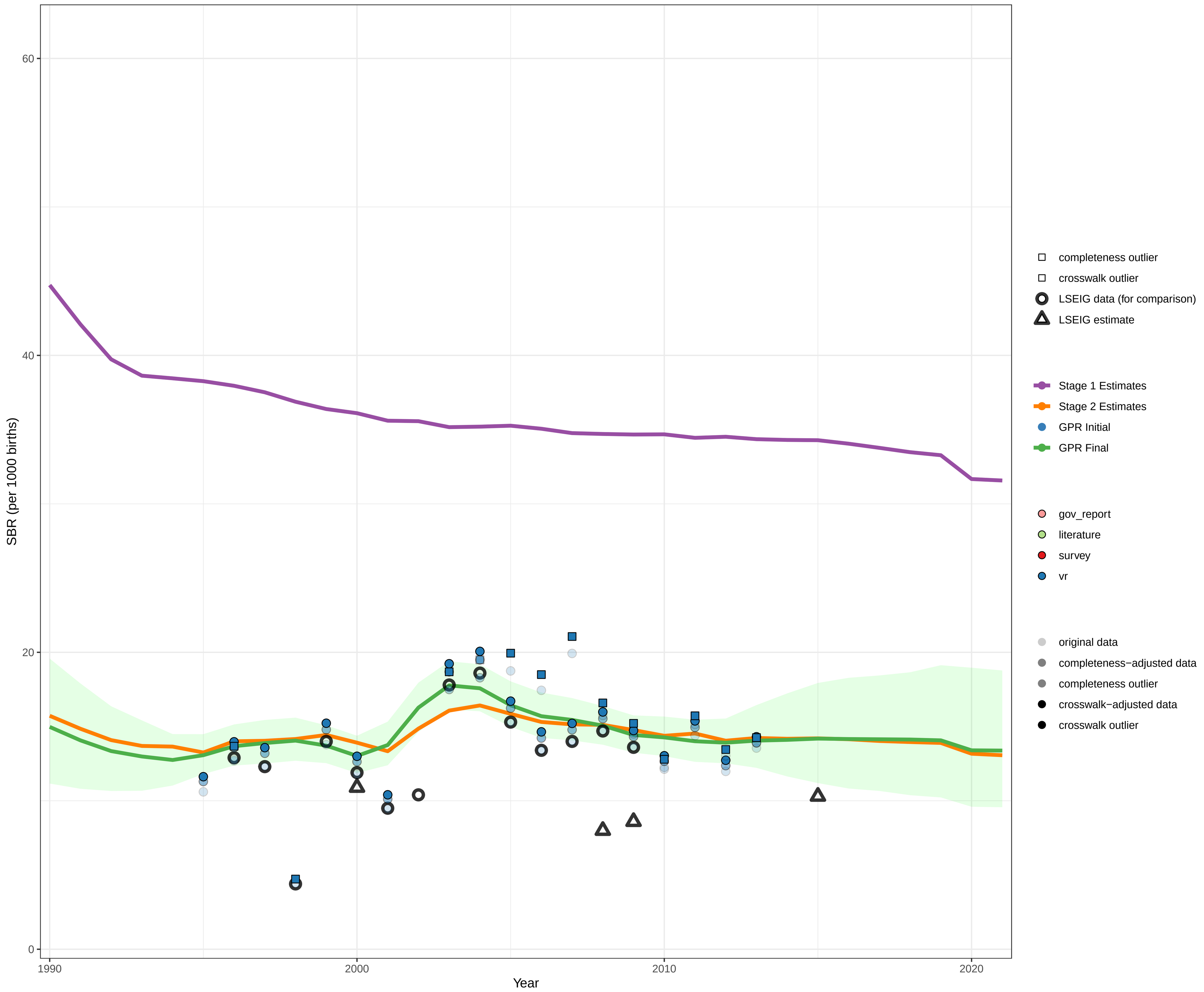

Bahamas   BHS   Caribbean   Stillbirth Rate (SBR) − 28 weeks
lambda = 0.4 ; zeta = 0.9 ; scale = 10 ; amp = 0.028

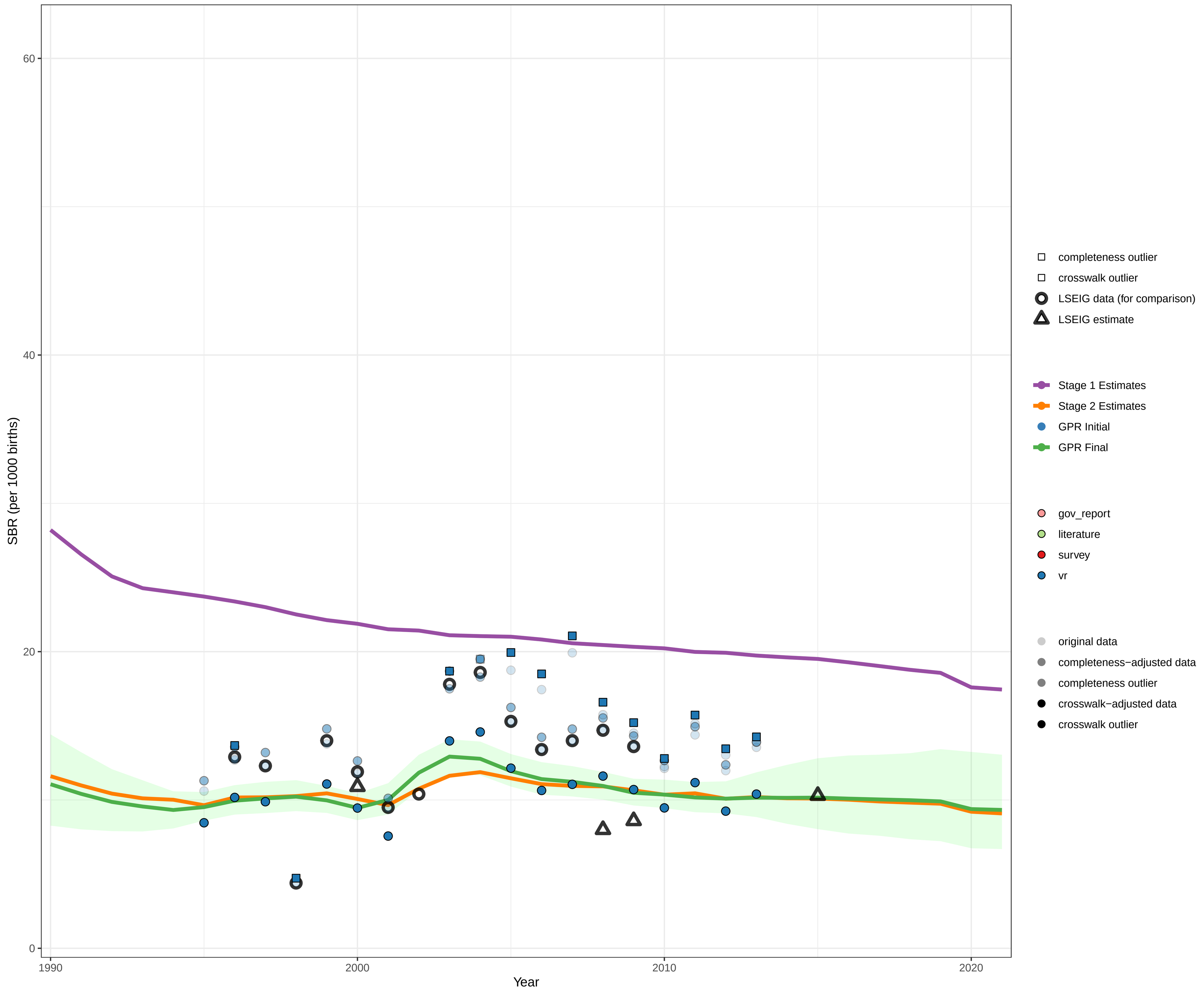

Year	Total Stillbirths (in thousands)	Total Stillbirths (in thousands)	SBR (per 1000 births)	SBR (per 1000 births)	SBR/NMR	SBR/NMR
	20 weeks	28 weeks	20 weeks	28 weeks	20 weeks	28 weeks
1990	0.1 (0.1 to 0.1)	0.1 (0.0 to 0.1)	15.0 (11.2 to 19.6)	11.1 (8.3 to 14.4)	1.30 (0.97 to 1.70)	0.96 (0.72 to 1.25)
1995	0.1 (0.1 to 0.1)	0.0 (0.0 to 0.0)	13.1 (11.8 to 14.5)	9.5 (8.6 to 10.5)	1.39 (1.25 to 1.54)	1.01 (0.91 to 1.12)
2000	0.1 (0.1 to 0.1)	0.0 (0.0 to 0.0)	13.0 (11.9 to 14.4)	9.5 (8.6 to 10.4)	1.57 (1.43 to 1.73)	1.14 (1.04 to 1.26)
2005	0.1 (0.1 to 0.1)	0.1 (0.1 to 0.1)	16.4 (15.0 to 18.0)	11.9 (10.9 to 13.1)	2.12 (1.93 to 2.33)	1.54 (1.41 to 1.69)
2010	0.1 (0.1 to 0.1)	0.1 (0.0 to 0.1)	14.3 (13.0 to 15.7)	10.4 (9.5 to 11.4)	1.98 (1.81 to 2.18)	1.44 (1.31 to 1.58)
2015	0.1 (0.0 to 0.1)	0.0 (0.0 to 0.1)	14.2 (11.2 to 17.9)	10.2 (8.0 to 12.8)	2.13 (1.68 to 2.69)	1.52 (1.20 to 1.92)
2020	0.1 (0.0 to 0.1)	0.0 (0.0 to 0.1)	13.4 (9.6 to 19.0)	9.4 (6.7 to 13.2)	2.32 (1.66 to 3.29)	1.63 (1.17 to 2.30)
2021	0.1 (0.0 to 0.1)	0.0 (0.0 to 0.1)	13.4 (9.6 to 18.8)	9.3 (6.7 to 13.1)	2.36 (1.69 to 3.31)	1.65 (1.18 to 2.30)

Barbados BRB Caribbean Stillbirth Rate / Neonatal Mortality Rate (SBR/NMR) − 20 weeks
lambda = 0.8 ; zeta = 0.7 ; scale = 15 ; amp = 0.028

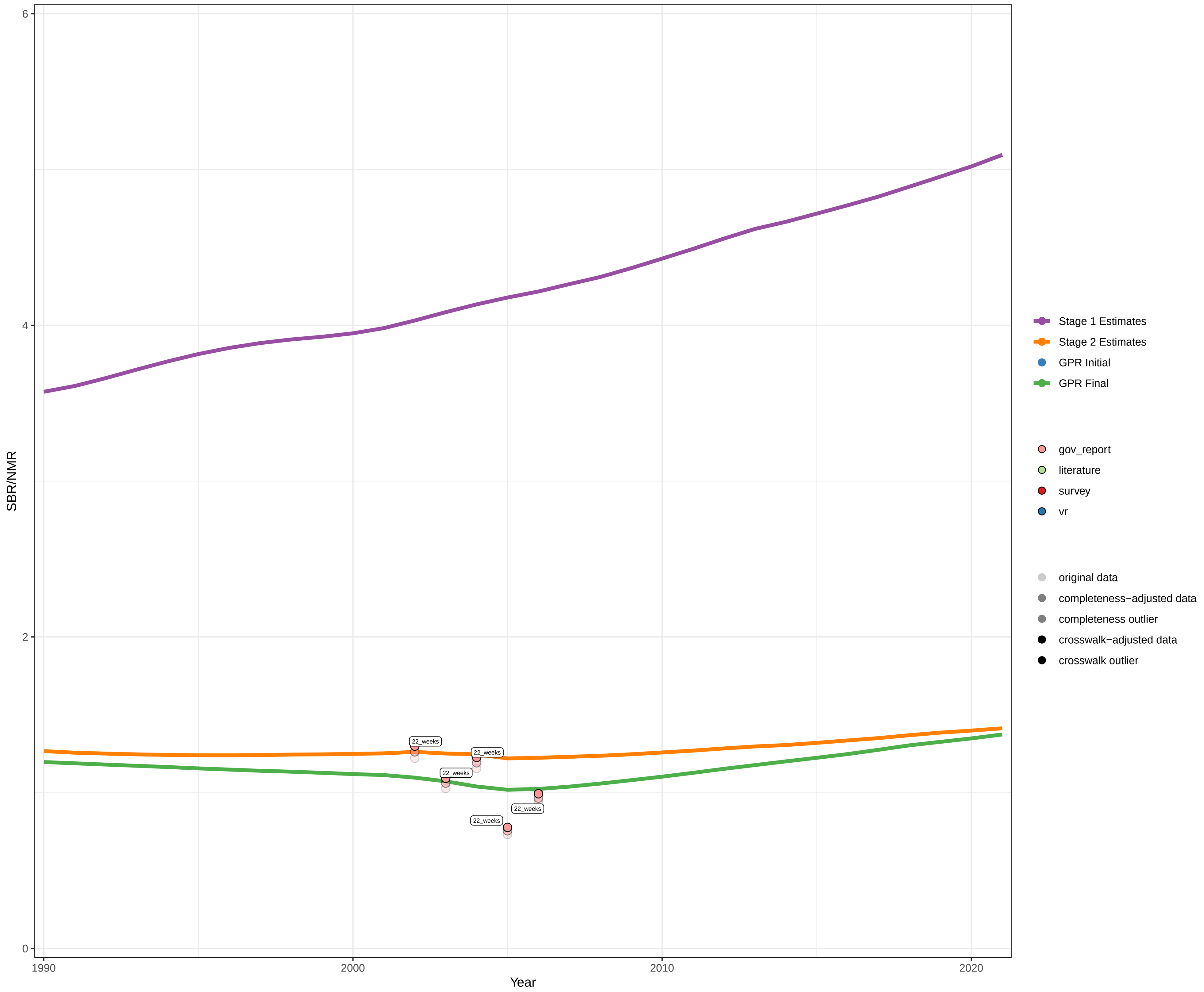

Barbados BRB Caribbean Stillbirth Rate / Neonatal Mortality Rate (SBR/NMR) − 28 weeks
lambda = 0.8 ; zeta = 0.7 ; scale = 15 ; amp = 0.028

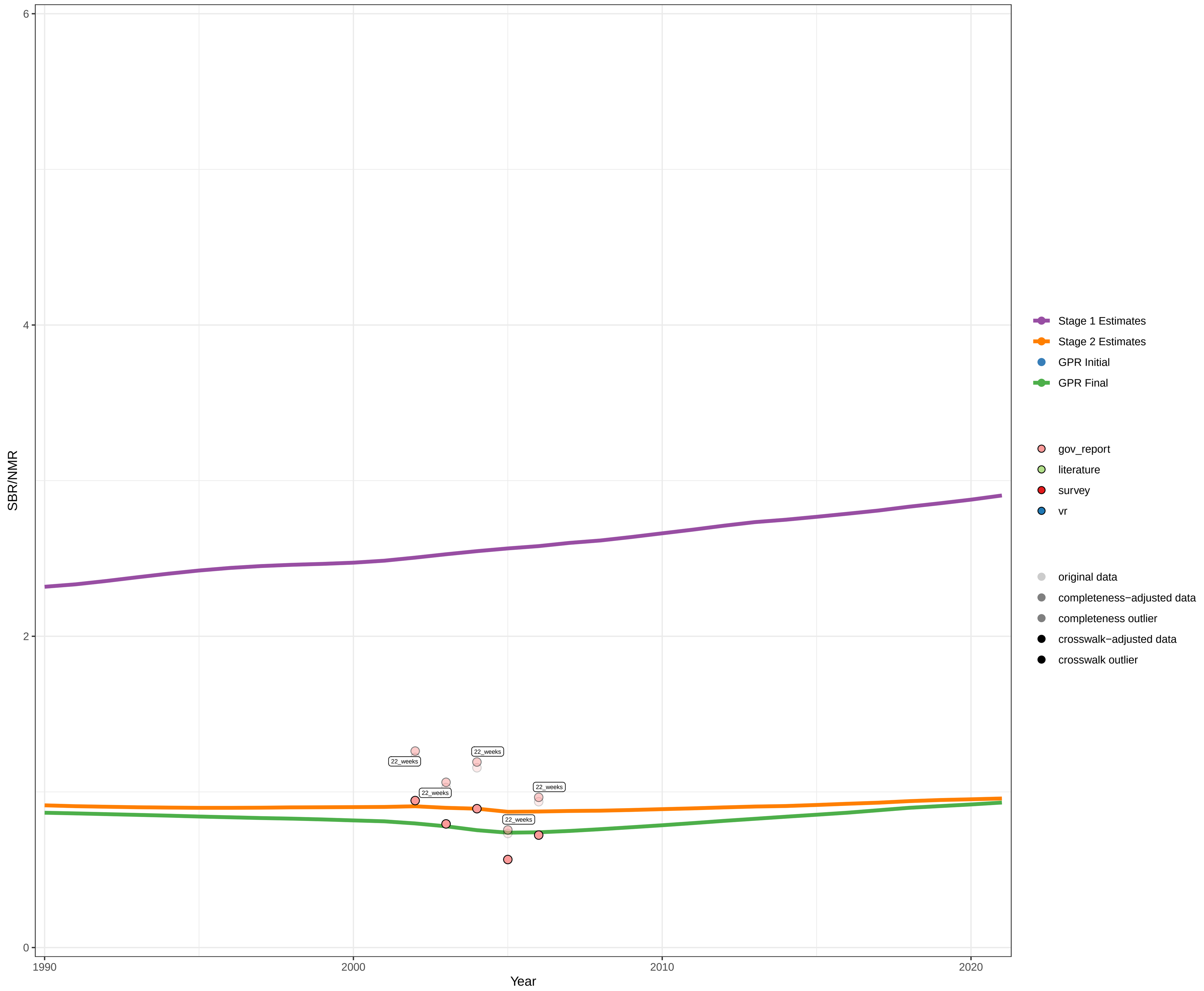

Definition	Years	Outlier	Source.Type	Title
22_weeks	2002−2006	0	Statistical Report	Barbados Economic and Social Report

Barbados BRB Caribbean Stillbirth Rate (SBR) − 20 weeks
lambda = 0.8 ; zeta = 0.7 ; scale = 15 ; amp = 0.028

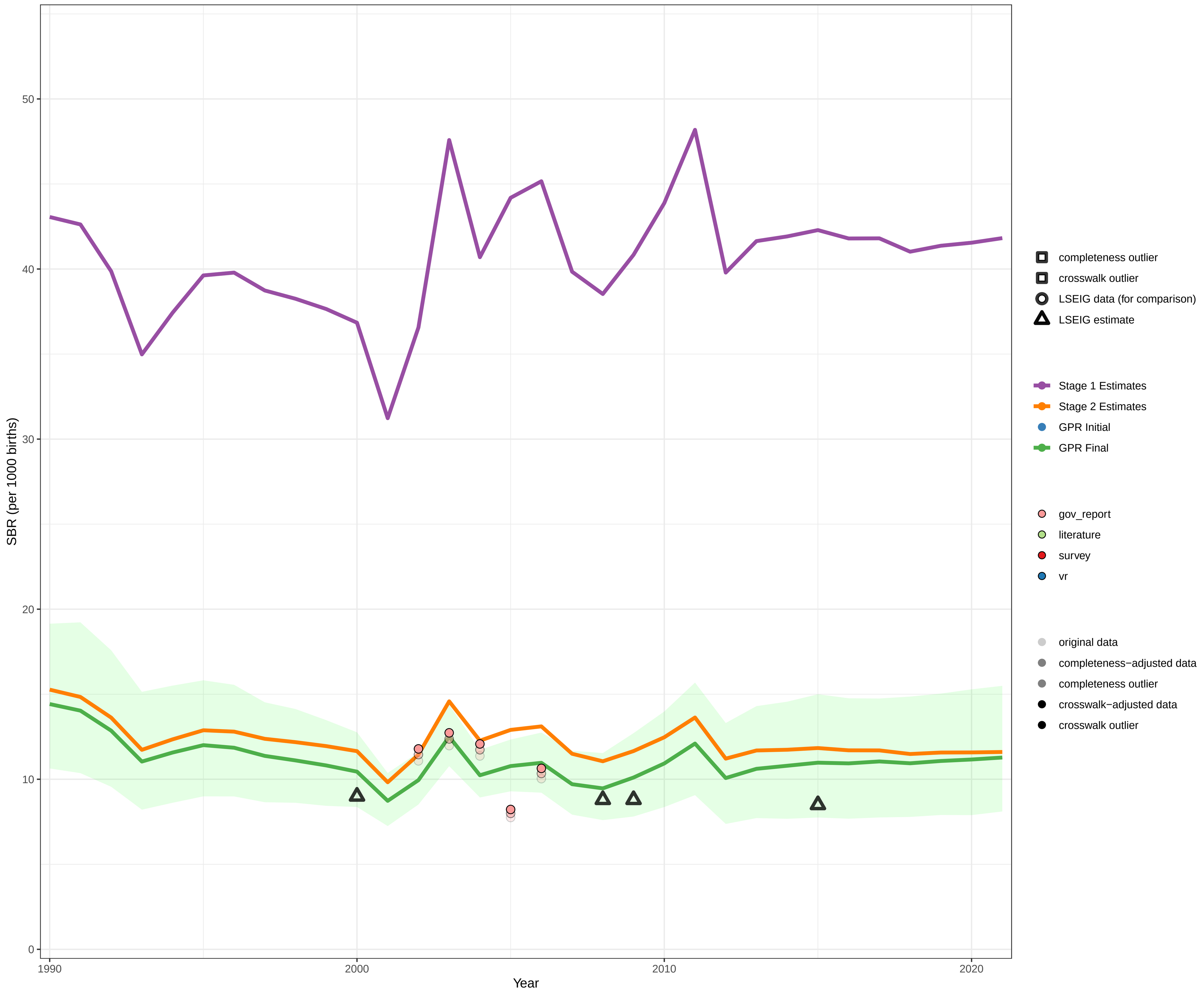

Barbados BRB Caribbean Stillbirth Rate (SBR) − 28 weeks
lambda = 0.8 ; zeta = 0.7 ; scale = 15 ; amp = 0.028

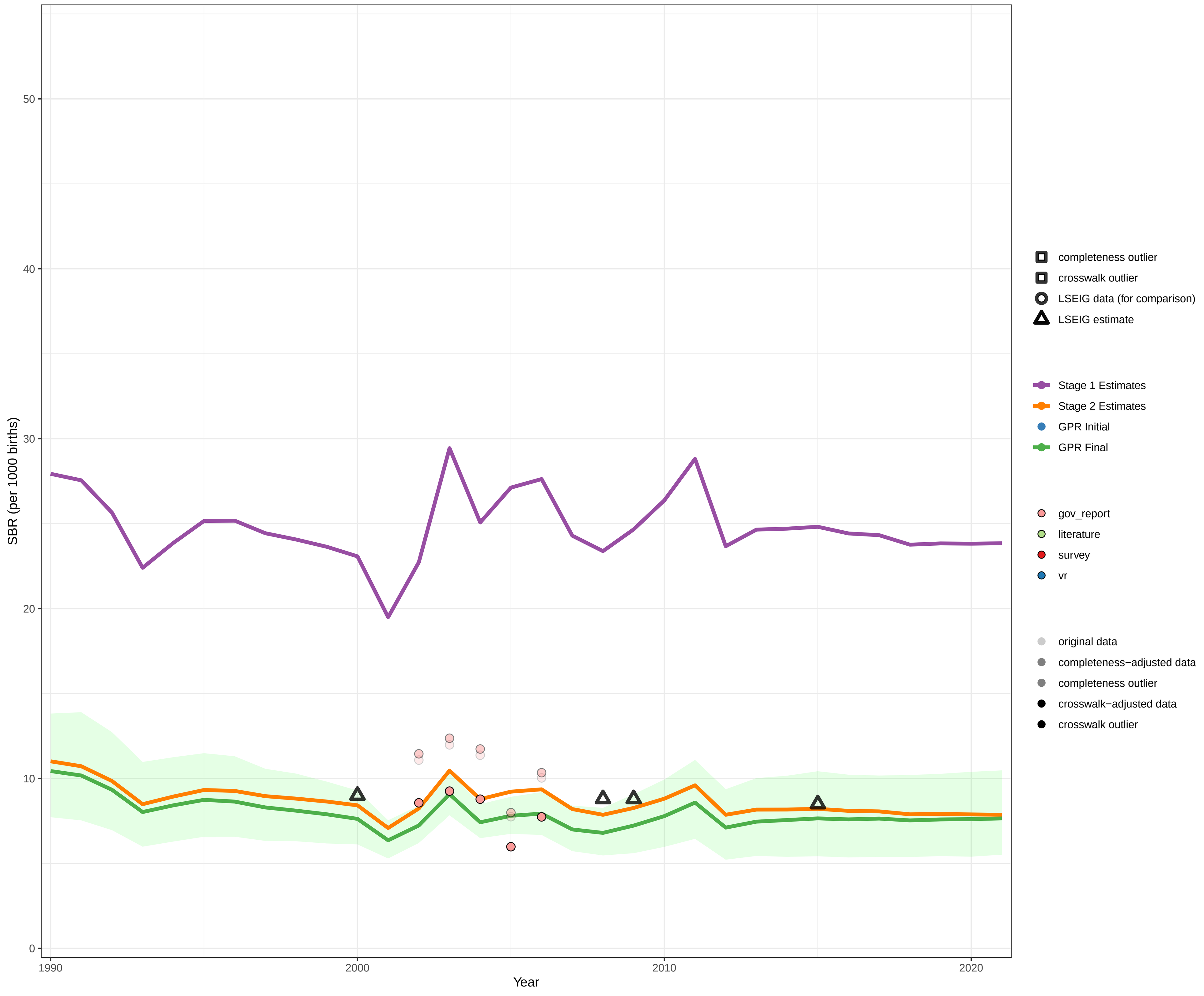

Year	Total Stillbirths (in thousands)	Total Stillbirths (in thousands)	SBR (per 1000 births)	SBR (per 1000 births)	SBR/NMR	SBR/NMR
	20 weeks	28 weeks	20 weeks	28 weeks	20 weeks	28 weeks
1990	0.1<br>(0.0 to 0.1)	0.0<br>(0.0 to 0.1)	14.4<br>(10.6 to 19.1)	10.4<br>(7.7 to 13.8)	1.20<br>(0.88 to 1.59)	0.87<br>(0.64 to 1.15)
1995	0.0<br>(0.0 to 0.1)	0.0<br>(0.0 to 0.0)	12.0<br>(9.0 to 15.8)	8.7<br>(6.6 to 11.5)	1.16<br>(0.87 to 1.52)	0.84<br>(0.63 to 1.11)
2000	0.0<br>(0.0 to 0.1)	0.0<br>(0.0 to 0.0)	10.4<br>(8.4 to 12.8)	7.6<br>(6.1 to 9.3)	1.12<br>(0.90 to 1.37)	0.82<br>(0.66 to 1.00)
2005	0.0<br>(0.0 to 0.0)	0.0<br>(0.0 to 0.0)	10.8<br>(9.3 to 12.3)	7.8<br>(6.7 to 8.9)	1.02<br>(0.88 to 1.17)	0.74<br>(0.64 to 0.84)
2010	0.0<br>(0.0 to 0.0)	0.0<br>(0.0 to 0.0)	10.9<br>(8.4 to 14.0)	7.8<br>(6.0 to 9.9)	1.10<br>(0.84 to 1.41)	0.79<br>(0.60 to 1.00)
2015	0.0<br>(0.0 to 0.0)	0.0<br>(0.0 to 0.0)	11.0<br>(7.7 to 15.0)	7.7<br>(5.4 to 10.4)	1.22<br>(0.86 to 1.67)	0.85<br>(0.60 to 1.16)
2020	0.0<br>(0.0 to 0.0)	0.0<br>(0.0 to 0.0)	11.2<br>(7.9 to 15.3)	7.6<br>(5.4 to 10.4)	1.35<br>(0.95 to 1.85)	0.92<br>(0.65 to 1.26)
2021	0.0<br>(0.0 to 0.0)	0.0<br>(0.0 to 0.0)	11.3<br>(8.1 to 15.5)	7.6<br>(5.5 to 10.5)	1.37<br>(0.99 to 1.89)	0.93<br>(0.67 to 1.28)

Belize BLZ Caribbean Stillbirth Rate / Neonatal Mortality Rate (SBR/NMR) − 20 weeks
lambda = 0.6 ; zeta = 0.8 ; scale = 15 ; amp = 0.028

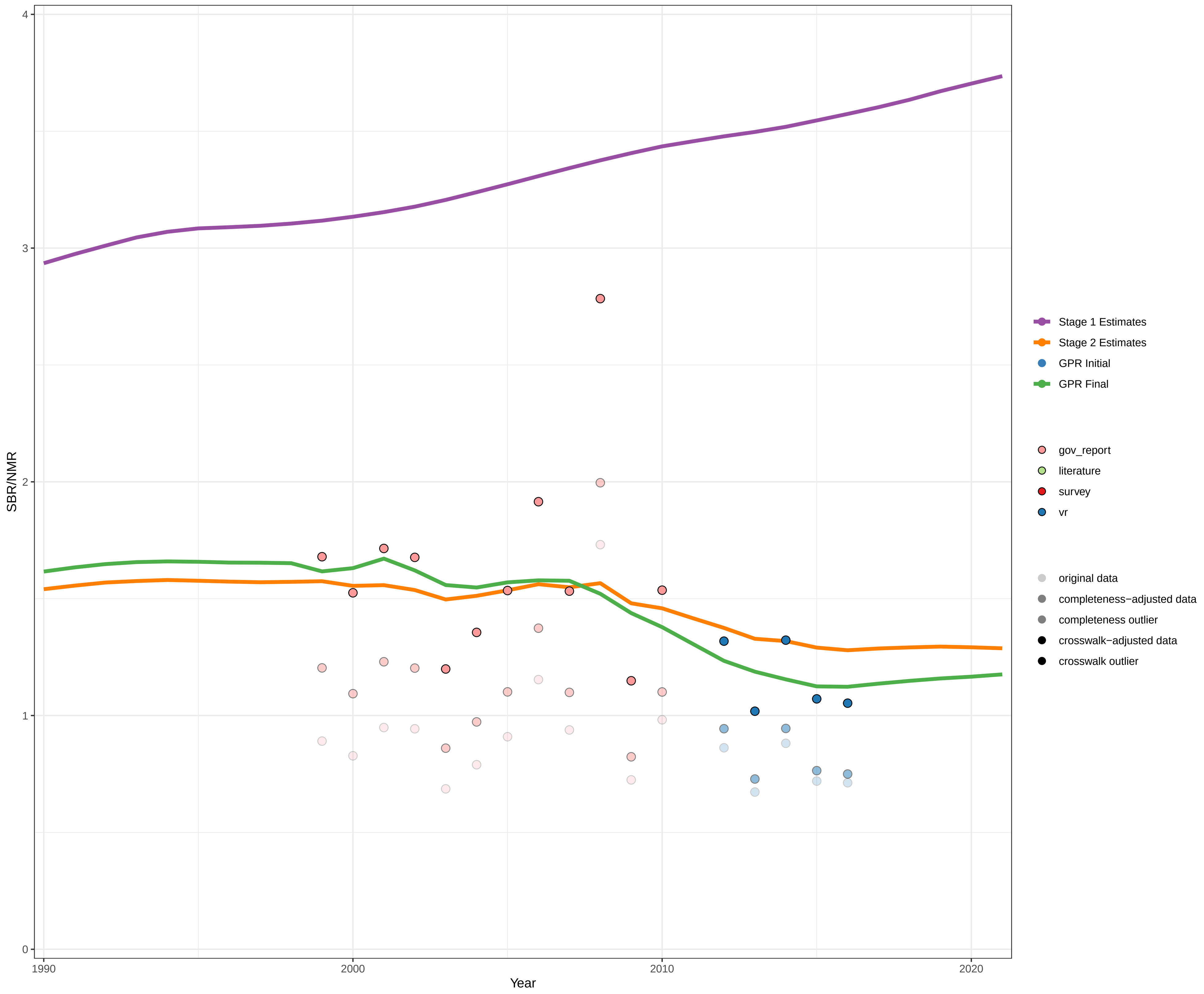

Belize BLZ Caribbean Stillbirth Rate / Neonatal Mortality Rate (SBR/NMR) − 28 weeks
lambda = 0.6 ; zeta = 0.8 ; scale = 15 ; amp = 0.028

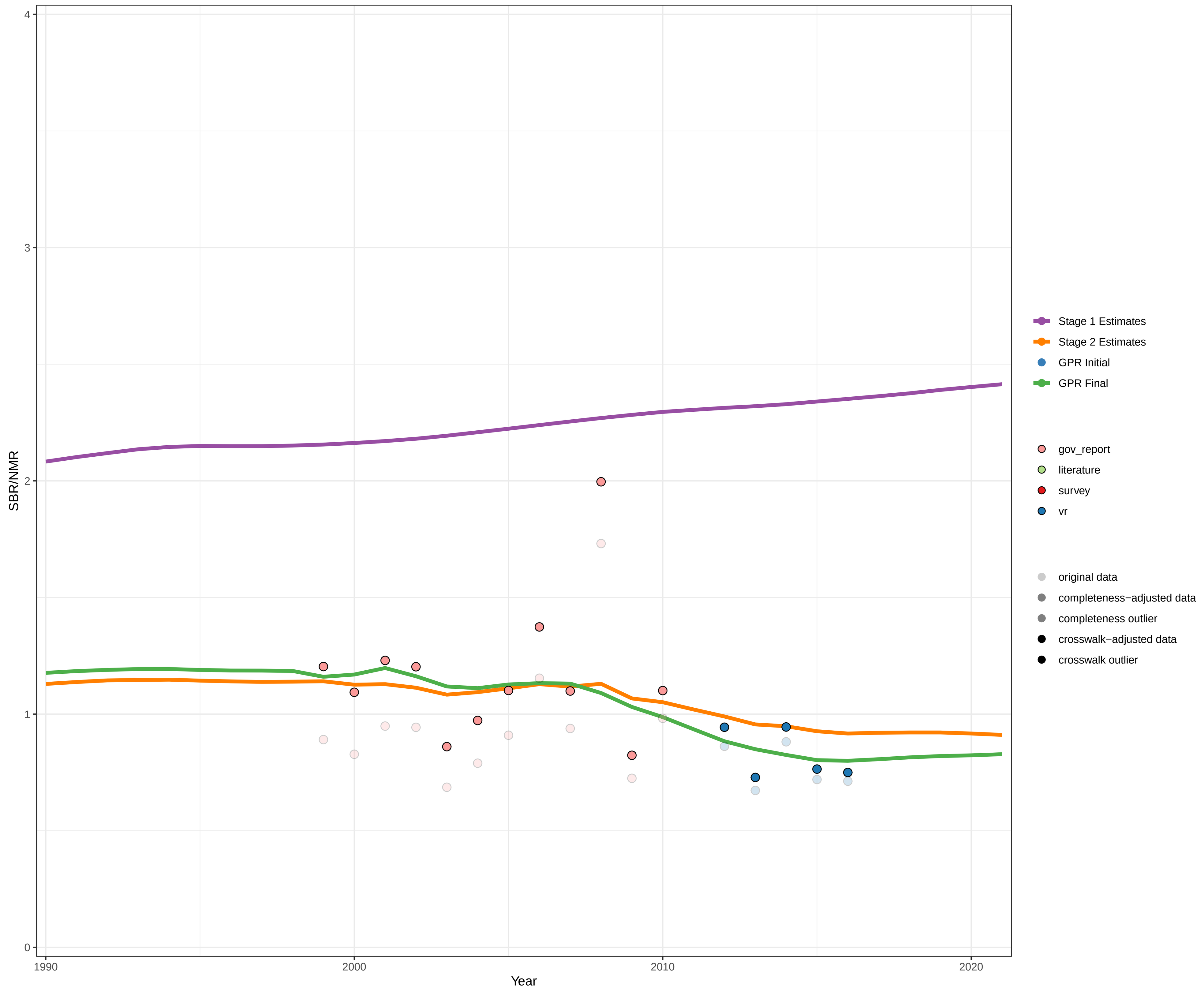

Definition	Years	Outlier	Source.Type	Title
28_weeks	1999−2010	0	Statistical Report	Health Statistics of Belize
28_weeks	2012−2016	0	VR	United Nations Demographic Yearbook

Belize BLZ Caribbean Stillbirth Rate (SBR) − 20 weeks
lambda = 0.6 ; zeta = 0.8 ; scale = 15 ; amp = 0.028

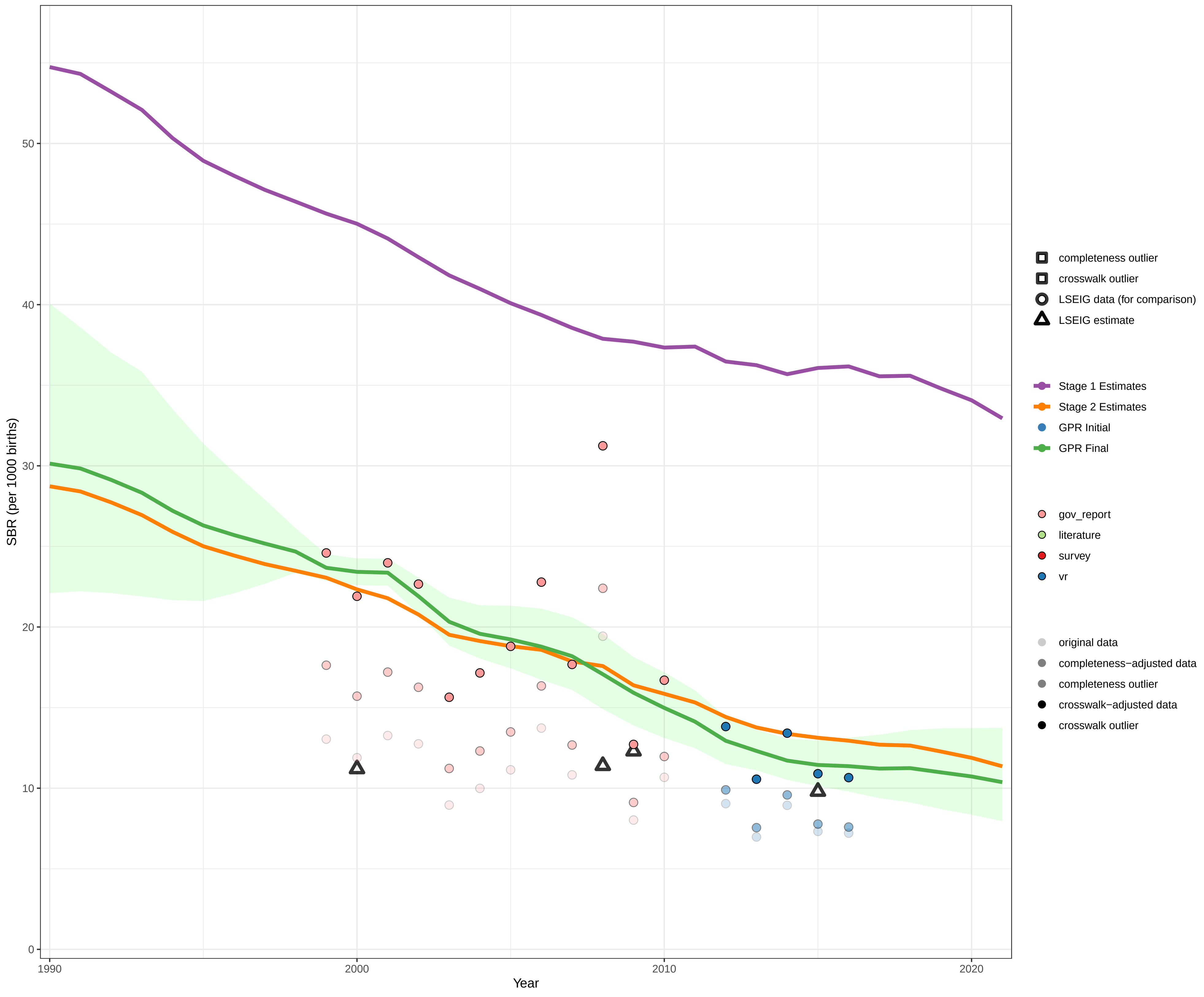

Belize BLZ Caribbean Stillbirth Rate (SBR) − 28 weeks
lambda = 0.6 ; zeta = 0.8 ; scale = 15 ; amp = 0.028

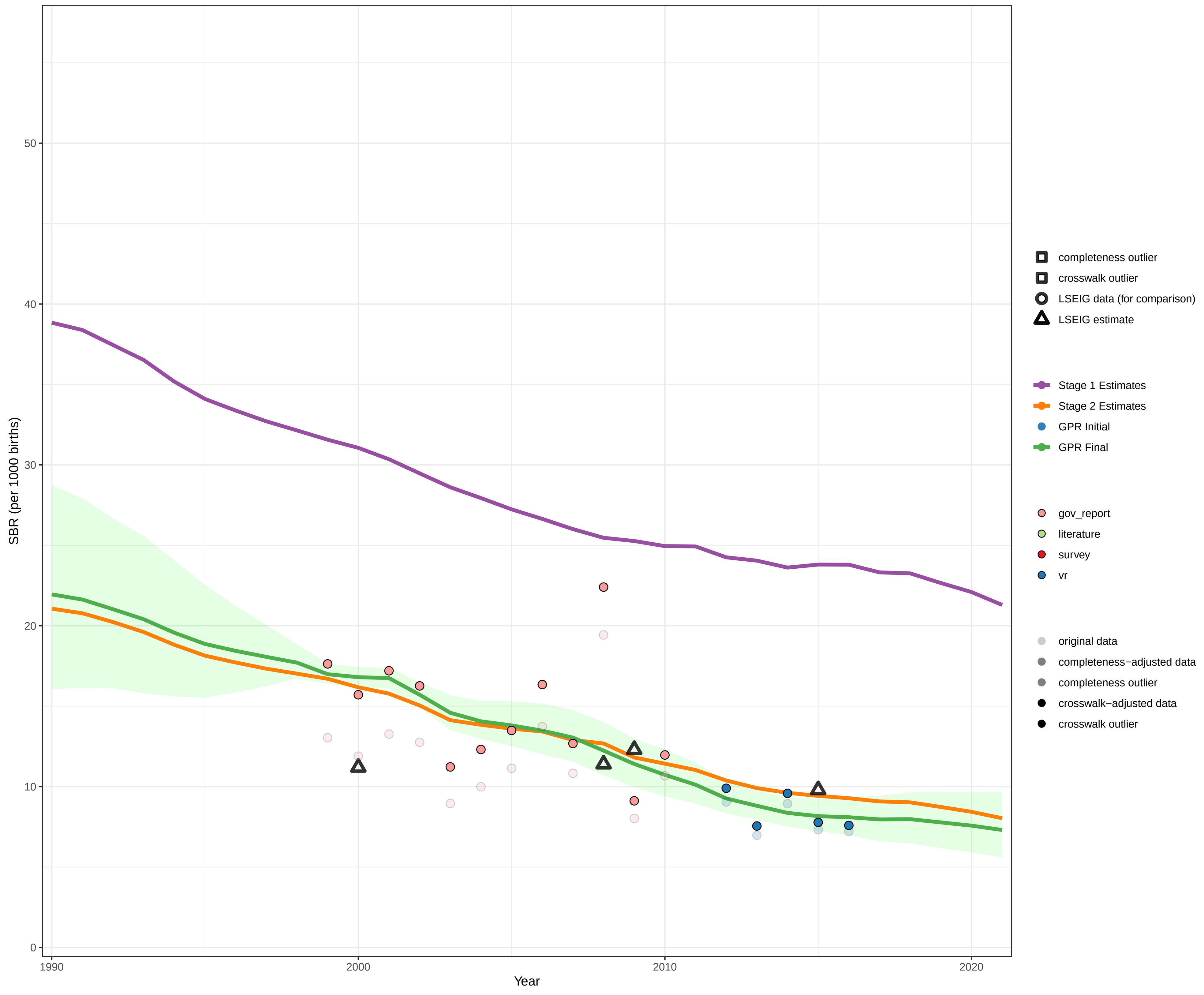

Year	Total Stillbirths (in thousands)	Total Stillbirths (in thousands)	SBR (per 1000 births)	SBR (per 1000 births)	SBR/NMR	SBR/NMR
	20 weeks	28 weeks	20 weeks	28 weeks	20 weeks	28 weeks
1990	0.2 (0.1 to 0.3)	0.1 (0.1 to 0.2)	30.1 (22.1 to 40.1)	21.9 (16.1 to 28.7)	1.62 (1.19 to 2.15)	1.18 (0.86 to 1.54)
1995	0.2 (0.1 to 0.2)	0.1 (0.1 to 0.1)	26.3 (21.6 to 31.4)	18.9 (15.5 to 22.5)	1.66 (1.36 to 1.98)	1.19 (0.98 to 1.42)
2000	0.2 (0.2 to 0.2)	0.1 (0.1 to 0.1)	23.4 (22.6 to 24.3)	16.8 (16.2 to 17.4)	1.63 (1.57 to 1.69)	1.17 (1.13 to 1.21)
2005	0.1 (0.1 to 0.2)	0.1 (0.1 to 0.1)	19.2 (17.4 to 21.3)	13.8 (12.5 to 15.3)	1.57 (1.42 to 1.74)	1.13 (1.02 to 1.25)
2010	0.1 (0.1 to 0.1)	0.1 (0.1 to 0.1)	15.0 (13.1 to 17.2)	10.7 (9.4 to 12.3)	1.38 (1.21 to 1.58)	0.99 (0.87 to 1.13)
2015	0.1 (0.1 to 0.1)	0.1 (0.1 to 0.1)	11.4 (10.1 to 13.0)	8.2 (7.2 to 9.2)	1.12 (1.00 to 1.27)	0.80 (0.71 to 0.91)
2020	0.1 (0.1 to 0.1)	0.1 (0.0 to 0.1)	10.7 (8.4 to 13.7)	7.6 (5.9 to 9.7)	1.17 (0.91 to 1.49)	0.82 (0.64 to 1.05)
2021	0.1 (0.1 to 0.1)	0.1 (0.0 to 0.1)	10.4 (8.0 to 13.8)	7.3 (5.6 to 9.7)	1.18 (0.90 to 1.56)	0.83 (0.64 to 1.09)

Bermuda BMU Caribbean Stillbirth Rate / Neonatal Mortality Rate (SBR/NMR) − 20 weeks
lambda = 0.6 ; zeta = 0.8 ; scale = 15 ; amp = 0.028

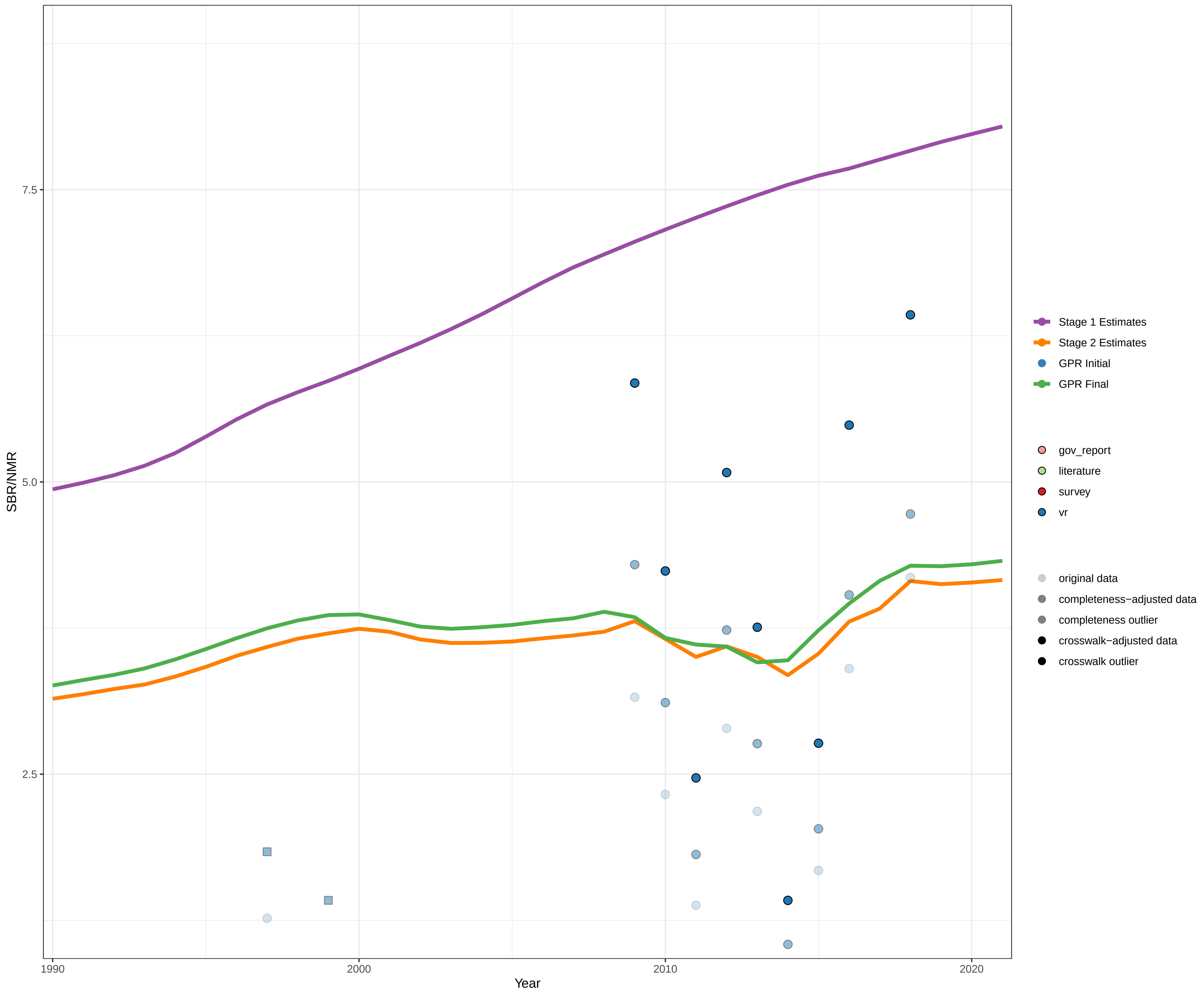

Bermuda BMU Caribbean Stillbirth Rate / Neonatal Mortality Rate (SBR/NMR) − 28 weeks
lambda = 0.6 ; zeta = 0.8 ; scale = 15 ; amp = 0.028

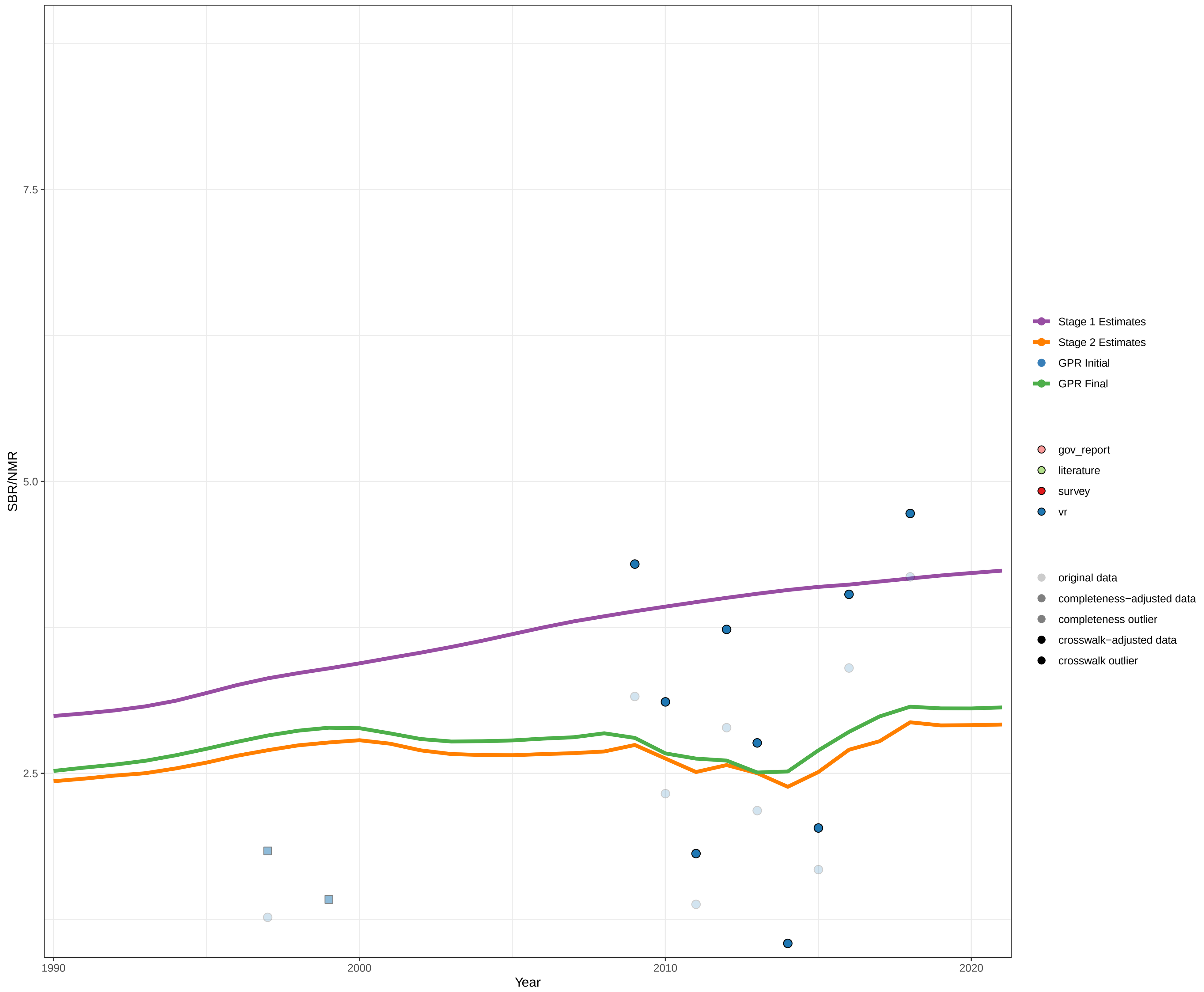

Definition	Years	Outlier	Source.Type	Title
28_weeks	2009−2016, 2018	0	VR	United Nations Demographic Yearbook
28_weeks	1997, 1999	1	VR	United Nations Demographic Yearbook

Bermuda BMU Caribbean Stillbirth Rate (SBR) − 20 weeks
lambda = 0.6 ; zeta = 0.8 ; scale = 15 ; amp = 0.028

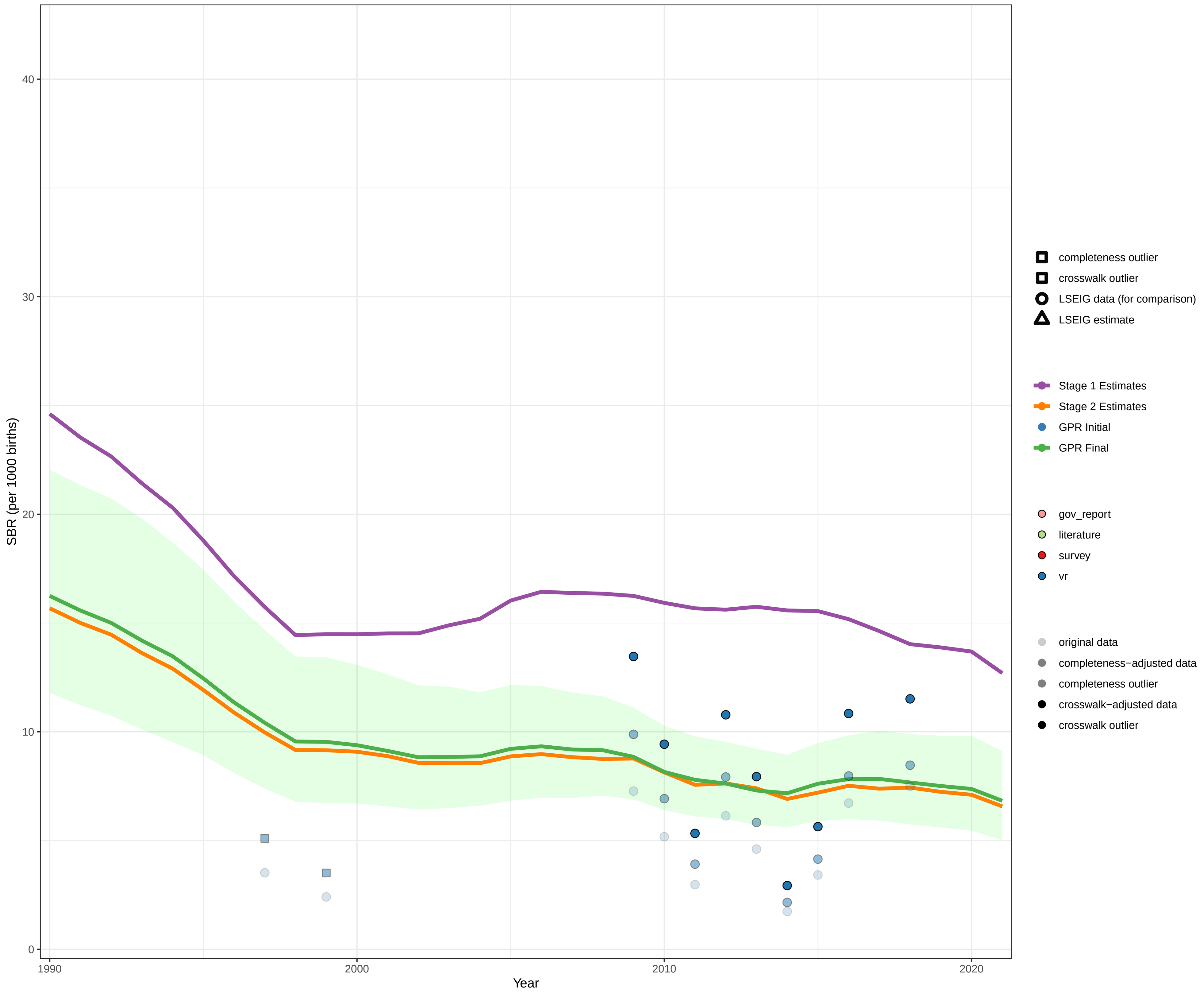

Bermuda BMU Caribbean Stillbirth Rate (SBR) − 28 weeks
lambda = 0.6 ; zeta = 0.8 ; scale = 15 ; amp = 0.028

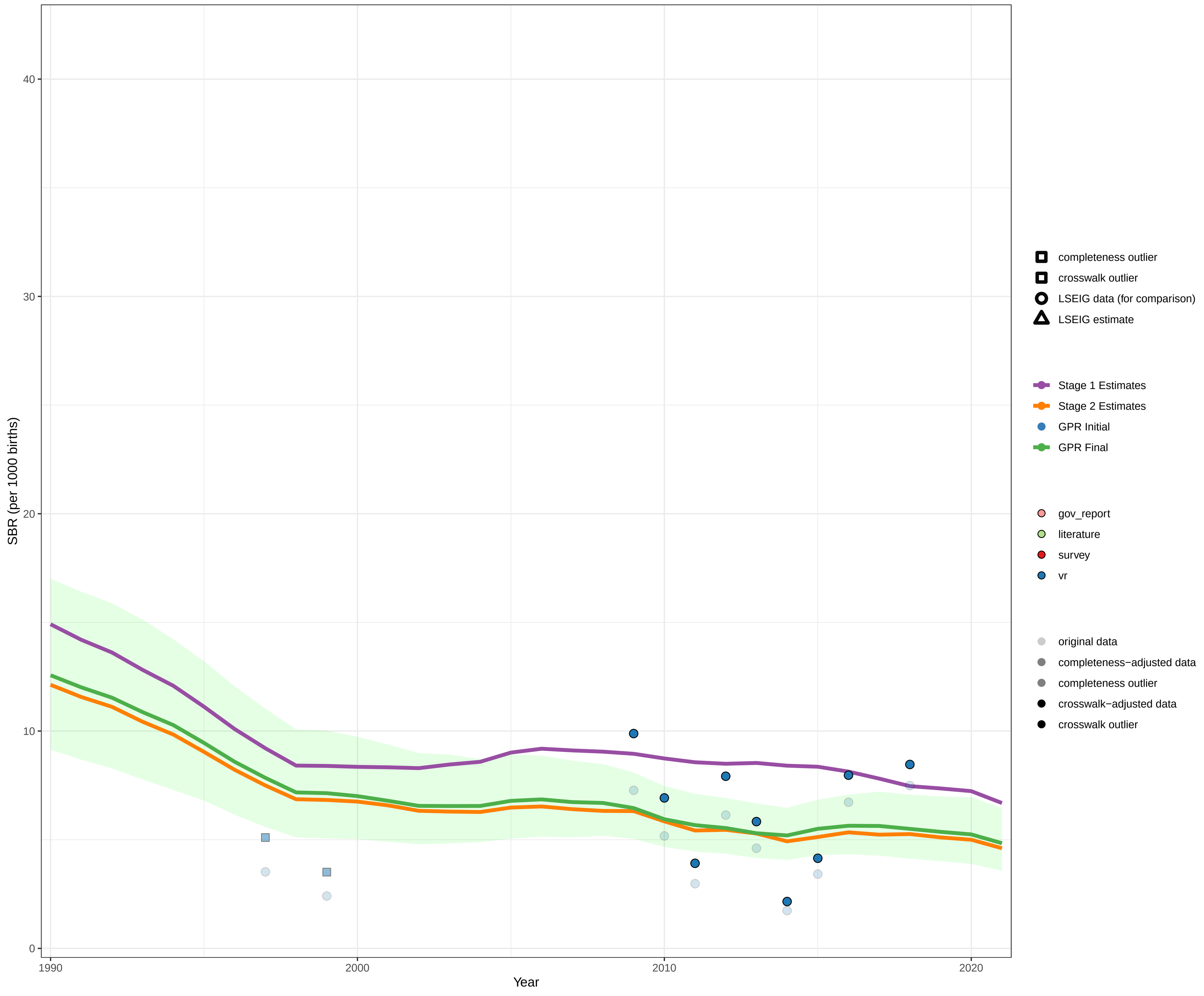

Year	Total Stillbirths (in thousands)	Total Stillbirths (in thousands)	SBR (per 1000 births)	SBR (per 1000 births)	SBR/NMR	SBR/NMR
	20 weeks	28 weeks	20 weeks	28 weeks	20 weeks	28 weeks
1990	0.0 (0.0 to 0.0)	0.0 (0.0 to 0.0)	16.2 (11.8 to 22.0)	12.6 (9.1 to 17.0)	3.26 (2.36 to 4.42)	2.52 (1.83 to 3.41)
1995	0.0 (0.0 to 0.0)	0.0 (0.0 to 0.0)	12.5 (8.9 to 17.5)	9.5 (6.8 to 13.2)	3.57 (2.56 to 5.01)	2.71 (1.95 to 3.79)
2000	0.0 (0.0 to 0.0)	0.0 (0.0 to 0.0)	9.4 (6.7 to 13.1)	7.0 (5.0 to 9.7)	3.87 (2.76 to 5.39)	2.89 (2.07 to 4.01)
2005	0.0 (0.0 to 0.0)	0.0 (0.0 to 0.0)	9.2 (6.8 to 12.2)	6.8 (5.0 to 8.9)	3.78 (2.80 to 4.98)	2.78 (2.07 to 3.66)
2010	0.0 (0.0 to 0.0)	0.0 (0.0 to 0.0)	8.2 (6.4 to 10.3)	5.9 (4.7 to 7.5)	3.67 (2.87 to 4.62)	2.67 (2.10 to 3.36)
2015	0.0 (0.0 to 0.0)	0.0 (0.0 to 0.0)	7.6 (5.9 to 9.5)	5.5 (4.3 to 6.8)	3.73 (2.89 to 4.65)	2.70 (2.09 to 3.35)
2020	0.0 (0.0 to 0.0)	0.0 (0.0 to 0.0)	7.4 (5.4 to 9.8)	5.2 (3.9 to 7.0)	4.30 (3.17 to 5.71)	3.06 (2.27 to 4.05)
2021	0.0 (0.0 to 0.0)	0.0 (0.0 to 0.0)	6.8 (5.0 to 9.1)	4.8 (3.6 to 6.4)	4.32 (3.18 to 5.78)	3.06 (2.26 to 4.08)

Cuba CUB Caribbean Stillbirth Rate / Neonatal Mortality Rate (SBR/NMR) − 20 weeks
lambda = 0.6 ; zeta = 0.8 ; scale = 15 ; amp = 0.028

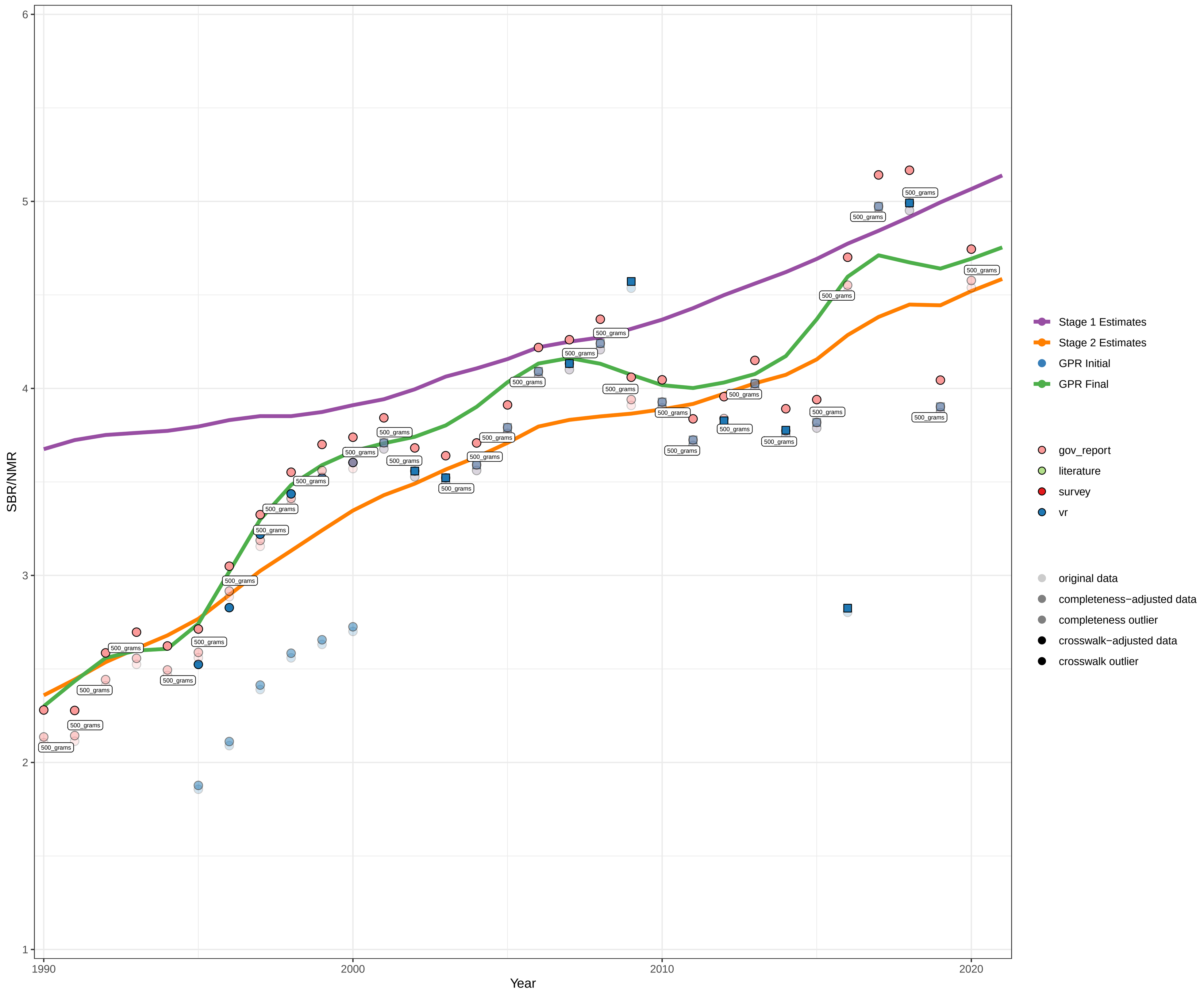

Cuba CUB Caribbean Stillbirth Rate / Neonatal Mortality Rate (SBR/NMR) − 28 weeks
lambda = 0.6 ; zeta = 0.8 ; scale = 15 ; amp = 0.028

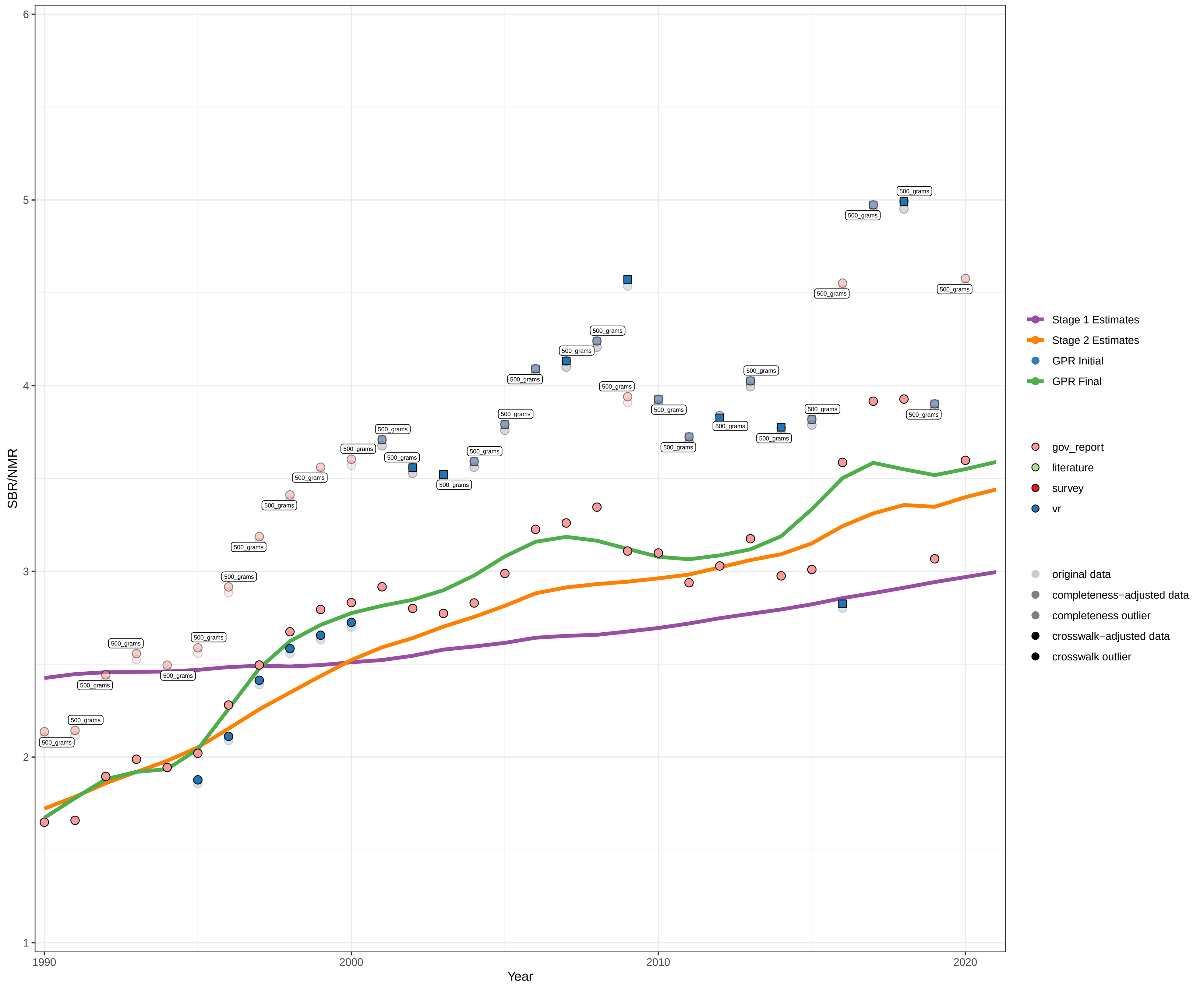

Definition	Years	Outlier	Source.Type	Title
28_weeks	1995−2000	0	VR	United Nations Demographic Yearbook
500_grams	1990−2020	0	Statistical Report	Cuba Health Statistics Yearbook
500_grams	2002, 2003, 2007, 2009, 2012, 2014, 2016, 2018	0	VR	United Nations Demographic Yearbook
500_grams	2001, 2004−2006, 2008, 2010, 2011, 2013, 2015, 2017, 2019	1	VR	United Nations Demographic Yearbook

Cuba CUB Caribbean Stillbirth Rate (SBR) − 20 weeks
lambda = 0.6 ; zeta = 0.8 ; scale = 15 ; amp = 0.028

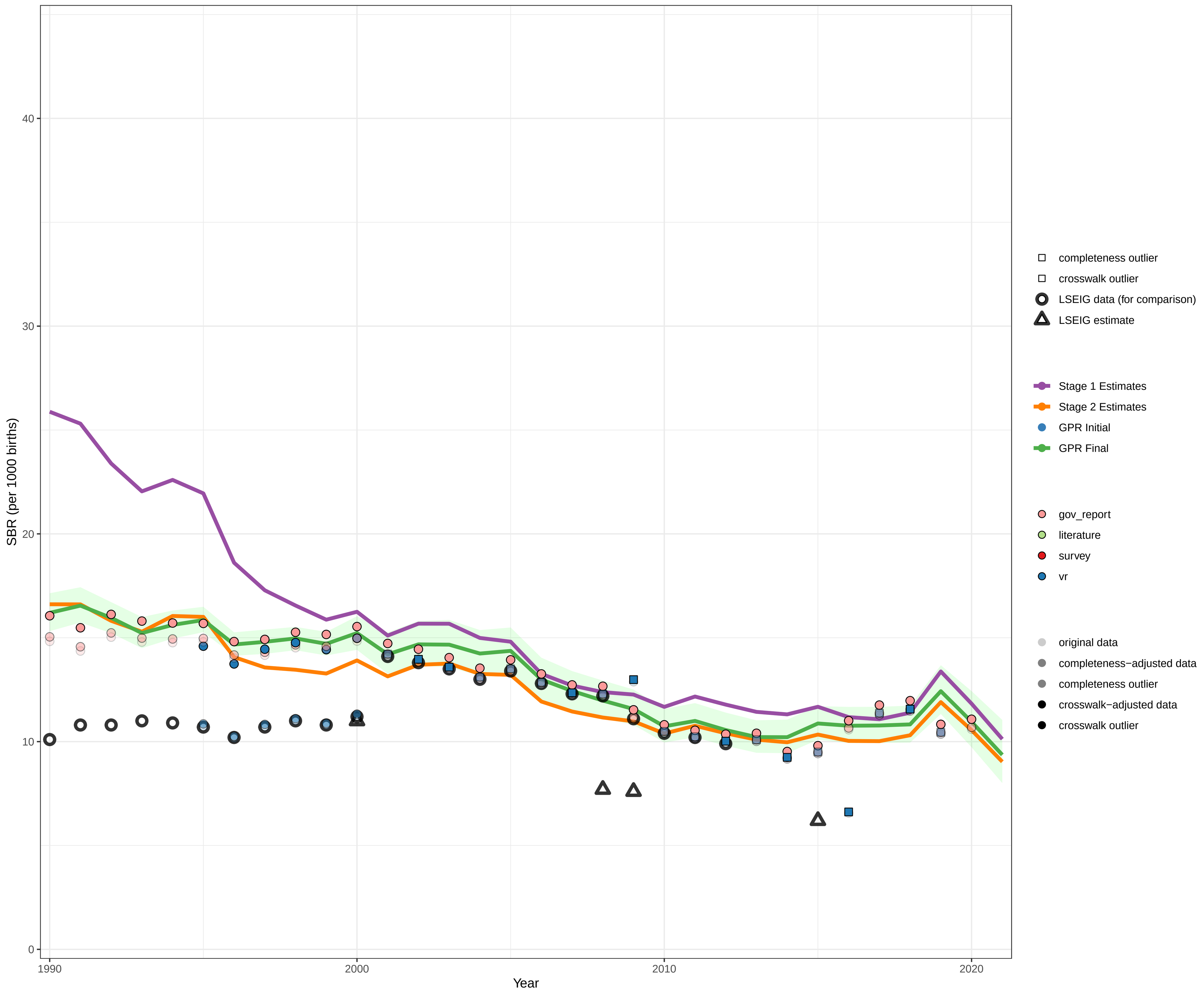

Cuba CUB Caribbean Stillbirth Rate (SBR) − 28 weeks
lambda = 0.6 ; zeta = 0.8 ; scale = 15 ; amp = 0.028

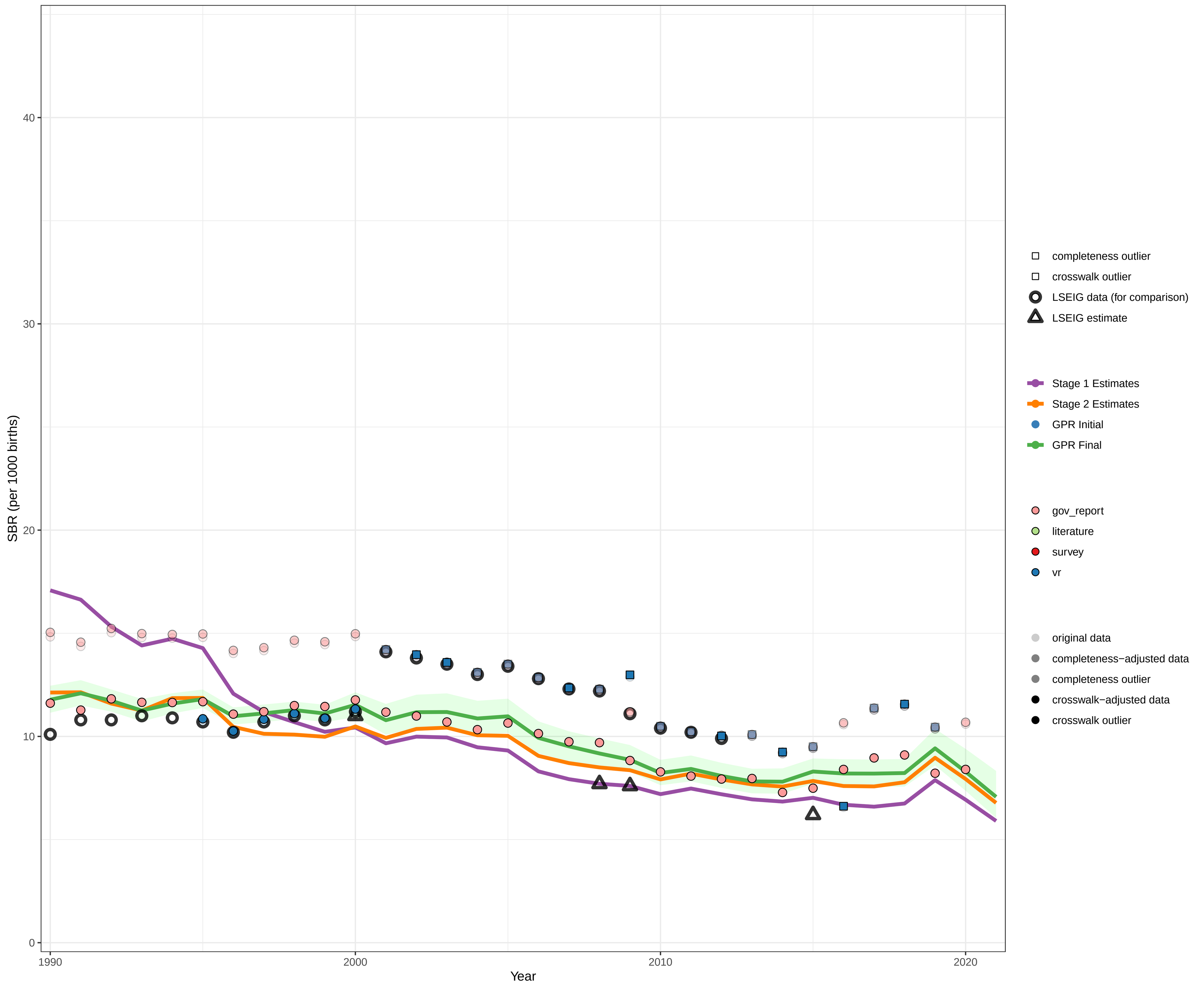

Year	Total Stillbirths (in thousands)	Total Stillbirths (in thousands)	SBR (per 1000 births)	SBR (per 1000 births)	SBR/NMR	SBR/NMR
	20 weeks	28 weeks	20 weeks	28 weeks	20 weeks	28 weeks
1990	2.9 (2.7 to 3.1)	2.1 (2.0 to 2.2)	16.2 (15.3 to 17.1)	11.8 (11.1 to 12.5)	2.30 (2.18 to 2.43)	1.67 (1.58 to 1.77)
1995	2.4 (2.3 to 2.5)	1.8 (1.7 to 1.8)	15.9 (15.3 to 16.5)	11.8 (11.4 to 12.3)	2.74 (2.64 to 2.85)	2.04 (1.97 to 2.12)
2000	2.3 (2.2 to 2.4)	1.7 (1.6 to 1.8)	15.2 (14.4 to 16.0)	11.5 (10.9 to 12.1)	3.66 (3.47 to 3.86)	2.78 (2.63 to 2.92)
2005	1.7 (1.6 to 1.9)	1.3 (1.2 to 1.4)	14.4 (13.3 to 15.5)	11.0 (10.2 to 11.8)	4.03 (3.74 to 4.35)	3.08 (2.85 to 3.32)
2010	1.4 (1.3 to 1.5)	1.1 (1.0 to 1.2)	10.7 (10.0 to 11.6)	8.2 (7.6 to 8.9)	4.02 (3.73 to 4.33)	3.08 (2.86 to 3.32)
2015	1.3 (1.2 to 1.4)	1.0 (0.9 to 1.1)	10.9 (10.1 to 11.7)	8.3 (7.7 to 8.9)	4.37 (4.06 to 4.70)	3.33 (3.10 to 3.59)
2020	1.1 (1.0 to 1.3)	0.9 (0.8 to 1.0)	11.0 (9.7 to 12.4)	8.3 (7.4 to 9.4)	4.69 (4.17 to 5.33)	3.55 (3.16 to 4.03)
2021	0.9 (0.8 to 1.1)	0.7 (0.6 to 0.8)	9.4 (8.0 to 11.0)	7.1 (6.0 to 8.3)	4.75 (4.06 to 5.61)	3.59 (3.07 to 4.23)

Dominica DMA Caribbean Stillbirth Rate / Neonatal Mortality Rate (SBR/NMR) − 20 weeks
lambda = 1 ; zeta = 0.6 ; scale = 15 ; amp = 0.028

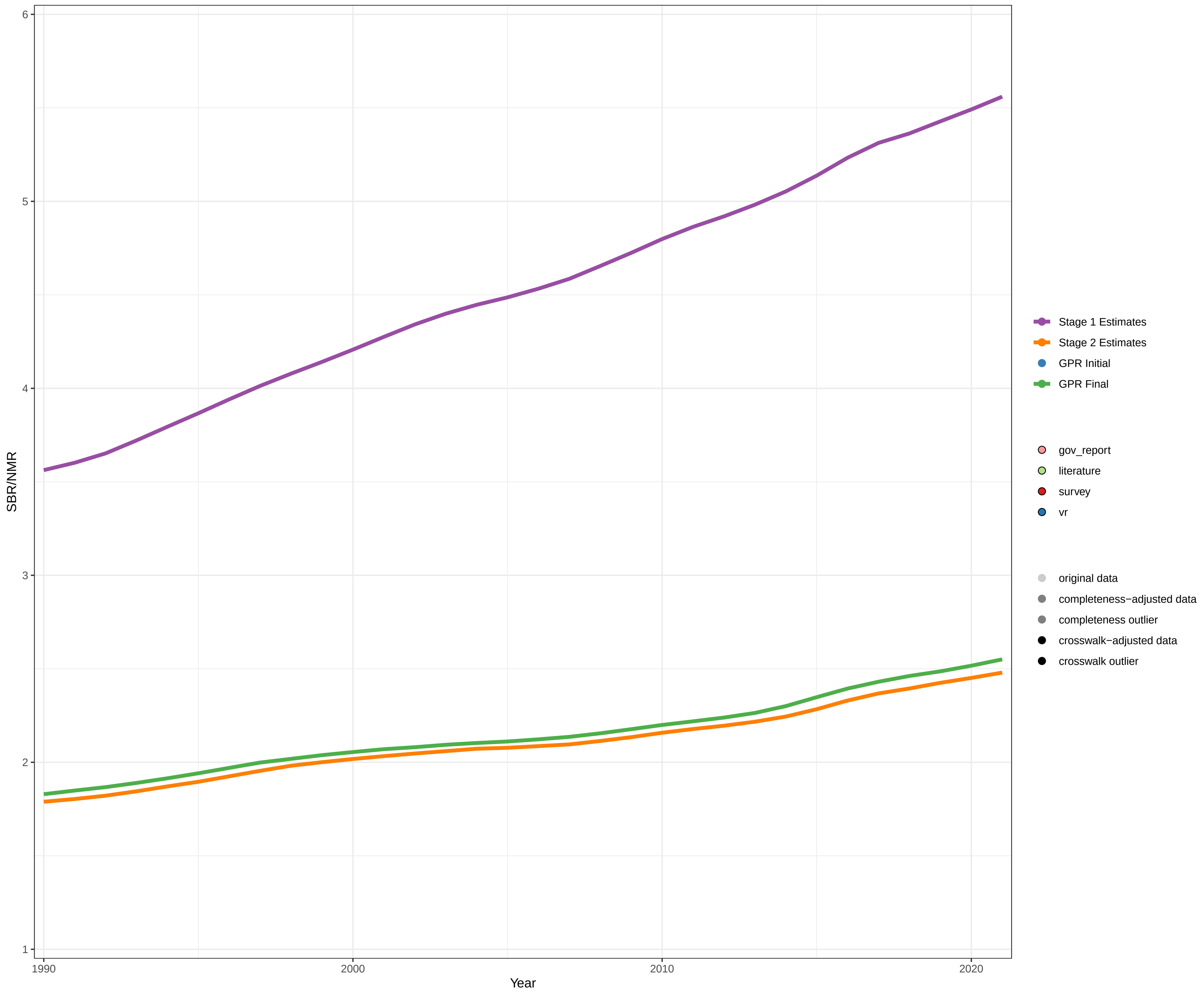

Dominica DMA Caribbean Stillbirth Rate / Neonatal Mortality Rate (SBR/NMR) − 28 weeks
lambda = 1 ; zeta = 0.6 ; scale = 15 ; amp = 0.028

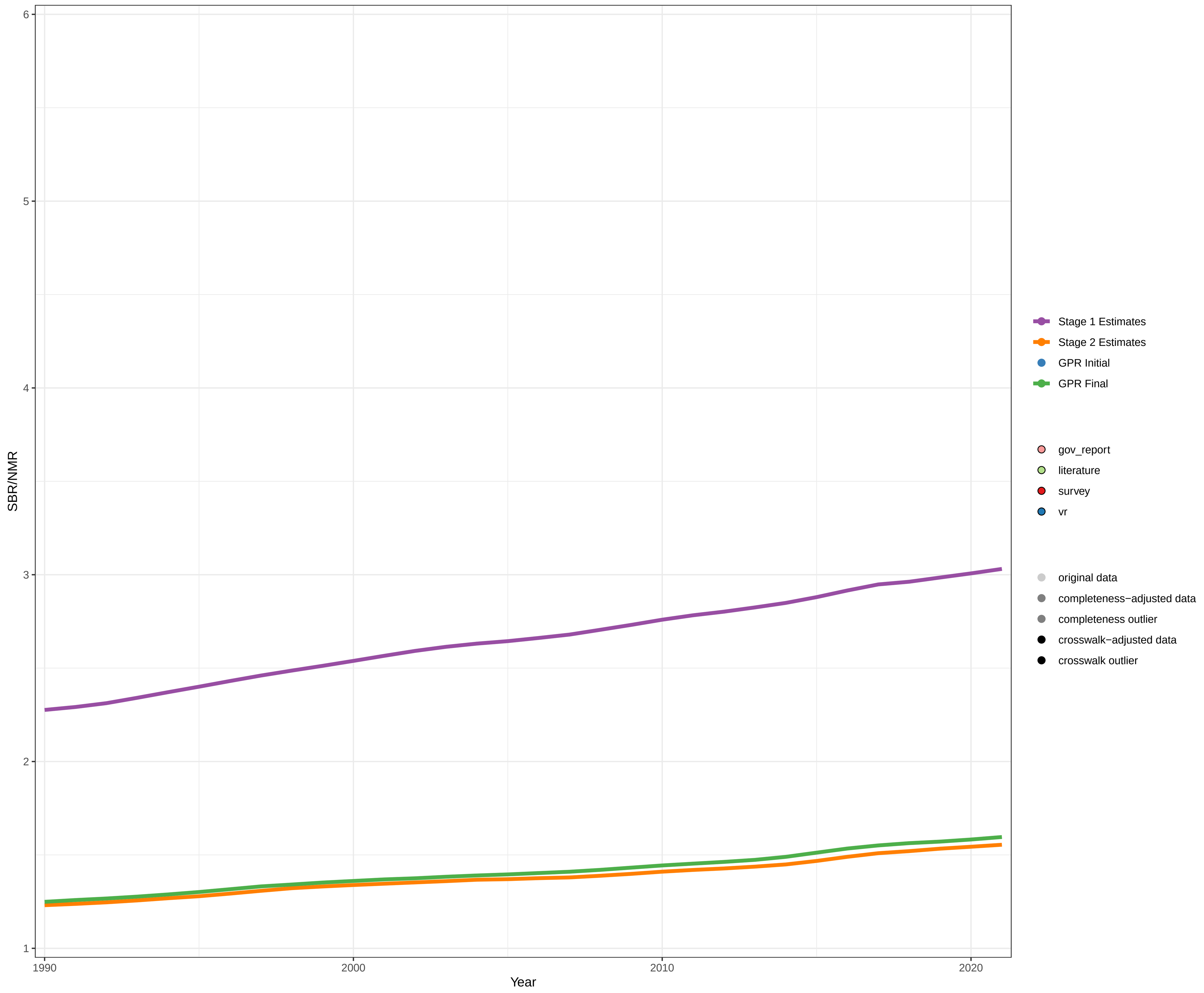

Definition	Years	Outlier	Source.Type	Title

Dominica DMA Caribbean Stillbirth Rate (SBR) − 20 weeks
lambda = 1 ; zeta = 0.6 ; scale = 15 ; amp = 0.028

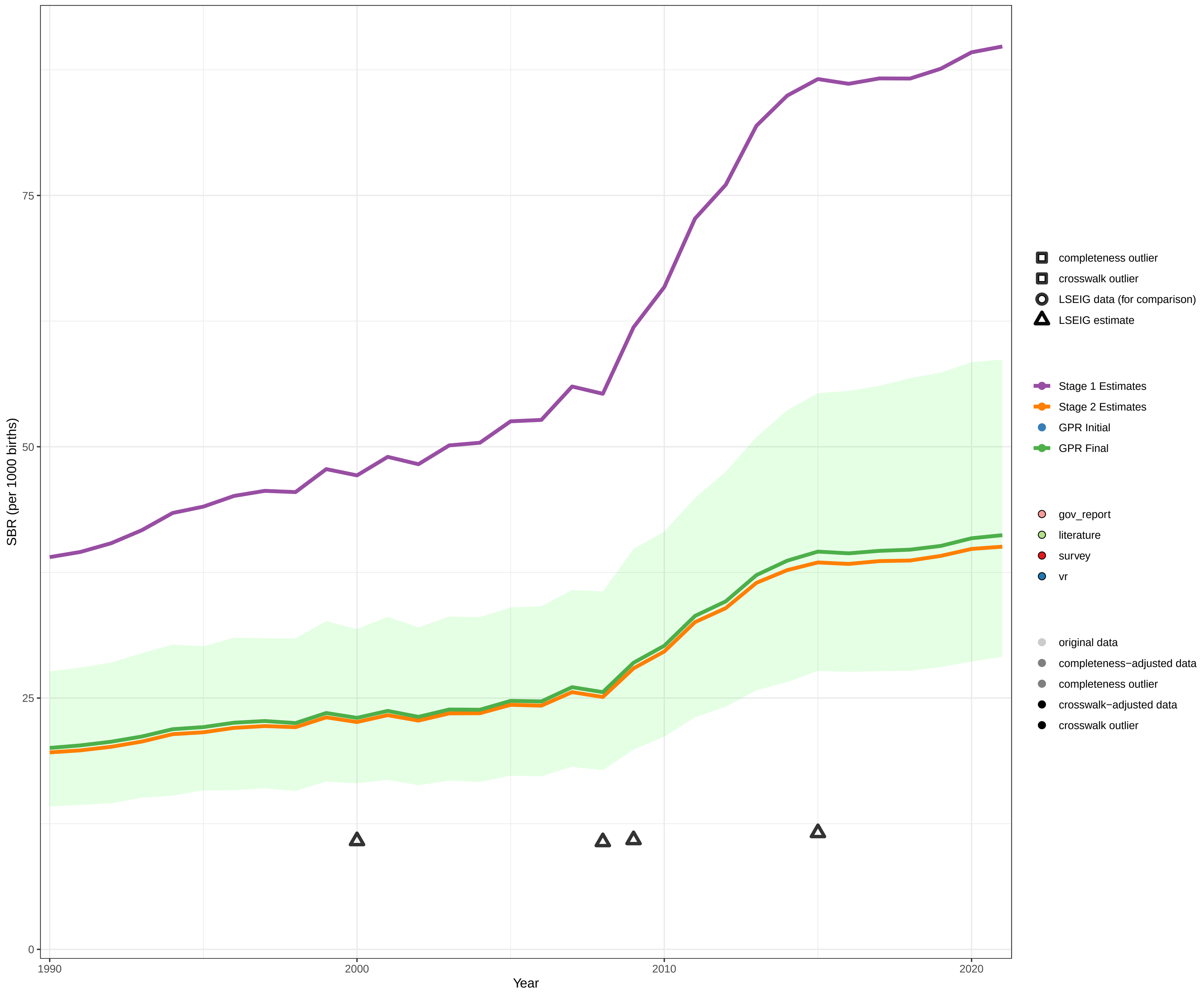

Dominica DMA Caribbean Stillbirth Rate (SBR) − 28 weeks
lambda = 1 ; zeta = 0.6 ; scale = 15 ; amp = 0.028

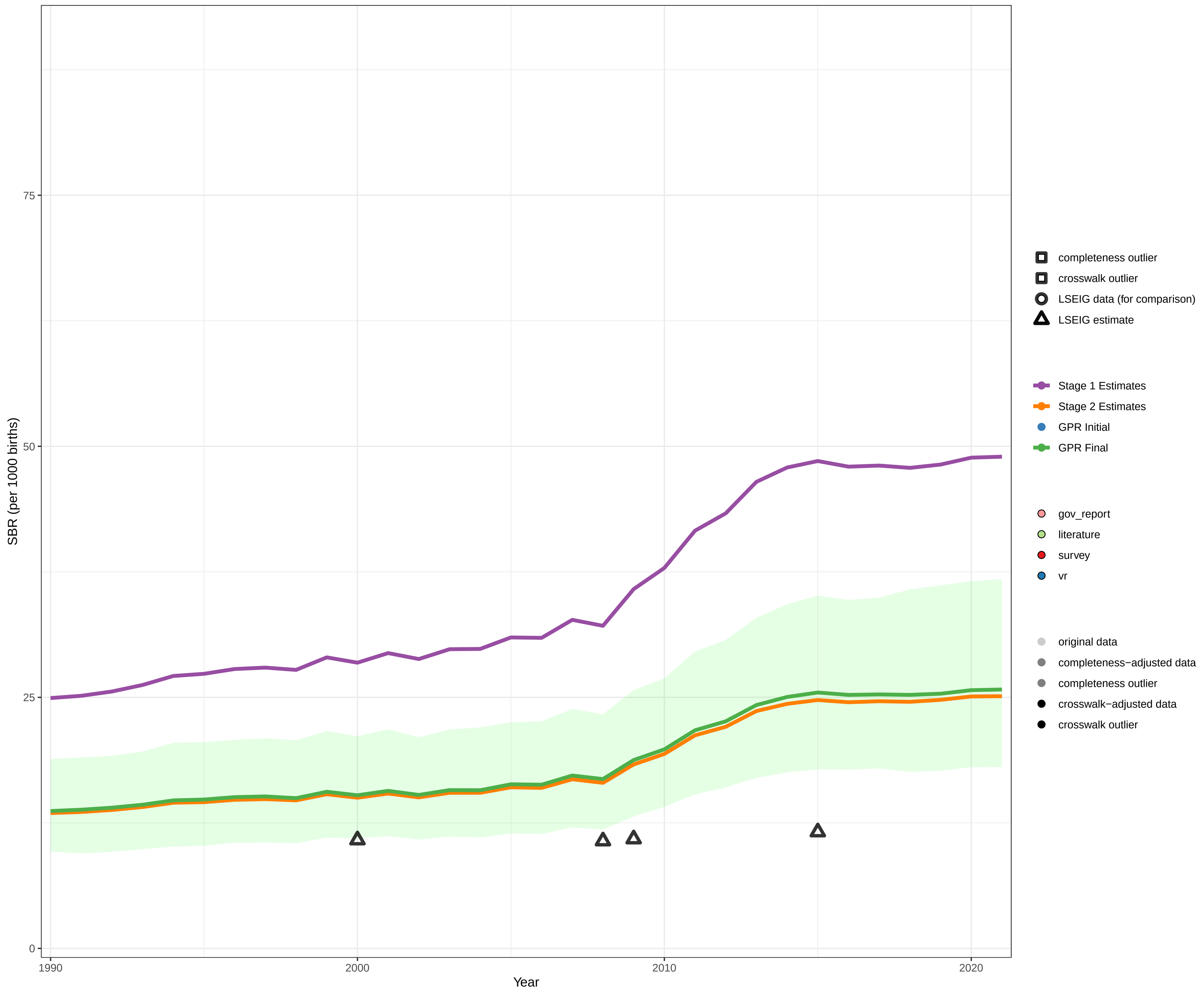

Year	Total Stillbirths (in thousands)	Total Stillbirths (in thousands)	SBR (per 1000 births)	SBR (per 1000 births)	SBR/NMR	SBR/NMR
	20 weeks	28 weeks	20 weeks	28 weeks	20 weeks	28 weeks
1990	0.0 (0.0 to 0.1)	0.0 (0.0 to 0.0)	20.0 (14.2 to 27.7)	13.7 (9.6 to 18.9)	1.83 (1.30 to 2.53)	1.25 (0.88 to 1.72)
1995	0.0 (0.0 to 0.0)	0.0 (0.0 to 0.0)	22.1 (15.8 to 30.2)	14.8 (10.2 to 20.5)	1.94 (1.39 to 2.65)	1.30 (0.90 to 1.80)
2000	0.0 (0.0 to 0.0)	0.0 (0.0 to 0.0)	23.0 (16.5 to 31.9)	15.3 (11.0 to 21.1)	2.05 (1.47 to 2.84)	1.36 (0.98 to 1.89)
2005	0.0 (0.0 to 0.0)	0.0 (0.0 to 0.0)	24.7 (17.3 to 34.0)	16.3 (11.5 to 22.5)	2.11 (1.48 to 2.90)	1.40 (0.98 to 1.92)
2010	0.0 (0.0 to 0.0)	0.0 (0.0 to 0.0)	30.2 (21.2 to 41.6)	19.8 (14.1 to 26.9)	2.20 (1.54 to 3.03)	1.44 (1.03 to 1.96)
2015	0.0 (0.0 to 0.0)	0.0 (0.0 to 0.0)	39.6 (27.7 to 55.3)	25.5 (17.8 to 35.1)	2.35 (1.64 to 3.28)	1.51 (1.06 to 2.08)
2020	0.0 (0.0 to 0.0)	0.0 (0.0 to 0.0)	40.9 (28.6 to 58.4)	25.7 (18.0 to 36.6)	2.52 (1.76 to 3.60)	1.58 (1.11 to 2.25)
2021	0.0 (0.0 to 0.0)	0.0 (0.0 to 0.0)	41.2 (29.1 to 58.7)	25.8 (18.1 to 36.8)	2.55 (1.80 to 3.63)	1.60 (1.12 to 2.28)

Dominican Republic DOM Caribbean Stillbirth Rate / Neonatal Mortality Rate (SBR/NMR) − 20 weeks
lambda = 0.4 ; zeta = 0.9 ; scale = 10 ; amp = 0.028

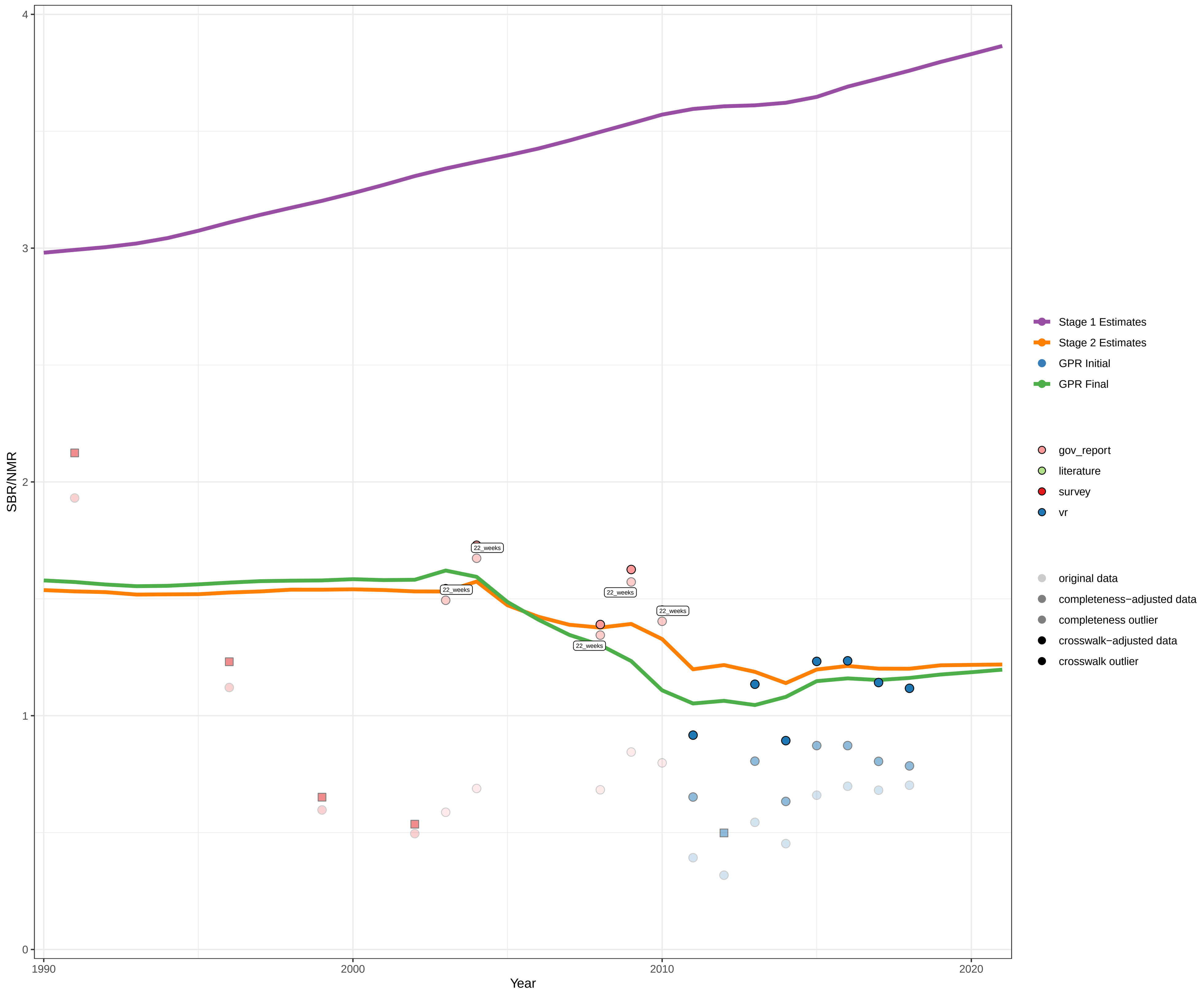

Dominican Republic DOM Caribbean Stillbirth Rate / Neonatal Mortality Rate (SBR/NMR) − 28 weeks
lambda = 0.4 ; zeta = 0.9 ; scale = 10 ; amp = 0.028

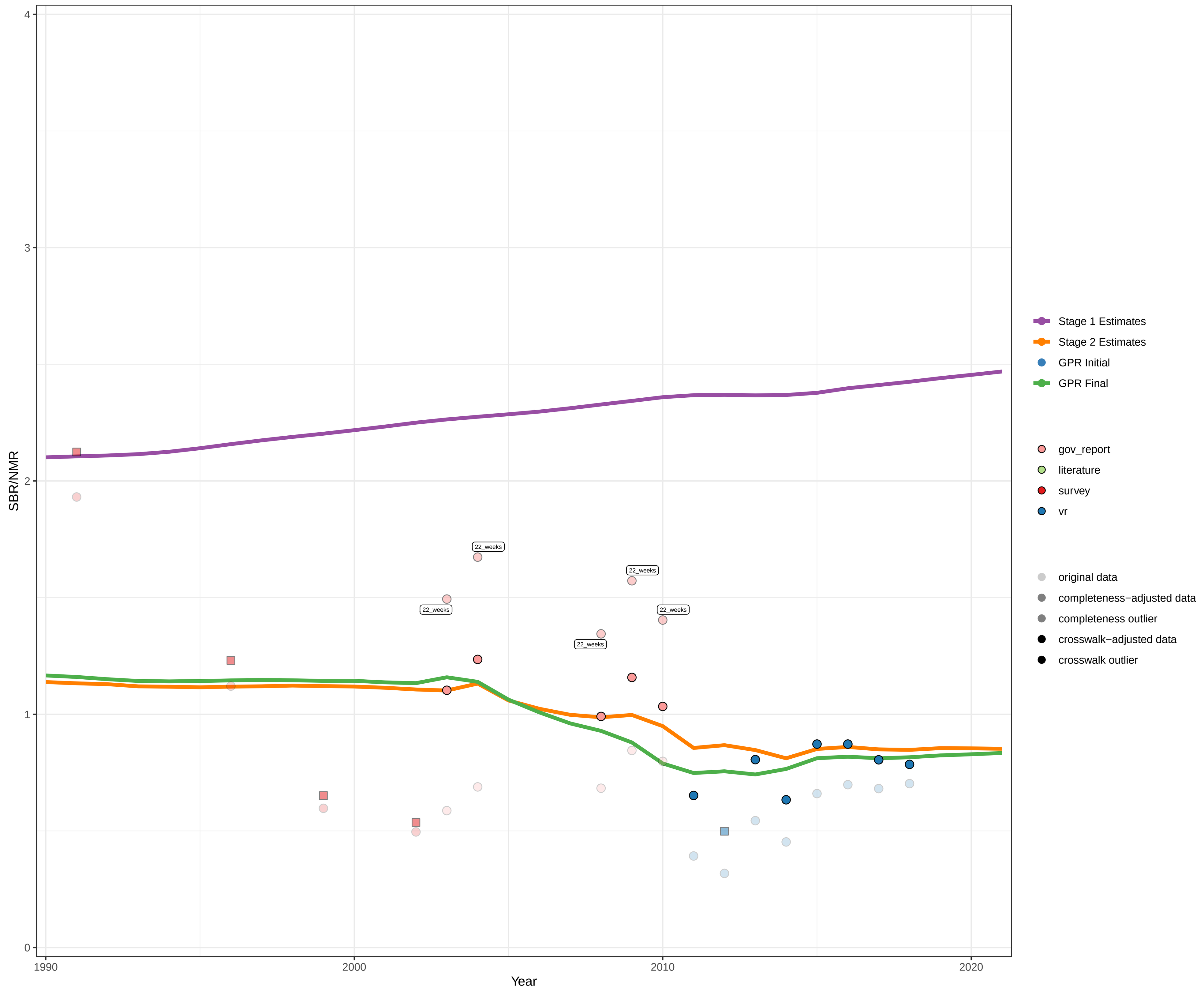

Definition	Years	Outlier	Source.Type	Title
22_weeks	2003, 2004	0	Statistical Report	Dominican Republic Health Statistics
22_weeks	2008	0	Statistical Report	Dominican Republic Statistical Yearbook
22_weeks	2009, 2010	0	Statistical Report	Dominican Republic Health Statistical Yearbook
28_weeks	2011, 2013−2018	0	VR	United Nations Demographic Yearbook
28_weeks	1991	1	Survey	Dominican Republic Demographic and Health Survey 1991
28_weeks	1996	1	Survey	Dominican Republic Demographic and Health Survey 1996
28_weeks	1999	1	Survey	Dominican Republic Experimental Demographic and Health Survey 1999
28_weeks	2002	1	Survey	Dominican Republic Demographic and Health Survey 2002
28_weeks	2012	1	VR	United Nations Demographic Yearbook

Dominican Republic DOM Caribbean Stillbirth Rate (SBR) − 20 weeks
lambda = 0.4 ; zeta = 0.9 ; scale = 10 ; amp = 0.028

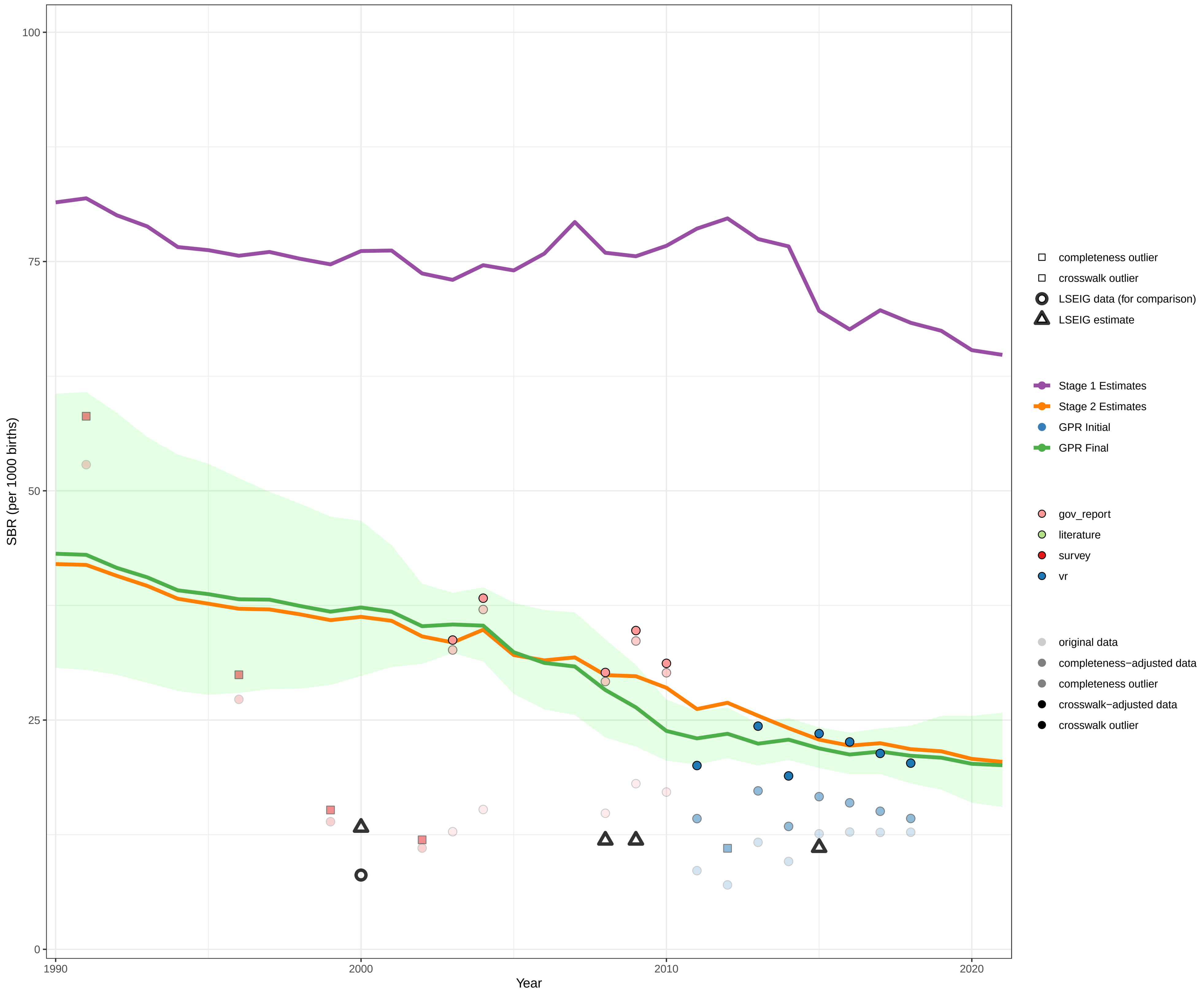

Dominican Republic DOM Caribbean Stillbirth Rate (SBR) − 28 weeks
lambda = 0.4 ; zeta = 0.9 ; scale = 10 ; amp = 0.028

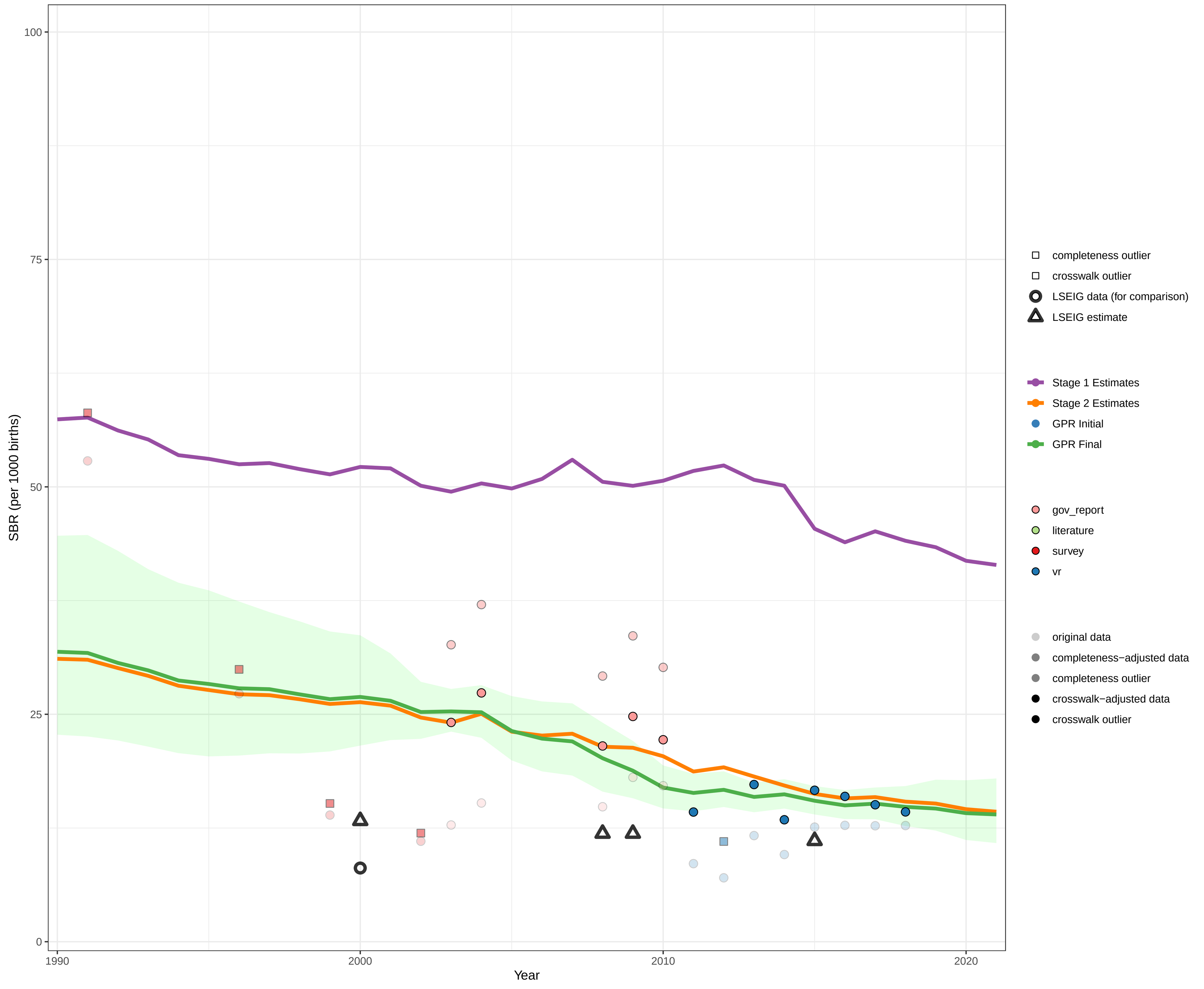

Year	Total Stillbirths (in thousands)	Total Stillbirths (in thousands)	SBR (per 1000 births)	SBR (per 1000 births)	SBR/NMR	SBR/NMR
	20 weeks	28 weeks	20 weeks	28 weeks	20 weeks	28 weeks
1990	10.4 (7.3 to 14.8)	7.6 (5.3 to 10.7)	43.1 (30.7 to 60.6)	31.9 (22.8 to 44.6)	1.58 (1.12 to 2.22)	1.17 (0.83 to 1.63)
1995	9.6 (6.8 to 13.2)	6.9 (4.9 to 9.5)	38.7 (27.8 to 53.0)	28.3 (20.4 to 38.6)	1.56 (1.12 to 2.14)	1.14 (0.82 to 1.56)
2000	8.6 (6.8 to 10.9)	6.1 (4.9 to 7.7)	37.3 (29.8 to 46.7)	26.9 (21.6 to 33.7)	1.58 (1.27 to 1.99)	1.14 (0.92 to 1.43)
2005	6.8 (5.8 to 8.0)	4.8 (4.2 to 5.7)	32.4 (27.9 to 37.8)	23.2 (19.9 to 27.0)	1.49 (1.28 to 1.73)	1.06 (0.91 to 1.24)
2010	4.9 (4.2 to 5.6)	3.5 (3.0 to 4.0)	23.8 (20.6 to 27.2)	17.0 (14.6 to 19.4)	1.11 (0.96 to 1.27)	0.79 (0.68 to 0.90)
2015	4.7 (4.2 to 5.2)	3.3 (3.0 to 3.7)	21.9 (19.8 to 24.2)	15.5 (14.0 to 17.1)	1.15 (1.04 to 1.27)	0.81 (0.73 to 0.90)
2020	4.4 (3.5 to 5.6)	3.1 (2.4 to 3.9)	20.2 (16.0 to 25.5)	14.1 (11.2 to 17.8)	1.19 (0.94 to 1.49)	0.83 (0.66 to 1.04)
2021	4.4 (3.4 to 5.7)	3.0 (2.3 to 3.9)	20.1 (15.5 to 25.8)	14.0 (10.8 to 17.9)	1.20 (0.93 to 1.54)	0.83 (0.65 to 1.07)

Grenada GRD Caribbean Stillbirth Rate / Neonatal Mortality Rate (SBR/NMR) − 20 weeks
lambda = 0.8 ; zeta = 0.7 ; scale = 15 ; amp = 0.028

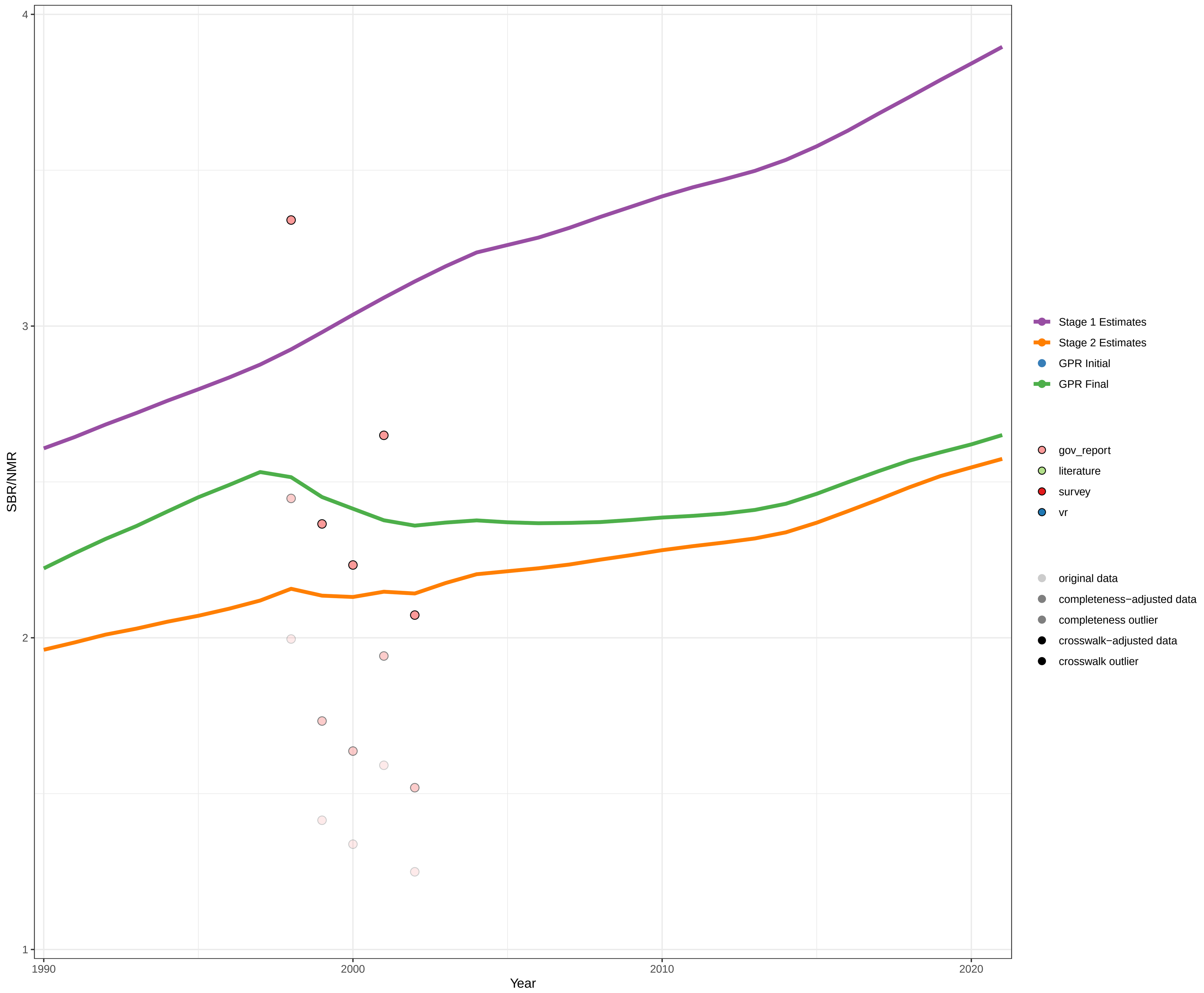

Grenada GRD Caribbean Stillbirth Rate / Neonatal Mortality Rate (SBR/NMR) − 28 weeks
lambda = 0.8 ; zeta = 0.7 ; scale = 15 ; amp = 0.028

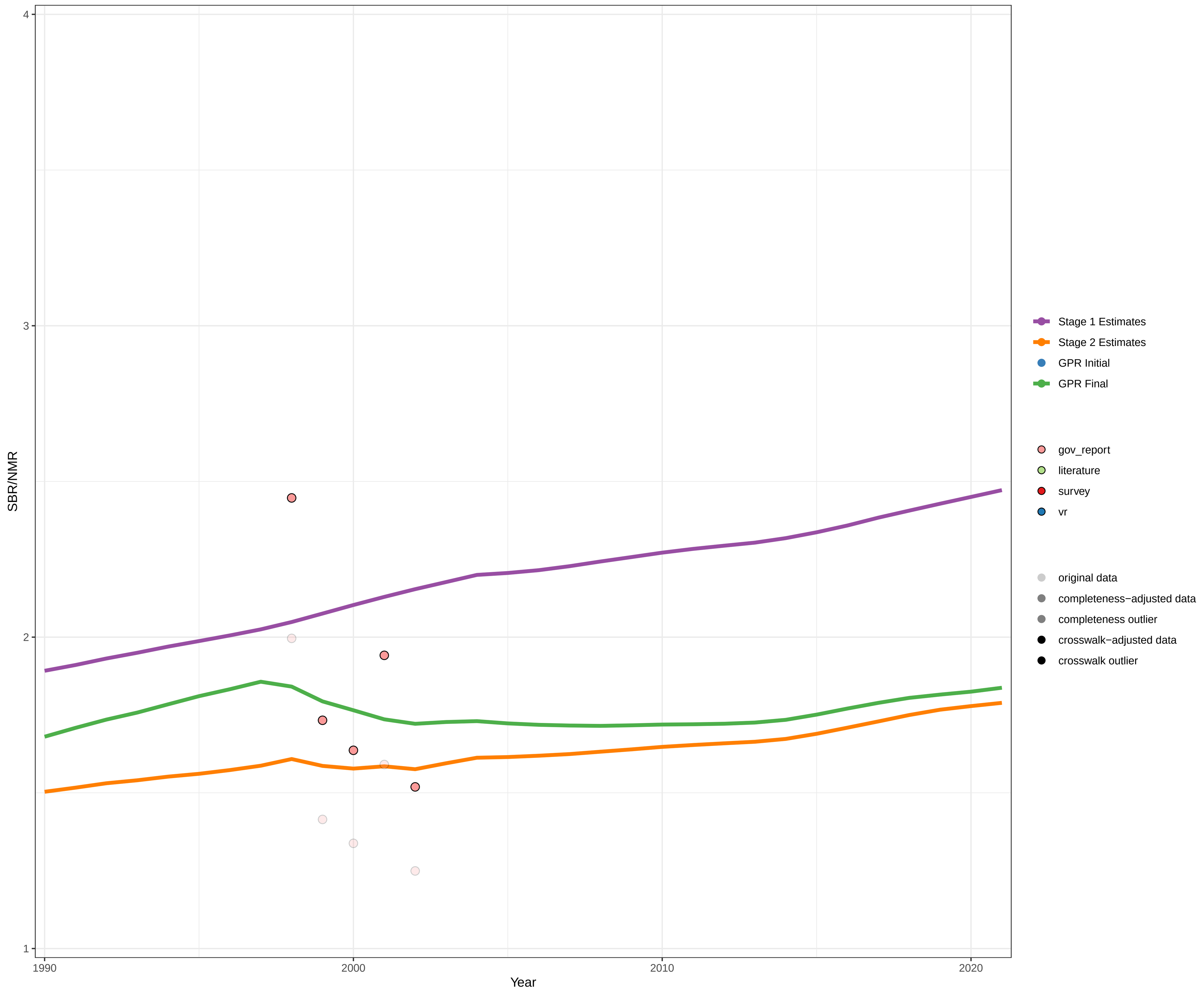

Definition	Years	Outlier	Source.Type	Title
28_weeks	1998−2002	0	Statistical Report	Grenada National Strategic Plan for Health

Grenada GRD Caribbean Stillbirth Rate (SBR) − 20 weeks
lambda = 0.8 ; zeta = 0.7 ; scale = 15 ; amp = 0.028

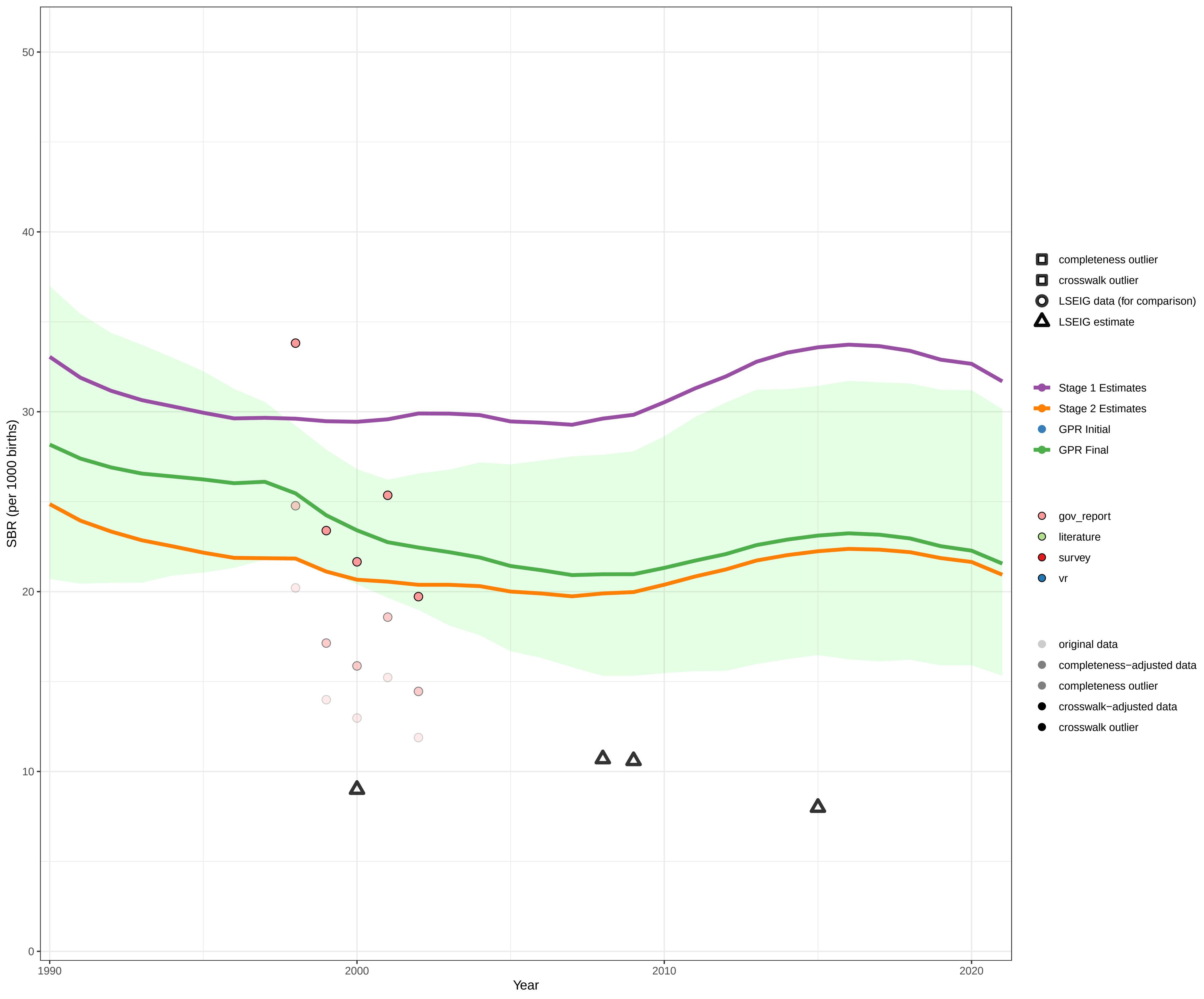

Grenada GRD Caribbean Stillbirth Rate (SBR) − 28 weeks
lambda = 0.8 ; zeta = 0.7 ; scale = 15 ; amp = 0.028

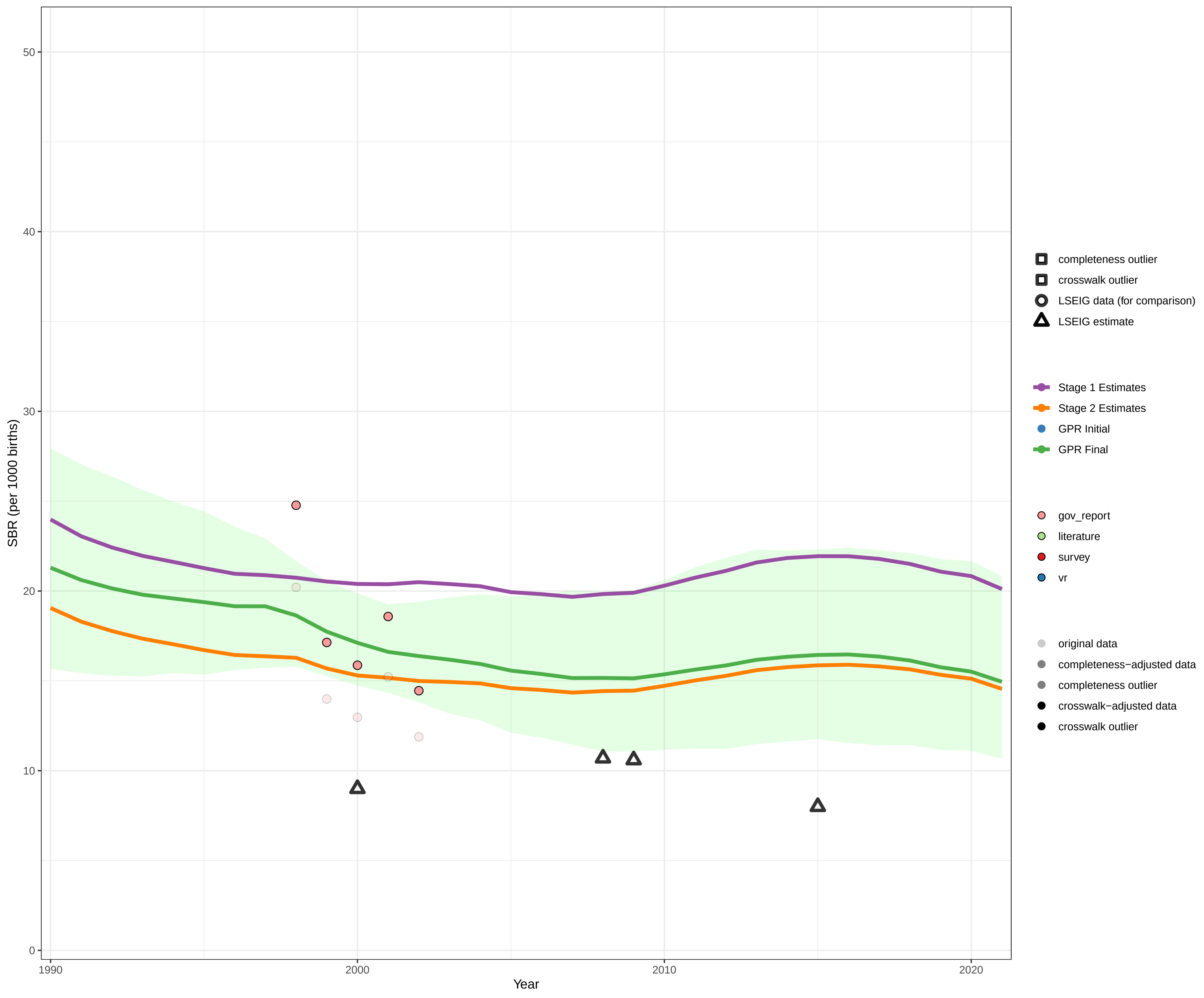

Year	Total Stillbirths (in thousands)	Total Stillbirths (in thousands)	SBR (per 1000 births)	SBR (per 1000 births)	SBR/NMR	SBR/NMR
	20 weeks	28 weeks	20 weeks	28 weeks	20 weeks	28 weeks
1990	0.1<br>(0.1 to 0.1)	0.1<br>(0.0 to 0.1)	28.2<br>(20.7 to 37.0)	21.3<br>(15.7 to 27.9)	2.22<br>(1.63 to 2.92)	1.68<br>(1.24 to 2.20)
1995	0.1<br>(0.0 to 0.1)	0.0<br>(0.0 to 0.1)	26.2<br>(21.1 to 32.3)	19.4<br>(15.3 to 24.4)	2.45<br>(1.97 to 3.01)	1.81<br>(1.43 to 2.28)
2000	0.1<br>(0.0 to 0.1)	0.0<br>(0.0 to 0.0)	23.4<br>(20.4 to 26.8)	17.1<br>(14.7 to 19.9)	2.41<br>(2.11 to 2.76)	1.77<br>(1.52 to 2.05)
2005	0.0<br>(0.0 to 0.1)	0.0<br>(0.0 to 0.0)	21.4<br>(16.7 to 27.1)	15.6<br>(12.1 to 19.7)	2.37<br>(1.85 to 3.00)	1.72<br>(1.34 to 2.18)
2010	0.0<br>(0.0 to 0.1)	0.0<br>(0.0 to 0.0)	21.3<br>(15.5 to 28.6)	15.4<br>(11.2 to 20.6)	2.39<br>(1.73 to 3.20)	1.72<br>(1.25 to 2.31)
2015	0.0<br>(0.0 to 0.1)	0.0<br>(0.0 to 0.0)	23.1<br>(16.5 to 31.4)	16.4<br>(11.7 to 22.3)	2.46<br>(1.75 to 3.35)	1.75<br>(1.25 to 2.38)
2020	0.0<br>(0.0 to 0.0)	0.0<br>(0.0 to 0.0)	22.3<br>(15.9 to 31.2)	15.5<br>(11.1 to 21.7)	2.62<br>(1.87 to 3.67)	1.82<br>(1.31 to 2.55)
2021	0.0<br>(0.0 to 0.0)	0.0<br>(0.0 to 0.0)	21.6<br>(15.3 to 30.1)	14.9<br>(10.7 to 20.8)	2.65<br>(1.89 to 3.71)	1.84<br>(1.31 to 2.56)

Guyana GUY Caribbean Stillbirth Rate / Neonatal Mortality Rate (SBR/NMR) − 20 weeks
lambda = 0.8 ; zeta = 0.7 ; scale = 15 ; amp = 0.028

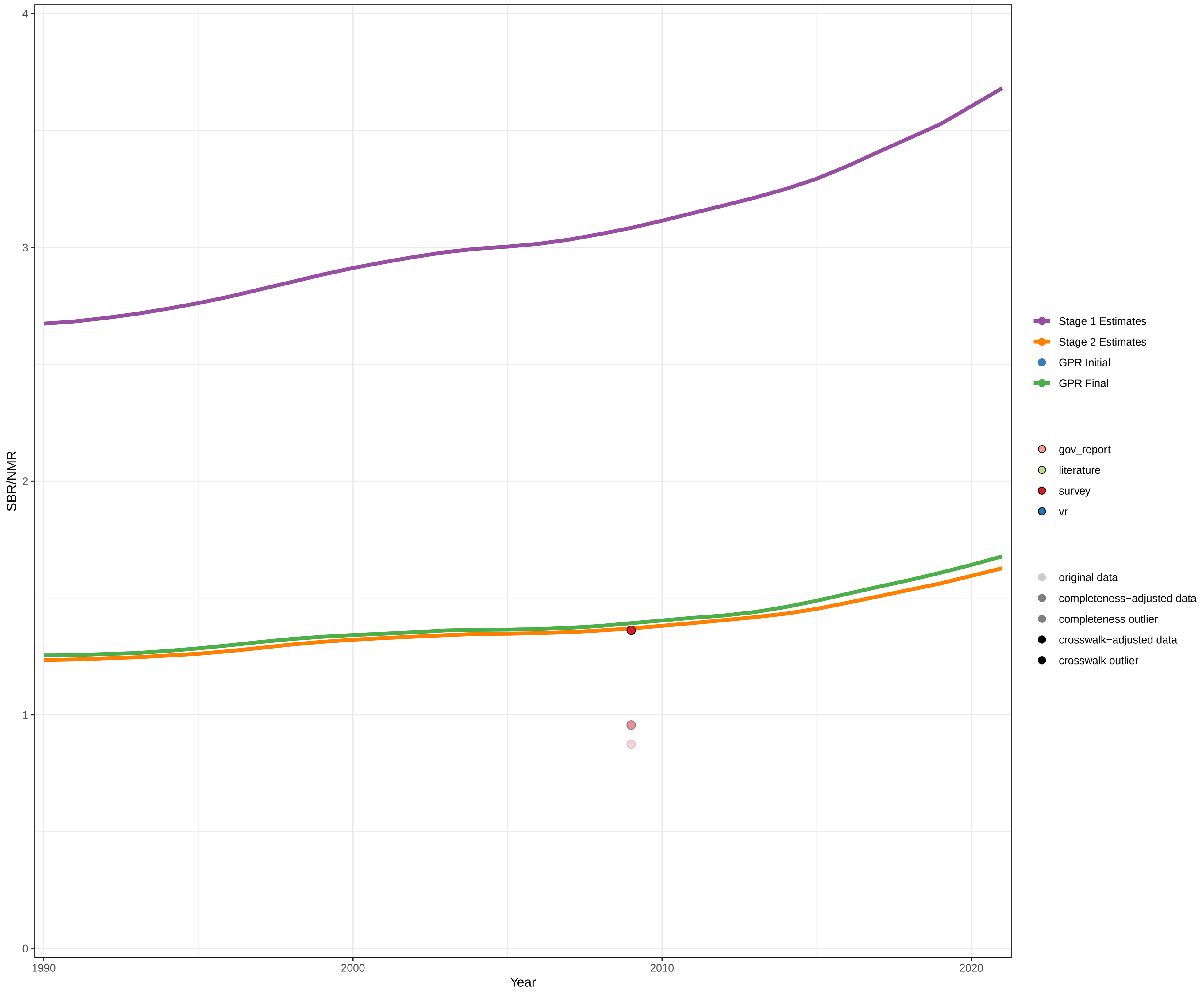

Guyana GUY Caribbean Stillbirth Rate / Neonatal Mortality Rate (SBR/NMR) − 28 weeks
lambda = 0.8 ; zeta = 0.7 ; scale = 15 ; amp = 0.028

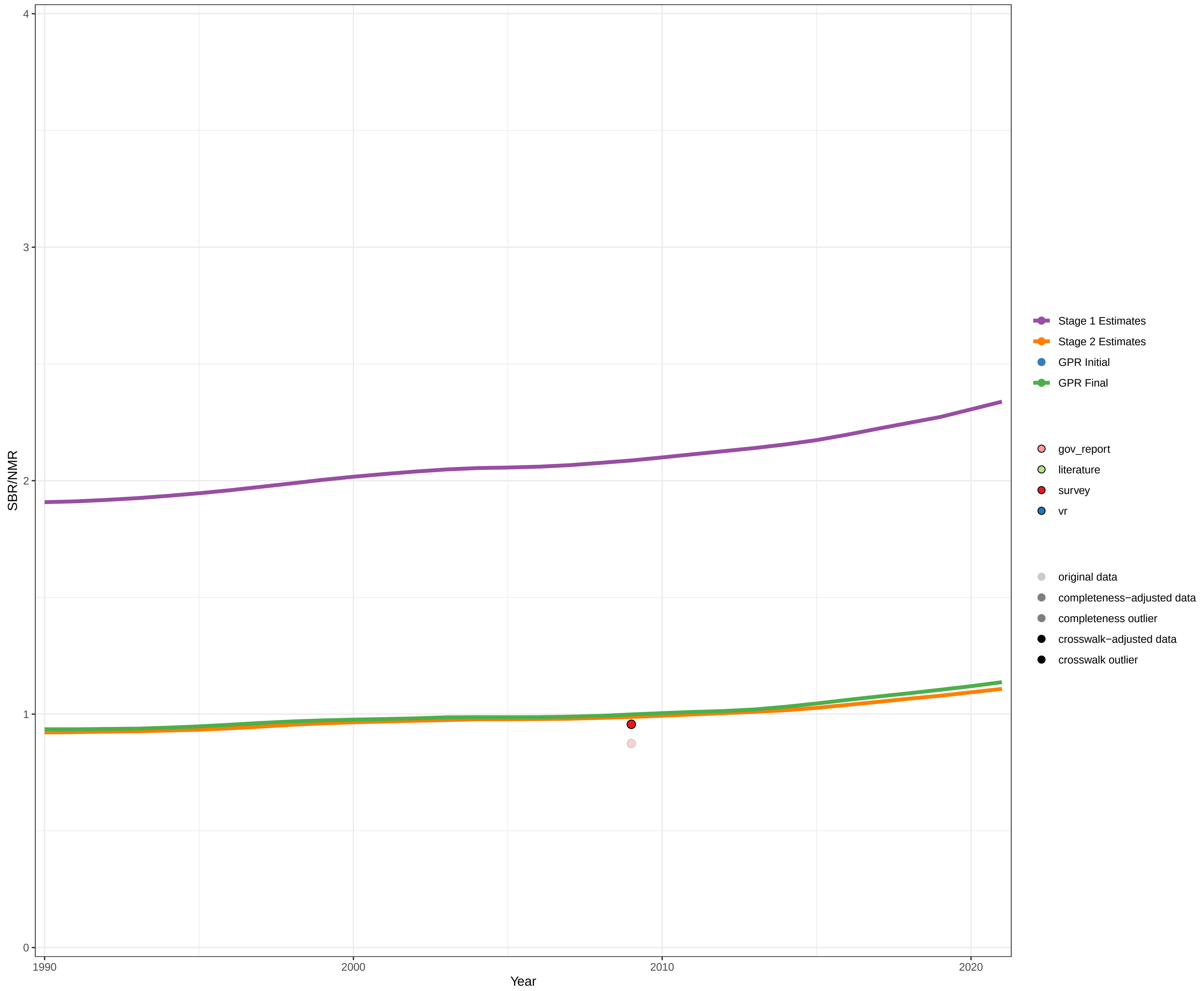

Definition	Years	Outlier	Source.Type	Title
28_weeks	2009	0	Survey	Guyana Demographic and Health Survey 2009

Guyana GUY Caribbean Stillbirth Rate (SBR) − 20 weeks
lambda = 0.8 ; zeta = 0.7 ; scale = 15 ; amp = 0.028

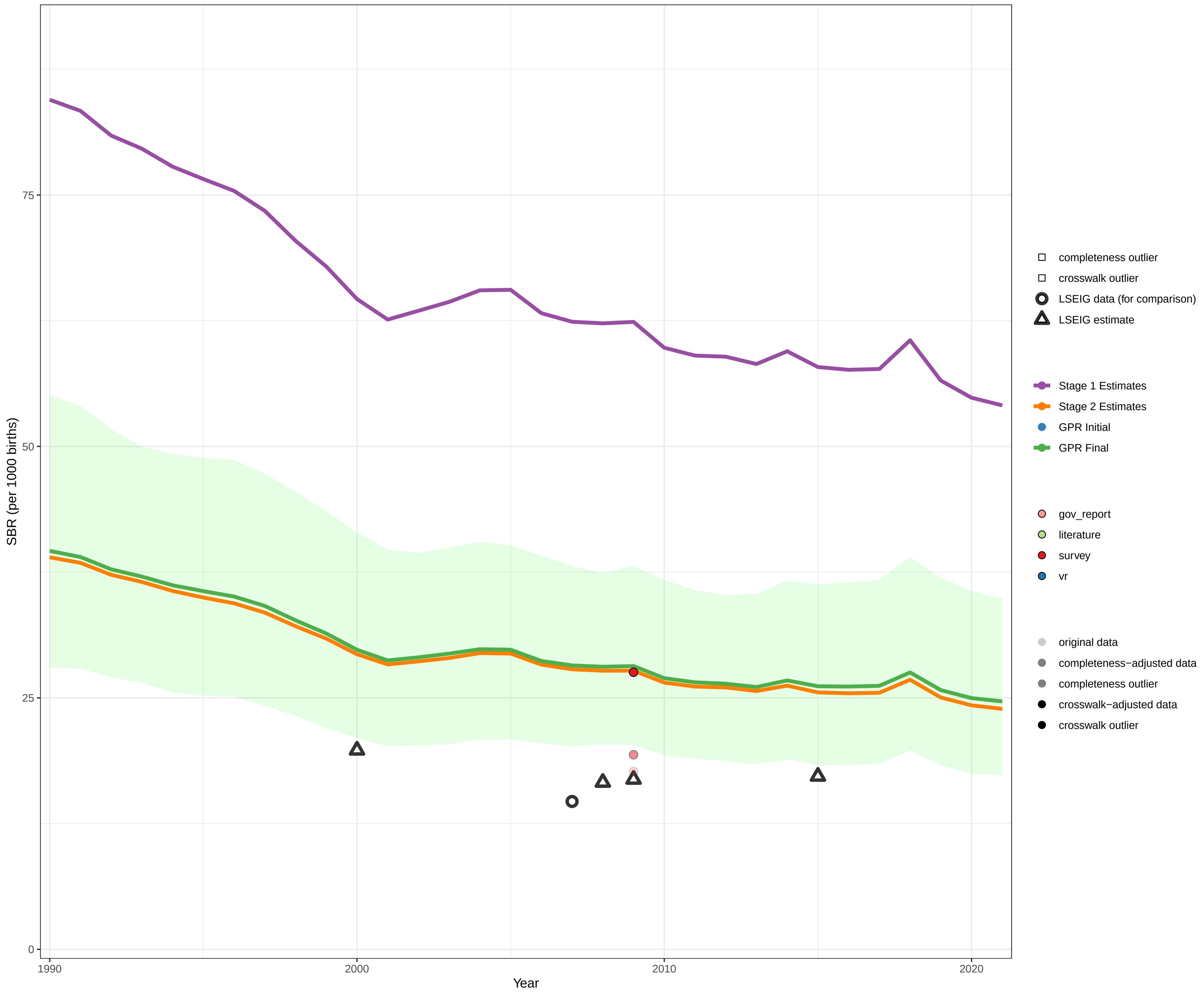

Guyana GUY Caribbean Stillbirth Rate (SBR) − 28 weeks
lambda = 0.8 ; zeta = 0.7 ; scale = 15 ; amp = 0.028

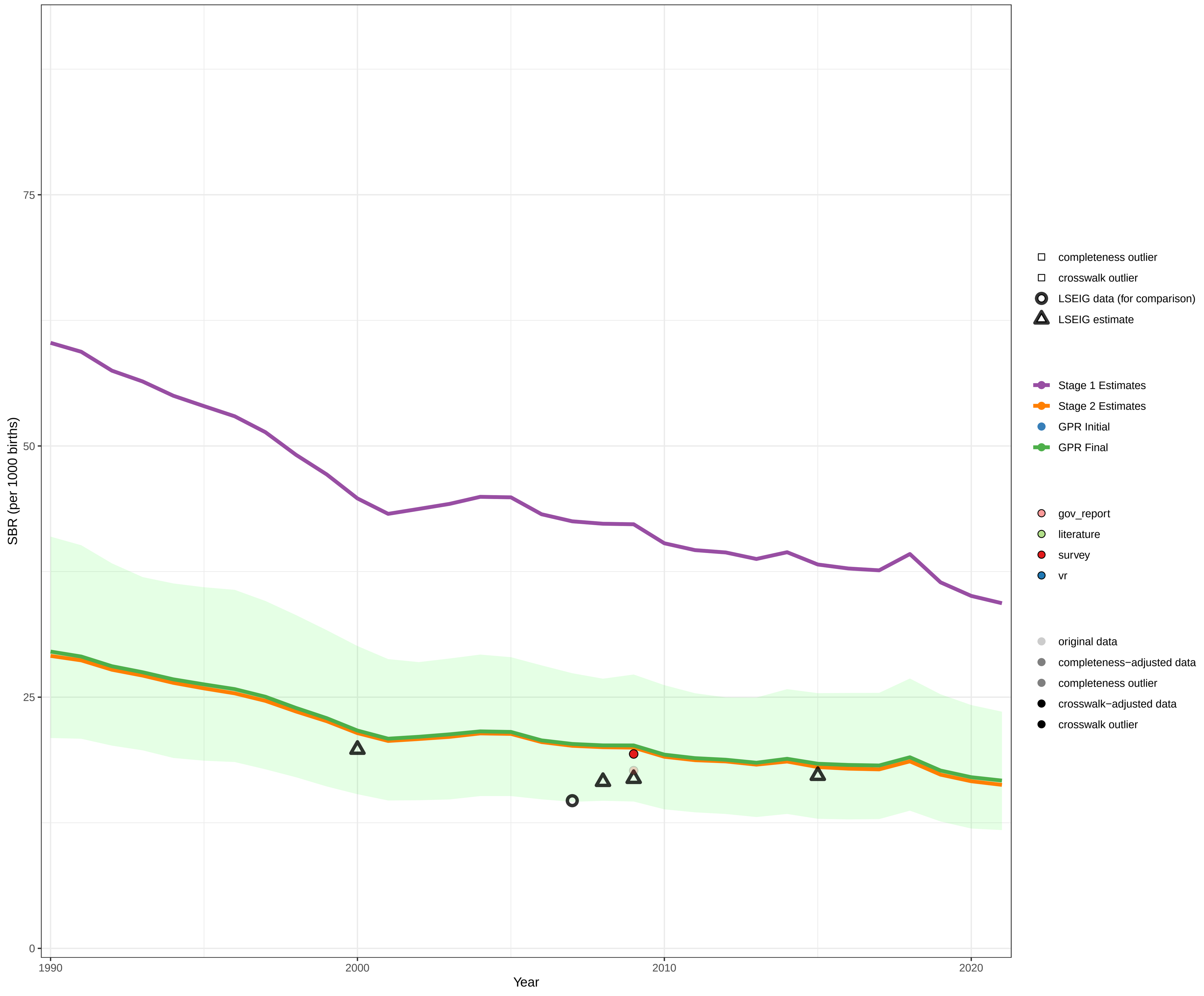

Year	Total Stillbirths (in thousands)	Total Stillbirths (in thousands)	SBR (per 1000 births)	SBR (per 1000 births)	SBR/NMR	SBR/NMR
	20 weeks	28 weeks	20 weeks	28 weeks	20 weeks	28 weeks
1990	1.1 (0.8 to 1.6)	0.8 (0.6 to 1.2)	39.6 (28.0 to 55.1)	29.5 (20.9 to 41.0)	1.25 (0.89 to 1.74)	0.94 (0.66 to 1.30)
1995	0.9 (0.6 to 1.3)	0.7 (0.5 to 0.9)	35.6 (25.2 to 48.9)	26.3 (18.7 to 35.9)	1.28 (0.91 to 1.76)	0.95 (0.67 to 1.30)
2000	0.6 (0.4 to 0.9)	0.4 (0.3 to 0.6)	29.8 (21.0 to 41.5)	21.7 (15.3 to 30.1)	1.34 (0.95 to 1.87)	0.98 (0.69 to 1.36)
2005	0.5 (0.4 to 0.7)	0.4 (0.3 to 0.5)	29.8 (20.9 to 40.2)	21.5 (15.2 to 29.0)	1.36 (0.96 to 1.84)	0.99 (0.69 to 1.33)
2010	0.4 (0.3 to 0.6)	0.3 (0.2 to 0.4)	27.0 (19.3 to 36.7)	19.3 (13.8 to 26.2)	1.40 (1.00 to 1.91)	1.00 (0.72 to 1.36)
2015	0.4 (0.3 to 0.6)	0.3 (0.2 to 0.4)	26.2 (18.3 to 36.3)	18.4 (12.9 to 25.4)	1.49 (1.04 to 2.06)	1.05 (0.73 to 1.45)
2020	0.4 (0.3 to 0.6)	0.3 (0.2 to 0.4)	25.0 (17.4 to 35.6)	17.0 (11.9 to 24.2)	1.64 (1.15 to 2.34)	1.12 (0.78 to 1.59)
2021	0.4 (0.3 to 0.6)	0.3 (0.2 to 0.4)	24.6 (17.3 to 34.9)	16.7 (11.8 to 23.6)	1.68 (1.18 to 2.37)	1.14 (0.80 to 1.60)

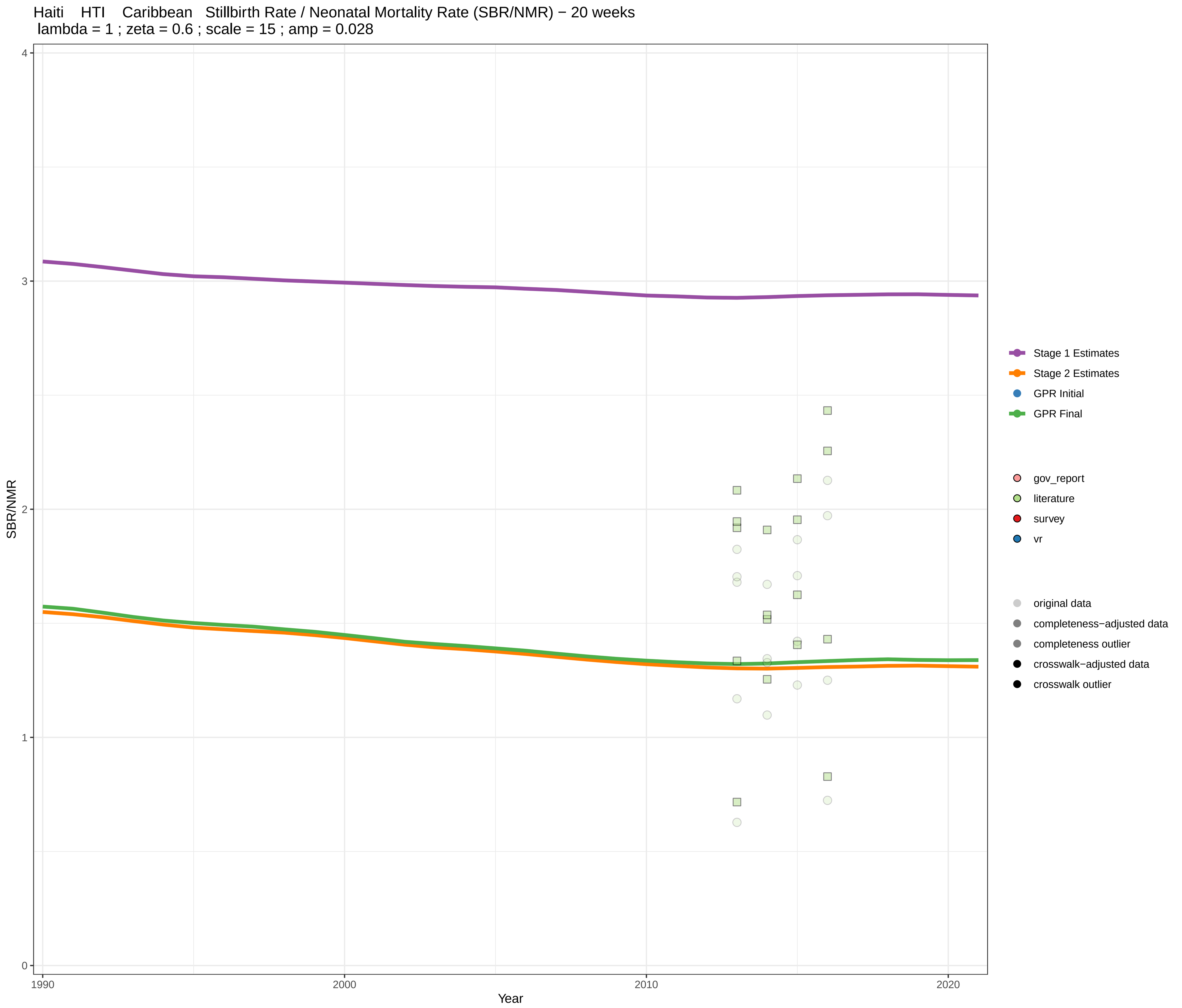

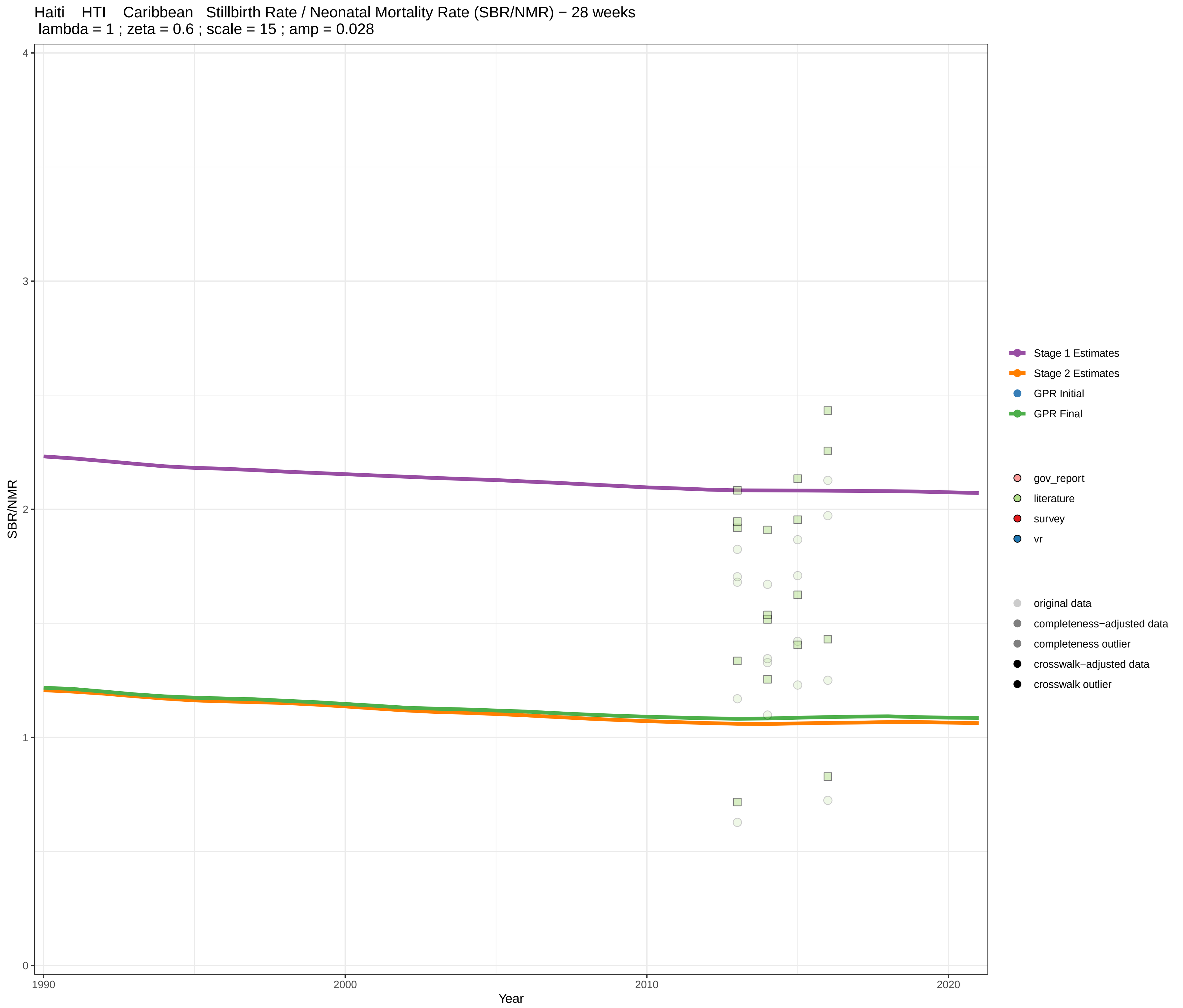

Definition	Years	Outlier	Source.Type	Title
28_weeks	2013	1	Sci Lit	Hypertensive disorders in pregnancy and maternal and neonatal outcomes in Haiti: the importance of surveillance and data collection
28_weeks	2013−2016	1	Sci Lit	Retrospective review of facility−level obstetric complications and stillbirths in southern Haiti, 2013 − 2016

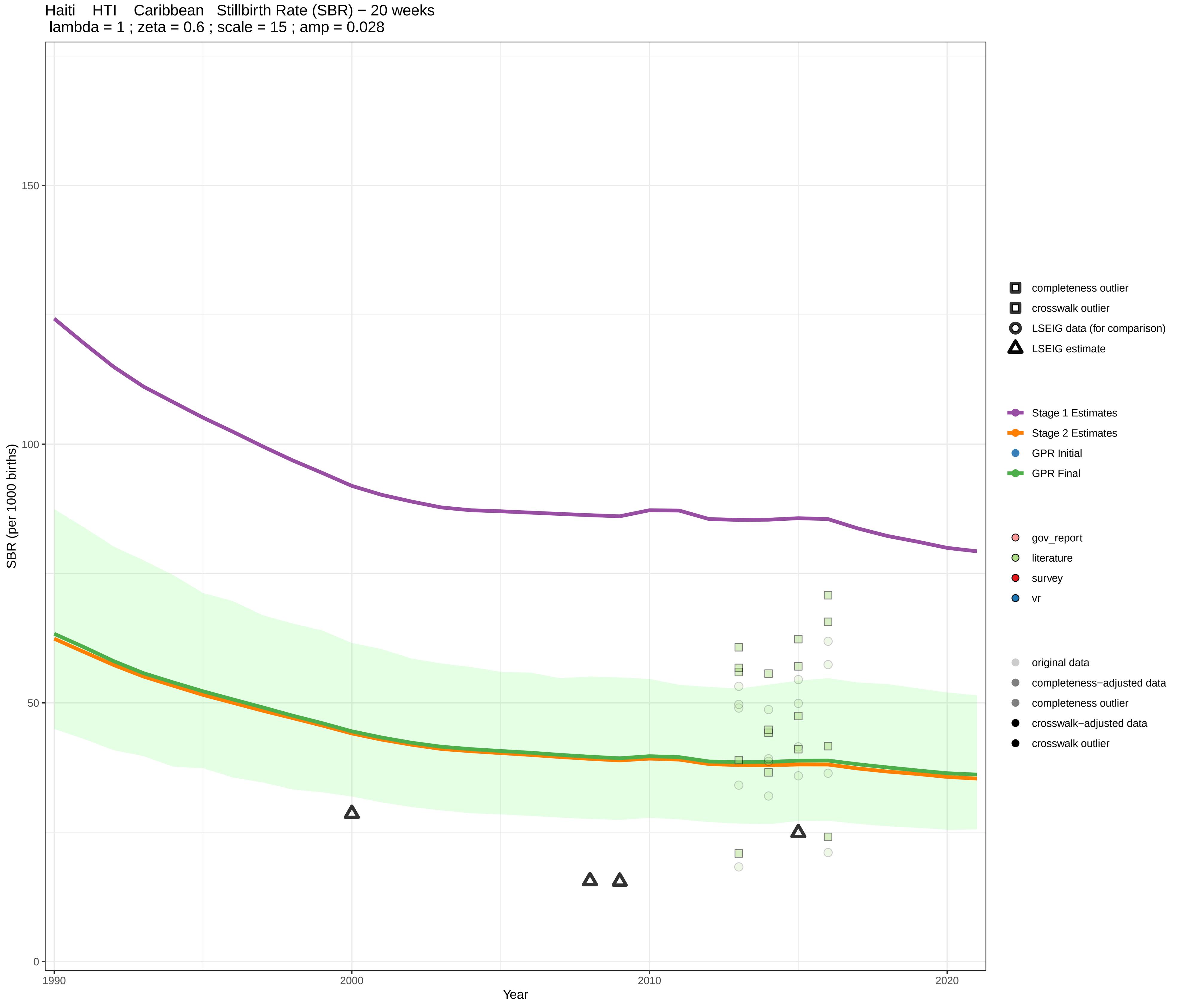

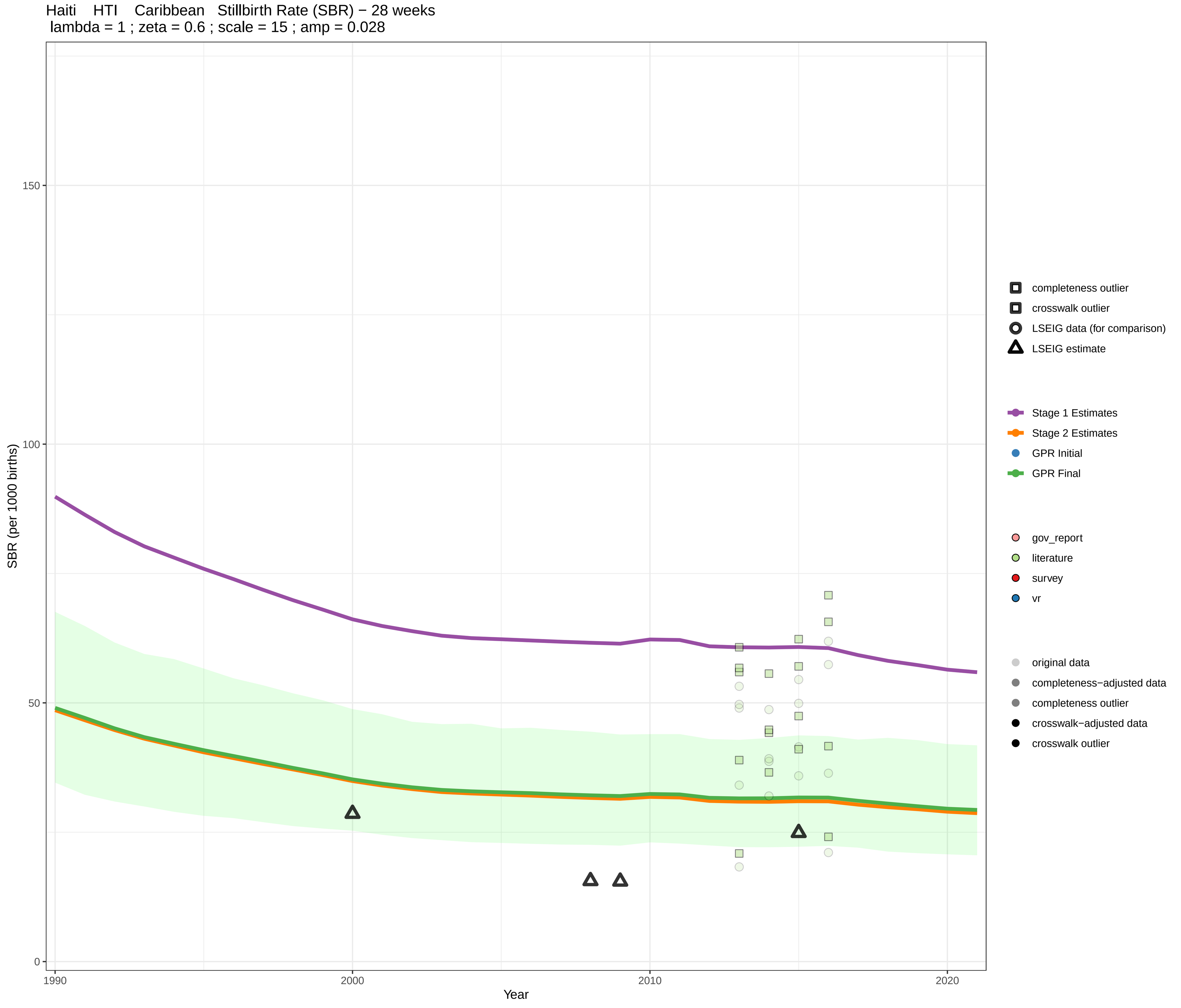

Year	Total Stillbirths (in thousands)	Total Stillbirths (in thousands)	SBR (per 1000 births)	SBR (per 1000 births)	SBR/NMR	SBR/NMR
	20 weeks	28 weeks	20 weeks	28 weeks	20 weeks	28 weeks
1990	17.3 (12.0 to 24.4)	13.1 (9.1 to 18.5)	63.3 (44.9 to 87.4)	49.0 (34.6 to 67.6)	1.57 (1.12 to 2.17)	1.22 (0.86 to 1.68)
1995	14.5 (10.2 to 20.1)	11.2 (7.6 to 15.7)	52.2 (37.4 to 71.2)	40.9 (28.2 to 56.6)	1.50 (1.07 to 2.05)	1.17 (0.81 to 1.63)
2000	12.9 (9.1 to 18.1)	10.1 (7.2 to 14.2)	44.5 (31.9 to 61.6)	35.2 (25.3 to 48.8)	1.45 (1.04 to 2.01)	1.15 (0.82 to 1.59)
2005	12.6 (8.7 to 17.7)	10.1 (7.0 to 14.1)	40.7 (28.4 to 56.0)	32.7 (22.9 to 45.1)	1.39 (0.97 to 1.91)	1.12 (0.78 to 1.54)
2010	13.1 (9.1 to 18.3)	10.6 (7.5 to 14.6)	39.7 (27.8 to 54.6)	32.4 (23.0 to 44.0)	1.34 (0.94 to 1.84)	1.09 (0.78 to 1.48)
2015	13.4 (9.2 to 19.0)	10.9 (7.5 to 15.1)	38.8 (27.2 to 54.3)	31.7 (22.2 to 43.7)	1.33 (0.93 to 1.86)	1.09 (0.76 to 1.50)
2020	13.0 (9.0 to 18.8)	10.4 (7.3 to 15.0)	36.4 (25.5 to 52.0)	29.6 (20.7 to 42.0)	1.34 (0.94 to 1.91)	1.09 (0.76 to 1.55)
2021	12.9 (9.0 to 18.7)	10.4 (7.2 to 15.0)	36.1 (25.5 to 51.5)	29.3 (20.5 to 41.8)	1.34 (0.95 to 1.91)	1.09 (0.76 to 1.55)

Jamaica JAM Caribbean Stillbirth Rate / Neonatal Mortality Rate (SBR/NMR) − 20 weeks
lambda = 0.4 ; zeta = 0.9 ; scale = 10 ; amp = 0.028

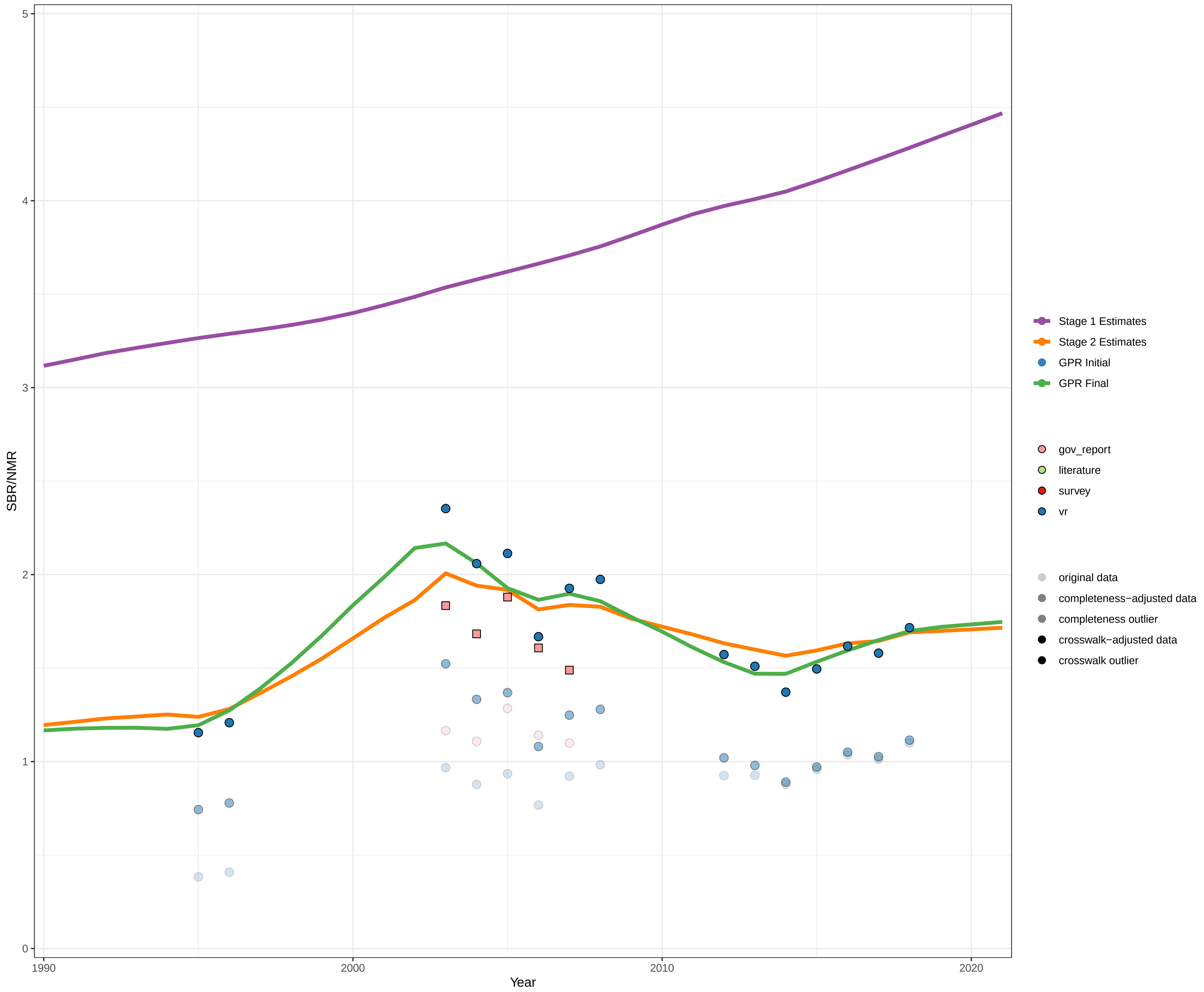

Jamaica JAM Caribbean Stillbirth Rate / Neonatal Mortality Rate (SBR/NMR) − 28 weeks
lambda = 0.4 ; zeta = 0.9 ; scale = 10 ; amp = 0.028

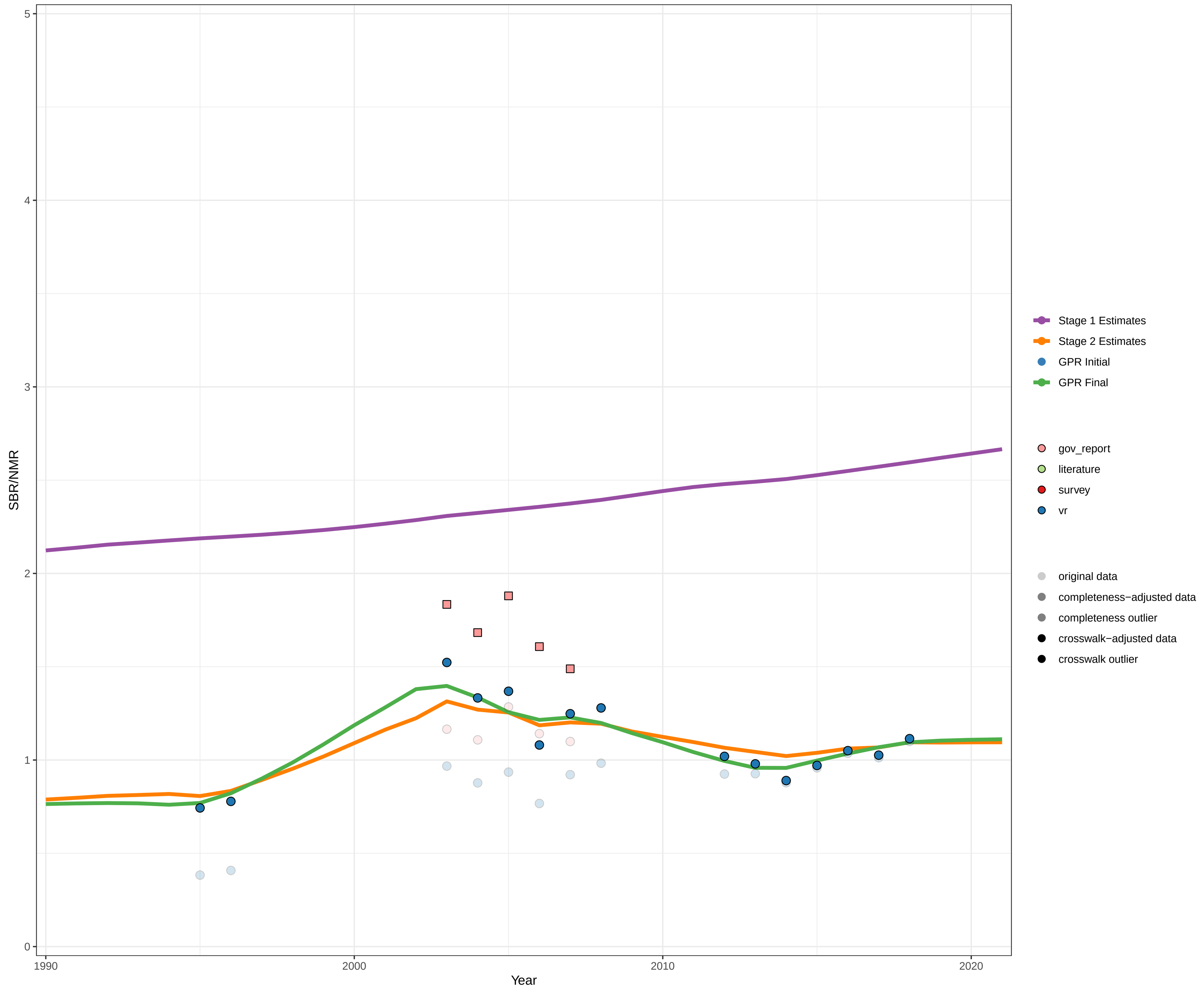

Definition	Years	Outlier	Source.Type	Title
28_weeks	1995, 1996	0	VR	United Nations Demographic Yearbook
28_weeks	2003−2008	0	VR	Jamaica Vital Statistics Report
28_weeks	2012−2014	0	VR	Jamaica Vital Registration − Deaths
28_weeks	2015−2018	0	VR	Jamaica Vital Registration Tables
28_weeks	2003−2007	2	Statistical Report	Jamaica Ministry of Health and Environment Annual Report

Jamaica JAM Caribbean Stillbirth Rate (SBR) − 20 weeks
lambda = 0.4 ; zeta = 0.9 ; scale = 10 ; amp = 0.028

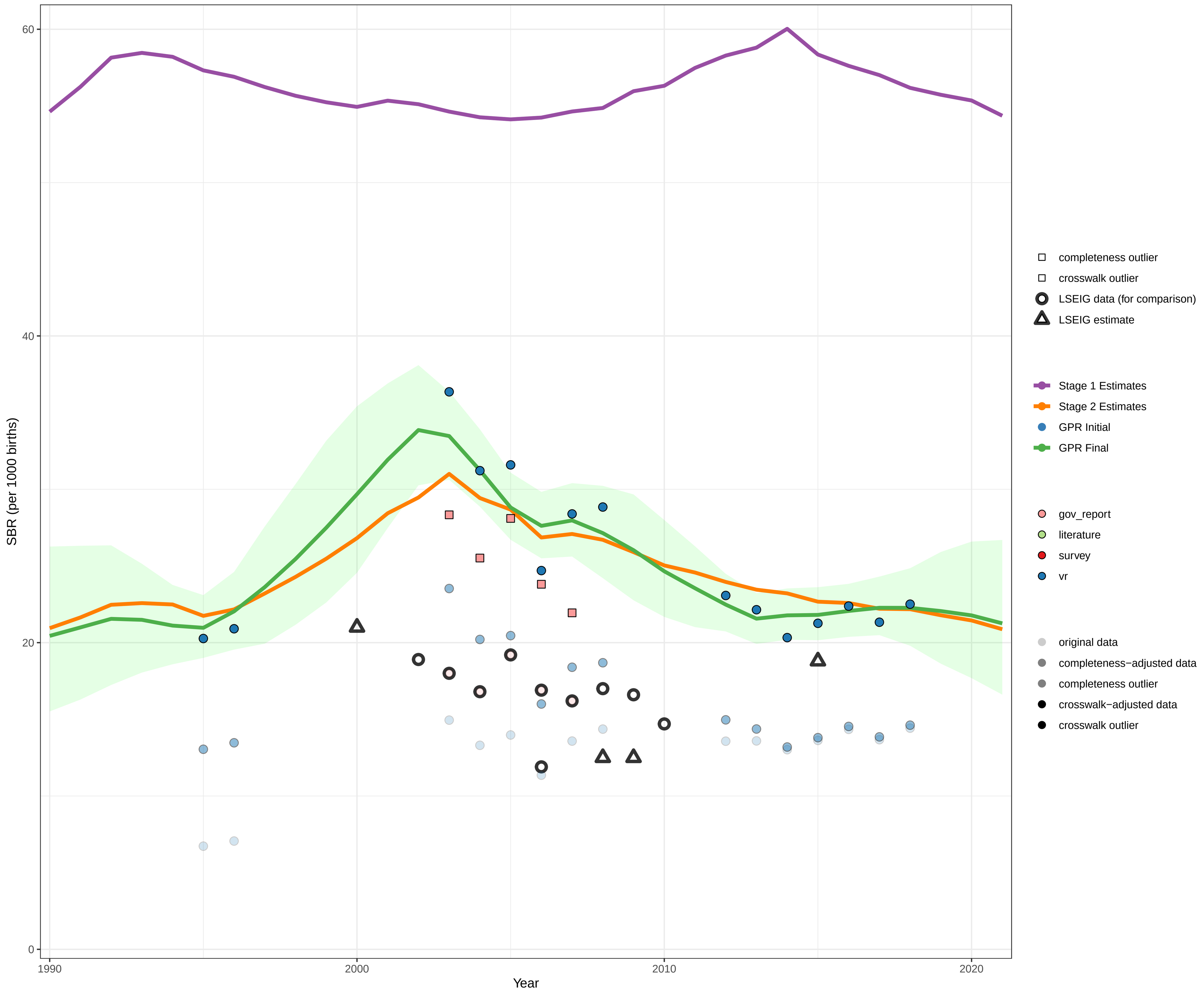

Jamaica JAM Caribbean Stillbirth Rate (SBR) − 28 weeks
lambda = 0.4 ; zeta = 0.9 ; scale = 10 ; amp = 0.028

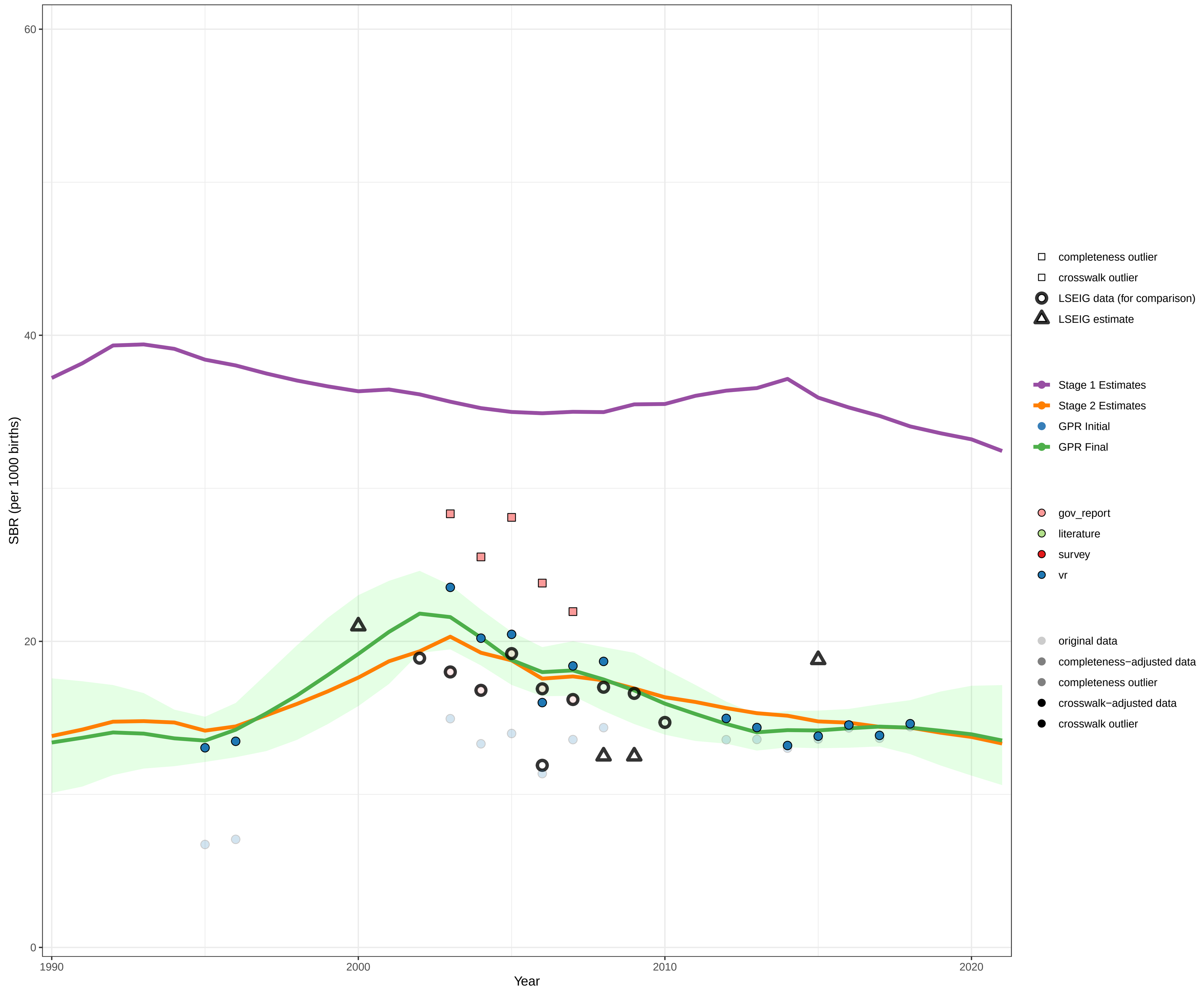

Year	Total Stillbirths (in thousands)	Total Stillbirths (in thousands)	SBR (per 1000 births)	SBR (per 1000 births)	SBR/NMR	SBR/NMR
	20 weeks	28 weeks	20 weeks	28 weeks	20 weeks	28 weeks
1990	1.2<br>(0.9 to 1.5)	0.8<br>(0.6 to 1.0)	20.4<br>(15.5 to 26.3)	13.4<br>(10.1 to 17.6)	1.17<br>(0.89 to 1.50)	0.76<br>(0.58 to 1.00)
1995	1.2<br>(1.1 to 1.3)	0.8<br>(0.7 to 0.9)	21.0<br>(19.0 to 23.1)	13.5<br>(12.1 to 15.1)	1.19<br>(1.08 to 1.32)	0.77<br>(0.69 to 0.86)
2000	1.5<br>(1.2 to 1.8)	1.0<br>(0.8 to 1.2)	29.7<br>(24.6 to 35.4)	19.2<br>(15.8 to 23.0)	1.84<br>(1.52 to 2.19)	1.19<br>(0.98 to 1.42)
2005	1.3<br>(1.2 to 1.4)	0.8<br>(0.8 to 0.9)	28.8<br>(26.7 to 31.1)	18.8<br>(17.2 to 20.6)	1.93<br>(1.79 to 2.08)	1.26<br>(1.15 to 1.38)
2010	1.1<br>(0.9 to 1.2)	0.7<br>(0.6 to 0.8)	24.7<br>(21.7 to 28.0)	15.9<br>(13.9 to 18.2)	1.70<br>(1.49 to 1.93)	1.10<br>(0.96 to 1.25)
2015	0.8<br>(0.8 to 0.9)	0.5<br>(0.5 to 0.6)	21.8<br>(20.2 to 23.6)	14.2<br>(13.0 to 15.5)	1.53<br>(1.42 to 1.66)	1.00<br>(0.92 to 1.09)
2020	0.7<br>(0.6 to 0.9)	0.5<br>(0.4 to 0.6)	21.8<br>(17.7 to 26.6)	13.9<br>(11.2 to 17.1)	1.73<br>(1.41 to 2.12)	1.11<br>(0.89 to 1.36)
2021	0.7<br>(0.6 to 0.9)	0.5<br>(0.4 to 0.6)	21.3<br>(16.6 to 26.7)	13.5<br>(10.6 to 17.1)	1.75<br>(1.36 to 2.19)	1.11<br>(0.87 to 1.41)

Puerto Rico PRI Caribbean Stillbirth Rate / Neonatal Mortality Rate (SBR/NMR) − 20 weeks
lambda = 0.2 ; zeta = 0.99 ; scale = 5 ; amp = 0.028

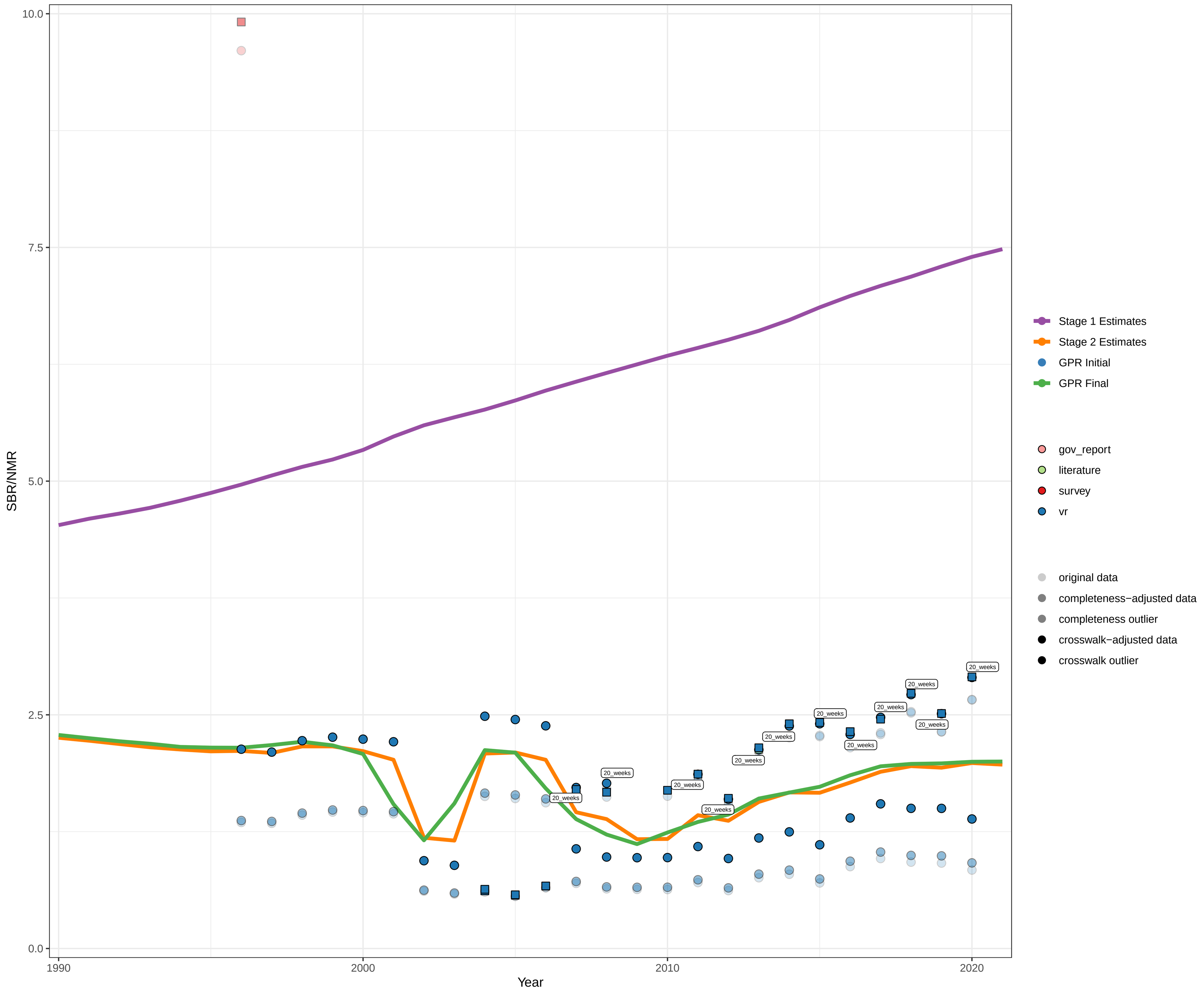

Puerto Rico PRI Caribbean Stillbirth Rate / Neonatal Mortality Rate (SBR/NMR) − 28 weeks
lambda = 0.2 ; zeta = 0.99 ; scale = 5 ; amp = 0.028

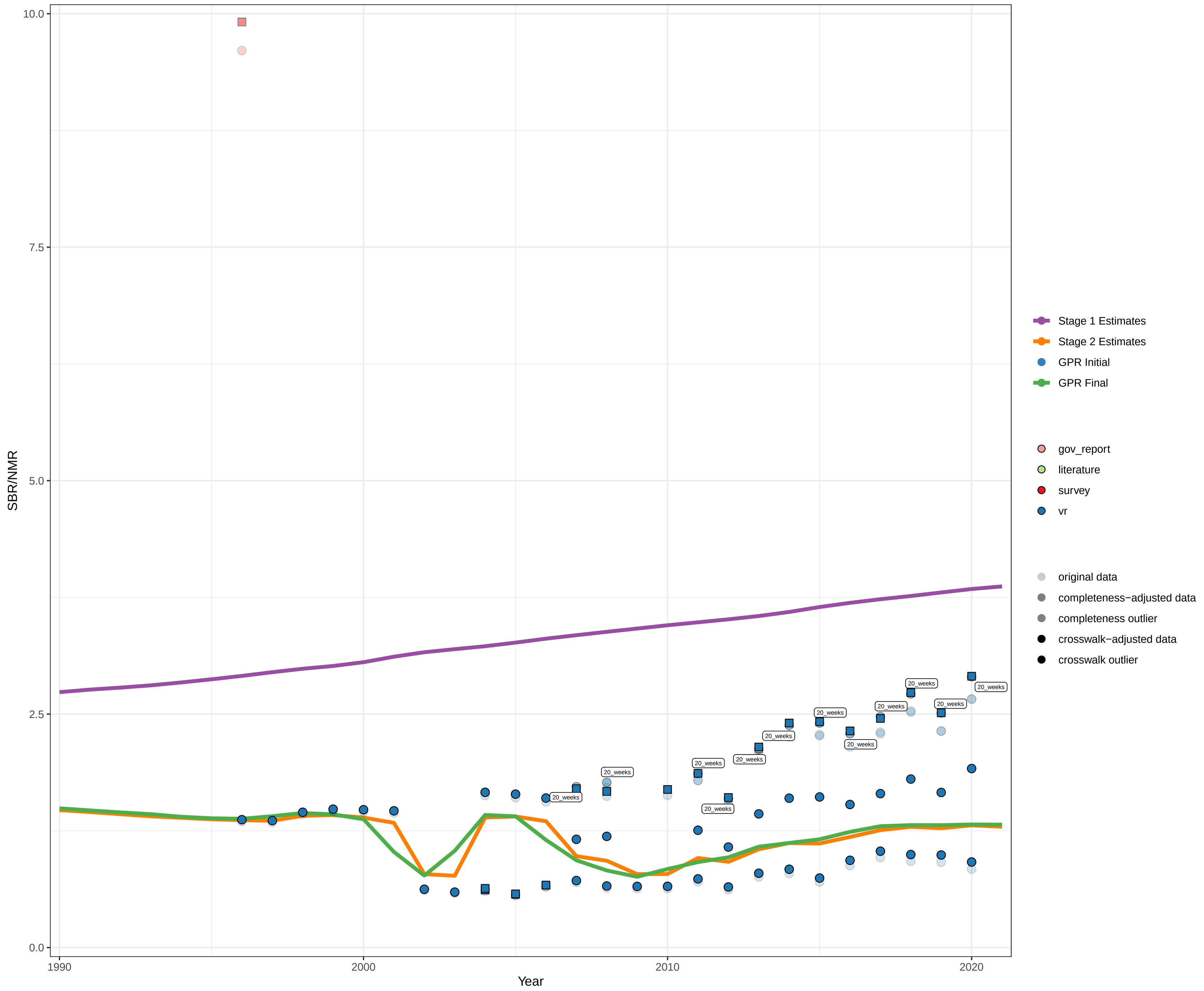

Definition	Years	Outlier	Source.Type	Title
20_weeks	2007, 2008, 2011−2020	0	VR	United States NVSS Custom Fetal Death Data
28_weeks	1996−2003	0	VR	United Nations Demographic Yearbook
28_weeks	2004−2020	0	VR	United States NVSS Custom Fetal Death Data
20_weeks	1996	1	Survey	Puerto Rico Reproductive Health Survey 1995−1996
20_weeks	2013	1	Sci Lit	United States Fetal and Perinatal Mortality 2013
28_weeks	2004−2006	2	VR	United States NVSS Custom Fetal Death Data
28_weeks	2004−2008, 2010−2020	2	VR	United Nations Demographic Yearbook

Puerto Rico PRI Caribbean Stillbirth Rate (SBR) − 20 weeks
lambda = 0.2 ; zeta = 0.99 ; scale = 5 ; amp = 0.028

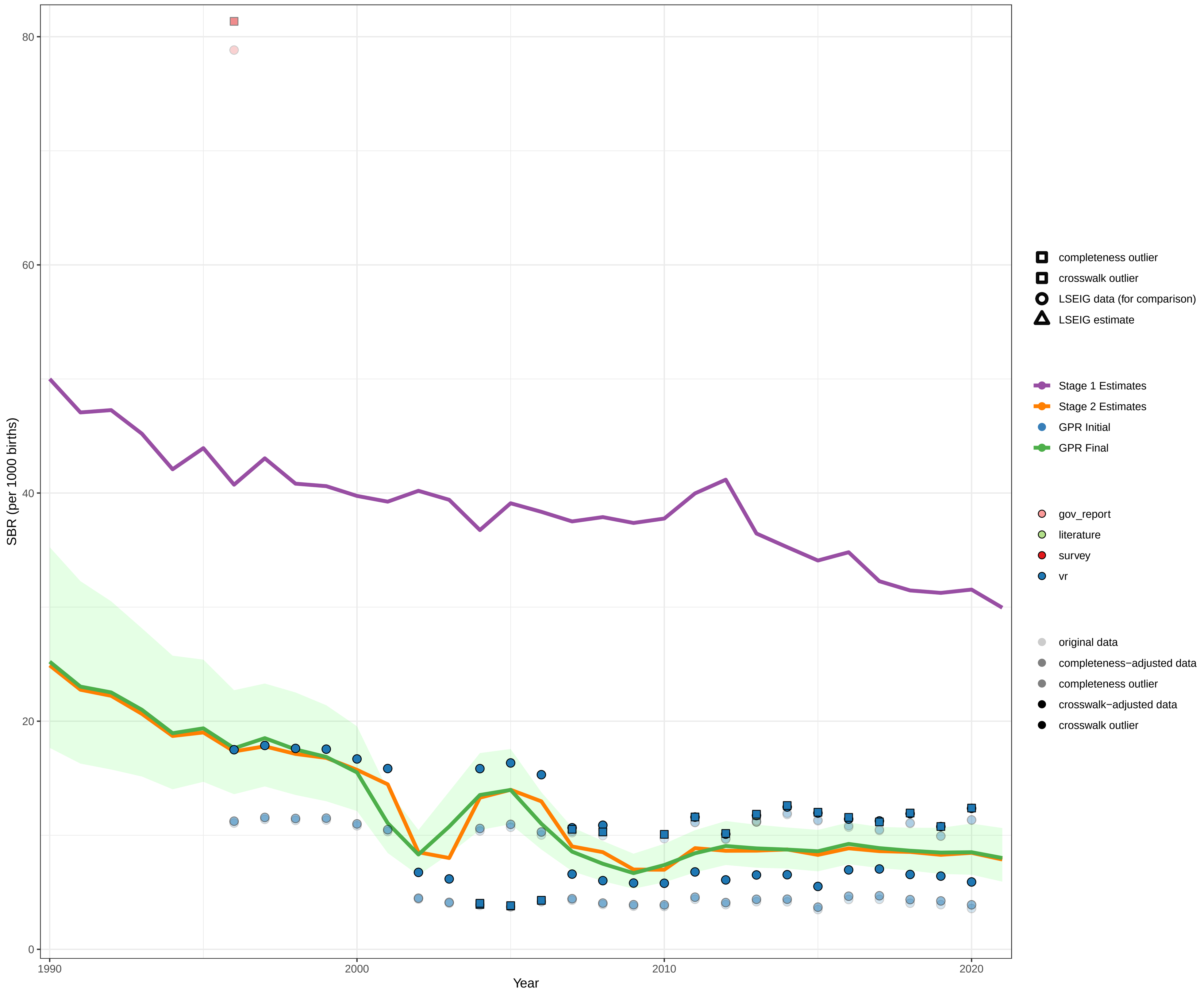

Puerto Rico PRI Caribbean Stillbirth Rate (SBR) − 28 weeks
lambda = 0.2 ; zeta = 0.99 ; scale = 5 ; amp = 0.028

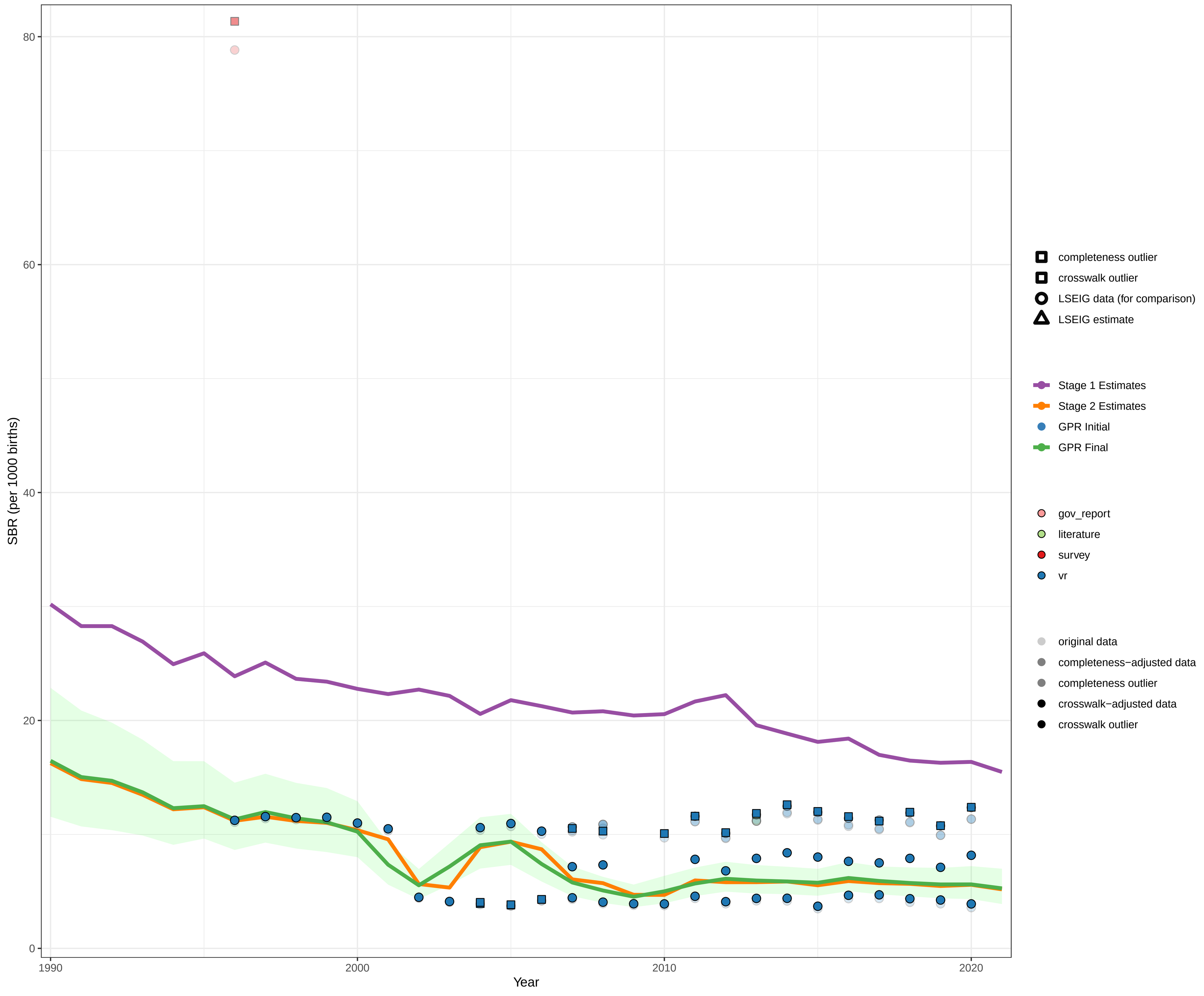

Year	Total Stillbirths (in thousands)	Total Stillbirths (in thousands)	SBR (per 1000 births)	SBR (per 1000 births)	SBR/NMR	SBR/NMR
	20 weeks	28 weeks	20 weeks	28 weeks	20 weeks	28 weeks
1990	1.7 (1.2 to 2.4)	1.1 (0.8 to 1.5)	25.2 (17.7 to 35.3)	16.5 (11.6 to 22.9)	2.28 (1.60 to 3.19)	1.49 (1.05 to 2.07)
1995	1.3 (0.9 to 1.7)	0.8 (0.6 to 1.1)	19.4 (14.7 to 25.4)	12.5 (9.6 to 16.4)	2.15 (1.63 to 2.82)	1.38 (1.07 to 1.82)
2000	0.9 (0.7 to 1.1)	0.6 (0.5 to 0.8)	15.5 (12.1 to 19.6)	10.2 (8.0 to 12.9)	2.08 (1.63 to 2.62)	1.37 (1.07 to 1.73)
2005	0.7 (0.6 to 0.9)	0.5 (0.4 to 0.6)	14.0 (11.0 to 17.6)	9.4 (7.3 to 11.8)	2.10 (1.64 to 2.63)	1.41 (1.10 to 1.77)
2010	0.3 (0.2 to 0.4)	0.2 (0.2 to 0.3)	7.4 (5.9 to 9.3)	5.0 (4.0 to 6.4)	1.24 (0.99 to 1.55)	0.84 (0.66 to 1.07)
2015	0.3 (0.2 to 0.3)	0.2 (0.1 to 0.2)	8.6 (6.8 to 10.5)	5.8 (4.6 to 7.0)	1.73 (1.38 to 2.11)	1.16 (0.93 to 1.41)
2020	0.2 (0.1 to 0.2)	0.1 (0.1 to 0.1)	8.5 (6.5 to 11.0)	5.6 (4.3 to 7.2)	2.00 (1.53 to 2.59)	1.32 (1.02 to 1.69)
2021	0.2 (0.1 to 0.2)	0.1 (0.1 to 0.1)	8.0 (5.9 to 10.6)	5.3 (3.9 to 7.0)	2.00 (1.48 to 2.66)	1.32 (0.97 to 1.75)

Saint Kitts and Nevis KNA Caribbean Stillbirth Rate / Neonatal Mortality Rate (SBR/NMR) − 20 weeks
lambda = 0.8 ; zeta = 0.7 ; scale = 15 ; amp = 0.028

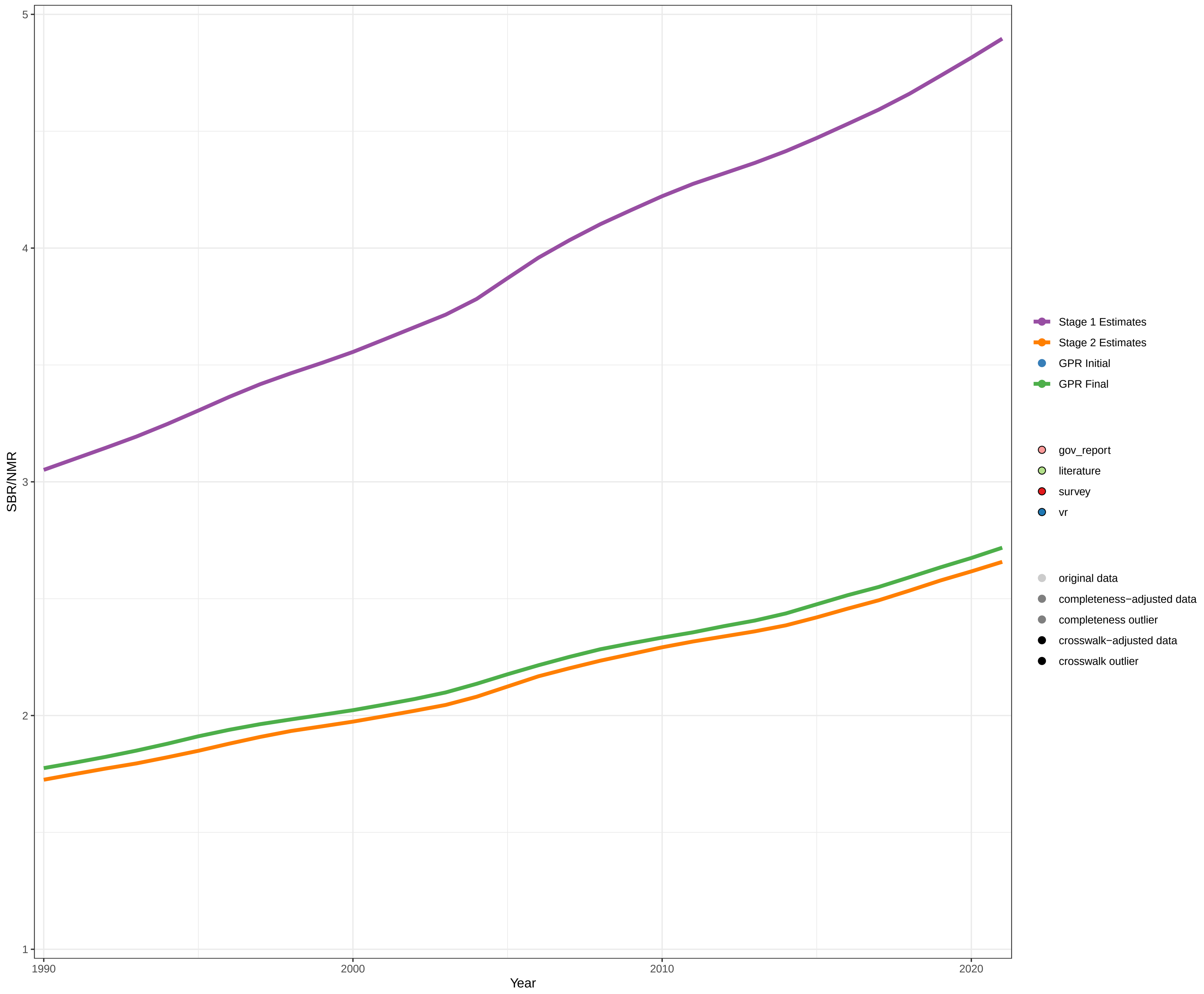

Saint Kitts and Nevis KNA Caribbean Stillbirth Rate / Neonatal Mortality Rate (SBR/NMR) − 28 weeks
lambda = 0.8 ; zeta = 0.7 ; scale = 15 ; amp = 0.028

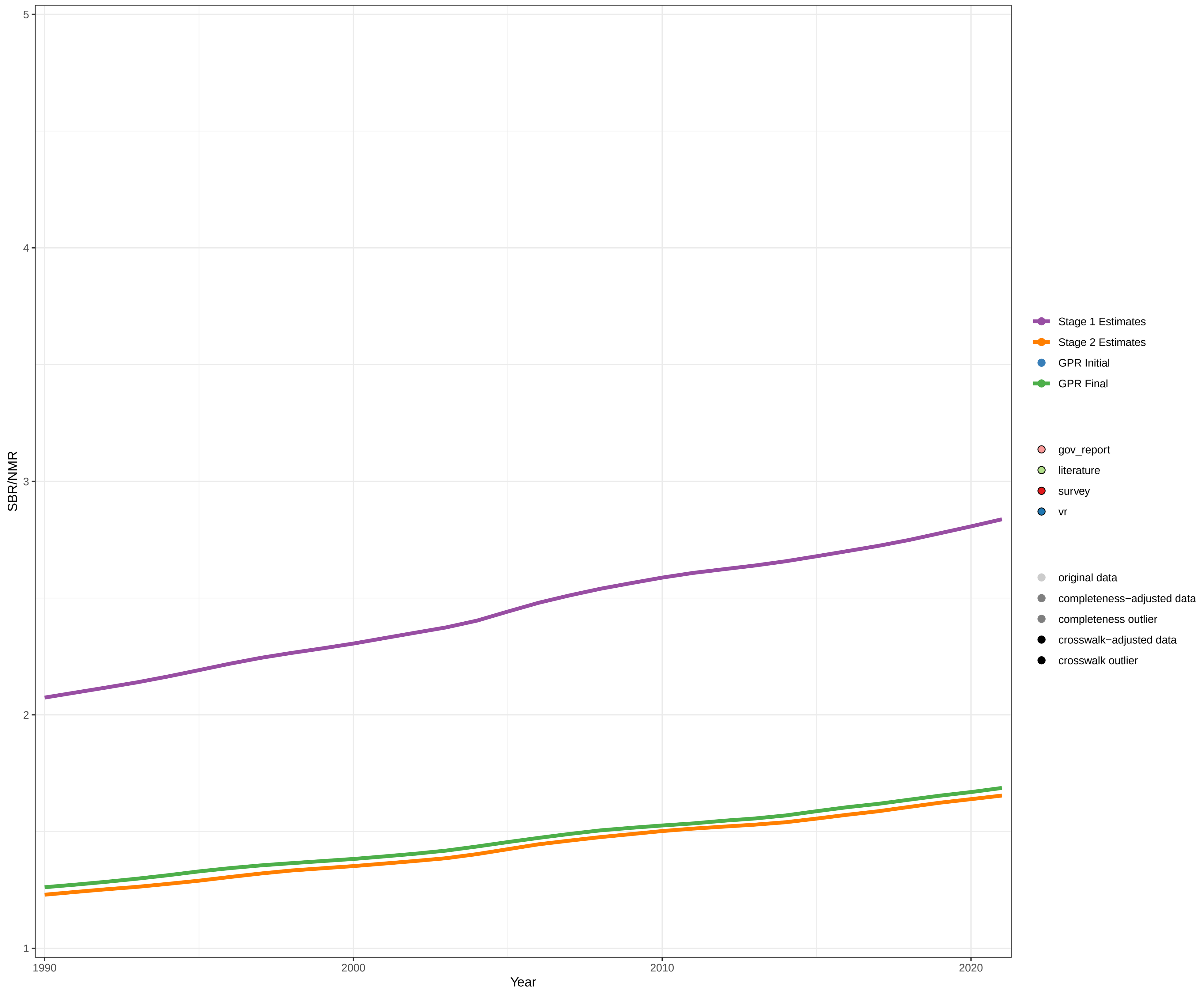

Definition	Years	Outlier	Source.Type	Title

Saint Kitts and Nevis KNA Caribbean Stillbirth Rate (SBR) − 20 weeks
lambda = 0.8 ; zeta = 0.7 ; scale = 15 ; amp = 0.028

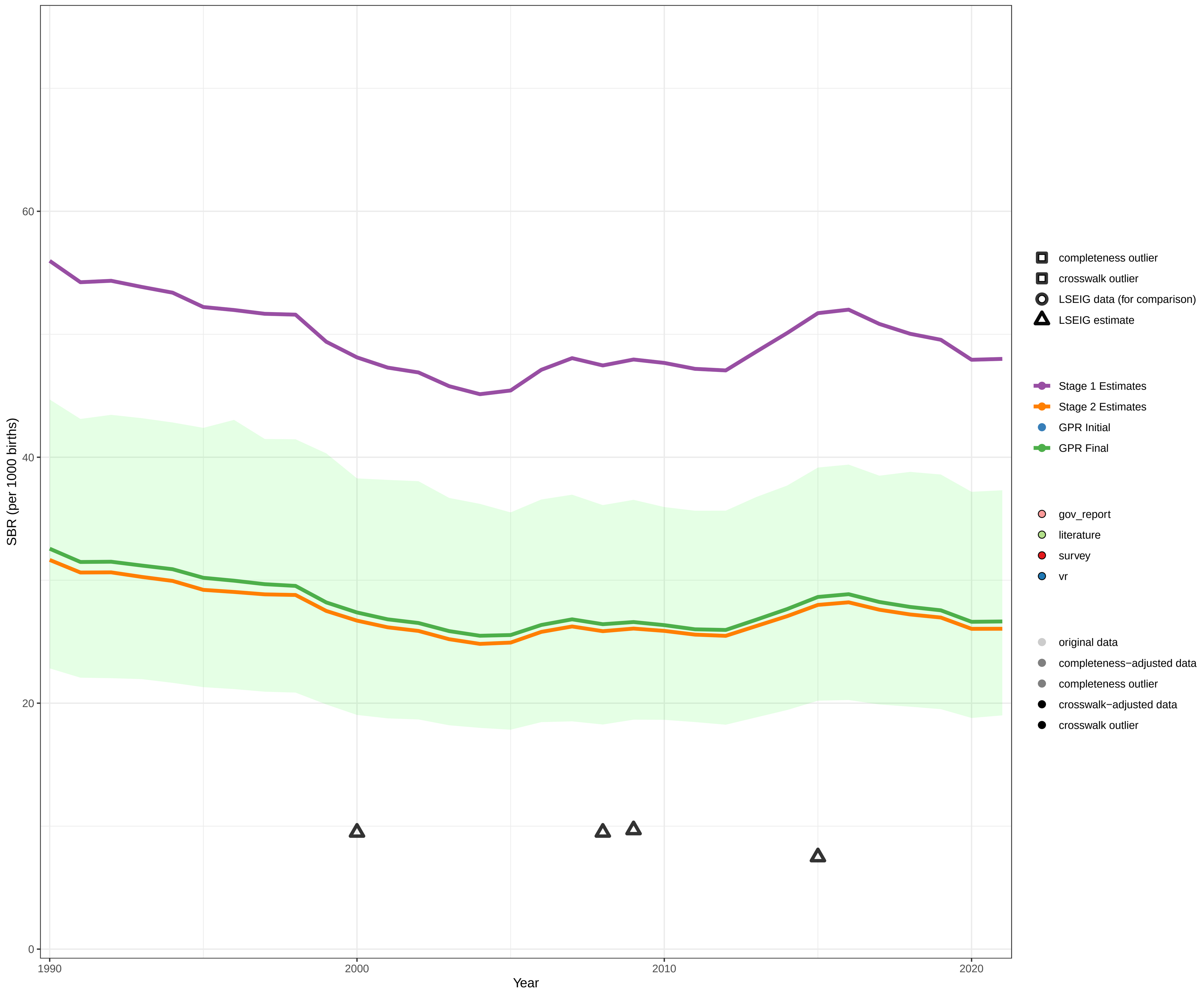

Saint Kitts and Nevis KNA Caribbean Stillbirth Rate (SBR) − 28 weeks
lambda = 0.8 ; zeta = 0.7 ; scale = 15 ; amp = 0.028

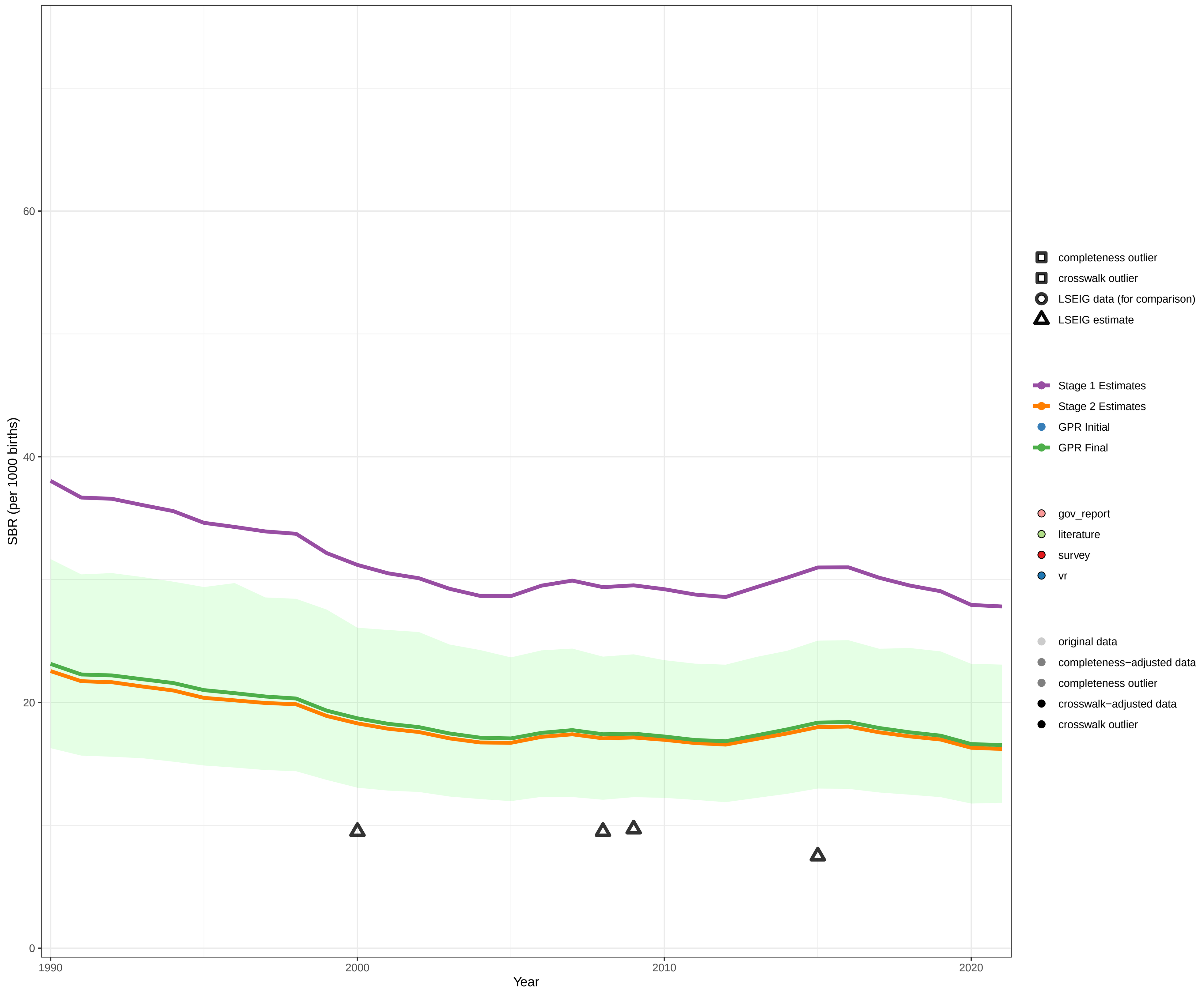

Year	Total Stillbirths (in thousands)	Total Stillbirths (in thousands)	SBR (per 1000 births)	SBR (per 1000 births)	SBR/NMR	SBR/NMR
	20 weeks	28 weeks	20 weeks	28 weeks	20 weeks	28 weeks
1990	0.0 (0.0 to 0.0)	0.0 (0.0 to 0.0)	32.6 (22.8 to 44.7)	23.1 (16.3 to 31.7)	1.78 (1.24 to 2.44)	1.26 (0.89 to 1.73)
1995	0.0 (0.0 to 0.0)	0.0 (0.0 to 0.0)	30.2 (21.3 to 42.4)	21.0 (14.9 to 29.4)	1.91 (1.35 to 2.68)	1.33 (0.94 to 1.86)
2000	0.0 (0.0 to 0.0)	0.0 (0.0 to 0.0)	27.4 (19.0 to 38.3)	18.7 (13.1 to 26.1)	2.02 (1.41 to 2.83)	1.38 (0.97 to 1.93)
2005	0.0 (0.0 to 0.0)	0.0 (0.0 to 0.0)	25.5 (17.8 to 35.5)	17.1 (12.0 to 23.7)	2.18 (1.52 to 3.03)	1.45 (1.02 to 2.02)
2010	0.0 (0.0 to 0.0)	0.0 (0.0 to 0.0)	26.3 (18.6 to 35.9)	17.2 (12.2 to 23.4)	2.33 (1.65 to 3.18)	1.53 (1.08 to 2.08)
2015	0.0 (0.0 to 0.0)	0.0 (0.0 to 0.0)	28.6 (20.2 to 39.2)	18.4 (13.0 to 25.0)	2.48 (1.75 to 3.39)	1.59 (1.12 to 2.16)
2020	0.0 (0.0 to 0.0)	0.0 (0.0 to 0.0)	26.6 (18.8 to 37.2)	16.6 (11.8 to 23.1)	2.67 (1.89 to 3.74)	1.67 (1.18 to 2.32)
2021	0.0 (0.0 to 0.0)	0.0 (0.0 to 0.0)	26.6 (19.0 to 37.3)	16.5 (11.8 to 23.1)	2.72 (1.94 to 3.81)	1.69 (1.21 to 2.35)

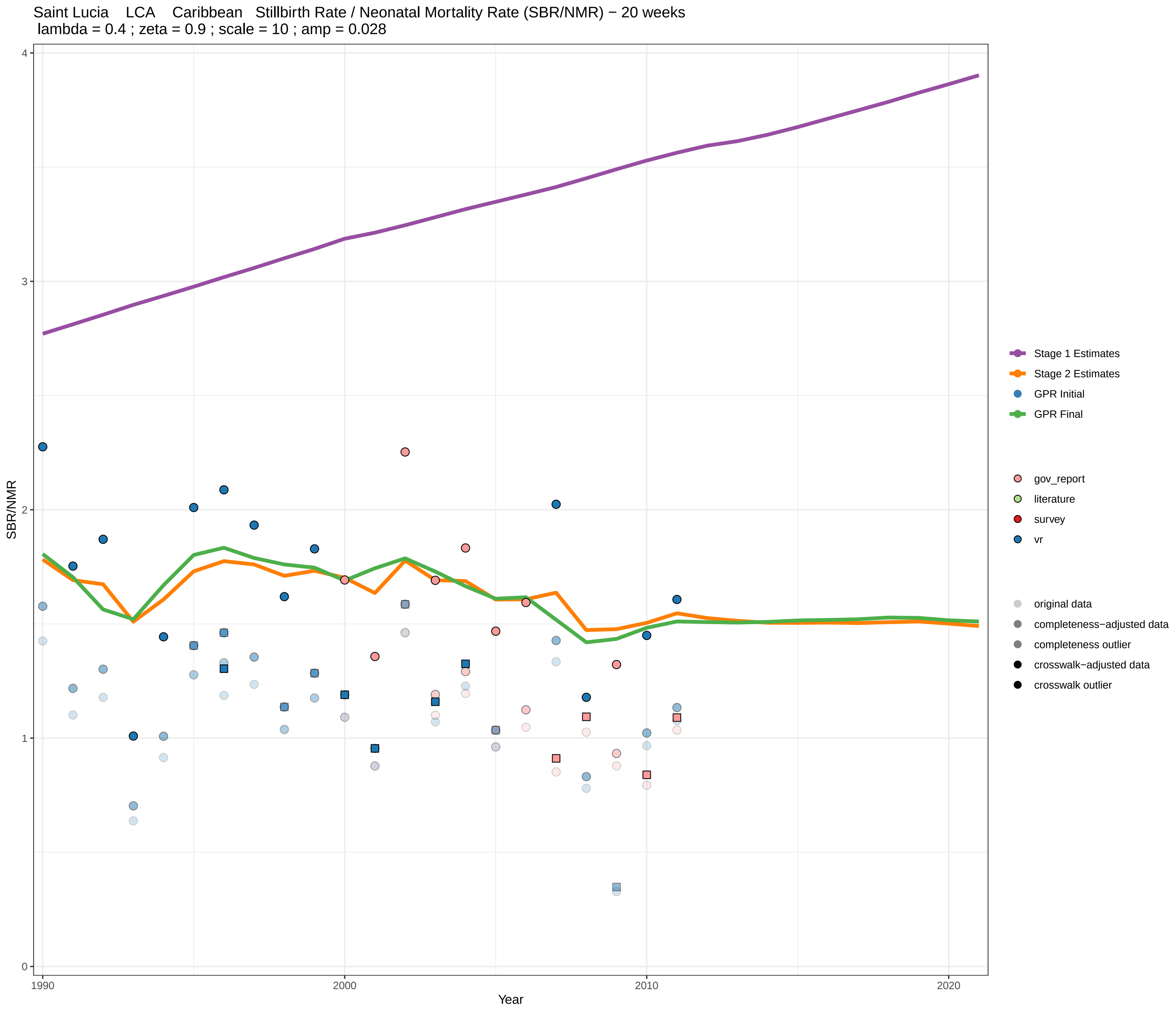

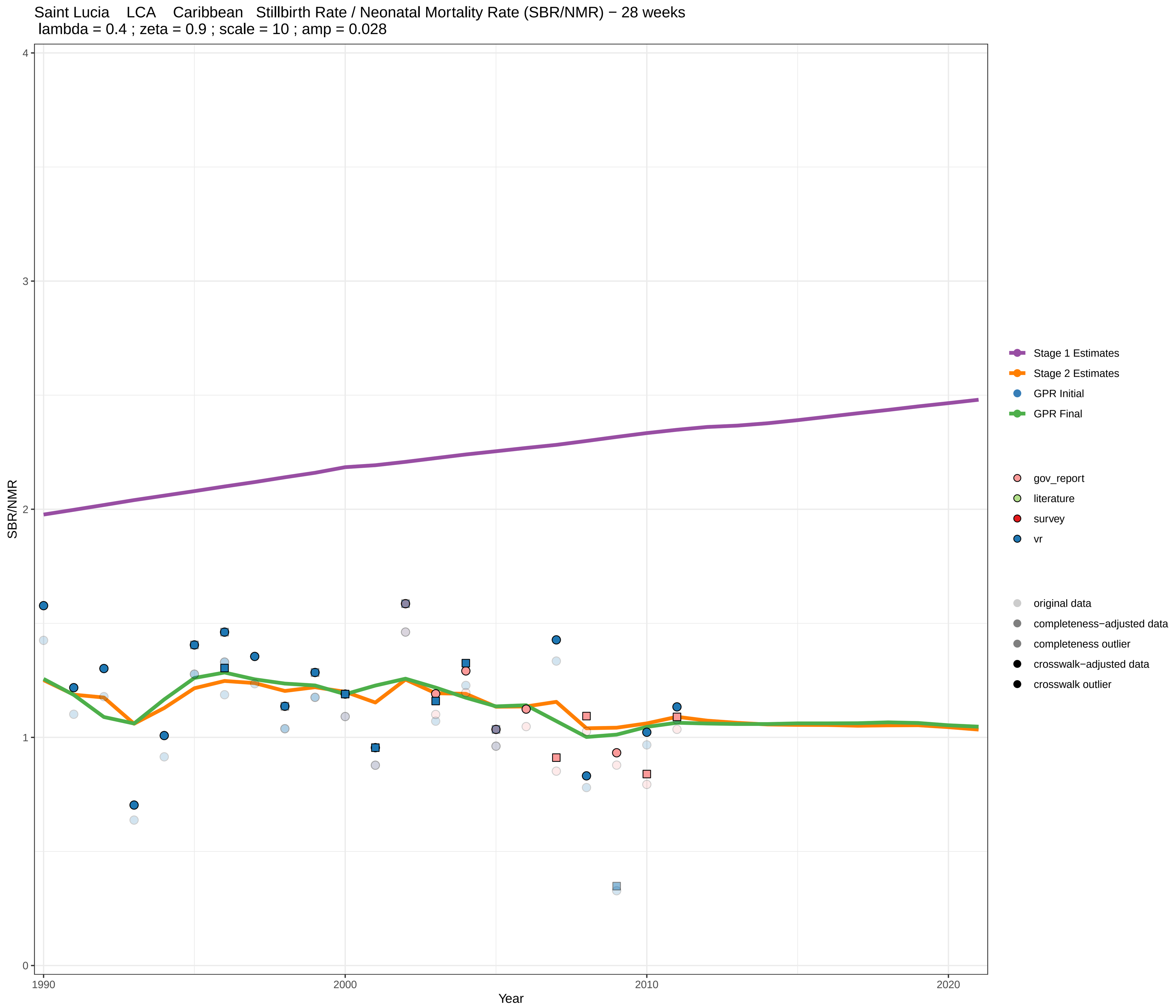

Definition	Years	Outlier	Source.Type	Title
28_weeks	1990−1996, 1998, 1999, 2007, 2008, 2010, 2011	0	VR	Saint Lucia Vital Statistics Report
28_weeks	1997	0	VR	United Nations Demographic Yearbook
28_weeks	2000−2006, 2009	0	Statistical Report	Saint Lucia Economic and Social Review
28_weeks	1995, 1996, 1998, 1999, 2002, 2005	1	VR	United Nations Demographic Yearbook
28_weeks	2009	1	VR	Saint Lucia Vital Statistics Report
28_weeks	1996	2	VR	Saint Lucia Vital Statistics Report
28_weeks	2000, 2001, 2003, 2004	2	VR	United Nations Demographic Yearbook
28_weeks	2007, 2008, 2010, 2011	2	Statistical Report	Saint Lucia Economic and Social Review

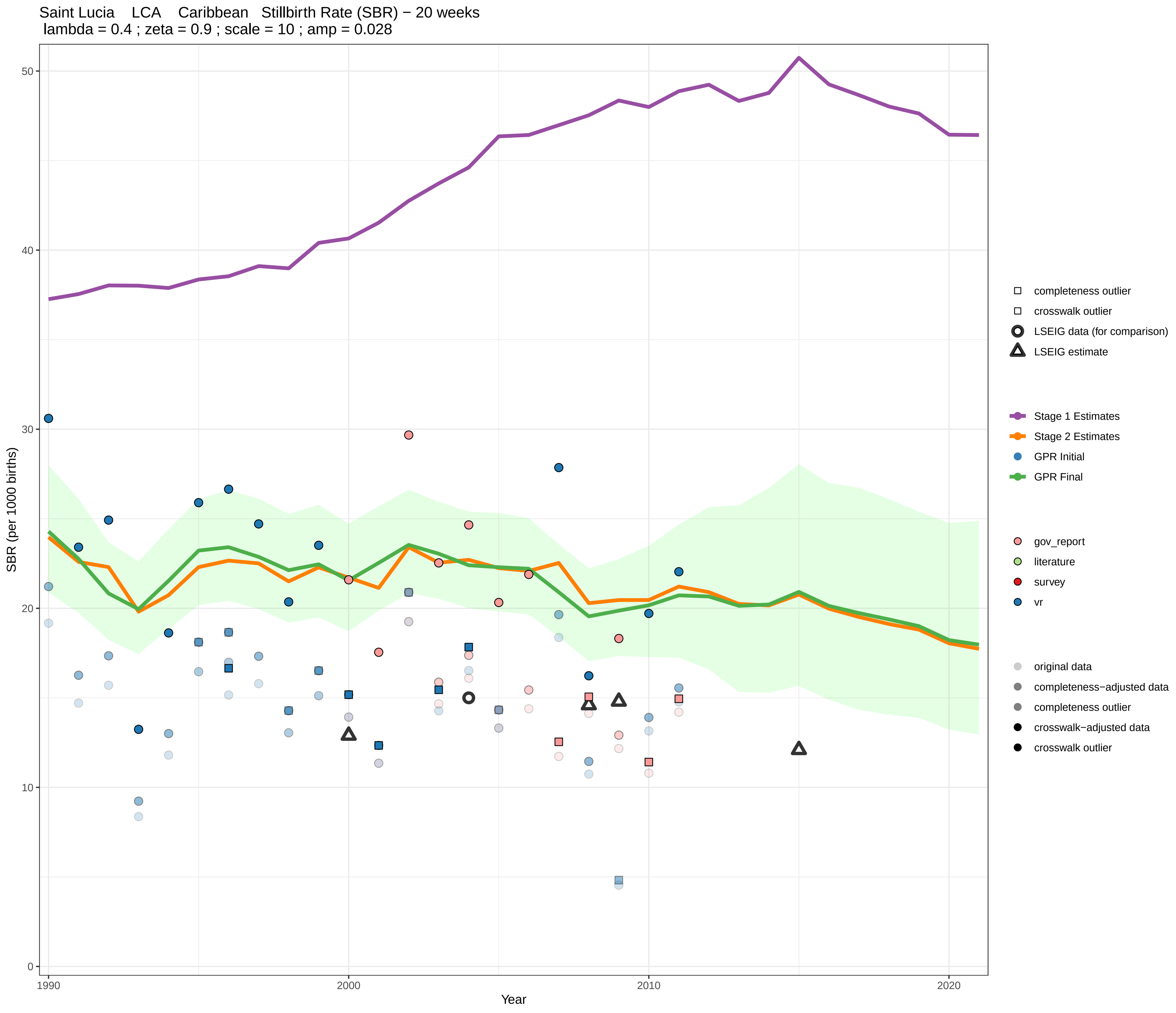

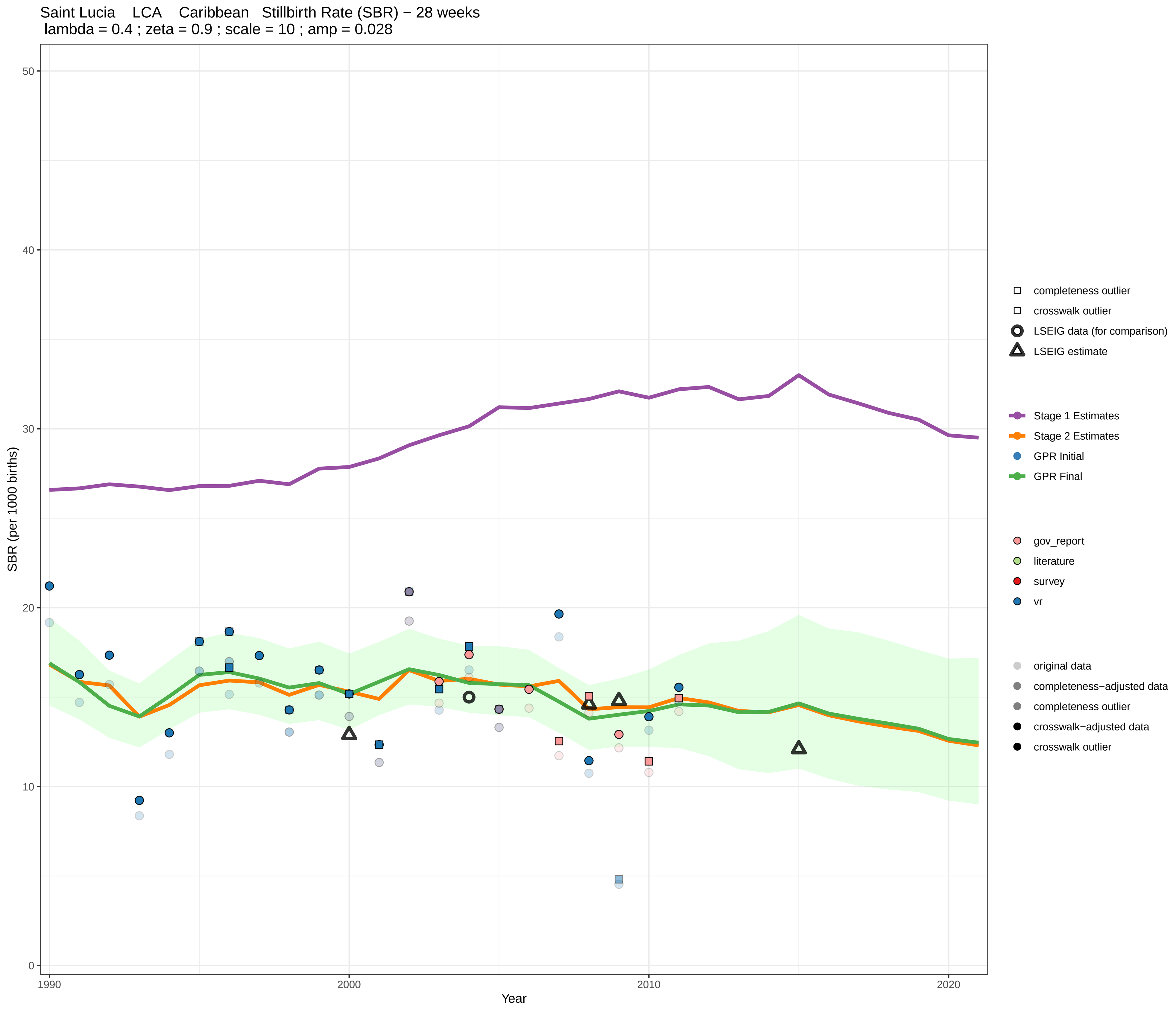

Year	Total Stillbirths (in thousands)	Total Stillbirths (in thousands)	SBR (per 1000 births)	SBR (per 1000 births)	SBR/NMR	SBR/NMR
	20 weeks	28 weeks	20 weeks	28 weeks	20 weeks	28 weeks
1990	0.1 (0.1 to 0.1)	0.1 (0.1 to 0.1)	24.3 (20.9 to 28.0)	16.9 (14.5 to 19.4)	1.81 (1.55 to 2.08)	1.26 (1.08 to 1.45)
1995	0.1 (0.1 to 0.1)	0.1 (0.0 to 0.1)	23.2 (20.2 to 26.1)	16.2 (14.1 to 18.2)	1.80 (1.57 to 2.03)	1.26 (1.10 to 1.42)
2000	0.1 (0.1 to 0.1)	0.0 (0.0 to 0.0)	21.6 (18.7 to 24.7)	15.2 (13.2 to 17.4)	1.69 (1.47 to 1.94)	1.19 (1.03 to 1.37)
2005	0.1 (0.0 to 0.1)	0.0 (0.0 to 0.0)	22.3 (19.8 to 25.3)	15.7 (14.0 to 17.9)	1.61 (1.43 to 1.83)	1.14 (1.01 to 1.29)
2010	0.0 (0.0 to 0.1)	0.0 (0.0 to 0.0)	20.2 (17.3 to 23.5)	14.2 (12.2 to 16.6)	1.48 (1.27 to 1.73)	1.05 (0.90 to 1.22)
2015	0.0 (0.0 to 0.1)	0.0 (0.0 to 0.0)	20.9 (15.7 to 28.1)	14.7 (11.0 to 19.6)	1.52 (1.14 to 2.03)	1.06 (0.80 to 1.42)
2020	0.0 (0.0 to 0.0)	0.0 (0.0 to 0.0)	18.2 (13.2 to 24.8)	12.7 (9.2 to 17.2)	1.52 (1.10 to 2.06)	1.05 (0.77 to 1.43)
2021	0.0 (0.0 to 0.0)	0.0 (0.0 to 0.0)	18.0 (12.9 to 24.9)	12.5 (9.0 to 17.2)	1.51 (1.09 to 2.09)	1.05 (0.76 to 1.45)

Saint Vincent and the Grenadines VCT Caribbean Stillbirth Rate / Neonatal Mortality Rate (SBR/NMR) − 20 weeks
lambda = 0.2 ; zeta = 0.99 ; scale = 5 ; amp = 0.028

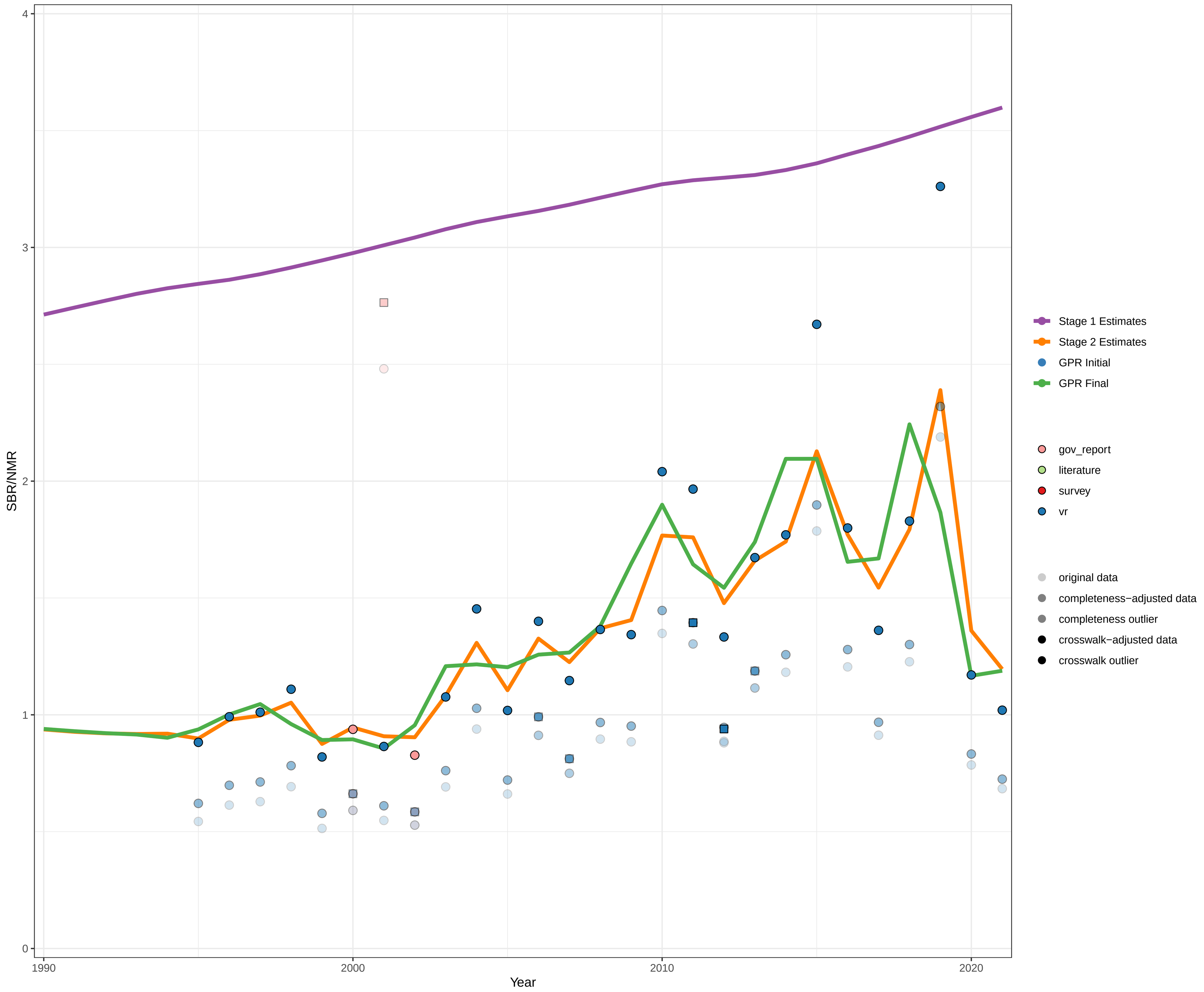

Saint Vincent and the Grenadines VCT Caribbean Stillbirth Rate / Neonatal Mortality Rate (SBR/NMR) − 28 weeks
lambda = 0.2 ; zeta = 0.99 ; scale = 5 ; amp = 0.028

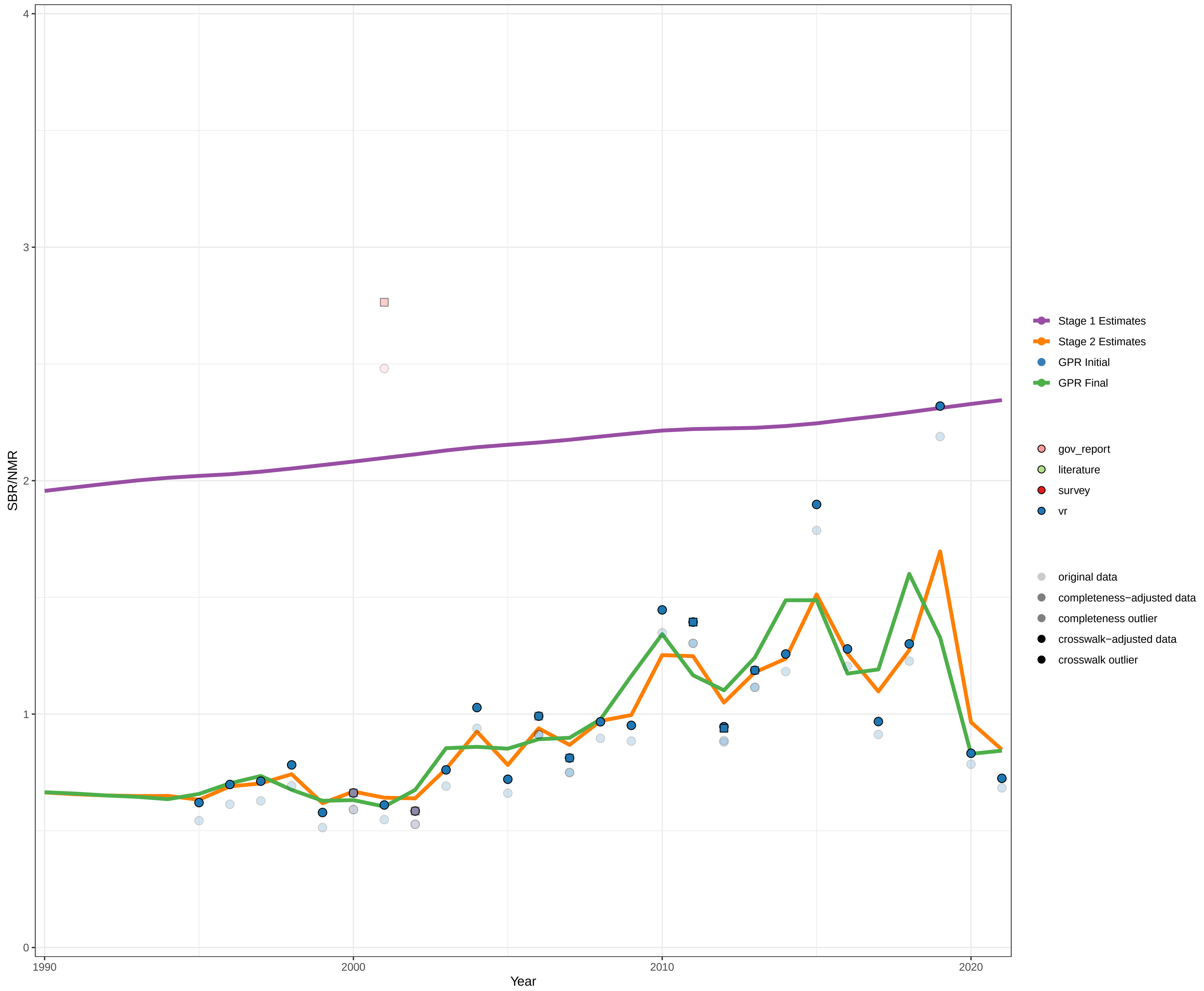

Definition	Years	Outlier	Source.Type	Title
28_weeks	1995−1999, 2001	0	VR	United Nations Demographic Yearbook
28_weeks	2000, 2002	0	Statistical Report	Saint Vincent and the Grenadines Statistics Digest
28_weeks	2003−2021	0	VR	Saint Vincent and the Grenadines Population and Vital Statistics Report
28_weeks	2000, 2002, 2006, 2007, 2013	1	VR	United Nations Demographic Yearbook
28_weeks	2001	1	Statistical Report	Saint Vincent and the Grenadines Statistics Digest
28_weeks	2011, 2012	2	VR	United Nations Demographic Yearbook

Saint Vincent and the Grenadines VCT Caribbean Stillbirth Rate (SBR) − 20 weeks
lambda = 0.2 ; zeta = 0.99 ; scale = 5 ; amp = 0.028

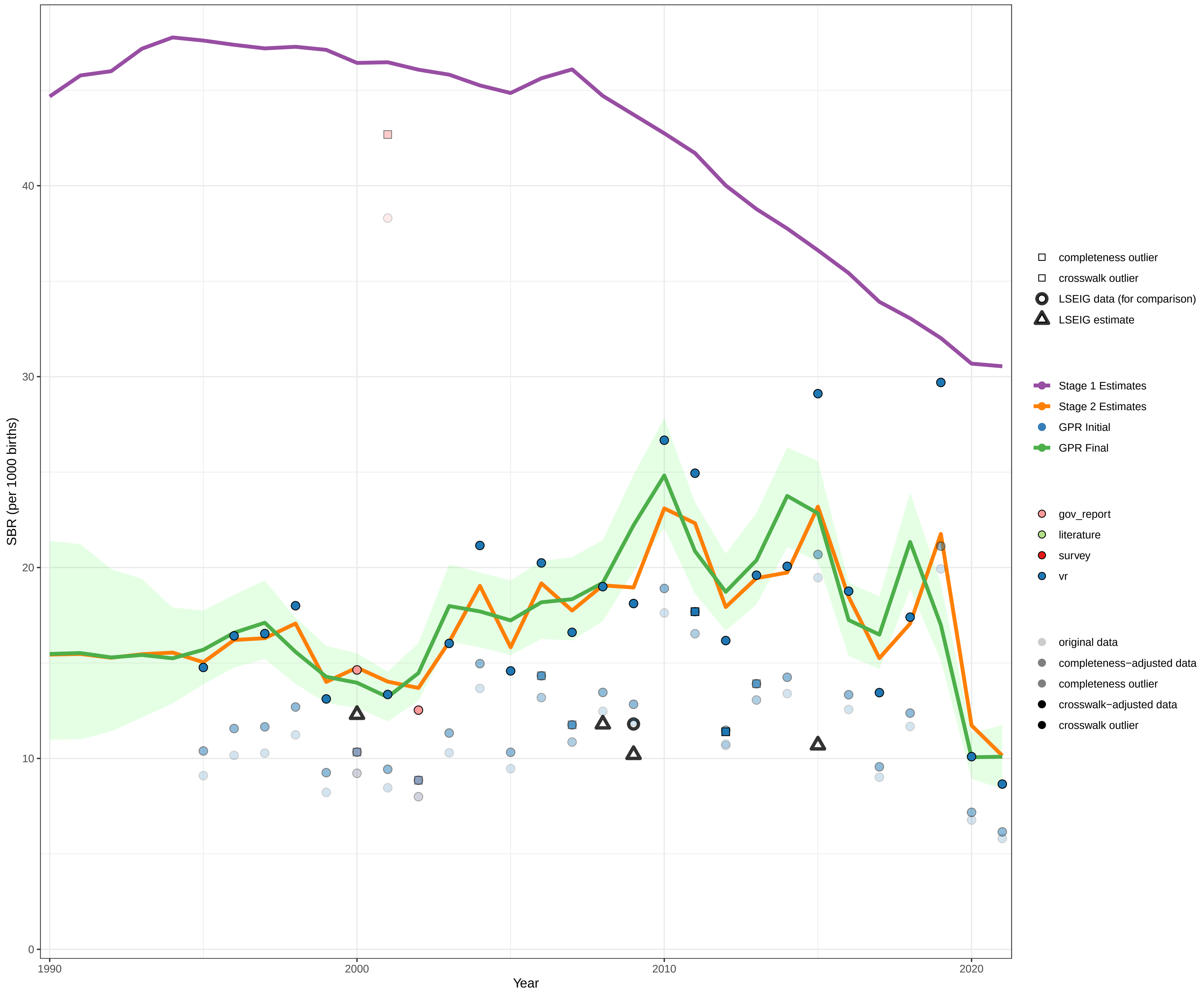

Saint Vincent and the Grenadines VCT Caribbean Stillbirth Rate (SBR) − 28 weeks
lambda = 0.2 ; zeta = 0.99 ; scale = 5 ; amp = 0.028

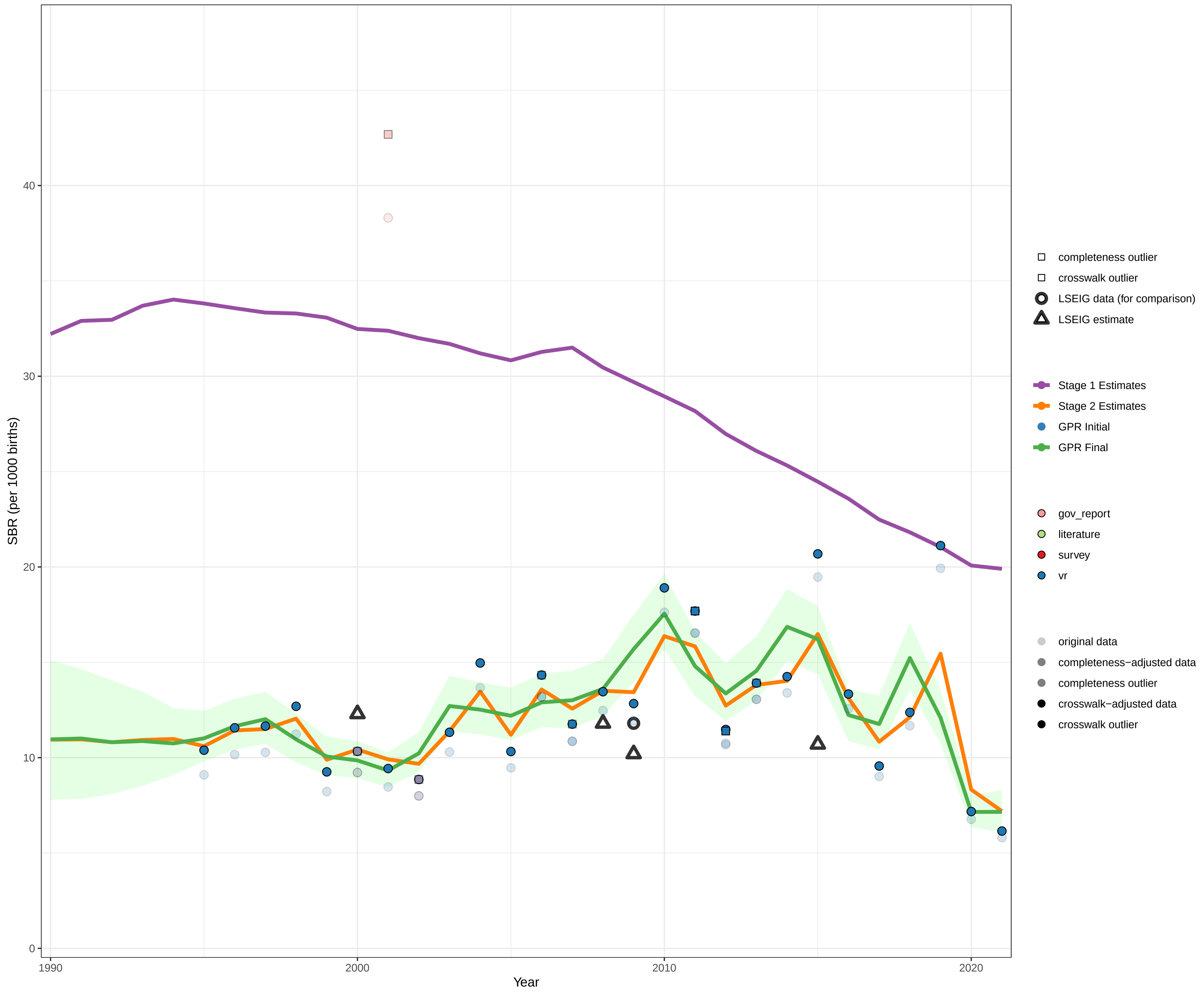

Year	Total Stillbirths (in thousands)	Total Stillbirths (in thousands)	SBR (per 1000 births)	SBR (per 1000 births)	SBR/NMR	SBR/NMR
	20 weeks	28 weeks	20 weeks	28 weeks	20 weeks	28 weeks
1990	0.0 (0.0 to 0.1)	0.0 (0.0 to 0.0)	15.5 (11.0 to 21.4)	11.0 (7.8 to 15.1)	0.94 (0.67 to 1.30)	0.67 (0.47 to 0.92)
1995	0.0 (0.0 to 0.0)	0.0 (0.0 to 0.0)	15.7 (13.9 to 17.8)	11.0 (9.8 to 12.5)	0.94 (0.83 to 1.06)	0.66 (0.59 to 0.74)
2000	0.0 (0.0 to 0.0)	0.0 (0.0 to 0.0)	14.0 (12.6 to 15.5)	9.9 (8.9 to 10.9)	0.90 (0.81 to 0.99)	0.63 (0.57 to 0.70)
2005	0.0 (0.0 to 0.0)	0.0 (0.0 to 0.0)	17.2 (15.4 to 19.3)	12.2 (10.9 to 13.7)	1.20 (1.08 to 1.35)	0.85 (0.76 to 0.95)
2010	0.0 (0.0 to 0.1)	0.0 (0.0 to 0.0)	24.8 (22.1 to 27.8)	17.5 (15.7 to 19.6)	1.90 (1.69 to 2.13)	1.34 (1.20 to 1.50)
2015	0.0 (0.0 to 0.0)	0.0 (0.0 to 0.0)	22.8 (20.3 to 25.6)	16.2 (14.4 to 18.0)	2.10 (1.86 to 2.35)	1.49 (1.32 to 1.65)
2020	0.0 (0.0 to 0.0)	0.0 (0.0 to 0.0)	10.1 (8.9 to 11.3)	7.2 (6.4 to 8.1)	1.17 (1.04 to 1.31)	0.83 (0.74 to 0.93)
2021	0.0 (0.0 to 0.0)	0.0 (0.0 to 0.0)	10.1 (8.4 to 11.7)	7.2 (6.1 to 8.3)	1.19 (0.99 to 1.38)	0.84 (0.72 to 0.98)

Suriname SUR Caribbean Stillbirth Rate / Neonatal Mortality Rate (SBR/NMR) − 20 weeks
lambda = 0.6 ; zeta = 0.8 ; scale = 15 ; amp = 0.028

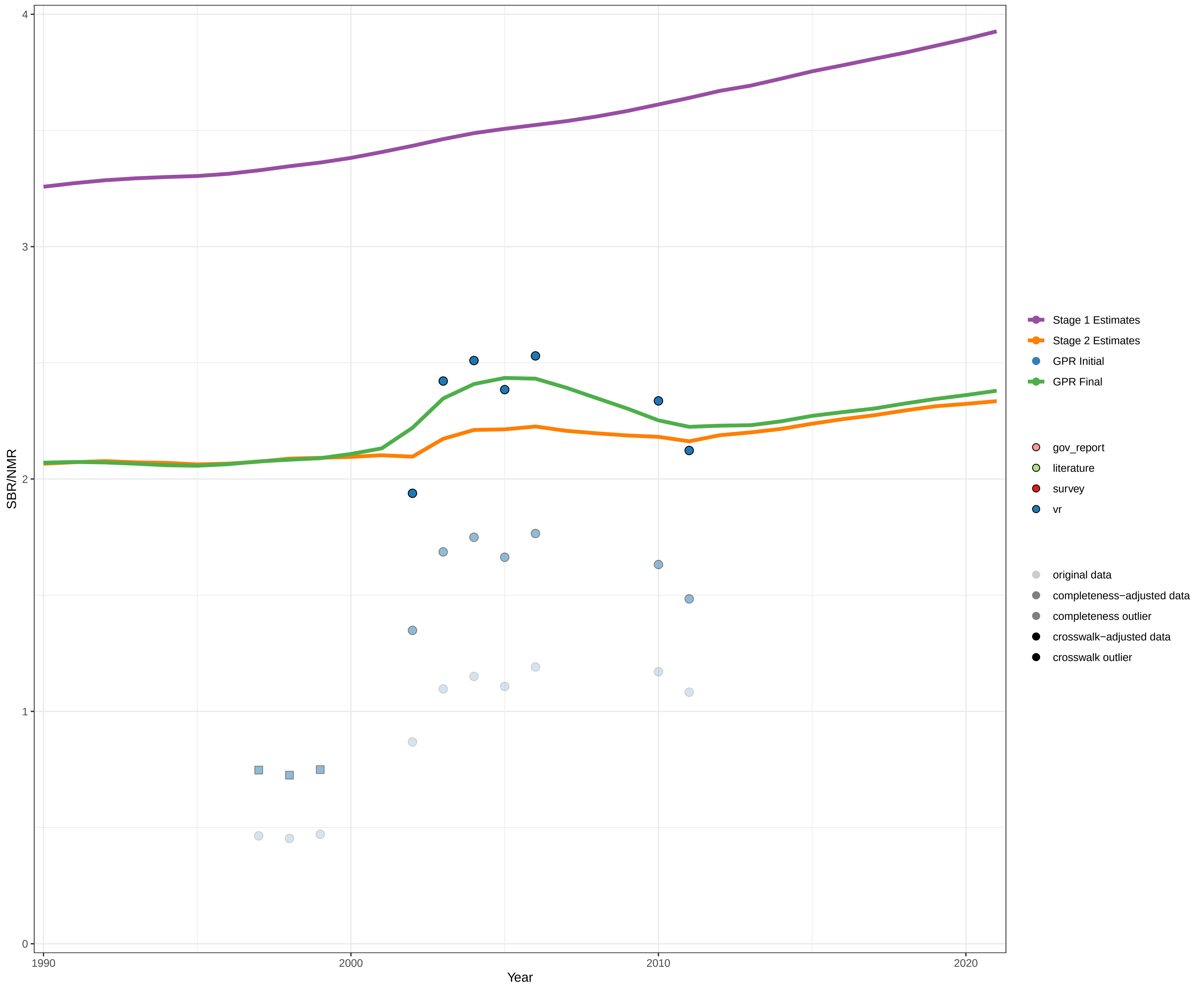

Suriname SUR Caribbean Stillbirth Rate / Neonatal Mortality Rate (SBR/NMR) − 28 weeks
lambda = 0.6 ; zeta = 0.8 ; scale = 15 ; amp = 0.028

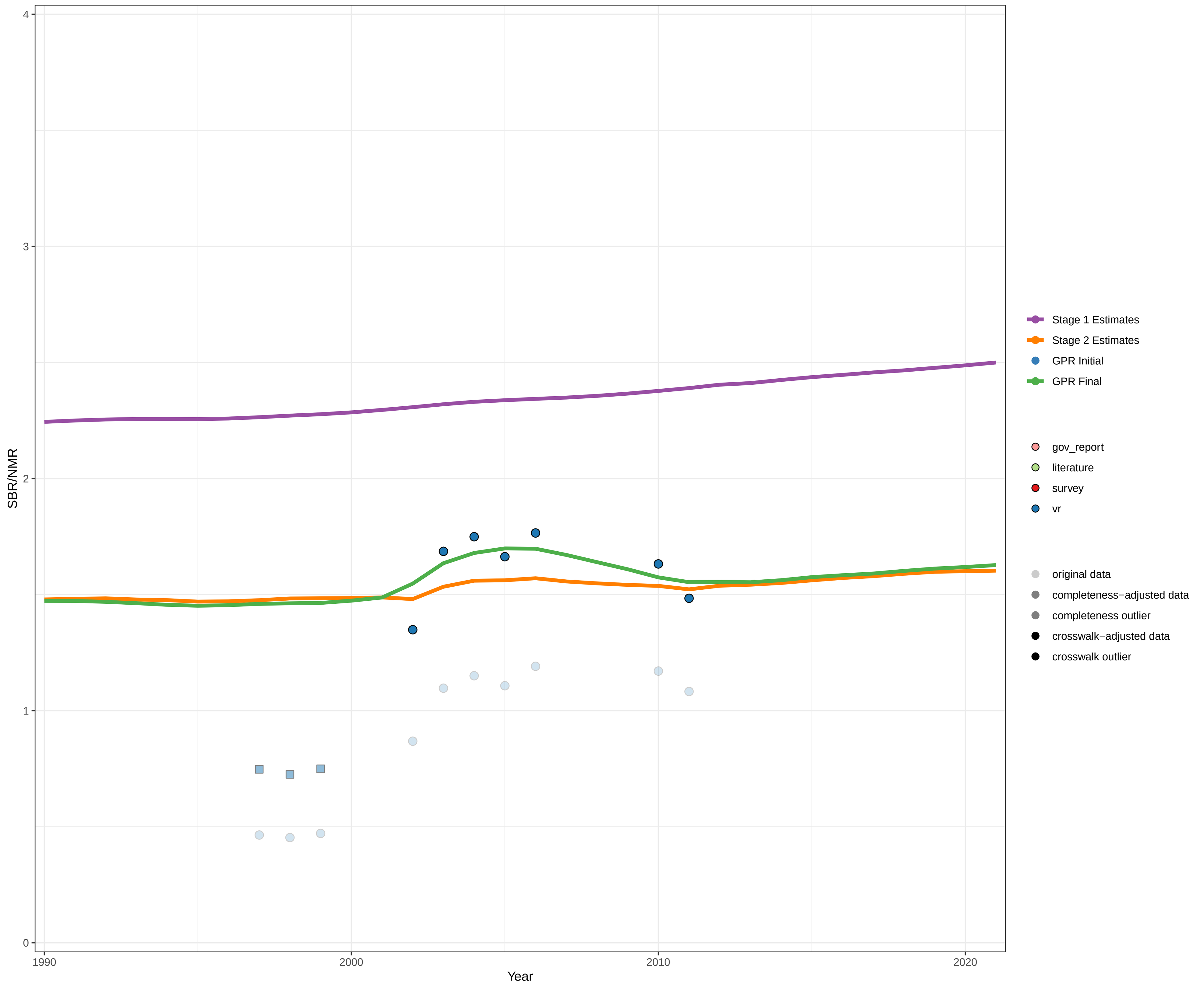

Definition	Years	Outlier	Source.Type	Title
28_weeks	2002−2006, 2010, 2011	0	VR	United Nations Demographic Yearbook
28_weeks	1997−1999	1	VR	United Nations Demographic Yearbook

Suriname SUR Caribbean Stillbirth Rate (SBR) − 20 weeks
lambda = 0.6 ; zeta = 0.8 ; scale = 15 ; amp = 0.028

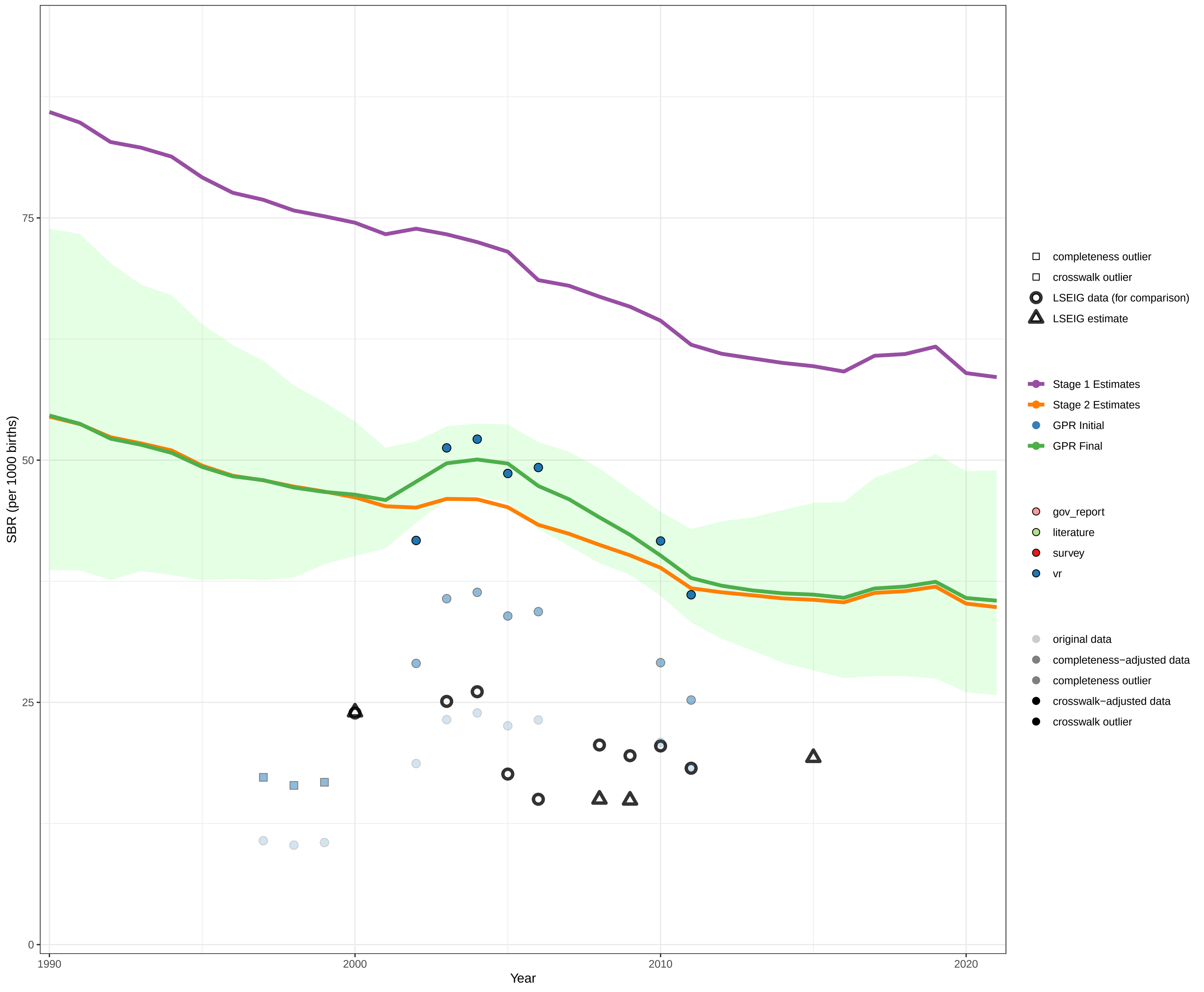

Suriname SUR Caribbean Stillbirth Rate (SBR) − 28 weeks
lambda = 0.6 ; zeta = 0.8 ; scale = 15 ; amp = 0.028

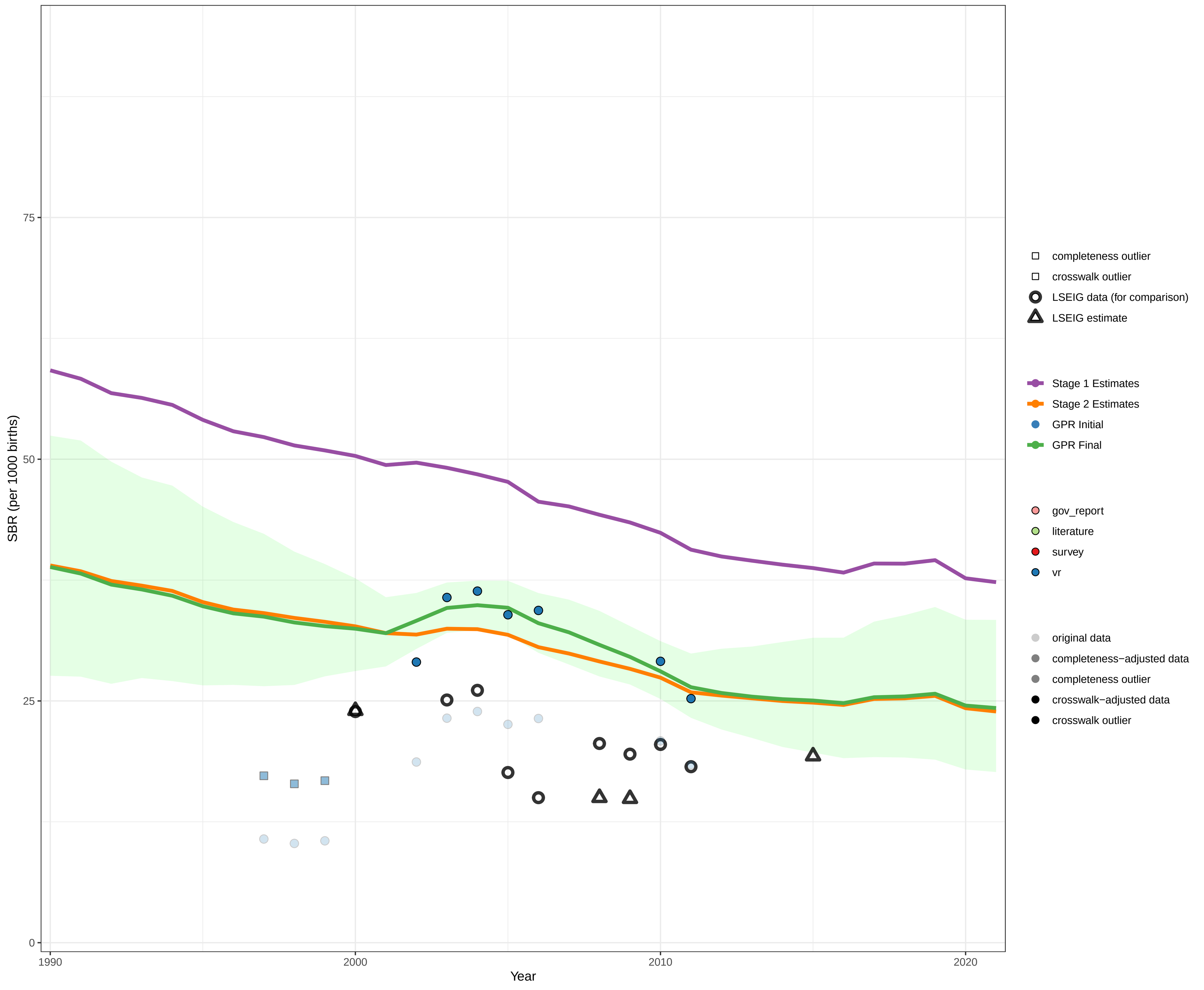

Year	Total Stillbirths (in thousands)	Total Stillbirths (in thousands)	SBR (per 1000 births)	SBR (per 1000 births)	SBR/NMR	SBR/NMR
	20 weeks	28 weeks	20 weeks	28 weeks	20 weeks	28 weeks
1990	0.6<br>(0.4 to 0.8)	0.4<br>(0.3 to 0.5)	54.6<br>(38.7 to 73.9)	38.9<br>(27.6 to 52.4)	2.07<br>(1.47 to 2.80)	1.47<br>(1.05 to 1.99)
1995	0.5<br>(0.4 to 0.6)	0.3<br>(0.2 to 0.4)	49.3<br>(37.6 to 64.1)	34.8<br>(26.6 to 45.1)	2.06<br>(1.57 to 2.67)	1.45<br>(1.11 to 1.88)
2000	0.5<br>(0.4 to 0.5)	0.3<br>(0.3 to 0.4)	46.4<br>(40.1 to 54.0)	32.5<br>(28.1 to 37.7)	2.11<br>(1.82 to 2.45)	1.47<br>(1.28 to 1.71)
2005	0.5<br>(0.5 to 0.6)	0.4<br>(0.3 to 0.4)	49.7<br>(45.6 to 53.7)	34.6<br>(31.9 to 37.4)	2.44<br>(2.24 to 2.63)	1.70<br>(1.56 to 1.84)
2010	0.4<br>(0.4 to 0.5)	0.3<br>(0.3 to 0.3)	40.2<br>(36.0 to 44.7)	28.1<br>(25.2 to 31.2)	2.25<br>(2.02 to 2.51)	1.57<br>(1.41 to 1.75)
2015	0.4<br>(0.3 to 0.5)	0.3<br>(0.2 to 0.3)	36.1<br>(28.3 to 45.6)	25.0<br>(19.7 to 31.5)	2.27<br>(1.78 to 2.87)	1.58<br>(1.24 to 1.98)
2020	0.3<br>(0.2 to 0.5)	0.2<br>(0.2 to 0.3)	35.8<br>(26.0 to 48.9)	24.5<br>(17.9 to 33.4)	2.36<br>(1.72 to 3.23)	1.62<br>(1.18 to 2.20)
2021	0.3<br>(0.2 to 0.5)	0.2<br>(0.2 to 0.3)	35.5<br>(25.7 to 49.0)	24.3<br>(17.6 to 33.4)	2.38<br>(1.73 to 3.28)	1.63<br>(1.18 to 2.24)

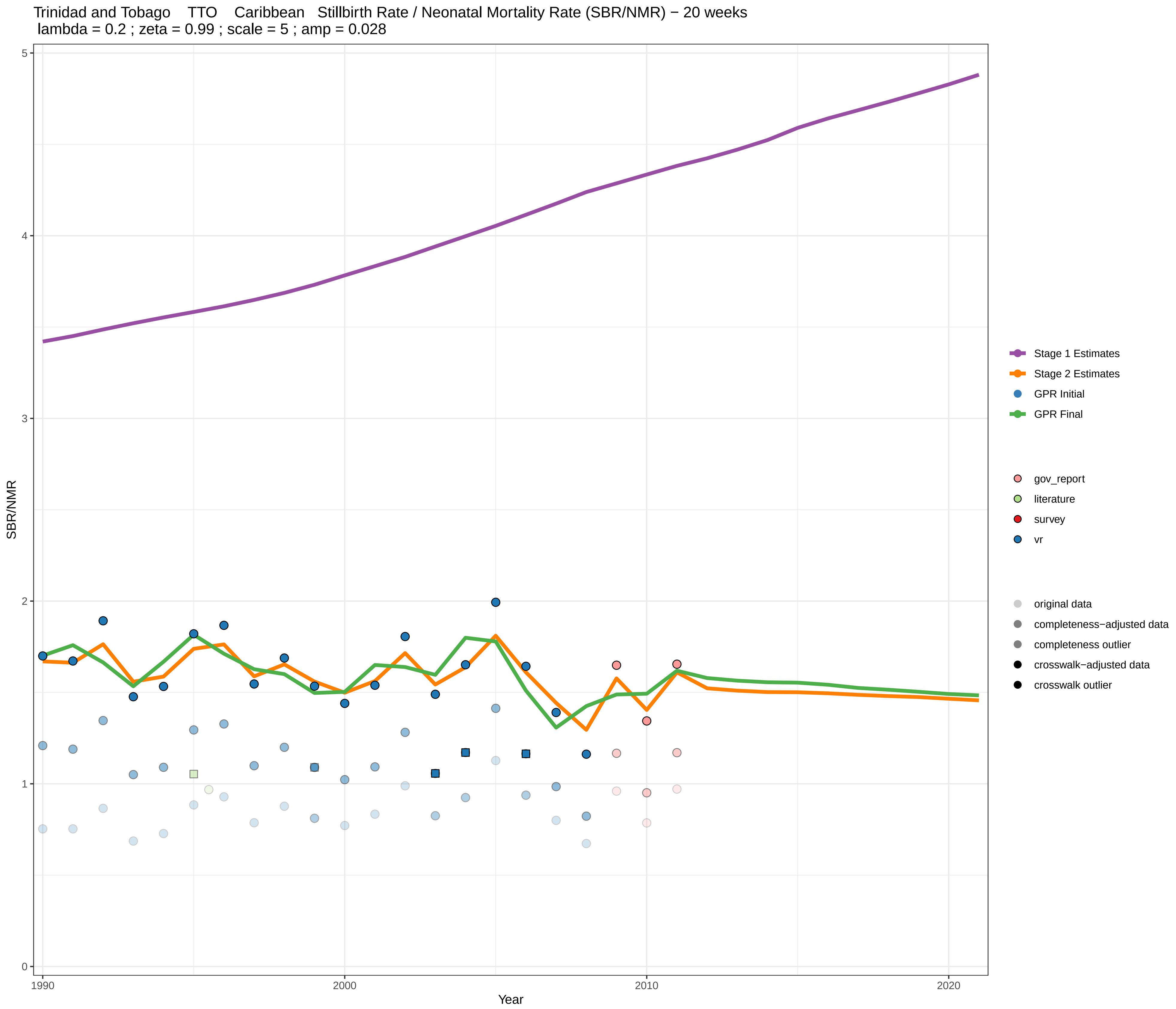

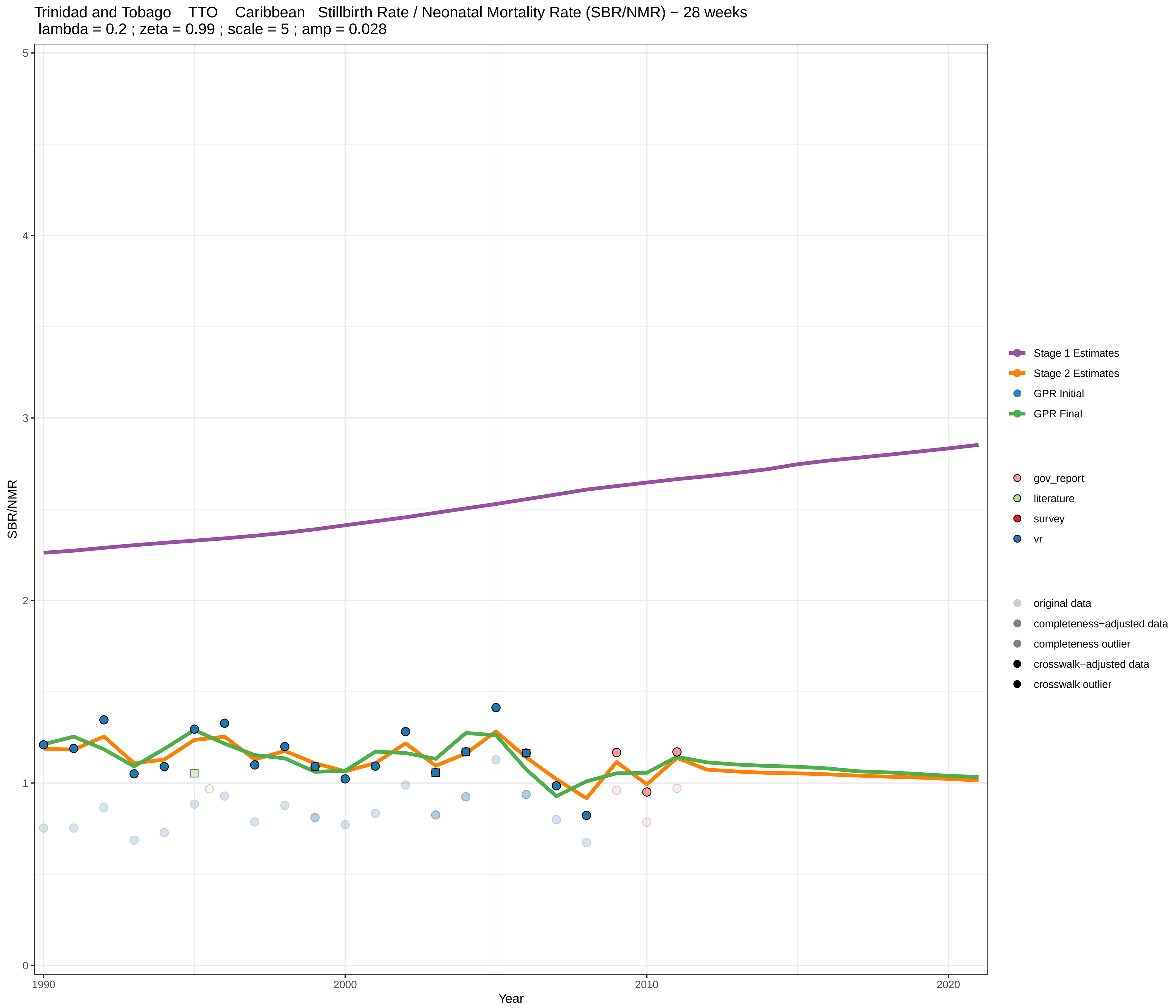

Definition	Years	Outlier	Source.Type	Title
28_weeks	1990−2008	0	VR	Trinidad and Tobago Review of Vital Statistics
28_weeks	2009−2011	0	Statistical Report	Trinidad and Tobago Health Statistical Report
24_weeks	1995	1	Sci Lit	An audit of perinatal mortality
28_weeks	1999	1	VR	United Nations Demographic Yearbook
28_weeks	2003, 2004, 2006	2	VR	United Nations Demographic Yearbook

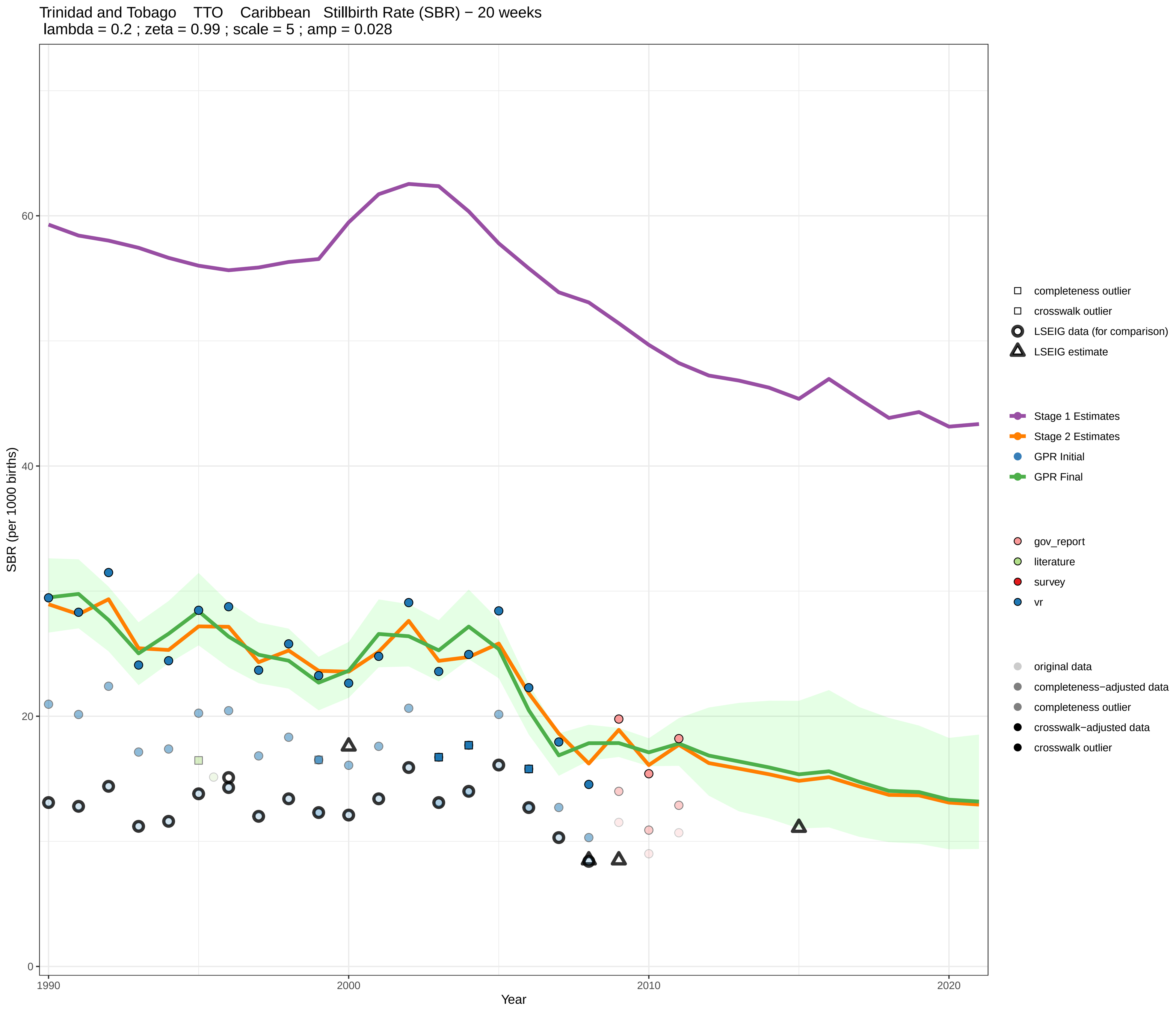

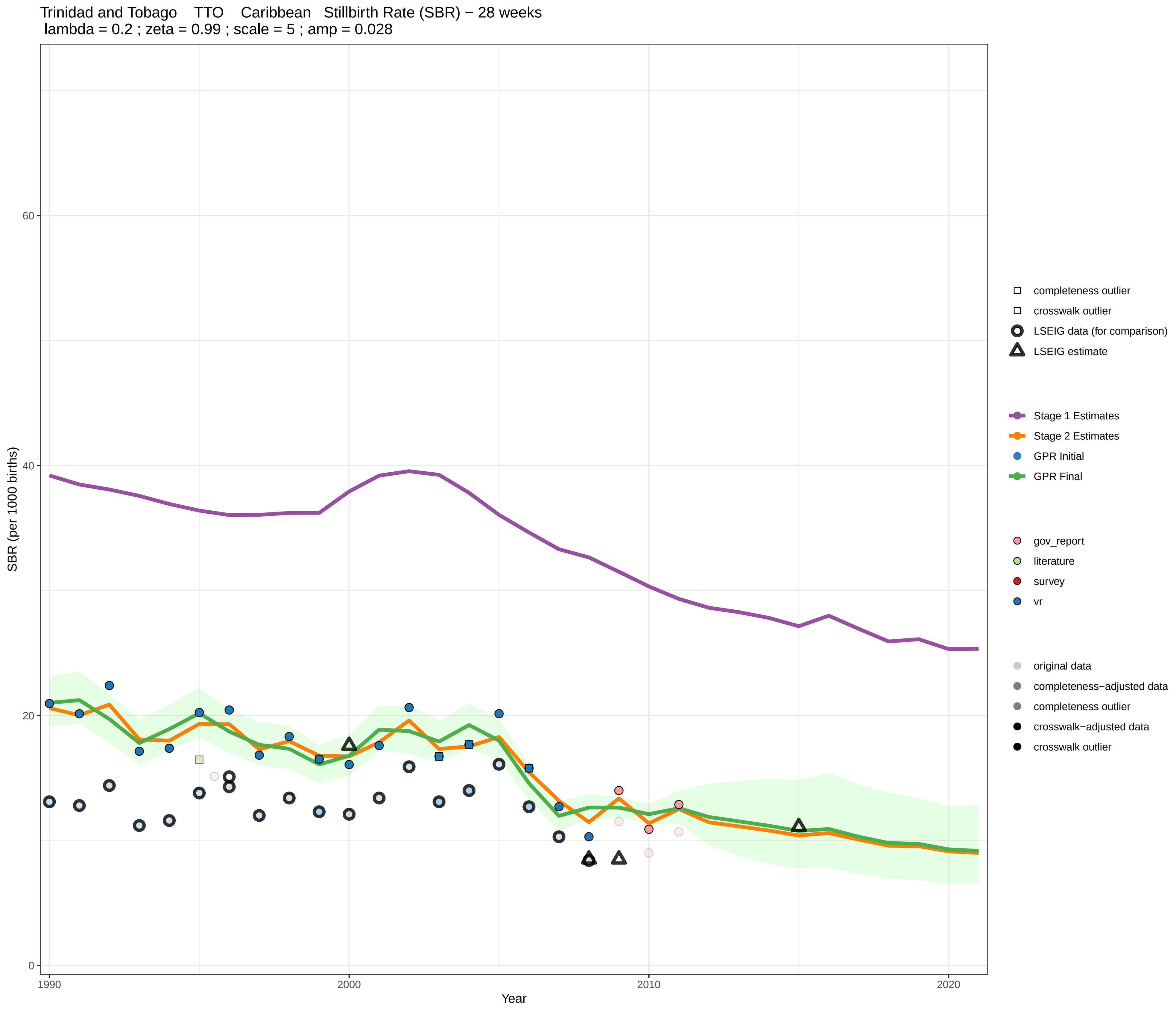

Year	Total Stillbirths (in thousands)	Total Stillbirths (in thousands)	SBR (per 1000 births)	SBR (per 1000 births)	SBR/NMR	SBR/NMR
	20 weeks	28 weeks	20 weeks	28 weeks	20 weeks	28 weeks
1990	0.7 (0.7 to 0.8)	0.5 (0.5 to 0.6)	29.5 (26.7 to 32.6)	21.0 (19.1 to 23.1)	1.70 (1.54 to 1.88)	1.21 (1.10 to 1.34)
1995	0.6 (0.5 to 0.6)	0.4 (0.4 to 0.4)	28.4 (25.7 to 31.5)	20.2 (18.3 to 22.2)	1.82 (1.64 to 2.01)	1.29 (1.17 to 1.42)
2000	0.4 (0.4 to 0.5)	0.3 (0.3 to 0.3)	23.6 (21.5 to 25.9)	16.8 (15.2 to 18.5)	1.50 (1.37 to 1.65)	1.07 (0.97 to 1.18)
2005	0.5 (0.4 to 0.5)	0.3 (0.3 to 0.4)	25.4 (23.0 to 27.7)	18.0 (16.4 to 19.7)	1.78 (1.62 to 1.94)	1.26 (1.15 to 1.38)
2010	0.3 (0.3 to 0.4)	0.2 (0.2 to 0.2)	17.1 (16.0 to 18.2)	12.1 (11.3 to 12.9)	1.49 (1.40 to 1.59)	1.06 (0.99 to 1.13)
2015	0.3 (0.2 to 0.4)	0.2 (0.1 to 0.3)	15.4 (11.0 to 21.2)	10.8 (7.8 to 14.8)	1.55 (1.12 to 2.15)	1.09 (0.78 to 1.50)
2020	0.2 (0.1 to 0.3)	0.1 (0.1 to 0.2)	13.3 (9.4 to 18.3)	9.3 (6.5 to 12.7)	1.49 (1.05 to 2.04)	1.04 (0.72 to 1.43)
2021	0.2 (0.1 to 0.3)	0.1 (0.1 to 0.2)	13.2 (9.4 to 18.5)	9.2 (6.6 to 12.9)	1.48 (1.06 to 2.09)	1.03 (0.74 to 1.45)

United States Virgin Islands   VIR   Caribbean   Stillbirth Rate / Neonatal Mortality Rate (SBR/NMR) − 20 weeks
lambda = 0.4 ; zeta = 0.9 ; scale = 10 ; amp = 0.028

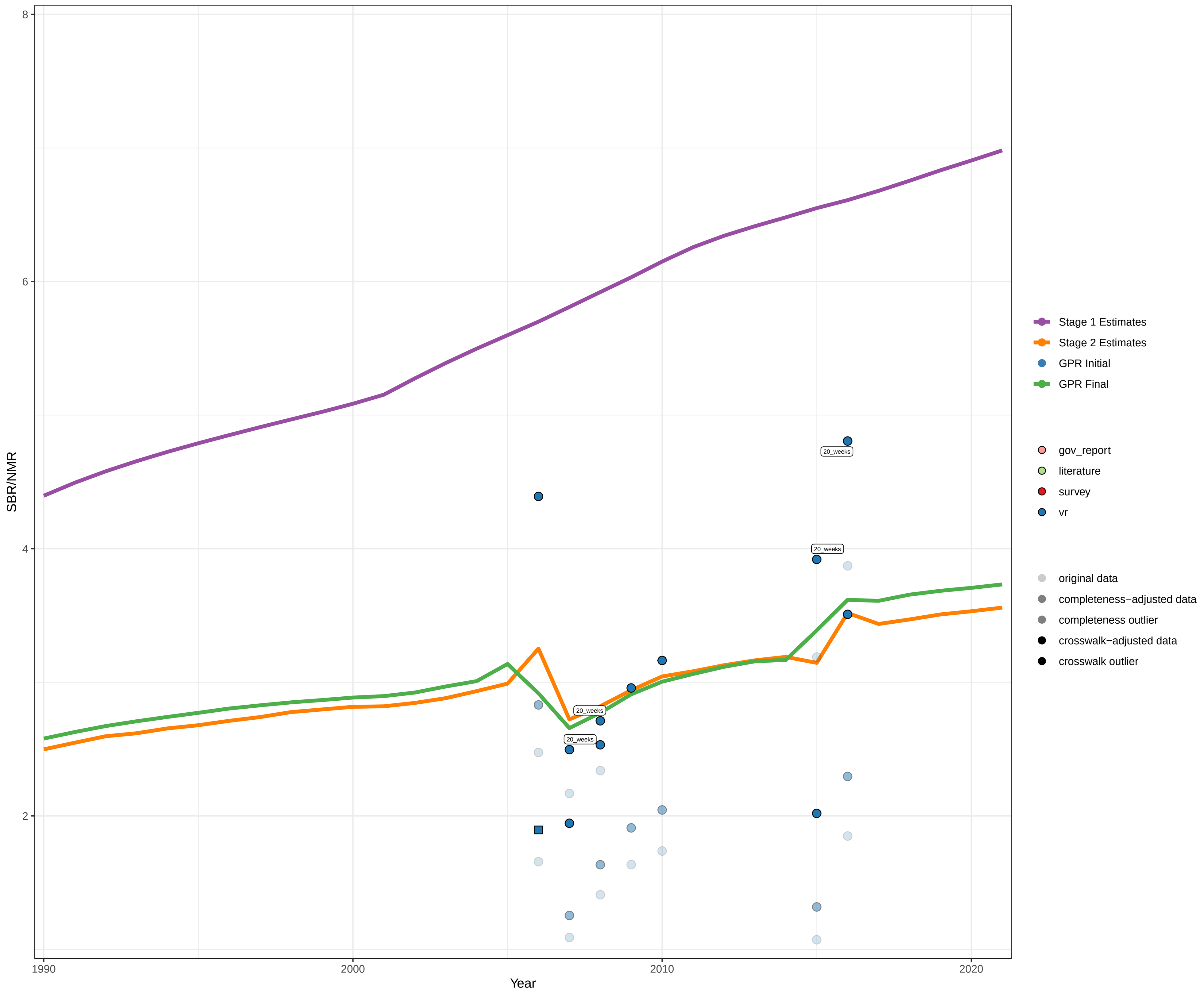

United States Virgin Islands   VIR   Caribbean   Stillbirth Rate / Neonatal Mortality Rate (SBR/NMR) − 28 weeks
lambda = 0.4 ; zeta = 0.9 ; scale = 10 ; amp = 0.028

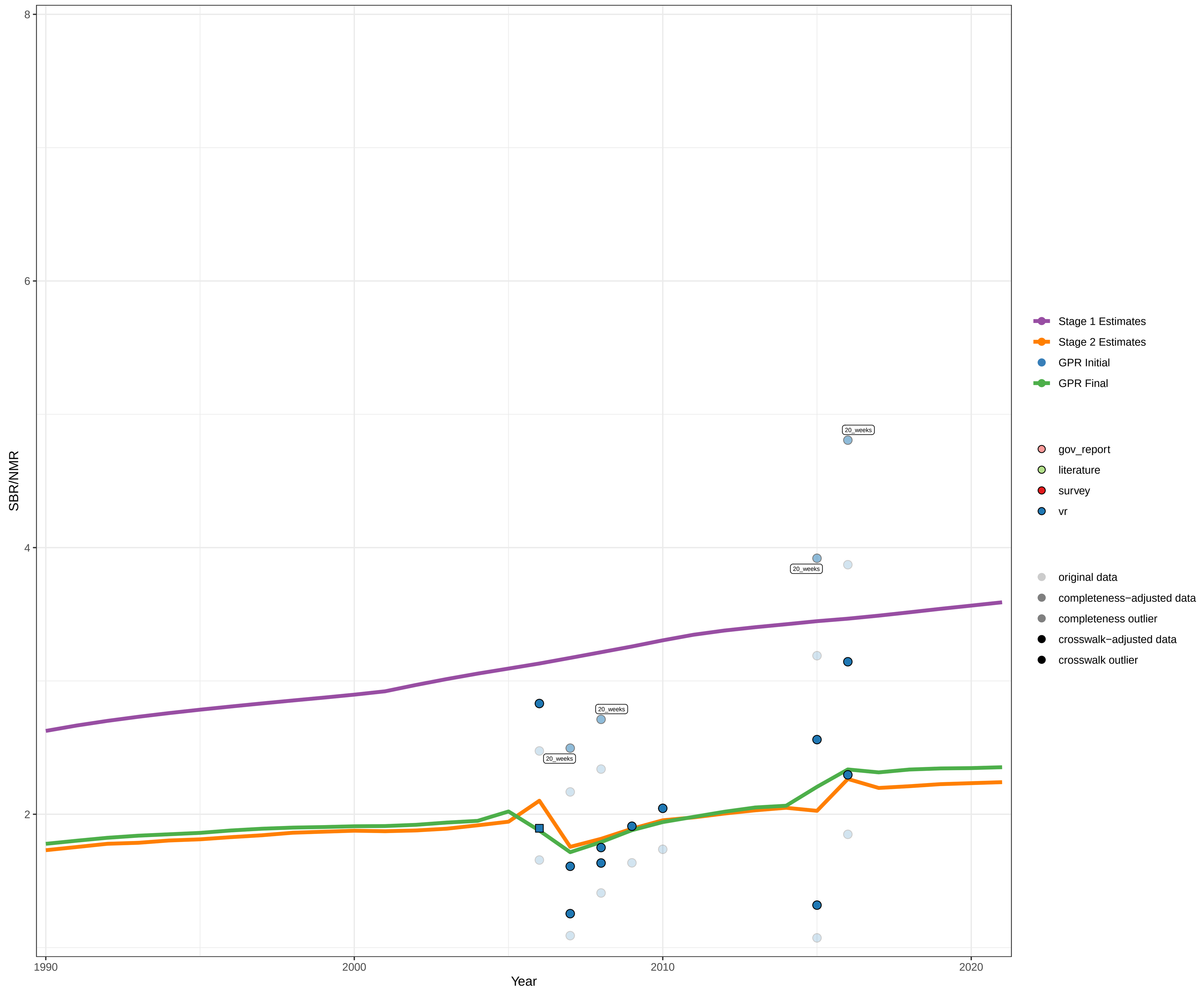

Definition	Years	Outlier	Source.Type	Title
20_weeks	2007, 2008, 2015, 2016	0	VR	United States NVSS Custom Fetal Death Data
28_weeks	2006−2010, 2015, 2016	0	VR	United States NVSS Custom Fetal Death Data
28_weeks	2006	2	VR	United States NVSS Custom Fetal Death Data

United States Virgin Islands   VIR   Caribbean   Stillbirth Rate (SBR) − 20 weeks
lambda = 0.4 ; zeta = 0.9 ; scale = 10 ; amp = 0.028

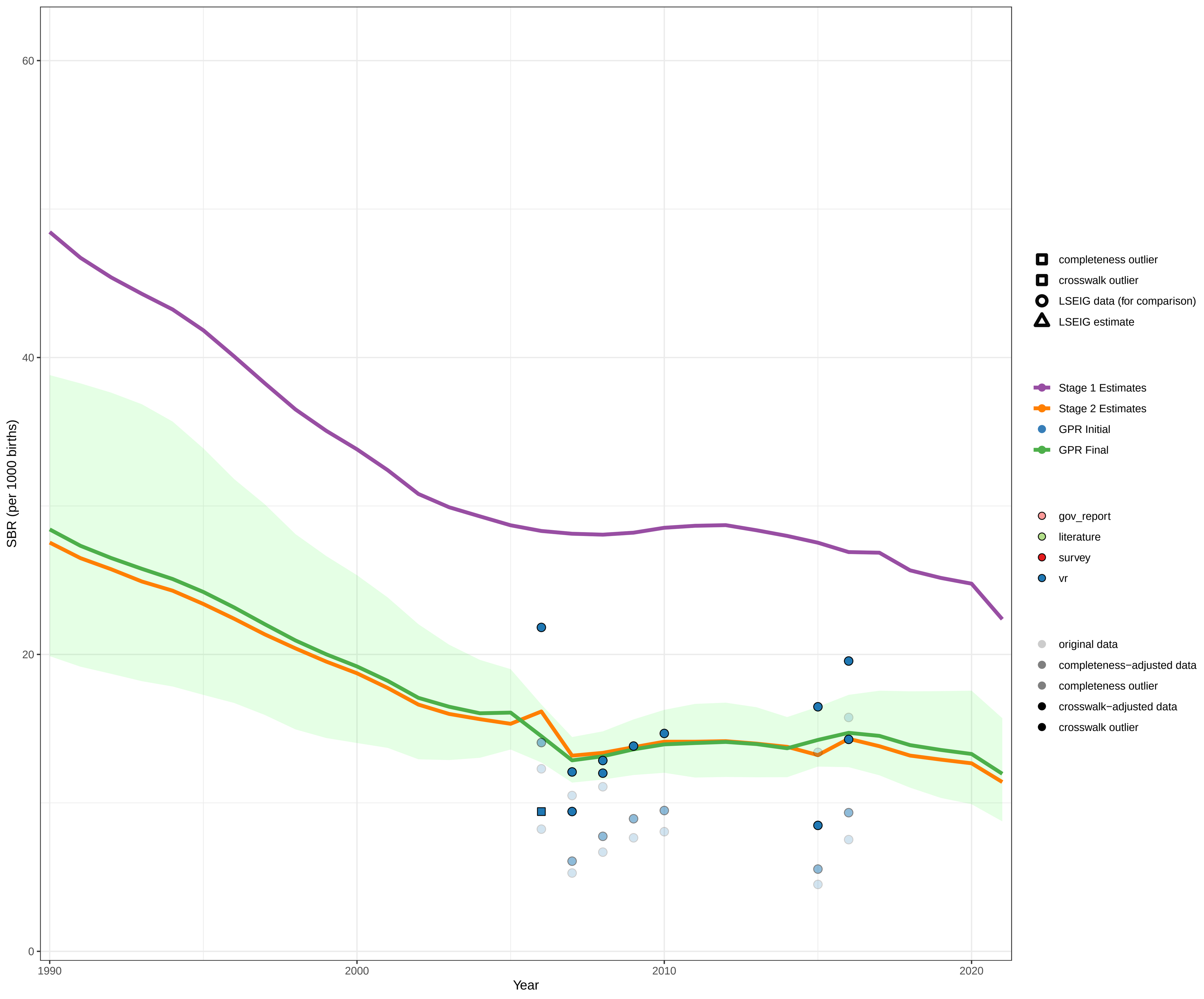

United States Virgin Islands   VIR   Caribbean   Stillbirth Rate (SBR) − 28 weeks
lambda = 0.4 ; zeta = 0.9 ; scale = 10 ; amp = 0.028

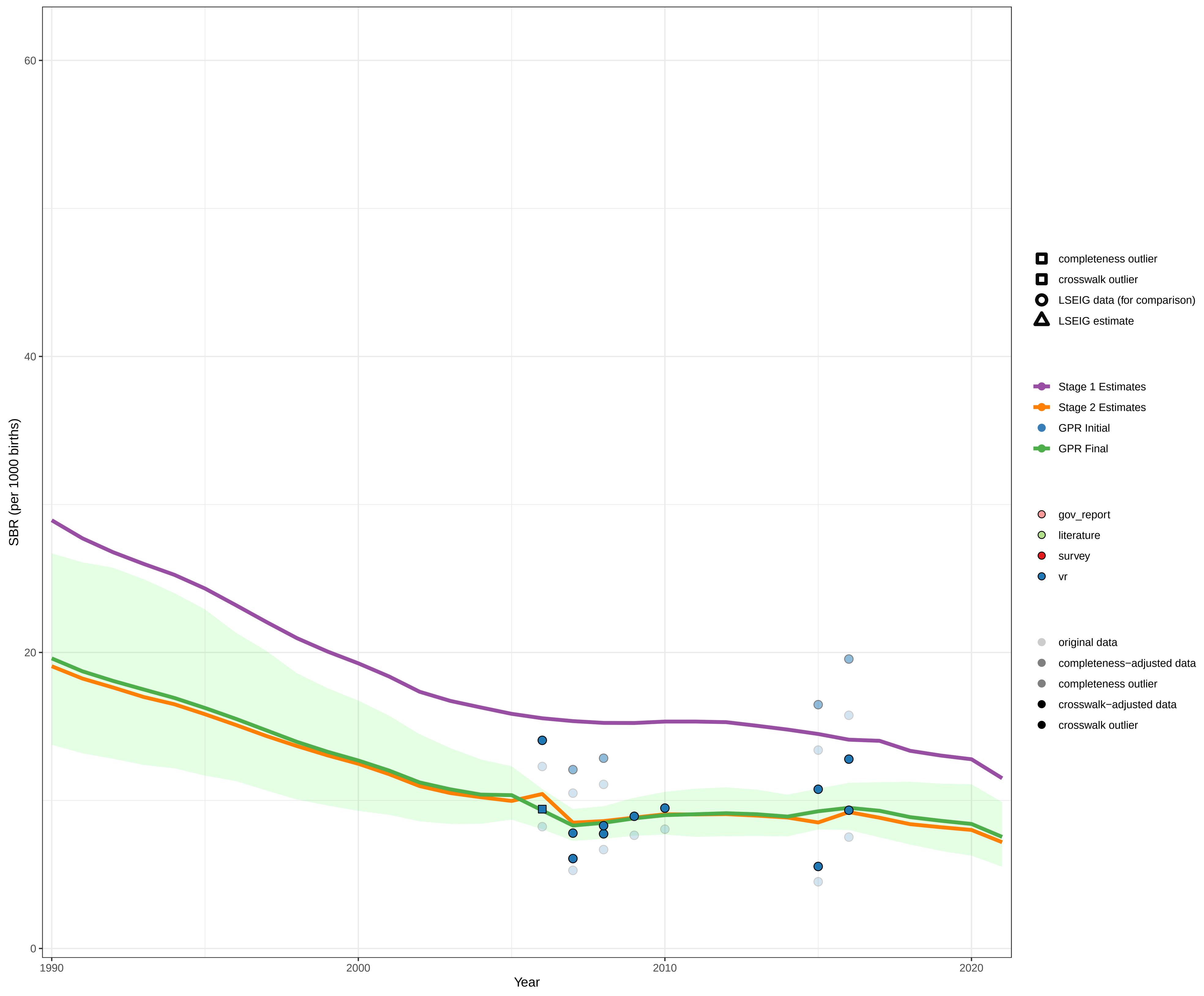

Year	Total Stillbirths (in thousands)	Total Stillbirths (in thousands)	SBR (per 1000 births)	SBR (per 1000 births)	SBR/NMR	SBR/NMR
	20 weeks	28 weeks	20 weeks	28 weeks	20 weeks	28 weeks
1990	0.1 (0.0 to 0.1)	0.0 (0.0 to 0.1)	28.4 (19.9 to 38.8)	19.6 (13.8 to 26.7)	2.58 (1.80 to 3.52)	1.78 (1.25 to 2.42)
1995	0.1 (0.0 to 0.1)	0.0 (0.0 to 0.1)	24.2 (17.3 to 33.9)	16.2 (11.7 to 22.9)	2.77 (1.98 to 3.88)	1.86 (1.34 to 2.62)
2000	0.0 (0.0 to 0.1)	0.0 (0.0 to 0.0)	19.2 (14.0 to 25.3)	12.7 (9.3 to 16.8)	2.89 (2.11 to 3.81)	1.91 (1.40 to 2.52)
2005	0.0 (0.0 to 0.0)	0.0 (0.0 to 0.0)	16.1 (13.6 to 19.0)	10.4 (8.7 to 12.3)	3.14 (2.65 to 3.71)	2.02 (1.70 to 2.40)
2010	0.0 (0.0 to 0.0)	0.0 (0.0 to 0.0)	13.9 (12.0 to 16.3)	9.0 (7.7 to 10.6)	3.00 (2.59 to 3.51)	1.94 (1.66 to 2.28)
2015	0.0 (0.0 to 0.0)	0.0 (0.0 to 0.0)	14.2 (12.4 to 16.5)	9.3 (8.0 to 10.8)	3.39 (2.96 to 3.92)	2.21 (1.91 to 2.57)
2020	0.0 (0.0 to 0.0)	0.0 (0.0 to 0.0)	13.3 (9.9 to 17.6)	8.4 (6.3 to 11.1)	3.71 (2.76 to 4.90)	2.35 (1.75 to 3.09)
2021	0.0 (0.0 to 0.0)	0.0 (0.0 to 0.0)	12.0 (8.8 to 15.7)	7.5 (5.5 to 9.9)	3.73 (2.73 to 4.90)	2.35 (1.72 to 3.09)

Central Latin America R12 Central Latin America Stillbirth Rate / Neonatal Mortality Rate (SBR/NMR) − 20 weeks
lambda = 1 ; zeta = 0.6 ; scale = 15 ; amp = 0.028

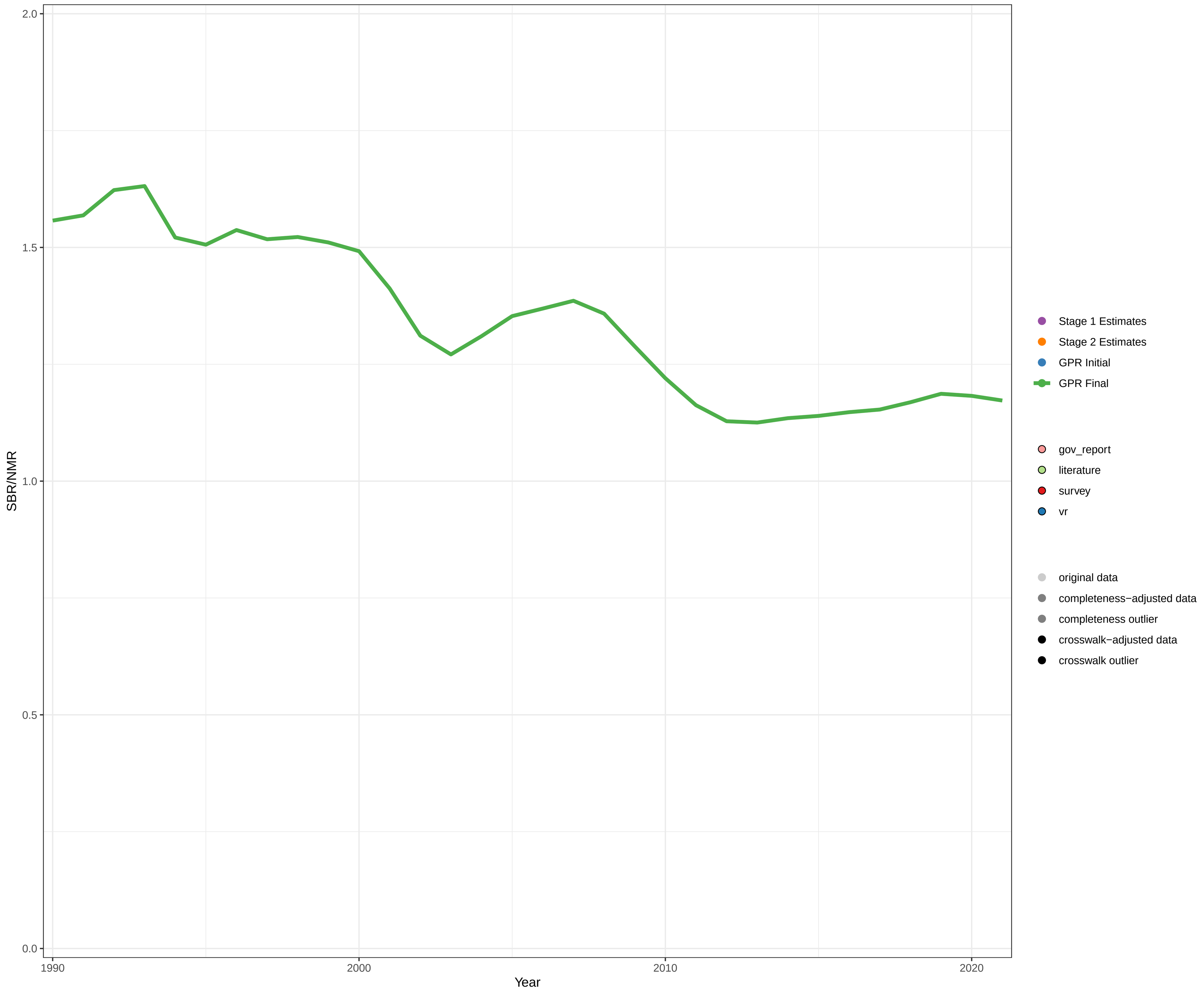

Central Latin America R12 Central Latin America Stillbirth Rate / Neonatal Mortality Rate (SBR/NMR) − 28 weeks
lambda = 1 ; zeta = 0.6 ; scale = 15 ; amp = 0.028

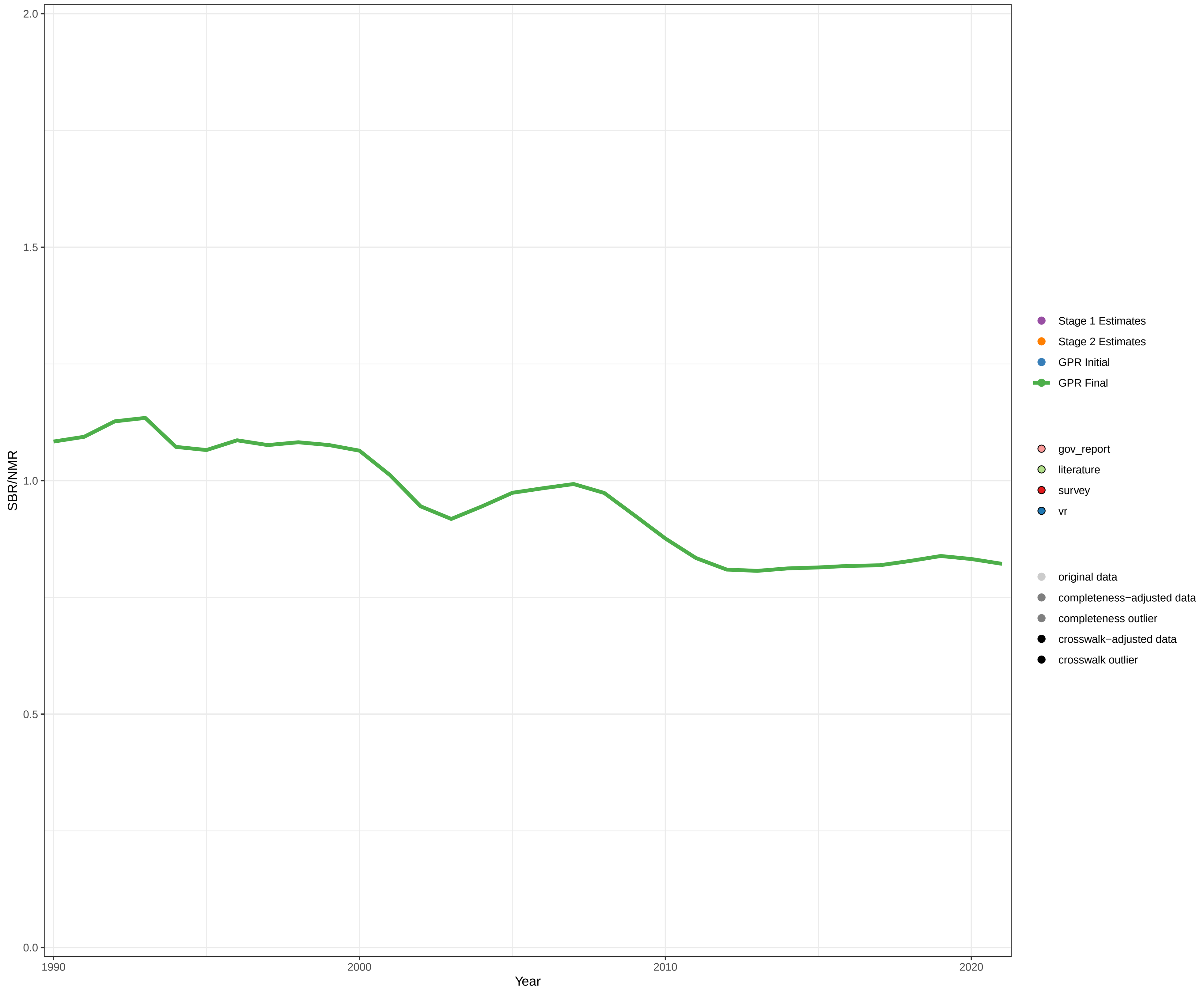

Definition	Years	Outlier	Source.Type	Title

Central Latin America R12 Central Latin America Stillbirth Rate (SBR) − 20 weeks
lambda = 1 ; zeta = 0.6 ; scale = 15 ; amp = 0.028

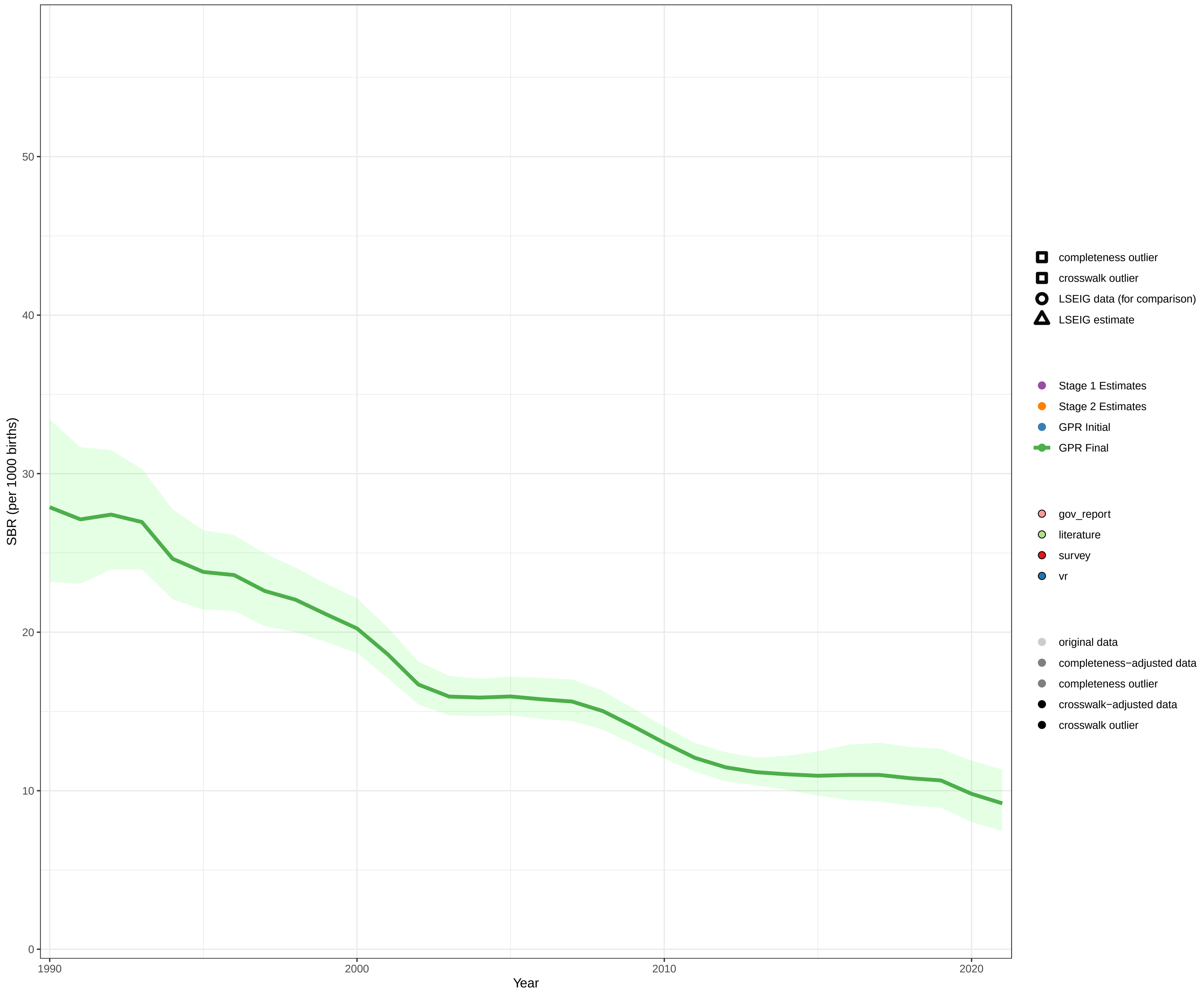

Central Latin America R12 Central Latin America Stillbirth Rate (SBR) − 28 weeks
lambda = 1 ; zeta = 0.6 ; scale = 15 ; amp = 0.028

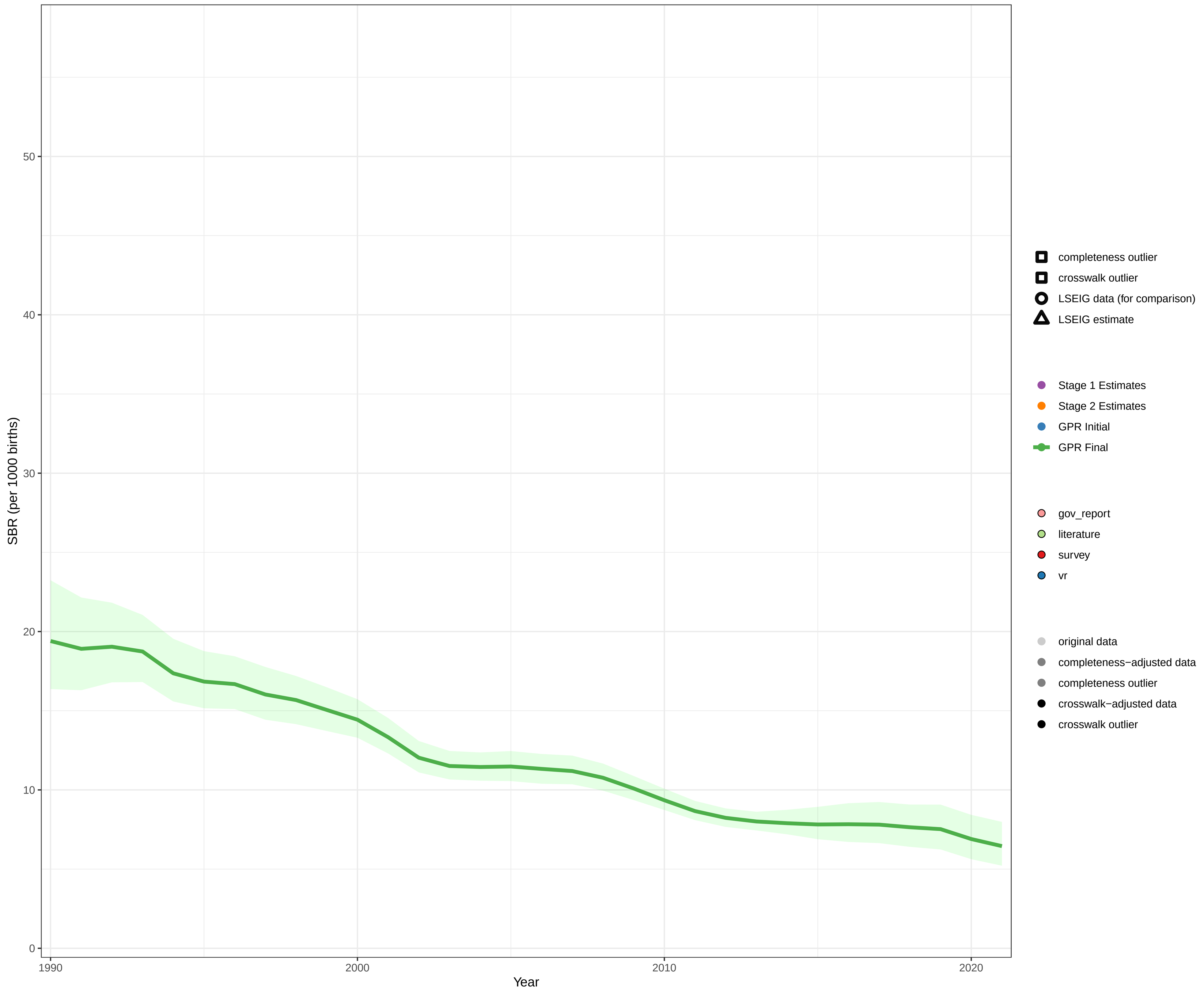

Year	Total Stillbirths (in thousands)	Total Stillbirths (in thousands)	SBR (per 1000 births)	SBR (per 1000 births)	SBR/NMR	SBR/NMR
	20 weeks	28 weeks	20 weeks	28 weeks	20 weeks	28 weeks
1990	143.9 (118.9 to 173.4)	99.3 (83.5 to 119.4)	27.9 (23.2 to 33.4)	19.4 (16.4 to 23.2)	1.56 (1.29 to 1.87)	1.08 (0.91 to 1.30)
1995	124.1 (111.5 to 138.3)	87.2 (78.3 to 97.4)	23.8 (21.4 to 26.4)	16.8 (15.2 to 18.8)	1.51 (1.36 to 1.67)	1.07 (0.96 to 1.19)
2000	101.5 (93.5 to 111.2)	72.0 (66.2 to 78.5)	20.2 (18.7 to 22.1)	14.4 (13.3 to 15.7)	1.49 (1.38 to 1.63)	1.06 (0.98 to 1.16)
2005	78.2 (72.3 to 84.5)	56.0 (51.5 to 60.9)	15.9 (14.8 to 17.2)	11.5 (10.6 to 12.5)	1.35 (1.25 to 1.46)	0.97 (0.90 to 1.06)
2010	63.9 (58.9 to 69.1)	45.7 (42.7 to 49.3)	13.0 (12.0 to 14.1)	9.3 (8.7 to 10.1)	1.22 (1.13 to 1.32)	0.88 (0.82 to 0.94)
2015	51.3 (45.3 to 58.6)	36.5 (32.1 to 41.7)	10.9 (9.7 to 12.5)	7.8 (6.9 to 8.9)	1.14 (1.01 to 1.30)	0.81 (0.72 to 0.93)
2020	39.4 (32.2 to 47.9)	27.6 (22.5 to 33.8)	9.8 (8.0 to 11.9)	6.9 (5.6 to 8.4)	1.18 (0.97 to 1.44)	0.83 (0.68 to 1.02)
2021	36.0 (29.1 to 44.5)	25.2 (20.3 to 31.2)	9.2 (7.4 to 11.3)	6.5 (5.2 to 8.0)	1.17 (0.95 to 1.44)	0.82 (0.66 to 1.02)

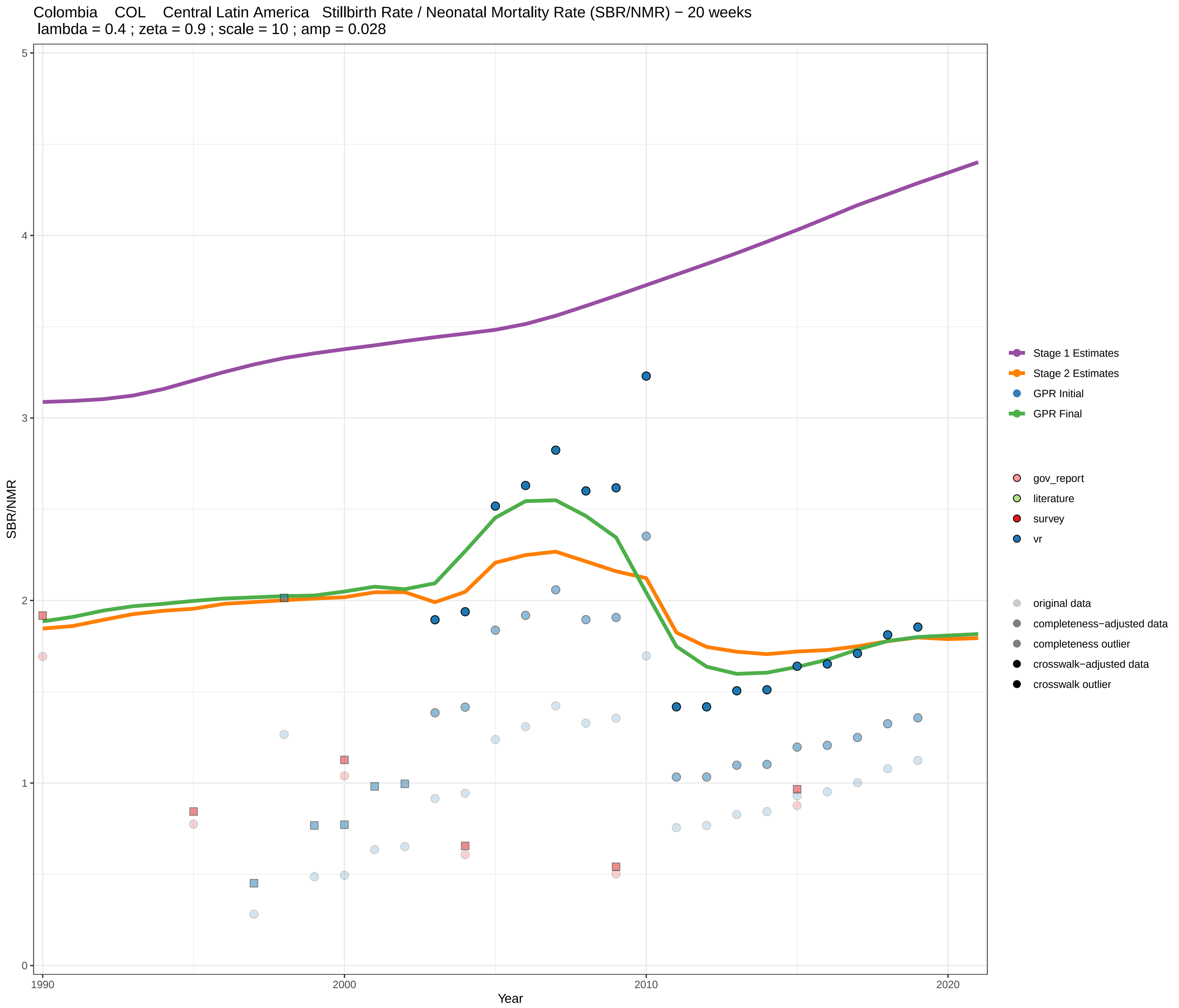

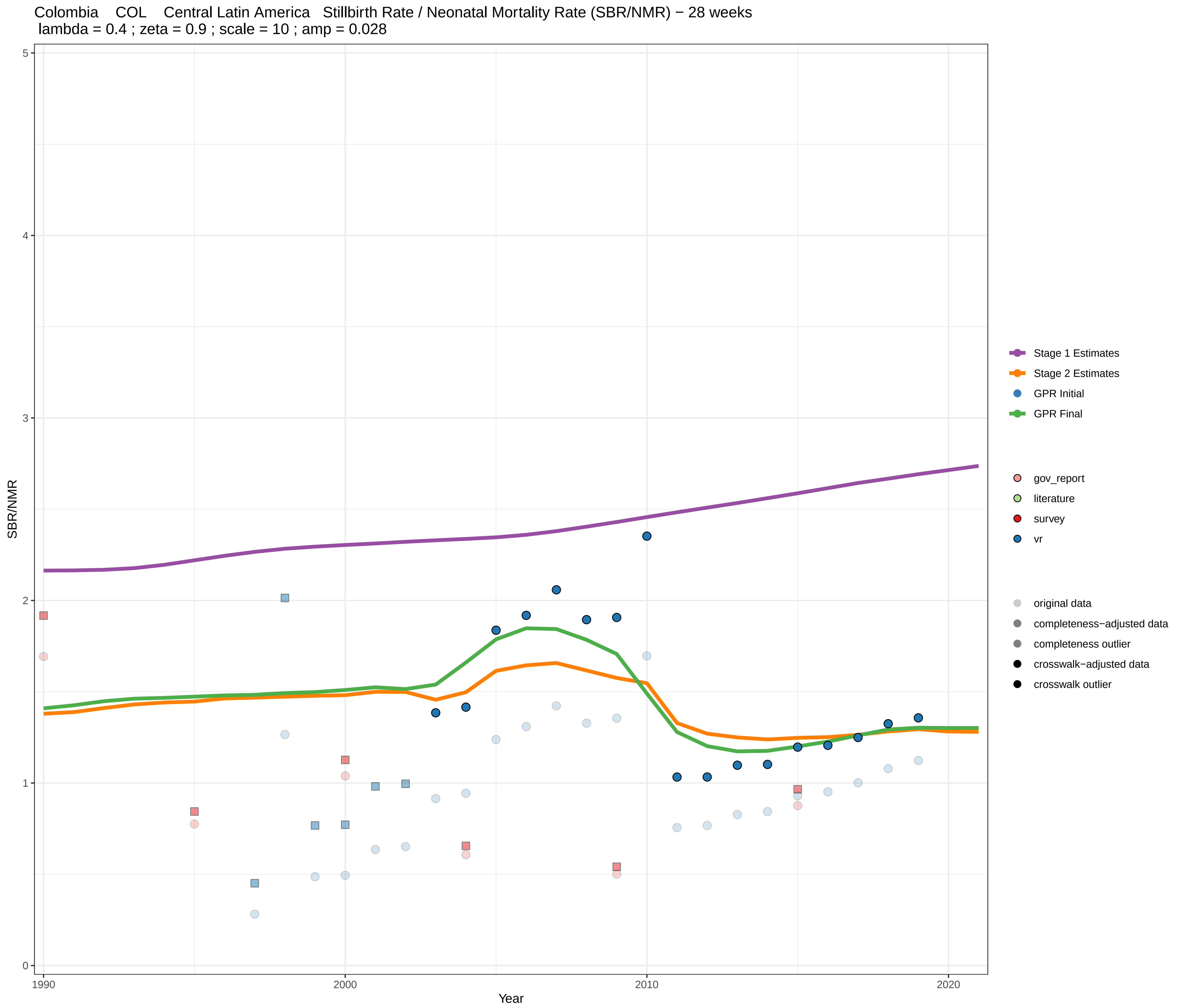

Definition	Years	Outlier	Source.Type	Title
28_weeks	2003−2019	0	VR	United Nations Demographic Yearbook
28_weeks	1990	1	Survey	Colombia Demographic and Health Survey 1990
28_weeks	1995	1	Survey	Colombia Demographic and Health Survey 1995
28_weeks	1997−2002	1	VR	United Nations Demographic Yearbook
28_weeks	2000	1	Survey	Colombia Demographic and Health Survey 2000
28_weeks	2004	1	Survey	Colombia Demographic and Health Survey 2004−2005
28_weeks	2009	1	Survey	Colombia Demographic and Health Survey 2009−2010
28_weeks	2015	1	Survey	Colombia Demographic and Health Survey 2015−2016

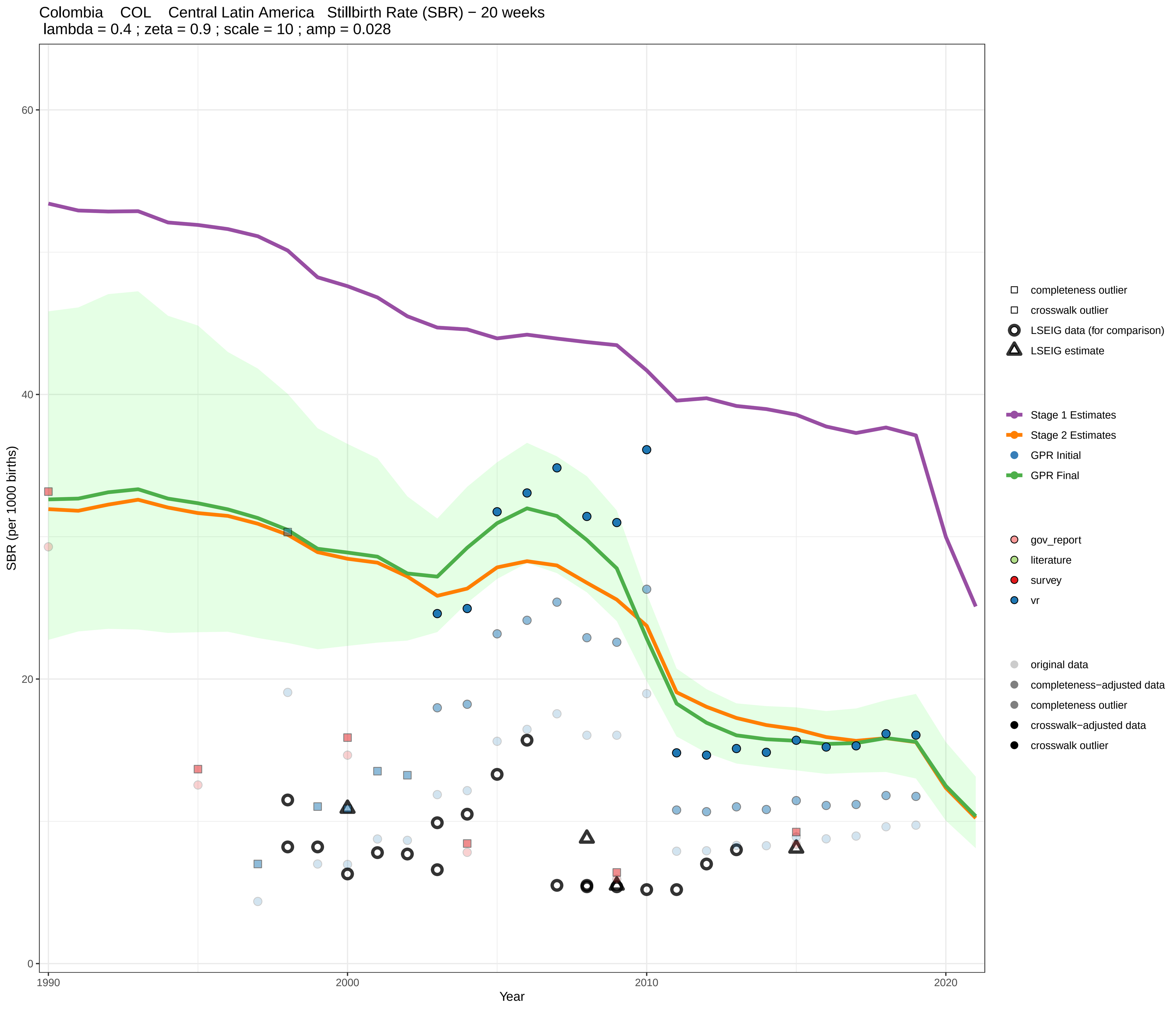

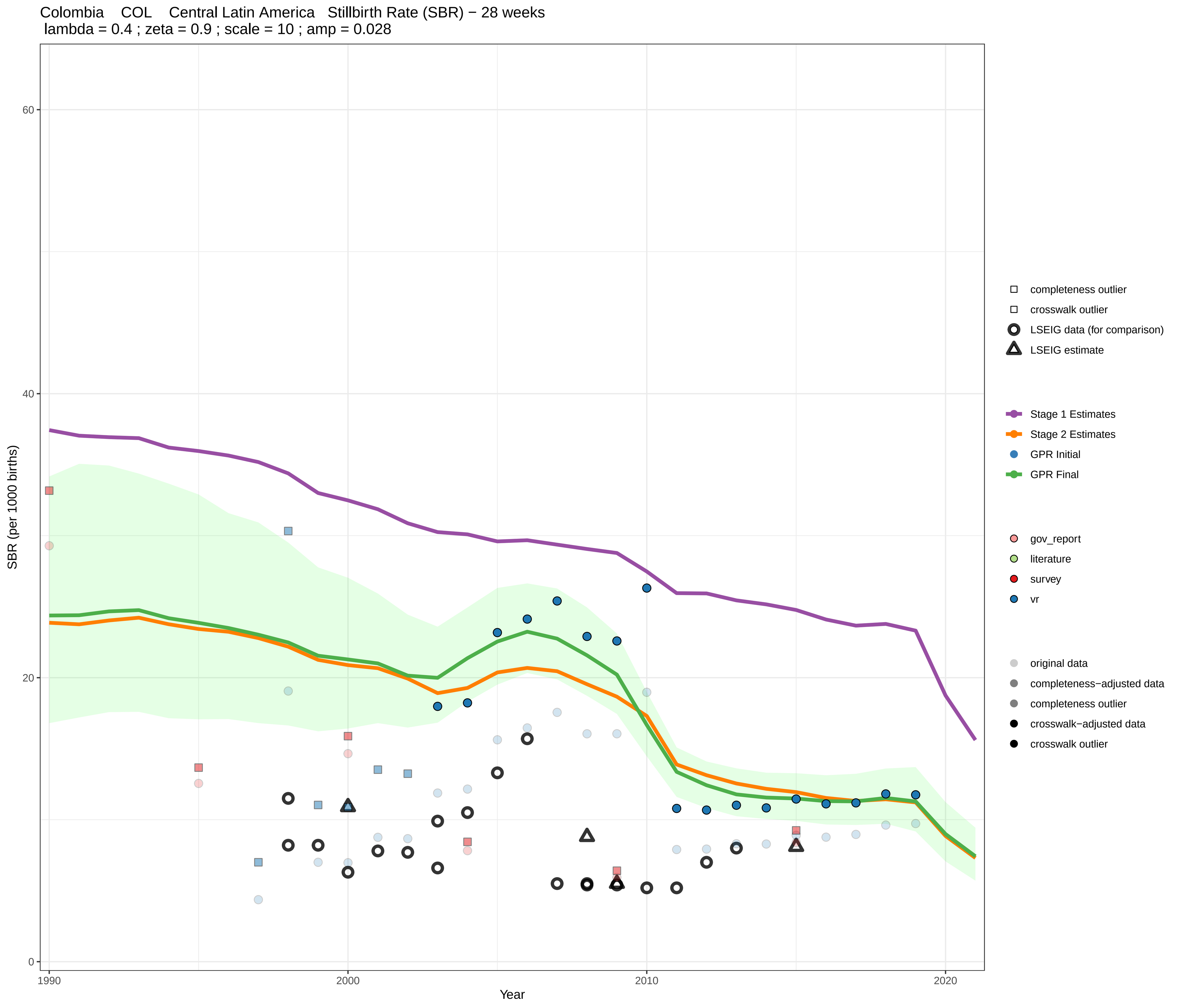

Year	Total Stillbirths (in thousands)	Total Stillbirths (in thousands)	SBR (per 1000 births)	SBR (per 1000 births)	SBR/NMR	SBR/NMR
	20 weeks	28 weeks	20 weeks	28 weeks	20 weeks	28 weeks
1990	31.2 (21.5 to 44.4)	23.1 (15.8 to 32.7)	32.6 (22.8 to 45.8)	24.4 (16.8 to 34.2)	1.89 (1.32 to 2.65)	1.41 (0.97 to 1.98)
1995	32.0 (22.8 to 44.9)	23.4 (16.6 to 32.5)	32.4 (23.3 to 44.8)	23.9 (17.1 to 32.9)	2.00 (1.44 to 2.77)	1.47 (1.05 to 2.03)
2000	26.8 (20.5 to 34.1)	19.6 (15.0 to 25.0)	28.9 (22.3 to 36.5)	21.3 (16.4 to 27.0)	2.05 (1.58 to 2.59)	1.51 (1.16 to 1.92)
2005	26.6 (23.2 to 30.5)	19.2 (16.6 to 22.5)	31.0 (27.0 to 35.2)	22.5 (19.5 to 26.3)	2.45 (2.14 to 2.79)	1.79 (1.55 to 2.09)
2010	18.1 (15.7 to 20.7)	13.1 (11.4 to 15.0)	22.9 (19.9 to 26.0)	16.7 (14.5 to 19.0)	2.04 (1.78 to 2.32)	1.49 (1.29 to 1.70)
2015	11.4 (9.9 to 13.2)	8.4 (7.2 to 9.7)	15.7 (13.6 to 18.0)	11.5 (9.9 to 13.3)	1.64 (1.42 to 1.88)	1.20 (1.04 to 1.39)
2020	8.7 (7.0 to 10.9)	6.2 (4.9 to 7.8)	12.5 (10.0 to 15.6)	9.0 (7.1 to 11.2)	1.81 (1.45 to 2.26)	1.30 (1.02 to 1.63)
2021	7.1 (5.5 to 9.0)	5.0 (3.9 to 6.4)	10.4 (8.1 to 13.1)	7.4 (5.7 to 9.4)	1.82 (1.42 to 2.30)	1.30 (1.00 to 1.65)

Costa Rica CRI Central Latin America Stillbirth Rate / Neonatal Mortality Rate (SBR/NMR) − 20 weeks
lambda = 0.2 ; zeta = 0.99 ; scale = 5 ; amp = 0.028

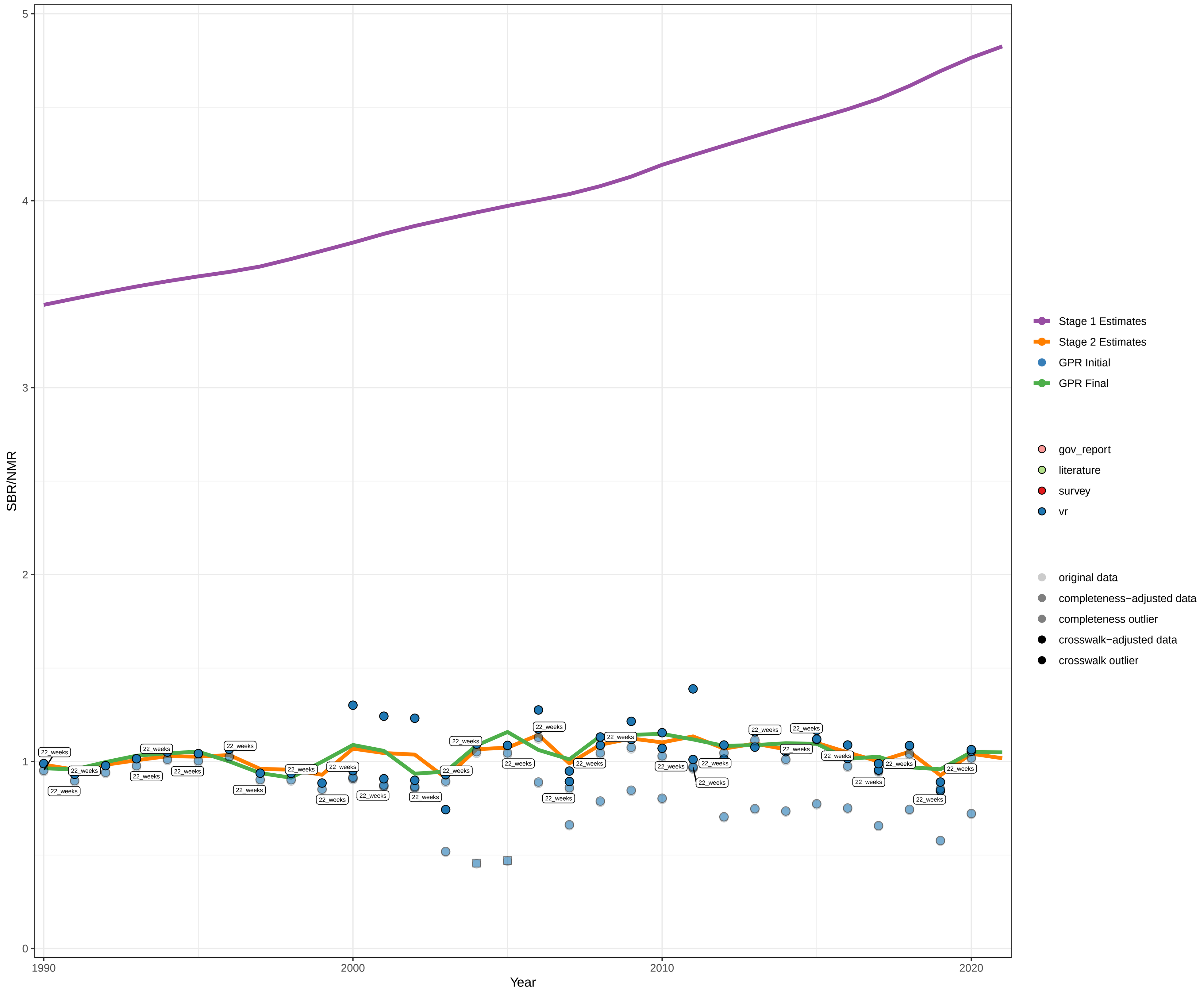

Costa Rica CRI Central Latin America Stillbirth Rate / Neonatal Mortality Rate (SBR/NMR) − 28 weeks
lambda = 0.2 ; zeta = 0.99 ; scale = 5 ; amp = 0.028

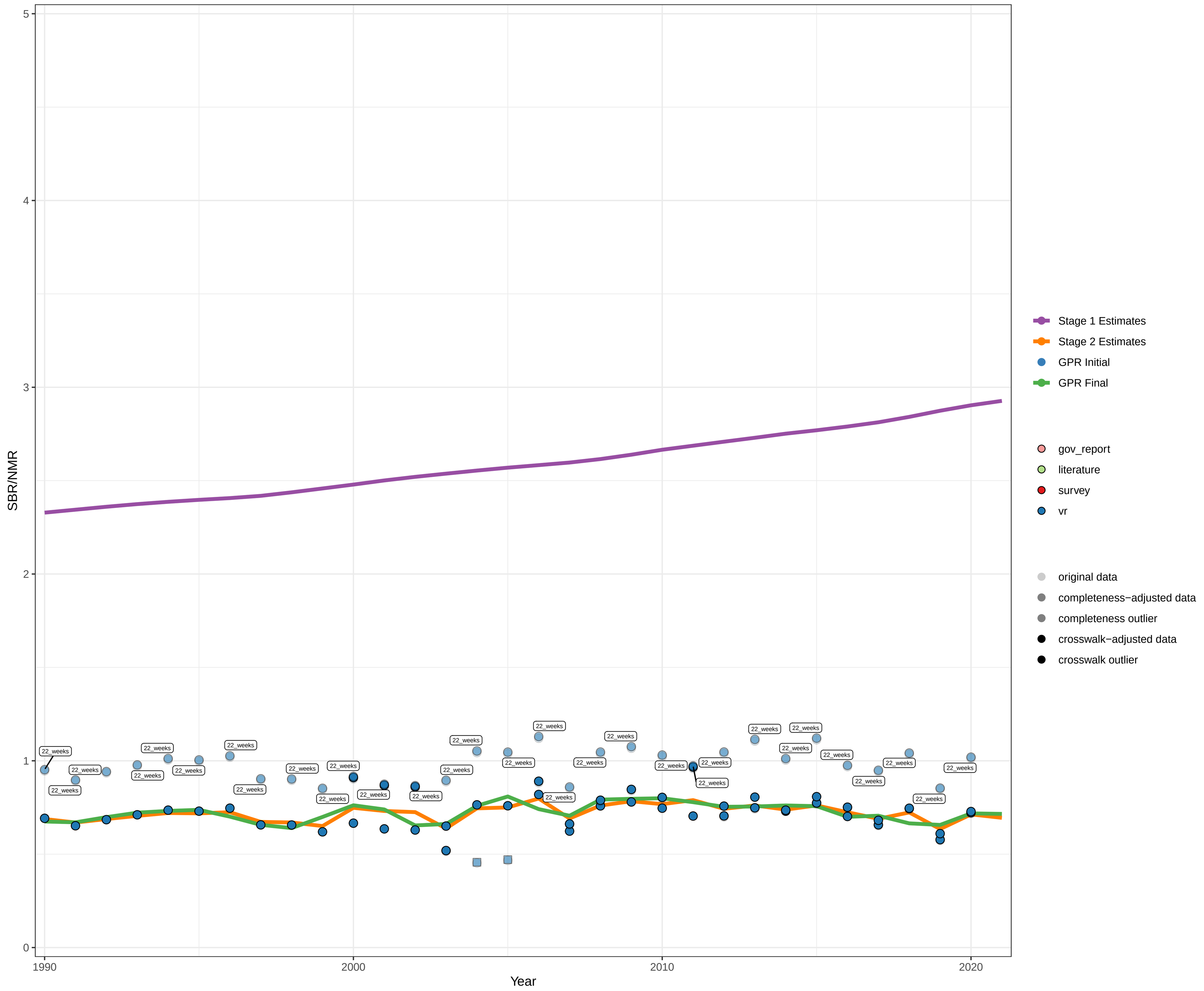

Definition	Years	Outlier	Source.Type	Title
22_weeks	1990−2020	0	VR	Costa Rica Main Demographic Indicators 1950−
28_weeks	2000−2003, 2006−2020	0	VR	United Nations Demographic Yearbook
28_weeks	2004, 2005	1	VR	United Nations Demographic Yearbook

Costa Rica CRI Central Latin America Stillbirth Rate (SBR) − 20 weeks
lambda = 0.2 ; zeta = 0.99 ; scale = 5 ; amp = 0.028

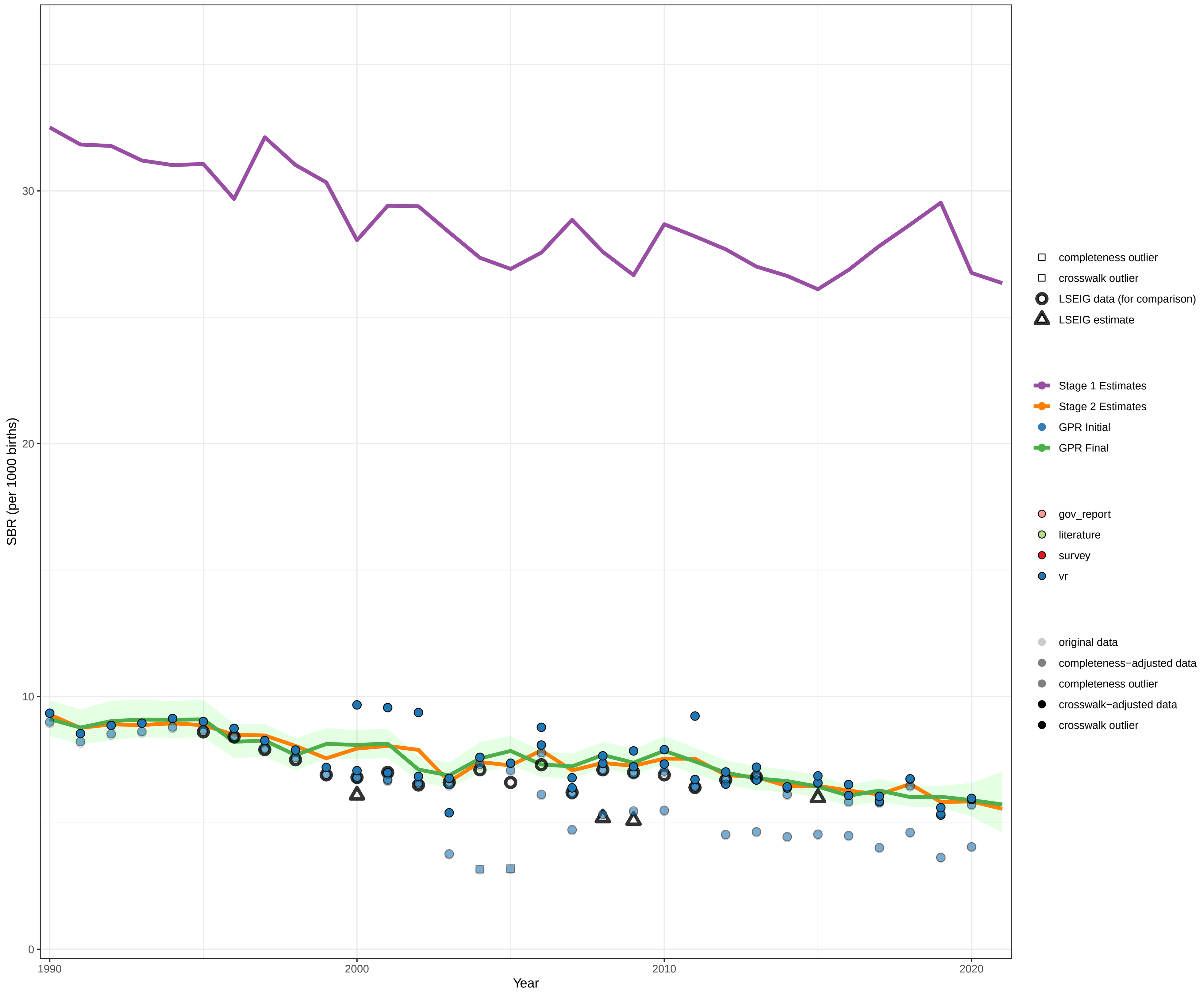

Costa Rica CRI Central Latin America Stillbirth Rate (SBR) − 28 weeks
lambda = 0.2 ; zeta = 0.99 ; scale = 5 ; amp = 0.028

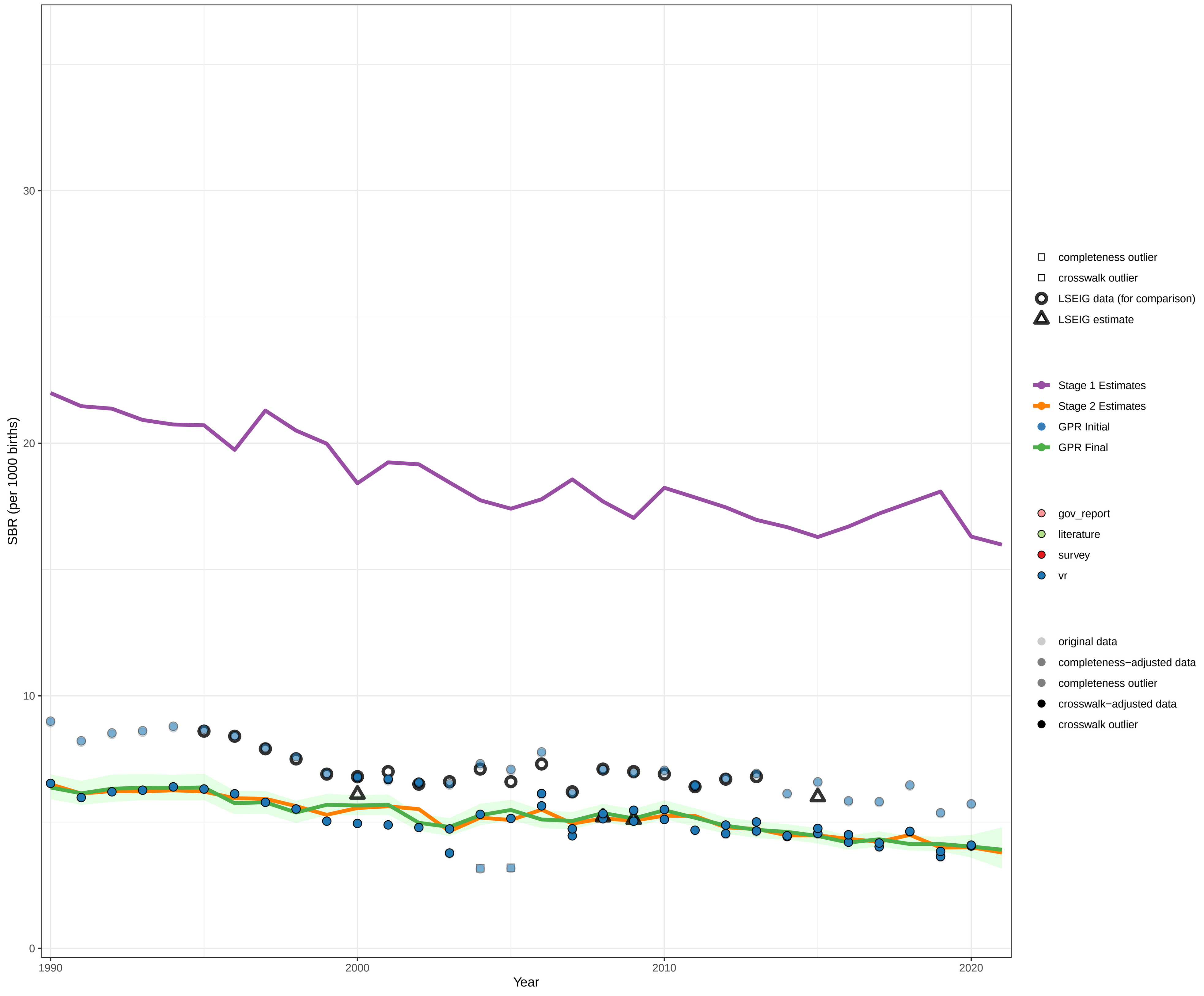

Year	Total Stillbirths (in thousands)	Total Stillbirths (in thousands)	SBR (per 1000 births)	SBR (per 1000 births)	SBR/NMR	SBR/NMR
	20 weeks	28 weeks	20 weeks	28 weeks	20 weeks	28 weeks
1990	0.7<br>(0.7 to 0.8)	0.5<br>(0.5 to 0.6)	9.1<br>(8.4 to 9.9)	6.4<br>(5.9 to 6.9)	0.96<br>(0.89 to 1.04)	0.67<br>(0.63 to 0.73)
1995	0.7<br>(0.7 to 0.8)	0.5<br>(0.5 to 0.5)	9.1<br>(8.4 to 9.9)	6.4<br>(5.9 to 6.9)	1.05<br>(0.97 to 1.14)	0.74<br>(0.68 to 0.80)
2000	0.6<br>(0.6 to 0.7)	0.4<br>(0.4 to 0.5)	8.1<br>(7.5 to 8.7)	5.7<br>(5.3 to 6.1)	1.09<br>(1.01 to 1.17)	0.76<br>(0.71 to 0.82)
2005	0.6<br>(0.5 to 0.6)	0.4<br>(0.4 to 0.4)	7.8<br>(7.3 to 8.4)	5.5<br>(5.1 to 5.9)	1.16<br>(1.07 to 1.25)	0.81<br>(0.75 to 0.87)
2010	0.6<br>(0.5 to 0.6)	0.4<br>(0.4 to 0.4)	7.9<br>(7.3 to 8.4)	5.5<br>(5.1 to 5.9)	1.15<br>(1.07 to 1.23)	0.80<br>(0.75 to 0.86)
2015	0.5<br>(0.4 to 0.5)	0.3<br>(0.3 to 0.3)	6.4<br>(6.0 to 6.9)	4.5<br>(4.2 to 4.8)	1.09<br>(1.02 to 1.17)	0.76<br>(0.71 to 0.81)
2020	0.3<br>(0.3 to 0.4)	0.2<br>(0.2 to 0.3)	5.9<br>(5.2 to 6.6)	4.0<br>(3.6 to 4.5)	1.05<br>(0.93 to 1.17)	0.72<br>(0.64 to 0.80)
2021	0.3<br>(0.3 to 0.4)	0.2<br>(0.2 to 0.3)	5.7<br>(4.6 to 7.0)	3.9<br>(3.2 to 4.8)	1.05<br>(0.84 to 1.29)	0.72<br>(0.58 to 0.88)

El Salvador SLV Central Latin America Stillbirth Rate / Neonatal Mortality Rate (SBR/NMR) − 20 weeks
lambda = 0.4 ; zeta = 0.9 ; scale = 10 ; amp = 0.028

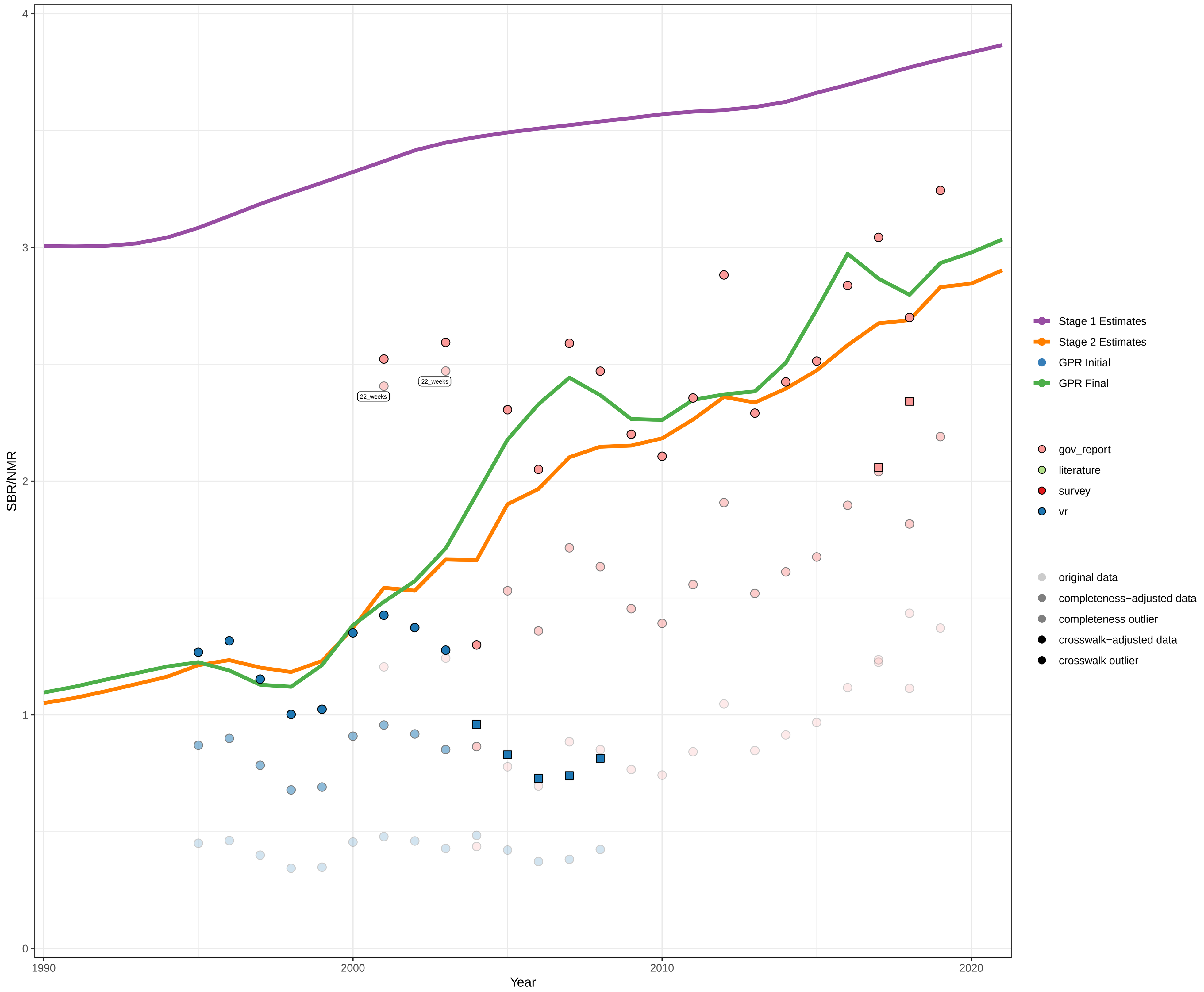

El Salvador SLV Central Latin America Stillbirth Rate / Neonatal Mortality Rate (SBR/NMR) − 28 weeks
lambda = 0.4 ; zeta = 0.9 ; scale = 10 ; amp = 0.028

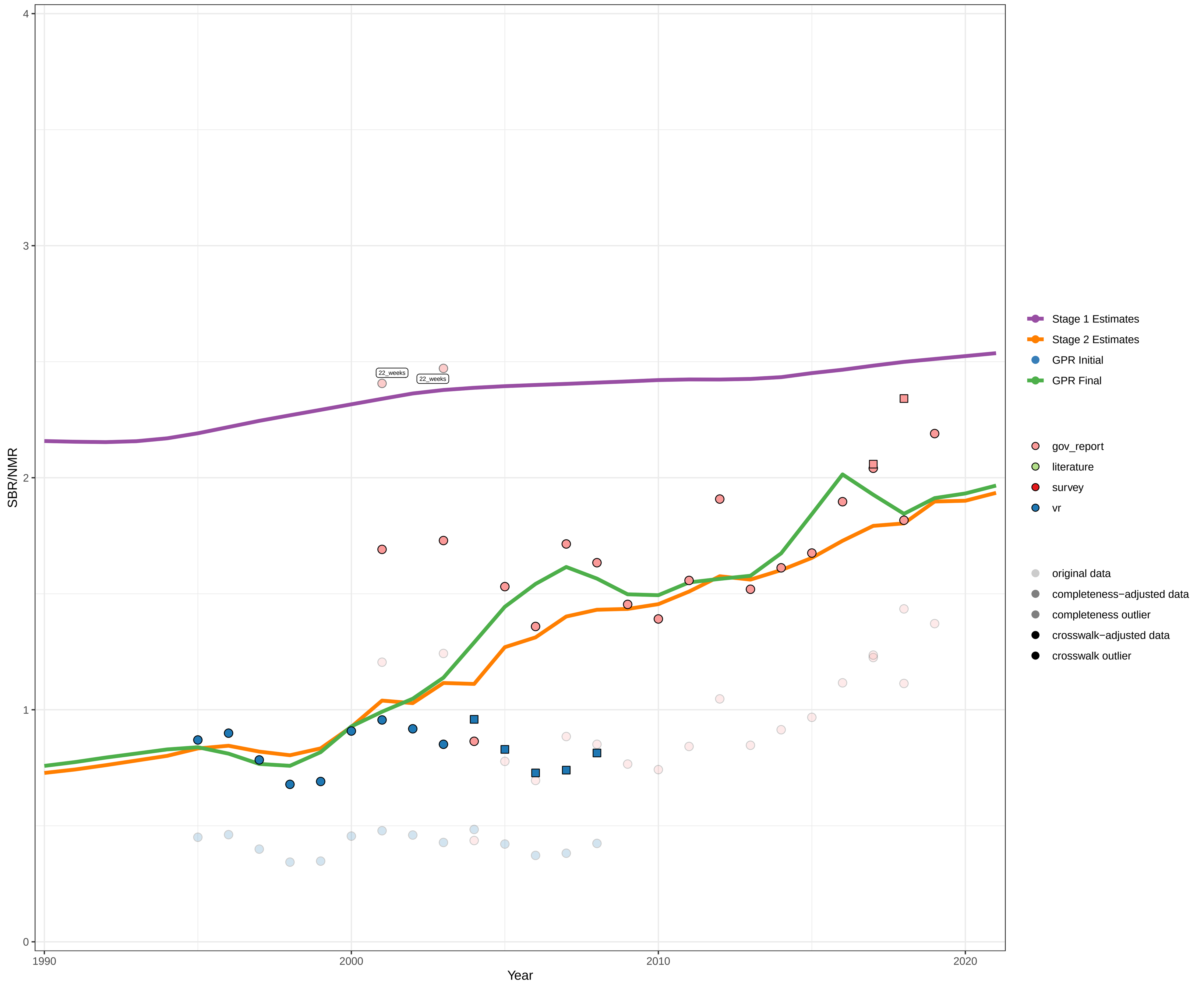

Definition	Years	Outlier	Source.Type	Title
22_weeks	2001, 2003	0	Statistical Report	El Salvador Health Statistical Bulletin
28_weeks	1995−2003	0	VR	United Nations Demographic Yearbook
28_weeks	2004−2009	0	Statistical Report	El Salvador Health Statistical Bulletin
28_weeks	2010−2019	0	Statistical Report	El Salvador Health Statistical Bulletin
28_weeks	2004−2008	2	VR	United Nations Demographic Yearbook
28_weeks	2017, 2018	2	Statistical Report	El Salvador Health Statistical Bulletin

El Salvador SLV Central Latin America Stillbirth Rate (SBR) − 20 weeks
lambda = 0.4 ; zeta = 0.9 ; scale = 10 ; amp = 0.028

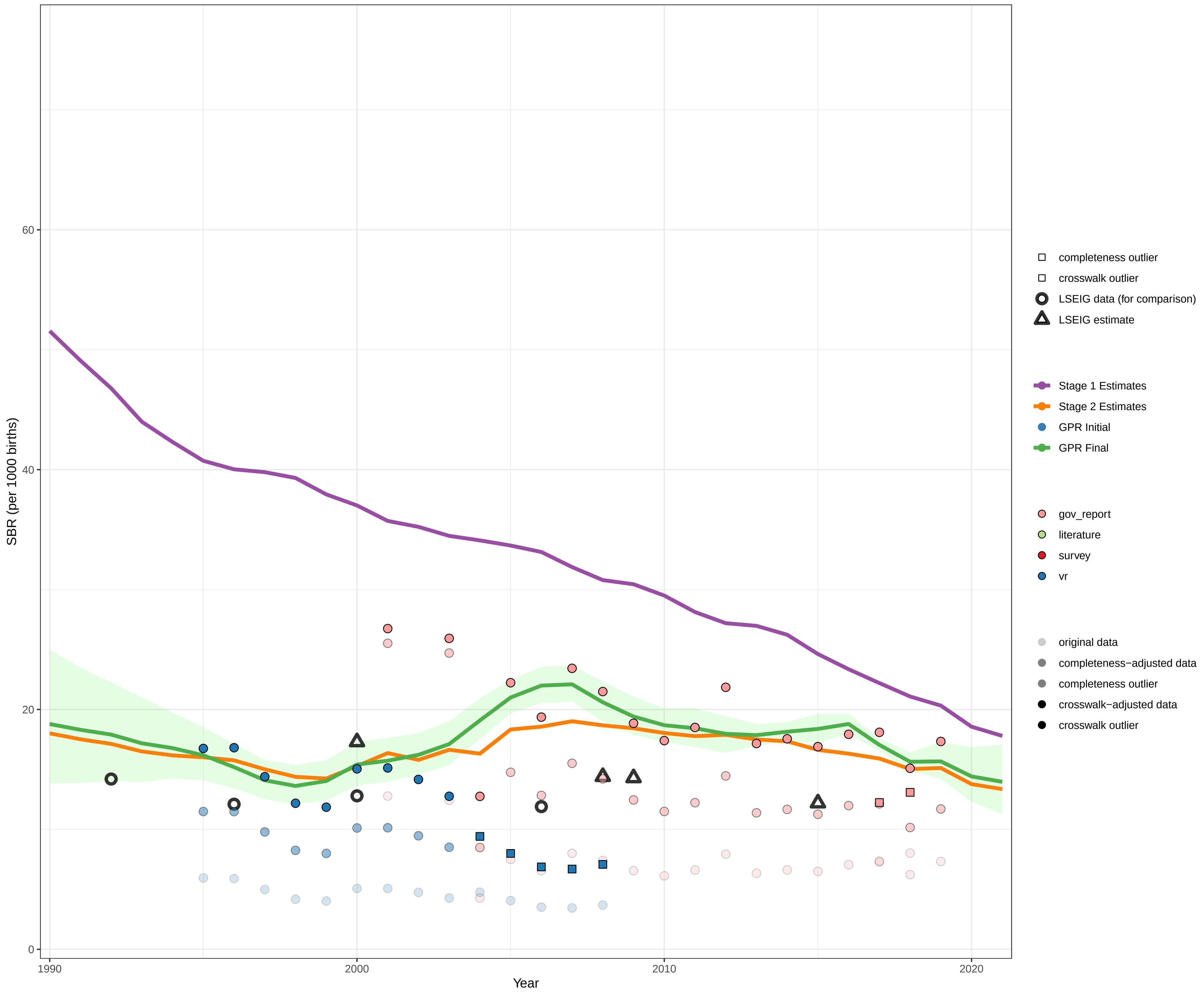

El Salvador SLV Central Latin America Stillbirth Rate (SBR) − 28 weeks
lambda = 0.4 ; zeta = 0.9 ; scale = 10 ; amp = 0.028

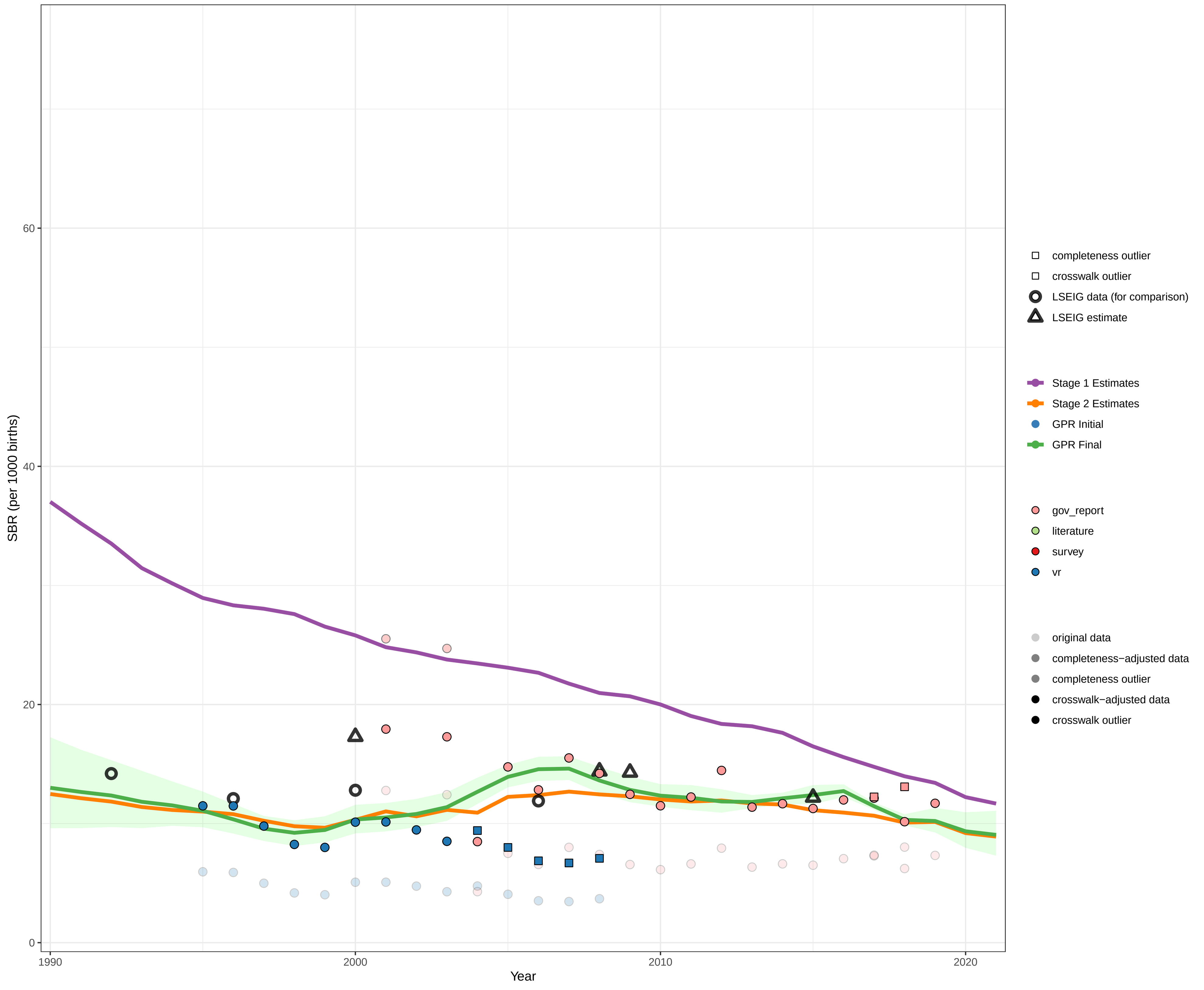

Year	Total Stillbirths (in thousands)	Total Stillbirths (in thousands)	SBR (per 1000 births)	SBR (per 1000 births)	SBR/NMR	SBR/NMR
	20 weeks	28 weeks	20 weeks	28 weeks	20 weeks	28 weeks
1990	3.4<br>(2.5 to 4.5)	2.3<br>(1.7 to 3.1)	18.8<br>(13.8 to 25.0)	13.0<br>(9.6 to 17.3)	1.10<br>(0.81 to 1.46)	0.76<br>(0.56 to 1.01)
1995	3.1<br>(2.7 to 3.6)	2.1<br>(1.8 to 2.4)	16.2<br>(14.1 to 18.5)	11.1<br>(9.7 to 12.7)	1.23<br>(1.07 to 1.40)	0.84<br>(0.73 to 0.96)
2000	2.4<br>(2.1 to 2.7)	1.6<br>(1.4 to 1.8)	15.4<br>(13.6 to 17.3)	10.3<br>(9.2 to 11.6)	1.38<br>(1.23 to 1.55)	0.93<br>(0.82 to 1.04)
2005	2.8<br>(2.7 to 3.0)	1.9<br>(1.8 to 2.0)	21.0<br>(19.7 to 22.5)	13.9<br>(13.0 to 14.9)	2.18<br>(2.04 to 2.33)	1.44<br>(1.35 to 1.55)
2010	2.5<br>(2.3 to 2.7)	1.7<br>(1.5 to 1.8)	18.7<br>(17.3 to 20.1)	12.3<br>(11.4 to 13.3)	2.26<br>(2.09 to 2.43)	1.49<br>(1.38 to 1.61)
2015	2.5<br>(2.3 to 2.6)	1.7<br>(1.5 to 1.8)	18.4<br>(17.3 to 19.6)	12.4<br>(11.6 to 13.2)	2.73<br>(2.57 to 2.92)	1.84<br>(1.73 to 1.97)
2020	1.7<br>(1.5 to 2.0)	1.1<br>(1.0 to 1.3)	14.4<br>(12.3 to 16.9)	9.4<br>(8.0 to 11.0)	2.98<br>(2.54 to 3.48)	1.93<br>(1.64 to 2.26)
2021	1.6<br>(1.3 to 2.0)	1.1<br>(0.9 to 1.3)	14.0<br>(11.3 to 17.1)	9.1<br>(7.3 to 11.1)	3.03<br>(2.45 to 3.71)	1.97<br>(1.59 to 2.41)

Guatemala GTM Central Latin America Stillbirth Rate / Neonatal Mortality Rate (SBR/NMR) − 20 weeks
lambda = 0.2 ; zeta = 0.99 ; scale = 5 ; amp = 0.028

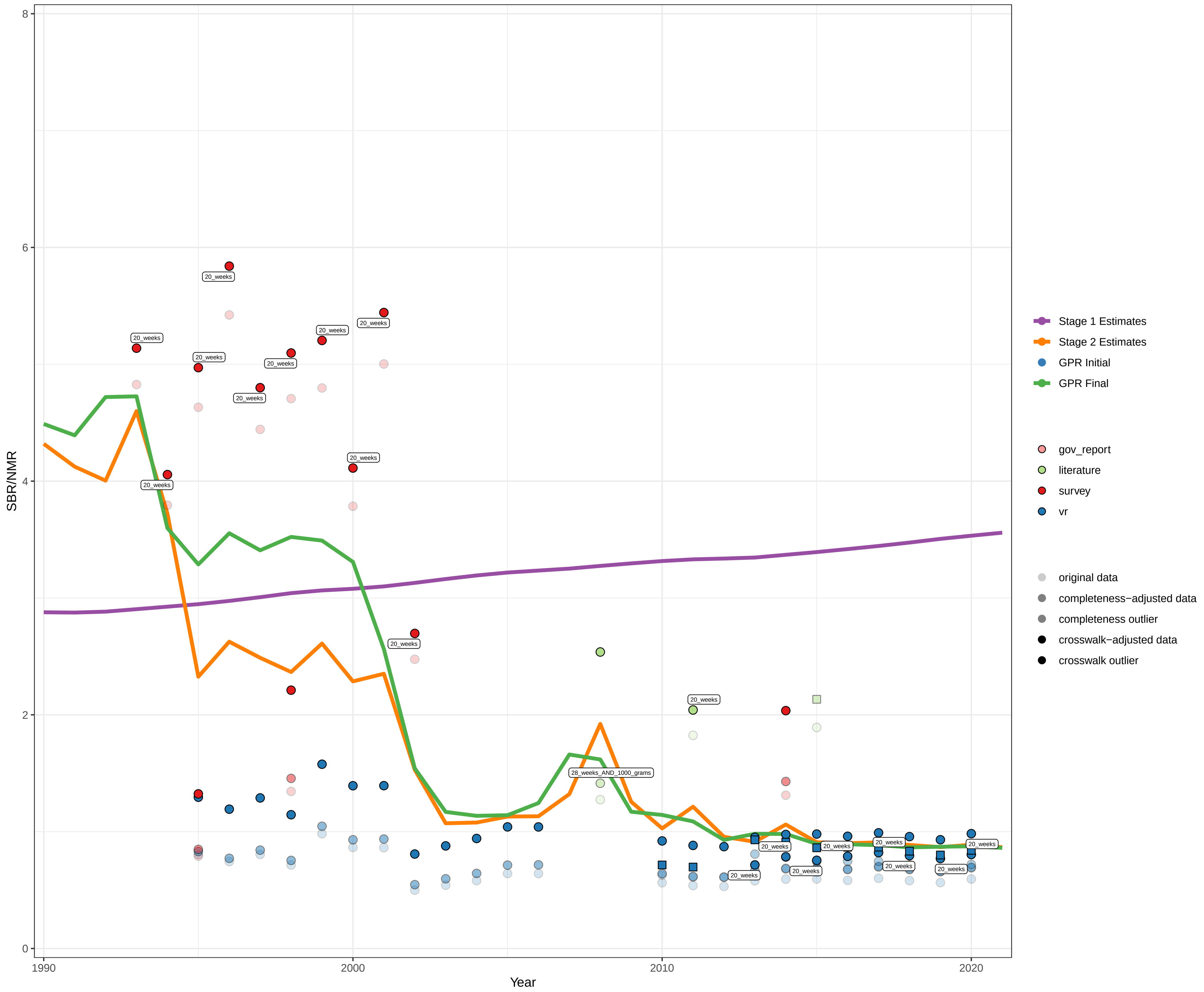

Guatemala GTM Central Latin America Stillbirth Rate / Neonatal Mortality Rate (SBR/NMR) − 28 weeks
lambda = 0.2 ; zeta = 0.99 ; scale = 5 ; amp = 0.028

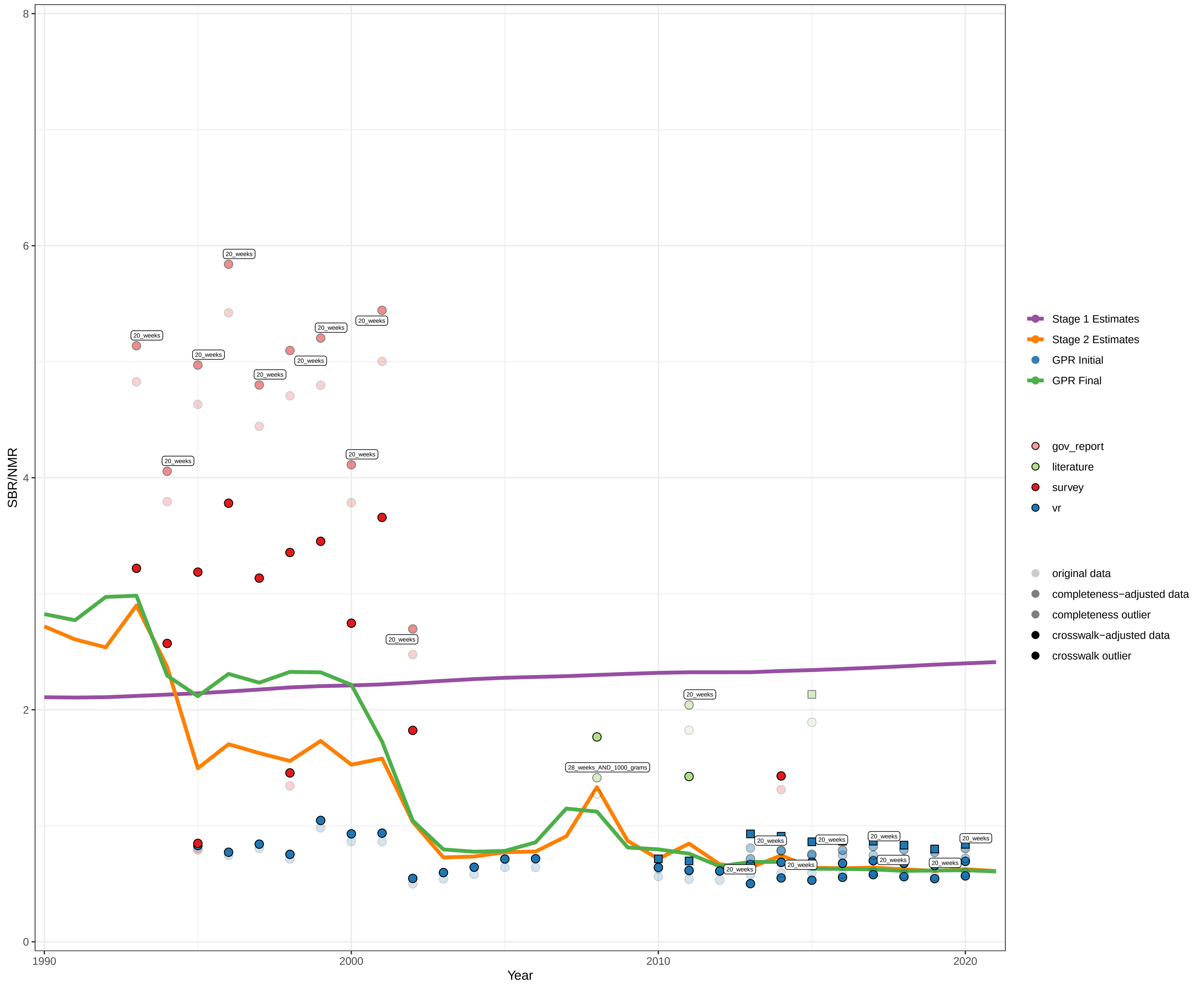

Definition	Years	Outlier	Source.Type	Title
20_weeks	1993−2002	0	Survey	Guatemala Reproductive Health Survey 2002
20_weeks	2011	0	Sci Lit	A prospective study of maternal, fetal and neonatal deaths in low− and middle−income countries
20_weeks	2012−2020	0	VR	Guatemala Vital Statistics
28_weeks	1995	0	Survey	Guatemala Demographic and Health Survey 1995
28_weeks	1995−2006	0	VR	United Nations Demographic Yearbook
28_weeks	1998	0	Survey	Guatemala Interim Demographic and Health Survey 1998−1999
28_weeks	2010−2020	0	VR	Guatemala Vital Statistics
28_weeks	2014	0	Survey	Guatemala Demographic and Health Survey 2014−2015
28_weeks_AND_1000_grams	2008	0	Sci Lit	Epidemiology of stillbirth in low−middle income countries: A Global Network Study
20_weeks	2015	1	Sci Lit	Global Network for Women's and Children's Health Research: probable causes of stillbirth in low− and middle−income countries using a prospectively defined classification system
other	2013−2020	1	VR	Guatemala Vital Statistics
20_weeks	2010, 2011	2	VR	Guatemala Vital Statistics
28_weeks	2013−2020	2	VR	United Nations Demographic Yearbook

Guatemala GTM Central Latin America Stillbirth Rate (SBR) − 20 weeks
lambda = 0.2 ; zeta = 0.99 ; scale = 5 ; amp = 0.028

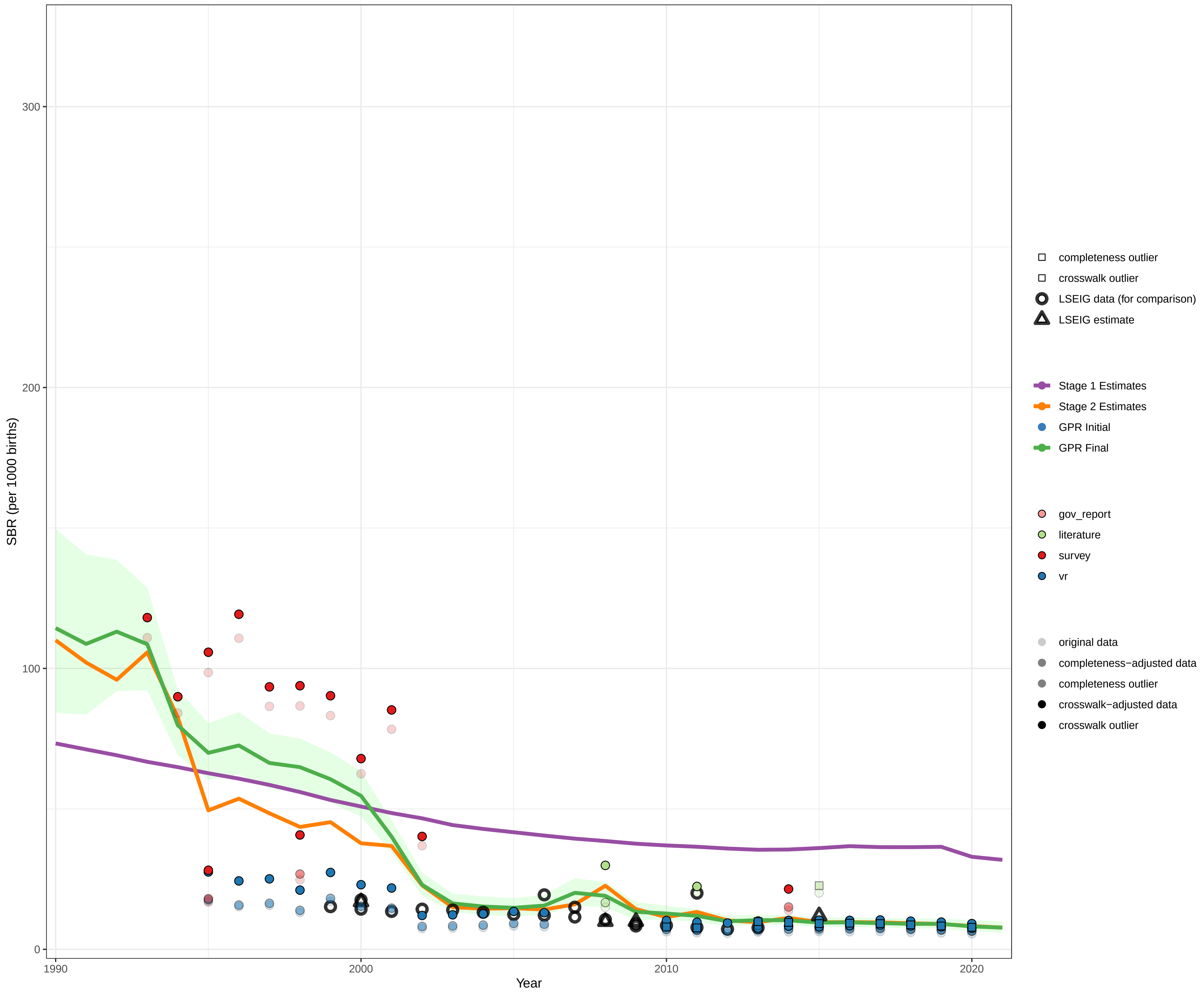

Guatemala GTM Central Latin America Stillbirth Rate (SBR) − 28 weeks
lambda = 0.2 ; zeta = 0.99 ; scale = 5 ; amp = 0.028

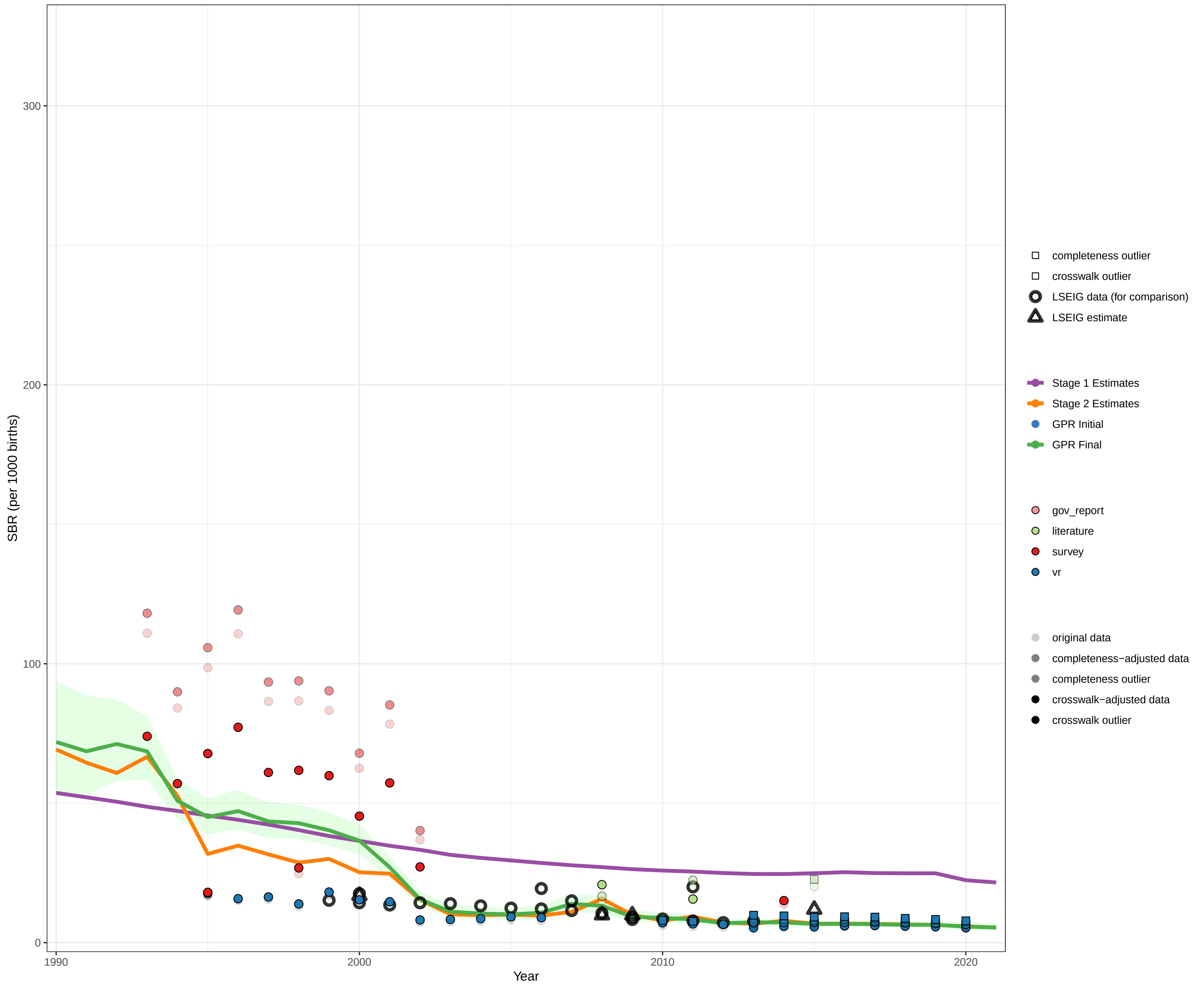

Year	Total Stillbirths (in thousands)	Total Stillbirths (in thousands)	SBR (per 1000 births)	SBR (per 1000 births)	SBR/NMR	SBR/NMR
	20 weeks	28 weeks	20 weeks	28 weeks	20 weeks	28 weeks
1990	44.3 (31.4 to 60.1)	26.5 (19.2 to 35.4)	114.3 (84.3 to 149.6)	72.0 (53.2 to 93.9)	4.49 (3.31 to 5.87)	2.82 (2.09 to 3.69)
1995	27.6 (23.5 to 32.1)	17.3 (14.8 to 20.0)	69.9 (60.4 to 80.5)	45.0 (38.9 to 51.8)	3.29 (2.84 to 3.79)	2.12 (1.83 to 2.44)
2000	22.2 (19.1 to 26.0)	14.6 (12.6 to 17.0)	54.6 (47.3 to 63.4)	36.6 (31.7 to 42.4)	3.31 (2.86 to 3.84)	2.22 (1.92 to 2.57)
2005	5.5 (4.4 to 6.8)	3.8 (3.0 to 4.7)	14.8 (11.7 to 18.2)	10.2 (8.0 to 12.5)	1.14 (0.90 to 1.41)	0.78 (0.62 to 0.96)
2010	4.7 (3.8 to 5.8)	3.3 (2.7 to 4.0)	12.8 (10.4 to 15.5)	8.9 (7.3 to 10.8)	1.14 (0.93 to 1.39)	0.80 (0.65 to 0.97)
2015	3.7 (3.1 to 4.4)	2.6 (2.2 to 3.1)	9.5 (7.9 to 11.3)	6.7 (5.6 to 7.9)	0.90 (0.74 to 1.06)	0.63 (0.52 to 0.75)
2020	2.9 (2.3 to 3.7)	2.0 (1.6 to 2.6)	8.2 (6.4 to 10.4)	5.8 (4.6 to 7.3)	0.88 (0.69 to 1.11)	0.62 (0.49 to 0.78)
2021	2.7 (2.0 to 3.5)	1.9 (1.4 to 2.4)	7.7 (5.8 to 10.0)	5.4 (4.1 to 7.0)	0.86 (0.65 to 1.12)	0.61 (0.46 to 0.78)

Honduras HND Central Latin America Stillbirth Rate / Neonatal Mortality Rate (SBR/NMR) − 20 weeks
lambda = 0.8 ; zeta = 0.7 ; scale = 15 ; amp = 0.028

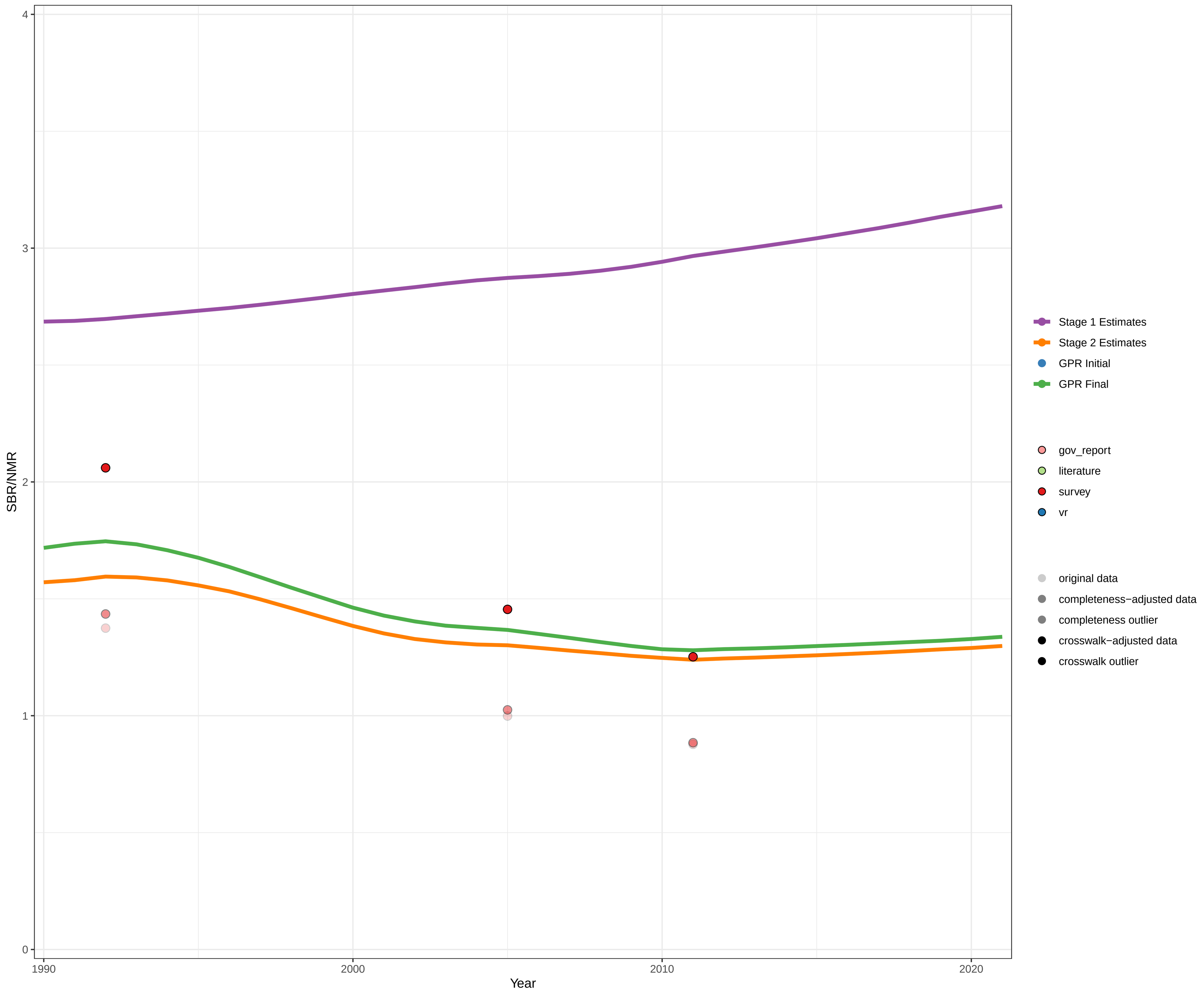

Honduras HND Central Latin America Stillbirth Rate / Neonatal Mortality Rate (SBR/NMR) − 28 weeks
lambda = 0.8 ; zeta = 0.7 ; scale = 15 ; amp = 0.028

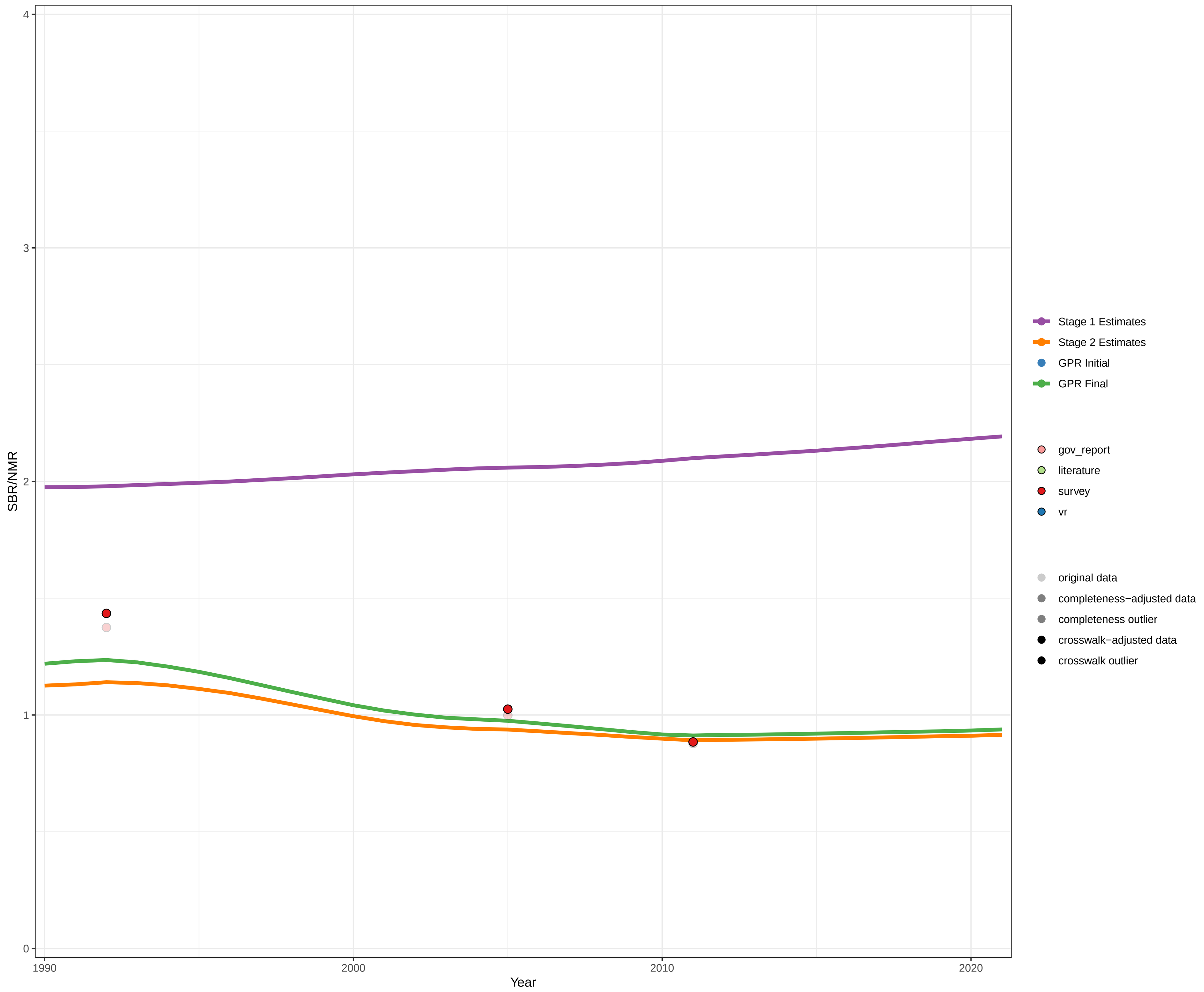

Definition	Years	Outlier	Source.Type	Title
28_weeks	1992	0	Survey	Honduras Family Planning/Maternal and Child Survey 1991−1992
28_weeks	2005	0	Survey	Honduras Demographic and Health Survey 2005−2006
28_weeks	2011	0	Survey	Honduras Demographic and Health Survey 2011−2012

Honduras HND Central Latin America Stillbirth Rate (SBR) − 20 weeks
lambda = 0.8 ; zeta = 0.7 ; scale = 15 ; amp = 0.028

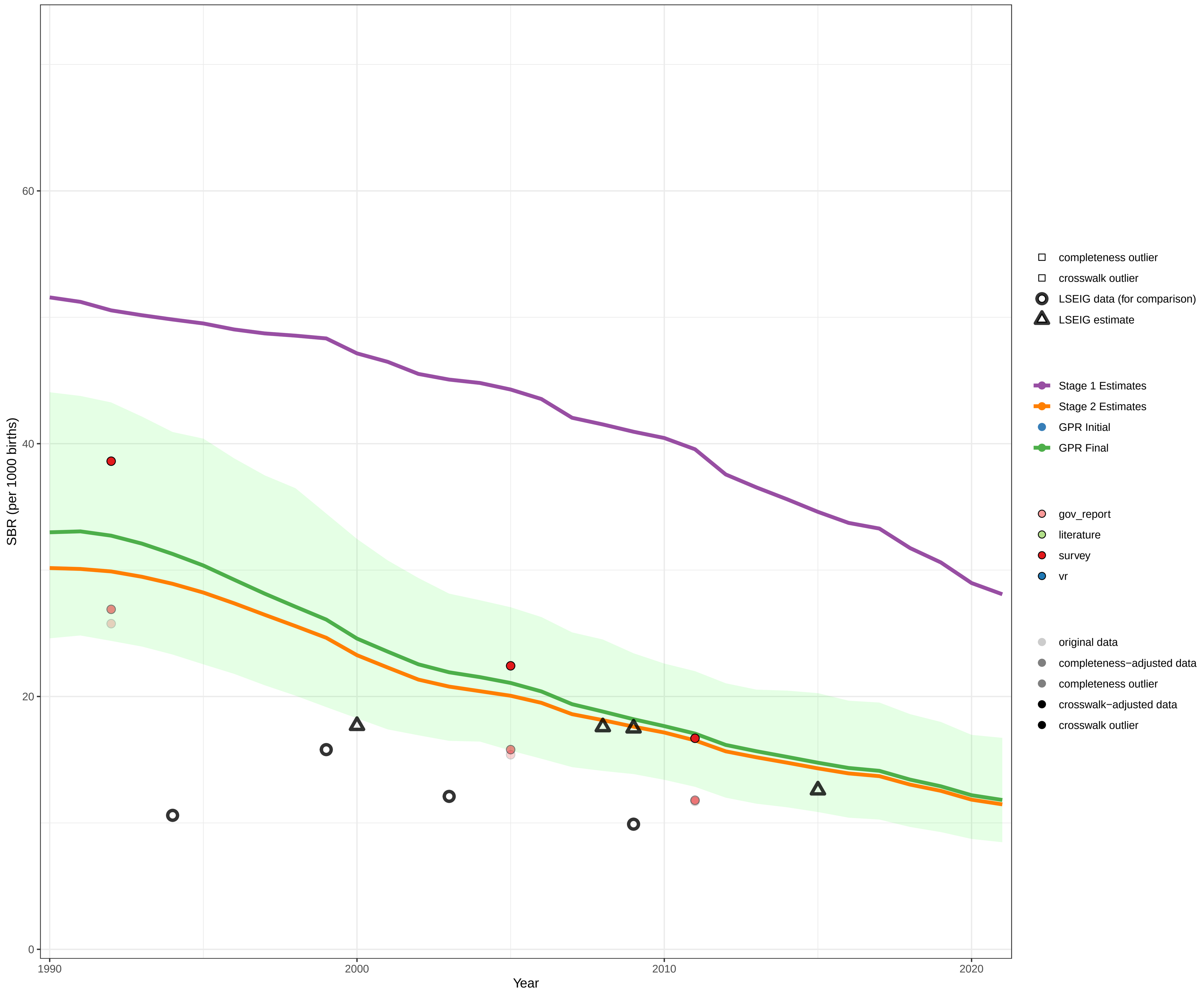

Honduras HND Central Latin America Stillbirth Rate (SBR) − 28 weeks
lambda = 0.8 ; zeta = 0.7 ; scale = 15 ; amp = 0.028

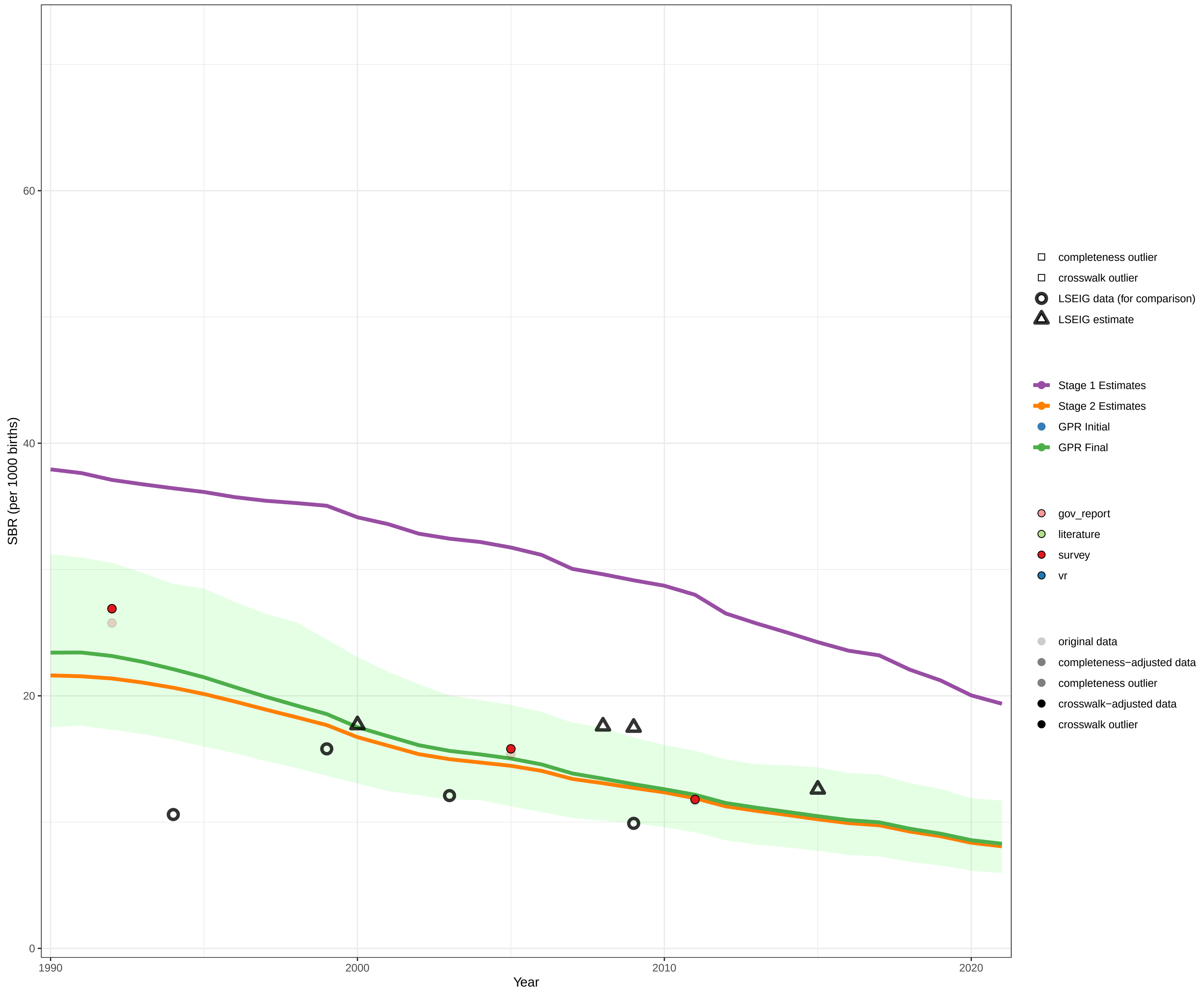

Year	Total Stillbirths (in thousands)	Total Stillbirths (in thousands)	SBR (per 1000 births)	SBR (per 1000 births)	SBR/NMR	SBR/NMR
	20 weeks	28 weeks	20 weeks	28 weeks	20 weeks	28 weeks
1990	6.1 (4.5 to 8.3)	4.3 (3.2 to 5.8)	33.0 (24.6 to 44.1)	23.4 (17.5 to 31.2)	1.72 (1.28 to 2.30)	1.22 (0.91 to 1.63)
1995	5.9 (4.4 to 7.9)	4.1 (3.1 to 5.5)	30.4 (22.5 to 40.4)	21.5 (16.0 to 28.5)	1.68 (1.24 to 2.23)	1.18 (0.88 to 1.57)
2000	4.9 (3.6 to 6.5)	3.5 (2.6 to 4.6)	24.6 (18.3 to 32.5)	17.5 (13.1 to 23.1)	1.46 (1.09 to 1.93)	1.04 (0.78 to 1.37)
2005	4.2 (3.1 to 5.5)	3.0 (2.2 to 3.9)	21.1 (15.7 to 27.1)	15.0 (11.2 to 19.3)	1.37 (1.02 to 1.76)	0.98 (0.73 to 1.25)
2010	3.7 (2.8 to 4.8)	2.6 (2.0 to 3.4)	17.7 (13.4 to 22.6)	12.6 (9.6 to 16.1)	1.28 (0.98 to 1.64)	0.92 (0.70 to 1.17)
2015	3.2 (2.4 to 4.5)	2.3 (1.7 to 3.1)	14.8 (10.9 to 20.3)	10.5 (7.7 to 14.3)	1.30 (0.95 to 1.78)	0.92 (0.68 to 1.26)
2020	2.7 (1.9 to 3.8)	1.9 (1.4 to 2.6)	12.2 (8.7 to 17.0)	8.6 (6.2 to 11.9)	1.33 (0.95 to 1.85)	0.93 (0.67 to 1.30)
2021	2.6 (1.9 to 3.8)	1.8 (1.3 to 2.6)	11.8 (8.5 to 16.7)	8.3 (6.0 to 11.7)	1.34 (0.96 to 1.89)	0.94 (0.67 to 1.32)

Mexico MEX Central Latin America Stillbirth Rate / Neonatal Mortality Rate (SBR/NMR) − 20 weeks
lambda = 0.2 ; zeta = 0.99 ; scale = 5 ; amp = 0.028

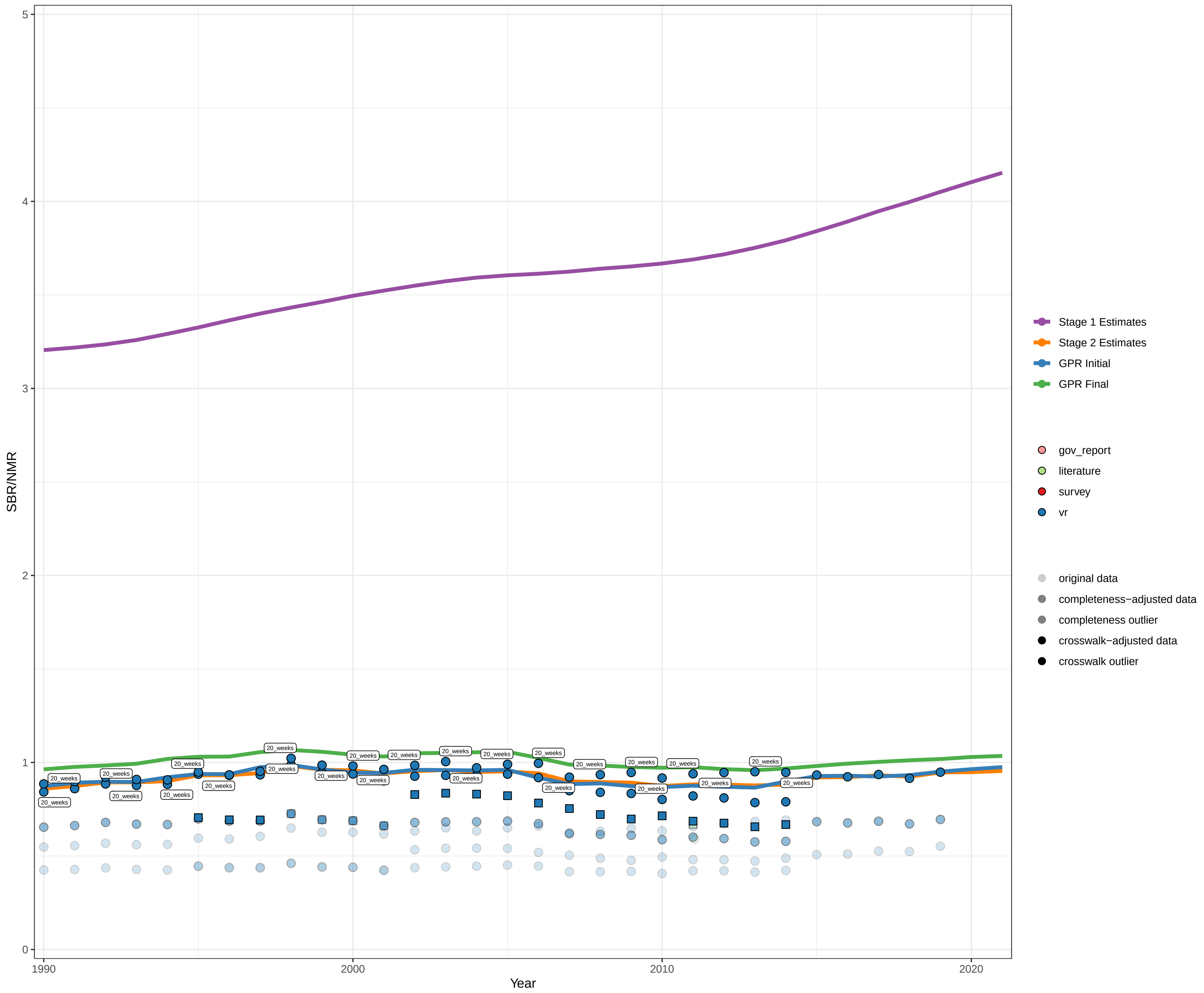

Mexico MEX Central Latin America Stillbirth Rate / Neonatal Mortality Rate (SBR/NMR) − 28 weeks
lambda = 0.2 ; zeta = 0.99 ; scale = 5 ; amp = 0.028

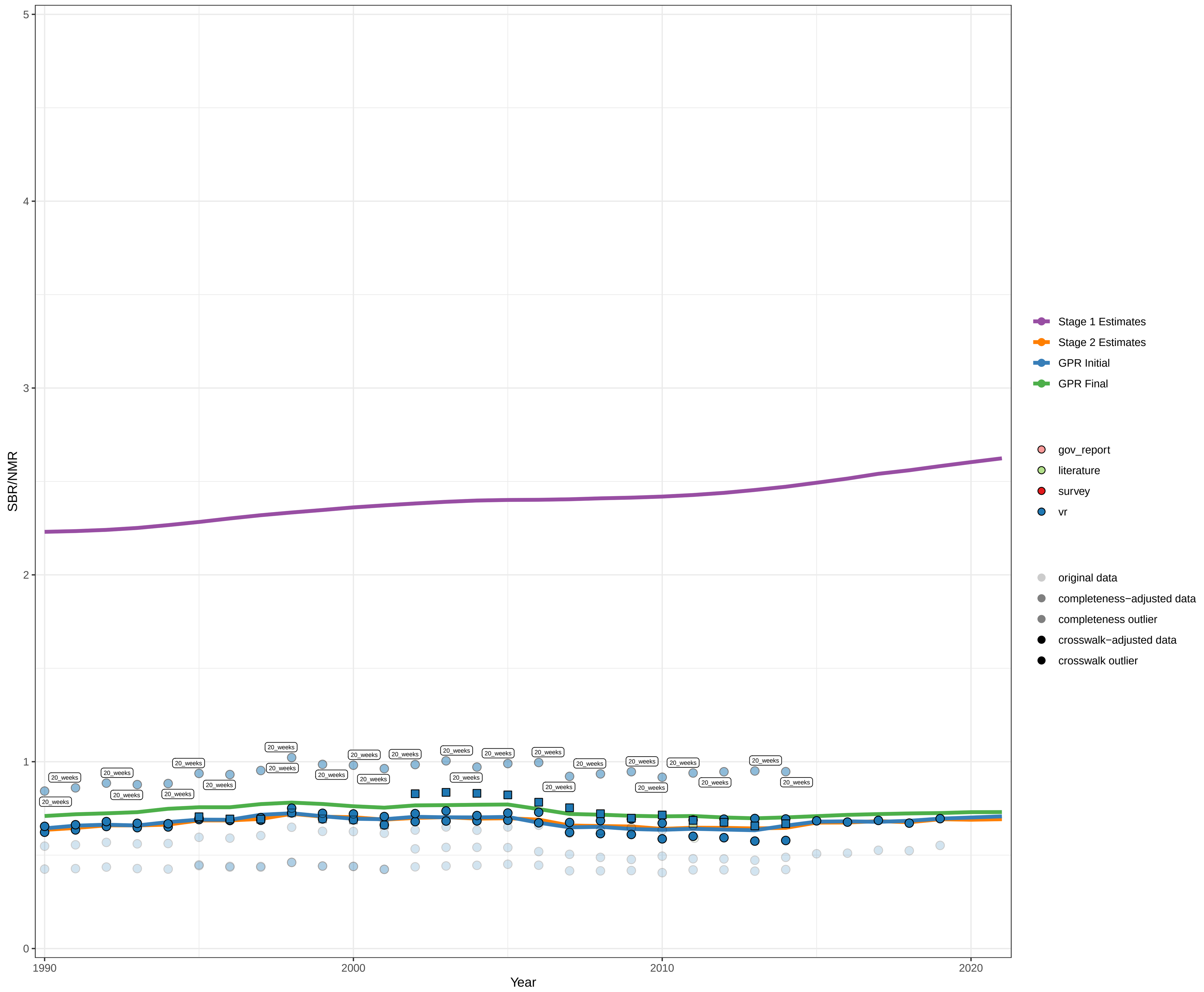

Definition	Years	Outlier	Source.Type	Title
20_weeks, 28_weeks	1990−2014	0	VR	Mexico INEGI Administrative Registers − Fetal Deaths by Municipality of Usual Residence of the Mother and Her Five−year Age Group According to Type of Fetal Death
28_weeks	2010, 2015−2019	0	VR	United Nations Demographic Yearbook
28_weeks	1998−2001	1	VR	United Nations Demographic Yearbook
28_weeks_OR_1000_grams	2011	1	Sci Lit	Maternal complications and perinatal mortality: findings of the World Health Organization Multicountry Survey on Maternal and Newborn Health
28_weeks	1995−1997, 2002−2009, 2011−2014	2	VR	United Nations Demographic Yearbook

Mexico MEX Central Latin America Stillbirth Rate (SBR) − 20 weeks
lambda = 0.2 ; zeta = 0.99 ; scale = 5 ; amp = 0.028

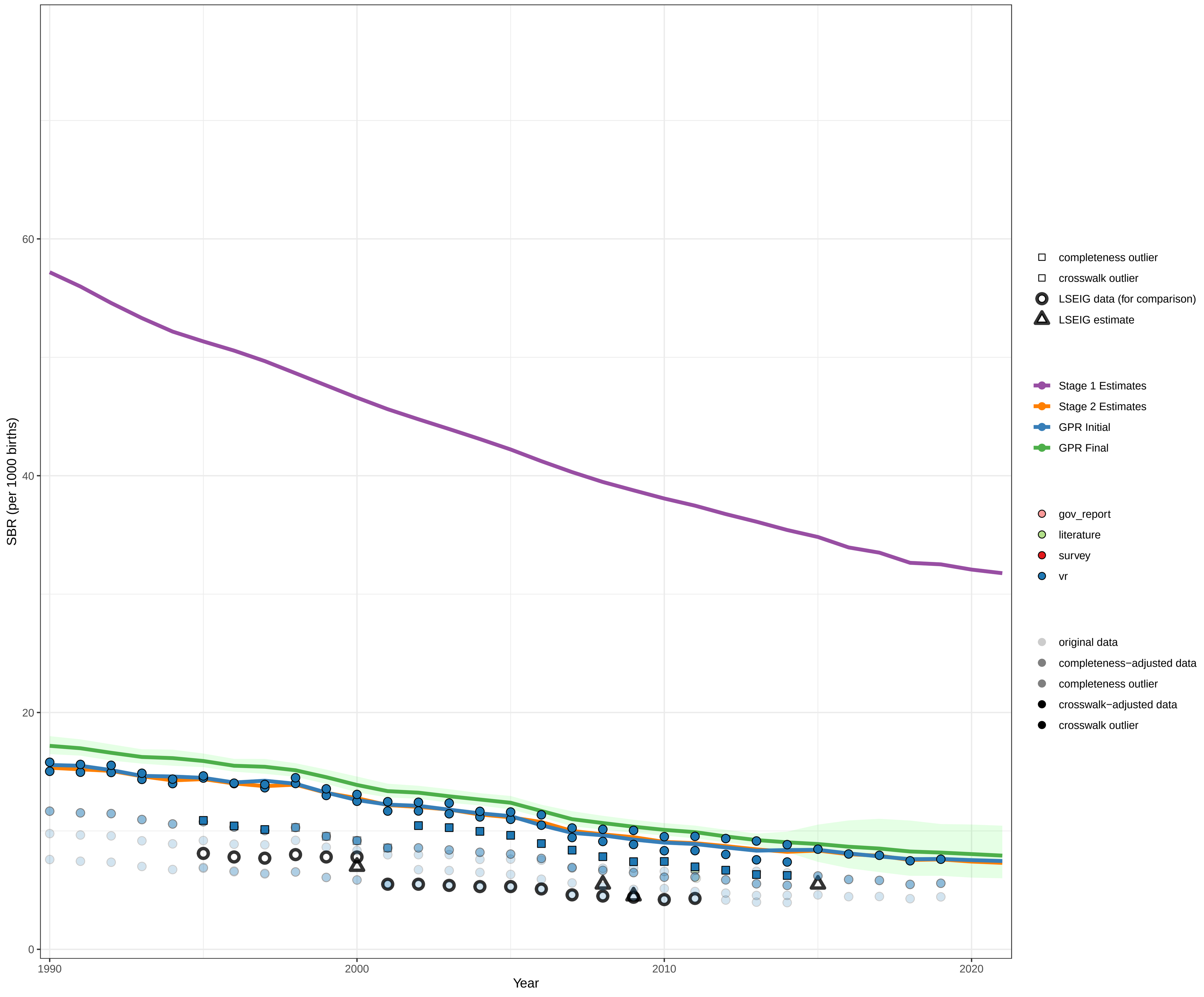

Mexico MEX Central Latin America Stillbirth Rate (SBR) − 28 weeks
lambda = 0.2 ; zeta = 0.99 ; scale = 5 ; amp = 0.028

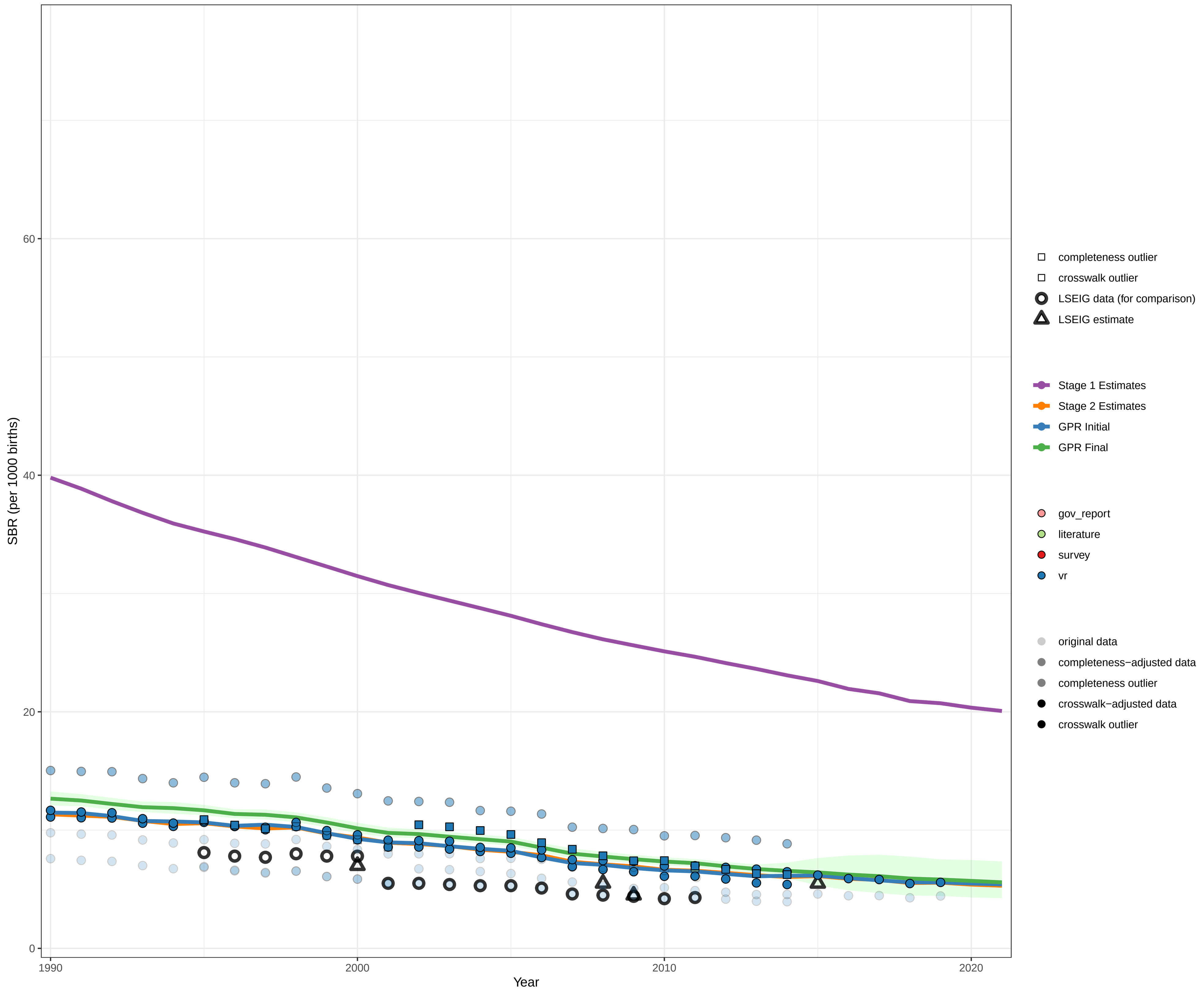

Year	Total Stillbirths (in thousands)	Total Stillbirths (in thousands)	SBR (per 1000 births)	SBR (per 1000 births)	SBR/NMR	SBR/NMR
	20 weeks	28 weeks	20 weeks	28 weeks	20 weeks	28 weeks
1990	44.9 (43.0 to 47.0)	32.9 (31.3 to 34.5)	17.2 (16.5 to 18.0)	12.7 (12.1 to 13.3)	0.96 (0.92 to 1.01)	0.71 (0.68 to 0.74)
1995	41.9 (40.5 to 43.5)	30.6 (29.5 to 31.8)	15.9 (15.4 to 16.5)	11.7 (11.3 to 12.1)	1.03 (1.00 to 1.07)	0.76 (0.73 to 0.79)
2000	35.1 (33.5 to 36.9)	25.5 (24.4 to 26.7)	13.9 (13.3 to 14.6)	10.2 (9.7 to 10.6)	1.04 (0.99 to 1.09)	0.76 (0.73 to 0.80)
2005	30.1 (28.7 to 31.5)	21.9 (21.0 to 22.8)	12.4 (11.8 to 13.0)	9.0 (8.7 to 9.4)	1.06 (1.01 to 1.11)	0.77 (0.74 to 0.81)
2010	25.4 (24.2 to 26.8)	18.4 (17.6 to 19.4)	10.1 (9.6 to 10.7)	7.3 (7.0 to 7.7)	0.97 (0.93 to 1.03)	0.71 (0.68 to 0.74)
2015	20.5 (17.0 to 24.4)	14.8 (12.2 to 17.6)	8.9 (7.4 to 10.5)	6.4 (5.3 to 7.6)	0.98 (0.81 to 1.16)	0.71 (0.59 to 0.84)
2020	15.5 (11.7 to 20.4)	11.0 (8.3 to 14.4)	8.0 (6.1 to 10.5)	5.7 (4.3 to 7.5)	1.03 (0.78 to 1.35)	0.73 (0.55 to 0.96)
2021	14.8 (11.2 to 19.6)	10.4 (7.9 to 13.8)	7.9 (6.0 to 10.4)	5.6 (4.2 to 7.4)	1.03 (0.78 to 1.37)	0.73 (0.55 to 0.96)

Aguascalientes MEX_4643 Central Latin America Stillbirth Rate / Neonatal Mortality Rate (SBR/NMR) − 20 weeks
lambda = 0.2 ; zeta = 0.99 ; scale = 5 ; amp = 0.028

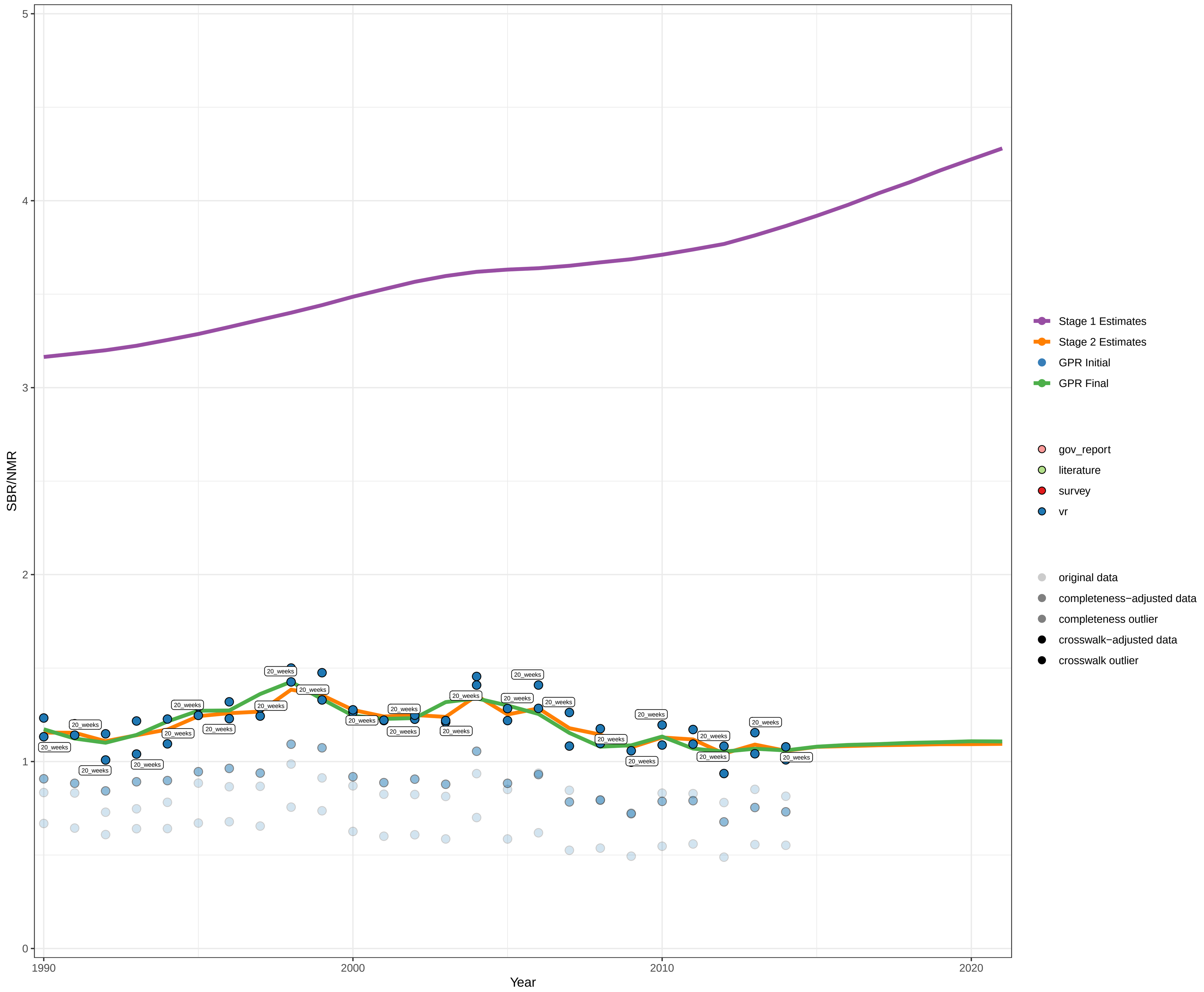

Aguascalientes MEX_4643 Central Latin America Stillbirth Rate / Neonatal Mortality Rate (SBR/NMR) − 28 weeks
lambda = 0.2 ; zeta = 0.99 ; scale = 5 ; amp = 0.028

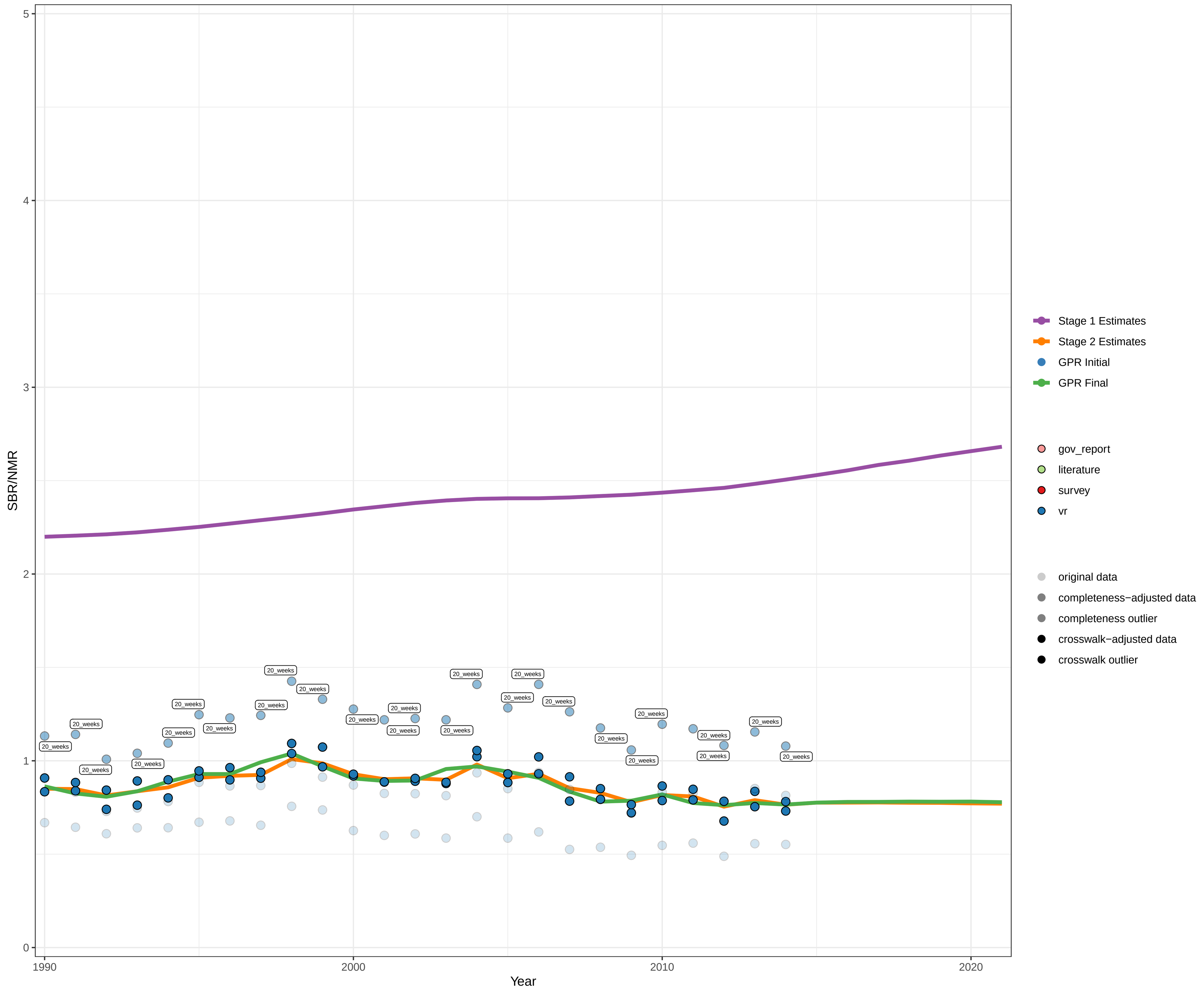

Definition	Years	Outlier	Source.Type	Title
20_weeks, 28_weeks	1990−2014	0	VR	Mexico INEGI Administrative Registers − Fetal Deaths by Municipality of Usual Residence of the Mother and Her Five−year Age Group According to Type of Fetal Death

Aguascalientes MEX_4643 Central Latin America Stillbirth Rate (SBR) − 20 weeks
lambda = 0.2 ; zeta = 0.99 ; scale = 5 ; amp = 0.028

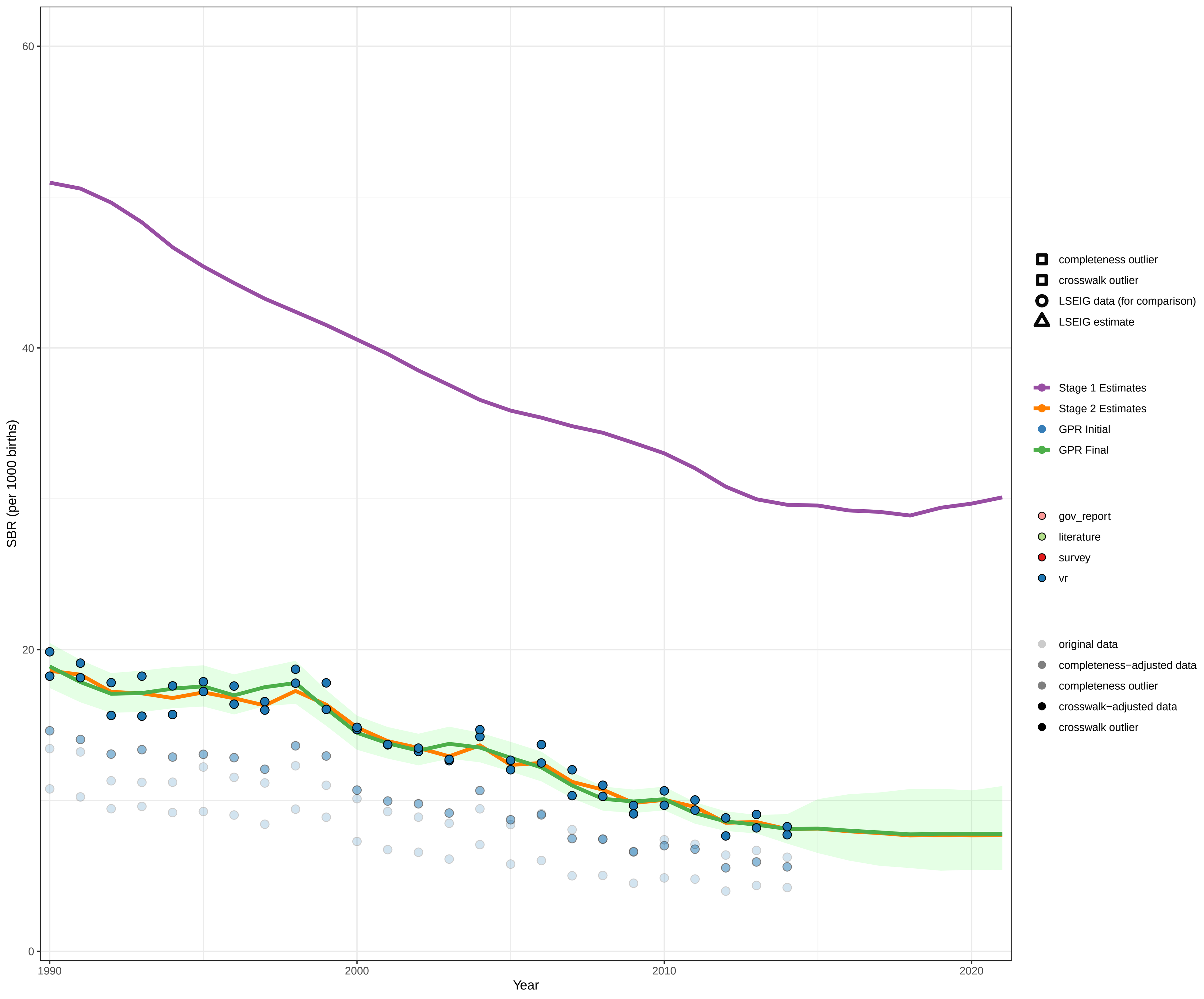

Aguascalientes MEX_4643 Central Latin America Stillbirth Rate (SBR) − 28 weeks
lambda = 0.2 ; zeta = 0.99 ; scale = 5 ; amp = 0.028

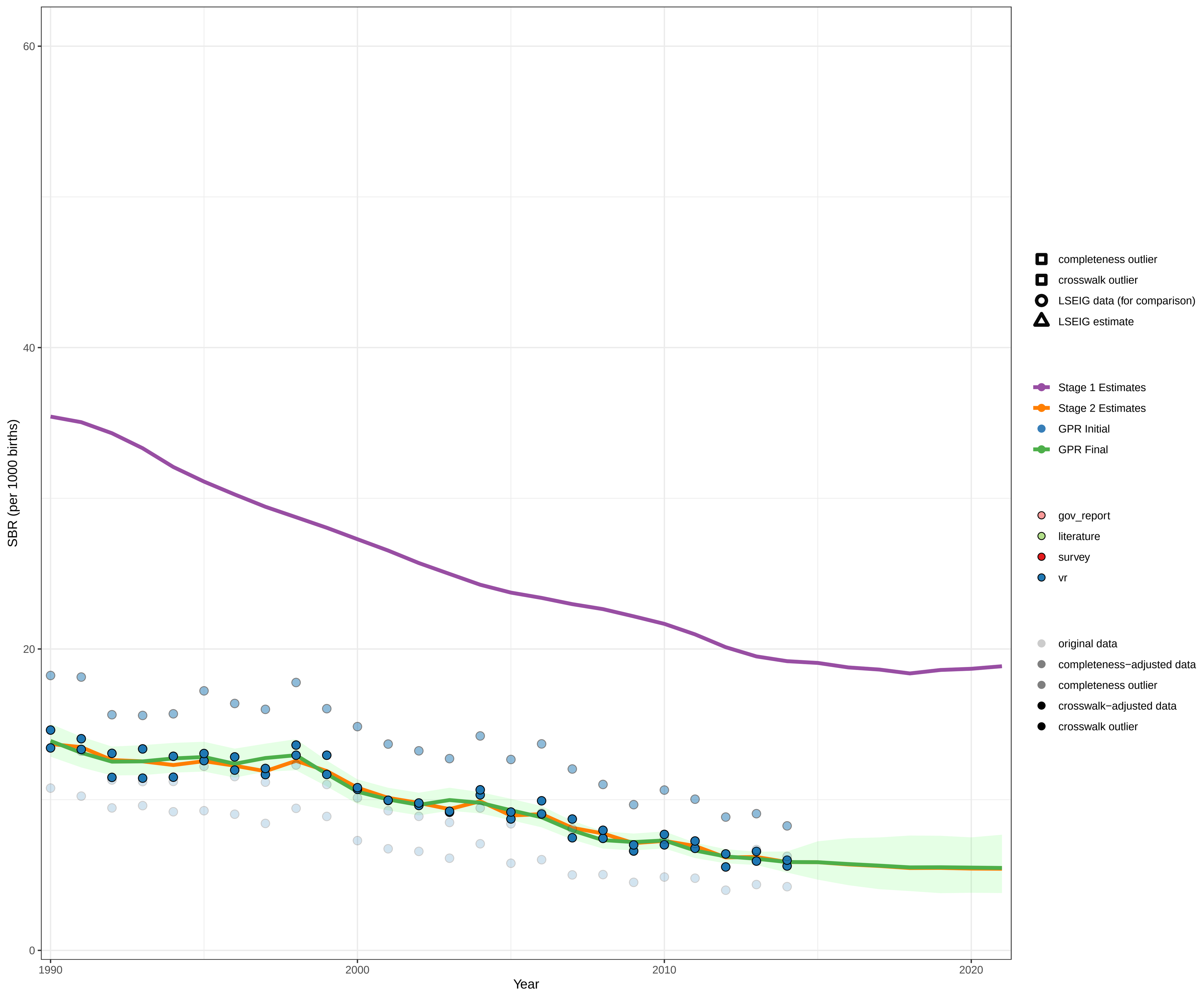

Year	Total Stillbirths (in thousands)	Total Stillbirths (in thousands)	SBR (per 1000 births)	SBR (per 1000 births)	SBR/NMR	SBR/NMR
	20 weeks	28 weeks	20 weeks	28 weeks	20 weeks	28 weeks
1990	0.5 (0.4 to 0.5)	0.3 (0.3 to 0.4)	18.9 (17.5 to 20.4)	13.9 (12.9 to 15.0)	1.17 (1.08 to 1.27)	0.86 (0.80 to 0.93)
1995	0.5 (0.4 to 0.5)	0.3 (0.3 to 0.4)	17.6 (16.2 to 19.0)	12.8 (11.9 to 13.8)	1.27 (1.18 to 1.37)	0.93 (0.86 to 1.00)
2000	0.4 (0.3 to 0.4)	0.3 (0.3 to 0.3)	14.5 (13.4 to 15.6)	10.5 (9.7 to 11.3)	1.25 (1.15 to 1.34)	0.91 (0.84 to 0.98)
2005	0.3 (0.3 to 0.4)	0.2 (0.2 to 0.3)	12.8 (11.9 to 13.9)	9.3 (8.6 to 10.1)	1.30 (1.21 to 1.41)	0.94 (0.88 to 1.02)
2010	0.3 (0.3 to 0.3)	0.2 (0.2 to 0.2)	10.1 (9.4 to 10.9)	7.3 (6.8 to 7.9)	1.13 (1.05 to 1.23)	0.82 (0.76 to 0.89)
2015	0.2 (0.2 to 0.3)	0.2 (0.1 to 0.2)	8.1 (6.5 to 10.1)	5.9 (4.7 to 7.2)	1.08 (0.86 to 1.34)	0.78 (0.62 to 0.96)
2020	0.2 (0.1 to 0.3)	0.1 (0.1 to 0.2)	7.8 (5.4 to 10.7)	5.5 (3.8 to 7.5)	1.11 (0.77 to 1.52)	0.78 (0.54 to 1.07)
2021	0.2 (0.1 to 0.3)	0.1 (0.1 to 0.2)	7.8 (5.4 to 11.0)	5.5 (3.8 to 7.7)	1.11 (0.77 to 1.56)	0.78 (0.54 to 1.09)

Baja California MEX_4644 Central Latin America Stillbirth Rate / Neonatal Mortality Rate (SBR/NMR) − 20 weeks
lambda = 0.2 ; zeta = 0.99 ; scale = 5 ; amp = 0.028

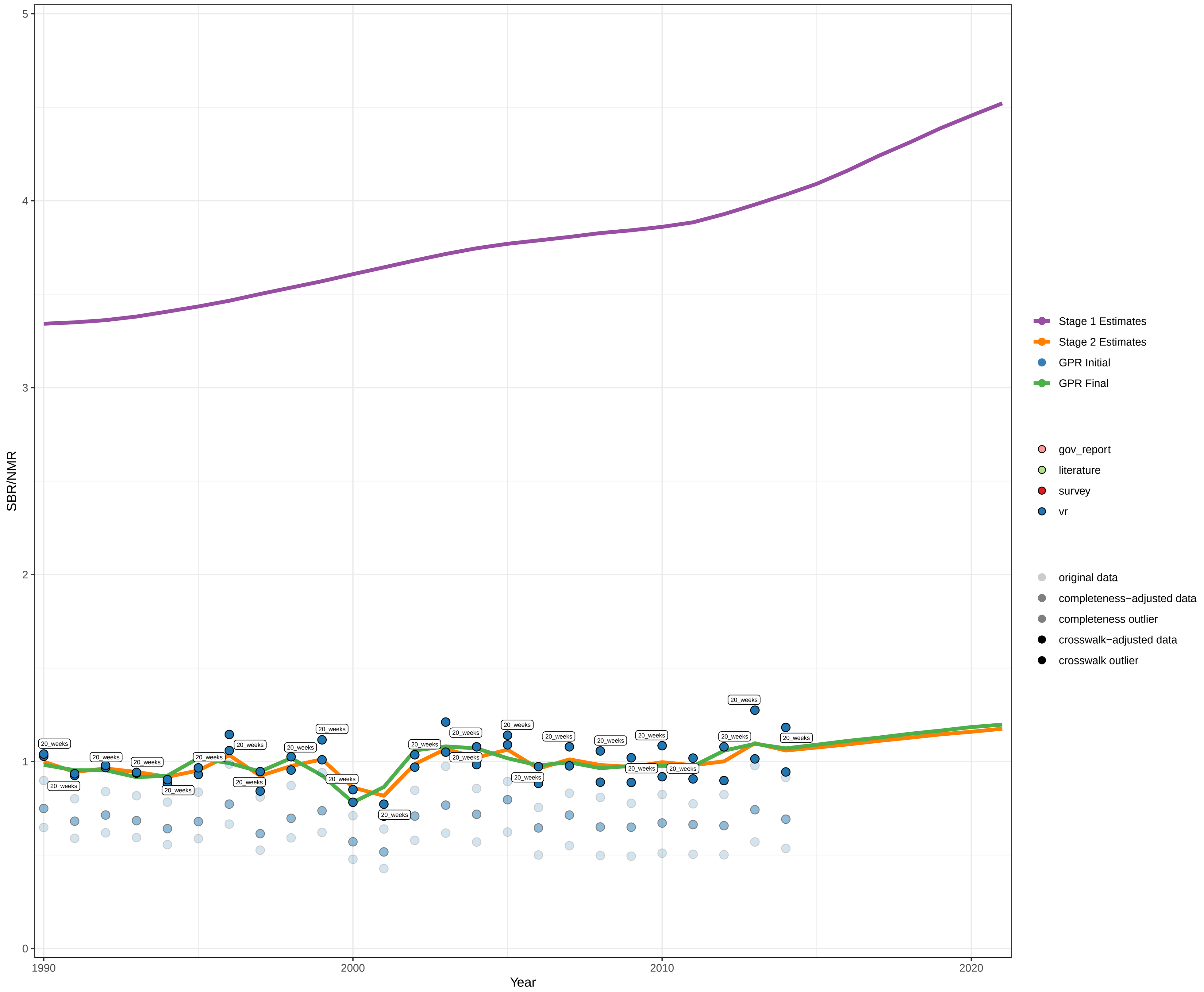

Baja California MEX_4644 Central Latin America Stillbirth Rate / Neonatal Mortality Rate (SBR/NMR) − 28 weeks
lambda = 0.2 ; zeta = 0.99 ; scale = 5 ; amp = 0.028

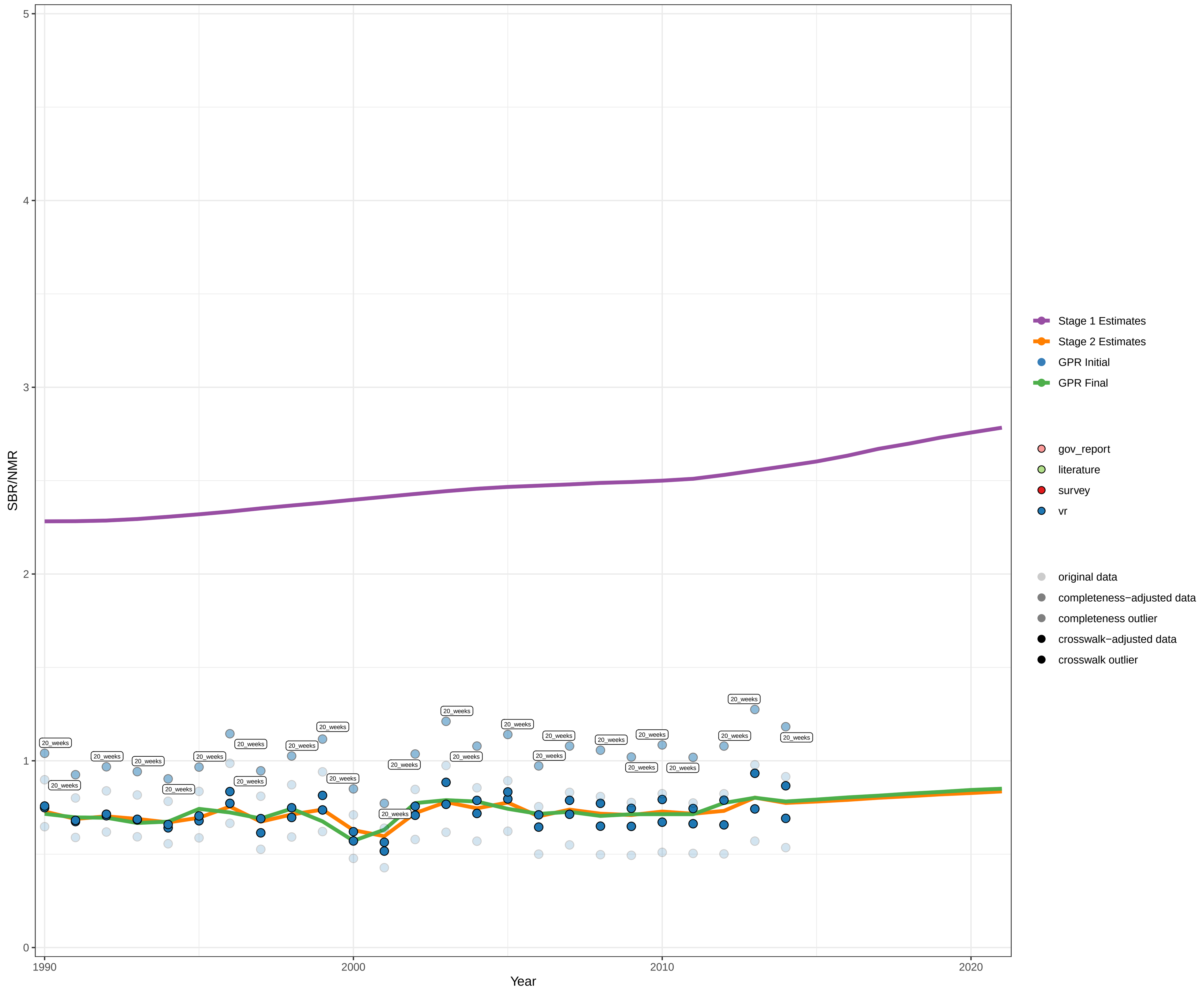

Definition	Years	Outlier	Source.Type	Title
20_weeks, 28_weeks	1990−2014	0	VR	Mexico INEGI Administrative Registers − Fetal Deaths by Municipality of Usual Residence of the Mother and Her Five−year Age Group According to Type of Fetal Death

Baja California MEX_4644 Central Latin America Stillbirth Rate (SBR) − 20 weeks
lambda = 0.2 ; zeta = 0.99 ; scale = 5 ; amp = 0.028

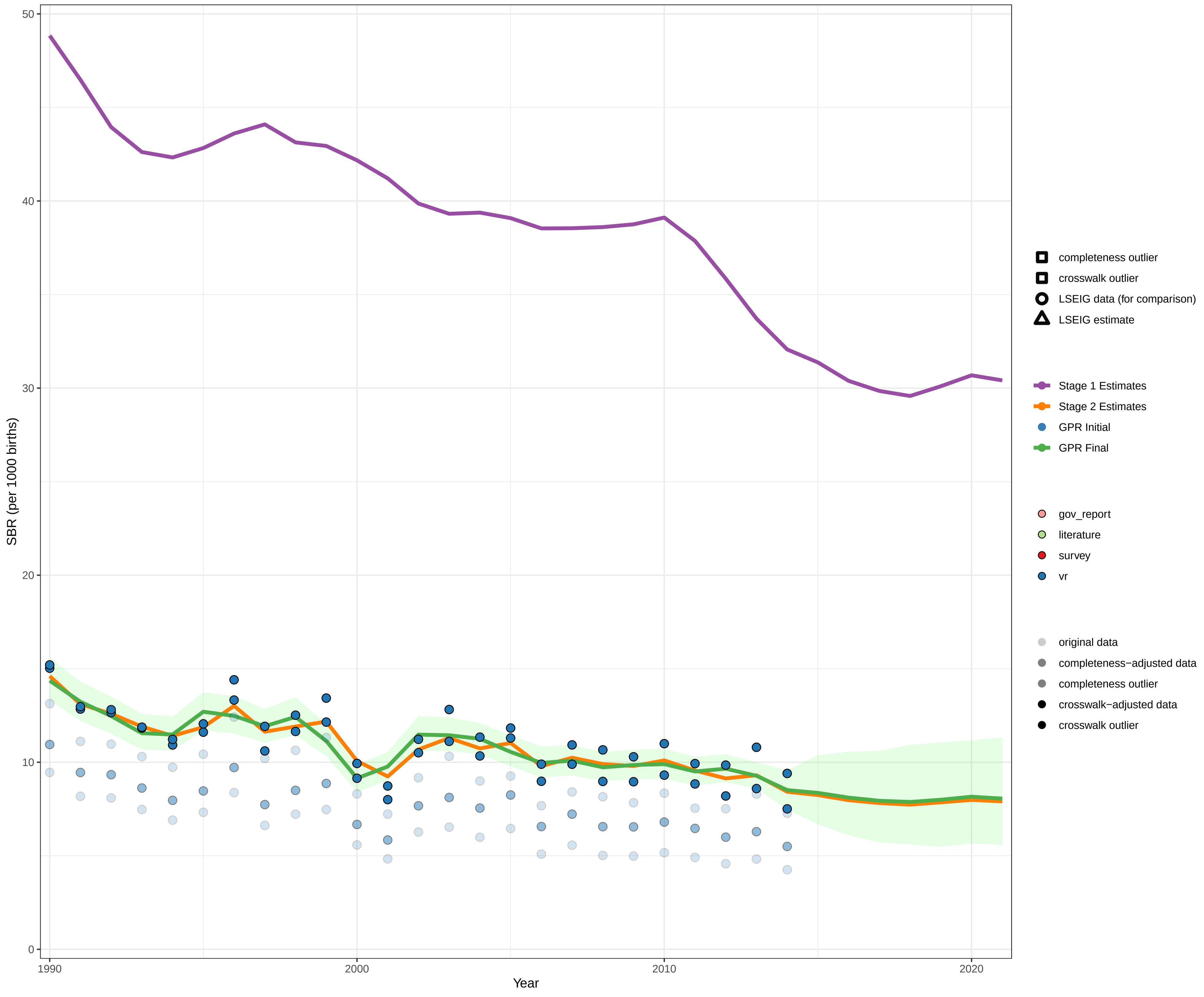

Baja California MEX_4644 Central Latin America Stillbirth Rate (SBR) − 28 weeks
lambda = 0.2 ; zeta = 0.99 ; scale = 5 ; amp = 0.028

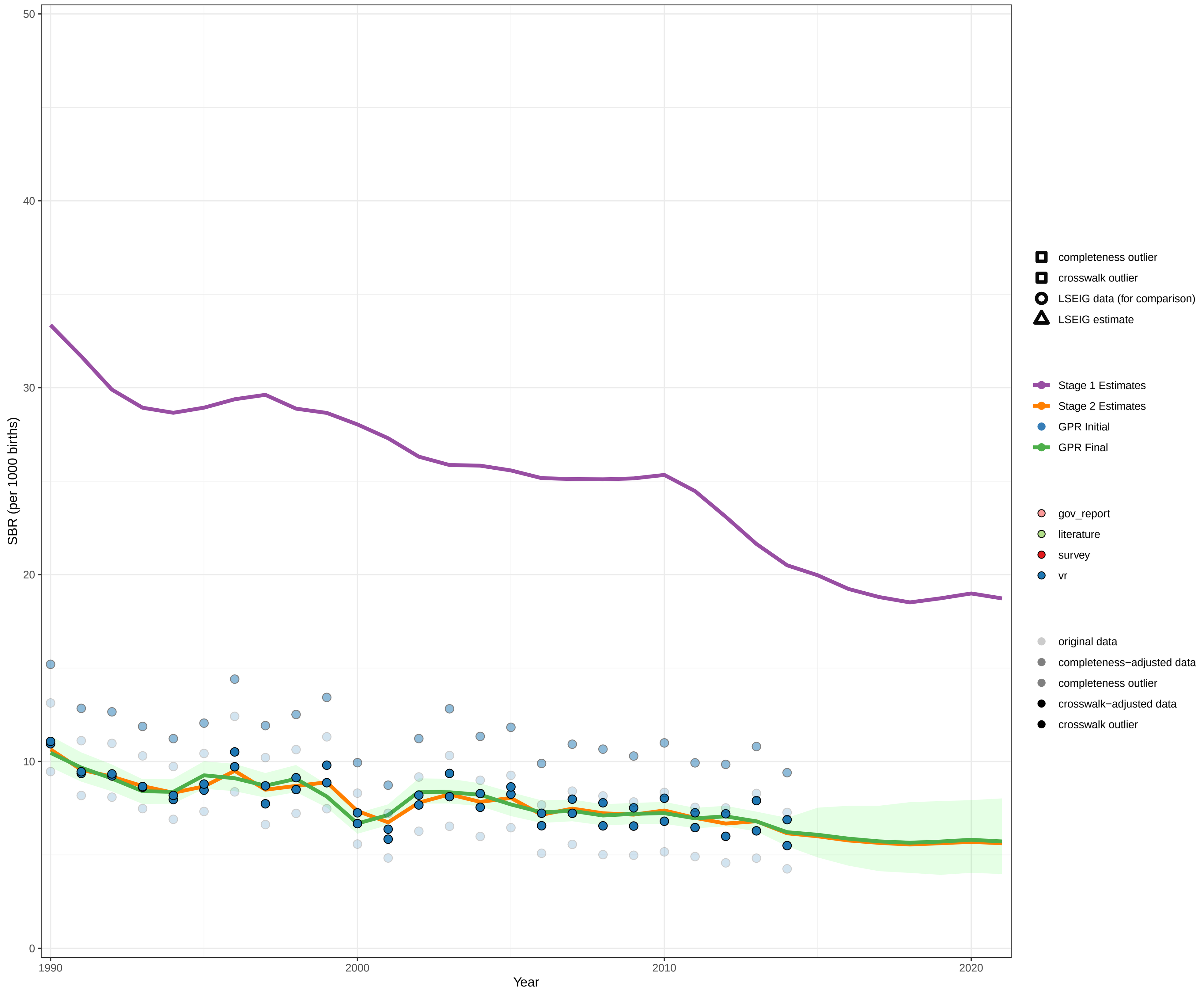

Year	Total Stillbirths (in thousands)	Total Stillbirths (in thousands)	SBR (per 1000 births)	SBR (per 1000 births)	SBR/NMR	SBR/NMR
	20 weeks	28 weeks	20 weeks	28 weeks	20 weeks	28 weeks
1990	0.7<br>(0.7 to 0.8)	0.5<br>(0.5 to 0.6)	14.4<br>(13.3 to 15.6)	10.5<br>(9.7 to 11.4)	0.98<br>(0.91 to 1.07)	0.72<br>(0.66 to 0.78)
1995	0.7<br>(0.7 to 0.8)	0.5<br>(0.5 to 0.6)	12.7<br>(11.7 to 13.7)	9.3<br>(8.5 to 10.0)	1.02<br>(0.94 to 1.10)	0.74<br>(0.68 to 0.80)
2000	0.5<br>(0.5 to 0.6)	0.4<br>(0.4 to 0.4)	9.1<br>(8.4 to 9.9)	6.7<br>(6.1 to 7.2)	0.78<br>(0.72 to 0.84)	0.57<br>(0.52 to 0.62)
2005	0.6<br>(0.6 to 0.7)	0.5<br>(0.4 to 0.5)	10.6<br>(9.8 to 11.4)	7.7<br>(7.1 to 8.4)	1.02<br>(0.94 to 1.10)	0.74<br>(0.68 to 0.81)
2010	0.6<br>(0.6 to 0.7)	0.5<br>(0.4 to 0.5)	9.9<br>(9.1 to 10.7)	7.2<br>(6.7 to 7.8)	0.98<br>(0.90 to 1.06)	0.71<br>(0.66 to 0.77)
2015	0.5<br>(0.4 to 0.7)	0.4<br>(0.3 to 0.5)	8.4<br>(6.7 to 10.4)	6.1<br>(4.9 to 7.5)	1.09<br>(0.87 to 1.35)	0.79<br>(0.63 to 0.98)
2020	0.4<br>(0.3 to 0.6)	0.3<br>(0.2 to 0.4)	8.2<br>(5.7 to 11.2)	5.8<br>(4.0 to 7.9)	1.18<br>(0.82 to 1.62)	0.84<br>(0.59 to 1.15)
2021	0.4<br>(0.3 to 0.6)	0.3<br>(0.2 to 0.4)	8.1<br>(5.6 to 11.3)	5.7<br>(4.0 to 8.0)	1.20<br>(0.83 to 1.68)	0.85<br>(0.59 to 1.19)

Baja California Sur MEX_4645 Central Latin America Stillbirth Rate / Neonatal Mortality Rate (SBR/NMR) − 20 weeks
lambda = 0.2 ; zeta = 0.99 ; scale = 5 ; amp = 0.028

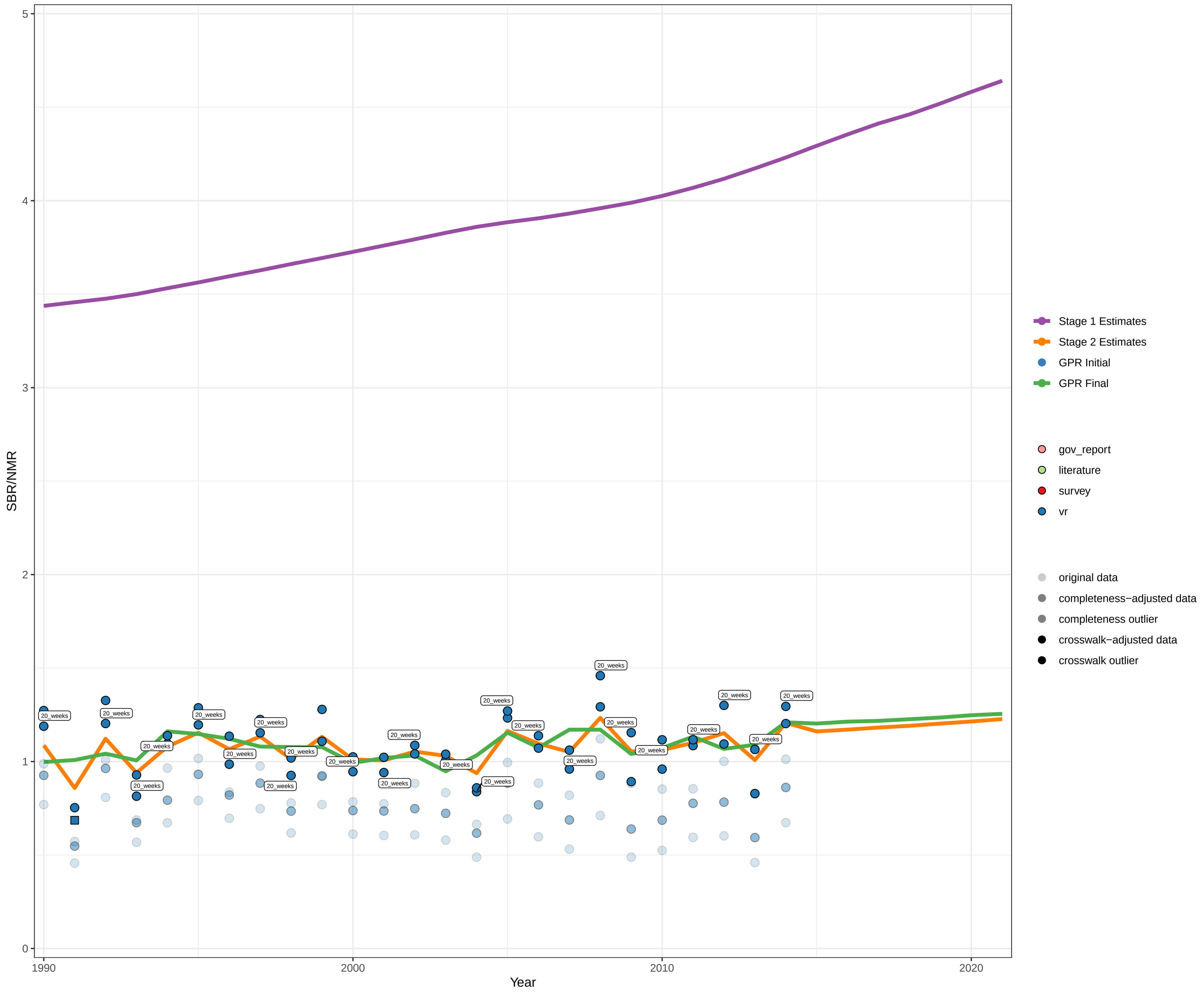

Baja California Sur MEX_4645 Central Latin America Stillbirth Rate / Neonatal Mortality Rate (SBR/NMR) − 28 weeks
lambda = 0.2 ; zeta = 0.99 ; scale = 5 ; amp = 0.028

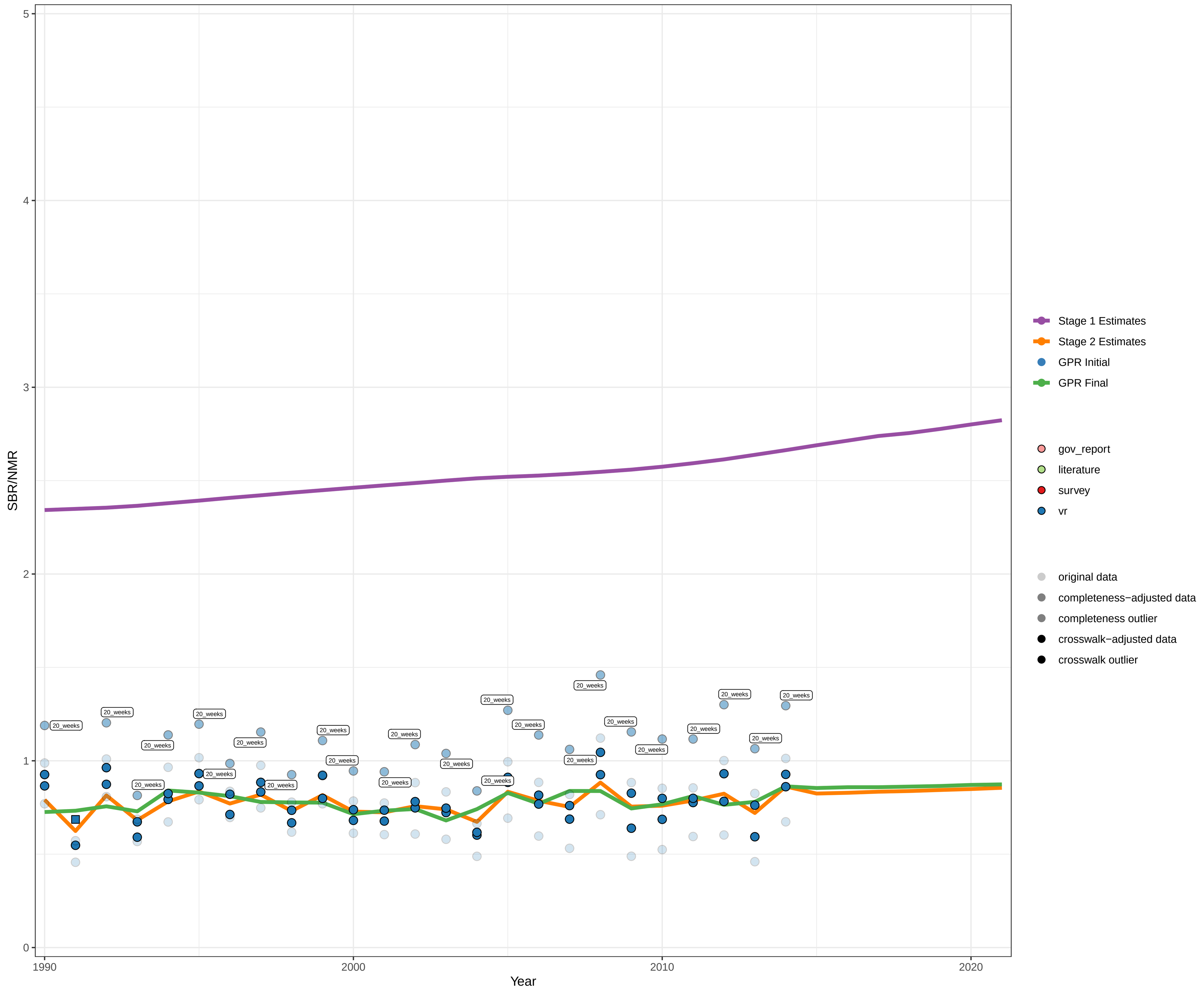

Definition	Years	Outlier	Source.Type	Title
20_weeks	1990, 1992−2014	0	VR	Mexico INEGI Administrative Registers − Fetal Deaths by Municipality of Usual Residence of the Mother and Her Five−year Age Group According to Type of Fetal Death
28_weeks	1990−2014	0	VR	Mexico INEGI Administrative Registers − Fetal Deaths by Municipality of Usual Residence of the Mother and Her Five−year Age Group According to Type of Fetal Death
20_weeks	1991	2	VR	Mexico INEGI Administrative Registers − Fetal Deaths by Municipality of Usual Residence of the Mother and Her Five−year Age Group According to Type of Fetal Death

Baja California Sur MEX_4645 Central Latin America Stillbirth Rate (SBR) − 20 weeks
lambda = 0.2 ; zeta = 0.99 ; scale = 5 ; amp = 0.028

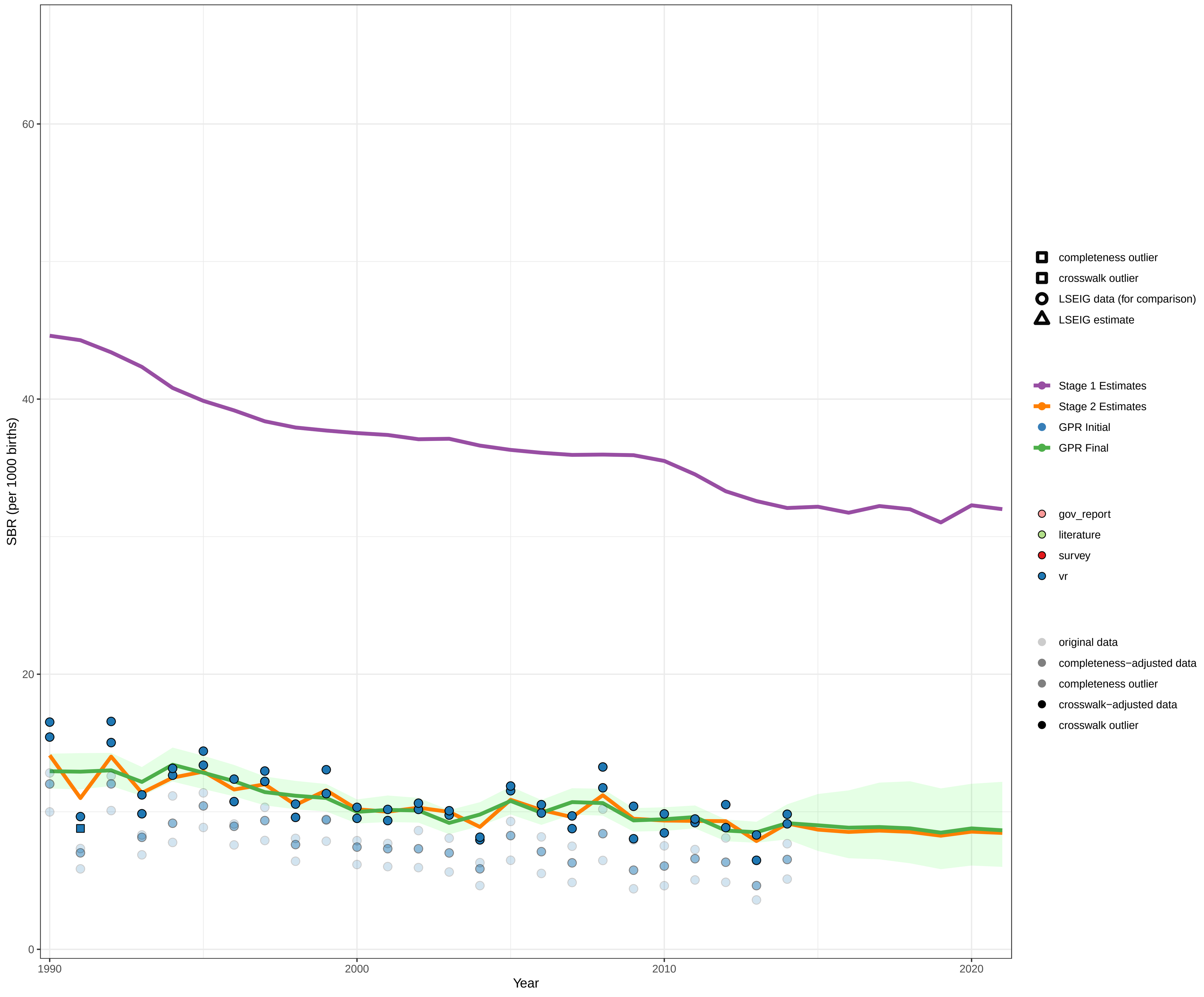

Baja California Sur MEX_4645 Central Latin America Stillbirth Rate (SBR) − 28 weeks
lambda = 0.2 ; zeta = 0.99 ; scale = 5 ; amp = 0.028

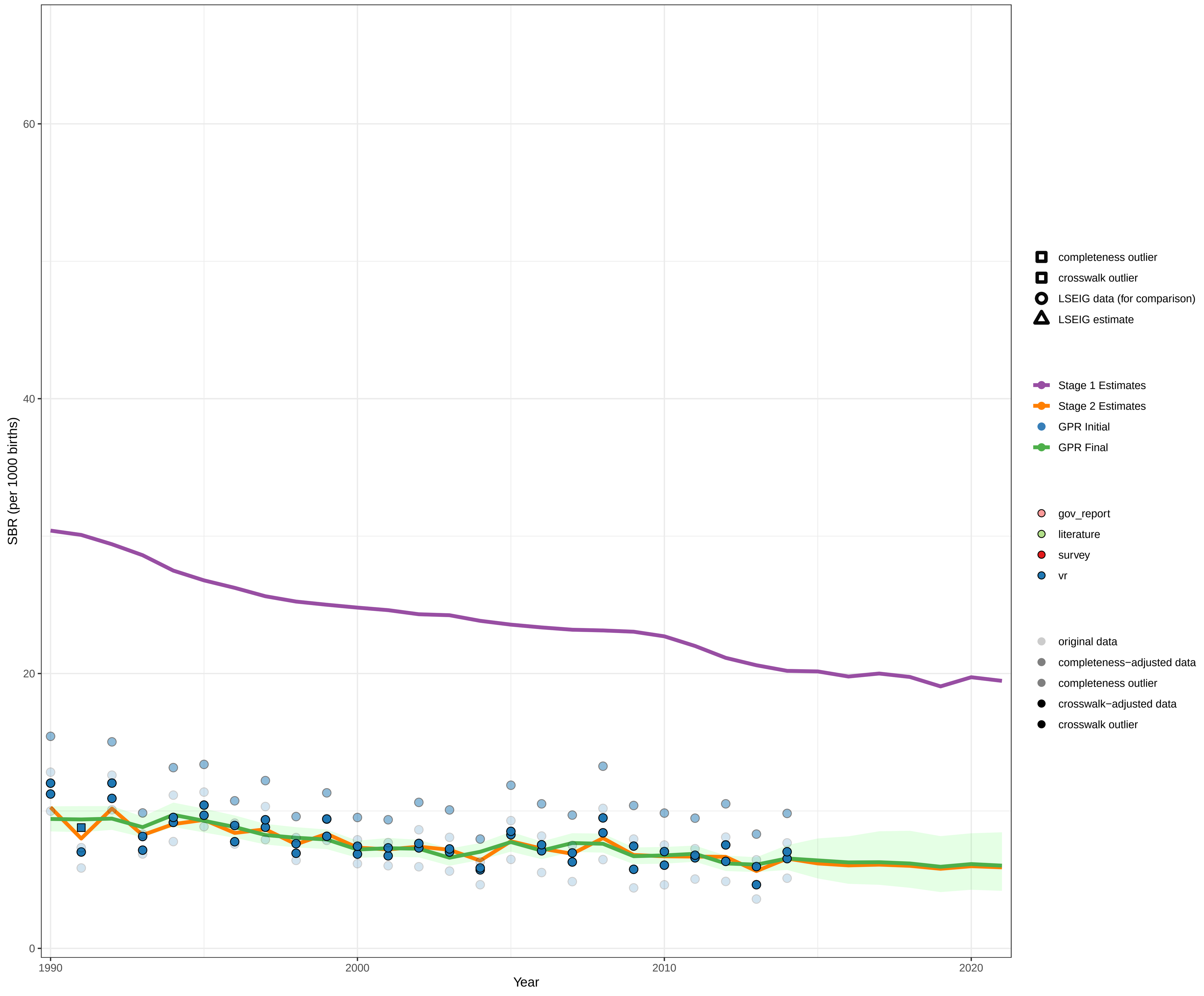

Year	Total Stillbirths (in thousands)	Total Stillbirths (in thousands)	SBR (per 1000 births)	SBR (per 1000 births)	SBR/NMR	SBR/NMR
	20 weeks	28 weeks	20 weeks	28 weeks	20 weeks	28 weeks
1990	0.1<br>(0.1 to 0.1)	0.1<br>(0.1 to 0.1)	12.9<br>(11.7 to 14.2)	9.4<br>(8.5 to 10.3)	1.00<br>(0.90 to 1.10)	0.73<br>(0.66 to 0.80)
1995	0.1<br>(0.1 to 0.1)	0.1<br>(0.1 to 0.1)	12.8<br>(11.7 to 14.1)	9.3<br>(8.4 to 10.2)	1.15<br>(1.04 to 1.26)	0.83<br>(0.75 to 0.91)
2000	0.1<br>(0.1 to 0.1)	0.1<br>(0.1 to 0.1)	10.0<br>(9.1 to 10.9)	7.2<br>(6.6 to 7.8)	0.99<br>(0.91 to 1.08)	0.71<br>(0.65 to 0.78)
2005	0.1<br>(0.1 to 0.1)	0.1<br>(0.1 to 0.1)	10.8<br>(9.8 to 11.8)	7.7<br>(7.0 to 8.5)	1.15<br>(1.05 to 1.27)	0.83<br>(0.75 to 0.91)
2010	0.1<br>(0.1 to 0.1)	0.1<br>(0.1 to 0.1)	9.5<br>(8.6 to 10.3)	6.8<br>(6.2 to 7.4)	1.07<br>(0.98 to 1.17)	0.77<br>(0.70 to 0.84)
2015	0.1<br>(0.1 to 0.1)	0.1<br>(0.1 to 0.1)	9.0<br>(7.2 to 11.3)	6.4<br>(5.1 to 8.0)	1.20<br>(0.95 to 1.51)	0.85<br>(0.68 to 1.07)
2020	0.1<br>(0.1 to 0.1)	0.1<br>(0.0 to 0.1)	8.8<br>(6.1 to 12.0)	6.1<br>(4.3 to 8.4)	1.25<br>(0.86 to 1.71)	0.87<br>(0.61 to 1.19)
2021	0.1<br>(0.1 to 0.1)	0.1<br>(0.0 to 0.1)	8.7<br>(6.0 to 12.2)	6.0<br>(4.2 to 8.4)	1.26<br>(0.87 to 1.77)	0.87<br>(0.61 to 1.23)

Campeche MEX_4646 Central Latin America Stillbirth Rate / Neonatal Mortality Rate (SBR/NMR) − 20 weeks
lambda = 0.2 ; zeta = 0.99 ; scale = 5 ; amp = 0.028

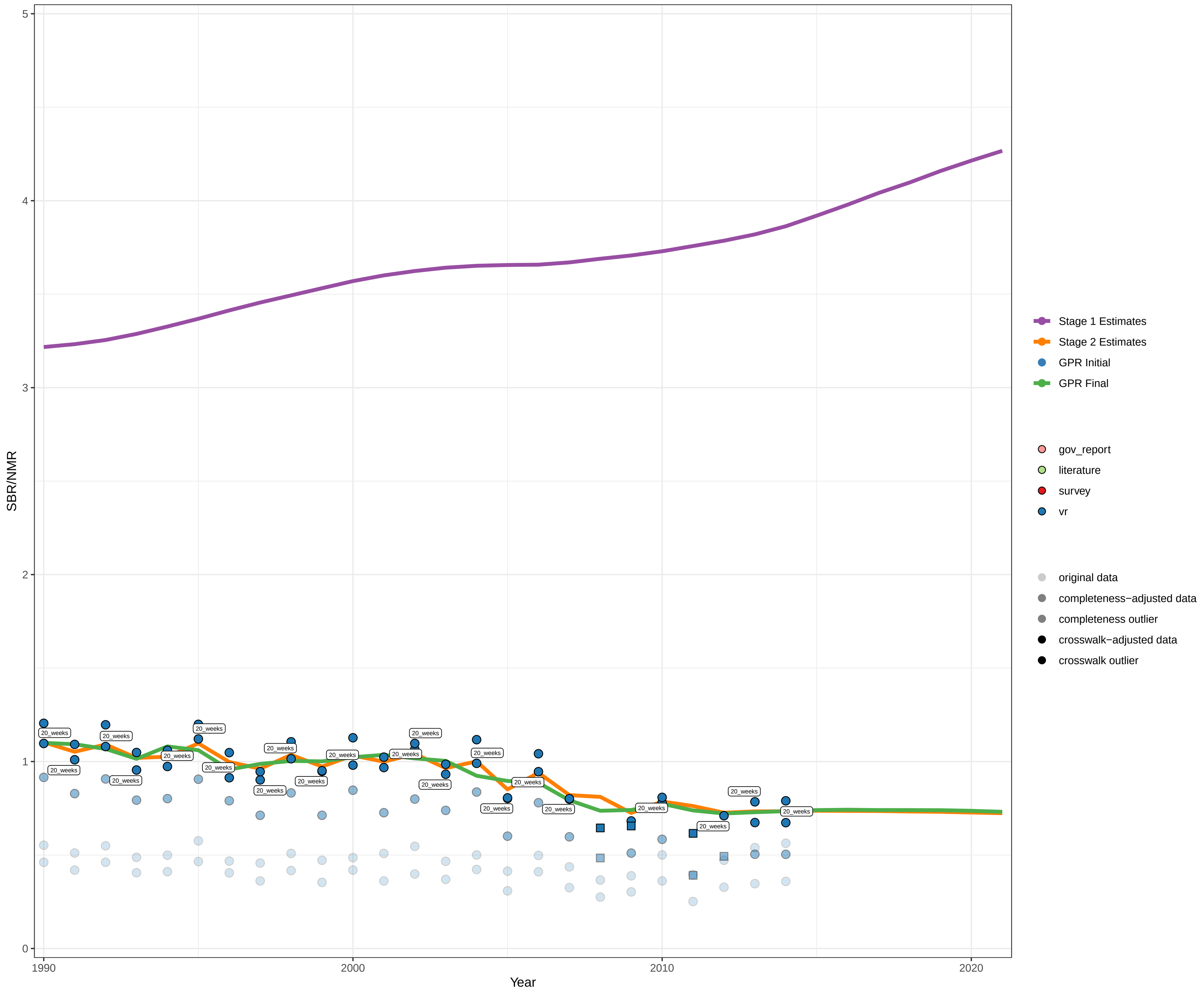

Campeche MEX_4646 Central Latin America Stillbirth Rate / Neonatal Mortality Rate (SBR/NMR) − 28 weeks
lambda = 0.2 ; zeta = 0.99 ; scale = 5 ; amp = 0.028

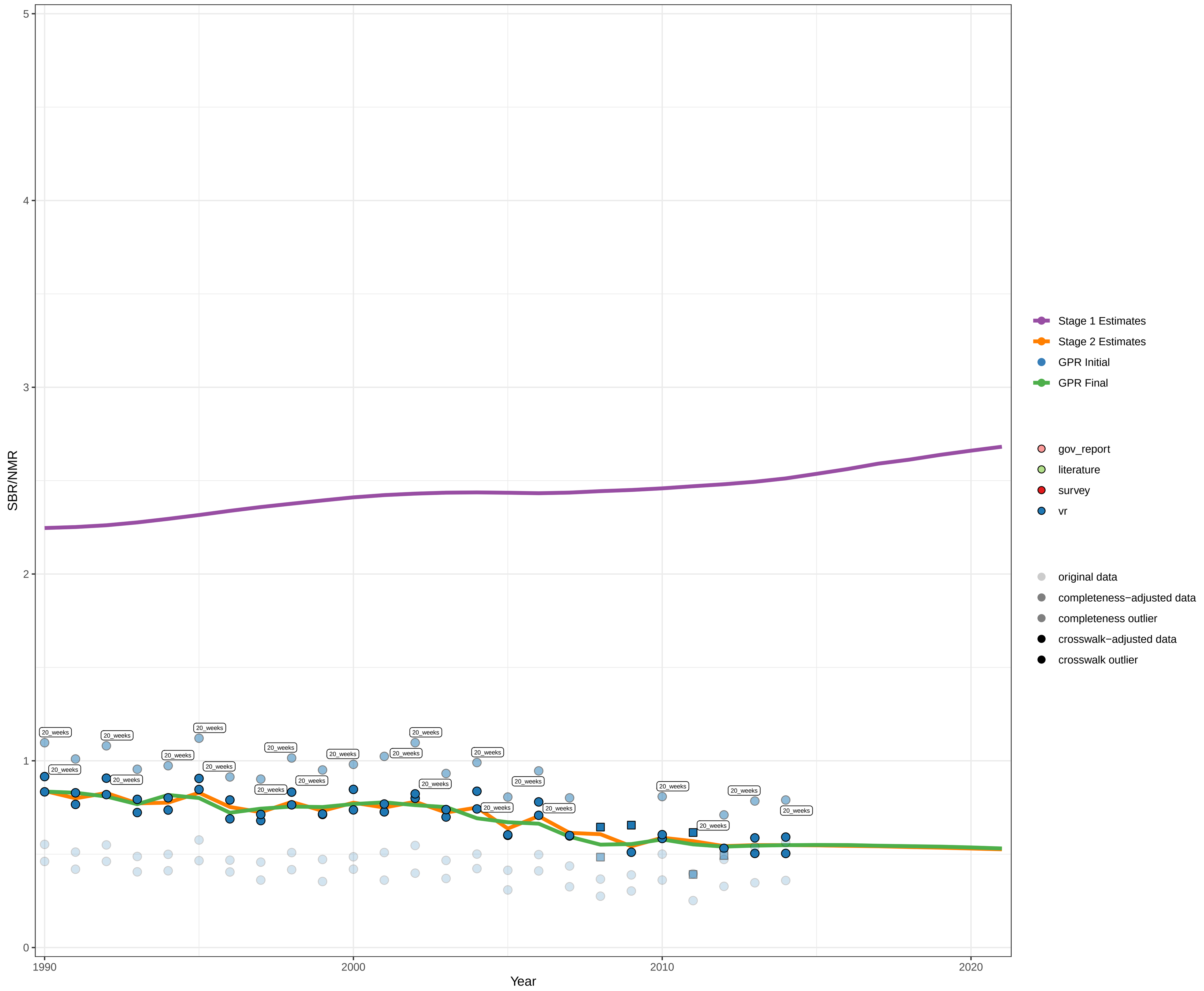

Definition	Years	Outlier	Source.Type	Title
20_weeks	1990−2007, 2010, 2012−2014	0	VR	Mexico INEGI Administrative Registers − Fetal Deaths by Municipality of Usual Residence of the Mother and Her Five−year Age Group According to Type of Fetal Death
28_weeks	1990−2007, 2009, 2010, 2013, 2014	0	VR	Mexico INEGI Administrative Registers − Fetal Deaths by Municipality of Usual Residence of the Mother and Her Five−year Age Group According to Type of Fetal Death
28_weeks	2008, 2011, 2012	1	VR	Mexico INEGI Administrative Registers − Fetal Deaths by Municipality of Usual Residence of the Mother and Her Five−year Age Group According to Type of Fetal Death
20_weeks	2008, 2009, 2011	2	VR	Mexico INEGI Administrative Registers − Fetal Deaths by Municipality of Usual Residence of the Mother and Her Five−year Age Group According to Type of Fetal Death

Campeche MEX_4646 Central Latin America Stillbirth Rate (SBR) − 20 weeks
lambda = 0.2 ; zeta = 0.99 ; scale = 5 ; amp = 0.028

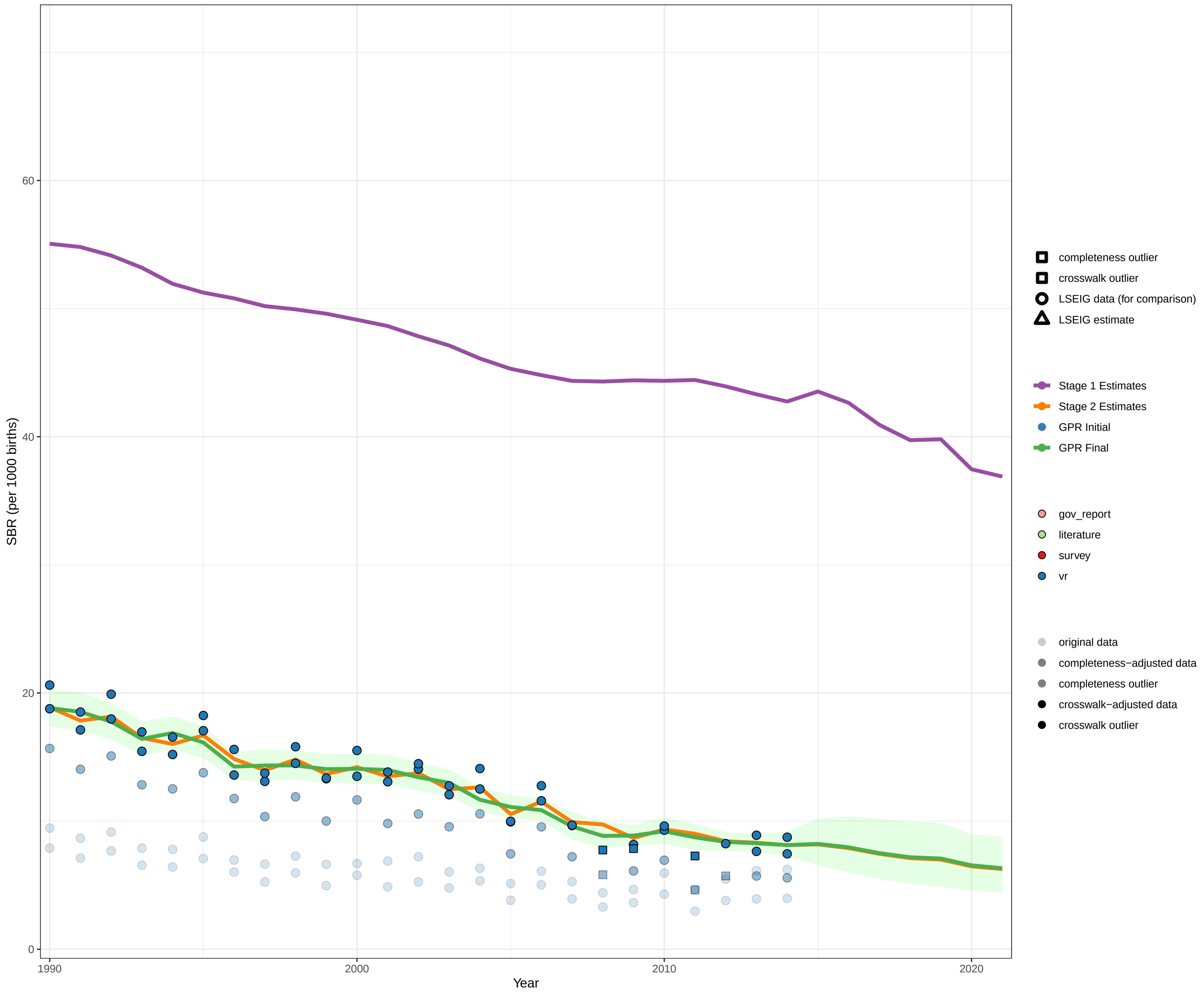

Campeche MEX_4646 Central Latin America Stillbirth Rate (SBR) − 28 weeks
lambda = 0.2 ; zeta = 0.99 ; scale = 5 ; amp = 0.028

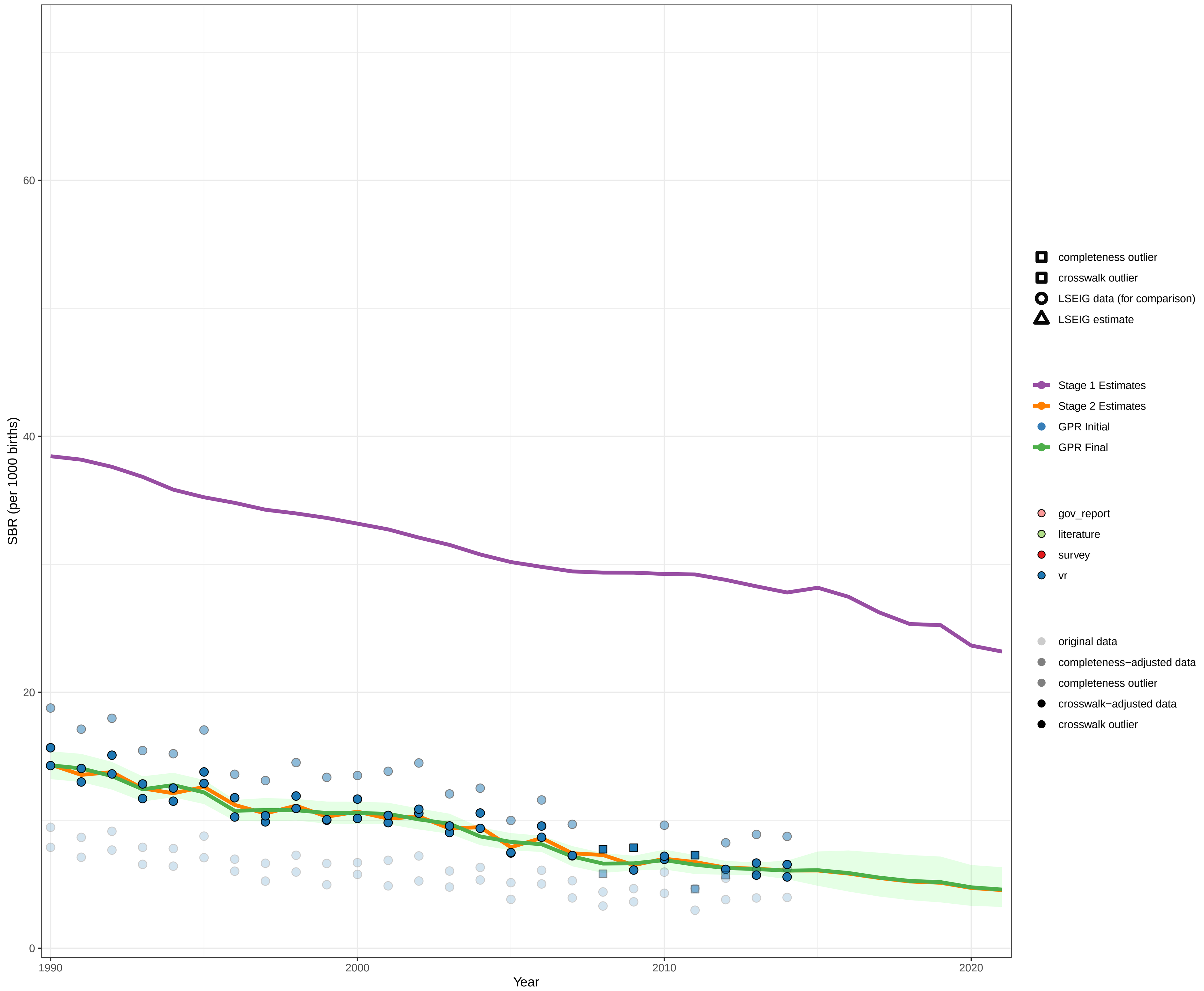

Year	Total Stillbirths (in thousands)	Total Stillbirths (in thousands)	SBR (per 1000 births)	SBR (per 1000 births)	SBR/NMR	SBR/NMR
	20 weeks	28 weeks	20 weeks	28 weeks	20 weeks	28 weeks
1990	0.3 (0.3 to 0.4)	0.3 (0.2 to 0.3)	18.8 (17.4 to 20.3)	14.3 (13.2 to 15.4)	1.10 (1.02 to 1.18)	0.84 (0.77 to 0.90)
1995	0.3 (0.3 to 0.3)	0.2 (0.2 to 0.2)	16.1 (14.9 to 17.4)	12.2 (11.3 to 13.2)	1.06 (0.98 to 1.15)	0.80 (0.74 to 0.86)
2000	0.2 (0.2 to 0.3)	0.2 (0.2 to 0.2)	14.1 (12.9 to 15.3)	10.6 (9.7 to 11.5)	1.02 (0.94 to 1.11)	0.77 (0.71 to 0.83)
2005	0.2 (0.2 to 0.2)	0.1 (0.1 to 0.2)	11.1 (10.2 to 12.0)	8.3 (7.7 to 9.0)	0.90 (0.82 to 0.97)	0.67 (0.62 to 0.73)
2010	0.2 (0.2 to 0.2)	0.1 (0.1 to 0.1)	9.2 (8.2 to 10.3)	6.9 (6.2 to 7.7)	0.77 (0.69 to 0.87)	0.58 (0.52 to 0.65)
2015	0.1 (0.1 to 0.2)	0.1 (0.1 to 0.1)	8.2 (6.6 to 10.2)	6.1 (4.9 to 7.6)	0.74 (0.59 to 0.92)	0.55 (0.44 to 0.68)
2020	0.1 (0.1 to 0.1)	0.1 (0.0 to 0.1)	6.5 (4.5 to 9.0)	4.8 (3.3 to 6.5)	0.74 (0.51 to 1.01)	0.54 (0.37 to 0.73)
2021	0.1 (0.1 to 0.1)	0.1 (0.0 to 0.1)	6.3 (4.4 to 8.8)	4.6 (3.2 to 6.3)	0.73 (0.51 to 1.01)	0.53 (0.37 to 0.73)

Chiapas MEX_4649 Central Latin America Stillbirth Rate / Neonatal Mortality Rate (SBR/NMR) − 20 weeks
lambda = 0.2 ; zeta = 0.99 ; scale = 5 ; amp = 0.028

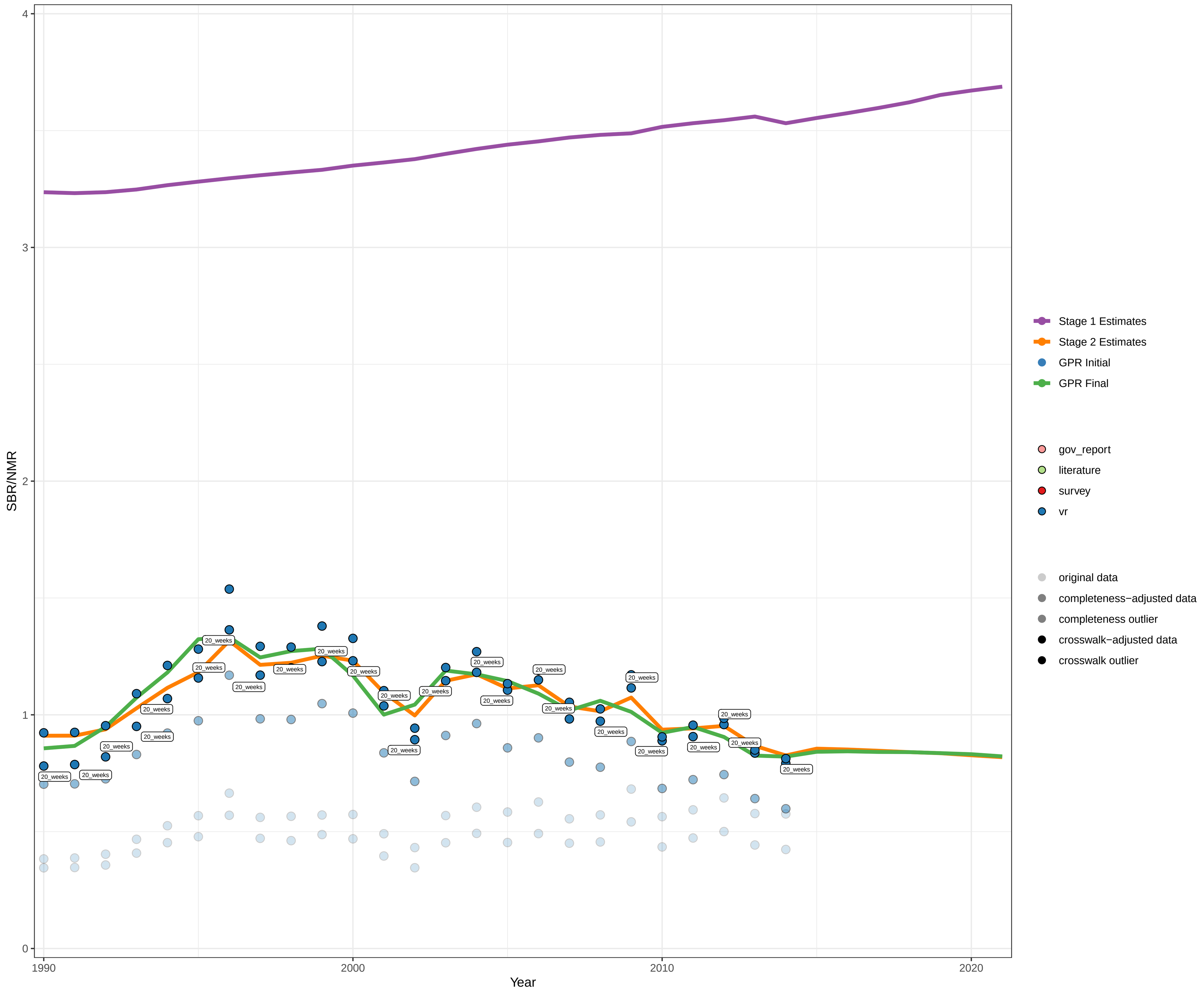

Chiapas MEX_4649 Central Latin America Stillbirth Rate / Neonatal Mortality Rate (SBR/NMR) − 28 weeks
lambda = 0.2 ; zeta = 0.99 ; scale = 5 ; amp = 0.028

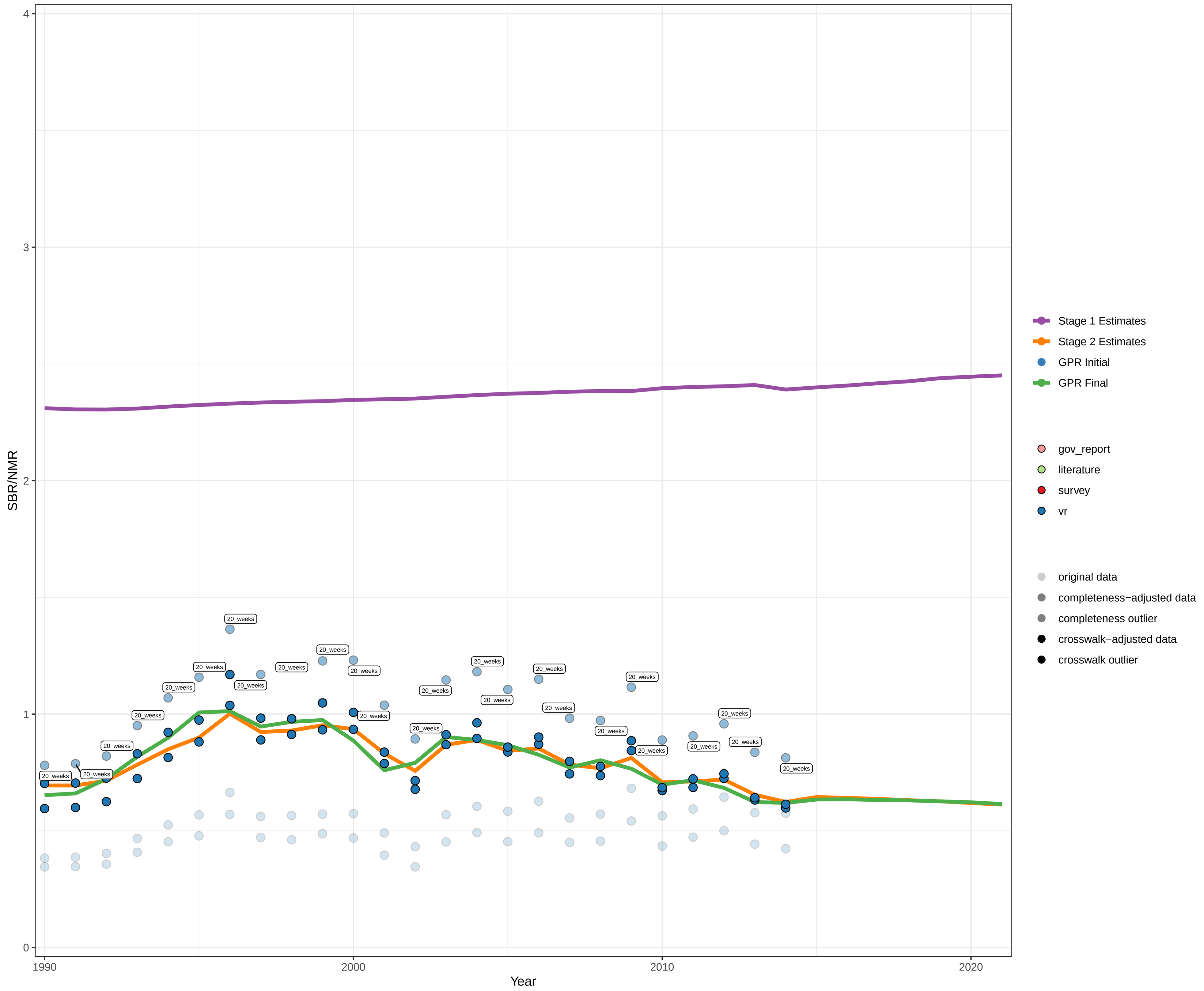

Definition	Years	Outlier	Source.Type	Title
20_weeks, 28_weeks	1990−2014	0	VR	Mexico INEGI Administrative Registers − Fetal Deaths by Municipality of Usual Residence of the Mother and Her Five−year Age Group According to Type of Fetal Death

Chiapas MEX_4649 Central Latin America Stillbirth Rate (SBR) − 20 weeks
lambda = 0.2 ; zeta = 0.99 ; scale = 5 ; amp = 0.028

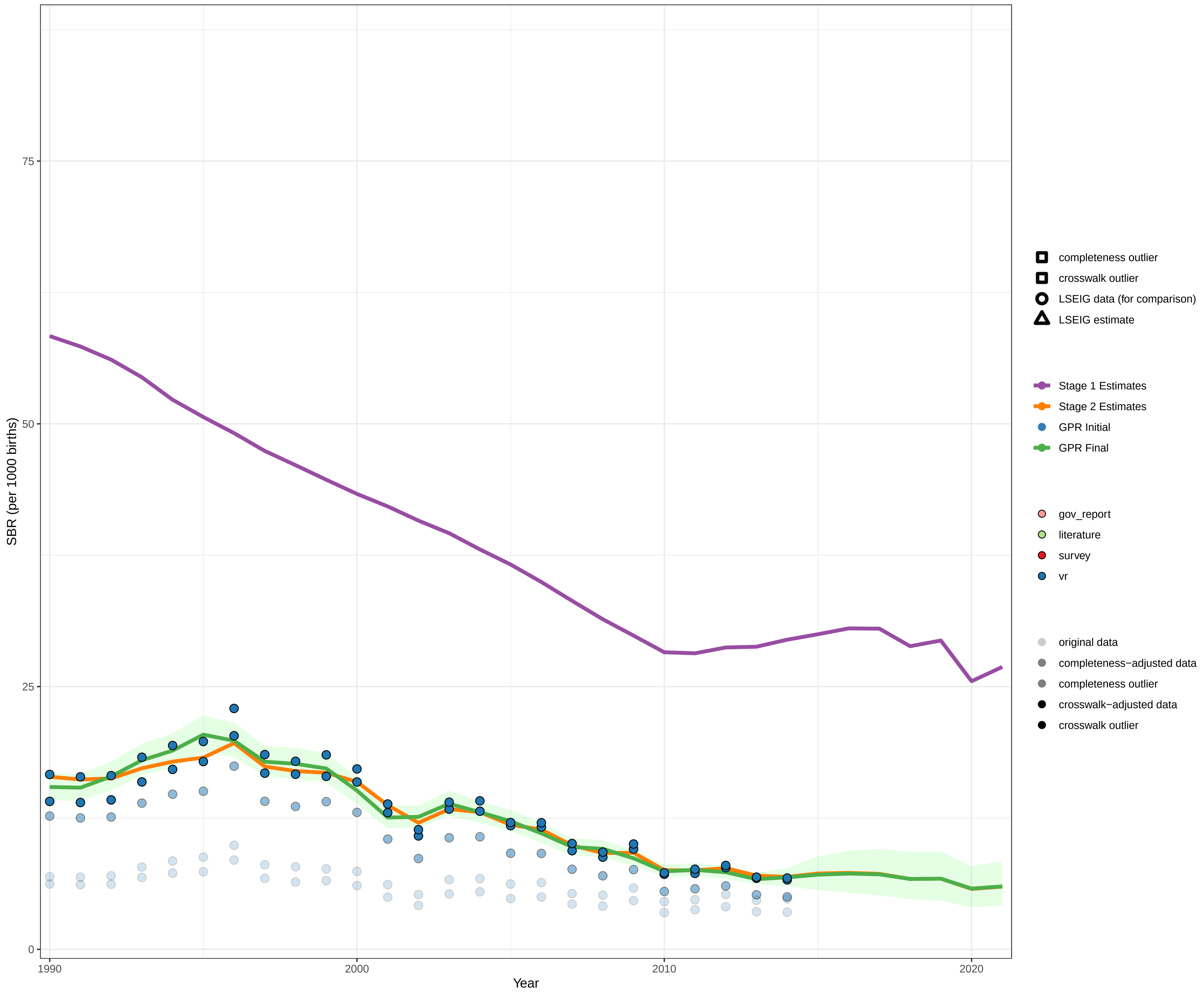

Chiapas MEX_4649 Central Latin America Stillbirth Rate (SBR) − 28 weeks
lambda = 0.2 ; zeta = 0.99 ; scale = 5 ; amp = 0.028

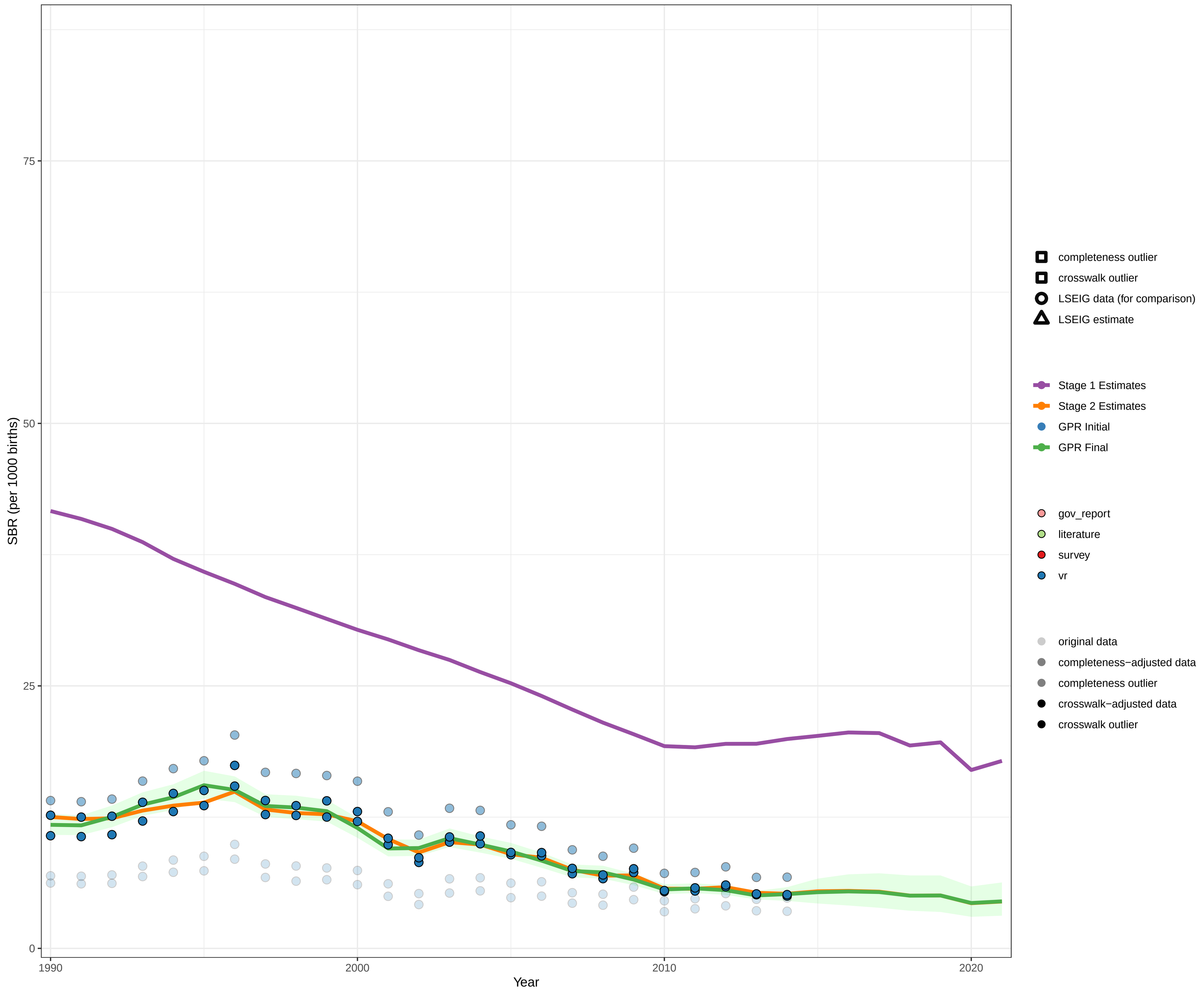

Year	Total Stillbirths (in thousands)	Total Stillbirths (in thousands)	SBR (per 1000 births)	SBR (per 1000 births)	SBR/NMR	SBR/NMR
	20 weeks	28 weeks	20 weeks	28 weeks	20 weeks	28 weeks
1990	2.1 (2.0 to 2.3)	1.6 (1.5 to 1.8)	15.4 (14.2 to 16.8)	11.8 (10.8 to 12.8)	0.86 (0.79 to 0.93)	0.65 (0.60 to 0.71)
1995	2.8 (2.6 to 3.1)	2.1 (1.9 to 2.3)	20.4 (18.8 to 22.3)	15.5 (14.3 to 16.9)	1.32 (1.21 to 1.44)	1.01 (0.93 to 1.10)
2000	2.0 (1.8 to 2.1)	1.5 (1.4 to 1.6)	15.1 (13.9 to 16.4)	11.5 (10.5 to 12.4)	1.17 (1.07 to 1.27)	0.89 (0.81 to 0.96)
2005	1.6 (1.5 to 1.8)	1.2 (1.1 to 1.3)	12.2 (11.3 to 13.3)	9.2 (8.5 to 10.0)	1.14 (1.06 to 1.25)	0.87 (0.80 to 0.94)
2010	1.1 (1.0 to 1.2)	0.8 (0.8 to 0.9)	7.4 (6.8 to 8.1)	5.6 (5.2 to 6.1)	0.92 (0.85 to 1.01)	0.70 (0.64 to 0.76)
2015	1.0 (0.8 to 1.3)	0.8 (0.6 to 0.9)	7.1 (5.7 to 8.8)	5.3 (4.3 to 6.6)	0.84 (0.67 to 1.05)	0.63 (0.51 to 0.79)
2020	0.7 (0.5 to 1.0)	0.5 (0.4 to 0.7)	5.8 (4.0 to 7.9)	4.3 (3.0 to 5.9)	0.83 (0.58 to 1.14)	0.62 (0.43 to 0.85)
2021	0.7 (0.5 to 1.0)	0.5 (0.4 to 0.8)	6.0 (4.2 to 8.4)	4.5 (3.1 to 6.3)	0.82 (0.57 to 1.16)	0.61 (0.43 to 0.86)

Chihuahua MEX_4650 Central Latin America Stillbirth Rate / Neonatal Mortality Rate (SBR/NMR) − 20 weeks
lambda = 0.2 ; zeta = 0.99 ; scale = 5 ; amp = 0.028

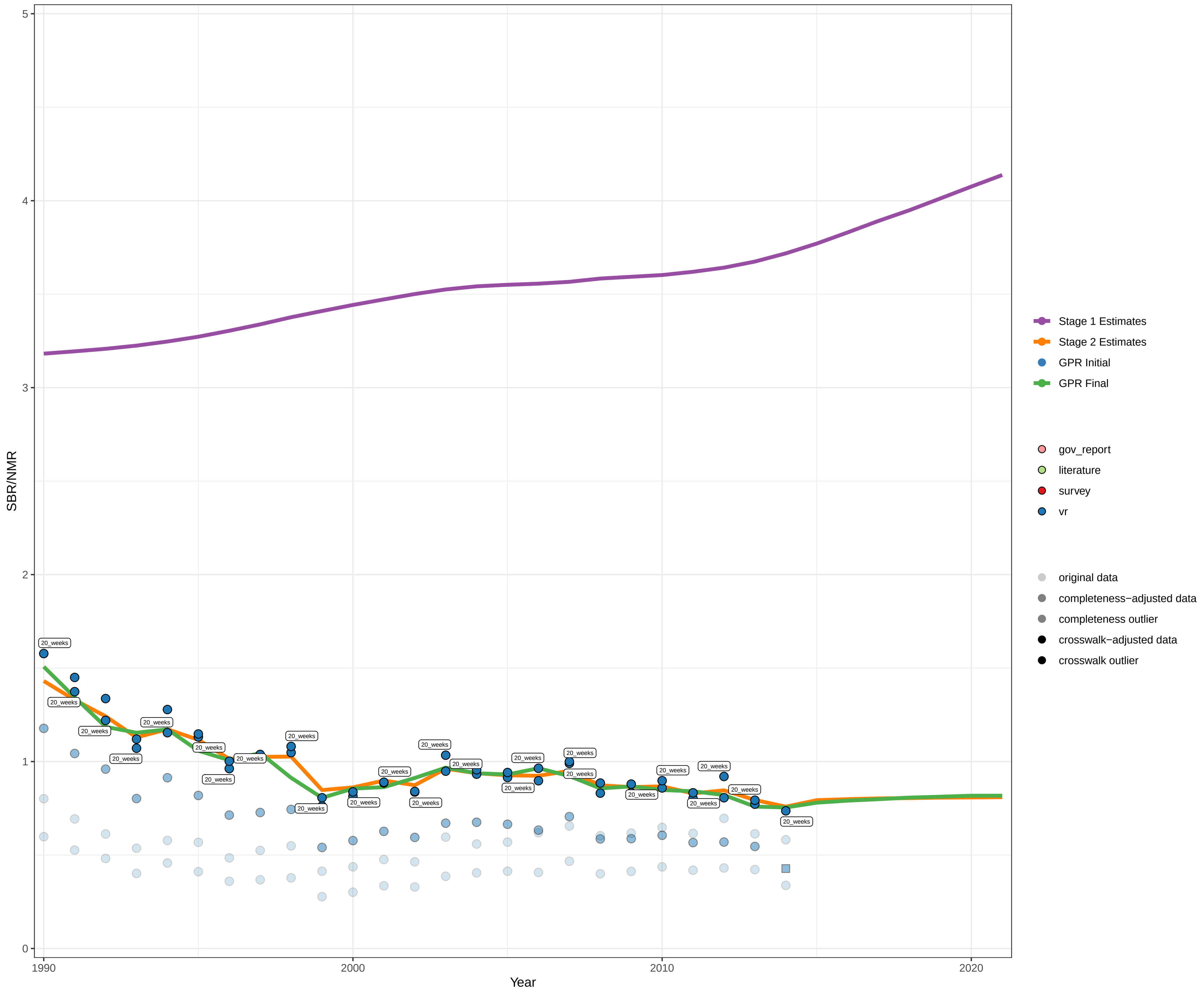

Chihuahua MEX_4650 Central Latin America Stillbirth Rate / Neonatal Mortality Rate (SBR/NMR) − 28 weeks
lambda = 0.2 ; zeta = 0.99 ; scale = 5 ; amp = 0.028

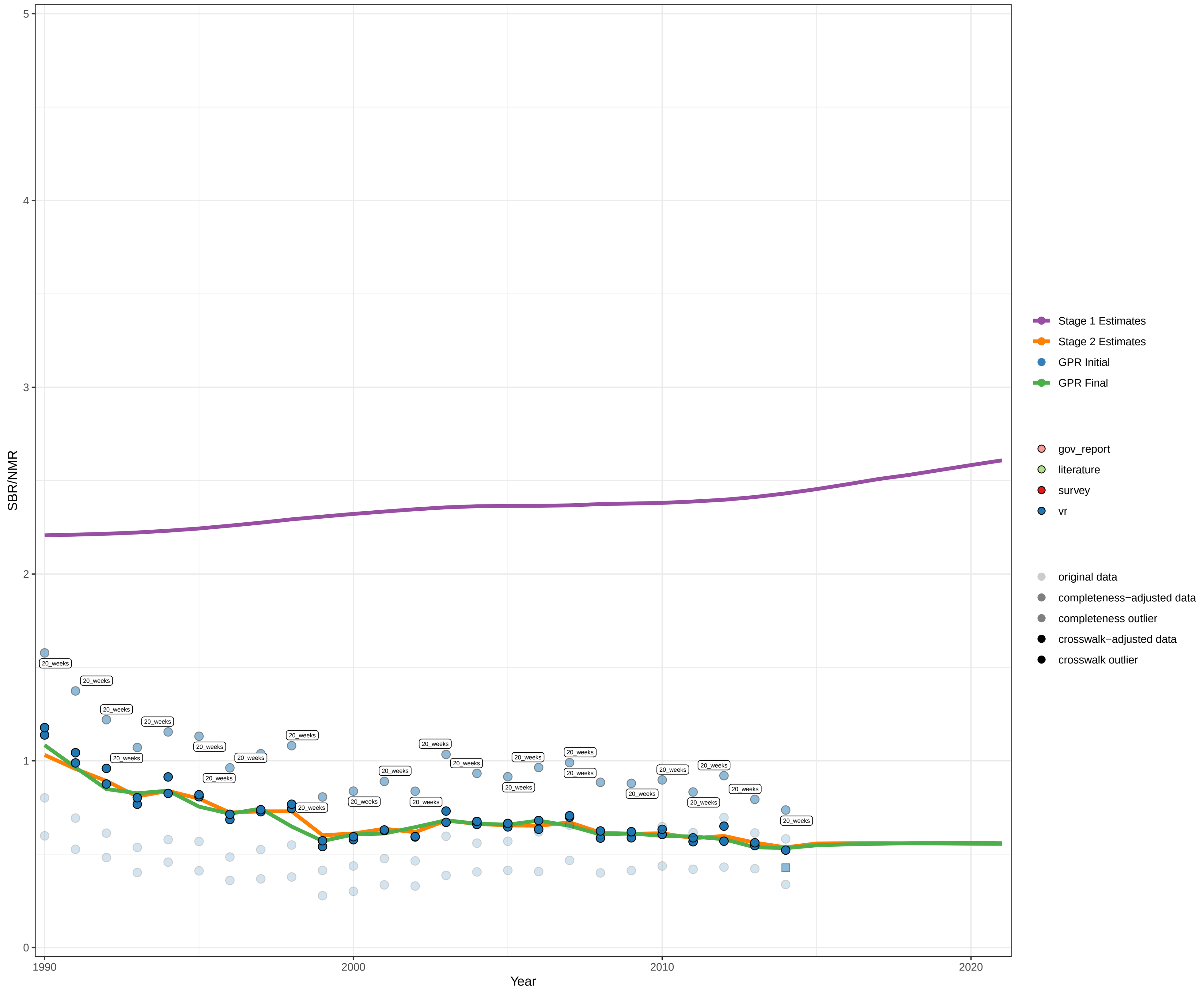

Definition	Years	Outlier	Source.Type	Title
20_weeks	1990−2014	0	VR	Mexico INEGI Administrative Registers − Fetal Deaths by Municipality of Usual Residence of the Mother and Her Five−year Age Group According to Type of Fetal Death
28_weeks	1990−2013	0	VR	Mexico INEGI Administrative Registers − Fetal Deaths by Municipality of Usual Residence of the Mother and Her Five−year Age Group According to Type of Fetal Death
28_weeks	2014	1	VR	Mexico INEGI Administrative Registers − Fetal Deaths by Municipality of Usual Residence of the Mother and Her Five−year Age Group According to Type of Fetal Death

Chihuahua MEX_4650 Central Latin America Stillbirth Rate (SBR) − 20 weeks
lambda = 0.2 ; zeta = 0.99 ; scale = 5 ; amp = 0.028

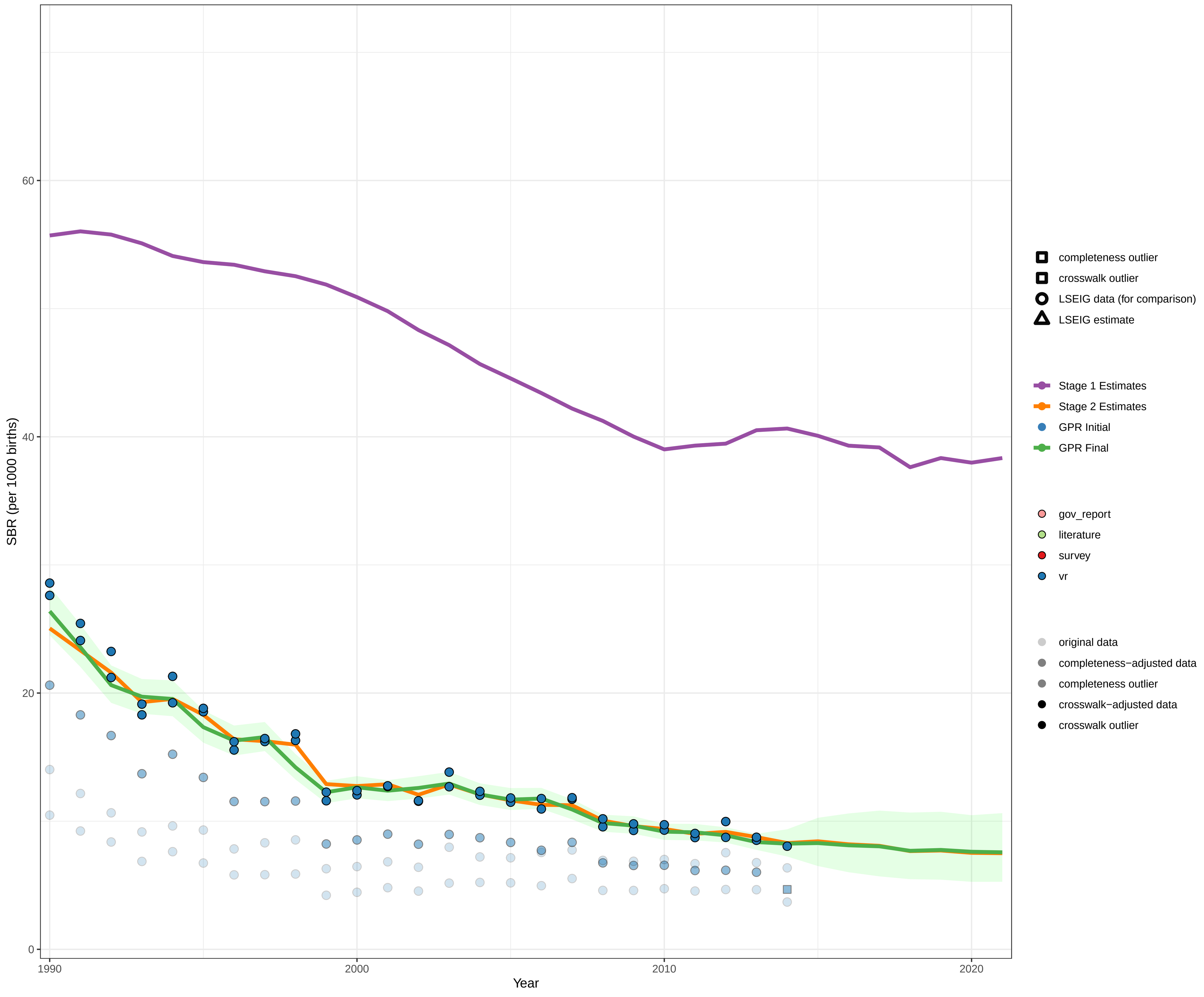

Chihuahua MEX_4650 Central Latin America Stillbirth Rate (SBR) − 28 weeks
lambda = 0.2 ; zeta = 0.99 ; scale = 5 ; amp = 0.028

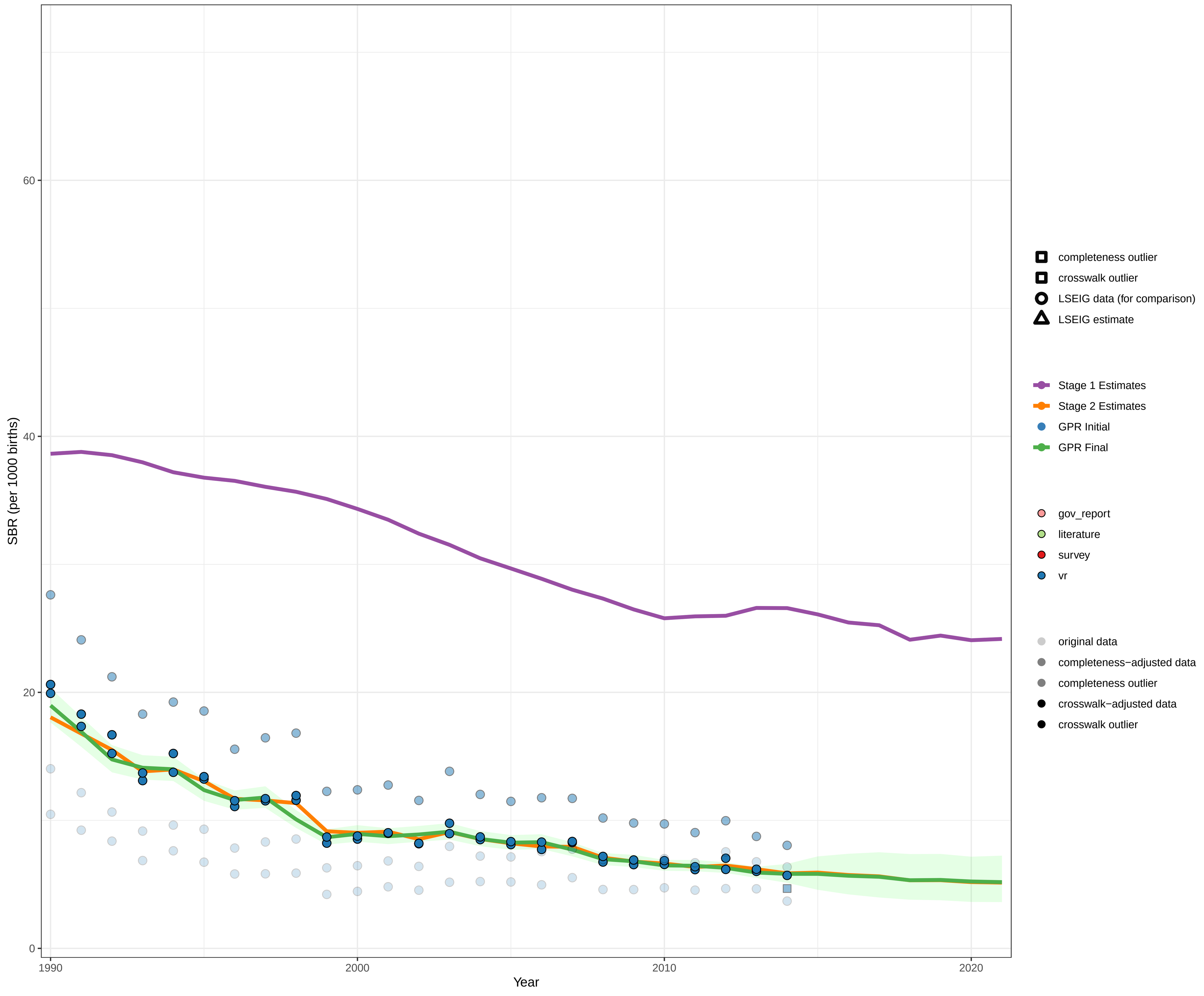

Year	Total Stillbirths (in thousands)	Total Stillbirths (in thousands)	SBR (per 1000 births)	SBR (per 1000 births)	SBR/NMR	SBR/NMR
	20 weeks	28 weeks	20 weeks	28 weeks	20 weeks	28 weeks
1990	1.9<br>(1.7 to 2.0)	1.3<br>(1.3 to 1.4)	26.4<br>(24.5 to 28.3)	19.0<br>(17.6 to 20.3)	1.51<br>(1.40 to 1.62)	1.08<br>(1.01 to 1.16)
1995	1.4<br>(1.3 to 1.5)	1.0<br>(0.9 to 1.0)	17.3<br>(16.1 to 18.7)	12.4<br>(11.5 to 13.2)	1.06<br>(0.98 to 1.14)	0.75<br>(0.70 to 0.81)
2000	1.0<br>(0.9 to 1.1)	0.7<br>(0.7 to 0.8)	12.6<br>(11.8 to 13.5)	8.9<br>(8.3 to 9.6)	0.86<br>(0.80 to 0.91)	0.61<br>(0.56 to 0.65)
2005	0.8<br>(0.8 to 0.9)	0.6<br>(0.6 to 0.6)	11.7<br>(10.9 to 12.6)	8.3<br>(7.7 to 8.9)	0.93<br>(0.87 to 1.00)	0.66<br>(0.61 to 0.71)
2010	0.7<br>(0.6 to 0.7)	0.5<br>(0.4 to 0.5)	9.2<br>(8.5 to 9.8)	6.5<br>(6.1 to 6.9)	0.85<br>(0.79 to 0.91)	0.60<br>(0.56 to 0.64)
2015	0.5<br>(0.4 to 0.7)	0.4<br>(0.3 to 0.5)	8.3<br>(6.5 to 10.3)	5.8<br>(4.6 to 7.2)	0.78<br>(0.61 to 0.97)	0.55<br>(0.43 to 0.68)
2020	0.4<br>(0.3 to 0.6)	0.3<br>(0.2 to 0.4)	7.6<br>(5.3 to 10.5)	5.2<br>(3.6 to 7.2)	0.82<br>(0.57 to 1.12)	0.56<br>(0.39 to 0.77)
2021	0.4<br>(0.3 to 0.6)	0.3<br>(0.2 to 0.4)	7.6<br>(5.3 to 10.6)	5.2<br>(3.6 to 7.2)	0.82<br>(0.57 to 1.15)	0.56<br>(0.39 to 0.78)

Coahuila MEX_4647 Central Latin America Stillbirth Rate / Neonatal Mortality Rate (SBR/NMR) − 20 weeks
lambda = 0.2 ; zeta = 0.99 ; scale = 5 ; amp = 0.028

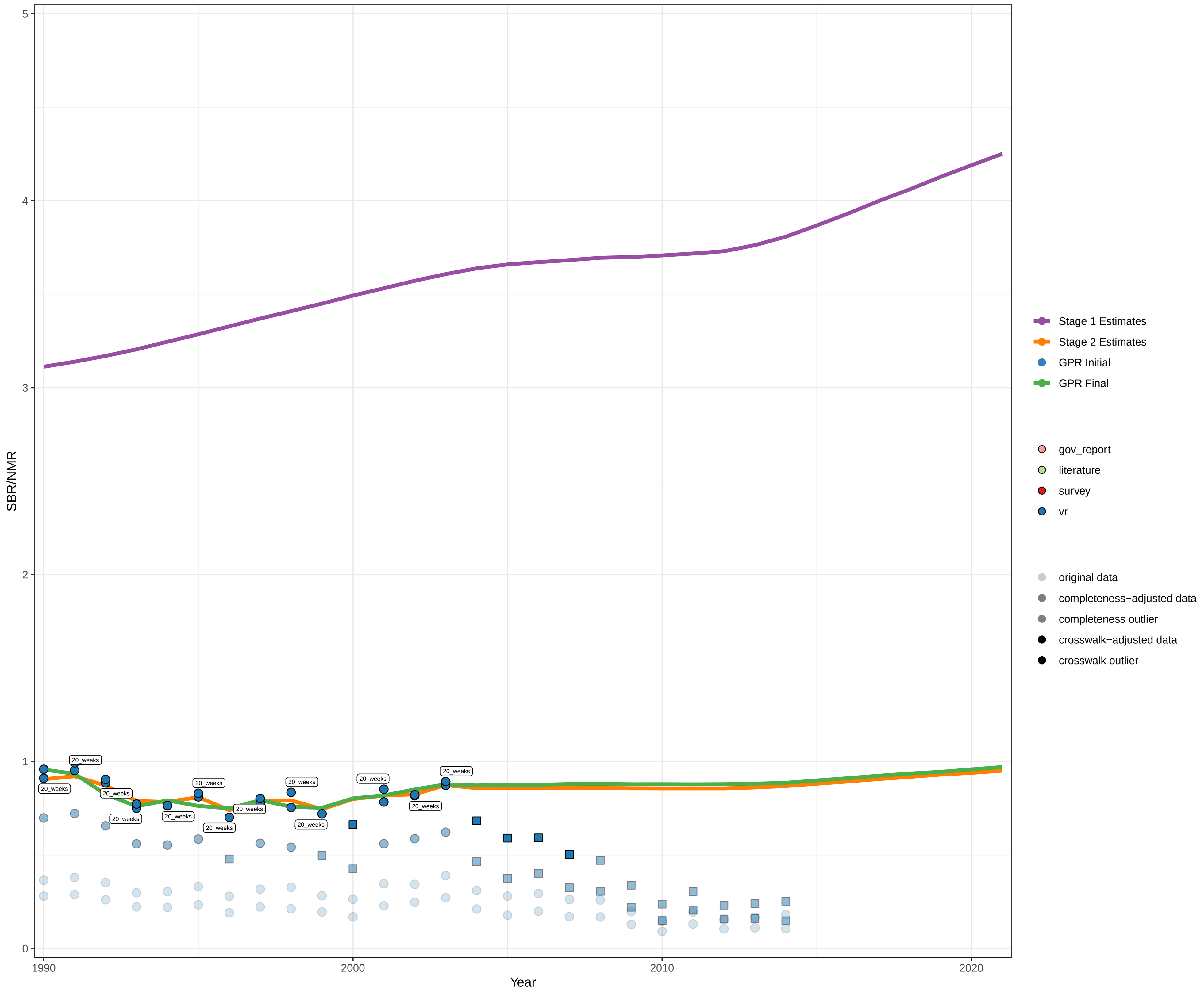

Coahuila MEX_4647 Central Latin America Stillbirth Rate / Neonatal Mortality Rate (SBR/NMR) − 28 weeks
lambda = 0.2 ; zeta = 0.99 ; scale = 5 ; amp = 0.028

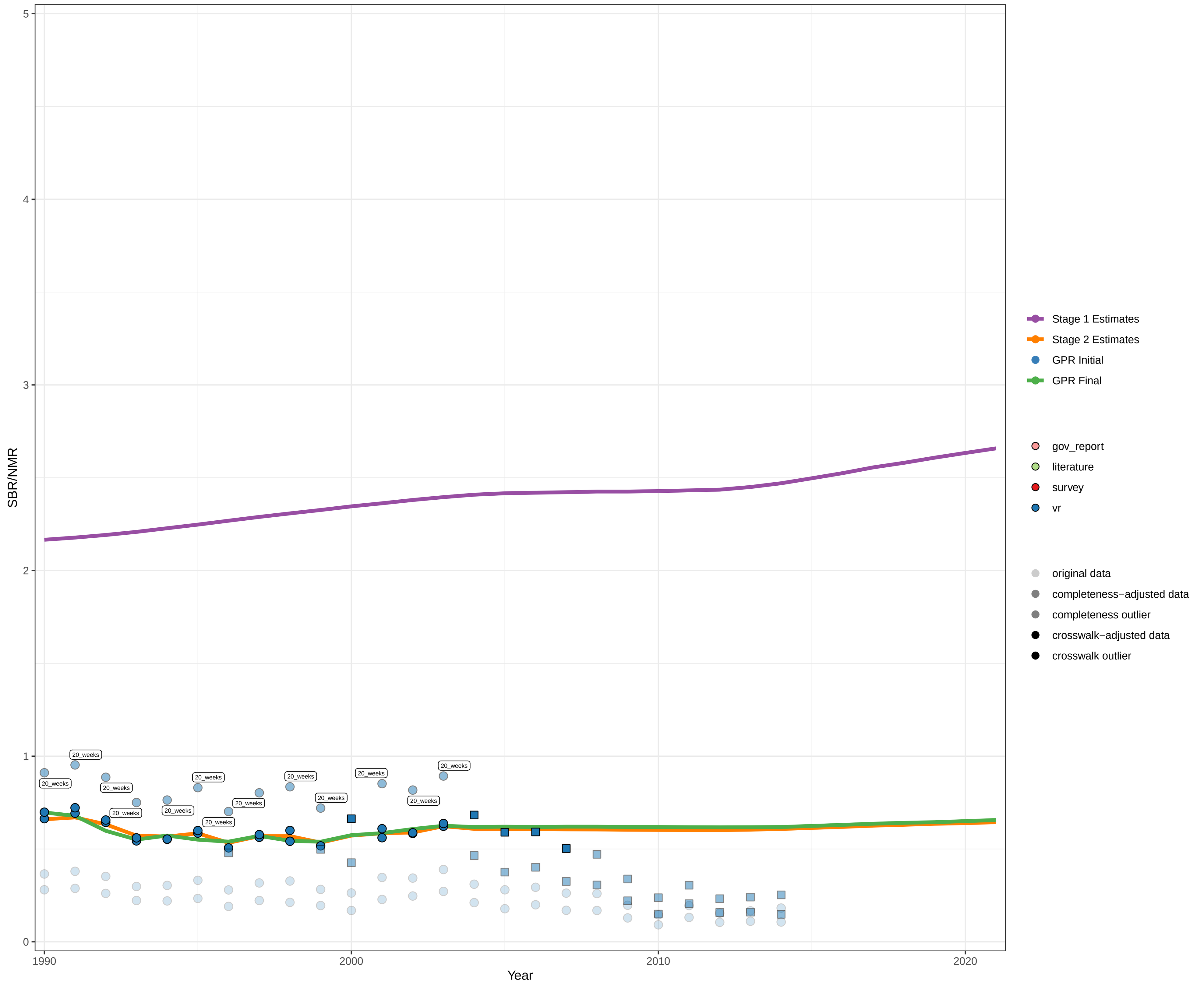

Definition	Years	Outlier	Source.Type	Title
20_weeks	1990−1999, 2001−2003	0	VR	Mexico INEGI Administrative Registers − Fetal Deaths by Municipality of Usual Residence of the Mother and Her Five−year Age Group According to Type of Fetal Death
28_weeks	1990−1995, 1997, 1998, 2001−2003	0	VR	Mexico INEGI Administrative Registers − Fetal Deaths by Municipality of Usual Residence of the Mother and Her Five−year Age Group According to Type of Fetal Death
20_weeks	2008−2014	1	VR	Mexico INEGI Administrative Registers − Fetal Deaths by Municipality of Usual Residence of the Mother and Her Five−year Age Group According to Type of Fetal Death
28_weeks	1996, 1999, 2000, 2004−2014	1	VR	Mexico INEGI Administrative Registers − Fetal Deaths by Municipality of Usual Residence of the Mother and Her Five−year Age Group According to Type of Fetal Death
20_weeks	2000, 2004−2007	2	VR	Mexico INEGI Administrative Registers − Fetal Deaths by Municipality of Usual Residence of the Mother and Her Five−year Age Group According to Type of Fetal Death

Coahuila MEX_4647 Central Latin America Stillbirth Rate (SBR) − 20 weeks
lambda = 0.2 ; zeta = 0.99 ; scale = 5 ; amp = 0.028

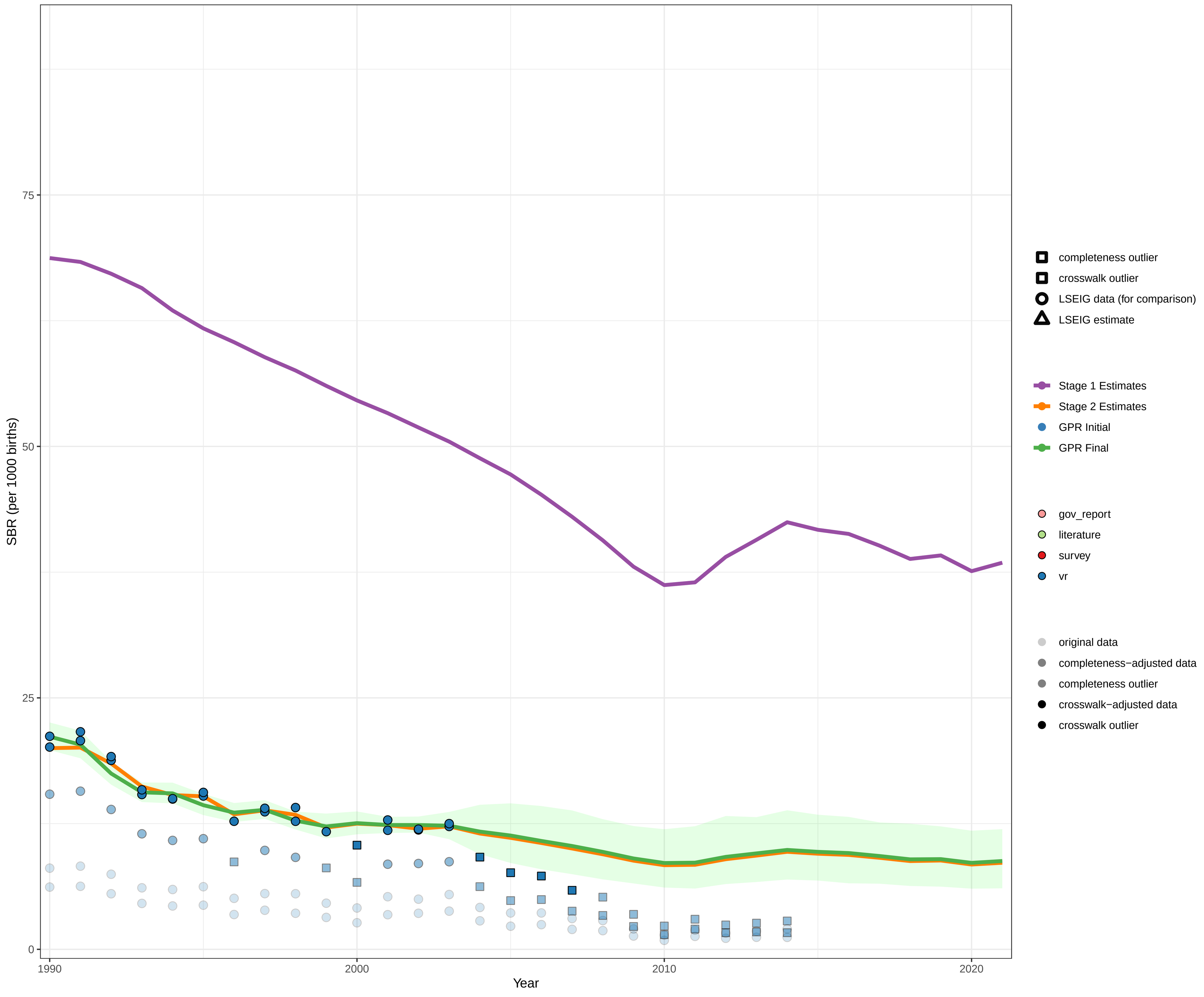

Coahuila MEX_4647 Central Latin America Stillbirth Rate (SBR) − 28 weeks
lambda = 0.2 ; zeta = 0.99 ; scale = 5 ; amp = 0.028

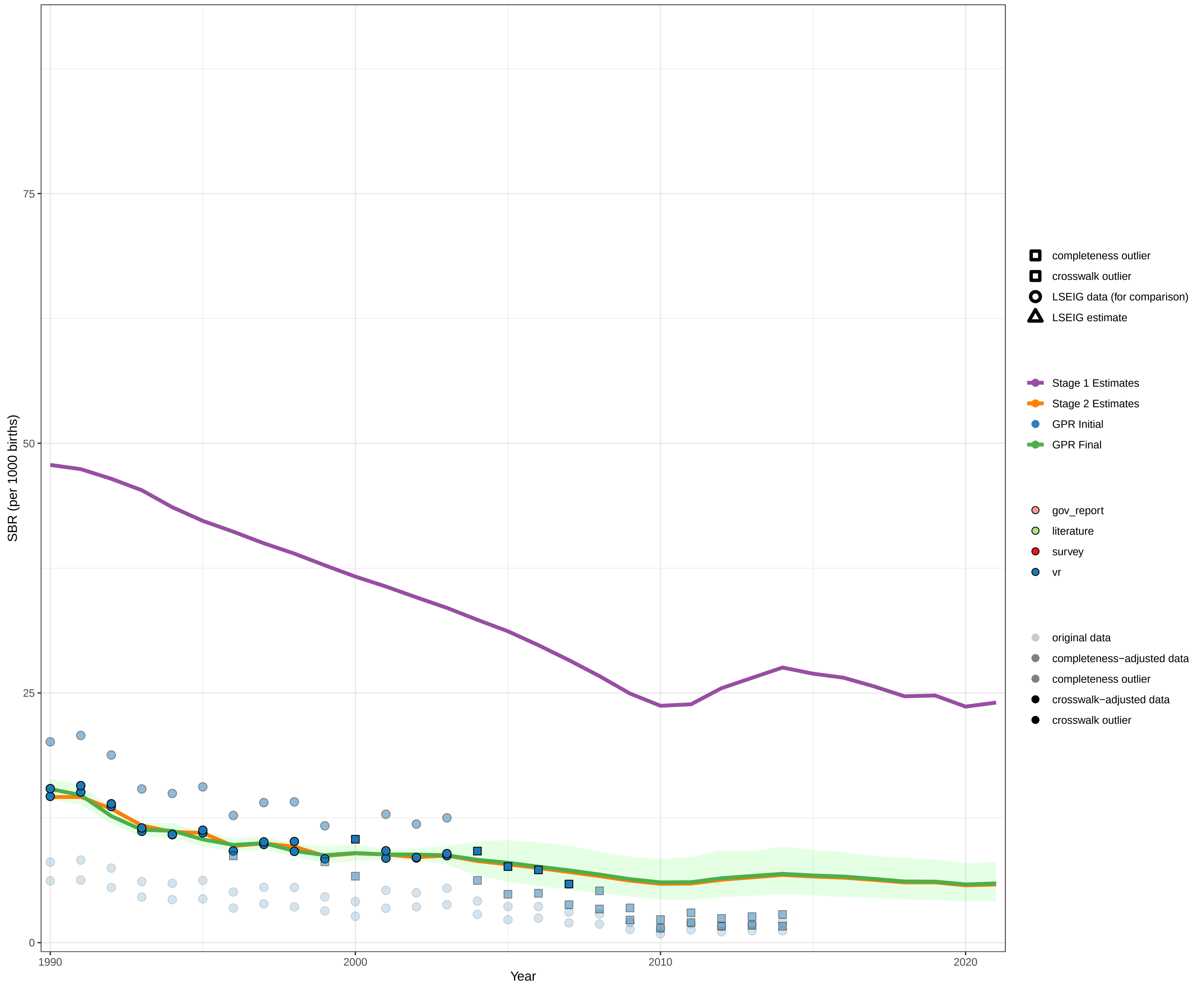

Year	Total Stillbirths (in thousands)	Total Stillbirths (in thousands)	SBR (per 1000 births)	SBR (per 1000 births)	SBR/NMR	SBR/NMR
	20 weeks	28 weeks	20 weeks	28 weeks	20 weeks	28 weeks
1990	1.2 (1.2 to 1.3)	0.9 (0.8 to 1.0)	21.1 (19.8 to 22.6)	15.4 (14.4 to 16.4)	0.96 (0.90 to 1.02)	0.70 (0.65 to 0.74)
1995	0.8 (0.8 to 0.9)	0.6 (0.6 to 0.6)	14.3 (13.3 to 15.5)	10.3 (9.6 to 11.1)	0.76 (0.71 to 0.82)	0.55 (0.51 to 0.59)
2000	0.7 (0.6 to 0.8)	0.5 (0.5 to 0.5)	12.5 (11.4 to 13.7)	9.0 (8.2 to 9.8)	0.80 (0.73 to 0.88)	0.57 (0.52 to 0.63)
2005	0.6 (0.5 to 0.8)	0.4 (0.3 to 0.6)	11.3 (8.6 to 14.5)	8.0 (6.1 to 10.2)	0.88 (0.67 to 1.13)	0.62 (0.47 to 0.79)
2010	0.5 (0.4 to 0.7)	0.4 (0.3 to 0.5)	8.6 (6.1 to 11.9)	6.0 (4.3 to 8.4)	0.88 (0.63 to 1.22)	0.62 (0.44 to 0.86)
2015	0.6 (0.4 to 0.8)	0.4 (0.3 to 0.6)	9.7 (6.8 to 13.4)	6.7 (4.8 to 9.3)	0.90 (0.63 to 1.24)	0.62 (0.44 to 0.86)
2020	0.4 (0.3 to 0.6)	0.3 (0.2 to 0.4)	8.6 (6.0 to 11.8)	5.8 (4.1 to 8.0)	0.96 (0.67 to 1.31)	0.65 (0.46 to 0.89)
2021	0.4 (0.3 to 0.6)	0.3 (0.2 to 0.4)	8.8 (6.1 to 11.9)	5.9 (4.1 to 8.1)	0.97 (0.67 to 1.32)	0.66 (0.45 to 0.89)

Colima MEX_4648 Central Latin America Stillbirth Rate / Neonatal Mortality Rate (SBR/NMR) − 20 weeks
lambda = 0.2 ; zeta = 0.99 ; scale = 5 ; amp = 0.028

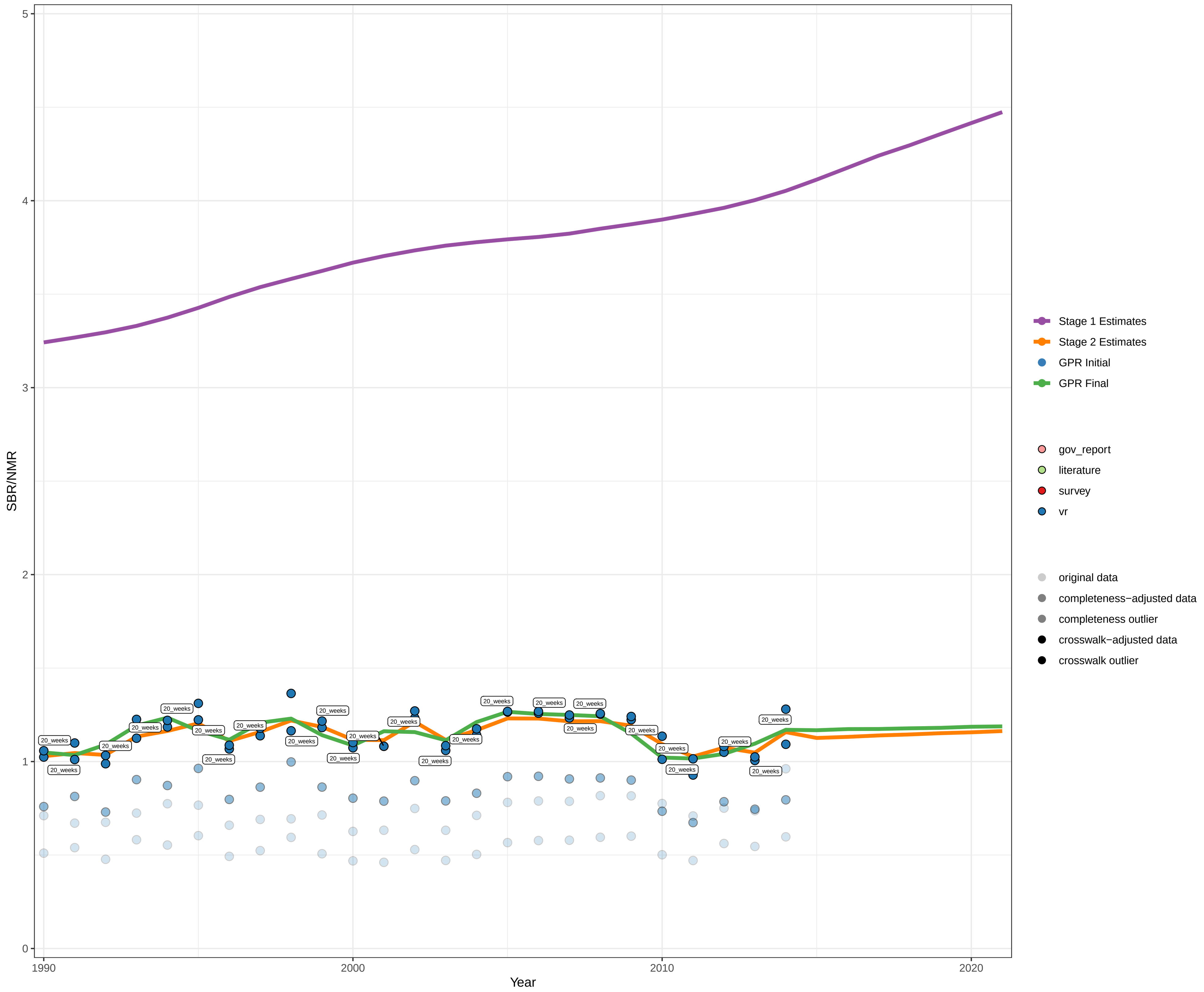

Colima MEX_4648 Central Latin America Stillbirth Rate / Neonatal Mortality Rate (SBR/NMR) − 28 weeks
lambda = 0.2 ; zeta = 0.99 ; scale = 5 ; amp = 0.028

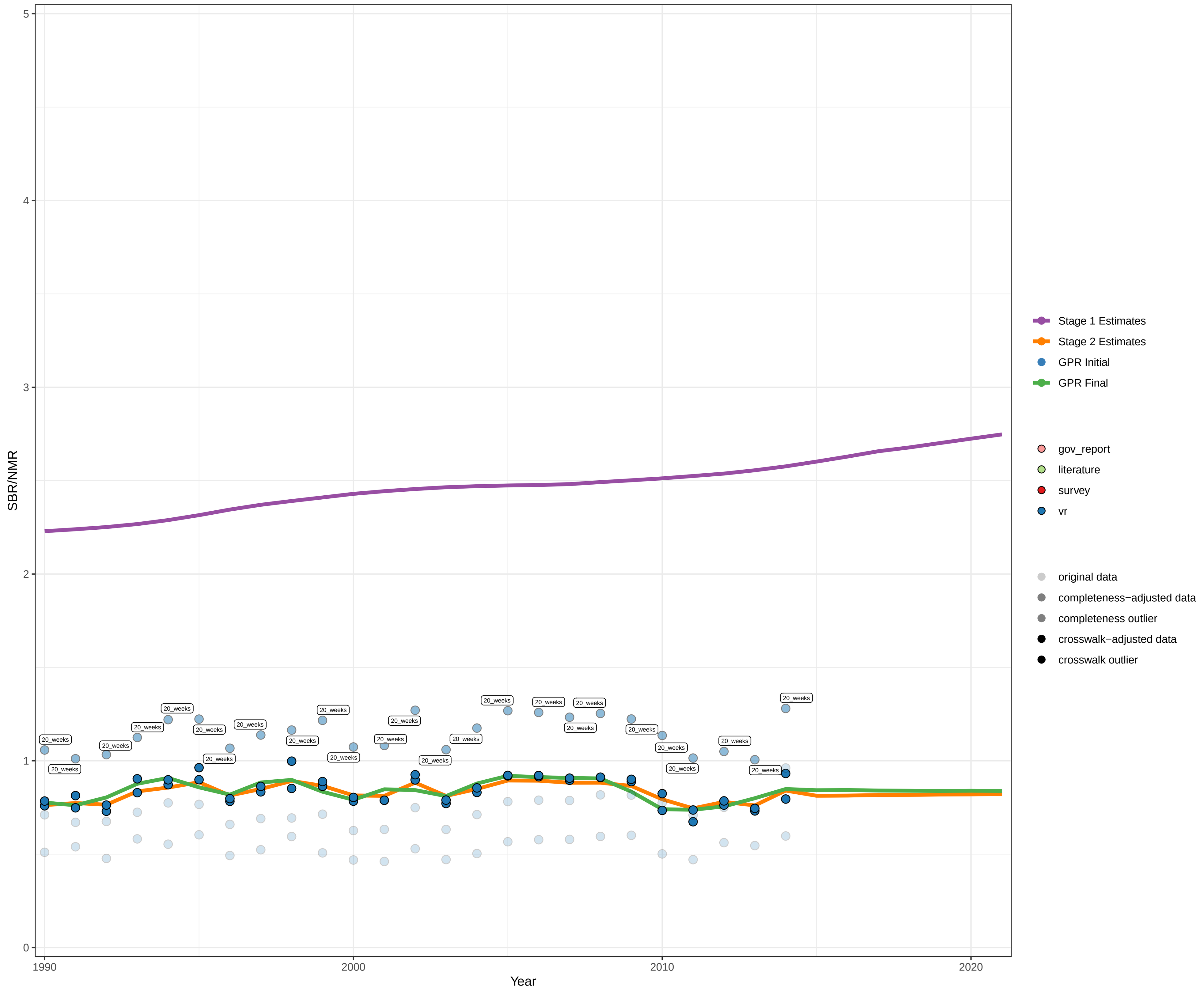

Definition	Years	Outlier	Source.Type	Title
20_weeks, 28_weeks	1990−2014	0	VR	Mexico INEGI Administrative Registers − Fetal Deaths by Municipality of Usual Residence of the Mother and Her Five−year Age Group According to Type of Fetal Death

Colima MEX_4648 Central Latin America Stillbirth Rate (SBR) − 20 weeks
lambda = 0.2 ; zeta = 0.99 ; scale = 5 ; amp = 0.028

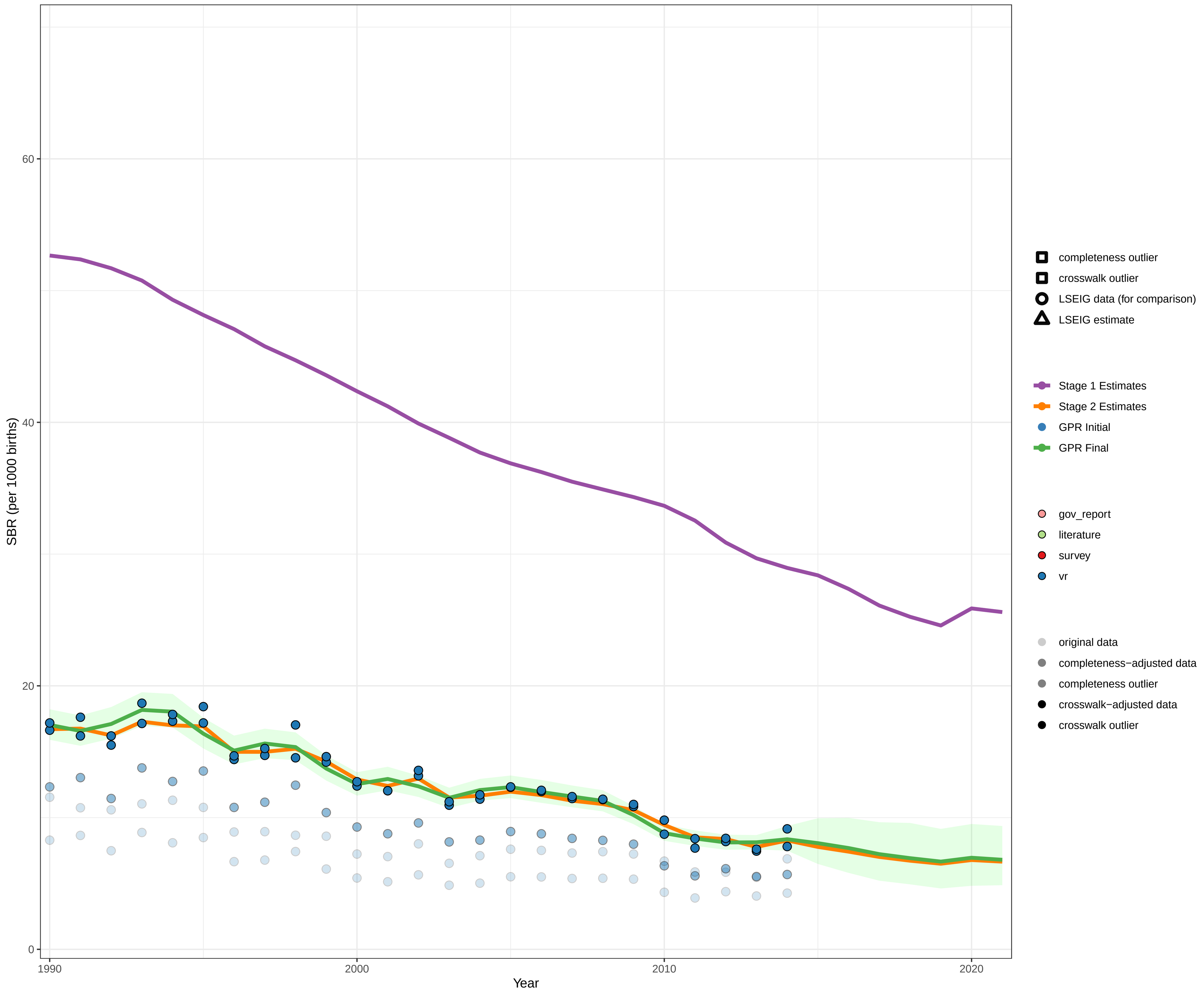

Colima MEX_4648 Central Latin America Stillbirth Rate (SBR) − 28 weeks
lambda = 0.2 ; zeta = 0.99 ; scale = 5 ; amp = 0.028

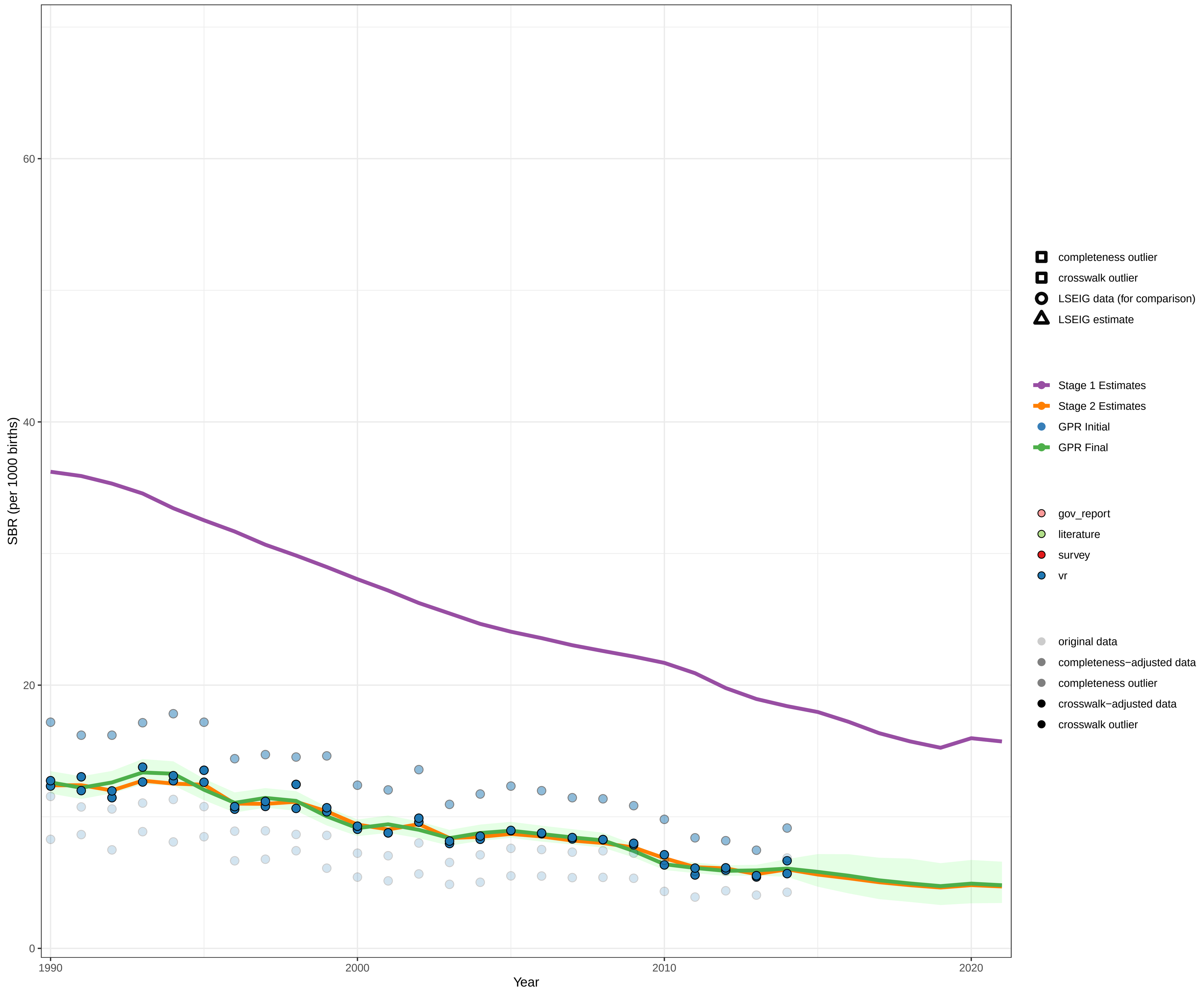

Year	Total Stillbirths (in thousands)	Total Stillbirths (in thousands)	SBR (per 1000 births)	SBR (per 1000 births)	SBR/NMR	SBR/NMR
	20 weeks	28 weeks	20 weeks	28 weeks	20 weeks	28 weeks
1990	0.2 (0.2 to 0.2)	0.2 (0.1 to 0.2)	17.0 (15.9 to 18.2)	12.6 (11.8 to 13.5)	1.05 (0.98 to 1.12)	0.78 (0.72 to 0.83)
1995	0.2 (0.2 to 0.2)	0.1 (0.1 to 0.2)	16.4 (15.2 to 17.6)	12.1 (11.3 to 12.9)	1.17 (1.09 to 1.25)	0.86 (0.80 to 0.92)
2000	0.1 (0.1 to 0.2)	0.1 (0.1 to 0.1)	12.5 (11.7 to 13.4)	9.1 (8.5 to 9.8)	1.09 (1.01 to 1.16)	0.79 (0.74 to 0.85)
2005	0.1 (0.1 to 0.2)	0.1 (0.1 to 0.1)	12.3 (11.5 to 13.2)	8.9 (8.4 to 9.6)	1.27 (1.18 to 1.36)	0.92 (0.86 to 0.99)
2010	0.1 (0.1 to 0.1)	0.1 (0.1 to 0.1)	8.8 (8.2 to 9.5)	6.4 (6.0 to 6.8)	1.02 (0.95 to 1.10)	0.74 (0.69 to 0.79)
2015	0.1 (0.1 to 0.1)	0.1 (0.1 to 0.1)	8.1 (6.5 to 10.0)	5.8 (4.7 to 7.2)	1.17 (0.94 to 1.44)	0.84 (0.68 to 1.04)
2020	0.1 (0.0 to 0.1)	0.1 (0.0 to 0.1)	7.0 (4.8 to 9.5)	4.9 (3.4 to 6.7)	1.19 (0.82 to 1.62)	0.84 (0.58 to 1.15)
2021	0.1 (0.0 to 0.1)	0.0 (0.0 to 0.1)	6.8 (4.9 to 9.4)	4.8 (3.4 to 6.6)	1.19 (0.85 to 1.64)	0.84 (0.60 to 1.15)

Durango MEX_4652 Central Latin America Stillbirth Rate / Neonatal Mortality Rate (SBR/NMR) − 20 weeks
lambda = 0.2 ; zeta = 0.99 ; scale = 5 ; amp = 0.028

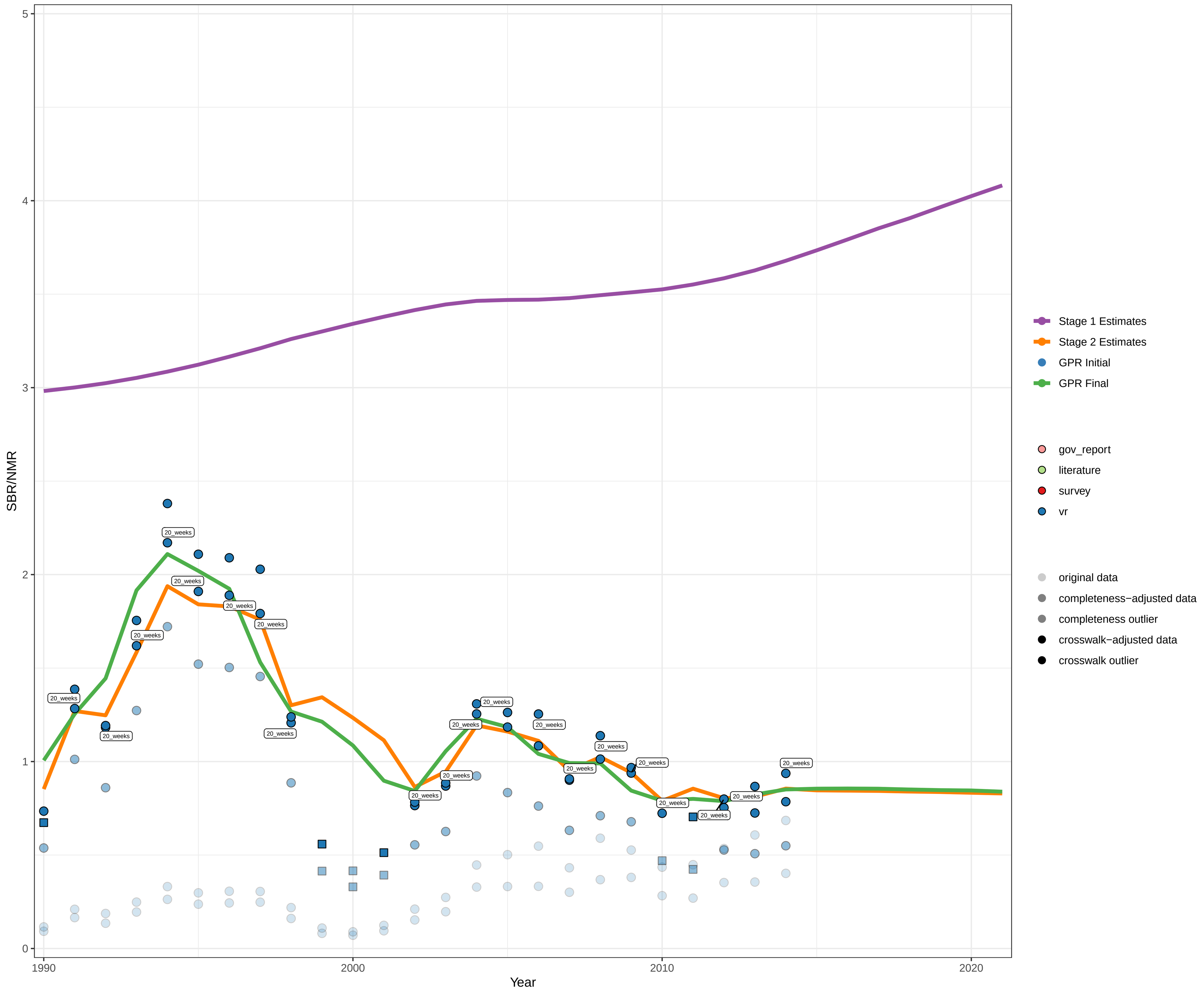

Durango MEX_4652 Central Latin America Stillbirth Rate / Neonatal Mortality Rate (SBR/NMR) − 28 weeks
lambda = 0.2 ; zeta = 0.99 ; scale = 5 ; amp = 0.028

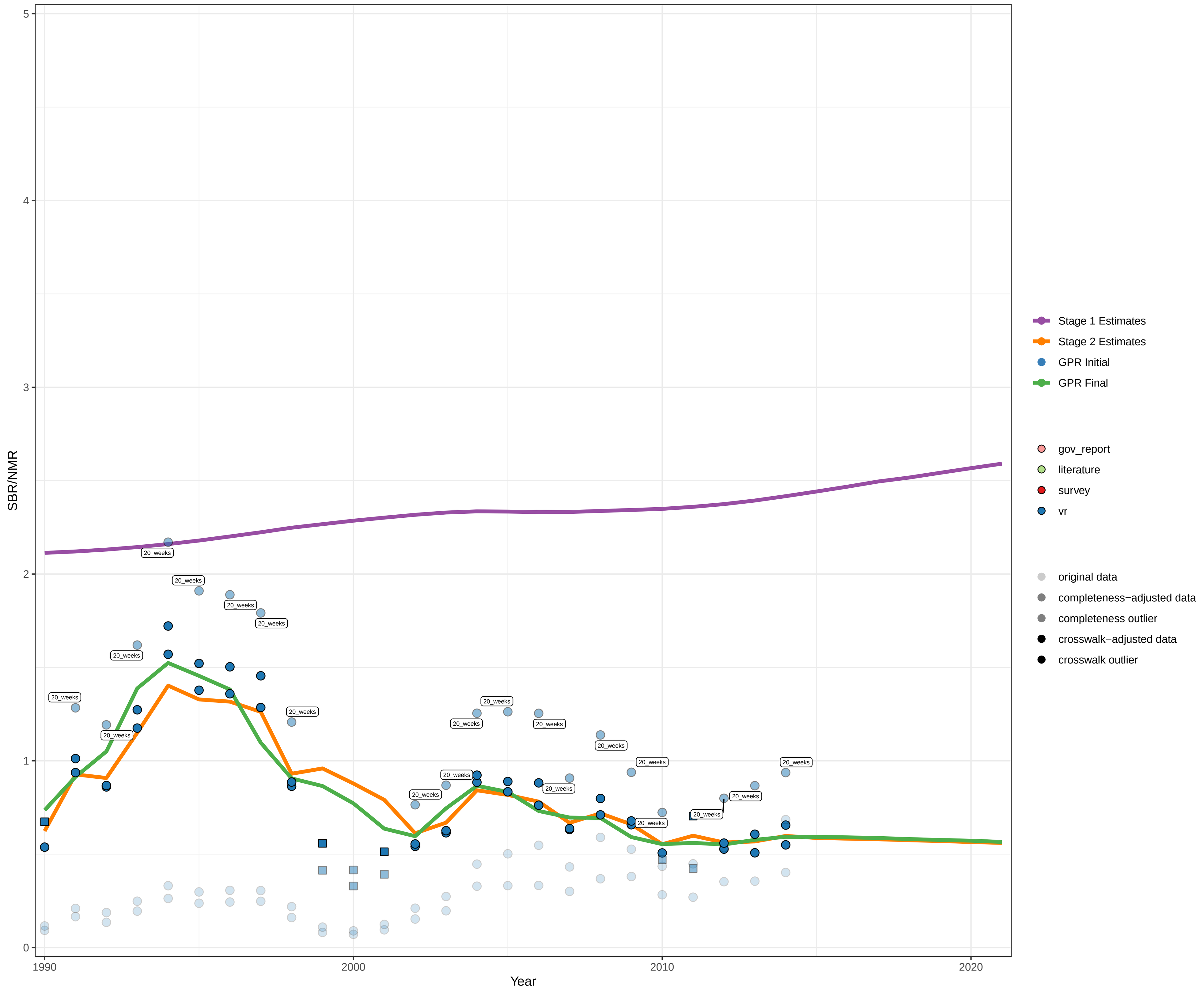

Definition	Years	Outlier	Source.Type	Title
20_weeks	1991−1998, 2002−2010, 2012−2014	0	VR	Mexico INEGI Administrative Registers − Fetal Deaths by Municipality of Usual Residence of the Mother and Her Five−year Age Group According to Type of Fetal Death
28_weeks	1990−1998, 2002−2009, 2012−2014	0	VR	Mexico INEGI Administrative Registers − Fetal Deaths by Municipality of Usual Residence of the Mother and Her Five−year Age Group According to Type of Fetal Death
20_weeks	2000	1	VR	Mexico INEGI Administrative Registers − Fetal Deaths by Municipality of Usual Residence of the Mother and Her Five−year Age Group According to Type of Fetal Death
28_weeks	1999−2001, 2010, 2011	1	VR	Mexico INEGI Administrative Registers − Fetal Deaths by Municipality of Usual Residence of the Mother and Her Five−year Age Group According to Type of Fetal Death
20_weeks	1990, 1999, 2001, 2011	2	VR	Mexico INEGI Administrative Registers − Fetal Deaths by Municipality of Usual Residence of the Mother and Her Five−year Age Group According to Type of Fetal Death

Durango MEX_4652 Central Latin America Stillbirth Rate (SBR) − 20 weeks
lambda = 0.2 ; zeta = 0.99 ; scale = 5 ; amp = 0.028

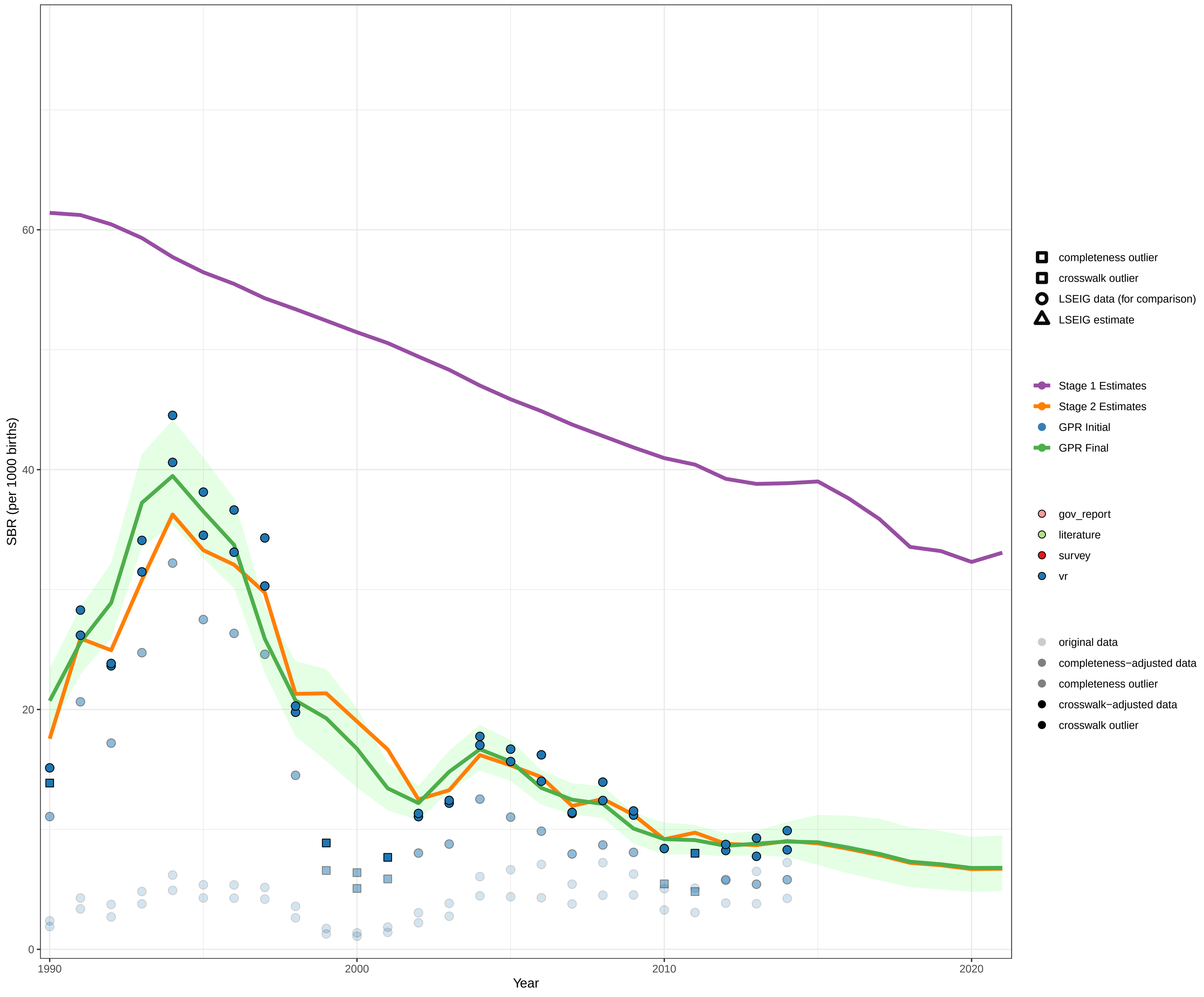

Durango MEX_4652 Central Latin America Stillbirth Rate (SBR) − 28 weeks
lambda = 0.2 ; zeta = 0.99 ; scale = 5 ; amp = 0.028

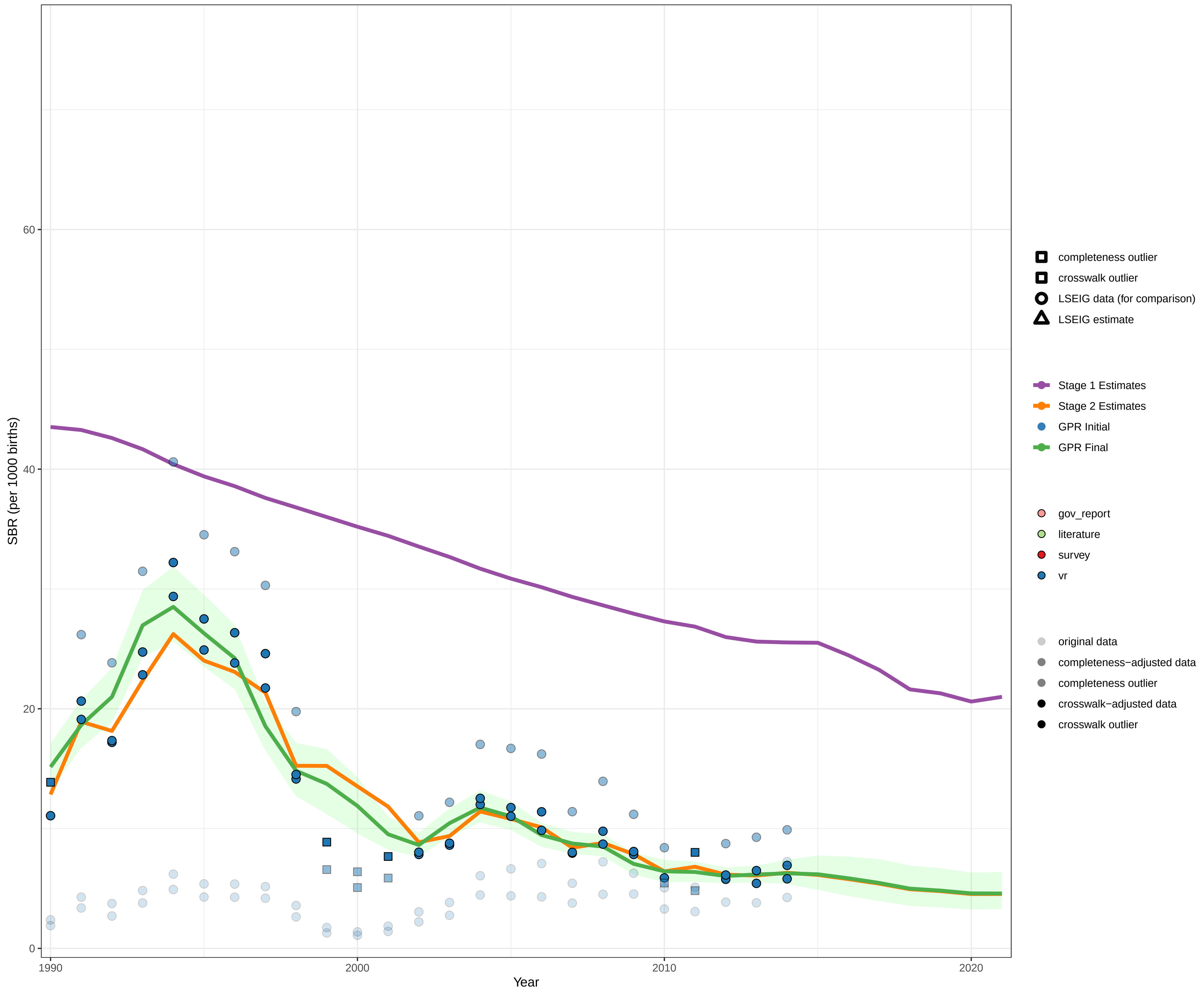

Year	Total Stillbirths (in thousands)	Total Stillbirths (in thousands)	SBR (per 1000 births)	SBR (per 1000 births)	SBR/NMR	SBR/NMR
	20 weeks	28 weeks	20 weeks	28 weeks	20 weeks	28 weeks
1990	1.0 (0.8 to 1.1)	0.7 (0.6 to 0.8)	20.7 (18.1 to 23.4)	15.1 (13.2 to 17.1)	1.01 (0.88 to 1.14)	0.74 (0.64 to 0.83)
1995	1.7 (1.6 to 2.0)	1.2 (1.1 to 1.4)	36.5 (32.6 to 41.0)	26.3 (23.5 to 29.5)	2.02 (1.80 to 2.27)	1.45 (1.30 to 1.63)
2000	0.7 (0.6 to 0.9)	0.5 (0.4 to 0.6)	16.7 (13.5 to 20.1)	11.9 (9.6 to 14.3)	1.09 (0.87 to 1.31)	0.77 (0.62 to 0.93)
2005	0.6 (0.5 to 0.7)	0.4 (0.4 to 0.5)	15.7 (14.0 to 17.4)	11.0 (9.9 to 12.3)	1.18 (1.06 to 1.32)	0.83 (0.75 to 0.93)
2010	0.4 (0.3 to 0.4)	0.3 (0.2 to 0.3)	9.2 (7.9 to 10.6)	6.4 (5.5 to 7.4)	0.79 (0.68 to 0.91)	0.55 (0.48 to 0.64)
2015	0.3 (0.3 to 0.4)	0.2 (0.2 to 0.3)	8.9 (7.0 to 11.2)	6.2 (4.9 to 7.7)	0.85 (0.67 to 1.07)	0.59 (0.47 to 0.74)
2020	0.2 (0.2 to 0.3)	0.1 (0.1 to 0.2)	6.8 (4.8 to 9.4)	4.6 (3.3 to 6.3)	0.85 (0.60 to 1.17)	0.57 (0.41 to 0.79)
2021	0.2 (0.1 to 0.3)	0.1 (0.1 to 0.2)	6.8 (4.8 to 9.5)	4.6 (3.3 to 6.4)	0.84 (0.60 to 1.17)	0.57 (0.40 to 0.79)

Guanajuato   MEX_4653   Central Latin America   Stillbirth Rate / Neonatal Mortality Rate (SBR/NMR) − 20 weeks
lambda = 0.2 ; zeta = 0.99 ; scale = 5 ; amp = 0.028

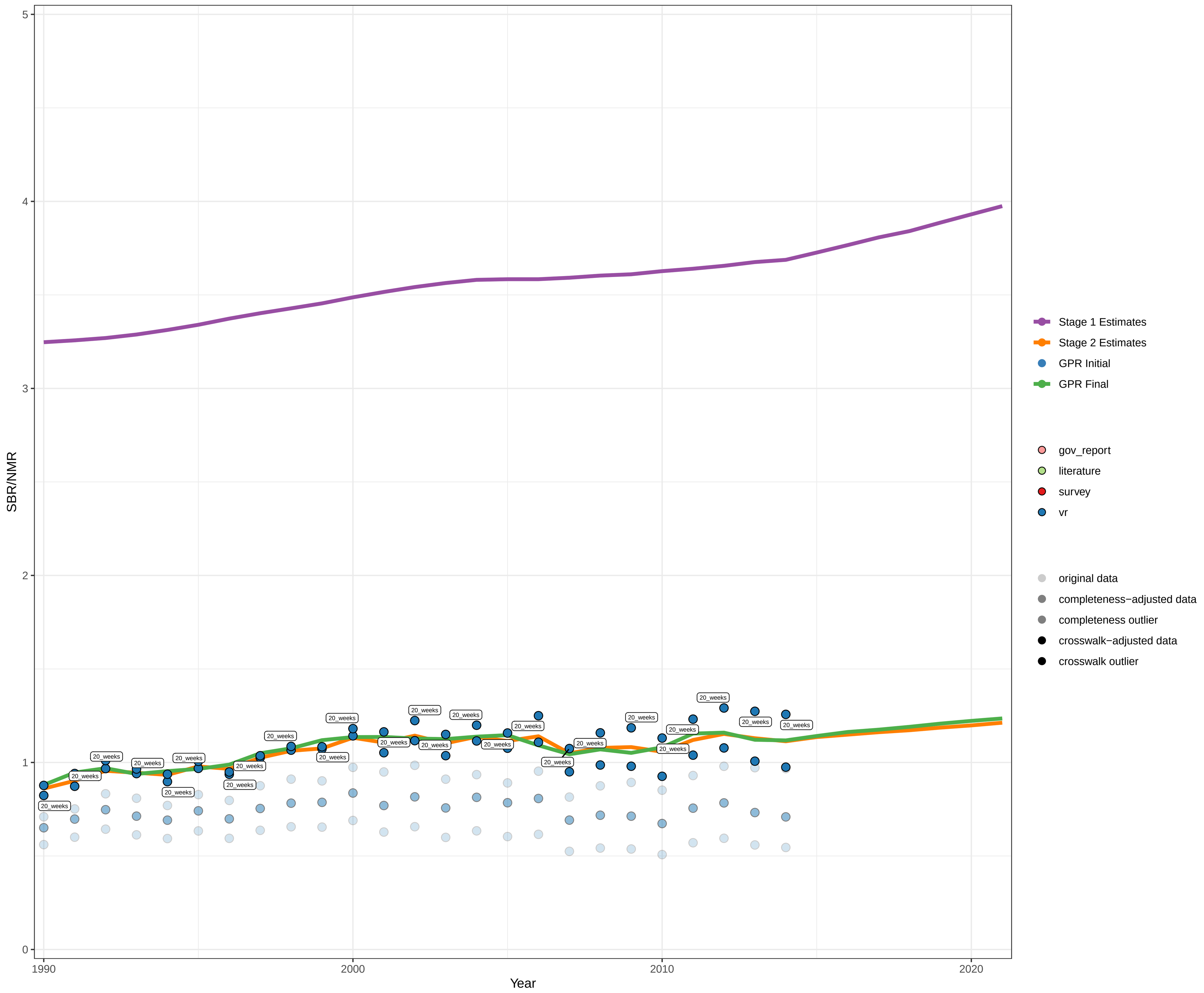

Guanajuato   MEX_4653   Central Latin America   Stillbirth Rate / Neonatal Mortality Rate (SBR/NMR) − 28 weeks
lambda = 0.2 ; zeta = 0.99 ; scale = 5 ; amp = 0.028

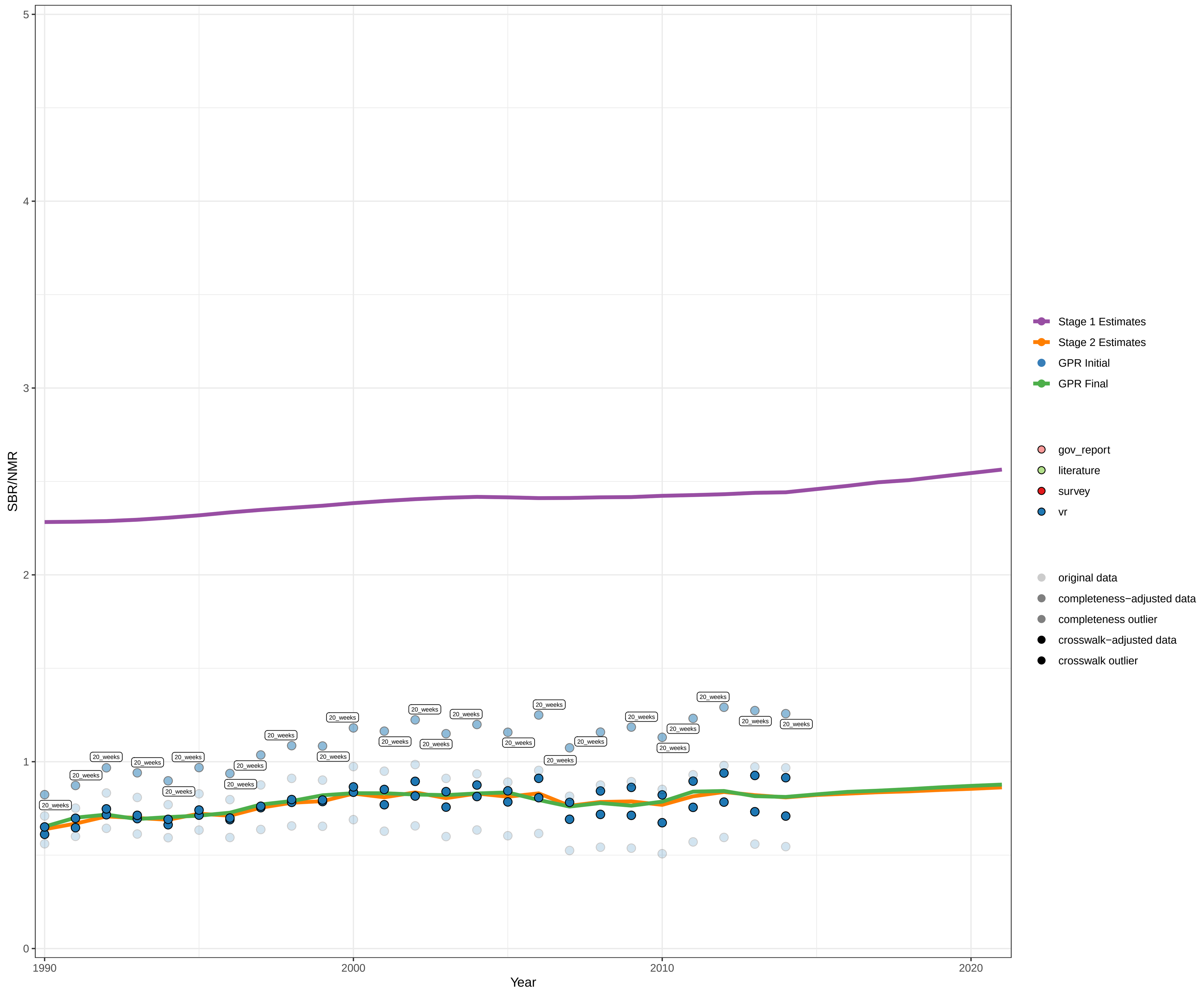

Definition	Years	Outlier	Source.Type	Title
20_weeks, 28_weeks	1990−2014	0	VR	Mexico INEGI Administrative Registers − Fetal Deaths by Municipality of Usual Residence of the Mother and Her Five−year Age Group According to Type of Fetal Death

Guanajuato   MEX_4653   Central Latin America   Stillbirth Rate (SBR) − 20 weeks
lambda = 0.2 ; zeta = 0.99 ; scale = 5 ; amp = 0.028

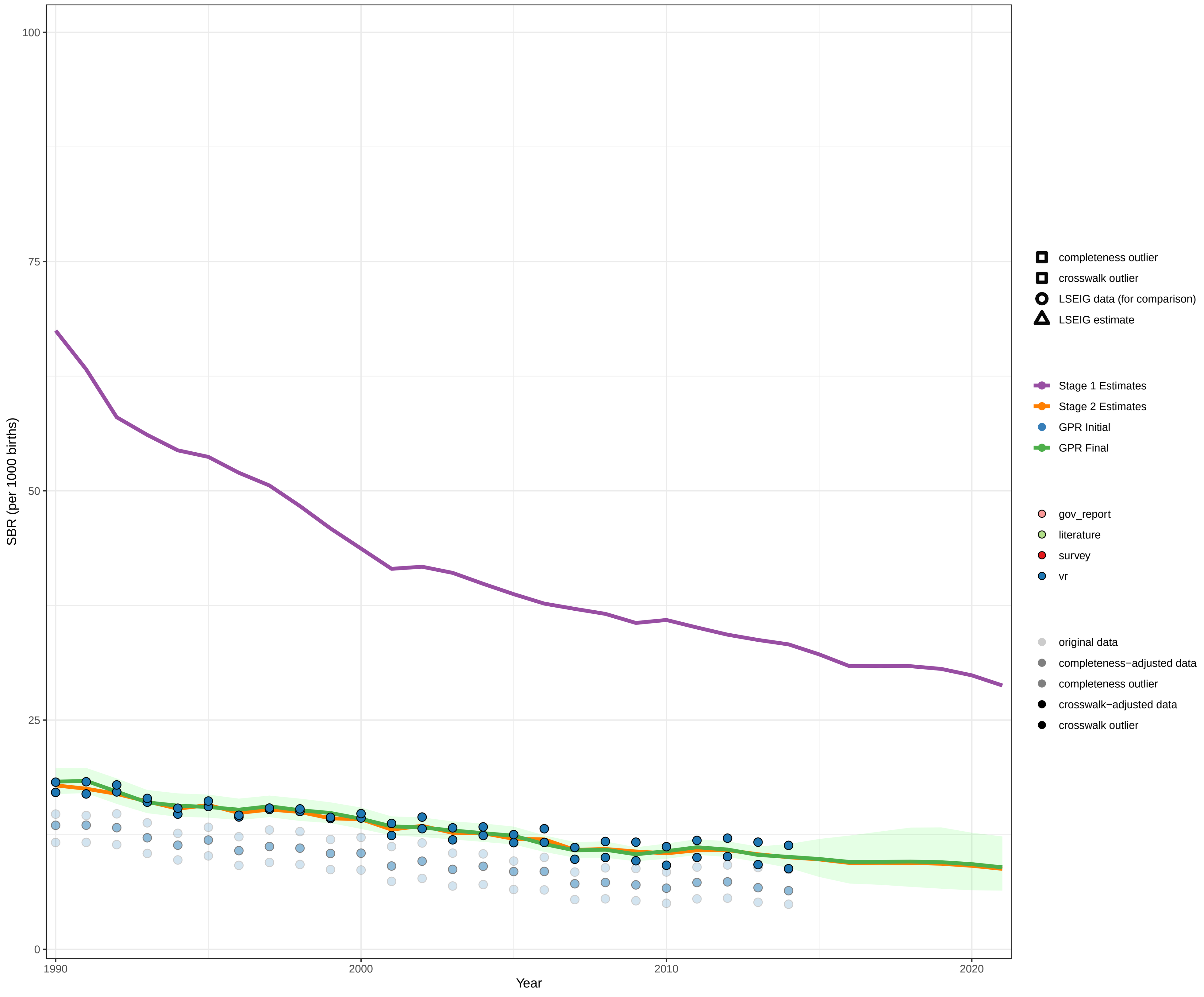

Guanajuato   MEX_4653   Central Latin America   Stillbirth Rate (SBR) − 28 weeks
lambda = 0.2 ; zeta = 0.99 ; scale = 5 ; amp = 0.028

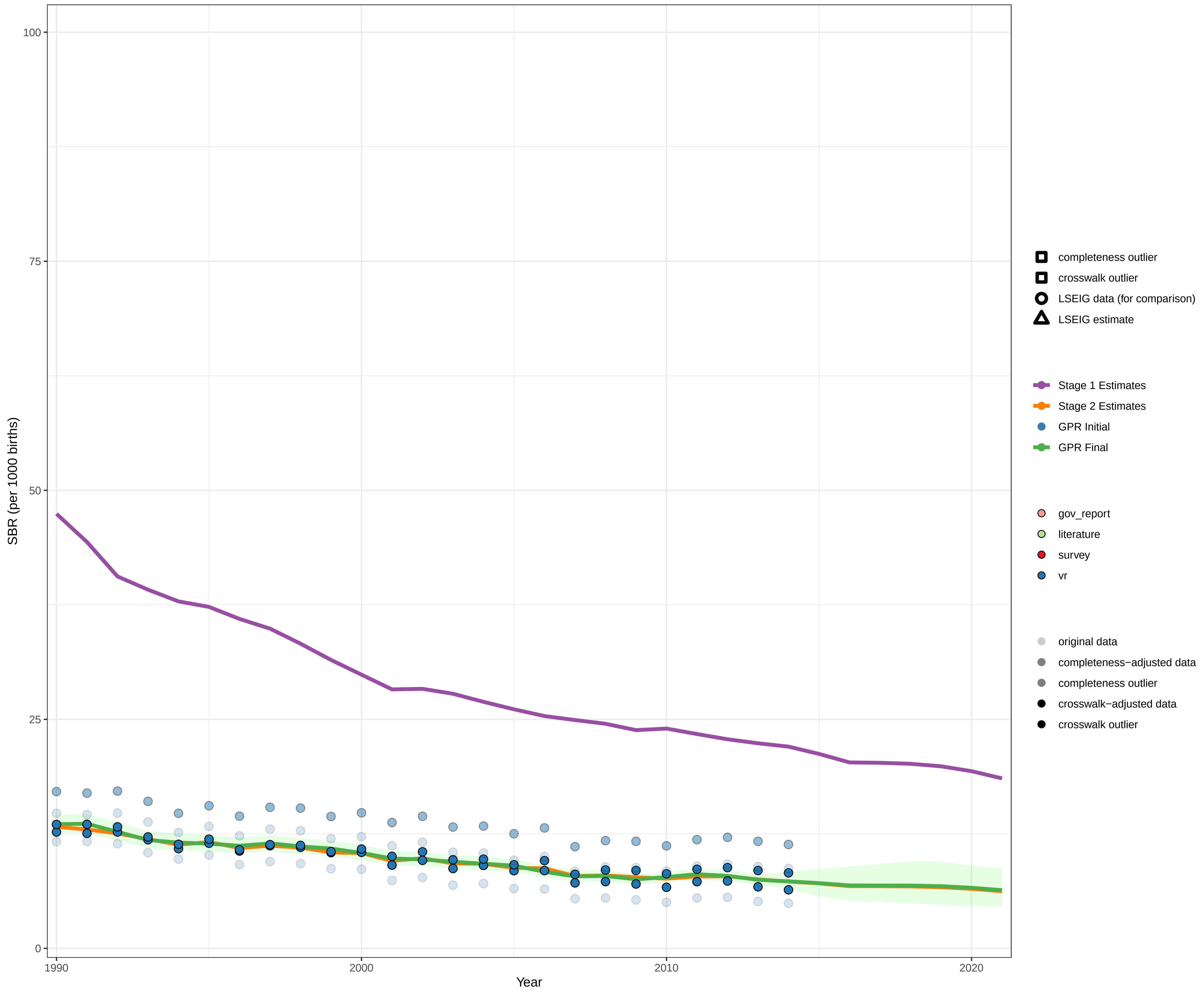

Year	Total Stillbirths (in thousands)	Total Stillbirths (in thousands)	SBR (per 1000 births)	SBR (per 1000 births)	SBR/NMR	SBR/NMR
	20 weeks	28 weeks	20 weeks	28 weeks	20 weeks	28 weeks
1990	2.5 (2.3 to 2.7)	1.8 (1.7 to 2.0)	18.3 (16.9 to 19.7)	13.6 (12.6 to 14.6)	0.88 (0.82 to 0.95)	0.65 (0.60 to 0.70)
1995	2.1 (2.0 to 2.3)	1.6 (1.4 to 1.7)	15.5 (14.4 to 16.9)	11.4 (10.6 to 12.4)	0.97 (0.89 to 1.05)	0.71 (0.66 to 0.77)
2000	1.8 (1.7 to 2.0)	1.3 (1.2 to 1.5)	14.2 (13.1 to 15.5)	10.4 (9.6 to 11.3)	1.14 (1.05 to 1.23)	0.83 (0.77 to 0.90)
2005	1.5 (1.4 to 1.7)	1.1 (1.0 to 1.2)	12.4 (11.4 to 13.4)	9.0 (8.3 to 9.7)	1.15 (1.06 to 1.24)	0.84 (0.77 to 0.90)
2010	1.4 (1.3 to 1.5)	1.0 (0.9 to 1.1)	10.7 (9.9 to 11.5)	7.8 (7.2 to 8.4)	1.08 (1.00 to 1.16)	0.79 (0.73 to 0.85)
2015	1.2 (0.9 to 1.4)	0.9 (0.7 to 1.0)	9.9 (7.9 to 12.0)	7.1 (5.7 to 8.7)	1.14 (0.91 to 1.39)	0.83 (0.66 to 1.01)
2020	0.9 (0.7 to 1.3)	0.7 (0.5 to 0.9)	9.3 (6.4 to 12.7)	6.6 (4.6 to 9.0)	1.22 (0.85 to 1.67)	0.87 (0.61 to 1.19)
2021	0.9 (0.6 to 1.2)	0.6 (0.5 to 0.9)	8.9 (6.4 to 12.3)	6.4 (4.6 to 8.7)	1.24 (0.88 to 1.70)	0.88 (0.63 to 1.20)

Guerrero MEX_4654 Central Latin America Stillbirth Rate / Neonatal Mortality Rate (SBR/NMR) − 20 weeks
lambda = 0.4 ; zeta = 0.9 ; scale = 10 ; amp = 0.028

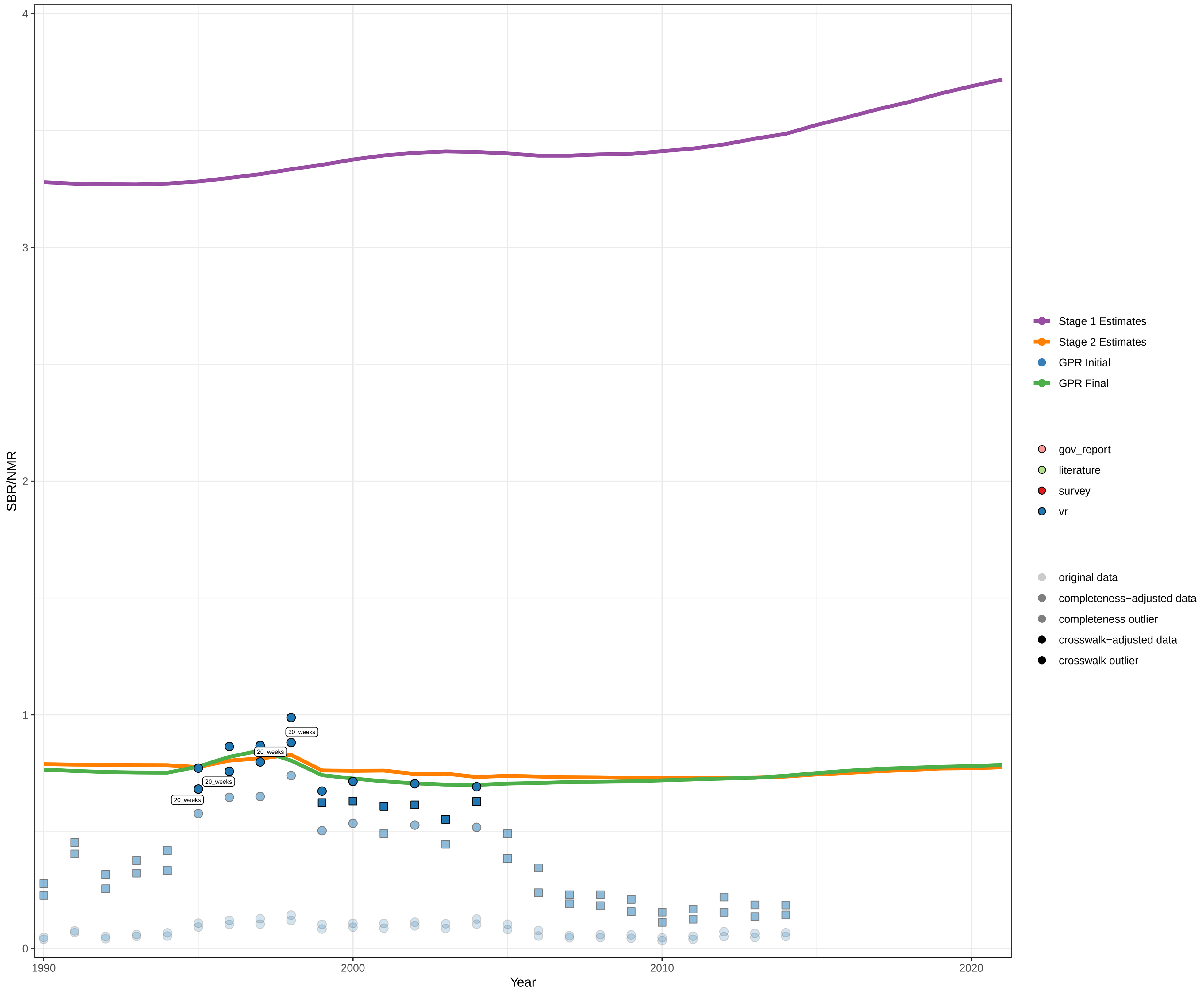

Guerrero MEX_4654 Central Latin America Stillbirth Rate / Neonatal Mortality Rate (SBR/NMR) − 28 weeks
lambda = 0.4 ; zeta = 0.9 ; scale = 10 ; amp = 0.028

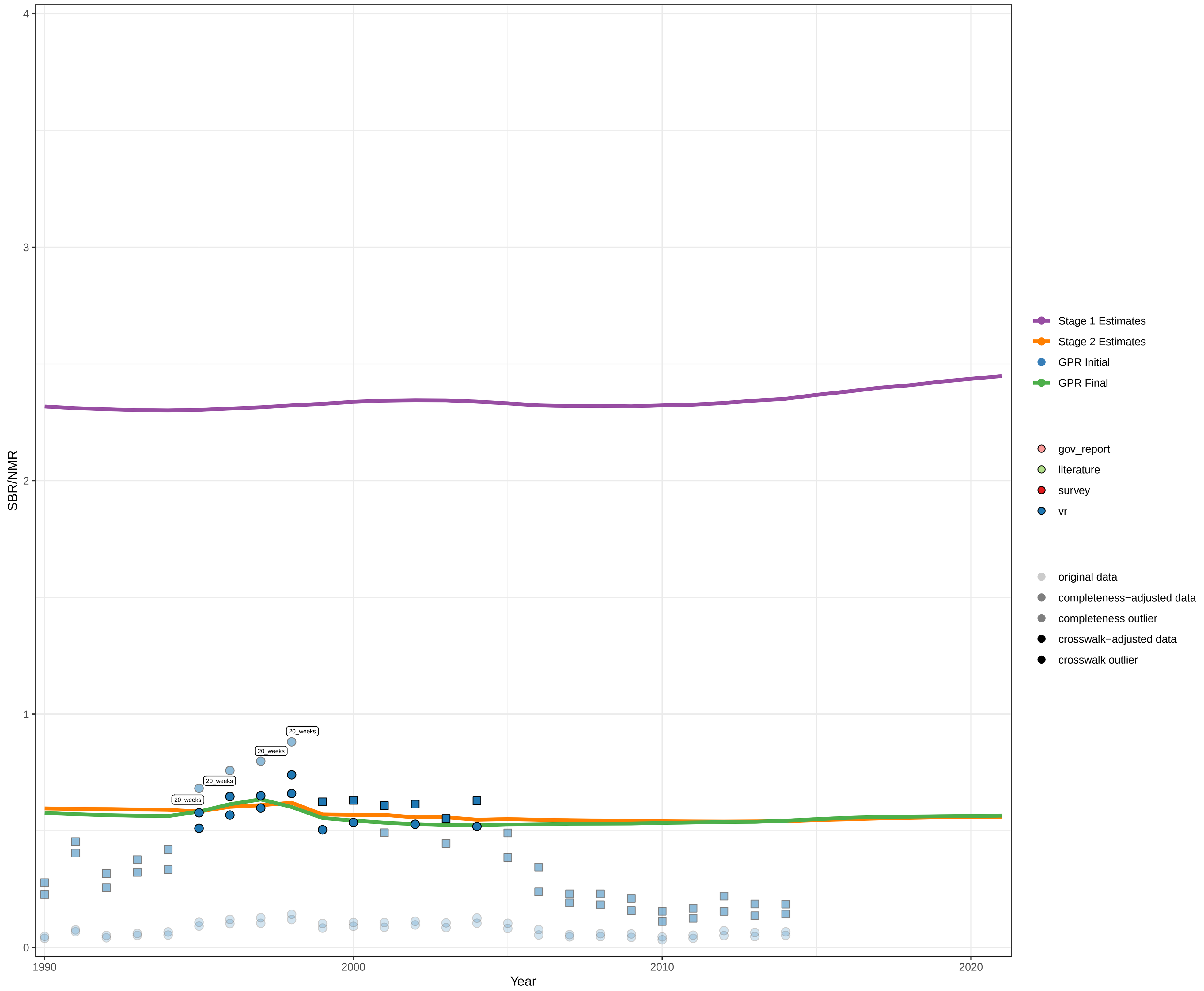

Definition	Years	Outlier	Source.Type	Title
20_weeks	1995−1998	0	VR	Mexico INEGI Administrative Registers − Fetal Deaths by Municipality of Usual Residence of the Mother and Her Five−year Age Group According to Type of Fetal Death
28_weeks	1995−2000, 2002, 2004	0	VR	Mexico INEGI Administrative Registers − Fetal Deaths by Municipality of Usual Residence of the Mother and Her Five−year Age Group According to Type of Fetal Death
20_weeks	1990−1994, 2005−2014	1	VR	Mexico INEGI Administrative Registers − Fetal Deaths by Municipality of Usual Residence of the Mother and Her Five−year Age Group According to Type of Fetal Death
28_weeks	1990−1994, 2001, 2003, 2005−2014	1	VR	Mexico INEGI Administrative Registers − Fetal Deaths by Municipality of Usual Residence of the Mother and Her Five−year Age Group According to Type of Fetal Death
20_weeks	1999−2004	2	VR	Mexico INEGI Administrative Registers − Fetal Deaths by Municipality of Usual Residence of the Mother and Her Five−year Age Group According to Type of Fetal Death

Guerrero MEX_4654 Central Latin America Stillbirth Rate (SBR) − 20 weeks
lambda = 0.4 ; zeta = 0.9 ; scale = 10 ; amp = 0.028

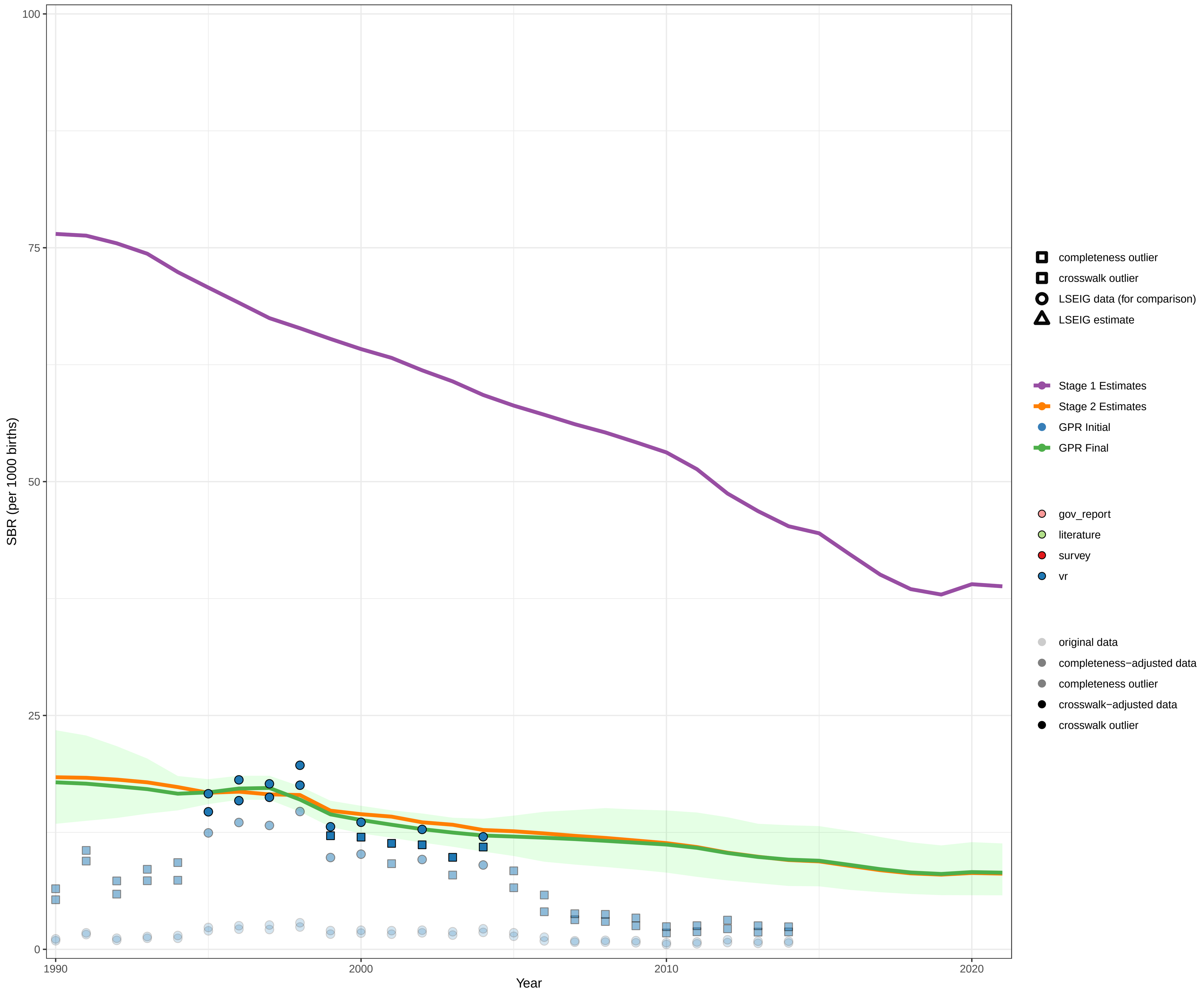

Guerrero MEX_4654 Central Latin America Stillbirth Rate (SBR) − 28 weeks
lambda = 0.4 ; zeta = 0.9 ; scale = 10 ; amp = 0.028

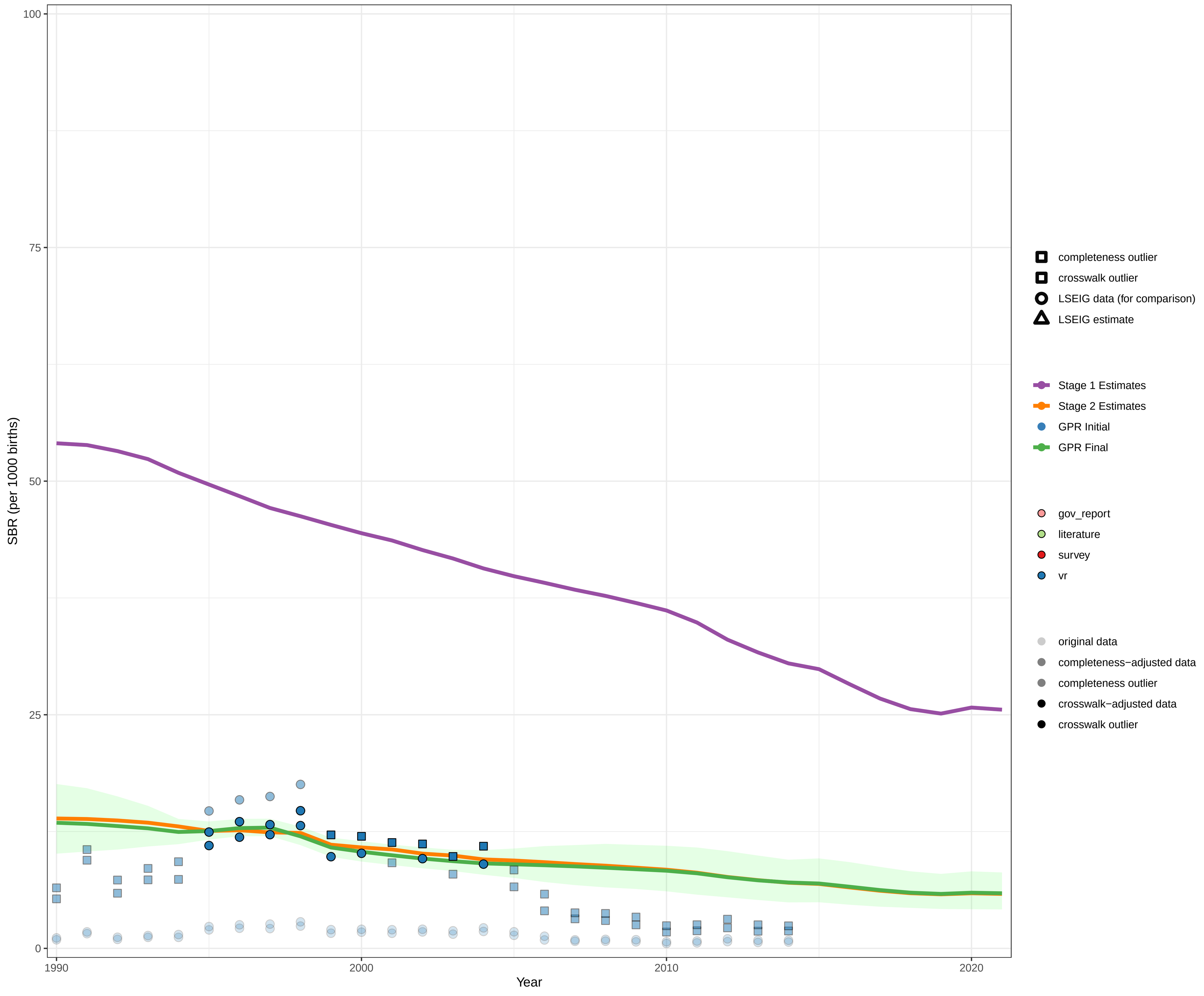

Year	Total Stillbirths (in thousands)	Total Stillbirths (in thousands)	SBR (per 1000 births)	SBR (per 1000 births)	SBR/NMR	SBR/NMR
	20 weeks	28 weeks	20 weeks	28 weeks	20 weeks	28 weeks
1990	1.9 (1.4 to 2.5)	1.4 (1.1 to 1.8)	17.9 (13.4 to 23.4)	13.4 (10.1 to 17.6)	0.77 (0.58 to 1.00)	0.58 (0.43 to 0.75)
1995	1.9 (1.7 to 2.0)	1.4 (1.3 to 1.5)	16.8 (15.5 to 18.2)	12.6 (11.6 to 13.6)	0.78 (0.72 to 0.84)	0.58 (0.54 to 0.63)
2000	1.5 (1.3 to 1.6)	1.1 (1.0 to 1.2)	13.8 (12.4 to 15.3)	10.3 (9.3 to 11.5)	0.73 (0.65 to 0.81)	0.54 (0.49 to 0.60)
2005	1.2 (1.0 to 1.4)	0.9 (0.7 to 1.0)	12.1 (10.0 to 14.3)	9.0 (7.5 to 10.7)	0.71 (0.58 to 0.84)	0.53 (0.44 to 0.63)
2010	1.1 (0.8 to 1.5)	0.8 (0.6 to 1.1)	11.2 (8.2 to 14.8)	8.3 (6.1 to 11.0)	0.72 (0.53 to 0.95)	0.53 (0.39 to 0.71)
2015	0.8 (0.6 to 1.2)	0.6 (0.4 to 0.8)	9.5 (6.7 to 13.2)	6.9 (4.9 to 9.6)	0.75 (0.53 to 1.04)	0.55 (0.39 to 0.76)
2020	0.6 (0.4 to 0.8)	0.4 (0.3 to 0.6)	8.3 (5.8 to 11.5)	6.0 (4.2 to 8.2)	0.78 (0.55 to 1.08)	0.56 (0.40 to 0.78)
2021	0.6 (0.4 to 0.8)	0.4 (0.3 to 0.6)	8.2 (5.8 to 11.3)	5.9 (4.2 to 8.1)	0.79 (0.55 to 1.08)	0.57 (0.40 to 0.78)

Hidalgo MEX_4655 Central Latin America Stillbirth Rate / Neonatal Mortality Rate (SBR/NMR) − 20 weeks
lambda = 0.2 ; zeta = 0.99 ; scale = 5 ; amp = 0.028

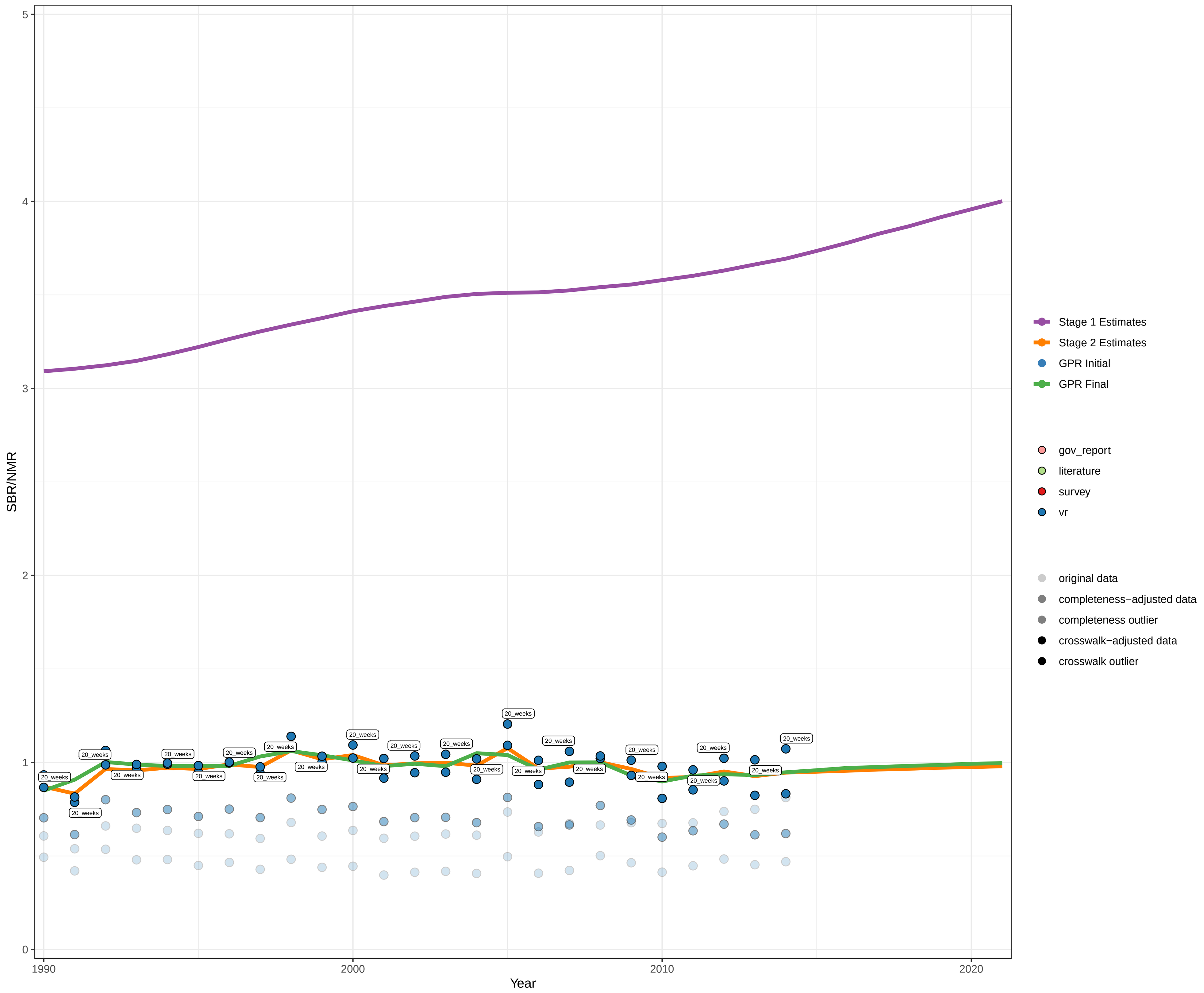

Hidalgo MEX_4655 Central Latin America Stillbirth Rate / Neonatal Mortality Rate (SBR/NMR) − 28 weeks
lambda = 0.2 ; zeta = 0.99 ; scale = 5 ; amp = 0.028

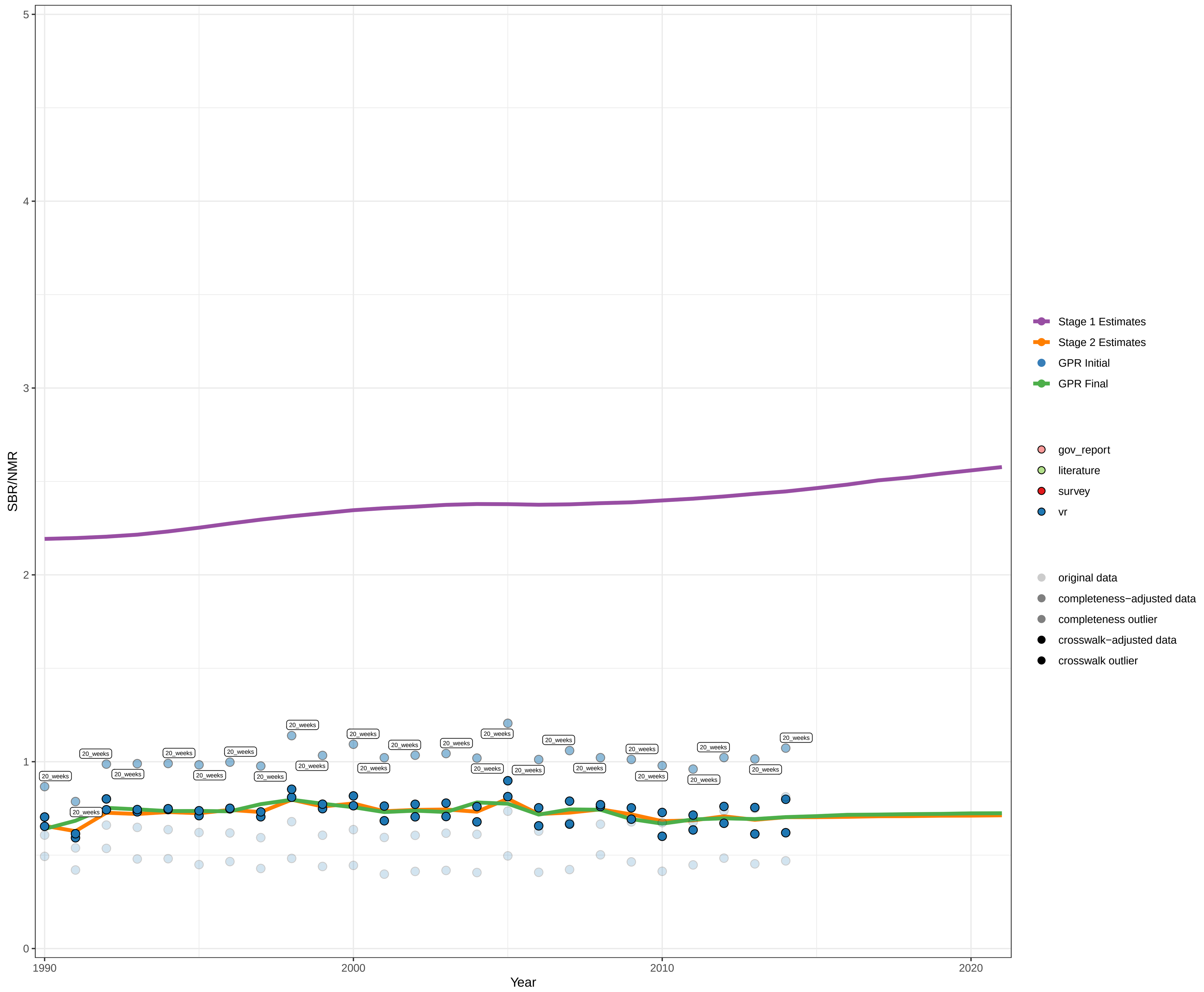

Definition	Years	Outlier	Source.Type	Title
20_weeks, 28_weeks	1990−2014	0	VR	Mexico INEGI Administrative Registers − Fetal Deaths by Municipality of Usual Residence of the Mother and Her Five−year Age Group According to Type of Fetal Death

Hidalgo MEX_4655 Central Latin America Stillbirth Rate (SBR) − 20 weeks
lambda = 0.2 ; zeta = 0.99 ; scale = 5 ; amp = 0.028

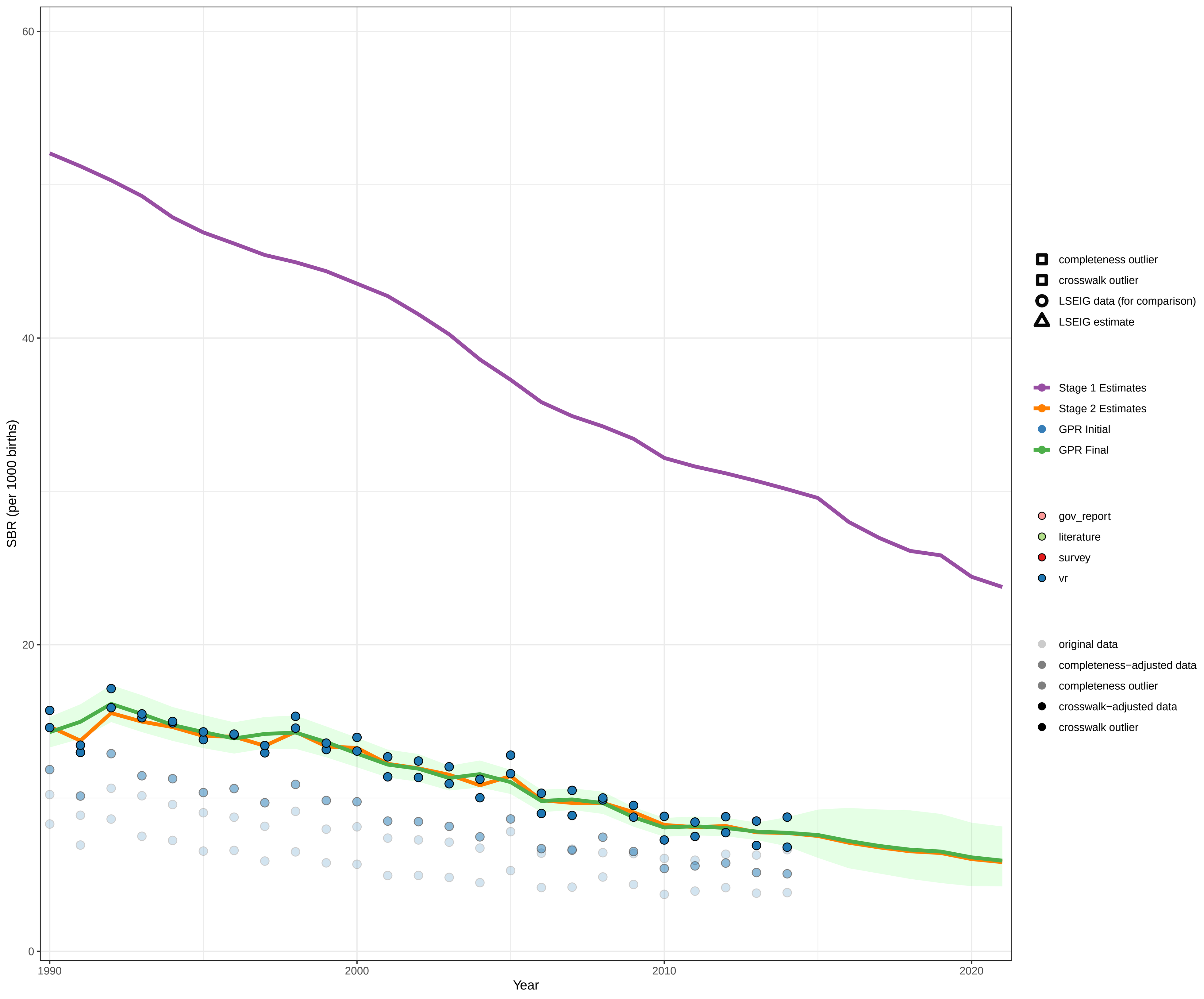

Hidalgo MEX_4655 Central Latin America Stillbirth Rate (SBR) − 28 weeks
lambda = 0.2 ; zeta = 0.99 ; scale = 5 ; amp = 0.028

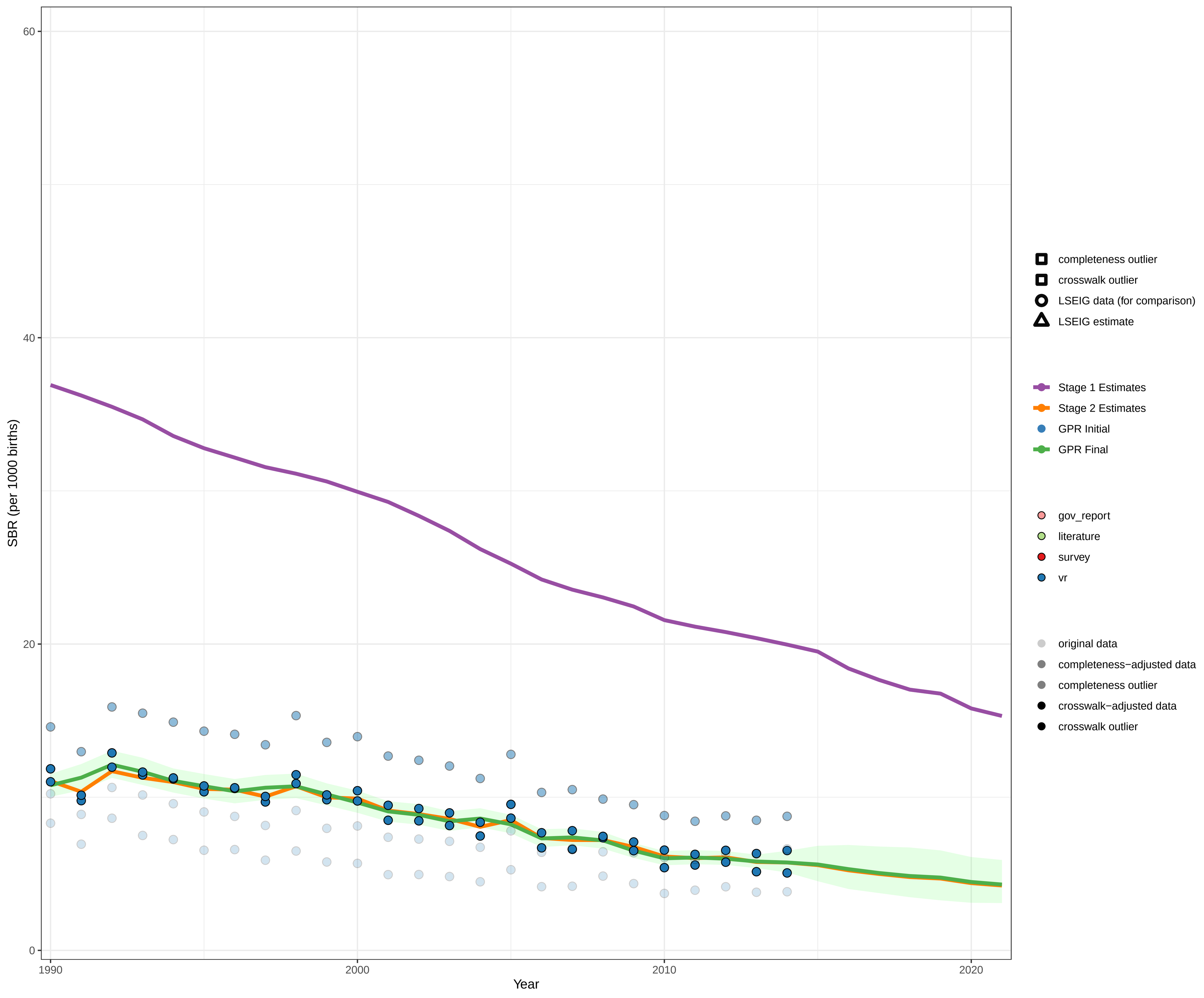

Year	Total Stillbirths (in thousands)	Total Stillbirths (in thousands)	SBR (per 1000 births)	SBR (per 1000 births)	SBR/NMR	SBR/NMR
	20 weeks	28 weeks	20 weeks	28 weeks	20 weeks	28 weeks
1990	0.9<br>(0.9 to 1.0)	0.7<br>(0.6 to 0.7)	14.3<br>(13.3 to 15.3)	10.8<br>(10.0 to 11.5)	0.85<br>(0.79 to 0.91)	0.64<br>(0.60 to 0.68)
1995	0.9<br>(0.8 to 1.0)	0.7<br>(0.6 to 0.7)	14.3<br>(13.3 to 15.4)	10.7<br>(9.9 to 11.5)	0.98<br>(0.91 to 1.06)	0.74<br>(0.68 to 0.79)
2000	0.8<br>(0.7 to 0.8)	0.6<br>(0.5 to 0.6)	12.9<br>(12.0 to 13.9)	9.7<br>(9.0 to 10.4)	1.01<br>(0.94 to 1.09)	0.76<br>(0.70 to 0.82)
2005	0.6<br>(0.6 to 0.7)	0.5<br>(0.4 to 0.5)	11.0<br>(10.3 to 11.8)	8.2<br>(7.6 to 8.9)	1.04<br>(0.97 to 1.12)	0.78<br>(0.72 to 0.83)
2010	0.5<br>(0.5 to 0.5)	0.4<br>(0.3 to 0.4)	8.1<br>(7.5 to 8.7)	6.0<br>(5.6 to 6.5)	0.90<br>(0.83 to 0.97)	0.67<br>(0.62 to 0.72)
2015	0.4<br>(0.3 to 0.5)	0.3<br>(0.2 to 0.4)	7.6<br>(6.1 to 9.2)	5.6<br>(4.5 to 6.8)	0.96<br>(0.77 to 1.17)	0.71<br>(0.57 to 0.86)
2020	0.3<br>(0.2 to 0.4)	0.2<br>(0.1 to 0.3)	6.1<br>(4.3 to 8.4)	4.5<br>(3.1 to 6.1)	0.99<br>(0.69 to 1.36)	0.72<br>(0.50 to 0.99)
2021	0.3<br>(0.2 to 0.4)	0.2<br>(0.1 to 0.3)	5.9<br>(4.2 to 8.1)	4.3<br>(3.1 to 5.9)	1.00<br>(0.71 to 1.37)	0.72<br>(0.52 to 0.99)

Jalisco MEX_4656 Central Latin America Stillbirth Rate / Neonatal Mortality Rate (SBR/NMR) − 20 weeks
lambda = 0.2 ; zeta = 0.99 ; scale = 5 ; amp = 0.028

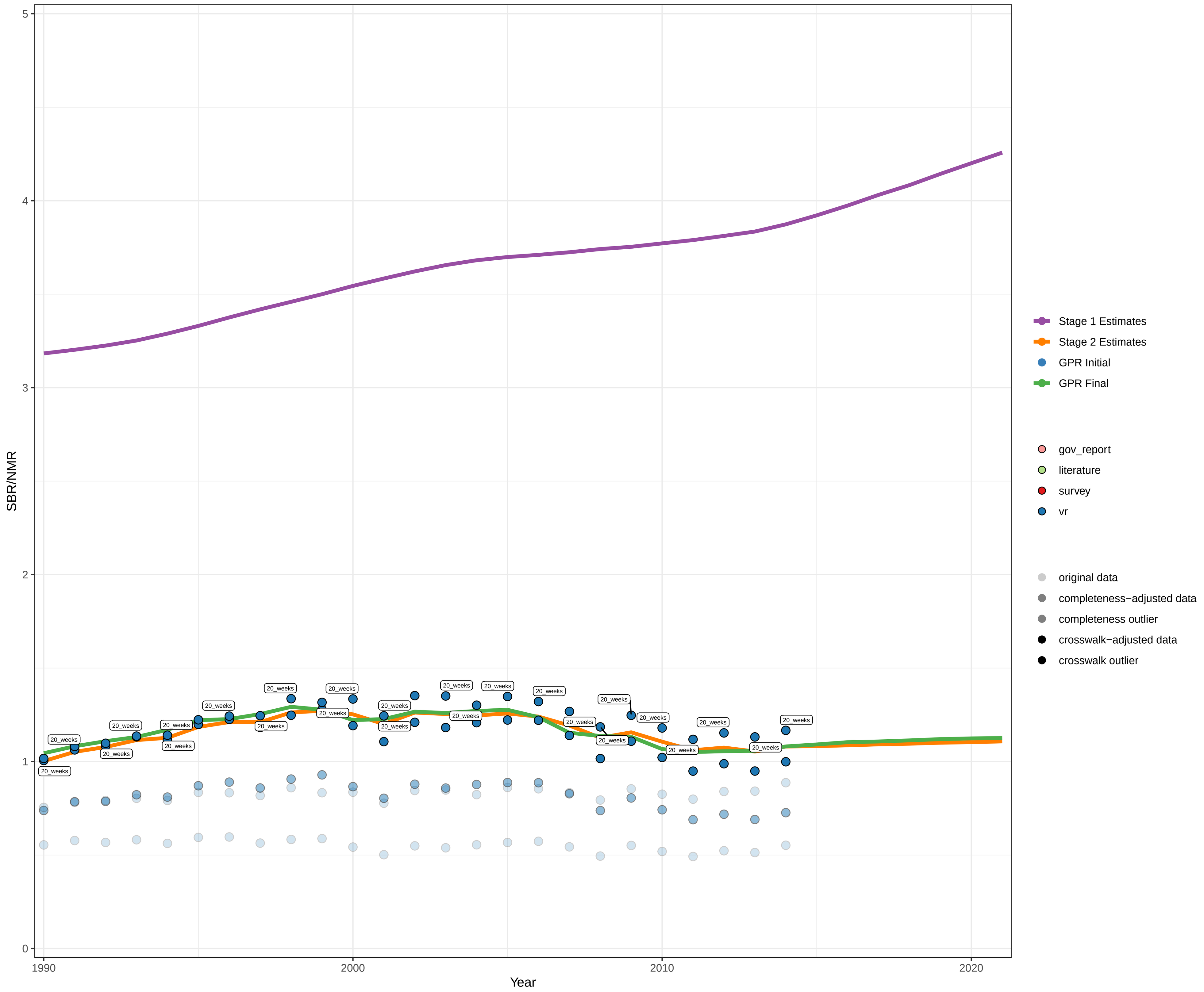

Jalisco MEX_4656 Central Latin America Stillbirth Rate / Neonatal Mortality Rate (SBR/NMR) − 28 weeks
lambda = 0.2 ; zeta = 0.99 ; scale = 5 ; amp = 0.028

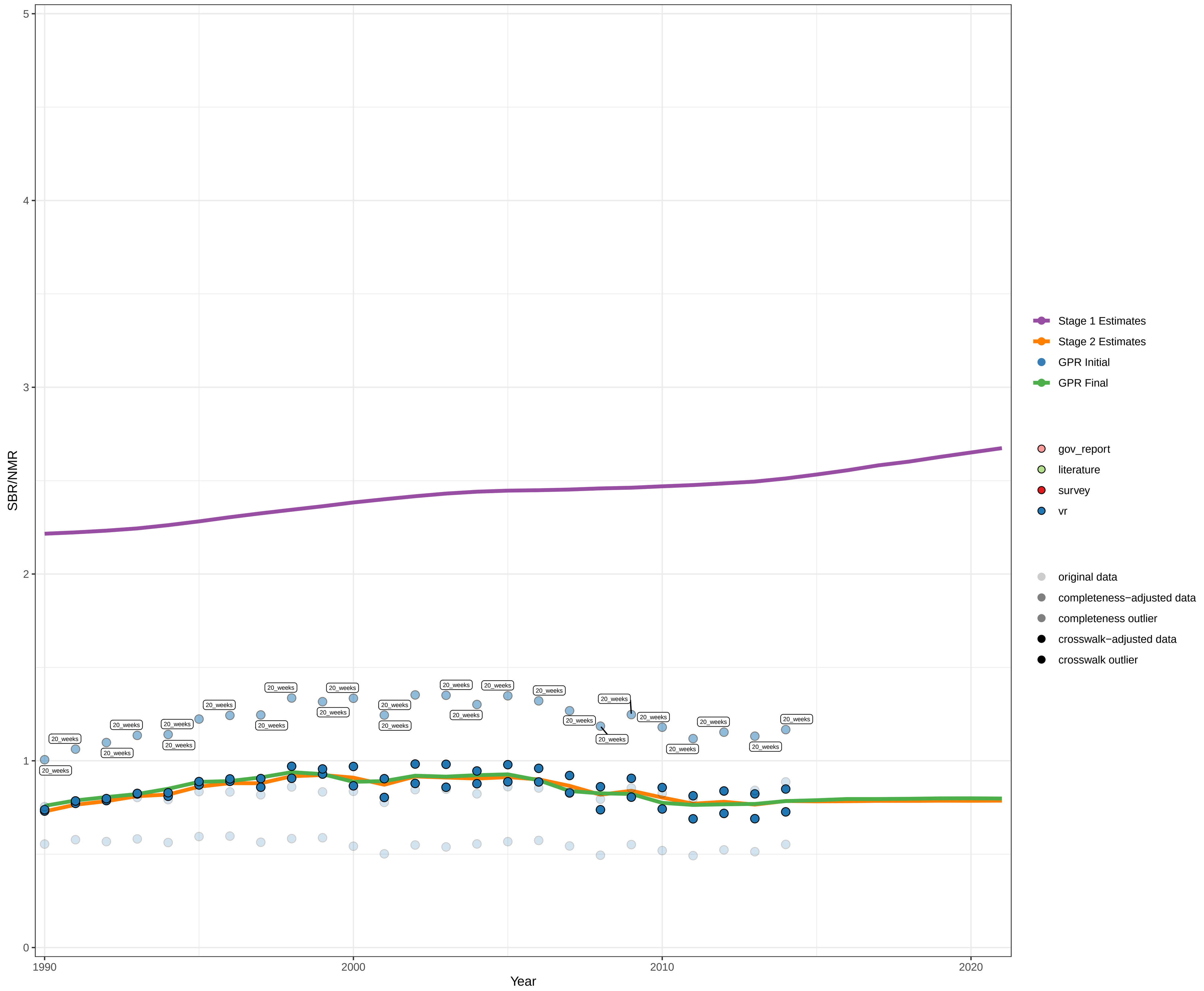

Definition	Years	Outlier	Source.Type	Title
20_weeks, 28_weeks	1990−2014	0	VR	Mexico INEGI Administrative Registers − Fetal Deaths by Municipality of Usual Residence of the Mother and Her Five−year Age Group According to Type of Fetal Death

Jalisco MEX_4656 Central Latin America Stillbirth Rate (SBR) − 20 weeks
lambda = 0.2 ; zeta = 0.99 ; scale = 5 ; amp = 0.028

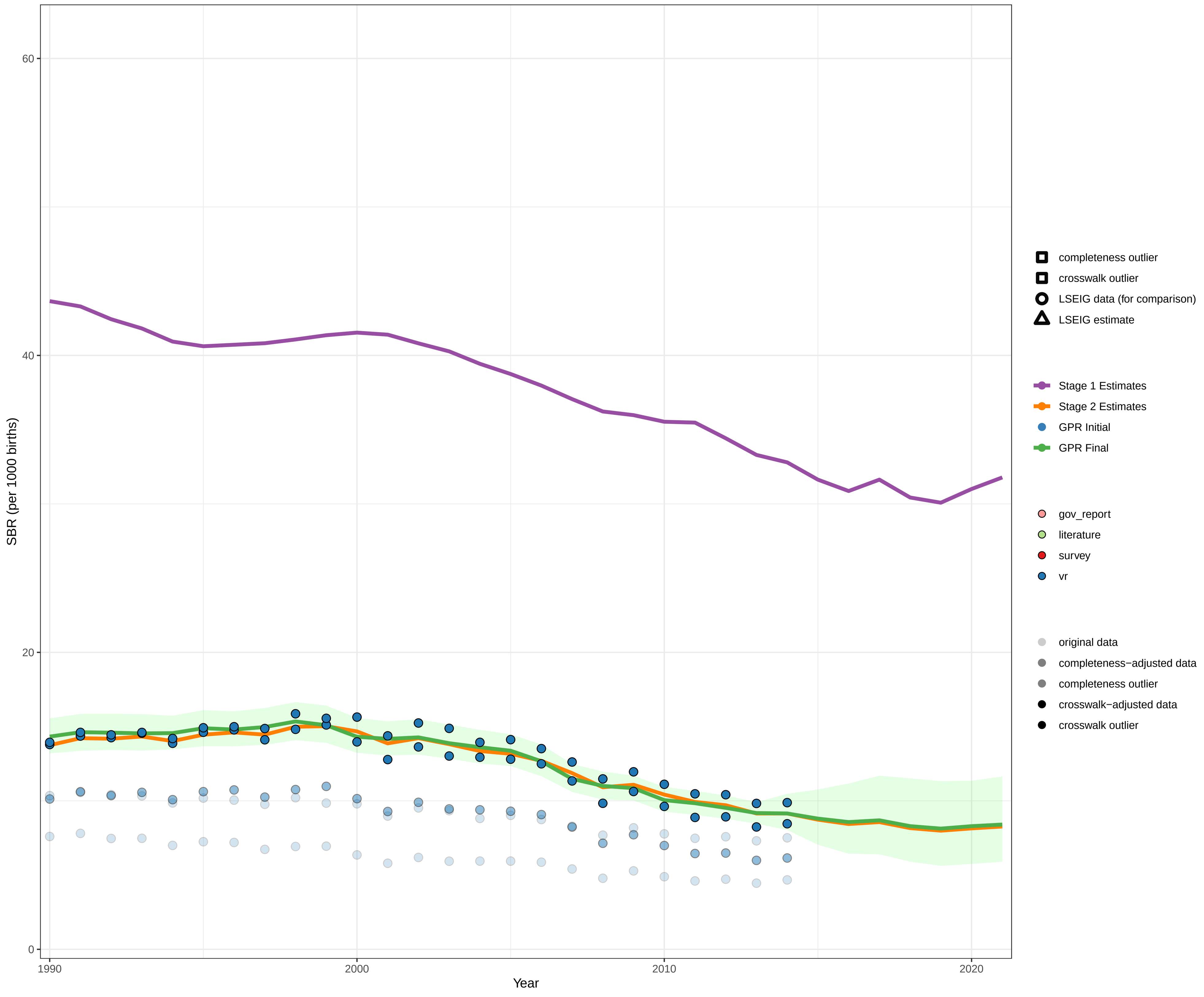

Jalisco MEX_4656 Central Latin America Stillbirth Rate (SBR) − 28 weeks
lambda = 0.2 ; zeta = 0.99 ; scale = 5 ; amp = 0.028

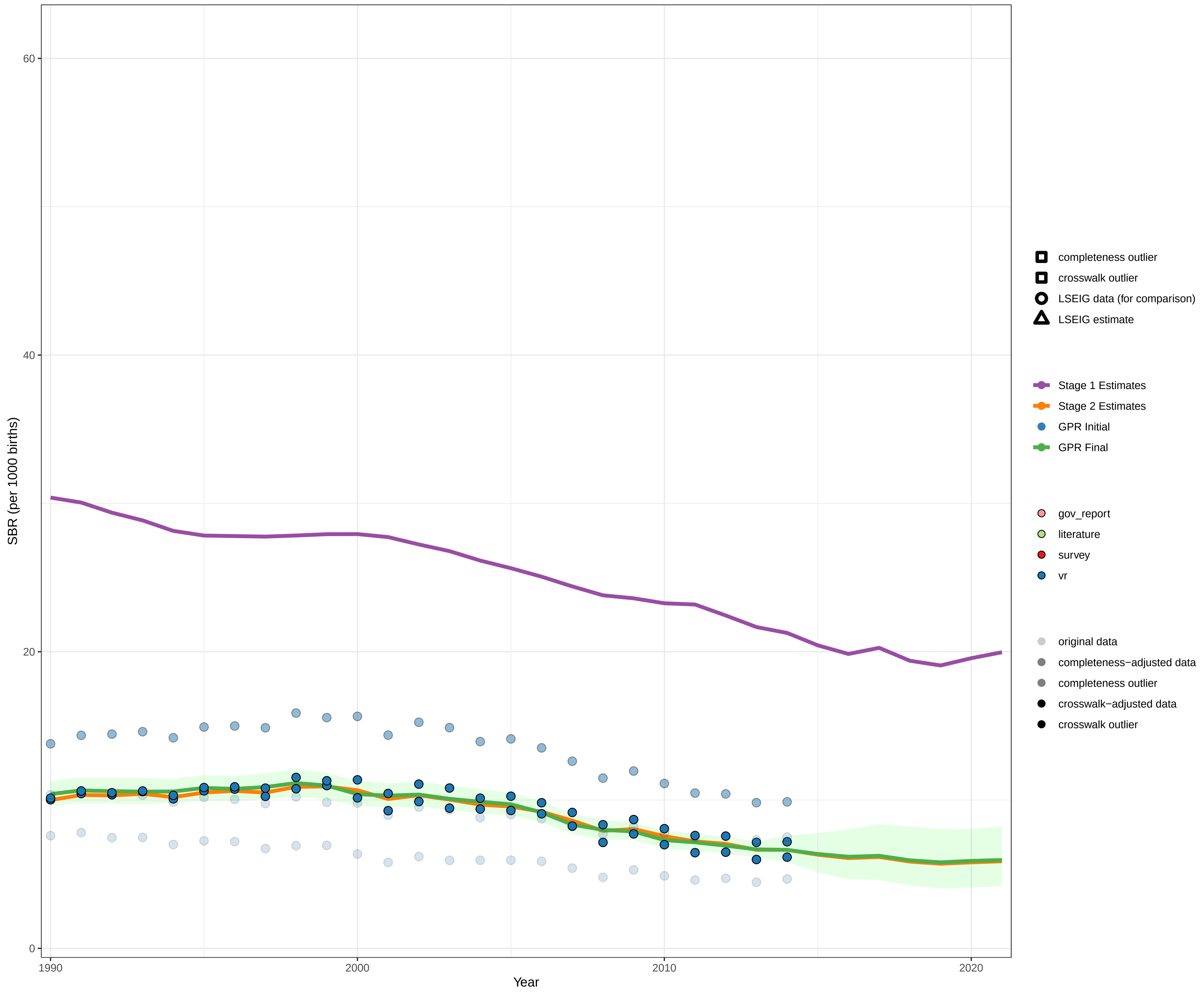

Year	Total Stillbirths (in thousands)	Total Stillbirths (in thousands)	SBR (per 1000 births)	SBR (per 1000 births)	SBR/NMR	SBR/NMR
	20 weeks	28 weeks	20 weeks	28 weeks	20 weeks	28 weeks
1990	2.4 (2.2 to 2.6)	1.7 (1.6 to 1.9)	14.3 (13.2 to 15.6)	10.4 (9.6 to 11.3)	1.04 (0.96 to 1.13)	0.76 (0.70 to 0.82)
1995	2.5 (2.3 to 2.7)	1.8 (1.7 to 2.0)	14.9 (13.7 to 16.1)	10.8 (9.9 to 11.7)	1.22 (1.12 to 1.32)	0.89 (0.82 to 0.96)
2000	2.3 (2.1 to 2.5)	1.7 (1.5 to 1.8)	14.3 (13.2 to 15.6)	10.4 (9.6 to 11.3)	1.22 (1.13 to 1.33)	0.89 (0.82 to 0.96)
2005	2.1 (1.9 to 2.2)	1.5 (1.4 to 1.6)	13.4 (12.3 to 14.5)	9.7 (9.0 to 10.5)	1.28 (1.18 to 1.38)	0.93 (0.86 to 1.00)
2010	1.6 (1.5 to 1.8)	1.2 (1.1 to 1.3)	10.0 (9.3 to 10.9)	7.3 (6.7 to 7.9)	1.07 (0.98 to 1.16)	0.77 (0.72 to 0.84)
2015	1.4 (1.1 to 1.7)	1.0 (0.8 to 1.2)	8.8 (7.0 to 10.8)	6.4 (5.1 to 7.8)	1.09 (0.87 to 1.34)	0.79 (0.63 to 0.96)
2020	1.1 (0.8 to 1.5)	0.8 (0.5 to 1.1)	8.3 (5.7 to 11.4)	5.9 (4.1 to 8.1)	1.12 (0.78 to 1.54)	0.80 (0.56 to 1.09)
2021	1.1 (0.8 to 1.5)	0.8 (0.5 to 1.1)	8.4 (5.9 to 11.6)	6.0 (4.2 to 8.2)	1.13 (0.79 to 1.56)	0.80 (0.56 to 1.10)

Mexico MEX_4657 Central Latin America Stillbirth Rate / Neonatal Mortality Rate (SBR/NMR) − 20 weeks
lambda = 0.2 ; zeta = 0.99 ; scale = 5 ; amp = 0.028

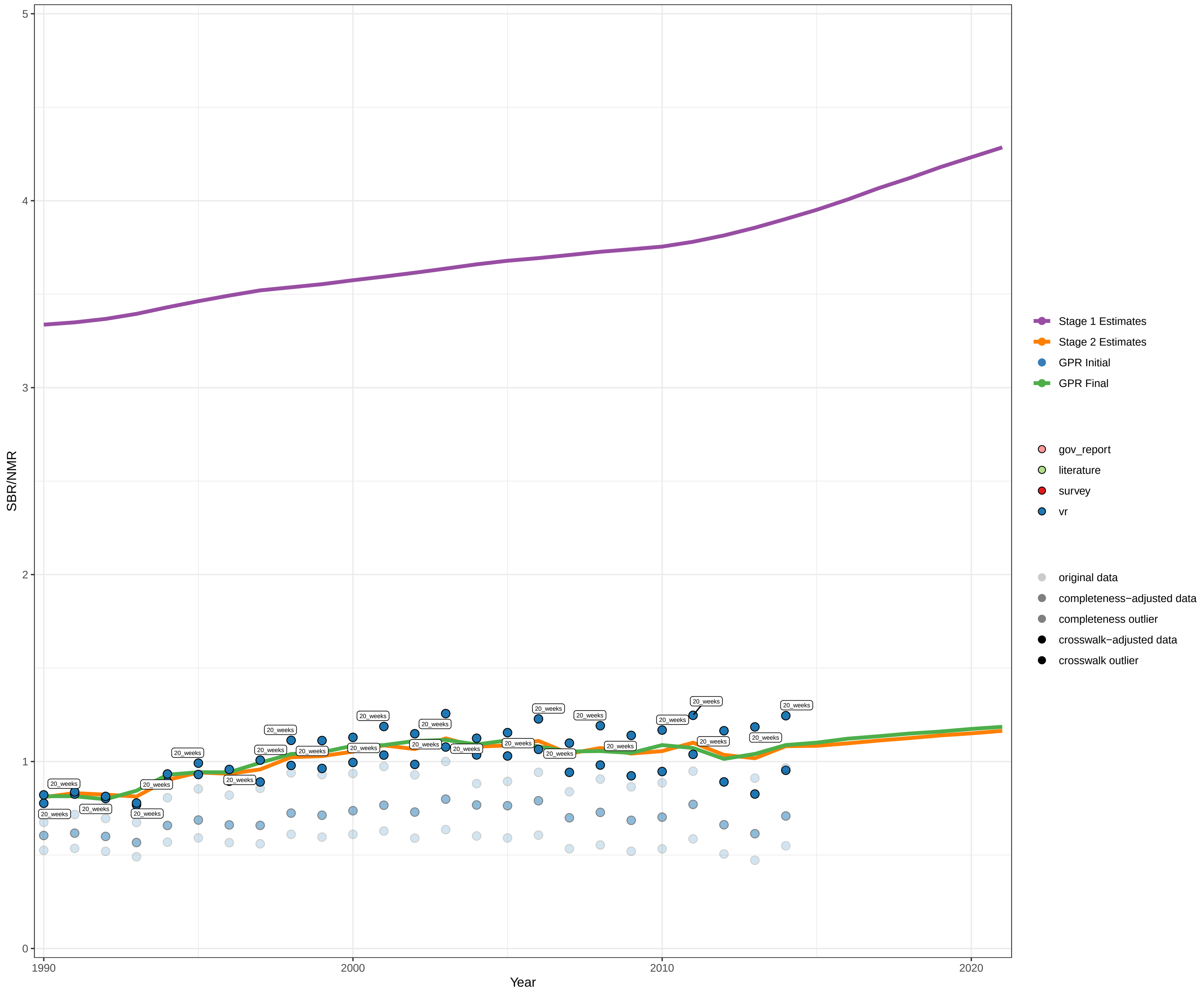

Mexico MEX_4657 Central Latin America Stillbirth Rate / Neonatal Mortality Rate (SBR/NMR) − 28 weeks
lambda = 0.2 ; zeta = 0.99 ; scale = 5 ; amp = 0.028

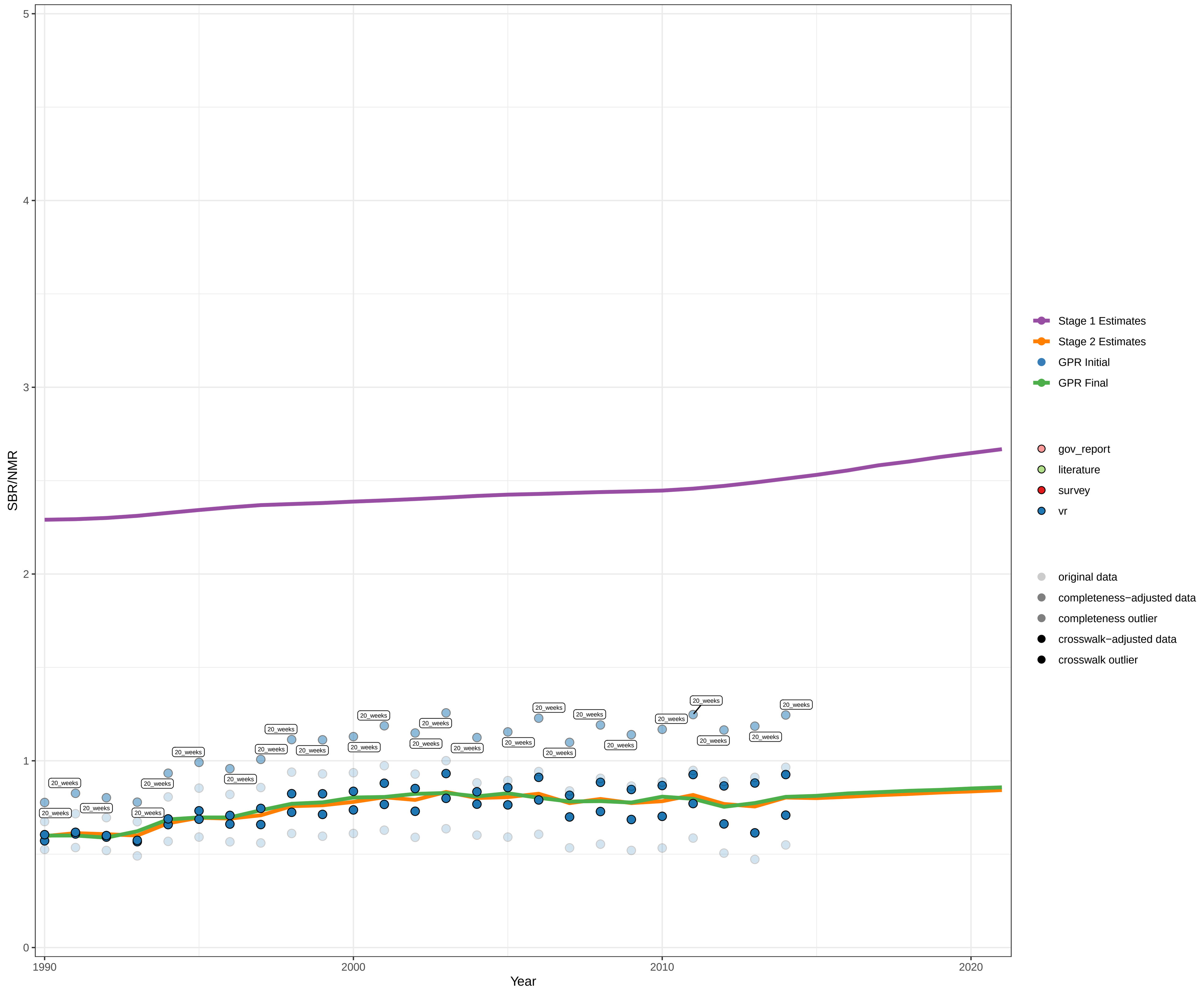

Definition	Years	Outlier	Source.Type	Title
20_weeks, 28_weeks	1990−2014	0	VR	Mexico INEGI Administrative Registers − Fetal Deaths by Municipality of Usual Residence of the Mother and Her Five−year Age Group According to Type of Fetal Death

Mexico MEX_4657 Central Latin America Stillbirth Rate (SBR) − 20 weeks
lambda = 0.2 ; zeta = 0.99 ; scale = 5 ; amp = 0.028

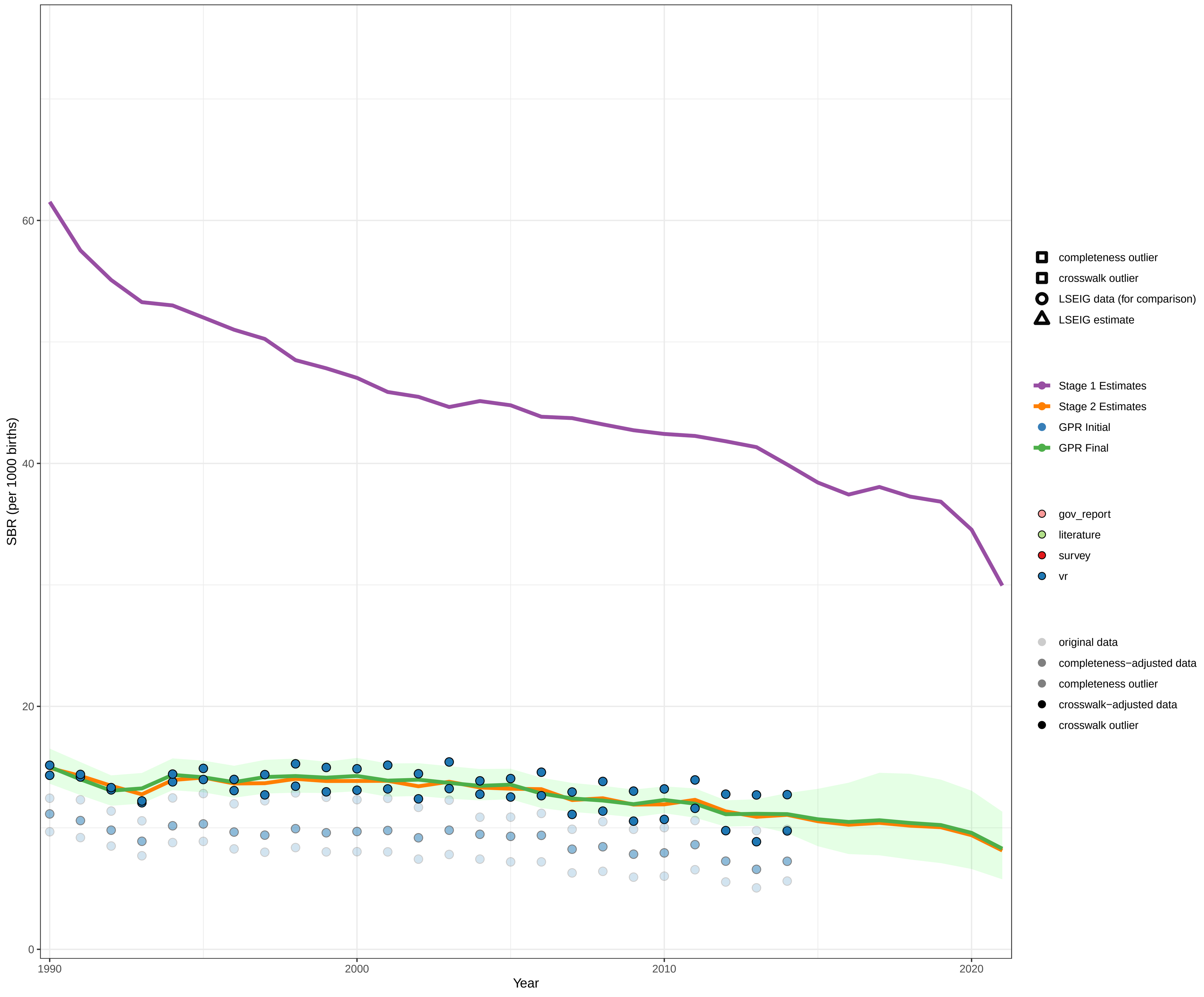

Mexico MEX_4657 Central Latin America Stillbirth Rate (SBR) − 28 weeks
lambda = 0.2 ; zeta = 0.99 ; scale = 5 ; amp = 0.028

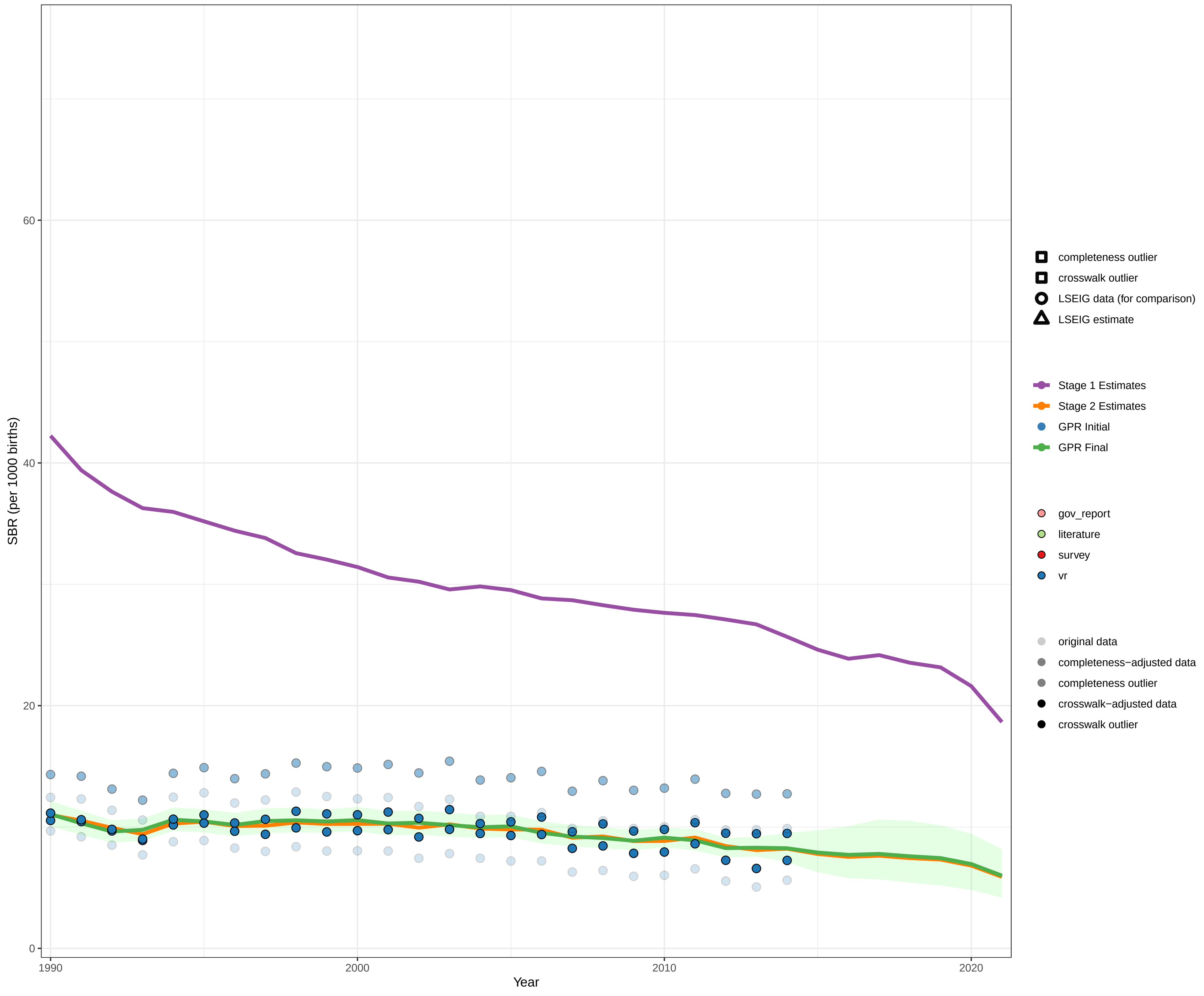

Year	Total Stillbirths (in thousands)	Total Stillbirths (in thousands)	SBR (per 1000 births)	SBR (per 1000 births)	SBR/NMR	SBR/NMR
	20 weeks	28 weeks	20 weeks	28 weeks	20 weeks	28 weeks
1990	4.6 (4.2 to 5.0)	3.4 (3.0 to 3.7)	15.0 (13.6 to 16.5)	11.1 (10.0 to 12.2)	0.81 (0.74 to 0.90)	0.60 (0.54 to 0.66)
1995	4.6 (4.2 to 5.0)	3.4 (3.1 to 3.7)	14.2 (12.9 to 15.5)	10.5 (9.6 to 11.5)	0.94 (0.86 to 1.03)	0.70 (0.64 to 0.76)
2000	4.7 (4.3 to 5.2)	3.4 (3.1 to 3.8)	14.3 (13.0 to 15.8)	10.6 (9.6 to 11.7)	1.08 (0.99 to 1.20)	0.80 (0.73 to 0.89)
2005	4.4 (4.0 to 4.8)	3.2 (2.9 to 3.5)	13.6 (12.4 to 14.9)	10.1 (9.2 to 11.0)	1.11 (1.01 to 1.22)	0.83 (0.75 to 0.91)
2010	4.0 (3.6 to 4.4)	3.0 (2.7 to 3.2)	12.3 (11.2 to 13.4)	9.1 (8.3 to 10.0)	1.09 (0.99 to 1.19)	0.81 (0.74 to 0.88)
2015	3.1 (2.5 to 3.8)	2.3 (1.8 to 2.8)	10.7 (8.5 to 13.2)	7.9 (6.3 to 9.7)	1.10 (0.87 to 1.36)	0.81 (0.65 to 1.00)
2020	2.3 (1.6 to 3.1)	1.6 (1.1 to 2.2)	9.6 (6.6 to 13.1)	7.0 (4.8 to 9.4)	1.17 (0.81 to 1.60)	0.85 (0.59 to 1.16)
2021	1.9 (1.3 to 2.6)	1.4 (1.0 to 1.9)	8.3 (5.8 to 11.3)	6.0 (4.2 to 8.2)	1.19 (0.82 to 1.62)	0.86 (0.60 to 1.17)

Mexico City MEX_4651 Central Latin America Stillbirth Rate / Neonatal Mortality Rate (SBR/NMR) − 20 weeks
lambda = 1 ; zeta = 0.6 ; scale = 15 ; amp = 0.028

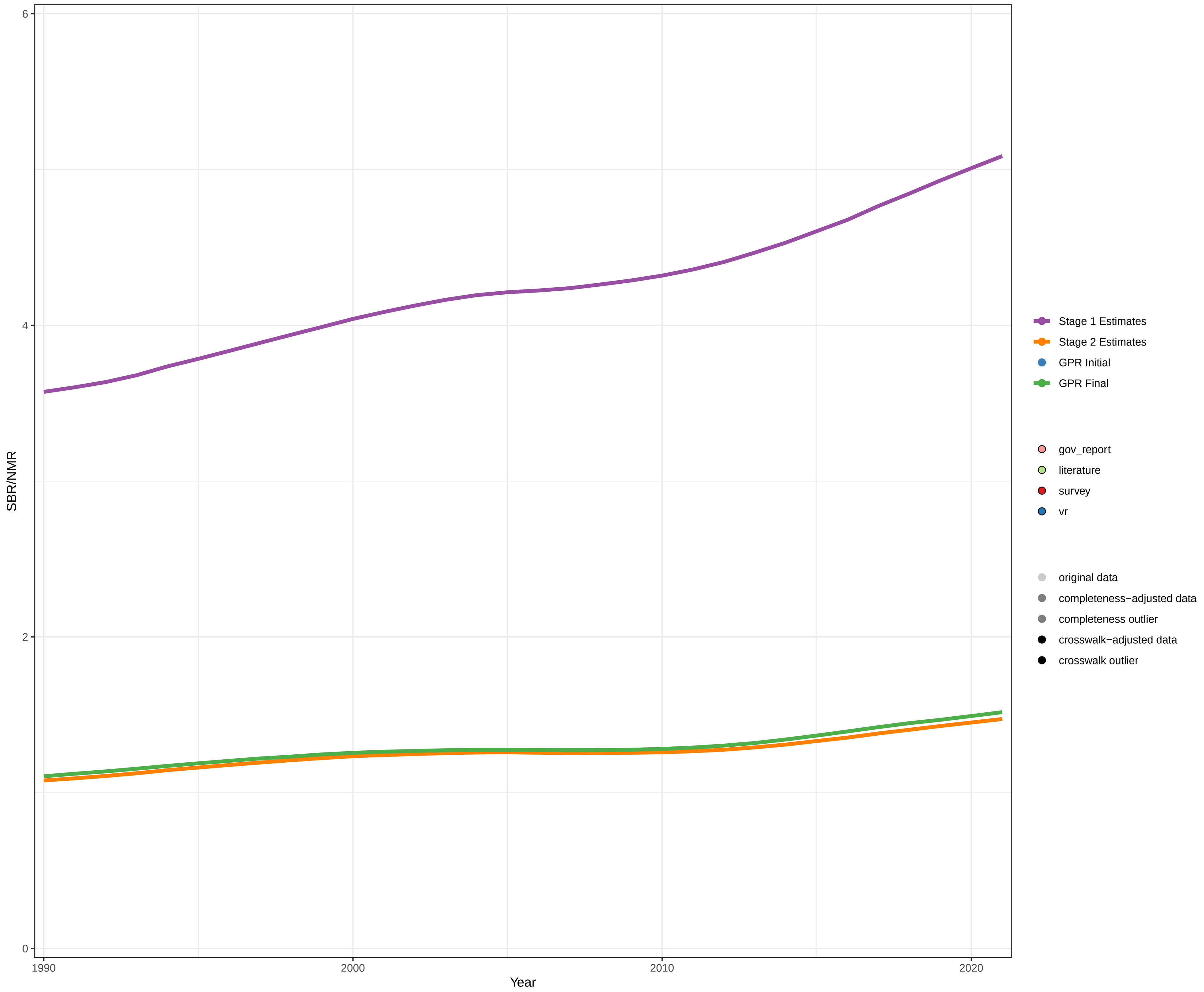

Mexico City MEX_4651 Central Latin America Stillbirth Rate / Neonatal Mortality Rate (SBR/NMR) − 28 weeks
lambda = 1 ; zeta = 0.6 ; scale = 15 ; amp = 0.028

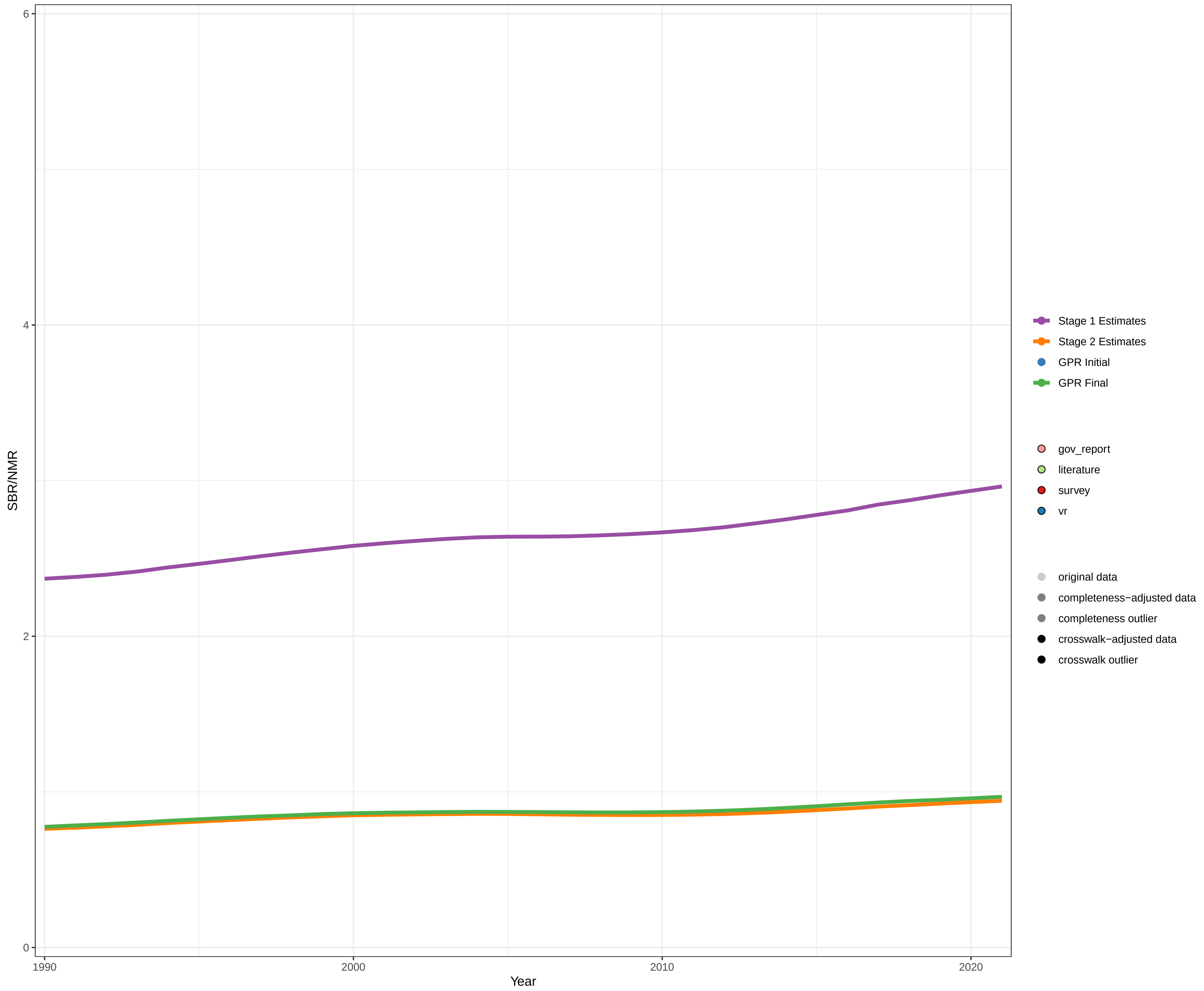

Definition	Years	Outlier	Source.Type	Title

Mexico City MEX_4651 Central Latin America Stillbirth Rate (SBR) − 20 weeks
lambda = 1 ; zeta = 0.6 ; scale = 15 ; amp = 0.028

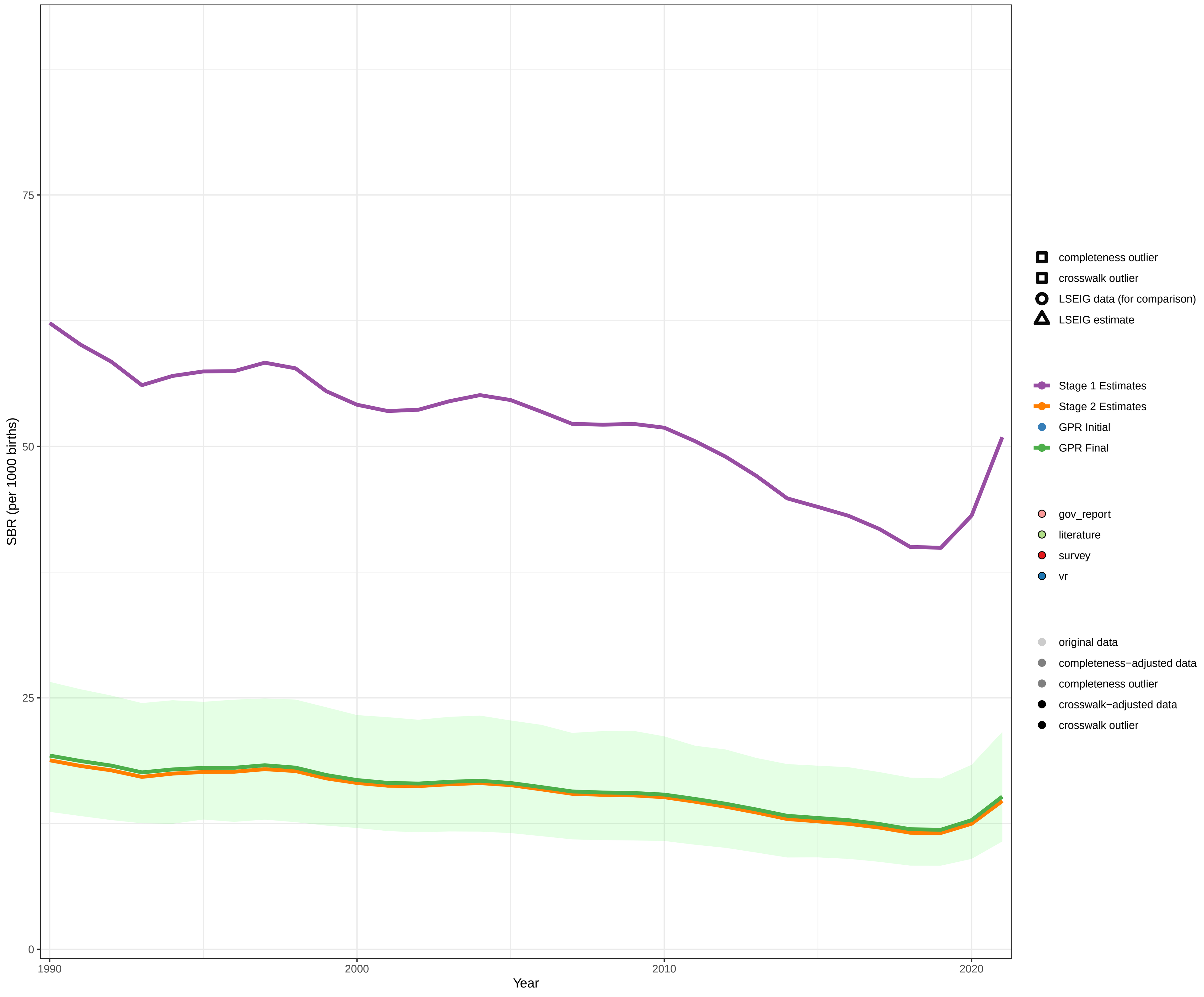

Mexico City MEX_4651 Central Latin America Stillbirth Rate (SBR) − 28 weeks
lambda = 1 ; zeta = 0.6 ; scale = 15 ; amp = 0.028

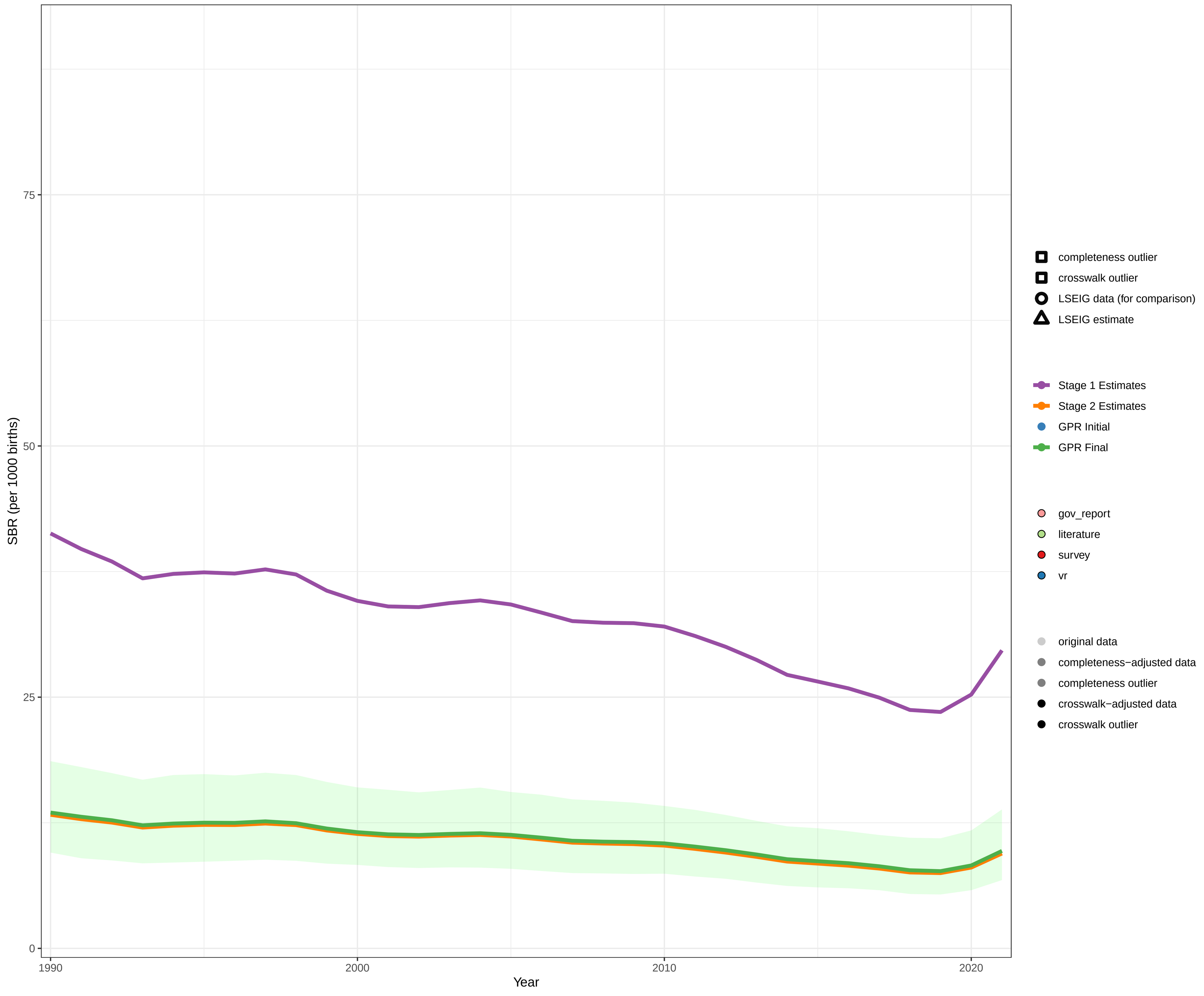

Year	Total Stillbirths (in thousands)	Total Stillbirths (in thousands)	SBR (per 1000 births)	SBR (per 1000 births)	SBR/NMR	SBR/NMR
	20 weeks	28 weeks	20 weeks	28 weeks	20 weeks	28 weeks
1990	4.0 (2.8 to 5.5)	2.8 (2.0 to 3.9)	19.3 (13.7 to 26.6)	13.5 (9.5 to 18.6)	1.11 (0.78 to 1.53)	0.78 (0.55 to 1.07)
1995	3.3 (2.4 to 4.6)	2.3 (1.6 to 3.2)	18.0 (12.9 to 24.6)	12.5 (8.6 to 17.3)	1.19 (0.85 to 1.62)	0.82 (0.57 to 1.14)
2000	2.8 (2.0 to 3.9)	1.9 (1.4 to 2.6)	16.8 (12.1 to 23.3)	11.6 (8.3 to 16.0)	1.26 (0.90 to 1.74)	0.86 (0.62 to 1.20)
2005	2.5 (1.7 to 3.4)	1.7 (1.2 to 2.3)	16.5 (11.6 to 22.8)	11.3 (7.9 to 15.6)	1.28 (0.89 to 1.76)	0.87 (0.61 to 1.20)
2010	2.2 (1.6 to 3.1)	1.5 (1.1 to 2.0)	15.4 (10.8 to 21.2)	10.4 (7.4 to 14.2)	1.28 (0.90 to 1.76)	0.87 (0.62 to 1.18)
2015	1.6 (1.1 to 2.3)	1.1 (0.7 to 1.5)	13.1 (9.1 to 18.3)	8.7 (6.1 to 12.0)	1.37 (0.96 to 1.91)	0.91 (0.64 to 1.25)
2020	1.2 (0.8 to 1.7)	0.8 (0.5 to 1.1)	12.8 (9.0 to 18.3)	8.3 (5.8 to 11.7)	1.49 (1.04 to 2.13)	0.96 (0.67 to 1.36)
2021	1.4 (1.0 to 1.9)	0.9 (0.6 to 1.2)	15.2 (10.7 to 21.6)	9.7 (6.8 to 13.8)	1.52 (1.07 to 2.16)	0.97 (0.68 to 1.38)

Michoacan de Ocampo MEX_4658 Central Latin America Stillbirth Rate / Neonatal Mortality Rate (SBR/NMR) − 20 weeks
lambda = 0.2 ; zeta = 0.99 ; scale = 5 ; amp = 0.028

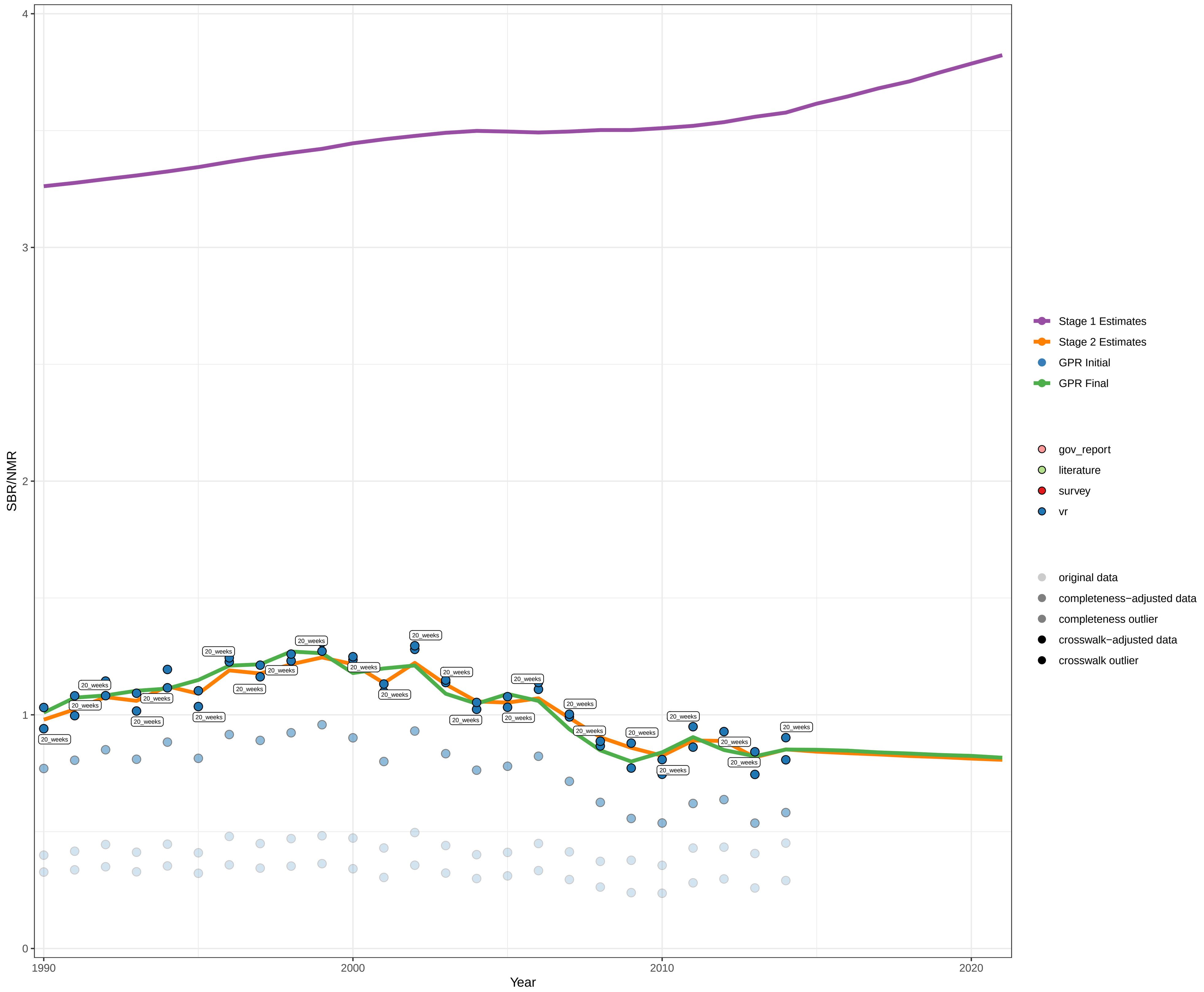

Michoacan de Ocampo MEX_4658 Central Latin America Stillbirth Rate / Neonatal Mortality Rate (SBR/NMR) − 28 weeks
lambda = 0.2 ; zeta = 0.99 ; scale = 5 ; amp = 0.028

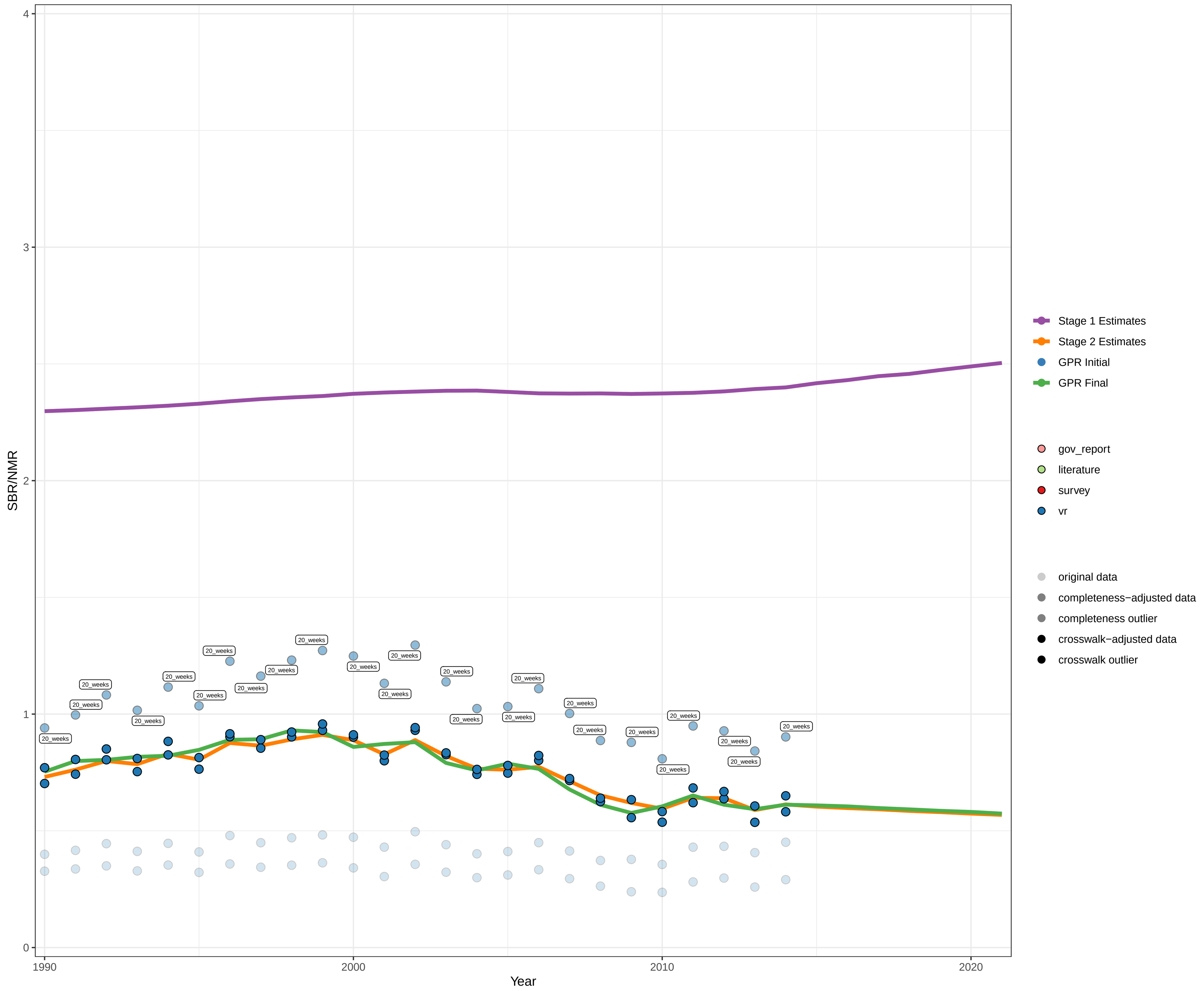

Definition	Years	Outlier	Source.Type	Title
20_weeks, 28_weeks	1990−2014	0	VR	Mexico INEGI Administrative Registers − Fetal Deaths by Municipality of Usual Residence of the Mother and Her Five−year Age Group According to Type of Fetal Death

Michoacan de Ocampo MEX_4658 Central Latin America Stillbirth Rate (SBR) − 20 weeks
lambda = 0.2 ; zeta = 0.99 ; scale = 5 ; amp = 0.028

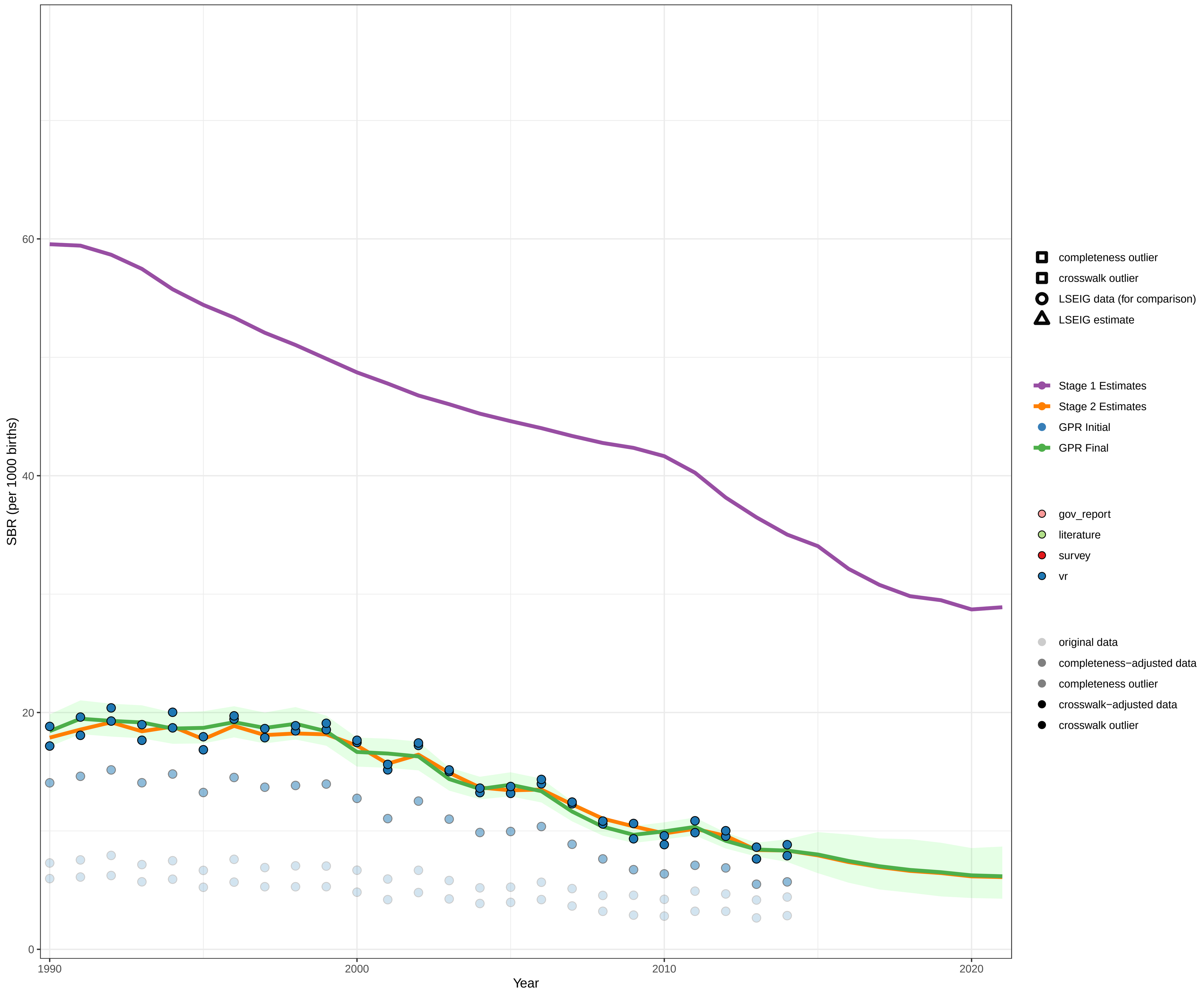

Michoacan de Ocampo MEX_4658 Central Latin America Stillbirth Rate (SBR) − 28 weeks
lambda = 0.2 ; zeta = 0.99 ; scale = 5 ; amp = 0.028

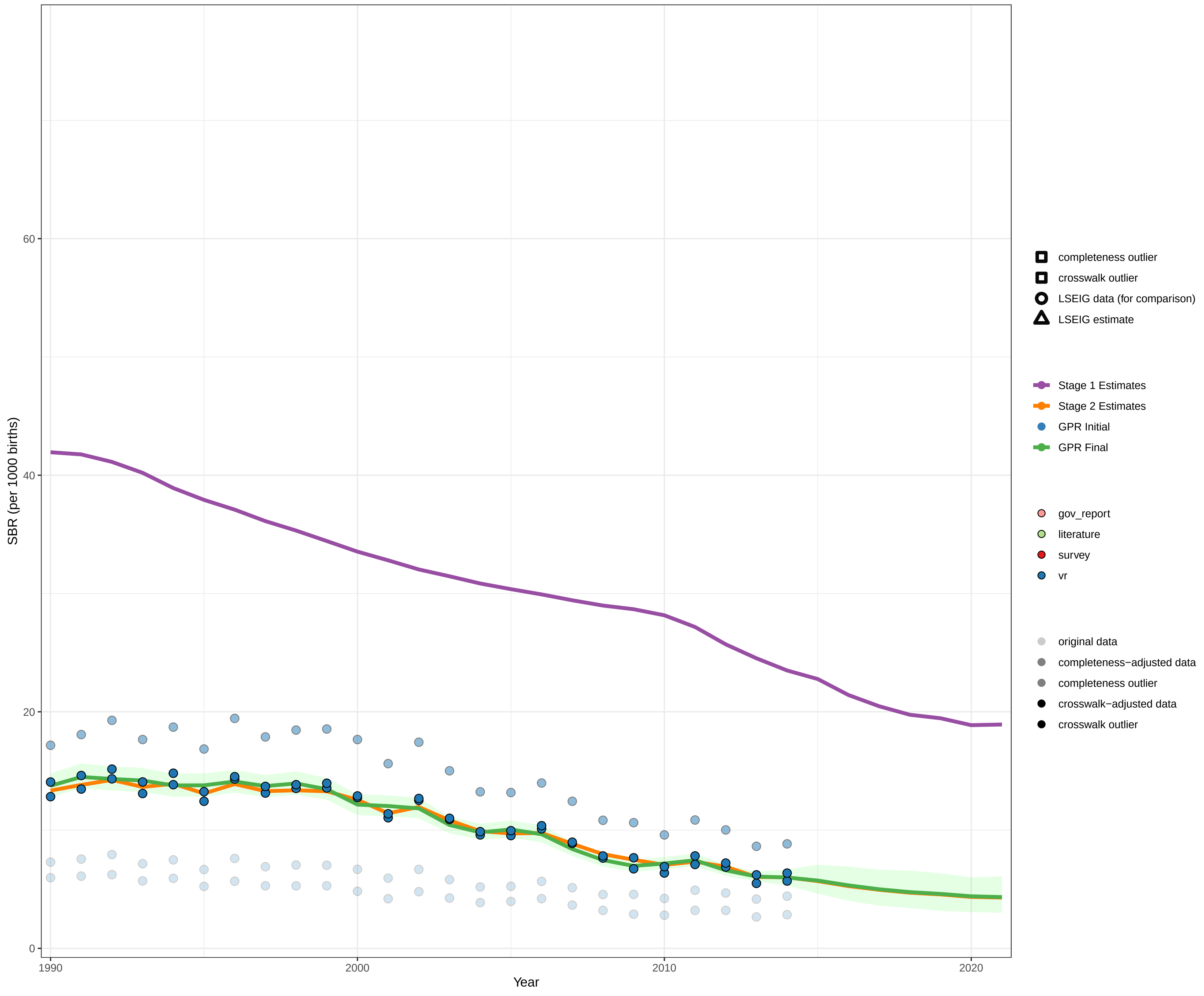

Year	Total Stillbirths (in thousands)	Total Stillbirths (in thousands)	SBR (per 1000 births)	SBR (per 1000 births)	SBR/NMR	SBR/NMR
	20 weeks	28 weeks	20 weeks	28 weeks	20 weeks	28 weeks
1990	2.3<br>(2.2 to 2.5)	1.7<br>(1.6 to 1.9)	18.4<br>(17.1 to 19.8)	13.7<br>(12.8 to 14.8)	1.01<br>(0.94 to 1.09)	0.75<br>(0.70 to 0.81)
1995	2.3<br>(2.1 to 2.4)	1.7<br>(1.5 to 1.8)	18.7<br>(17.4 to 20.1)	13.8<br>(12.8 to 14.8)	1.15<br>(1.07 to 1.24)	0.85<br>(0.79 to 0.91)
2000	1.8<br>(1.7 to 2.0)	1.3<br>(1.2 to 1.4)	16.7<br>(15.4 to 17.9)	12.2<br>(11.3 to 13.0)	1.18<br>(1.09 to 1.26)	0.86<br>(0.80 to 0.92)
2005	1.4<br>(1.3 to 1.5)	1.0<br>(1.0 to 1.1)	13.9<br>(12.9 to 15.0)	10.1<br>(9.3 to 10.8)	1.09<br>(1.01 to 1.17)	0.79<br>(0.73 to 0.85)
2010	1.1<br>(1.0 to 1.1)	0.8<br>(0.7 to 0.8)	10.0<br>(9.3 to 10.7)	7.2<br>(6.7 to 7.7)	0.84<br>(0.78 to 0.90)	0.60<br>(0.56 to 0.65)
2015	0.8<br>(0.6 to 1.0)	0.6<br>(0.5 to 0.7)	8.0<br>(6.4 to 9.9)	5.7<br>(4.6 to 7.1)	0.85<br>(0.68 to 1.05)	0.61<br>(0.49 to 0.75)
2020	0.5<br>(0.4 to 0.7)	0.4<br>(0.3 to 0.5)	6.2<br>(4.3 to 8.6)	4.4<br>(3.1 to 6.0)	0.82<br>(0.57 to 1.13)	0.58<br>(0.40 to 0.79)
2021	0.5<br>(0.3 to 0.7)	0.3<br>(0.2 to 0.5)	6.2<br>(4.3 to 8.7)	4.3<br>(3.0 to 6.1)	0.82<br>(0.57 to 1.15)	0.57<br>(0.40 to 0.80)

Morelos   MEX_4659   Central Latin America   Stillbirth Rate / Neonatal Mortality Rate (SBR/NMR) − 20 weeks
lambda = 0.2 ; zeta = 0.99 ; scale = 5 ; amp = 0.028

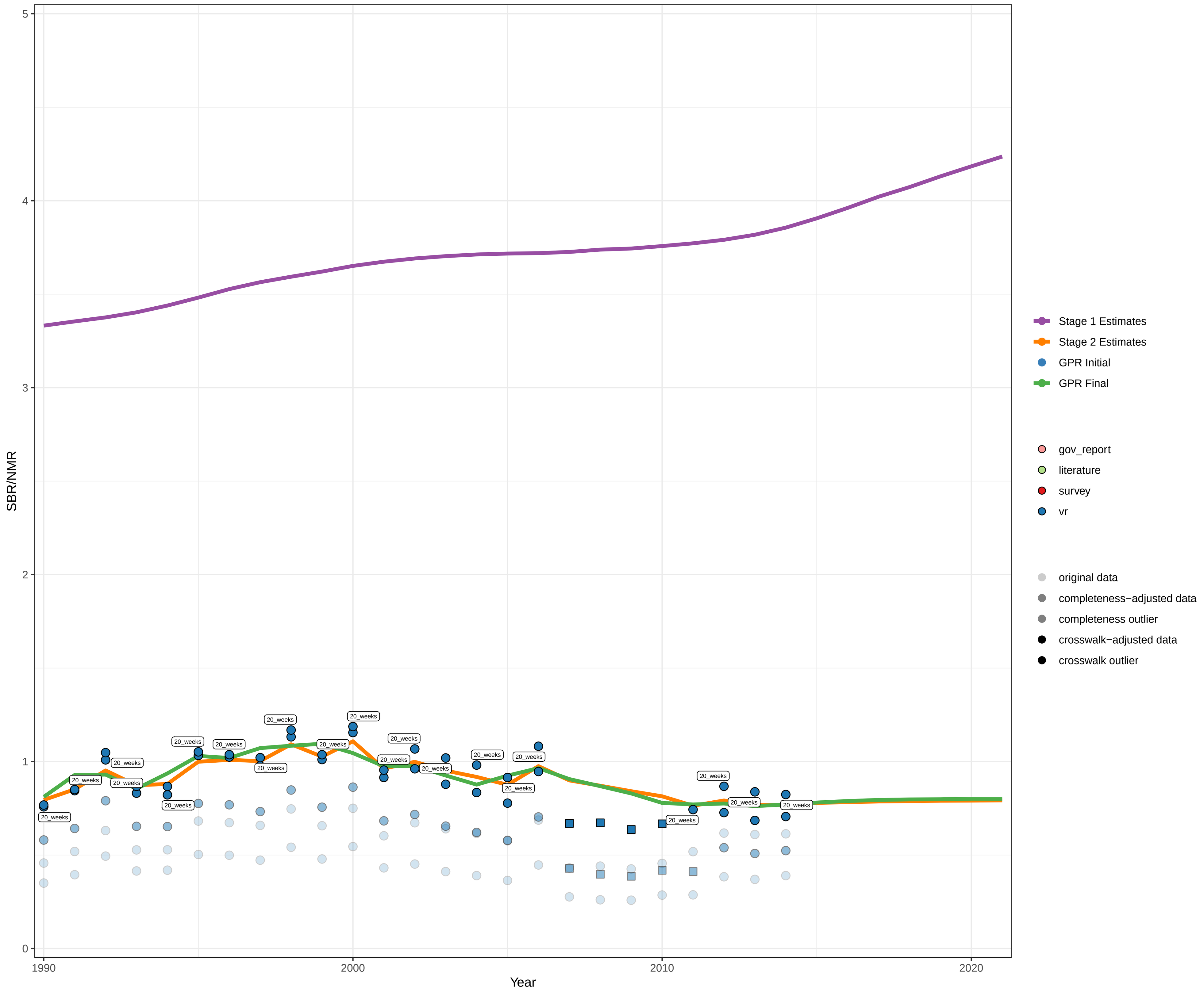

Morelos   MEX_4659   Central Latin America   Stillbirth Rate / Neonatal Mortality Rate (SBR/NMR) − 28 weeks
lambda = 0.2 ; zeta = 0.99 ; scale = 5 ; amp = 0.028

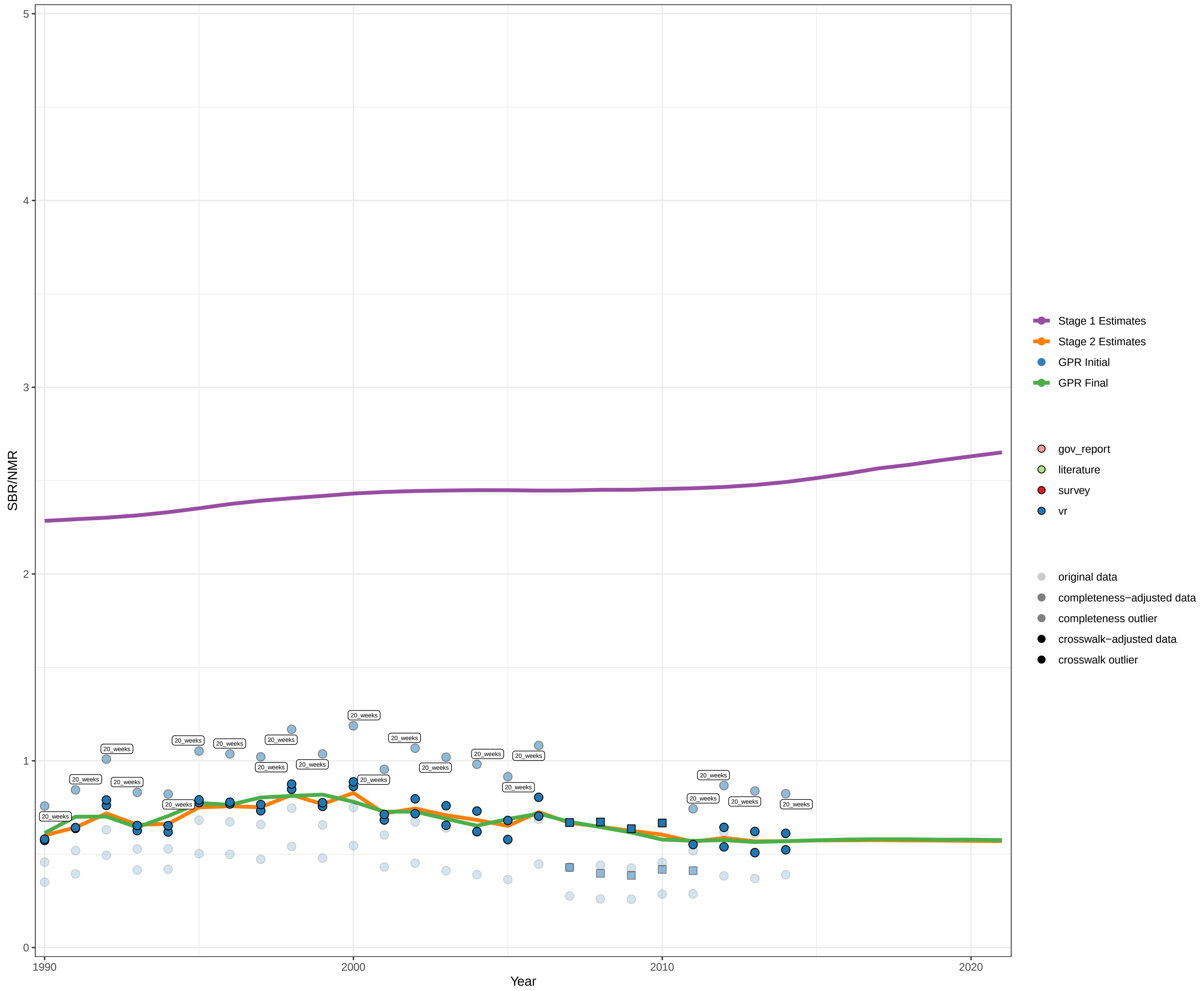

Definition	Years	Outlier	Source.Type	Title
20_weeks	1990−2006, 2011−2014	0	VR	Mexico INEGI Administrative Registers − Fetal Deaths by Municipality of Usual Residence of the Mother and Her Five−year Age Group According to Type of Fetal Death
28_weeks	1990−2006, 2012−2014	0	VR	Mexico INEGI Administrative Registers − Fetal Deaths by Municipality of Usual Residence of the Mother and Her Five−year Age Group According to Type of Fetal Death
28_weeks	2007−2011	1	VR	Mexico INEGI Administrative Registers − Fetal Deaths by Municipality of Usual Residence of the Mother and Her Five−year Age Group According to Type of Fetal Death
20_weeks	2007−2010	2	VR	Mexico INEGI Administrative Registers − Fetal Deaths by Municipality of Usual Residence of the Mother and Her Five−year Age Group According to Type of Fetal Death

Morelos   MEX_4659   Central Latin America   Stillbirth Rate (SBR) − 20 weeks
lambda = 0.2 ; zeta = 0.99 ; scale = 5 ; amp = 0.028

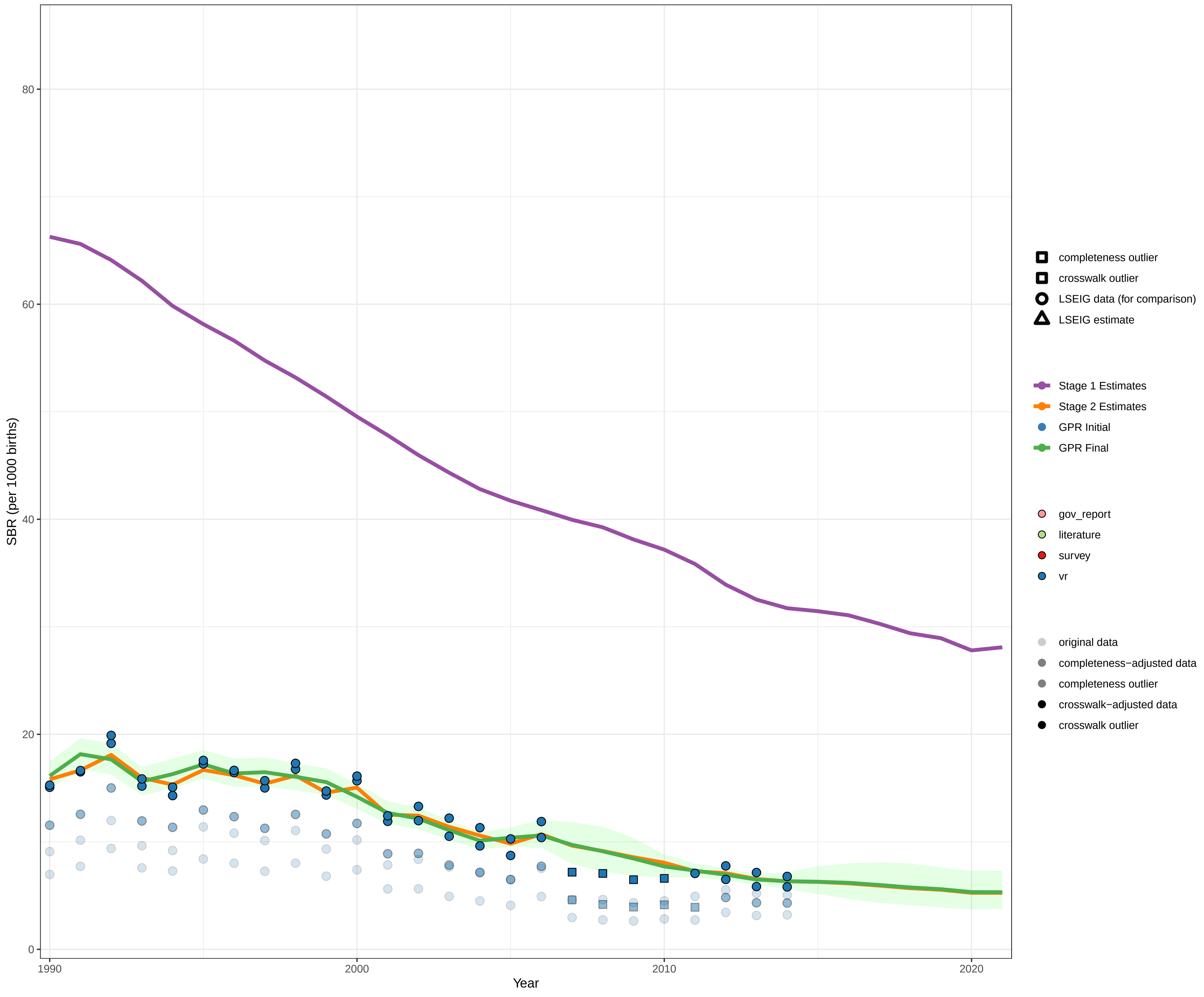

Morelos   MEX_4659   Central Latin America   Stillbirth Rate (SBR) − 28 weeks
lambda = 0.2 ; zeta = 0.99 ; scale = 5 ; amp = 0.028

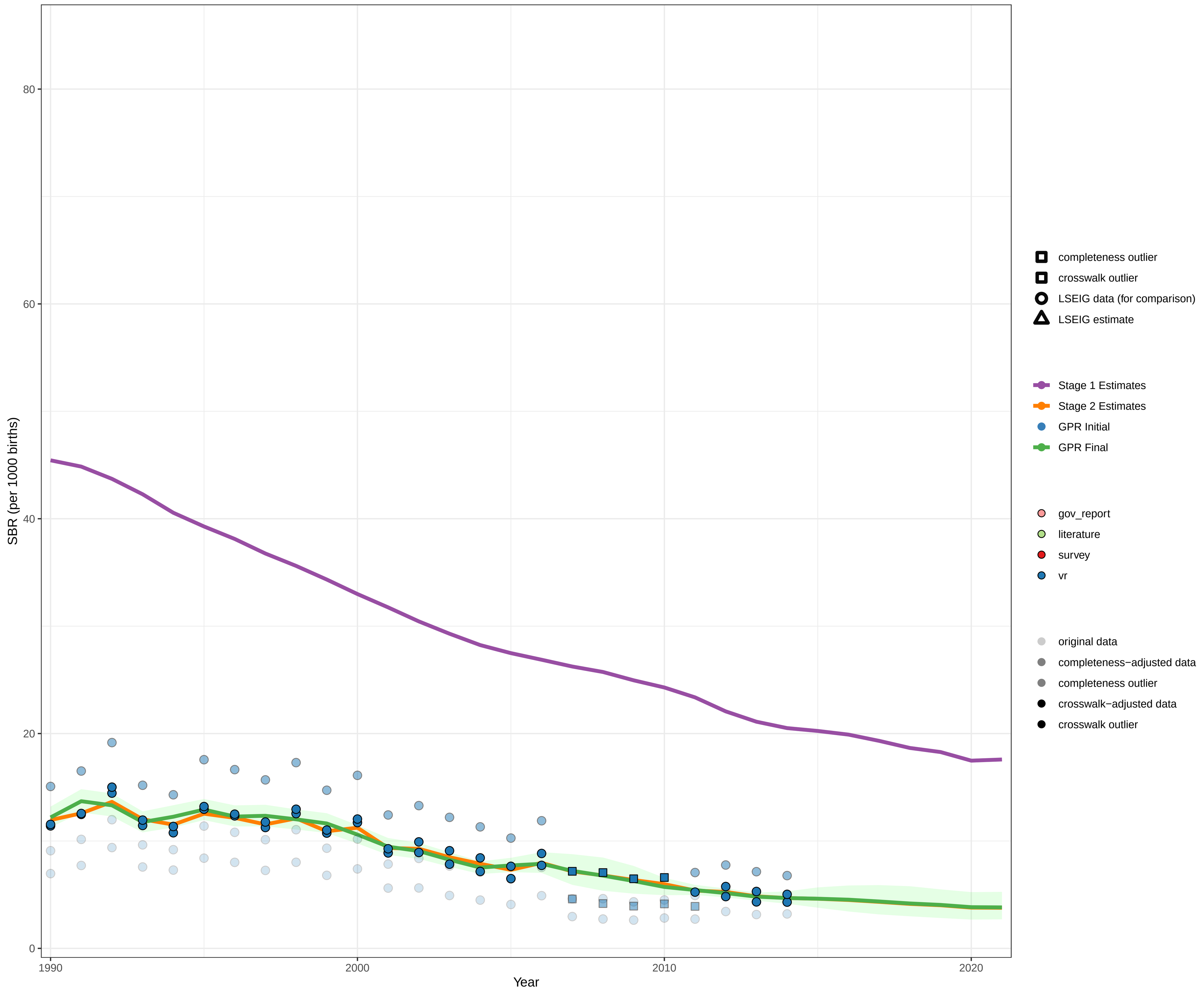

Year	Total Stillbirths (in thousands)	Total Stillbirths (in thousands)	SBR (per 1000 births)	SBR (per 1000 births)	SBR/NMR	SBR/NMR
	20 weeks	28 weeks	20 weeks	28 weeks	20 weeks	28 weeks
1990	0.6 (0.5 to 0.6)	0.4 (0.4 to 0.5)	16.1 (14.9 to 17.5)	12.2 (11.3 to 13.2)	0.81 (0.75 to 0.88)	0.61 (0.57 to 0.66)
1995	0.6 (0.6 to 0.7)	0.5 (0.4 to 0.5)	17.2 (15.9 to 18.5)	12.9 (11.9 to 13.9)	1.03 (0.95 to 1.11)	0.77 (0.72 to 0.83)
2000	0.5 (0.5 to 0.5)	0.4 (0.3 to 0.4)	14.2 (13.1 to 15.4)	10.6 (9.8 to 11.5)	1.05 (0.96 to 1.13)	0.78 (0.72 to 0.85)
2005	0.4 (0.3 to 0.4)	0.3 (0.2 to 0.3)	10.4 (9.5 to 11.3)	7.7 (7.1 to 8.4)	0.92 (0.85 to 1.01)	0.69 (0.63 to 0.75)
2010	0.3 (0.3 to 0.3)	0.2 (0.2 to 0.2)	7.7 (6.7 to 8.9)	5.7 (5.0 to 6.6)	0.78 (0.67 to 0.90)	0.58 (0.50 to 0.66)
2015	0.2 (0.2 to 0.3)	0.2 (0.1 to 0.2)	6.3 (5.1 to 7.7)	4.6 (3.8 to 5.7)	0.78 (0.64 to 0.96)	0.57 (0.47 to 0.71)
2020	0.2 (0.1 to 0.2)	0.1 (0.1 to 0.1)	5.3 (3.7 to 7.3)	3.8 (2.7 to 5.2)	0.80 (0.56 to 1.10)	0.58 (0.40 to 0.79)
2021	0.1 (0.1 to 0.2)	0.1 (0.1 to 0.1)	5.3 (3.8 to 7.3)	3.8 (2.7 to 5.3)	0.80 (0.57 to 1.11)	0.58 (0.41 to 0.79)

Nayarit MEX_4660 Central Latin America Stillbirth Rate / Neonatal Mortality Rate (SBR/NMR) − 20 weeks
lambda = 0.2 ; zeta = 0.99 ; scale = 5 ; amp = 0.028

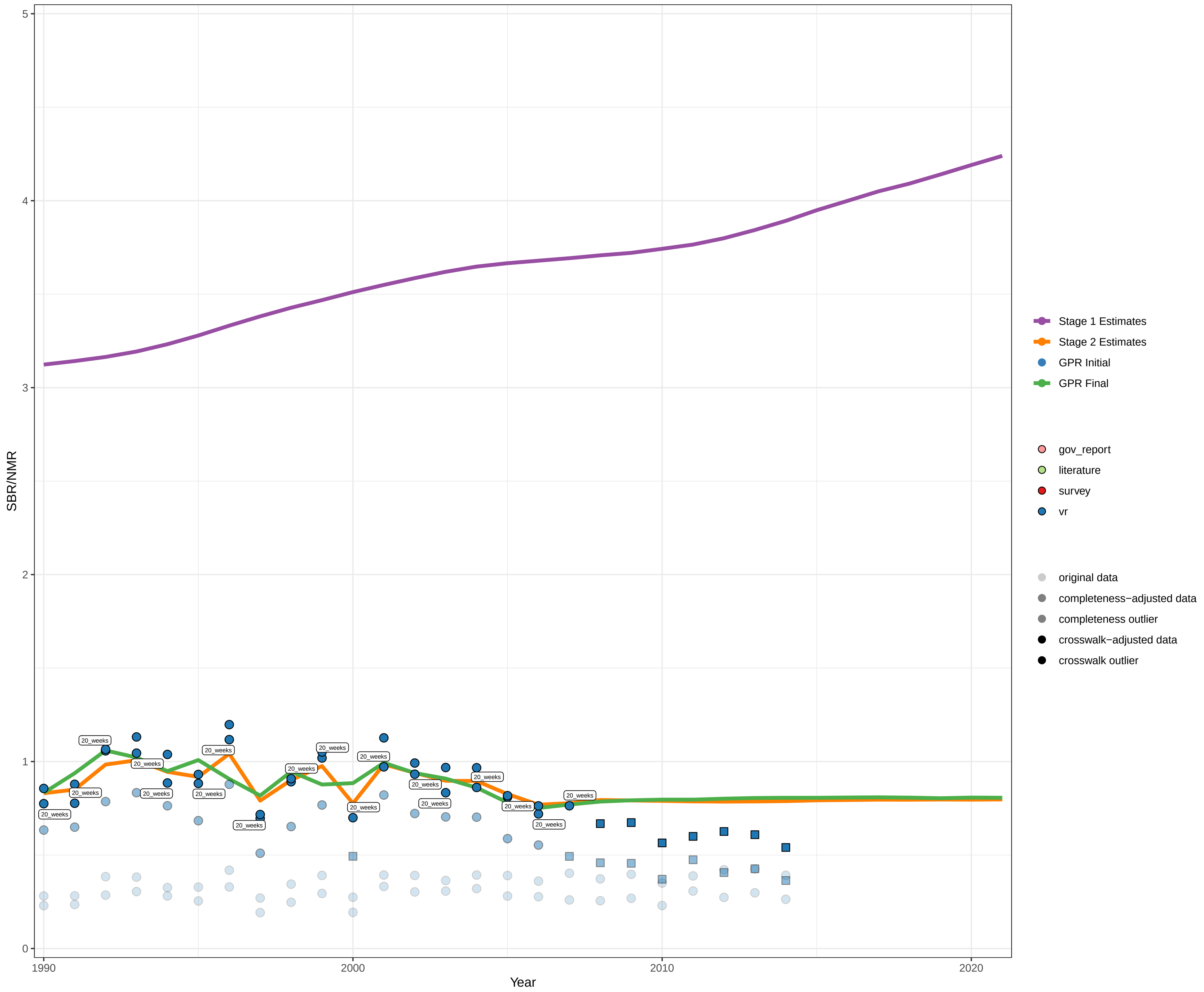

Nayarit MEX_4660 Central Latin America Stillbirth Rate / Neonatal Mortality Rate (SBR/NMR) − 28 weeks
lambda = 0.2 ; zeta = 0.99 ; scale = 5 ; amp = 0.028

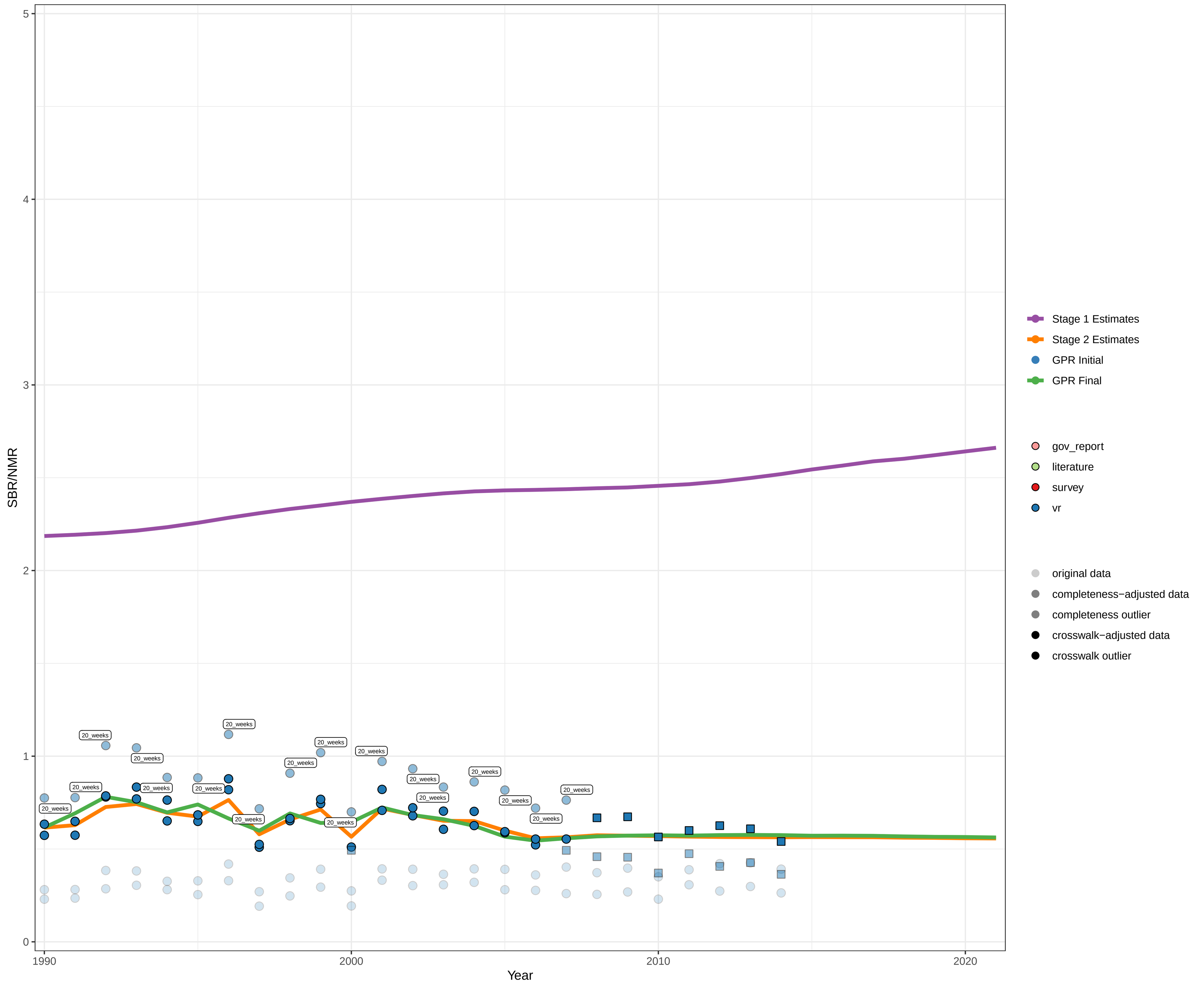

Definition	Years	Outlier	Source.Type	Title
20_weeks	1990−2007	0	VR	Mexico INEGI Administrative Registers − Fetal Deaths by Municipality of Usual Residence of the Mother and Her Five−year Age Group According to Type of Fetal Death
28_weeks	1990−1999, 2001−2006	0	VR	Mexico INEGI Administrative Registers − Fetal Deaths by Municipality of Usual Residence of the Mother and Her Five−year Age Group According to Type of Fetal Death
28_weeks	2000, 2007−2014	1	VR	Mexico INEGI Administrative Registers − Fetal Deaths by Municipality of Usual Residence of the Mother and Her Five−year Age Group According to Type of Fetal Death
20_weeks	2008−2014	2	VR	Mexico INEGI Administrative Registers − Fetal Deaths by Municipality of Usual Residence of the Mother and Her Five−year Age Group According to Type of Fetal Death

Nayarit MEX_4660 Central Latin America Stillbirth Rate (SBR) − 20 weeks
lambda = 0.2 ; zeta = 0.99 ; scale = 5 ; amp = 0.028

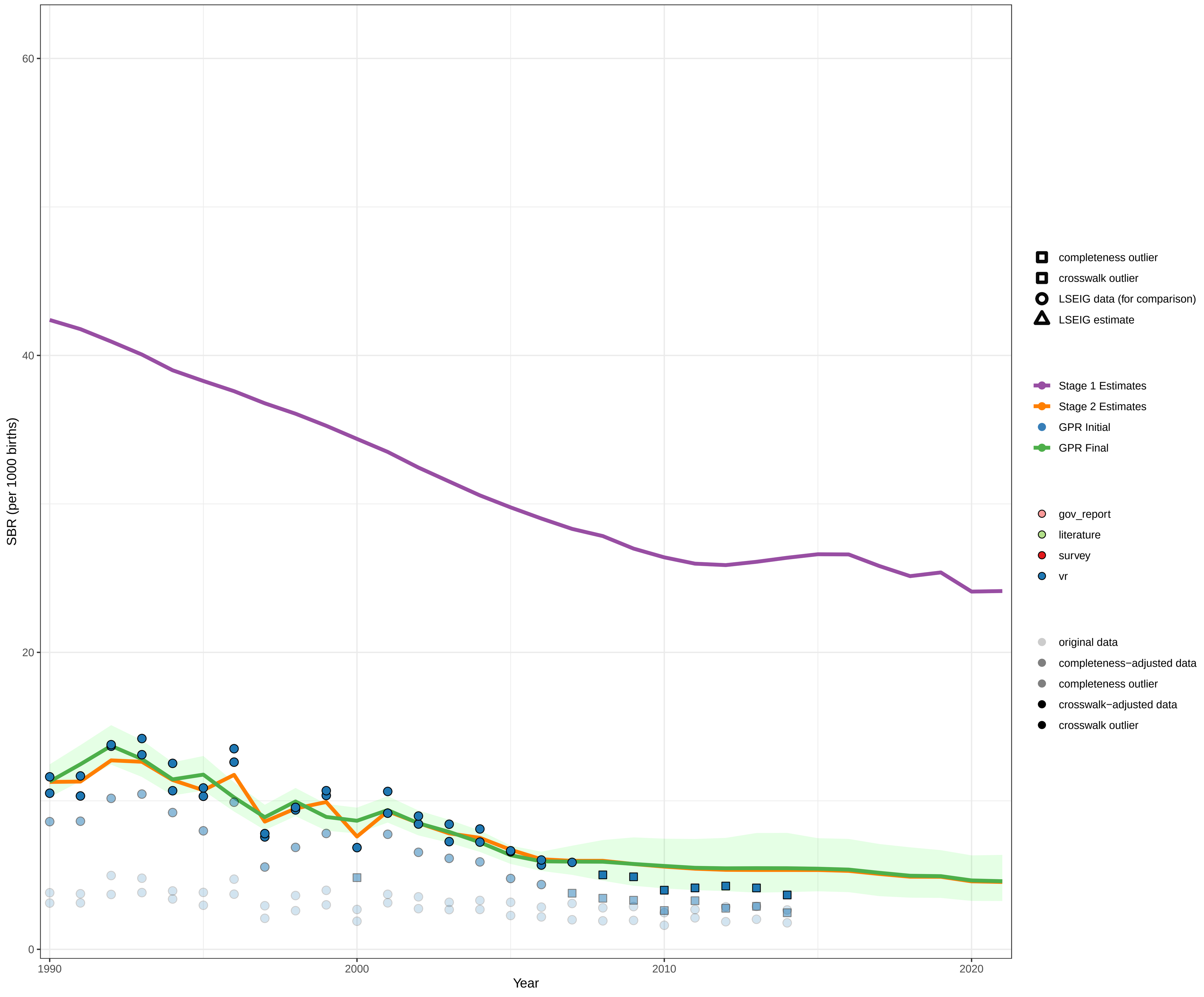

Nayarit MEX_4660 Central Latin America Stillbirth Rate (SBR) − 28 weeks
lambda = 0.2 ; zeta = 0.99 ; scale = 5 ; amp = 0.028

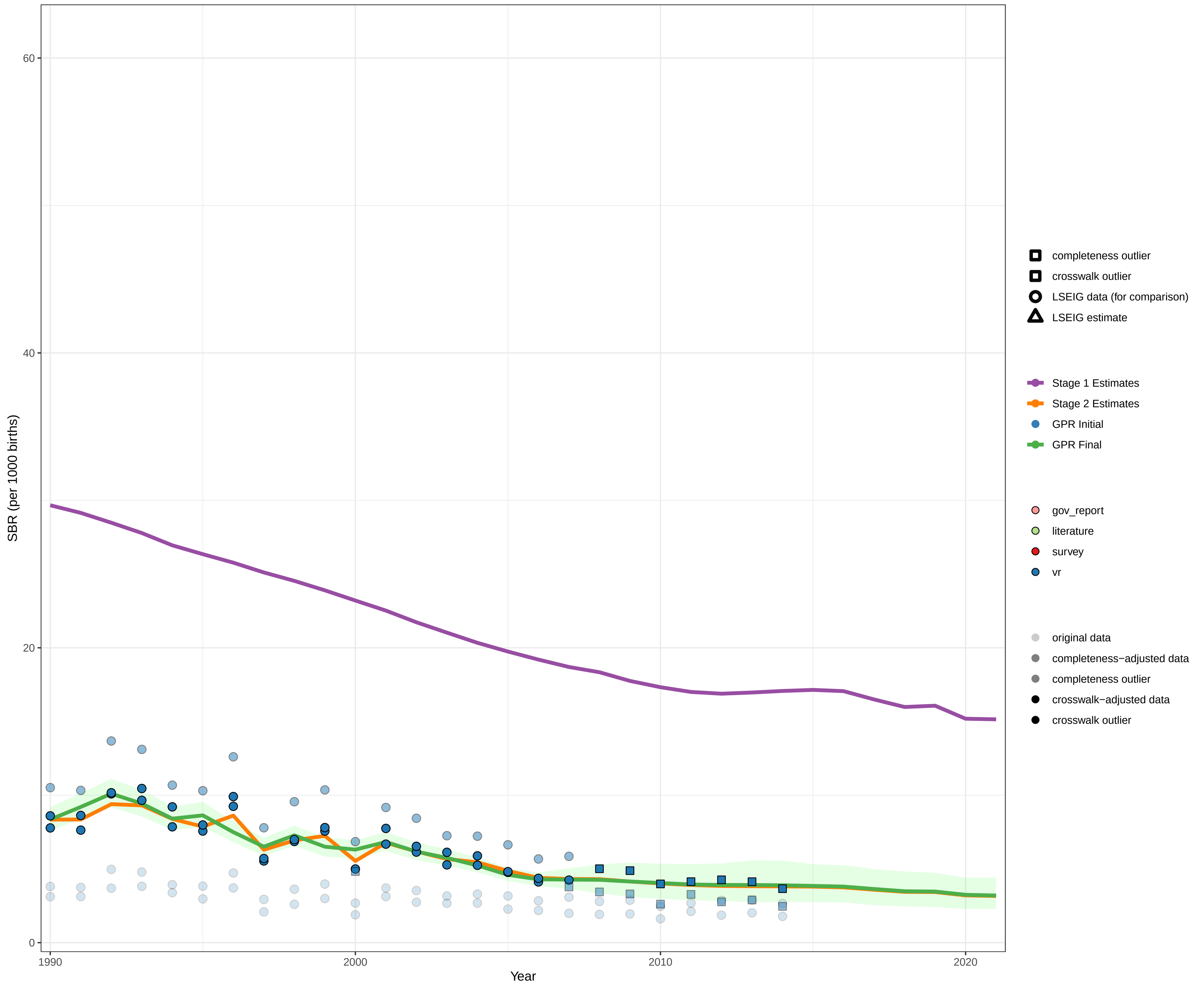

Year	Total Stillbirths (in thousands)	Total Stillbirths (in thousands)	SBR (per 1000 births)	SBR (per 1000 births)	SBR/NMR	SBR/NMR
	20 weeks	28 weeks	20 weeks	28 weeks	20 weeks	28 weeks
1990	0.3 (0.3 to 0.3)	0.2 (0.2 to 0.2)	11.3 (10.2 to 12.5)	8.4 (7.6 to 9.2)	0.83 (0.75 to 0.92)	0.62 (0.56 to 0.68)
1995	0.3 (0.3 to 0.3)	0.2 (0.2 to 0.2)	11.8 (10.7 to 13.0)	8.6 (7.9 to 9.5)	1.01 (0.92 to 1.12)	0.74 (0.67 to 0.82)
2000	0.2 (0.2 to 0.2)	0.1 (0.1 to 0.2)	8.7 (7.8 to 9.5)	6.3 (5.7 to 7.0)	0.88 (0.80 to 0.97)	0.64 (0.58 to 0.71)
2005	0.1 (0.1 to 0.2)	0.1 (0.1 to 0.1)	6.3 (5.8 to 7.0)	4.6 (4.2 to 5.1)	0.78 (0.71 to 0.86)	0.57 (0.51 to 0.62)
2010	0.1 (0.1 to 0.2)	0.1 (0.1 to 0.1)	5.6 (4.1 to 7.5)	4.0 (3.0 to 5.4)	0.80 (0.58 to 1.06)	0.57 (0.42 to 0.76)
2015	0.1 (0.1 to 0.2)	0.1 (0.1 to 0.1)	5.4 (3.9 to 7.5)	3.9 (2.7 to 5.3)	0.81 (0.58 to 1.11)	0.57 (0.41 to 0.79)
2020	0.1 (0.1 to 0.1)	0.1 (0.0 to 0.1)	4.6 (3.3 to 6.3)	3.2 (2.3 to 4.4)	0.81 (0.57 to 1.10)	0.56 (0.40 to 0.77)
2021	0.1 (0.1 to 0.1)	0.1 (0.0 to 0.1)	4.6 (3.3 to 6.4)	3.2 (2.3 to 4.4)	0.81 (0.57 to 1.12)	0.56 (0.40 to 0.78)

Nuevo Leon MEX_4661 Central Latin America Stillbirth Rate / Neonatal Mortality Rate (SBR/NMR) − 20 weeks
lambda = 0.2 ; zeta = 0.99 ; scale = 5 ; amp = 0.028

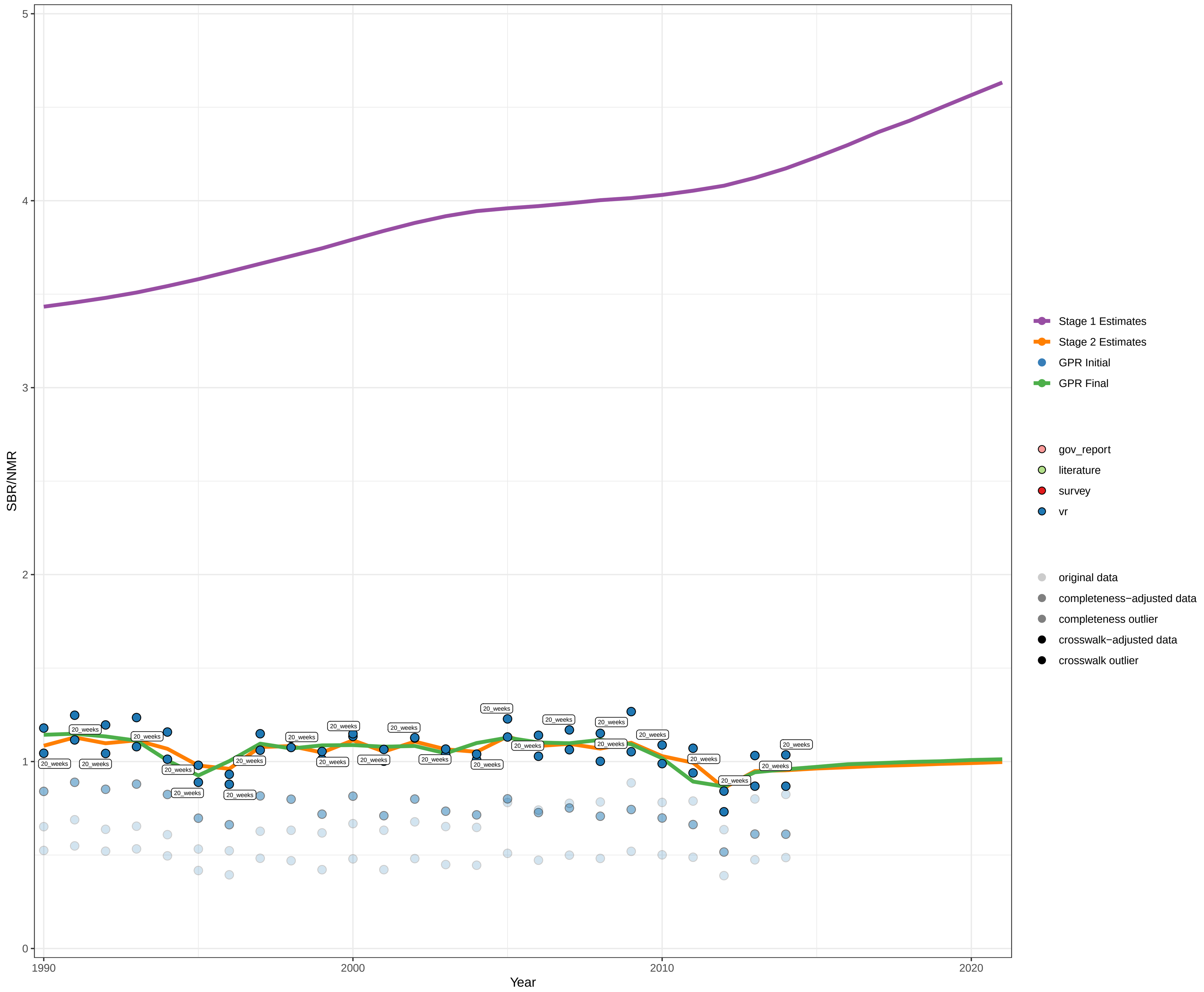

Nuevo Leon MEX_4661 Central Latin America Stillbirth Rate / Neonatal Mortality Rate (SBR/NMR) − 28 weeks
lambda = 0.2 ; zeta = 0.99 ; scale = 5 ; amp = 0.028

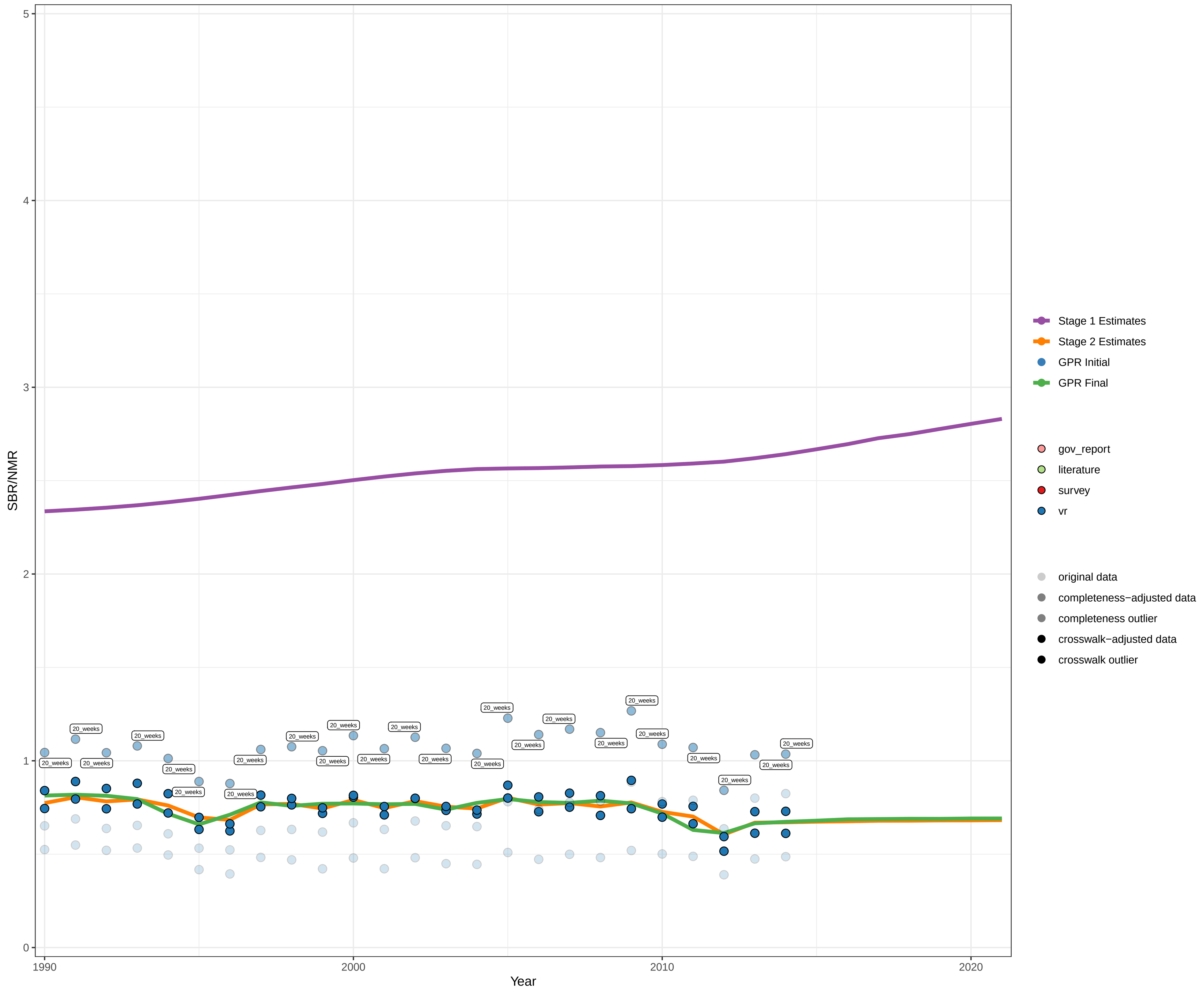

Definition	Years	Outlier	Source.Type	Title
20_weeks, 28_weeks	1990−2014	0	VR	Mexico INEGI Administrative Registers − Fetal Deaths by Municipality of Usual Residence of the Mother and Her Five−year Age Group According to Type of Fetal Death

Nuevo Leon MEX_4661 Central Latin America Stillbirth Rate (SBR) − 20 weeks
lambda = 0.2 ; zeta = 0.99 ; scale = 5 ; amp = 0.028

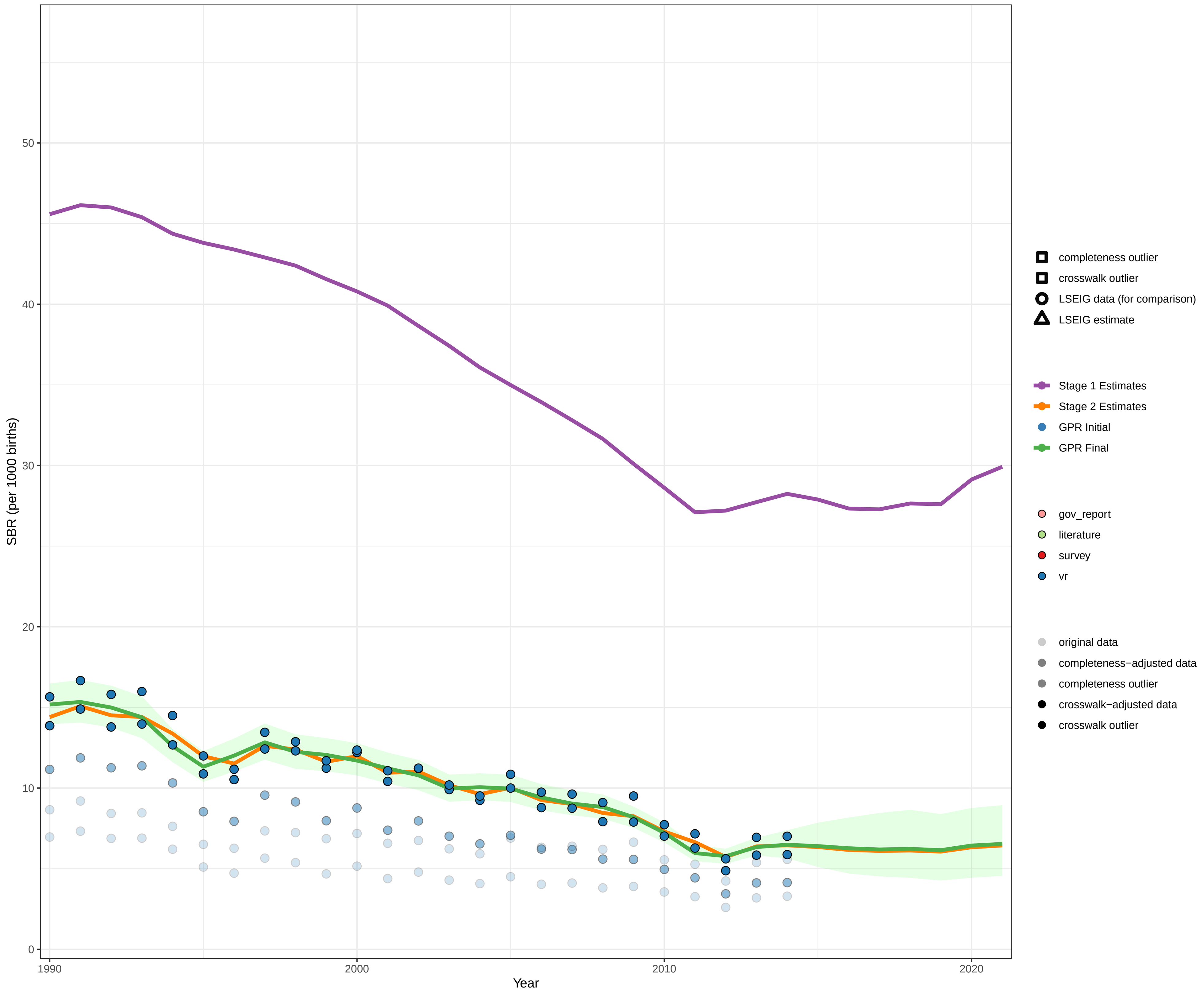

Nuevo Leon MEX_4661 Central Latin America Stillbirth Rate (SBR) − 28 weeks
lambda = 0.2 ; zeta = 0.99 ; scale = 5 ; amp = 0.028

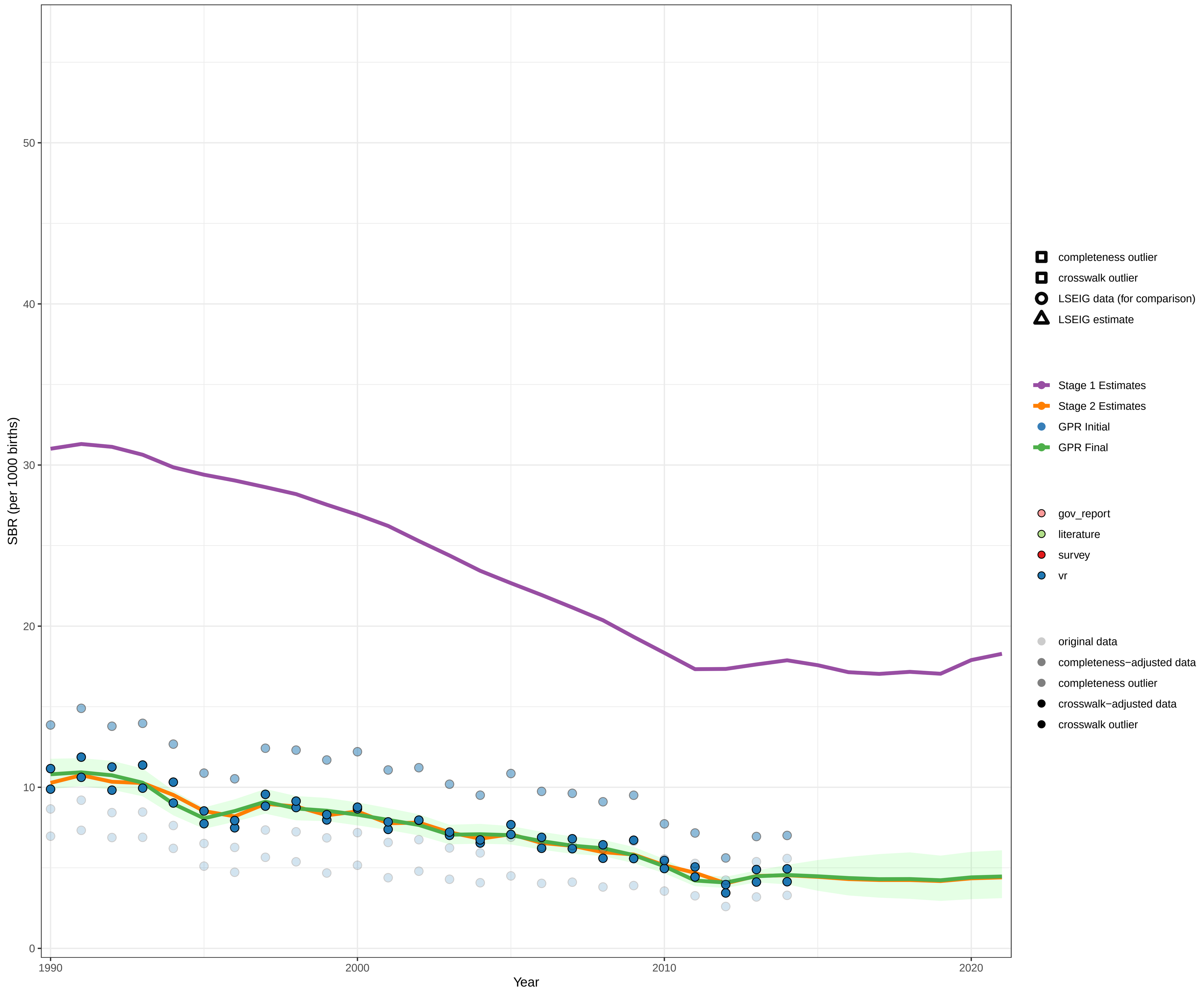

Year	Total Stillbirths (in thousands)	Total Stillbirths (in thousands)	SBR (per 1000 births)	SBR (per 1000 births)	SBR/NMR	SBR/NMR
	20 weeks	28 weeks	20 weeks	28 weeks	20 weeks	28 weeks
1990	1.3 (1.2 to 1.4)	0.9 (0.8 to 1.0)	15.2 (14.0 to 16.5)	10.8 (9.9 to 11.8)	1.14 (1.05 to 1.24)	0.81 (0.74 to 0.89)
1995	1.0 (0.9 to 1.1)	0.7 (0.7 to 0.8)	11.3 (10.4 to 12.3)	8.1 (7.4 to 8.7)	0.93 (0.85 to 1.00)	0.66 (0.61 to 0.71)
2000	1.1 (1.0 to 1.1)	0.7 (0.7 to 0.8)	11.7 (10.8 to 12.8)	8.3 (7.6 to 9.1)	1.09 (1.00 to 1.19)	0.77 (0.71 to 0.84)
2005	0.9 (0.8 to 1.0)	0.6 (0.6 to 0.7)	10.0 (9.1 to 10.8)	7.0 (6.4 to 7.6)	1.13 (1.03 to 1.23)	0.80 (0.73 to 0.86)
2010	0.7 (0.6 to 0.7)	0.5 (0.4 to 0.5)	7.2 (6.6 to 7.9)	5.1 (4.7 to 5.5)	1.02 (0.93 to 1.11)	0.72 (0.66 to 0.78)
2015	0.6 (0.5 to 0.7)	0.4 (0.3 to 0.5)	6.4 (5.1 to 7.9)	4.5 (3.6 to 5.5)	0.97 (0.77 to 1.19)	0.68 (0.54 to 0.83)
2020	0.5 (0.4 to 0.7)	0.4 (0.3 to 0.5)	6.4 (4.4 to 8.8)	4.4 (3.1 to 6.0)	1.01 (0.70 to 1.37)	0.69 (0.48 to 0.94)
2021	0.5 (0.4 to 0.7)	0.4 (0.3 to 0.5)	6.5 (4.5 to 8.9)	4.5 (3.1 to 6.1)	1.01 (0.70 to 1.38)	0.69 (0.48 to 0.94)

Oaxaca MEX_4662 Central Latin America Stillbirth Rate / Neonatal Mortality Rate (SBR/NMR) − 20 weeks
lambda = 0.2 ; zeta = 0.99 ; scale = 5 ; amp = 0.028

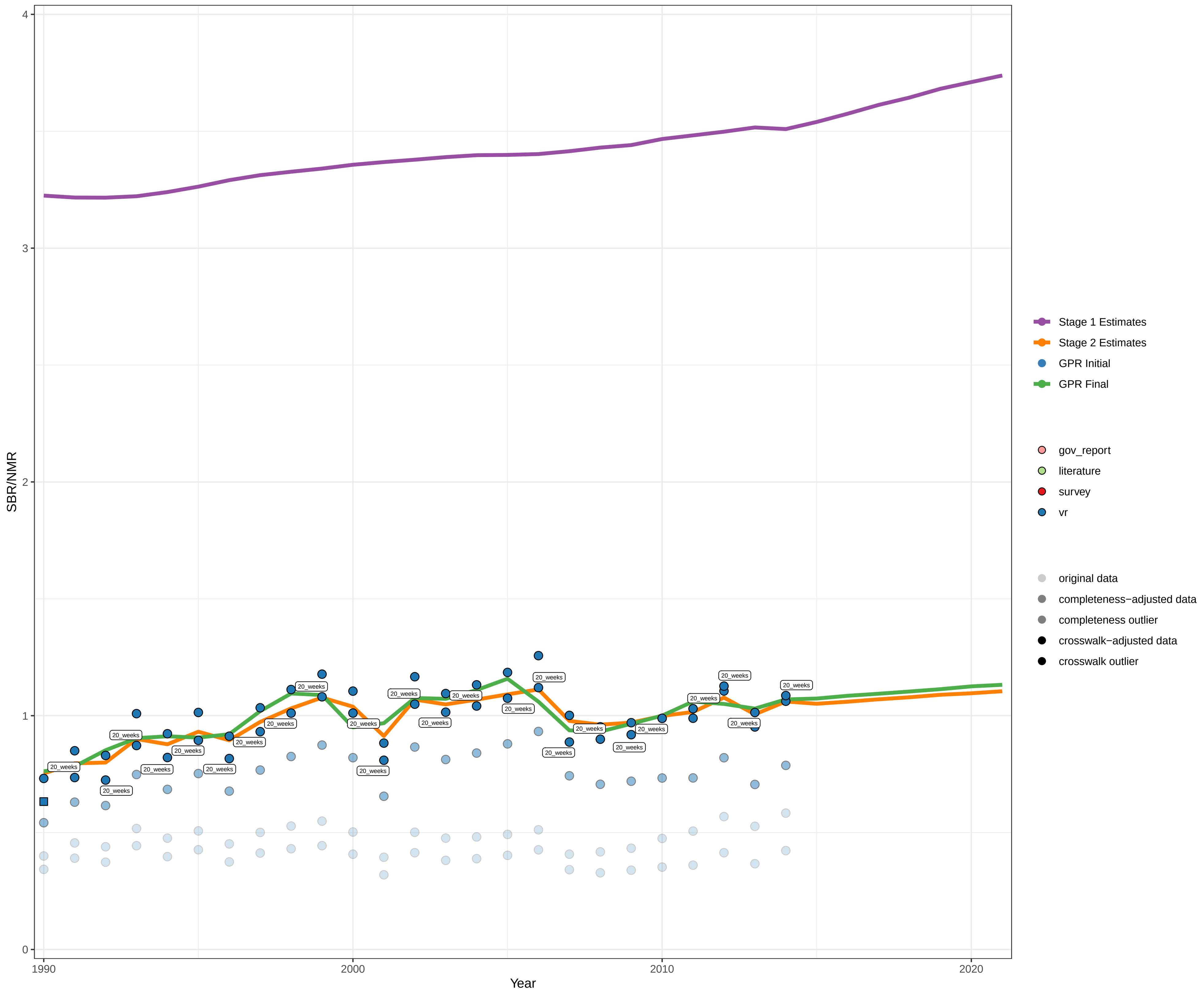

Oaxaca MEX_4662 Central Latin America Stillbirth Rate / Neonatal Mortality Rate (SBR/NMR) − 28 weeks
lambda = 0.2 ; zeta = 0.99 ; scale = 5 ; amp = 0.028

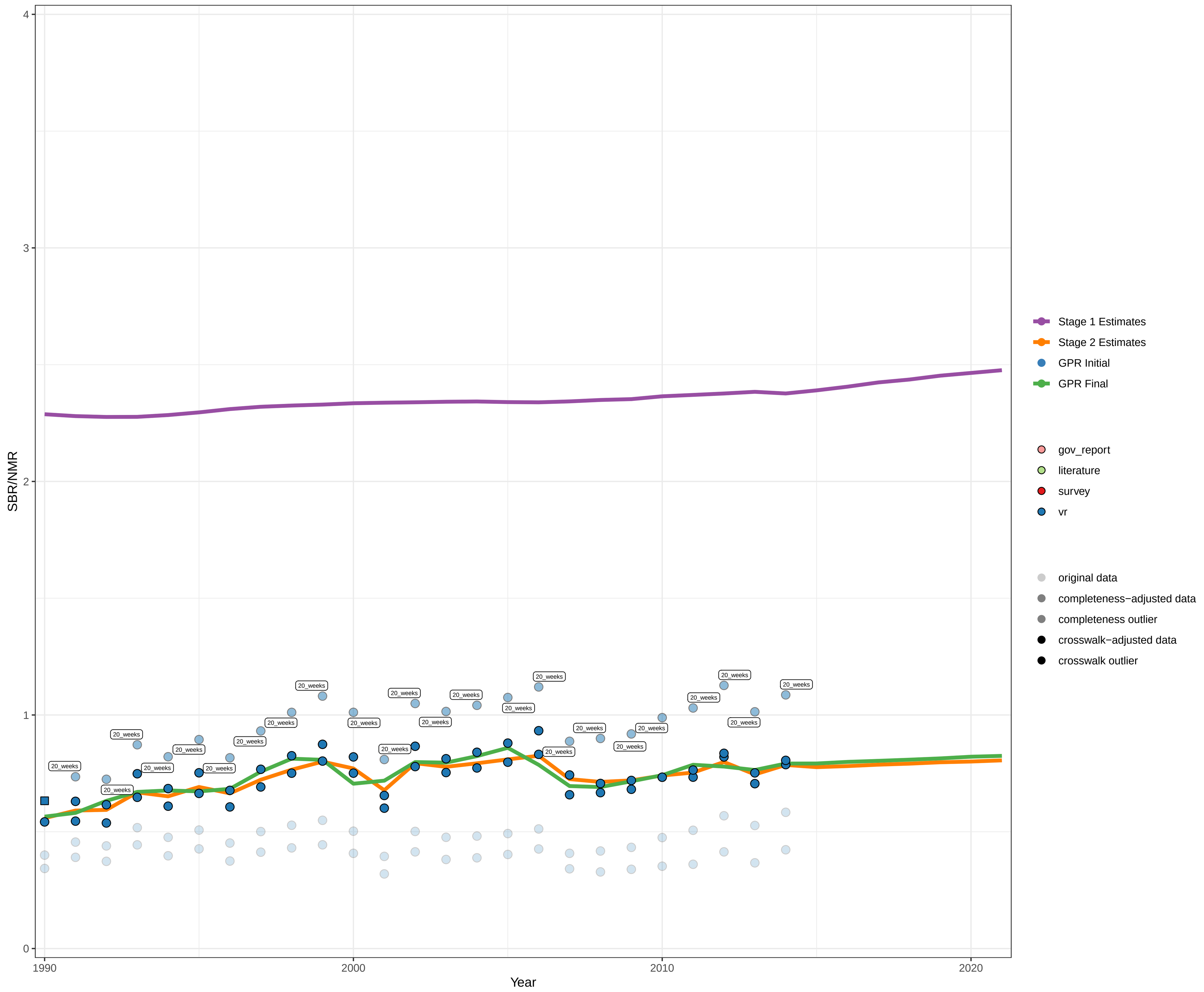

Definition	Years	Outlier	Source.Type	Title
20_weeks	1991−2014	0	VR	Mexico INEGI Administrative Registers − Fetal Deaths by Municipality of Usual Residence of the Mother and Her Five−year Age Group According to Type of Fetal Death
28_weeks	1990−2014	0	VR	Mexico INEGI Administrative Registers − Fetal Deaths by Municipality of Usual Residence of the Mother and Her Five−year Age Group According to Type of Fetal Death
20_weeks	1990	2	VR	Mexico INEGI Administrative Registers − Fetal Deaths by Municipality of Usual Residence of the Mother and Her Five−year Age Group According to Type of Fetal Death

Oaxaca MEX_4662 Central Latin America Stillbirth Rate (SBR) − 20 weeks
lambda = 0.2 ; zeta = 0.99 ; scale = 5 ; amp = 0.028

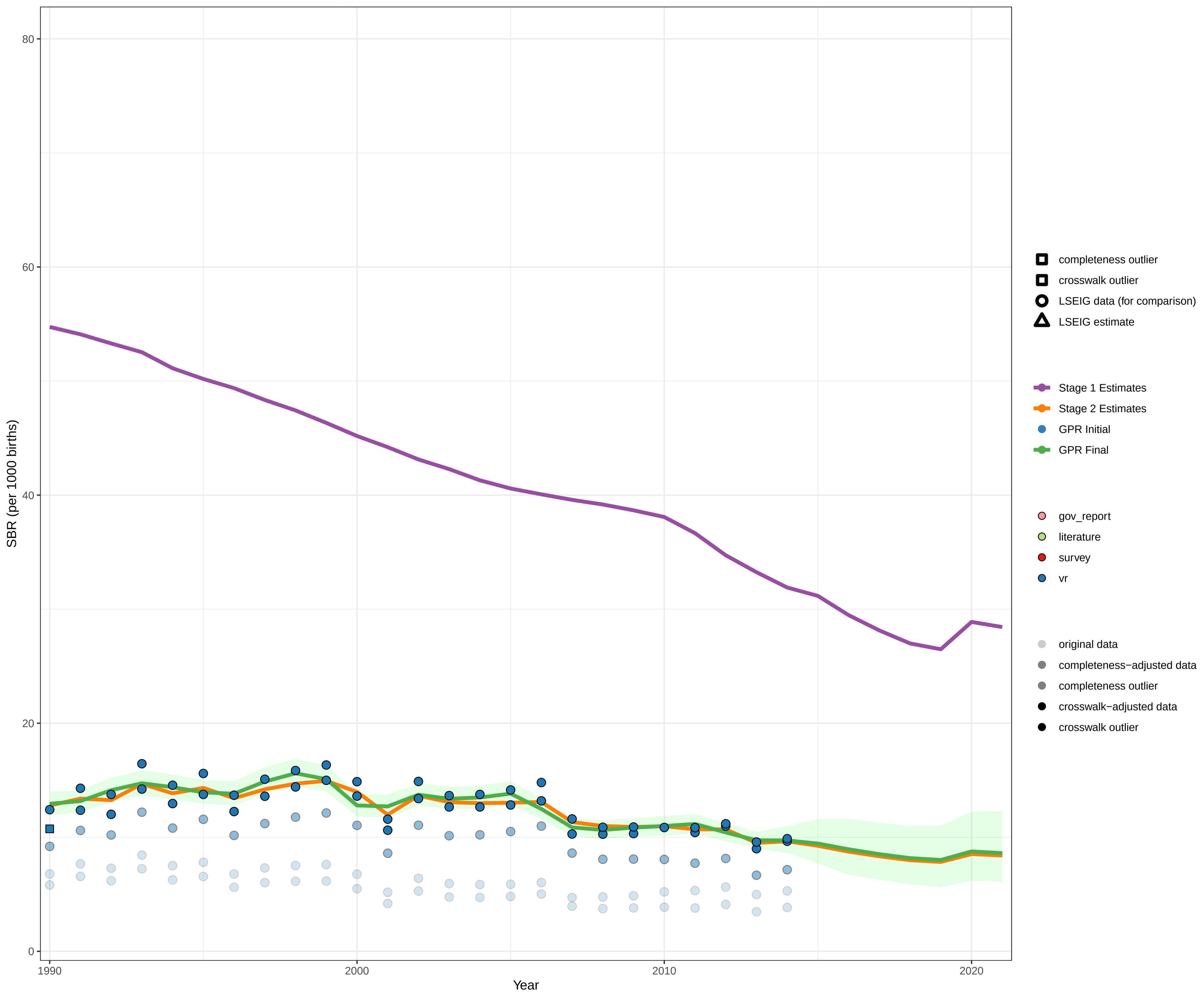

Oaxaca MEX_4662 Central Latin America Stillbirth Rate (SBR) − 28 weeks
lambda = 0.2 ; zeta = 0.99 ; scale = 5 ; amp = 0.028

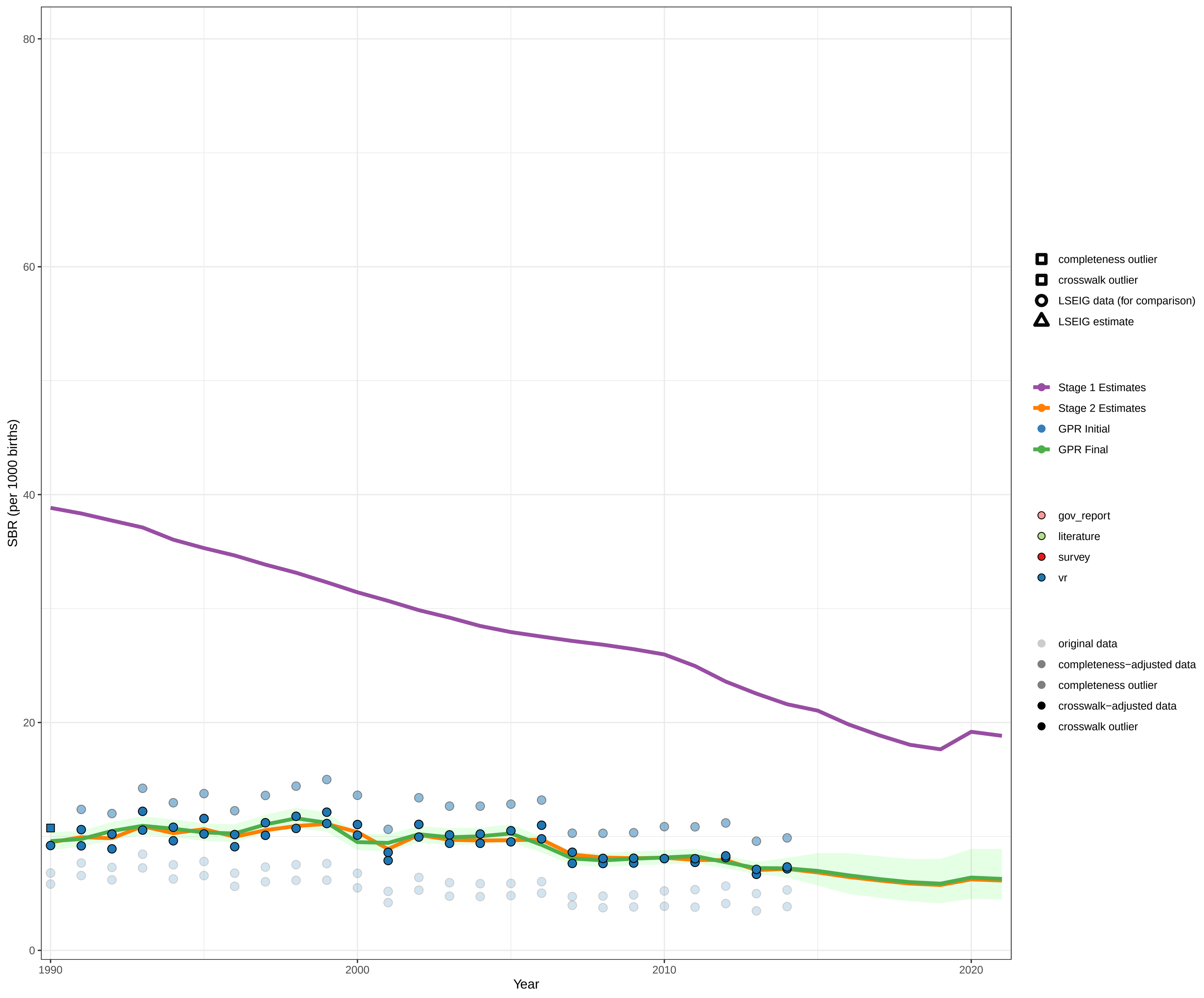

Year	Total Stillbirths (in thousands)	Total Stillbirths (in thousands)	SBR (per 1000 births)	SBR (per 1000 births)	SBR/NMR	SBR/NMR
	20 weeks	28 weeks	20 weeks	28 weeks	20 weeks	28 weeks
1990	1.3 (1.2 to 1.4)	1.0 (0.9 to 1.1)	12.9 (11.9 to 14.0)	9.6 (8.8 to 10.4)	0.76 (0.70 to 0.82)	0.56 (0.52 to 0.61)
1995	1.5 (1.3 to 1.6)	1.1 (1.0 to 1.2)	13.9 (12.9 to 15.0)	10.3 (9.6 to 11.1)	0.91 (0.84 to 0.98)	0.67 (0.62 to 0.72)
2000	1.3 (1.2 to 1.4)	0.9 (0.9 to 1.0)	12.8 (11.8 to 13.8)	9.5 (8.8 to 10.2)	0.95 (0.88 to 1.02)	0.71 (0.65 to 0.76)
2005	1.3 (1.2 to 1.4)	1.0 (0.9 to 1.1)	13.8 (12.8 to 14.9)	10.3 (9.5 to 11.1)	1.16 (1.07 to 1.25)	0.86 (0.80 to 0.93)
2010	1.1 (1.0 to 1.2)	0.8 (0.7 to 0.9)	11.0 (10.1 to 11.9)	8.2 (7.5 to 8.8)	1.00 (0.92 to 1.08)	0.74 (0.69 to 0.80)
2015	0.8 (0.7 to 1.0)	0.6 (0.5 to 0.7)	9.4 (7.7 to 11.6)	7.0 (5.7 to 8.5)	1.07 (0.87 to 1.32)	0.79 (0.65 to 0.97)
2020	0.6 (0.4 to 0.9)	0.5 (0.3 to 0.6)	8.8 (6.2 to 12.2)	6.4 (4.5 to 8.9)	1.13 (0.79 to 1.57)	0.82 (0.58 to 1.14)
2021	0.6 (0.4 to 0.9)	0.4 (0.3 to 0.6)	8.6 (6.1 to 12.3)	6.3 (4.5 to 8.9)	1.13 (0.80 to 1.61)	0.82 (0.59 to 1.17)

Puebla MEX_4663 Central Latin America Stillbirth Rate / Neonatal Mortality Rate (SBR/NMR) − 20 weeks
lambda = 0.2 ; zeta = 0.99 ; scale = 5 ; amp = 0.028

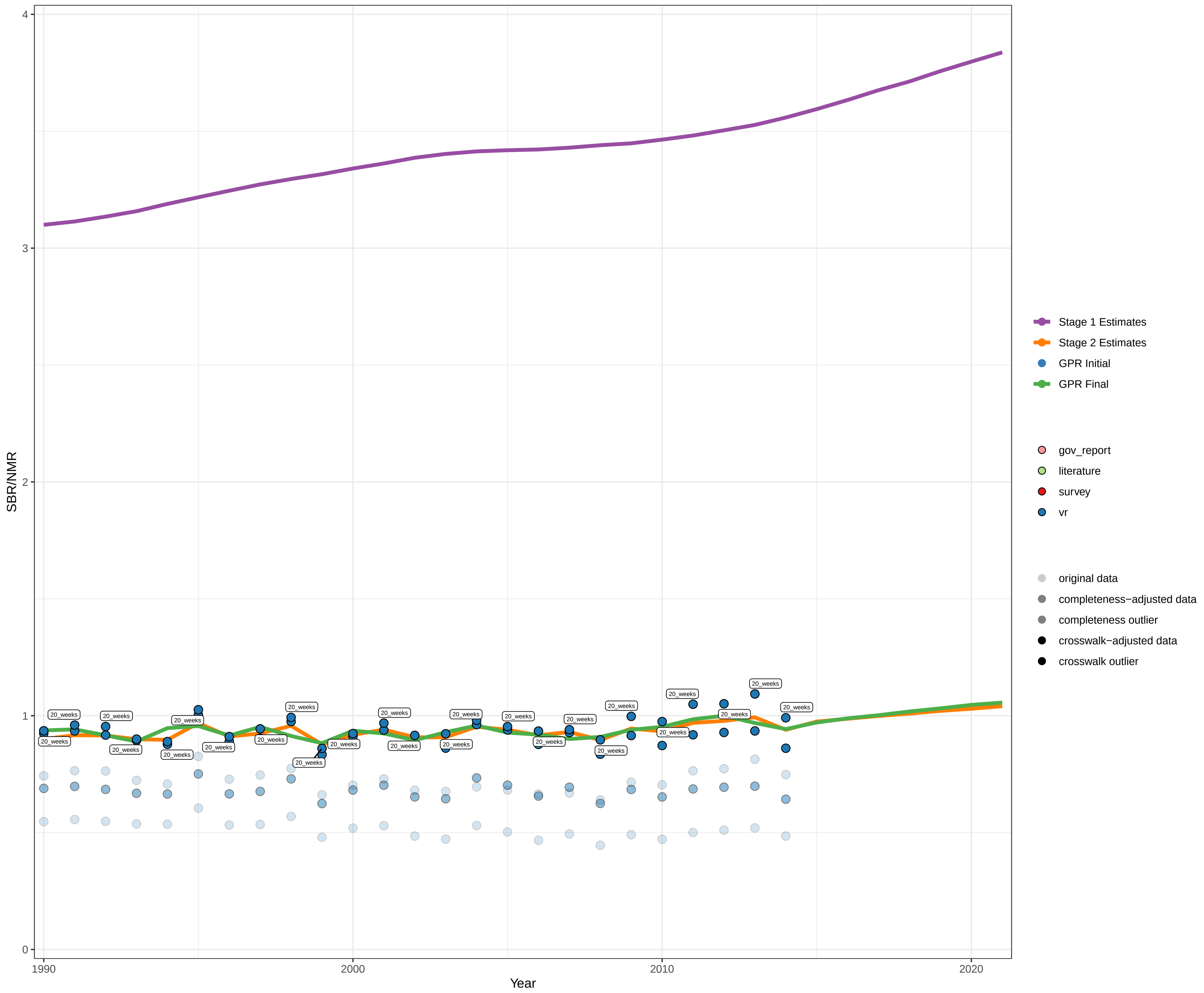

Puebla MEX_4663 Central Latin America Stillbirth Rate / Neonatal Mortality Rate (SBR/NMR) − 28 weeks
lambda = 0.2 ; zeta = 0.99 ; scale = 5 ; amp = 0.028

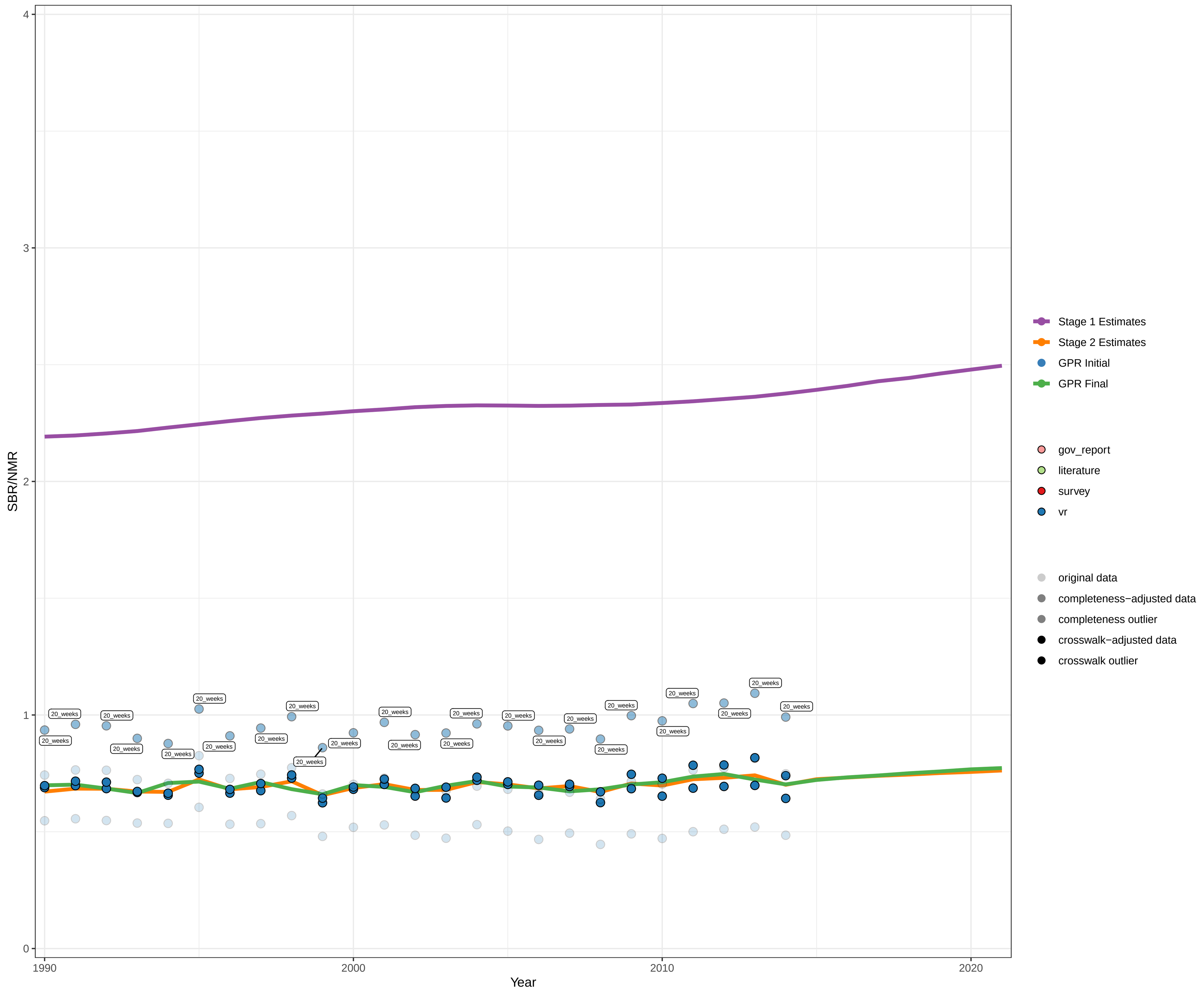

Definition	Years	Outlier	Source.Type	Title
20_weeks, 28_weeks	1990−2014	0	VR	Mexico INEGI Administrative Registers − Fetal Deaths by Municipality of Usual Residence of the Mother and Her Five−year Age Group According to Type of Fetal Death

Puebla MEX_4663 Central Latin America Stillbirth Rate (SBR) − 20 weeks
lambda = 0.2 ; zeta = 0.99 ; scale = 5 ; amp = 0.028

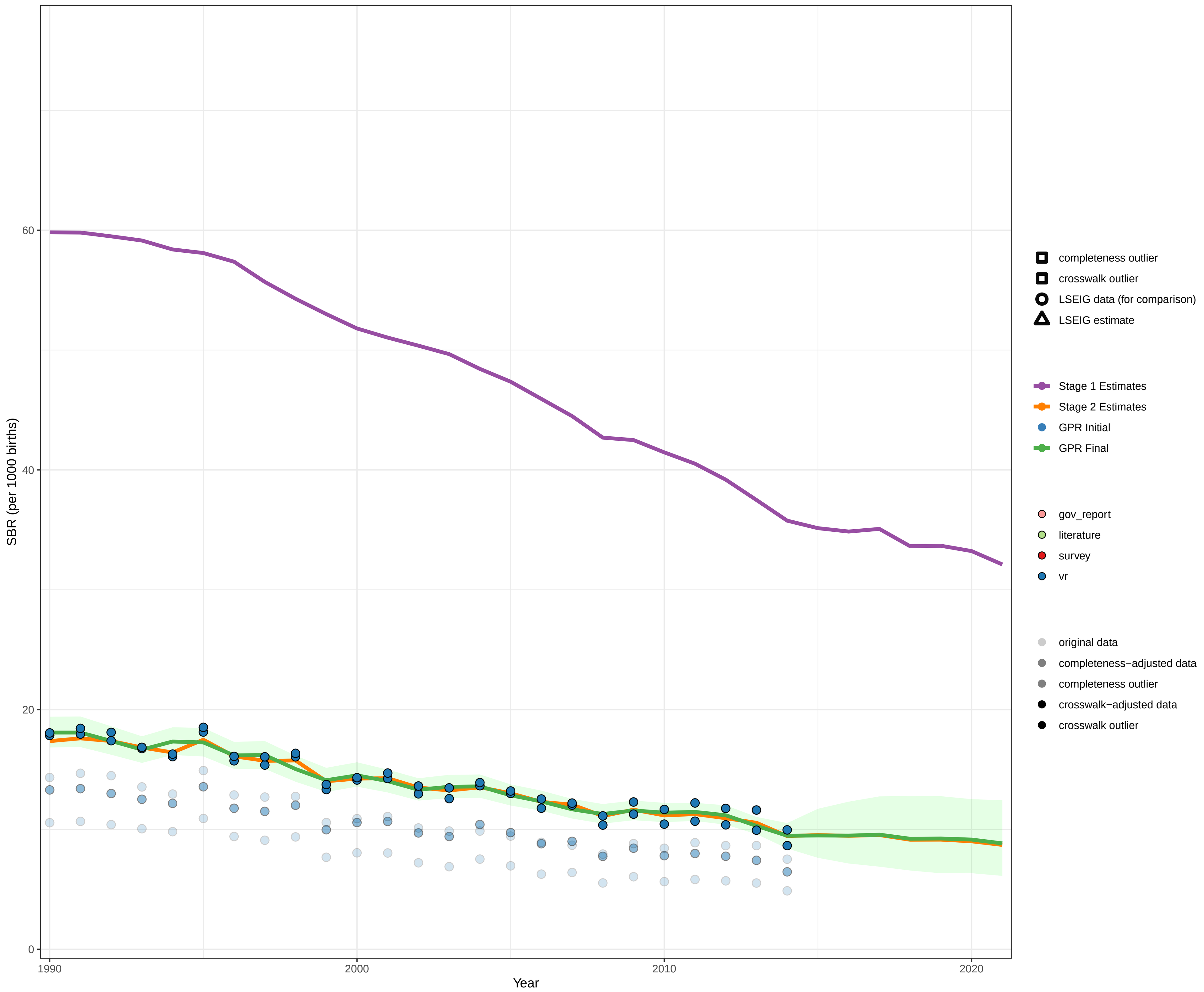

Puebla MEX_4663 Central Latin America Stillbirth Rate (SBR) − 28 weeks
lambda = 0.2 ; zeta = 0.99 ; scale = 5 ; amp = 0.028

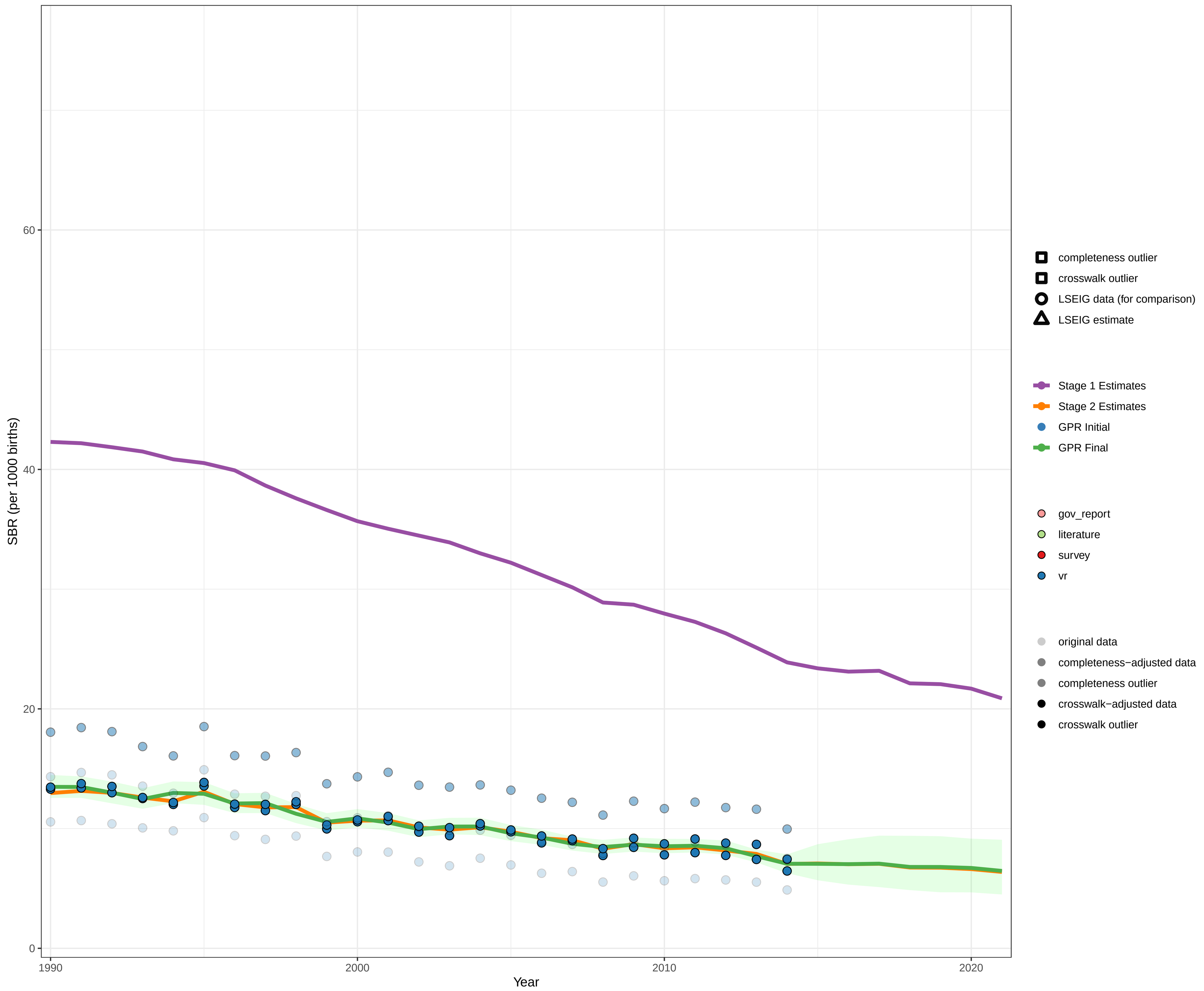

Year	Total Stillbirths (in thousands)	Total Stillbirths (in thousands)	SBR (per 1000 births)	SBR (per 1000 births)	SBR/NMR	SBR/NMR
	20 weeks	28 weeks	20 weeks	28 weeks	20 weeks	28 weeks
1990	2.6 (2.4 to 2.8)	1.9 (1.8 to 2.1)	18.1 (16.8 to 19.4)	13.5 (12.6 to 14.5)	0.94 (0.87 to 1.01)	0.70 (0.65 to 0.75)
1995	2.5 (2.4 to 2.7)	1.9 (1.7 to 2.0)	17.3 (16.1 to 18.5)	12.9 (12.0 to 13.9)	0.96 (0.89 to 1.02)	0.71 (0.66 to 0.77)
2000	2.1 (2.0 to 2.3)	1.6 (1.5 to 1.7)	14.5 (13.6 to 15.6)	10.9 (10.1 to 11.6)	0.94 (0.88 to 1.01)	0.70 (0.65 to 0.75)
2005	1.8 (1.7 to 2.0)	1.4 (1.3 to 1.5)	12.8 (12.0 to 13.8)	9.6 (9.0 to 10.3)	0.93 (0.87 to 0.99)	0.69 (0.65 to 0.74)
2010	1.7 (1.6 to 1.8)	1.3 (1.2 to 1.4)	11.4 (10.6 to 12.2)	8.5 (7.9 to 9.1)	0.95 (0.89 to 1.02)	0.71 (0.66 to 0.76)
2015	1.3 (1.0 to 1.6)	1.0 (0.8 to 1.2)	9.5 (7.6 to 11.7)	7.1 (5.7 to 8.7)	0.97 (0.78 to 1.20)	0.72 (0.58 to 0.89)
2020	1.1 (0.7 to 1.5)	0.8 (0.5 to 1.1)	9.2 (6.3 to 12.5)	6.7 (4.7 to 9.2)	1.05 (0.73 to 1.43)	0.77 (0.53 to 1.05)
2021	1.0 (0.7 to 1.4)	0.7 (0.5 to 1.0)	8.8 (6.1 to 12.4)	6.5 (4.5 to 9.1)	1.06 (0.73 to 1.49)	0.77 (0.54 to 1.08)

Queretaro MEX_4664 Central Latin America Stillbirth Rate / Neonatal Mortality Rate (SBR/NMR) − 20 weeks
lambda = 0.2 ; zeta = 0.99 ; scale = 5 ; amp = 0.028

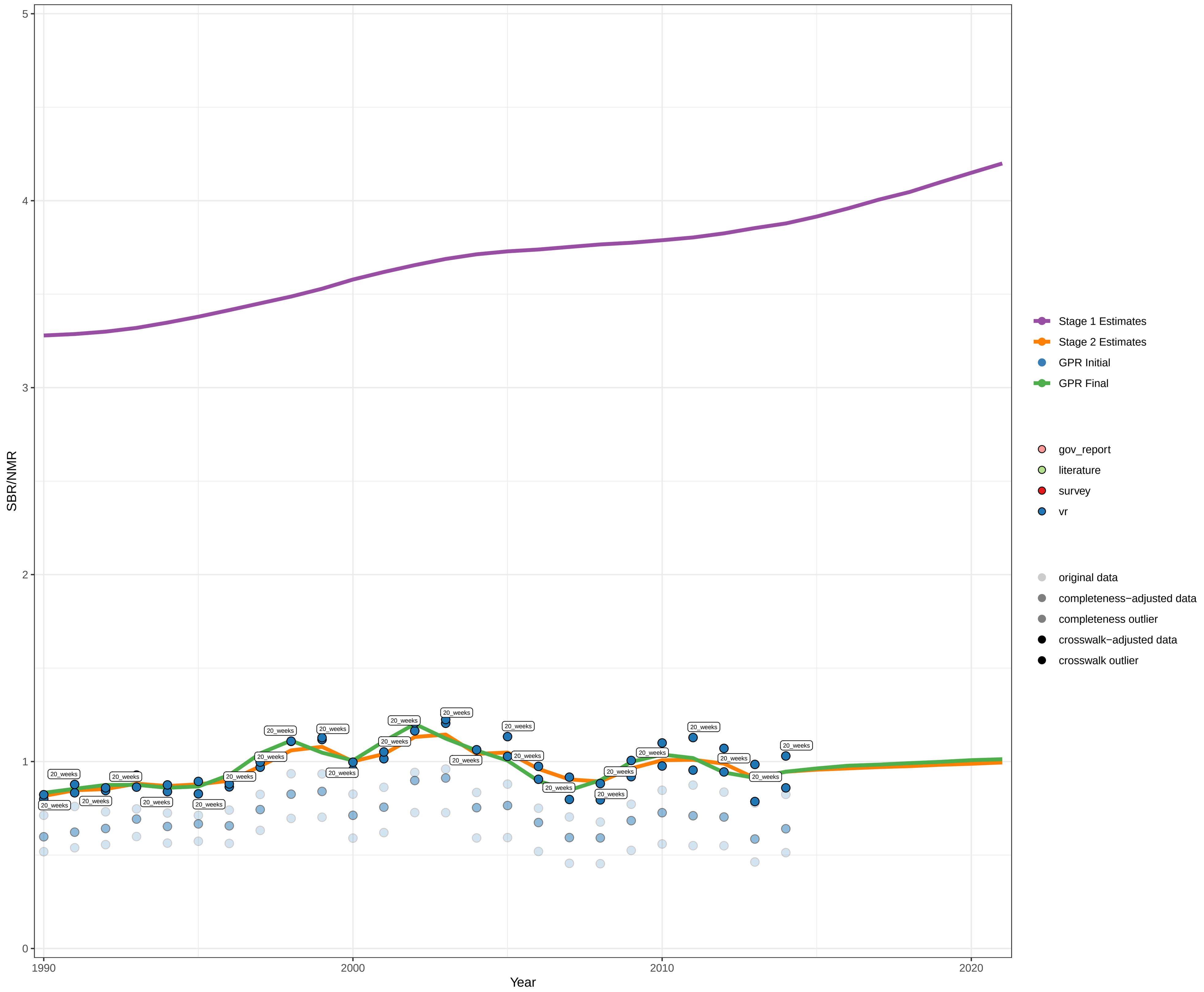

Queretaro MEX_4664 Central Latin America Stillbirth Rate / Neonatal Mortality Rate (SBR/NMR) − 28 weeks
lambda = 0.2 ; zeta = 0.99 ; scale = 5 ; amp = 0.028

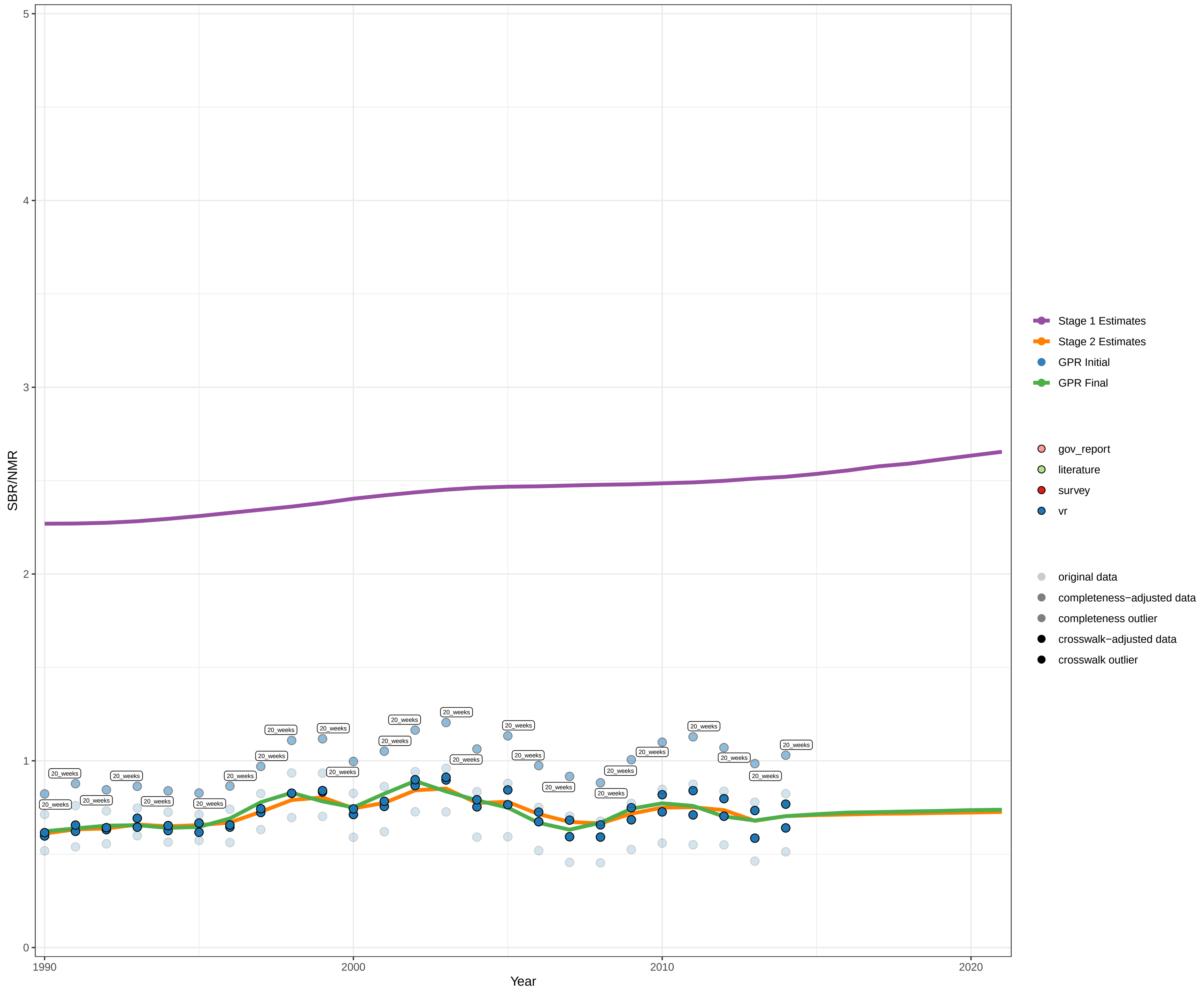

Definition	Years	Outlier	Source.Type	Title
20_weeks, 28_weeks	1990−2014	0	VR	Mexico INEGI Administrative Registers − Fetal Deaths by Municipality of Usual Residence of the Mother and Her Five−year Age Group According to Type of Fetal Death

Queretaro MEX_4664 Central Latin America Stillbirth Rate (SBR) − 20 weeks
lambda = 0.2 ; zeta = 0.99 ; scale = 5 ; amp = 0.028

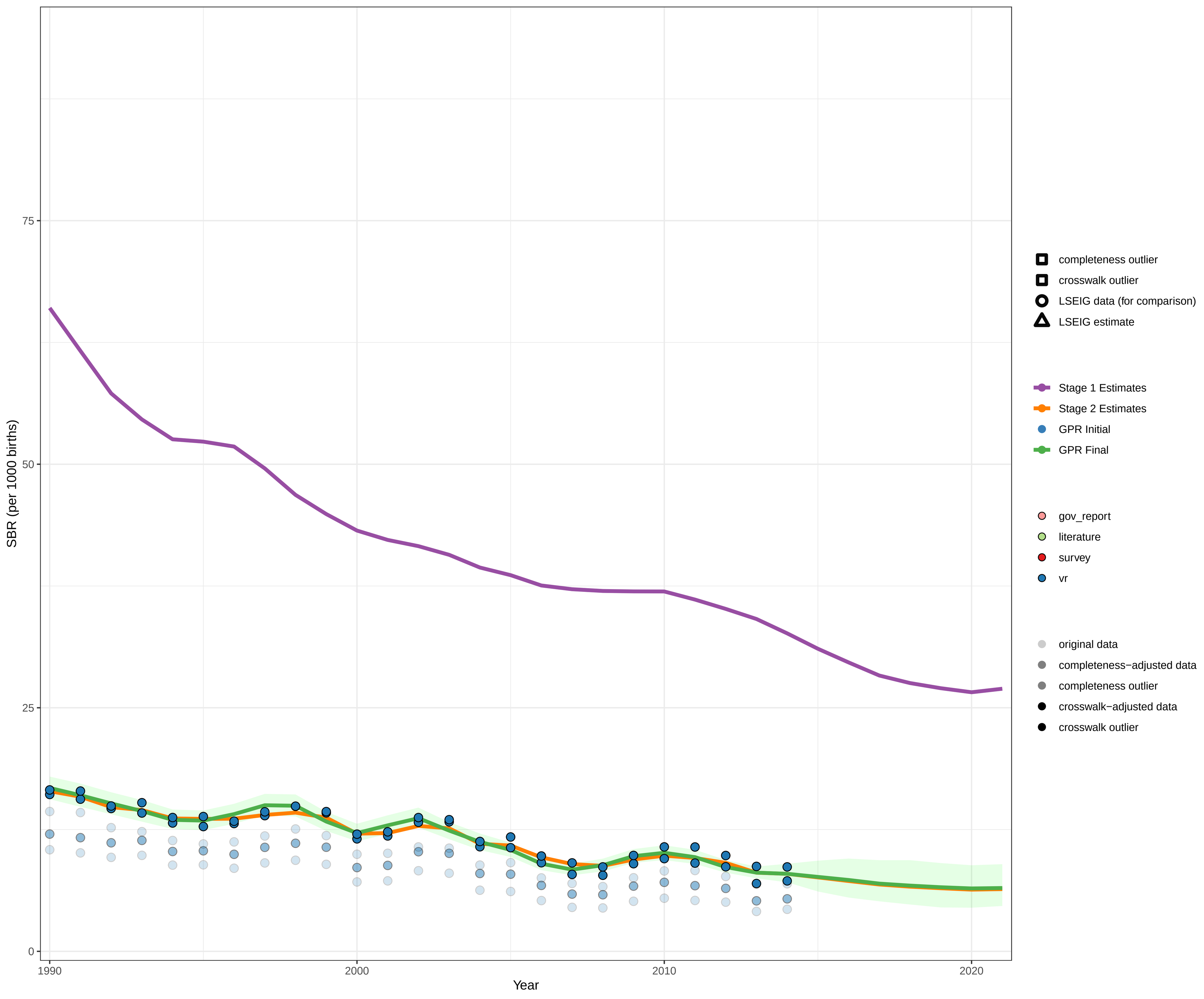

Queretaro MEX_4664 Central Latin America Stillbirth Rate (SBR) − 28 weeks
lambda = 0.2 ; zeta = 0.99 ; scale = 5 ; amp = 0.028

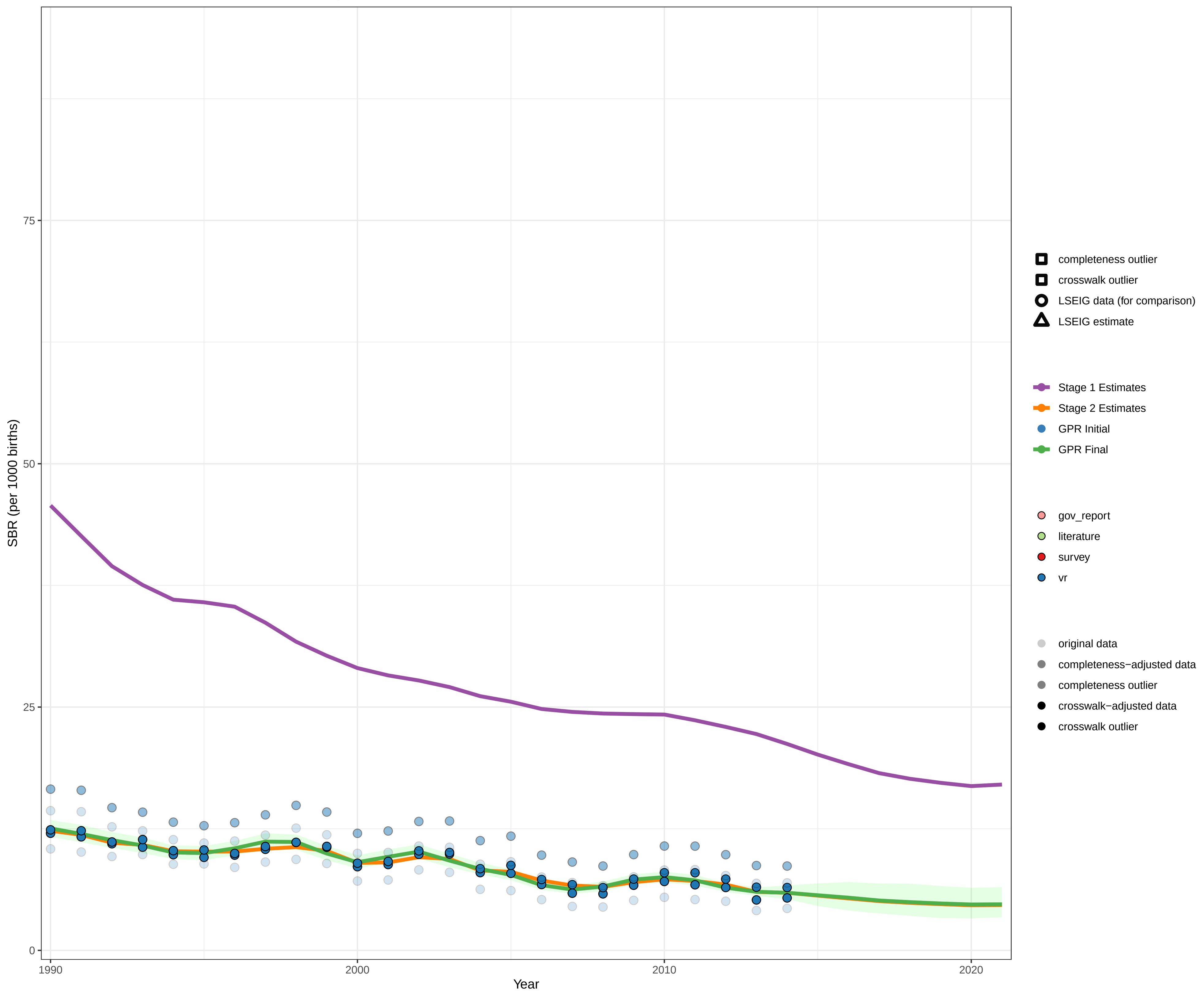

Year	Total Stillbirths (in thousands)	Total Stillbirths (in thousands)	SBR (per 1000 births)	SBR (per 1000 births)	SBR/NMR	SBR/NMR
	20 weeks	28 weeks	20 weeks	28 weeks	20 weeks	28 weeks
1990	0.6 (0.6 to 0.7)	0.5 (0.4 to 0.5)	16.8 (15.6 to 17.9)	12.5 (11.7 to 13.4)	0.83 (0.78 to 0.89)	0.62 (0.58 to 0.67)
1995	0.5 (0.5 to 0.6)	0.4 (0.4 to 0.4)	13.4 (12.4 to 14.5)	10.0 (9.3 to 10.7)	0.87 (0.80 to 0.93)	0.65 (0.60 to 0.69)
2000	0.5 (0.4 to 0.5)	0.3 (0.3 to 0.4)	12.1 (11.3 to 13.1)	9.1 (8.4 to 9.8)	1.01 (0.94 to 1.09)	0.75 (0.70 to 0.81)
2005	0.4 (0.4 to 0.4)	0.3 (0.3 to 0.3)	10.4 (9.7 to 11.2)	7.8 (7.2 to 8.3)	1.01 (0.94 to 1.08)	0.75 (0.70 to 0.81)
2010	0.4 (0.4 to 0.4)	0.3 (0.3 to 0.3)	10.1 (9.4 to 10.9)	7.5 (7.0 to 8.1)	1.04 (0.96 to 1.12)	0.77 (0.72 to 0.83)
2015	0.3 (0.2 to 0.4)	0.2 (0.2 to 0.3)	7.6 (6.1 to 9.3)	5.7 (4.6 to 6.9)	0.96 (0.77 to 1.17)	0.71 (0.58 to 0.87)
2020	0.2 (0.2 to 0.3)	0.2 (0.1 to 0.2)	6.5 (4.5 to 8.8)	4.7 (3.3 to 6.4)	1.01 (0.70 to 1.38)	0.74 (0.51 to 1.00)
2021	0.2 (0.2 to 0.3)	0.2 (0.1 to 0.2)	6.5 (4.7 to 8.9)	4.7 (3.4 to 6.5)	1.01 (0.73 to 1.39)	0.74 (0.53 to 1.01)

Quintana Roo   MEX_4665   Central Latin America   Stillbirth Rate / Neonatal Mortality Rate (SBR/NMR) − 20 weeks
lambda = 0.2 ; zeta = 0.99 ; scale = 5 ; amp = 0.028

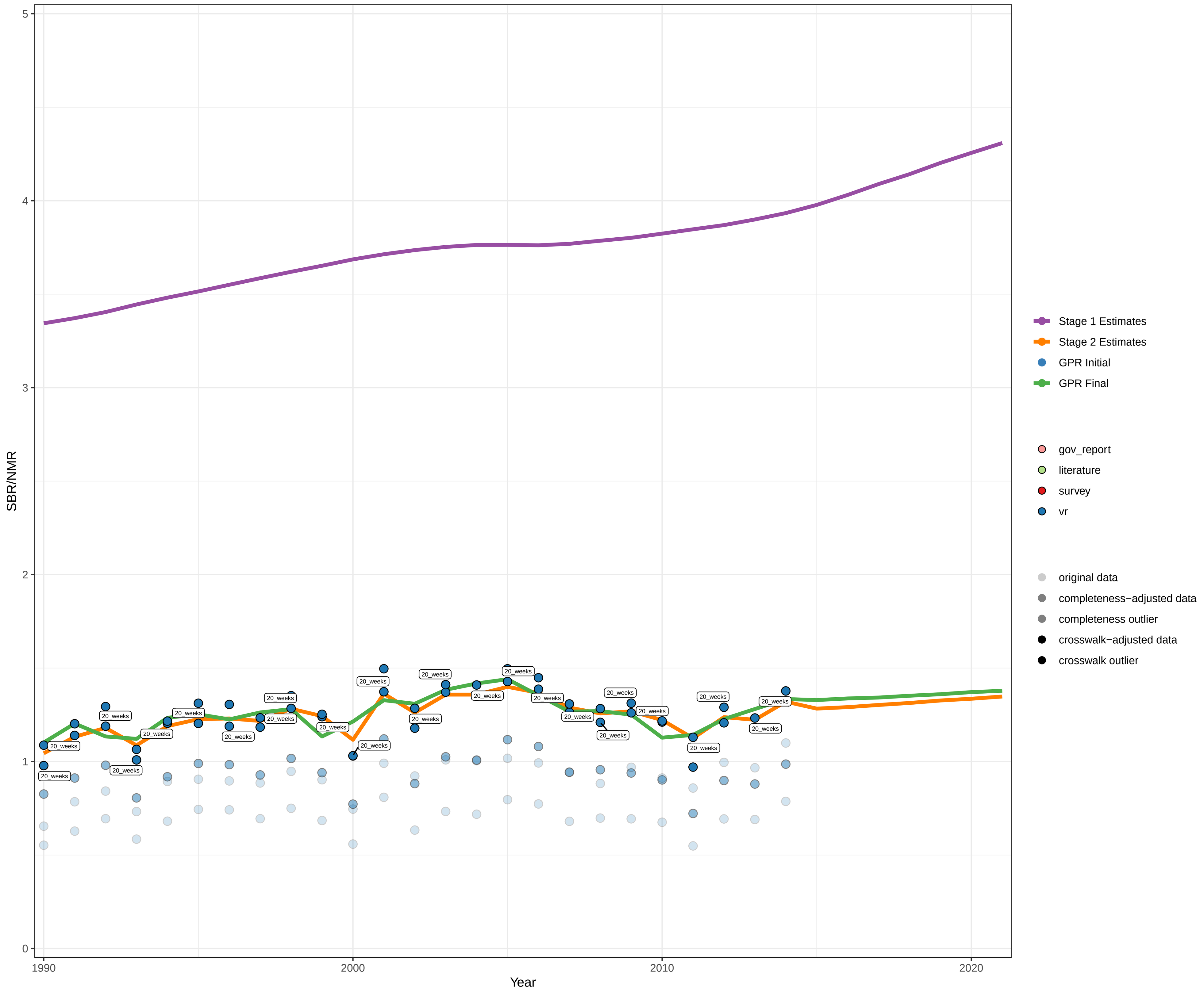

Quintana Roo   MEX_4665   Central Latin America   Stillbirth Rate / Neonatal Mortality Rate (SBR/NMR) − 28 weeks
lambda = 0.2 ; zeta = 0.99 ; scale = 5 ; amp = 0.028

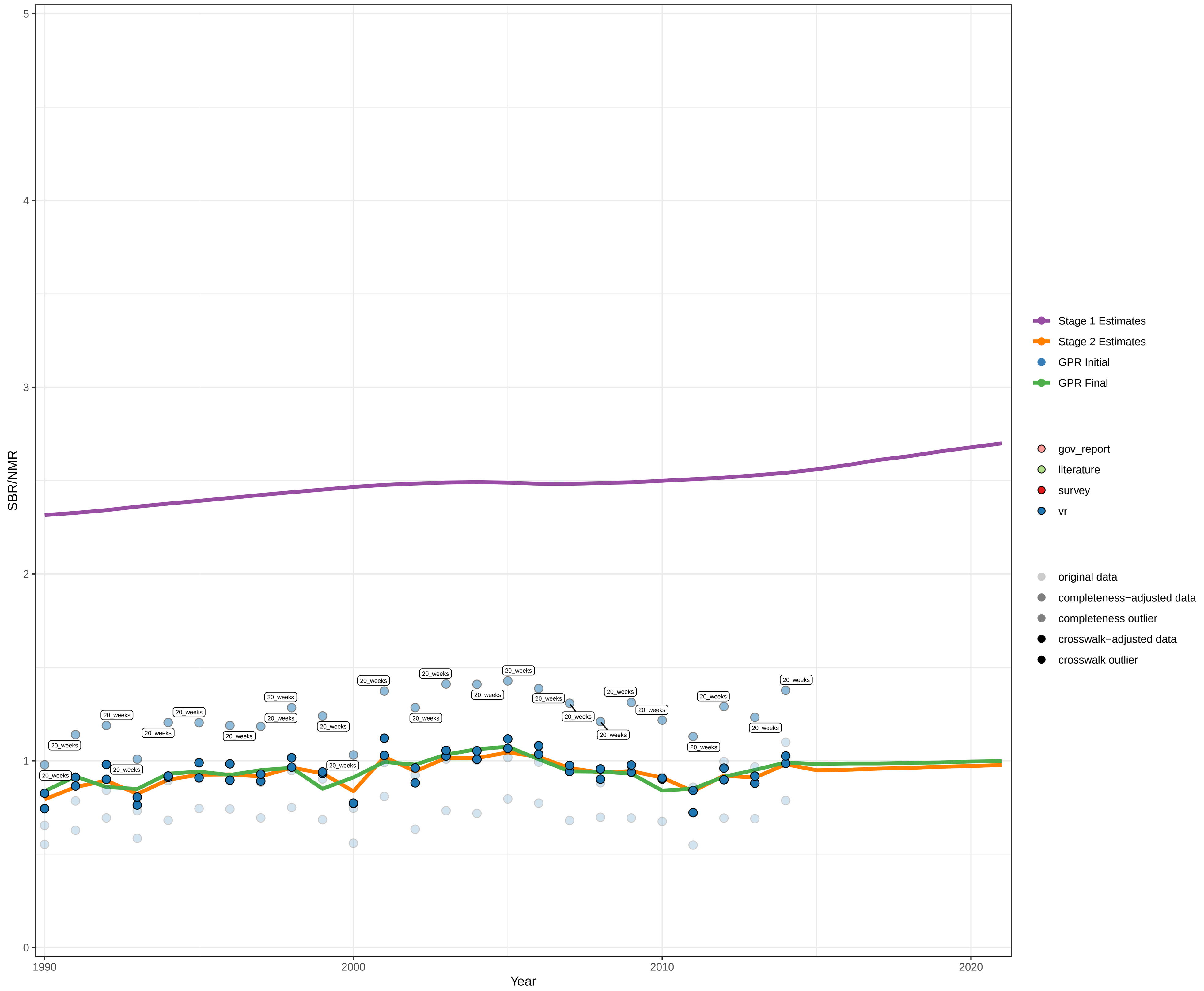

Definition	Years	Outlier	Source.Type	Title
20_weeks, 28_weeks	1990−2014	0	VR	Mexico INEGI Administrative Registers − Fetal Deaths by Municipality of Usual Residence of the Mother and Her Five−year Age Group According to Type of Fetal Death

Quintana Roo   MEX_4665   Central Latin America   Stillbirth Rate (SBR) − 20 weeks
lambda = 0.2 ; zeta = 0.99 ; scale = 5 ; amp = 0.028

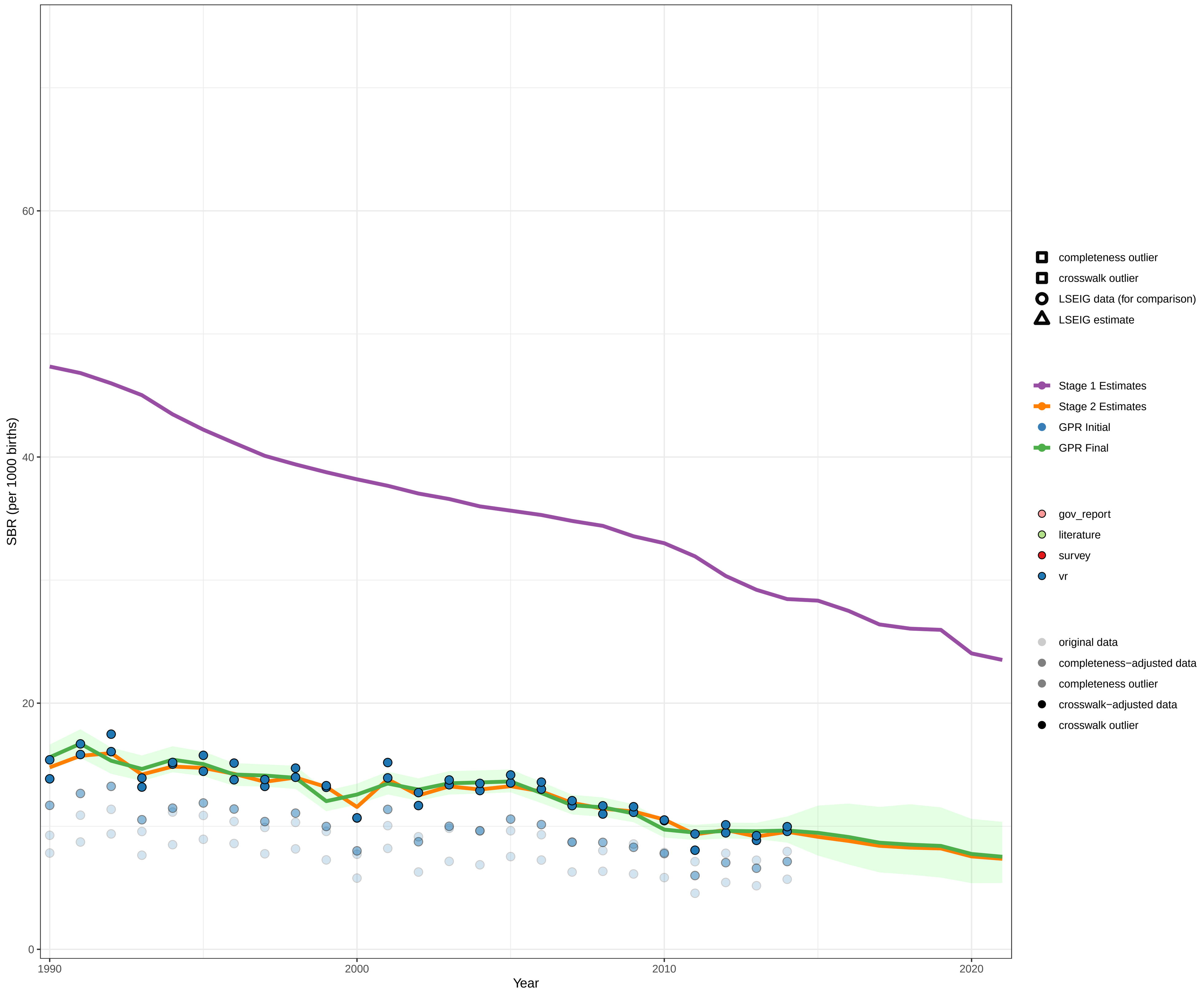

Quintana Roo   MEX_4665   Central Latin America   Stillbirth Rate (SBR) − 28 weeks
lambda = 0.2 ; zeta = 0.99 ; scale = 5 ; amp = 0.028

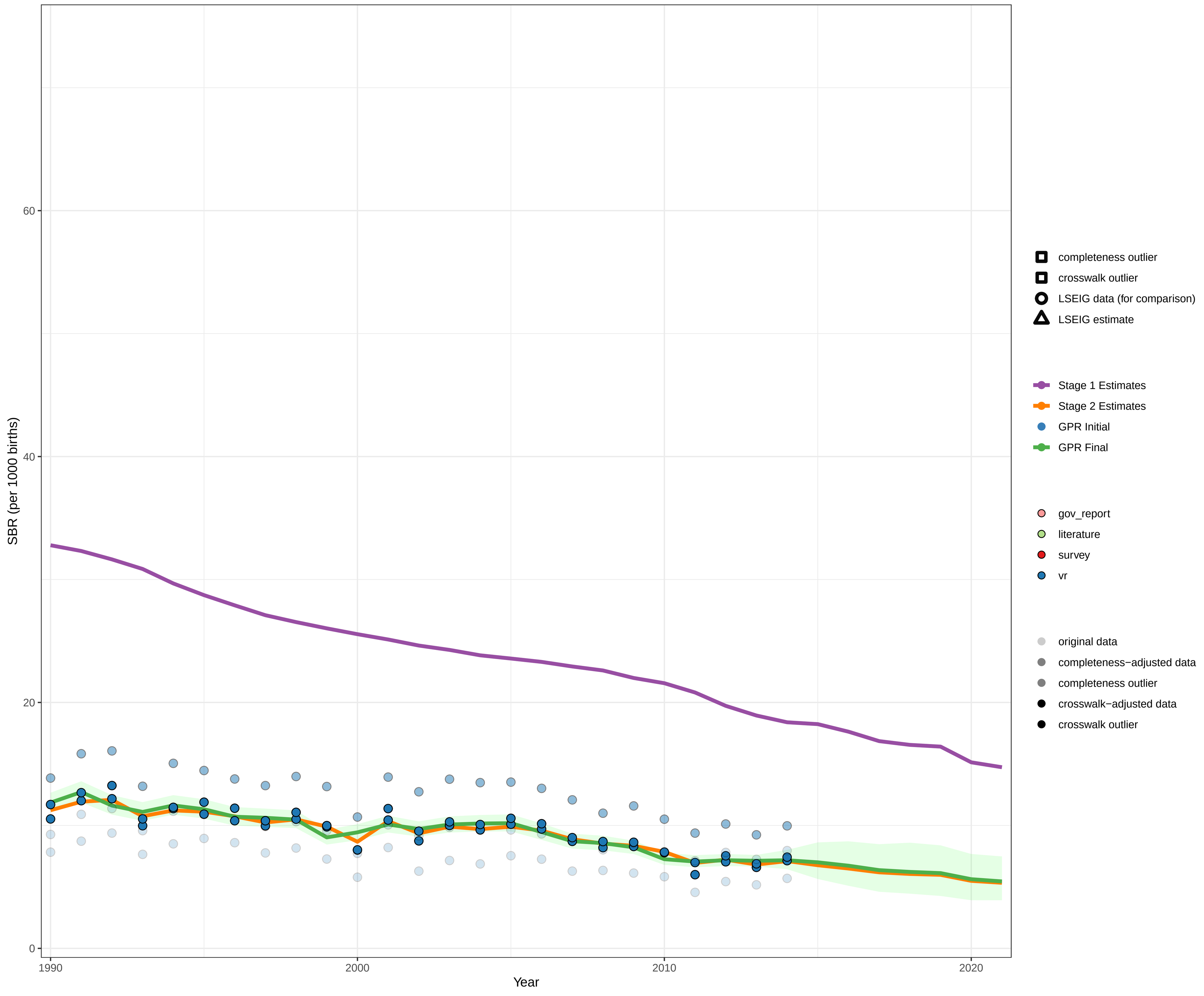

Year	Total Stillbirths (in thousands)	Total Stillbirths (in thousands)	SBR (per 1000 births)	SBR (per 1000 births)	SBR/NMR	SBR/NMR
	20 weeks	28 weeks	20 weeks	28 weeks	20 weeks	28 weeks
1990	0.3 (0.2 to 0.3)	0.2 (0.2 to 0.2)	15.6 (14.6 to 16.6)	11.9 (11.1 to 12.7)	1.10 (1.03 to 1.17)	0.84 (0.78 to 0.89)
1995	0.3 (0.3 to 0.3)	0.2 (0.2 to 0.2)	15.1 (14.1 to 16.1)	11.3 (10.6 to 12.1)	1.25 (1.17 to 1.34)	0.94 (0.88 to 1.01)
2000	0.3 (0.3 to 0.3)	0.2 (0.2 to 0.2)	12.6 (11.8 to 13.5)	9.4 (8.8 to 10.1)	1.21 (1.14 to 1.30)	0.91 (0.85 to 0.98)
2005	0.3 (0.3 to 0.4)	0.3 (0.2 to 0.3)	13.6 (12.8 to 14.6)	10.2 (9.5 to 10.9)	1.44 (1.35 to 1.54)	1.08 (1.00 to 1.15)
2010	0.3 (0.3 to 0.3)	0.2 (0.2 to 0.2)	9.7 (9.1 to 10.4)	7.3 (6.8 to 7.8)	1.13 (1.05 to 1.21)	0.84 (0.79 to 0.90)
2015	0.3 (0.2 to 0.4)	0.2 (0.2 to 0.3)	9.5 (7.6 to 11.7)	7.0 (5.6 to 8.6)	1.33 (1.07 to 1.64)	0.98 (0.79 to 1.21)
2020	0.2 (0.2 to 0.3)	0.2 (0.1 to 0.2)	7.7 (5.4 to 10.6)	5.6 (3.9 to 7.7)	1.37 (0.95 to 1.88)	1.00 (0.69 to 1.36)
2021	0.2 (0.2 to 0.3)	0.2 (0.1 to 0.2)	7.5 (5.4 to 10.4)	5.4 (3.9 to 7.5)	1.38 (0.99 to 1.90)	1.00 (0.72 to 1.37)

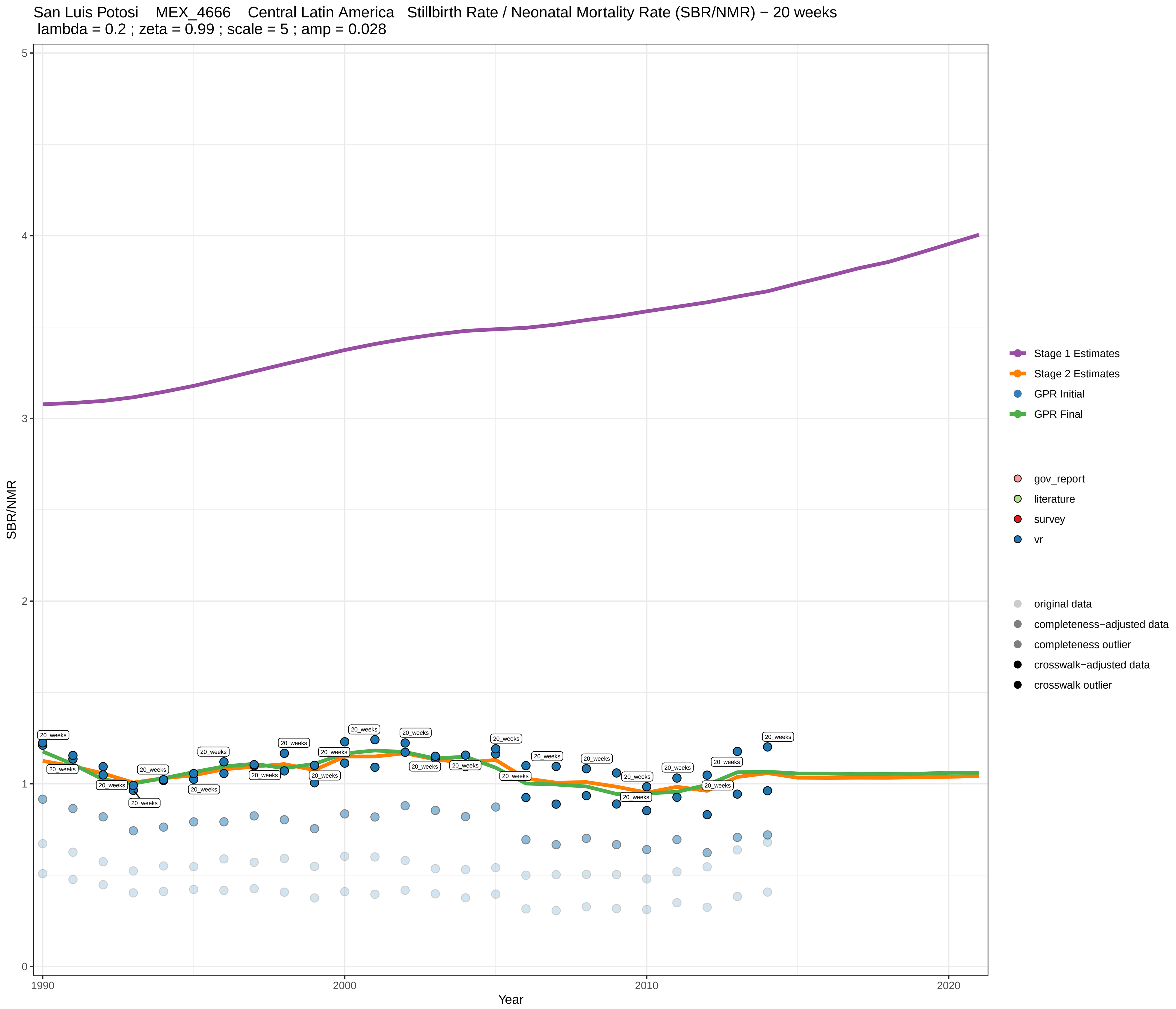

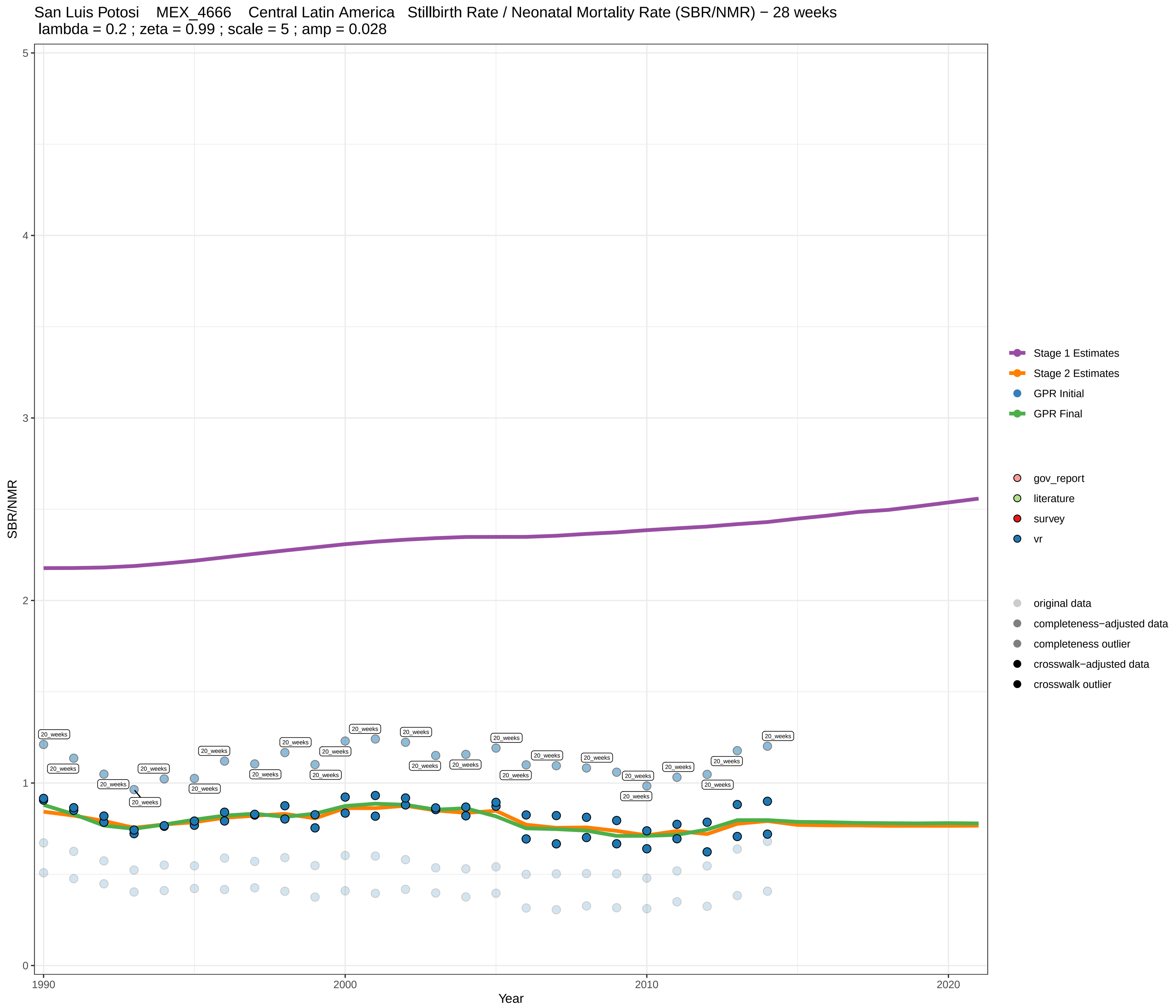

Definition	Years	Outlier	Source.Type	Title
20_weeks, 28_weeks	1990−2014	0	VR	Mexico INEGI Administrative Registers − Fetal Deaths by Municipality of Usual Residence of the Mother and Her Five−year Age Group According to Type of Fetal Death

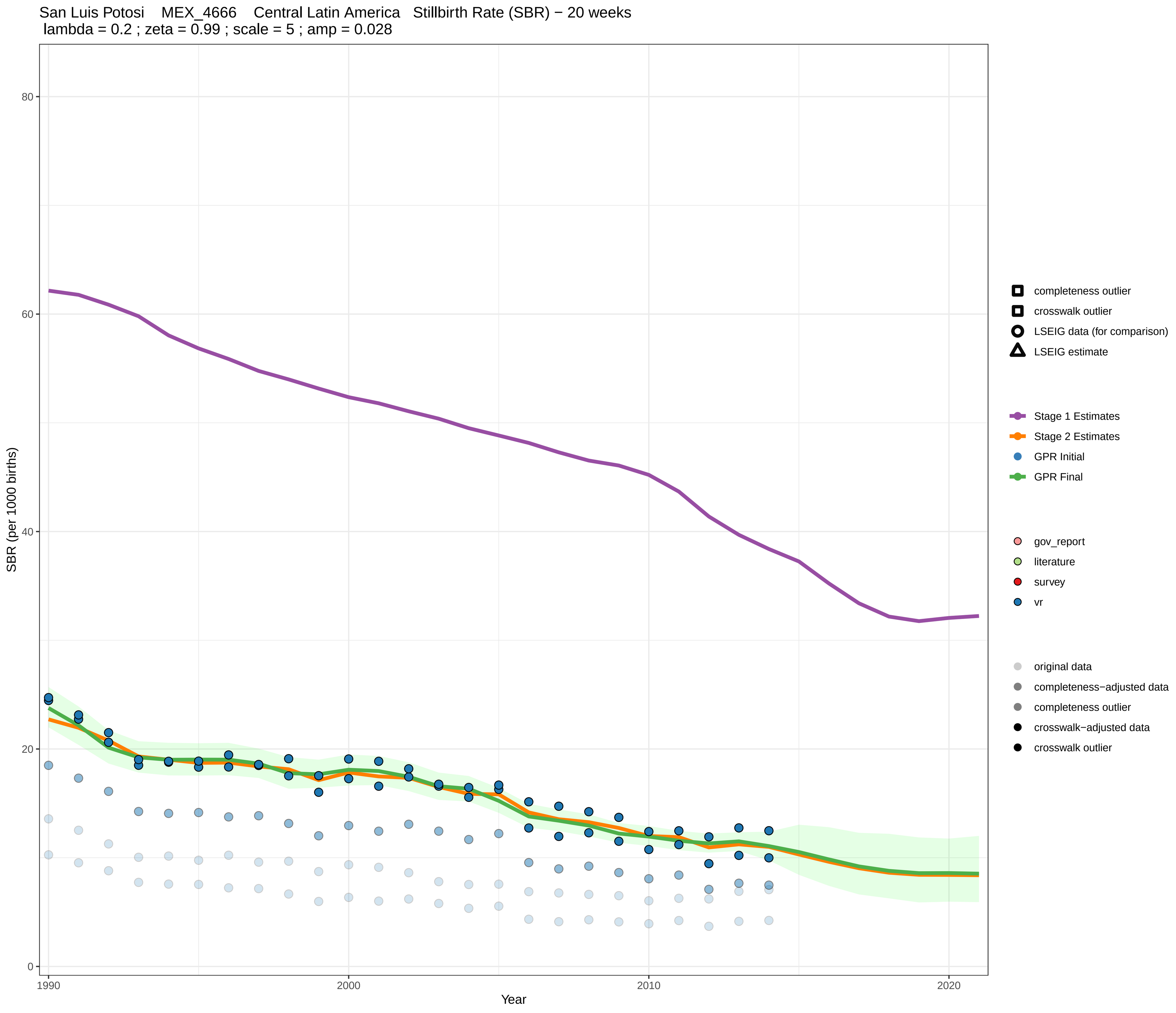

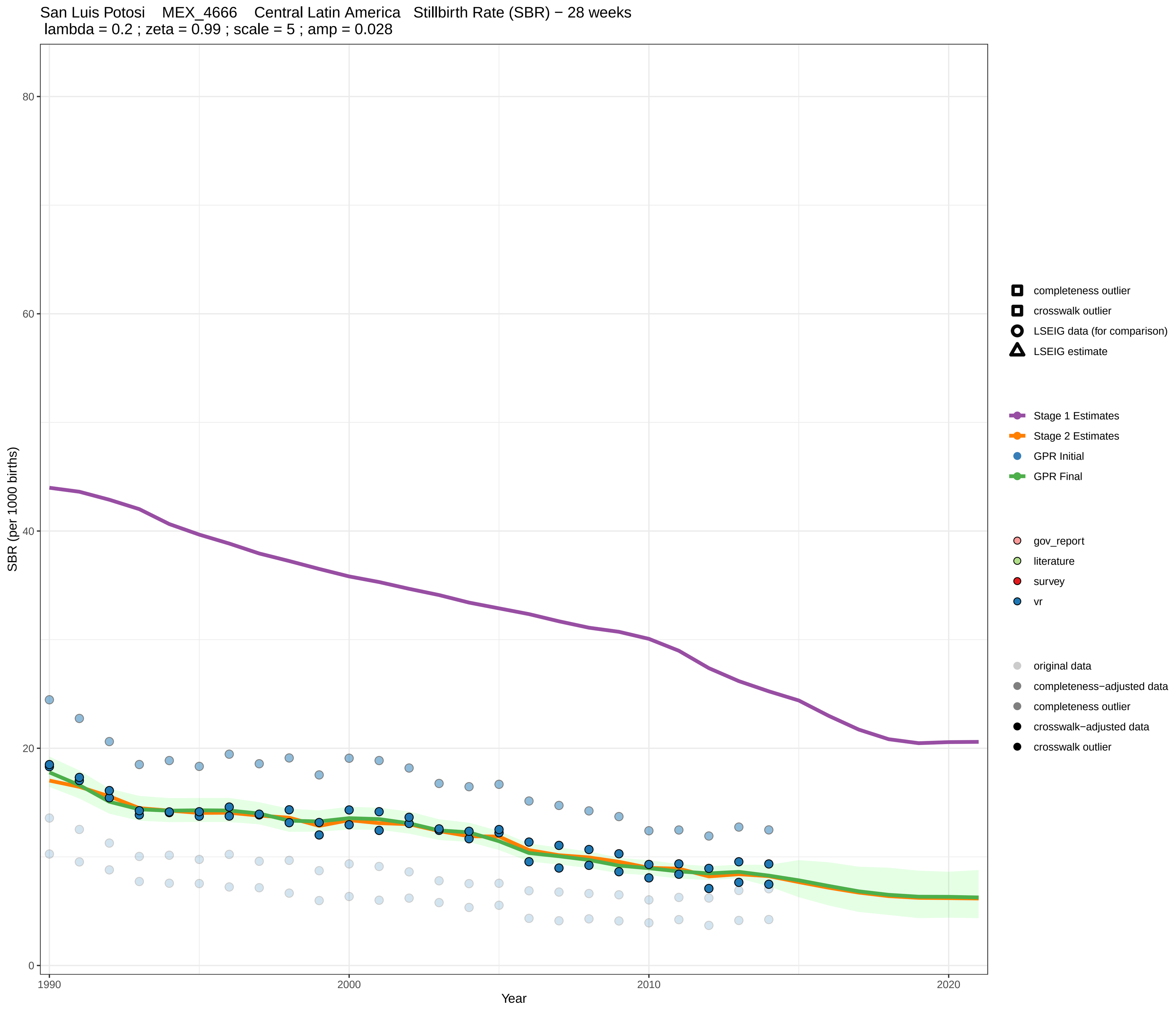

Year	Total Stillbirths (in thousands)	Total Stillbirths (in thousands)	SBR (per 1000 births)	SBR (per 1000 births)	SBR/NMR	SBR/NMR
	20 weeks	28 weeks	20 weeks	28 weeks	20 weeks	28 weeks
1990	1.6 (1.5 to 1.8)	1.2 (1.1 to 1.3)	23.8 (22.0 to 25.7)	17.8 (16.4 to 19.2)	1.18 (1.09 to 1.27)	0.88 (0.81 to 0.95)
1995	1.3 (1.2 to 1.4)	1.0 (0.9 to 1.0)	19.0 (17.6 to 20.5)	14.3 (13.2 to 15.4)	1.06 (0.98 to 1.15)	0.80 (0.74 to 0.86)
2000	1.1 (1.0 to 1.2)	0.8 (0.8 to 0.9)	18.1 (16.6 to 19.5)	13.6 (12.5 to 14.6)	1.17 (1.07 to 1.26)	0.87 (0.81 to 0.94)
2005	0.9 (0.8 to 1.0)	0.7 (0.6 to 0.7)	15.2 (14.1 to 16.4)	11.4 (10.6 to 12.4)	1.09 (1.01 to 1.17)	0.82 (0.76 to 0.88)
2010	0.7 (0.6 to 0.8)	0.5 (0.5 to 0.6)	11.9 (11.1 to 12.9)	9.0 (8.3 to 9.7)	0.95 (0.88 to 1.02)	0.71 (0.66 to 0.77)
2015	0.6 (0.4 to 0.7)	0.4 (0.3 to 0.5)	10.5 (8.4 to 13.0)	7.8 (6.3 to 9.7)	1.06 (0.85 to 1.31)	0.79 (0.63 to 0.97)
2020	0.4 (0.3 to 0.5)	0.3 (0.2 to 0.4)	8.6 (6.0 to 11.8)	6.3 (4.4 to 8.6)	1.06 (0.74 to 1.45)	0.78 (0.54 to 1.06)
2021	0.4 (0.3 to 0.5)	0.3 (0.2 to 0.4)	8.5 (5.9 to 12.0)	6.3 (4.4 to 8.8)	1.06 (0.73 to 1.49)	0.78 (0.54 to 1.09)

Sinaloa MEX_4667 Central Latin America Stillbirth Rate / Neonatal Mortality Rate (SBR/NMR) − 20 weeks
lambda = 0.6 ; zeta = 0.8 ; scale = 15 ; amp = 0.028

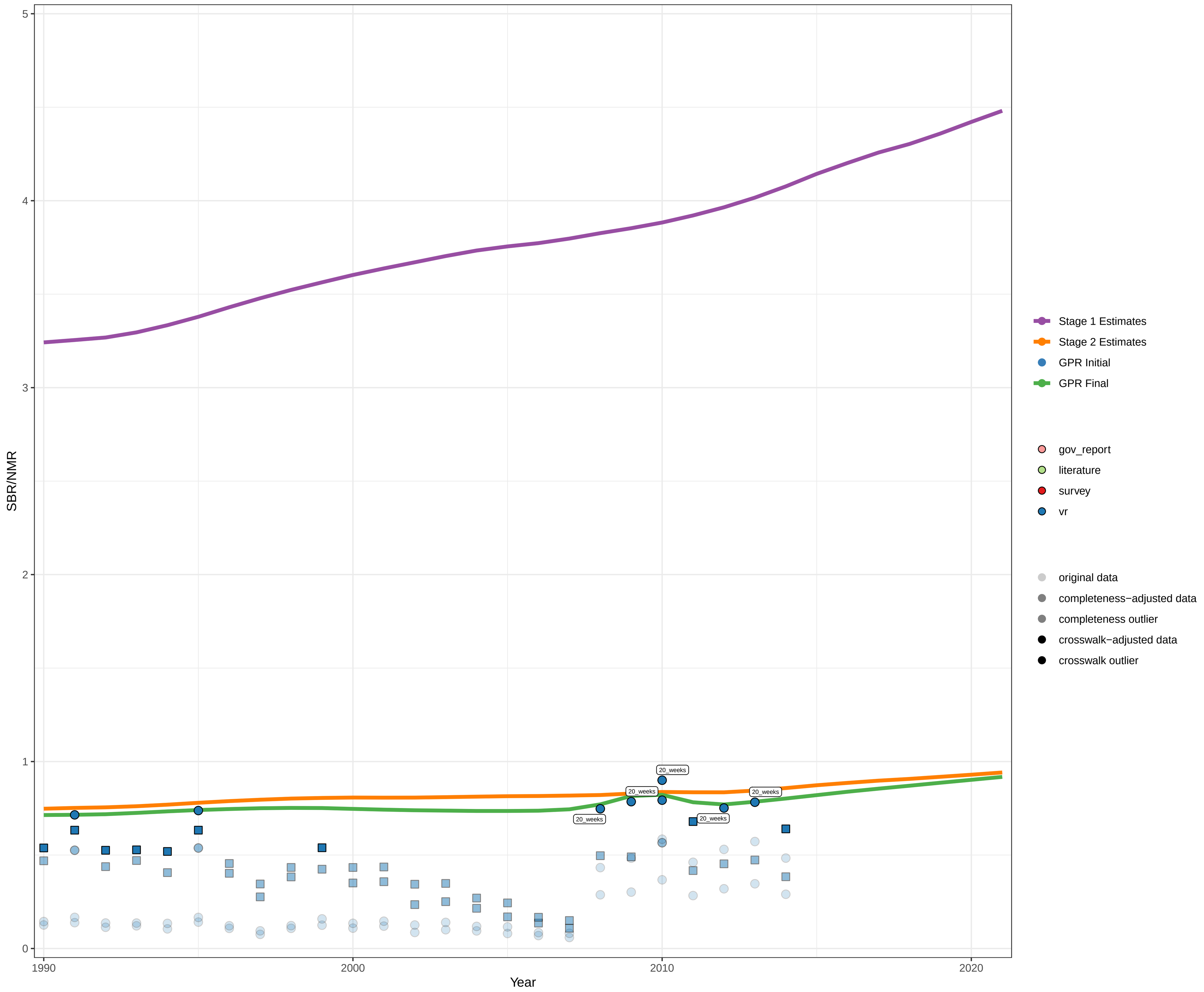

Sinaloa MEX_4667 Central Latin America Stillbirth Rate / Neonatal Mortality Rate (SBR/NMR) − 28 weeks
lambda = 0.6 ; zeta = 0.8 ; scale = 15 ; amp = 0.028

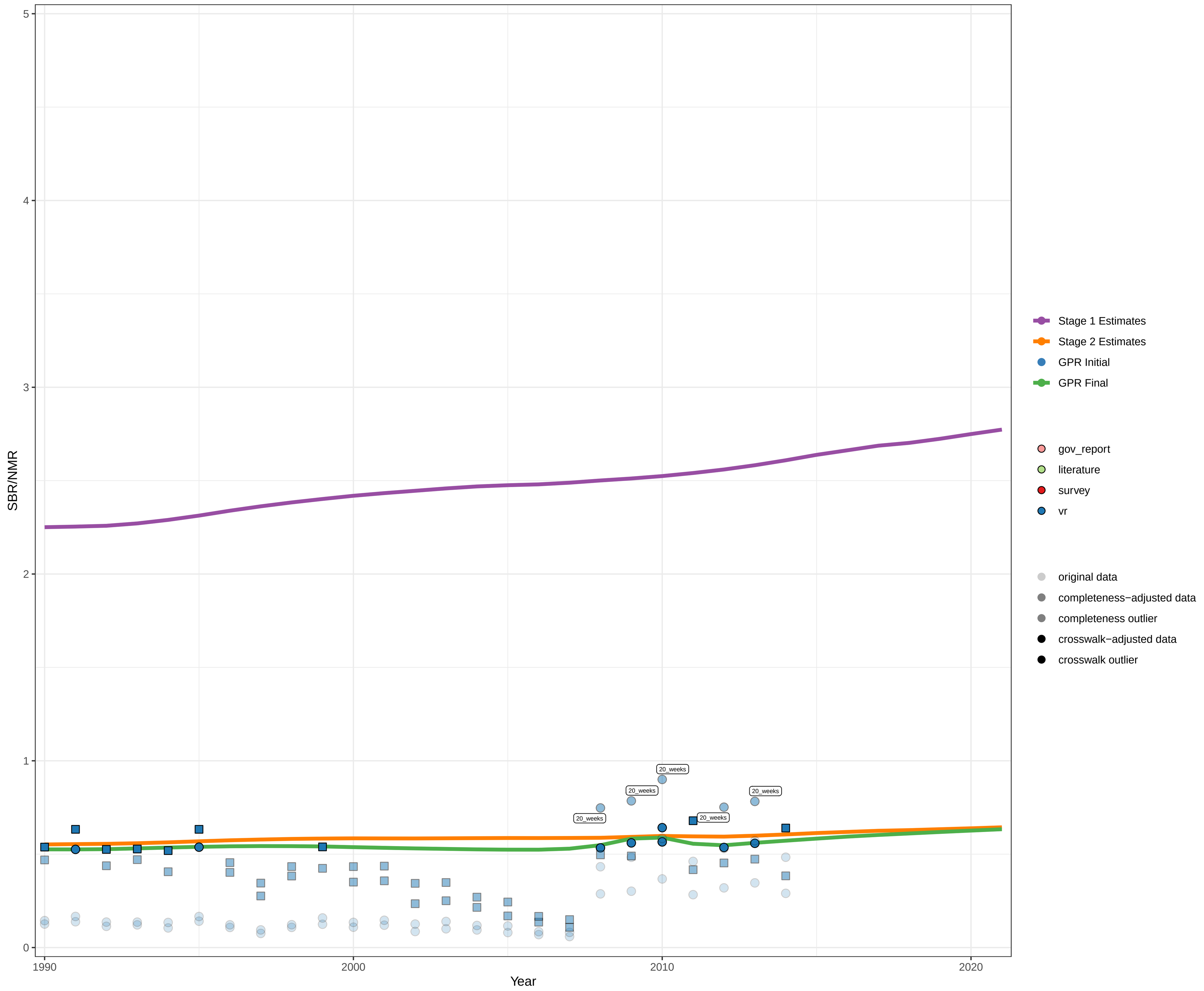

Definition	Years	Outlier	Source.Type	Title
20_weeks	2008−2010, 2012, 2013	0	VR	Mexico INEGI Administrative Registers − Fetal Deaths by Municipality of Usual Residence of the Mother and Her Five−year Age Group According to Type of Fetal Death
28_weeks	1991, 1995, 2010	0	VR	Mexico INEGI Administrative Registers − Fetal Deaths by Municipality of Usual Residence of the Mother and Her Five−year Age Group According to Type of Fetal Death
20_weeks	1996−1998, 2000−2007	1	VR	Mexico INEGI Administrative Registers − Fetal Deaths by Municipality of Usual Residence of the Mother and Her Five−year Age Group According to Type of Fetal Death
28_weeks	1990, 1992−1994, 1996−2009, 2011−2014	1	VR	Mexico INEGI Administrative Registers − Fetal Deaths by Municipality of Usual Residence of the Mother and Her Five−year Age Group According to Type of Fetal Death
20_weeks	1990−1995, 1999, 2011, 2014	2	VR	Mexico INEGI Administrative Registers − Fetal Deaths by Municipality of Usual Residence of the Mother and Her Five−year Age Group According to Type of Fetal Death

Sinaloa MEX_4667 Central Latin America Stillbirth Rate (SBR) − 20 weeks
lambda = 0.6 ; zeta = 0.8 ; scale = 15 ; amp = 0.028

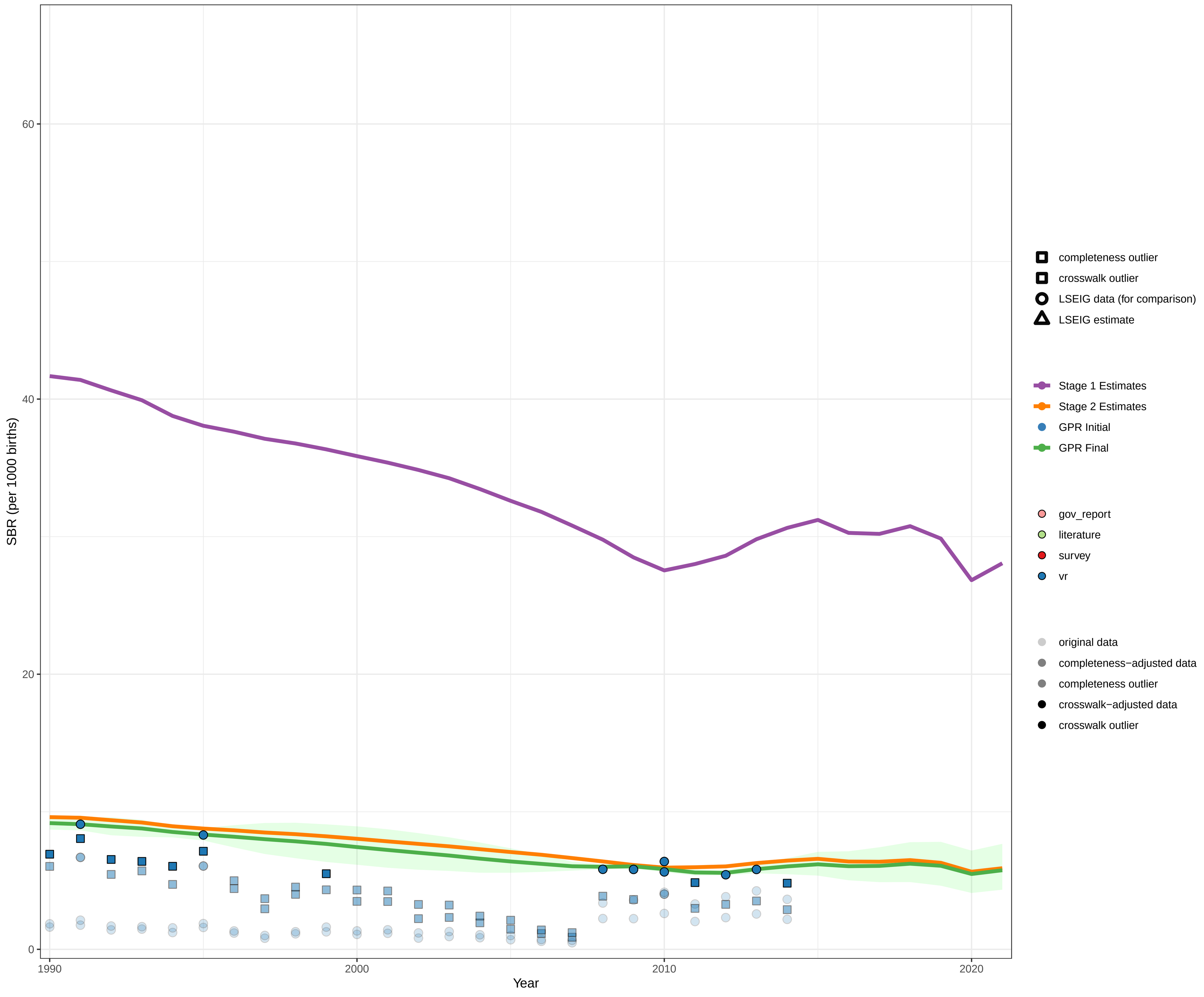

Sinaloa MEX_4667 Central Latin America Stillbirth Rate (SBR) − 28 weeks
lambda = 0.6 ; zeta = 0.8 ; scale = 15 ; amp = 0.028

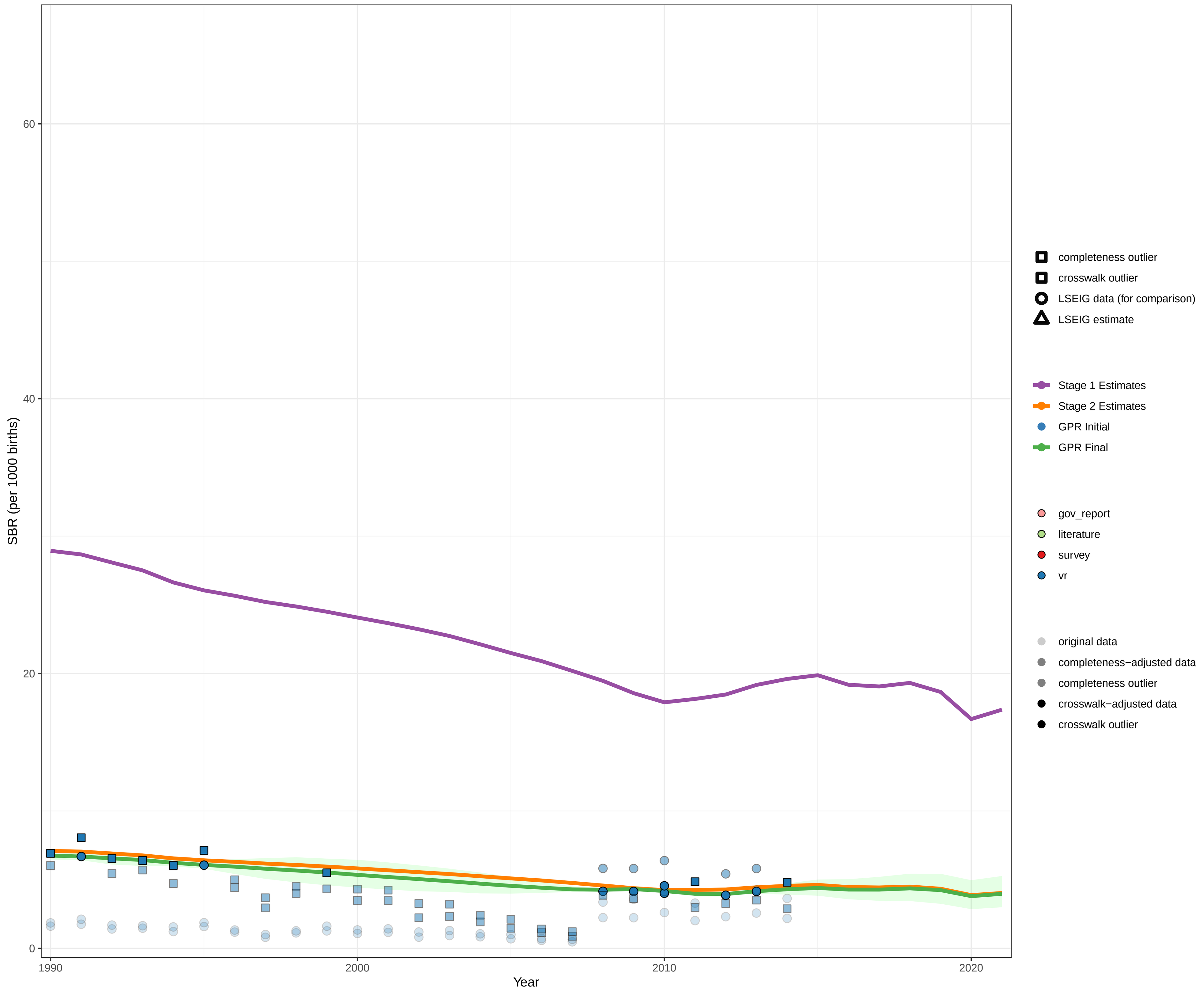

Year	Total Stillbirths (in thousands)	Total Stillbirths (in thousands)	SBR (per 1000 births)	SBR (per 1000 births)	SBR/NMR	SBR/NMR
	20 weeks	28 weeks	20 weeks	28 weeks	20 weeks	28 weeks
1990	0.6 (0.6 to 0.7)	0.5 (0.4 to 0.5)	9.2 (8.7 to 9.7)	6.8 (6.5 to 7.1)	0.71 (0.68 to 0.75)	0.53 (0.50 to 0.55)
1995	0.6 (0.6 to 0.6)	0.4 (0.4 to 0.5)	8.3 (7.9 to 8.8)	6.1 (5.8 to 6.4)	0.74 (0.70 to 0.78)	0.54 (0.51 to 0.56)
2000	0.5 (0.4 to 0.6)	0.4 (0.3 to 0.4)	7.4 (6.1 to 8.9)	5.3 (4.4 to 6.4)	0.75 (0.62 to 0.90)	0.54 (0.44 to 0.65)
2005	0.4 (0.3 to 0.4)	0.3 (0.2 to 0.3)	6.4 (5.6 to 7.3)	4.6 (4.0 to 5.2)	0.74 (0.64 to 0.85)	0.52 (0.46 to 0.60)
2010	0.3 (0.3 to 0.4)	0.2 (0.2 to 0.3)	5.8 (5.6 to 6.0)	4.2 (4.0 to 4.3)	0.82 (0.79 to 0.85)	0.59 (0.57 to 0.61)
2015	0.3 (0.3 to 0.4)	0.2 (0.2 to 0.3)	6.2 (5.4 to 7.1)	4.4 (3.8 to 5.0)	0.82 (0.71 to 0.94)	0.58 (0.51 to 0.67)
2020	0.2 (0.2 to 0.3)	0.2 (0.1 to 0.2)	5.5 (4.1 to 7.2)	3.8 (2.9 to 5.0)	0.90 (0.67 to 1.18)	0.63 (0.47 to 0.82)
2021	0.3 (0.2 to 0.3)	0.2 (0.1 to 0.2)	5.7 (4.3 to 7.7)	4.0 (3.0 to 5.3)	0.92 (0.69 to 1.22)	0.63 (0.48 to 0.84)

Sonora MEX_4668 Central Latin America Stillbirth Rate / Neonatal Mortality Rate (SBR/NMR) − 20 weeks
lambda = 0.2 ; zeta = 0.99 ; scale = 5 ; amp = 0.028

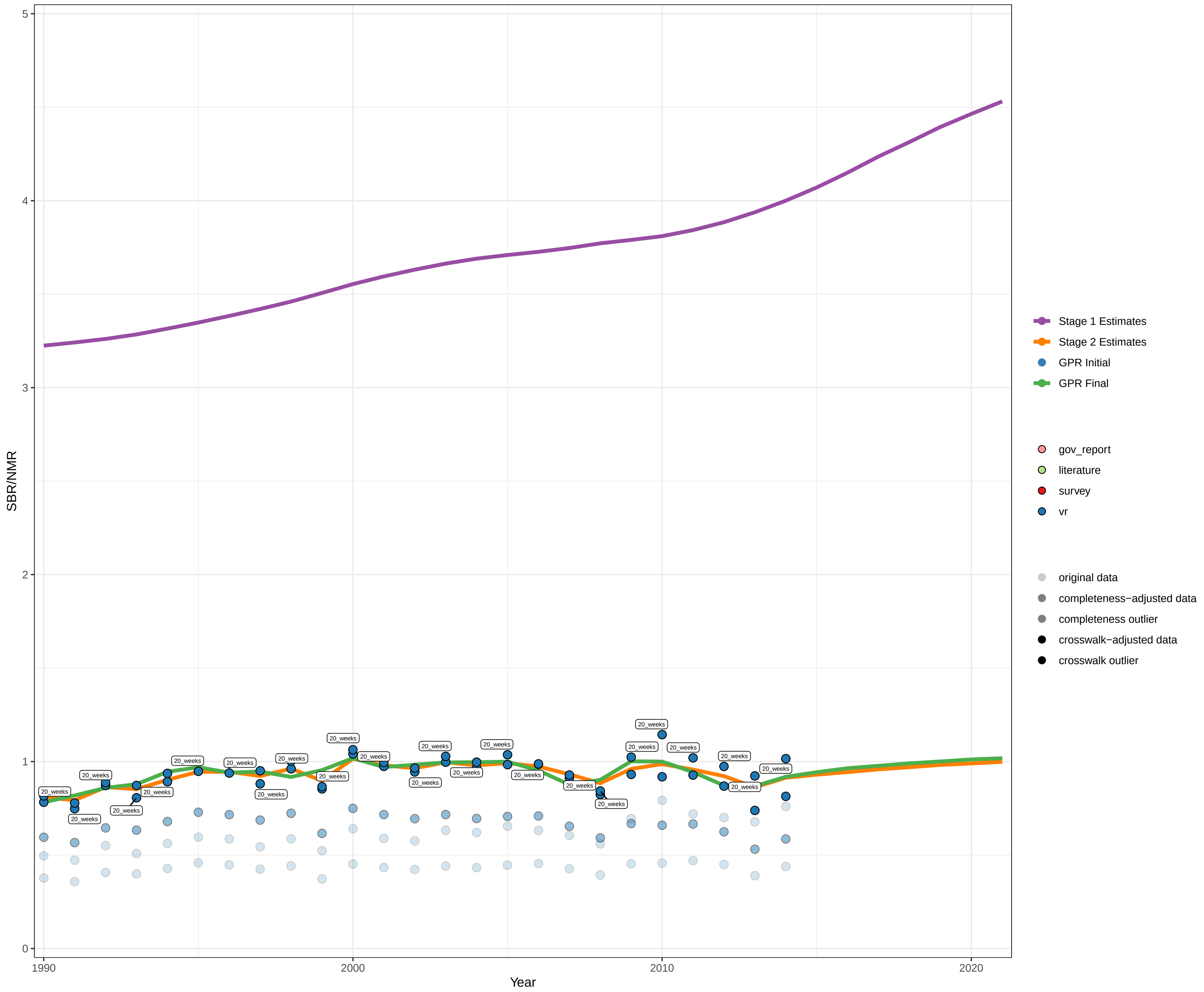

Sonora MEX_4668 Central Latin America Stillbirth Rate / Neonatal Mortality Rate (SBR/NMR) − 28 weeks
lambda = 0.2 ; zeta = 0.99 ; scale = 5 ; amp = 0.028

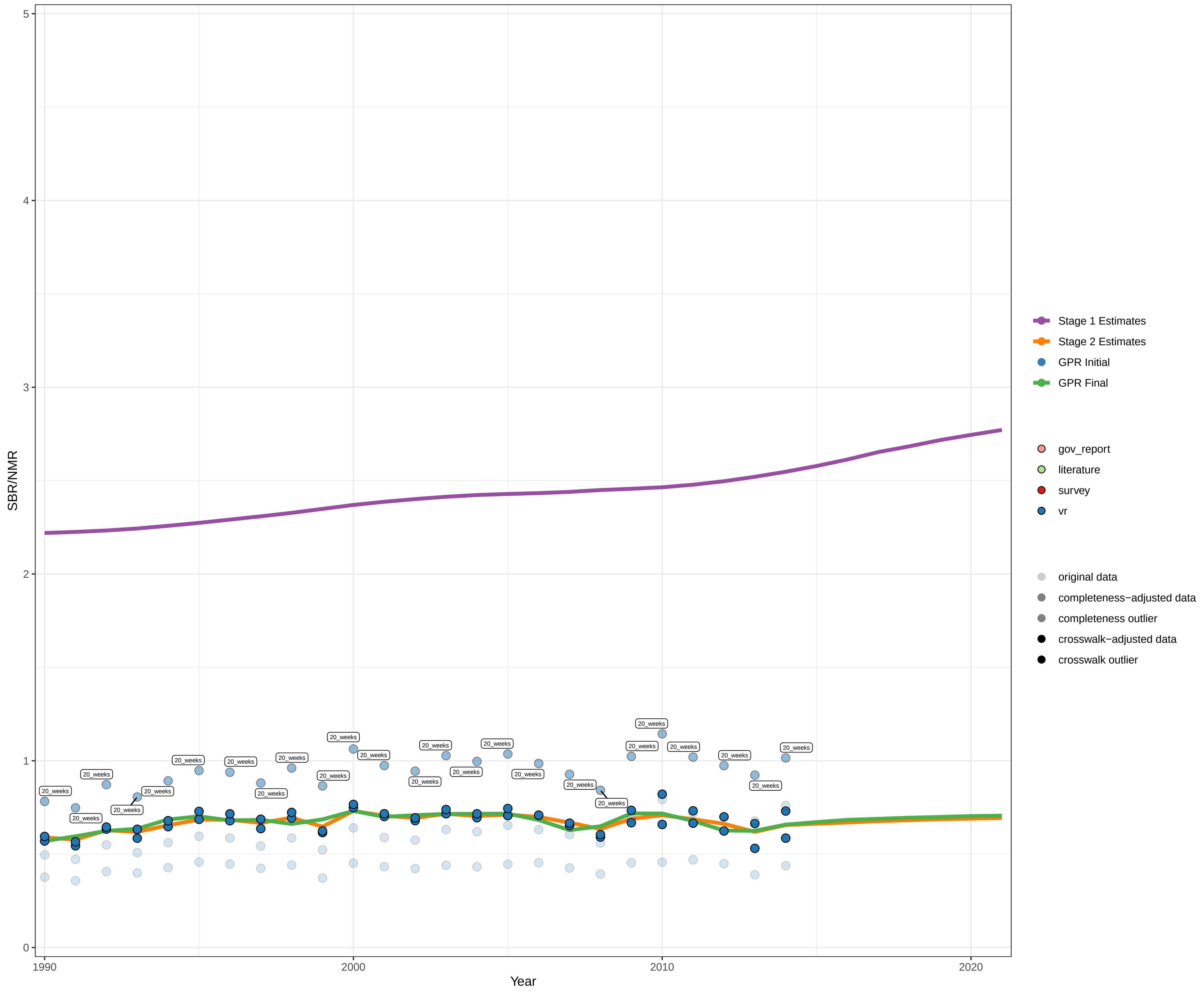

Definition	Years	Outlier	Source.Type	Title
20_weeks, 28_weeks	1990−2014	0	VR	Mexico INEGI Administrative Registers − Fetal Deaths by Municipality of Usual Residence of the Mother and Her Five−year Age Group According to Type of Fetal Death

Sonora MEX_4668 Central Latin America Stillbirth Rate (SBR) − 20 weeks
lambda = 0.2 ; zeta = 0.99 ; scale = 5 ; amp = 0.028

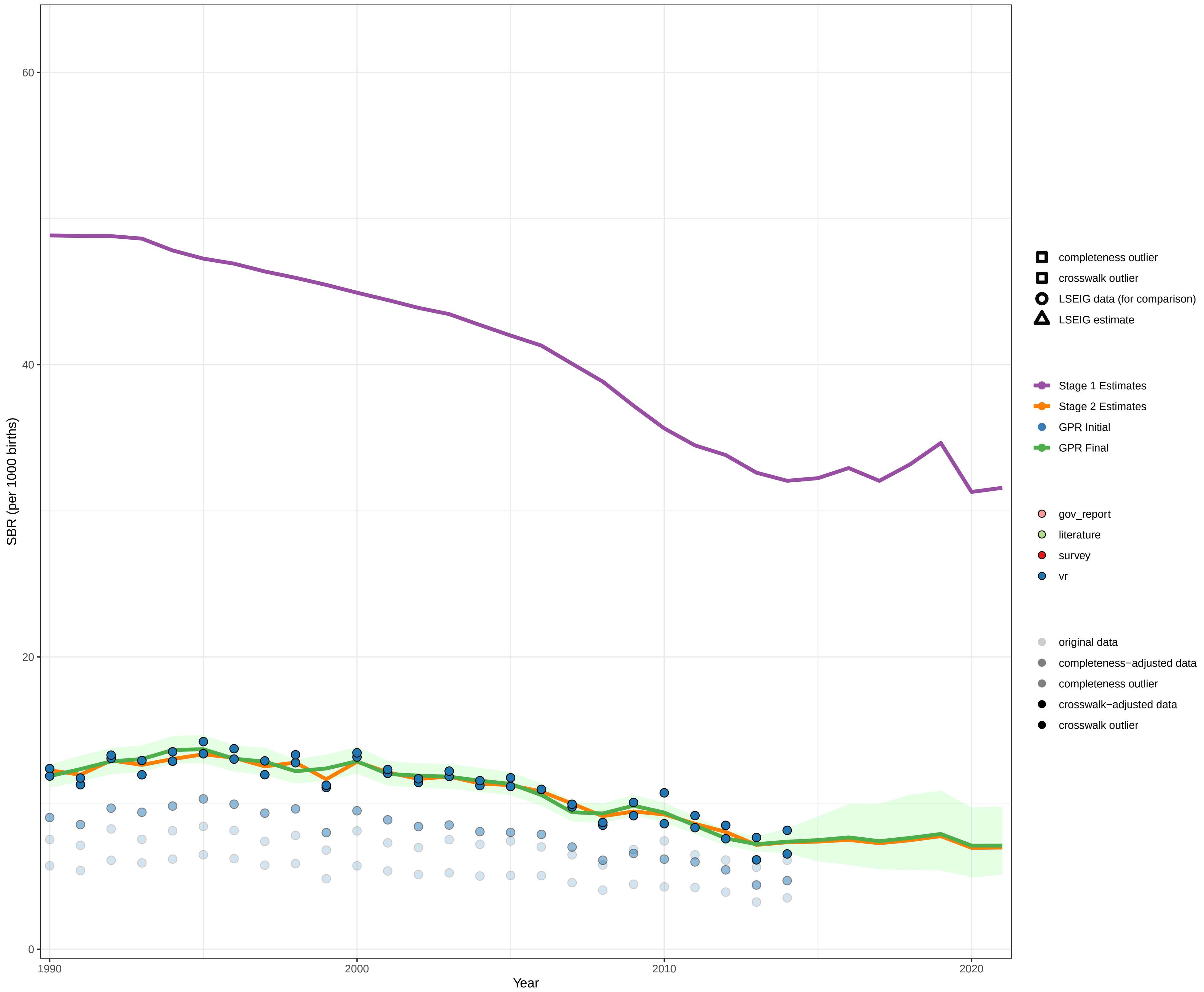

Sonora MEX_4668 Central Latin America Stillbirth Rate (SBR) − 28 weeks
lambda = 0.2 ; zeta = 0.99 ; scale = 5 ; amp = 0.028

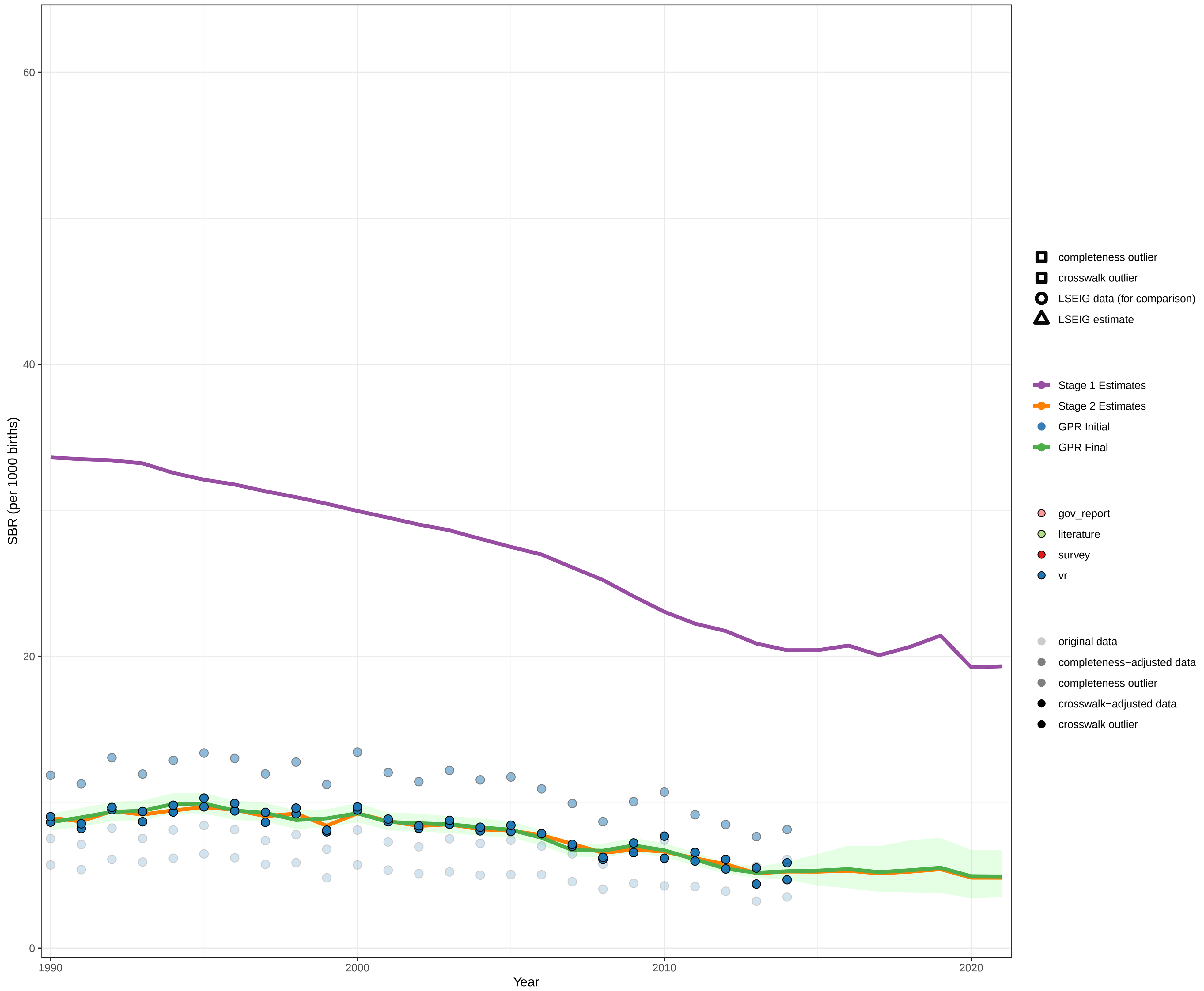

Year	Total Stillbirths (in thousands)	Total Stillbirths (in thousands)	SBR (per 1000 births)	SBR (per 1000 births)	SBR/NMR	SBR/NMR
	20 weeks	28 weeks	20 weeks	28 weeks	20 weeks	28 weeks
1990	0.6 (0.6 to 0.7)	0.5 (0.4 to 0.5)	11.8 (11.1 to 12.6)	8.6 (8.1 to 9.2)	0.78 (0.73 to 0.83)	0.57 (0.53 to 0.61)
1995	0.8 (0.7 to 0.8)	0.6 (0.5 to 0.6)	13.7 (12.7 to 14.7)	9.9 (9.2 to 10.6)	0.97 (0.90 to 1.04)	0.70 (0.65 to 0.75)
2000	0.7 (0.7 to 0.8)	0.5 (0.5 to 0.5)	12.9 (12.0 to 13.8)	9.2 (8.6 to 9.9)	1.02 (0.95 to 1.10)	0.73 (0.68 to 0.79)
2005	0.6 (0.6 to 0.6)	0.4 (0.4 to 0.5)	11.3 (10.6 to 12.1)	8.1 (7.6 to 8.7)	1.00 (0.93 to 1.07)	0.72 (0.67 to 0.77)
2010	0.5 (0.5 to 0.6)	0.4 (0.3 to 0.4)	9.4 (8.7 to 10.0)	6.7 (6.2 to 7.2)	1.00 (0.93 to 1.07)	0.72 (0.67 to 0.77)
2015	0.4 (0.3 to 0.5)	0.3 (0.2 to 0.3)	7.5 (6.0 to 9.1)	5.3 (4.3 to 6.5)	0.94 (0.76 to 1.15)	0.67 (0.54 to 0.82)
2020	0.3 (0.2 to 0.4)	0.2 (0.1 to 0.3)	7.1 (4.9 to 9.7)	4.9 (3.4 to 6.7)	1.01 (0.70 to 1.38)	0.70 (0.49 to 0.96)
2021	0.3 (0.2 to 0.4)	0.2 (0.1 to 0.3)	7.1 (5.1 to 9.8)	4.9 (3.5 to 6.8)	1.02 (0.73 to 1.40)	0.71 (0.51 to 0.97)

Tabasco MEX_4669 Central Latin America Stillbirth Rate / Neonatal Mortality Rate (SBR/NMR) − 20 weeks
lambda = 0.2 ; zeta = 0.99 ; scale = 5 ; amp = 0.028

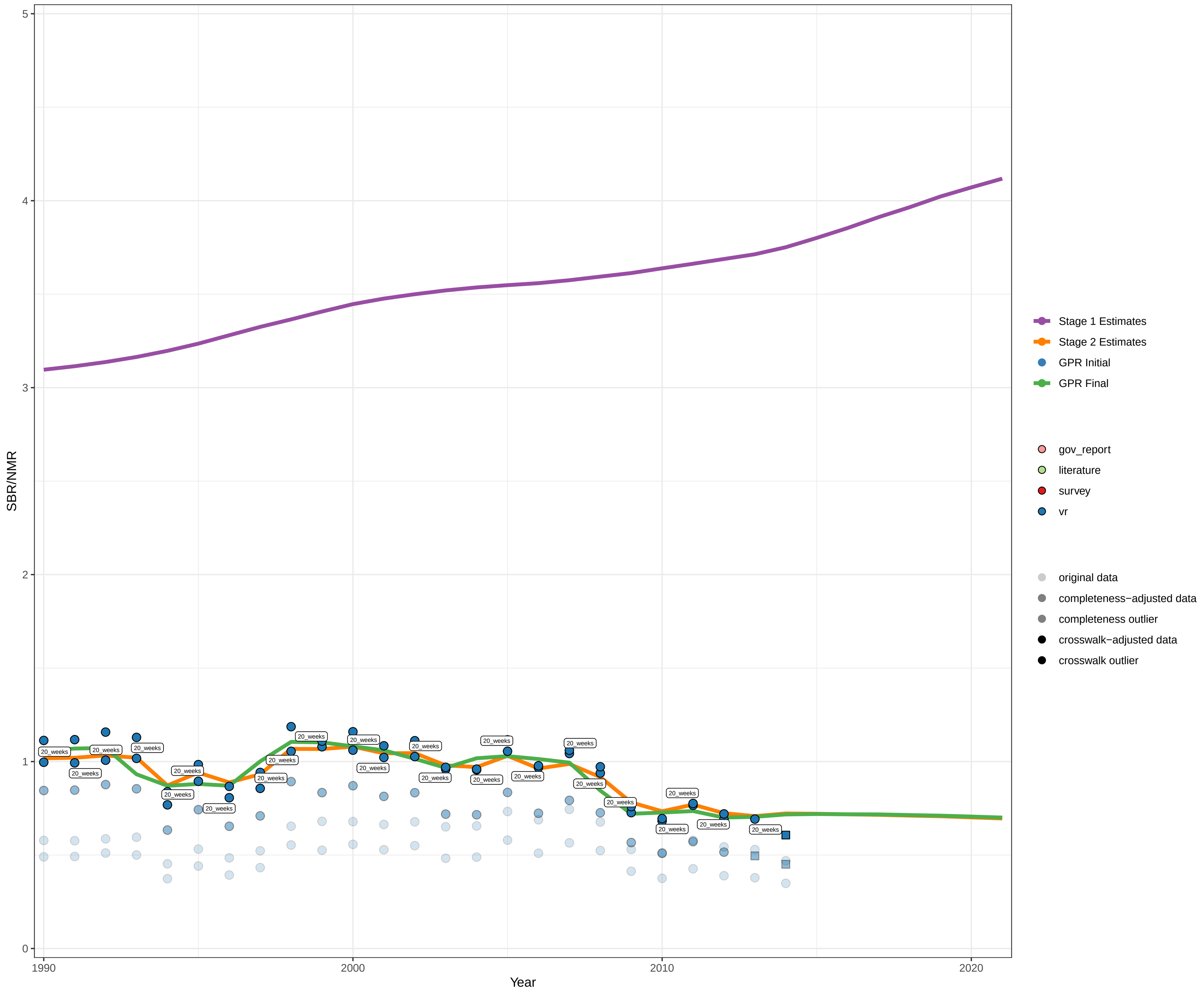

Tabasco MEX_4669 Central Latin America Stillbirth Rate / Neonatal Mortality Rate (SBR/NMR) − 28 weeks
lambda = 0.2 ; zeta = 0.99 ; scale = 5 ; amp = 0.028

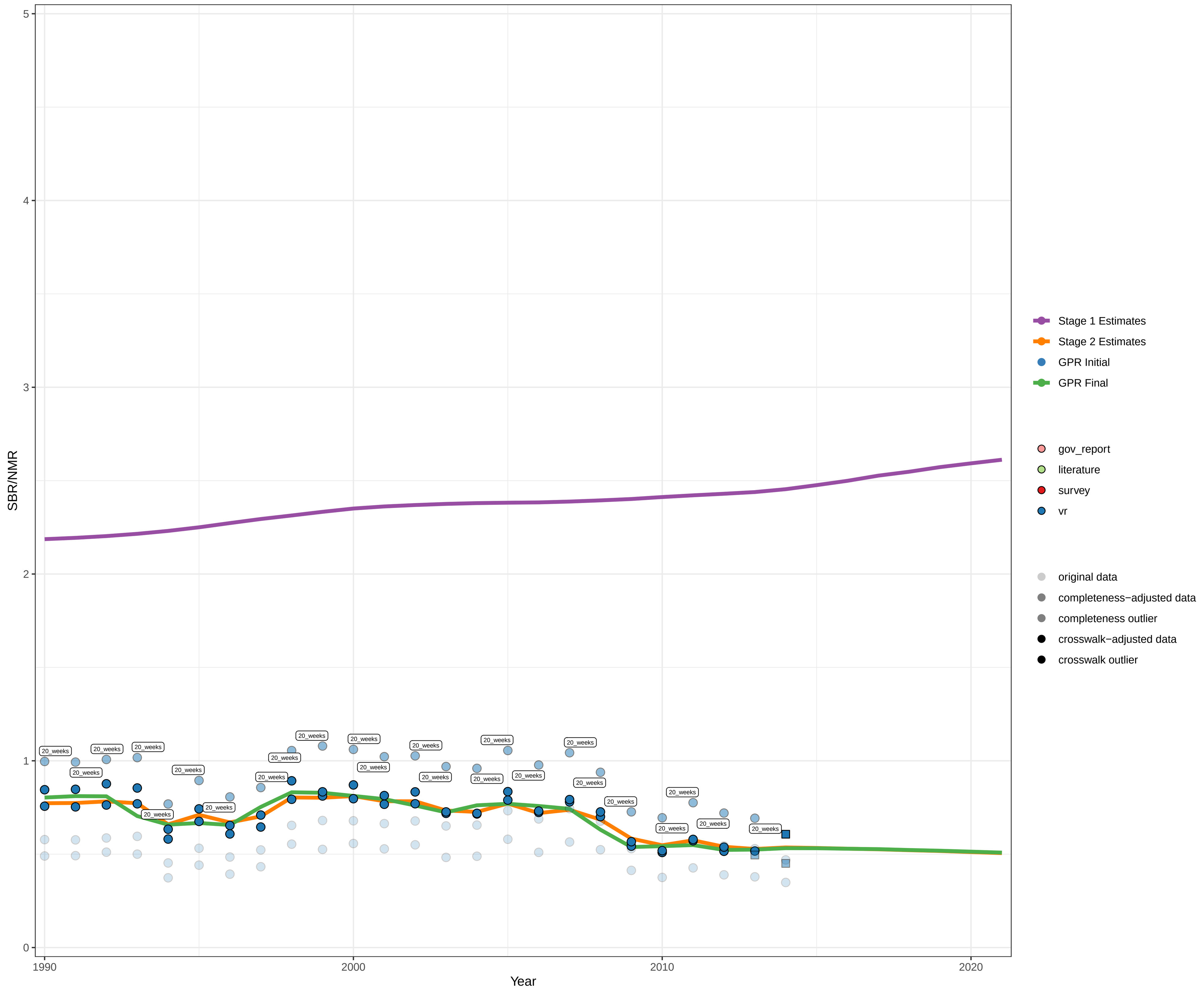

Definition	Years	Outlier	Source.Type	Title
20_weeks	1990−2013	0	VR	Mexico INEGI Administrative Registers − Fetal Deaths by Municipality of Usual Residence of the Mother and Her Five−year Age Group According to Type of Fetal Death
28_weeks	1990−2012	0	VR	Mexico INEGI Administrative Registers − Fetal Deaths by Municipality of Usual Residence of the Mother and Her Five−year Age Group According to Type of Fetal Death
28_weeks	2013, 2014	1	VR	Mexico INEGI Administrative Registers − Fetal Deaths by Municipality of Usual Residence of the Mother and Her Five−year Age Group According to Type of Fetal Death
20_weeks	2014	2	VR	Mexico INEGI Administrative Registers − Fetal Deaths by Municipality of Usual Residence of the Mother and Her Five−year Age Group According to Type of Fetal Death

Tabasco MEX_4669 Central Latin America Stillbirth Rate (SBR) − 20 weeks
lambda = 0.2 ; zeta = 0.99 ; scale = 5 ; amp = 0.028

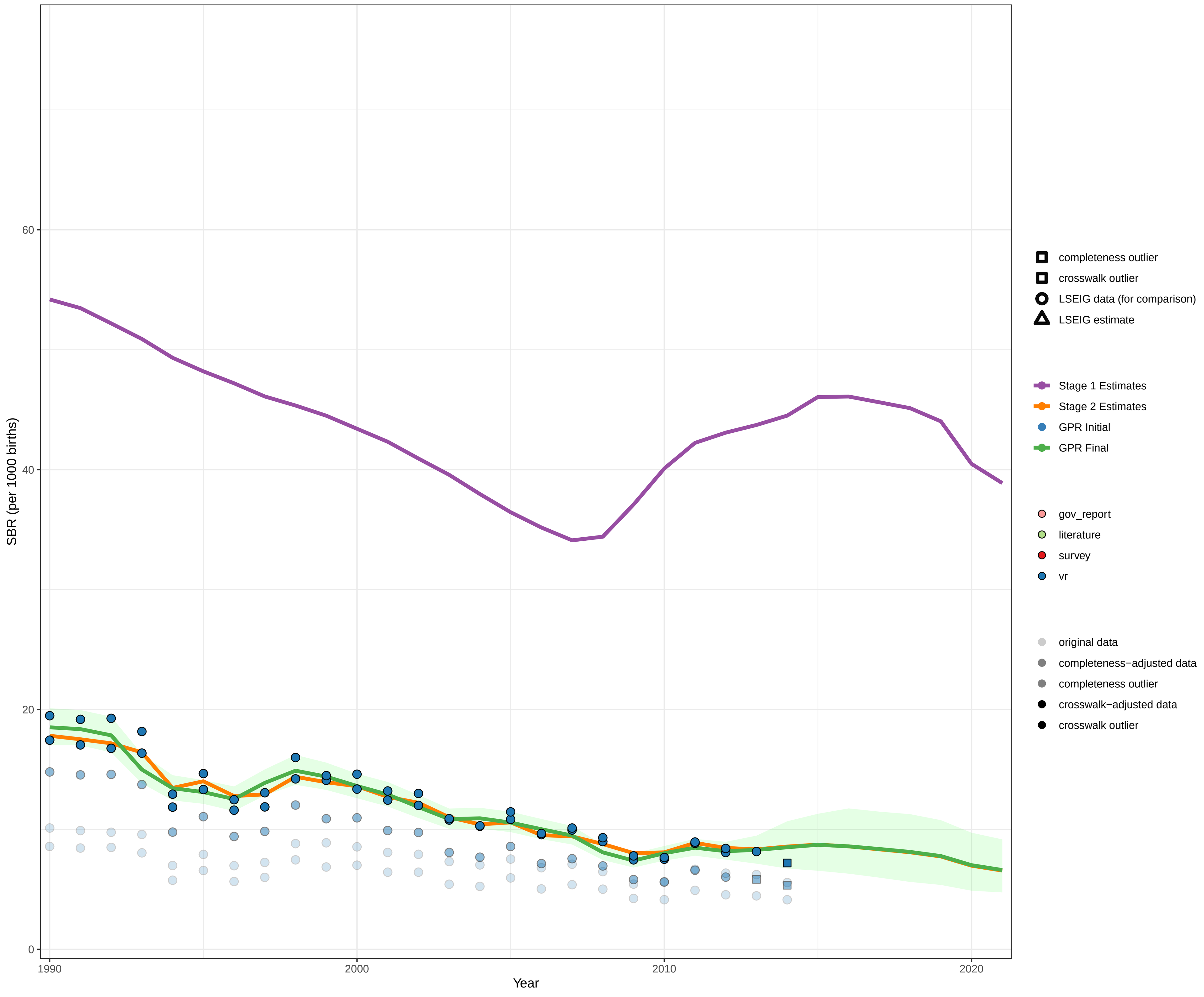

Tabasco MEX_4669 Central Latin America Stillbirth Rate (SBR) − 28 weeks
lambda = 0.2 ; zeta = 0.99 ; scale = 5 ; amp = 0.028

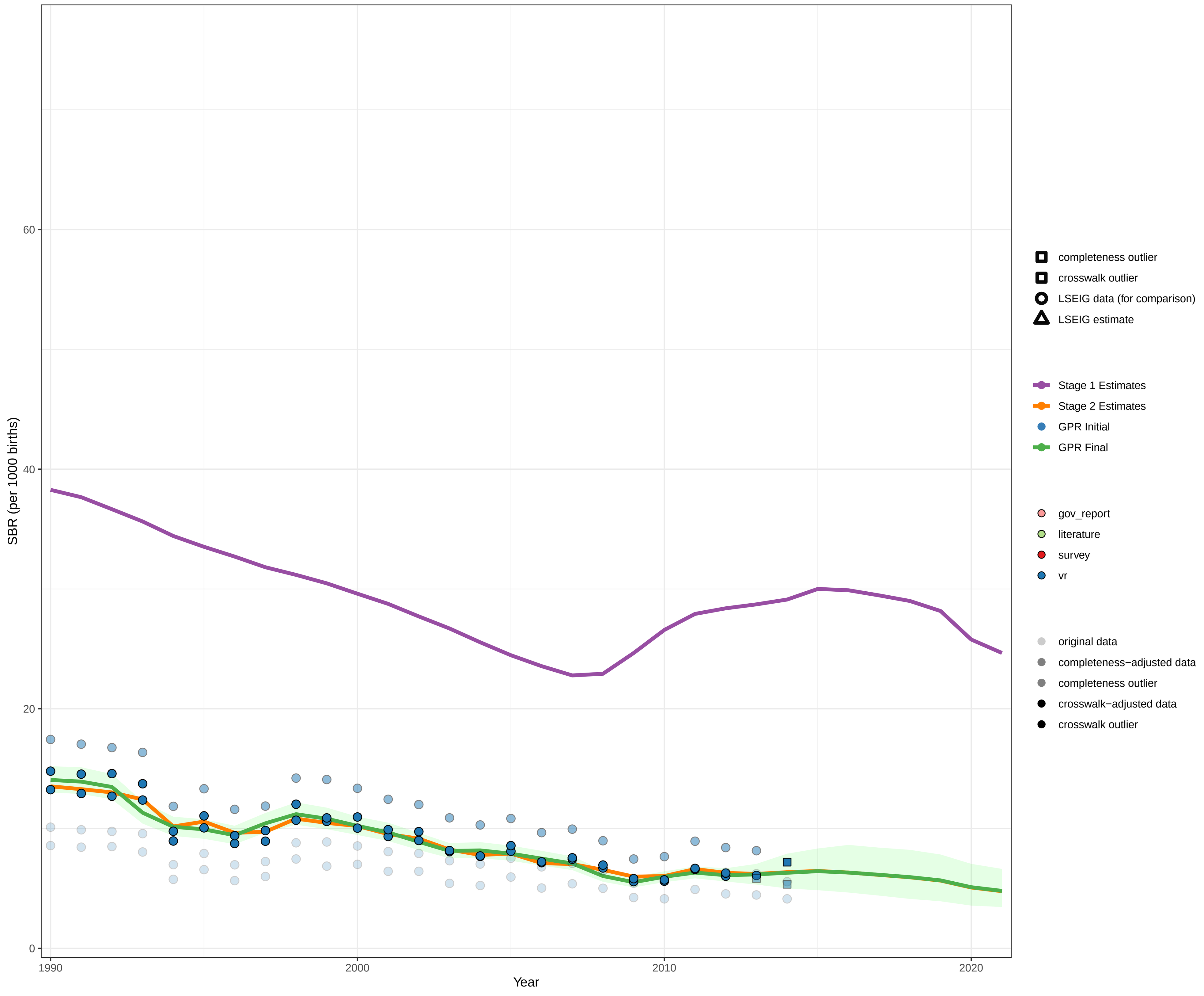

Year	Total Stillbirths (in thousands)	Total Stillbirths (in thousands)	SBR (per 1000 births)	SBR (per 1000 births)	SBR/NMR	SBR/NMR
	20 weeks	28 weeks	20 weeks	28 weeks	20 weeks	28 weeks
1990	1.0 (0.9 to 1.1)	0.7 (0.7 to 0.8)	18.5 (17.0 to 20.1)	14.1 (13.0 to 15.2)	1.06 (0.97 to 1.15)	0.80 (0.74 to 0.87)
1995	0.7 (0.6 to 0.8)	0.5 (0.5 to 0.6)	13.1 (12.1 to 14.1)	9.9 (9.1 to 10.8)	0.88 (0.81 to 0.95)	0.67 (0.61 to 0.72)
2000	0.7 (0.6 to 0.7)	0.5 (0.5 to 0.6)	13.6 (12.6 to 14.6)	10.2 (9.5 to 11.0)	1.08 (1.00 to 1.16)	0.81 (0.75 to 0.87)
2005	0.5 (0.5 to 0.6)	0.4 (0.4 to 0.4)	10.6 (9.8 to 11.5)	7.9 (7.3 to 8.6)	1.03 (0.95 to 1.12)	0.77 (0.71 to 0.84)
2010	0.4 (0.4 to 0.4)	0.3 (0.3 to 0.3)	8.0 (7.4 to 8.6)	6.0 (5.5 to 6.4)	0.73 (0.67 to 0.78)	0.54 (0.50 to 0.58)
2015	0.4 (0.3 to 0.5)	0.3 (0.2 to 0.4)	8.7 (6.6 to 11.3)	6.4 (4.9 to 8.3)	0.72 (0.54 to 0.93)	0.53 (0.40 to 0.69)
2020	0.3 (0.2 to 0.4)	0.2 (0.1 to 0.3)	7.0 (4.9 to 9.7)	5.1 (3.6 to 7.1)	0.71 (0.49 to 0.98)	0.51 (0.36 to 0.71)
2021	0.2 (0.2 to 0.3)	0.2 (0.1 to 0.2)	6.6 (4.7 to 9.2)	4.8 (3.5 to 6.6)	0.70 (0.50 to 0.97)	0.51 (0.37 to 0.70)

Tamaulipas   MEX_4670   Central Latin America   Stillbirth Rate / Neonatal Mortality Rate (SBR/NMR) − 20 weeks
lambda = 0.2 ; zeta = 0.99 ; scale = 5 ; amp = 0.028

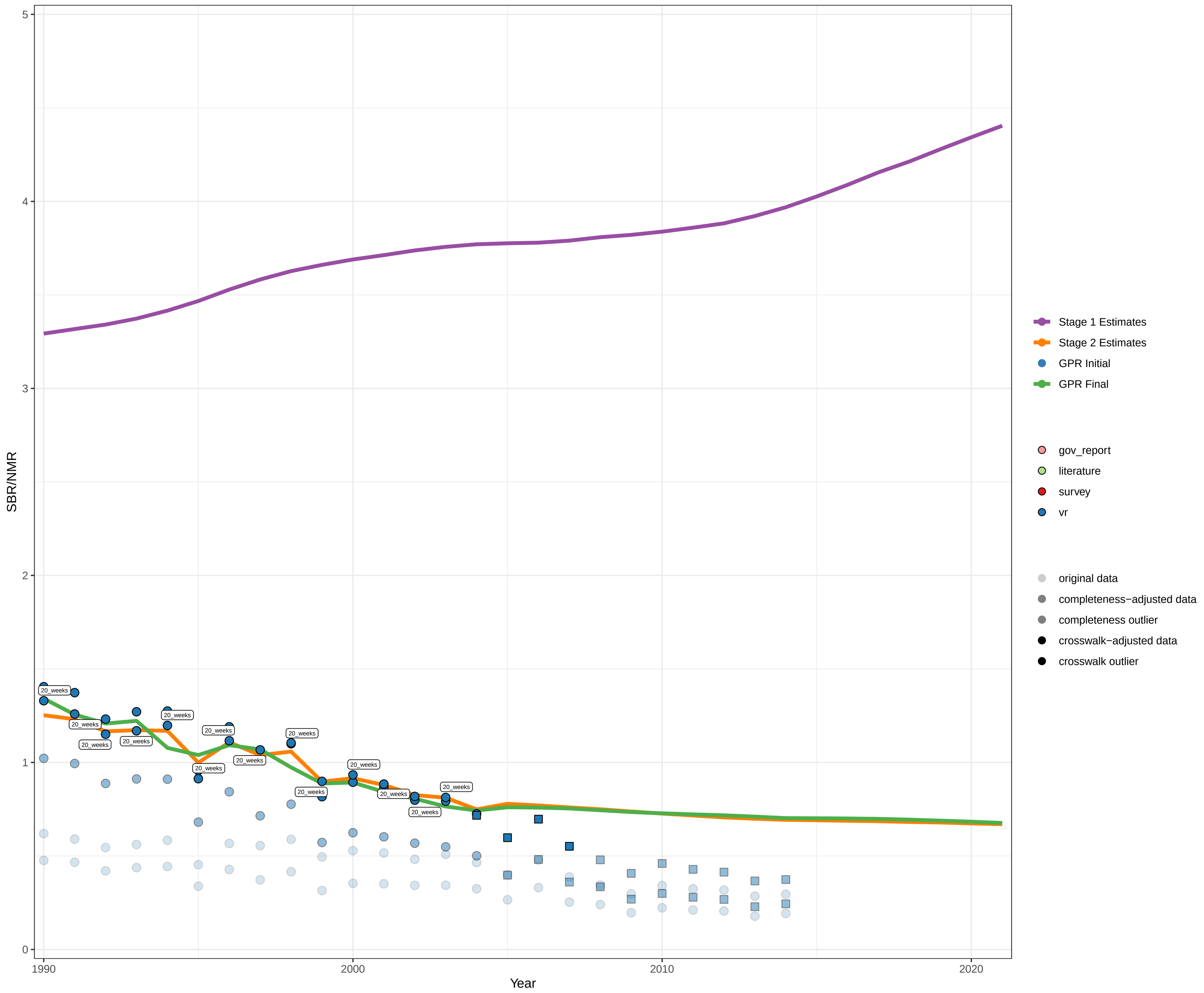

Tamaulipas   MEX_4670   Central Latin America   Stillbirth Rate / Neonatal Mortality Rate (SBR/NMR) − 28 weeks
lambda = 0.2 ; zeta = 0.99 ; scale = 5 ; amp = 0.028

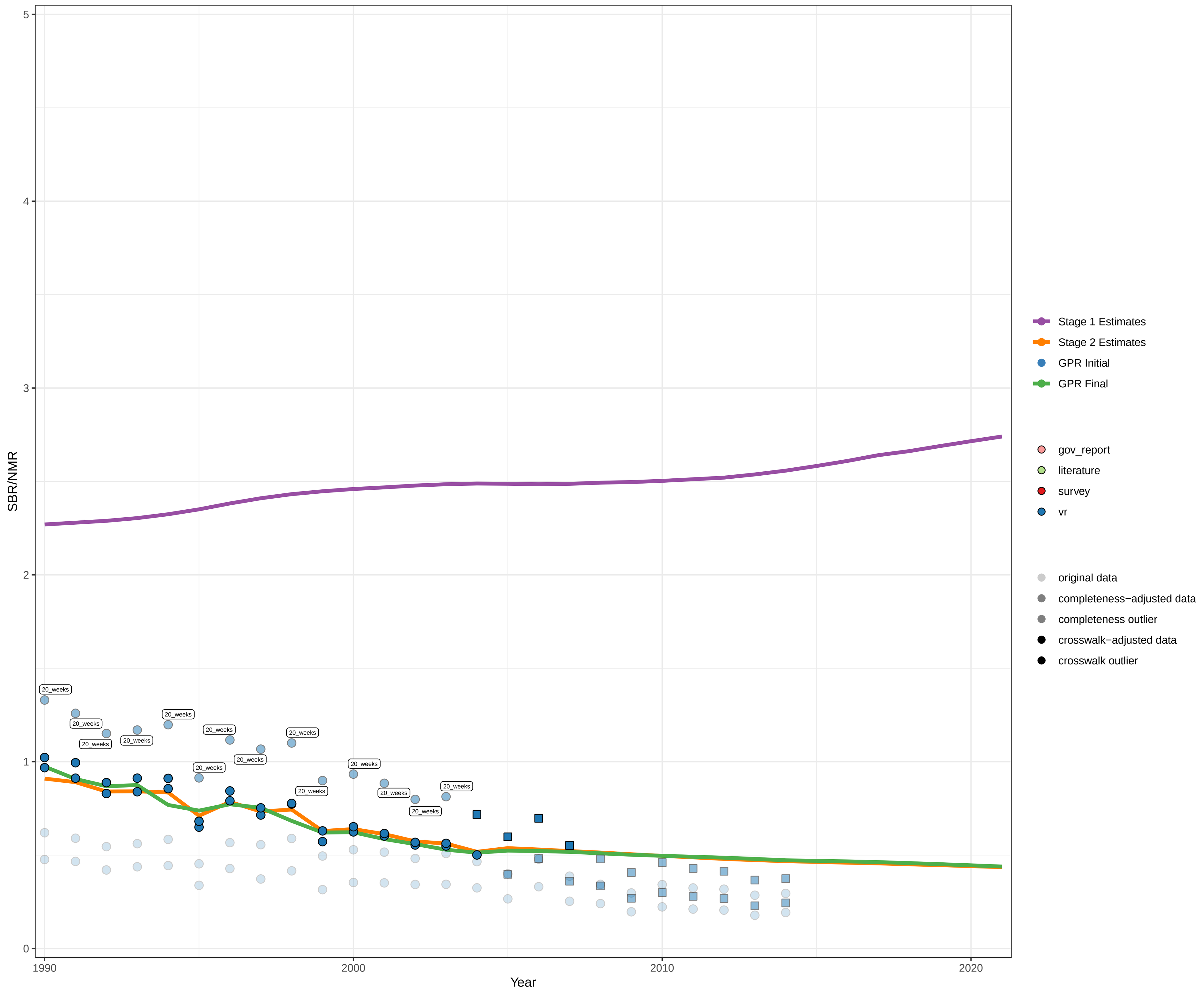

Definition	Years	Outlier	Source.Type	Title
20_weeks	1990−2003	0	VR	Mexico INEGI Administrative Registers − Fetal Deaths by Municipality of Usual Residence of the Mother and Her Five−year Age Group According to Type of Fetal Death
28_weeks	1990−2004	0	VR	Mexico INEGI Administrative Registers − Fetal Deaths by Municipality of Usual Residence of the Mother and Her Five−year Age Group According to Type of Fetal Death
20_weeks	2008−2014	1	VR	Mexico INEGI Administrative Registers − Fetal Deaths by Municipality of Usual Residence of the Mother and Her Five−year Age Group According to Type of Fetal Death
28_weeks	2005−2014	1	VR	Mexico INEGI Administrative Registers − Fetal Deaths by Municipality of Usual Residence of the Mother and Her Five−year Age Group According to Type of Fetal Death
20_weeks	2004−2007	2	VR	Mexico INEGI Administrative Registers − Fetal Deaths by Municipality of Usual Residence of the Mother and Her Five−year Age Group According to Type of Fetal Death

Tamaulipas   MEX_4670   Central Latin America   Stillbirth Rate (SBR) − 20 weeks
lambda = 0.2 ; zeta = 0.99 ; scale = 5 ; amp = 0.028

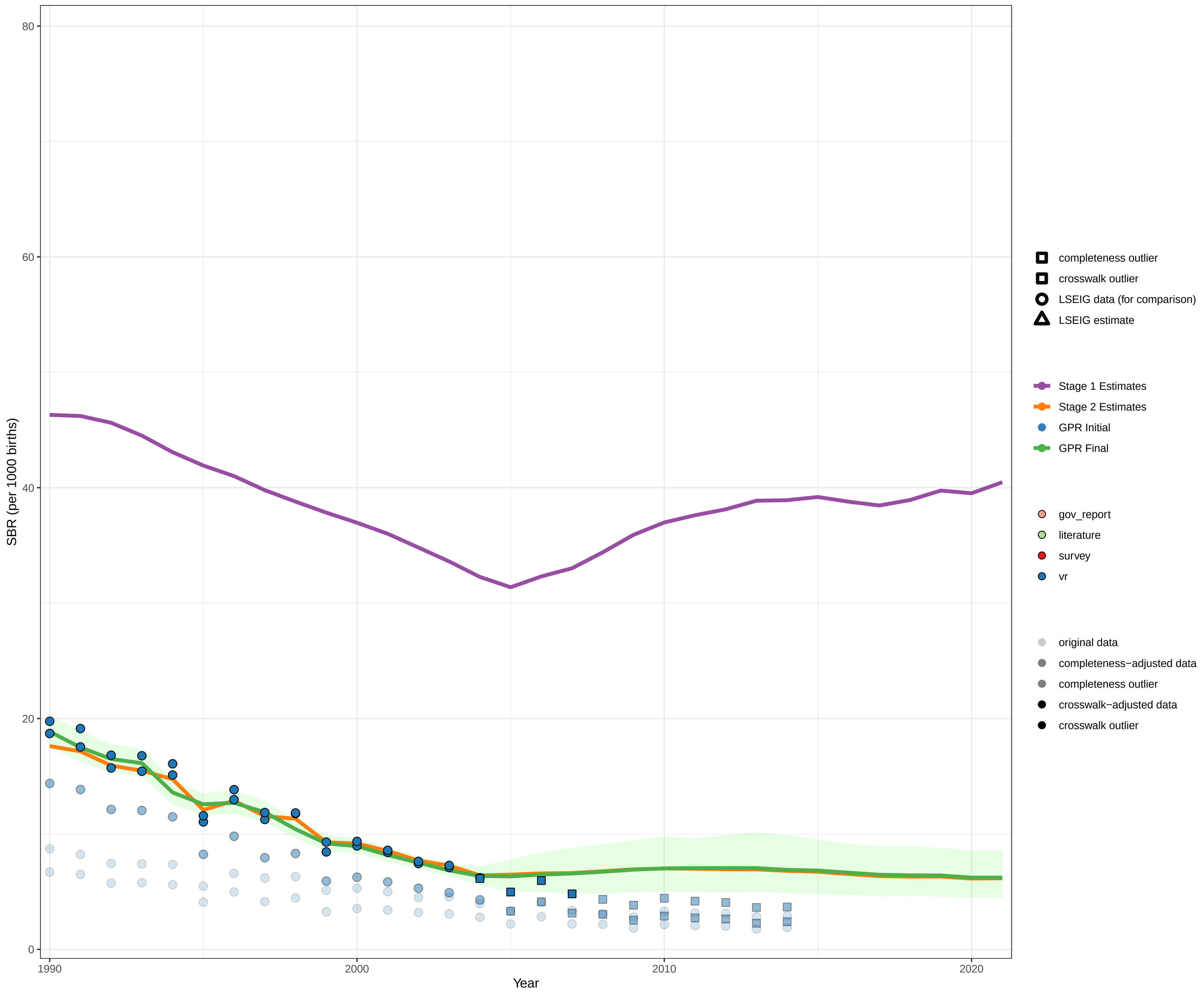

Tamaulipas   MEX_4670   Central Latin America   Stillbirth Rate (SBR) − 28 weeks
lambda = 0.2 ; zeta = 0.99 ; scale = 5 ; amp = 0.028

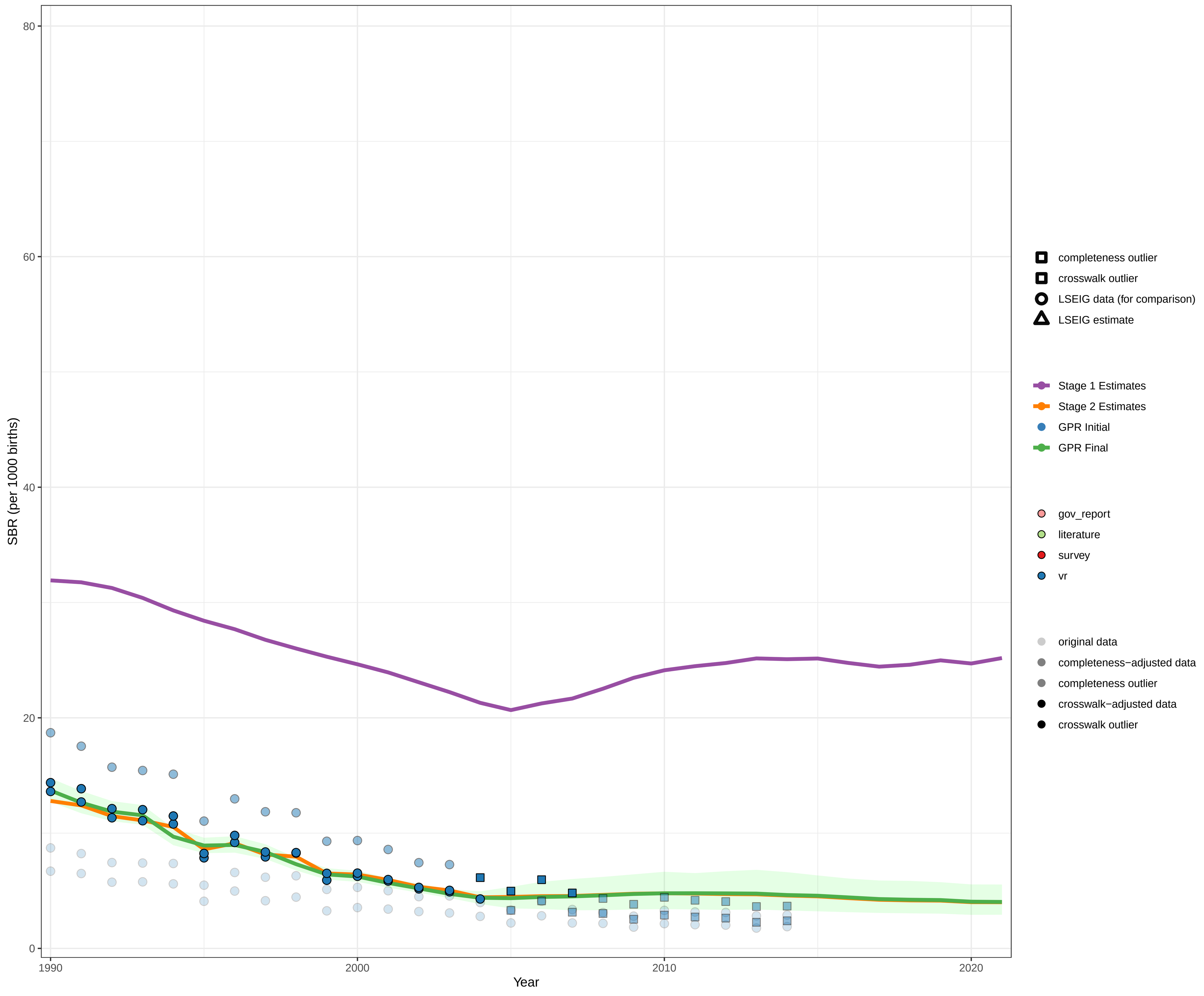

Year	Total Stillbirths (in thousands)	Total Stillbirths (in thousands)	SBR (per 1000 births)	SBR (per 1000 births)	SBR/NMR	SBR/NMR
	20 weeks	28 weeks	20 weeks	28 weeks	20 weeks	28 weeks
1990	1.2 (1.1 to 1.3)	0.9 (0.8 to 0.9)	18.9 (17.5 to 20.3)	13.7 (12.7 to 14.8)	1.34 (1.25 to 1.45)	0.97 (0.90 to 1.05)
1995	0.8 (0.8 to 0.9)	0.6 (0.5 to 0.6)	12.6 (11.6 to 13.5)	8.9 (8.3 to 9.6)	1.04 (0.96 to 1.12)	0.74 (0.68 to 0.79)
2000	0.6 (0.5 to 0.6)	0.4 (0.4 to 0.4)	8.9 (8.3 to 9.7)	6.2 (5.8 to 6.7)	0.89 (0.83 to 0.96)	0.62 (0.58 to 0.67)
2005	0.4 (0.3 to 0.5)	0.3 (0.2 to 0.4)	6.3 (5.0 to 7.8)	4.4 (3.5 to 5.3)	0.76 (0.61 to 0.93)	0.52 (0.42 to 0.64)
2010	0.5 (0.3 to 0.7)	0.3 (0.2 to 0.5)	7.0 (5.0 to 9.8)	4.8 (3.4 to 6.6)	0.73 (0.52 to 1.01)	0.50 (0.35 to 0.69)
2015	0.4 (0.3 to 0.6)	0.3 (0.2 to 0.4)	6.8 (4.8 to 9.5)	4.6 (3.2 to 6.3)	0.70 (0.49 to 0.97)	0.47 (0.33 to 0.65)
2020	0.3 (0.2 to 0.4)	0.2 (0.1 to 0.3)	6.2 (4.4 to 8.5)	4.1 (2.9 to 5.6)	0.68 (0.49 to 0.94)	0.45 (0.32 to 0.61)
2021	0.3 (0.2 to 0.4)	0.2 (0.1 to 0.3)	6.2 (4.5 to 8.6)	4.0 (2.9 to 5.5)	0.68 (0.49 to 0.93)	0.44 (0.32 to 0.60)

Tlaxcala MEX_4671 Central Latin America Stillbirth Rate / Neonatal Mortality Rate (SBR/NMR) − 20 weeks
lambda = 0.2 ; zeta = 0.99 ; scale = 5 ; amp = 0.028

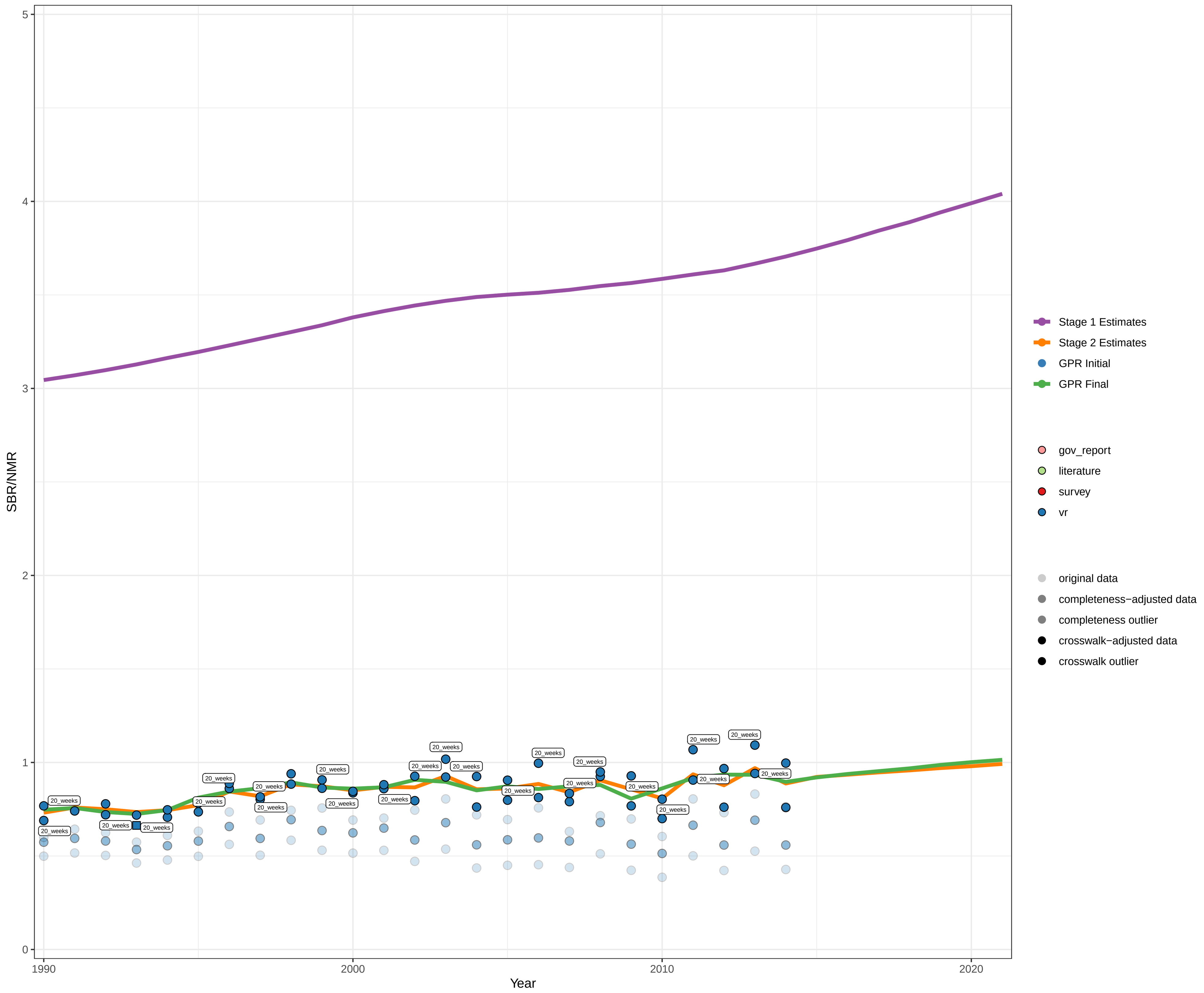

Tlaxcala MEX_4671 Central Latin America Stillbirth Rate / Neonatal Mortality Rate (SBR/NMR) − 28 weeks
lambda = 0.2 ; zeta = 0.99 ; scale = 5 ; amp = 0.028

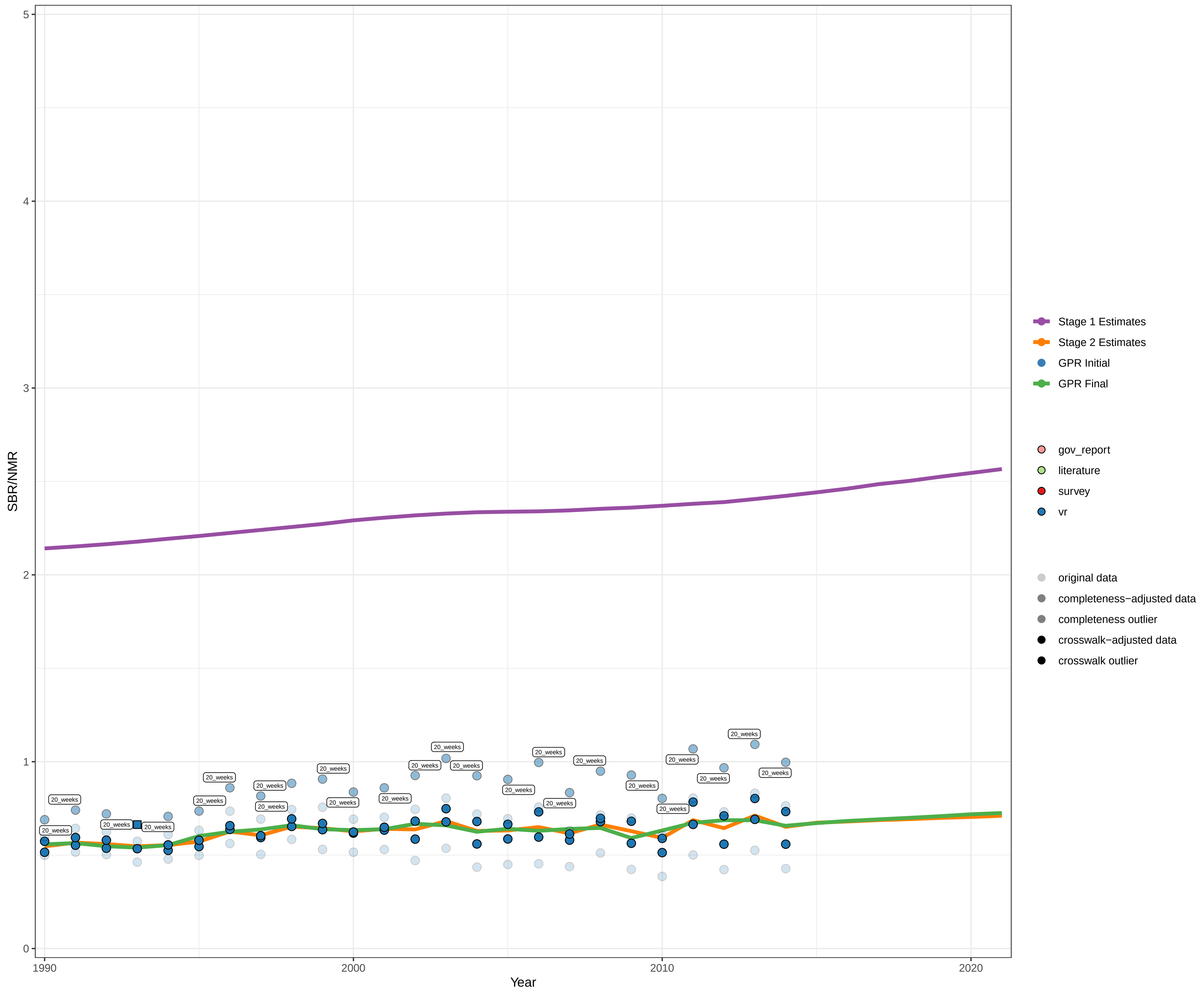

Definition	Years	Outlier	Source.Type	Title
20_weeks	1990−1992, 1994−2014	0	VR	Mexico INEGI Administrative Registers − Fetal Deaths by Municipality of Usual Residence of the Mother and Her Five−year Age Group According to Type of Fetal Death
28_weeks	1990−2014	0	VR	Mexico INEGI Administrative Registers − Fetal Deaths by Municipality of Usual Residence of the Mother and Her Five−year Age Group According to Type of Fetal Death
20_weeks	1993	2	VR	Mexico INEGI Administrative Registers − Fetal Deaths by Municipality of Usual Residence of the Mother and Her Five−year Age Group According to Type of Fetal Death

Tlaxcala MEX_4671 Central Latin America Stillbirth Rate (SBR) − 20 weeks
lambda = 0.2 ; zeta = 0.99 ; scale = 5 ; amp = 0.028

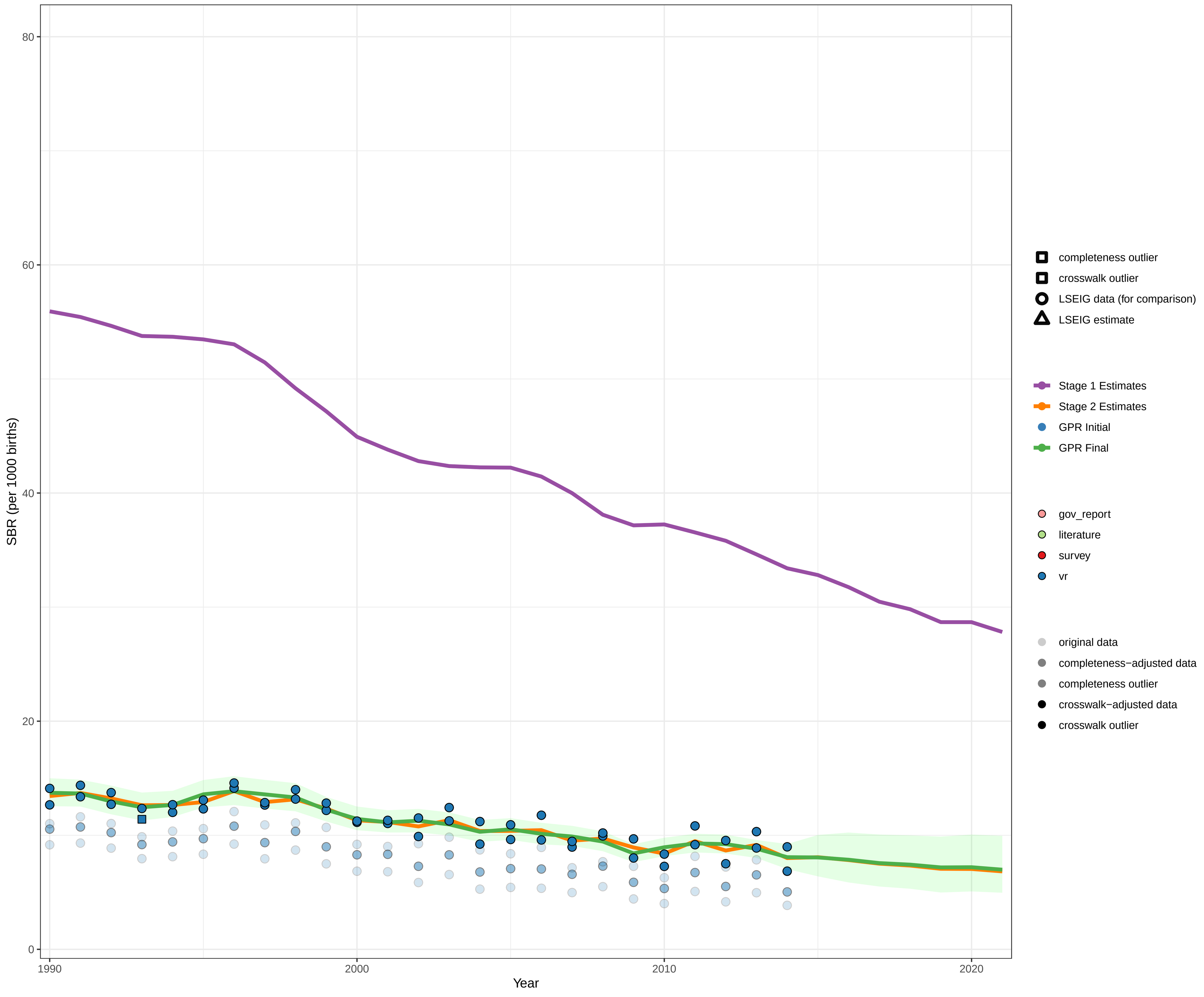

Tlaxcala MEX_4671 Central Latin America Stillbirth Rate (SBR) − 28 weeks
lambda = 0.2 ; zeta = 0.99 ; scale = 5 ; amp = 0.028

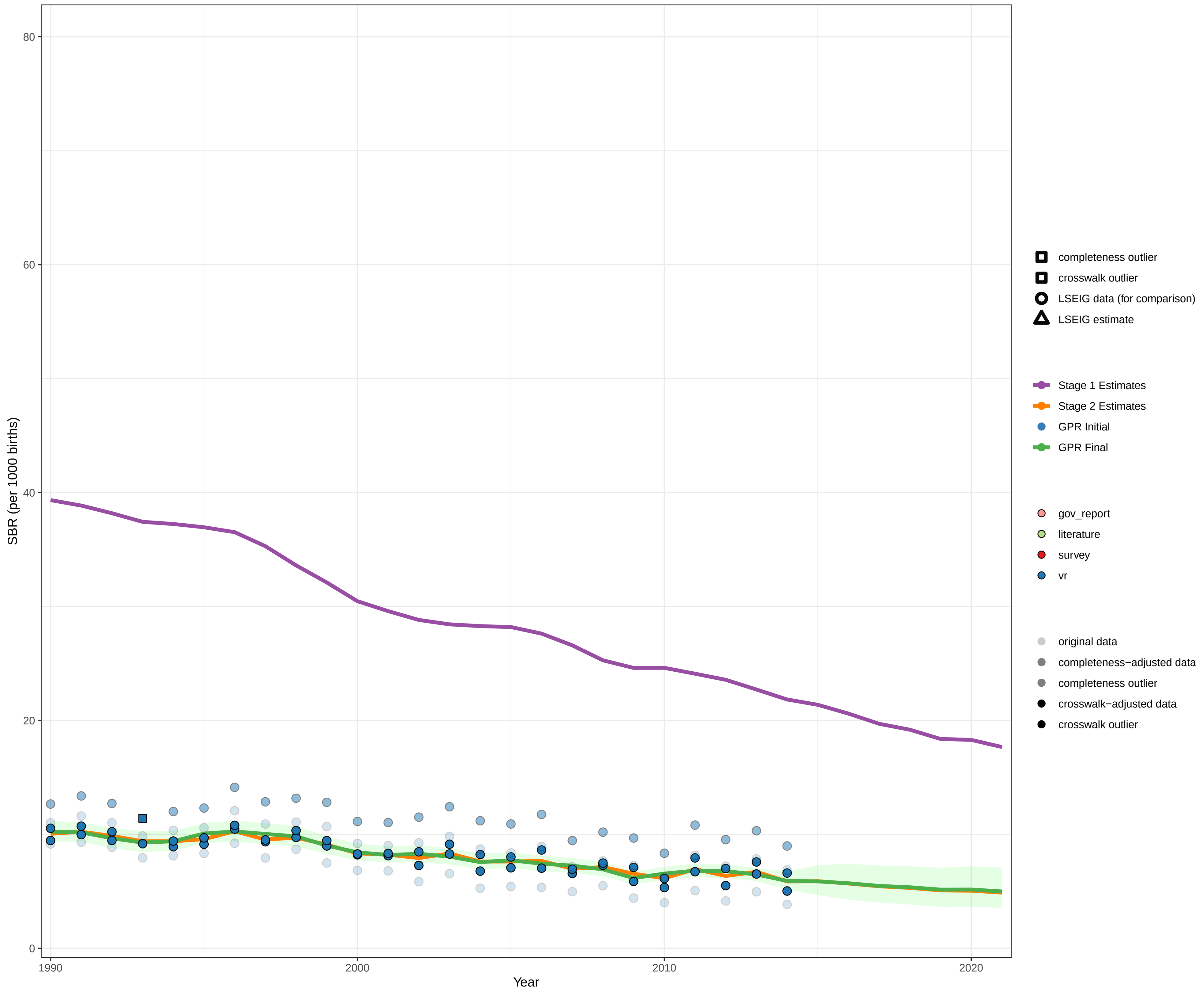

Year	Total Stillbirths (in thousands)	Total Stillbirths (in thousands)	SBR (per 1000 births)	SBR (per 1000 births)	SBR/NMR	SBR/NMR
	20 weeks	28 weeks	20 weeks	28 weeks	20 weeks	28 weeks
1990	0.3 (0.3 to 0.4)	0.3 (0.2 to 0.3)	13.7 (12.5 to 15.0)	10.2 (9.4 to 11.2)	0.75 (0.68 to 0.82)	0.56 (0.51 to 0.61)
1995	0.4 (0.3 to 0.4)	0.3 (0.2 to 0.3)	13.6 (12.4 to 14.8)	10.1 (9.2 to 11.0)	0.81 (0.74 to 0.89)	0.60 (0.55 to 0.66)
2000	0.3 (0.3 to 0.3)	0.2 (0.2 to 0.2)	11.4 (10.4 to 12.5)	8.4 (7.7 to 9.2)	0.86 (0.79 to 0.94)	0.63 (0.58 to 0.69)
2005	0.3 (0.3 to 0.3)	0.2 (0.2 to 0.2)	10.5 (9.6 to 11.5)	7.7 (7.1 to 8.4)	0.87 (0.80 to 0.95)	0.64 (0.59 to 0.70)
2010	0.2 (0.2 to 0.3)	0.2 (0.2 to 0.2)	8.9 (8.2 to 9.8)	6.6 (6.0 to 7.2)	0.86 (0.79 to 0.94)	0.63 (0.58 to 0.69)
2015	0.2 (0.2 to 0.3)	0.2 (0.1 to 0.2)	8.1 (6.4 to 10.0)	5.9 (4.7 to 7.3)	0.92 (0.73 to 1.15)	0.67 (0.53 to 0.83)
2020	0.2 (0.1 to 0.2)	0.1 (0.1 to 0.2)	7.2 (5.1 to 10.1)	5.2 (3.7 to 7.2)	1.00 (0.71 to 1.40)	0.72 (0.51 to 1.00)
2021	0.2 (0.1 to 0.2)	0.1 (0.1 to 0.2)	7.0 (5.0 to 9.9)	5.0 (3.6 to 7.1)	1.01 (0.72 to 1.44)	0.73 (0.52 to 1.03)

Veracruz de Ignacio de la Llave MEX_4672 Central Latin America Stillbirth Rate / Neonatal Mortality Rate (SBR/NMR) − 20 weeks
lambda = 0.2 ; zeta = 0.99 ; scale = 5 ; amp = 0.028

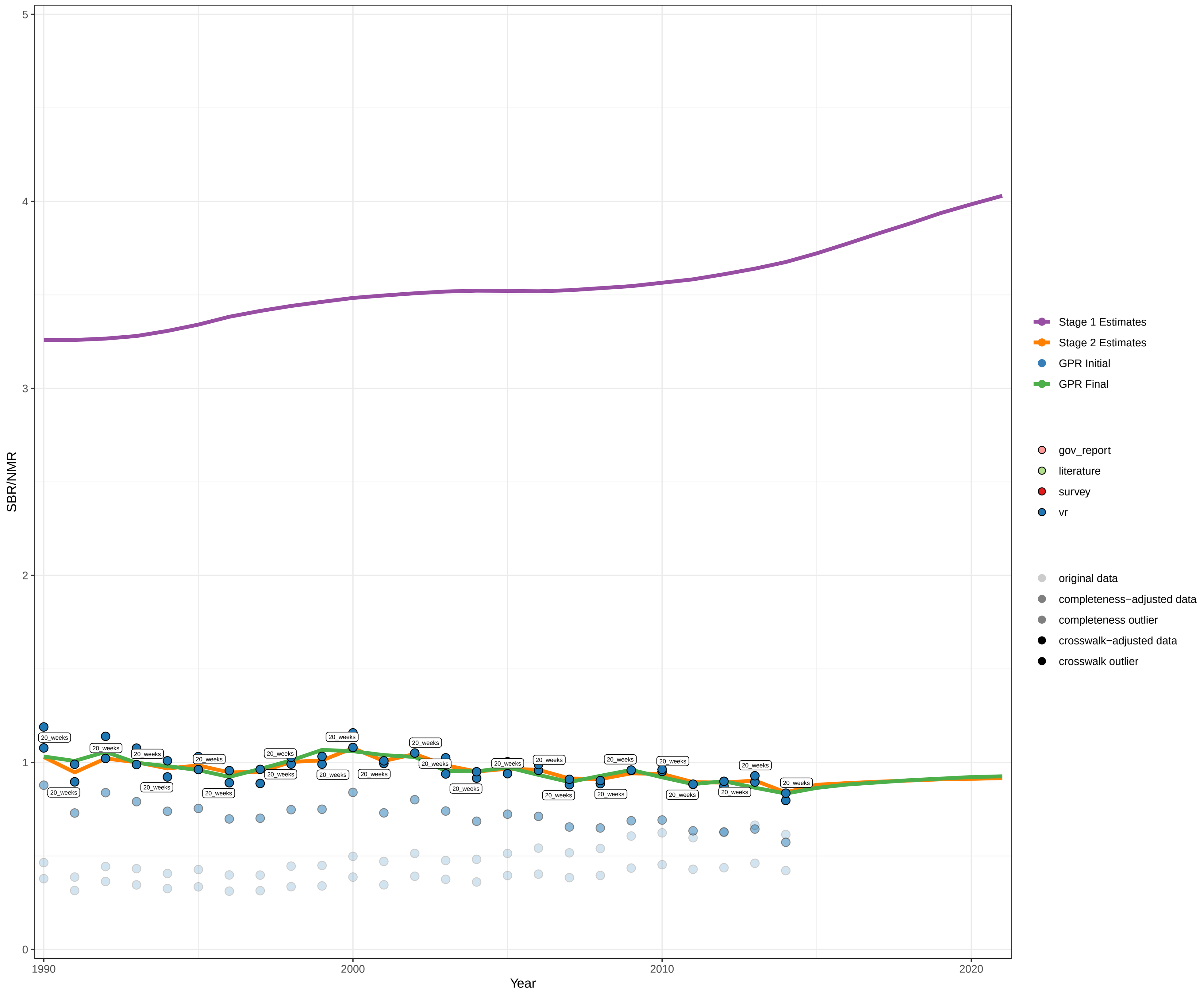

Veracruz de Ignacio de la Llave MEX_4672 Central Latin America Stillbirth Rate / Neonatal Mortality Rate (SBR/NMR) − 28 weeks
lambda = 0.2 ; zeta = 0.99 ; scale = 5 ; amp = 0.028

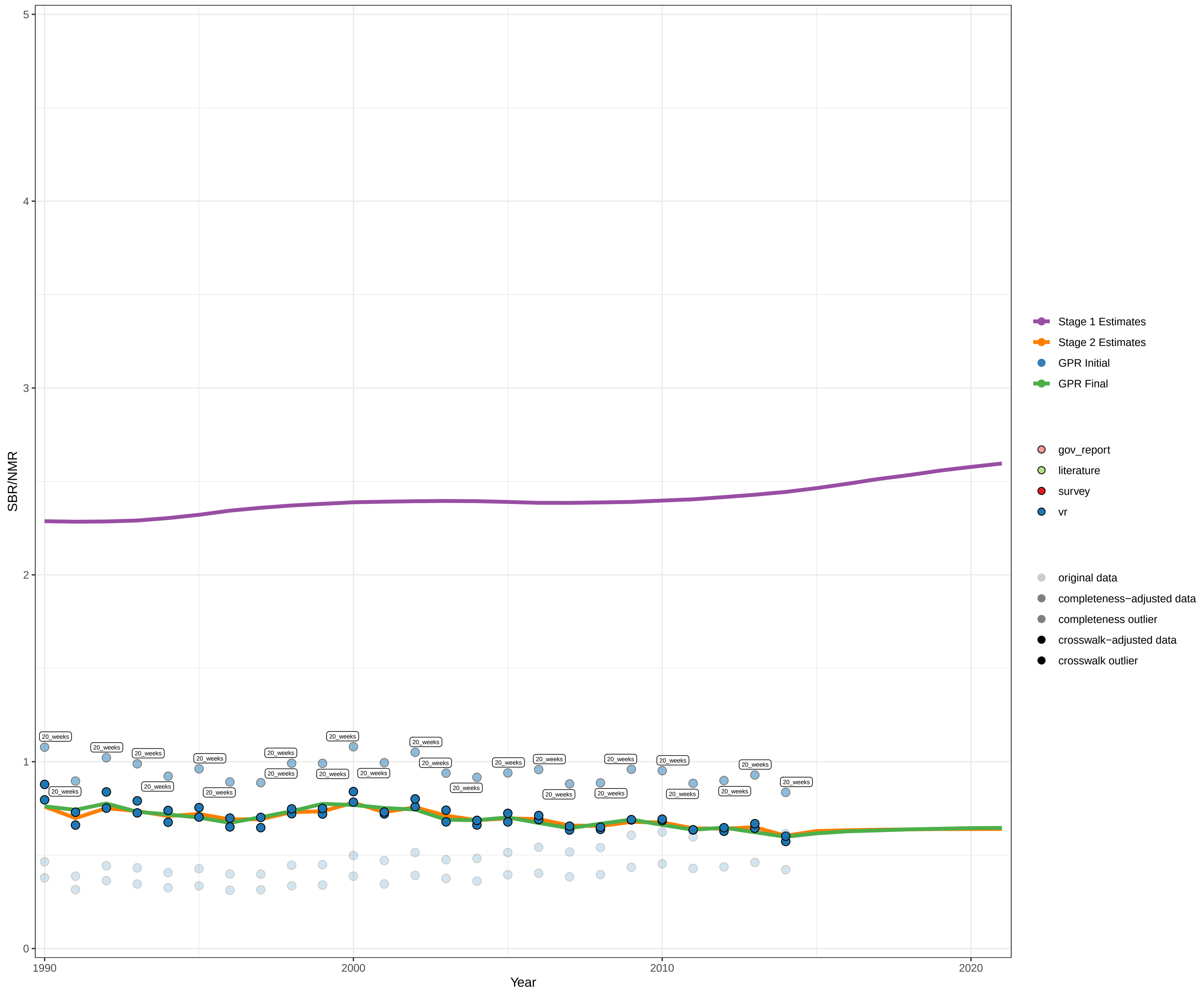

Definition	Years	Outlier	Source.Type	Title
20_weeks, 28_weeks	1990−2014	0	VR	Mexico INEGI Administrative Registers − Fetal Deaths by Municipality of Usual Residence of the Mother and Her Five−year Age Group According to Type of Fetal Death

Veracruz de Ignacio de la Llave MEX_4672 Central Latin America Stillbirth Rate (SBR) − 20 weeks
lambda = 0.2 ; zeta = 0.99 ; scale = 5 ; amp = 0.028

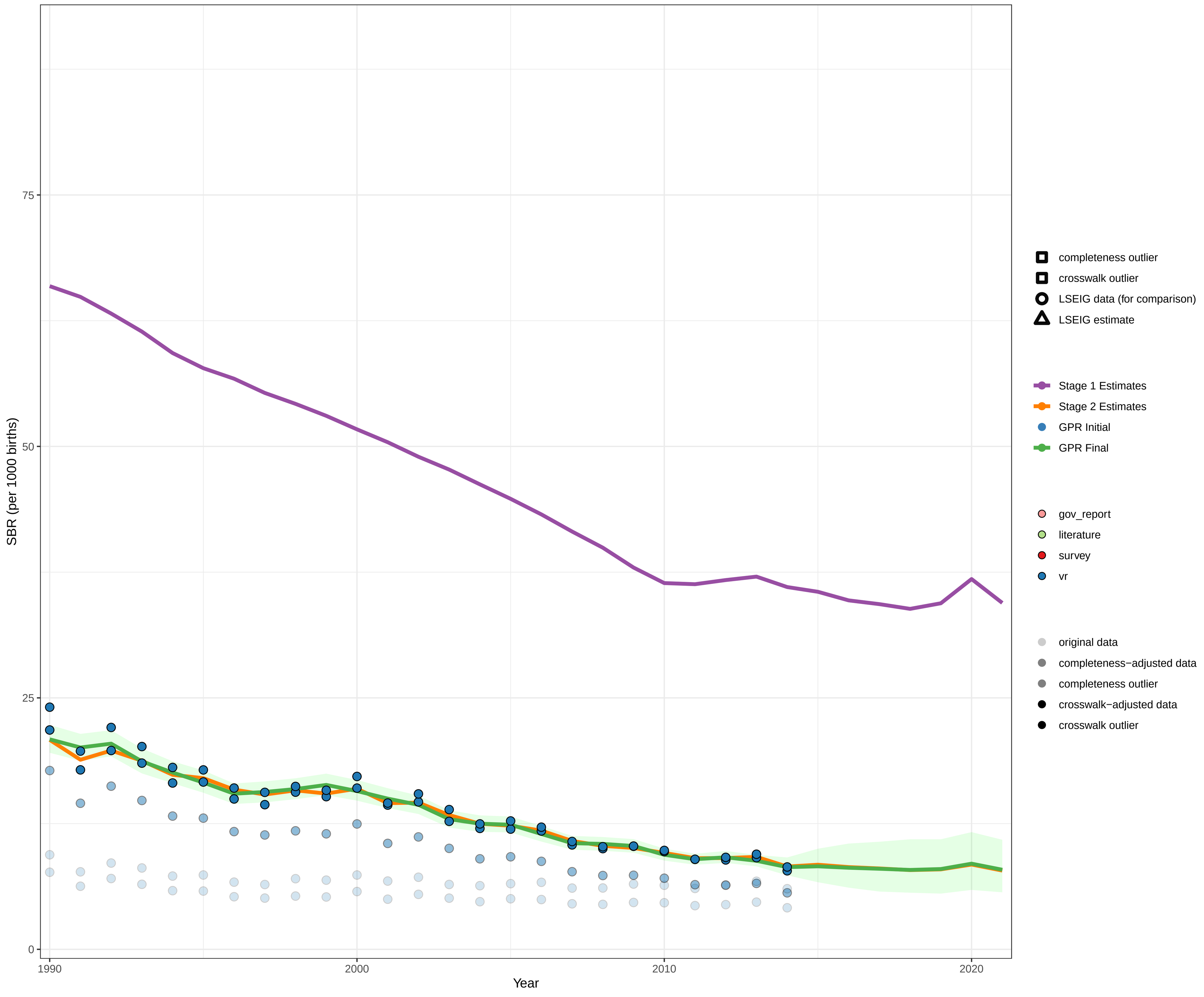

Veracruz de Ignacio de la Llave MEX_4672 Central Latin America Stillbirth Rate (SBR) − 28 weeks
lambda = 0.2 ; zeta = 0.99 ; scale = 5 ; amp = 0.028

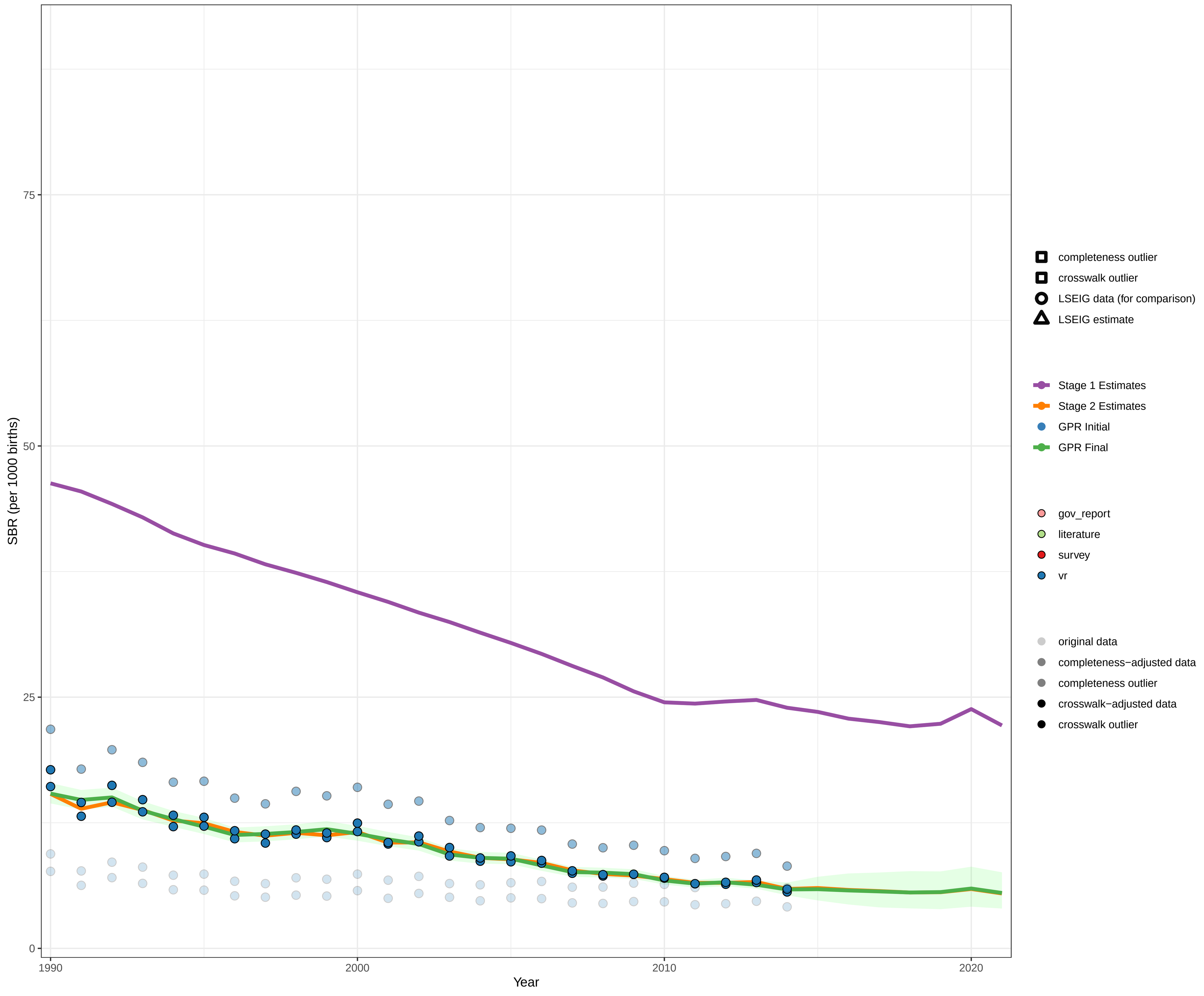

Year	Total Stillbirths (in thousands)	Total Stillbirths (in thousands)	SBR (per 1000 births)	SBR (per 1000 births)	SBR/NMR	SBR/NMR
	20 weeks	28 weeks	20 weeks	28 weeks	20 weeks	28 weeks
1990	4.3 (4.0 to 4.6)	3.2 (3.0 to 3.4)	20.9 (19.5 to 22.3)	15.4 (14.4 to 16.5)	1.03 (0.97 to 1.10)	0.76 (0.71 to 0.81)
1995	3.3 (3.1 to 3.5)	2.4 (2.3 to 2.6)	16.6 (15.6 to 17.8)	12.1 (11.4 to 13.0)	0.96 (0.90 to 1.03)	0.70 (0.66 to 0.75)
2000	2.8 (2.7 to 3.0)	2.0 (1.9 to 2.2)	15.7 (14.8 to 16.8)	11.4 (10.7 to 12.2)	1.06 (1.00 to 1.13)	0.77 (0.72 to 0.82)
2005	2.1 (1.9 to 2.2)	1.5 (1.4 to 1.6)	12.4 (11.6 to 13.2)	8.9 (8.4 to 9.5)	0.97 (0.91 to 1.04)	0.70 (0.66 to 0.75)
2010	1.6 (1.5 to 1.7)	1.1 (1.0 to 1.2)	9.4 (8.8 to 10.0)	6.8 (6.3 to 7.2)	0.92 (0.86 to 0.98)	0.66 (0.62 to 0.70)
2015	1.2 (0.9 to 1.4)	0.8 (0.7 to 1.0)	8.3 (6.7 to 10.0)	5.9 (4.8 to 7.1)	0.86 (0.70 to 1.05)	0.62 (0.50 to 0.75)
2020	0.9 (0.7 to 1.3)	0.7 (0.5 to 0.9)	8.5 (5.9 to 11.7)	6.0 (4.1 to 8.1)	0.92 (0.64 to 1.26)	0.64 (0.45 to 0.88)
2021	0.8 (0.6 to 1.2)	0.6 (0.4 to 0.8)	7.9 (5.7 to 10.9)	5.5 (4.0 to 7.6)	0.93 (0.66 to 1.27)	0.65 (0.46 to 0.89)

Yucatan MEX_4673 Central Latin America Stillbirth Rate / Neonatal Mortality Rate (SBR/NMR) − 20 weeks
lambda = 0.2 ; zeta = 0.99 ; scale = 5 ; amp = 0.028

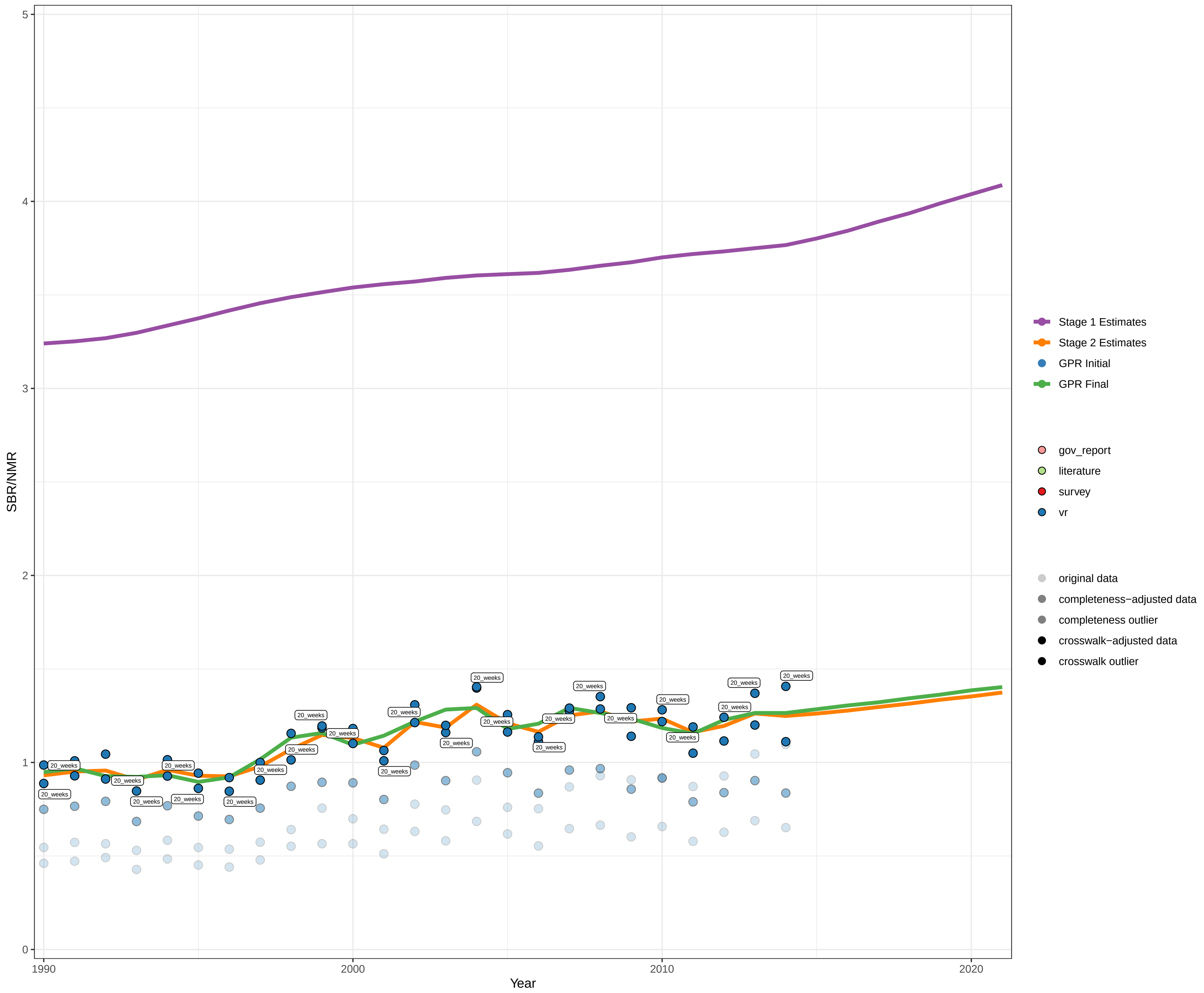

Yucatan MEX_4673 Central Latin America Stillbirth Rate / Neonatal Mortality Rate (SBR/NMR) − 28 weeks
lambda = 0.2 ; zeta = 0.99 ; scale = 5 ; amp = 0.028

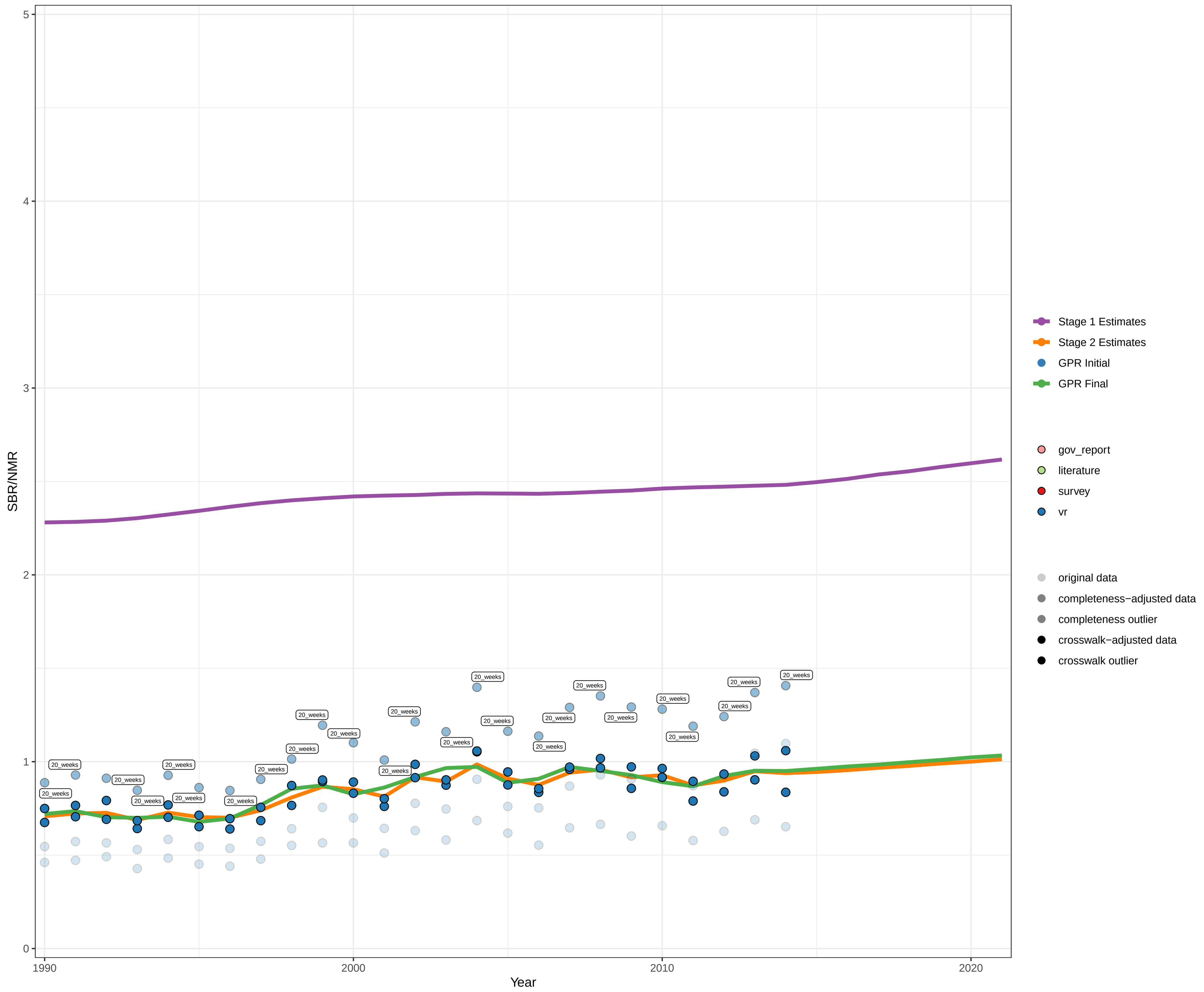

Definition	Years	Outlier	Source.Type	Title
20_weeks, 28_weeks	1990−2014	0	VR	Mexico INEGI Administrative Registers − Fetal Deaths by Municipality of Usual Residence of the Mother and Her Five−year Age Group According to Type of Fetal Death

Yucatan MEX_4673 Central Latin America Stillbirth Rate (SBR) − 20 weeks
lambda = 0.2 ; zeta = 0.99 ; scale = 5 ; amp = 0.028

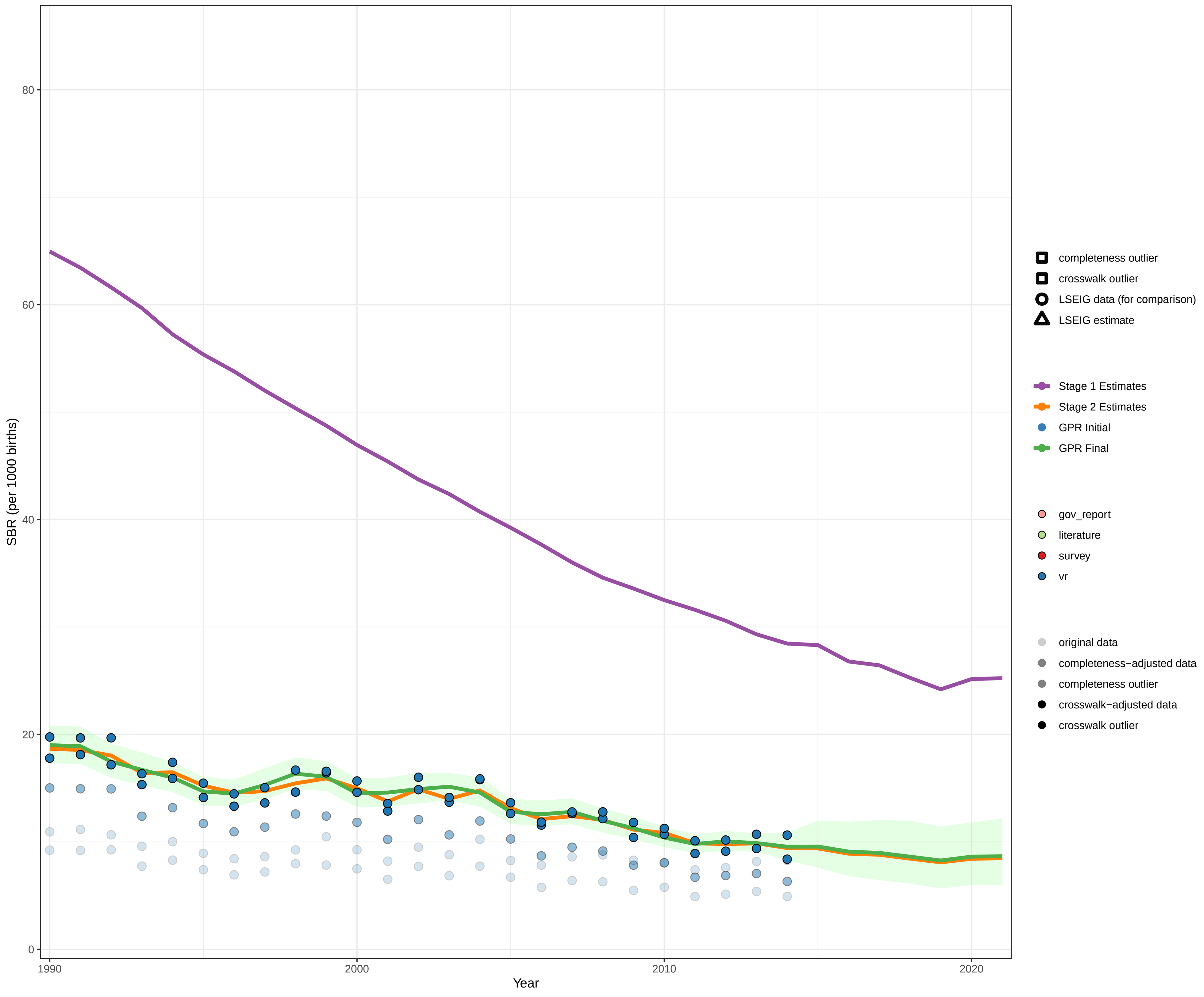

Yucatan MEX_4673 Central Latin America Stillbirth Rate (SBR) − 28 weeks
lambda = 0.2 ; zeta = 0.99 ; scale = 5 ; amp = 0.028

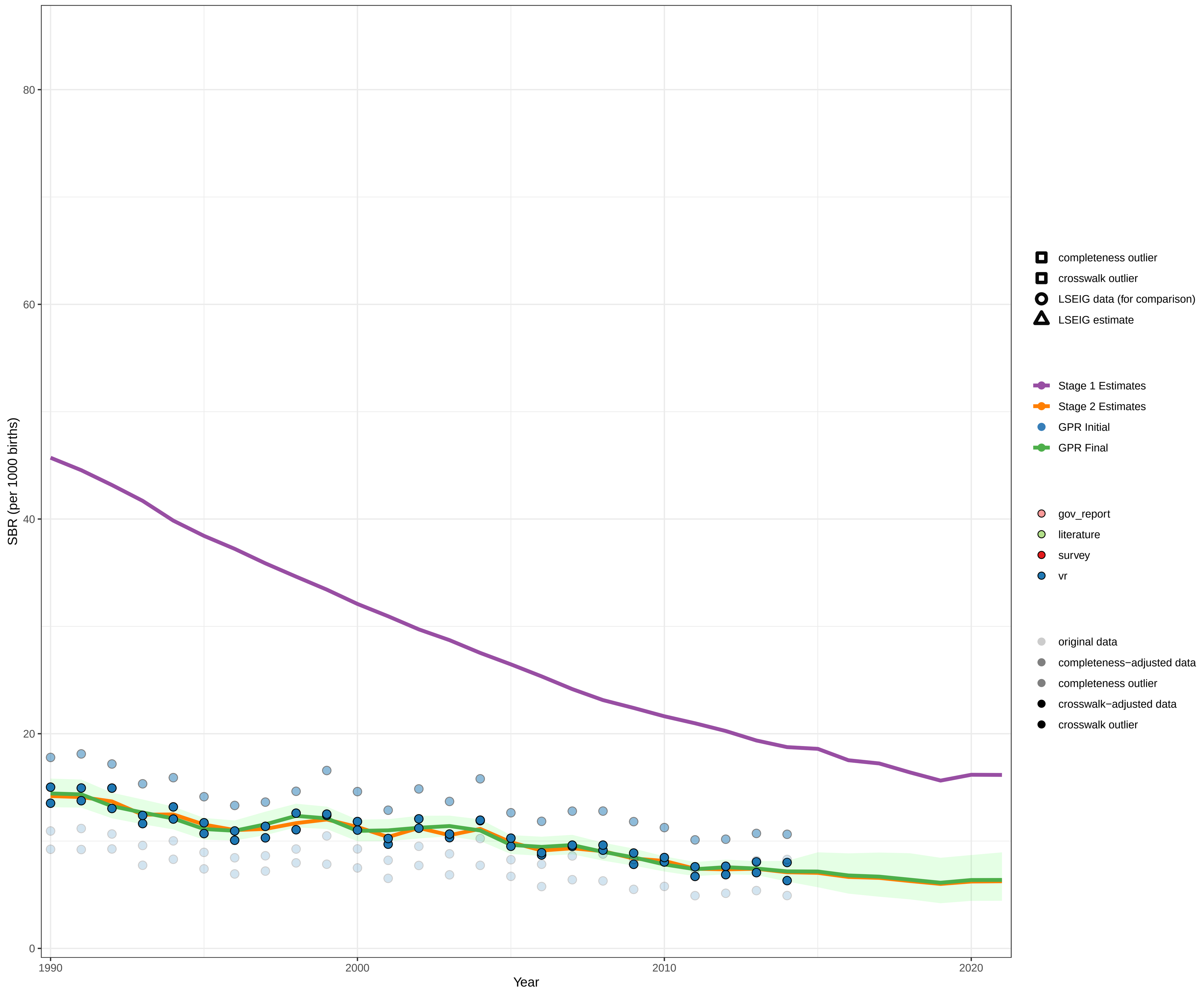

Year	Total Stillbirths (in thousands)	Total Stillbirths (in thousands)	SBR (per 1000 births)	SBR (per 1000 births)	SBR/NMR	SBR/NMR
	20 weeks	28 weeks	20 weeks	28 weeks	20 weeks	28 weeks
1990	0.8 (0.7 to 0.9)	0.6 (0.5 to 0.7)	19.0 (17.3 to 20.8)	14.4 (13.2 to 15.8)	0.95 (0.86 to 1.04)	0.72 (0.66 to 0.79)
1995	0.6 (0.5 to 0.7)	0.5 (0.4 to 0.5)	14.7 (13.4 to 16.1)	11.1 (10.1 to 12.2)	0.90 (0.82 to 0.98)	0.68 (0.62 to 0.74)
2000	0.6 (0.5 to 0.6)	0.4 (0.4 to 0.5)	14.5 (13.2 to 15.9)	10.9 (10.0 to 12.0)	1.09 (1.00 to 1.20)	0.83 (0.75 to 0.90)
2005	0.5 (0.4 to 0.5)	0.4 (0.3 to 0.4)	12.8 (11.7 to 14.0)	9.6 (8.8 to 10.6)	1.18 (1.07 to 1.29)	0.89 (0.81 to 0.97)
2010	0.4 (0.4 to 0.4)	0.3 (0.3 to 0.3)	10.4 (9.5 to 11.4)	7.8 (7.2 to 8.6)	1.18 (1.08 to 1.30)	0.89 (0.82 to 0.98)
2015	0.4 (0.3 to 0.4)	0.3 (0.2 to 0.3)	9.6 (7.6 to 12.0)	7.2 (5.7 to 9.0)	1.28 (1.02 to 1.61)	0.96 (0.76 to 1.20)
2020	0.3 (0.2 to 0.4)	0.2 (0.1 to 0.3)	8.6 (6.0 to 11.8)	6.4 (4.4 to 8.7)	1.39 (0.96 to 1.90)	1.02 (0.71 to 1.40)
2021	0.3 (0.2 to 0.4)	0.2 (0.1 to 0.3)	8.7 (6.0 to 12.2)	6.4 (4.4 to 8.9)	1.40 (0.97 to 1.97)	1.03 (0.72 to 1.45)

Zacatecas MEX_4674 Central Latin America Stillbirth Rate / Neonatal Mortality Rate (SBR/NMR) − 20 weeks
lambda = 0.2 ; zeta = 0.99 ; scale = 5 ; amp = 0.028

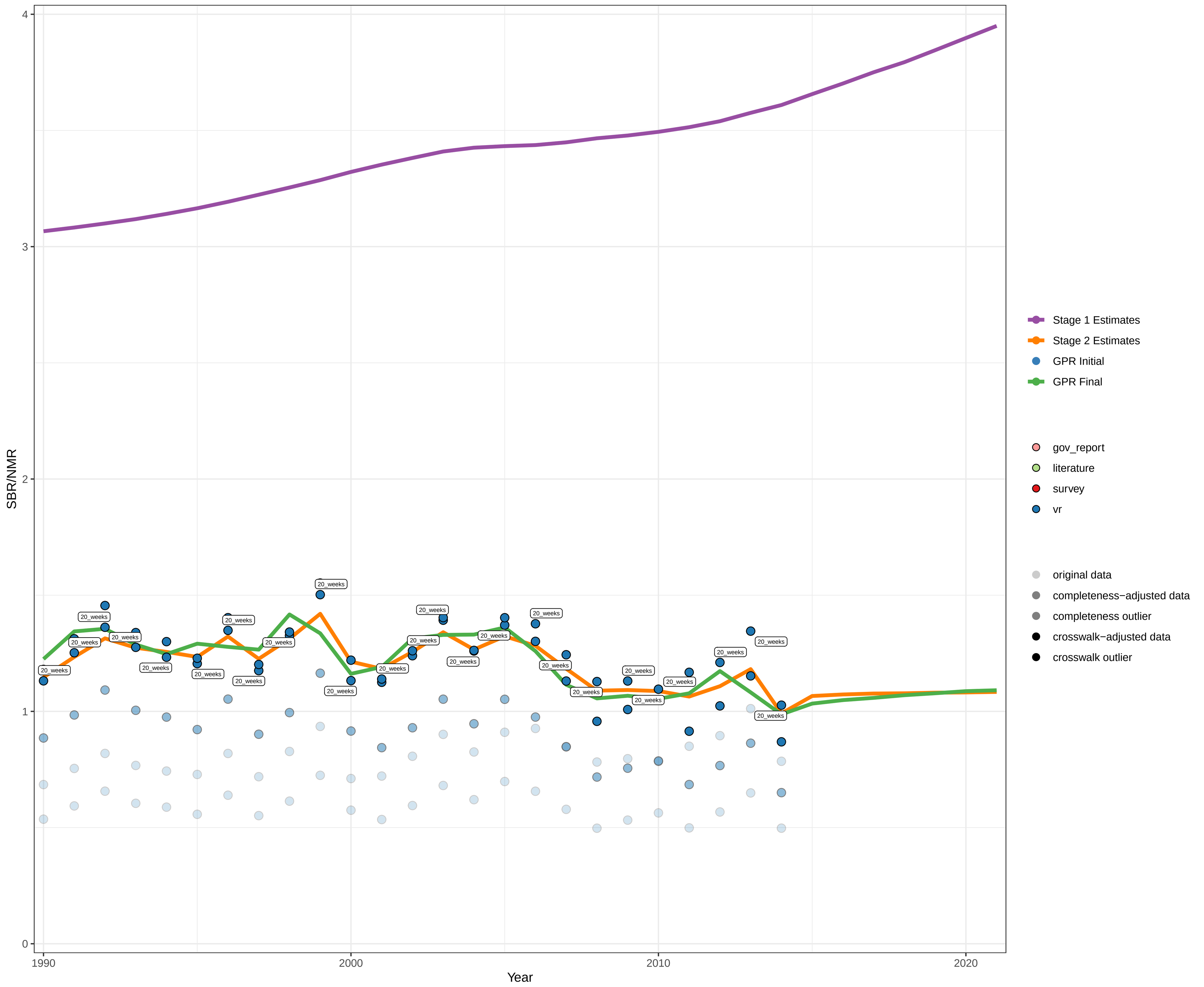

Zacatecas MEX_4674 Central Latin America Stillbirth Rate / Neonatal Mortality Rate (SBR/NMR) − 28 weeks
lambda = 0.2 ; zeta = 0.99 ; scale = 5 ; amp = 0.028

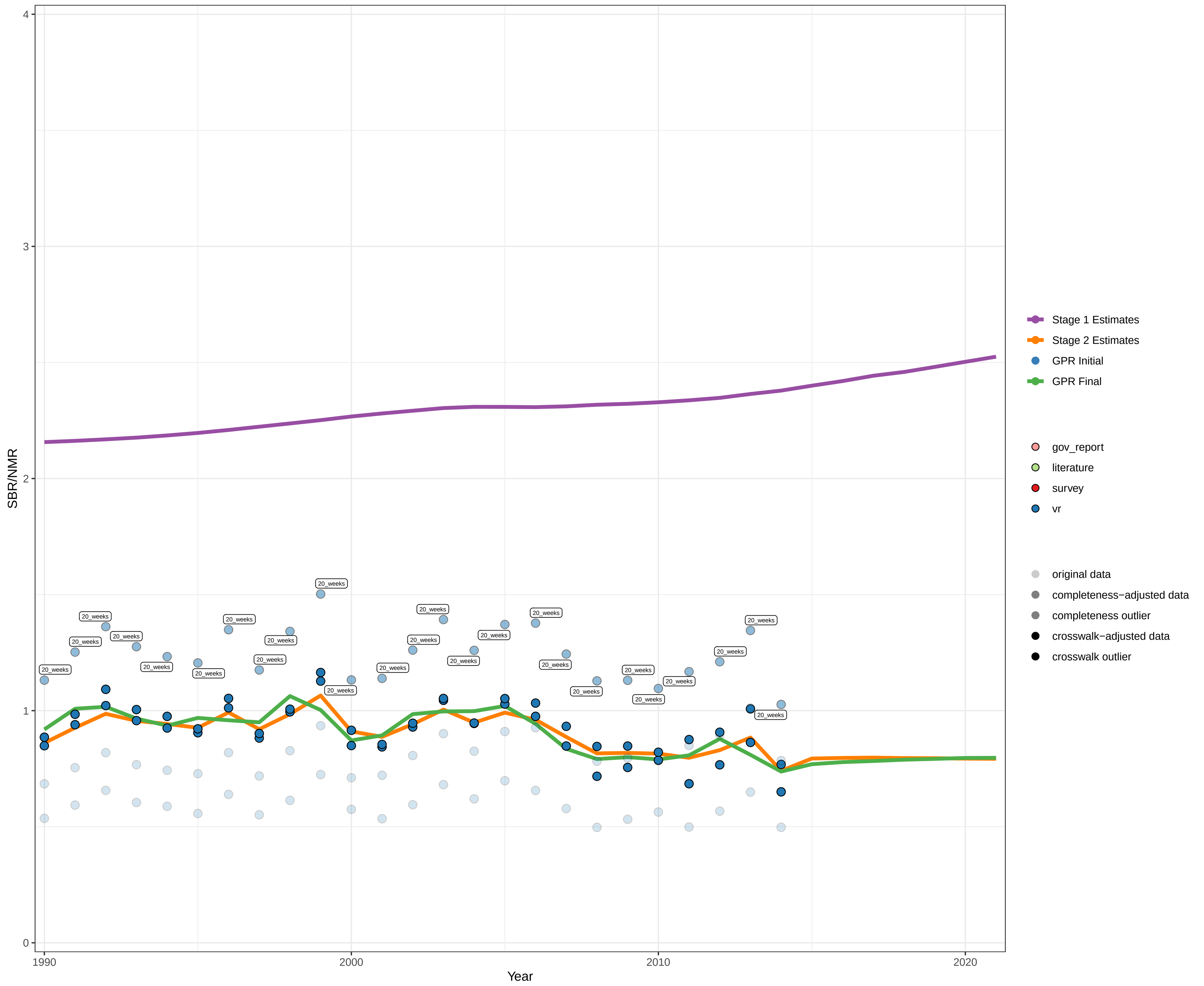

Definition	Years	Outlier	Source.Type	Title
20_weeks, 28_weeks	1990−2014	0	VR	Mexico INEGI Administrative Registers − Fetal Deaths by Municipality of Usual Residence of the Mother and Her Five−year Age Group According to Type of Fetal Death

Zacatecas MEX_4674 Central Latin America Stillbirth Rate (SBR) − 20 weeks
lambda = 0.2 ; zeta = 0.99 ; scale = 5 ; amp = 0.028

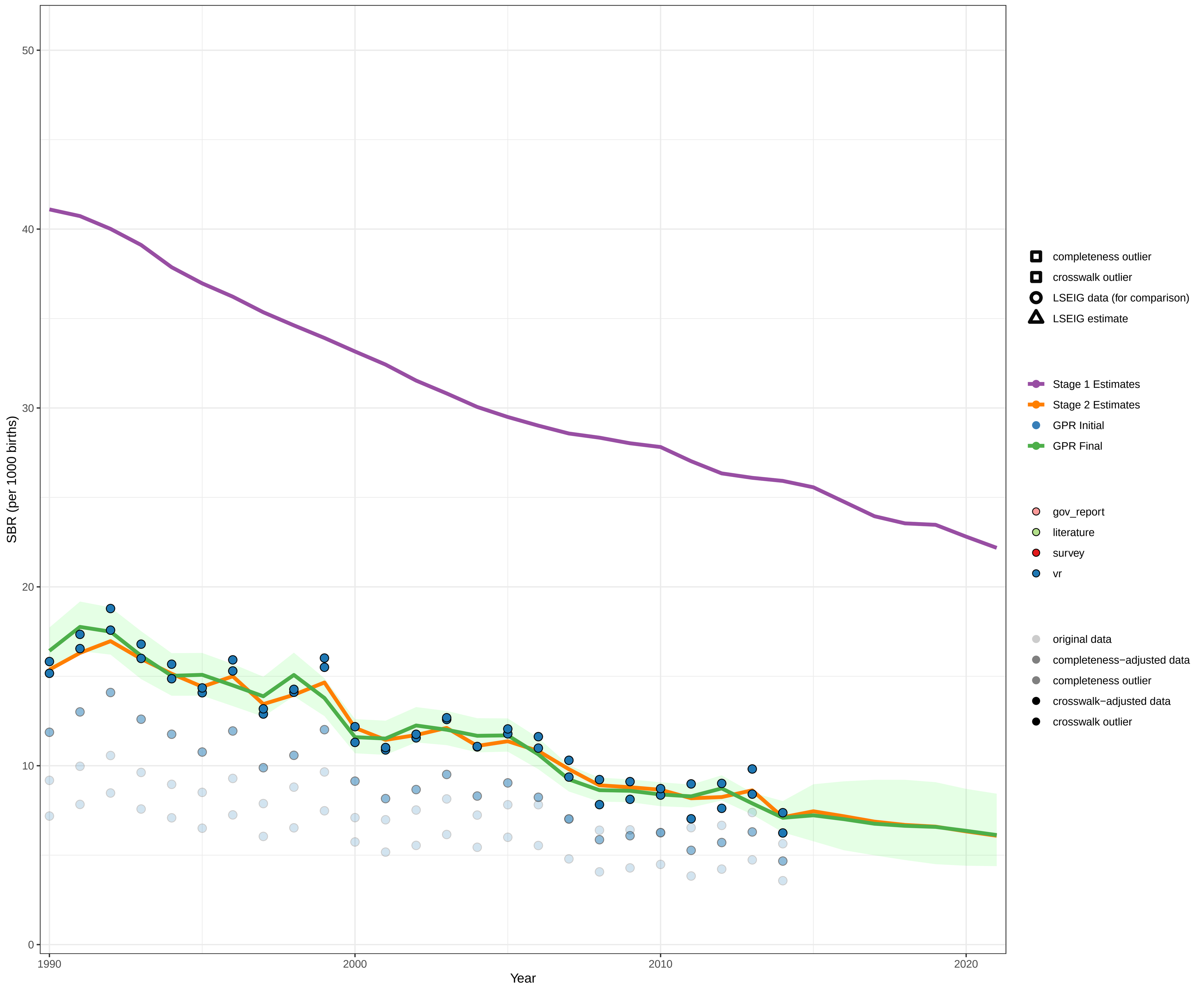

Zacatecas MEX_4674 Central Latin America Stillbirth Rate (SBR) − 28 weeks
lambda = 0.2 ; zeta = 0.99 ; scale = 5 ; amp = 0.028

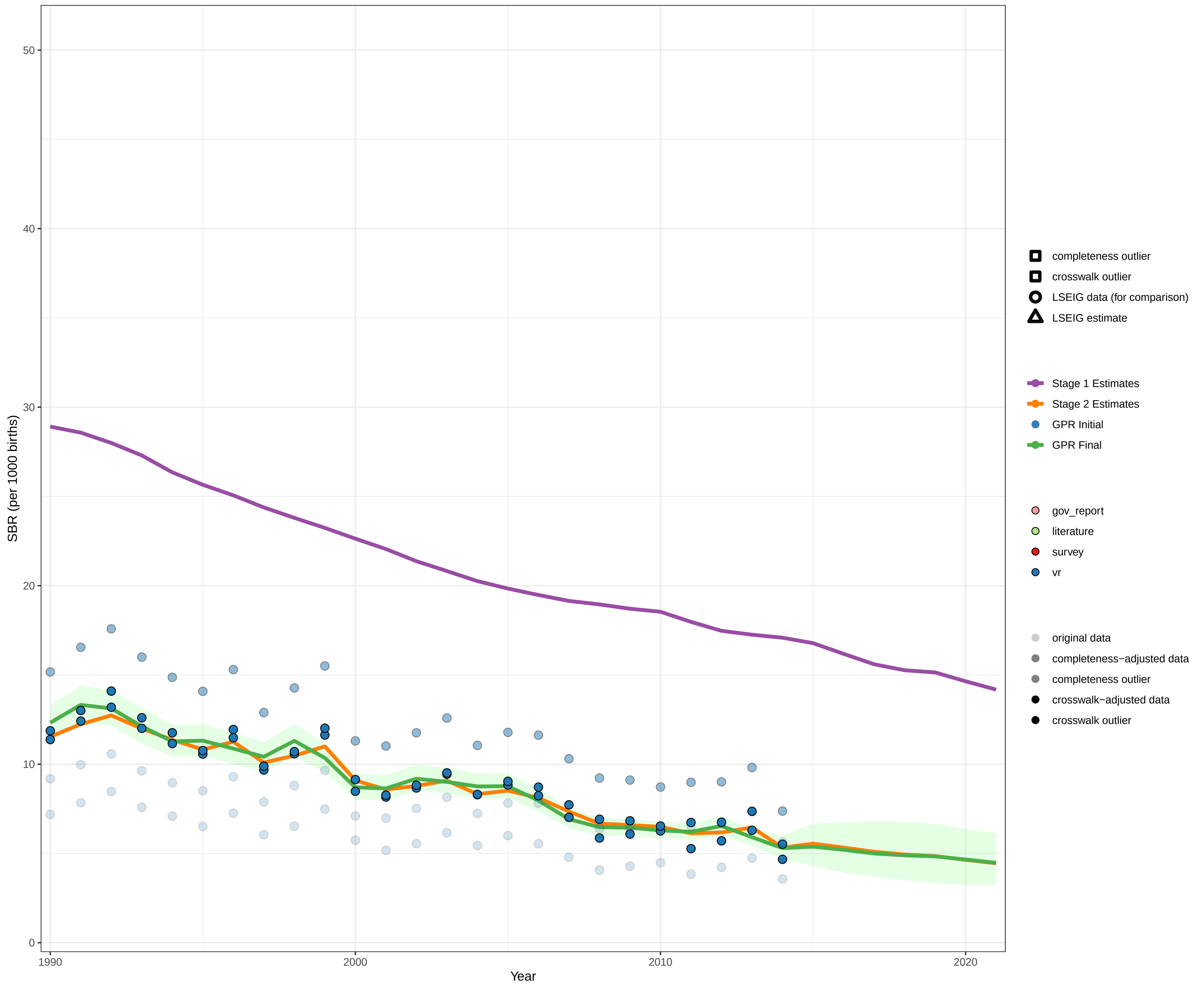

Year	Total Stillbirths (in thousands)	Total Stillbirths (in thousands)	SBR (per 1000 births)	SBR (per 1000 births)	SBR/NMR	SBR/NMR
	20 weeks	28 weeks	20 weeks	28 weeks	20 weeks	28 weeks
1990	0.7 (0.6 to 0.8)	0.5 (0.5 to 0.6)	16.4 (15.2 to 17.7)	12.3 (11.4 to 13.3)	1.23 (1.13 to 1.32)	0.92 (0.85 to 0.99)
1995	0.6 (0.6 to 0.7)	0.5 (0.4 to 0.5)	15.1 (13.9 to 16.3)	11.3 (10.4 to 12.2)	1.29 (1.19 to 1.40)	0.97 (0.89 to 1.05)
2000	0.4 (0.4 to 0.5)	0.3 (0.3 to 0.3)	11.6 (10.7 to 12.6)	8.7 (8.0 to 9.5)	1.16 (1.07 to 1.26)	0.87 (0.80 to 0.95)
2005	0.4 (0.4 to 0.4)	0.3 (0.3 to 0.3)	11.7 (10.8 to 12.6)	8.8 (8.1 to 9.5)	1.36 (1.26 to 1.47)	1.02 (0.94 to 1.10)
2010	0.3 (0.3 to 0.3)	0.2 (0.2 to 0.2)	8.4 (7.7 to 9.1)	6.3 (5.8 to 6.8)	1.05 (0.97 to 1.14)	0.79 (0.73 to 0.85)
2015	0.2 (0.2 to 0.3)	0.2 (0.1 to 0.2)	7.2 (5.8 to 9.0)	5.4 (4.3 to 6.7)	1.03 (0.83 to 1.28)	0.77 (0.62 to 0.95)
2020	0.2 (0.1 to 0.2)	0.1 (0.1 to 0.2)	6.4 (4.4 to 8.7)	4.7 (3.2 to 6.4)	1.09 (0.75 to 1.49)	0.80 (0.55 to 1.09)
2021	0.2 (0.1 to 0.2)	0.1 (0.1 to 0.2)	6.1 (4.4 to 8.4)	4.5 (3.2 to 6.1)	1.09 (0.78 to 1.50)	0.80 (0.57 to 1.09)

Nicaragua NIC Central Latin America Stillbirth Rate / Neonatal Mortality Rate (SBR/NMR) − 20 weeks
lambda = 0.8 ; zeta = 0.7 ; scale = 15 ; amp = 0.028

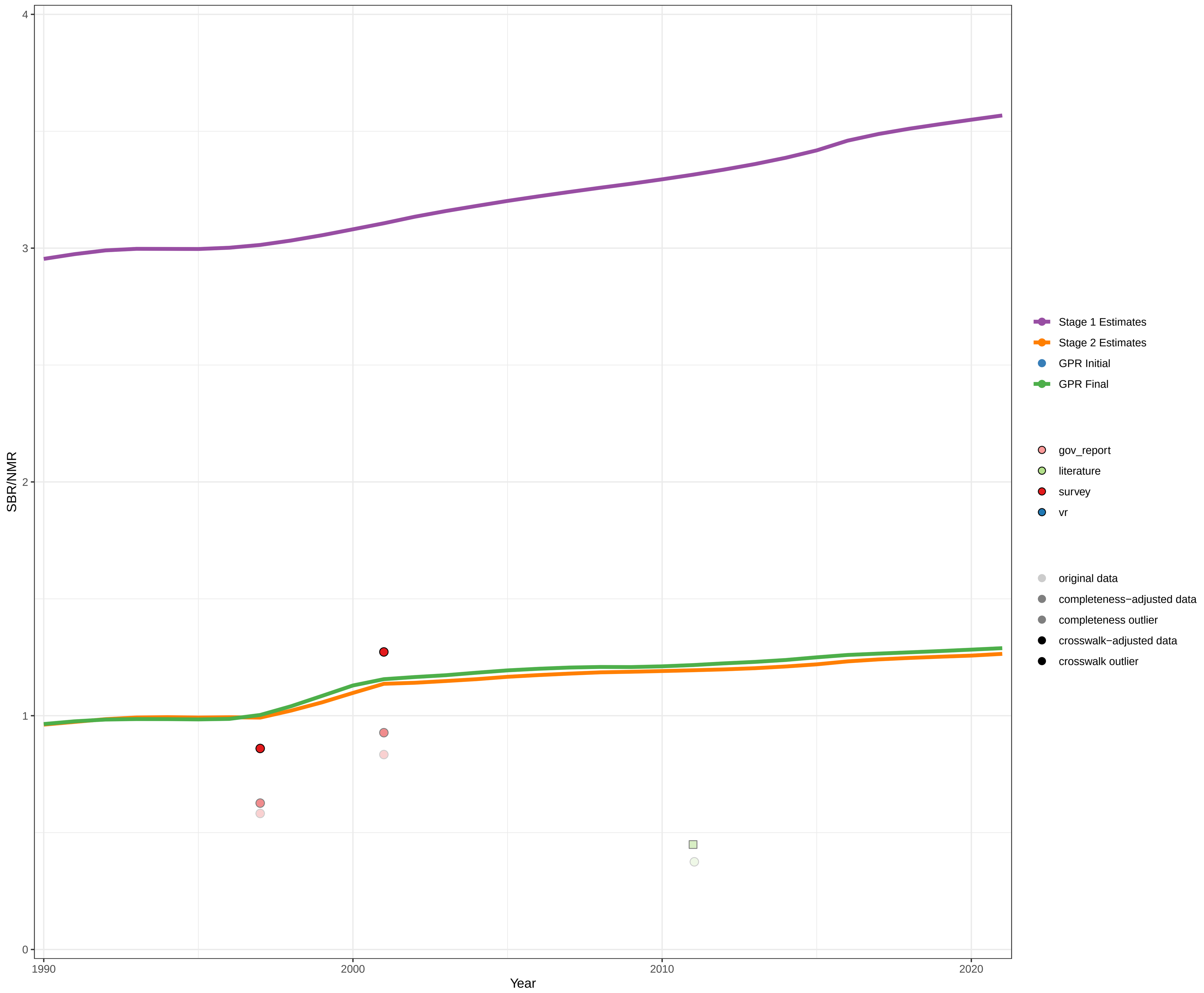

Nicaragua NIC Central Latin America Stillbirth Rate / Neonatal Mortality Rate (SBR/NMR) − 28 weeks
lambda = 0.8 ; zeta = 0.7 ; scale = 15 ; amp = 0.028

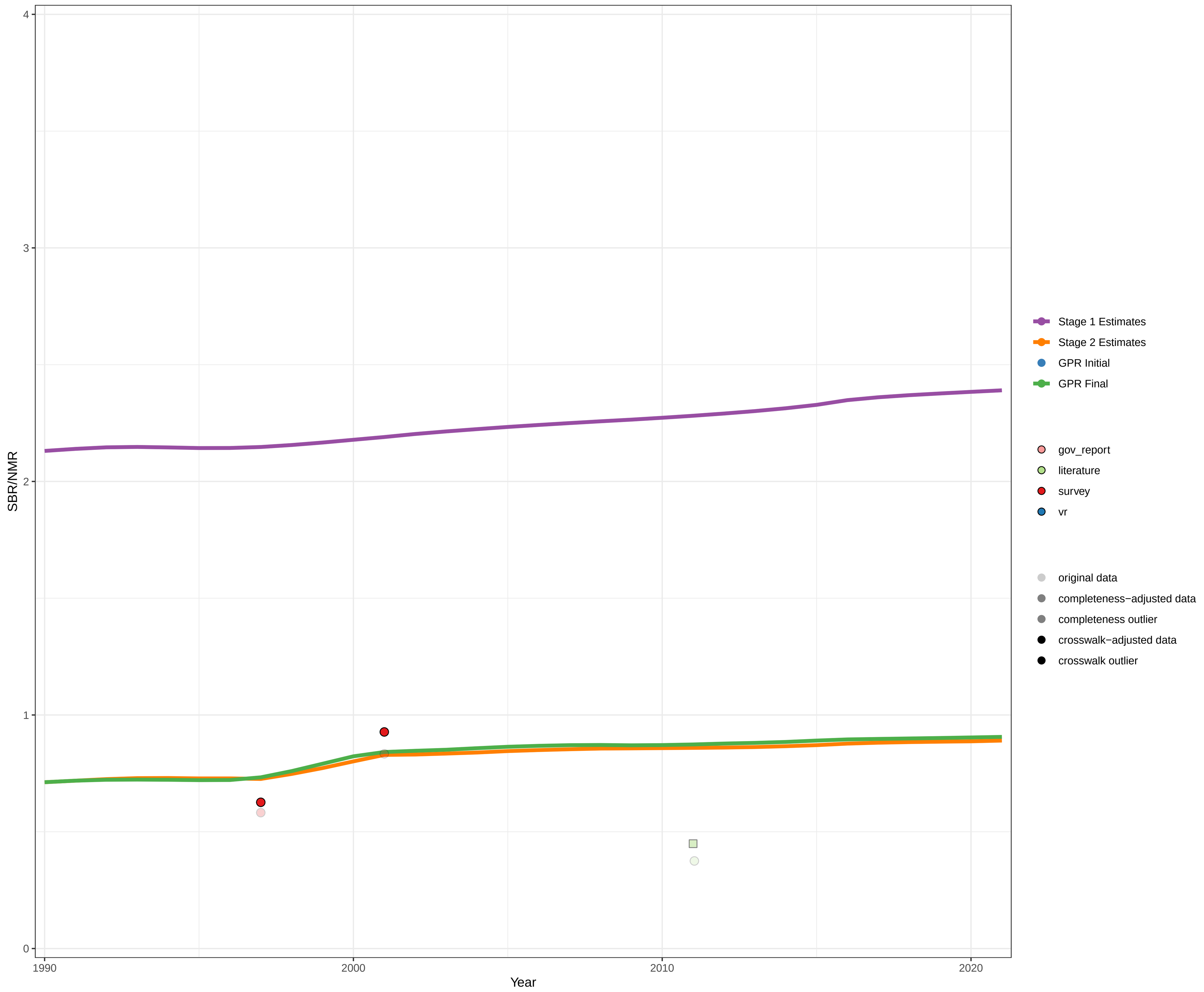

Definition	Years	Outlier	Source.Type	Title
28_weeks	1997	0	Survey	Nicaragua Demographic and Health Survey 1997−1998
28_weeks	2001	0	Survey	Nicaragua Demographic and Health Survey 2001
28_weeks_OR_1000_grams	2011	1	Sci Lit	Maternal complications and perinatal mortality: findings of the World Health Organization Multicountry Survey on Maternal and Newborn Health

Nicaragua NIC Central Latin America Stillbirth Rate (SBR) − 20 weeks
lambda = 0.8 ; zeta = 0.7 ; scale = 15 ; amp = 0.028

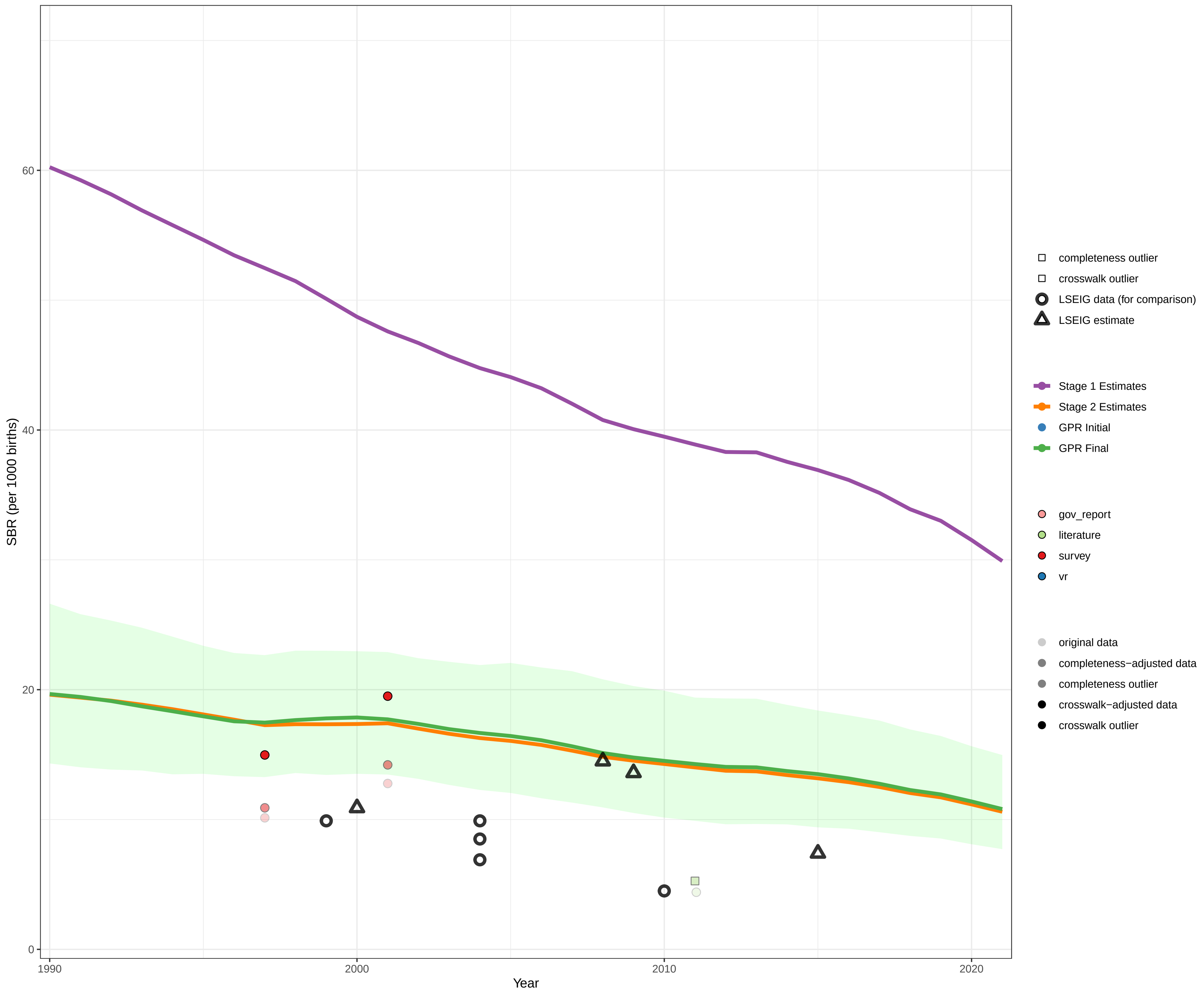

Nicaragua NIC Central Latin America Stillbirth Rate (SBR) − 28 weeks
lambda = 0.8 ; zeta = 0.7 ; scale = 15 ; amp = 0.028

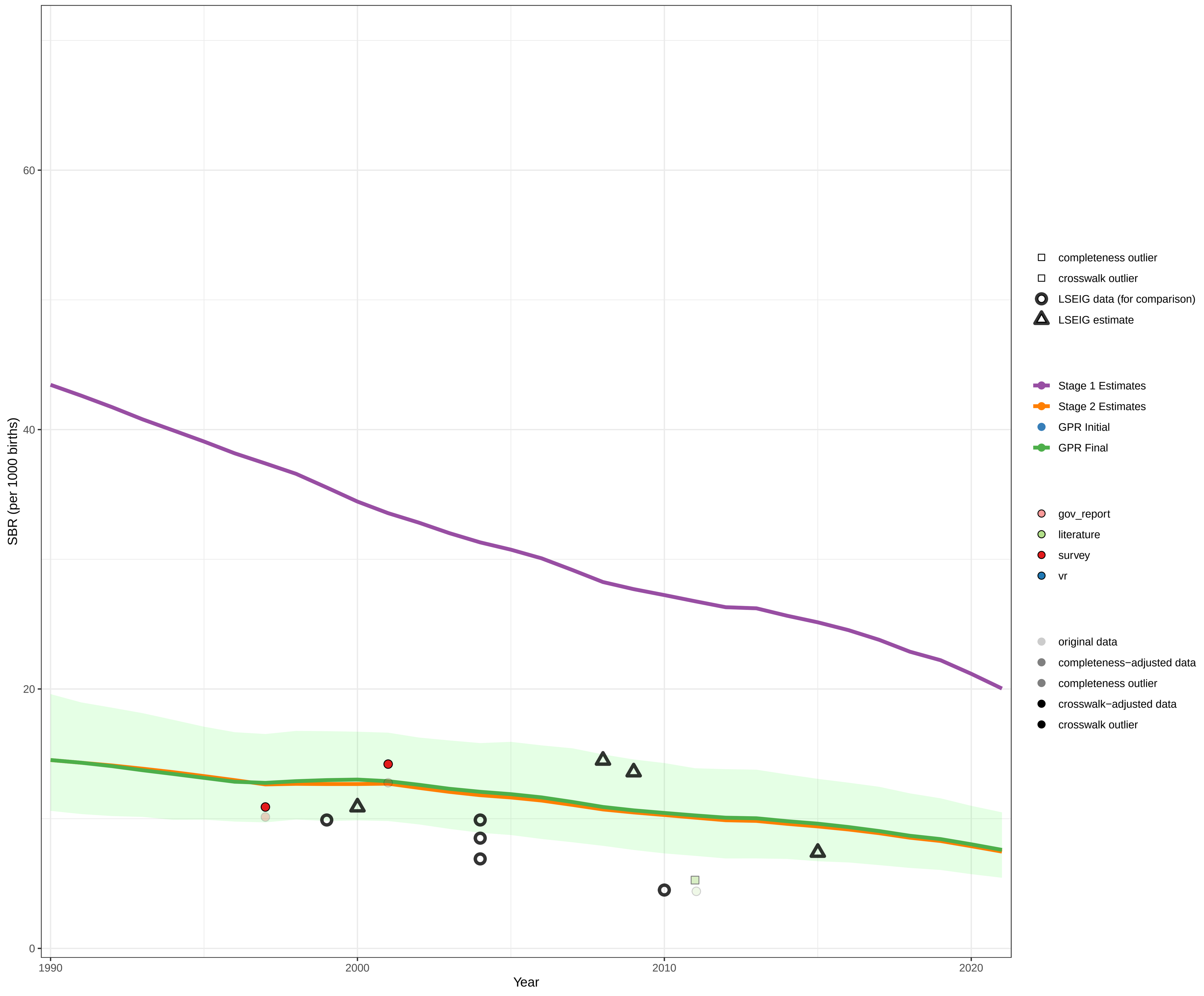

Year	Total Stillbirths (in thousands)	Total Stillbirths (in thousands)	SBR (per 1000 births)	SBR (per 1000 births)	SBR/NMR	SBR/NMR
	20 weeks	28 weeks	20 weeks	28 weeks	20 weeks	28 weeks
1990	2.8 (2.1 to 3.9)	2.1 (1.5 to 2.8)	19.7 (14.3 to 26.6)	14.5 (10.6 to 19.6)	0.96 (0.70 to 1.31)	0.71 (0.52 to 0.96)
1995	2.5 (1.9 to 3.3)	1.9 (1.4 to 2.4)	17.9 (13.5 to 23.4)	13.1 (9.9 to 17.1)	0.98 (0.74 to 1.28)	0.72 (0.54 to 0.94)
2000	2.4 (1.8 to 3.1)	1.7 (1.3 to 2.2)	17.9 (13.5 to 23.0)	13.0 (9.9 to 16.7)	1.13 (0.85 to 1.45)	0.82 (0.62 to 1.06)
2005	2.2 (1.6 to 2.9)	1.6 (1.1 to 2.1)	16.4 (12.0 to 22.1)	11.9 (8.7 to 15.9)	1.19 (0.88 to 1.60)	0.86 (0.63 to 1.16)
2010	2.0 (1.4 to 2.7)	1.4 (1.0 to 1.9)	14.5 (10.1 to 19.9)	10.4 (7.3 to 14.3)	1.21 (0.85 to 1.66)	0.87 (0.61 to 1.19)
2015	1.9 (1.3 to 2.5)	1.3 (0.9 to 1.8)	13.5 (9.4 to 18.4)	9.6 (6.7 to 13.1)	1.25 (0.87 to 1.70)	0.89 (0.62 to 1.21)
2020	1.5 (1.0 to 2.0)	1.0 (0.7 to 1.4)	11.4 (8.1 to 15.6)	8.0 (5.7 to 11.0)	1.28 (0.91 to 1.76)	0.90 (0.64 to 1.24)
2021	1.4 (1.0 to 1.9)	1.0 (0.7 to 1.3)	10.8 (7.7 to 15.0)	7.6 (5.4 to 10.5)	1.29 (0.92 to 1.79)	0.91 (0.65 to 1.25)

Panama PAN Central Latin America Stillbirth Rate / Neonatal Mortality Rate (SBR/NMR) − 20 weeks
lambda = 0.2 ; zeta = 0.99 ; scale = 5 ; amp = 0.028

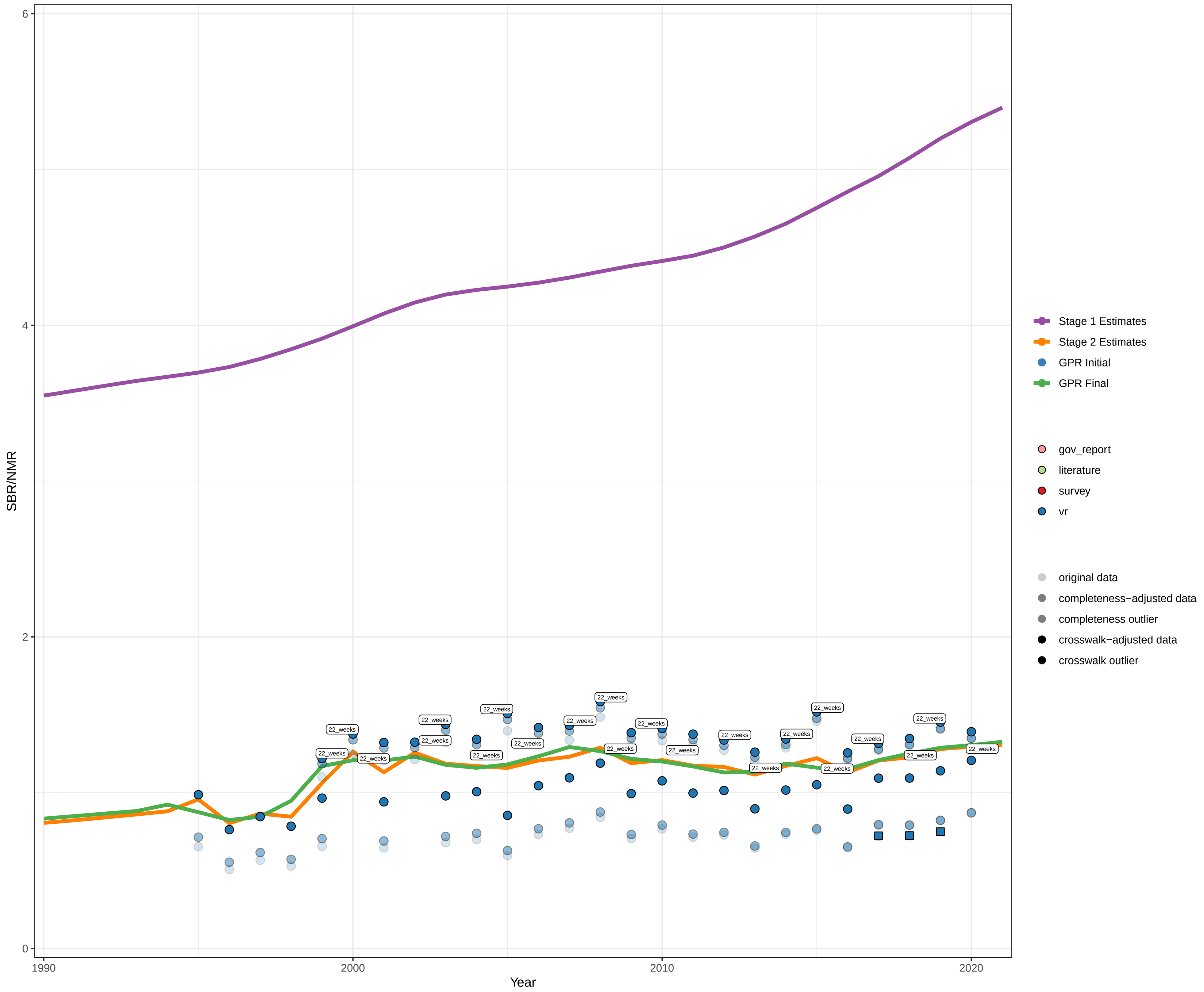

Panama PAN Central Latin America Stillbirth Rate / Neonatal Mortality Rate (SBR/NMR) − 28 weeks
lambda = 0.2 ; zeta = 0.99 ; scale = 5 ; amp = 0.028

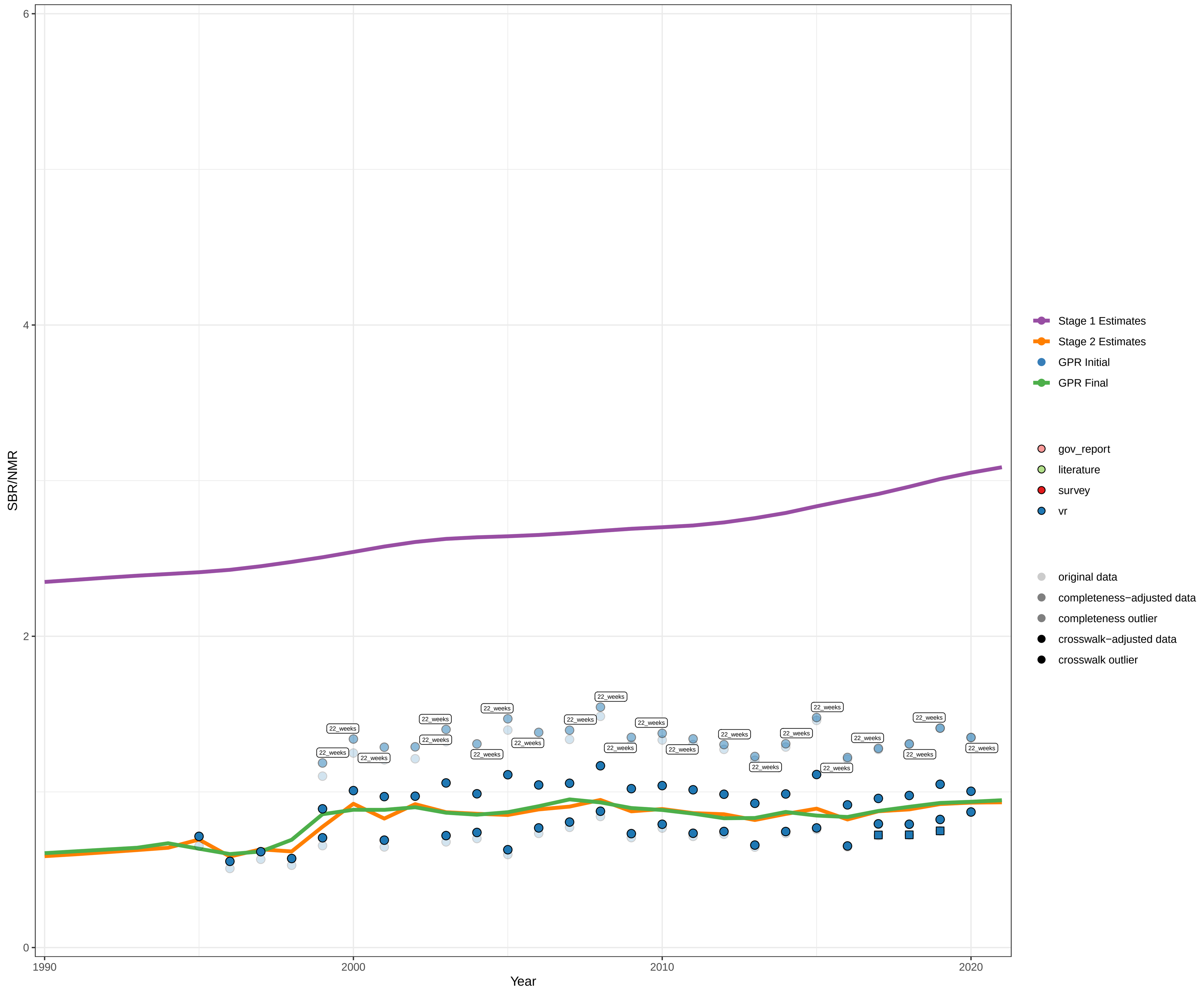

Definition	Years	Outlier	Source.Type	Title
22_weeks	1999−2020	0	VR	Panama Vital Statistics − Live Births and Fetal Deaths
28_weeks	1995−1999, 2001, 2003−2016	0	VR	United Nations Demographic Yearbook
28_weeks	2017−2020	0	VR	Panama Vital Statistics − Live Births and Fetal Deaths
28_weeks	2017−2019	2	VR	United Nations Demographic Yearbook

Panama PAN Central Latin America Stillbirth Rate (SBR) − 20 weeks
lambda = 0.2 ; zeta = 0.99 ; scale = 5 ; amp = 0.028

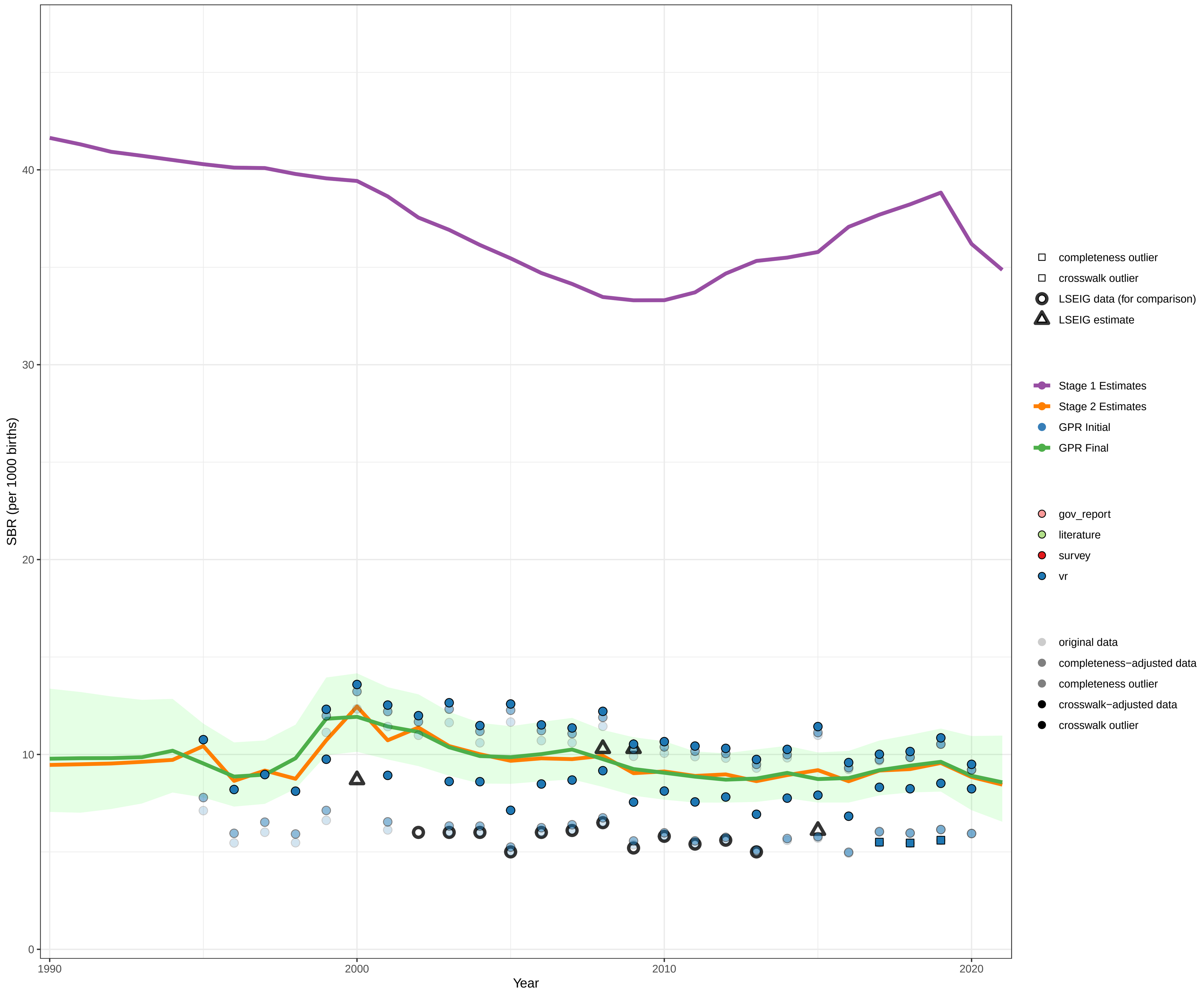

Panama PAN Central Latin America Stillbirth Rate (SBR) − 28 weeks
lambda = 0.2 ; zeta = 0.99 ; scale = 5 ; amp = 0.028

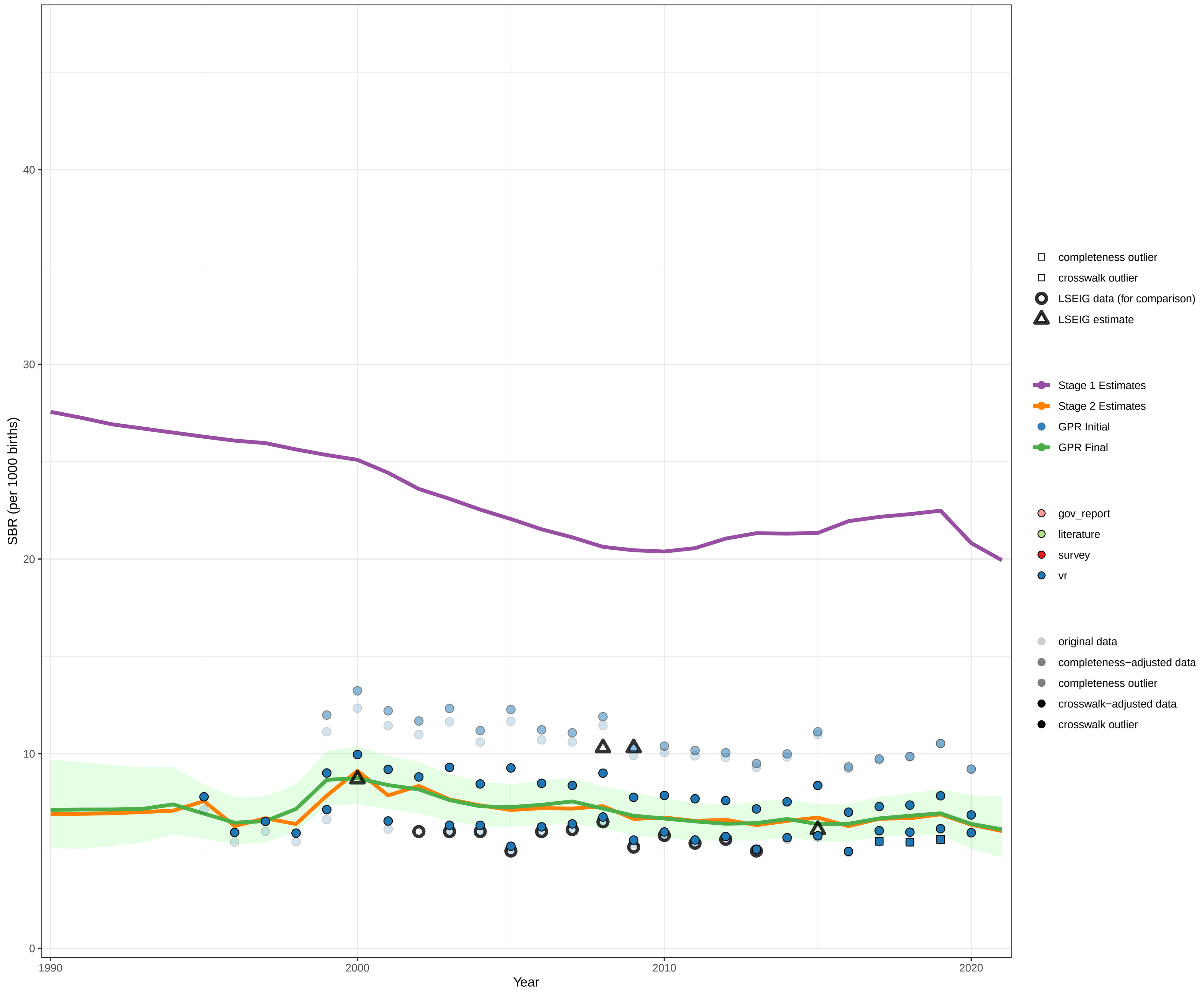

Year	Total Stillbirths (in thousands)	Total Stillbirths (in thousands)	SBR (per 1000 births)	SBR (per 1000 births)	SBR/NMR	SBR/NMR
	20 weeks	28 weeks	20 weeks	28 weeks	20 weeks	28 weeks
1990	0.6 (0.4 to 0.8)	0.4 (0.3 to 0.6)	9.8 (7.1 to 13.4)	7.1 (5.1 to 9.7)	0.83 (0.60 to 1.14)	0.61 (0.44 to 0.83)
1995	0.6 (0.5 to 0.7)	0.4 (0.4 to 0.5)	9.5 (7.8 to 11.6)	6.9 (5.6 to 8.4)	0.88 (0.71 to 1.06)	0.63 (0.52 to 0.77)
2000	0.8 (0.6 to 0.9)	0.6 (0.5 to 0.7)	11.9 (10.1 to 14.2)	8.7 (7.4 to 10.4)	1.21 (1.03 to 1.43)	0.89 (0.75 to 1.05)
2005	0.6 (0.6 to 0.7)	0.5 (0.4 to 0.5)	9.9 (8.5 to 11.5)	7.3 (6.2 to 8.4)	1.18 (1.02 to 1.37)	0.87 (0.75 to 1.01)
2010	0.6 (0.5 to 0.8)	0.5 (0.4 to 0.5)	9.1 (7.7 to 10.7)	6.7 (5.7 to 7.7)	1.20 (1.02 to 1.41)	0.88 (0.75 to 1.02)
2015	0.7 (0.6 to 0.8)	0.5 (0.4 to 0.6)	8.7 (7.5 to 10.1)	6.4 (5.5 to 7.4)	1.16 (1.00 to 1.34)	0.85 (0.73 to 0.98)
2020	0.6 (0.5 to 0.8)	0.5 (0.4 to 0.6)	8.9 (7.1 to 10.9)	6.4 (5.1 to 7.9)	1.31 (1.05 to 1.60)	0.94 (0.75 to 1.15)
2021	0.6 (0.5 to 0.8)	0.4 (0.3 to 0.6)	8.6 (6.5 to 11.0)	6.1 (4.7 to 7.8)	1.33 (1.01 to 1.70)	0.95 (0.72 to 1.21)

Venezuela (Bolivarian Republic of) VEN Central Latin America Stillbirth Rate / Neonatal Mortality Rate (SBR/NMR) − 20 weeks
lambda = 0.6 ; zeta = 0.8 ; scale = 15 ; amp = 0.028

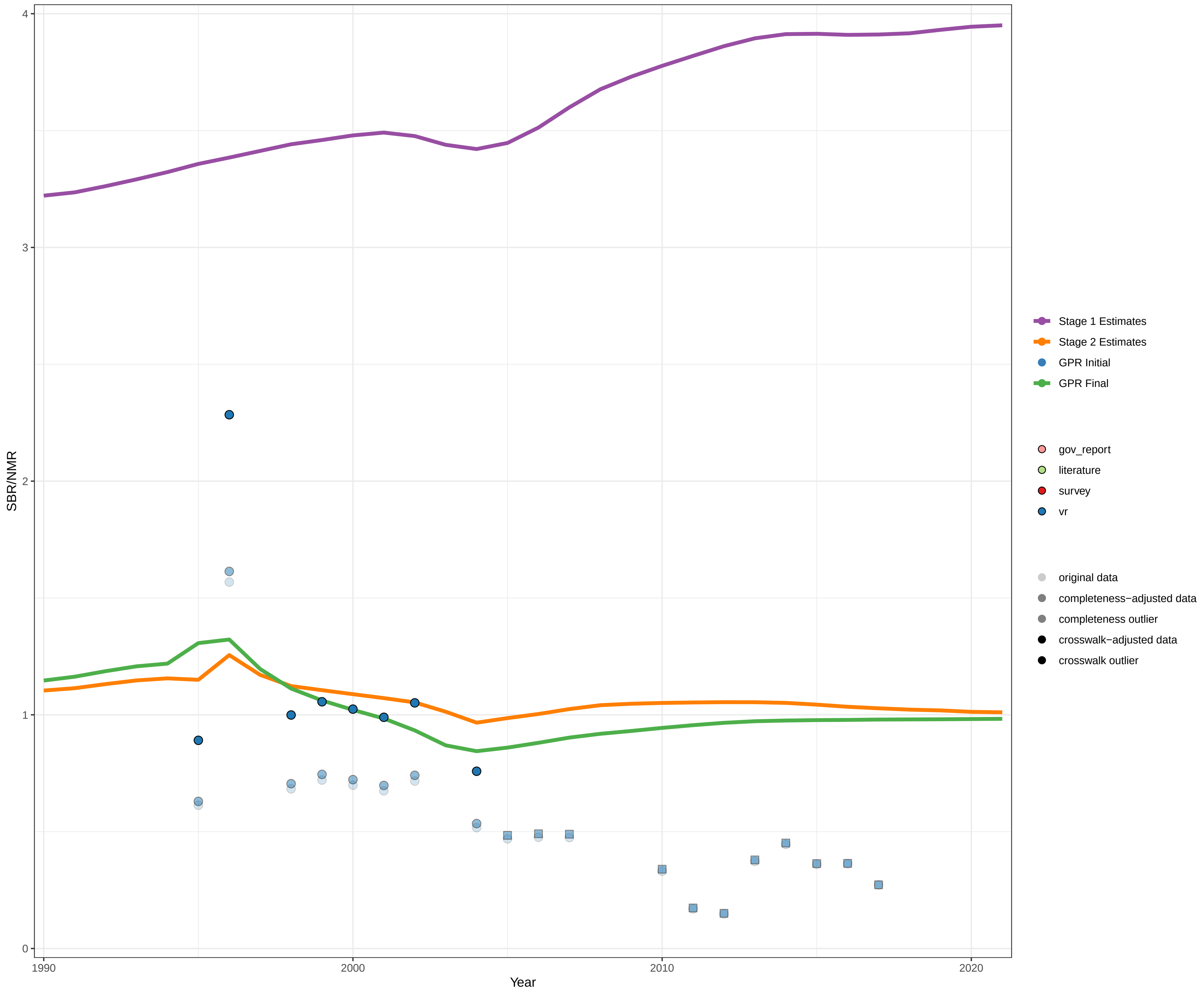

Venezuela (Bolivarian Republic of) VEN Central Latin America Stillbirth Rate / Neonatal Mortality Rate (SBR/NMR) − 28 weeks
lambda = 0.6 ; zeta = 0.8 ; scale = 15 ; amp = 0.028

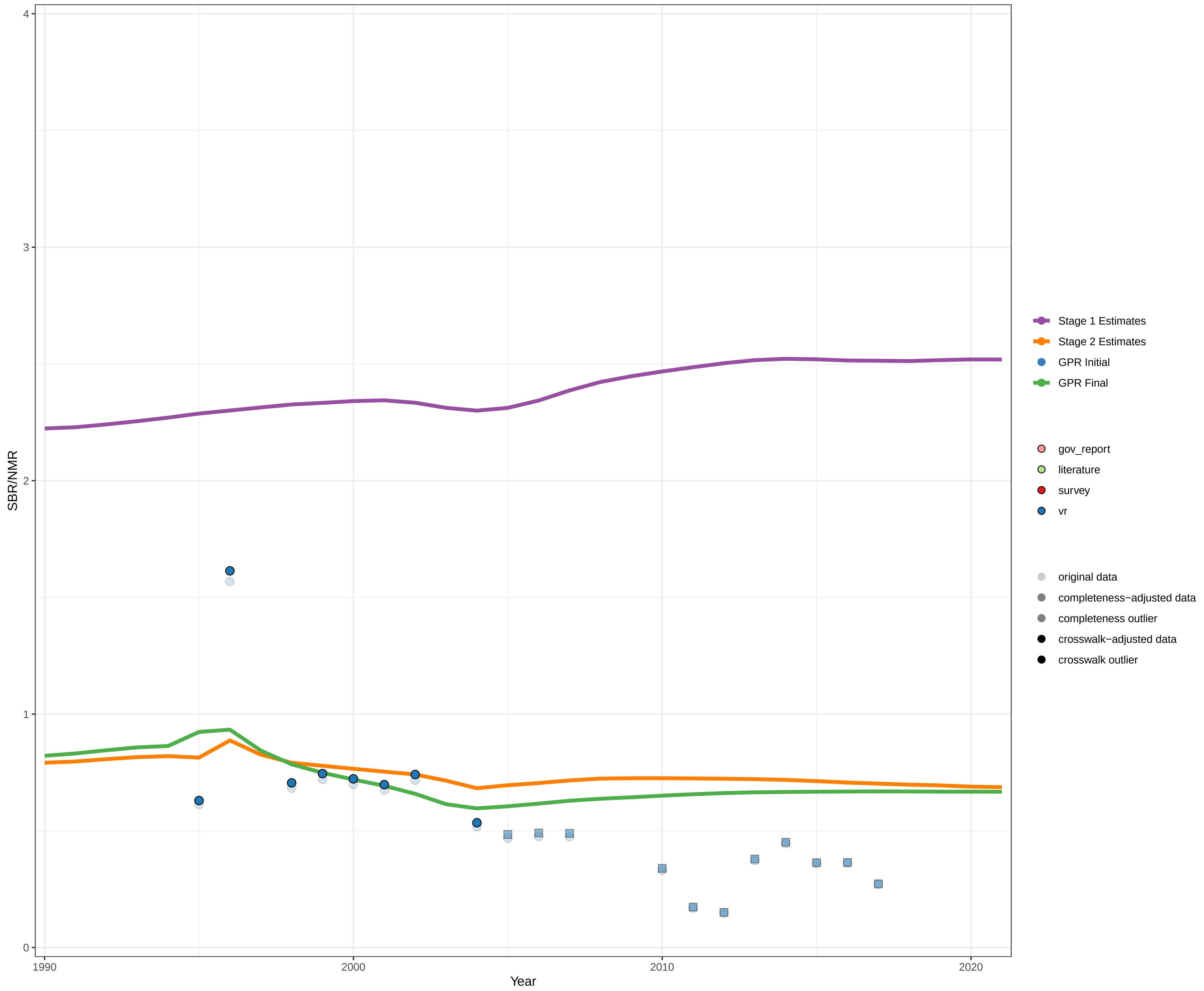

Definition	Years	Outlier	Source.Type	Title
28_weeks	1995, 1996, 1998−2002, 2004	0	VR	United Nations Demographic Yearbook
28_weeks	2005−2007, 2010−2017	1	VR	United Nations Demographic Yearbook

Venezuela (Bolivarian Republic of) VEN Central Latin America Stillbirth Rate (SBR) − 20 weeks
lambda = 0.6 ; zeta = 0.8 ; scale = 15 ; amp = 0.028

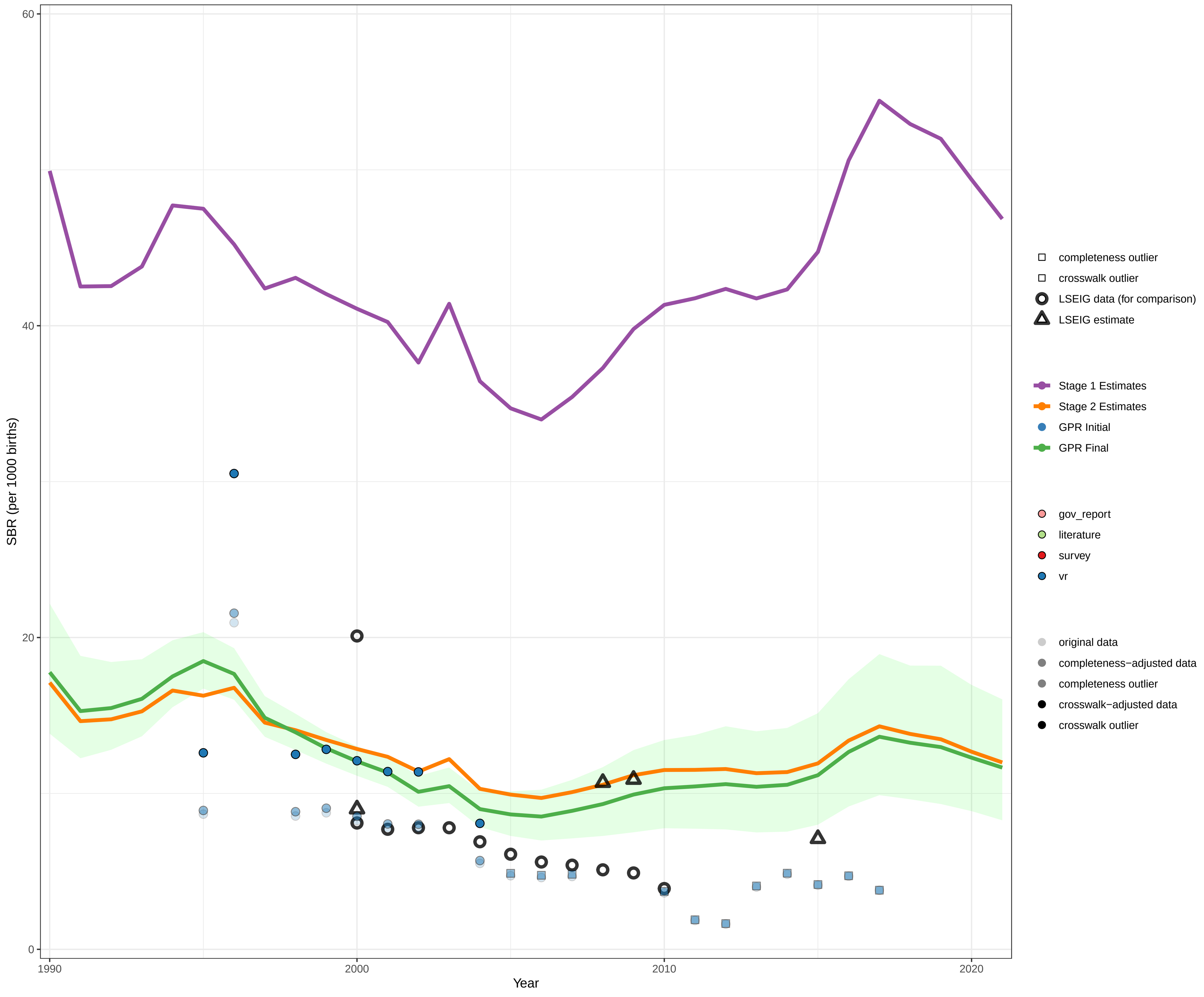

Venezuela (Bolivarian Republic of) VEN Central Latin America Stillbirth Rate (SBR) − 28 weeks
lambda = 0.6 ; zeta = 0.8 ; scale = 15 ; amp = 0.028

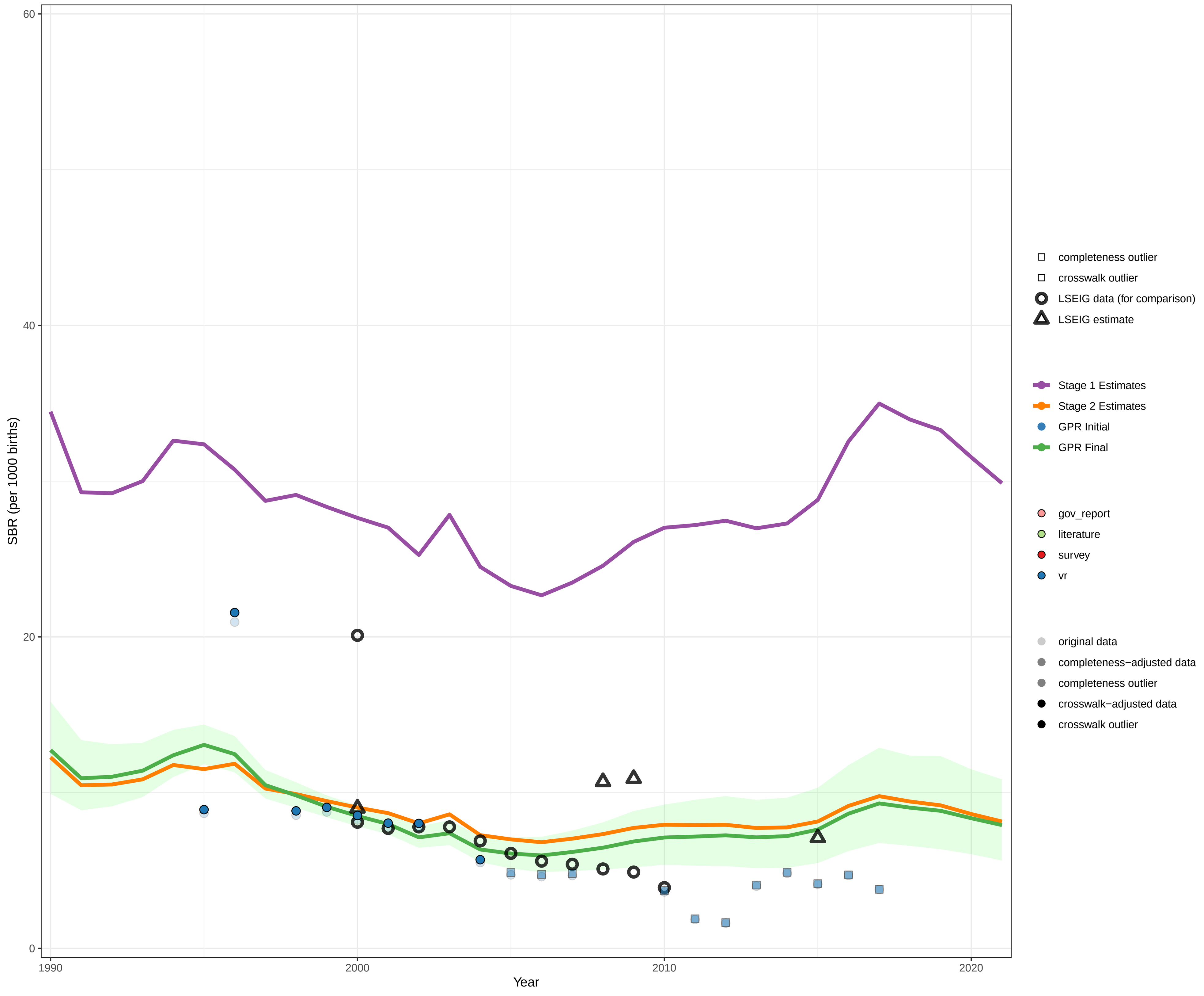

Year	Total Stillbirths (in thousands)	Total Stillbirths (in thousands)	SBR (per 1000 births)	SBR (per 1000 births)	SBR/NMR	SBR/NMR
	20 weeks	28 weeks	20 weeks	28 weeks	20 weeks	28 weeks
1990	9.9 (7.7 to 12.4)	7.1 (5.5 to 8.8)	17.8 (13.8 to 22.2)	12.7 (9.9 to 15.9)	1.15 (0.89 to 1.43)	0.82 (0.64 to 1.02)
1995	9.8 (8.9 to 10.8)	6.9 (6.2 to 7.6)	18.5 (16.7 to 20.3)	13.1 (11.8 to 14.4)	1.31 (1.18 to 1.44)	0.92 (0.84 to 1.02)
2000	6.3 (5.8 to 6.9)	4.5 (4.1 to 4.8)	12.1 (11.1 to 13.0)	8.5 (7.8 to 9.2)	1.02 (0.94 to 1.10)	0.72 (0.66 to 0.78)
2005	5.5 (4.6 to 6.4)	3.9 (3.2 to 4.5)	8.7 (7.3 to 10.1)	6.1 (5.1 to 7.1)	0.86 (0.72 to 1.01)	0.61 (0.51 to 0.71)
2010	6.2 (4.6 to 8.1)	4.3 (3.2 to 5.5)	10.3 (7.8 to 13.4)	7.1 (5.4 to 9.2)	0.94 (0.71 to 1.23)	0.65 (0.49 to 0.84)
2015	6.9 (4.9 to 9.4)	4.7 (3.4 to 6.4)	11.2 (8.0 to 15.1)	7.6 (5.5 to 10.3)	0.98 (0.70 to 1.33)	0.67 (0.48 to 0.90)
2020	5.4 (3.9 to 7.5)	3.7 (2.6 to 5.1)	12.3 (8.9 to 17.0)	8.4 (6.0 to 11.5)	0.98 (0.71 to 1.36)	0.67 (0.48 to 0.92)
2021	4.9 (3.4 to 6.7)	3.3 (2.3 to 4.5)	11.7 (8.3 to 16.0)	7.9 (5.6 to 10.9)	0.98 (0.70 to 1.35)	0.67 (0.48 to 0.92)

Tropical Latin America R14 Tropical Latin America Stillbirth Rate / Neonatal Mortality Rate (SBR/NMR) − 20 weeks
lambda = 1 ; zeta = 0.6 ; scale = 15 ; amp = 0.028

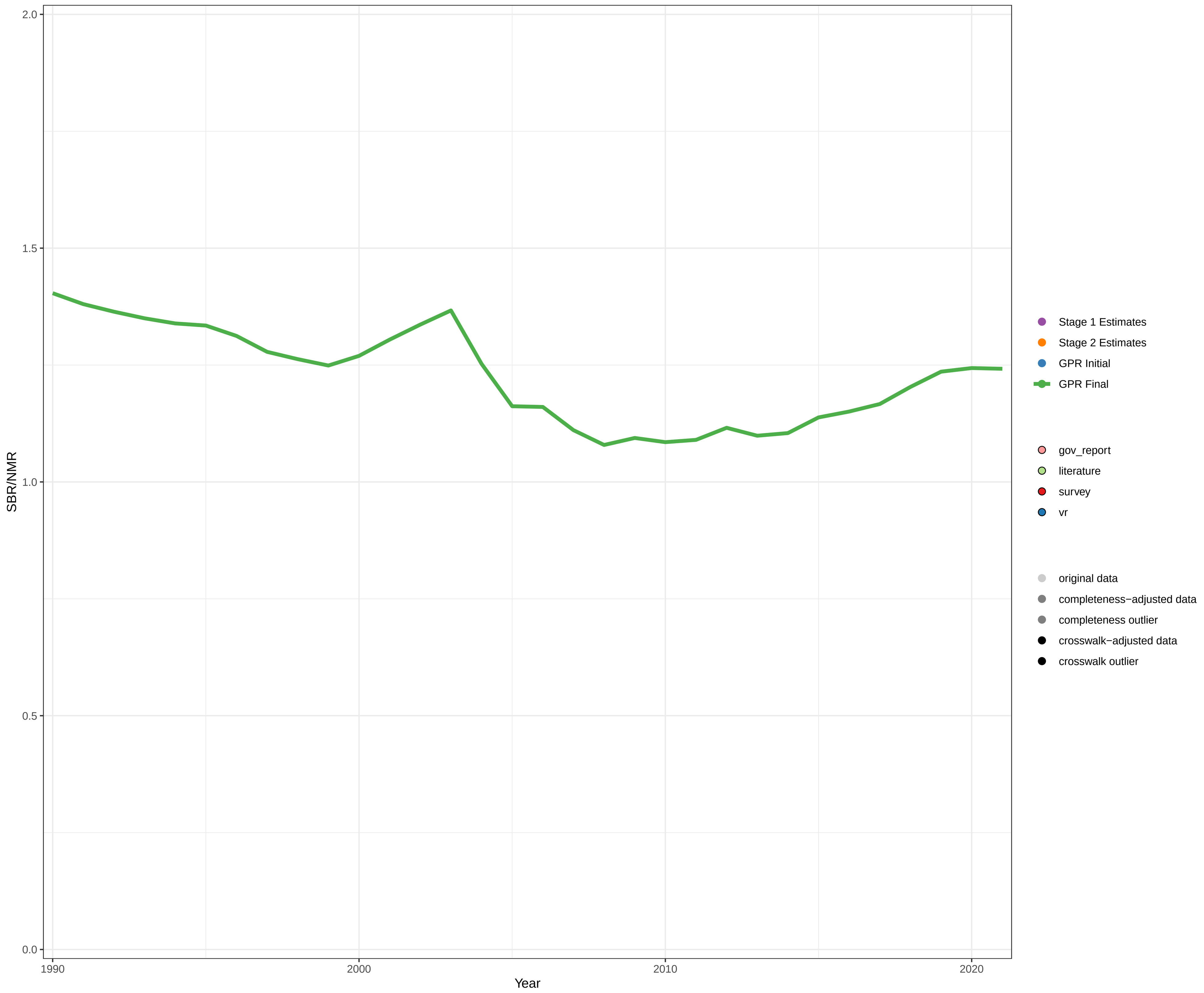

Tropical Latin America R14 Tropical Latin America Stillbirth Rate / Neonatal Mortality Rate (SBR/NMR) − 28 weeks
lambda = 1 ; zeta = 0.6 ; scale = 15 ; amp = 0.028

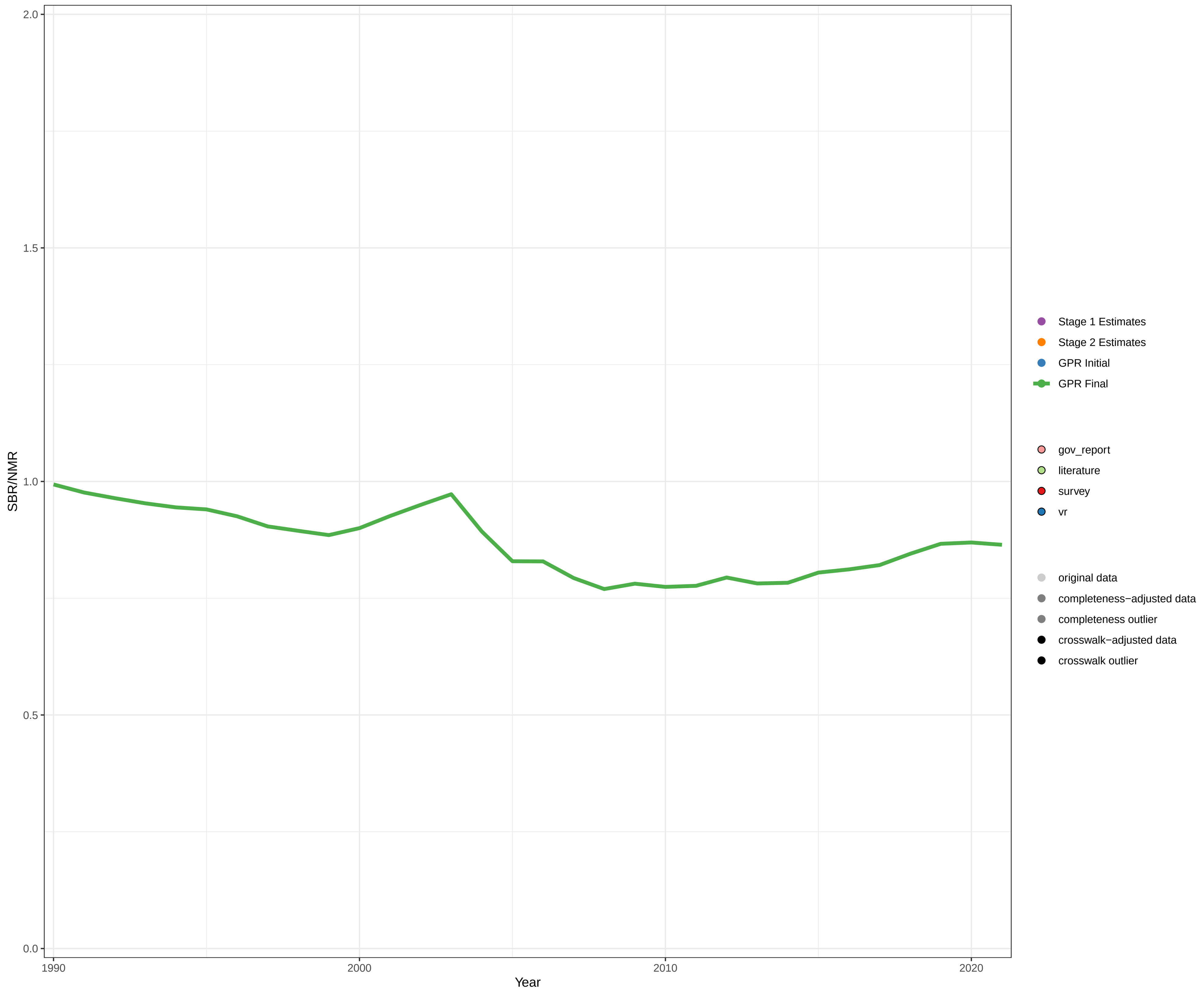

Definition	Years	Outlier	Source.Type	Title

Tropical Latin America R14 Tropical Latin America Stillbirth Rate (SBR) − 20 weeks
lambda = 1 ; zeta = 0.6 ; scale = 15 ; amp = 0.028

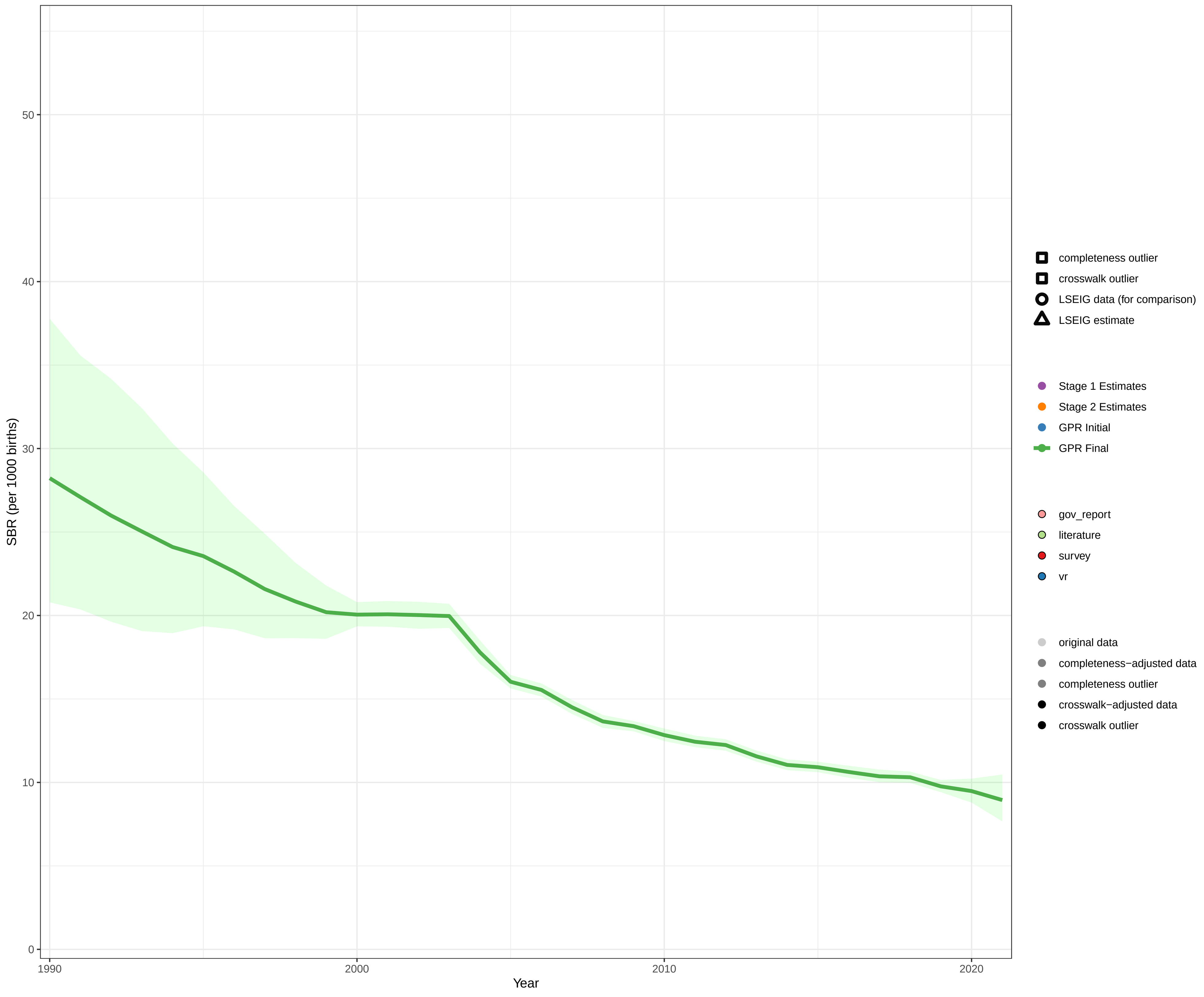

Tropical Latin America R14 Tropical Latin America Stillbirth Rate (SBR) − 28 weeks
lambda = 1 ; zeta = 0.6 ; scale = 15 ; amp = 0.028

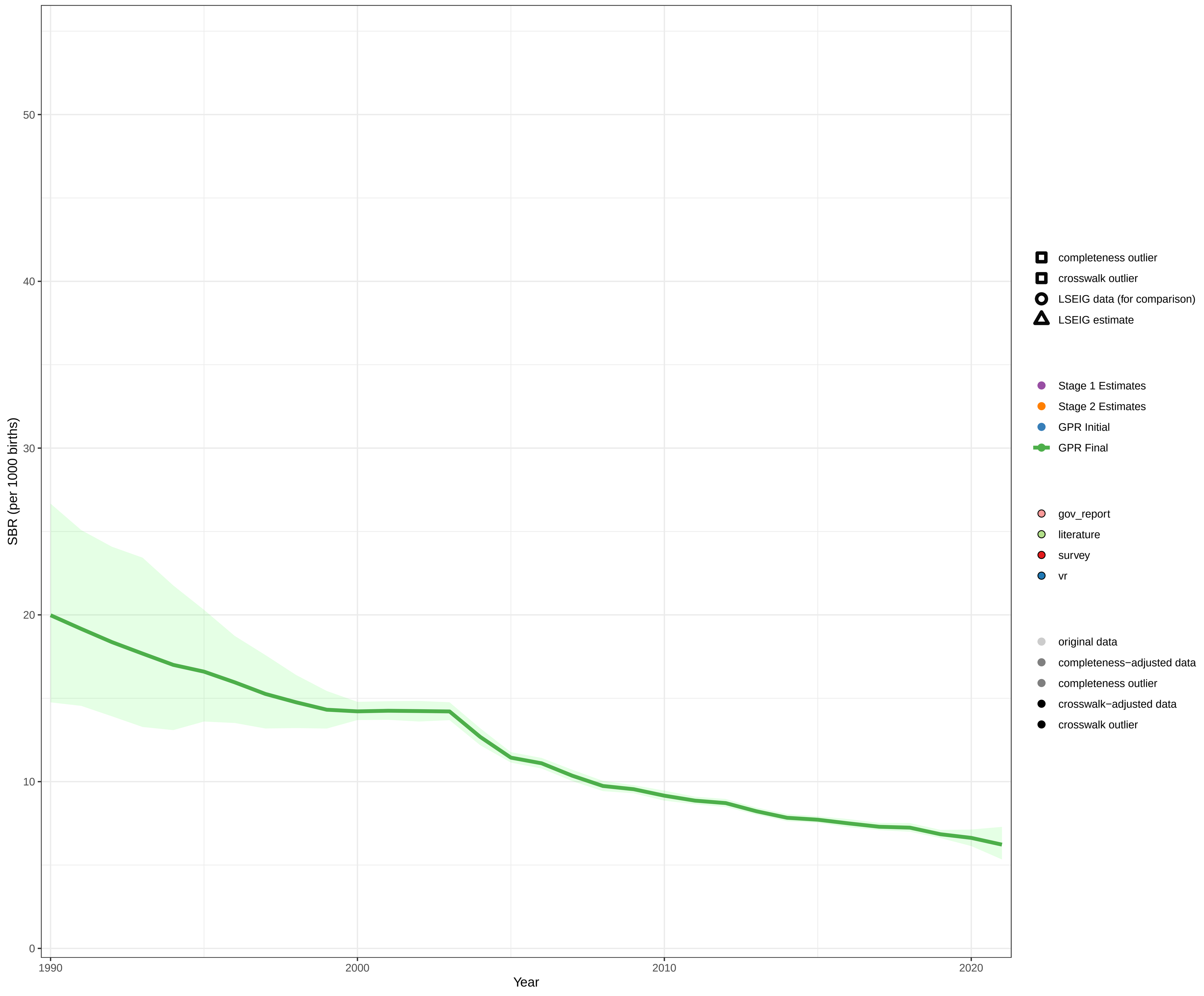

Year	Total Stillbirths (in thousands)	Total Stillbirths (in thousands)	SBR (per 1000 births)	SBR (per 1000 births)	SBR/NMR	SBR/NMR
	20 weeks	28 weeks	20 weeks	28 weeks	20 weeks	28 weeks
1990	96.4 (70.4 to 130.3)	67.7 (49.7 to 90.9)	28.2 (20.8 to 37.8)	20.0 (14.8 to 26.7)	1.40 (1.03 to 1.88)	0.99 (0.73 to 1.33)
1995	84.6 (69.2 to 103.1)	59.2 (48.4 to 72.7)	23.6 (19.4 to 28.6)	16.6 (13.6 to 20.3)	1.33 (1.10 to 1.62)	0.94 (0.77 to 1.15)
2000	75.4 (72.7 to 78.3)	53.1 (51.2 to 55.3)	20.1 (19.4 to 20.8)	14.2 (13.7 to 14.8)	1.27 (1.23 to 1.32)	0.90 (0.87 to 0.94)
2005	58.4 (56.9 to 59.9)	41.5 (40.4 to 42.7)	16.0 (15.6 to 16.4)	11.4 (11.1 to 11.8)	1.16 (1.13 to 1.19)	0.83 (0.81 to 0.85)
2010	46.2 (44.9 to 47.6)	32.8 (31.7 to 33.9)	12.8 (12.5 to 13.2)	9.2 (8.9 to 9.5)	1.09 (1.05 to 1.12)	0.77 (0.75 to 0.80)
2015	40.1 (39.0 to 41.3)	28.3 (27.6 to 29.0)	10.9 (10.6 to 11.2)	7.7 (7.5 to 7.9)	1.14 (1.11 to 1.17)	0.80 (0.79 to 0.83)
2020	33.6 (31.1 to 36.3)	23.4 (21.7 to 25.2)	9.5 (8.8 to 10.2)	6.6 (6.1 to 7.1)	1.24 (1.15 to 1.34)	0.87 (0.80 to 0.93)
2021	31.2 (26.7 to 36.6)	21.7 (18.6 to 25.4)	8.9 (7.7 to 10.5)	6.2 (5.3 to 7.3)	1.24 (1.06 to 1.45)	0.86 (0.74 to 1.01)

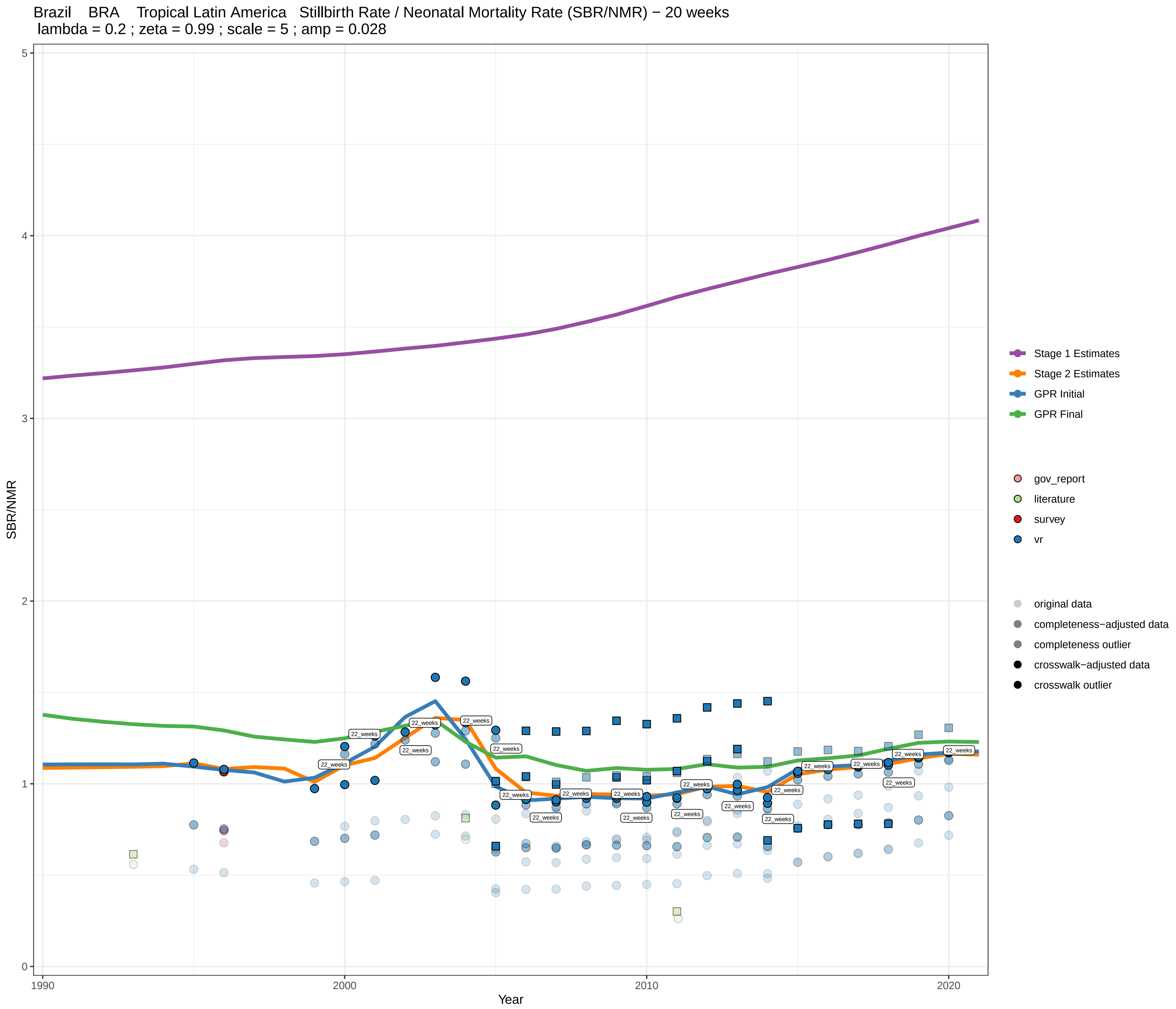

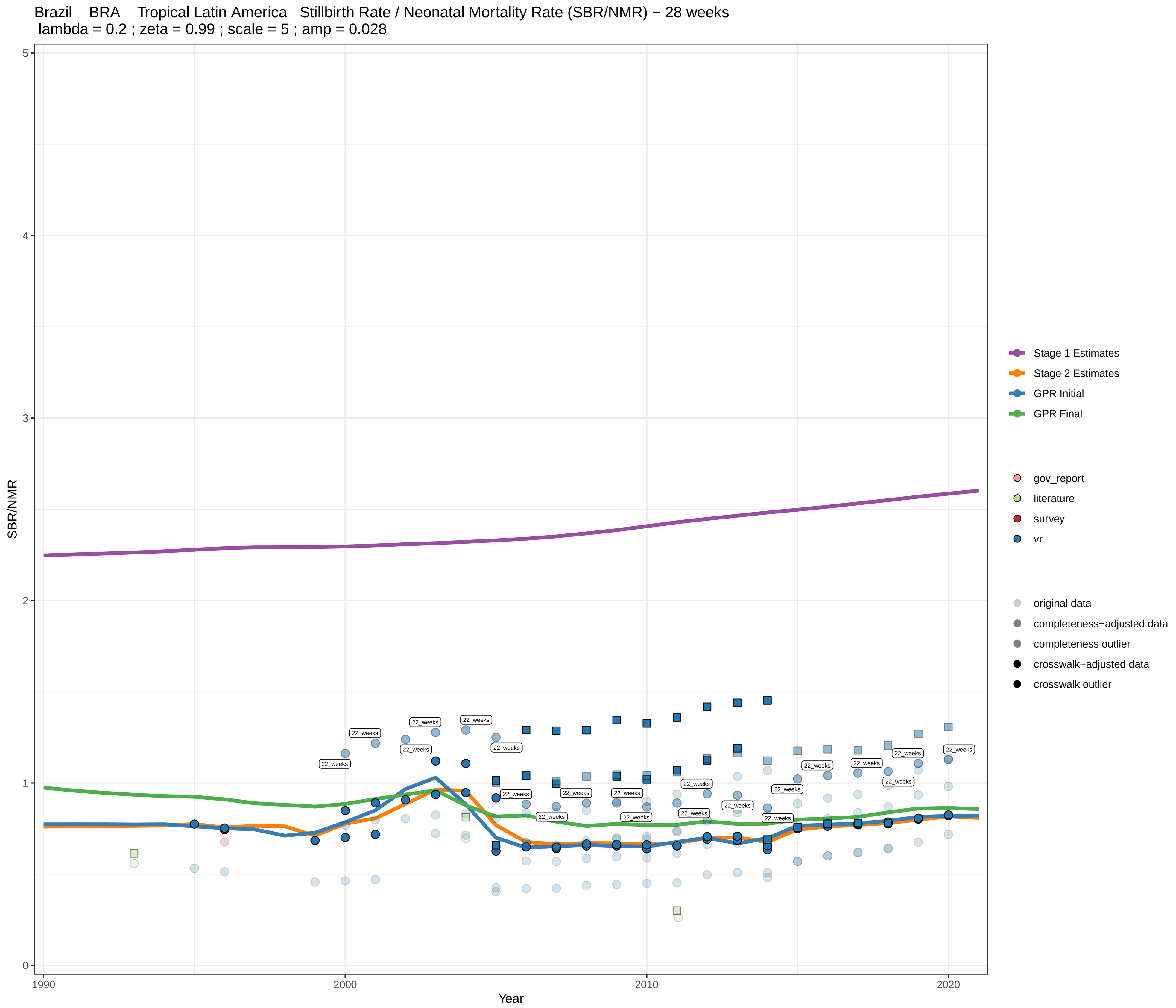

Definition	Years	Outlier	Source.Type	Title
22_weeks	2000−2014	0	VR	Brazil Mortality Information System − Stillbirths
22_weeks, 28_weeks	2005−2020	0	VR	Brazil Civil Registry Statistics − Fetal Deaths at 28 Weeks or More, by Place of Birth, Number of Births by Birth and Sex, and Residence of Mother
28_weeks	1995, 1996, 1999−2001, 2003, 2004, 2008	0	VR	United Nations Demographic Yearbook
28_weeks	1996	0	Survey	Brazil Demographic and Health Survey 1996
28_weeks	1993, 2004	1	Sci Lit	The challenge of reducing neonatal mortality in middle−income countries: findings from three Brazilian birth cohorts in 1982, 1993, and 2004
28_weeks_OR_1000_grams	2011	1	Sci Lit	Maternal complications and perinatal mortality: findings of the World Health Organization Multicountry Survey on Maternal and Newborn Health
other	2005−2020	1	VR	Brazil Civil Registry Statistics − Fetal Deaths at 28 Weeks or More, by Place of Birth, Number of Births by Birth and Sex, and Residence of Mother
28_weeks	2005−2007, 2009−2018	2	VR	United Nations Demographic Yearbook

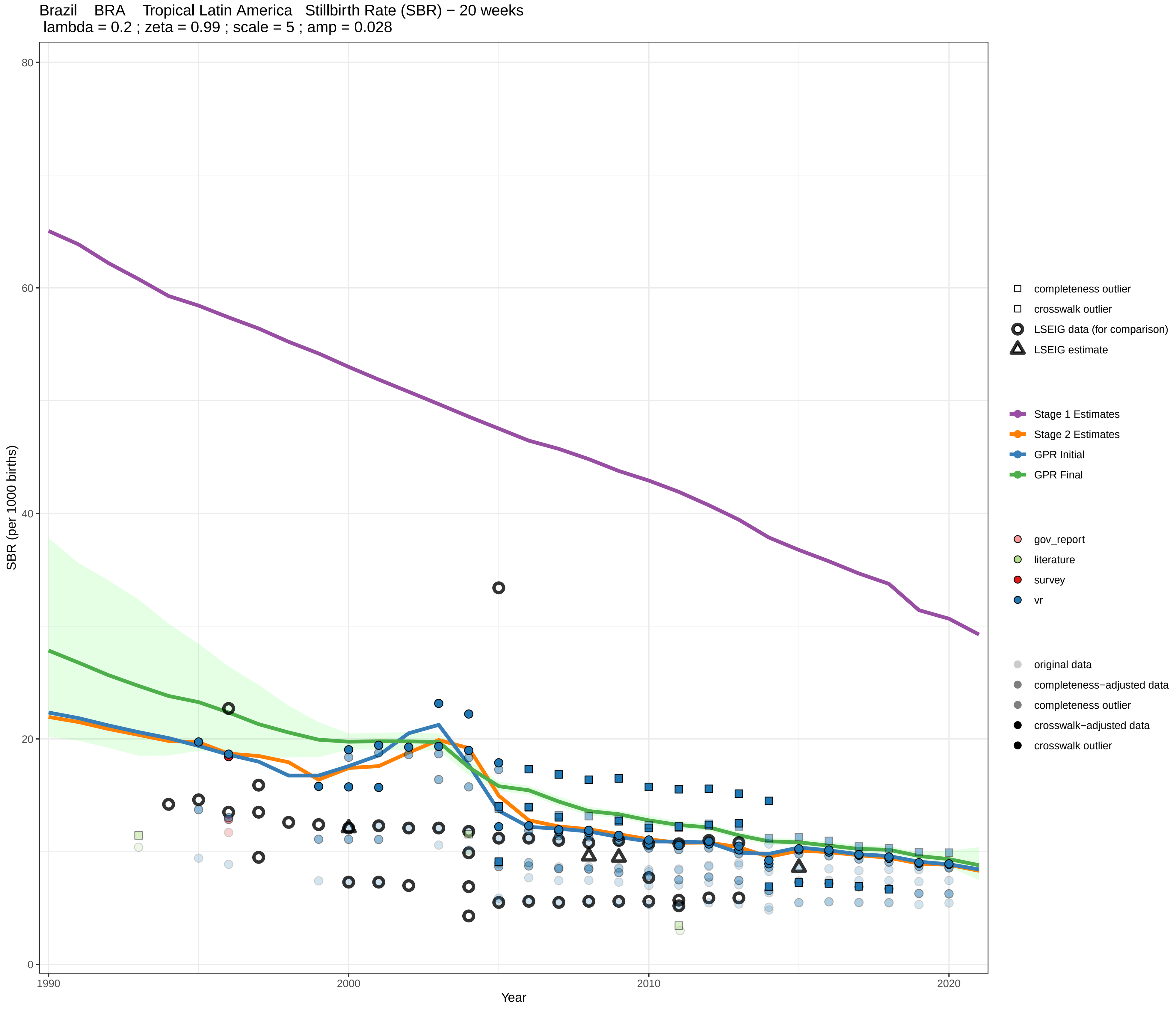

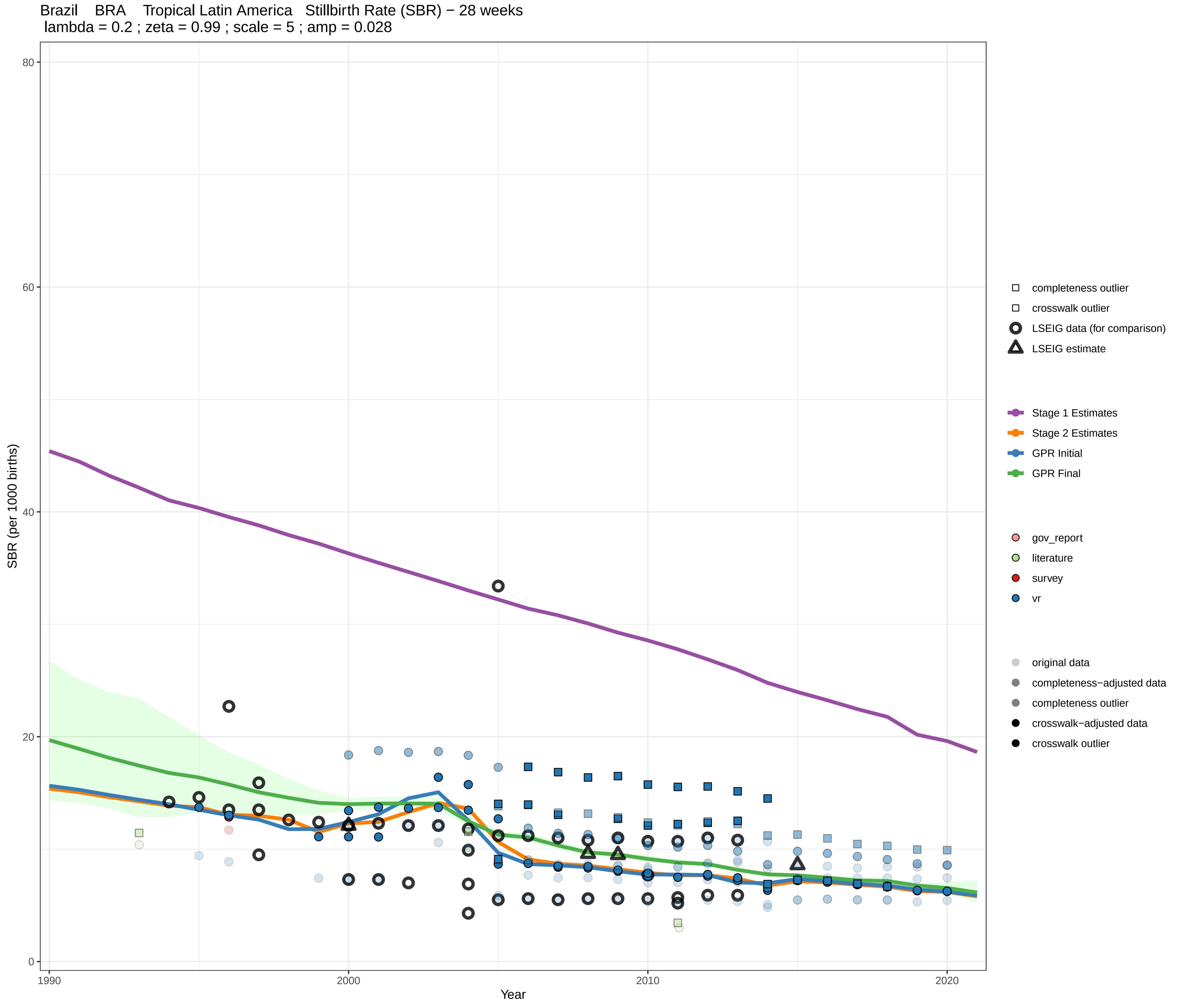

Year	Total Stillbirths (in thousands)	Total Stillbirths (in thousands)	SBR (per 1000 births)	SBR (per 1000 births)	SBR/NMR	SBR/NMR
	20 weeks	28 weeks	20 weeks	28 weeks	20 weeks	28 weeks
1990	91.4 (65.7 to 125.3)	64.1 (46.4 to 87.6)	27.8 (20.2 to 37.8)	19.7 (14.3 to 26.7)	1.38 (1.00 to 1.87)	0.97 (0.71 to 1.32)
1995	80.4 (65.2 to 98.8)	56.2 (45.3 to 69.4)	23.3 (19.0 to 28.4)	16.4 (13.2 to 20.1)	1.31 (1.07 to 1.61)	0.92 (0.75 to 1.14)
2000	71.6 (69.0 to 74.3)	50.5 (48.6 to 52.5)	19.8 (19.1 to 20.5)	14.0 (13.5 to 14.6)	1.25 (1.20 to 1.30)	0.89 (0.85 to 0.92)
2005	55.4 (54.0 to 56.9)	39.4 (38.3 to 40.6)	15.8 (15.4 to 16.2)	11.3 (11.0 to 11.6)	1.14 (1.11 to 1.17)	0.82 (0.79 to 0.84)
2010	44.2 (42.9 to 45.7)	31.5 (30.4 to 32.6)	12.8 (12.4 to 13.2)	9.1 (8.8 to 9.4)	1.08 (1.05 to 1.11)	0.77 (0.74 to 0.80)
2015	38.3 (37.3 to 39.5)	27.1 (26.5 to 27.8)	10.8 (10.5 to 11.1)	7.7 (7.5 to 7.9)	1.13 (1.10 to 1.16)	0.80 (0.78 to 0.82)
2020	31.9 (29.5 to 34.5)	22.3 (20.6 to 24.1)	9.3 (8.6 to 10.1)	6.5 (6.1 to 7.1)	1.23 (1.14 to 1.33)	0.86 (0.80 to 0.93)
2021	29.6 (25.1 to 35.0)	20.6 (17.6 to 24.4)	8.8 (7.5 to 10.4)	6.1 (5.3 to 7.3)	1.23 (1.05 to 1.45)	0.86 (0.73 to 1.01)

Acre BRA_4750 Tropical Latin America Stillbirth Rate / Neonatal Mortality Rate (SBR/NMR) − 20 weeks
lambda = 0.2 ; zeta = 0.99 ; scale = 5 ; amp = 0.028

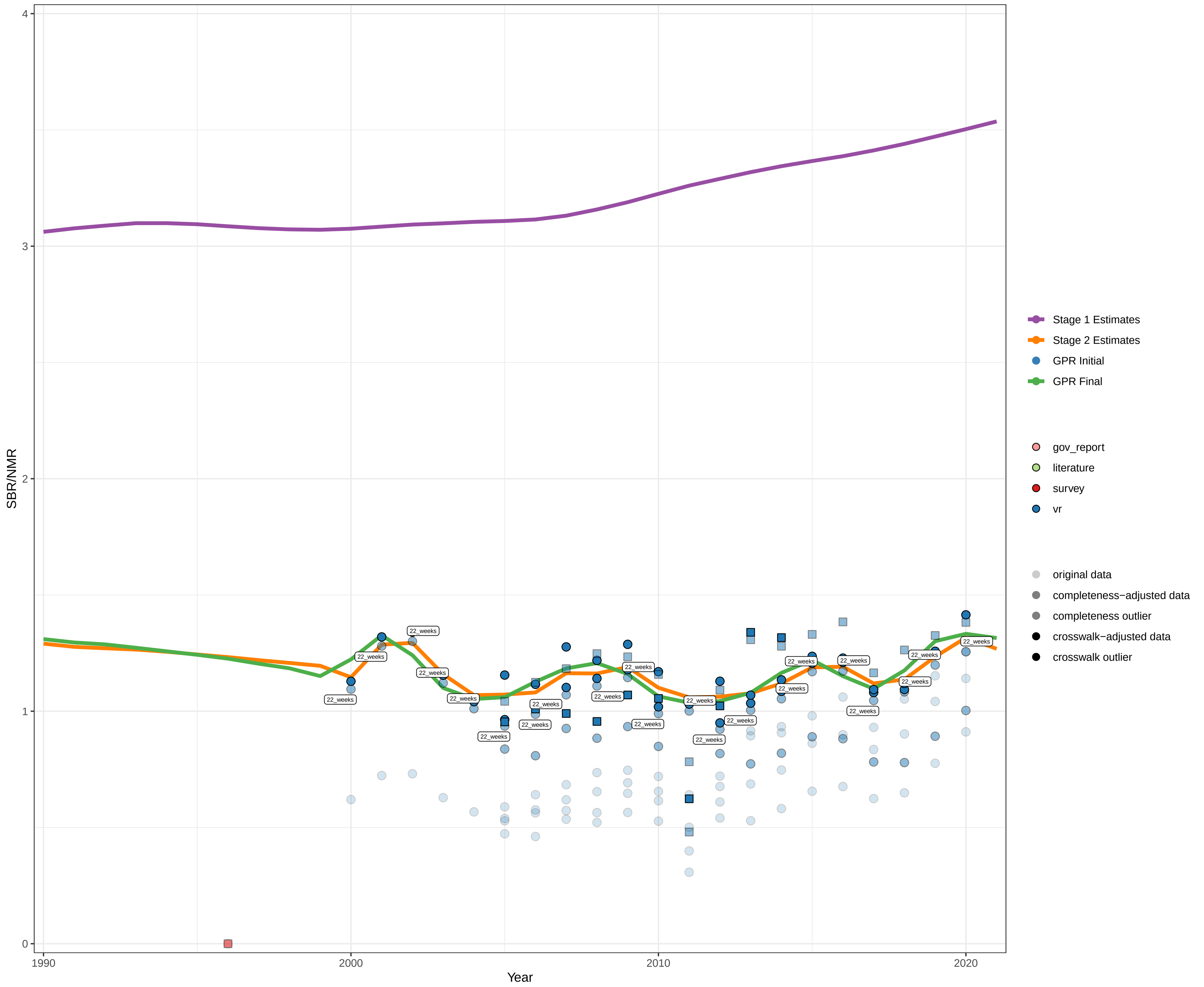

Acre BRA_4750 Tropical Latin America Stillbirth Rate / Neonatal Mortality Rate (SBR/NMR) − 28 weeks
lambda = 0.2 ; zeta = 0.99 ; scale = 5 ; amp = 0.028

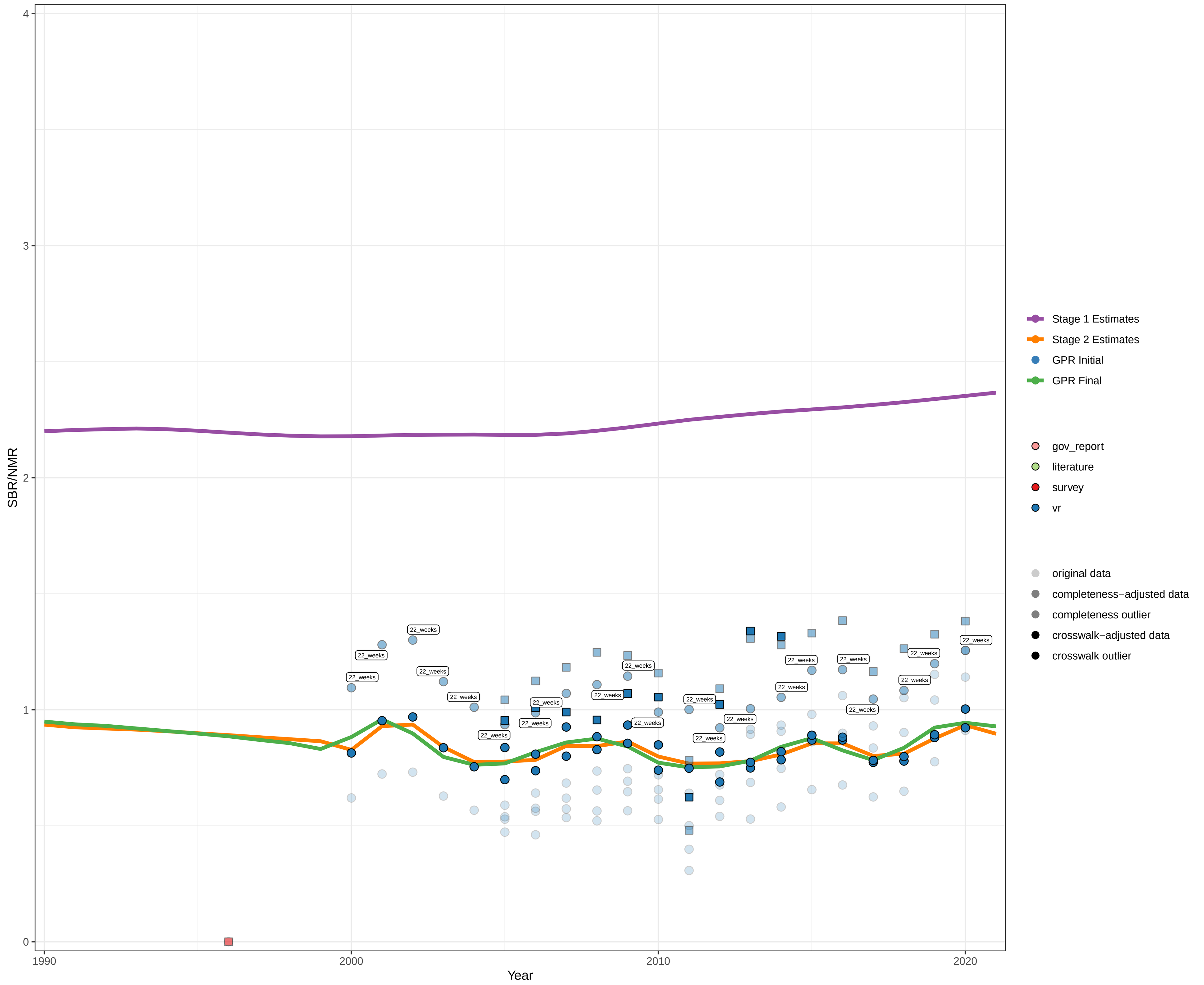

Definition	Years	Outlier	Source.Type	Title
22_weeks	2000−2014	0	VR	Brazil Mortality Information System − Stillbirths
22_weeks	2005−2020	0	VR	Brazil Civil Registry Statistics − Fetal Deaths at 28 Weeks or More, by Place of Birth, Number of Births by Birth and Sex, and Residence of Mother
28_weeks	2005−2010, 2012−2020	0	VR	Brazil Civil Registry Statistics − Fetal Deaths at 28 Weeks or More, by Place of Birth, Number of Births by Birth and Sex, and Residence of Mother
28_weeks	1996	1	Survey	Brazil Demographic and Health Survey 1996
28_weeks	2011	1	VR	Brazil Civil Registry Statistics − Fetal Deaths at 28 Weeks or More, by Place of Birth, Number of Births by Birth and Sex, and Residence of Mother
other	2005−2020	1	VR	Brazil Civil Registry Statistics − Fetal Deaths at 28 Weeks or More, by Place of Birth, Number of Births by Birth and Sex, and Residence of Mother

Acre BRA_4750 Tropical Latin America Stillbirth Rate (SBR) − 20 weeks
lambda = 0.2 ; zeta = 0.99 ; scale = 5 ; amp = 0.028

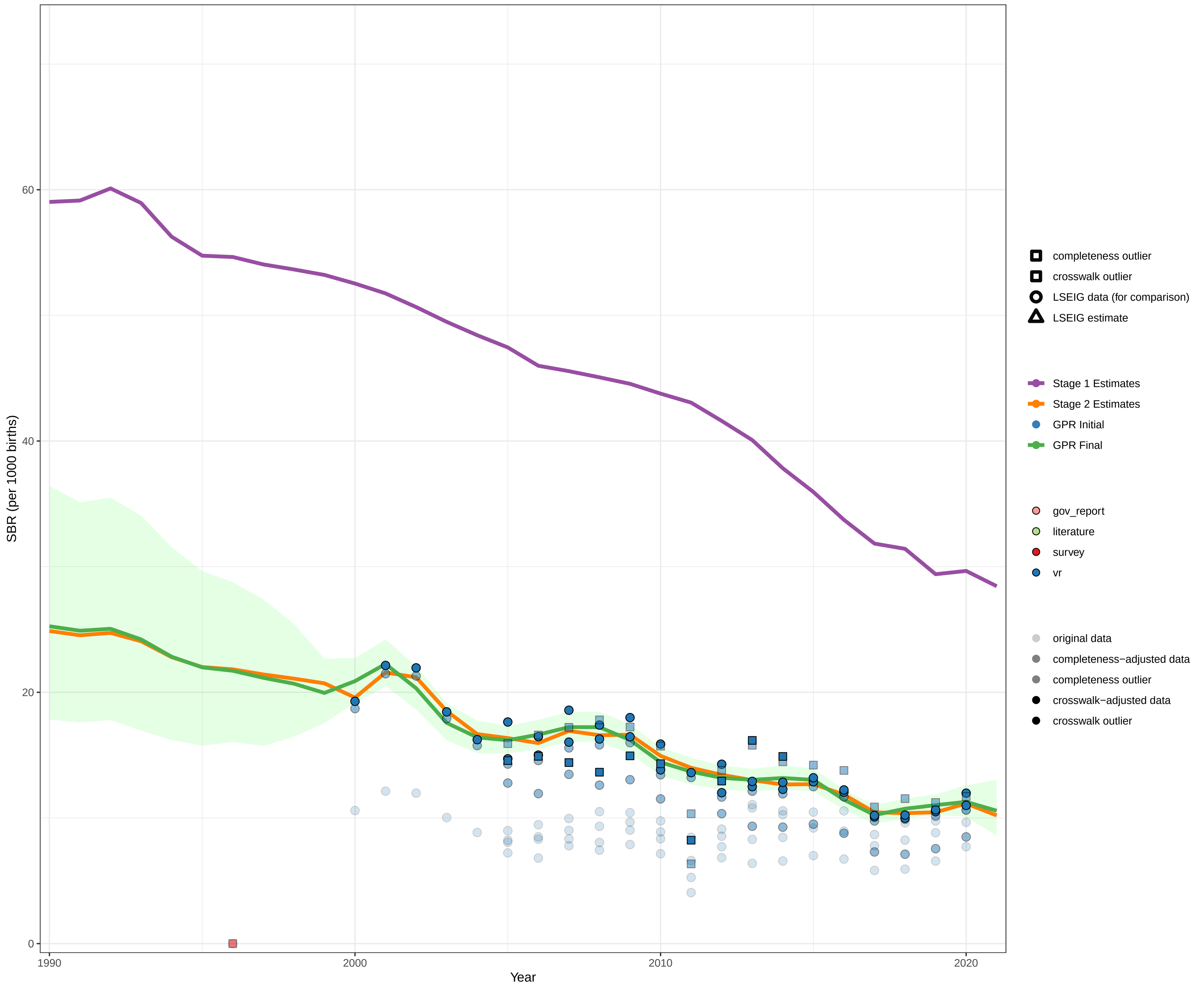

Acre BRA_4750 Tropical Latin America Stillbirth Rate (SBR) − 28 weeks
lambda = 0.2 ; zeta = 0.99 ; scale = 5 ; amp = 0.028

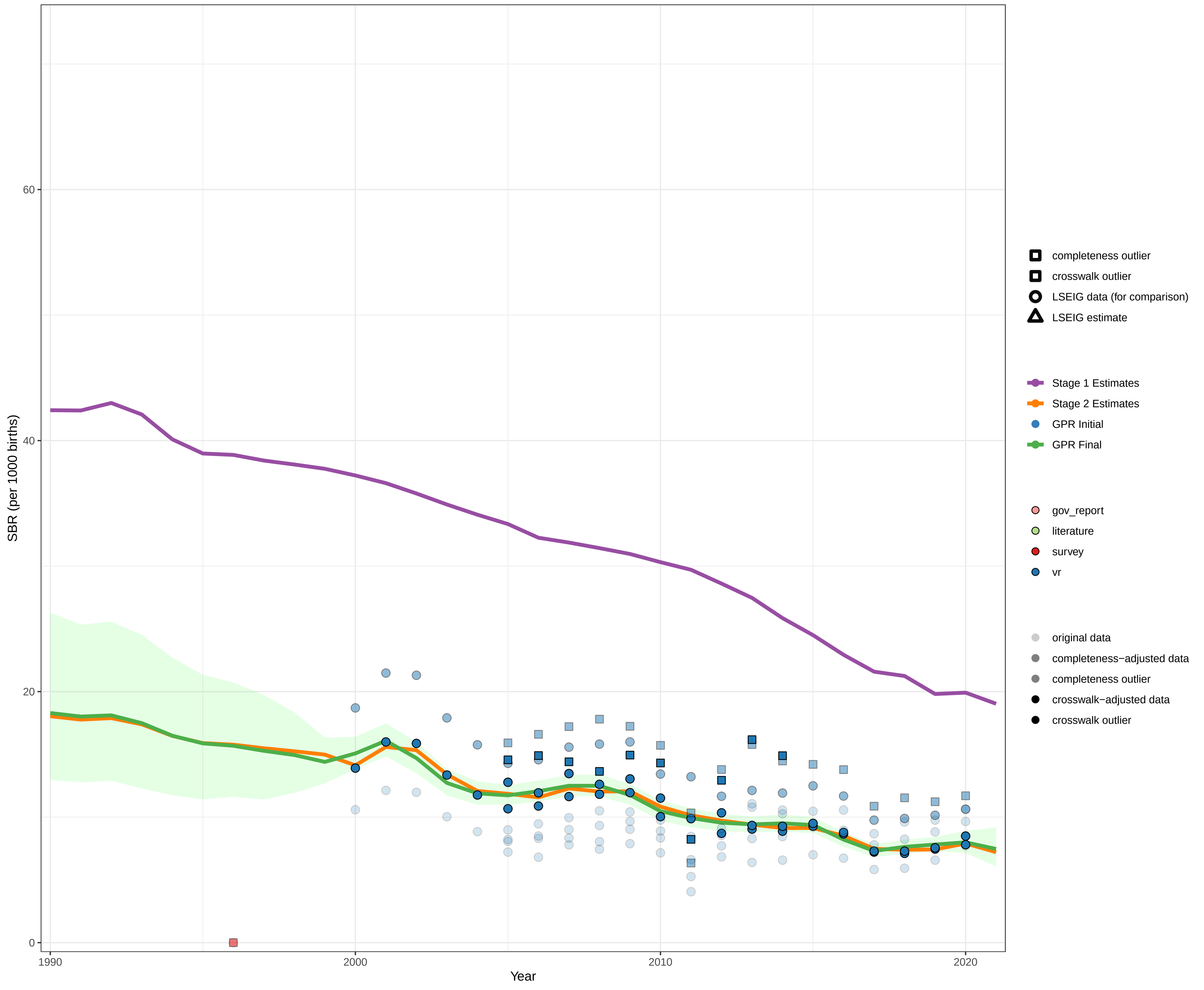

Year	Total Stillbirths (in thousands)	Total Stillbirths (in thousands)	SBR (per 1000 births)	SBR (per 1000 births)	SBR/NMR	SBR/NMR
	20 weeks	28 weeks	20 weeks	28 weeks	20 weeks	28 weeks
1990	0.3 (0.2 to 0.4)	0.2 (0.2 to 0.3)	25.3 (17.8 to 36.4)	18.3 (13.0 to 26.3)	1.31 (0.92 to 1.89)	0.95 (0.67 to 1.36)
1995	0.3 (0.2 to 0.4)	0.2 (0.2 to 0.3)	22.0 (15.8 to 29.6)	15.9 (11.4 to 21.3)	1.24 (0.89 to 1.68)	0.90 (0.65 to 1.21)
2000	0.4 (0.3 to 0.4)	0.3 (0.2 to 0.3)	20.9 (19.1 to 22.7)	15.1 (13.8 to 16.4)	1.22 (1.12 to 1.33)	0.88 (0.81 to 0.96)
2005	0.3 (0.3 to 0.3)	0.2 (0.2 to 0.2)	16.2 (15.1 to 17.3)	11.7 (11.0 to 12.6)	1.06 (0.99 to 1.14)	0.77 (0.72 to 0.82)
2010	0.3 (0.3 to 0.3)	0.2 (0.2 to 0.2)	14.4 (13.4 to 15.6)	10.5 (9.7 to 11.3)	1.06 (0.99 to 1.15)	0.77 (0.72 to 0.84)
2015	0.3 (0.2 to 0.3)	0.2 (0.2 to 0.2)	13.0 (12.1 to 13.9)	9.4 (8.7 to 10.0)	1.22 (1.14 to 1.31)	0.88 (0.82 to 0.94)
2020	0.2 (0.2 to 0.3)	0.2 (0.1 to 0.2)	11.3 (10.1 to 12.6)	8.0 (7.1 to 8.9)	1.33 (1.19 to 1.49)	0.94 (0.84 to 1.05)
2021	0.2 (0.2 to 0.3)	0.2 (0.1 to 0.2)	10.6 (8.6 to 13.0)	7.5 (6.1 to 9.2)	1.31 (1.07 to 1.62)	0.93 (0.76 to 1.14)

Alagoas BRA_4751 Tropical Latin America Stillbirth Rate / Neonatal Mortality Rate (SBR/NMR) − 20 weeks
lambda = 0.2 ; zeta = 0.99 ; scale = 5 ; amp = 0.028

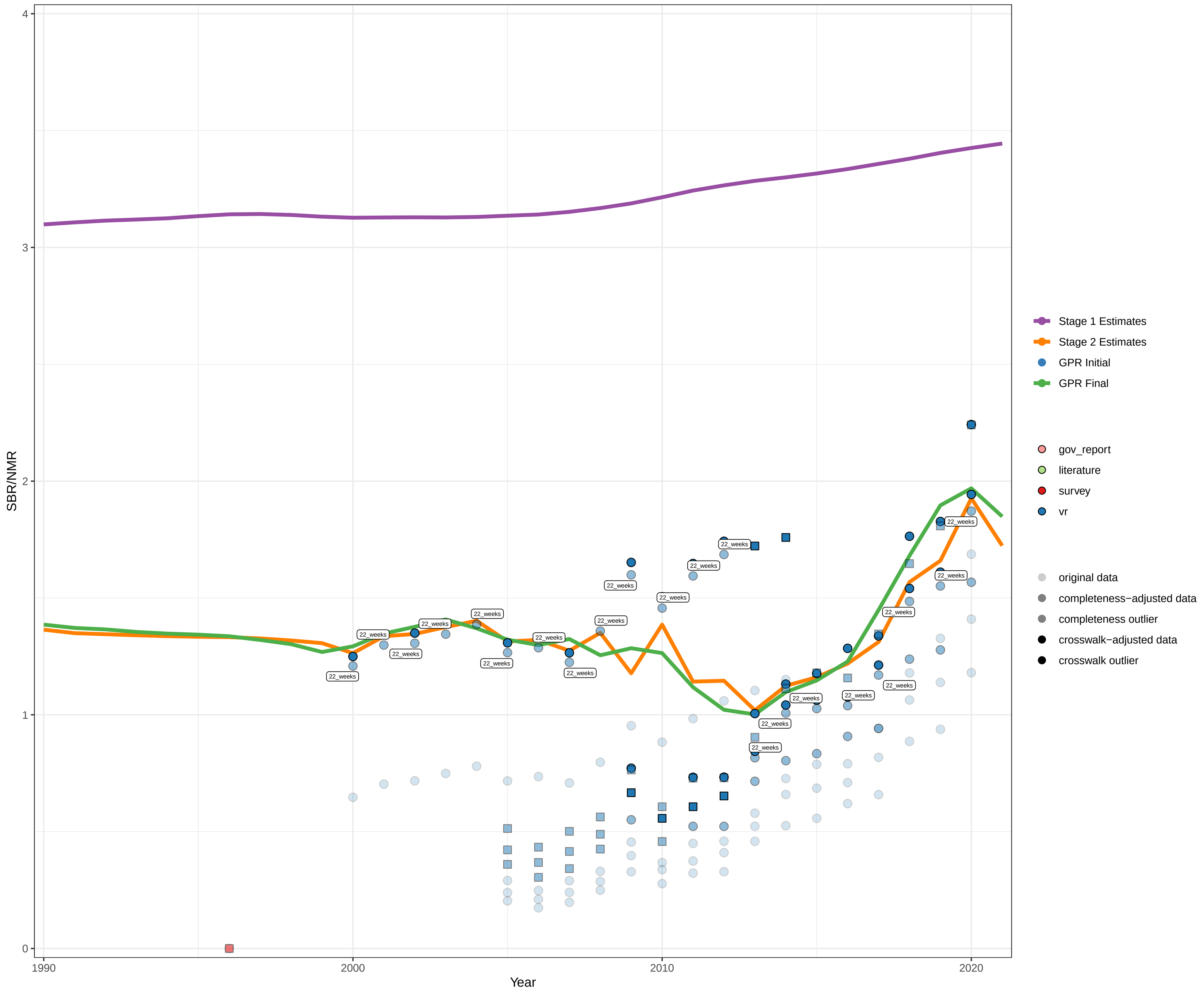

Alagoas BRA_4751 Tropical Latin America Stillbirth Rate / Neonatal Mortality Rate (SBR/NMR) − 28 weeks
lambda = 0.2 ; zeta = 0.99 ; scale = 5 ; amp = 0.028

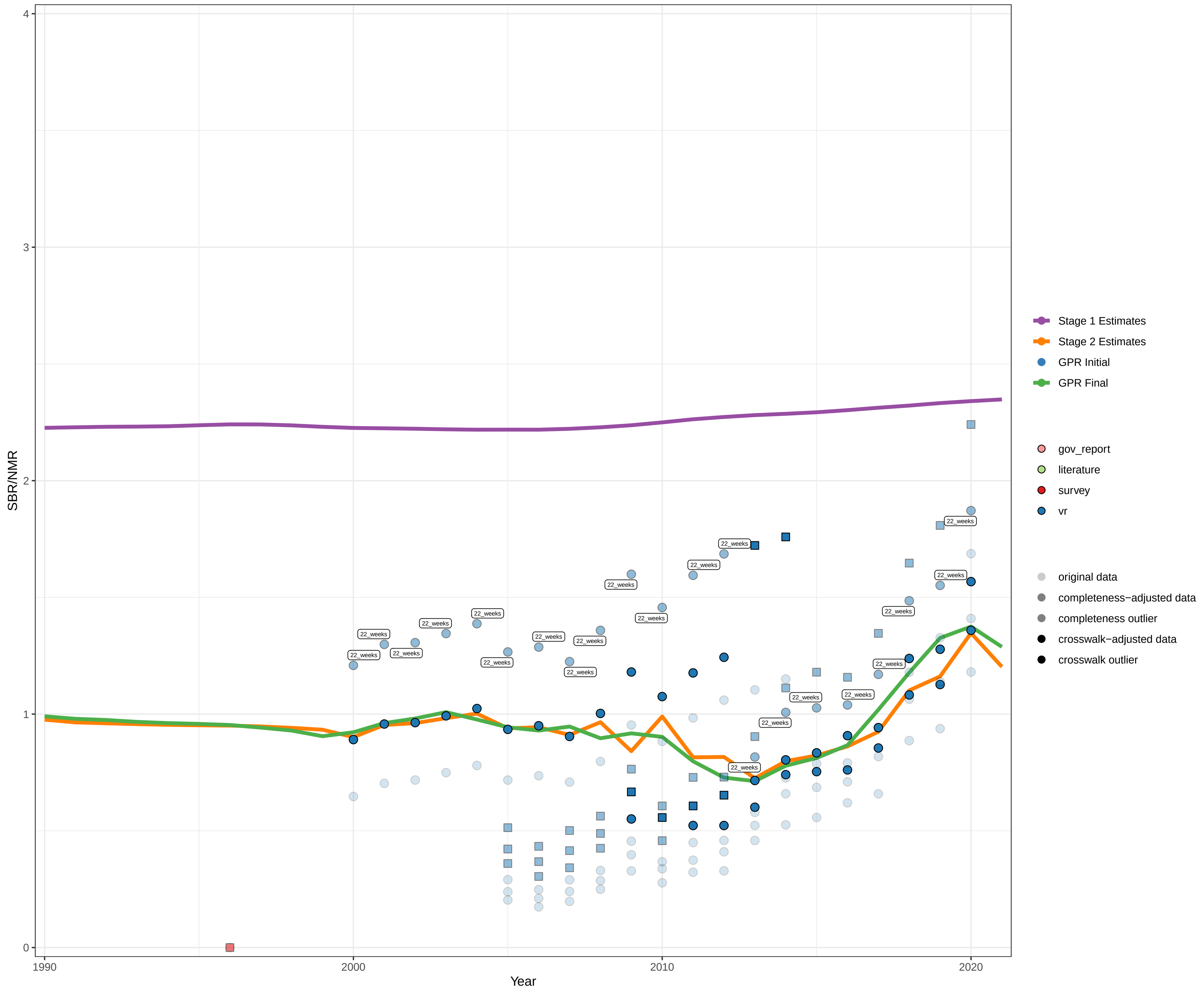

Definition	Years	Outlier	Source.Type	Title
22_weeks	2000−2014	0	VR	Brazil Mortality Information System − Stillbirths
22_weeks	2009−2020	0	VR	Brazil Civil Registry Statistics − Fetal Deaths at 28 Weeks or More, by Place of Birth, Number of Births by Birth and Sex, and Residence of Mother
28_weeks	2009, 2011−2020	0	VR	Brazil Civil Registry Statistics − Fetal Deaths at 28 Weeks or More, by Place of Birth, Number of Births by Birth and Sex, and Residence of Mother
22_weeks	2005−2008	1	VR	Brazil Civil Registry Statistics − Fetal Deaths at 28 Weeks or More, by Place of Birth, Number of Births by Birth and Sex, and Residence of Mother
28_weeks	1996	1	Survey	Brazil Demographic and Health Survey 1996
28_weeks	2005−2008, 2010	1	VR	Brazil Civil Registry Statistics − Fetal Deaths at 28 Weeks or More, by Place of Birth, Number of Births by Birth and Sex, and Residence of Mother
other	2005−2020	1	VR	Brazil Civil Registry Statistics − Fetal Deaths at 28 Weeks or More, by Place of Birth, Number of Births by Birth and Sex, and Residence of Mother

Alagoas BRA_4751 Tropical Latin America Stillbirth Rate (SBR) − 20 weeks
lambda = 0.2 ; zeta = 0.99 ; scale = 5 ; amp = 0.028

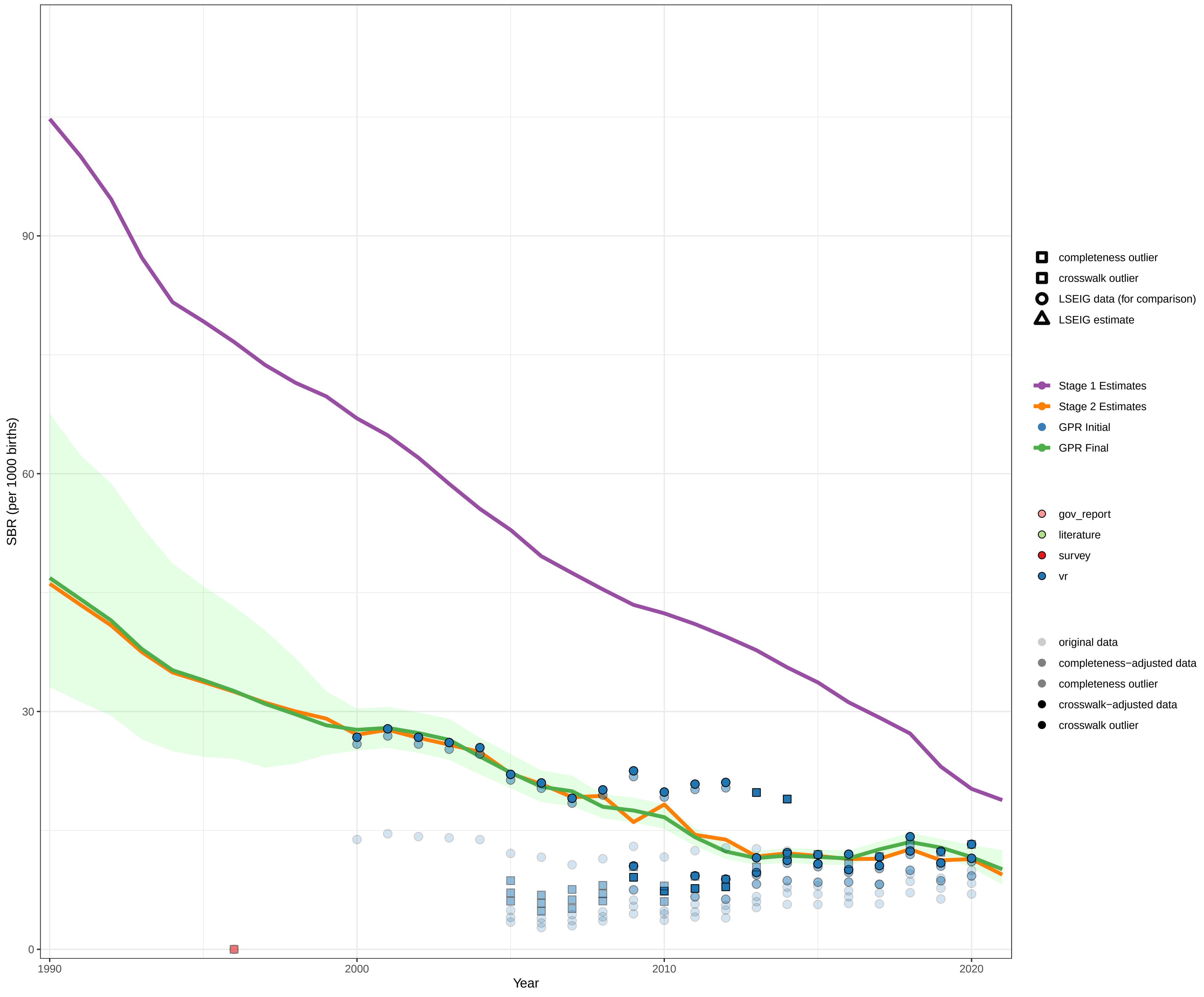

Alagoas BRA_4751 Tropical Latin America Stillbirth Rate (SBR) − 28 weeks
lambda = 0.2 ; zeta = 0.99 ; scale = 5 ; amp = 0.028

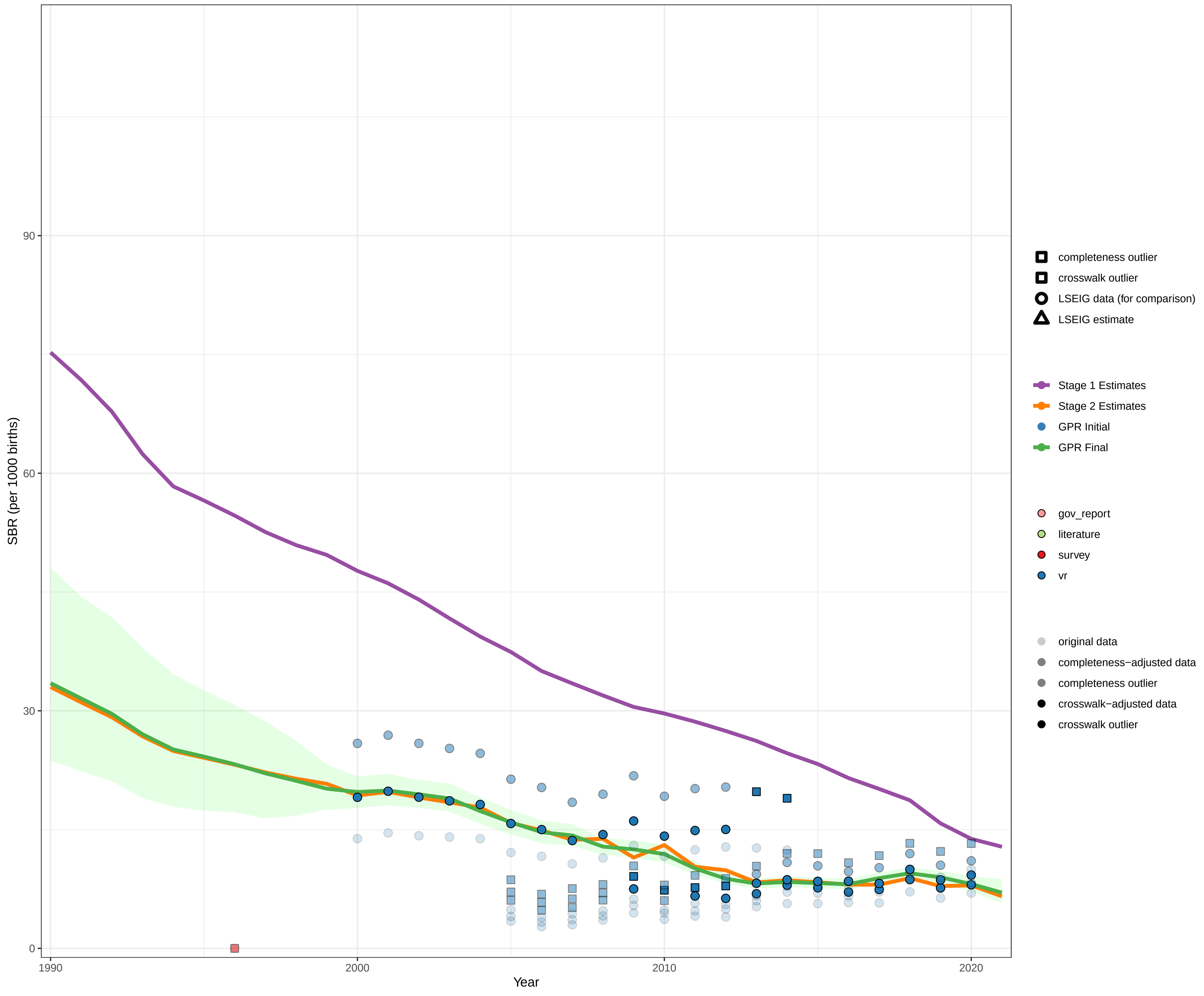

Year	Total Stillbirths (in thousands)	Total Stillbirths (in thousands)	SBR (per 1000 births)	SBR (per 1000 births)	SBR/NMR	SBR/NMR
	20 weeks	28 weeks	20 weeks	28 weeks	20 weeks	28 weeks
1990	3.7 (2.6 to 5.5)	2.6 (1.8 to 3.8)	46.9 (33.1 to 67.6)	33.5 (23.7 to 48.1)	1.39 (0.98 to 2.00)	0.99 (0.70 to 1.42)
1995	2.9 (2.1 to 4.0)	2.1 (1.5 to 2.8)	33.9 (24.3 to 45.8)	24.2 (17.4 to 32.6)	1.34 (0.96 to 1.81)	0.96 (0.69 to 1.29)
2000	2.6 (2.3 to 2.8)	1.8 (1.6 to 2.0)	27.7 (25.1 to 30.3)	19.8 (17.7 to 21.7)	1.29 (1.17 to 1.42)	0.92 (0.83 to 1.01)
2005	2.0 (1.8 to 2.2)	1.4 (1.3 to 1.6)	22.3 (20.3 to 24.6)	15.9 (14.4 to 17.5)	1.32 (1.20 to 1.46)	0.94 (0.85 to 1.04)
2010	1.5 (1.3 to 1.6)	1.1 (1.0 to 1.2)	16.7 (15.2 to 18.3)	11.9 (10.8 to 13.1)	1.26 (1.15 to 1.39)	0.90 (0.82 to 0.99)
2015	1.0 (0.9 to 1.1)	0.7 (0.7 to 0.8)	11.6 (10.7 to 12.6)	8.2 (7.6 to 8.9)	1.15 (1.05 to 1.24)	0.81 (0.75 to 0.88)
2020	1.0 (0.9 to 1.1)	0.7 (0.6 to 0.8)	11.6 (10.3 to 13.1)	8.1 (7.2 to 9.2)	1.97 (1.74 to 2.23)	1.37 (1.22 to 1.55)
2021	0.9 (0.7 to 1.1)	0.6 (0.5 to 0.7)	10.1 (8.2 to 12.5)	7.0 (5.7 to 8.7)	1.85 (1.49 to 2.29)	1.29 (1.04 to 1.59)

Amapa BRA_4753 Tropical Latin America Stillbirth Rate / Neonatal Mortality Rate (SBR/NMR) − 20 weeks
lambda = 0.4 ; zeta = 0.9 ; scale = 10 ; amp = 0.028

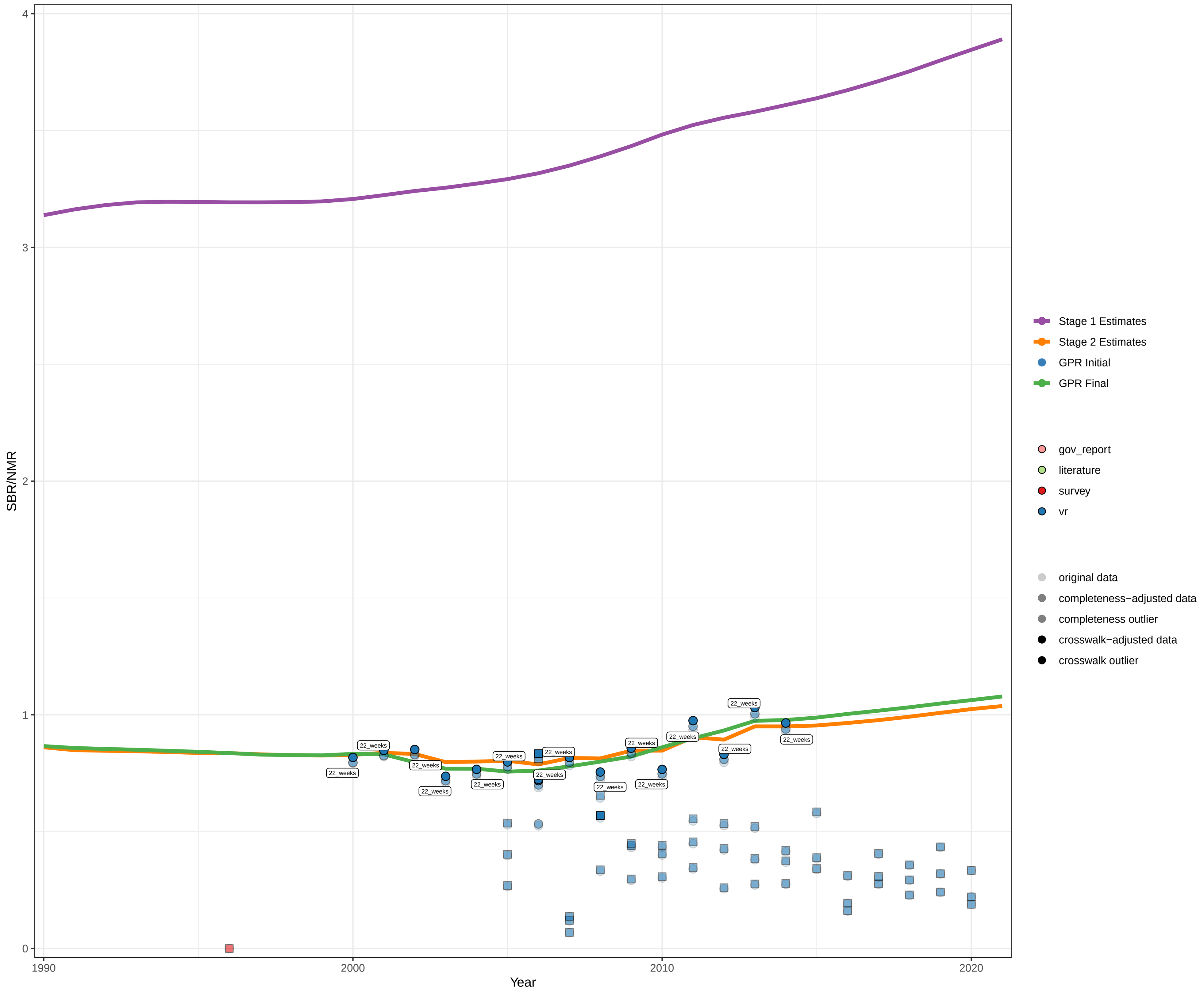

Amapa BRA_4753 Tropical Latin America Stillbirth Rate / Neonatal Mortality Rate (SBR/NMR) − 28 weeks
lambda = 0.4 ; zeta = 0.9 ; scale = 10 ; amp = 0.028

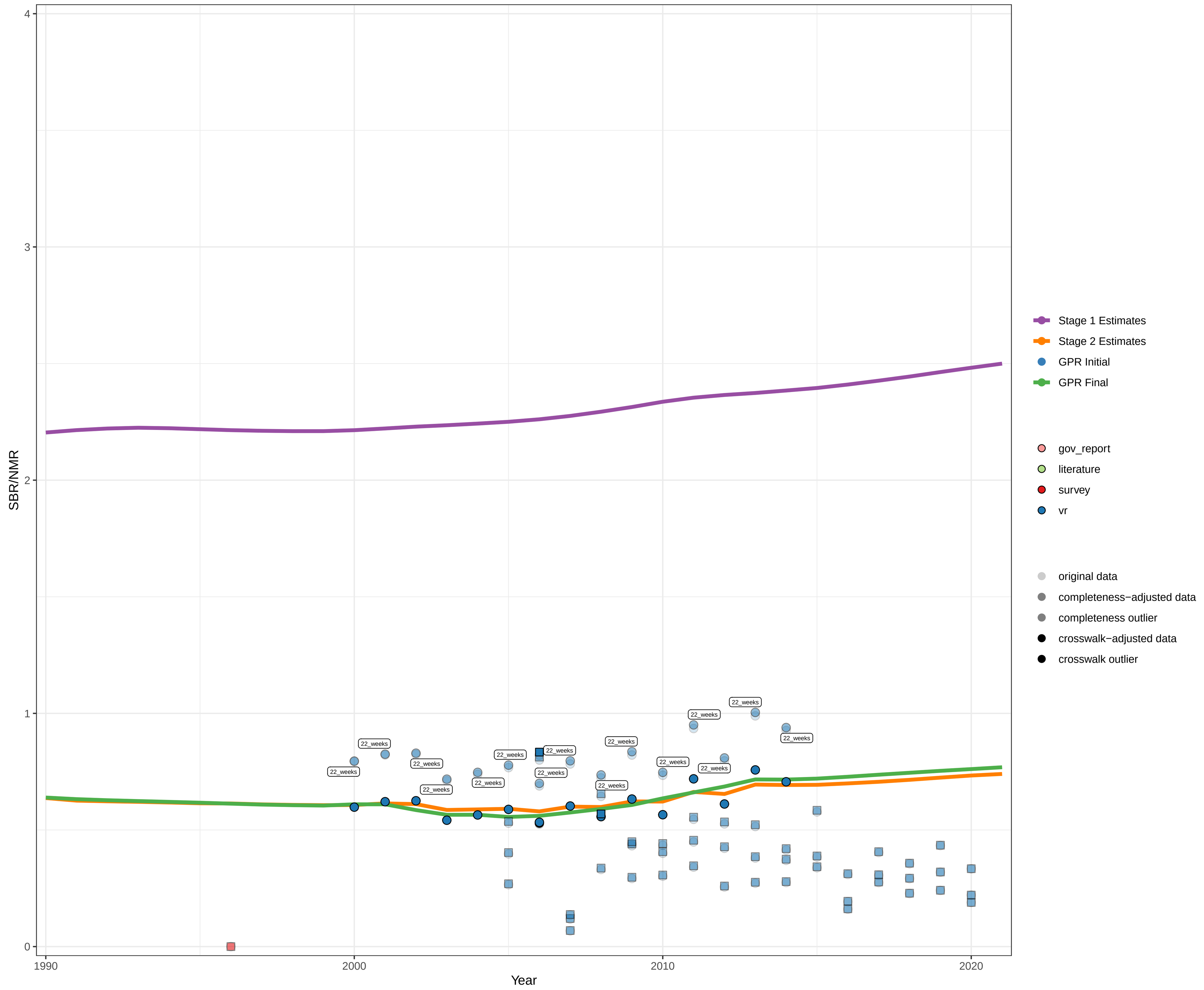

Definition	Years	Outlier	Source.Type	Title
22_weeks	2000−2014	0	VR	Brazil Mortality Information System − Stillbirths
22_weeks	2006, 2008	0	VR	Brazil Civil Registry Statistics − Fetal Deaths at 28 Weeks or More, by Place of Birth, Number of Births by Birth and Sex, and Residence of Mother
28_weeks	2006	0	VR	Brazil Civil Registry Statistics − Fetal Deaths at 28 Weeks or More, by Place of Birth, Number of Births by Birth and Sex, and Residence of Mother
22_weeks	2005, 2007, 2009−2020	1	VR	Brazil Civil Registry Statistics − Fetal Deaths at 28 Weeks or More, by Place of Birth, Number of Births by Birth and Sex, and Residence of Mother
28_weeks	1996	1	Survey	Brazil Demographic and Health Survey 1996
28_weeks	2005, 2007−2020	1	VR	Brazil Civil Registry Statistics − Fetal Deaths at 28 Weeks or More, by Place of Birth, Number of Births by Birth and Sex, and Residence of Mother
other	2005−2020	1	VR	Brazil Civil Registry Statistics − Fetal Deaths at 28 Weeks or More, by Place of Birth, Number of Births by Birth and Sex, and Residence of Mother

Amapa BRA_4753 Tropical Latin America Stillbirth Rate (SBR) − 20 weeks
lambda = 0.4 ; zeta = 0.9 ; scale = 10 ; amp = 0.028

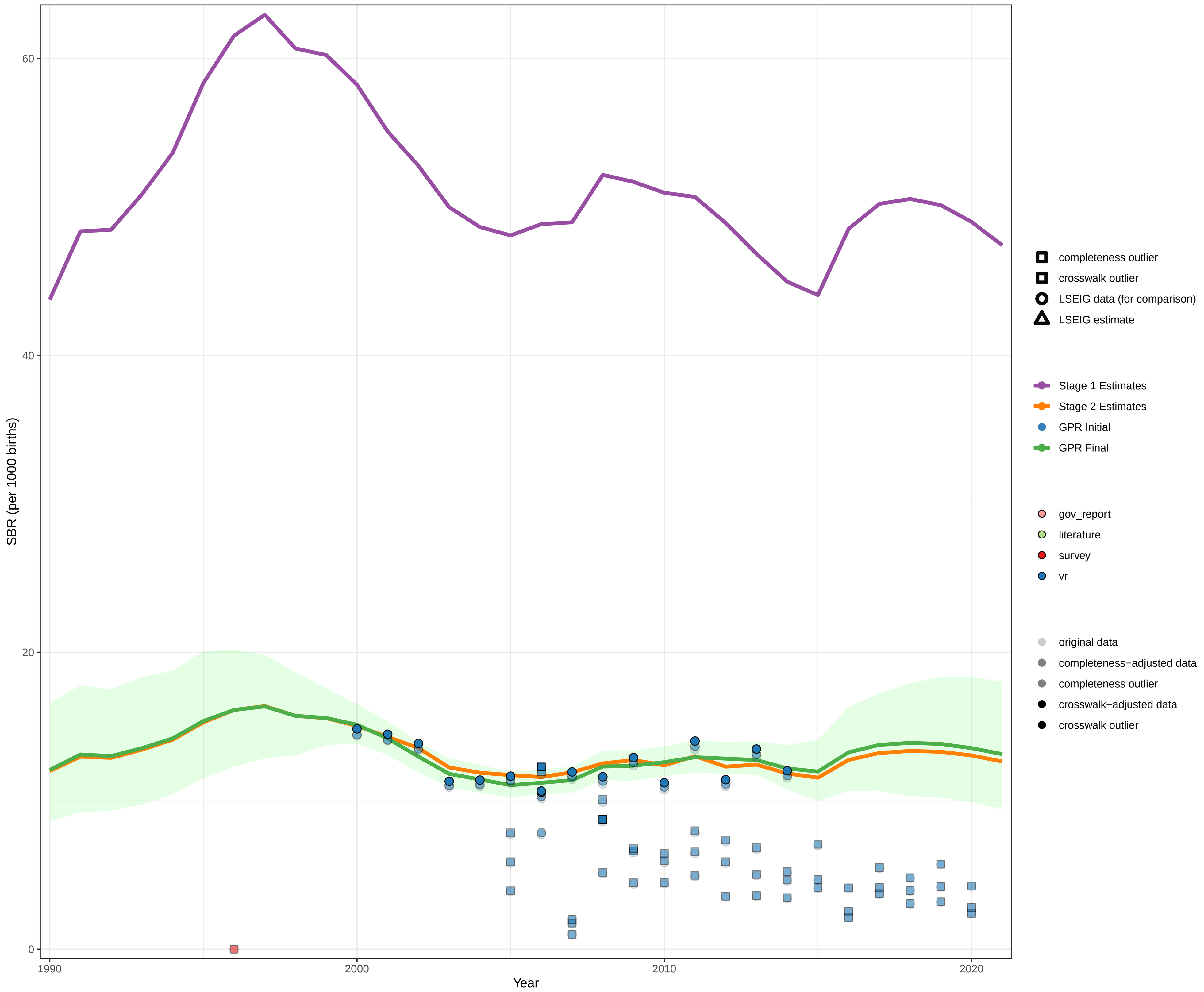

Amapa BRA_4753 Tropical Latin America Stillbirth Rate (SBR) − 28 weeks
lambda = 0.4 ; zeta = 0.9 ; scale = 10 ; amp = 0.028

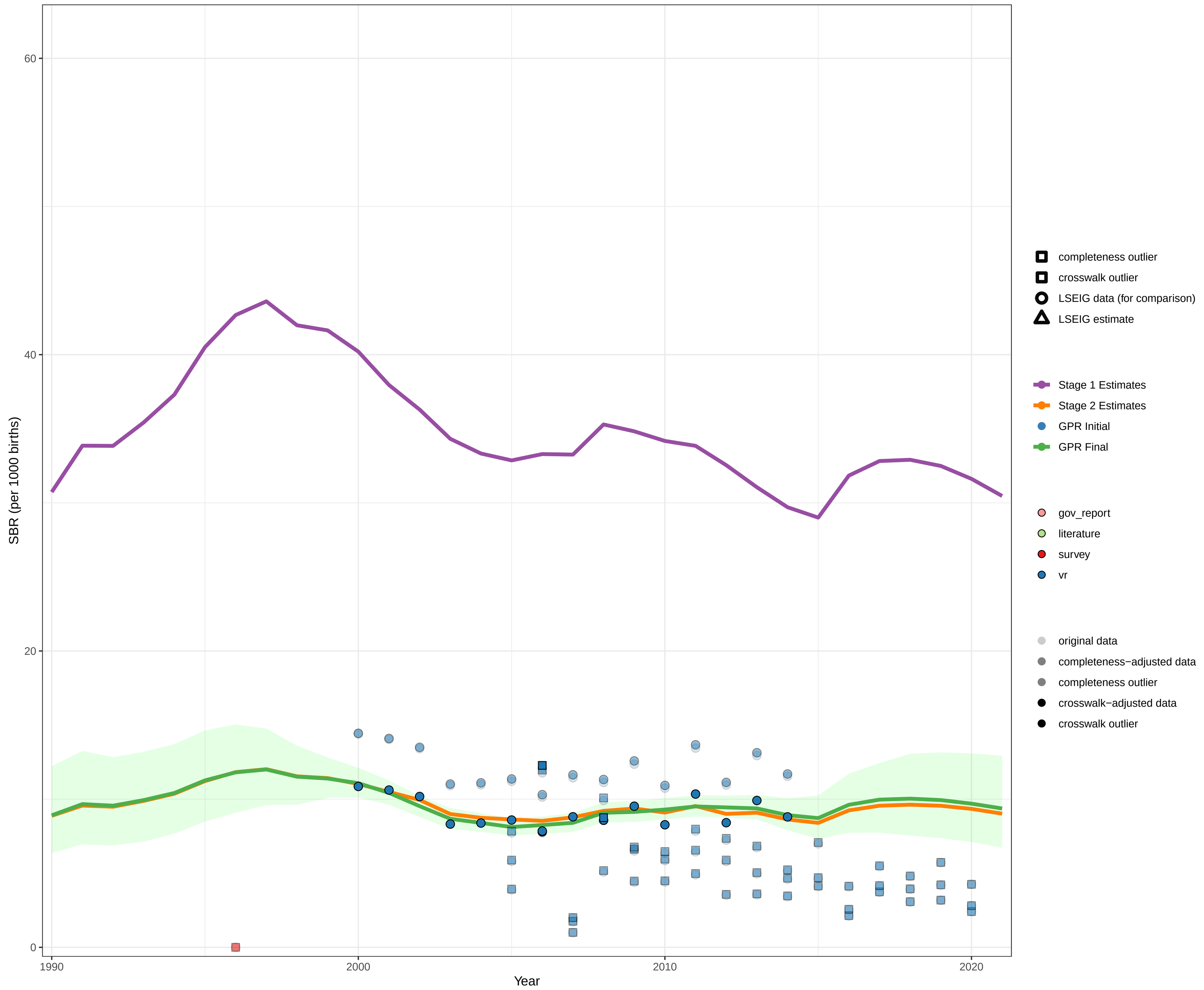

Year	Total Stillbirths (in thousands)	Total Stillbirths (in thousands)	SBR (per 1000 births)	SBR (per 1000 births)	SBR/NMR	SBR/NMR
	20 weeks	28 weeks	20 weeks	28 weeks	20 weeks	28 weeks
1990	0.1 (0.1 to 0.1)	0.1 (0.0 to 0.1)	12.1 (8.6 to 16.6)	8.9 (6.4 to 12.2)	0.87 (0.62 to 1.19)	0.64 (0.46 to 0.88)
1995	0.2 (0.1 to 0.2)	0.1 (0.1 to 0.1)	15.4 (11.5 to 20.0)	11.3 (8.5 to 14.6)	0.84 (0.63 to 1.10)	0.62 (0.46 to 0.80)
2000	0.2 (0.2 to 0.2)	0.1 (0.1 to 0.2)	15.1 (13.8 to 16.5)	11.1 (10.1 to 12.1)	0.83 (0.76 to 0.91)	0.61 (0.56 to 0.67)
2005	0.1 (0.1 to 0.2)	0.1 (0.1 to 0.1)	11.1 (10.2 to 11.9)	8.1 (7.6 to 8.7)	0.76 (0.70 to 0.82)	0.56 (0.52 to 0.60)
2010	0.2 (0.2 to 0.2)	0.1 (0.1 to 0.1)	12.6 (11.7 to 13.7)	9.3 (8.6 to 10.1)	0.86 (0.80 to 0.93)	0.64 (0.59 to 0.69)
2015	0.2 (0.1 to 0.2)	0.1 (0.1 to 0.2)	12.0 (10.0 to 14.1)	8.7 (7.3 to 10.3)	0.99 (0.83 to 1.17)	0.72 (0.60 to 0.85)
2020	0.2 (0.2 to 0.3)	0.1 (0.1 to 0.2)	13.5 (9.9 to 18.3)	9.7 (7.1 to 13.1)	1.06 (0.78 to 1.44)	0.76 (0.56 to 1.03)
2021	0.2 (0.1 to 0.3)	0.1 (0.1 to 0.2)	13.1 (9.4 to 18.0)	9.4 (6.7 to 12.9)	1.08 (0.77 to 1.48)	0.77 (0.55 to 1.06)

Amazonas BRA_4752 Tropical Latin America Stillbirth Rate / Neonatal Mortality Rate (SBR/NMR) − 20 weeks
lambda = 0.2 ; zeta = 0.99 ; scale = 5 ; amp = 0.028

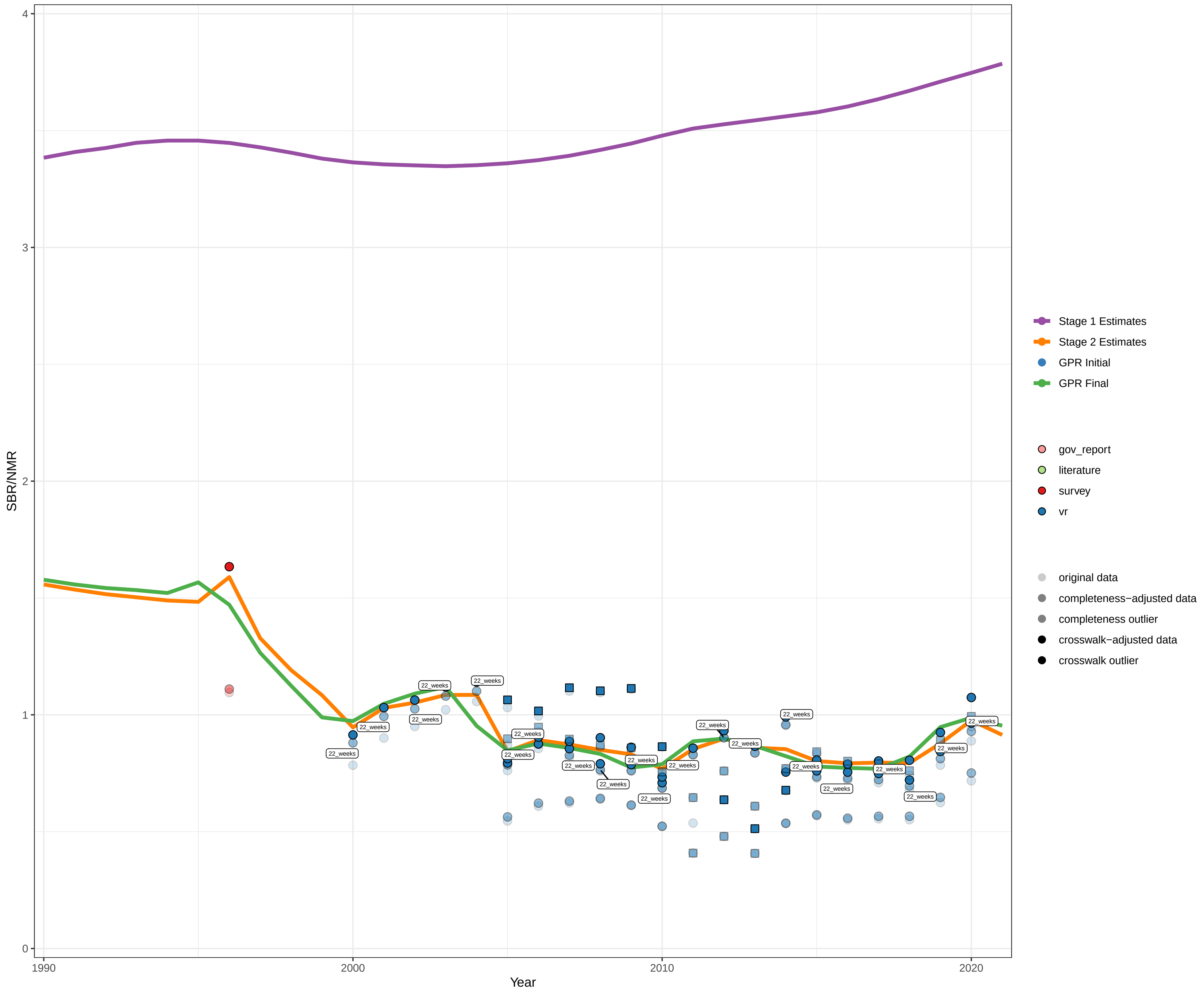

Amazonas BRA_4752 Tropical Latin America Stillbirth Rate / Neonatal Mortality Rate (SBR/NMR) − 28 weeks
lambda = 0.2 ; zeta = 0.99 ; scale = 5 ; amp = 0.028

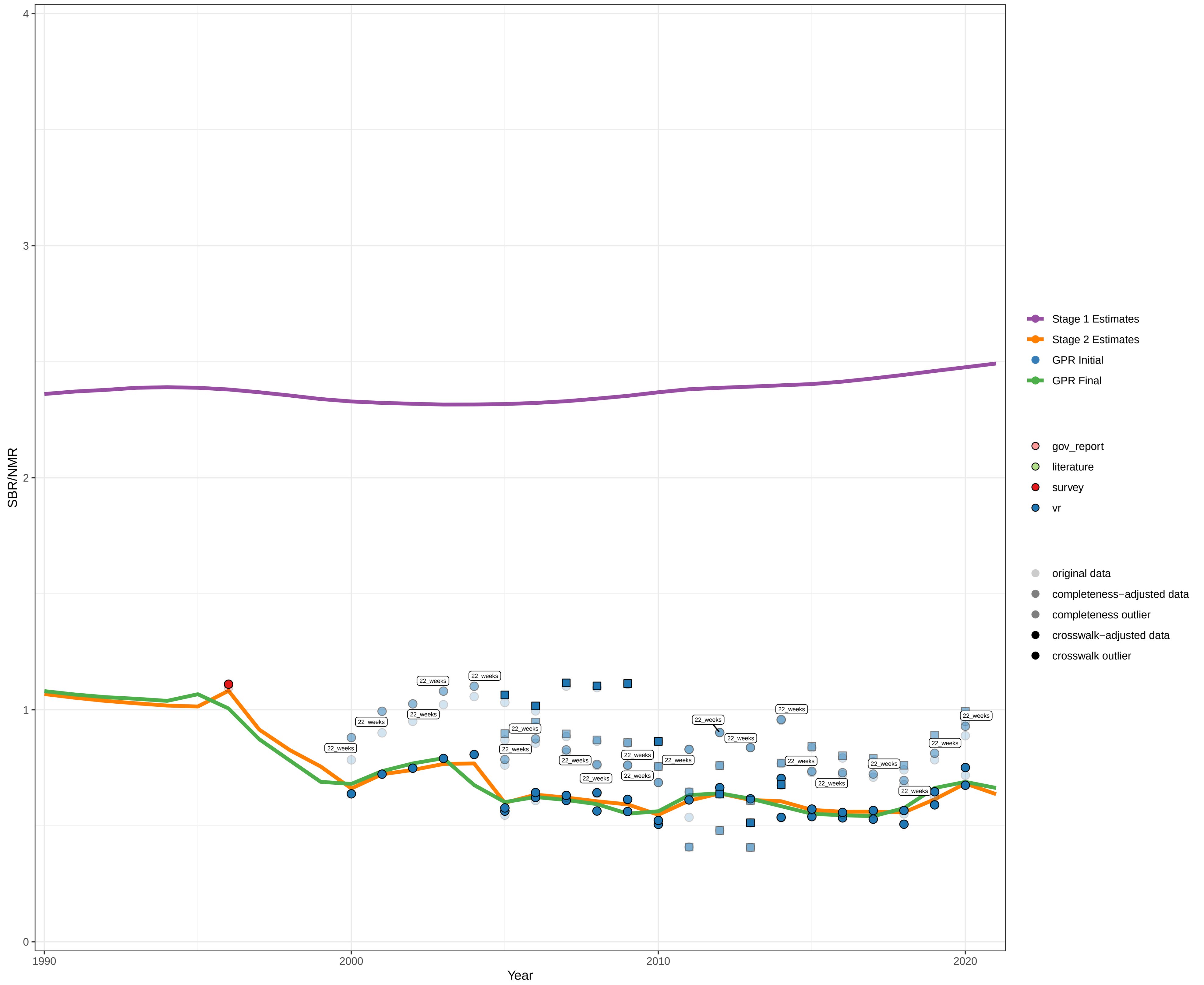

Definition	Years	Outlier	Source.Type	Title
22_weeks	2000−2014	0	VR	Brazil Mortality Information System − Stillbirths
22_weeks	2005−2020	0	VR	Brazil Civil Registry Statistics − Fetal Deaths at 28 Weeks or More, by Place of Birth, Number of Births by Birth and Sex, and Residence of Mother
28_weeks	1996	0	Survey	Brazil Demographic and Health Survey 1996
28_weeks	2005−2010, 2014−2020	0	VR	Brazil Civil Registry Statistics − Fetal Deaths at 28 Weeks or More, by Place of Birth, Number of Births by Birth and Sex, and Residence of Mother
28_weeks	2011−2013	1	VR	Brazil Civil Registry Statistics − Fetal Deaths at 28 Weeks or More, by Place of Birth, Number of Births by Birth and Sex, and Residence of Mother
other	2005−2020	1	VR	Brazil Civil Registry Statistics − Fetal Deaths at 28 Weeks or More, by Place of Birth, Number of Births by Birth and Sex, and Residence of Mother

Amazonas BRA_4752 Tropical Latin America Stillbirth Rate (SBR) − 20 weeks
lambda = 0.2 ; zeta = 0.99 ; scale = 5 ; amp = 0.028

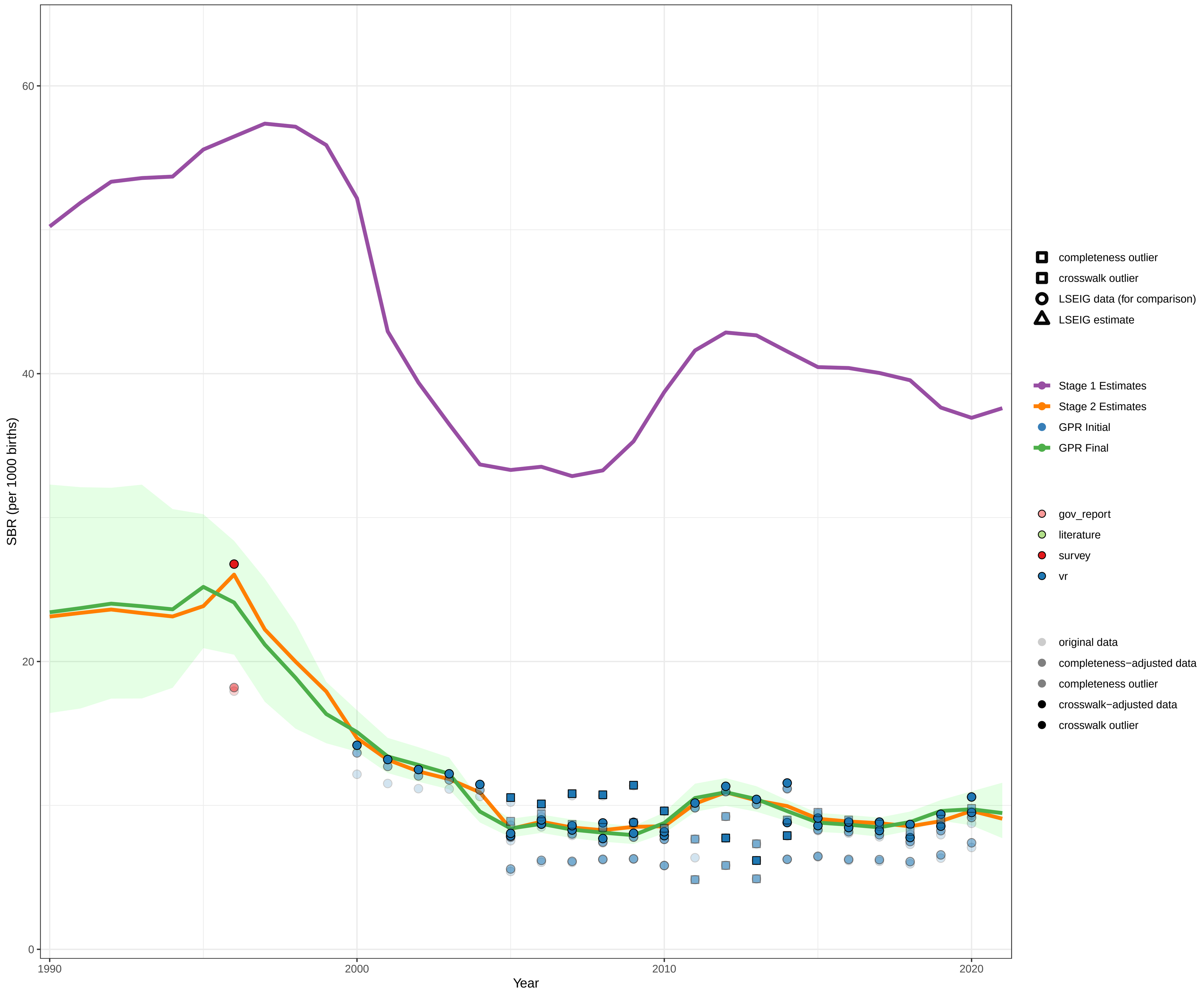

Amazonas BRA_4752 Tropical Latin America Stillbirth Rate (SBR) − 28 weeks
lambda = 0.2 ; zeta = 0.99 ; scale = 5 ; amp = 0.028

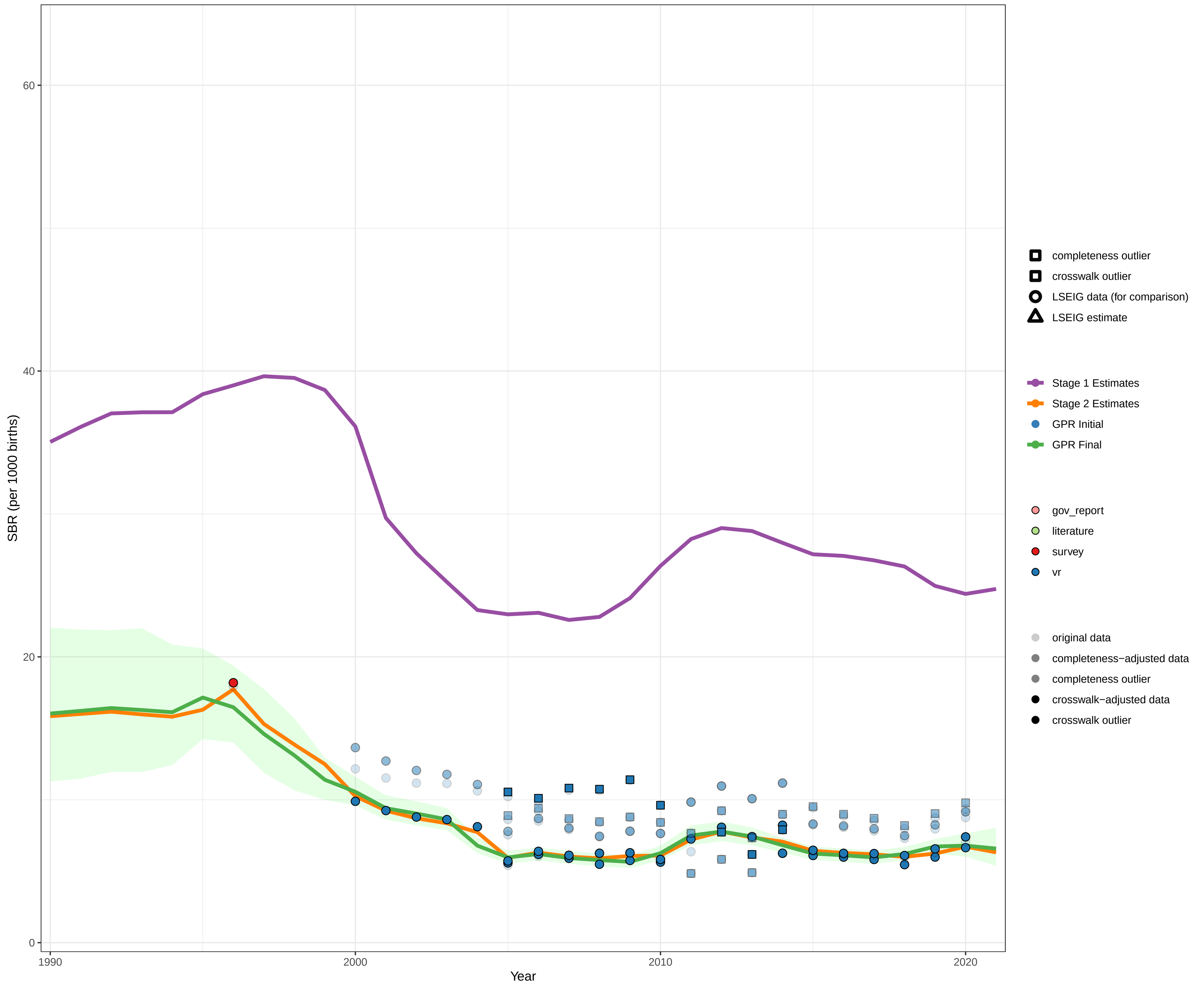

Year	Total Stillbirths (in thousands)	Total Stillbirths (in thousands)	SBR (per 1000 births)	SBR (per 1000 births)	SBR/NMR	SBR/NMR
	20 weeks	28 weeks	20 weeks	28 weeks	20 weeks	28 weeks
1990	1.1 (0.8 to 1.5)	0.8 (0.5 to 1.0)	23.4 (16.4 to 32.3)	16.0 (11.3 to 22.0)	1.58 (1.11 to 2.18)	1.08 (0.76 to 1.49)
1995	1.4 (1.2 to 1.7)	1.0 (0.8 to 1.1)	25.2 (20.9 to 30.2)	17.2 (14.2 to 20.6)	1.57 (1.30 to 1.88)	1.07 (0.89 to 1.28)
2000	1.0 (0.9 to 1.1)	0.7 (0.6 to 0.8)	15.1 (13.7 to 16.6)	10.6 (9.6 to 11.6)	0.97 (0.89 to 1.07)	0.68 (0.62 to 0.75)
2005	0.6 (0.6 to 0.7)	0.4 (0.4 to 0.5)	8.4 (7.8 to 9.1)	6.0 (5.5 to 6.4)	0.85 (0.78 to 0.92)	0.60 (0.56 to 0.65)
2010	0.7 (0.6 to 0.7)	0.5 (0.4 to 0.5)	8.8 (8.1 to 9.5)	6.3 (5.8 to 6.8)	0.79 (0.72 to 0.85)	0.56 (0.52 to 0.61)
2015	0.7 (0.7 to 0.8)	0.5 (0.5 to 0.5)	8.8 (8.2 to 9.5)	6.2 (5.8 to 6.7)	0.78 (0.72 to 0.84)	0.55 (0.51 to 0.59)
2020	0.8 (0.7 to 0.9)	0.6 (0.5 to 0.6)	9.7 (8.6 to 11.0)	6.8 (6.0 to 7.7)	0.99 (0.87 to 1.11)	0.69 (0.61 to 0.78)
2021	0.8 (0.6 to 0.9)	0.5 (0.4 to 0.7)	9.5 (7.7 to 11.6)	6.6 (5.4 to 8.0)	0.95 (0.78 to 1.17)	0.66 (0.54 to 0.81)

Bahia   BRA_4754   Tropical Latin America   Stillbirth Rate / Neonatal Mortality Rate (SBR/NMR) − 20 weeks
lambda = 0.2 ; zeta = 0.99 ; scale = 5 ; amp = 0.028

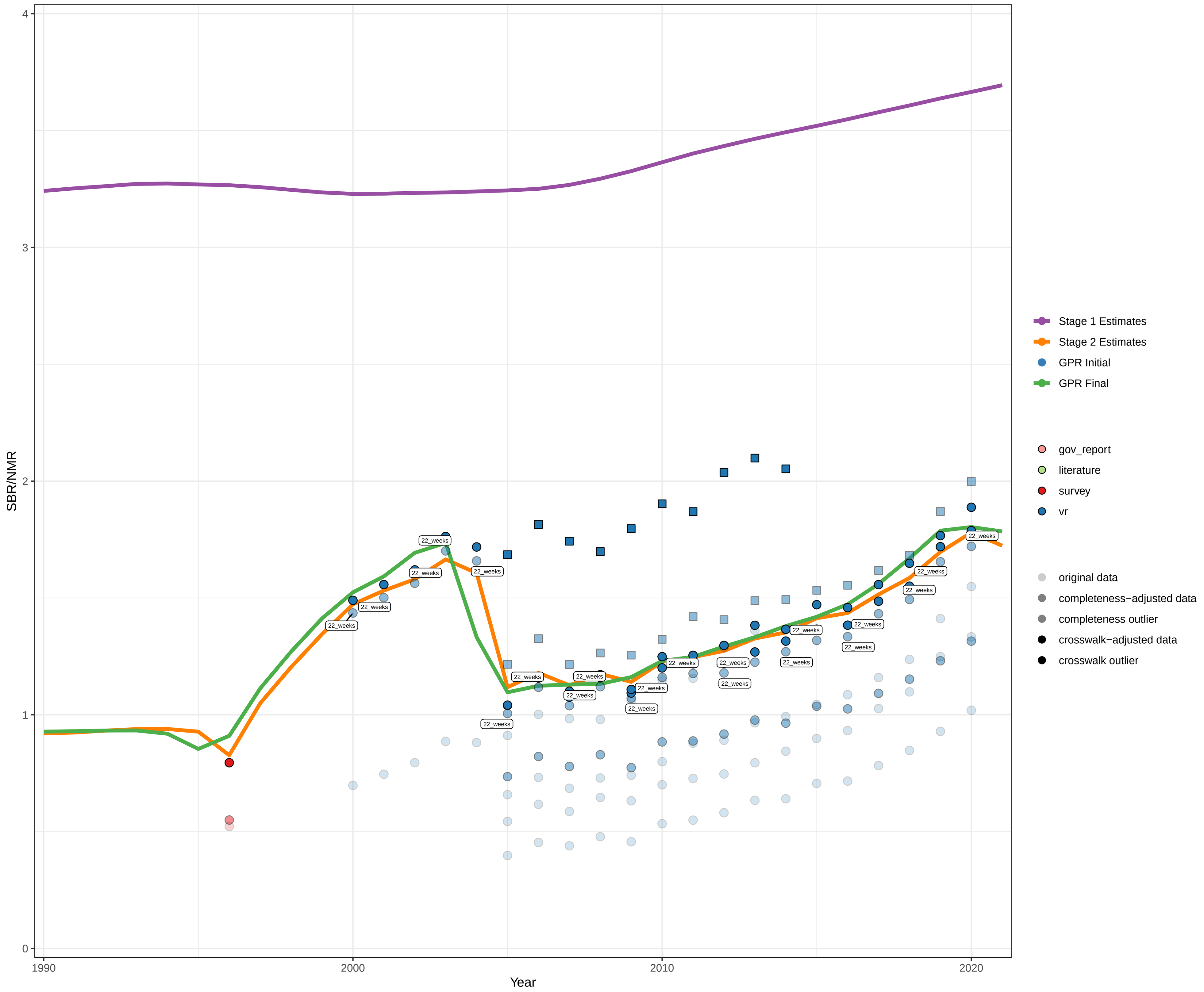

Bahia   BRA_4754   Tropical Latin America   Stillbirth Rate / Neonatal Mortality Rate (SBR/NMR) − 28 weeks
lambda = 0.2 ; zeta = 0.99 ; scale = 5 ; amp = 0.028

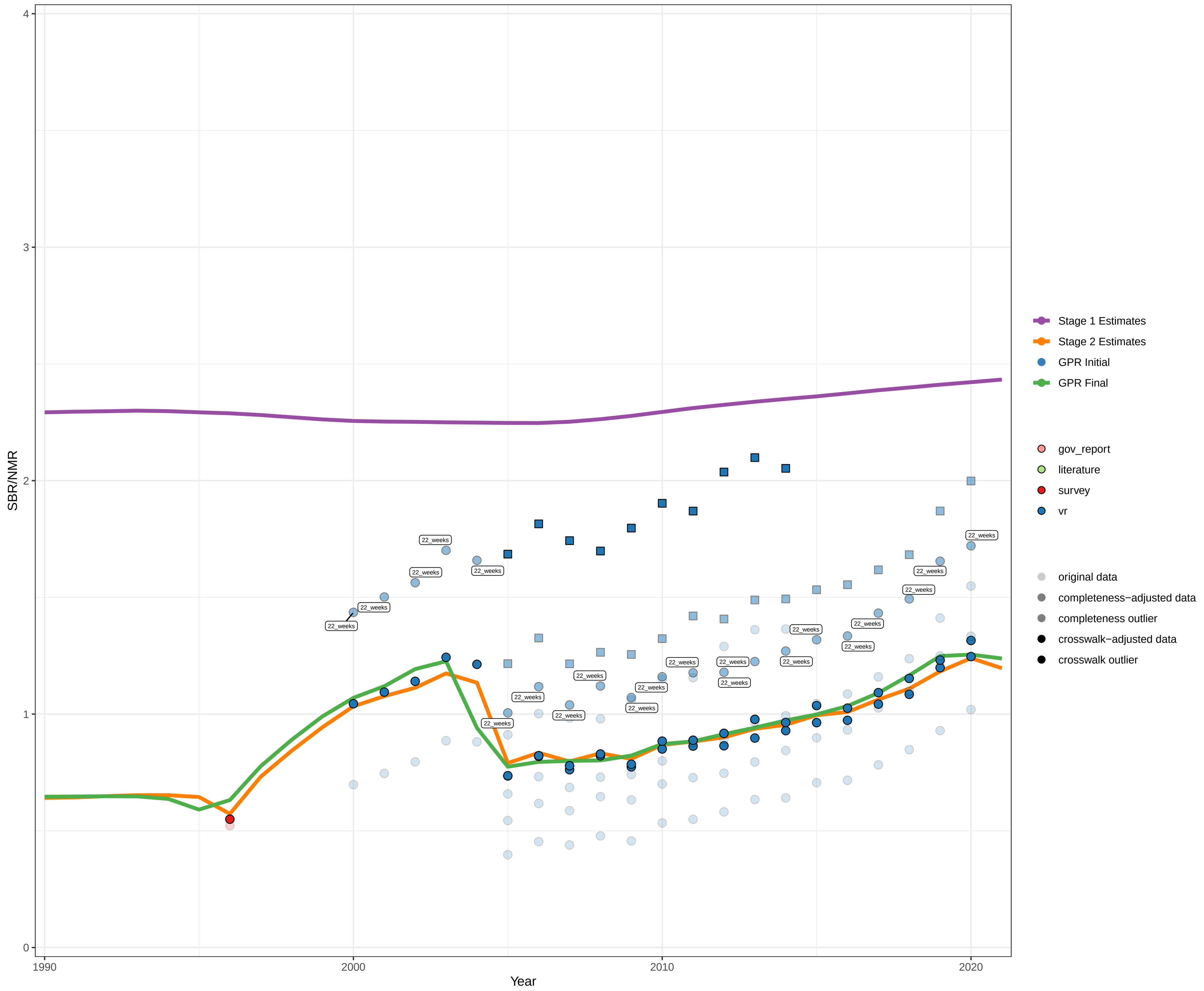

Definition	Years	Outlier	Source.Type	Title
22_weeks	2000−2014	0	VR	Brazil Mortality Information System − Stillbirths
22_weeks, 28_weeks	2005−2020	0	VR	Brazil Civil Registry Statistics − Fetal Deaths at 28 Weeks or More, by Place of Birth, Number of Births by Birth and Sex, and Residence of Mother
28_weeks	1996	0	Survey	Brazil Demographic and Health Survey 1996
other	2005−2020	1	VR	Brazil Civil Registry Statistics − Fetal Deaths at 28 Weeks or More, by Place of Birth, Number of Births by Birth and Sex, and Residence of Mother

Bahia   BRA_4754   Tropical Latin America   Stillbirth Rate (SBR) − 20 weeks
lambda = 0.2 ; zeta = 0.99 ; scale = 5 ; amp = 0.028

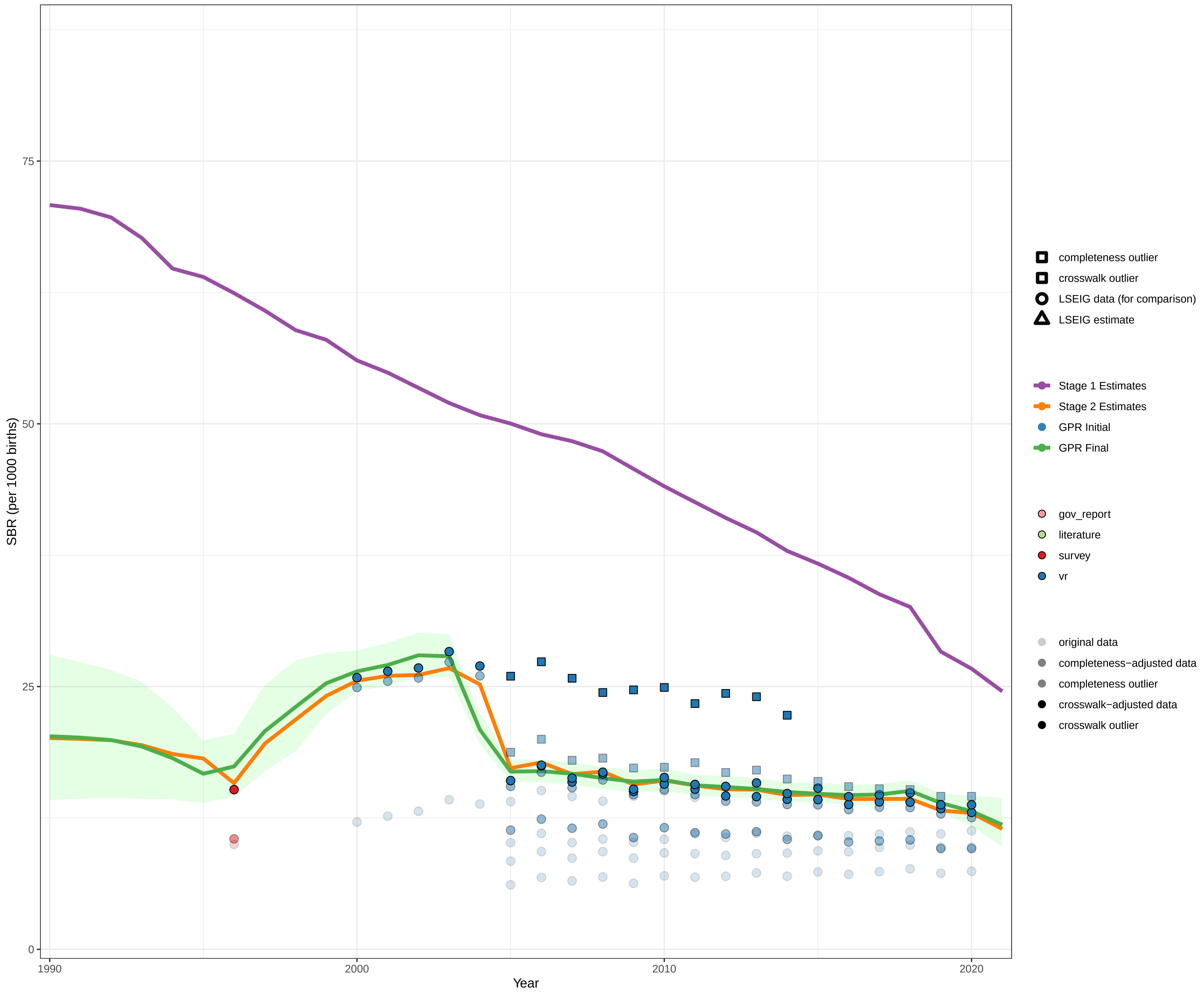

Bahia   BRA_4754   Tropical Latin America   Stillbirth Rate (SBR) − 28 weeks
lambda = 0.2 ; zeta = 0.99 ; scale = 5 ; amp = 0.028

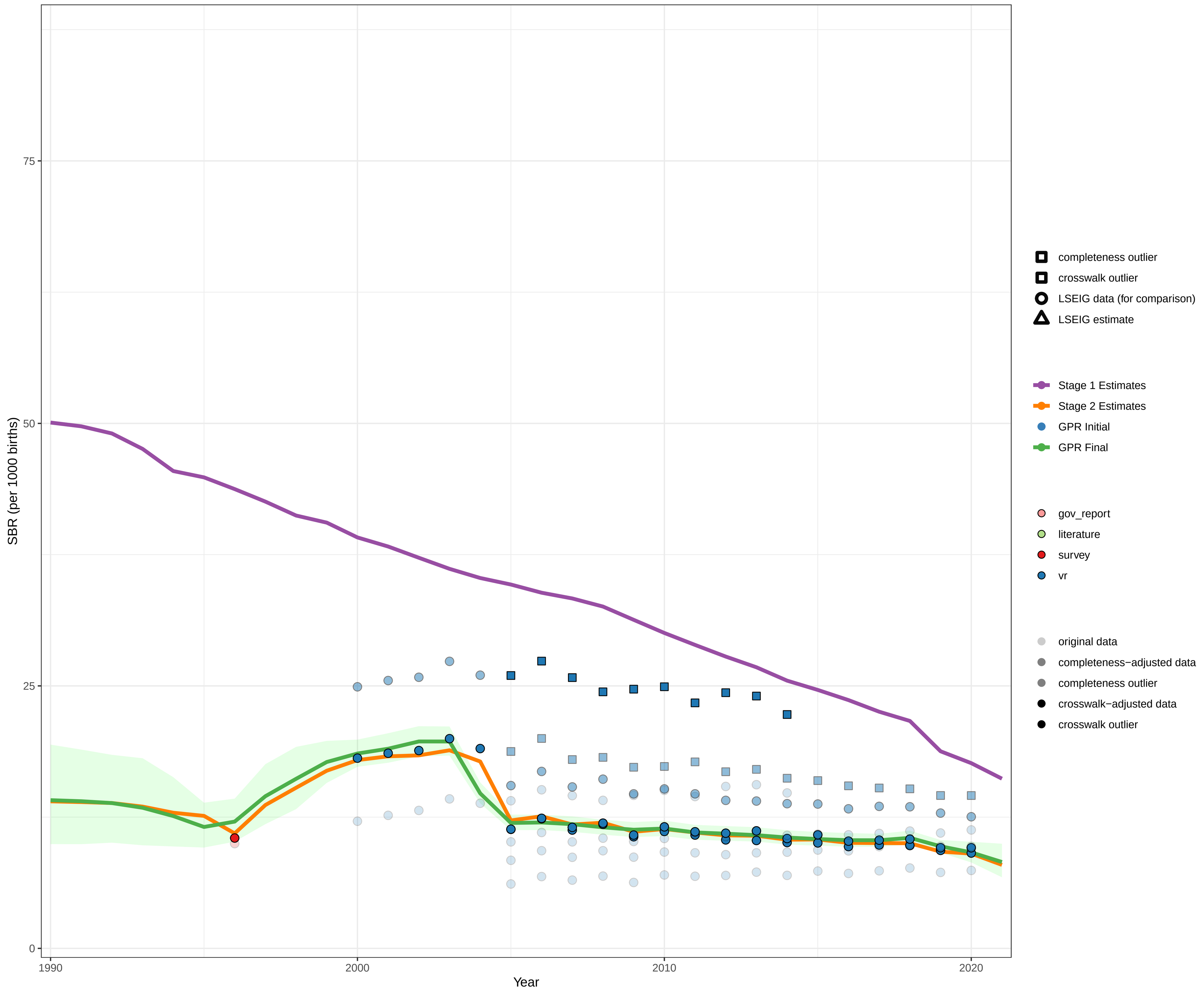

Year	Total Stillbirths (in thousands)	Total Stillbirths (in thousands)	SBR (per 1000 births)	SBR (per 1000 births)	SBR/NMR	SBR/NMR
	20 weeks	28 weeks	20 weeks	28 weeks	20 weeks	28 weeks
1990	6.0 (4.2 to 8.3)	4.1 (2.9 to 5.7)	20.3 (14.2 to 28.0)	14.1 (9.9 to 19.4)	0.93 (0.65 to 1.28)	0.65 (0.45 to 0.89)
1995	5.1 (4.2 to 6.1)	3.5 (2.9 to 4.2)	16.7 (13.9 to 19.9)	11.6 (9.6 to 13.9)	0.85 (0.71 to 1.02)	0.59 (0.49 to 0.71)
2000	8.9 (8.2 to 9.6)	6.2 (5.8 to 6.6)	26.4 (24.6 to 28.4)	18.6 (17.3 to 19.9)	1.52 (1.42 to 1.64)	1.07 (1.00 to 1.15)
2005	5.6 (5.3 to 6.0)	3.9 (3.7 to 4.2)	16.9 (15.9 to 18.1)	11.9 (11.3 to 12.7)	1.10 (1.03 to 1.17)	0.77 (0.73 to 0.83)
2010	5.2 (4.8 to 5.5)	3.6 (3.4 to 3.9)	16.1 (15.1 to 17.2)	11.4 (10.7 to 12.2)	1.23 (1.15 to 1.31)	0.87 (0.82 to 0.93)
2015	4.7 (4.4 to 5.0)	3.3 (3.1 to 3.5)	14.8 (13.9 to 15.9)	10.4 (9.8 to 11.1)	1.42 (1.34 to 1.52)	1.00 (0.94 to 1.06)
2020	3.9 (3.5 to 4.4)	2.7 (2.4 to 3.0)	13.1 (11.7 to 14.6)	9.1 (8.2 to 10.2)	1.80 (1.61 to 2.01)	1.25 (1.12 to 1.39)
2021	3.5 (2.9 to 4.3)	2.4 (2.0 to 2.9)	11.9 (9.7 to 14.4)	8.2 (6.8 to 10.0)	1.78 (1.46 to 2.17)	1.24 (1.02 to 1.50)

Ceara BRA_4755 Tropical Latin America Stillbirth Rate / Neonatal Mortality Rate (SBR/NMR) − 20 weeks
lambda = 0.2 ; zeta = 0.99 ; scale = 5 ; amp = 0.028

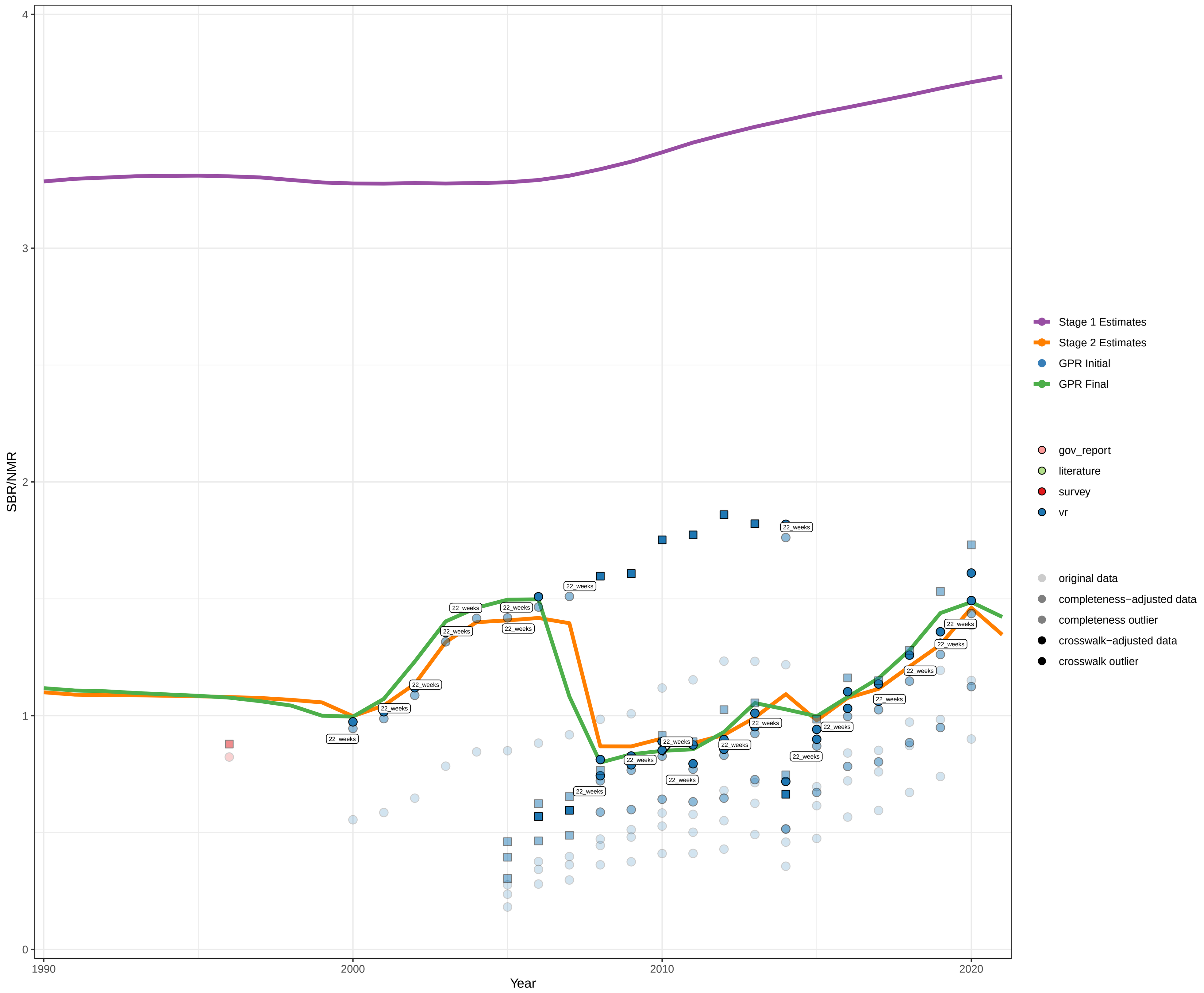

Ceara BRA_4755 Tropical Latin America Stillbirth Rate / Neonatal Mortality Rate (SBR/NMR) − 28 weeks
lambda = 0.2 ; zeta = 0.99 ; scale = 5 ; amp = 0.028

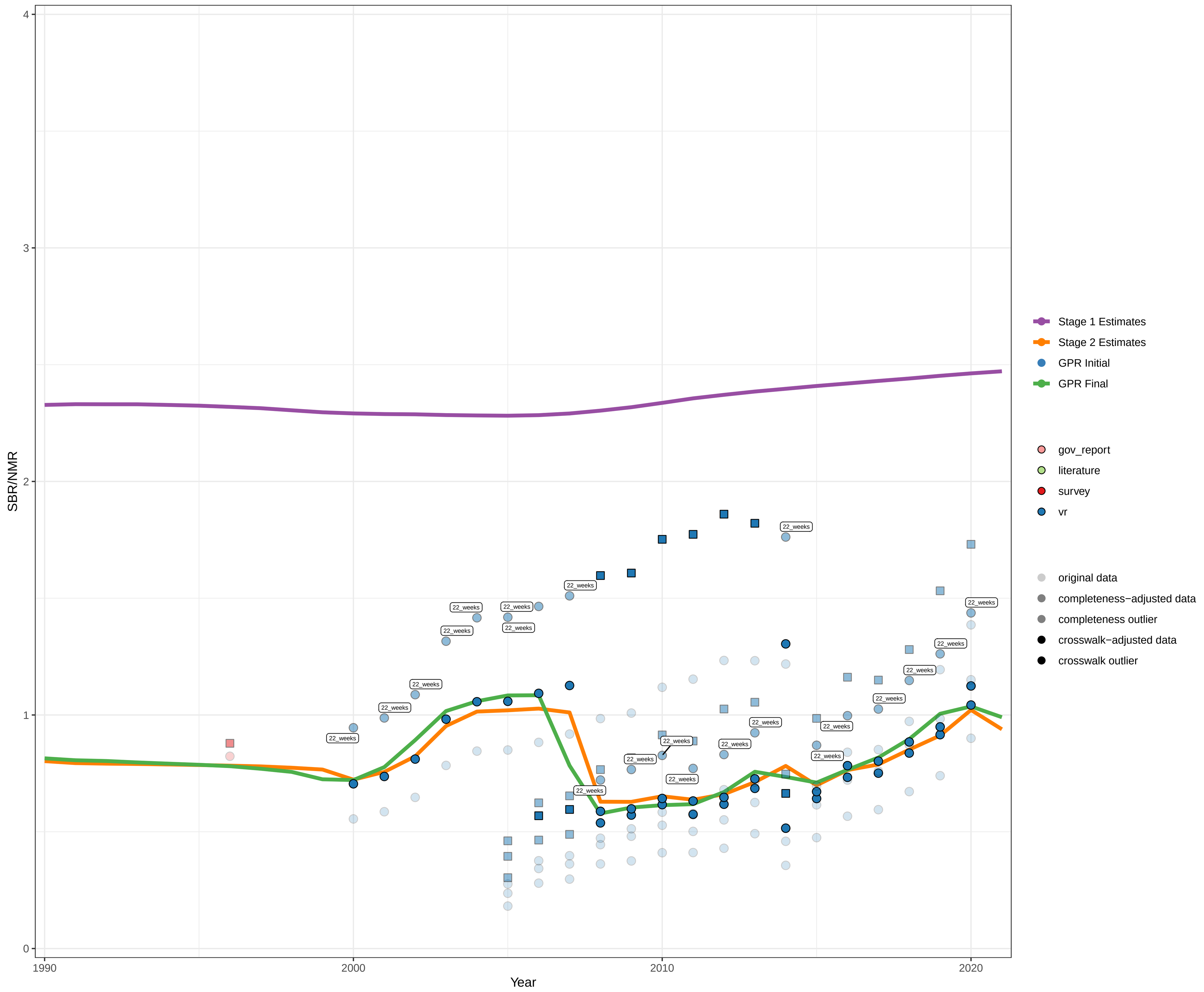

Definition	Years	Outlier	Source.Type	Title
22_weeks	2000−2014	0	VR	Brazil Mortality Information System − Stillbirths
22_weeks	2006−2020	0	VR	Brazil Civil Registry Statistics − Fetal Deaths at 28 Weeks or More, by Place of Birth, Number of Births by Birth and Sex, and Residence of Mother
28_weeks	2008−2020	0	VR	Brazil Civil Registry Statistics − Fetal Deaths at 28 Weeks or More, by Place of Birth, Number of Births by Birth and Sex, and Residence of Mother
22_weeks	2005	1	VR	Brazil Civil Registry Statistics − Fetal Deaths at 28 Weeks or More, by Place of Birth, Number of Births by Birth and Sex, and Residence of Mother
28_weeks	1996	1	Survey	Brazil Demographic and Health Survey 1996
28_weeks	2005−2007	1	VR	Brazil Civil Registry Statistics − Fetal Deaths at 28 Weeks or More, by Place of Birth, Number of Births by Birth and Sex, and Residence of Mother
other	2005−2020	1	VR	Brazil Civil Registry Statistics − Fetal Deaths at 28 Weeks or More, by Place of Birth, Number of Births by Birth and Sex, and Residence of Mother

Ceara BRA_4755 Tropical Latin America Stillbirth Rate (SBR) − 20 weeks
lambda = 0.2 ; zeta = 0.99 ; scale = 5 ; amp = 0.028

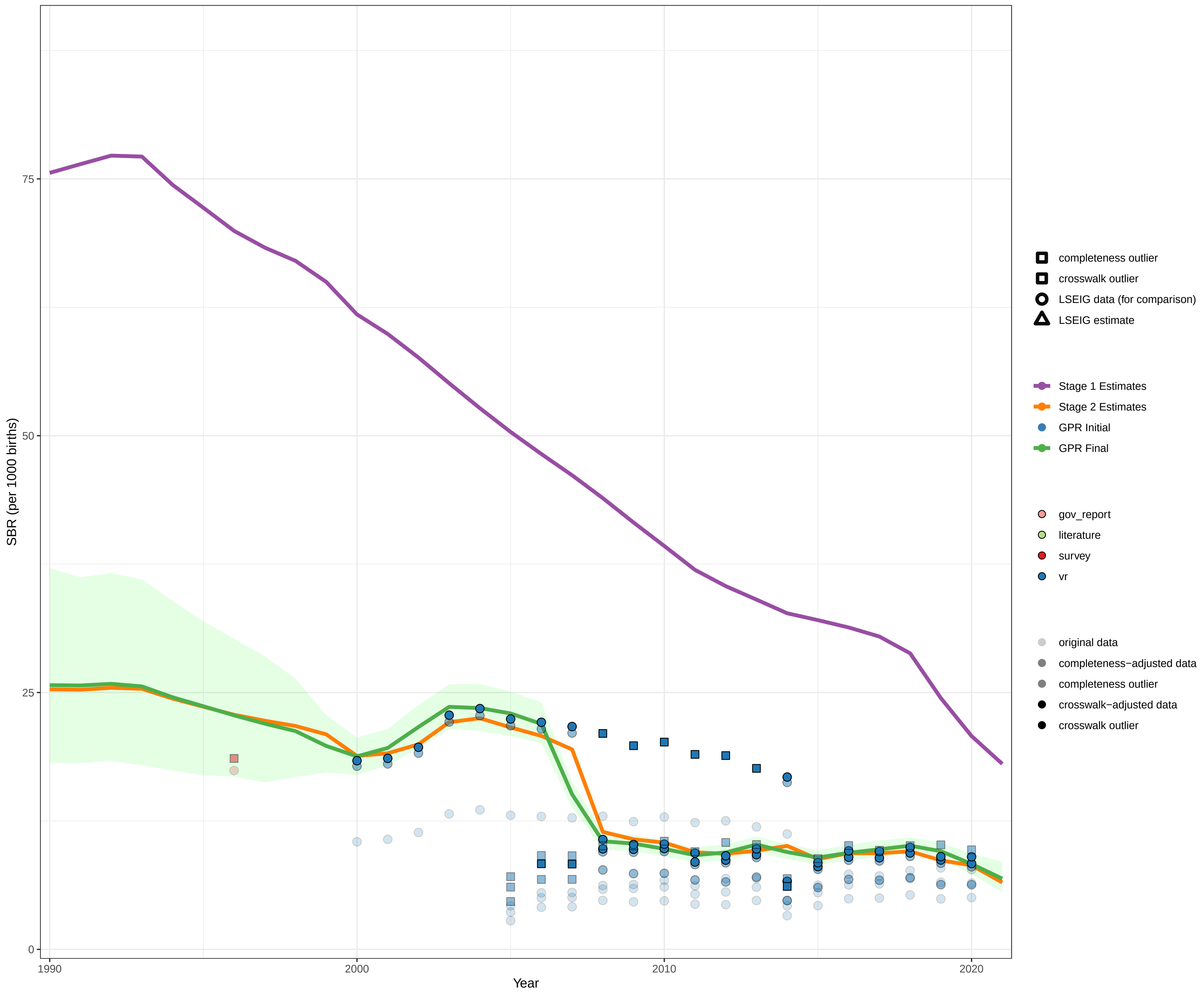

Ceara BRA_4755 Tropical Latin America Stillbirth Rate (SBR) − 28 weeks
lambda = 0.2 ; zeta = 0.99 ; scale = 5 ; amp = 0.028

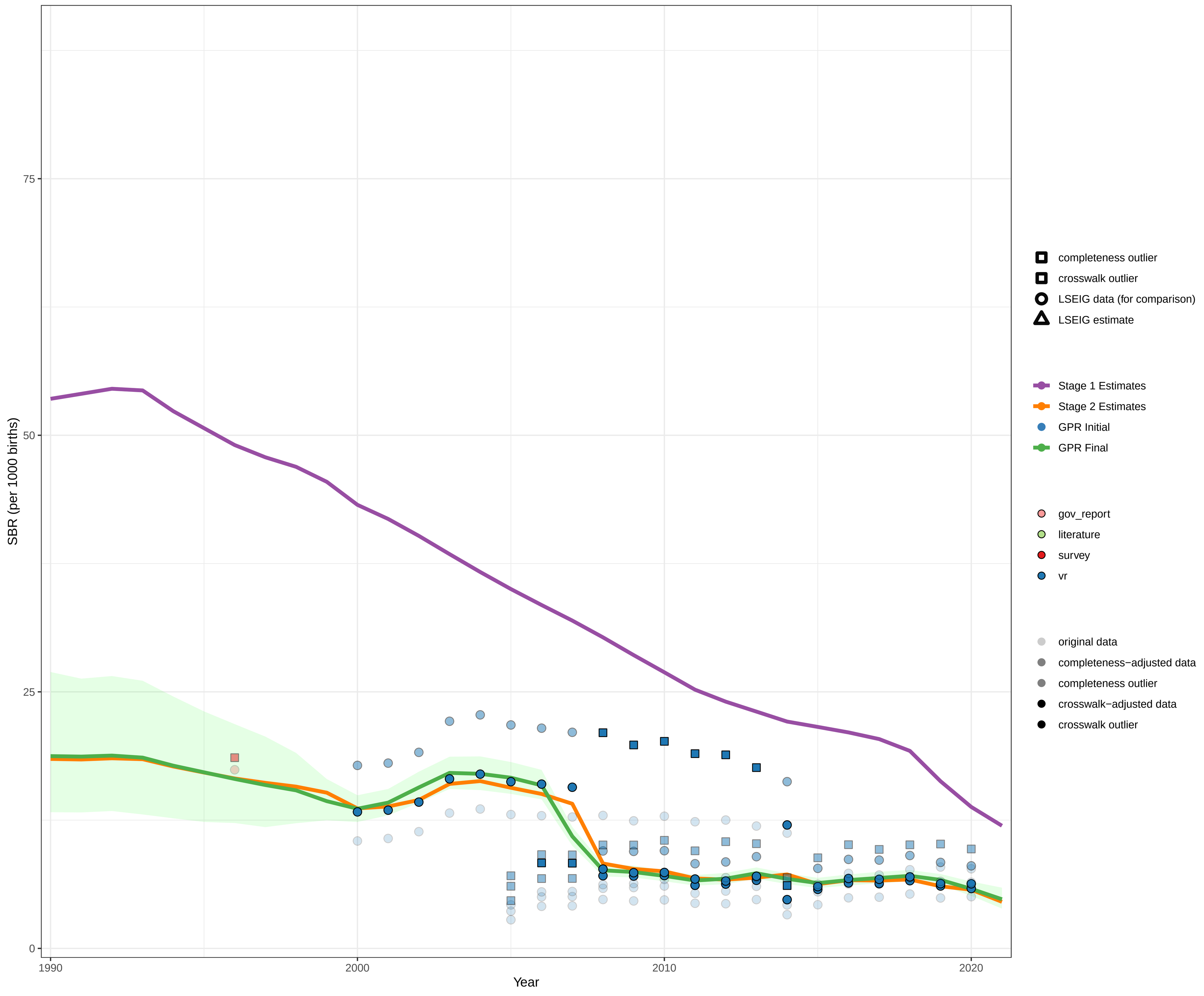

Year	Total Stillbirths (in thousands)	Total Stillbirths (in thousands)	SBR (per 1000 births)	SBR (per 1000 births)	SBR/NMR	SBR/NMR
	20 weeks	28 weeks	20 weeks	28 weeks	20 weeks	28 weeks
1990	4.4 (3.1 to 6.4)	3.2 (2.2 to 4.6)	25.7 (18.2 to 37.1)	18.7 (13.3 to 26.9)	1.12 (0.79 to 1.61)	0.81 (0.58 to 1.17)
1995	4.4 (3.1 to 5.9)	3.1 (2.2 to 4.3)	23.7 (16.9 to 31.9)	17.2 (12.3 to 23.1)	1.09 (0.78 to 1.46)	0.79 (0.56 to 1.06)
2000	3.8 (3.5 to 4.2)	2.8 (2.5 to 3.0)	18.8 (17.0 to 20.6)	13.6 (12.3 to 14.9)	1.00 (0.90 to 1.09)	0.72 (0.65 to 0.79)
2005	4.8 (4.3 to 5.3)	3.5 (3.1 to 3.8)	23.0 (20.8 to 25.1)	16.6 (15.1 to 18.2)	1.50 (1.35 to 1.64)	1.08 (0.98 to 1.18)
2010	2.0 (1.9 to 2.2)	1.5 (1.4 to 1.6)	9.8 (9.0 to 10.5)	7.1 (6.5 to 7.6)	0.85 (0.79 to 0.92)	0.61 (0.57 to 0.66)
2015	1.9 (1.8 to 2.1)	1.4 (1.3 to 1.5)	8.9 (8.2 to 9.7)	6.4 (5.9 to 6.9)	1.00 (0.92 to 1.08)	0.71 (0.66 to 0.77)
2020	1.8 (1.5 to 2.0)	1.2 (1.1 to 1.4)	8.3 (7.3 to 9.4)	5.8 (5.1 to 6.5)	1.49 (1.31 to 1.67)	1.04 (0.91 to 1.16)
2021	1.4 (1.2 to 1.8)	1.0 (0.8 to 1.2)	6.9 (5.6 to 8.5)	4.8 (3.9 to 5.9)	1.42 (1.15 to 1.76)	0.99 (0.80 to 1.22)

Distrito Federal BRA_4756 Tropical Latin America Stillbirth Rate / Neonatal Mortality Rate (SBR/NMR) − 20 weeks
lambda = 0.2 ; zeta = 0.99 ; scale = 5 ; amp = 0.028

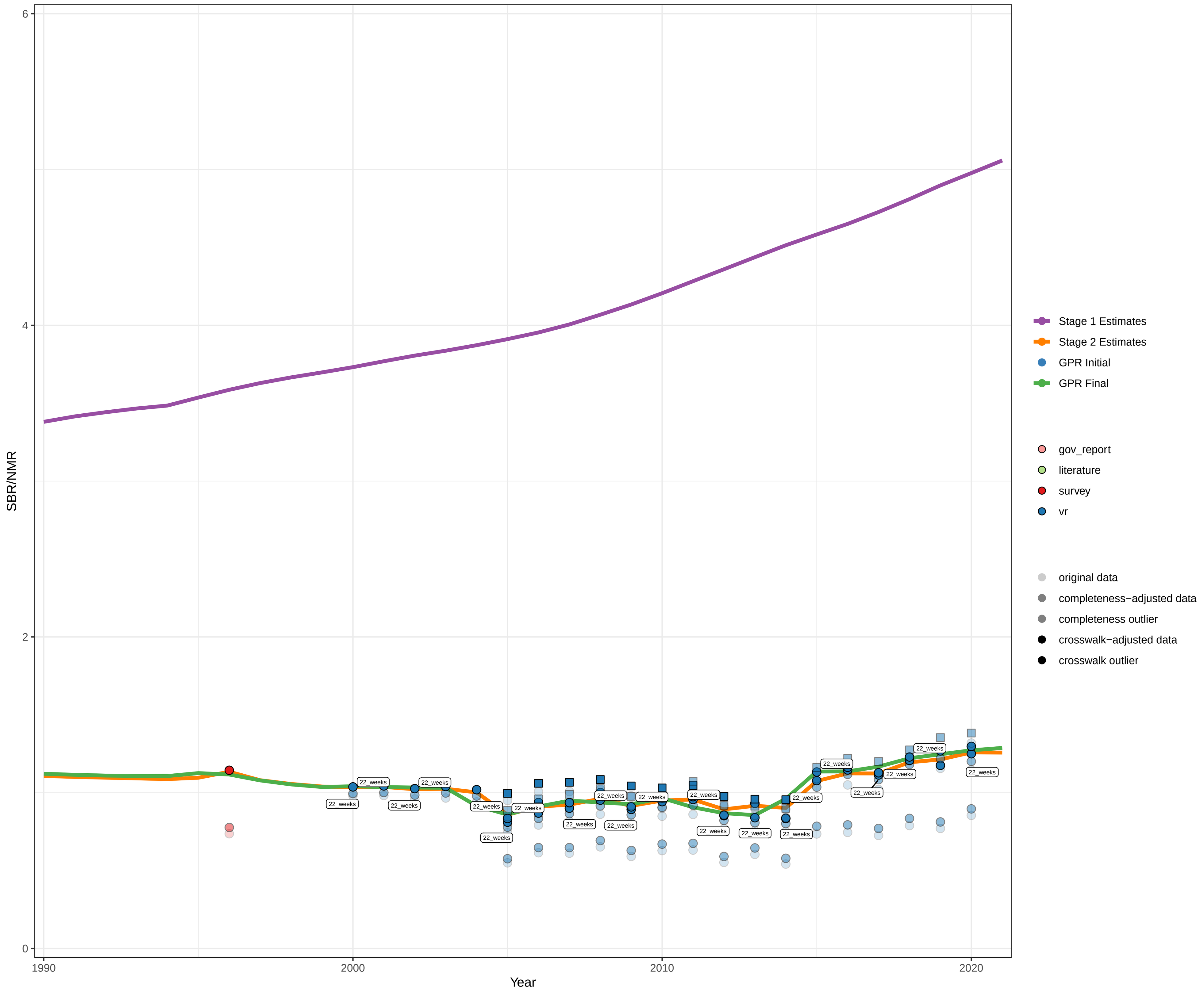

Distrito Federal BRA_4756 Tropical Latin America Stillbirth Rate / Neonatal Mortality Rate (SBR/NMR) − 28 weeks
lambda = 0.2 ; zeta = 0.99 ; scale = 5 ; amp = 0.028

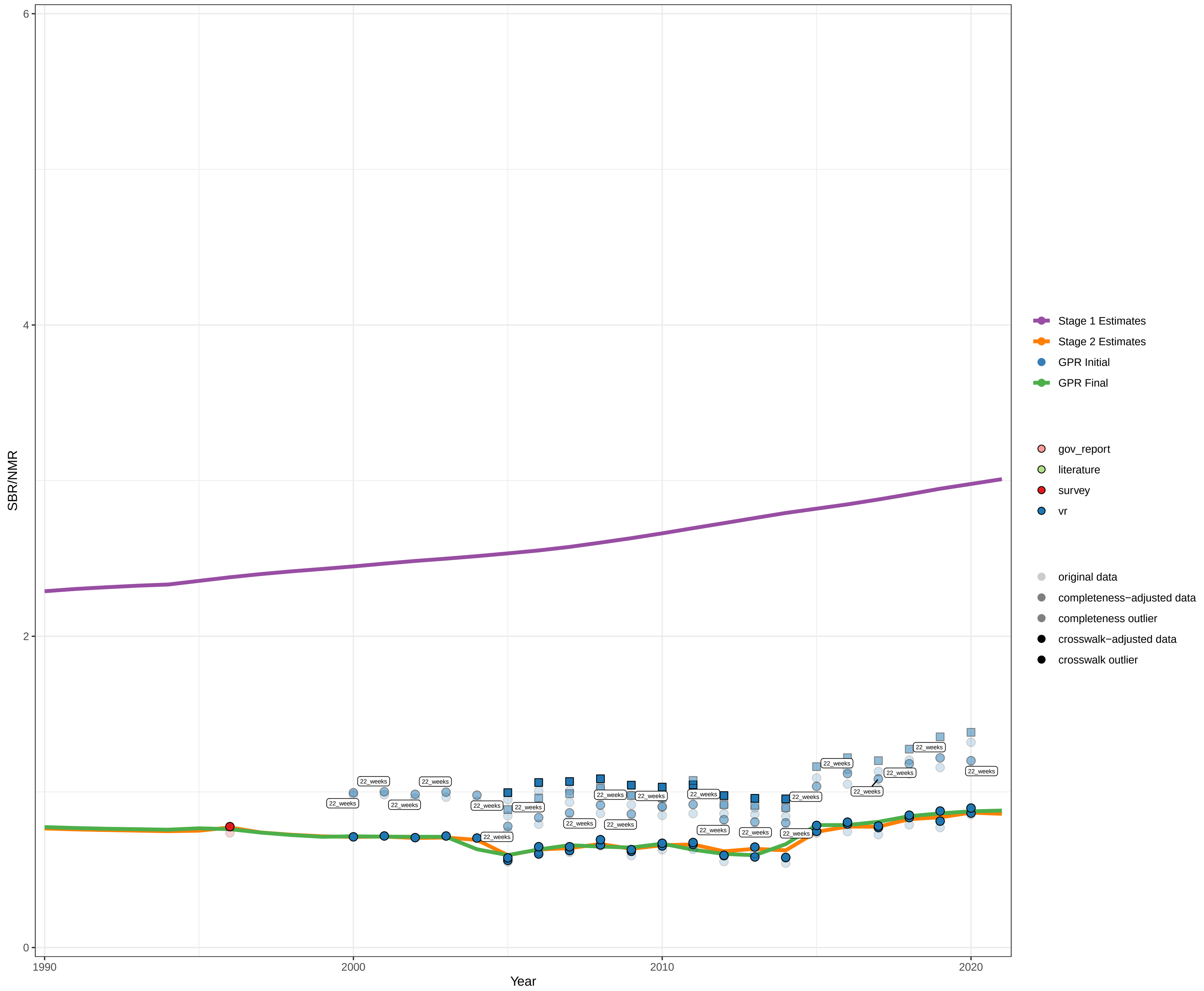

Definition	Years	Outlier	Source.Type	Title
22_weeks	2000−2014	0	VR	Brazil Mortality Information System − Stillbirths
22_weeks, 28_weeks	2005−2020	0	VR	Brazil Civil Registry Statistics − Fetal Deaths at 28 Weeks or More, by Place of Birth, Number of Births by Birth and Sex, and Residence of Mother
28_weeks	1996	0	Survey	Brazil Demographic and Health Survey 1996
other	2005−2020	1	VR	Brazil Civil Registry Statistics − Fetal Deaths at 28 Weeks or More, by Place of Birth, Number of Births by Birth and Sex, and Residence of Mother

Distrito Federal BRA_4756 Tropical Latin America Stillbirth Rate (SBR) − 20 weeks
lambda = 0.2 ; zeta = 0.99 ; scale = 5 ; amp = 0.028

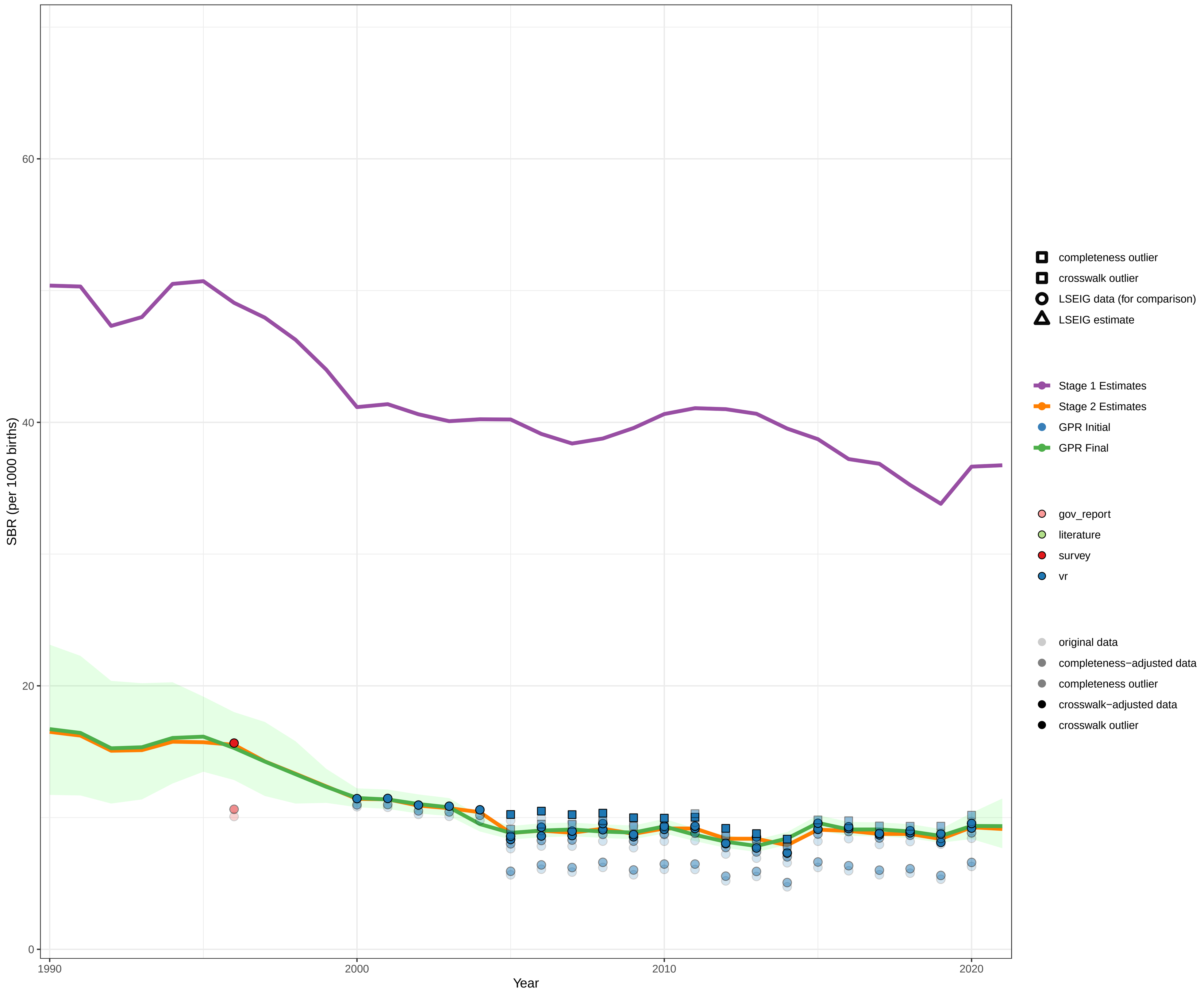

Distrito Federal BRA_4756 Tropical Latin America Stillbirth Rate (SBR) − 28 weeks
lambda = 0.2 ; zeta = 0.99 ; scale = 5 ; amp = 0.028

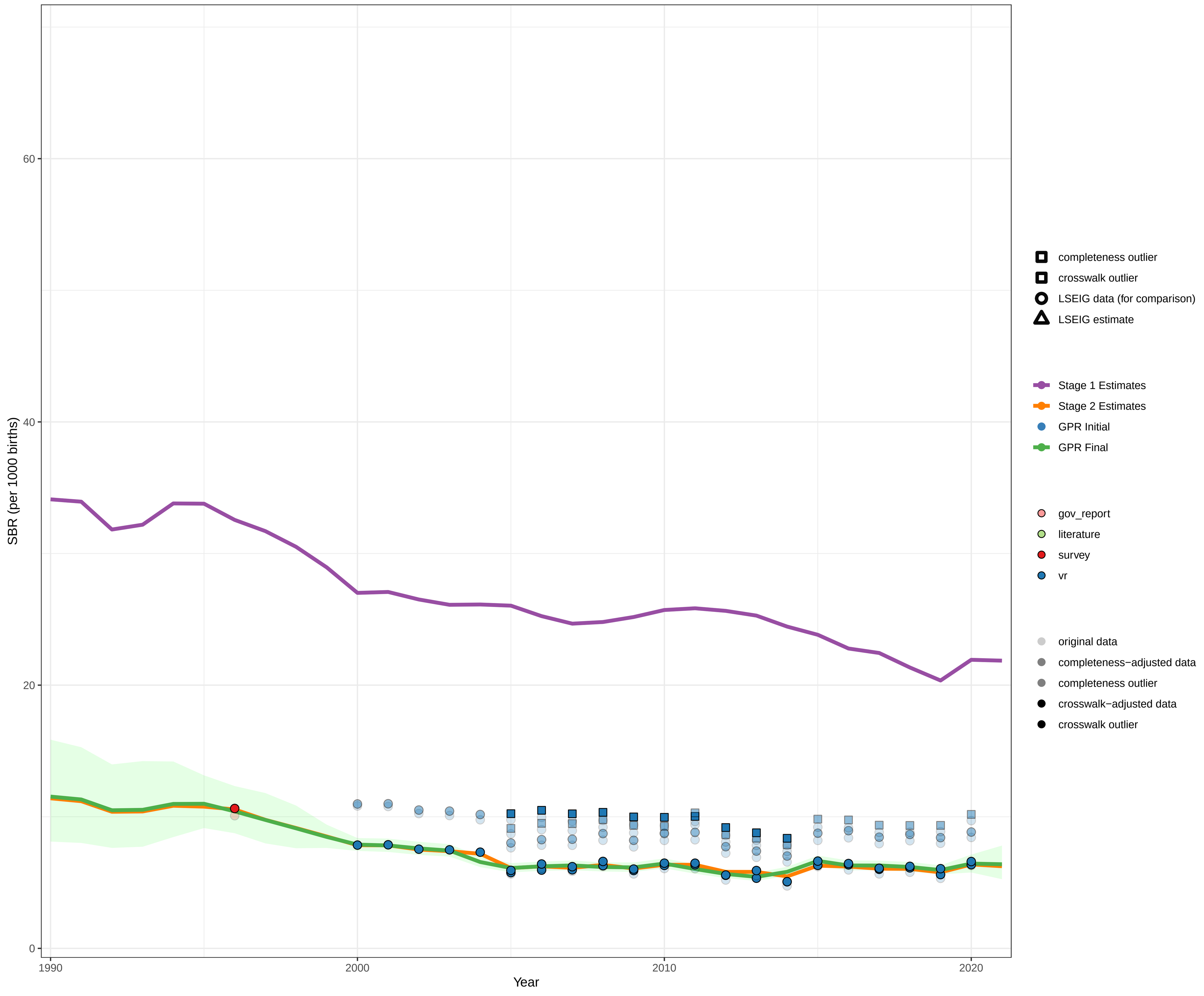

Year	Total Stillbirths (in thousands)	Total Stillbirths (in thousands)	SBR (per 1000 births)	SBR (per 1000 births)	SBR/NMR	SBR/NMR
	20 weeks	28 weeks	20 weeks	28 weeks	20 weeks	28 weeks
1990	0.7 (0.5 to 0.9)	0.4 (0.3 to 0.6)	16.7 (11.7 to 23.1)	11.5 (8.1 to 15.9)	1.12 (0.79 to 1.55)	0.77 (0.54 to 1.06)
1995	0.7 (0.6 to 0.8)	0.5 (0.4 to 0.6)	16.1 (13.5 to 19.2)	11.0 (9.1 to 13.1)	1.13 (0.94 to 1.34)	0.77 (0.64 to 0.92)
2000	0.5 (0.5 to 0.6)	0.4 (0.3 to 0.4)	11.5 (10.8 to 12.2)	7.9 (7.4 to 8.4)	1.04 (0.98 to 1.11)	0.71 (0.67 to 0.76)
2005	0.4 (0.4 to 0.4)	0.3 (0.3 to 0.3)	8.8 (8.3 to 9.4)	6.1 (5.8 to 6.5)	0.86 (0.81 to 0.91)	0.59 (0.56 to 0.63)
2010	0.4 (0.4 to 0.4)	0.3 (0.3 to 0.3)	9.3 (8.8 to 9.9)	6.4 (6.1 to 6.8)	0.97 (0.91 to 1.03)	0.67 (0.63 to 0.71)
2015	0.4 (0.4 to 0.5)	0.3 (0.3 to 0.3)	9.6 (9.0 to 10.2)	6.6 (6.3 to 7.1)	1.14 (1.07 to 1.21)	0.79 (0.74 to 0.83)
2020	0.4 (0.4 to 0.4)	0.3 (0.2 to 0.3)	9.4 (8.4 to 10.4)	6.4 (5.8 to 7.1)	1.27 (1.14 to 1.41)	0.88 (0.78 to 0.97)
2021	0.4 (0.3 to 0.5)	0.3 (0.2 to 0.3)	9.4 (7.7 to 11.4)	6.4 (5.3 to 7.8)	1.29 (1.06 to 1.58)	0.88 (0.72 to 1.08)

Espirito Santo BRA_4757 Tropical Latin America Stillbirth Rate / Neonatal Mortality Rate (SBR/NMR) − 20 weeks
lambda = 0.2 ; zeta = 0.99 ; scale = 5 ; amp = 0.028

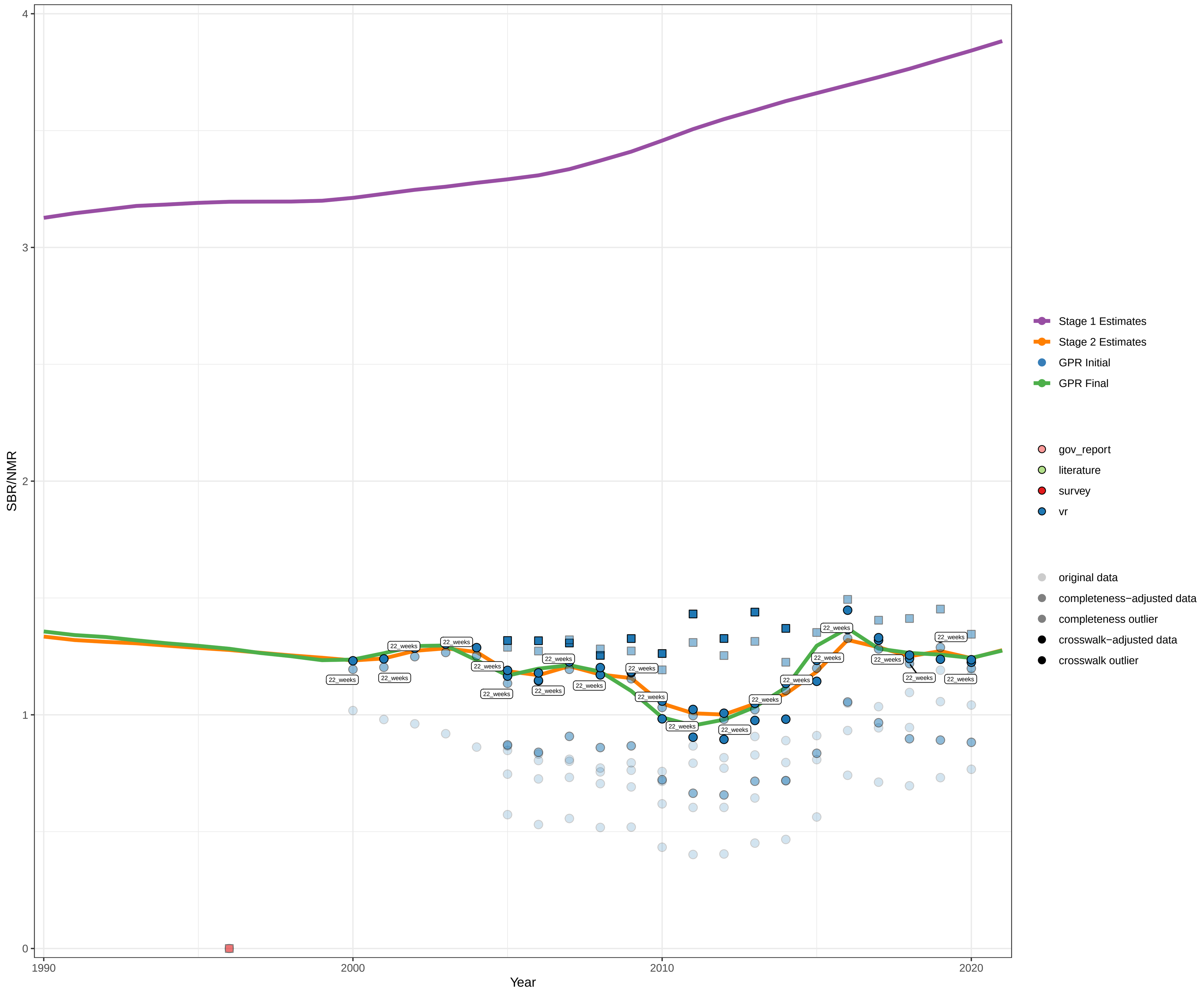

Espirito Santo BRA_4757 Tropical Latin America Stillbirth Rate / Neonatal Mortality Rate (SBR/NMR) − 28 weeks
lambda = 0.2 ; zeta = 0.99 ; scale = 5 ; amp = 0.028

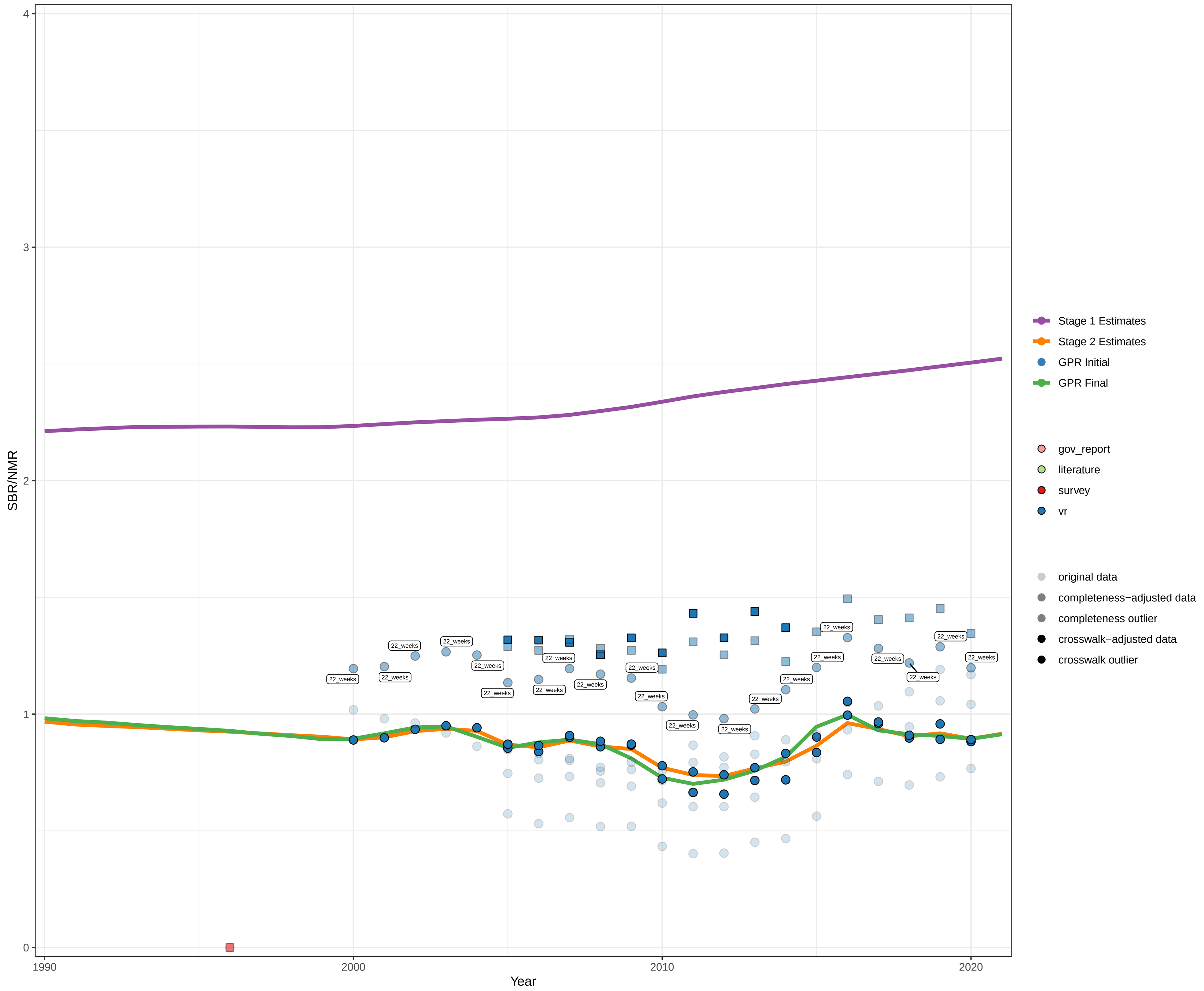

Definition	Years	Outlier	Source.Type	Title
22_weeks	2000−2014	0	VR	Brazil Mortality Information System − Stillbirths
22_weeks, 28_weeks	2005−2020	0	VR	Brazil Civil Registry Statistics − Fetal Deaths at 28 Weeks or More, by Place of Birth, Number of Births by Birth and Sex, and Residence of Mother
28_weeks	1996	1	Survey	Brazil Demographic and Health Survey 1996
other	2005−2020	1	VR	Brazil Civil Registry Statistics − Fetal Deaths at 28 Weeks or More, by Place of Birth, Number of Births by Birth and Sex, and Residence of Mother

Espirito Santo BRA_4757 Tropical Latin America Stillbirth Rate (SBR) − 20 weeks
lambda = 0.2 ; zeta = 0.99 ; scale = 5 ; amp = 0.028

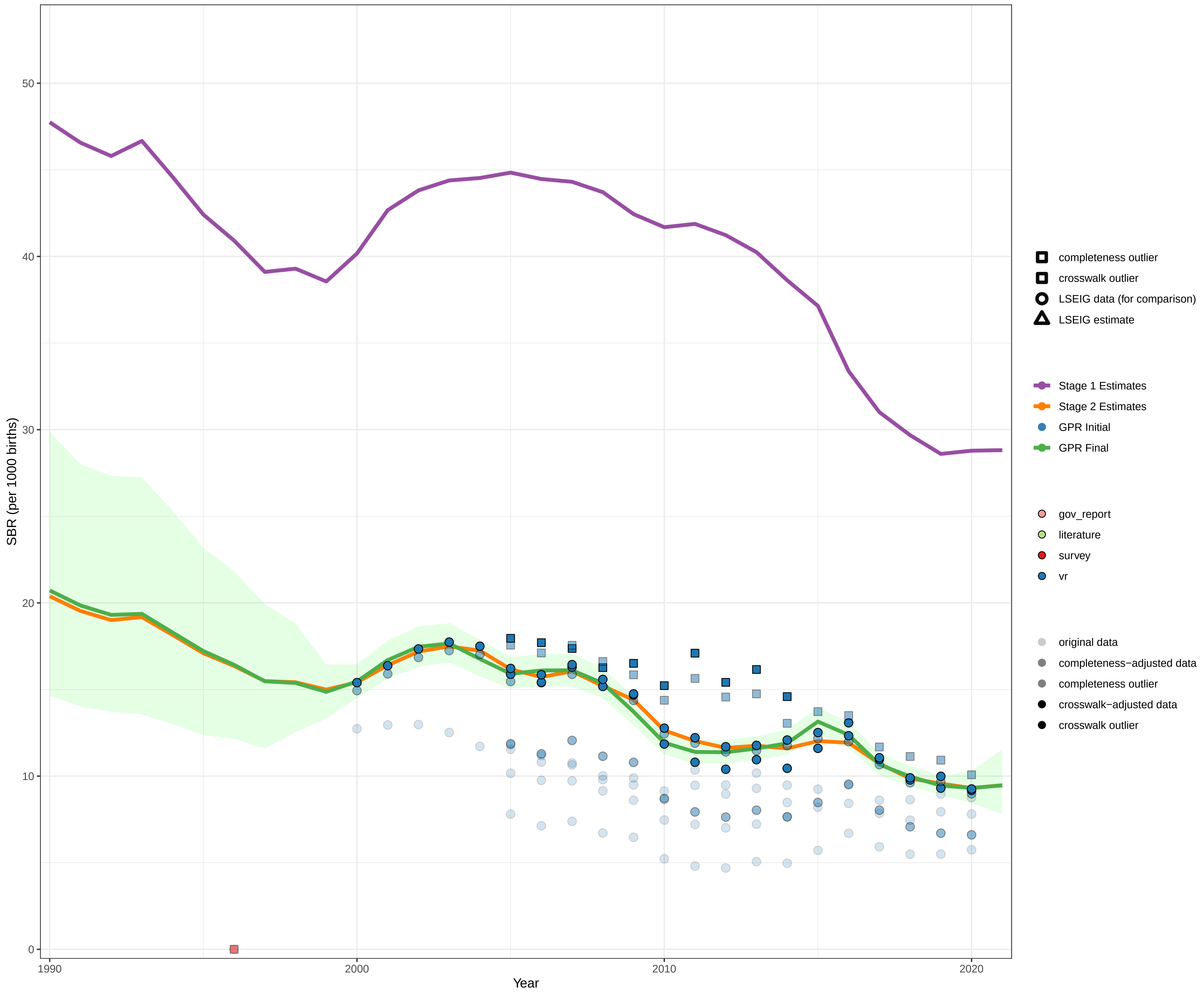

Espirito Santo BRA_4757 Tropical Latin America Stillbirth Rate (SBR) − 28 weeks
lambda = 0.2 ; zeta = 0.99 ; scale = 5 ; amp = 0.028

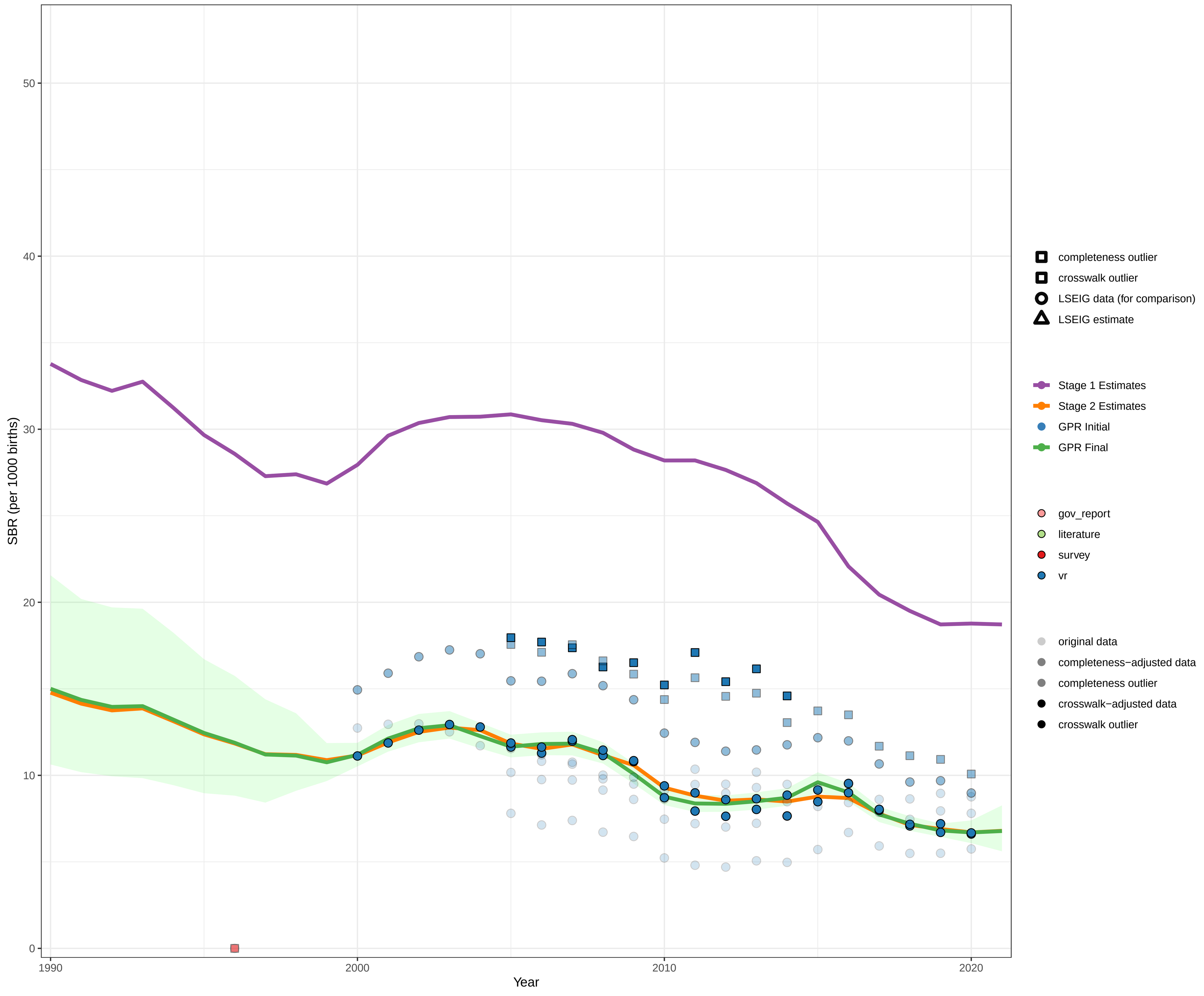

Year	Total Stillbirths (in thousands)	Total Stillbirths (in thousands)	SBR (per 1000 births)	SBR (per 1000 births)	SBR/NMR	SBR/NMR
	20 weeks	28 weeks	20 weeks	28 weeks	20 weeks	28 weeks
1990	1.2 (0.8 to 1.8)	0.9 (0.6 to 1.3)	20.7 (14.6 to 29.9)	15.0 (10.6 to 21.6)	1.36 (0.96 to 1.96)	0.98 (0.70 to 1.41)
1995	1.0 (0.7 to 1.4)	0.8 (0.5 to 1.0)	17.2 (12.4 to 23.2)	12.4 (9.0 to 16.7)	1.30 (0.93 to 1.74)	0.94 (0.67 to 1.26)
2000	1.0 (0.9 to 1.1)	0.7 (0.7 to 0.8)	15.5 (14.5 to 16.5)	11.2 (10.5 to 11.9)	1.24 (1.16 to 1.32)	0.89 (0.84 to 0.95)
2005	1.0 (0.9 to 1.0)	0.7 (0.7 to 0.7)	15.9 (15.0 to 16.9)	11.7 (11.0 to 12.3)	1.17 (1.10 to 1.24)	0.86 (0.81 to 0.91)
2010	0.7 (0.7 to 0.8)	0.5 (0.5 to 0.6)	11.9 (11.2 to 12.7)	8.8 (8.3 to 9.3)	0.99 (0.93 to 1.05)	0.73 (0.69 to 0.77)
2015	0.8 (0.8 to 0.9)	0.6 (0.6 to 0.6)	13.1 (12.4 to 14.0)	9.6 (9.0 to 10.2)	1.30 (1.22 to 1.38)	0.95 (0.89 to 1.00)
2020	0.6 (0.5 to 0.6)	0.4 (0.4 to 0.4)	9.3 (8.4 to 10.3)	6.7 (6.1 to 7.4)	1.24 (1.13 to 1.38)	0.89 (0.81 to 0.99)
2021	0.6 (0.5 to 0.7)	0.4 (0.3 to 0.5)	9.5 (7.8 to 11.6)	6.8 (5.6 to 8.3)	1.27 (1.05 to 1.56)	0.91 (0.76 to 1.11)

Goias BRA_4758 Tropical Latin America Stillbirth Rate / Neonatal Mortality Rate (SBR/NMR) − 20 weeks
lambda = 0.2 ; zeta = 0.99 ; scale = 5 ; amp = 0.028

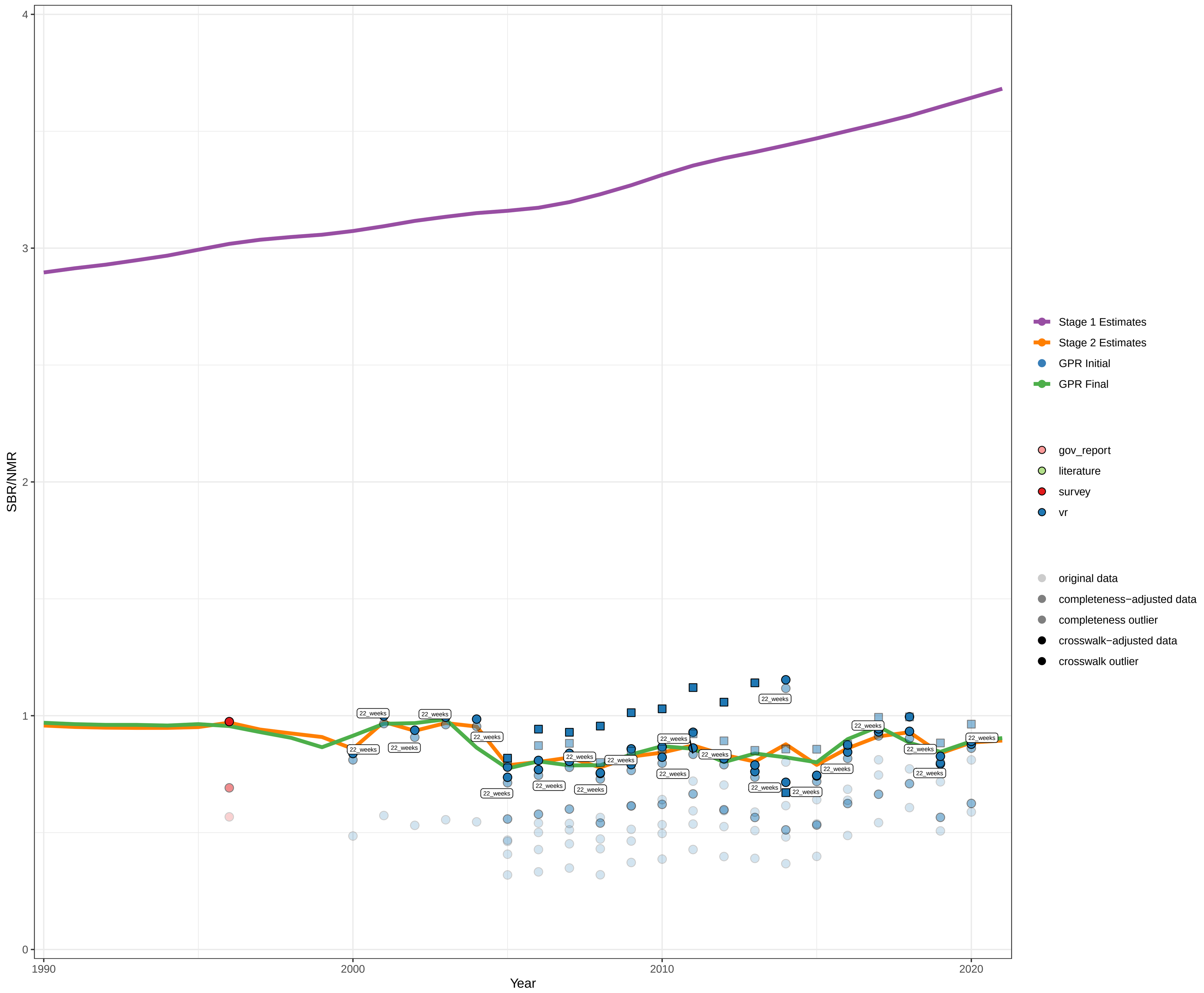

Goias BRA_4758 Tropical Latin America Stillbirth Rate / Neonatal Mortality Rate (SBR/NMR) − 28 weeks
lambda = 0.2 ; zeta = 0.99 ; scale = 5 ; amp = 0.028

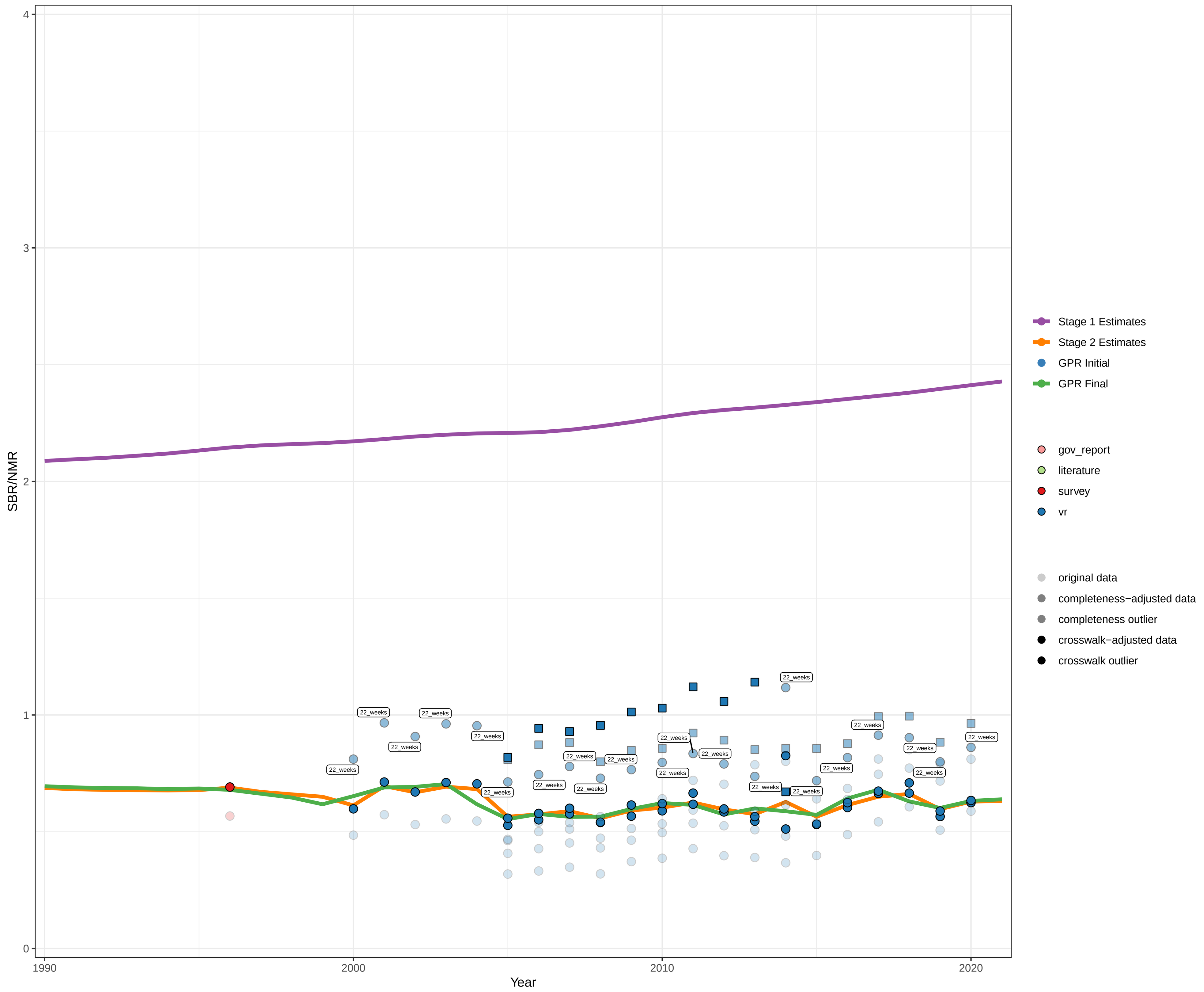

Definition	Years	Outlier	Source.Type	Title
22_weeks	2000−2014	0	VR	Brazil Mortality Information System − Stillbirths
22_weeks, 28_weeks	2005−2020	0	VR	Brazil Civil Registry Statistics − Fetal Deaths at 28 Weeks or More, by Place of Birth, Number of Births by Birth and Sex, and Residence of Mother
28_weeks	1996	0	Survey	Brazil Demographic and Health Survey 1996
other	2005−2020	1	VR	Brazil Civil Registry Statistics − Fetal Deaths at 28 Weeks or More, by Place of Birth, Number of Births by Birth and Sex, and Residence of Mother

Goias BRA_4758 Tropical Latin America Stillbirth Rate (SBR) − 20 weeks
lambda = 0.2 ; zeta = 0.99 ; scale = 5 ; amp = 0.028

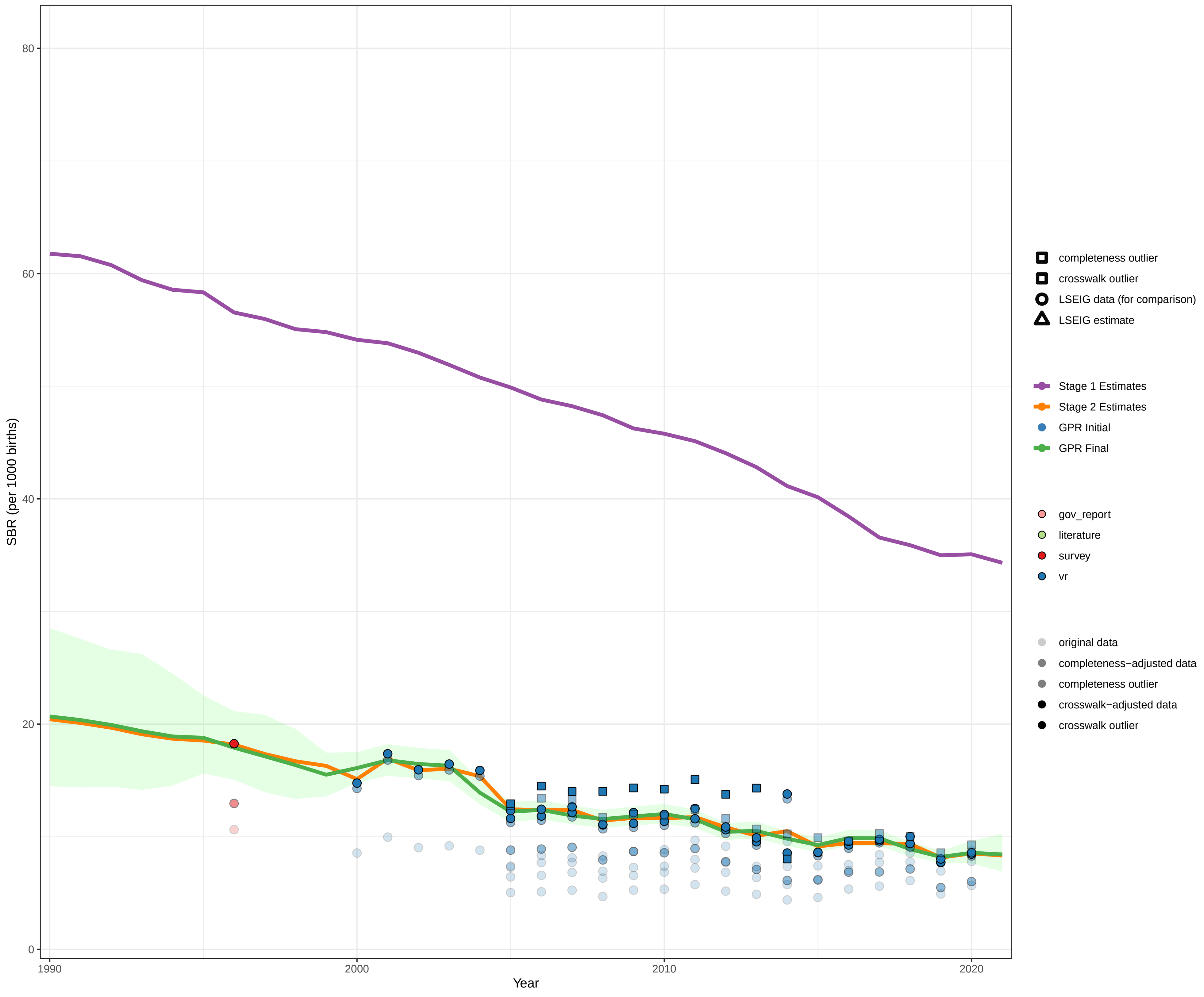

Goias BRA_4758 Tropical Latin America Stillbirth Rate (SBR) − 28 weeks
lambda = 0.2 ; zeta = 0.99 ; scale = 5 ; amp = 0.028

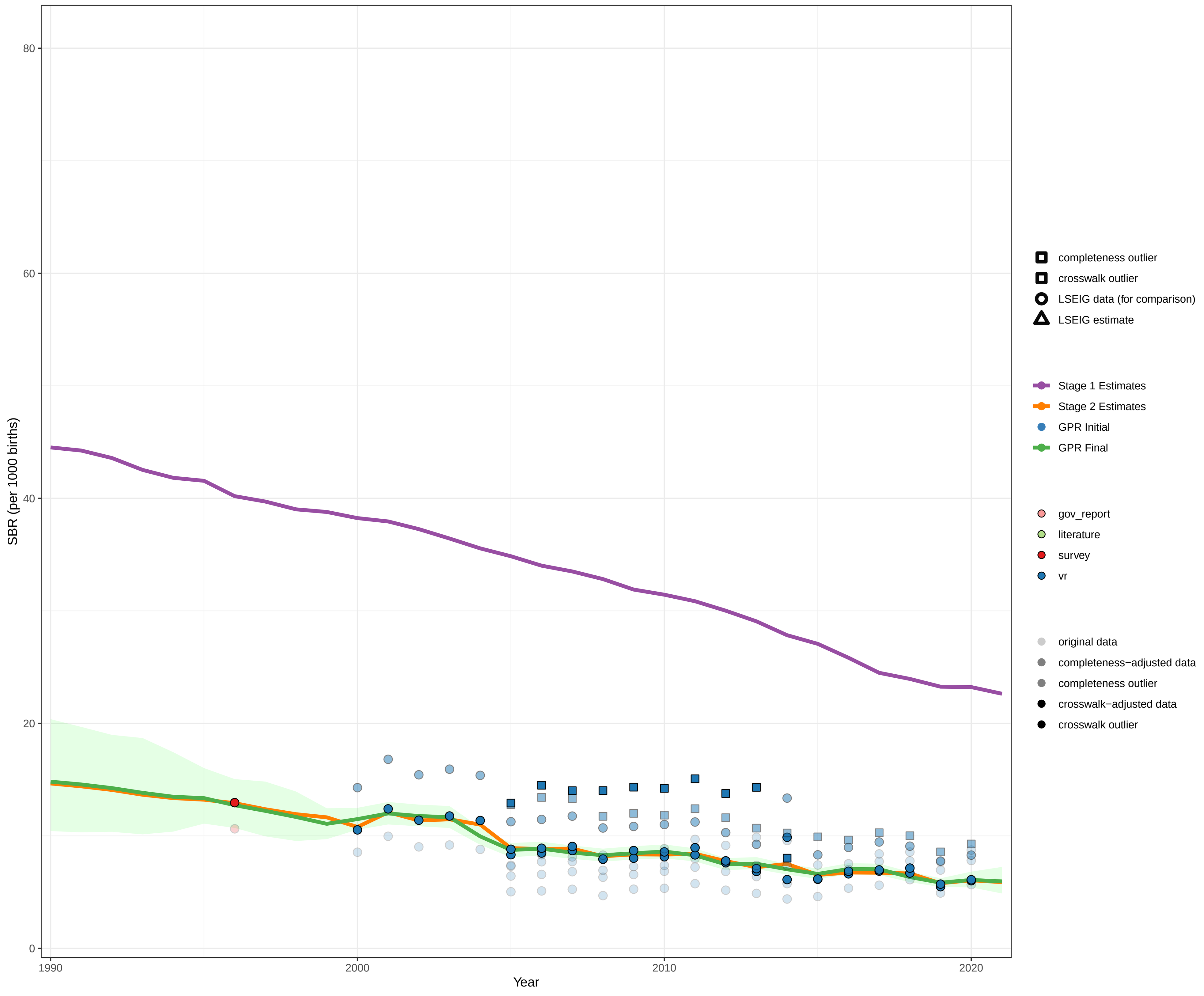

Year	Total Stillbirths (in thousands)	Total Stillbirths (in thousands)	SBR (per 1000 births)	SBR (per 1000 births)	SBR/NMR	SBR/NMR
	20 weeks	28 weeks	20 weeks	28 weeks	20 weeks	28 weeks
1990	1.8 (1.2 to 2.4)	1.2 (0.9 to 1.7)	20.7 (14.5 to 28.5)	14.8 (10.4 to 20.4)	0.97 (0.68 to 1.34)	0.69 (0.49 to 0.96)
1995	1.7 (1.4 to 2.1)	1.2 (1.0 to 1.4)	18.8 (15.6 to 22.6)	13.4 (11.1 to 16.0)	0.96 (0.80 to 1.16)	0.69 (0.57 to 0.82)
2000	1.5 (1.4 to 1.7)	1.1 (1.0 to 1.2)	16.1 (14.8 to 17.5)	11.5 (10.6 to 12.5)	0.91 (0.84 to 0.99)	0.65 (0.60 to 0.71)
2005	1.1 (1.0 to 1.2)	0.8 (0.7 to 0.8)	12.2 (11.3 to 13.1)	8.8 (8.1 to 9.4)	0.77 (0.72 to 0.83)	0.55 (0.51 to 0.59)
2010	1.1 (1.0 to 1.1)	0.8 (0.7 to 0.8)	12.0 (11.2 to 12.9)	8.6 (8.0 to 9.2)	0.87 (0.81 to 0.93)	0.62 (0.58 to 0.67)
2015	0.9 (0.8 to 0.9)	0.6 (0.6 to 0.7)	9.3 (8.6 to 9.9)	6.6 (6.2 to 7.1)	0.80 (0.75 to 0.86)	0.57 (0.53 to 0.61)
2020	0.8 (0.7 to 0.9)	0.6 (0.5 to 0.6)	8.6 (7.6 to 9.6)	6.1 (5.4 to 6.8)	0.89 (0.79 to 1.00)	0.63 (0.56 to 0.71)
2021	0.8 (0.6 to 0.9)	0.5 (0.4 to 0.7)	8.4 (6.9 to 10.3)	6.0 (4.9 to 7.2)	0.90 (0.74 to 1.10)	0.64 (0.52 to 0.78)

Maranhao BRA_4759 Tropical Latin America Stillbirth Rate / Neonatal Mortality Rate (SBR/NMR) − 20 weeks
lambda = 0.2 ; zeta = 0.99 ; scale = 5 ; amp = 0.028

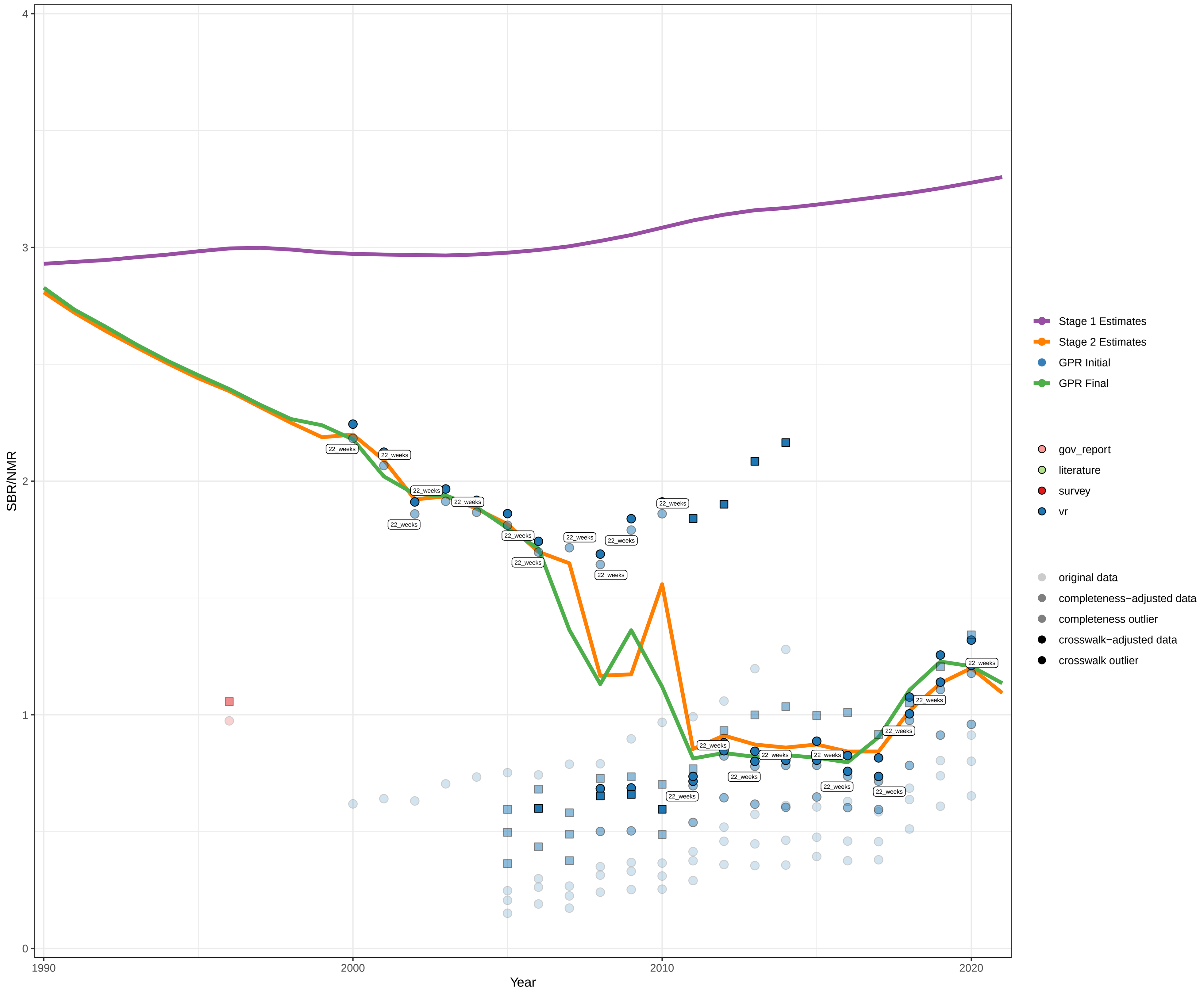

Maranhao BRA_4759 Tropical Latin America Stillbirth Rate / Neonatal Mortality Rate (SBR/NMR) − 28 weeks
lambda = 0.2 ; zeta = 0.99 ; scale = 5 ; amp = 0.028

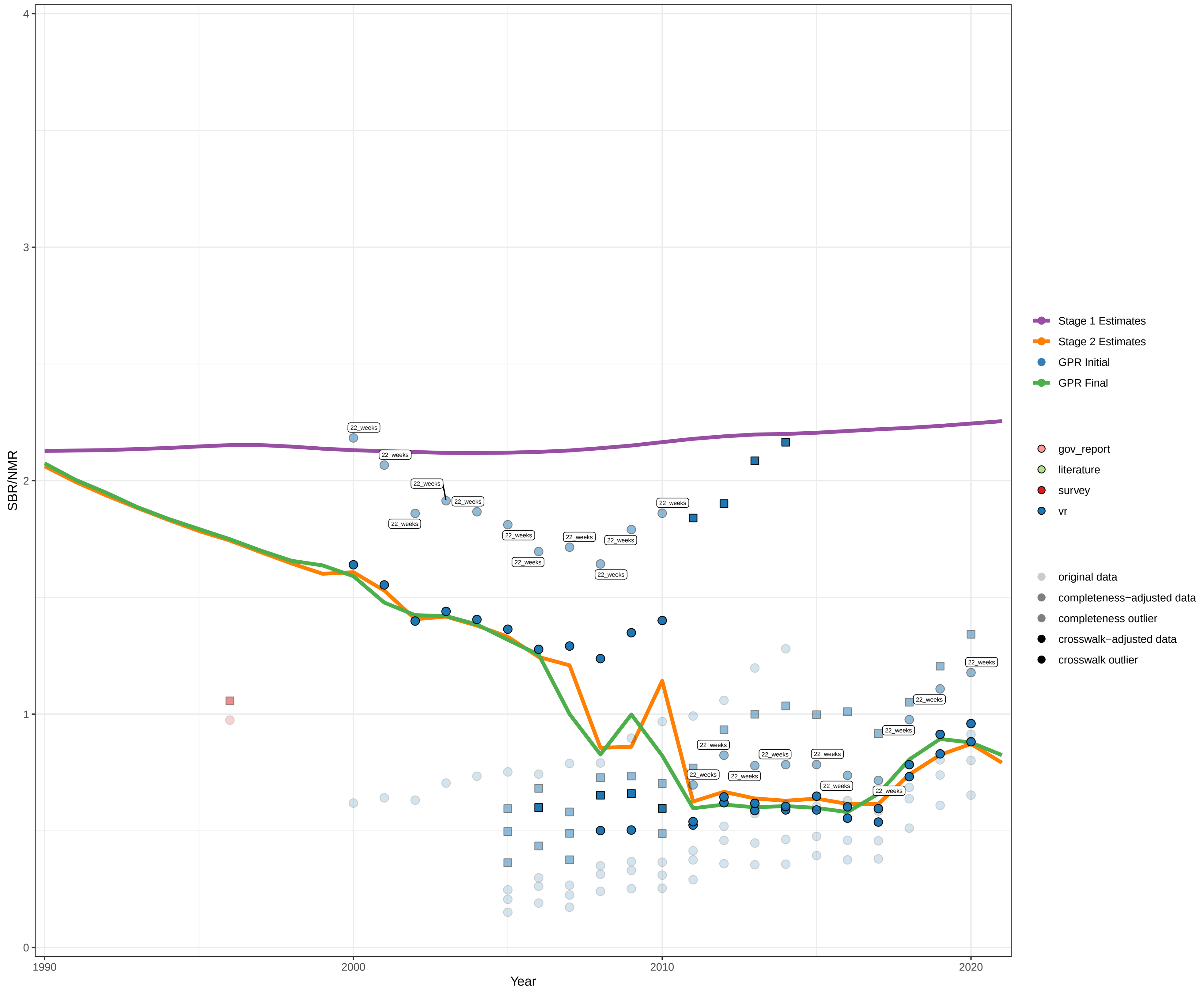

Definition	Years	Outlier	Source.Type	Title
22_weeks	2000−2014	0	VR	Brazil Mortality Information System − Stillbirths
22_weeks	2006, 2008−2020	0	VR	Brazil Civil Registry Statistics − Fetal Deaths at 28 Weeks or More, by Place of Birth, Number of Births by Birth and Sex, and Residence of Mother
28_weeks	2008, 2009, 2011−2020	0	VR	Brazil Civil Registry Statistics − Fetal Deaths at 28 Weeks or More, by Place of Birth, Number of Births by Birth and Sex, and Residence of Mother
22_weeks	2005, 2007	1	VR	Brazil Civil Registry Statistics − Fetal Deaths at 28 Weeks or More, by Place of Birth, Number of Births by Birth and Sex, and Residence of Mother
28_weeks	1996	1	Survey	Brazil Demographic and Health Survey 1996
28_weeks	2005−2007, 2010	1	VR	Brazil Civil Registry Statistics − Fetal Deaths at 28 Weeks or More, by Place of Birth, Number of Births by Birth and Sex, and Residence of Mother
other	2005−2020	1	VR	Brazil Civil Registry Statistics − Fetal Deaths at 28 Weeks or More, by Place of Birth, Number of Births by Birth and Sex, and Residence of Mother

Maranhao BRA_4759 Tropical Latin America Stillbirth Rate (SBR) − 20 weeks
lambda = 0.2 ; zeta = 0.99 ; scale = 5 ; amp = 0.028

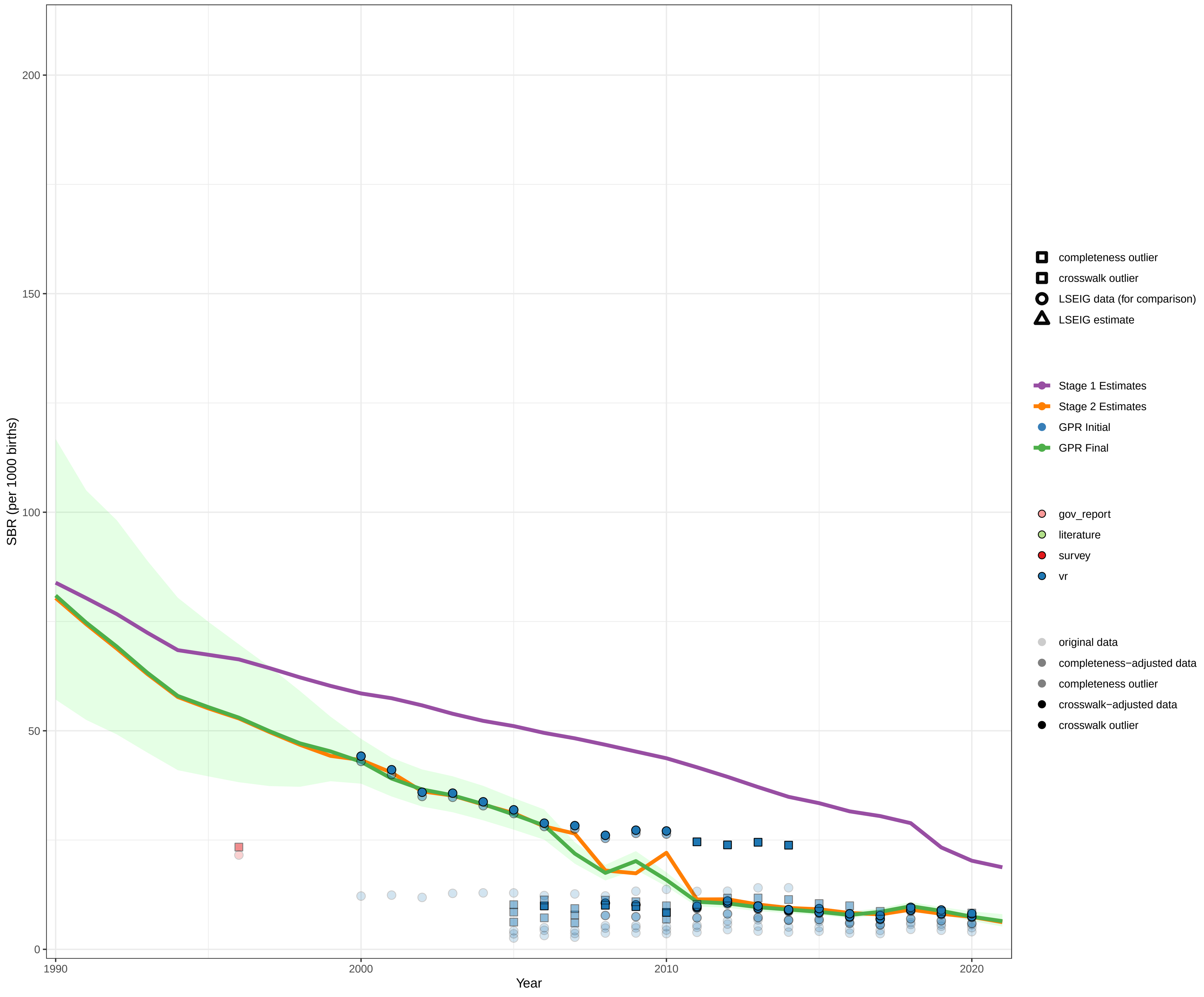

Maranhao BRA_4759 Tropical Latin America Stillbirth Rate (SBR) − 28 weeks
lambda = 0.2 ; zeta = 0.99 ; scale = 5 ; amp = 0.028

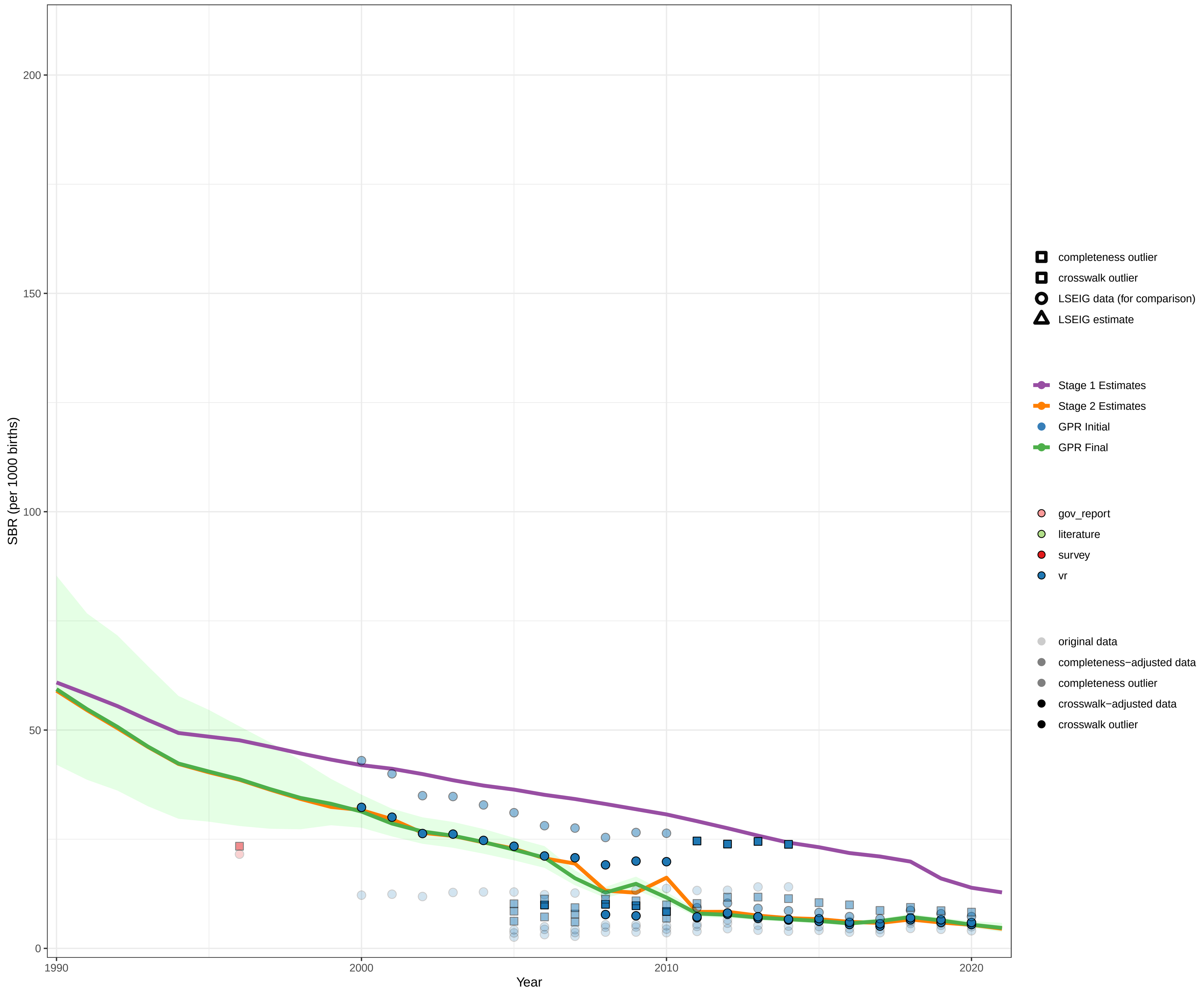

Year	Total Stillbirths (in thousands)	Total Stillbirths (in thousands)	SBR (per 1000 births)	SBR (per 1000 births)	SBR/NMR	SBR/NMR
	20 weeks	28 weeks	20 weeks	28 weeks	20 weeks	28 weeks
1990	13.9 (9.5 to 20.7)	9.9 (6.9 to 14.6)	80.9 (57.1 to 116.7)	59.4 (42.1 to 85.4)	2.83 (2.00 to 4.08)	2.07 (1.47 to 2.98)
1995	9.7 (6.8 to 13.4)	7.0 (4.9 to 9.6)	55.4 (39.6 to 74.9)	40.5 (29.0 to 54.6)	2.45 (1.75 to 3.32)	1.79 (1.28 to 2.42)
2000	8.6 (7.6 to 9.7)	6.2 (5.5 to 7.0)	42.9 (37.9 to 48.2)	31.3 (27.6 to 35.2)	2.18 (1.92 to 2.45)	1.59 (1.40 to 1.79)
2005	6.7 (5.9 to 7.5)	4.8 (4.3 to 5.4)	30.8 (27.4 to 34.6)	22.6 (20.2 to 25.3)	1.80 (1.60 to 2.02)	1.32 (1.18 to 1.48)
2010	3.5 (3.1 to 3.9)	2.5 (2.3 to 2.8)	15.9 (14.4 to 17.6)	11.7 (10.5 to 12.9)	1.12 (1.01 to 1.24)	0.82 (0.74 to 0.91)
2015	1.9 (1.8 to 2.1)	1.4 (1.3 to 1.5)	8.6 (7.8 to 9.5)	6.3 (5.7 to 6.9)	0.82 (0.75 to 0.90)	0.60 (0.54 to 0.66)
2020	1.7 (1.5 to 2.0)	1.2 (1.1 to 1.4)	7.5 (6.5 to 8.7)	5.4 (4.7 to 6.2)	1.21 (1.05 to 1.41)	0.88 (0.76 to 1.01)
2021	1.4 (1.2 to 1.8)	1.0 (0.8 to 1.3)	6.5 (5.2 to 8.1)	4.7 (3.8 to 5.8)	1.14 (0.92 to 1.42)	0.82 (0.66 to 1.03)

Mato Grosso BRA_4762 Tropical Latin America Stillbirth Rate / Neonatal Mortality Rate (SBR/NMR) − 20 weeks
lambda = 0.2 ; zeta = 0.99 ; scale = 5 ; amp = 0.028

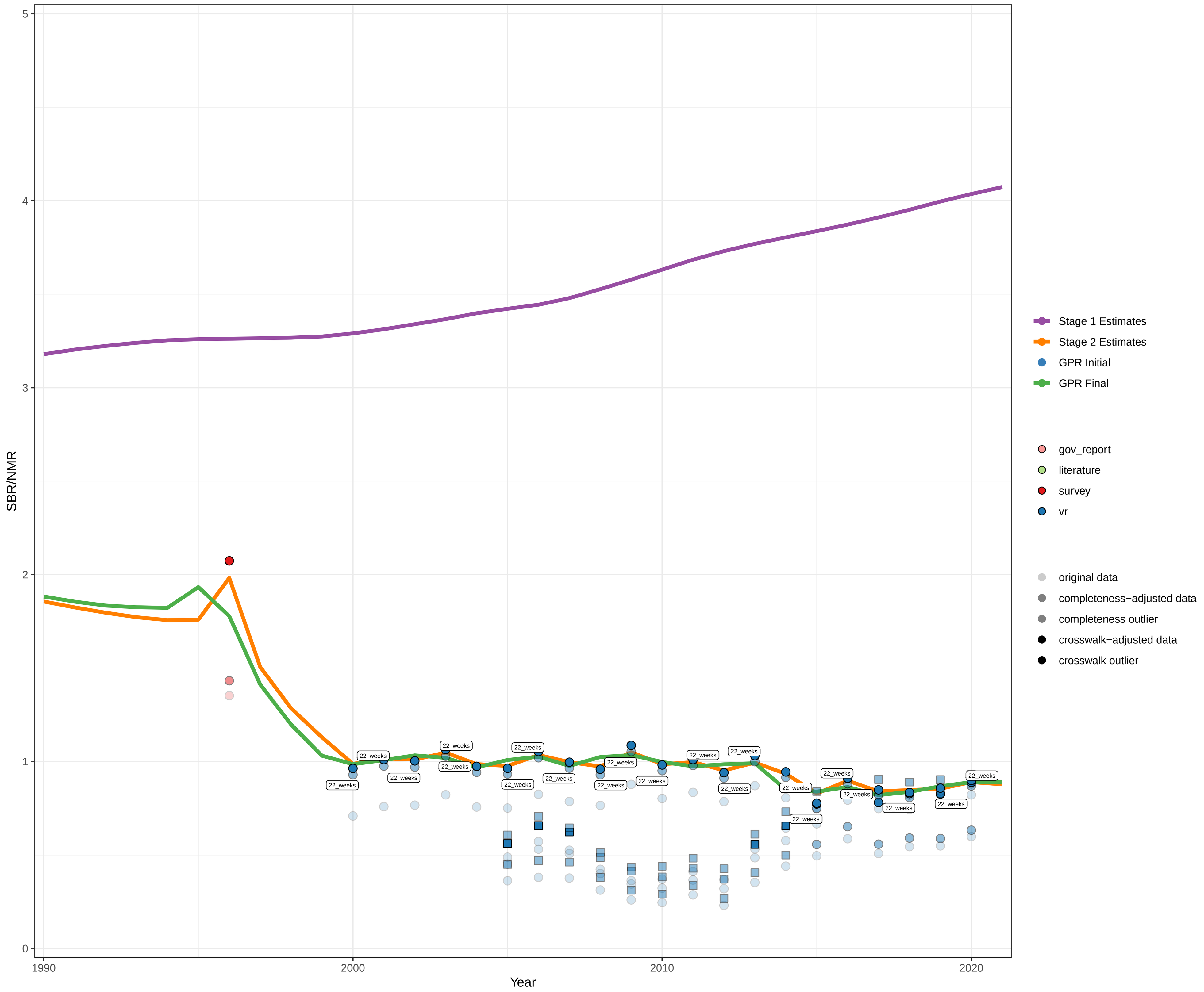

Mato Grosso BRA_4762 Tropical Latin America Stillbirth Rate / Neonatal Mortality Rate (SBR/NMR) − 28 weeks
lambda = 0.2 ; zeta = 0.99 ; scale = 5 ; amp = 0.028

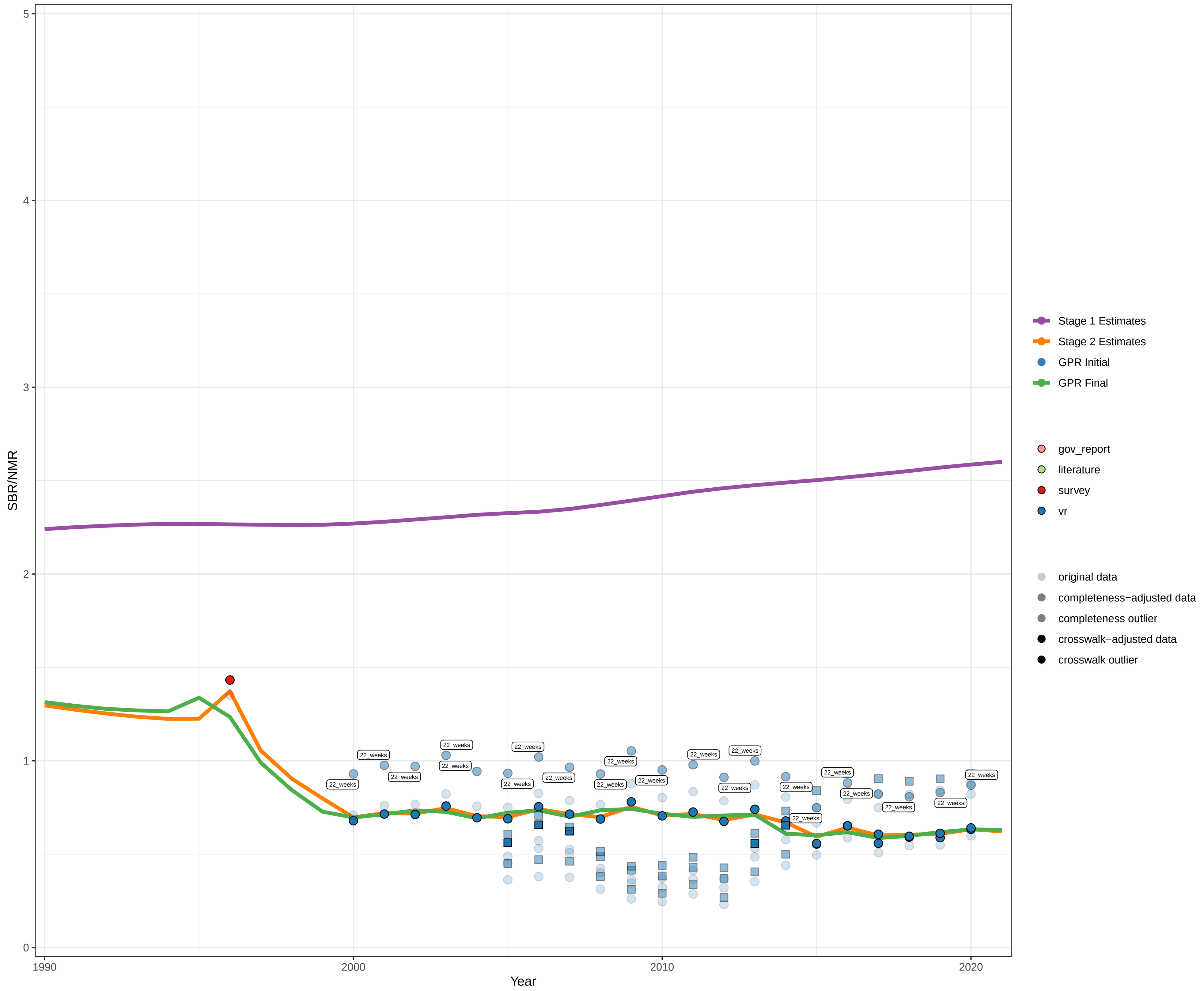

Definition	Years	Outlier	Source.Type	Title
22_weeks	2000−2014	0	VR	Brazil Mortality Information System − Stillbirths
22_weeks	2005−2007, 2013−2020	0	VR	Brazil Civil Registry Statistics − Fetal Deaths at 28 Weeks or More, by Place of Birth, Number of Births by Birth and Sex, and Residence of Mother
28_weeks	1996	0	Survey	Brazil Demographic and Health Survey 1996
28_weeks	2015−2020	0	VR	Brazil Civil Registry Statistics − Fetal Deaths at 28 Weeks or More, by Place of Birth, Number of Births by Birth and Sex, and Residence of Mother
22_weeks	2008−2012	1	VR	Brazil Civil Registry Statistics − Fetal Deaths at 28 Weeks or More, by Place of Birth, Number of Births by Birth and Sex, and Residence of Mother
28_weeks	2005−2014	1	VR	Brazil Civil Registry Statistics − Fetal Deaths at 28 Weeks or More, by Place of Birth, Number of Births by Birth and Sex, and Residence of Mother
other	2005−2020	1	VR	Brazil Civil Registry Statistics − Fetal Deaths at 28 Weeks or More, by Place of Birth, Number of Births by Birth and Sex, and Residence of Mother

Mato Grosso BRA_4762 Tropical Latin America Stillbirth Rate (SBR) − 20 weeks
lambda = 0.2 ; zeta = 0.99 ; scale = 5 ; amp = 0.028

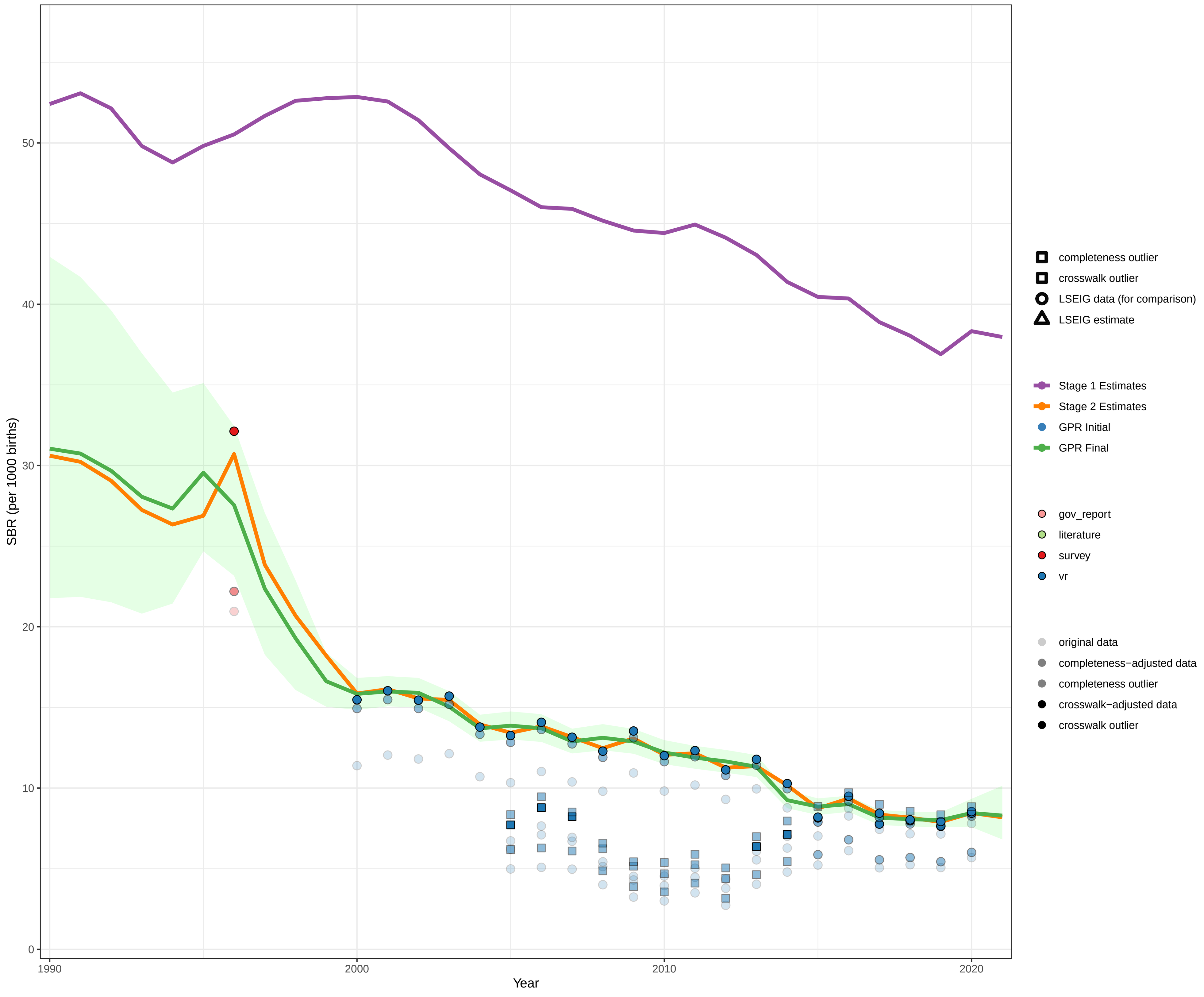

Mato Grosso BRA_4762 Tropical Latin America Stillbirth Rate (SBR) − 28 weeks
lambda = 0.2 ; zeta = 0.99 ; scale = 5 ; amp = 0.028

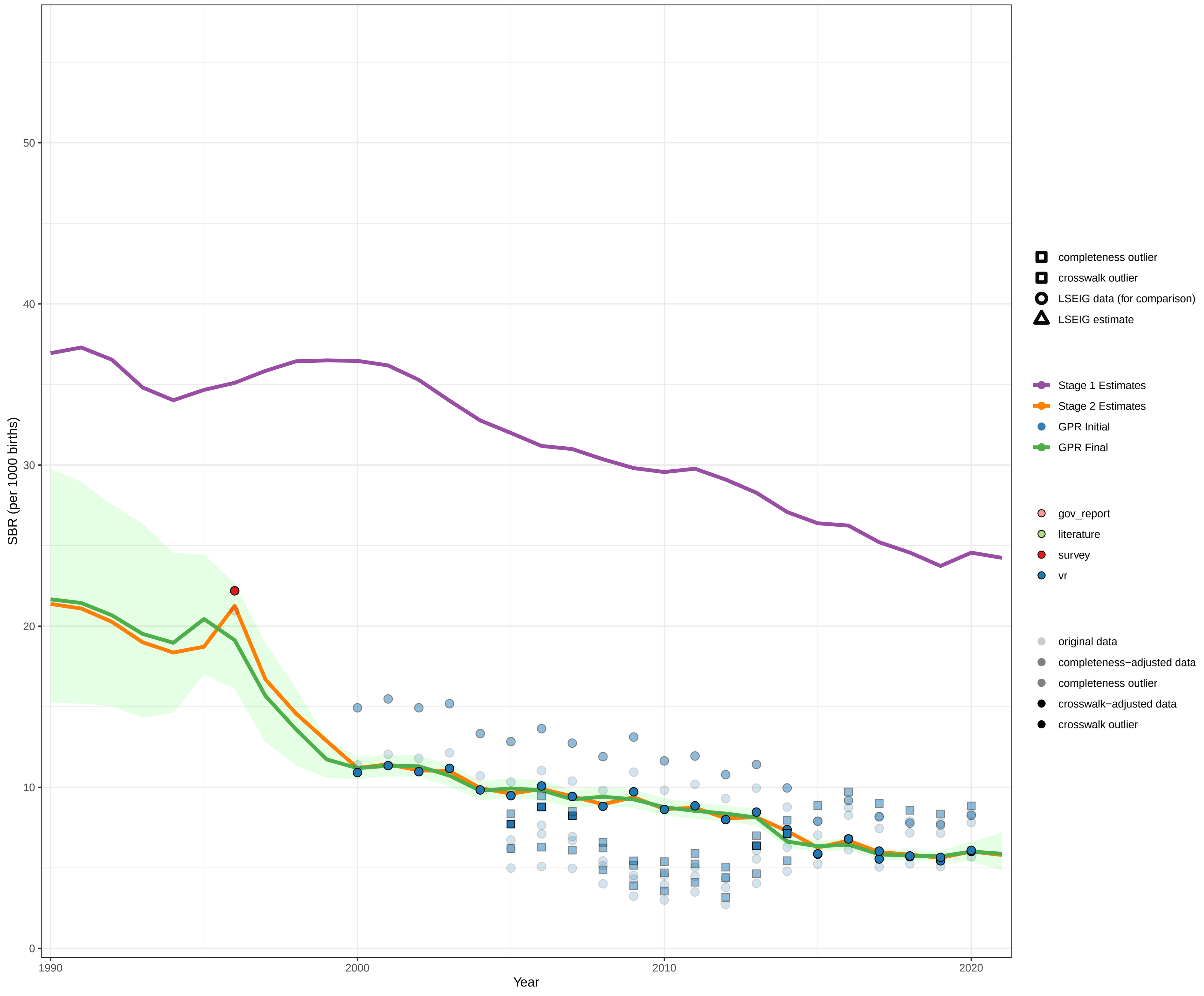

Year	Total Stillbirths (in thousands)	Total Stillbirths (in thousands)	SBR (per 1000 births)	SBR (per 1000 births)	SBR/NMR	SBR/NMR
	20 weeks	28 weeks	20 weeks	28 weeks	20 weeks	28 weeks
1990	1.3 (0.9 to 1.9)	0.9 (0.6 to 1.3)	31.0 (21.8 to 42.9)	21.7 (15.2 to 29.8)	1.88 (1.32 to 2.60)	1.31 (0.92 to 1.81)
1995	1.4 (1.2 to 1.7)	1.0 (0.8 to 1.1)	29.5 (24.7 to 35.1)	20.4 (17.0 to 24.5)	1.93 (1.61 to 2.30)	1.34 (1.11 to 1.60)
2000	0.8 (0.7 to 0.8)	0.5 (0.5 to 0.6)	15.8 (14.8 to 16.8)	11.2 (10.5 to 11.9)	0.99 (0.92 to 1.05)	0.70 (0.65 to 0.74)
2005	0.7 (0.6 to 0.7)	0.5 (0.4 to 0.5)	13.9 (13.0 to 14.8)	9.9 (9.3 to 10.5)	1.01 (0.95 to 1.07)	0.72 (0.68 to 0.77)
2010	0.6 (0.6 to 0.6)	0.4 (0.4 to 0.5)	12.2 (11.5 to 13.0)	8.8 (8.2 to 9.3)	1.00 (0.94 to 1.06)	0.72 (0.67 to 0.76)
2015	0.5 (0.4 to 0.5)	0.3 (0.3 to 0.4)	8.8 (8.3 to 9.4)	6.3 (6.0 to 6.7)	0.84 (0.79 to 0.89)	0.60 (0.57 to 0.64)
2020	0.5 (0.4 to 0.5)	0.3 (0.3 to 0.4)	8.5 (7.6 to 9.3)	6.0 (5.4 to 6.6)	0.89 (0.80 to 0.98)	0.63 (0.57 to 0.70)
2021	0.5 (0.4 to 0.6)	0.3 (0.3 to 0.4)	8.3 (6.8 to 10.1)	5.9 (4.8 to 7.2)	0.89 (0.73 to 1.09)	0.63 (0.52 to 0.77)

Mato Grosso do Sul   BRA_4761   Tropical Latin America   Stillbirth Rate / Neonatal Mortality Rate (SBR/NMR) − 20 weeks
lambda = 0.2 ; zeta = 0.99 ; scale = 5 ; amp = 0.028

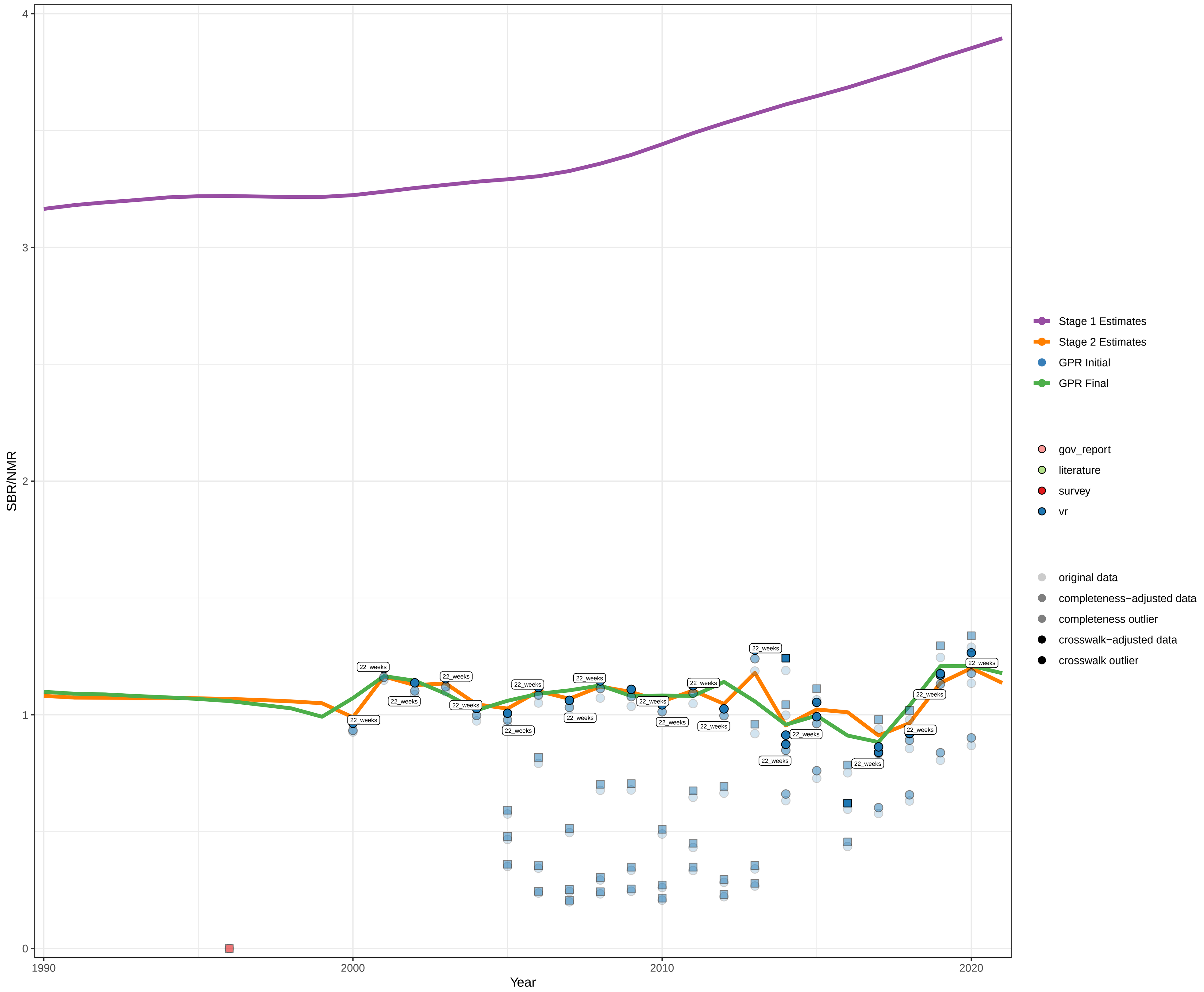

Mato Grosso do Sul   BRA_4761   Tropical Latin America   Stillbirth Rate / Neonatal Mortality Rate (SBR/NMR) − 28 weeks
lambda = 0.2 ; zeta = 0.99 ; scale = 5 ; amp = 0.028

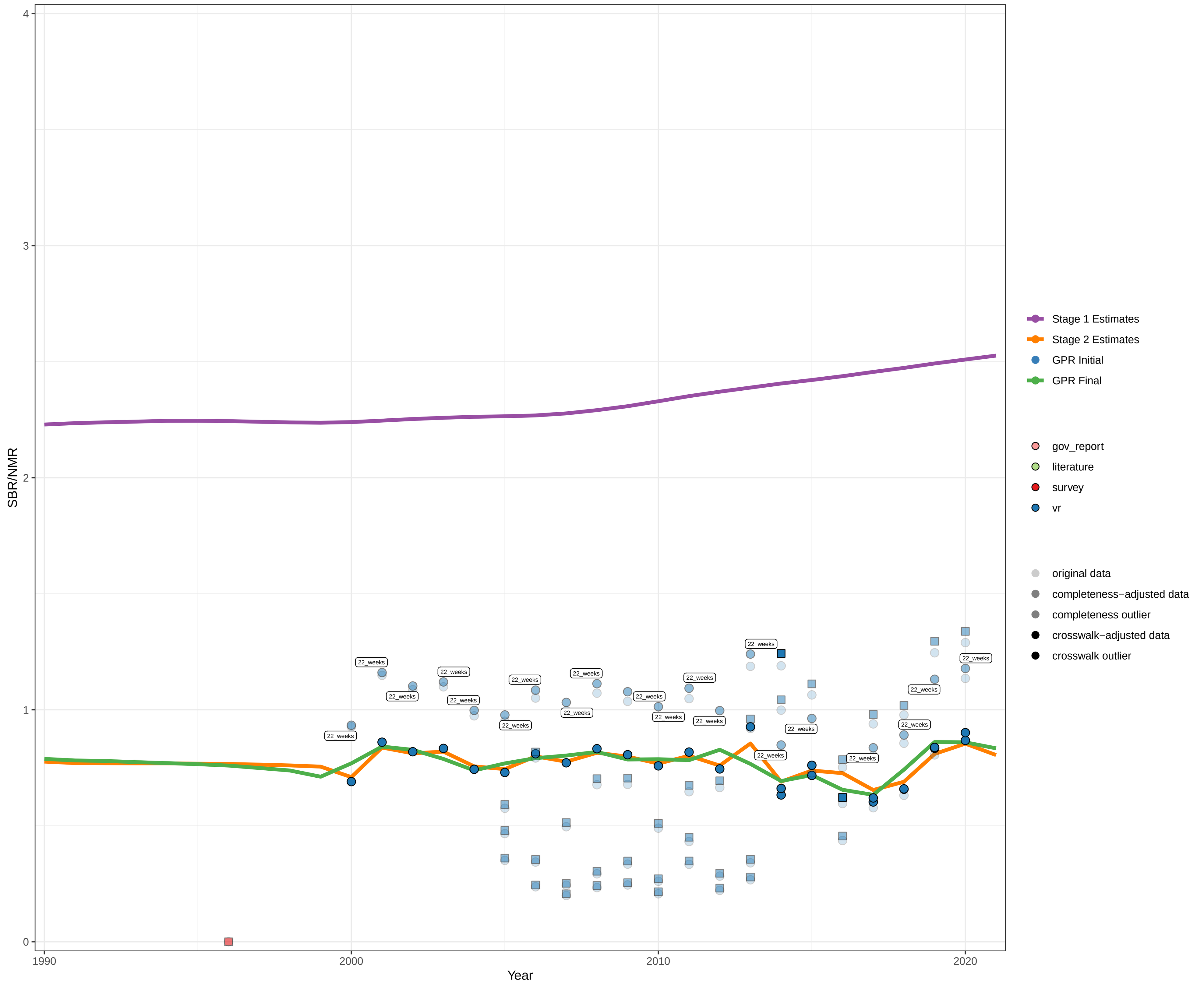

Definition	Years	Outlier	Source.Type	Title
22_weeks	2000−2014	0	VR	Brazil Mortality Information System − Stillbirths
22_weeks	2014−2020	0	VR	Brazil Civil Registry Statistics − Fetal Deaths at 28 Weeks or More, by Place of Birth, Number of Births by Birth and Sex, and Residence of Mother
28_weeks	2014, 2015, 2017−2020	0	VR	Brazil Civil Registry Statistics − Fetal Deaths at 28 Weeks or More, by Place of Birth, Number of Births by Birth and Sex, and Residence of Mother
22_weeks	2005−2013	1	VR	Brazil Civil Registry Statistics − Fetal Deaths at 28 Weeks or More, by Place of Birth, Number of Births by Birth and Sex, and Residence of Mother
28_weeks	1996	1	Survey	Brazil Demographic and Health Survey 1996
28_weeks	2005−2013, 2016	1	VR	Brazil Civil Registry Statistics − Fetal Deaths at 28 Weeks or More, by Place of Birth, Number of Births by Birth and Sex, and Residence of Mother
other	2005−2020	1	VR	Brazil Civil Registry Statistics − Fetal Deaths at 28 Weeks or More, by Place of Birth, Number of Births by Birth and Sex, and Residence of Mother

Mato Grosso do Sul   BRA_4761   Tropical Latin America   Stillbirth Rate (SBR) − 20 weeks
lambda = 0.2 ; zeta = 0.99 ; scale = 5 ; amp = 0.028

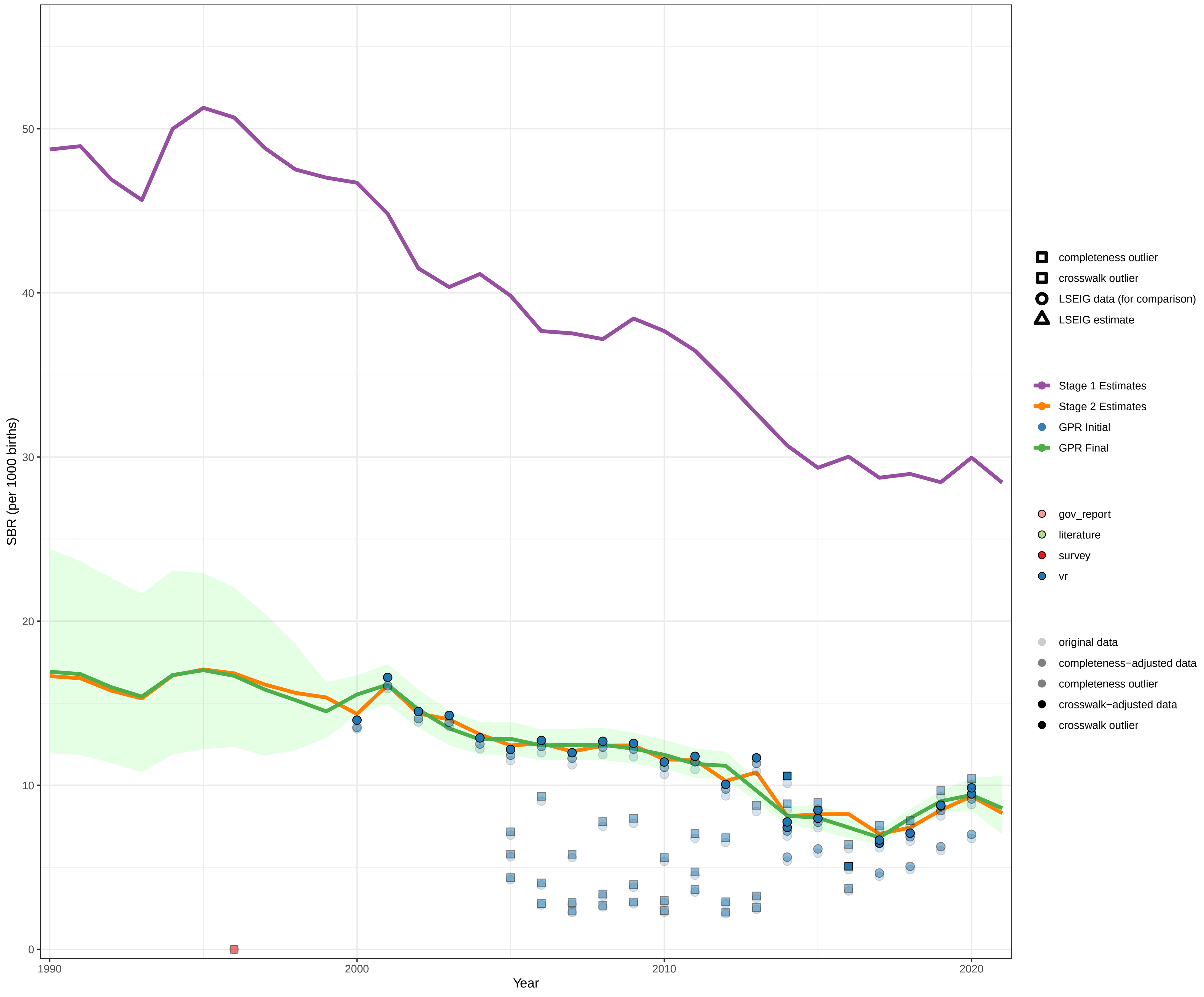

Mato Grosso do Sul   BRA_4761   Tropical Latin America   Stillbirth Rate (SBR) − 28 weeks
lambda = 0.2 ; zeta = 0.99 ; scale = 5 ; amp = 0.028

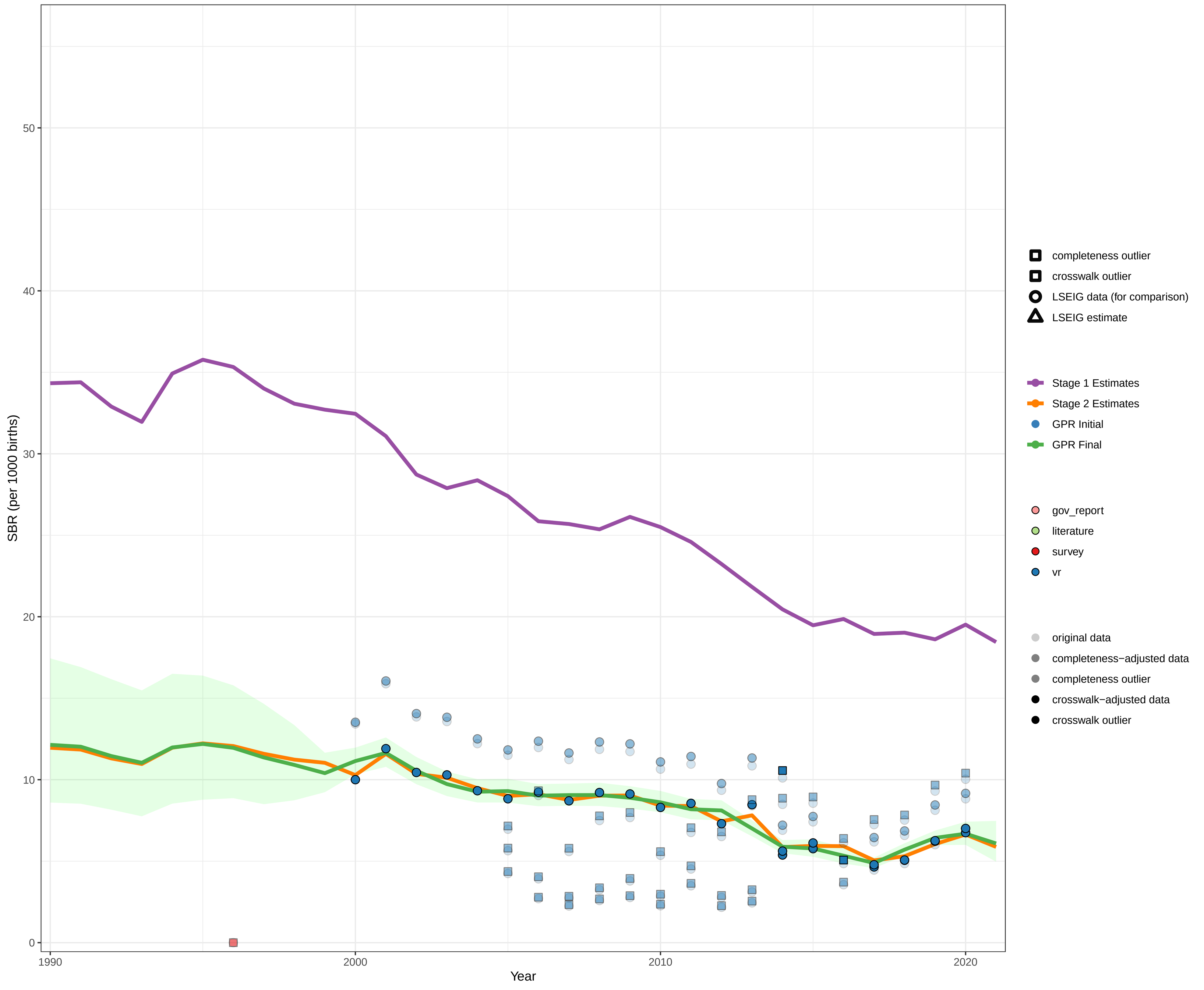

Year	Total Stillbirths (in thousands)	Total Stillbirths (in thousands)	SBR (per 1000 births)	SBR (per 1000 births)	SBR/NMR	SBR/NMR
	20 weeks	28 weeks	20 weeks	28 weeks	20 weeks	28 weeks
1990	0.7 (0.5 to 1.0)	0.5 (0.3 to 0.7)	16.9 (11.9 to 24.4)	12.1 (8.6 to 17.4)	1.10 (0.78 to 1.58)	0.79 (0.56 to 1.13)
1995	0.7 (0.5 to 1.0)	0.5 (0.4 to 0.7)	17.0 (12.2 to 22.9)	12.2 (8.8 to 16.4)	1.07 (0.77 to 1.44)	0.77 (0.55 to 1.03)
2000	0.7 (0.6 to 0.7)	0.5 (0.4 to 0.5)	15.5 (14.3 to 16.7)	11.1 (10.3 to 12.0)	1.07 (0.99 to 1.15)	0.77 (0.71 to 0.83)
2005	0.5 (0.5 to 0.6)	0.4 (0.4 to 0.4)	12.8 (11.9 to 13.9)	9.3 (8.6 to 10.1)	1.06 (0.98 to 1.15)	0.77 (0.71 to 0.83)
2010	0.5 (0.5 to 0.5)	0.4 (0.3 to 0.4)	11.9 (11.0 to 12.8)	8.6 (8.0 to 9.3)	1.08 (1.01 to 1.17)	0.79 (0.73 to 0.85)
2015	0.4 (0.3 to 0.4)	0.3 (0.2 to 0.3)	8.0 (7.3 to 8.8)	5.8 (5.3 to 6.3)	1.00 (0.91 to 1.09)	0.72 (0.65 to 0.79)
2020	0.4 (0.4 to 0.5)	0.3 (0.3 to 0.3)	9.4 (8.4 to 10.4)	6.7 (6.0 to 7.4)	1.21 (1.08 to 1.34)	0.86 (0.77 to 0.95)
2021	0.4 (0.3 to 0.5)	0.3 (0.2 to 0.3)	8.6 (7.0 to 10.6)	6.1 (5.0 to 7.5)	1.18 (0.96 to 1.45)	0.83 (0.68 to 1.02)

Minas Gerais   BRA_4760   Tropical Latin America   Stillbirth Rate / Neonatal Mortality Rate (SBR/NMR) − 20 weeks
lambda = 0.2 ; zeta = 0.99 ; scale = 5 ; amp = 0.028

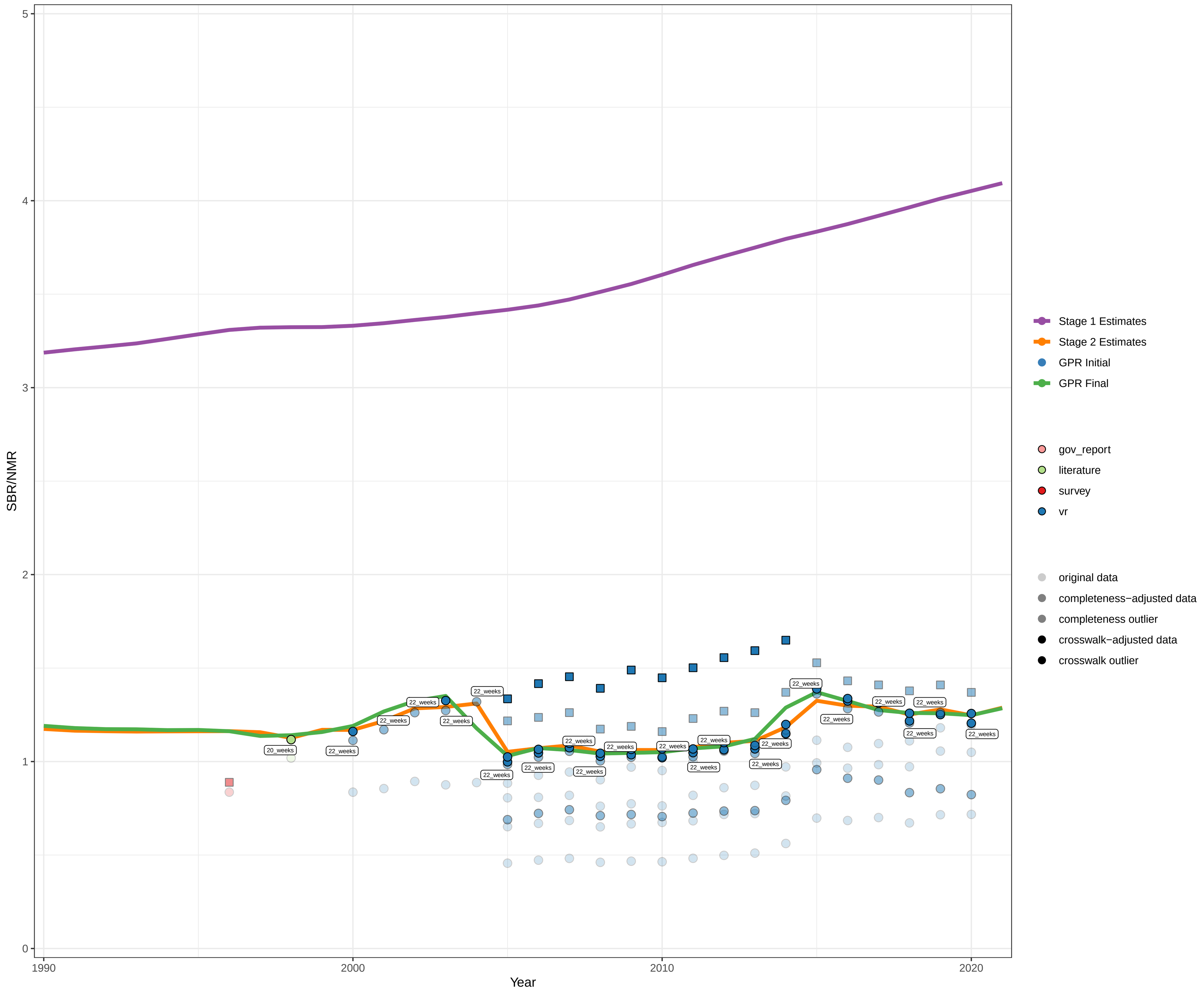

Minas Gerais   BRA_4760   Tropical Latin America   Stillbirth Rate / Neonatal Mortality Rate (SBR/NMR) − 28 weeks
lambda = 0.2 ; zeta = 0.99 ; scale = 5 ; amp = 0.028

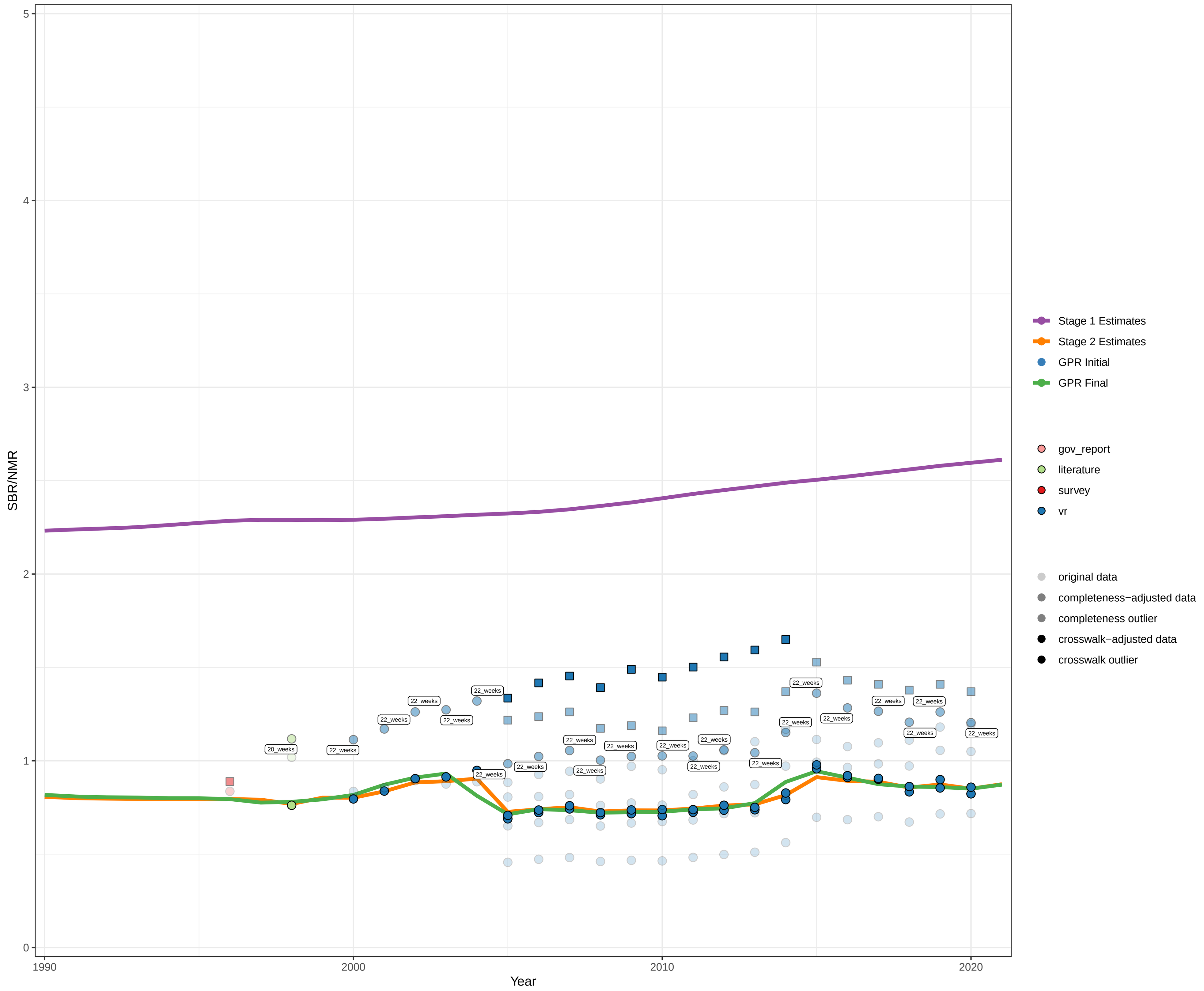

Definition	Years	Outlier	Source.Type	Title
20_weeks	1998	0	Sci Lit	Stillbirth in a microrregion of Minas Gerais State: causes and associated factors
22_weeks	2000−2014	0	VR	Brazil Mortality Information System − Stillbirths
22_weeks, 28_weeks	2005−2020	0	VR	Brazil Civil Registry Statistics − Fetal Deaths at 28 Weeks or More, by Place of Birth, Number of Births by Birth and Sex, and Residence of Mother
28_weeks	1996	1	Survey	Brazil Demographic and Health Survey 1996
other	2005−2020	1	VR	Brazil Civil Registry Statistics − Fetal Deaths at 28 Weeks or More, by Place of Birth, Number of Births by Birth and Sex, and Residence of Mother

Minas Gerais   BRA_4760   Tropical Latin America   Stillbirth Rate (SBR) − 20 weeks
lambda = 0.2 ; zeta = 0.99 ; scale = 5 ; amp = 0.028

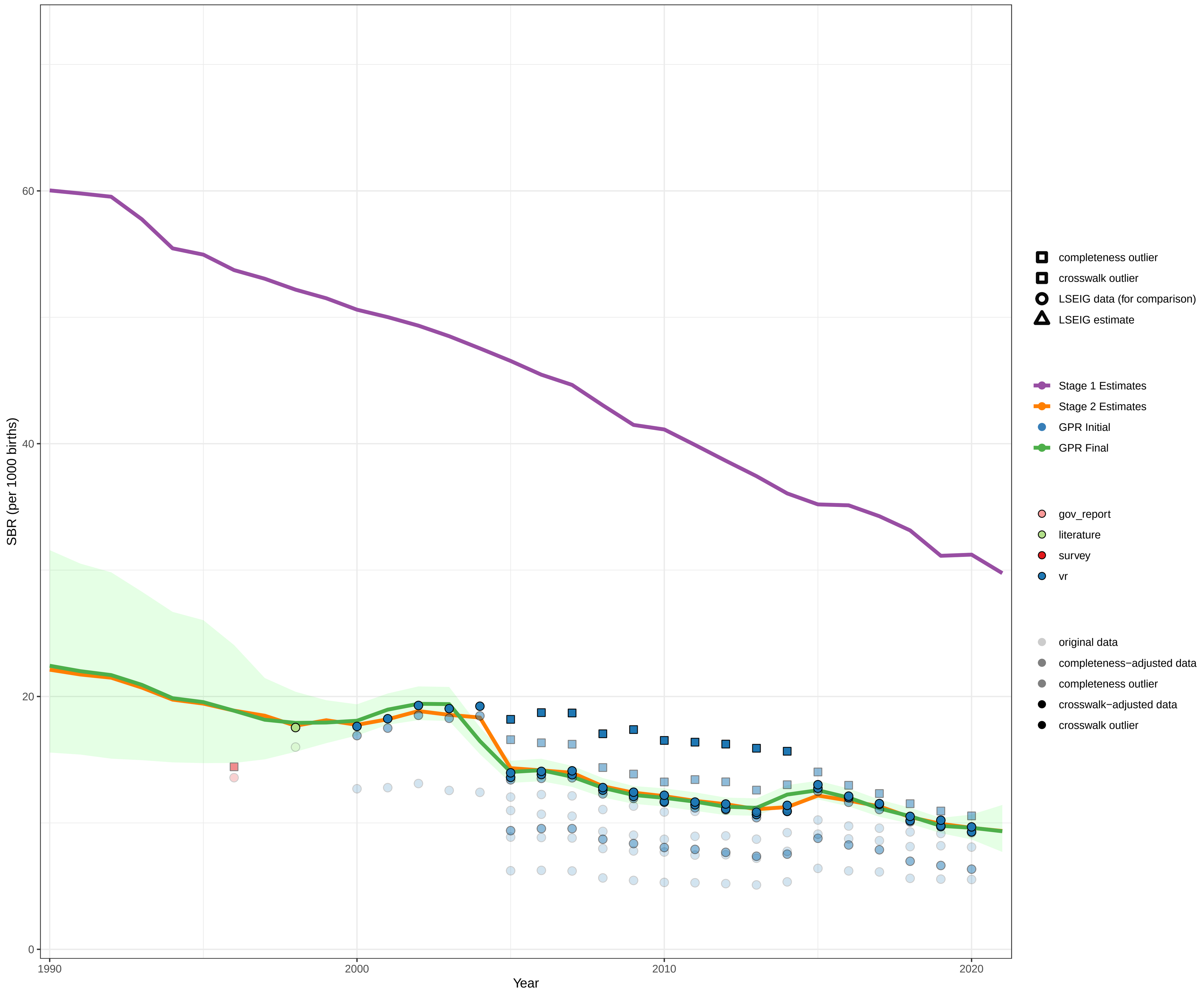

Minas Gerais   BRA_4760   Tropical Latin America   Stillbirth Rate (SBR) − 28 weeks
lambda = 0.2 ; zeta = 0.99 ; scale = 5 ; amp = 0.028

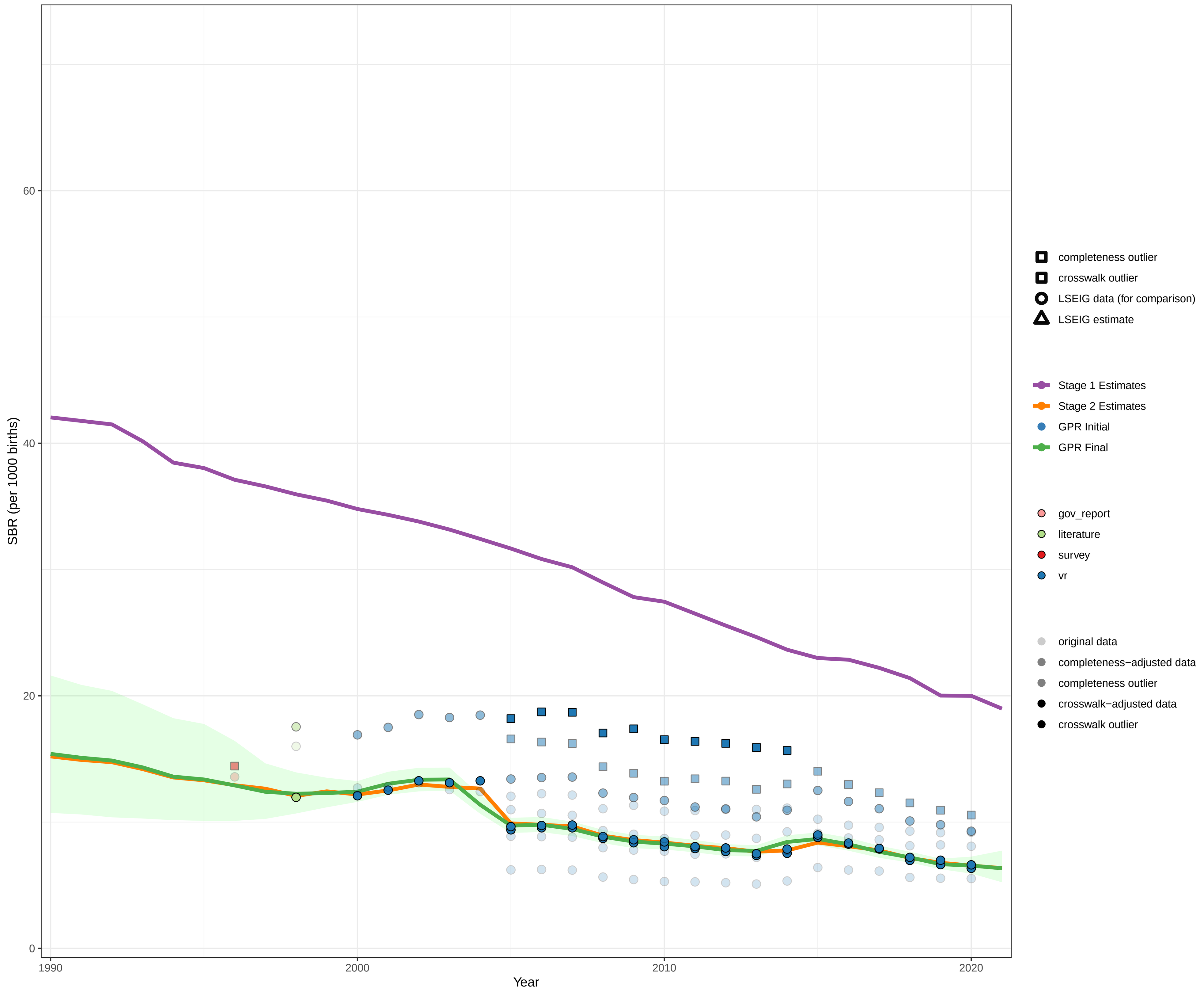

Year	Total Stillbirths (in thousands)	Total Stillbirths (in thousands)	SBR (per 1000 births)	SBR (per 1000 births)	SBR/NMR	SBR/NMR
	20 weeks	28 weeks	20 weeks	28 weeks	20 weeks	28 weeks
1990	7.6 (5.2 to 10.8)	5.2 (3.6 to 7.3)	22.4 (15.6 to 31.6)	15.4 (10.7 to 21.6)	1.19 (0.83 to 1.68)	0.82 (0.57 to 1.15)
1995	6.7 (5.0 to 8.9)	4.5 (3.4 to 6.0)	19.6 (14.7 to 26.0)	13.4 (10.1 to 17.8)	1.17 (0.88 to 1.56)	0.80 (0.60 to 1.06)
2000	6.3 (5.8 to 6.7)	4.3 (4.0 to 4.6)	18.1 (16.9 to 19.4)	12.4 (11.6 to 13.2)	1.19 (1.11 to 1.28)	0.82 (0.76 to 0.87)
2005	4.5 (4.2 to 4.7)	3.1 (2.9 to 3.3)	14.0 (13.2 to 14.9)	9.7 (9.1 to 10.3)	1.03 (0.97 to 1.09)	0.71 (0.67 to 0.76)
2010	3.7 (3.5 to 3.9)	2.5 (2.4 to 2.7)	12.0 (11.3 to 12.7)	8.3 (7.8 to 8.9)	1.05 (0.99 to 1.12)	0.73 (0.69 to 0.78)
2015	3.9 (3.7 to 4.1)	2.7 (2.5 to 2.8)	12.6 (11.9 to 13.3)	8.7 (8.2 to 9.2)	1.37 (1.29 to 1.45)	0.94 (0.89 to 1.00)
2020	2.8 (2.6 to 3.2)	1.9 (1.7 to 2.1)	9.6 (8.7 to 10.7)	6.6 (5.9 to 7.3)	1.25 (1.13 to 1.38)	0.85 (0.77 to 0.94)
2021	2.7 (2.2 to 3.3)	1.8 (1.5 to 2.2)	9.3 (7.7 to 11.4)	6.3 (5.2 to 7.7)	1.28 (1.06 to 1.57)	0.87 (0.72 to 1.07)

Para BRA_4763 Tropical Latin America Stillbirth Rate / Neonatal Mortality Rate (SBR/NMR) − 20 weeks
lambda = 0.4 ; zeta = 0.9 ; scale = 10 ; amp = 0.028

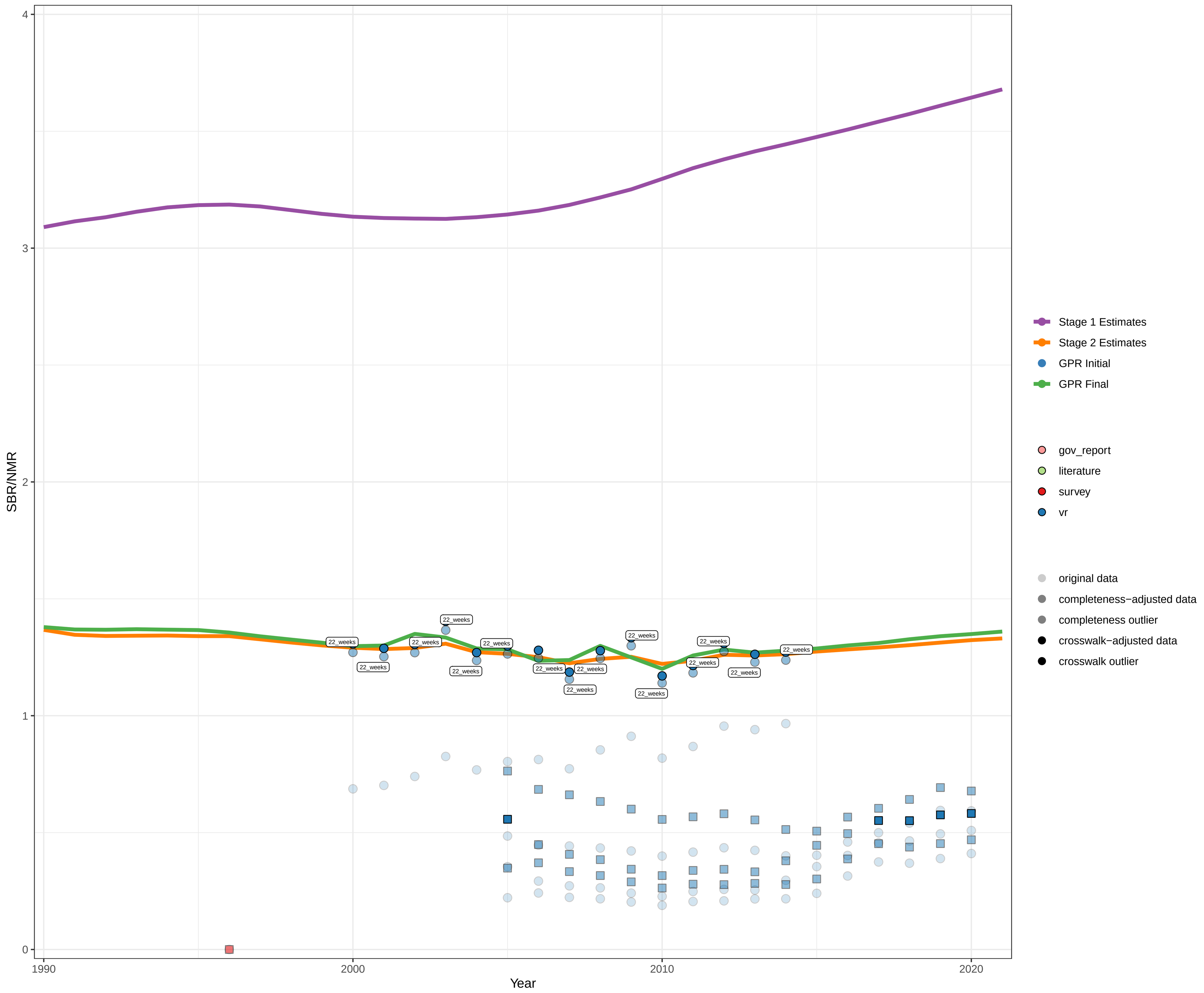

Para BRA_4763 Tropical Latin America Stillbirth Rate / Neonatal Mortality Rate (SBR/NMR) − 28 weeks
lambda = 0.4 ; zeta = 0.9 ; scale = 10 ; amp = 0.028

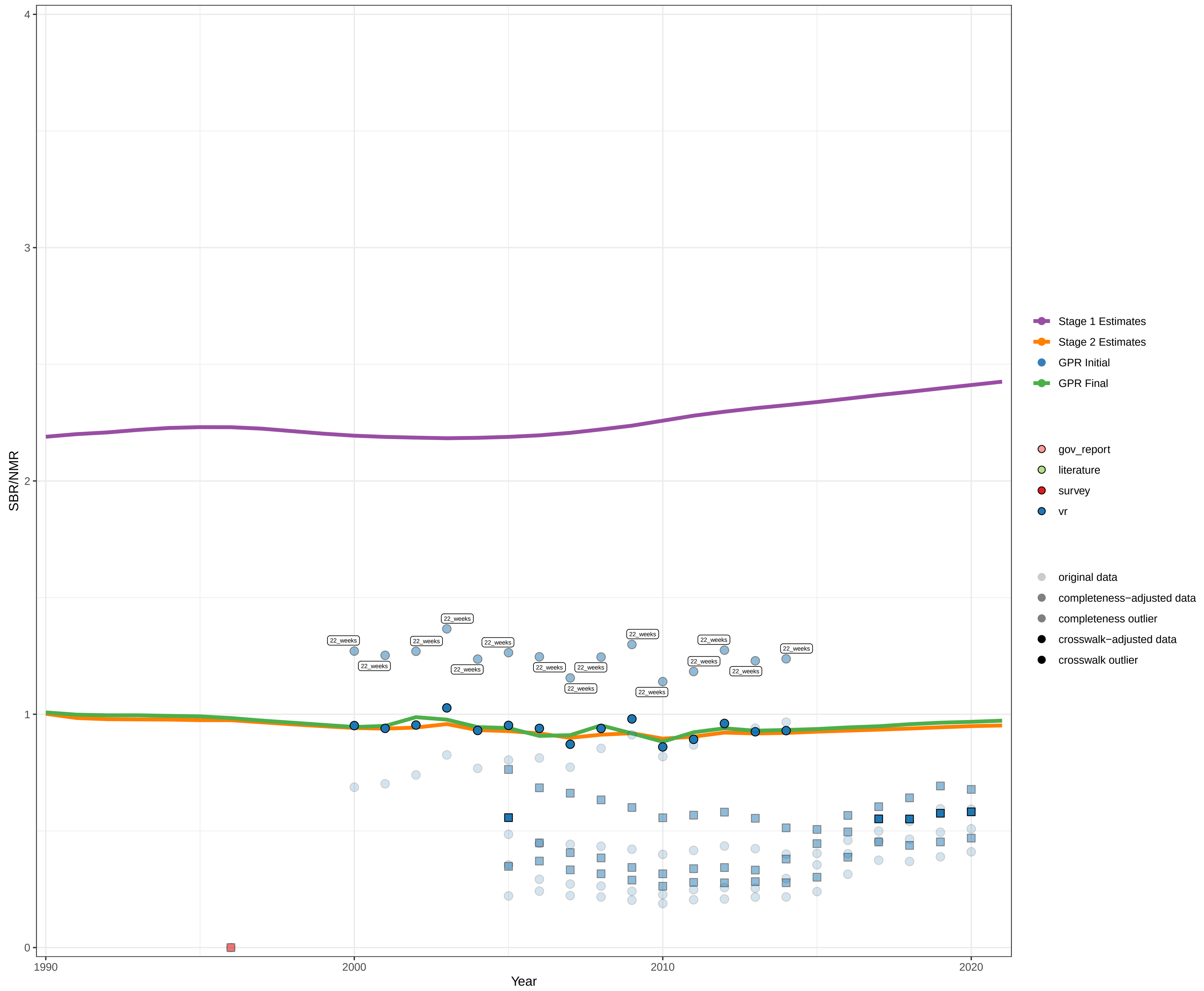

Definition	Years	Outlier	Source.Type	Title
22_weeks	2000−2014	0	VR	Brazil Mortality Information System − Stillbirths
22_weeks	2005, 2017−2020	0	VR	Brazil Civil Registry Statistics − Fetal Deaths at 28 Weeks or More, by Place of Birth, Number of Births by Birth and Sex, and Residence of Mother
22_weeks	2006−2016	1	VR	Brazil Civil Registry Statistics − Fetal Deaths at 28 Weeks or More, by Place of Birth, Number of Births by Birth and Sex, and Residence of Mother
28_weeks	1996	1	Survey	Brazil Demographic and Health Survey 1996
28_weeks, other	2005−2020	1	VR	Brazil Civil Registry Statistics − Fetal Deaths at 28 Weeks or More, by Place of Birth, Number of Births by Birth and Sex, and Residence of Mother

Para BRA_4763 Tropical Latin America Stillbirth Rate (SBR) − 20 weeks
lambda = 0.4 ; zeta = 0.9 ; scale = 10 ; amp = 0.028

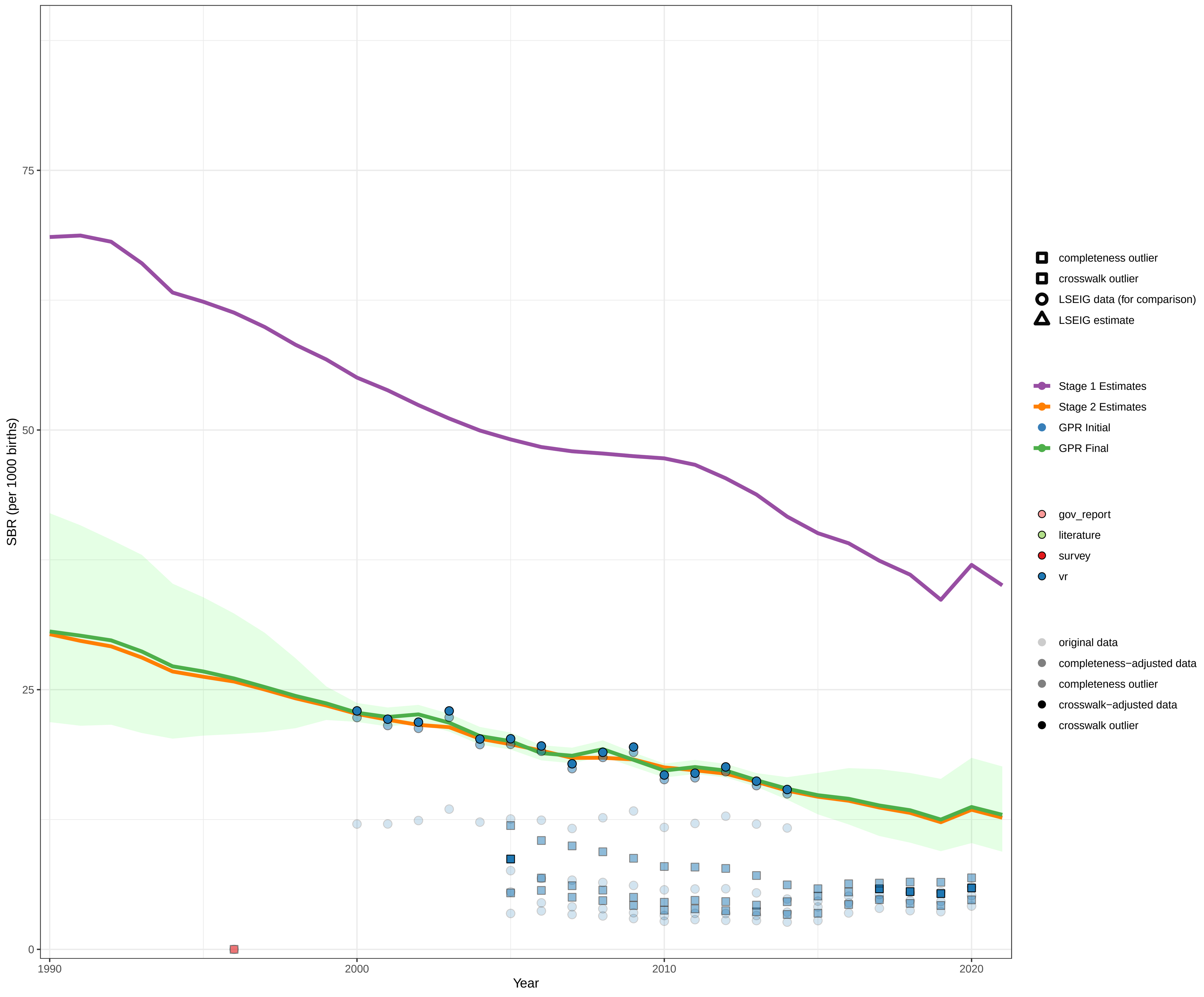

Para BRA_4763 Tropical Latin America Stillbirth Rate (SBR) − 28 weeks
lambda = 0.4 ; zeta = 0.9 ; scale = 10 ; amp = 0.028

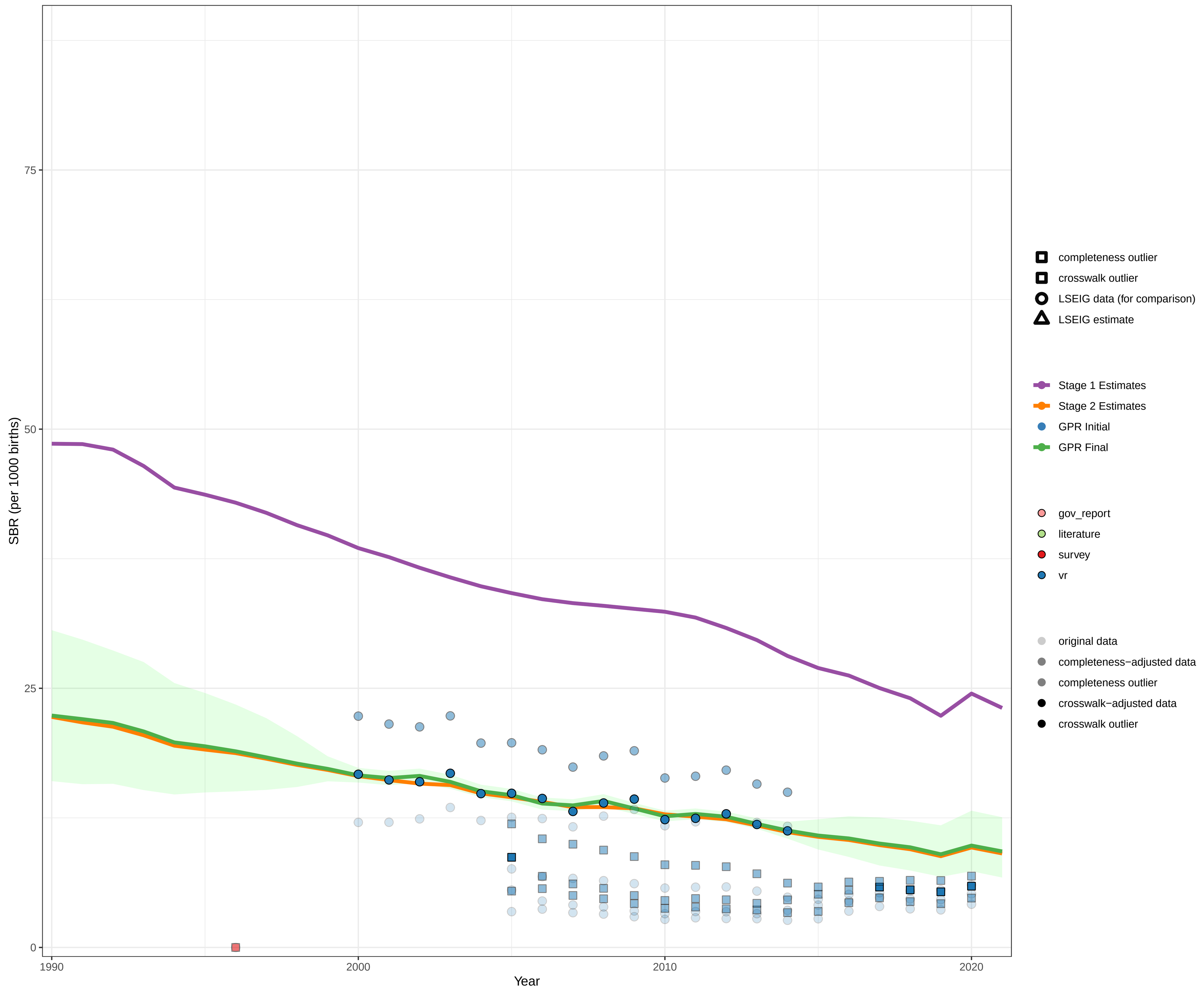

Year	Total Stillbirths (in thousands)	Total Stillbirths (in thousands)	SBR (per 1000 births)	SBR (per 1000 births)	SBR/NMR	SBR/NMR
	20 weeks	28 weeks	20 weeks	28 weeks	20 weeks	28 weeks
1990	3.4 (2.4 to 4.8)	2.5 (1.8 to 3.4)	30.6 (21.9 to 42.0)	22.4 (16.0 to 30.6)	1.38 (0.99 to 1.89)	1.01 (0.72 to 1.38)
1995	3.4 (2.6 to 4.3)	2.4 (1.9 to 3.1)	26.8 (20.6 to 33.9)	19.4 (15.0 to 24.5)	1.37 (1.05 to 1.73)	0.99 (0.76 to 1.25)
2000	3.4 (3.2 to 3.5)	2.4 (2.3 to 2.5)	22.8 (21.9 to 23.7)	16.6 (15.9 to 17.3)	1.30 (1.25 to 1.35)	0.95 (0.91 to 0.99)
2005	3.2 (3.1 to 3.3)	2.3 (2.2 to 2.4)	20.0 (19.2 to 20.9)	14.7 (14.1 to 15.3)	1.28 (1.23 to 1.34)	0.94 (0.90 to 0.98)
2010	2.8 (2.7 to 2.9)	2.0 (1.9 to 2.1)	17.2 (16.5 to 17.9)	12.7 (12.2 to 13.2)	1.20 (1.15 to 1.25)	0.88 (0.85 to 0.92)
2015	2.5 (2.2 to 2.8)	1.8 (1.6 to 2.0)	14.8 (13.0 to 17.0)	10.8 (9.4 to 12.4)	1.29 (1.13 to 1.47)	0.94 (0.82 to 1.07)
2020	2.3 (1.7 to 3.1)	1.6 (1.2 to 2.2)	13.7 (10.2 to 18.4)	9.8 (7.4 to 13.2)	1.35 (1.01 to 1.82)	0.97 (0.72 to 1.30)
2021	2.1 (1.5 to 2.9)	1.5 (1.1 to 2.1)	13.0 (9.4 to 17.6)	9.3 (6.7 to 12.6)	1.36 (0.99 to 1.85)	0.97 (0.71 to 1.32)

Paraiba BRA_4764 Tropical Latin America Stillbirth Rate / Neonatal Mortality Rate (SBR/NMR) − 20 weeks
lambda = 0.2 ; zeta = 0.99 ; scale = 5 ; amp = 0.028

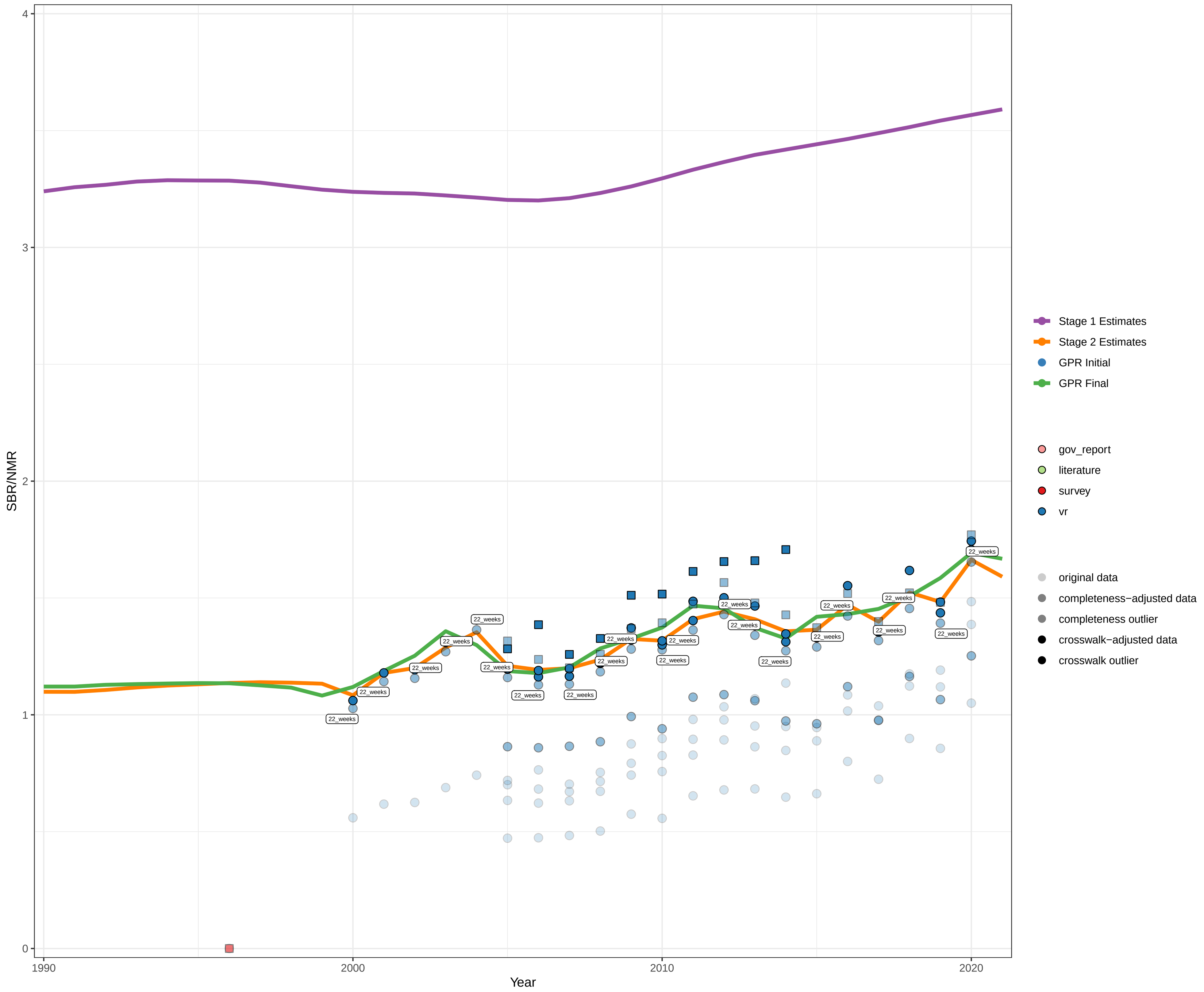

Paraiba BRA_4764 Tropical Latin America Stillbirth Rate / Neonatal Mortality Rate (SBR/NMR) − 28 weeks
lambda = 0.2 ; zeta = 0.99 ; scale = 5 ; amp = 0.028

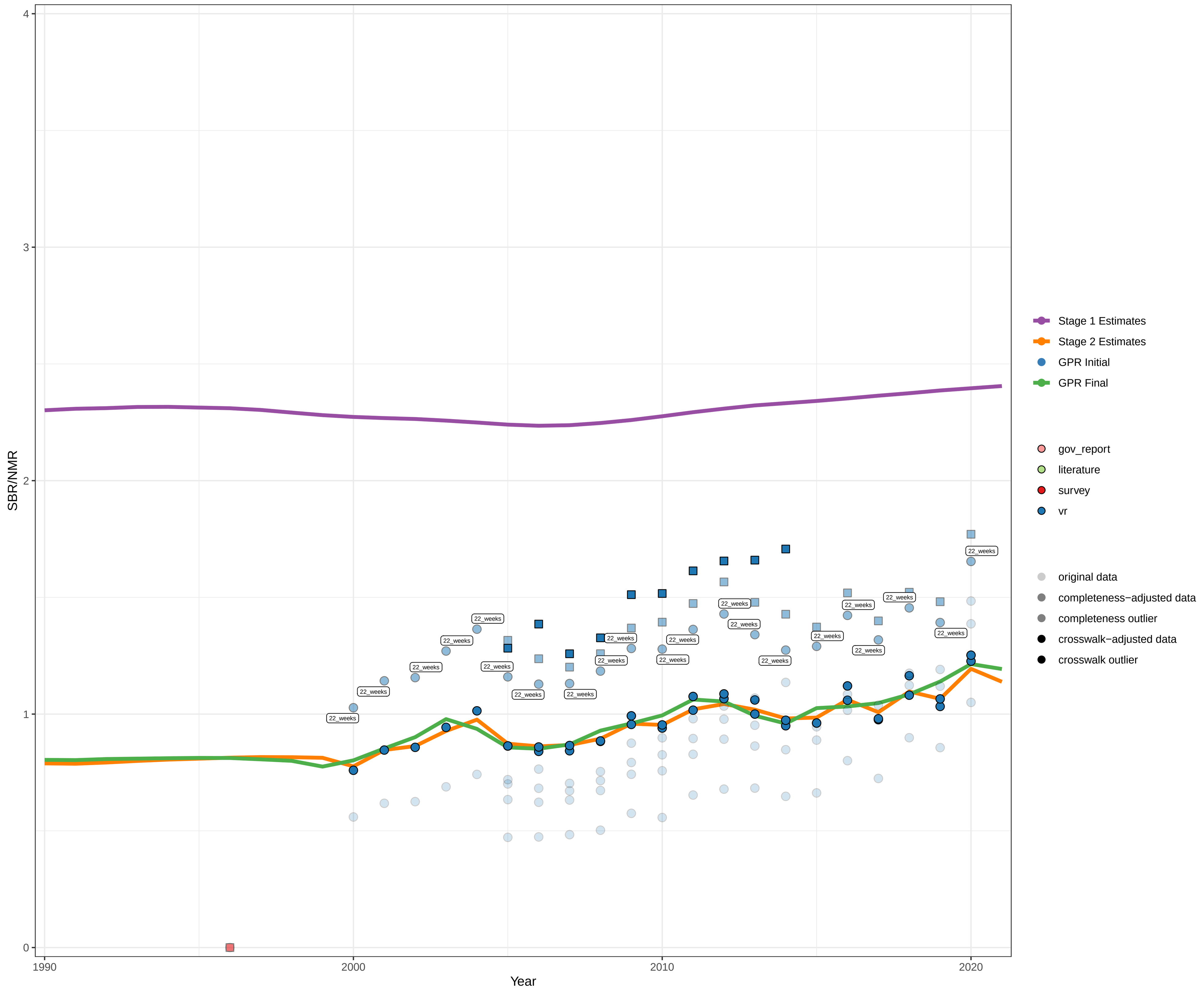

Definition	Years	Outlier	Source.Type	Title
22_weeks	2000−2014	0	VR	Brazil Mortality Information System − Stillbirths
22_weeks, 28_weeks	2005−2020	0	VR	Brazil Civil Registry Statistics − Fetal Deaths at 28 Weeks or More, by Place of Birth, Number of Births by Birth and Sex, and Residence of Mother
28_weeks	1996	1	Survey	Brazil Demographic and Health Survey 1996
other	2005−2020	1	VR	Brazil Civil Registry Statistics − Fetal Deaths at 28 Weeks or More, by Place of Birth, Number of Births by Birth and Sex, and Residence of Mother

Paraiba BRA_4764 Tropical Latin America Stillbirth Rate (SBR) − 20 weeks
lambda = 0.2 ; zeta = 0.99 ; scale = 5 ; amp = 0.028

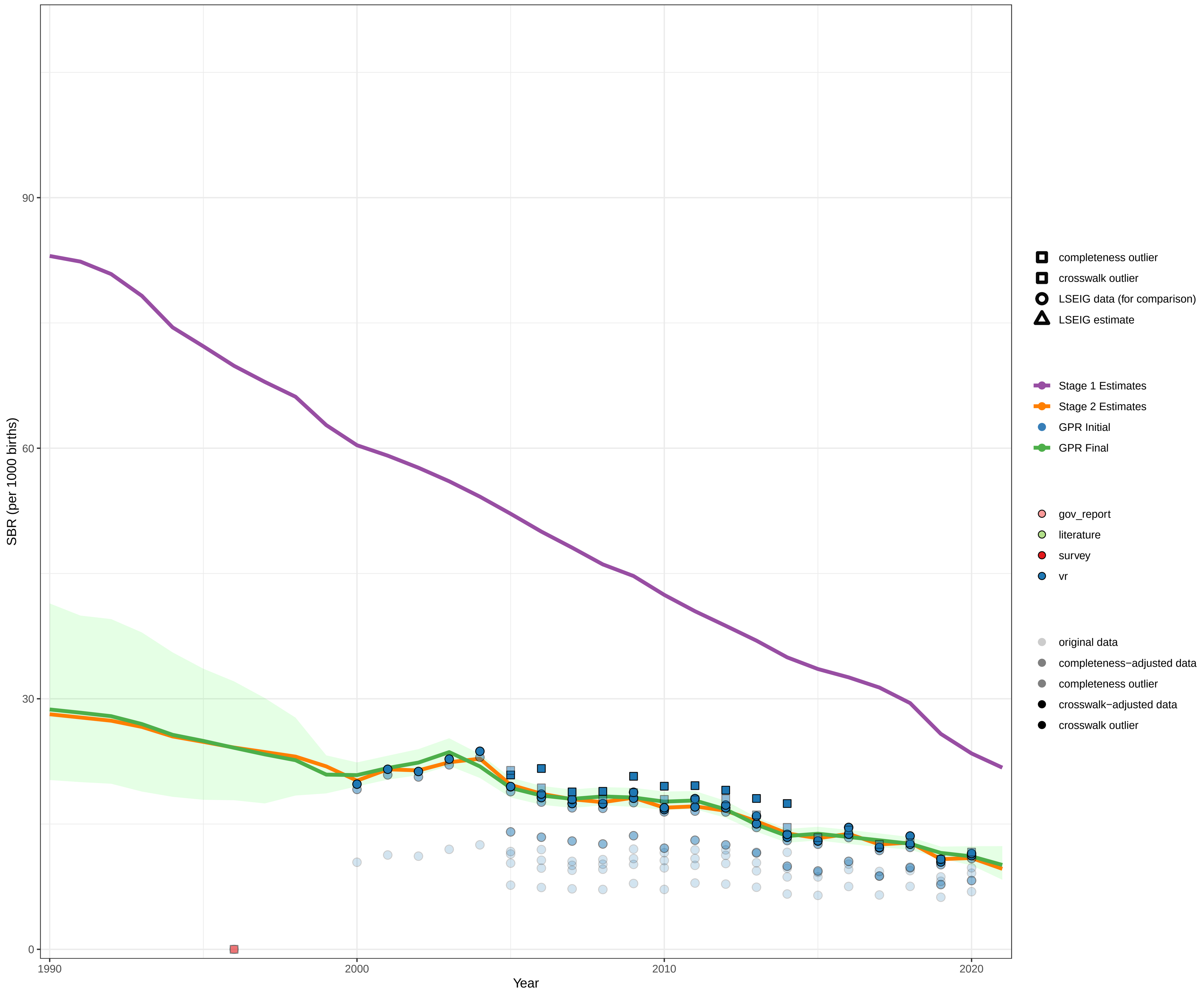

Paraiba BRA_4764 Tropical Latin America Stillbirth Rate (SBR) − 28 weeks
lambda = 0.2 ; zeta = 0.99 ; scale = 5 ; amp = 0.028

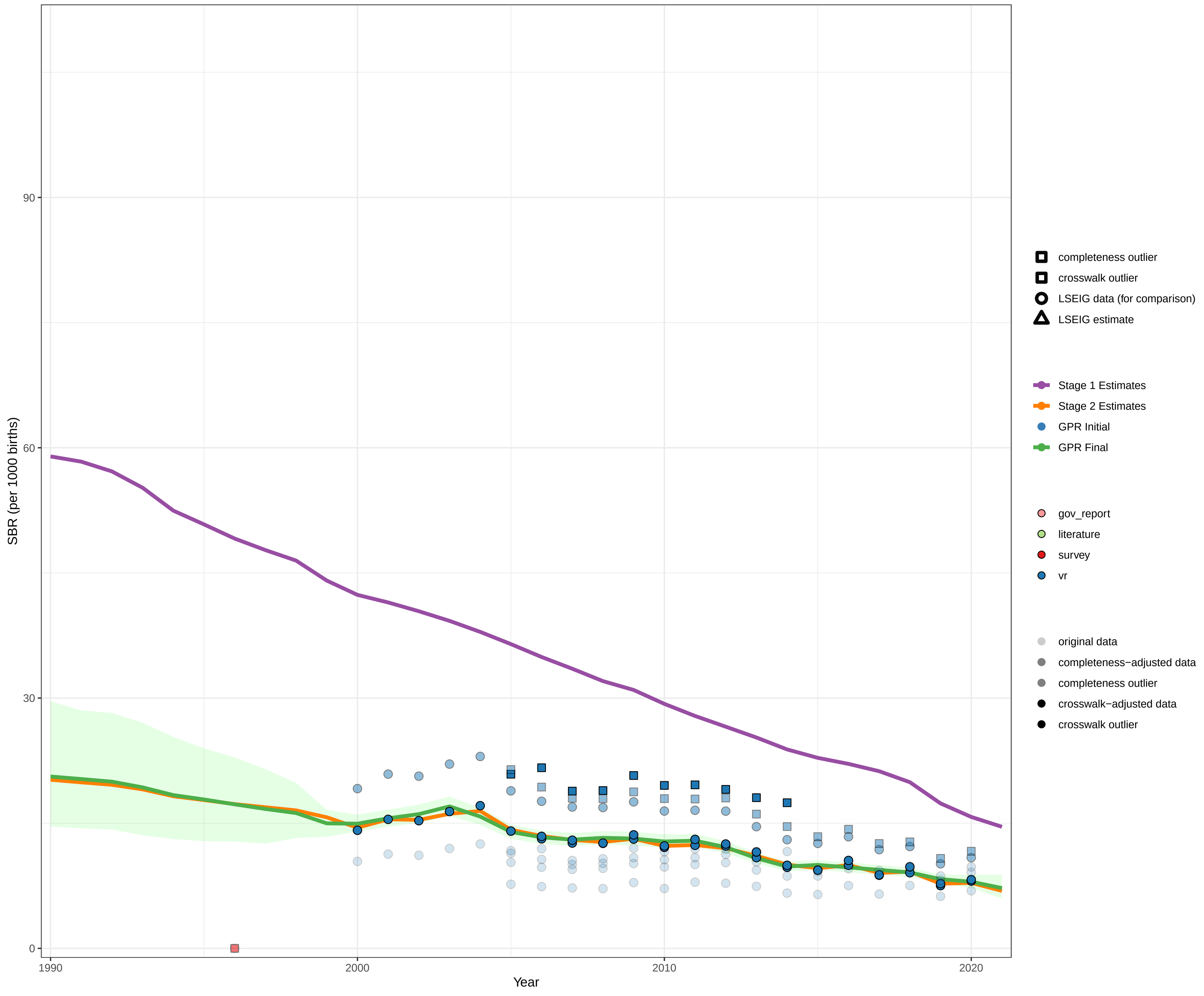

Year	Total Stillbirths (in thousands)	Total Stillbirths (in thousands)	SBR (per 1000 births)	SBR (per 1000 births)	SBR/NMR	SBR/NMR
	20 weeks	28 weeks	20 weeks	28 weeks	20 weeks	28 weeks
1990	2.1 (1.5 to 3.1)	1.5 (1.1 to 2.2)	28.7 (20.3 to 41.4)	20.6 (14.6 to 29.6)	1.12 (0.79 to 1.62)	0.80 (0.57 to 1.16)
1995	1.9 (1.3 to 2.6)	1.3 (1.0 to 1.8)	25.0 (17.9 to 33.6)	17.8 (12.8 to 24.0)	1.14 (0.82 to 1.53)	0.81 (0.58 to 1.09)
2000	1.8 (1.7 to 1.9)	1.3 (1.2 to 1.4)	20.9 (19.5 to 22.4)	14.9 (14.0 to 16.0)	1.12 (1.05 to 1.20)	0.80 (0.75 to 0.86)
2005	1.7 (1.6 to 1.8)	1.2 (1.2 to 1.3)	19.3 (18.2 to 20.6)	14.0 (13.1 to 14.8)	1.19 (1.12 to 1.26)	0.86 (0.81 to 0.91)
2010	1.6 (1.5 to 1.7)	1.1 (1.1 to 1.2)	17.7 (16.7 to 18.9)	12.8 (12.1 to 13.7)	1.37 (1.30 to 1.47)	0.99 (0.94 to 1.06)
2015	1.2 (1.2 to 1.3)	0.9 (0.8 to 1.0)	13.8 (13.0 to 14.7)	10.0 (9.4 to 10.6)	1.42 (1.34 to 1.50)	1.03 (0.97 to 1.09)
2020	1.0 (0.9 to 1.1)	0.7 (0.6 to 0.8)	11.1 (10.1 to 12.3)	8.0 (7.2 to 8.8)	1.69 (1.53 to 1.88)	1.21 (1.10 to 1.35)
2021	0.9 (0.7 to 1.1)	0.6 (0.5 to 0.8)	10.1 (8.3 to 12.4)	7.2 (6.0 to 8.8)	1.67 (1.38 to 2.04)	1.19 (0.99 to 1.46)

Parana   BRA_4765   Tropical Latin America   Stillbirth Rate / Neonatal Mortality Rate (SBR/NMR) − 20 weeks
lambda = 0.2 ; zeta = 0.99 ; scale = 5 ; amp = 0.028

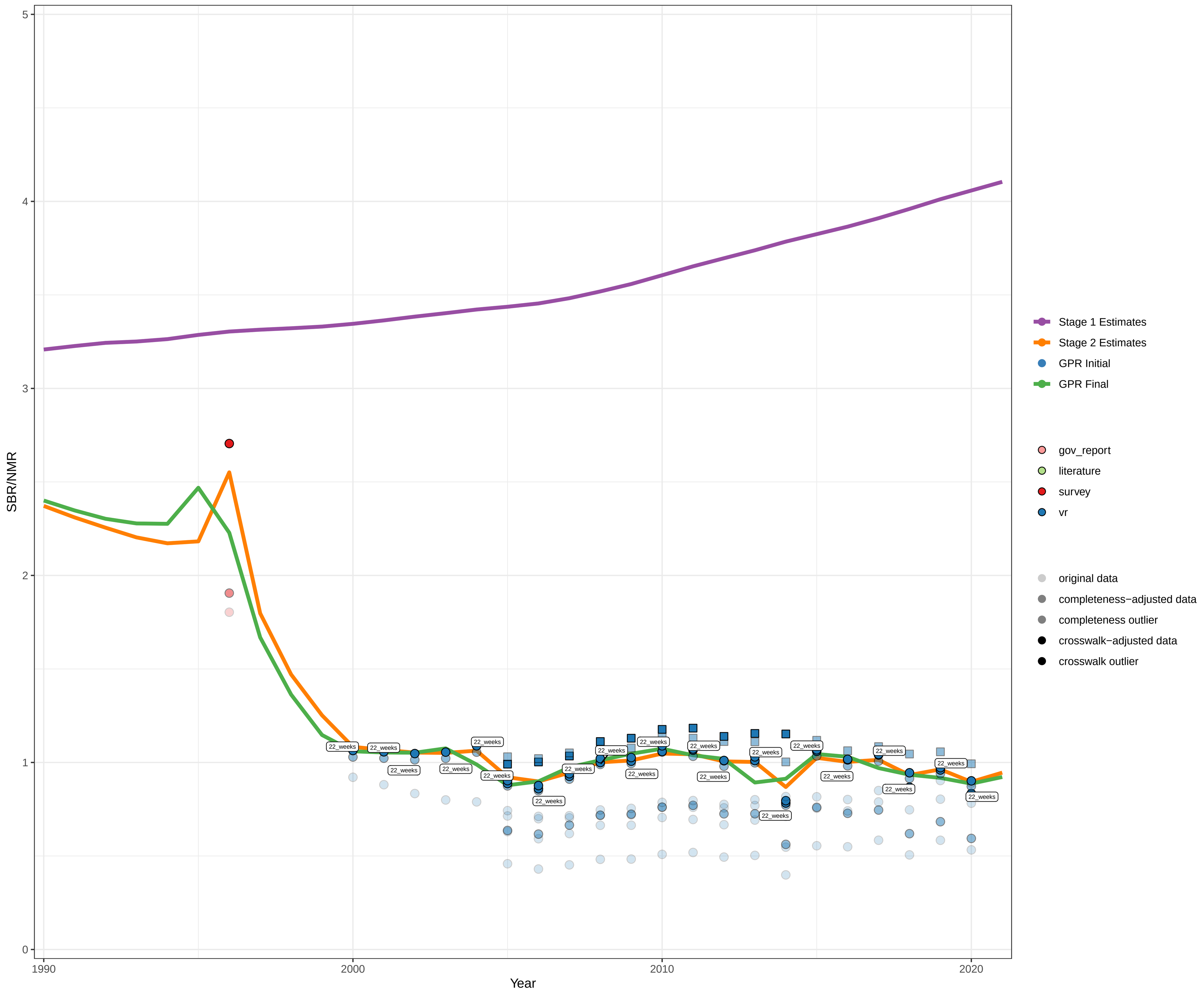

Parana   BRA_4765   Tropical Latin America   Stillbirth Rate / Neonatal Mortality Rate (SBR/NMR) − 28 weeks
lambda = 0.2 ; zeta = 0.99 ; scale = 5 ; amp = 0.028

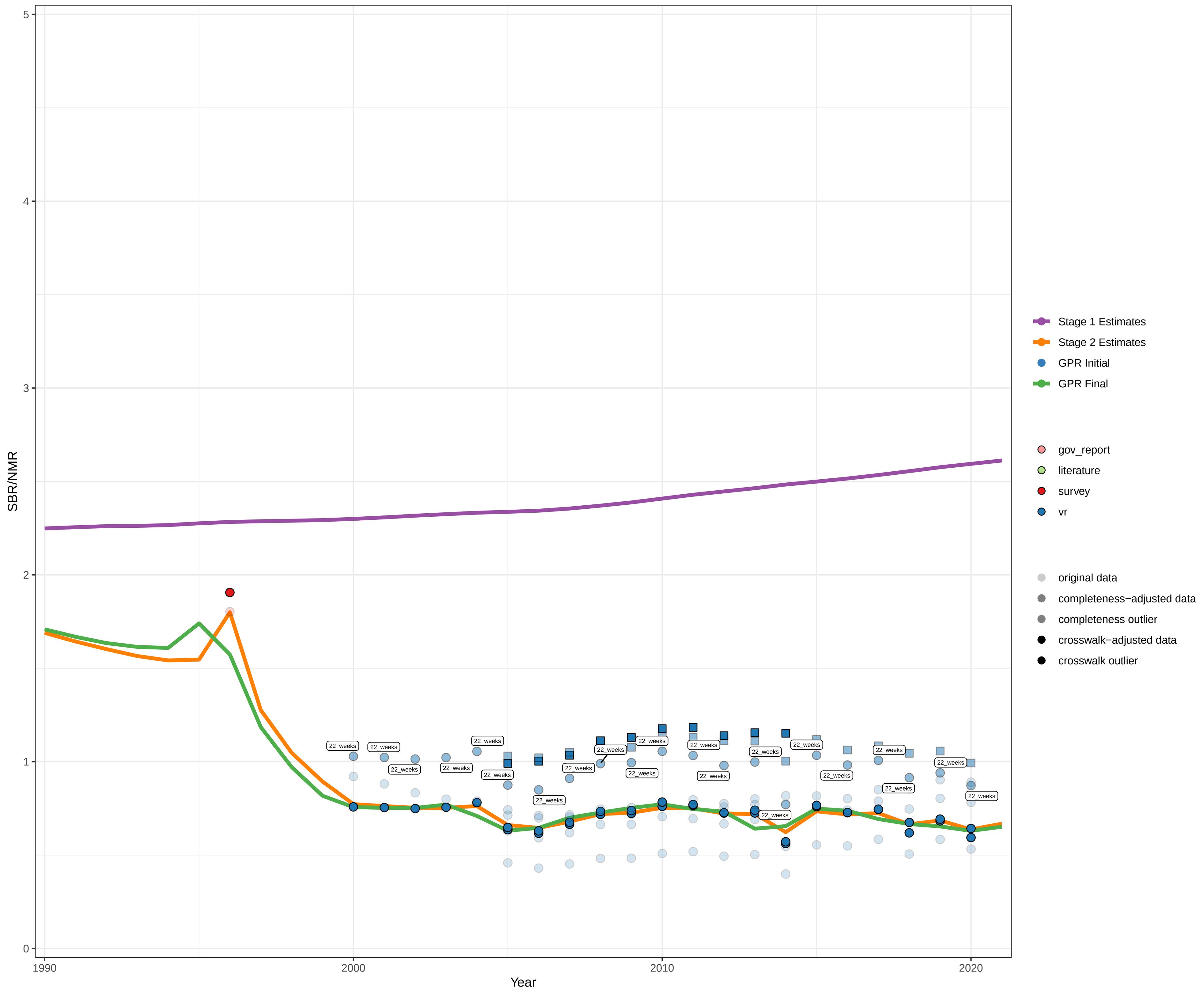

Definition	Years	Outlier	Source.Type	Title
22_weeks	2000−2014	0	VR	Brazil Mortality Information System − Stillbirths
22_weeks, 28_weeks	2005−2020	0	VR	Brazil Civil Registry Statistics − Fetal Deaths at 28 Weeks or More, by Place of Birth, Number of Births by Birth and Sex, and Residence of Mother
28_weeks	1996	0	Survey	Brazil Demographic and Health Survey 1996
other	2005−2020	1	VR	Brazil Civil Registry Statistics − Fetal Deaths at 28 Weeks or More, by Place of Birth, Number of Births by Birth and Sex, and Residence of Mother

Parana   BRA_4765   Tropical Latin America   Stillbirth Rate (SBR) − 20 weeks
lambda = 0.2 ; zeta = 0.99 ; scale = 5 ; amp = 0.028

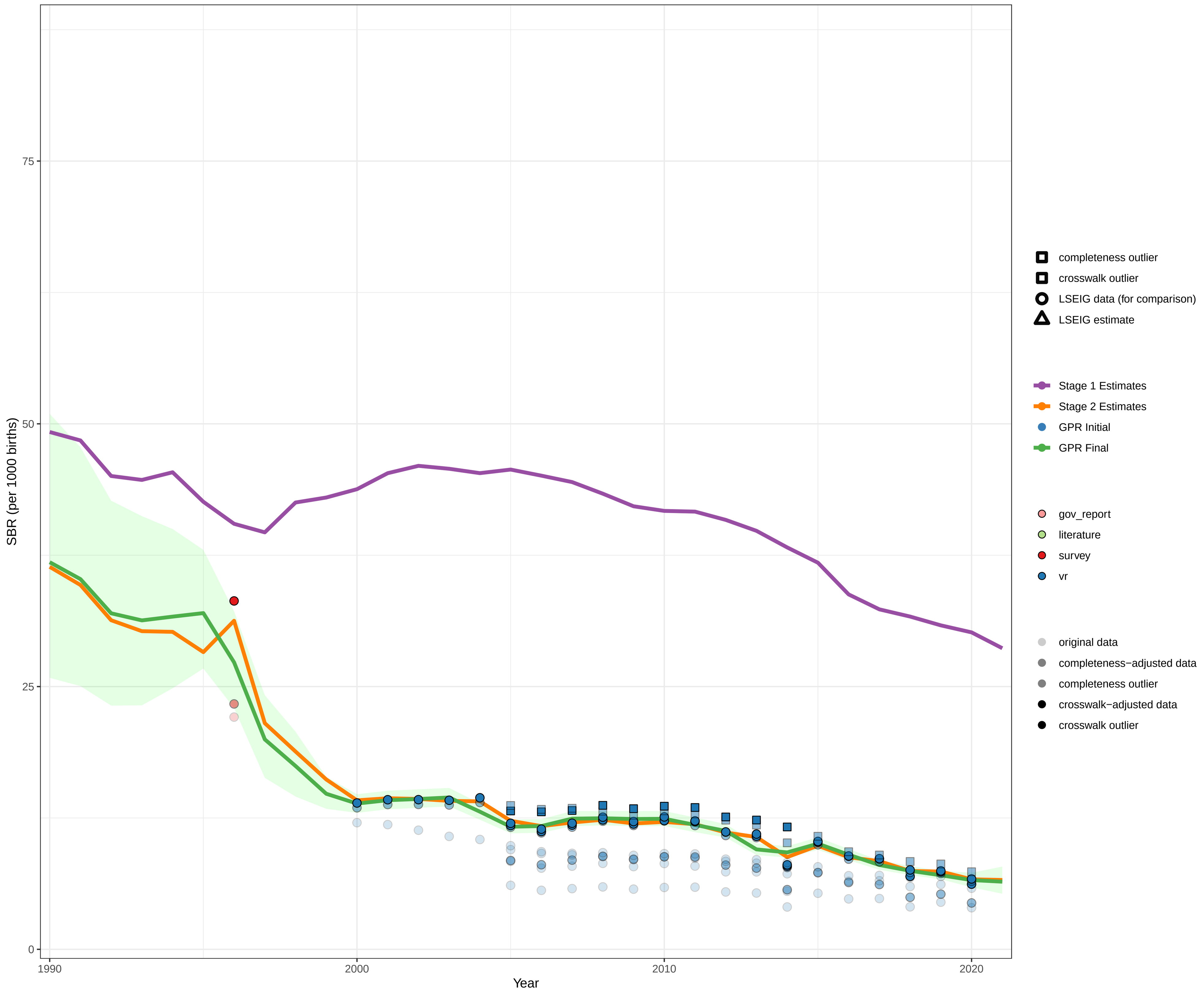

Parana   BRA_4765   Tropical Latin America   Stillbirth Rate (SBR) − 28 weeks
lambda = 0.2 ; zeta = 0.99 ; scale = 5 ; amp = 0.028

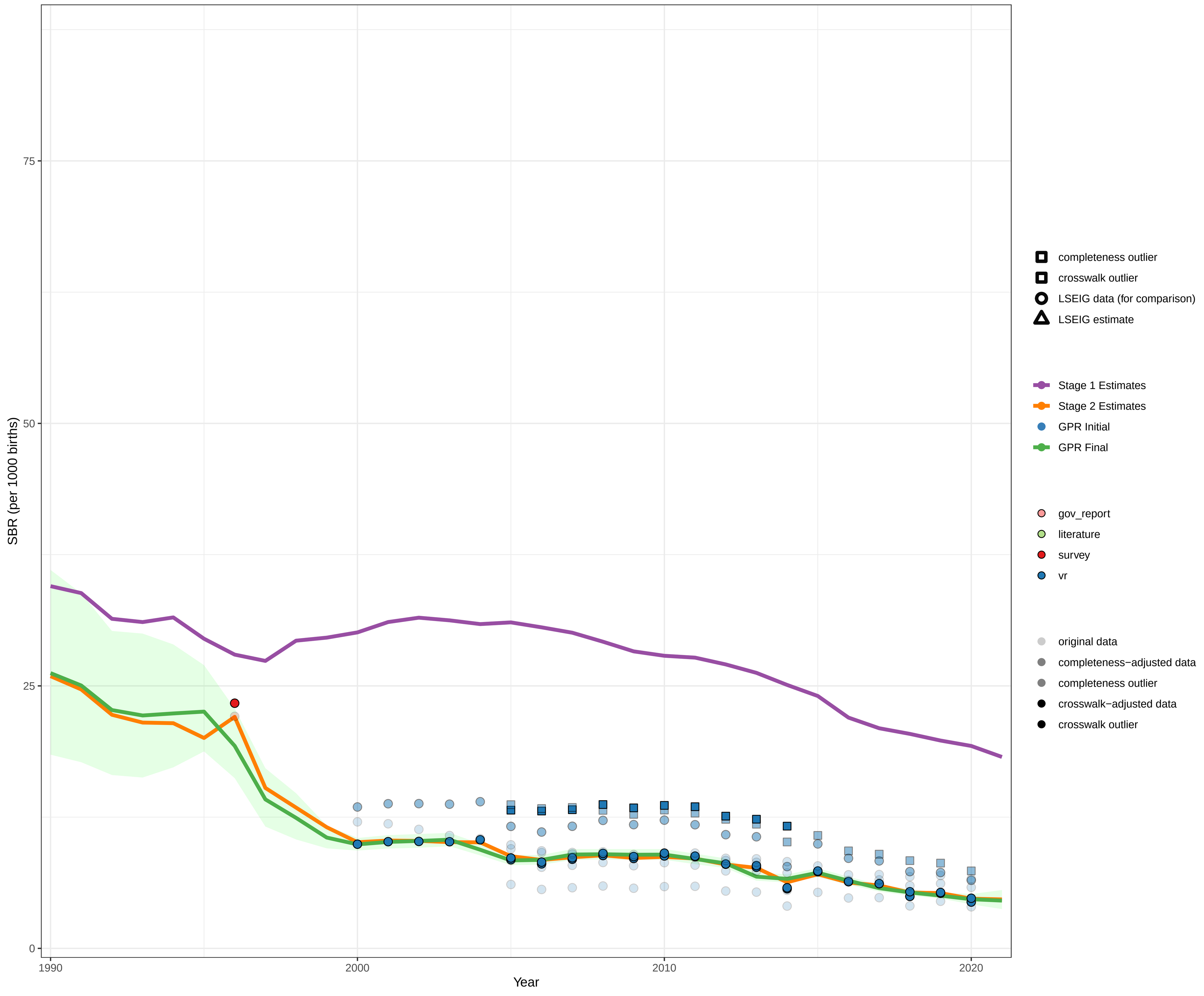

Year	Total Stillbirths (in thousands)	Total Stillbirths (in thousands)	SBR (per 1000 births)	SBR (per 1000 births)	SBR/NMR	SBR/NMR
	20 weeks	28 weeks	20 weeks	28 weeks	20 weeks	28 weeks
1990	6.9 (4.8 to 9.6)	4.8 (3.4 to 6.7)	36.8 (25.8 to 50.9)	26.2 (18.4 to 36.0)	2.40 (1.68 to 3.32)	1.71 (1.20 to 2.35)
1995	6.1 (5.1 to 7.3)	4.3 (3.6 to 5.2)	32.0 (26.7 to 38.0)	22.5 (18.8 to 27.0)	2.47 (2.06 to 2.93)	1.74 (1.45 to 2.08)
2000	2.5 (2.3 to 2.6)	1.8 (1.7 to 1.9)	13.9 (13.0 to 14.8)	9.9 (9.3 to 10.5)	1.06 (0.99 to 1.13)	0.76 (0.71 to 0.80)
2005	1.9 (1.8 to 2.0)	1.3 (1.3 to 1.4)	11.7 (11.0 to 12.4)	8.4 (7.9 to 8.9)	0.88 (0.83 to 0.93)	0.63 (0.60 to 0.67)
2010	1.9 (1.8 to 2.1)	1.4 (1.3 to 1.5)	12.4 (11.7 to 13.2)	8.9 (8.4 to 9.5)	1.07 (1.01 to 1.14)	0.77 (0.73 to 0.82)
2015	1.6 (1.5 to 1.7)	1.2 (1.1 to 1.2)	10.1 (9.5 to 10.7)	7.2 (6.8 to 7.6)	1.05 (0.98 to 1.11)	0.75 (0.71 to 0.79)
2020	1.0 (0.9 to 1.1)	0.7 (0.7 to 0.8)	6.6 (5.9 to 7.3)	4.7 (4.2 to 5.2)	0.89 (0.79 to 0.98)	0.63 (0.56 to 0.70)
2021	1.0 (0.8 to 1.2)	0.7 (0.6 to 0.9)	6.4 (5.3 to 7.9)	4.6 (3.7 to 5.6)	0.92 (0.76 to 1.13)	0.65 (0.54 to 0.80)

Pernambuco BRA_4766 Tropical Latin America Stillbirth Rate / Neonatal Mortality Rate (SBR/NMR) − 20 weeks
lambda = 0.2 ; zeta = 0.99 ; scale = 5 ; amp = 0.028

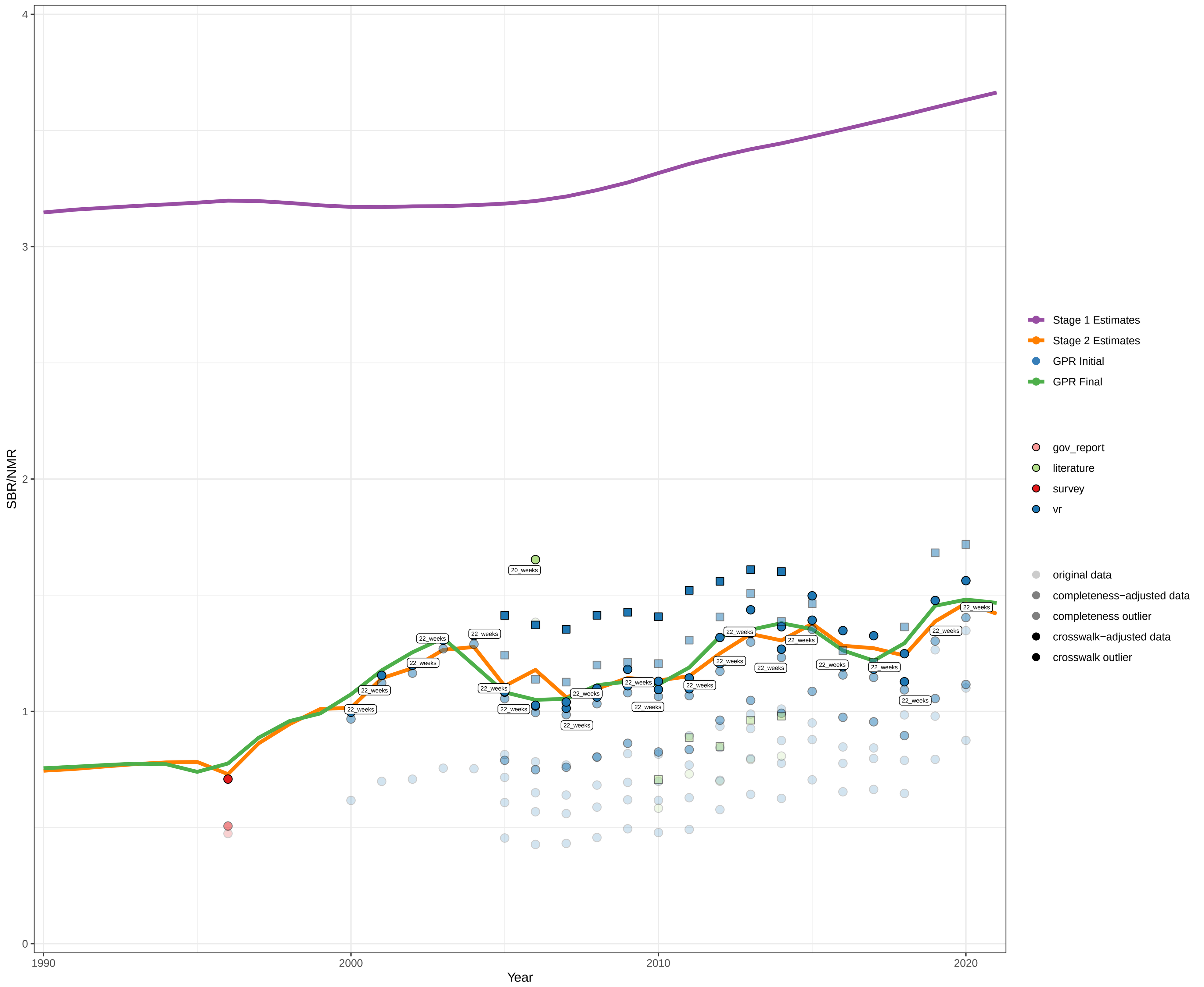

Pernambuco BRA_4766 Tropical Latin America Stillbirth Rate / Neonatal Mortality Rate (SBR/NMR) − 28 weeks
lambda = 0.2 ; zeta = 0.99 ; scale = 5 ; amp = 0.028

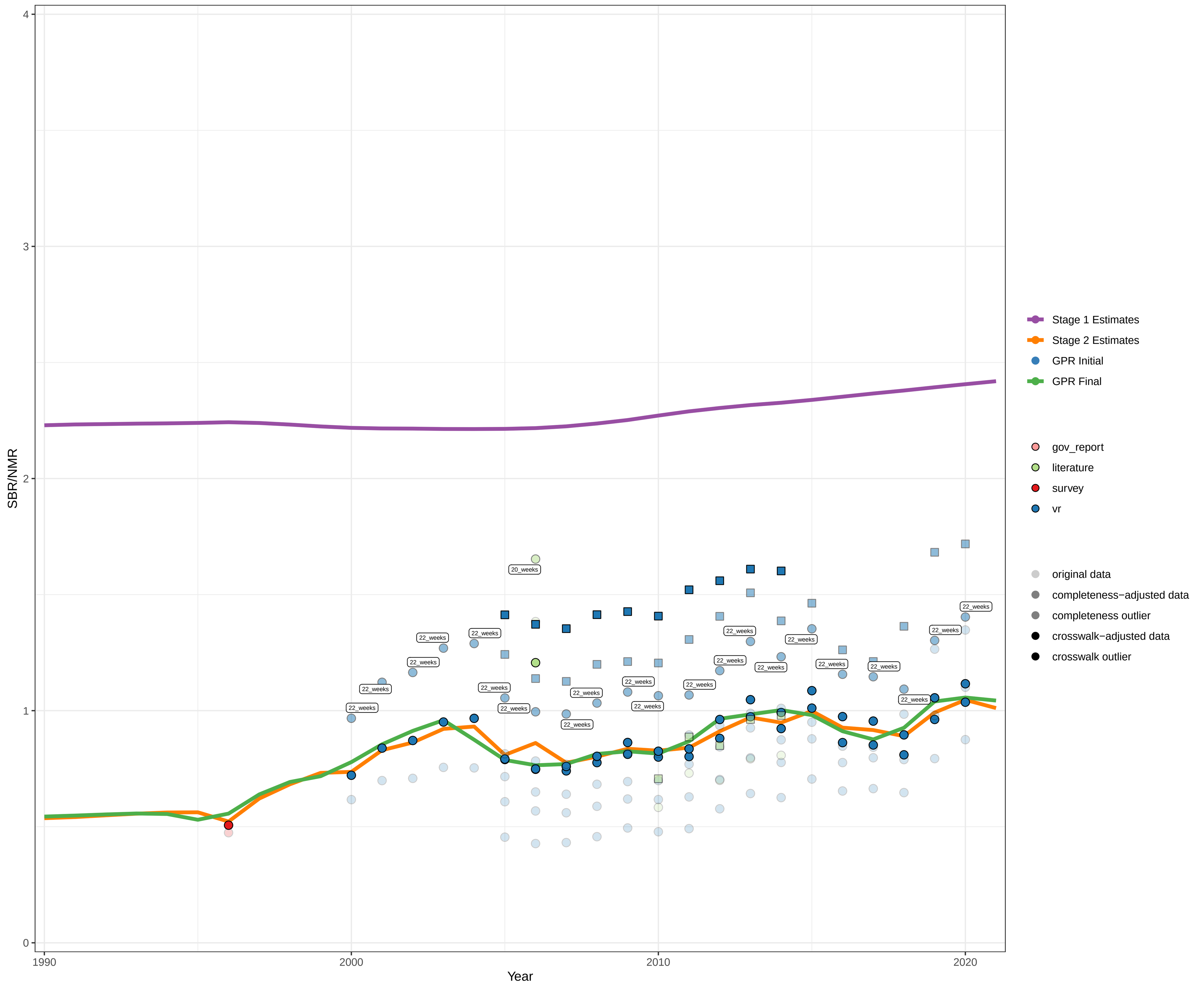

Definition	Years	Outlier	Source.Type	Title
20_weeks	2006	0	Sci Lit	Cesarean section in fetal death
22_weeks	2000−2014	0	VR	Brazil Mortality Information System − Stillbirths
22_weeks, 28_weeks	2005−2020	0	VR	Brazil Civil Registry Statistics − Fetal Deaths at 28 Weeks or More, by Place of Birth, Number of Births by Birth and Sex, and Residence of Mother
28_weeks	1996	0	Survey	Brazil Demographic and Health Survey 1996
22_weeks_OR_500_grams	2010−2014	1	Sci Lit	Perinatal deaths preventable by intervention of the Unified Health System of Brazil
other	2005−2020	1	VR	Brazil Civil Registry Statistics − Fetal Deaths at 28 Weeks or More, by Place of Birth, Number of Births by Birth and Sex, and Residence of Mother

Pernambuco BRA_4766 Tropical Latin America Stillbirth Rate (SBR) − 20 weeks
lambda = 0.2 ; zeta = 0.99 ; scale = 5 ; amp = 0.028

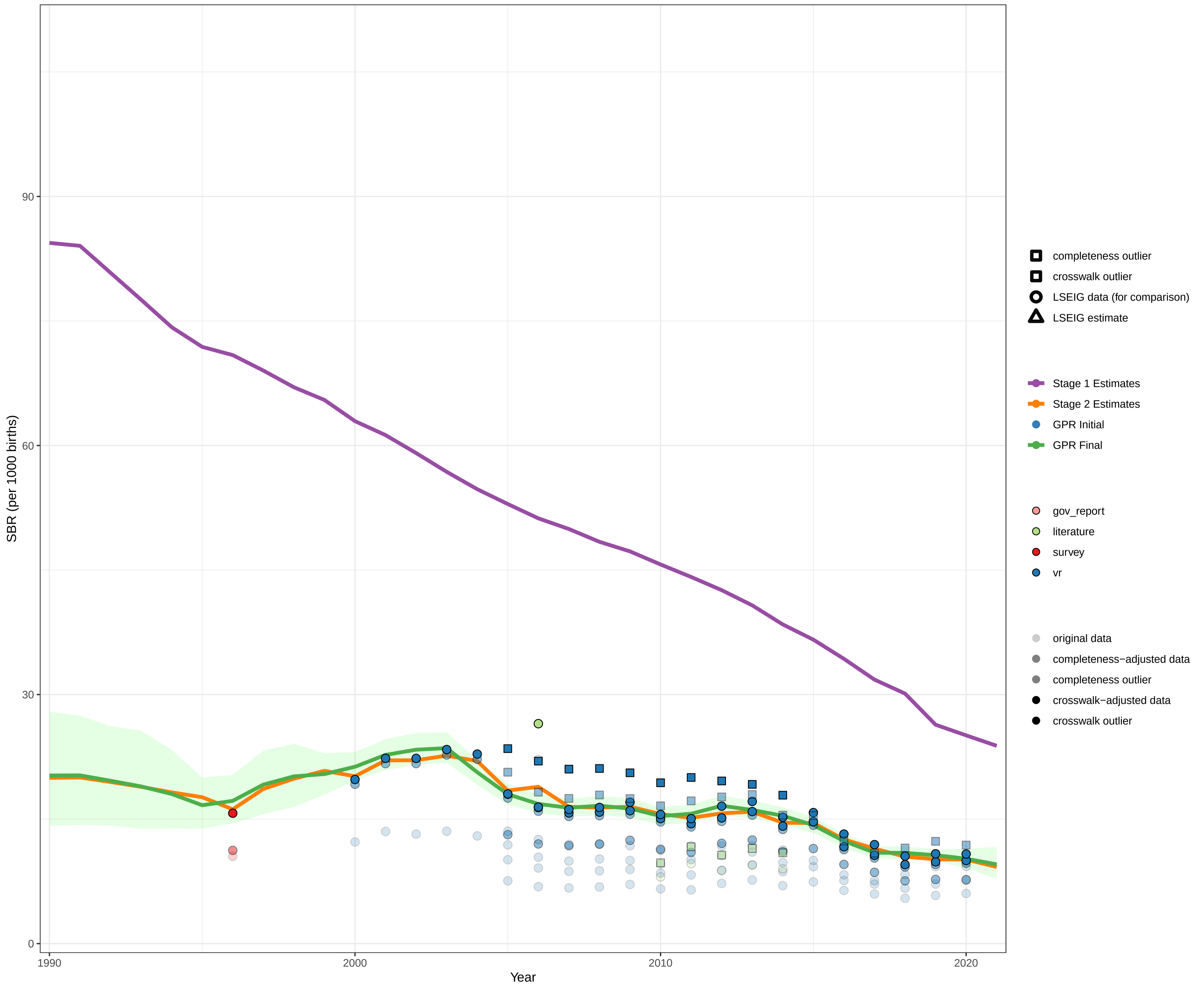

Pernambuco BRA_4766 Tropical Latin America Stillbirth Rate (SBR) − 28 weeks
lambda = 0.2 ; zeta = 0.99 ; scale = 5 ; amp = 0.028

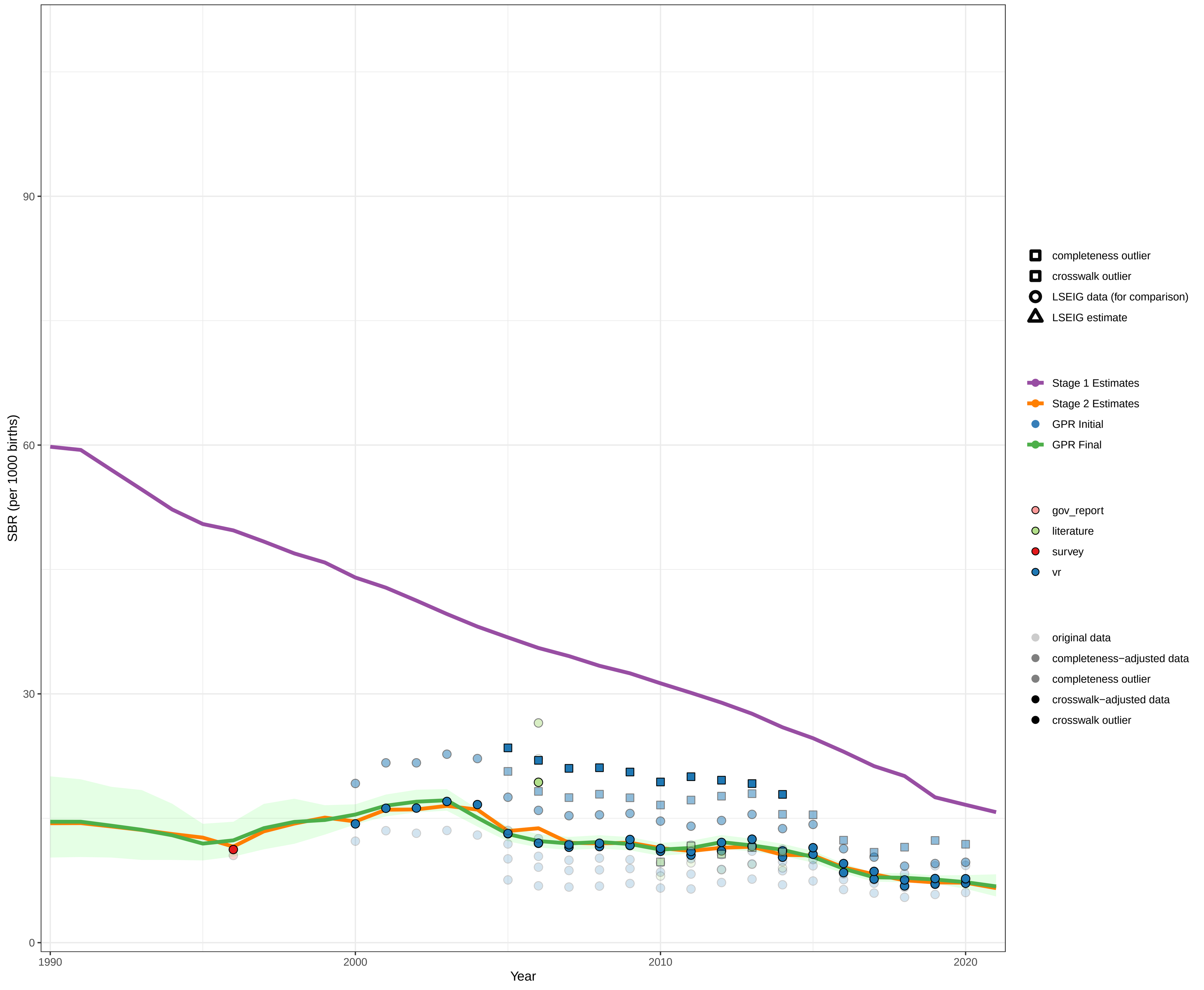

Year	Total Stillbirths (in thousands)	Total Stillbirths (in thousands)	SBR (per 1000 births)	SBR (per 1000 births)	SBR/NMR	SBR/NMR
	20 weeks	28 weeks	20 weeks	28 weeks	20 weeks	28 weeks
1990	3.7 (2.6 to 5.2)	2.7 (1.9 to 3.7)	20.3 (14.2 to 27.9)	14.6 (10.3 to 20.1)	0.76 (0.53 to 1.04)	0.54 (0.38 to 0.75)
1995	3.3 (2.7 to 3.9)	2.3 (1.9 to 2.8)	16.7 (13.9 to 20.0)	11.9 (9.9 to 14.3)	0.74 (0.61 to 0.89)	0.53 (0.44 to 0.64)
2000	4.5 (4.2 to 4.9)	3.3 (3.0 to 3.5)	21.3 (19.6 to 23.1)	15.4 (14.3 to 16.7)	1.07 (0.99 to 1.16)	0.78 (0.72 to 0.84)
2005	3.8 (3.5 to 4.1)	2.7 (2.6 to 2.9)	18.0 (16.8 to 19.2)	13.1 (12.2 to 14.0)	1.08 (1.01 to 1.16)	0.79 (0.73 to 0.84)
2010	3.1 (2.9 to 3.4)	2.3 (2.1 to 2.4)	15.4 (14.3 to 16.5)	11.2 (10.5 to 12.0)	1.12 (1.04 to 1.20)	0.82 (0.76 to 0.87)
2015	2.9 (2.7 to 3.1)	2.1 (1.9 to 2.2)	14.3 (13.3 to 15.3)	10.3 (9.6 to 11.0)	1.35 (1.26 to 1.45)	0.98 (0.92 to 1.05)
2020	2.0 (1.7 to 2.2)	1.4 (1.2 to 1.6)	10.2 (9.1 to 11.4)	7.3 (6.5 to 8.1)	1.48 (1.31 to 1.66)	1.06 (0.94 to 1.18)
2021	1.8 (1.5 to 2.2)	1.3 (1.0 to 1.5)	9.5 (7.8 to 11.6)	6.8 (5.6 to 8.2)	1.47 (1.20 to 1.79)	1.04 (0.86 to 1.27)

Piaui BRA_4767 Tropical Latin America Stillbirth Rate / Neonatal Mortality Rate (SBR/NMR) − 20 weeks
lambda = 0.2 ; zeta = 0.99 ; scale = 5 ; amp = 0.028

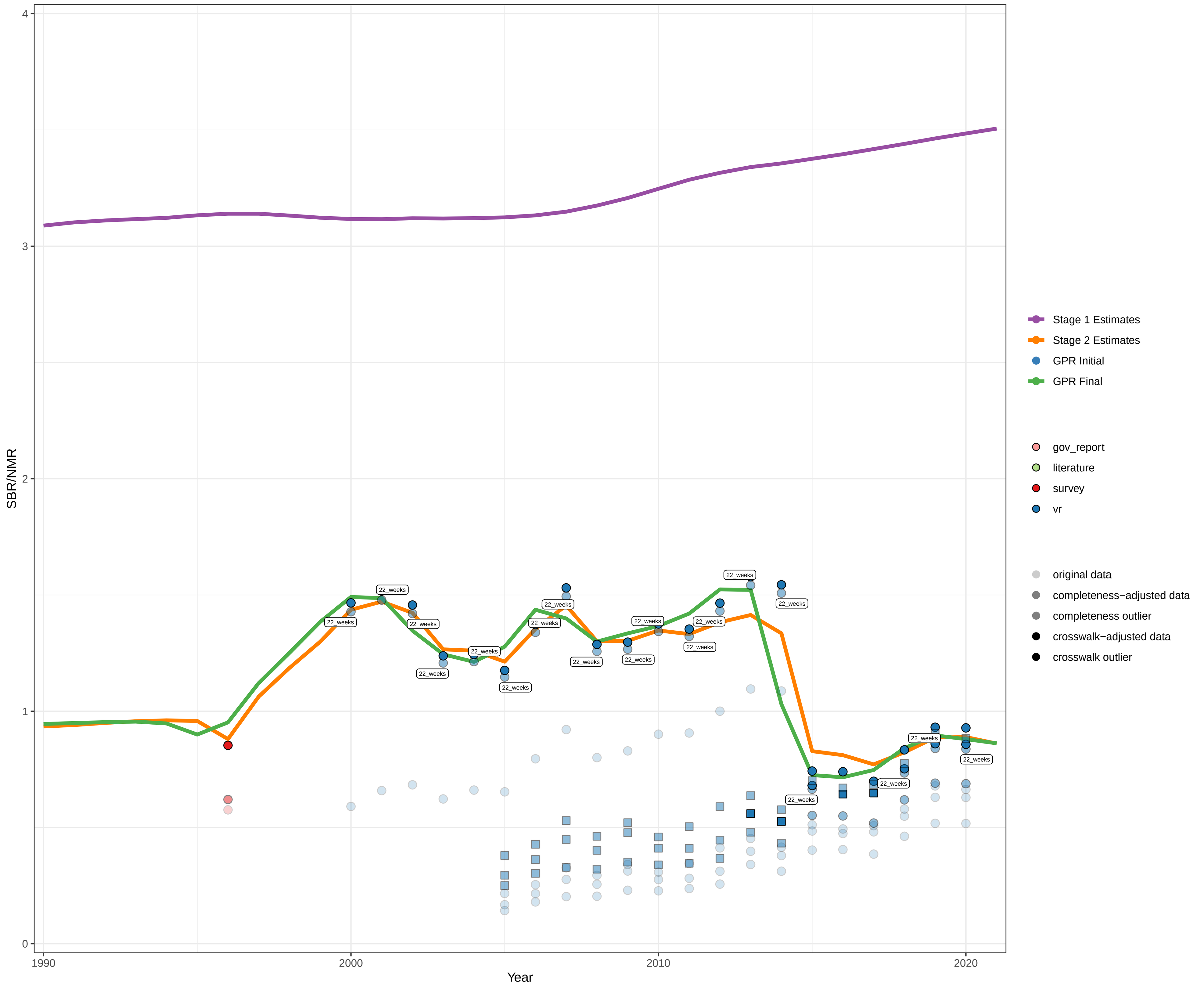

Piaui BRA_4767 Tropical Latin America Stillbirth Rate / Neonatal Mortality Rate (SBR/NMR) − 28 weeks
lambda = 0.2 ; zeta = 0.99 ; scale = 5 ; amp = 0.028

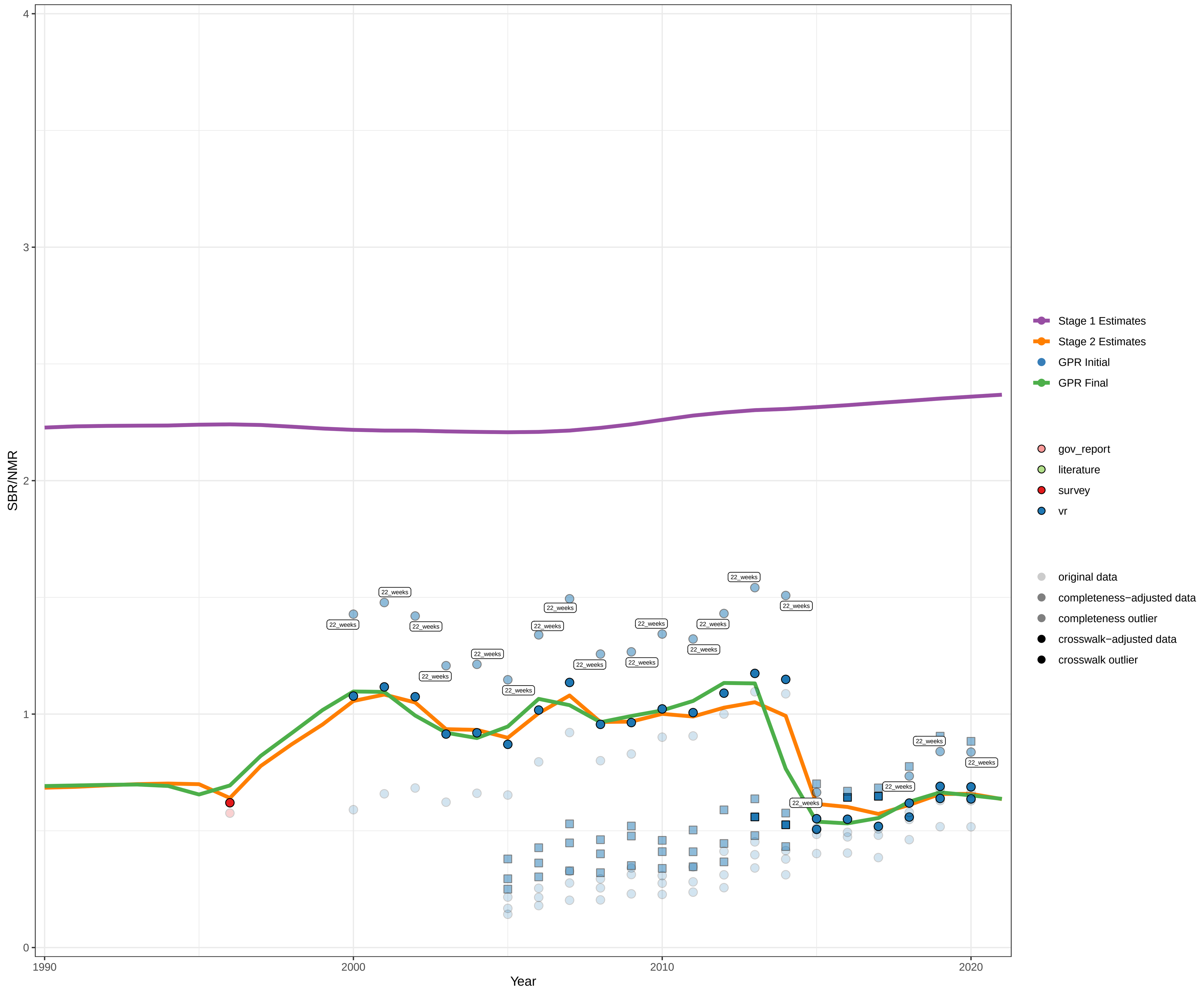

Definition	Years	Outlier	Source.Type	Title
22_weeks	2000−2014	0	VR	Brazil Mortality Information System − Stillbirths
22_weeks	2013−2020	0	VR	Brazil Civil Registry Statistics − Fetal Deaths at 28 Weeks or More, by Place of Birth, Number of Births by Birth and Sex, and Residence of Mother
28_weeks	1996	0	Survey	Brazil Demographic and Health Survey 1996
28_weeks	2015−2020	0	VR	Brazil Civil Registry Statistics − Fetal Deaths at 28 Weeks or More, by Place of Birth, Number of Births by Birth and Sex, and Residence of Mother
22_weeks	2005−2012	1	VR	Brazil Civil Registry Statistics − Fetal Deaths at 28 Weeks or More, by Place of Birth, Number of Births by Birth and Sex, and Residence of Mother
28_weeks	2005−2014	1	VR	Brazil Civil Registry Statistics − Fetal Deaths at 28 Weeks or More, by Place of Birth, Number of Births by Birth and Sex, and Residence of Mother
other	2005−2020	1	VR	Brazil Civil Registry Statistics − Fetal Deaths at 28 Weeks or More, by Place of Birth, Number of Births by Birth and Sex, and Residence of Mother

Piaui BRA_4767 Tropical Latin America Stillbirth Rate (SBR) − 20 weeks
lambda = 0.2 ; zeta = 0.99 ; scale = 5 ; amp = 0.028

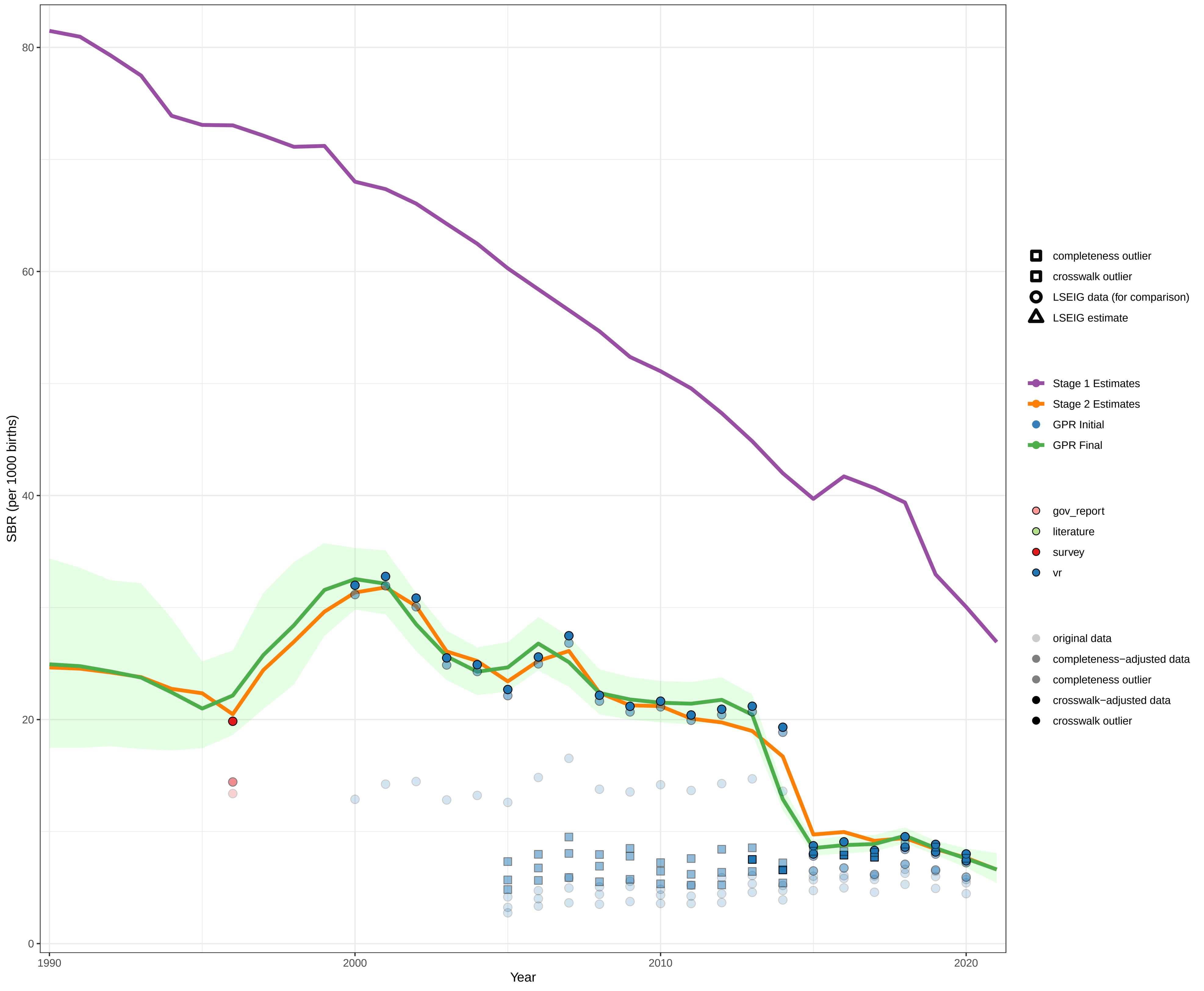

Piaui BRA_4767 Tropical Latin America Stillbirth Rate (SBR) − 28 weeks
lambda = 0.2 ; zeta = 0.99 ; scale = 5 ; amp = 0.028

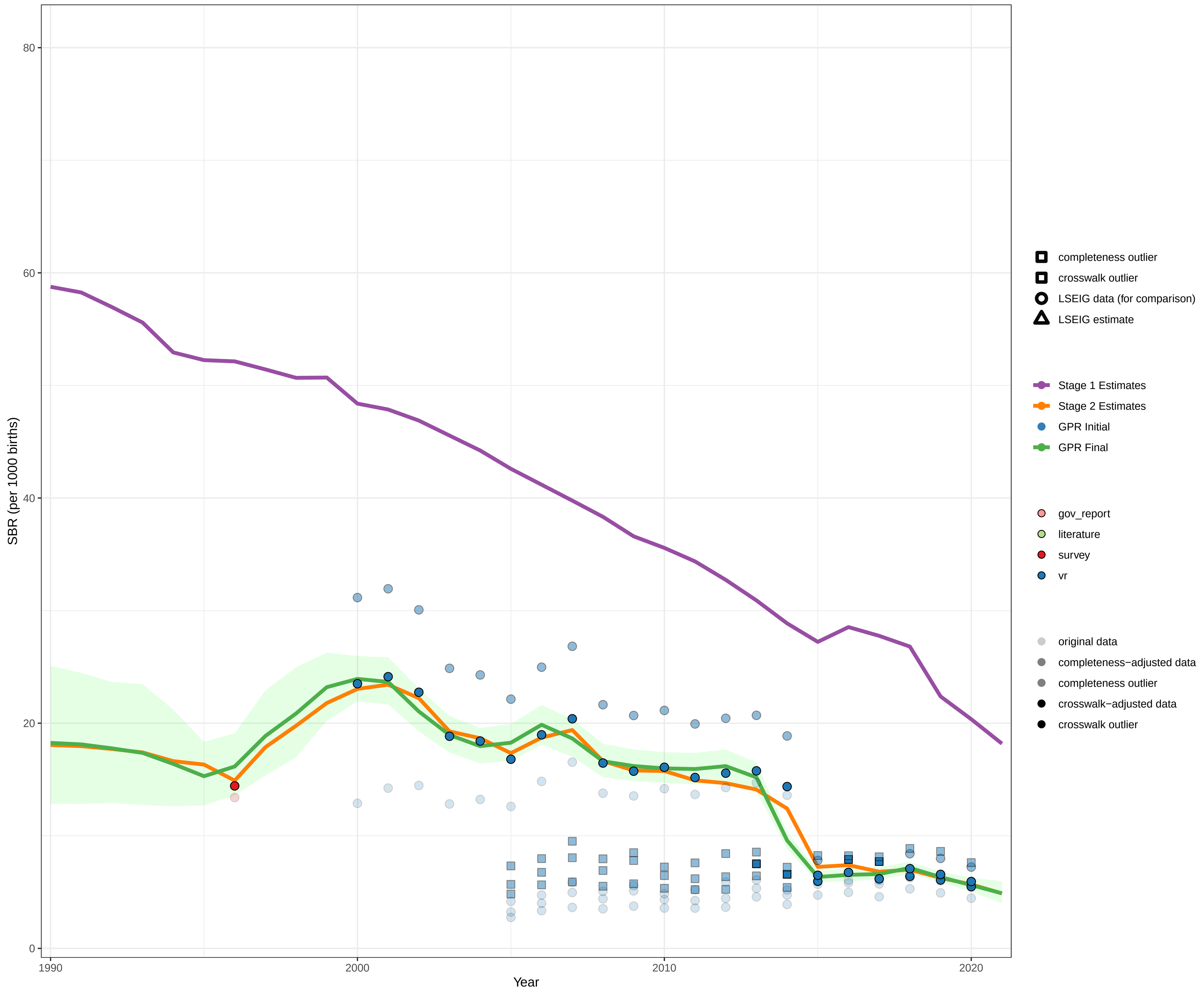

Year	Total Stillbirths (in thousands)	Total Stillbirths (in thousands)	SBR (per 1000 births)	SBR (per 1000 births)	SBR/NMR	SBR/NMR
	20 weeks	28 weeks	20 weeks	28 weeks	20 weeks	28 weeks
1990	1.7 (1.2 to 2.4)	1.2 (0.9 to 1.7)	24.9 (17.5 to 34.4)	18.3 (12.8 to 25.1)	0.95 (0.66 to 1.30)	0.69 (0.49 to 0.95)
1995	1.5 (1.2 to 1.8)	1.1 (0.9 to 1.3)	21.0 (17.4 to 25.2)	15.3 (12.7 to 18.4)	0.90 (0.75 to 1.08)	0.66 (0.54 to 0.79)
2000	2.6 (2.4 to 2.8)	1.9 (1.7 to 2.1)	32.5 (29.8 to 35.3)	23.9 (21.9 to 26.0)	1.49 (1.37 to 1.62)	1.10 (1.01 to 1.19)
2005	2.0 (1.8 to 2.2)	1.5 (1.4 to 1.6)	24.7 (22.5 to 26.9)	18.3 (16.7 to 19.9)	1.28 (1.17 to 1.39)	0.95 (0.86 to 1.03)
2010	1.8 (1.6 to 1.9)	1.3 (1.2 to 1.4)	21.5 (19.7 to 23.4)	16.0 (14.7 to 17.4)	1.37 (1.25 to 1.49)	1.02 (0.93 to 1.11)
2015	0.7 (0.6 to 0.8)	0.5 (0.5 to 0.6)	8.5 (7.9 to 9.3)	6.3 (5.9 to 6.9)	0.73 (0.67 to 0.79)	0.54 (0.50 to 0.59)
2020	0.6 (0.5 to 0.7)	0.4 (0.4 to 0.5)	7.6 (6.8 to 8.5)	5.6 (5.0 to 6.3)	0.88 (0.79 to 0.99)	0.65 (0.58 to 0.73)
2021	0.5 (0.4 to 0.6)	0.4 (0.3 to 0.5)	6.6 (5.4 to 8.1)	4.9 (4.0 to 6.0)	0.86 (0.70 to 1.05)	0.64 (0.52 to 0.78)

Rio de Janeiro   BRA_4768   Tropical Latin America   Stillbirth Rate / Neonatal Mortality Rate (SBR/NMR) − 20 weeks
lambda = 0.2 ; zeta = 0.99 ; scale = 5 ; amp = 0.028

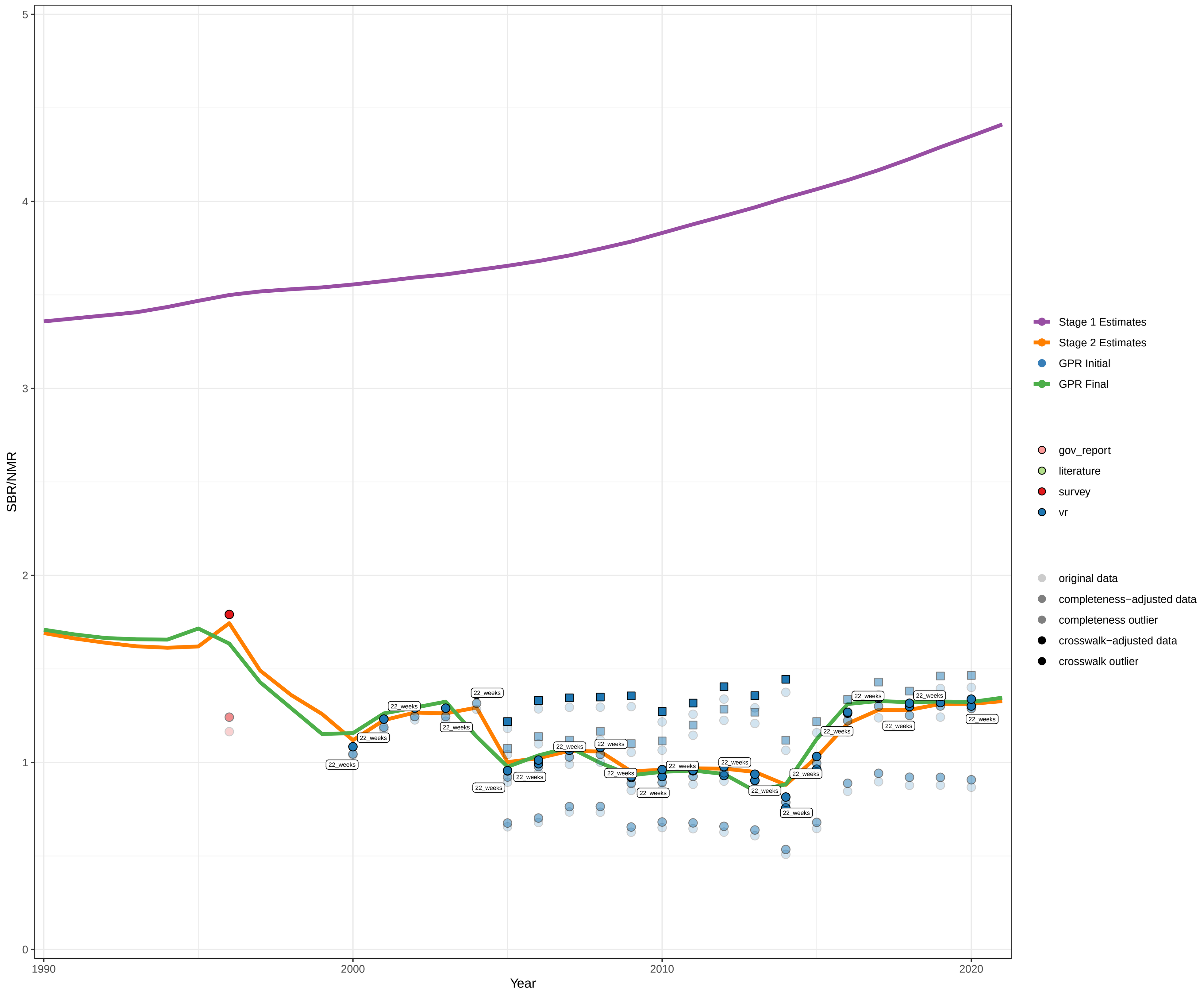

Rio de Janeiro   BRA_4768   Tropical Latin America   Stillbirth Rate / Neonatal Mortality Rate (SBR/NMR) − 28 weeks
lambda = 0.2 ; zeta = 0.99 ; scale = 5 ; amp = 0.028

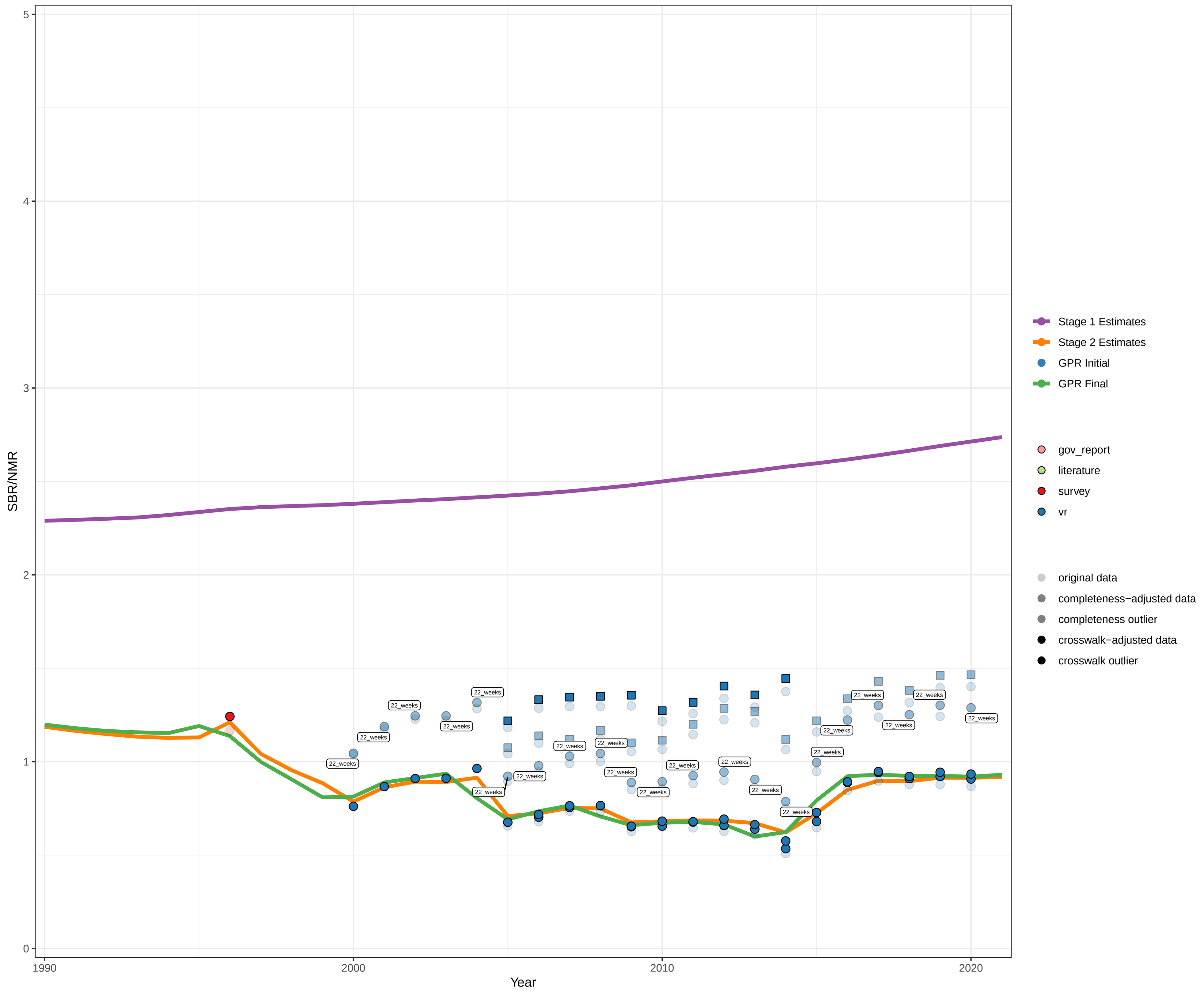

Definition	Years	Outlier	Source.Type	Title
22_weeks	2000−2014	0	VR	Brazil Mortality Information System − Stillbirths
22_weeks, 28_weeks	2005−2020	0	VR	Brazil Civil Registry Statistics − Fetal Deaths at 28 Weeks or More, by Place of Birth, Number of Births by Birth and Sex, and Residence of Mother
28_weeks	1996	0	Survey	Brazil Demographic and Health Survey 1996
other	2005−2020	1	VR	Brazil Civil Registry Statistics − Fetal Deaths at 28 Weeks or More, by Place of Birth, Number of Births by Birth and Sex, and Residence of Mother

Rio de Janeiro   BRA_4768   Tropical Latin America   Stillbirth Rate (SBR) − 20 weeks
lambda = 0.2 ; zeta = 0.99 ; scale = 5 ; amp = 0.028

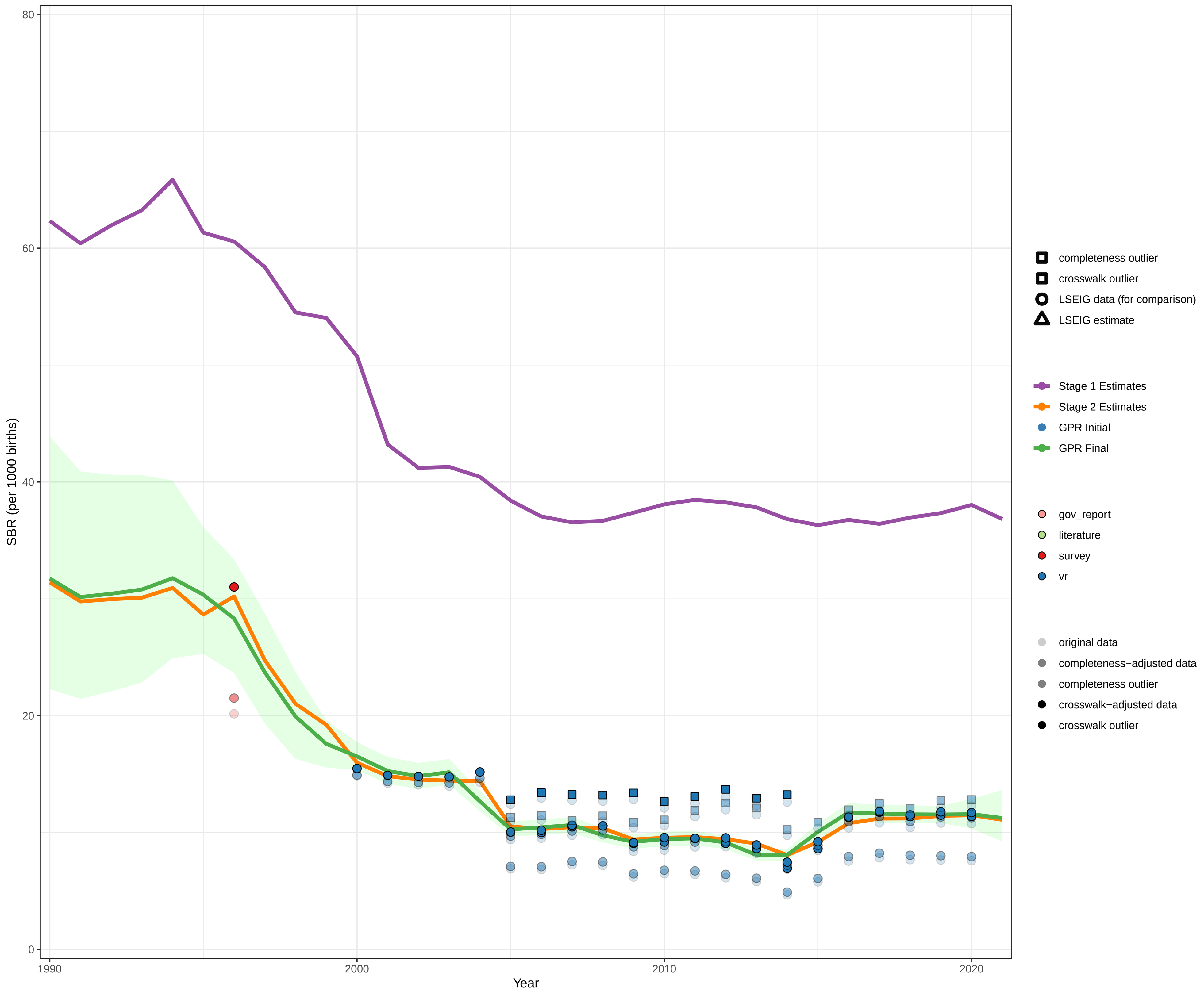

Rio de Janeiro   BRA_4768   Tropical Latin America   Stillbirth Rate (SBR) − 28 weeks
lambda = 0.2 ; zeta = 0.99 ; scale = 5 ; amp = 0.028

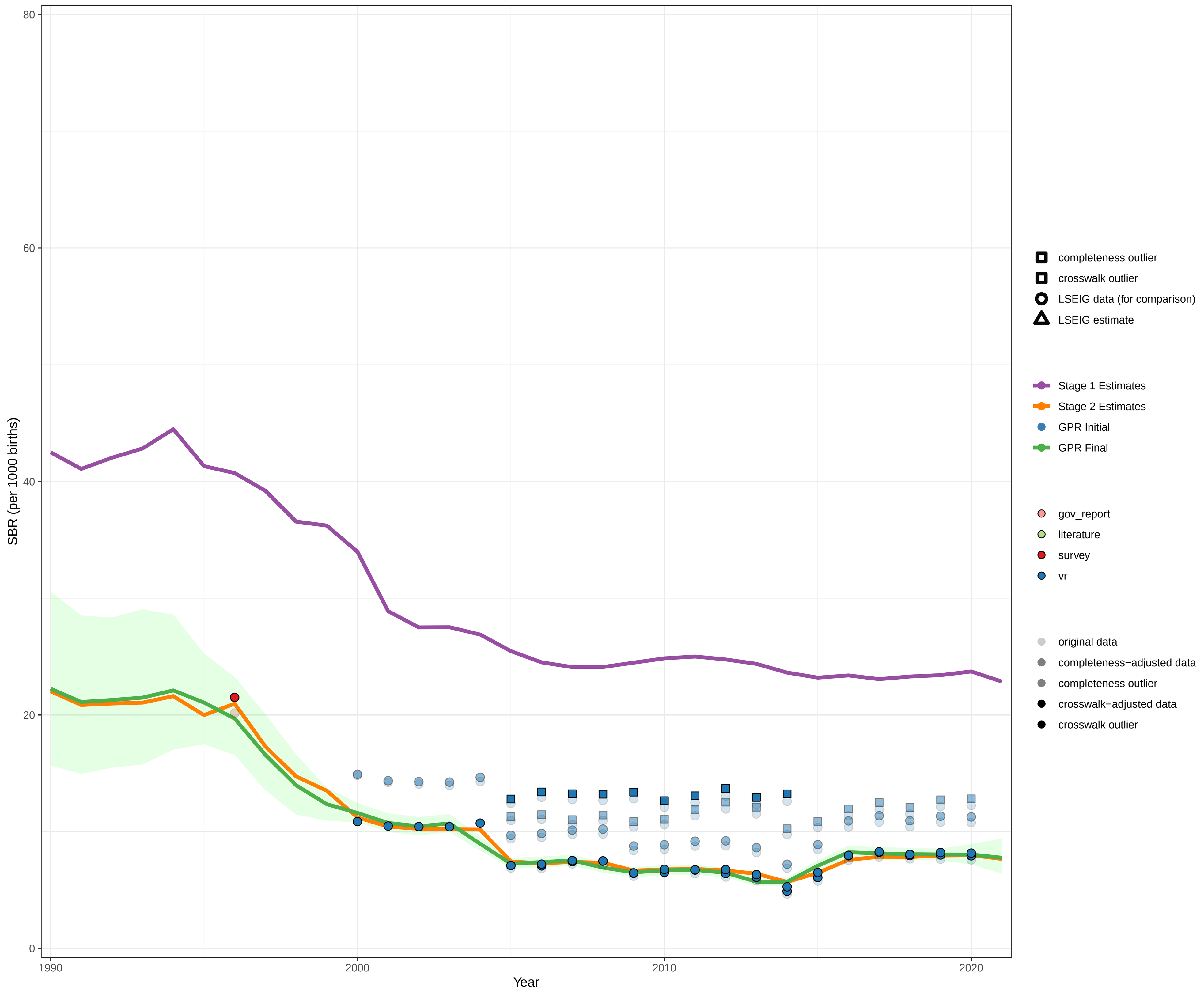

Year	Total Stillbirths (in thousands)	Total Stillbirths (in thousands)	SBR (per 1000 births)	SBR (per 1000 births)	SBR/NMR	SBR/NMR
	20 weeks	28 weeks	20 weeks	28 weeks	20 weeks	28 weeks
1990	7.5 (5.2 to 10.5)	5.2 (3.6 to 7.2)	31.7 (22.3 to 43.9)	22.2 (15.7 to 30.6)	1.71 (1.20 to 2.37)	1.20 (0.84 to 1.65)
1995	7.6 (6.3 to 9.1)	5.2 (4.3 to 6.3)	30.4 (25.3 to 36.2)	21.1 (17.5 to 25.3)	1.72 (1.43 to 2.04)	1.19 (0.99 to 1.43)
2000	4.1 (3.8 to 4.4)	2.8 (2.6 to 3.0)	16.5 (15.3 to 17.7)	11.6 (10.8 to 12.5)	1.16 (1.08 to 1.24)	0.81 (0.76 to 0.87)
2005	2.3 (2.2 to 2.4)	1.6 (1.5 to 1.7)	10.3 (9.6 to 11.0)	7.3 (6.8 to 7.8)	0.98 (0.92 to 1.04)	0.69 (0.65 to 0.74)
2010	2.1 (2.0 to 2.2)	1.5 (1.4 to 1.6)	9.4 (8.8 to 10.1)	6.7 (6.3 to 7.1)	0.95 (0.89 to 1.01)	0.67 (0.63 to 0.72)
2015	2.3 (2.2 to 2.5)	1.6 (1.5 to 1.7)	10.1 (9.5 to 10.8)	7.1 (6.7 to 7.6)	1.13 (1.06 to 1.21)	0.79 (0.75 to 0.85)
2020	2.5 (2.2 to 2.8)	1.7 (1.5 to 1.9)	11.6 (10.3 to 12.9)	8.0 (7.2 to 8.9)	1.32 (1.18 to 1.47)	0.92 (0.82 to 1.02)
2021	2.4 (1.9 to 2.9)	1.6 (1.3 to 2.0)	11.2 (9.2 to 13.6)	7.8 (6.4 to 9.4)	1.35 (1.11 to 1.63)	0.93 (0.77 to 1.13)

Rio Grande do Norte BRA_4769 Tropical Latin America Stillbirth Rate / Neonatal Mortality Rate (SBR/NMR) − 20 weeks
lambda = 0.2 ; zeta = 0.99 ; scale = 5 ; amp = 0.028

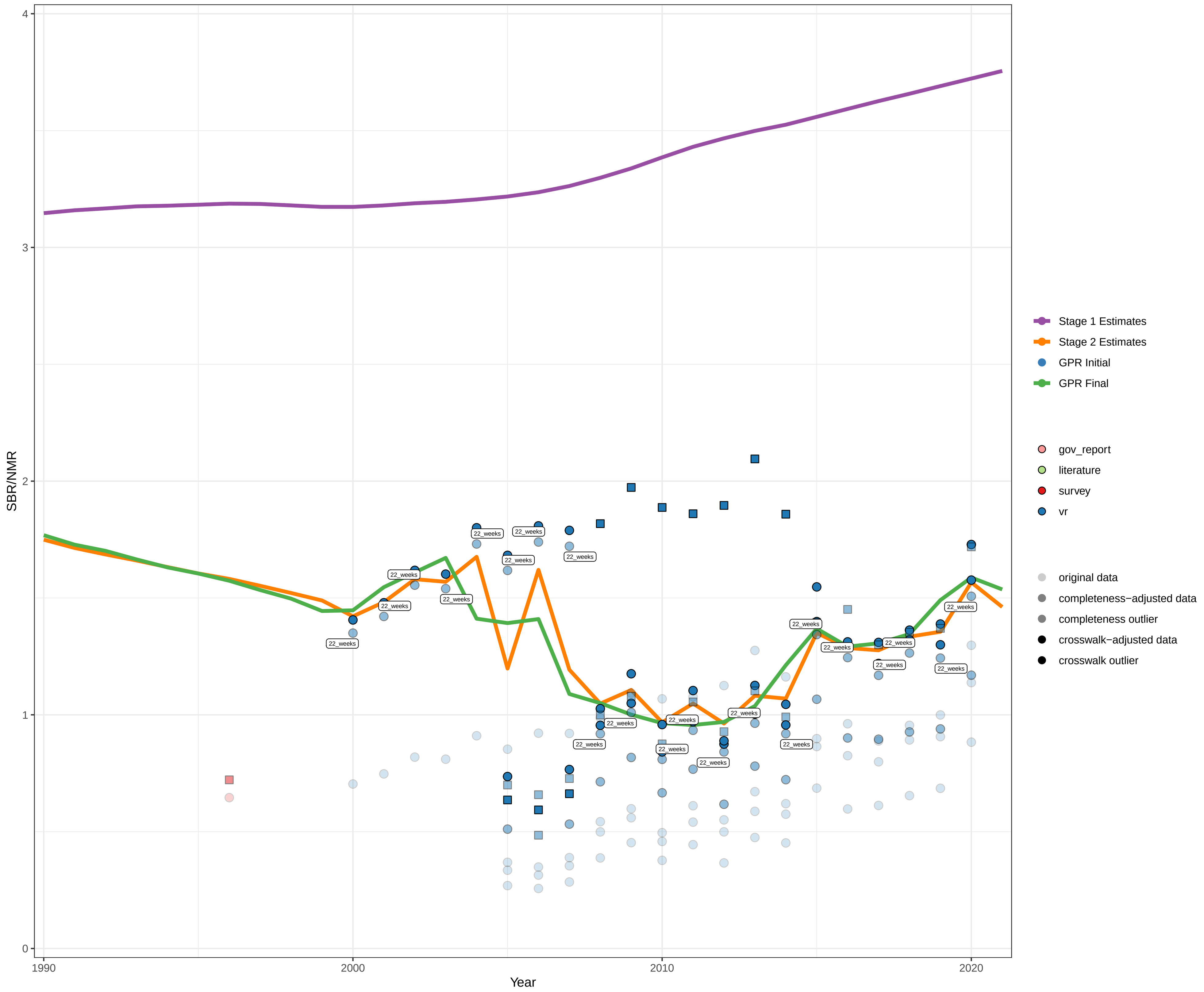

Rio Grande do Norte BRA_4769 Tropical Latin America Stillbirth Rate / Neonatal Mortality Rate (SBR/NMR) − 28 weeks
lambda = 0.2 ; zeta = 0.99 ; scale = 5 ; amp = 0.028

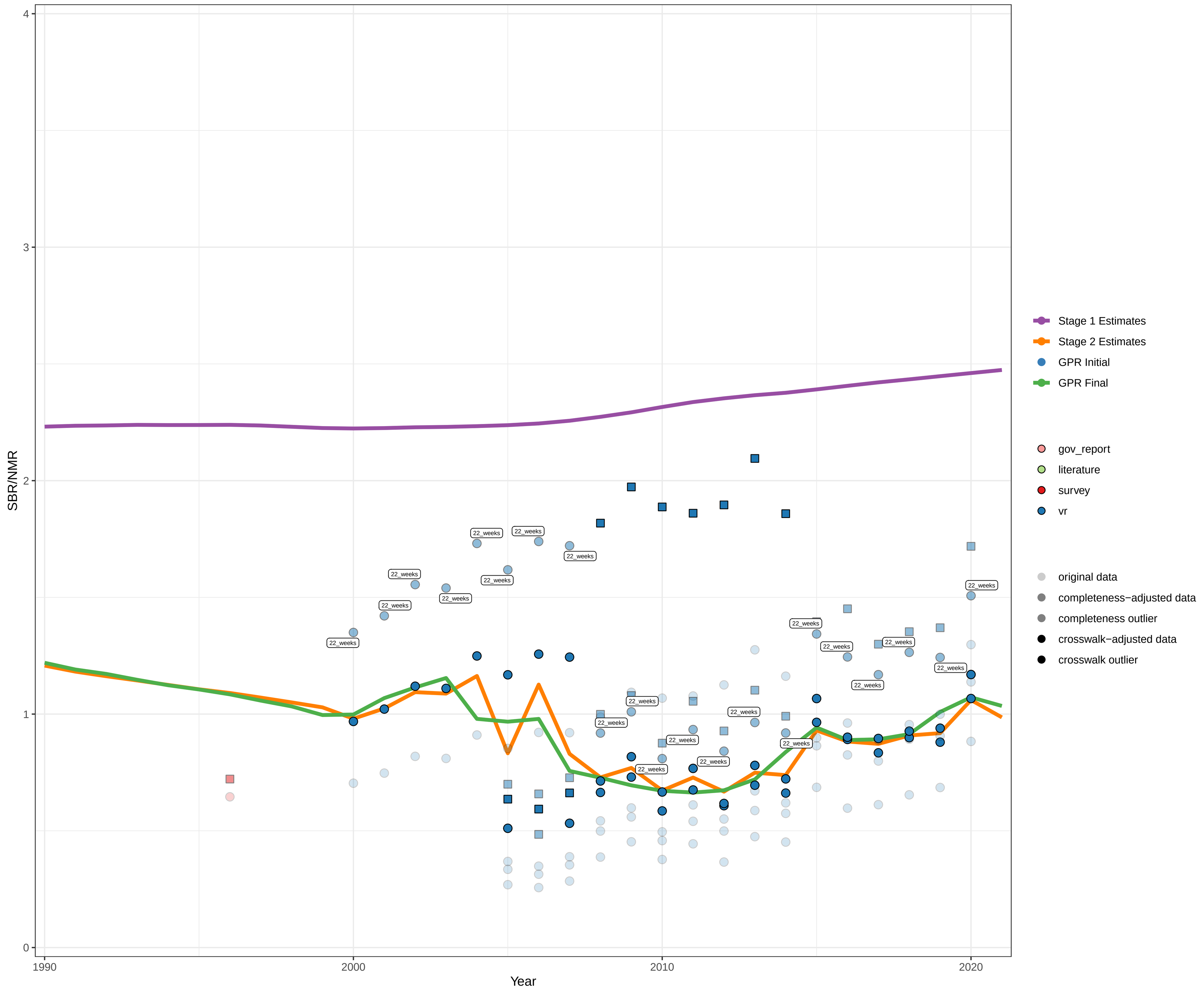

Definition	Years	Outlier	Source.Type	Title
22_weeks	2000−2014	0	VR	Brazil Mortality Information System − Stillbirths
22_weeks	2005−2020	0	VR	Brazil Civil Registry Statistics − Fetal Deaths at 28 Weeks or More, by Place of Birth, Number of Births by Birth and Sex, and Residence of Mother
28_weeks	2005, 2007−2020	0	VR	Brazil Civil Registry Statistics − Fetal Deaths at 28 Weeks or More, by Place of Birth, Number of Births by Birth and Sex, and Residence of Mother
28_weeks	1996	1	Survey	Brazil Demographic and Health Survey 1996
28_weeks	2006	1	VR	Brazil Civil Registry Statistics − Fetal Deaths at 28 Weeks or More, by Place of Birth, Number of Births by Birth and Sex, and Residence of Mother
other	2005−2020	1	VR	Brazil Civil Registry Statistics − Fetal Deaths at 28 Weeks or More, by Place of Birth, Number of Births by Birth and Sex, and Residence of Mother

Rio Grande do Norte BRA_4769 Tropical Latin America Stillbirth Rate (SBR) − 20 weeks
lambda = 0.2 ; zeta = 0.99 ; scale = 5 ; amp = 0.028

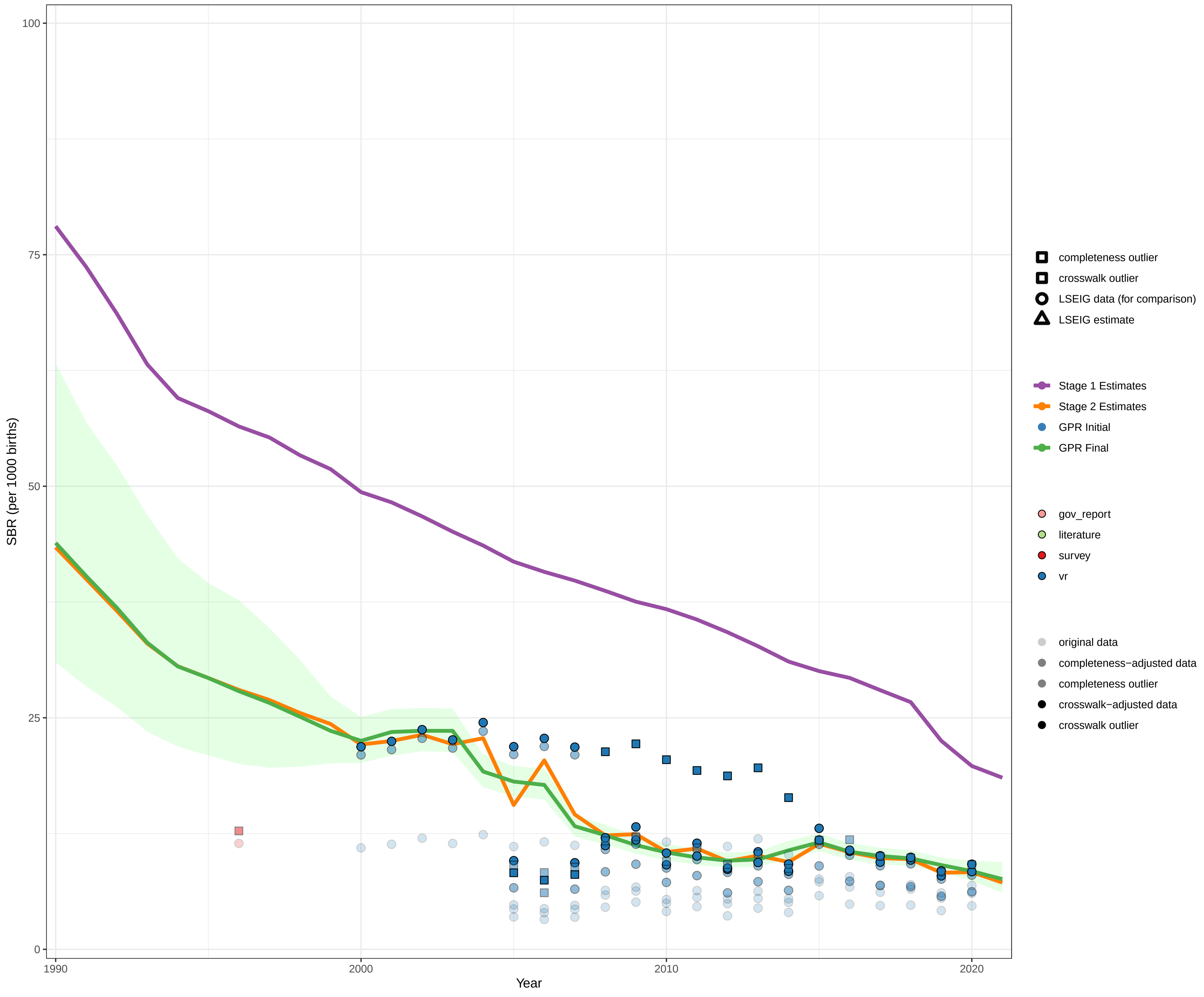

Rio Grande do Norte BRA_4769 Tropical Latin America Stillbirth Rate (SBR) − 28 weeks
lambda = 0.2 ; zeta = 0.99 ; scale = 5 ; amp = 0.028

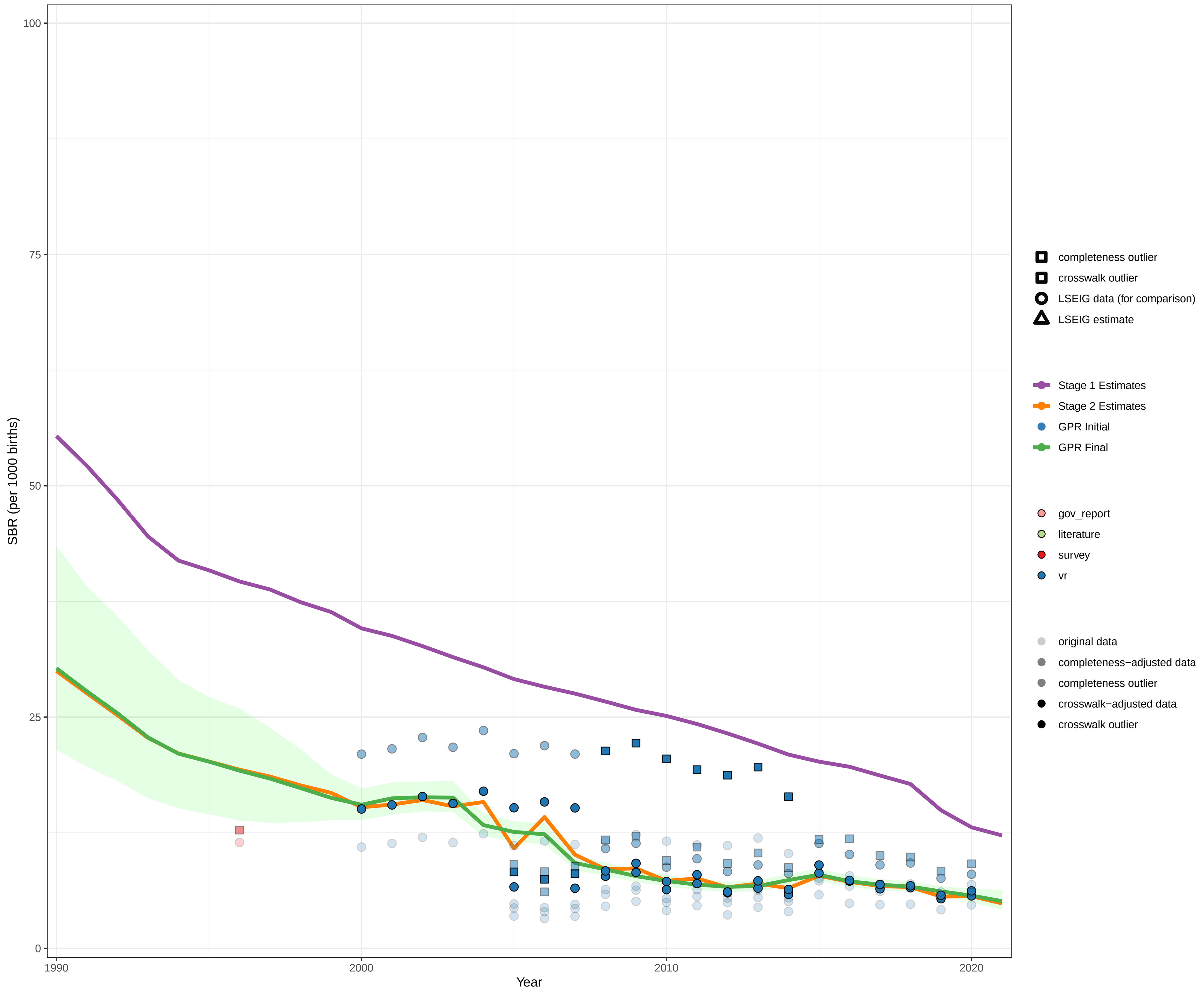

Year	Total Stillbirths (in thousands)	Total Stillbirths (in thousands)	SBR (per 1000 births)	SBR (per 1000 births)	SBR/NMR	SBR/NMR
	20 weeks	28 weeks	20 weeks	28 weeks	20 weeks	28 weeks
1990	2.9 (2.0 to 4.3)	2.0 (1.4 to 2.9)	43.9 (31.0 to 63.3)	30.3 (21.4 to 43.5)	1.77 (1.25 to 2.55)	1.22 (0.86 to 1.75)
1995	2.1 (1.5 to 2.9)	1.4 (1.0 to 2.0)	29.3 (20.9 to 39.6)	20.2 (14.5 to 27.2)	1.60 (1.15 to 2.17)	1.11 (0.79 to 1.49)
2000	1.8 (1.6 to 2.0)	1.2 (1.1 to 1.3)	22.5 (20.1 to 25.1)	15.5 (13.9 to 17.3)	1.45 (1.29 to 1.61)	1.00 (0.89 to 1.11)
2005	1.4 (1.3 to 1.5)	1.0 (0.9 to 1.1)	18.1 (16.5 to 19.8)	12.6 (11.5 to 13.7)	1.39 (1.27 to 1.52)	0.97 (0.88 to 1.06)
2010	0.8 (0.7 to 0.9)	0.6 (0.5 to 0.6)	10.5 (9.6 to 11.4)	7.3 (6.7 to 7.9)	0.96 (0.89 to 1.05)	0.67 (0.61 to 0.73)
2015	0.9 (0.8 to 1.0)	0.6 (0.6 to 0.7)	11.6 (10.6 to 12.5)	8.0 (7.3 to 8.6)	1.37 (1.26 to 1.48)	0.94 (0.86 to 1.02)
2020	0.6 (0.5 to 0.7)	0.4 (0.4 to 0.5)	8.4 (7.4 to 9.6)	5.7 (5.0 to 6.5)	1.59 (1.39 to 1.80)	1.07 (0.94 to 1.21)
2021	0.5 (0.4 to 0.7)	0.4 (0.3 to 0.5)	7.6 (6.1 to 9.4)	5.1 (4.1 to 6.3)	1.54 (1.24 to 1.91)	1.03 (0.83 to 1.28)

Rio Grande do Sul BRA_4772 Tropical Latin America Stillbirth Rate / Neonatal Mortality Rate (SBR/NMR) − 20 weeks
lambda = 0.2 ; zeta = 0.99 ; scale = 5 ; amp = 0.028

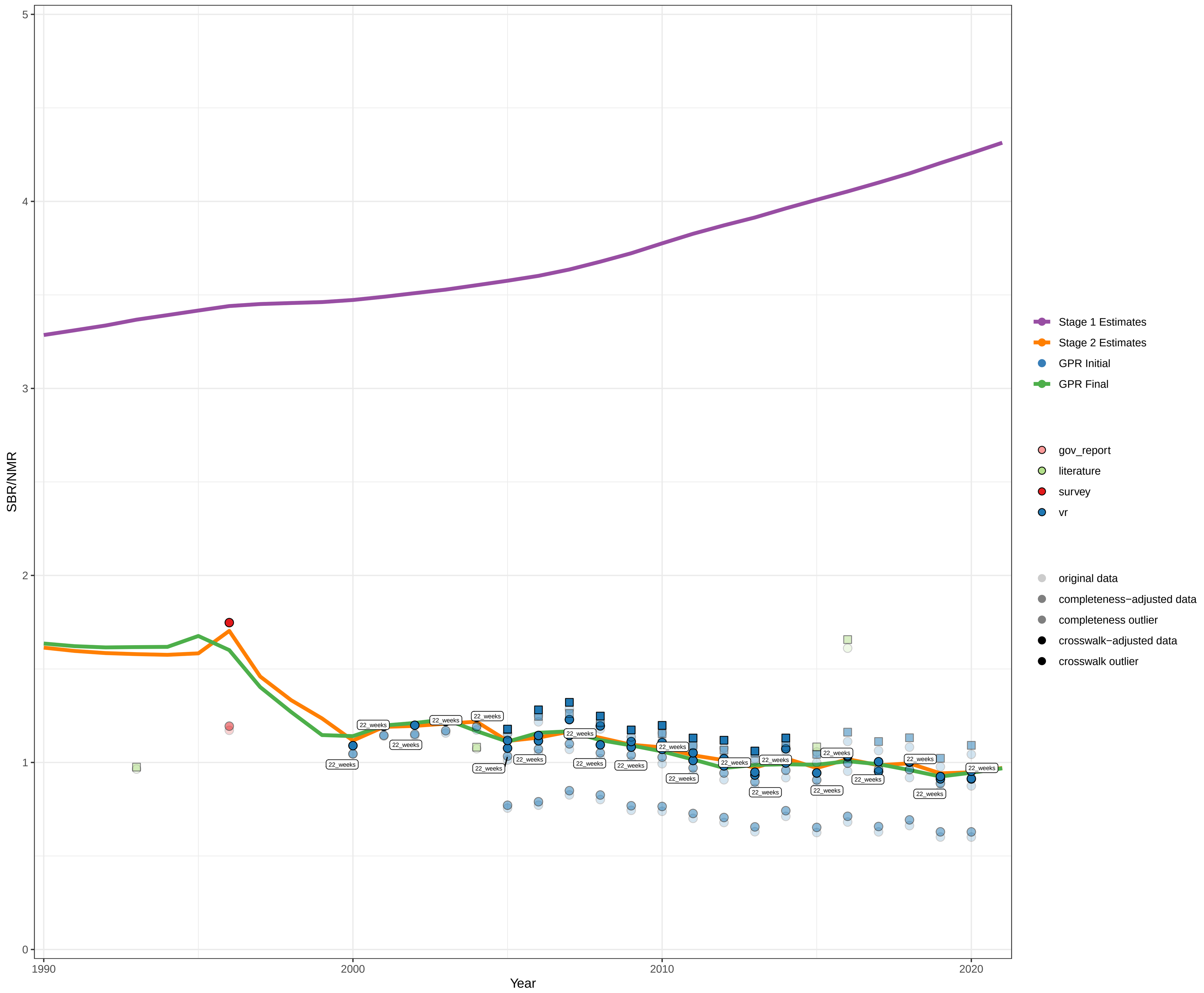

Rio Grande do Sul BRA_4772 Tropical Latin America Stillbirth Rate / Neonatal Mortality Rate (SBR/NMR) − 28 weeks
lambda = 0.2 ; zeta = 0.99 ; scale = 5 ; amp = 0.028

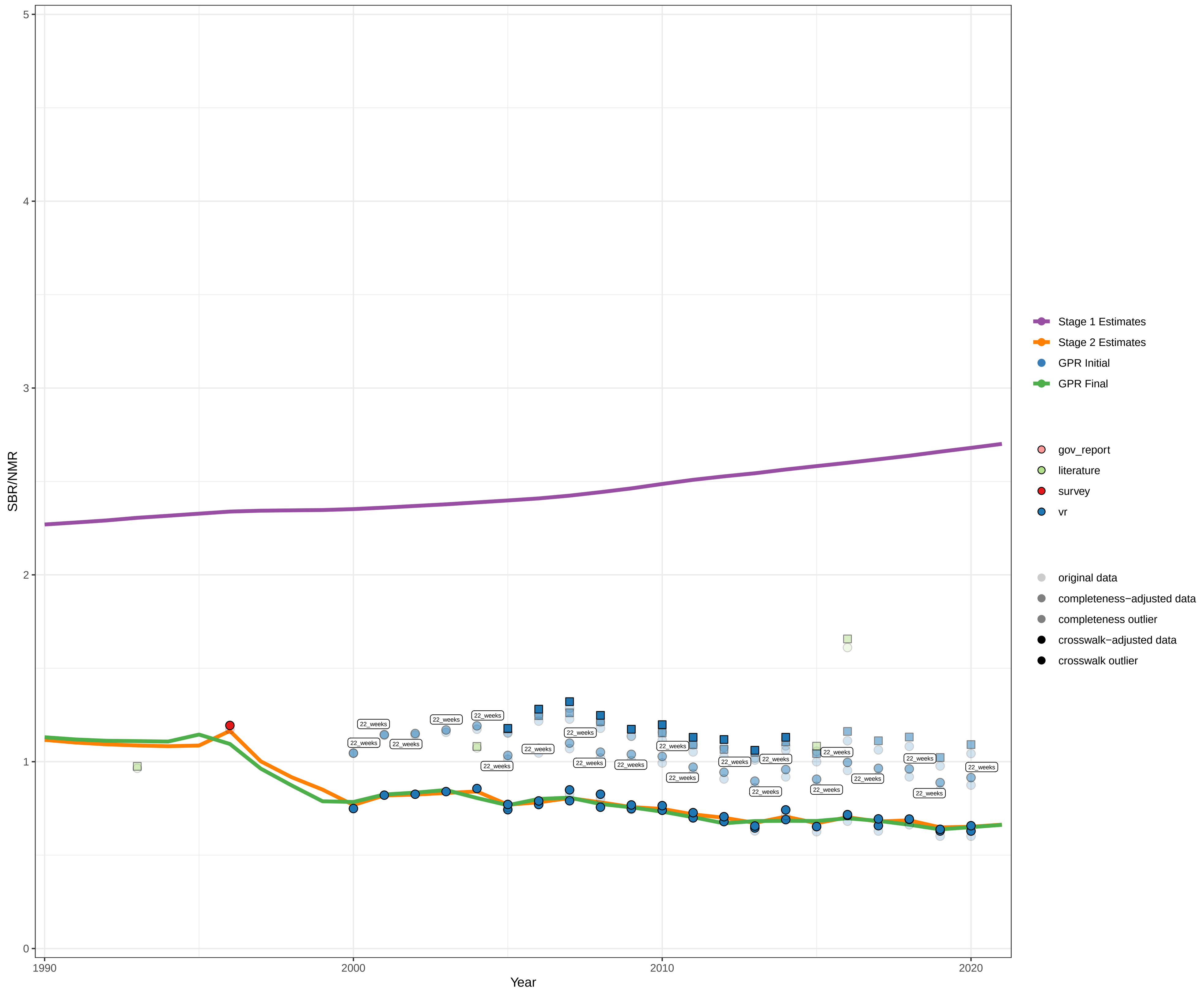

Definition	Years	Outlier	Source.Type	Title
22_weeks	2000−2014	0	VR	Brazil Mortality Information System − Stillbirths
22_weeks, 28_weeks	2005−2020	0	VR	Brazil Civil Registry Statistics − Fetal Deaths at 28 Weeks or More, by Place of Birth, Number of Births by Birth and Sex, and Residence of Mother
28_weeks	1996	0	Survey	Brazil Demographic and Health Survey 1996
28_weeks	2004, 2015	1	Sci Lit	Stillbirth, newborn and infant mortality: trends and inequalities in four population−based birth cohorts in Pelotas, Brazil, 1982−2015
28_weeks_OR_1000_grams	1993	1	Sci Lit	Stillbirth, newborn and infant mortality: trends and inequalities in four population−based birth cohorts in Pelotas, Brazil, 1982−2015
500_grams	2016	1	Sci Lit	Fetal, neonatal, and post−neonatal mortality in the 2015 Pelotas (Brazil) birth cohort and associated factors
other	2005−2020	1	VR	Brazil Civil Registry Statistics − Fetal Deaths at 28 Weeks or More, by Place of Birth, Number of Births by Birth and Sex, and Residence of Mother

Rio Grande do Sul BRA_4772 Tropical Latin America Stillbirth Rate (SBR) − 20 weeks
lambda = 0.2 ; zeta = 0.99 ; scale = 5 ; amp = 0.028

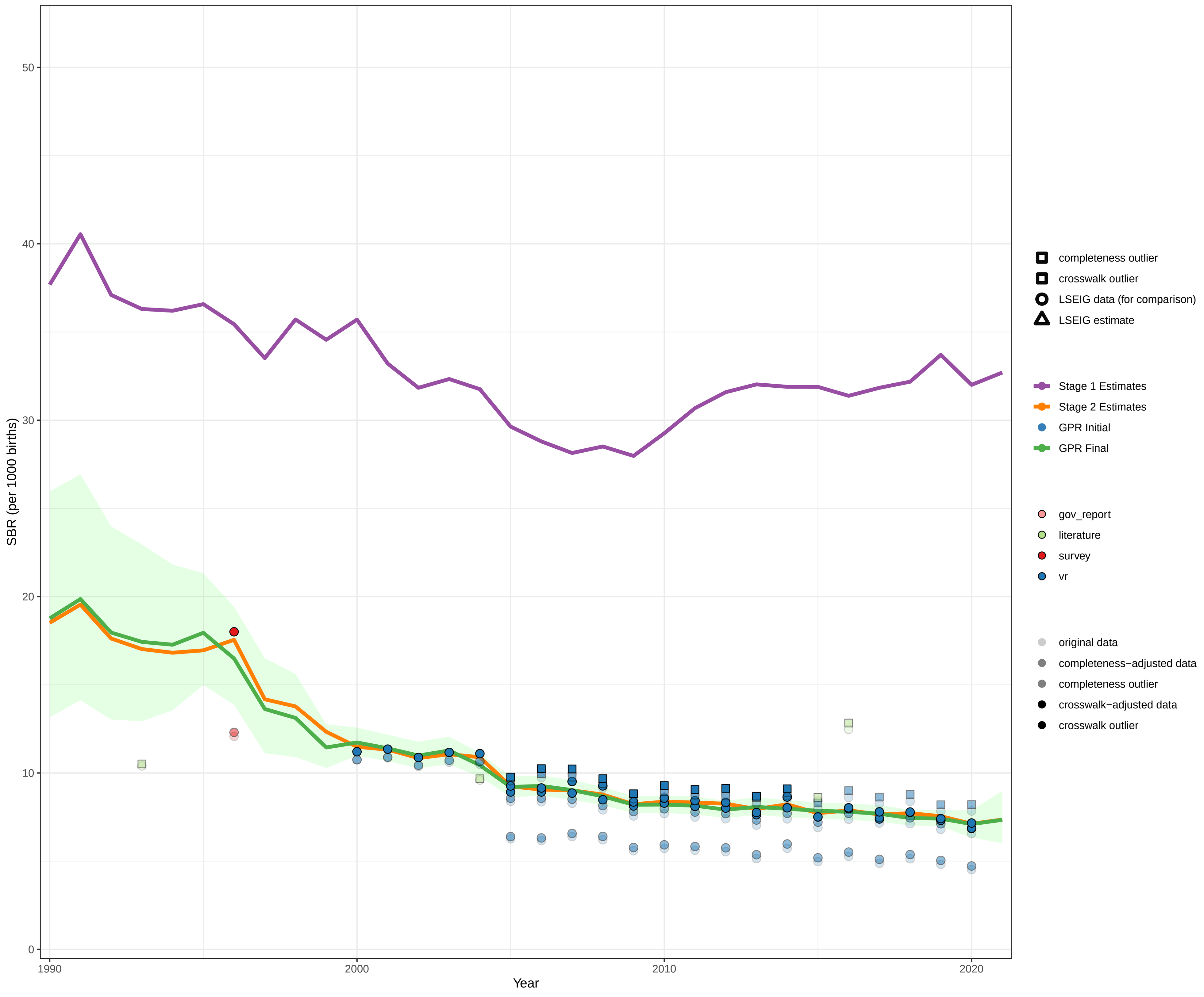

Rio Grande do Sul BRA_4772 Tropical Latin America Stillbirth Rate (SBR) − 28 weeks
lambda = 0.2 ; zeta = 0.99 ; scale = 5 ; amp = 0.028

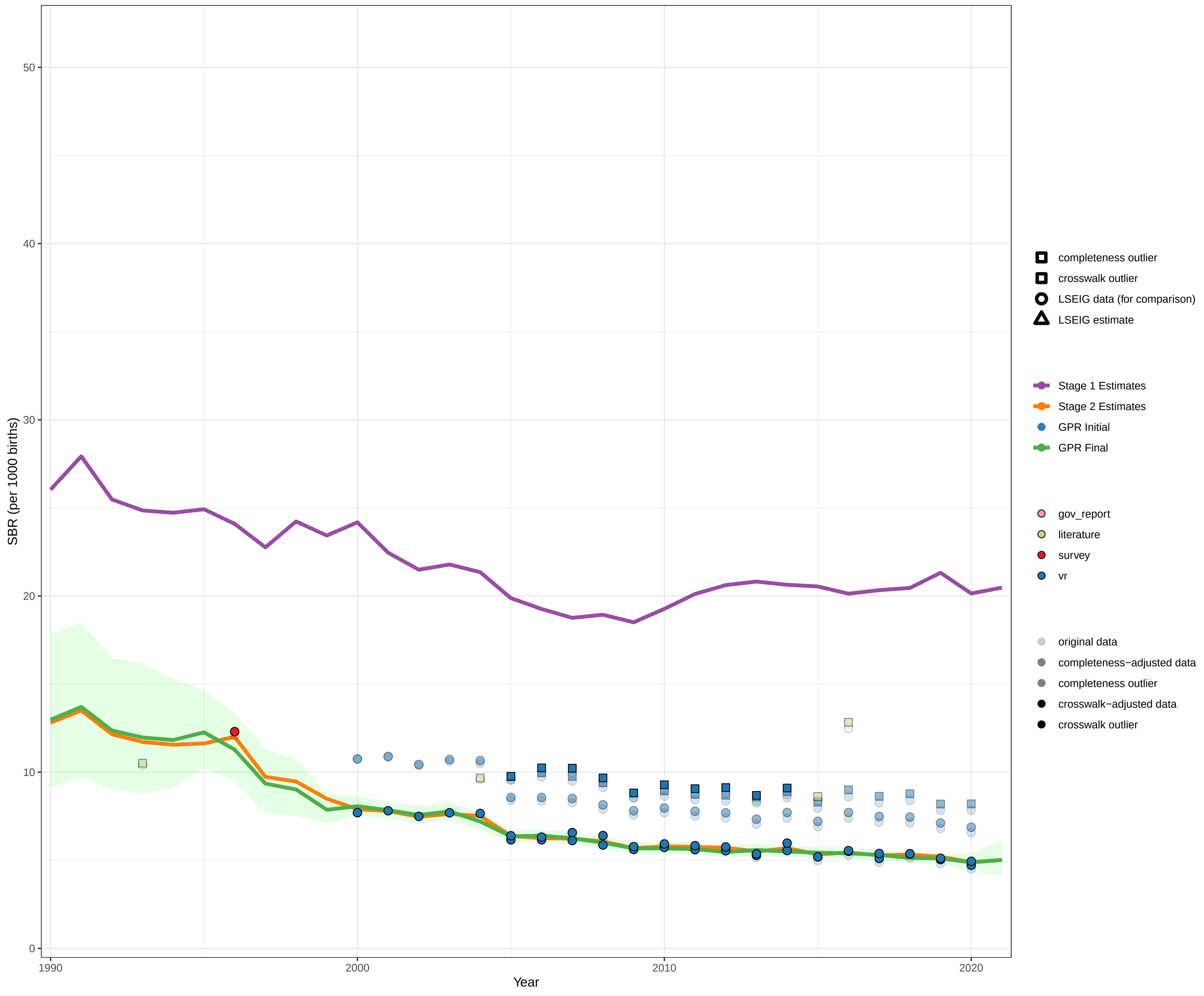

Year	Total Stillbirths (in thousands)	Total Stillbirths (in thousands)	SBR (per 1000 births)	SBR (per 1000 births)	SBR/NMR	SBR/NMR
	20 weeks	28 weeks	20 weeks	28 weeks	20 weeks	28 weeks
1990	3.0 (2.1 to 4.2)	2.1 (1.4 to 2.8)	18.8 (13.2 to 26.0)	13.0 (9.1 to 17.8)	1.64 (1.15 to 2.26)	1.13 (0.80 to 1.55)
1995	2.9 (2.4 to 3.5)	2.0 (1.7 to 2.4)	17.9 (15.0 to 21.3)	12.3 (10.2 to 14.7)	1.68 (1.40 to 1.99)	1.15 (0.95 to 1.37)
2000	1.8 (1.7 to 2.0)	1.3 (1.2 to 1.3)	11.7 (11.0 to 12.6)	8.1 (7.6 to 8.6)	1.14 (1.07 to 1.22)	0.78 (0.74 to 0.84)
2005	1.2 (1.2 to 1.3)	0.8 (0.8 to 0.9)	9.2 (8.6 to 9.8)	6.4 (6.0 to 6.8)	1.11 (1.04 to 1.18)	0.77 (0.72 to 0.81)
2010	1.0 (1.0 to 1.1)	0.7 (0.7 to 0.8)	8.2 (7.7 to 8.7)	5.7 (5.3 to 6.0)	1.06 (1.00 to 1.13)	0.73 (0.69 to 0.78)
2015	1.0 (1.0 to 1.1)	0.7 (0.7 to 0.8)	7.9 (7.4 to 8.3)	5.4 (5.1 to 5.8)	0.99 (0.93 to 1.05)	0.68 (0.64 to 0.72)
2020	0.9 (0.8 to 1.0)	0.6 (0.5 to 0.7)	7.1 (6.3 to 7.9)	4.9 (4.4 to 5.4)	0.95 (0.84 to 1.05)	0.65 (0.58 to 0.72)
2021	0.9 (0.7 to 1.1)	0.6 (0.5 to 0.7)	7.3 (6.0 to 9.0)	5.0 (4.1 to 6.1)	0.97 (0.79 to 1.19)	0.66 (0.54 to 0.81)

Rondonia BRA_4770 Tropical Latin America Stillbirth Rate / Neonatal Mortality Rate (SBR/NMR) − 20 weeks
lambda = 0.2 ; zeta = 0.99 ; scale = 5 ; amp = 0.028

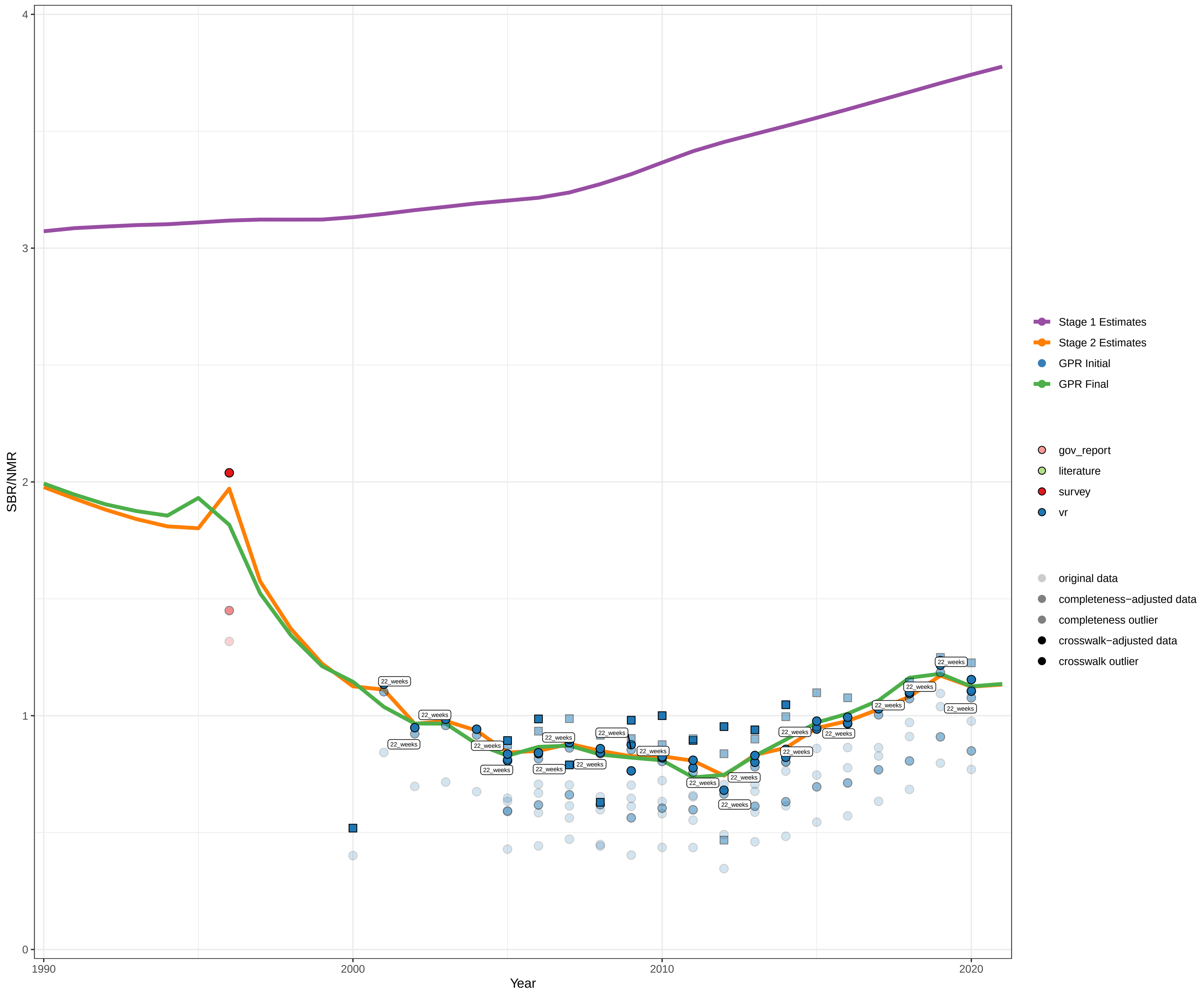

Rondonia BRA_4770 Tropical Latin America Stillbirth Rate / Neonatal Mortality Rate (SBR/NMR) − 28 weeks
lambda = 0.2 ; zeta = 0.99 ; scale = 5 ; amp = 0.028

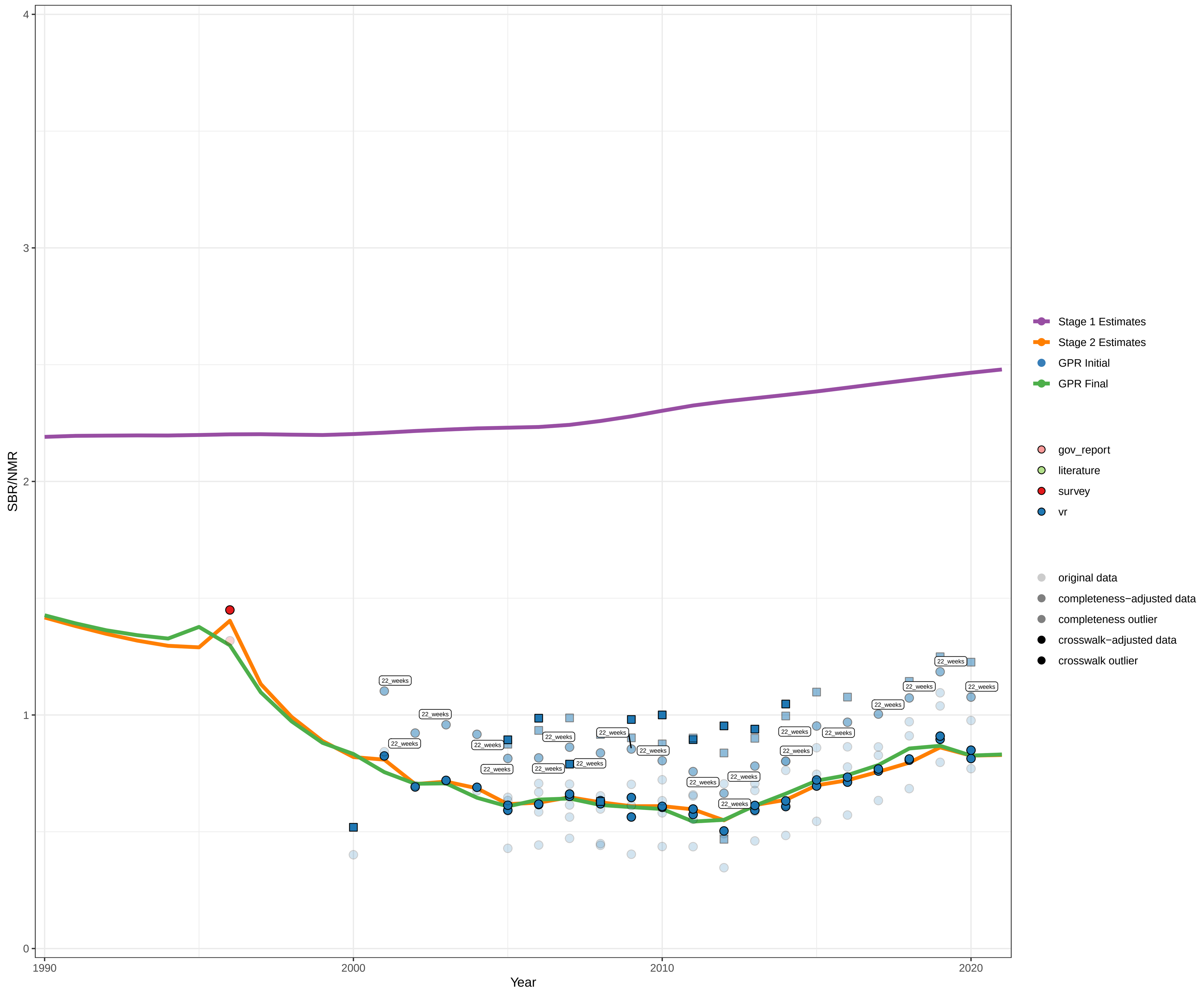

Definition	Years	Outlier	Source.Type	Title
22_weeks	2000−2014	0	VR	Brazil Mortality Information System − Stillbirths
22_weeks	2005−2020	0	VR	Brazil Civil Registry Statistics − Fetal Deaths at 28 Weeks or More, by Place of Birth, Number of Births by Birth and Sex, and Residence of Mother
28_weeks	1996	0	Survey	Brazil Demographic and Health Survey 1996
28_weeks	2005−2011, 2013−2020	0	VR	Brazil Civil Registry Statistics − Fetal Deaths at 28 Weeks or More, by Place of Birth, Number of Births by Birth and Sex, and Residence of Mother
28_weeks	2012	1	VR	Brazil Civil Registry Statistics − Fetal Deaths at 28 Weeks or More, by Place of Birth, Number of Births by Birth and Sex, and Residence of Mother
other	2005−2020	1	VR	Brazil Civil Registry Statistics − Fetal Deaths at 28 Weeks or More, by Place of Birth, Number of Births by Birth and Sex, and Residence of Mother

Rondonia BRA_4770 Tropical Latin America Stillbirth Rate (SBR) − 20 weeks
lambda = 0.2 ; zeta = 0.99 ; scale = 5 ; amp = 0.028

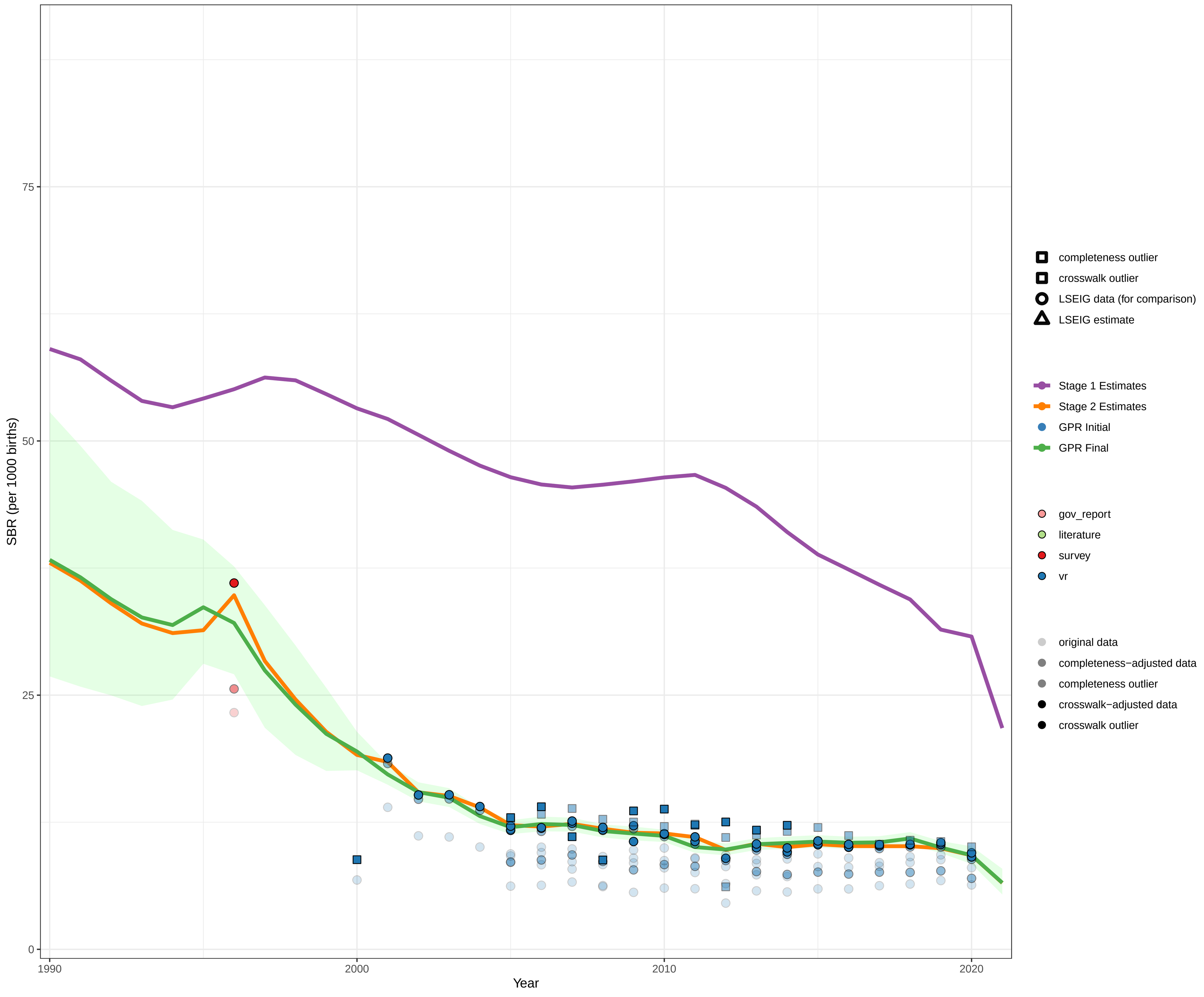

Rondonia BRA_4770 Tropical Latin America Stillbirth Rate (SBR) − 28 weeks
lambda = 0.2 ; zeta = 0.99 ; scale = 5 ; amp = 0.028

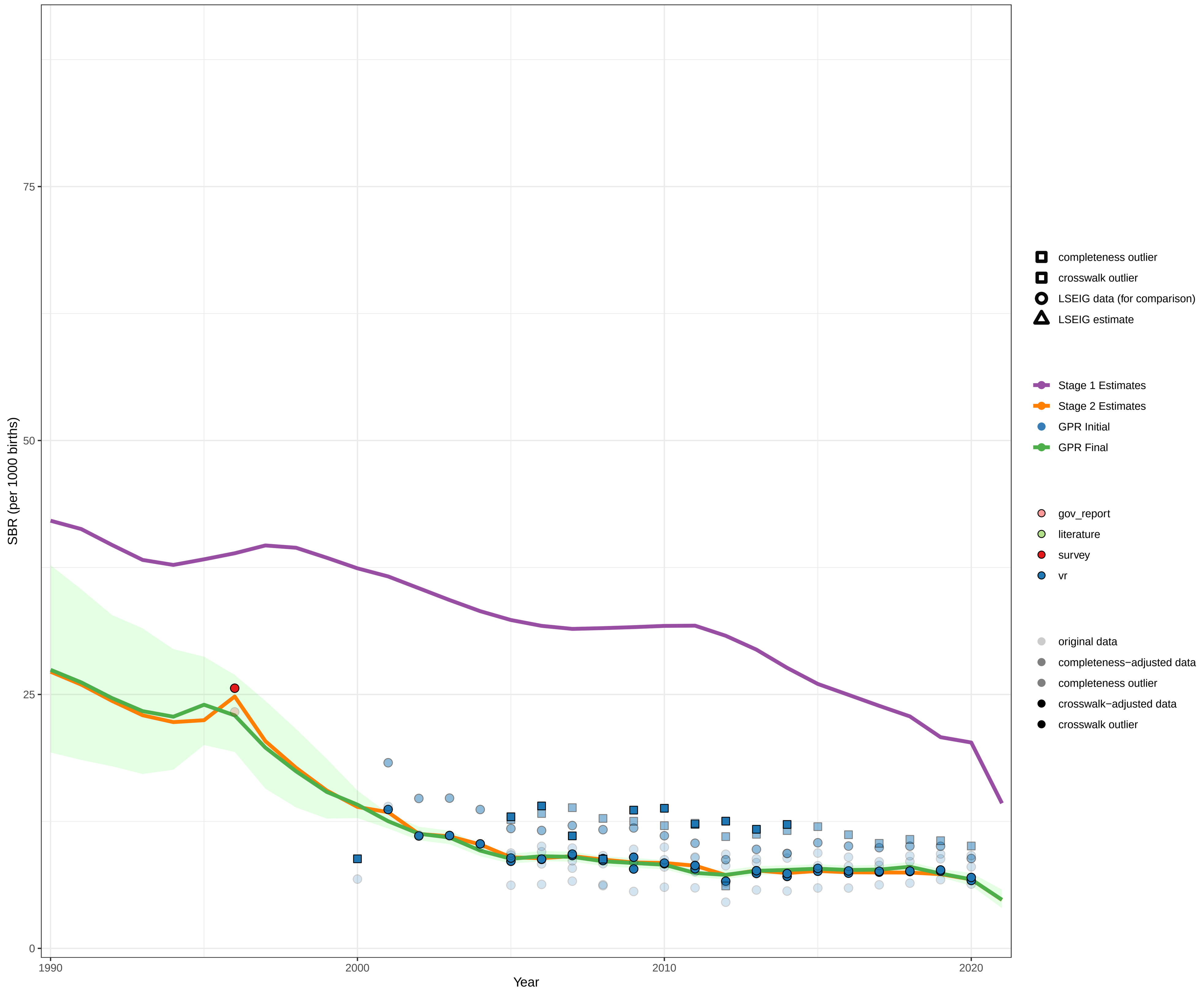

Year	Total Stillbirths (in thousands)	Total Stillbirths (in thousands)	SBR (per 1000 births)	SBR (per 1000 births)	SBR/NMR	SBR/NMR
	20 weeks	28 weeks	20 weeks	28 weeks	20 weeks	28 weeks
1990	1.1 (0.7 to 1.5)	0.7 (0.5 to 1.0)	38.3 (26.8 to 52.8)	27.4 (19.3 to 37.7)	1.99 (1.40 to 2.75)	1.43 (1.00 to 1.96)
1995	1.1 (0.9 to 1.3)	0.7 (0.6 to 0.9)	33.6 (28.1 to 40.3)	24.0 (20.0 to 28.7)	1.93 (1.61 to 2.31)	1.38 (1.15 to 1.65)
2000	0.6 (0.6 to 0.7)	0.5 (0.4 to 0.5)	19.5 (17.6 to 21.4)	14.1 (12.8 to 15.5)	1.15 (1.04 to 1.26)	0.83 (0.76 to 0.91)
2005	0.4 (0.4 to 0.4)	0.3 (0.3 to 0.3)	12.0 (11.3 to 12.7)	8.8 (8.3 to 9.3)	0.83 (0.78 to 0.88)	0.61 (0.58 to 0.64)
2010	0.4 (0.3 to 0.4)	0.3 (0.2 to 0.3)	11.2 (10.6 to 11.8)	8.2 (7.8 to 8.7)	0.81 (0.77 to 0.85)	0.60 (0.57 to 0.63)
2015	0.3 (0.3 to 0.4)	0.3 (0.2 to 0.3)	10.6 (10.0 to 11.2)	7.8 (7.4 to 8.3)	0.97 (0.91 to 1.03)	0.72 (0.68 to 0.76)
2020	0.3 (0.3 to 0.3)	0.2 (0.2 to 0.2)	9.3 (8.4 to 10.1)	6.8 (6.2 to 7.4)	1.13 (1.02 to 1.23)	0.83 (0.75 to 0.90)
2021	0.2 (0.2 to 0.2)	0.1 (0.1 to 0.2)	6.5 (5.4 to 7.9)	4.8 (4.0 to 5.8)	1.14 (0.94 to 1.37)	0.83 (0.69 to 1.00)

Roraima BRA_4771 Tropical Latin America Stillbirth Rate / Neonatal Mortality Rate (SBR/NMR) − 20 weeks
lambda = 0.2 ; zeta = 0.99 ; scale = 5 ; amp = 0.028

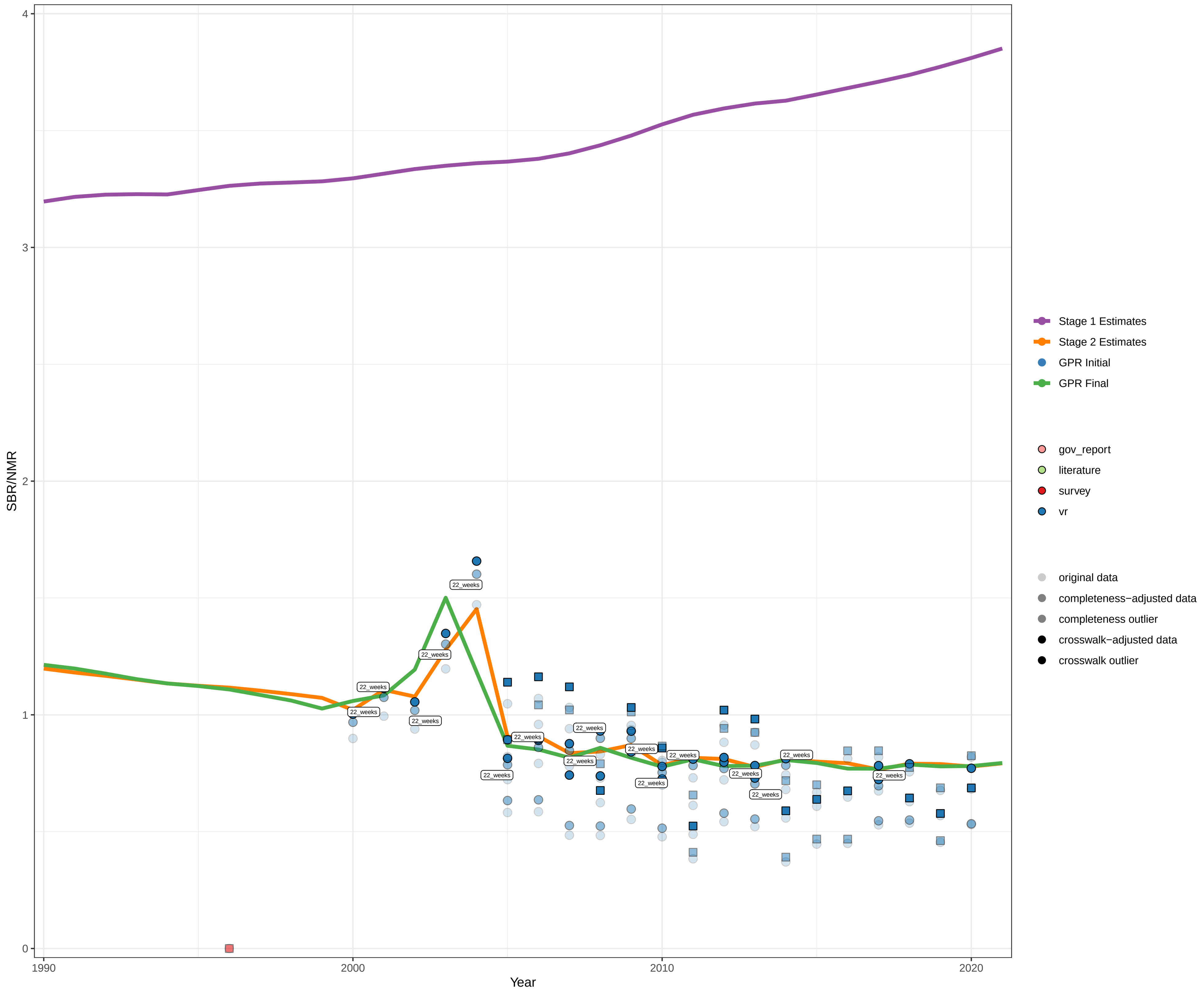

Roraima BRA_4771 Tropical Latin America Stillbirth Rate / Neonatal Mortality Rate (SBR/NMR) − 28 weeks
lambda = 0.2 ; zeta = 0.99 ; scale = 5 ; amp = 0.028

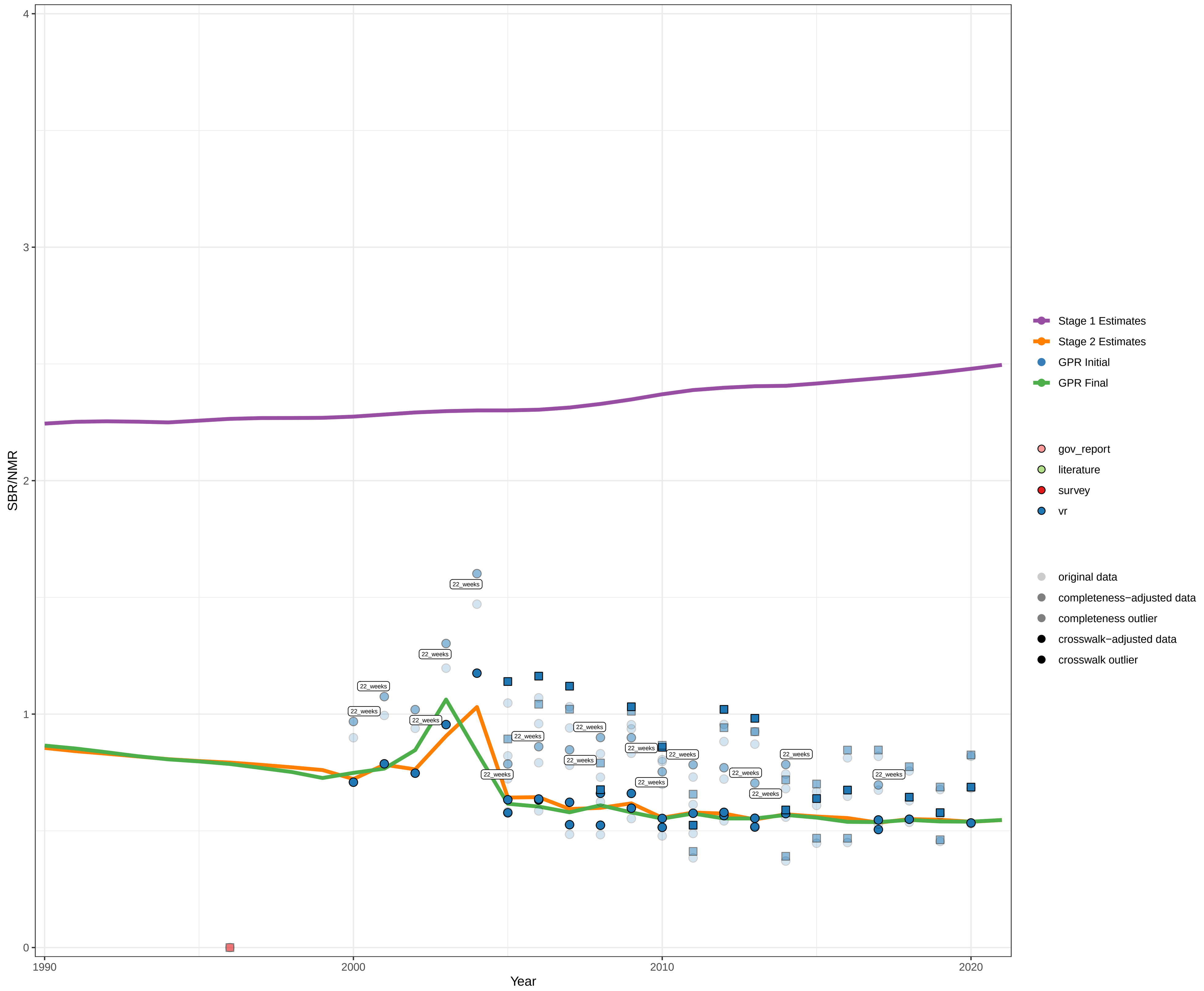

Definition	Years	Outlier	Source.Type	Title
22_weeks	2000−2014	0	VR	Brazil Mortality Information System − Stillbirths
22_weeks	2005−2020	0	VR	Brazil Civil Registry Statistics − Fetal Deaths at 28 Weeks or More, by Place of Birth, Number of Births by Birth and Sex, and Residence of Mother
28_weeks	2005−2010, 2012, 2013, 2017, 2018, 2020	0	VR	Brazil Civil Registry Statistics − Fetal Deaths at 28 Weeks or More, by Place of Birth, Number of Births by Birth and Sex, and Residence of Mother
28_weeks	1996	1	Survey	Brazil Demographic and Health Survey 1996
28_weeks	2011, 2014−2016, 2019	1	VR	Brazil Civil Registry Statistics − Fetal Deaths at 28 Weeks or More, by Place of Birth, Number of Births by Birth and Sex, and Residence of Mother
other	2005−2020	1	VR	Brazil Civil Registry Statistics − Fetal Deaths at 28 Weeks or More, by Place of Birth, Number of Births by Birth and Sex, and Residence of Mother

Roraima BRA_4771 Tropical Latin America Stillbirth Rate (SBR) − 20 weeks
lambda = 0.2 ; zeta = 0.99 ; scale = 5 ; amp = 0.028

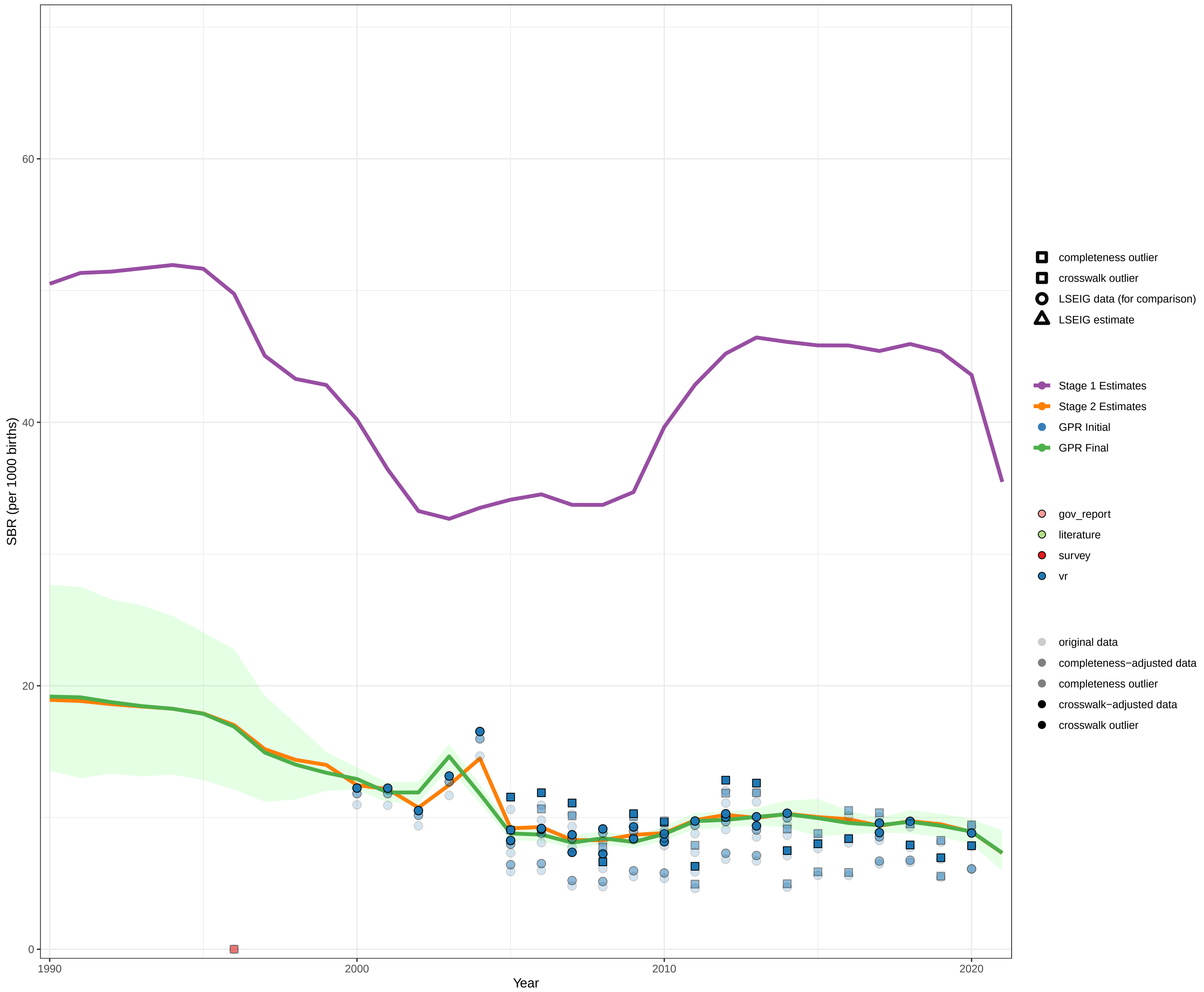

Roraima BRA_4771 Tropical Latin America Stillbirth Rate (SBR) − 28 weeks
lambda = 0.2 ; zeta = 0.99 ; scale = 5 ; amp = 0.028

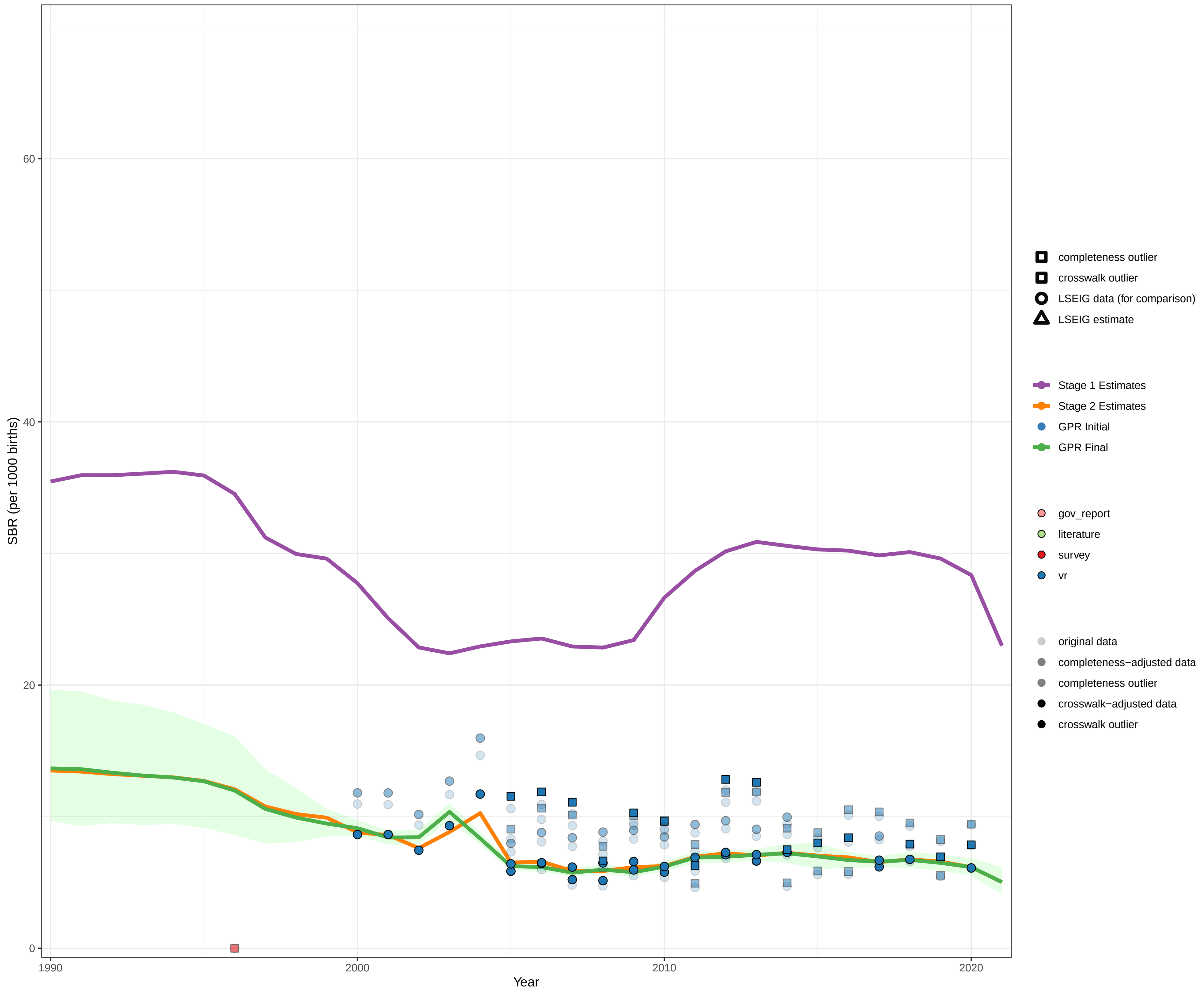

Year	Total Stillbirths (in thousands)	Total Stillbirths (in thousands)	SBR (per 1000 births)	SBR (per 1000 births)	SBR/NMR	SBR/NMR
	20 weeks	28 weeks	20 weeks	28 weeks	20 weeks	28 weeks
1990	0.1 (0.1 to 0.1)	0.1 (0.1 to 0.1)	19.2 (13.5 to 27.7)	13.7 (9.7 to 19.6)	1.21 (0.86 to 1.75)	0.87 (0.61 to 1.24)
1995	0.1 (0.1 to 0.2)	0.1 (0.1 to 0.1)	17.9 (12.8 to 24.1)	12.7 (9.1 to 17.0)	1.12 (0.81 to 1.51)	0.80 (0.57 to 1.07)
2000	0.1 (0.1 to 0.1)	0.1 (0.1 to 0.1)	12.9 (12.1 to 13.8)	9.1 (8.5 to 9.8)	1.06 (0.99 to 1.13)	0.75 (0.70 to 0.80)
2005	0.1 (0.1 to 0.1)	0.1 (0.1 to 0.1)	8.8 (8.3 to 9.3)	6.2 (5.9 to 6.6)	0.87 (0.82 to 0.92)	0.62 (0.58 to 0.65)
2010	0.1 (0.1 to 0.1)	0.1 (0.1 to 0.1)	8.7 (8.2 to 9.3)	6.2 (5.9 to 6.6)	0.78 (0.73 to 0.83)	0.55 (0.52 to 0.59)
2015	0.1 (0.1 to 0.1)	0.1 (0.1 to 0.1)	10.0 (8.6 to 11.4)	7.0 (6.0 to 8.0)	0.79 (0.68 to 0.91)	0.56 (0.48 to 0.64)
2020	0.1 (0.1 to 0.1)	0.1 (0.1 to 0.1)	8.9 (8.0 to 9.9)	6.2 (5.5 to 6.8)	0.78 (0.70 to 0.87)	0.54 (0.48 to 0.60)
2021	0.1 (0.1 to 0.1)	0.1 (0.1 to 0.1)	7.3 (6.0 to 9.0)	5.0 (4.1 to 6.2)	0.79 (0.65 to 0.98)	0.55 (0.45 to 0.67)

Santa Catarina BRA_4773 Tropical Latin America Stillbirth Rate / Neonatal Mortality Rate (SBR/NMR) − 20 weeks
lambda = 0.2 ; zeta = 0.99 ; scale = 5 ; amp = 0.028

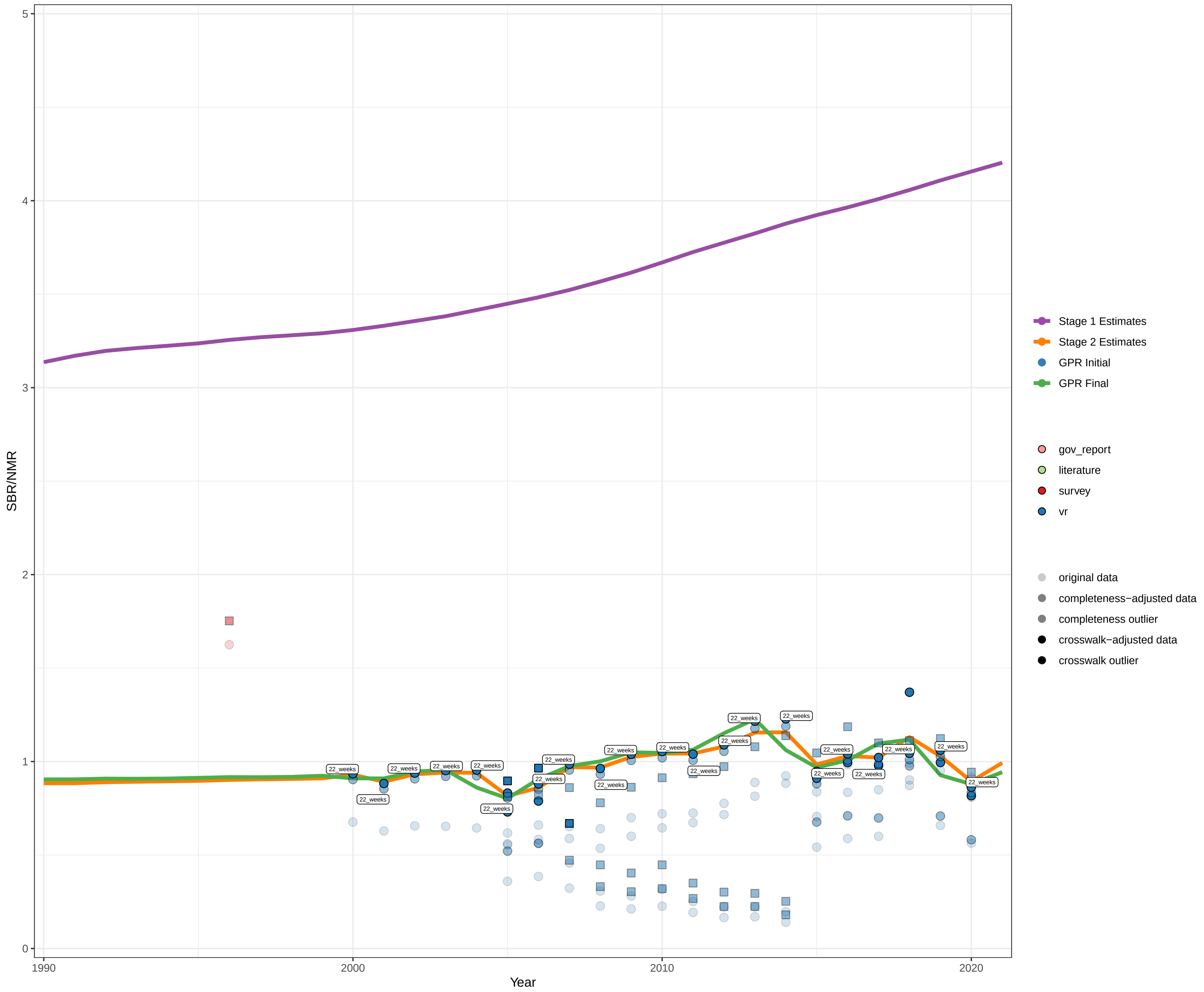

Santa Catarina BRA_4773 Tropical Latin America Stillbirth Rate / Neonatal Mortality Rate (SBR/NMR) − 28 weeks
lambda = 0.2 ; zeta = 0.99 ; scale = 5 ; amp = 0.028

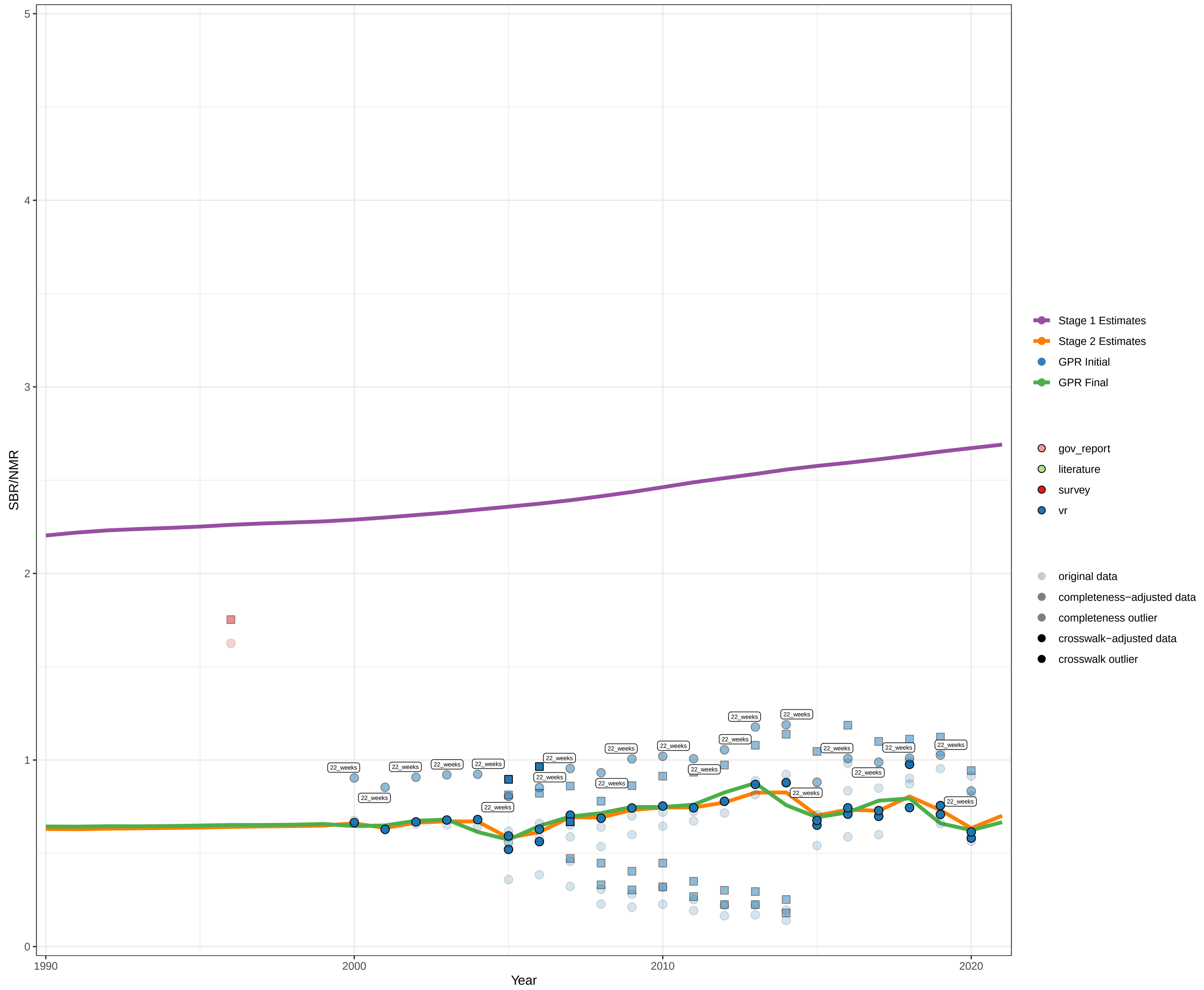

Definition	Years	Outlier	Source.Type	Title
22_weeks	2000−2014	0	VR	Brazil Mortality Information System − Stillbirths
22_weeks	2005−2007, 2015−2020	0	VR	Brazil Civil Registry Statistics − Fetal Deaths at 28 Weeks or More, by Place of Birth, Number of Births by Birth and Sex, and Residence of Mother
28_weeks	2005, 2006, 2015−2020	0	VR	Brazil Civil Registry Statistics − Fetal Deaths at 28 Weeks or More, by Place of Birth, Number of Births by Birth and Sex, and Residence of Mother
22_weeks	2008−2014	1	VR	Brazil Civil Registry Statistics − Fetal Deaths at 28 Weeks or More, by Place of Birth, Number of Births by Birth and Sex, and Residence of Mother
28_weeks	1996	1	Survey	Brazil Demographic and Health Survey 1996
28_weeks	2007−2014	1	VR	Brazil Civil Registry Statistics − Fetal Deaths at 28 Weeks or More, by Place of Birth, Number of Births by Birth and Sex, and Residence of Mother
other	2005−2020	1	VR	Brazil Civil Registry Statistics − Fetal Deaths at 28 Weeks or More, by Place of Birth, Number of Births by Birth and Sex, and Residence of Mother

Santa Catarina BRA_4773 Tropical Latin America Stillbirth Rate (SBR) − 20 weeks
lambda = 0.2 ; zeta = 0.99 ; scale = 5 ; amp = 0.028

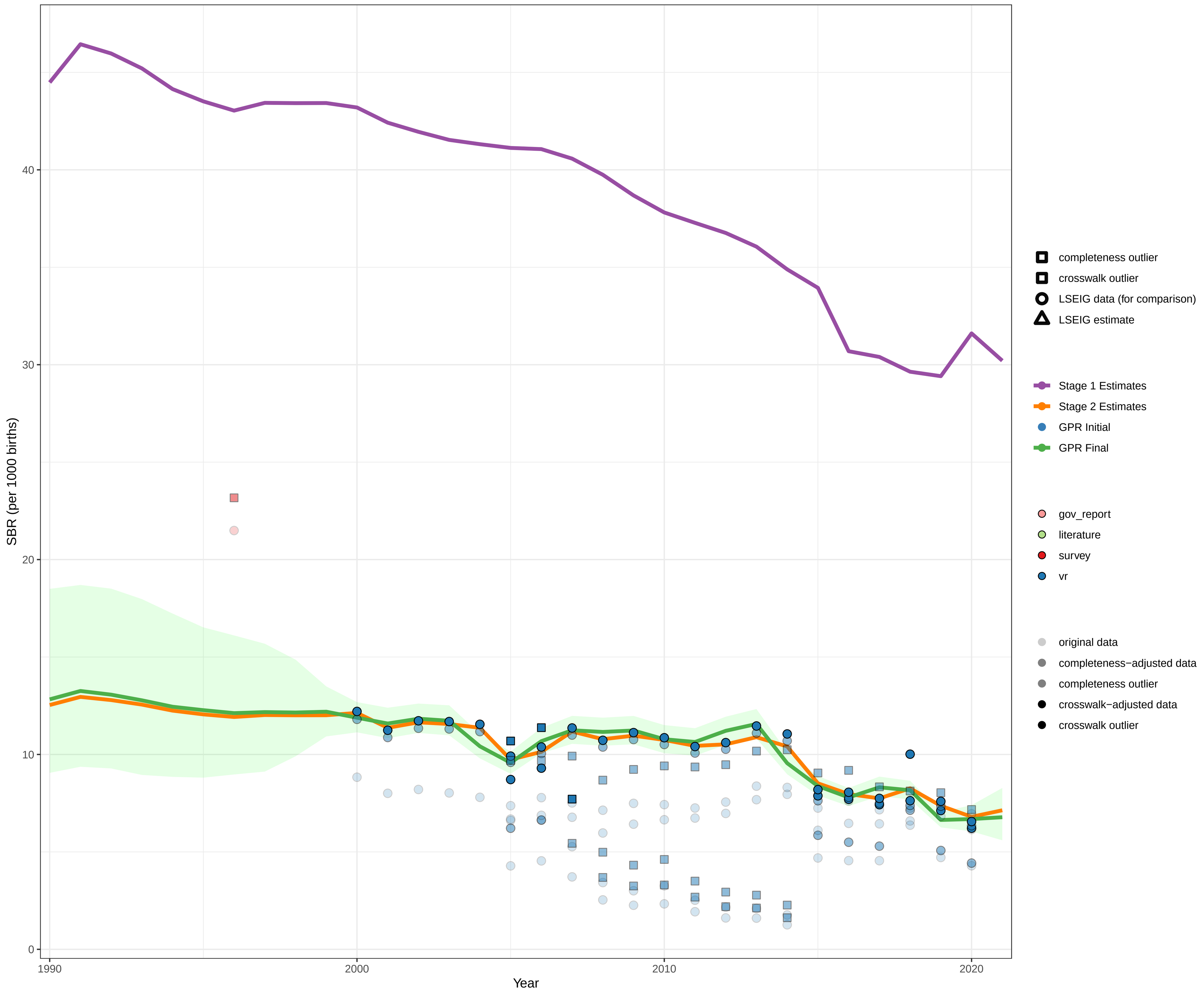

Santa Catarina BRA_4773 Tropical Latin America Stillbirth Rate (SBR) − 28 weeks
lambda = 0.2 ; zeta = 0.99 ; scale = 5 ; amp = 0.028

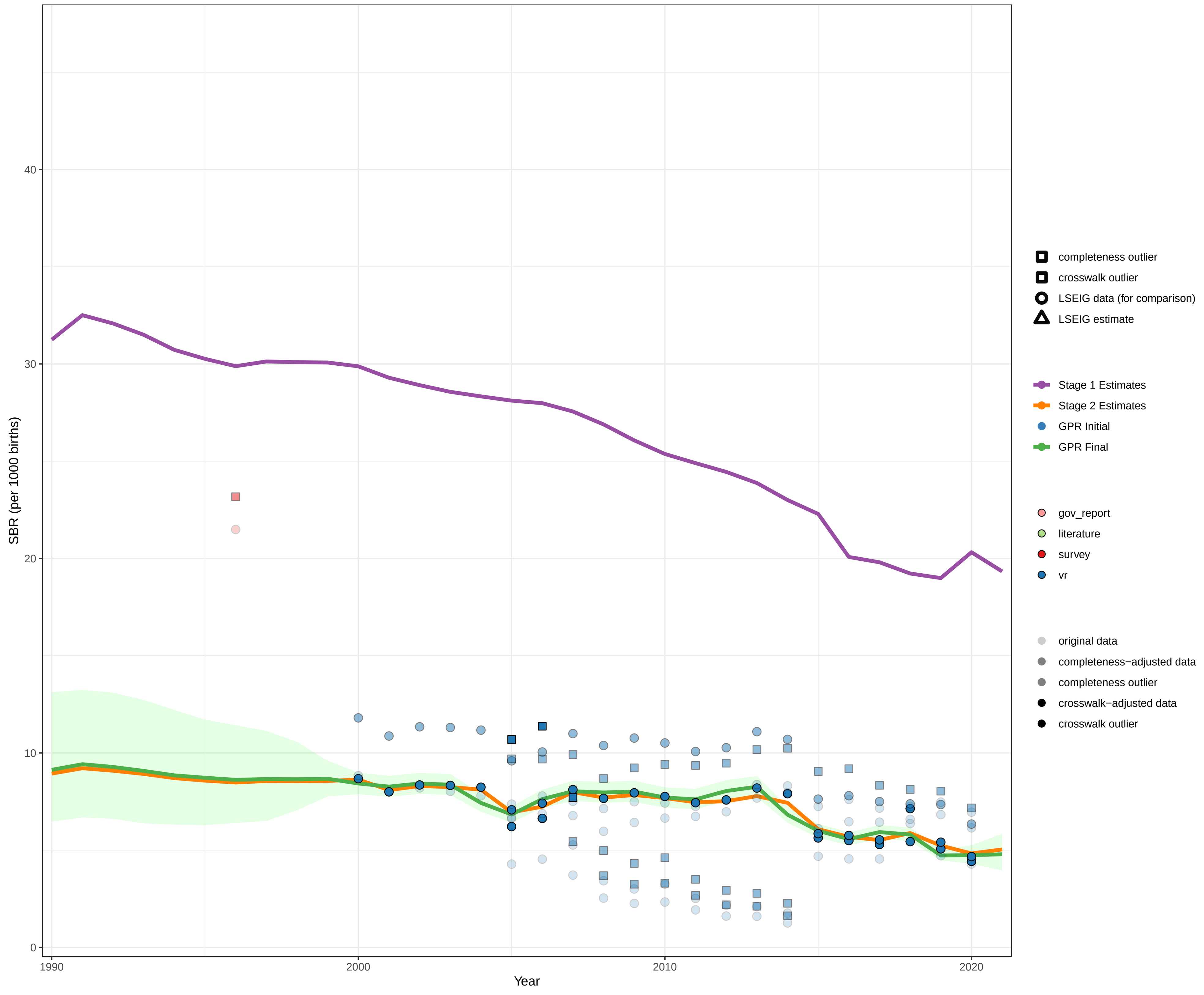

Year	Total Stillbirths (in thousands)	Total Stillbirths (in thousands)	SBR (per 1000 births)	SBR (per 1000 births)	SBR/NMR	SBR/NMR
	20 weeks	28 weeks	20 weeks	28 weeks	20 weeks	28 weeks
1990	1.1 (0.7 to 1.5)	0.7 (0.5 to 1.1)	12.8 (9.1 to 18.5)	9.1 (6.5 to 13.1)	0.90 (0.64 to 1.30)	0.64 (0.46 to 0.93)
1995	1.1 (0.8 to 1.4)	0.8 (0.5 to 1.0)	12.3 (8.8 to 16.5)	8.7 (6.3 to 11.7)	0.91 (0.66 to 1.23)	0.65 (0.47 to 0.87)
2000	1.0 (1.0 to 1.1)	0.7 (0.7 to 0.8)	11.9 (11.1 to 12.7)	8.4 (7.9 to 9.0)	0.91 (0.85 to 0.97)	0.65 (0.60 to 0.69)
2005	0.8 (0.7 to 0.8)	0.5 (0.5 to 0.6)	9.6 (9.0 to 10.2)	6.8 (6.4 to 7.3)	0.80 (0.76 to 0.85)	0.57 (0.54 to 0.61)
2010	0.9 (0.8 to 0.9)	0.6 (0.6 to 0.7)	10.8 (10.1 to 11.5)	7.7 (7.2 to 8.2)	1.05 (0.98 to 1.12)	0.75 (0.70 to 0.80)
2015	0.8 (0.7 to 0.8)	0.5 (0.5 to 0.6)	8.4 (7.9 to 8.9)	6.0 (5.6 to 6.4)	0.97 (0.91 to 1.03)	0.69 (0.65 to 0.73)
2020	0.6 (0.6 to 0.7)	0.5 (0.4 to 0.5)	6.7 (6.1 to 7.4)	4.7 (4.3 to 5.3)	0.88 (0.80 to 0.97)	0.62 (0.57 to 0.69)
2021	0.6 (0.5 to 0.8)	0.5 (0.4 to 0.6)	6.8 (5.6 to 8.3)	4.8 (4.0 to 5.8)	0.94 (0.78 to 1.15)	0.67 (0.55 to 0.81)

Sao Paulo BRA_4775 Tropical Latin America Stillbirth Rate / Neonatal Mortality Rate (SBR/NMR) − 20 weeks
lambda = 0.2 ; zeta = 0.99 ; scale = 5 ; amp = 0.028

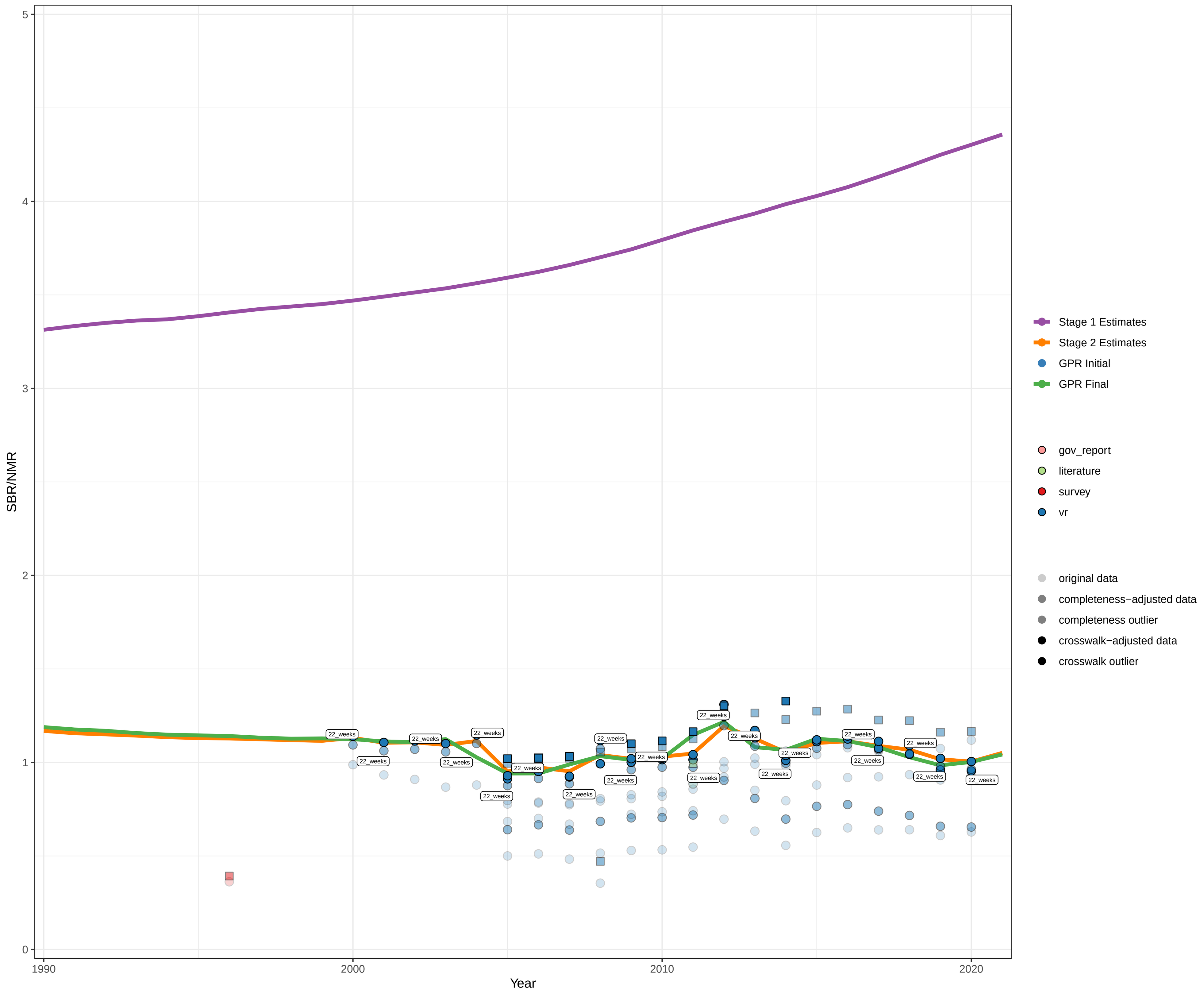

Sao Paulo BRA_4775 Tropical Latin America Stillbirth Rate / Neonatal Mortality Rate (SBR/NMR) − 28 weeks
lambda = 0.2 ; zeta = 0.99 ; scale = 5 ; amp = 0.028

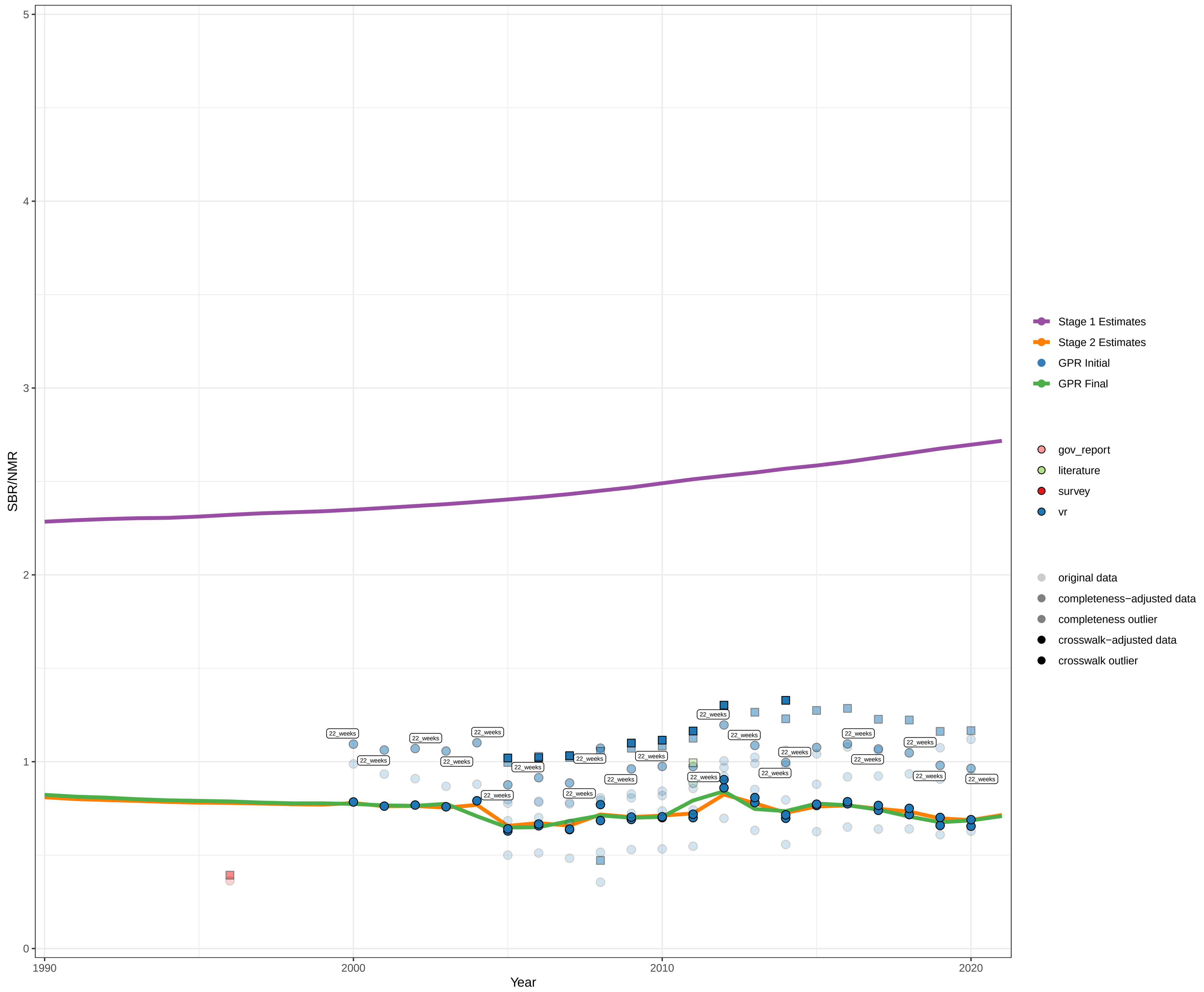

Definition	Years	Outlier	Source.Type	Title
22_weeks	2000−2014	0	VR	Brazil Mortality Information System − Stillbirths
22_weeks	2005−2007, 2009−2020	0	VR	Brazil Civil Registry Statistics − Fetal Deaths at 28 Weeks or More, by Place of Birth, Number of Births by Birth and Sex, and Residence of Mother
28_weeks	2005−2020	0	VR	Brazil Civil Registry Statistics − Fetal Deaths at 28 Weeks or More, by Place of Birth, Number of Births by Birth and Sex, and Residence of Mother
22_weeks	2008	1	VR	Brazil Civil Registry Statistics − Fetal Deaths at 28 Weeks or More, by Place of Birth, Number of Births by Birth and Sex, and Residence of Mother
28_weeks	1996	1	Survey	Brazil Demographic and Health Survey 1996
other	2005−2020	1	VR	Brazil Civil Registry Statistics − Fetal Deaths at 28 Weeks or More, by Place of Birth, Number of Births by Birth and Sex, and Residence of Mother
other	2011	1	Sci Lit	The study of the mother−child binomium: description and general results

Sao Paulo BRA_4775 Tropical Latin America Stillbirth Rate (SBR) − 20 weeks
lambda = 0.2 ; zeta = 0.99 ; scale = 5 ; amp = 0.028

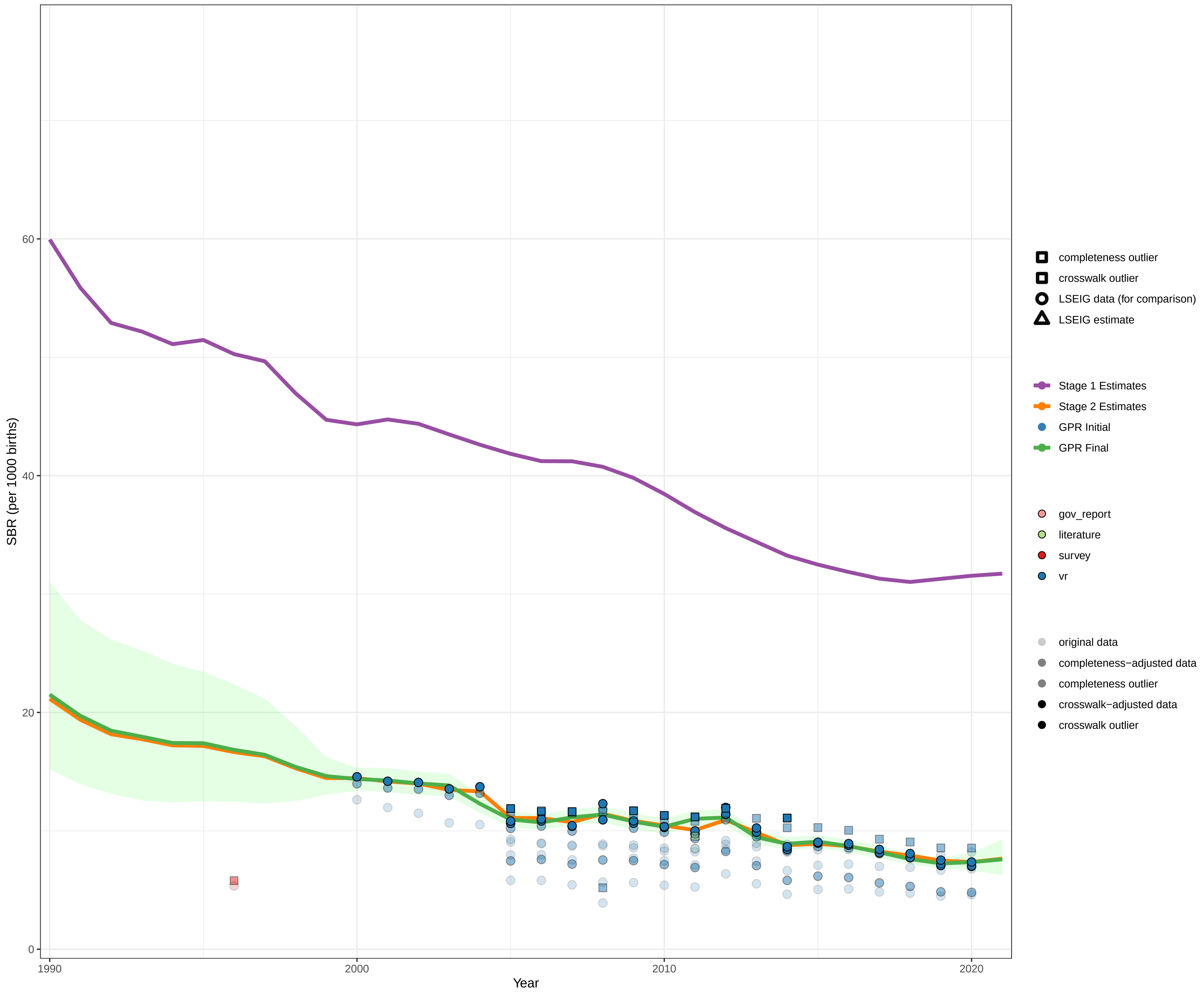

Sao Paulo BRA_4775 Tropical Latin America Stillbirth Rate (SBR) − 28 weeks
lambda = 0.2 ; zeta = 0.99 ; scale = 5 ; amp = 0.028

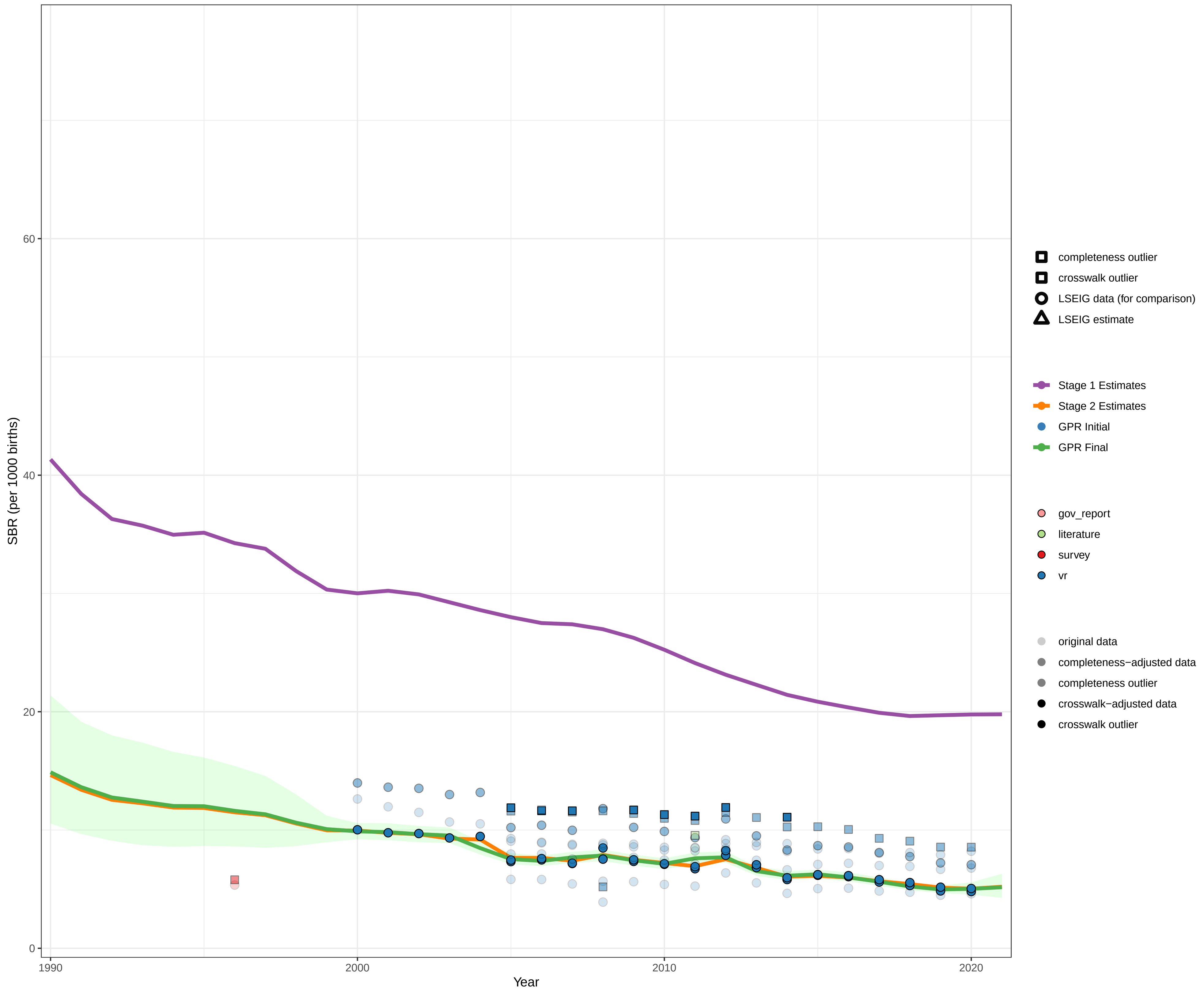

Year	Total Stillbirths (in thousands)	Total Stillbirths (in thousands)	SBR (per 1000 births)	SBR (per 1000 births)	SBR/NMR	SBR/NMR
	20 weeks	28 weeks	20 weeks	28 weeks	20 weeks	28 weeks
1990	13.6 (9.5 to 19.8)	9.4 (6.6 to 13.5)	21.5 (15.2 to 31.0)	14.9 (10.5 to 21.4)	1.19 (0.84 to 1.71)	0.82 (0.58 to 1.18)
1995	11.4 (8.2 to 15.5)	7.9 (5.6 to 10.6)	17.4 (12.5 to 23.4)	12.0 (8.6 to 16.1)	1.14 (0.82 to 1.54)	0.79 (0.57 to 1.06)
2000	9.5 (8.8 to 10.1)	6.5 (6.0 to 6.9)	14.4 (13.4 to 15.3)	9.9 (9.2 to 10.6)	1.12 (1.05 to 1.20)	0.77 (0.72 to 0.83)
2005	6.7 (6.3 to 7.1)	4.6 (4.3 to 4.9)	11.0 (10.3 to 11.7)	7.5 (7.1 to 8.0)	0.94 (0.88 to 1.00)	0.65 (0.61 to 0.69)
2010	6.2 (5.9 to 6.7)	4.3 (4.0 to 4.6)	10.3 (9.8 to 11.1)	7.1 (6.7 to 7.6)	1.02 (0.96 to 1.09)	0.70 (0.66 to 0.75)
2015	5.6 (5.2 to 5.9)	3.8 (3.6 to 4.1)	9.1 (8.6 to 9.6)	6.3 (5.9 to 6.6)	1.13 (1.06 to 1.19)	0.78 (0.73 to 0.82)
2020	4.2 (3.8 to 4.7)	2.9 (2.6 to 3.2)	7.4 (6.6 to 8.2)	5.0 (4.5 to 5.6)	1.00 (0.91 to 1.11)	0.69 (0.62 to 0.76)
2021	4.2 (3.5 to 5.2)	2.9 (2.4 to 3.5)	7.6 (6.3 to 9.3)	5.2 (4.3 to 6.3)	1.04 (0.86 to 1.28)	0.71 (0.58 to 0.87)

Sergipe BRA_4774 Tropical Latin America Stillbirth Rate / Neonatal Mortality Rate (SBR/NMR) − 20 weeks
lambda = 0.2 ; zeta = 0.99 ; scale = 5 ; amp = 0.028

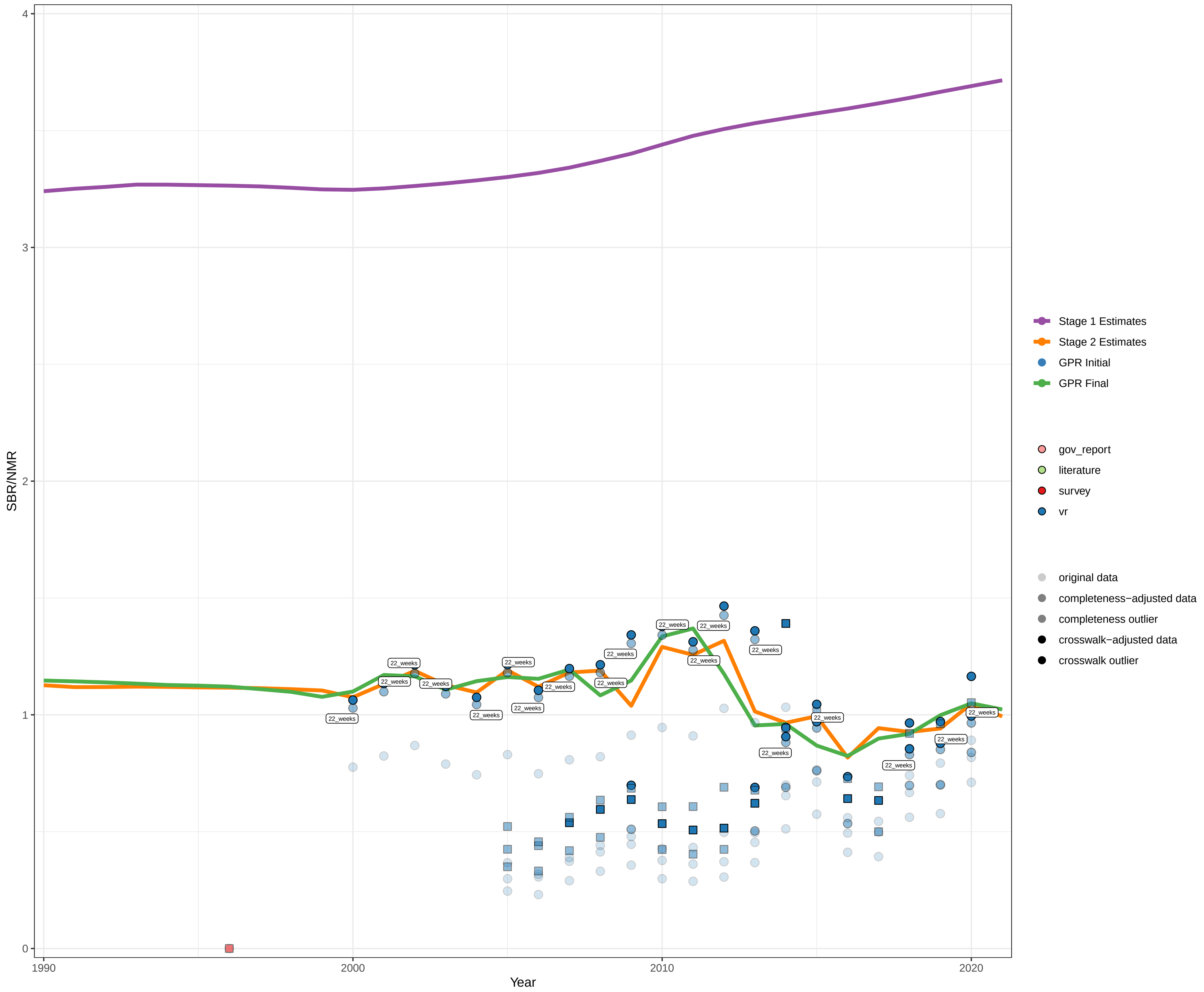

Sergipe BRA_4774 Tropical Latin America Stillbirth Rate / Neonatal Mortality Rate (SBR/NMR) − 28 weeks
lambda = 0.2 ; zeta = 0.99 ; scale = 5 ; amp = 0.028

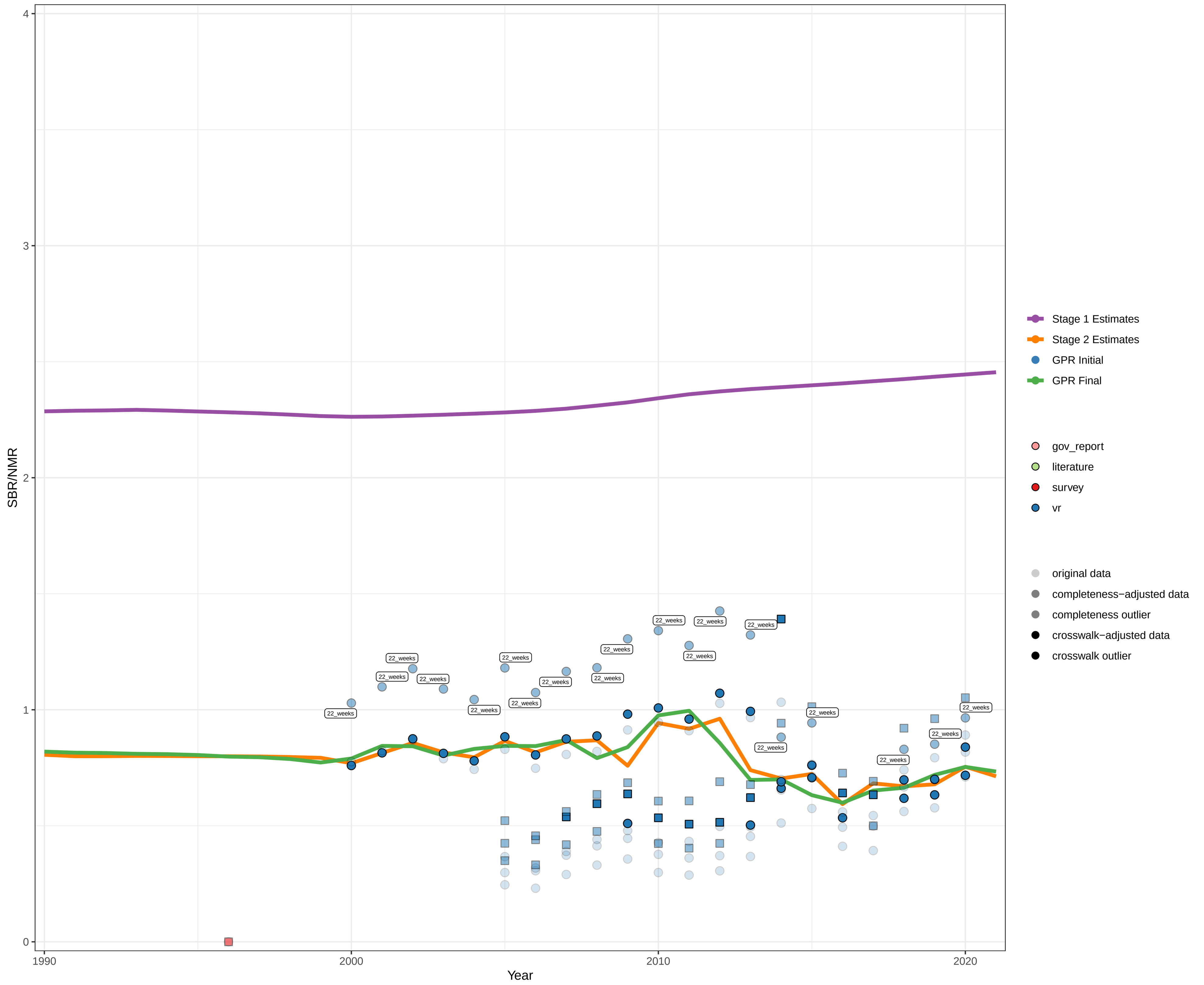

Definition	Years	Outlier	Source.Type	Title
22_weeks	2000−2014	0	VR	Brazil Mortality Information System − Stillbirths
22_weeks	2007−2020	0	VR	Brazil Civil Registry Statistics − Fetal Deaths at 28 Weeks or More, by Place of Birth, Number of Births by Birth and Sex, and Residence of Mother
28_weeks	2009, 2013−2016, 2018−2020	0	VR	Brazil Civil Registry Statistics − Fetal Deaths at 28 Weeks or More, by Place of Birth, Number of Births by Birth and Sex, and Residence of Mother
22_weeks	2005, 2006	1	VR	Brazil Civil Registry Statistics − Fetal Deaths at 28 Weeks or More, by Place of Birth, Number of Births by Birth and Sex, and Residence of Mother
28_weeks	1996	1	Survey	Brazil Demographic and Health Survey 1996
28_weeks	2005−2008, 2010−2012, 2017	1	VR	Brazil Civil Registry Statistics − Fetal Deaths at 28 Weeks or More, by Place of Birth, Number of Births by Birth and Sex, and Residence of Mother
other	2005−2020	1	VR	Brazil Civil Registry Statistics − Fetal Deaths at 28 Weeks or More, by Place of Birth, Number of Births by Birth and Sex, and Residence of Mother

Sergipe BRA_4774 Tropical Latin America Stillbirth Rate (SBR) − 20 weeks
lambda = 0.2 ; zeta = 0.99 ; scale = 5 ; amp = 0.028

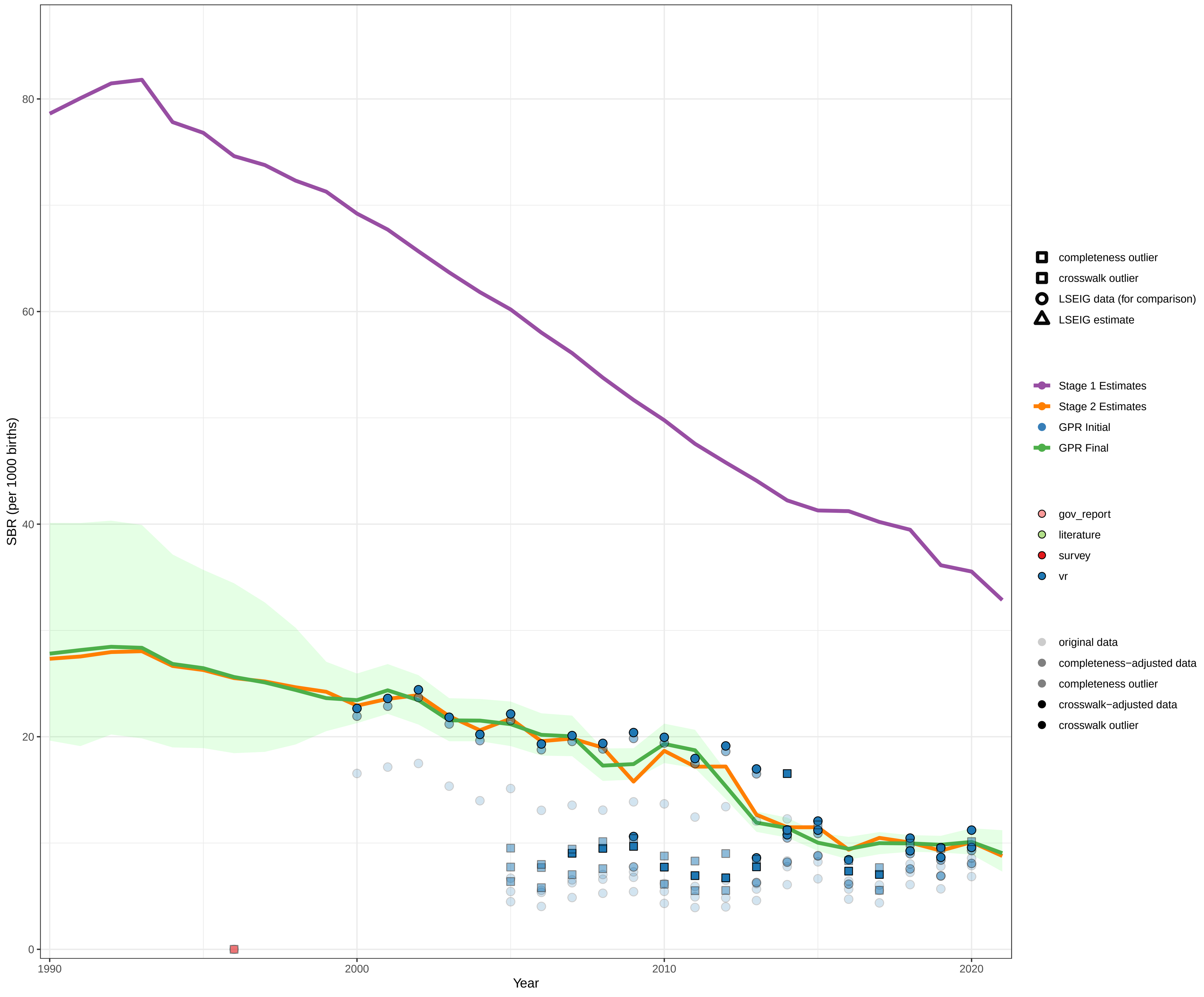

Sergipe BRA_4774 Tropical Latin America Stillbirth Rate (SBR) − 28 weeks
lambda = 0.2 ; zeta = 0.99 ; scale = 5 ; amp = 0.028

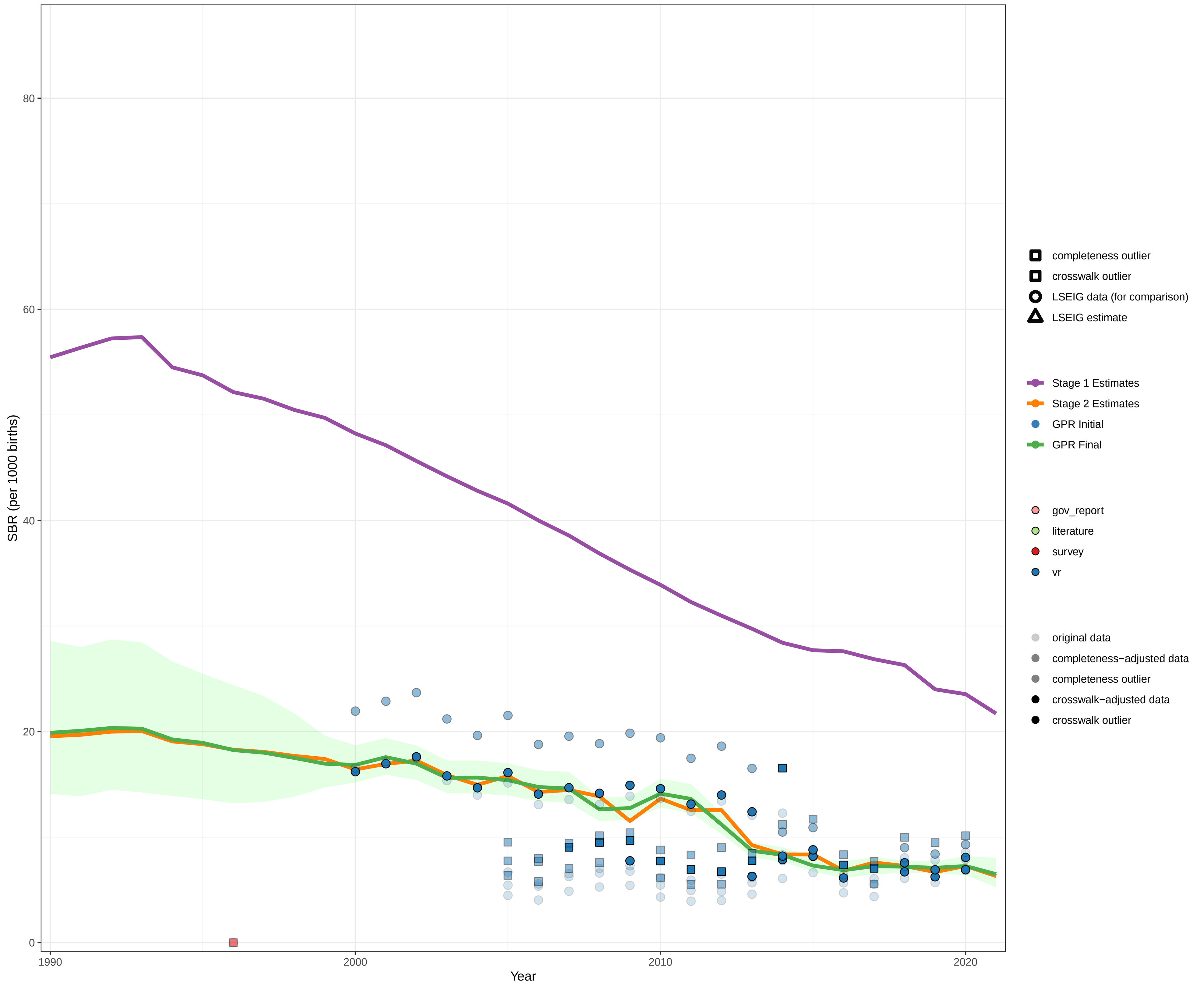

Year	Total Stillbirths (in thousands)	Total Stillbirths (in thousands)	SBR (per 1000 births)	SBR (per 1000 births)	SBR/NMR	SBR/NMR
	20 weeks	28 weeks	20 weeks	28 weeks	20 weeks	28 weeks
1990	1.0 (0.7 to 1.5)	0.7 (0.5 to 1.1)	27.8 (19.6 to 40.1)	19.9 (14.1 to 28.6)	1.15 (0.81 to 1.65)	0.82 (0.58 to 1.18)
1995	1.1 (0.8 to 1.5)	0.8 (0.6 to 1.1)	26.4 (18.9 to 35.7)	18.9 (13.6 to 25.5)	1.12 (0.80 to 1.52)	0.81 (0.58 to 1.08)
2000	1.1 (1.0 to 1.2)	0.8 (0.7 to 0.9)	23.4 (21.3 to 25.9)	16.8 (15.2 to 18.7)	1.10 (1.00 to 1.22)	0.79 (0.71 to 0.88)
2005	1.0 (0.9 to 1.1)	0.7 (0.6 to 0.8)	21.2 (19.1 to 23.3)	15.4 (13.9 to 17.0)	1.16 (1.05 to 1.28)	0.84 (0.76 to 0.93)
2010	0.9 (0.8 to 1.0)	0.6 (0.6 to 0.7)	19.3 (17.5 to 21.2)	14.1 (12.8 to 15.5)	1.34 (1.21 to 1.47)	0.98 (0.88 to 1.07)
2015	0.5 (0.4 to 0.5)	0.3 (0.3 to 0.4)	10.0 (9.3 to 11.0)	7.3 (6.7 to 8.0)	0.87 (0.80 to 0.95)	0.63 (0.58 to 0.69)
2020	0.5 (0.4 to 0.5)	0.3 (0.3 to 0.4)	10.1 (8.9 to 11.4)	7.3 (6.4 to 8.2)	1.05 (0.93 to 1.18)	0.75 (0.66 to 0.85)
2021	0.4 (0.3 to 0.5)	0.3 (0.2 to 0.4)	9.0 (7.3 to 11.2)	6.5 (5.2 to 8.0)	1.02 (0.83 to 1.27)	0.73 (0.59 to 0.91)

Tocantins   BRA_4776   Tropical Latin America   Stillbirth Rate / Neonatal Mortality Rate (SBR/NMR) − 20 weeks
lambda = 0.2 ; zeta = 0.99 ; scale = 5 ; amp = 0.028

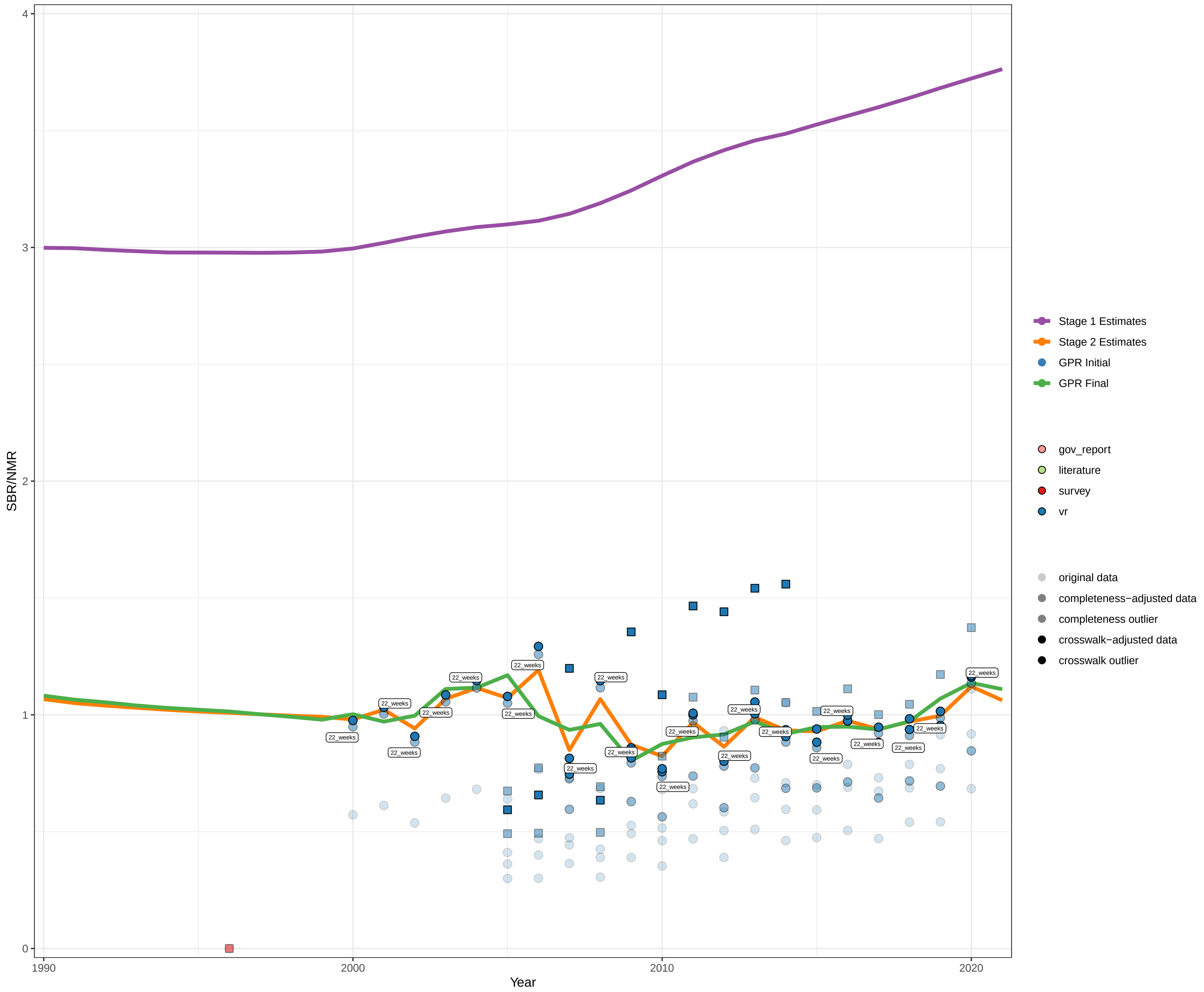

Tocantins   BRA_4776   Tropical Latin America   Stillbirth Rate / Neonatal Mortality Rate (SBR/NMR) − 28 weeks
lambda = 0.2 ; zeta = 0.99 ; scale = 5 ; amp = 0.028

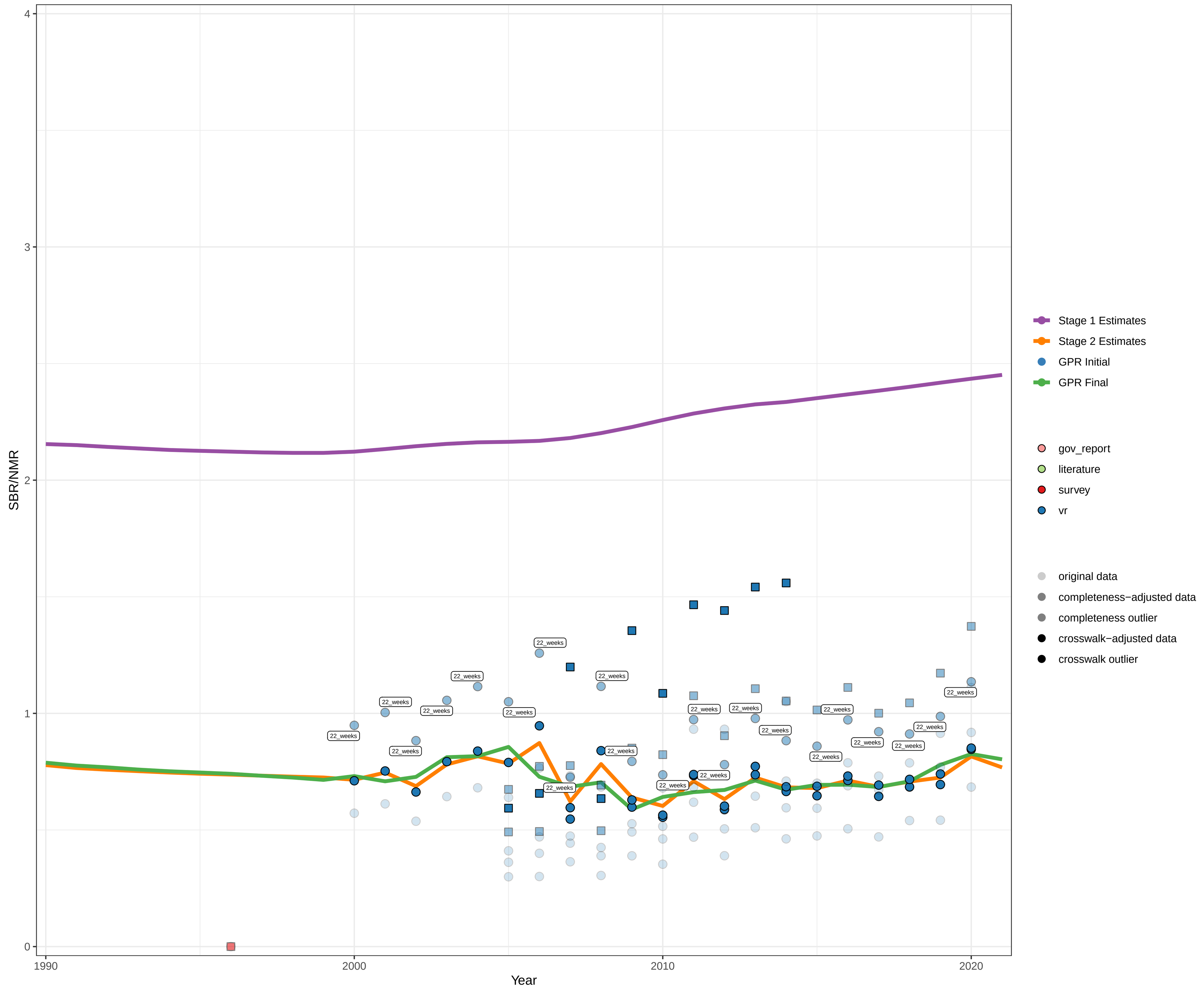

Definition	Years	Outlier	Source.Type	Title
22_weeks	2000−2014	0	VR	Brazil Mortality Information System − Stillbirths
22_weeks	2005−2020	0	VR	Brazil Civil Registry Statistics − Fetal Deaths at 28 Weeks or More, by Place of Birth, Number of Births by Birth and Sex, and Residence of Mother
28_weeks	2007, 2009−2020	0	VR	Brazil Civil Registry Statistics − Fetal Deaths at 28 Weeks or More, by Place of Birth, Number of Births by Birth and Sex, and Residence of Mother
28_weeks	1996	1	Survey	Brazil Demographic and Health Survey 1996
28_weeks	2005, 2006, 2008	1	VR	Brazil Civil Registry Statistics − Fetal Deaths at 28 Weeks or More, by Place of Birth, Number of Births by Birth and Sex, and Residence of Mother
other	2005−2020	1	VR	Brazil Civil Registry Statistics − Fetal Deaths at 28 Weeks or More, by Place of Birth, Number of Births by Birth and Sex, and Residence of Mother

Tocantins   BRA_4776   Tropical Latin America   Stillbirth Rate (SBR) − 20 weeks
lambda = 0.2 ; zeta = 0.99 ; scale = 5 ; amp = 0.028

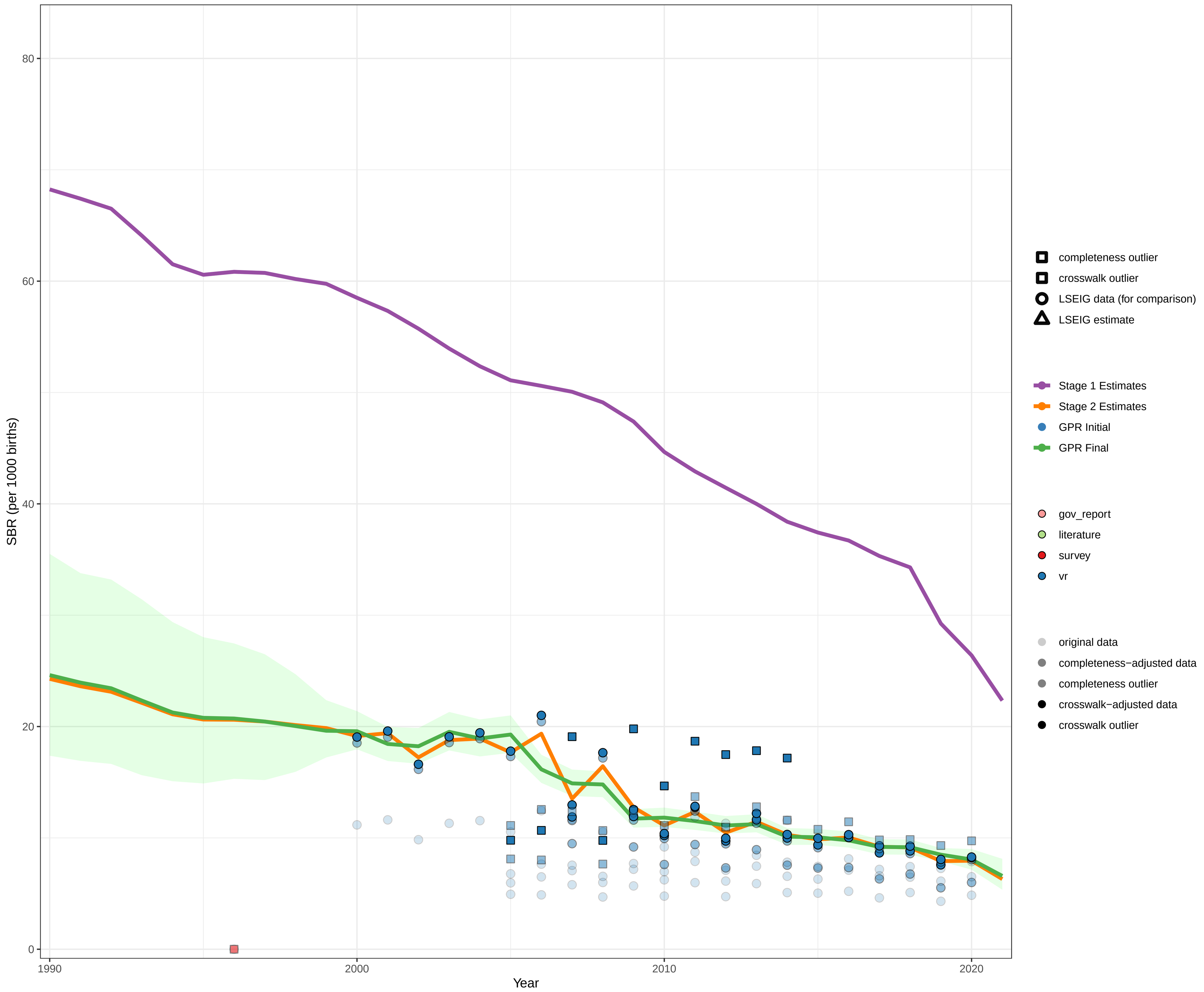

Tocantins   BRA_4776   Tropical Latin America   Stillbirth Rate (SBR) − 28 weeks
lambda = 0.2 ; zeta = 0.99 ; scale = 5 ; amp = 0.028

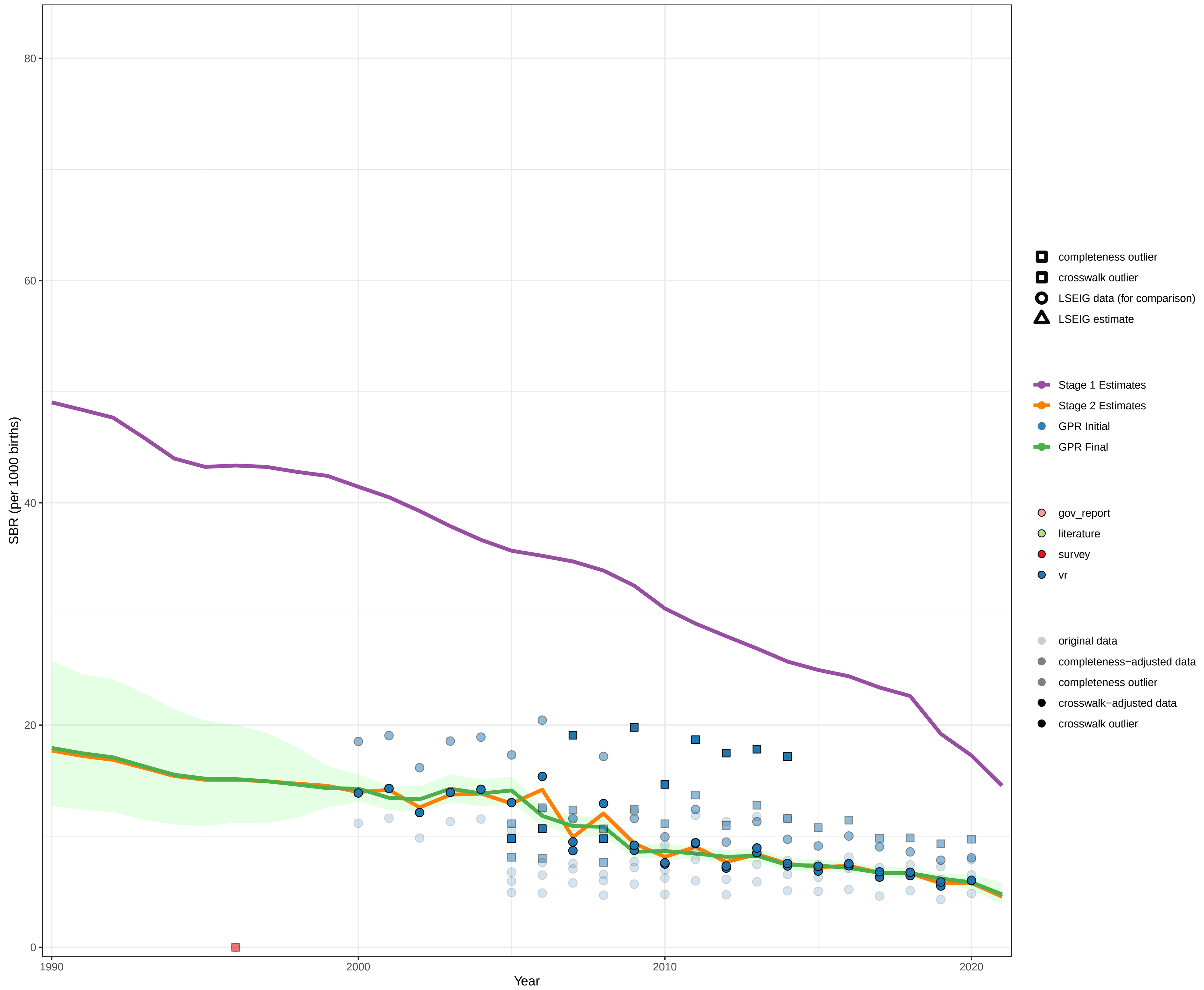

Year	Total Stillbirths (in thousands)	Total Stillbirths (in thousands)	SBR (per 1000 births)	SBR (per 1000 births)	SBR/NMR	SBR/NMR
	20 weeks	28 weeks	20 weeks	28 weeks	20 weeks	28 weeks
1990	0.6 (0.4 to 0.9)	0.4 (0.3 to 0.7)	24.6 (17.4 to 35.5)	17.9 (12.7 to 25.8)	1.08 (0.76 to 1.56)	0.79 (0.56 to 1.13)
1995	0.6 (0.5 to 0.9)	0.5 (0.3 to 0.6)	20.8 (14.9 to 28.0)	15.2 (10.9 to 20.4)	1.02 (0.73 to 1.38)	0.75 (0.54 to 1.00)
2000	0.7 (0.6 to 0.8)	0.5 (0.5 to 0.6)	19.6 (18.0 to 21.4)	14.3 (13.1 to 15.6)	1.00 (0.92 to 1.09)	0.73 (0.67 to 0.80)
2005	0.7 (0.6 to 0.8)	0.5 (0.5 to 0.6)	19.3 (17.6 to 21.0)	14.1 (12.9 to 15.4)	1.17 (1.07 to 1.27)	0.86 (0.78 to 0.93)
2010	0.4 (0.4 to 0.5)	0.3 (0.3 to 0.3)	11.8 (11.0 to 12.7)	8.7 (8.1 to 9.3)	0.88 (0.81 to 0.94)	0.64 (0.60 to 0.69)
2015	0.4 (0.4 to 0.4)	0.3 (0.3 to 0.3)	10.1 (9.4 to 10.8)	7.4 (6.9 to 7.9)	0.95 (0.88 to 1.02)	0.69 (0.65 to 0.74)
2020	0.3 (0.3 to 0.3)	0.2 (0.2 to 0.2)	8.1 (7.1 to 9.0)	5.9 (5.2 to 6.5)	1.14 (1.01 to 1.27)	0.83 (0.73 to 0.92)
2021	0.2 (0.2 to 0.3)	0.2 (0.1 to 0.2)	6.6 (5.3 to 8.1)	4.8 (3.9 to 5.9)	1.11 (0.90 to 1.37)	0.80 (0.65 to 0.99)

Paraguay PRY Tropical Latin America Stillbirth Rate / Neonatal Mortality Rate (SBR/NMR) − 20 weeks
lambda = 0.6 ; zeta = 0.8 ; scale = 15 ; amp = 0.028

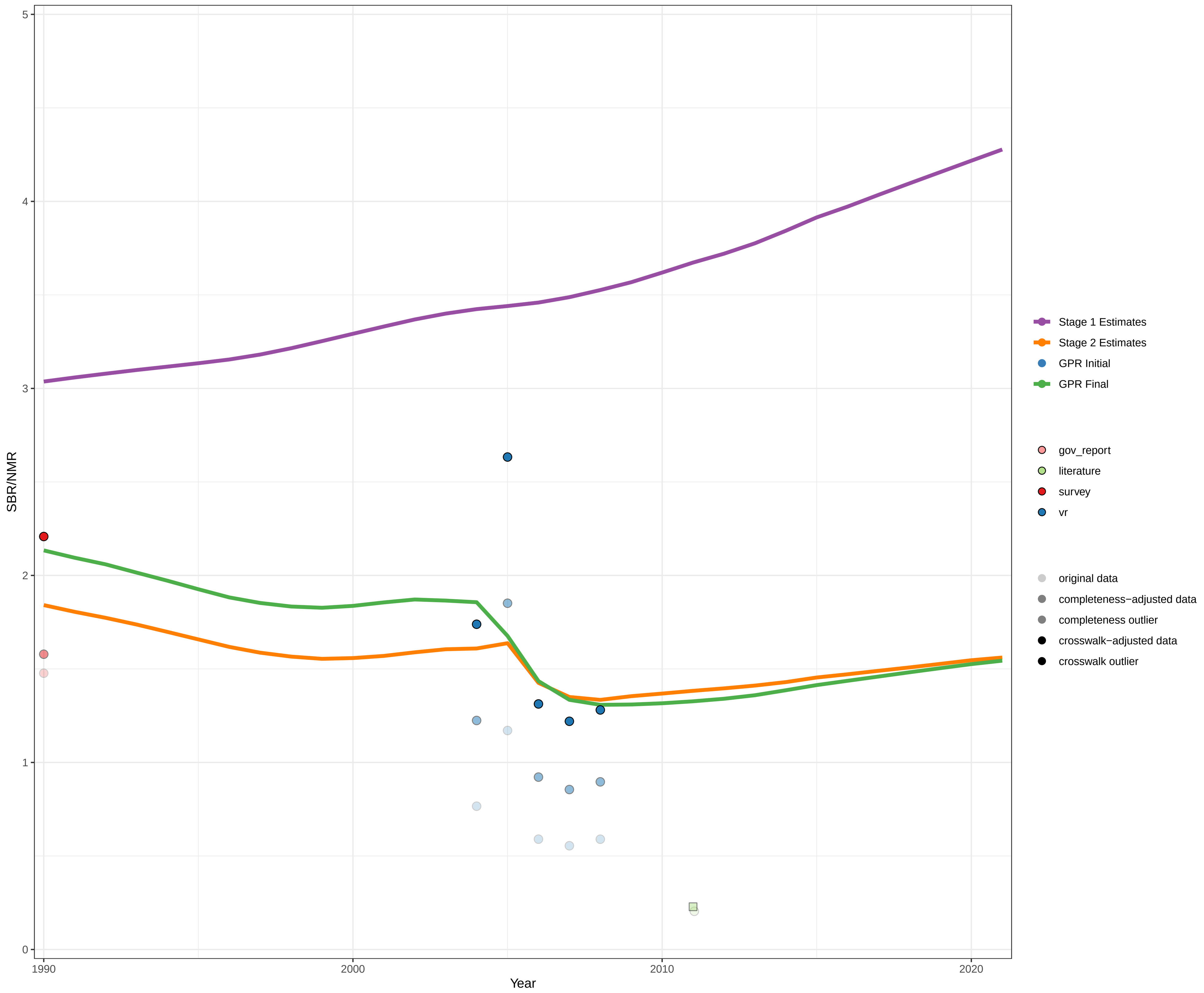

Paraguay PRY Tropical Latin America Stillbirth Rate / Neonatal Mortality Rate (SBR/NMR) − 28 weeks
lambda = 0.6 ; zeta = 0.8 ; scale = 15 ; amp = 0.028

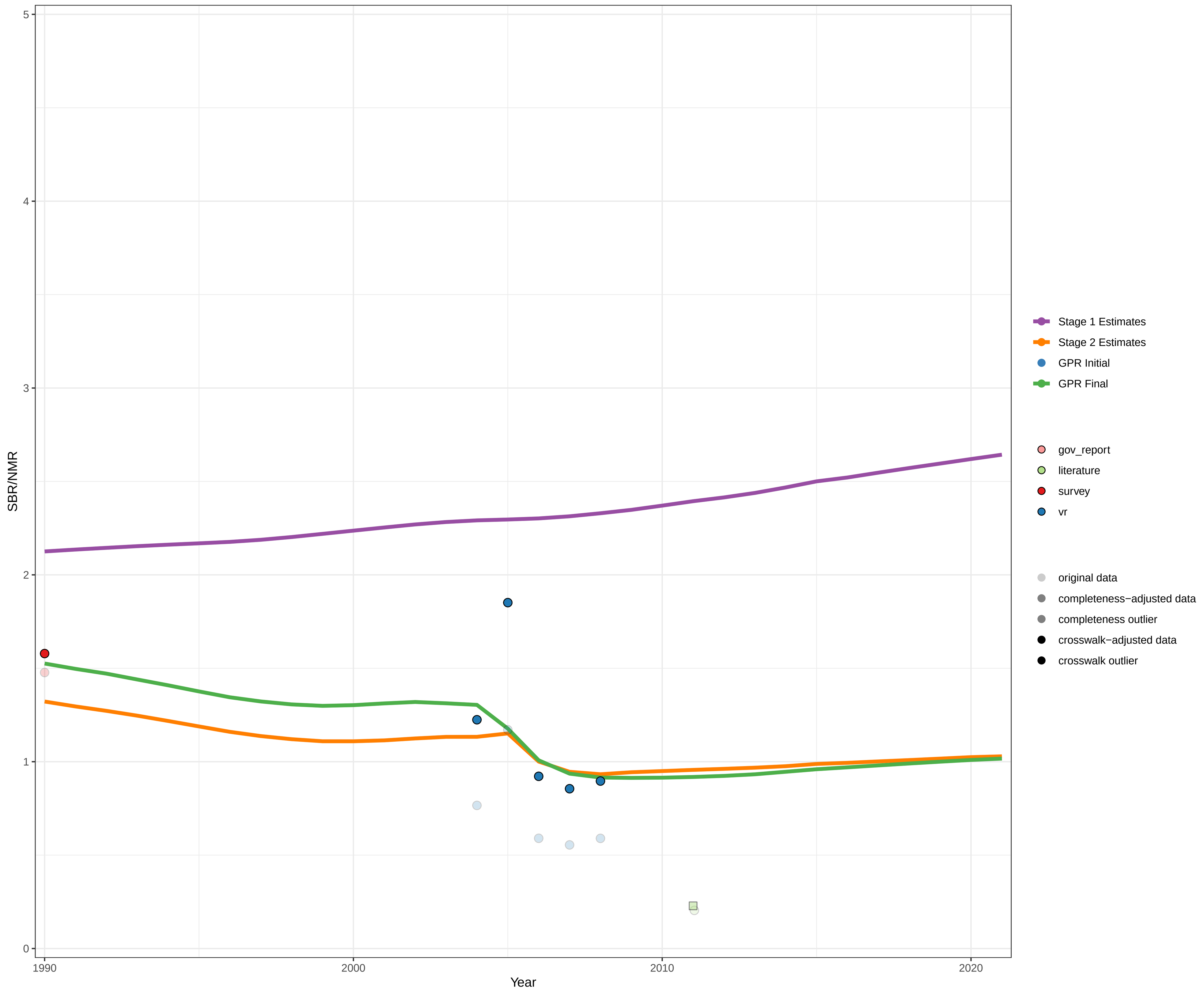

Definition	Years	Outlier	Source.Type	Title
28_weeks	1990	0	Survey	Paraguay Demographic and Health Survey 1990
28_weeks	2004−2008	0	VR	United Nations Demographic Yearbook
28_weeks_OR_1000_grams	2011	1	Sci Lit	Maternal complications and perinatal mortality: findings of the World Health Organization Multicountry Survey on Maternal and Newborn Health

Paraguay PRY Tropical Latin America Stillbirth Rate (SBR) − 20 weeks
lambda = 0.6 ; zeta = 0.8 ; scale = 15 ; amp = 0.028

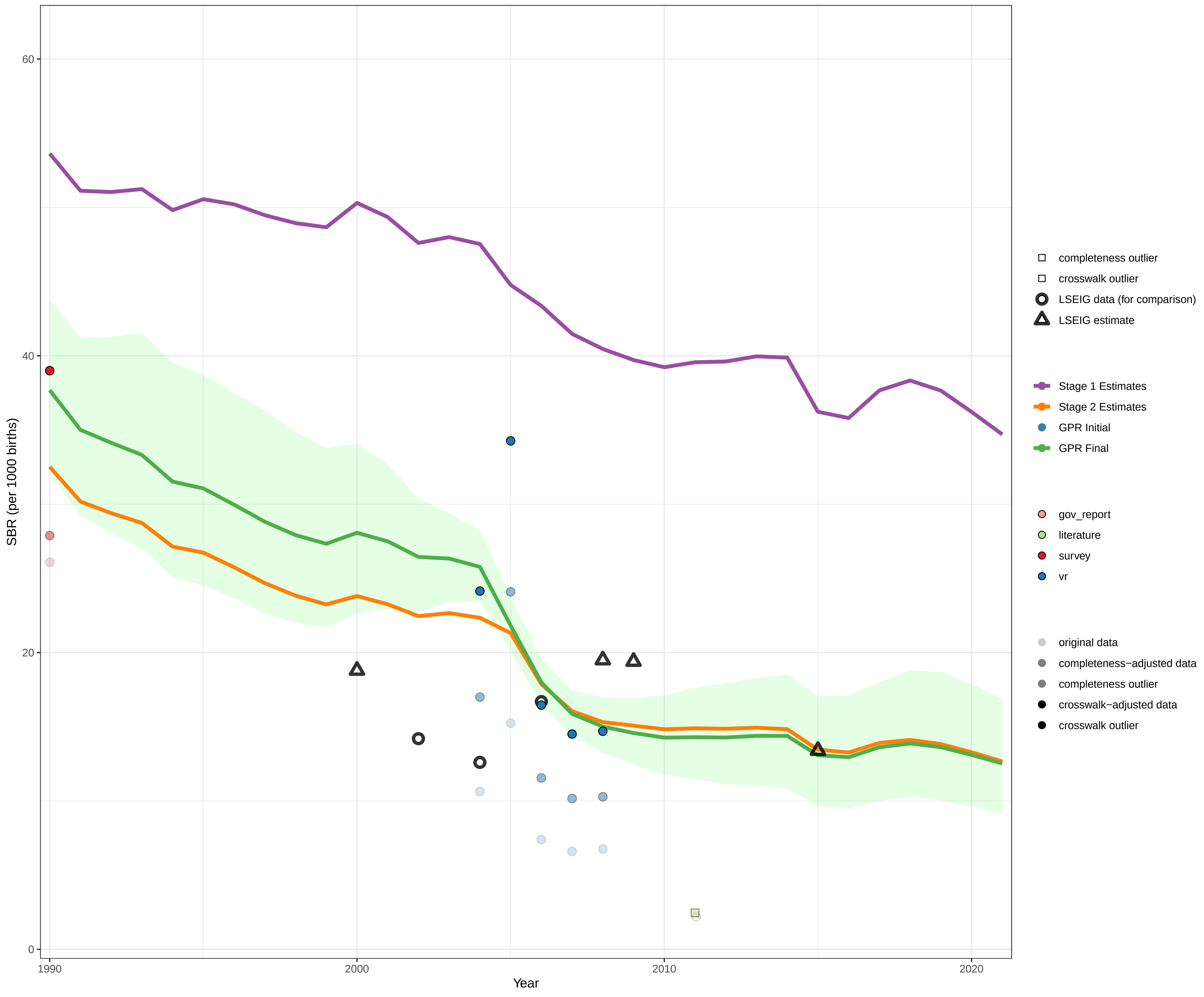

Paraguay PRY Tropical Latin America Stillbirth Rate (SBR) − 28 weeks
lambda = 0.6 ; zeta = 0.8 ; scale = 15 ; amp = 0.028

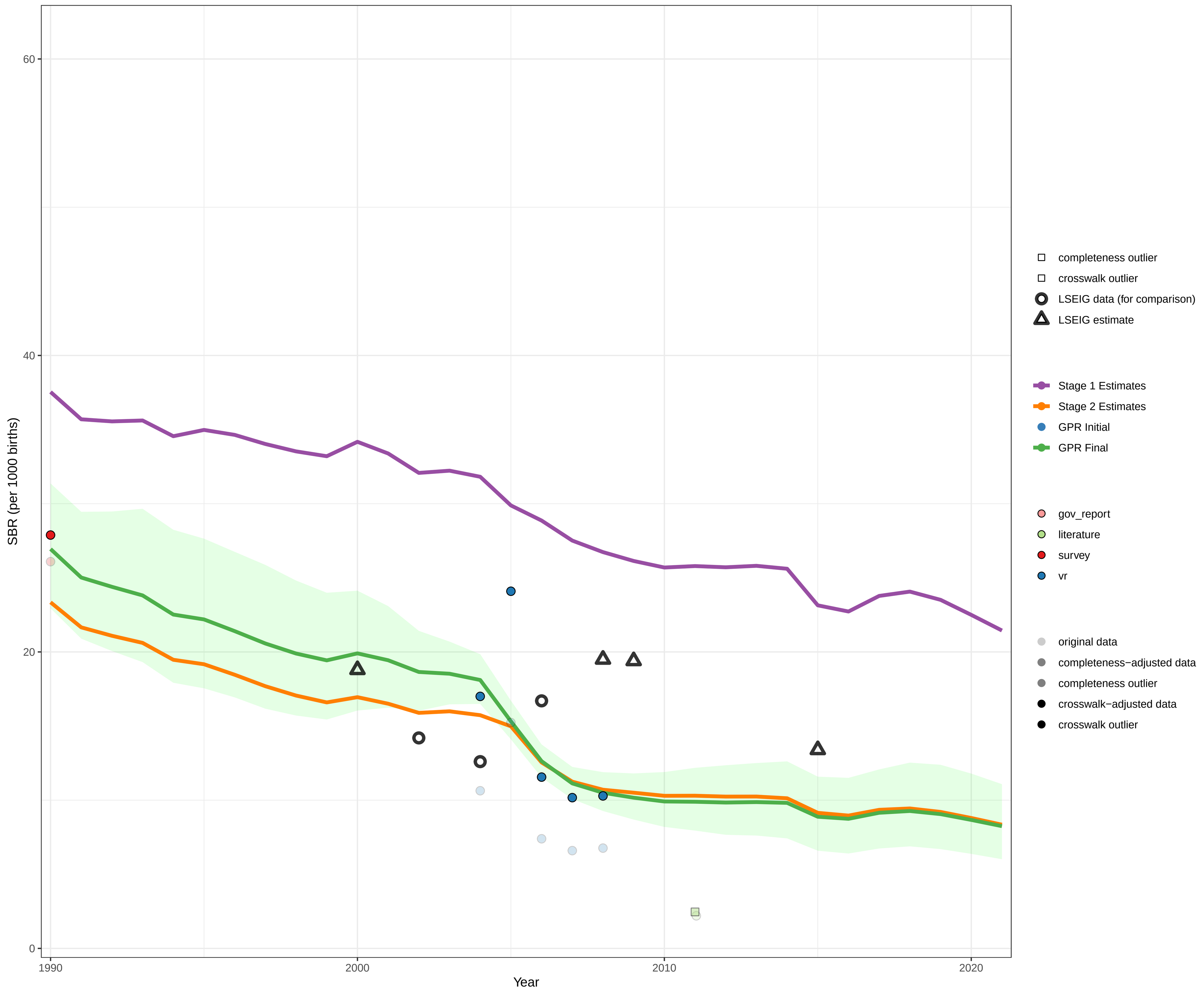

Year	Total Stillbirths (in thousands)	Total Stillbirths (in thousands)	SBR (per 1000 births)	SBR (per 1000 births)	SBR/NMR	SBR/NMR
	20 weeks	28 weeks	20 weeks	28 weeks	20 weeks	28 weeks
1990	5.0 (4.3 to 5.9)	3.6 (3.0 to 4.2)	37.7 (32.1 to 43.8)	26.9 (22.9 to 31.4)	2.13 (1.82 to 2.48)	1.53 (1.30 to 1.78)
1995	4.2 (3.3 to 5.3)	3.0 (2.3 to 3.7)	31.1 (24.5 to 38.7)	22.2 (17.5 to 27.6)	1.93 (1.52 to 2.40)	1.38 (1.09 to 1.71)
2000	3.8 (3.0 to 4.6)	2.7 (2.1 to 3.2)	28.1 (22.6 to 34.1)	19.9 (16.0 to 24.1)	1.84 (1.48 to 2.23)	1.30 (1.05 to 1.58)
2005	3.0 (2.7 to 3.2)	2.1 (1.9 to 2.3)	21.8 (20.1 to 23.8)	15.3 (14.1 to 16.7)	1.68 (1.54 to 1.83)	1.18 (1.08 to 1.28)
2010	2.0 (1.6 to 2.4)	1.4 (1.1 to 1.6)	14.3 (11.8 to 17.1)	9.9 (8.2 to 11.9)	1.32 (1.09 to 1.58)	0.91 (0.76 to 1.10)
2015	1.8 (1.3 to 2.3)	1.2 (0.9 to 1.6)	13.1 (9.7 to 17.1)	8.9 (6.6 to 11.6)	1.41 (1.05 to 1.85)	0.96 (0.71 to 1.25)
2020	1.7 (1.3 to 2.4)	1.1 (0.8 to 1.5)	13.1 (9.6 to 17.9)	8.7 (6.4 to 11.8)	1.53 (1.12 to 2.08)	1.01 (0.74 to 1.37)
2021	1.6 (1.2 to 2.2)	1.1 (0.8 to 1.4)	12.5 (9.1 to 16.9)	8.2 (6.0 to 11.1)	1.54 (1.12 to 2.08)	1.02 (0.74 to 1.37)

North Africa and Middle East   S4   NA   Stillbirth Rate / Neonatal Mortality Rate (SBR/NMR) − 20 weeks
lambda = 1 ; zeta = 0.6 ; scale = 15 ; amp = 0.028

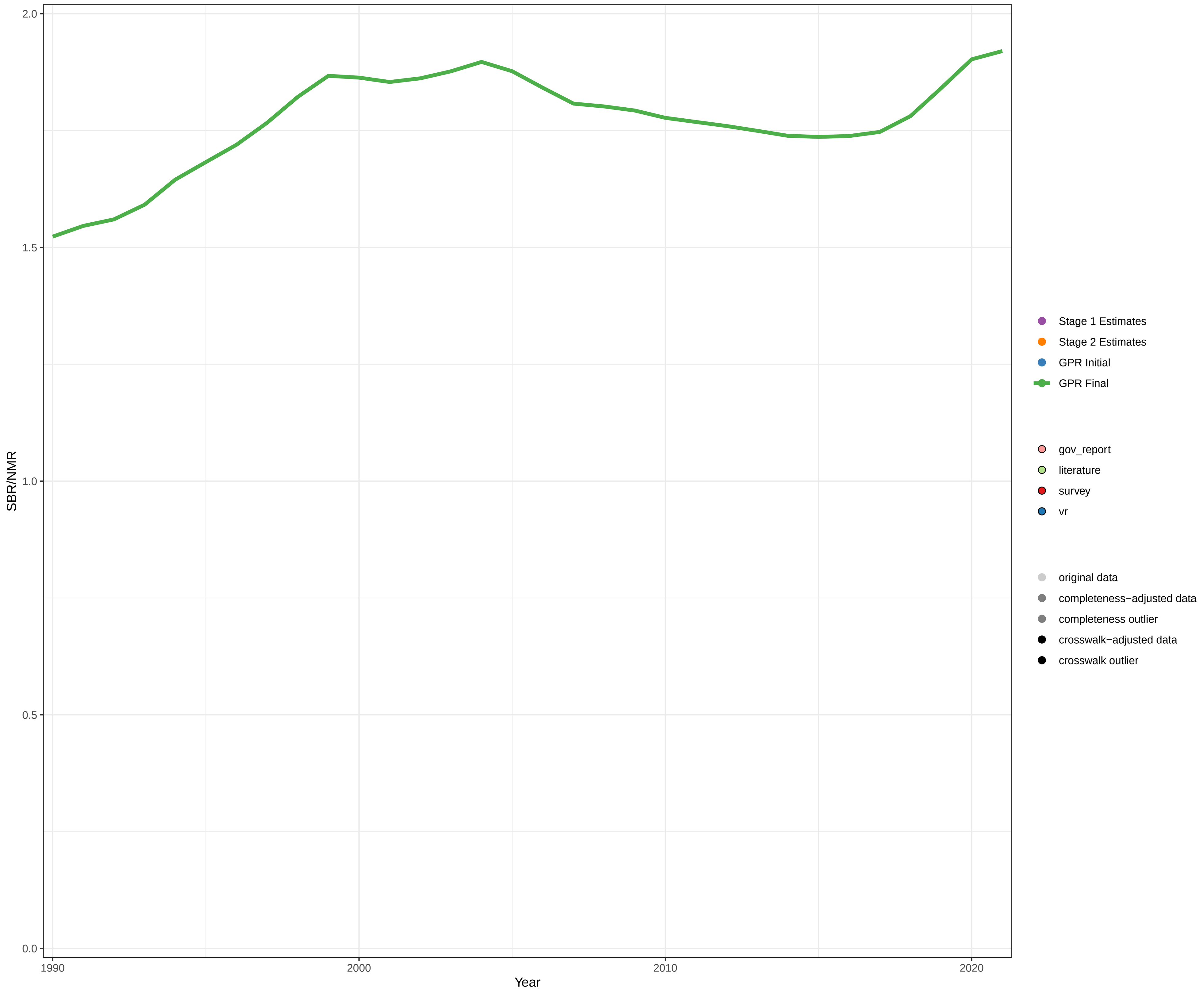

North Africa and Middle East   S4   NA   Stillbirth Rate / Neonatal Mortality Rate (SBR/NMR) − 28 weeks
lambda = 1 ; zeta = 0.6 ; scale = 15 ; amp = 0.028

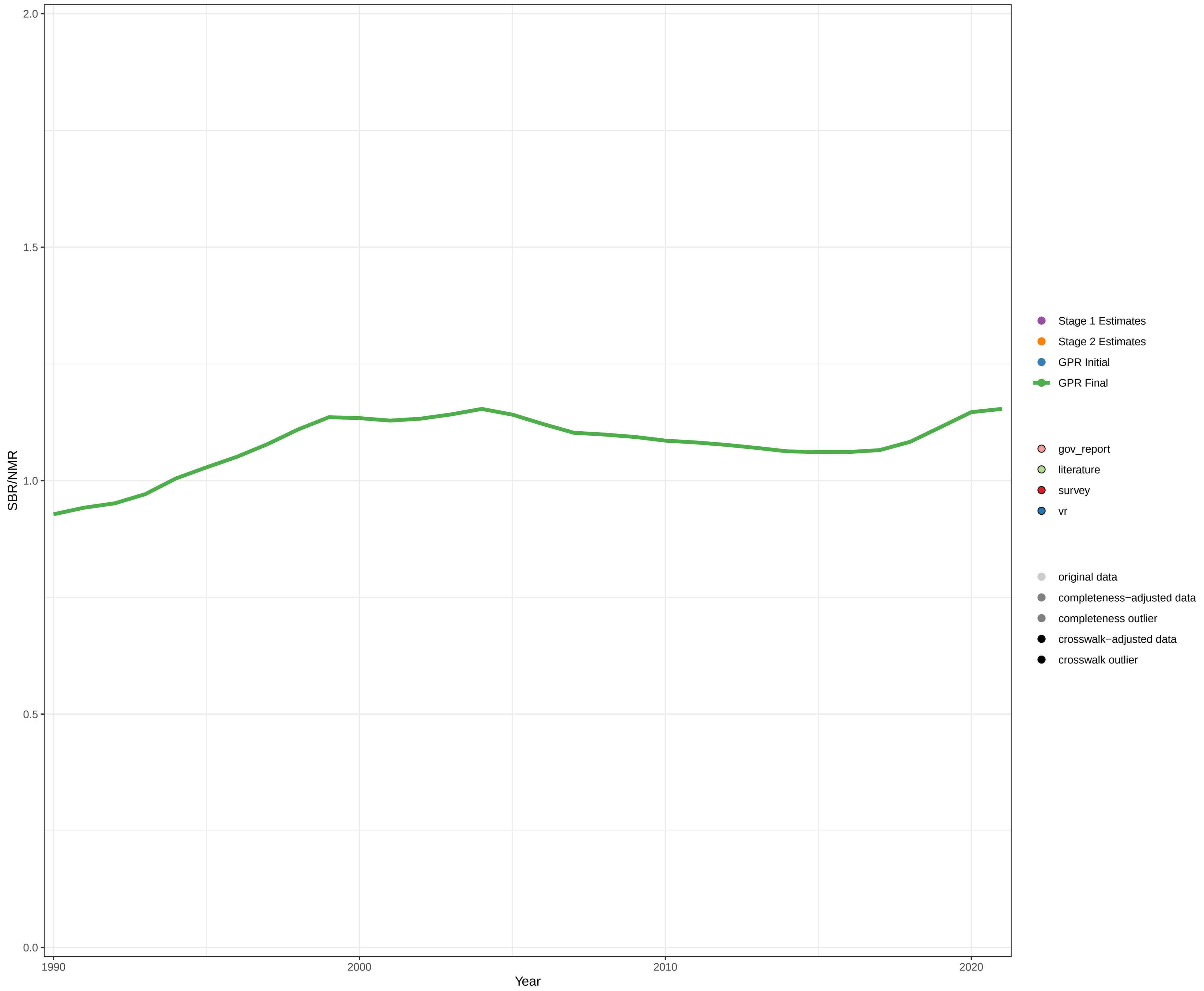

Definition	Years	Outlier	Source.Type	Title

North Africa and Middle East   S4   NA   Stillbirth Rate (SBR) − 20 weeks
lambda = 1 ; zeta = 0.6 ; scale = 15 ; amp = 0.028

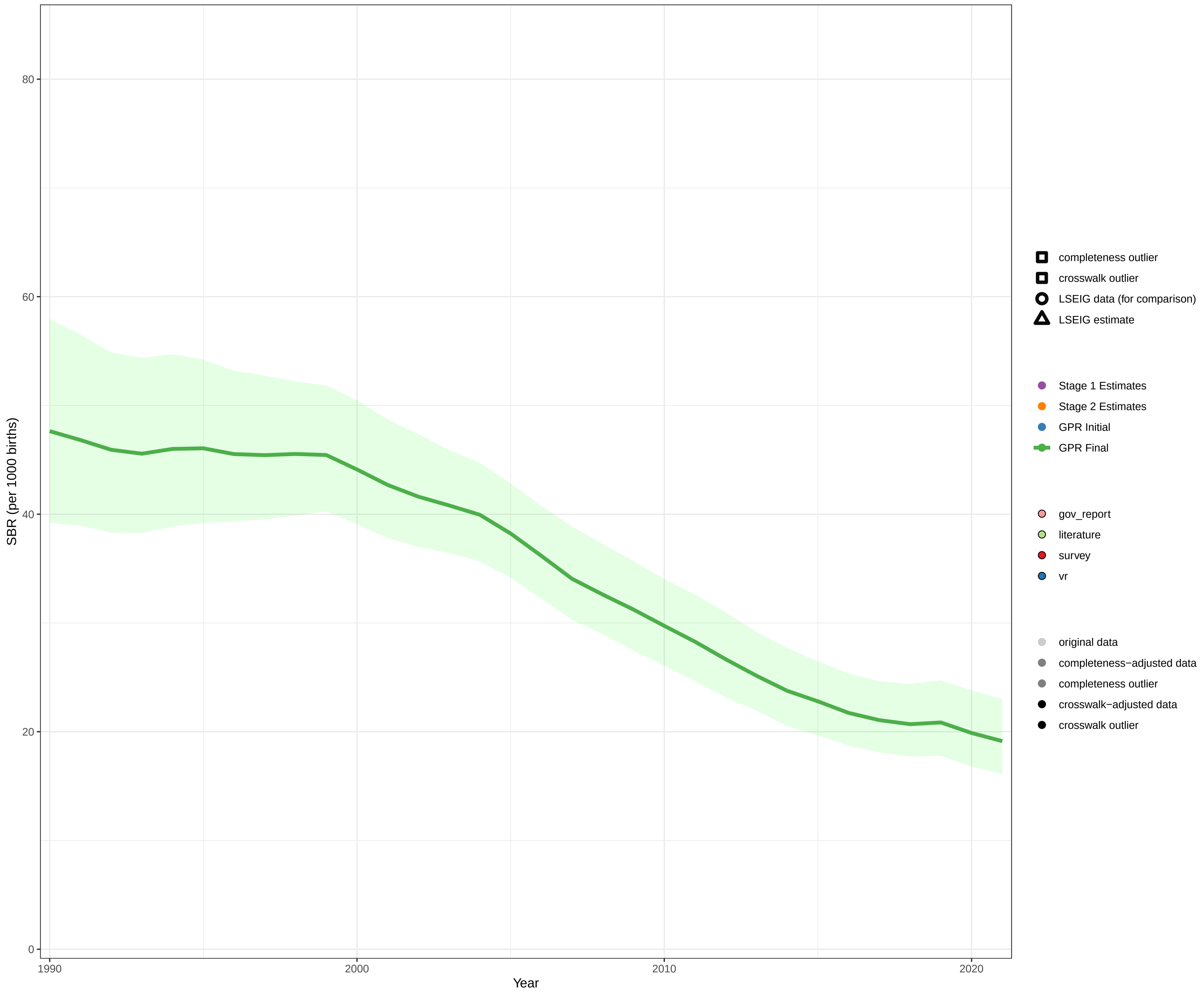

North Africa and Middle East   S4   NA   Stillbirth Rate (SBR) − 28 weeks
lambda = 1 ; zeta = 0.6 ; scale = 15 ; amp = 0.028

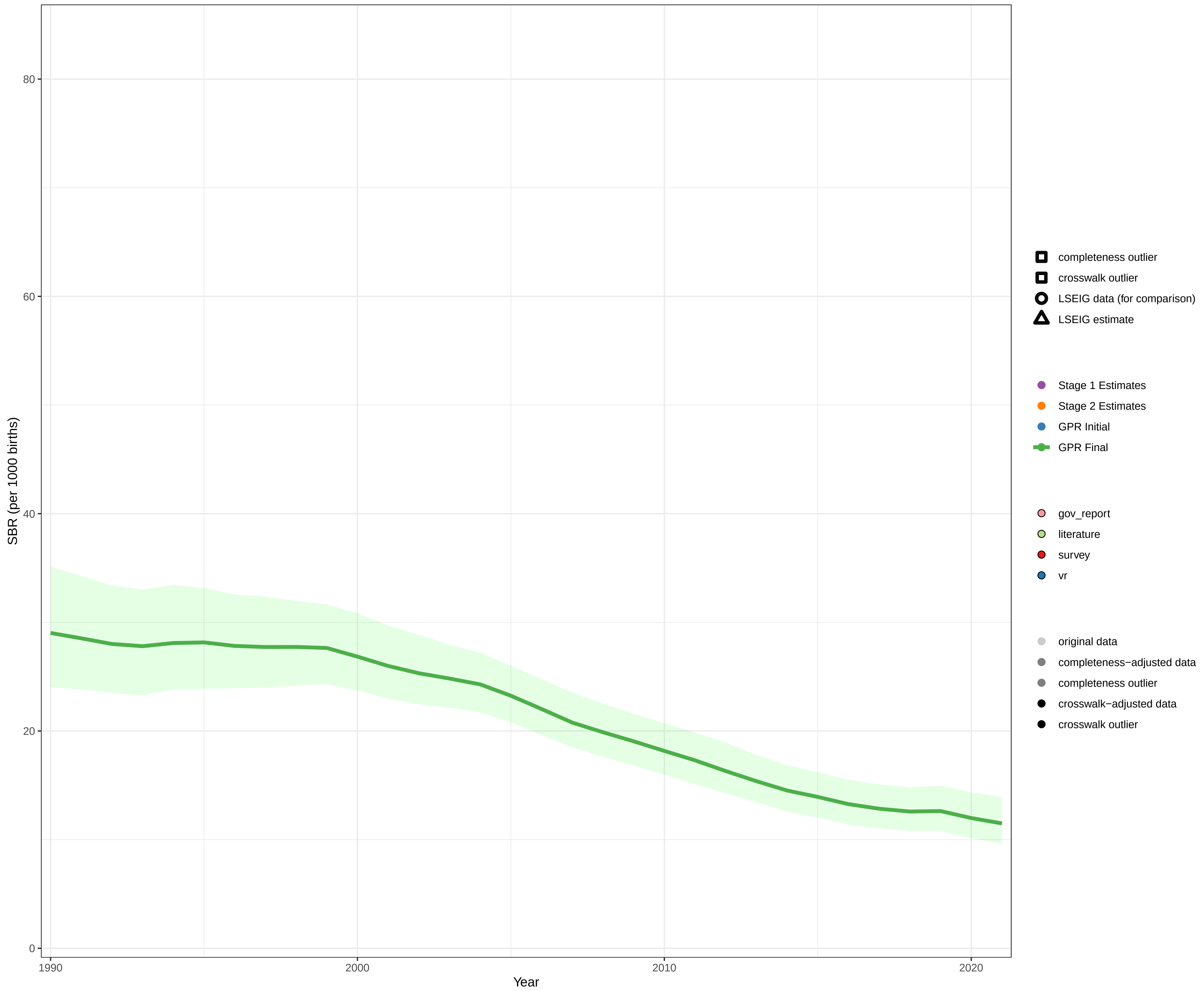

Year	Total Stillbirths (in thousands)	Total Stillbirths (in thousands)	SBR (per 1000 births)	SBR (per 1000 births)	SBR/NMR	SBR/NMR
	20 weeks	28 weeks	20 weeks	28 weeks	20 weeks	28 weeks
1990	556.8 (454.1 to 684.4)	332.6 (273.7 to 405.3)	47.6 (39.2 to 58.0)	29.0 (24.0 to 35.2)	1.52 (1.25 to 1.85)	0.93 (0.77 to 1.12)
1995	539.3 (455.6 to 640.1)	323.5 (273.0 to 383.0)	46.1 (39.2 to 54.2)	28.2 (23.9 to 33.2)	1.68 (1.43 to 1.98)	1.03 (0.87 to 1.21)
2000	533.3 (470.0 to 614.4)	318.7 (280.6 to 367.7)	44.1 (39.1 to 50.5)	26.8 (23.7 to 30.8)	1.86 (1.65 to 2.13)	1.13 (1.00 to 1.30)
2005	486.2 (432.3 to 547.6)	291.1 (259.7 to 326.4)	38.2 (34.1 to 42.8)	23.2 (20.8 to 26.0)	1.88 (1.68 to 2.10)	1.14 (1.02 to 1.28)
2010	422.9 (369.3 to 486.2)	255.3 (224.0 to 291.9)	29.7 (26.1 to 34.0)	18.2 (16.0 to 20.7)	1.78 (1.56 to 2.03)	1.09 (0.95 to 1.24)
2015	325.9 (280.2 to 380.0)	197.4 (170.1 to 230.0)	22.8 (19.7 to 26.5)	13.9 (12.0 to 16.2)	1.74 (1.50 to 2.02)	1.06 (0.92 to 1.23)
2020	251.8 (212.0 to 302.7)	150.6 (126.7 to 181.0)	19.9 (16.8 to 23.8)	12.0 (10.1 to 14.4)	1.90 (1.61 to 2.28)	1.15 (0.97 to 1.38)
2021	236.8 (198.5 to 286.2)	141.2 (118.3 to 171.3)	19.1 (16.1 to 23.0)	11.5 (9.7 to 13.9)	1.92 (1.61 to 2.31)	1.15 (0.97 to 1.40)

North Africa and Middle East R15 North Africa and Middle East Stillbirth Rate / Neonatal Mortality Rate (SBR/NMR) − 20 weeks
lambda = 1 ; zeta = 0.6 ; scale = 15 ; amp = 0.028

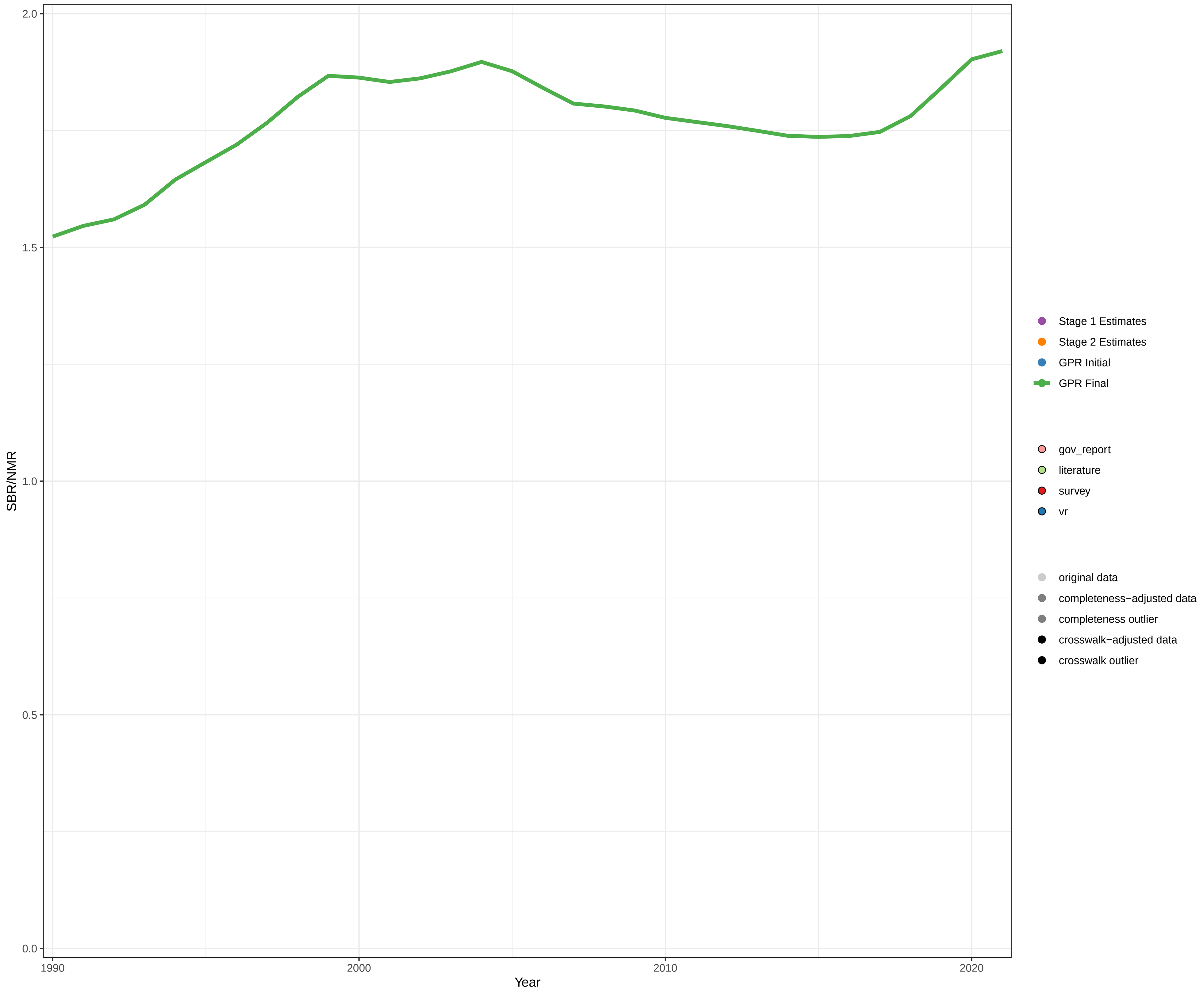

North Africa and Middle East R15 North Africa and Middle East Stillbirth Rate / Neonatal Mortality Rate (SBR/NMR) − 28 weeks
lambda = 1 ; zeta = 0.6 ; scale = 15 ; amp = 0.028

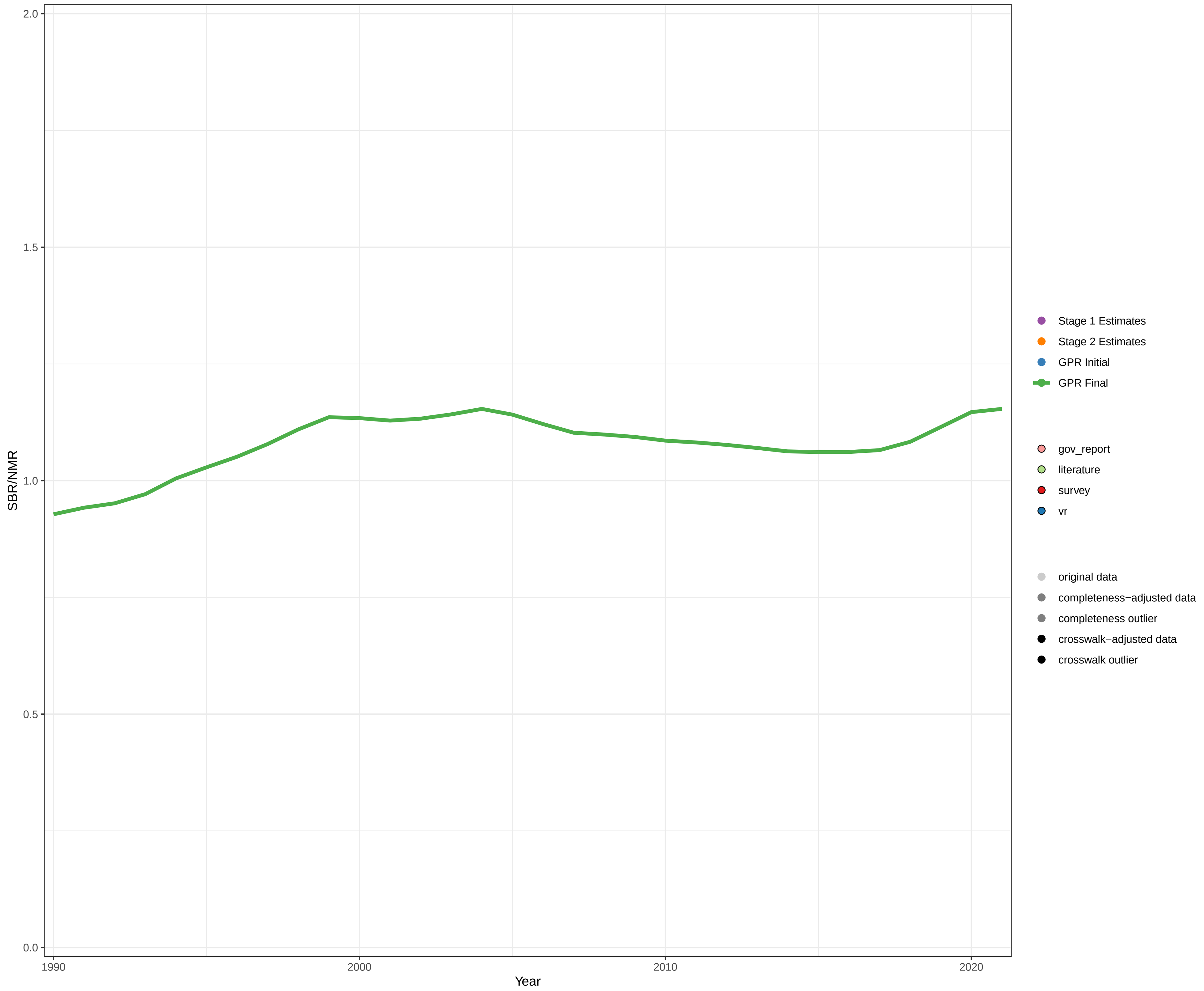

Definition	Years	Outlier	Source.Type	Title

North Africa and Middle East R15 North Africa and Middle East Stillbirth Rate (SBR) − 20 weeks
lambda = 1 ; zeta = 0.6 ; scale = 15 ; amp = 0.028

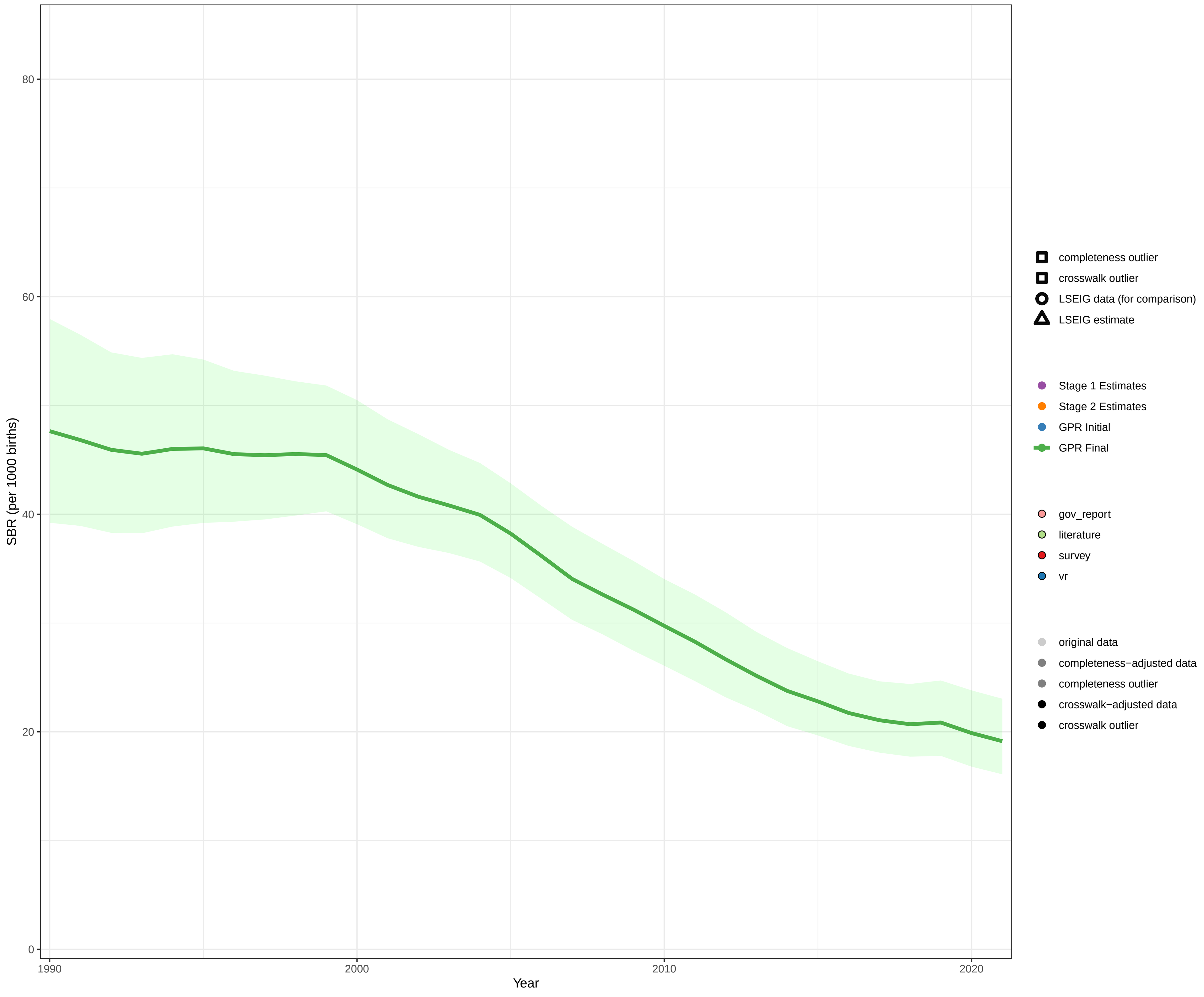

North Africa and Middle East R15 North Africa and Middle East Stillbirth Rate (SBR) − 28 weeks
lambda = 1 ; zeta = 0.6 ; scale = 15 ; amp = 0.028

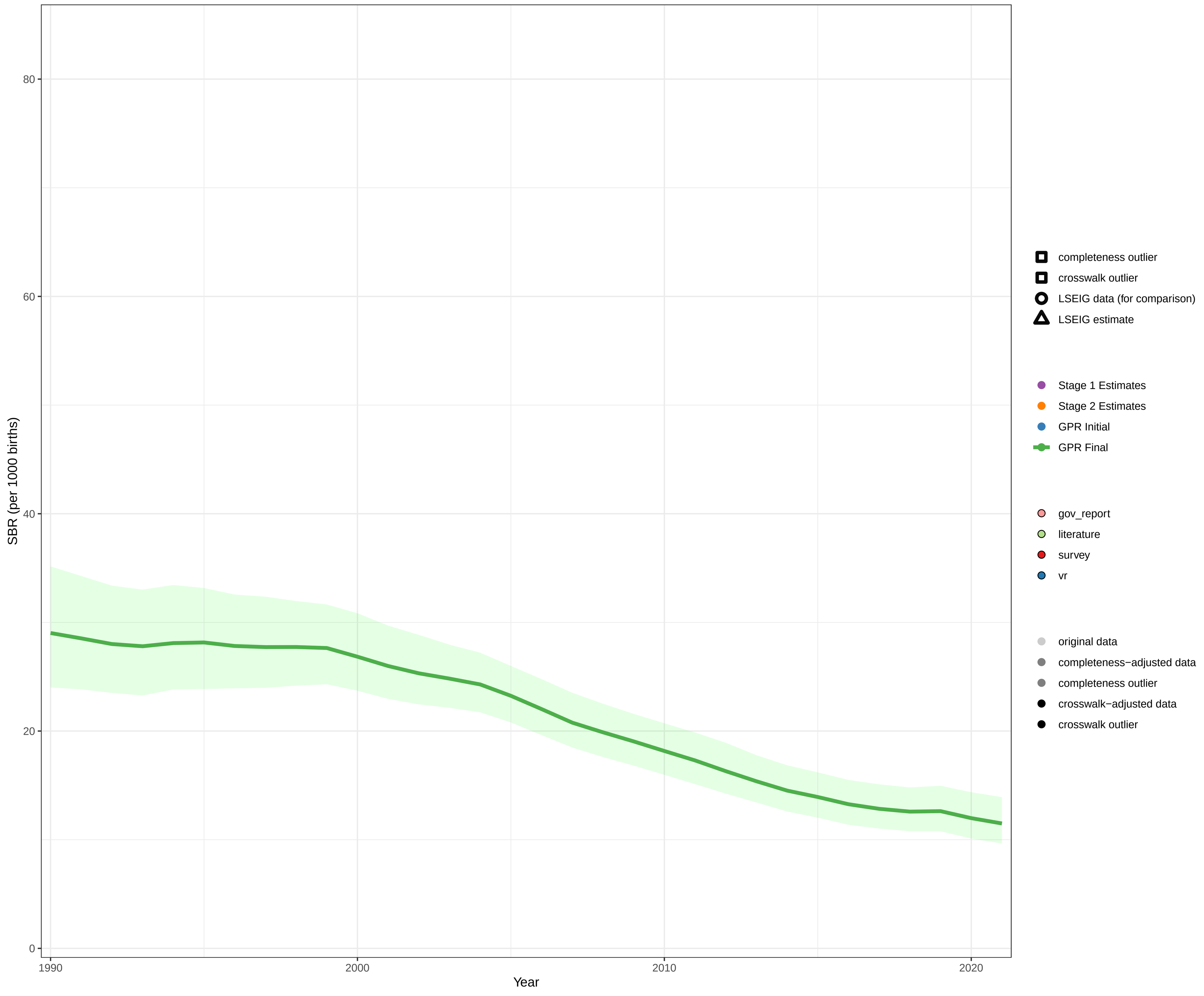

Year	Total Stillbirths (in thousands)	Total Stillbirths (in thousands)	SBR (per 1000 births)	SBR (per 1000 births)	SBR/NMR	SBR/NMR
	20 weeks	28 weeks	20 weeks	28 weeks	20 weeks	28 weeks
1990	556.8 (454.1 to 684.4)	332.6 (273.7 to 405.3)	47.6 (39.2 to 58.0)	29.0 (24.0 to 35.2)	1.52 (1.25 to 1.85)	0.93 (0.77 to 1.12)
1995	539.3 (455.6 to 640.1)	323.5 (273.0 to 383.0)	46.1 (39.2 to 54.2)	28.2 (23.9 to 33.2)	1.68 (1.43 to 1.98)	1.03 (0.87 to 1.21)
2000	533.3 (470.0 to 614.4)	318.7 (280.6 to 367.7)	44.1 (39.1 to 50.5)	26.8 (23.7 to 30.8)	1.86 (1.65 to 2.13)	1.13 (1.00 to 1.30)
2005	486.2 (432.3 to 547.6)	291.1 (259.7 to 326.4)	38.2 (34.1 to 42.8)	23.2 (20.8 to 26.0)	1.88 (1.68 to 2.10)	1.14 (1.02 to 1.28)
2010	422.9 (369.3 to 486.2)	255.3 (224.0 to 291.9)	29.7 (26.1 to 34.0)	18.2 (16.0 to 20.7)	1.78 (1.56 to 2.03)	1.09 (0.95 to 1.24)
2015	325.9 (280.2 to 380.0)	197.4 (170.1 to 230.0)	22.8 (19.7 to 26.5)	13.9 (12.0 to 16.2)	1.74 (1.50 to 2.02)	1.06 (0.92 to 1.23)
2020	251.8 (212.0 to 302.7)	150.6 (126.7 to 181.0)	19.9 (16.8 to 23.8)	12.0 (10.1 to 14.4)	1.90 (1.61 to 2.28)	1.15 (0.97 to 1.38)
2021	236.8 (198.5 to 286.2)	141.2 (118.3 to 171.3)	19.1 (16.1 to 23.0)	11.5 (9.7 to 13.9)	1.92 (1.61 to 2.31)	1.15 (0.97 to 1.40)

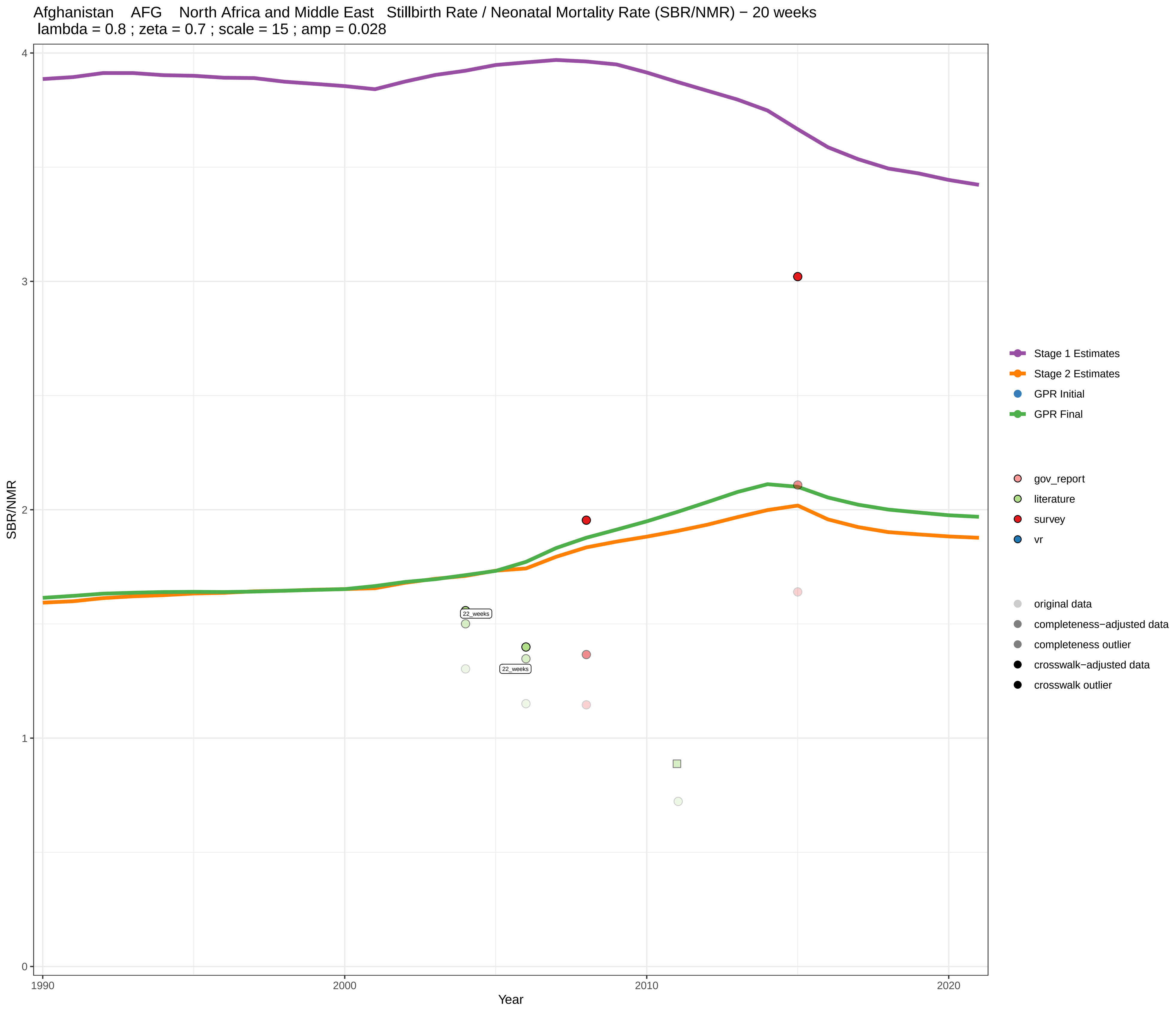

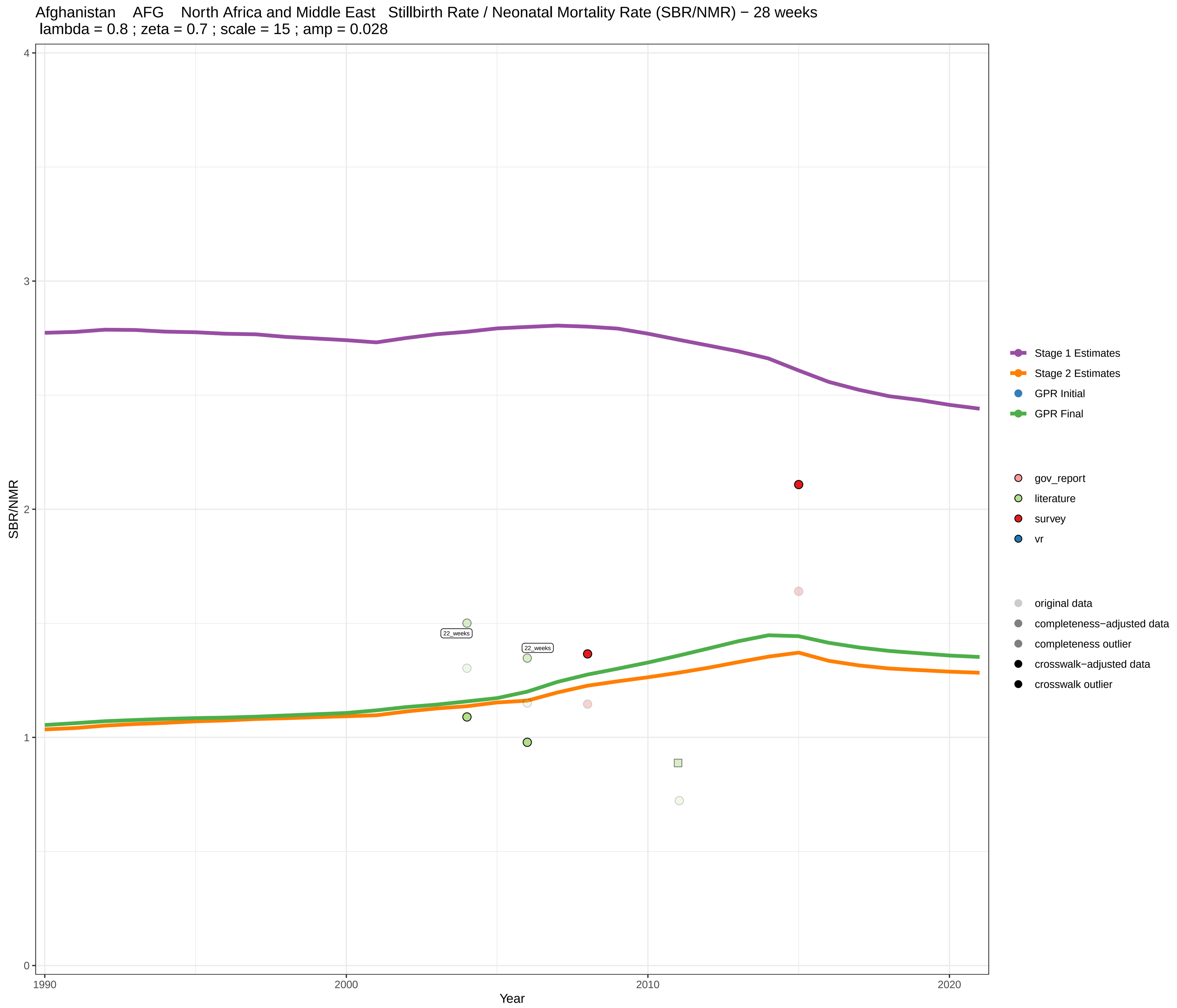

Definition	Years	Outlier	Source.Type	Title
22_weeks	2004	0	Sci Lit	Implementing a facility−based maternal and perinatal health care surveillance system in Afghanistan
22_weeks	2006	0	Sci Lit	Monitoring perinatal outcomes in hospitals in Kabul, Afghanistan: The first step of a quality assurance process
28_weeks	2008	0	Survey	Afghanistan Special Demographic and Health Survey 2010
28_weeks	2015	0	Survey	Afghanistan Demographic and Health Survey 2015−2016
28_weeks_OR_1000_grams	2011	1	Sci Lit	Maternal complications and perinatal mortality: findings of the World Health Organization Multicountry Survey on Maternal and Newborn Health

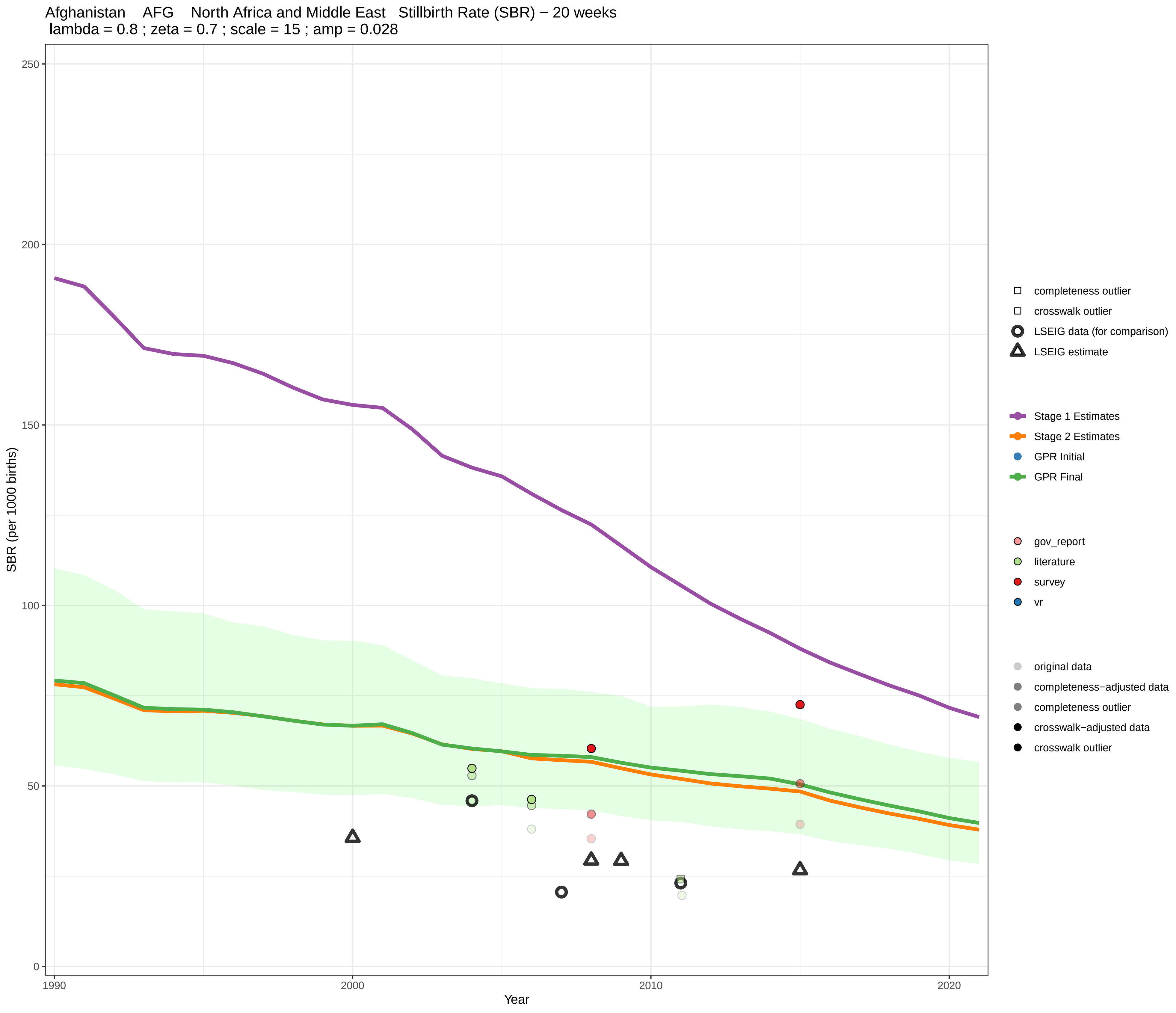

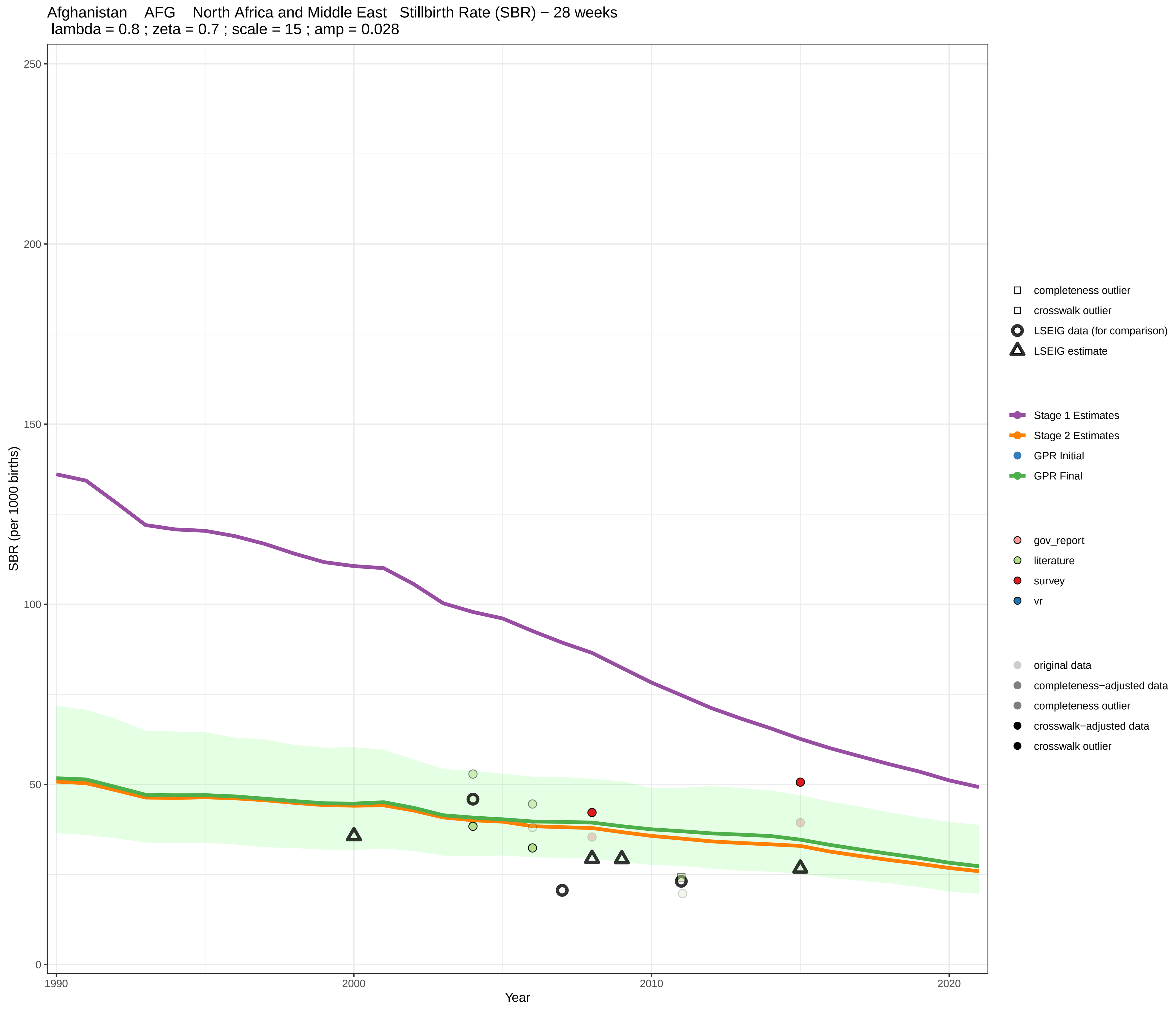

Year	Total Stillbirths (in thousands)	Total Stillbirths (in thousands)	SBR (per 1000 births)	SBR (per 1000 births)	SBR/NMR	SBR/NMR
	20 weeks	28 weeks	20 weeks	28 weeks	20 weeks	28 weeks
1990	38.7 (26.5 to 55.7)	24.5 (17.0 to 34.7)	79.2 (55.7 to 110.3)	51.7 (36.5 to 71.8)	1.61 (1.13 to 2.25)	1.05 (0.74 to 1.46)
1995	53.6 (37.5 to 75.7)	34.5 (24.4 to 48.1)	71.2 (51.0 to 97.8)	47.0 (33.8 to 64.5)	1.64 (1.17 to 2.26)	1.08 (0.78 to 1.49)
2000	56.0 (39.0 to 77.7)	36.7 (25.8 to 50.3)	66.7 (47.4 to 90.2)	44.7 (31.9 to 60.3)	1.65 (1.18 to 2.24)	1.11 (0.79 to 1.49)
2005	54.3 (40.0 to 72.9)	36.0 (26.7 to 47.9)	59.6 (44.7 to 78.4)	40.3 (30.3 to 53.0)	1.73 (1.30 to 2.28)	1.17 (0.88 to 1.54)
2010	53.1 (38.4 to 70.6)	35.5 (25.9 to 46.8)	55.1 (40.5 to 72.0)	37.5 (27.6 to 48.9)	1.95 (1.43 to 2.55)	1.33 (0.98 to 1.73)
2015	56.4 (40.4 to 78.1)	38.1 (27.5 to 52.3)	50.4 (36.7 to 68.6)	34.7 (25.3 to 47.0)	2.10 (1.53 to 2.86)	1.44 (1.05 to 1.96)
2020	50.8 (35.9 to 72.6)	34.5 (24.5 to 48.8)	41.1 (29.4 to 57.8)	28.3 (20.3 to 39.6)	1.98 (1.41 to 2.78)	1.36 (0.98 to 1.90)
2021	50.2 (35.4 to 72.7)	34.0 (24.2 to 48.9)	39.7 (28.4 to 56.6)	27.3 (19.6 to 38.8)	1.97 (1.41 to 2.80)	1.35 (0.97 to 1.92)

Algeria DZA North Africa and Middle East Stillbirth Rate / Neonatal Mortality Rate (SBR/NMR) − 20 weeks
lambda = 0.2 ; zeta = 0.99 ; scale = 5 ; amp = 0.028

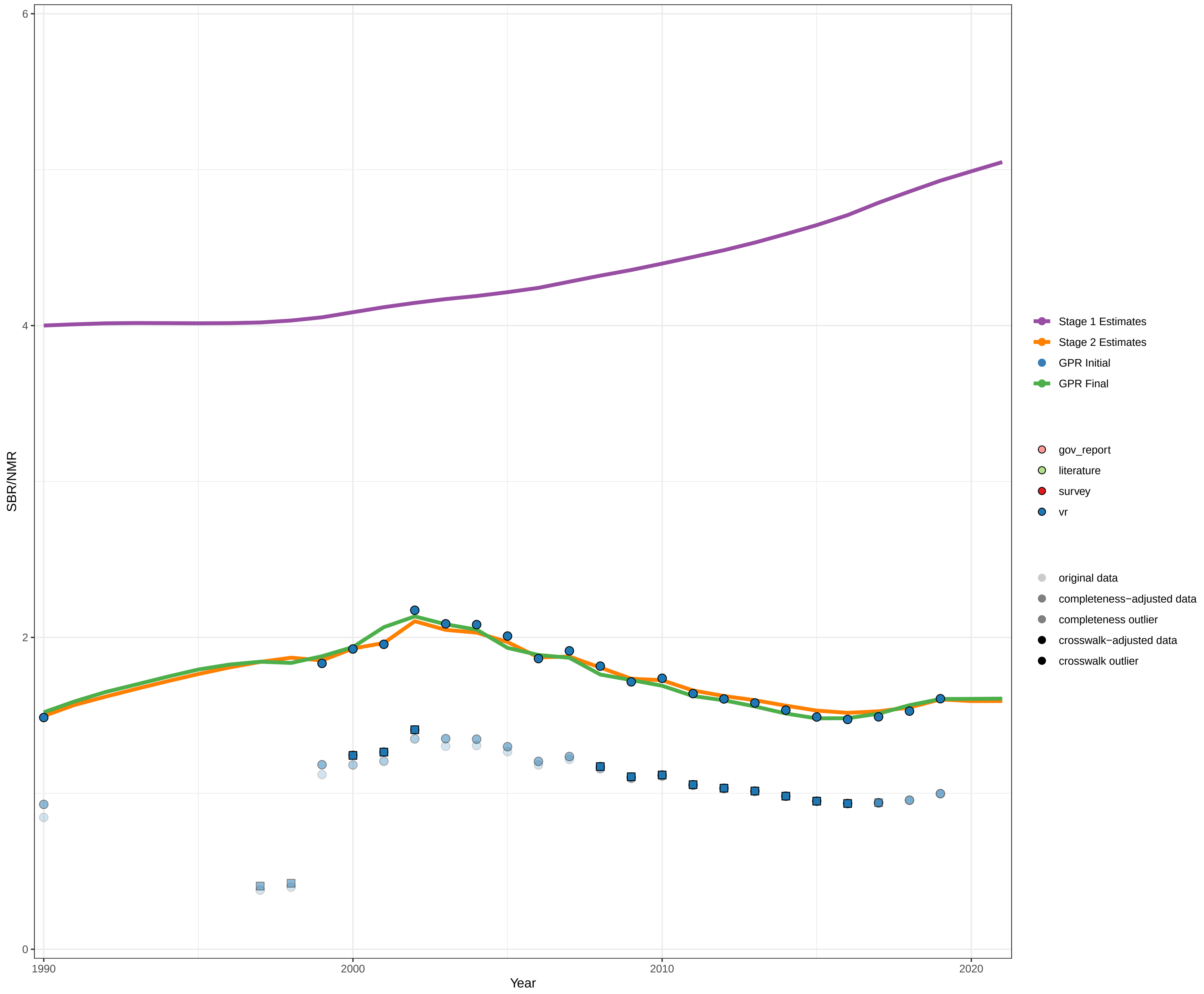

Algeria DZA North Africa and Middle East Stillbirth Rate / Neonatal Mortality Rate (SBR/NMR) − 28 weeks
lambda = 0.2 ; zeta = 0.99 ; scale = 5 ; amp = 0.028

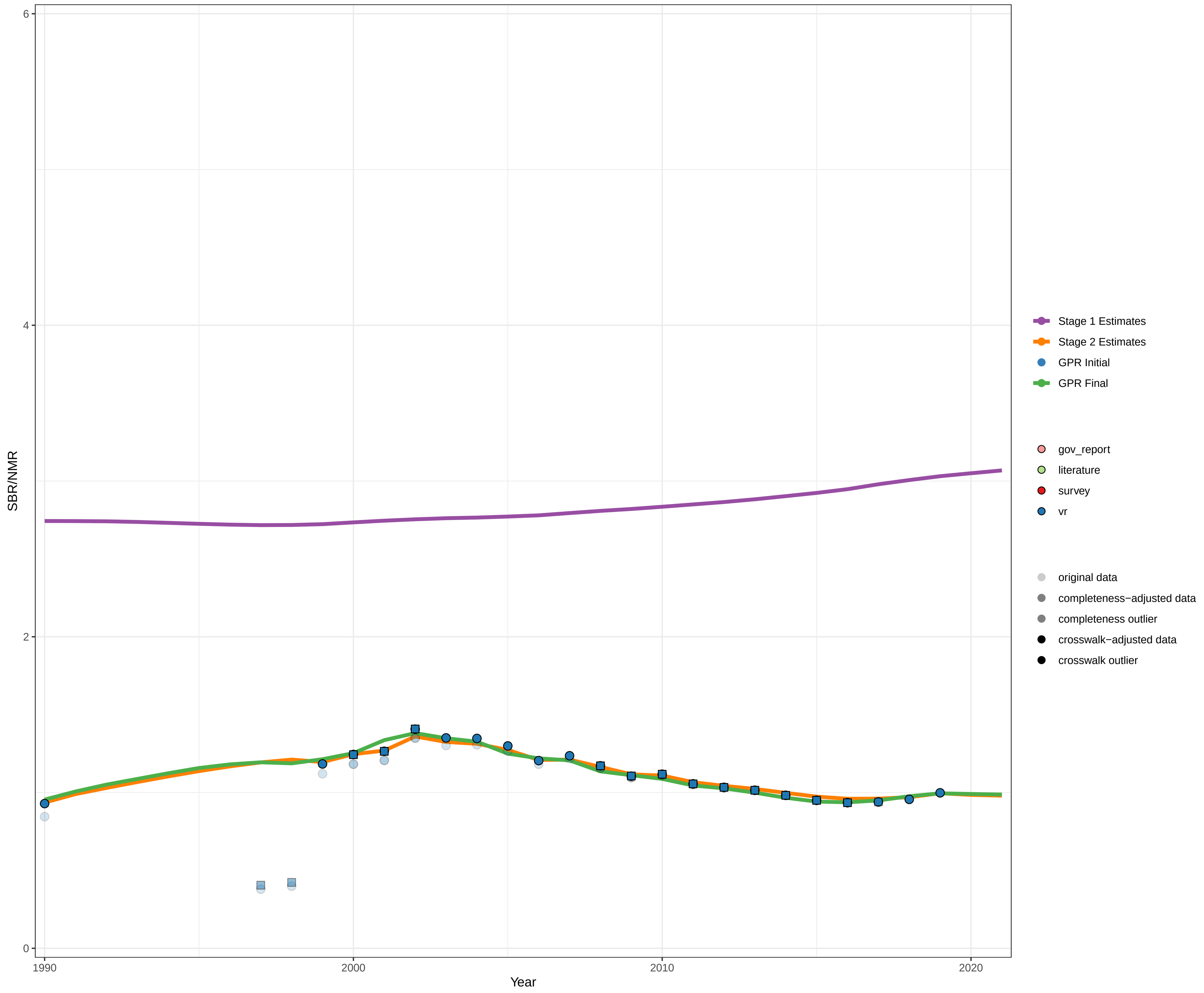

Definition	Years	Outlier	Source.Type	Title
28_weeks	1990, 2000−2019	0	VR	Algeria Demography
28_weeks	1999	0	VR	United Nations Demographic Yearbook
28_weeks	1997, 1998, 2017	1	VR	United Nations Demographic Yearbook
28_weeks	2000−2002, 2008−2016	2	VR	United Nations Demographic Yearbook

Algeria DZA North Africa and Middle East Stillbirth Rate (SBR) − 20 weeks
lambda = 0.2 ; zeta = 0.99 ; scale = 5 ; amp = 0.028

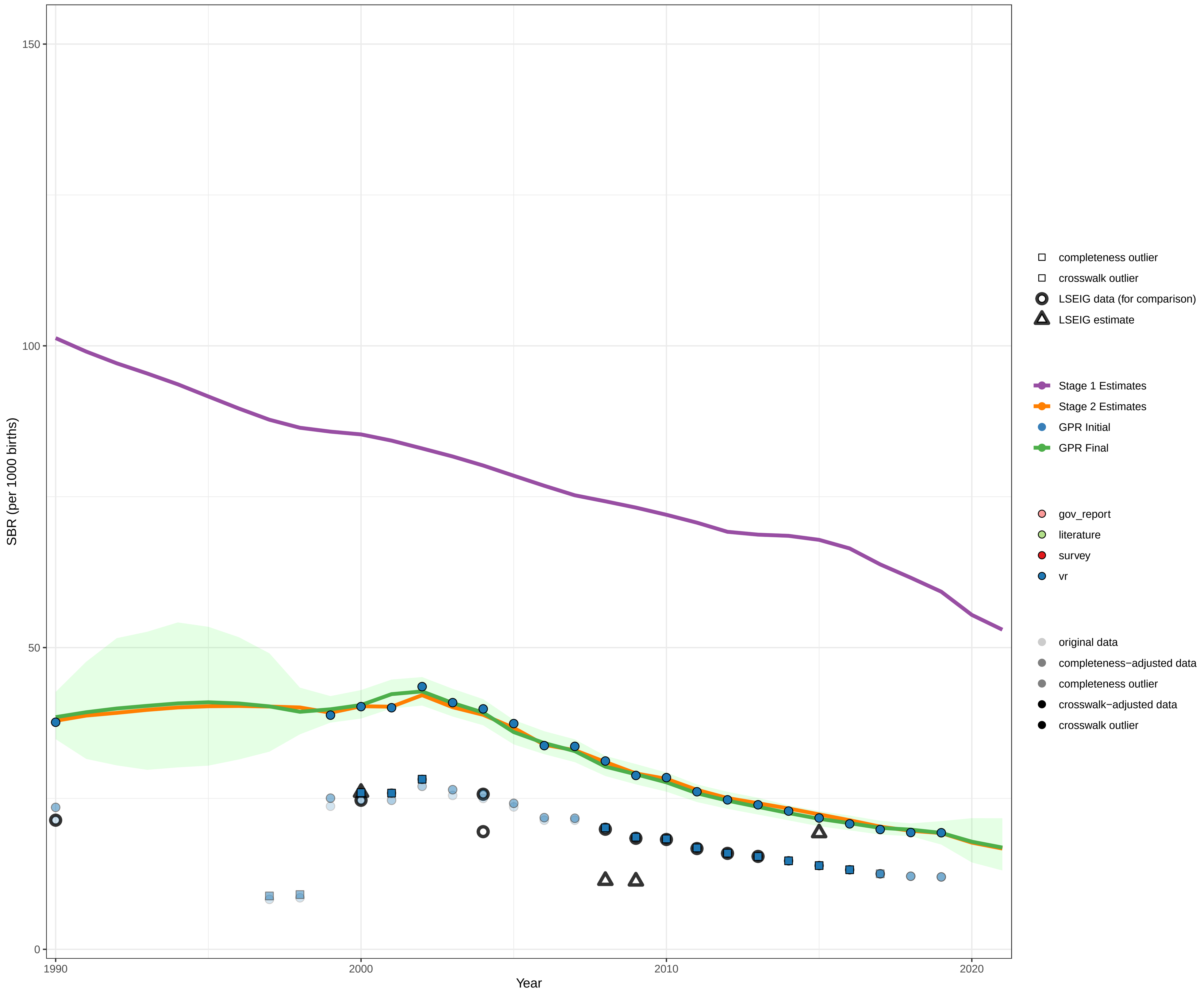

Algeria DZA North Africa and Middle East Stillbirth Rate (SBR) − 28 weeks
lambda = 0.2 ; zeta = 0.99 ; scale = 5 ; amp = 0.028

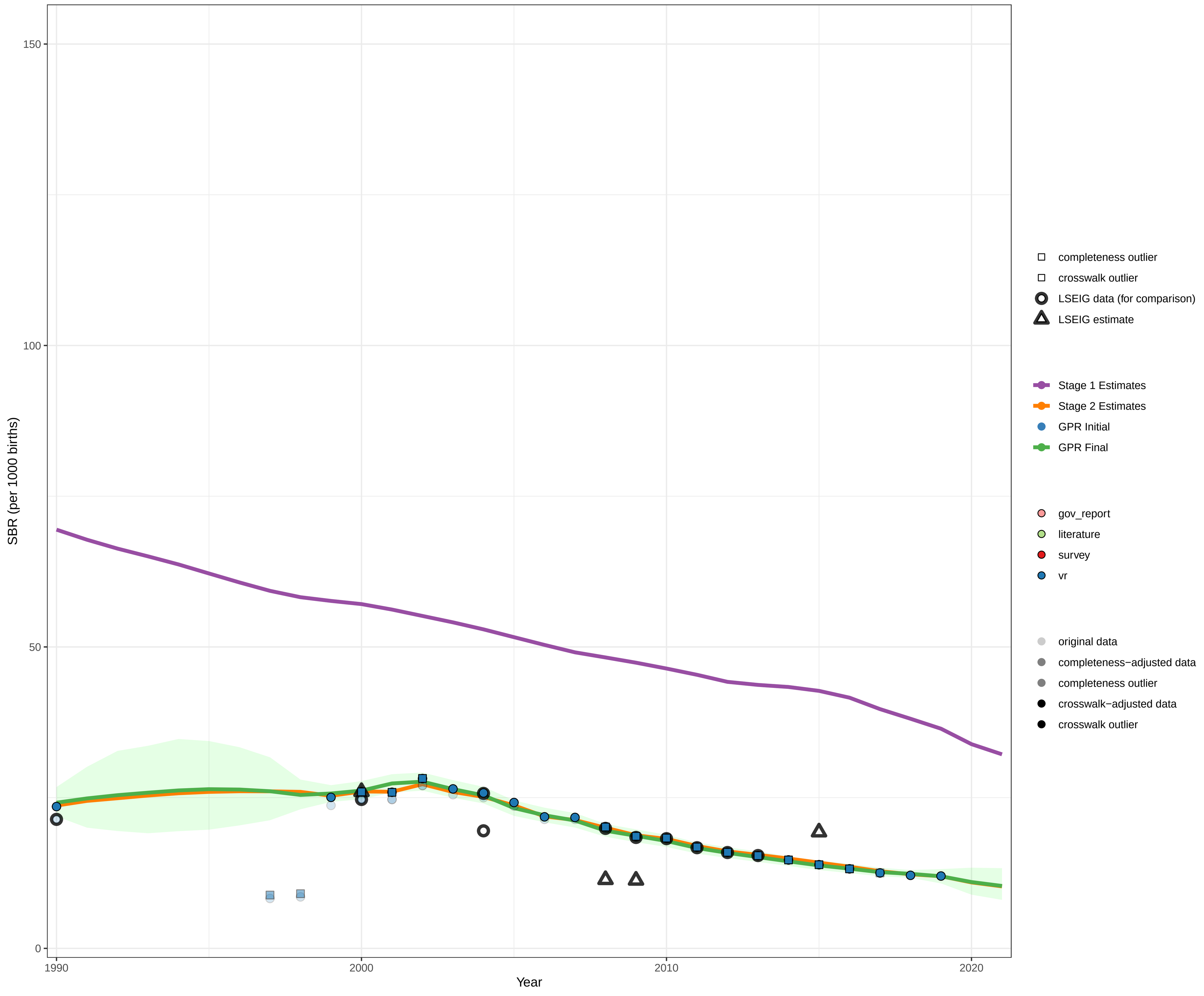

Year	Total Stillbirths (in thousands)	Total Stillbirths (in thousands)	SBR (per 1000 births)	SBR (per 1000 births)	SBR/NMR	SBR/NMR
	20 weeks	28 weeks	20 weeks	28 weeks	20 weeks	28 weeks
1990	30.9 (27.9 to 34.4)	19.1 (17.3 to 21.2)	38.5 (34.8 to 42.7)	24.2 (21.9 to 26.8)	1.52 (1.38 to 1.68)	0.95 (0.86 to 1.06)
1995	31.1 (22.8 to 41.1)	19.7 (14.6 to 25.9)	40.9 (30.4 to 53.4)	26.4 (19.7 to 34.4)	1.79 (1.33 to 2.34)	1.16 (0.86 to 1.51)
2000	27.6 (26.0 to 29.3)	17.6 (16.6 to 18.6)	40.5 (38.2 to 43.0)	26.2 (24.7 to 27.7)	1.94 (1.83 to 2.06)	1.25 (1.18 to 1.33)
2005	27.1 (25.5 to 28.7)	17.3 (16.3 to 18.3)	36.0 (34.0 to 38.0)	23.3 (22.0 to 24.5)	1.93 (1.82 to 2.04)	1.25 (1.18 to 1.32)
2010	25.7 (24.2 to 27.2)	16.3 (15.4 to 17.3)	27.7 (26.1 to 29.3)	17.8 (16.8 to 18.8)	1.69 (1.60 to 1.79)	1.09 (1.03 to 1.15)
2015	22.3 (21.0 to 23.6)	14.1 (13.3 to 14.9)	21.6 (20.4 to 22.9)	13.8 (13.0 to 14.6)	1.48 (1.40 to 1.57)	0.94 (0.89 to 1.00)
2020	17.0 (13.7 to 20.8)	10.4 (8.4 to 12.7)	17.8 (14.4 to 21.7)	11.0 (8.9 to 13.4)	1.61 (1.30 to 1.96)	0.99 (0.80 to 1.21)
2021	15.6 (12.0 to 20.2)	9.5 (7.4 to 12.2)	16.9 (13.1 to 21.7)	10.4 (8.0 to 13.3)	1.61 (1.25 to 2.07)	0.99 (0.77 to 1.27)

Bahrain BHR North Africa and Middle East Stillbirth Rate / Neonatal Mortality Rate (SBR/NMR) − 20 weeks
lambda = 0.4 ; zeta = 0.9 ; scale = 10 ; amp = 0.028

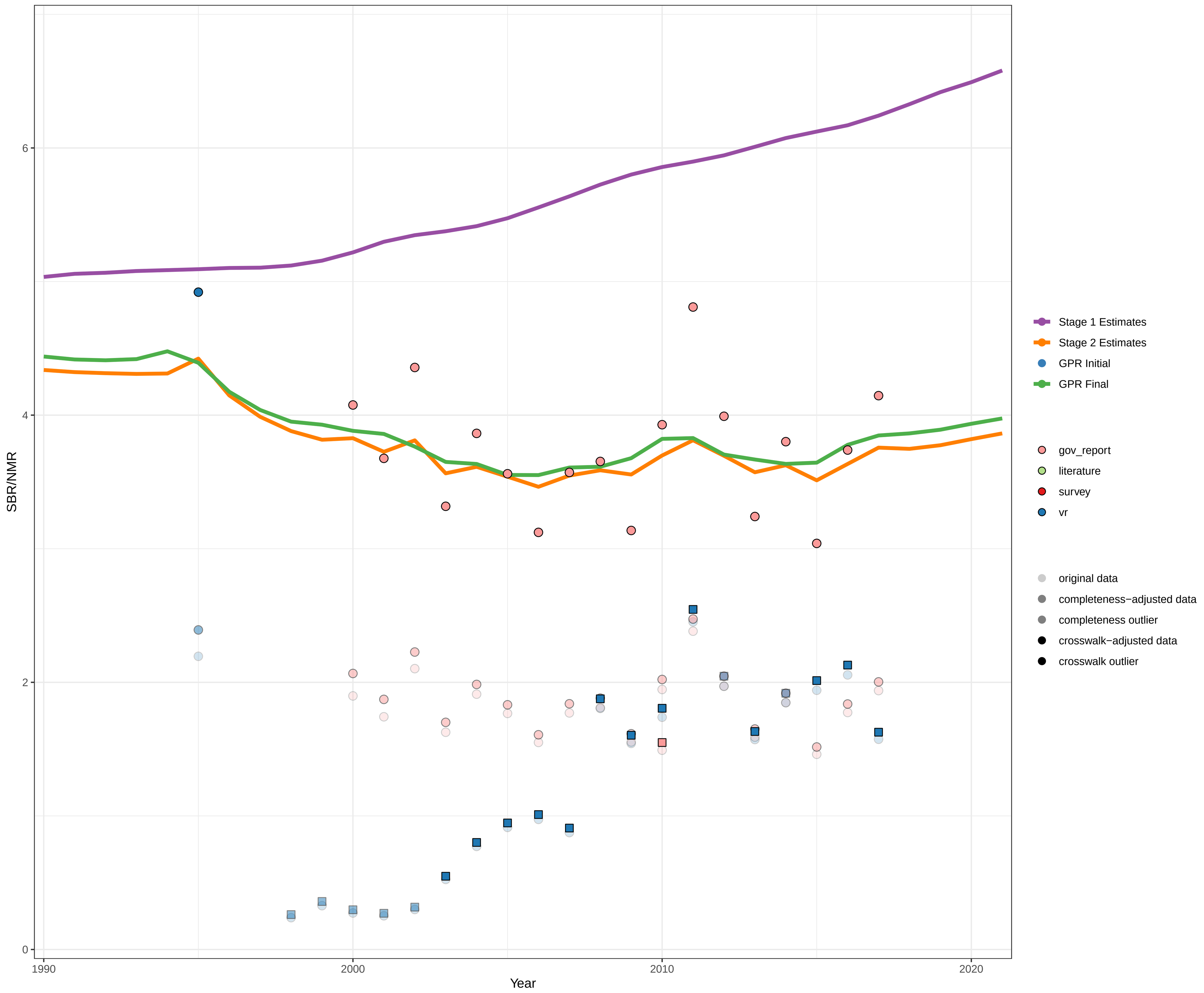

Bahrain BHR North Africa and Middle East Stillbirth Rate / Neonatal Mortality Rate (SBR/NMR) − 28 weeks
lambda = 0.4 ; zeta = 0.9 ; scale = 10 ; amp = 0.028

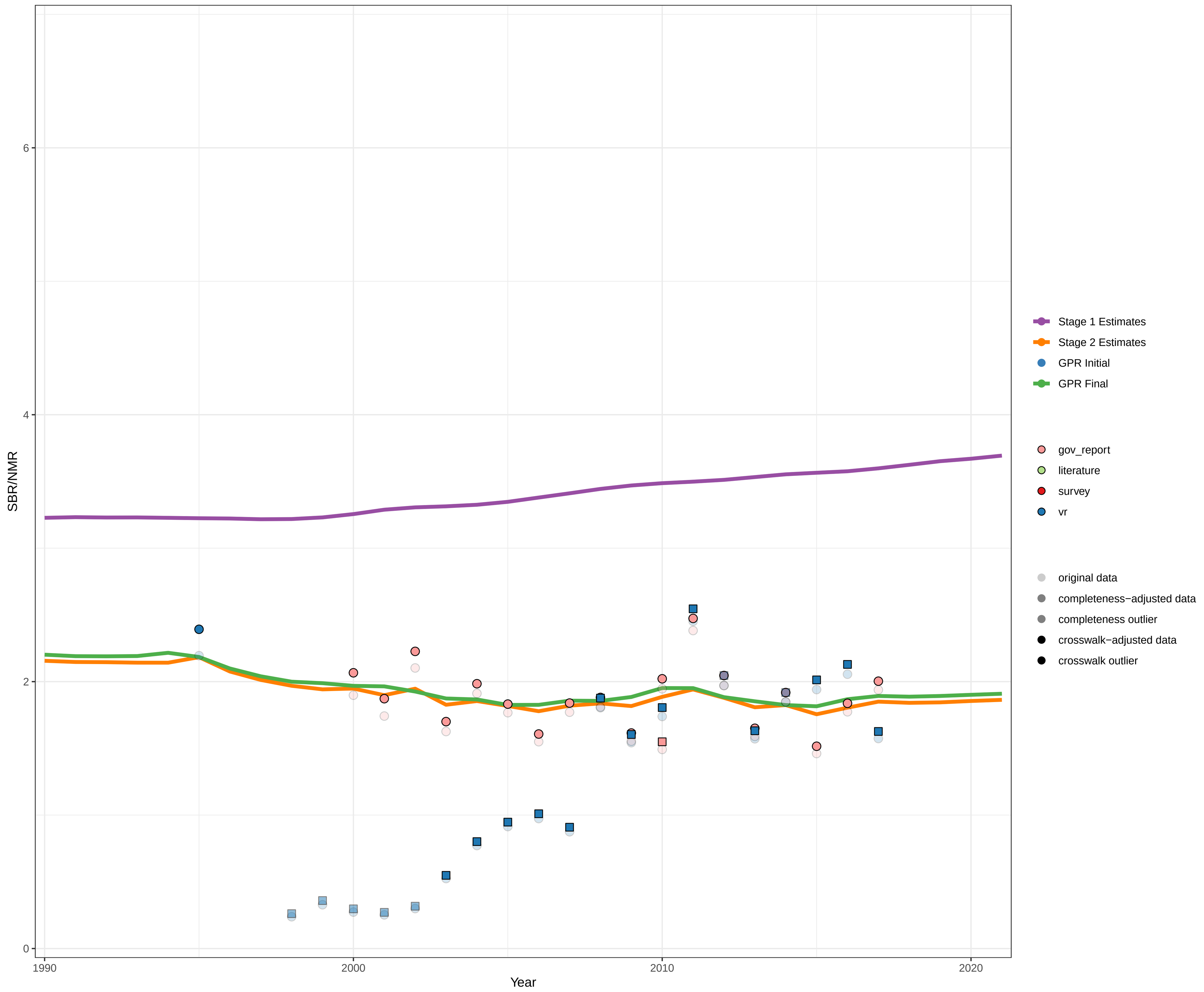

Definition	Years	Outlier	Source.Type	Title
28_weeks	1995	0	VR	United Nations Demographic Yearbook
28_weeks	2000−2017	0	Statistical Report	Bahrain Health Statistics
28_weeks	1998−2002, 2012, 2014	1	VR	United Nations Demographic Yearbook
28_weeks	2003−2011, 2013, 2015−2017	2	VR	United Nations Demographic Yearbook
28_weeks	2010	2	Statistical Report	Bahrain Health Statistics

Bahrain BHR North Africa and Middle East Stillbirth Rate (SBR) − 20 weeks
lambda = 0.4 ; zeta = 0.9 ; scale = 10 ; amp = 0.028

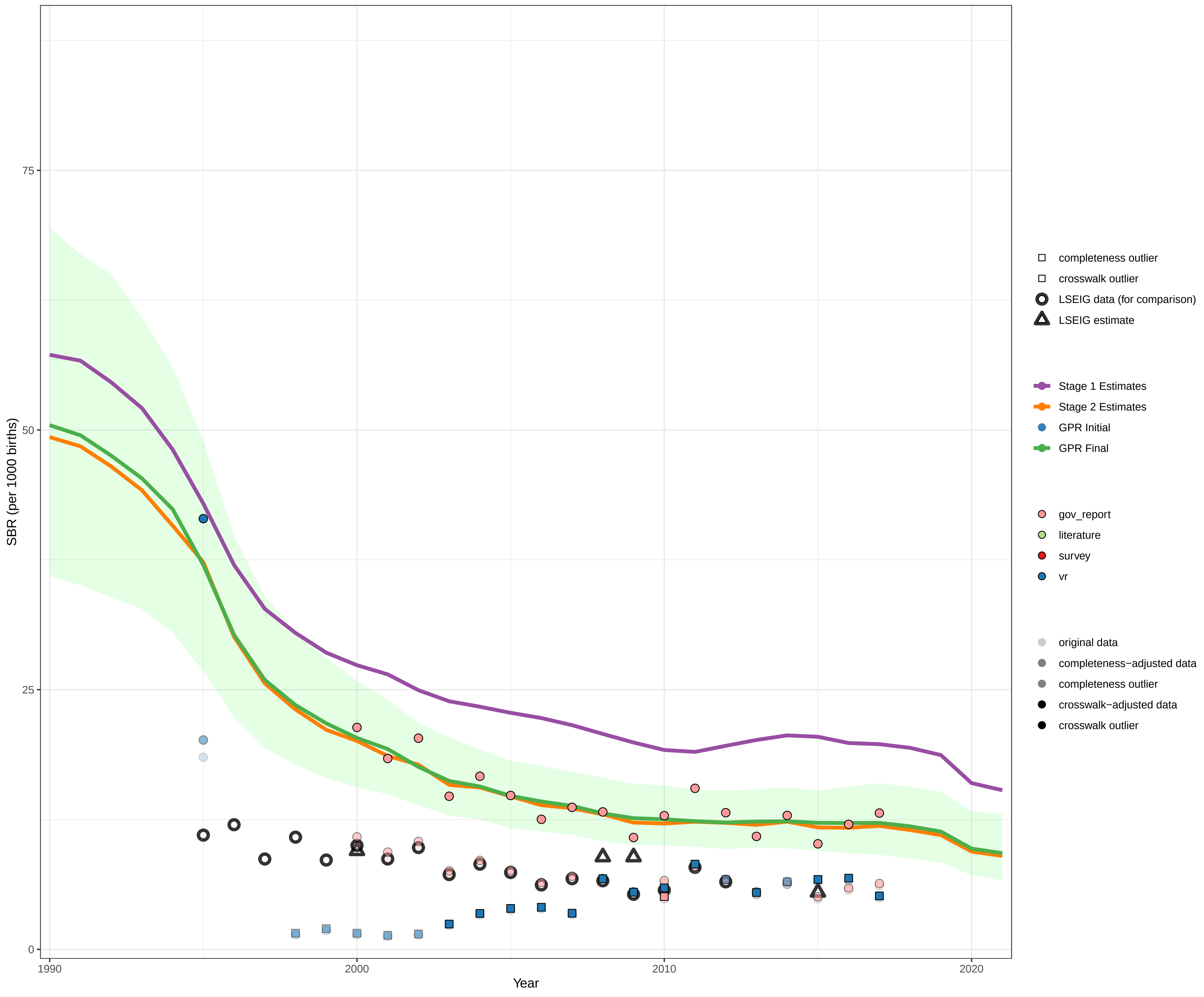

Bahrain BHR North Africa and Middle East Stillbirth Rate (SBR) − 28 weeks
lambda = 0.4 ; zeta = 0.9 ; scale = 10 ; amp = 0.028

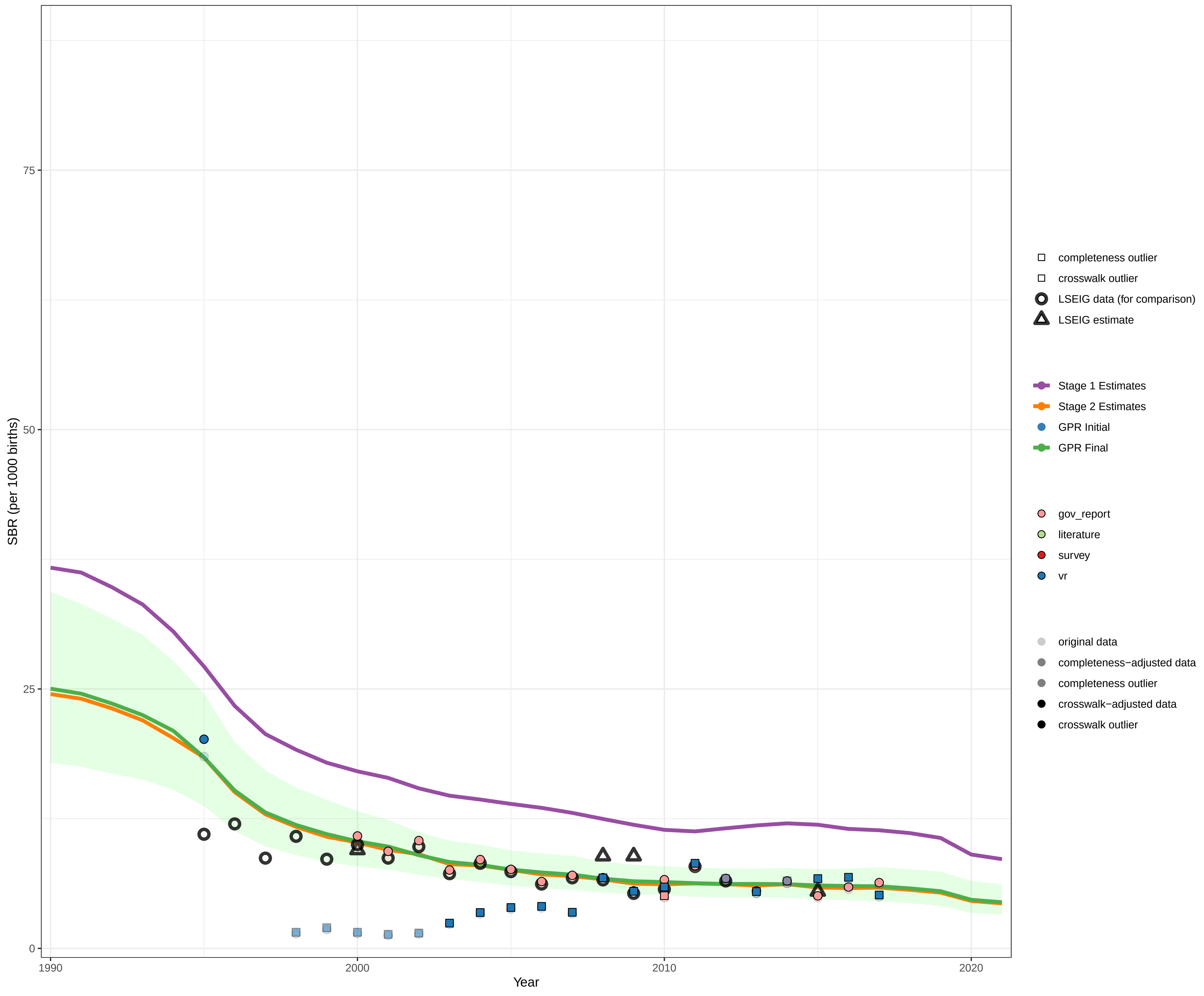

Year	Total Stillbirths (in thousands)	Total Stillbirths (in thousands)	SBR (per 1000 births)	SBR (per 1000 births)	SBR/NMR	SBR/NMR
	20 weeks	28 weeks	20 weeks	28 weeks	20 weeks	28 weeks
1990	0.7 (0.5 to 1.0)	0.3 (0.2 to 0.5)	50.5 (35.9 to 69.5)	25.0 (17.9 to 34.4)	4.44 (3.16 to 6.11)	2.20 (1.57 to 3.02)
1995	0.5 (0.4 to 0.7)	0.3 (0.2 to 0.3)	37.0 (26.8 to 48.9)	18.4 (13.7 to 24.6)	4.39 (3.18 to 5.81)	2.18 (1.63 to 2.92)
2000	0.3 (0.2 to 0.3)	0.1 (0.1 to 0.2)	20.4 (15.6 to 25.8)	10.3 (7.9 to 13.2)	3.88 (2.97 to 4.93)	1.97 (1.51 to 2.53)
2005	0.2 (0.2 to 0.3)	0.1 (0.1 to 0.1)	14.8 (11.7 to 18.2)	7.6 (6.0 to 9.5)	3.55 (2.80 to 4.36)	1.83 (1.45 to 2.27)
2010	0.3 (0.2 to 0.3)	0.1 (0.1 to 0.2)	12.5 (10.0 to 15.8)	6.4 (5.2 to 7.9)	3.82 (3.05 to 4.82)	1.95 (1.57 to 2.42)
2015	0.3 (0.2 to 0.3)	0.1 (0.1 to 0.2)	12.2 (9.5 to 15.3)	6.1 (4.7 to 7.6)	3.64 (2.84 to 4.58)	1.82 (1.42 to 2.29)
2020	0.2 (0.1 to 0.2)	0.1 (0.1 to 0.1)	9.7 (7.1 to 13.3)	4.7 (3.4 to 6.5)	3.94 (2.90 to 5.39)	1.90 (1.39 to 2.64)
2021	0.2 (0.1 to 0.2)	0.1 (0.1 to 0.1)	9.3 (6.6 to 13.0)	4.4 (3.2 to 6.2)	3.98 (2.85 to 5.59)	1.91 (1.39 to 2.66)

Egypt EGY North Africa and Middle East Stillbirth Rate / Neonatal Mortality Rate (SBR/NMR) − 20 weeks
lambda = 0.6 ; zeta = 0.8 ; scale = 15 ; amp = 0.028

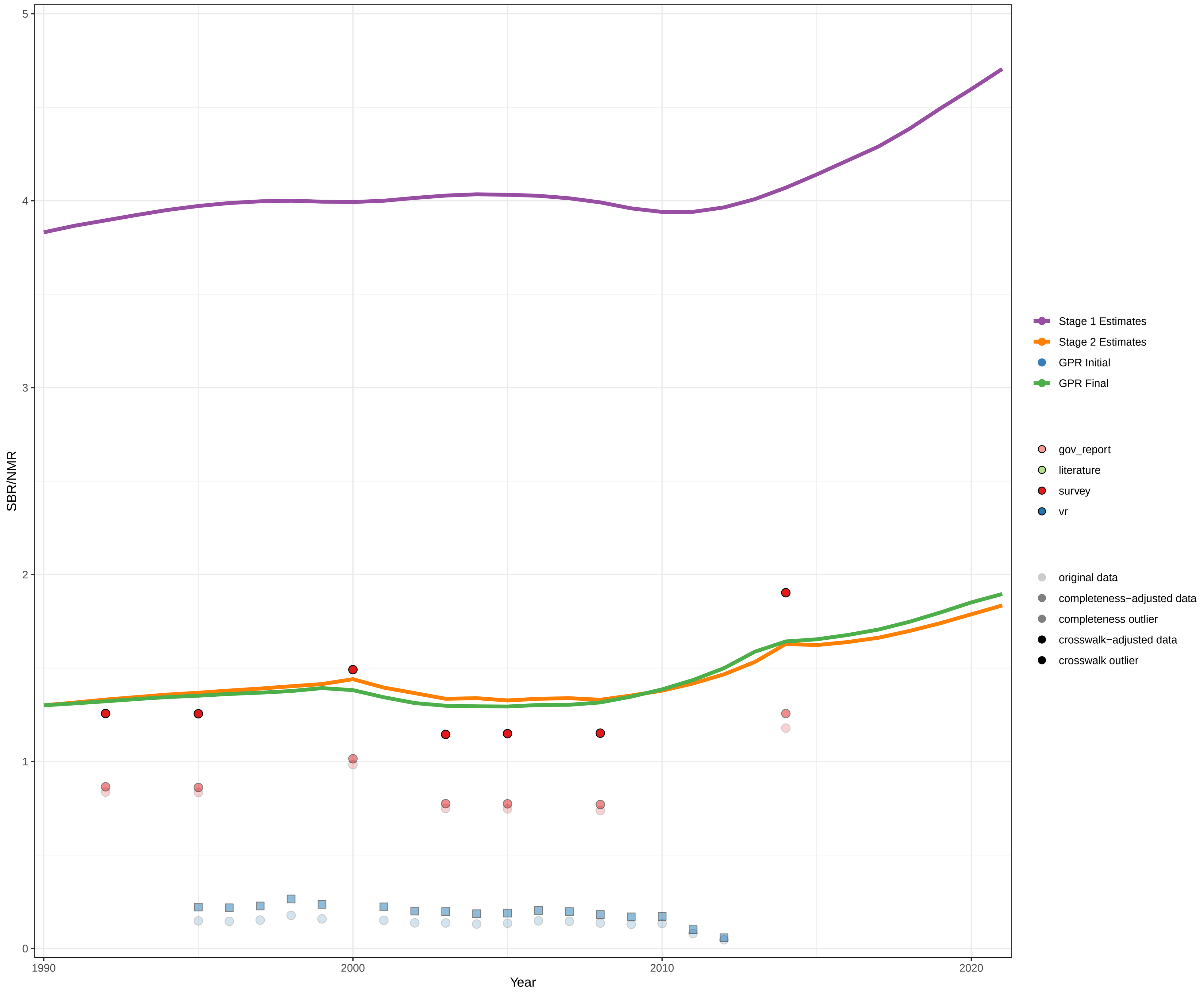

Egypt EGY North Africa and Middle East Stillbirth Rate / Neonatal Mortality Rate (SBR/NMR) − 28 weeks
lambda = 0.6 ; zeta = 0.8 ; scale = 15 ; amp = 0.028

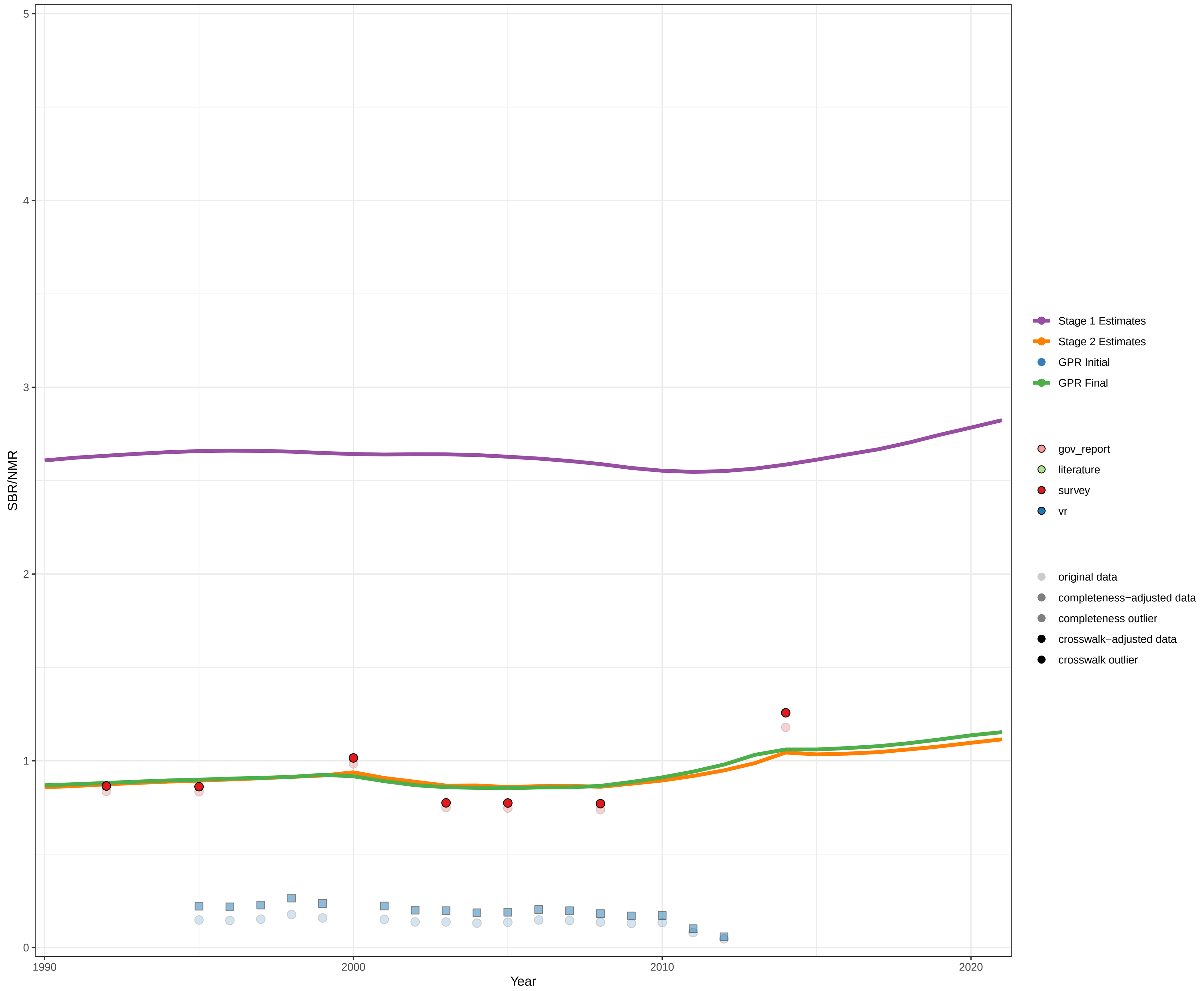

Definition	Years	Outlier	Source.Type	Title
28_weeks	1992	0	Survey	Egypt Demographic and Health Survey 1992−1993
28_weeks	1995	0	Survey	Egypt Demographic and Health Survey 1995−1996
28_weeks	2000	0	Survey	Egypt Demographic and Health Survey 2000
28_weeks	2003	0	Survey	Egypt Interim Demographic and Health Survey 2003
28_weeks	2005	0	Survey	Egypt Demographic and Health Survey 2005
28_weeks	2008	0	Survey	Egypt Demographic and Health Survey 2008
28_weeks	2014	0	Survey	Egypt Demographic and Health Survey 2014
28_weeks	1995−1999, 2001−2012	1	VR	United Nations Demographic Yearbook

Egypt EGY North Africa and Middle East Stillbirth Rate (SBR) − 20 weeks
lambda = 0.6 ; zeta = 0.8 ; scale = 15 ; amp = 0.028

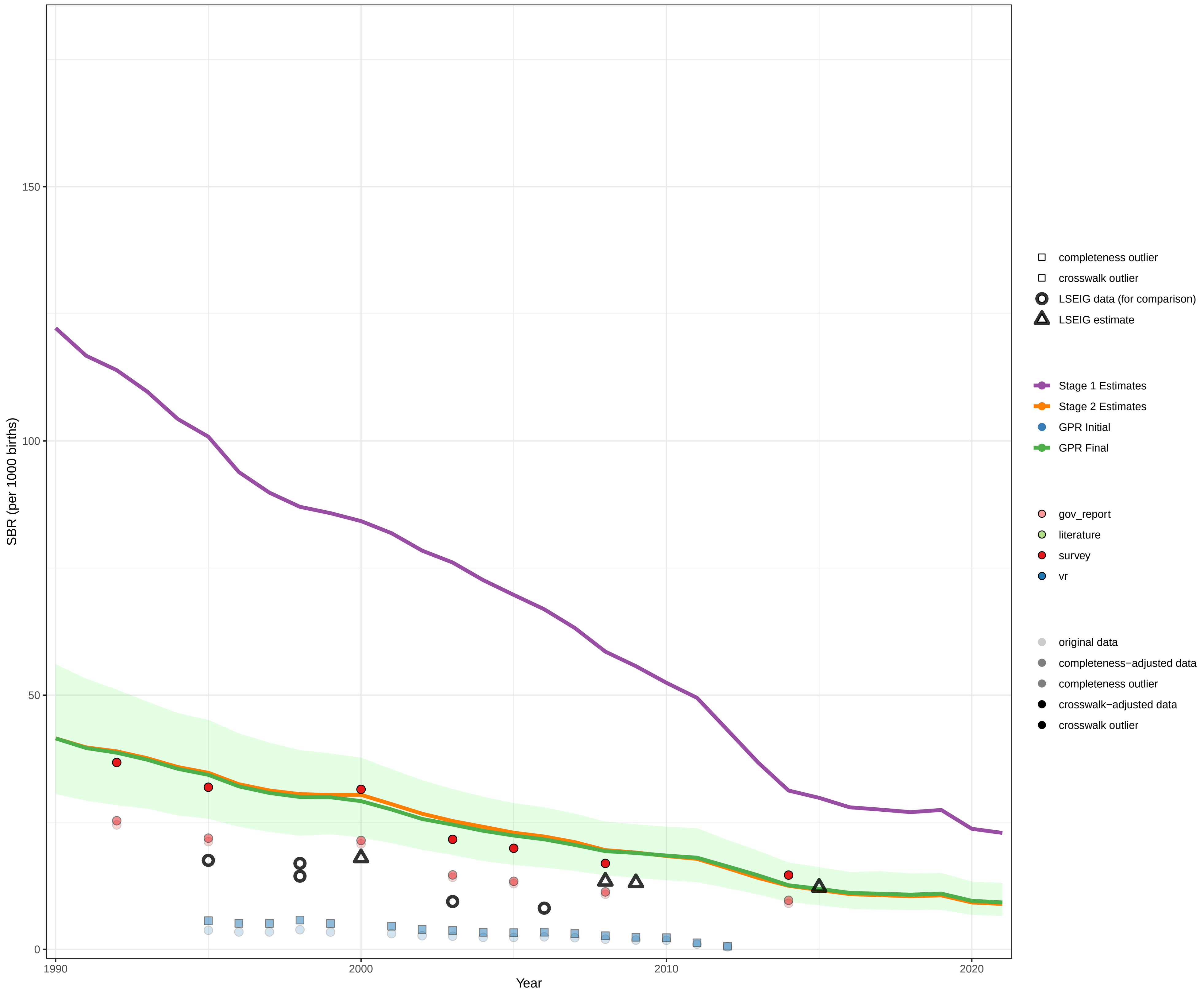

Egypt EGY North Africa and Middle East Stillbirth Rate (SBR) − 28 weeks
lambda = 0.6 ; zeta = 0.8 ; scale = 15 ; amp = 0.028

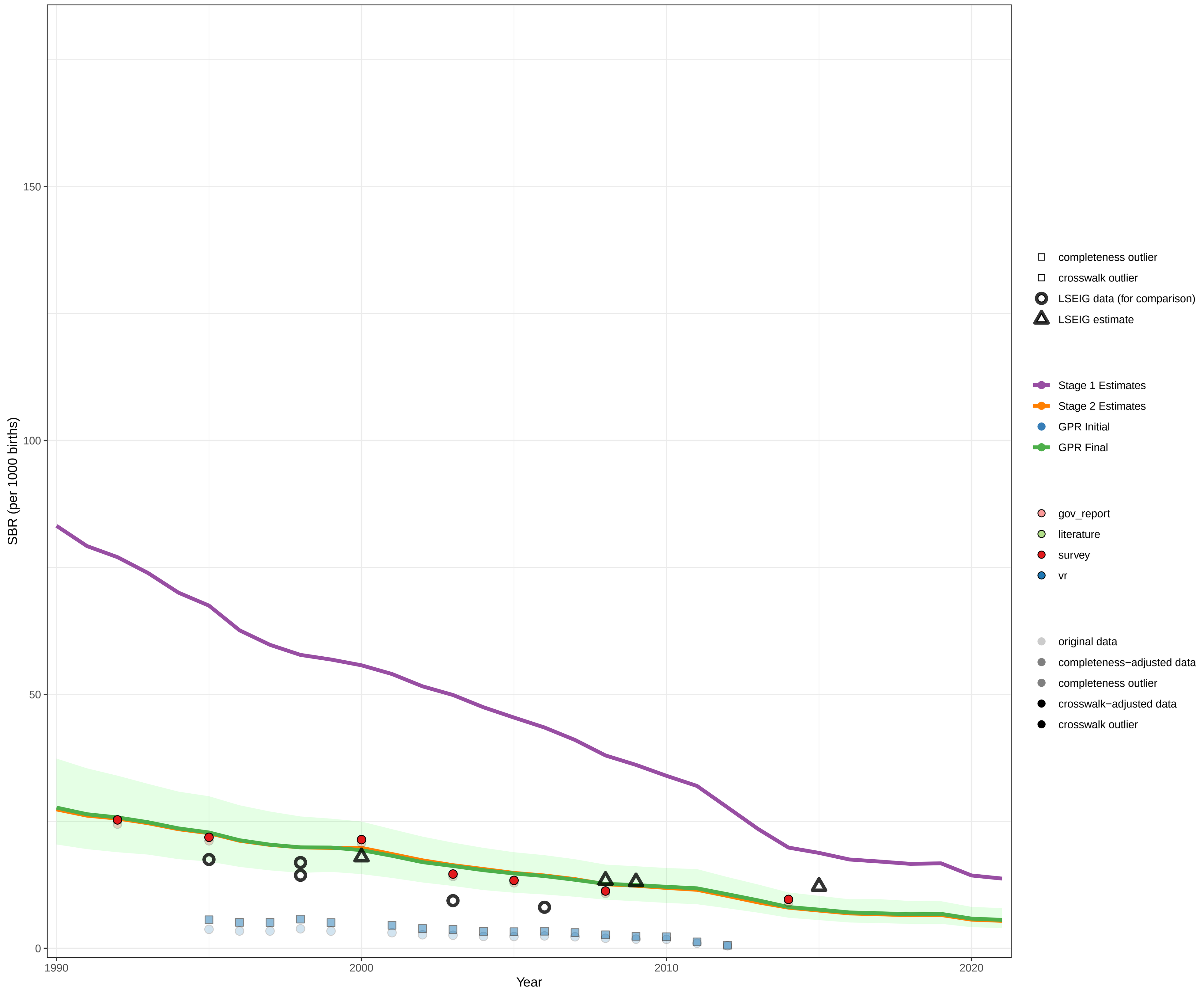

Year	Total Stillbirths (in thousands)	Total Stillbirths (in thousands)	SBR (per 1000 births)	SBR (per 1000 births)	SBR/NMR	SBR/NMR
	20 weeks	28 weeks	20 weeks	28 weeks	20 weeks	28 weeks
1990	82.4 (59.9 to 113.0)	54.3 (39.7 to 73.8)	41.5 (30.5 to 56.1)	27.7 (20.5 to 37.4)	1.30 (0.96 to 1.76)	0.87 (0.64 to 1.17)
1995	62.7 (46.4 to 83.4)	41.2 (30.7 to 54.4)	34.3 (25.7 to 45.2)	22.8 (17.1 to 30.0)	1.35 (1.01 to 1.78)	0.90 (0.67 to 1.18)
2000	59.0 (44.1 to 76.9)	38.7 (29.1 to 50.3)	29.2 (22.0 to 37.7)	19.3 (14.6 to 25.0)	1.38 (1.04 to 1.79)	0.92 (0.69 to 1.18)
2005	50.3 (37.0 to 65.0)	32.9 (24.3 to 42.4)	22.4 (16.6 to 28.8)	14.8 (11.0 to 18.9)	1.29 (0.96 to 1.66)	0.85 (0.63 to 1.09)
2010	56.9 (41.7 to 74.9)	37.2 (27.4 to 48.7)	18.4 (13.6 to 24.1)	12.1 (9.0 to 15.8)	1.39 (1.02 to 1.81)	0.91 (0.67 to 1.19)
2015	37.8 (27.4 to 51.5)	24.1 (17.5 to 32.8)	11.9 (8.7 to 16.2)	7.6 (5.6 to 10.3)	1.65 (1.20 to 2.25)	1.06 (0.77 to 1.44)
2020	25.8 (18.2 to 36.2)	15.8 (11.2 to 22.0)	9.5 (6.8 to 13.3)	5.9 (4.2 to 8.2)	1.85 (1.31 to 2.59)	1.14 (0.81 to 1.58)
2021	24.3 (17.3 to 34.6)	14.7 (10.5 to 20.9)	9.2 (6.6 to 13.1)	5.6 (4.0 to 7.9)	1.90 (1.35 to 2.69)	1.15 (0.82 to 1.63)

Iran (Islamic Republic of) IRN North Africa and Middle East Stillbirth Rate / Neonatal Mortality Rate (SBR/NMR) − 20 weeks
lambda = 0.8 ; zeta = 0.7 ; scale = 15 ; amp = 0.028

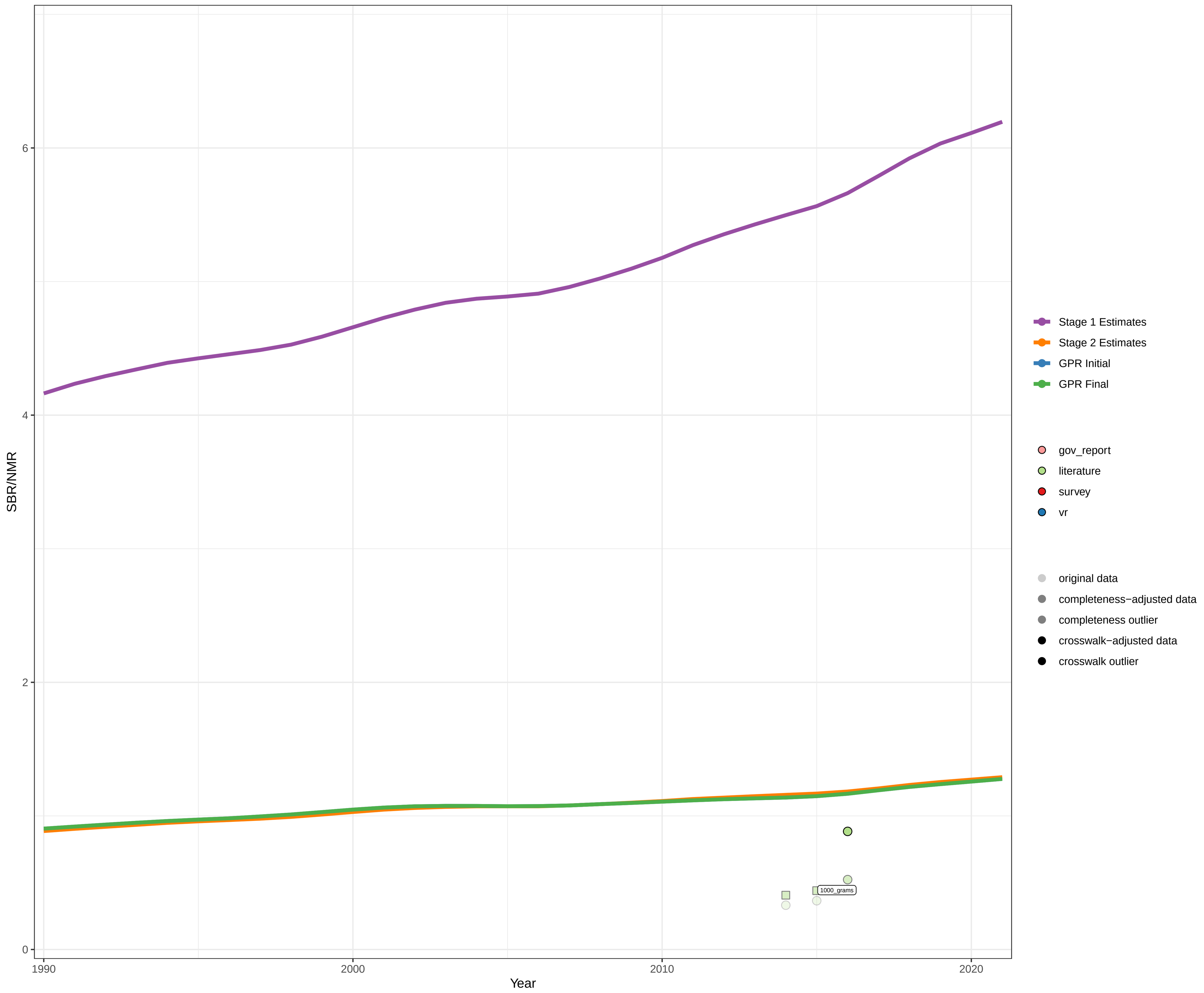

Iran (Islamic Republic of) IRN North Africa and Middle East Stillbirth Rate / Neonatal Mortality Rate (SBR/NMR) − 28 weeks
lambda = 0.8 ; zeta = 0.7 ; scale = 15 ; amp = 0.028

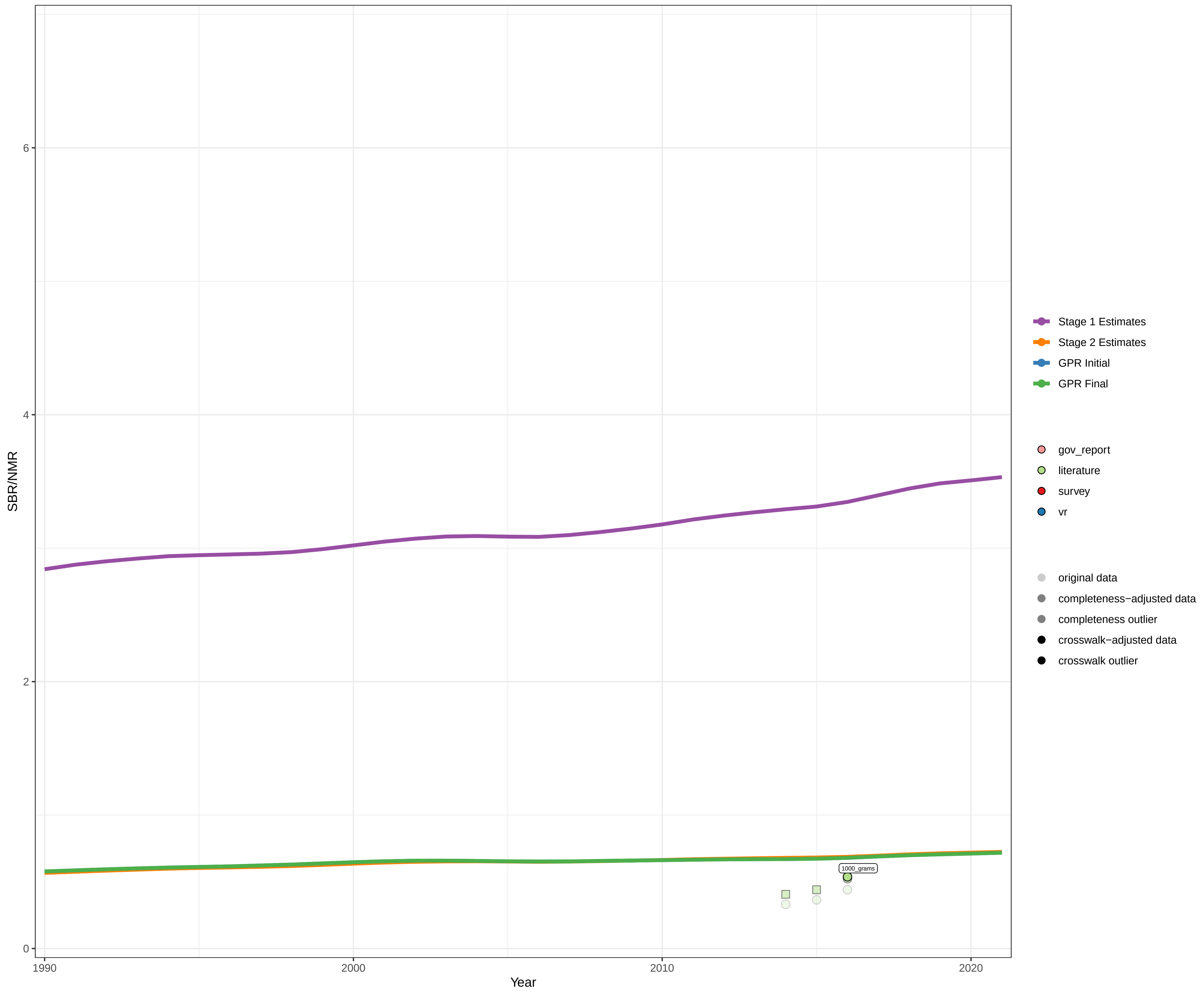

Definition	Years	Outlier	Source.Type	Title
1000_grams	2016	0	Sci Lit	Stillbirth in Iran and associated factors (2014−2016): A population−based study
1000_grams	2014, 2015	1	Sci Lit	Stillbirth in Iran and associated factors (2014−2016): A population−based study

Iran (Islamic Republic of) IRN North Africa and Middle East Stillbirth Rate (SBR) − 20 weeks
lambda = 0.8 ; zeta = 0.7 ; scale = 15 ; amp = 0.028

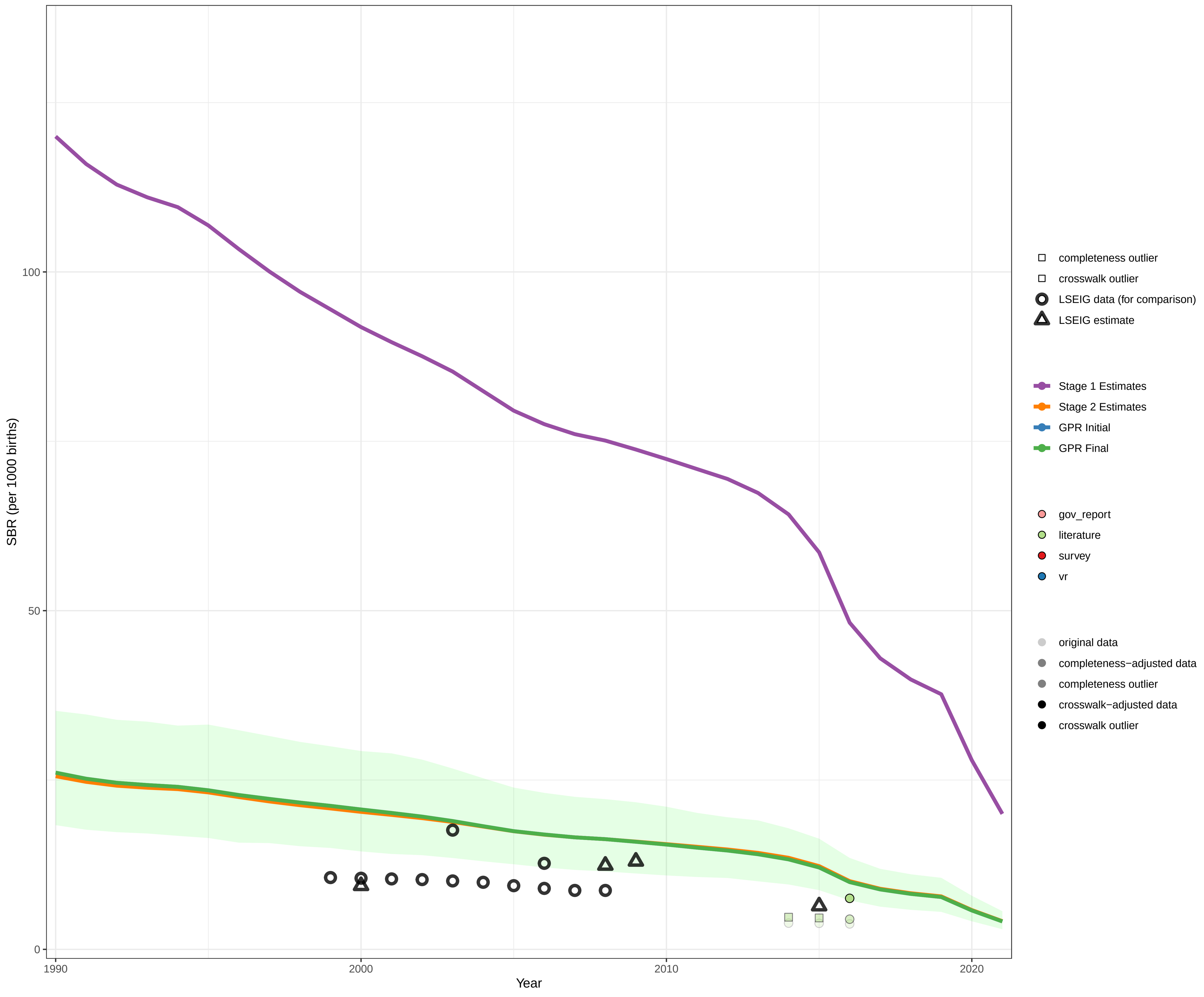

Iran (Islamic Republic of) IRN North Africa and Middle East Stillbirth Rate (SBR) − 28 weeks
lambda = 0.8 ; zeta = 0.7 ; scale = 15 ; amp = 0.028

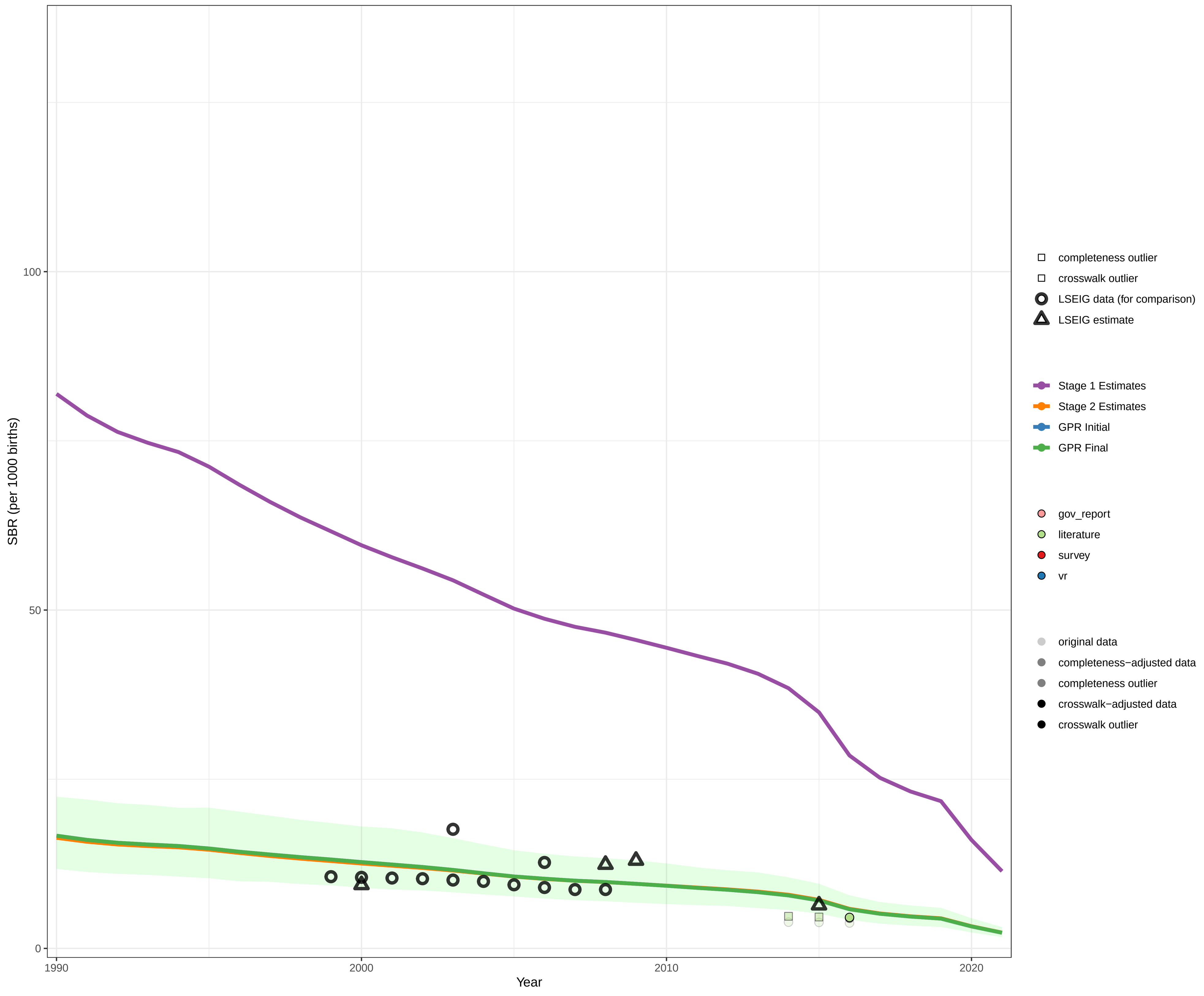

Year	Total Stillbirths (in thousands)	Total Stillbirths (in thousands)	SBR (per 1000 births)	SBR (per 1000 births)	SBR/NMR	SBR/NMR
	20 weeks	28 weeks	20 weeks	28 weeks	20 weeks	28 weeks
1990	41.2 (28.7 to 56.2)	26.1 (18.3 to 35.3)	26.1 (18.3 to 35.2)	16.7 (11.7 to 22.4)	0.90 (0.64 to 1.22)	0.58 (0.41 to 0.78)
1995	29.2 (20.3 to 41.7)	18.2 (12.7 to 25.8)	23.5 (16.4 to 33.2)	14.8 (10.4 to 20.8)	0.97 (0.68 to 1.37)	0.61 (0.43 to 0.86)
2000	23.4 (16.3 to 33.5)	14.3 (10.0 to 20.4)	20.6 (14.5 to 29.3)	12.8 (9.0 to 18.0)	1.05 (0.73 to 1.48)	0.65 (0.45 to 0.91)
2005	20.0 (14.3 to 27.5)	12.1 (8.7 to 16.6)	17.4 (12.6 to 23.9)	10.6 (7.7 to 14.5)	1.07 (0.77 to 1.47)	0.65 (0.47 to 0.89)
2010	21.8 (15.3 to 29.8)	12.9 (9.1 to 17.6)	15.5 (10.9 to 21.1)	9.3 (6.5 to 12.6)	1.11 (0.78 to 1.51)	0.66 (0.47 to 0.90)
2015	18.4 (13.2 to 24.9)	10.7 (7.7 to 14.5)	12.1 (8.7 to 16.3)	7.1 (5.1 to 9.6)	1.15 (0.83 to 1.55)	0.67 (0.49 to 0.91)
2020	6.4 (4.6 to 8.8)	3.6 (2.6 to 4.9)	5.7 (4.1 to 7.9)	3.3 (2.4 to 4.5)	1.26 (0.91 to 1.73)	0.71 (0.52 to 0.98)
2021	4.3 (3.1 to 5.8)	2.4 (1.7 to 3.3)	4.1 (3.0 to 5.6)	2.3 (1.7 to 3.2)	1.28 (0.92 to 1.75)	0.72 (0.52 to 0.98)

Alborz IRN_44864 North Africa and Middle East Stillbirth Rate / Neonatal Mortality Rate (SBR/NMR) − 20 weeks
lambda = 1 ; zeta = 0.6 ; scale = 15 ; amp = 0.028

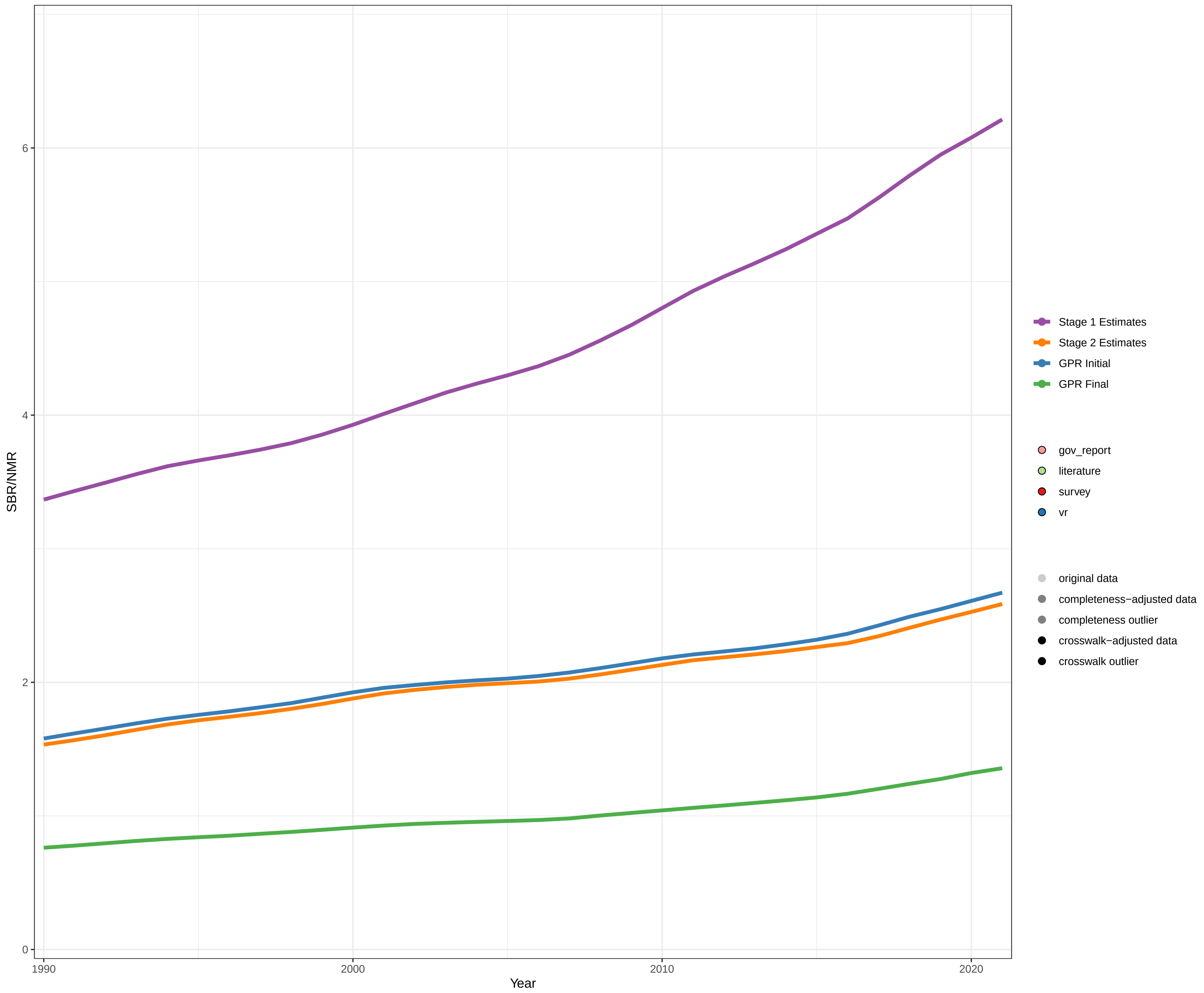

Alborz IRN_44864 North Africa and Middle East Stillbirth Rate / Neonatal Mortality Rate (SBR/NMR) − 28 weeks
lambda = 1 ; zeta = 0.6 ; scale = 15 ; amp = 0.028

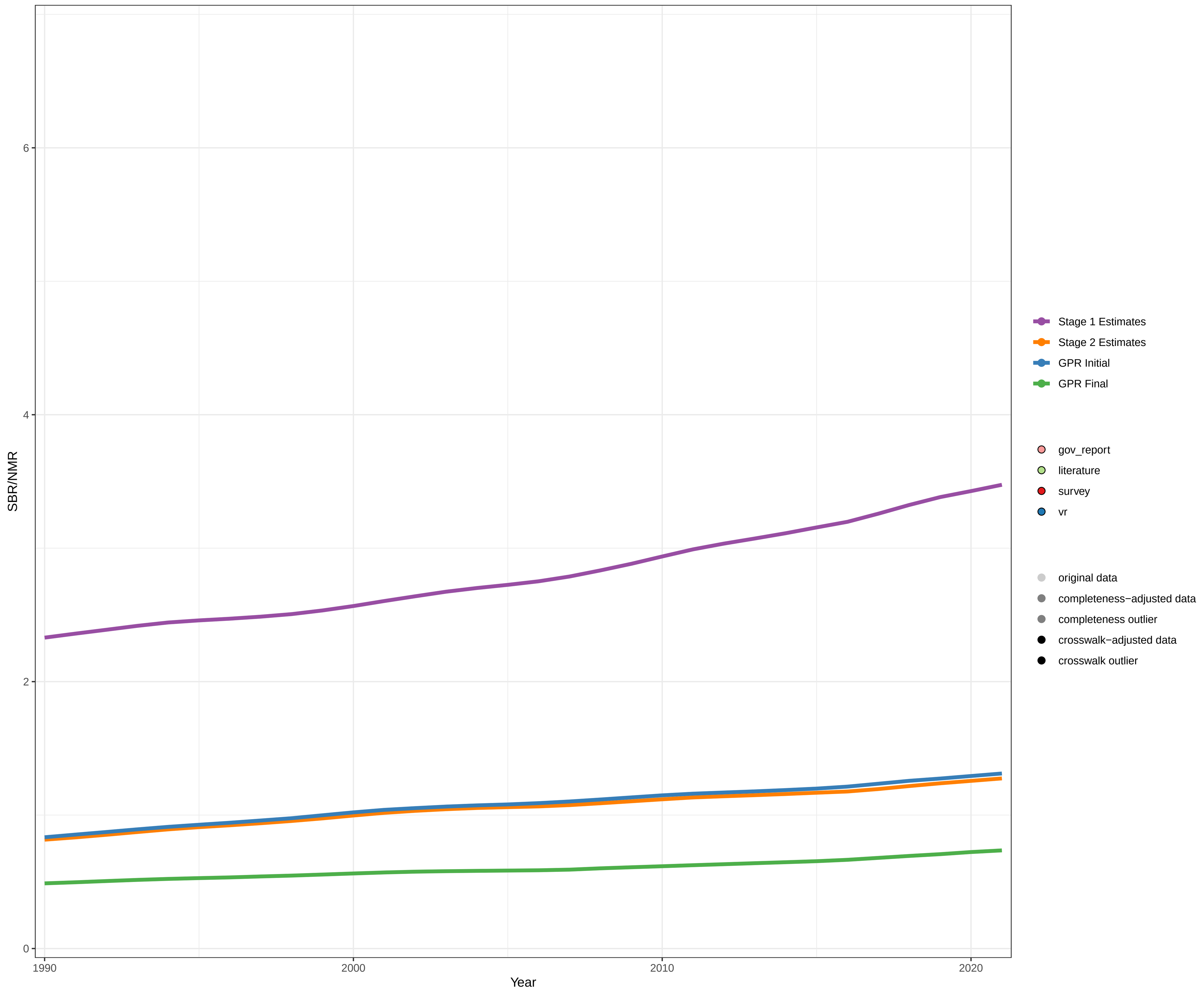

Definition	Years	Outlier	Source.Type	Title

Alborz IRN_44864 North Africa and Middle East Stillbirth Rate (SBR) − 20 weeks
lambda = 1 ; zeta = 0.6 ; scale = 15 ; amp = 0.028

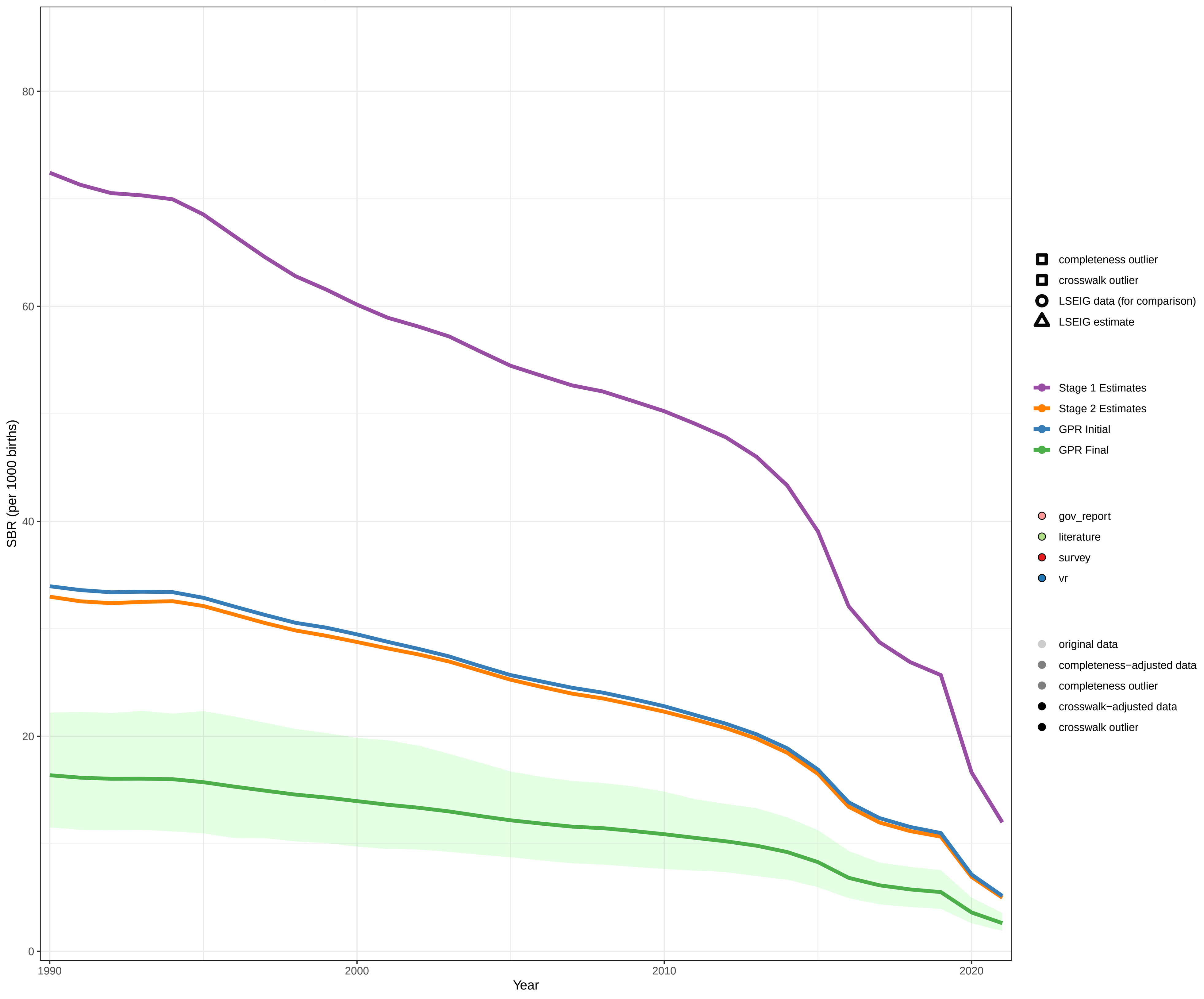

Alborz IRN_44864 North Africa and Middle East Stillbirth Rate (SBR) − 28 weeks
lambda = 1 ; zeta = 0.6 ; scale = 15 ; amp = 0.028

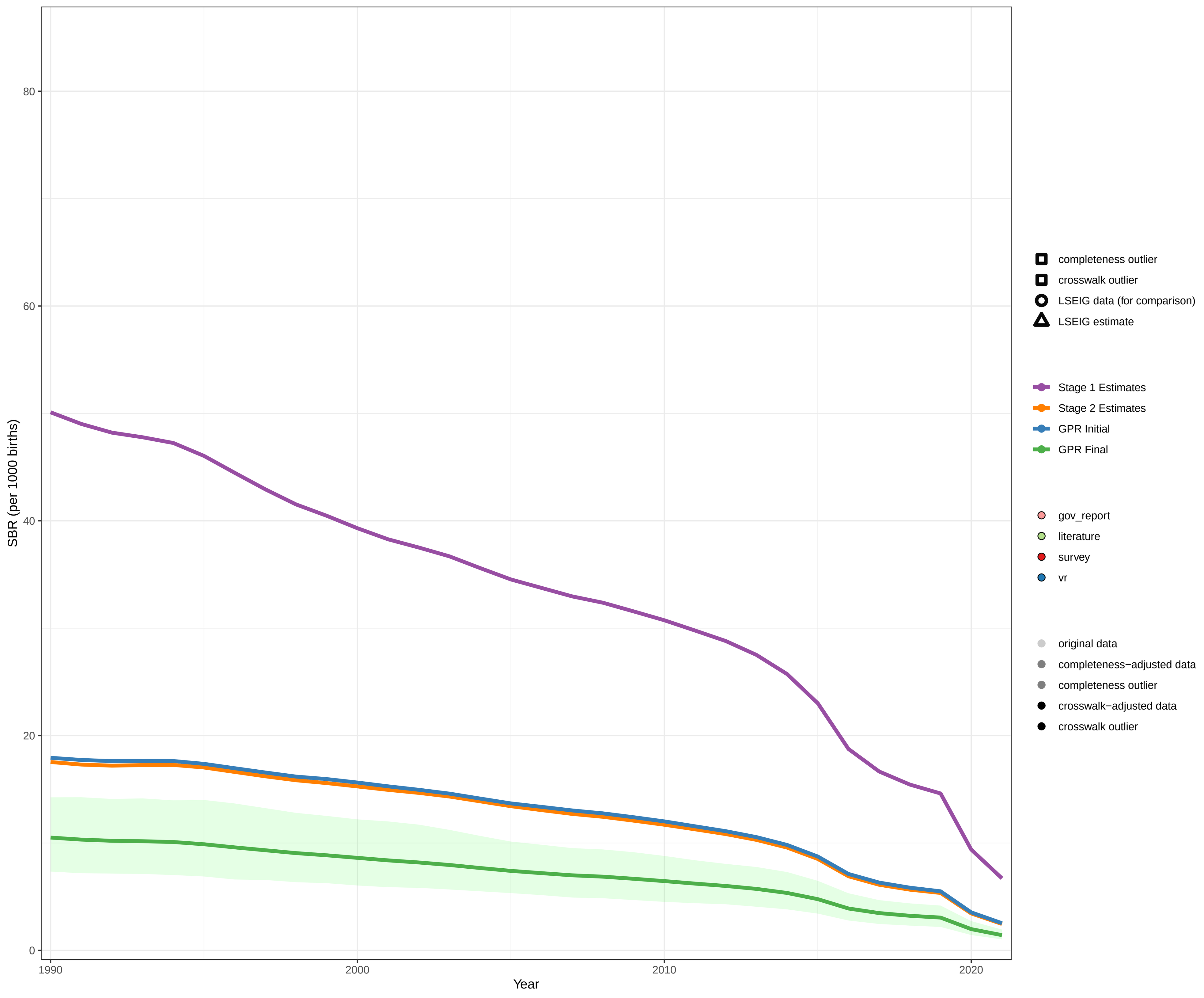

Year	Total Stillbirths (in thousands)	Total Stillbirths (in thousands)	SBR (per 1000 births)	SBR (per 1000 births)	SBR/NMR	SBR/NMR
	20 weeks	28 weeks	20 weeks	28 weeks	20 weeks	28 weeks
1990	0.5 (0.4 to 0.7)	0.3 (0.2 to 0.5)	16.4 (11.5 to 22.2)	10.5 (7.3 to 14.3)	0.76 (0.54 to 1.03)	0.49 (0.34 to 0.66)
1995	0.4 (0.3 to 0.6)	0.3 (0.2 to 0.4)	15.7 (11.0 to 22.4)	9.9 (6.9 to 14.0)	0.84 (0.59 to 1.19)	0.53 (0.37 to 0.75)
2000	0.4 (0.3 to 0.6)	0.2 (0.2 to 0.3)	14.0 (9.7 to 19.9)	8.6 (6.0 to 12.2)	0.91 (0.64 to 1.30)	0.56 (0.39 to 0.80)
2005	0.4 (0.3 to 0.5)	0.2 (0.2 to 0.3)	12.2 (8.8 to 16.7)	7.4 (5.3 to 10.1)	0.96 (0.69 to 1.32)	0.58 (0.42 to 0.80)
2010	0.4 (0.3 to 0.6)	0.2 (0.2 to 0.3)	10.9 (7.7 to 14.9)	6.5 (4.5 to 8.8)	1.04 (0.73 to 1.42)	0.62 (0.43 to 0.84)
2015	0.3 (0.2 to 0.5)	0.2 (0.1 to 0.3)	8.3 (6.0 to 11.3)	4.8 (3.4 to 6.5)	1.14 (0.82 to 1.55)	0.65 (0.47 to 0.89)
2020	0.1 (0.1 to 0.1)	0.1 (0.0 to 0.1)	3.6 (2.6 to 5.0)	2.0 (1.4 to 2.7)	1.32 (0.95 to 1.83)	0.72 (0.52 to 1.00)
2021	0.1 (0.1 to 0.1)	0.0 (0.0 to 0.1)	2.6 (1.9 to 3.6)	1.4 (1.0 to 1.9)	1.36 (0.98 to 1.86)	0.74 (0.53 to 1.00)

Ardebil IRN_44865 North Africa and Middle East Stillbirth Rate / Neonatal Mortality Rate (SBR/NMR) − 20 weeks
lambda = 1 ; zeta = 0.6 ; scale = 15 ; amp = 0.028

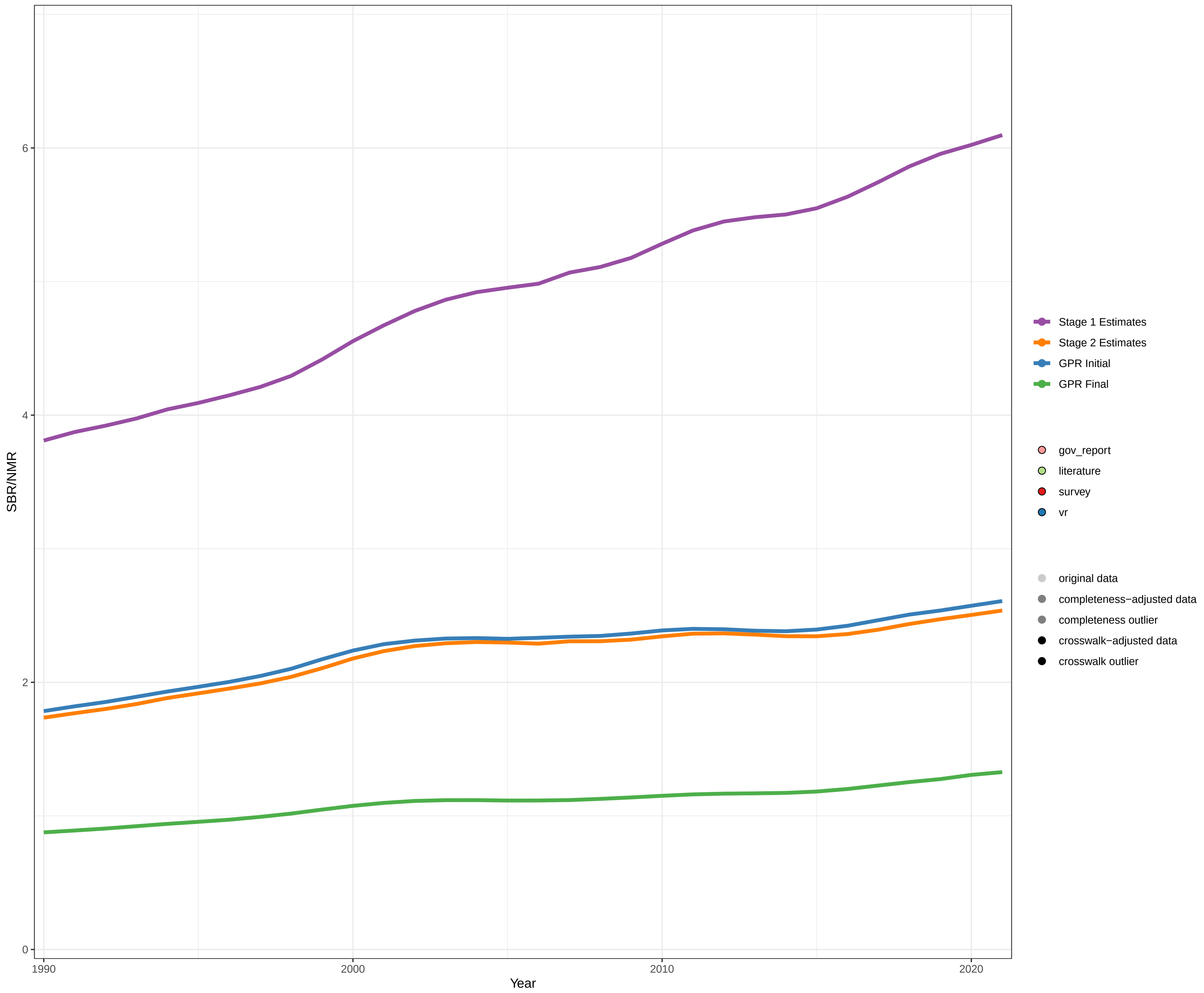

Ardebil IRN_44865 North Africa and Middle East Stillbirth Rate / Neonatal Mortality Rate (SBR/NMR) − 28 weeks
lambda = 1 ; zeta = 0.6 ; scale = 15 ; amp = 0.028

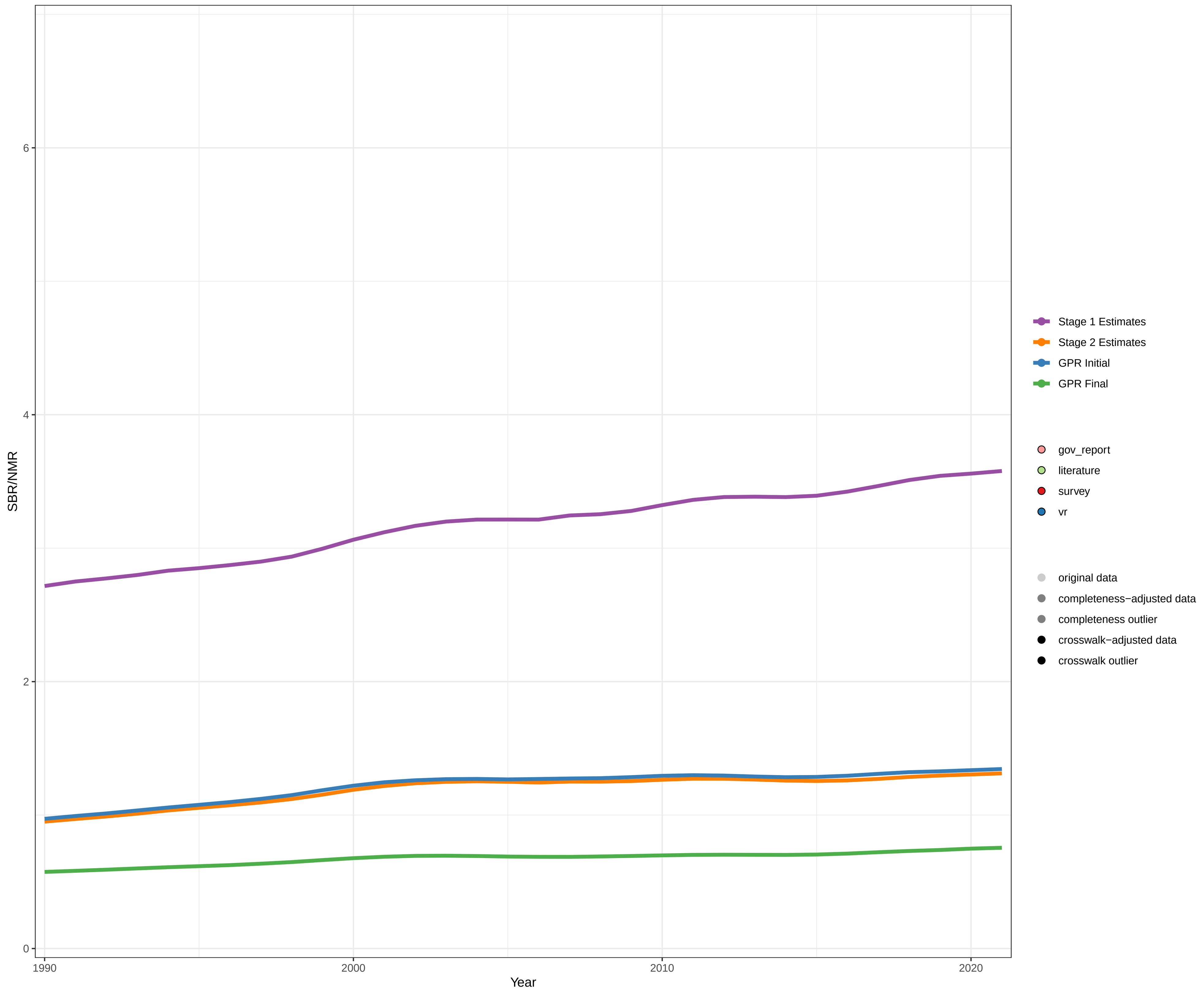

Definition	Years	Outlier	Source.Type	Title

Ardebil IRN_44865 North Africa and Middle East Stillbirth Rate (SBR) − 20 weeks
lambda = 1 ; zeta = 0.6 ; scale = 15 ; amp = 0.028

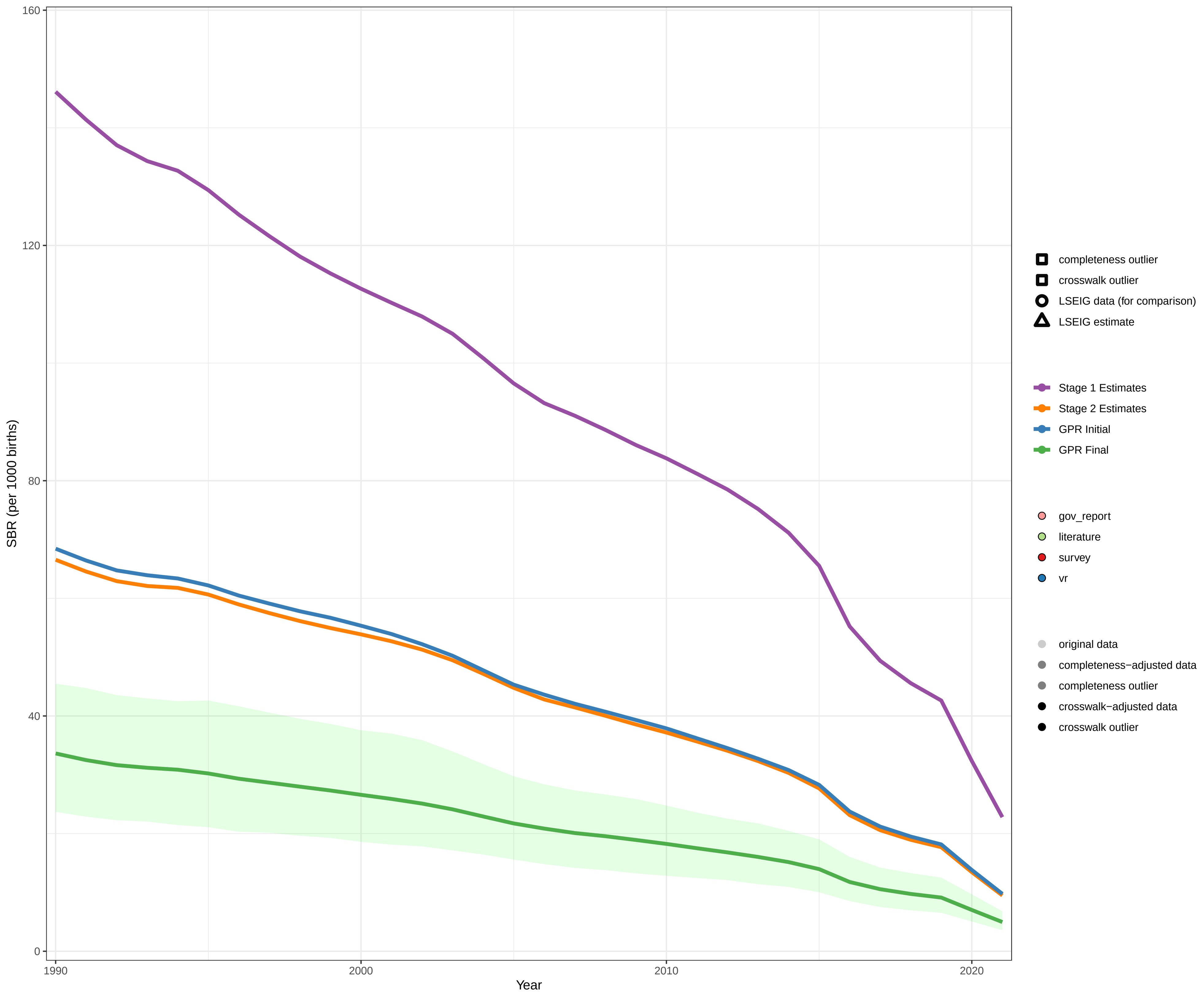

Ardebil IRN_44865 North Africa and Middle East Stillbirth Rate (SBR) − 28 weeks
lambda = 1 ; zeta = 0.6 ; scale = 15 ; amp = 0.028

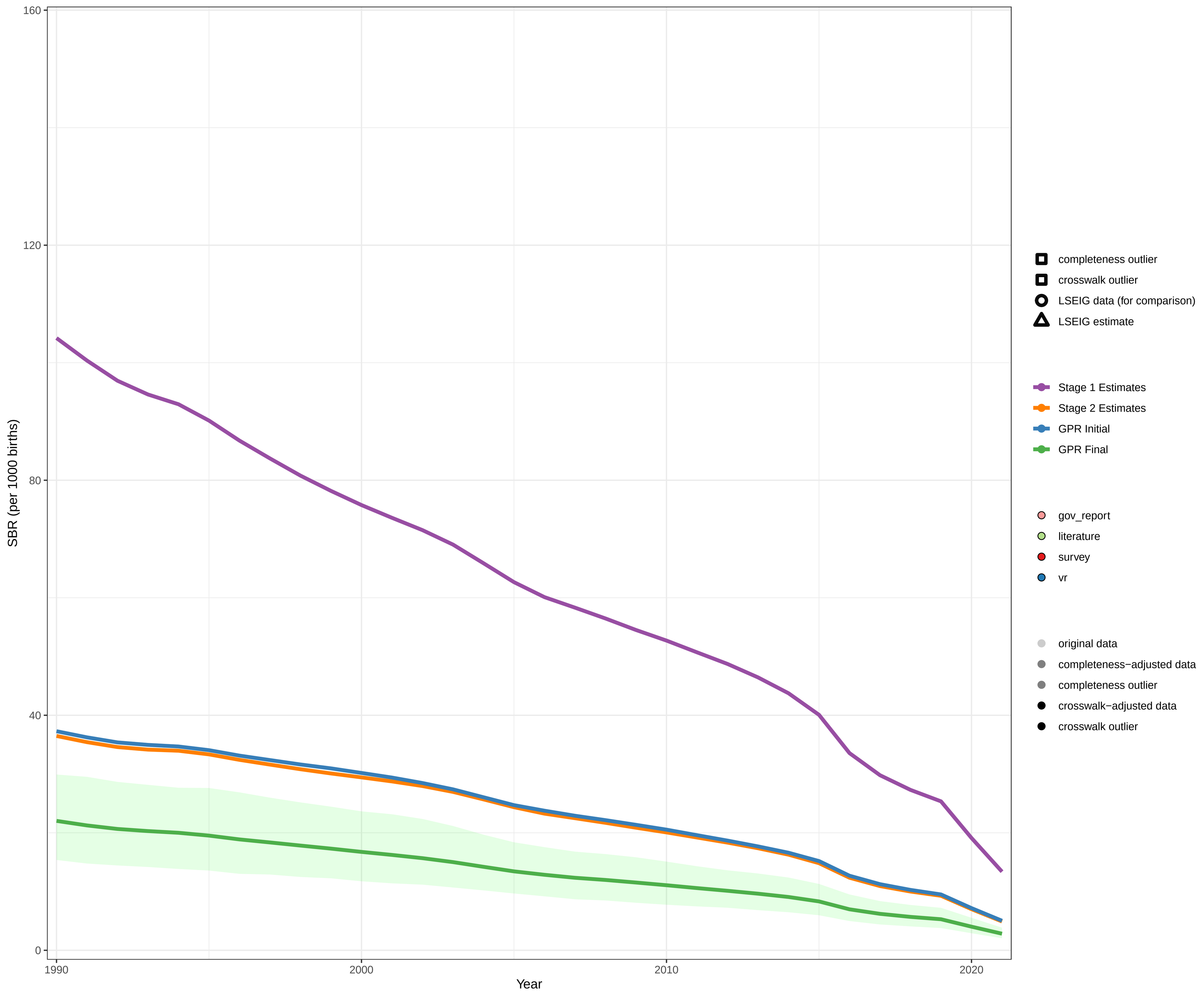

Year	Total Stillbirths (in thousands)	Total Stillbirths (in thousands)	SBR (per 1000 births)	SBR (per 1000 births)	SBR/NMR	SBR/NMR
	20 weeks	28 weeks	20 weeks	28 weeks	20 weeks	28 weeks
1990	1.2 (0.9 to 1.7)	0.8 (0.6 to 1.1)	33.6 (23.7 to 45.5)	22.0 (15.4 to 29.9)	0.88 (0.62 to 1.19)	0.57 (0.40 to 0.78)
1995	0.8 (0.6 to 1.2)	0.5 (0.4 to 0.8)	30.2 (21.1 to 42.6)	19.5 (13.6 to 27.6)	0.96 (0.67 to 1.35)	0.62 (0.43 to 0.87)
2000	0.6 (0.4 to 0.9)	0.4 (0.3 to 0.6)	26.6 (18.6 to 37.6)	16.7 (11.7 to 23.6)	1.08 (0.75 to 1.52)	0.68 (0.47 to 0.96)
2005	0.5 (0.3 to 0.6)	0.3 (0.2 to 0.4)	21.7 (15.6 to 29.8)	13.4 (9.7 to 18.4)	1.11 (0.80 to 1.53)	0.69 (0.50 to 0.94)
2010	0.4 (0.3 to 0.6)	0.3 (0.2 to 0.4)	18.3 (12.8 to 24.8)	11.1 (7.8 to 15.1)	1.15 (0.81 to 1.56)	0.70 (0.49 to 0.95)
2015	0.3 (0.2 to 0.5)	0.2 (0.1 to 0.3)	14.0 (10.0 to 19.0)	8.3 (6.0 to 11.3)	1.18 (0.85 to 1.61)	0.70 (0.50 to 0.96)
2020	0.1 (0.1 to 0.2)	0.1 (0.0 to 0.1)	7.0 (5.1 to 9.7)	4.0 (2.9 to 5.5)	1.31 (0.94 to 1.80)	0.75 (0.54 to 1.03)
2021	0.1 (0.1 to 0.1)	0.0 (0.0 to 0.1)	5.0 (3.6 to 6.8)	2.8 (2.0 to 3.9)	1.33 (0.96 to 1.82)	0.75 (0.55 to 1.03)

Bushehr IRN_44868 North Africa and Middle East Stillbirth Rate / Neonatal Mortality Rate (SBR/NMR) − 20 weeks
lambda = 1 ; zeta = 0.6 ; scale = 15 ; amp = 0.028

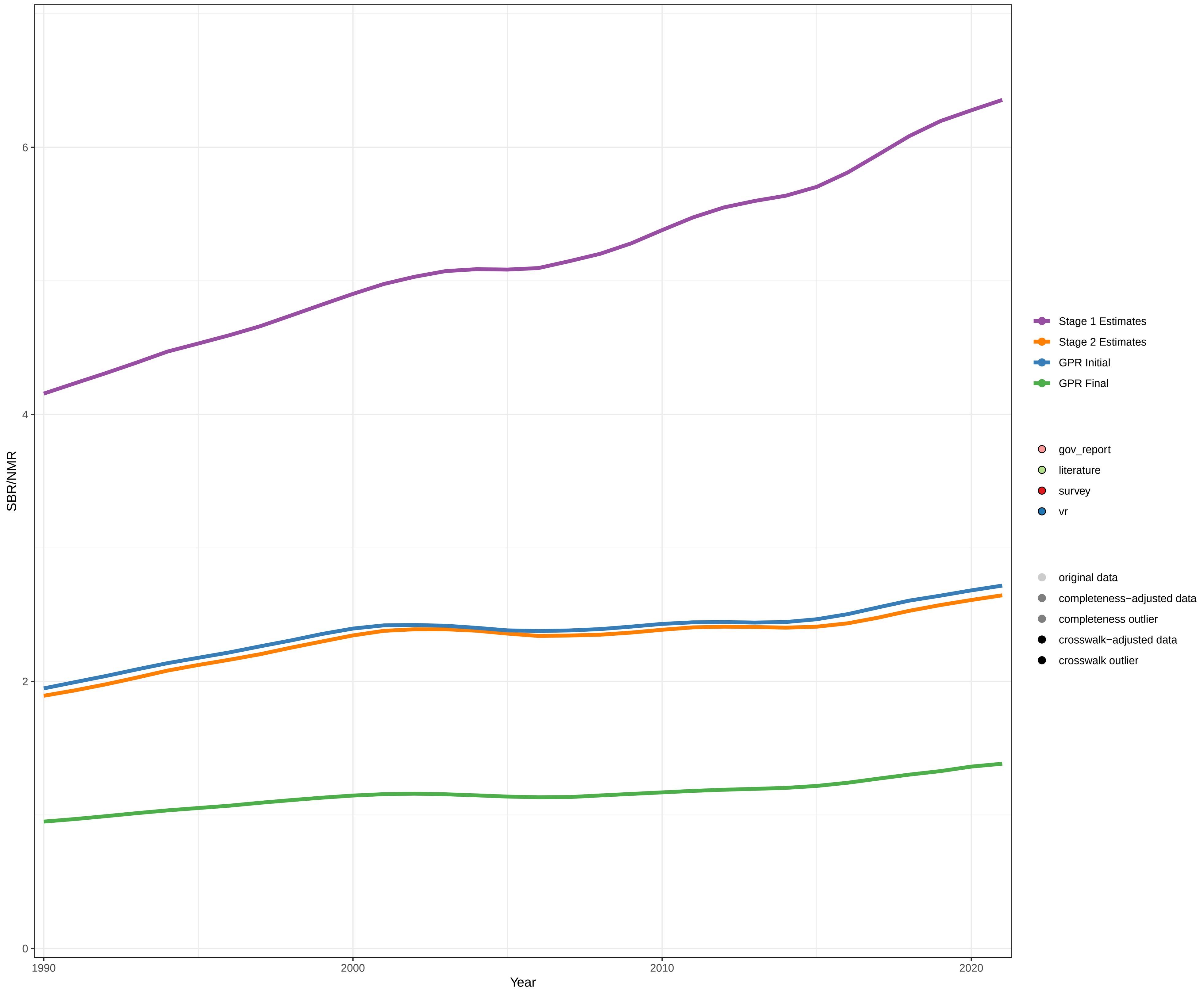

Bushehr IRN_44868 North Africa and Middle East Stillbirth Rate / Neonatal Mortality Rate (SBR/NMR) − 28 weeks
lambda = 1 ; zeta = 0.6 ; scale = 15 ; amp = 0.028

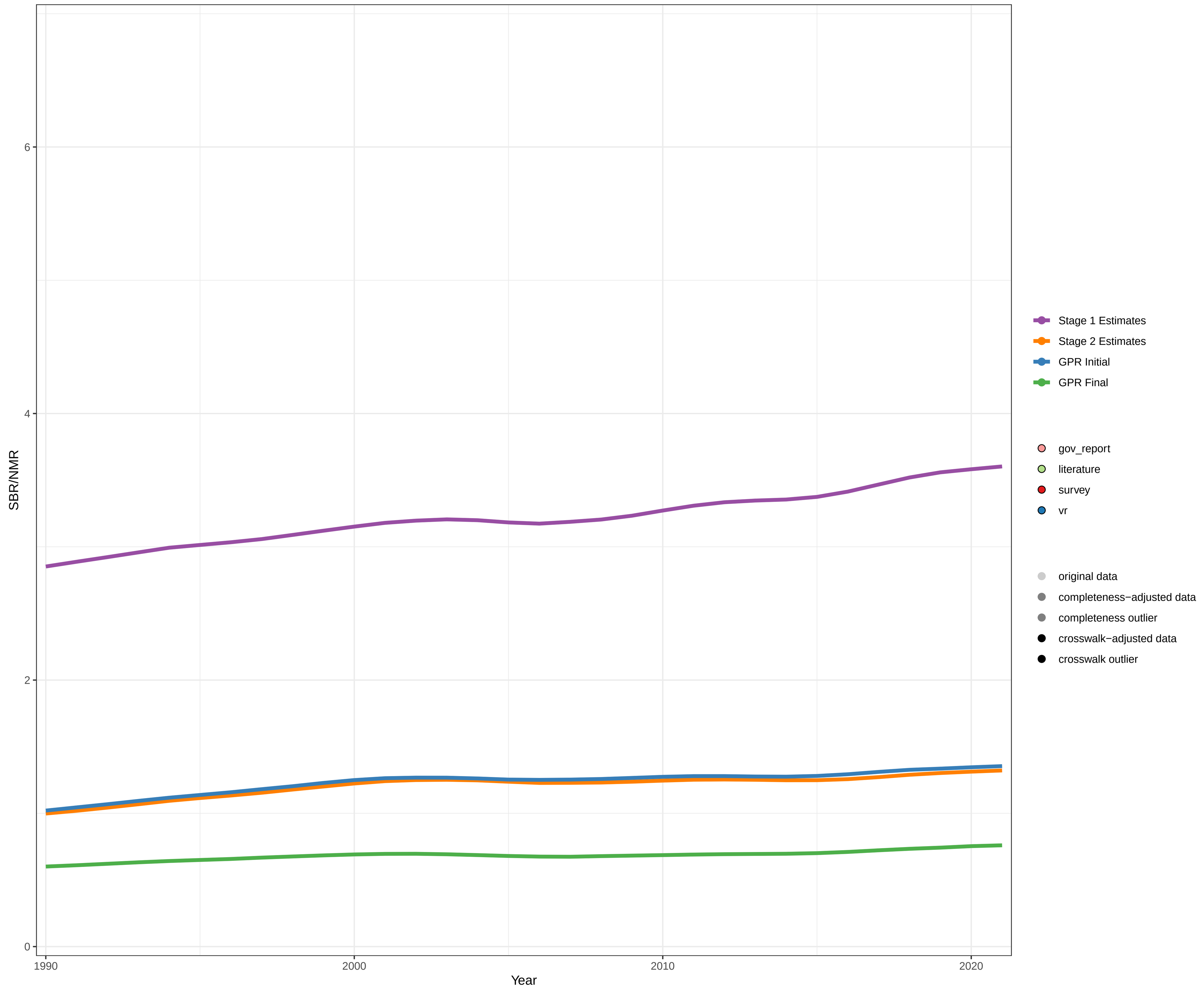

Definition	Years	Outlier	Source.Type	Title

Bushehr IRN_44868 North Africa and Middle East Stillbirth Rate (SBR) − 20 weeks
lambda = 1 ; zeta = 0.6 ; scale = 15 ; amp = 0.028

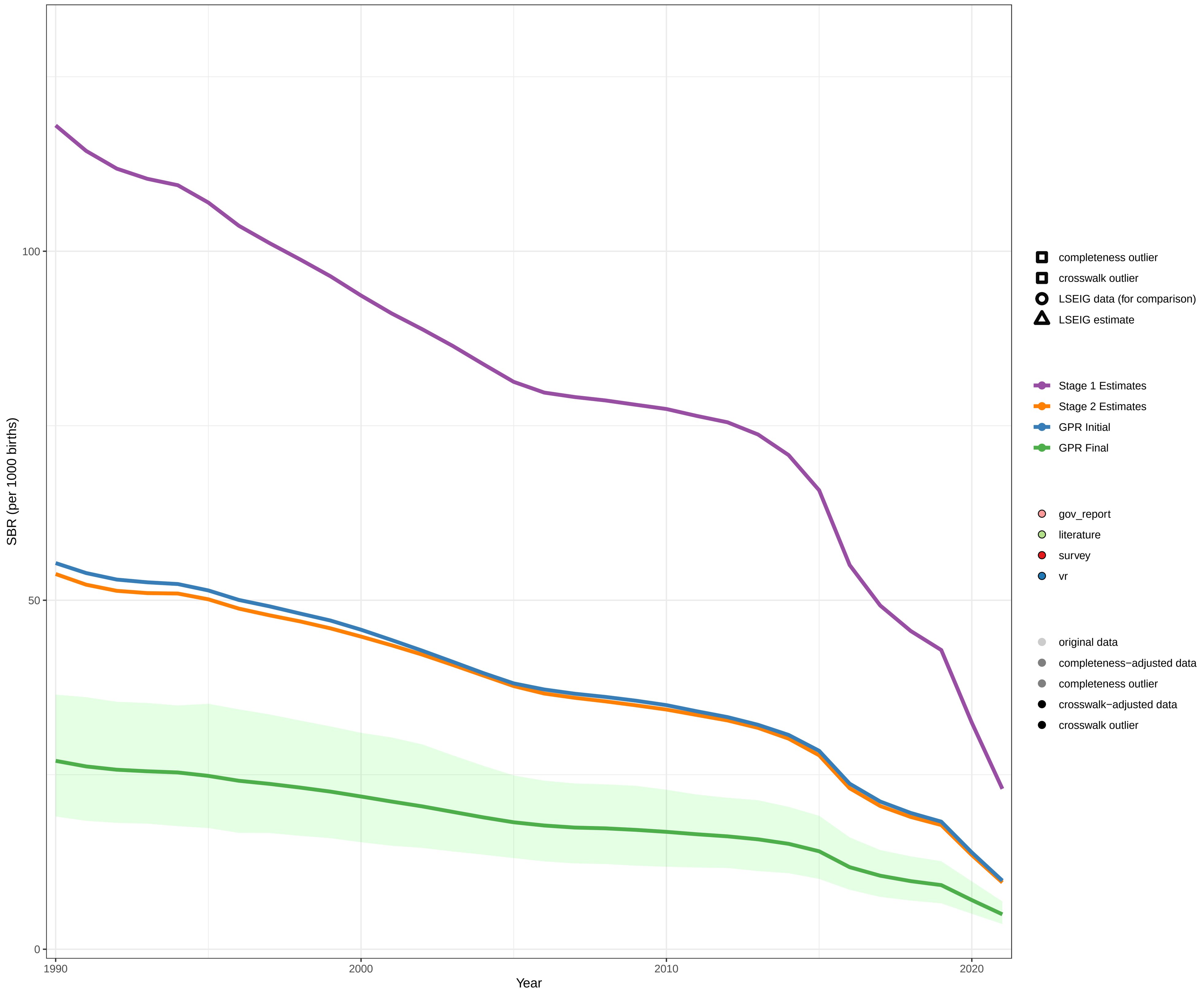

Bushehr IRN_44868 North Africa and Middle East Stillbirth Rate (SBR) − 28 weeks
lambda = 1 ; zeta = 0.6 ; scale = 15 ; amp = 0.028

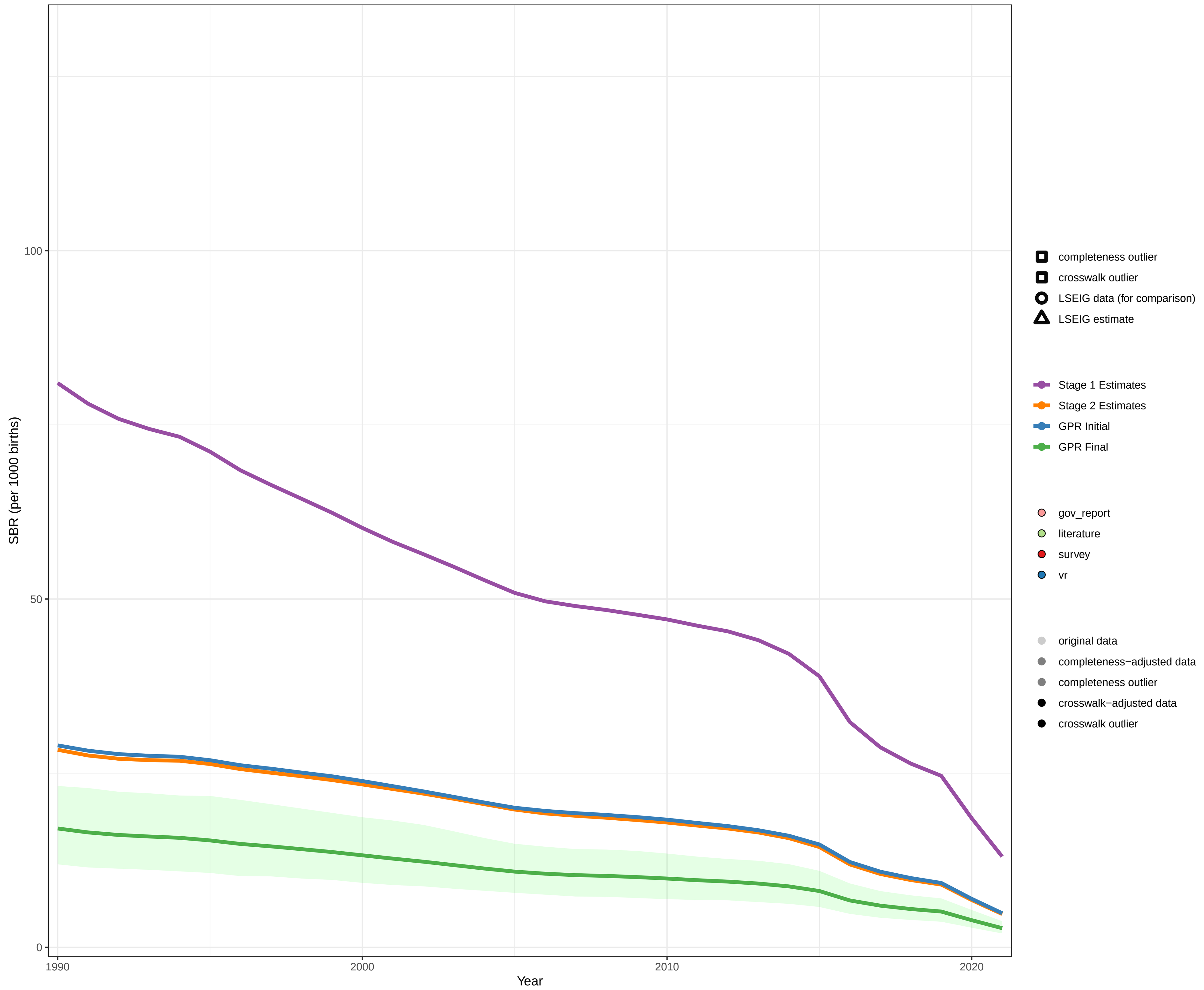

Year	Total Stillbirths (in thousands)	Total Stillbirths (in thousands)	SBR (per 1000 births)	SBR (per 1000 births)	SBR/NMR	SBR/NMR
	20 weeks	28 weeks	20 weeks	28 weeks	20 weeks	28 weeks
1990	0.6 (0.4 to 0.8)	0.4 (0.2 to 0.5)	27.0 (19.0 to 36.5)	17.1 (11.9 to 23.2)	0.95 (0.67 to 1.28)	0.60 (0.42 to 0.82)
1995	0.4 (0.3 to 0.6)	0.2 (0.2 to 0.4)	24.8 (17.4 to 35.2)	15.3 (10.7 to 21.7)	1.05 (0.74 to 1.49)	0.65 (0.45 to 0.92)
2000	0.3 (0.2 to 0.4)	0.2 (0.1 to 0.3)	21.9 (15.3 to 31.0)	13.2 (9.3 to 18.7)	1.15 (0.80 to 1.62)	0.69 (0.48 to 0.98)
2005	0.3 (0.2 to 0.3)	0.1 (0.1 to 0.2)	18.2 (13.1 to 24.9)	10.9 (7.8 to 14.9)	1.14 (0.82 to 1.56)	0.68 (0.49 to 0.93)
2010	0.3 (0.2 to 0.4)	0.2 (0.1 to 0.2)	16.8 (11.8 to 22.9)	9.9 (6.9 to 13.5)	1.17 (0.82 to 1.59)	0.69 (0.48 to 0.94)
2015	0.3 (0.2 to 0.4)	0.2 (0.1 to 0.2)	14.0 (10.1 to 19.1)	8.1 (5.8 to 11.0)	1.22 (0.87 to 1.66)	0.70 (0.50 to 0.95)
2020	0.1 (0.1 to 0.2)	0.1 (0.0 to 0.1)	7.0 (5.1 to 9.7)	3.9 (2.8 to 5.4)	1.36 (0.98 to 1.88)	0.75 (0.54 to 1.04)
2021	0.1 (0.1 to 0.1)	0.0 (0.0 to 0.1)	5.0 (3.6 to 6.8)	2.7 (2.0 to 3.8)	1.38 (1.00 to 1.89)	0.76 (0.55 to 1.04)

Chahar Mahaal and Bakhtiari IRN_44869 North Africa and Middle East Stillbirth Rate / Neonatal Mortality Rate (SBR/NMR) − 20 weeks
lambda = 1 ; zeta = 0.6 ; scale = 15 ; amp = 0.028

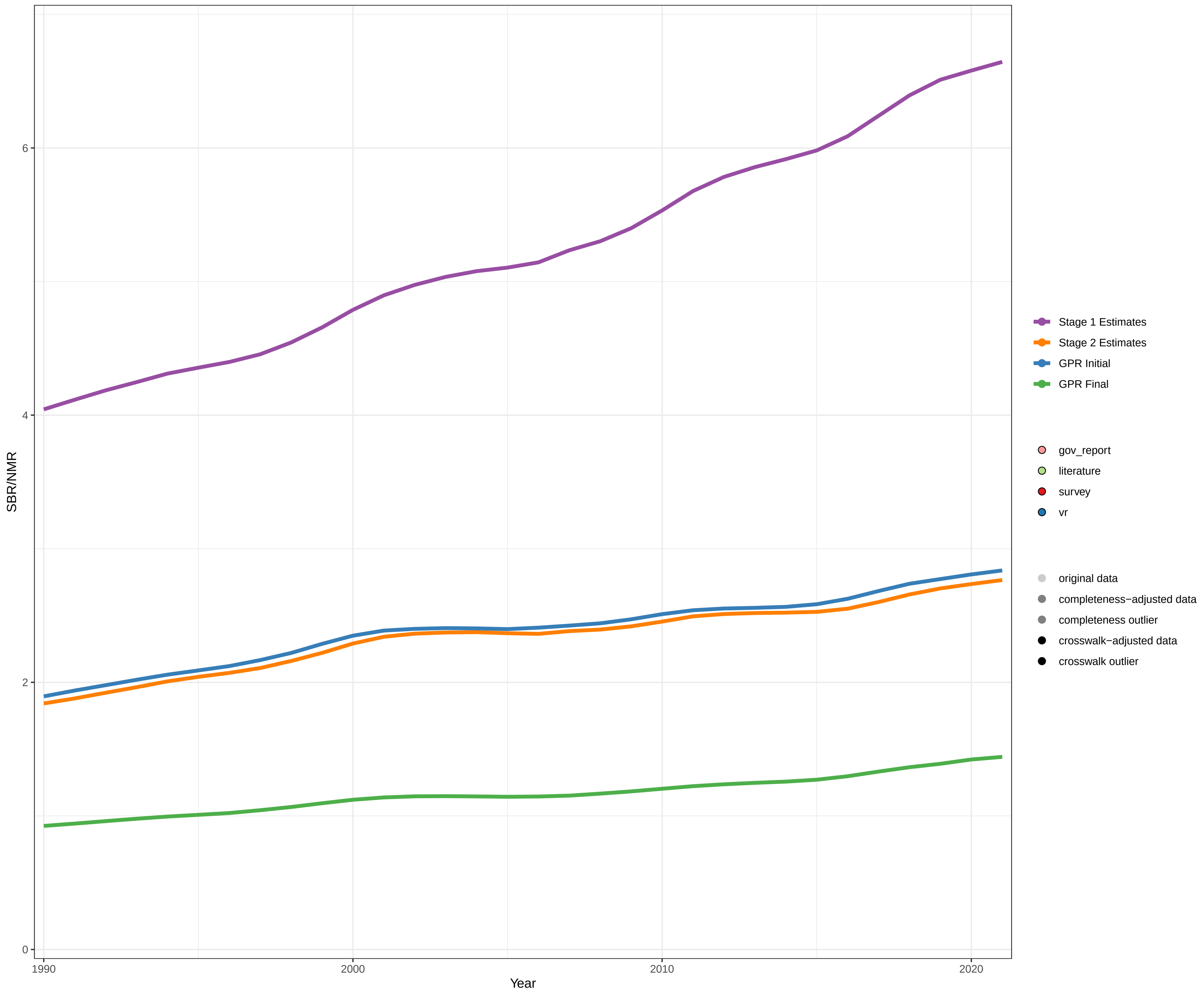

Chahar Mahaal and Bakhtiari IRN_44869 North Africa and Middle East Stillbirth Rate / Neonatal Mortality Rate (SBR/NMR) − 28 weeks
lambda = 1 ; zeta = 0.6 ; scale = 15 ; amp = 0.028

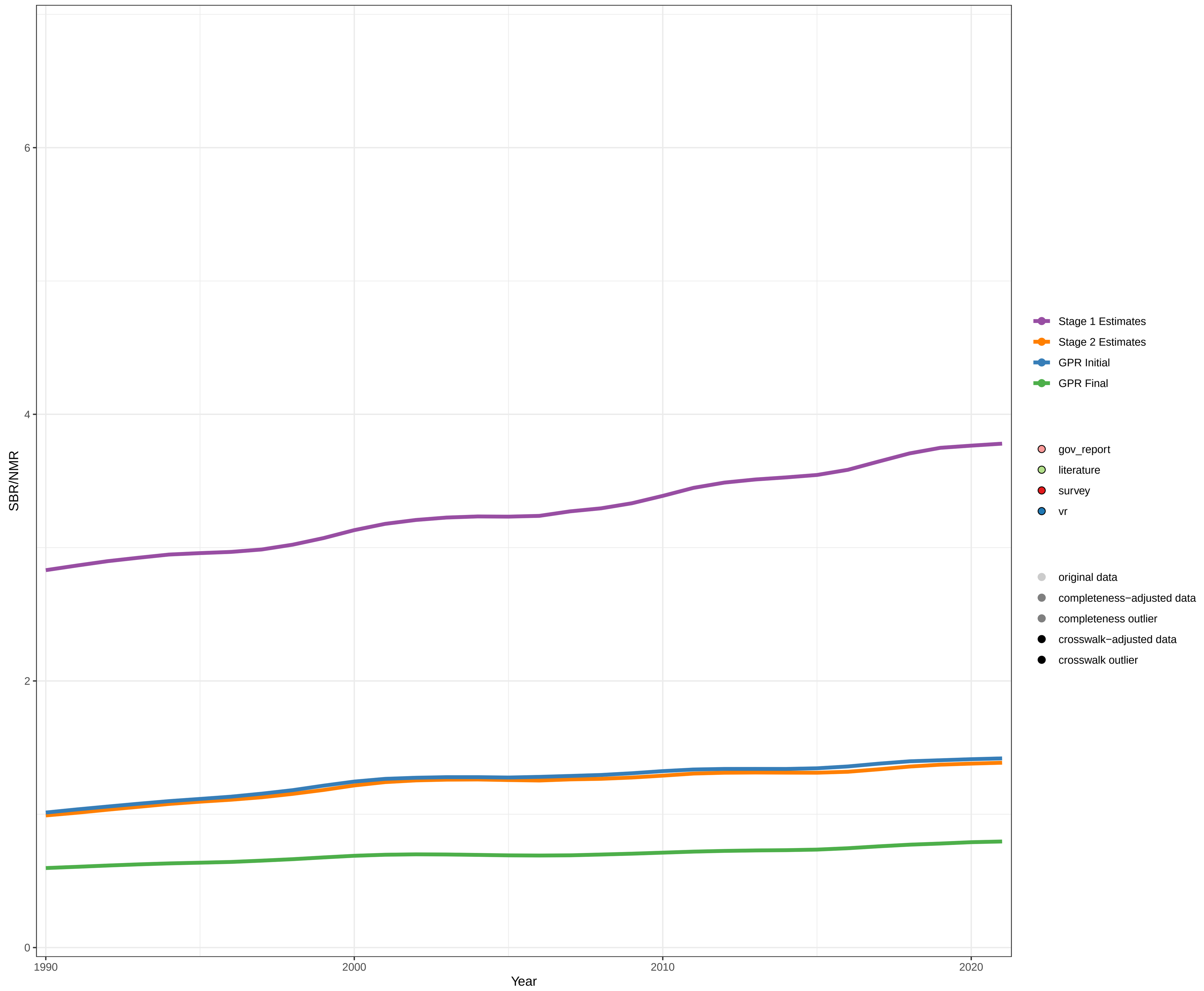

Definition	Years	Outlier	Source.Type	Title

Chahar Mahaal and Bakhtiari IRN_44869 North Africa and Middle East Stillbirth Rate (SBR) − 20 weeks
lambda = 1 ; zeta = 0.6 ; scale = 15 ; amp = 0.028

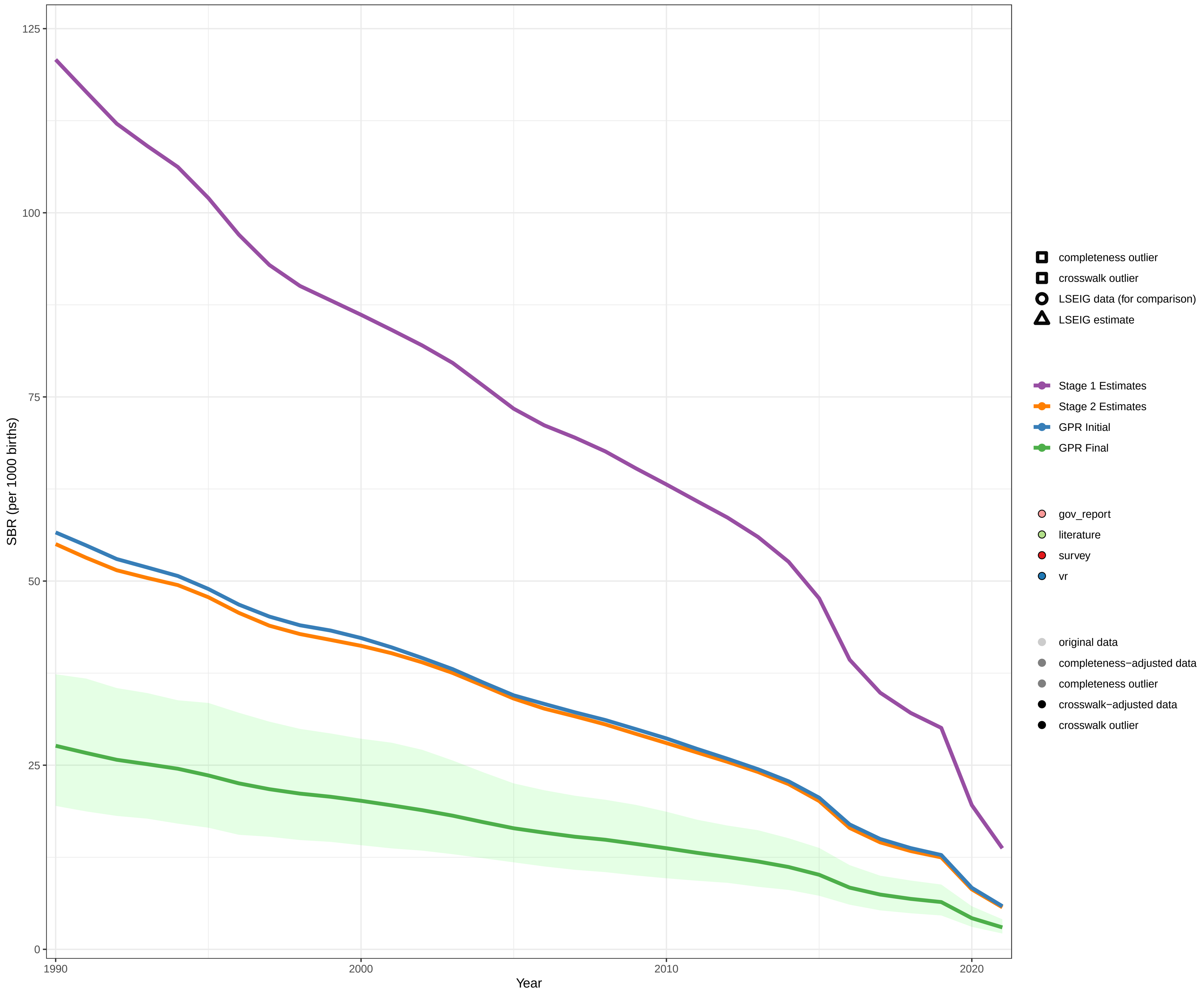

Chahar Mahaal and Bakhtiari IRN_44869 North Africa and Middle East Stillbirth Rate (SBR) − 28 weeks
lambda = 1 ; zeta = 0.6 ; scale = 15 ; amp = 0.028

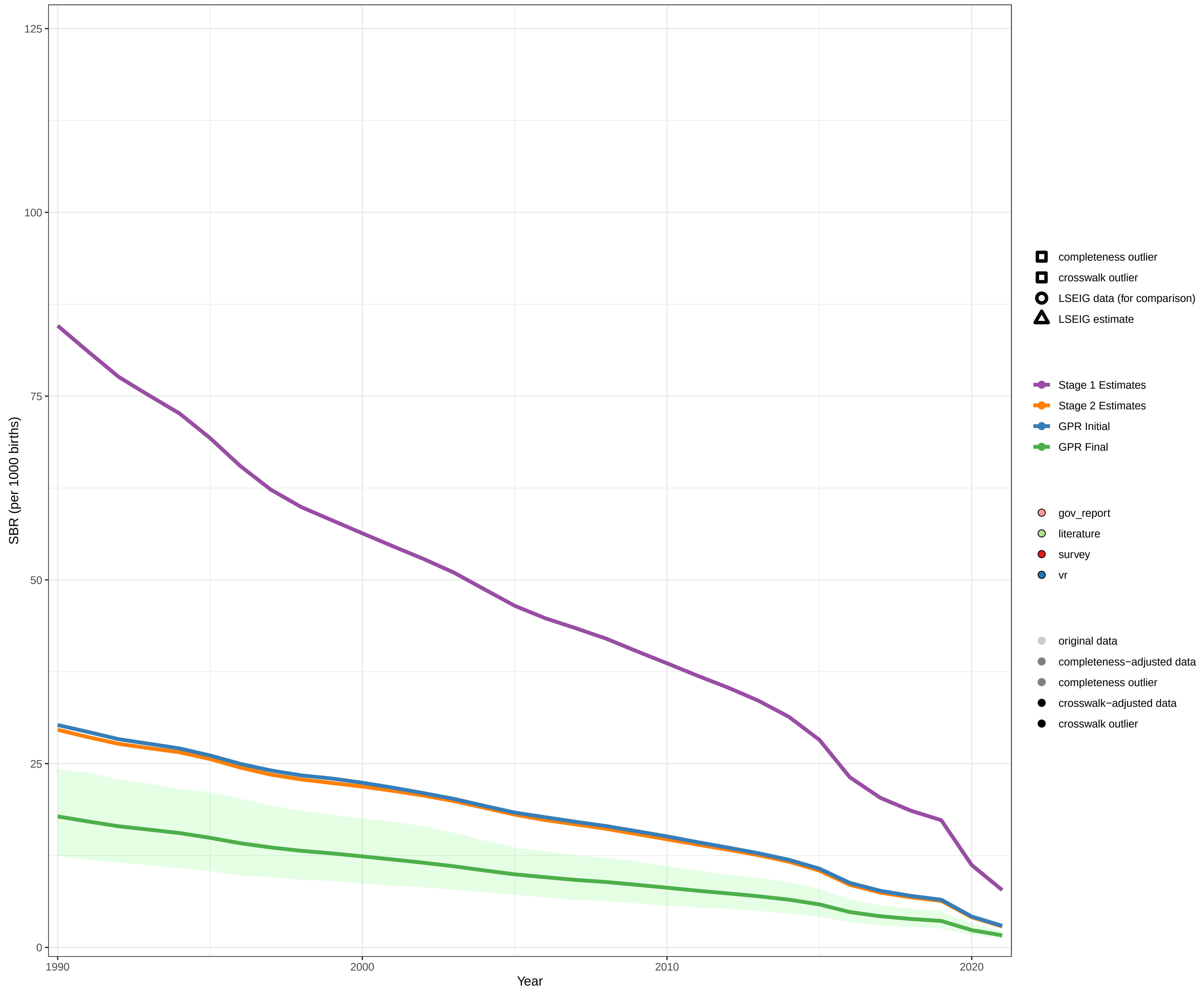

Year	Total Stillbirths (in thousands)	Total Stillbirths (in thousands)	SBR (per 1000 births)	SBR (per 1000 births)	SBR/NMR	SBR/NMR
	20 weeks	28 weeks	20 weeks	28 weeks	20 weeks	28 weeks
1990	0.6 (0.4 to 0.9)	0.4 (0.3 to 0.5)	27.6 (19.5 to 37.3)	17.8 (12.4 to 24.2)	0.93 (0.65 to 1.25)	0.60 (0.42 to 0.81)
1995	0.4 (0.3 to 0.6)	0.3 (0.2 to 0.4)	23.6 (16.5 to 33.5)	14.9 (10.4 to 21.1)	1.01 (0.71 to 1.43)	0.64 (0.44 to 0.90)
2000	0.3 (0.2 to 0.4)	0.2 (0.1 to 0.3)	20.2 (14.1 to 28.6)	12.4 (8.7 to 17.5)	1.12 (0.79 to 1.59)	0.69 (0.48 to 0.97)
2005	0.2 (0.2 to 0.3)	0.1 (0.1 to 0.2)	16.4 (11.8 to 22.5)	9.9 (7.2 to 13.6)	1.14 (0.82 to 1.57)	0.69 (0.50 to 0.95)
2010	0.3 (0.2 to 0.4)	0.2 (0.1 to 0.2)	13.7 (9.6 to 18.7)	8.1 (5.7 to 11.1)	1.20 (0.85 to 1.64)	0.71 (0.50 to 0.97)
2015	0.2 (0.2 to 0.3)	0.1 (0.1 to 0.2)	10.1 (7.3 to 13.8)	5.9 (4.2 to 7.9)	1.27 (0.91 to 1.73)	0.73 (0.53 to 1.00)
2020	0.1 (0.0 to 0.1)	0.0 (0.0 to 0.1)	4.2 (3.0 to 5.9)	2.4 (1.7 to 3.2)	1.42 (1.02 to 1.97)	0.79 (0.57 to 1.09)
2021	0.0 (0.0 to 0.1)	0.0 (0.0 to 0.0)	3.0 (2.1 to 4.1)	1.6 (1.2 to 2.2)	1.44 (1.04 to 1.97)	0.80 (0.58 to 1.09)

East Azarbayejan IRN_44866 North Africa and Middle East Stillbirth Rate / Neonatal Mortality Rate (SBR/NMR) − 20 weeks
lambda = 1 ; zeta = 0.6 ; scale = 15 ; amp = 0.028

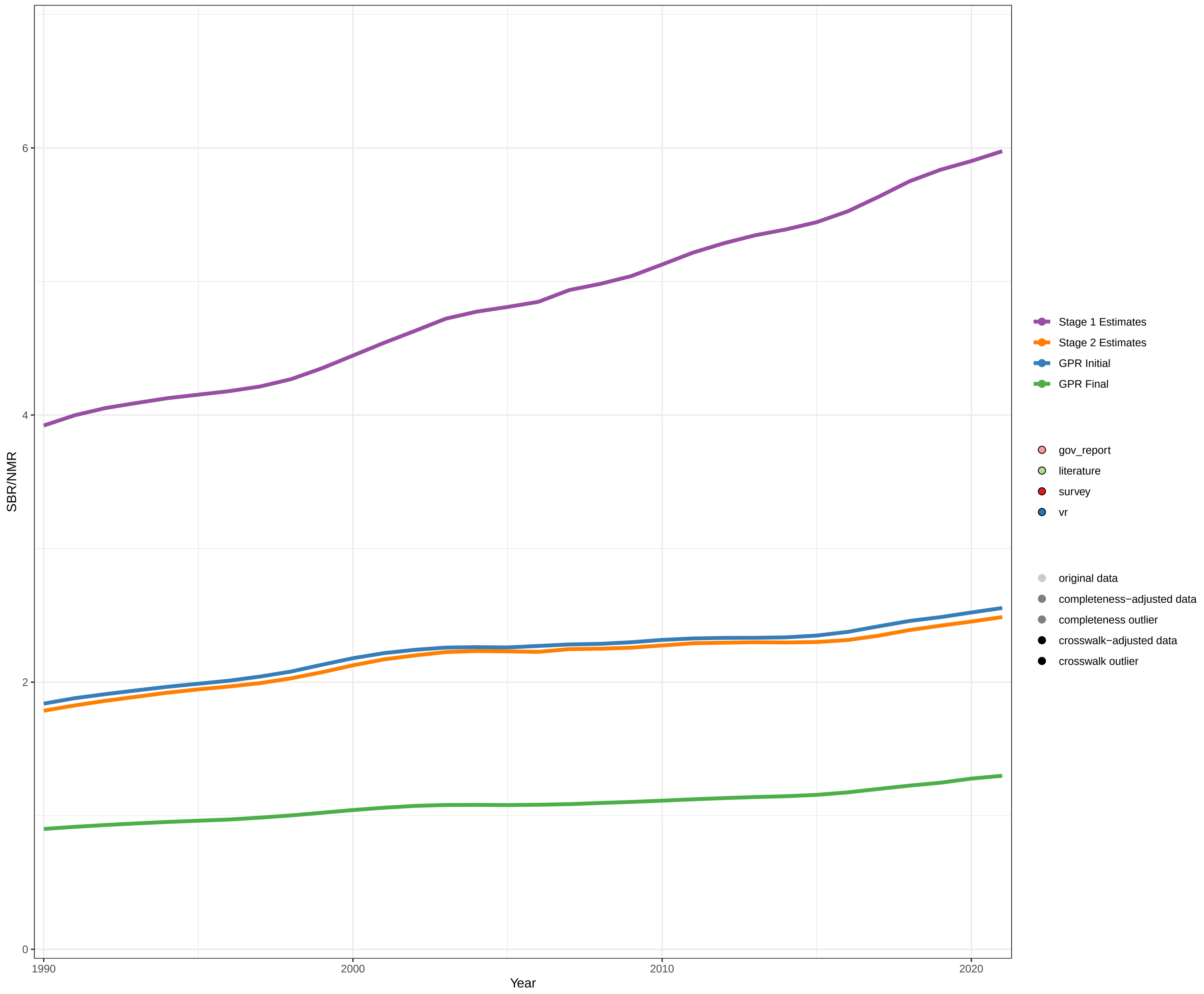

East Azarbayejan IRN_44866 North Africa and Middle East Stillbirth Rate / Neonatal Mortality Rate (SBR/NMR) − 28 weeks
lambda = 1 ; zeta = 0.6 ; scale = 15 ; amp = 0.028

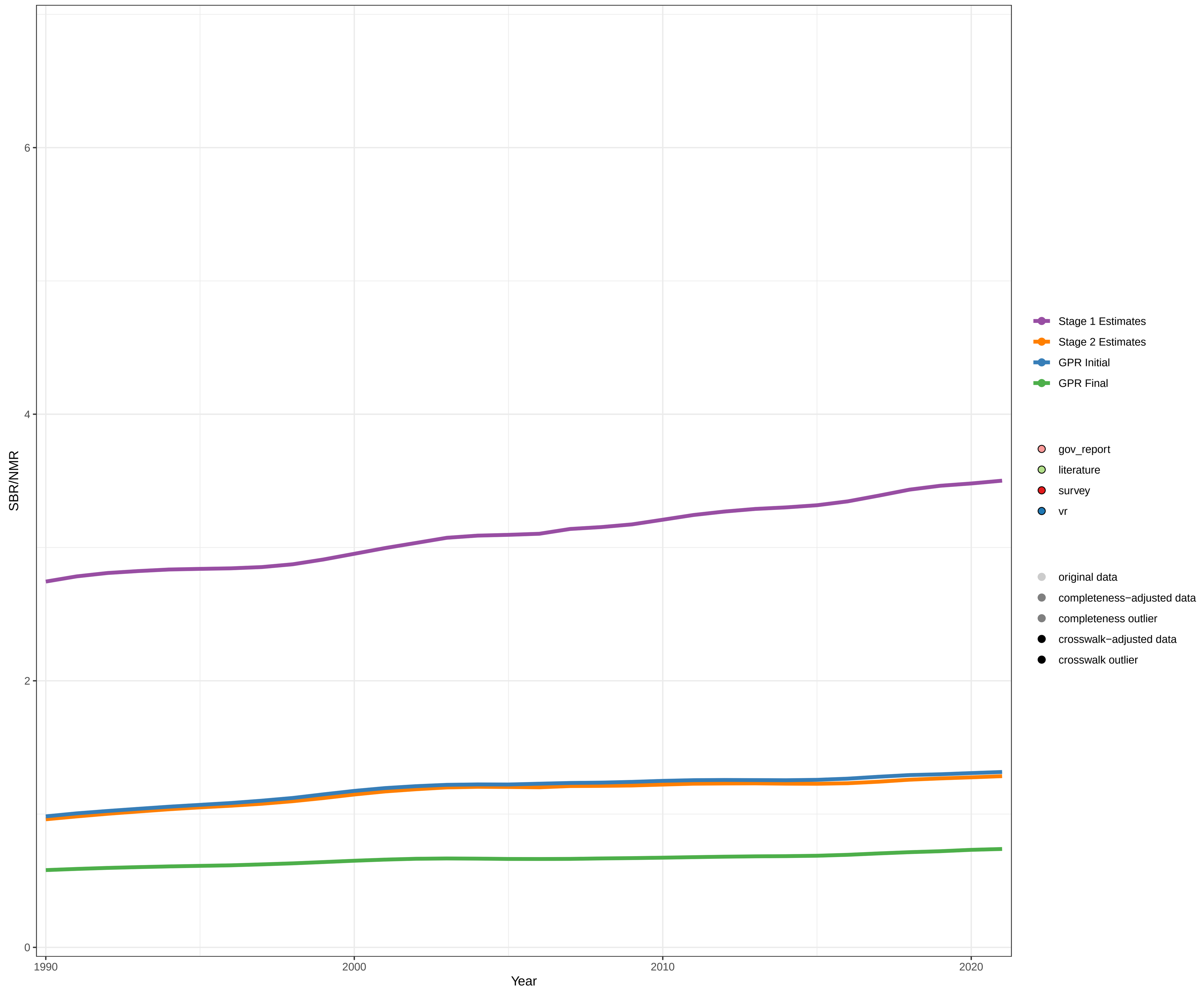

Definition	Years	Outlier	Source.Type	Title

East Azarbayejan IRN_44866 North Africa and Middle East Stillbirth Rate (SBR) − 20 weeks
lambda = 1 ; zeta = 0.6 ; scale = 15 ; amp = 0.028

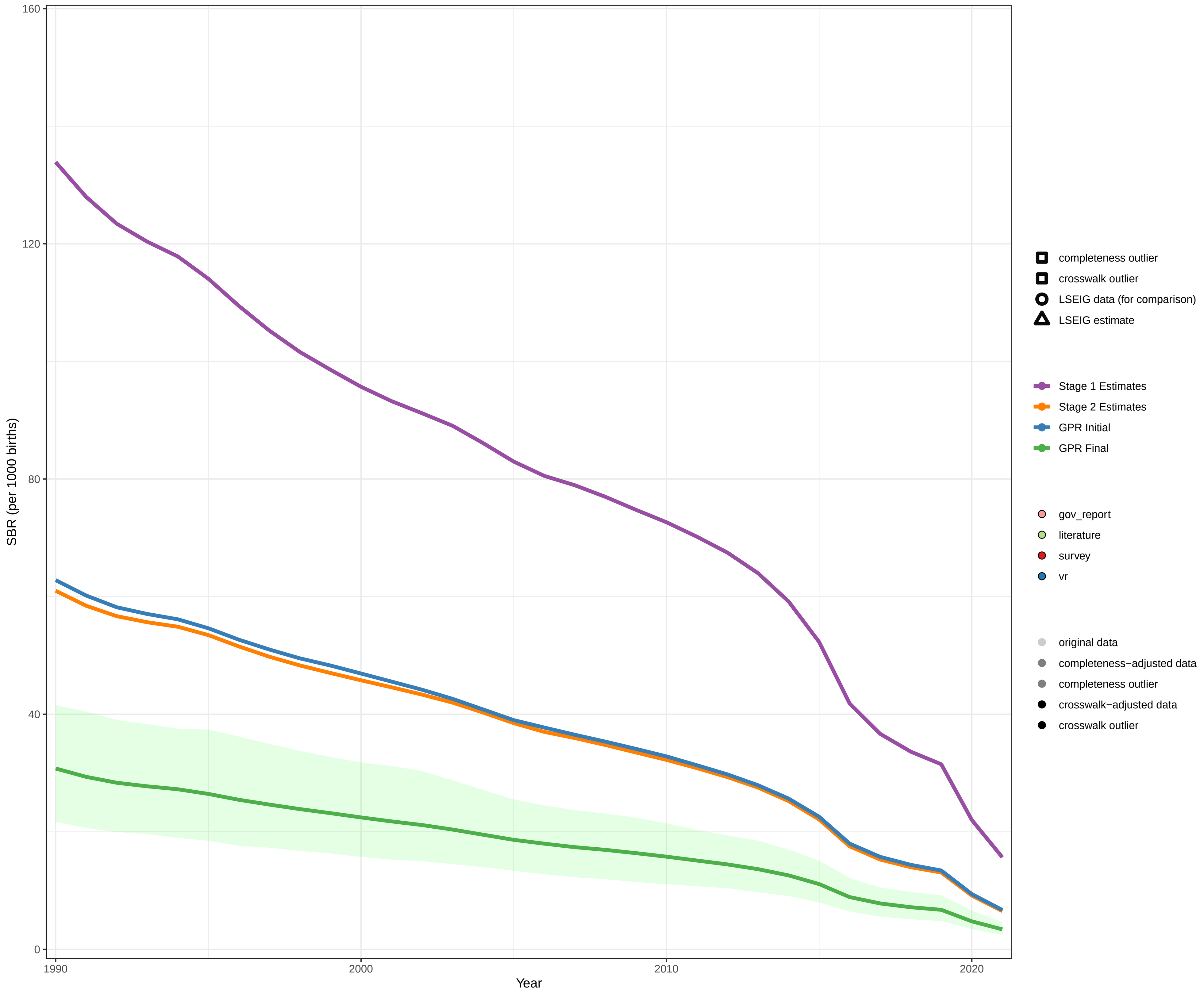

East Azarbayejan IRN_44866 North Africa and Middle East Stillbirth Rate (SBR) − 28 weeks
lambda = 1 ; zeta = 0.6 ; scale = 15 ; amp = 0.028

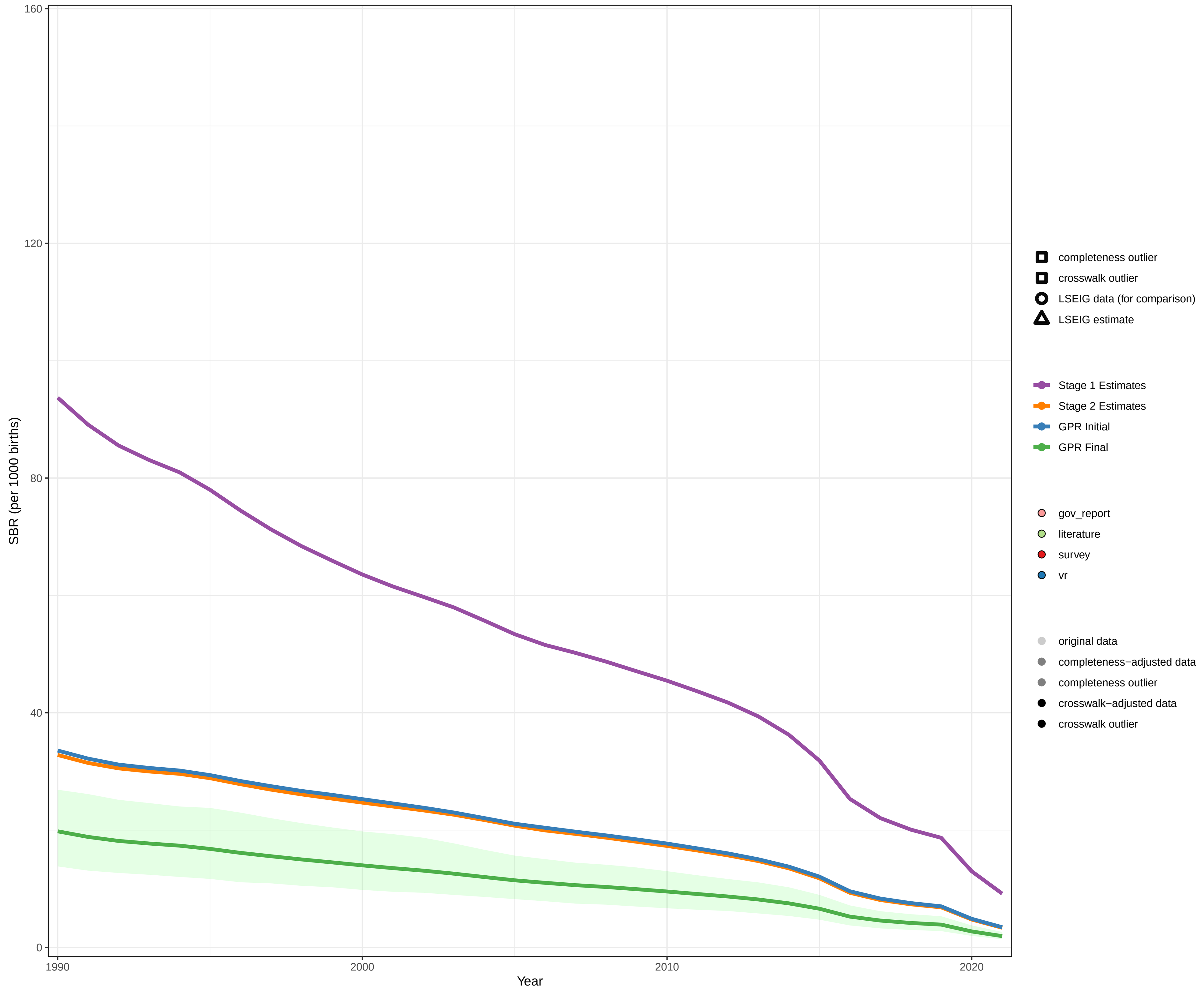

Year	Total Stillbirths (in thousands)	Total Stillbirths (in thousands)	SBR (per 1000 births)	SBR (per 1000 births)	SBR/NMR	SBR/NMR
	20 weeks	28 weeks	20 weeks	28 weeks	20 weeks	28 weeks
1990	3.0 (2.1 to 4.1)	1.9 (1.3 to 2.6)	30.8 (21.7 to 41.5)	19.8 (13.8 to 26.9)	0.90 (0.63 to 1.22)	0.58 (0.40 to 0.79)
1995	1.9 (1.3 to 2.8)	1.2 (0.8 to 1.7)	26.4 (18.5 to 37.4)	16.8 (11.7 to 23.8)	0.96 (0.67 to 1.36)	0.61 (0.43 to 0.87)
2000	1.4 (1.0 to 2.1)	0.9 (0.6 to 1.3)	22.4 (15.7 to 31.8)	14.0 (9.8 to 19.8)	1.04 (0.73 to 1.48)	0.65 (0.46 to 0.92)
2005	1.1 (0.8 to 1.6)	0.7 (0.5 to 1.0)	18.6 (13.4 to 25.5)	11.4 (8.2 to 15.7)	1.08 (0.78 to 1.48)	0.66 (0.48 to 0.91)
2010	1.1 (0.8 to 1.5)	0.7 (0.5 to 0.9)	15.8 (11.1 to 21.4)	9.5 (6.7 to 13.0)	1.11 (0.78 to 1.51)	0.67 (0.47 to 0.92)
2015	0.8 (0.6 to 1.1)	0.5 (0.3 to 0.6)	11.1 (8.0 to 15.1)	6.6 (4.7 to 9.0)	1.16 (0.83 to 1.57)	0.69 (0.49 to 0.93)
2020	0.2 (0.2 to 0.3)	0.1 (0.1 to 0.2)	4.8 (3.4 to 6.6)	2.7 (2.0 to 3.8)	1.28 (0.92 to 1.77)	0.73 (0.53 to 1.01)
2021	0.2 (0.1 to 0.2)	0.1 (0.1 to 0.1)	3.4 (2.5 to 4.7)	1.9 (1.4 to 2.6)	1.30 (0.94 to 1.78)	0.74 (0.53 to 1.01)

Fars IRN_44870 North Africa and Middle East Stillbirth Rate / Neonatal Mortality Rate (SBR/NMR) − 20 weeks
lambda = 1 ; zeta = 0.6 ; scale = 15 ; amp = 0.028

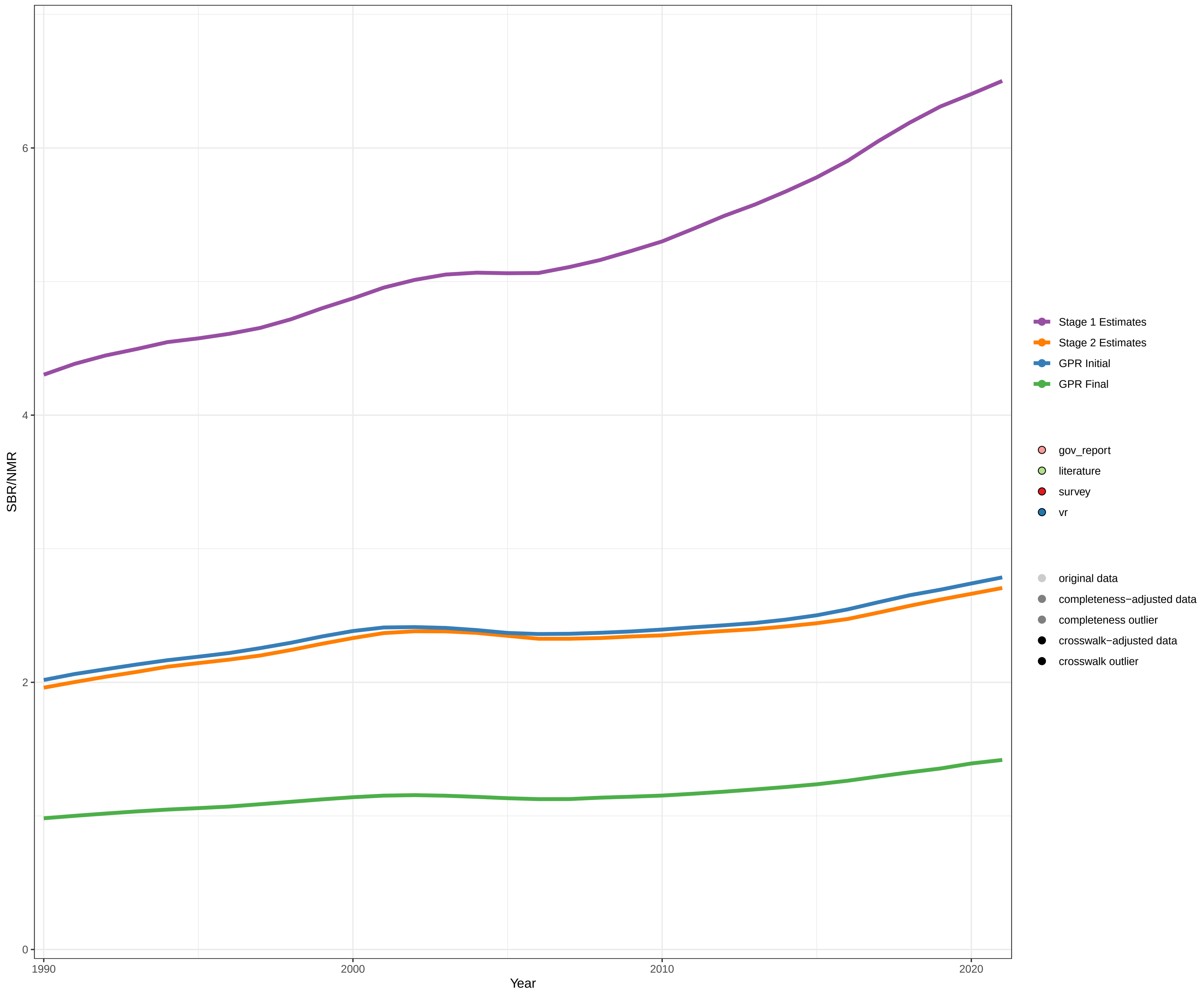

Fars IRN_44870 North Africa and Middle East Stillbirth Rate / Neonatal Mortality Rate (SBR/NMR) − 28 weeks
lambda = 1 ; zeta = 0.6 ; scale = 15 ; amp = 0.028

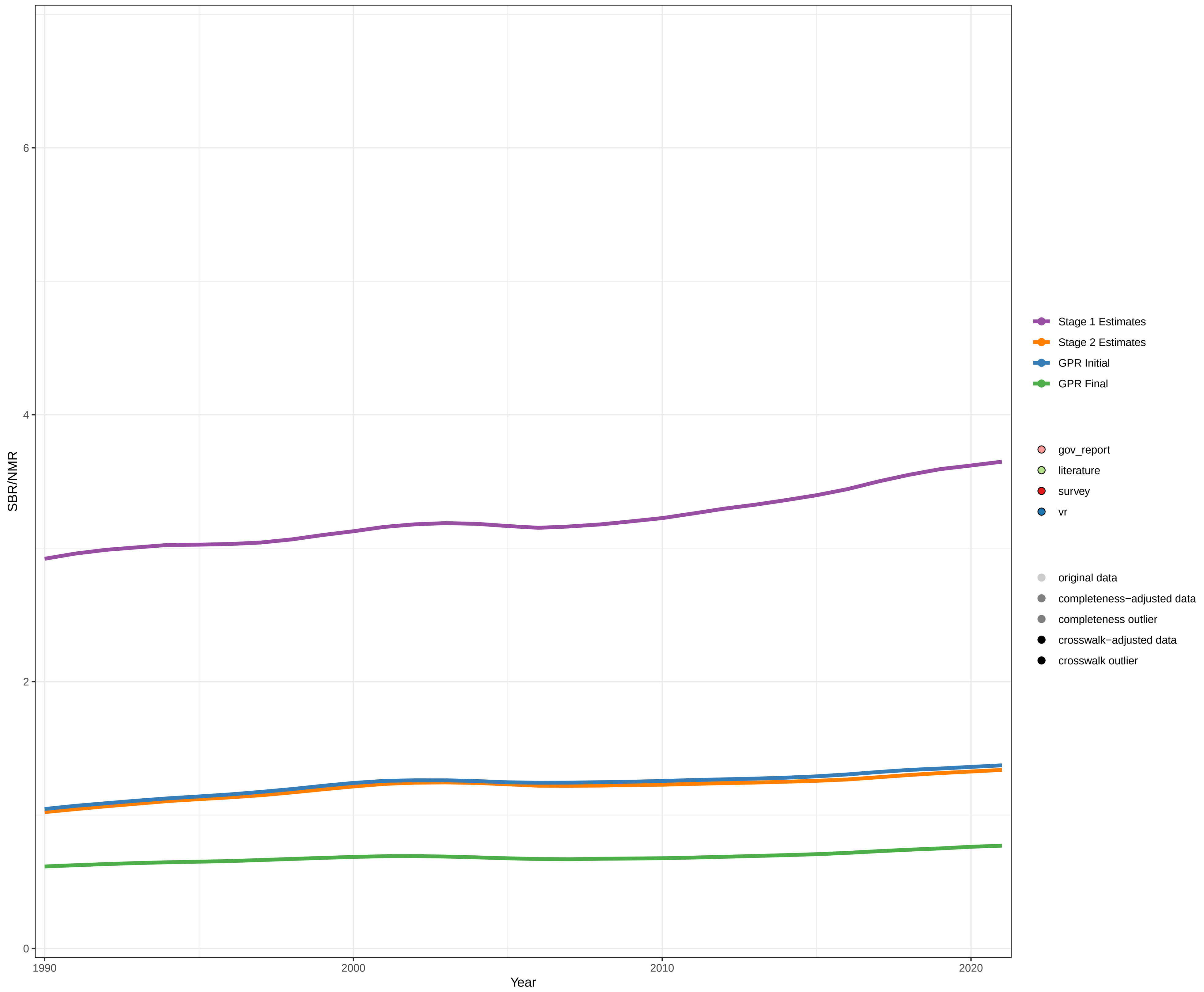

Definition	Years	Outlier	Source.Type	Title

Fars IRN_44870 North Africa and Middle East Stillbirth Rate (SBR) − 20 weeks
lambda = 1 ; zeta = 0.6 ; scale = 15 ; amp = 0.028

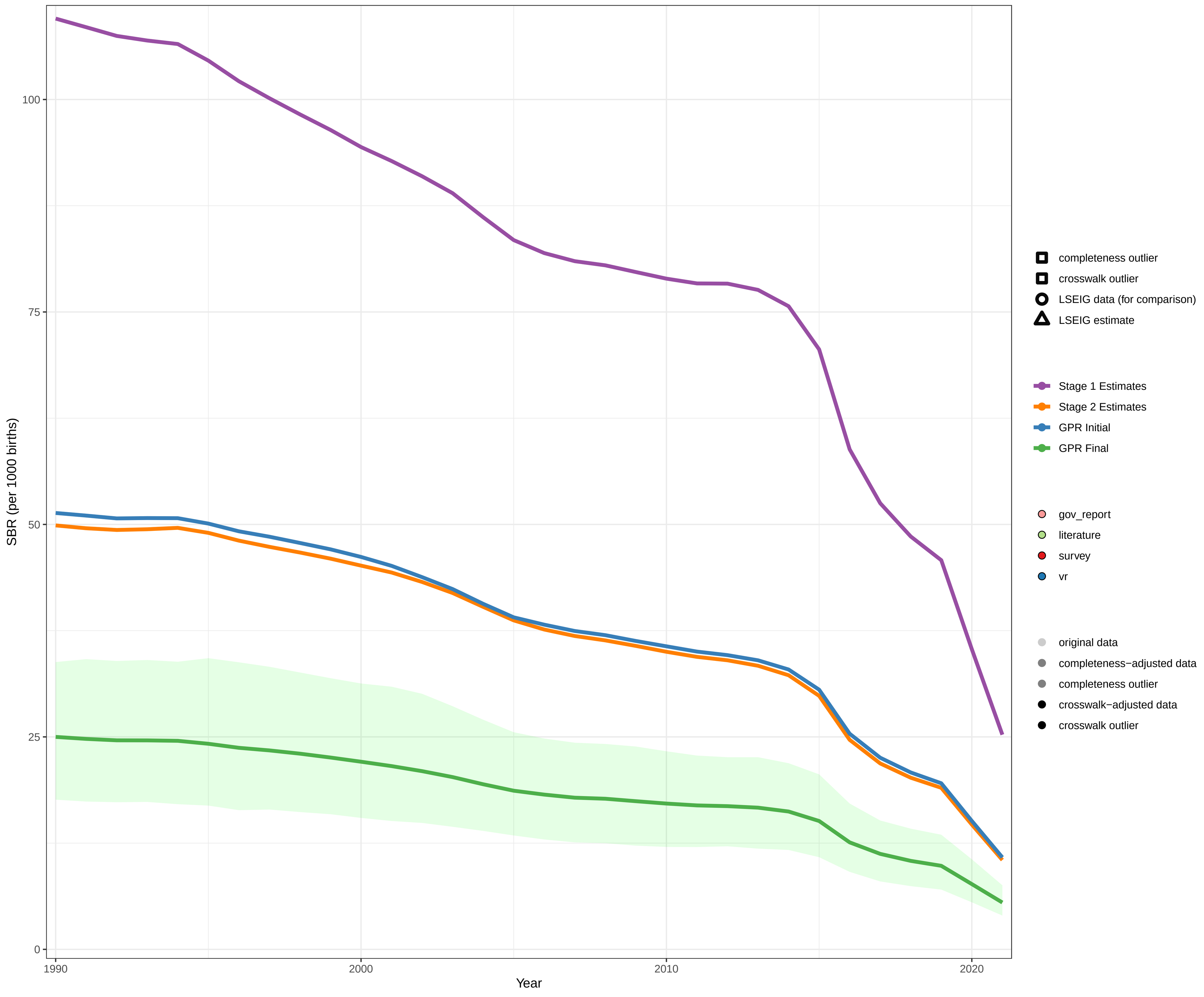

Fars IRN_44870 North Africa and Middle East Stillbirth Rate (SBR) − 28 weeks
lambda = 1 ; zeta = 0.6 ; scale = 15 ; amp = 0.028

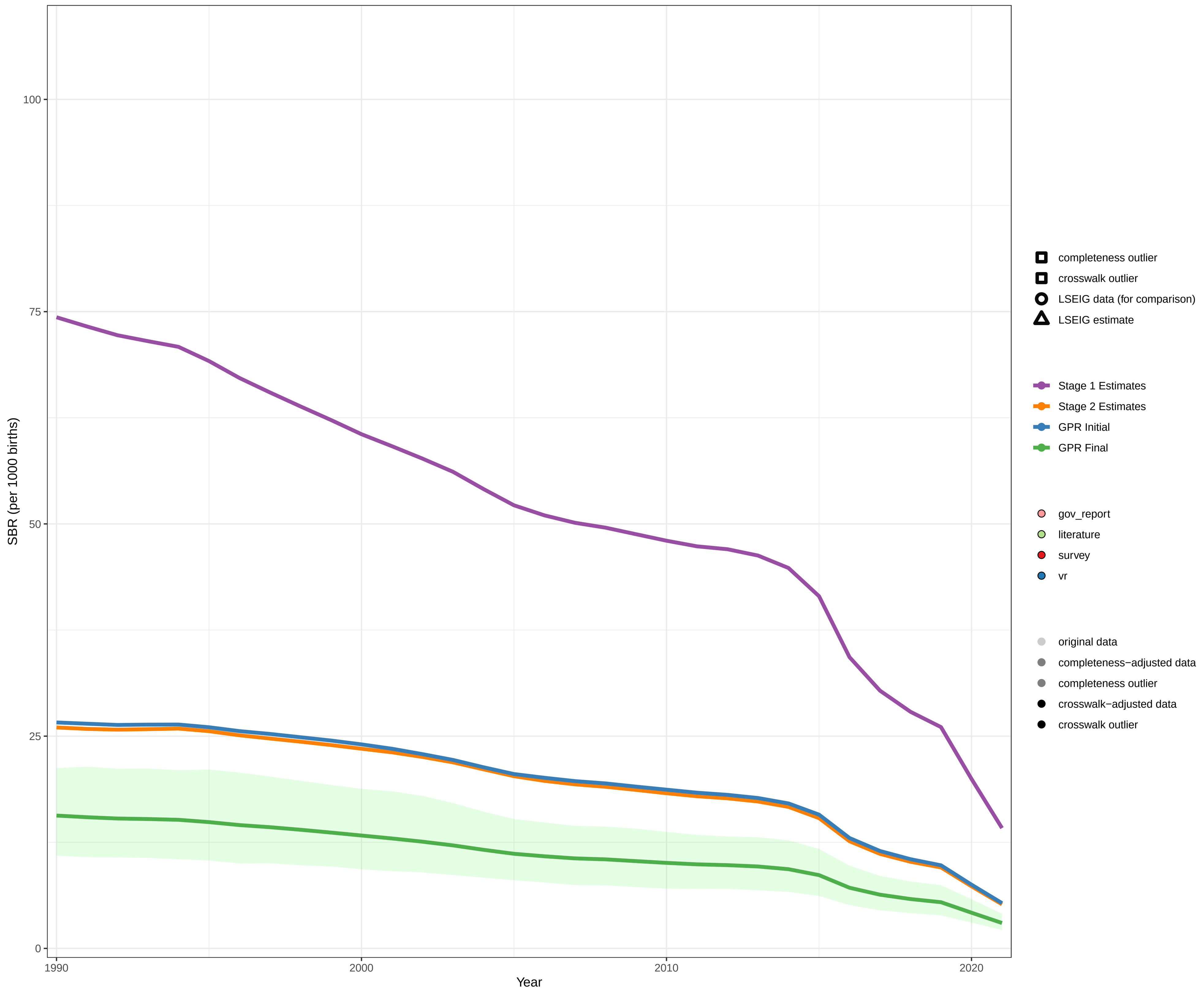

Year	Total Stillbirths (in thousands)	Total Stillbirths (in thousands)	SBR (per 1000 births)	SBR (per 1000 births)	SBR/NMR	SBR/NMR
	20 weeks	28 weeks	20 weeks	28 weeks	20 weeks	28 weeks
1990	2.4<br>(1.7 to 3.2)	1.5<br>(1.0 to 2.0)	25.0<br>(17.6 to 33.8)	15.6<br>(10.9 to 21.2)	0.98<br>(0.69 to 1.33)	0.61<br>(0.43 to 0.83)
1995	1.8<br>(1.2 to 2.5)	1.1<br>(0.7 to 1.5)	24.2<br>(16.9 to 34.3)	14.9<br>(10.4 to 21.1)	1.06<br>(0.74 to 1.50)	0.65<br>(0.45 to 0.92)
2000	1.5<br>(1.0 to 2.1)	0.9<br>(0.6 to 1.2)	22.1<br>(15.5 to 31.3)	13.3<br>(9.3 to 18.8)	1.14<br>(0.80 to 1.62)	0.69<br>(0.48 to 0.97)
2005	1.3<br>(0.9 to 1.7)	0.7<br>(0.5 to 1.0)	18.7<br>(13.4 to 25.6)	11.1<br>(8.0 to 15.2)	1.13<br>(0.81 to 1.55)	0.68<br>(0.49 to 0.92)
2010	1.4<br>(1.0 to 1.9)	0.8<br>(0.6 to 1.1)	17.2<br>(12.1 to 23.3)	10.1<br>(7.1 to 13.7)	1.15<br>(0.81 to 1.57)	0.68<br>(0.47 to 0.92)
2015	1.3<br>(0.9 to 1.7)	0.7<br>(0.5 to 1.0)	15.1<br>(10.8 to 20.6)	8.6<br>(6.2 to 11.7)	1.24<br>(0.89 to 1.69)	0.71<br>(0.51 to 0.96)
2020	0.5<br>(0.3 to 0.6)	0.2<br>(0.2 to 0.3)	7.7<br>(5.5 to 10.6)	4.2<br>(3.0 to 5.8)	1.39<br>(1.00 to 1.92)	0.76<br>(0.55 to 1.05)
2021	0.3<br>(0.2 to 0.4)	0.2<br>(0.1 to 0.2)	5.5<br>(4.0 to 7.5)	3.0<br>(2.2 to 4.1)	1.42<br>(1.02 to 1.94)	0.77<br>(0.56 to 1.05)

Gilan   IRN_44871   North Africa and Middle East   Stillbirth Rate / Neonatal Mortality Rate (SBR/NMR) − 20 weeks
lambda = 1 ; zeta = 0.6 ; scale = 15 ; amp = 0.028

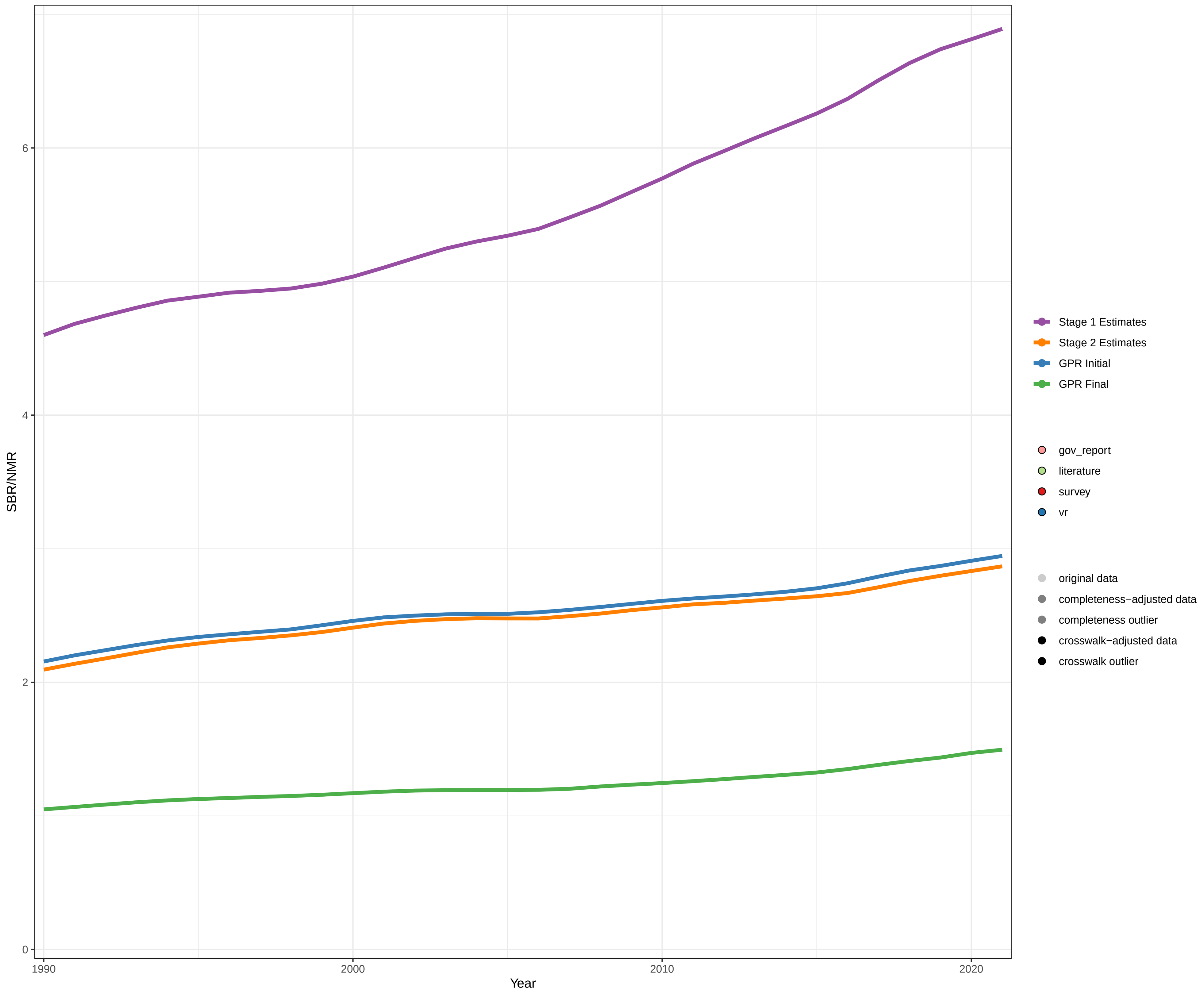

Gilan   IRN_44871   North Africa and Middle East   Stillbirth Rate / Neonatal Mortality Rate (SBR/NMR) − 28 weeks
lambda = 1 ; zeta = 0.6 ; scale = 15 ; amp = 0.028

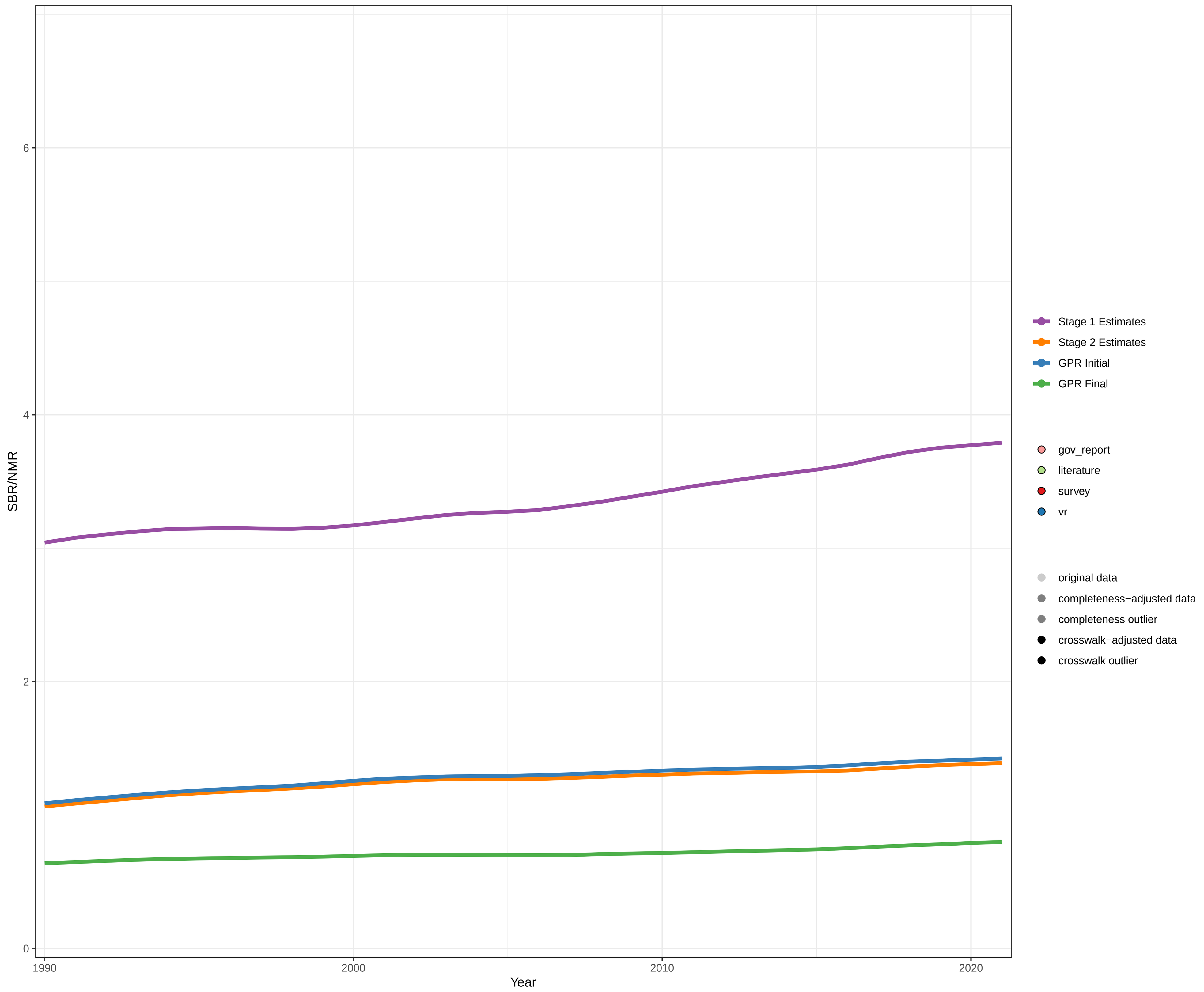

Definition	Years	Outlier	Source.Type	Title

Gilan   IRN_44871   North Africa and Middle East   Stillbirth Rate (SBR) − 20 weeks
lambda = 1 ; zeta = 0.6 ; scale = 15 ; amp = 0.028

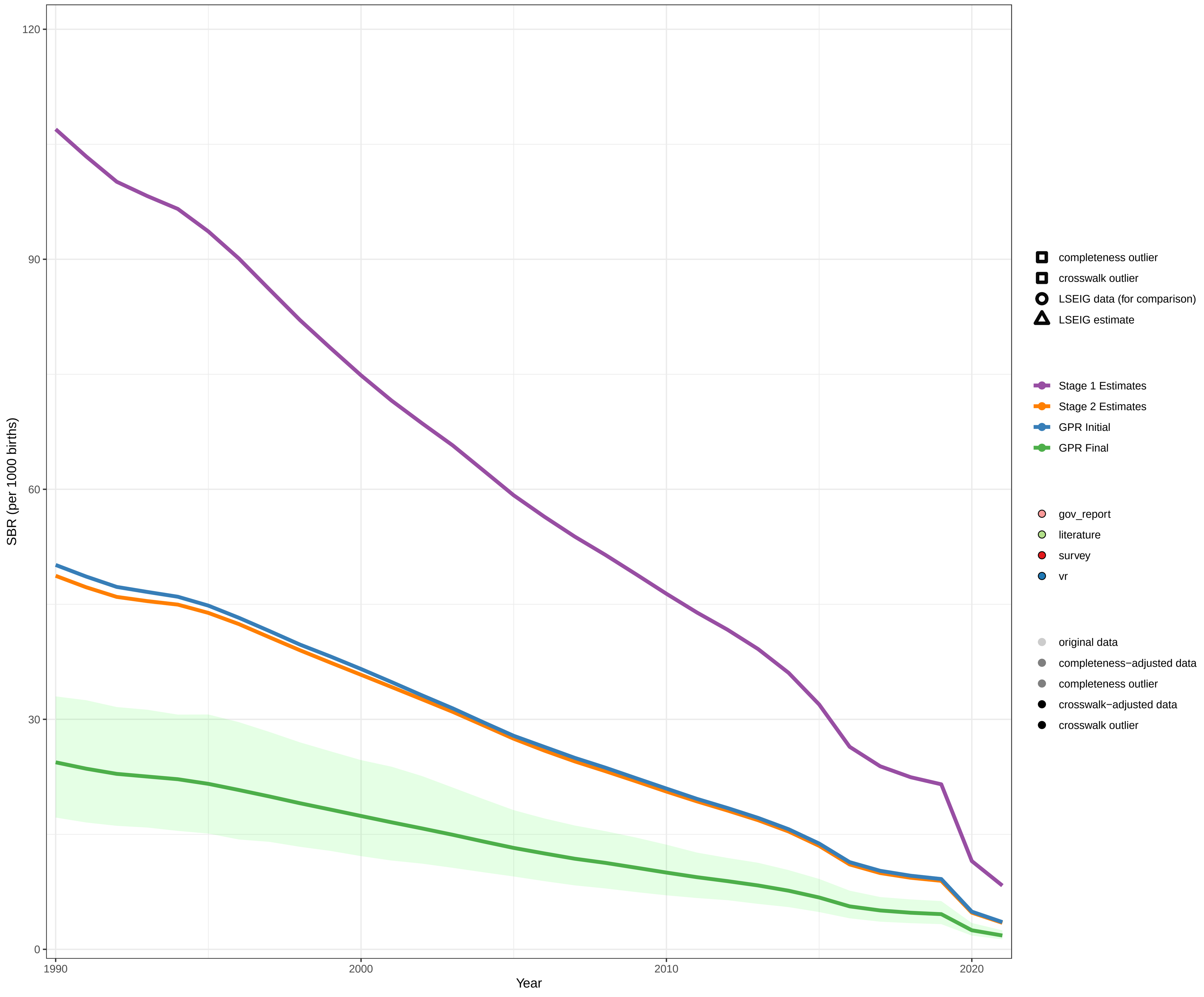

Gilan   IRN_44871   North Africa and Middle East   Stillbirth Rate (SBR) − 28 weeks
lambda = 1 ; zeta = 0.6 ; scale = 15 ; amp = 0.028

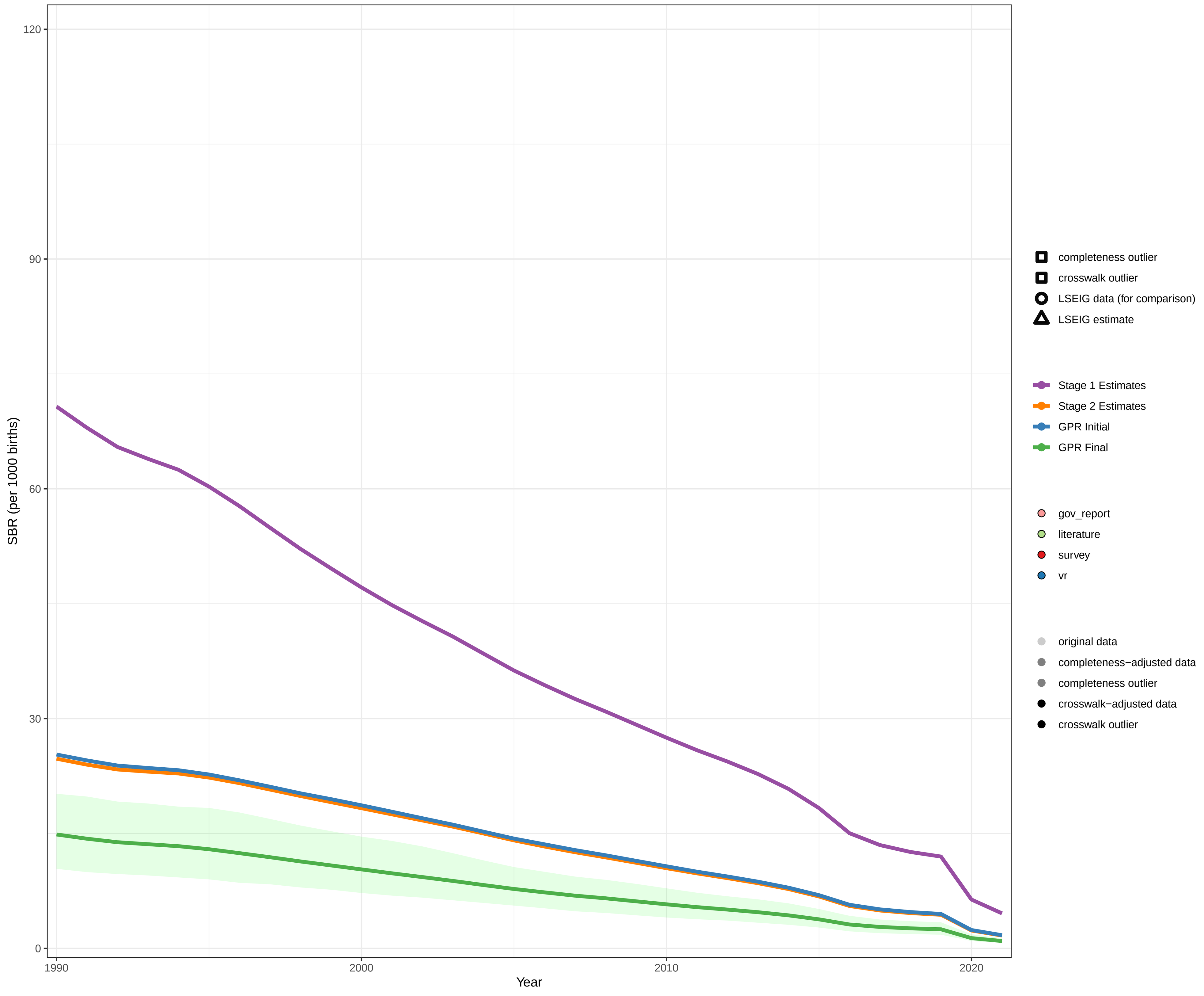

Year	Total Stillbirths (in thousands)	Total Stillbirths (in thousands)	SBR (per 1000 births)	SBR (per 1000 births)	SBR/NMR	SBR/NMR
	20 weeks	28 weeks	20 weeks	28 weeks	20 weeks	28 weeks
1990	1.3 (0.9 to 1.7)	0.8 (0.5 to 1.0)	24.4 (17.2 to 33.0)	14.9 (10.4 to 20.2)	1.05 (0.74 to 1.42)	0.64 (0.45 to 0.87)
1995	0.8 (0.6 to 1.1)	0.5 (0.3 to 0.7)	21.6 (15.1 to 30.6)	12.9 (9.0 to 18.3)	1.13 (0.79 to 1.60)	0.68 (0.47 to 0.96)
2000	0.5 (0.4 to 0.8)	0.3 (0.2 to 0.4)	17.4 (12.2 to 24.7)	10.3 (7.2 to 14.6)	1.17 (0.82 to 1.66)	0.69 (0.49 to 0.98)
2005	0.4 (0.3 to 0.5)	0.2 (0.2 to 0.3)	13.2 (9.5 to 18.2)	7.8 (5.6 to 10.6)	1.19 (0.86 to 1.64)	0.70 (0.50 to 0.96)
2010	0.3 (0.2 to 0.4)	0.2 (0.1 to 0.2)	10.0 (7.0 to 13.7)	5.8 (4.0 to 7.8)	1.25 (0.88 to 1.70)	0.72 (0.50 to 0.98)
2015	0.2 (0.2 to 0.3)	0.1 (0.1 to 0.2)	6.8 (4.9 to 9.2)	3.8 (2.7 to 5.1)	1.32 (0.95 to 1.80)	0.74 (0.53 to 1.01)
2020	0.1 (0.0 to 0.1)	0.0 (0.0 to 0.0)	2.5 (1.8 to 3.4)	1.3 (1.0 to 1.8)	1.47 (1.06 to 2.04)	0.79 (0.57 to 1.09)
2021	0.0 (0.0 to 0.1)	0.0 (0.0 to 0.0)	1.8 (1.3 to 2.5)	1.0 (0.7 to 1.3)	1.50 (1.08 to 2.05)	0.80 (0.58 to 1.09)

Golestan   IRN_44872   North Africa and Middle East   Stillbirth Rate / Neonatal Mortality Rate (SBR/NMR) − 20 weeks
lambda = 1 ; zeta = 0.6 ; scale = 15 ; amp = 0.028

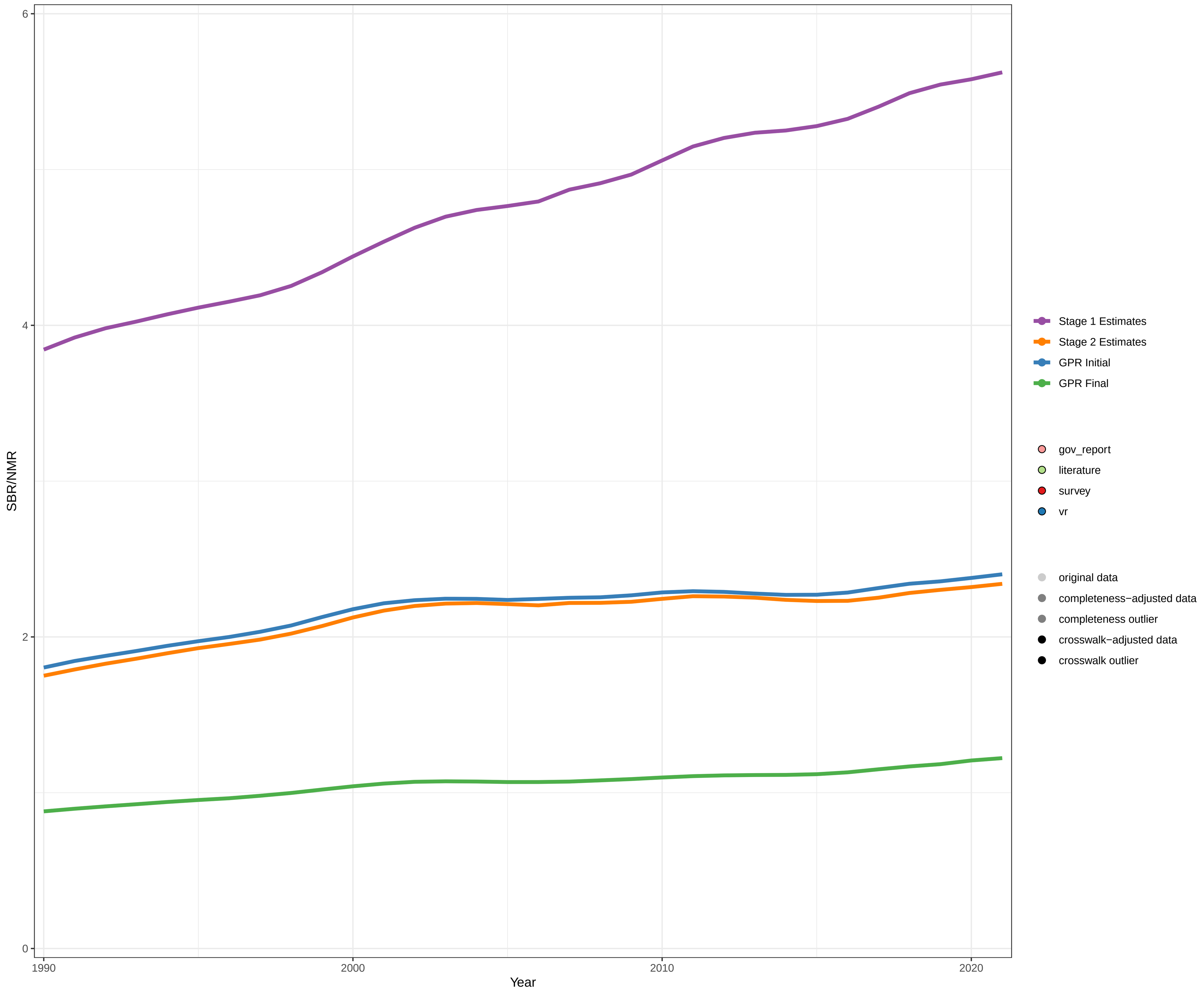

Golestan   IRN_44872   North Africa and Middle East   Stillbirth Rate / Neonatal Mortality Rate (SBR/NMR) − 28 weeks
lambda = 1 ; zeta = 0.6 ; scale = 15 ; amp = 0.028

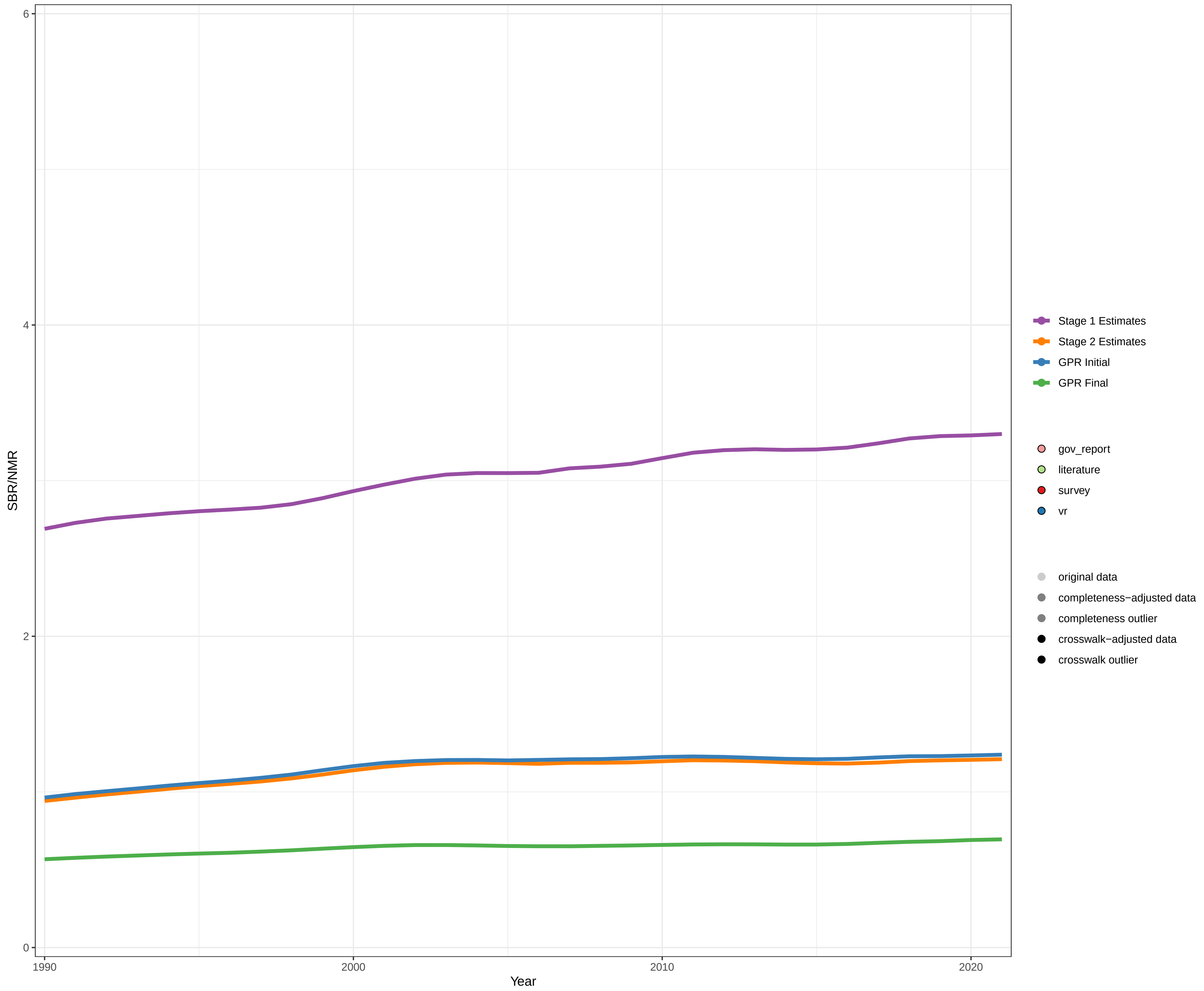

Definition	Years	Outlier	Source.Type	Title

Golestan   IRN_44872   North Africa and Middle East   Stillbirth Rate (SBR) − 20 weeks
lambda = 1 ; zeta = 0.6 ; scale = 15 ; amp = 0.028

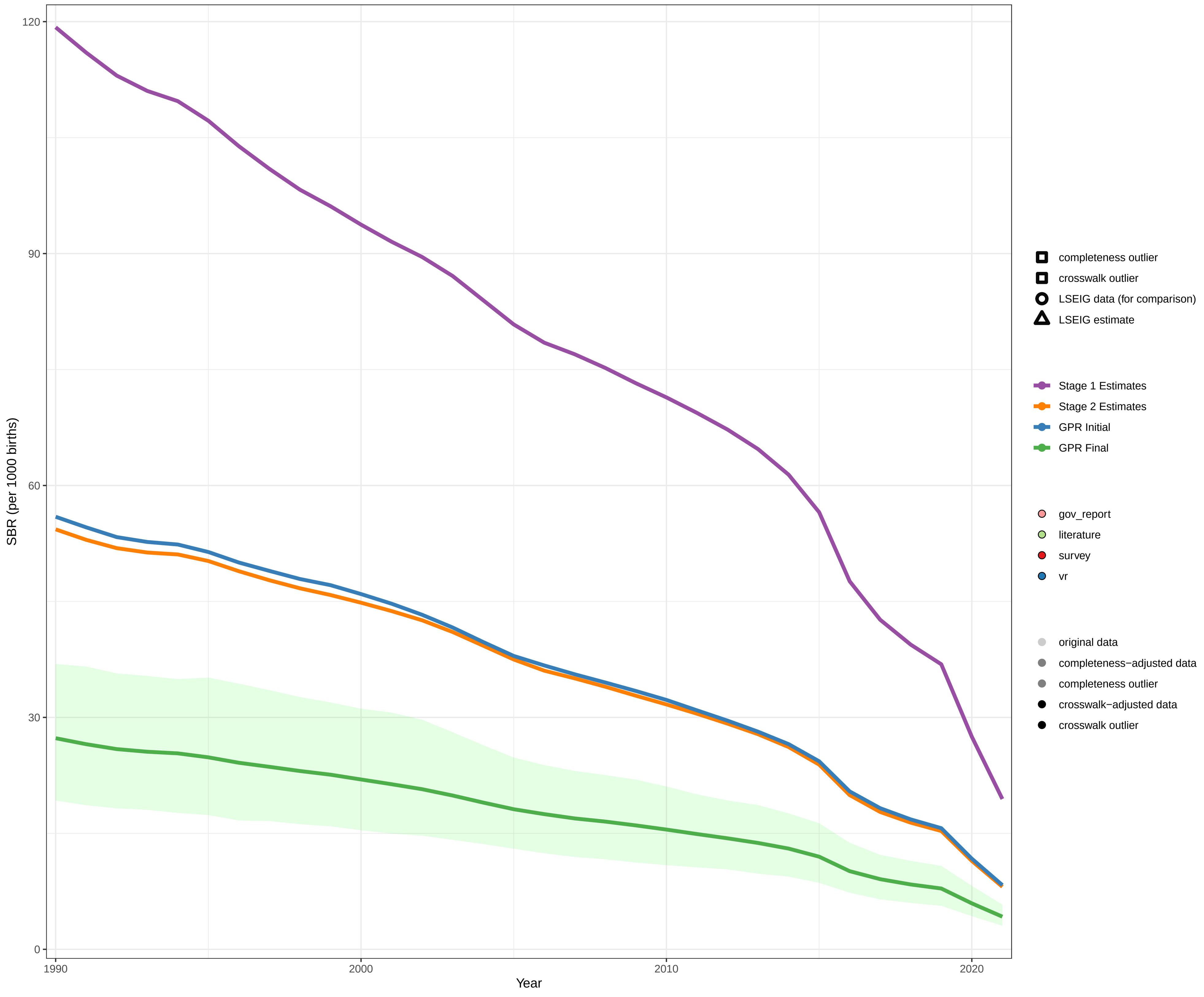

Golestan   IRN_44872   North Africa and Middle East   Stillbirth Rate (SBR) − 28 weeks
lambda = 1 ; zeta = 0.6 ; scale = 15 ; amp = 0.028

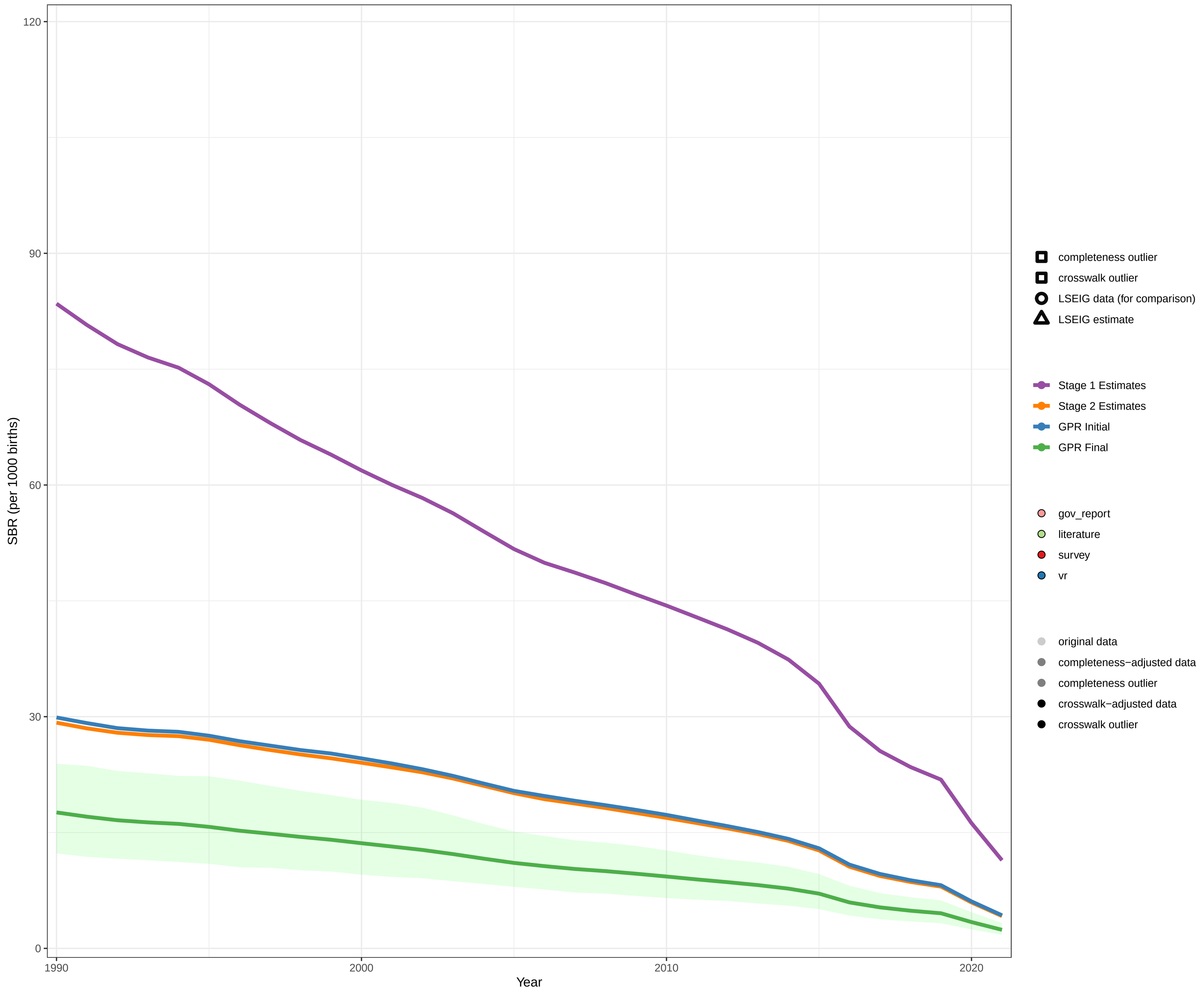

Year	Total Stillbirths (in thousands)	Total Stillbirths (in thousands)	SBR (per 1000 births)	SBR (per 1000 births)	SBR/NMR	SBR/NMR
	20 weeks	28 weeks	20 weeks	28 weeks	20 weeks	28 weeks
1990	1.2 (0.8 to 1.6)	0.8 (0.5 to 1.0)	27.3 (19.2 to 36.9)	17.6 (12.3 to 23.9)	0.88 (0.62 to 1.19)	0.57 (0.40 to 0.77)
1995	0.8 (0.6 to 1.2)	0.5 (0.4 to 0.7)	24.8 (17.4 to 35.2)	15.7 (10.9 to 22.3)	0.95 (0.67 to 1.35)	0.60 (0.42 to 0.86)
2000	0.7 (0.5 to 0.9)	0.4 (0.3 to 0.6)	22.0 (15.4 to 31.1)	13.6 (9.5 to 19.2)	1.04 (0.73 to 1.48)	0.65 (0.45 to 0.91)
2005	0.5 (0.4 to 0.7)	0.3 (0.2 to 0.4)	18.1 (13.0 to 24.8)	11.1 (8.0 to 15.1)	1.07 (0.77 to 1.46)	0.65 (0.47 to 0.89)
2010	0.6 (0.4 to 0.8)	0.4 (0.2 to 0.5)	15.5 (10.9 to 21.1)	9.3 (6.5 to 12.7)	1.10 (0.77 to 1.49)	0.66 (0.46 to 0.90)
2015	0.5 (0.4 to 0.7)	0.3 (0.2 to 0.4)	12.0 (8.6 to 16.3)	7.1 (5.1 to 9.6)	1.12 (0.80 to 1.52)	0.66 (0.47 to 0.90)
2020	0.2 (0.1 to 0.2)	0.1 (0.1 to 0.1)	5.9 (4.3 to 8.2)	3.4 (2.5 to 4.7)	1.21 (0.87 to 1.67)	0.69 (0.50 to 0.95)
2021	0.1 (0.1 to 0.2)	0.1 (0.0 to 0.1)	4.2 (3.0 to 5.8)	2.4 (1.7 to 3.3)	1.22 (0.88 to 1.67)	0.70 (0.50 to 0.95)

Hamadan IRN_44873 North Africa and Middle East Stillbirth Rate / Neonatal Mortality Rate (SBR/NMR) − 20 weeks
lambda = 1 ; zeta = 0.6 ; scale = 15 ; amp = 0.028

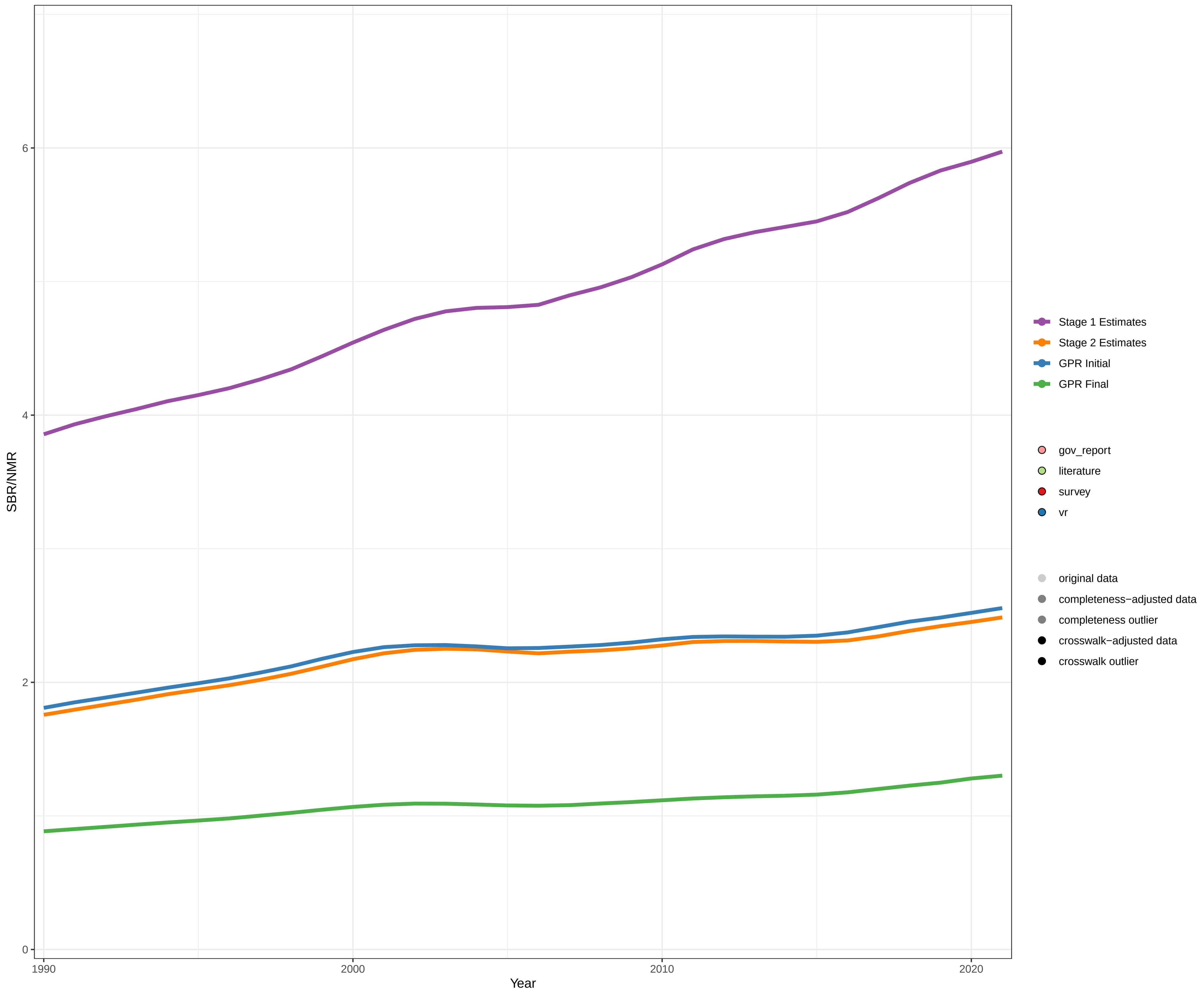

Hamadan IRN_44873 North Africa and Middle East Stillbirth Rate / Neonatal Mortality Rate (SBR/NMR) − 28 weeks
lambda = 1 ; zeta = 0.6 ; scale = 15 ; amp = 0.028

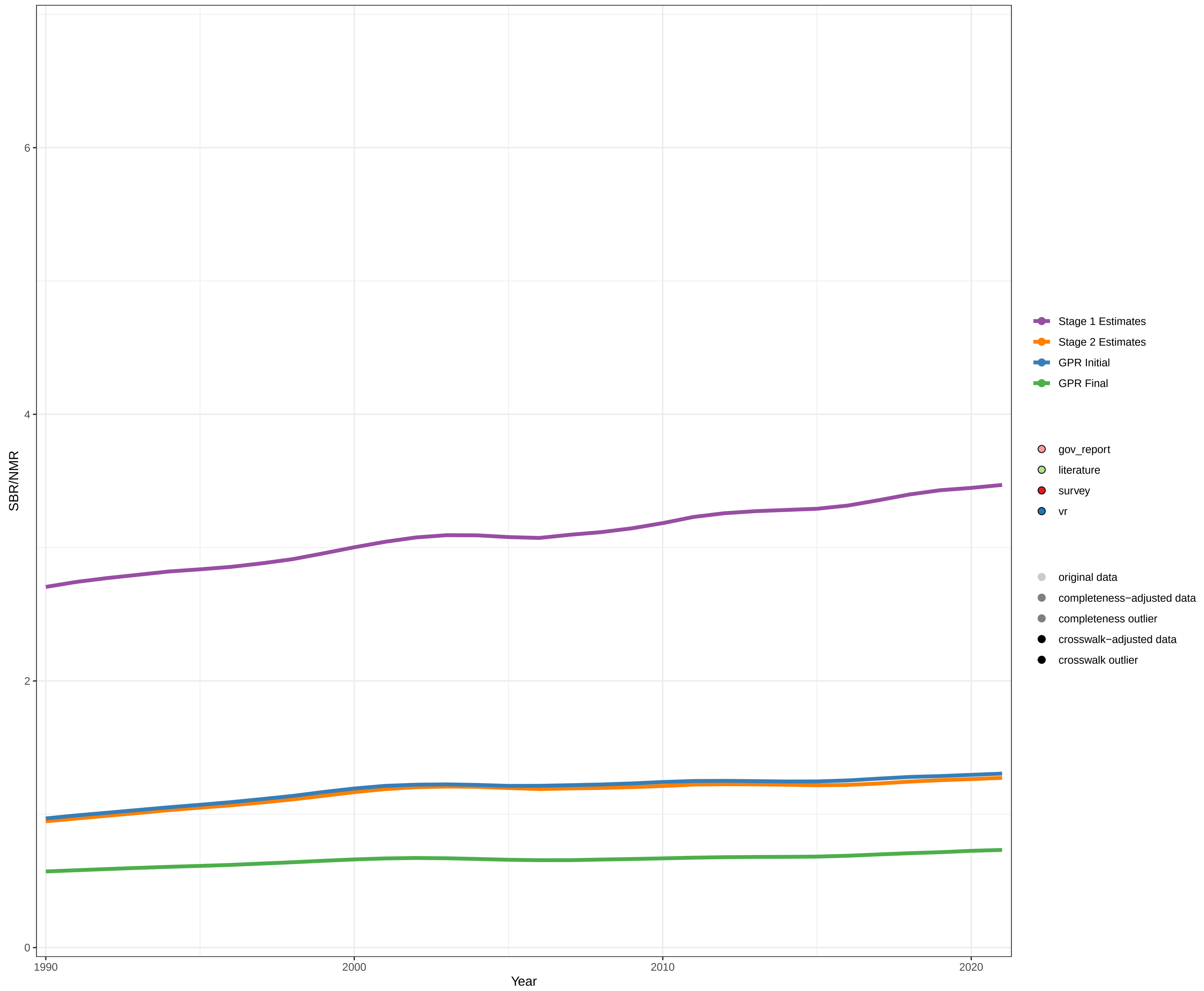

Definition	Years	Outlier	Source.Type	Title

Hamadan IRN_44873 North Africa and Middle East Stillbirth Rate (SBR) − 20 weeks
lambda = 1 ; zeta = 0.6 ; scale = 15 ; amp = 0.028

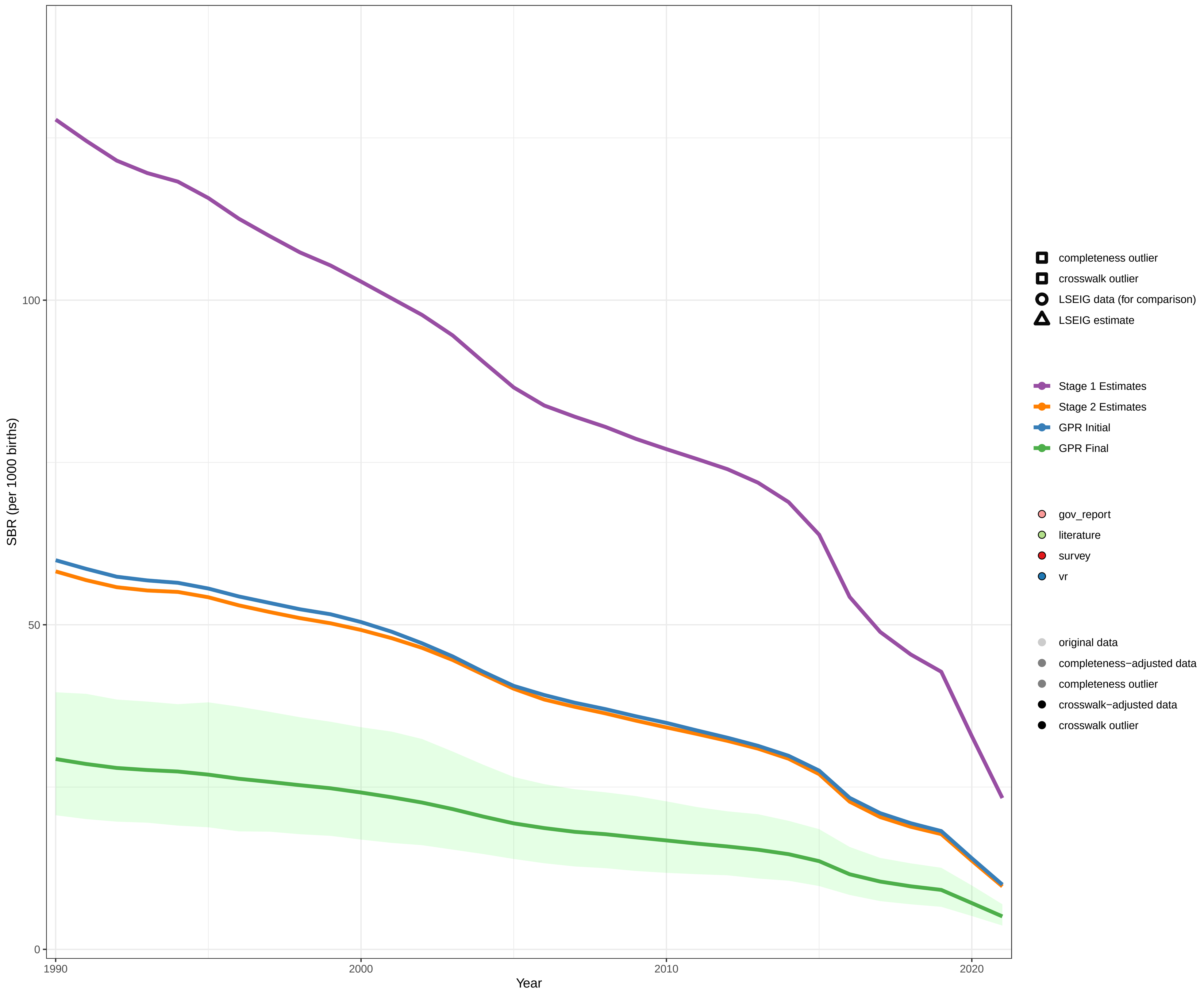

Hamadan IRN_44873 North Africa and Middle East Stillbirth Rate (SBR) − 28 weeks
lambda = 1 ; zeta = 0.6 ; scale = 15 ; amp = 0.028

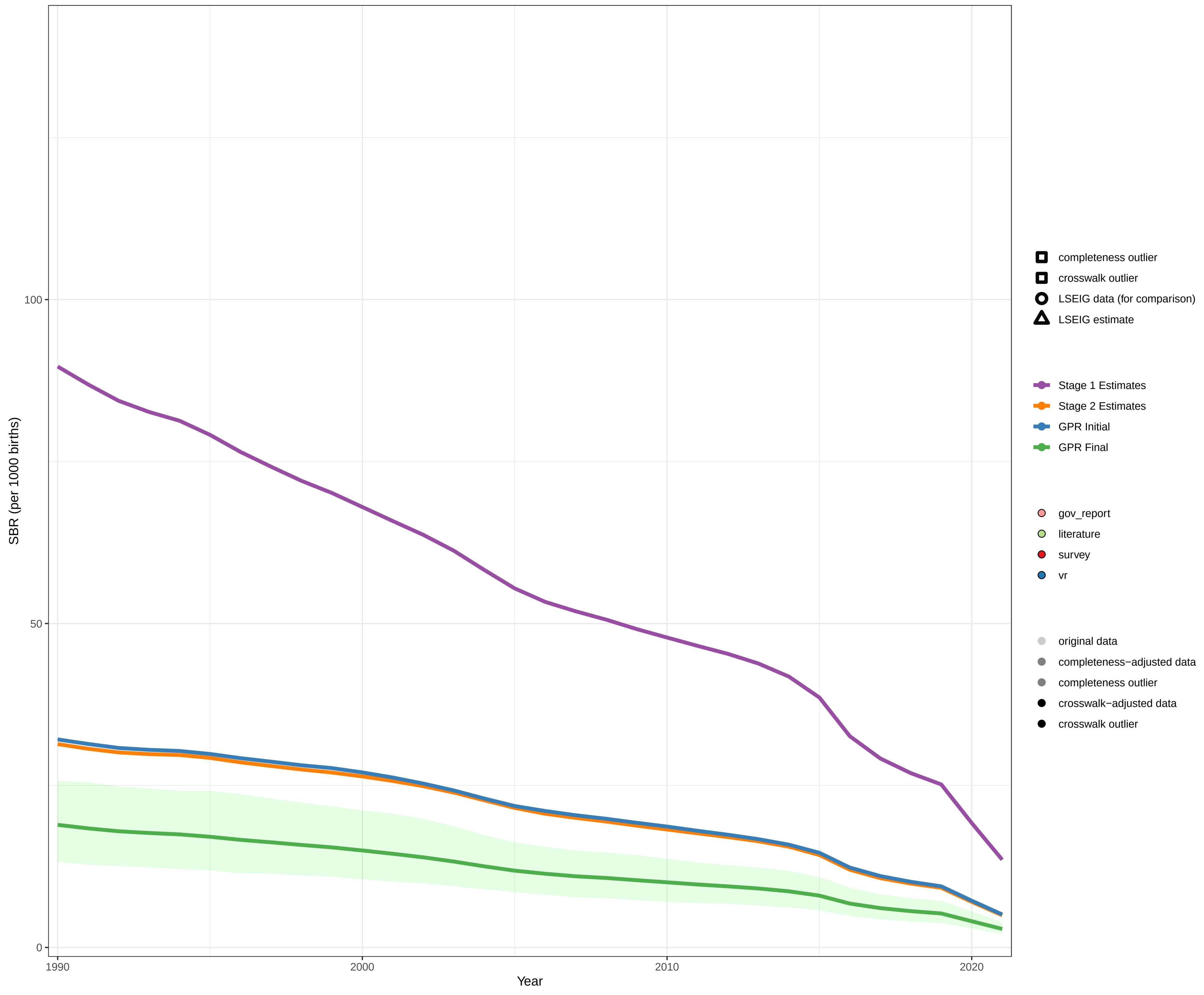

Year	Total Stillbirths (in thousands)	Total Stillbirths (in thousands)	SBR (per 1000 births)	SBR (per 1000 births)	SBR/NMR	SBR/NMR
	20 weeks	28 weeks	20 weeks	28 weeks	20 weeks	28 weeks
1990	1.5 (1.0 to 2.0)	0.9 (0.6 to 1.3)	29.3 (20.7 to 39.6)	18.9 (13.2 to 25.7)	0.88 (0.62 to 1.19)	0.57 (0.40 to 0.78)
1995	1.0 (0.7 to 1.5)	0.6 (0.4 to 0.9)	26.9 (18.8 to 38.1)	17.1 (11.9 to 24.2)	0.97 (0.67 to 1.36)	0.61 (0.43 to 0.87)
2000	0.7 (0.5 to 1.1)	0.5 (0.3 to 0.6)	24.2 (16.9 to 34.2)	15.0 (10.5 to 21.2)	1.07 (0.75 to 1.51)	0.66 (0.46 to 0.93)
2005	0.5 (0.4 to 0.7)	0.3 (0.2 to 0.4)	19.4 (13.9 to 26.6)	11.9 (8.5 to 16.2)	1.08 (0.77 to 1.48)	0.66 (0.47 to 0.90)
2010	0.5 (0.4 to 0.7)	0.3 (0.2 to 0.4)	16.8 (11.8 to 22.8)	10.1 (7.0 to 13.7)	1.12 (0.78 to 1.52)	0.67 (0.47 to 0.91)
2015	0.4 (0.3 to 0.6)	0.3 (0.2 to 0.4)	13.6 (9.8 to 18.5)	8.0 (5.7 to 10.9)	1.16 (0.83 to 1.58)	0.68 (0.49 to 0.93)
2020	0.2 (0.1 to 0.2)	0.1 (0.1 to 0.1)	7.1 (5.1 to 9.8)	4.0 (2.9 to 5.6)	1.28 (0.92 to 1.77)	0.73 (0.52 to 1.00)
2021	0.1 (0.1 to 0.1)	0.1 (0.0 to 0.1)	5.1 (3.7 to 6.9)	2.9 (2.1 to 3.9)	1.30 (0.94 to 1.78)	0.73 (0.53 to 1.00)

Hormozgan IRN_44874 North Africa and Middle East Stillbirth Rate / Neonatal Mortality Rate (SBR/NMR) − 20 weeks
lambda = 1 ; zeta = 0.6 ; scale = 15 ; amp = 0.028

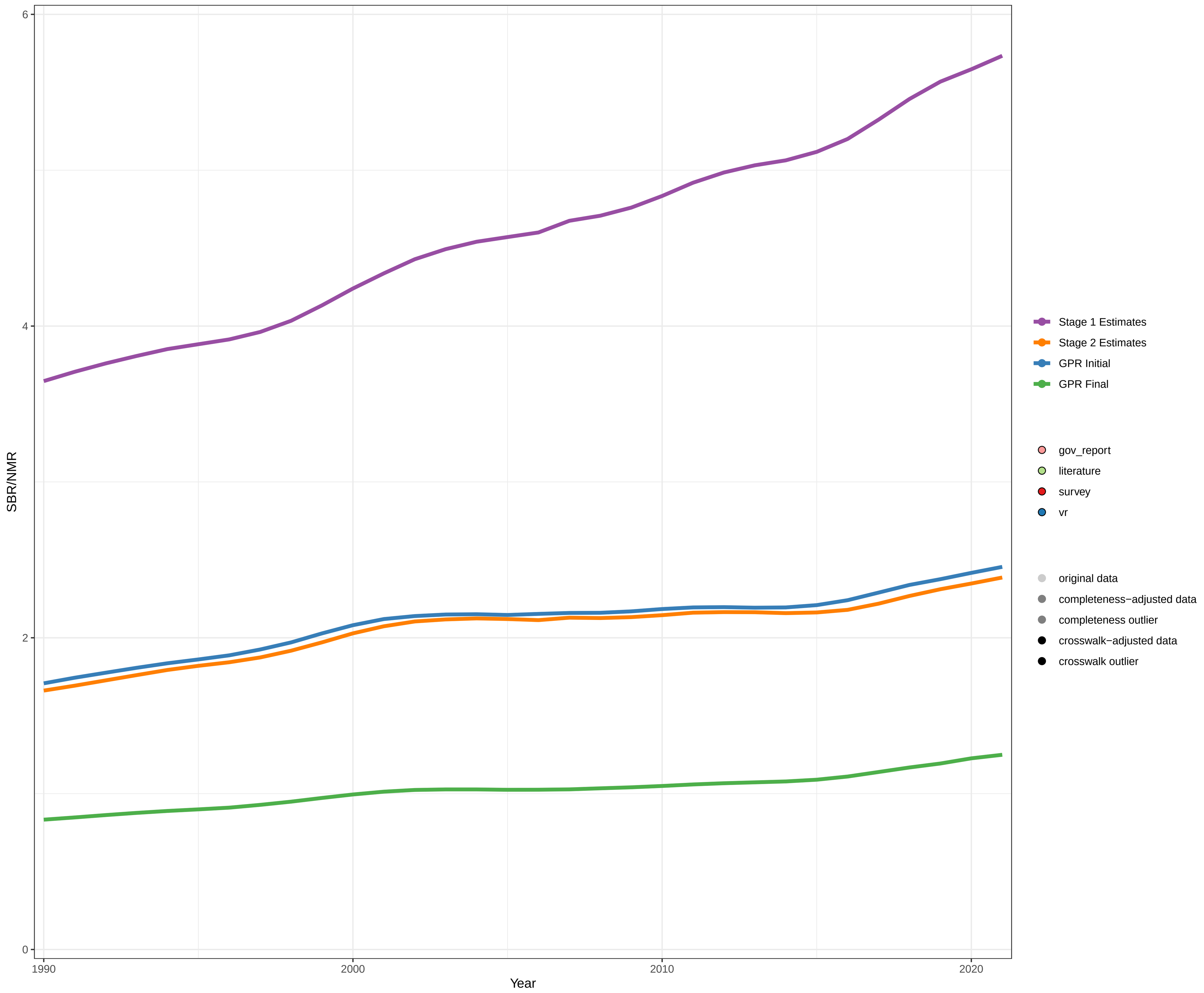

Hormozgan IRN_44874 North Africa and Middle East Stillbirth Rate / Neonatal Mortality Rate (SBR/NMR) − 28 weeks
lambda = 1 ; zeta = 0.6 ; scale = 15 ; amp = 0.028

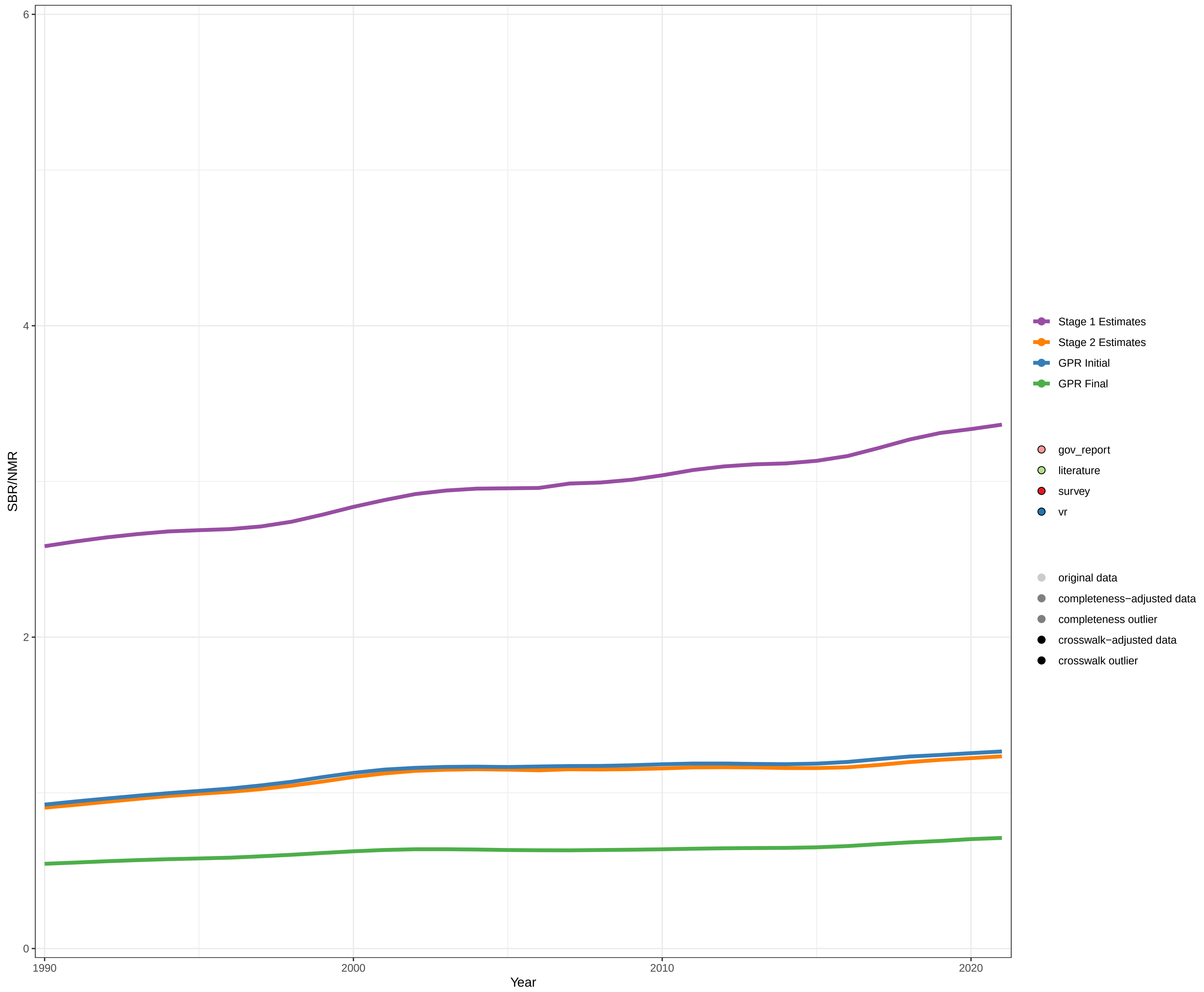

Definition	Years	Outlier	Source.Type	Title

Hormozgan IRN_44874 North Africa and Middle East Stillbirth Rate (SBR) − 20 weeks
lambda = 1 ; zeta = 0.6 ; scale = 15 ; amp = 0.028

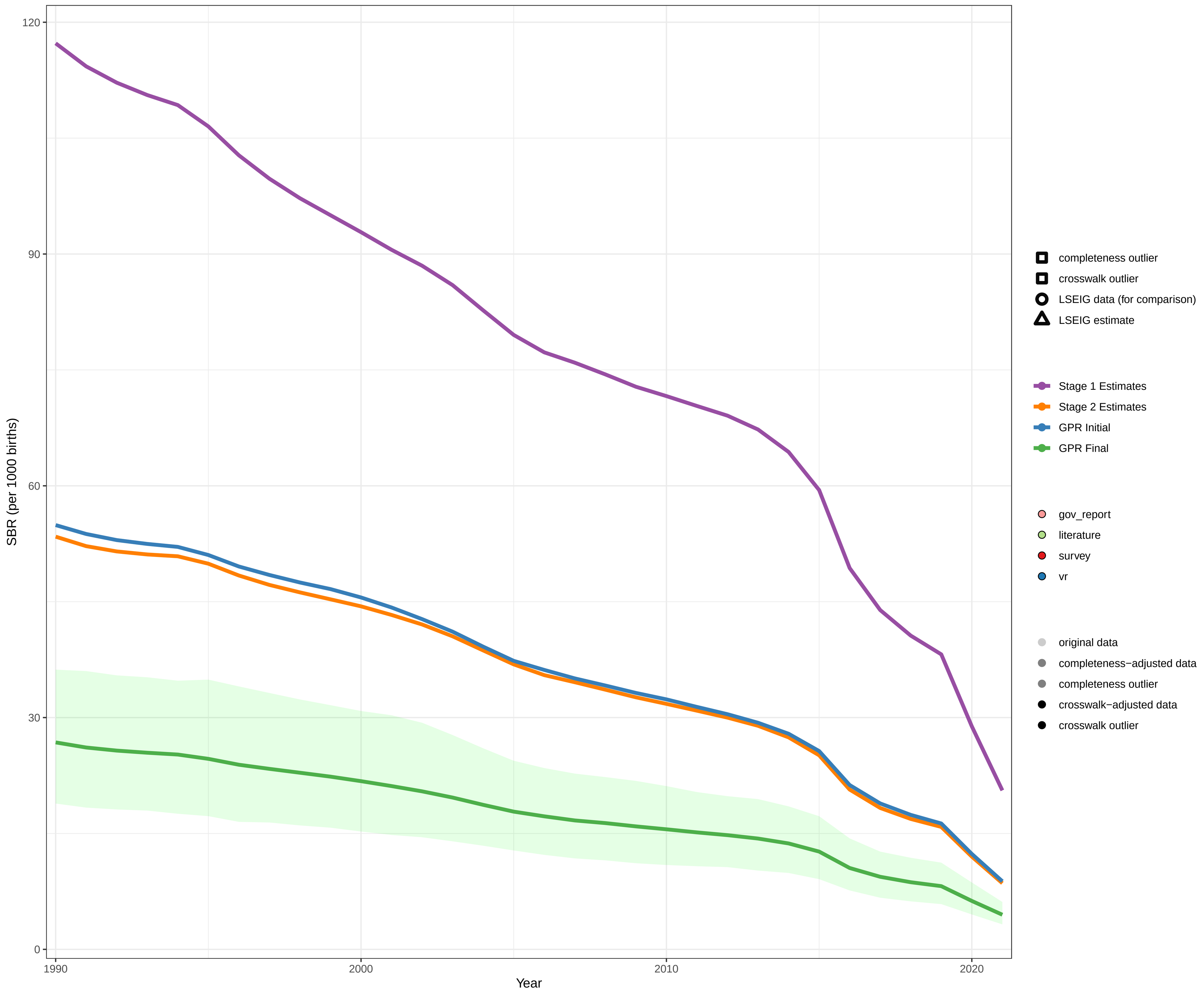

Hormozgan IRN_44874 North Africa and Middle East Stillbirth Rate (SBR) − 28 weeks
lambda = 1 ; zeta = 0.6 ; scale = 15 ; amp = 0.028

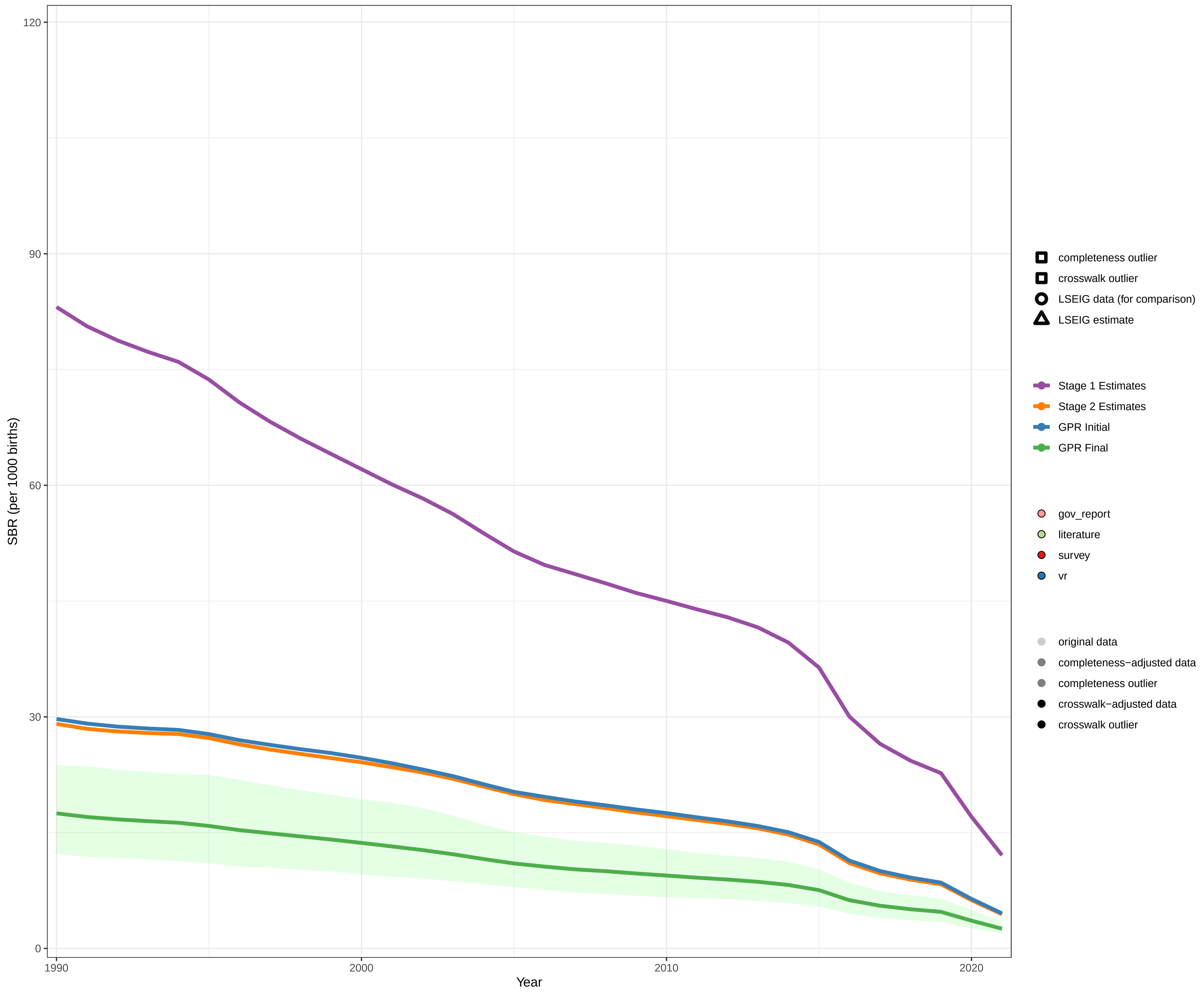

Year	Total Stillbirths (in thousands)	Total Stillbirths (in thousands)	SBR (per 1000 births)	SBR (per 1000 births)	SBR/NMR	SBR/NMR
	20 weeks	28 weeks	20 weeks	28 weeks	20 weeks	28 weeks
1990	0.8 (0.6 to 1.1)	0.5 (0.4 to 0.7)	26.8 (18.9 to 36.2)	17.5 (12.2 to 23.8)	0.83 (0.59 to 1.13)	0.54 (0.38 to 0.74)
1995	0.6 (0.4 to 0.9)	0.4 (0.3 to 0.6)	24.7 (17.2 to 34.9)	15.9 (11.0 to 22.5)	0.90 (0.63 to 1.27)	0.58 (0.40 to 0.82)
2000	0.5 (0.4 to 0.8)	0.3 (0.2 to 0.5)	21.8 (15.3 to 30.9)	13.7 (9.6 to 19.3)	0.99 (0.70 to 1.41)	0.62 (0.44 to 0.88)
2005	0.5 (0.3 to 0.6)	0.3 (0.2 to 0.4)	17.8 (12.8 to 24.4)	11.0 (7.9 to 15.1)	1.02 (0.74 to 1.40)	0.63 (0.46 to 0.87)
2010	0.5 (0.4 to 0.7)	0.3 (0.2 to 0.4)	15.5 (10.9 to 21.1)	9.4 (6.6 to 12.9)	1.05 (0.74 to 1.43)	0.64 (0.45 to 0.87)
2015	0.5 (0.4 to 0.7)	0.3 (0.2 to 0.4)	12.7 (9.1 to 17.2)	7.6 (5.4 to 10.3)	1.09 (0.78 to 1.48)	0.65 (0.47 to 0.88)
2020	0.2 (0.1 to 0.3)	0.1 (0.1 to 0.2)	6.3 (4.5 to 8.7)	3.6 (2.6 to 4.9)	1.23 (0.88 to 1.69)	0.70 (0.51 to 0.97)
2021	0.1 (0.1 to 0.2)	0.1 (0.1 to 0.1)	4.5 (3.2 to 6.1)	2.5 (1.8 to 3.5)	1.25 (0.90 to 1.71)	0.71 (0.51 to 0.97)

Ilam IRN_44875 North Africa and Middle East Stillbirth Rate / Neonatal Mortality Rate (SBR/NMR) − 20 weeks
lambda = 1 ; zeta = 0.6 ; scale = 15 ; amp = 0.028

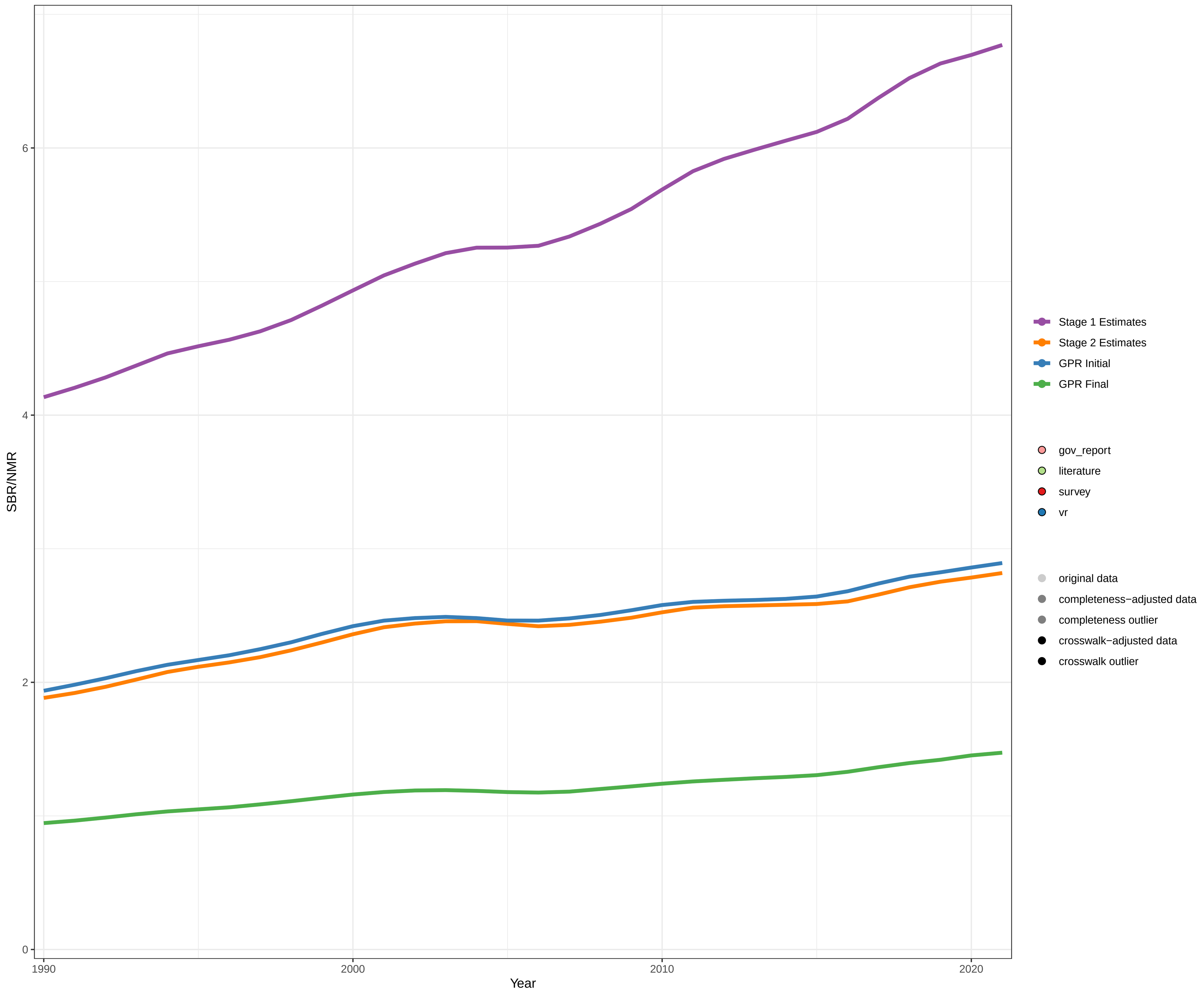

Ilam IRN_44875 North Africa and Middle East Stillbirth Rate / Neonatal Mortality Rate (SBR/NMR) − 28 weeks
lambda = 1 ; zeta = 0.6 ; scale = 15 ; amp = 0.028

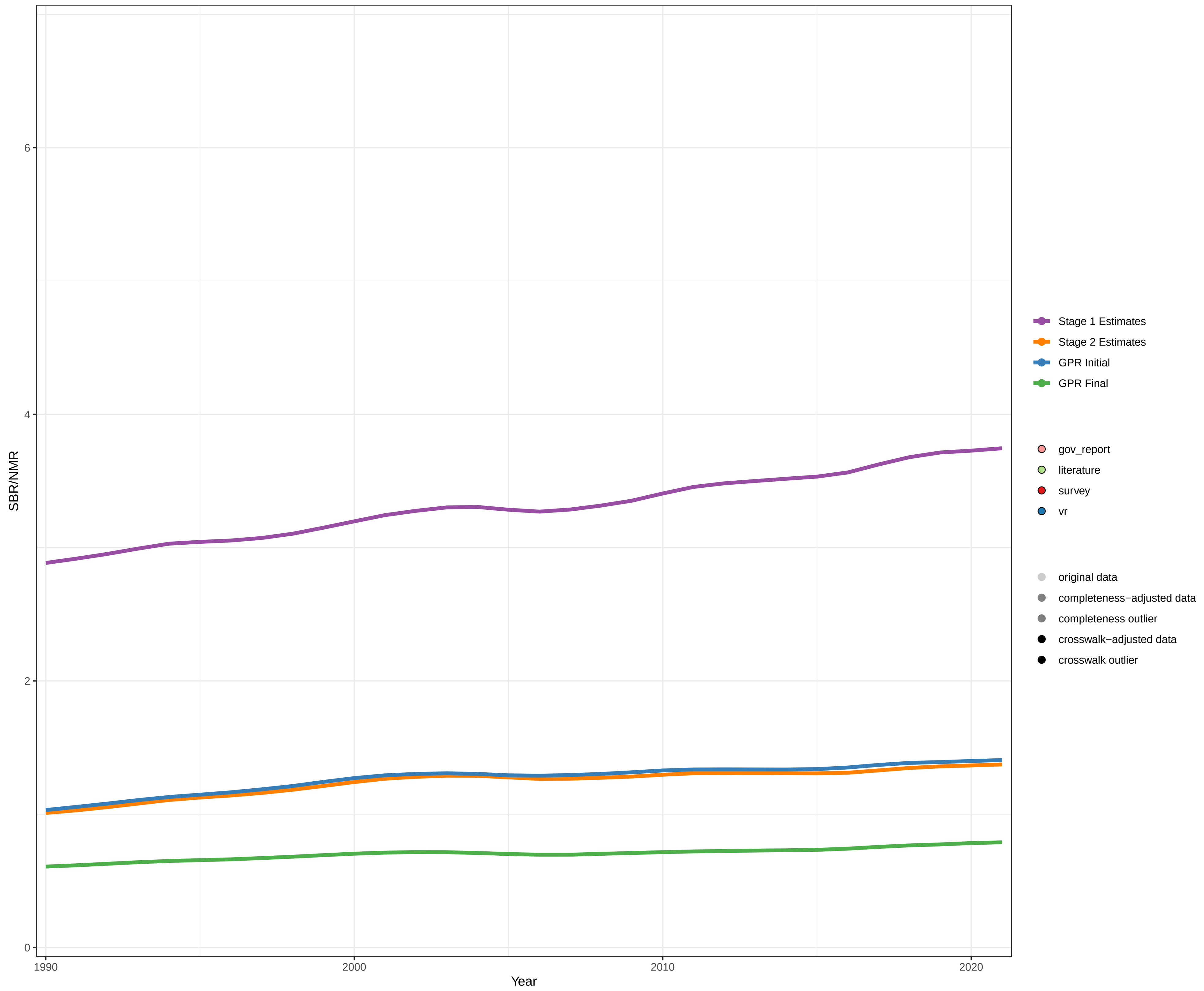

Definition	Years	Outlier	Source.Type	Title

Ilam IRN_44875 North Africa and Middle East Stillbirth Rate (SBR) − 20 weeks
lambda = 1 ; zeta = 0.6 ; scale = 15 ; amp = 0.028

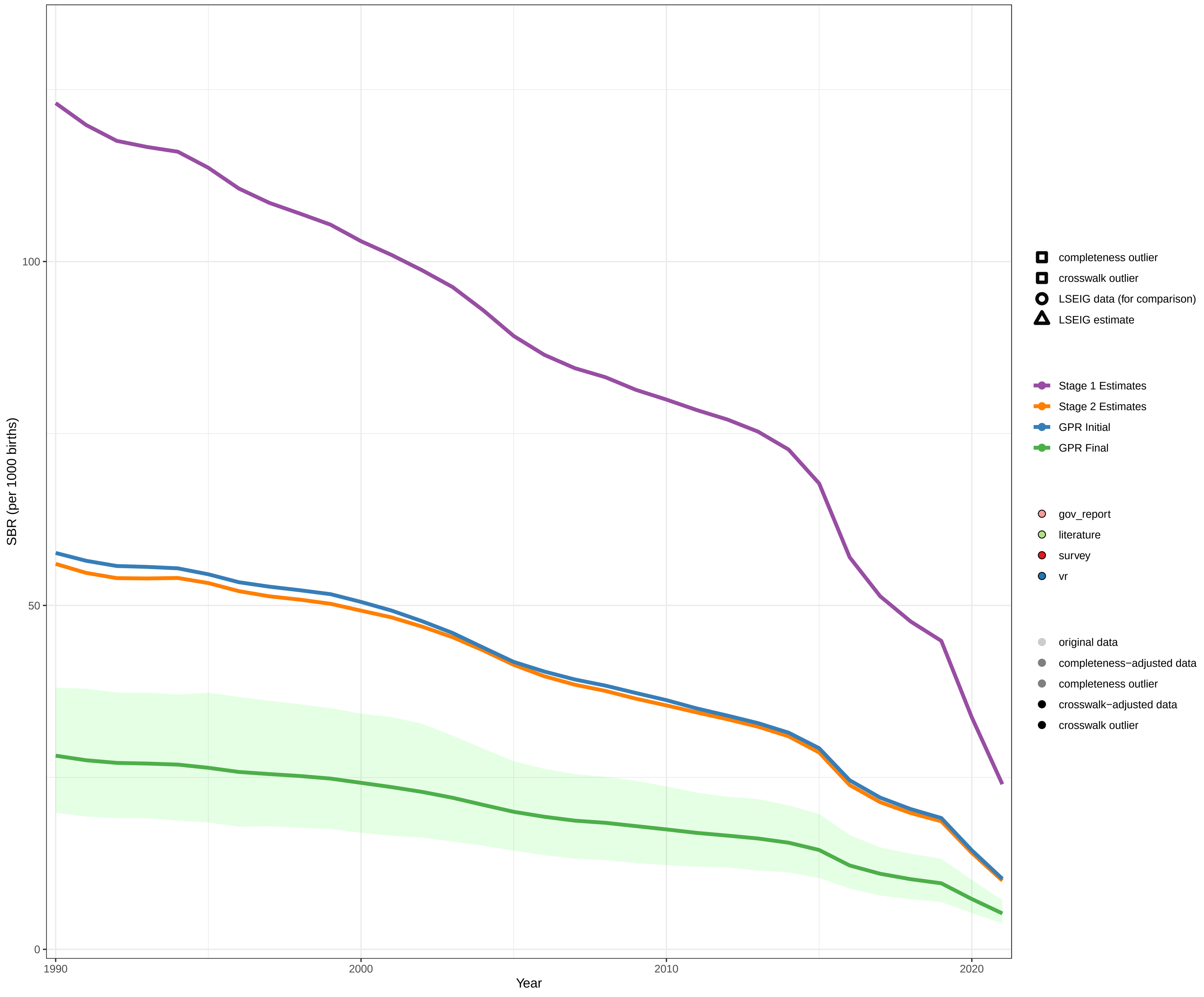

Ilam IRN_44875 North Africa and Middle East Stillbirth Rate (SBR) − 28 weeks
lambda = 1 ; zeta = 0.6 ; scale = 15 ; amp = 0.028

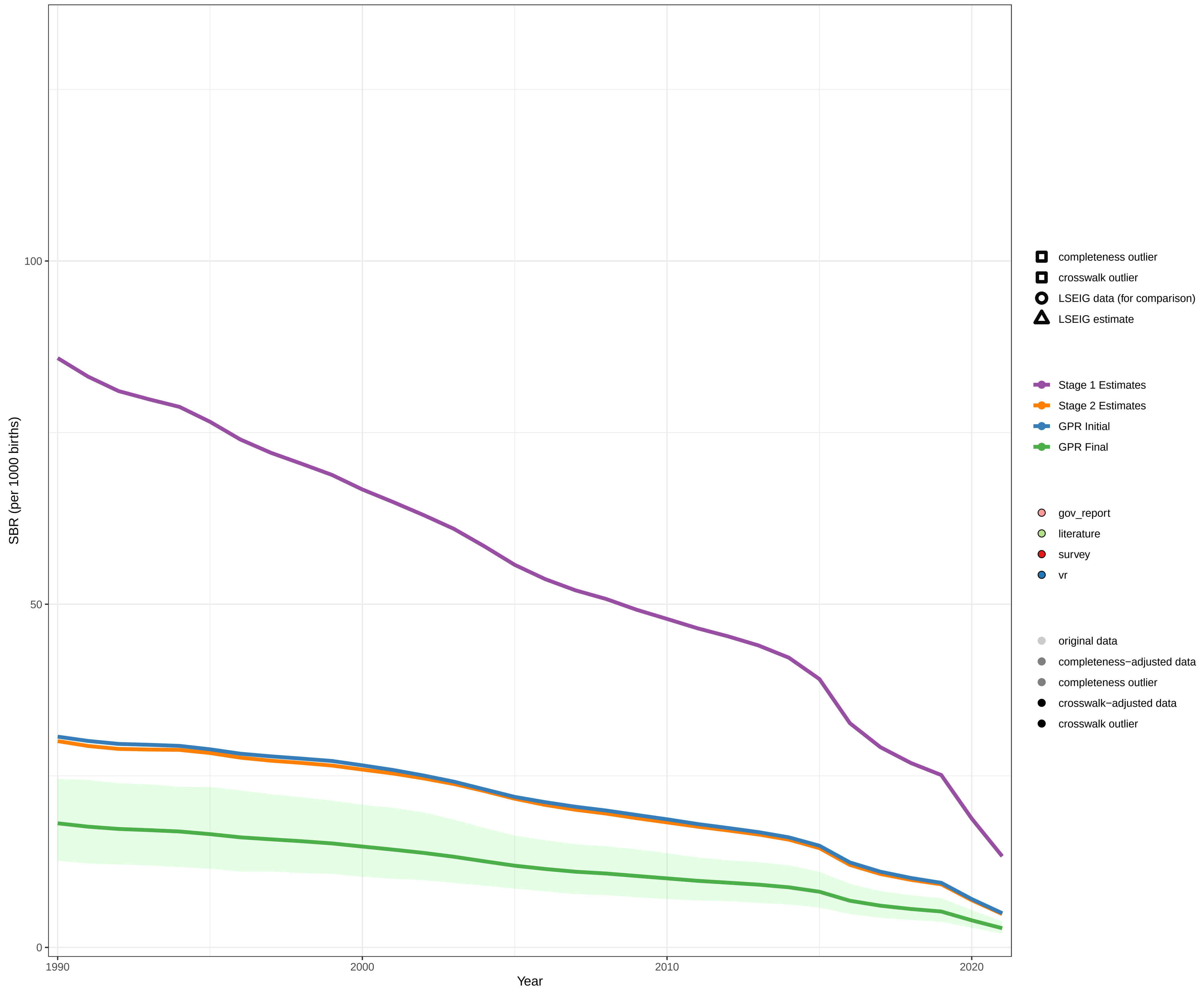

Year	Total Stillbirths (in thousands)	Total Stillbirths (in thousands)	SBR (per 1000 births)	SBR (per 1000 births)	SBR/NMR	SBR/NMR
	20 weeks	28 weeks	20 weeks	28 weeks	20 weeks	28 weeks
1990	0.4 (0.3 to 0.5)	0.2 (0.2 to 0.3)	28.2 (19.8 to 38.0)	18.1 (12.6 to 24.6)	0.95 (0.67 to 1.28)	0.61 (0.42 to 0.83)
1995	0.3 (0.2 to 0.4)	0.2 (0.1 to 0.3)	26.4 (18.4 to 37.3)	16.5 (11.5 to 23.4)	1.05 (0.73 to 1.48)	0.66 (0.46 to 0.93)
2000	0.2 (0.2 to 0.3)	0.1 (0.1 to 0.2)	24.2 (16.9 to 34.3)	14.7 (10.3 to 20.8)	1.16 (0.81 to 1.64)	0.70 (0.49 to 1.00)
2005	0.2 (0.1 to 0.2)	0.1 (0.1 to 0.1)	20.0 (14.3 to 27.4)	11.9 (8.6 to 16.3)	1.18 (0.85 to 1.61)	0.70 (0.51 to 0.96)
2010	0.2 (0.1 to 0.2)	0.1 (0.1 to 0.1)	17.4 (12.3 to 23.7)	10.1 (7.0 to 13.7)	1.24 (0.87 to 1.69)	0.72 (0.50 to 0.98)
2015	0.2 (0.1 to 0.2)	0.1 (0.1 to 0.1)	14.4 (10.4 to 19.7)	8.1 (5.8 to 11.0)	1.31 (0.94 to 1.78)	0.73 (0.53 to 1.00)
2020	0.1 (0.0 to 0.1)	0.0 (0.0 to 0.0)	7.3 (5.3 to 10.1)	3.9 (2.8 to 5.4)	1.45 (1.05 to 2.01)	0.78 (0.57 to 1.08)
2021	0.0 (0.0 to 0.1)	0.0 (0.0 to 0.0)	5.2 (3.8 to 7.1)	2.8 (2.0 to 3.8)	1.47 (1.06 to 2.02)	0.79 (0.57 to 1.08)

Isfahan IRN_44876 North Africa and Middle East Stillbirth Rate / Neonatal Mortality Rate (SBR/NMR) − 20 weeks
lambda = 1 ; zeta = 0.6 ; scale = 15 ; amp = 0.028

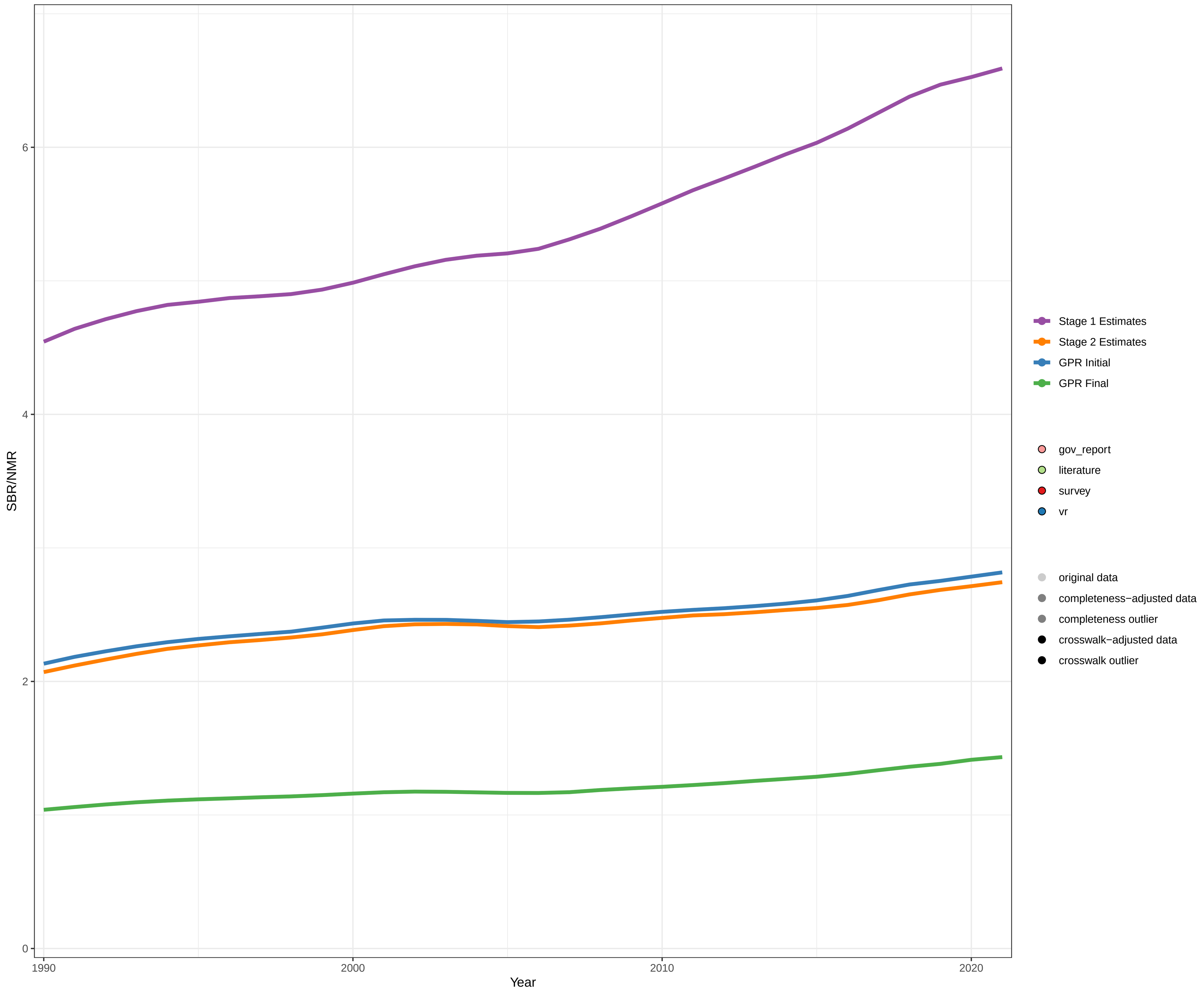

Isfahan IRN_44876 North Africa and Middle East Stillbirth Rate / Neonatal Mortality Rate (SBR/NMR) − 28 weeks
lambda = 1 ; zeta = 0.6 ; scale = 15 ; amp = 0.028

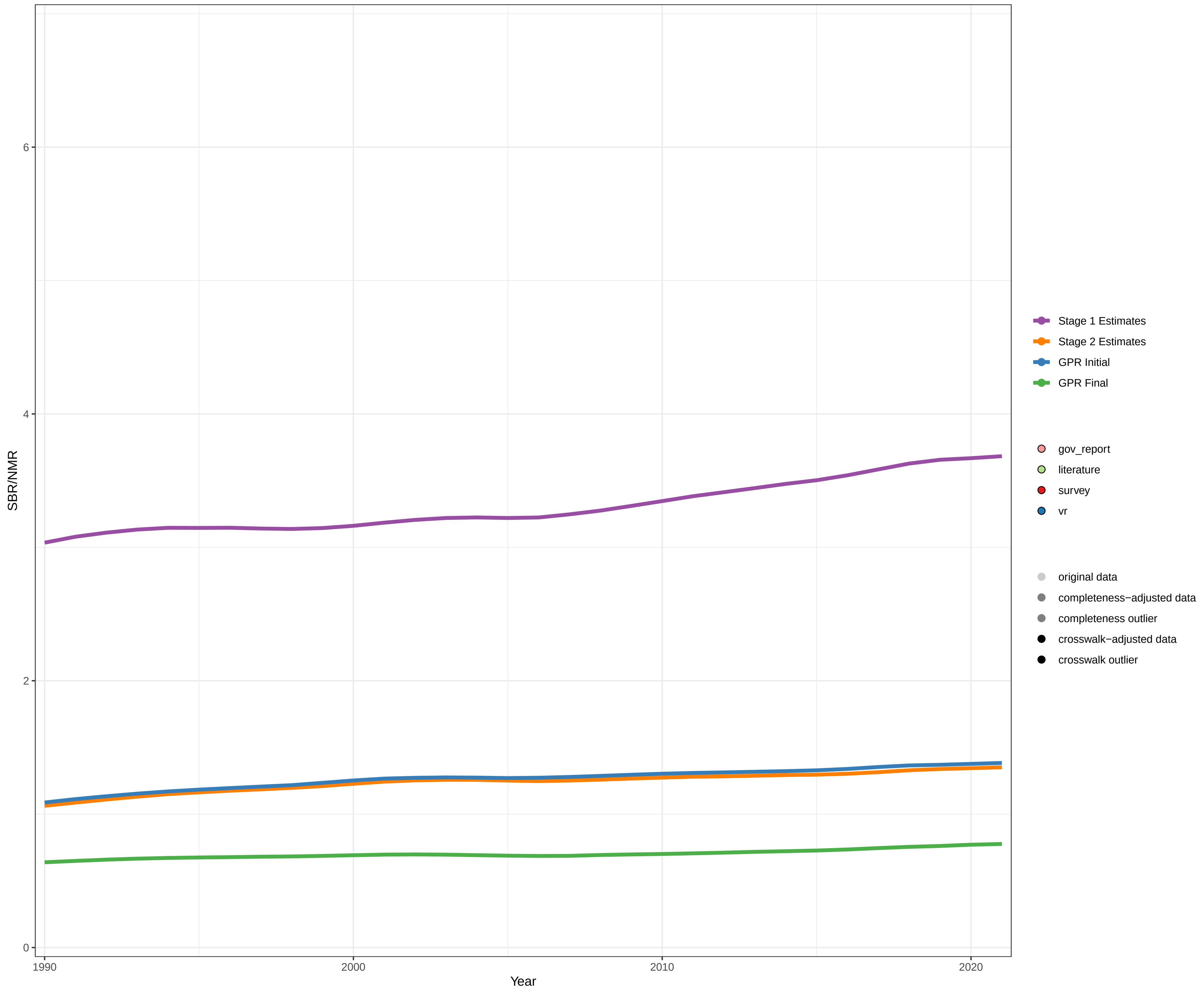

Definition	Years	Outlier	Source.Type	Title

Isfahan IRN_44876 North Africa and Middle East Stillbirth Rate (SBR) − 20 weeks
lambda = 1 ; zeta = 0.6 ; scale = 15 ; amp = 0.028

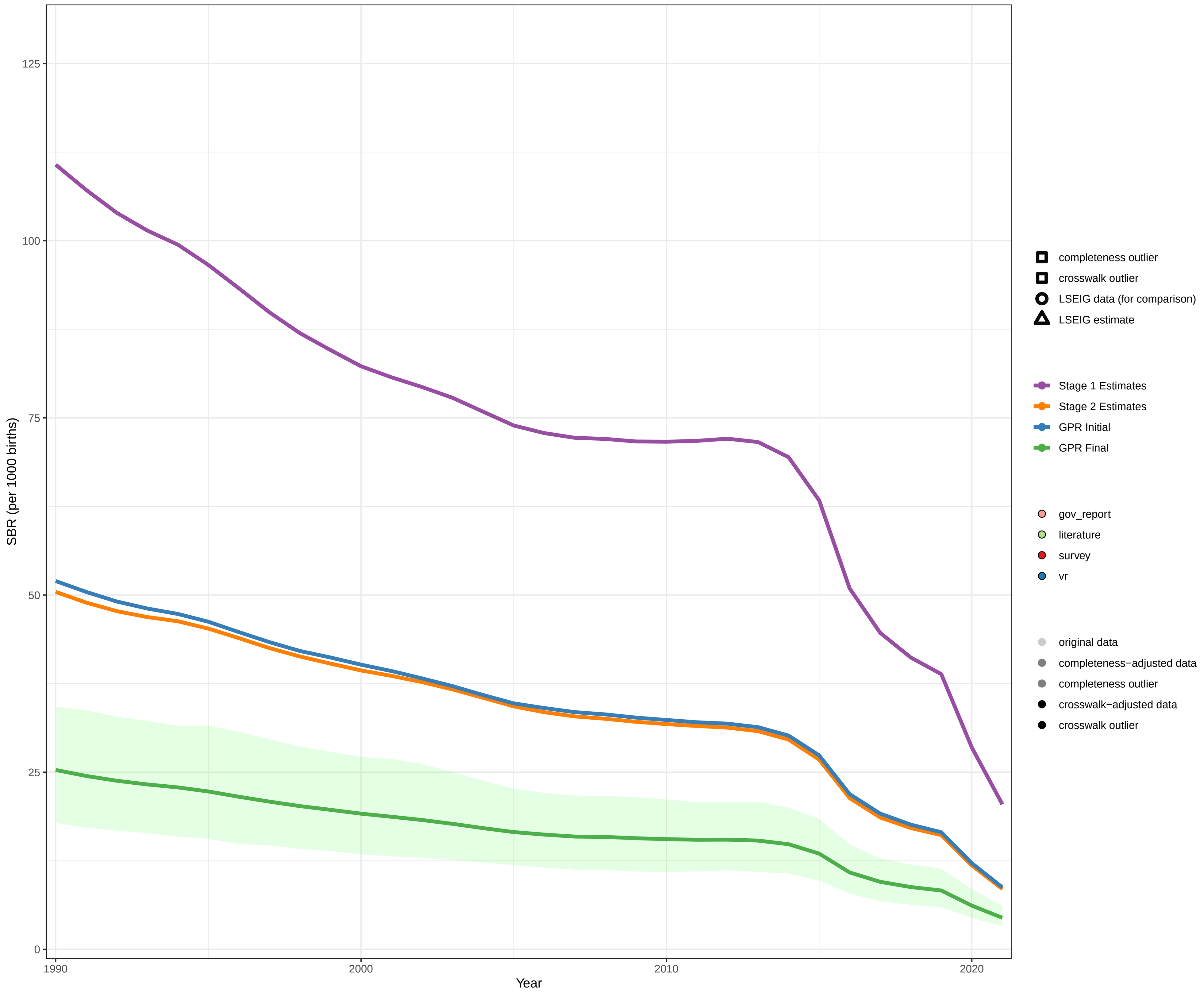

Isfahan IRN_44876 North Africa and Middle East Stillbirth Rate (SBR) − 28 weeks
lambda = 1 ; zeta = 0.6 ; scale = 15 ; amp = 0.028

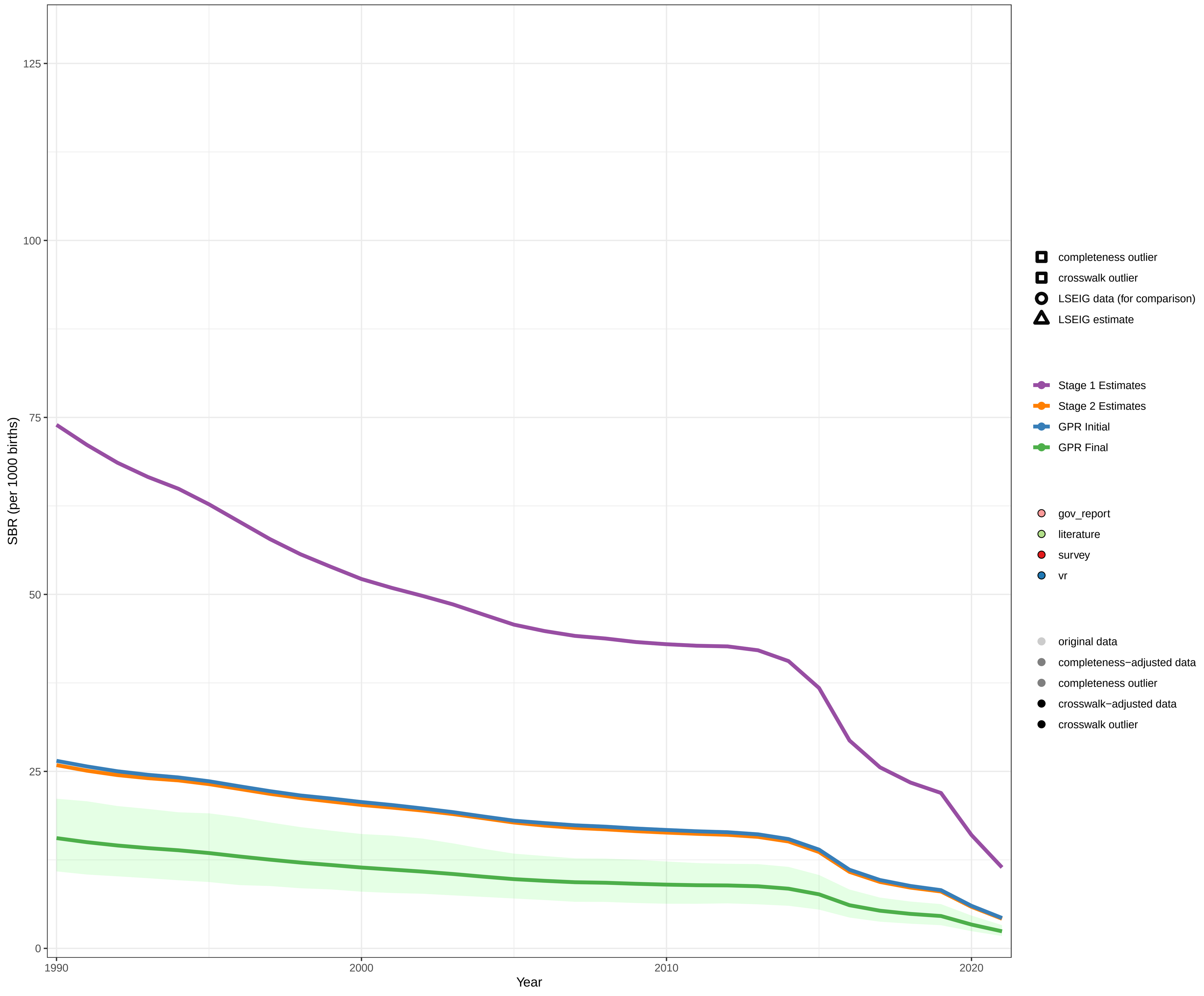

Year	Total Stillbirths (in thousands)	Total Stillbirths (in thousands)	SBR (per 1000 births)	SBR (per 1000 births)	SBR/NMR	SBR/NMR
	20 weeks	28 weeks	20 weeks	28 weeks	20 weeks	28 weeks
1990	2.3 (1.6 to 3.1)	1.4 (1.0 to 1.9)	25.3 (17.8 to 34.2)	15.6 (10.9 to 21.1)	1.04 (0.73 to 1.40)	0.64 (0.45 to 0.87)
1995	1.5 (1.0 to 2.2)	0.9 (0.6 to 1.3)	22.3 (15.6 to 31.6)	13.5 (9.4 to 19.1)	1.12 (0.78 to 1.58)	0.68 (0.47 to 0.96)
2000	1.2 (0.8 to 1.7)	0.7 (0.5 to 1.0)	19.1 (13.4 to 27.2)	11.4 (8.0 to 16.2)	1.16 (0.81 to 1.65)	0.69 (0.49 to 0.98)
2005	1.0 (0.7 to 1.4)	0.6 (0.4 to 0.8)	16.5 (11.9 to 22.7)	9.8 (7.0 to 13.4)	1.17 (0.84 to 1.60)	0.69 (0.50 to 0.94)
2010	1.2 (0.8 to 1.6)	0.7 (0.5 to 0.9)	15.5 (10.9 to 21.1)	9.0 (6.3 to 12.3)	1.21 (0.85 to 1.65)	0.70 (0.49 to 0.96)
2015	1.1 (0.8 to 1.5)	0.6 (0.4 to 0.8)	13.5 (9.7 to 18.4)	7.6 (5.5 to 10.4)	1.29 (0.92 to 1.75)	0.73 (0.52 to 0.99)
2020	0.4 (0.3 to 0.5)	0.2 (0.1 to 0.3)	6.2 (4.4 to 8.5)	3.4 (2.4 to 4.6)	1.41 (1.02 to 1.95)	0.77 (0.56 to 1.06)
2021	0.2 (0.2 to 0.3)	0.1 (0.1 to 0.2)	4.5 (3.2 to 6.1)	2.4 (1.7 to 3.3)	1.43 (1.03 to 1.96)	0.78 (0.56 to 1.06)

Kerman IRN_44877 North Africa and Middle East Stillbirth Rate / Neonatal Mortality Rate (SBR/NMR) − 20 weeks
lambda = 0.8 ; zeta = 0.7 ; scale = 15 ; amp = 0.028

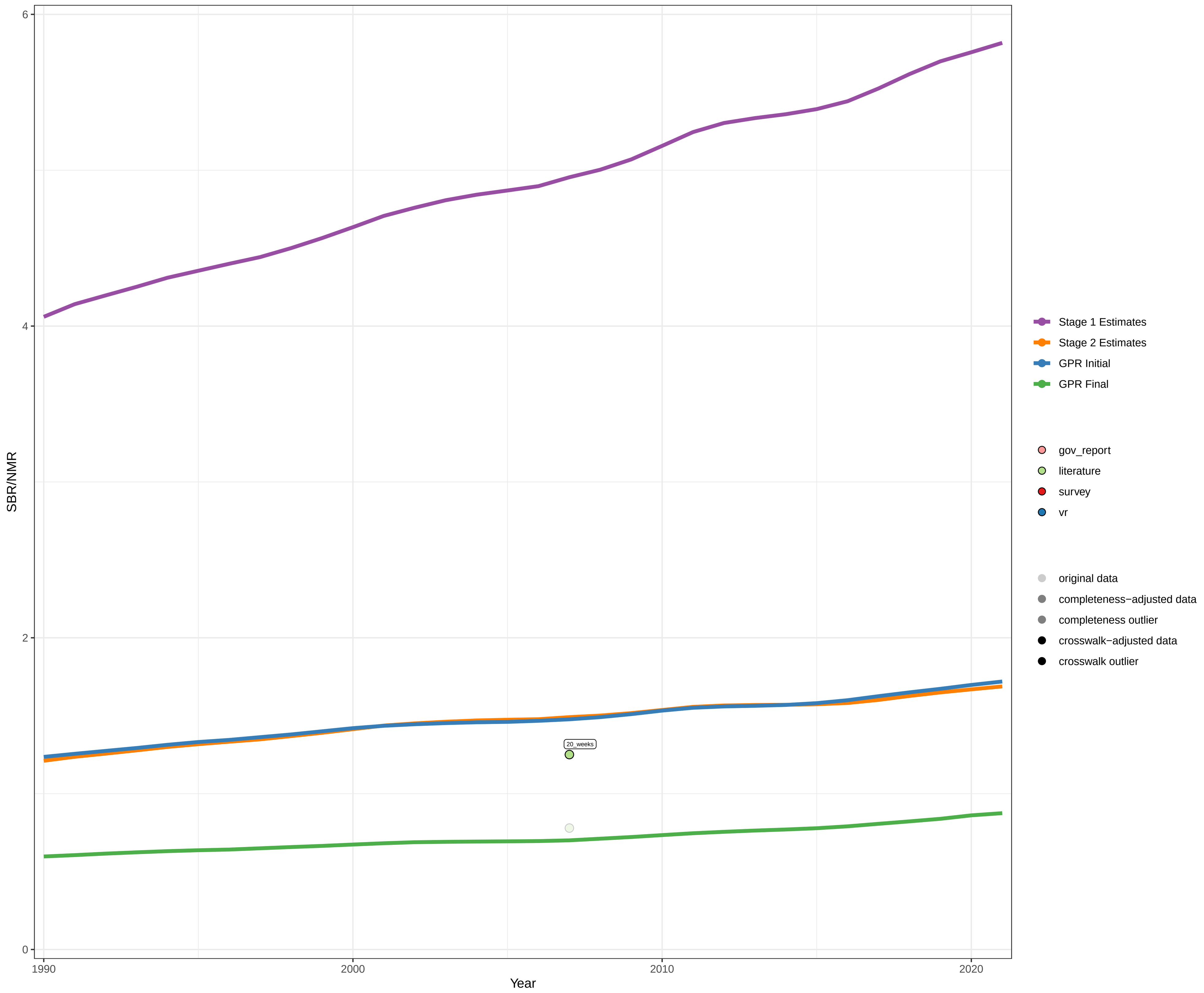

Kerman IRN_44877 North Africa and Middle East Stillbirth Rate / Neonatal Mortality Rate (SBR/NMR) − 28 weeks
lambda = 0.8 ; zeta = 0.7 ; scale = 15 ; amp = 0.028

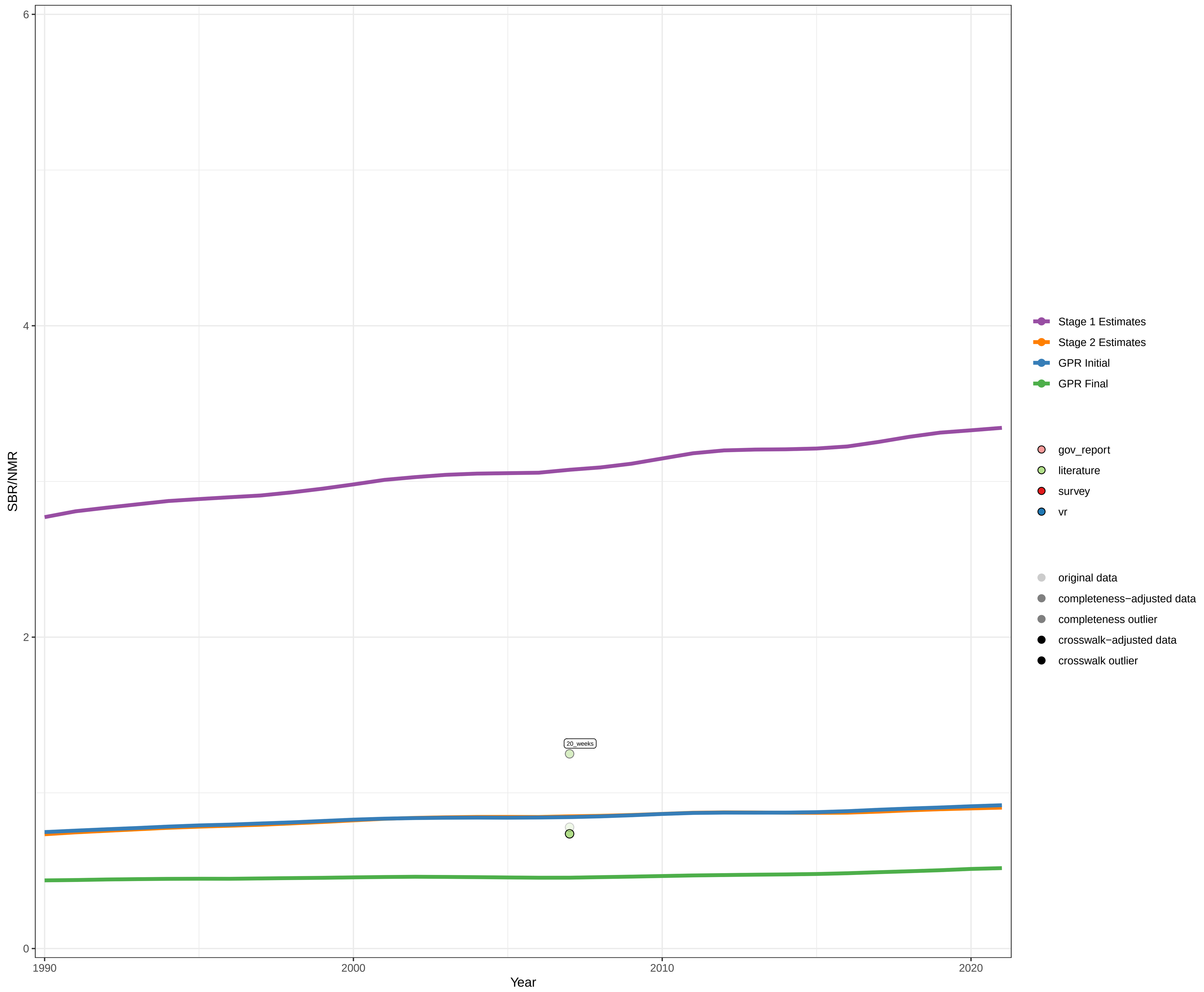

Definition	Years	Outlier	Source.Type	Title
20_weeks	2007	0	Sci Lit	Etiologies and contributing factors of perinatal mortality: A report from southeast of Iran

Kerman IRN_44877 North Africa and Middle East Stillbirth Rate (SBR) − 20 weeks
lambda = 0.8 ; zeta = 0.7 ; scale = 15 ; amp = 0.028

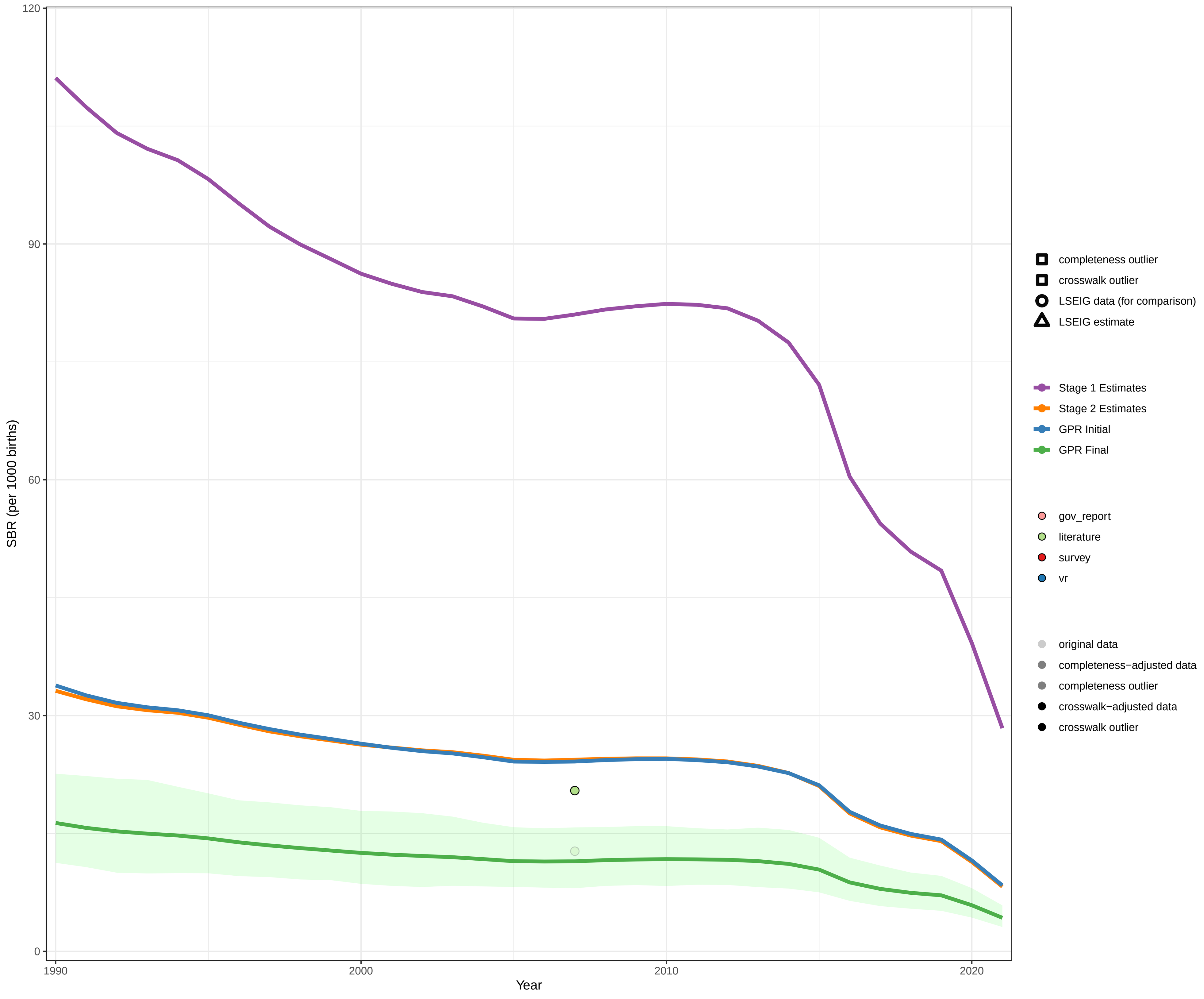

Kerman IRN_44877 North Africa and Middle East Stillbirth Rate (SBR) − 28 weeks
lambda = 0.8 ; zeta = 0.7 ; scale = 15 ; amp = 0.028

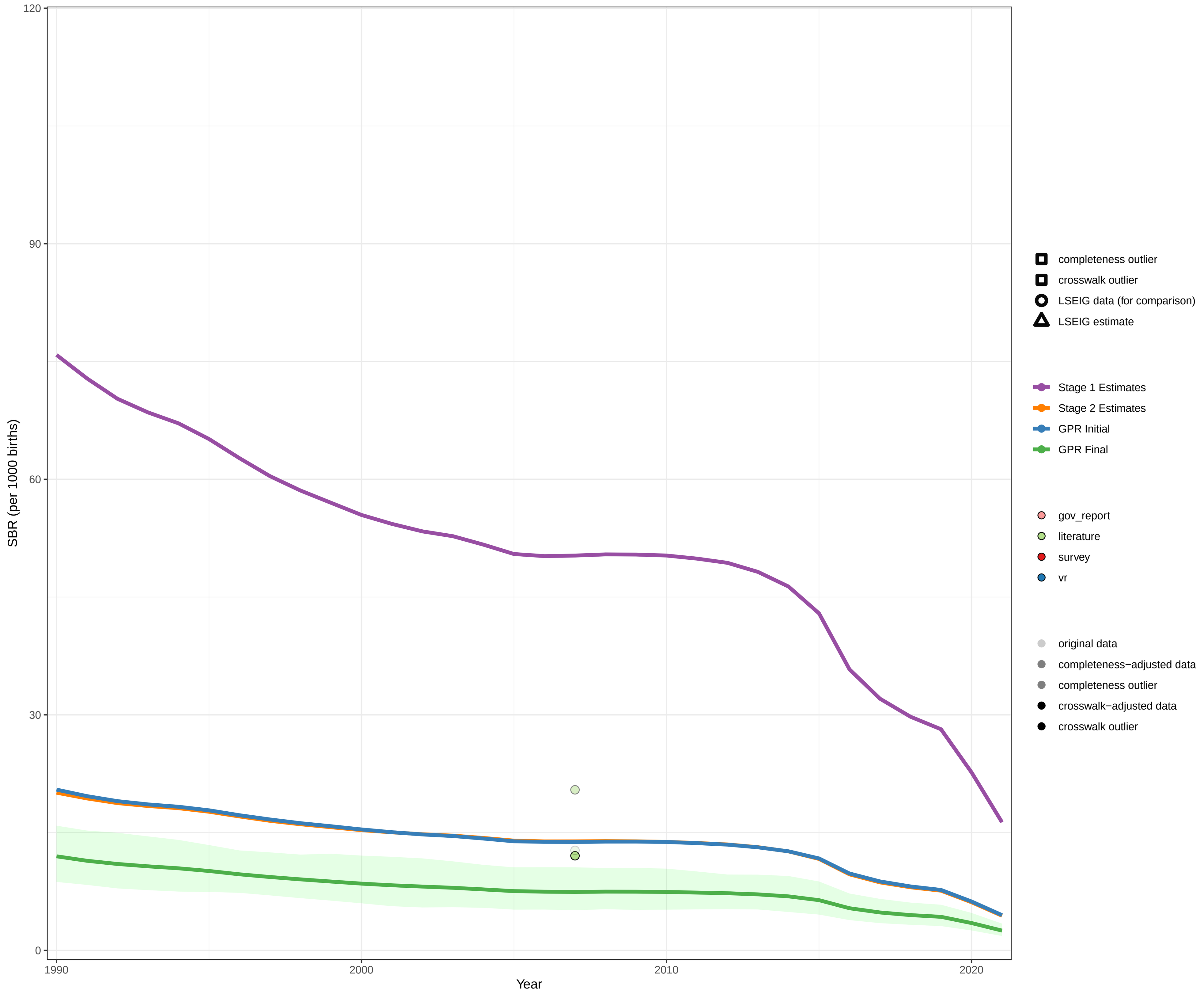

Year	Total Stillbirths (in thousands)	Total Stillbirths (in thousands)	SBR (per 1000 births)	SBR (per 1000 births)	SBR/NMR	SBR/NMR
	20 weeks	28 weeks	20 weeks	28 weeks	20 weeks	28 weeks
1990	0.8 (0.6 to 1.2)	0.6 (0.4 to 0.8)	16.3 (11.3 to 22.6)	12.0 (8.7 to 15.9)	0.60 (0.41 to 0.83)	0.44 (0.32 to 0.58)
1995	0.6 (0.4 to 0.8)	0.4 (0.3 to 0.5)	14.4 (9.9 to 20.1)	10.1 (7.4 to 13.4)	0.64 (0.44 to 0.89)	0.45 (0.33 to 0.59)
2000	0.5 (0.3 to 0.7)	0.3 (0.2 to 0.5)	12.5 (8.6 to 17.9)	8.5 (6.0 to 12.1)	0.67 (0.46 to 0.96)	0.46 (0.32 to 0.65)
2005	0.5 (0.3 to 0.7)	0.3 (0.2 to 0.4)	11.5 (8.2 to 15.8)	7.5 (5.2 to 10.6)	0.69 (0.50 to 0.96)	0.46 (0.31 to 0.64)
2010	0.7 (0.5 to 0.9)	0.4 (0.3 to 0.6)	11.7 (8.3 to 15.9)	7.4 (5.2 to 10.4)	0.73 (0.52 to 1.00)	0.47 (0.32 to 0.65)
2015	0.7 (0.5 to 0.9)	0.4 (0.3 to 0.6)	10.4 (7.5 to 14.5)	6.4 (4.6 to 8.8)	0.78 (0.56 to 1.08)	0.48 (0.34 to 0.66)
2020	0.3 (0.2 to 0.4)	0.2 (0.1 to 0.2)	5.9 (4.3 to 8.0)	3.5 (2.5 to 4.8)	0.86 (0.63 to 1.18)	0.51 (0.37 to 0.70)
2021	0.2 (0.1 to 0.3)	0.1 (0.1 to 0.2)	4.3 (3.1 to 5.8)	2.5 (1.8 to 3.4)	0.87 (0.63 to 1.20)	0.52 (0.38 to 0.70)

Kermanshah IRN_44878 North Africa and Middle East Stillbirth Rate / Neonatal Mortality Rate (SBR/NMR) − 20 weeks
lambda = 1 ; zeta = 0.6 ; scale = 15 ; amp = 0.028

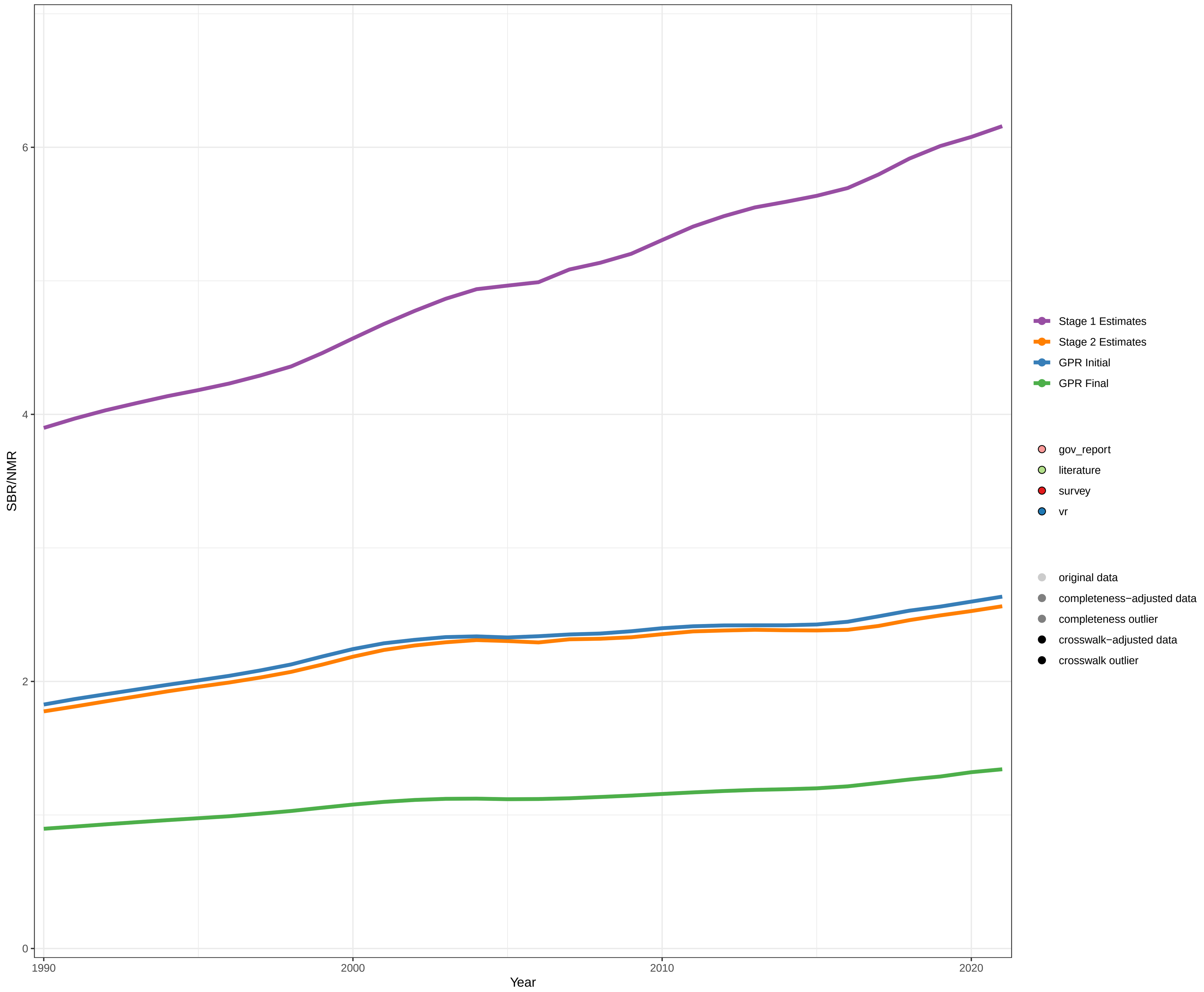

Kermanshah IRN_44878 North Africa and Middle East Stillbirth Rate / Neonatal Mortality Rate (SBR/NMR) − 28 weeks
lambda = 1 ; zeta = 0.6 ; scale = 15 ; amp = 0.028

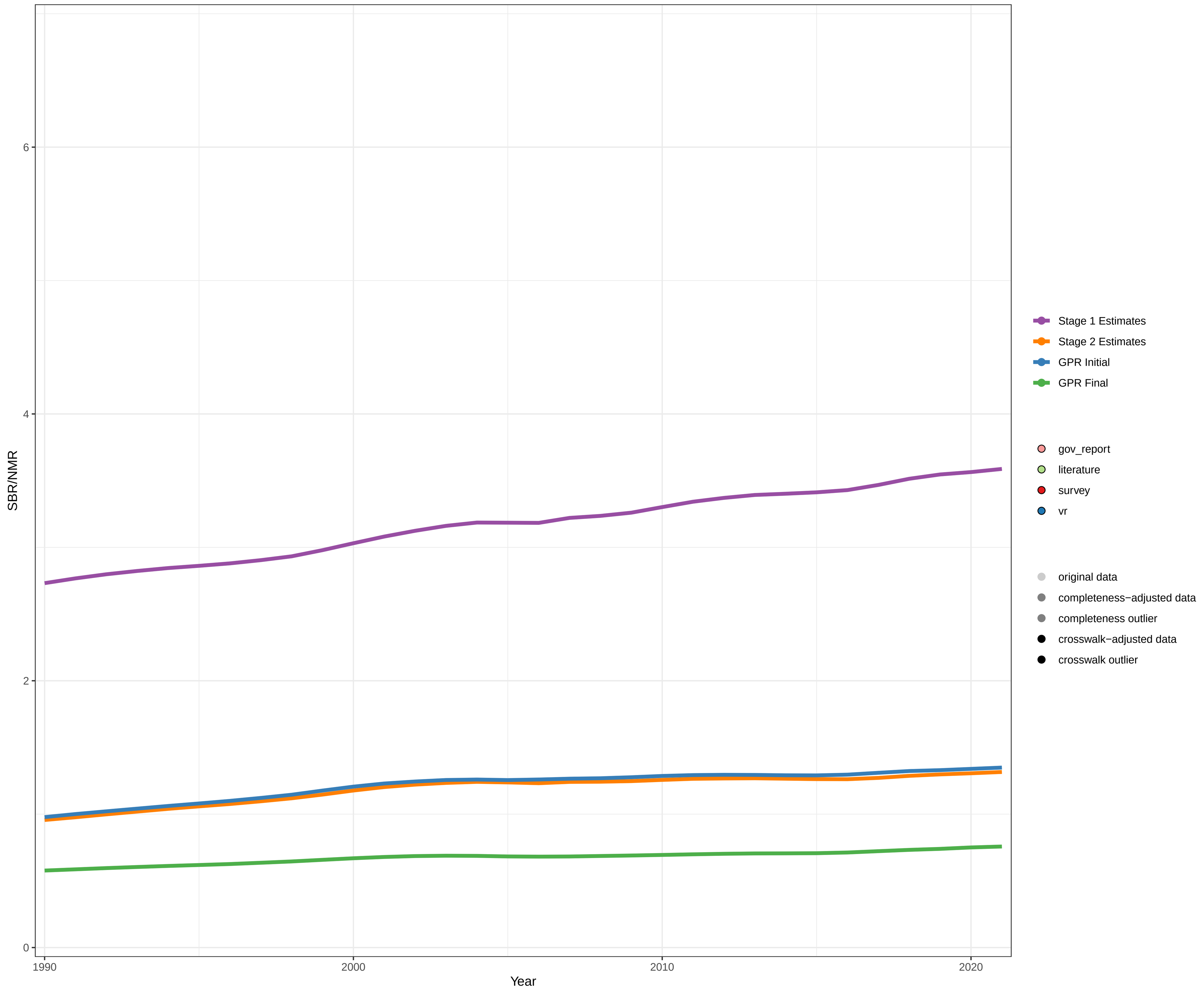

Definition	Years	Outlier	Source.Type	Title

Kermanshah IRN_44878 North Africa and Middle East Stillbirth Rate (SBR) − 20 weeks
lambda = 1 ; zeta = 0.6 ; scale = 15 ; amp = 0.028

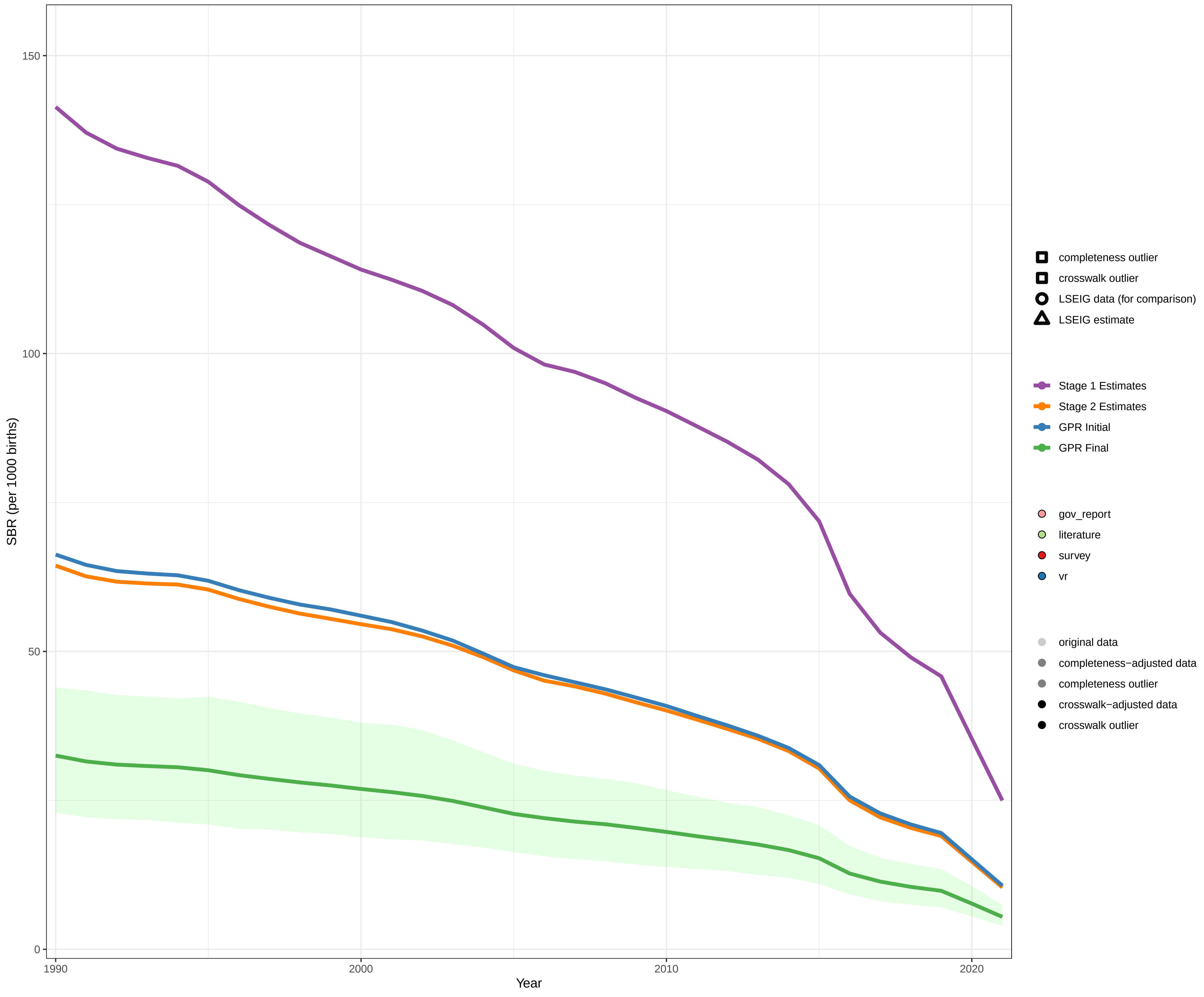

Kermanshah IRN_44878 North Africa and Middle East Stillbirth Rate (SBR) − 28 weeks
lambda = 1 ; zeta = 0.6 ; scale = 15 ; amp = 0.028

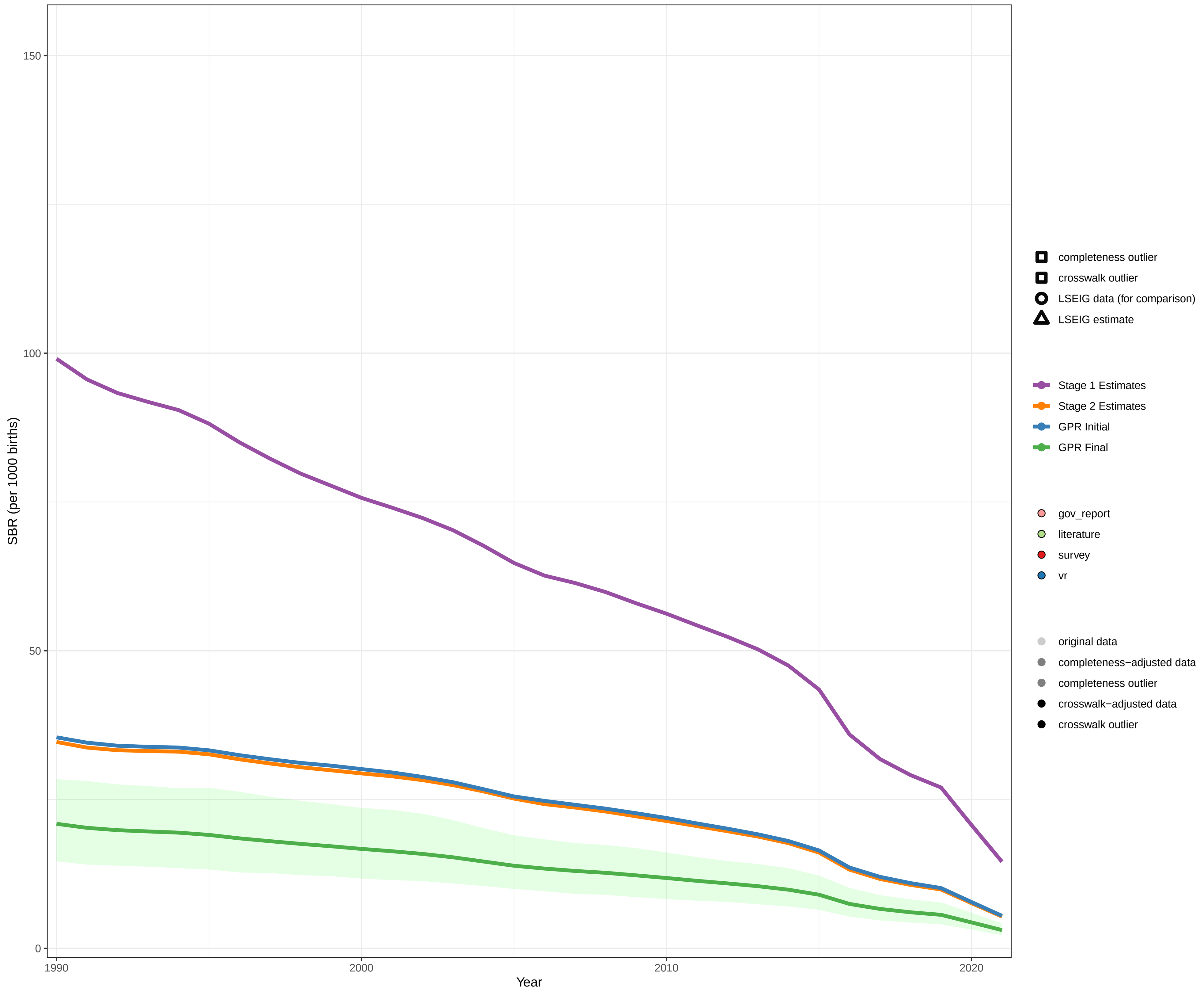

Year	Total Stillbirths (in thousands)	Total Stillbirths (in thousands)	SBR (per 1000 births)	SBR (per 1000 births)	SBR/NMR	SBR/NMR
	20 weeks	28 weeks	20 weeks	28 weeks	20 weeks	28 weeks
1990	1.6 (1.1 to 2.2)	1.0 (0.7 to 1.4)	32.5 (22.9 to 44.0)	20.9 (14.6 to 28.4)	0.90 (0.63 to 1.21)	0.58 (0.40 to 0.78)
1995	1.1 (0.8 to 1.6)	0.7 (0.5 to 1.0)	30.1 (21.0 to 42.4)	19.1 (13.3 to 27.0)	0.98 (0.68 to 1.38)	0.62 (0.43 to 0.88)
2000	0.9 (0.6 to 1.2)	0.5 (0.4 to 0.7)	26.9 (18.8 to 38.0)	16.7 (11.7 to 23.6)	1.08 (0.75 to 1.52)	0.67 (0.47 to 0.95)
2005	0.7 (0.5 to 0.9)	0.4 (0.3 to 0.6)	22.7 (16.3 to 31.1)	13.9 (10.0 to 19.0)	1.12 (0.80 to 1.53)	0.68 (0.49 to 0.93)
2010	0.7 (0.5 to 0.9)	0.4 (0.3 to 0.6)	19.7 (13.8 to 26.7)	11.8 (8.3 to 16.1)	1.16 (0.81 to 1.57)	0.69 (0.49 to 0.95)
2015	0.6 (0.4 to 0.8)	0.3 (0.2 to 0.4)	15.3 (11.0 to 20.8)	9.0 (6.5 to 12.2)	1.20 (0.86 to 1.64)	0.71 (0.51 to 0.96)
2020	0.2 (0.1 to 0.3)	0.1 (0.1 to 0.1)	7.7 (5.5 to 10.6)	4.4 (3.2 to 6.0)	1.32 (0.95 to 1.82)	0.75 (0.54 to 1.03)
2021	0.1 (0.1 to 0.2)	0.1 (0.1 to 0.1)	5.4 (3.9 to 7.5)	3.1 (2.2 to 4.2)	1.34 (0.97 to 1.84)	0.76 (0.55 to 1.03)

Khorasan−e−Razavi IRN_44880 North Africa and Middle East Stillbirth Rate / Neonatal Mortality Rate (SBR/NMR) − 20 weeks
lambda = 1 ; zeta = 0.6 ; scale = 15 ; amp = 0.028

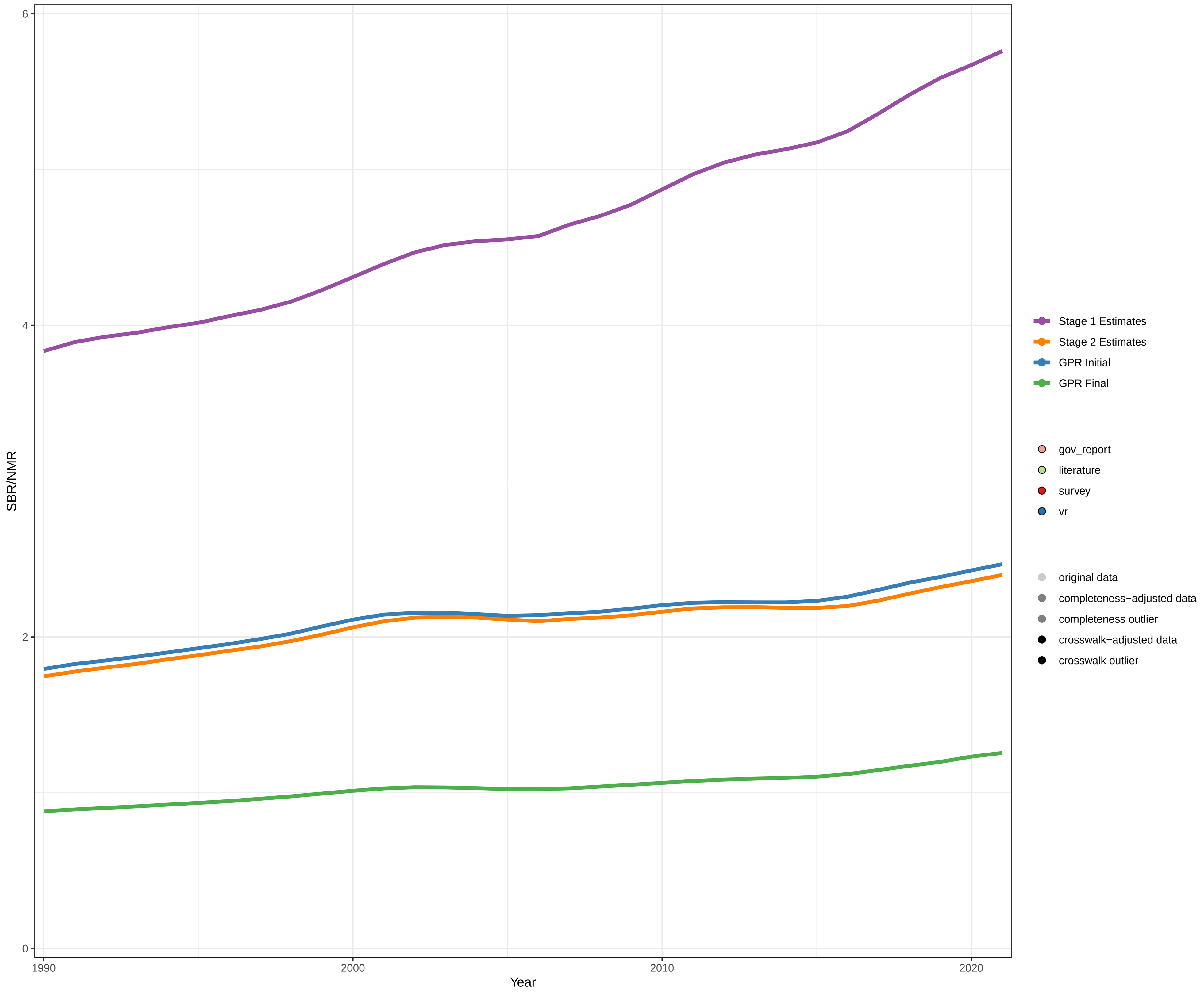

Khorasan−e−Razavi IRN_44880 North Africa and Middle East Stillbirth Rate / Neonatal Mortality Rate (SBR/NMR) − 28 weeks
lambda = 1 ; zeta = 0.6 ; scale = 15 ; amp = 0.028

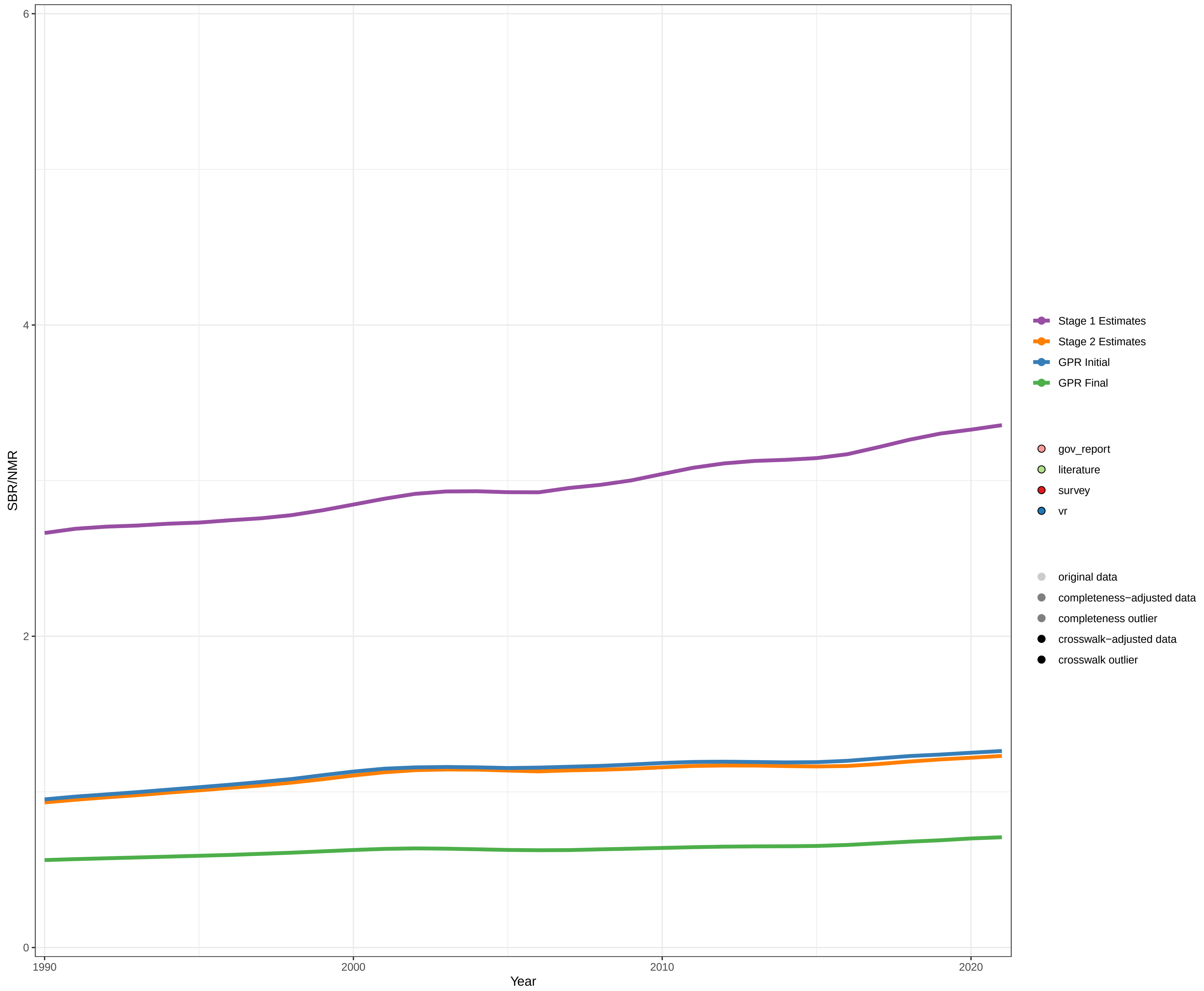

Definition	Years	Outlier	Source.Type	Title

Khorasan−e−Razavi IRN_44880 North Africa and Middle East Stillbirth Rate (SBR) − 20 weeks
lambda = 1 ; zeta = 0.6 ; scale = 15 ; amp = 0.028

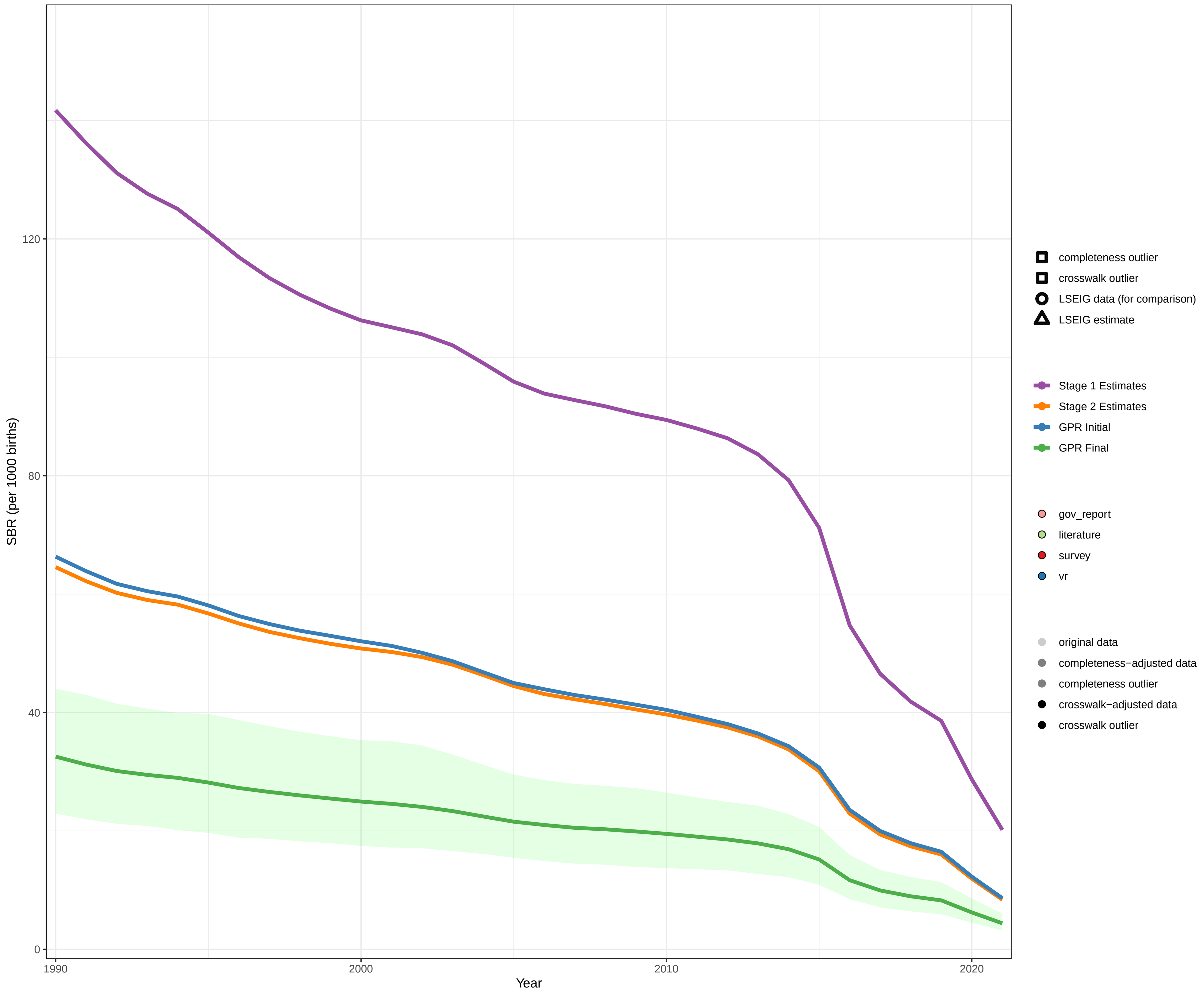

Khorasan−e−Razavi IRN_44880 North Africa and Middle East Stillbirth Rate (SBR) − 28 weeks
lambda = 1 ; zeta = 0.6 ; scale = 15 ; amp = 0.028

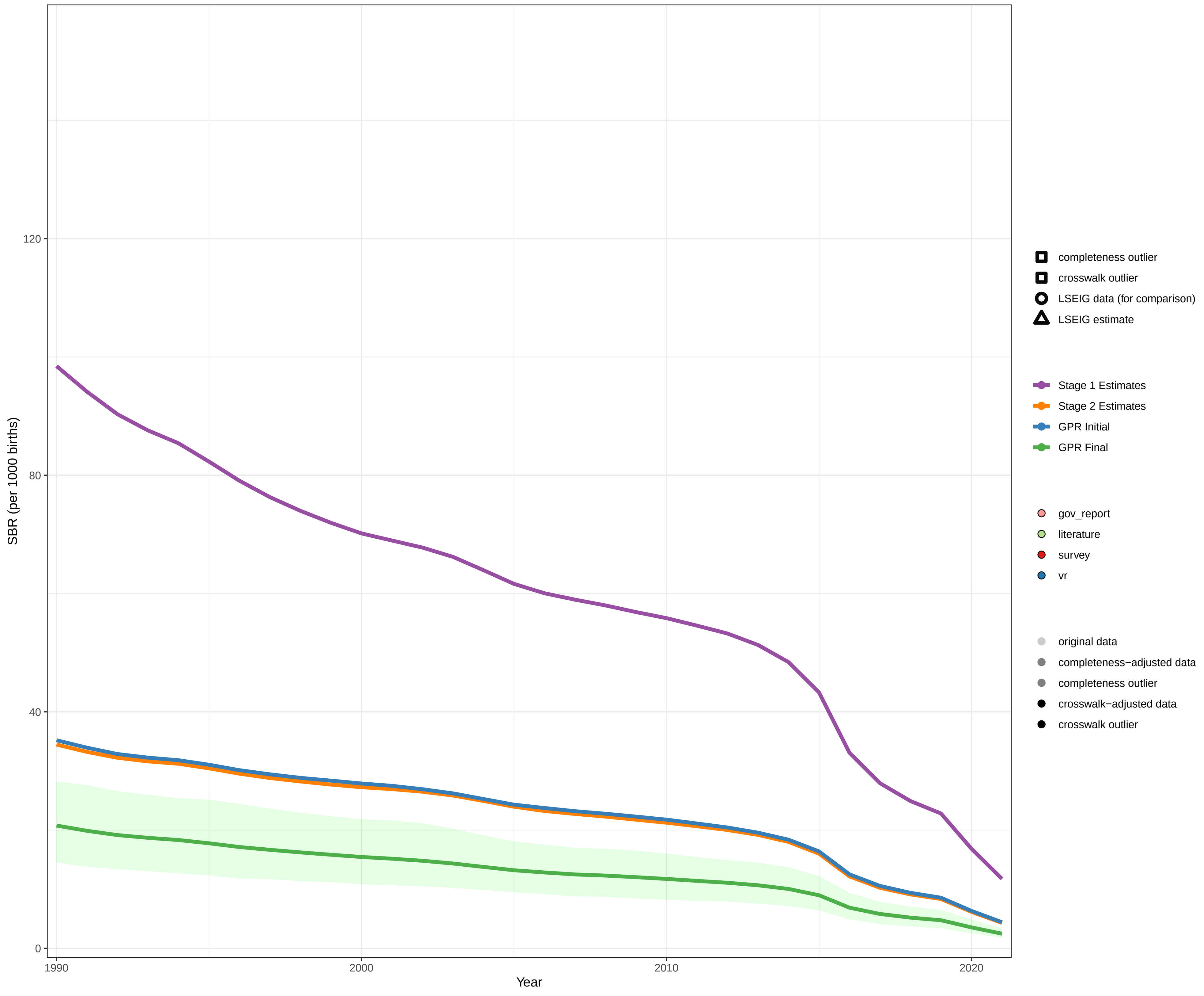

Year	Total Stillbirths (in thousands)	Total Stillbirths (in thousands)	SBR (per 1000 births)	SBR (per 1000 births)	SBR/NMR	SBR/NMR
	20 weeks	28 weeks	20 weeks	28 weeks	20 weeks	28 weeks
1990	4.6 (3.2 to 6.4)	2.9 (2.0 to 4.0)	32.6 (22.9 to 44.0)	20.8 (14.5 to 28.2)	0.88 (0.62 to 1.19)	0.56 (0.39 to 0.76)
1995	3.0 (2.1 to 4.3)	1.9 (1.3 to 2.7)	28.2 (19.7 to 39.8)	17.8 (12.4 to 25.2)	0.93 (0.65 to 1.32)	0.59 (0.41 to 0.83)
2000	2.5 (1.8 to 3.6)	1.5 (1.1 to 2.2)	25.0 (17.5 to 35.3)	15.5 (10.8 to 21.8)	1.01 (0.71 to 1.43)	0.63 (0.44 to 0.89)
2005	2.3 (1.6 to 3.2)	1.4 (1.0 to 1.9)	21.6 (15.5 to 29.5)	13.2 (9.5 to 18.1)	1.02 (0.73 to 1.40)	0.63 (0.45 to 0.86)
2010	2.6 (1.8 to 3.5)	1.5 (1.1 to 2.1)	19.5 (13.7 to 26.5)	11.7 (8.2 to 16.0)	1.06 (0.75 to 1.44)	0.64 (0.45 to 0.87)
2015	2.2 (1.5 to 3.0)	1.3 (0.9 to 1.7)	15.2 (10.9 to 20.7)	9.0 (6.4 to 12.2)	1.10 (0.79 to 1.50)	0.65 (0.47 to 0.89)
2020	0.6 (0.5 to 0.9)	0.4 (0.3 to 0.5)	6.2 (4.5 to 8.6)	3.5 (2.6 to 4.9)	1.23 (0.89 to 1.70)	0.70 (0.51 to 0.97)
2021	0.4 (0.3 to 0.6)	0.2 (0.2 to 0.3)	4.4 (3.2 to 6.0)	2.5 (1.8 to 3.4)	1.26 (0.91 to 1.72)	0.71 (0.51 to 0.97)

Khuzestan IRN_44882 North Africa and Middle East Stillbirth Rate / Neonatal Mortality Rate (SBR/NMR) − 20 weeks
lambda = 1 ; zeta = 0.6 ; scale = 15 ; amp = 0.028

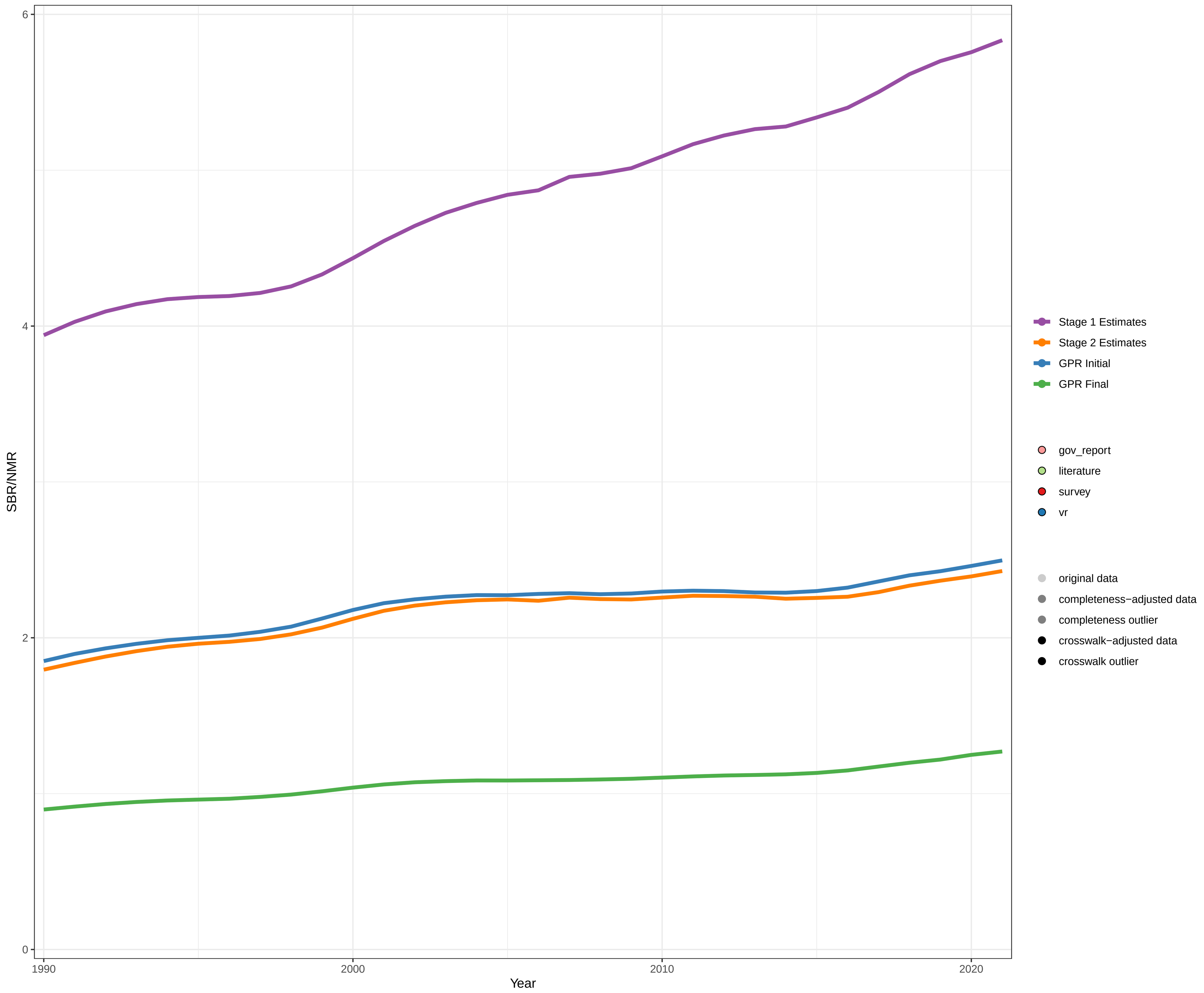

Khuzestan IRN_44882 North Africa and Middle East Stillbirth Rate / Neonatal Mortality Rate (SBR/NMR) − 28 weeks
lambda = 1 ; zeta = 0.6 ; scale = 15 ; amp = 0.028

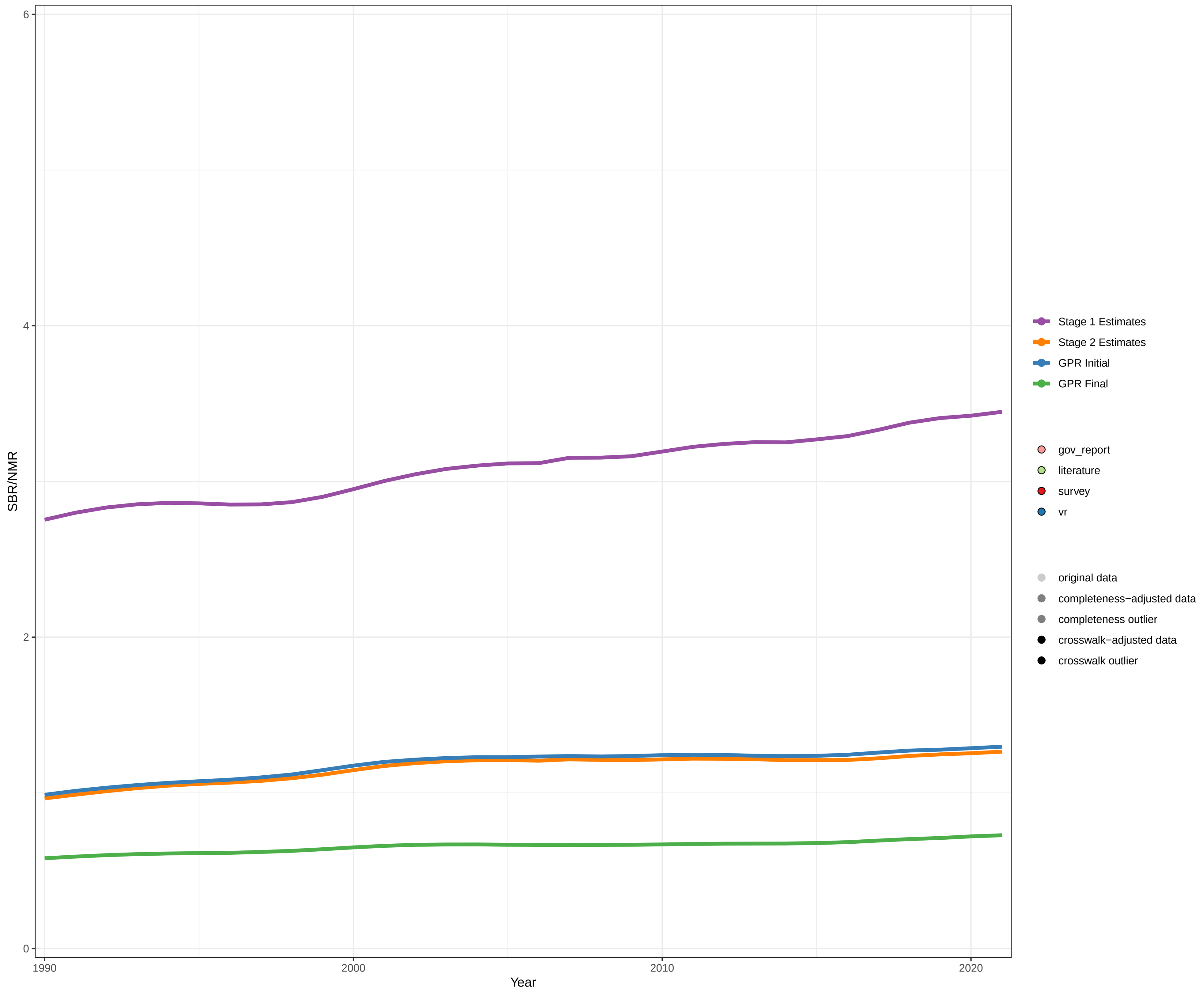

Definition	Years	Outlier	Source.Type	Title

Khuzestan IRN_44882 North Africa and Middle East Stillbirth Rate (SBR) − 20 weeks
lambda = 1 ; zeta = 0.6 ; scale = 15 ; amp = 0.028

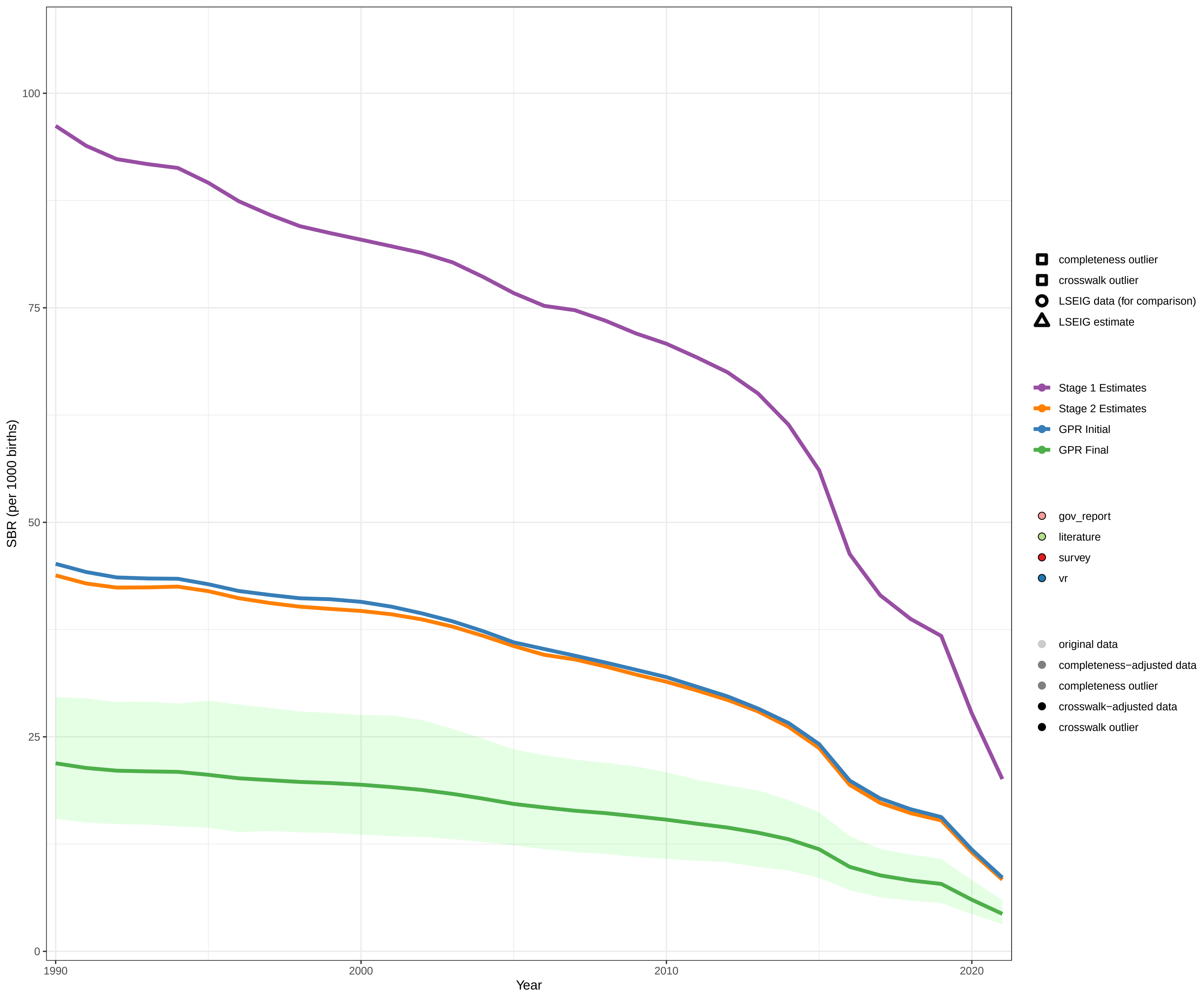

Khuzestan IRN_44882 North Africa and Middle East Stillbirth Rate (SBR) − 28 weeks
lambda = 1 ; zeta = 0.6 ; scale = 15 ; amp = 0.028

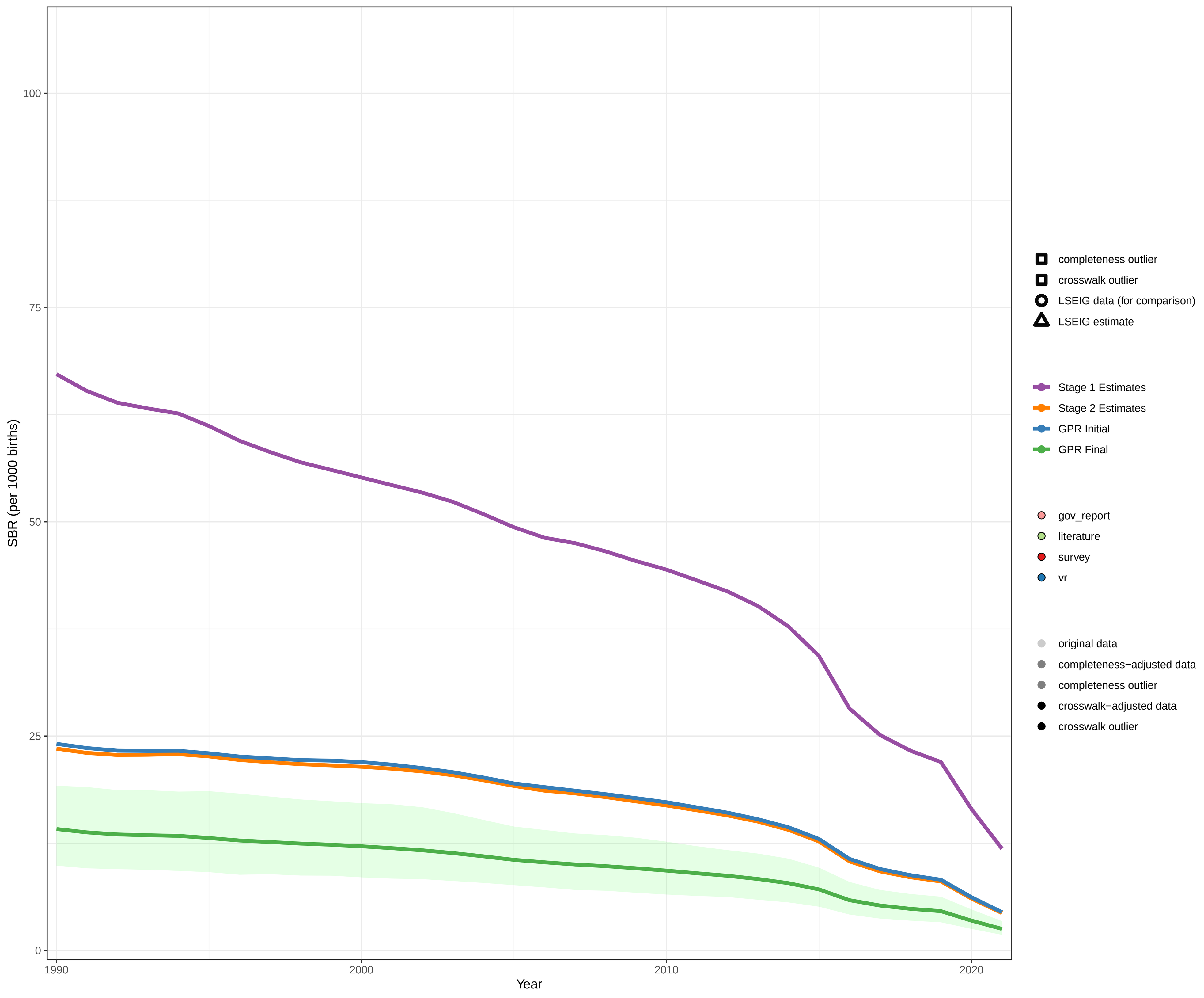

Year	Total Stillbirths (in thousands)	Total Stillbirths (in thousands)	SBR (per 1000 births)	SBR (per 1000 births)	SBR/NMR	SBR/NMR
	20 weeks	28 weeks	20 weeks	28 weeks	20 weeks	28 weeks
1990	2.1 (1.5 to 2.9)	1.4 (0.9 to 1.9)	21.9 (15.4 to 29.7)	14.2 (9.9 to 19.2)	0.90 (0.63 to 1.22)	0.58 (0.41 to 0.79)
1995	1.7 (1.2 to 2.5)	1.1 (0.8 to 1.5)	20.6 (14.4 to 29.2)	13.1 (9.1 to 18.6)	0.96 (0.67 to 1.37)	0.61 (0.43 to 0.87)
2000	1.6 (1.1 to 2.3)	1.0 (0.7 to 1.4)	19.4 (13.6 to 27.5)	12.1 (8.5 to 17.2)	1.04 (0.73 to 1.47)	0.65 (0.46 to 0.92)
2005	1.5 (1.0 to 2.0)	0.9 (0.6 to 1.2)	17.2 (12.3 to 23.5)	10.6 (7.6 to 14.4)	1.08 (0.78 to 1.49)	0.67 (0.48 to 0.91)
2010	1.6 (1.1 to 2.2)	1.0 (0.7 to 1.3)	15.3 (10.8 to 20.9)	9.3 (6.5 to 12.7)	1.10 (0.77 to 1.50)	0.67 (0.47 to 0.91)
2015	1.3 (1.0 to 1.8)	0.8 (0.6 to 1.1)	11.9 (8.5 to 16.2)	7.1 (5.1 to 9.7)	1.13 (0.81 to 1.54)	0.68 (0.49 to 0.92)
2020	0.5 (0.4 to 0.7)	0.3 (0.2 to 0.4)	6.0 (4.3 to 8.3)	3.5 (2.5 to 4.8)	1.25 (0.90 to 1.73)	0.72 (0.52 to 0.99)
2021	0.3 (0.2 to 0.5)	0.2 (0.1 to 0.3)	4.4 (3.2 to 6.0)	2.5 (1.8 to 3.4)	1.27 (0.92 to 1.74)	0.73 (0.53 to 0.99)

Kohgiluyeh and Boyer−Ahmad IRN_44883 North Africa and Middle East Stillbirth Rate / Neonatal Mortality Rate (SBR/NMR) − 20 weeks
lambda = 1 ; zeta = 0.6 ; scale = 15 ; amp = 0.028

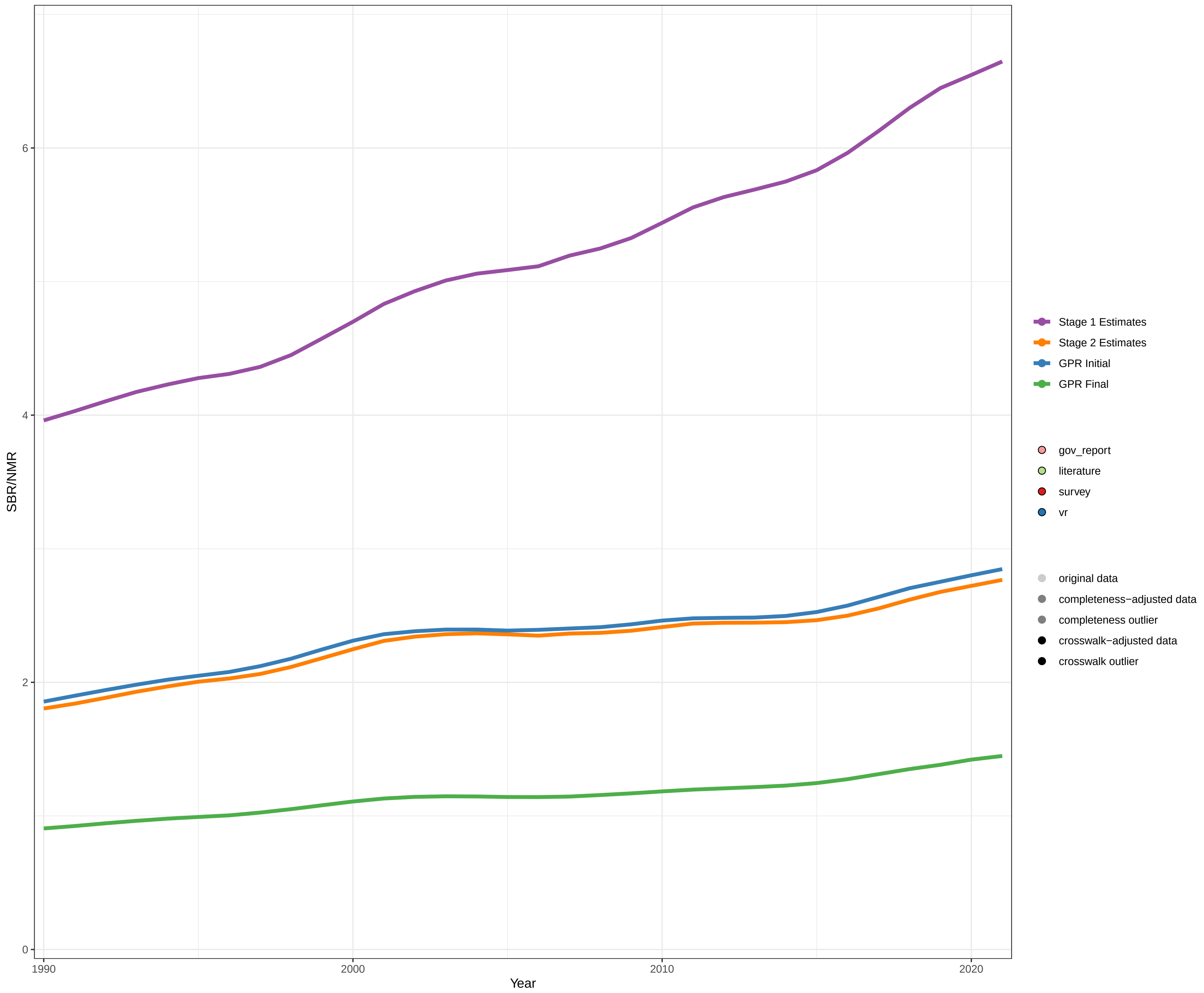

Kohgiluyeh and Boyer−Ahmad IRN_44883 North Africa and Middle East Stillbirth Rate / Neonatal Mortality Rate (SBR/NMR) − 28 weeks
lambda = 1 ; zeta = 0.6 ; scale = 15 ; amp = 0.028

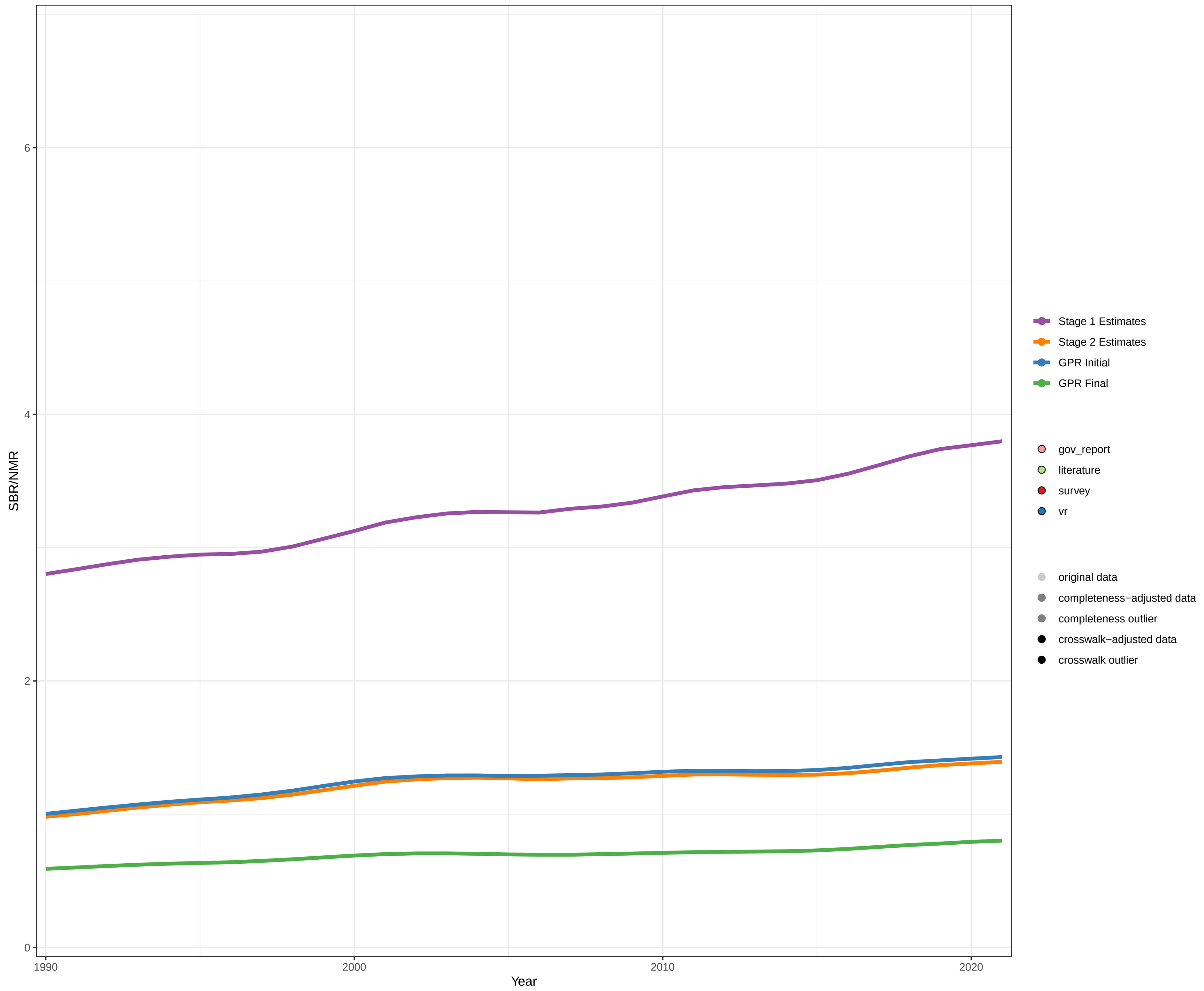

Definition	Years	Outlier	Source.Type	Title

Kohgiluyeh and Boyer−Ahmad IRN_44883 North Africa and Middle East Stillbirth Rate (SBR) − 20 weeks
lambda = 1 ; zeta = 0.6 ; scale = 15 ; amp = 0.028

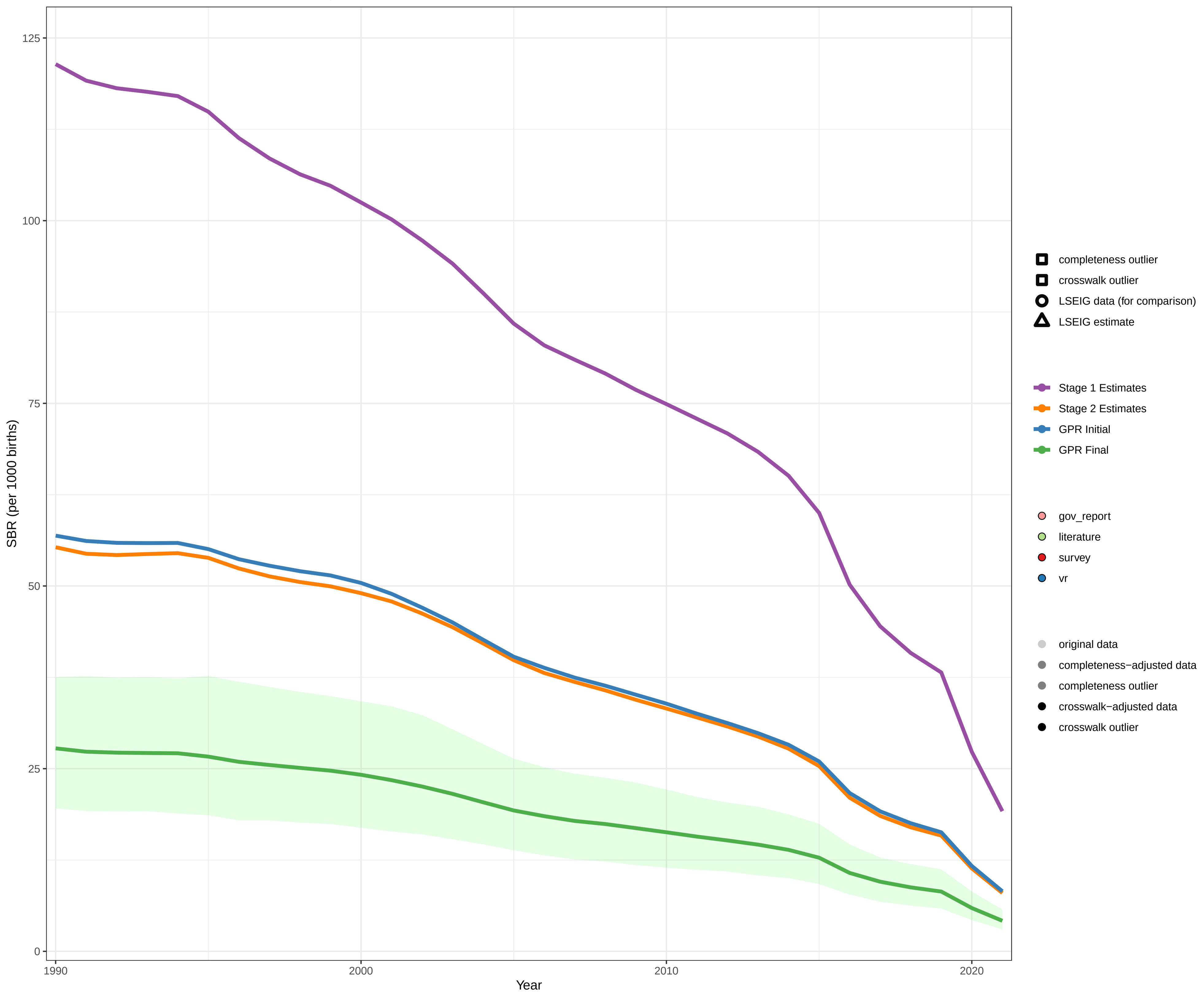

Kohgiluyeh and Boyer−Ahmad IRN_44883 North Africa and Middle East Stillbirth Rate (SBR) − 28 weeks
lambda = 1 ; zeta = 0.6 ; scale = 15 ; amp = 0.028

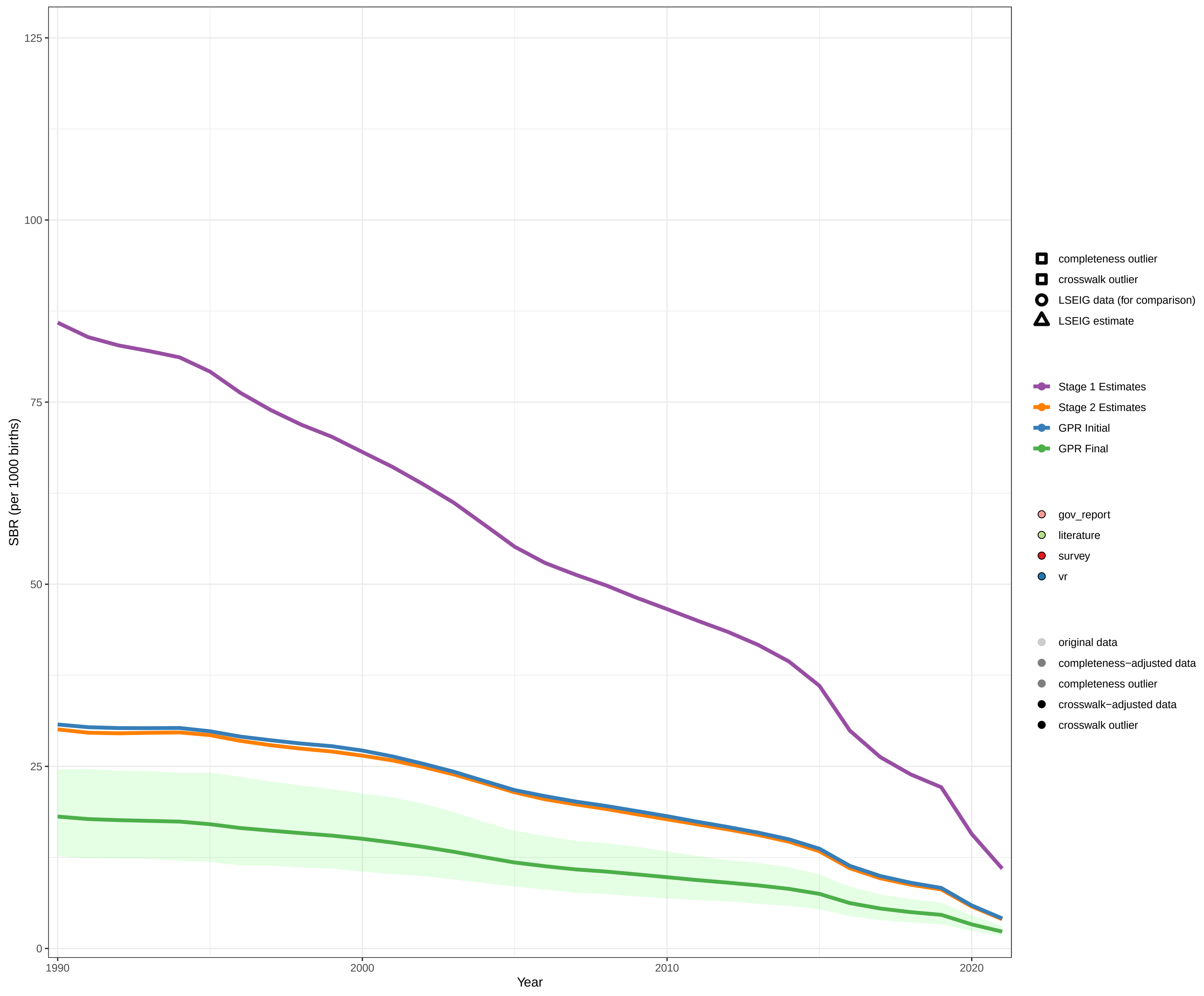

Year	Total Stillbirths (in thousands)	Total Stillbirths (in thousands)	SBR (per 1000 births)	SBR (per 1000 births)	SBR/NMR	SBR/NMR
	20 weeks	28 weeks	20 weeks	28 weeks	20 weeks	28 weeks
1990	0.5 (0.3 to 0.6)	0.3 (0.2 to 0.4)	27.8 (19.6 to 37.5)	18.1 (12.6 to 24.6)	0.91 (0.64 to 1.22)	0.59 (0.41 to 0.80)
1995	0.4 (0.3 to 0.5)	0.2 (0.2 to 0.3)	26.7 (18.6 to 37.7)	17.1 (11.9 to 24.2)	0.99 (0.69 to 1.40)	0.63 (0.44 to 0.90)
2000	0.3 (0.2 to 0.4)	0.2 (0.1 to 0.3)	24.2 (16.9 to 34.2)	15.1 (10.6 to 21.3)	1.11 (0.78 to 1.57)	0.69 (0.48 to 0.98)
2005	0.2 (0.2 to 0.3)	0.1 (0.1 to 0.2)	19.3 (13.8 to 26.4)	11.8 (8.5 to 16.2)	1.14 (0.82 to 1.56)	0.70 (0.50 to 0.96)
2010	0.2 (0.1 to 0.3)	0.1 (0.1 to 0.2)	16.3 (11.4 to 22.1)	9.8 (6.9 to 13.3)	1.18 (0.83 to 1.61)	0.71 (0.50 to 0.97)
2015	0.2 (0.1 to 0.3)	0.1 (0.1 to 0.2)	12.8 (9.2 to 17.5)	7.5 (5.4 to 10.2)	1.25 (0.89 to 1.70)	0.73 (0.52 to 0.99)
2020	0.1 (0.1 to 0.1)	0.0 (0.0 to 0.1)	5.9 (4.3 to 8.2)	3.3 (2.4 to 4.6)	1.42 (1.02 to 1.96)	0.79 (0.57 to 1.09)
2021	0.0 (0.0 to 0.1)	0.0 (0.0 to 0.0)	4.2 (3.0 to 5.7)	2.3 (1.7 to 3.2)	1.45 (1.04 to 1.98)	0.80 (0.58 to 1.09)

Kurdistan   IRN_44884   North Africa and Middle East   Stillbirth Rate / Neonatal Mortality Rate (SBR/NMR) − 20 weeks
lambda = 1 ; zeta = 0.6 ; scale = 15 ; amp = 0.028

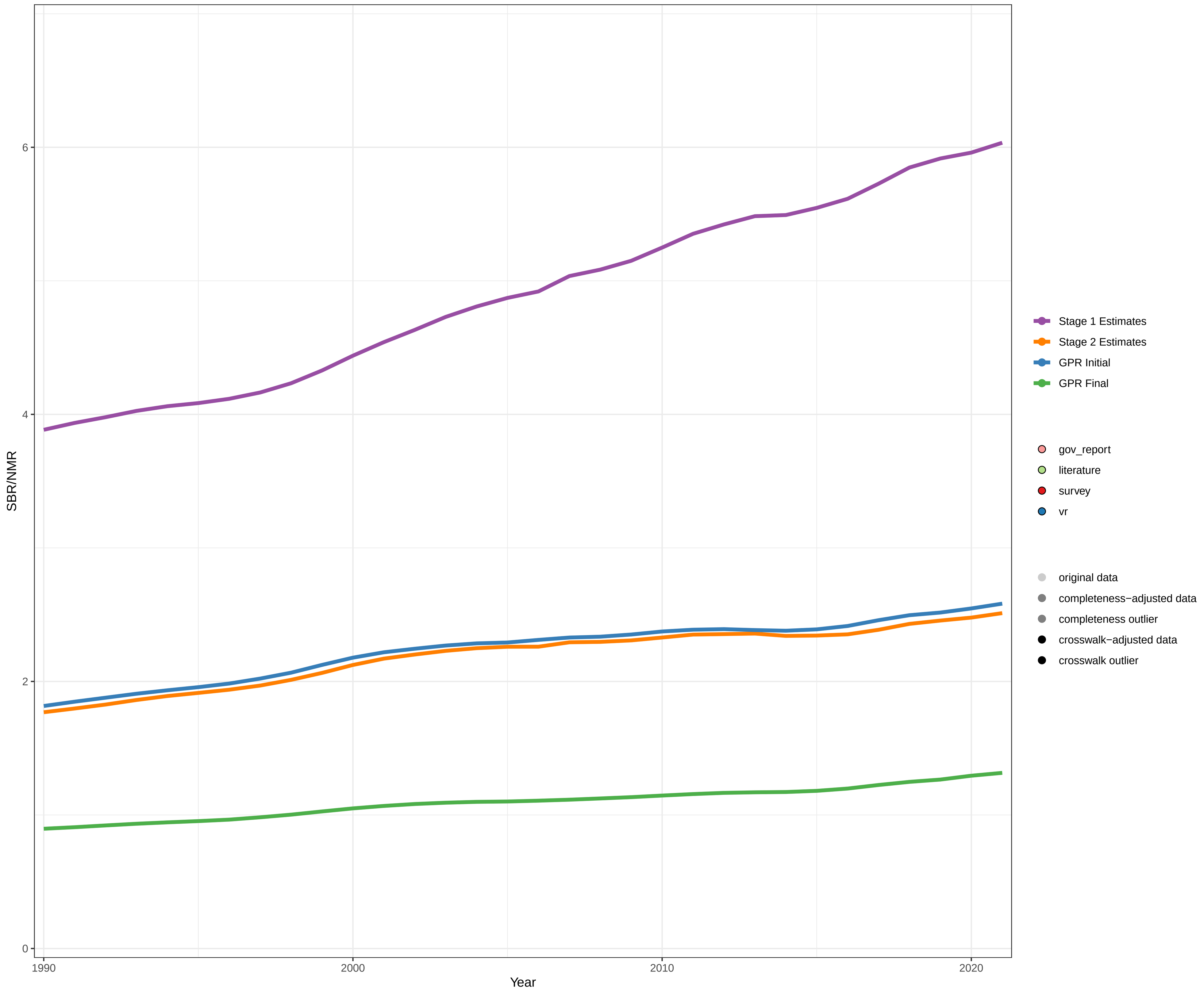

Kurdistan   IRN_44884   North Africa and Middle East   Stillbirth Rate / Neonatal Mortality Rate (SBR/NMR) − 28 weeks
lambda = 1 ; zeta = 0.6 ; scale = 15 ; amp = 0.028

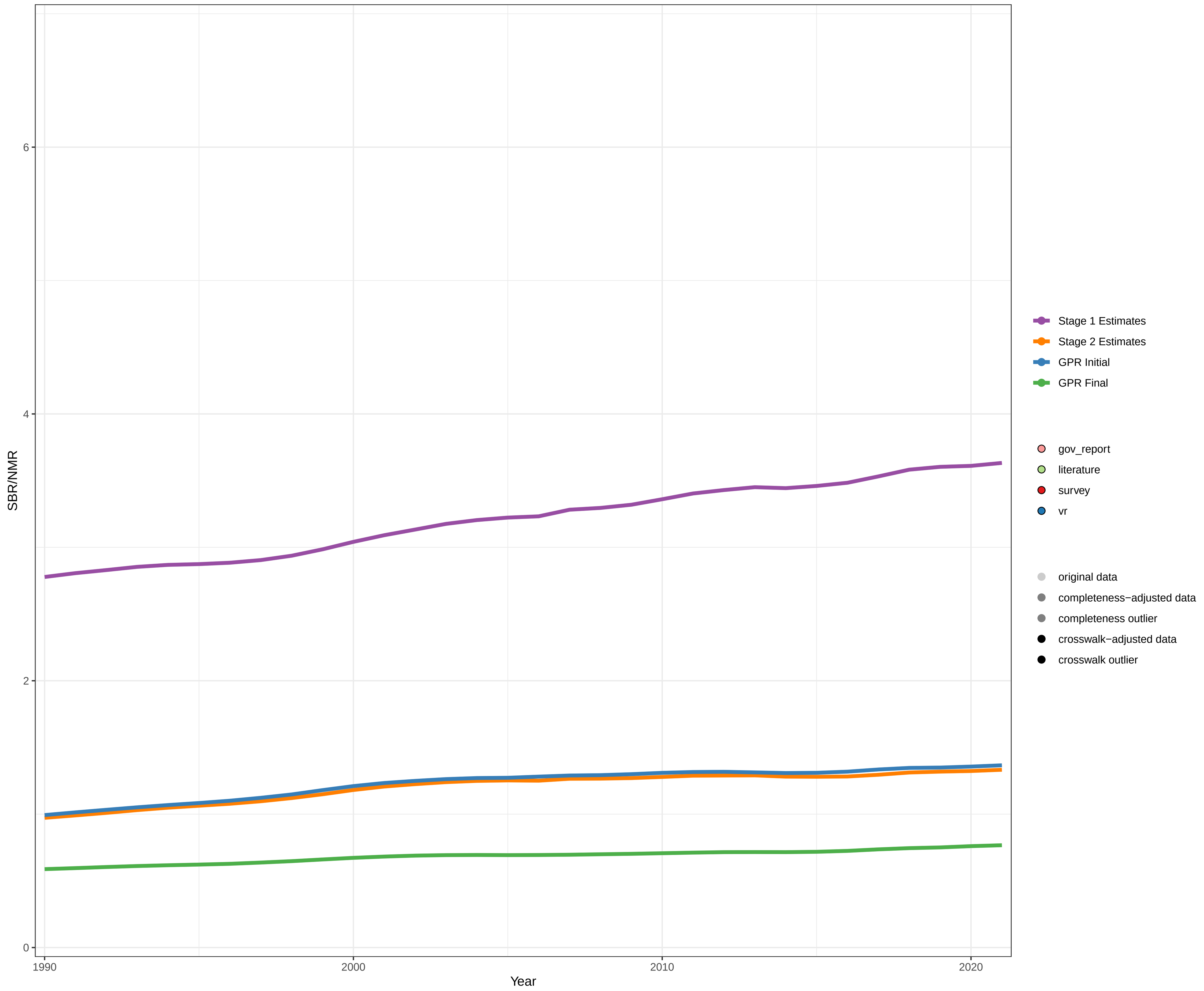

Definition	Years	Outlier	Source.Type	Title

Kurdistan   IRN_44884   North Africa and Middle East   Stillbirth Rate (SBR) − 20 weeks
lambda = 1 ; zeta = 0.6 ; scale = 15 ; amp = 0.028

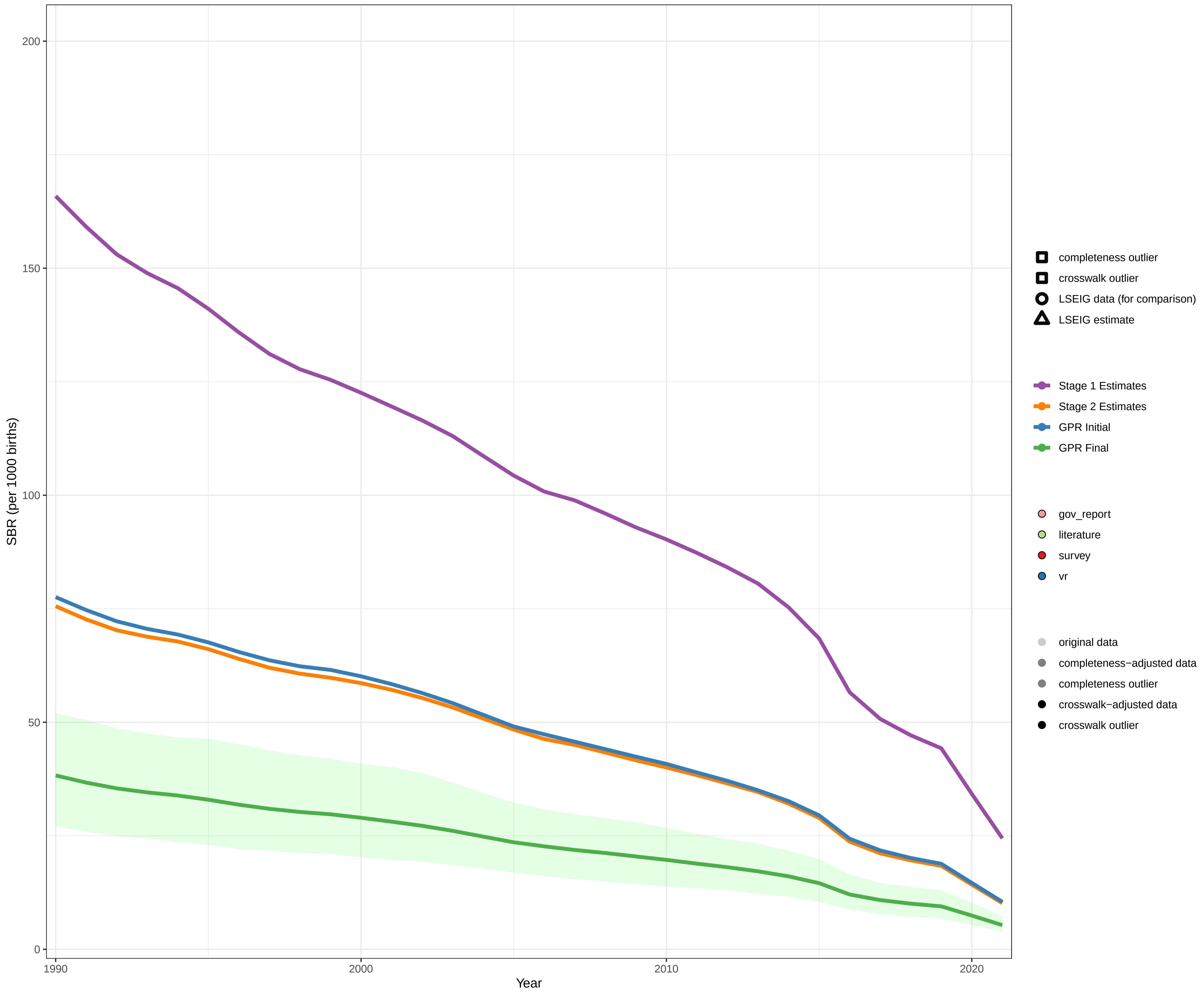

Kurdistan   IRN_44884   North Africa and Middle East   Stillbirth Rate (SBR) − 28 weeks
lambda = 1 ; zeta = 0.6 ; scale = 15 ; amp = 0.028

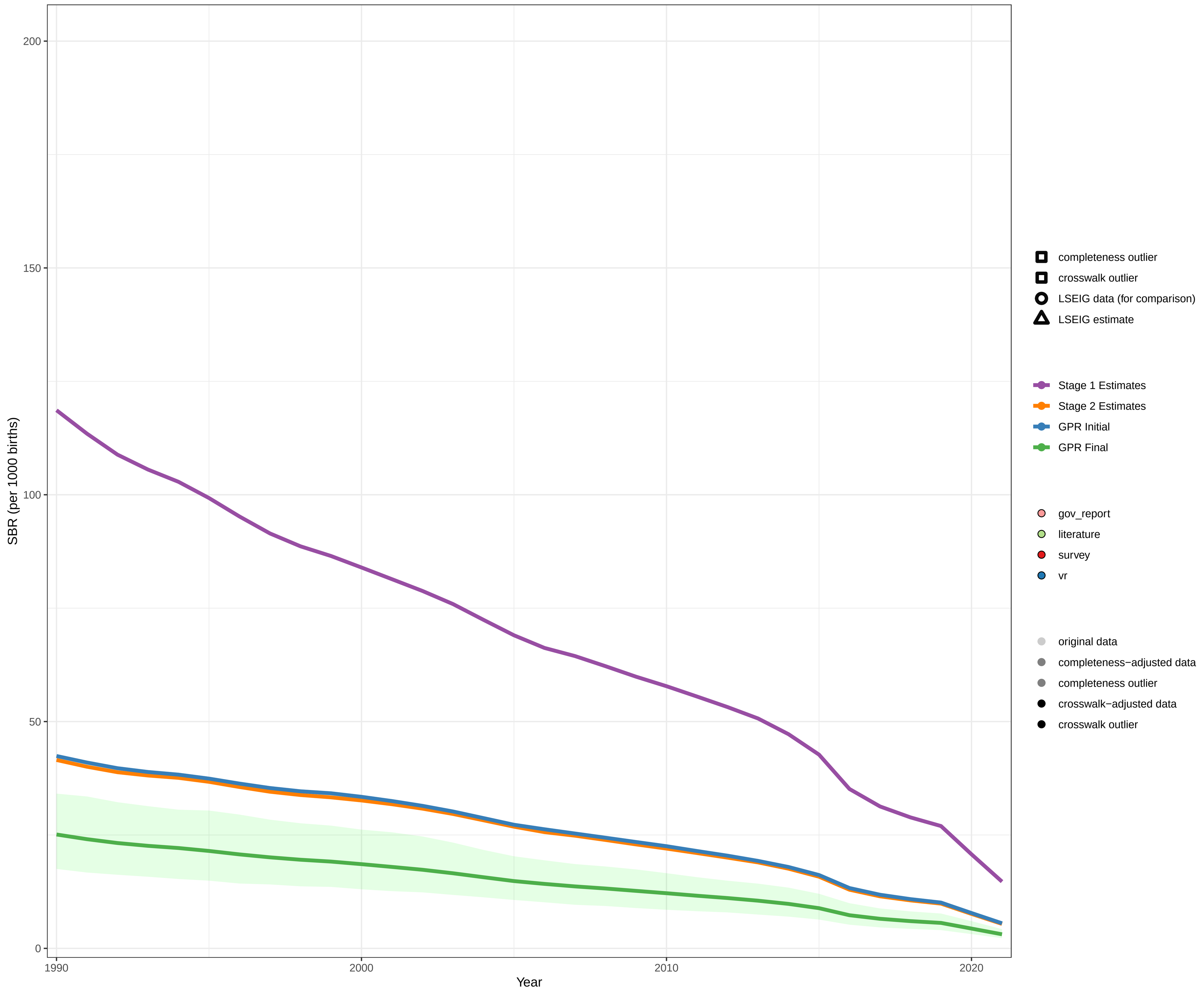

Year	Total Stillbirths (in thousands)	Total Stillbirths (in thousands)	SBR (per 1000 births)	SBR (per 1000 births)	SBR/NMR	SBR/NMR
	20 weeks	28 weeks	20 weeks	28 weeks	20 weeks	28 weeks
1990	1.4 (1.0 to 2.0)	0.9 (0.6 to 1.3)	38.3 (27.0 to 52.0)	25.1 (17.5 to 34.1)	0.90 (0.63 to 1.22)	0.59 (0.41 to 0.80)
1995	1.0 (0.7 to 1.4)	0.6 (0.4 to 0.9)	33.0 (23.0 to 46.4)	21.5 (14.9 to 30.4)	0.95 (0.66 to 1.34)	0.62 (0.43 to 0.88)
2000	0.8 (0.5 to 1.1)	0.5 (0.3 to 0.7)	29.0 (20.2 to 40.9)	18.6 (13.0 to 26.2)	1.05 (0.73 to 1.48)	0.67 (0.47 to 0.95)
2005	0.6 (0.4 to 0.8)	0.4 (0.3 to 0.5)	23.6 (16.9 to 32.3)	14.8 (10.7 to 20.3)	1.10 (0.79 to 1.51)	0.69 (0.50 to 0.95)
2010	0.6 (0.4 to 0.8)	0.3 (0.2 to 0.5)	19.7 (13.8 to 26.7)	12.2 (8.5 to 16.6)	1.15 (0.80 to 1.55)	0.71 (0.49 to 0.96)
2015	0.4 (0.3 to 0.6)	0.3 (0.2 to 0.4)	14.6 (10.5 to 19.9)	8.9 (6.4 to 12.0)	1.18 (0.85 to 1.61)	0.72 (0.51 to 0.97)
2020	0.2 (0.1 to 0.2)	0.1 (0.1 to 0.1)	7.4 (5.4 to 10.3)	4.4 (3.2 to 6.0)	1.29 (0.93 to 1.79)	0.76 (0.55 to 1.05)
2021	0.1 (0.1 to 0.2)	0.1 (0.0 to 0.1)	5.3 (3.8 to 7.3)	3.1 (2.3 to 4.2)	1.32 (0.95 to 1.80)	0.77 (0.56 to 1.05)

Lorestan IRN_44885 North Africa and Middle East Stillbirth Rate / Neonatal Mortality Rate (SBR/NMR) − 20 weeks
lambda = 1 ; zeta = 0.6 ; scale = 15 ; amp = 0.028

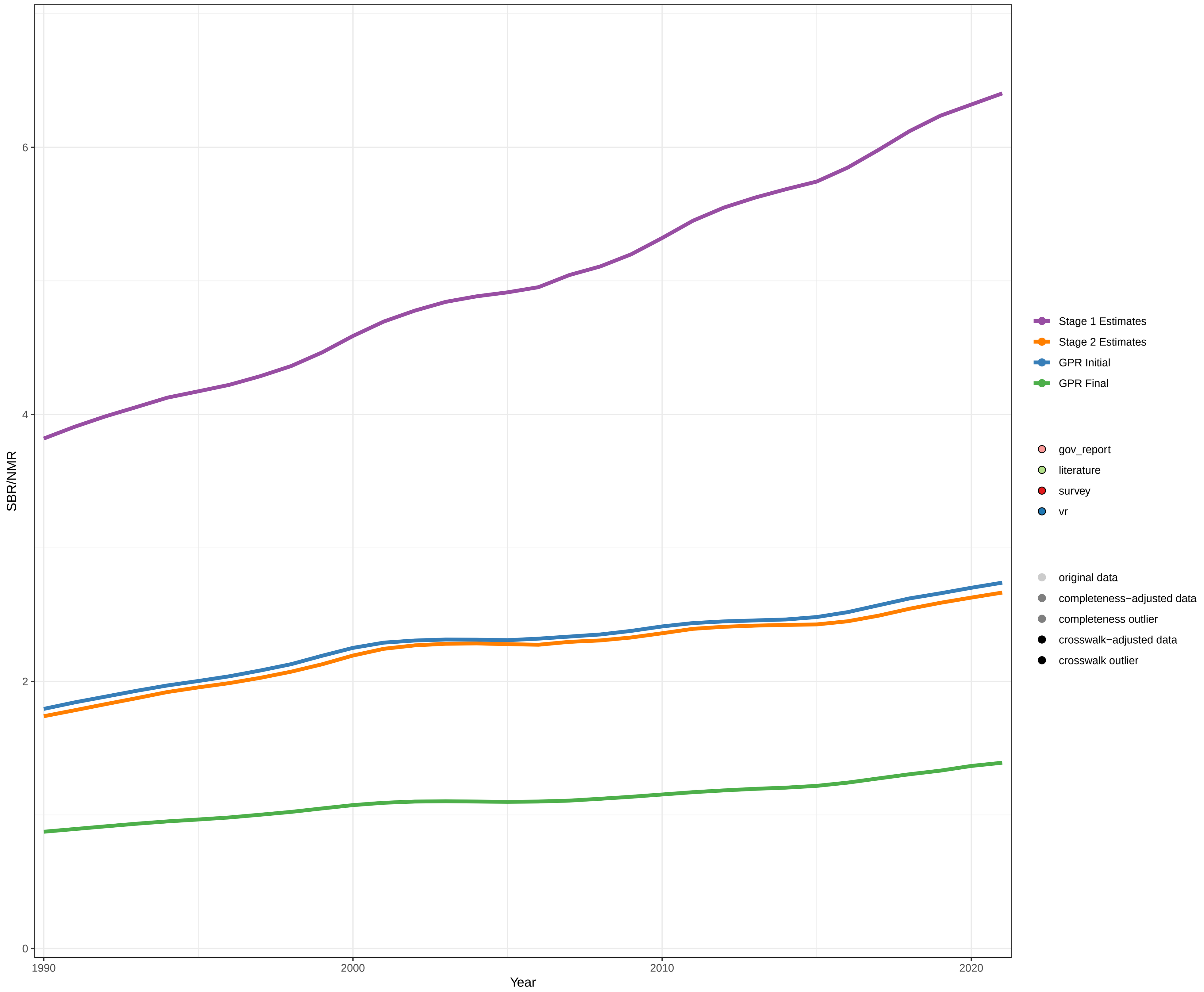

Lorestan IRN_44885 North Africa and Middle East Stillbirth Rate / Neonatal Mortality Rate (SBR/NMR) − 28 weeks
lambda = 1 ; zeta = 0.6 ; scale = 15 ; amp = 0.028

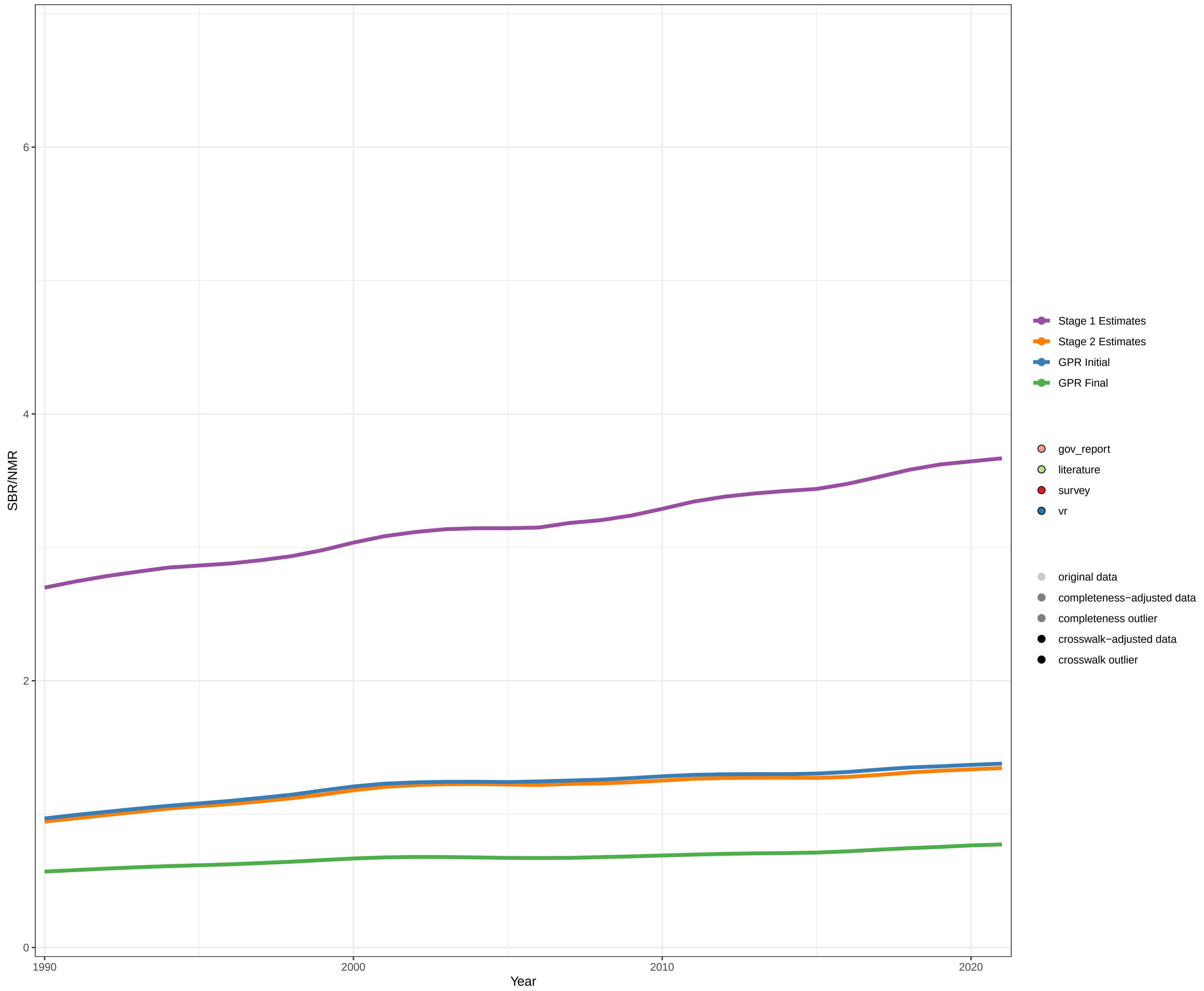

Definition	Years	Outlier	Source.Type	Title

Lorestan IRN_44885 North Africa and Middle East Stillbirth Rate (SBR) − 20 weeks
lambda = 1 ; zeta = 0.6 ; scale = 15 ; amp = 0.028

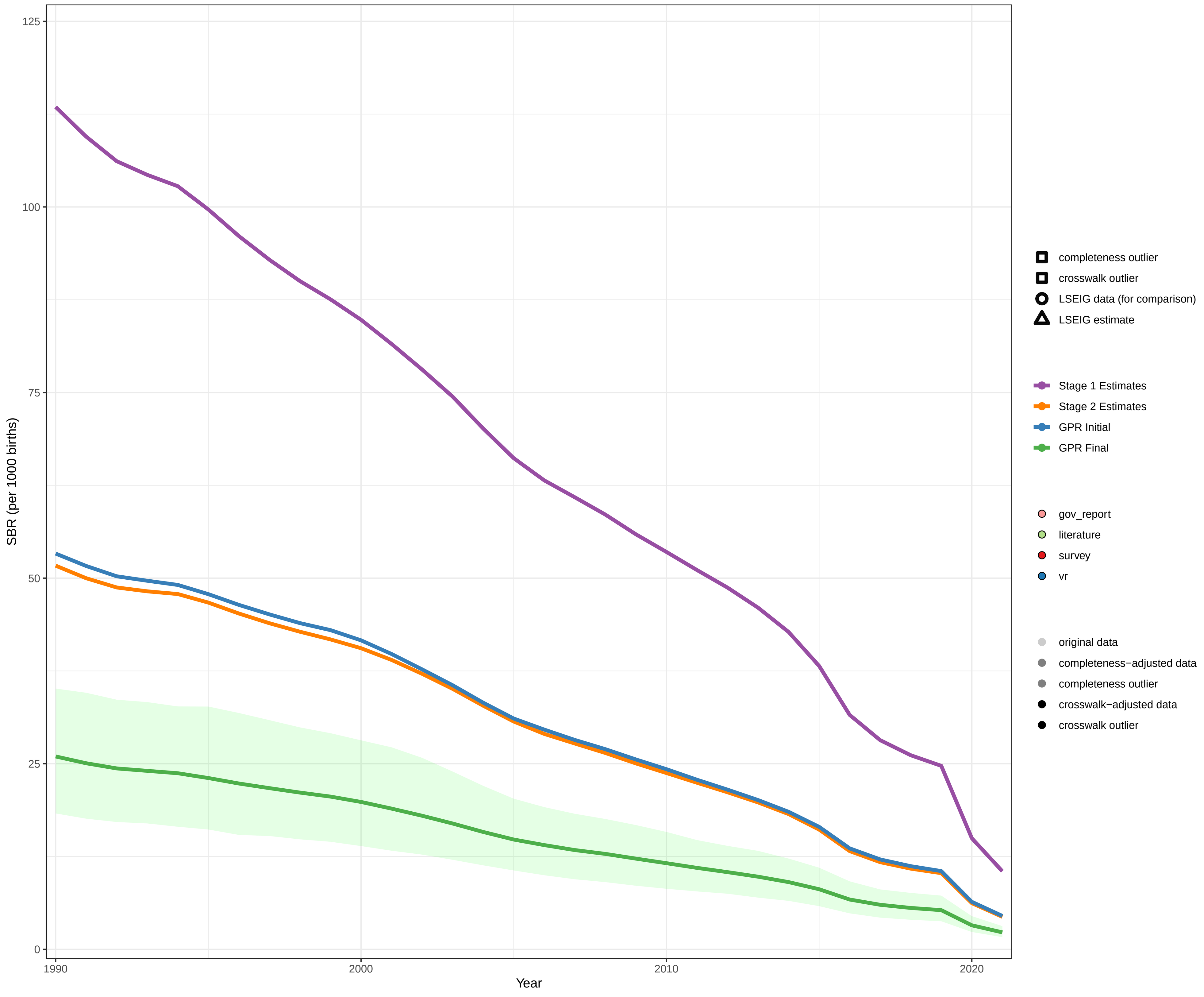

Lorestan IRN_44885 North Africa and Middle East Stillbirth Rate (SBR) − 28 weeks
lambda = 1 ; zeta = 0.6 ; scale = 15 ; amp = 0.028

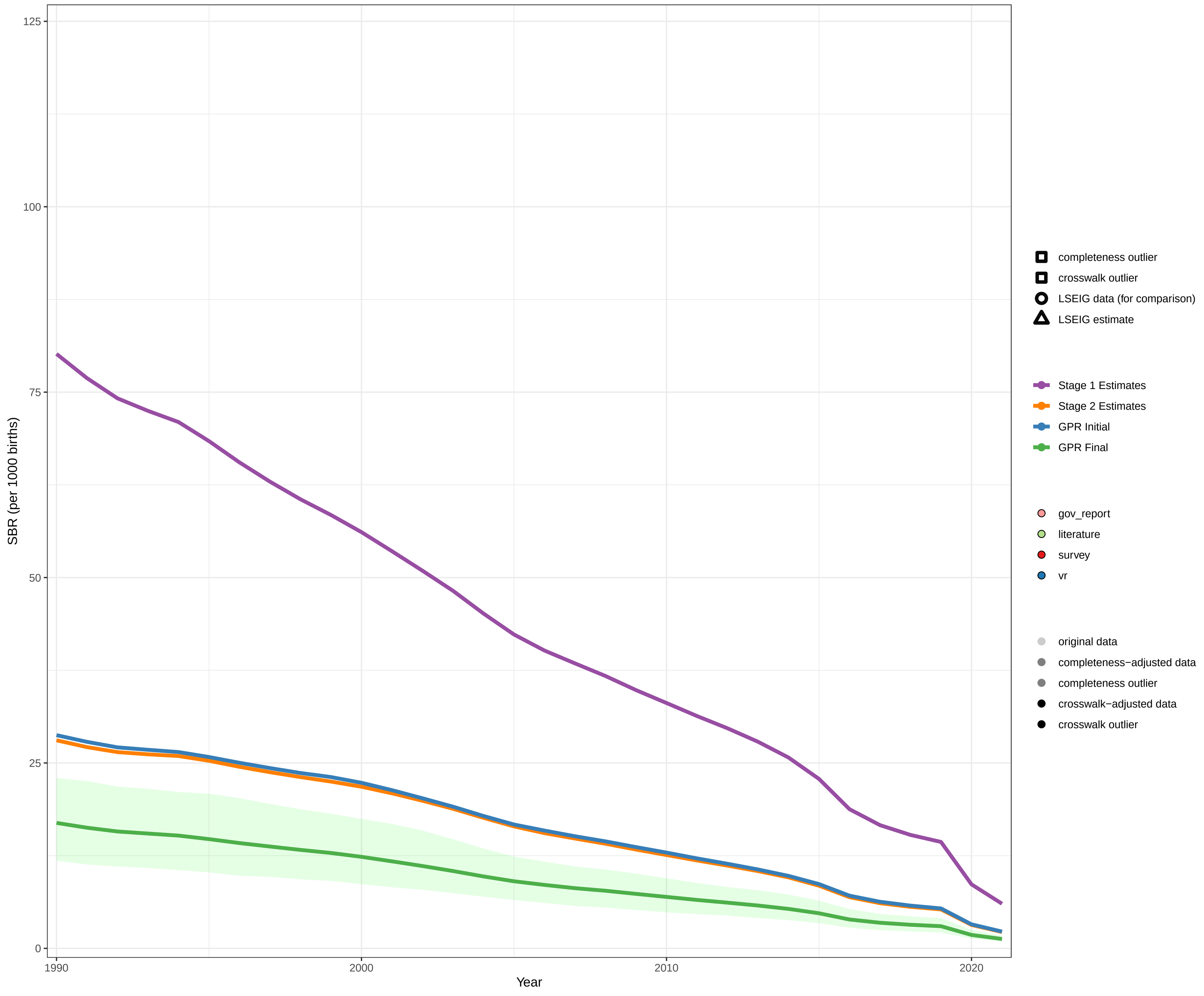

Year	Total Stillbirths (in thousands)	Total Stillbirths (in thousands)	SBR (per 1000 births)	SBR (per 1000 births)	SBR/NMR	SBR/NMR
	20 weeks	28 weeks	20 weeks	28 weeks	20 weeks	28 weeks
1990	1.4 (0.9 to 1.8)	0.9 (0.6 to 1.2)	26.0 (18.3 to 35.1)	16.9 (11.8 to 23.0)	0.87 (0.62 to 1.18)	0.57 (0.40 to 0.77)
1995	0.9 (0.6 to 1.3)	0.6 (0.4 to 0.8)	23.1 (16.1 to 32.7)	14.7 (10.3 to 20.9)	0.97 (0.68 to 1.37)	0.62 (0.43 to 0.87)
2000	0.7 (0.5 to 0.9)	0.4 (0.3 to 0.6)	19.9 (13.9 to 28.2)	12.3 (8.7 to 17.5)	1.07 (0.75 to 1.52)	0.67 (0.47 to 0.94)
2005	0.4 (0.3 to 0.6)	0.3 (0.2 to 0.4)	14.8 (10.6 to 20.3)	9.0 (6.5 to 12.4)	1.10 (0.79 to 1.51)	0.67 (0.48 to 0.92)
2010	0.4 (0.3 to 0.6)	0.2 (0.2 to 0.3)	11.6 (8.2 to 15.8)	6.9 (4.9 to 9.5)	1.15 (0.81 to 1.57)	0.69 (0.48 to 0.94)
2015	0.3 (0.2 to 0.4)	0.2 (0.1 to 0.2)	8.1 (5.8 to 11.0)	4.7 (3.4 to 6.4)	1.22 (0.88 to 1.66)	0.71 (0.51 to 0.97)
2020	0.1 (0.1 to 0.1)	0.0 (0.0 to 0.1)	3.2 (2.3 to 4.5)	1.8 (1.3 to 2.5)	1.37 (0.99 to 1.89)	0.77 (0.55 to 1.06)
2021	0.1 (0.0 to 0.1)	0.0 (0.0 to 0.0)	2.3 (1.6 to 3.1)	1.3 (0.9 to 1.7)	1.39 (1.00 to 1.91)	0.77 (0.56 to 1.06)

Markazi IRN_44886 North Africa and Middle East Stillbirth Rate / Neonatal Mortality Rate (SBR/NMR) − 20 weeks
lambda = 1 ; zeta = 0.6 ; scale = 15 ; amp = 0.028

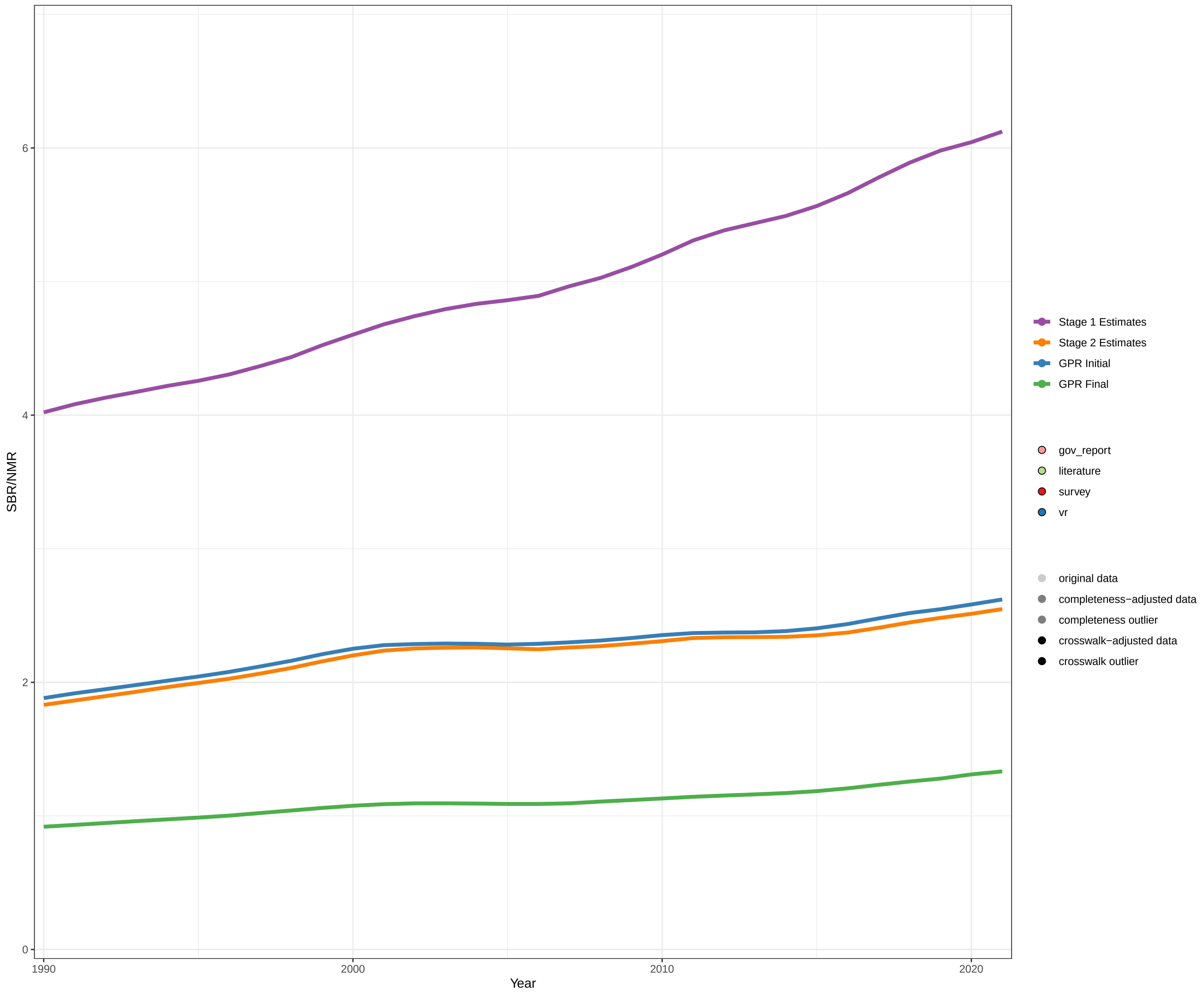

Markazi IRN_44886 North Africa and Middle East Stillbirth Rate / Neonatal Mortality Rate (SBR/NMR) − 28 weeks
lambda = 1 ; zeta = 0.6 ; scale = 15 ; amp = 0.028

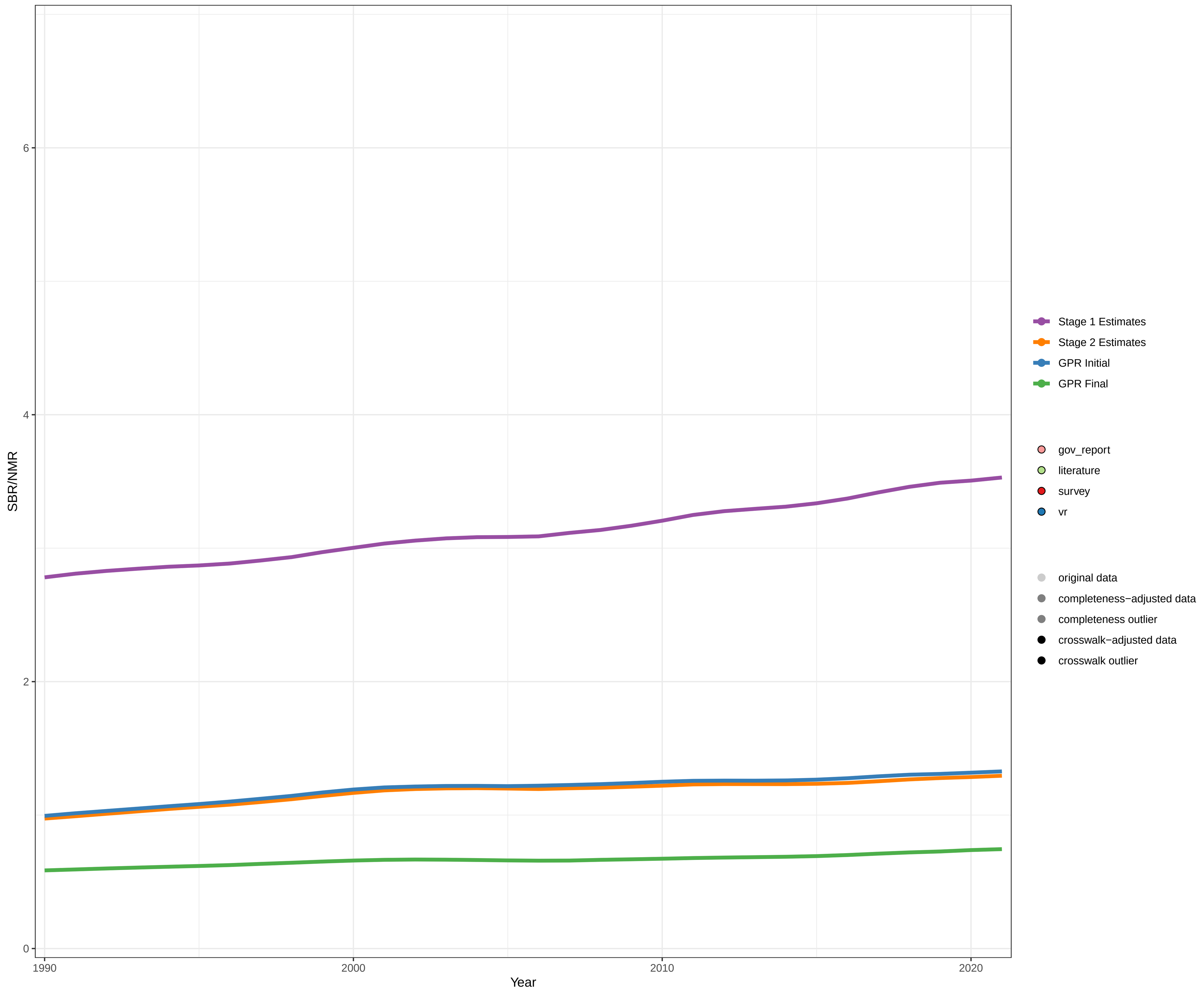

Definition	Years	Outlier	Source.Type	Title

Markazi IRN_44886 North Africa and Middle East Stillbirth Rate (SBR) − 20 weeks
lambda = 1 ; zeta = 0.6 ; scale = 15 ; amp = 0.028

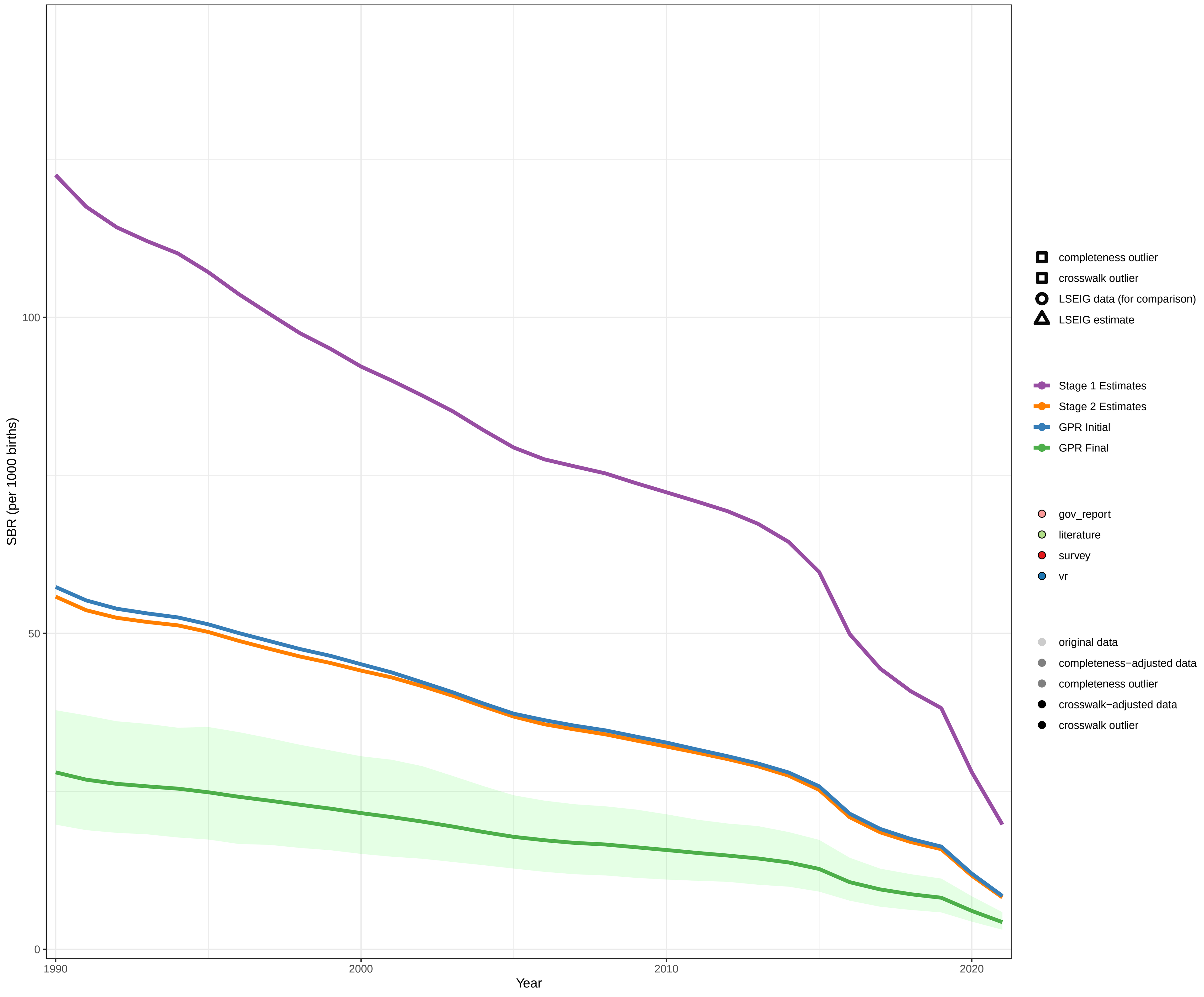

Markazi IRN_44886 North Africa and Middle East Stillbirth Rate (SBR) − 28 weeks
lambda = 1 ; zeta = 0.6 ; scale = 15 ; amp = 0.028

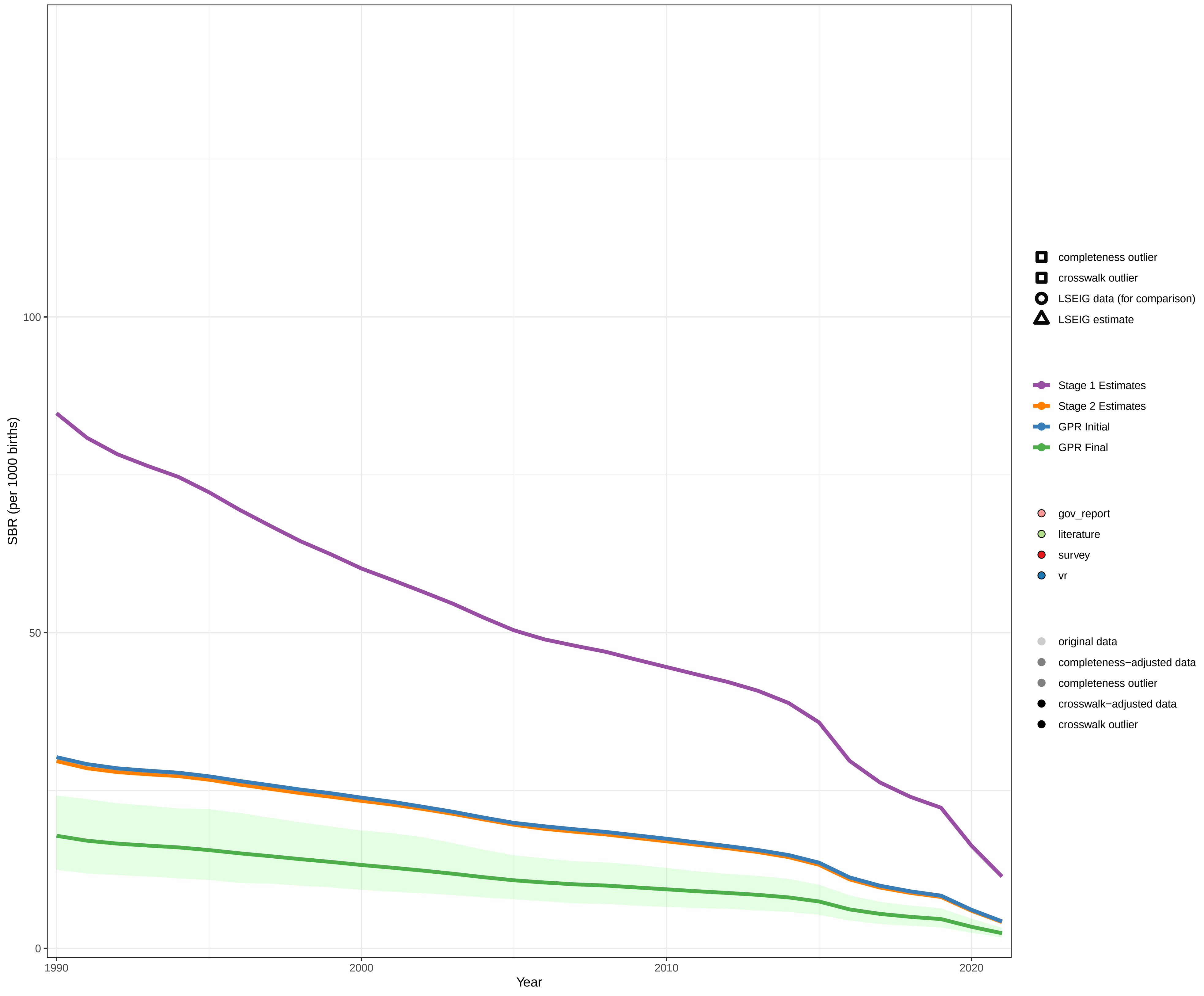

Year	Total Stillbirths (in thousands)	Total Stillbirths (in thousands)	SBR (per 1000 births)	SBR (per 1000 births)	SBR/NMR	SBR/NMR
	20 weeks	28 weeks	20 weeks	28 weeks	20 weeks	28 weeks
1990	0.8 (0.6 to 1.1)	0.5 (0.4 to 0.7)	28.0 (19.7 to 37.8)	17.8 (12.5 to 24.2)	0.92 (0.65 to 1.24)	0.59 (0.41 to 0.79)
1995	0.5 (0.4 to 0.8)	0.3 (0.2 to 0.5)	24.8 (17.4 to 35.2)	15.6 (10.8 to 22.0)	0.99 (0.69 to 1.40)	0.62 (0.43 to 0.88)
2000	0.4 (0.3 to 0.6)	0.2 (0.2 to 0.3)	21.6 (15.1 to 30.6)	13.2 (9.3 to 18.7)	1.08 (0.75 to 1.52)	0.66 (0.46 to 0.93)
2005	0.3 (0.2 to 0.4)	0.2 (0.1 to 0.3)	17.8 (12.8 to 24.4)	10.8 (7.8 to 14.8)	1.09 (0.78 to 1.49)	0.66 (0.48 to 0.90)
2010	0.3 (0.2 to 0.5)	0.2 (0.1 to 0.3)	15.7 (11.0 to 21.4)	9.4 (6.5 to 12.8)	1.13 (0.79 to 1.54)	0.67 (0.47 to 0.92)
2015	0.3 (0.2 to 0.4)	0.2 (0.1 to 0.2)	12.7 (9.1 to 17.3)	7.4 (5.3 to 10.1)	1.19 (0.85 to 1.61)	0.69 (0.50 to 0.94)
2020	0.1 (0.1 to 0.1)	0.1 (0.0 to 0.1)	6.1 (4.4 to 8.4)	3.4 (2.5 to 4.7)	1.31 (0.94 to 1.81)	0.74 (0.53 to 1.02)
2021	0.1 (0.0 to 0.1)	0.0 (0.0 to 0.0)	4.3 (3.1 to 5.9)	2.4 (1.7 to 3.3)	1.33 (0.96 to 1.82)	0.74 (0.54 to 1.02)

Mazandaran IRN_44887 North Africa and Middle East Stillbirth Rate / Neonatal Mortality Rate (SBR/NMR) − 20 weeks
lambda = 0.8 ; zeta = 0.7 ; scale = 15 ; amp = 0.028

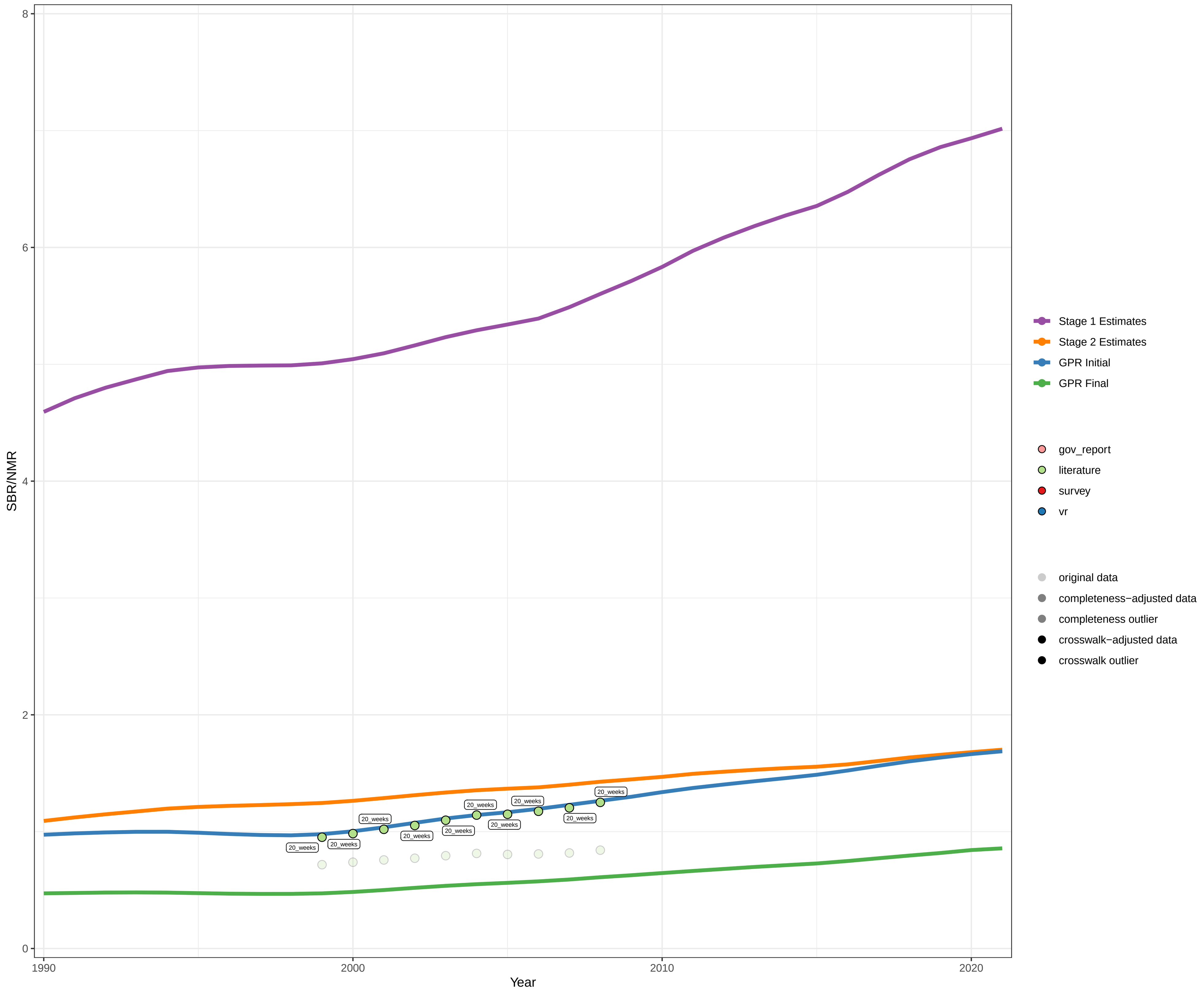

Mazandaran IRN_44887 North Africa and Middle East Stillbirth Rate / Neonatal Mortality Rate (SBR/NMR) − 28 weeks
lambda = 0.8 ; zeta = 0.7 ; scale = 15 ; amp = 0.028

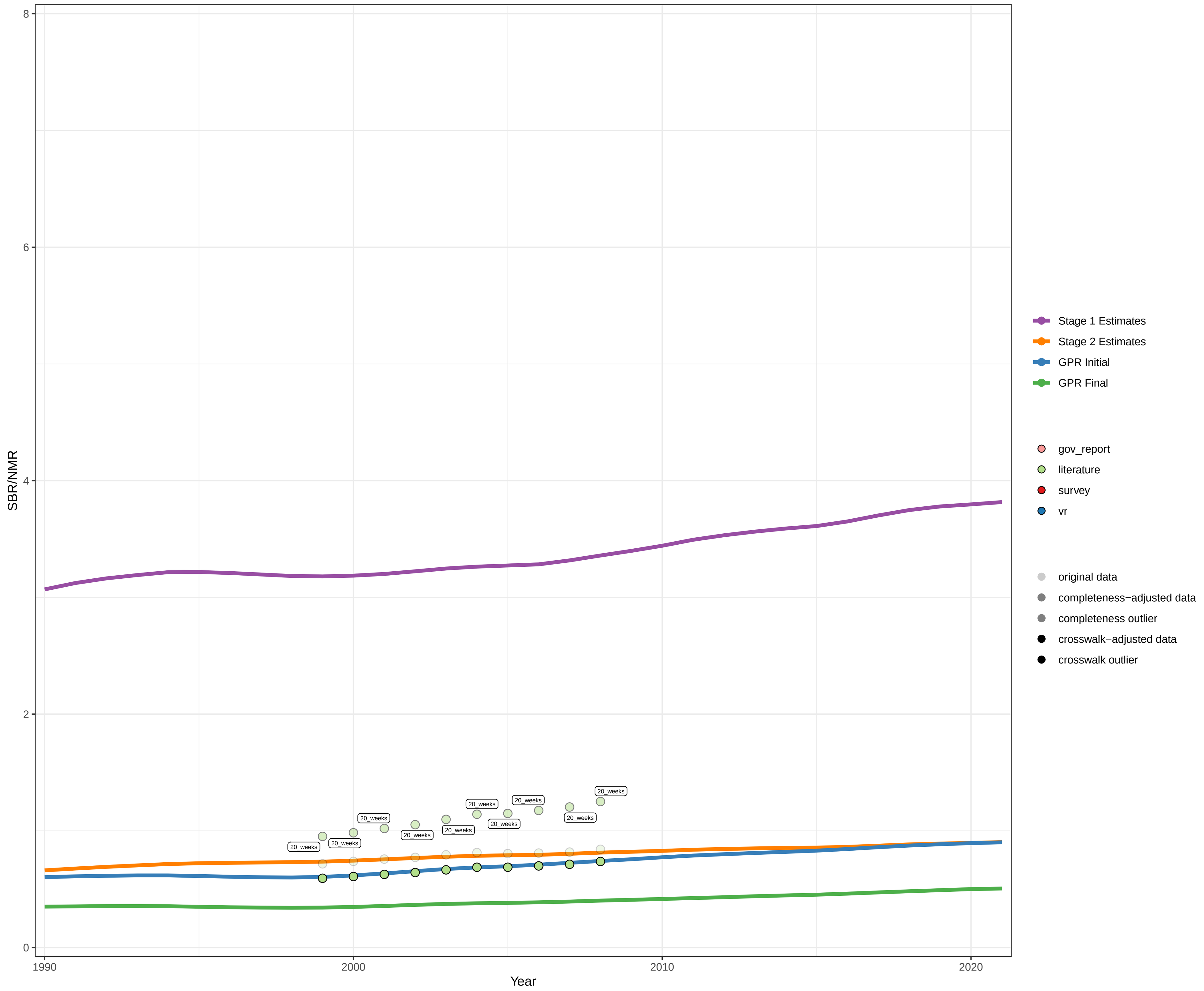

Definition	Years	Outlier	Source.Type	Title
20_weeks	1999−2008	0	Sci Lit	Trend of Stillbirth Rates and the Associated Risk Factors in Babol, Northern Iran

Mazandaran IRN_44887 North Africa and Middle East Stillbirth Rate (SBR) − 20 weeks
lambda = 0.8 ; zeta = 0.7 ; scale = 15 ; amp = 0.028

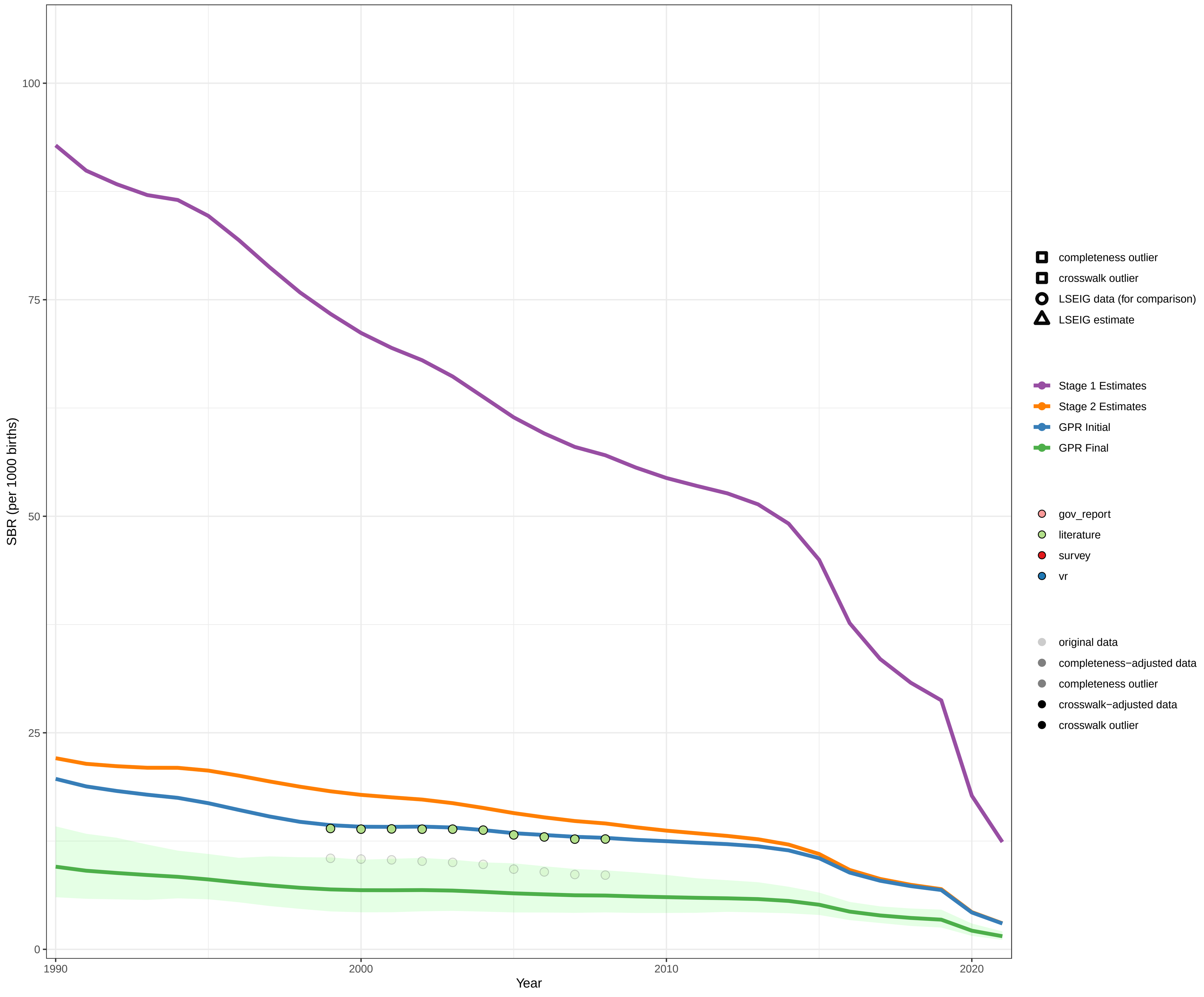

Mazandaran IRN_44887 North Africa and Middle East Stillbirth Rate (SBR) − 28 weeks
lambda = 0.8 ; zeta = 0.7 ; scale = 15 ; amp = 0.028

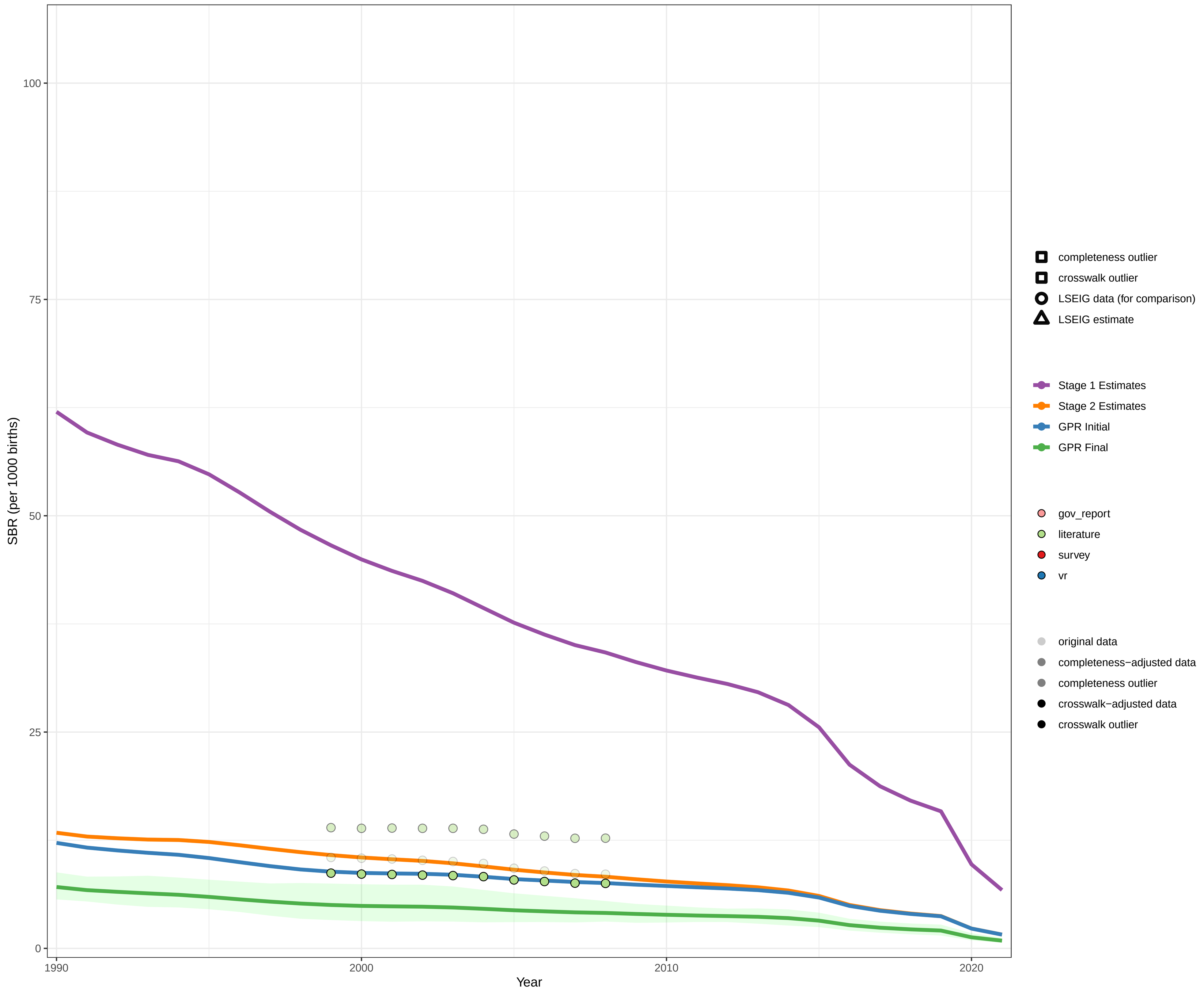

Year	Total Stillbirths (in thousands)	Total Stillbirths (in thousands)	SBR (per 1000 births)	SBR (per 1000 births)	SBR/NMR	SBR/NMR
	20 weeks	28 weeks	20 weeks	28 weeks	20 weeks	28 weeks
1990	0.6 (0.4 to 0.9)	0.4 (0.4 to 0.6)	9.5 (6.0 to 14.2)	7.1 (5.7 to 8.8)	0.47 (0.30 to 0.70)	0.35 (0.28 to 0.43)
1995	0.4 (0.3 to 0.5)	0.3 (0.2 to 0.4)	8.1 (5.8 to 11.0)	6.0 (4.5 to 7.9)	0.47 (0.34 to 0.65)	0.35 (0.27 to 0.47)
2000	0.3 (0.2 to 0.4)	0.2 (0.1 to 0.3)	6.8 (4.3 to 10.3)	4.9 (3.2 to 7.4)	0.48 (0.30 to 0.73)	0.35 (0.22 to 0.53)
2005	0.2 (0.2 to 0.4)	0.2 (0.1 to 0.2)	6.5 (4.3 to 9.9)	4.4 (3.0 to 6.4)	0.56 (0.37 to 0.86)	0.38 (0.26 to 0.55)
2010	0.3 (0.2 to 0.4)	0.2 (0.1 to 0.2)	6.0 (4.2 to 8.6)	3.9 (3.0 to 4.9)	0.65 (0.45 to 0.92)	0.42 (0.32 to 0.53)
2015	0.2 (0.2 to 0.3)	0.1 (0.1 to 0.2)	5.2 (4.0 to 6.6)	3.2 (2.5 to 4.1)	0.73 (0.56 to 0.93)	0.45 (0.35 to 0.58)
2020	0.1 (0.1 to 0.1)	0.0 (0.0 to 0.1)	2.2 (1.6 to 3.0)	1.3 (0.9 to 1.8)	0.84 (0.61 to 1.16)	0.50 (0.36 to 0.69)
2021	0.0 (0.0 to 0.1)	0.0 (0.0 to 0.0)	1.5 (1.1 to 2.1)	0.9 (0.6 to 1.2)	0.86 (0.61 to 1.17)	0.51 (0.36 to 0.69)

North Khorasan IRN_44879 North Africa and Middle East Stillbirth Rate / Neonatal Mortality Rate (SBR/NMR) − 20 weeks
lambda = 1 ; zeta = 0.6 ; scale = 15 ; amp = 0.028

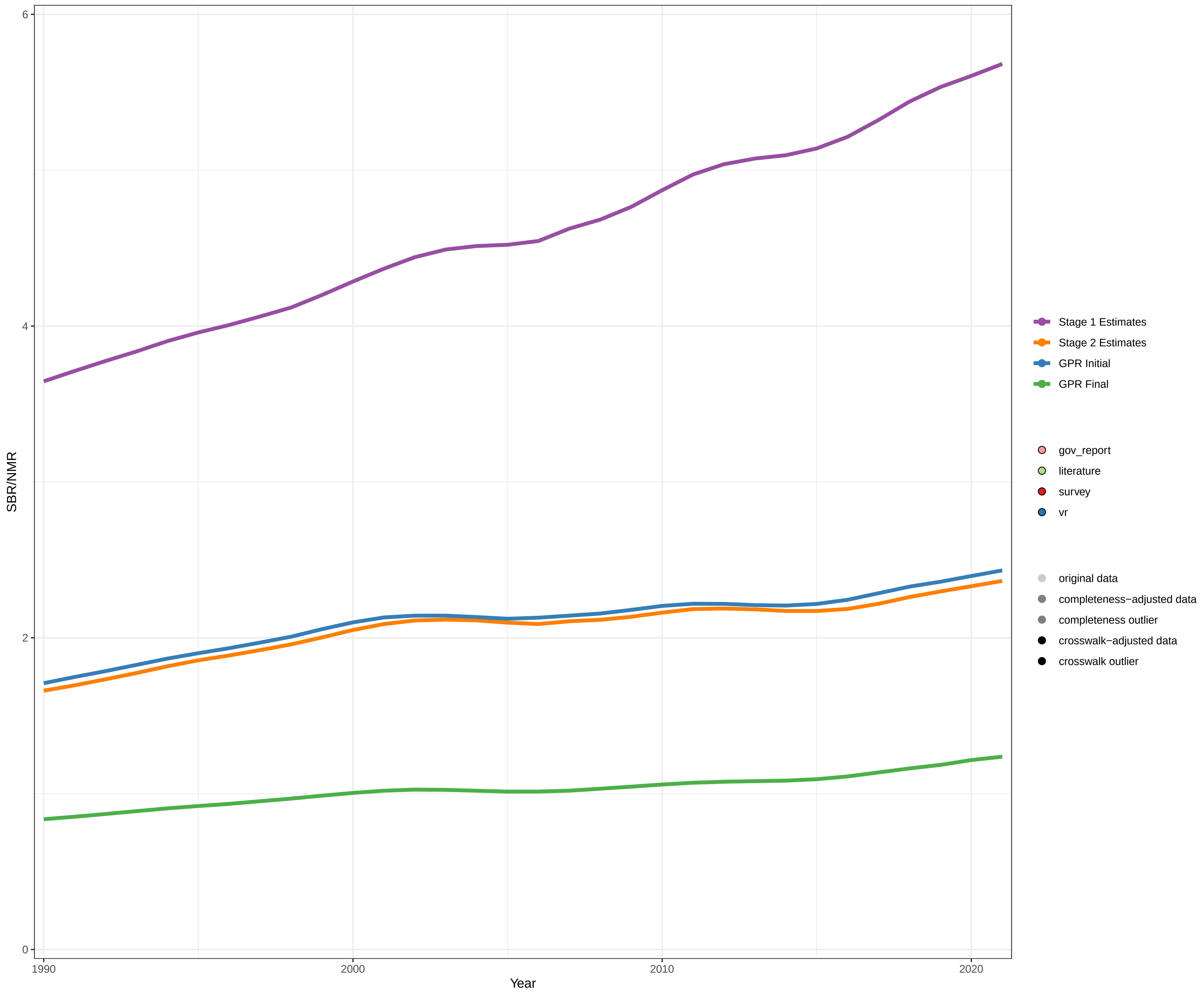

North Khorasan IRN_44879 North Africa and Middle East Stillbirth Rate / Neonatal Mortality Rate (SBR/NMR) − 28 weeks
lambda = 1 ; zeta = 0.6 ; scale = 15 ; amp = 0.028

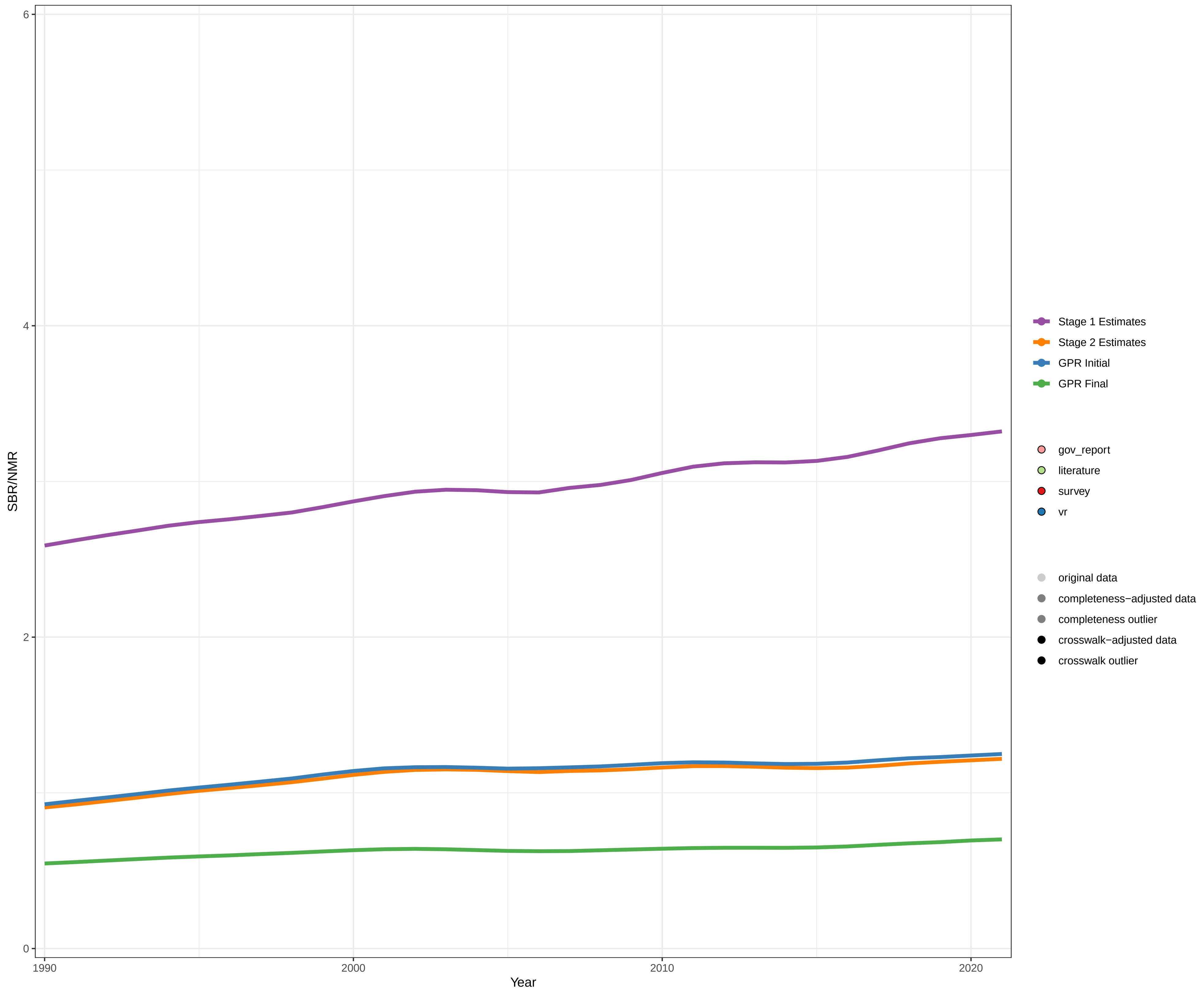

Definition	Years	Outlier	Source.Type	Title

North Khorasan IRN_44879 North Africa and Middle East Stillbirth Rate (SBR) − 20 weeks
lambda = 1 ; zeta = 0.6 ; scale = 15 ; amp = 0.028

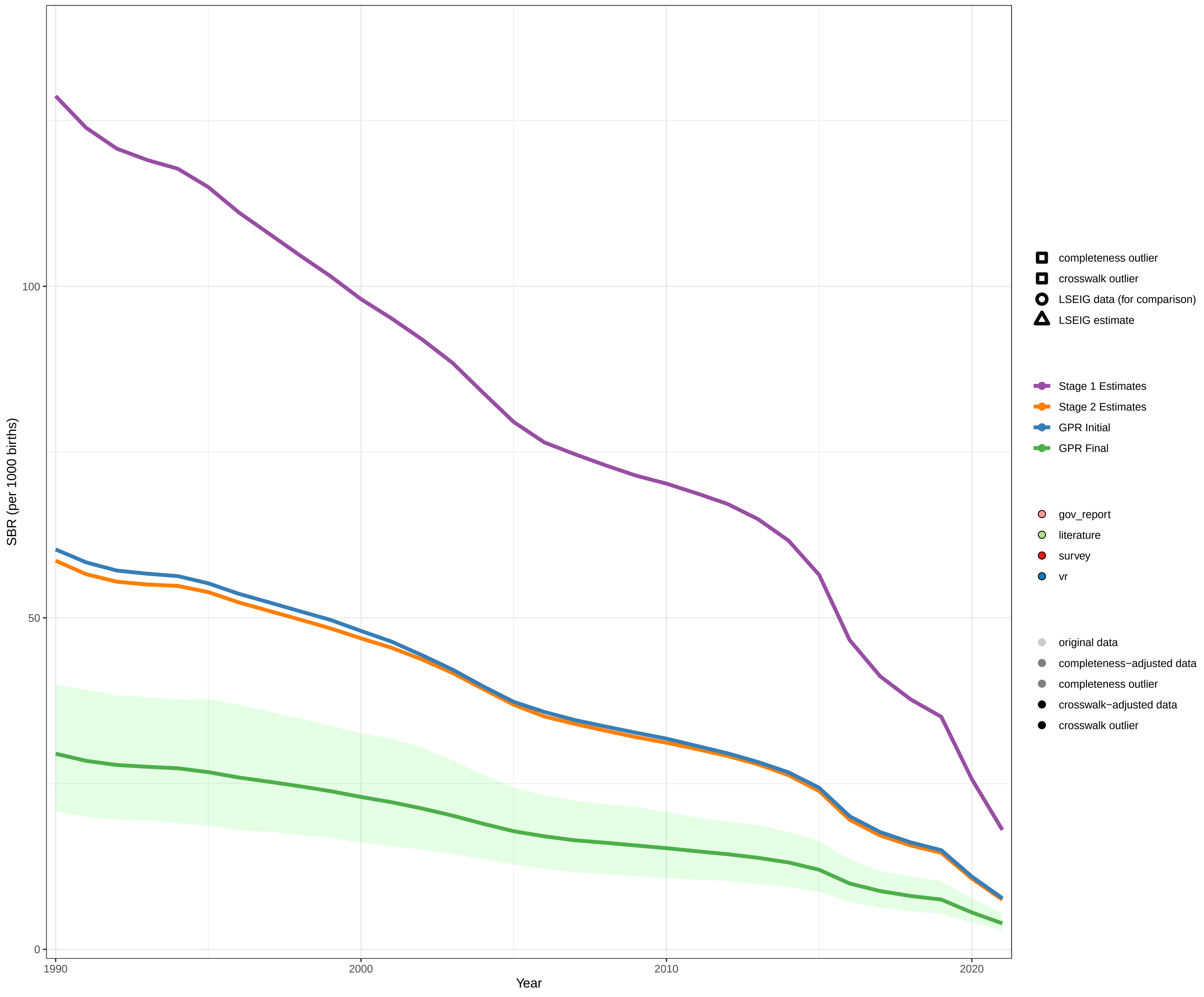

North Khorasan IRN_44879 North Africa and Middle East Stillbirth Rate (SBR) − 28 weeks
lambda = 1 ; zeta = 0.6 ; scale = 15 ; amp = 0.028

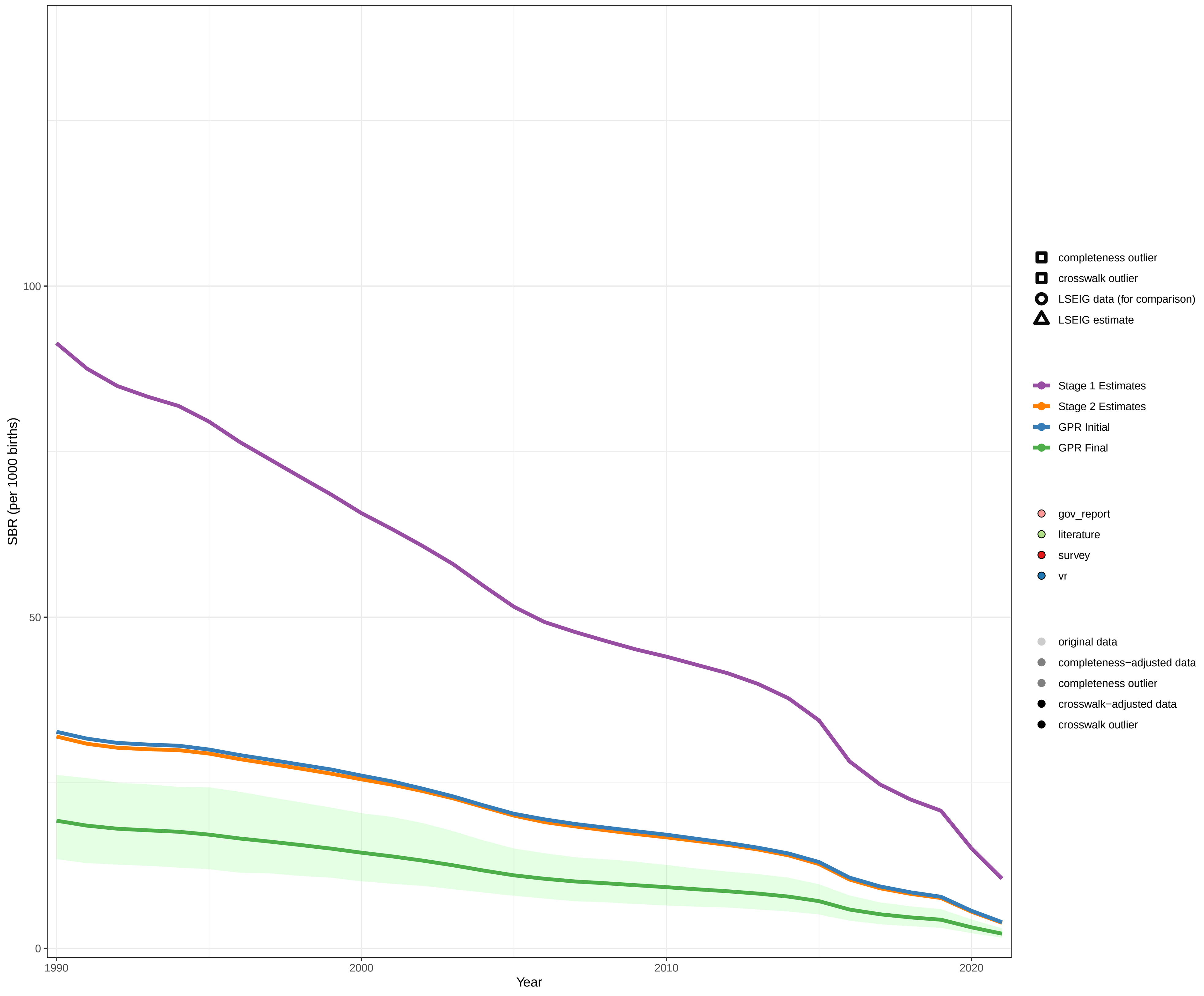

Year	Total Stillbirths (in thousands)	Total Stillbirths (in thousands)	SBR (per 1000 births)	SBR (per 1000 births)	SBR/NMR	SBR/NMR
	20 weeks	28 weeks	20 weeks	28 weeks	20 weeks	28 weeks
1990	0.7 (0.5 to 1.0)	0.5 (0.3 to 0.6)	29.5 (20.8 to 39.9)	19.3 (13.5 to 26.2)	0.84 (0.59 to 1.13)	0.55 (0.38 to 0.74)
1995	0.5 (0.3 to 0.7)	0.3 (0.2 to 0.5)	26.7 (18.7 to 37.8)	17.2 (11.9 to 24.3)	0.92 (0.64 to 1.30)	0.59 (0.41 to 0.84)
2000	0.4 (0.3 to 0.5)	0.2 (0.2 to 0.3)	23.0 (16.1 to 32.6)	14.5 (10.1 to 20.4)	1.00 (0.70 to 1.42)	0.63 (0.44 to 0.89)
2005	0.3 (0.2 to 0.4)	0.2 (0.1 to 0.2)	17.8 (12.8 to 24.4)	11.0 (7.9 to 15.1)	1.01 (0.73 to 1.39)	0.63 (0.45 to 0.86)
2010	0.3 (0.2 to 0.4)	0.2 (0.1 to 0.2)	15.3 (10.7 to 20.8)	9.2 (6.5 to 12.6)	1.06 (0.74 to 1.44)	0.64 (0.45 to 0.87)
2015	0.2 (0.2 to 0.3)	0.1 (0.1 to 0.2)	12.0 (8.6 to 16.3)	7.1 (5.1 to 9.7)	1.09 (0.78 to 1.49)	0.65 (0.47 to 0.88)
2020	0.1 (0.1 to 0.1)	0.0 (0.0 to 0.1)	5.6 (4.0 to 7.7)	3.2 (2.3 to 4.4)	1.22 (0.88 to 1.68)	0.69 (0.50 to 0.96)
2021	0.0 (0.0 to 0.1)	0.0 (0.0 to 0.0)	3.9 (2.8 to 5.4)	2.2 (1.6 to 3.0)	1.24 (0.89 to 1.69)	0.70 (0.51 to 0.96)

Qazvin IRN_44888 North Africa and Middle East Stillbirth Rate / Neonatal Mortality Rate (SBR/NMR) − 20 weeks
lambda = 1 ; zeta = 0.6 ; scale = 15 ; amp = 0.028

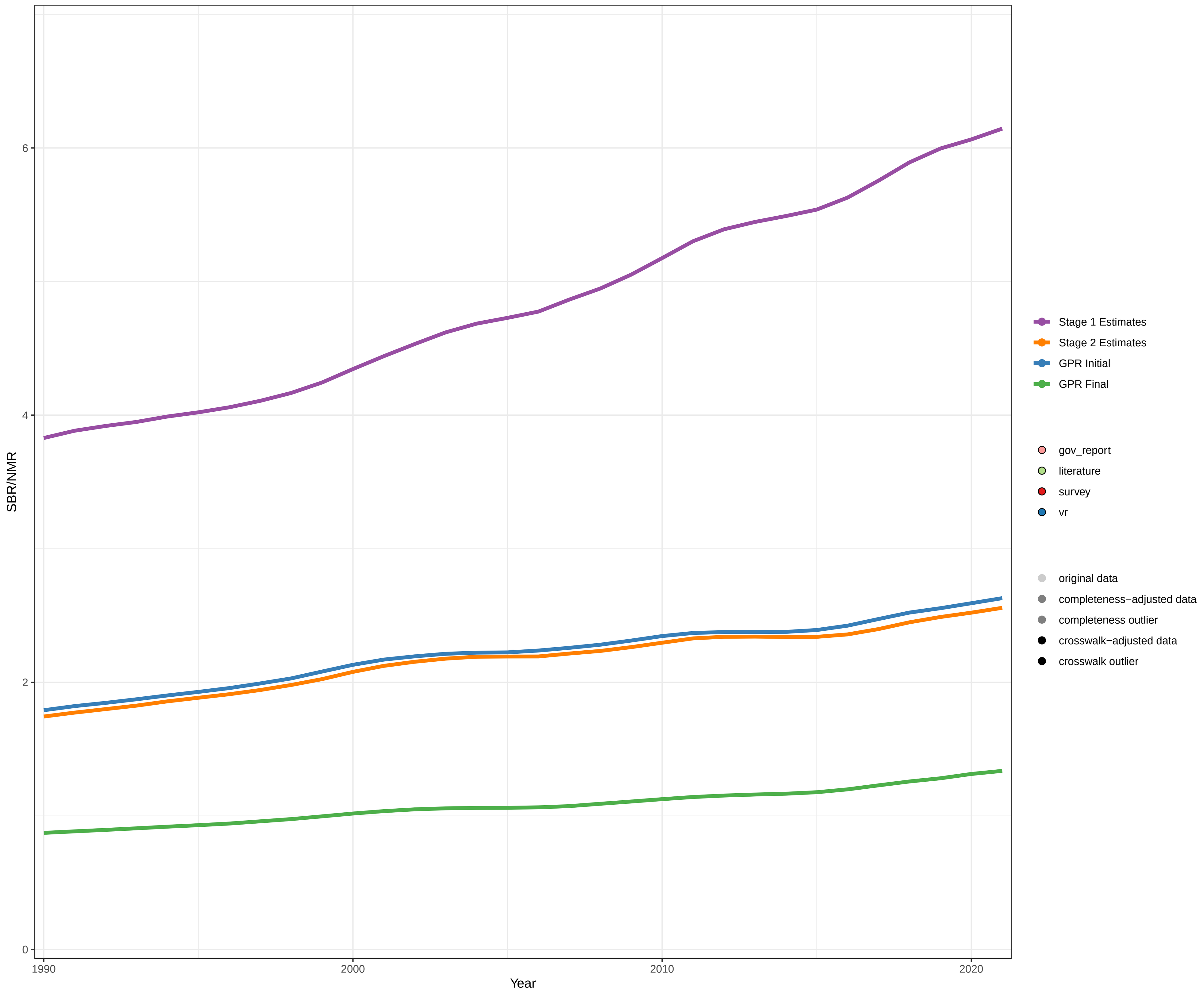

Qazvin IRN_44888 North Africa and Middle East Stillbirth Rate / Neonatal Mortality Rate (SBR/NMR) − 28 weeks
lambda = 1 ; zeta = 0.6 ; scale = 15 ; amp = 0.028

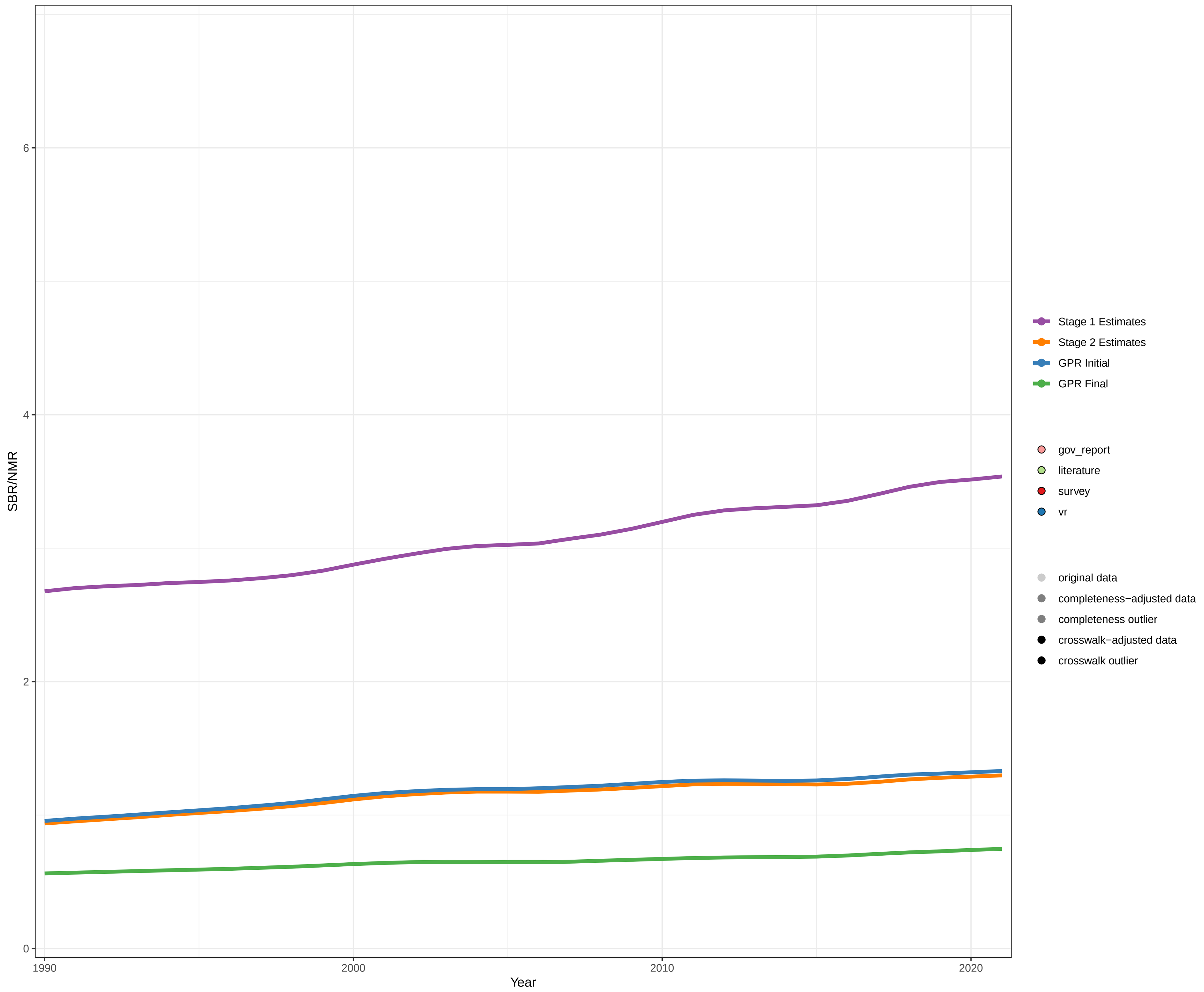

Definition	Years	Outlier	Source.Type	Title

Qazvin IRN_44888 North Africa and Middle East Stillbirth Rate (SBR) − 20 weeks
lambda = 1 ; zeta = 0.6 ; scale = 15 ; amp = 0.028

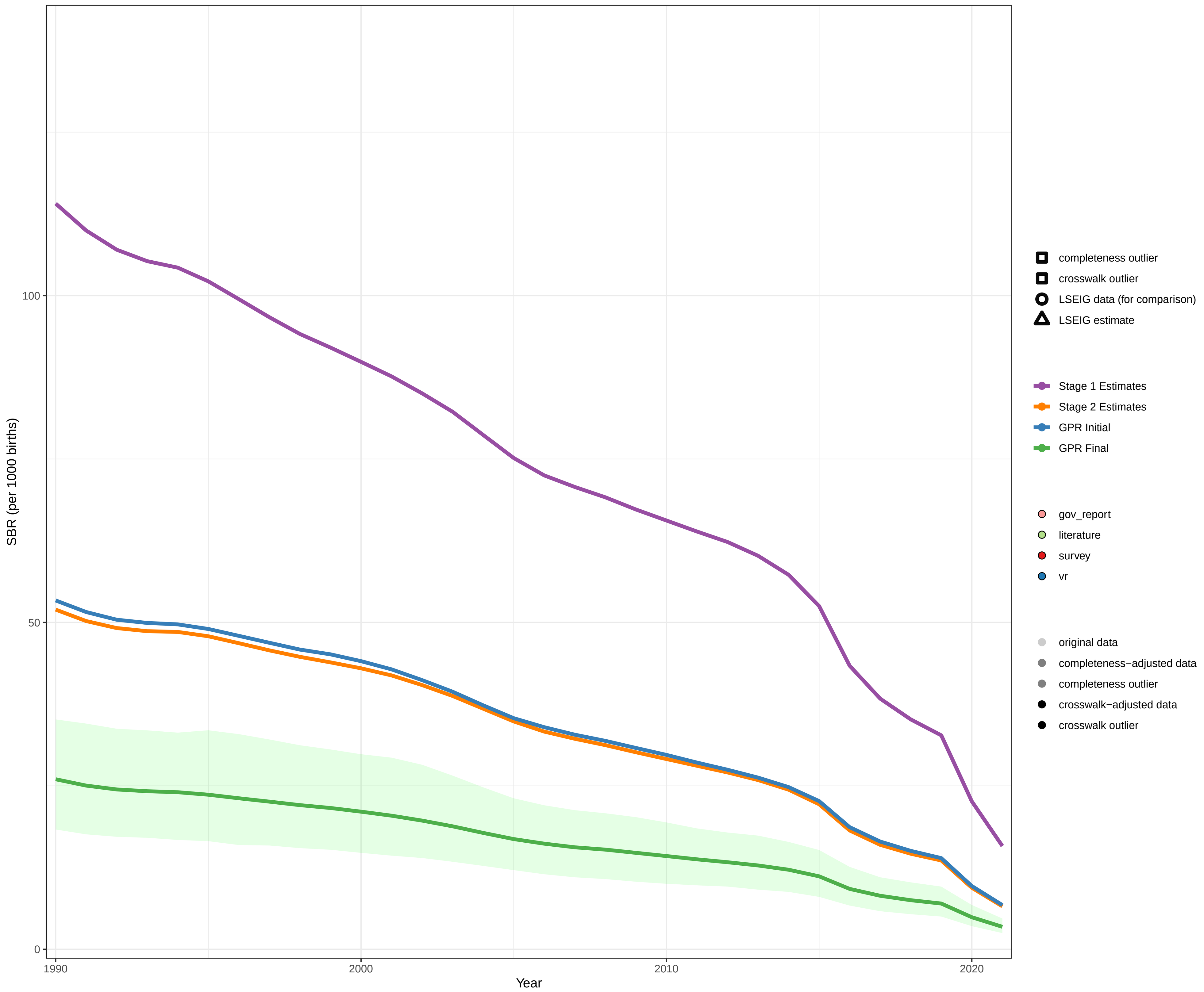

Qazvin IRN_44888 North Africa and Middle East Stillbirth Rate (SBR) − 28 weeks
lambda = 1 ; zeta = 0.6 ; scale = 15 ; amp = 0.028

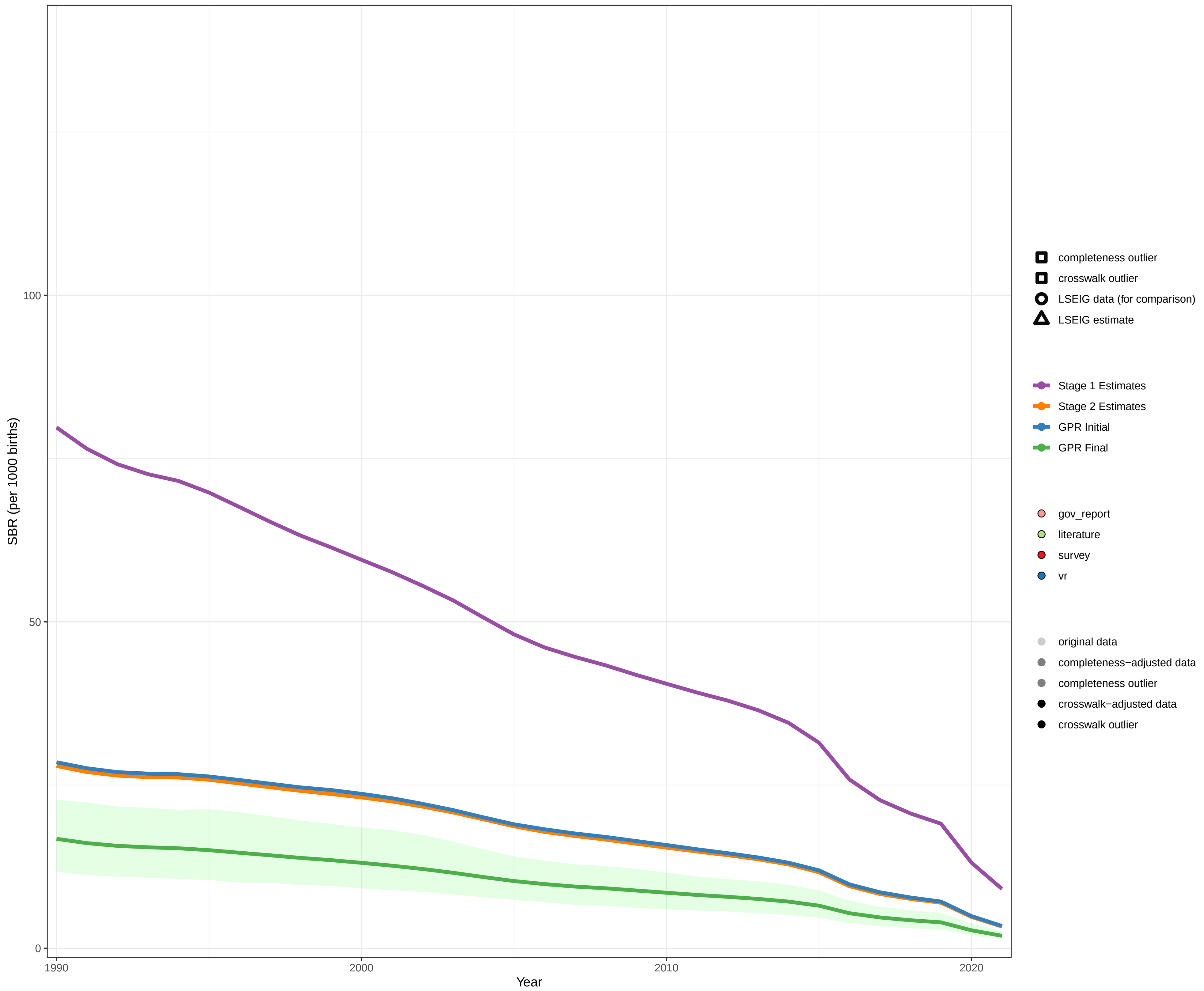

Year	Total Stillbirths (in thousands)	Total Stillbirths (in thousands)	SBR (per 1000 births)	SBR (per 1000 births)	SBR/NMR	SBR/NMR
	20 weeks	28 weeks	20 weeks	28 weeks	20 weeks	28 weeks
1990	0.7 (0.5 to 0.9)	0.4 (0.3 to 0.6)	26.0 (18.3 to 35.2)	16.8 (11.7 to 22.8)	0.87 (0.62 to 1.18)	0.56 (0.39 to 0.76)
1995	0.5 (0.3 to 0.7)	0.3 (0.2 to 0.4)	23.6 (16.5 to 33.5)	15.0 (10.5 to 21.3)	0.93 (0.65 to 1.32)	0.59 (0.41 to 0.84)
2000	0.4 (0.3 to 0.5)	0.2 (0.2 to 0.3)	21.1 (14.8 to 29.8)	13.1 (9.2 to 18.5)	1.02 (0.71 to 1.44)	0.63 (0.44 to 0.89)
2005	0.3 (0.2 to 0.4)	0.2 (0.1 to 0.2)	16.9 (12.1 to 23.1)	10.3 (7.4 to 14.1)	1.06 (0.76 to 1.45)	0.65 (0.47 to 0.89)
2010	0.3 (0.2 to 0.4)	0.2 (0.1 to 0.2)	14.3 (10.0 to 19.4)	8.5 (6.0 to 11.6)	1.13 (0.79 to 1.53)	0.67 (0.47 to 0.92)
2015	0.2 (0.2 to 0.3)	0.1 (0.1 to 0.2)	11.2 (8.0 to 15.2)	6.5 (4.7 to 8.9)	1.18 (0.85 to 1.60)	0.69 (0.49 to 0.94)
2020	0.1 (0.1 to 0.1)	0.0 (0.0 to 0.1)	4.9 (3.5 to 6.8)	2.8 (2.0 to 3.8)	1.31 (0.95 to 1.82)	0.74 (0.53 to 1.02)
2021	0.1 (0.0 to 0.1)	0.0 (0.0 to 0.0)	3.4 (2.5 to 4.7)	1.9 (1.4 to 2.6)	1.34 (0.96 to 1.83)	0.75 (0.54 to 1.02)

Qom IRN_44889 North Africa and Middle East Stillbirth Rate / Neonatal Mortality Rate (SBR/NMR) − 20 weeks
lambda = 1 ; zeta = 0.6 ; scale = 15 ; amp = 0.028

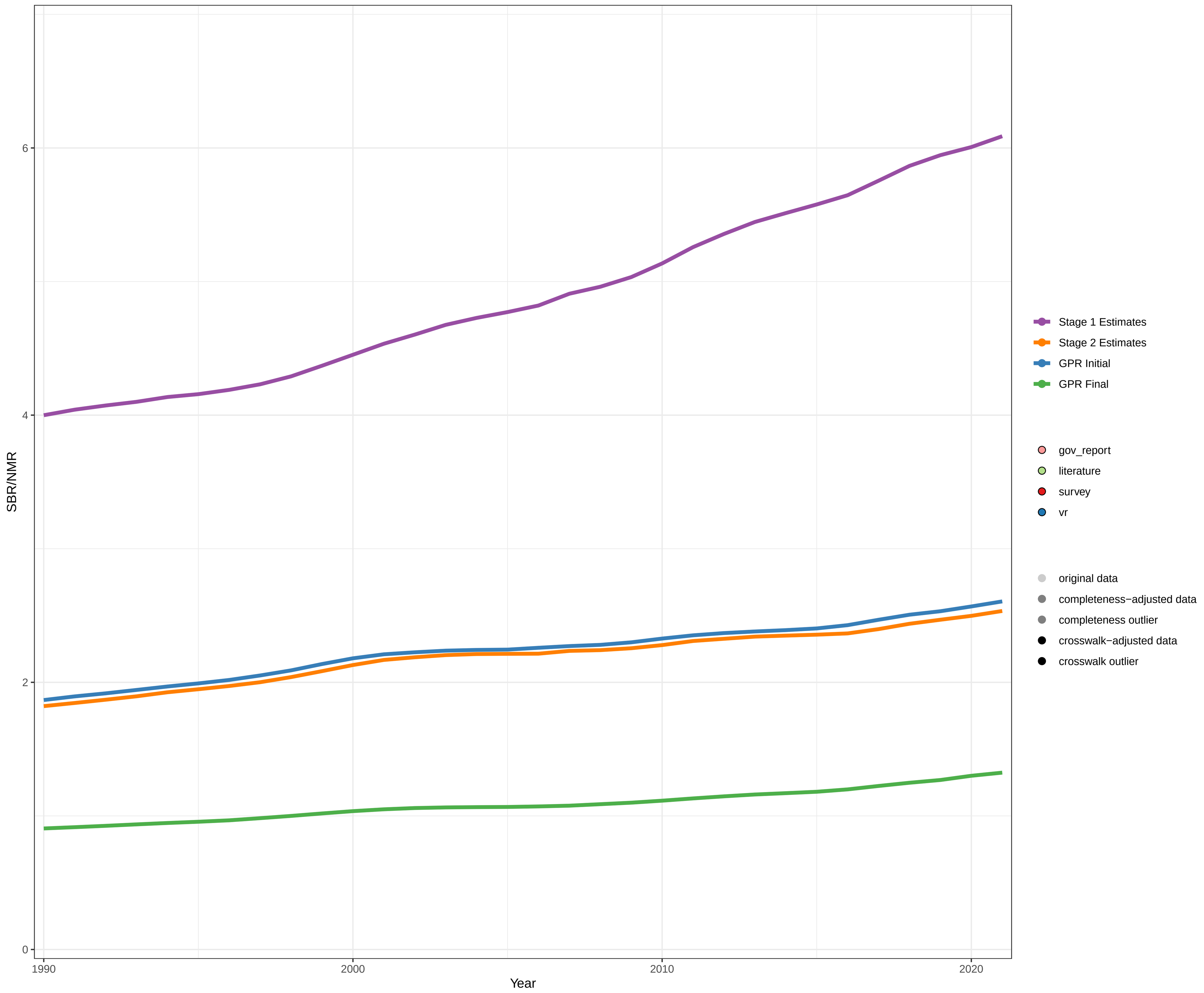

Qom IRN_44889 North Africa and Middle East Stillbirth Rate / Neonatal Mortality Rate (SBR/NMR) − 28 weeks
lambda = 1 ; zeta = 0.6 ; scale = 15 ; amp = 0.028

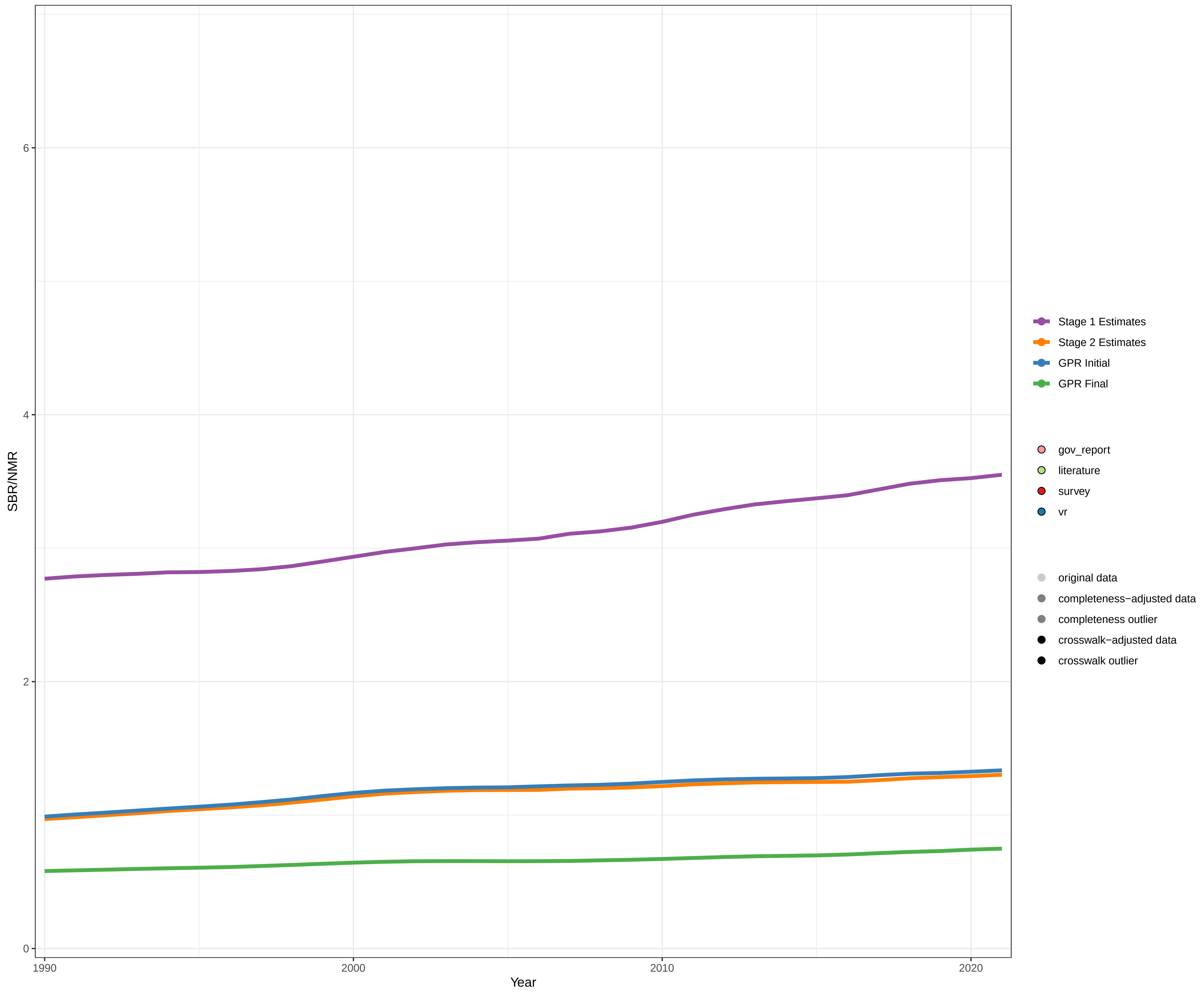

Definition	Years	Outlier	Source.Type	Title

Qom IRN_44889 North Africa and Middle East Stillbirth Rate (SBR) − 20 weeks
lambda = 1 ; zeta = 0.6 ; scale = 15 ; amp = 0.028

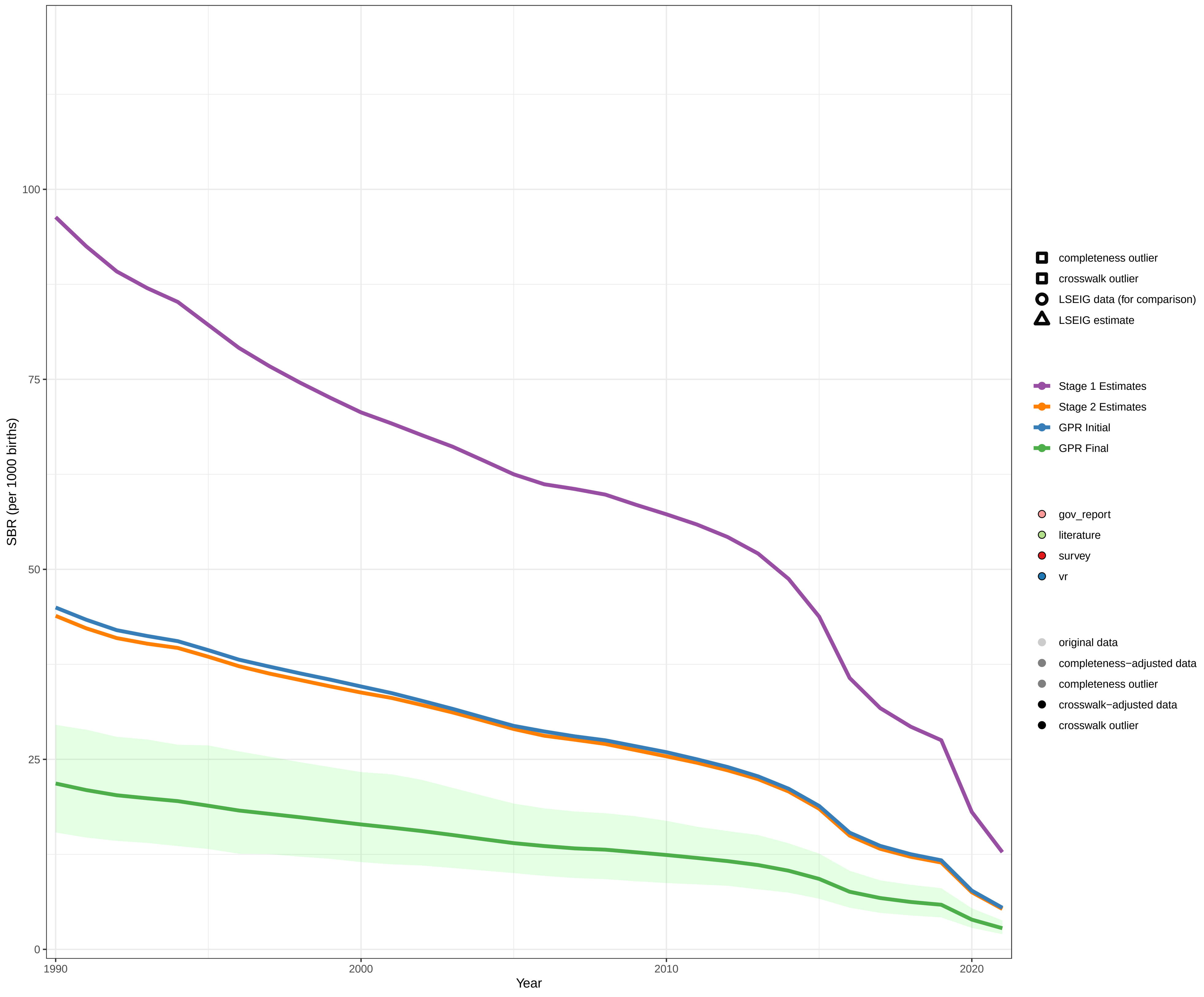

Qom IRN_44889 North Africa and Middle East Stillbirth Rate (SBR) − 28 weeks
lambda = 1 ; zeta = 0.6 ; scale = 15 ; amp = 0.028

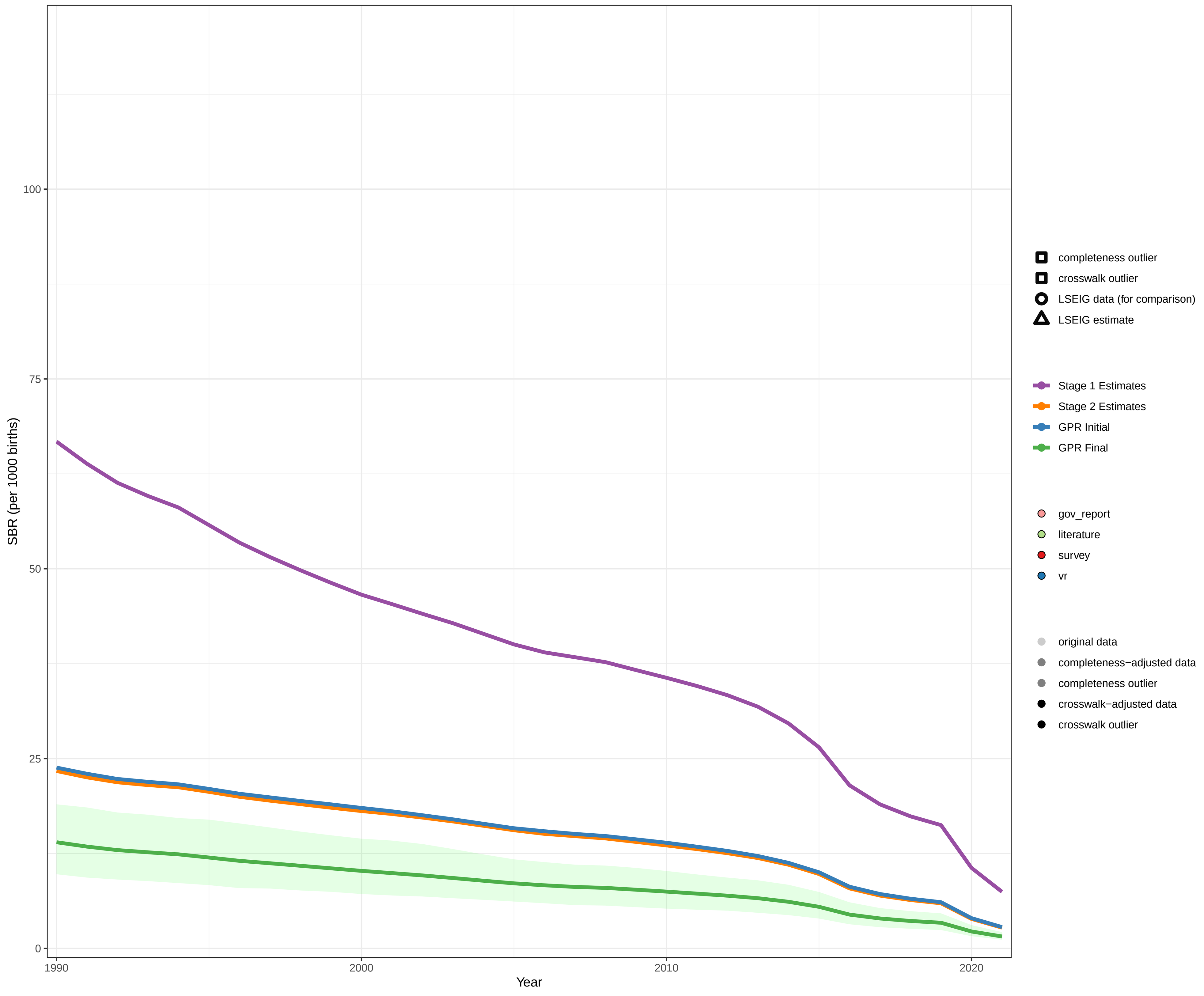

Year	Total Stillbirths (in thousands)	Total Stillbirths (in thousands)	SBR (per 1000 births)	SBR (per 1000 births)	SBR/NMR	SBR/NMR
	20 weeks	28 weeks	20 weeks	28 weeks	20 weeks	28 weeks
1990	0.4<br>(0.3 to 0.6)	0.3<br>(0.2 to 0.4)	21.8<br>(15.4 to 29.5)	14.0<br>(9.8 to 19.0)	0.91<br>(0.64 to 1.23)	0.58<br>(0.41 to 0.79)
1995	0.3<br>(0.2 to 0.4)	0.2<br>(0.1 to 0.3)	18.9<br>(13.2 to 26.8)	12.0<br>(8.3 to 17.0)	0.96<br>(0.67 to 1.36)	0.61<br>(0.42 to 0.86)
2000	0.3<br>(0.2 to 0.4)	0.2<br>(0.1 to 0.2)	16.4<br>(11.5 to 23.3)	10.2<br>(7.2 to 14.5)	1.04<br>(0.72 to 1.47)	0.64<br>(0.45 to 0.91)
2005	0.2<br>(0.2 to 0.3)	0.1<br>(0.1 to 0.2)	14.0<br>(10.0 to 19.2)	8.6<br>(6.2 to 11.7)	1.07<br>(0.77 to 1.46)	0.65<br>(0.47 to 0.90)
2010	0.3<br>(0.2 to 0.4)	0.2<br>(0.1 to 0.2)	12.4<br>(8.7 to 16.9)	7.5<br>(5.2 to 10.2)	1.11<br>(0.78 to 1.52)	0.67<br>(0.47 to 0.91)
2015	0.2<br>(0.2 to 0.3)	0.1<br>(0.1 to 0.2)	9.3<br>(6.7 to 12.6)	5.5<br>(3.9 to 7.4)	1.18<br>(0.85 to 1.61)	0.70<br>(0.50 to 0.95)
2020	0.1<br>(0.1 to 0.1)	0.0<br>(0.0 to 0.1)	3.9<br>(2.8 to 5.4)	2.2<br>(1.6 to 3.1)	1.30<br>(0.94 to 1.80)	0.74<br>(0.54 to 1.02)
2021	0.1<br>(0.0 to 0.1)	0.0<br>(0.0 to 0.0)	2.8<br>(2.0 to 3.8)	1.6<br>(1.1 to 2.1)	1.32<br>(0.95 to 1.81)	0.75<br>(0.54 to 1.02)

Semnan IRN_44890 North Africa and Middle East Stillbirth Rate / Neonatal Mortality Rate (SBR/NMR) − 20 weeks
lambda = 1 ; zeta = 0.6 ; scale = 15 ; amp = 0.028

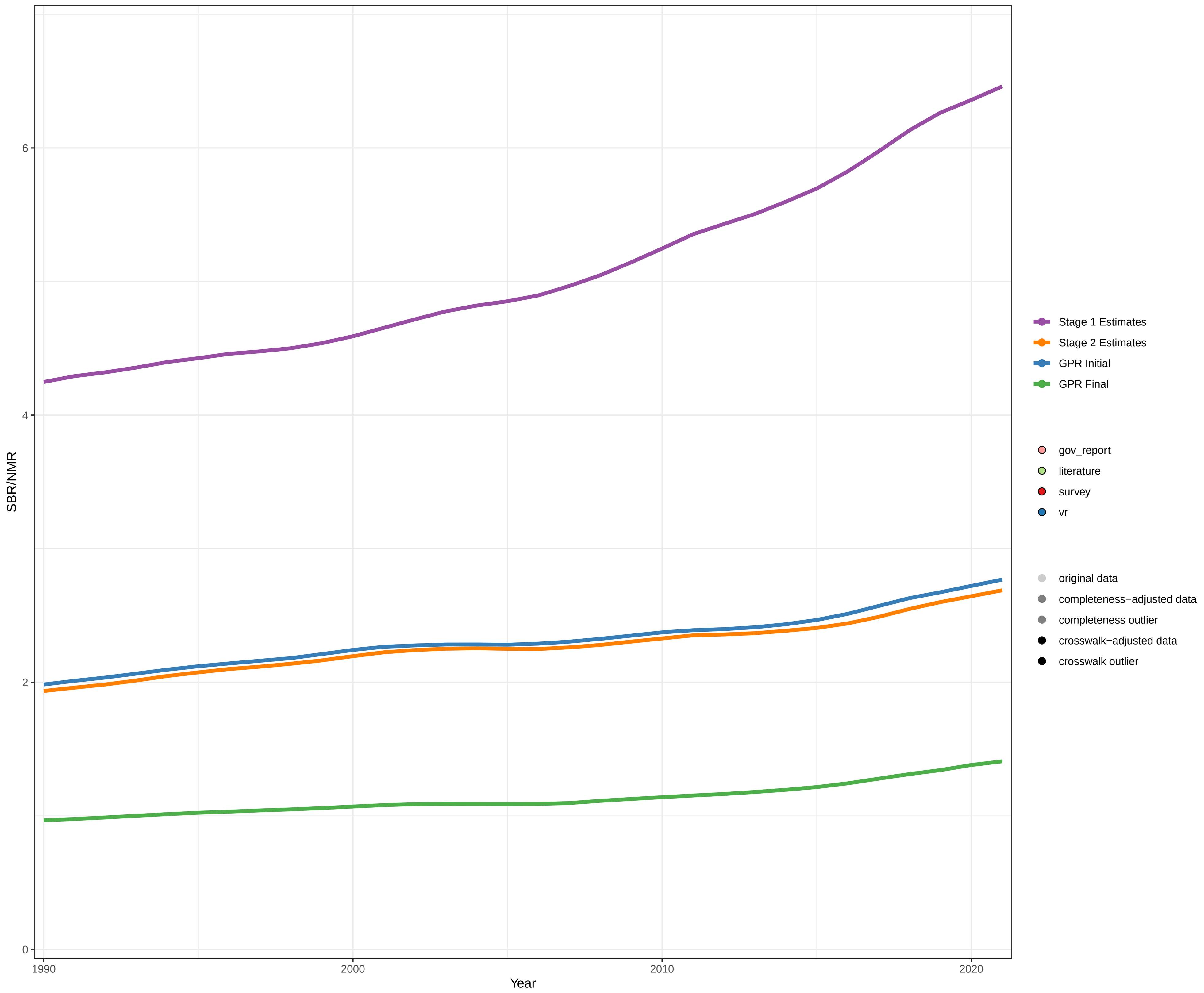

Semnan IRN_44890 North Africa and Middle East Stillbirth Rate / Neonatal Mortality Rate (SBR/NMR) − 28 weeks
lambda = 1 ; zeta = 0.6 ; scale = 15 ; amp = 0.028

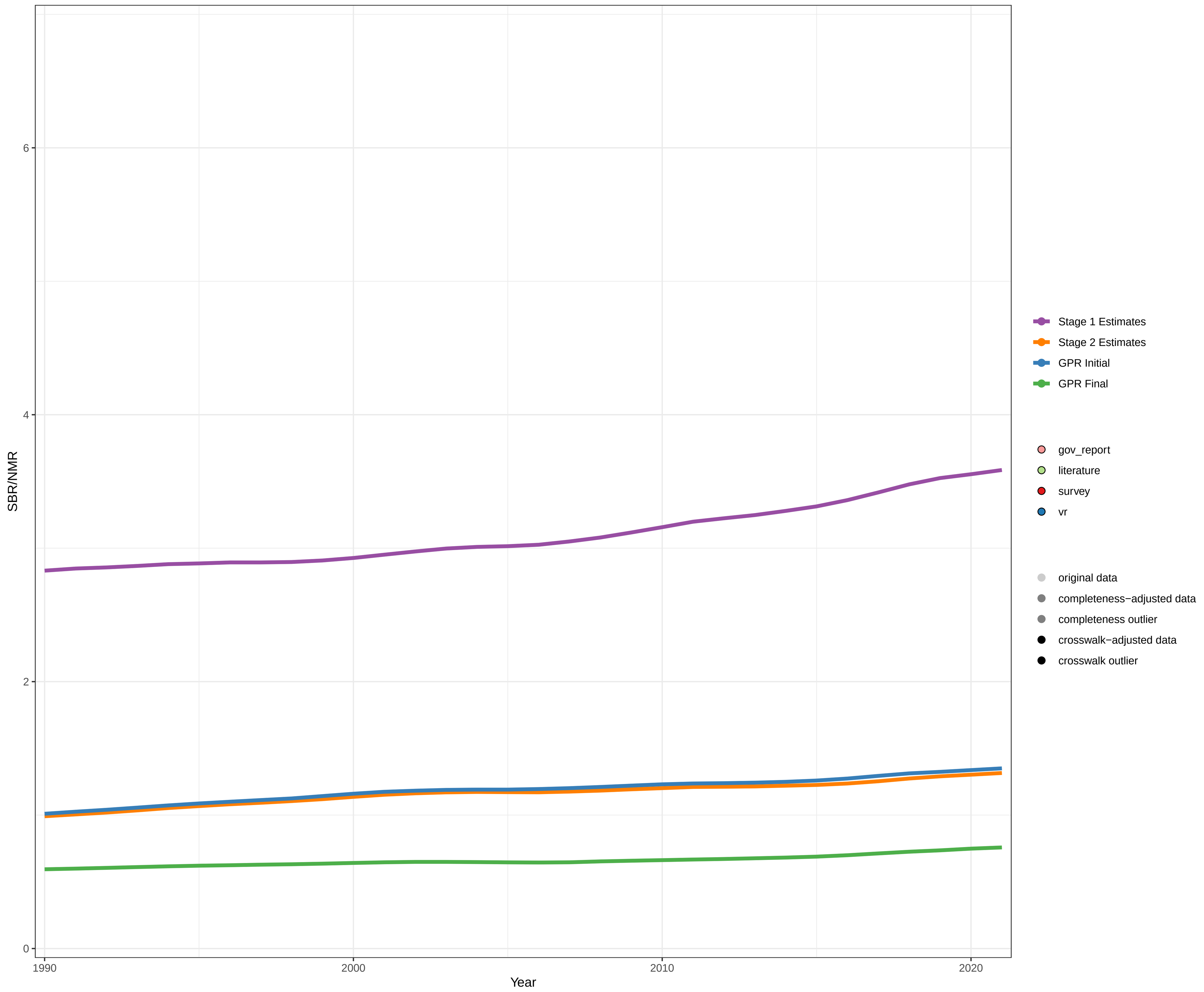

Definition	Years	Outlier	Source.Type	Title

Semnan IRN_44890 North Africa and Middle East Stillbirth Rate (SBR) − 20 weeks
lambda = 1 ; zeta = 0.6 ; scale = 15 ; amp = 0.028

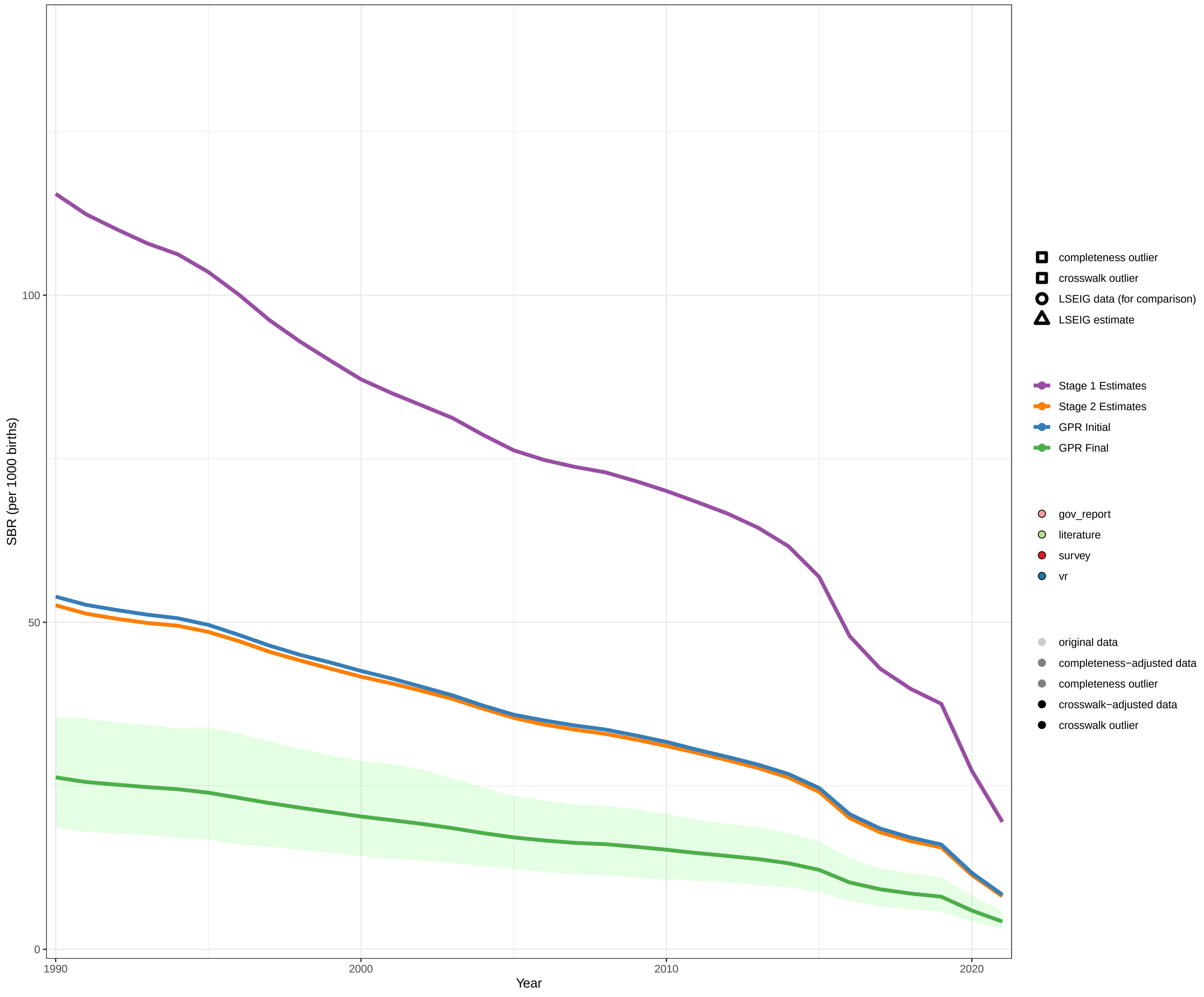

Semnan IRN_44890 North Africa and Middle East Stillbirth Rate (SBR) − 28 weeks
lambda = 1 ; zeta = 0.6 ; scale = 15 ; amp = 0.028

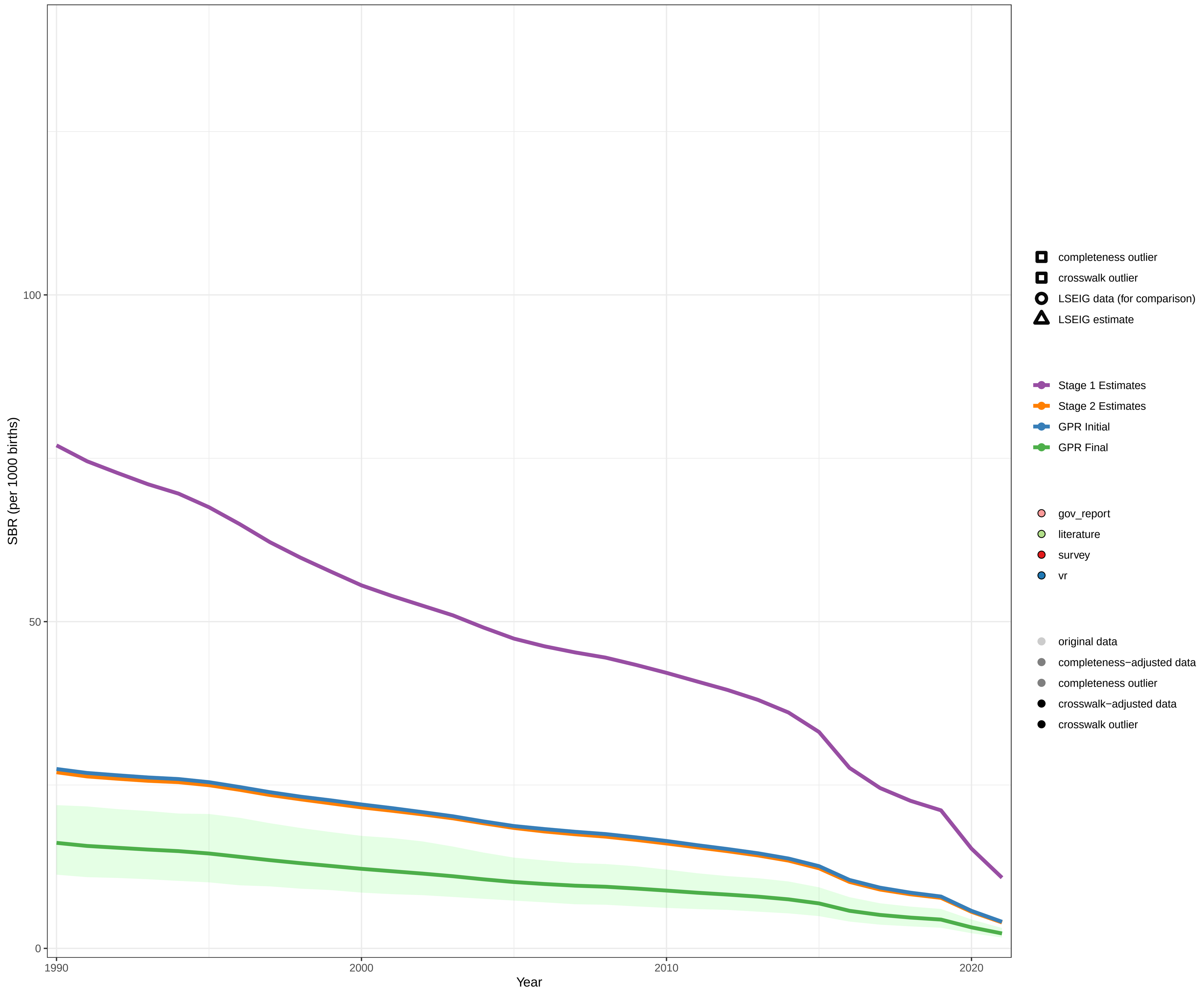

Year	Total Stillbirths (in thousands)	Total Stillbirths (in thousands)	SBR (per 1000 births)	SBR (per 1000 births)	SBR/NMR	SBR/NMR
	20 weeks	28 weeks	20 weeks	28 weeks	20 weeks	28 weeks
1990	0.3 (0.2 to 0.4)	0.2 (0.1 to 0.2)	26.3 (18.5 to 35.5)	16.1 (11.3 to 21.9)	0.97 (0.68 to 1.31)	0.59 (0.41 to 0.81)
1995	0.2 (0.1 to 0.3)	0.1 (0.1 to 0.2)	23.9 (16.7 to 33.9)	14.5 (10.1 to 20.6)	1.02 (0.72 to 1.45)	0.62 (0.43 to 0.88)
2000	0.1 (0.1 to 0.2)	0.1 (0.1 to 0.1)	20.3 (14.2 to 28.8)	12.2 (8.5 to 17.2)	1.07 (0.75 to 1.52)	0.64 (0.45 to 0.91)
2005	0.1 (0.1 to 0.2)	0.1 (0.1 to 0.1)	17.1 (12.3 to 23.4)	10.2 (7.3 to 13.9)	1.09 (0.78 to 1.49)	0.65 (0.47 to 0.88)
2010	0.1 (0.1 to 0.2)	0.1 (0.1 to 0.1)	15.2 (10.7 to 20.7)	8.8 (6.2 to 12.1)	1.14 (0.80 to 1.55)	0.66 (0.46 to 0.90)
2015	0.1 (0.1 to 0.2)	0.1 (0.1 to 0.1)	12.2 (8.7 to 16.5)	6.9 (4.9 to 9.3)	1.22 (0.87 to 1.66)	0.69 (0.49 to 0.93)
2020	0.0 (0.0 to 0.1)	0.0 (0.0 to 0.0)	5.9 (4.3 to 8.2)	3.2 (2.3 to 4.4)	1.38 (1.00 to 1.91)	0.75 (0.54 to 1.03)
2021	0.0 (0.0 to 0.0)	0.0 (0.0 to 0.0)	4.2 (3.1 to 5.8)	2.3 (1.7 to 3.1)	1.41 (1.02 to 1.93)	0.76 (0.55 to 1.03)

Sistan and Baluchistan IRN_44891 North Africa and Middle East Stillbirth Rate / Neonatal Mortality Rate (SBR/NMR) − 20 weeks
lambda = 1 ; zeta = 0.6 ; scale = 15 ; amp = 0.028

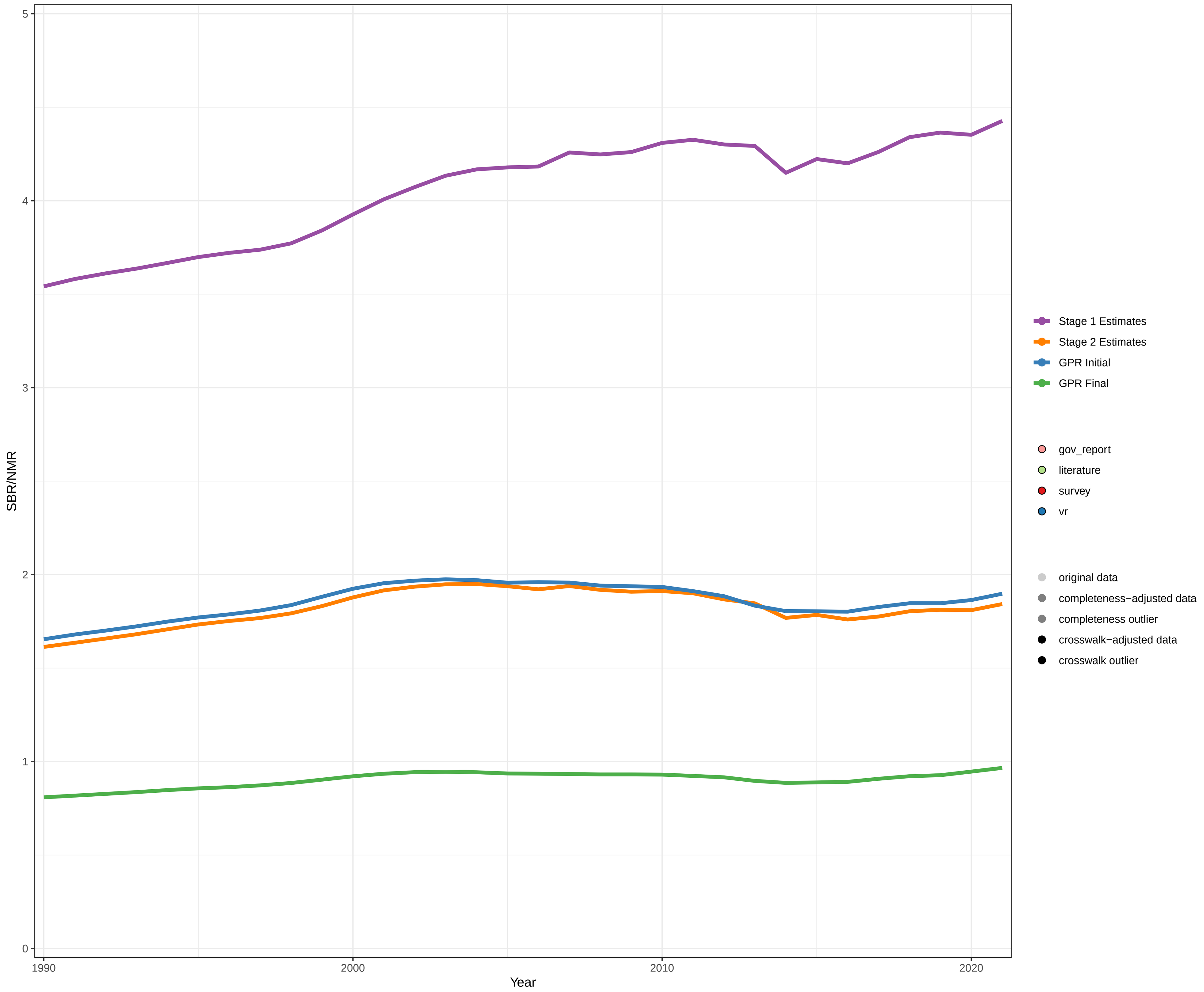

Sistan and Baluchistan IRN_44891 North Africa and Middle East Stillbirth Rate / Neonatal Mortality Rate (SBR/NMR) − 28 weeks
lambda = 1 ; zeta = 0.6 ; scale = 15 ; amp = 0.028

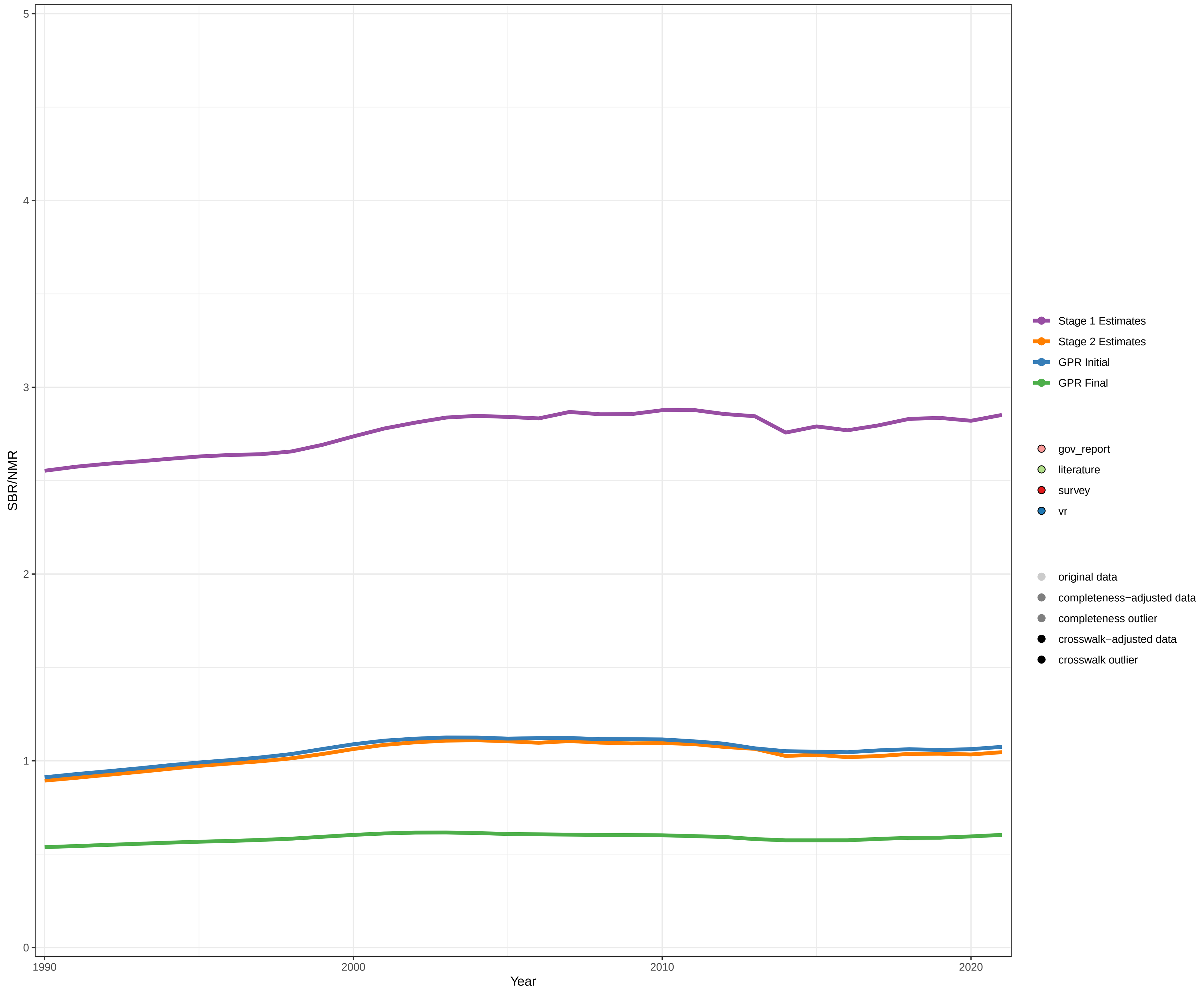

Definition	Years	Outlier	Source.Type	Title

Sistan and Baluchistan IRN_44891 North Africa and Middle East Stillbirth Rate (SBR) − 20 weeks
lambda = 1 ; zeta = 0.6 ; scale = 15 ; amp = 0.028

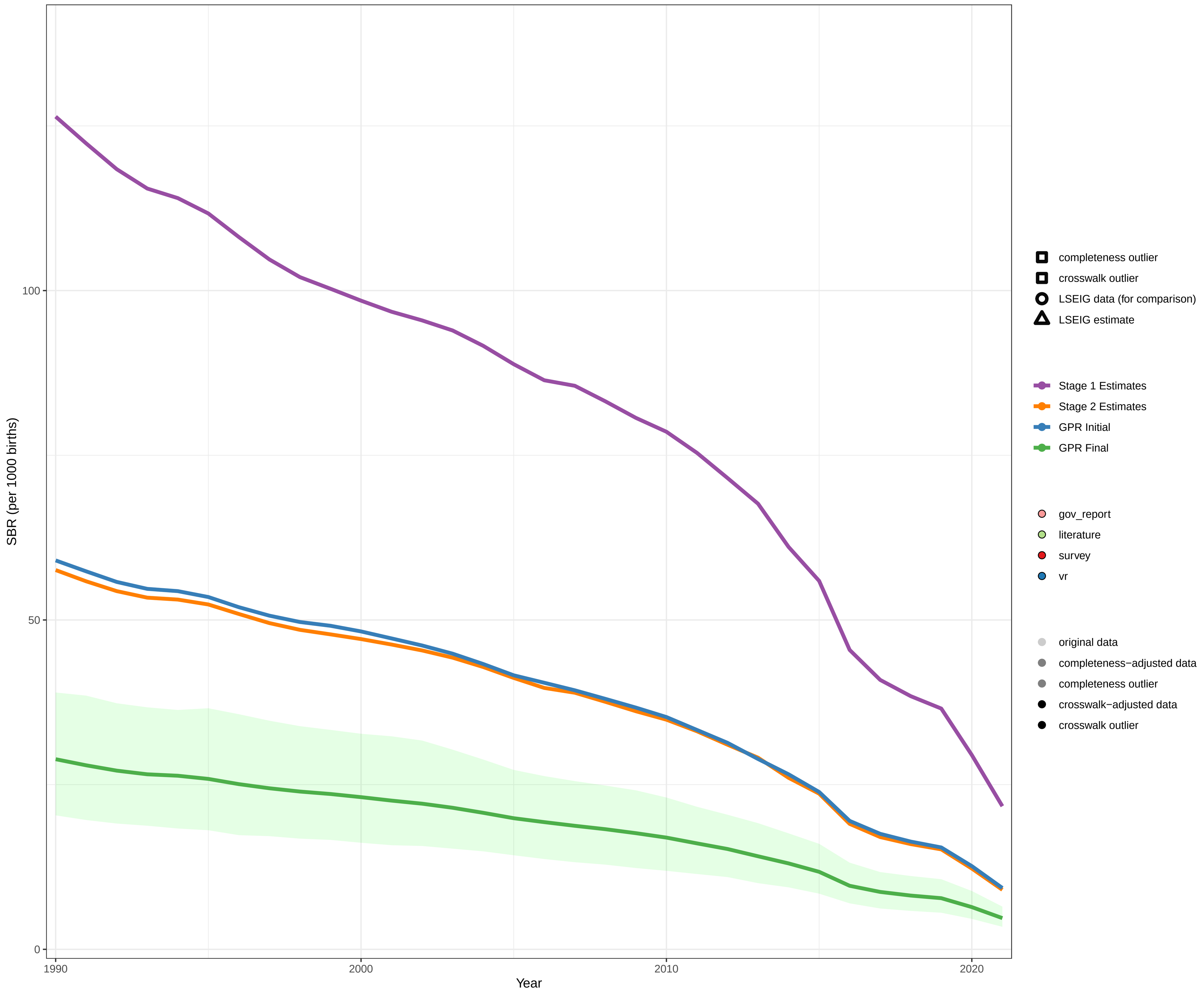

Sistan and Baluchistan IRN_44891 North Africa and Middle East Stillbirth Rate (SBR) − 28 weeks
lambda = 1 ; zeta = 0.6 ; scale = 15 ; amp = 0.028

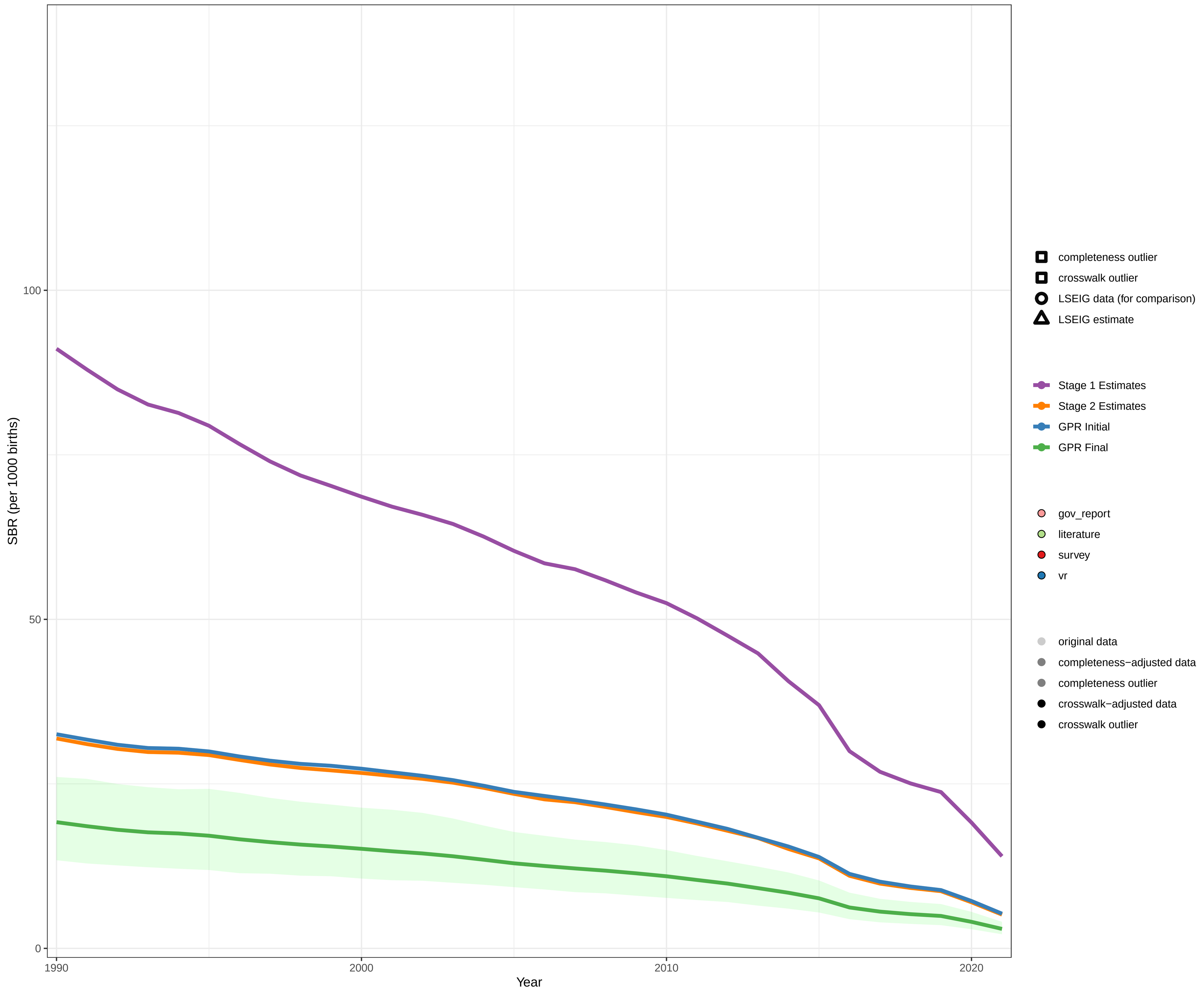

Year	Total Stillbirths (in thousands)	Total Stillbirths (in thousands)	SBR (per 1000 births)	SBR (per 1000 births)	SBR/NMR	SBR/NMR
	20 weeks	28 weeks	20 weeks	28 weeks	20 weeks	28 weeks
1990	1.6 (1.1 to 2.2)	1.1 (0.7 to 1.4)	28.9 (20.3 to 39.0)	19.2 (13.4 to 26.0)	0.81 (0.57 to 1.09)	0.54 (0.38 to 0.73)
1995	1.3 (0.9 to 1.8)	0.8 (0.6 to 1.2)	25.9 (18.1 to 36.6)	17.1 (11.9 to 24.2)	0.86 (0.60 to 1.21)	0.57 (0.39 to 0.80)
2000	1.3 (0.9 to 1.8)	0.8 (0.6 to 1.2)	23.1 (16.2 to 32.7)	15.1 (10.6 to 21.4)	0.92 (0.65 to 1.30)	0.60 (0.42 to 0.85)
2005	1.3 (0.9 to 1.8)	0.8 (0.6 to 1.1)	19.9 (14.3 to 27.2)	12.9 (9.3 to 17.7)	0.94 (0.67 to 1.28)	0.61 (0.44 to 0.83)
2010	1.5 (1.0 to 2.0)	1.0 (0.7 to 1.3)	17.0 (11.9 to 23.0)	11.0 (7.7 to 14.9)	0.93 (0.65 to 1.26)	0.60 (0.42 to 0.82)
2015	1.2 (0.9 to 1.6)	0.8 (0.6 to 1.1)	11.8 (8.5 to 16.0)	7.6 (5.5 to 10.3)	0.89 (0.64 to 1.21)	0.57 (0.41 to 0.78)
2020	0.5 (0.4 to 0.7)	0.3 (0.2 to 0.4)	6.4 (4.6 to 8.9)	4.0 (2.9 to 5.6)	0.95 (0.68 to 1.31)	0.60 (0.43 to 0.82)
2021	0.4 (0.3 to 0.5)	0.2 (0.2 to 0.3)	4.7 (3.4 to 6.5)	3.0 (2.1 to 4.0)	0.97 (0.70 to 1.32)	0.60 (0.44 to 0.82)

South Khorasan IRN_44881 North Africa and Middle East Stillbirth Rate / Neonatal Mortality Rate (SBR/NMR) − 20 weeks
lambda = 1 ; zeta = 0.6 ; scale = 15 ; amp = 0.028

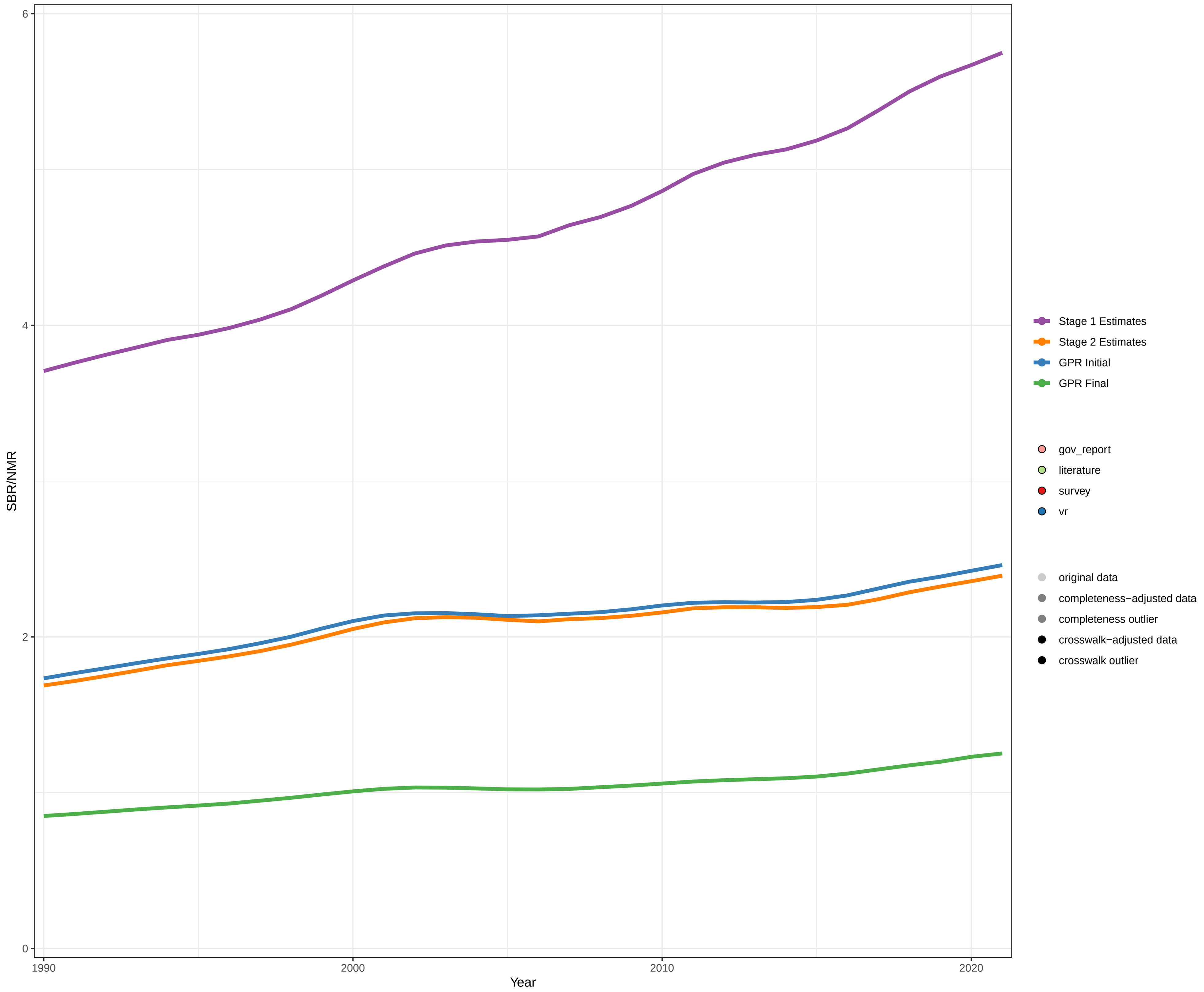

South Khorasan IRN_44881 North Africa and Middle East Stillbirth Rate / Neonatal Mortality Rate (SBR/NMR) − 28 weeks
lambda = 1 ; zeta = 0.6 ; scale = 15 ; amp = 0.028

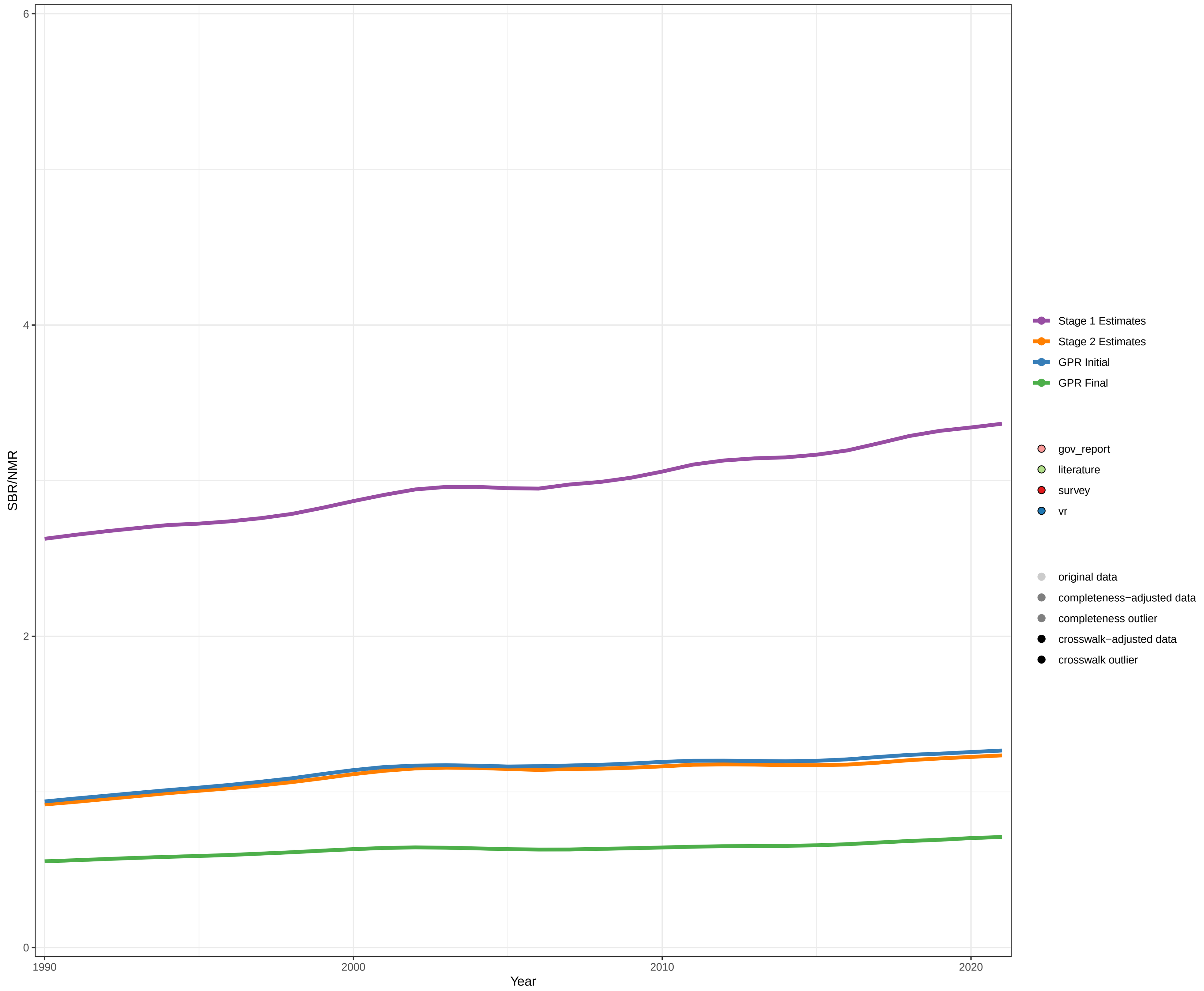

Definition	Years	Outlier	Source.Type	Title

South Khorasan IRN_44881 North Africa and Middle East Stillbirth Rate (SBR) − 20 weeks
lambda = 1 ; zeta = 0.6 ; scale = 15 ; amp = 0.028

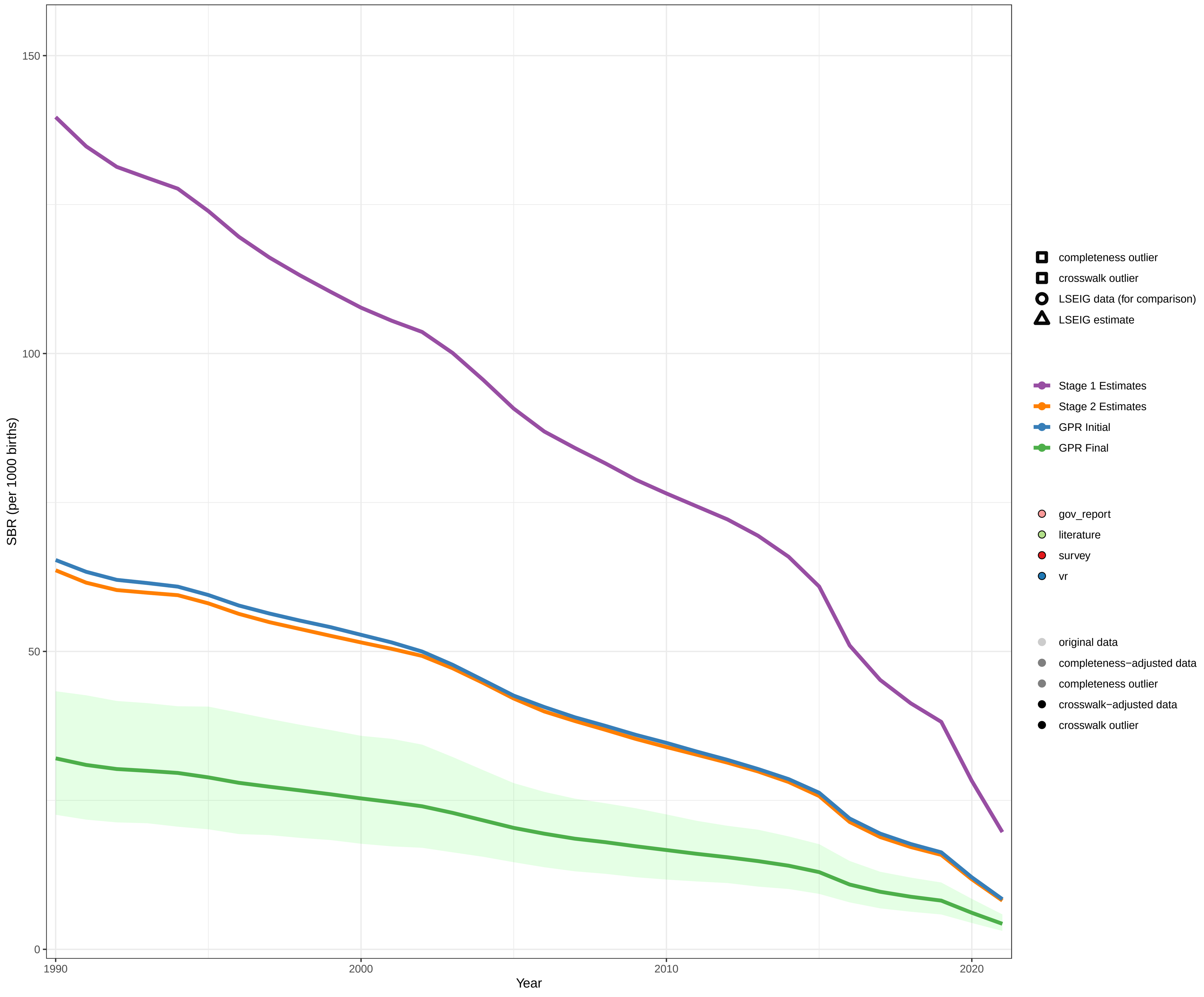

South Khorasan IRN_44881 North Africa and Middle East Stillbirth Rate (SBR) − 28 weeks
lambda = 1 ; zeta = 0.6 ; scale = 15 ; amp = 0.028

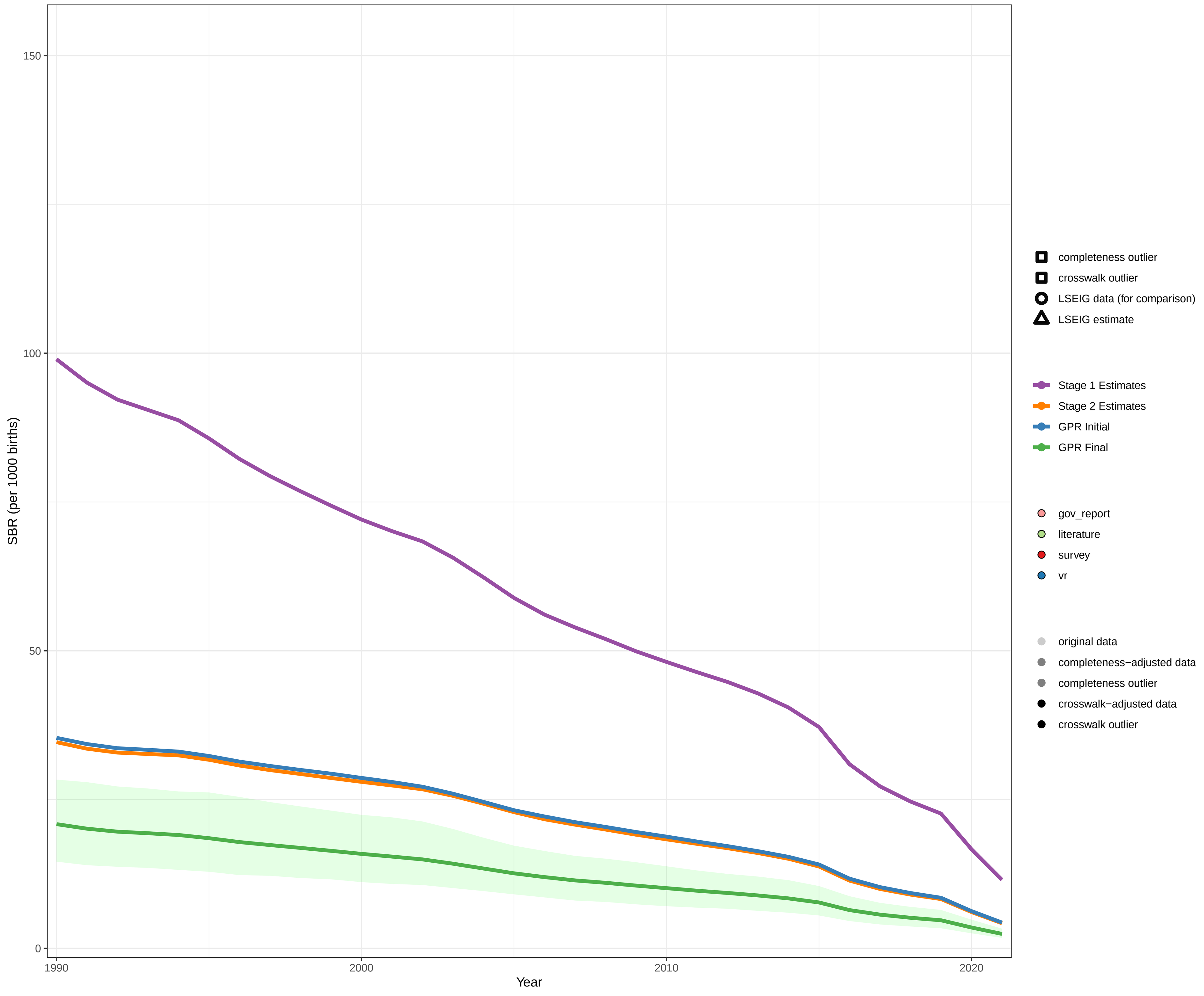

Year	Total Stillbirths (in thousands)	Total Stillbirths (in thousands)	SBR (per 1000 births)	SBR (per 1000 births)	SBR/NMR	SBR/NMR
	20 weeks	28 weeks	20 weeks	28 weeks	20 weeks	28 weeks
1990	0.7 (0.5 to 1.0)	0.5 (0.3 to 0.6)	32.1 (22.6 to 43.3)	20.9 (14.6 to 28.4)	0.85 (0.60 to 1.15)	0.55 (0.39 to 0.75)
1995	0.4 (0.3 to 0.6)	0.3 (0.2 to 0.4)	28.9 (20.1 to 40.7)	18.5 (12.9 to 26.2)	0.92 (0.64 to 1.30)	0.59 (0.41 to 0.83)
2000	0.3 (0.2 to 0.5)	0.2 (0.1 to 0.3)	25.3 (17.7 to 35.8)	15.9 (11.1 to 22.4)	1.01 (0.71 to 1.43)	0.63 (0.44 to 0.89)
2005	0.2 (0.2 to 0.3)	0.1 (0.1 to 0.2)	20.4 (14.6 to 27.9)	12.6 (9.1 to 17.3)	1.02 (0.73 to 1.40)	0.63 (0.46 to 0.87)
2010	0.2 (0.2 to 0.3)	0.1 (0.1 to 0.2)	16.7 (11.7 to 22.6)	10.1 (7.1 to 13.8)	1.06 (0.74 to 1.44)	0.64 (0.45 to 0.88)
2015	0.2 (0.2 to 0.3)	0.1 (0.1 to 0.2)	13.0 (9.3 to 17.7)	7.7 (5.5 to 10.5)	1.10 (0.79 to 1.50)	0.66 (0.47 to 0.89)
2020	0.1 (0.1 to 0.1)	0.1 (0.0 to 0.1)	6.1 (4.4 to 8.5)	3.5 (2.5 to 4.8)	1.23 (0.89 to 1.70)	0.70 (0.51 to 0.97)
2021	0.1 (0.0 to 0.1)	0.0 (0.0 to 0.0)	4.3 (3.1 to 5.9)	2.4 (1.8 to 3.3)	1.25 (0.90 to 1.71)	0.71 (0.51 to 0.97)

Tehran IRN_44892 North Africa and Middle East Stillbirth Rate / Neonatal Mortality Rate (SBR/NMR) − 20 weeks
lambda = 1 ; zeta = 0.6 ; scale = 15 ; amp = 0.028

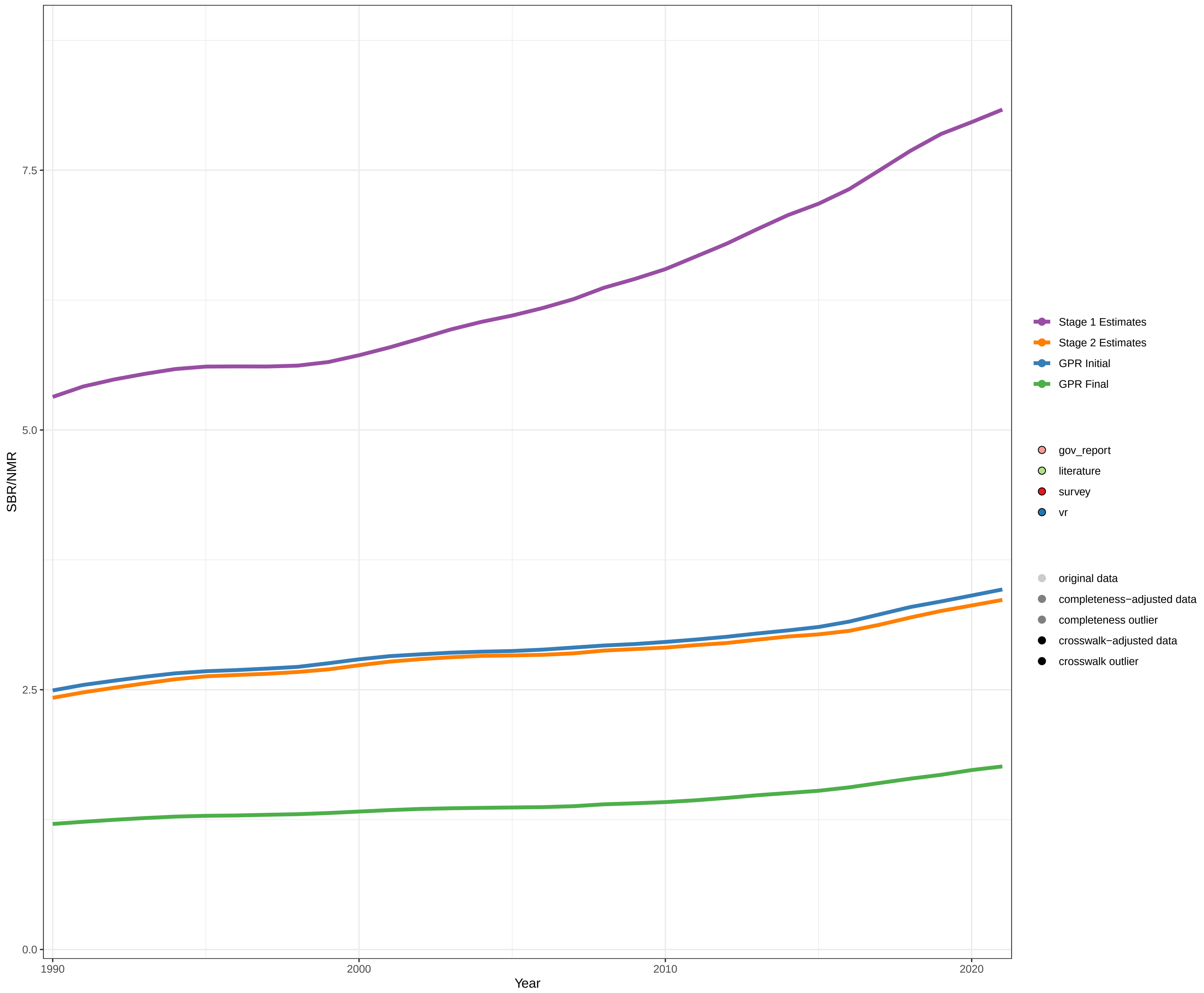

Tehran IRN_44892 North Africa and Middle East Stillbirth Rate / Neonatal Mortality Rate (SBR/NMR) − 28 weeks
lambda = 1 ; zeta = 0.6 ; scale = 15 ; amp = 0.028

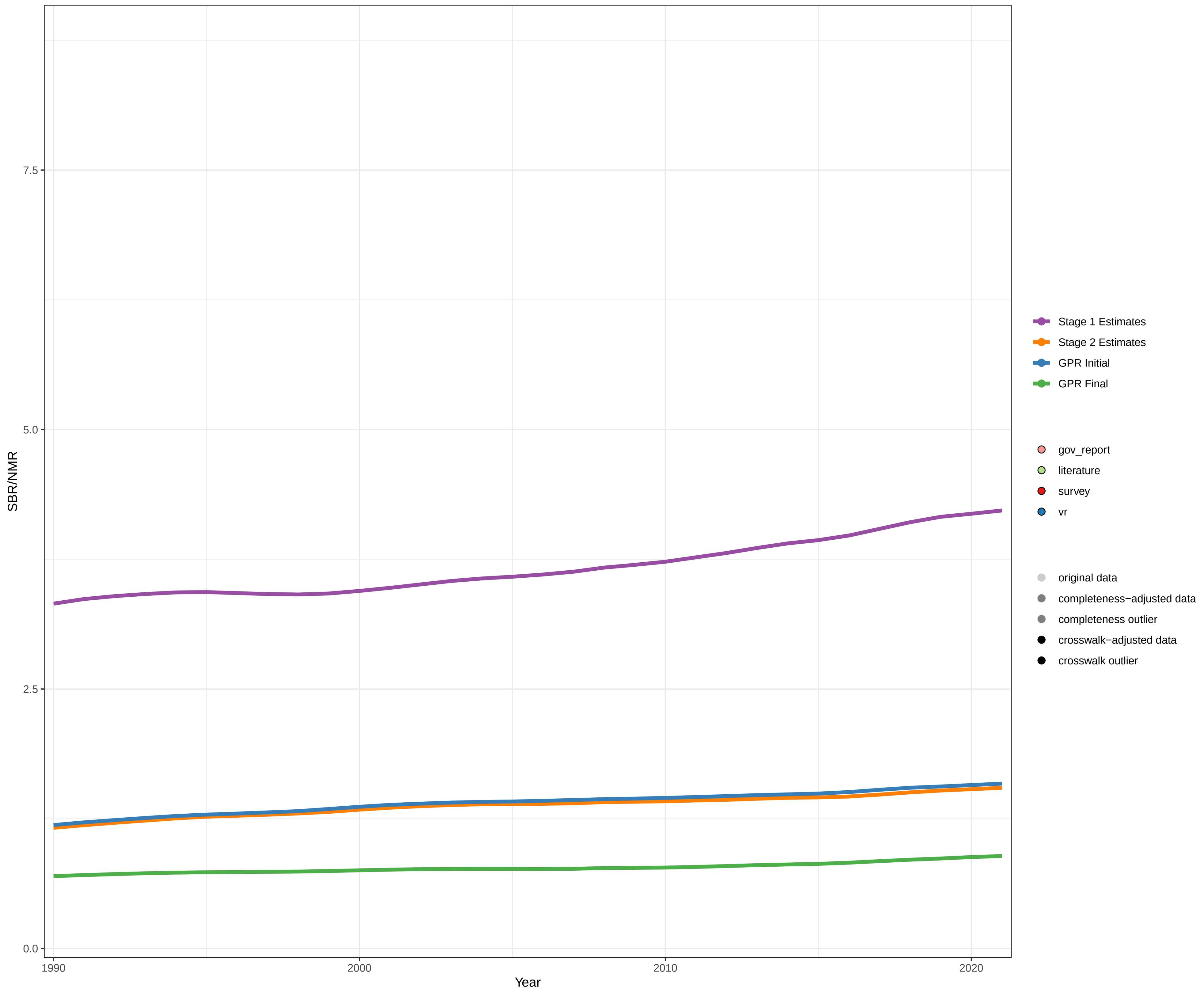

Definition	Years	Outlier	Source.Type	Title

Tehran IRN_44892 North Africa and Middle East Stillbirth Rate (SBR) − 20 weeks
lambda = 1 ; zeta = 0.6 ; scale = 15 ; amp = 0.028

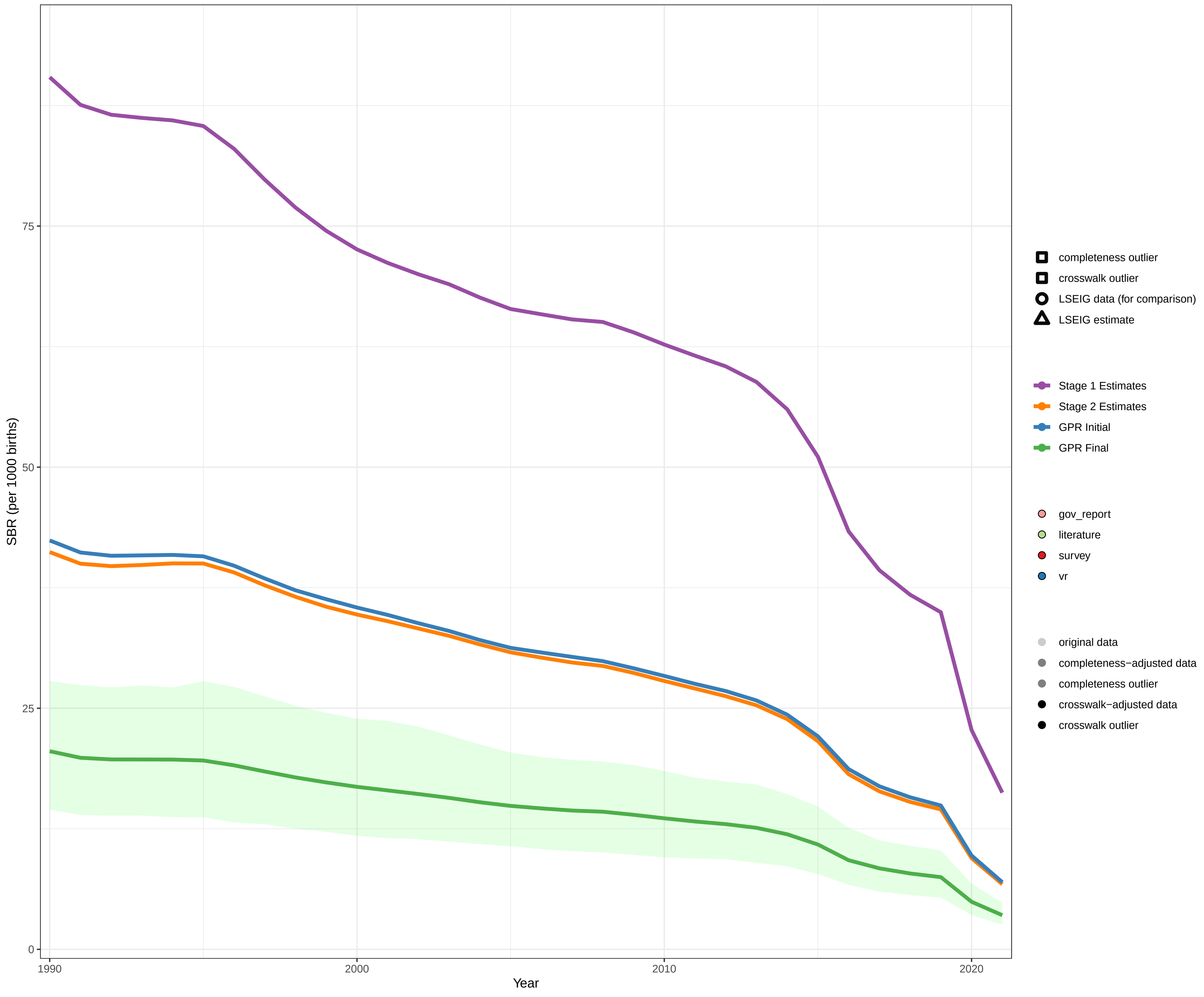

Tehran IRN_44892 North Africa and Middle East Stillbirth Rate (SBR) − 28 weeks
lambda = 1 ; zeta = 0.6 ; scale = 15 ; amp = 0.028

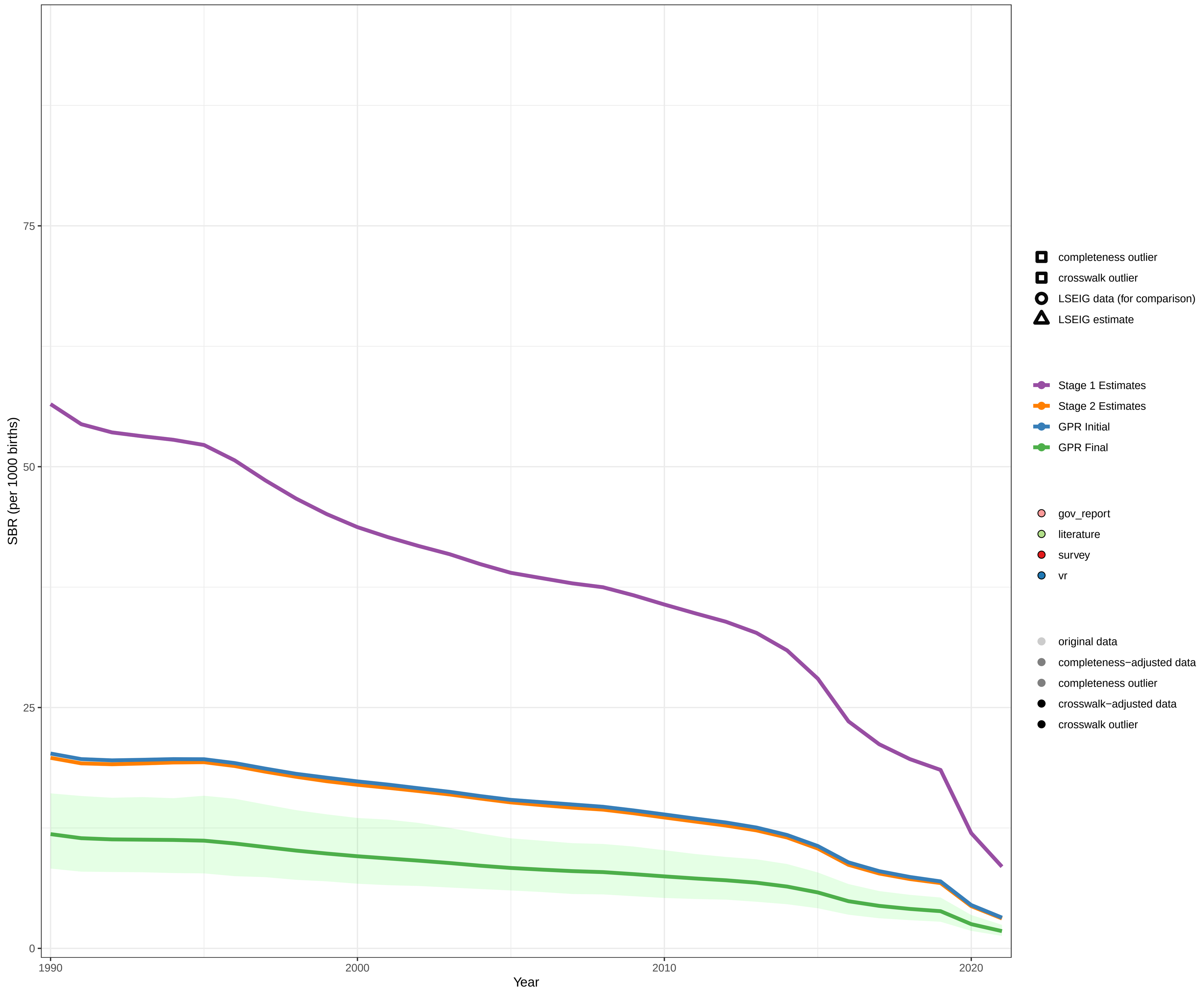

Year	Total Stillbirths (in thousands)	Total Stillbirths (in thousands)	SBR (per 1000 births)	SBR (per 1000 births)	SBR/NMR	SBR/NMR
	20 weeks	28 weeks	20 weeks	28 weeks	20 weeks	28 weeks
1990	3.9 (2.7 to 5.3)	2.2 (1.5 to 3.0)	20.5 (14.5 to 27.8)	11.9 (8.3 to 16.1)	1.21 (0.85 to 1.64)	0.70 (0.49 to 0.95)
1995	3.1 (2.1 to 4.4)	1.7 (1.2 to 2.5)	19.6 (13.7 to 27.8)	11.2 (7.8 to 15.8)	1.29 (0.90 to 1.83)	0.73 (0.51 to 1.04)
2000	2.5 (1.7 to 3.5)	1.4 (1.0 to 2.0)	16.8 (11.8 to 23.9)	9.6 (6.7 to 13.5)	1.33 (0.93 to 1.88)	0.75 (0.53 to 1.07)
2005	2.3 (1.6 to 3.2)	1.3 (0.9 to 1.8)	14.9 (10.7 to 20.4)	8.3 (6.0 to 11.4)	1.37 (0.98 to 1.88)	0.77 (0.55 to 1.05)
2010	2.5 (1.8 to 3.5)	1.4 (1.0 to 1.9)	13.6 (9.5 to 18.5)	7.5 (5.2 to 10.2)	1.42 (1.00 to 1.93)	0.78 (0.55 to 1.06)
2015	2.2 (1.6 to 3.0)	1.2 (0.8 to 1.6)	10.9 (7.8 to 14.8)	5.8 (4.2 to 7.9)	1.53 (1.10 to 2.08)	0.82 (0.59 to 1.11)
2020	0.7 (0.5 to 1.0)	0.4 (0.3 to 0.5)	4.9 (3.6 to 6.8)	2.5 (1.8 to 3.5)	1.73 (1.24 to 2.39)	0.88 (0.64 to 1.21)
2021	0.5 (0.3 to 0.7)	0.2 (0.2 to 0.3)	3.5 (2.6 to 4.8)	1.8 (1.3 to 2.4)	1.76 (1.27 to 2.41)	0.89 (0.65 to 1.22)

West Azarbayejan IRN_44867 North Africa and Middle East Stillbirth Rate / Neonatal Mortality Rate (SBR/NMR) − 20 weeks
lambda = 1 ; zeta = 0.6 ; scale = 15 ; amp = 0.028

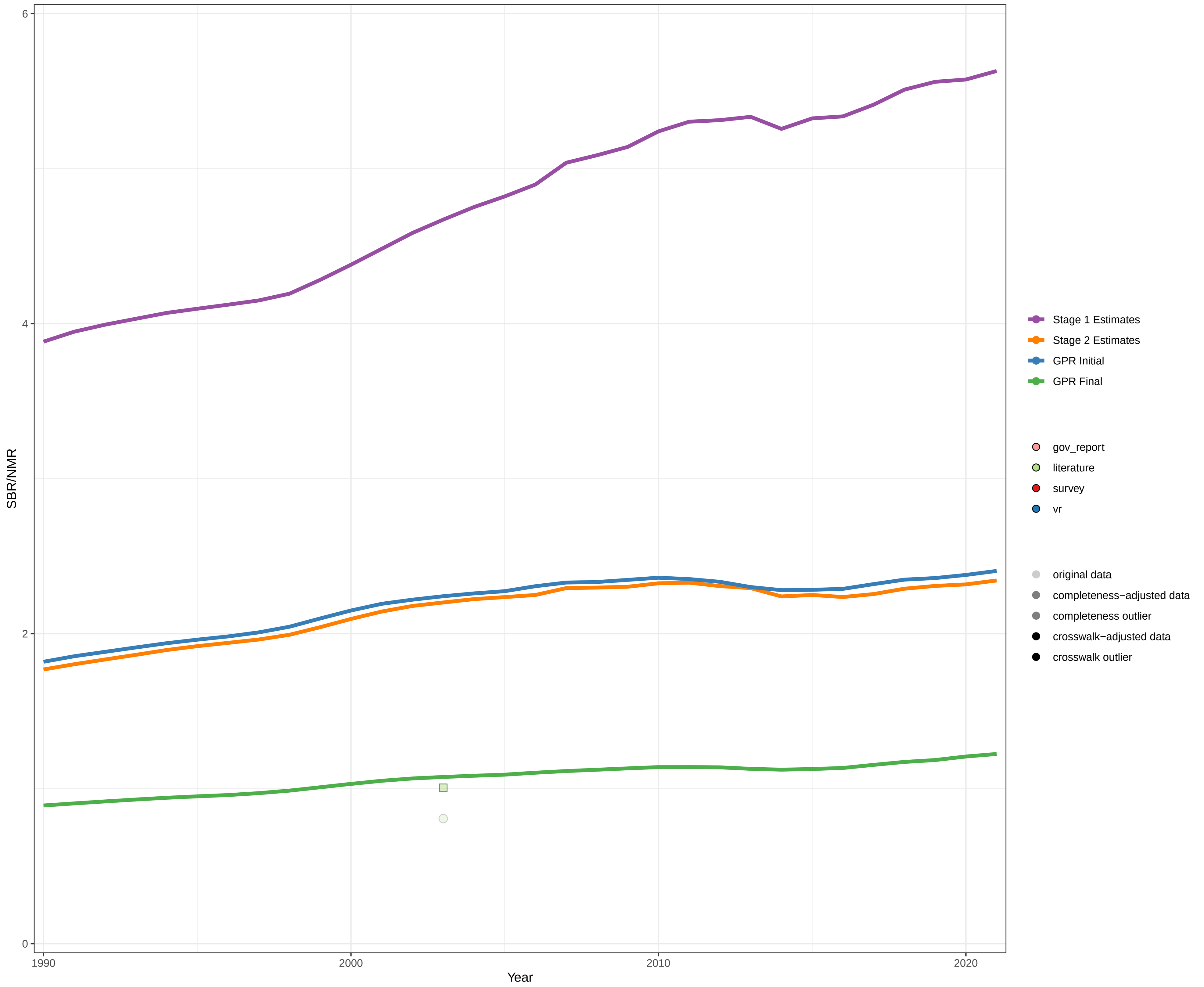

West Azarbayejan IRN_44867 North Africa and Middle East Stillbirth Rate / Neonatal Mortality Rate (SBR/NMR) − 28 weeks
lambda = 1 ; zeta = 0.6 ; scale = 15 ; amp = 0.028

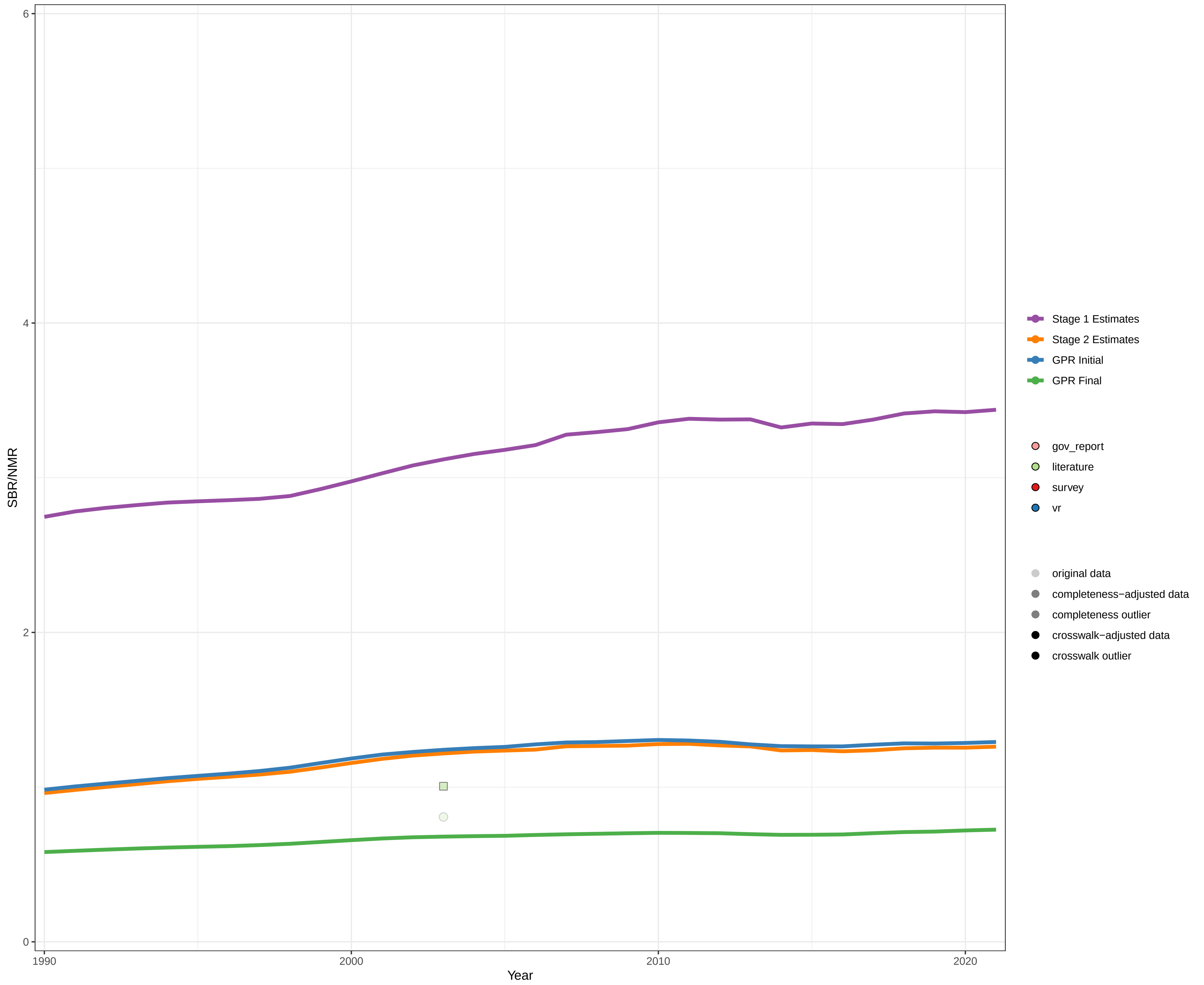

Definition	Years	Outlier	Source.Type	Title
28_weeks	2003	1	Sci Lit	The prevalence at birth of overt congenital anomalies in Urmia, Northwestern Iran

West Azarbayejan IRN_44867 North Africa and Middle East Stillbirth Rate (SBR) − 20 weeks
lambda = 1 ; zeta = 0.6 ; scale = 15 ; amp = 0.028

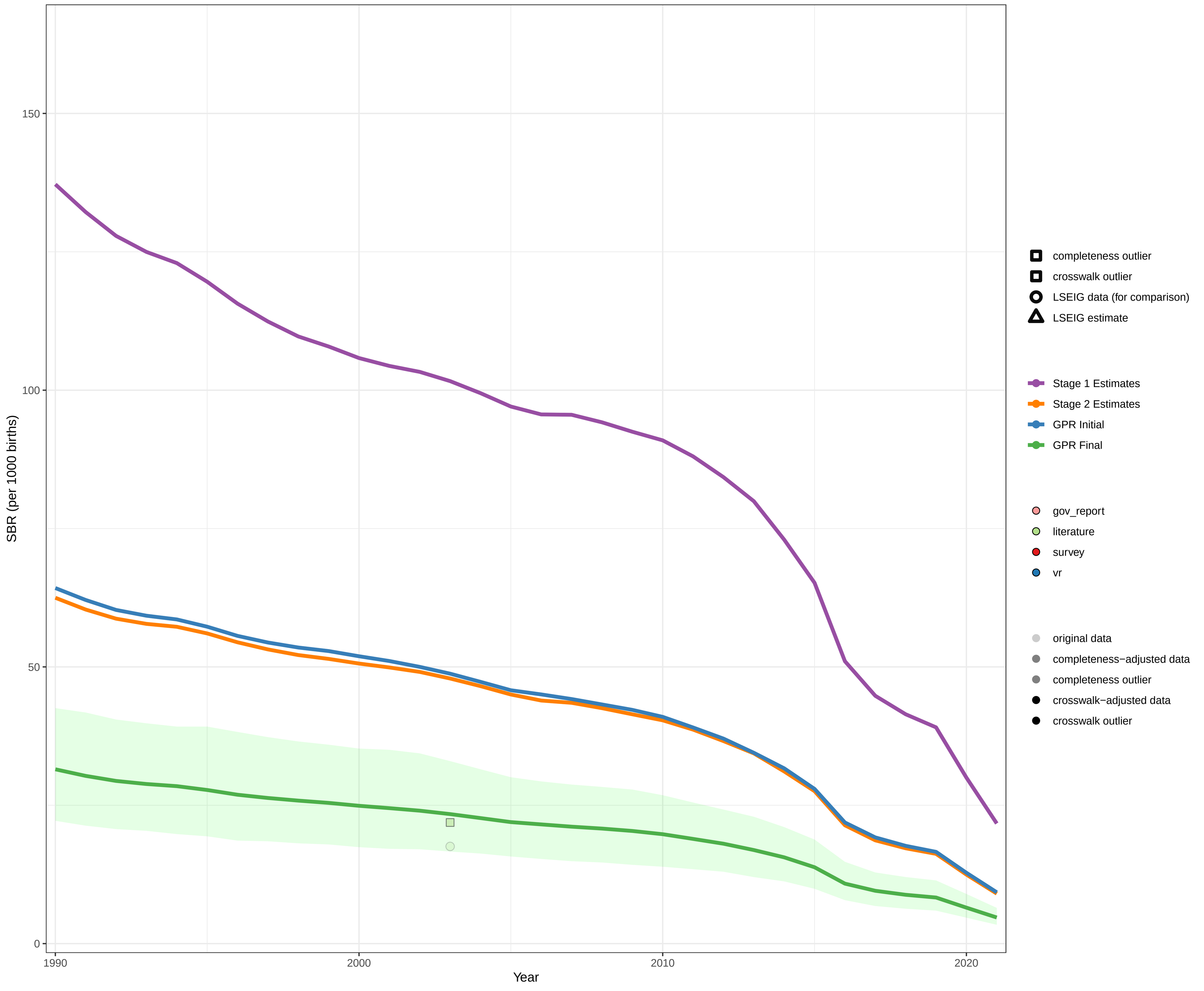

West Azarbayejan IRN_44867 North Africa and Middle East Stillbirth Rate (SBR) − 28 weeks
lambda = 1 ; zeta = 0.6 ; scale = 15 ; amp = 0.028

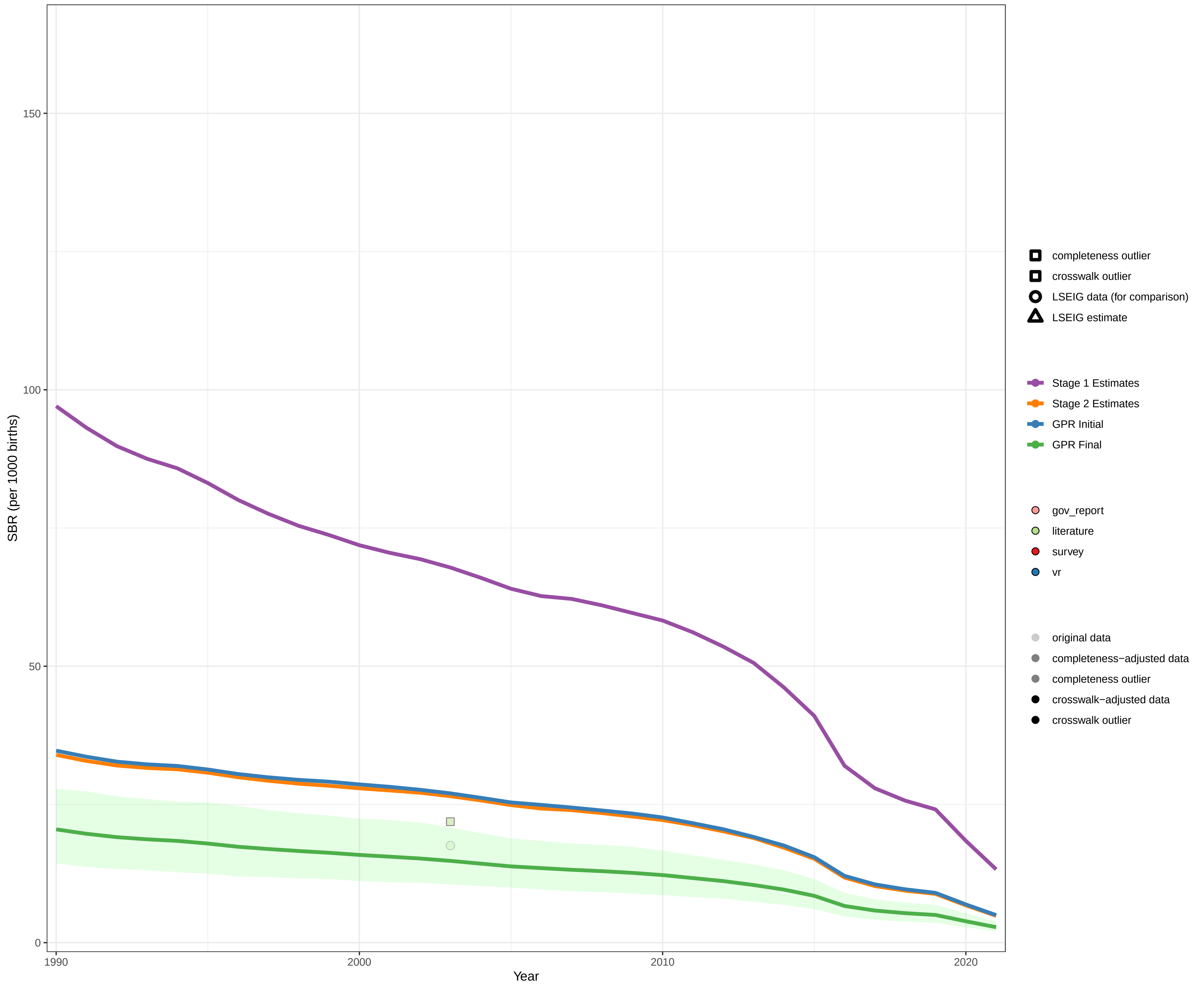

Year	Total Stillbirths (in thousands)	Total Stillbirths (in thousands)	SBR (per 1000 births)	SBR (per 1000 births)	SBR/NMR	SBR/NMR
	20 weeks	28 weeks	20 weeks	28 weeks	20 weeks	28 weeks
1990	2.2 (1.5 to 3.0)	1.4 (1.0 to 1.9)	31.5 (22.2 to 42.6)	20.5 (14.3 to 27.8)	0.89 (0.63 to 1.20)	0.58 (0.40 to 0.79)
1995	1.5 (1.1 to 2.2)	1.0 (0.7 to 1.4)	27.8 (19.4 to 39.2)	17.9 (12.5 to 25.4)	0.95 (0.66 to 1.34)	0.61 (0.43 to 0.87)
2000	1.3 (0.9 to 1.8)	0.8 (0.6 to 1.2)	24.9 (17.4 to 35.2)	15.9 (11.1 to 22.4)	1.03 (0.72 to 1.46)	0.66 (0.46 to 0.93)
2005	1.2 (0.8 to 1.6)	0.7 (0.5 to 1.0)	22.0 (15.7 to 30.1)	13.8 (9.9 to 18.9)	1.09 (0.78 to 1.49)	0.69 (0.49 to 0.94)
2010	1.3 (0.9 to 1.8)	0.8 (0.6 to 1.1)	19.8 (13.9 to 26.8)	12.2 (8.6 to 16.7)	1.14 (0.80 to 1.55)	0.70 (0.49 to 0.96)
2015	1.0 (0.7 to 1.3)	0.6 (0.4 to 0.8)	13.8 (9.9 to 18.8)	8.5 (6.1 to 11.5)	1.13 (0.81 to 1.54)	0.69 (0.50 to 0.94)
2020	0.3 (0.2 to 0.5)	0.2 (0.1 to 0.3)	6.5 (4.7 to 9.0)	3.9 (2.8 to 5.3)	1.21 (0.87 to 1.67)	0.72 (0.52 to 0.99)
2021	0.2 (0.2 to 0.3)	0.1 (0.1 to 0.2)	4.7 (3.4 to 6.5)	2.8 (2.0 to 3.8)	1.22 (0.88 to 1.68)	0.73 (0.53 to 0.99)

Yazd IRN_44893 North Africa and Middle East Stillbirth Rate / Neonatal Mortality Rate (SBR/NMR) − 20 weeks
lambda = 1 ; zeta = 0.6 ; scale = 15 ; amp = 0.028

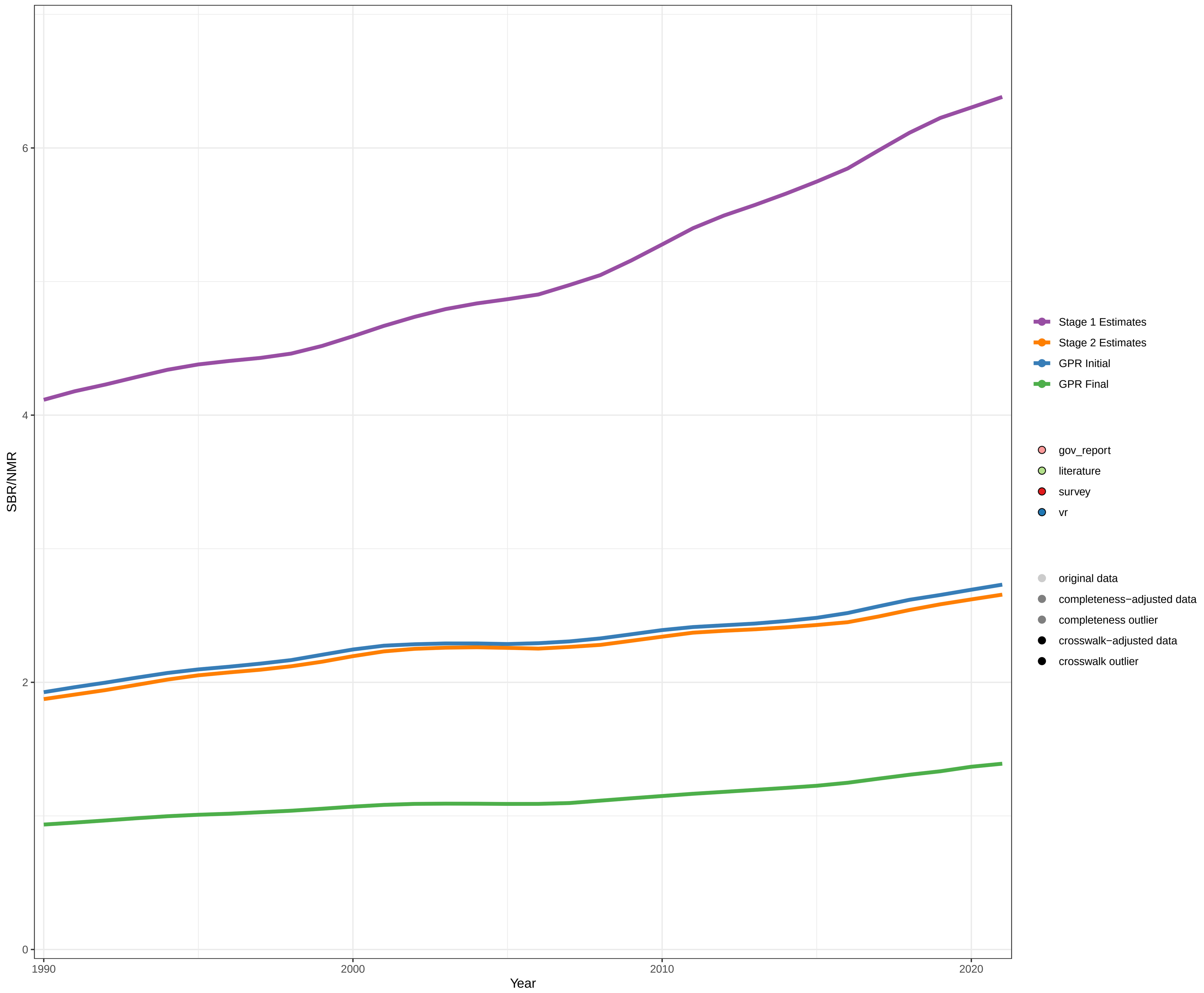

Yazd IRN_44893 North Africa and Middle East Stillbirth Rate / Neonatal Mortality Rate (SBR/NMR) − 28 weeks
lambda = 1 ; zeta = 0.6 ; scale = 15 ; amp = 0.028

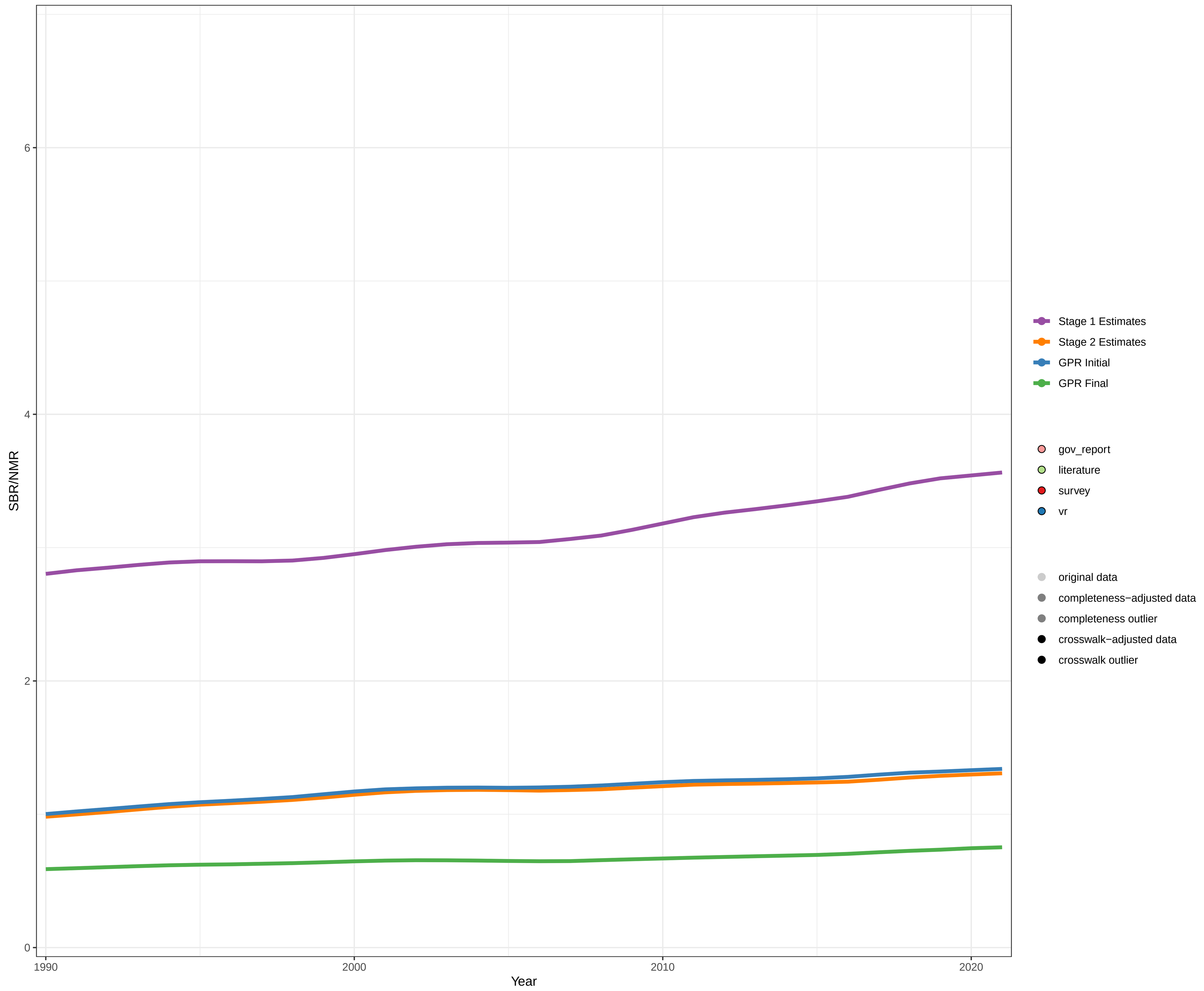

Definition	Years	Outlier	Source.Type	Title

Yazd IRN_44893 North Africa and Middle East Stillbirth Rate (SBR) − 20 weeks
lambda = 1 ; zeta = 0.6 ; scale = 15 ; amp = 0.028

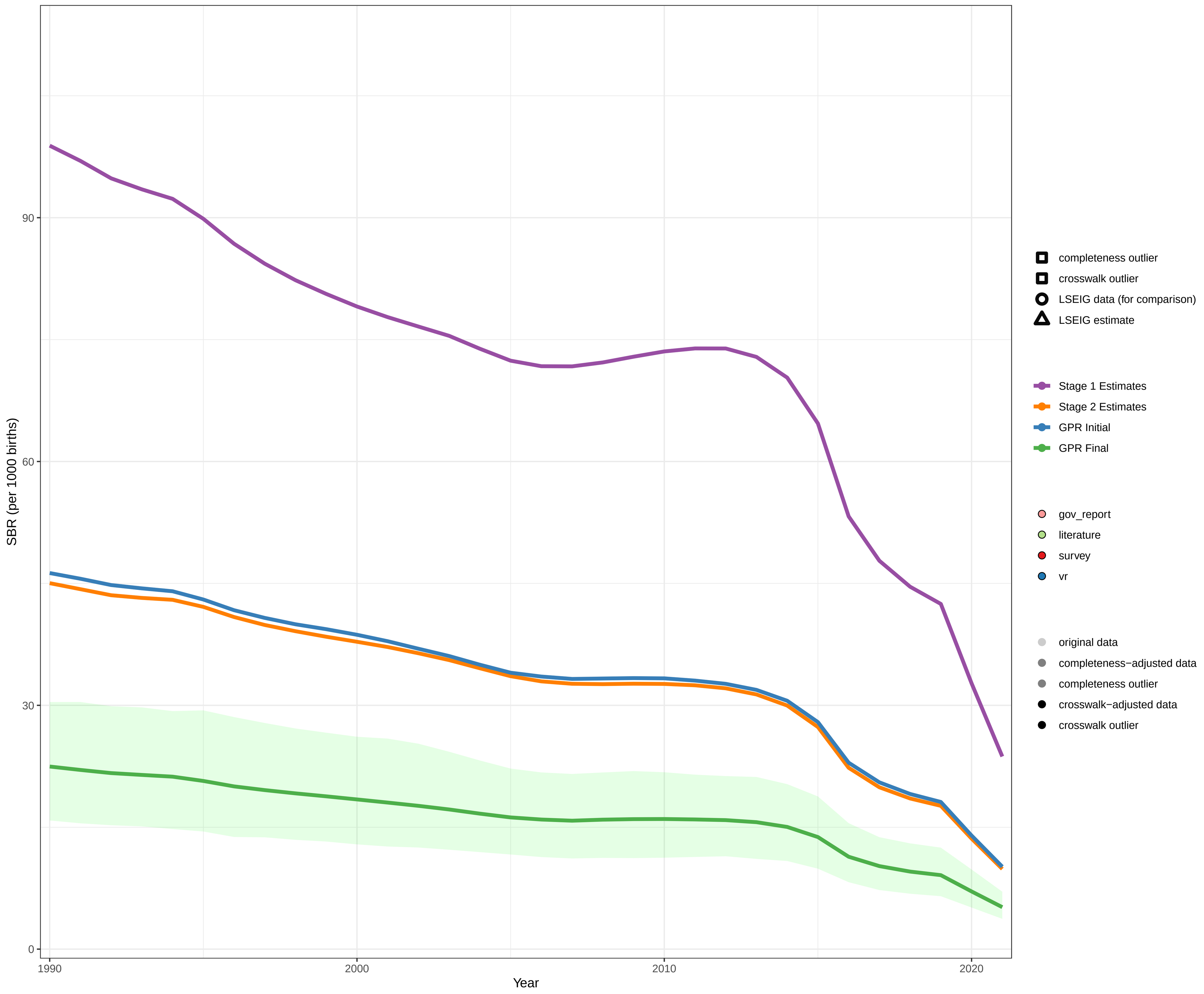

Yazd IRN_44893 North Africa and Middle East Stillbirth Rate (SBR) − 28 weeks
lambda = 1 ; zeta = 0.6 ; scale = 15 ; amp = 0.028

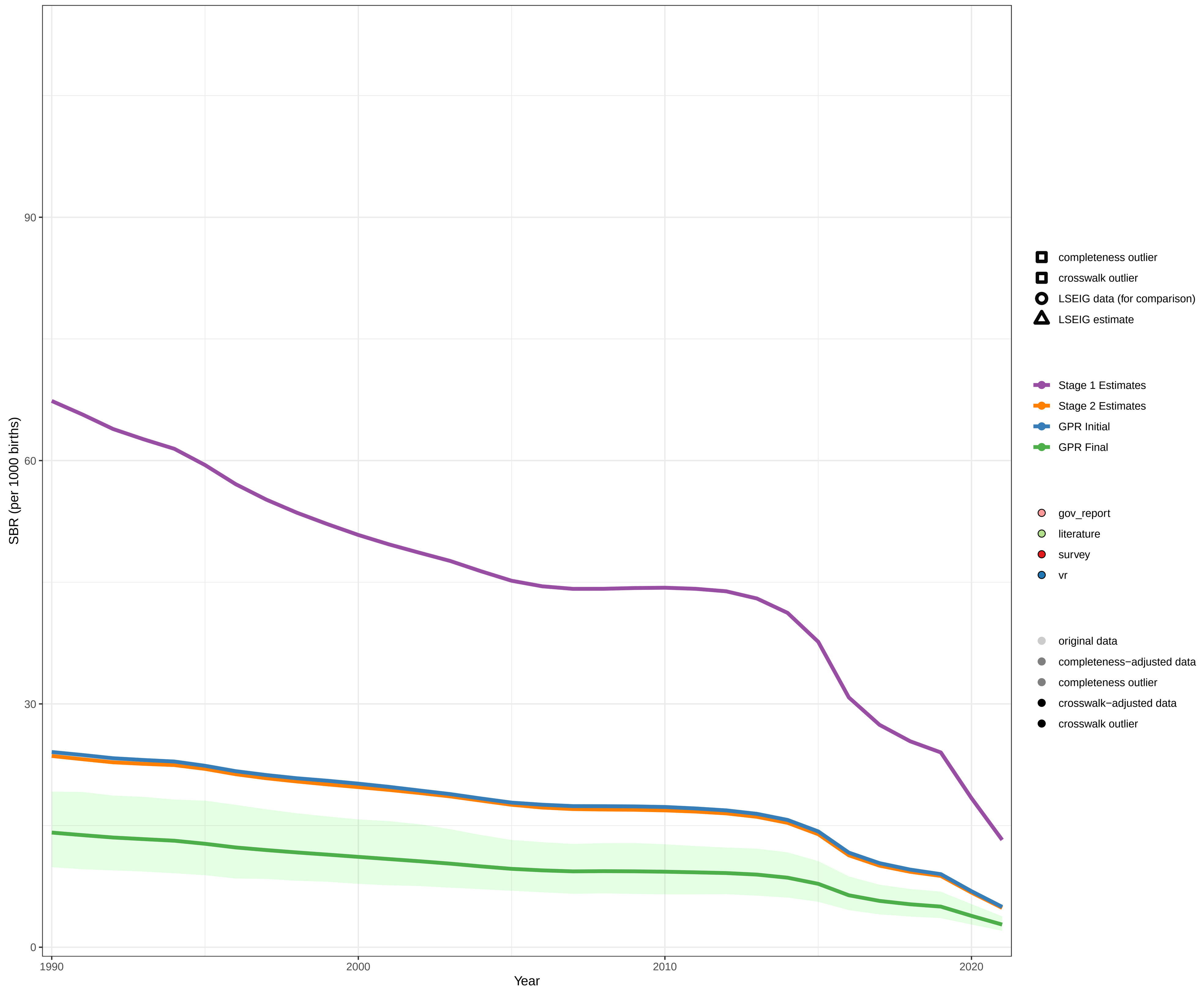

Year	Total Stillbirths (in thousands)	Total Stillbirths (in thousands)	SBR (per 1000 births)	SBR (per 1000 births)	SBR/NMR	SBR/NMR
	20 weeks	28 weeks	20 weeks	28 weeks	20 weeks	28 weeks
1990	0.4 (0.3 to 0.6)	0.3 (0.2 to 0.4)	22.5 (15.8 to 30.4)	14.1 (9.9 to 19.2)	0.94 (0.66 to 1.27)	0.59 (0.41 to 0.80)
1995	0.3 (0.2 to 0.4)	0.2 (0.1 to 0.3)	20.7 (14.5 to 29.4)	12.8 (8.9 to 18.1)	1.01 (0.71 to 1.43)	0.62 (0.43 to 0.88)
2000	0.3 (0.2 to 0.4)	0.2 (0.1 to 0.2)	18.4 (12.9 to 26.1)	11.1 (7.8 to 15.8)	1.07 (0.75 to 1.52)	0.65 (0.45 to 0.92)
2005	0.3 (0.2 to 0.4)	0.2 (0.1 to 0.2)	16.2 (11.6 to 22.2)	9.7 (7.0 to 13.2)	1.09 (0.78 to 1.49)	0.65 (0.47 to 0.89)
2010	0.3 (0.2 to 0.5)	0.2 (0.1 to 0.3)	16.0 (11.2 to 21.8)	9.3 (6.5 to 12.7)	1.15 (0.81 to 1.56)	0.67 (0.47 to 0.91)
2015	0.3 (0.2 to 0.4)	0.2 (0.1 to 0.2)	13.8 (9.9 to 18.8)	7.8 (5.6 to 10.6)	1.23 (0.88 to 1.67)	0.69 (0.50 to 0.94)
2020	0.1 (0.1 to 0.2)	0.1 (0.0 to 0.1)	7.1 (5.1 to 9.8)	3.9 (2.8 to 5.3)	1.37 (0.99 to 1.89)	0.75 (0.54 to 1.03)
2021	0.1 (0.1 to 0.1)	0.0 (0.0 to 0.1)	5.2 (3.7 to 7.1)	2.8 (2.0 to 3.8)	1.39 (1.00 to 1.90)	0.75 (0.54 to 1.03)

Zanjan IRN_44894 North Africa and Middle East Stillbirth Rate / Neonatal Mortality Rate (SBR/NMR) − 20 weeks
lambda = 1 ; zeta = 0.6 ; scale = 15 ; amp = 0.028

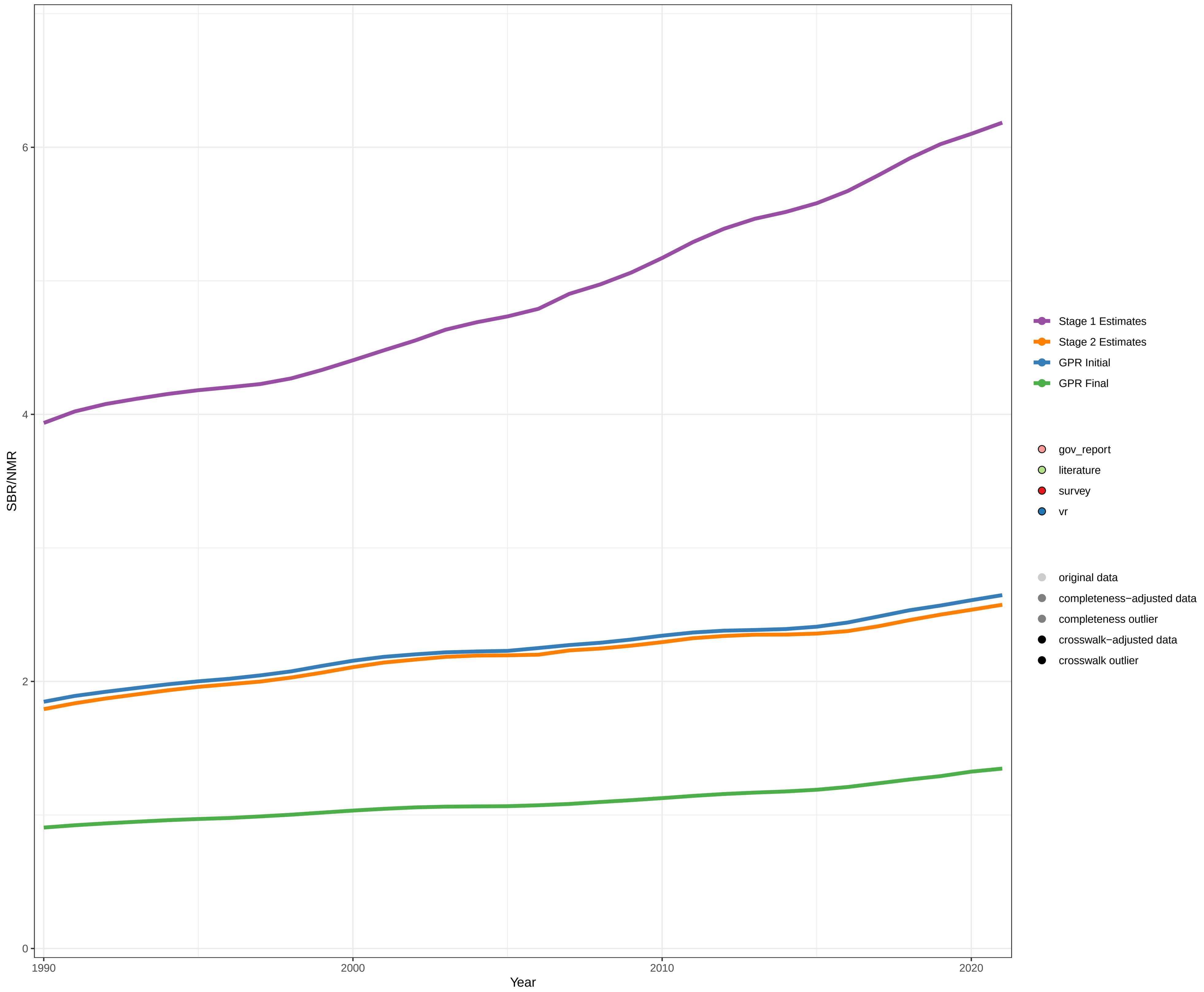

Zanjan IRN_44894 North Africa and Middle East Stillbirth Rate / Neonatal Mortality Rate (SBR/NMR) − 28 weeks
lambda = 1 ; zeta = 0.6 ; scale = 15 ; amp = 0.028

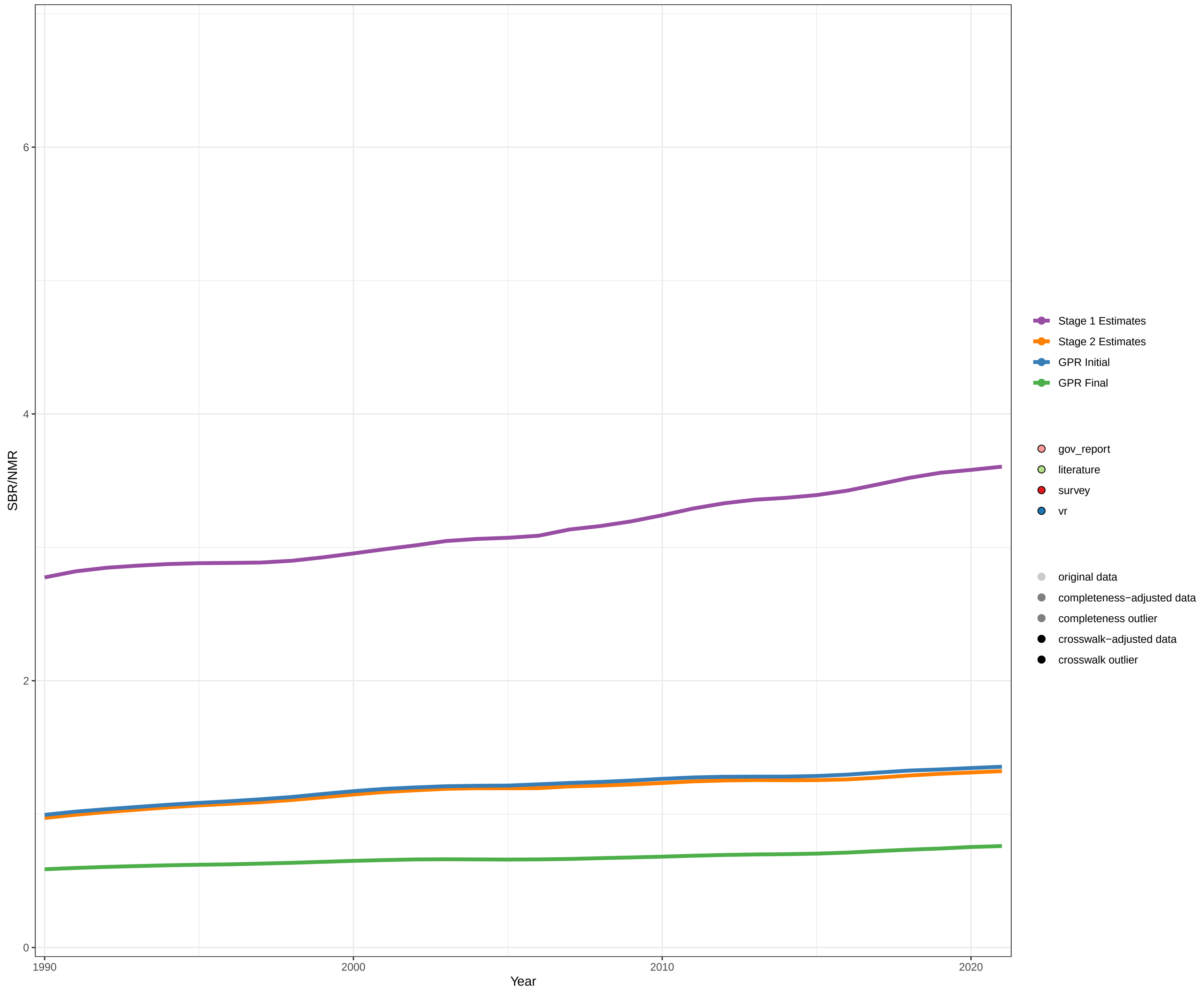

Definition	Years	Outlier	Source.Type	Title

Zanjan IRN_44894 North Africa and Middle East Stillbirth Rate (SBR) − 20 weeks
lambda = 1 ; zeta = 0.6 ; scale = 15 ; amp = 0.028

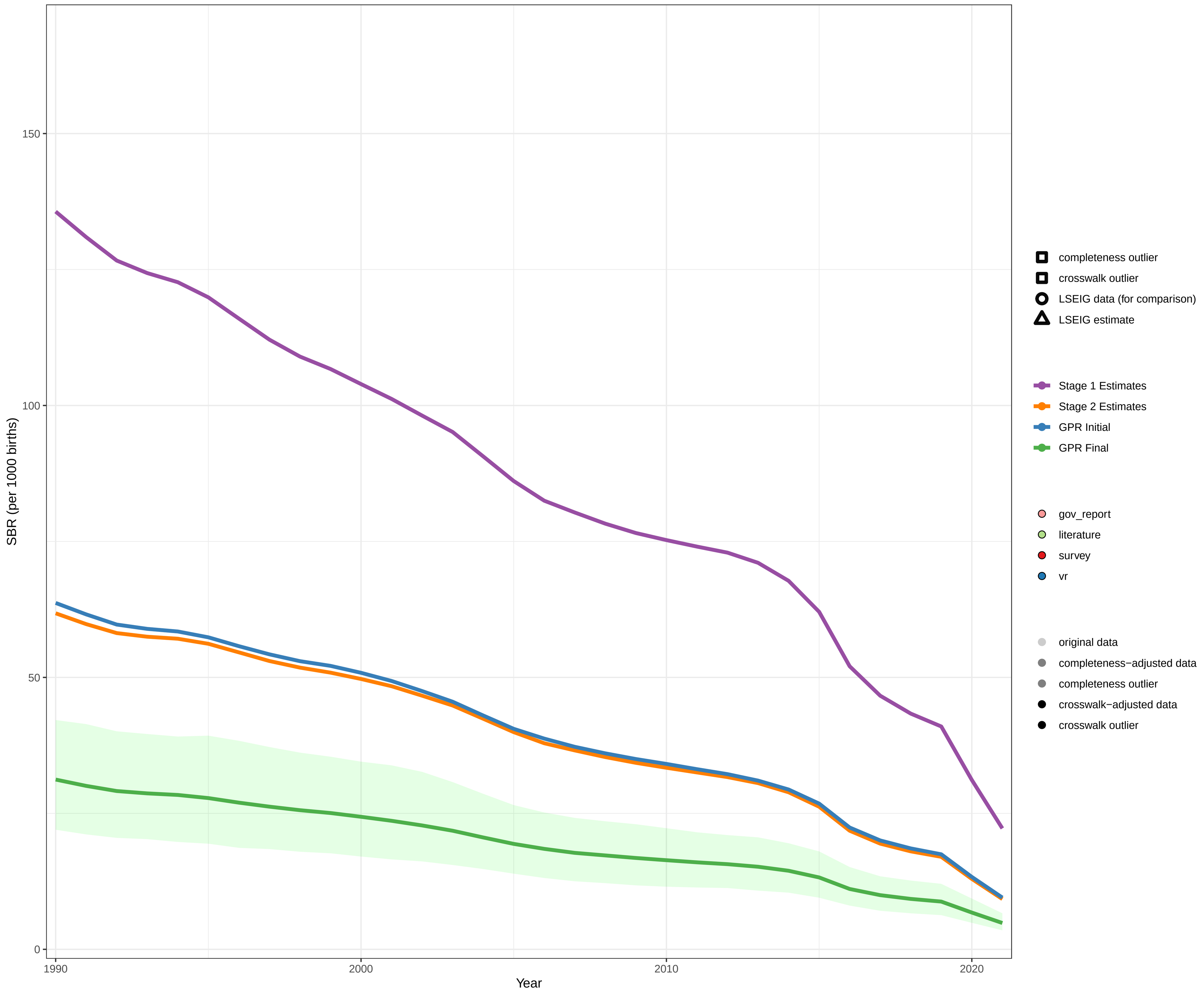

Zanjan IRN_44894 North Africa and Middle East Stillbirth Rate (SBR) − 28 weeks
lambda = 1 ; zeta = 0.6 ; scale = 15 ; amp = 0.028

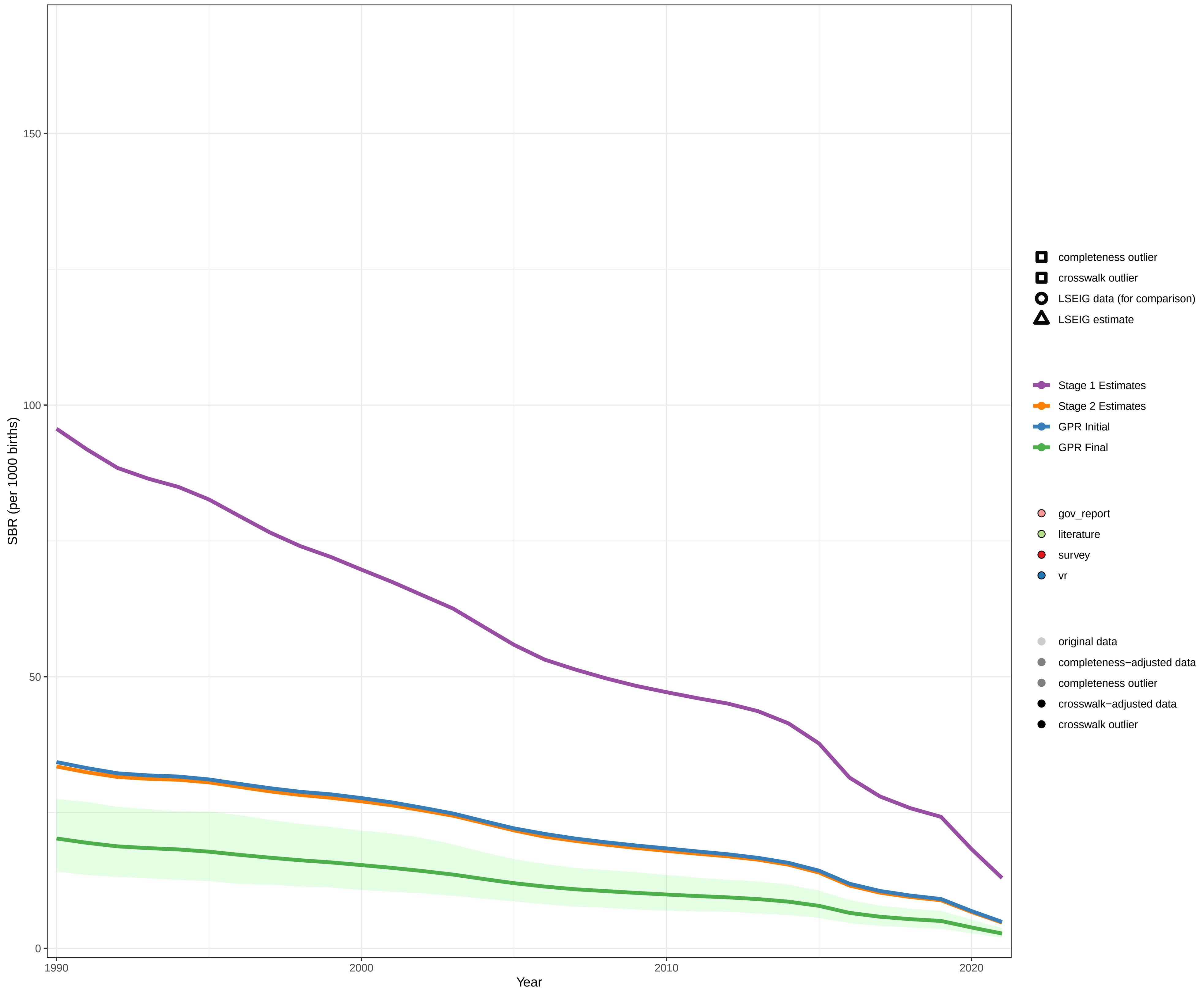

Year	Total Stillbirths (in thousands)	Total Stillbirths (in thousands)	SBR (per 1000 births)	SBR (per 1000 births)	SBR/NMR	SBR/NMR
	20 weeks	28 weeks	20 weeks	28 weeks	20 weeks	28 weeks
1990	0.8 (0.6 to 1.1)	0.5 (0.4 to 0.7)	31.2 (22.0 to 42.2)	20.2 (14.1 to 27.5)	0.91 (0.64 to 1.22)	0.59 (0.41 to 0.80)
1995	0.6 (0.4 to 0.8)	0.4 (0.3 to 0.5)	27.8 (19.4 to 39.3)	17.8 (12.4 to 25.2)	0.97 (0.68 to 1.37)	0.62 (0.43 to 0.88)
2000	0.4 (0.3 to 0.6)	0.3 (0.2 to 0.4)	24.4 (17.1 to 34.5)	15.3 (10.7 to 21.7)	1.03 (0.72 to 1.46)	0.65 (0.46 to 0.92)
2005	0.3 (0.2 to 0.5)	0.2 (0.1 to 0.3)	19.4 (13.9 to 26.5)	12.0 (8.6 to 16.4)	1.07 (0.76 to 1.46)	0.66 (0.47 to 0.90)
2010	0.3 (0.2 to 0.4)	0.2 (0.1 to 0.3)	16.4 (11.5 to 22.3)	9.9 (6.9 to 13.5)	1.13 (0.79 to 1.53)	0.68 (0.48 to 0.93)
2015	0.3 (0.2 to 0.4)	0.2 (0.1 to 0.2)	13.2 (9.5 to 18.0)	7.8 (5.6 to 10.6)	1.19 (0.85 to 1.62)	0.70 (0.50 to 0.96)
2020	0.1 (0.1 to 0.1)	0.1 (0.0 to 0.1)	6.8 (4.9 to 9.3)	3.8 (2.8 to 5.3)	1.32 (0.95 to 1.83)	0.75 (0.54 to 1.04)
2021	0.1 (0.0 to 0.1)	0.0 (0.0 to 0.0)	4.8 (3.5 to 6.6)	2.7 (2.0 to 3.7)	1.35 (0.97 to 1.84)	0.76 (0.55 to 1.04)

Iraq IRQ North Africa and Middle East Stillbirth Rate / Neonatal Mortality Rate (SBR/NMR) − 20 weeks
lambda = 0.6 ; zeta = 0.8 ; scale = 15 ; amp = 0.028

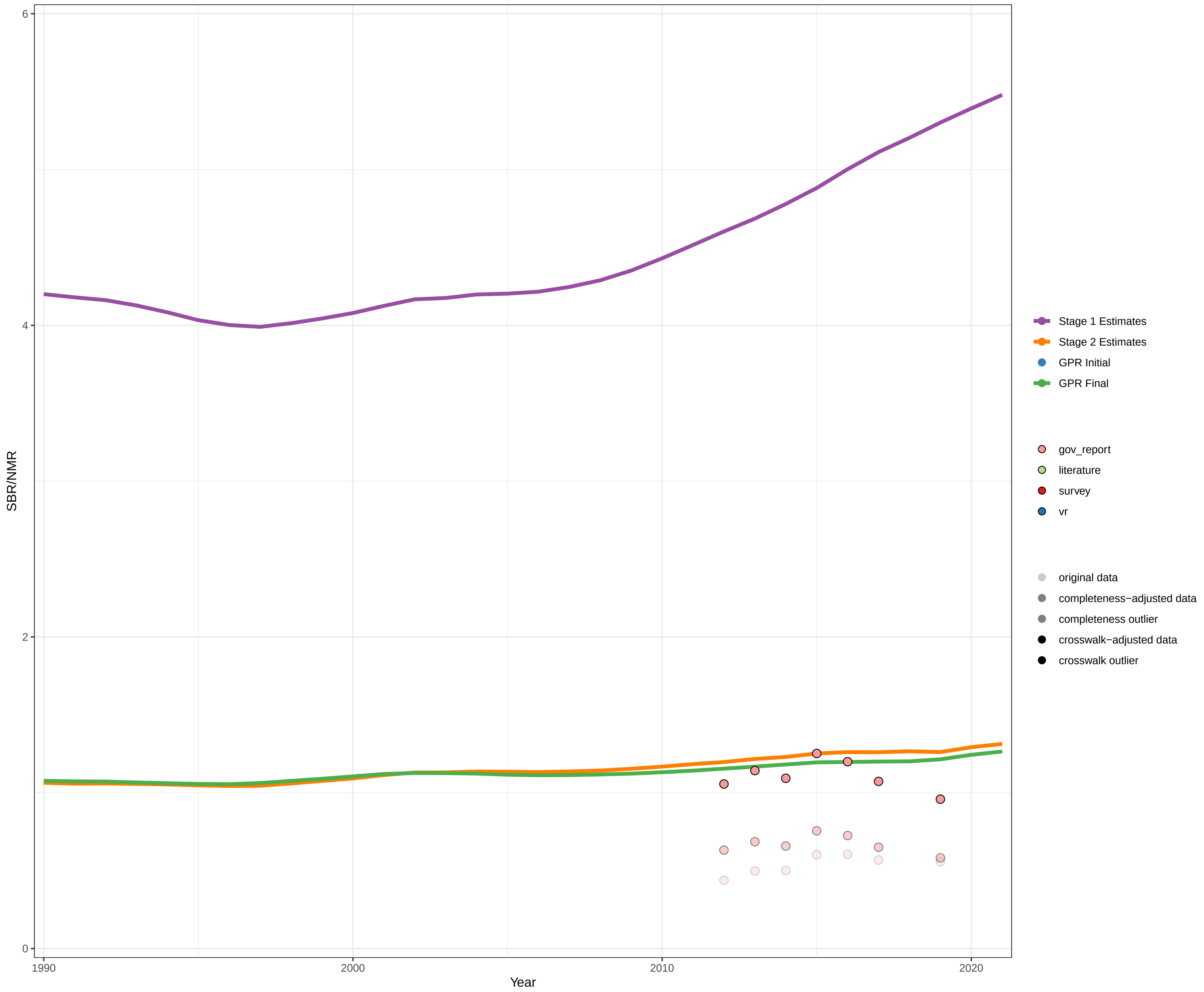

Iraq IRQ North Africa and Middle East Stillbirth Rate / Neonatal Mortality Rate (SBR/NMR) − 28 weeks
lambda = 0.6 ; zeta = 0.8 ; scale = 15 ; amp = 0.028

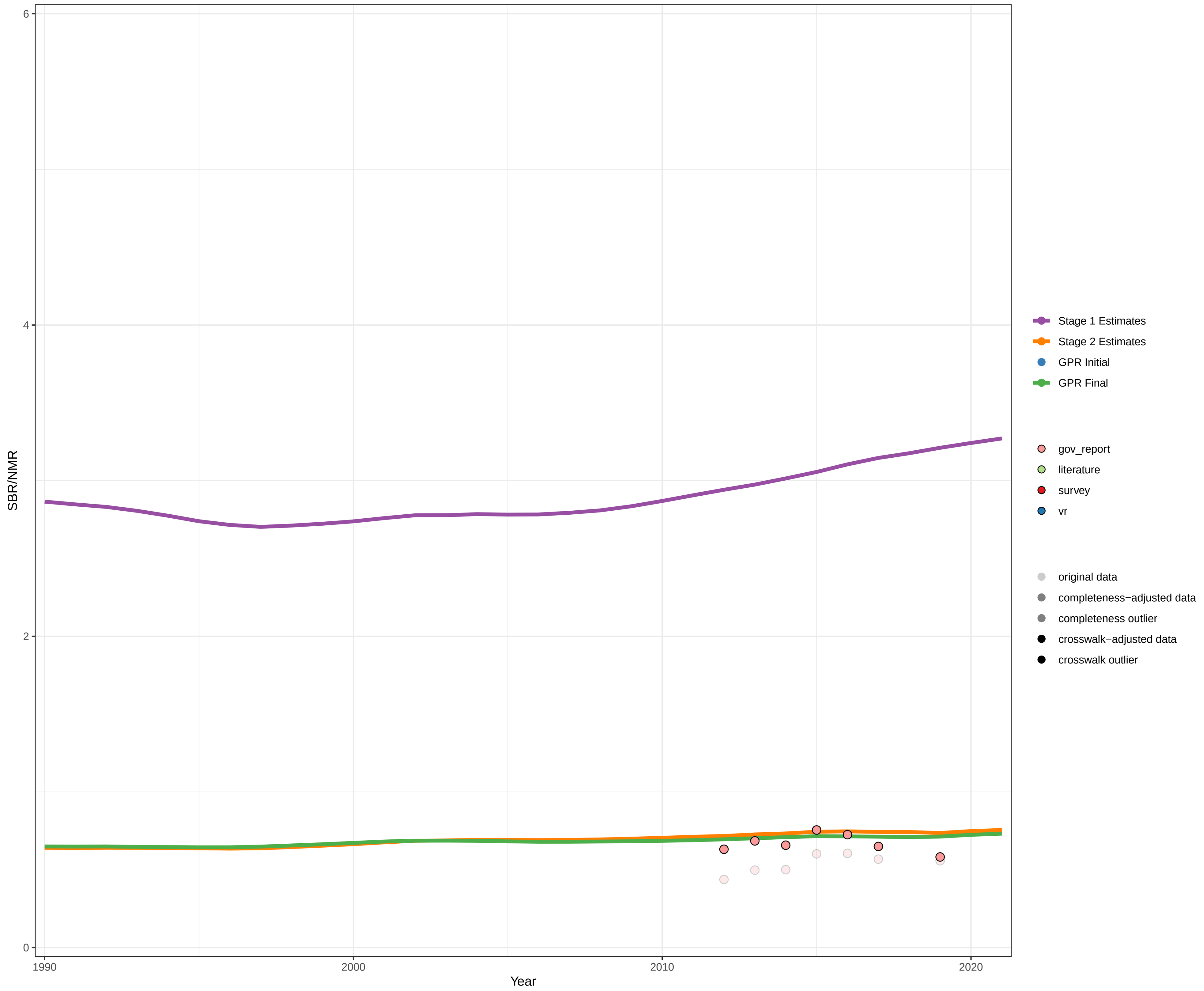

Definition	Years	Outlier	Source.Type	Title
28_weeks	2012−2017, 2019	0	Statistical Report	Iraq Annual Statistical Report

Iraq IRQ North Africa and Middle East Stillbirth Rate (SBR) − 20 weeks
lambda = 0.6 ; zeta = 0.8 ; scale = 15 ; amp = 0.028

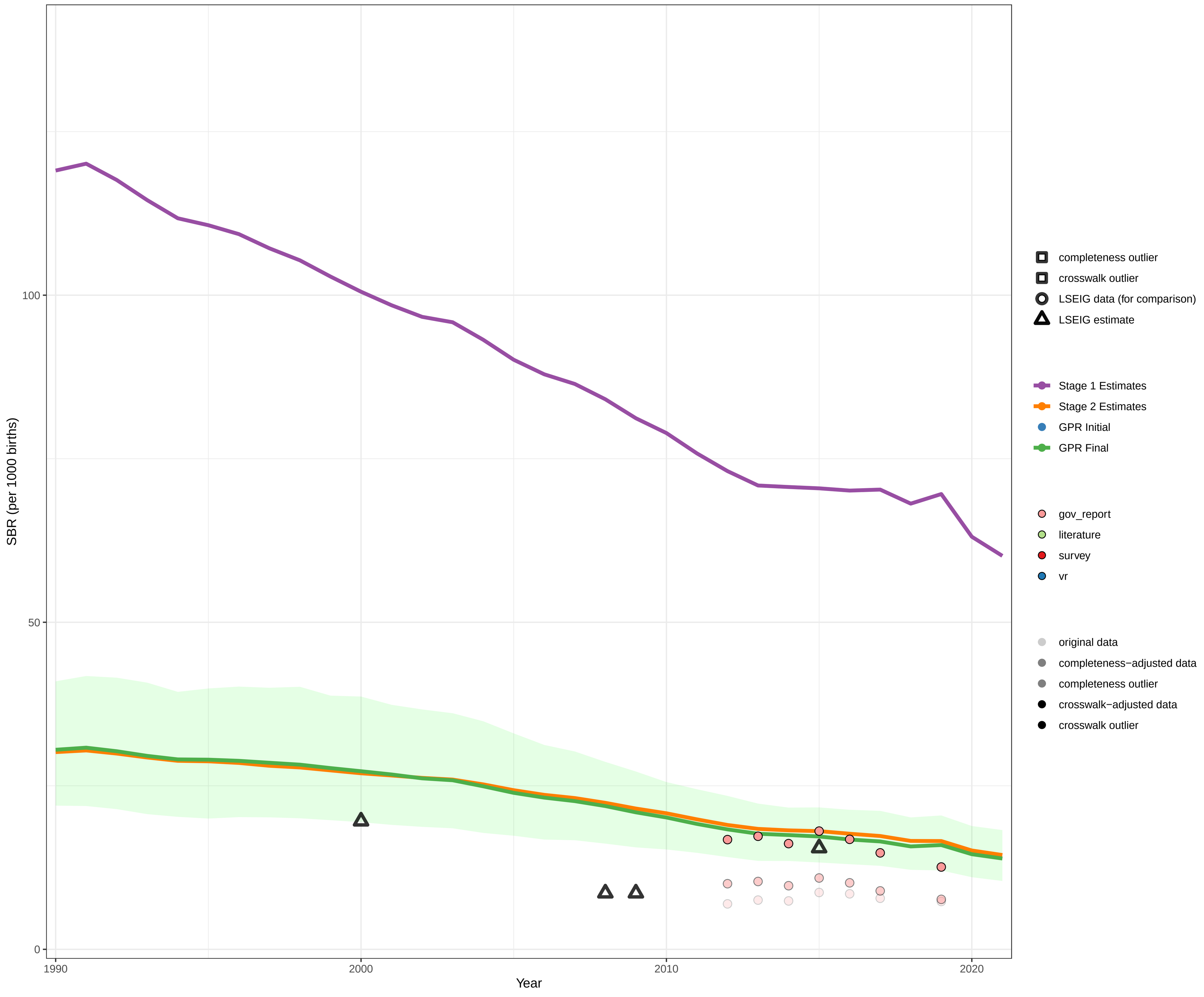

Iraq IRQ North Africa and Middle East Stillbirth Rate (SBR) − 28 weeks
lambda = 0.6 ; zeta = 0.8 ; scale = 15 ; amp = 0.028

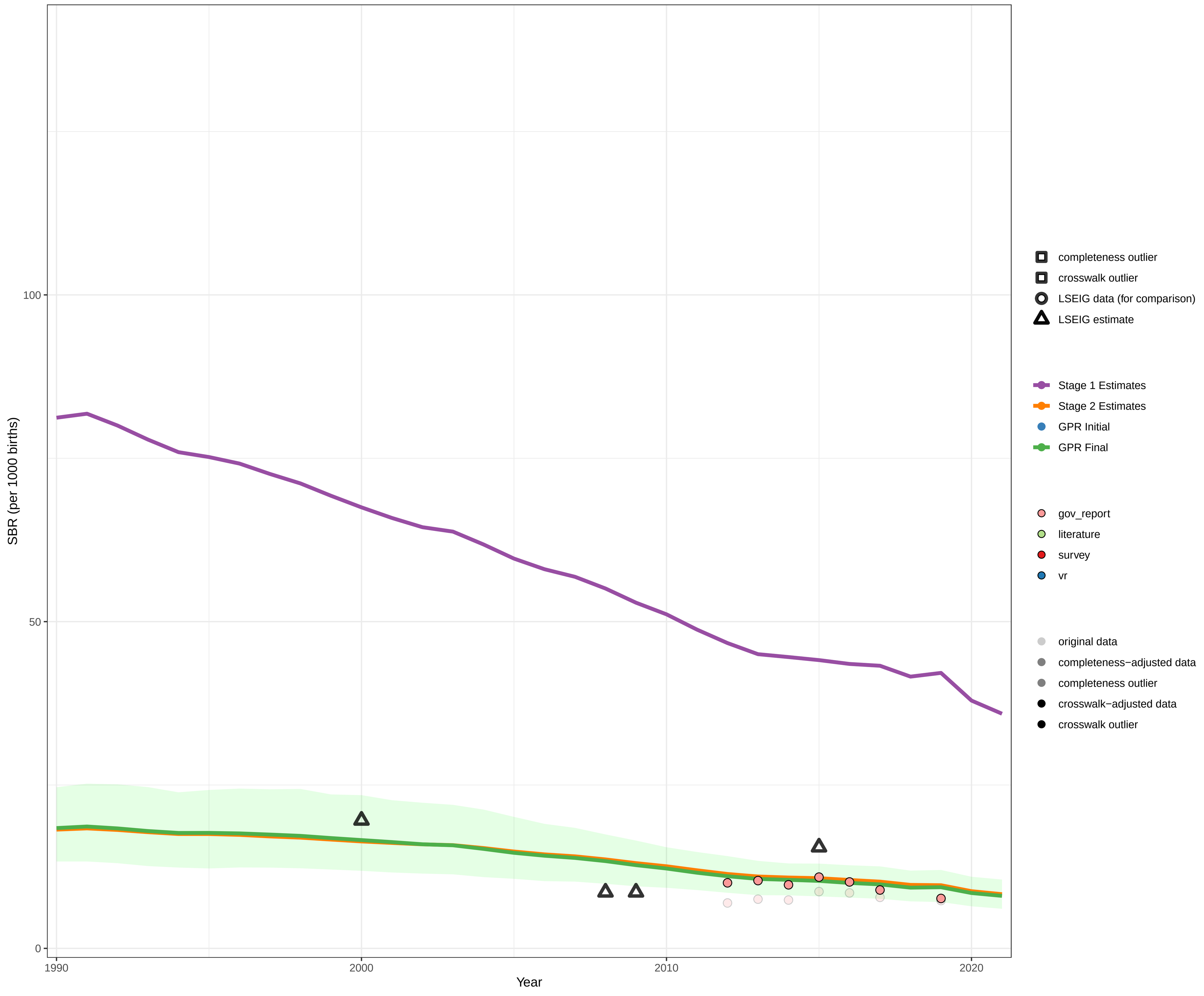

Year	Total Stillbirths (in thousands)	Total Stillbirths (in thousands)	SBR (per 1000 births)	SBR (per 1000 births)	SBR/NMR	SBR/NMR
	20 weeks	28 weeks	20 weeks	28 weeks	20 weeks	28 weeks
1990	23.7 (16.9 to 32.1)	14.1 (10.1 to 19.0)	30.5 (22.0 to 41.0)	18.4 (13.3 to 24.7)	1.08 (0.77 to 1.45)	0.65 (0.47 to 0.87)
1995	25.0 (17.1 to 34.8)	15.1 (10.4 to 20.8)	29.0 (20.0 to 39.9)	17.7 (12.2 to 24.2)	1.06 (0.73 to 1.45)	0.64 (0.45 to 0.88)
2000	26.1 (18.5 to 37.5)	15.7 (11.2 to 22.4)	27.2 (19.4 to 38.6)	16.6 (11.9 to 23.4)	1.10 (0.79 to 1.57)	0.67 (0.48 to 0.95)
2005	25.4 (18.3 to 35.4)	15.4 (11.2 to 21.3)	23.9 (17.4 to 33.0)	14.6 (10.6 to 20.1)	1.12 (0.81 to 1.54)	0.68 (0.50 to 0.94)
2010	21.2 (16.0 to 27.0)	12.7 (9.6 to 16.2)	20.2 (15.3 to 25.6)	12.2 (9.3 to 15.5)	1.13 (0.86 to 1.44)	0.69 (0.52 to 0.87)
2015	18.4 (14.1 to 23.2)	10.9 (8.4 to 13.8)	17.3 (13.3 to 21.7)	10.3 (8.0 to 13.0)	1.19 (0.92 to 1.50)	0.72 (0.55 to 0.90)
2020	14.0 (10.6 to 18.2)	8.1 (6.2 to 10.5)	14.5 (11.0 to 18.9)	8.5 (6.4 to 11.0)	1.24 (0.94 to 1.61)	0.72 (0.55 to 0.94)
2021	13.1 (9.8 to 17.3)	7.6 (5.7 to 9.9)	13.9 (10.4 to 18.2)	8.0 (6.1 to 10.5)	1.26 (0.95 to 1.66)	0.73 (0.55 to 0.96)

Jordan JOR North Africa and Middle East Stillbirth Rate / Neonatal Mortality Rate (SBR/NMR) − 20 weeks
lambda = 0.6 ; zeta = 0.8 ; scale = 15 ; amp = 0.028

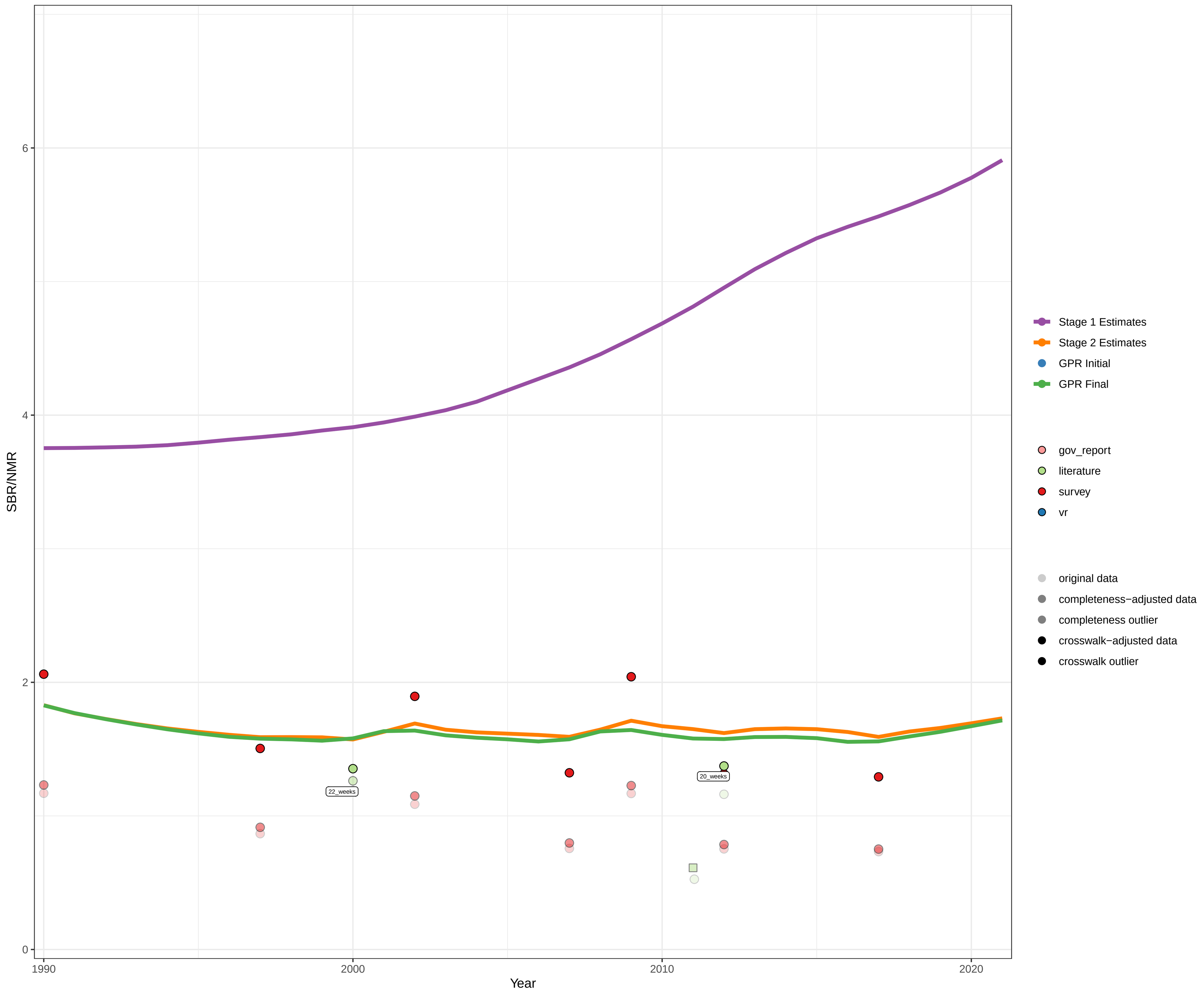

Jordan JOR North Africa and Middle East Stillbirth Rate / Neonatal Mortality Rate (SBR/NMR) − 28 weeks
lambda = 0.6 ; zeta = 0.8 ; scale = 15 ; amp = 0.028

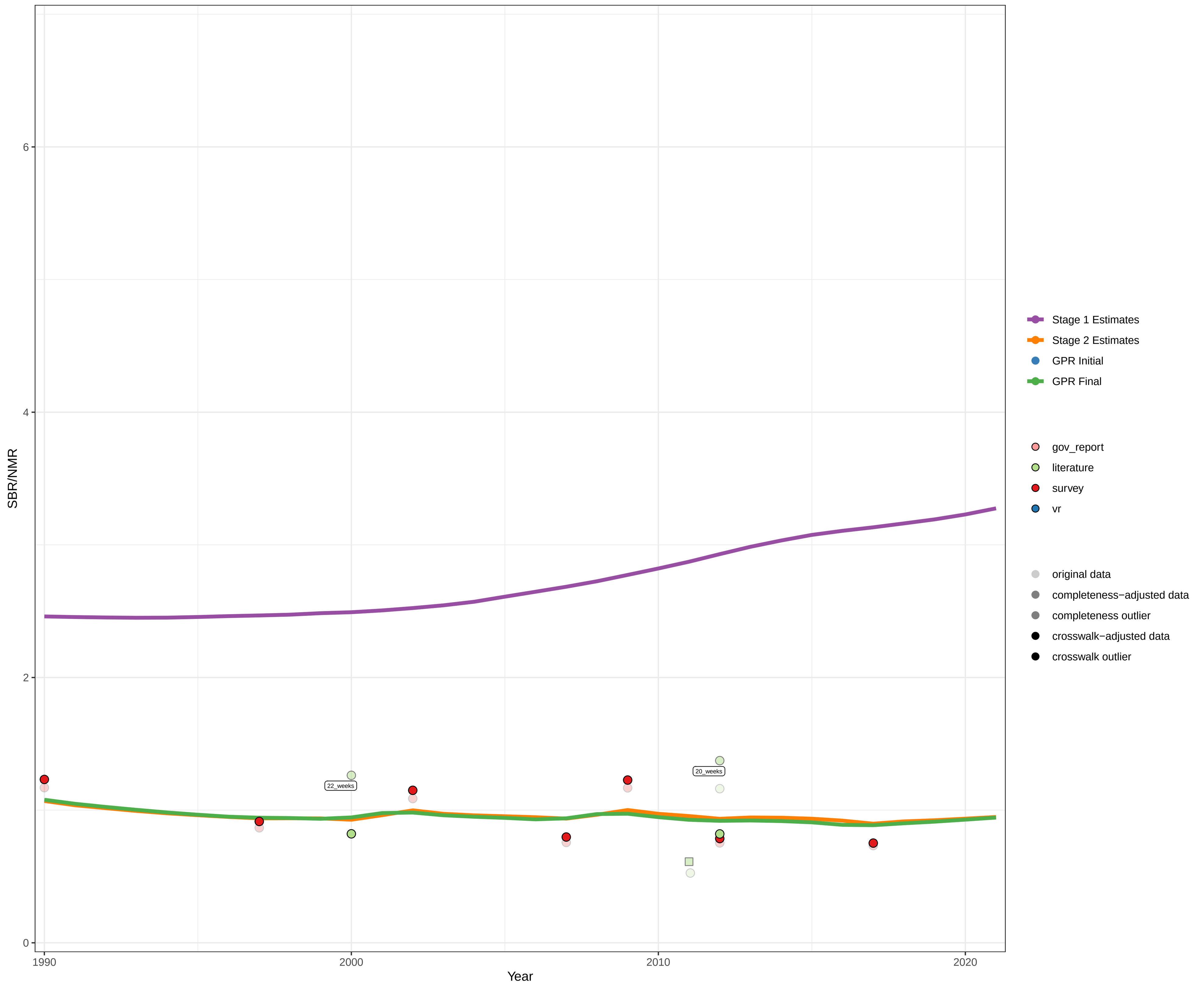

Definition	Years	Outlier	Source.Type	Title
20_weeks	2012	0	Sci Lit	Stillbirths in Jordan: rate, causes, and preventability
22_weeks	2000	0	Sci Lit	Evaluation of child deaths registration in a Jordanian community
28_weeks	1990	0	Survey	Jordan Demographic and Health Survey 1990
28_weeks	1997	0	Survey	Jordan Demographic and Health Survey 1997
28_weeks	2002	0	Survey	Jordan Demographic and Health Survey 2002
28_weeks	2007	0	Survey	Jordan Demographic and Health Survey 2007
28_weeks	2009	0	Survey	Jordan Interim Demographic and Health Survey 2009
28_weeks	2012	0	Survey	Jordan Demographic and Health Survey 2012
28_weeks	2017	0	Survey	Jordan Demographic and Health Survey 2017−2018
28_weeks_OR_1000_grams	2011	1	Sci Lit	Maternal complications and perinatal mortality: findings of the World Health Organization Multicountry Survey on Maternal and Newborn Health

Jordan JOR North Africa and Middle East Stillbirth Rate (SBR) − 20 weeks
lambda = 0.6 ; zeta = 0.8 ; scale = 15 ; amp = 0.028

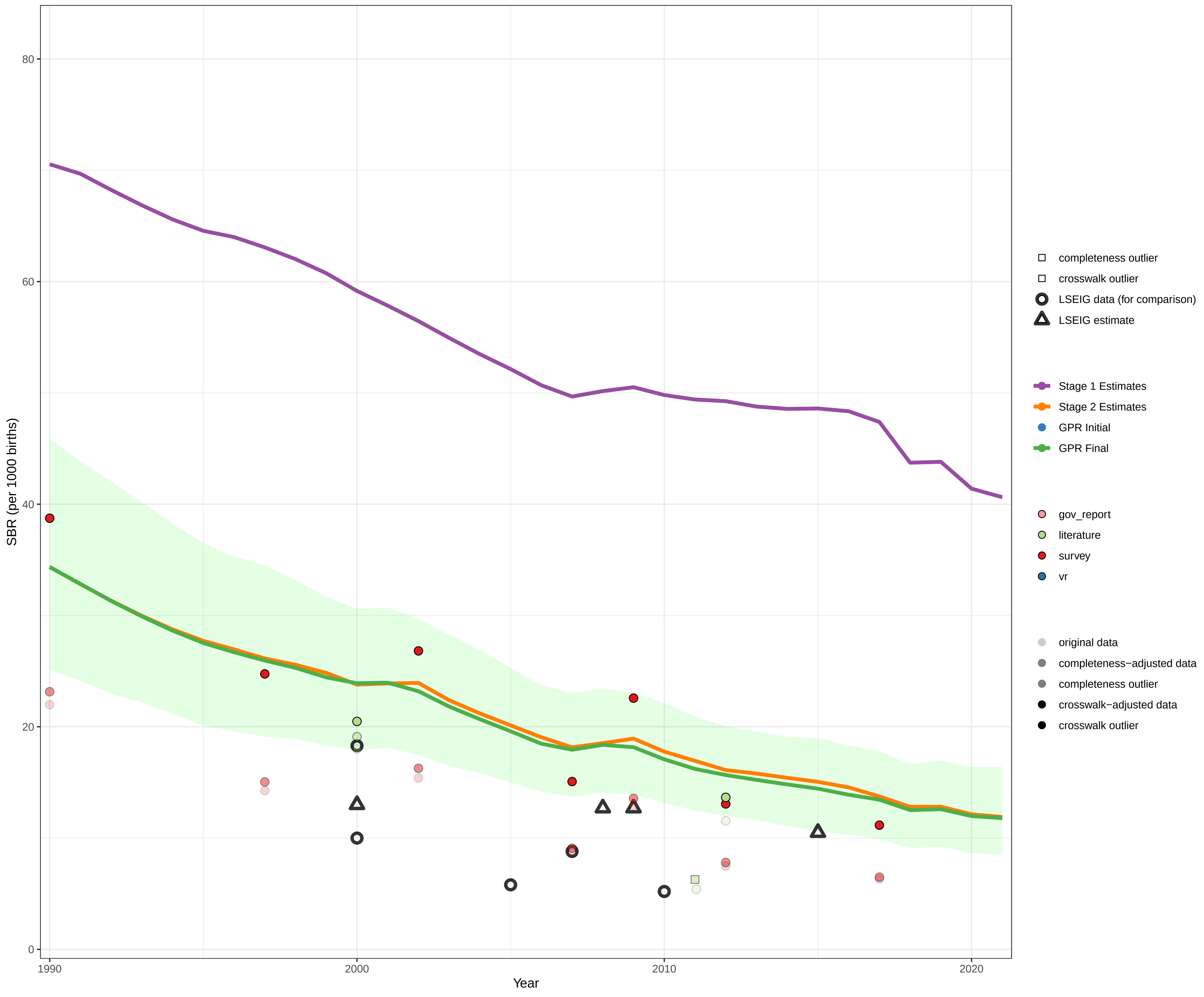

Jordan JOR North Africa and Middle East Stillbirth Rate (SBR) − 28 weeks
lambda = 0.6 ; zeta = 0.8 ; scale = 15 ; amp = 0.028

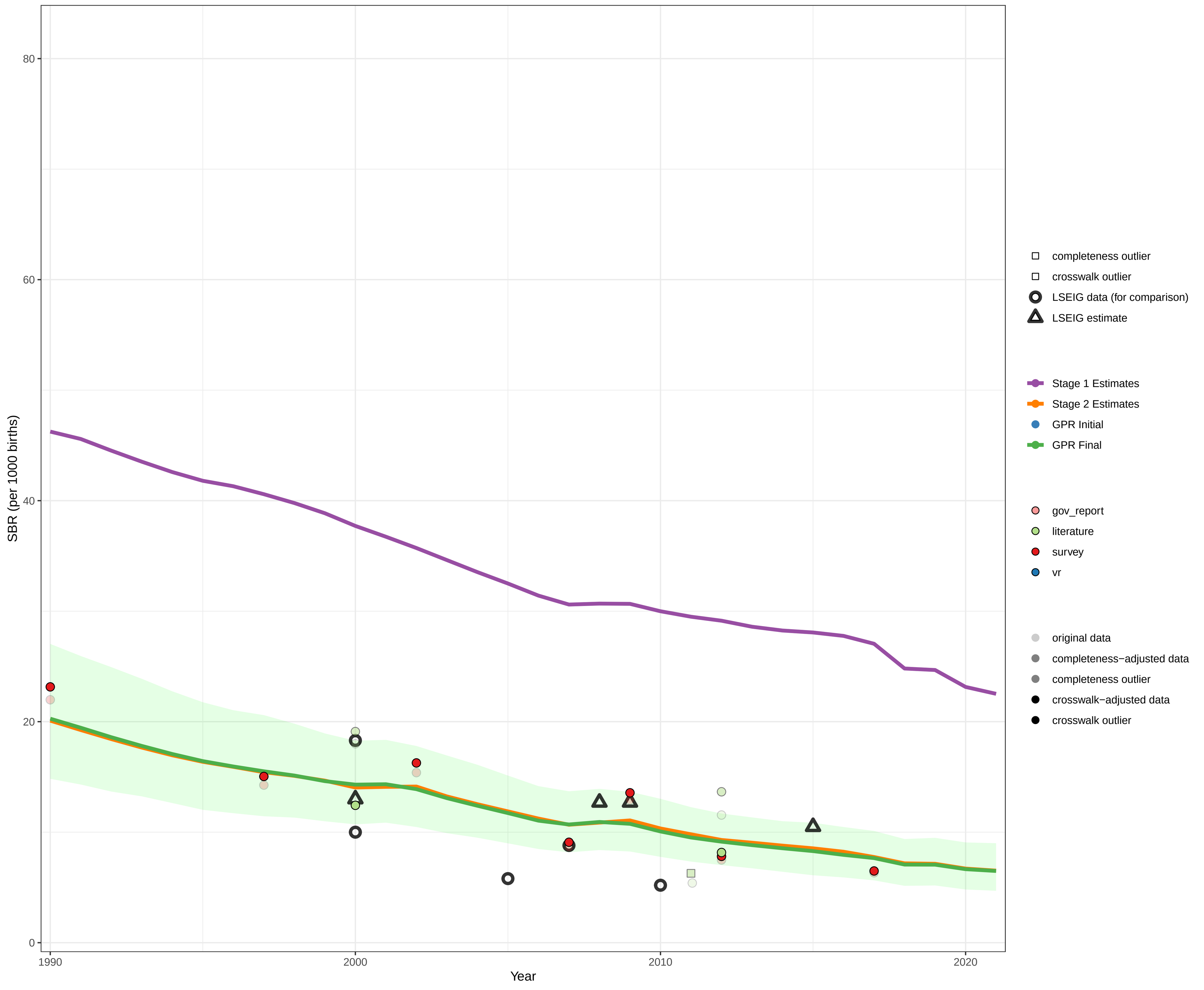

Year	Total Stillbirths (in thousands)	Total Stillbirths (in thousands)	SBR (per 1000 births)	SBR (per 1000 births)	SBR/NMR	SBR/NMR
	20 weeks	28 weeks	20 weeks	28 weeks	20 weeks	28 weeks
1990	4.7 (3.4 to 6.4)	2.8 (2.0 to 3.7)	34.3 (25.1 to 45.9)	20.3 (14.8 to 27.0)	1.83 (1.33 to 2.44)	1.08 (0.79 to 1.44)
1995	4.1 (3.0 to 5.5)	2.4 (1.8 to 3.2)	27.5 (20.1 to 36.5)	16.4 (12.0 to 21.8)	1.62 (1.18 to 2.15)	0.97 (0.71 to 1.28)
2000	3.7 (2.7 to 4.7)	2.2 (1.6 to 2.8)	23.9 (17.9 to 30.6)	14.3 (10.7 to 18.3)	1.58 (1.18 to 2.02)	0.95 (0.71 to 1.21)
2005	3.3 (2.5 to 4.2)	1.9 (1.5 to 2.5)	19.6 (15.0 to 25.3)	11.7 (9.0 to 15.1)	1.57 (1.20 to 2.03)	0.94 (0.72 to 1.21)
2010	3.4 (2.6 to 4.5)	2.0 (1.5 to 2.6)	17.1 (13.1 to 22.1)	10.1 (7.8 to 13.0)	1.61 (1.24 to 2.08)	0.95 (0.73 to 1.23)
2015	3.1 (2.3 to 4.1)	1.8 (1.3 to 2.3)	14.4 (10.6 to 19.0)	8.3 (6.1 to 10.9)	1.58 (1.16 to 2.08)	0.91 (0.67 to 1.19)
2020	2.6 (1.8 to 3.5)	1.4 (1.0 to 1.9)	12.0 (8.6 to 16.4)	6.7 (4.8 to 9.1)	1.67 (1.21 to 2.29)	0.93 (0.67 to 1.27)
2021	2.5 (1.8 to 3.5)	1.4 (1.0 to 1.9)	11.8 (8.5 to 16.4)	6.5 (4.7 to 9.0)	1.71 (1.24 to 2.39)	0.94 (0.68 to 1.31)

Kuwait KWT North Africa and Middle East Stillbirth Rate / Neonatal Mortality Rate (SBR/NMR) − 20 weeks
lambda = 0.2 ; zeta = 0.99 ; scale = 5 ; amp = 0.028

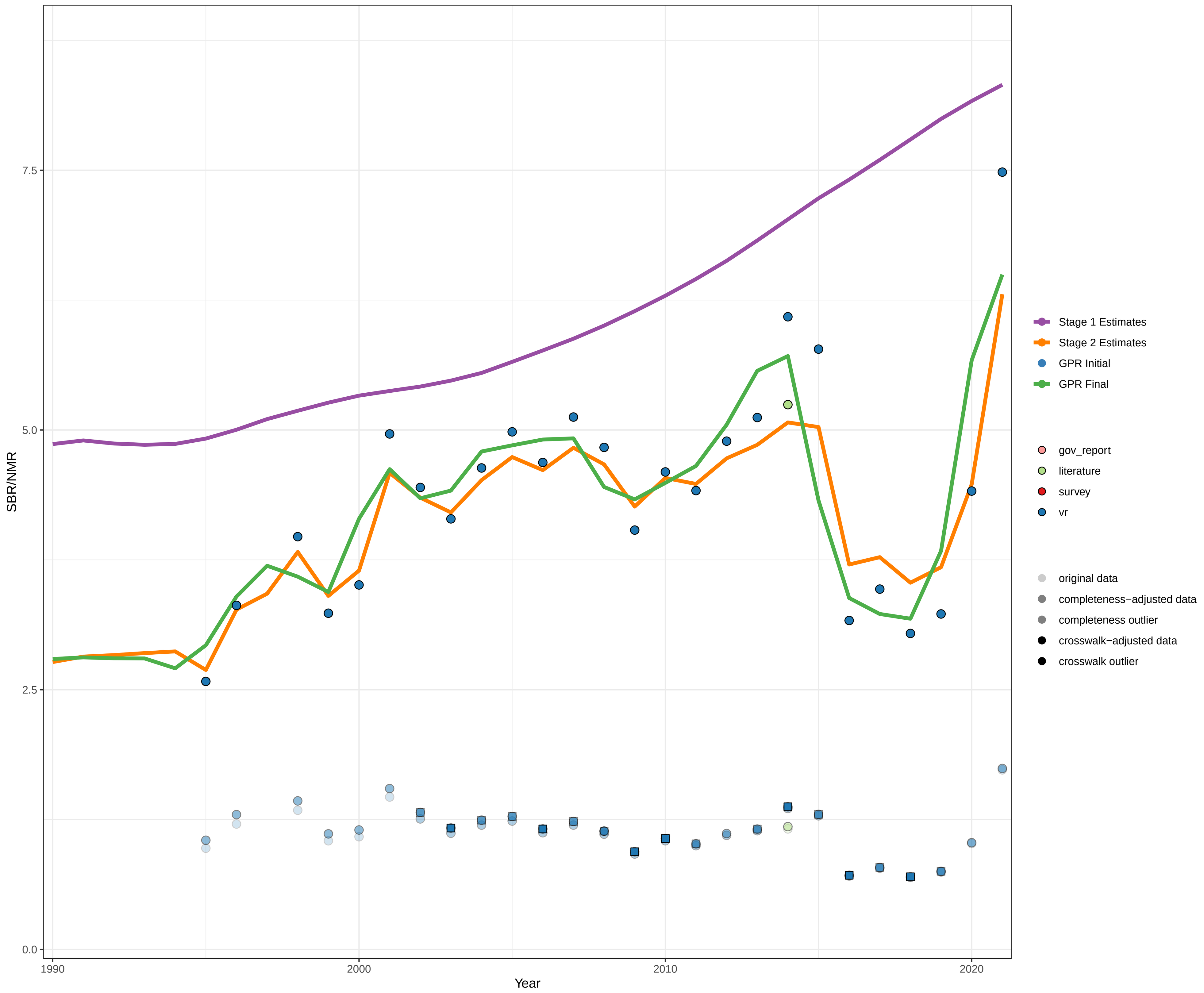

Kuwait KWT North Africa and Middle East Stillbirth Rate / Neonatal Mortality Rate (SBR/NMR) − 28 weeks
lambda = 0.2 ; zeta = 0.99 ; scale = 5 ; amp = 0.028

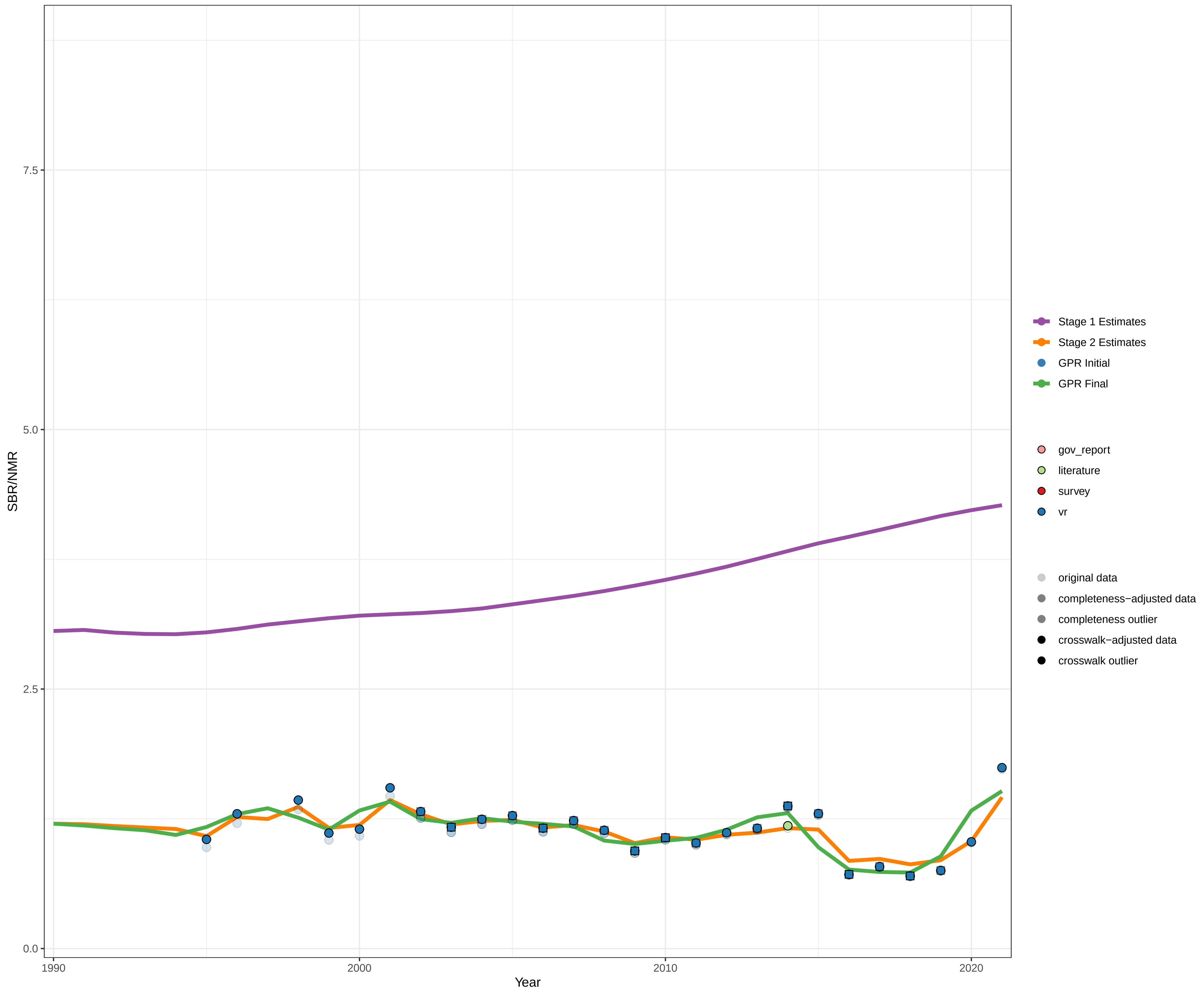

Definition	Years	Outlier	Source.Type	Title
28_weeks	1995, 1996, 1998−2001, 2012	0	VR	United Nations Demographic Yearbook
28_weeks	2002−2021	0	VR	Kuwait Annual Bulletin for Vital Statistics − Births and Deaths
28_weeks	2014	0	Sci Lit	Birth Outcomes in a Prospective Pregnancy−Birth Cohort Study of Environmental Risk Factors in Kuwait: The TRACER Study
28_weeks	2002, 2004, 2005, 2007, 2008, 2011, 2013, 2015, 2017, 2019	1	VR	United Nations Demographic Yearbook
28_weeks	2003, 2006, 2009, 2010, 2014, 2016, 2018	2	VR	United Nations Demographic Yearbook

Kuwait KWT North Africa and Middle East Stillbirth Rate (SBR) − 20 weeks
lambda = 0.2 ; zeta = 0.99 ; scale = 5 ; amp = 0.028

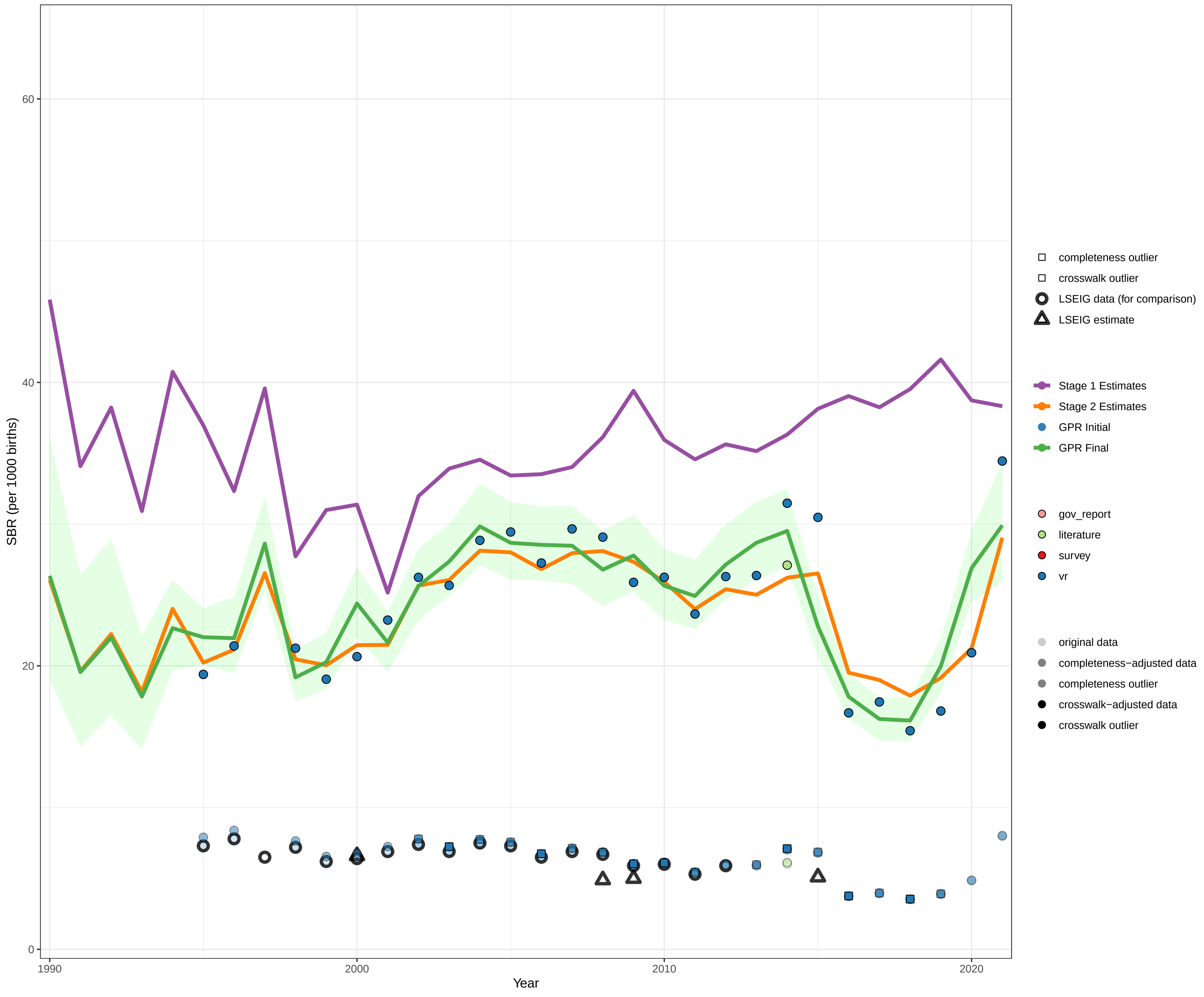

Kuwait KWT North Africa and Middle East Stillbirth Rate (SBR) − 28 weeks
lambda = 0.2 ; zeta = 0.99 ; scale = 5 ; amp = 0.028

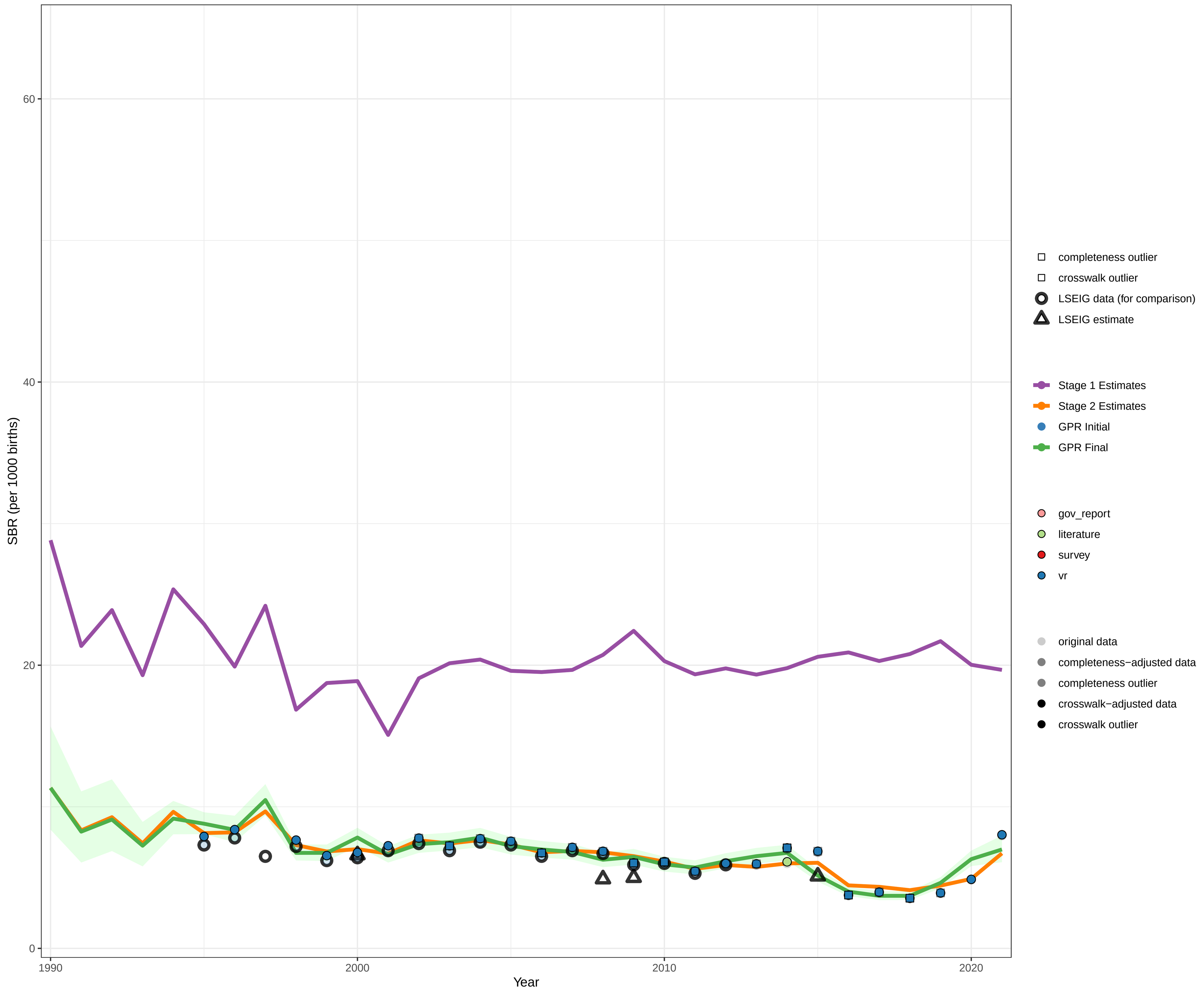

Year	Total Stillbirths (in thousands)	Total Stillbirths (in thousands)	SBR (per 1000 births)	SBR (per 1000 births)	SBR/NMR	SBR/NMR
	20 weeks	28 weeks	20 weeks	28 weeks	20 weeks	28 weeks
1990	0.9 (0.7 to 1.3)	0.4 (0.3 to 0.5)	26.3 (19.0 to 36.1)	11.3 (8.4 to 15.7)	2.80 (2.02 to 3.83)	1.20 (0.89 to 1.66)
1995	0.9 (0.9 to 1.0)	0.4 (0.3 to 0.4)	22.0 (20.1 to 24.1)	8.8 (8.1 to 9.6)	2.93 (2.67 to 3.20)	1.17 (1.07 to 1.28)
2000	1.0 (0.9 to 1.1)	0.3 (0.3 to 0.4)	24.4 (22.0 to 27.0)	7.8 (7.2 to 8.5)	4.15 (3.74 to 4.58)	1.33 (1.22 to 1.45)
2005	1.4 (1.3 to 1.6)	0.4 (0.3 to 0.4)	28.7 (26.1 to 31.6)	7.2 (6.6 to 7.9)	4.85 (4.41 to 5.34)	1.22 (1.12 to 1.33)
2010	1.5 (1.3 to 1.6)	0.3 (0.3 to 0.4)	25.7 (23.3 to 28.3)	5.9 (5.4 to 6.5)	4.49 (4.07 to 4.95)	1.04 (0.95 to 1.13)
2015	1.4 (1.2 to 1.5)	0.3 (0.3 to 0.3)	22.8 (20.6 to 25.0)	5.1 (4.7 to 5.6)	4.32 (3.91 to 4.73)	0.98 (0.89 to 1.06)
2020	1.4 (1.3 to 1.6)	0.3 (0.3 to 0.4)	26.9 (24.5 to 29.6)	6.3 (5.8 to 6.9)	5.67 (5.17 to 6.23)	1.33 (1.22 to 1.46)
2021	1.6 (1.4 to 1.8)	0.4 (0.3 to 0.4)	29.9 (25.9 to 34.4)	7.0 (6.1 to 8.0)	6.50 (5.63 to 7.46)	1.52 (1.32 to 1.73)

Lebanon LBN North Africa and Middle East Stillbirth Rate / Neonatal Mortality Rate (SBR/NMR) − 20 weeks
lambda = 0.6 ; zeta = 0.8 ; scale = 15 ; amp = 0.028

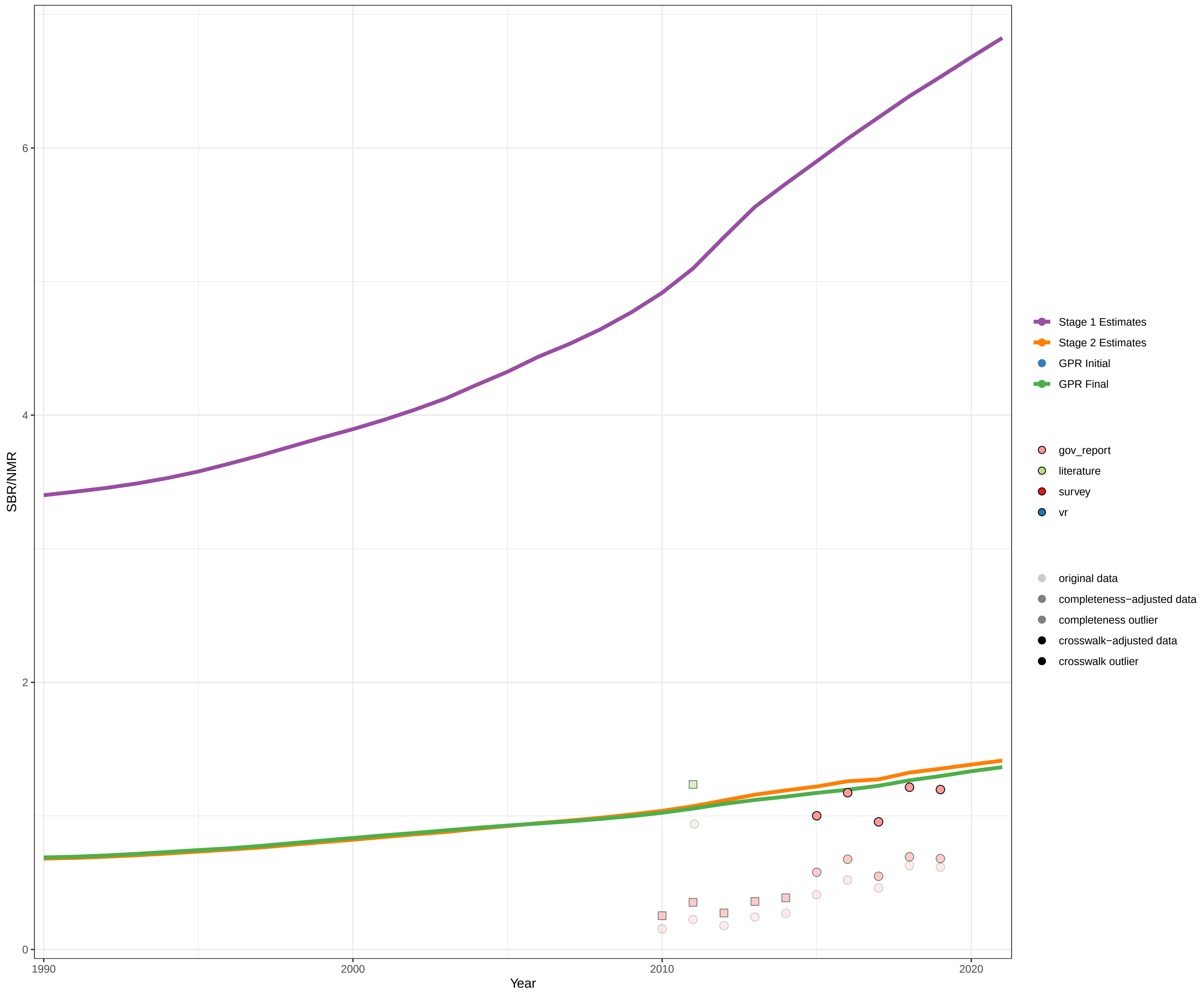

Lebanon LBN North Africa and Middle East Stillbirth Rate / Neonatal Mortality Rate (SBR/NMR) − 28 weeks
lambda = 0.6 ; zeta = 0.8 ; scale = 15 ; amp = 0.028

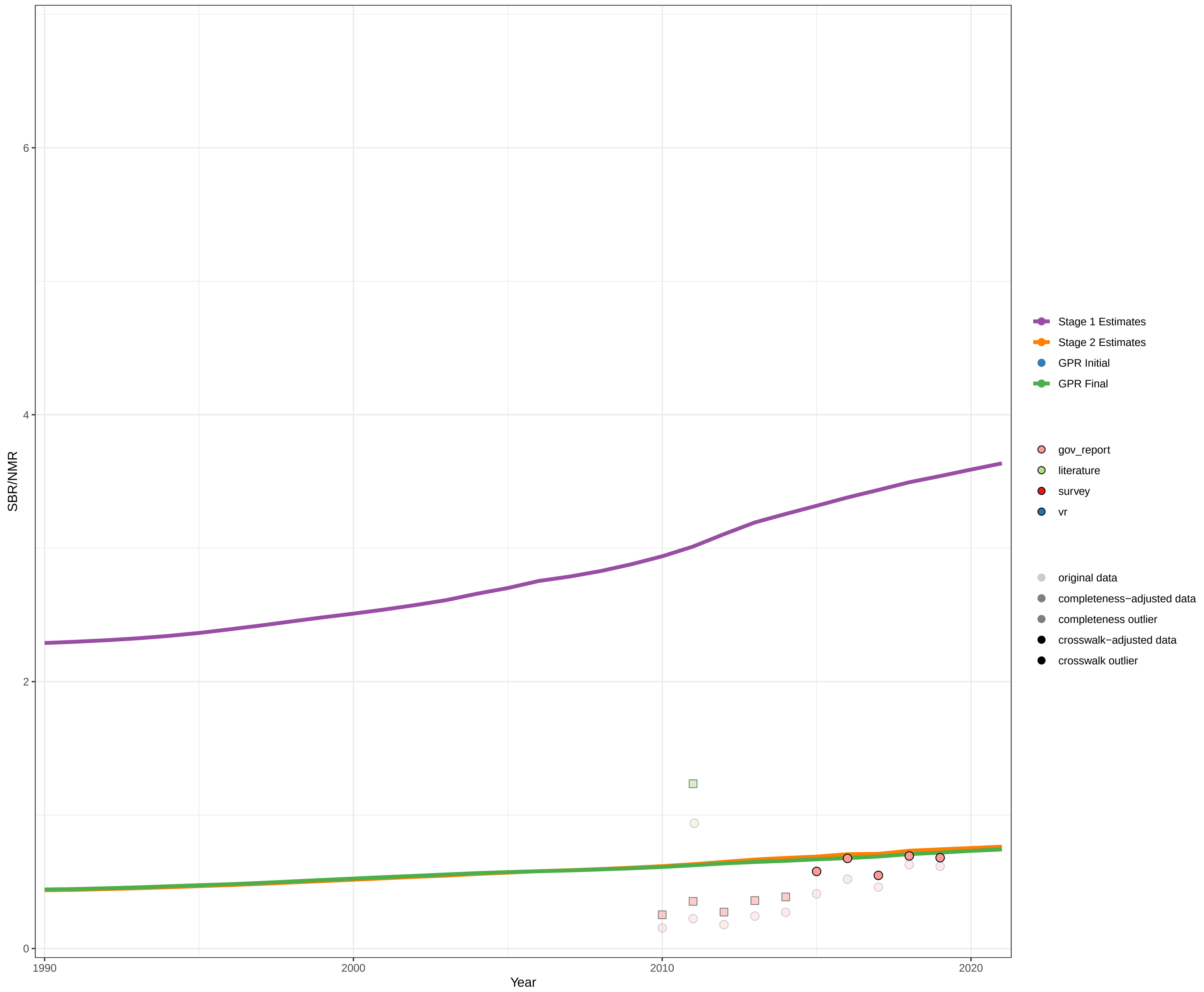

Definition	Years	Outlier	Source.Type	Title
28_weeks	2015−2019	0	Statistical Report	Lebanon Statistical Bulletin
28_weeks	2010−2014	1	Statistical Report	Lebanon Statistical Bulletin
28_weeks_OR_1000_grams	2011	1	Sci Lit	Maternal complications and perinatal mortality: findings of the World Health Organization Multicountry Survey on Maternal and Newborn Health

Lebanon LBN North Africa and Middle East Stillbirth Rate (SBR) − 20 weeks
lambda = 0.6 ; zeta = 0.8 ; scale = 15 ; amp = 0.028

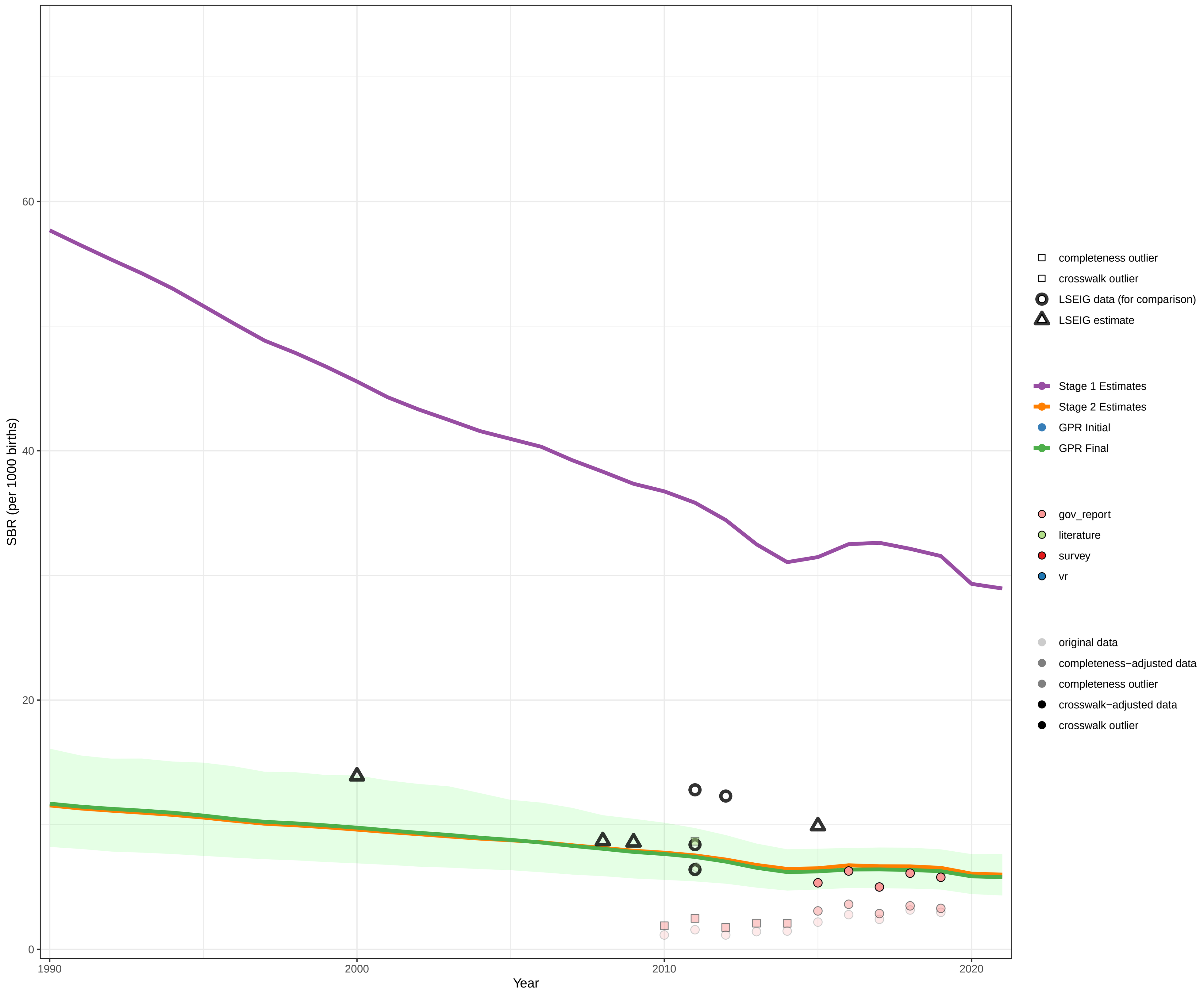

Lebanon LBN North Africa and Middle East Stillbirth Rate (SBR) − 28 weeks
lambda = 0.6 ; zeta = 0.8 ; scale = 15 ; amp = 0.028

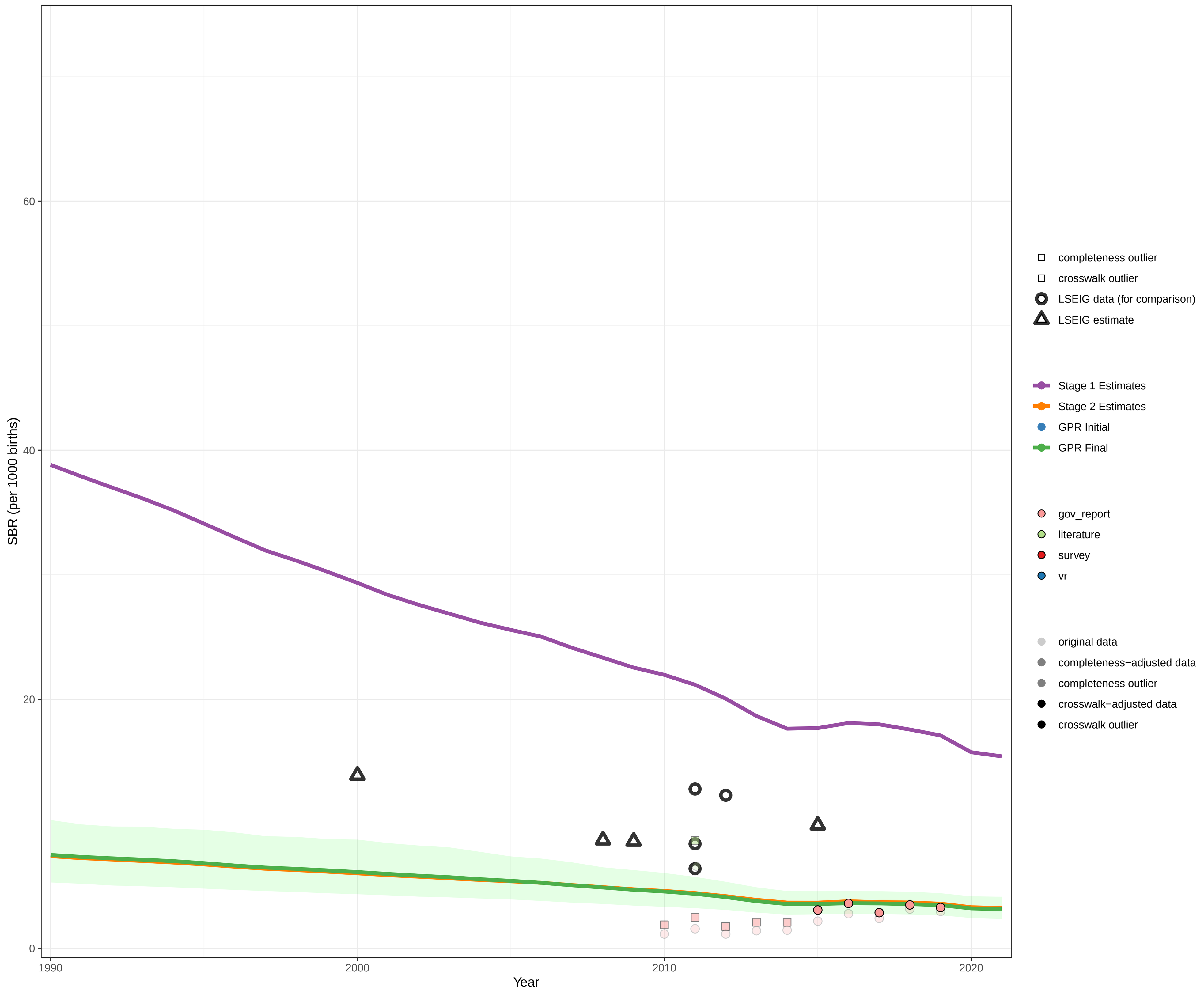

Year	Total Stillbirths (in thousands)	Total Stillbirths (in thousands)	SBR (per 1000 births)	SBR (per 1000 births)	SBR/NMR	SBR/NMR
	20 weeks	28 weeks	20 weeks	28 weeks	20 weeks	28 weeks
1990	1.0 (0.7 to 1.4)	0.7 (0.5 to 0.9)	11.7 (8.2 to 16.1)	7.5 (5.3 to 10.3)	0.69 (0.48 to 0.95)	0.44 (0.31 to 0.61)
1995	1.0 (0.7 to 1.4)	0.6 (0.4 to 0.9)	10.7 (7.5 to 15.0)	6.8 (4.8 to 9.5)	0.74 (0.52 to 1.04)	0.47 (0.33 to 0.66)
2000	0.9 (0.6 to 1.3)	0.6 (0.4 to 0.8)	9.8 (6.9 to 14.0)	6.1 (4.3 to 8.7)	0.83 (0.59 to 1.19)	0.52 (0.37 to 0.75)
2005	0.8 (0.6 to 1.1)	0.5 (0.4 to 0.7)	8.8 (6.3 to 12.0)	5.4 (3.9 to 7.4)	0.93 (0.67 to 1.27)	0.57 (0.42 to 0.78)
2010	0.7 (0.5 to 0.9)	0.4 (0.3 to 0.5)	7.6 (5.6 to 10.2)	4.6 (3.3 to 6.1)	1.02 (0.75 to 1.36)	0.61 (0.45 to 0.81)
2015	0.7 (0.5 to 0.9)	0.4 (0.3 to 0.5)	6.3 (4.8 to 8.1)	3.6 (2.7 to 4.6)	1.17 (0.90 to 1.51)	0.67 (0.51 to 0.86)
2020	0.5 (0.4 to 0.7)	0.3 (0.2 to 0.4)	5.9 (4.4 to 7.6)	3.2 (2.4 to 4.2)	1.33 (1.01 to 1.74)	0.73 (0.56 to 0.95)
2021	0.5 (0.4 to 0.6)	0.3 (0.2 to 0.3)	5.8 (4.3 to 7.6)	3.1 (2.4 to 4.2)	1.36 (1.02 to 1.80)	0.74 (0.56 to 0.98)

Libya LBY North Africa and Middle East Stillbirth Rate / Neonatal Mortality Rate (SBR/NMR) − 20 weeks
lambda = 1 ; zeta = 0.6 ; scale = 15 ; amp = 0.028

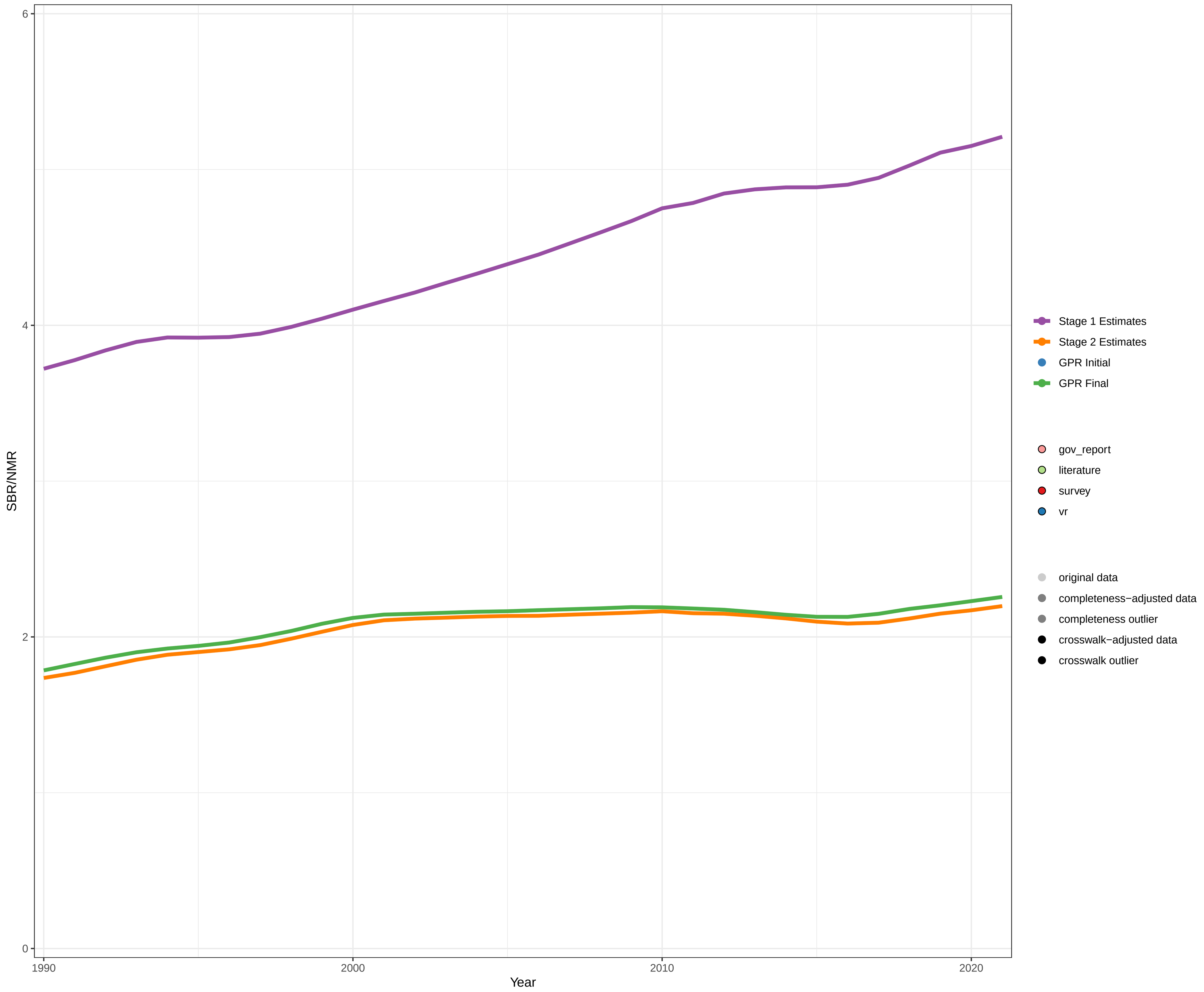

Libya LBY North Africa and Middle East Stillbirth Rate / Neonatal Mortality Rate (SBR/NMR) − 28 weeks
lambda = 1 ; zeta = 0.6 ; scale = 15 ; amp = 0.028

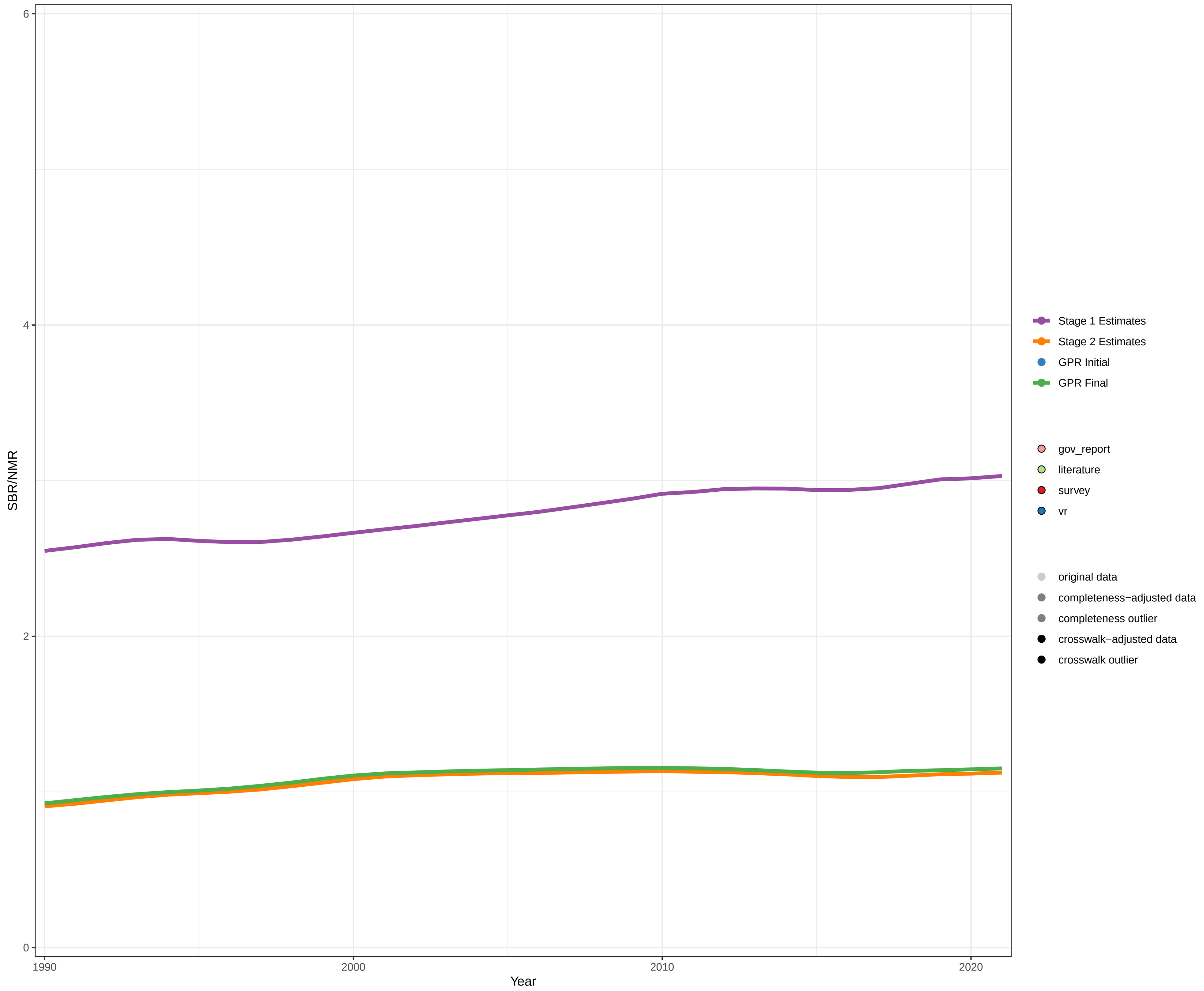

Definition	Years	Outlier	Source.Type	Title

Libya LBY North Africa and Middle East Stillbirth Rate (SBR) − 20 weeks
lambda = 1 ; zeta = 0.6 ; scale = 15 ; amp = 0.028

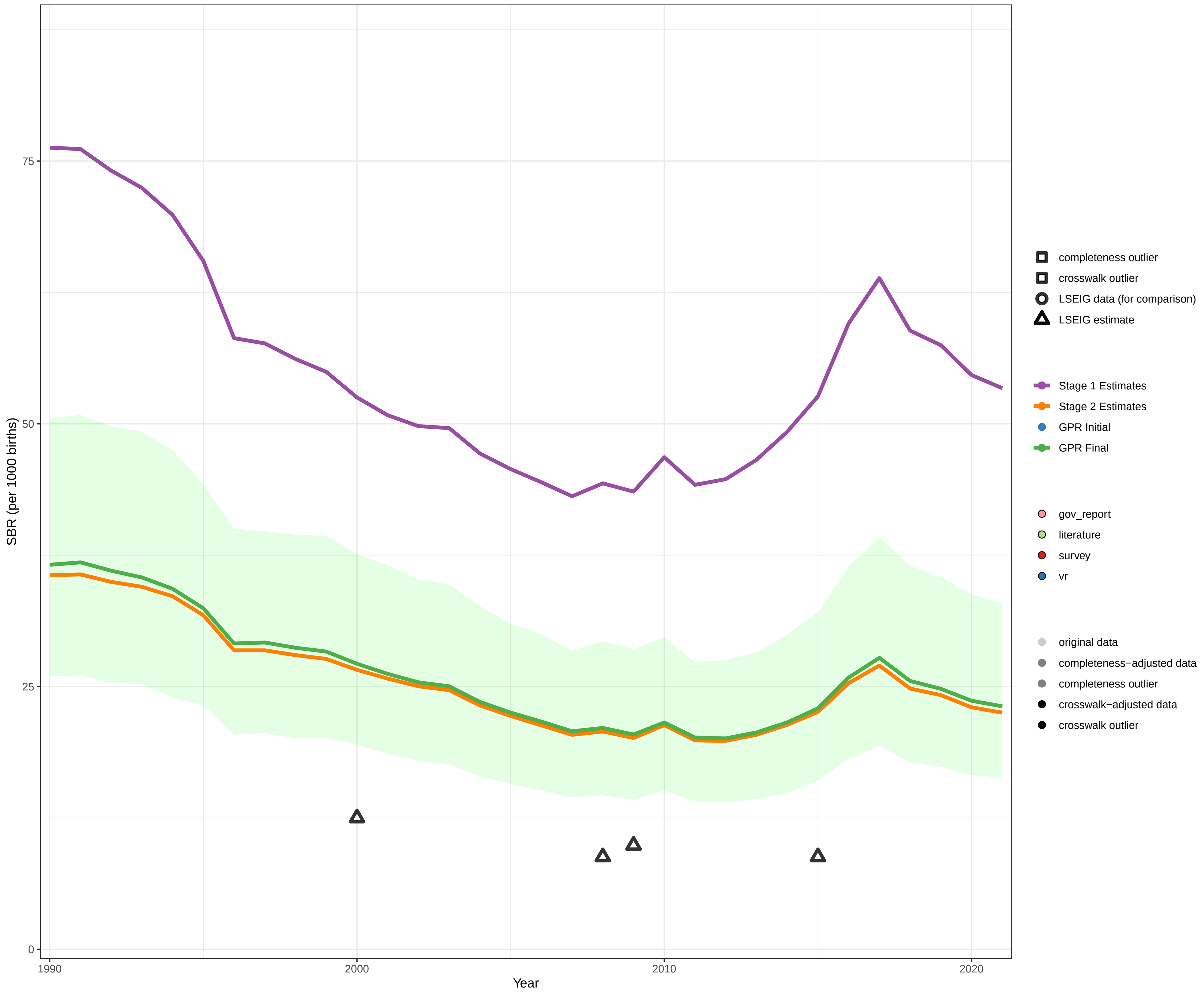

Libya LBY North Africa and Middle East Stillbirth Rate (SBR) − 28 weeks
lambda = 1 ; zeta = 0.6 ; scale = 15 ; amp = 0.028

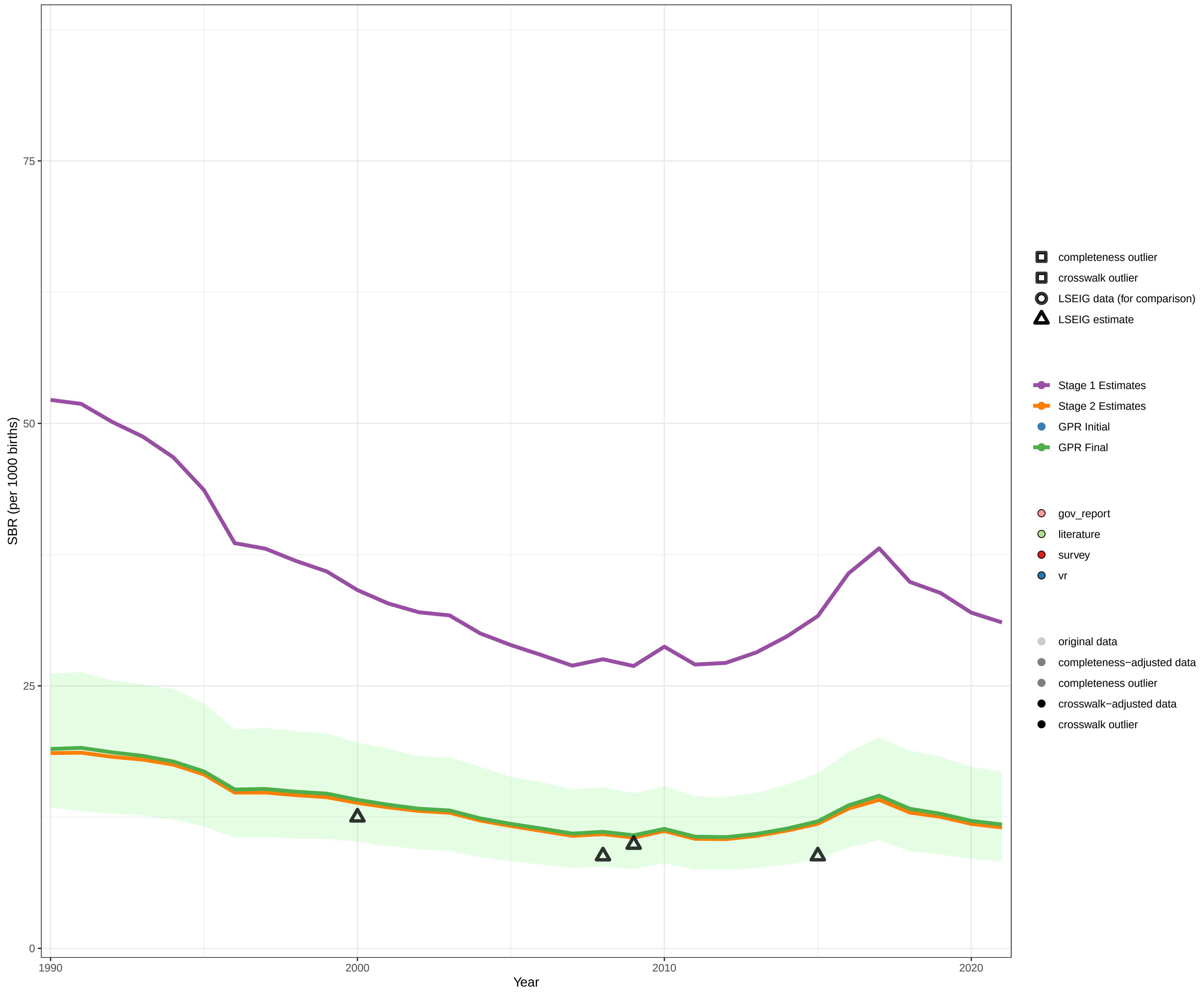

Year	Total Stillbirths (in thousands)	Total Stillbirths (in thousands)	SBR (per 1000 births)	SBR (per 1000 births)	SBR/NMR	SBR/NMR
	20 weeks	28 weeks	20 weeks	28 weeks	20 weeks	28 weeks
1990	5.1 (3.6 to 7.1)	2.6 (1.8 to 3.6)	36.6 (26.0 to 50.5)	19.0 (13.4 to 26.2)	1.79 (1.27 to 2.46)	0.93 (0.65 to 1.28)
1995	4.2 (3.0 to 5.8)	2.2 (1.5 to 3.0)	32.4 (23.2 to 44.2)	16.9 (11.6 to 23.4)	1.94 (1.39 to 2.65)	1.01 (0.70 to 1.40)
2000	3.5 (2.5 to 4.8)	1.8 (1.3 to 2.5)	27.2 (19.5 to 37.6)	14.2 (10.2 to 19.6)	2.12 (1.52 to 2.94)	1.11 (0.79 to 1.53)
2005	2.8 (1.9 to 3.9)	1.5 (1.0 to 2.0)	22.5 (15.7 to 31.0)	11.9 (8.3 to 16.3)	2.16 (1.51 to 2.98)	1.14 (0.80 to 1.57)
2010	2.5 (1.7 to 3.4)	1.3 (0.9 to 1.8)	21.6 (15.1 to 29.7)	11.4 (8.1 to 15.4)	2.19 (1.53 to 3.01)	1.15 (0.82 to 1.57)
2015	2.3 (1.6 to 3.2)	1.2 (0.8 to 1.6)	22.9 (16.0 to 32.1)	12.1 (8.5 to 16.7)	2.13 (1.49 to 2.98)	1.12 (0.79 to 1.55)
2020	2.0 (1.4 to 2.9)	1.0 (0.7 to 1.4)	23.6 (16.6 to 33.8)	12.1 (8.5 to 17.3)	2.23 (1.56 to 3.19)	1.14 (0.80 to 1.63)
2021	1.9 (1.3 to 2.7)	0.9 (0.7 to 1.4)	23.1 (16.3 to 32.9)	11.8 (8.3 to 16.8)	2.26 (1.59 to 3.21)	1.15 (0.81 to 1.64)

Morocco MAR North Africa and Middle East Stillbirth Rate / Neonatal Mortality Rate (SBR/NMR) − 20 weeks
lambda = 0.4 ; zeta = 0.9 ; scale = 10 ; amp = 0.028

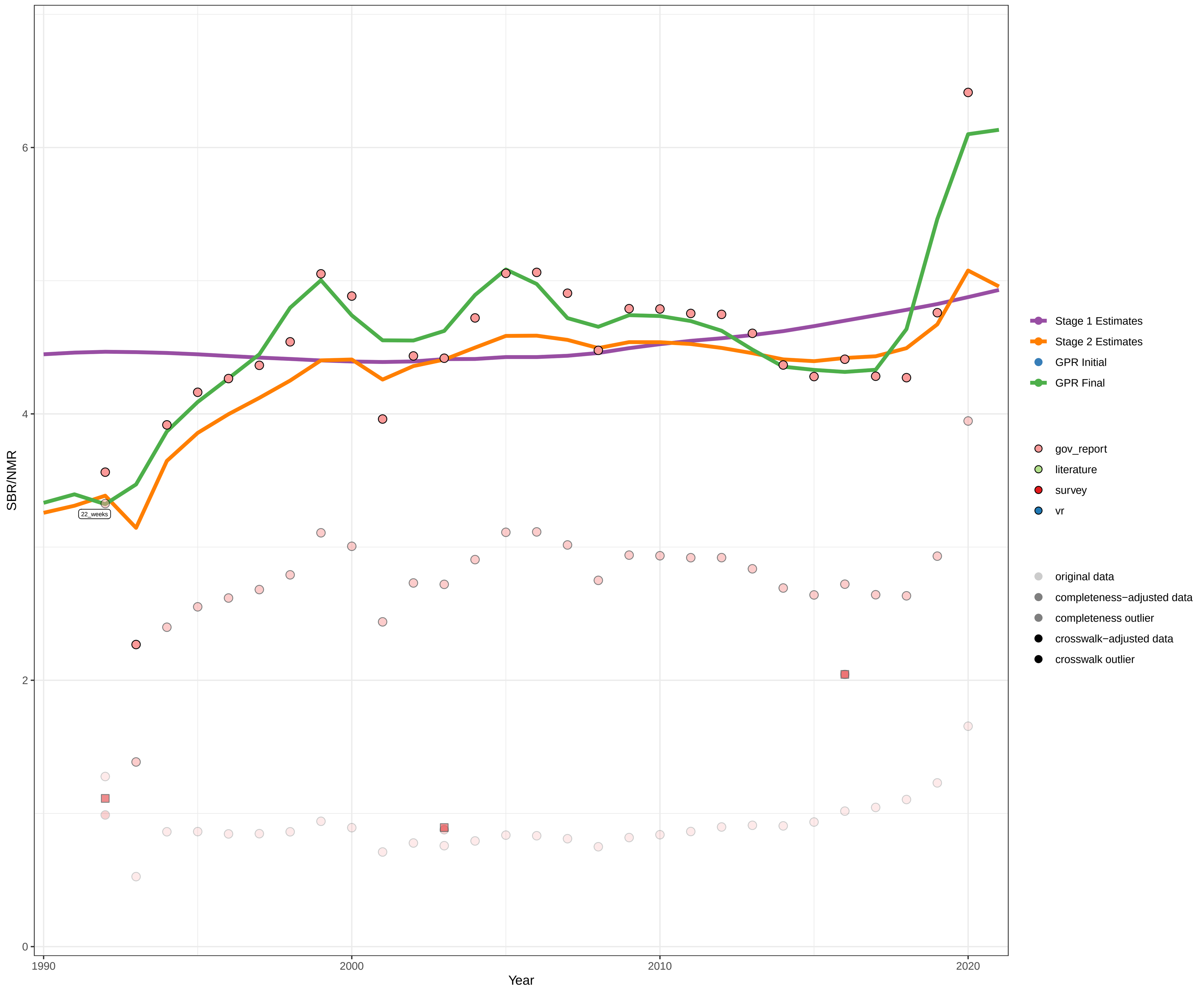

Morocco MAR North Africa and Middle East Stillbirth Rate / Neonatal Mortality Rate (SBR/NMR) − 28 weeks
lambda = 0.4 ; zeta = 0.9 ; scale = 10 ; amp = 0.028

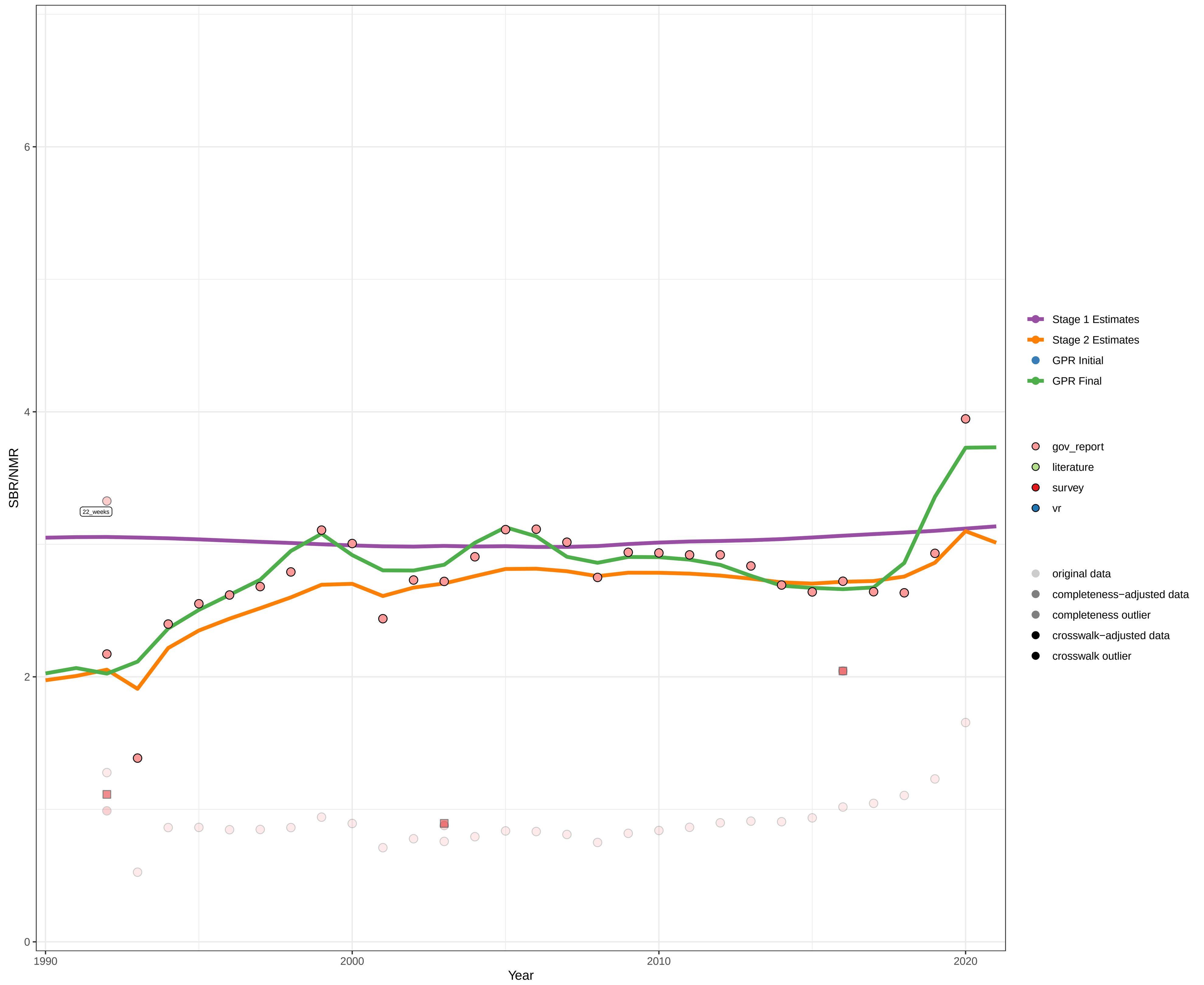

Definition	Years	Outlier	Source.Type	Title
22_weeks	1992	0	Statistical Report	Morocco Health in Figures
28_weeks	1993−2020	0	Statistical Report	Morocco Health in Figures
28_weeks	1992	1	Survey	Morocco Demographic and Health Survey 1992
28_weeks	2003	1	Survey	Morocco Demographic and Health Survey 2003−2004
28_weeks	2016	1	Survey	Morocco National Survey on Population and Family Health 2017−2018

Morocco MAR North Africa and Middle East Stillbirth Rate (SBR) − 20 weeks
lambda = 0.4 ; zeta = 0.9 ; scale = 10 ; amp = 0.028

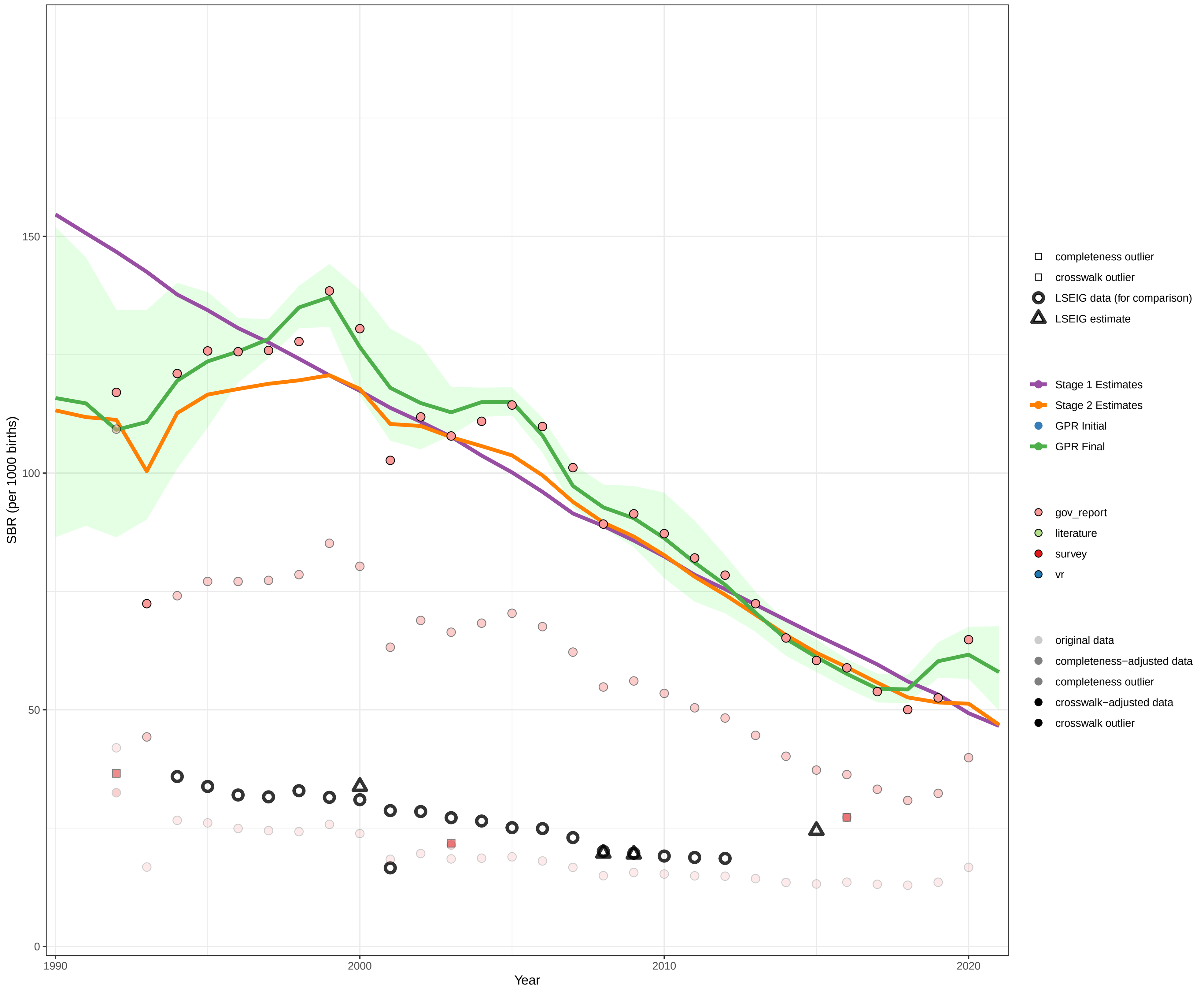

Morocco MAR North Africa and Middle East Stillbirth Rate (SBR) − 28 weeks
lambda = 0.4 ; zeta = 0.9 ; scale = 10 ; amp = 0.028

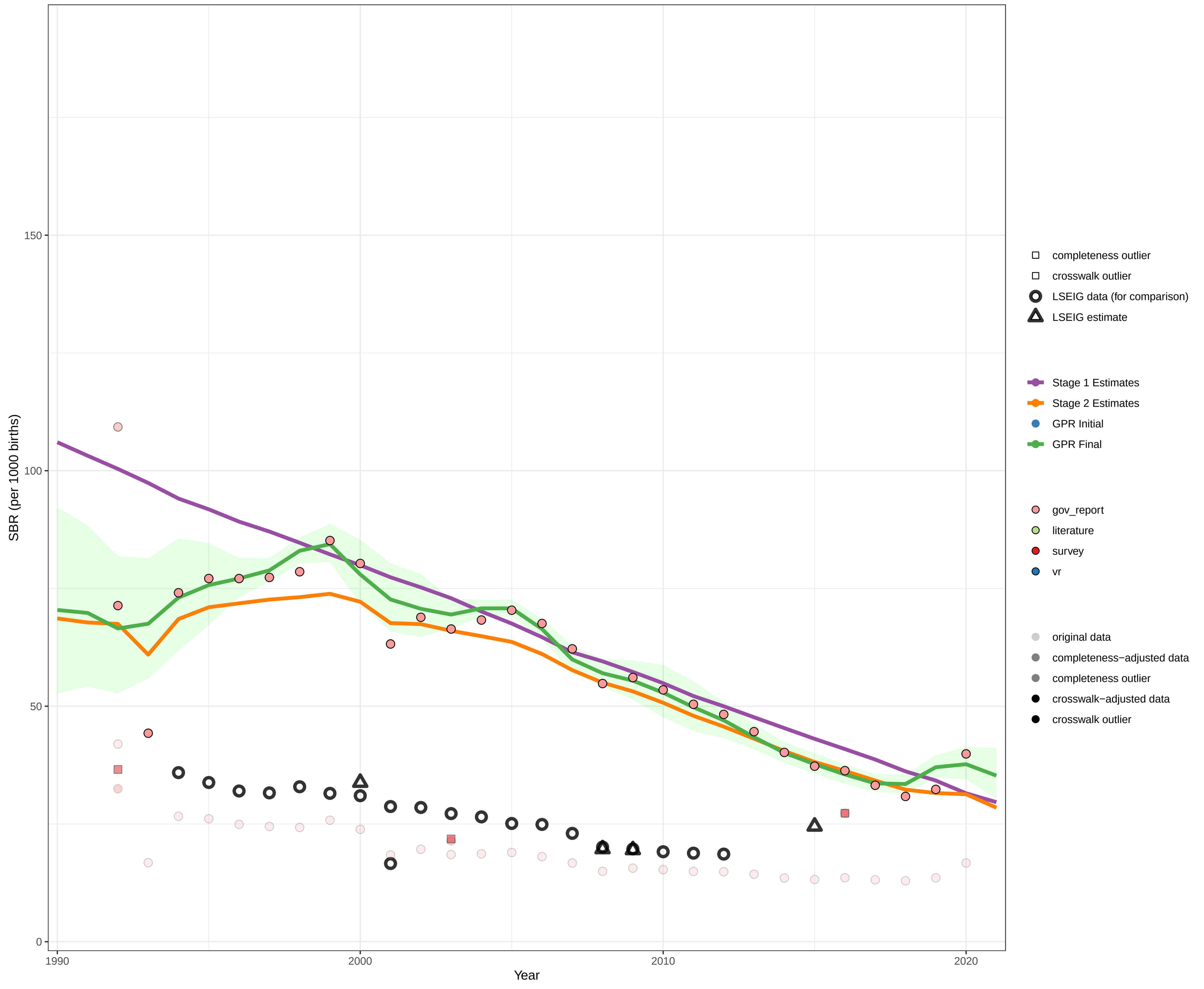

Year	Total Stillbirths (in thousands)	Total Stillbirths (in thousands)	SBR (per 1000 births)	SBR (per 1000 births)	SBR/NMR	SBR/NMR
	20 weeks	28 weeks	20 weeks	28 weeks	20 weeks	28 weeks
1990	105.6 (76.1 to 144.0)	61.0 (44.7 to 81.6)	115.9 (86.5 to 152.0)	70.4 (52.7 to 92.2)	3.33 (2.49 to 4.37)	2.03 (1.52 to 2.65)
1995	115.2 (100.5 to 131.0)	66.9 (58.8 to 75.5)	123.6 (109.6 to 138.2)	75.7 (67.2 to 84.6)	4.09 (3.63 to 4.57)	2.50 (2.22 to 2.80)
2000	121.1 (109.9 to 134.5)	70.6 (64.5 to 77.9)	126.6 (116.3 to 138.7)	78.0 (71.7 to 85.4)	4.74 (4.35 to 5.19)	2.92 (2.68 to 3.19)
2005	111.2 (108.1 to 114.6)	65.1 (63.4 to 67.0)	115.0 (112.2 to 118.1)	70.8 (69.0 to 72.6)	5.08 (4.96 to 5.22)	3.13 (3.05 to 3.21)
2010	79.2 (70.7 to 88.9)	46.8 (42.0 to 52.4)	86.2 (77.8 to 95.9)	52.9 (47.7 to 58.8)	4.73 (4.27 to 5.26)	2.90 (2.62 to 3.23)
2015	50.5 (47.7 to 53.8)	30.4 (28.7 to 32.3)	61.1 (57.9 to 64.8)	37.7 (35.7 to 40.0)	4.33 (4.11 to 4.59)	2.67 (2.53 to 2.83)
2020	43.8 (40.0 to 48.3)	26.1 (23.8 to 28.8)	61.6 (56.5 to 67.5)	37.7 (34.5 to 41.3)	6.10 (5.59 to 6.68)	3.73 (3.42 to 4.09)
2021	39.8 (33.9 to 46.9)	23.6 (20.2 to 27.8)	58.0 (49.8 to 67.6)	35.3 (30.3 to 41.2)	6.13 (5.27 to 7.16)	3.73 (3.21 to 4.36)

Oman OMN North Africa and Middle East Stillbirth Rate / Neonatal Mortality Rate (SBR/NMR) − 20 weeks
lambda = 0.4 ; zeta = 0.9 ; scale = 10 ; amp = 0.028

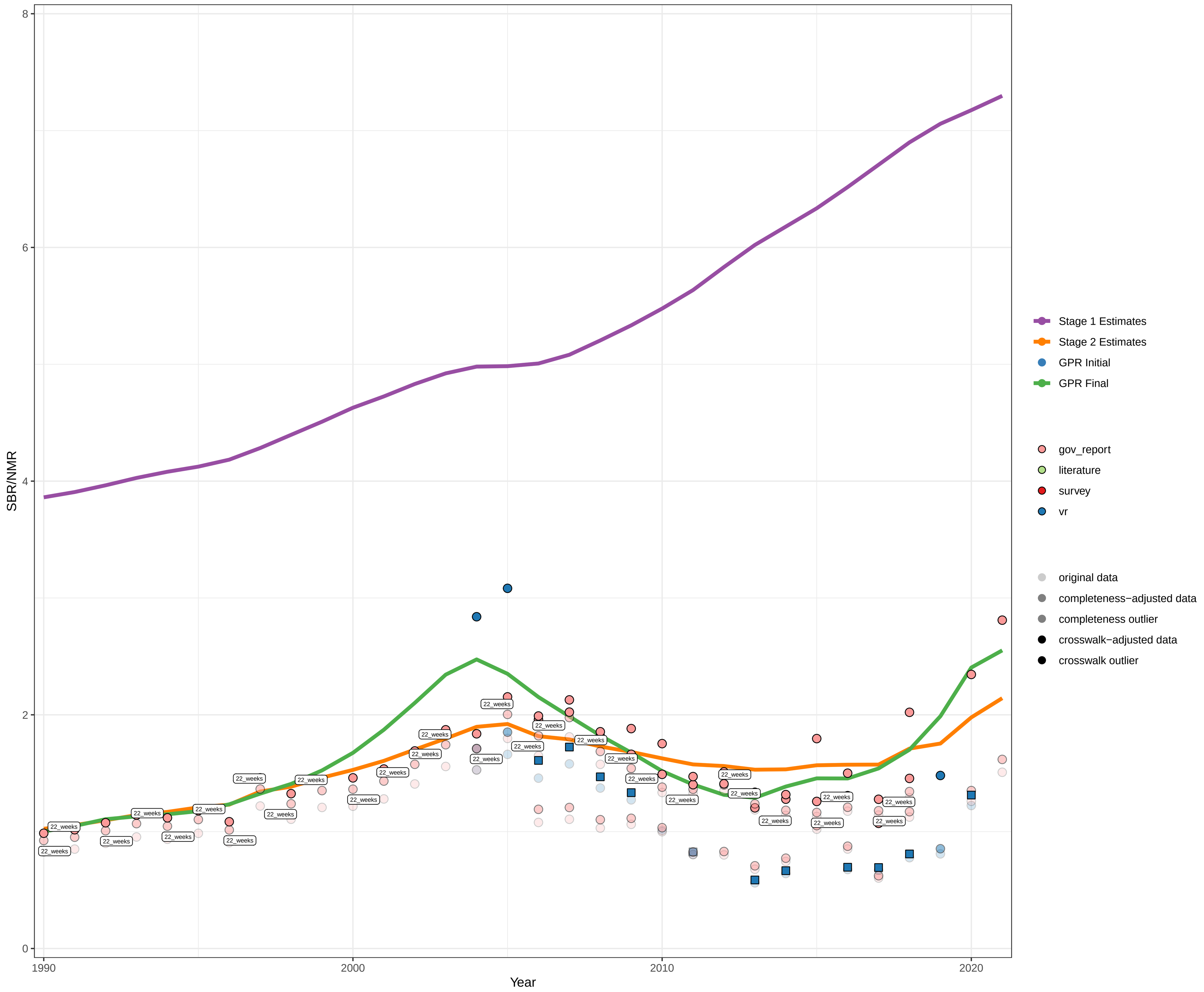

Oman OMN North Africa and Middle East Stillbirth Rate / Neonatal Mortality Rate (SBR/NMR) − 28 weeks
lambda = 0.4 ; zeta = 0.9 ; scale = 10 ; amp = 0.028

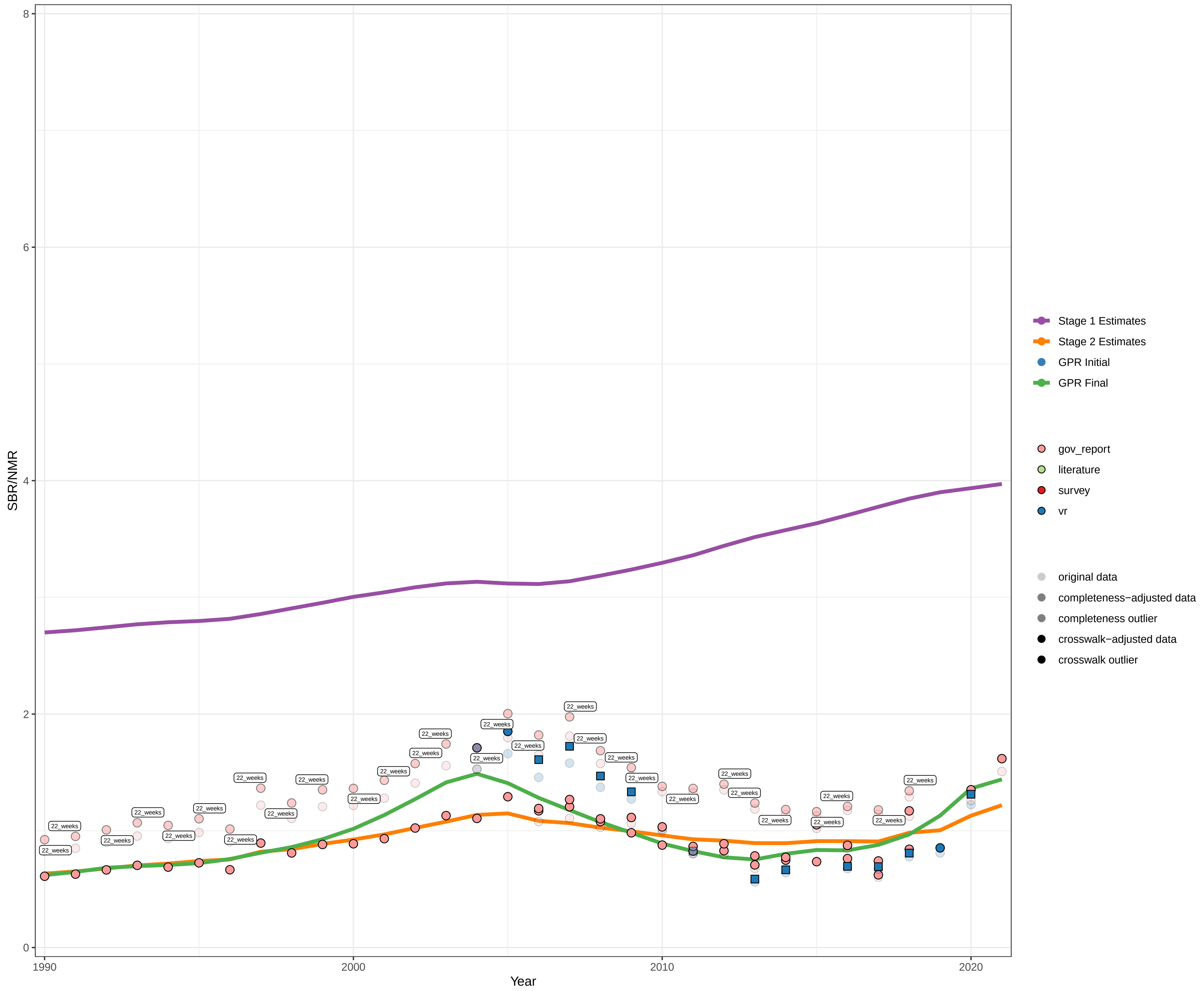

Definition	Years	Outlier	Source.Type	Title
22_weeks	1990−1996, 1999−2018	0	Statistical Report	Oman Annual Health Report
22_weeks	1997, 1998	0	Statistical Report	Oman Annual Statistical Report
28_weeks	2004, 2005, 2010, 2019	0	VR	United Nations Demographic Yearbook
28_weeks	2006−2018, 2020, 2021	0	Statistical Report	Oman Birth and Death Facts
28_weeks	2011	1	VR	United Nations Demographic Yearbook
28_weeks	2006−2009, 2013, 2014, 2016−2018, 2020	2	VR	United Nations Demographic Yearbook

Oman OMN North Africa and Middle East Stillbirth Rate (SBR) − 20 weeks
lambda = 0.4 ; zeta = 0.9 ; scale = 10 ; amp = 0.028

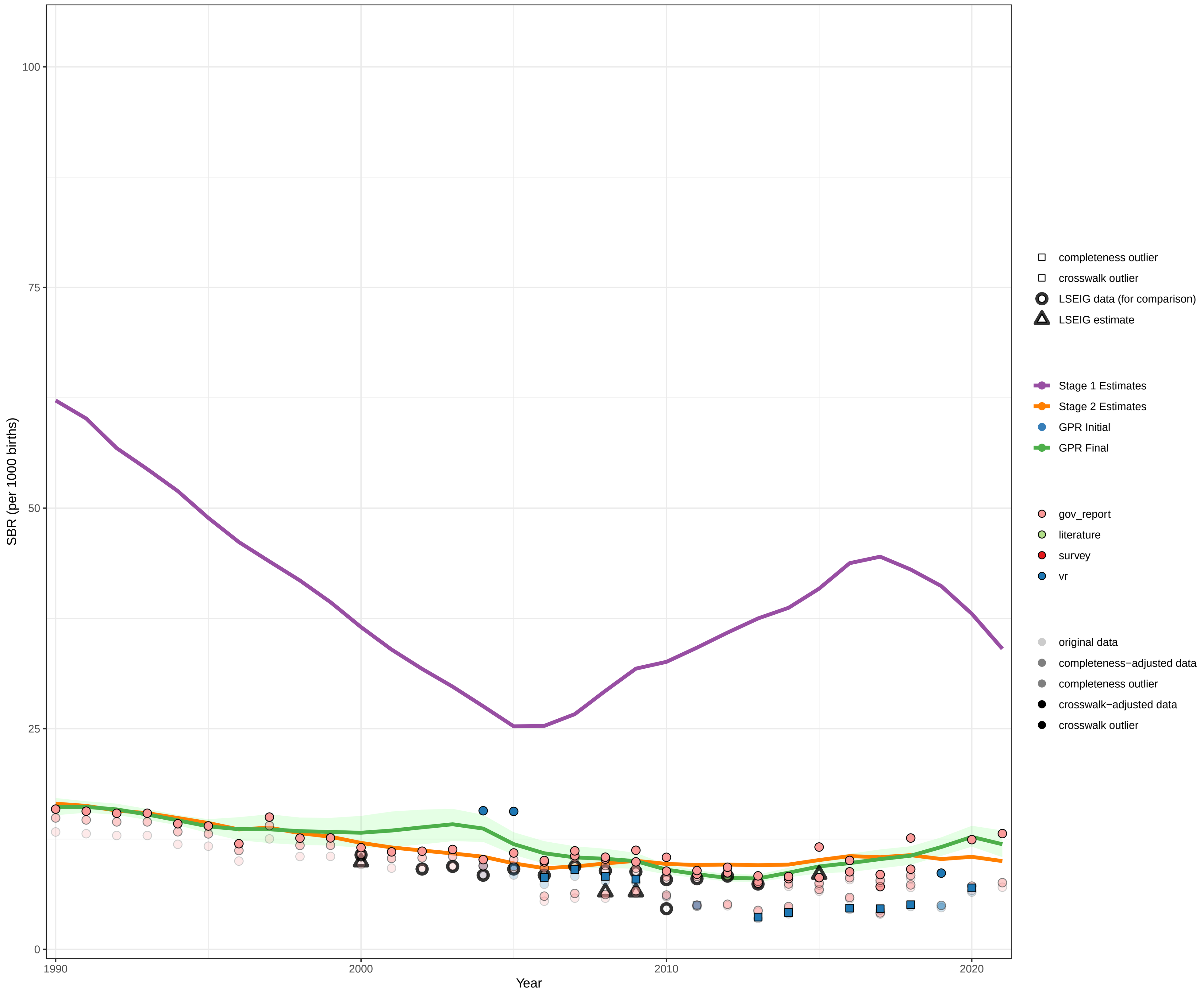

Oman OMN North Africa and Middle East Stillbirth Rate (SBR) − 28 weeks
lambda = 0.4 ; zeta = 0.9 ; scale = 10 ; amp = 0.028

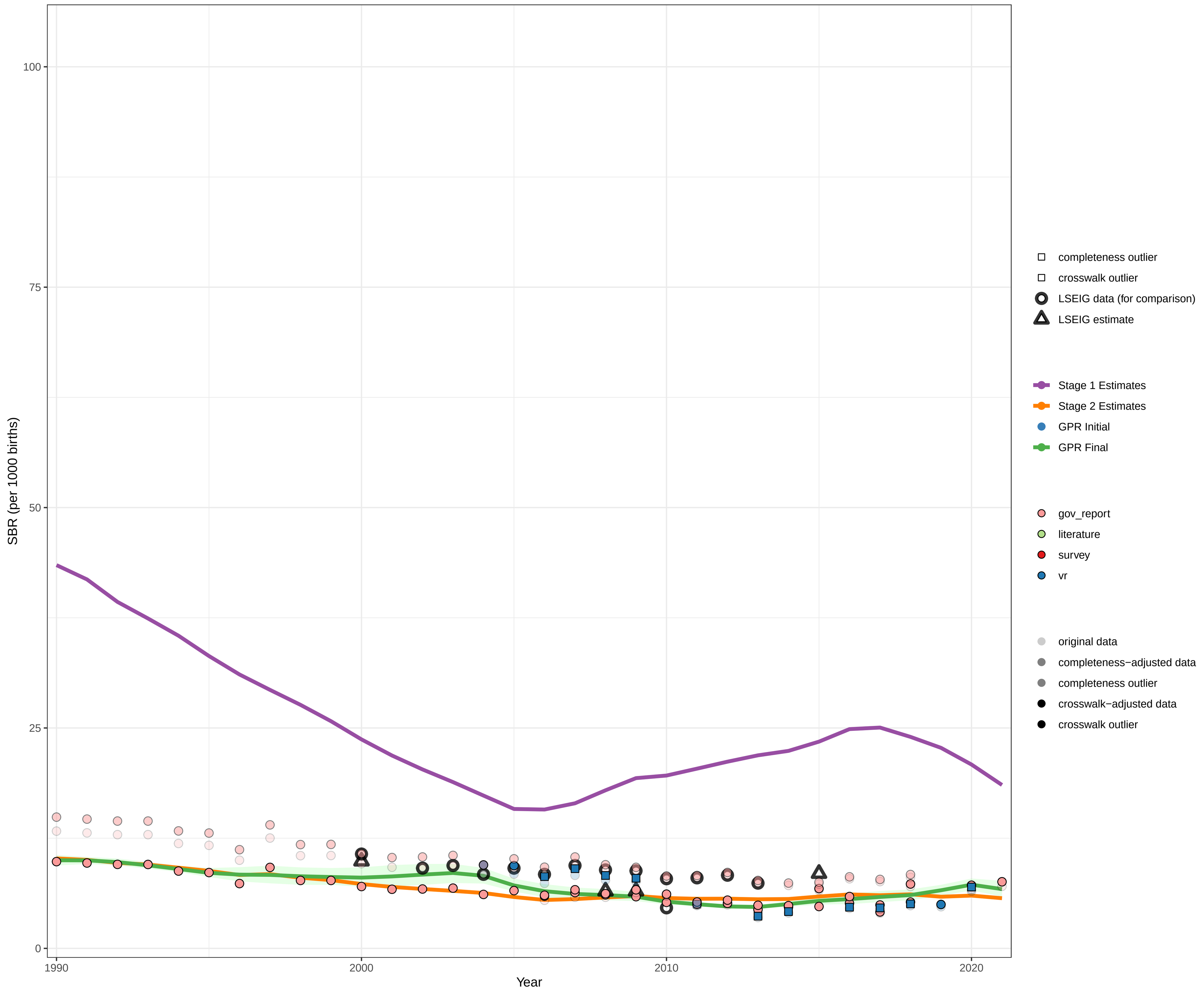

Year	Total Stillbirths (in thousands)	Total Stillbirths (in thousands)	SBR (per 1000 births)	SBR (per 1000 births)	SBR/NMR	SBR/NMR
	20 weeks	28 weeks	20 weeks	28 weeks	20 weeks	28 weeks
1990	1.2 (1.1 to 1.3)	0.7 (0.7 to 0.8)	16.1 (15.2 to 17.1)	10.0 (9.4 to 10.6)	1.00 (0.94 to 1.06)	0.62 (0.58 to 0.66)
1995	1.0 (0.9 to 1.0)	0.6 (0.6 to 0.6)	13.9 (13.2 to 14.7)	8.6 (8.1 to 9.1)	1.18 (1.11 to 1.24)	0.72 (0.68 to 0.77)
2000	0.8 (0.7 to 0.9)	0.5 (0.4 to 0.5)	13.2 (11.5 to 15.1)	8.0 (7.0 to 9.2)	1.67 (1.46 to 1.92)	1.02 (0.89 to 1.16)
2005	0.7 (0.6 to 0.7)	0.4 (0.4 to 0.4)	11.9 (10.7 to 13.3)	7.1 (6.5 to 7.9)	2.35 (2.11 to 2.62)	1.41 (1.27 to 1.56)
2010	0.6 (0.6 to 0.6)	0.4 (0.3 to 0.4)	9.0 (8.5 to 9.6)	5.3 (5.0 to 5.6)	1.52 (1.43 to 1.62)	0.89 (0.84 to 0.95)
2015	0.8 (0.7 to 0.8)	0.4 (0.4 to 0.5)	9.4 (8.6 to 10.3)	5.4 (5.0 to 5.9)	1.46 (1.34 to 1.59)	0.84 (0.77 to 0.91)
2020	1.1 (1.0 to 1.2)	0.6 (0.5 to 0.7)	12.7 (11.6 to 14.0)	7.2 (6.6 to 7.9)	2.40 (2.20 to 2.64)	1.36 (1.24 to 1.50)
2021	1.0 (0.9 to 1.1)	0.6 (0.5 to 0.6)	11.9 (10.4 to 13.5)	6.7 (5.9 to 7.7)	2.55 (2.24 to 2.89)	1.44 (1.26 to 1.64)

Palestine PSE North Africa and Middle East Stillbirth Rate / Neonatal Mortality Rate (SBR/NMR) − 20 weeks
lambda = 0.4 ; zeta = 0.9 ; scale = 10 ; amp = 0.028

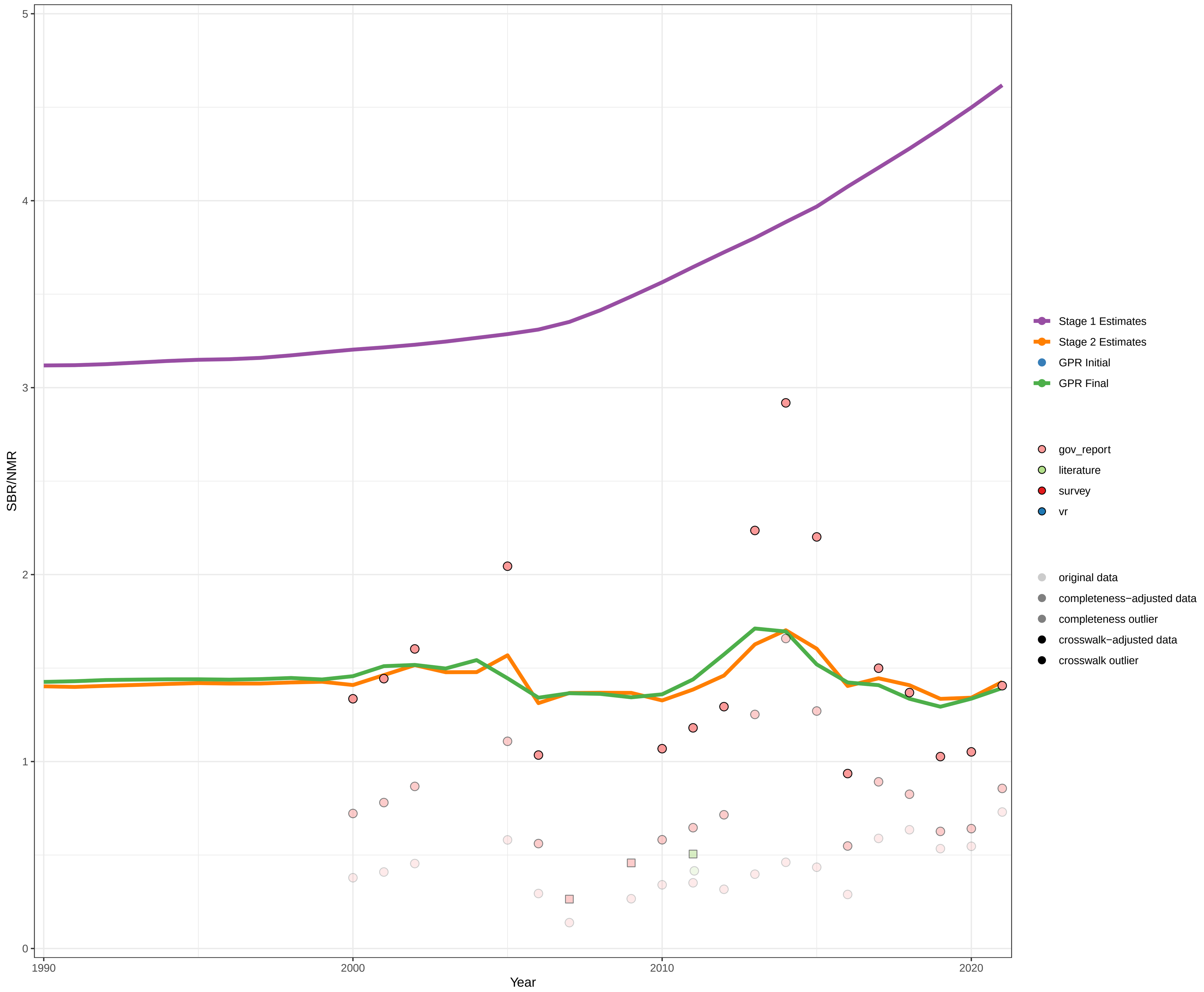

Palestine PSE North Africa and Middle East Stillbirth Rate / Neonatal Mortality Rate (SBR/NMR) − 28 weeks
lambda = 0.4 ; zeta = 0.9 ; scale = 10 ; amp = 0.028

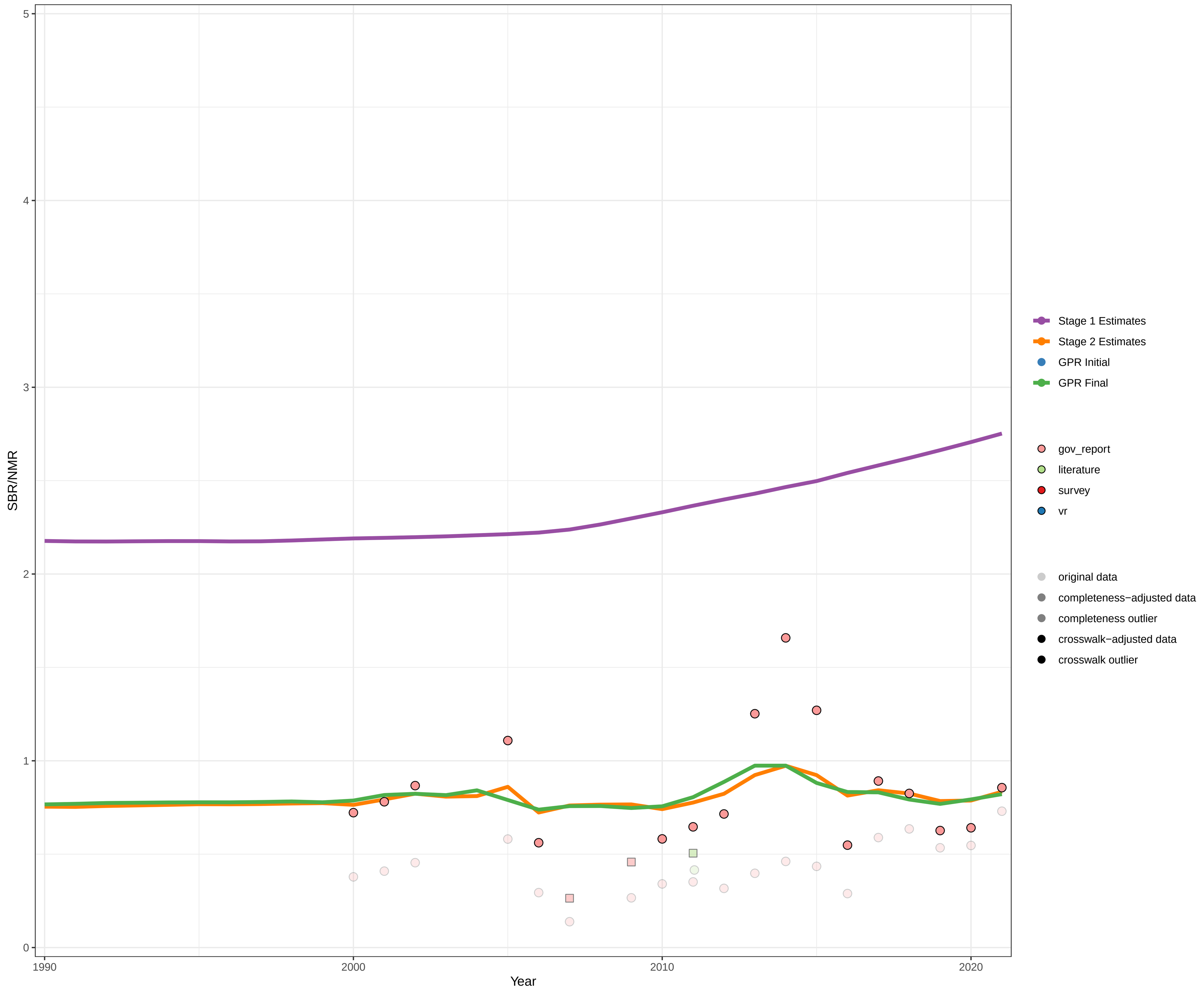

Definition	Years	Outlier	Source.Type	Title
28_weeks	2000−2002, 2005, 2006, 2010−2019	0	Statistical Report	Palestine Health Status Annual Report
28_weeks	2020, 2021	0	Statistical Report	Palestine Health Annual Report
28_weeks	2007, 2009	1	Statistical Report	Palestine Health Status Annual Report
28_weeks_OR_1000_grams	2011	1	Sci Lit	Maternal complications and perinatal mortality: findings of the World Health Organization Multicountry Survey on Maternal and Newborn Health

Palestine PSE North Africa and Middle East Stillbirth Rate (SBR) − 20 weeks
lambda = 0.4 ; zeta = 0.9 ; scale = 10 ; amp = 0.028

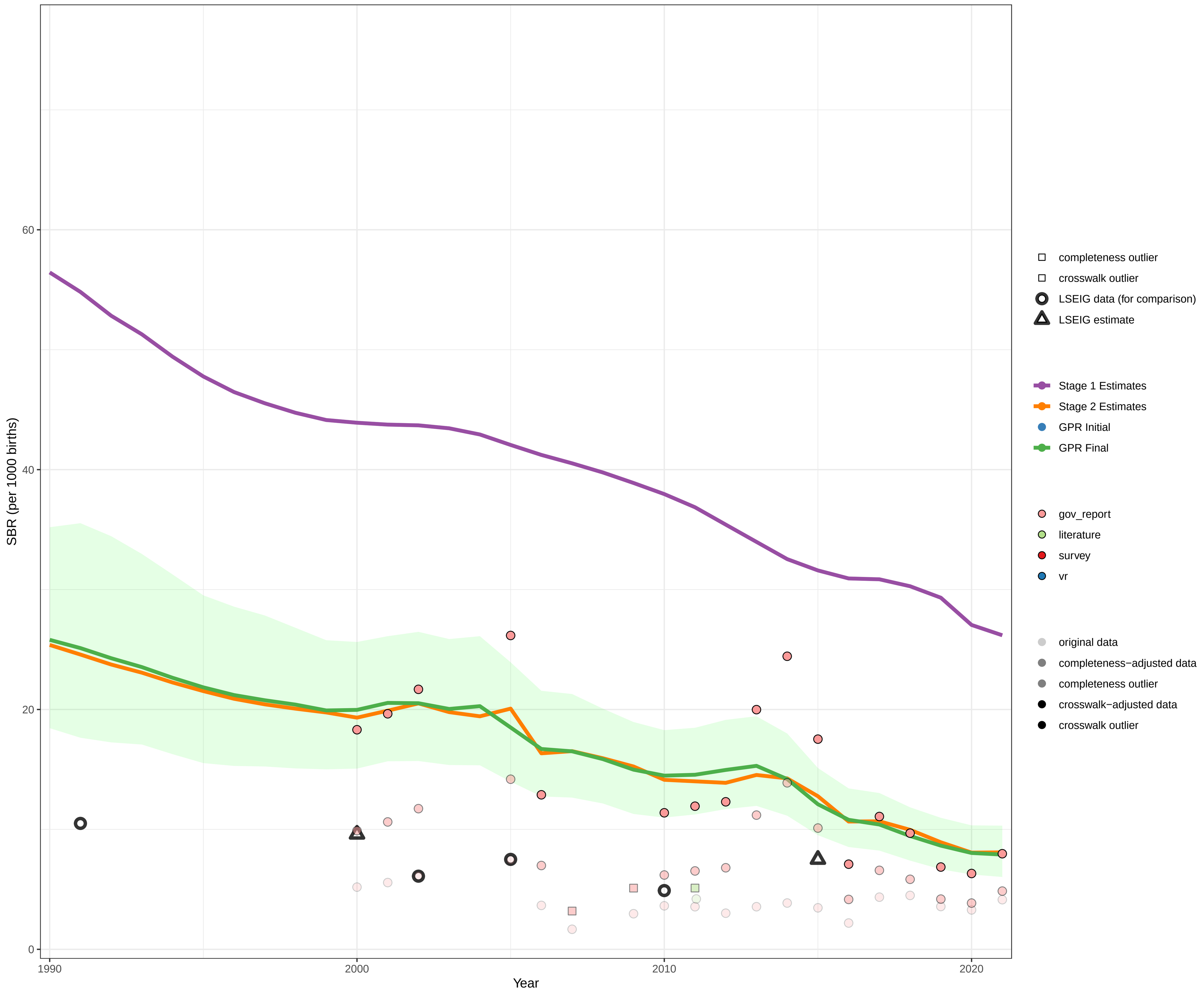

Palestine PSE North Africa and Middle East Stillbirth Rate (SBR) − 28 weeks
lambda = 0.4 ; zeta = 0.9 ; scale = 10 ; amp = 0.028

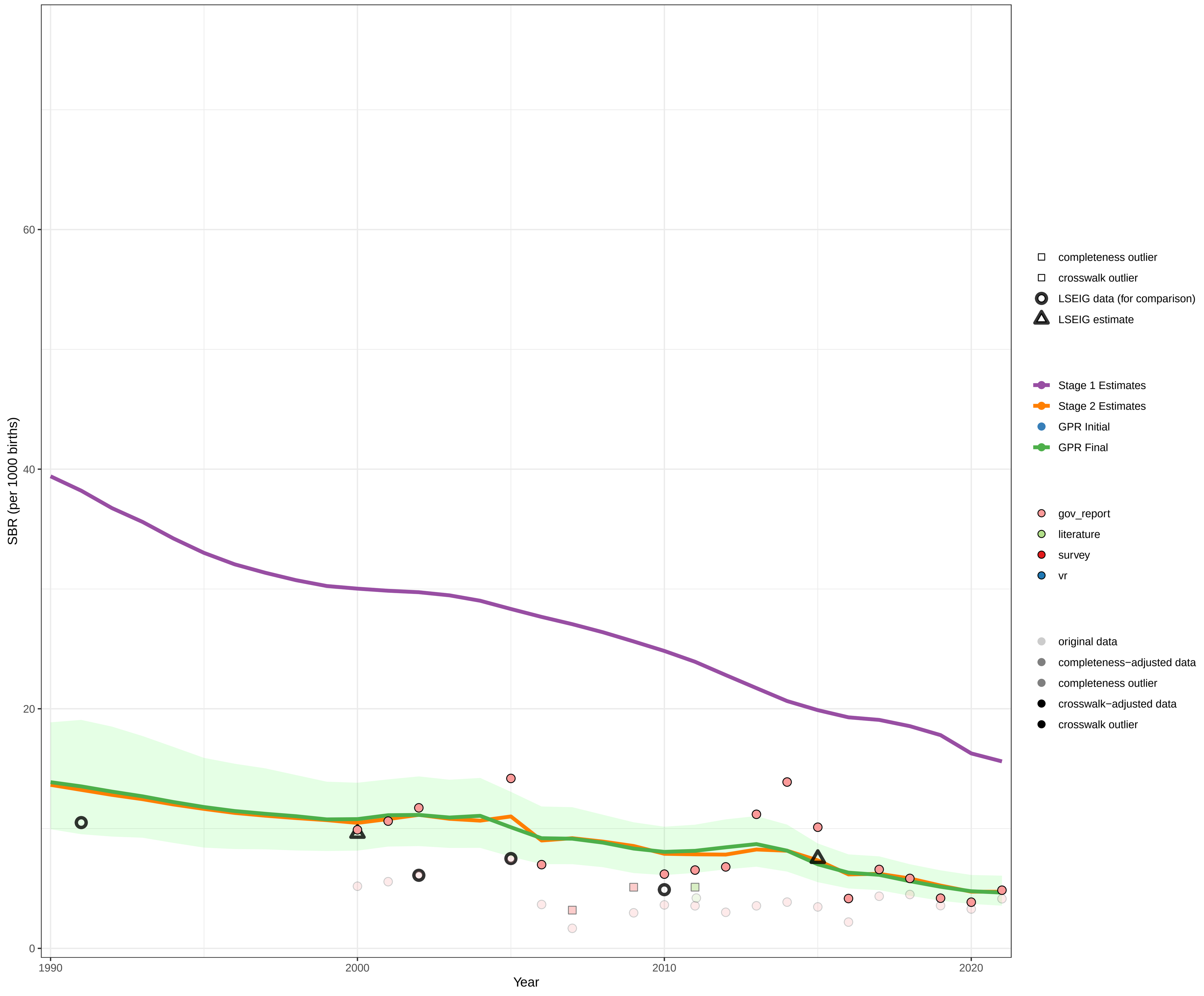

Year	Total Stillbirths (in thousands)	Total Stillbirths (in thousands)	SBR (per 1000 births)	SBR (per 1000 births)	SBR/NMR	SBR/NMR
	20 weeks	28 weeks	20 weeks	28 weeks	20 weeks	28 weeks
1990	2.3 (1.6 to 3.2)	1.2 (0.9 to 1.7)	25.8 (18.4 to 35.2)	13.9 (9.9 to 18.9)	1.43 (1.02 to 1.94)	0.77 (0.55 to 1.04)
1995	2.3 (1.6 to 3.1)	1.2 (0.9 to 1.7)	21.8 (15.5 to 29.5)	11.8 (8.4 to 15.9)	1.44 (1.02 to 1.95)	0.78 (0.55 to 1.05)
2000	2.4 (1.8 to 3.1)	1.3 (1.0 to 1.6)	20.0 (15.1 to 25.6)	10.8 (8.2 to 13.8)	1.46 (1.10 to 1.87)	0.79 (0.60 to 1.01)
2005	2.4 (1.8 to 3.2)	1.3 (1.0 to 1.7)	18.5 (14.0 to 23.9)	10.1 (7.7 to 13.1)	1.45 (1.09 to 1.87)	0.79 (0.60 to 1.02)
2010	2.0 (1.5 to 2.5)	1.1 (0.8 to 1.4)	14.5 (11.0 to 18.3)	8.1 (6.1 to 10.2)	1.36 (1.03 to 1.72)	0.76 (0.57 to 0.95)
2015	1.6 (1.2 to 2.0)	0.9 (0.7 to 1.1)	12.1 (9.5 to 15.1)	7.0 (5.5 to 8.8)	1.52 (1.20 to 1.90)	0.88 (0.69 to 1.10)
2020	1.0 (0.8 to 1.3)	0.6 (0.5 to 0.7)	8.0 (6.2 to 10.3)	4.8 (3.7 to 6.1)	1.34 (1.04 to 1.72)	0.79 (0.62 to 1.02)
2021	1.0 (0.7 to 1.2)	0.6 (0.4 to 0.7)	7.9 (6.0 to 10.3)	4.7 (3.6 to 6.1)	1.39 (1.06 to 1.82)	0.82 (0.63 to 1.07)

Qatar QAT North Africa and Middle East Stillbirth Rate / Neonatal Mortality Rate (SBR/NMR) − 20 weeks
lambda = 0.2 ; zeta = 0.99 ; scale = 5 ; amp = 0.028

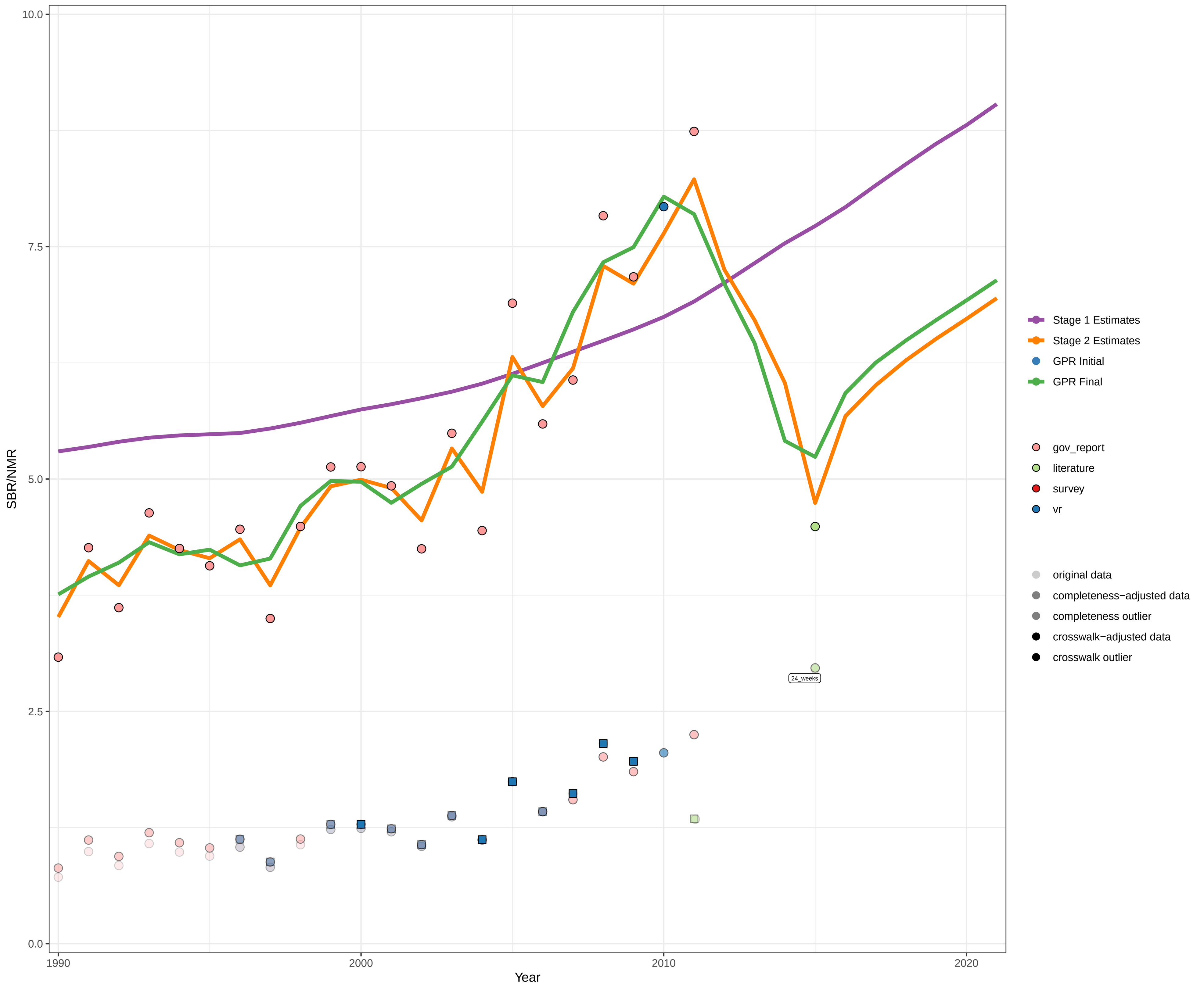

Qatar QAT North Africa and Middle East Stillbirth Rate / Neonatal Mortality Rate (SBR/NMR) − 28 weeks
lambda = 0.2 ; zeta = 0.99 ; scale = 5 ; amp = 0.028

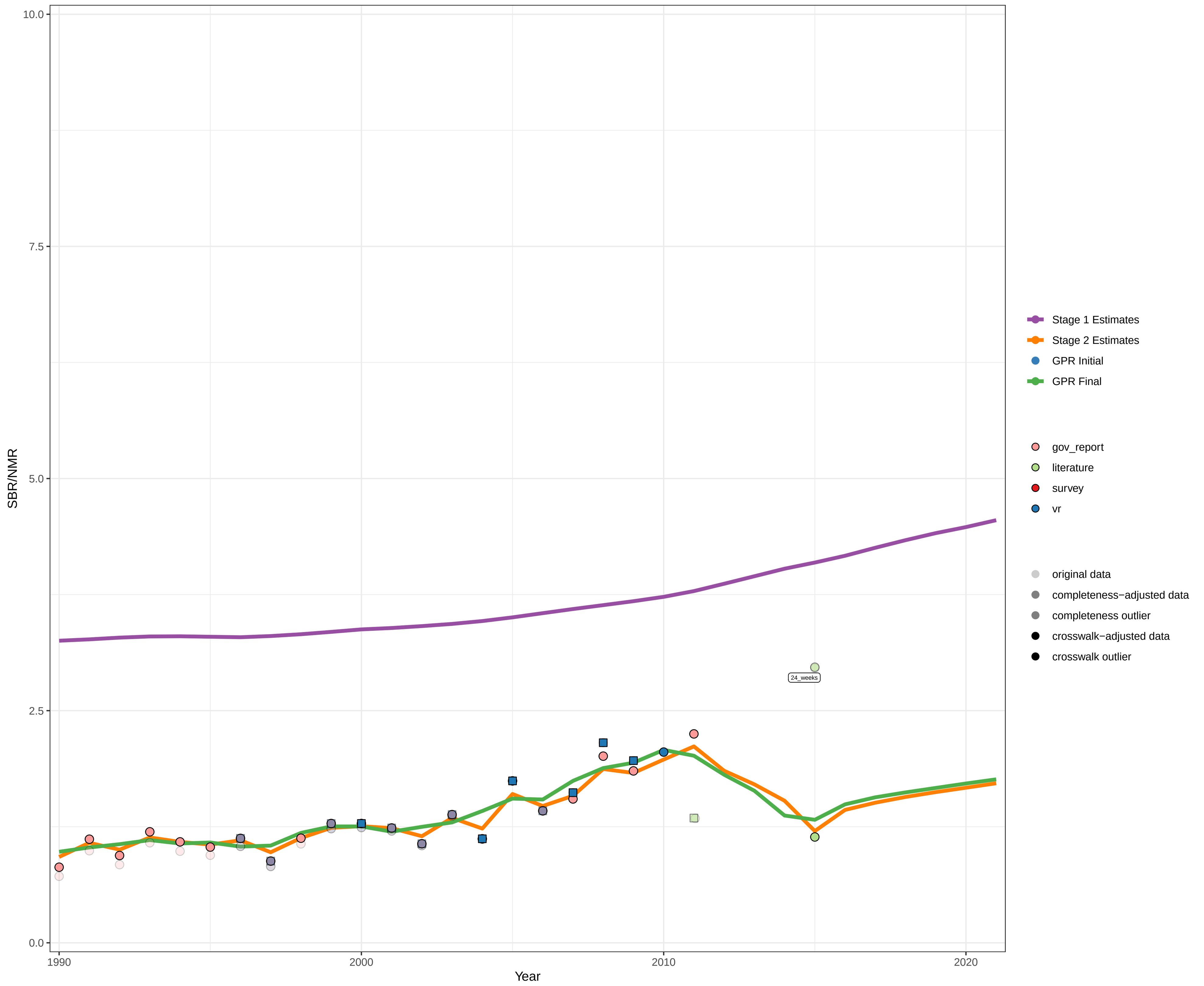

Definition	Years	Outlier	Source.Type	Title
24_weeks	2015	0	Sci Lit	Risk factors and classification of stillbirth in a Middle Eastern population: a retrospective study
28_weeks	1990−2009, 2011	0	Statistical Report	Qatar Annual Statistical Abstract
28_weeks	2010	0	VR	United Nations Demographic Yearbook
28_weeks	1996, 1997, 1999, 2001−2003, 2006	1	VR	United Nations Demographic Yearbook
28_weeks_OR_1000_grams	2011	1	Sci Lit	Maternal complications and perinatal mortality: findings of the World Health Organization Multicountry Survey on Maternal and Newborn Health
28_weeks	2000, 2004, 2005, 2007−2009	2	VR	United Nations Demographic Yearbook

Qatar QAT North Africa and Middle East Stillbirth Rate (SBR) − 20 weeks
lambda = 0.2 ; zeta = 0.99 ; scale = 5 ; amp = 0.028

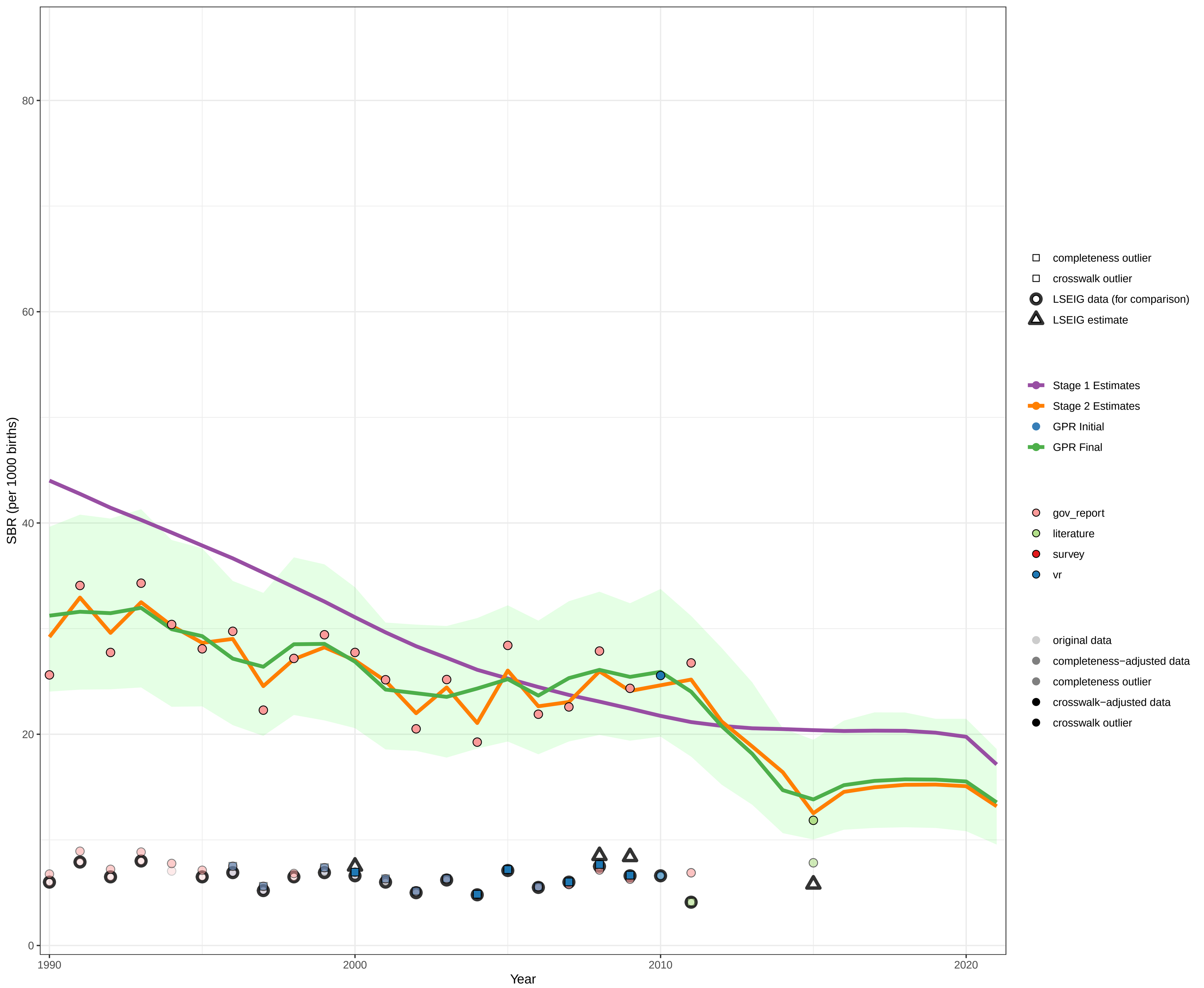

Qatar QAT North Africa and Middle East Stillbirth Rate (SBR) − 28 weeks
lambda = 0.2 ; zeta = 0.99 ; scale = 5 ; amp = 0.028

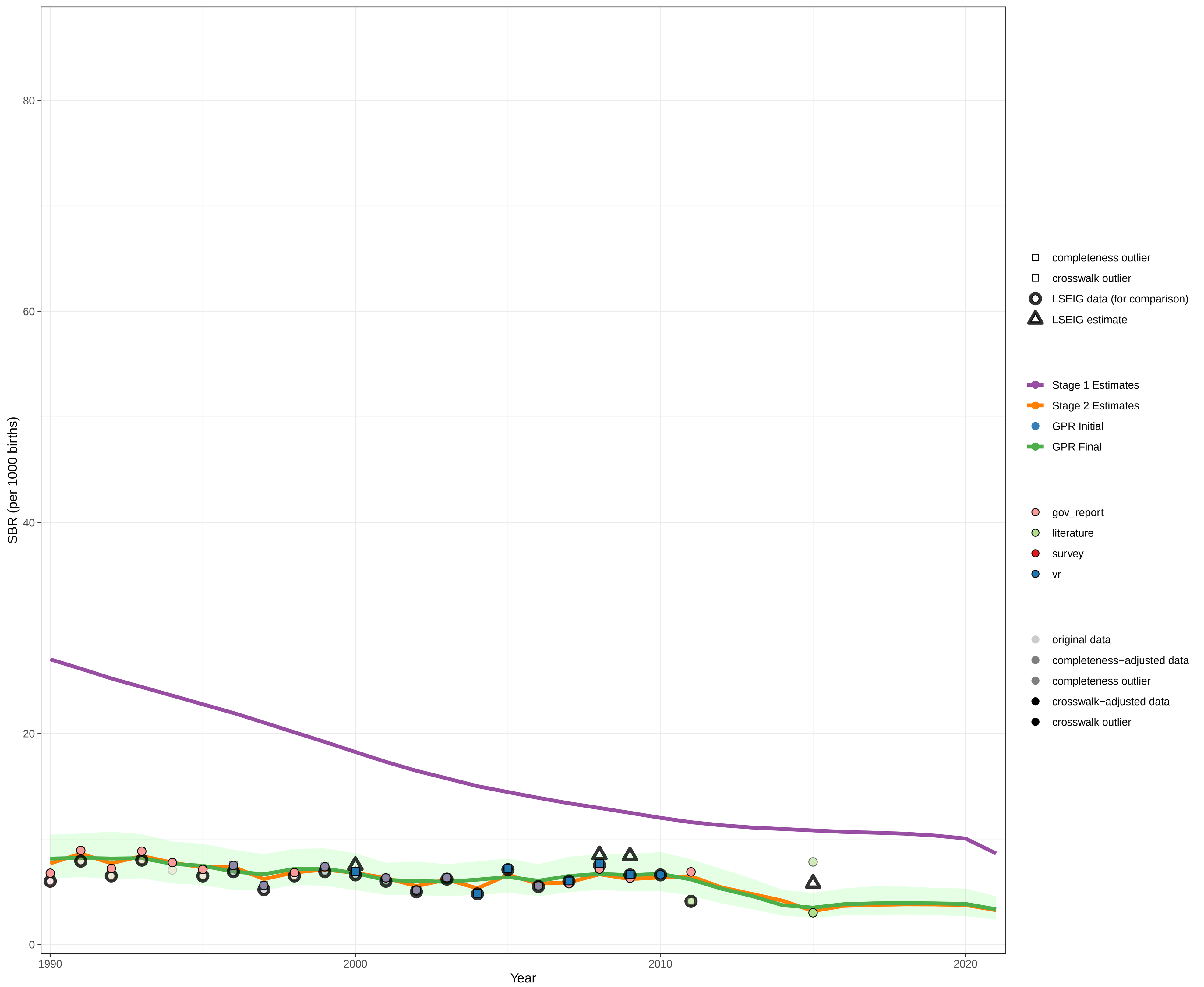

Year	Total Stillbirths (in thousands)	Total Stillbirths (in thousands)	SBR (per 1000 births)	SBR (per 1000 births)	SBR/NMR	SBR/NMR
	20 weeks	28 weeks	20 weeks	28 weeks	20 weeks	28 weeks
1990	0.4 (0.3 to 0.5)	0.1 (0.1 to 0.1)	31.2 (24.0 to 39.6)	8.1 (6.2 to 10.4)	3.76 (2.89 to 4.77)	0.98 (0.75 to 1.25)
1995	0.4 (0.3 to 0.5)	0.1 (0.1 to 0.1)	29.3 (22.6 to 37.6)	7.5 (5.6 to 9.5)	4.24 (3.28 to 5.44)	1.08 (0.81 to 1.38)
2000	0.4 (0.3 to 0.5)	0.1 (0.1 to 0.1)	26.9 (20.6 to 33.9)	6.8 (5.2 to 8.7)	4.97 (3.81 to 6.27)	1.25 (0.95 to 1.60)
2005	0.4 (0.3 to 0.6)	0.1 (0.1 to 0.1)	25.2 (19.3 to 32.2)	6.4 (4.9 to 8.1)	6.12 (4.68 to 7.81)	1.55 (1.19 to 1.98)
2010	0.7 (0.5 to 0.9)	0.2 (0.1 to 0.2)	25.9 (19.8 to 33.8)	6.7 (5.0 to 8.8)	8.04 (6.13 to 10.47)	2.08 (1.56 to 2.72)
2015	0.5 (0.3 to 0.7)	0.1 (0.1 to 0.2)	13.8 (10.0 to 19.5)	3.5 (2.5 to 4.9)	5.24 (3.80 to 7.38)	1.32 (0.96 to 1.86)
2020	0.6 (0.4 to 0.8)	0.1 (0.1 to 0.2)	15.5 (10.8 to 21.5)	3.8 (2.7 to 5.3)	6.92 (4.82 to 9.57)	1.72 (1.20 to 2.36)
2021	0.5 (0.4 to 0.7)	0.1 (0.1 to 0.2)	13.5 (9.6 to 18.6)	3.3 (2.4 to 4.6)	7.14 (5.03 to 9.80)	1.76 (1.25 to 2.41)

Saudi Arabia SAU North Africa and Middle East Stillbirth Rate / Neonatal Mortality Rate (SBR/NMR) − 20 weeks
lambda = 0.2 ; zeta = 0.99 ; scale = 5 ; amp = 0.028

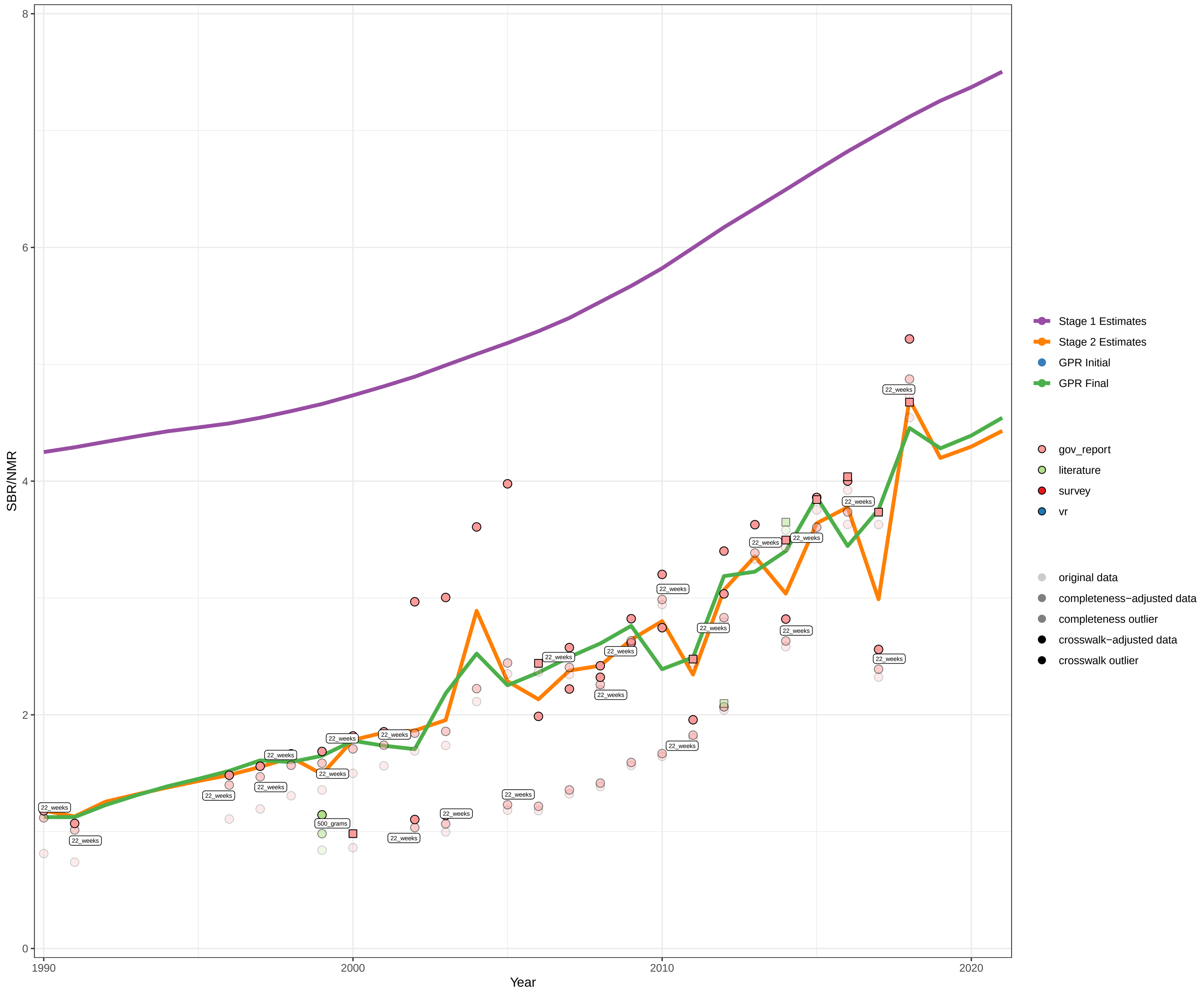

Saudi Arabia SAU North Africa and Middle East Stillbirth Rate / Neonatal Mortality Rate (SBR/NMR) − 28 weeks
lambda = 0.2 ; zeta = 0.99 ; scale = 5 ; amp = 0.028

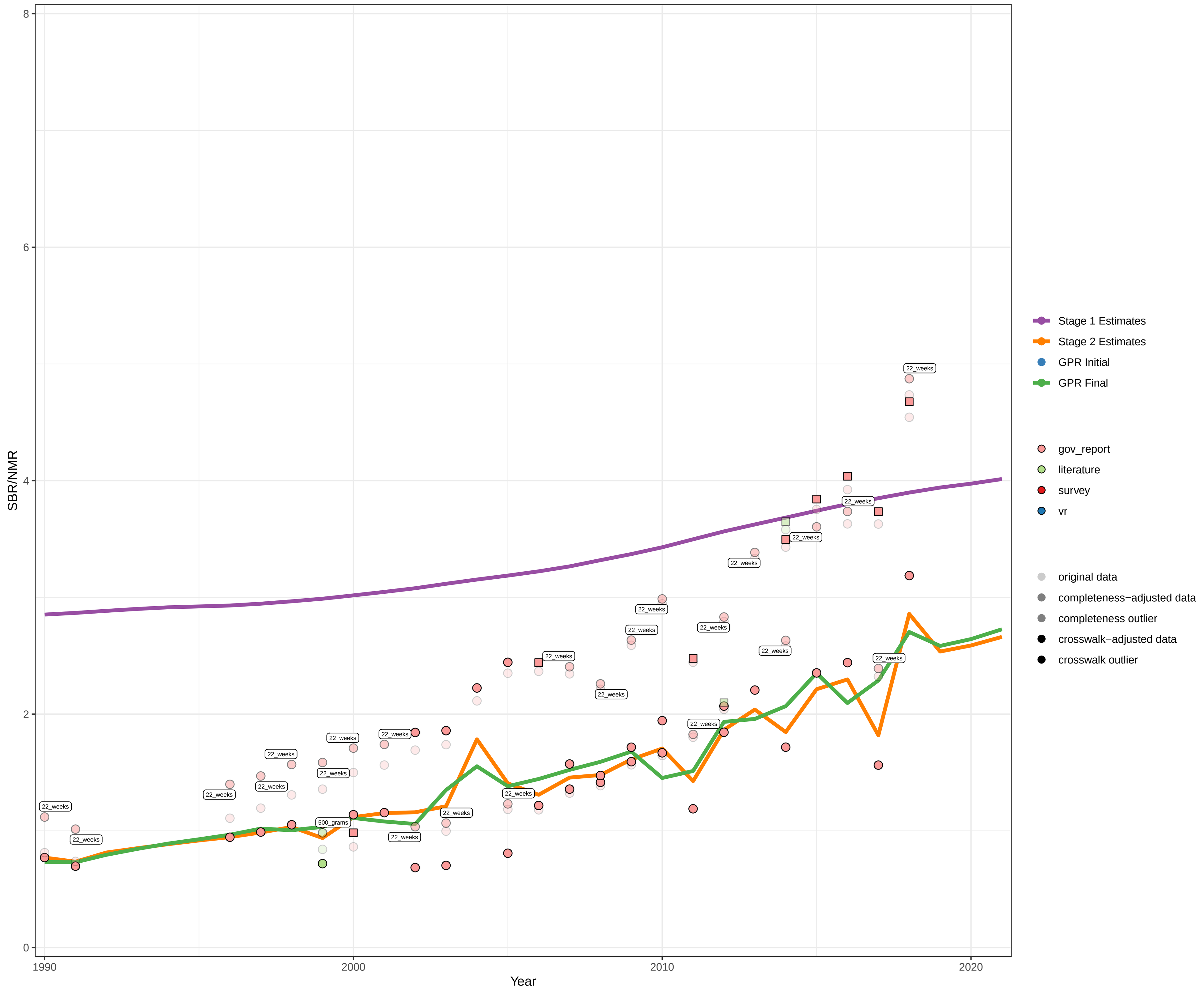

Definition	Years	Outlier	Source.Type	Title
22_weeks	1990, 1991	0	Statistical Report	Saudi Arabia Statistical Yearbook
22_weeks	1996−2003, 2005, 2007−2018	0	Statistical Report	Saudi Arabia Health Statistical Yearbook
28_weeks	2002−2010, 2012	0	Statistical Report	Saudi Arabia Health Statistical Yearbook
500_grams	1999	0	Sci Lit	Antenatal intrauterine fetal death: a prospective study in a tertiary hospital in south−western Saudi Arabia
24_weeks	2014	1	Sci Lit	Riyadh Mother and Baby Multicenter Cohort Study: The Cohort Profile
28_weeks	2012	1	Sci Lit	Patterns of folic acid use in pregnant Saudi women and prevalence of neural tube defects − Results from a nested case−control study
28_weeks	2006	2	Statistical Report	Saudi Arabia Health Statistical Yearbook

Saudi Arabia SAU North Africa and Middle East Stillbirth Rate (SBR) − 20 weeks
lambda = 0.2 ; zeta = 0.99 ; scale = 5 ; amp = 0.028

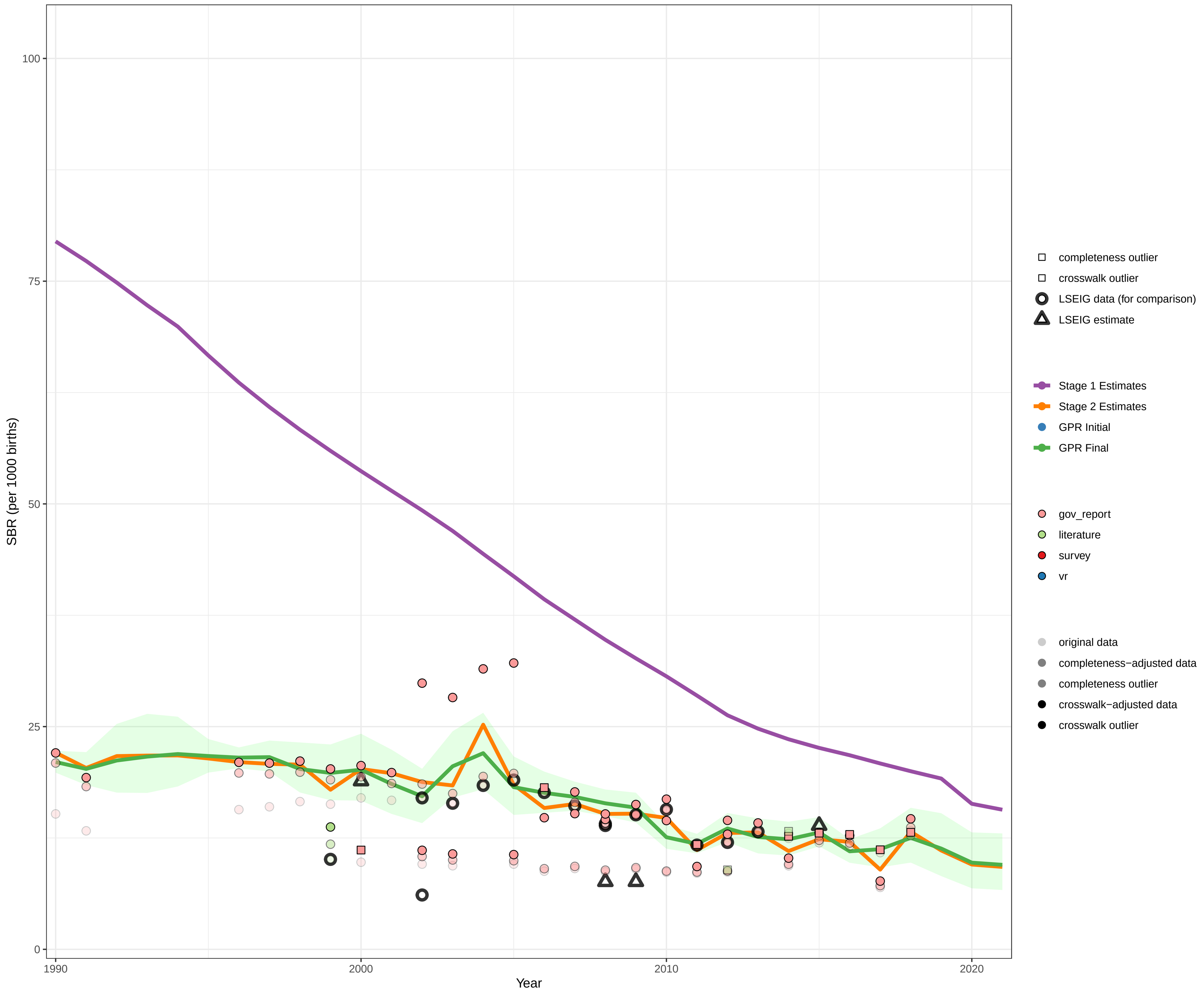

Saudi Arabia SAU North Africa and Middle East Stillbirth Rate (SBR) − 28 weeks
lambda = 0.2 ; zeta = 0.99 ; scale = 5 ; amp = 0.028

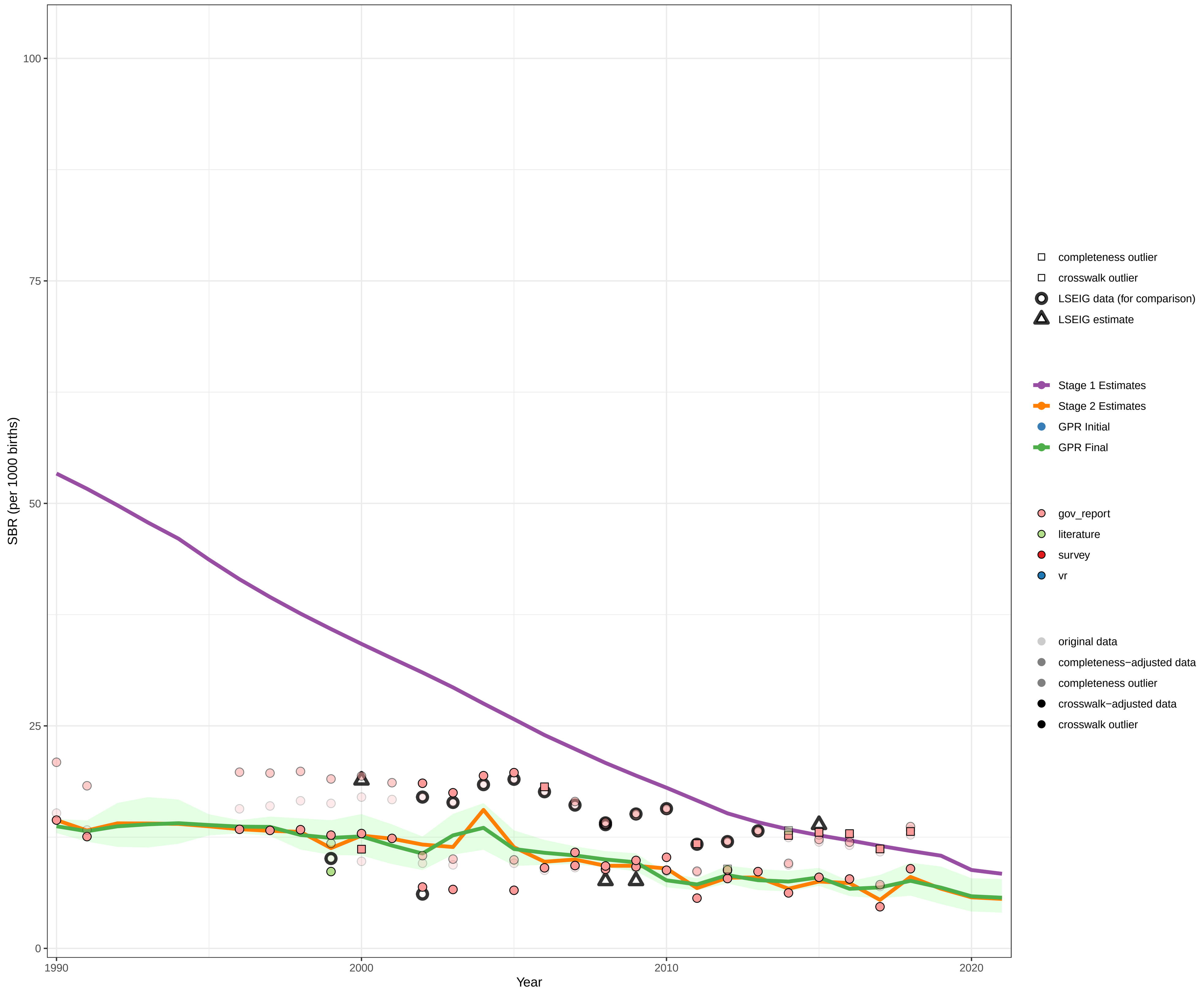

Year	Total Stillbirths (in thousands)	Total Stillbirths (in thousands)	SBR (per 1000 births)	SBR (per 1000 births)	SBR/NMR	SBR/NMR
	20 weeks	28 weeks	20 weeks	28 weeks	20 weeks	28 weeks
1990	11.0 (10.4 to 11.7)	7.1 (6.7 to 7.6)	21.0 (19.8 to 22.3)	13.7 (13.0 to 14.5)	1.12 (1.06 to 1.19)	0.73 (0.69 to 0.78)
1995	11.5 (10.5 to 12.5)	7.3 (6.6 to 7.9)	21.7 (19.8 to 23.6)	13.9 (12.7 to 15.1)	1.45 (1.33 to 1.58)	0.93 (0.85 to 1.01)
2000	10.2 (8.4 to 12.3)	6.3 (5.2 to 7.6)	20.2 (16.7 to 24.2)	12.6 (10.4 to 15.1)	1.78 (1.47 to 2.13)	1.11 (0.92 to 1.33)
2005	9.1 (7.5 to 10.9)	5.5 (4.6 to 6.6)	18.2 (15.1 to 21.7)	11.2 (9.3 to 13.3)	2.25 (1.87 to 2.68)	1.38 (1.15 to 1.64)
2010	6.6 (5.9 to 7.4)	4.0 (3.6 to 4.4)	12.6 (11.3 to 14.0)	7.6 (6.9 to 8.5)	2.39 (2.14 to 2.66)	1.45 (1.30 to 1.61)
2015	6.9 (6.1 to 7.8)	4.2 (3.7 to 4.8)	13.1 (11.6 to 14.8)	8.0 (7.0 to 9.0)	3.86 (3.40 to 4.37)	2.35 (2.07 to 2.66)
2020	4.7 (3.3 to 6.3)	2.8 (2.0 to 3.8)	9.7 (6.9 to 13.1)	5.9 (4.1 to 7.9)	4.39 (3.09 to 5.92)	2.64 (1.87 to 3.56)
2021	4.4 (3.1 to 6.1)	2.6 (1.9 to 3.6)	9.5 (6.7 to 13.0)	5.7 (4.0 to 7.8)	4.54 (3.19 to 6.23)	2.73 (1.92 to 3.73)

Sudan SDN North Africa and Middle East Stillbirth Rate / Neonatal Mortality Rate (SBR/NMR) − 20 weeks
lambda = 0.4 ; zeta = 0.9 ; scale = 10 ; amp = 0.028

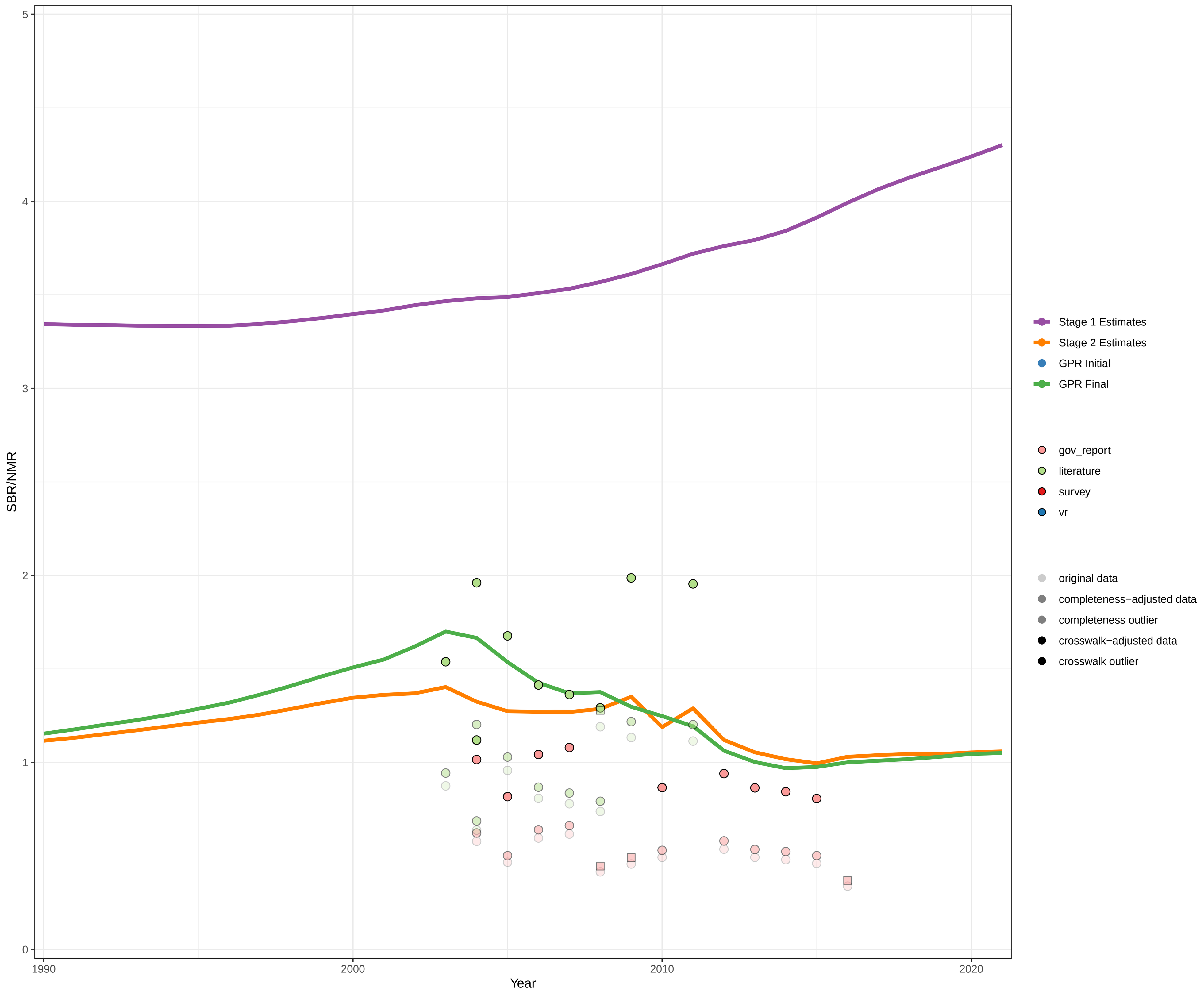

Sudan SDN North Africa and Middle East Stillbirth Rate / Neonatal Mortality Rate (SBR/NMR) − 28 weeks
lambda = 0.4 ; zeta = 0.9 ; scale = 10 ; amp = 0.028

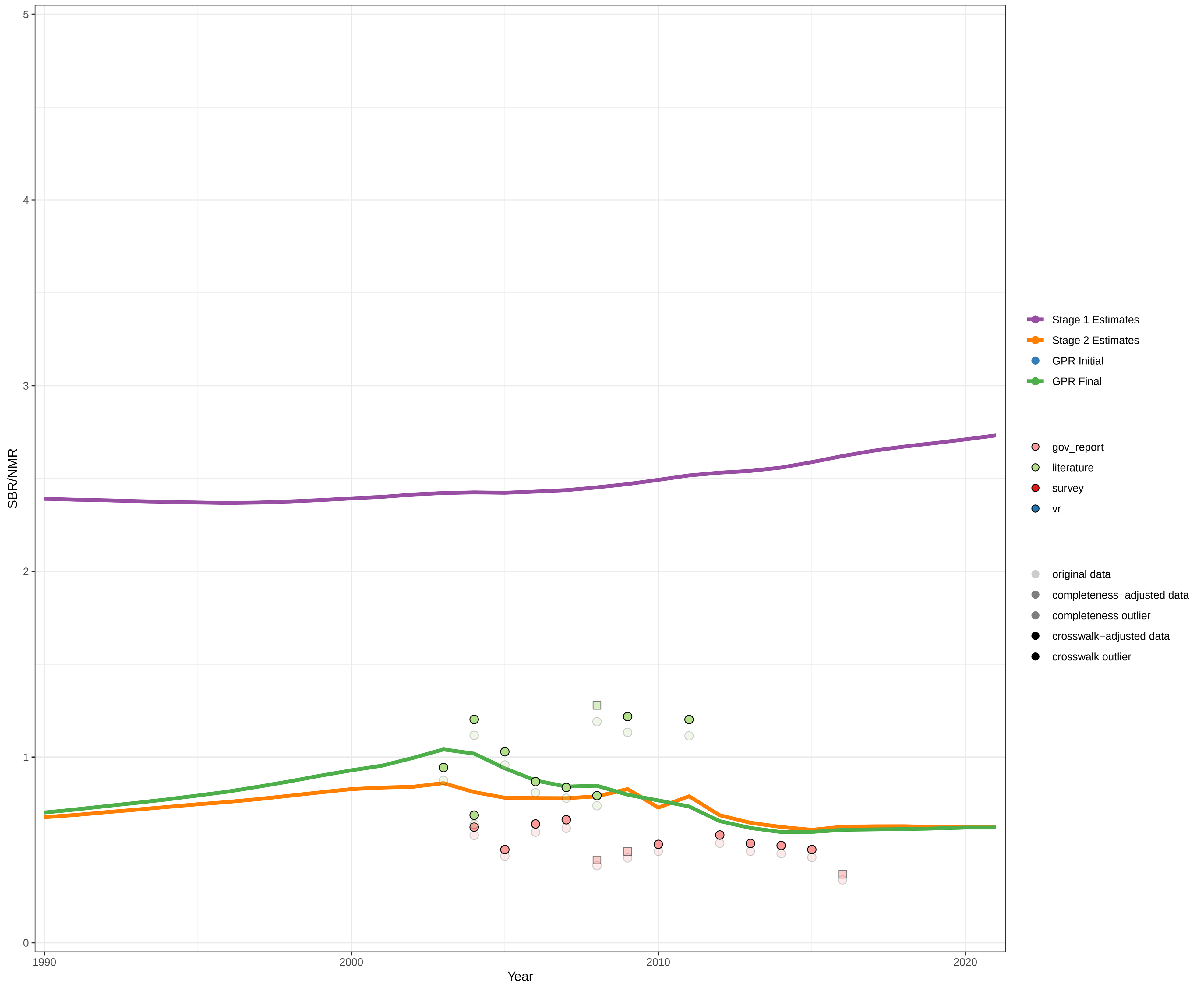

Definition	Years	Outlier	Source.Type	Title
28_weeks	2003−2007	0	Sci Lit	High maternal mortality and stillbirth in the Wad Medani Hospital, Central Sudan, 2003−2007
28_weeks	2004	0	Sci Lit	High perinatal mortality in Darfur, Sudan
28_weeks	2004−2007	0	Statistical Report	Sudan Health Statistical Report
28_weeks	2008	0	Sci Lit	Malaria and stillbirth in Omdurman Maternity Hospital, Sudan
28_weeks	2009	0	Sci Lit	Anaemia and stillbirth in Kassala Hospital, Eastern Sudan
28_weeks	2010, 2012	0	Statistical Report	Sudan Annual Health Statistics Report
28_weeks	2011	0	Sci Lit	Factors associated with perinatal mortality in Kassala, Eastern Sudan: a community−based study 2010−2011
28_weeks	2013−2015	0	Statistical Report	Sudan Annual Health Statistical Report
28_weeks	2008	1	Sci Lit	Education, prenatal care, and poor perinatal outcome in Khartoum, Sudan
28_weeks	2008, 2009	1	Statistical Report	Sudan Statistical Yearbook
28_weeks	2016	1	Statistical Report	Sudan Annual Health Statistical Report

Sudan SDN North Africa and Middle East Stillbirth Rate (SBR) − 20 weeks
lambda = 0.4 ; zeta = 0.9 ; scale = 10 ; amp = 0.028

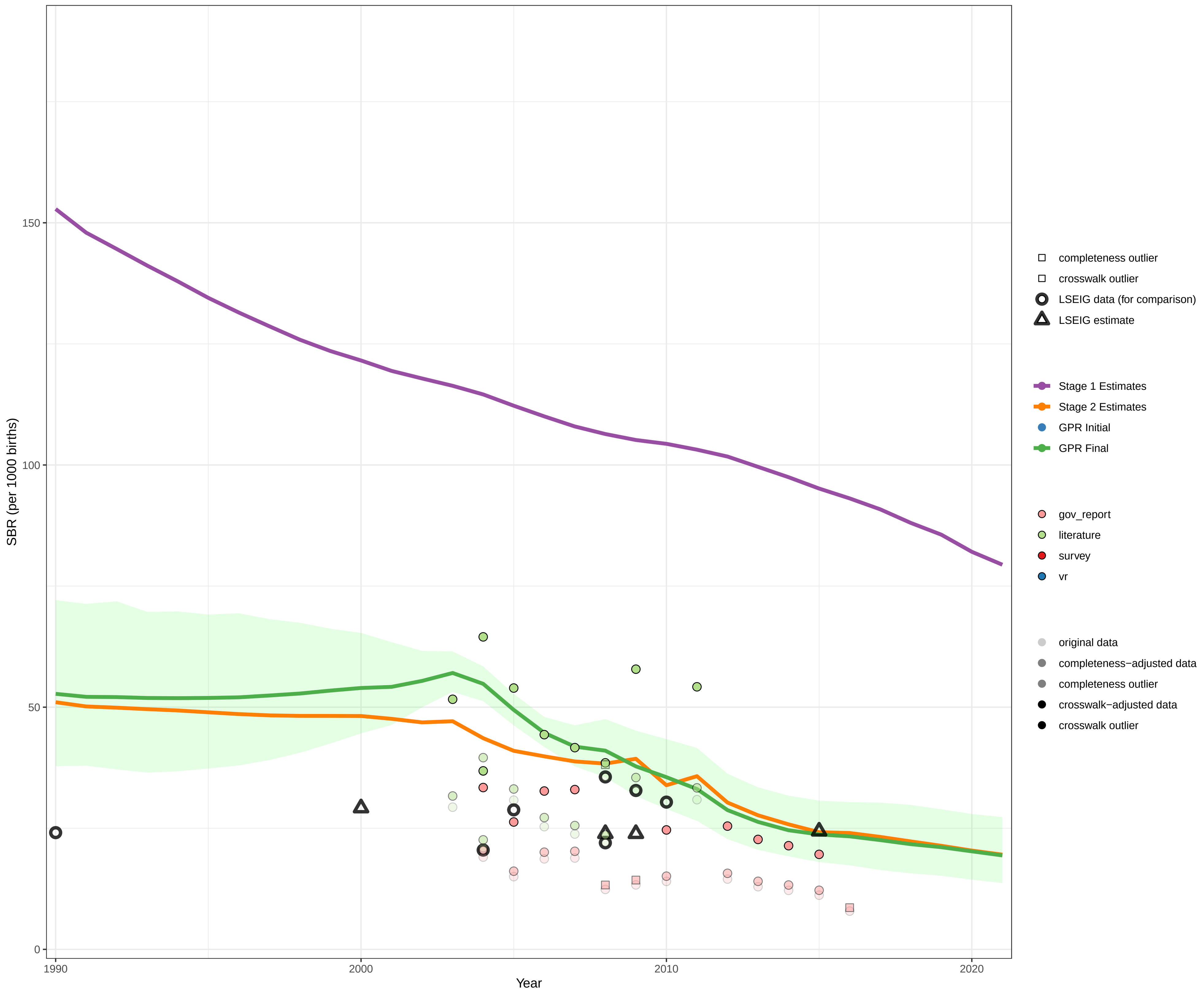

Sudan SDN North Africa and Middle East Stillbirth Rate (SBR) − 28 weeks
lambda = 0.4 ; zeta = 0.9 ; scale = 10 ; amp = 0.028

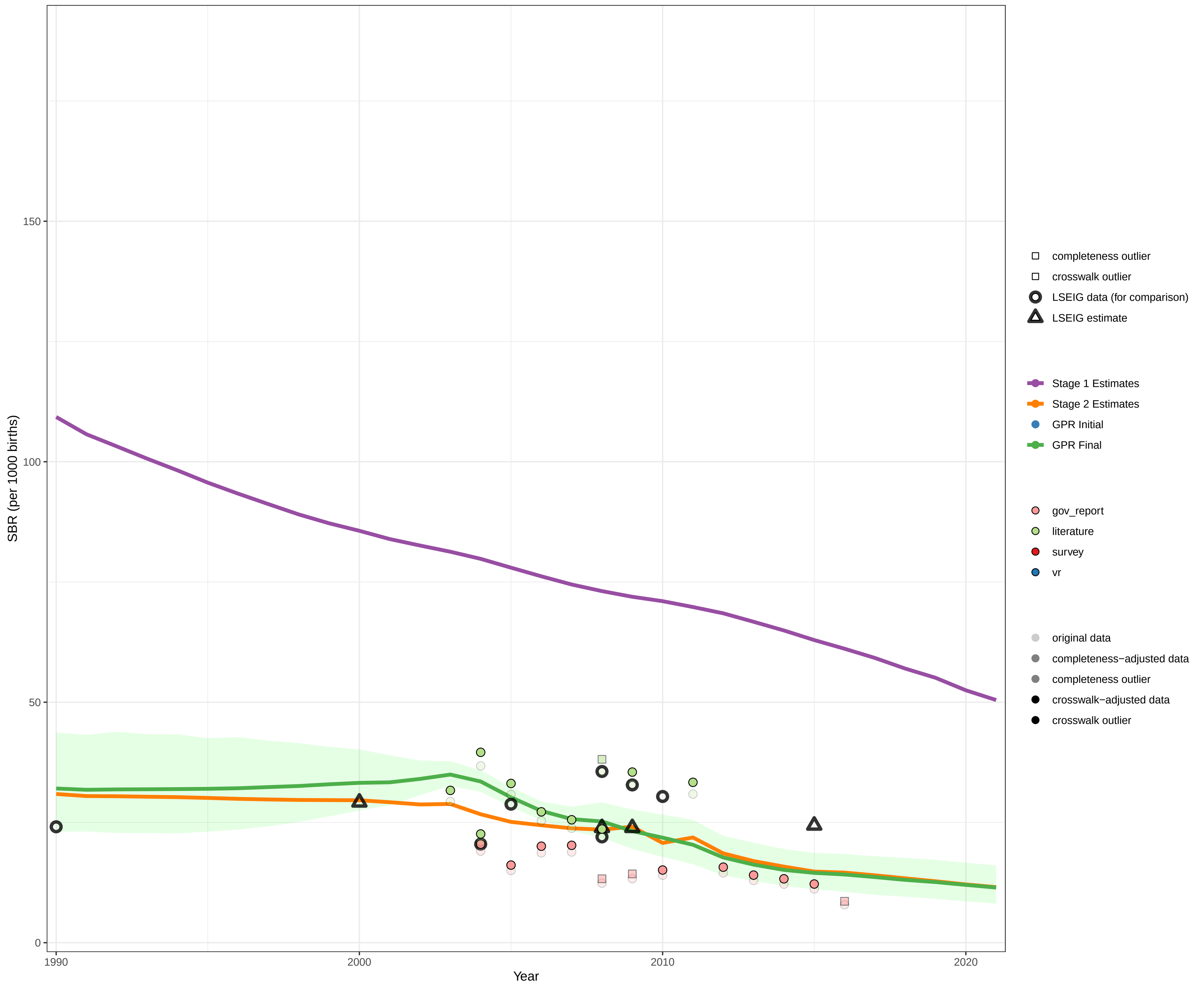

Year	Total Stillbirths (in thousands)	Total Stillbirths (in thousands)	SBR (per 1000 births)	SBR (per 1000 births)	SBR/NMR	SBR/NMR
	20 weeks	28 weeks	20 weeks	28 weeks	20 weeks	28 weeks
1990	51.0 (36.0 to 71.1)	30.3 (21.6 to 41.8)	52.8 (37.8 to 72.1)	32.1 (23.1 to 43.7)	1.15 (0.83 to 1.58)	0.70 (0.50 to 0.96)
1995	57.3 (40.5 to 77.6)	34.5 (24.7 to 46.4)	51.9 (37.3 to 69.1)	32.0 (23.1 to 42.5)	1.29 (0.93 to 1.71)	0.79 (0.57 to 1.05)
2000	65.2 (53.3 to 79.9)	39.3 (32.3 to 47.8)	54.0 (44.6 to 65.3)	33.2 (27.5 to 40.2)	1.51 (1.25 to 1.83)	0.93 (0.77 to 1.12)
2005	62.9 (58.7 to 67.4)	37.7 (35.2 to 40.5)	49.4 (46.2 to 52.7)	30.2 (28.2 to 32.4)	1.54 (1.44 to 1.64)	0.94 (0.88 to 1.01)
2010	46.7 (37.8 to 57.5)	28.3 (23.0 to 34.7)	35.5 (29.0 to 43.4)	21.8 (17.8 to 26.6)	1.25 (1.02 to 1.52)	0.77 (0.63 to 0.94)
2015	30.0 (22.7 to 39.1)	18.2 (13.9 to 23.5)	23.7 (18.0 to 30.7)	14.5 (11.1 to 18.7)	0.98 (0.74 to 1.26)	0.60 (0.46 to 0.77)
2020	24.5 (17.3 to 34.1)	14.4 (10.2 to 20.0)	20.2 (14.4 to 28.0)	12.0 (8.6 to 16.6)	1.05 (0.74 to 1.44)	0.62 (0.44 to 0.86)
2021	23.1 (16.2 to 32.8)	13.6 (9.6 to 19.1)	19.4 (13.7 to 27.3)	11.5 (8.1 to 16.1)	1.05 (0.74 to 1.48)	0.62 (0.44 to 0.87)

Syrian Arab Republic SYR North Africa and Middle East Stillbirth Rate / Neonatal Mortality Rate (SBR/NMR) − 20 weeks
lambda = 1 ; zeta = 0.6 ; scale = 15 ; amp = 0.028

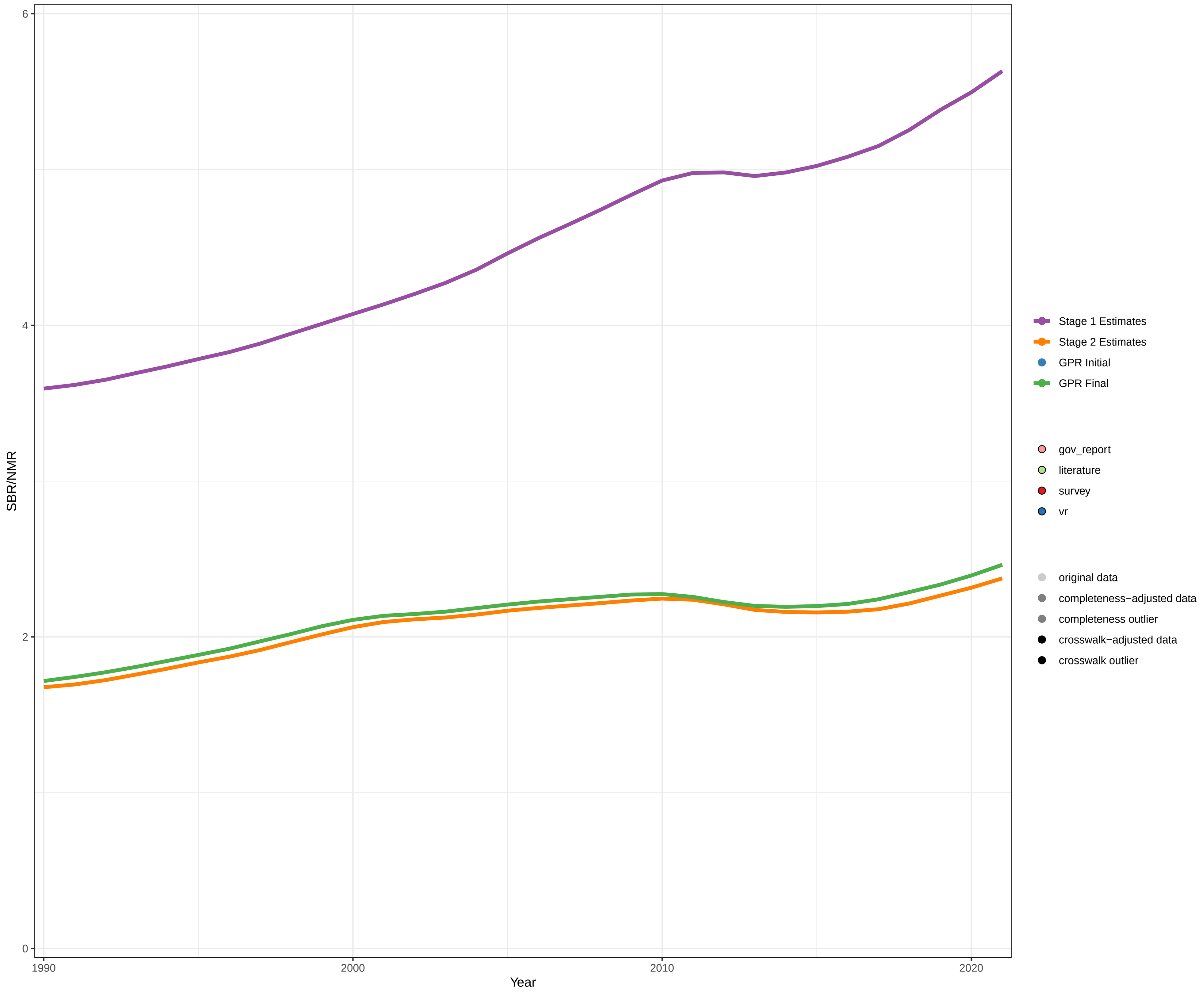

Syrian Arab Republic SYR North Africa and Middle East Stillbirth Rate / Neonatal Mortality Rate (SBR/NMR) − 28 weeks
lambda = 1 ; zeta = 0.6 ; scale = 15 ; amp = 0.028

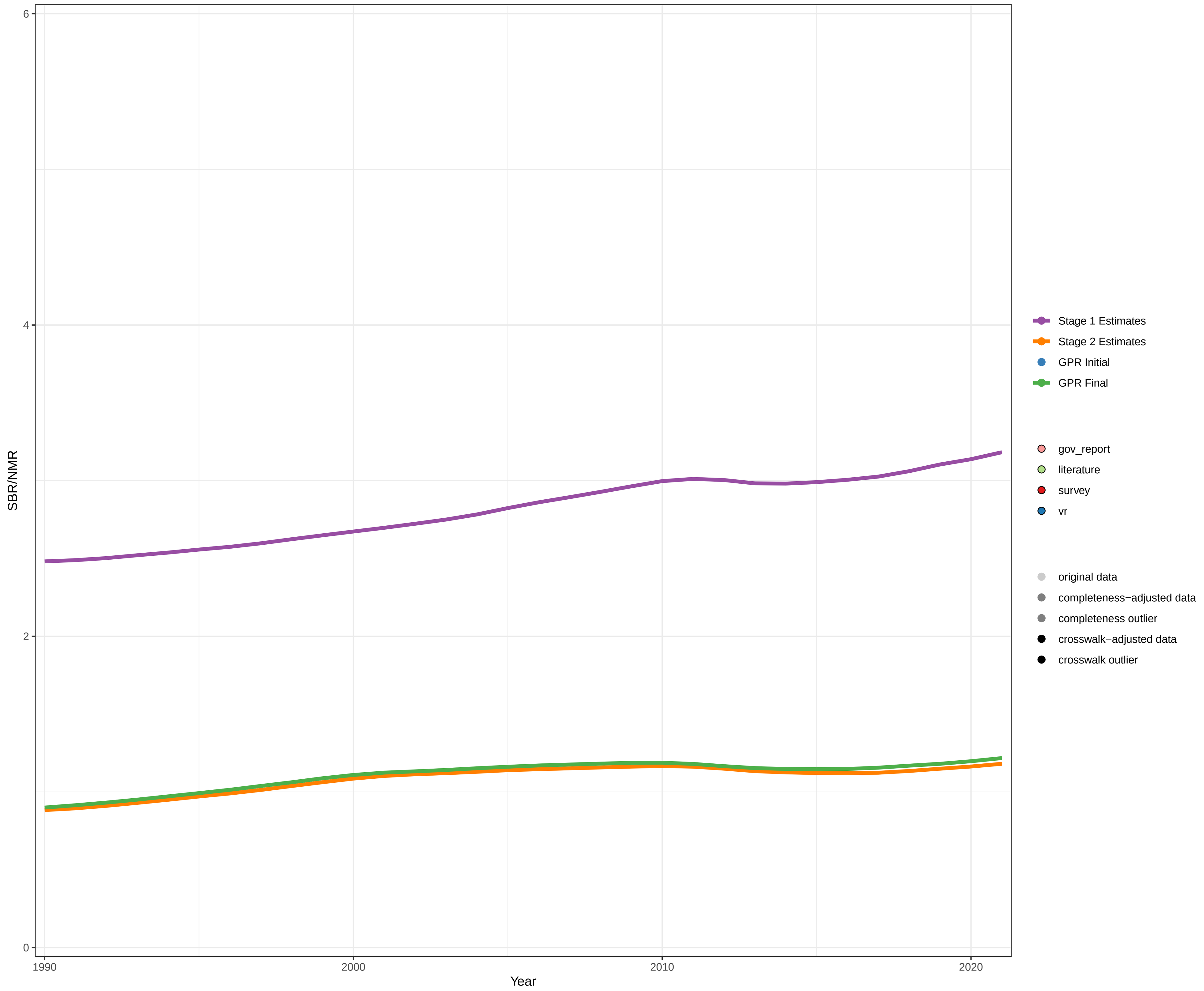

Definition	Years	Outlier	Source.Type	Title

Syrian Arab Republic SYR North Africa and Middle East Stillbirth Rate (SBR) − 20 weeks
lambda = 1 ; zeta = 0.6 ; scale = 15 ; amp = 0.028

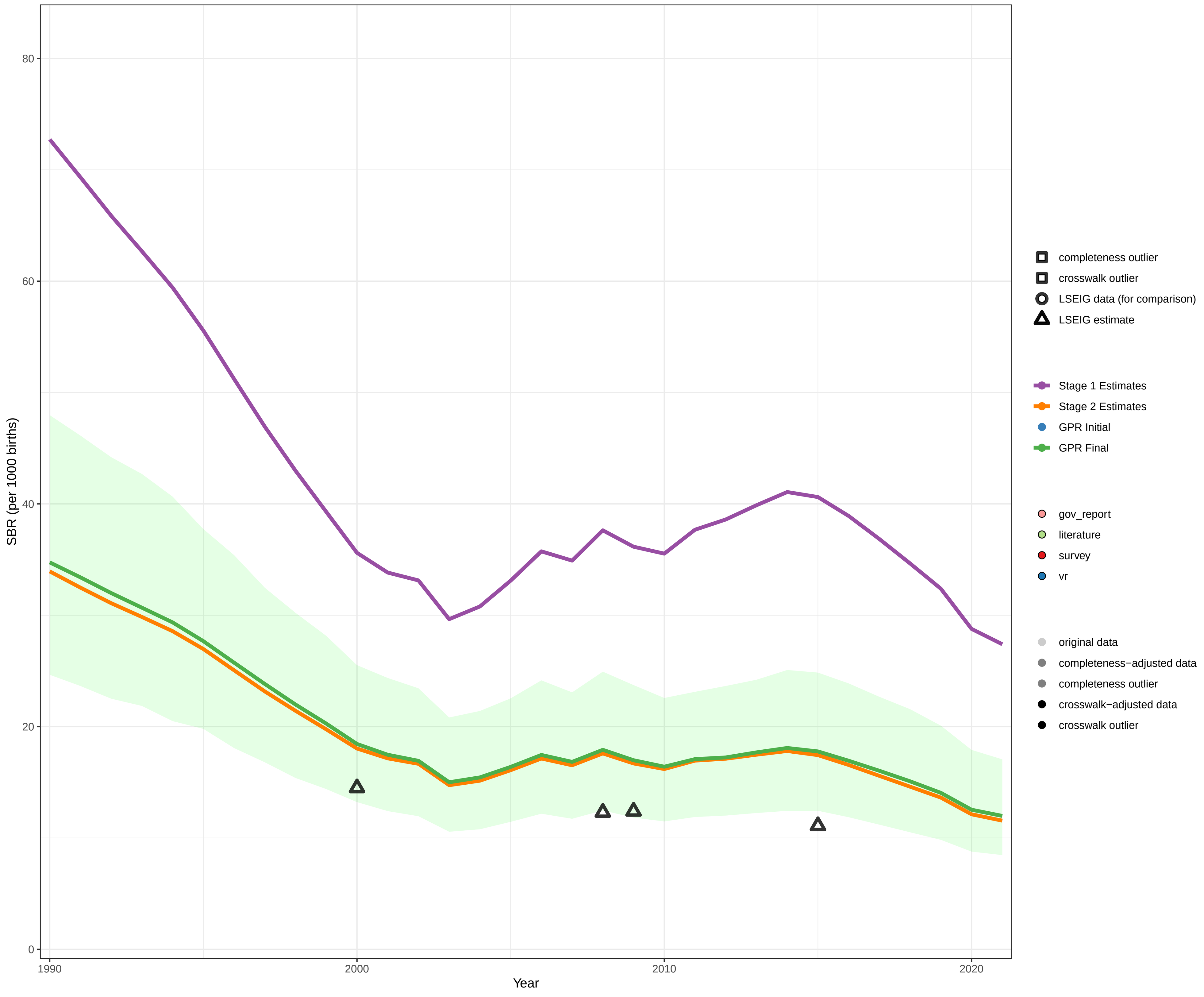

Syrian Arab Republic SYR North Africa and Middle East Stillbirth Rate (SBR) − 28 weeks
lambda = 1 ; zeta = 0.6 ; scale = 15 ; amp = 0.028

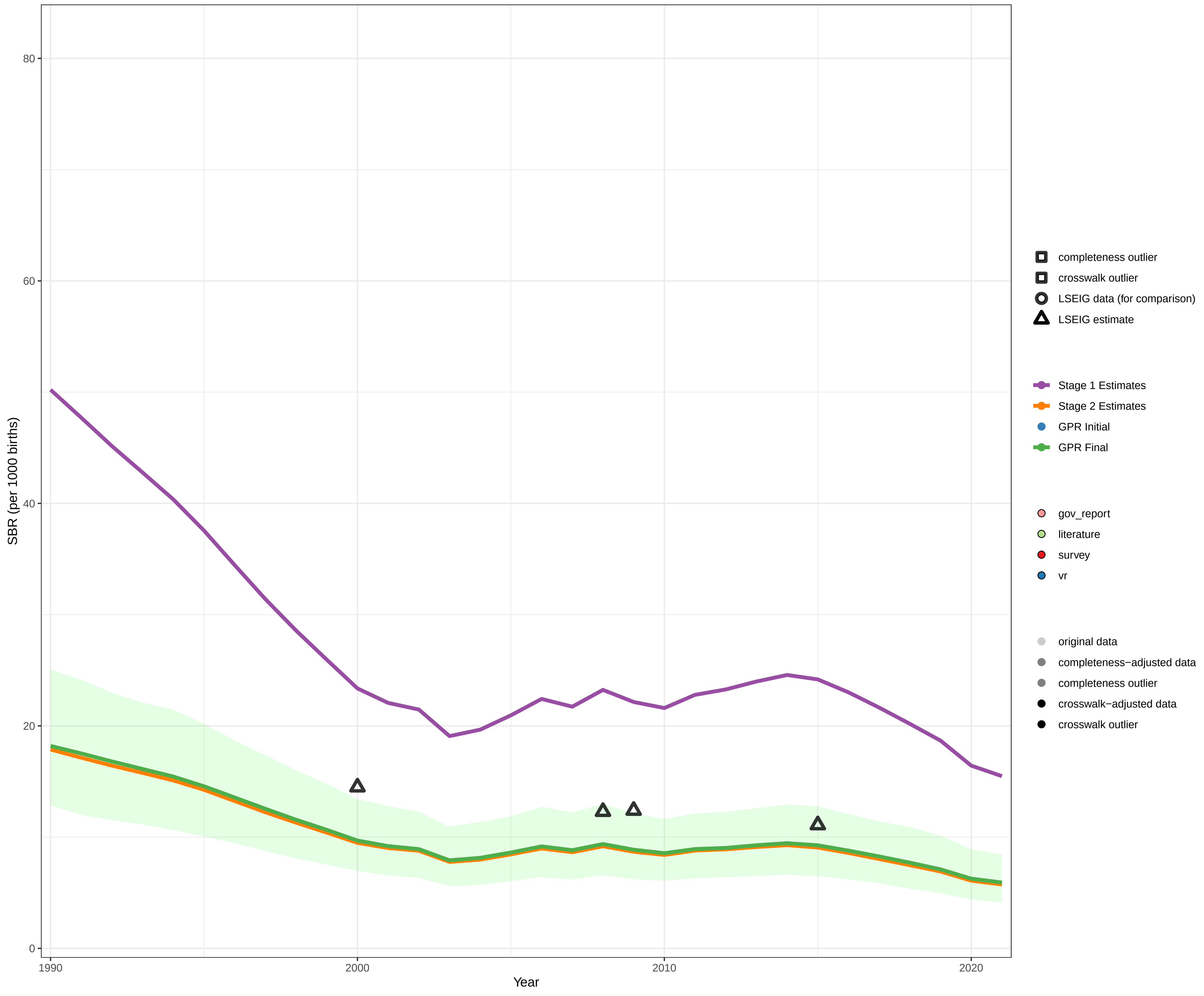

Year	Total Stillbirths (in thousands)	Total Stillbirths (in thousands)	SBR (per 1000 births)	SBR (per 1000 births)	SBR/NMR	SBR/NMR
	20 weeks	28 weeks	20 weeks	28 weeks	20 weeks	28 weeks
1990	16.4 (11.5 to 23.0)	8.5 (5.9 to 11.7)	34.7 (24.7 to 48.0)	18.2 (12.8 to 25.1)	1.72 (1.22 to 2.37)	0.90 (0.63 to 1.24)
1995	13.7 (9.7 to 18.8)	7.1 (4.9 to 9.9)	27.7 (19.8 to 37.7)	14.6 (10.0 to 20.2)	1.88 (1.35 to 2.57)	0.99 (0.68 to 1.38)
2000	9.3 (6.6 to 13.0)	4.8 (3.5 to 6.7)	18.4 (13.2 to 25.5)	9.7 (7.0 to 13.4)	2.11 (1.51 to 2.92)	1.11 (0.80 to 1.54)
2005	8.5 (5.9 to 11.7)	4.4 (3.1 to 6.1)	16.4 (11.4 to 22.5)	8.6 (6.0 to 11.9)	2.21 (1.54 to 3.04)	1.16 (0.81 to 1.60)
2010	8.8 (6.1 to 12.1)	4.5 (3.2 to 6.2)	16.4 (11.5 to 22.6)	8.6 (6.1 to 11.6)	2.28 (1.59 to 3.13)	1.19 (0.84 to 1.61)
2015	5.5 (3.9 to 7.8)	2.9 (2.0 to 4.0)	17.8 (12.4 to 24.9)	9.3 (6.5 to 12.8)	2.20 (1.54 to 3.07)	1.15 (0.80 to 1.58)
2020	2.5 (1.8 to 3.6)	1.3 (0.9 to 1.8)	12.5 (8.8 to 17.9)	6.3 (4.4 to 8.9)	2.39 (1.68 to 3.42)	1.20 (0.84 to 1.70)
2021	2.4 (1.7 to 3.4)	1.2 (0.8 to 1.7)	12.0 (8.5 to 17.1)	5.9 (4.1 to 8.4)	2.46 (1.74 to 3.51)	1.22 (0.85 to 1.74)

Tunisia TUN North Africa and Middle East Stillbirth Rate / Neonatal Mortality Rate (SBR/NMR) − 20 weeks
lambda = 0.2 ; zeta = 0.99 ; scale = 5 ; amp = 0.028

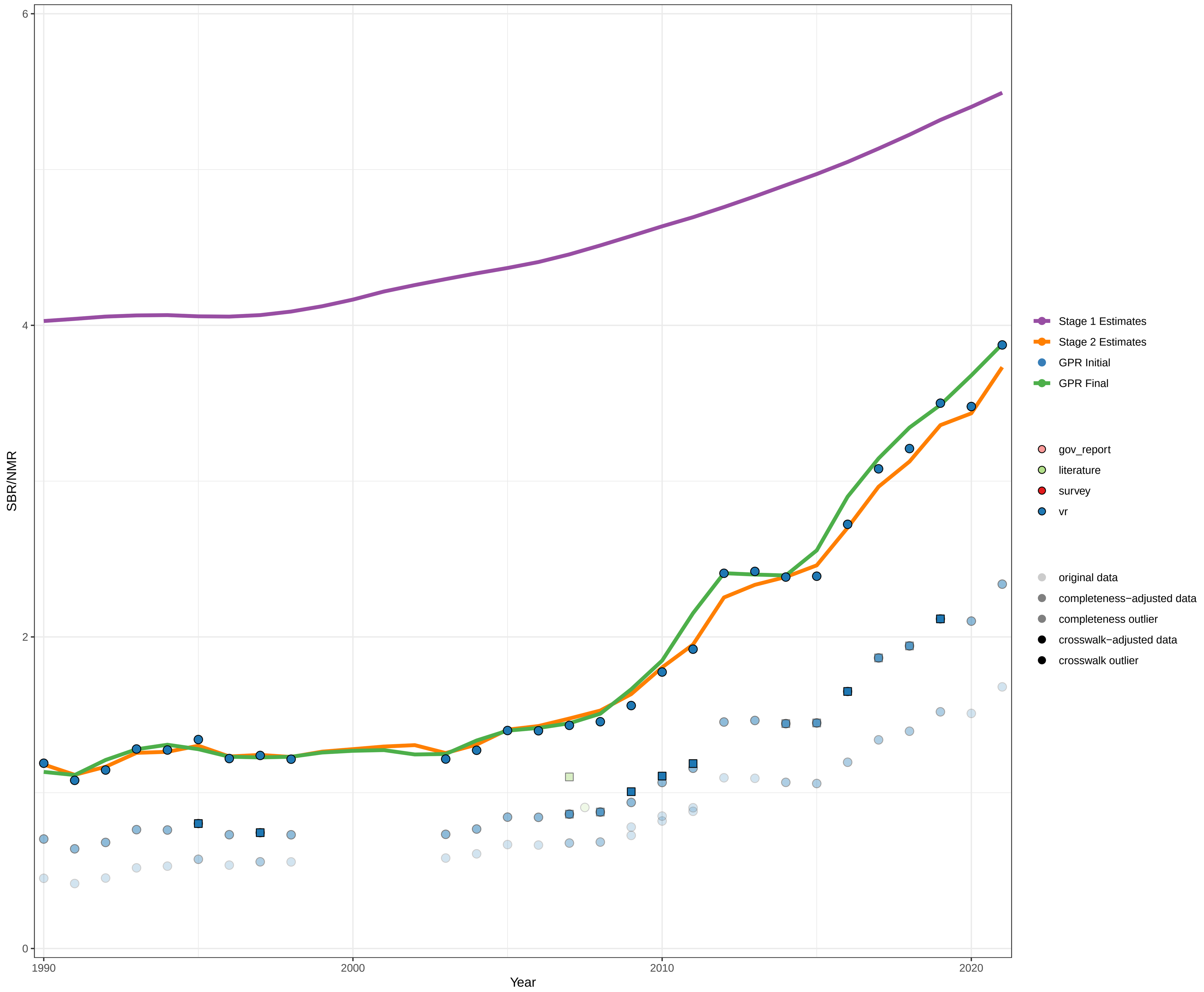

Tunisia TUN North Africa and Middle East Stillbirth Rate / Neonatal Mortality Rate (SBR/NMR) − 28 weeks
lambda = 0.2 ; zeta = 0.99 ; scale = 5 ; amp = 0.028

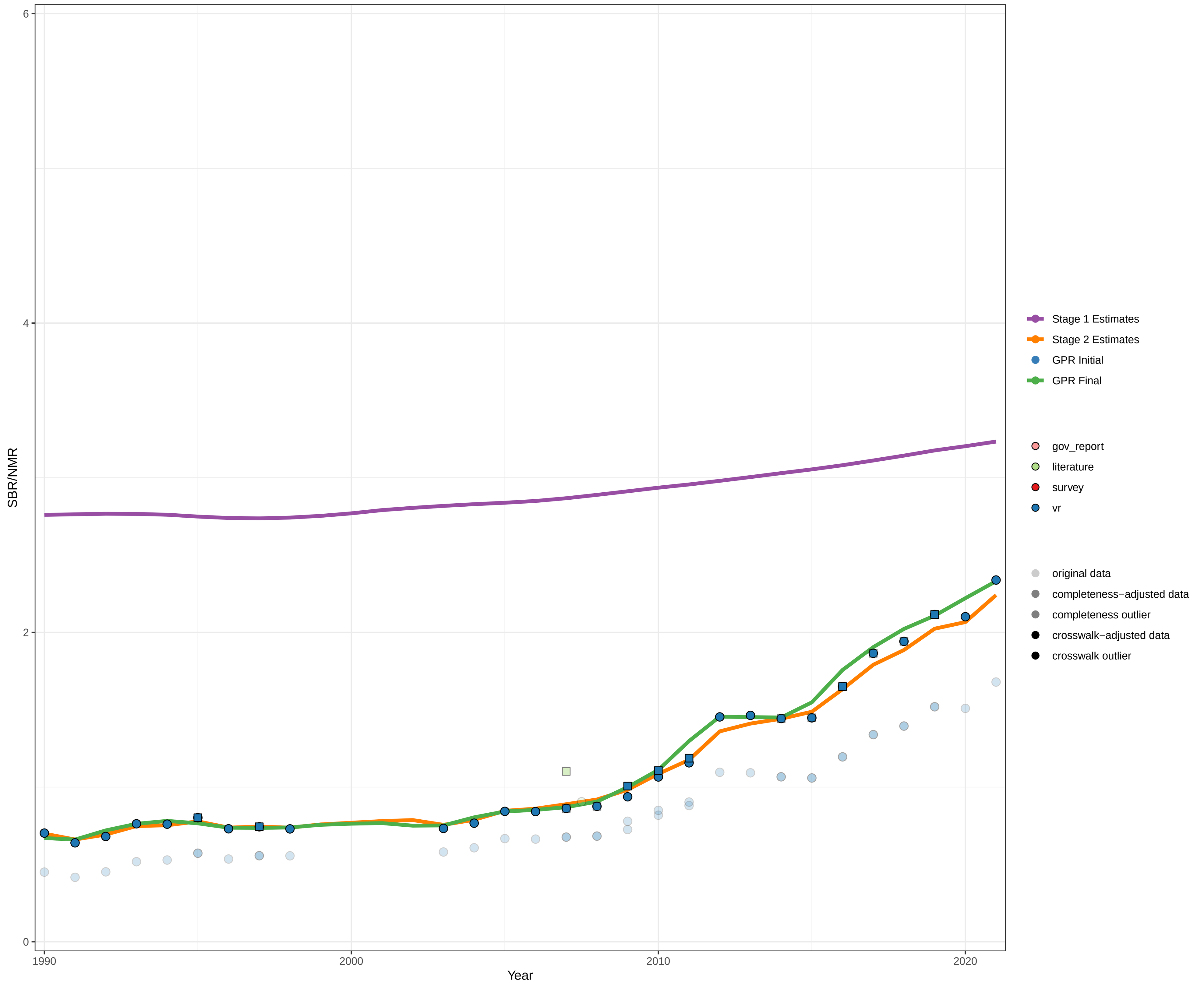

Definition	Years	Outlier	Source.Type	Title
28_weeks	1990−1998, 2007−2021	0	VR	Tunisia National Institute of Statistics Database − Births and Stillbirths by Governate
28_weeks	2003−2006	0	VR	United Nations Demographic Yearbook
22_weeks	2007	1	Sci Lit	Risk factors for perinatal mortality in a Tunisian population
28_weeks	2007, 2008, 2014, 2015, 2017, 2018	1	VR	United Nations Demographic Yearbook
28_weeks	1995, 1997, 2009−2011, 2016, 2019	2	VR	United Nations Demographic Yearbook

Tunisia TUN North Africa and Middle East Stillbirth Rate (SBR) − 20 weeks
lambda = 0.2 ; zeta = 0.99 ; scale = 5 ; amp = 0.028

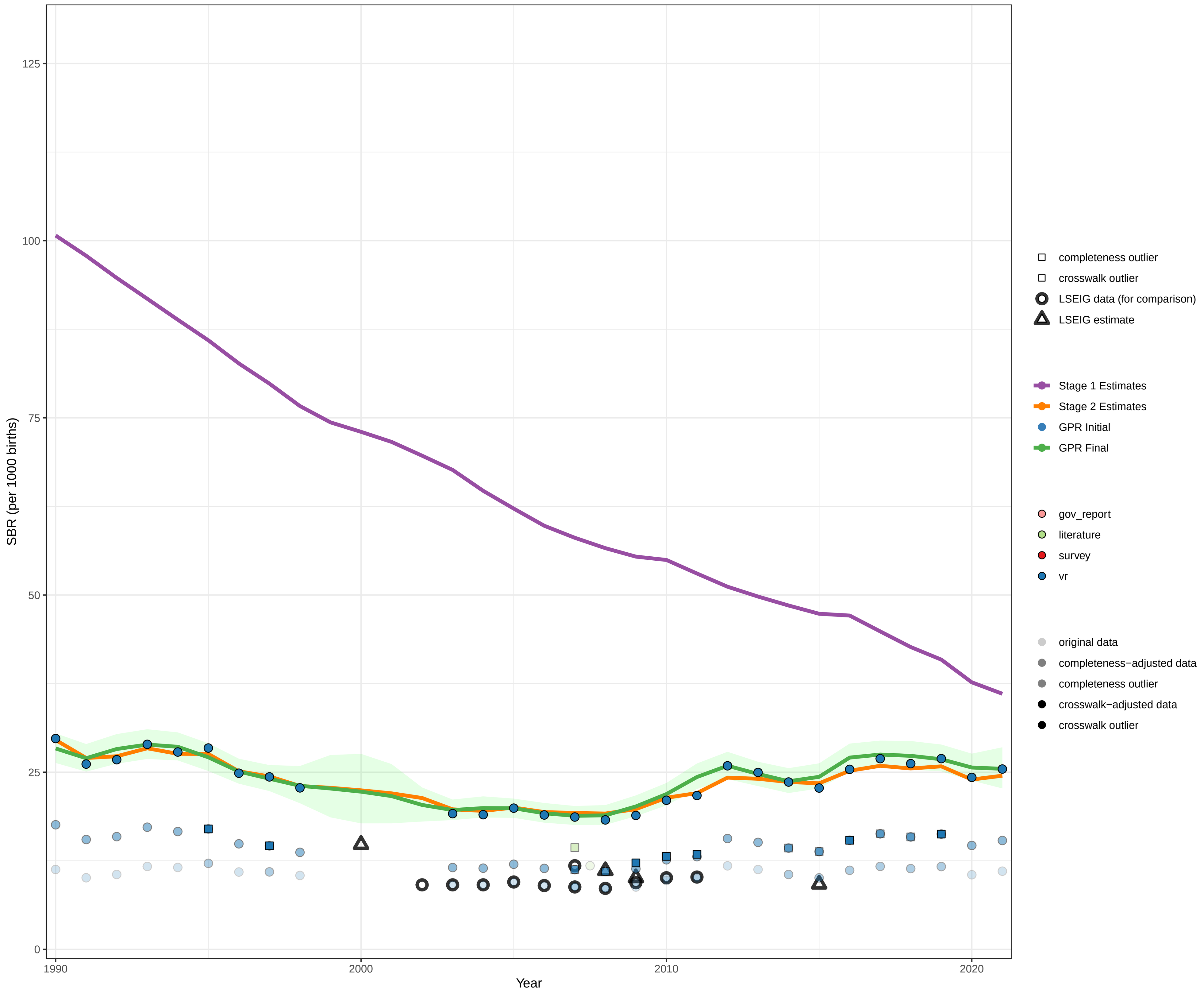

Tunisia TUN North Africa and Middle East Stillbirth Rate (SBR) − 28 weeks
lambda = 0.2 ; zeta = 0.99 ; scale = 5 ; amp = 0.028

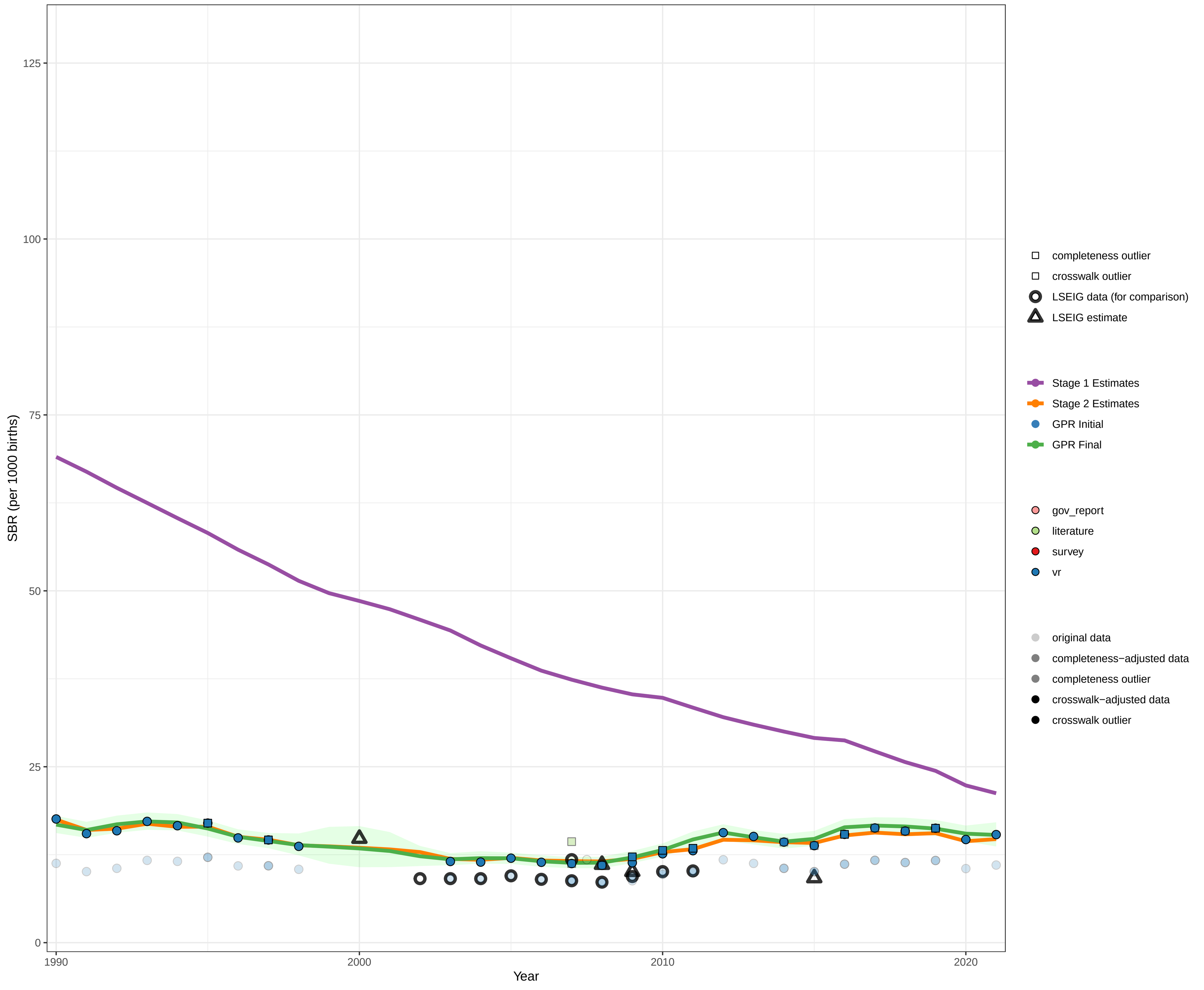

Year	Total Stillbirths (in thousands)	Total Stillbirths (in thousands)	SBR (per 1000 births)	SBR (per 1000 births)	SBR/NMR	SBR/NMR
	20 weeks	28 weeks	20 weeks	28 weeks	20 weeks	28 weeks
1990	6.4 (5.9 to 6.9)	3.7 (3.5 to 4.0)	28.3 (26.3 to 30.5)	16.8 (15.6 to 18.0)	1.13 (1.05 to 1.22)	0.67 (0.62 to 0.72)
1995	5.6 (5.2 to 6.1)	3.3 (3.1 to 3.6)	27.1 (25.2 to 29.1)	16.2 (15.1 to 17.4)	1.28 (1.19 to 1.37)	0.77 (0.71 to 0.82)
2000	4.2 (3.4 to 5.3)	2.5 (2.0 to 3.1)	22.2 (17.8 to 27.6)	13.4 (10.7 to 16.6)	1.27 (1.01 to 1.57)	0.76 (0.61 to 0.94)
2005	3.8 (3.5 to 4.1)	2.3 (2.1 to 2.4)	19.9 (18.6 to 21.3)	12.0 (11.2 to 12.8)	1.40 (1.30 to 1.49)	0.84 (0.79 to 0.90)
2010	4.5 (4.1 to 4.8)	2.7 (2.5 to 2.9)	21.9 (20.3 to 23.5)	13.2 (12.2 to 14.1)	1.85 (1.71 to 1.98)	1.11 (1.03 to 1.19)
2015	5.0 (4.6 to 5.4)	3.0 (2.8 to 3.2)	24.3 (22.7 to 26.2)	14.7 (13.8 to 15.9)	2.56 (2.38 to 2.76)	1.55 (1.45 to 1.67)
2020	4.6 (4.2 to 4.9)	2.7 (2.5 to 2.9)	25.7 (23.8 to 27.6)	15.5 (14.4 to 16.6)	3.68 (3.41 to 3.96)	2.22 (2.06 to 2.39)
2021	4.4 (3.9 to 4.9)	2.6 (2.3 to 2.9)	25.5 (22.7 to 28.5)	15.3 (13.7 to 17.1)	3.88 (3.46 to 4.34)	2.33 (2.08 to 2.61)

Turkiye TUR North Africa and Middle East Stillbirth Rate / Neonatal Mortality Rate (SBR/NMR) − 20 weeks
lambda = 0.4 ; zeta = 0.9 ; scale = 10 ; amp = 0.028

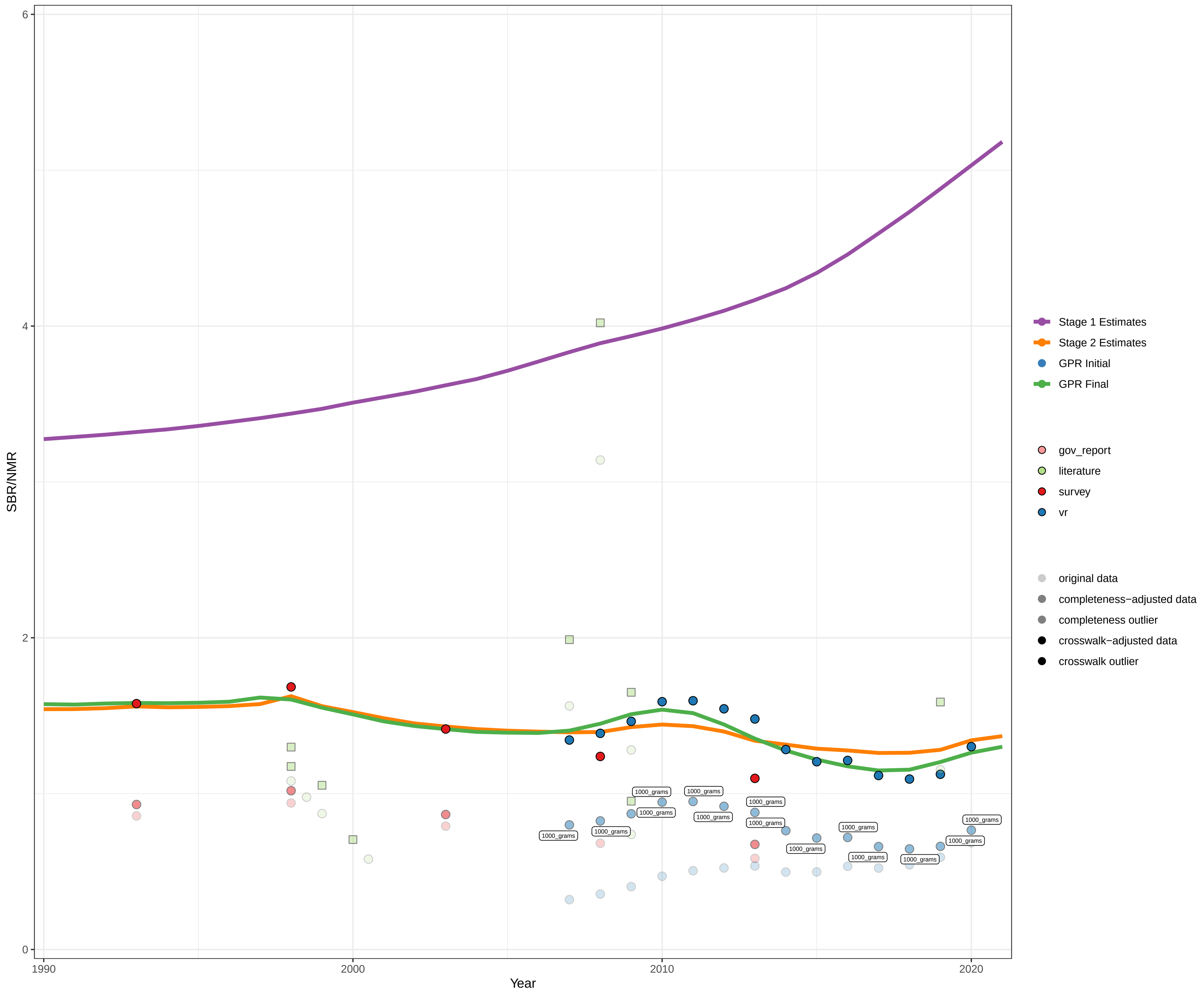

Turkiye TUR North Africa and Middle East Stillbirth Rate / Neonatal Mortality Rate (SBR/NMR) − 28 weeks
lambda = 0.4 ; zeta = 0.9 ; scale = 10 ; amp = 0.028

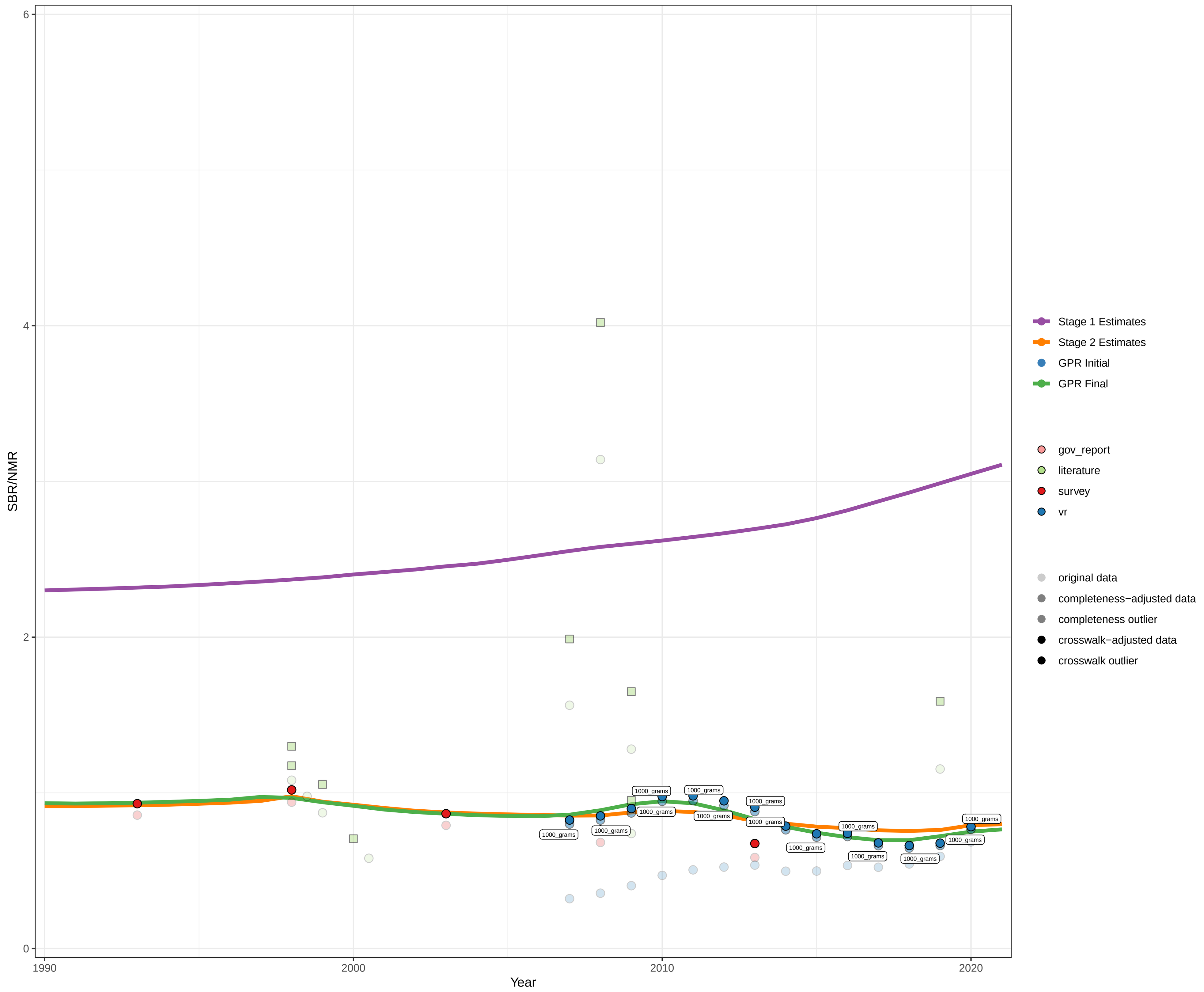

Definition	Years	Outlier	Source.Type	Title
1000_grams	2007−2020	0	VR	European Health for All Database
28_weeks	1993	0	Survey	Turkey Demographic and Health Survey 1993
28_weeks	1998	0	Survey	Turkey Demographic and Health Survey 1998
28_weeks	2003	0	Survey	Turkey Demographic and Health Survey 2003−2004
28_weeks	2008	0	Survey	Turkey Demographic and Health Survey 2008
28_weeks	2013	0	Survey	Turkey Demographic and Health Survey 2013−2014
20_weeks	2008	1	Sci Lit	The Change of Perinatal Mortality Over Three Decades in a Reference Centre in the Aegean Region: Neonatal Mortality has decreased but Foetal Mortality Remains Unchanged
22_weeks	1998, 2009	1	Sci Lit	The changing pattern of perinatal mortality and causes of death in central Anatolian region of Turkey
22_weeks	2009	1	Sci Lit	Spatial analysis of perinatal mortality rates with geographic information systems in Kocaeli, Turkey
22_weeks_AND_500_grams	2007	1	Sci Lit	Assesment of perinatal mortality in two different periods: results of a single center
28_weeks	1998, 2000	1	Sci Lit	Perinatal mortality rate − hospital based study during 1998−2001 at Hacettepe University
28_weeks	2019	1	Sci Lit	Effect of Immigration on Adverse Perinatal Outcomes: Analysis of Experiences at a Turkish Tertiary Hospital
500_grams	1999	1	Sci Lit	Perinatal mortality in Turkey

Turkiye TUR North Africa and Middle East Stillbirth Rate (SBR) − 20 weeks
lambda = 0.4 ; zeta = 0.9 ; scale = 10 ; amp = 0.028

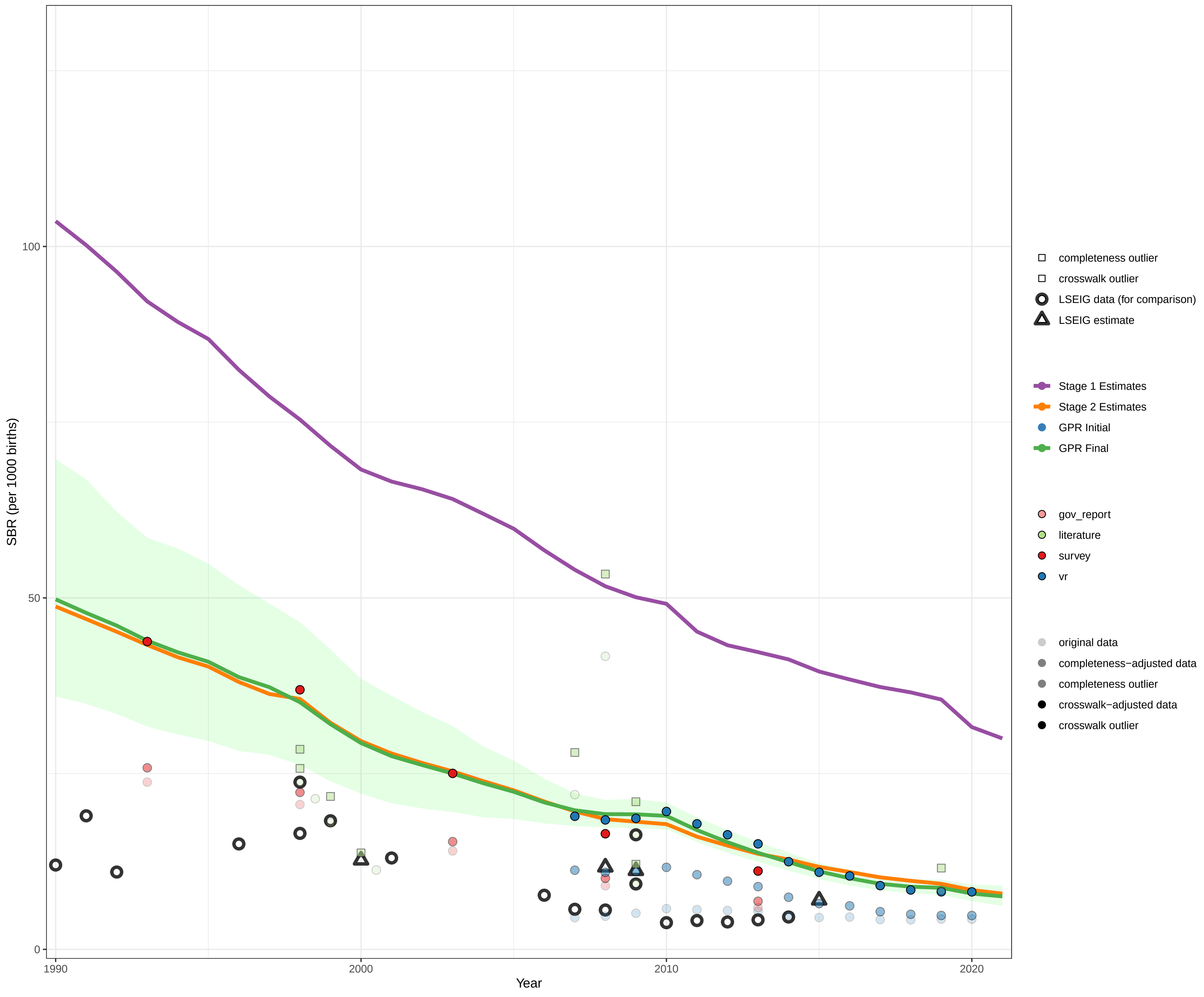

Turkiye TUR North Africa and Middle East Stillbirth Rate (SBR) − 28 weeks
lambda = 0.4 ; zeta = 0.9 ; scale = 10 ; amp = 0.028

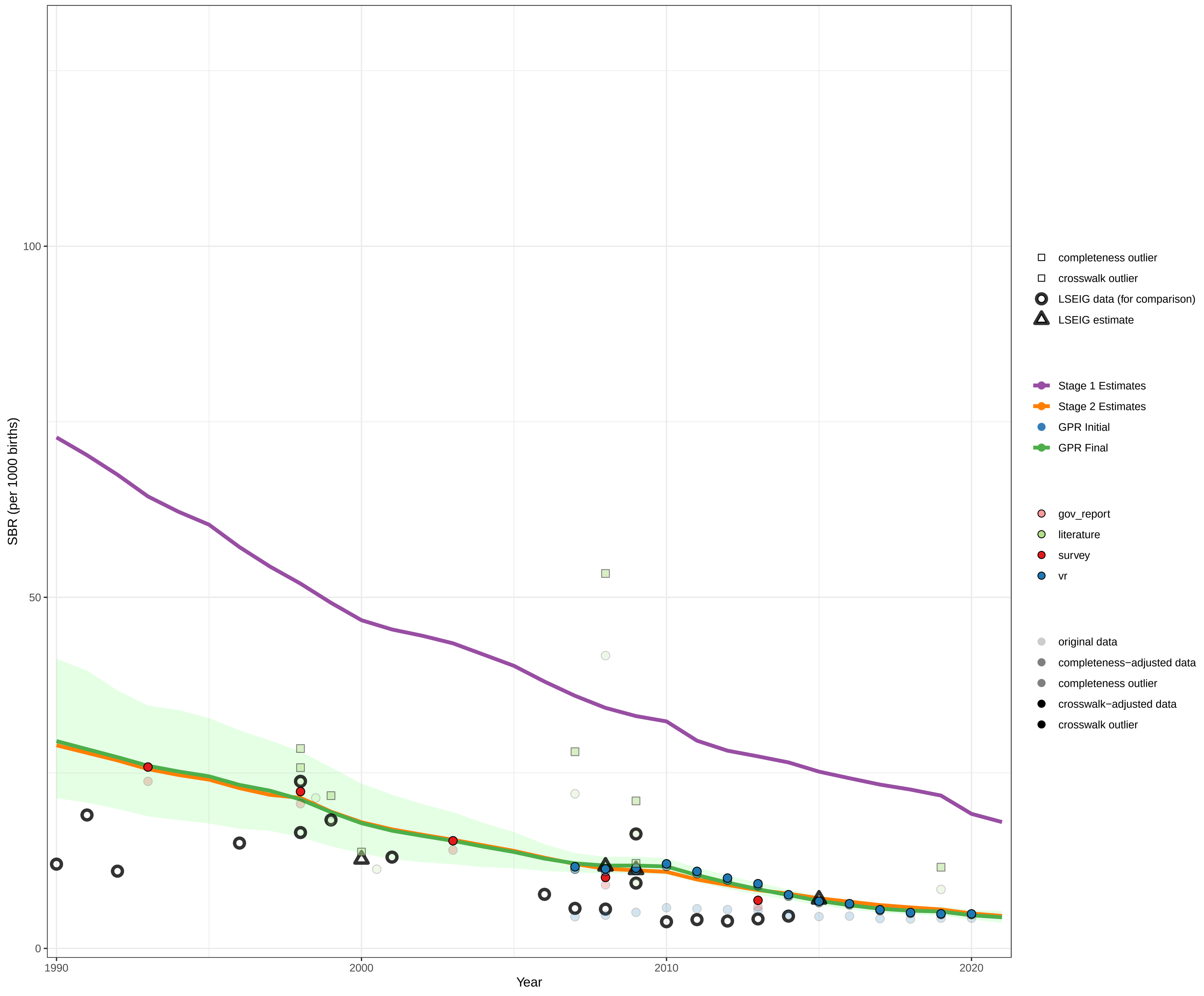

Year	Total Stillbirths (in thousands)	Total Stillbirths (in thousands)	SBR (per 1000 births)	SBR (per 1000 births)	SBR/NMR	SBR/NMR
	20 weeks	28 weeks	20 weeks	28 weeks	20 weeks	28 weeks
1990	79.5 (56.5 to 113.6)	46.1 (33.1 to 65.1)	49.8 (36.0 to 69.7)	29.5 (21.4 to 41.2)	1.57 (1.14 to 2.20)	0.93 (0.68 to 1.30)
1995	62.9 (44.9 to 85.5)	37.0 (26.6 to 49.9)	40.9 (29.6 to 54.9)	24.5 (17.8 to 32.8)	1.58 (1.15 to 2.12)	0.95 (0.69 to 1.27)
2000	43.6 (32.6 to 57.7)	26.2 (19.8 to 34.7)	29.3 (22.1 to 38.5)	17.8 (13.5 to 23.5)	1.51 (1.14 to 1.98)	0.92 (0.70 to 1.21)
2005	31.1 (25.6 to 37.4)	18.9 (15.6 to 22.9)	22.4 (18.5 to 26.9)	13.7 (11.4 to 16.6)	1.39 (1.15 to 1.67)	0.85 (0.71 to 1.03)
2010	25.7 (23.0 to 28.3)	15.7 (14.1 to 17.2)	19.0 (17.1 to 20.9)	11.7 (10.5 to 12.8)	1.54 (1.38 to 1.69)	0.95 (0.85 to 1.04)
2015	14.4 (13.0 to 16.0)	8.8 (7.9 to 9.7)	11.1 (10.0 to 12.3)	6.8 (6.1 to 7.5)	1.22 (1.10 to 1.35)	0.74 (0.67 to 0.82)
2020	8.8 (7.6 to 10.2)	5.2 (4.5 to 6.0)	7.9 (6.9 to 9.2)	4.7 (4.1 to 5.5)	1.26 (1.09 to 1.47)	0.75 (0.65 to 0.87)
2021	8.0 (6.6 to 9.7)	4.7 (3.9 to 5.7)	7.5 (6.2 to 9.1)	4.4 (3.7 to 5.3)	1.30 (1.07 to 1.57)	0.77 (0.63 to 0.92)

United Arab Emirates ARE North Africa and Middle East Stillbirth Rate / Neonatal Mortality Rate (SBR/NMR) − 20 weeks
lambda = 0.4 ; zeta = 0.9 ; scale = 10 ; amp = 0.028

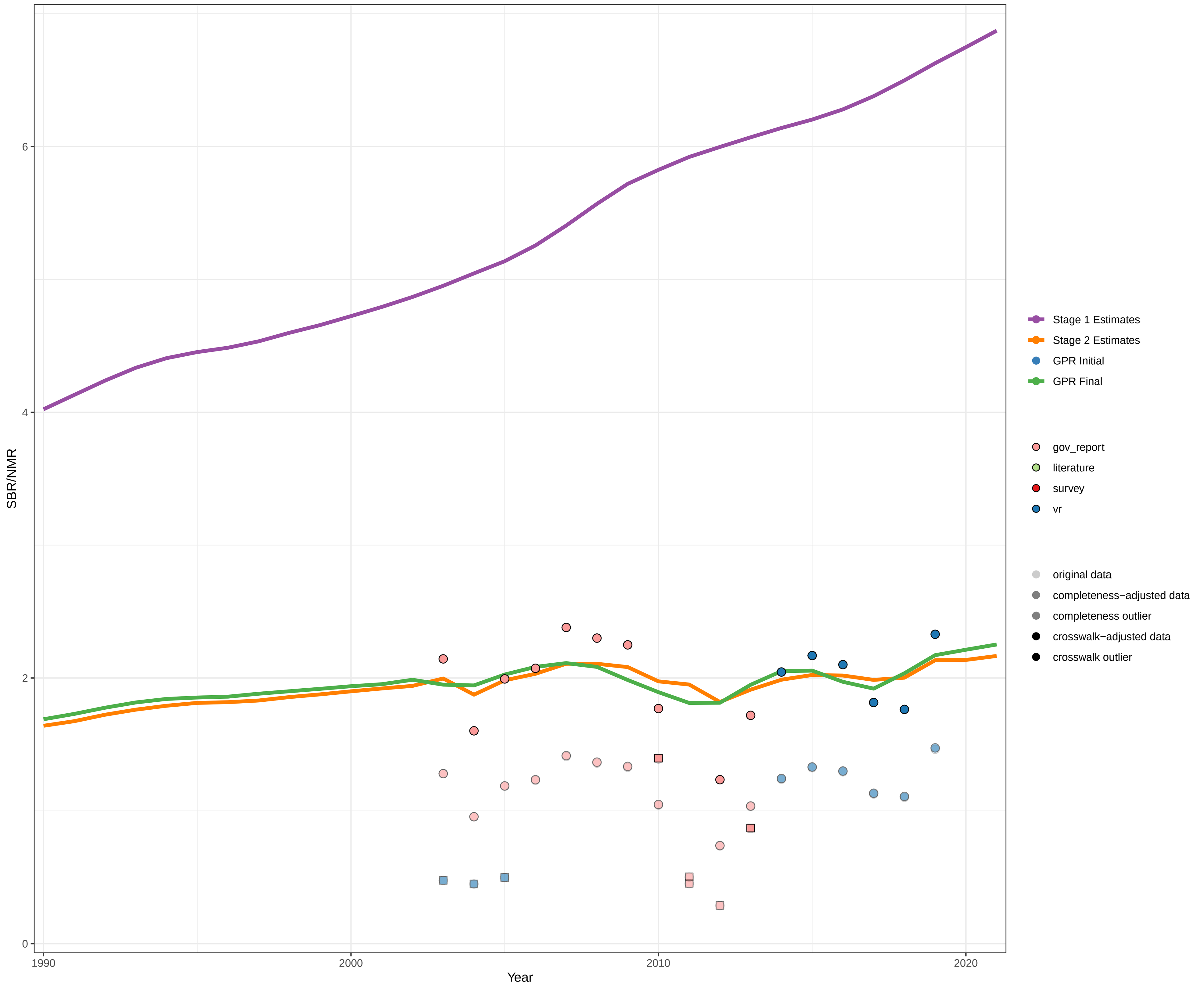

United Arab Emirates ARE North Africa and Middle East Stillbirth Rate / Neonatal Mortality Rate (SBR/NMR) − 28 weeks
lambda = 0.4 ; zeta = 0.9 ; scale = 10 ; amp = 0.028

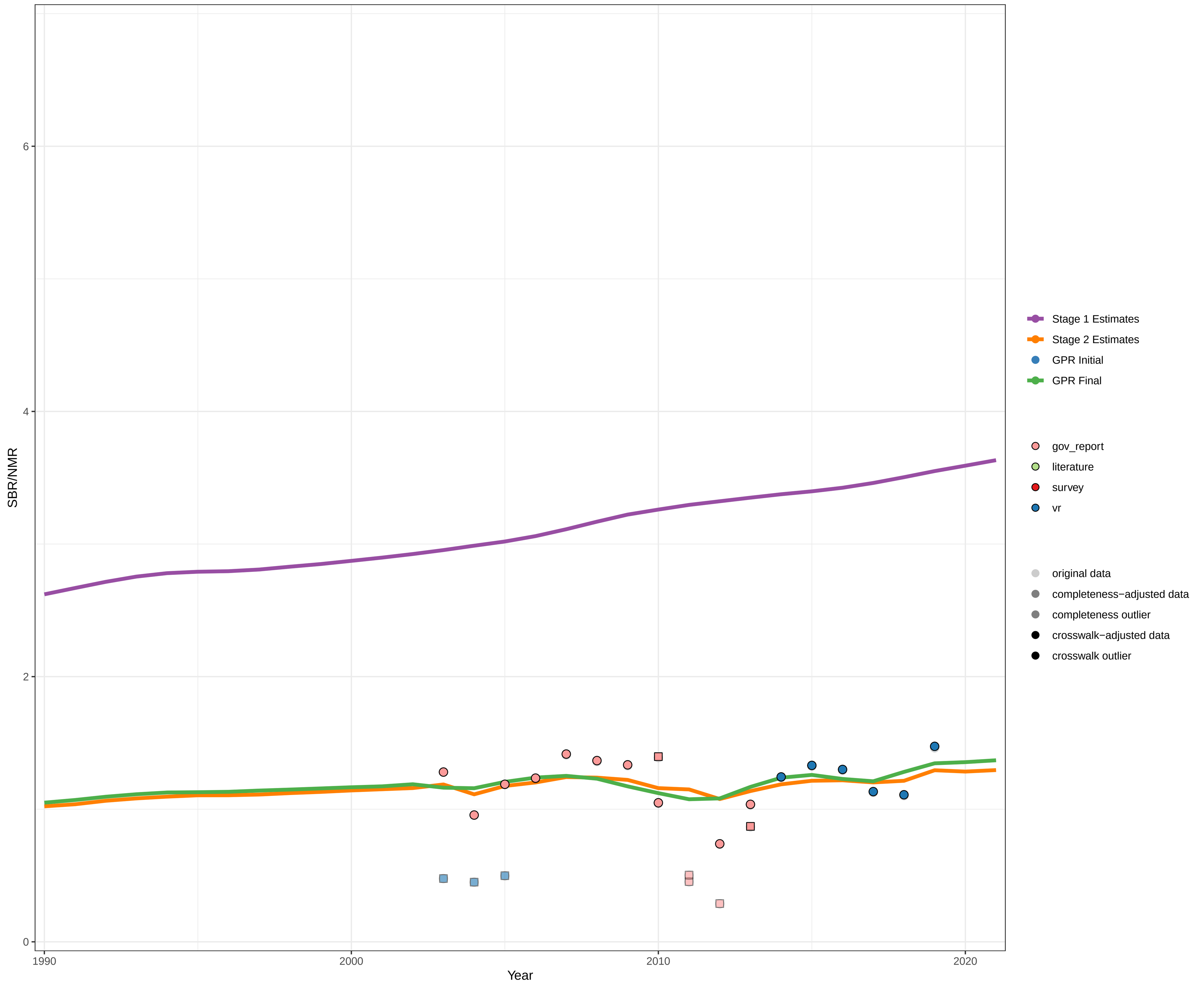

Definition	Years	Outlier	Source.Type	Title
28_weeks	2003−2007	0	Statistical Report	United Arab Emirates Health Statistical Yearbook
28_weeks	2008	0	Statistical Report	United Arab Emirates Health Statistics
28_weeks	2009, 2010, 2012, 2013	0	Statistical Report	United Arab Emirates − Abu Dhabi Health Statistics
28_weeks	2014−2019	0	VR	United Nations Demographic Yearbook
28_weeks	2003−2005	1	VR	United Nations Demographic Yearbook
28_weeks	2011, 2012	1	Statistical Report	United Arab Emirates − Abu Dhabi Health Statistics
28_weeks	2010, 2013	2	Statistical Report	United Arab Emirates − Abu Dhabi Health Statistics

United Arab Emirates ARE North Africa and Middle East Stillbirth Rate (SBR) − 20 weeks
lambda = 0.4 ; zeta = 0.9 ; scale = 10 ; amp = 0.028

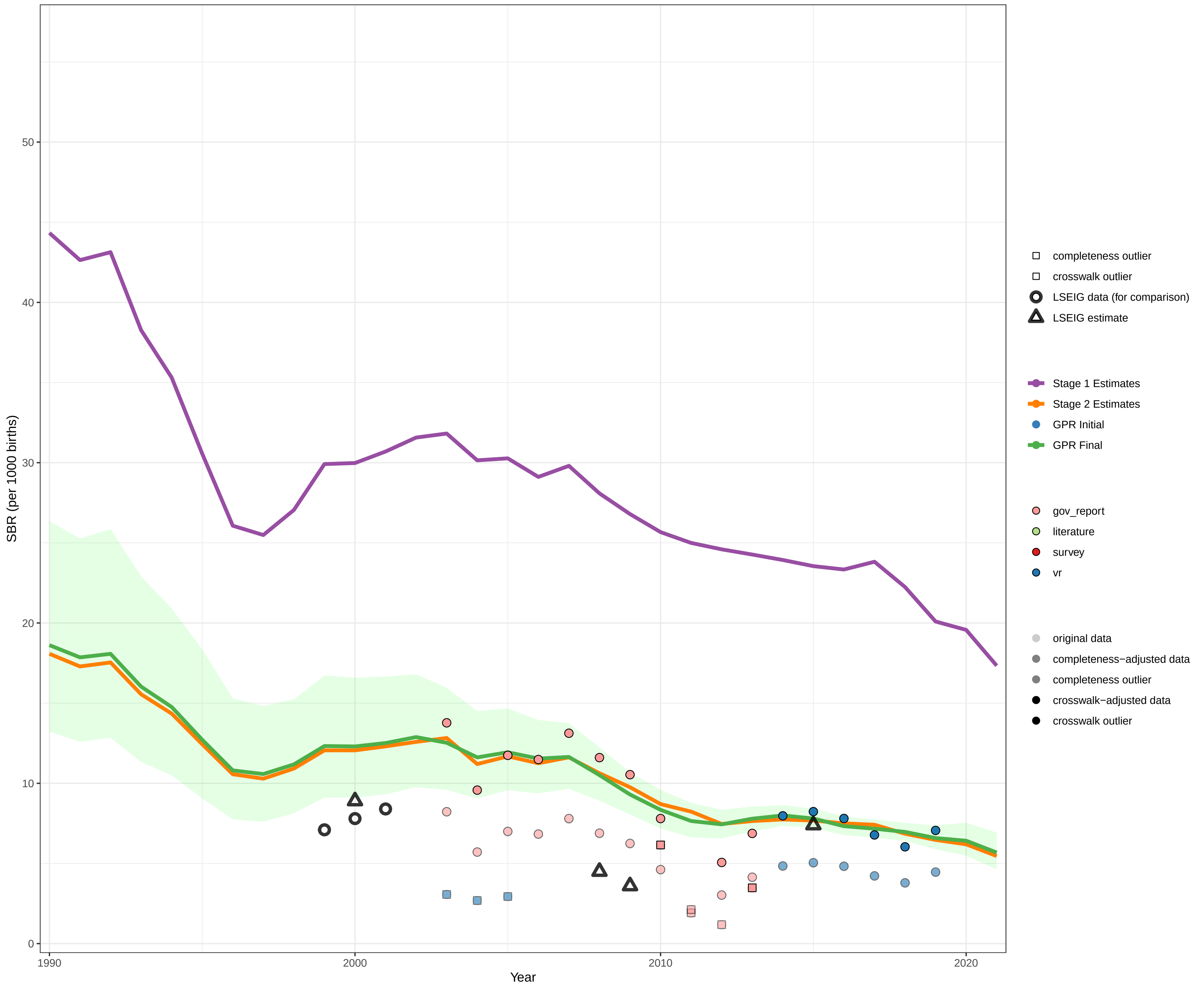

United Arab Emirates ARE North Africa and Middle East Stillbirth Rate (SBR) − 28 weeks
lambda = 0.4 ; zeta = 0.9 ; scale = 10 ; amp = 0.028

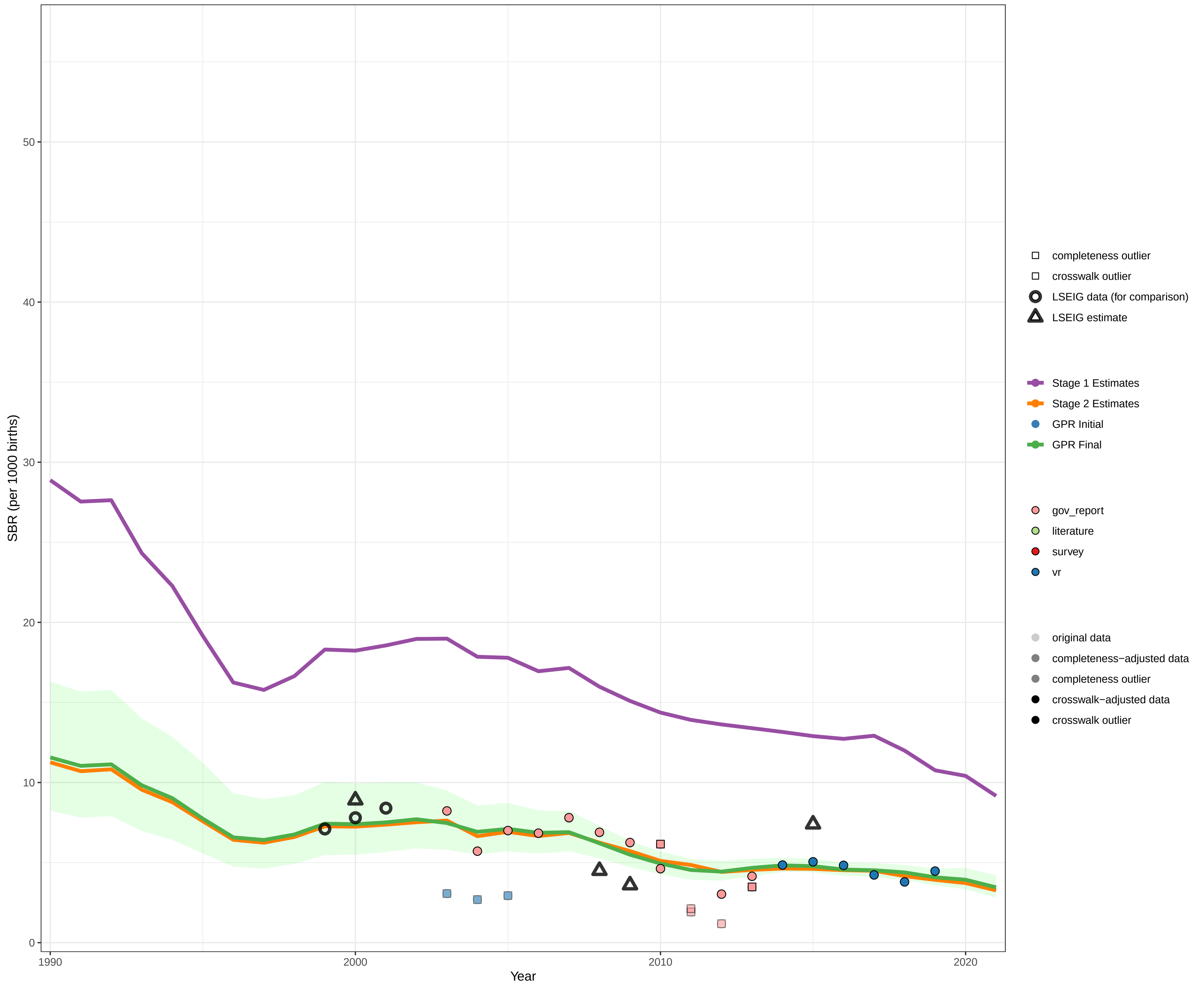

Year	Total Stillbirths (in thousands)	Total Stillbirths (in thousands)	SBR (per 1000 births)	SBR (per 1000 births)	SBR/NMR	SBR/NMR
	20 weeks	28 weeks	20 weeks	28 weeks	20 weeks	28 weeks
1990	0.9 (0.6 to 1.3)	0.6 (0.4 to 0.8)	18.6 (13.2 to 26.3)	11.6 (8.2 to 16.3)	1.69 (1.20 to 2.39)	1.05 (0.75 to 1.48)
1995	0.6 (0.4 to 0.9)	0.4 (0.3 to 0.5)	12.7 (9.1 to 18.4)	7.7 (5.6 to 11.2)	1.85 (1.32 to 2.67)	1.13 (0.81 to 1.64)
2000	0.6 (0.5 to 0.8)	0.4 (0.3 to 0.5)	12.3 (9.1 to 16.6)	7.4 (5.5 to 10.0)	1.94 (1.44 to 2.61)	1.17 (0.87 to 1.57)
2005	0.7 (0.5 to 0.8)	0.4 (0.3 to 0.5)	11.9 (9.6 to 14.7)	7.1 (5.7 to 8.7)	2.03 (1.62 to 2.49)	1.21 (0.97 to 1.48)
2010	0.7 (0.6 to 0.9)	0.4 (0.4 to 0.5)	8.3 (7.2 to 9.6)	4.9 (4.3 to 5.7)	1.89 (1.63 to 2.18)	1.12 (0.97 to 1.29)
2015	0.8 (0.7 to 0.8)	0.5 (0.4 to 0.5)	7.8 (7.2 to 8.4)	4.8 (4.4 to 5.2)	2.06 (1.90 to 2.22)	1.26 (1.16 to 1.38)
2020	0.5 (0.4 to 0.6)	0.3 (0.3 to 0.4)	6.4 (5.5 to 7.5)	3.9 (3.3 to 4.7)	2.21 (1.89 to 2.60)	1.35 (1.15 to 1.61)
2021	0.4 (0.3 to 0.5)	0.3 (0.2 to 0.3)	5.7 (4.6 to 6.9)	3.5 (2.8 to 4.2)	2.25 (1.83 to 2.75)	1.37 (1.11 to 1.67)

Yemen YEM North Africa and Middle East Stillbirth Rate / Neonatal Mortality Rate (SBR/NMR) − 20 weeks
lambda = 0.8 ; zeta = 0.7 ; scale = 15 ; amp = 0.028

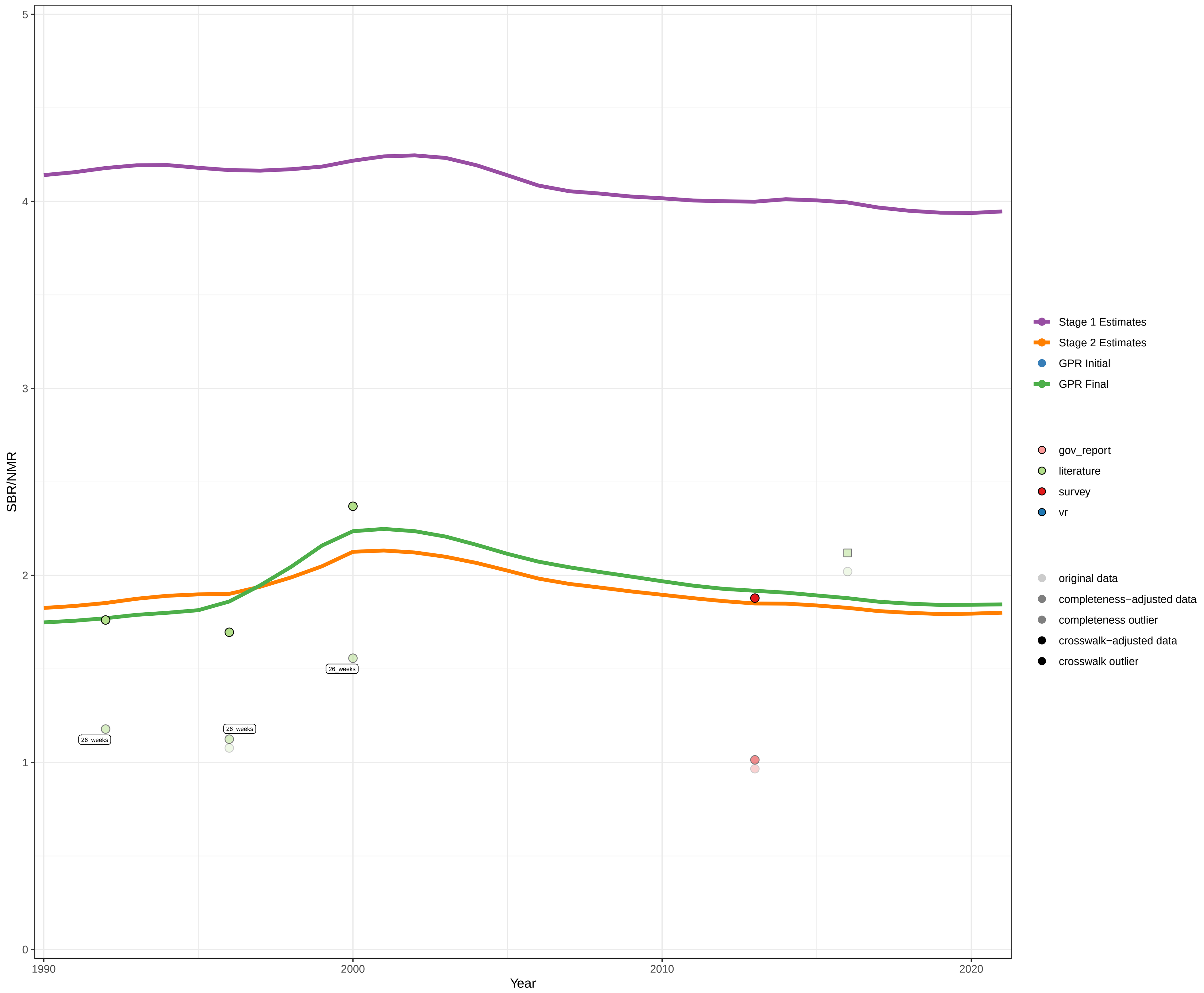

Yemen YEM North Africa and Middle East Stillbirth Rate / Neonatal Mortality Rate (SBR/NMR) − 28 weeks
lambda = 0.8 ; zeta = 0.7 ; scale = 15 ; amp = 0.028

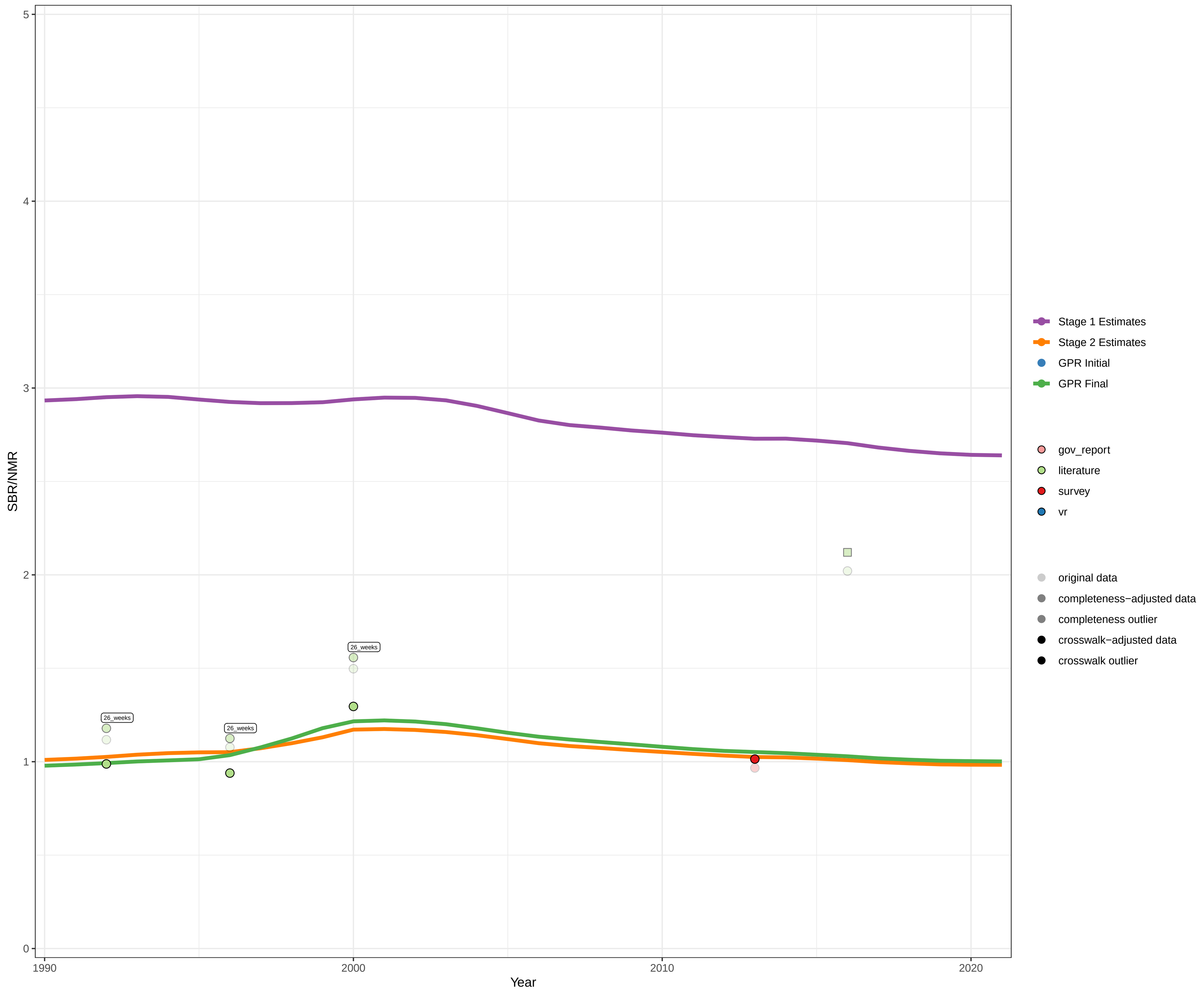

Definition	Years	Outlier	Source.Type	Title
26_weeks	1992, 1996, 2000	0	Sci Lit	Burden of perinatal conditions in Yemen: A 12−year hospital−based study
28_weeks	2013	0	Survey	Yemen Demographic and Health Survey 2013
28_weeks	2016	1	Sci Lit	Maternal, prenatal and traditional practice factors associated with perinatal mortality in Yemen

Yemen YEM North Africa and Middle East Stillbirth Rate (SBR) − 20 weeks
lambda = 0.8 ; zeta = 0.7 ; scale = 15 ; amp = 0.028

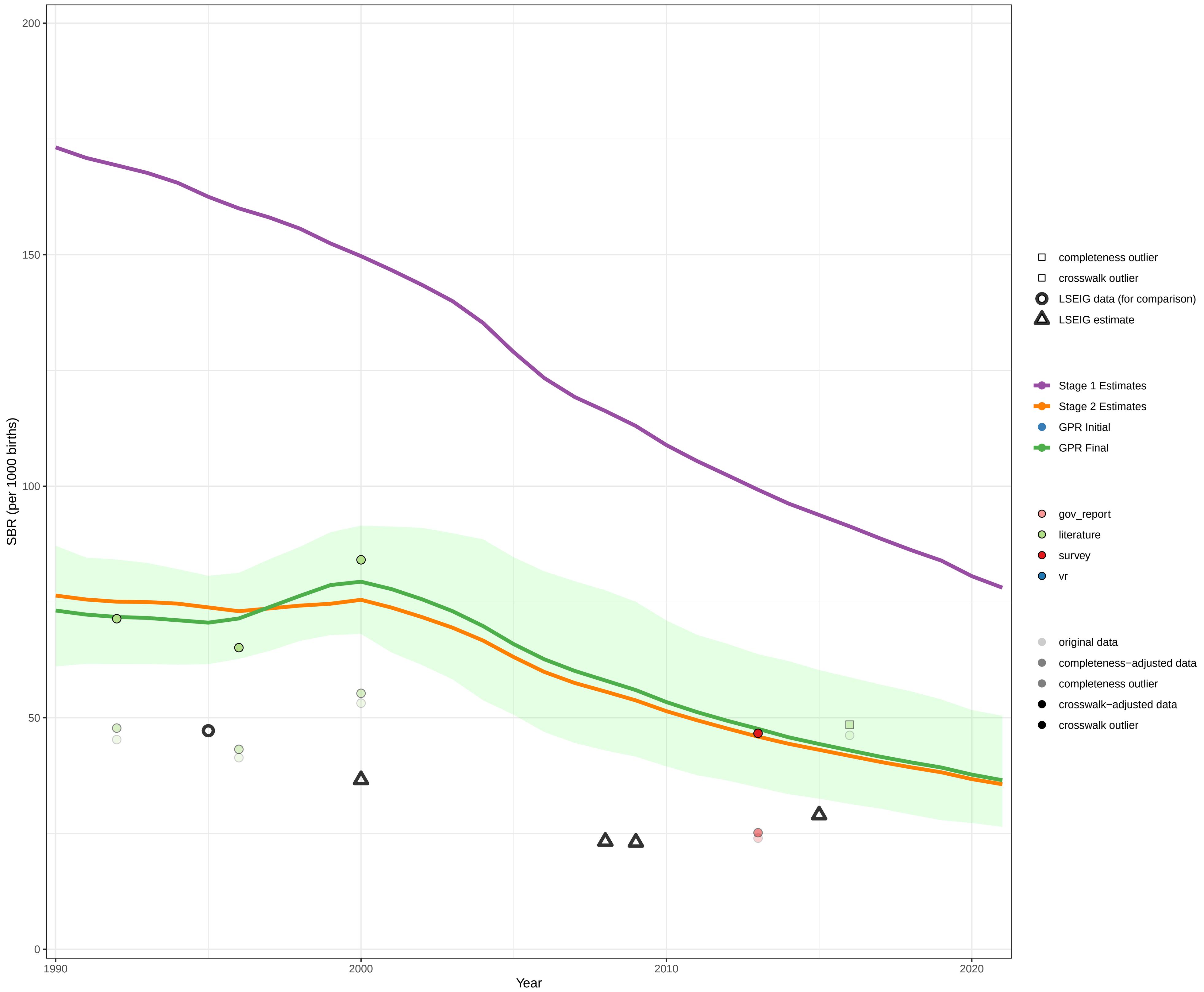

Yemen YEM North Africa and Middle East Stillbirth Rate (SBR) − 28 weeks
lambda = 0.8 ; zeta = 0.7 ; scale = 15 ; amp = 0.028

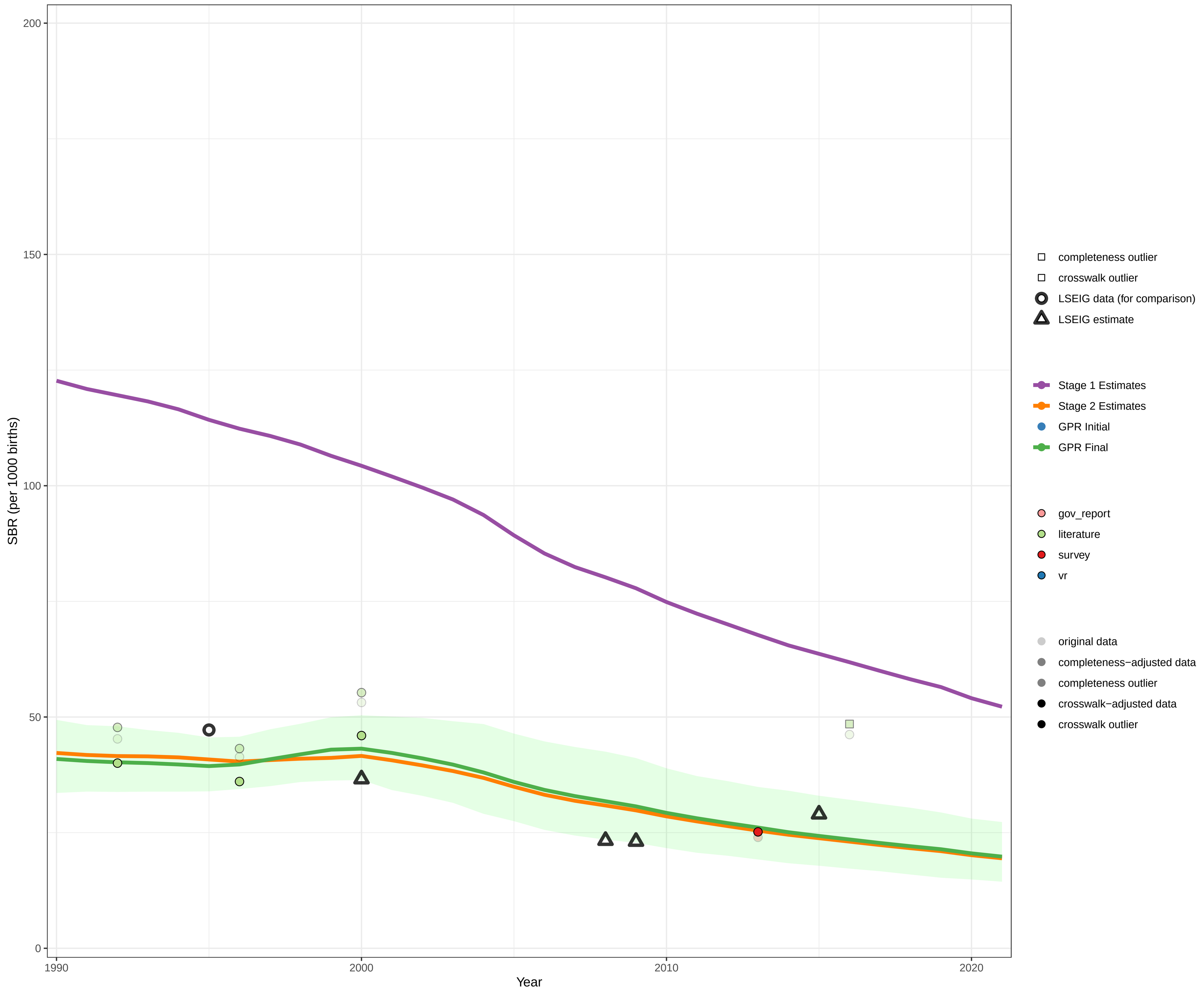

Year	Total Stillbirths (in thousands)	Total Stillbirths (in thousands)	SBR (per 1000 births)	SBR (per 1000 births)	SBR/NMR	SBR/NMR
	20 weeks	28 weeks	20 weeks	28 weeks	20 weeks	28 weeks
1990	52.2 (43.0 to 63.1)	28.2 (23.0 to 34.3)	73.2 (61.1 to 87.2)	40.9 (33.6 to 49.4)	1.75 (1.46 to 2.08)	0.98 (0.80 to 1.18)
1995	56.2 (48.5 to 64.9)	30.3 (26.0 to 35.3)	70.5 (61.6 to 80.7)	39.4 (33.9 to 45.6)	1.81 (1.58 to 2.07)	1.01 (0.87 to 1.17)
2000	73.7 (62.4 to 86.1)	38.6 (32.3 to 45.3)	79.4 (68.1 to 91.5)	43.2 (36.4 to 50.4)	2.24 (1.92 to 2.58)	1.22 (1.03 to 1.42)
2005	69.4 (52.4 to 90.9)	36.7 (27.8 to 47.8)	65.9 (50.6 to 84.7)	36.0 (27.5 to 46.4)	2.12 (1.63 to 2.72)	1.15 (0.88 to 1.49)
2010	60.1 (43.8 to 81.4)	32.1 (23.6 to 43.1)	53.4 (39.5 to 71.0)	29.3 (21.7 to 38.9)	1.97 (1.46 to 2.62)	1.08 (0.80 to 1.44)
2015	48.8 (35.3 to 67.4)	26.2 (19.1 to 35.8)	44.3 (32.5 to 60.3)	24.3 (17.8 to 33.0)	1.89 (1.39 to 2.58)	1.04 (0.76 to 1.41)
2020	39.0 (27.9 to 54.2)	20.9 (15.0 to 28.7)	37.7 (27.3 to 51.7)	20.5 (14.9 to 28.0)	1.84 (1.33 to 2.53)	1.00 (0.73 to 1.37)
2021	37.5 (26.9 to 52.5)	20.0 (14.4 to 27.8)	36.5 (26.4 to 50.4)	19.8 (14.4 to 27.3)	1.85 (1.34 to 2.55)	1.00 (0.73 to 1.38)

South Asia S5 NA Stillbirth Rate / Neonatal Mortality Rate (SBR/NMR) − 20 weeks
lambda = 1 ; zeta = 0.6 ; scale = 15 ; amp = 0.028

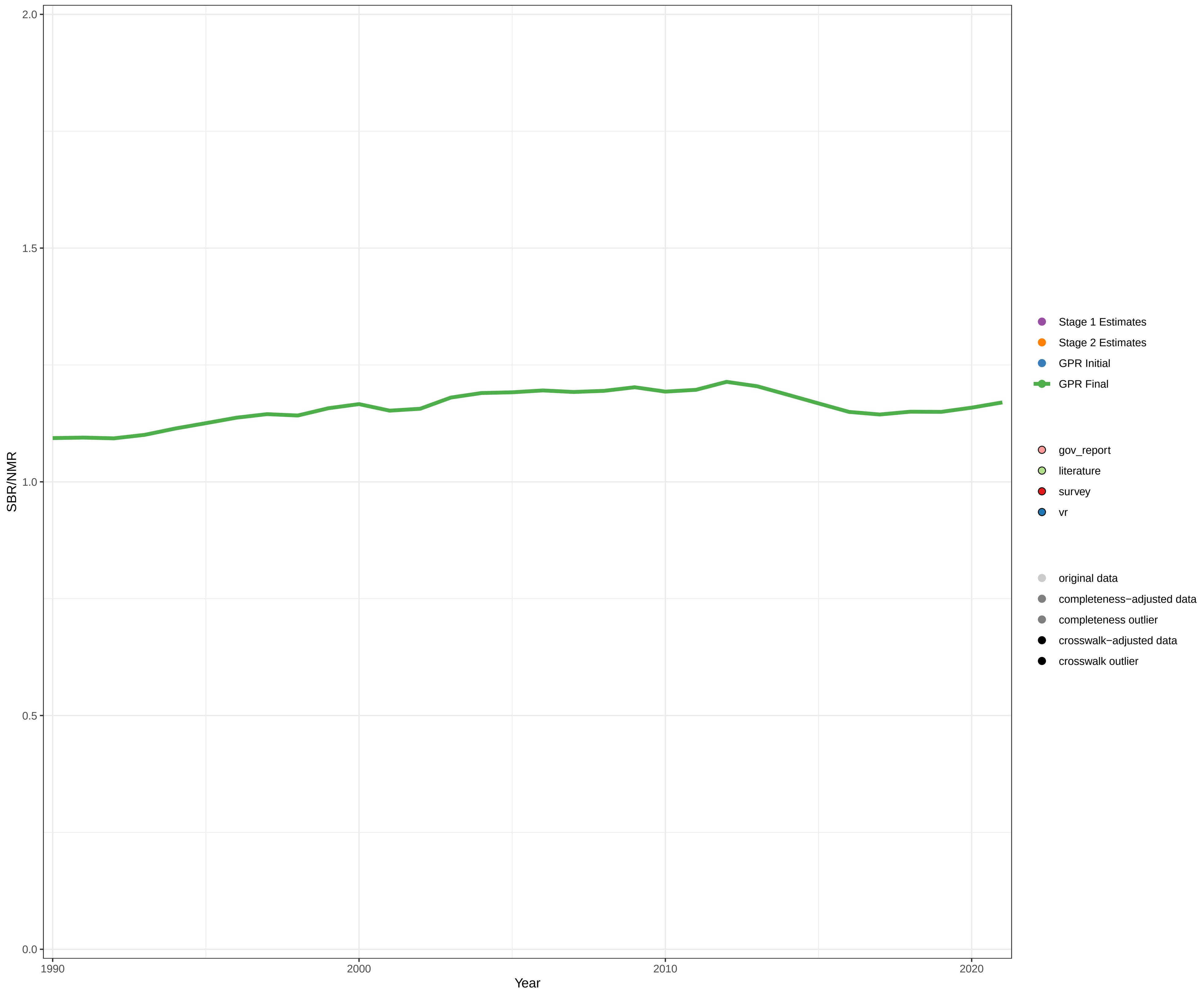

South Asia S5 NA Stillbirth Rate / Neonatal Mortality Rate (SBR/NMR) − 28 weeks
lambda = 1 ; zeta = 0.6 ; scale = 15 ; amp = 0.028

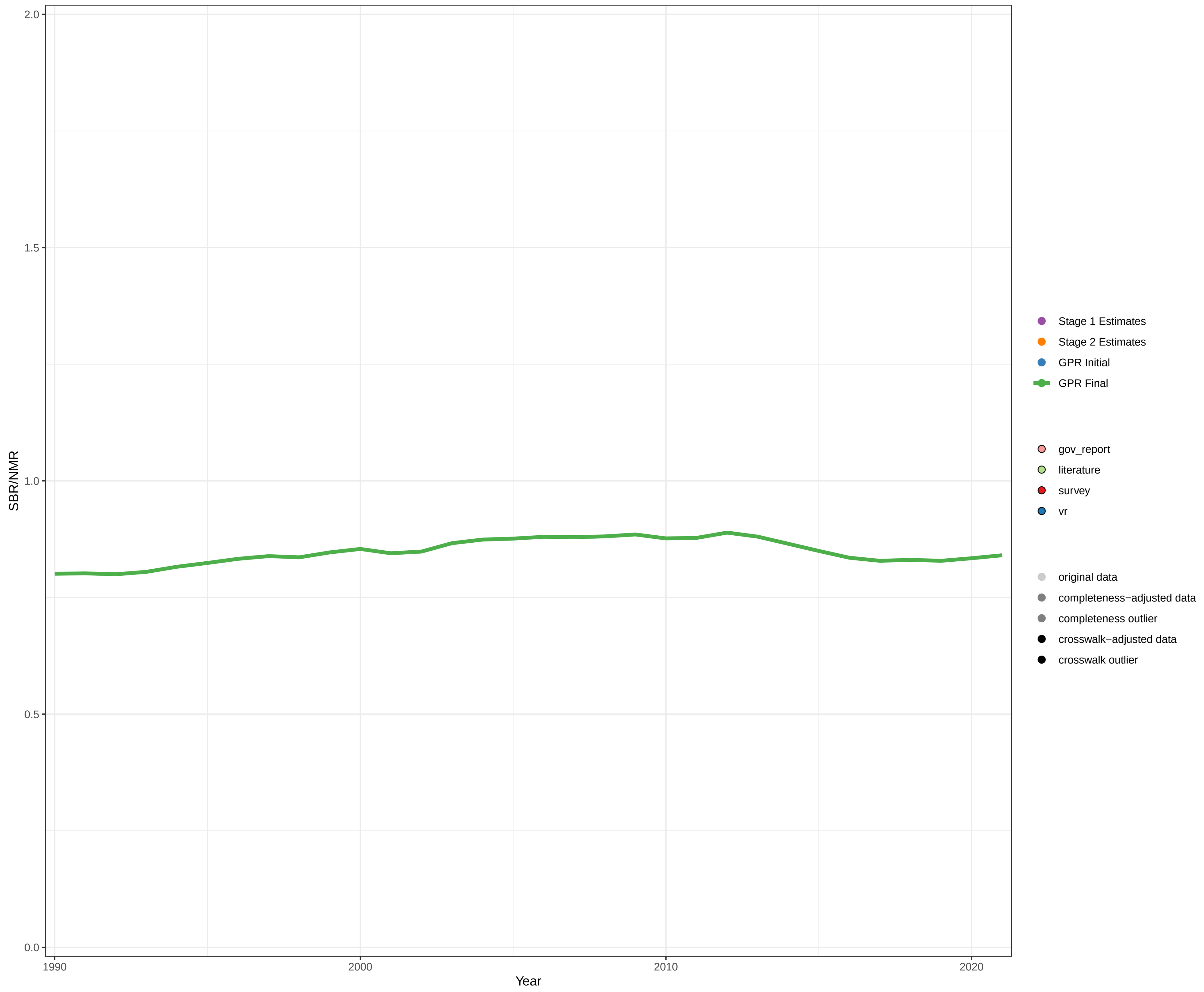

Definition	Years	Outlier	Source.Type	Title

South Asia S5 NA Stillbirth Rate (SBR) − 20 weeks
lambda = 1 ; zeta = 0.6 ; scale = 15 ; amp = 0.028

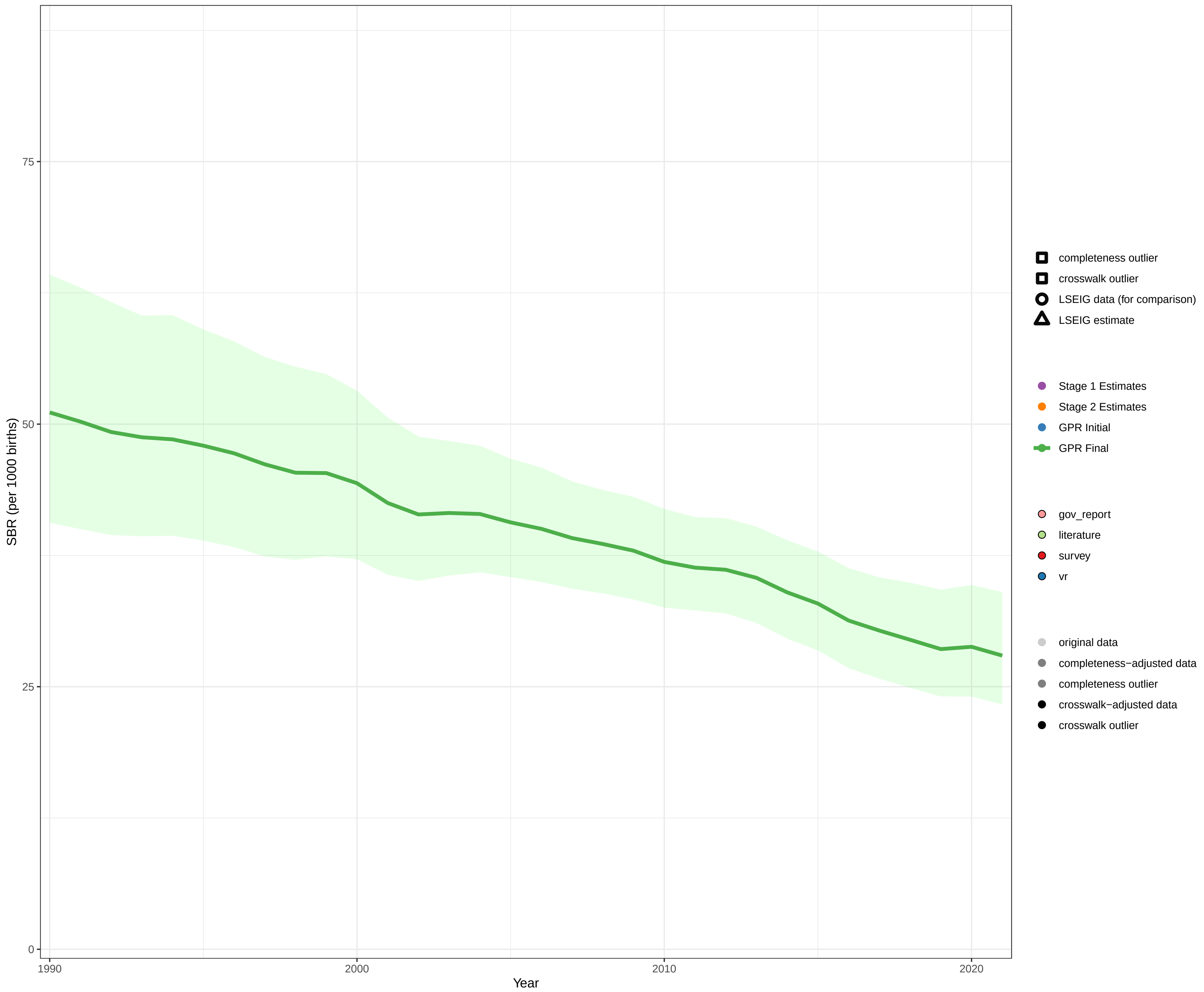

South Asia S5 NA Stillbirth Rate (SBR) − 28 weeks
lambda = 1 ; zeta = 0.6 ; scale = 15 ; amp = 0.028

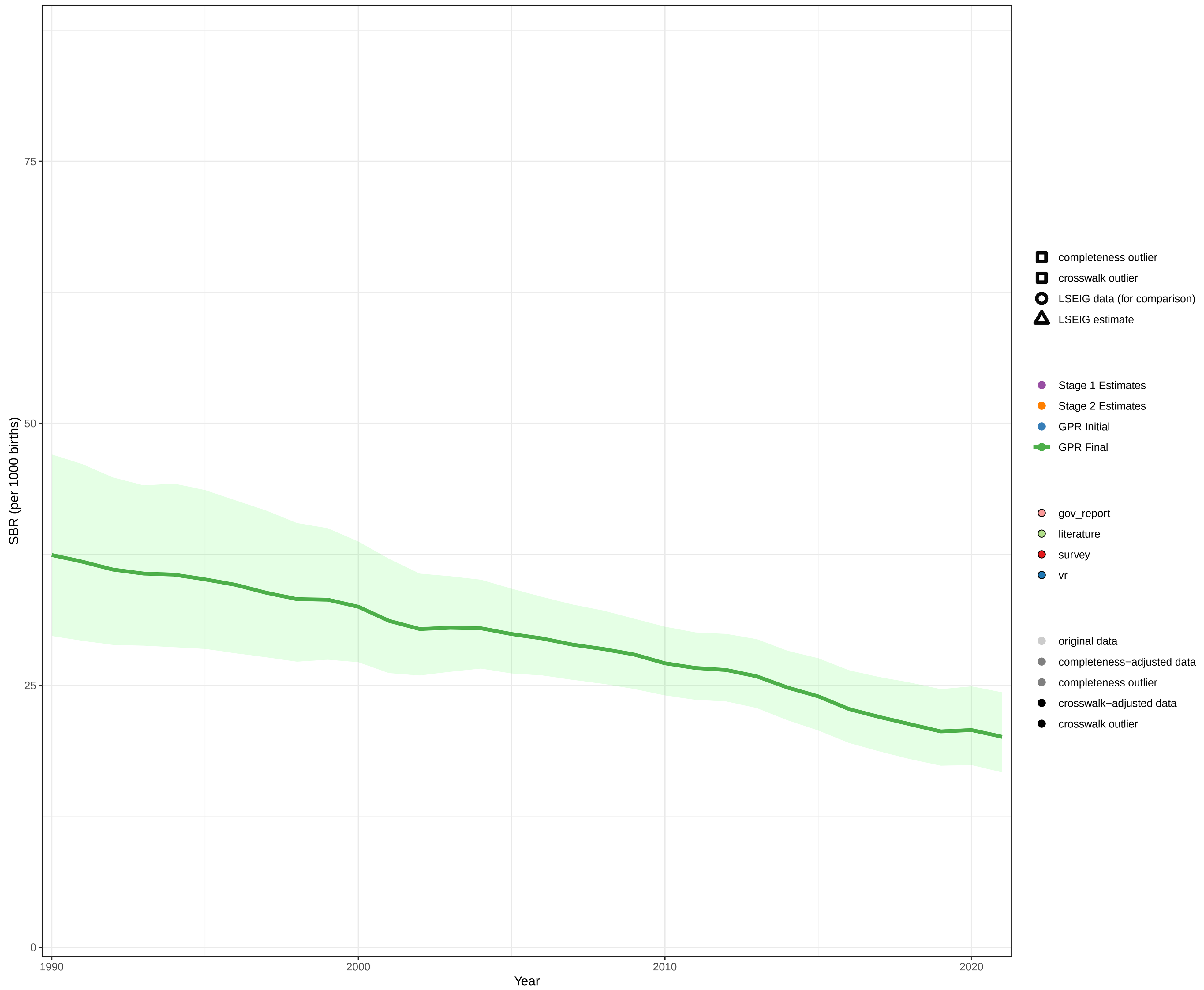

Year	Total Stillbirths (in thousands)	Total Stillbirths (in thousands)	SBR (per 1000 births)	SBR (per 1000 births)	SBR/NMR	SBR/NMR
	20 weeks	28 weeks	20 weeks	28 weeks	20 weeks	28 weeks
1990	1757.7 (1379.6 to 2238.5)	1268.6 (998.1 to 1609.0)	51.1 (40.6 to 64.3)	37.4 (29.7 to 47.0)	1.09 (0.87 to 1.37)	0.80 (0.64 to 1.01)
1995	1744.3 (1401.3 to 2171.0)	1259.9 (1014.6 to 1579.0)	47.9 (38.9 to 59.0)	35.1 (28.5 to 43.6)	1.13 (0.91 to 1.39)	0.82 (0.67 to 1.02)
2000	1617.3 (1342.5 to 1955.4)	1169.6 (973.6 to 1402.3)	44.4 (37.1 to 53.2)	32.5 (27.2 to 38.7)	1.17 (0.98 to 1.40)	0.85 (0.71 to 1.02)
2005	1497.6 (1298.2 to 1731.3)	1089.0 (948.2 to 1252.2)	40.6 (35.4 to 46.7)	29.9 (26.1 to 34.2)	1.19 (1.04 to 1.37)	0.88 (0.77 to 1.00)
2010	1358.3 (1192.3 to 1552.0)	988.0 (873.6 to 1119.3)	36.9 (32.5 to 41.9)	27.1 (24.0 to 30.6)	1.19 (1.05 to 1.36)	0.88 (0.78 to 0.99)
2015	1167.0 (1004.1 to 1349.6)	841.4 (724.4 to 972.6)	32.9 (28.5 to 37.9)	24.0 (20.7 to 27.6)	1.17 (1.01 to 1.34)	0.85 (0.73 to 0.98)
2020	962.6 (800.2 to 1166.2)	687.2 (574.5 to 828.9)	28.8 (24.1 to 34.7)	20.7 (17.4 to 24.9)	1.16 (0.97 to 1.40)	0.83 (0.70 to 1.00)
2021	922.1 (764.4 to 1128.3)	657.1 (544.1 to 799.1)	28.0 (23.3 to 34.0)	20.1 (16.7 to 24.3)	1.17 (0.97 to 1.42)	0.84 (0.70 to 1.02)

South Asia R4 South Asia Stillbirth Rate / Neonatal Mortality Rate (SBR/NMR) − 20 weeks
lambda = 1 ; zeta = 0.6 ; scale = 15 ; amp = 0.028

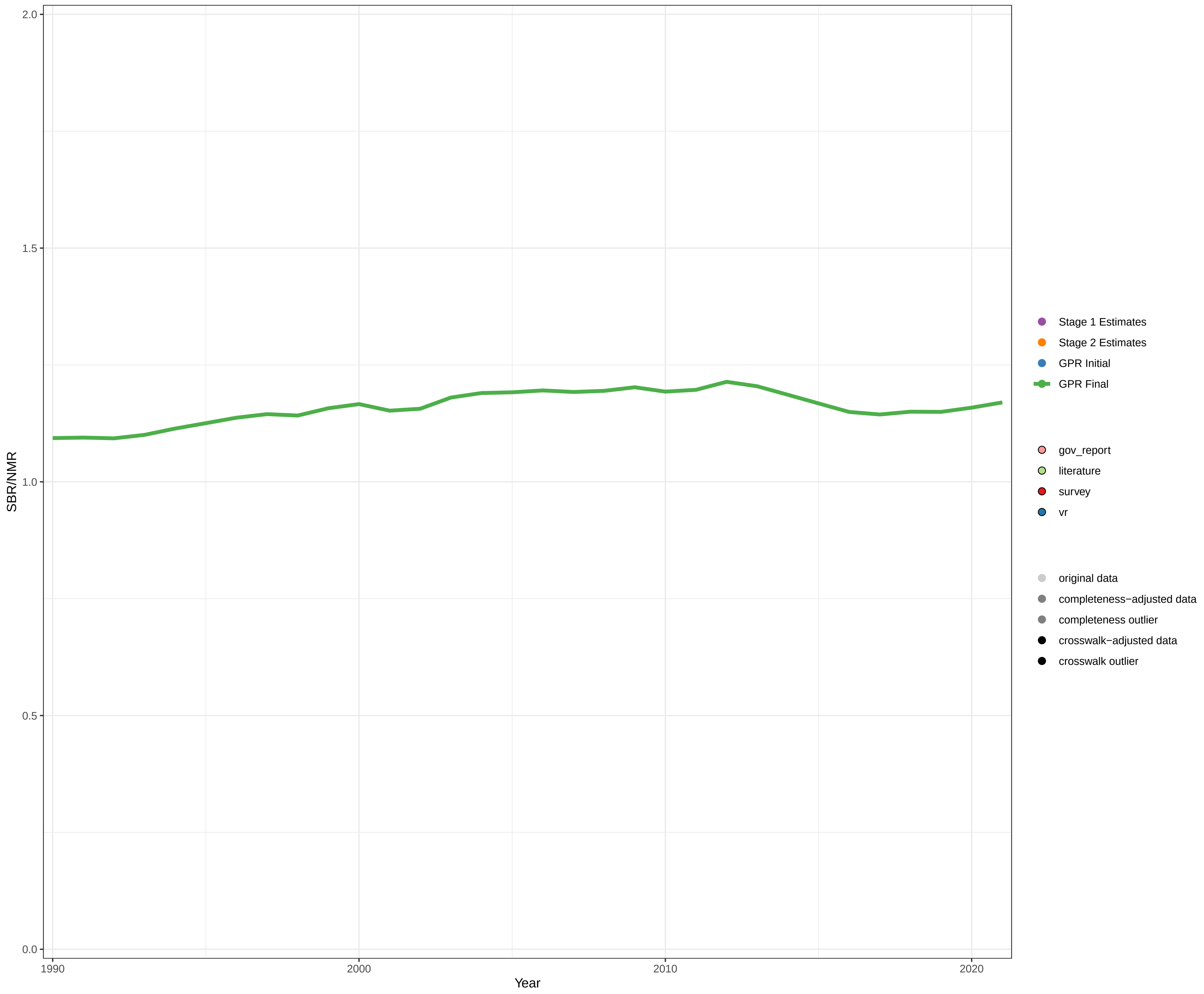

South Asia R4 South Asia Stillbirth Rate / Neonatal Mortality Rate (SBR/NMR) − 28 weeks
lambda = 1 ; zeta = 0.6 ; scale = 15 ; amp = 0.028

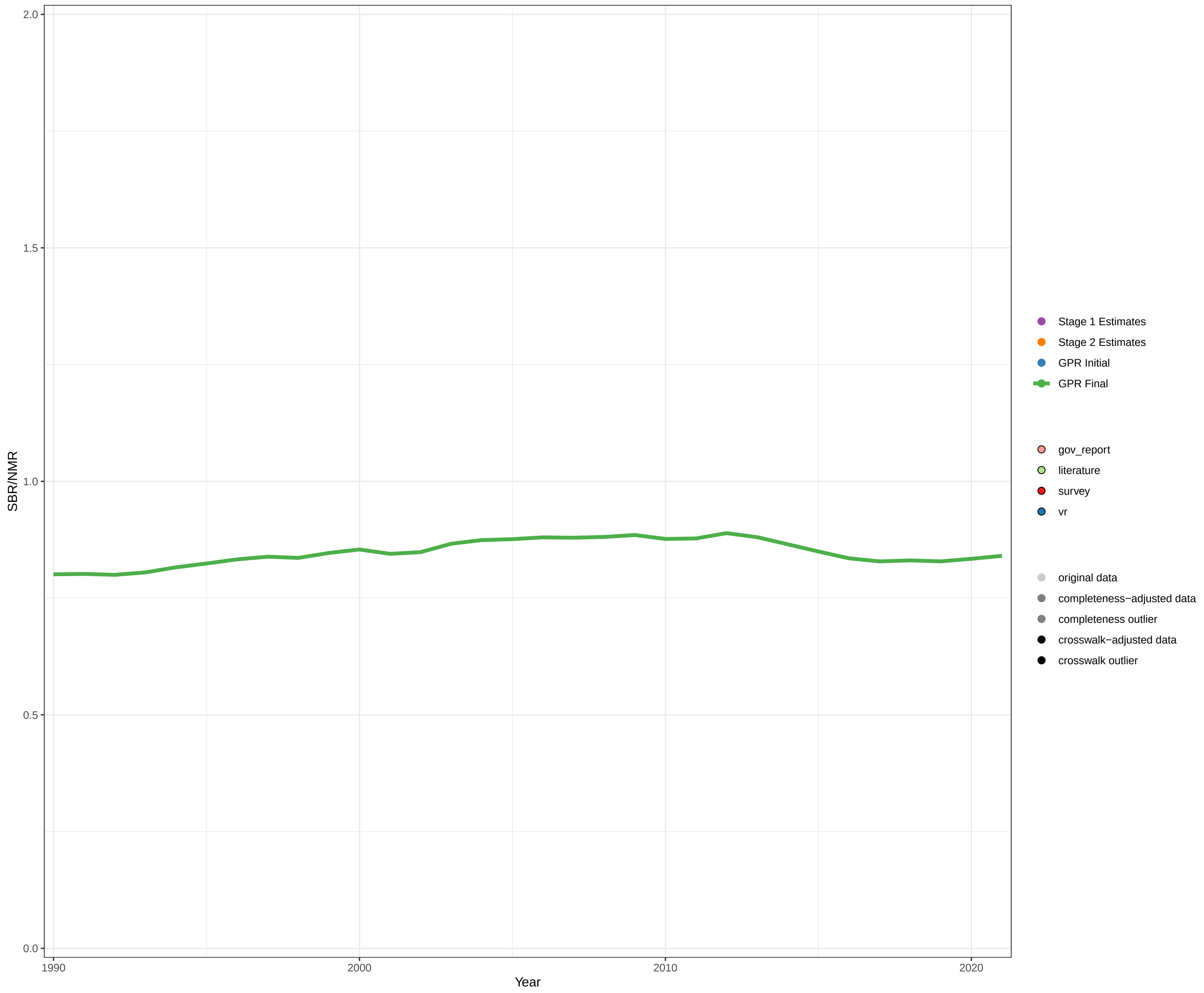

Definition	Years	Outlier	Source.Type	Title

South Asia R4 South Asia Stillbirth Rate (SBR) − 20 weeks
lambda = 1 ; zeta = 0.6 ; scale = 15 ; amp = 0.028

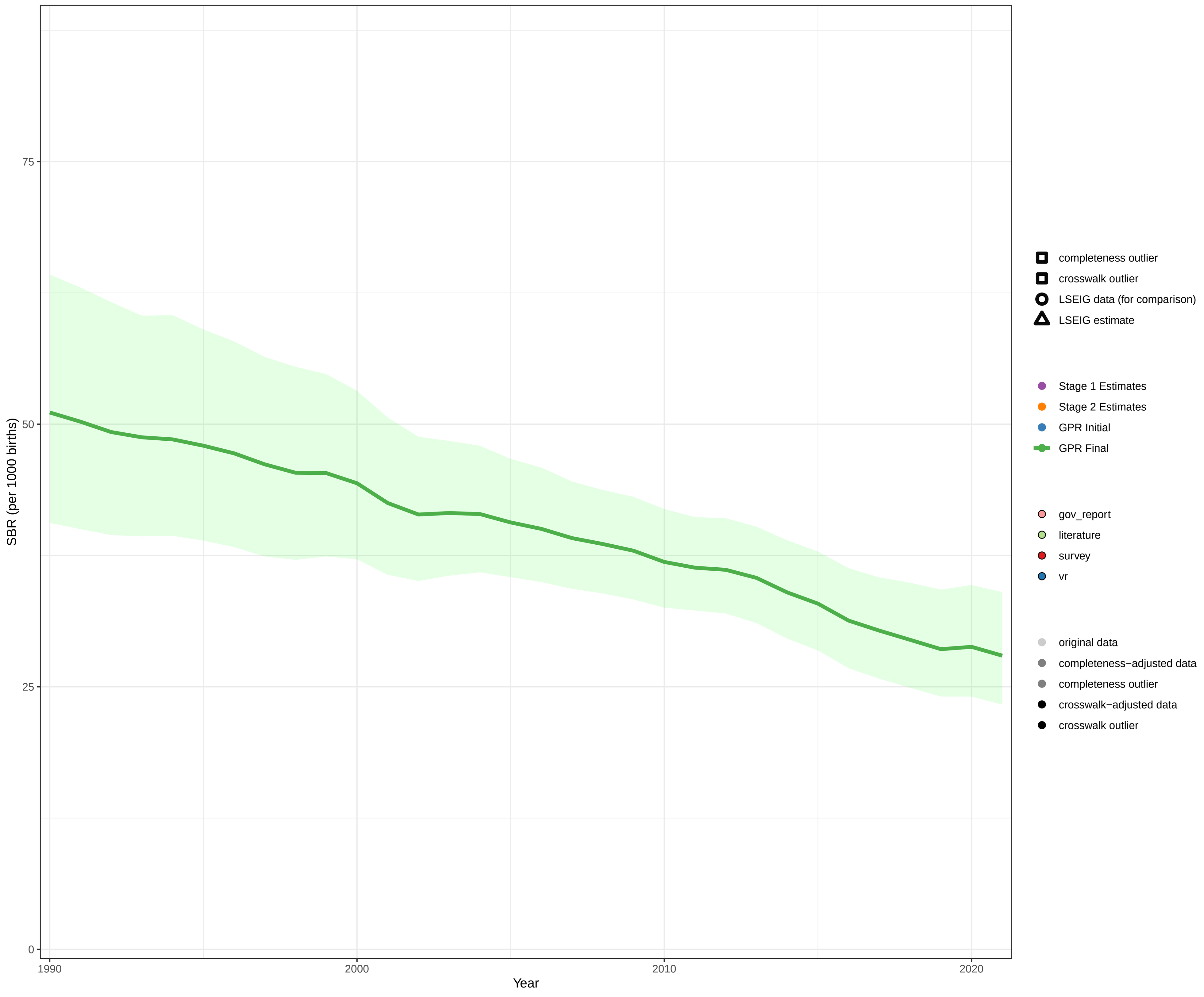

South Asia R4 South Asia Stillbirth Rate (SBR) − 28 weeks
lambda = 1 ; zeta = 0.6 ; scale = 15 ; amp = 0.028

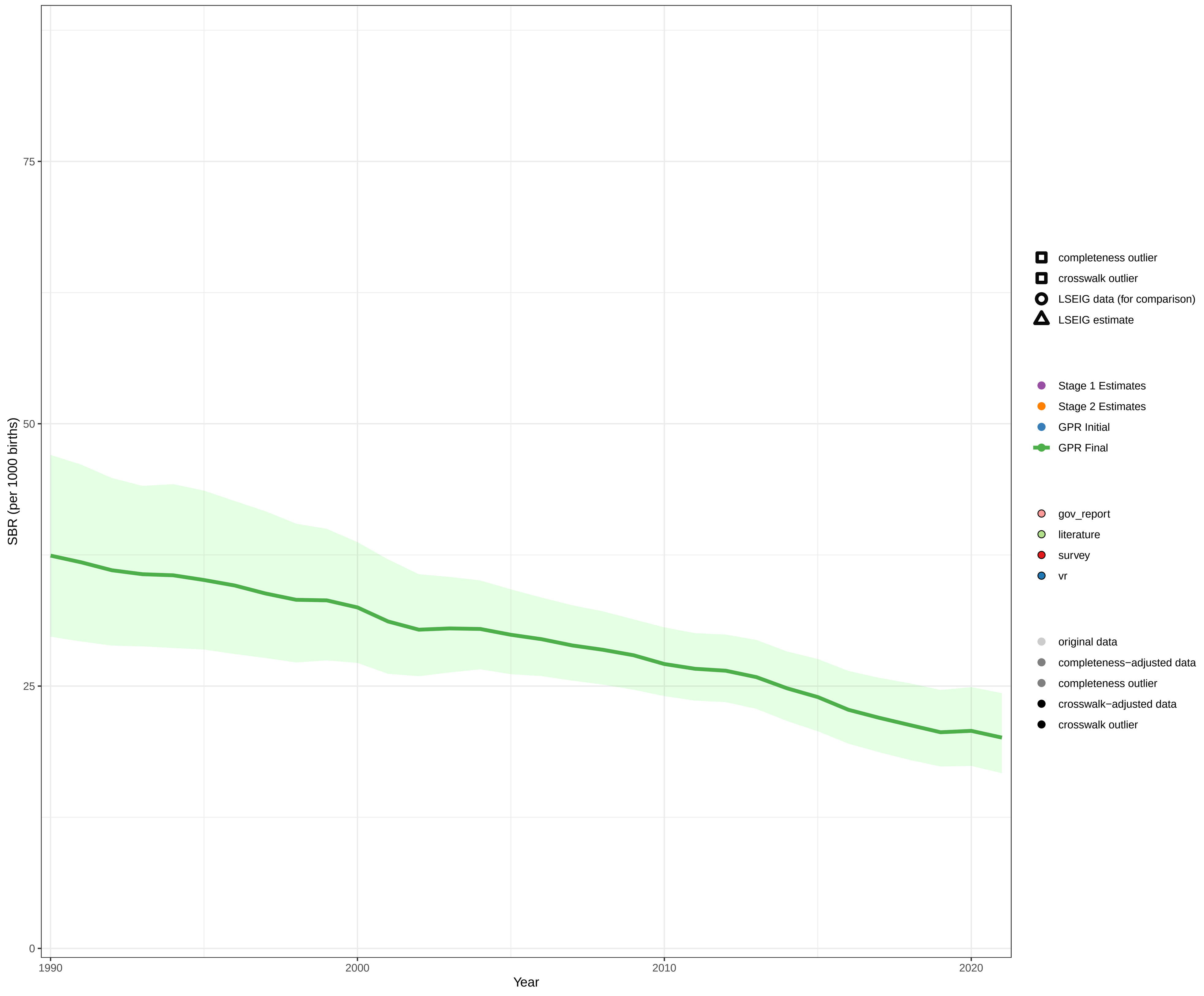

Year	Total Stillbirths (in thousands)	Total Stillbirths (in thousands)	SBR (per 1000 births)	SBR (per 1000 births)	SBR/NMR	SBR/NMR
	20 weeks	28 weeks	20 weeks	28 weeks	20 weeks	28 weeks
1990	1757.7<br>(1379.6 to 2238.5)	1268.6<br>(998.1 to 1609.0)	51.1<br>(40.6 to 64.3)	37.4<br>(29.7 to 47.0)	1.09<br>(0.87 to 1.37)	0.80<br>(0.64 to 1.01)
1995	1744.3<br>(1401.3 to 2171.0)	1259.9<br>(1014.6 to 1579.0)	47.9<br>(38.9 to 59.0)	35.1<br>(28.5 to 43.6)	1.13<br>(0.91 to 1.39)	0.82<br>(0.67 to 1.02)
2000	1617.3<br>(1342.5 to 1955.4)	1169.6<br>(973.6 to 1402.3)	44.4<br>(37.1 to 53.2)	32.5<br>(27.2 to 38.7)	1.17<br>(0.98 to 1.40)	0.85<br>(0.71 to 1.02)
2005	1497.6<br>(1298.2 to 1731.3)	1089.0<br>(948.2 to 1252.2)	40.6<br>(35.4 to 46.7)	29.9<br>(26.1 to 34.2)	1.19<br>(1.04 to 1.37)	0.88<br>(0.77 to 1.00)
2010	1358.3<br>(1192.3 to 1552.0)	988.0<br>(873.6 to 1119.3)	36.9<br>(32.5 to 41.9)	27.1<br>(24.0 to 30.6)	1.19<br>(1.05 to 1.36)	0.88<br>(0.78 to 0.99)
2015	1167.0<br>(1004.1 to 1349.6)	841.4<br>(724.4 to 972.6)	32.9<br>(28.5 to 37.9)	24.0<br>(20.7 to 27.6)	1.17<br>(1.01 to 1.34)	0.85<br>(0.73 to 0.98)
2020	962.6<br>(800.2 to 1166.2)	687.2<br>(574.5 to 828.9)	28.8<br>(24.1 to 34.7)	20.7<br>(17.4 to 24.9)	1.16<br>(0.97 to 1.40)	0.83<br>(0.70 to 1.00)
2021	922.1<br>(764.4 to 1128.3)	657.1<br>(544.1 to 799.1)	28.0<br>(23.3 to 34.0)	20.1<br>(16.7 to 24.3)	1.17<br>(0.97 to 1.42)	0.84<br>(0.70 to 1.02)

Bangladesh   BGD   South Asia   Stillbirth Rate / Neonatal Mortality Rate (SBR/NMR) − 20 weeks
lambda = 0.2 ; zeta = 0.99 ; scale = 5 ; amp = 0.028

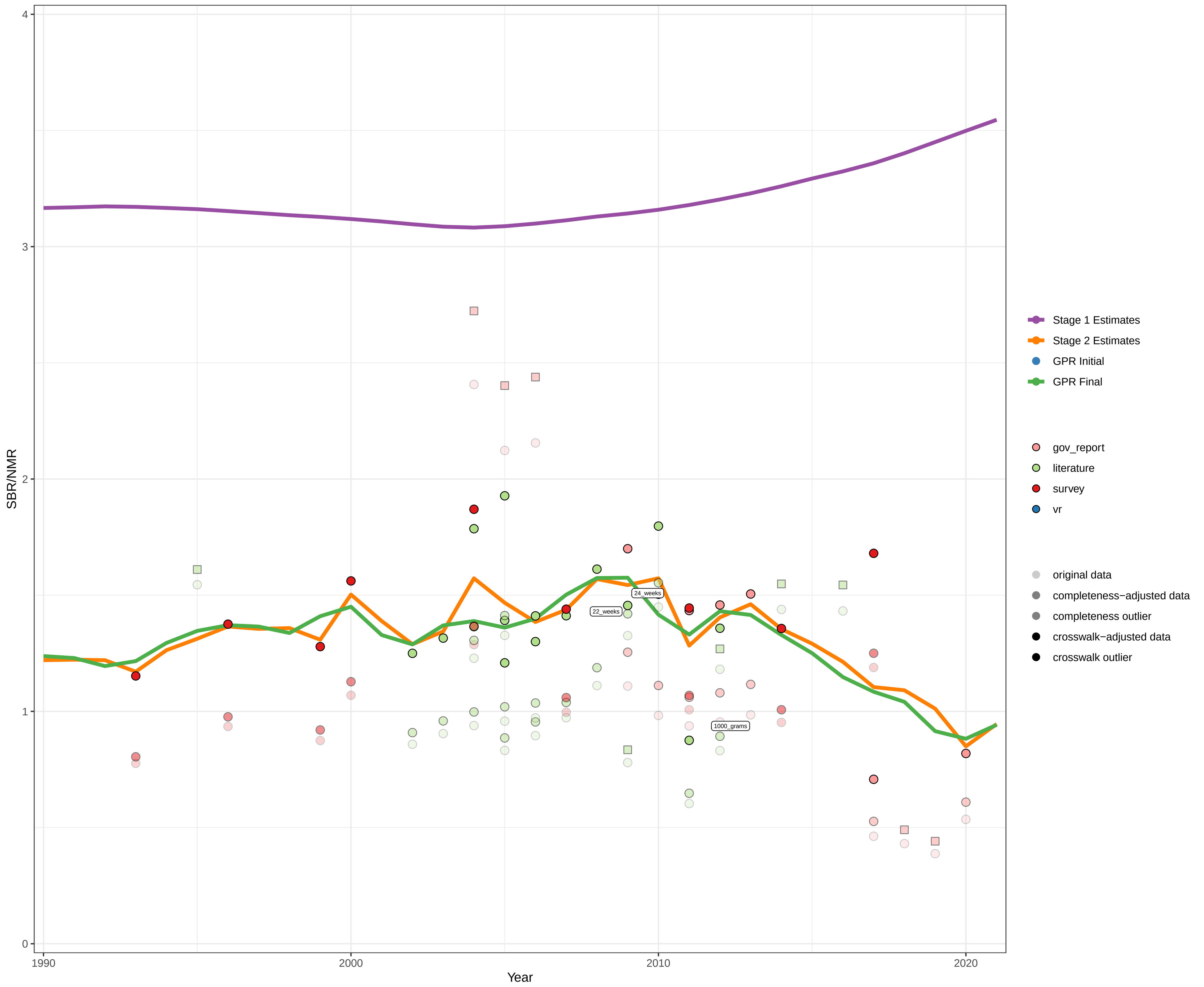

Bangladesh   BGD   South Asia   Stillbirth Rate / Neonatal Mortality Rate (SBR/NMR) − 28 weeks
lambda = 0.2 ; zeta = 0.99 ; scale = 5 ; amp = 0.028

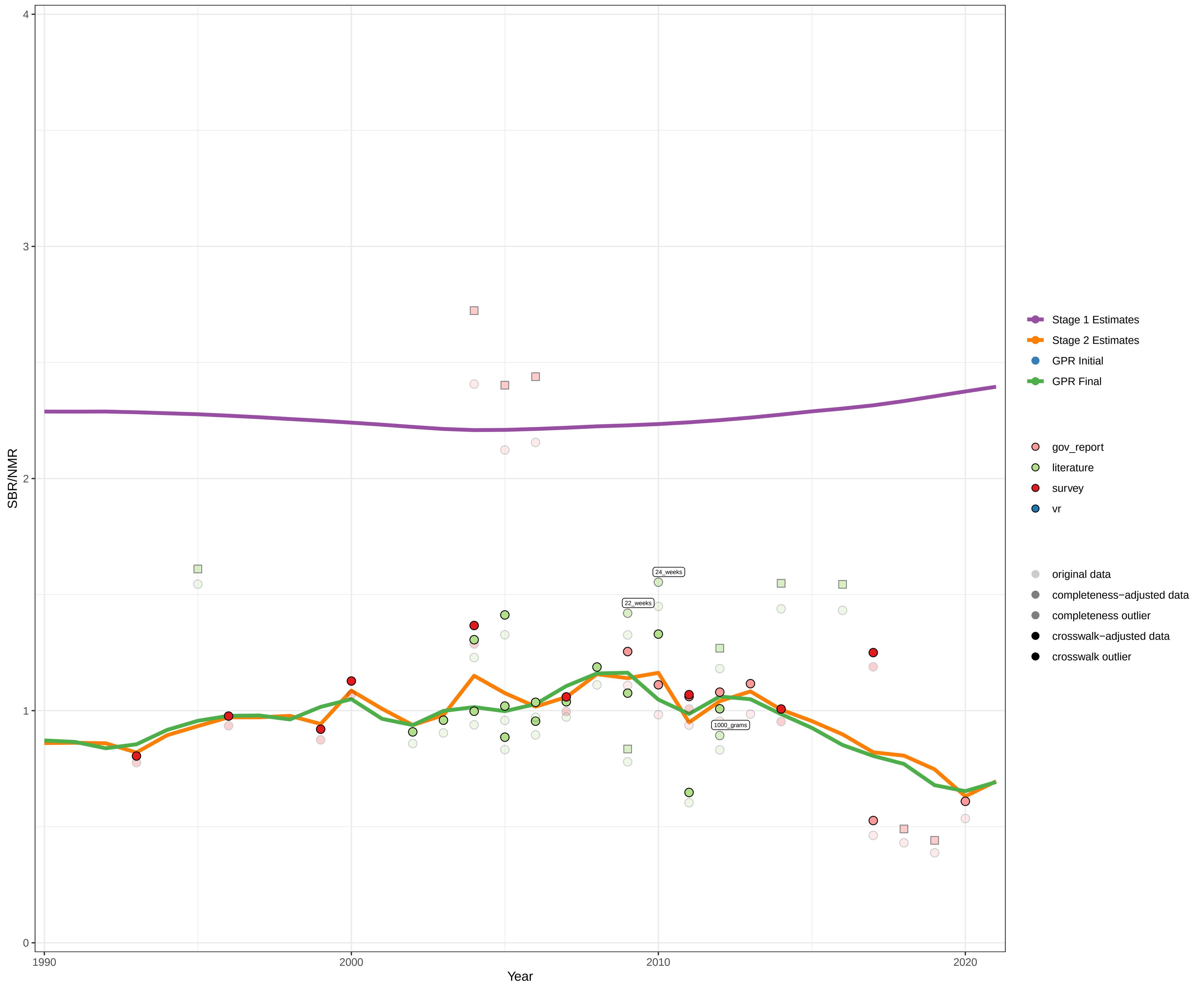

Definition	Years	Outlier	Source.Type	Title
1000_grams	2012	0	Sci Lit	Perinatal mortality associated with use of uterotonics outside of Comprehensive Emergency Obstetric and Neonatal Care: a cross−sectional study
22_weeks	2009	0	Sci Lit	Risk factors for reported obstetric complications and near misses in rural northwest Bangladesh: analysis from a prospective cohort study
24_weeks	2010	0	Sci Lit	Effect of maternal multiple micronutrient vs iron−folic acid supplementation on infant mortality and adverse birth outcomes in rural Bangladesh: the JiVitA−3 randomized trial
28_weeks	1993	0	Survey	Bangladesh Demographic and Health Survey 1993−1994
28_weeks	1996	0	Survey	Bangladesh Demographic and Health Survey 1996−1997
28_weeks	1999	0	Survey	Bangladesh Demographic and Health Survey 1999−2000
28_weeks	2000	0	Survey	Bangladesh Maternal Health Services and Maternal Mortality Survey 2001
28_weeks	2002	0	Sci Lit	Stillbirth in rural Bangladesh: arsenic exposure and other etiological factors: a report from Gonoshasthaya Kendra
28_weeks	2003−2005	0	Sci Lit	Levels, timing, and etiology of stillbirths in Sylhet district of Bangladesh
28_weeks	2004	0	Survey	Bangladesh Demographic and Health Survey 2004
28_weeks	2004	0	Sci Lit	Effects of vitamin A or beta carotene supplementation on pregnancy−related mortality and infant mortality in rural Bangladesh: a cluster randomized trial
28_weeks	2005	0	Sci Lit	Effect of timing of first postnatal care home visit on neonatal mortality in Bangladesh: a observational cohort study
28_weeks	2005−2007	0	Sci Lit	Effect of scaling up women's groups on birth outcomes in three rural districts in Bangladesh: a cluster−randomised controlled trial
28_weeks	2006	0	Sci Lit	Intrapartum−Related Stillbirths and Neonatal Deaths in Rural Bangladesh: A Prospective, Community−Based Cohort Study
28_weeks	2007	0	Survey	Bangladesh Demographic and Health Survey 2007
28_weeks	2008	0	Sci Lit	Neonatal Mortality Risks Among Preterm Births in a Rural Bangladeshi Cohort
28_weeks	2009−2013	0	Statistical Report	Bangladesh Health Bulletin
28_weeks	2011	0	Survey	Bangladesh Demographic and Health Survey 2011−2012
28_weeks	2011	0	Sci Lit	Maternal and Antenatal Risk Factors for Stillbirths and Neonatal Mortality in Rural Bangladesh: A Case−Control Study
28_weeks	2014	0	Survey	Bangladesh Demographic and Health Survey 2014
28_weeks	2017	0	Survey	Bangladesh Demographic and Health Survey 2017−2018
28_weeks	2017, 2020	0	Statistical Report	Bangladesh Sample Vital Registration System
22_weeks	1995	1	Sci Lit	A cost effective small hospital in Bangladesh: what it can mean for emergency obstetric care
22_weeks	2004−2006	1	Statistical Report	Bangladesh Health Bulletin
22_weeks	2016	1	Sci Lit	Feasibility and effectiveness of electronic vs. paper partograph on improving birth outcomes: A prospective crossover study design
28_weeks	2009	1	Sci Lit	Factors Influencing Stillbirth in Bangladesh: A Case−Control Study
28_weeks	2012	1	Sci Lit	Causes and incidence of community−acquired serious infections among young children in south Asia (ANISA): an observational cohort study
28_weeks	2014	1	Sci Lit	Population−based rates, timing, and causes of maternal deaths, stillbirths, and neonatal deaths in south Asia and sub−Saharan Africa: a multi−country prospective cohort study
28_weeks	2018, 2019	1	Statistical Report	Bangladesh Sample Vital Registration System

Bangladesh   BGD   South Asia   Stillbirth Rate (SBR) − 20 weeks
lambda = 0.2 ; zeta = 0.99 ; scale = 5 ; amp = 0.028

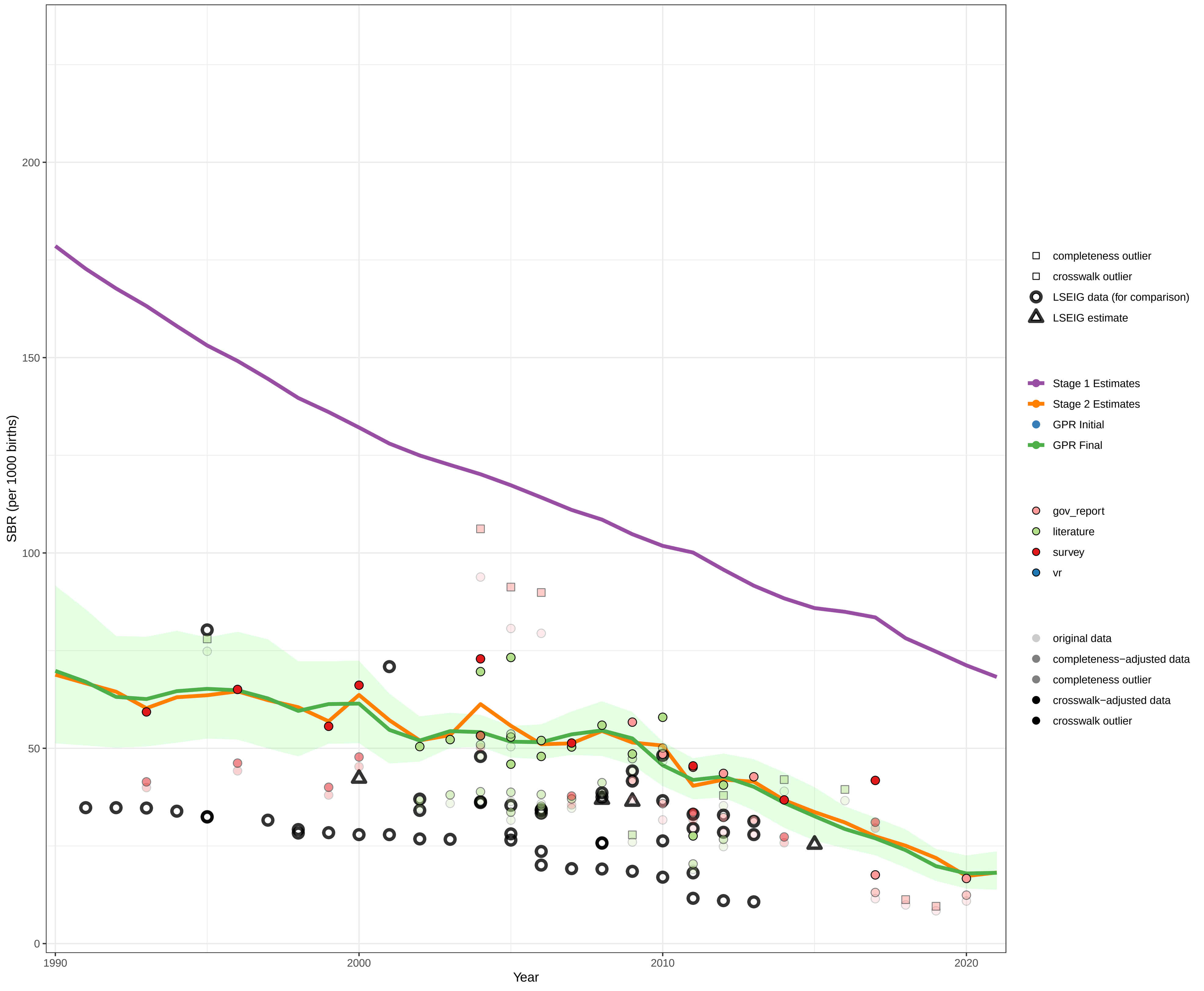

Bangladesh   BGD   South Asia   Stillbirth Rate (SBR) − 28 weeks
lambda = 0.2 ; zeta = 0.99 ; scale = 5 ; amp = 0.028

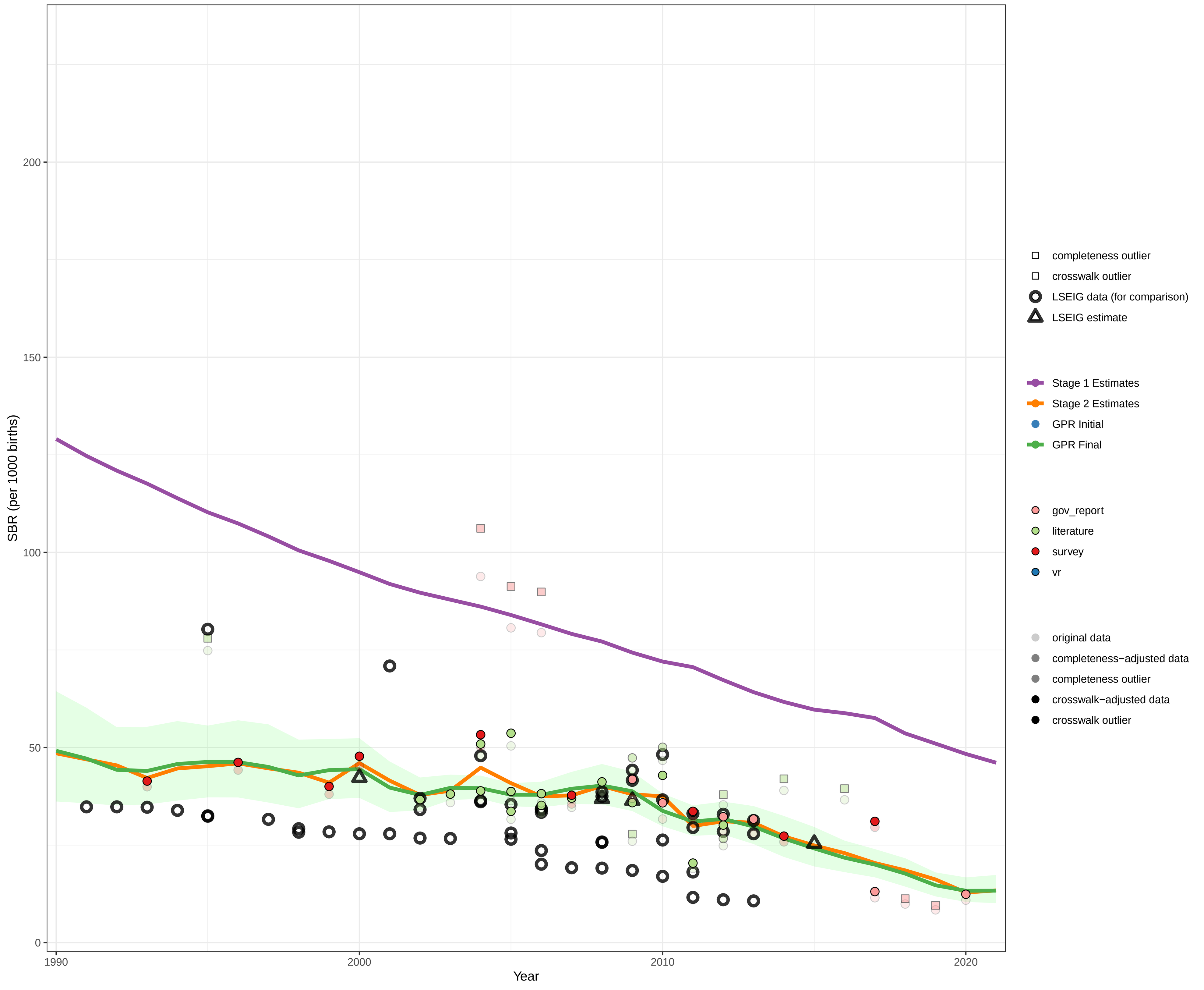

Year	Total Stillbirths (in thousands)	Total Stillbirths (in thousands)	SBR (per 1000 births)	SBR (per 1000 births)	SBR/NMR	SBR/NMR
	20 weeks	28 weeks	20 weeks	28 weeks	20 weeks	28 weeks
1990	312.6 (224.6 to 419.3)	215.2 (156.0 to 286.2)	69.8 (51.3 to 91.6)	49.2 (36.2 to 64.4)	1.24 (0.91 to 1.62)	0.87 (0.64 to 1.14)
1995	287.5 (228.1 to 350.1)	200.1 (159.2 to 242.6)	65.2 (52.5 to 78.4)	46.3 (37.2 to 55.6)	1.35 (1.08 to 1.62)	0.96 (0.77 to 1.15)
2000	266.5 (220.0 to 317.7)	189.4 (156.7 to 224.9)	61.4 (51.3 to 72.4)	44.5 (37.1 to 52.4)	1.45 (1.21 to 1.71)	1.05 (0.88 to 1.24)
2005	220.1 (201.8 to 238.3)	159.1 (146.2 to 172.0)	51.7 (47.6 to 55.7)	37.9 (34.9 to 40.9)	1.36 (1.25 to 1.47)	1.00 (0.92 to 1.08)
2010	181.0 (158.9 to 206.1)	132.2 (116.3 to 150.3)	45.6 (40.3 to 51.7)	33.8 (29.8 to 38.2)	1.42 (1.25 to 1.60)	1.05 (0.92 to 1.19)
2015	111.9 (90.0 to 138.5)	82.2 (66.1 to 101.4)	32.6 (26.4 to 40.0)	24.1 (19.5 to 29.6)	1.25 (1.01 to 1.54)	0.93 (0.75 to 1.14)
2020	52.7 (41.1 to 66.5)	38.8 (30.4 to 49.0)	18.0 (14.1 to 22.6)	13.3 (10.4 to 16.7)	0.88 (0.69 to 1.11)	0.65 (0.51 to 0.82)
2021	51.9 (39.2 to 67.9)	37.9 (28.8 to 49.5)	18.1 (13.8 to 23.6)	13.3 (10.1 to 17.3)	0.94 (0.72 to 1.23)	0.69 (0.53 to 0.90)

Bhutan BTN South Asia Stillbirth Rate / Neonatal Mortality Rate (SBR/NMR) − 20 weeks
lambda = 1 ; zeta = 0.6 ; scale = 15 ; amp = 0.028

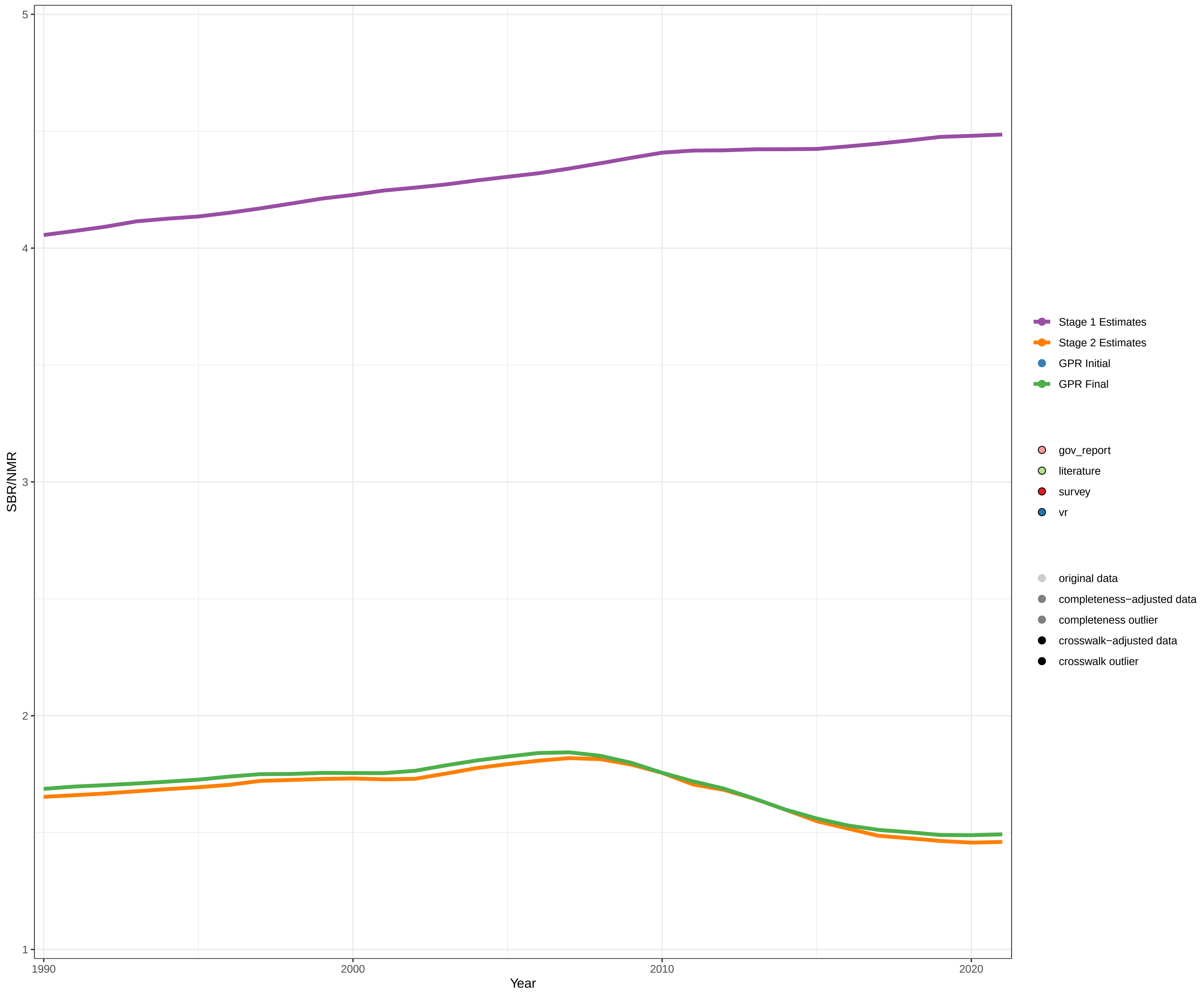

Bhutan BTN South Asia Stillbirth Rate / Neonatal Mortality Rate (SBR/NMR) − 28 weeks
lambda = 1 ; zeta = 0.6 ; scale = 15 ; amp = 0.028

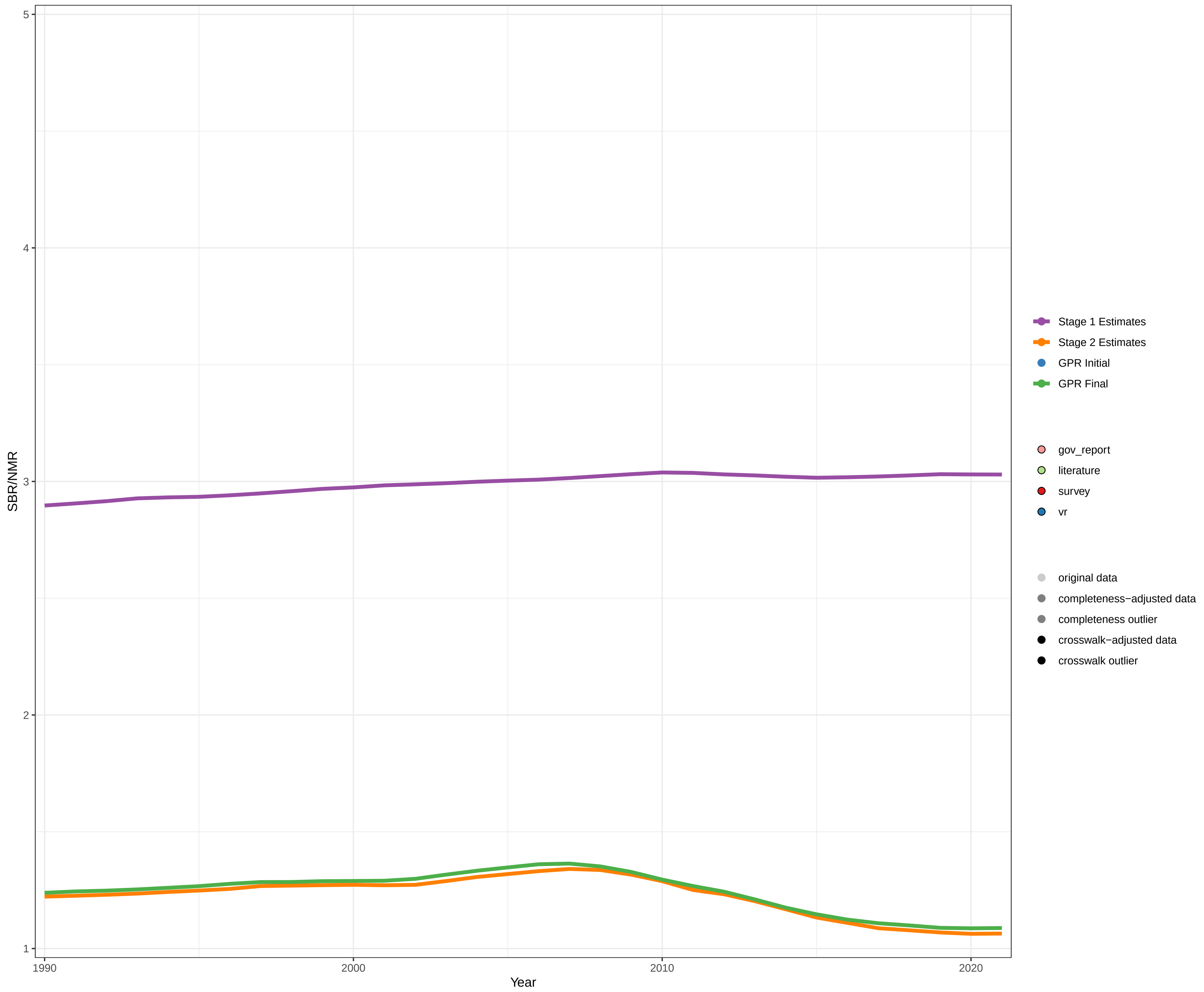

Definition	Years	Outlier	Source.Type	Title

Bhutan BTN South Asia Stillbirth Rate (SBR) − 20 weeks
lambda = 1 ; zeta = 0.6 ; scale = 15 ; amp = 0.028

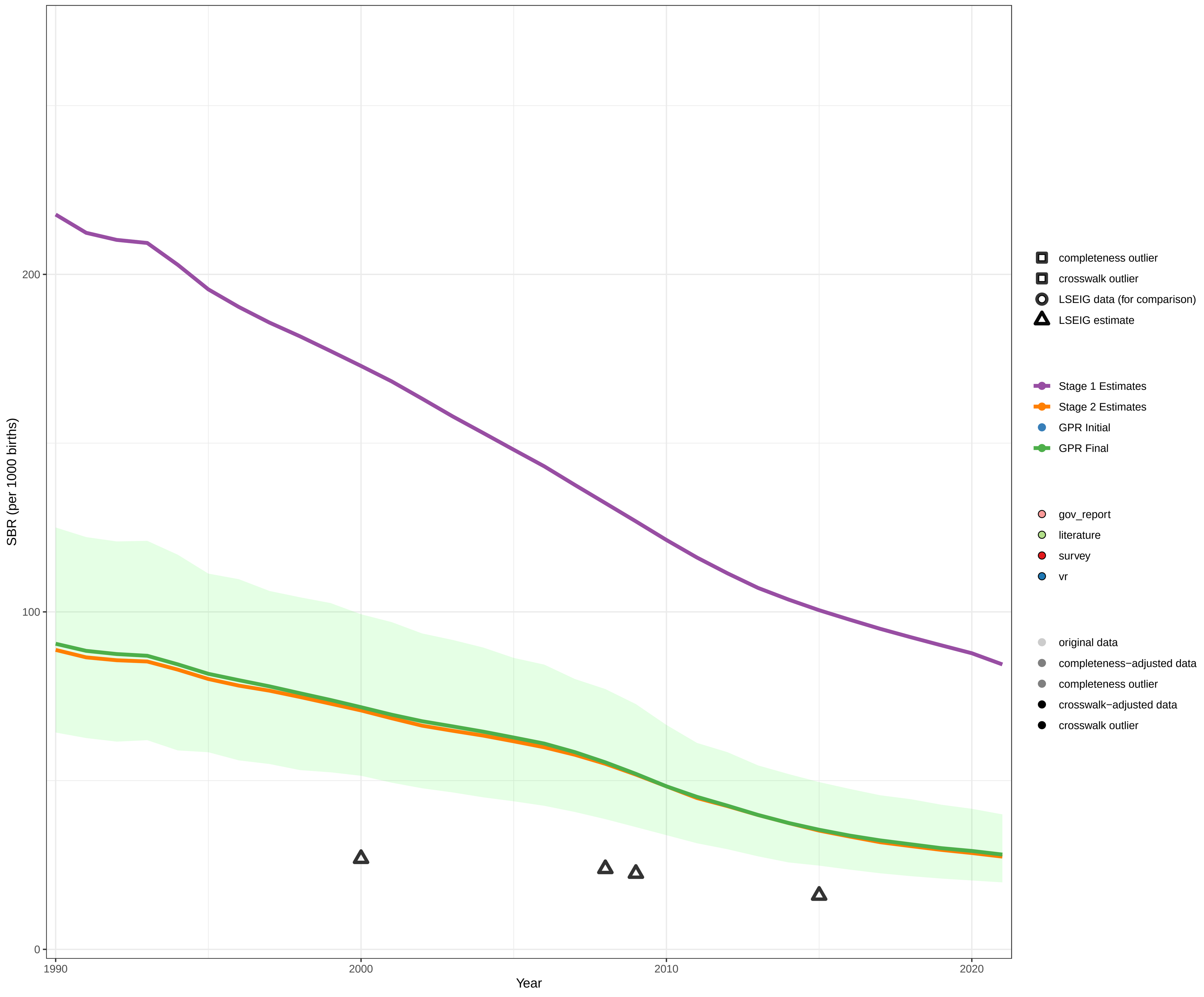

Bhutan BTN South Asia Stillbirth Rate (SBR) − 28 weeks
lambda = 1 ; zeta = 0.6 ; scale = 15 ; amp = 0.028

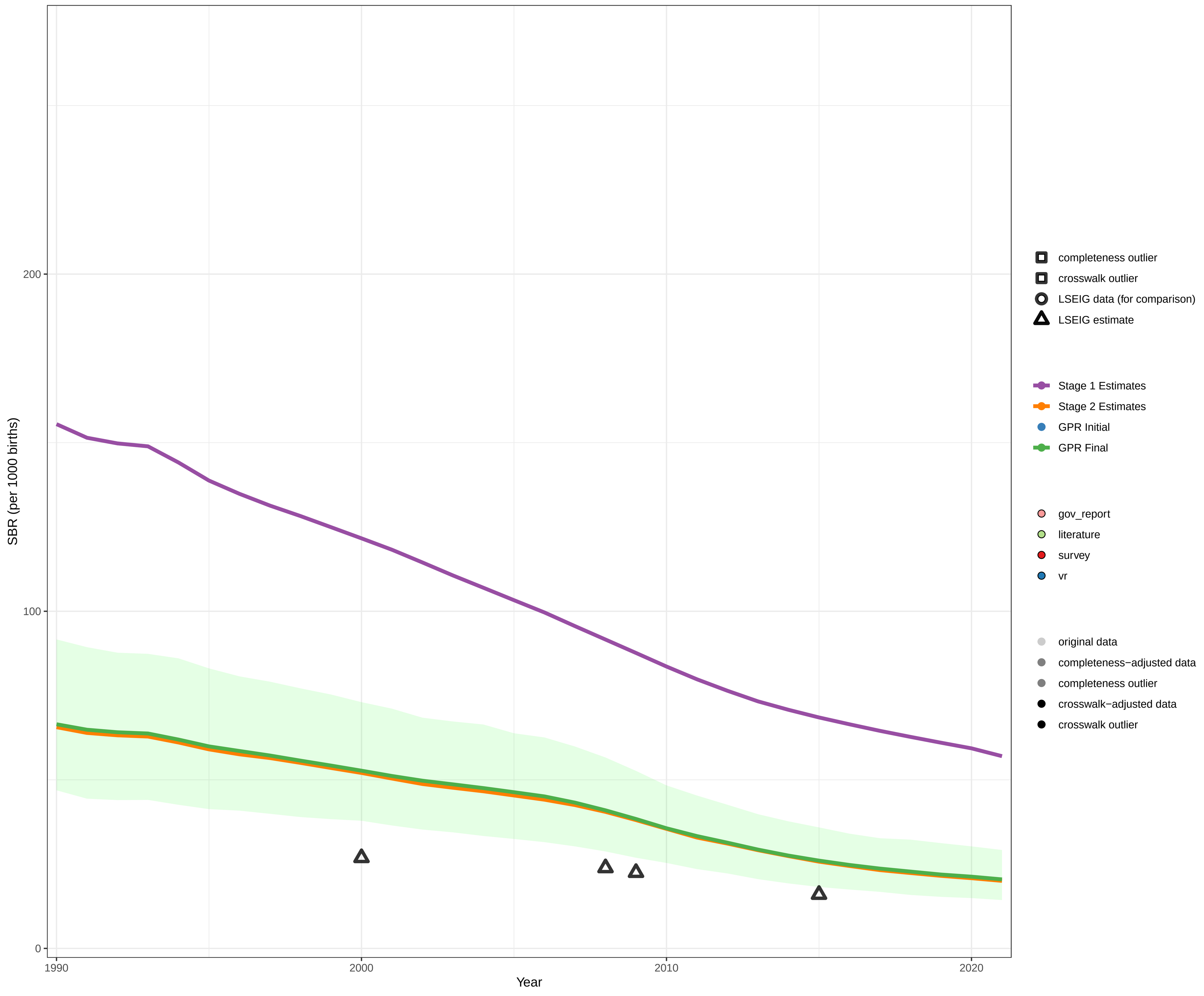

Year	Total Stillbirths (in thousands)	Total Stillbirths (in thousands)	SBR (per 1000 births)	SBR (per 1000 births)	SBR/NMR	SBR/NMR
	20 weeks	28 weeks	20 weeks	28 weeks	20 weeks	28 weeks
1990	2.2<br>(1.5 to 3.2)	1.6<br>(1.1 to 2.3)	90.6<br>(64.3 to 125.0)	66.5<br>(46.9 to 91.7)	1.69<br>(1.20 to 2.33)	1.24<br>(0.87 to 1.71)
1995	1.6<br>(1.1 to 2.3)	1.2<br>(0.8 to 1.7)	81.6<br>(58.4 to 111.3)	59.9<br>(41.3 to 83.1)	1.73<br>(1.24 to 2.35)	1.27<br>(0.87 to 1.76)
2000	1.4<br>(1.0 to 2.0)	1.0<br>(0.7 to 1.4)	71.8<br>(51.4 to 99.3)	52.7<br>(37.9 to 73.0)	1.75<br>(1.26 to 2.43)	1.29<br>(0.93 to 1.79)
2005	1.1<br>(0.7 to 1.5)	0.8<br>(0.5 to 1.1)	62.8<br>(43.9 to 86.4)	46.3<br>(32.5 to 63.8)	1.83<br>(1.28 to 2.51)	1.35<br>(0.94 to 1.86)
2010	0.7<br>(0.5 to 1.0)	0.5<br>(0.4 to 0.7)	48.3<br>(33.8 to 66.5)	35.6<br>(25.3 to 48.4)	1.76<br>(1.23 to 2.42)	1.30<br>(0.92 to 1.76)
2015	0.5<br>(0.4 to 0.7)	0.4<br>(0.3 to 0.5)	35.4<br>(24.8 to 49.6)	26.0<br>(18.2 to 35.9)	1.56<br>(1.09 to 2.18)	1.15<br>(0.80 to 1.58)
2020	0.4<br>(0.3 to 0.6)	0.3<br>(0.2 to 0.4)	29.2<br>(20.4 to 41.7)	21.3<br>(14.9 to 30.3)	1.49<br>(1.04 to 2.13)	1.09<br>(0.76 to 1.55)
2021	0.4<br>(0.3 to 0.5)	0.3<br>(0.2 to 0.4)	28.1<br>(19.8 to 40.0)	20.5<br>(14.3 to 29.2)	1.49<br>(1.05 to 2.13)	1.09<br>(0.76 to 1.55)

India IND South Asia Stillbirth Rate / Neonatal Mortality Rate (SBR/NMR) − 20 weeks
lambda = 0.8 ; zeta = 0.7 ; scale = 15 ; amp = 0.028

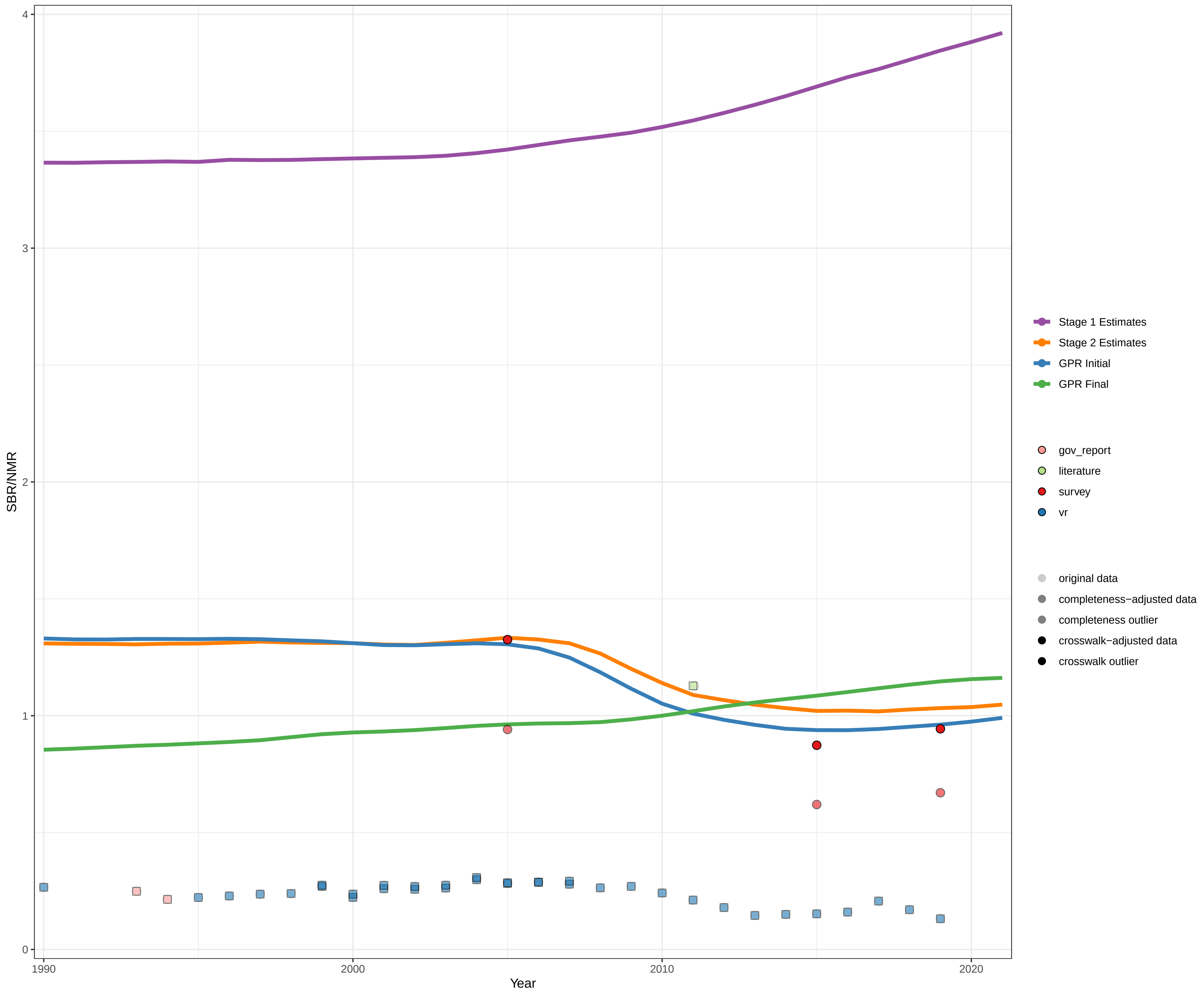

India IND South Asia Stillbirth Rate / Neonatal Mortality Rate (SBR/NMR) − 28 weeks
lambda = 0.8 ; zeta = 0.7 ; scale = 15 ; amp = 0.028

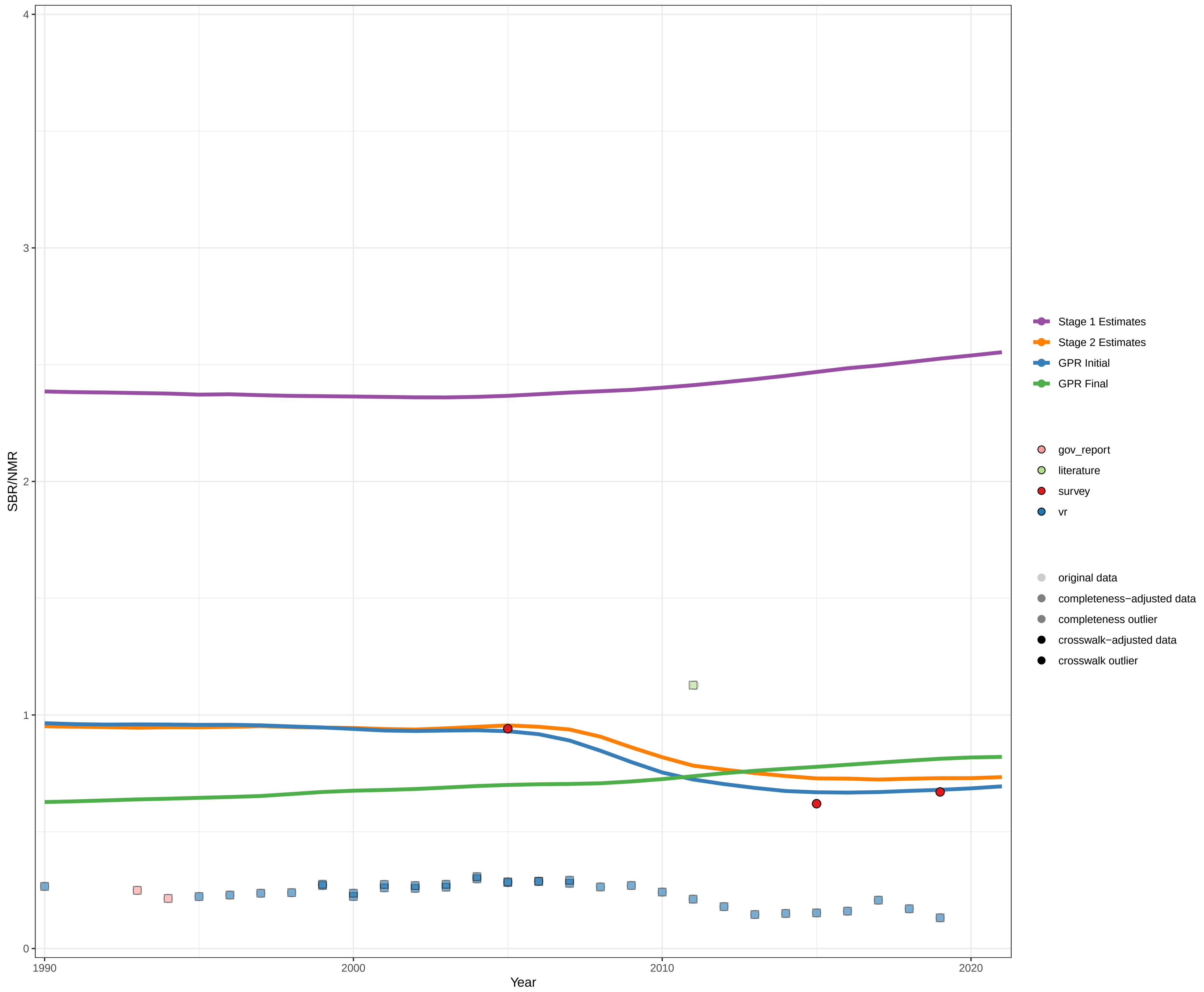

Definition	Years	Outlier	Source.Type	Title
28_weeks	2005	0	Survey	India Demographic and Health Survey 2005−2006
28_weeks	2015	0	Survey	India Demographic and Health Survey 2015−2016
28_weeks	2019	0	Survey	India Demographic and Health Survey 2019−2020
1000_grams	1993, 1994	1	Statistical Report	India National Health Profile
28_weeks	1990, 1999−2007	1	VR	India SRS Compendium of Fertility and Mortality Indicators 1971−2007
28_weeks	1995−2019	1	VR	India SRS Statistical Report
28_weeks_OR_1000_grams	2011	1	Sci Lit	Maternal complications and perinatal mortality: findings of the World Health Organization Multicountry Survey on Maternal and Newborn Health

India IND South Asia Stillbirth Rate (SBR) − 20 weeks
lambda = 0.8 ; zeta = 0.7 ; scale = 15 ; amp = 0.028

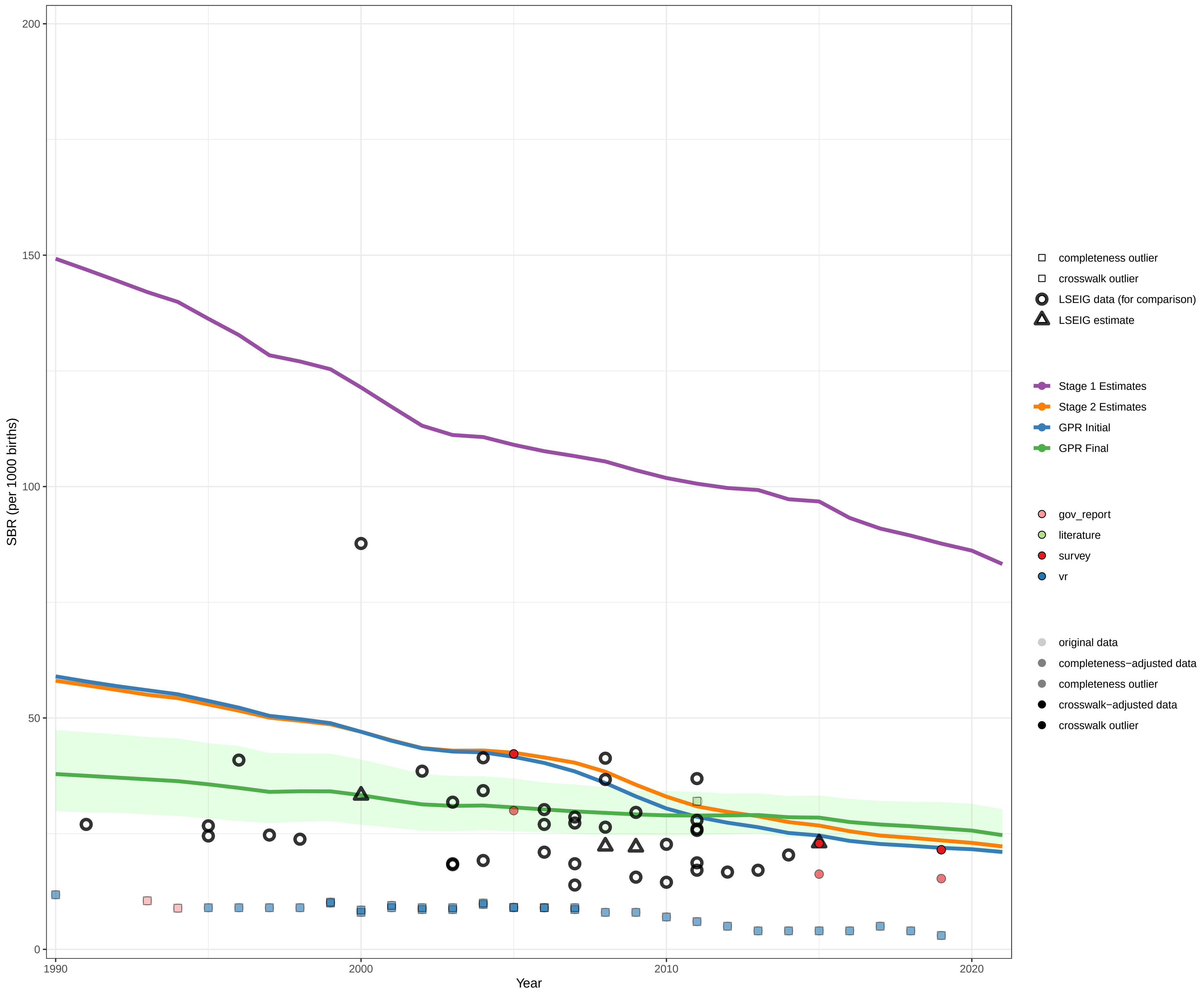

India IND South Asia Stillbirth Rate (SBR) − 28 weeks
lambda = 0.8 ; zeta = 0.7 ; scale = 15 ; amp = 0.028

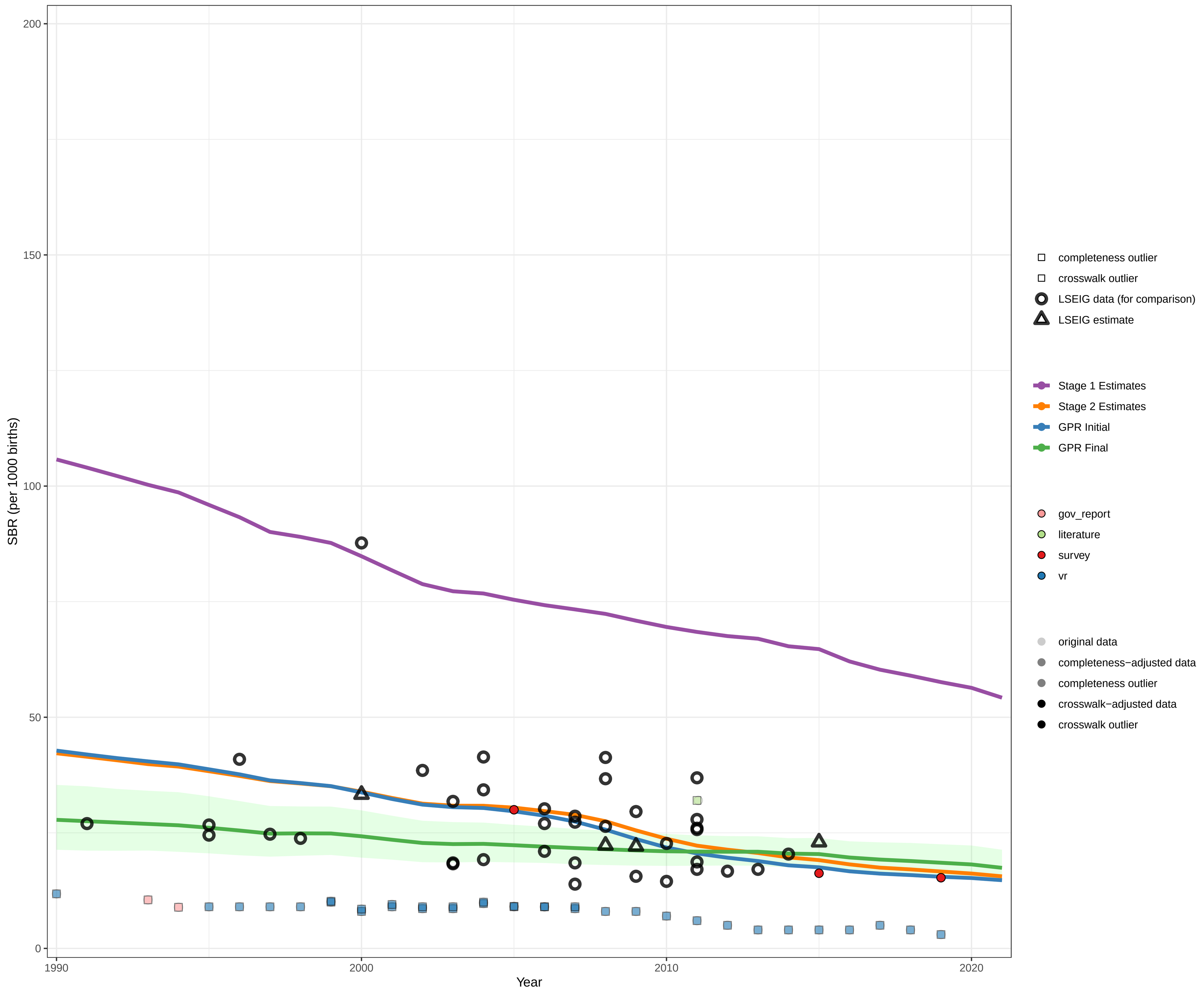

Year	Total Stillbirths (in thousands)	Total Stillbirths (in thousands)	SBR (per 1000 births)	SBR (per 1000 births)	SBR/NMR	SBR/NMR
	20 weeks	28 weeks	20 weeks	28 weeks	20 weeks	28 weeks
1990	922.7 (720.6 to 1165.9)	670.3 (510.7 to 858.5)	37.9 (29.8 to 47.4)	27.8 (21.3 to 35.4)	0.85 (0.67 to 1.07)	0.63 (0.48 to 0.80)
1995	931.5 (733.1 to 1174.3)	675.0 (528.5 to 856.7)	35.7 (28.3 to 44.6)	26.1 (20.6 to 32.9)	0.88 (0.70 to 1.10)	0.65 (0.51 to 0.81)
2000	874.3 (701.6 to 1085.7)	630.5 (507.9 to 780.5)	33.3 (26.9 to 41.1)	24.3 (19.6 to 29.9)	0.93 (0.75 to 1.14)	0.68 (0.55 to 0.83)
2005	810.7 (669.7 to 980.9)	584.7 (485.4 to 703.3)	30.7 (25.5 to 36.9)	22.3 (18.6 to 26.7)	0.96 (0.80 to 1.16)	0.70 (0.58 to 0.84)
2010	757.1 (638.3 to 898.7)	544.8 (461.4 to 644.7)	28.9 (24.5 to 34.2)	21.0 (17.8 to 24.8)	1.00 (0.85 to 1.18)	0.73 (0.62 to 0.86)
2015	711.3 (599.1 to 834.2)	505.5 (426.4 to 593.8)	28.5 (24.1 to 33.2)	20.4 (17.3 to 23.9)	1.09 (0.92 to 1.27)	0.78 (0.66 to 0.91)
2020	599.0 (495.4 to 738.1)	420.5 (348.5 to 517.5)	25.7 (21.3 to 31.5)	18.2 (15.1 to 22.3)	1.16 (0.96 to 1.42)	0.82 (0.68 to 1.00)
2021	566.7 (465.8 to 700.3)	397.3 (328.3 to 488.4)	24.7 (20.4 to 30.3)	17.4 (14.4 to 21.3)	1.16 (0.96 to 1.43)	0.82 (0.68 to 1.01)

Nepal NPL South Asia Stillbirth Rate / Neonatal Mortality Rate (SBR/NMR) − 20 weeks
lambda = 0.4 ; zeta = 0.9 ; scale = 10 ; amp = 0.028

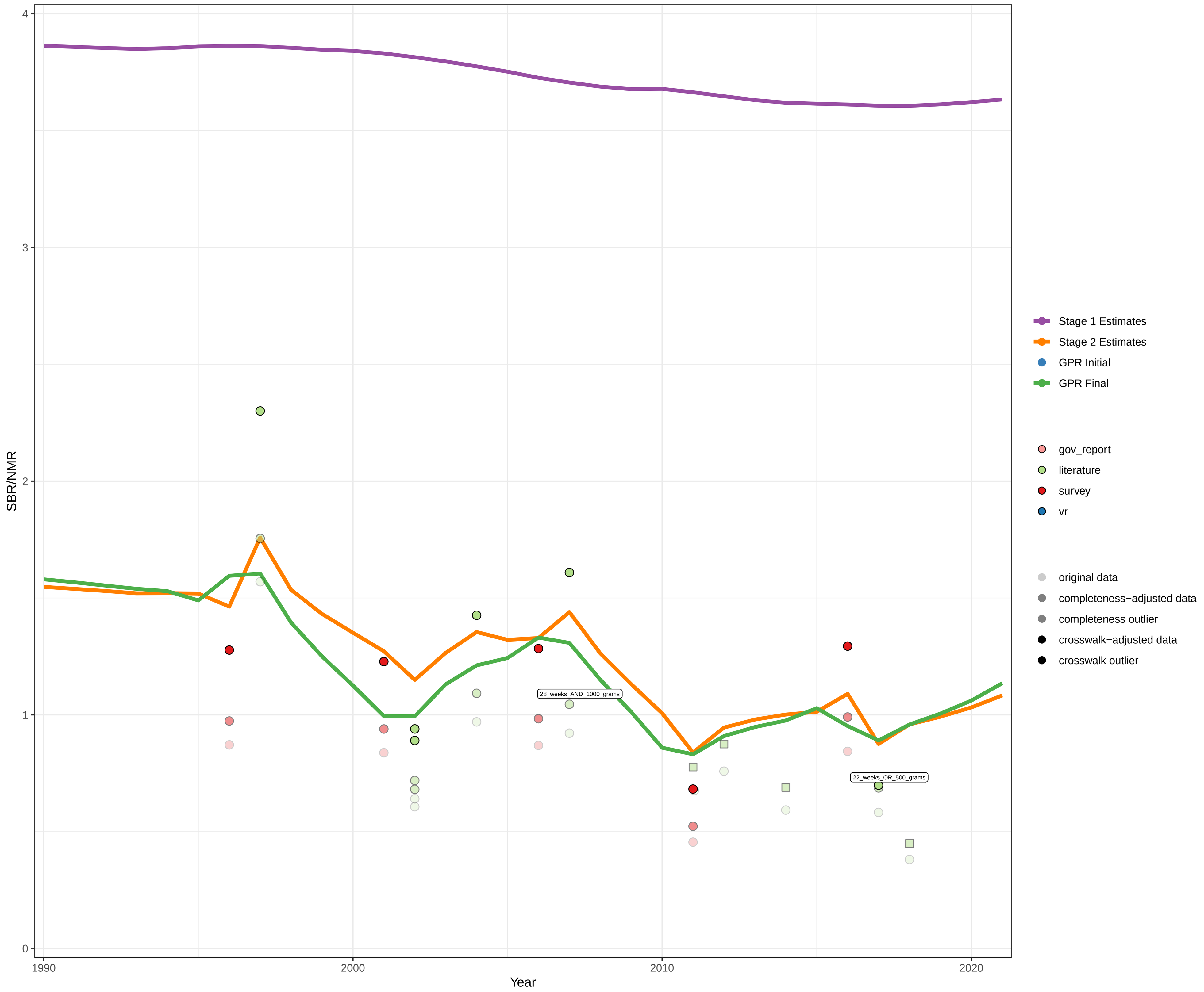

Nepal NPL South Asia Stillbirth Rate / Neonatal Mortality Rate (SBR/NMR) − 28 weeks
lambda = 0.4 ; zeta = 0.9 ; scale = 10 ; amp = 0.028

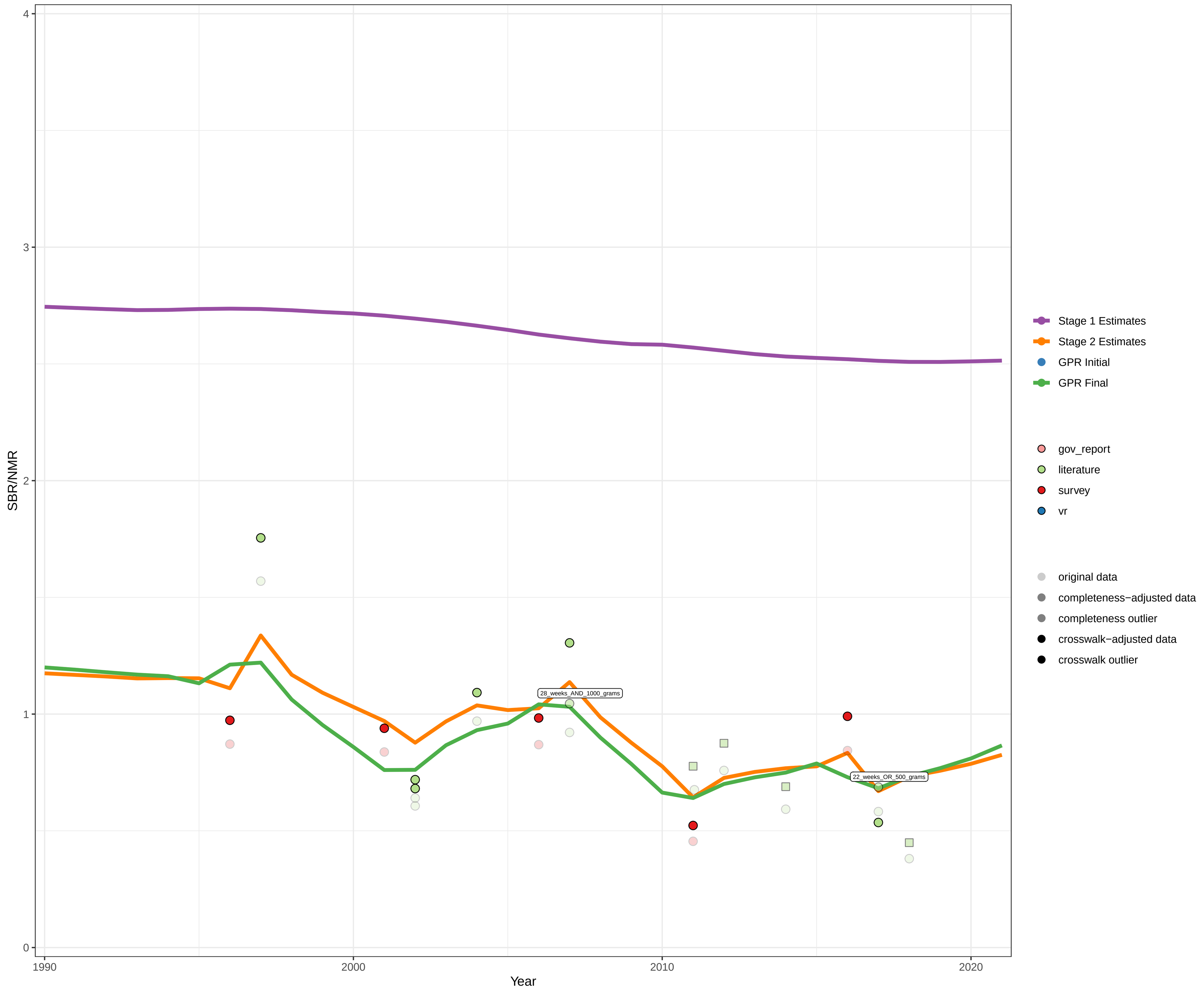

Definition	Years	Outlier	Source.Type	Title
22_weeks_OR_500_grams	2017	0	Sci Lit	Burden of misclassification of antepartum stillbirth in Nepal
28_weeks	1996	0	Survey	Nepal Demographic and Health Survey 1996
28_weeks	1997	0	Sci Lit	Maternity care in rural Nepal: a health service analysis
28_weeks	2001	0	Survey	Nepal Demographic and Health Survey 2001
28_weeks	2002	0	Sci Lit	Effect of a participatory intervention with women's groups on birth outcomes in Nepal: cluster−randomised controlled trial
28_weeks	2004	0	Sci Lit	Community−based stillbirth rates and risk factors in rural Sarlahi, Nepal
28_weeks	2006	0	Survey	Nepal Demographic and Health Survey 2006
28_weeks	2011	0	Survey	Nepal Demographic and Health Survey 2011
28_weeks	2016	0	Survey	Nepal Demographic and Health Survey 2016−2017
28_weeks	2022	0	Survey	Nepal Demographic and Health Survey 2022
28_weeks_AND_1000_grams	2007	0	Sci Lit	Causes of stillbirths and neonatal deaths in Dhanusha district, Nepal: A verbal autopsy study
22_weeks	2012	1	Sci Lit	Reducing Perinatal Mortality in Nepal Using Helping Babies Breathe
22_weeks_OR_500_grams	2018	1	Sci Lit	Prevalence of Perinatal Deaths in a Tertiary Care Hospital of Nepal
28_weeks	2014	1	Sci Lit	Risk Factors Related to Intrauterine Fetal Death
28_weeks_OR_1000_grams	2011	1	Sci Lit	Maternal complications and perinatal mortality: findings of the World Health Organization Multicountry Survey on Maternal and Newborn Health

Nepal NPL South Asia Stillbirth Rate (SBR) − 20 weeks
lambda = 0.4 ; zeta = 0.9 ; scale = 10 ; amp = 0.028

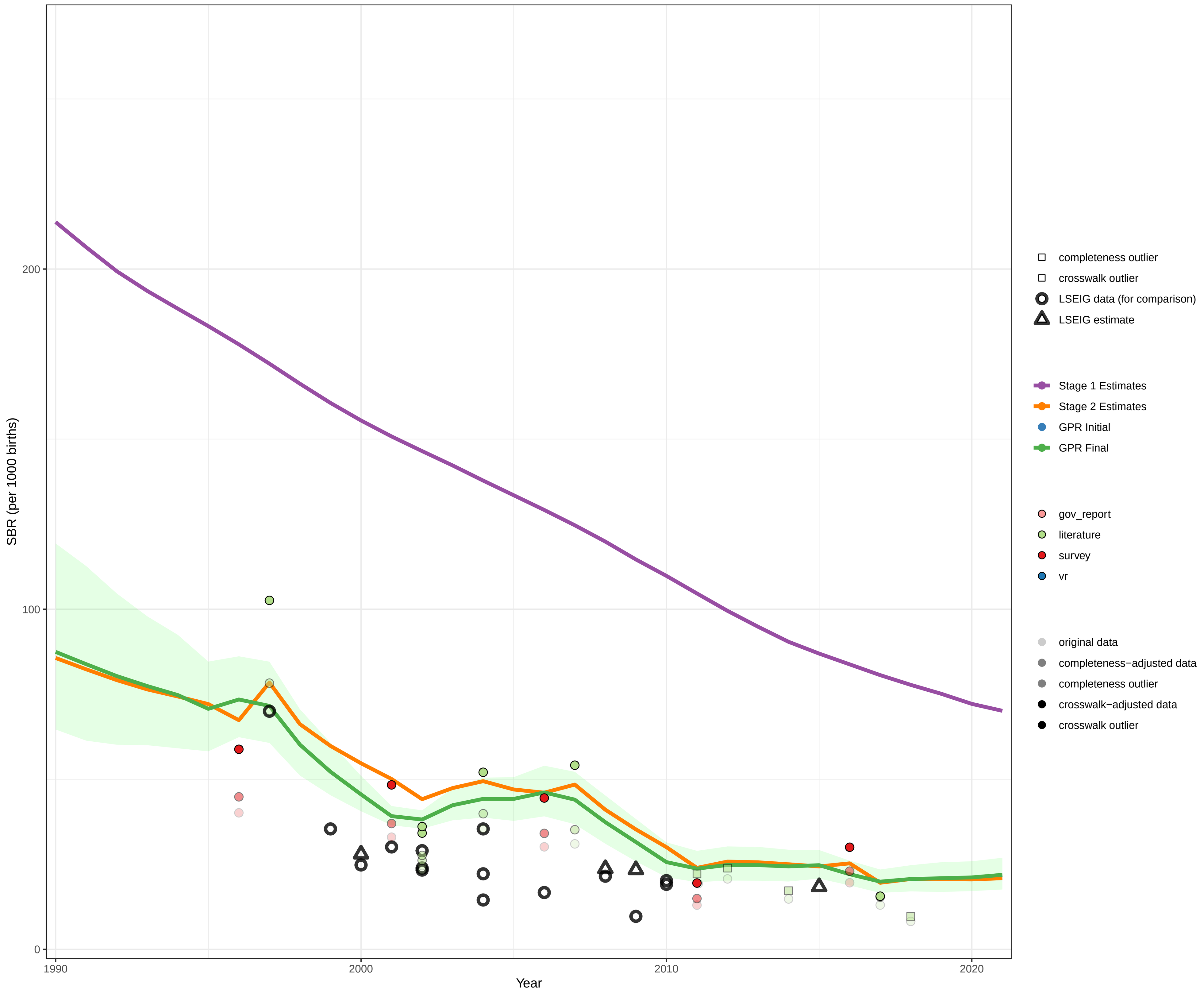

Nepal NPL South Asia Stillbirth Rate (SBR) − 28 weeks
lambda = 0.4 ; zeta = 0.9 ; scale = 10 ; amp = 0.028

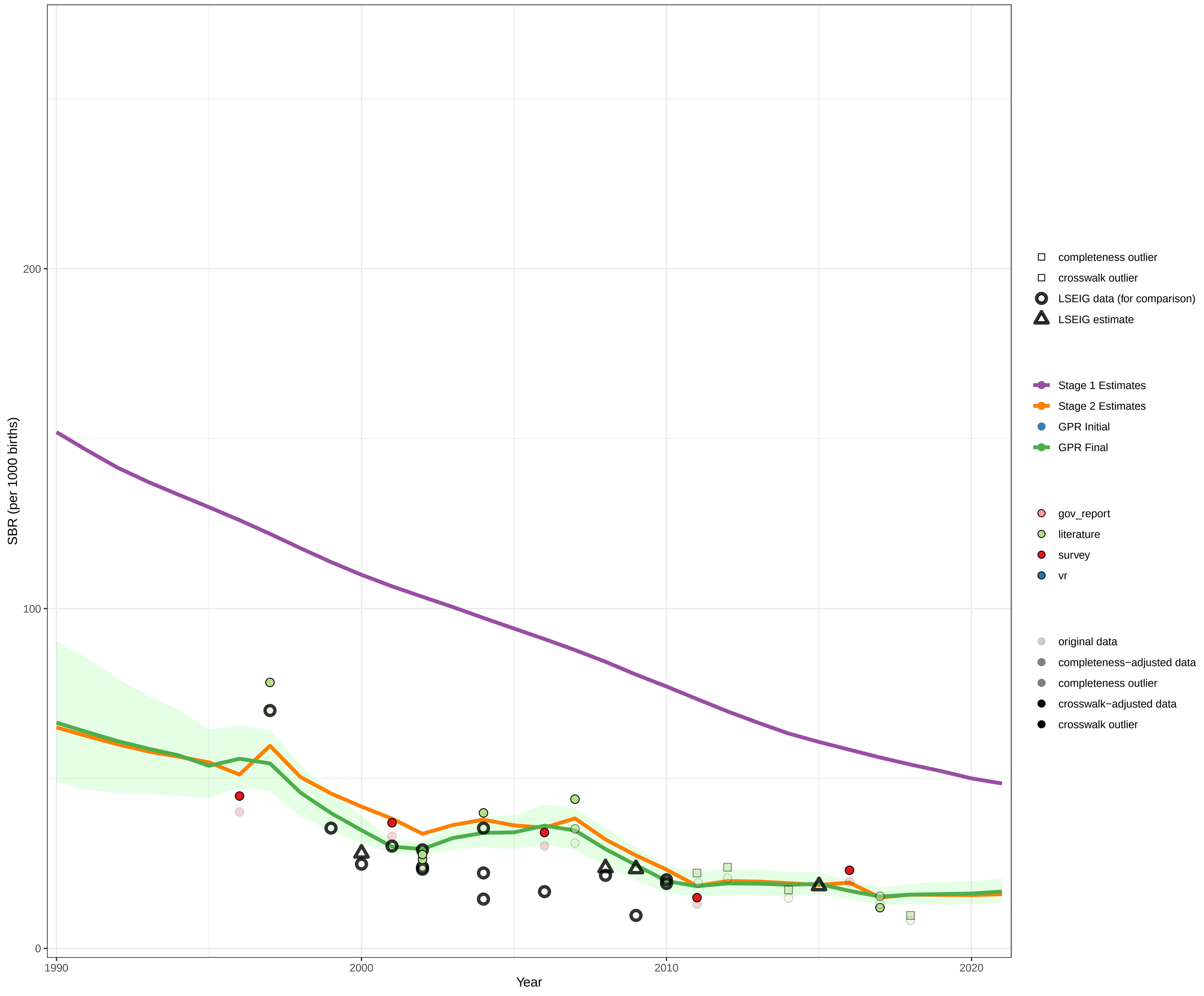

Year	Total Stillbirths (in thousands)	Total Stillbirths (in thousands)	SBR (per 1000 births)	SBR (per 1000 births)	SBR/NMR	SBR/NMR
	20 weeks	28 weeks	20 weeks	28 weeks	20 weeks	28 weeks
1990	76.3 (54.8 to 107.5)	56.6 (41.1 to 78.9)	87.4 (64.6 to 119.3)	66.4 (49.2 to 90.4)	1.58 (1.17 to 2.16)	1.20 (0.89 to 1.63)
1995	61.7 (50.1 to 74.9)	46.0 (37.5 to 55.8)	70.7 (58.2 to 84.6)	53.7 (44.2 to 64.4)	1.49 (1.23 to 1.78)	1.13 (0.93 to 1.36)
2000	37.3 (33.1 to 42.0)	28.2 (25.0 to 31.7)	45.6 (40.6 to 51.0)	34.8 (31.0 to 38.9)	1.13 (1.00 to 1.26)	0.86 (0.77 to 0.96)
2005	32.6 (27.6 to 37.5)	24.9 (21.1 to 28.7)	44.2 (37.8 to 50.6)	34.1 (29.2 to 39.1)	1.24 (1.06 to 1.42)	0.96 (0.82 to 1.10)
2010	17.1 (14.1 to 21.1)	13.1 (10.8 to 16.2)	25.6 (21.2 to 31.5)	19.8 (16.4 to 24.3)	0.86 (0.71 to 1.05)	0.66 (0.55 to 0.82)
2015	16.3 (13.7 to 19.3)	12.4 (10.4 to 14.8)	24.7 (20.8 to 29.2)	19.0 (15.9 to 22.4)	1.03 (0.86 to 1.21)	0.79 (0.66 to 0.93)
2020	13.9 (11.2 to 17.1)	10.6 (8.5 to 13.0)	21.1 (17.1 to 25.9)	16.1 (13.1 to 19.8)	1.06 (0.86 to 1.30)	0.81 (0.66 to 0.99)
2021	14.4 (11.5 to 17.8)	10.9 (8.7 to 13.5)	21.9 (17.6 to 26.9)	16.7 (13.4 to 20.6)	1.13 (0.91 to 1.39)	0.87 (0.69 to 1.06)

Pakistan PAK South Asia Stillbirth Rate / Neonatal Mortality Rate (SBR/NMR) − 20 weeks
lambda = 0.8 ; zeta = 0.7 ; scale = 15 ; amp = 0.028

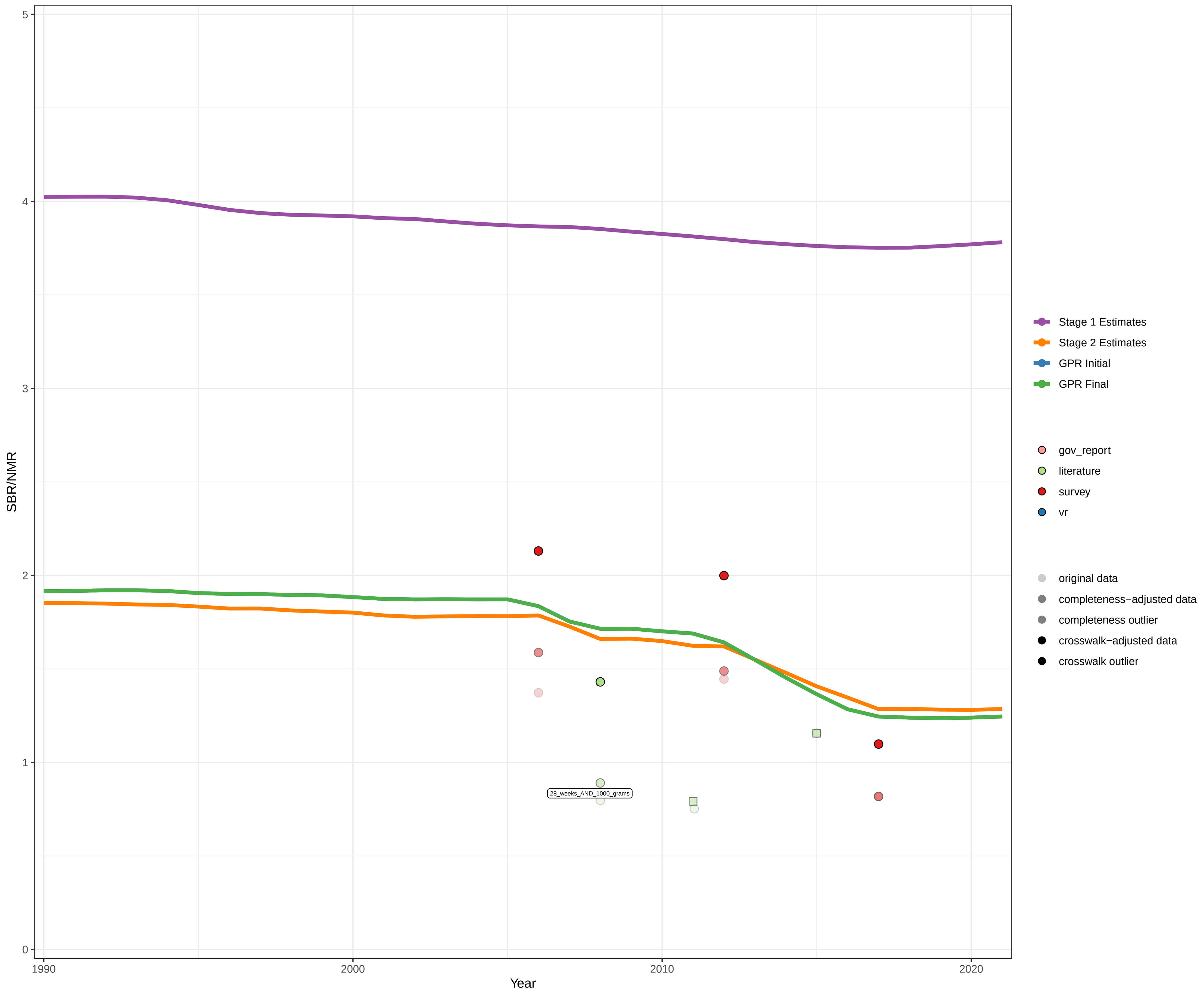

Pakistan PAK South Asia Stillbirth Rate / Neonatal Mortality Rate (SBR/NMR) − 28 weeks
lambda = 0.8 ; zeta = 0.7 ; scale = 15 ; amp = 0.028

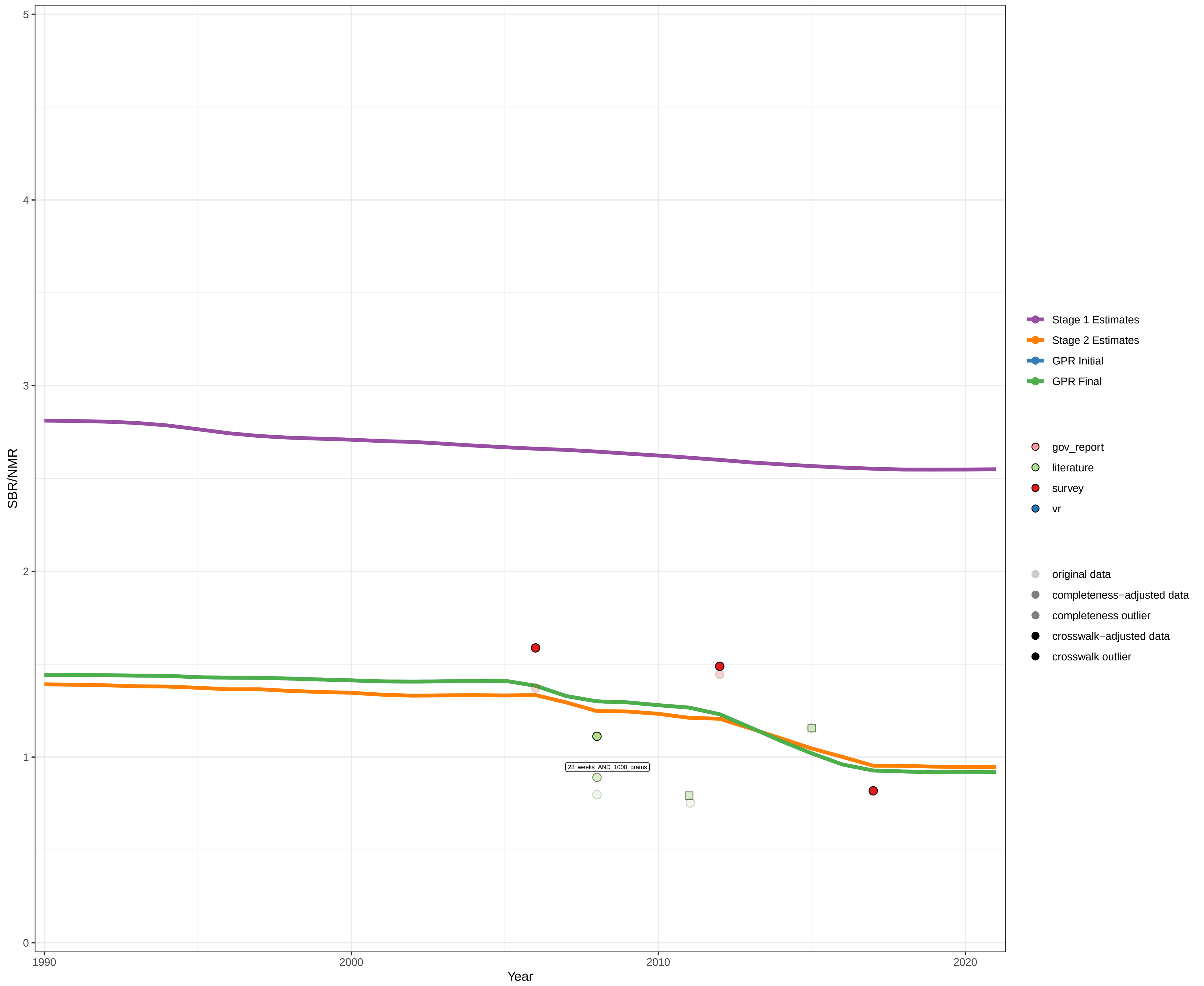

Definition	Years	Outlier	Source.Type	Title
28_weeks	2006	0	Survey	Pakistan Demographic and Health Survey 2006−2007
28_weeks	2012	0	Survey	Pakistan Demographic and Health Survey 2012−2013
28_weeks	2017	0	Survey	Pakistan Demographic and Health Survey 2017−2018
28_weeks_AND_1000_grams	2008	0	Sci Lit	Epidemiology of stillbirth in low−middle income countries: A Global Network Study
20_weeks	2015	1	Sci Lit	Global Network for Women's and Children's Health Research: probable causes of stillbirth in low− and middle−income countries using a prospectively defined classification system
28_weeks_OR_1000_grams	2011	1	Sci Lit	Maternal complications and perinatal mortality: findings of the World Health Organization Multicountry Survey on Maternal and Newborn Health

Pakistan PAK South Asia Stillbirth Rate (SBR) − 20 weeks
lambda = 0.8 ; zeta = 0.7 ; scale = 15 ; amp = 0.028

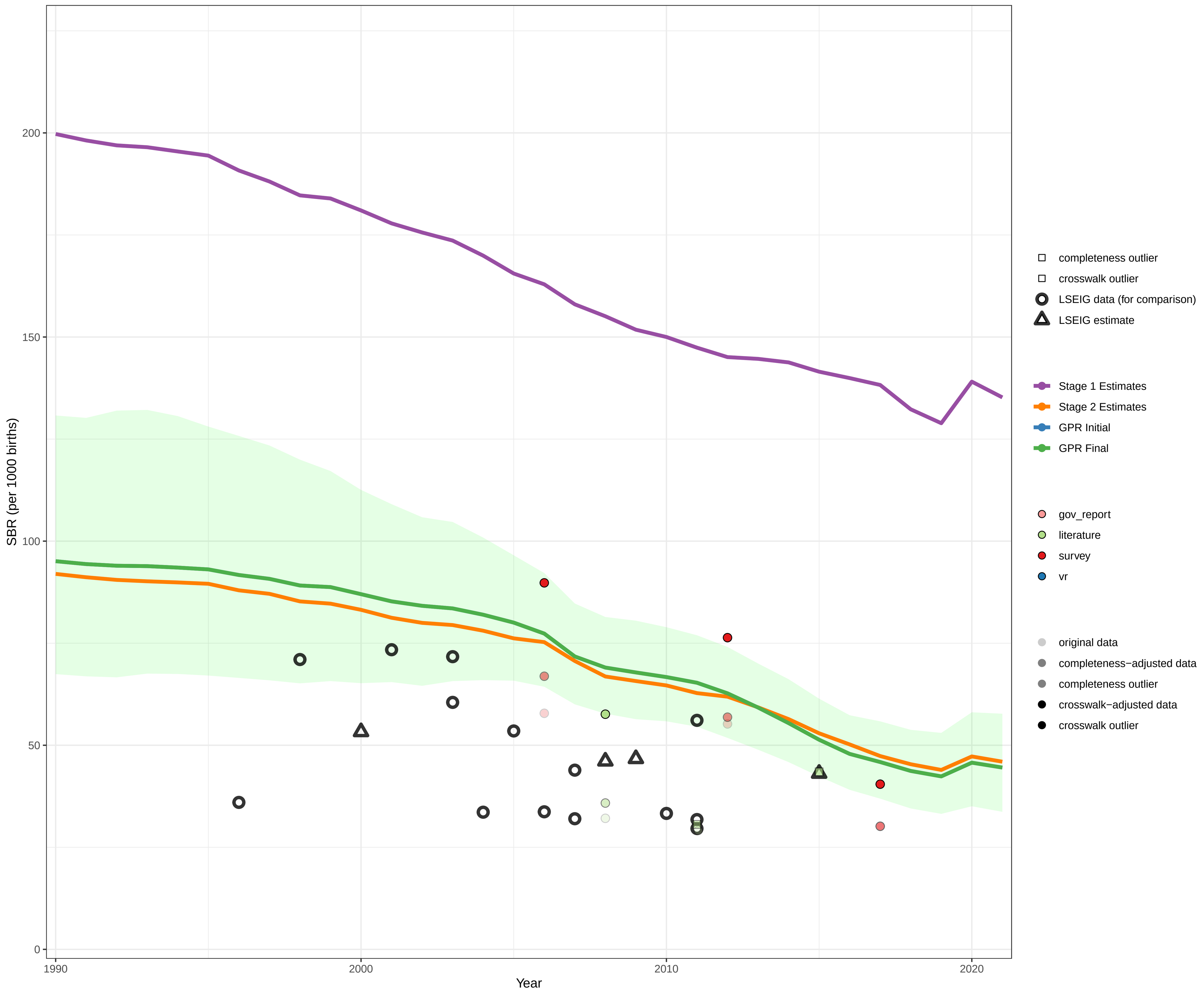

Pakistan PAK South Asia Stillbirth Rate (SBR) − 28 weeks
lambda = 0.8 ; zeta = 0.7 ; scale = 15 ; amp = 0.028

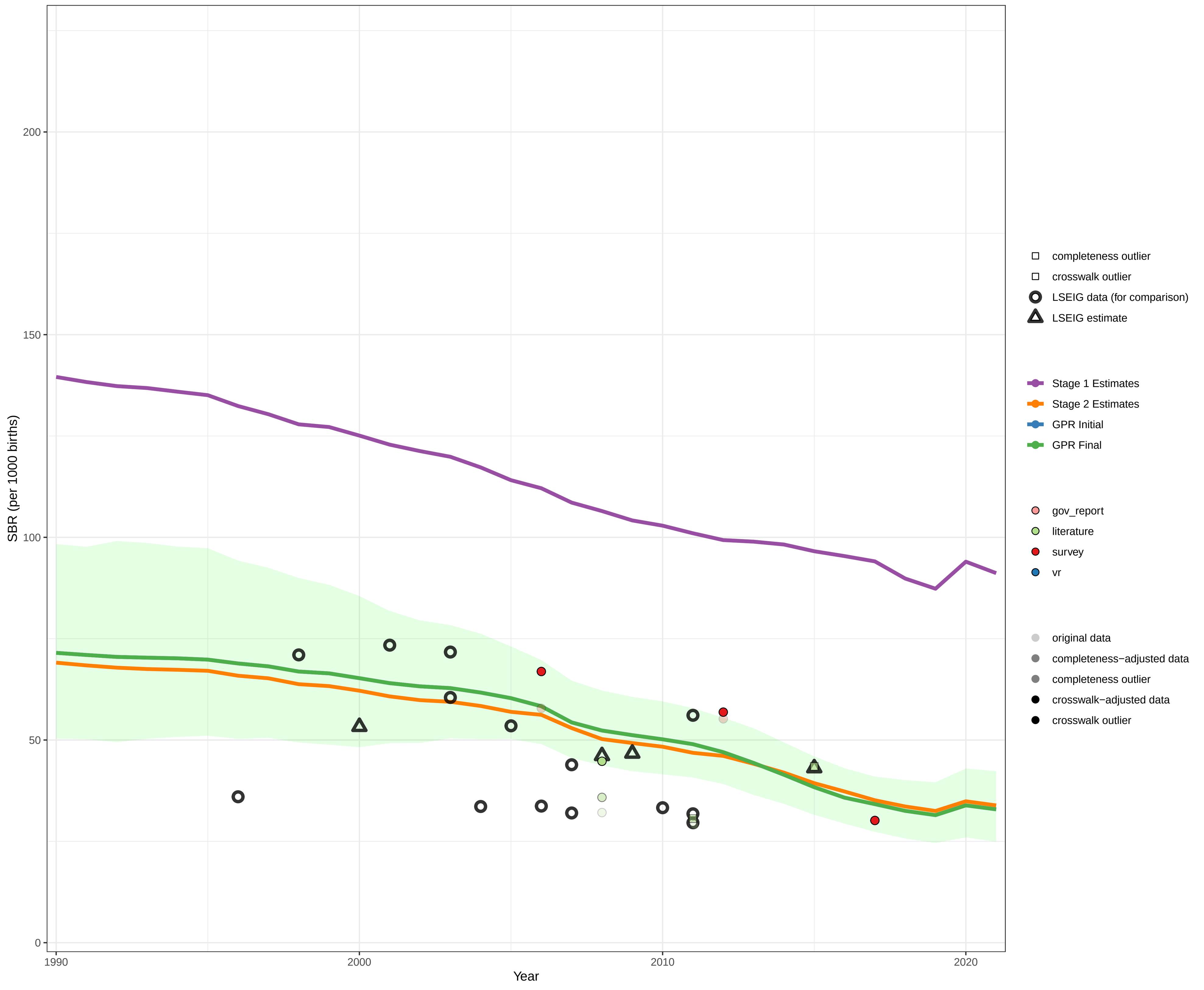

Year	Total Stillbirths (in thousands)	Total Stillbirths (in thousands)	SBR (per 1000 births)	SBR (per 1000 births)	SBR/NMR	SBR/NMR
	20 weeks	28 weeks	20 weeks	28 weeks	20 weeks	28 weeks
1990	443.9 (304.4 to 633.4)	325.0 (223.1 to 459.1)	95.1 (67.4 to 130.8)	71.5 (50.3 to 98.3)	1.92 (1.36 to 2.64)	1.44 (1.01 to 1.98)
1995	461.9 (322.5 to 658.9)	337.6 (241.4 to 483.7)	93.1 (67.1 to 128.1)	69.8 (51.1 to 97.3)	1.91 (1.37 to 2.62)	1.43 (1.05 to 1.99)
2000	437.9 (319.8 to 581.5)	320.5 (232.4 to 428.8)	87.0 (65.2 to 112.6)	65.3 (48.2 to 85.5)	1.88 (1.41 to 2.44)	1.41 (1.04 to 1.85)
2005	433.2 (350.4 to 531.5)	319.5 (263.2 to 392.1)	80.0 (65.8 to 96.5)	60.3 (50.3 to 73.1)	1.87 (1.54 to 2.26)	1.41 (1.18 to 1.71)
2010	402.4 (332.9 to 482.1)	297.3 (243.9 to 356.2)	66.7 (55.9 to 78.9)	50.2 (41.5 to 59.5)	1.70 (1.42 to 2.01)	1.28 (1.06 to 1.52)
2015	326.9 (266.8 to 394.9)	240.9 (196.5 to 290.7)	51.4 (42.3 to 61.4)	38.4 (31.5 to 46.0)	1.37 (1.13 to 1.63)	1.02 (0.84 to 1.22)
2020	296.6 (224.6 to 381.3)	217.0 (165.0 to 277.6)	45.7 (35.0 to 58.1)	33.9 (26.0 to 43.0)	1.24 (0.95 to 1.57)	0.92 (0.70 to 1.16)
2021	288.7 (215.8 to 379.1)	210.7 (157.9 to 273.6)	44.5 (33.7 to 57.7)	32.9 (24.9 to 42.3)	1.25 (0.94 to 1.61)	0.92 (0.70 to 1.18)

Azad Jammu & Kashmir   PAK_53615   South Asia   Stillbirth Rate / Neonatal Mortality Rate (SBR/NMR) − 20 weeks
lambda = 1 ; zeta = 0.6 ; scale = 15 ; amp = 0.028

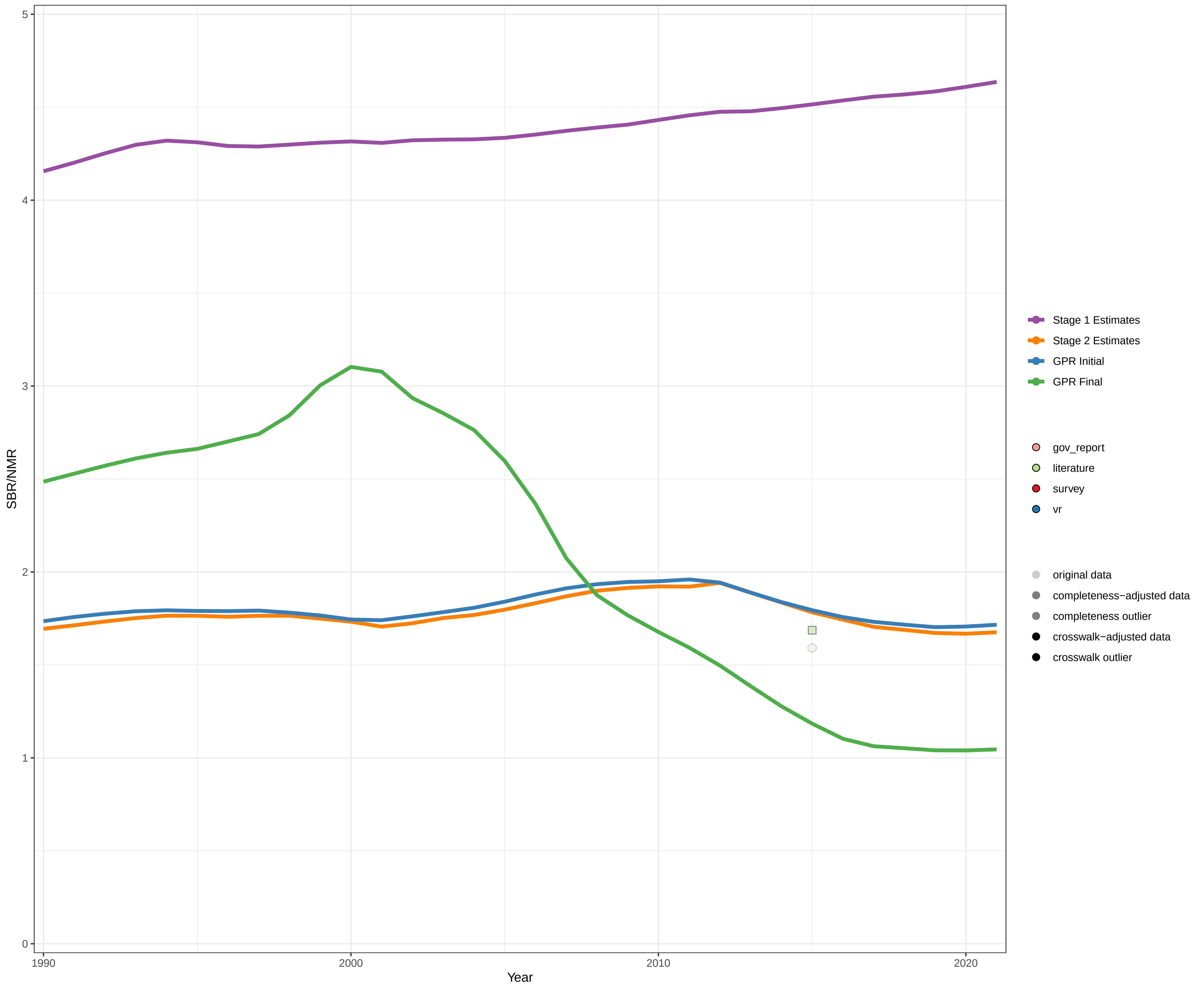

Azad Jammu & Kashmir   PAK_53615   South Asia   Stillbirth Rate / Neonatal Mortality Rate (SBR/NMR) − 28 weeks
lambda = 1 ; zeta = 0.6 ; scale = 15 ; amp = 0.028

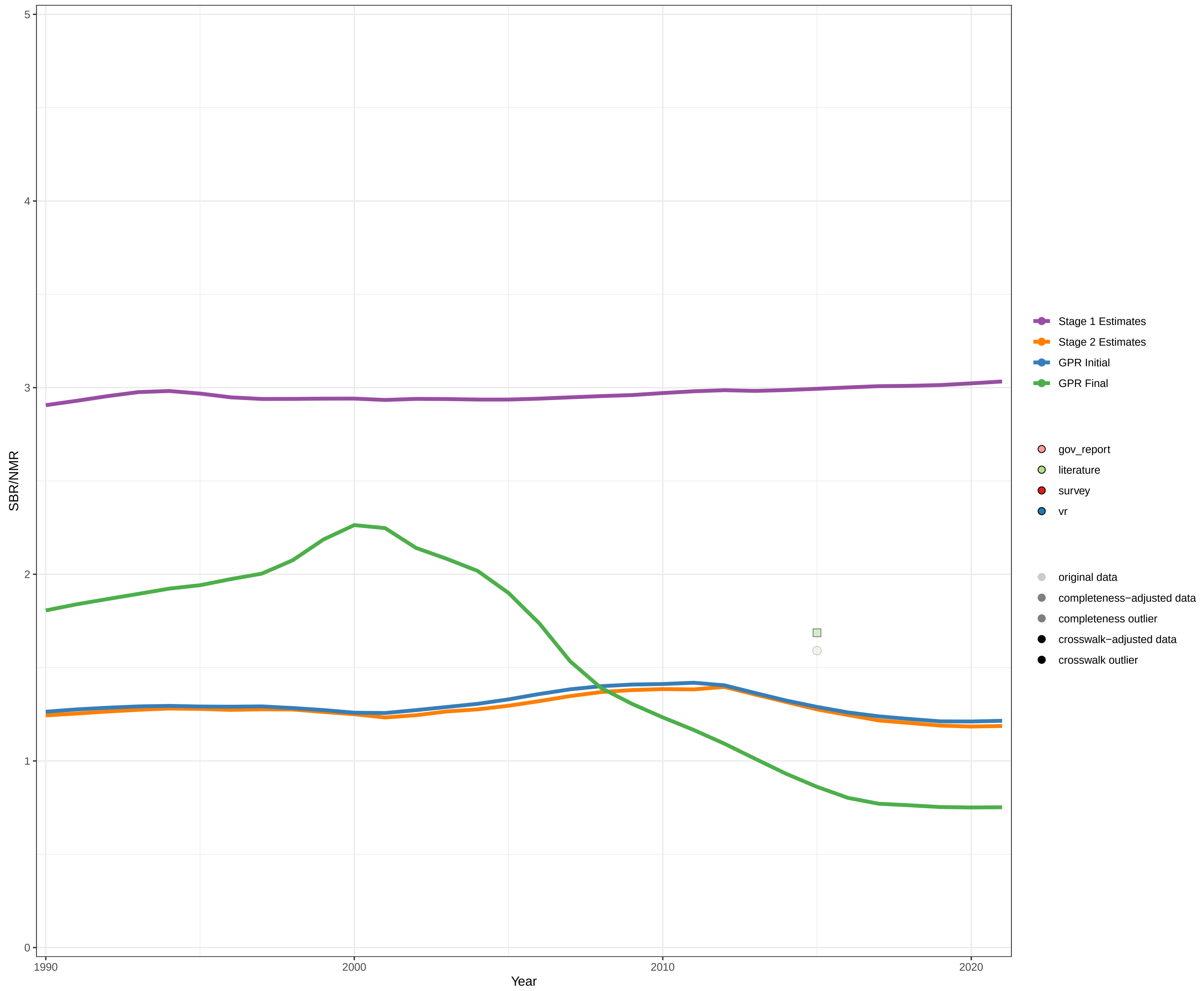

Definition	Years	Outlier	Source.Type	Title
28_weeks	2015	1	Sci Lit	Incidence and associated risk factors of low birth weight babies born in Shaikh Khalifa Bin Zayad Al−Nayan Hospital Muzaffarabad, Azad Jammu and Kashmir

Azad Jammu & Kashmir   PAK_53615   South Asia   Stillbirth Rate (SBR) − 20 weeks
lambda = 1 ; zeta = 0.6 ; scale = 15 ; amp = 0.028

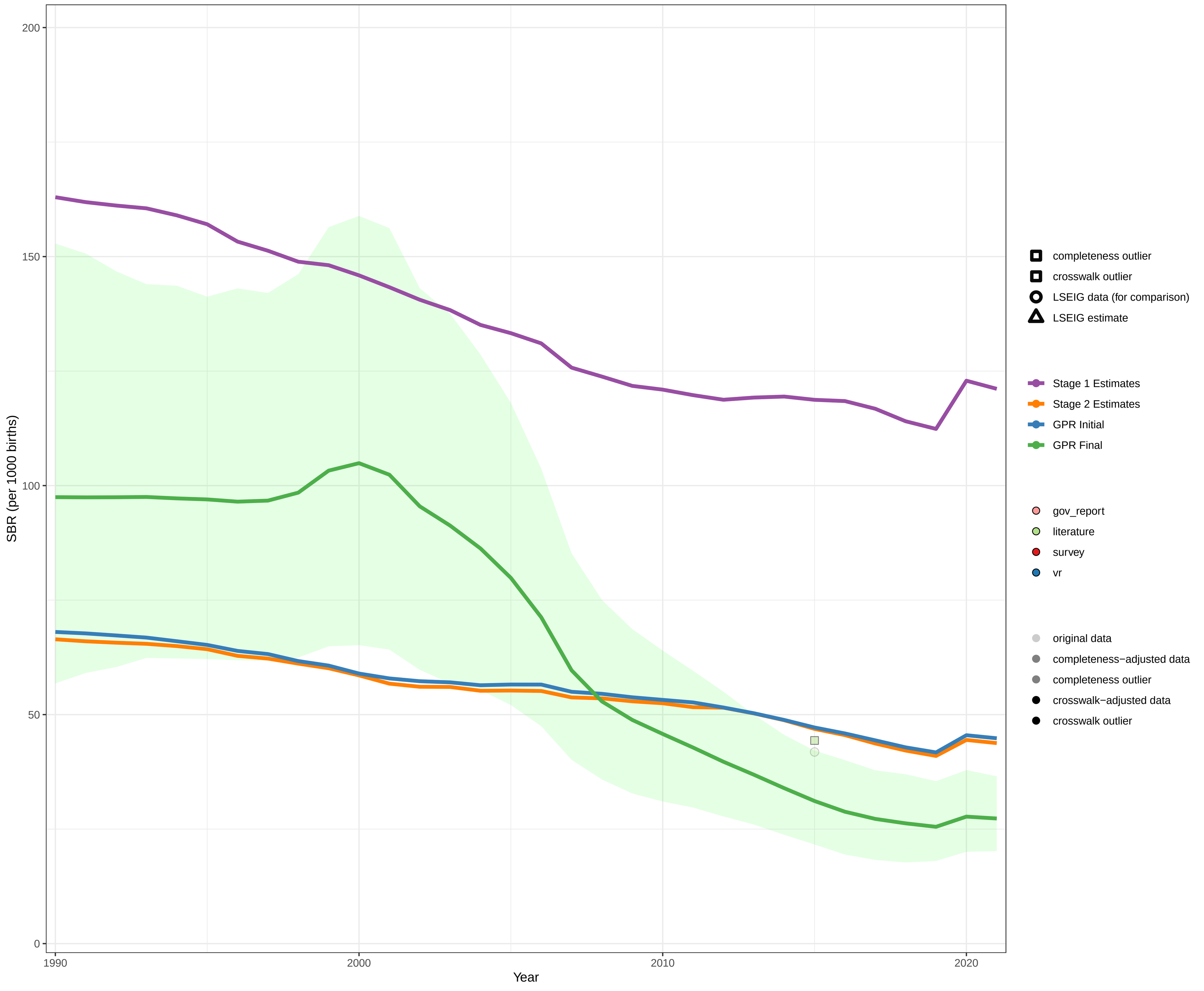

Azad Jammu & Kashmir   PAK_53615   South Asia   Stillbirth Rate (SBR) − 28 weeks
lambda = 1 ; zeta = 0.6 ; scale = 15 ; amp = 0.028

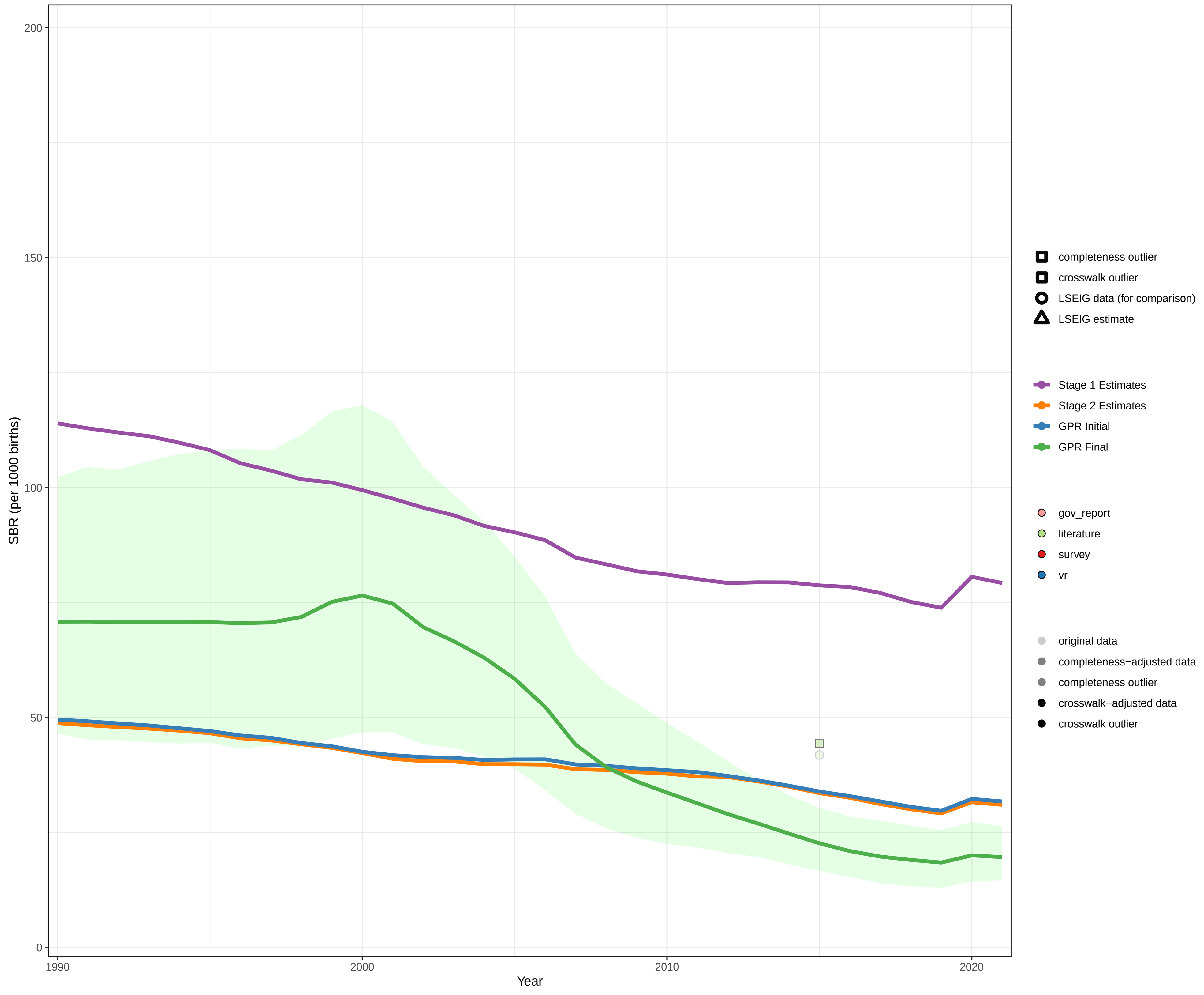

Year	Total Stillbirths (in thousands)	Total Stillbirths (in thousands)	SBR (per 1000 births)	SBR (per 1000 births)	SBR/NMR	SBR/NMR
	20 weeks	28 weeks	20 weeks	28 weeks	20 weeks	28 weeks
1990	9.3<br>(5.1 to 15.4)	6.5<br>(4.2 to 9.7)	97.5<br>(56.8 to 152.8)	70.8<br>(46.5 to 102.3)	2.49<br>(1.45 to 3.90)	1.81<br>(1.19 to 2.61)
1995	9.0<br>(5.5 to 13.8)	6.4<br>(3.9 to 10.1)	97.0<br>(62.1 to 141.2)	70.7<br>(44.5 to 108.1)	2.66<br>(1.71 to 3.88)	1.94<br>(1.22 to 2.97)
2000	9.3<br>(5.5 to 14.8)	6.5<br>(3.8 to 10.5)	104.9<br>(65.2 to 158.9)	76.5<br>(46.8 to 117.9)	3.10<br>(1.93 to 4.70)	2.26<br>(1.38 to 3.49)
2005	7.0<br>(4.4 to 10.7)	5.0<br>(3.2 to 7.4)	79.8<br>(52.1 to 118.1)	58.4<br>(38.9 to 85.0)	2.60<br>(1.69 to 3.84)	1.90<br>(1.26 to 2.76)
2010	4.1<br>(2.7 to 5.8)	3.0<br>(2.0 to 4.4)	45.8<br>(31.0 to 64.0)	33.7<br>(22.5 to 48.9)	1.68<br>(1.14 to 2.34)	1.23<br>(0.82 to 1.79)
2015	2.8<br>(1.9 to 3.8)	2.0<br>(1.5 to 2.7)	31.1<br>(21.6 to 42.1)	22.6<br>(16.7 to 30.4)	1.18<br>(0.82 to 1.60)	0.86<br>(0.63 to 1.16)
2020	2.3<br>(1.6 to 3.2)	1.6<br>(1.2 to 2.3)	27.7<br>(20.0 to 37.9)	20.0<br>(14.3 to 27.3)	1.04<br>(0.75 to 1.42)	0.75<br>(0.54 to 1.02)
2021	2.2<br>(1.6 to 3.0)	1.6<br>(1.2 to 2.1)	27.3<br>(20.2 to 36.5)	19.6<br>(14.6 to 26.4)	1.05<br>(0.77 to 1.40)	0.75<br>(0.56 to 1.01)

Balochistan PAK_53616 South Asia Stillbirth Rate / Neonatal Mortality Rate (SBR/NMR) − 20 weeks
lambda = 0.8 ; zeta = 0.7 ; scale = 15 ; amp = 0.028

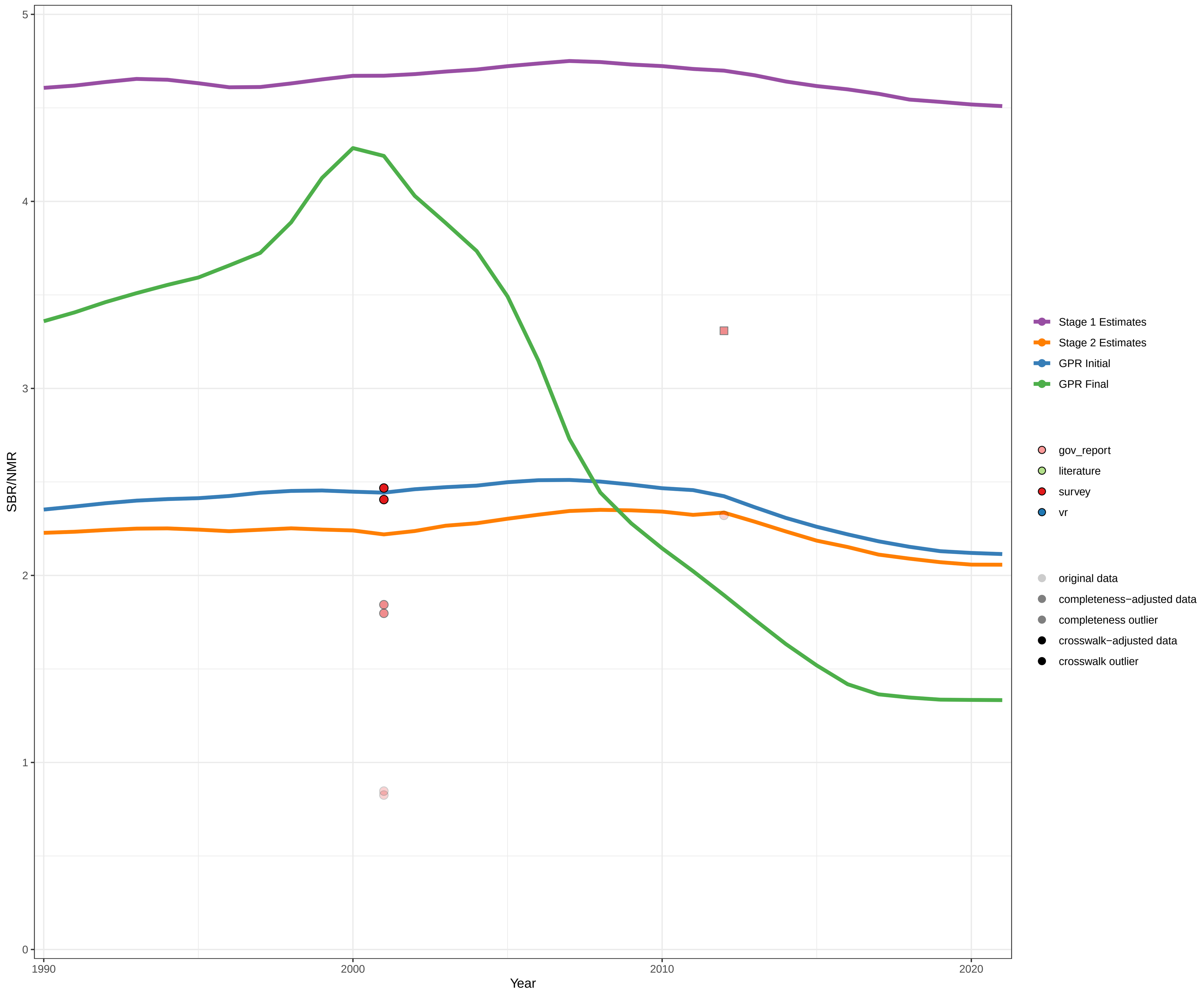

Balochistan PAK_53616 South Asia Stillbirth Rate / Neonatal Mortality Rate (SBR/NMR) − 28 weeks
lambda = 0.8 ; zeta = 0.7 ; scale = 15 ; amp = 0.028

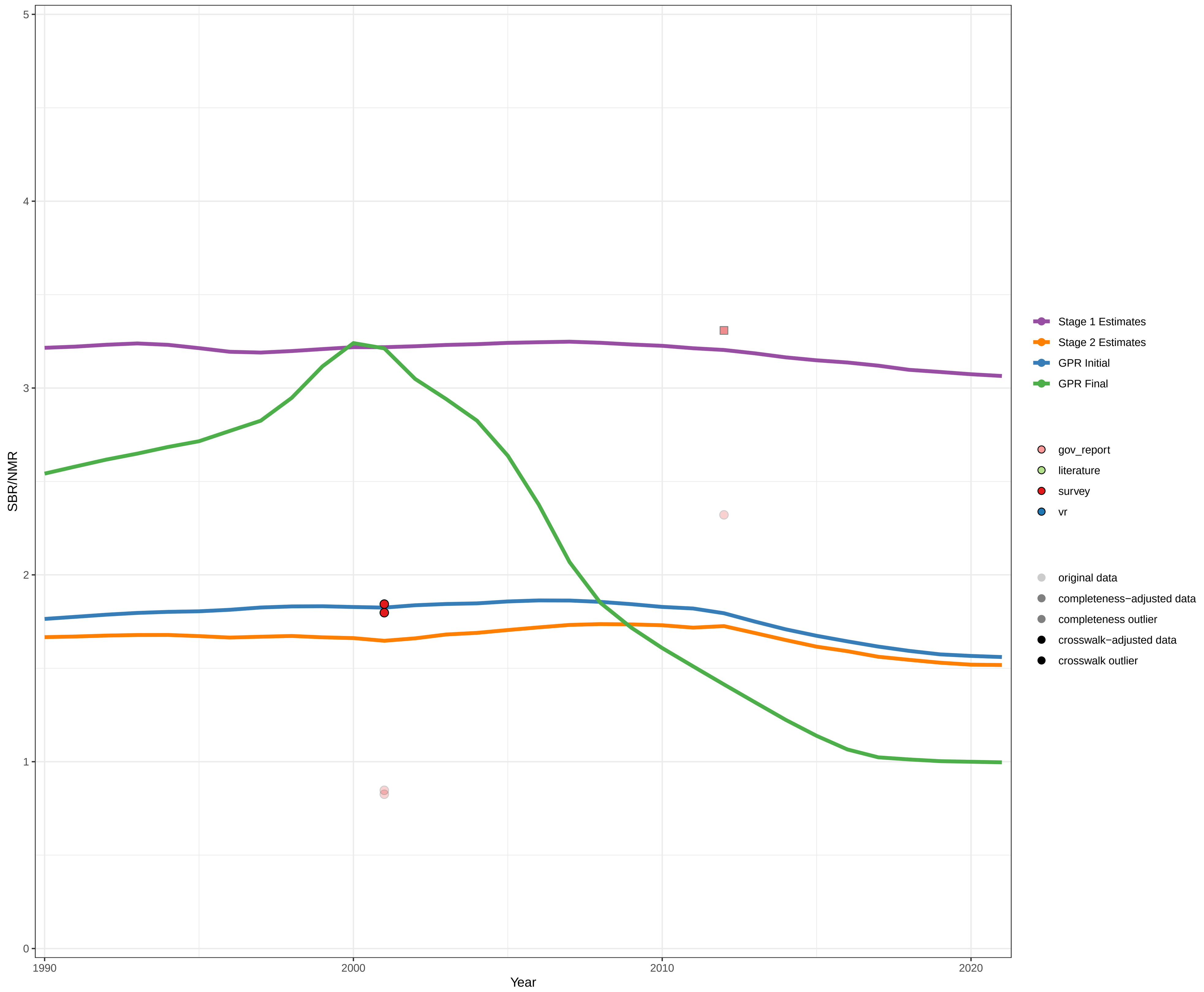

Definition	Years	Outlier	Source.Type	Title
28_weeks	2001	0	Survey	Pakistan Reproductive Health and Family Planning Survey 2000−2001
28_weeks	2012	1	Survey	Pakistan Demographic and Health Survey 2012−2013

Balochistan PAK_53616 South Asia Stillbirth Rate (SBR) − 20 weeks
lambda = 0.8 ; zeta = 0.7 ; scale = 15 ; amp = 0.028

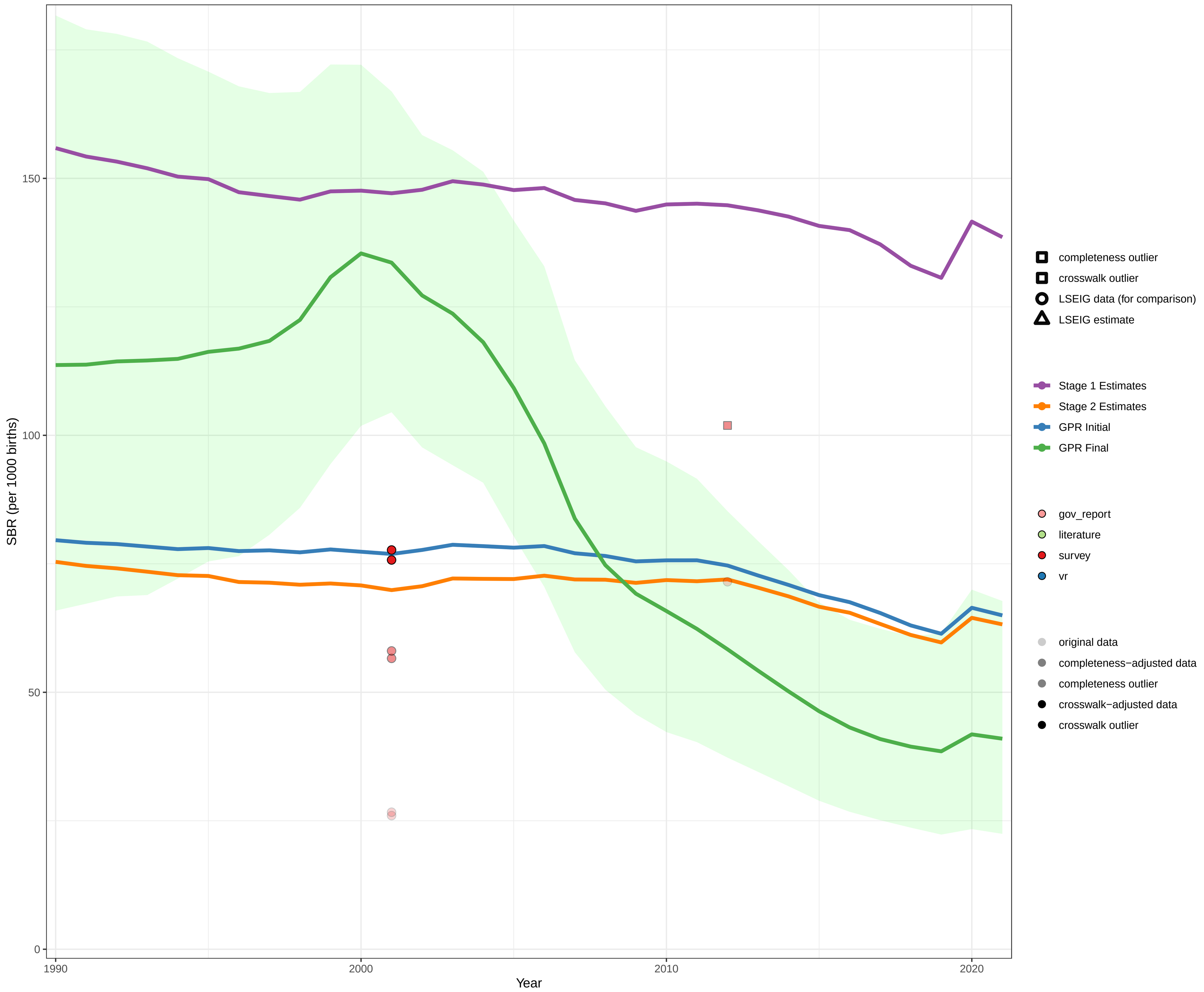

Balochistan PAK_53616 South Asia Stillbirth Rate (SBR) − 28 weeks
lambda = 0.8 ; zeta = 0.7 ; scale = 15 ; amp = 0.028

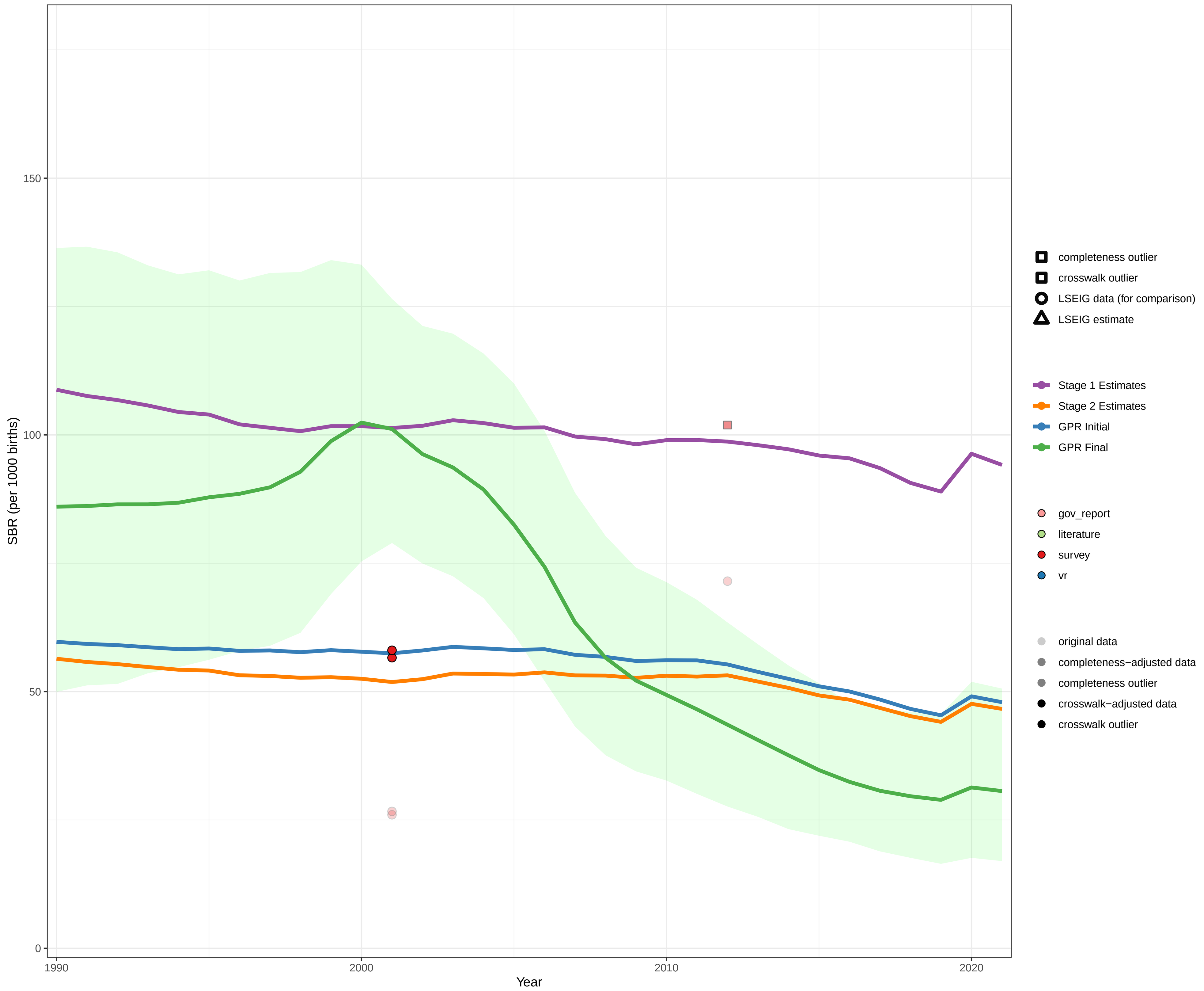

Year	Total Stillbirths (in thousands)	Total Stillbirths (in thousands)	SBR (per 1000 births)	SBR (per 1000 births)	SBR/NMR	SBR/NMR
	20 weeks	28 weeks	20 weeks	28 weeks	20 weeks	28 weeks
1990	26.8 (14.6 to 46.0)	19.6 (10.9 to 32.7)	113.7 (65.9 to 181.7)	86.0 (50.0 to 136.4)	3.36 (1.95 to 5.37)	2.54 (1.48 to 4.03)
1995	29.7 (18.3 to 46.2)	21.7 (13.4 to 34.1)	116.2 (75.5 to 170.8)	87.8 (56.2 to 132.1)	3.59 (2.33 to 5.28)	2.72 (1.74 to 4.08)
2000	37.7 (27.2 to 49.8)	27.4 (19.5 to 36.8)	135.4 (101.8 to 172.1)	102.4 (75.4 to 133.2)	4.29 (3.22 to 5.45)	3.24 (2.38 to 4.21)
2005	33.8 (24.0 to 45.5)	24.8 (17.9 to 34.0)	109.2 (80.3 to 141.8)	82.5 (61.1 to 110.0)	3.49 (2.57 to 4.53)	2.64 (1.96 to 3.52)
2010	23.8 (14.9 to 35.3)	17.5 (11.4 to 25.9)	65.8 (42.3 to 94.9)	49.3 (32.7 to 71.3)	2.14 (1.38 to 3.09)	1.61 (1.06 to 2.32)
2015	19.3 (11.8 to 28.7)	14.3 (8.9 to 21.5)	46.3 (28.9 to 67.5)	34.7 (21.9 to 51.6)	1.52 (0.95 to 2.21)	1.14 (0.72 to 1.69)
2020	19.8 (10.8 to 34.0)	14.7 (8.1 to 24.8)	41.8 (23.4 to 70.0)	31.3 (17.6 to 51.9)	1.33 (0.75 to 2.23)	1.00 (0.56 to 1.66)
2021	19.8 (10.6 to 33.6)	14.6 (8.0 to 24.6)	41.0 (22.5 to 67.8)	30.6 (17.0 to 50.6)	1.33 (0.73 to 2.21)	1.00 (0.55 to 1.65)

Gilgit−Baltistan   PAK_53617   South Asia   Stillbirth Rate / Neonatal Mortality Rate (SBR/NMR) − 20 weeks
lambda = 0.8 ; zeta = 0.7 ; scale = 15 ; amp = 0.028

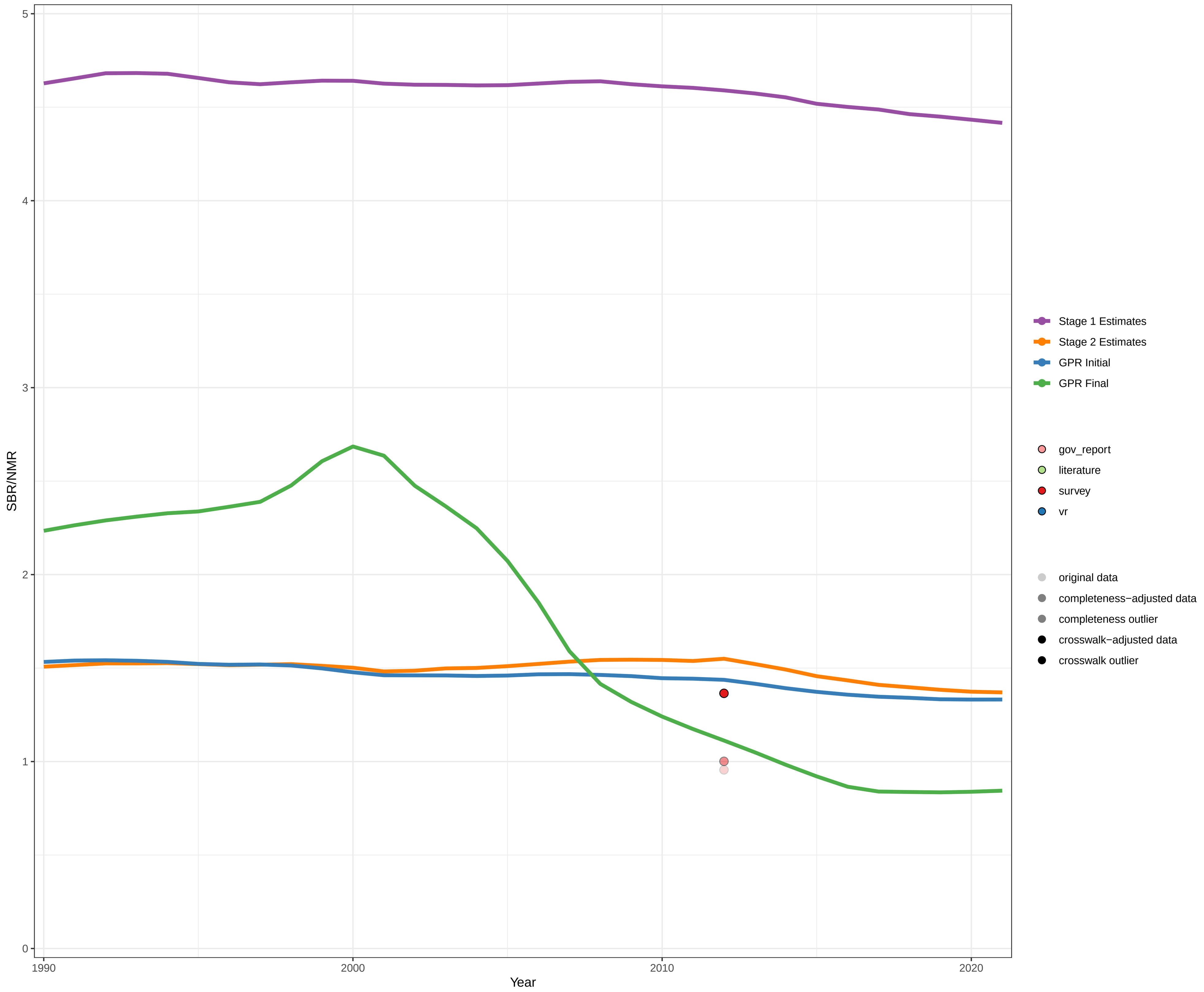

Gilgit−Baltistan   PAK_53617   South Asia   Stillbirth Rate / Neonatal Mortality Rate (SBR/NMR) − 28 weeks
lambda = 0.8 ; zeta = 0.7 ; scale = 15 ; amp = 0.028

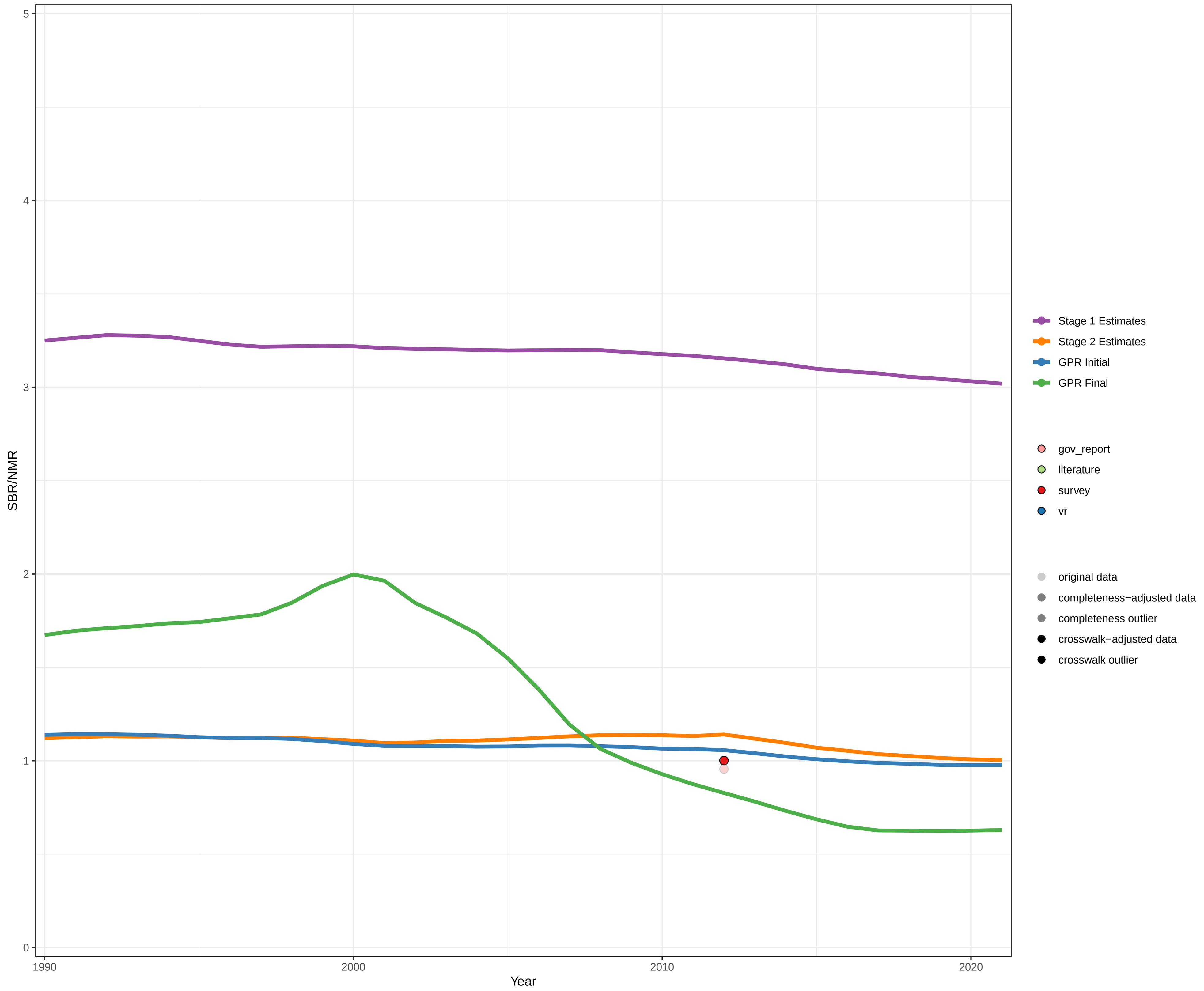

Definition	Years	Outlier	Source.Type	Title
28_weeks	2012	0	Survey	Pakistan Demographic and Health Survey 2012−2013

Gilgit−Baltistan   PAK_53617   South Asia   Stillbirth Rate (SBR) − 20 weeks
lambda = 0.8 ; zeta = 0.7 ; scale = 15 ; amp = 0.028

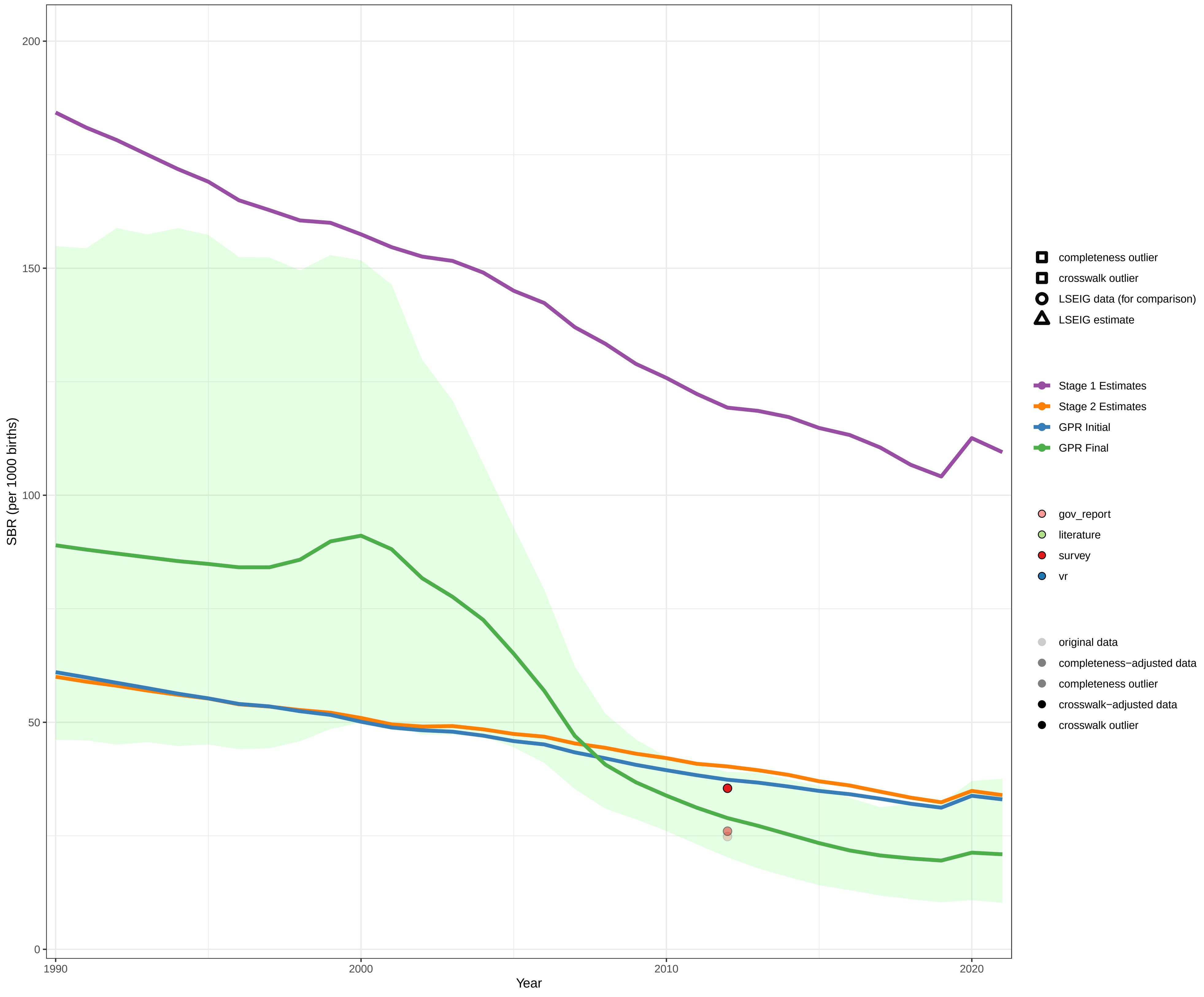

Gilgit−Baltistan   PAK_53617   South Asia   Stillbirth Rate (SBR) − 28 weeks
lambda = 0.8 ; zeta = 0.7 ; scale = 15 ; amp = 0.028

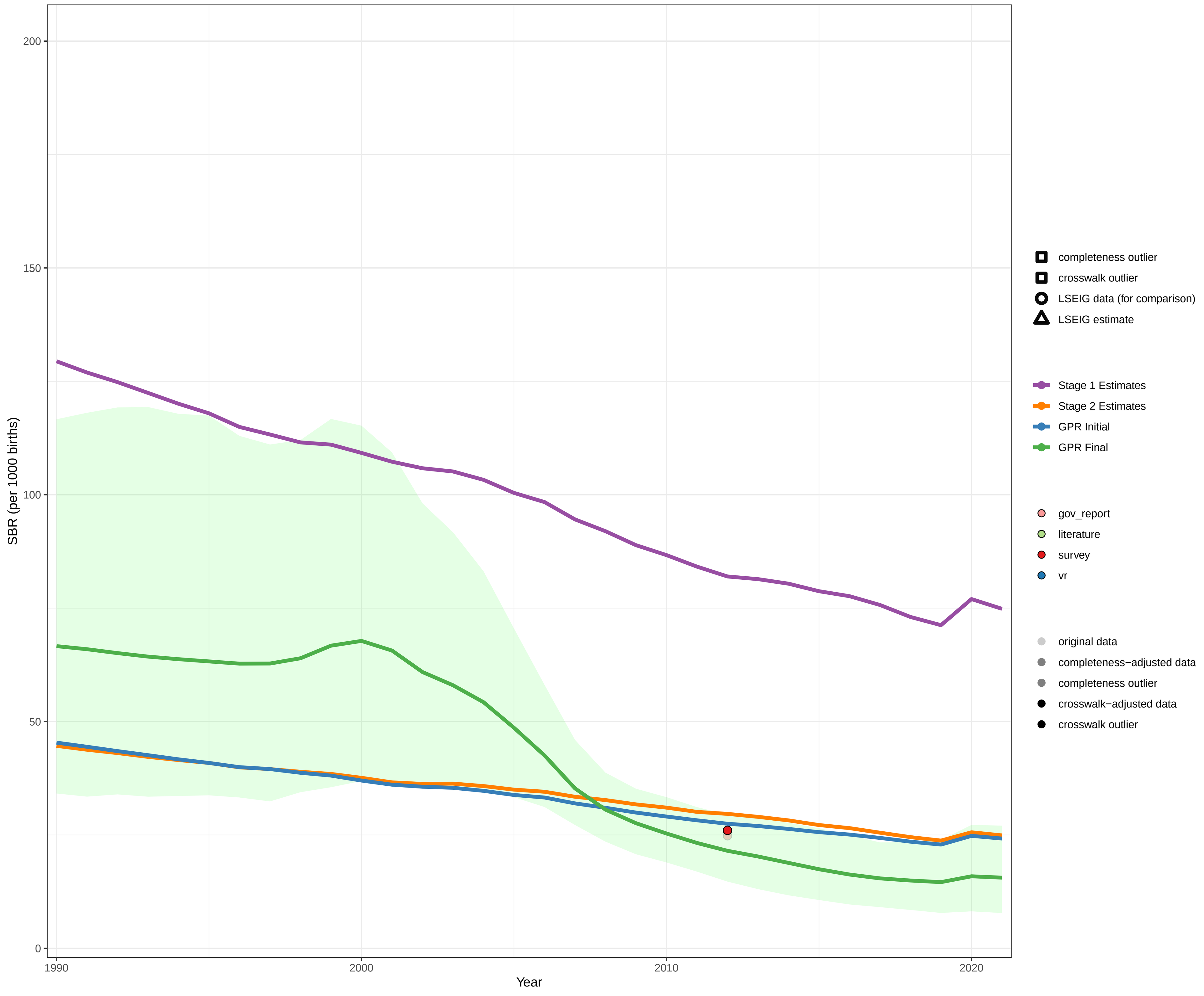

Year	Total Stillbirths (in thousands)	Total Stillbirths (in thousands)	SBR (per 1000 births)	SBR (per 1000 births)	SBR/NMR	SBR/NMR
	20 weeks	28 weeks	20 weeks	28 weeks	20 weeks	28 weeks
1990	3.1 (1.5 to 5.7)	2.2 (1.1 to 4.1)	89.0 (46.1 to 154.9)	66.6 (34.1 to 116.6)	2.23 (1.16 to 3.89)	1.67 (0.86 to 2.93)
1995	3.2 (1.6 to 6.4)	2.3 (1.2 to 4.6)	84.9 (45.1 to 157.4)	63.3 (33.7 to 117.5)	2.34 (1.24 to 4.33)	1.74 (0.93 to 3.24)
2000	3.7 (1.9 to 6.6)	2.7 (1.4 to 4.8)	91.1 (49.9 to 151.8)	67.8 (36.9 to 115.2)	2.69 (1.47 to 4.47)	2.00 (1.09 to 3.40)
2005	2.9 (1.9 to 4.2)	2.1 (1.4 to 3.1)	65.1 (44.5 to 93.0)	48.6 (33.3 to 70.5)	2.07 (1.42 to 2.96)	1.55 (1.06 to 2.25)
2010	1.7 (1.3 to 2.1)	1.3 (0.9 to 1.7)	33.9 (26.0 to 42.4)	25.3 (18.9 to 33.3)	1.24 (0.95 to 1.56)	0.93 (0.69 to 1.22)
2015	1.3 (0.8 to 2.0)	1.0 (0.6 to 1.5)	23.4 (14.1 to 36.0)	17.4 (10.7 to 26.5)	0.92 (0.56 to 1.42)	0.69 (0.42 to 1.04)
2020	1.3 (0.6 to 2.3)	0.9 (0.5 to 1.6)	21.3 (10.8 to 37.1)	15.9 (8.2 to 27.2)	0.84 (0.43 to 1.46)	0.63 (0.32 to 1.07)
2021	1.3 (0.6 to 2.3)	0.9 (0.5 to 1.6)	20.9 (10.2 to 37.5)	15.6 (7.8 to 27.1)	0.84 (0.41 to 1.51)	0.63 (0.31 to 1.09)

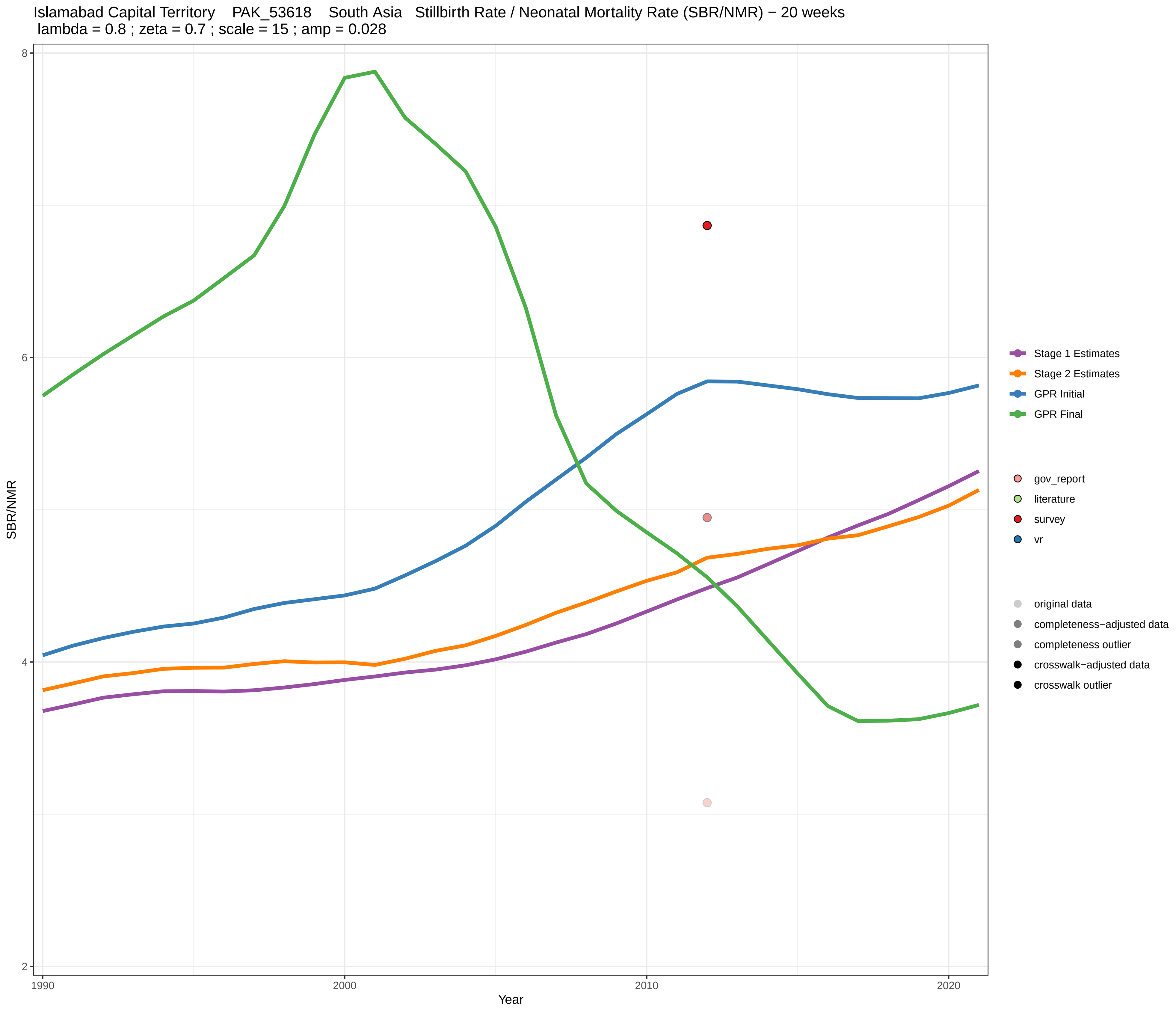

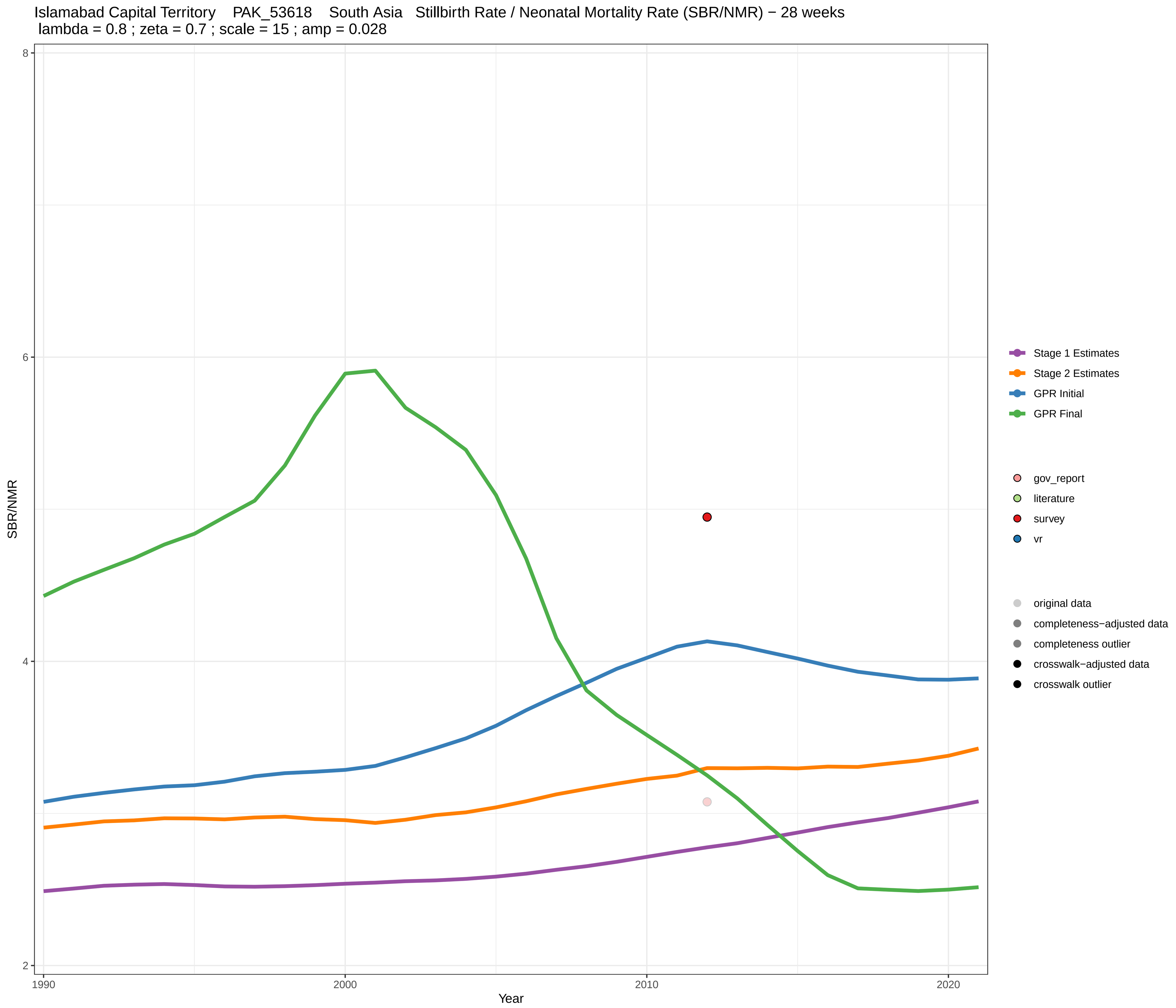

Definition	Years	Outlier	Source.Type	Title
28_weeks	2012	0	Survey	Pakistan Demographic and Health Survey 2012−2013

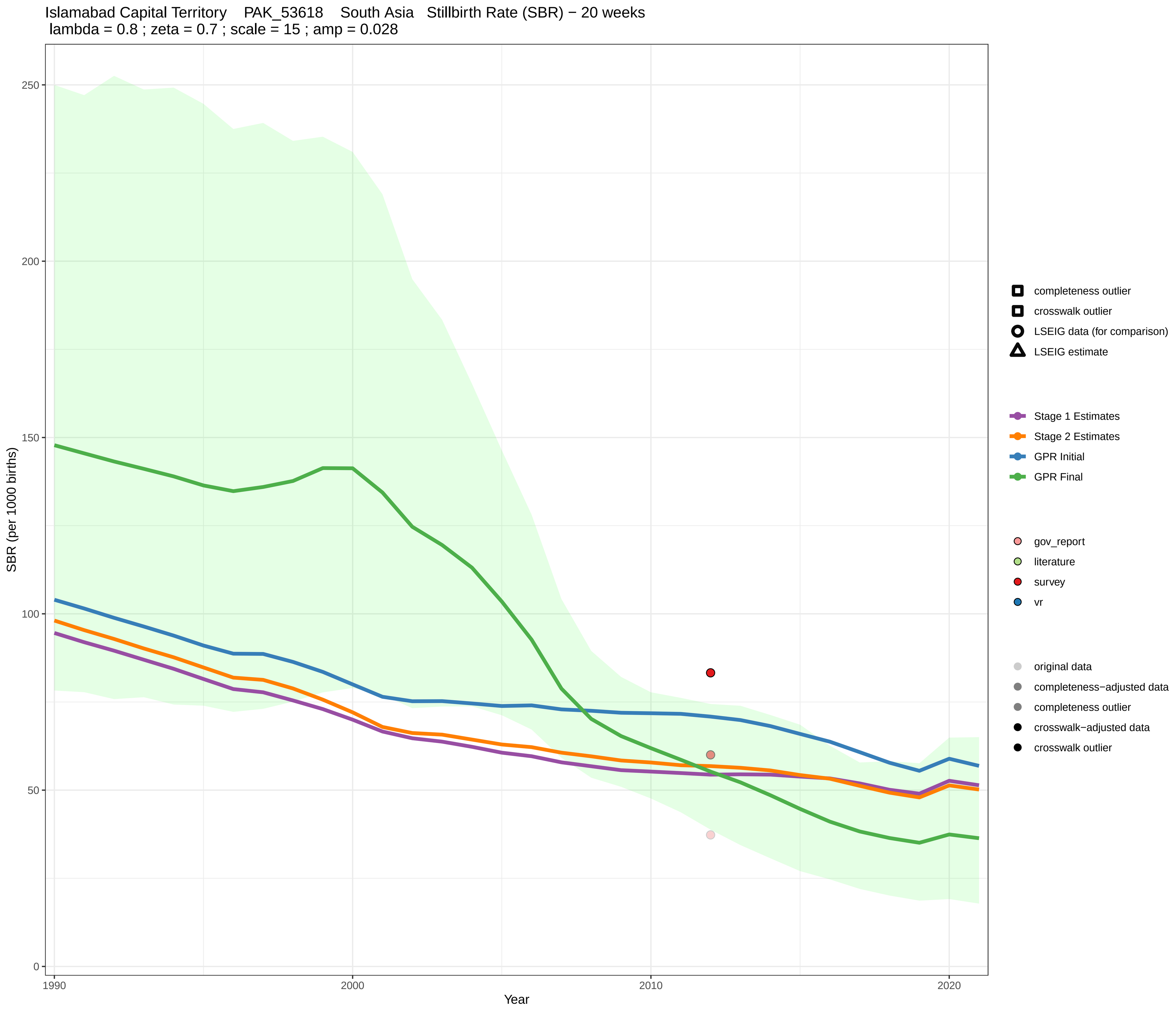

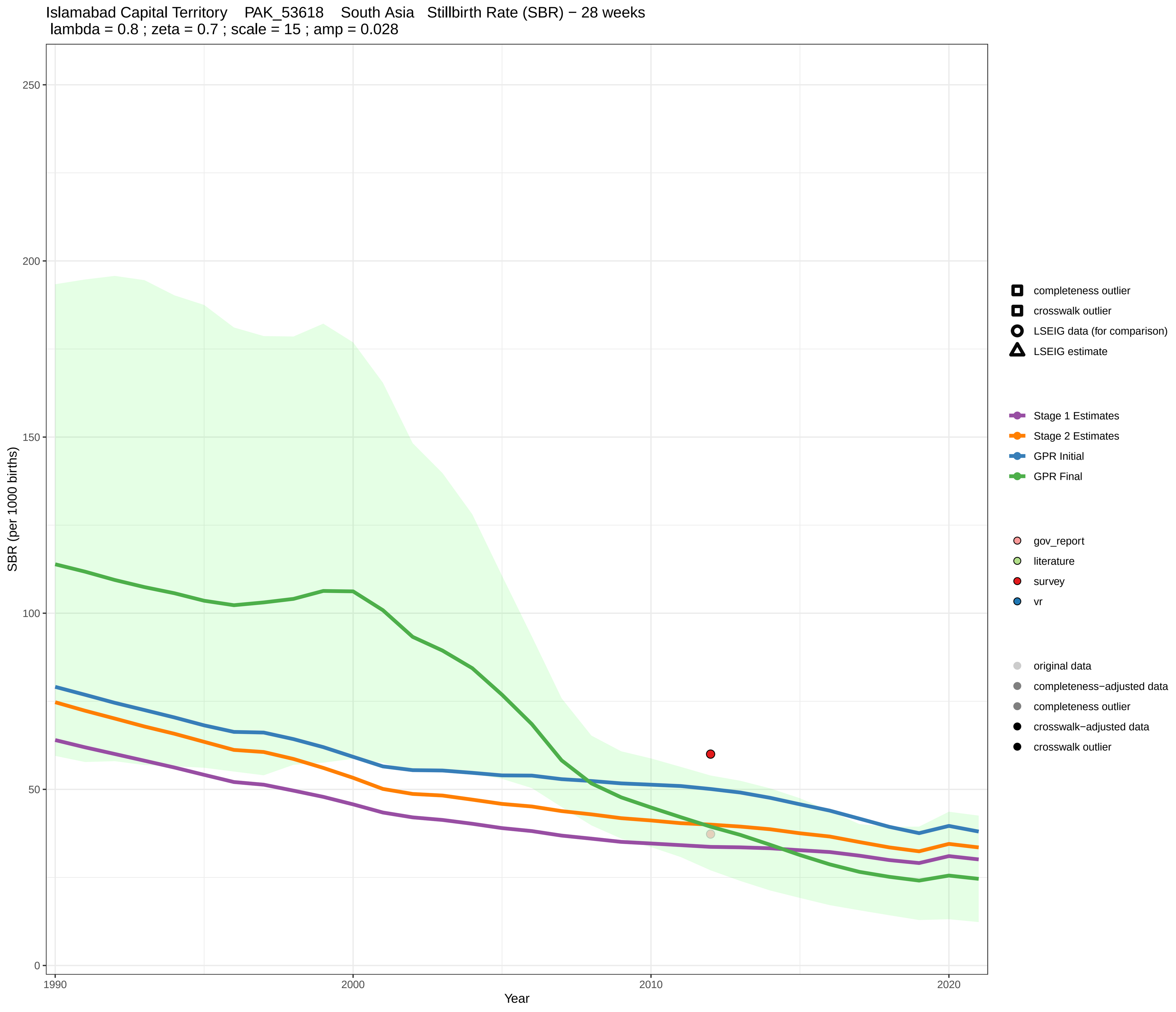

Year	Total Stillbirths (in thousands)	Total Stillbirths (in thousands)	SBR (per 1000 births)	SBR (per 1000 births)	SBR/NMR	SBR/NMR
	20 weeks	28 weeks	20 weeks	28 weeks	20 weeks	28 weeks
1990	2.8 (1.3 to 5.2)	2.0 (1.0 to 3.7)	147.8 (78.3 to 250.0)	113.9 (59.5 to 193.4)	5.75 (3.04 to 9.72)	4.43 (2.31 to 7.52)
1995	3.0 (1.5 to 6.1)	2.2 (1.1 to 4.3)	136.4 (73.9 to 244.6)	103.5 (56.1 to 187.5)	6.37 (3.46 to 11.43)	4.84 (2.62 to 8.76)
2000	3.9 (2.0 to 6.9)	2.8 (1.4 to 5.0)	141.3 (79.0 to 231.0)	106.2 (58.7 to 176.9)	7.84 (4.38 to 12.81)	5.89 (3.26 to 9.81)
2005	3.4 (2.2 to 5.0)	2.5 (1.6 to 3.6)	103.5 (71.2 to 146.4)	76.9 (53.0 to 110.6)	6.86 (4.72 to 9.70)	5.09 (3.51 to 7.33)
2010	2.4 (1.8 to 3.1)	1.7 (1.3 to 2.3)	61.9 (47.6 to 77.8)	44.9 (33.6 to 58.8)	4.85 (3.73 to 6.09)	3.52 (2.64 to 4.61)
2015	1.9 (1.1 to 3.0)	1.3 (0.8 to 2.1)	44.7 (27.0 to 68.6)	31.4 (19.2 to 47.5)	3.92 (2.37 to 6.02)	2.75 (1.68 to 4.17)
2020	1.7 (0.9 to 3.0)	1.1 (0.6 to 2.0)	37.4 (19.1 to 64.9)	25.5 (13.1 to 43.7)	3.66 (1.87 to 6.35)	2.50 (1.29 to 4.27)
2021	1.7 (0.8 to 3.1)	1.1 (0.6 to 2.0)	36.4 (17.8 to 65.0)	24.6 (12.3 to 42.5)	3.72 (1.82 to 6.65)	2.52 (1.26 to 4.35)

Khyber Pakhtunkhwa PAK_53619 South Asia Stillbirth Rate / Neonatal Mortality Rate (SBR/NMR) − 20 weeks
lambda = 0.8 ; zeta = 0.7 ; scale = 15 ; amp = 0.028

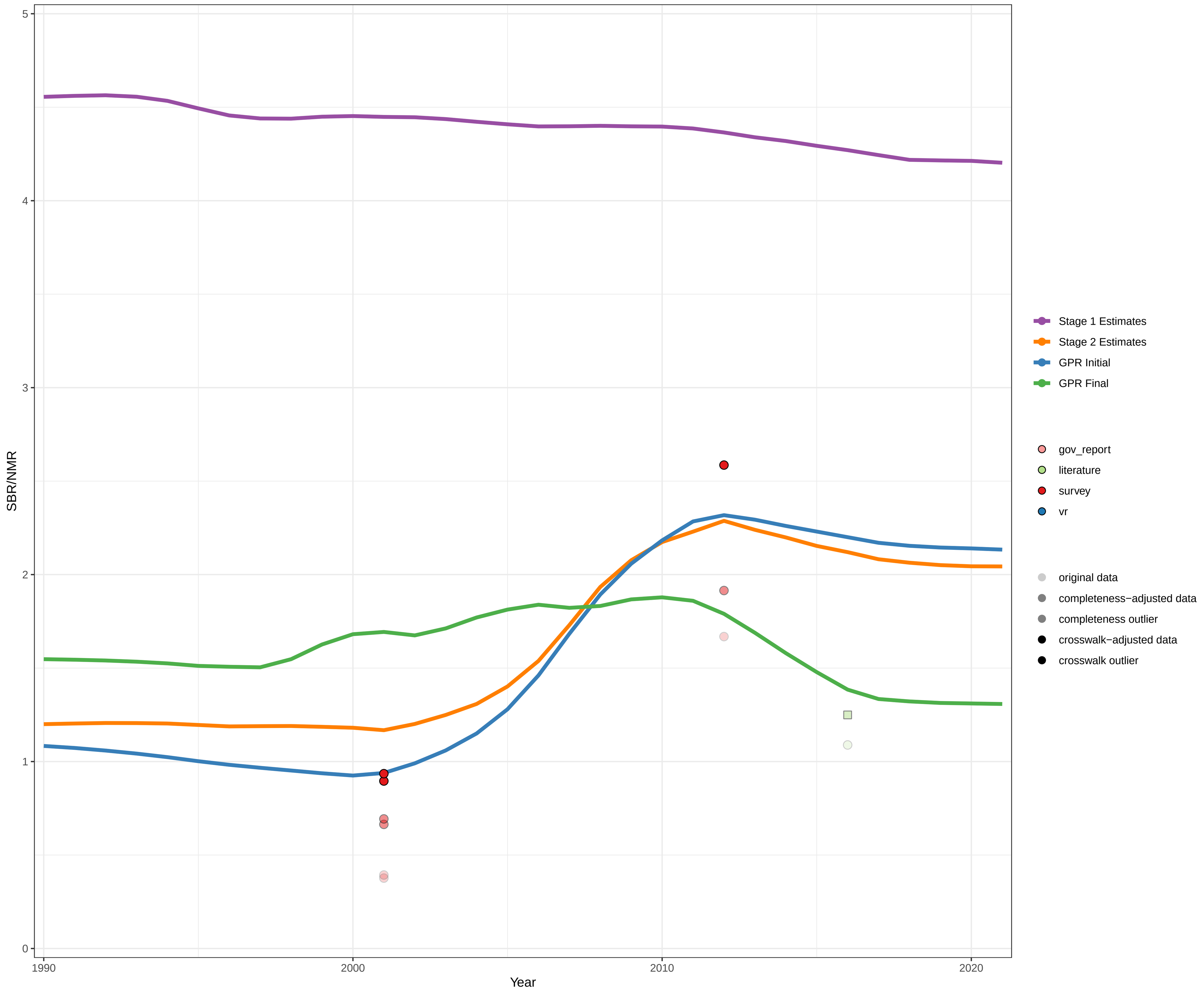

Khyber Pakhtunkhwa PAK_53619 South Asia Stillbirth Rate / Neonatal Mortality Rate (SBR/NMR) − 28 weeks
lambda = 0.8 ; zeta = 0.7 ; scale = 15 ; amp = 0.028

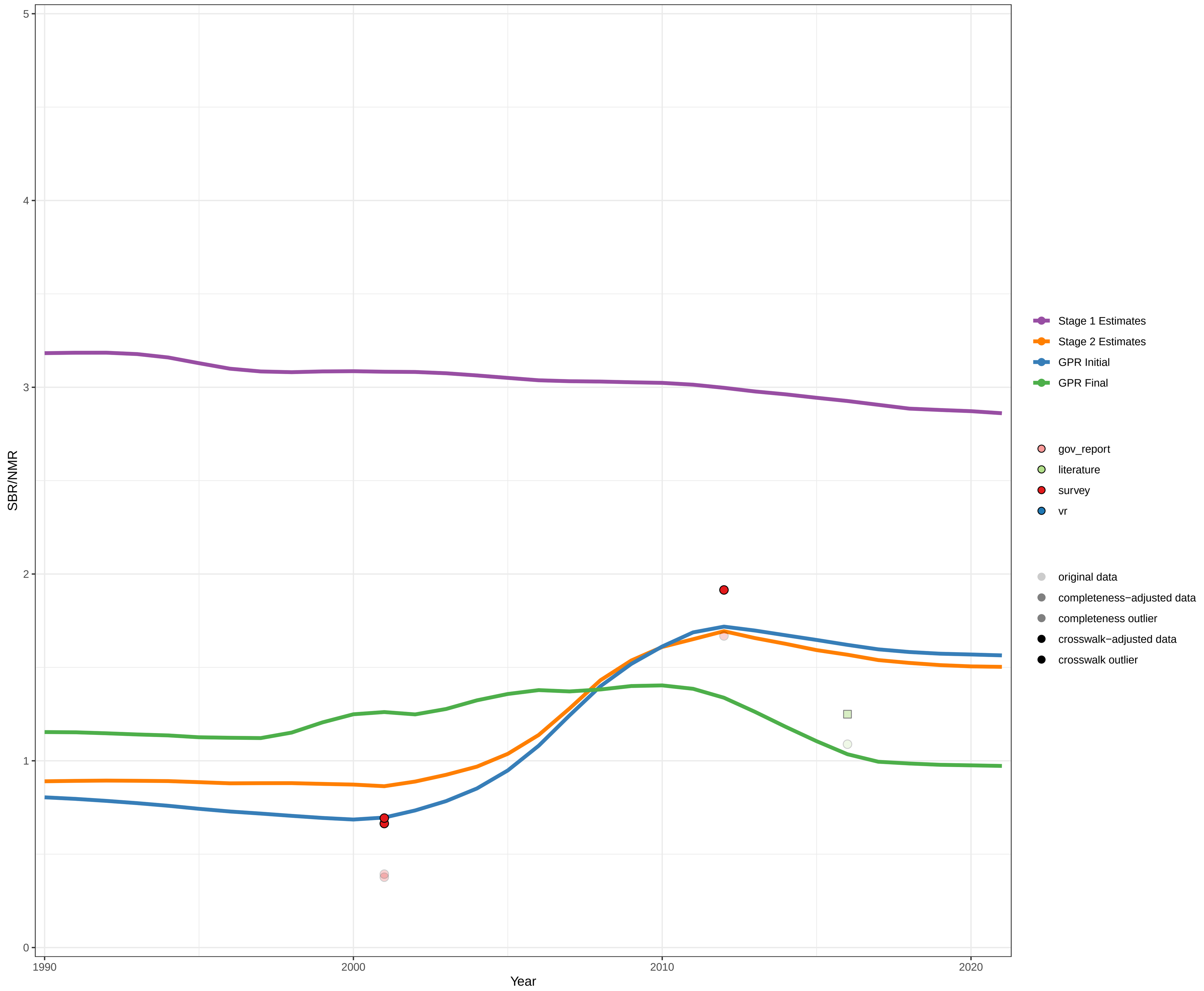

Definition	Years	Outlier	Source.Type	Title
28_weeks	2001	0	Survey	Pakistan Reproductive Health and Family Planning Survey 2000−2001
28_weeks	2012	0	Survey	Pakistan Demographic and Health Survey 2012−2013
28_weeks	2016	1	Sci Lit	Completeness of a Maternal and Perinatal Mortality Enhanced Surveillance System in Pakistan: Evidence from Capture−Recapture Methods

Khyber Pakhtunkhwa PAK_53619 South Asia Stillbirth Rate (SBR) − 20 weeks
lambda = 0.8 ; zeta = 0.7 ; scale = 15 ; amp = 0.028

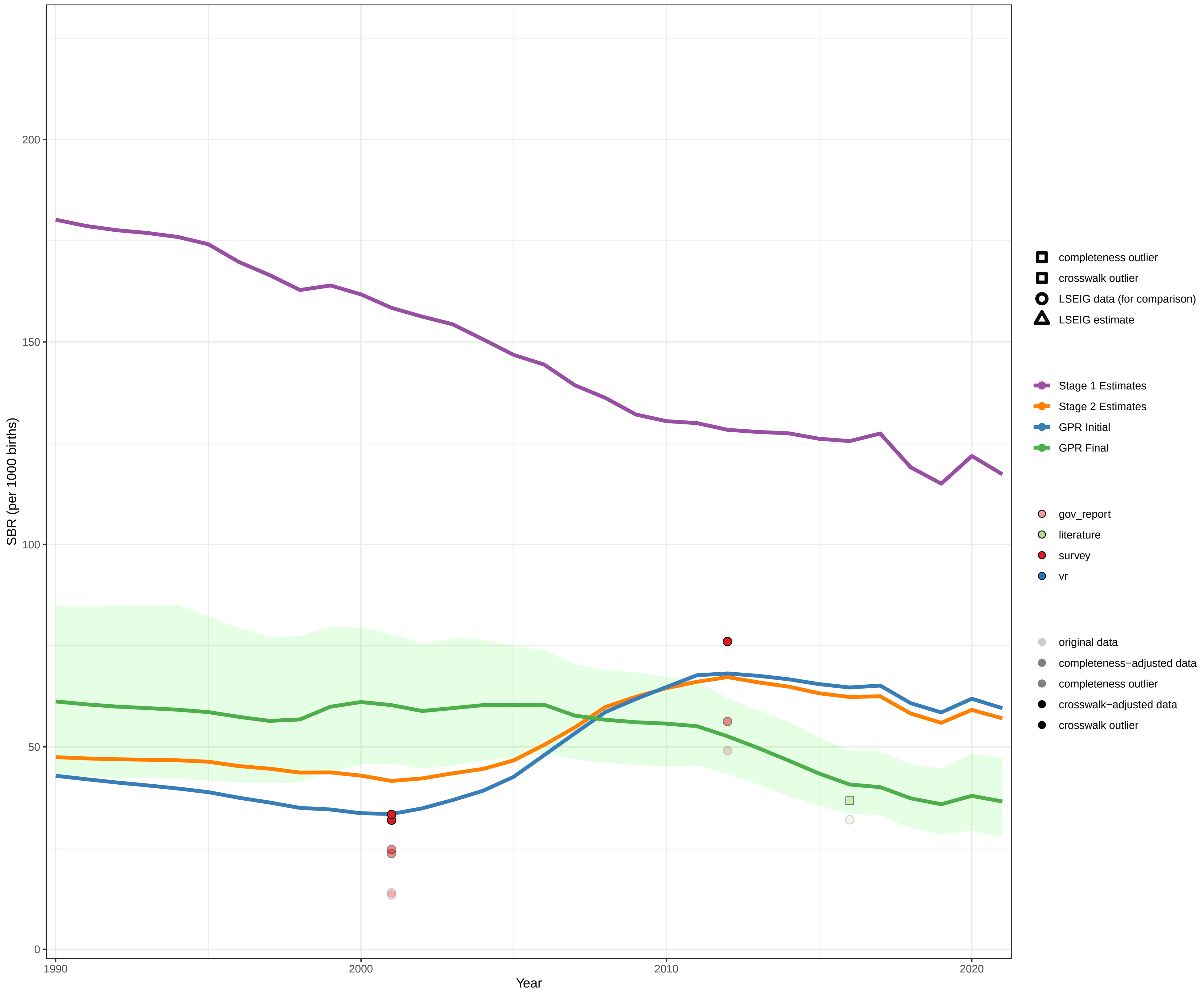

Khyber Pakhtunkhwa PAK_53619 South Asia Stillbirth Rate (SBR) − 28 weeks
lambda = 0.8 ; zeta = 0.7 ; scale = 15 ; amp = 0.028

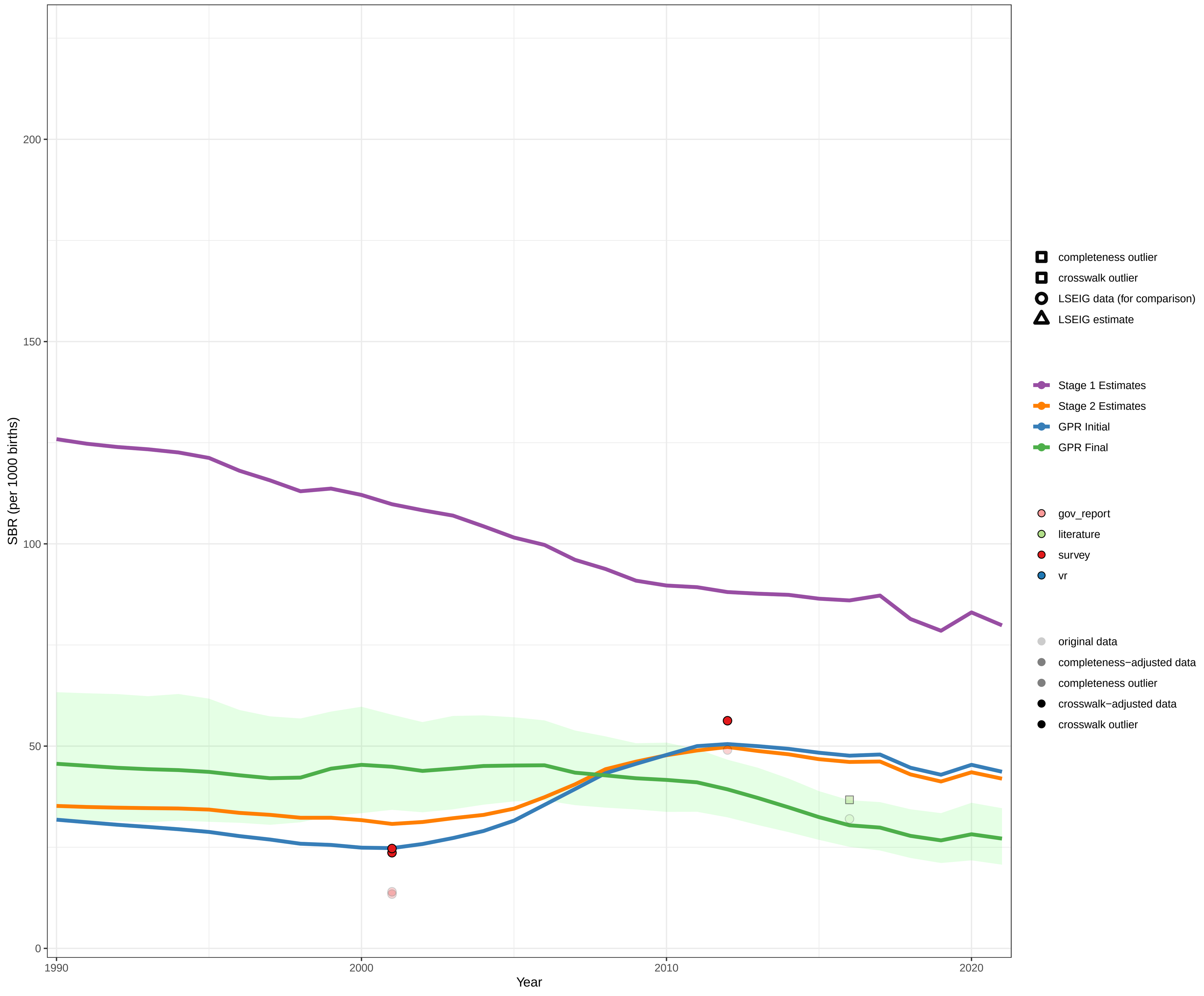

Year	Total Stillbirths (in thousands)	Total Stillbirths (in thousands)	SBR (per 1000 births)	SBR (per 1000 births)	SBR/NMR	SBR/NMR
	20 weeks	28 weeks	20 weeks	28 weeks	20 weeks	28 weeks
1990	41.7 (28.7 to 59.2)	30.6 (21.2 to 43.2)	61.2 (43.0 to 84.8)	45.6 (32.2 to 63.3)	1.55 (1.09 to 2.14)	1.15 (0.81 to 1.60)
1995	44.1 (30.8 to 63.5)	32.3 (22.9 to 46.6)	58.6 (41.7 to 82.3)	43.6 (31.3 to 61.7)	1.51 (1.08 to 2.12)	1.13 (0.81 to 1.59)
2000	50.2 (36.9 to 66.5)	36.7 (26.7 to 49.0)	61.1 (45.7 to 79.4)	45.4 (33.4 to 59.7)	1.68 (1.26 to 2.19)	1.25 (0.92 to 1.64)
2005	56.4 (43.4 to 71.0)	41.6 (33.1 to 53.1)	60.4 (47.1 to 74.8)	45.2 (36.3 to 57.1)	1.81 (1.41 to 2.25)	1.36 (1.09 to 1.72)
2010	60.8 (48.8 to 74.4)	44.8 (36.0 to 55.2)	55.7 (45.2 to 67.4)	41.6 (33.7 to 50.9)	1.88 (1.52 to 2.27)	1.40 (1.14 to 1.71)
2015	50.9 (41.1 to 61.9)	37.6 (30.9 to 45.3)	43.4 (35.4 to 52.3)	32.4 (26.8 to 38.9)	1.48 (1.21 to 1.78)	1.10 (0.91 to 1.32)
2020	45.9 (35.1 to 59.0)	33.8 (25.9 to 43.5)	37.9 (29.3 to 48.3)	28.2 (21.8 to 36.0)	1.31 (1.01 to 1.67)	0.98 (0.75 to 1.24)
2021	44.3 (33.3 to 57.8)	32.6 (24.7 to 41.9)	36.5 (27.7 to 47.2)	27.1 (20.7 to 34.7)	1.31 (0.99 to 1.69)	0.97 (0.74 to 1.24)

Punjab PAK_53620 South Asia Stillbirth Rate / Neonatal Mortality Rate (SBR/NMR) − 20 weeks
lambda = 0.8 ; zeta = 0.7 ; scale = 15 ; amp = 0.028

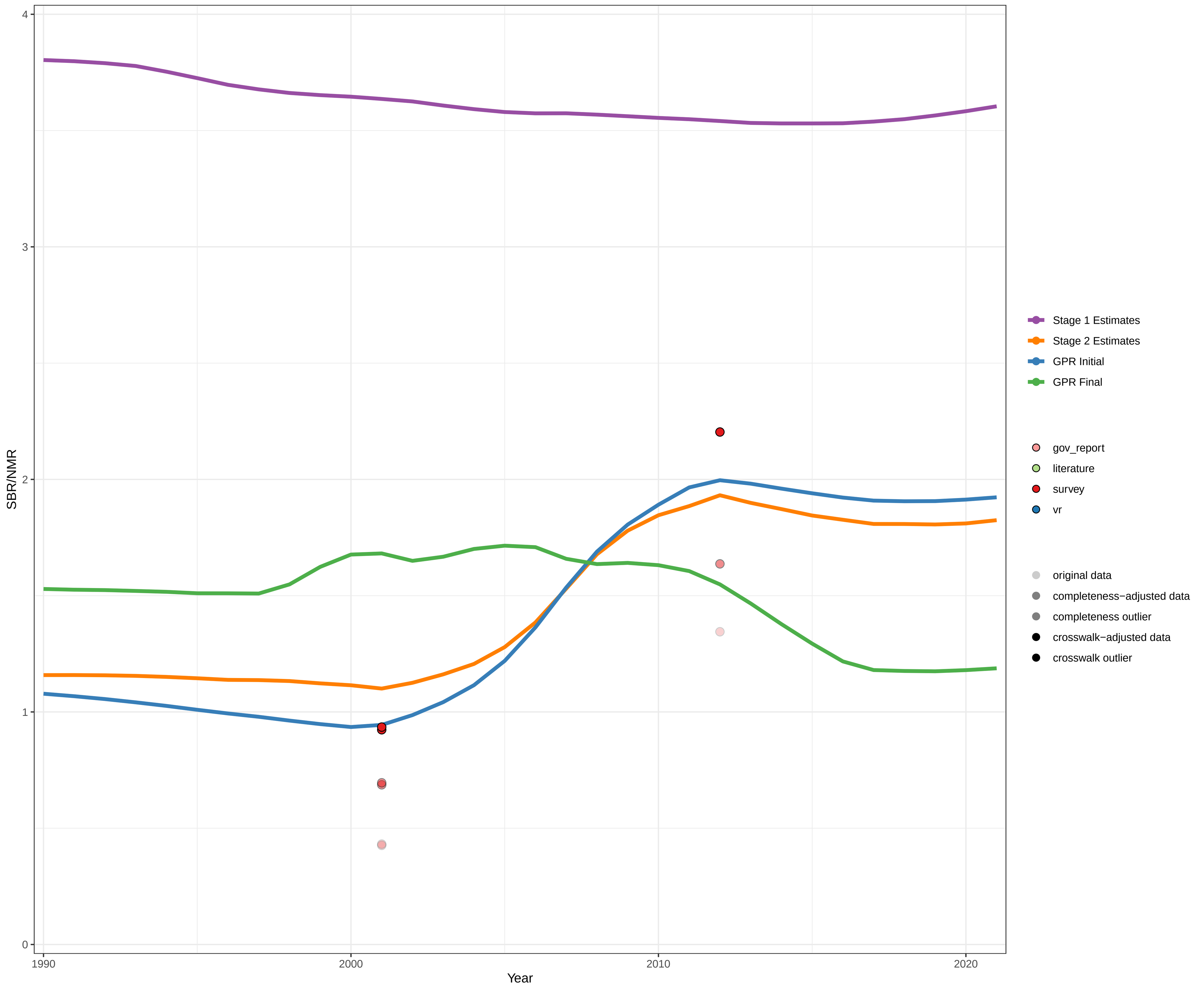

Punjab PAK_53620 South Asia Stillbirth Rate / Neonatal Mortality Rate (SBR/NMR) − 28 weeks
lambda = 0.8 ; zeta = 0.7 ; scale = 15 ; amp = 0.028

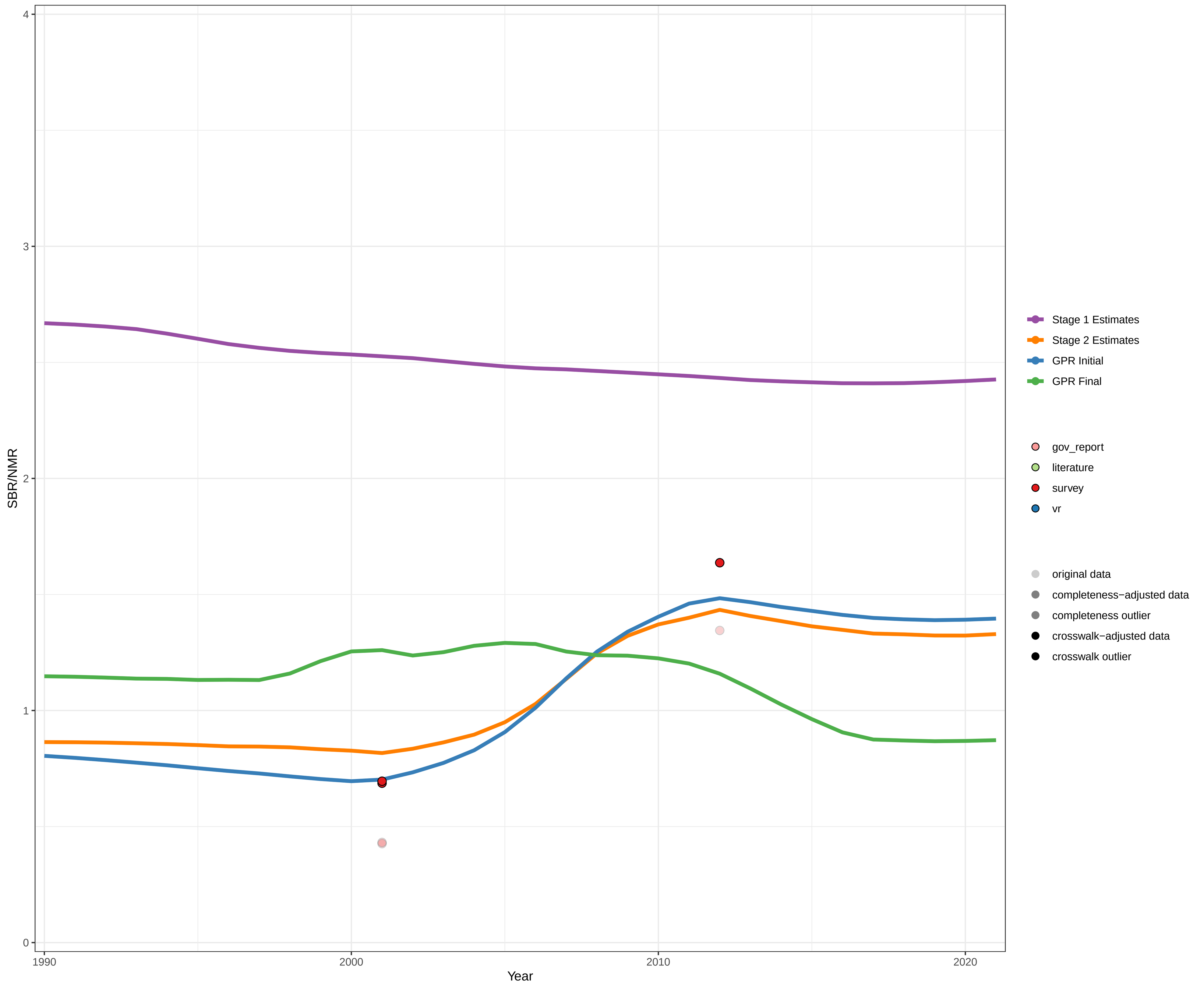

Definition	Years	Outlier	Source.Type	Title
28_weeks	2001	0	Survey	Pakistan Reproductive Health and Family Planning Survey 2000−2001
28_weeks	2012	0	Survey	Pakistan Demographic and Health Survey 2012−2013

Punjab PAK_53620 South Asia Stillbirth Rate (SBR) − 20 weeks
lambda = 0.8 ; zeta = 0.7 ; scale = 15 ; amp = 0.028

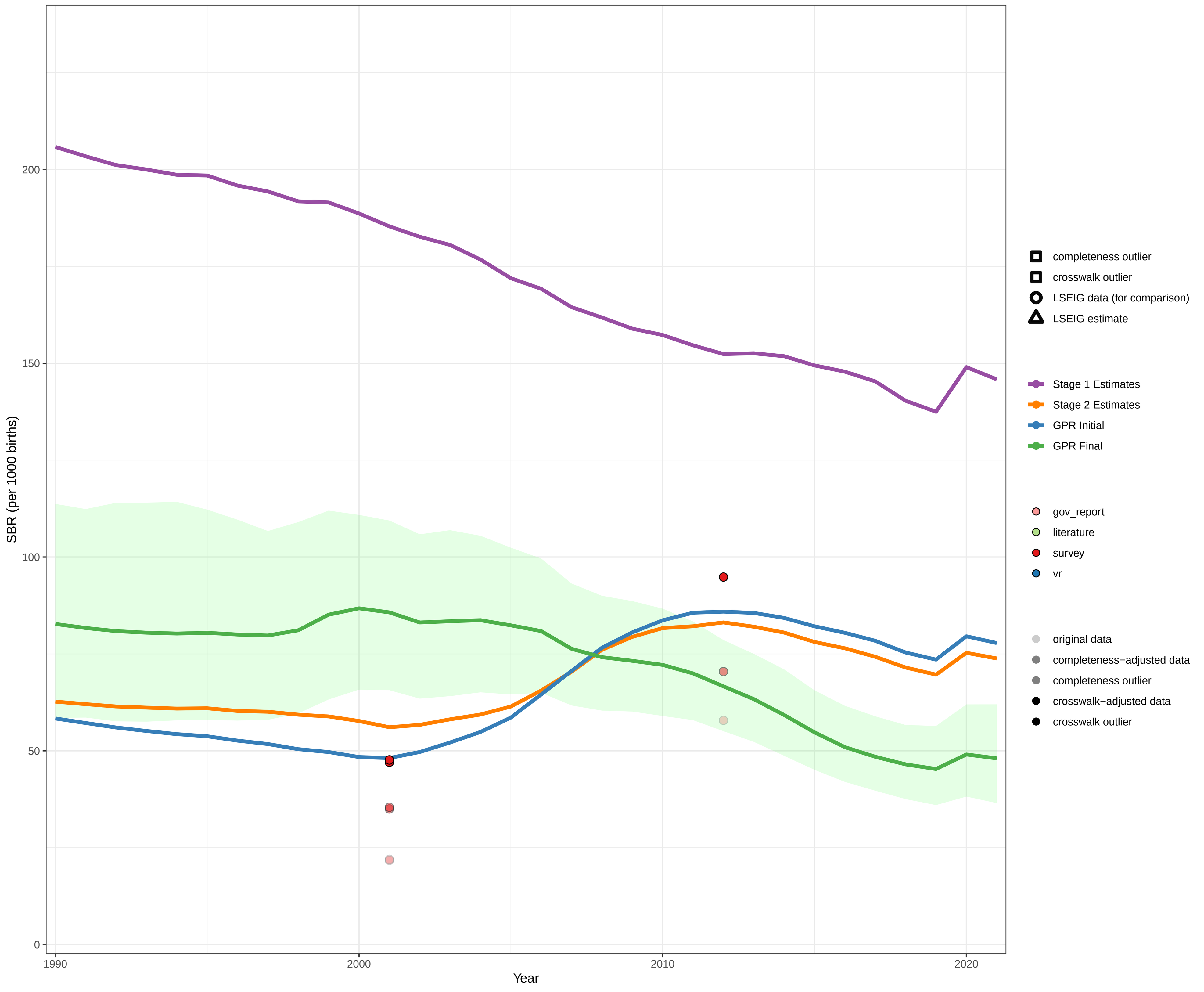

Punjab PAK_53620 South Asia Stillbirth Rate (SBR) − 28 weeks
lambda = 0.8 ; zeta = 0.7 ; scale = 15 ; amp = 0.028

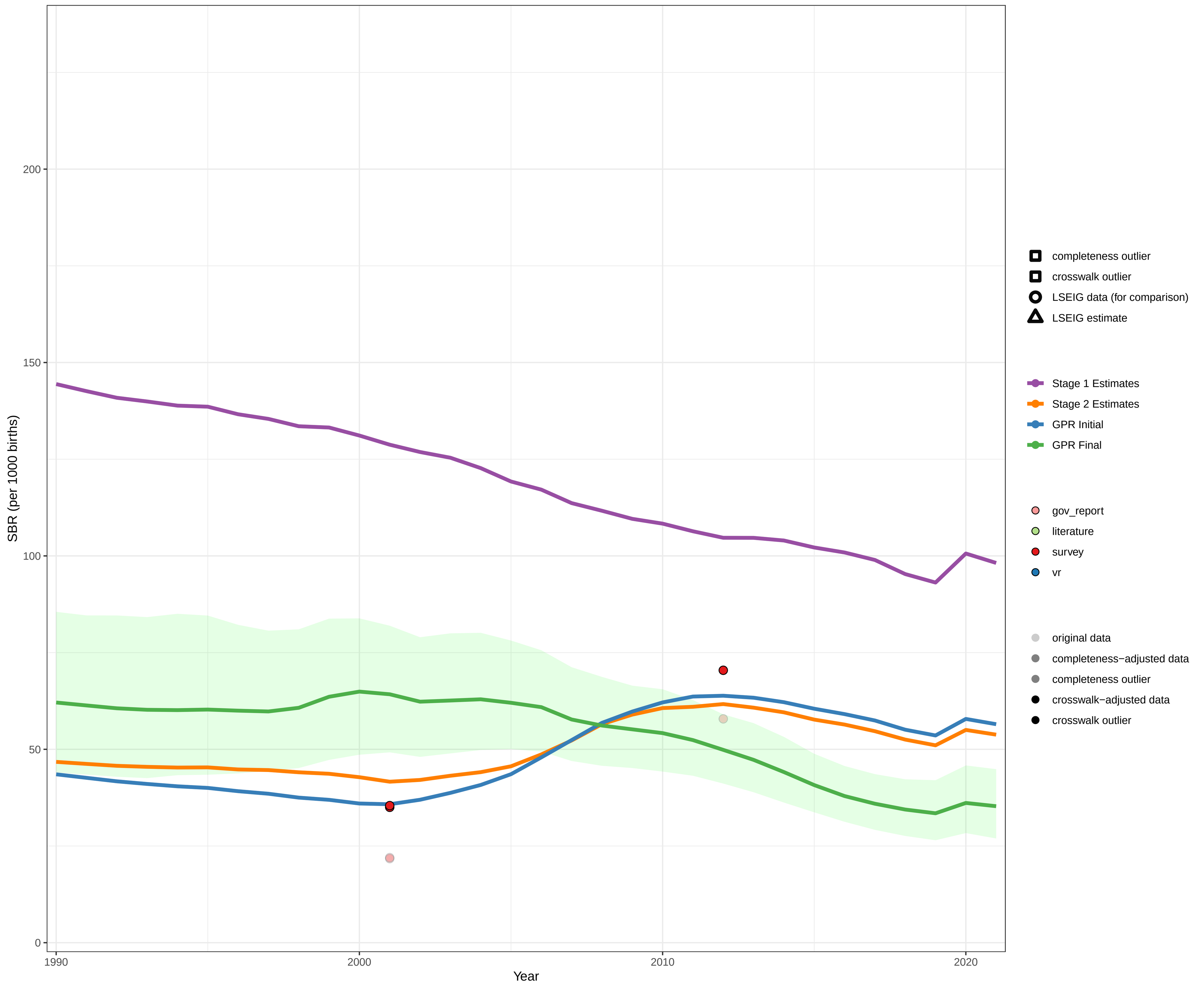

Year	Total Stillbirths (in thousands)	Total Stillbirths (in thousands)	SBR (per 1000 births)	SBR (per 1000 births)	SBR/NMR	SBR/NMR
	20 weeks	28 weeks	20 weeks	28 weeks	20 weeks	28 weeks
1990	205.8 (141.3 to 291.9)	150.9 (104.8 to 212.8)	82.7 (58.5 to 113.7)	62.1 (44.0 to 85.5)	1.53 (1.08 to 2.10)	1.15 (0.81 to 1.58)
1995	209.4 (146.7 to 301.7)	153.4 (108.3 to 220.5)	80.4 (57.9 to 112.2)	60.3 (43.4 to 84.6)	1.51 (1.09 to 2.11)	1.13 (0.82 to 1.59)
2000	226.4 (167.5 to 296.5)	165.3 (121.5 to 217.7)	86.8 (65.8 to 110.9)	64.9 (48.6 to 83.9)	1.68 (1.27 to 2.14)	1.25 (0.94 to 1.62)
2005	227.5 (174.6 to 288.8)	167.6 (133.4 to 214.5)	82.4 (64.5 to 102.4)	62.0 (50.0 to 78.1)	1.71 (1.34 to 2.13)	1.29 (1.04 to 1.63)
2010	220.3 (177.4 to 268.5)	162.2 (131.0 to 198.4)	72.2 (59.0 to 86.7)	54.2 (44.2 to 65.5)	1.63 (1.33 to 1.96)	1.22 (1.00 to 1.48)
2015	175.5 (143.0 to 212.6)	128.7 (105.6 to 155.5)	54.8 (45.1 to 65.6)	40.8 (33.7 to 48.8)	1.29 (1.07 to 1.55)	0.96 (0.80 to 1.15)
2020	158.3 (121.8 to 202.6)	115.0 (89.4 to 147.2)	49.1 (38.2 to 62.0)	36.1 (28.3 to 45.8)	1.18 (0.92 to 1.49)	0.87 (0.68 to 1.10)
2021	154.7 (116.0 to 202.3)	112.1 (84.8 to 143.8)	48.1 (36.5 to 62.0)	35.3 (26.9 to 44.9)	1.19 (0.90 to 1.53)	0.87 (0.67 to 1.11)

Sindh PAK_53621 South Asia Stillbirth Rate / Neonatal Mortality Rate (SBR/NMR) − 20 weeks
lambda = 0.6 ; zeta = 0.8 ; scale = 15 ; amp = 0.028

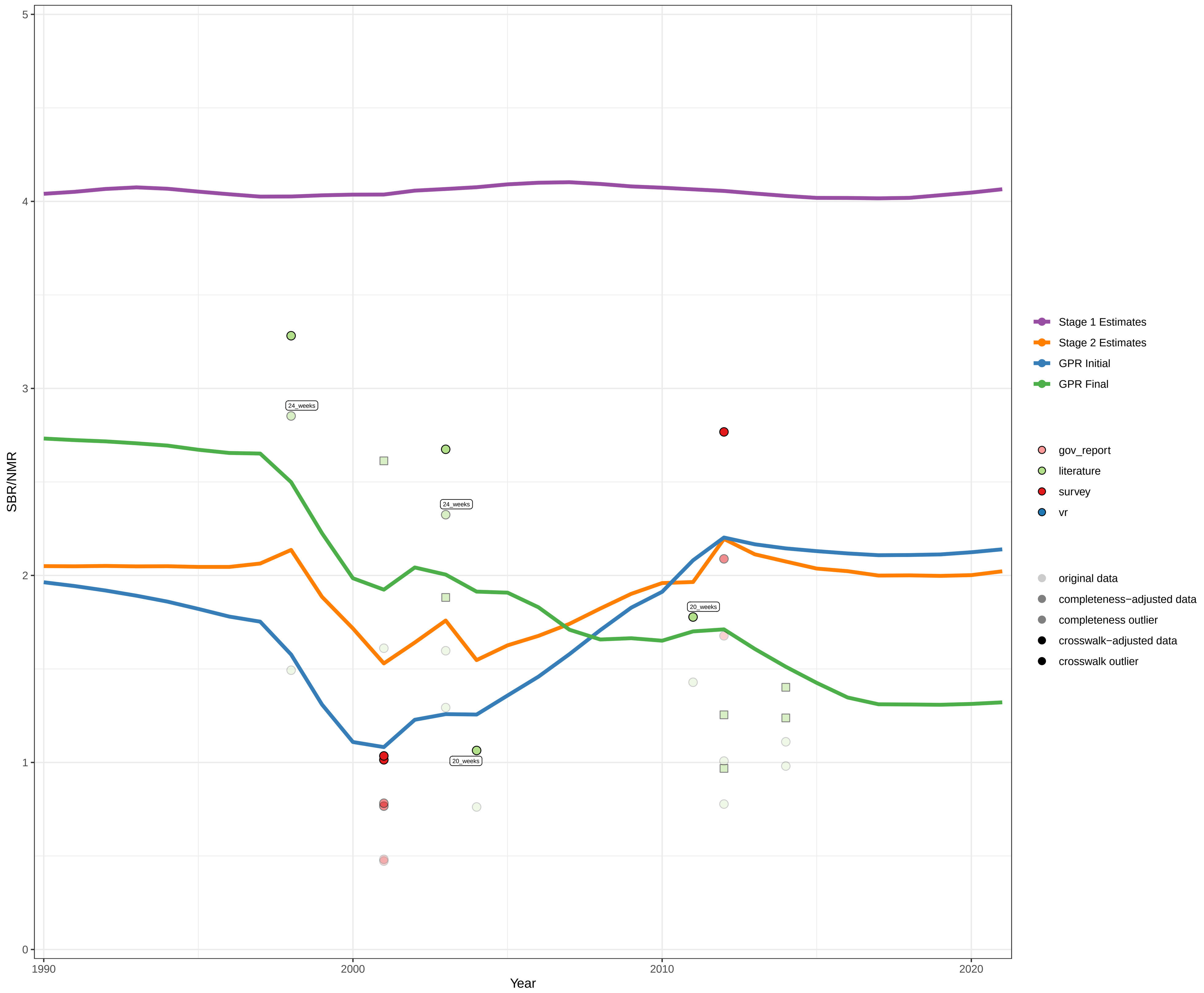

Sindh PAK_53621 South Asia Stillbirth Rate / Neonatal Mortality Rate (SBR/NMR) − 28 weeks
lambda = 0.6 ; zeta = 0.8 ; scale = 15 ; amp = 0.028

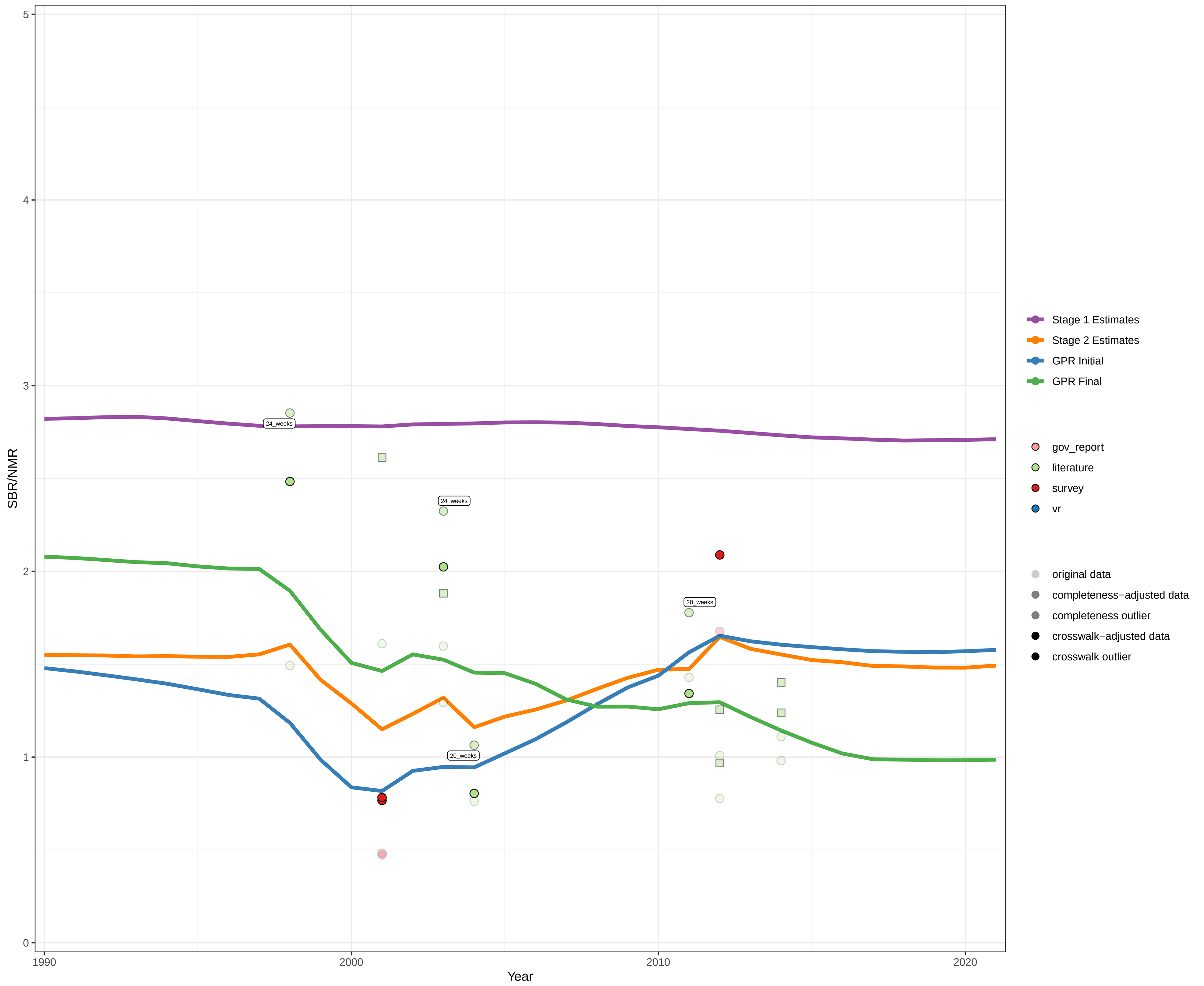

Definition	Years	Outlier	Source.Type	Title
20_weeks	2004	0	Sci Lit	Stillbirths in an urban community in Pakistan
20_weeks	2011	0	Sci Lit	A prospective study of maternal, fetal and neonatal deaths in low− and middle−income countries
24_weeks	1998	0	Sci Lit	An Intervention Involving Traditional Birth Attendants and Perinatal and Maternal Mortality in Pakistan
24_weeks	2003	0	Sci Lit	Pattern of fetal deaths at a university hospital of Sindh
28_weeks	2001	0	Survey	Pakistan Reproductive Health and Family Planning Survey 2000−2001
28_weeks	2012	0	Survey	Pakistan Demographic and Health Survey 2012−2013
28_weeks	2001	1	Sci Lit	An audit and trends of perinatal mortality at the Jinnah Postgraduate Medical Centre, Karachi
28_weeks	2003	1	Sci Lit	Implementing community−based perinatal care: results from a pilot study in rural Pakistan
28_weeks	2012	1	Sci Lit	Causes and incidence of community−acquired serious infections among young children in south Asia (ANISA): an observational cohort study
28_weeks	2014	1	Sci Lit	Population−based rates, timing, and causes of maternal deaths, stillbirths, and neonatal deaths in south Asia and sub−Saharan Africa: a multi−country prospective cohort study

Sindh PAK_53621 South Asia Stillbirth Rate (SBR) − 20 weeks
lambda = 0.6 ; zeta = 0.8 ; scale = 15 ; amp = 0.028

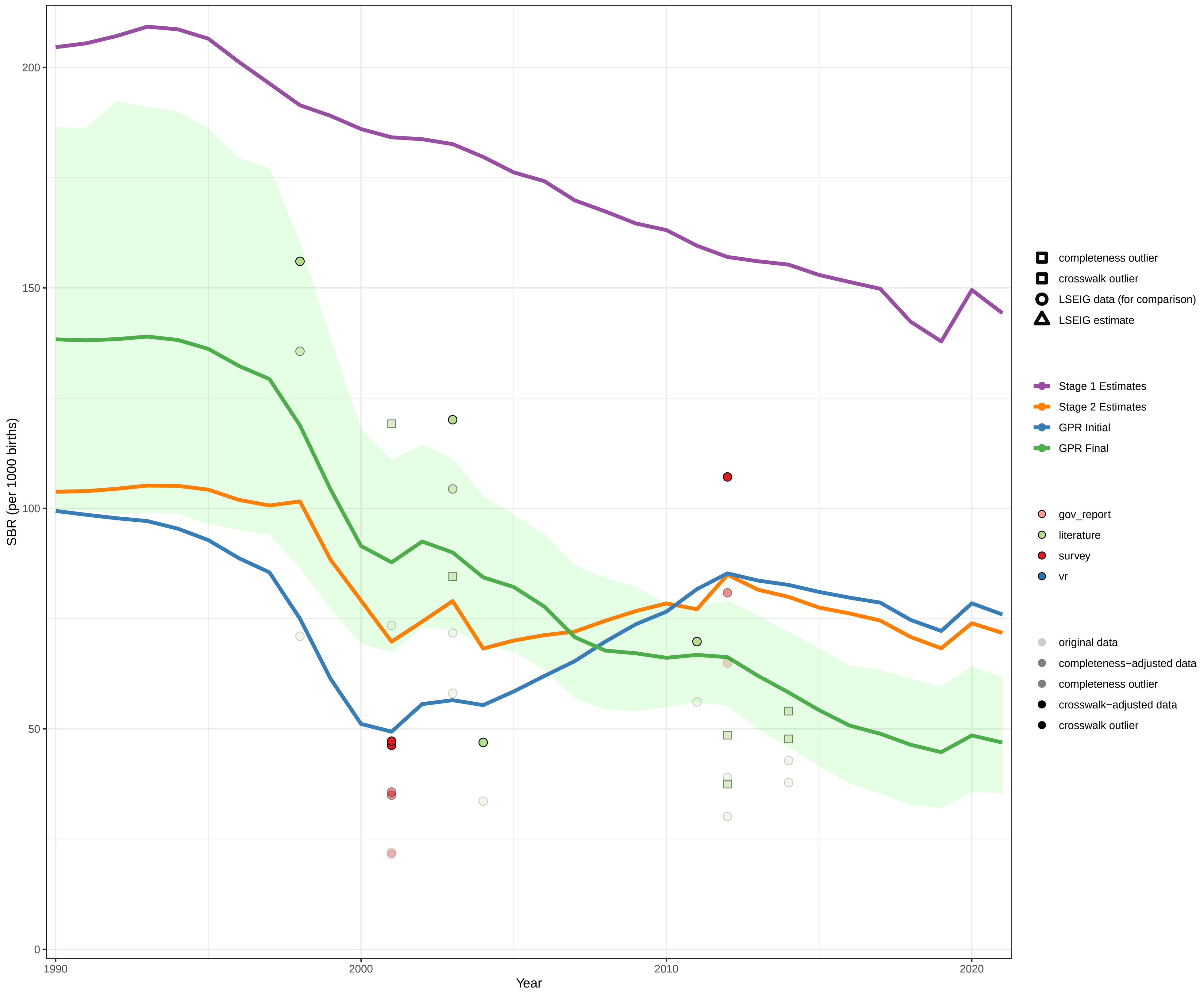

Sindh PAK_53621 South Asia Stillbirth Rate (SBR) − 28 weeks
lambda = 0.6 ; zeta = 0.8 ; scale = 15 ; amp = 0.028

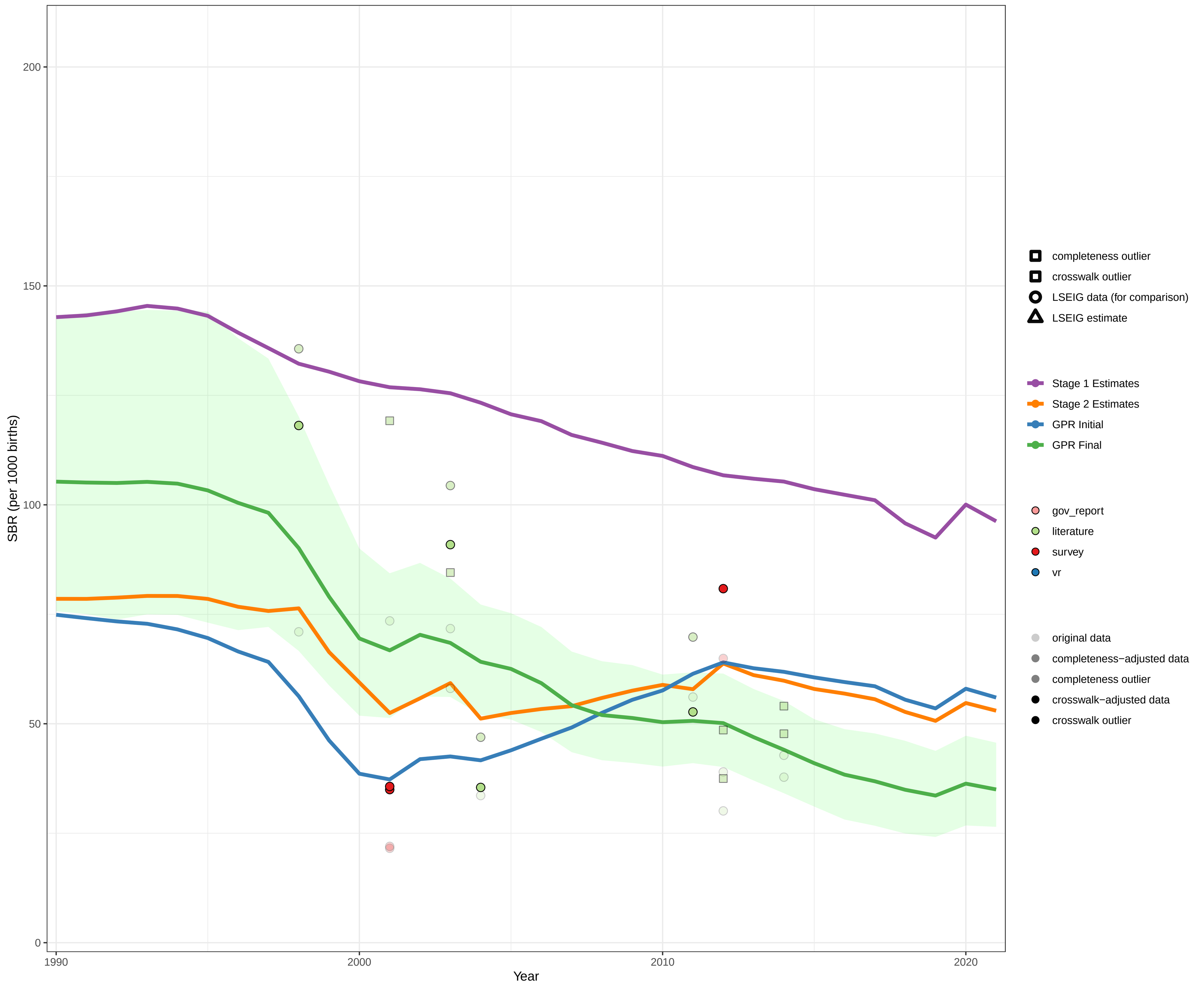

Year	Total Stillbirths (in thousands)	Total Stillbirths (in thousands)	SBR (per 1000 births)	SBR (per 1000 births)	SBR/NMR	SBR/NMR
	20 weeks	28 weeks	20 weeks	28 weeks	20 weeks	28 weeks
1990	154.4 (105.9 to 219.3)	113.0 (78.4 to 159.3)	138.3 (99.6 to 186.5)	105.3 (75.7 to 142.7)	2.73 (1.97 to 3.68)	2.08 (1.49 to 2.82)
1995	163.4 (110.1 to 236.0)	119.2 (81.3 to 173.6)	136.2 (96.5 to 186.3)	103.3 (73.1 to 144.1)	2.67 (1.89 to 3.66)	2.03 (1.43 to 2.83)
2000	106.7 (78.8 to 141.6)	79.1 (57.8 to 104.6)	91.5 (69.3 to 118.1)	69.5 (51.8 to 90.0)	1.98 (1.50 to 2.56)	1.51 (1.12 to 1.95)
2005	102.1 (82.5 to 124.5)	76.1 (61.2 to 92.7)	82.2 (67.5 to 98.5)	62.5 (51.0 to 75.3)	1.91 (1.57 to 2.29)	1.45 (1.18 to 1.75)
2010	89.3 (73.2 to 107.3)	66.9 (52.8 to 82.2)	66.1 (54.9 to 78.5)	50.4 (40.2 to 61.2)	1.65 (1.37 to 1.96)	1.26 (1.00 to 1.53)
2015	75.2 (56.8 to 96.2)	56.0 (42.0 to 70.5)	54.2 (41.6 to 68.4)	41.0 (31.1 to 51.0)	1.43 (1.09 to 1.80)	1.08 (0.82 to 1.34)
2020	67.4 (48.8 to 90.5)	49.8 (36.3 to 65.5)	48.5 (35.6 to 64.2)	36.3 (26.8 to 47.3)	1.31 (0.96 to 1.74)	0.98 (0.72 to 1.28)
2021	64.8 (48.4 to 86.6)	47.7 (35.7 to 63.0)	46.9 (35.5 to 61.8)	35.0 (26.5 to 45.7)	1.32 (1.00 to 1.74)	0.99 (0.75 to 1.29)

Southeast Asia, East Asia, and Oceania S6 NA Stillbirth Rate / Neonatal Mortality Rate (SBR/NMR) − 20 weeks
lambda = 1 ; zeta = 0.6 ; scale = 15 ; amp = 0.028

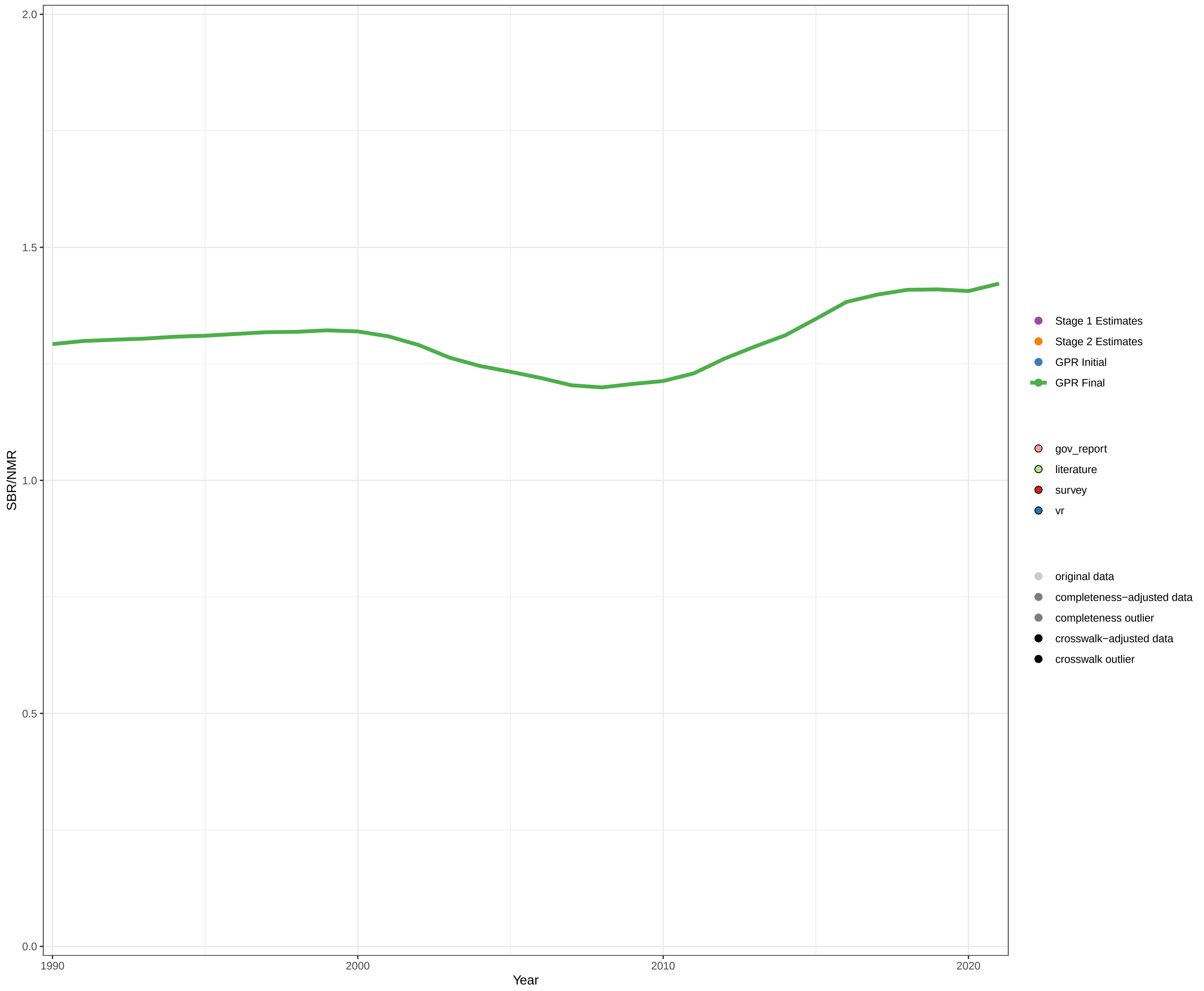

Southeast Asia, East Asia, and Oceania S6 NA Stillbirth Rate / Neonatal Mortality Rate (SBR/NMR) − 28 weeks
lambda = 1 ; zeta = 0.6 ; scale = 15 ; amp = 0.028

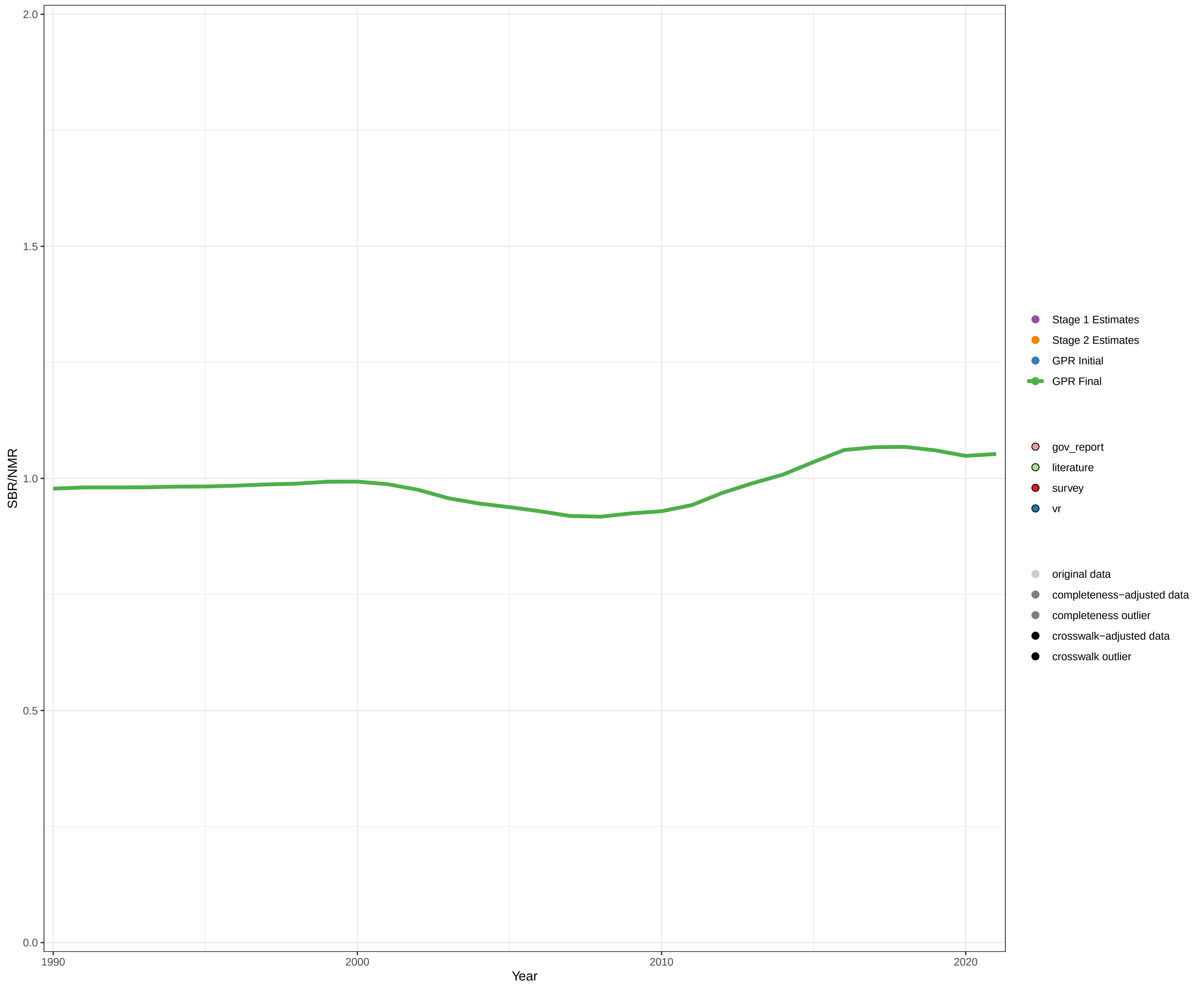

Definition	Years	Outlier	Source.Type	Title

Southeast Asia, East Asia, and Oceania S6 NA Stillbirth Rate (SBR) − 20 weeks
lambda = 1 ; zeta = 0.6 ; scale = 15 ; amp = 0.028

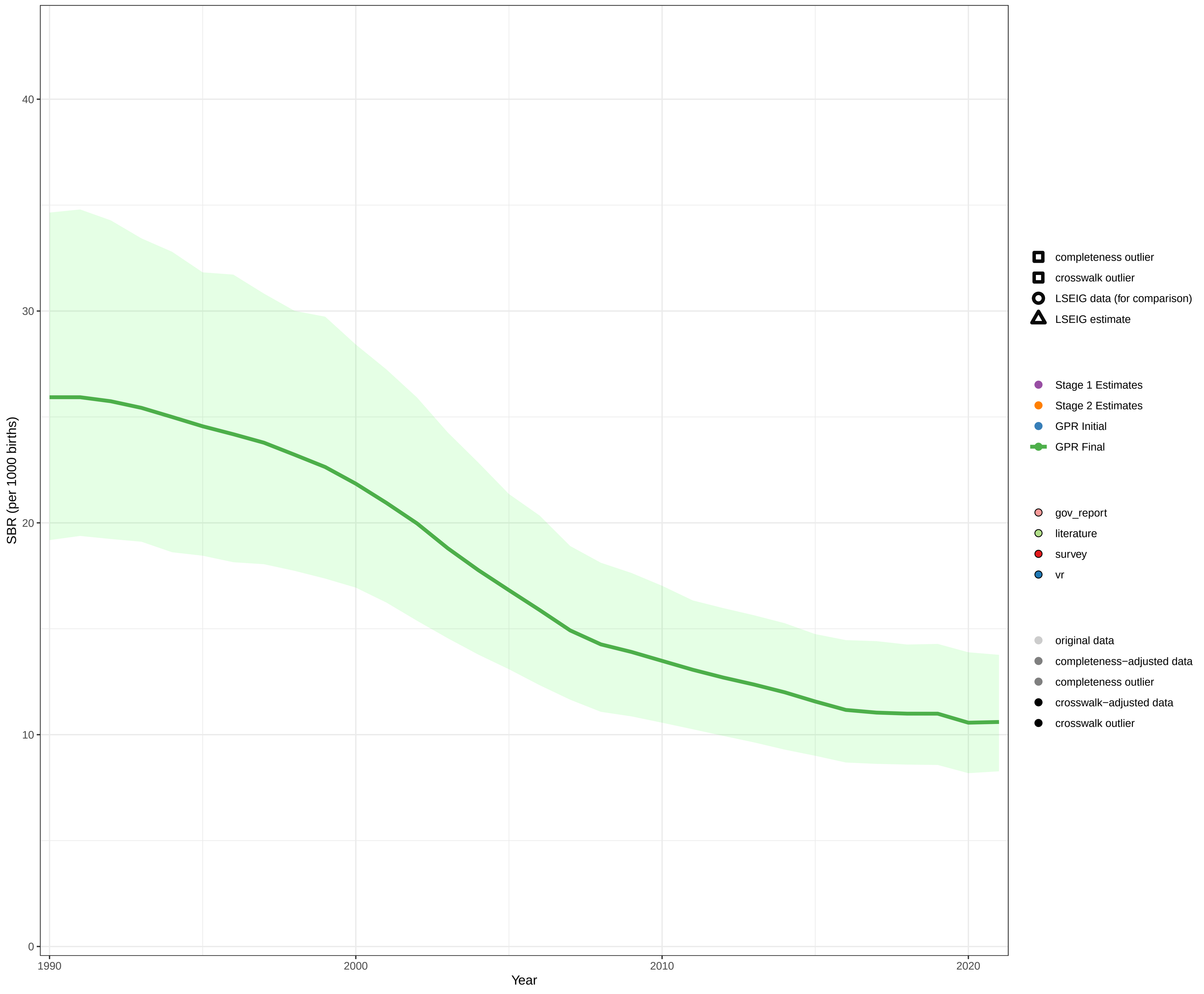

Southeast Asia, East Asia, and Oceania S6 NA Stillbirth Rate (SBR) − 28 weeks
lambda = 1 ; zeta = 0.6 ; scale = 15 ; amp = 0.028

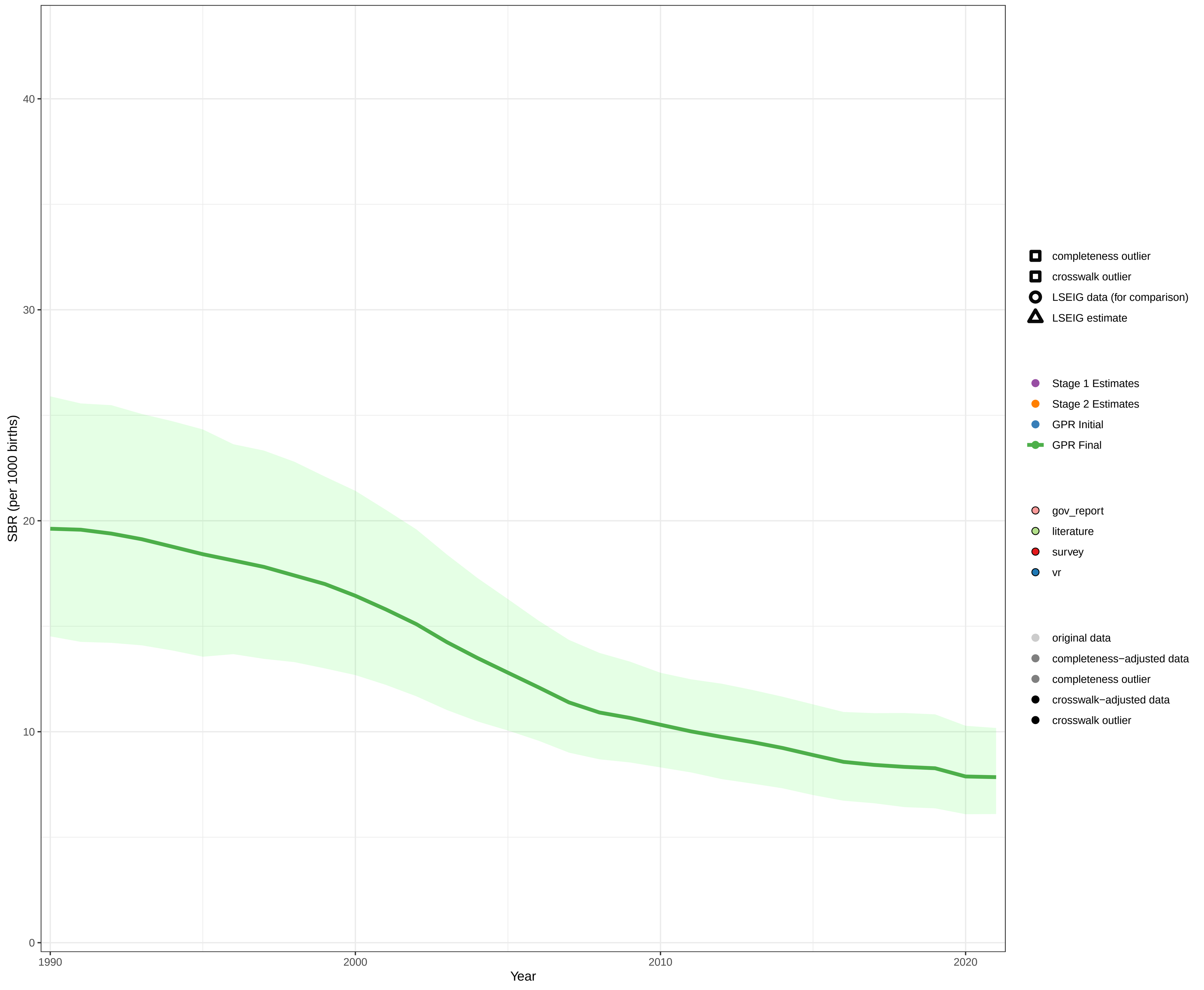

Year	Total Stillbirths (in thousands)	Total Stillbirths (in thousands)	SBR (per 1000 births)	SBR (per 1000 births)	SBR/NMR	SBR/NMR
	20 weeks	28 weeks	20 weeks	28 weeks	20 weeks	28 weeks
1990	966.1 (709.5 to 1301.8)	726.2 (534.4 to 964.2)	25.9 (19.2 to 34.6)	19.6 (14.5 to 25.9)	1.29 (0.96 to 1.73)	0.98 (0.72 to 1.29)
1995	805.2 (600.8 to 1050.8)	599.9 (439.3 to 797.1)	24.6 (18.4 to 31.8)	18.4 (13.6 to 24.3)	1.31 (0.98 to 1.70)	0.98 (0.72 to 1.30)
2000	634.1 (489.0 to 830.0)	474.6 (364.6 to 621.1)	21.8 (16.9 to 28.4)	16.4 (12.7 to 21.4)	1.32 (1.02 to 1.72)	0.99 (0.77 to 1.29)
2005	472.7 (366.3 to 602.9)	358.2 (280.6 to 457.3)	16.8 (13.1 to 21.4)	12.8 (10.1 to 16.3)	1.23 (0.96 to 1.57)	0.94 (0.74 to 1.19)
2010	358.4 (279.9 to 454.1)	273.6 (219.6 to 339.7)	13.5 (10.6 to 17.0)	10.3 (8.3 to 12.8)	1.21 (0.95 to 1.53)	0.93 (0.75 to 1.15)
2015	345.0 (267.8 to 441.3)	264.6 (207.7 to 336.8)	11.6 (9.0 to 14.7)	8.9 (7.0 to 11.3)	1.35 (1.05 to 1.72)	1.04 (0.81 to 1.31)
2020	256.2 (197.9 to 337.9)	190.5 (147.0 to 249.2)	10.6 (8.2 to 13.9)	7.9 (6.1 to 10.3)	1.41 (1.09 to 1.85)	1.05 (0.81 to 1.37)
2021	244.4 (190.2 to 318.4)	180.4 (139.9 to 234.5)	10.6 (8.3 to 13.8)	7.8 (6.1 to 10.2)	1.42 (1.11 to 1.85)	1.05 (0.82 to 1.37)

East Asia R3 East Asia Stillbirth Rate / Neonatal Mortality Rate (SBR/NMR) − 20 weeks
lambda = 1 ; zeta = 0.6 ; scale = 15 ; amp = 0.028

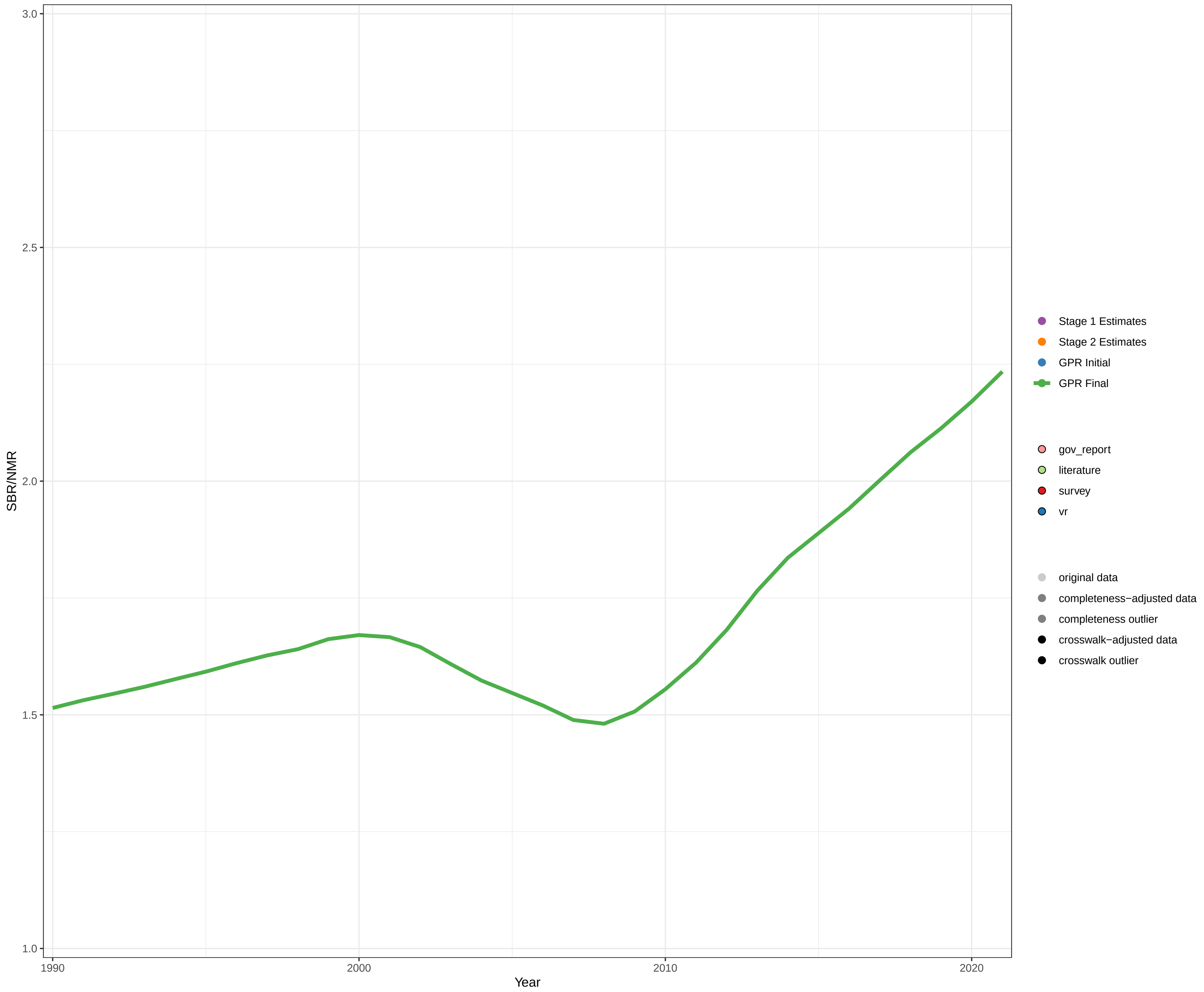

East Asia R3 East Asia Stillbirth Rate / Neonatal Mortality Rate (SBR/NMR) − 28 weeks
lambda = 1 ; zeta = 0.6 ; scale = 15 ; amp = 0.028

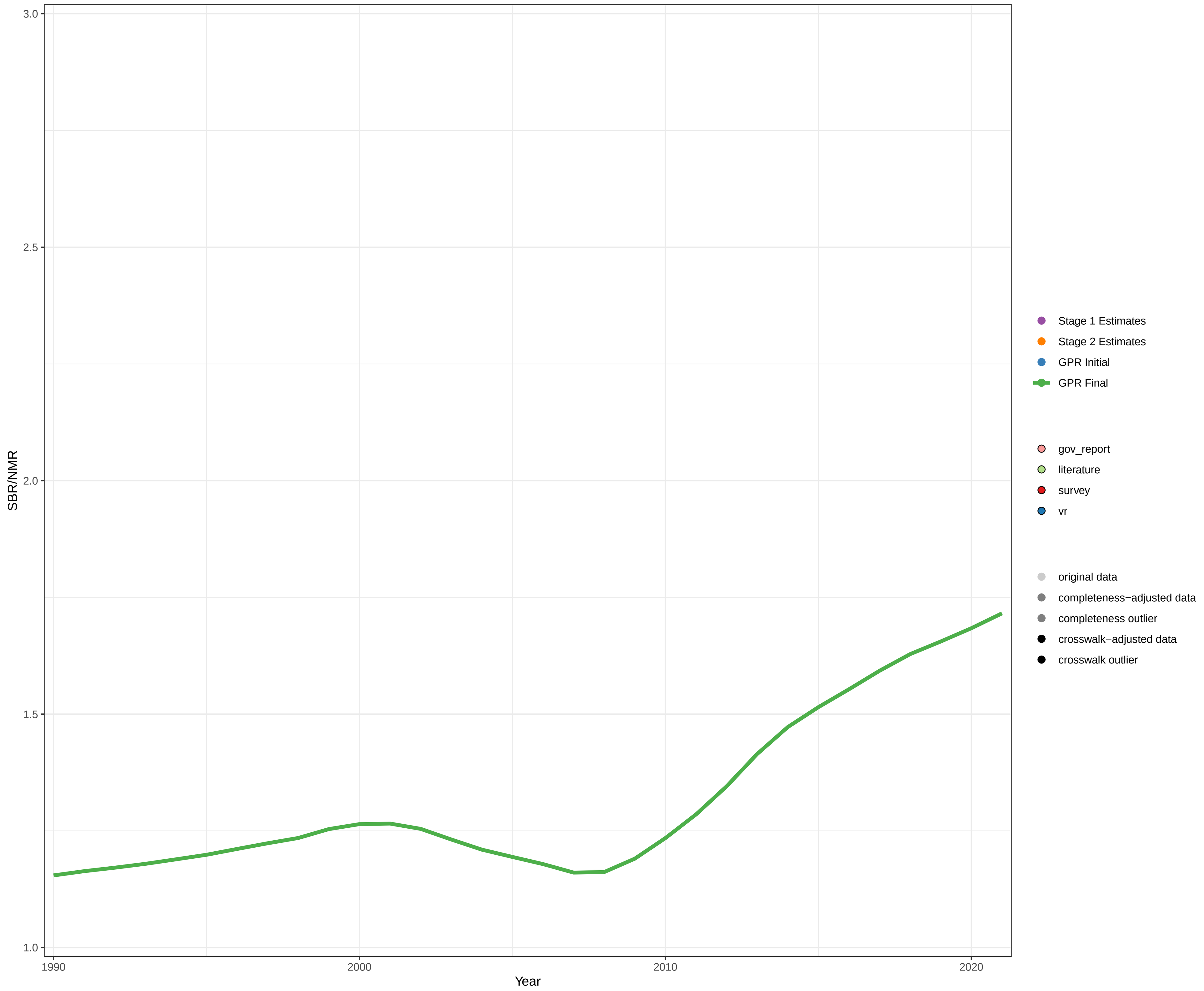

Definition	Years	Outlier	Source.Type	Title

East Asia R3 East Asia Stillbirth Rate (SBR) − 20 weeks
lambda = 1 ; zeta = 0.6 ; scale = 15 ; amp = 0.028

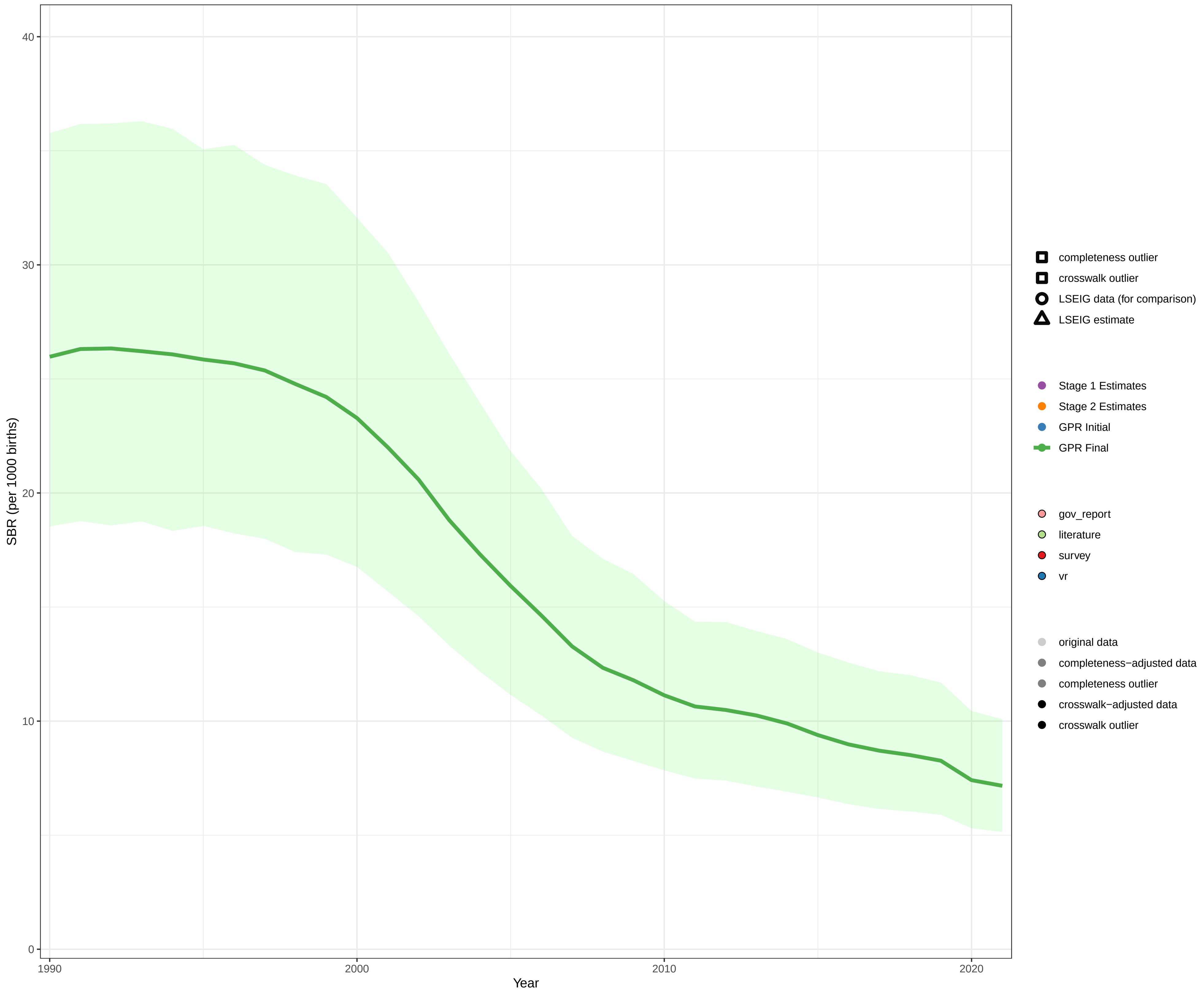

East Asia R3 East Asia Stillbirth Rate (SBR) − 28 weeks
lambda = 1 ; zeta = 0.6 ; scale = 15 ; amp = 0.028

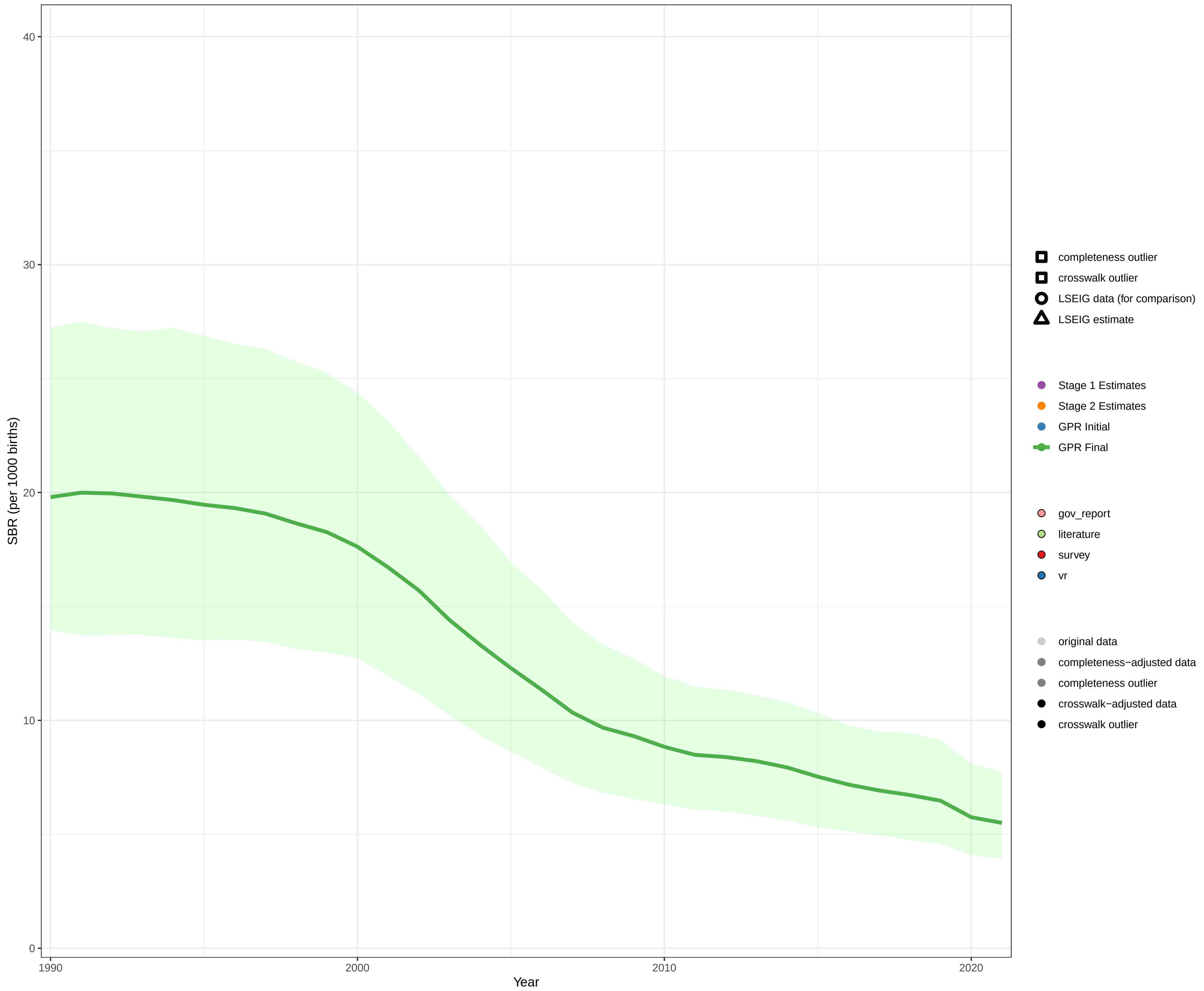

Year	Total Stillbirths (in thousands)	Total Stillbirths (in thousands)	SBR (per 1000 births)	SBR (per 1000 births)	SBR/NMR	SBR/NMR
	20 weeks	28 weeks	20 weeks	28 weeks	20 weeks	28 weeks
1990	634.0 (448.5 to 881.5)	480.1 (336.4 to 665.4)	26.0 (18.5 to 35.8)	19.8 (14.0 to 27.2)	1.51 (1.08 to 2.09)	1.15 (0.81 to 1.59)
1995	522.4 (371.8 to 714.7)	390.6 (269.3 to 543.3)	25.9 (18.6 to 35.1)	19.5 (13.5 to 26.9)	1.59 (1.14 to 2.16)	1.20 (0.83 to 1.66)
2000	386.8 (276.3 to 537.1)	291.0 (209.0 to 405.1)	23.3 (16.8 to 32.1)	17.6 (12.7 to 24.4)	1.67 (1.20 to 2.30)	1.26 (0.91 to 1.75)
2005	247.1 (172.0 to 340.7)	190.0 (132.6 to 262.6)	15.9 (11.1 to 21.8)	12.3 (8.6 to 16.9)	1.55 (1.08 to 2.12)	1.19 (0.84 to 1.64)
2010	153.2 (107.5 to 210.9)	121.4 (86.6 to 164.4)	11.1 (7.8 to 15.3)	8.8 (6.3 to 11.9)	1.55 (1.09 to 2.13)	1.23 (0.88 to 1.67)
2015	163.5 (115.4 to 227.2)	130.9 (92.1 to 180.0)	9.4 (6.7 to 13.0)	7.5 (5.3 to 10.3)	1.89 (1.34 to 2.62)	1.51 (1.07 to 2.08)
2020	91.8 (65.6 to 129.8)	71.1 (50.5 to 100.4)	7.4 (5.3 to 10.4)	5.8 (4.1 to 8.1)	2.17 (1.55 to 3.06)	1.68 (1.20 to 2.37)
2021	80.9 (57.8 to 114.1)	62.0 (44.2 to 87.5)	7.2 (5.1 to 10.1)	5.5 (3.9 to 7.7)	2.23 (1.60 to 3.14)	1.72 (1.22 to 2.42)

China CHN East Asia Stillbirth Rate / Neonatal Mortality Rate (SBR/NMR) − 20 weeks
lambda = 1 ; zeta = 0.6 ; scale = 15 ; amp = 0.028

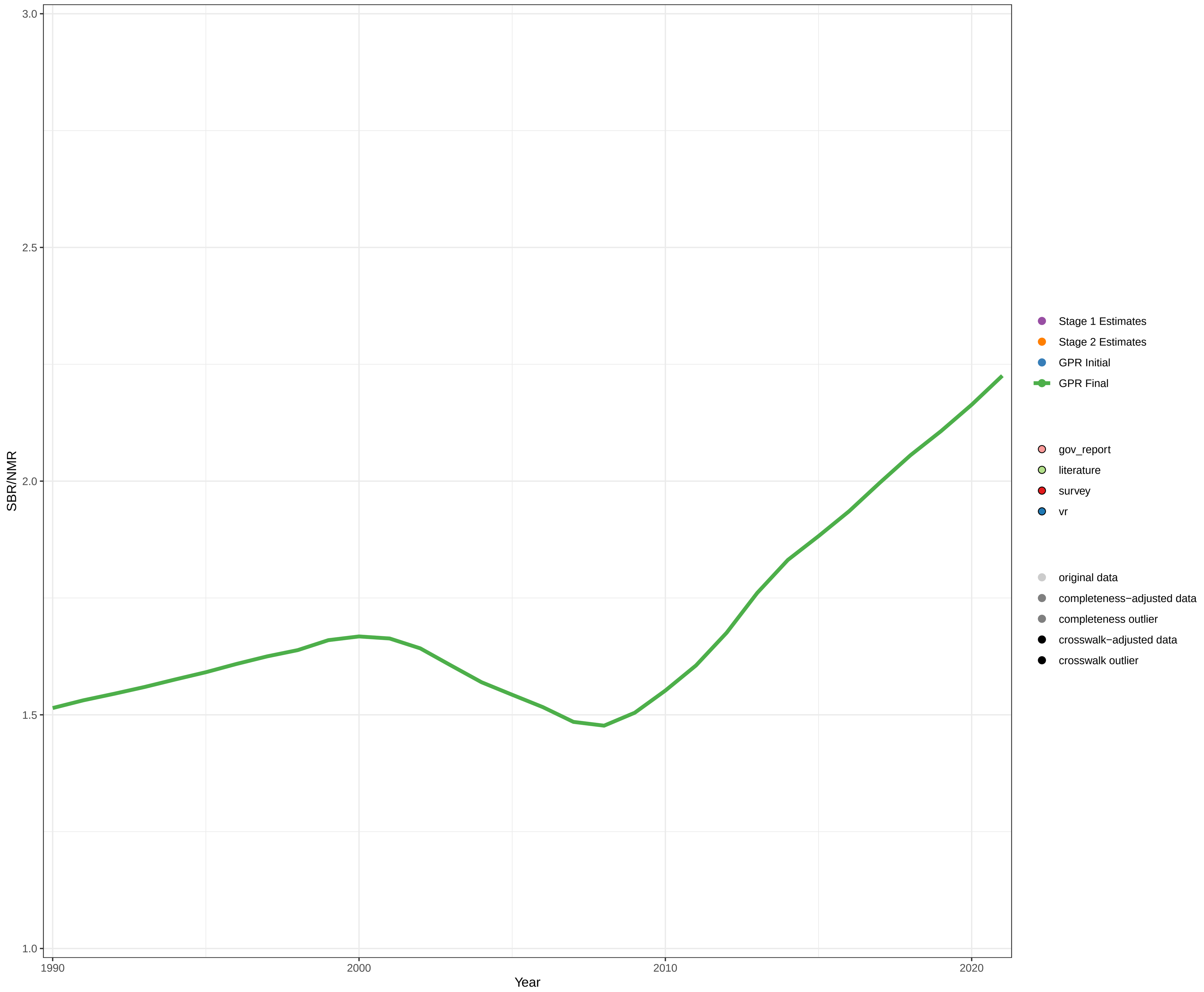

China CHN East Asia Stillbirth Rate / Neonatal Mortality Rate (SBR/NMR) − 28 weeks
lambda = 1 ; zeta = 0.6 ; scale = 15 ; amp = 0.028

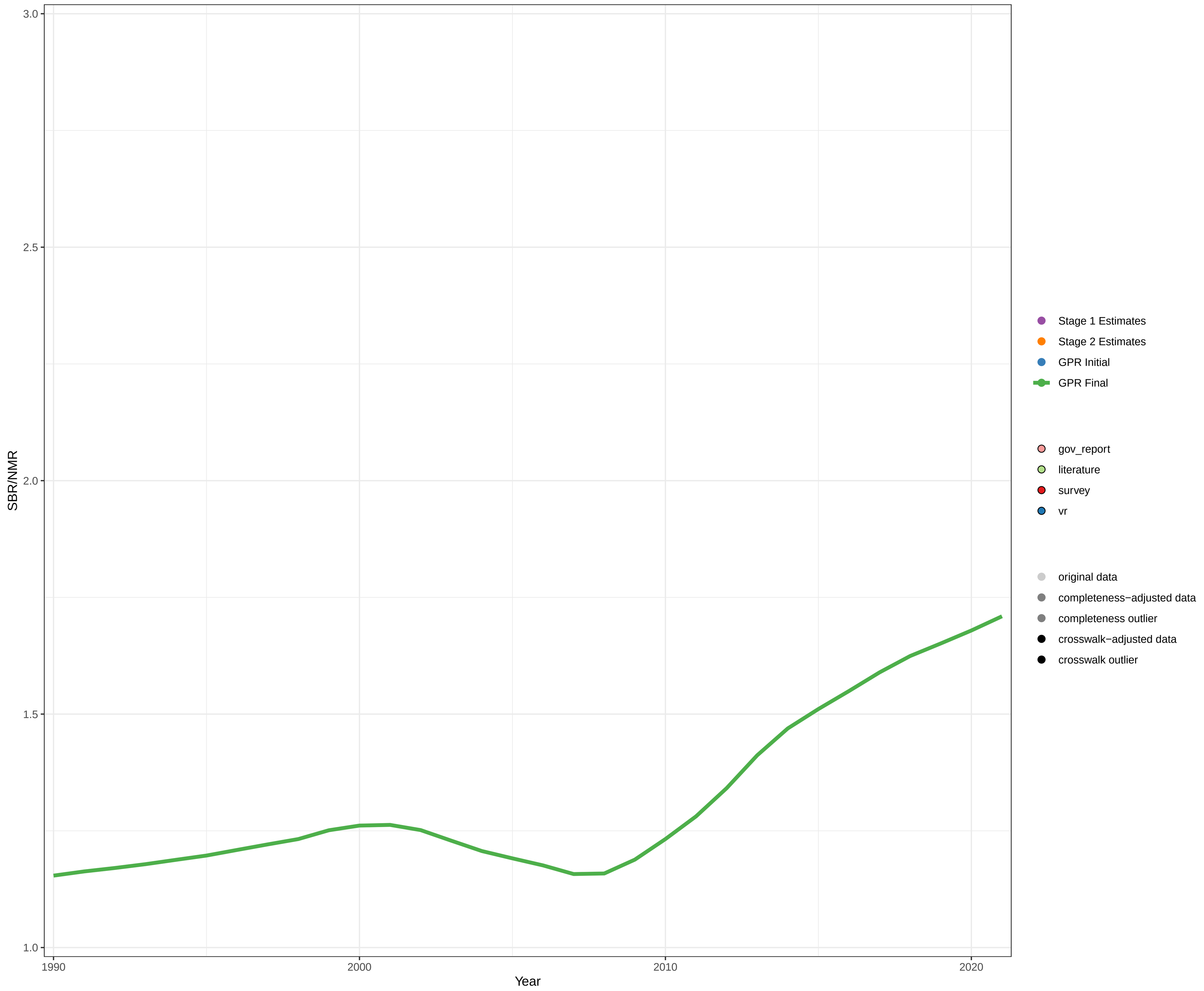

Definition	Years	Outlier	Source.Type	Title

China CHN East Asia Stillbirth Rate (SBR) − 20 weeks
lambda = 1 ; zeta = 0.6 ; scale = 15 ; amp = 0.028

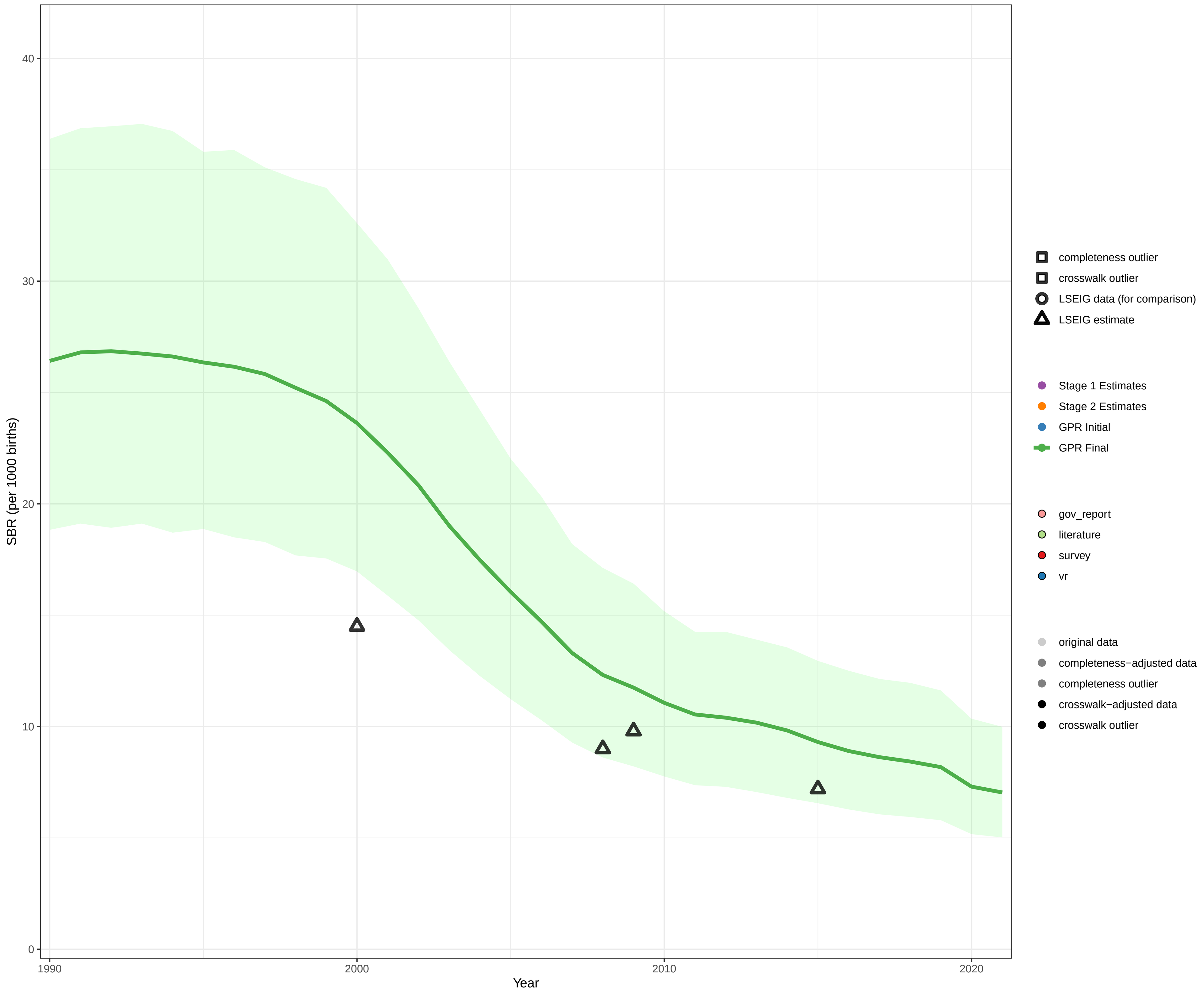

China CHN East Asia Stillbirth Rate (SBR) − 28 weeks
lambda = 1 ; zeta = 0.6 ; scale = 15 ; amp = 0.028

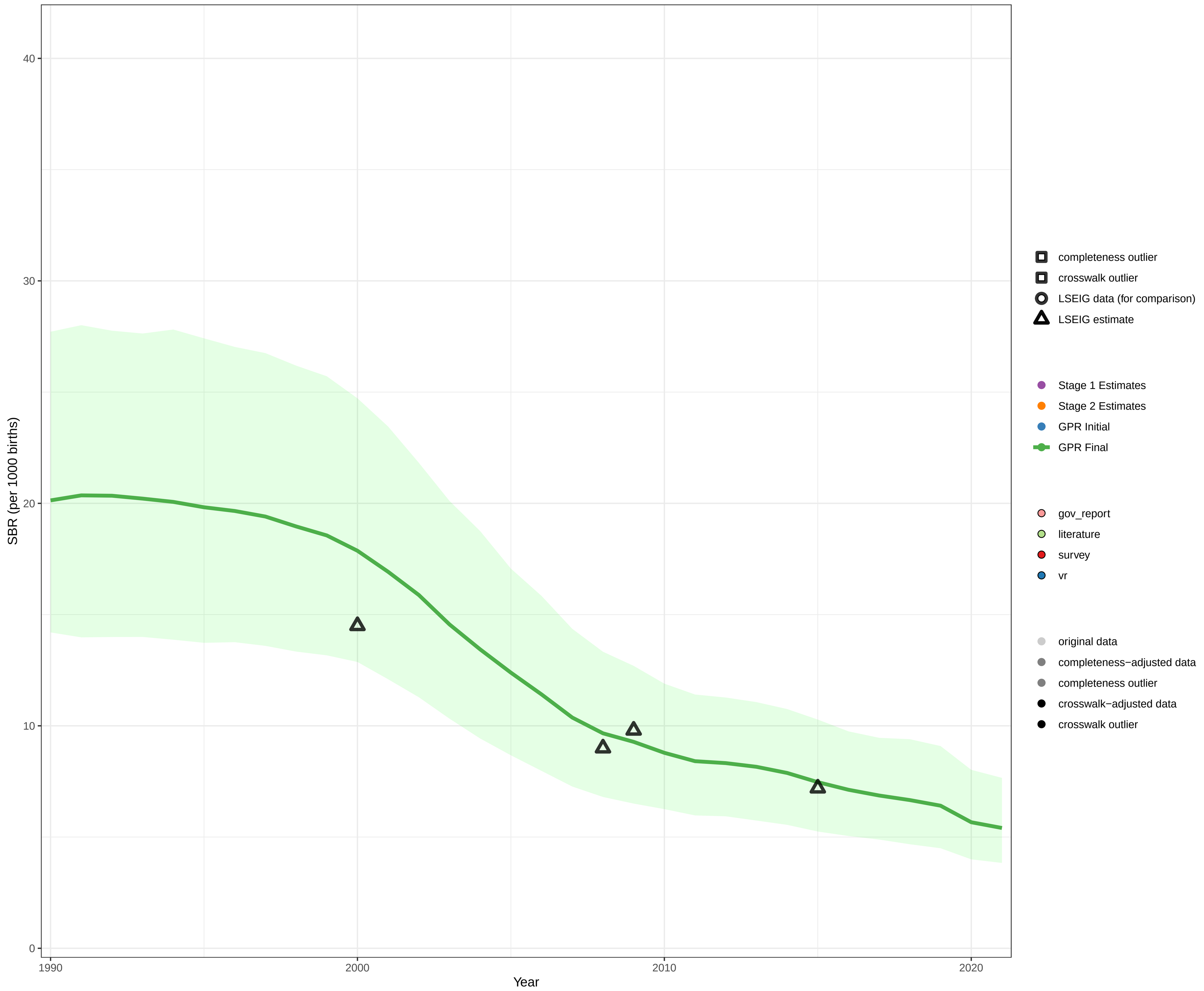

Year	Total Stillbirths (in thousands)	Total Stillbirths (in thousands)	SBR (per 1000 births)	SBR (per 1000 births)	SBR/NMR	SBR/NMR
	20 weeks	28 weeks	20 weeks	28 weeks	20 weeks	28 weeks
1990	621.4 (439.2 to 864.0)	470.4 (329.6 to 652.2)	26.4 (18.8 to 36.4)	20.1 (14.2 to 27.7)	1.51 (1.08 to 2.09)	1.15 (0.81 to 1.59)
1995	508.5 (361.1 to 697.3)	380.0 (261.4 to 529.3)	26.3 (18.9 to 35.8)	19.8 (13.7 to 27.4)	1.59 (1.14 to 2.16)	1.20 (0.83 to 1.66)
2000	373.4 (266.1 to 519.8)	280.7 (201.1 to 390.9)	23.6 (17.0 to 32.6)	17.9 (12.9 to 24.7)	1.67 (1.20 to 2.30)	1.26 (0.91 to 1.75)
2005	238.4 (165.9 to 329.1)	183.3 (127.8 to 253.6)	16.0 (11.2 to 22.0)	12.4 (8.7 to 17.1)	1.54 (1.08 to 2.12)	1.19 (0.83 to 1.64)
2010	145.8 (101.9 to 200.7)	115.5 (82.0 to 156.8)	11.1 (7.8 to 15.2)	8.8 (6.3 to 11.9)	1.55 (1.09 to 2.13)	1.23 (0.88 to 1.67)
2015	156.7 (110.1 to 218.9)	125.6 (88.0 to 173.3)	9.3 (6.6 to 12.9)	7.5 (5.2 to 10.3)	1.88 (1.33 to 2.62)	1.51 (1.06 to 2.08)
2020	87.0 (61.4 to 123.7)	67.4 (47.5 to 95.6)	7.3 (5.2 to 10.4)	5.7 (4.0 to 8.0)	2.16 (1.53 to 3.07)	1.68 (1.18 to 2.38)
2021	76.2 (54.2 to 108.4)	58.4 (41.4 to 83.0)	7.0 (5.0 to 10.0)	5.4 (3.8 to 7.7)	2.23 (1.59 to 3.16)	1.71 (1.21 to 2.42)

China (without Hong Kong and Macao)   CHN_44533   East Asia   Stillbirth Rate / Neonatal Mortality Rate (SBR/NMR) − 20 weeks
lambda = 0.8 ; zeta = 0.7 ; scale = 15 ; amp = 0.028

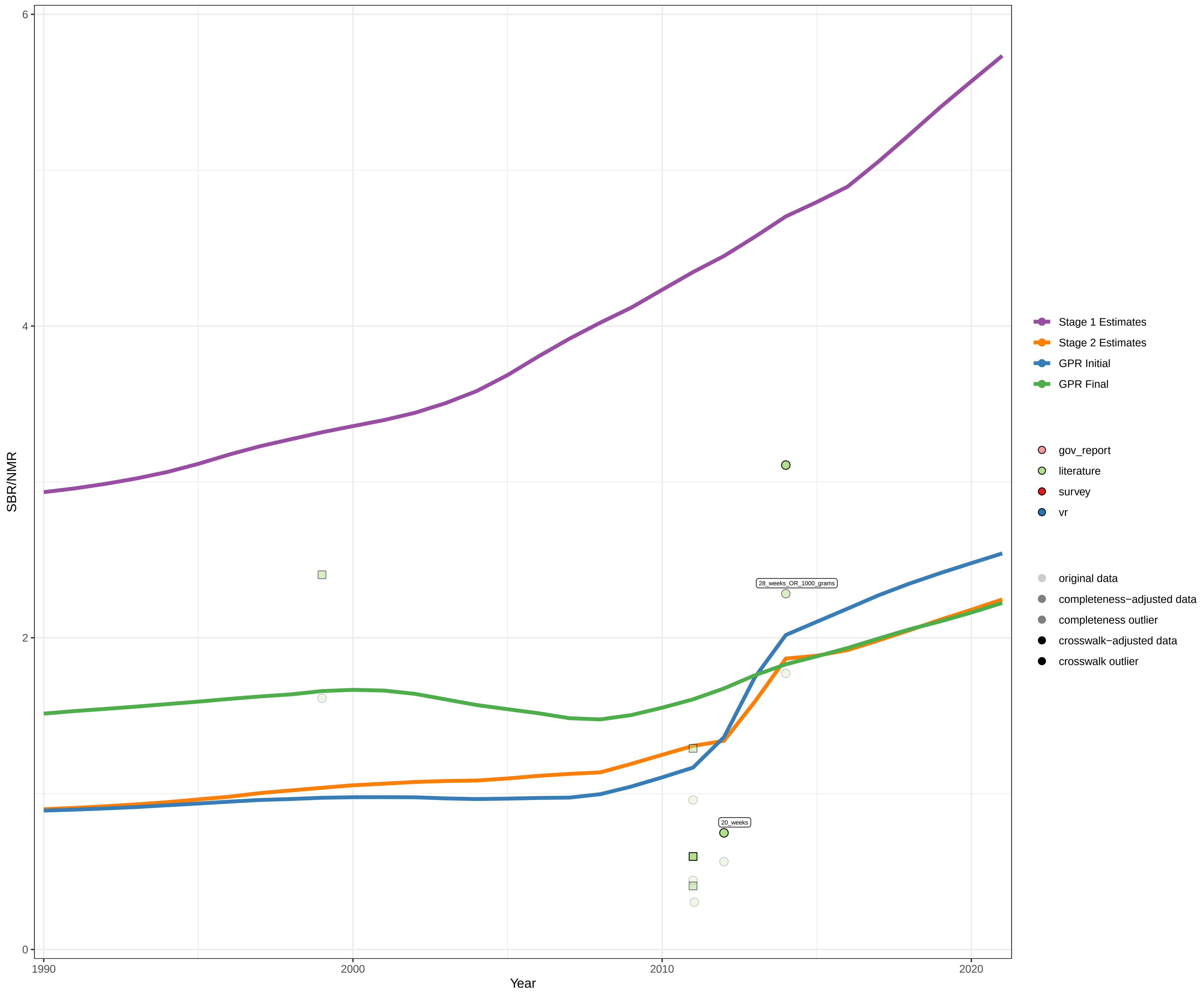

China (without Hong Kong and Macao)   CHN_44533   East Asia   Stillbirth Rate / Neonatal Mortality Rate (SBR/NMR) − 28 weeks
lambda = 0.8 ; zeta = 0.7 ; scale = 15 ; amp = 0.028

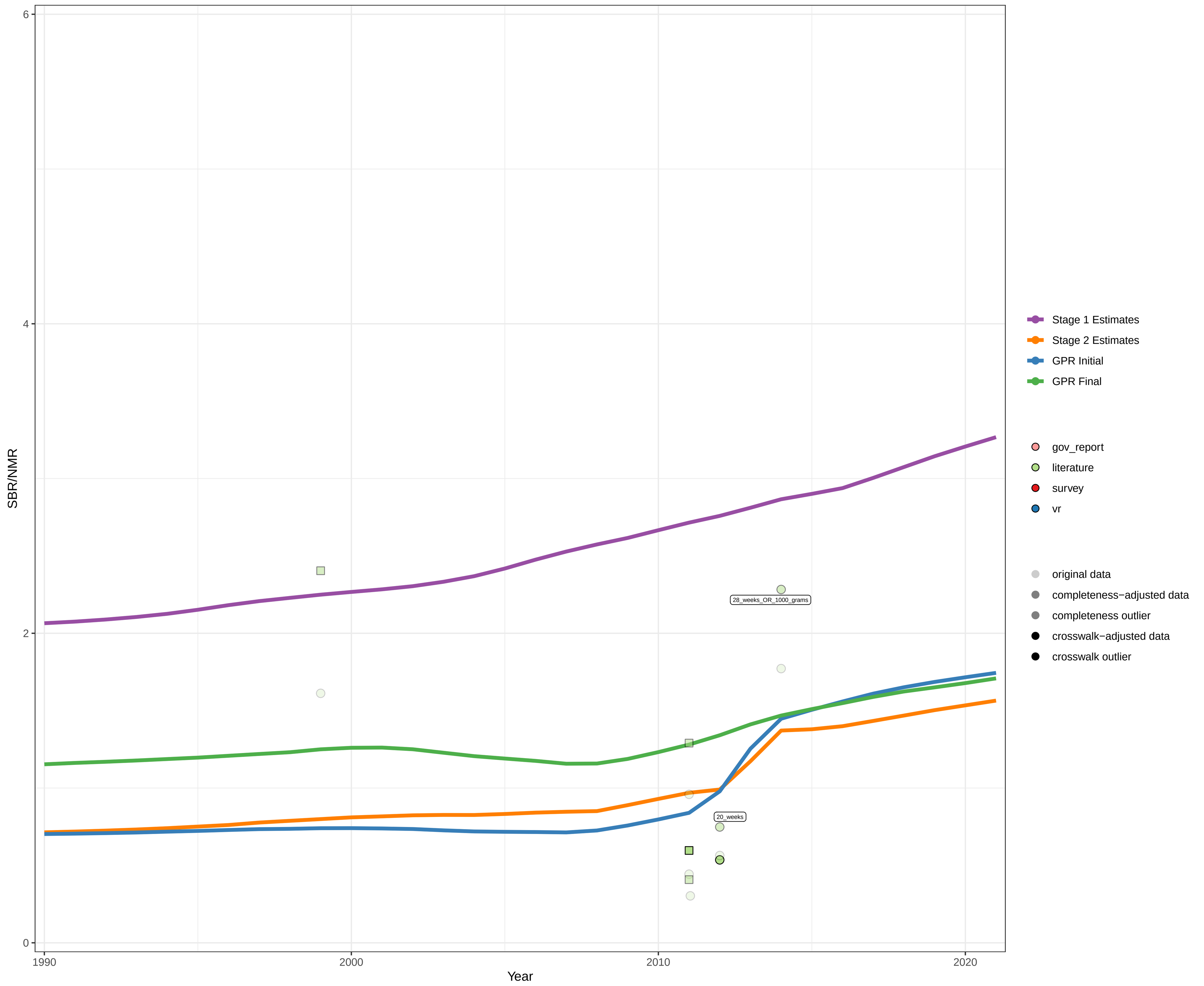

Definition	Years	Outlier	Source.Type	Title
20_weeks	2012	0	Sci Lit	Risk factors of stillbirth in rural China: A national cohort study
24_weeks	2011	0	Sci Lit	Preconception TSH and pregnancy outcomes: a population−based cohort study in 184611 women
28_weeks_OR_1000_grams	2014	0	Sci Lit	Hypertensive disorders in pregnancy and stillbirth rates: a facility−based study in China
22_weeks	1999	1	Sci Lit	Perinatal mortality in rural China: retrospective cohort study
28_weeks	2011	1	Sci Lit	Delivery modes and pregnancy outcomes of low birth weight infants in China
28_weeks_OR_1000_grams	2011	1	Sci Lit	Maternal complications and perinatal mortality: findings of the World Health Organization Multicountry Survey on Maternal and Newborn Health

China (without Hong Kong and Macao)   CHN_44533   East Asia   Stillbirth Rate (SBR) − 20 weeks
lambda = 0.8 ; zeta = 0.7 ; scale = 15 ; amp = 0.028

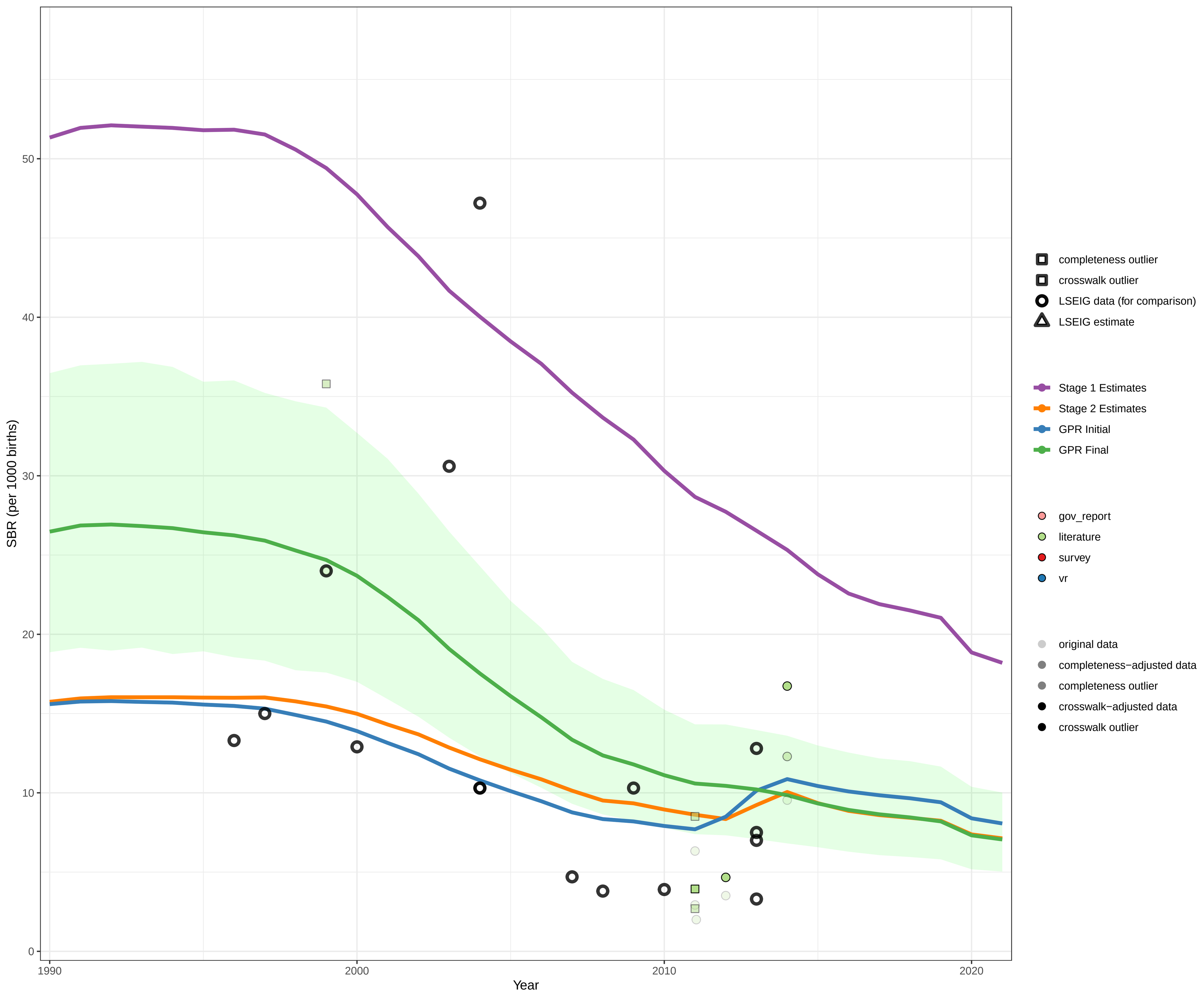

China (without Hong Kong and Macao)   CHN_44533   East Asia   Stillbirth Rate (SBR) − 28 weeks
lambda = 0.8 ; zeta = 0.7 ; scale = 15 ; amp = 0.028

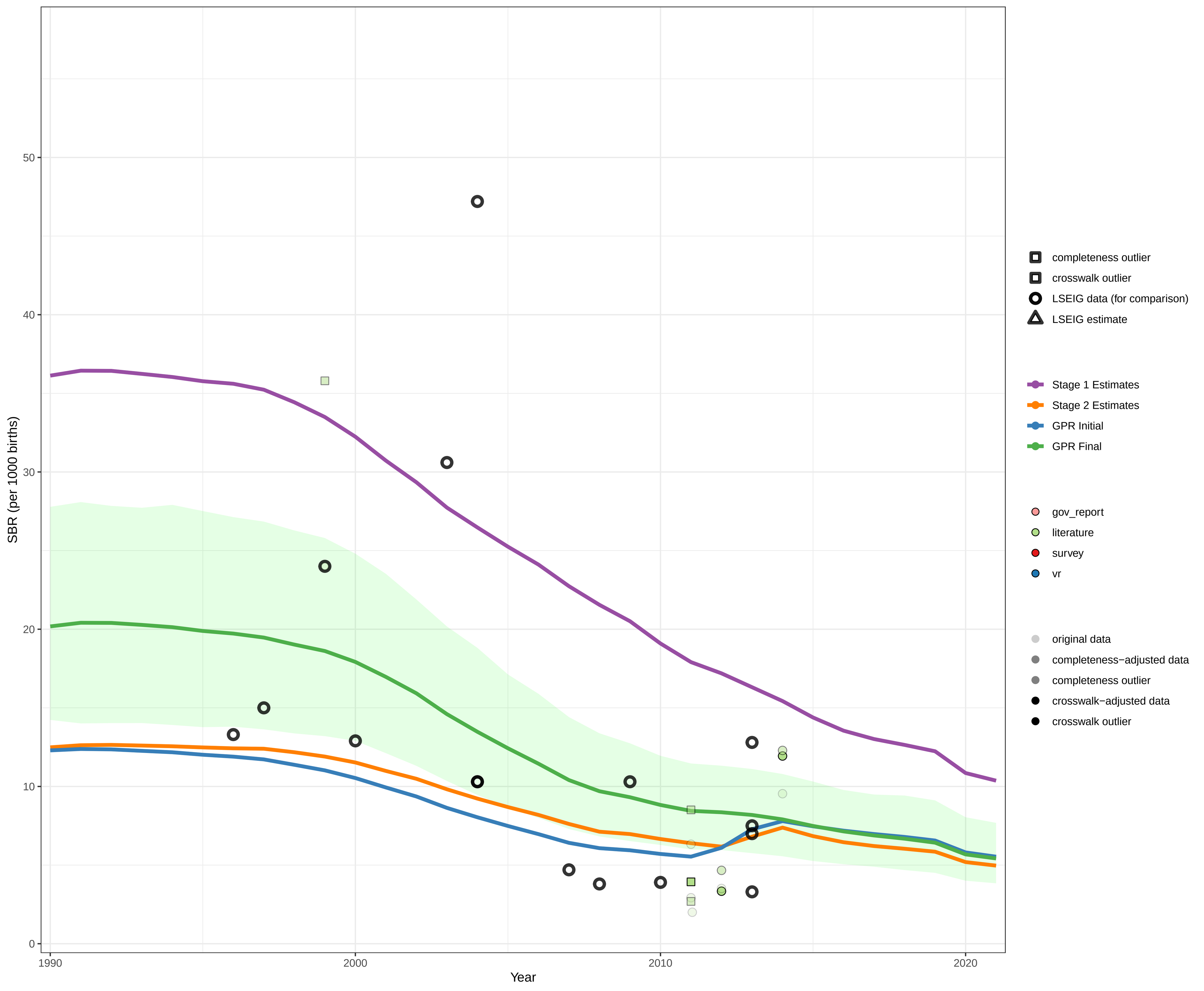

Year	Total Stillbirths (in thousands)	Total Stillbirths (in thousands)	SBR (per 1000 births)	SBR (per 1000 births)	SBR/NMR	SBR/NMR
	20 weeks	28 weeks	20 weeks	28 weeks	20 weeks	28 weeks
1990	620.6 (438.5 to 863.1)	469.9 (329.2 to 651.5)	26.5 (18.9 to 36.5)	20.2 (14.2 to 27.8)	1.51 (1.08 to 2.08)	1.15 (0.81 to 1.59)
1995	508.0 (360.7 to 696.8)	379.6 (261.1 to 529.0)	26.4 (18.9 to 35.9)	19.9 (13.8 to 27.5)	1.59 (1.14 to 2.16)	1.20 (0.83 to 1.66)
2000	372.9 (265.6 to 519.3)	280.4 (200.7 to 390.6)	23.7 (17.0 to 32.7)	17.9 (12.9 to 24.8)	1.67 (1.20 to 2.30)	1.26 (0.91 to 1.74)
2005	238.1 (165.6 to 328.8)	183.1 (127.6 to 253.4)	16.1 (11.3 to 22.1)	12.4 (8.7 to 17.1)	1.54 (1.08 to 2.12)	1.19 (0.83 to 1.64)
2010	145.6 (101.7 to 200.5)	115.4 (81.9 to 156.7)	11.1 (7.8 to 15.2)	8.8 (6.3 to 12.0)	1.55 (1.09 to 2.13)	1.23 (0.88 to 1.67)
2015	156.5 (109.8 to 218.6)	125.4 (87.8 to 173.1)	9.3 (6.6 to 13.0)	7.5 (5.3 to 10.3)	1.88 (1.32 to 2.62)	1.51 (1.06 to 2.08)
2020	86.8 (61.3 to 123.5)	67.3 (47.4 to 95.5)	7.3 (5.2 to 10.4)	5.7 (4.0 to 8.0)	2.16 (1.53 to 3.07)	1.68 (1.18 to 2.38)
2021	76.1 (54.1 to 108.2)	58.3 (41.3 to 82.9)	7.1 (5.0 to 10.0)	5.4 (3.8 to 7.7)	2.22 (1.58 to 3.16)	1.71 (1.21 to 2.42)

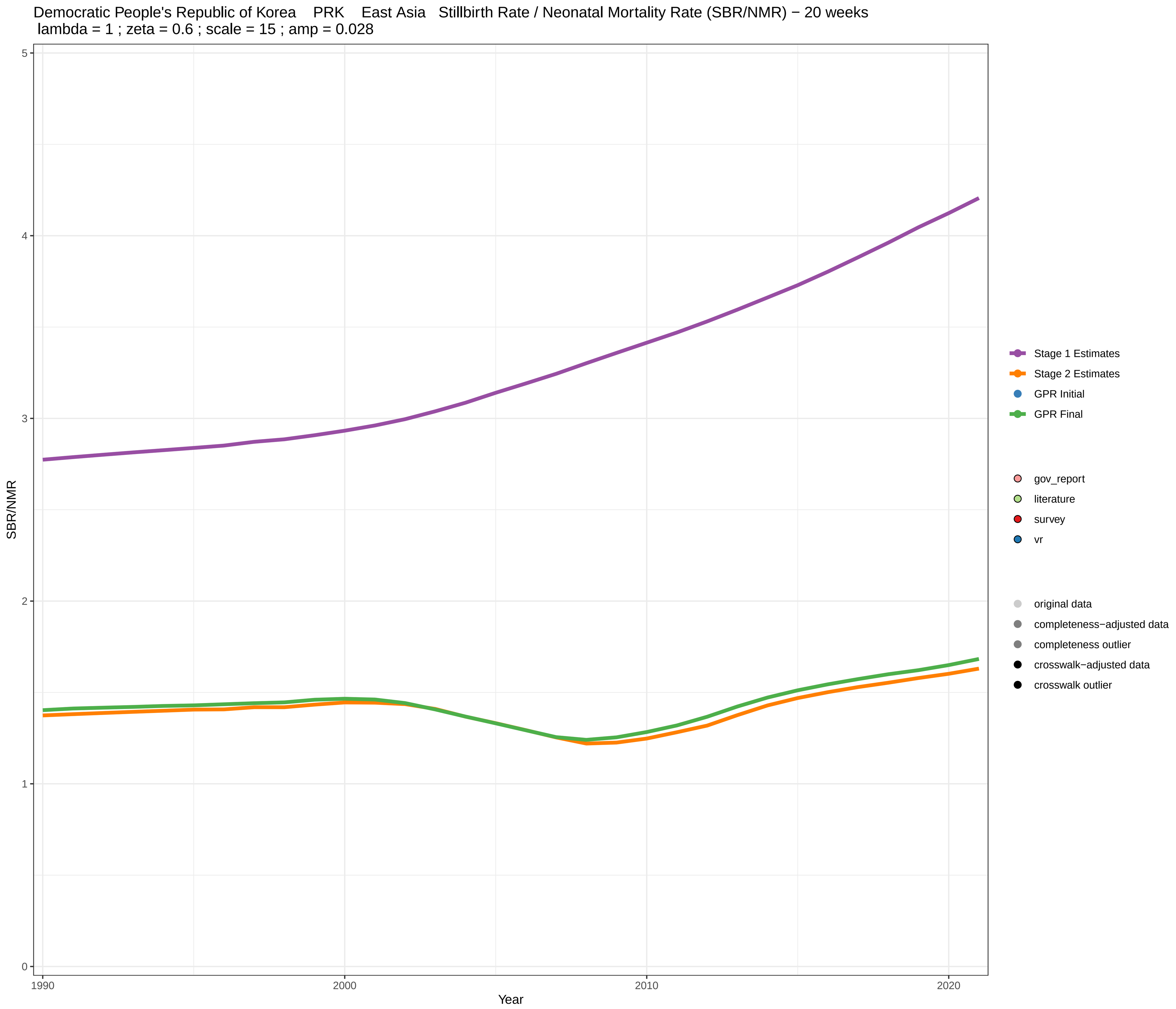

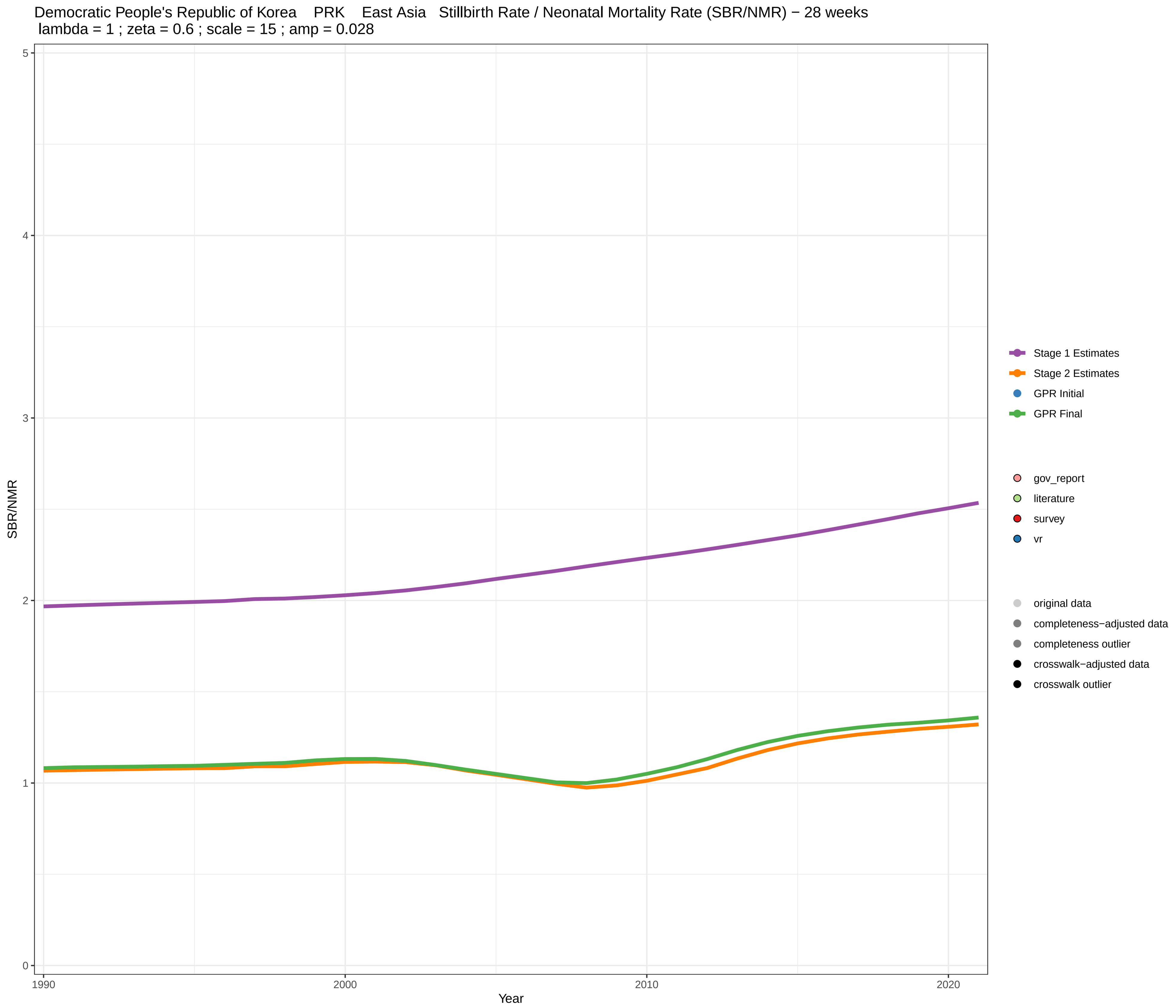

Definition	Years	Outlier	Source.Type	Title

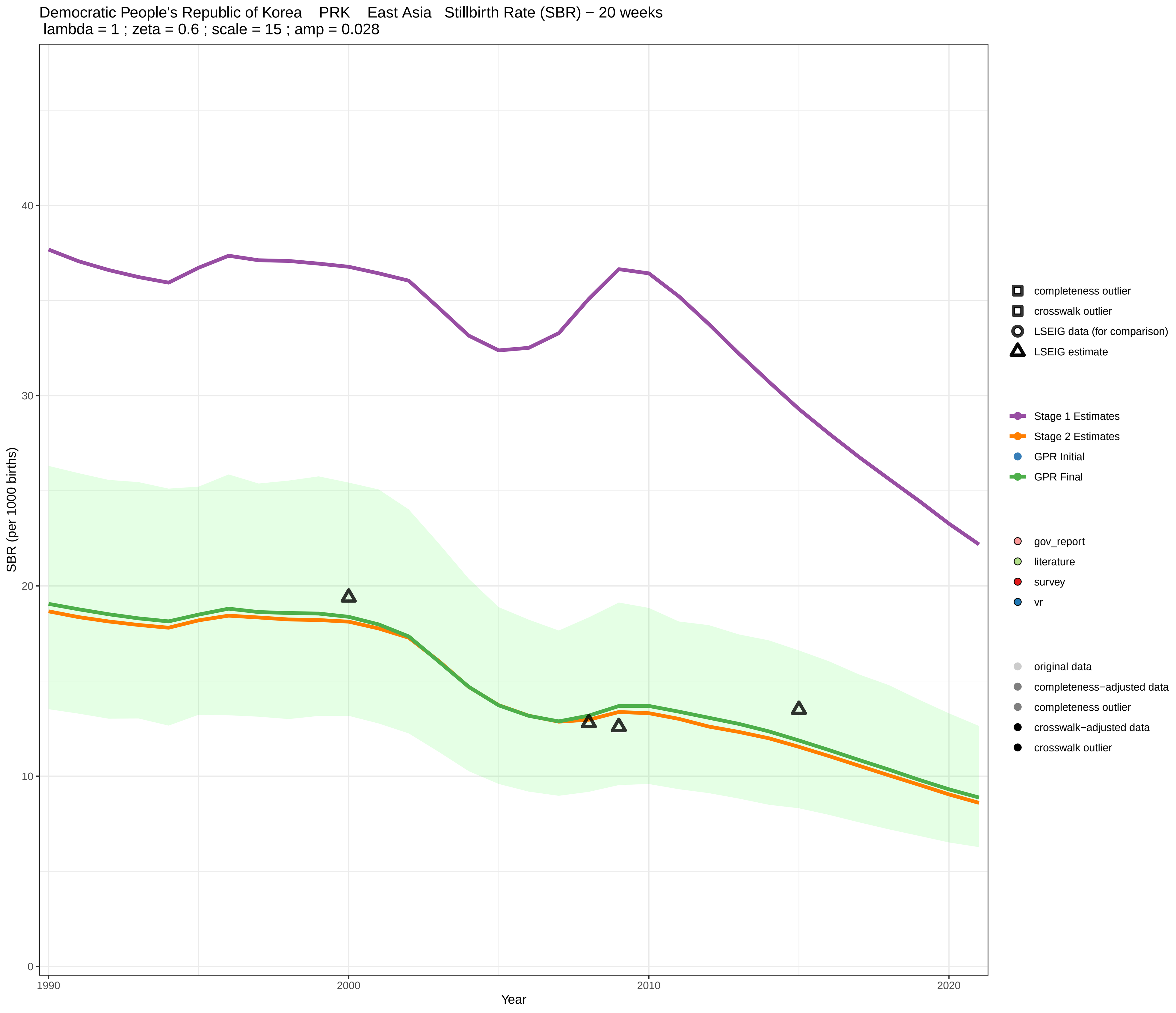

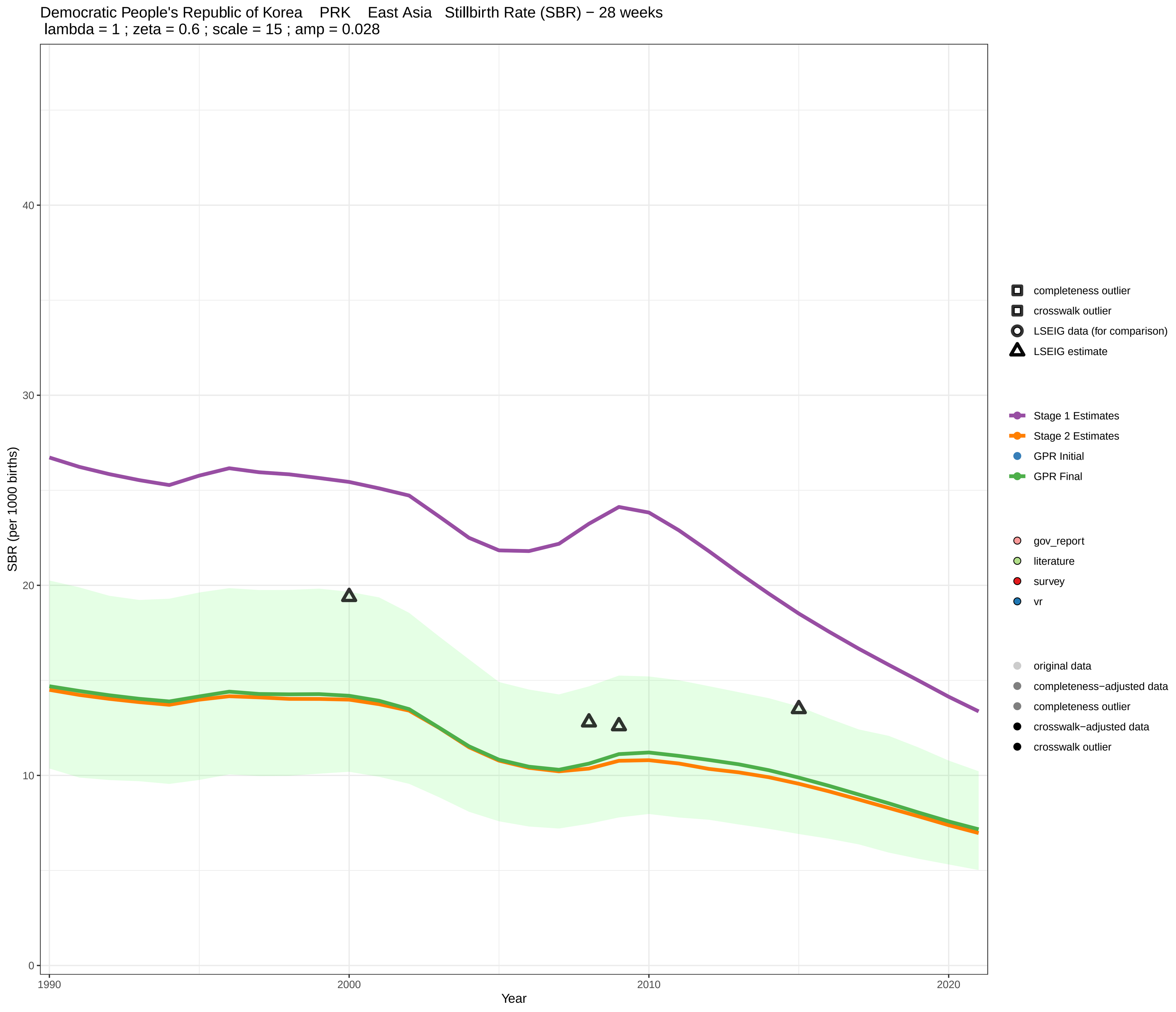

Year	Total Stillbirths (in thousands)	Total Stillbirths (in thousands)	SBR (per 1000 births)	SBR (per 1000 births)	SBR/NMR	SBR/NMR
	20 weeks	28 weeks	20 weeks	28 weeks	20 weeks	28 weeks
1990	10.7 (7.6 to 14.9)	8.2 (5.8 to 11.4)	19.1 (13.5 to 26.3)	14.7 (10.4 to 20.3)	1.40 (1.00 to 1.94)	1.08 (0.76 to 1.49)
1995	10.8 (7.7 to 14.8)	8.2 (5.6 to 11.5)	18.5 (13.2 to 25.2)	14.2 (9.8 to 19.6)	1.43 (1.02 to 1.95)	1.09 (0.75 to 1.52)
2000	9.8 (7.0 to 13.6)	7.5 (5.4 to 10.5)	18.4 (13.2 to 25.4)	14.2 (10.2 to 19.7)	1.47 (1.05 to 2.03)	1.13 (0.81 to 1.57)
2005	6.2 (4.3 to 8.5)	4.8 (3.4 to 6.7)	13.7 (9.6 to 18.9)	10.8 (7.6 to 14.9)	1.33 (0.93 to 1.83)	1.05 (0.74 to 1.45)
2010	5.4 (3.8 to 7.5)	4.4 (3.1 to 6.0)	13.7 (9.6 to 18.8)	11.2 (8.0 to 15.2)	1.28 (0.90 to 1.77)	1.05 (0.75 to 1.43)
2015	4.2 (2.9 to 5.9)	3.5 (2.4 to 4.8)	11.9 (8.3 to 16.6)	9.9 (6.9 to 13.6)	1.51 (1.06 to 2.11)	1.26 (0.88 to 1.73)
2020	2.9 (2.0 to 4.2)	2.4 (1.6 to 3.4)	9.3 (6.5 to 13.3)	7.6 (5.3 to 10.8)	1.65 (1.15 to 2.36)	1.34 (0.94 to 1.91)
2021	2.7 (1.9 to 3.8)	2.2 (1.5 to 3.1)	8.9 (6.3 to 12.6)	7.2 (5.0 to 10.2)	1.68 (1.19 to 2.40)	1.36 (0.95 to 1.94)

Taiwan TWN East Asia Stillbirth Rate / Neonatal Mortality Rate (SBR/NMR) − 20 weeks
lambda = 0.6 ; zeta = 0.8 ; scale = 15 ; amp = 0.028

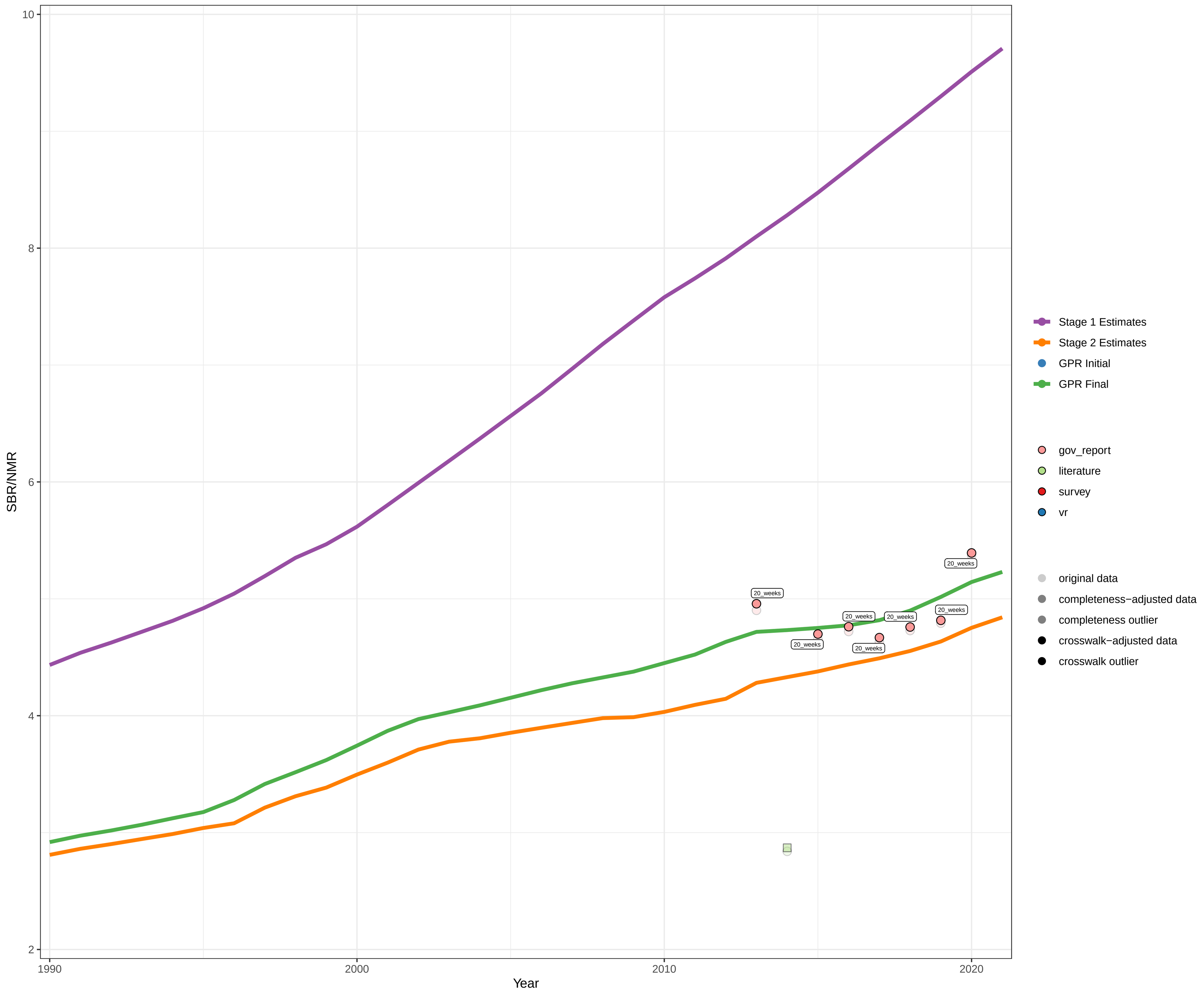

Taiwan TWN East Asia Stillbirth Rate / Neonatal Mortality Rate (SBR/NMR) − 28 weeks
lambda = 0.6 ; zeta = 0.8 ; scale = 15 ; amp = 0.028

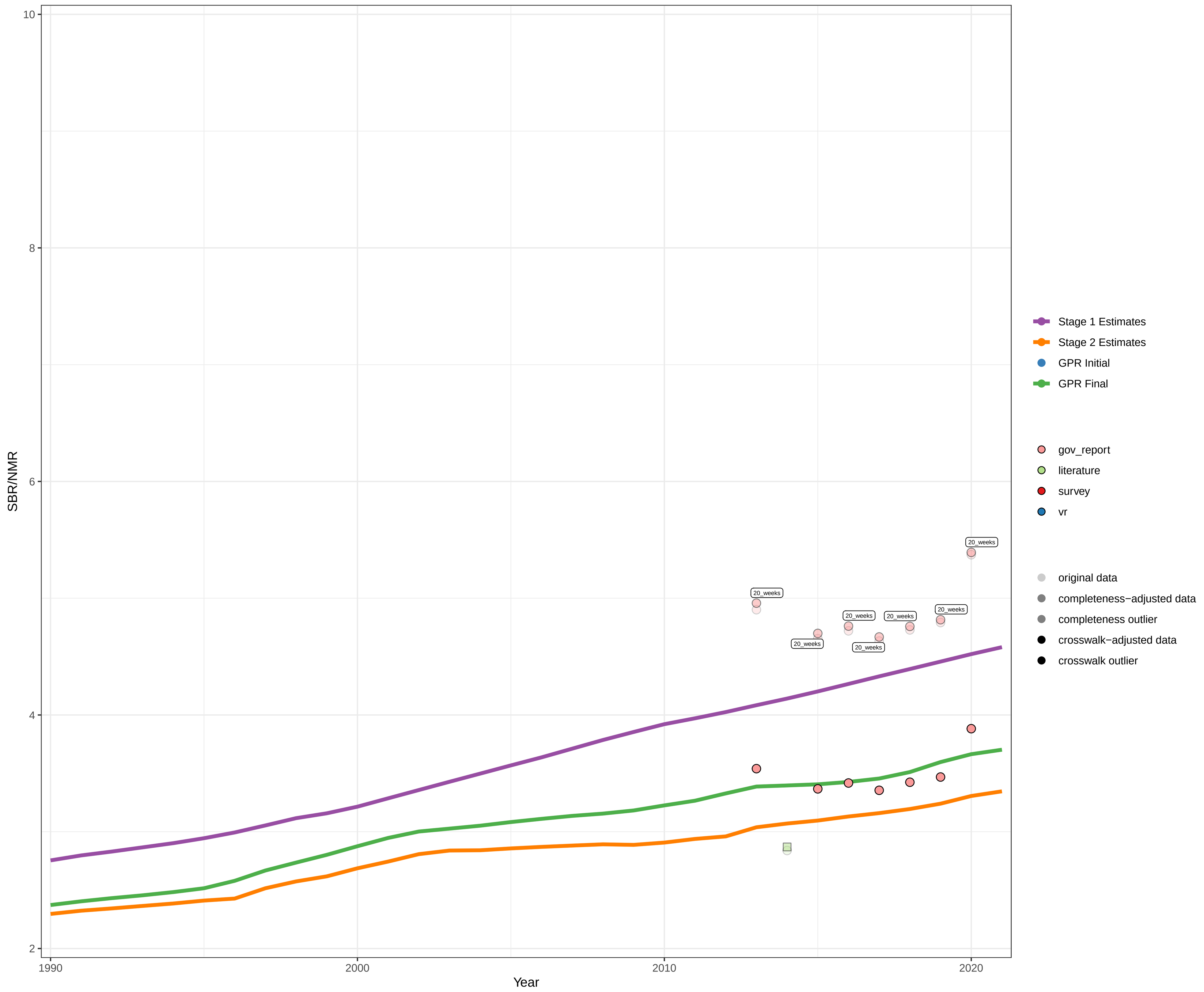

Definition	Years	Outlier	Source.Type	Title
20_weeks	2013, 2015−2020	0	Statistical Report	Taiwan Statistics of Birth Reporting System
28_weeks	2014	1	Sci Lit	The role of preterm placental calcification on assessing risks of stillbirth

Taiwan TWN East Asia Stillbirth Rate (SBR) − 20 weeks
lambda = 0.6 ; zeta = 0.8 ; scale = 15 ; amp = 0.028

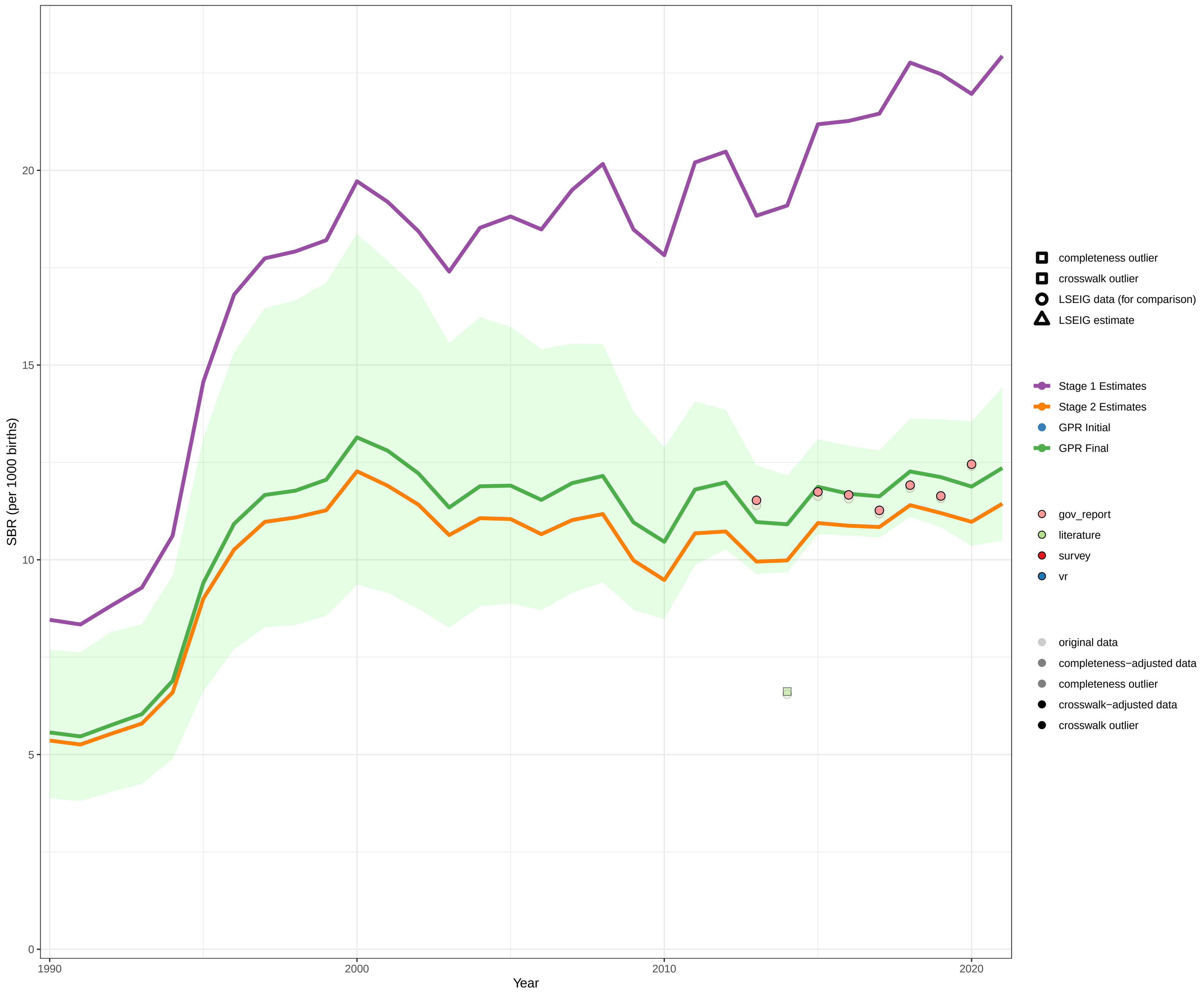

Taiwan TWN East Asia Stillbirth Rate (SBR) − 28 weeks
lambda = 0.6 ; zeta = 0.8 ; scale = 15 ; amp = 0.028

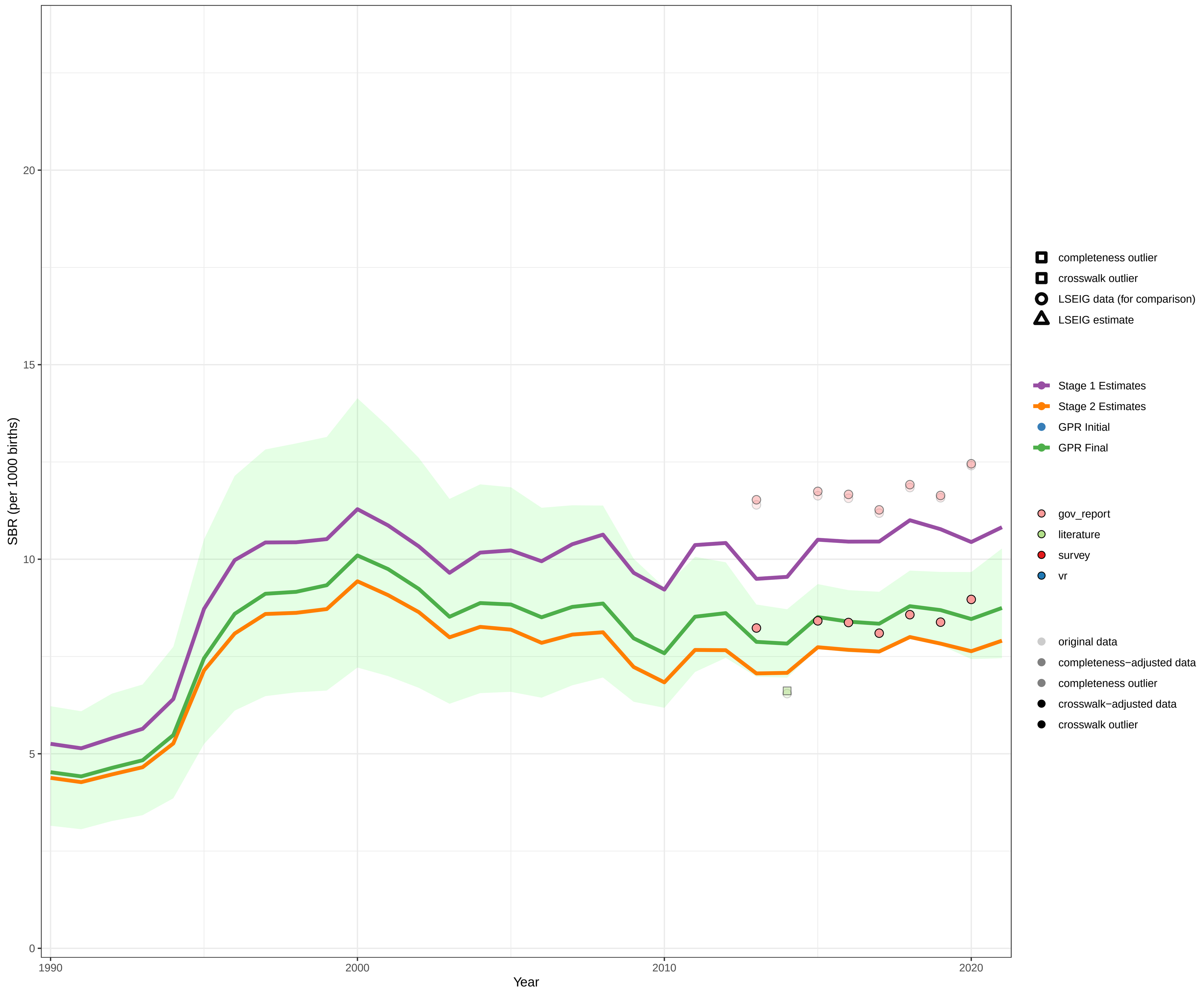

Year	Total Stillbirths (in thousands)	Total Stillbirths (in thousands)	SBR (per 1000 births)	SBR (per 1000 births)	SBR/NMR	SBR/NMR
	20 weeks	28 weeks	20 weeks	28 weeks	20 weeks	28 weeks
1990	1.8 (1.3 to 2.5)	1.5 (1.0 to 2.0)	5.6 (3.9 to 7.7)	4.5 (3.1 to 6.2)	2.92 (2.03 to 4.03)	2.37 (1.65 to 3.26)
1995	3.0 (2.1 to 4.3)	2.4 (1.7 to 3.4)	9.4 (6.6 to 13.1)	7.5 (5.2 to 10.5)	3.18 (2.24 to 4.43)	2.52 (1.77 to 3.55)
2000	3.6 (2.6 to 5.1)	2.8 (2.0 to 3.9)	13.1 (9.4 to 18.4)	10.1 (7.2 to 14.1)	3.74 (2.67 to 5.23)	2.88 (2.06 to 4.03)
2005	2.5 (1.9 to 3.4)	1.9 (1.4 to 2.5)	11.9 (8.9 to 16.0)	8.8 (6.6 to 11.8)	4.15 (3.10 to 5.58)	3.08 (2.30 to 4.13)
2010	2.0 (1.6 to 2.5)	1.5 (1.2 to 1.8)	10.5 (8.5 to 12.9)	7.6 (6.2 to 9.3)	4.45 (3.61 to 5.48)	3.23 (2.63 to 3.95)
2015	2.5 (2.2 to 2.8)	1.8 (1.6 to 2.0)	11.9 (10.7 to 13.1)	8.5 (7.7 to 9.4)	4.75 (4.26 to 5.24)	3.41 (3.08 to 3.75)
2020	2.0 (1.7 to 2.2)	1.4 (1.2 to 1.6)	11.9 (10.4 to 13.6)	8.5 (7.4 to 9.7)	5.14 (4.49 to 5.87)	3.66 (3.22 to 4.19)
2021	2.0 (1.7 to 2.3)	1.4 (1.2 to 1.6)	12.4 (10.5 to 14.4)	8.7 (7.5 to 10.3)	5.23 (4.44 to 6.11)	3.70 (3.16 to 4.35)

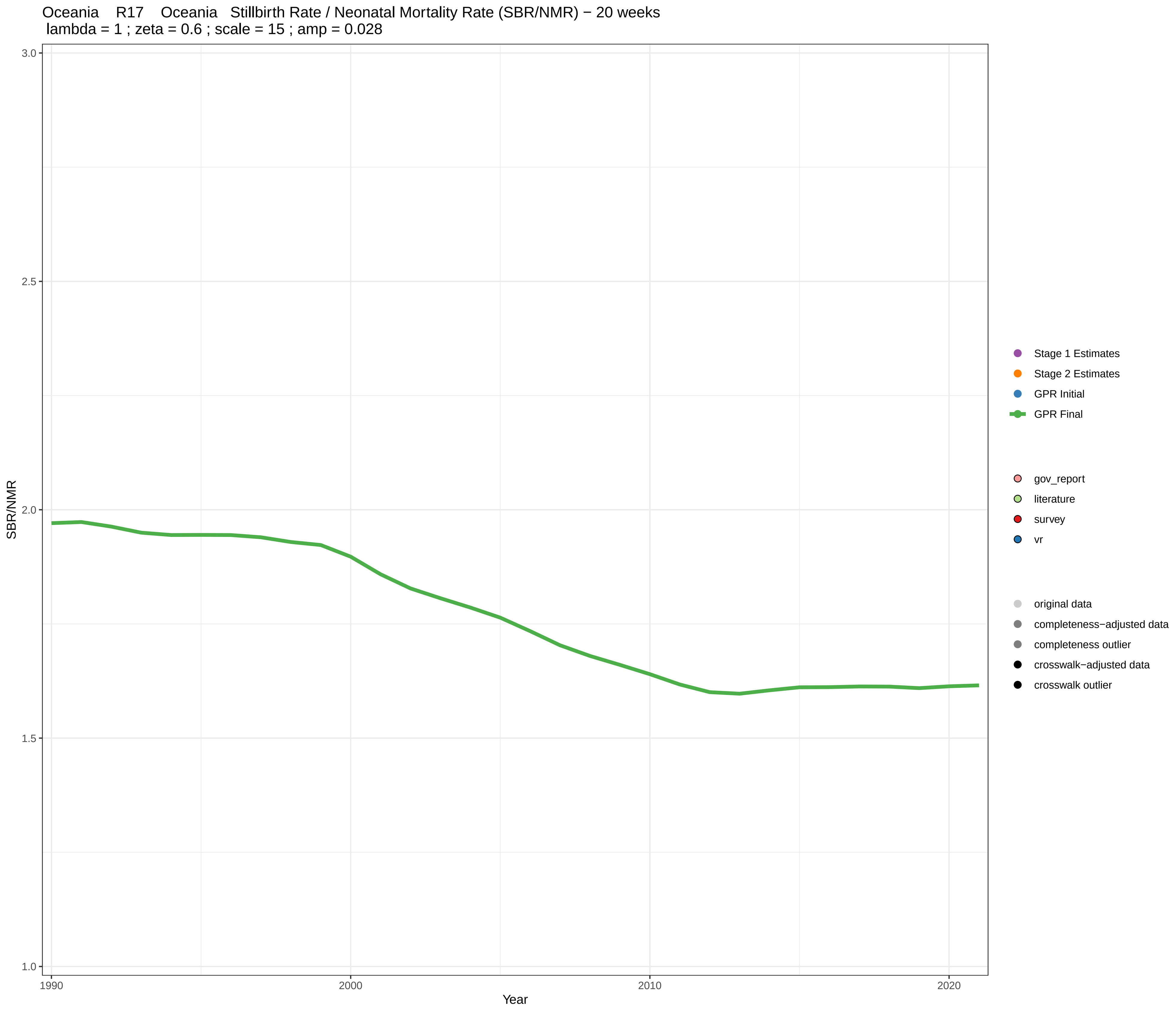

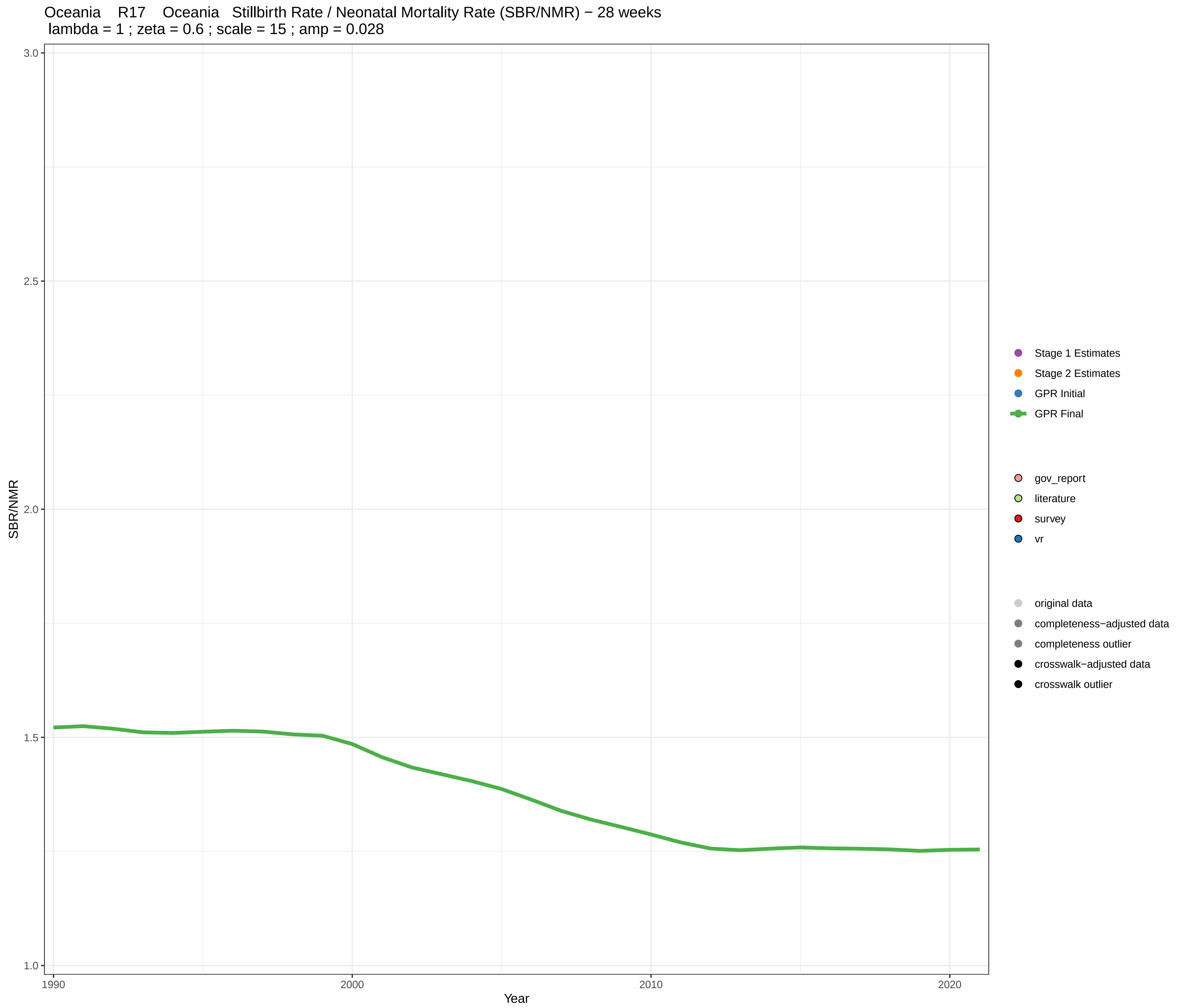

Definition	Years	Outlier	Source.Type	Title

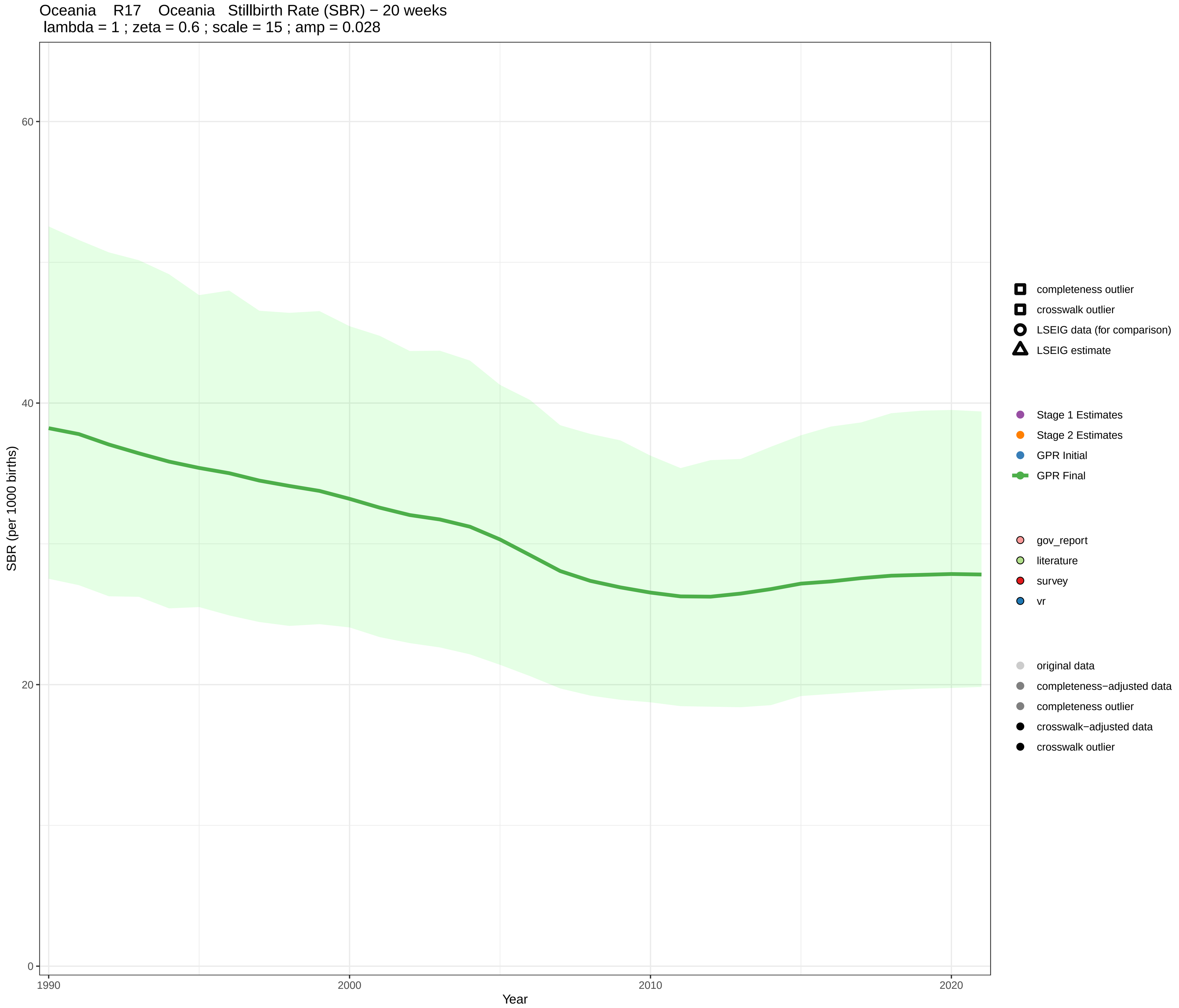

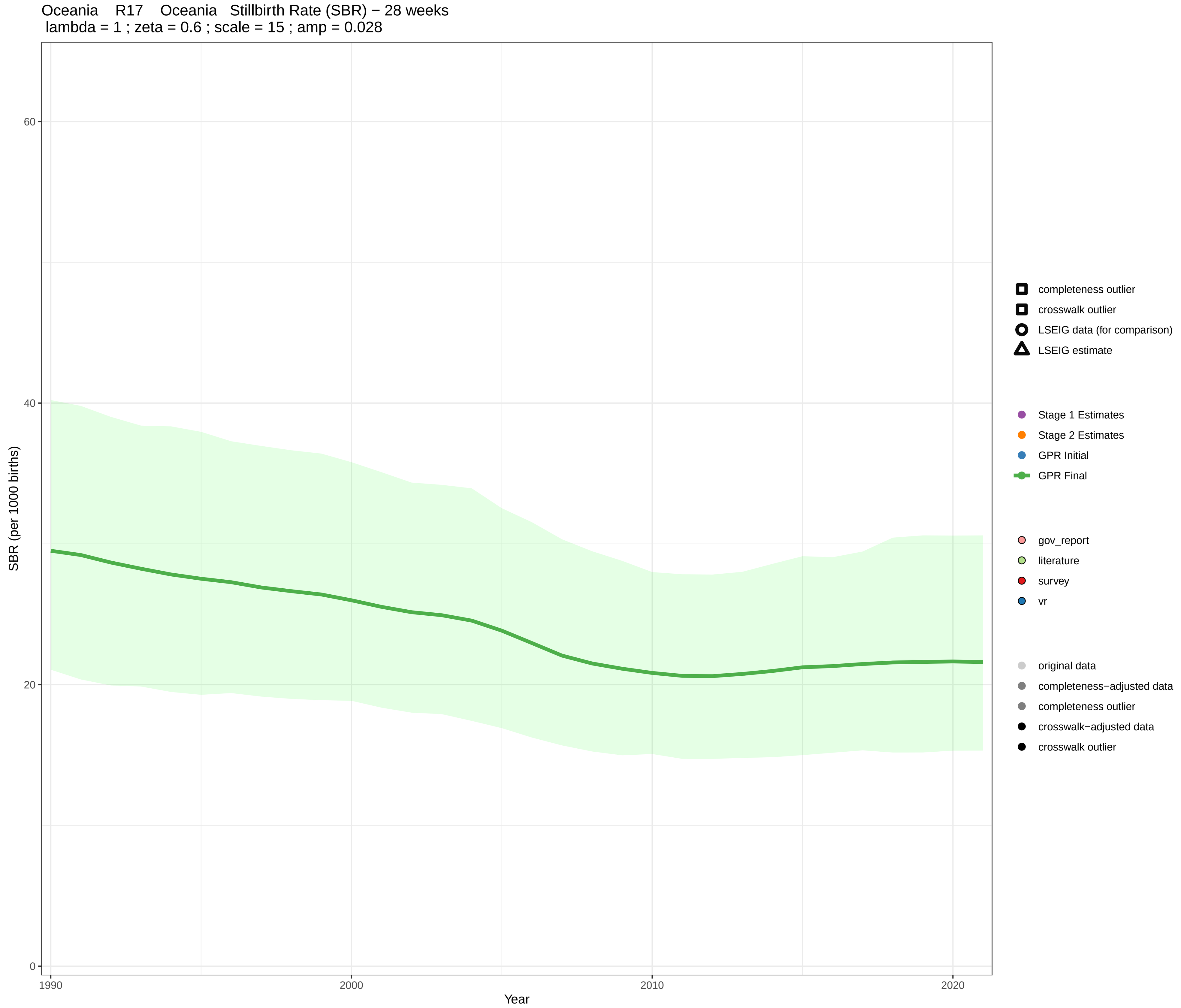

Year	Total Stillbirths (in thousands)	Total Stillbirths (in thousands)	SBR (per 1000 births)	SBR (per 1000 births)	SBR/NMR	SBR/NMR
	20 weeks	28 weeks	20 weeks	28 weeks	20 weeks	28 weeks
1990	9.0 (6.4 to 12.5)	6.9 (4.8 to 9.4)	38.2 (27.5 to 52.5)	29.5 (21.1 to 40.2)	1.97 (1.42 to 2.71)	1.52 (1.09 to 2.07)
1995	9.2 (6.6 to 12.6)	7.1 (4.9 to 9.9)	35.4 (25.5 to 47.7)	27.5 (19.3 to 38.0)	1.94 (1.40 to 2.62)	1.51 (1.06 to 2.09)
2000	9.5 (6.8 to 13.2)	7.4 (5.3 to 10.3)	33.2 (24.1 to 45.5)	26.0 (18.9 to 35.8)	1.90 (1.38 to 2.60)	1.49 (1.08 to 2.05)
2005	9.6 (6.7 to 13.2)	7.5 (5.3 to 10.3)	30.3 (21.4 to 41.3)	23.8 (16.9 to 32.5)	1.76 (1.25 to 2.40)	1.39 (0.98 to 1.89)
2010	9.5 (6.6 to 13.1)	7.4 (5.3 to 10.0)	26.5 (18.7 to 36.3)	20.8 (15.1 to 28.0)	1.64 (1.16 to 2.24)	1.29 (0.93 to 1.73)
2015	10.8 (7.6 to 15.1)	8.4 (5.9 to 11.6)	27.2 (19.2 to 37.7)	21.2 (15.0 to 29.1)	1.61 (1.14 to 2.24)	1.26 (0.89 to 1.73)
2020	12.1 (8.5 to 17.4)	9.4 (6.6 to 13.4)	27.9 (19.8 to 39.5)	21.6 (15.3 to 30.6)	1.61 (1.14 to 2.29)	1.25 (0.89 to 1.77)
2021	12.3 (8.7 to 17.7)	9.5 (6.7 to 13.6)	27.8 (19.8 to 39.4)	21.6 (15.3 to 30.6)	1.62 (1.15 to 2.29)	1.25 (0.89 to 1.78)

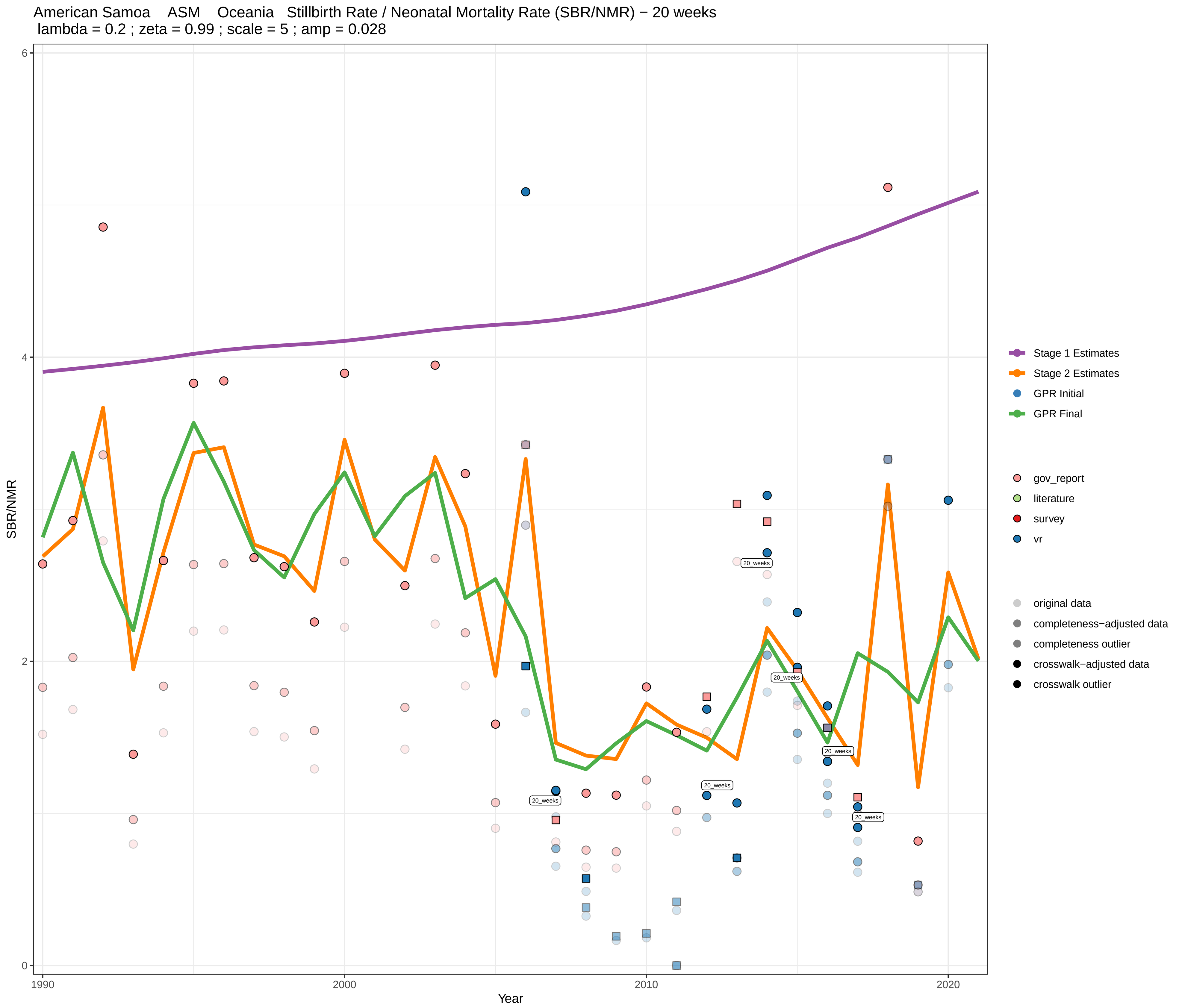

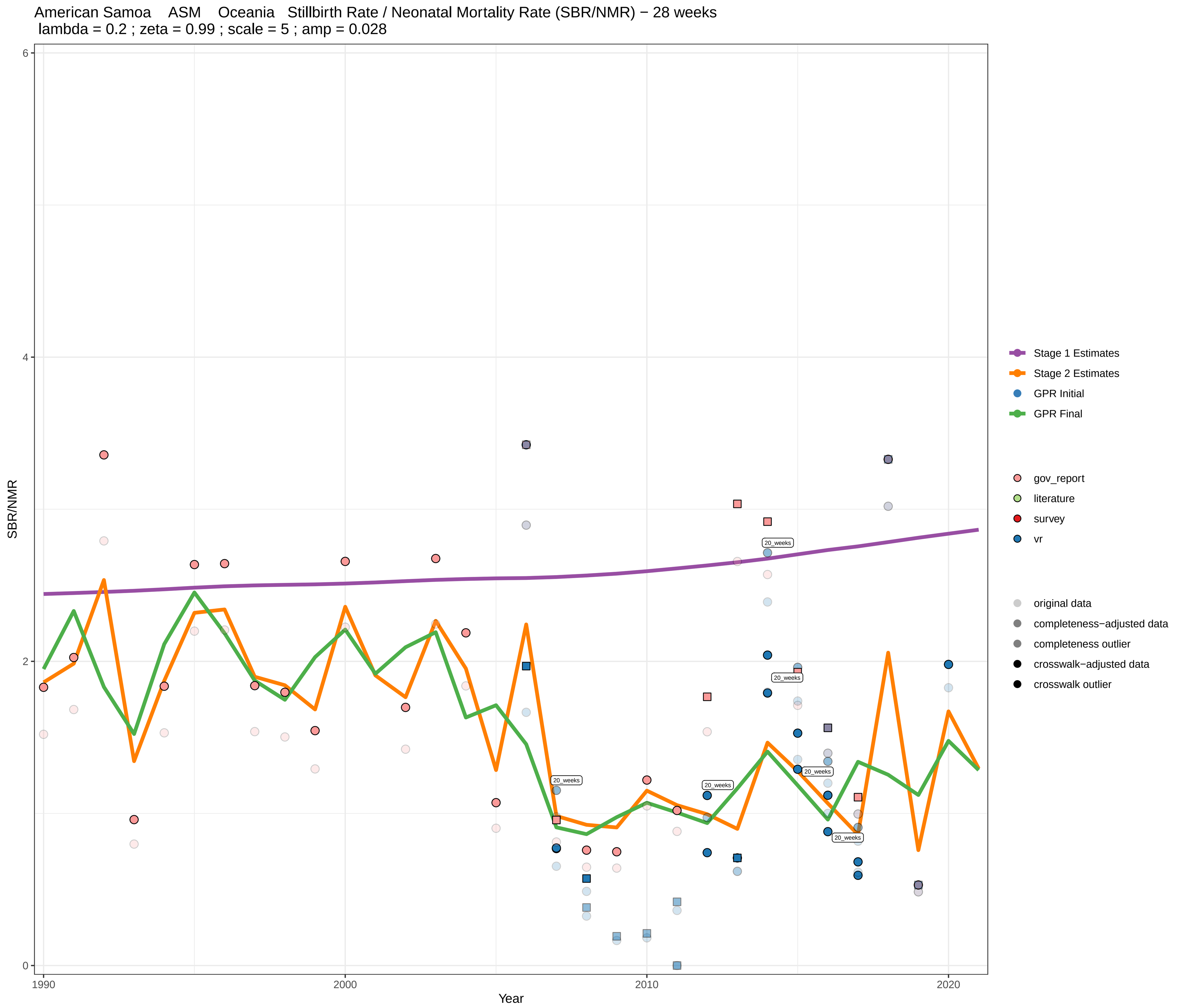

Definition	Years	Outlier	Source.Type	Title
20_weeks	2007, 2012, 2014−2017	0	VR	United States NVSS Custom Fetal Death Data
28_weeks	1990−2000, 2002−2005, 2008	0	Statistical Report	American Samoa Statistical Yearbook
28_weeks	2006, 2007, 2012−2017	0	VR	United States NVSS Custom Fetal Death Data
28_weeks	2009−2011, 2018, 2019	0	Statistical Report	American Samoa Statistical Yearbook
28_weeks	2020	0	VR	United Nations Demographic Yearbook
20_weeks	2011	1	VR	United States NVSS Custom Fetal Death Data
28_weeks	2006	1	Statistical Report	American Samoa Statistical Yearbook
28_weeks	2008−2011	1	VR	United States NVSS Custom Fetal Death Data
28_weeks	2016−2019	1	VR	United Nations Demographic Yearbook
20_weeks	2008, 2013	2	VR	United States NVSS Custom Fetal Death Data
28_weeks	2007	2	Statistical Report	American Samoa Statistical Yearbook
28_weeks	2012−2017	2	Statistical Report	American Samoa Statistical Yearbook

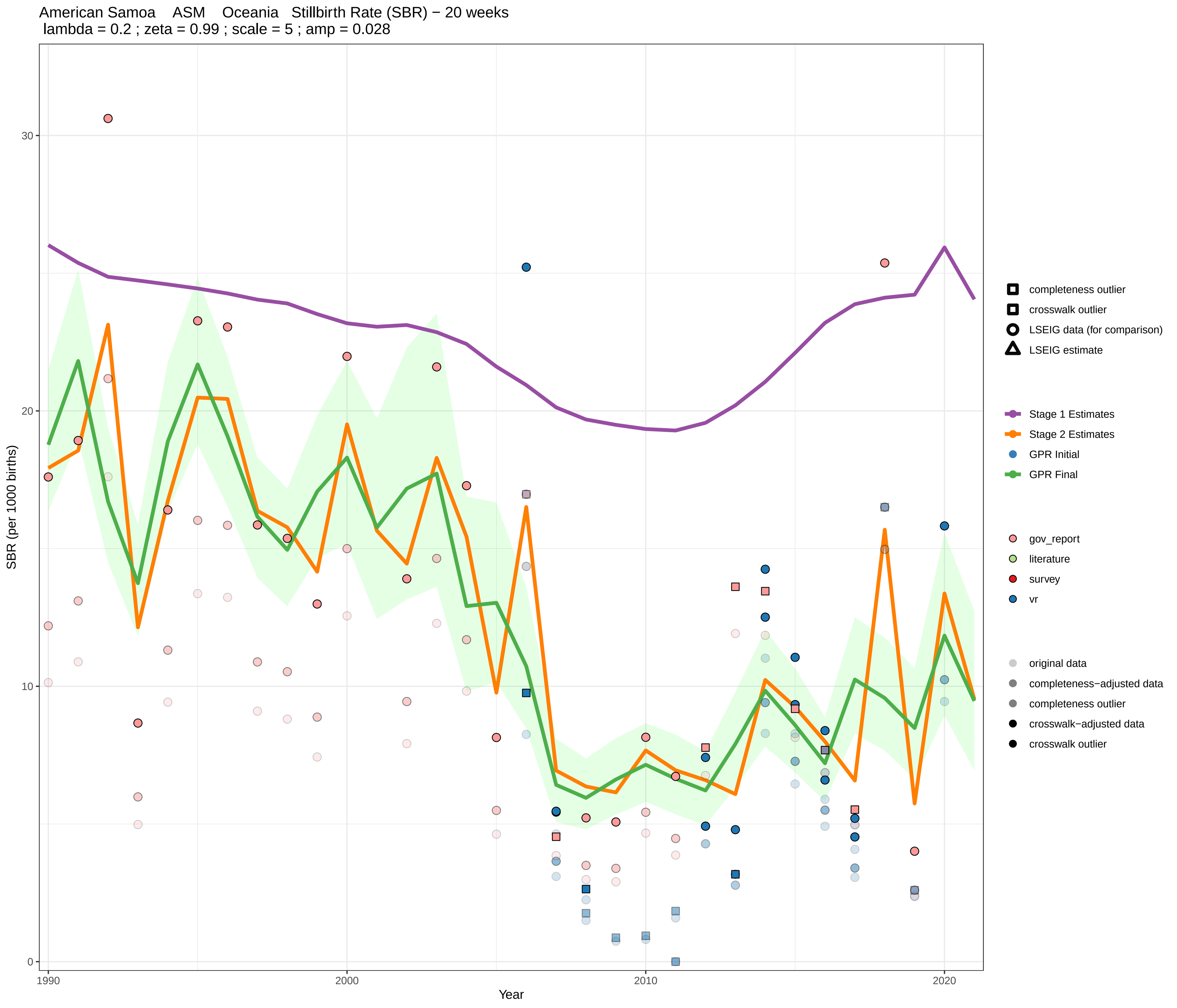

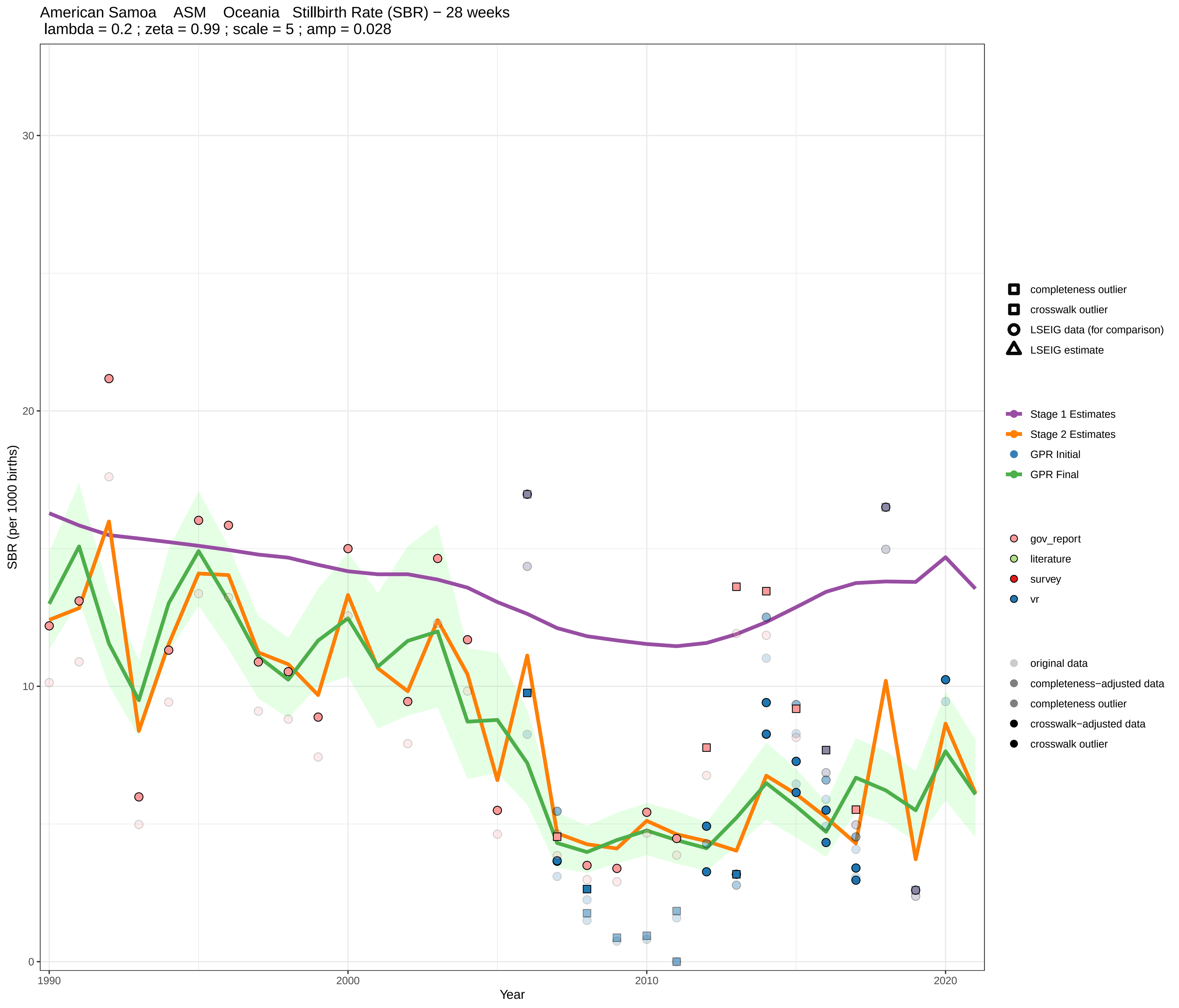

Year	Total Stillbirths (in thousands)	Total Stillbirths (in thousands)	SBR (per 1000 births)	SBR (per 1000 births)	SBR/NMR	SBR/NMR
	20 weeks	28 weeks	20 weeks	28 weeks	20 weeks	28 weeks
1990	0.0 (0.0 to 0.0)	0.0 (0.0 to 0.0)	18.8 (16.4 to 21.5)	13.0 (11.3 to 14.9)	2.82 (2.46 to 3.23)	1.95 (1.70 to 2.23)
1995	0.0 (0.0 to 0.0)	0.0 (0.0 to 0.0)	21.7 (18.8 to 24.8)	14.9 (12.9 to 17.1)	3.57 (3.09 to 4.09)	2.45 (2.13 to 2.81)
2000	0.0 (0.0 to 0.0)	0.0 (0.0 to 0.0)	18.3 (15.2 to 21.8)	12.5 (10.4 to 14.9)	3.24 (2.69 to 3.86)	2.21 (1.83 to 2.63)
2005	0.0 (0.0 to 0.0)	0.0 (0.0 to 0.0)	13.0 (10.1 to 16.7)	8.8 (6.8 to 11.2)	2.54 (1.98 to 3.25)	1.71 (1.33 to 2.19)
2010	0.0 (0.0 to 0.0)	0.0 (0.0 to 0.0)	7.2 (5.8 to 8.7)	4.8 (3.9 to 5.8)	1.61 (1.30 to 1.95)	1.07 (0.87 to 1.30)
2015	0.0 (0.0 to 0.0)	0.0 (0.0 to 0.0)	8.6 (6.9 to 10.7)	5.6 (4.5 to 7.0)	1.80 (1.44 to 2.24)	1.18 (0.95 to 1.47)
2020	0.0 (0.0 to 0.0)	0.0 (0.0 to 0.0)	11.8 (8.9 to 15.6)	7.6 (5.9 to 9.8)	2.29 (1.73 to 3.01)	1.48 (1.13 to 1.90)
2021	0.0 (0.0 to 0.0)	0.0 (0.0 to 0.0)	9.5 (7.0 to 12.7)	6.1 (4.5 to 8.1)	2.00 (1.47 to 2.69)	1.28 (0.95 to 1.71)

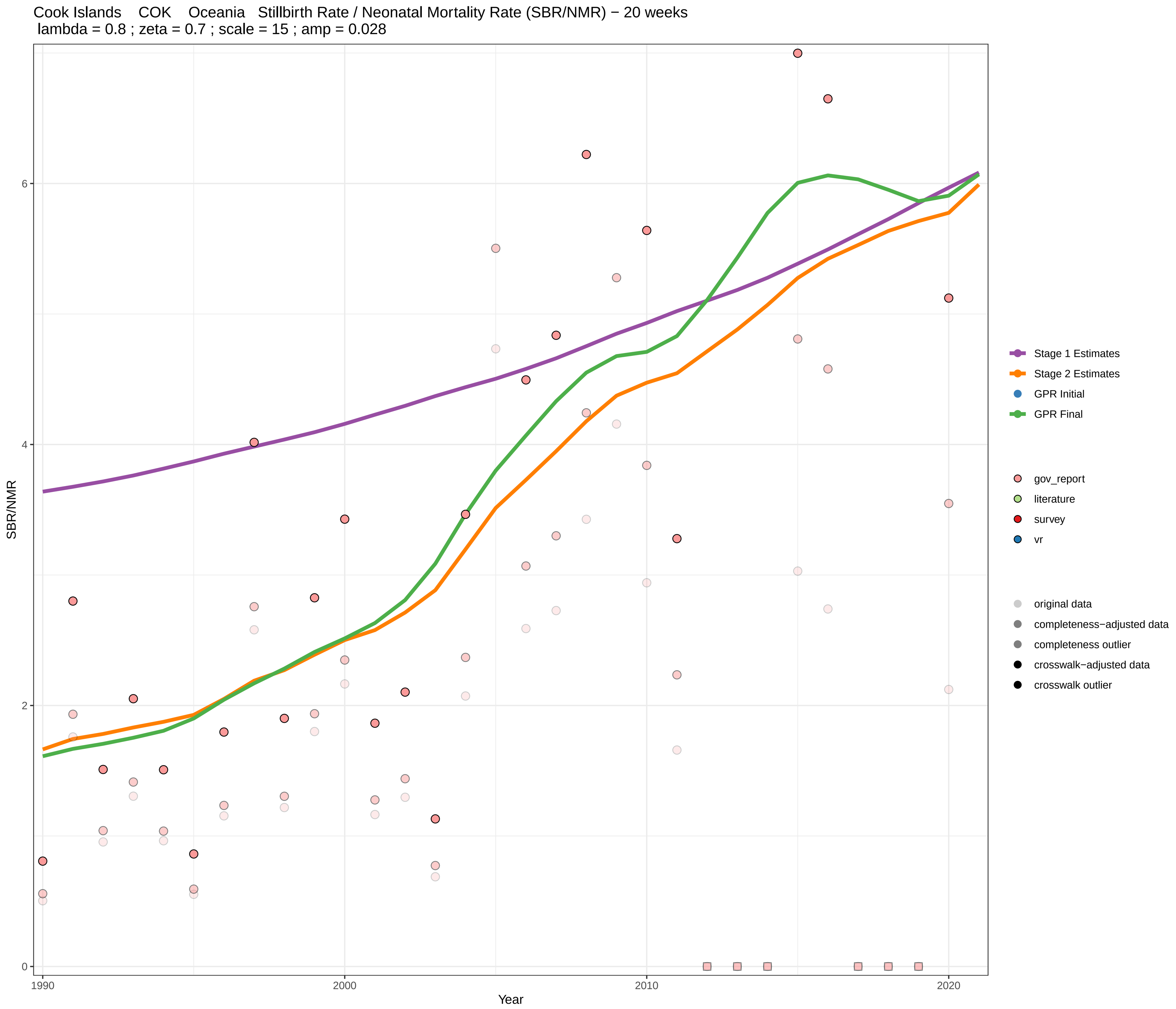

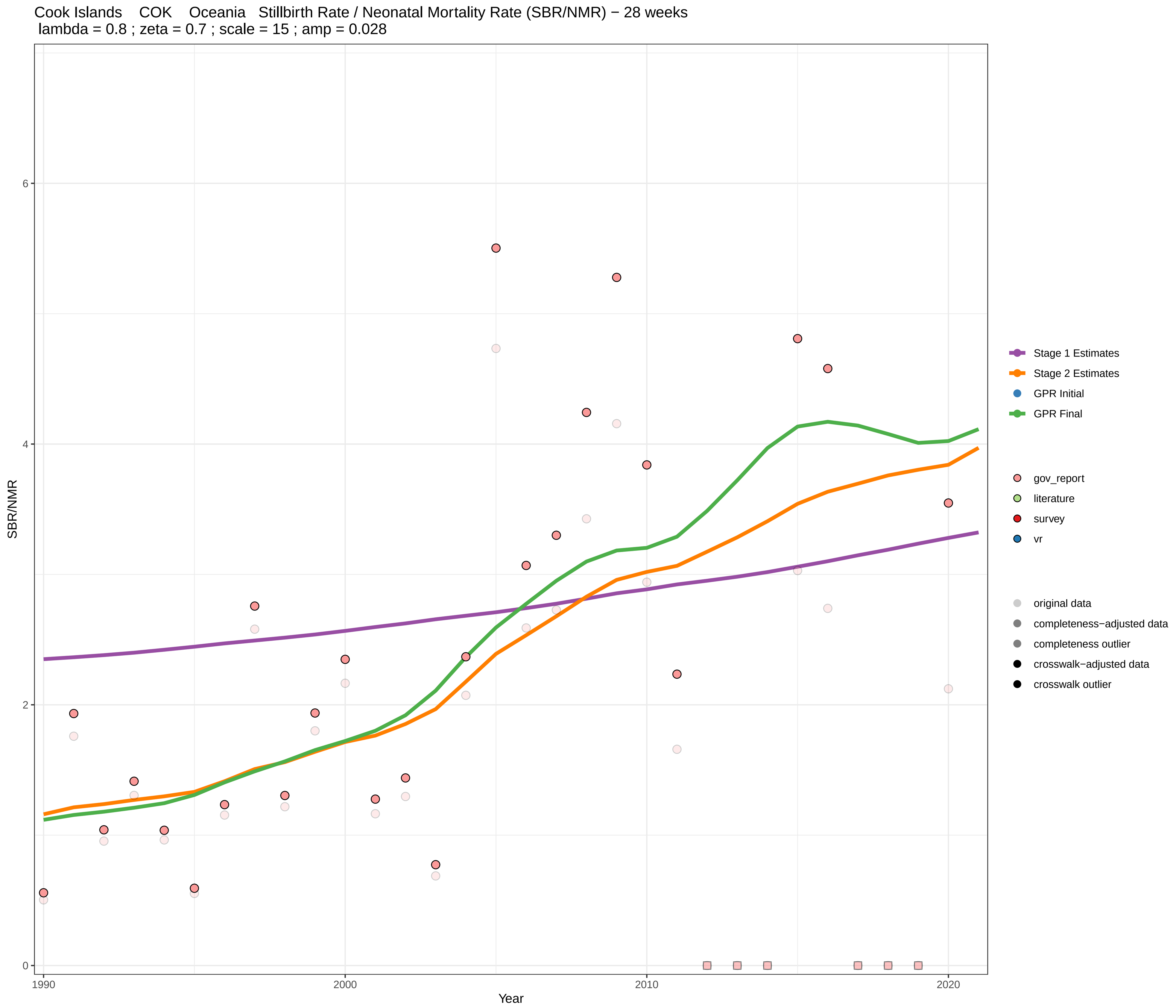

Definition	Years	Outlier	Source.Type	Title
28_weeks	1990−2009	0	Statistical Report	Cook Islands Ministry of Health Annual Statistical Bulletin
28_weeks	2010, 2011, 2015, 2016, 2020	0	Statistical Report	Cook Islands Ministry of Health Annual Statistical Bulletin
28_weeks	2012−2014, 2017−2019	1	Statistical Report	Cook Islands Ministry of Health Annual Statistical Bulletin

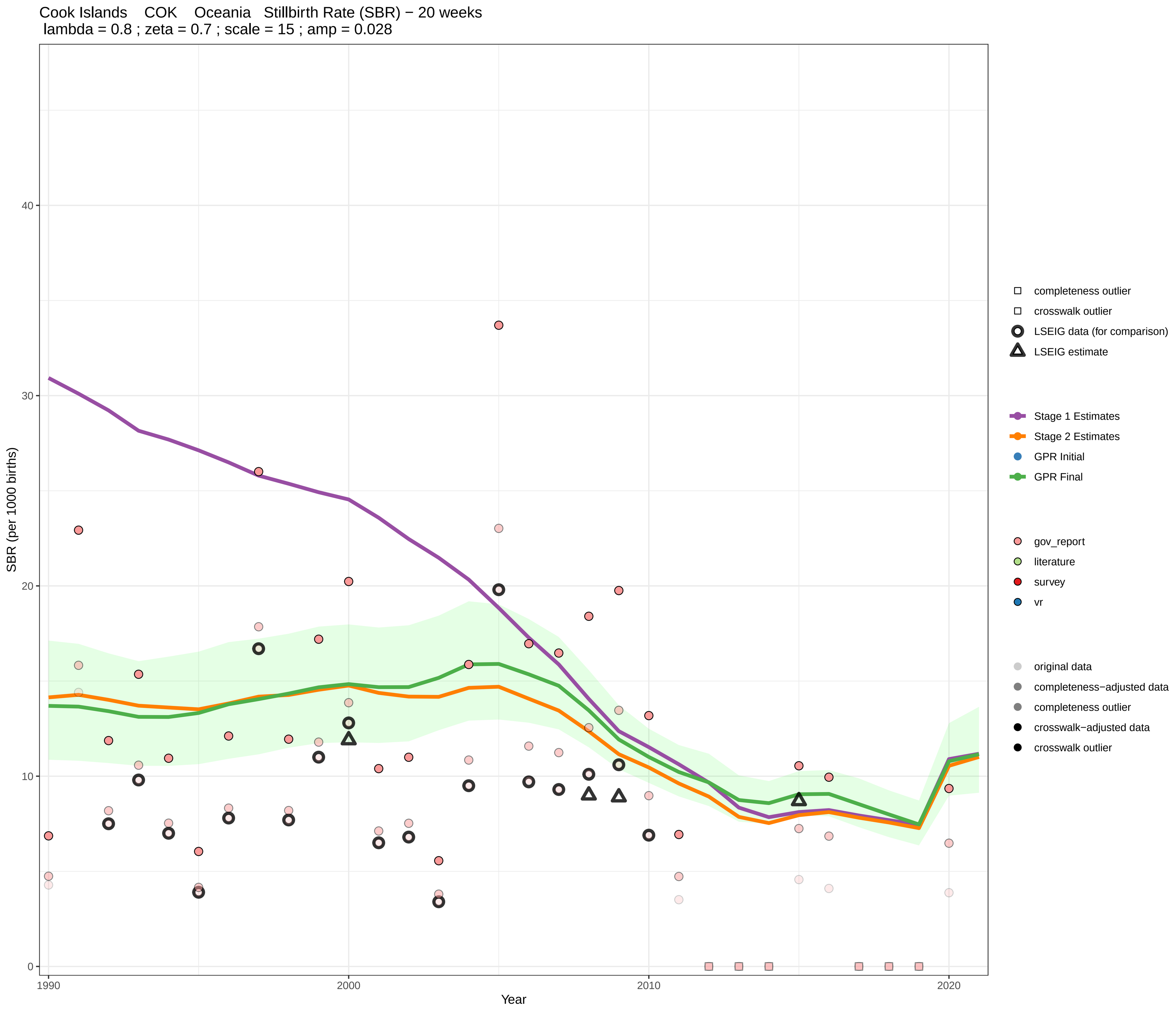

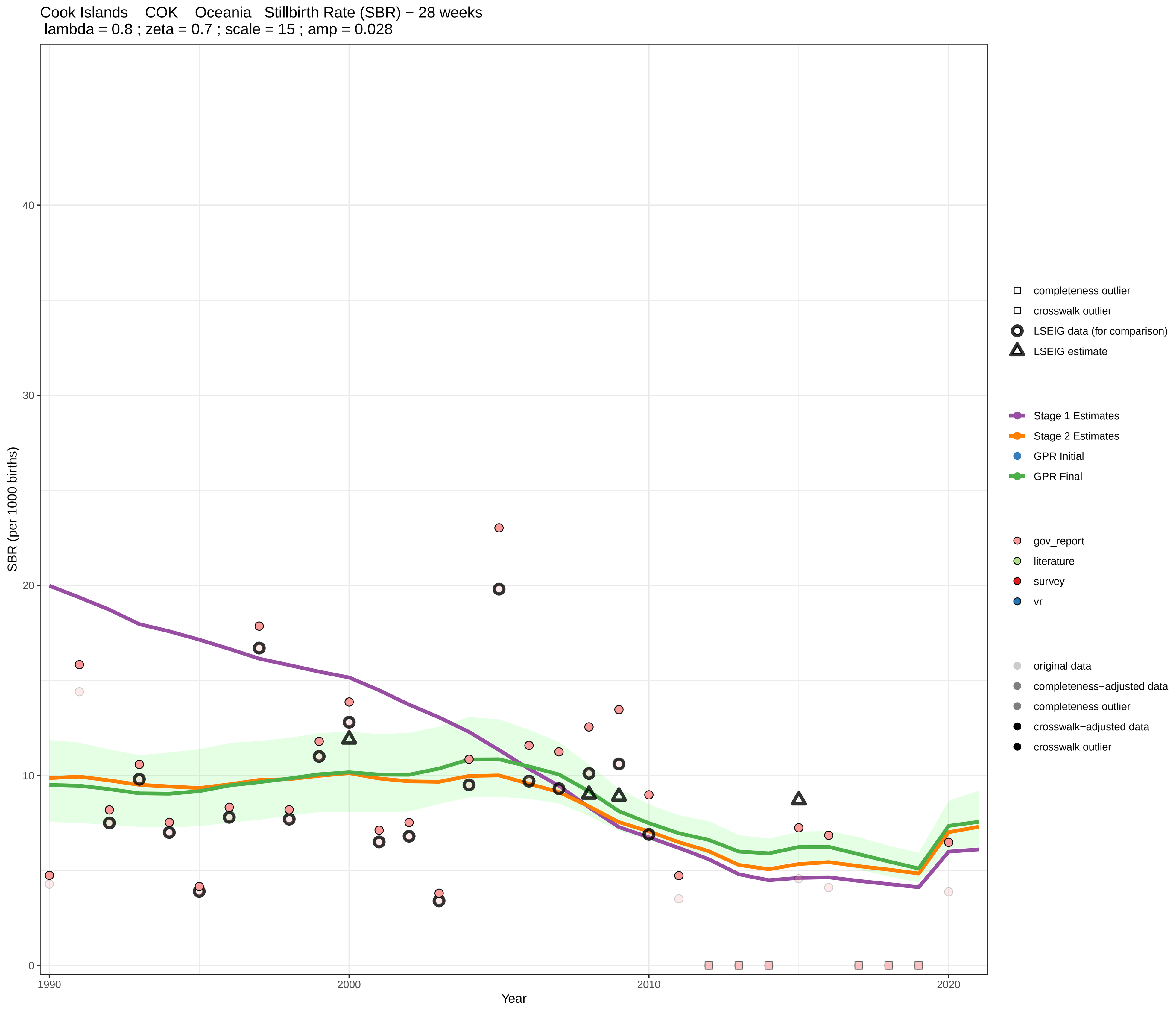

Year	Total Stillbirths (in thousands)	Total Stillbirths (in thousands)	SBR (per 1000 births)	SBR (per 1000 births)	SBR/NMR	SBR/NMR
	20 weeks	28 weeks	20 weeks	28 weeks	20 weeks	28 weeks
1990	0.0 (0.0 to 0.0)	0.0 (0.0 to 0.0)	13.7 (10.9 to 17.1)	9.5 (7.5 to 11.9)	1.61 (1.28 to 2.01)	1.12 (0.89 to 1.40)
1995	0.0 (0.0 to 0.0)	0.0 (0.0 to 0.0)	13.3 (10.6 to 16.5)	9.2 (7.3 to 11.4)	1.90 (1.52 to 2.36)	1.31 (1.05 to 1.62)
2000	0.0 (0.0 to 0.0)	0.0 (0.0 to 0.0)	14.8 (11.8 to 18.0)	10.2 (8.1 to 12.3)	2.51 (2.00 to 3.05)	1.72 (1.37 to 2.08)
2005	0.0 (0.0 to 0.0)	0.0 (0.0 to 0.0)	15.9 (13.0 to 19.0)	10.8 (8.9 to 13.0)	3.80 (3.10 to 4.55)	2.59 (2.12 to 3.10)
2010	0.0 (0.0 to 0.0)	0.0 (0.0 to 0.0)	11.0 (9.6 to 12.5)	7.5 (6.6 to 8.5)	4.71 (4.13 to 5.34)	3.20 (2.82 to 3.63)
2015	0.0 (0.0 to 0.0)	0.0 (0.0 to 0.0)	9.0 (7.9 to 10.3)	6.2 (5.5 to 7.0)	6.00 (5.26 to 6.81)	4.13 (3.64 to 4.67)
2020	0.0 (0.0 to 0.0)	0.0 (0.0 to 0.0)	10.8 (9.0 to 12.8)	7.3 (6.2 to 8.7)	5.91 (4.92 to 7.00)	4.02 (3.37 to 4.75)
2021	0.0 (0.0 to 0.0)	0.0 (0.0 to 0.0)	11.2 (9.1 to 13.6)	7.6 (6.2 to 9.2)	6.07 (4.97 to 7.43)	4.11 (3.38 to 5.00)

Fiji FJI Oceania Stillbirth Rate / Neonatal Mortality Rate (SBR/NMR) − 20 weeks
lambda = 0.8 ; zeta = 0.7 ; scale = 15 ; amp = 0.028

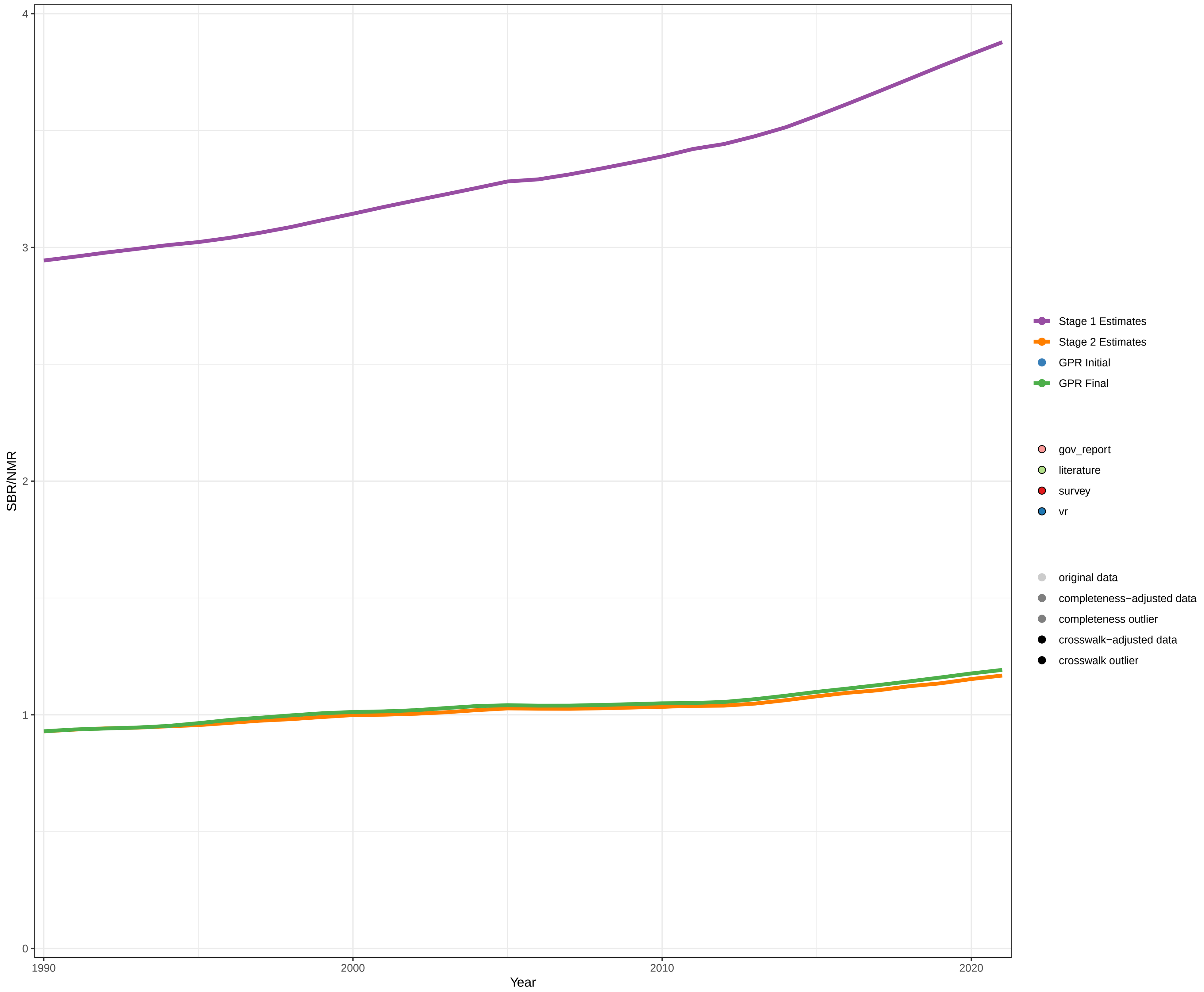

Fiji FJI Oceania Stillbirth Rate / Neonatal Mortality Rate (SBR/NMR) − 28 weeks
lambda = 0.8 ; zeta = 0.7 ; scale = 15 ; amp = 0.028

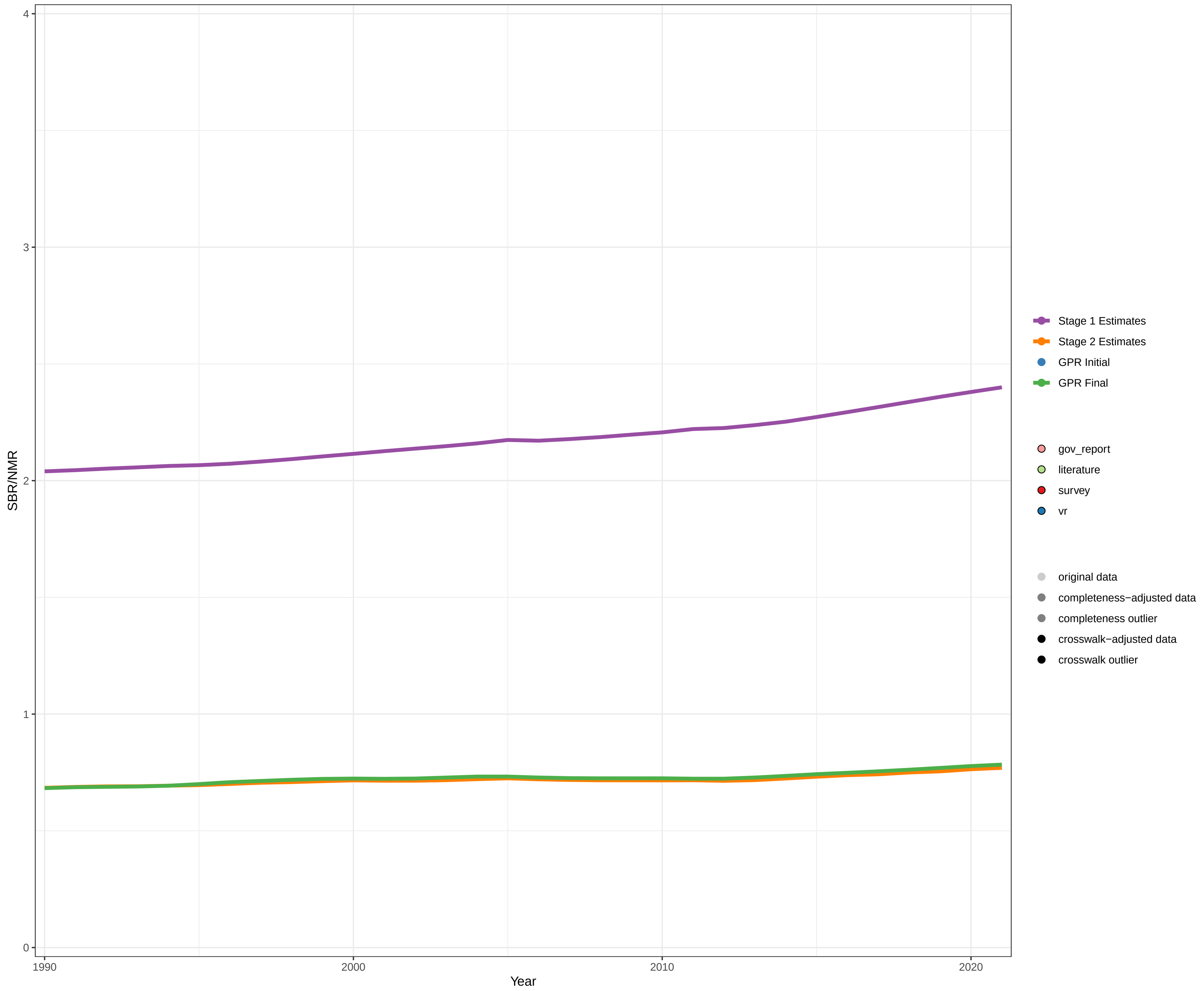

Definition	Years	Outlier	Source.Type	Title

Fiji FJI Oceania Stillbirth Rate (SBR) − 20 weeks
lambda = 0.8 ; zeta = 0.7 ; scale = 15 ; amp = 0.028

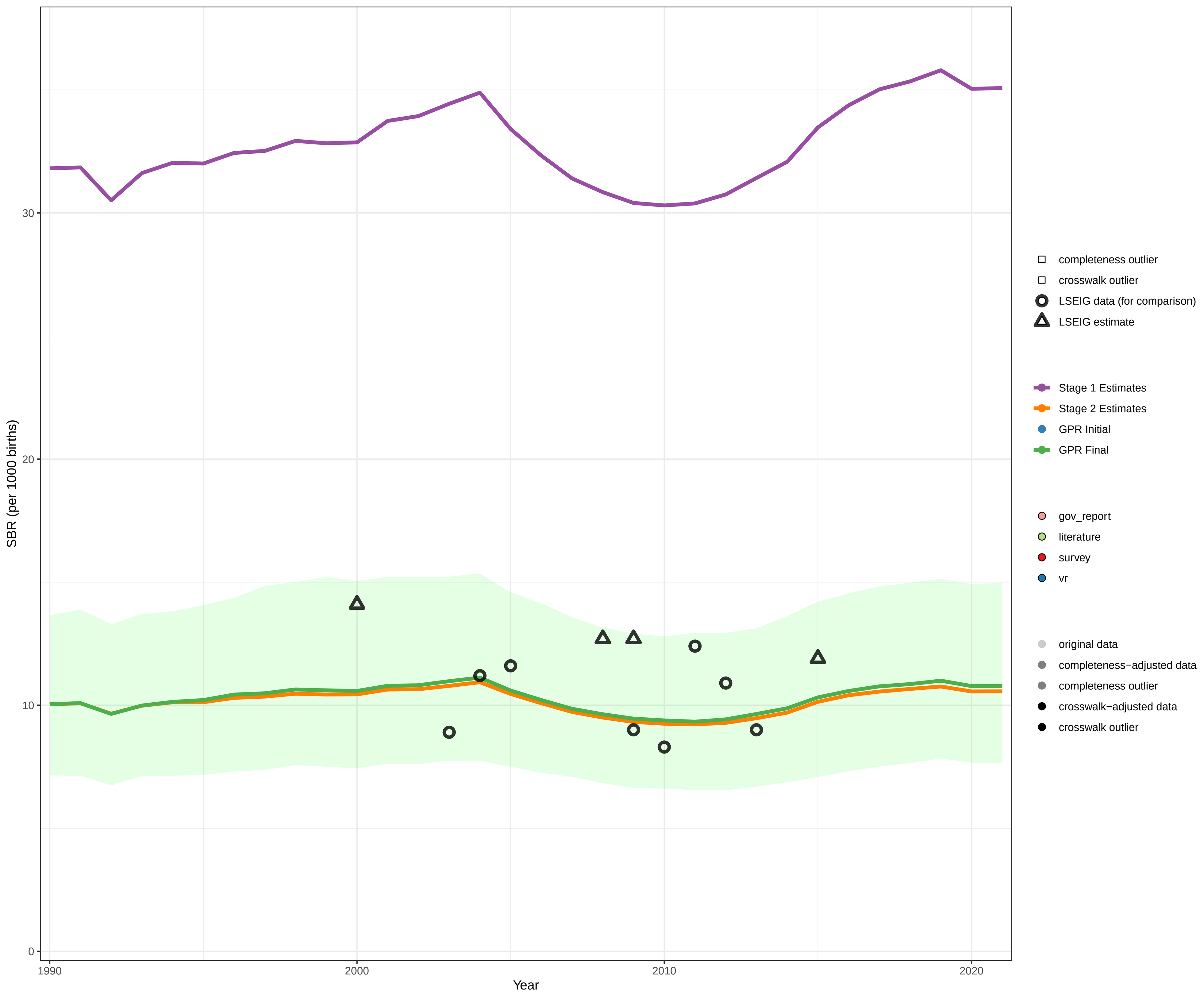

Fiji FJI Oceania Stillbirth Rate (SBR) − 28 weeks
lambda = 0.8 ; zeta = 0.7 ; scale = 15 ; amp = 0.028

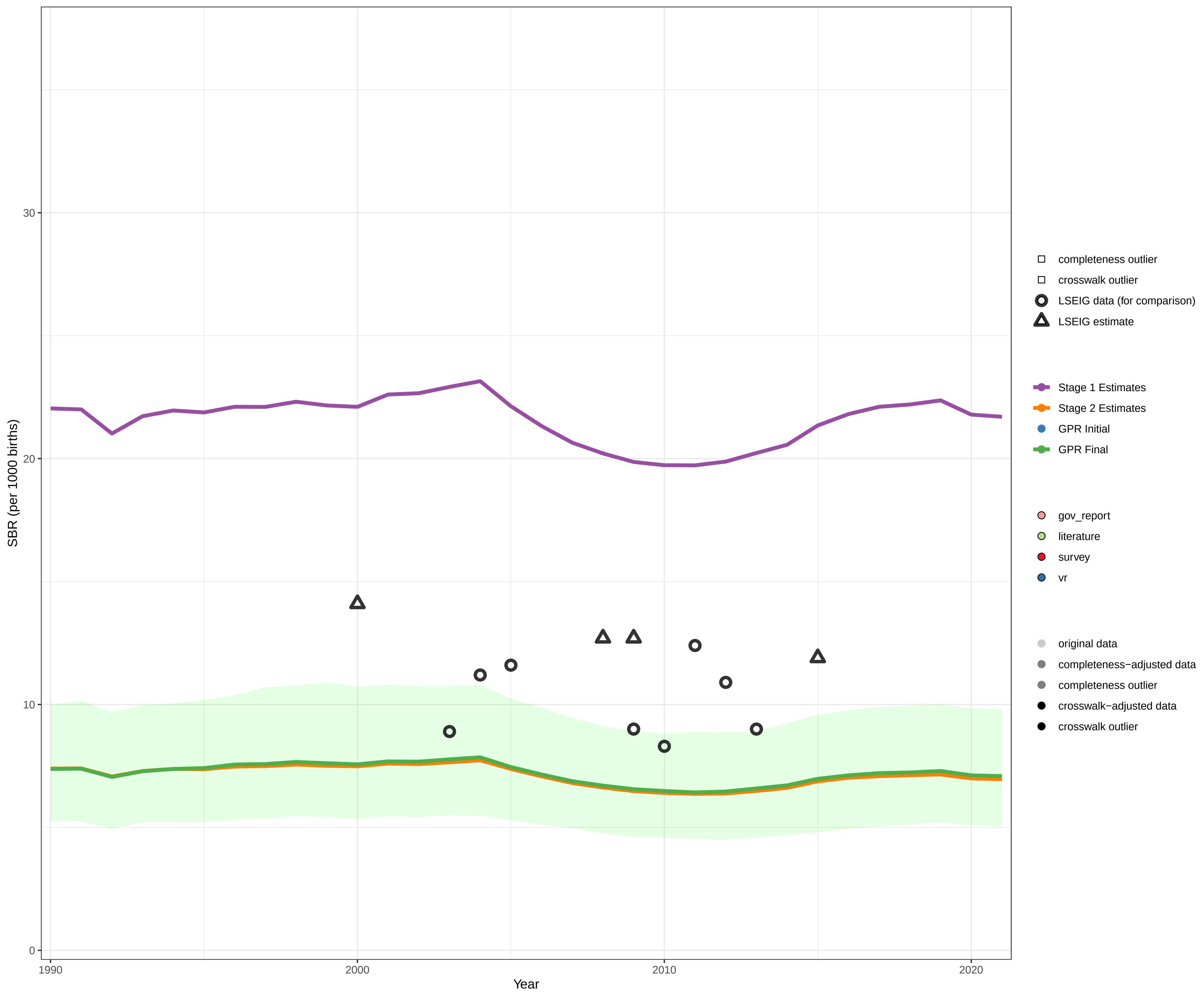

Year	Total Stillbirths (in thousands)	Total Stillbirths (in thousands)	SBR (per 1000 births)	SBR (per 1000 births)	SBR/NMR	SBR/NMR
	20 weeks	28 weeks	20 weeks	28 weeks	20 weeks	28 weeks
1990	0.2 (0.1 to 0.3)	0.1 (0.1 to 0.2)	10.0 (7.1 to 13.7)	7.4 (5.3 to 10.0)	0.93 (0.66 to 1.26)	0.68 (0.49 to 0.93)
1995	0.2 (0.1 to 0.3)	0.1 (0.1 to 0.2)	10.2 (7.2 to 14.1)	7.4 (5.2 to 10.2)	0.96 (0.68 to 1.33)	0.70 (0.49 to 0.96)
2000	0.2 (0.1 to 0.3)	0.1 (0.1 to 0.2)	10.6 (7.4 to 15.1)	7.6 (5.3 to 10.7)	1.01 (0.71 to 1.44)	0.72 (0.51 to 1.03)
2005	0.2 (0.1 to 0.3)	0.1 (0.1 to 0.2)	10.6 (7.5 to 14.6)	7.5 (5.3 to 10.2)	1.04 (0.74 to 1.43)	0.73 (0.52 to 1.01)
2010	0.2 (0.1 to 0.3)	0.1 (0.1 to 0.2)	9.4 (6.6 to 12.8)	6.5 (4.6 to 8.8)	1.05 (0.74 to 1.43)	0.73 (0.51 to 0.99)
2015	0.2 (0.1 to 0.3)	0.1 (0.1 to 0.2)	10.3 (7.1 to 14.2)	7.0 (4.8 to 9.6)	1.10 (0.75 to 1.51)	0.74 (0.51 to 1.02)
2020	0.2 (0.1 to 0.3)	0.1 (0.1 to 0.2)	10.8 (7.7 to 14.9)	7.1 (5.1 to 9.8)	1.18 (0.84 to 1.63)	0.78 (0.55 to 1.07)
2021	0.2 (0.1 to 0.3)	0.1 (0.1 to 0.2)	10.8 (7.7 to 15.0)	7.1 (5.0 to 9.8)	1.19 (0.85 to 1.65)	0.78 (0.56 to 1.08)

Guam GUM Oceania Stillbirth Rate / Neonatal Mortality Rate (SBR/NMR) − 20 weeks
lambda = 0.2 ; zeta = 0.99 ; scale = 5 ; amp = 0.028

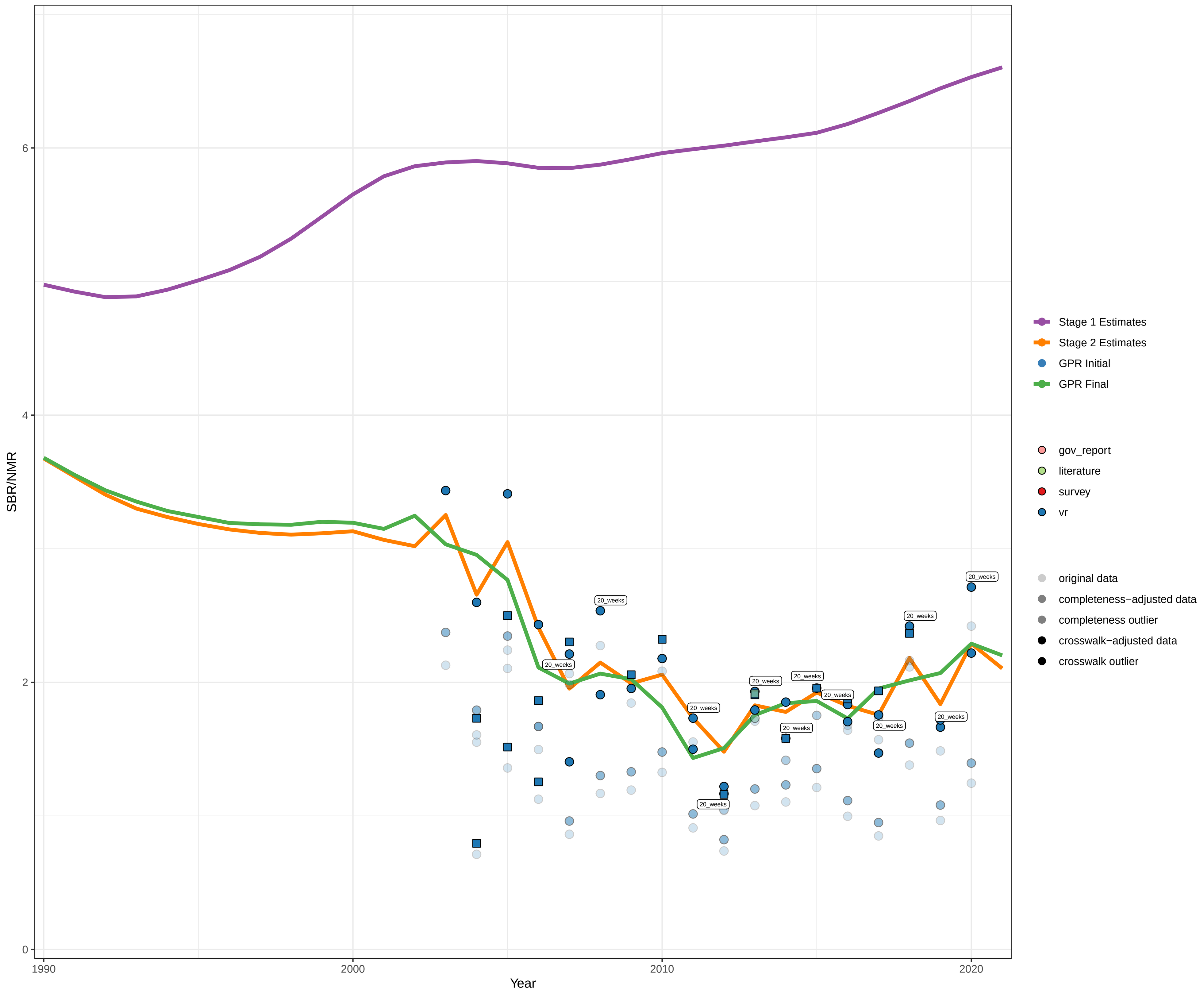

Guam GUM Oceania Stillbirth Rate / Neonatal Mortality Rate (SBR/NMR) − 28 weeks
lambda = 0.2 ; zeta = 0.99 ; scale = 5 ; amp = 0.028

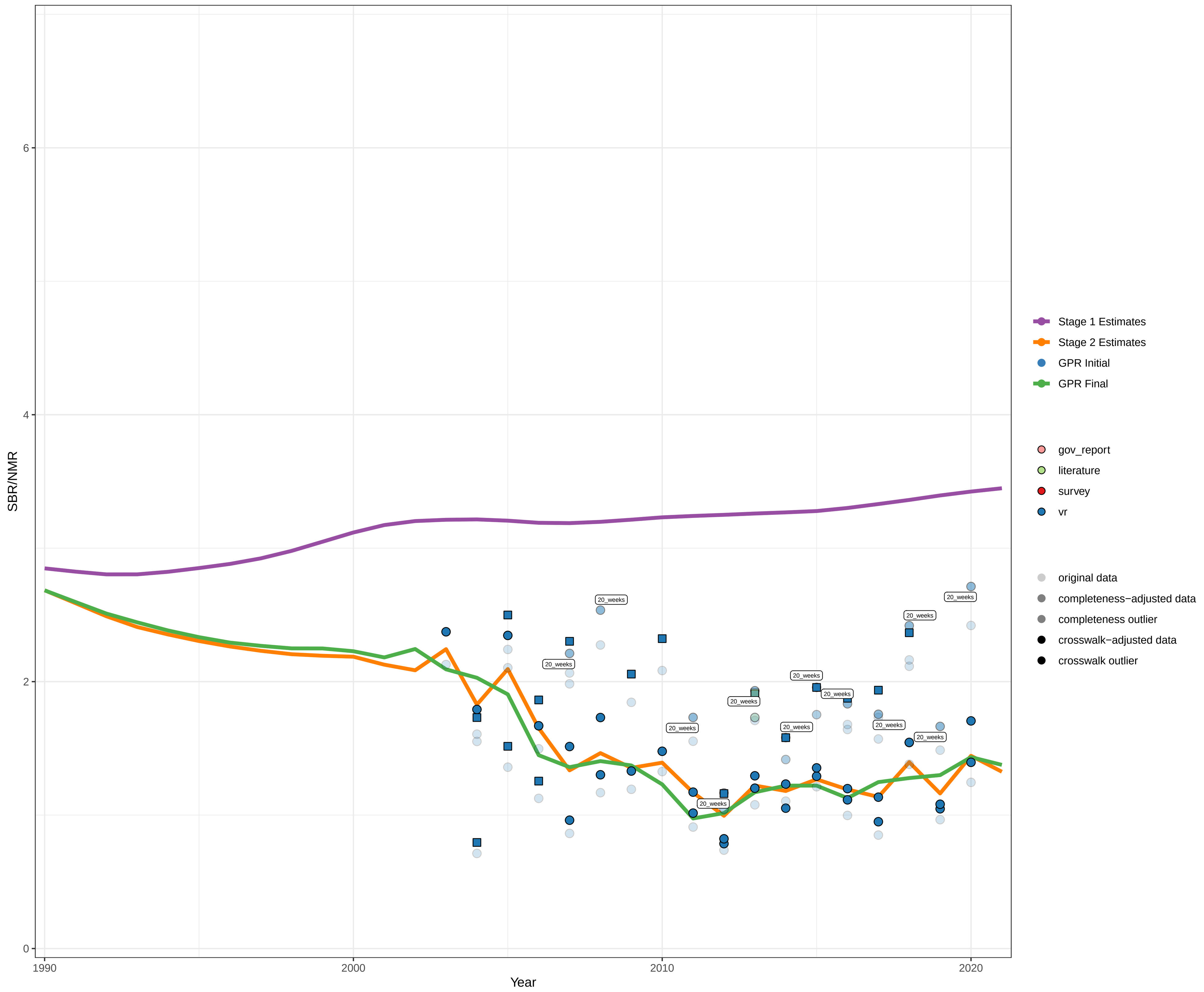

Definition	Years	Outlier	Source.Type	Title
20_weeks	2007, 2008, 2011−2020	0	VR	United States NVSS Custom Fetal Death Data
28_weeks	2003, 2007	0	VR	United Nations Demographic Yearbook
28_weeks	2004−2020	0	VR	United States NVSS Custom Fetal Death Data
20_weeks	2013	1	Sci Lit	United States Fetal and Perinatal Mortality 2013
28_weeks	2004−2006	2	VR	United States NVSS Custom Fetal Death Data
28_weeks	2004−2006, 2009, 2010, 2012−2018	2	VR	United Nations Demographic Yearbook

Guam GUM Oceania Stillbirth Rate (SBR) − 20 weeks
lambda = 0.2 ; zeta = 0.99 ; scale = 5 ; amp = 0.028

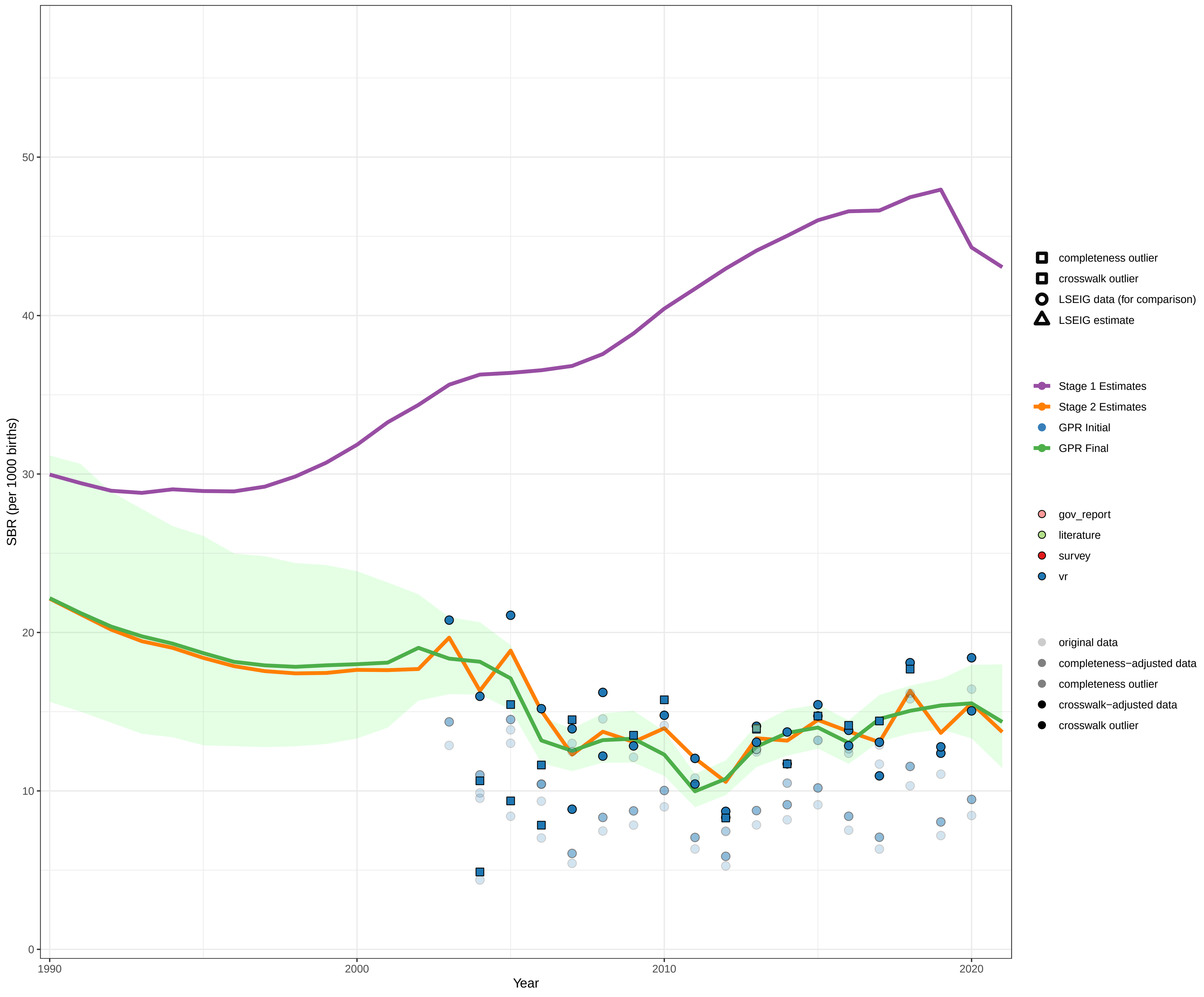

Guam GUM Oceania Stillbirth Rate (SBR) − 28 weeks
lambda = 0.2 ; zeta = 0.99 ; scale = 5 ; amp = 0.028

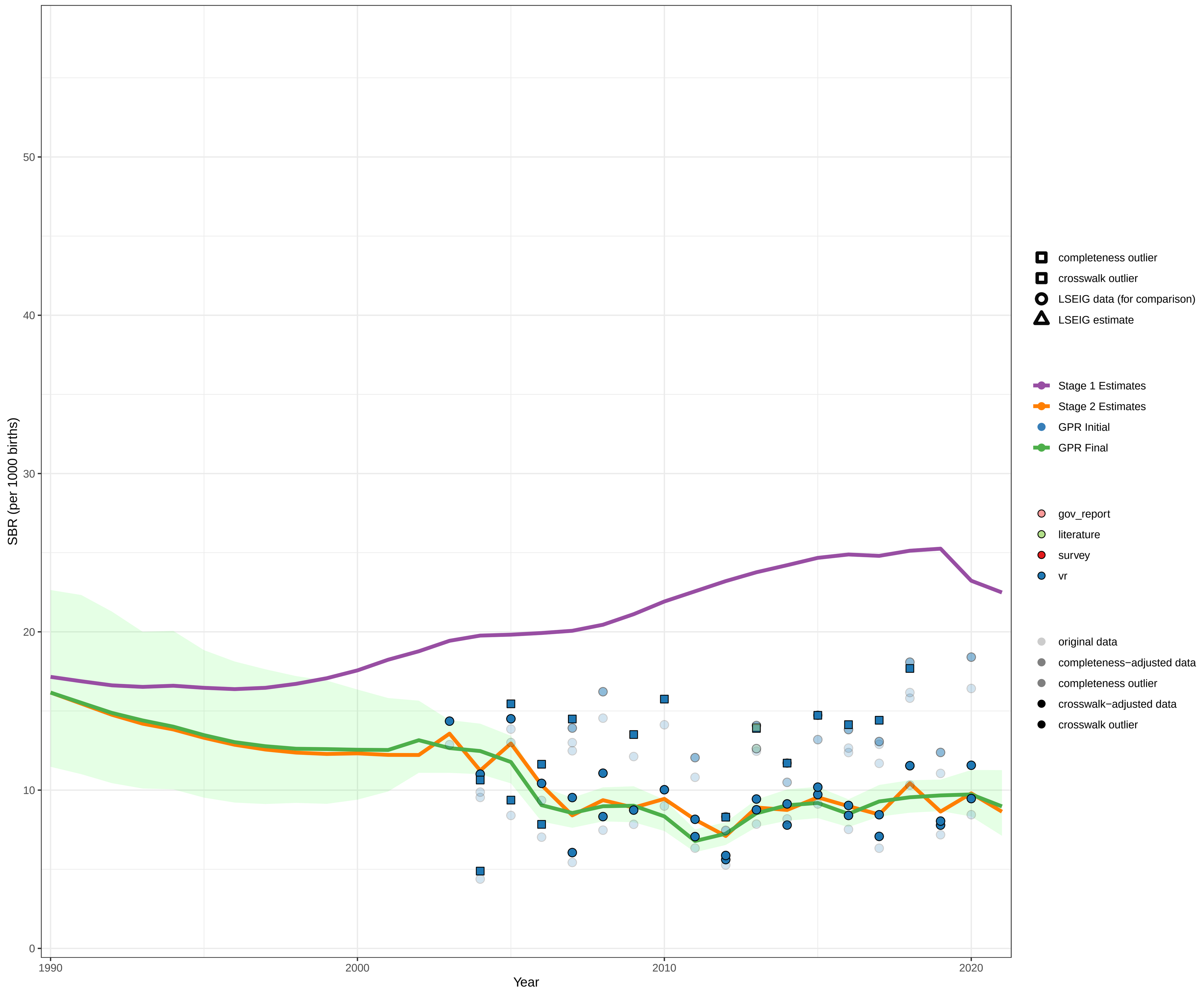

Year	Total Stillbirths (in thousands)	Total Stillbirths (in thousands)	SBR (per 1000 births)	SBR (per 1000 births)	SBR/NMR	SBR/NMR
	20 weeks	28 weeks	20 weeks	28 weeks	20 weeks	28 weeks
1990	0.1 (0.1 to 0.1)	0.1 (0.0 to 0.1)	22.2 (15.6 to 31.2)	16.2 (11.5 to 22.6)	3.68 (2.59 to 5.18)	2.68 (1.91 to 3.76)
1995	0.1 (0.1 to 0.1)	0.1 (0.0 to 0.1)	18.7 (12.9 to 26.1)	13.5 (9.5 to 18.8)	3.24 (2.23 to 4.52)	2.33 (1.65 to 3.26)
2000	0.1 (0.0 to 0.1)	0.0 (0.0 to 0.1)	18.0 (13.3 to 23.9)	12.6 (9.4 to 16.4)	3.19 (2.36 to 4.24)	2.23 (1.67 to 2.90)
2005	0.1 (0.1 to 0.1)	0.0 (0.0 to 0.0)	17.1 (15.1 to 19.2)	11.8 (10.4 to 13.4)	2.77 (2.45 to 3.11)	1.90 (1.69 to 2.17)
2010	0.0 (0.0 to 0.0)	0.0 (0.0 to 0.0)	12.3 (10.9 to 13.8)	8.3 (7.4 to 9.4)	1.81 (1.61 to 2.03)	1.23 (1.09 to 1.38)
2015	0.0 (0.0 to 0.1)	0.0 (0.0 to 0.0)	14.0 (12.7 to 15.4)	9.2 (8.2 to 10.2)	1.86 (1.68 to 2.05)	1.22 (1.09 to 1.36)
2020	0.0 (0.0 to 0.1)	0.0 (0.0 to 0.0)	15.5 (13.3 to 18.0)	9.7 (8.3 to 11.3)	2.29 (1.96 to 2.65)	1.43 (1.23 to 1.66)
2021	0.0 (0.0 to 0.0)	0.0 (0.0 to 0.0)	14.4 (11.4 to 18.0)	9.0 (7.1 to 11.3)	2.20 (1.75 to 2.76)	1.38 (1.09 to 1.73)

Kiribati KIR Oceania Stillbirth Rate / Neonatal Mortality Rate (SBR/NMR) − 20 weeks
lambda = 1 ; zeta = 0.6 ; scale = 15 ; amp = 0.028

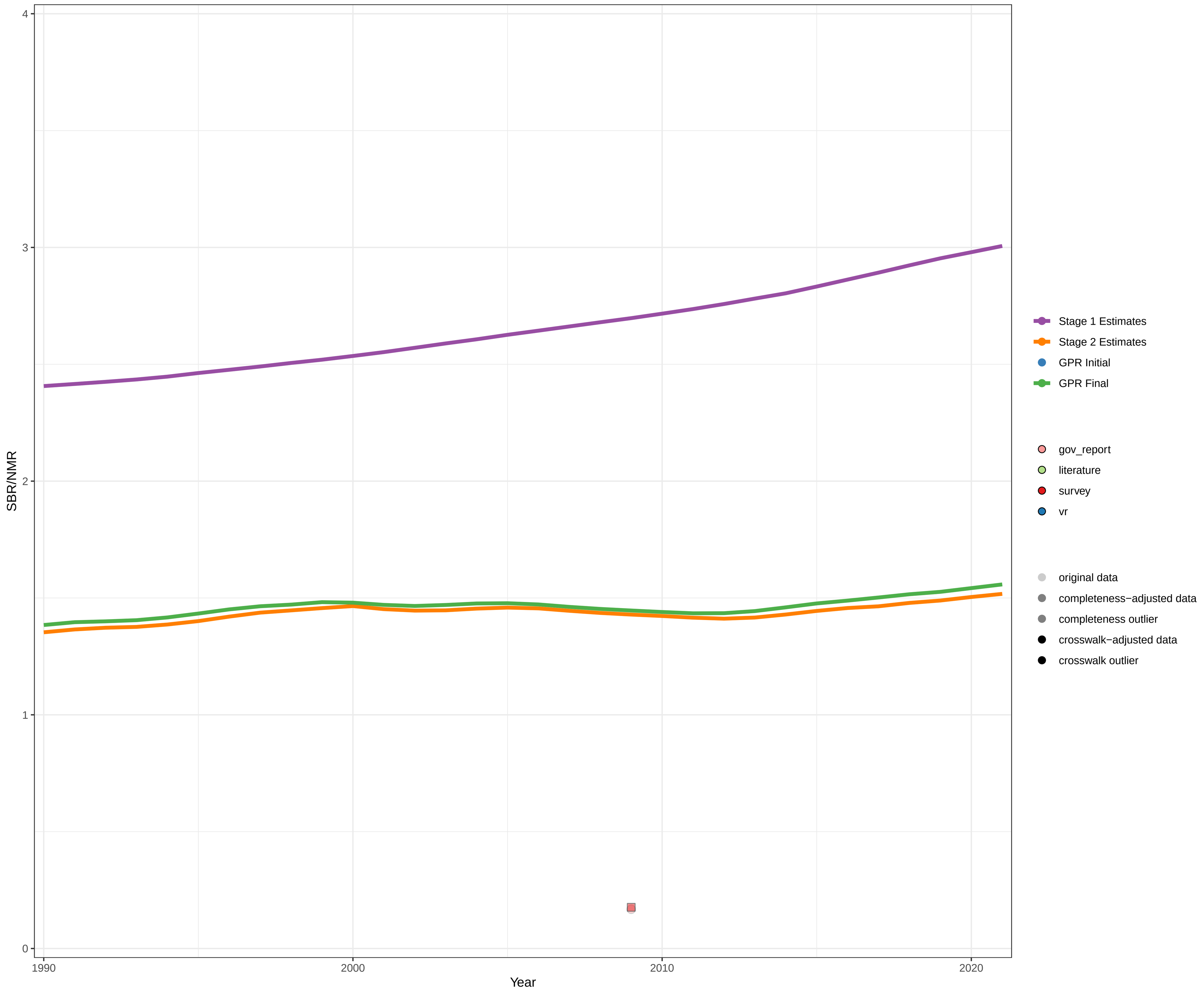

Kiribati KIR Oceania Stillbirth Rate / Neonatal Mortality Rate (SBR/NMR) − 28 weeks
lambda = 1 ; zeta = 0.6 ; scale = 15 ; amp = 0.028

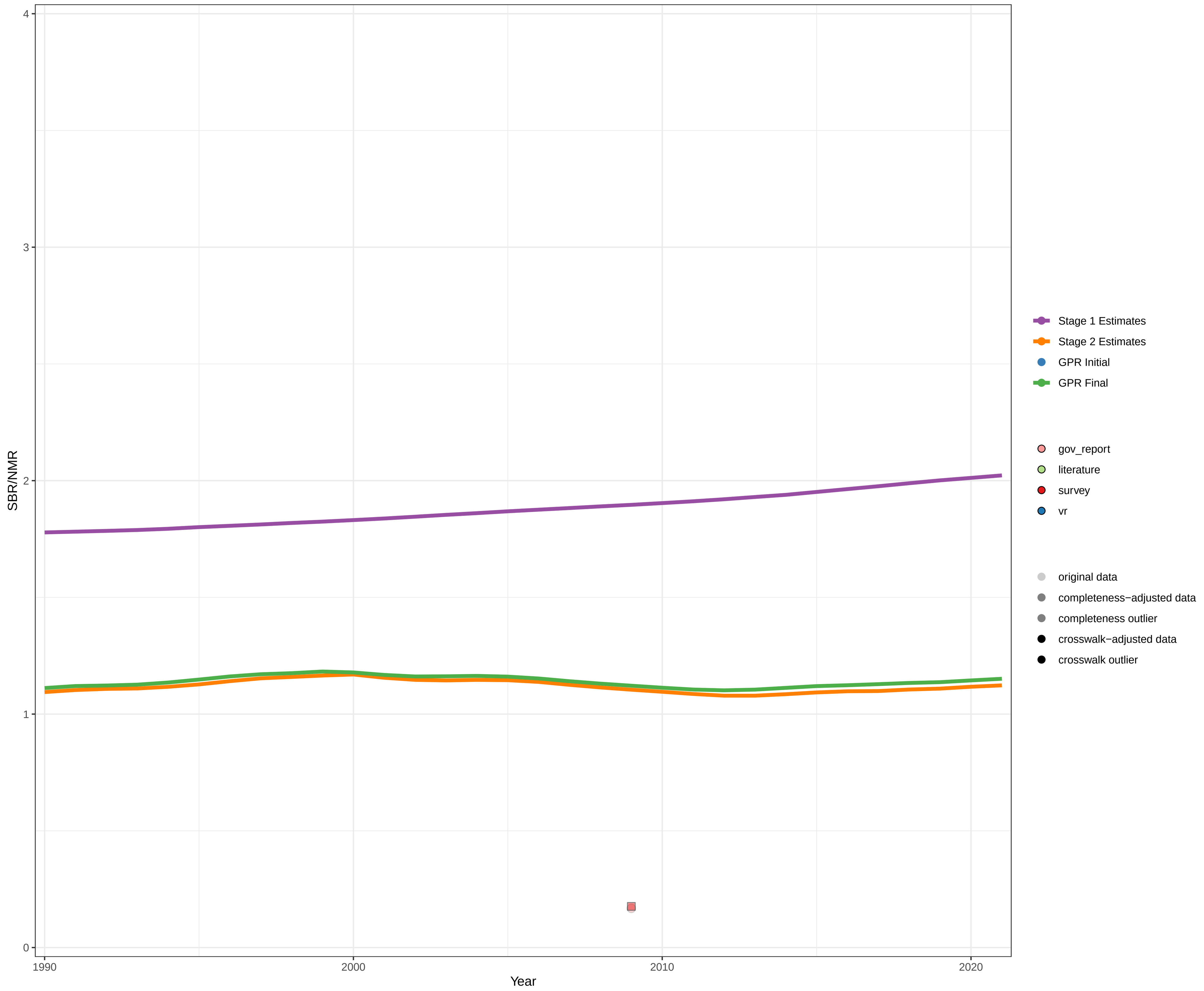

Definition	Years	Outlier	Source.Type	Title
28_weeks	2009	1	Survey	Kiribati Demographic and Health Survey 2009

Kiribati KIR Oceania Stillbirth Rate (SBR) − 20 weeks
lambda = 1 ; zeta = 0.6 ; scale = 15 ; amp = 0.028

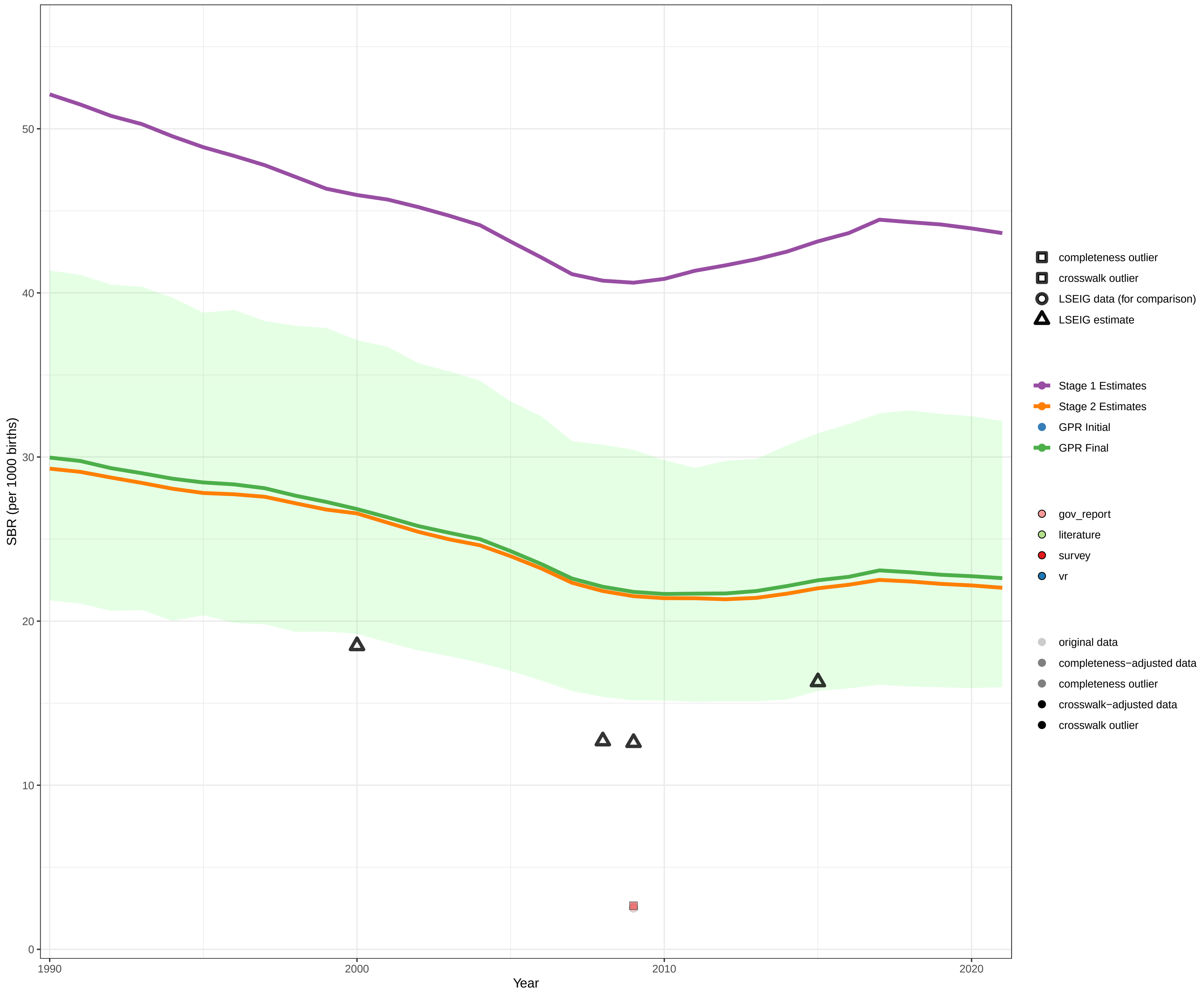

Kiribati KIR Oceania Stillbirth Rate (SBR) − 28 weeks
lambda = 1 ; zeta = 0.6 ; scale = 15 ; amp = 0.028

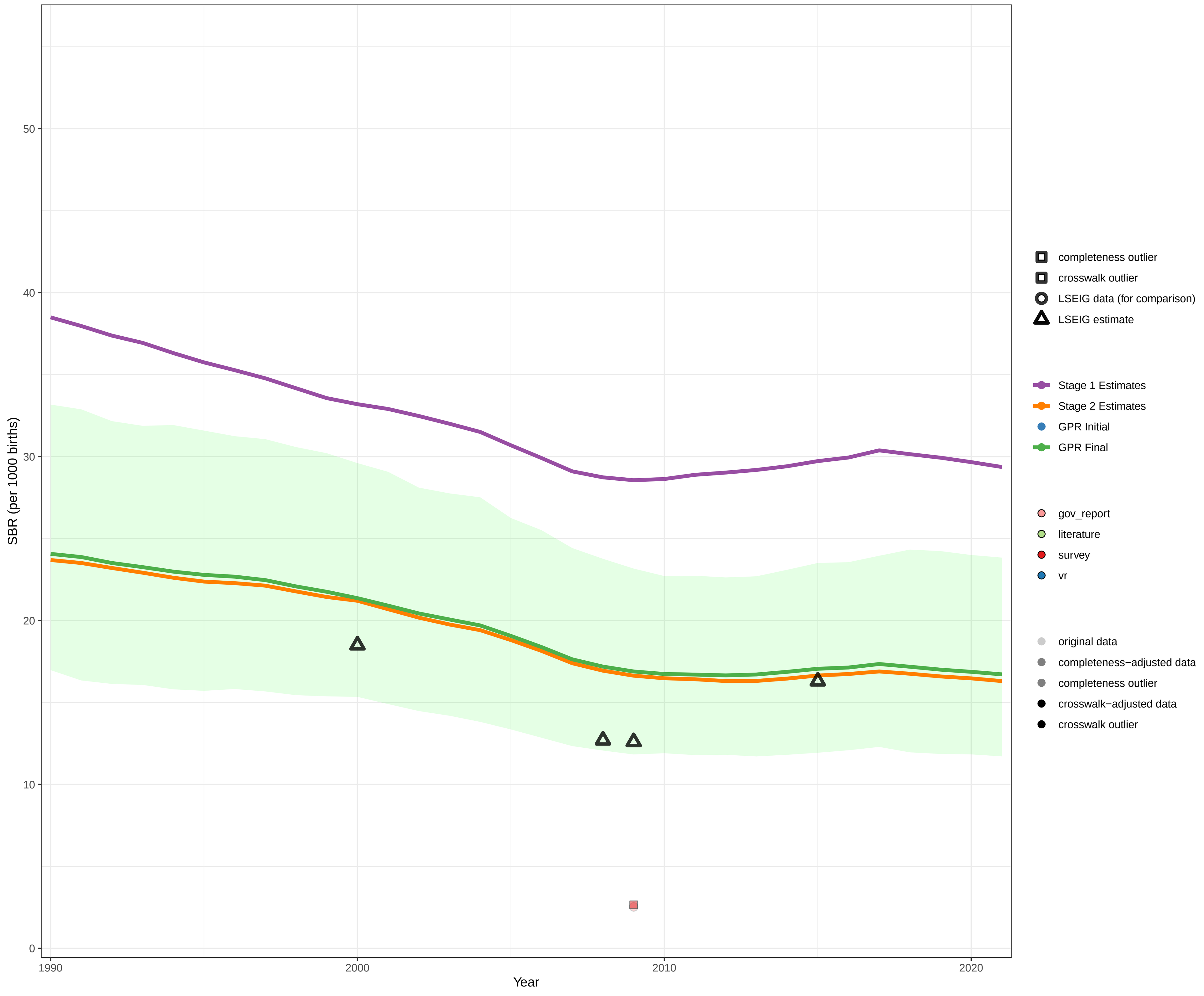

Year	Total Stillbirths (in thousands)	Total Stillbirths (in thousands)	SBR (per 1000 births)	SBR (per 1000 births)	SBR/NMR	SBR/NMR
	20 weeks	28 weeks	20 weeks	28 weeks	20 weeks	28 weeks
1990	0.1 (0.1 to 0.1)	0.1 (0.0 to 0.1)	30.0 (21.3 to 41.4)	24.1 (17.0 to 33.2)	1.38 (0.98 to 1.91)	1.11 (0.78 to 1.53)
1995	0.1 (0.1 to 0.1)	0.1 (0.0 to 0.1)	28.4 (20.4 to 38.8)	22.8 (15.7 to 31.6)	1.43 (1.03 to 1.95)	1.15 (0.79 to 1.59)
2000	0.1 (0.1 to 0.1)	0.1 (0.0 to 0.1)	26.8 (19.2 to 37.1)	21.4 (15.3 to 29.6)	1.48 (1.06 to 2.05)	1.18 (0.85 to 1.63)
2005	0.1 (0.0 to 0.1)	0.1 (0.0 to 0.1)	24.3 (17.0 to 33.4)	19.1 (13.4 to 26.3)	1.48 (1.03 to 2.03)	1.16 (0.81 to 1.60)
2010	0.1 (0.0 to 0.1)	0.0 (0.0 to 0.1)	21.7 (15.2 to 29.8)	16.7 (11.9 to 22.7)	1.44 (1.01 to 1.98)	1.11 (0.79 to 1.51)
2015	0.1 (0.0 to 0.1)	0.1 (0.0 to 0.1)	22.5 (15.7 to 31.4)	17.1 (11.9 to 23.5)	1.48 (1.03 to 2.06)	1.12 (0.78 to 1.54)
2020	0.1 (0.0 to 0.1)	0.1 (0.0 to 0.1)	22.7 (15.9 to 32.5)	16.9 (11.8 to 24.0)	1.54 (1.08 to 2.20)	1.14 (0.80 to 1.63)
2021	0.1 (0.0 to 0.1)	0.0 (0.0 to 0.1)	22.6 (16.0 to 32.2)	16.7 (11.7 to 23.8)	1.56 (1.10 to 2.22)	1.15 (0.81 to 1.64)

Marshall Islands MHL Oceania Stillbirth Rate / Neonatal Mortality Rate (SBR/NMR) − 20 weeks
lambda = 0.8 ; zeta = 0.7 ; scale = 15 ; amp = 0.028

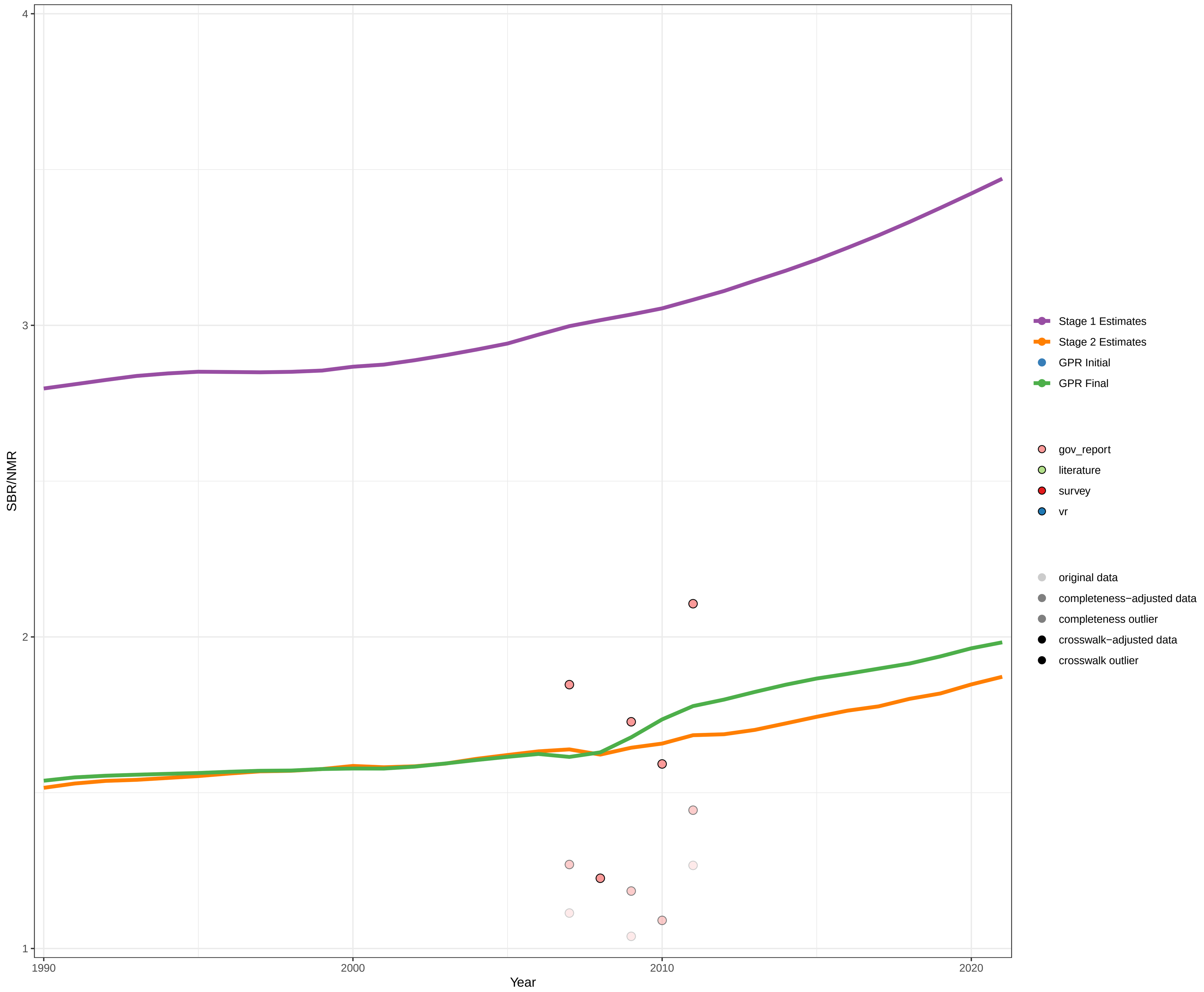

Marshall Islands MHL Oceania Stillbirth Rate / Neonatal Mortality Rate (SBR/NMR) − 28 weeks
lambda = 0.8 ; zeta = 0.7 ; scale = 15 ; amp = 0.028

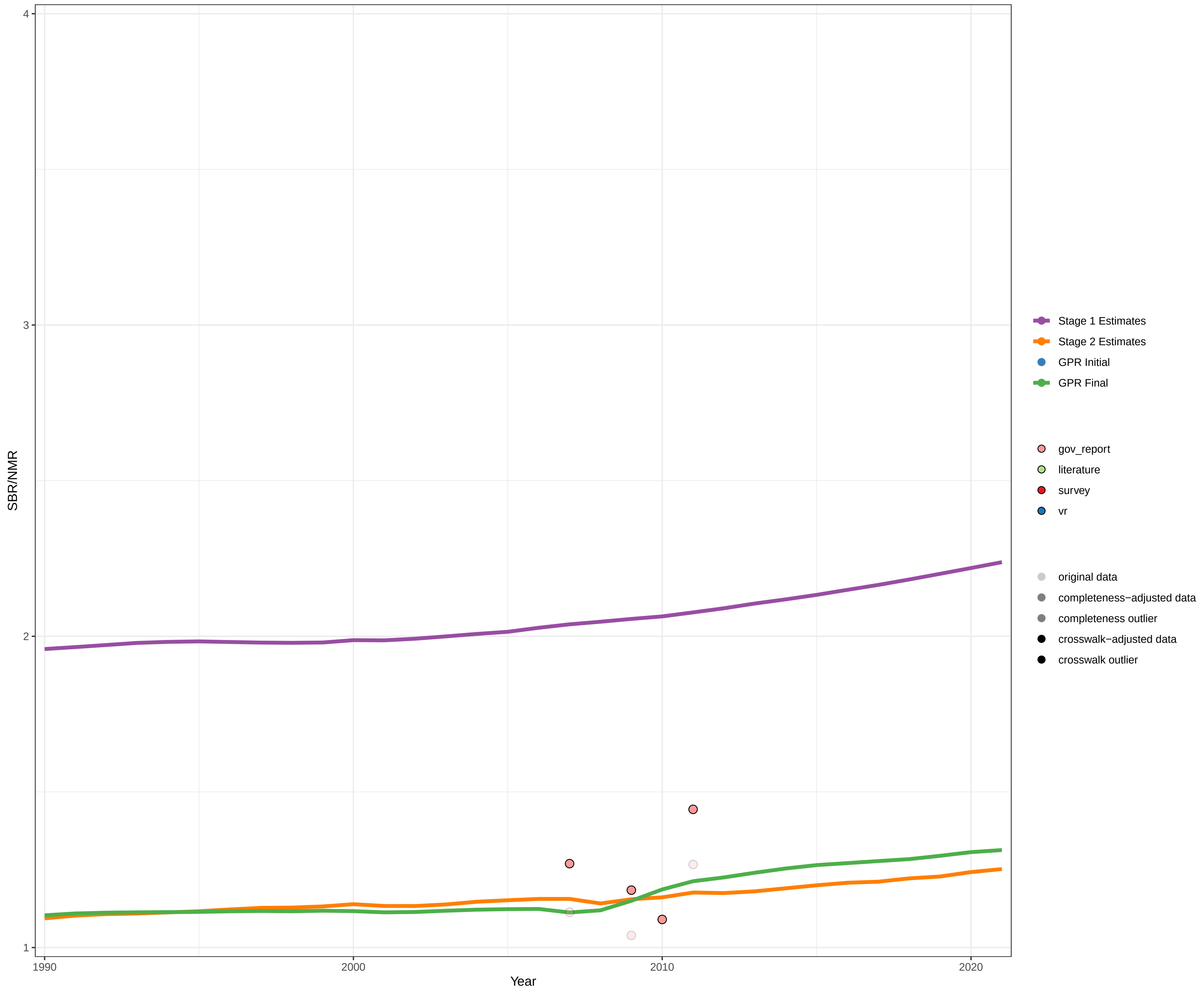

Definition	Years	Outlier	Source.Type	Title
28_weeks	2007−2011	0	Statistical Report	Marshall Islands Ministry of Health Annual Report

Marshall Islands MHL Oceania Stillbirth Rate (SBR) − 20 weeks
lambda = 0.8 ; zeta = 0.7 ; scale = 15 ; amp = 0.028

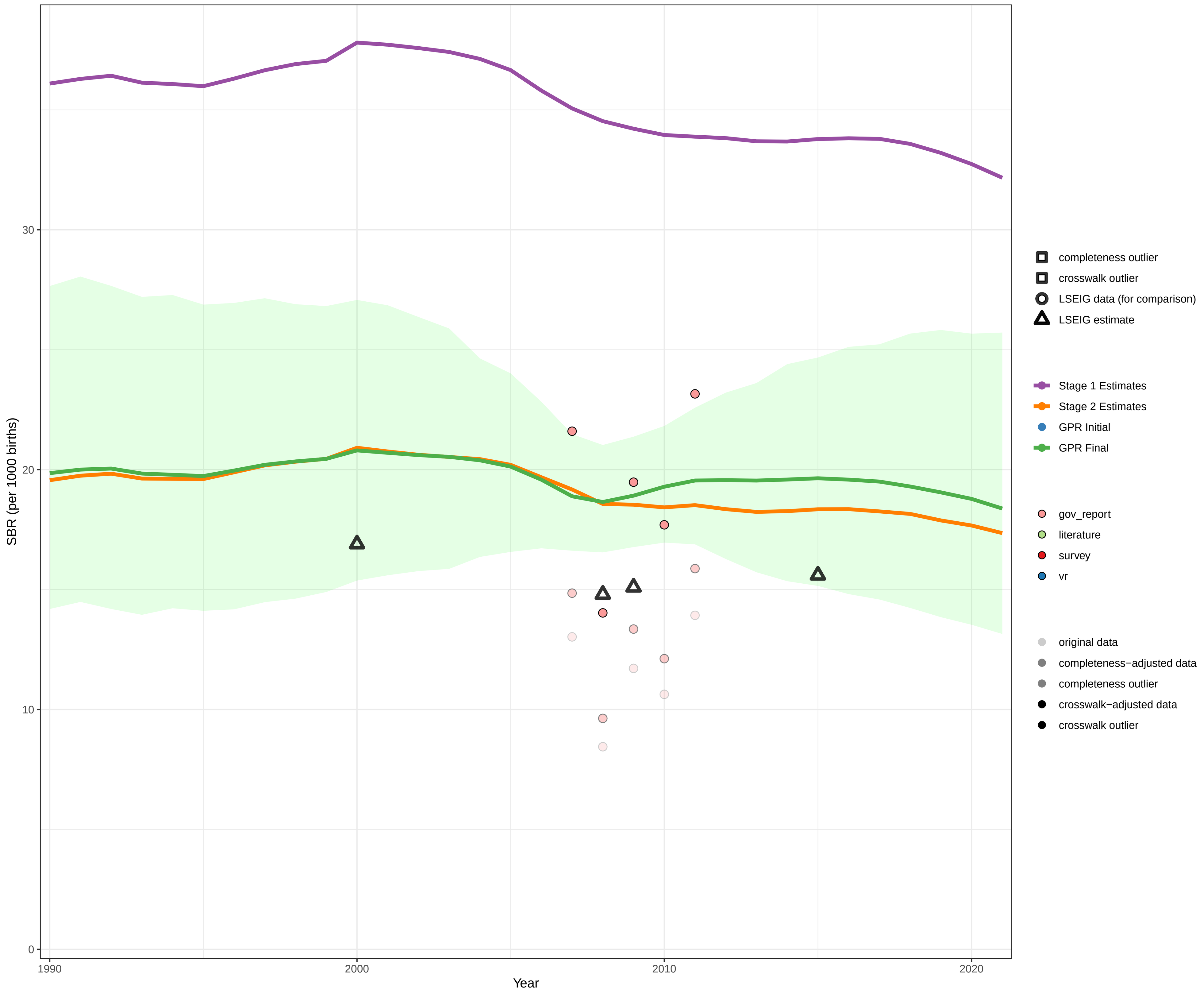

Marshall Islands MHL Oceania Stillbirth Rate (SBR) − 28 weeks
lambda = 0.8 ; zeta = 0.7 ; scale = 15 ; amp = 0.028

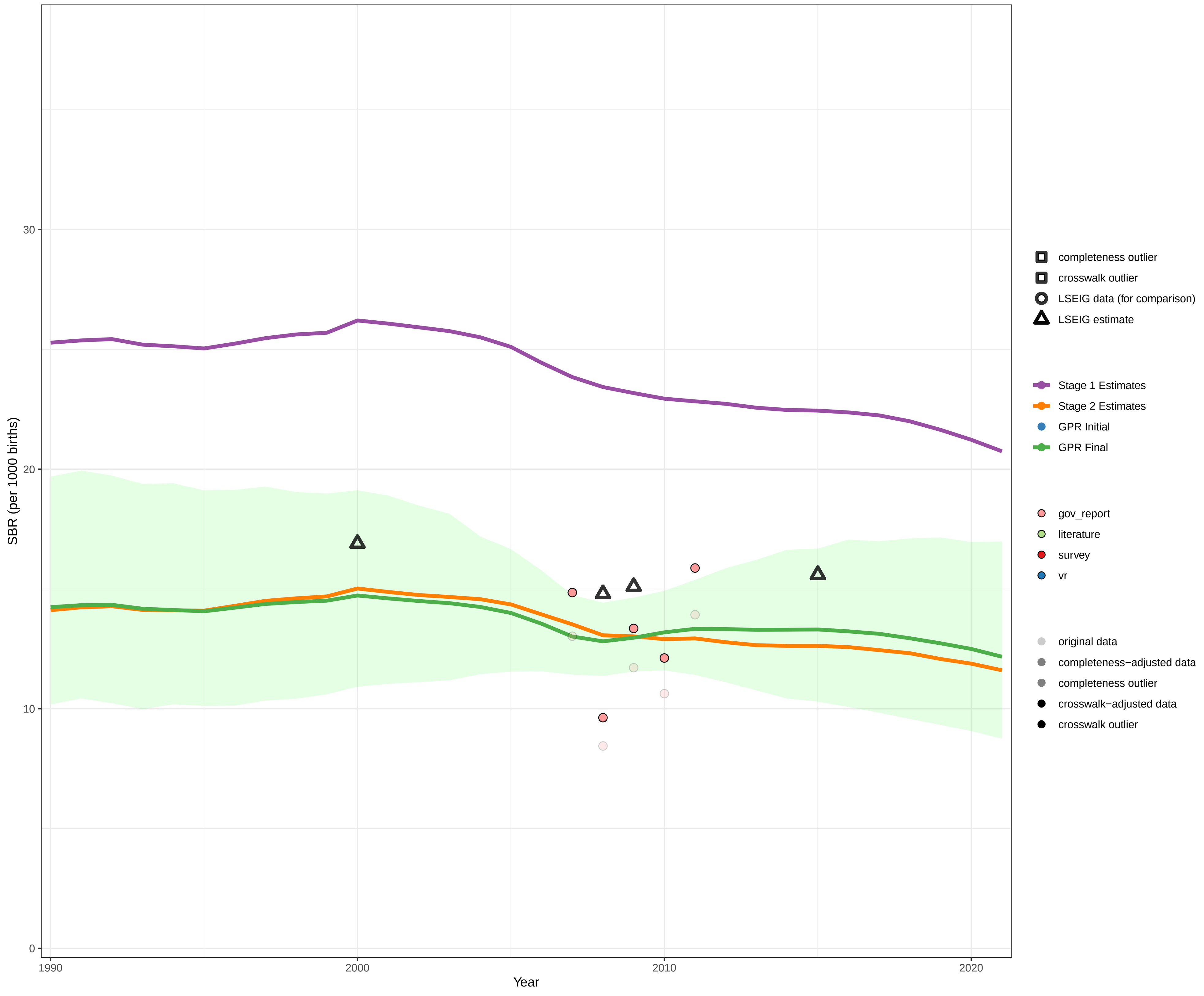

Year	Total Stillbirths (in thousands)	Total Stillbirths (in thousands)	SBR (per 1000 births)	SBR (per 1000 births)	SBR/NMR	SBR/NMR
	20 weeks	28 weeks	20 weeks	28 weeks	20 weeks	28 weeks
1990	0.0 (0.0 to 0.0)	0.0 (0.0 to 0.0)	19.9 (14.2 to 27.7)	14.2 (10.2 to 19.7)	1.54 (1.10 to 2.14)	1.10 (0.79 to 1.53)
1995	0.0 (0.0 to 0.0)	0.0 (0.0 to 0.0)	19.7 (14.1 to 26.9)	14.1 (10.1 to 19.1)	1.56 (1.12 to 2.13)	1.11 (0.80 to 1.51)
2000	0.0 (0.0 to 0.0)	0.0 (0.0 to 0.0)	20.8 (15.4 to 27.1)	14.7 (10.9 to 19.1)	1.58 (1.17 to 2.05)	1.12 (0.83 to 1.45)
2005	0.0 (0.0 to 0.0)	0.0 (0.0 to 0.0)	20.1 (16.6 to 24.0)	14.0 (11.6 to 16.7)	1.62 (1.33 to 1.93)	1.12 (0.93 to 1.34)
2010	0.0 (0.0 to 0.0)	0.0 (0.0 to 0.0)	19.3 (17.0 to 21.8)	13.2 (11.6 to 14.9)	1.74 (1.53 to 1.96)	1.19 (1.04 to 1.34)
2015	0.0 (0.0 to 0.0)	0.0 (0.0 to 0.0)	19.6 (15.2 to 24.7)	13.3 (10.3 to 16.7)	1.87 (1.44 to 2.35)	1.26 (0.98 to 1.59)
2020	0.0 (0.0 to 0.0)	0.0 (0.0 to 0.0)	18.8 (13.5 to 25.7)	12.5 (9.1 to 17.0)	1.96 (1.41 to 2.68)	1.31 (0.95 to 1.77)
2021	0.0 (0.0 to 0.0)	0.0 (0.0 to 0.0)	18.4 (13.1 to 25.7)	12.2 (8.8 to 17.0)	1.98 (1.42 to 2.77)	1.31 (0.94 to 1.83)

Micronesia (Federated States of) FSM Oceania Stillbirth Rate / Neonatal Mortality Rate (SBR/NMR) − 20 weeks
lambda = 1 ; zeta = 0.6 ; scale = 15 ; amp = 0.028

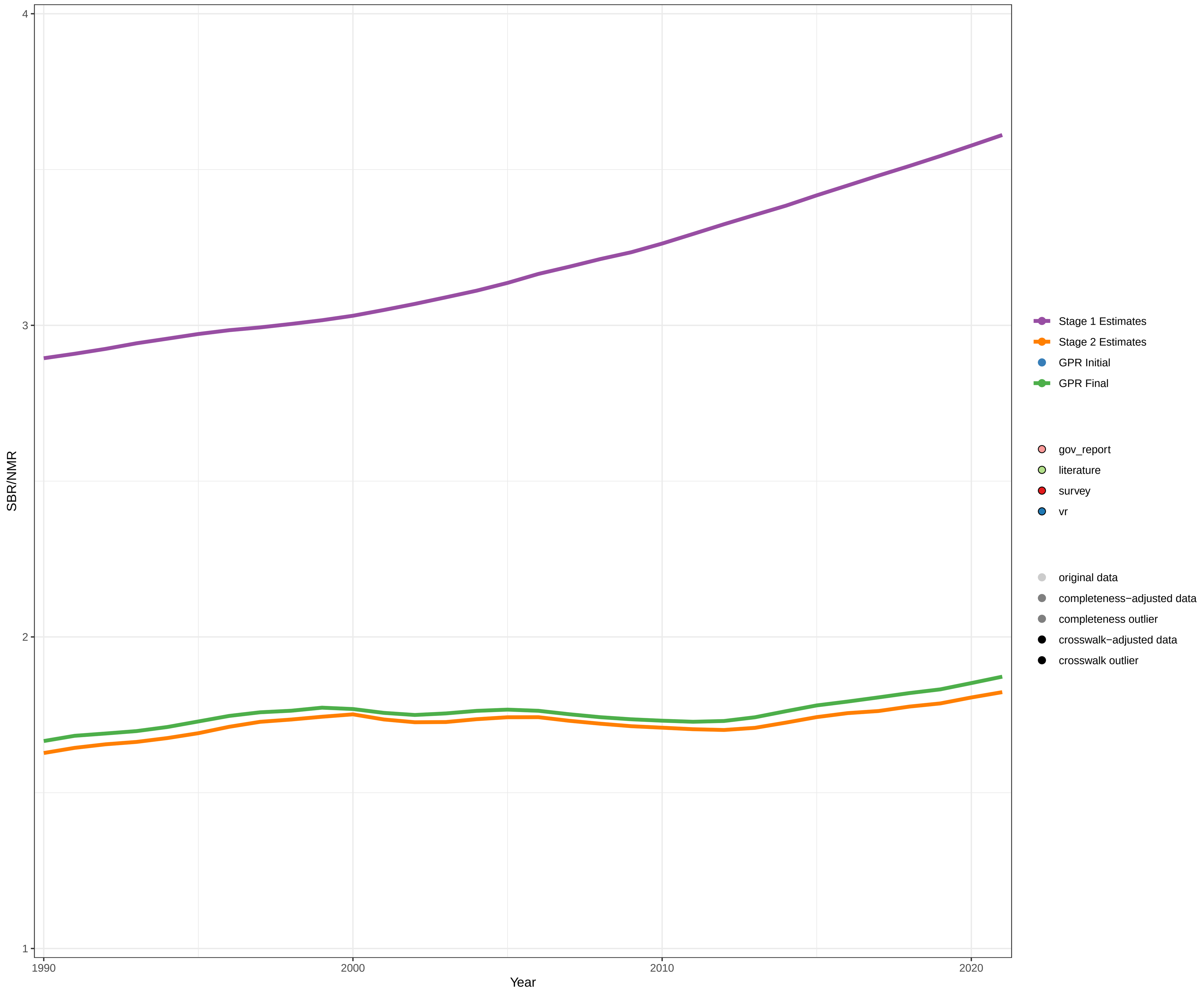

Micronesia (Federated States of) FSM Oceania Stillbirth Rate / Neonatal Mortality Rate (SBR/NMR) − 28 weeks
lambda = 1 ; zeta = 0.6 ; scale = 15 ; amp = 0.028

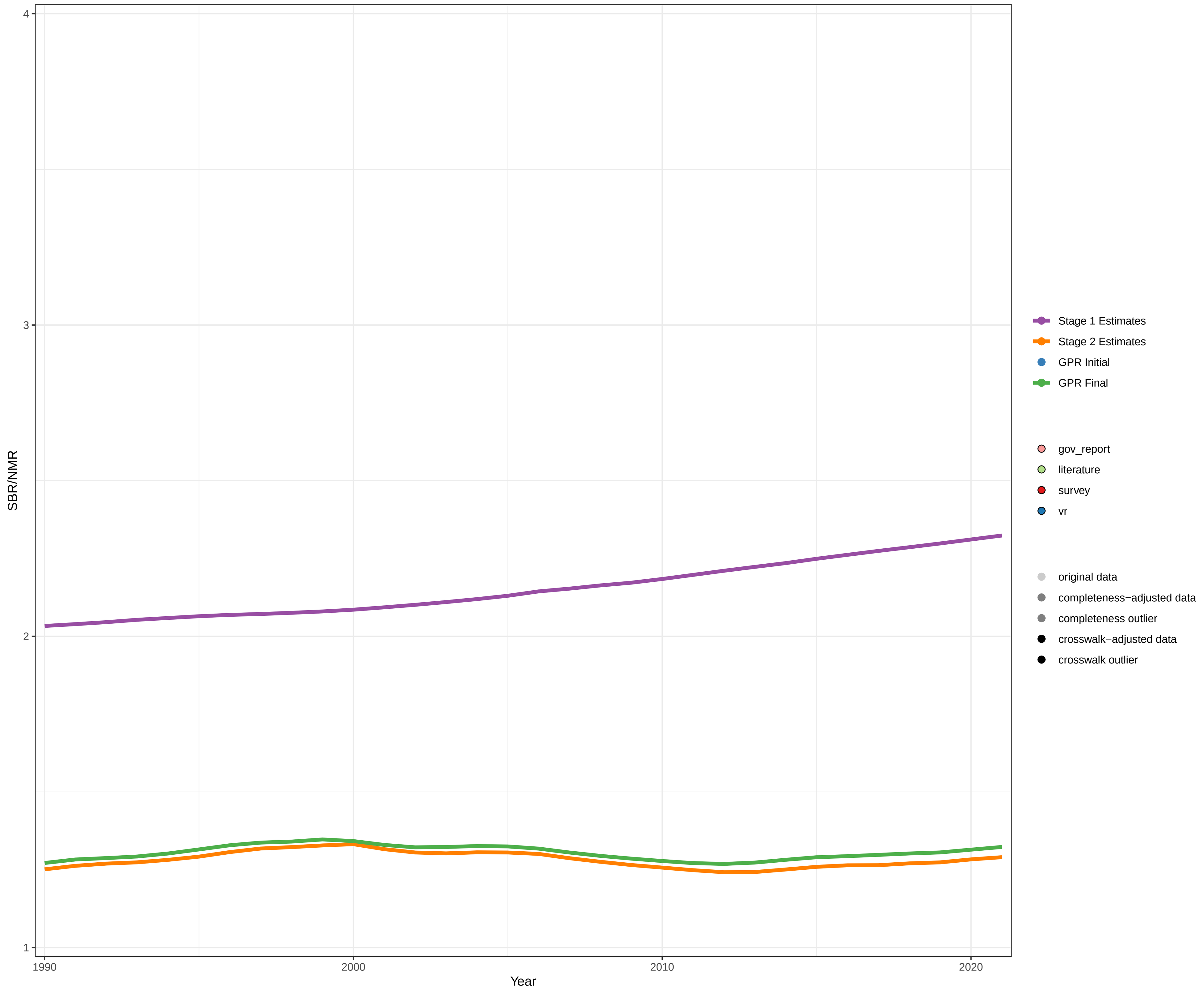

Definition	Years	Outlier	Source.Type	Title

Micronesia (Federated States of) FSM Oceania Stillbirth Rate (SBR) − 20 weeks
lambda = 1 ; zeta = 0.6 ; scale = 15 ; amp = 0.028

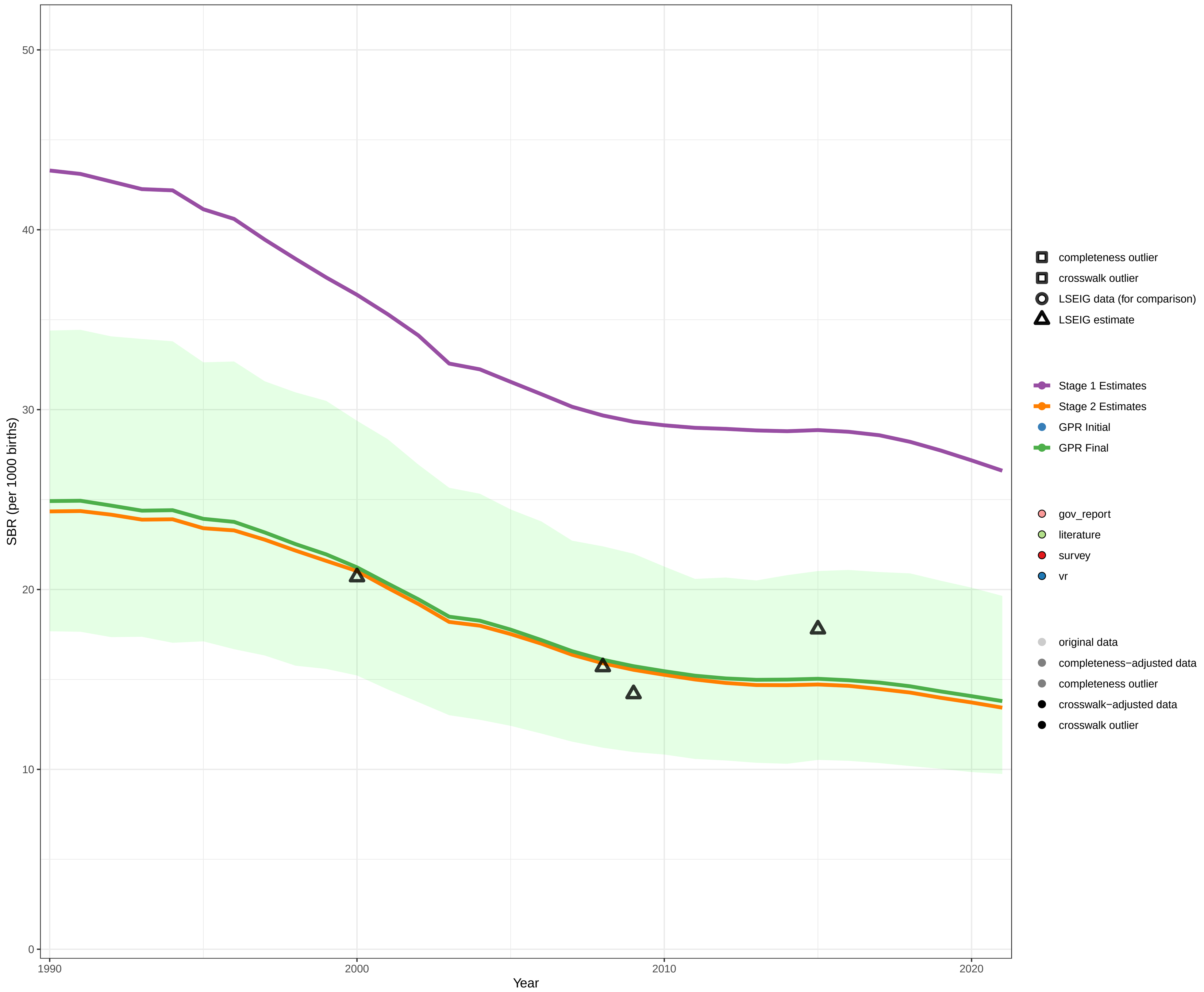

Micronesia (Federated States of) FSM Oceania Stillbirth Rate (SBR) − 28 weeks
lambda = 1 ; zeta = 0.6 ; scale = 15 ; amp = 0.028

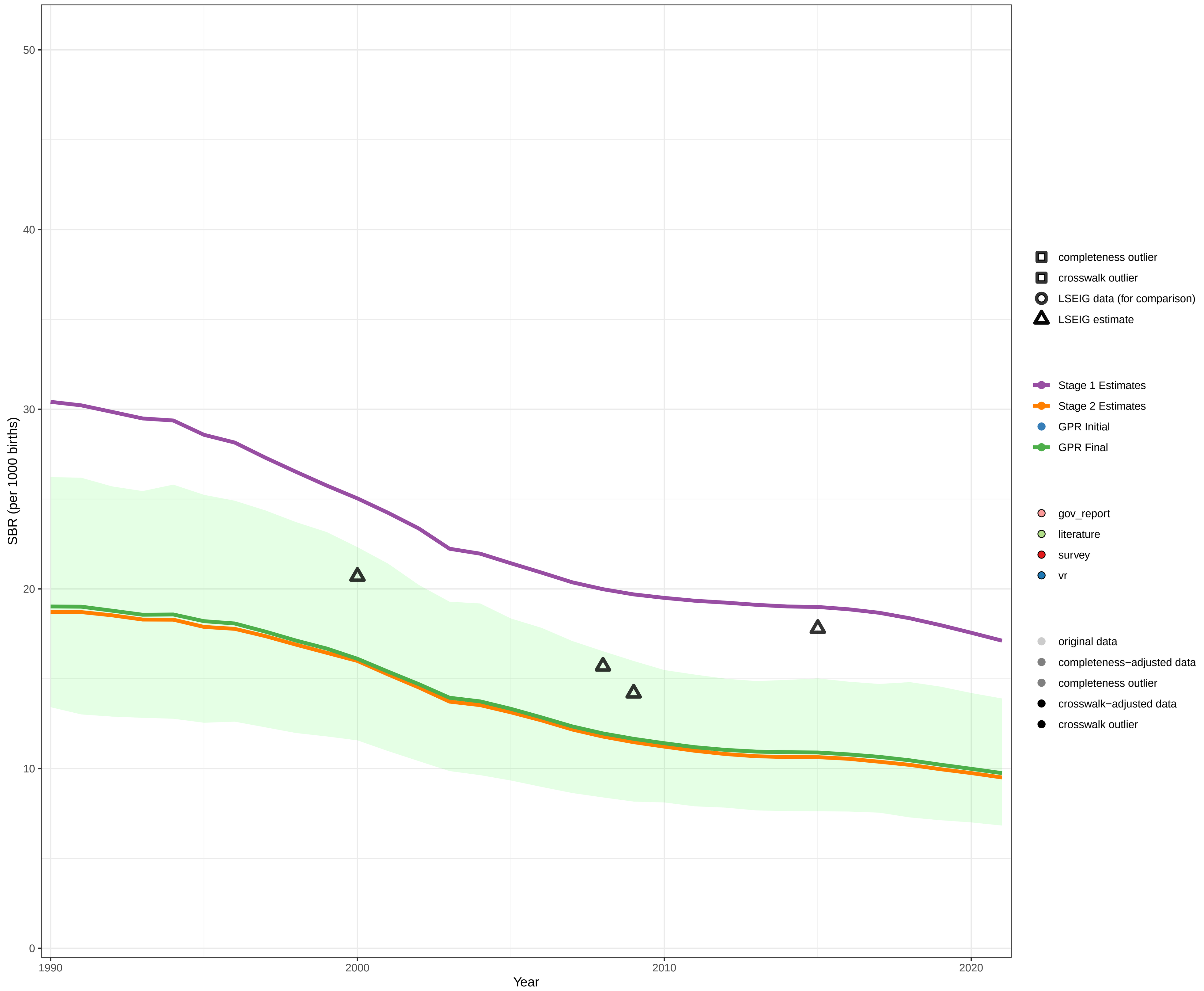

Year	Total Stillbirths (in thousands)	Total Stillbirths (in thousands)	SBR (per 1000 births)	SBR (per 1000 births)	SBR/NMR	SBR/NMR
	20 weeks	28 weeks	20 weeks	28 weeks	20 weeks	28 weeks
1990	0.1 (0.1 to 0.1)	0.1 (0.0 to 0.1)	24.9 (17.7 to 34.4)	19.0 (13.4 to 26.2)	1.67 (1.18 to 2.30)	1.27 (0.90 to 1.75)
1995	0.1 (0.1 to 0.1)	0.1 (0.0 to 0.1)	23.9 (17.1 to 32.6)	18.2 (12.6 to 25.2)	1.73 (1.24 to 2.36)	1.32 (0.91 to 1.82)
2000	0.1 (0.0 to 0.1)	0.0 (0.0 to 0.1)	21.2 (15.2 to 29.4)	16.1 (11.6 to 22.3)	1.77 (1.27 to 2.45)	1.34 (0.96 to 1.86)
2005	0.0 (0.0 to 0.1)	0.0 (0.0 to 0.0)	17.8 (12.4 to 24.5)	13.3 (9.3 to 18.4)	1.77 (1.23 to 2.43)	1.32 (0.93 to 1.82)
2010	0.0 (0.0 to 0.0)	0.0 (0.0 to 0.0)	15.5 (10.8 to 21.3)	11.4 (8.1 to 15.5)	1.73 (1.21 to 2.38)	1.28 (0.91 to 1.73)
2015	0.0 (0.0 to 0.0)	0.0 (0.0 to 0.0)	15.0 (10.5 to 21.0)	10.9 (7.6 to 15.0)	1.78 (1.25 to 2.49)	1.29 (0.90 to 1.78)
2020	0.0 (0.0 to 0.0)	0.0 (0.0 to 0.0)	14.1 (9.8 to 20.1)	10.0 (7.0 to 14.2)	1.85 (1.30 to 2.65)	1.31 (0.92 to 1.87)
2021	0.0 (0.0 to 0.0)	0.0 (0.0 to 0.0)	13.8 (9.7 to 19.6)	9.7 (6.8 to 13.9)	1.87 (1.32 to 2.67)	1.32 (0.93 to 1.89)

Nauru NRU Oceania Stillbirth Rate / Neonatal Mortality Rate (SBR/NMR) − 20 weeks
lambda = 1 ; zeta = 0.6 ; scale = 15 ; amp = 0.028

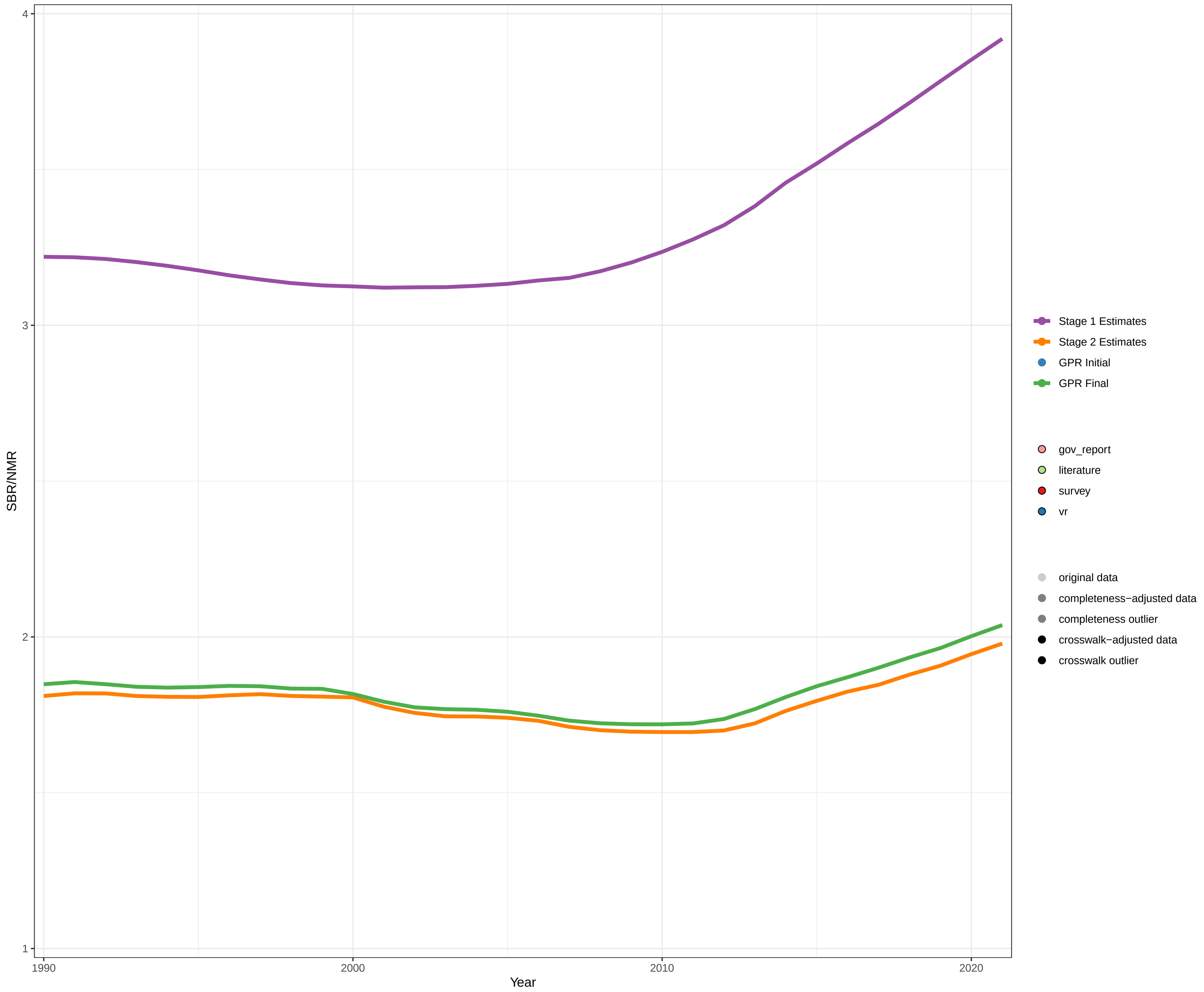

Nauru NRU Oceania Stillbirth Rate / Neonatal Mortality Rate (SBR/NMR) − 28 weeks
lambda = 1 ; zeta = 0.6 ; scale = 15 ; amp = 0.028

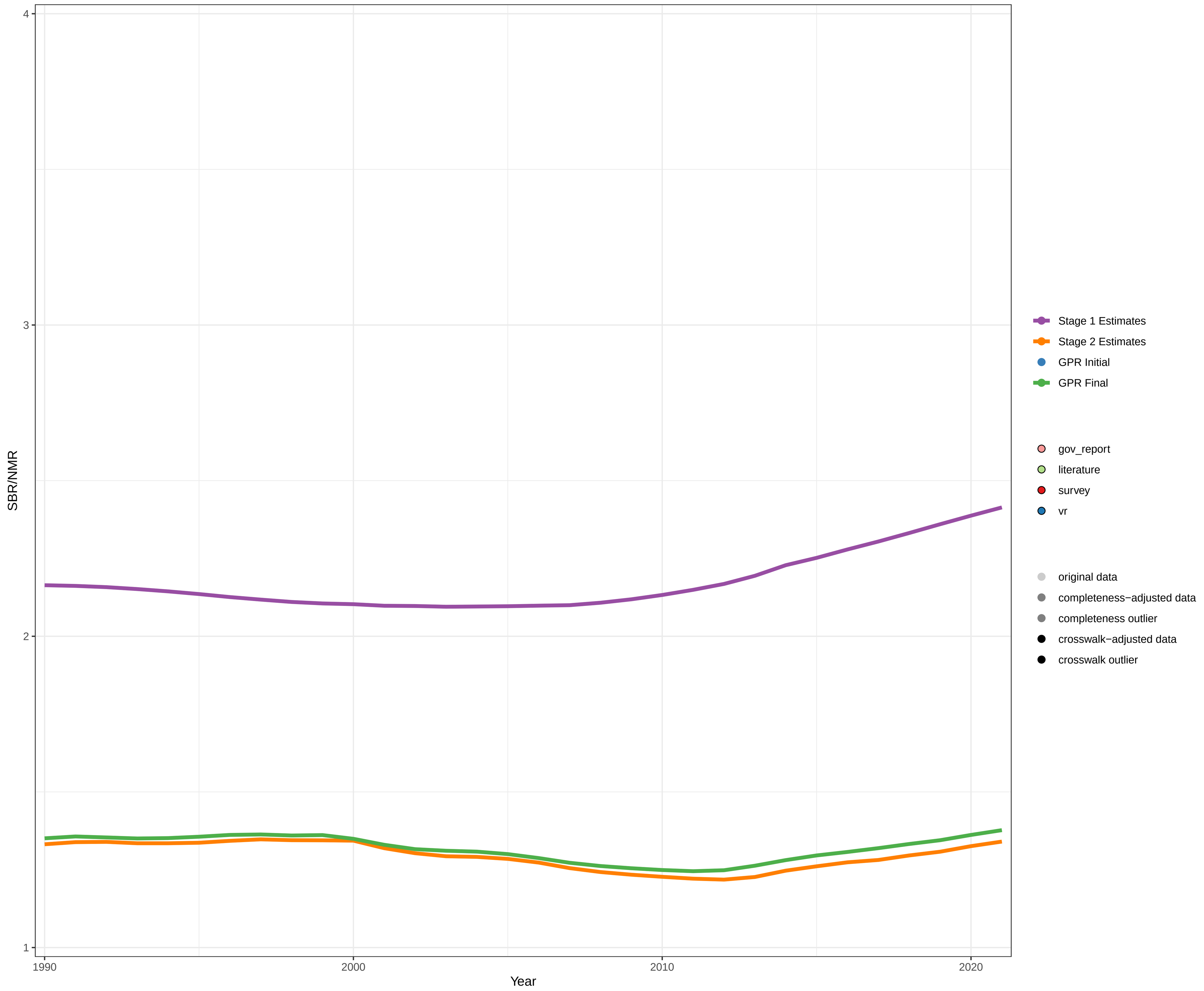

Definition	Years	Outlier	Source.Type	Title

Nauru NRU Oceania Stillbirth Rate (SBR) − 20 weeks
lambda = 1 ; zeta = 0.6 ; scale = 15 ; amp = 0.028

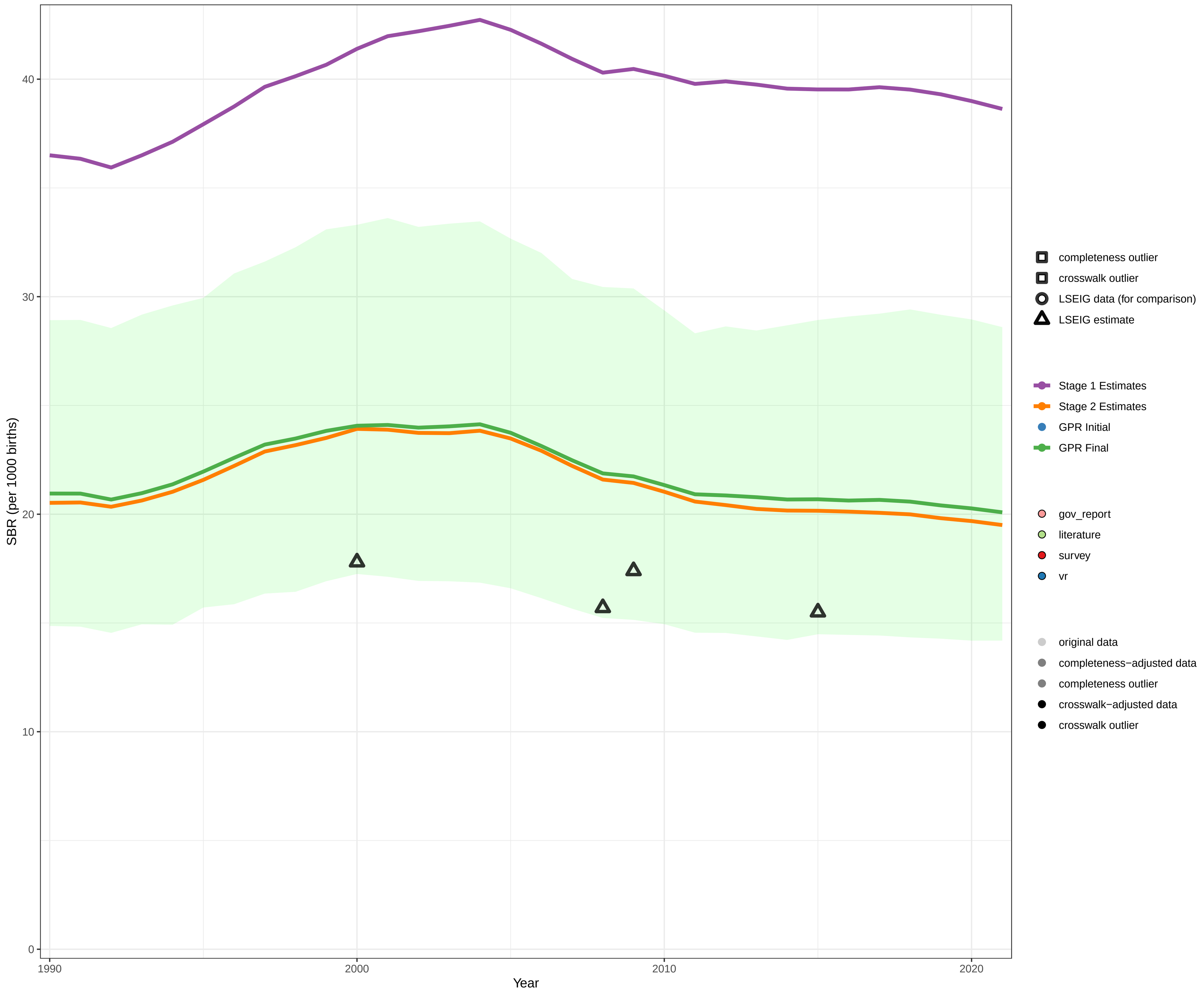

Nauru NRU Oceania Stillbirth Rate (SBR) − 28 weeks
lambda = 1 ; zeta = 0.6 ; scale = 15 ; amp = 0.028

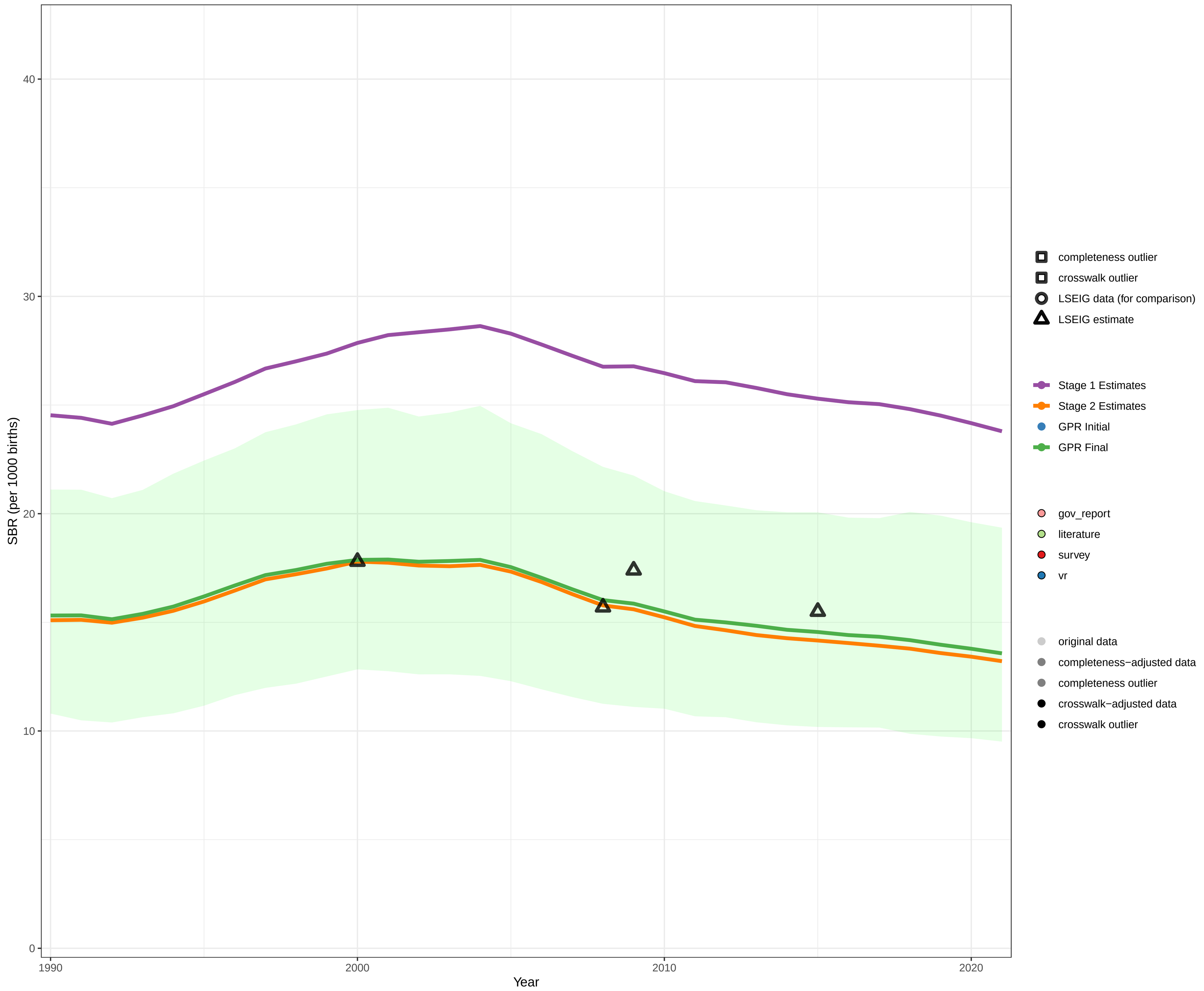

Year	Total Stillbirths (in thousands)	Total Stillbirths (in thousands)	SBR (per 1000 births)	SBR (per 1000 births)	SBR/NMR	SBR/NMR
	20 weeks	28 weeks	20 weeks	28 weeks	20 weeks	28 weeks
1990	0.0 (0.0 to 0.0)	0.0 (0.0 to 0.0)	21.0 (14.9 to 28.9)	15.3 (10.8 to 21.1)	1.85 (1.31 to 2.55)	1.35 (0.95 to 1.86)
1995	0.0 (0.0 to 0.0)	0.0 (0.0 to 0.0)	22.0 (15.7 to 29.9)	16.2 (11.2 to 22.4)	1.84 (1.32 to 2.51)	1.36 (0.93 to 1.88)
2000	0.0 (0.0 to 0.0)	0.0 (0.0 to 0.0)	24.1 (17.3 to 33.3)	17.9 (12.8 to 24.8)	1.82 (1.30 to 2.51)	1.35 (0.97 to 1.87)
2005	0.0 (0.0 to 0.0)	0.0 (0.0 to 0.0)	23.7 (16.6 to 32.7)	17.5 (12.3 to 24.2)	1.76 (1.23 to 2.42)	1.30 (0.91 to 1.79)
2010	0.0 (0.0 to 0.0)	0.0 (0.0 to 0.0)	21.3 (14.9 to 29.4)	15.5 (11.0 to 21.0)	1.72 (1.20 to 2.37)	1.25 (0.89 to 1.69)
2015	0.0 (0.0 to 0.0)	0.0 (0.0 to 0.0)	20.7 (14.5 to 28.9)	14.6 (10.2 to 20.1)	1.84 (1.29 to 2.58)	1.30 (0.91 to 1.79)
2020	0.0 (0.0 to 0.0)	0.0 (0.0 to 0.0)	20.3 (14.2 to 29.0)	13.8 (9.7 to 19.6)	2.00 (1.40 to 2.86)	1.36 (0.96 to 1.94)
2021	0.0 (0.0 to 0.0)	0.0 (0.0 to 0.0)	20.1 (14.2 to 28.6)	13.6 (9.5 to 19.4)	2.04 (1.44 to 2.90)	1.38 (0.96 to 1.96)

Niue NIU Oceania Stillbirth Rate / Neonatal Mortality Rate (SBR/NMR) − 20 weeks
lambda = 1 ; zeta = 0.6 ; scale = 15 ; amp = 0.028

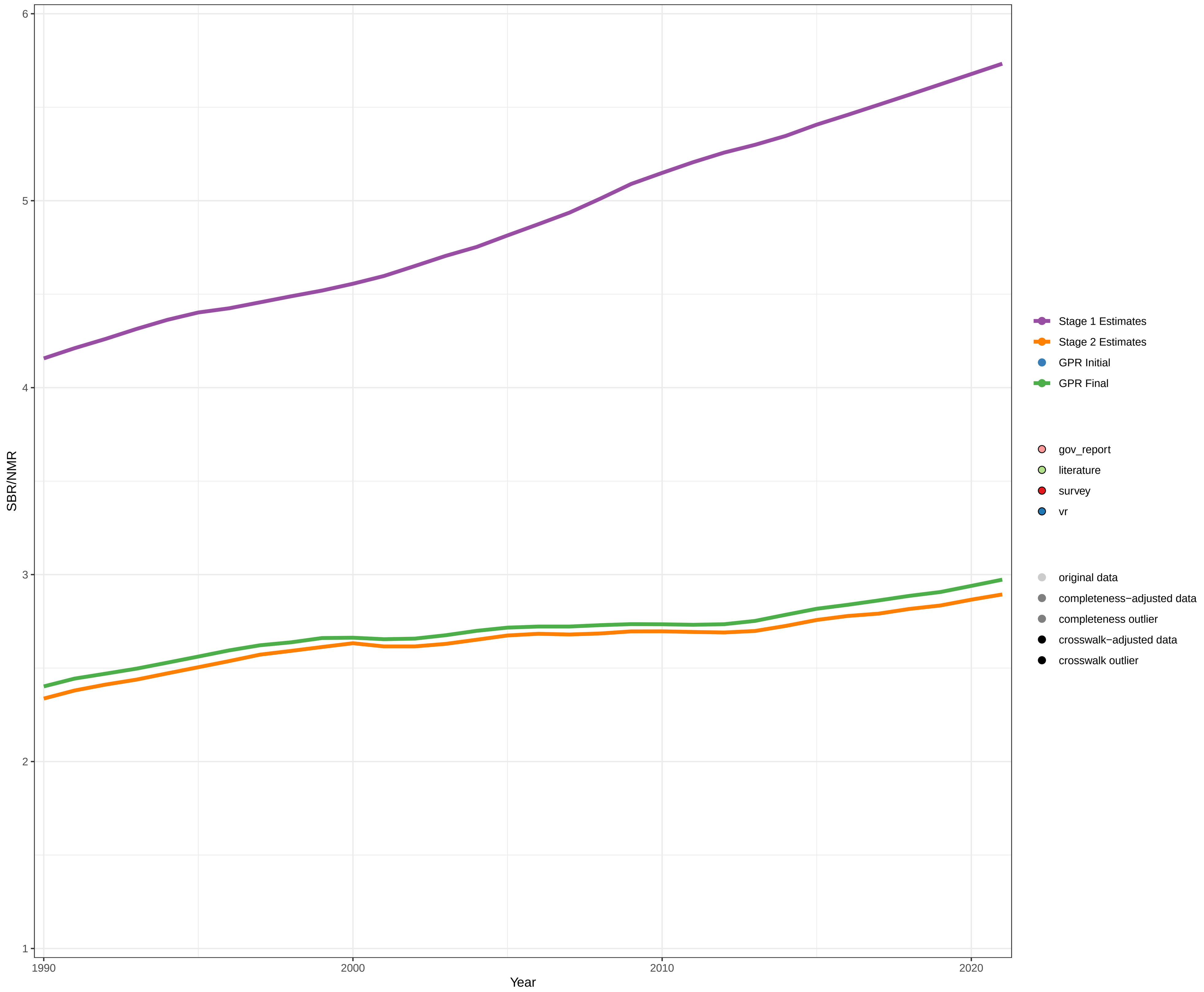

Niue NIU Oceania Stillbirth Rate / Neonatal Mortality Rate (SBR/NMR) − 28 weeks
lambda = 1 ; zeta = 0.6 ; scale = 15 ; amp = 0.028

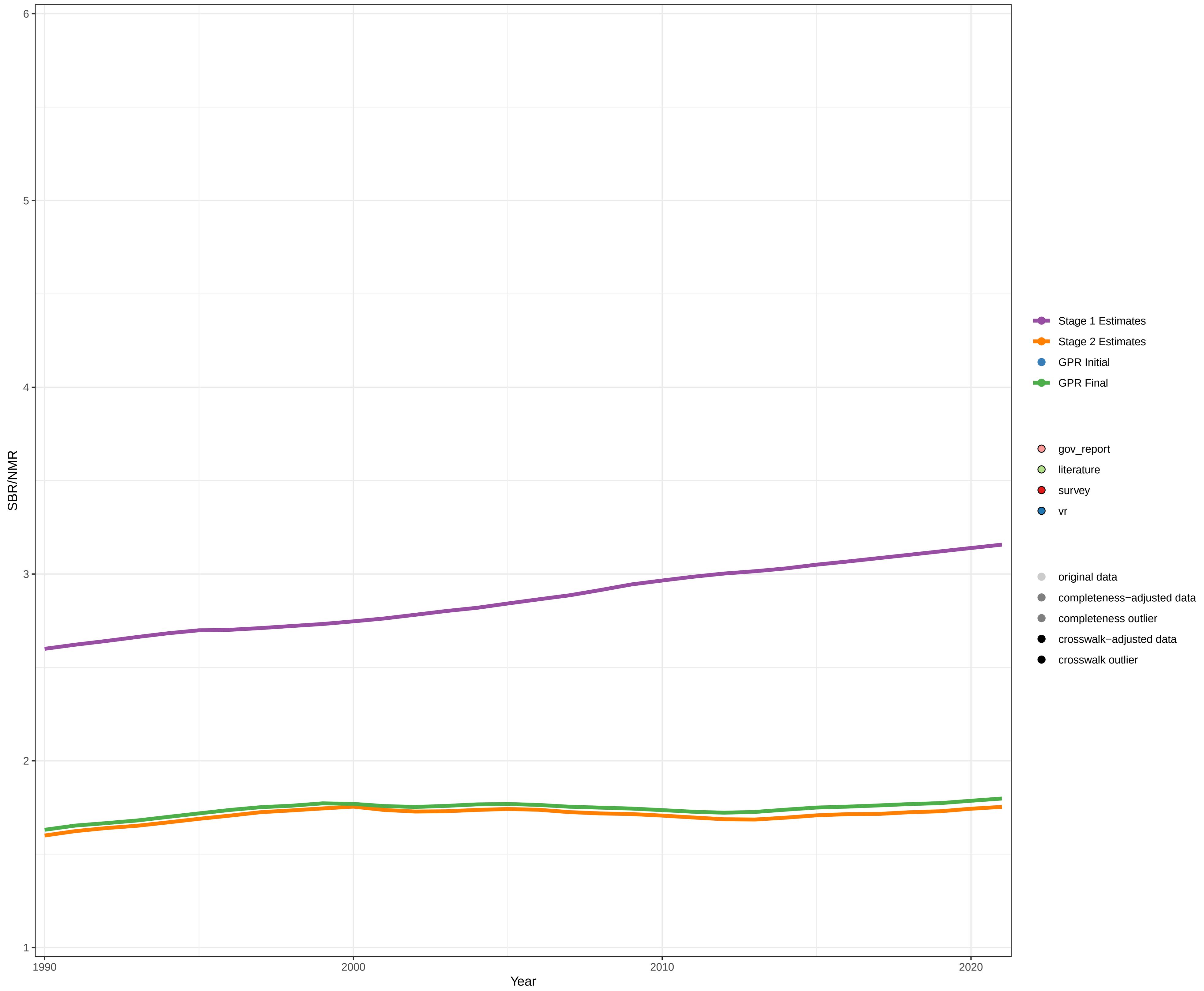

Definition	Years	Outlier	Source.Type	Title

Niue NIU Oceania Stillbirth Rate (SBR) − 20 weeks
lambda = 1 ; zeta = 0.6 ; scale = 15 ; amp = 0.028

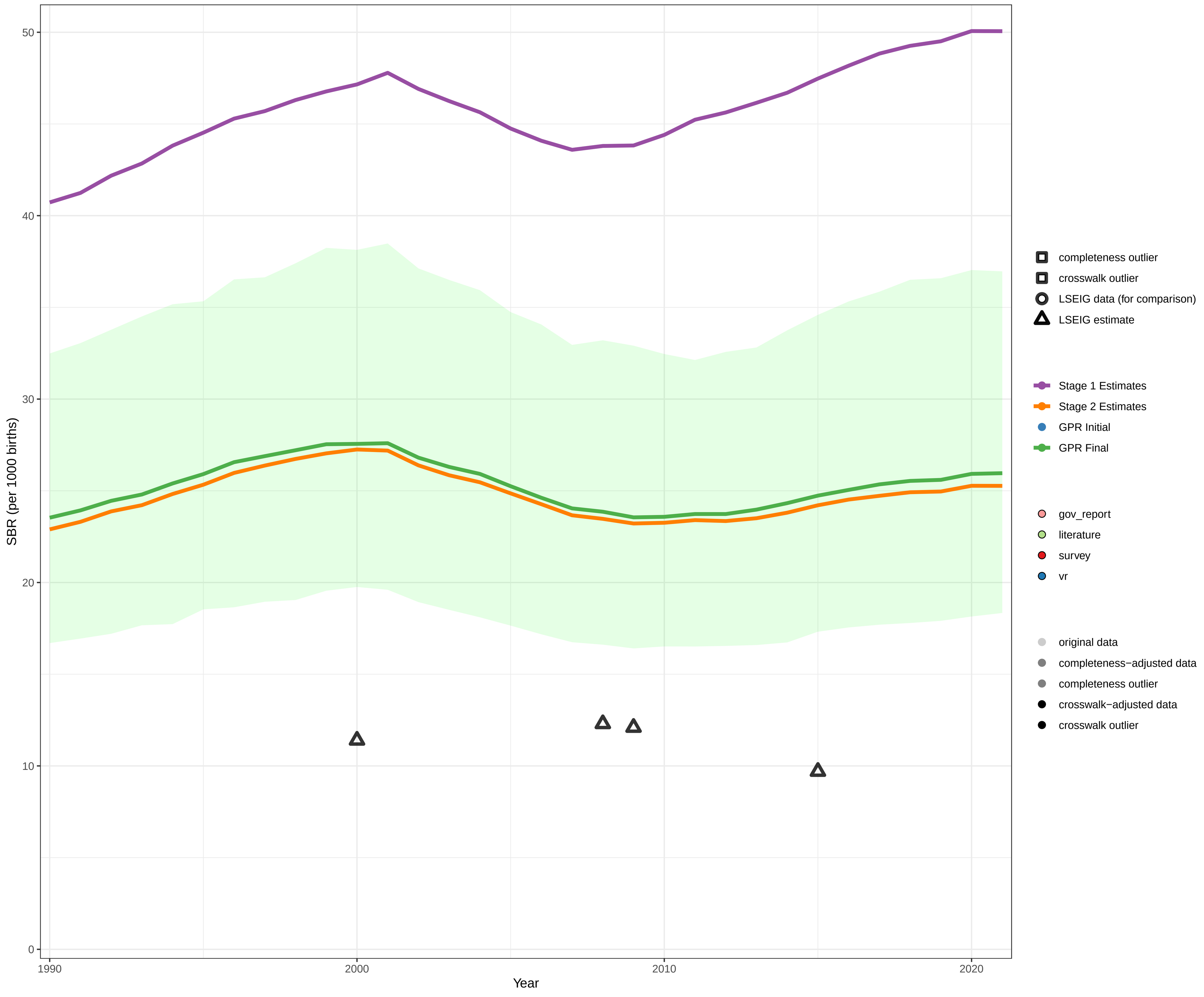

Niue NIU Oceania Stillbirth Rate (SBR) − 28 weeks
lambda = 1 ; zeta = 0.6 ; scale = 15 ; amp = 0.028

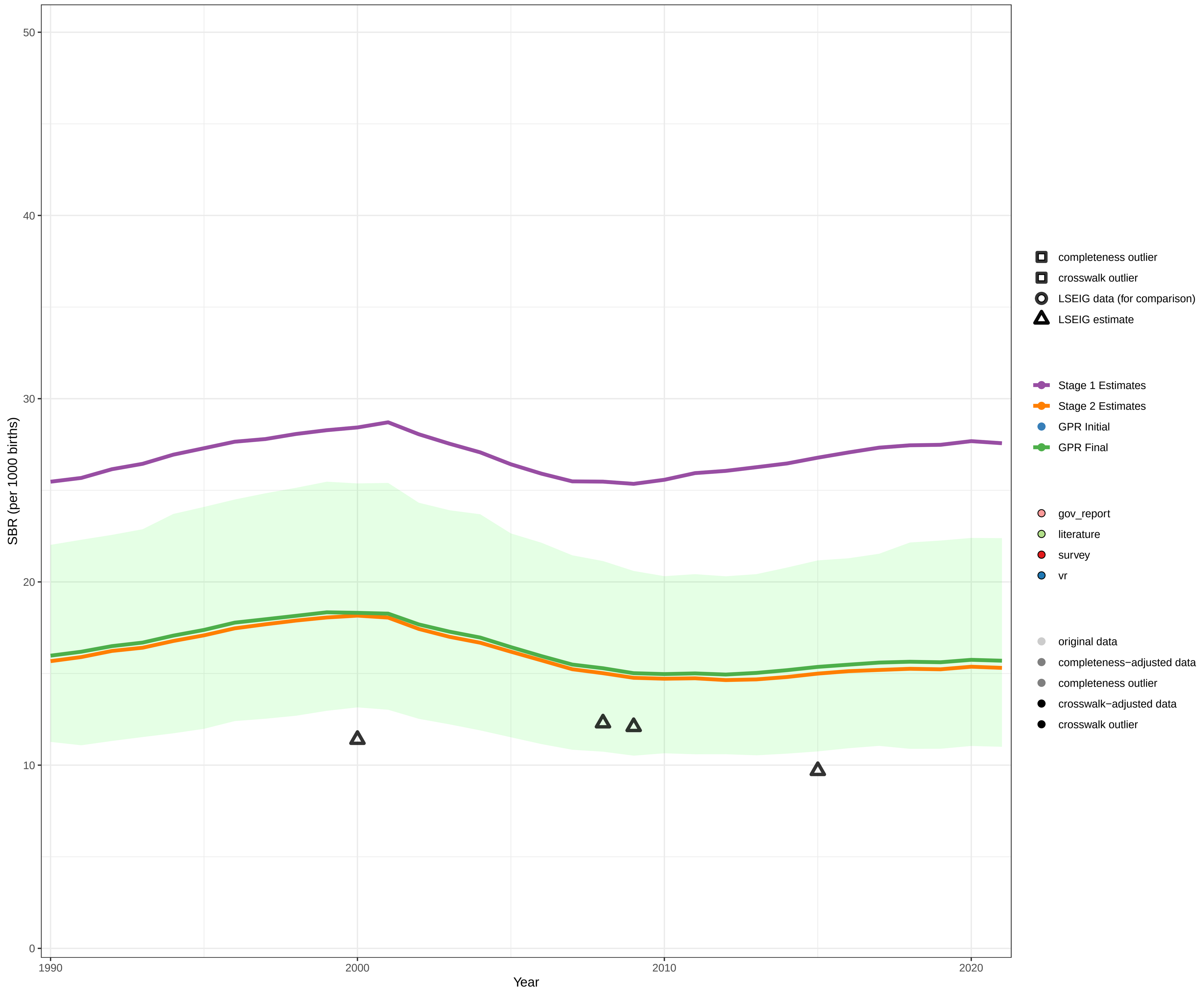

Year	Total Stillbirths (in thousands)	Total Stillbirths (in thousands)	SBR (per 1000 births)	SBR (per 1000 births)	SBR/NMR	SBR/NMR
	20 weeks	28 weeks	20 weeks	28 weeks	20 weeks	28 weeks
1990	0.0 (0.0 to 0.0)	0.0 (0.0 to 0.0)	23.5 (16.7 to 32.5)	16.0 (11.3 to 22.0)	2.40 (1.70 to 3.32)	1.63 (1.15 to 2.25)
1995	0.0 (0.0 to 0.0)	0.0 (0.0 to 0.0)	25.9 (18.5 to 35.3)	17.4 (12.0 to 24.1)	2.56 (1.83 to 3.49)	1.72 (1.18 to 2.38)
2000	0.0 (0.0 to 0.0)	0.0 (0.0 to 0.0)	27.6 (19.8 to 38.1)	18.3 (13.2 to 25.4)	2.66 (1.91 to 3.68)	1.77 (1.27 to 2.45)
2005	0.0 (0.0 to 0.0)	0.0 (0.0 to 0.0)	25.3 (17.6 to 34.7)	16.4 (11.5 to 22.6)	2.72 (1.90 to 3.74)	1.77 (1.24 to 2.44)
2010	0.0 (0.0 to 0.0)	0.0 (0.0 to 0.0)	23.6 (16.5 to 32.5)	15.0 (10.6 to 20.3)	2.73 (1.91 to 3.76)	1.74 (1.23 to 2.36)
2015	0.0 (0.0 to 0.0)	0.0 (0.0 to 0.0)	24.7 (17.3 to 34.6)	15.4 (10.7 to 21.2)	2.82 (1.97 to 3.94)	1.75 (1.22 to 2.41)
2020	0.0 (0.0 to 0.0)	0.0 (0.0 to 0.0)	25.9 (18.1 to 37.0)	15.7 (11.0 to 22.4)	1.49 (1.04 to 2.13)	0.91 (0.64 to 1.29)
2021	0.0 (0.0 to 0.0)	0.0 (0.0 to 0.0)	26.0 (18.3 to 37.0)	15.7 (11.0 to 22.4)	1.48 (1.05 to 2.11)	0.90 (0.63 to 1.28)

Northern Mariana Islands MNP Oceania Stillbirth Rate / Neonatal Mortality Rate (SBR/NMR) − 20 weeks
lambda = 0.2 ; zeta = 0.99 ; scale = 5 ; amp = 0.028

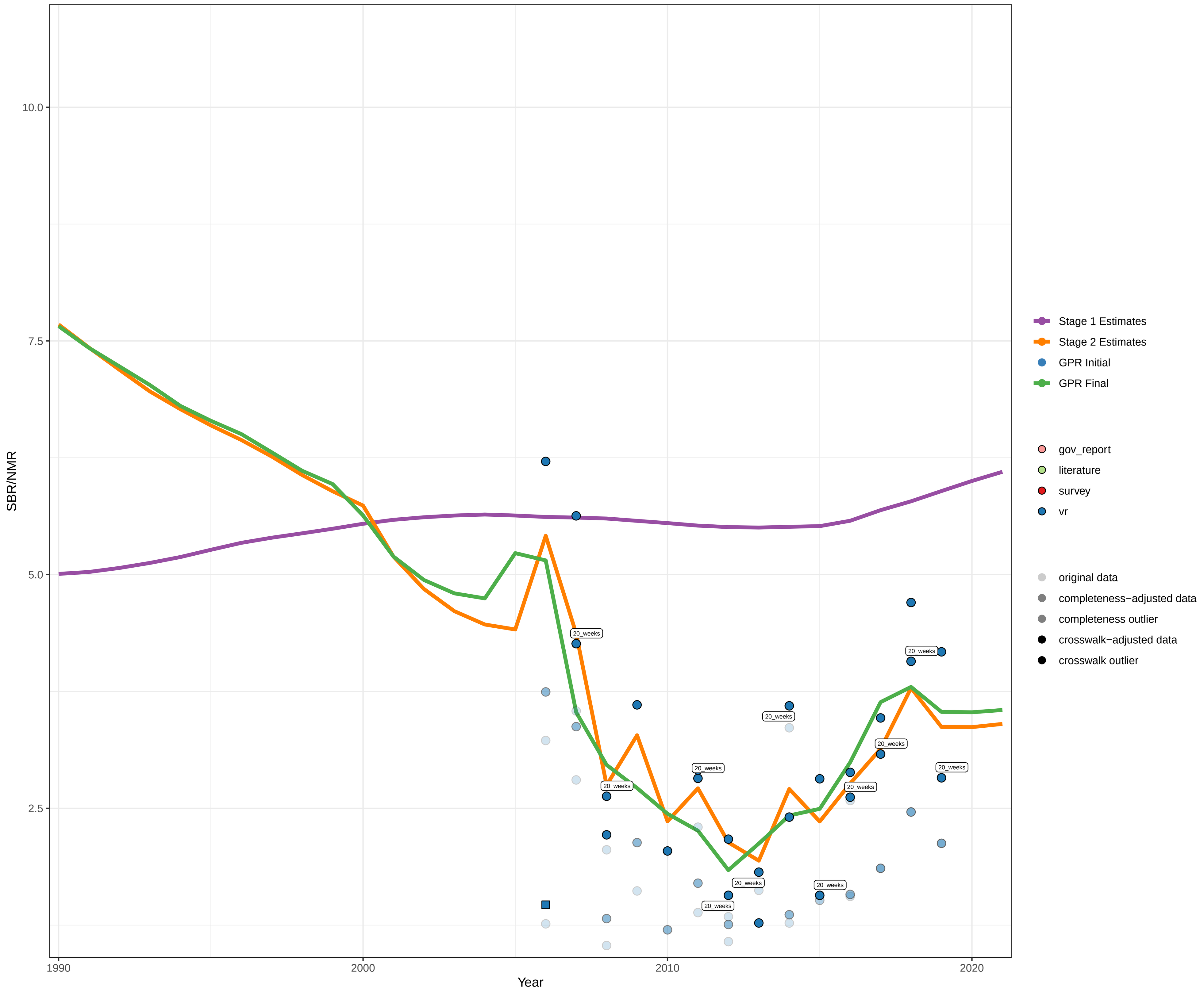

Northern Mariana Islands MNP Oceania Stillbirth Rate / Neonatal Mortality Rate (SBR/NMR) − 28 weeks
lambda = 0.2 ; zeta = 0.99 ; scale = 5 ; amp = 0.028

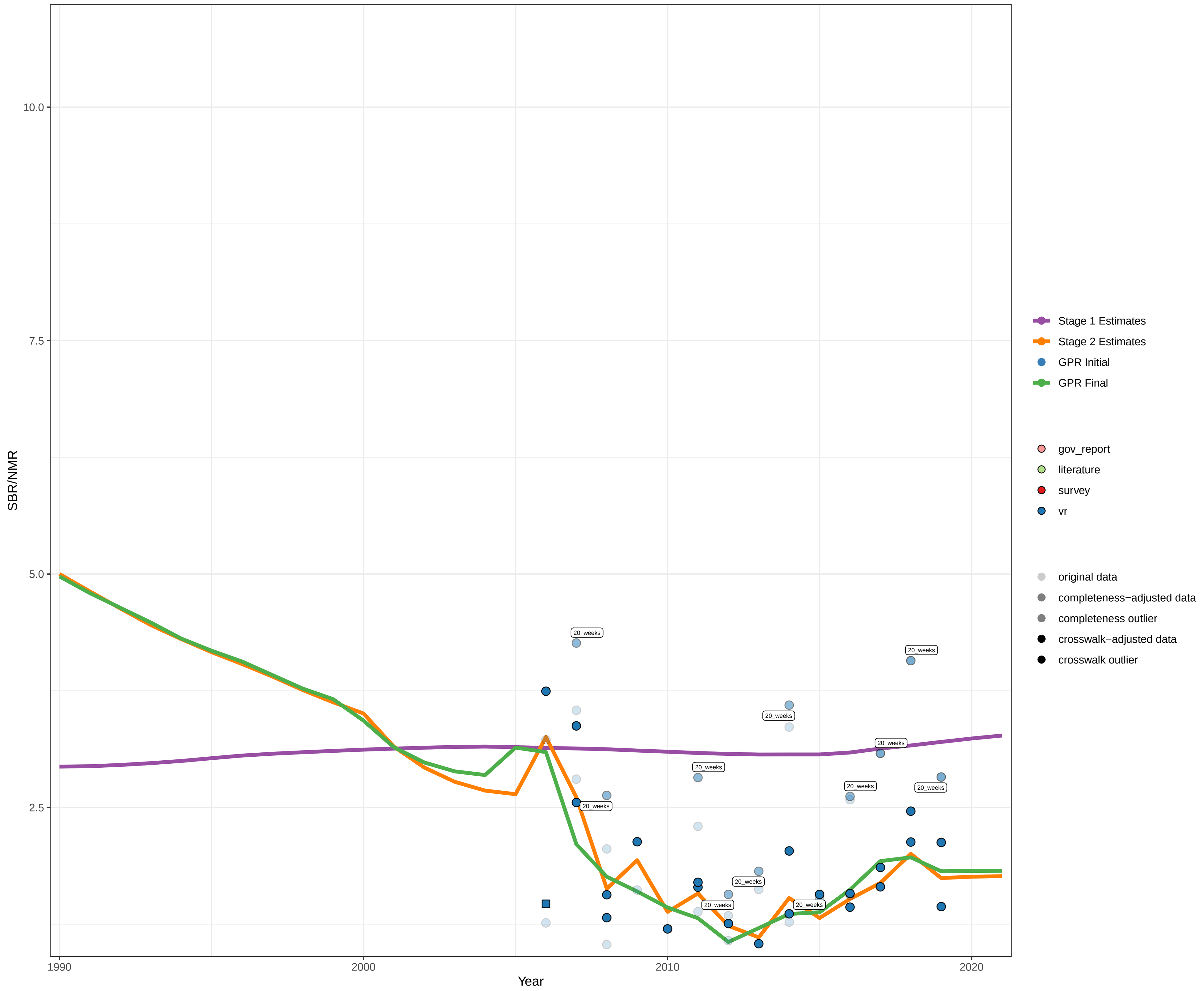

Definition	Years	Outlier	Source.Type	Title
20_weeks	2007, 2008, 2011−2019	0	VR	United States NVSS Custom Fetal Death Data
28_weeks	2006−2019	0	VR	United States NVSS Custom Fetal Death Data
28_weeks	2006	2	VR	United States NVSS Custom Fetal Death Data

Northern Mariana Islands MNP Oceania Stillbirth Rate (SBR) − 20 weeks
lambda = 0.2 ; zeta = 0.99 ; scale = 5 ; amp = 0.028

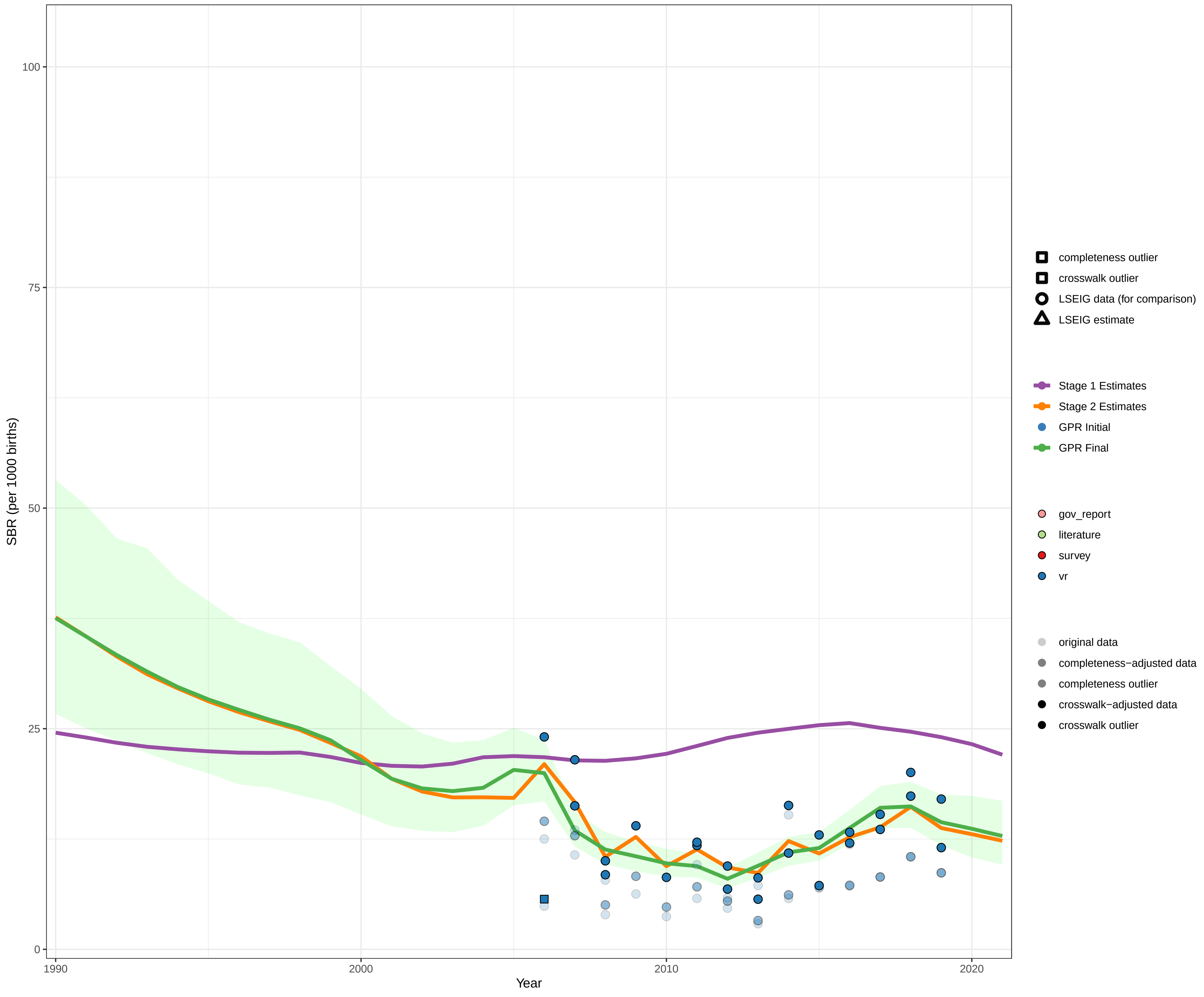

Northern Mariana Islands MNP Oceania Stillbirth Rate (SBR) − 28 weeks
lambda = 0.2 ; zeta = 0.99 ; scale = 5 ; amp = 0.028

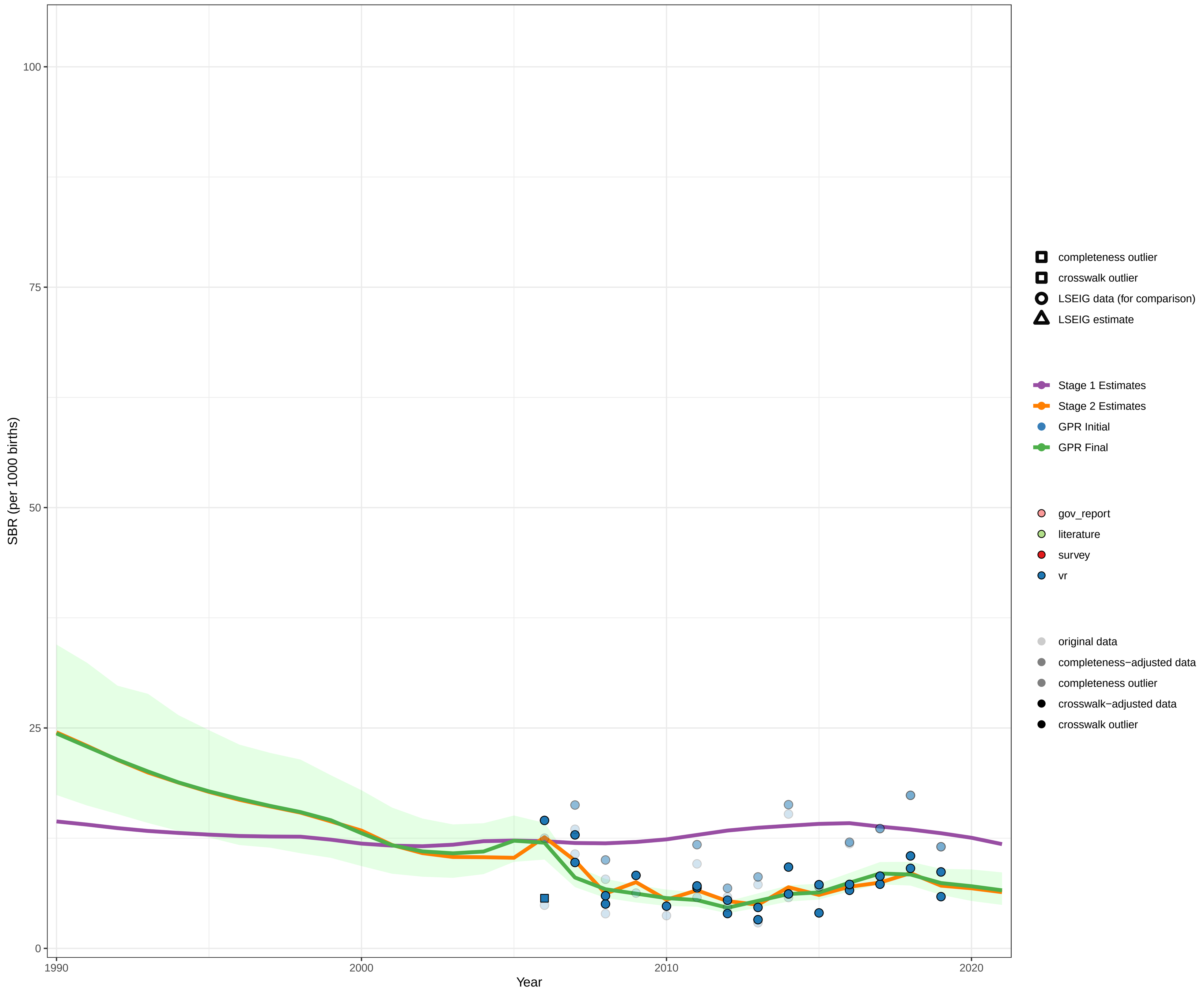

Year	Total Stillbirths (in thousands)	Total Stillbirths (in thousands)	SBR (per 1000 births)	SBR (per 1000 births)	SBR/NMR	SBR/NMR
	20 weeks	28 weeks	20 weeks	28 weeks	20 weeks	28 weeks
1990	0.0 (0.0 to 0.1)	0.0 (0.0 to 0.0)	37.5 (26.7 to 53.2)	24.4 (17.4 to 34.5)	7.66 (5.45 to 10.86)	4.97 (3.55 to 7.03)
1995	0.0 (0.0 to 0.0)	0.0 (0.0 to 0.0)	28.3 (19.9 to 39.5)	17.8 (12.6 to 24.7)	6.65 (4.68 to 9.26)	4.18 (2.95 to 5.81)
2000	0.1 (0.0 to 0.1)	0.0 (0.0 to 0.0)	21.5 (15.3 to 29.5)	13.1 (9.3 to 17.9)	5.63 (4.01 to 7.75)	3.43 (2.45 to 4.71)
2005	0.0 (0.0 to 0.0)	0.0 (0.0 to 0.0)	20.3 (16.3 to 25.1)	12.2 (9.8 to 15.1)	5.23 (4.20 to 6.46)	3.14 (2.52 to 3.88)
2010	0.0 (0.0 to 0.0)	0.0 (0.0 to 0.0)	9.7 (8.2 to 11.4)	5.7 (4.8 to 6.7)	2.44 (2.06 to 2.85)	1.43 (1.21 to 1.67)
2015	0.0 (0.0 to 0.0)	0.0 (0.0 to 0.0)	11.5 (10.0 to 13.3)	6.3 (5.5 to 7.3)	2.49 (2.18 to 2.89)	1.38 (1.20 to 1.59)
2020	0.0 (0.0 to 0.0)	0.0 (0.0 to 0.0)	13.7 (10.4 to 17.4)	7.0 (5.4 to 9.0)	3.53 (2.68 to 4.49)	1.82 (1.39 to 2.31)
2021	0.0 (0.0 to 0.0)	0.0 (0.0 to 0.0)	12.8 (9.6 to 16.8)	6.6 (4.9 to 8.6)	3.55 (2.65 to 4.66)	1.82 (1.36 to 2.38)

Palau PLW Oceania Stillbirth Rate / Neonatal Mortality Rate (SBR/NMR) − 20 weeks
lambda = 0.4 ; zeta = 0.9 ; scale = 10 ; amp = 0.028

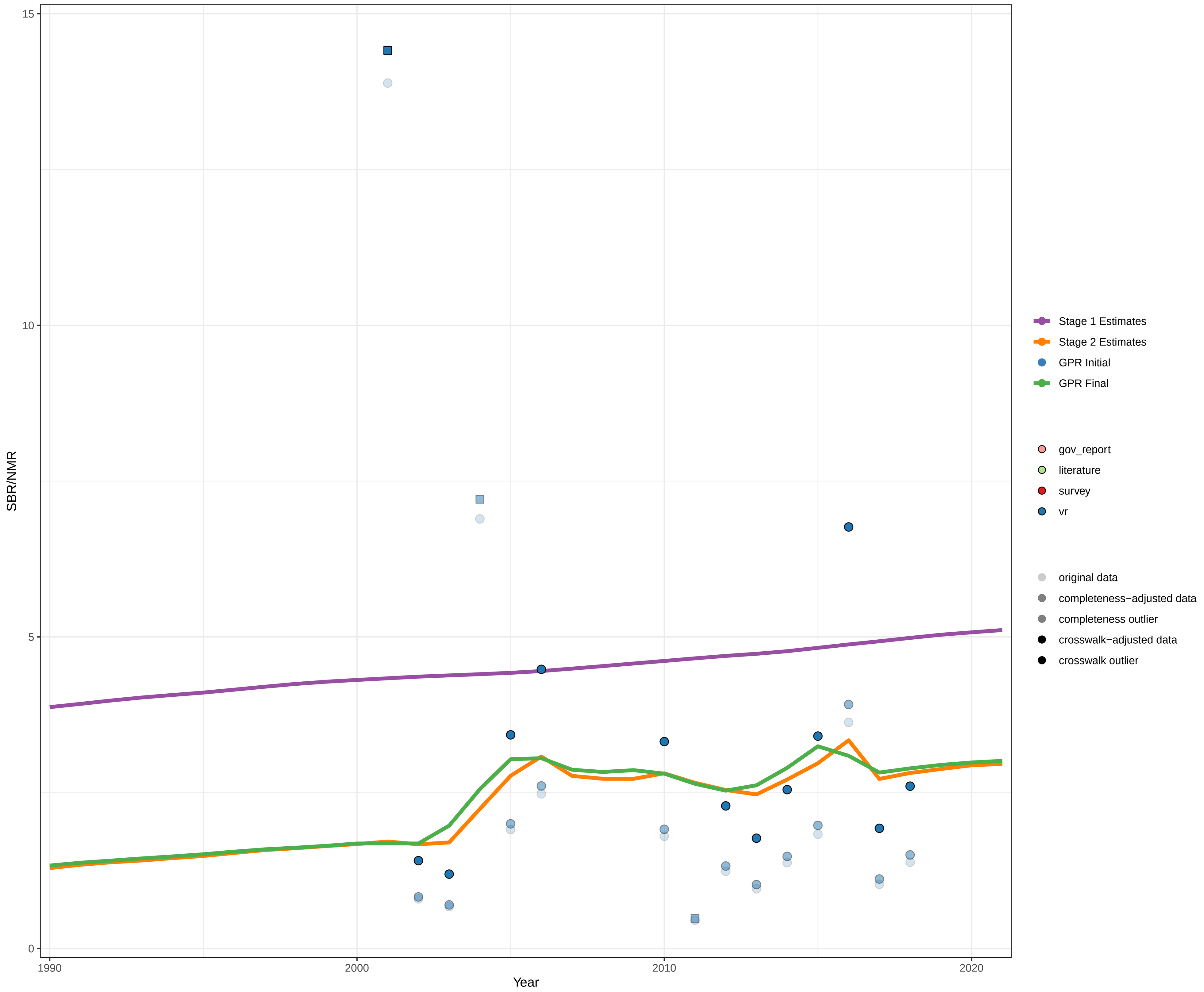

Palau PLW Oceania Stillbirth Rate / Neonatal Mortality Rate (SBR/NMR) − 28 weeks
lambda = 0.4 ; zeta = 0.9 ; scale = 10 ; amp = 0.028

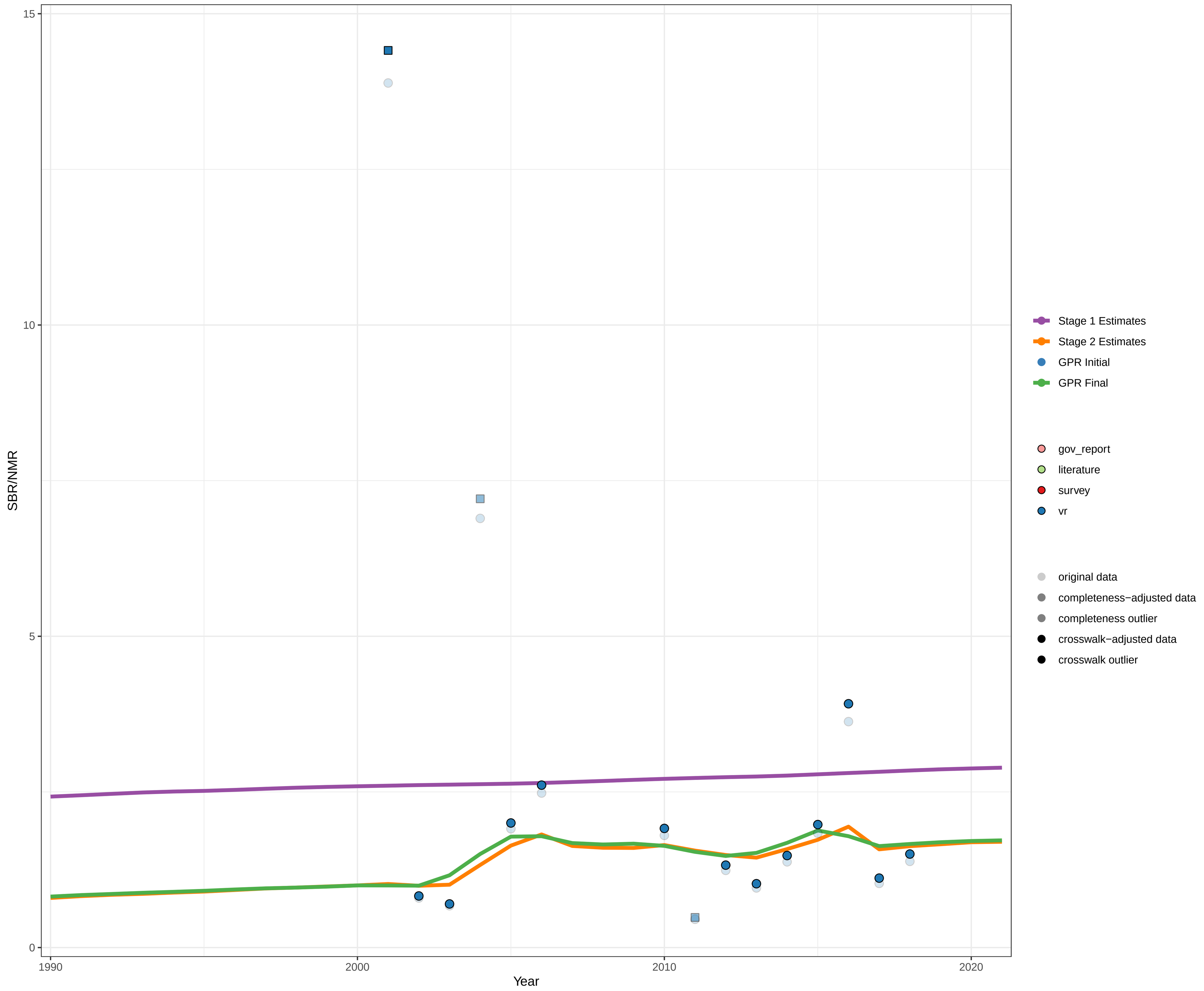

Definition	Years	Outlier	Source.Type	Title
28_weeks	2002, 2003, 2005, 2006, 2010, 2012−2018	0	VR	United Nations Demographic Yearbook
28_weeks	2004, 2011	1	VR	United Nations Demographic Yearbook
28_weeks	2001	2	VR	United Nations Demographic Yearbook

Palau PLW Oceania Stillbirth Rate (SBR) − 20 weeks
lambda = 0.4 ; zeta = 0.9 ; scale = 10 ; amp = 0.028

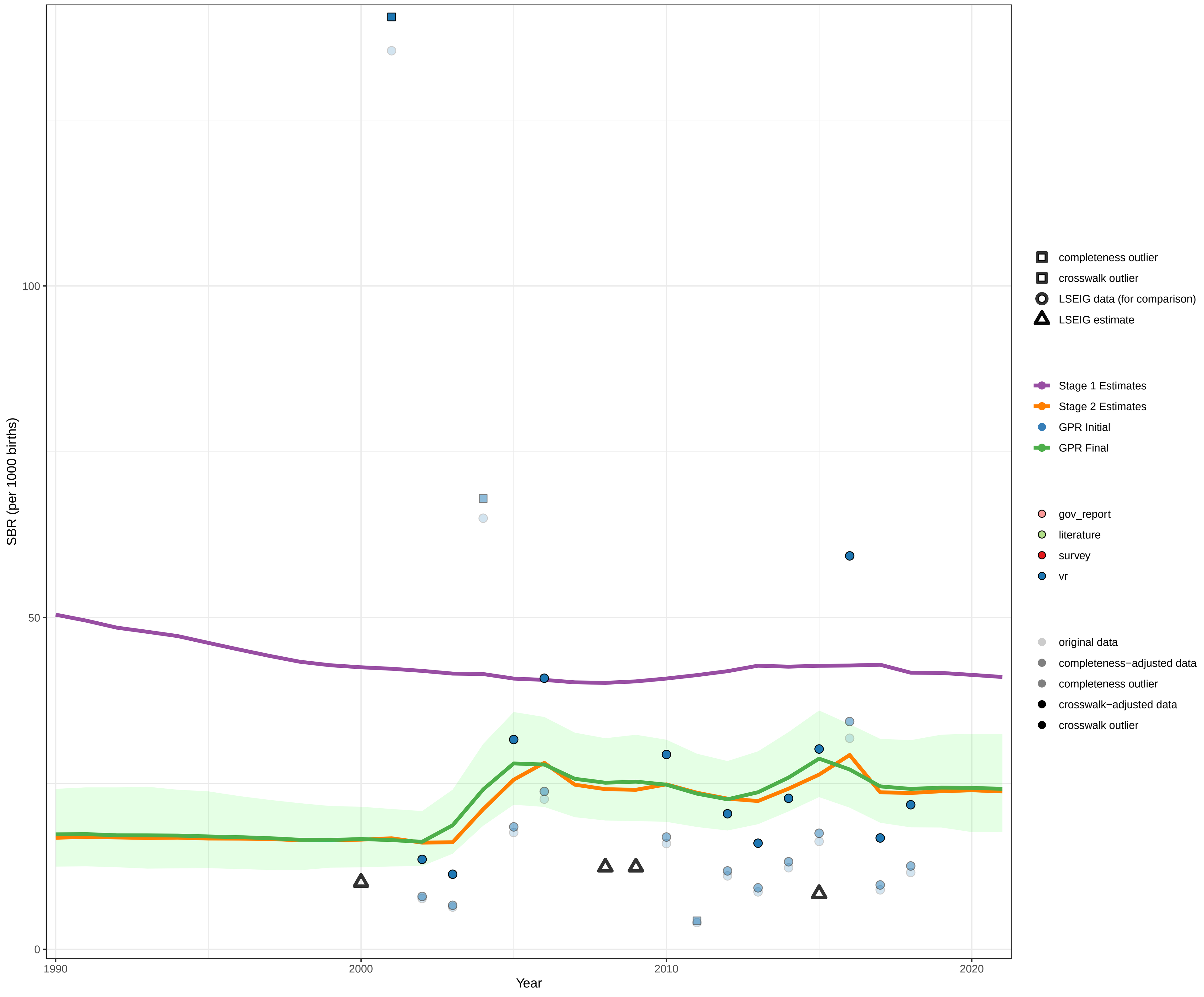

Palau PLW Oceania Stillbirth Rate (SBR) − 28 weeks
lambda = 0.4 ; zeta = 0.9 ; scale = 10 ; amp = 0.028

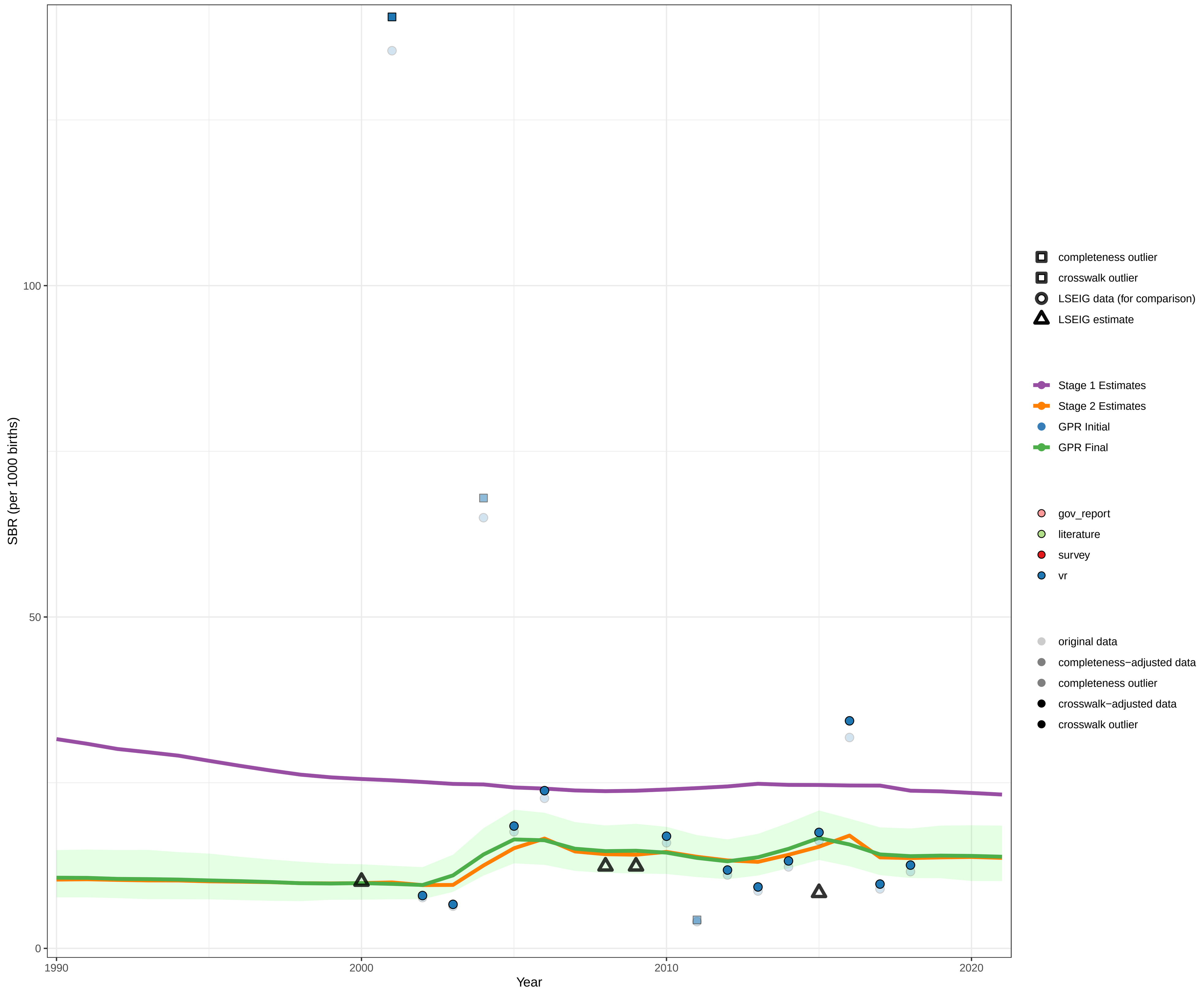

Year	Total Stillbirths (in thousands)	Total Stillbirths (in thousands)	SBR (per 1000 births)	SBR (per 1000 births)	SBR/NMR	SBR/NMR
	20 weeks	28 weeks	20 weeks	28 weeks	20 weeks	28 weeks
1990	0.0 (0.0 to 0.0)	0.0 (0.0 to 0.0)	17.3 (12.5 to 24.2)	10.7 (7.7 to 14.8)	1.33 (0.96 to 1.86)	0.82 (0.59 to 1.14)
1995	0.0 (0.0 to 0.0)	0.0 (0.0 to 0.0)	17.0 (12.2 to 23.8)	10.3 (7.4 to 14.3)	1.51 (1.09 to 2.12)	0.91 (0.66 to 1.27)
2000	0.0 (0.0 to 0.0)	0.0 (0.0 to 0.0)	16.6 (12.4 to 21.5)	9.8 (7.3 to 12.7)	1.69 (1.25 to 2.18)	1.00 (0.74 to 1.29)
2005	0.0 (0.0 to 0.0)	0.0 (0.0 to 0.0)	28.0 (21.8 to 35.8)	16.4 (12.8 to 20.9)	3.04 (2.36 to 3.88)	1.78 (1.39 to 2.27)
2010	0.0 (0.0 to 0.0)	0.0 (0.0 to 0.0)	24.8 (19.2 to 31.6)	14.4 (11.2 to 18.3)	2.81 (2.17 to 3.57)	1.63 (1.27 to 2.07)
2015	0.0 (0.0 to 0.0)	0.0 (0.0 to 0.0)	28.7 (22.9 to 36.0)	16.7 (13.3 to 20.8)	3.25 (2.59 to 4.06)	1.88 (1.51 to 2.35)
2020	0.0 (0.0 to 0.0)	0.0 (0.0 to 0.0)	24.3 (17.7 to 32.5)	14.0 (10.2 to 18.6)	2.99 (2.17 to 3.99)	1.71 (1.25 to 2.28)
2021	0.0 (0.0 to 0.0)	0.0 (0.0 to 0.0)	24.2 (17.7 to 32.5)	13.8 (10.2 to 18.5)	3.01 (2.20 to 4.04)	1.72 (1.26 to 2.31)

Papua New Guinea PNG Oceania Stillbirth Rate / Neonatal Mortality Rate (SBR/NMR) − 20 weeks
lambda = 1 ; zeta = 0.6 ; scale = 15 ; amp = 0.028

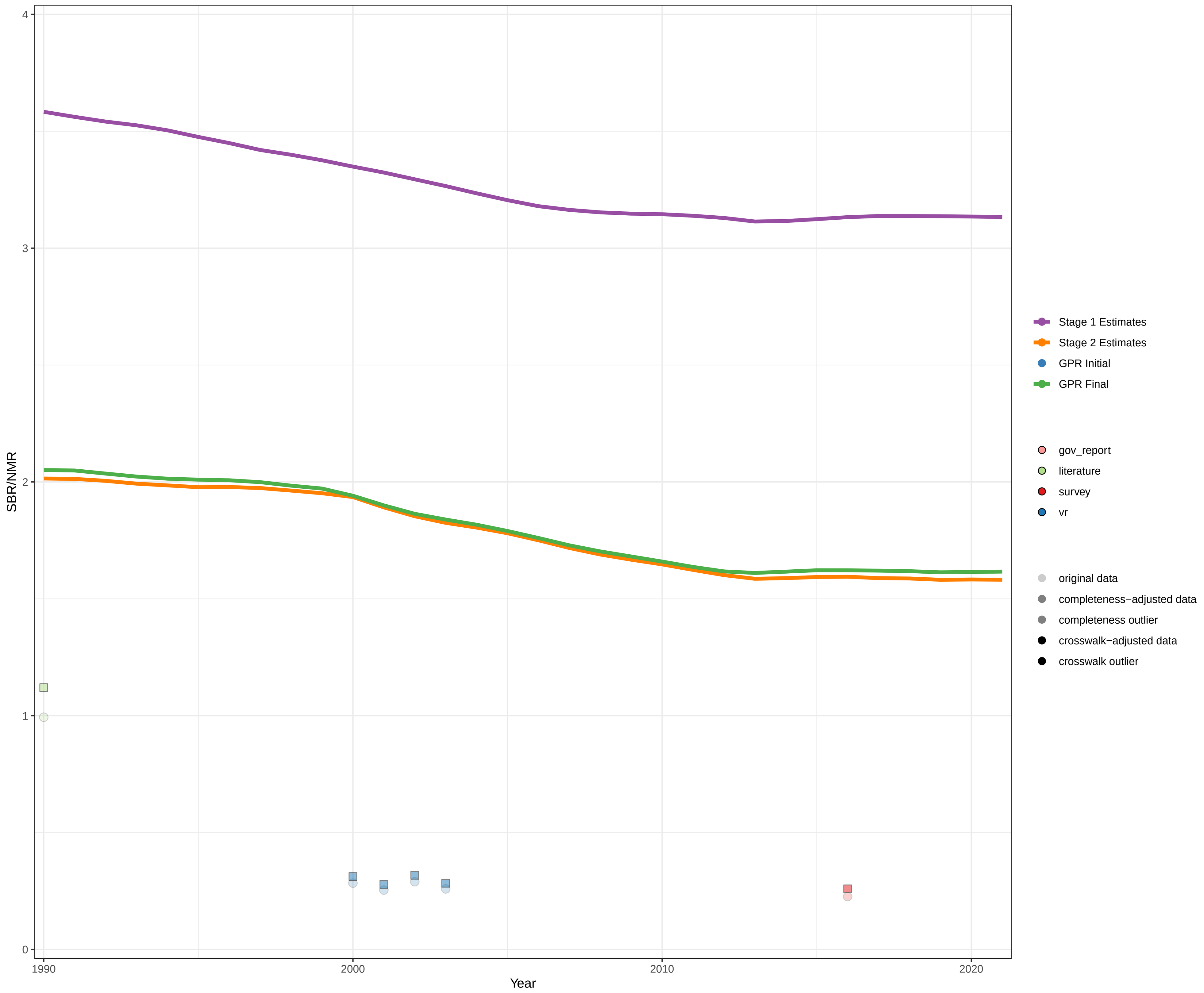

Papua New Guinea PNG Oceania Stillbirth Rate / Neonatal Mortality Rate (SBR/NMR) − 28 weeks
lambda = 1 ; zeta = 0.6 ; scale = 15 ; amp = 0.028

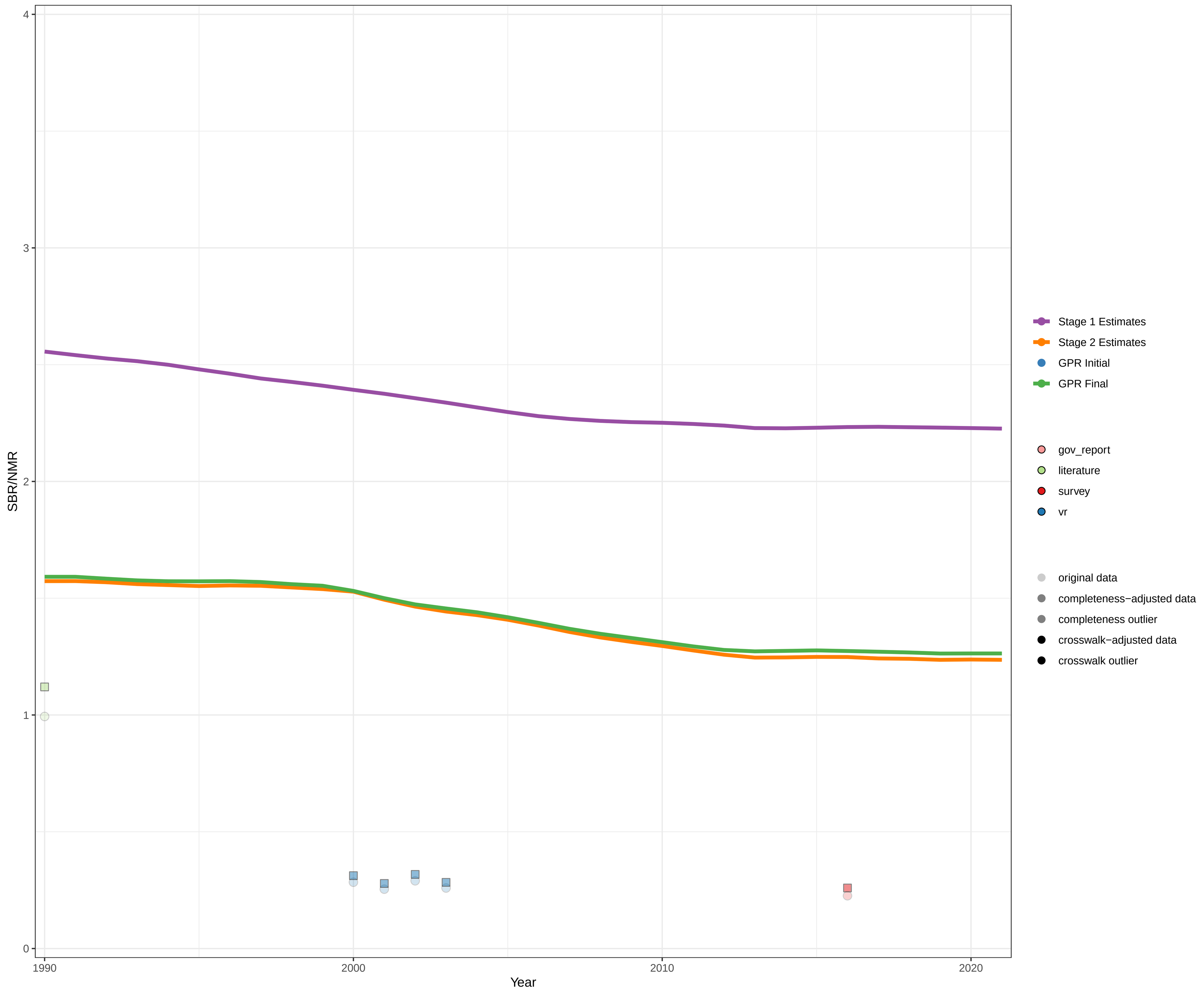

Definition	Years	Outlier	Source.Type	Title
22_weeks	1990	1	Sci Lit	A survey of Papua New Guinean parturients at the Port Moresby General Hospital: sociodemographic and reproductive characteristics
28_weeks	2000−2003	1	VR	United Nations Demographic Yearbook
28_weeks	2016	1	Survey	Papua New Guinea Demographic and Health Survey 2016−2018

Papua New Guinea PNG Oceania Stillbirth Rate (SBR) − 20 weeks
lambda = 1 ; zeta = 0.6 ; scale = 15 ; amp = 0.028

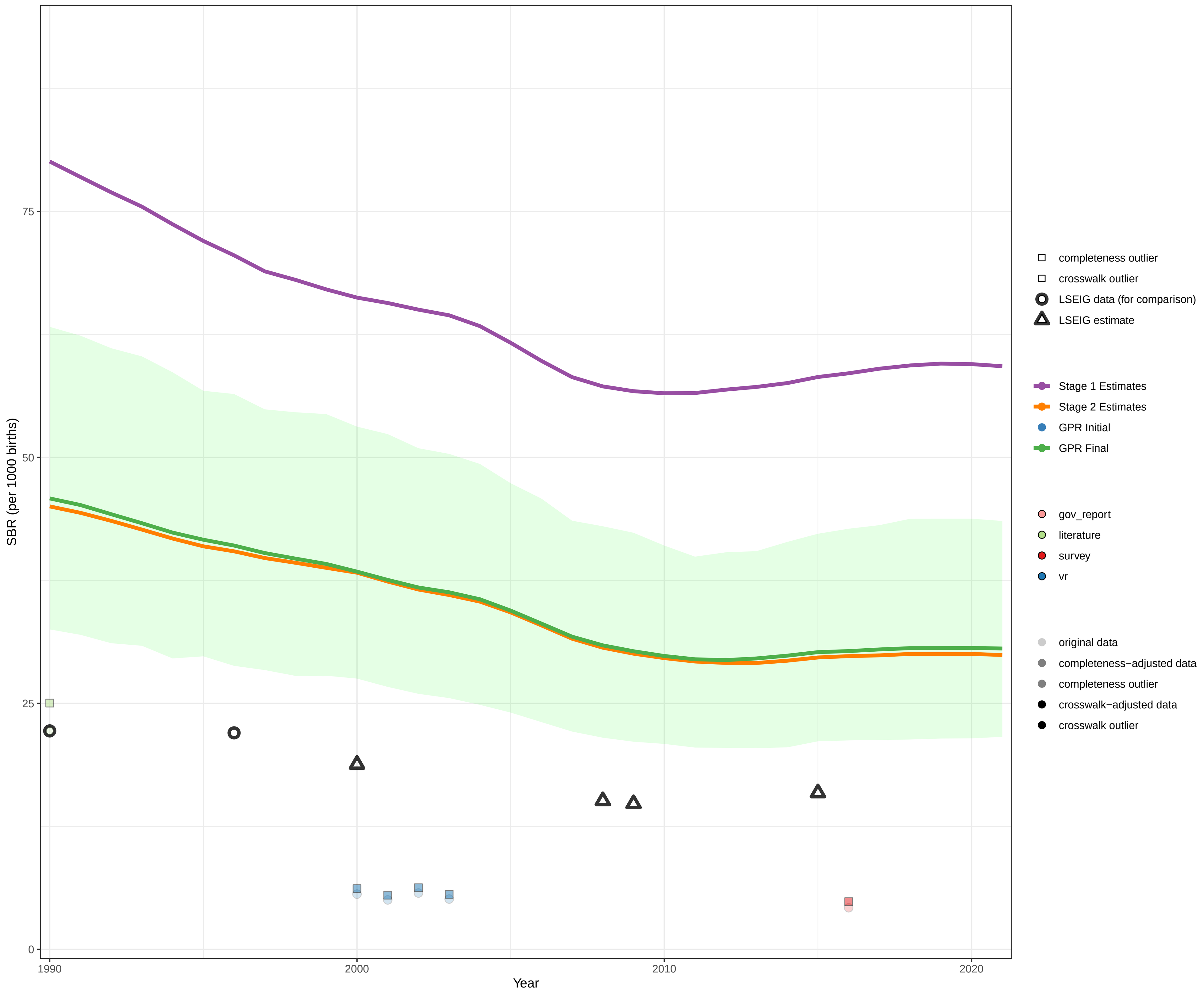

Papua New Guinea PNG Oceania Stillbirth Rate (SBR) − 28 weeks
lambda = 1 ; zeta = 0.6 ; scale = 15 ; amp = 0.028

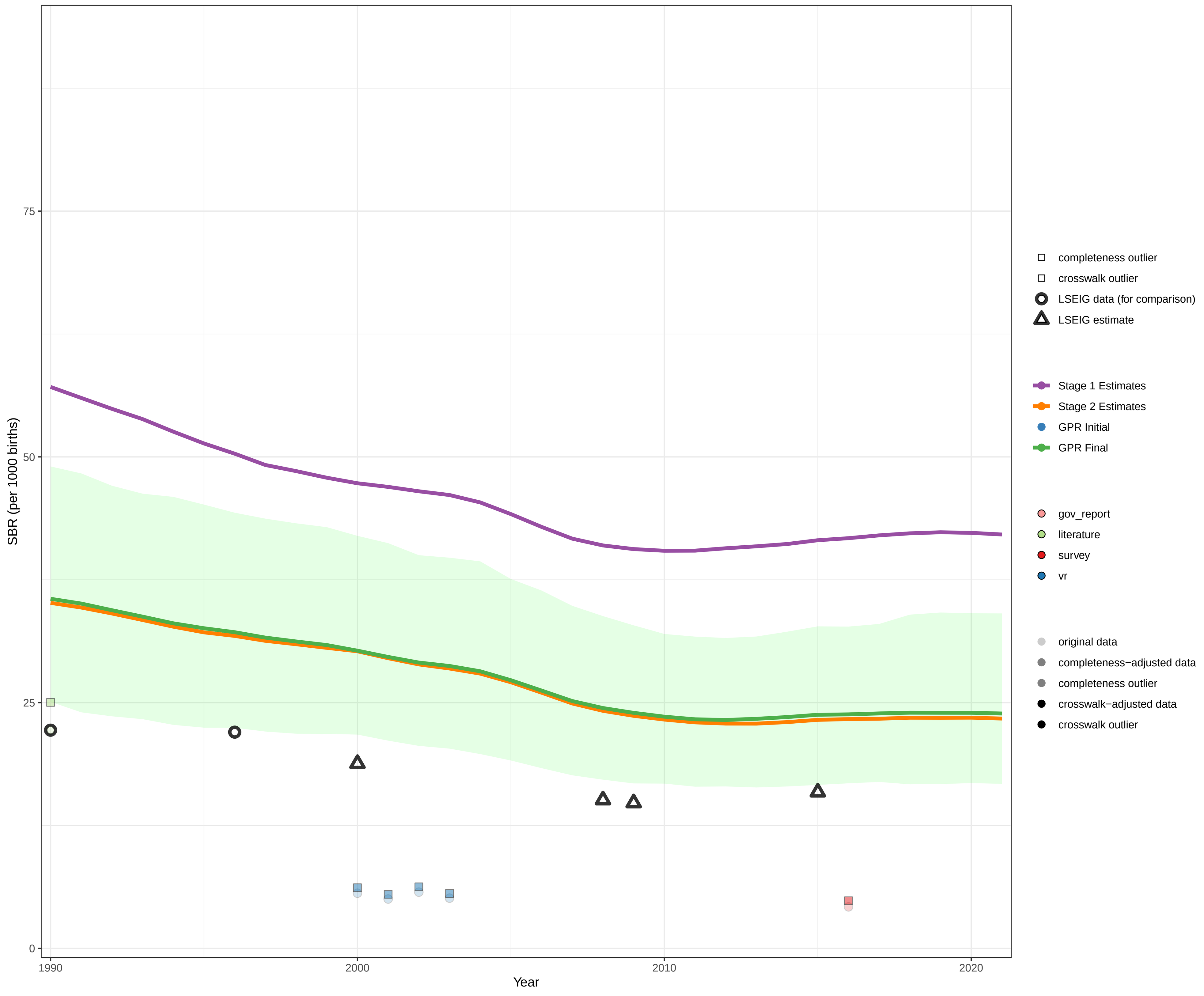

Year	Total Stillbirths (in thousands)	Total Stillbirths (in thousands)	SBR (per 1000 births)	SBR (per 1000 births)	SBR/NMR	SBR/NMR
	20 weeks	28 weeks	20 weeks	28 weeks	20 weeks	28 weeks
1990	7.1 (5.0 to 10.0)	5.4 (3.8 to 7.6)	45.8 (32.5 to 63.3)	35.6 (25.1 to 49.0)	2.05 (1.46 to 2.83)	1.59 (1.12 to 2.19)
1995	7.4 (5.2 to 10.2)	5.7 (3.9 to 8.0)	41.6 (29.8 to 56.8)	32.6 (22.5 to 45.1)	2.01 (1.44 to 2.74)	1.57 (1.08 to 2.18)
2000	7.7 (5.5 to 10.9)	6.1 (4.3 to 8.5)	38.4 (27.5 to 53.1)	30.3 (21.8 to 42.0)	1.94 (1.39 to 2.69)	1.53 (1.10 to 2.12)
2005	7.9 (5.5 to 11.1)	6.2 (4.3 to 8.7)	34.4 (24.1 to 47.4)	27.3 (19.1 to 37.6)	1.79 (1.25 to 2.46)	1.42 (0.99 to 1.95)
2010	8.0 (5.6 to 11.2)	6.3 (4.4 to 8.6)	29.8 (20.9 to 41.0)	23.6 (16.8 to 32.0)	1.66 (1.16 to 2.28)	1.31 (0.93 to 1.78)
2015	9.3 (6.5 to 13.2)	7.3 (5.1 to 10.1)	30.2 (21.1 to 42.2)	23.8 (16.6 to 32.8)	1.62 (1.14 to 2.27)	1.28 (0.89 to 1.76)
2020	10.7 (7.4 to 15.5)	8.3 (5.8 to 11.9)	30.6 (21.4 to 43.8)	24.0 (16.8 to 34.1)	1.61 (1.13 to 2.31)	1.26 (0.89 to 1.80)
2021	10.9 (7.6 to 15.7)	8.5 (5.9 to 12.2)	30.6 (21.6 to 43.5)	23.9 (16.7 to 34.1)	1.62 (1.14 to 2.30)	1.26 (0.89 to 1.80)

Samoa WSM Oceania Stillbirth Rate / Neonatal Mortality Rate (SBR/NMR) − 20 weeks
lambda = 0.8 ; zeta = 0.7 ; scale = 15 ; amp = 0.028

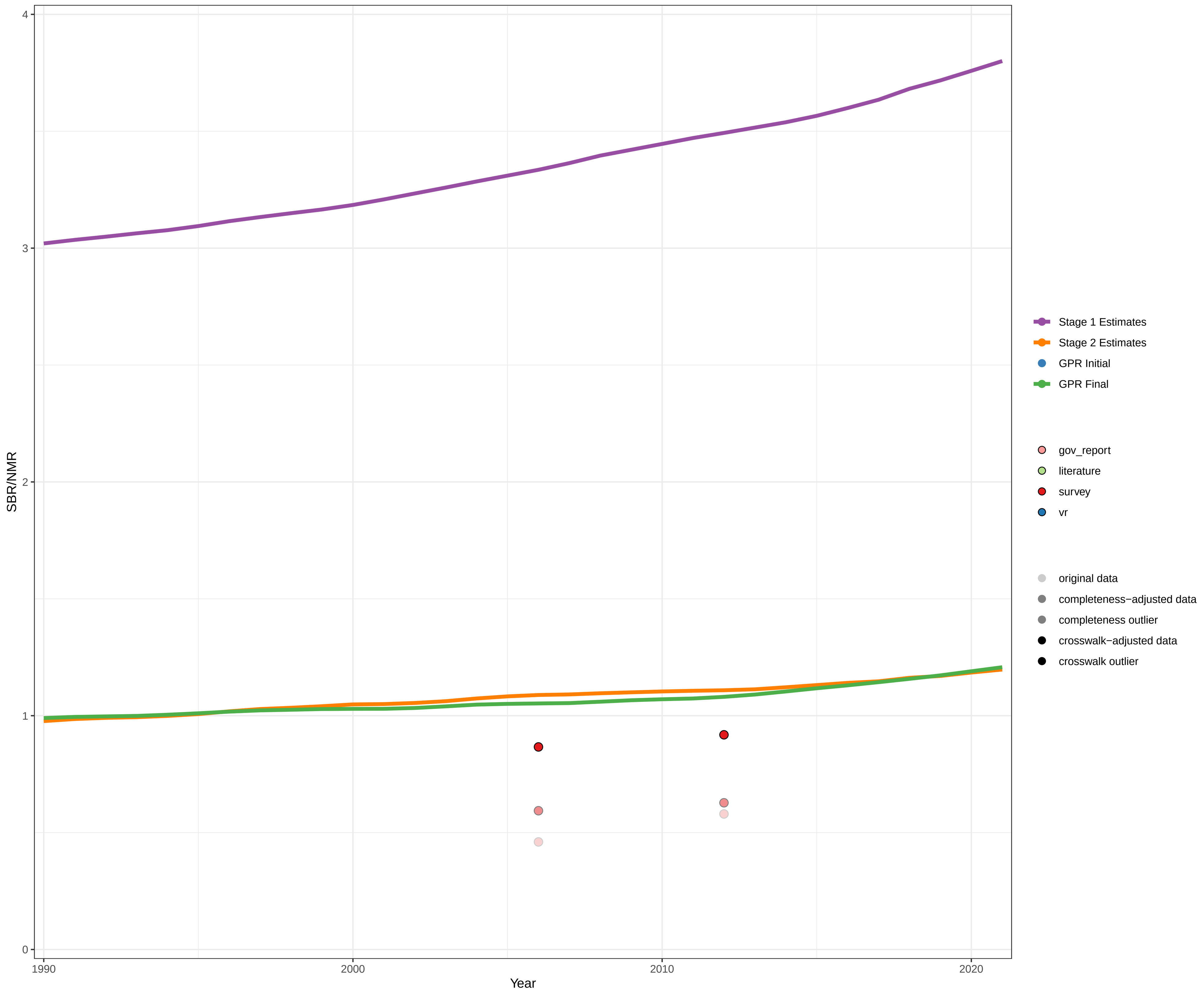

Samoa WSM Oceania Stillbirth Rate / Neonatal Mortality Rate (SBR/NMR) − 28 weeks
lambda = 0.8 ; zeta = 0.7 ; scale = 15 ; amp = 0.028

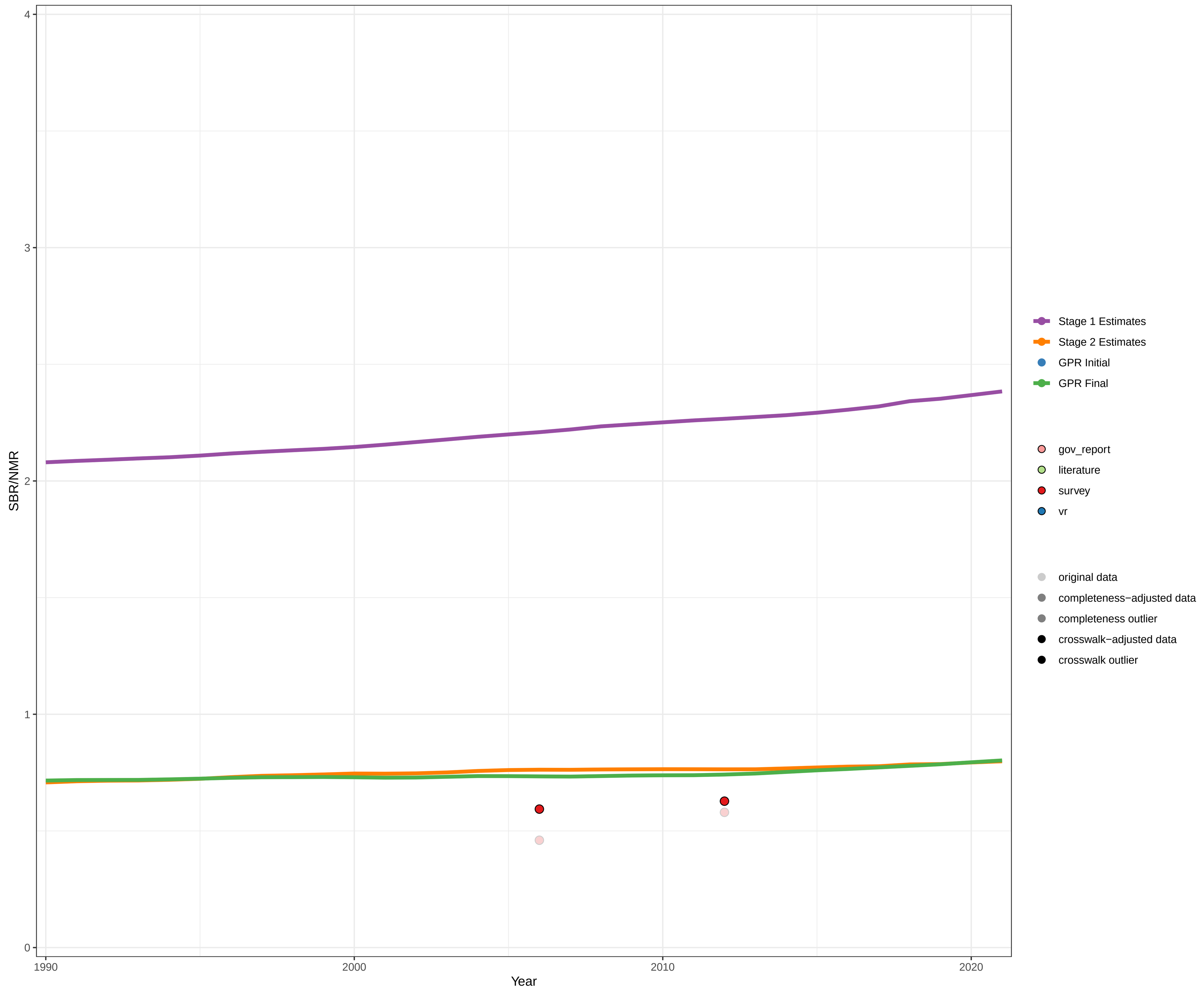

Definition	Years	Outlier	Source.Type	Title
28_weeks	2006	0	Survey	Samoa Demographic and Health Survey 2009
28_weeks	2012	0	Survey	Samoa Demographic and Health Survey 2014

Samoa WSM Oceania Stillbirth Rate (SBR) − 20 weeks
lambda = 0.8 ; zeta = 0.7 ; scale = 15 ; amp = 0.028

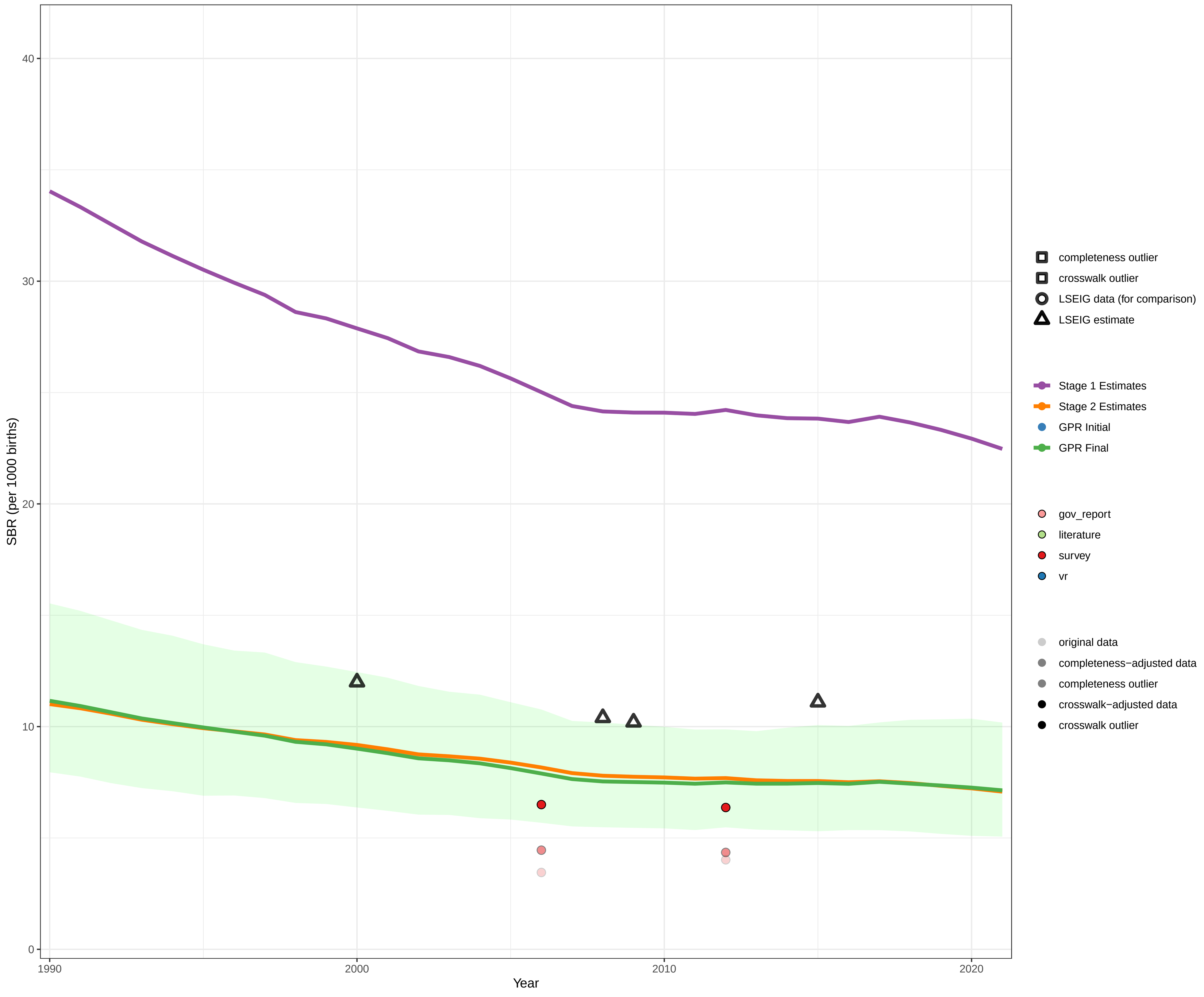

Samoa WSM Oceania Stillbirth Rate (SBR) − 28 weeks
lambda = 0.8 ; zeta = 0.7 ; scale = 15 ; amp = 0.028

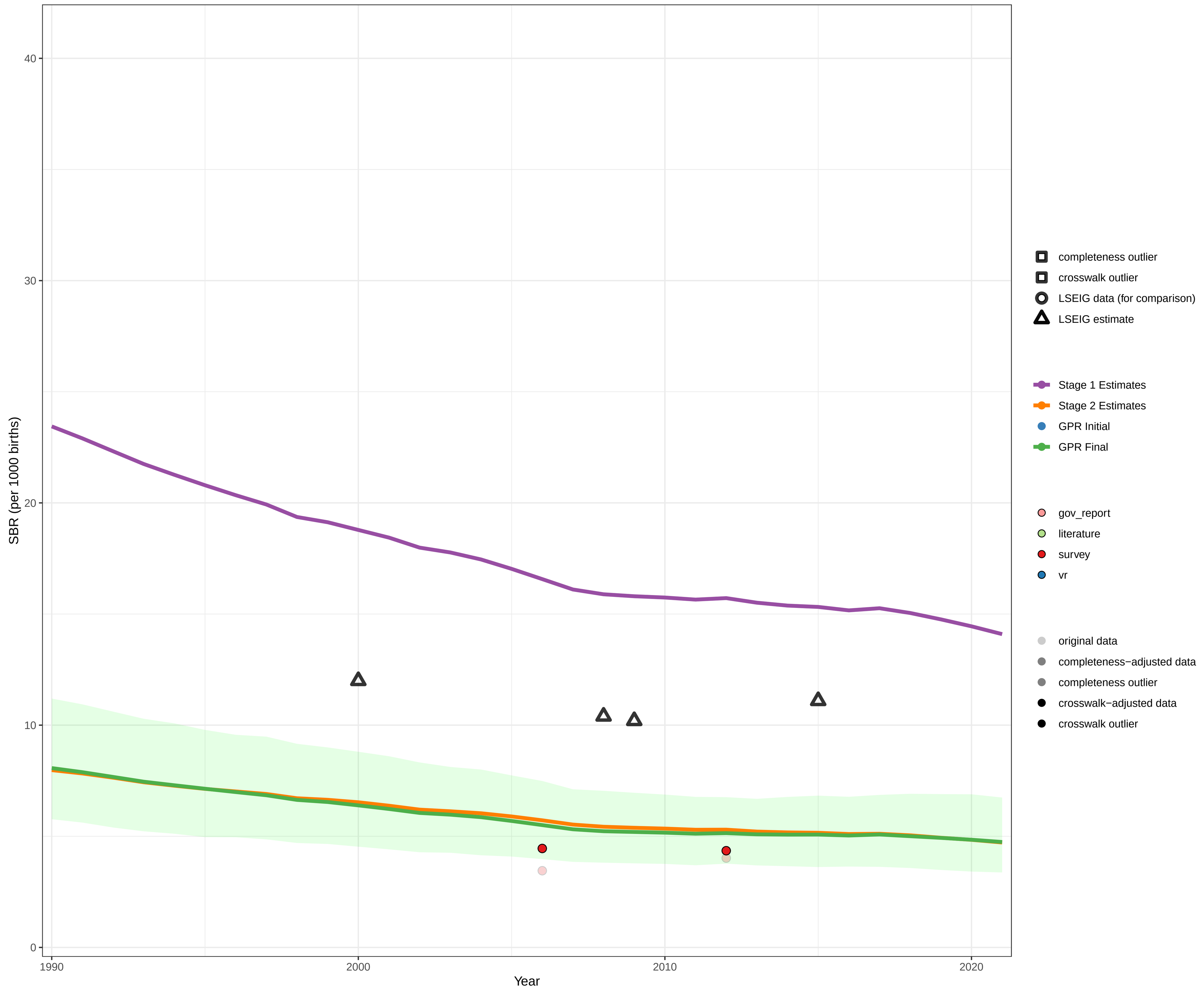

Year	Total Stillbirths (in thousands)	Total Stillbirths (in thousands)	SBR (per 1000 births)	SBR (per 1000 births)	SBR/NMR	SBR/NMR
	20 weeks	28 weeks	20 weeks	28 weeks	20 weeks	28 weeks
1990	0.1 (0.0 to 0.1)	0.0 (0.0 to 0.1)	11.2 (8.0 to 15.5)	8.1 (5.8 to 11.2)	0.99 (0.71 to 1.38)	0.72 (0.51 to 0.99)
1995	0.1 (0.0 to 0.1)	0.0 (0.0 to 0.1)	10.0 (6.9 to 13.7)	7.1 (5.0 to 9.8)	1.01 (0.70 to 1.39)	0.72 (0.50 to 0.99)
2000	0.1 (0.0 to 0.1)	0.0 (0.0 to 0.0)	9.0 (6.4 to 12.4)	6.4 (4.5 to 8.8)	1.03 (0.73 to 1.42)	0.73 (0.52 to 1.01)
2005	0.0 (0.0 to 0.1)	0.0 (0.0 to 0.0)	8.1 (5.8 to 11.1)	5.7 (4.1 to 7.7)	1.05 (0.75 to 1.43)	0.73 (0.53 to 1.00)
2010	0.0 (0.0 to 0.1)	0.0 (0.0 to 0.0)	7.5 (5.4 to 10.0)	5.2 (3.8 to 6.9)	1.07 (0.78 to 1.43)	0.74 (0.54 to 0.98)
2015	0.0 (0.0 to 0.1)	0.0 (0.0 to 0.0)	7.5 (5.3 to 10.1)	5.1 (3.6 to 6.8)	1.12 (0.79 to 1.51)	0.76 (0.54 to 1.02)
2020	0.0 (0.0 to 0.1)	0.0 (0.0 to 0.0)	7.3 (5.1 to 10.4)	4.8 (3.4 to 6.9)	1.19 (0.84 to 1.70)	0.79 (0.56 to 1.13)
2021	0.0 (0.0 to 0.1)	0.0 (0.0 to 0.0)	7.1 (5.1 to 10.2)	4.7 (3.4 to 6.7)	1.21 (0.86 to 1.72)	0.80 (0.57 to 1.14)

Solomon Islands SLB Oceania Stillbirth Rate / Neonatal Mortality Rate (SBR/NMR) − 20 weeks
lambda = 1 ; zeta = 0.6 ; scale = 15 ; amp = 0.028

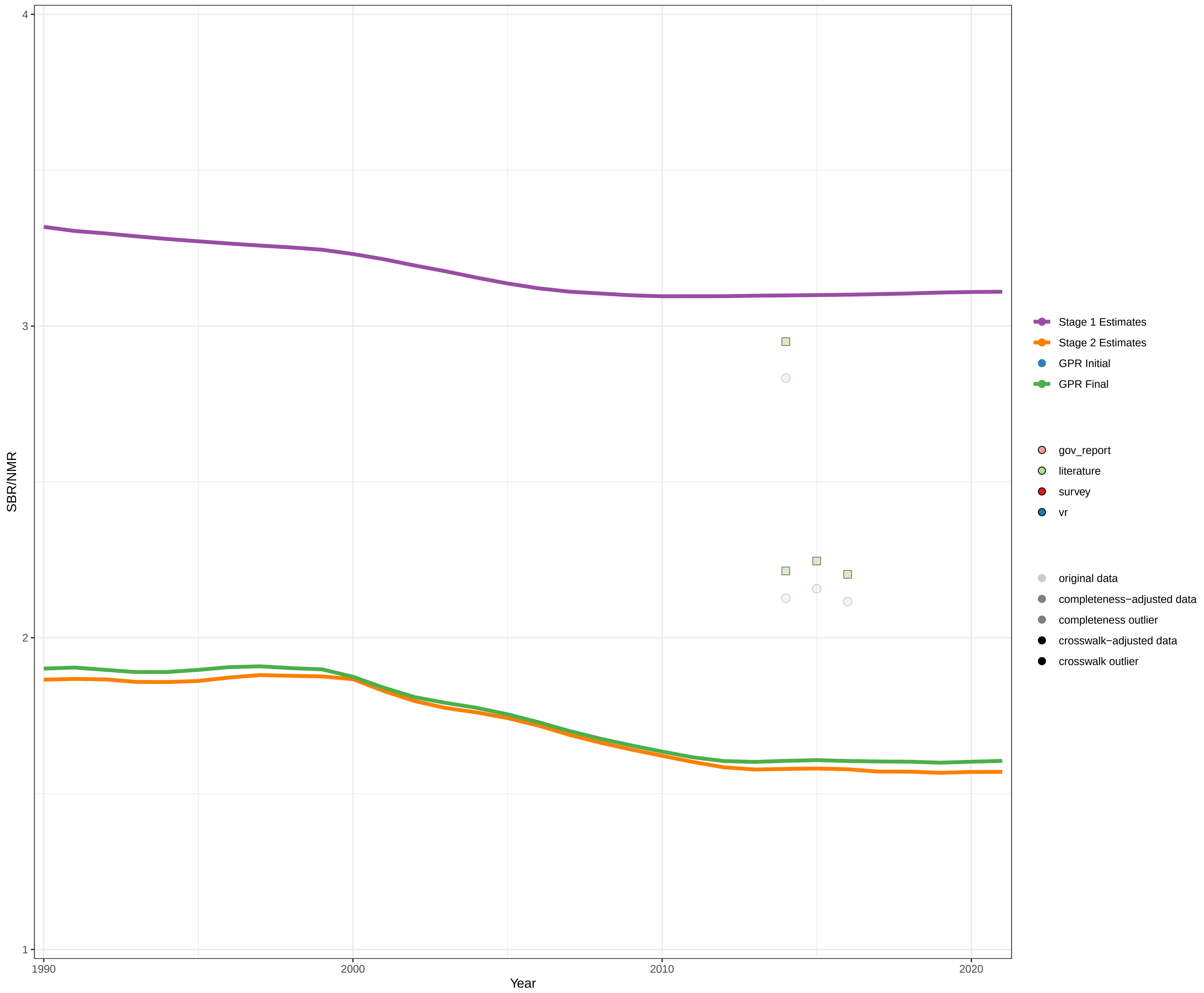

Solomon Islands SLB Oceania Stillbirth Rate / Neonatal Mortality Rate (SBR/NMR) − 28 weeks
lambda = 1 ; zeta = 0.6 ; scale = 15 ; amp = 0.028

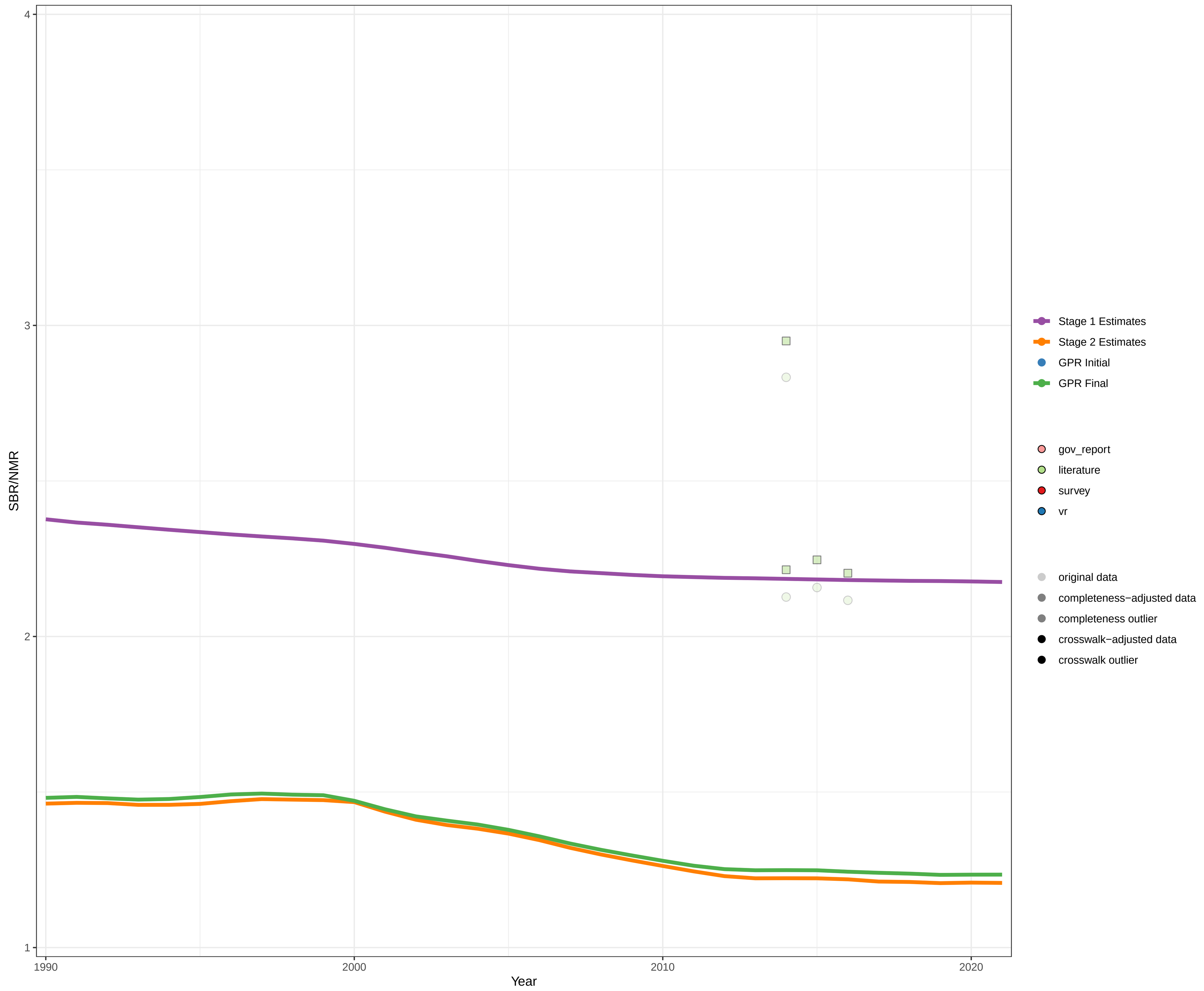

Definition	Years	Outlier	Source.Type	Title
28_weeks	2014	1	Sci Lit	Evaluating the process and outcomes of child death review in the Solomon Islands
28_weeks	2014−2016	1	Sci Lit	High perinatal mortality rates persist in Kirakira: The sustainable development goals for health remain out of reach in the provinces of Solomon Islands

Solomon Islands SLB Oceania Stillbirth Rate (SBR) − 20 weeks
lambda = 1 ; zeta = 0.6 ; scale = 15 ; amp = 0.028

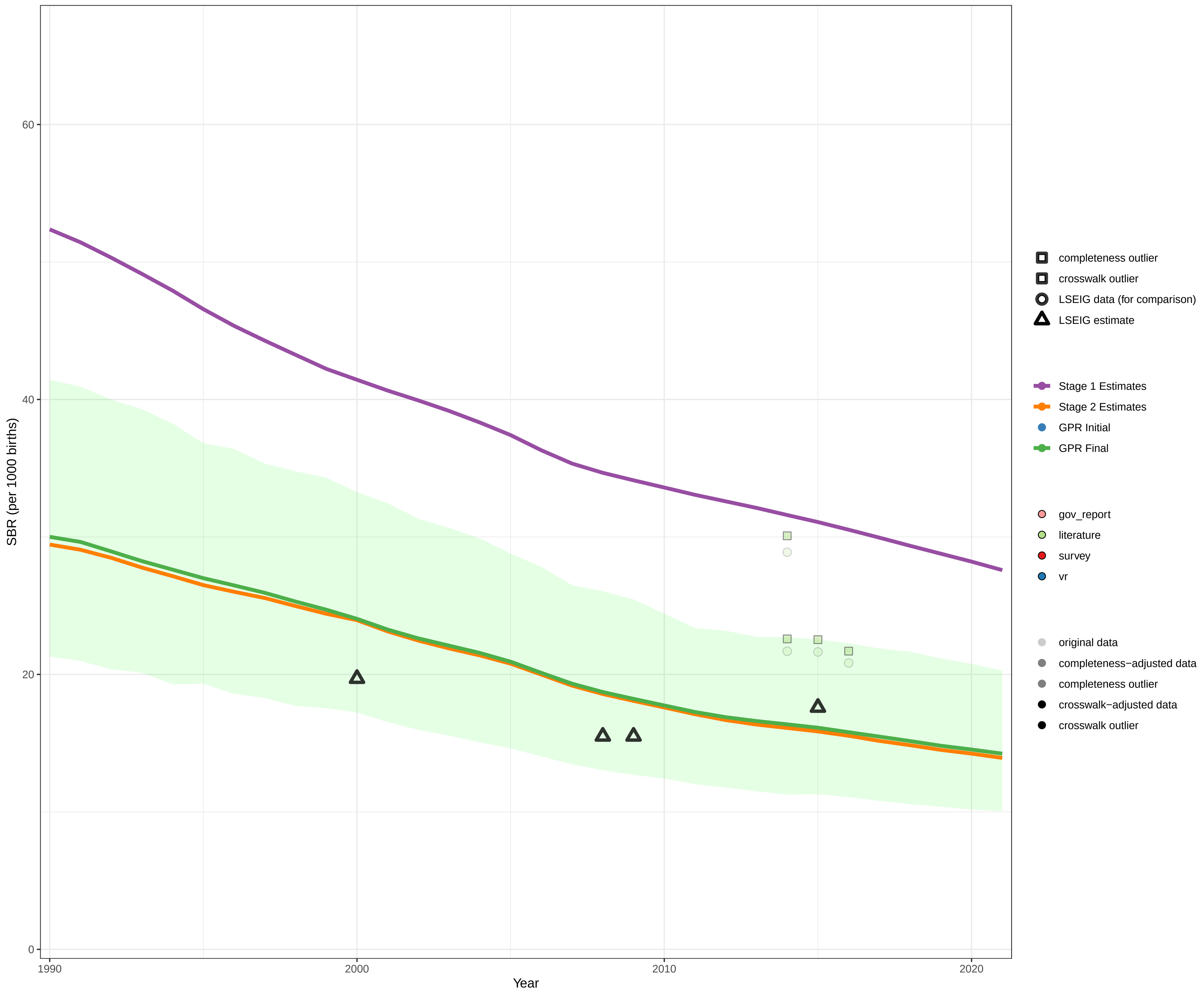

Solomon Islands SLB Oceania Stillbirth Rate (SBR) − 28 weeks
lambda = 1 ; zeta = 0.6 ; scale = 15 ; amp = 0.028

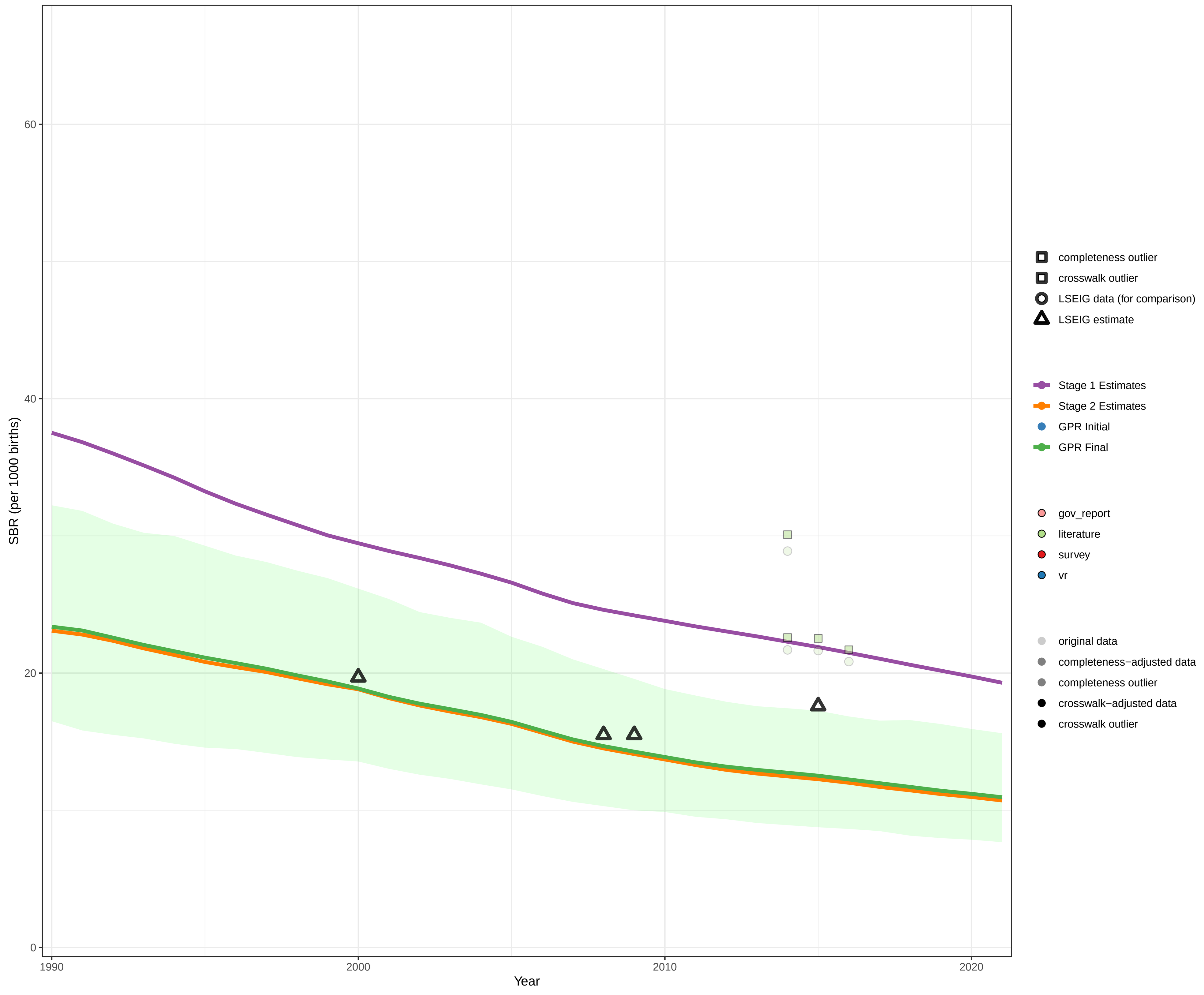

Year	Total Stillbirths (in thousands)	Total Stillbirths (in thousands)	SBR (per 1000 births)	SBR (per 1000 births)	SBR/NMR	SBR/NMR
	20 weeks	28 weeks	20 weeks	28 weeks	20 weeks	28 weeks
1990	0.4 (0.3 to 0.6)	0.3 (0.2 to 0.5)	30.0 (21.3 to 41.4)	23.4 (16.5 to 32.2)	1.90 (1.35 to 2.62)	1.48 (1.04 to 2.04)
1995	0.4 (0.3 to 0.6)	0.3 (0.2 to 0.5)	27.0 (19.3 to 36.8)	21.1 (14.6 to 29.3)	1.90 (1.36 to 2.59)	1.48 (1.02 to 2.06)
2000	0.4 (0.3 to 0.6)	0.3 (0.2 to 0.5)	24.0 (17.2 to 33.3)	18.9 (13.6 to 26.2)	1.87 (1.34 to 2.59)	1.47 (1.06 to 2.04)
2005	0.4 (0.3 to 0.5)	0.3 (0.2 to 0.4)	20.9 (14.6 to 28.8)	16.4 (11.5 to 22.6)	1.75 (1.23 to 2.41)	1.38 (0.97 to 1.90)
2010	0.3 (0.2 to 0.5)	0.3 (0.2 to 0.4)	17.7 (12.4 to 24.4)	13.9 (9.9 to 18.8)	1.63 (1.14 to 2.25)	1.28 (0.91 to 1.74)
2015	0.3 (0.2 to 0.5)	0.2 (0.2 to 0.3)	16.1 (11.3 to 22.5)	12.5 (8.8 to 17.3)	1.61 (1.13 to 2.25)	1.25 (0.87 to 1.72)
2020	0.3 (0.2 to 0.4)	0.2 (0.2 to 0.3)	14.5 (10.2 to 20.8)	11.2 (7.9 to 15.9)	1.60 (1.12 to 2.29)	1.23 (0.87 to 1.76)
2021	0.3 (0.2 to 0.4)	0.2 (0.2 to 0.3)	14.2 (10.1 to 20.3)	10.9 (7.7 to 15.6)	1.61 (1.13 to 2.29)	1.23 (0.87 to 1.76)

Tokelau TKL Oceania Stillbirth Rate / Neonatal Mortality Rate (SBR/NMR) − 20 weeks
lambda = 1 ; zeta = 0.6 ; scale = 15 ; amp = 0.028

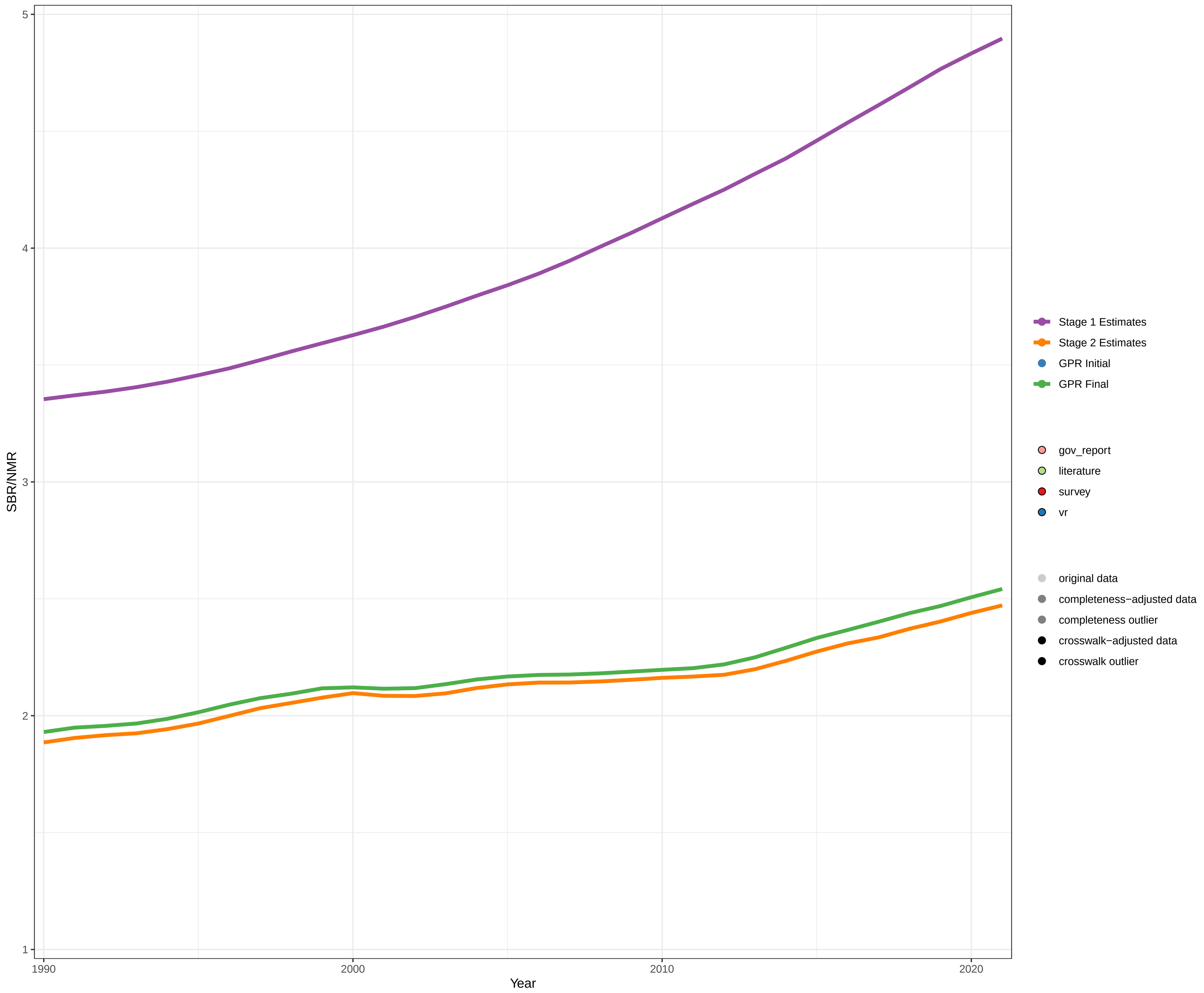

Tokelau TKL Oceania Stillbirth Rate / Neonatal Mortality Rate (SBR/NMR) − 28 weeks
lambda = 1 ; zeta = 0.6 ; scale = 15 ; amp = 0.028

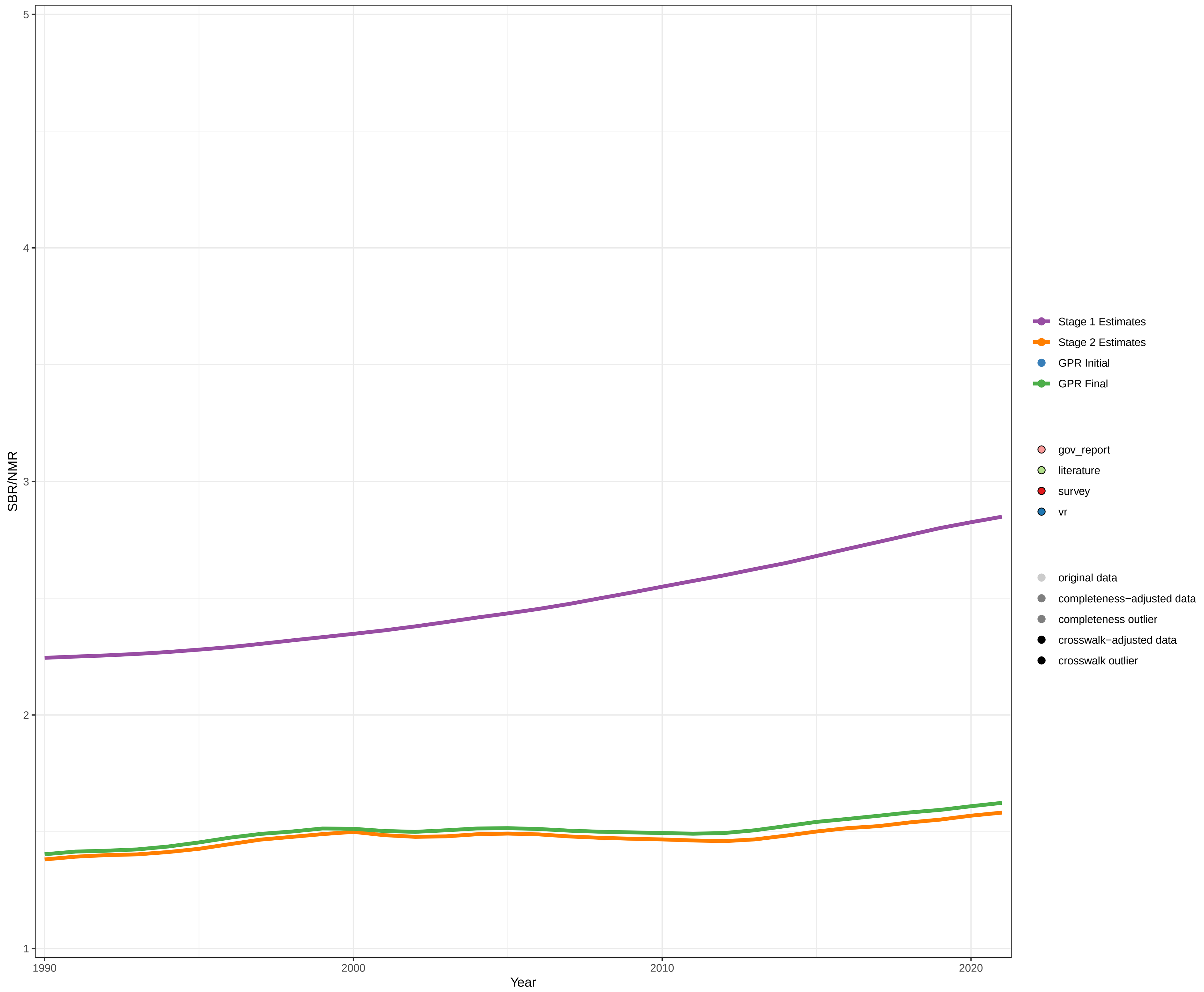

Definition	Years	Outlier	Source.Type	Title

Tokelau TKL Oceania Stillbirth Rate (SBR) − 20 weeks
lambda = 1 ; zeta = 0.6 ; scale = 15 ; amp = 0.028

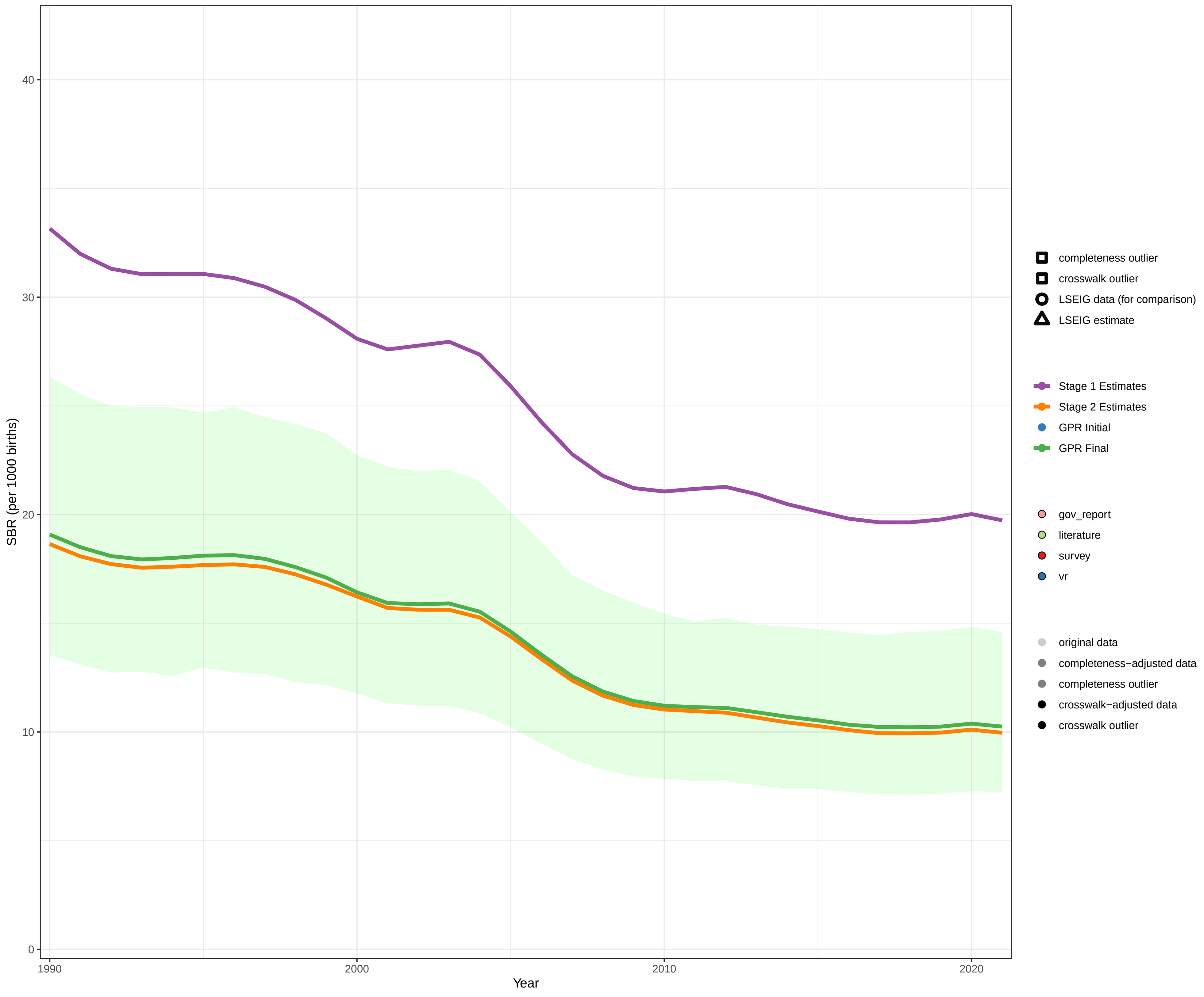

Tokelau TKL Oceania Stillbirth Rate (SBR) − 28 weeks
lambda = 1 ; zeta = 0.6 ; scale = 15 ; amp = 0.028

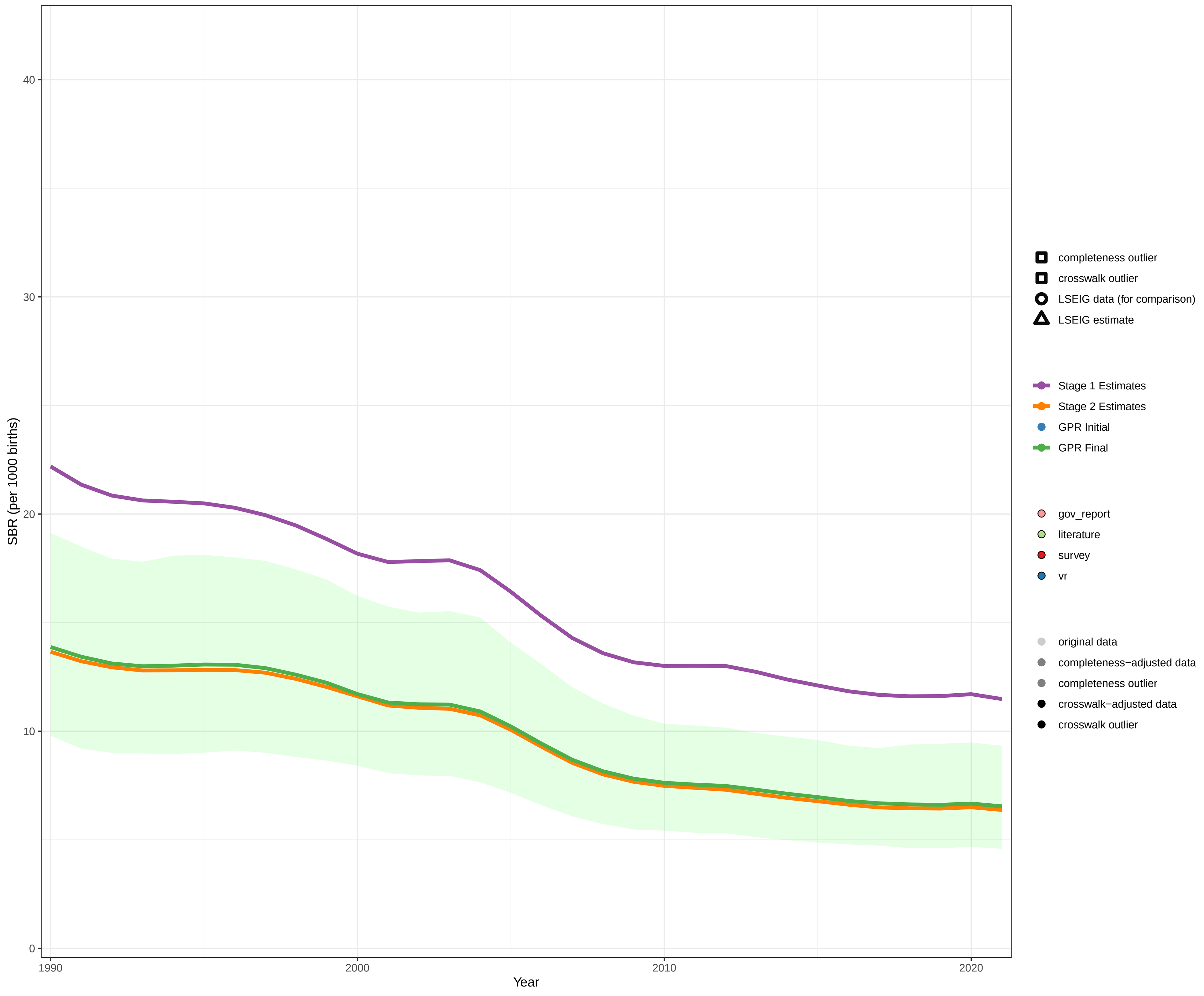

Year	Total Stillbirths (in thousands)	Total Stillbirths (in thousands)	SBR (per 1000 births)	SBR (per 1000 births)	SBR/NMR	SBR/NMR
	20 weeks	28 weeks	20 weeks	28 weeks	20 weeks	28 weeks
1990	0.0 (0.0 to 0.0)	0.0 (0.0 to 0.0)	19.1 (13.5 to 26.3)	13.9 (9.8 to 19.1)	1.93 (1.37 to 2.66)	1.40 (0.99 to 1.94)
1995	0.0 (0.0 to 0.0)	0.0 (0.0 to 0.0)	18.1 (13.0 to 24.7)	13.1 (9.0 to 18.1)	2.01 (1.44 to 2.75)	1.45 (1.00 to 2.02)
2000	0.0 (0.0 to 0.0)	0.0 (0.0 to 0.0)	16.4 (11.8 to 22.7)	11.7 (8.4 to 16.2)	2.12 (1.52 to 2.94)	1.51 (1.09 to 2.10)
2005	0.0 (0.0 to 0.0)	0.0 (0.0 to 0.0)	14.6 (10.2 to 20.1)	10.2 (7.2 to 14.1)	2.17 (1.52 to 2.98)	1.52 (1.06 to 2.09)
2010	0.0 (0.0 to 0.0)	0.0 (0.0 to 0.0)	11.2 (7.8 to 15.4)	7.6 (5.4 to 10.3)	2.20 (1.54 to 3.02)	1.49 (1.06 to 2.03)
2015	0.0 (0.0 to 0.0)	0.0 (0.0 to 0.0)	10.5 (7.4 to 14.7)	7.0 (4.9 to 9.6)	2.33 (1.63 to 3.26)	1.54 (1.08 to 2.13)
2020	0.0 (0.0 to 0.0)	0.0 (0.0 to 0.0)	10.4 (7.3 to 14.8)	6.7 (4.7 to 9.5)	0.47 (0.33 to 0.67)	0.30 (0.21 to 0.43)
2021	0.0 (0.0 to 0.0)	0.0 (0.0 to 0.0)	10.2 (7.2 to 14.6)	6.5 (4.6 to 9.3)	0.45 (0.32 to 0.65)	0.29 (0.20 to 0.41)

Tonga TON Oceania Stillbirth Rate / Neonatal Mortality Rate (SBR/NMR) − 20 weeks
lambda = 0.8 ; zeta = 0.7 ; scale = 15 ; amp = 0.028

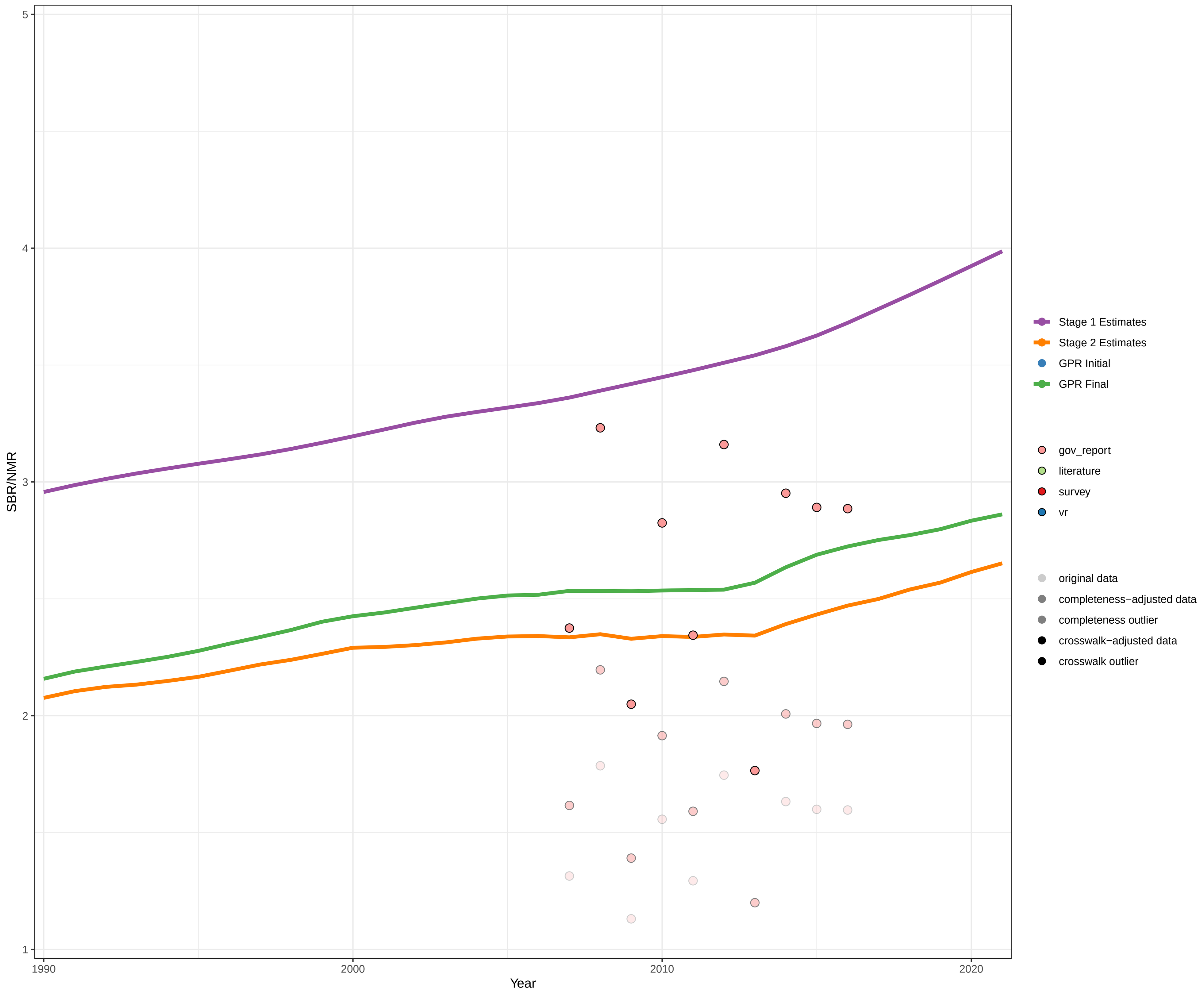

Tonga TON Oceania Stillbirth Rate / Neonatal Mortality Rate (SBR/NMR) − 28 weeks
lambda = 0.8 ; zeta = 0.7 ; scale = 15 ; amp = 0.028

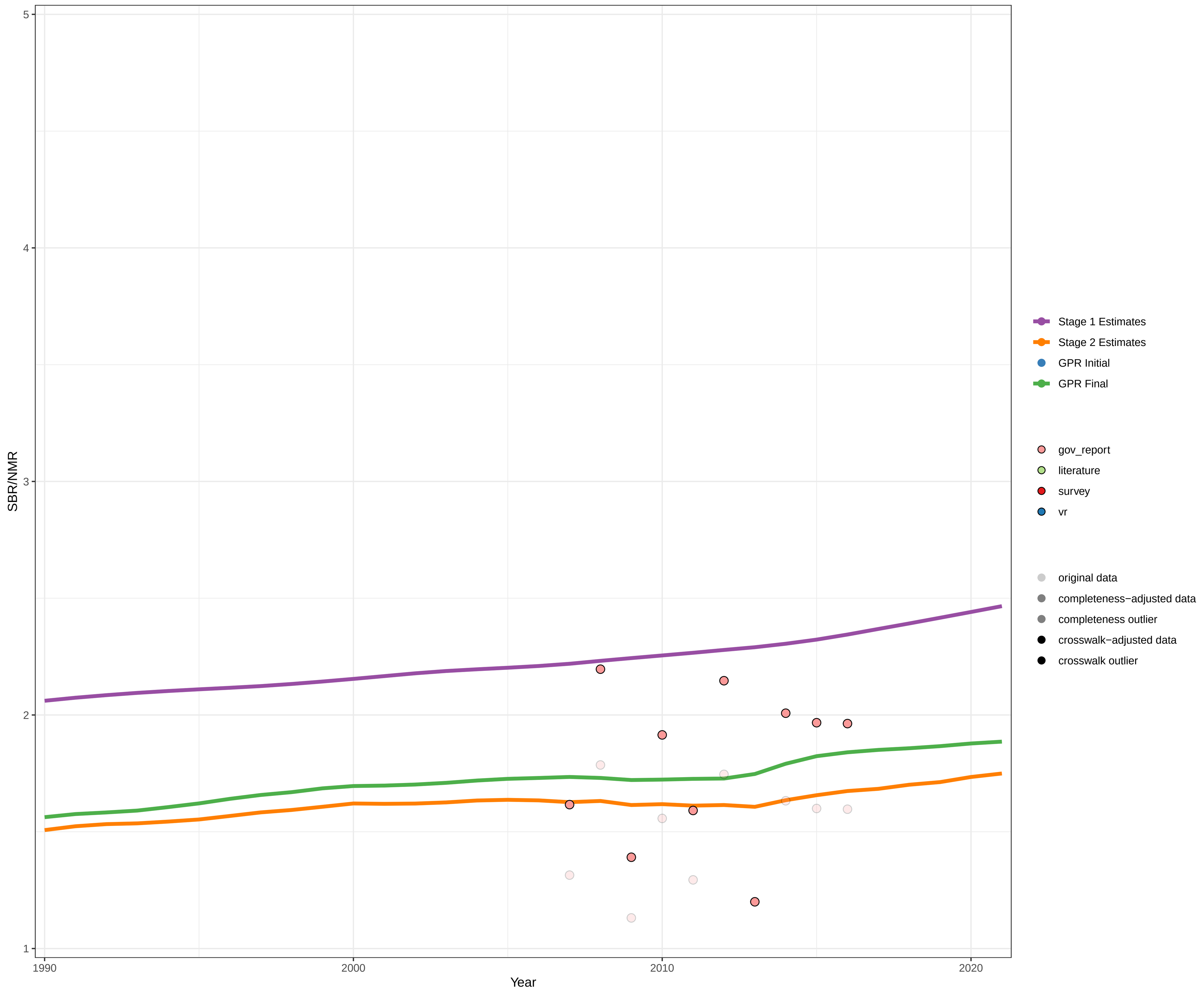

Definition	Years	Outlier	Source.Type	Title
28_weeks	2007–2016	0	Statistical Report	Tonga Report of the Minister for Health for the Year

Tonga TON Oceania Stillbirth Rate (SBR) − 20 weeks
lambda = 0.8 ; zeta = 0.7 ; scale = 15 ; amp = 0.028

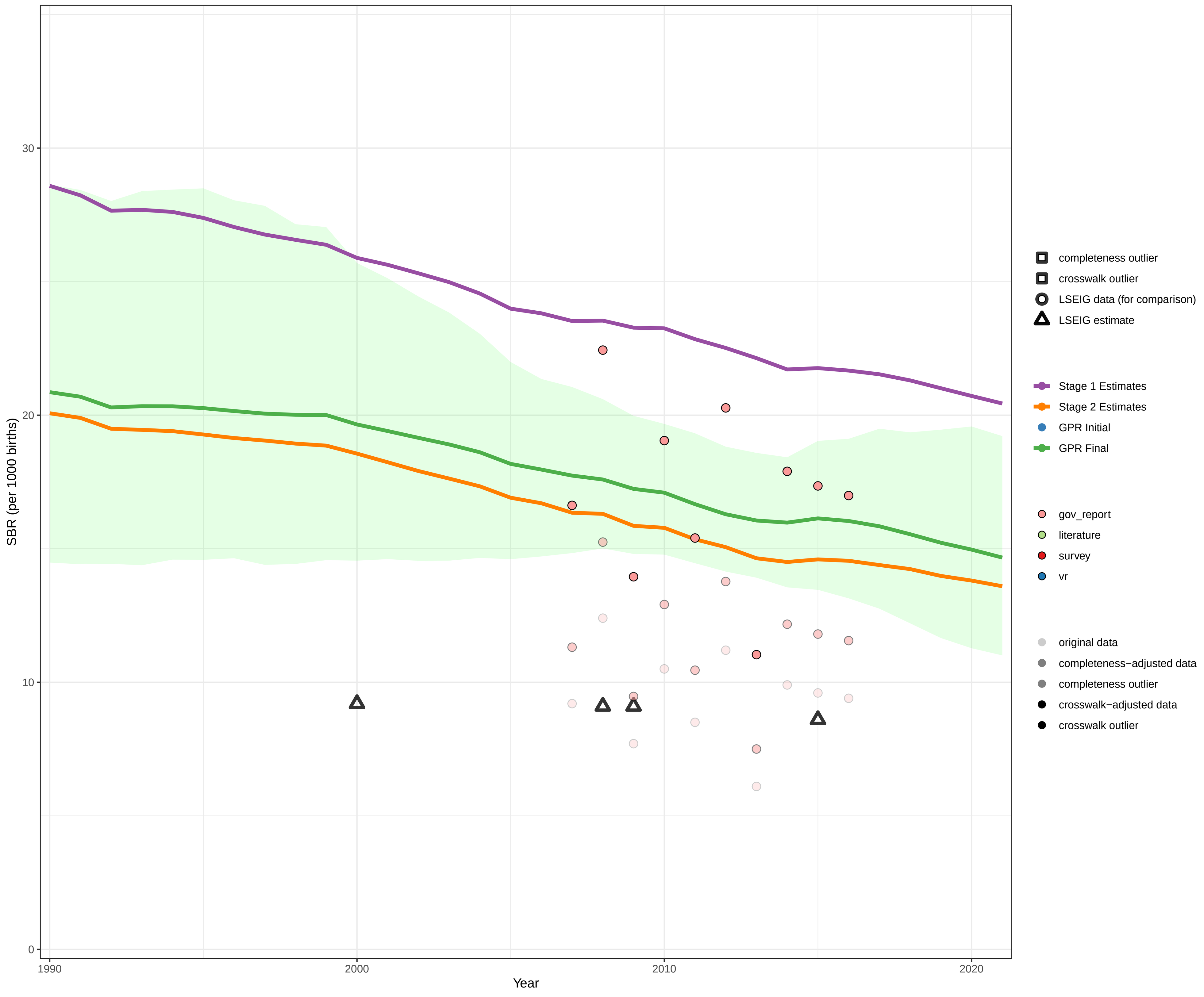

Tonga TON Oceania Stillbirth Rate (SBR) − 28 weeks
lambda = 0.8 ; zeta = 0.7 ; scale = 15 ; amp = 0.028

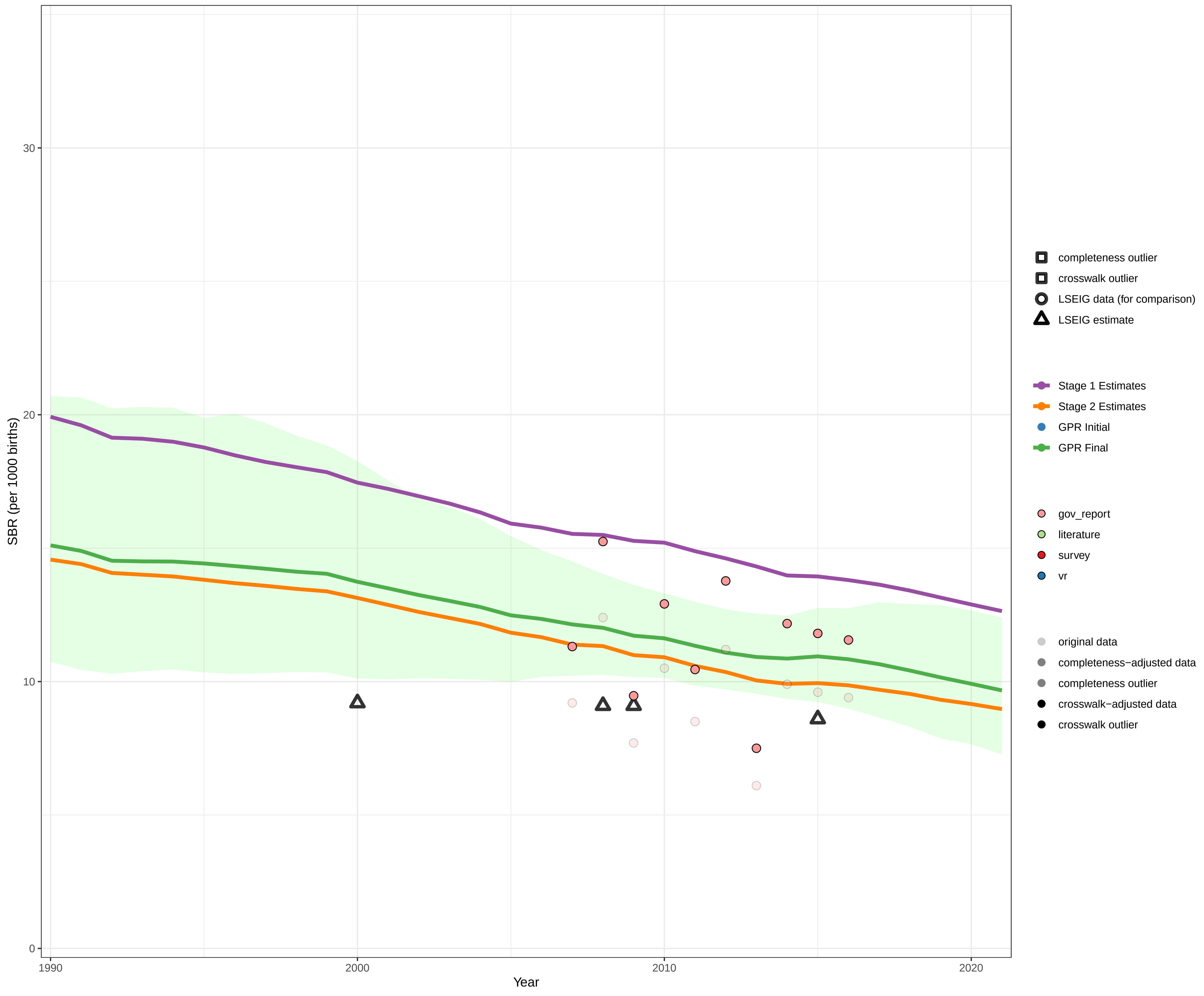

Year	Total Stillbirths (in thousands)	Total Stillbirths (in thousands)	SBR (per 1000 births)	SBR (per 1000 births)	SBR/NMR	SBR/NMR
	20 weeks	28 weeks	20 weeks	28 weeks	20 weeks	28 weeks
1990	0.1 (0.0 to 0.1)	0.1 (0.0 to 0.1)	20.9 (14.5 to 28.6)	15.1 (10.7 to 20.7)	2.16 (1.50 to 2.96)	1.56 (1.11 to 2.14)
1995	0.1 (0.0 to 0.1)	0.0 (0.0 to 0.1)	20.3 (14.6 to 28.5)	14.4 (10.3 to 19.9)	2.28 (1.64 to 3.20)	1.62 (1.16 to 2.23)
2000	0.1 (0.0 to 0.1)	0.0 (0.0 to 0.1)	19.7 (14.6 to 25.7)	13.7 (10.1 to 18.3)	2.43 (1.80 to 3.17)	1.70 (1.25 to 2.25)
2005	0.1 (0.0 to 0.1)	0.0 (0.0 to 0.1)	18.2 (14.6 to 22.0)	12.5 (10.0 to 15.5)	2.51 (2.02 to 3.04)	1.73 (1.38 to 2.14)
2010	0.1 (0.0 to 0.1)	0.0 (0.0 to 0.0)	17.1 (14.8 to 19.7)	11.6 (10.1 to 13.3)	2.54 (2.19 to 2.92)	1.72 (1.50 to 1.97)
2015	0.1 (0.0 to 0.1)	0.0 (0.0 to 0.0)	16.1 (13.5 to 19.0)	10.9 (9.2 to 12.8)	2.69 (2.24 to 3.17)	1.82 (1.54 to 2.13)
2020	0.0 (0.0 to 0.1)	0.0 (0.0 to 0.0)	15.0 (11.3 to 19.6)	9.9 (7.6 to 12.7)	2.83 (2.14 to 3.71)	1.88 (1.45 to 2.40)
2021	0.0 (0.0 to 0.1)	0.0 (0.0 to 0.0)	14.7 (11.0 to 19.2)	9.7 (7.3 to 12.4)	2.86 (2.15 to 3.75)	1.89 (1.42 to 2.42)

Tuvalu TUV Oceania Stillbirth Rate / Neonatal Mortality Rate (SBR/NMR) − 20 weeks
lambda = 1 ; zeta = 0.6 ; scale = 15 ; amp = 0.028

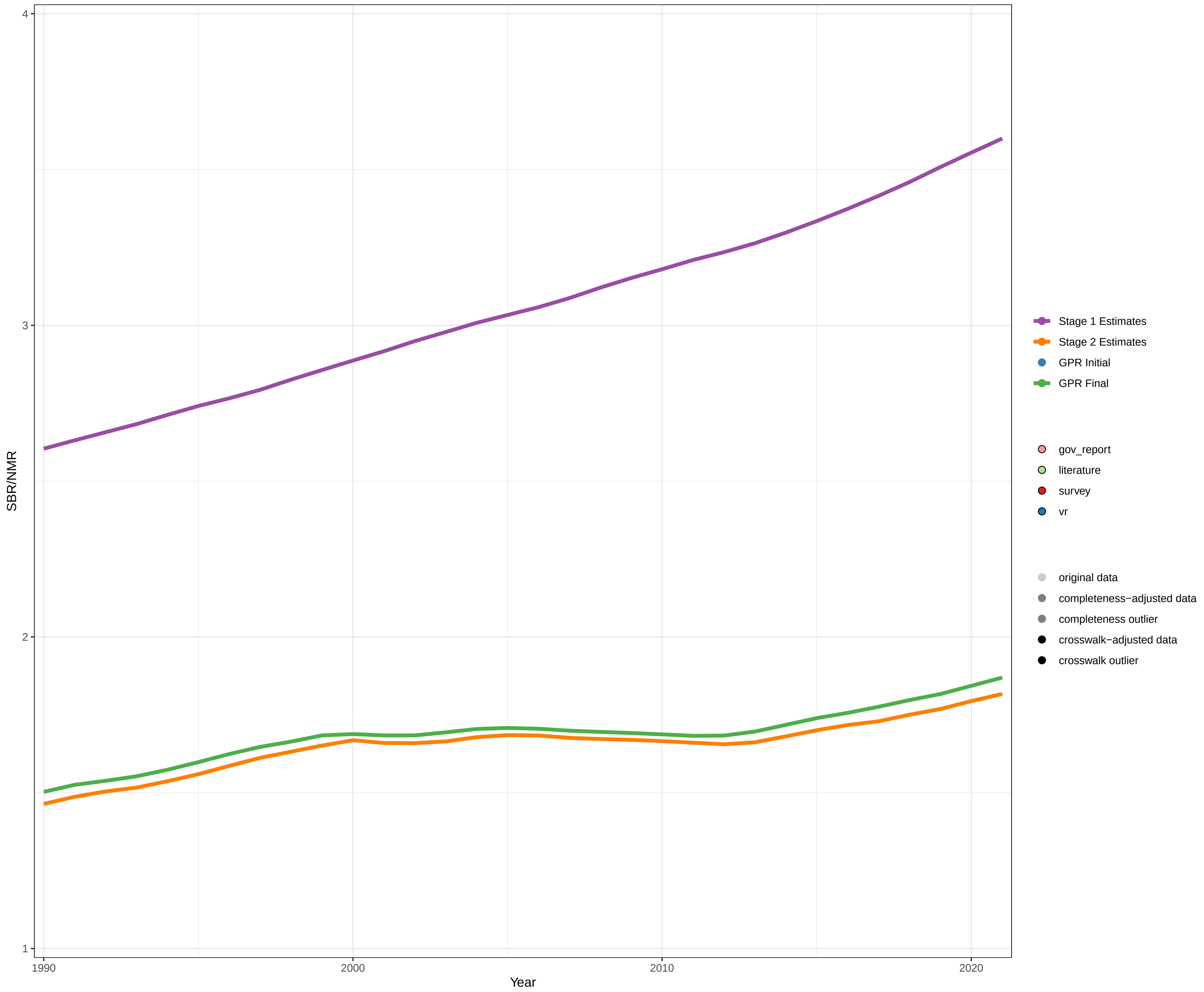

Tuvalu TUV Oceania Stillbirth Rate / Neonatal Mortality Rate (SBR/NMR) − 28 weeks
lambda = 1 ; zeta = 0.6 ; scale = 15 ; amp = 0.028

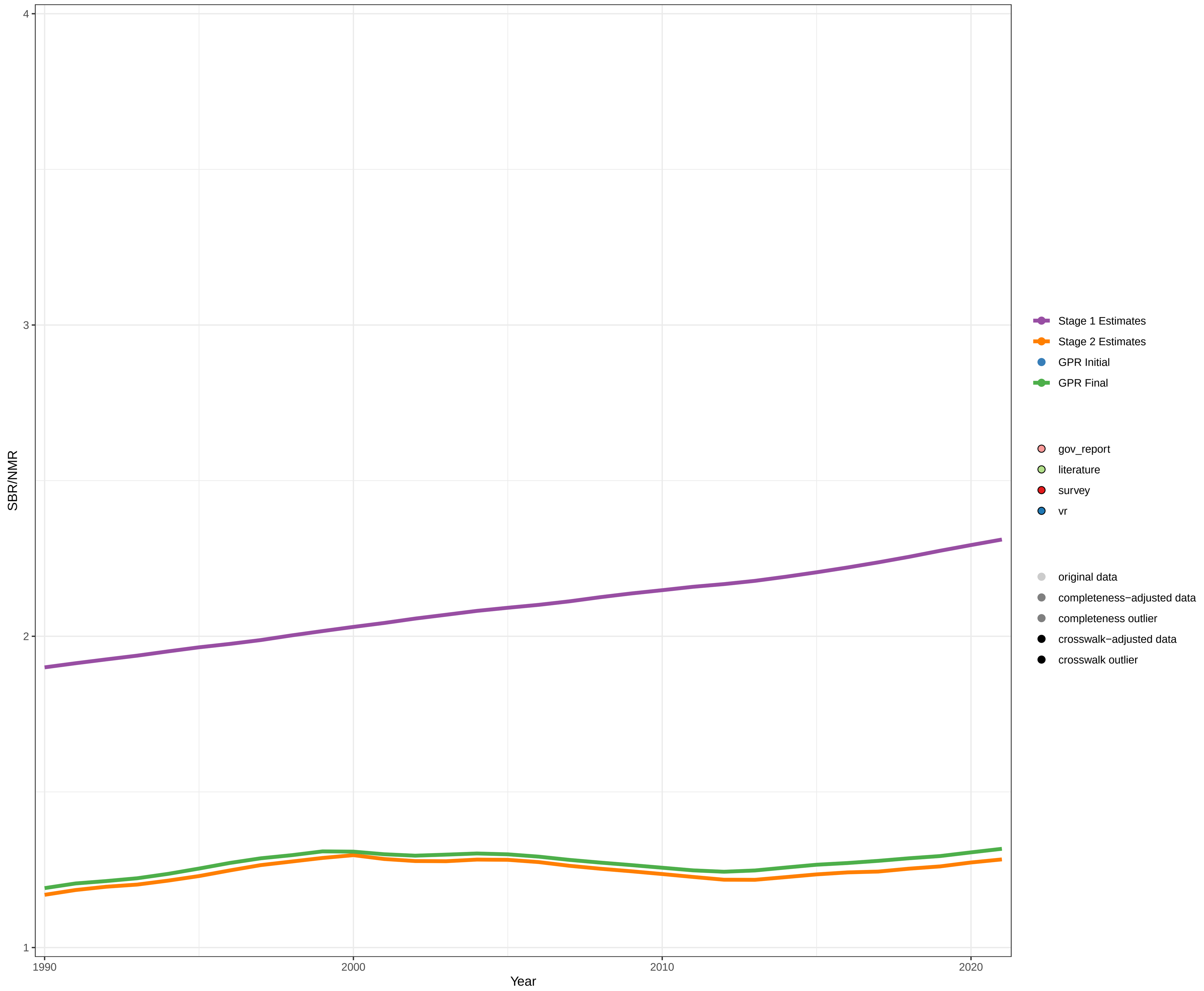

Definition	Years	Outlier	Source.Type	Title

Tuvalu TUV Oceania Stillbirth Rate (SBR) − 20 weeks
lambda = 1 ; zeta = 0.6 ; scale = 15 ; amp = 0.028

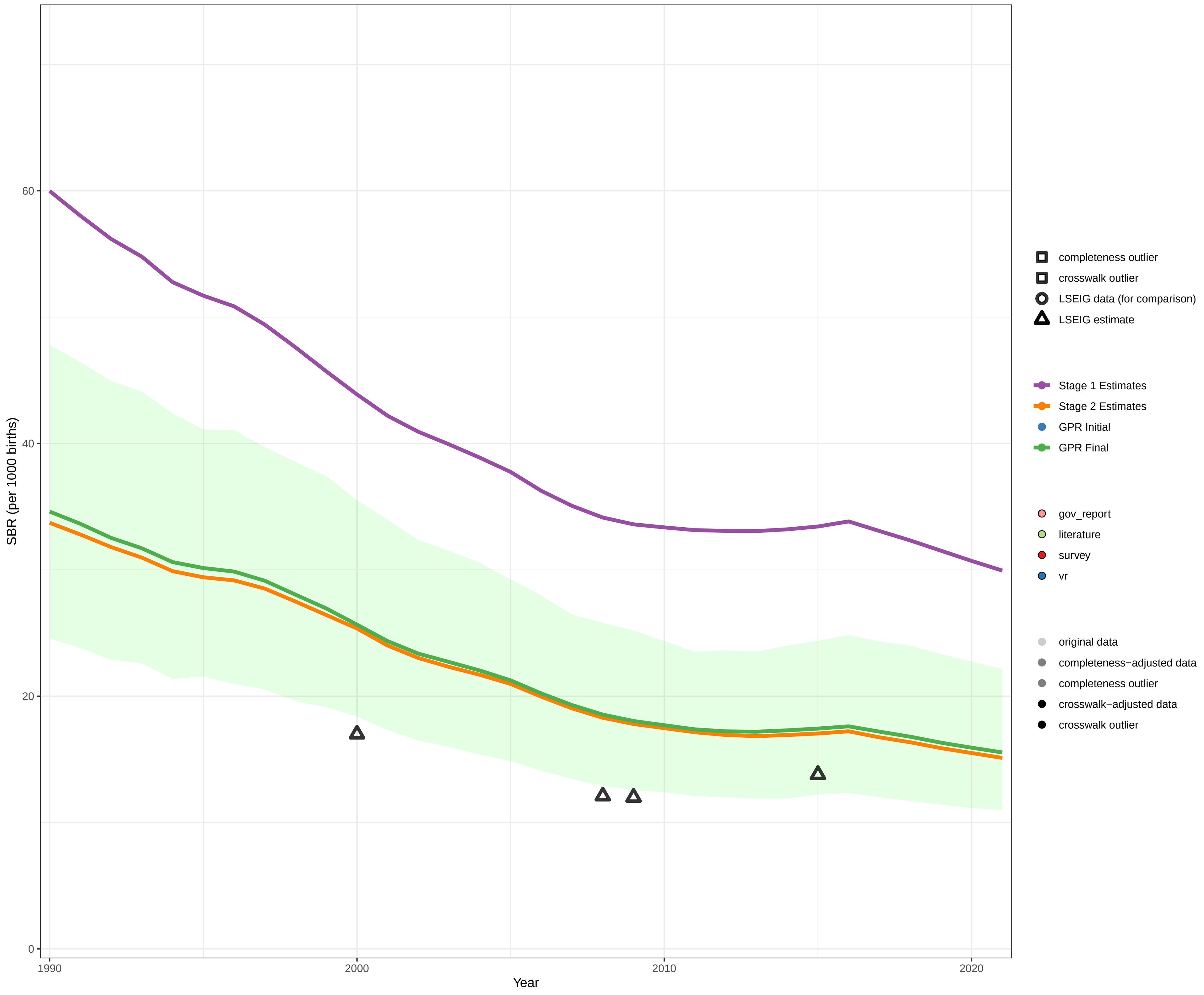

Tuvalu TUV Oceania Stillbirth Rate (SBR) − 28 weeks
lambda = 1 ; zeta = 0.6 ; scale = 15 ; amp = 0.028

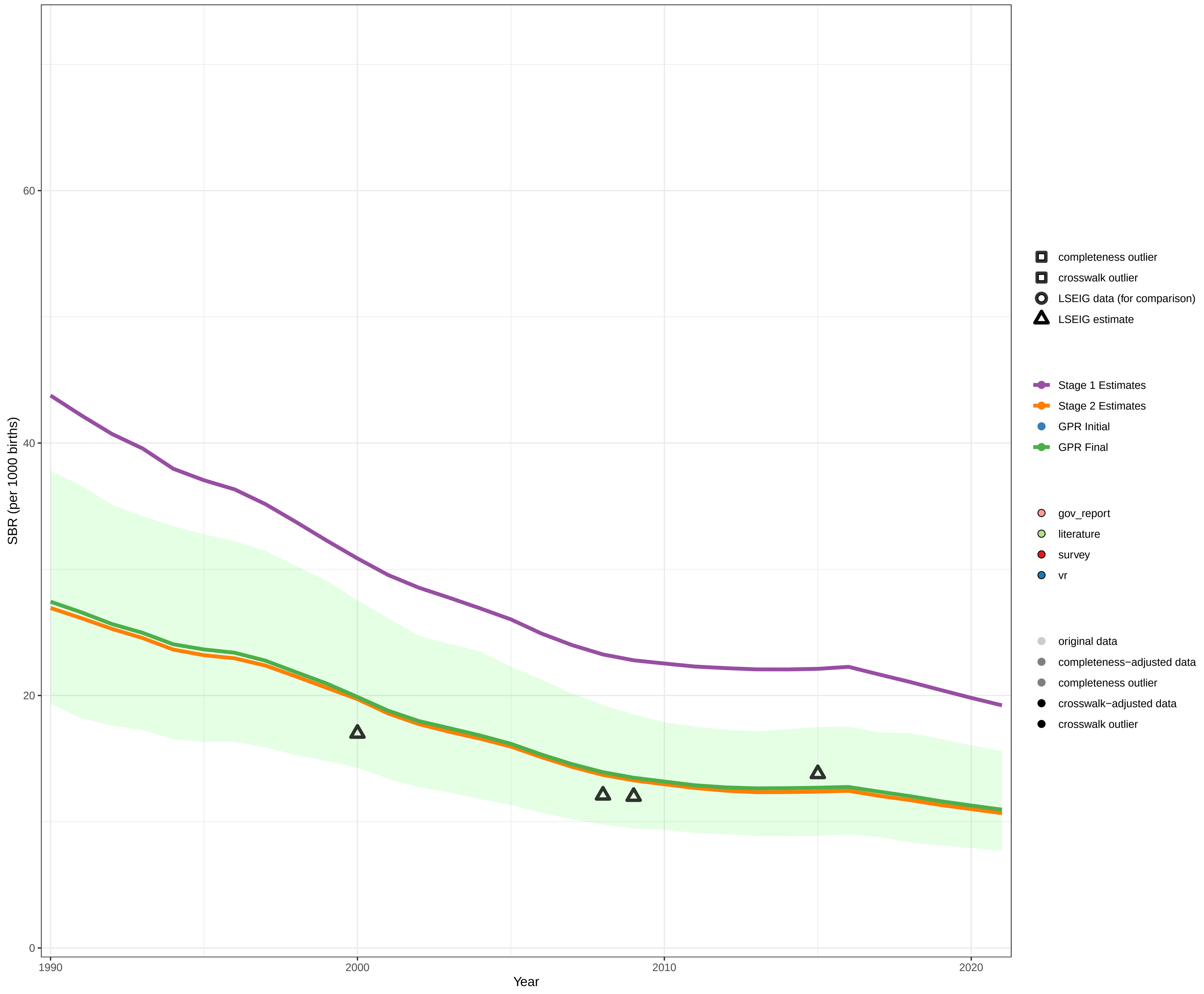

Year	Total Stillbirths (in thousands)	Total Stillbirths (in thousands)	SBR (per 1000 births)	SBR (per 1000 births)	SBR/NMR	SBR/NMR
	20 weeks	28 weeks	20 weeks	28 weeks	20 weeks	28 weeks
1990	0.0 (0.0 to 0.0)	0.0 (0.0 to 0.0)	34.6 (24.6 to 47.8)	27.4 (19.3 to 37.8)	1.50 (1.07 to 2.07)	1.19 (0.84 to 1.64)
1995	0.0 (0.0 to 0.0)	0.0 (0.0 to 0.0)	30.1 (21.6 to 41.1)	23.6 (16.3 to 32.8)	1.60 (1.14 to 2.18)	1.25 (0.86 to 1.74)
2000	0.0 (0.0 to 0.0)	0.0 (0.0 to 0.0)	25.7 (18.4 to 35.5)	19.9 (14.3 to 27.6)	1.69 (1.21 to 2.34)	1.31 (0.94 to 1.81)
2005	0.0 (0.0 to 0.0)	0.0 (0.0 to 0.0)	21.3 (14.9 to 29.2)	16.2 (11.3 to 22.3)	1.71 (1.19 to 2.35)	1.30 (0.91 to 1.79)
2010	0.0 (0.0 to 0.0)	0.0 (0.0 to 0.0)	17.7 (12.4 to 24.4)	13.2 (9.4 to 17.9)	1.69 (1.18 to 2.32)	1.26 (0.89 to 1.70)
2015	0.0 (0.0 to 0.0)	0.0 (0.0 to 0.0)	17.4 (12.2 to 24.4)	12.7 (8.9 to 17.5)	1.74 (1.22 to 2.43)	1.27 (0.89 to 1.75)
2020	0.0 (0.0 to 0.0)	0.0 (0.0 to 0.0)	15.9 (11.1 to 22.8)	11.3 (7.9 to 16.0)	1.84 (1.29 to 2.63)	1.31 (0.92 to 1.86)
2021	0.0 (0.0 to 0.0)	0.0 (0.0 to 0.0)	15.6 (11.0 to 22.1)	11.0 (7.7 to 15.6)	1.87 (1.32 to 2.66)	1.32 (0.92 to 1.88)

Vanuatu VUT Oceania Stillbirth Rate / Neonatal Mortality Rate (SBR/NMR) − 20 weeks
lambda = 1 ; zeta = 0.6 ; scale = 15 ; amp = 0.028

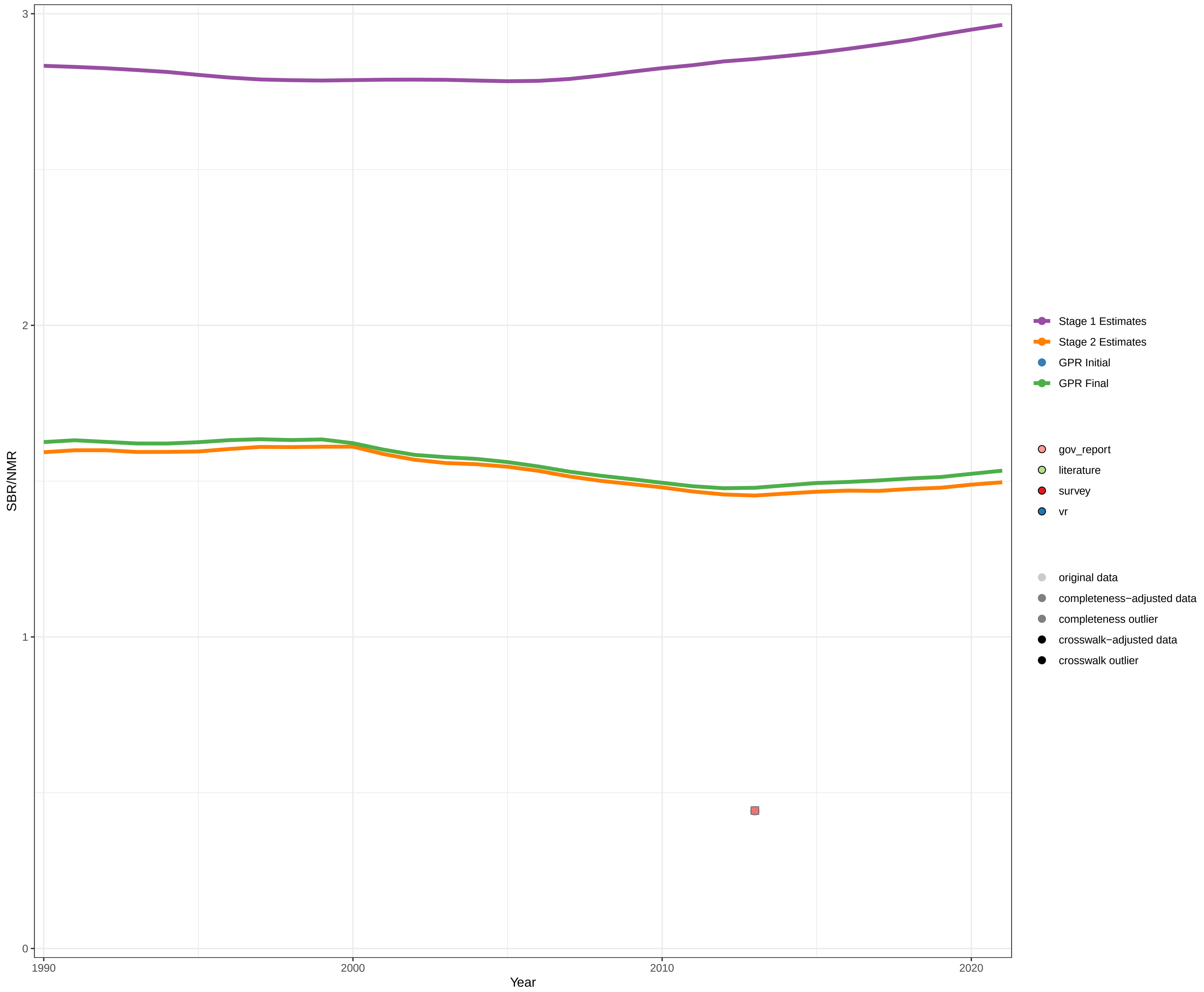

Vanuatu VUT Oceania Stillbirth Rate / Neonatal Mortality Rate (SBR/NMR) − 28 weeks
lambda = 1 ; zeta = 0.6 ; scale = 15 ; amp = 0.028

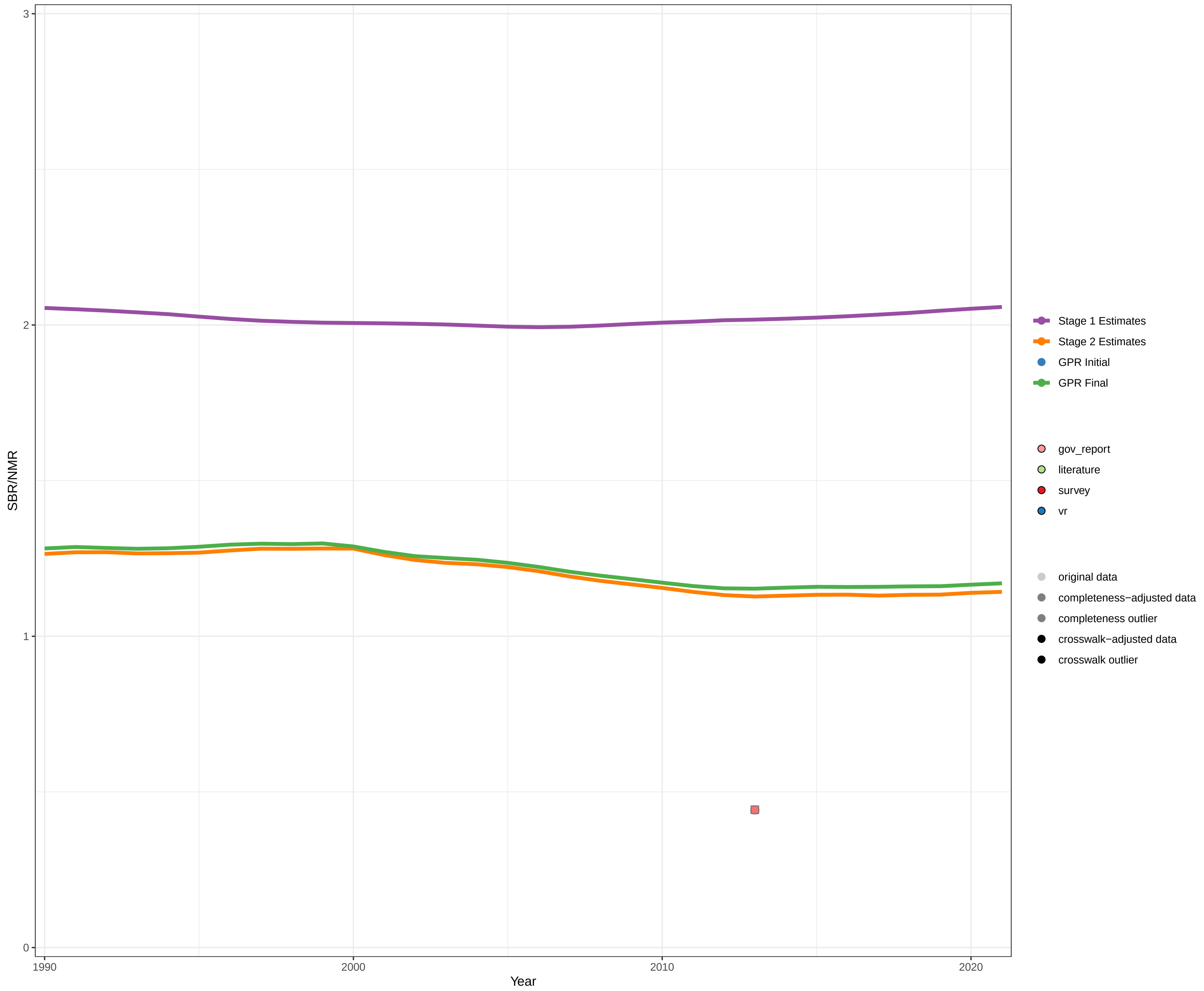

Definition	Years	Outlier	Source.Type	Title
28_weeks	2013	1	Survey	Vanuatu Demographic and Health Survey 2013

Vanuatu VUT Oceania Stillbirth Rate (SBR) − 20 weeks
lambda = 1 ; zeta = 0.6 ; scale = 15 ; amp = 0.028

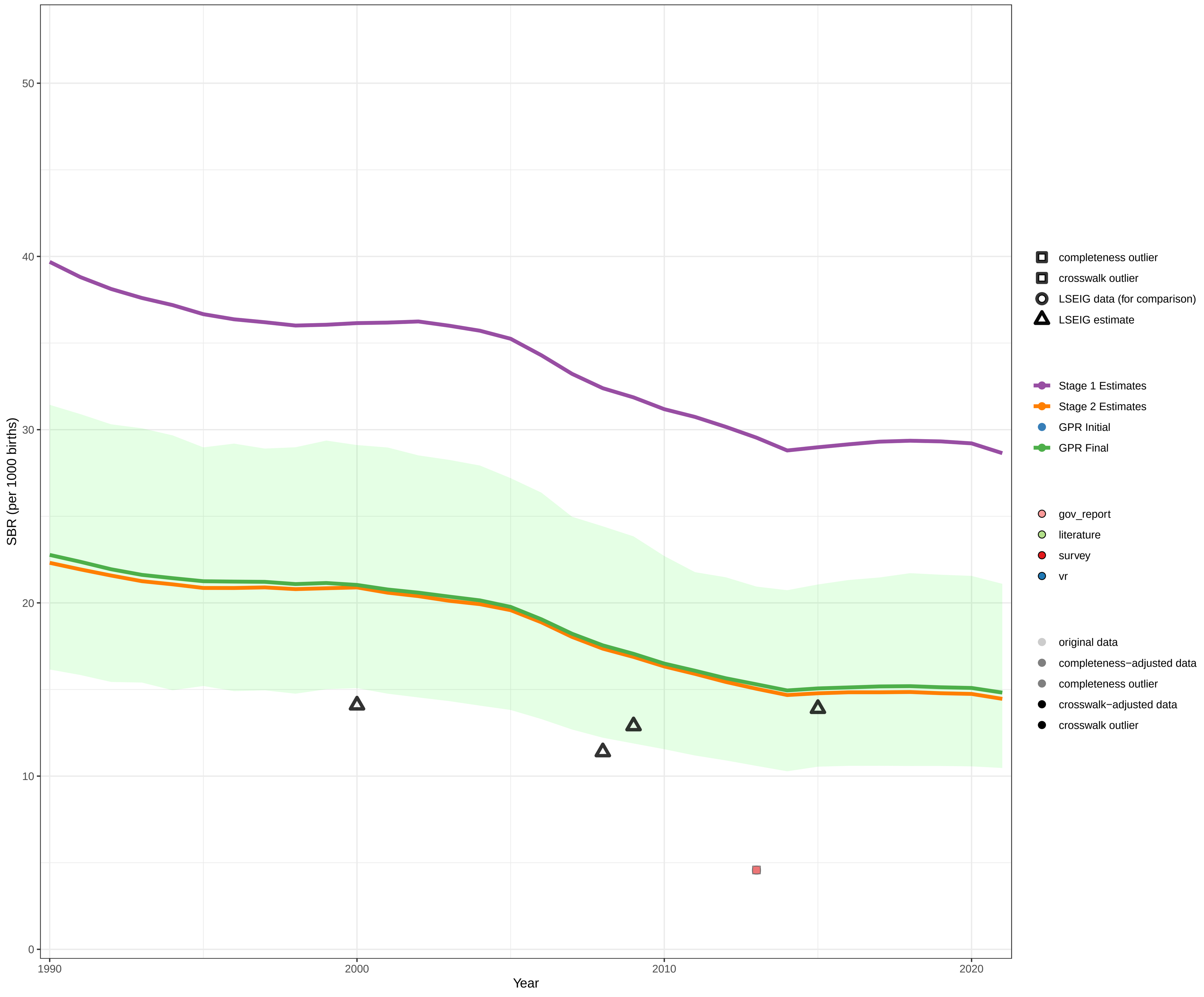

Vanuatu VUT Oceania Stillbirth Rate (SBR) − 28 weeks
lambda = 1 ; zeta = 0.6 ; scale = 15 ; amp = 0.028

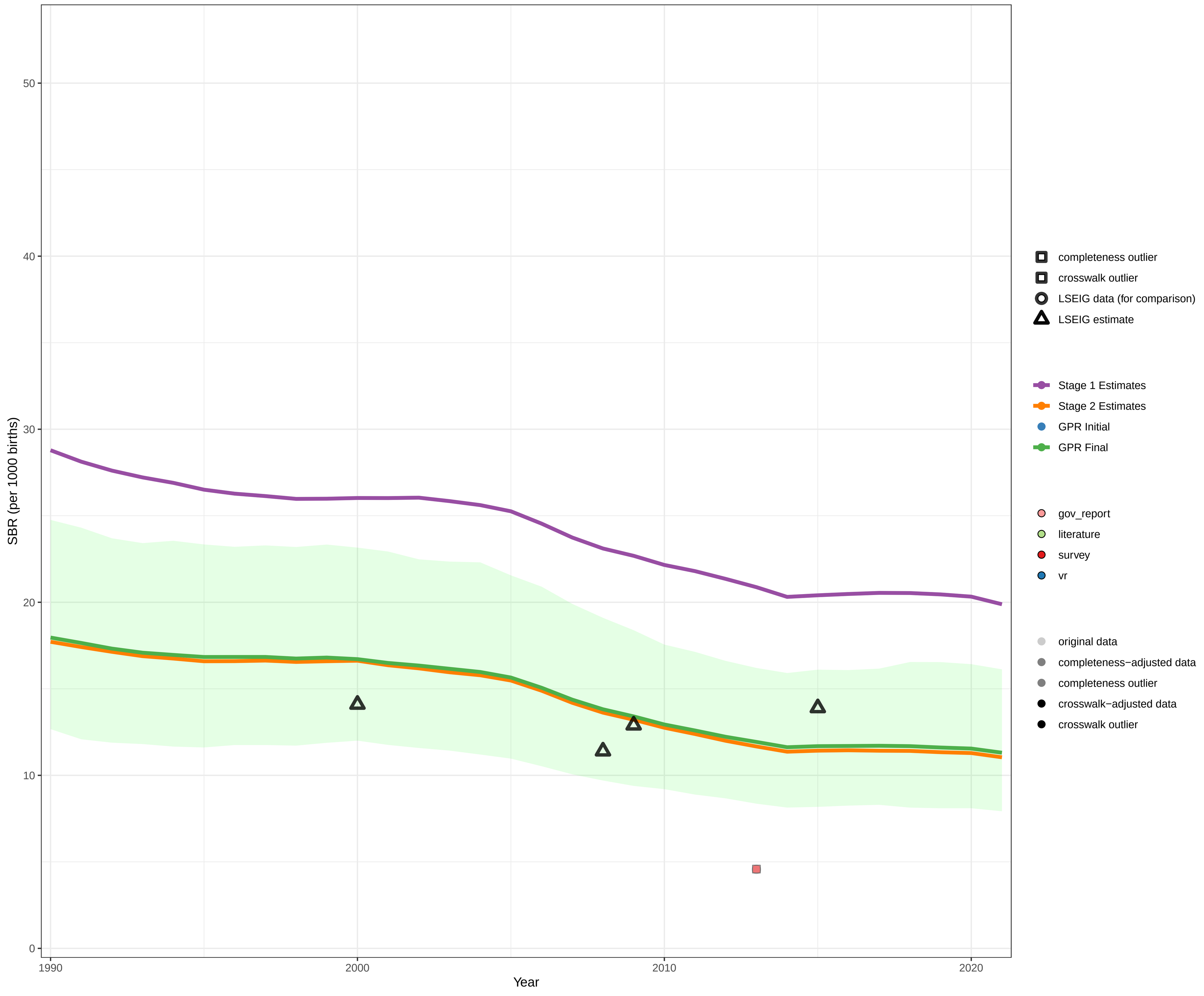

Year	Total Stillbirths (in thousands)	Total Stillbirths (in thousands)	SBR (per 1000 births)	SBR (per 1000 births)	SBR/NMR	SBR/NMR
	20 weeks	28 weeks	20 weeks	28 weeks	20 weeks	28 weeks
1990	0.1 (0.1 to 0.2)	0.1 (0.1 to 0.2)	22.8 (16.2 to 31.4)	18.0 (12.7 to 24.8)	1.63 (1.15 to 2.24)	1.28 (0.90 to 1.77)
1995	0.1 (0.1 to 0.2)	0.1 (0.1 to 0.2)	21.2 (15.2 to 29.0)	16.8 (11.6 to 23.3)	1.62 (1.16 to 2.22)	1.29 (0.89 to 1.78)
2000	0.2 (0.1 to 0.2)	0.1 (0.1 to 0.2)	21.0 (15.1 to 29.1)	16.7 (12.0 to 23.2)	1.62 (1.16 to 2.24)	1.29 (0.93 to 1.79)
2005	0.2 (0.1 to 0.2)	0.1 (0.1 to 0.2)	19.8 (13.8 to 27.2)	15.7 (11.0 to 21.6)	1.56 (1.09 to 2.15)	1.24 (0.87 to 1.70)
2010	0.1 (0.1 to 0.2)	0.1 (0.1 to 0.1)	16.5 (11.6 to 22.7)	12.9 (9.2 to 17.6)	1.49 (1.05 to 2.06)	1.17 (0.83 to 1.59)
2015	0.1 (0.1 to 0.2)	0.1 (0.1 to 0.1)	15.1 (10.5 to 21.1)	11.7 (8.2 to 16.1)	1.49 (1.05 to 2.09)	1.16 (0.81 to 1.60)
2020	0.1 (0.1 to 0.2)	0.1 (0.1 to 0.1)	15.1 (10.6 to 21.6)	11.5 (8.1 to 16.4)	1.52 (1.07 to 2.18)	1.17 (0.82 to 1.66)
2021	0.1 (0.1 to 0.2)	0.1 (0.1 to 0.1)	14.8 (10.5 to 21.1)	11.3 (7.9 to 16.1)	1.53 (1.08 to 2.18)	1.17 (0.82 to 1.67)

Southeast Asia R5 Southeast Asia Stillbirth Rate / Neonatal Mortality Rate (SBR/NMR) − 20 weeks
lambda = 1 ; zeta = 0.6 ; scale = 15 ; amp = 0.028

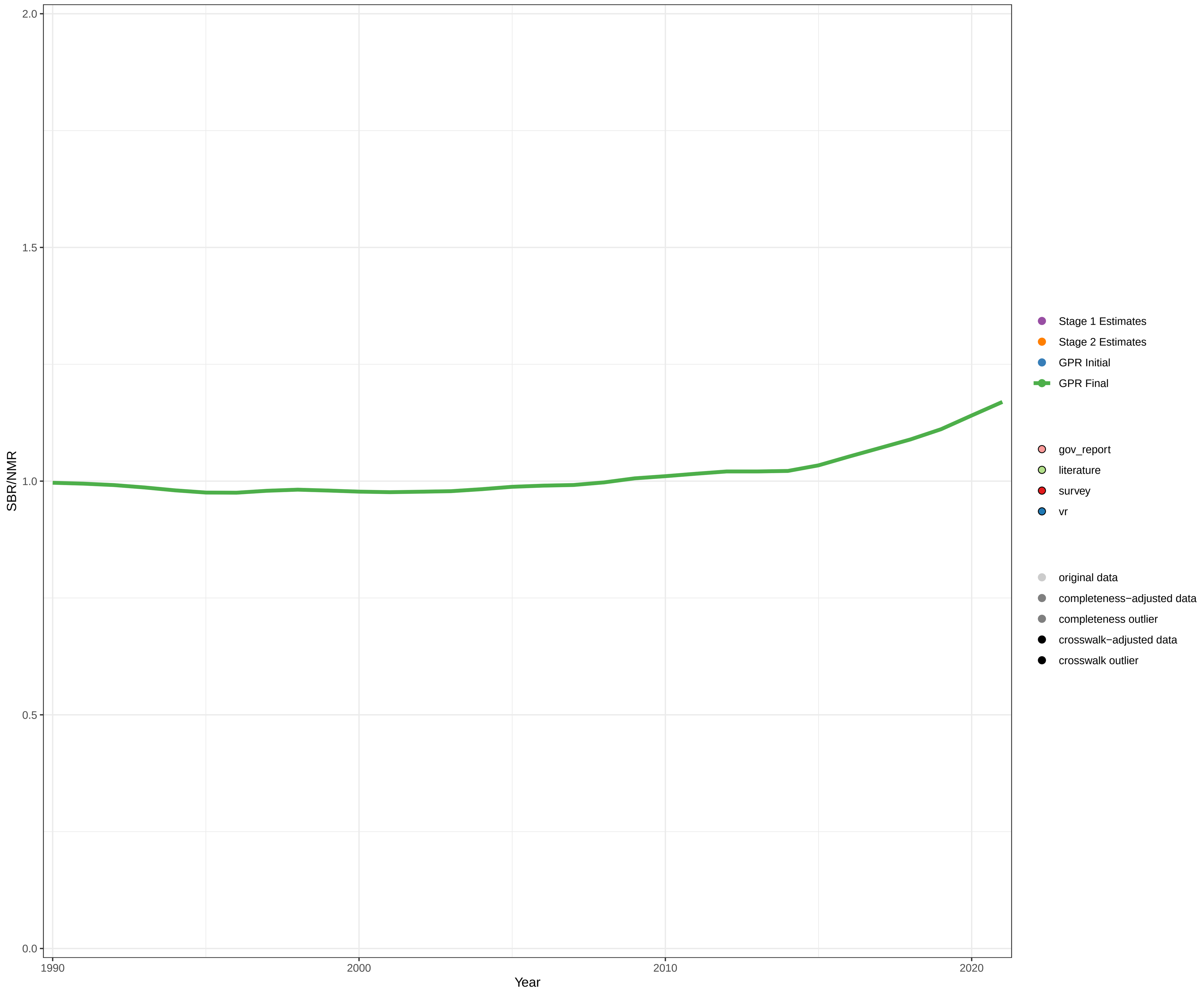

Southeast Asia R5 Southeast Asia Stillbirth Rate / Neonatal Mortality Rate (SBR/NMR) − 28 weeks
lambda = 1 ; zeta = 0.6 ; scale = 15 ; amp = 0.028

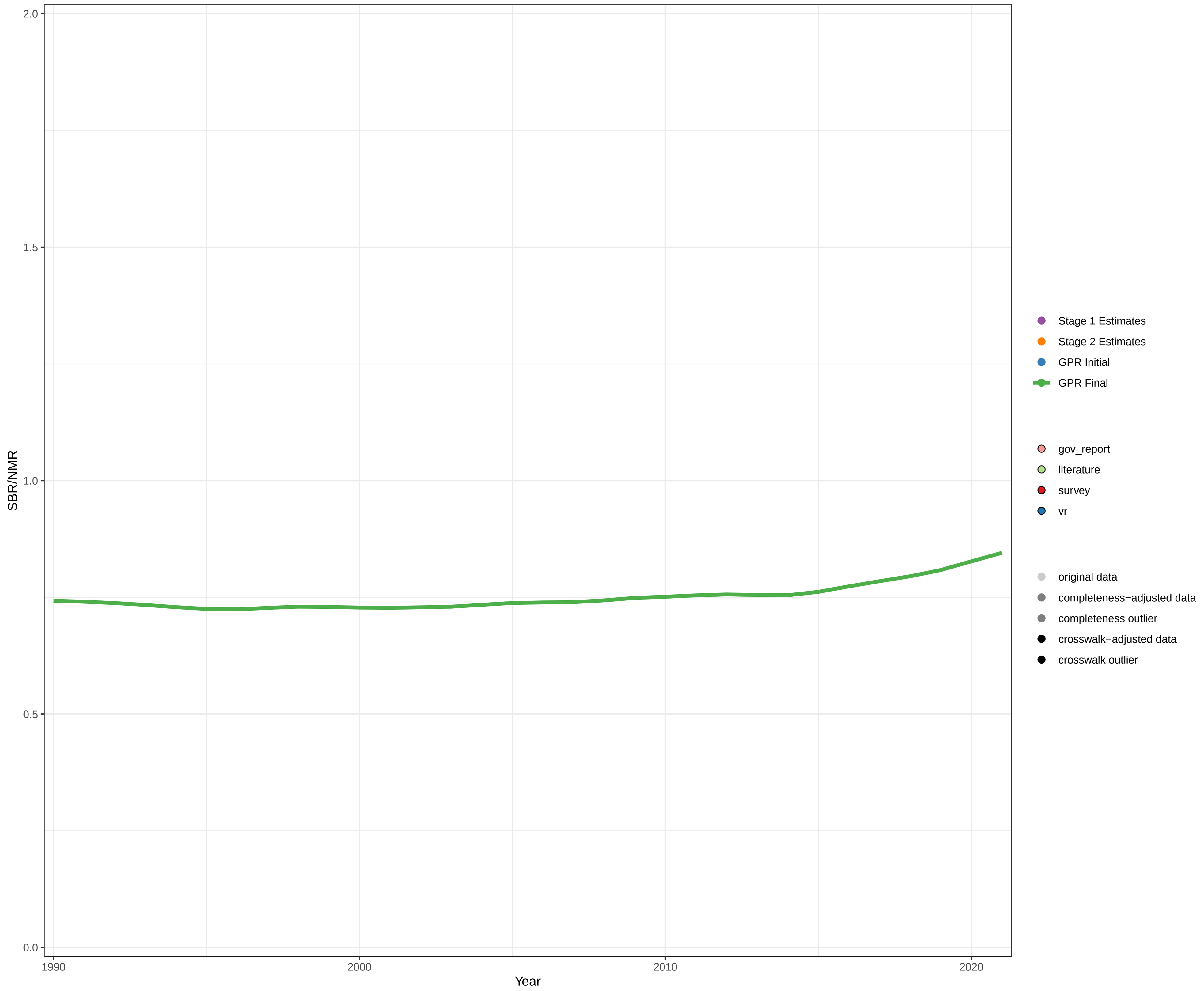

Definition	Years	Outlier	Source.Type	Title

Southeast Asia R5 Southeast Asia Stillbirth Rate (SBR) − 20 weeks
lambda = 1 ; zeta = 0.6 ; scale = 15 ; amp = 0.028

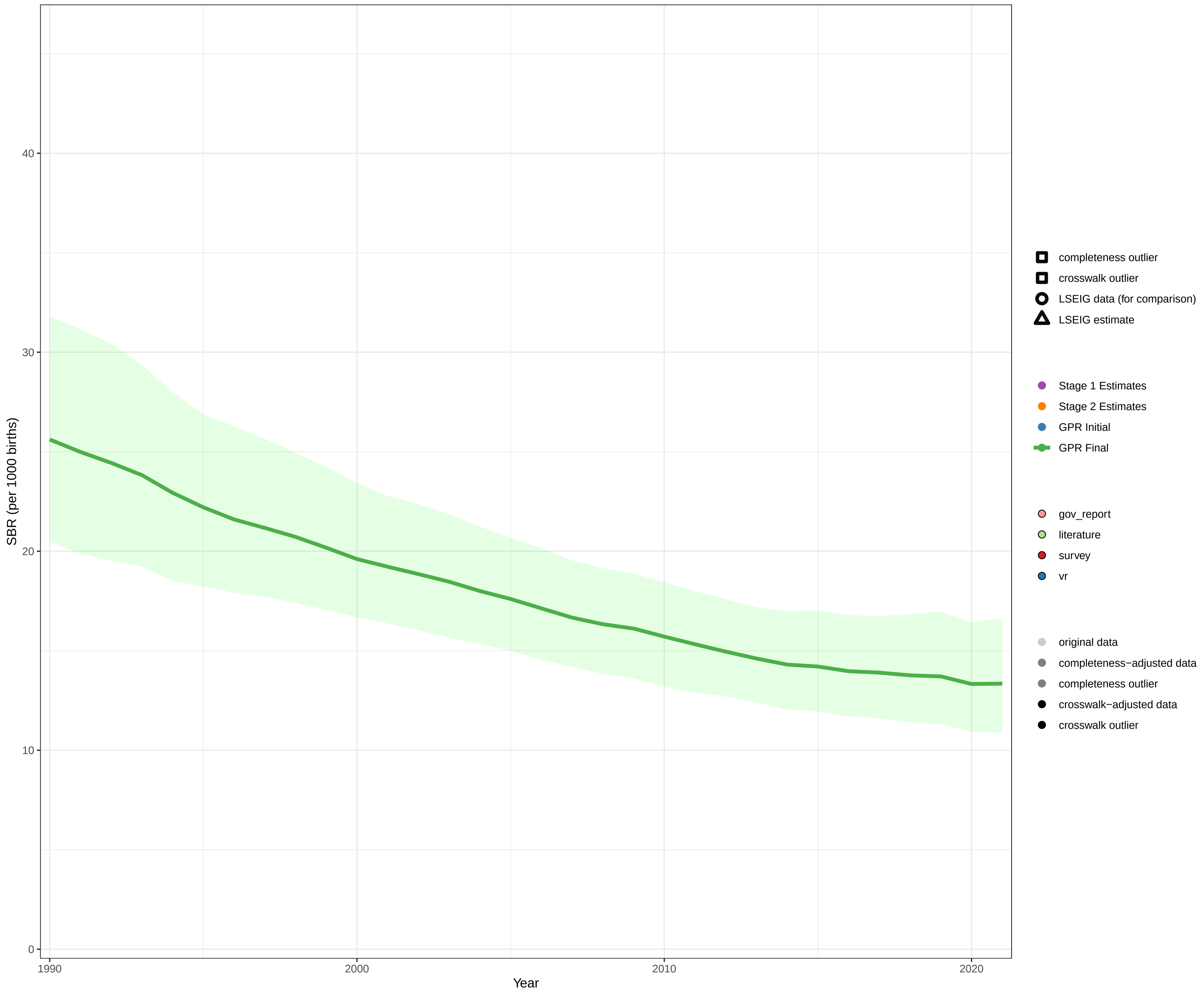

Southeast Asia R5 Southeast Asia Stillbirth Rate (SBR) − 28 weeks
lambda = 1 ; zeta = 0.6 ; scale = 15 ; amp = 0.028

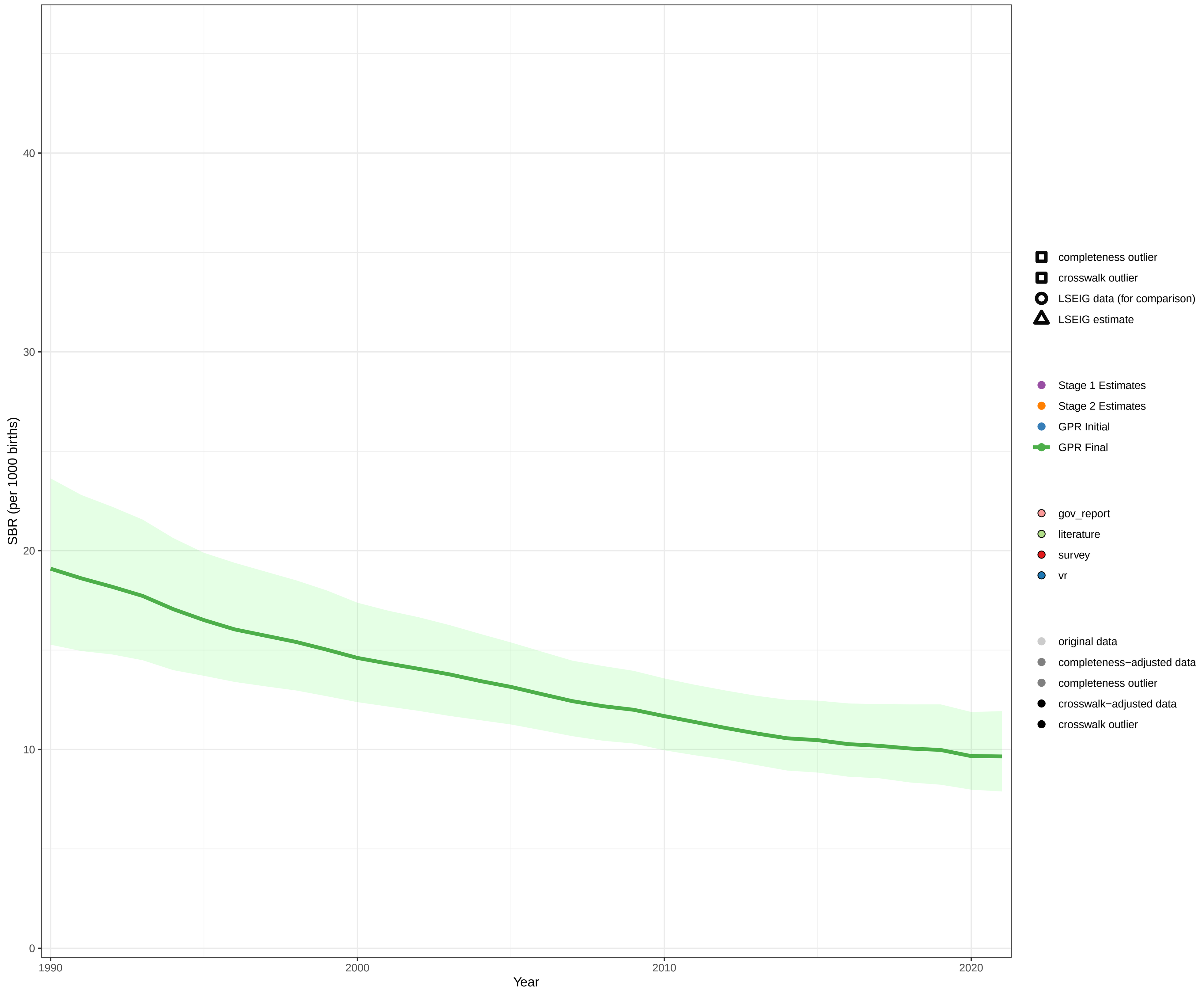

Year	Total Stillbirths (in thousands)	Total Stillbirths (in thousands)	SBR (per 1000 births)	SBR (per 1000 births)	SBR/NMR	SBR/NMR
	20 weeks	28 weeks	20 weeks	28 weeks	20 weeks	28 weeks
1990	323.2 (256.5 to 403.5)	239.2 (190.6 to 297.5)	25.6 (20.5 to 31.8)	19.1 (15.3 to 23.6)	1.00 (0.80 to 1.24)	0.74 (0.59 to 0.92)
1995	273.7 (223.6 to 332.8)	202.2 (167.4 to 244.5)	22.2 (18.2 to 26.9)	16.5 (13.7 to 19.9)	0.98 (0.80 to 1.18)	0.73 (0.60 to 0.87)
2000	237.7 (201.5 to 285.2)	176.2 (149.0 to 210.2)	19.6 (16.7 to 23.4)	14.6 (12.4 to 17.4)	0.98 (0.83 to 1.17)	0.73 (0.62 to 0.87)
2005	216.0 (183.4 to 254.5)	160.6 (137.3 to 188.4)	17.6 (15.0 to 20.7)	13.1 (11.3 to 15.4)	0.99 (0.84 to 1.16)	0.74 (0.63 to 0.86)
2010	195.7 (163.9 to 230.4)	144.9 (123.3 to 168.7)	15.7 (13.2 to 18.4)	11.7 (10.0 to 13.6)	1.01 (0.85 to 1.19)	0.75 (0.64 to 0.87)
2015	170.8 (143.3 to 205.0)	125.4 (105.7 to 149.5)	14.2 (11.9 to 17.0)	10.5 (8.8 to 12.5)	1.03 (0.87 to 1.24)	0.76 (0.64 to 0.91)
2020	152.2 (124.5 to 187.8)	110.0 (90.6 to 135.5)	13.3 (10.9 to 16.4)	9.7 (8.0 to 11.9)	1.14 (0.94 to 1.40)	0.83 (0.68 to 1.02)
2021	151.2 (122.7 to 189.1)	108.9 (88.8 to 134.9)	13.3 (10.9 to 16.6)	9.7 (7.9 to 11.9)	1.17 (0.95 to 1.46)	0.85 (0.69 to 1.05)

Cambodia KHM Southeast Asia Stillbirth Rate / Neonatal Mortality Rate (SBR/NMR) − 20 weeks
lambda = 0.8 ; zeta = 0.7 ; scale = 15 ; amp = 0.028

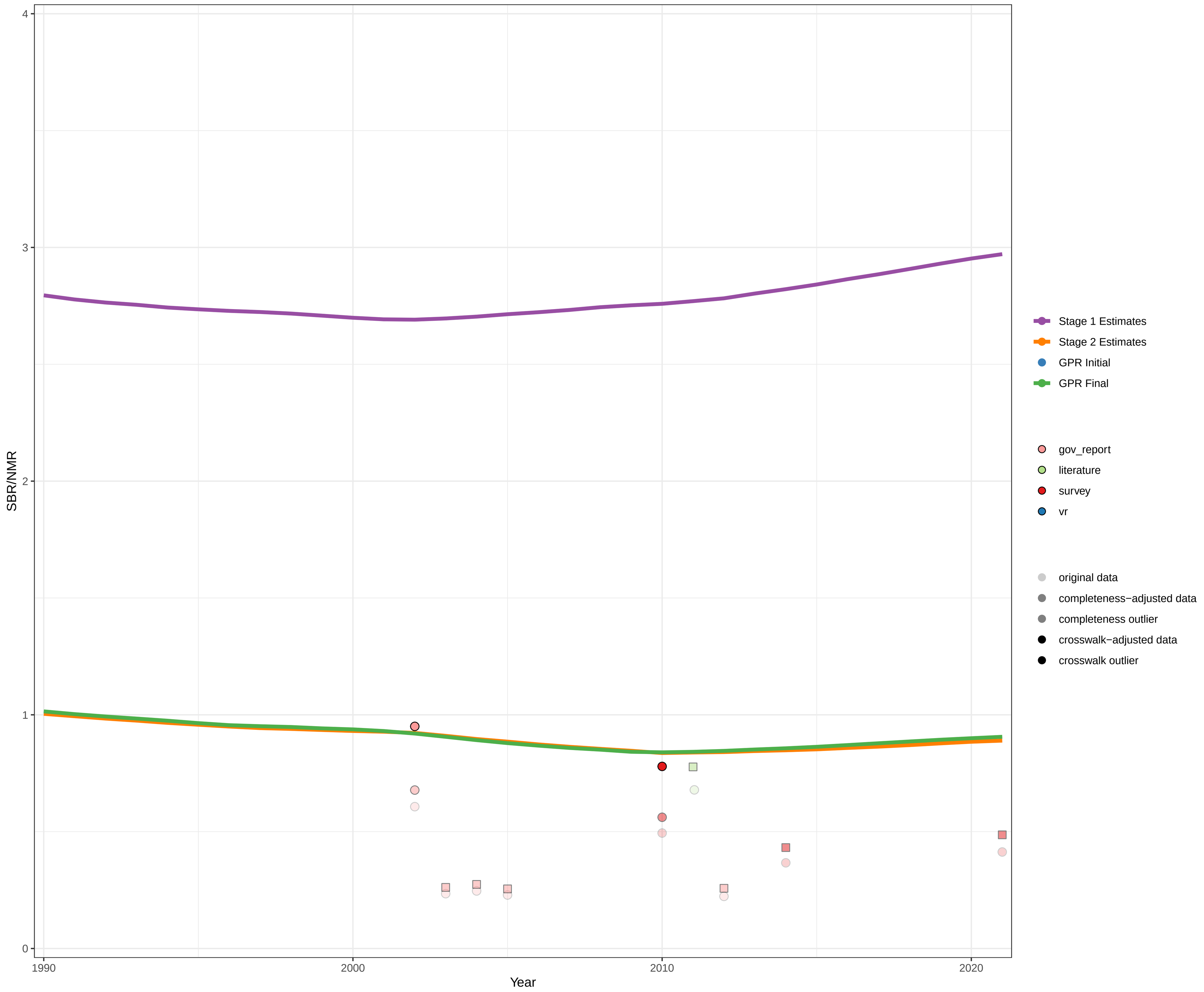

Cambodia KHM Southeast Asia Stillbirth Rate / Neonatal Mortality Rate (SBR/NMR) − 28 weeks
lambda = 0.8 ; zeta = 0.7 ; scale = 15 ; amp = 0.028

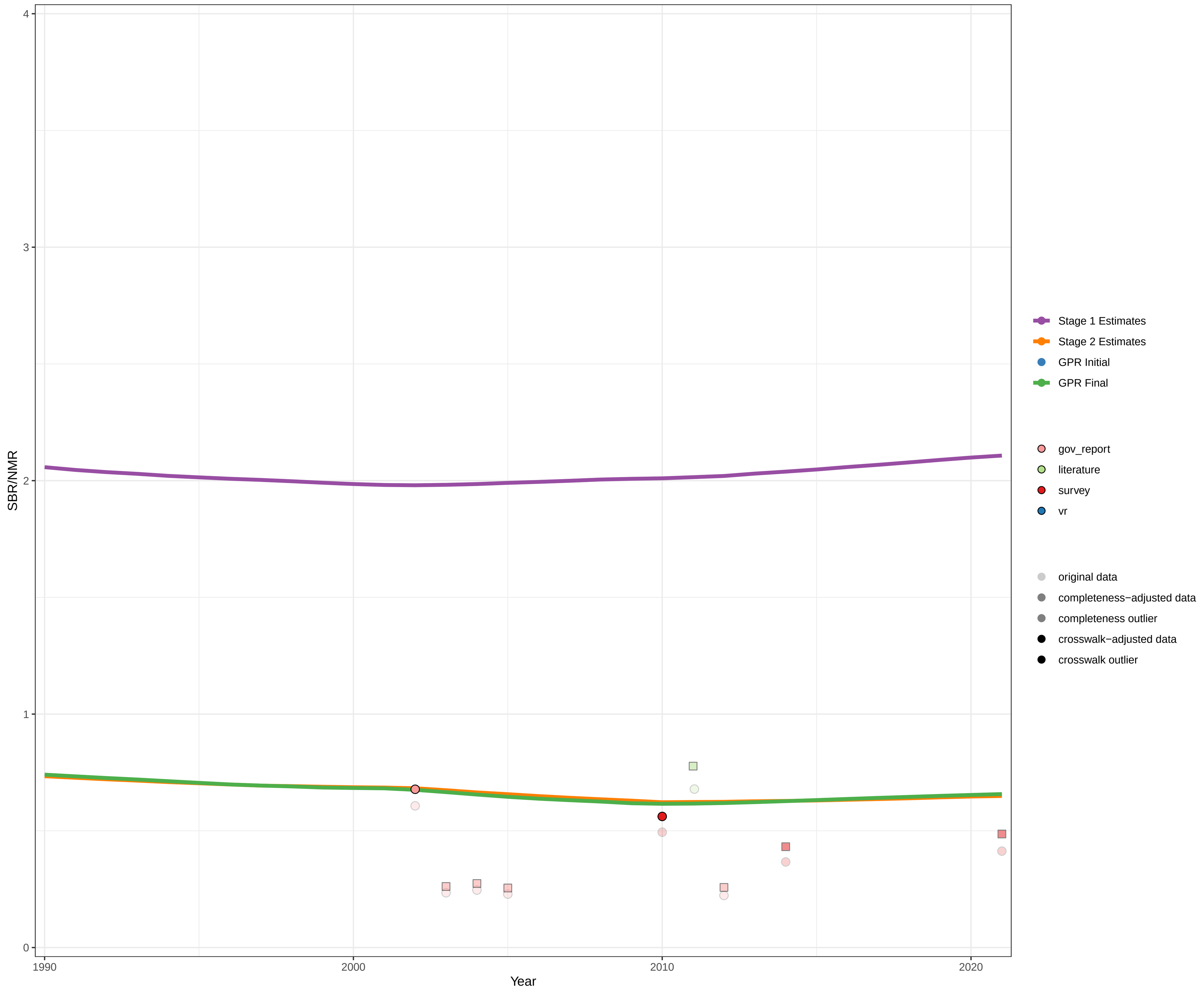

Definition	Years	Outlier	Source.Type	Title
28_weeks	2002	0	Statistical Report	Cambodia National Health Statistics
28_weeks	2010	0	Survey	Cambodia Demographic and Health Survey 2010−2011
28_weeks	2003−2005	1	Statistical Report	Cambodia National Health Statistics
28_weeks	2012	1	Statistical Report	Cambodia Annual Health Statistics Report
28_weeks	2014	1	Survey	Cambodia Demographic and Health Survey 2014
28_weeks	2021	1	Survey	Cambodia Demographic and Health Survey 2021−2022
28_weeks_OR_1000_grams	2011	1	Sci Lit	Maternal complications and perinatal mortality: findings of the World Health Organization Multicountry Survey on Maternal and Newborn Health

Cambodia KHM Southeast Asia Stillbirth Rate (SBR) − 20 weeks
lambda = 0.8 ; zeta = 0.7 ; scale = 15 ; amp = 0.028

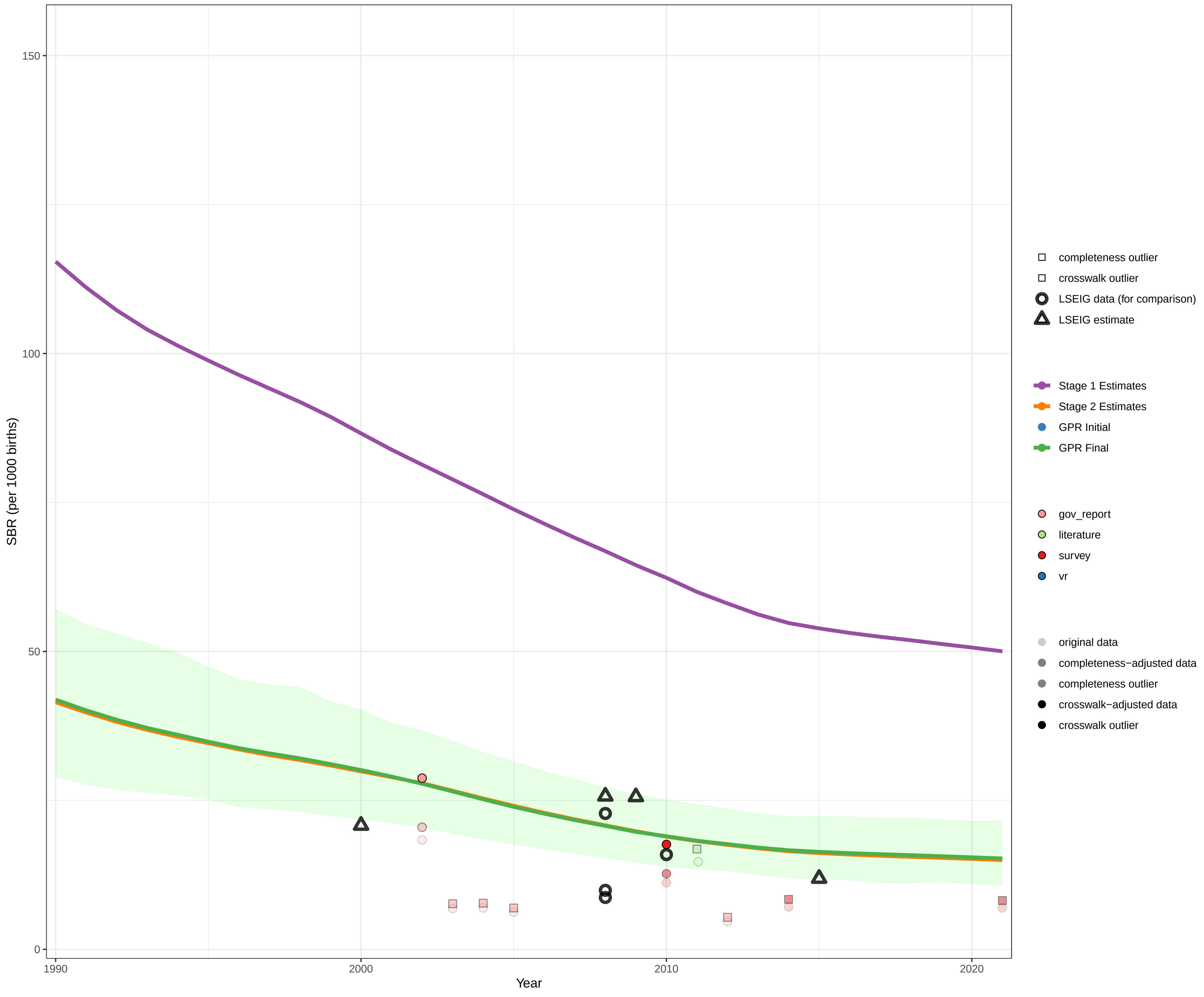

Cambodia KHM Southeast Asia Stillbirth Rate (SBR) − 28 weeks
lambda = 0.8 ; zeta = 0.7 ; scale = 15 ; amp = 0.028

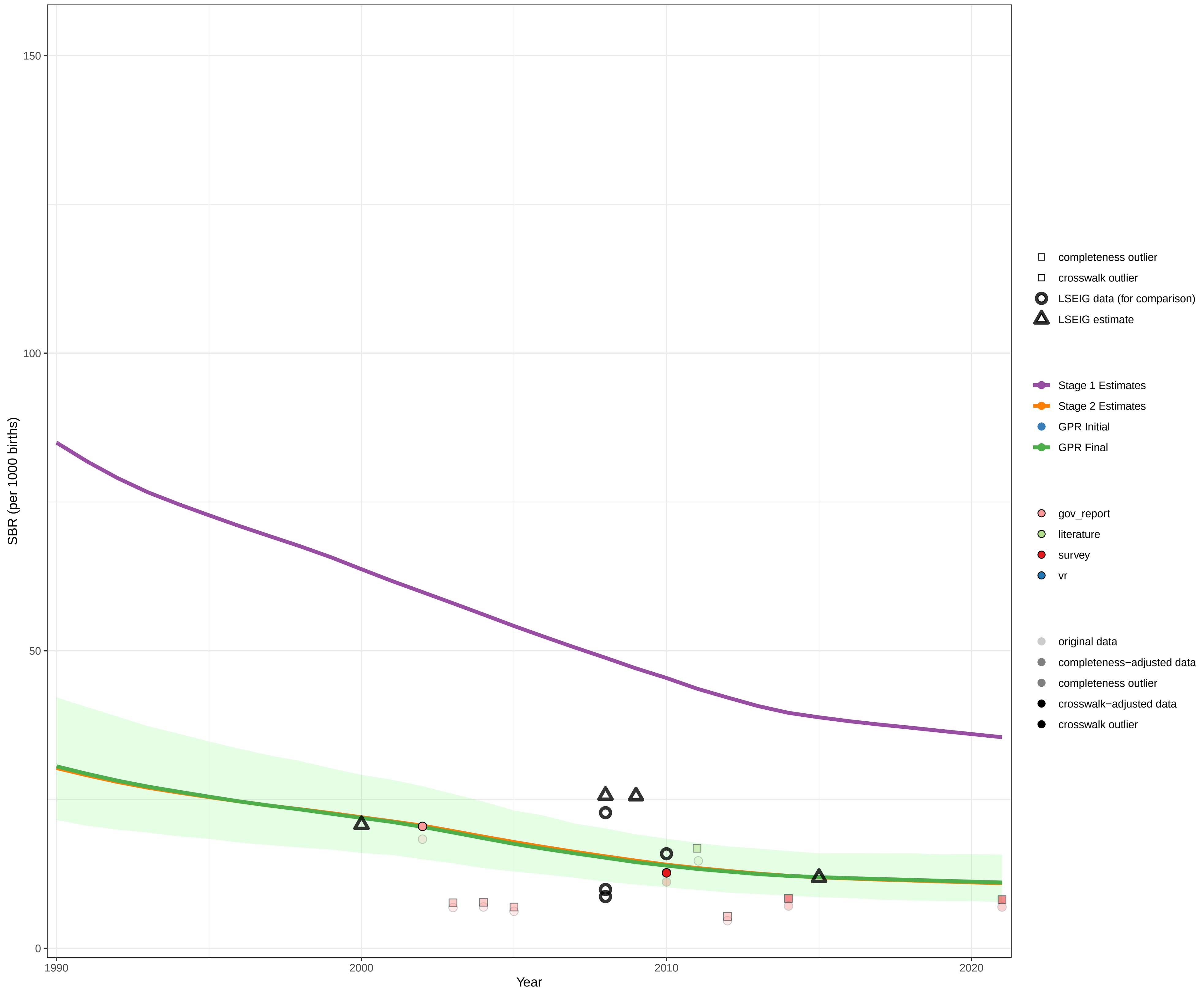

Year	Total Stillbirths (in thousands)	Total Stillbirths (in thousands)	SBR (per 1000 births)	SBR (per 1000 births)	SBR/NMR	SBR/NMR
	20 weeks	28 weeks	20 weeks	28 weeks	20 weeks	28 weeks
1990	18.9 (12.8 to 26.2)	13.6 (9.5 to 19.0)	41.9 (28.9 to 57.1)	30.6 (21.6 to 42.2)	1.01 (0.70 to 1.38)	0.74 (0.52 to 1.02)
1995	14.5 (10.3 to 19.9)	10.5 (7.5 to 14.4)	34.8 (25.1 to 47.4)	25.5 (18.4 to 34.8)	0.96 (0.70 to 1.31)	0.71 (0.51 to 0.96)
2000	11.3 (8.2 to 15.3)	8.2 (5.9 to 11.0)	30.1 (21.9 to 40.3)	21.9 (16.0 to 29.2)	0.94 (0.68 to 1.26)	0.68 (0.50 to 0.91)
2005	8.8 (6.4 to 11.7)	6.4 (4.7 to 8.5)	23.9 (17.6 to 31.6)	17.6 (12.9 to 23.2)	0.88 (0.65 to 1.16)	0.65 (0.47 to 0.85)
2010	7.1 (5.1 to 9.4)	5.1 (3.8 to 6.8)	19.0 (13.8 to 25.2)	13.9 (10.3 to 18.4)	0.84 (0.61 to 1.11)	0.62 (0.45 to 0.82)
2015	6.1 (4.3 to 8.4)	4.4 (3.2 to 5.9)	16.4 (11.7 to 22.4)	12.0 (8.6 to 16.0)	0.86 (0.62 to 1.18)	0.63 (0.45 to 0.84)
2020	5.8 (4.1 to 8.2)	4.2 (3.0 to 6.0)	15.4 (10.9 to 21.6)	11.2 (7.9 to 15.8)	0.90 (0.64 to 1.26)	0.65 (0.46 to 0.92)
2021	5.8 (4.0 to 8.2)	4.2 (2.9 to 6.0)	15.3 (10.7 to 21.6)	11.1 (7.8 to 15.8)	0.91 (0.63 to 1.28)	0.66 (0.46 to 0.94)

Indonesia IDN Southeast Asia Stillbirth Rate / Neonatal Mortality Rate (SBR/NMR) − 20 weeks
lambda = 0.6 ; zeta = 0.8 ; scale = 15 ; amp = 0.028

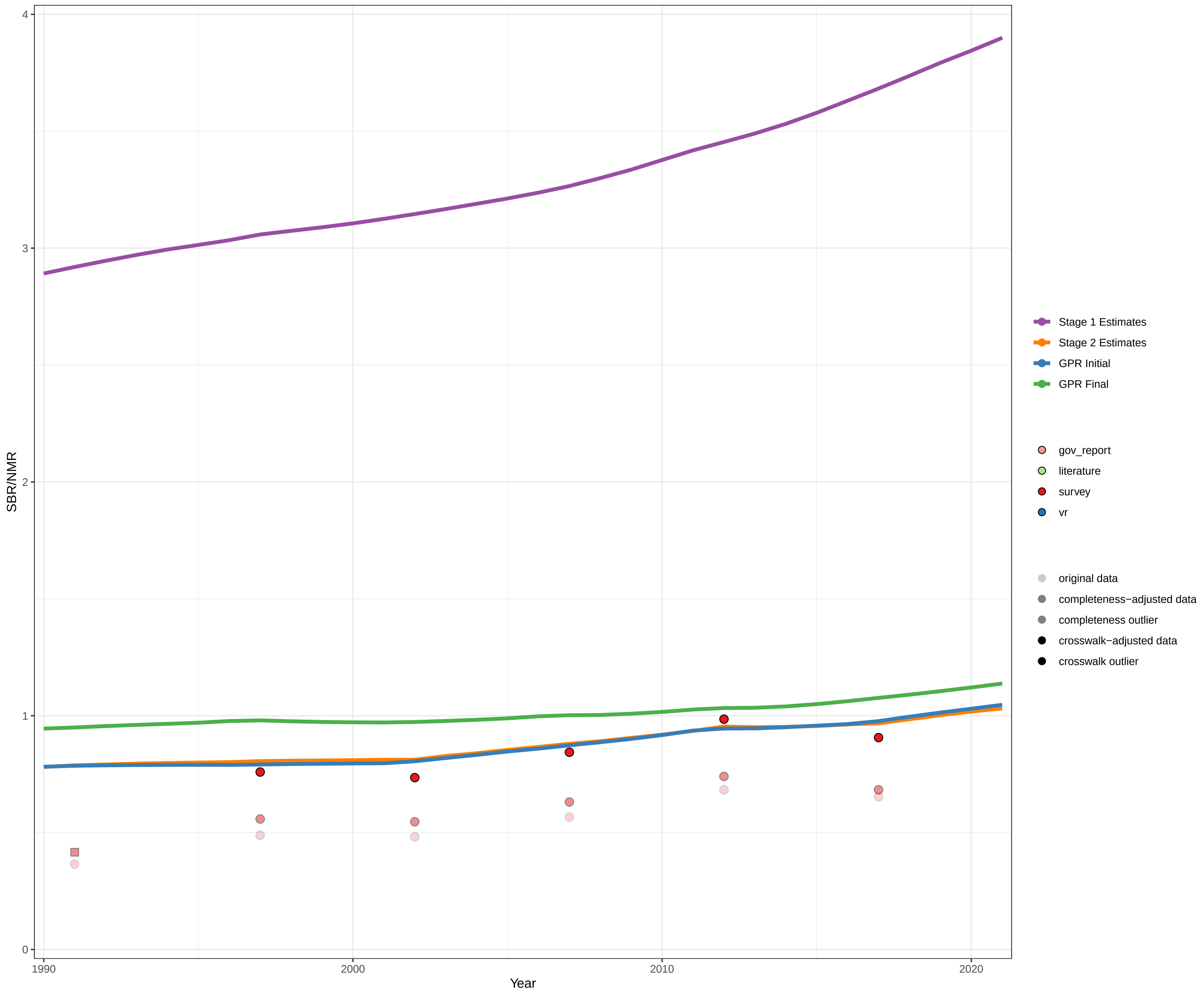

Indonesia IDN Southeast Asia Stillbirth Rate / Neonatal Mortality Rate (SBR/NMR) − 28 weeks
lambda = 0.6 ; zeta = 0.8 ; scale = 15 ; amp = 0.028

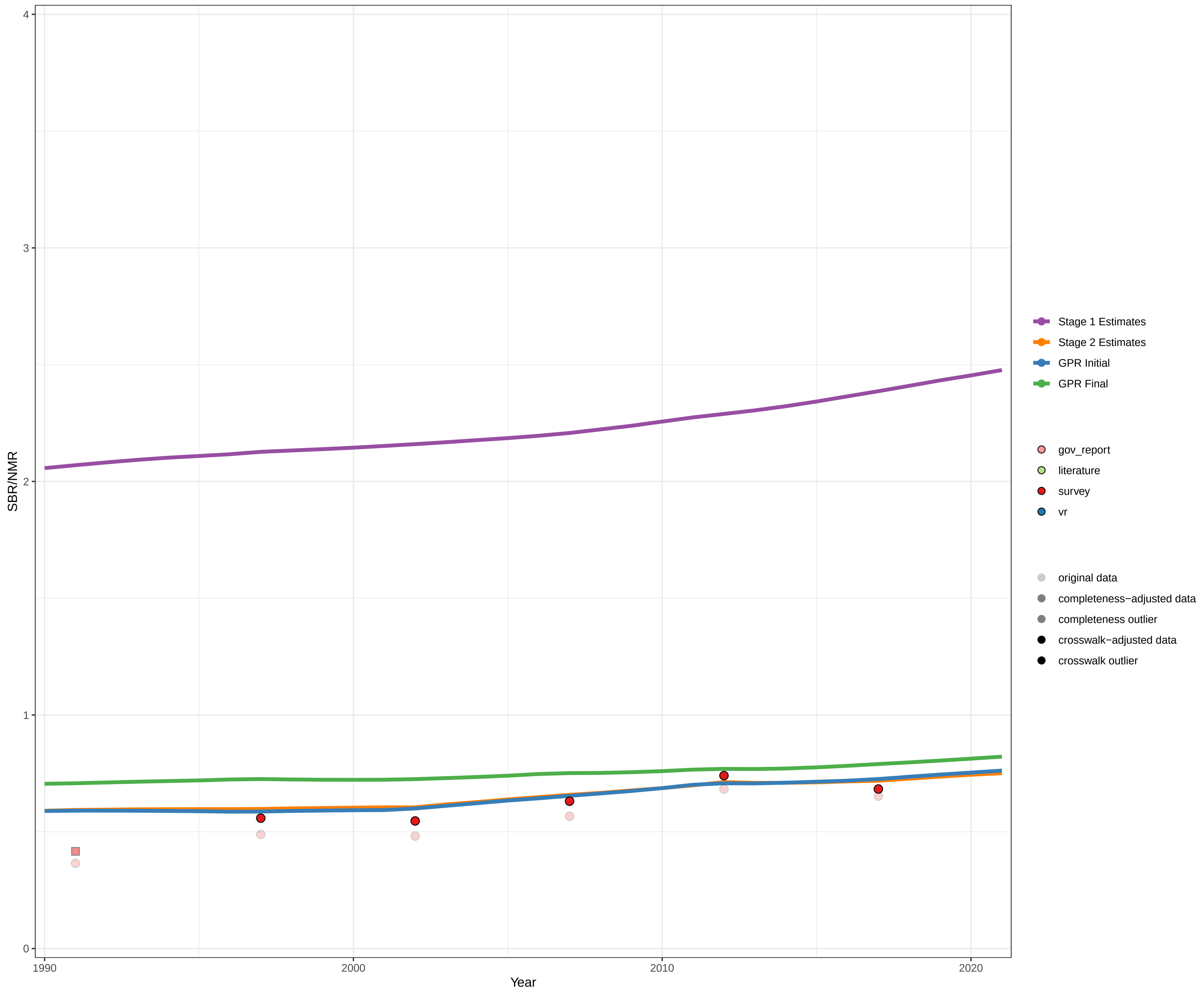

Definition	Years	Outlier	Source.Type	Title
28_weeks	1997	0	Survey	Indonesia Demographic and Health Survey 1997
28_weeks	2002	0	Survey	Indonesia Demographic and Health Survey 2002−2003
28_weeks	2007	0	Survey	Indonesia Demographic and Health Survey 2007
28_weeks	2012	0	Survey	Indonesia Demographic and Health Survey 2012
28_weeks	2017	0	Survey	Indonesia Demographic and Health Survey 2017
28_weeks	1991	1	Survey	Indonesia Demographic and Health Survey 1991

Indonesia IDN Southeast Asia Stillbirth Rate (SBR) − 20 weeks
lambda = 0.6 ; zeta = 0.8 ; scale = 15 ; amp = 0.028

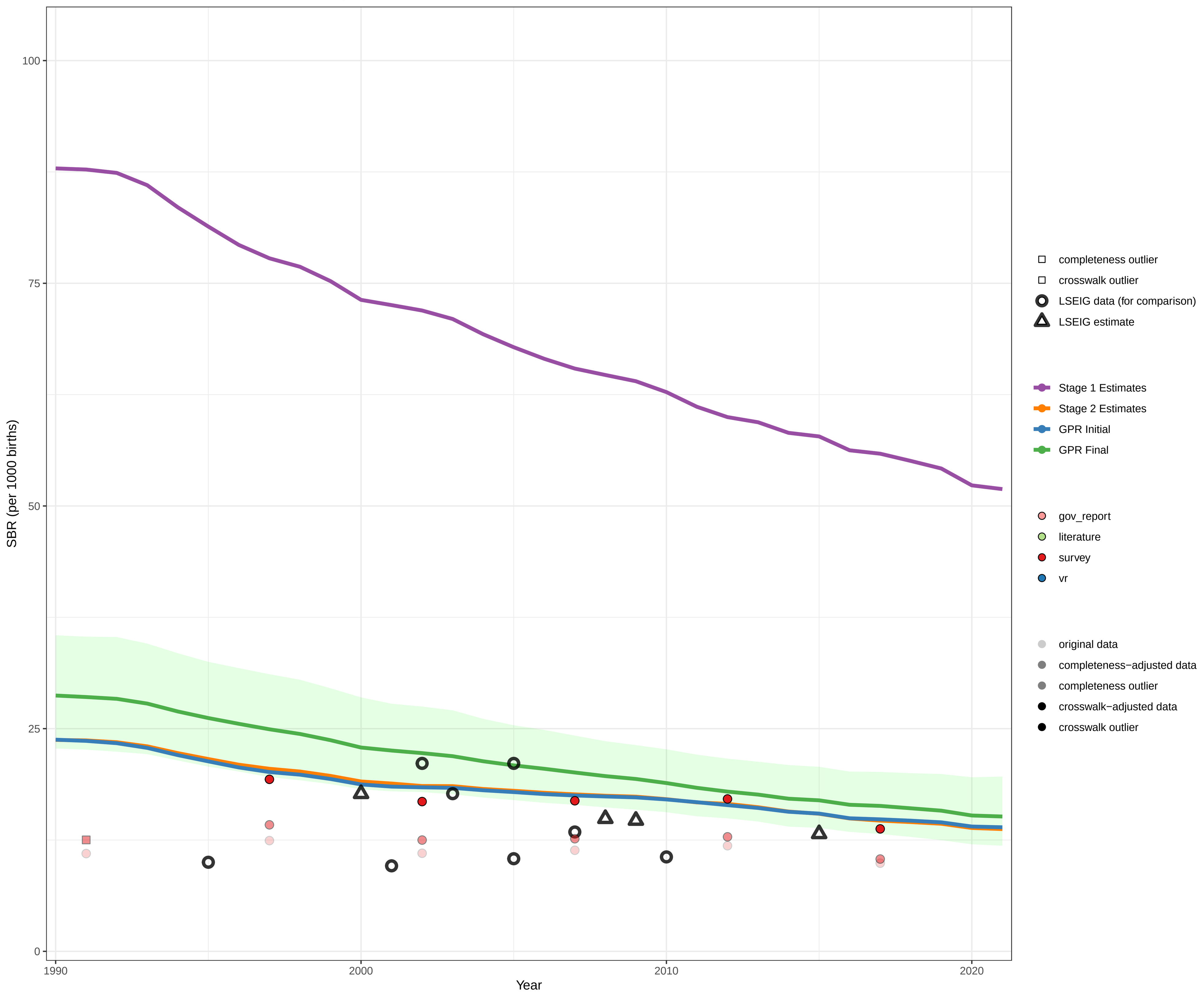

Indonesia IDN Southeast Asia Stillbirth Rate (SBR) − 28 weeks
lambda = 0.6 ; zeta = 0.8 ; scale = 15 ; amp = 0.028

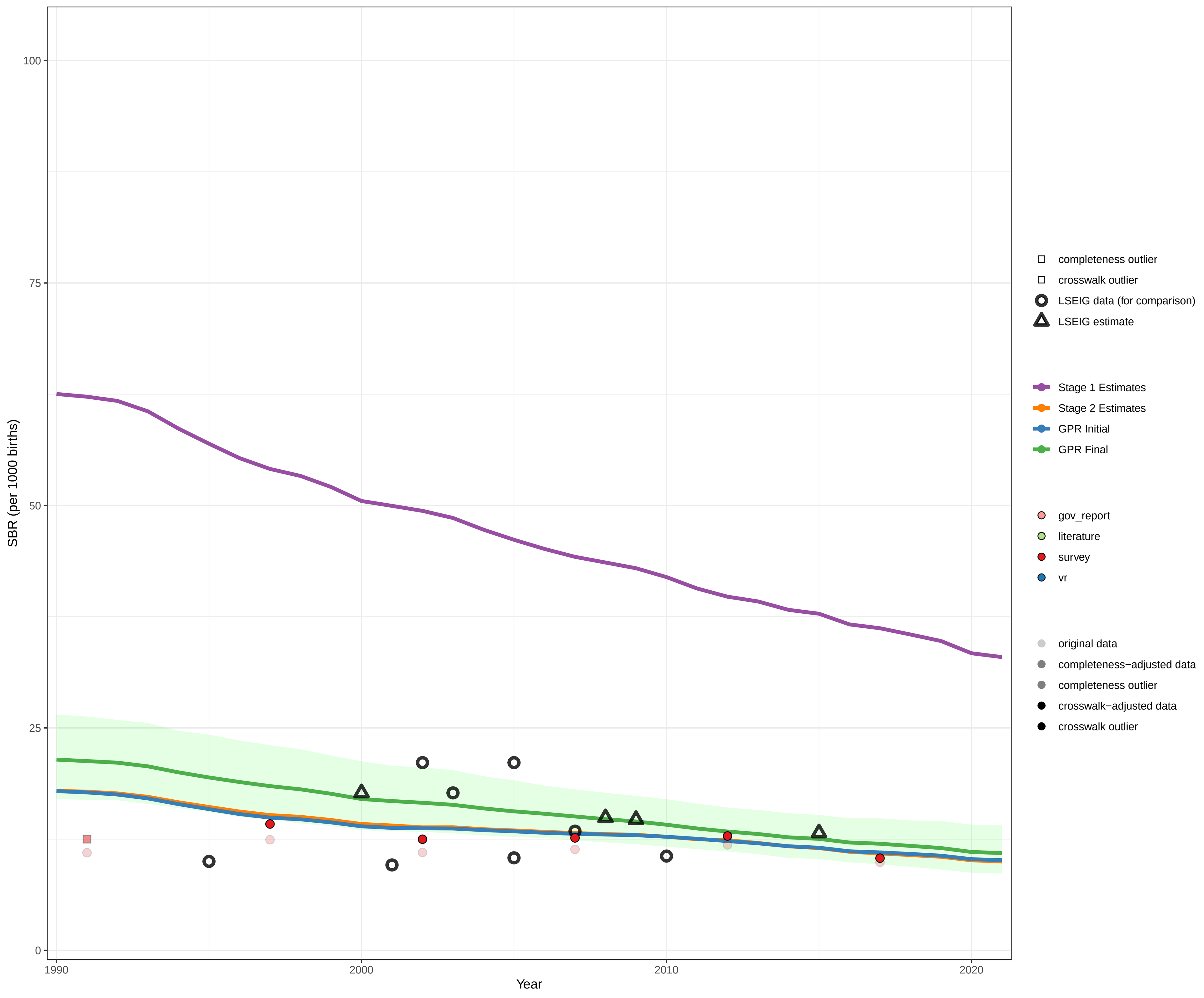

Year	Total Stillbirths (in thousands)	Total Stillbirths (in thousands)	SBR (per 1000 births)	SBR (per 1000 births)	SBR/NMR	SBR/NMR
	20 weeks	28 weeks	20 weeks	28 weeks	20 weeks	28 weeks
1990	137.7 (108.5 to 171.3)	102.0 (80.5 to 126.8)	28.7 (22.8 to 35.5)	21.4 (17.0 to 26.5)	0.94 (0.75 to 1.17)	0.71 (0.56 to 0.87)
1995	124.7 (98.1 to 155.6)	91.8 (73.2 to 115.1)	26.2 (20.7 to 32.5)	19.4 (15.6 to 24.2)	0.97 (0.77 to 1.20)	0.72 (0.58 to 0.90)
2000	110.1 (87.1 to 137.9)	81.3 (64.4 to 102.0)	22.9 (18.2 to 28.5)	17.0 (13.5 to 21.2)	0.97 (0.77 to 1.21)	0.72 (0.57 to 0.90)
2005	103.3 (83.7 to 126.2)	76.9 (62.6 to 94.4)	20.9 (17.0 to 25.4)	15.6 (12.8 to 19.1)	0.99 (0.80 to 1.20)	0.74 (0.60 to 0.90)
2010	93.0 (76.6 to 112.1)	69.2 (57.0 to 83.5)	18.9 (15.6 to 22.7)	14.1 (11.7 to 17.0)	1.02 (0.84 to 1.22)	0.76 (0.63 to 0.91)
2015	79.3 (64.5 to 97.3)	58.3 (47.7 to 71.0)	17.0 (13.8 to 20.7)	12.5 (10.3 to 15.2)	1.05 (0.86 to 1.28)	0.78 (0.64 to 0.94)
2020	68.5 (53.8 to 88.1)	49.5 (38.9 to 63.4)	15.3 (12.0 to 19.5)	11.1 (8.7 to 14.1)	1.12 (0.88 to 1.44)	0.81 (0.64 to 1.04)
2021	67.5 (52.7 to 87.9)	48.6 (38.3 to 62.7)	15.1 (11.9 to 19.6)	10.9 (8.6 to 14.1)	1.14 (0.89 to 1.47)	0.82 (0.65 to 1.06)

Aceh IDN_4709 Southeast Asia Stillbirth Rate / Neonatal Mortality Rate (SBR/NMR) − 20 weeks
lambda = 0.6 ; zeta = 0.8 ; scale = 15 ; amp = 0.028

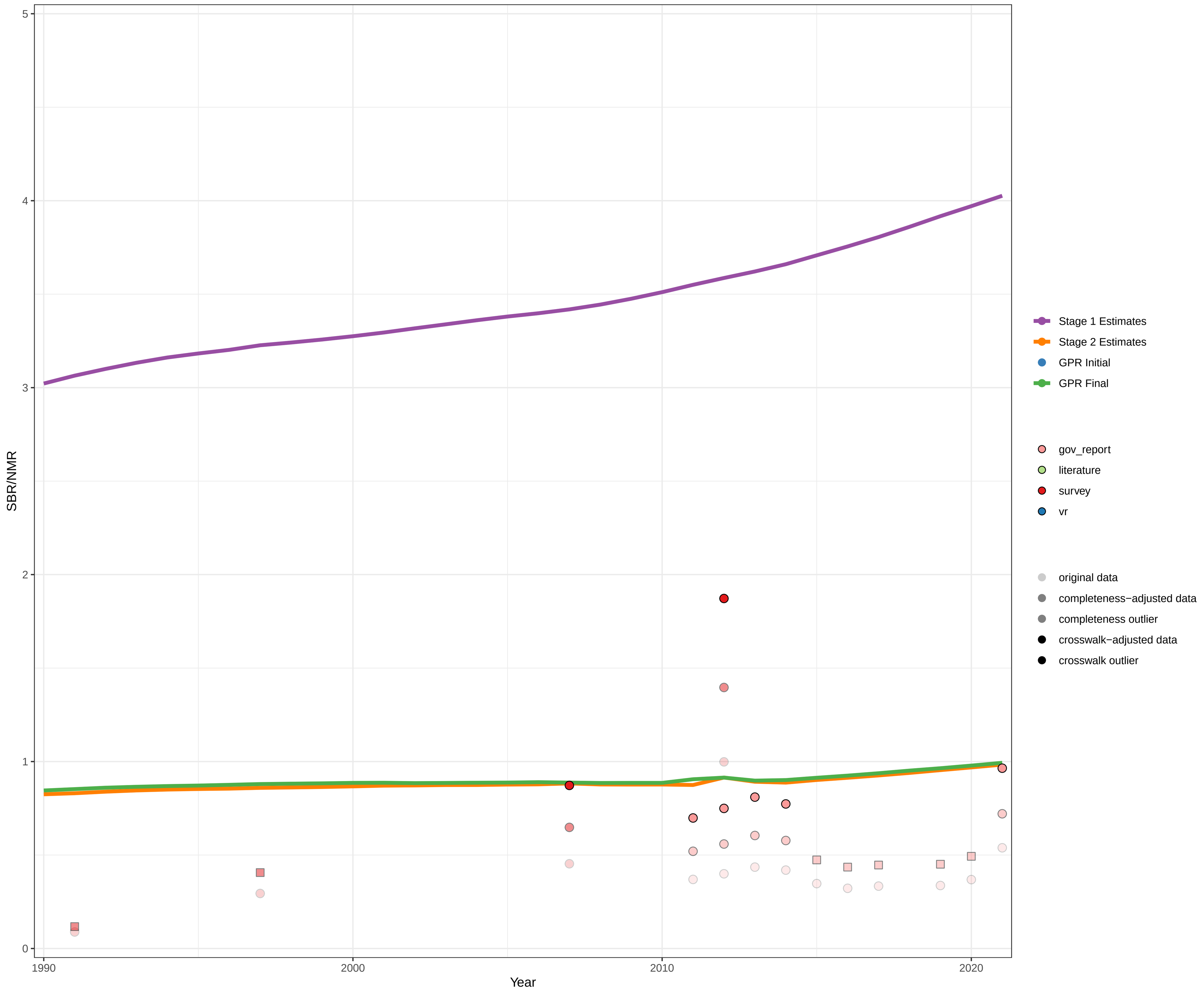

Aceh IDN_4709 Southeast Asia Stillbirth Rate / Neonatal Mortality Rate (SBR/NMR) − 28 weeks
lambda = 0.6 ; zeta = 0.8 ; scale = 15 ; amp = 0.028

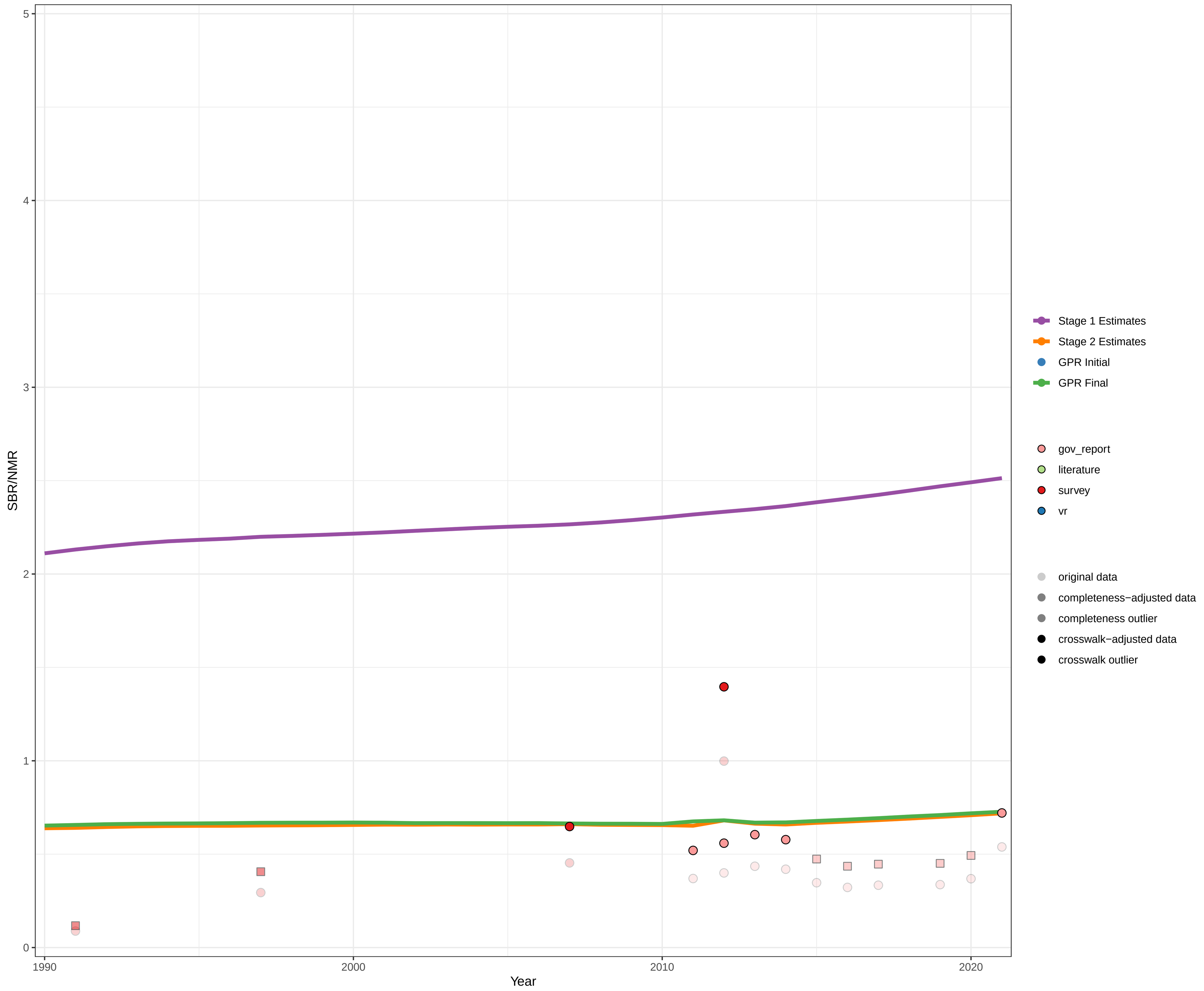

Definition	Years	Outlier	Source.Type	Title
28_weeks	2007	0	Survey	Indonesia Demographic and Health Survey 2007
28_weeks	2011−2014, 2021	0	Statistical Report	Indonesia − Aceh Province Health Profile
28_weeks	2012	0	Survey	Indonesia Demographic and Health Survey 2012
28_weeks	1991	1	Survey	Indonesia Demographic and Health Survey 1991
28_weeks	1997	1	Survey	Indonesia Demographic and Health Survey 1997
28_weeks	2015−2017, 2019, 2020	1	Statistical Report	Indonesia − Aceh Province Health Profile

Aceh IDN_4709 Southeast Asia Stillbirth Rate (SBR) − 20 weeks
lambda = 0.6 ; zeta = 0.8 ; scale = 15 ; amp = 0.028

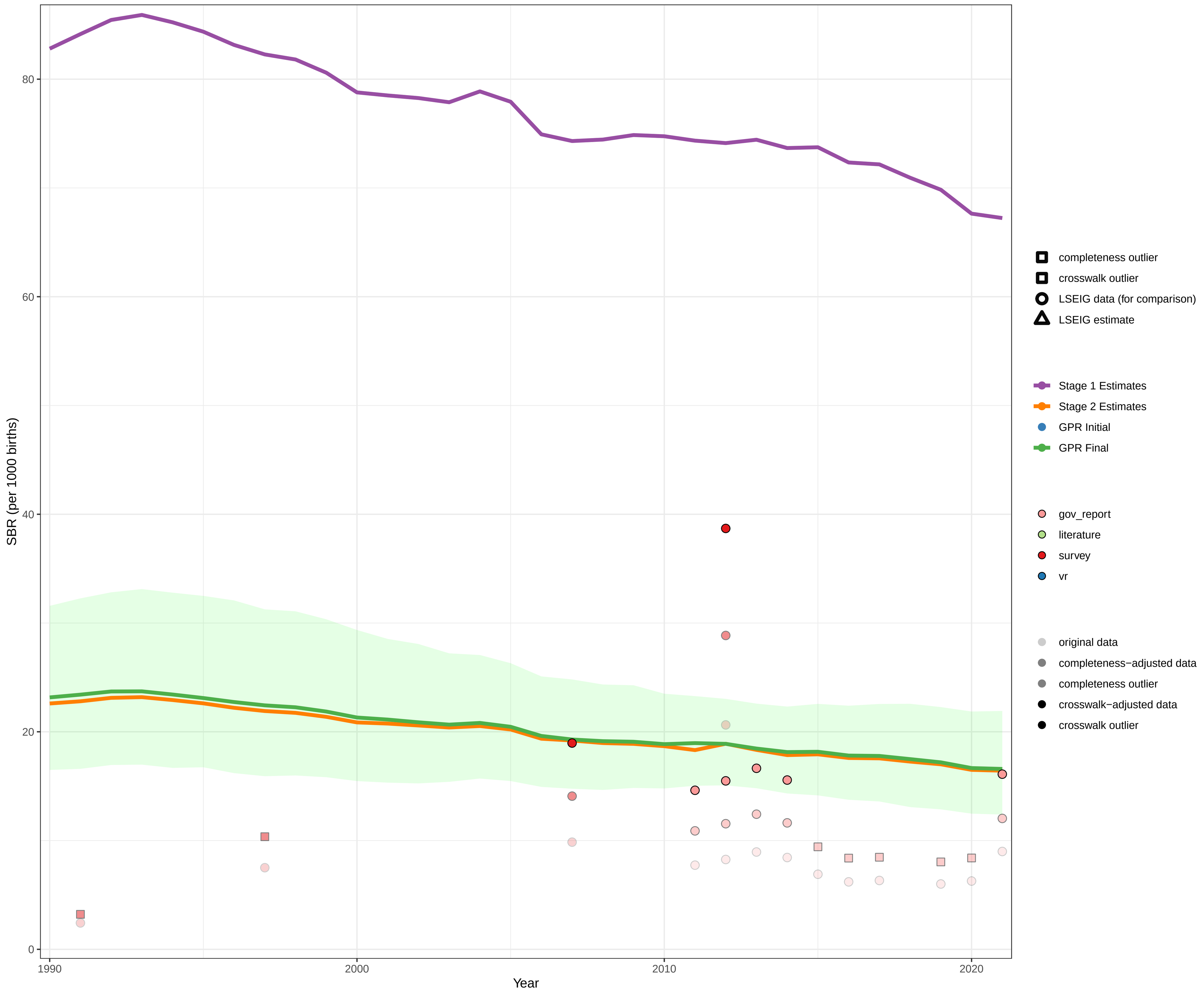

Aceh IDN_4709 Southeast Asia Stillbirth Rate (SBR) − 28 weeks
lambda = 0.6 ; zeta = 0.8 ; scale = 15 ; amp = 0.028

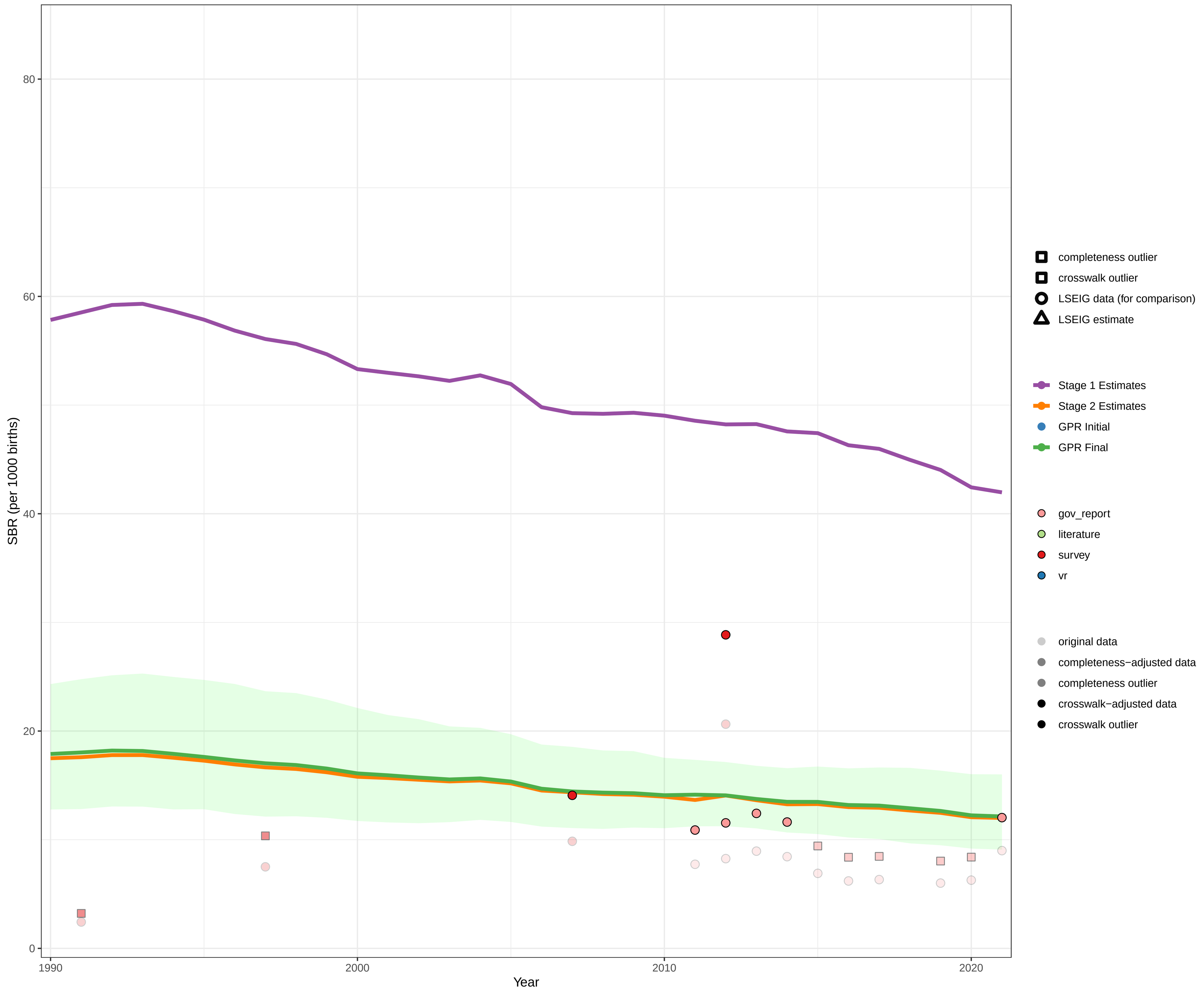

Year	Total Stillbirths (in thousands)	Total Stillbirths (in thousands)	SBR (per 1000 births)	SBR (per 1000 births)	SBR/NMR	SBR/NMR
	20 weeks	28 weeks	20 weeks	28 weeks	20 weeks	28 weeks
1990	2.4 (1.7 to 3.3)	1.9 (1.3 to 2.5)	23.2 (16.5 to 31.6)	17.9 (12.8 to 24.3)	0.85 (0.60 to 1.15)	0.65 (0.47 to 0.89)
1995	2.4 (1.7 to 3.4)	1.8 (1.3 to 2.6)	23.1 (16.7 to 32.5)	17.6 (12.8 to 24.7)	0.87 (0.63 to 1.23)	0.66 (0.48 to 0.93)
2000	2.3 (1.7 to 3.2)	1.7 (1.3 to 2.4)	21.3 (15.5 to 29.4)	16.1 (11.7 to 22.1)	0.89 (0.64 to 1.22)	0.67 (0.49 to 0.92)
2005	2.2 (1.6 to 2.8)	1.6 (1.2 to 2.1)	20.5 (15.5 to 26.3)	15.4 (11.6 to 19.7)	0.89 (0.67 to 1.14)	0.67 (0.50 to 0.85)
2010	1.9 (1.5 to 2.4)	1.4 (1.1 to 1.8)	18.9 (14.8 to 23.5)	14.1 (11.1 to 17.5)	0.89 (0.69 to 1.10)	0.66 (0.52 to 0.82)
2015	1.7 (1.3 to 2.1)	1.3 (1.0 to 1.6)	18.2 (14.2 to 22.6)	13.5 (10.5 to 16.7)	0.91 (0.71 to 1.13)	0.68 (0.53 to 0.84)
2020	1.5 (1.1 to 2.0)	1.1 (0.8 to 1.5)	16.7 (12.5 to 21.9)	12.2 (9.2 to 16.0)	0.98 (0.73 to 1.28)	0.72 (0.54 to 0.94)
2021	1.5 (1.1 to 2.0)	1.1 (0.8 to 1.5)	16.6 (12.4 to 21.9)	12.1 (9.1 to 16.0)	0.99 (0.74 to 1.31)	0.73 (0.54 to 0.96)

Bali IDN_4726 Southeast Asia Stillbirth Rate / Neonatal Mortality Rate (SBR/NMR) − 20 weeks
lambda = 0.6 ; zeta = 0.8 ; scale = 15 ; amp = 0.028

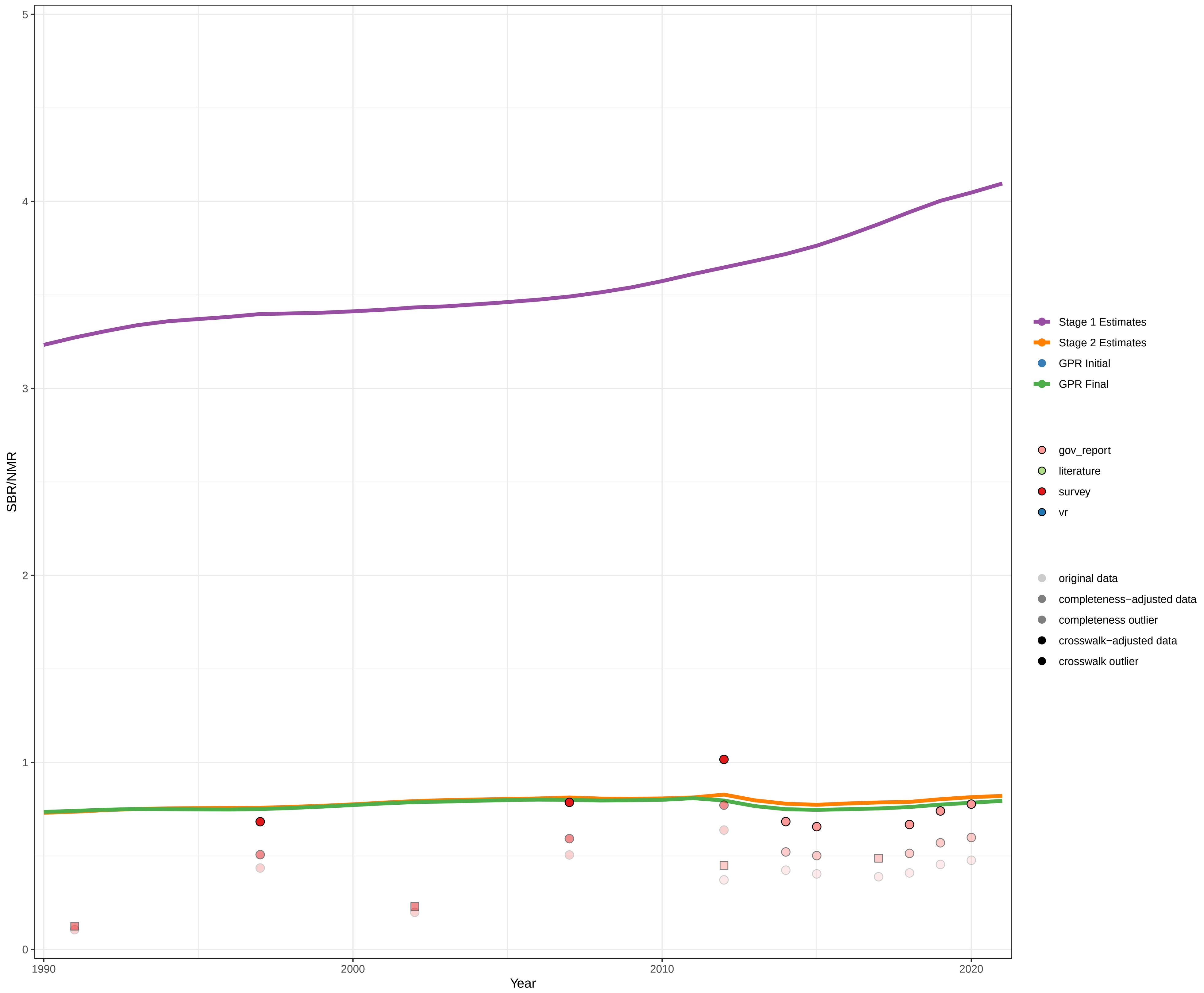

Bali IDN_4726 Southeast Asia Stillbirth Rate / Neonatal Mortality Rate (SBR/NMR) − 28 weeks
lambda = 0.6 ; zeta = 0.8 ; scale = 15 ; amp = 0.028

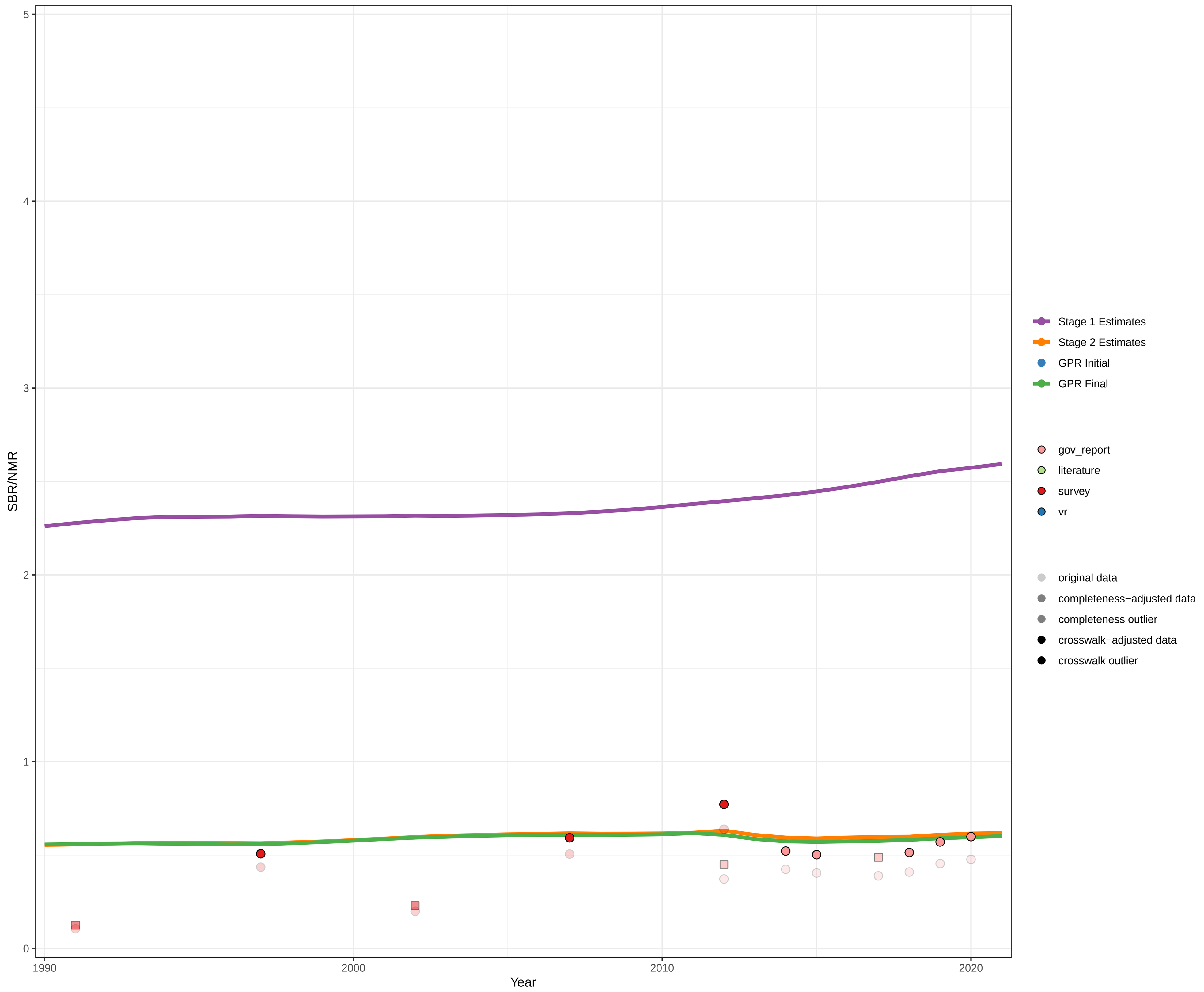

Definition	Years	Outlier	Source.Type	Title
28_weeks	1997	0	Survey	Indonesia Demographic and Health Survey 1997
28_weeks	2007	0	Survey	Indonesia Demographic and Health Survey 2007
28_weeks	2012	0	Survey	Indonesia Demographic and Health Survey 2012
28_weeks	2014, 2015, 2018−2020	0	Statistical Report	Indonesia − Bali Province Health Profile
28_weeks	1991	1	Survey	Indonesia Demographic and Health Survey 1991
28_weeks	2002	1	Survey	Indonesia Demographic and Health Survey 2002−2003
28_weeks	2012, 2017	1	Statistical Report	Indonesia − Bali Province Health Profile

Bali IDN_4726 Southeast Asia Stillbirth Rate (SBR) − 20 weeks
lambda = 0.6 ; zeta = 0.8 ; scale = 15 ; amp = 0.028

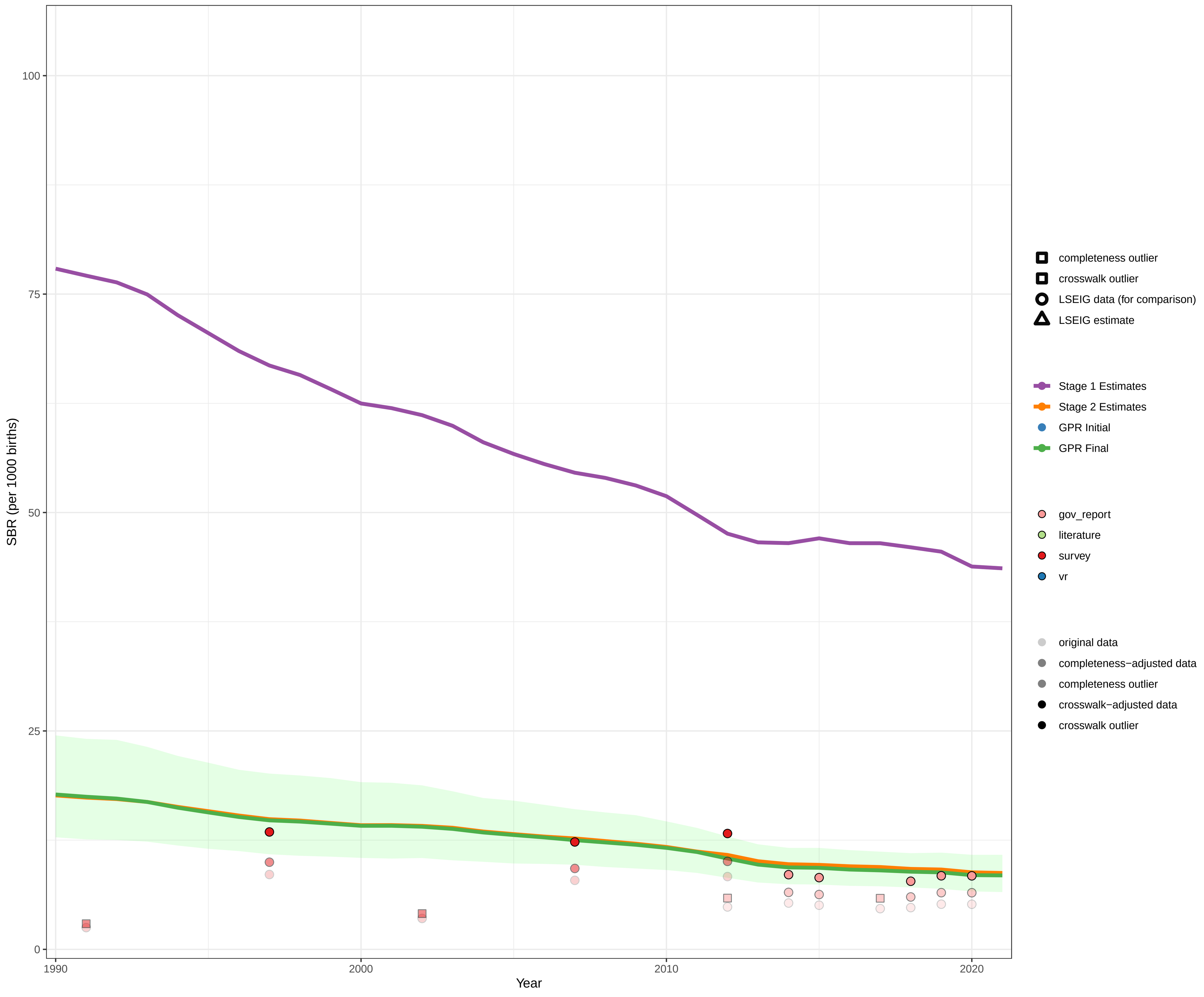

Bali IDN_4726 Southeast Asia Stillbirth Rate (SBR) − 28 weeks
lambda = 0.6 ; zeta = 0.8 ; scale = 15 ; amp = 0.028

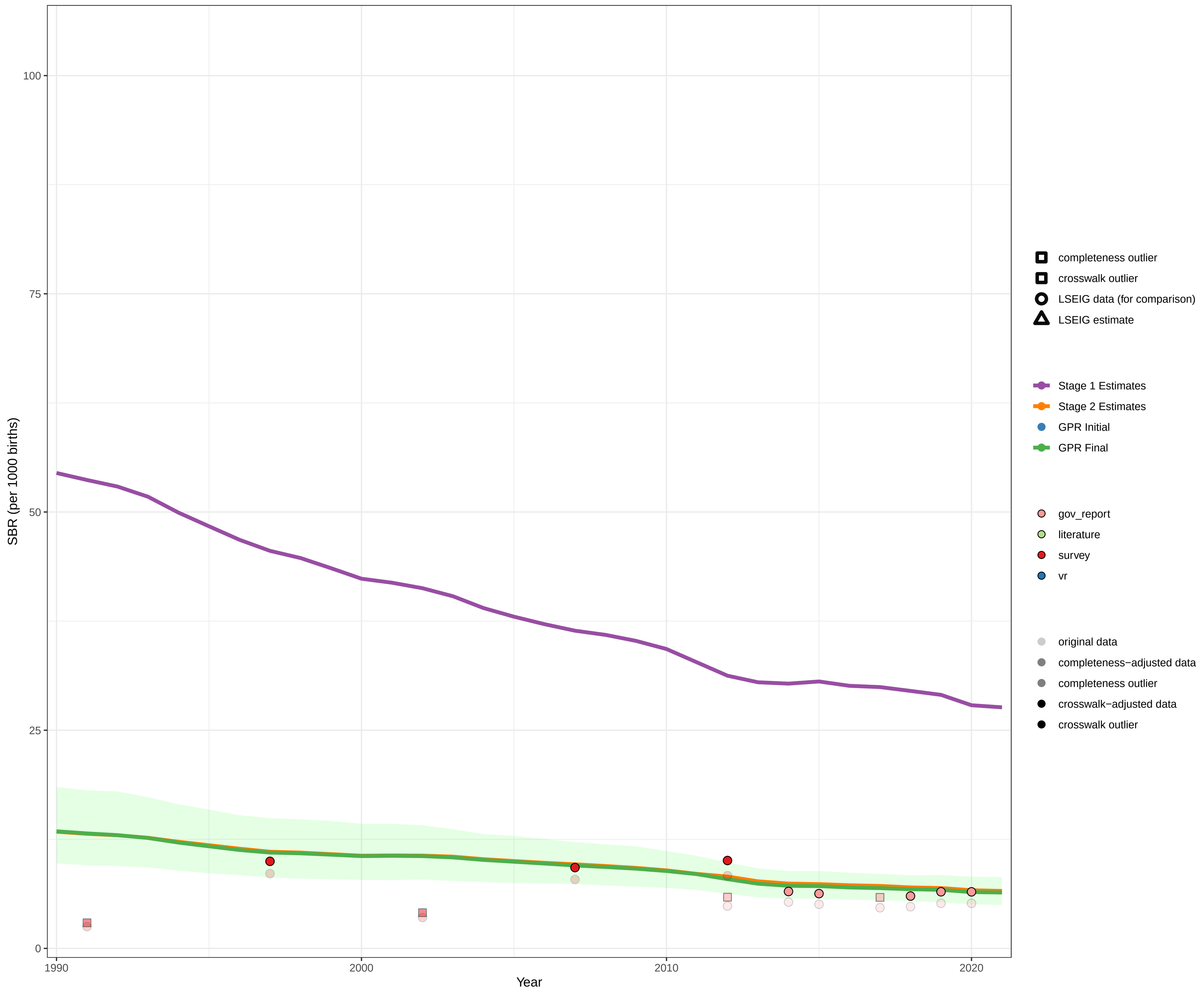

Year	Total Stillbirths (in thousands)	Total Stillbirths (in thousands)	SBR (per 1000 births)	SBR (per 1000 births)	SBR/NMR	SBR/NMR
	20 weeks	28 weeks	20 weeks	28 weeks	20 weeks	28 weeks
1990	1.1 (0.8 to 1.5)	0.8 (0.6 to 1.2)	17.7 (12.8 to 24.5)	13.4 (9.7 to 18.5)	0.74 (0.53 to 1.02)	0.56 (0.40 to 0.77)
1995	1.0 (0.7 to 1.3)	0.7 (0.5 to 1.0)	15.7 (11.5 to 21.4)	11.7 (8.6 to 15.9)	0.75 (0.55 to 1.02)	0.56 (0.41 to 0.76)
2000	0.9 (0.7 to 1.2)	0.7 (0.5 to 0.9)	14.1 (10.5 to 19.1)	10.6 (7.8 to 14.3)	0.77 (0.57 to 1.05)	0.58 (0.43 to 0.78)
2005	0.8 (0.6 to 1.1)	0.6 (0.5 to 0.8)	13.1 (9.8 to 17.0)	9.9 (7.5 to 12.9)	0.80 (0.60 to 1.04)	0.61 (0.46 to 0.79)
2010	0.7 (0.6 to 0.9)	0.5 (0.4 to 0.7)	11.6 (9.1 to 14.6)	8.9 (6.9 to 11.2)	0.80 (0.63 to 1.01)	0.61 (0.48 to 0.77)
2015	0.5 (0.4 to 0.7)	0.4 (0.3 to 0.5)	9.3 (7.4 to 11.6)	7.1 (5.7 to 8.9)	0.75 (0.59 to 0.93)	0.57 (0.45 to 0.71)
2020	0.4 (0.3 to 0.6)	0.3 (0.3 to 0.4)	8.5 (6.6 to 10.8)	6.5 (5.0 to 8.2)	0.78 (0.61 to 1.00)	0.60 (0.47 to 0.76)
2021	0.4 (0.3 to 0.6)	0.3 (0.3 to 0.4)	8.5 (6.6 to 10.8)	6.4 (5.0 to 8.2)	0.79 (0.62 to 1.02)	0.60 (0.47 to 0.77)

Bangka−Belitung Islands IDN_4717 Southeast Asia Stillbirth Rate / Neonatal Mortality Rate (SBR/NMR) − 20 weeks
lambda = 0.6 ; zeta = 0.8 ; scale = 15 ; amp = 0.028

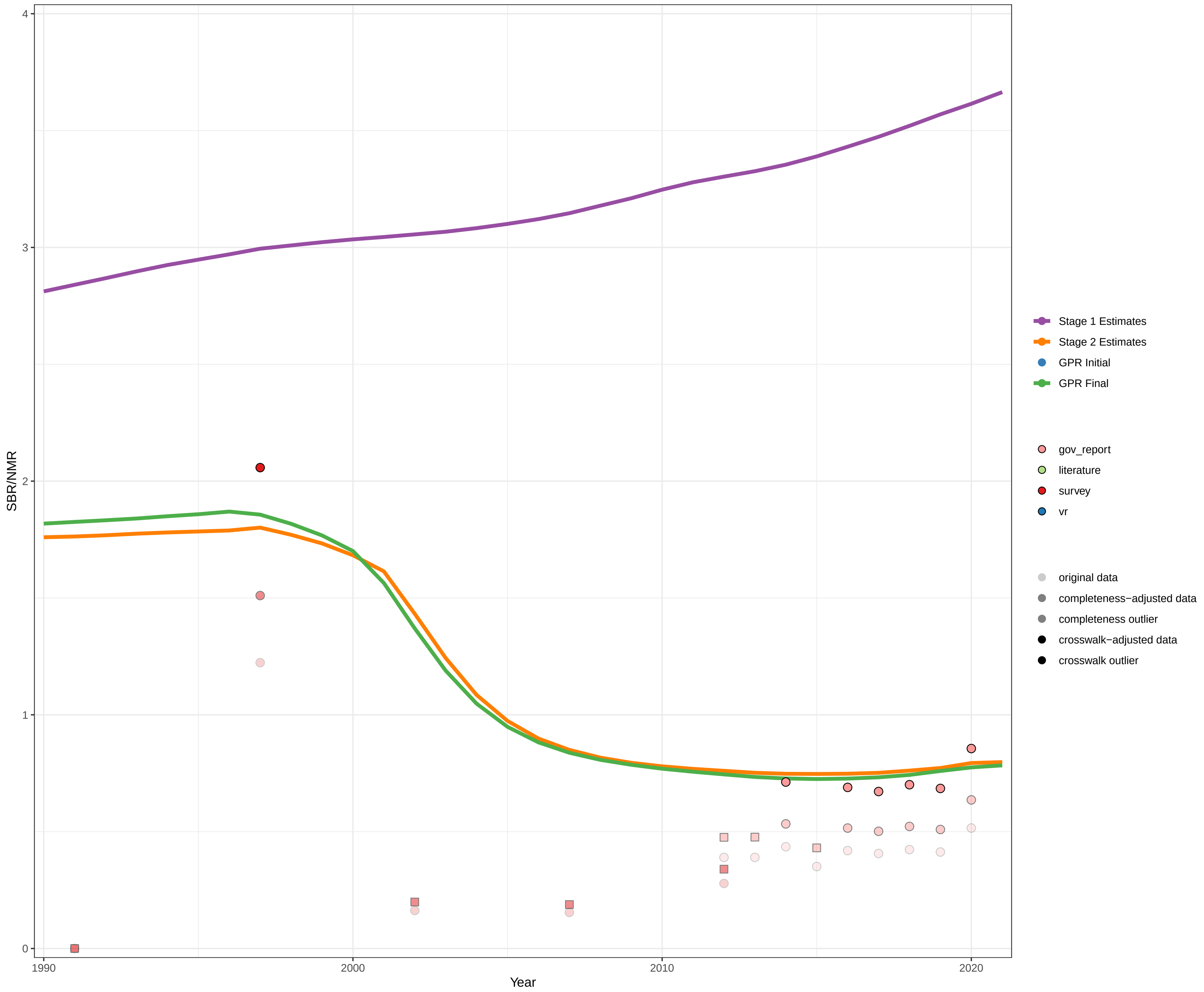

Bangka−Belitung Islands IDN_4717 Southeast Asia Stillbirth Rate / Neonatal Mortality Rate (SBR/NMR) − 28 weeks
lambda = 0.6 ; zeta = 0.8 ; scale = 15 ; amp = 0.028

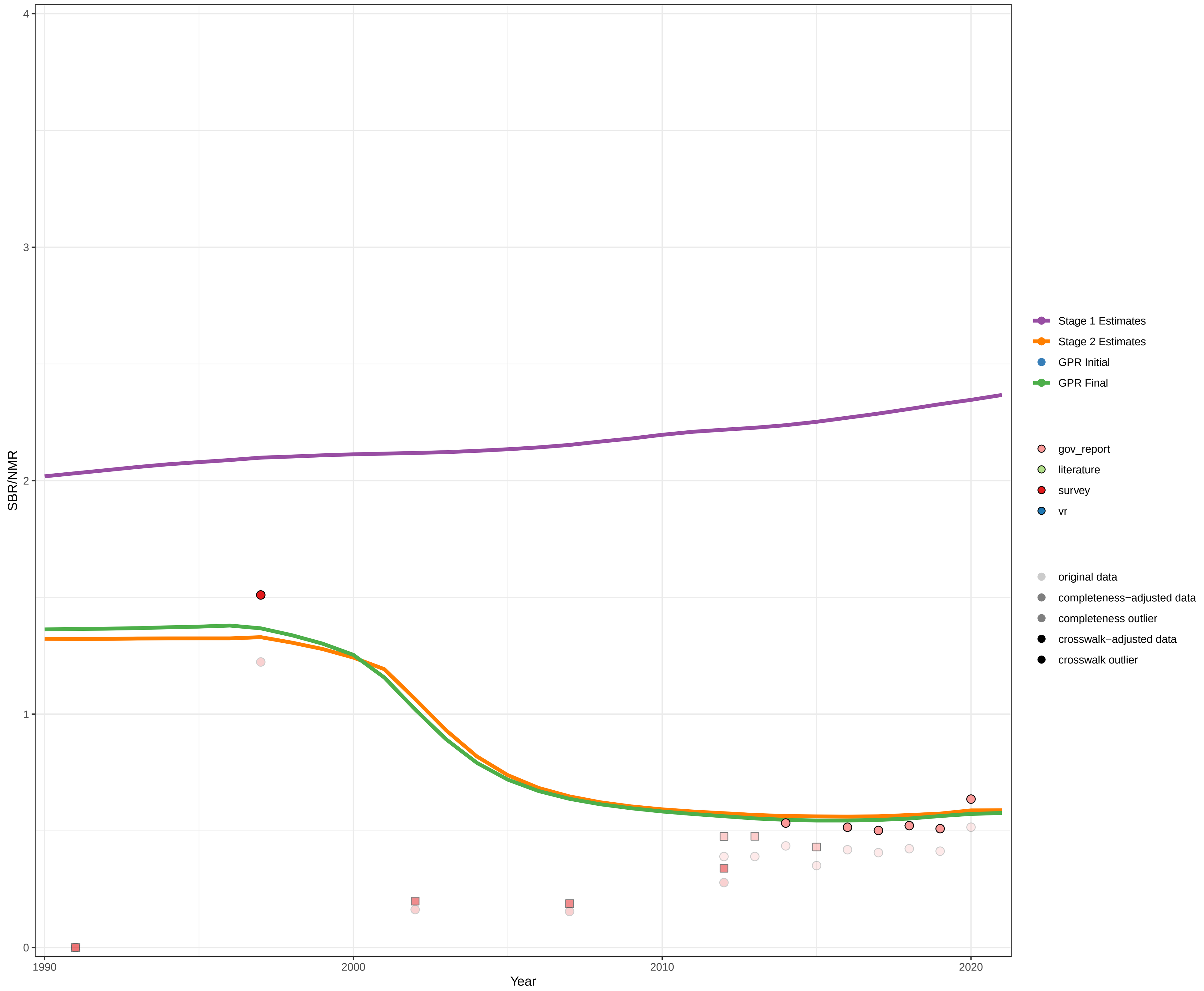

Definition	Years	Outlier	Source.Type	Title
28_weeks	1997	0	Survey	Indonesia Demographic and Health Survey 1997
28_weeks	2014, 2016−2020	0	Statistical Report	Indonesia − Kepulauan Bangka Belitung Province Health Profile
28_weeks	1991	1	Survey	Indonesia Demographic and Health Survey 1991
28_weeks	2002	1	Survey	Indonesia Demographic and Health Survey 2002−2003
28_weeks	2007	1	Survey	Indonesia Demographic and Health Survey 2007
28_weeks	2012	1	Survey	Indonesia Demographic and Health Survey 2012
28_weeks	2012, 2013, 2015	1	Statistical Report	Indonesia − Kepulauan Bangka Belitung Province Health Profile

Bangka−Belitung Islands IDN_4717 Southeast Asia Stillbirth Rate (SBR) − 20 weeks
lambda = 0.6 ; zeta = 0.8 ; scale = 15 ; amp = 0.028

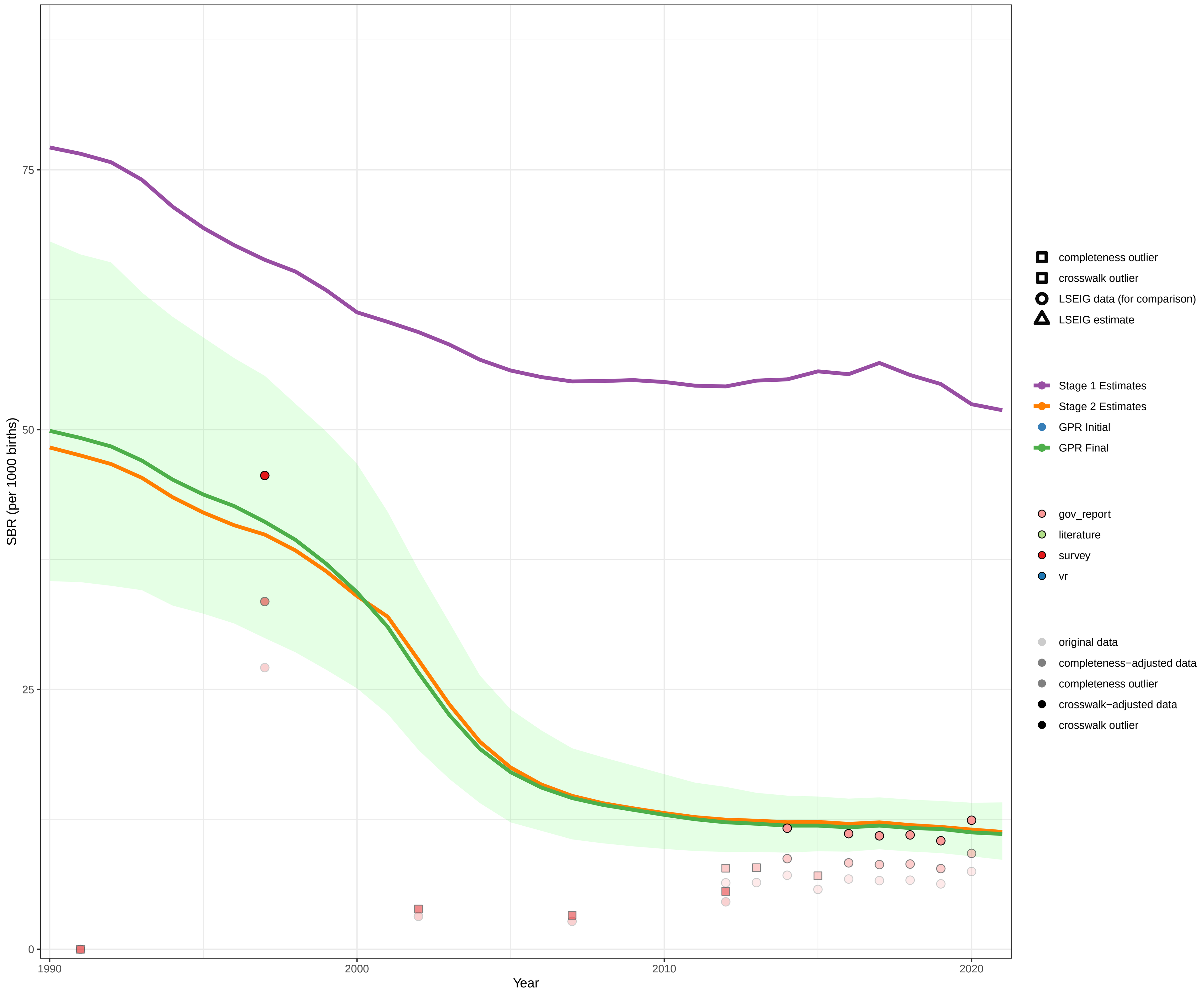

Bangka−Belitung Islands IDN_4717 Southeast Asia Stillbirth Rate (SBR) − 28 weeks
lambda = 0.6 ; zeta = 0.8 ; scale = 15 ; amp = 0.028

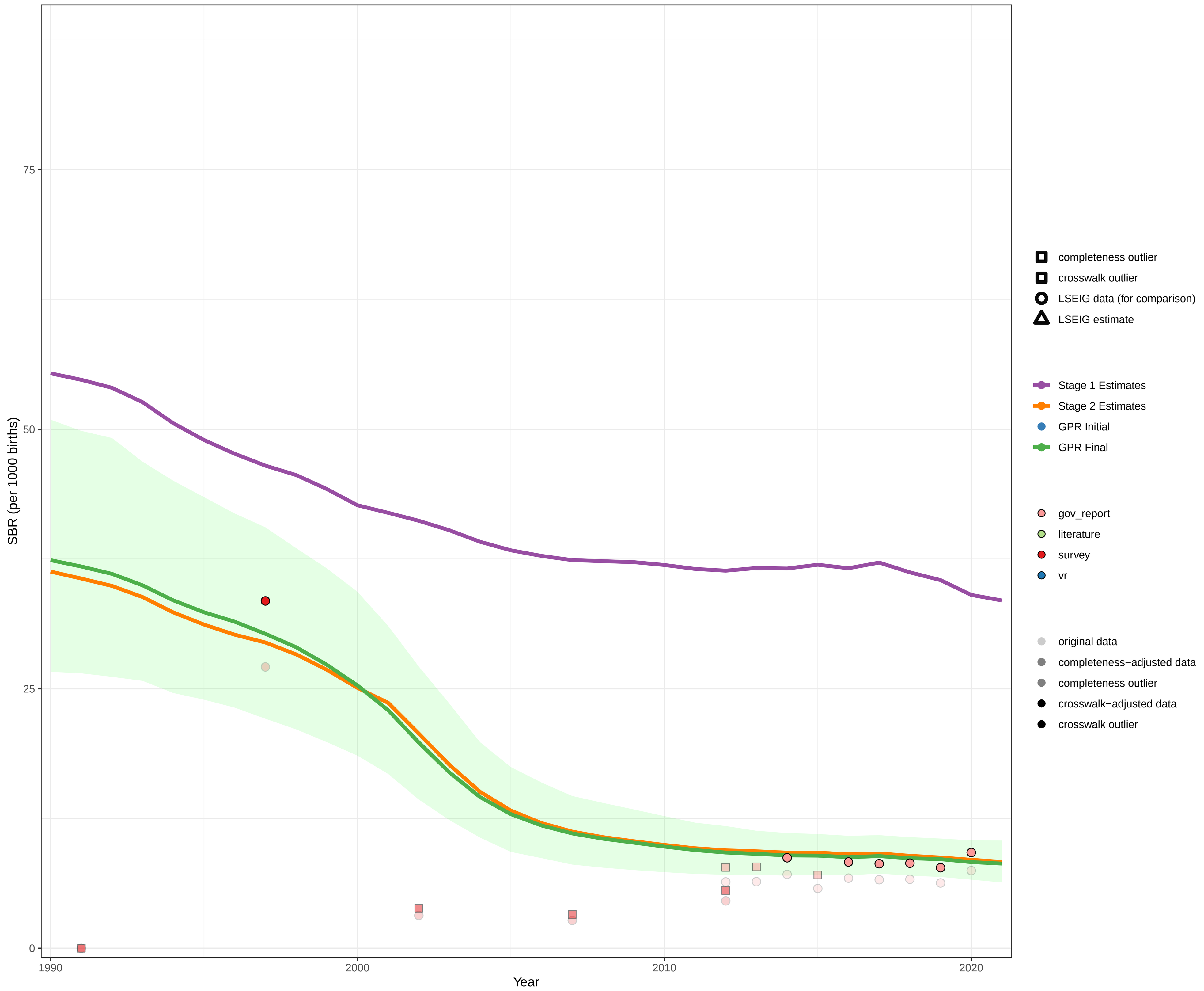

Year	Total Stillbirths (in thousands)	Total Stillbirths (in thousands)	SBR (per 1000 births)	SBR (per 1000 births)	SBR/NMR	SBR/NMR
	20 weeks	28 weeks	20 weeks	28 weeks	20 weeks	28 weeks
1990	1.2 (0.8 to 1.6)	0.9 (0.6 to 1.2)	49.9 (35.4 to 68.1)	37.4 (26.6 to 50.9)	1.82 (1.29 to 2.48)	1.36 (0.97 to 1.86)
1995	1.0 (0.7 to 1.4)	0.7 (0.5 to 1.0)	43.8 (32.3 to 58.9)	32.4 (23.9 to 43.5)	1.86 (1.37 to 2.50)	1.37 (1.02 to 1.85)
2000	0.8 (0.6 to 1.1)	0.6 (0.4 to 0.8)	34.3 (25.1 to 46.7)	25.3 (18.6 to 34.3)	1.70 (1.24 to 2.31)	1.25 (0.92 to 1.70)
2005	0.4 (0.3 to 0.6)	0.3 (0.2 to 0.5)	17.0 (12.2 to 23.1)	12.9 (9.3 to 17.5)	0.95 (0.68 to 1.29)	0.72 (0.52 to 0.97)
2010	0.4 (0.3 to 0.5)	0.3 (0.2 to 0.4)	12.9 (9.7 to 16.8)	9.8 (7.3 to 12.7)	0.77 (0.57 to 1.00)	0.58 (0.44 to 0.76)
2015	0.3 (0.3 to 0.4)	0.2 (0.2 to 0.3)	11.9 (9.4 to 14.7)	8.9 (7.1 to 11.0)	0.73 (0.57 to 0.90)	0.54 (0.43 to 0.67)
2020	0.3 (0.2 to 0.4)	0.2 (0.2 to 0.3)	11.2 (8.9 to 14.1)	8.3 (6.6 to 10.4)	0.77 (0.62 to 0.97)	0.57 (0.46 to 0.72)
2021	0.3 (0.2 to 0.4)	0.2 (0.2 to 0.3)	11.1 (8.6 to 14.1)	8.2 (6.3 to 10.4)	0.78 (0.61 to 1.00)	0.58 (0.45 to 0.73)

Banten IDN_4725 Southeast Asia Stillbirth Rate / Neonatal Mortality Rate (SBR/NMR) − 20 weeks
lambda = 0.8 ; zeta = 0.7 ; scale = 15 ; amp = 0.028

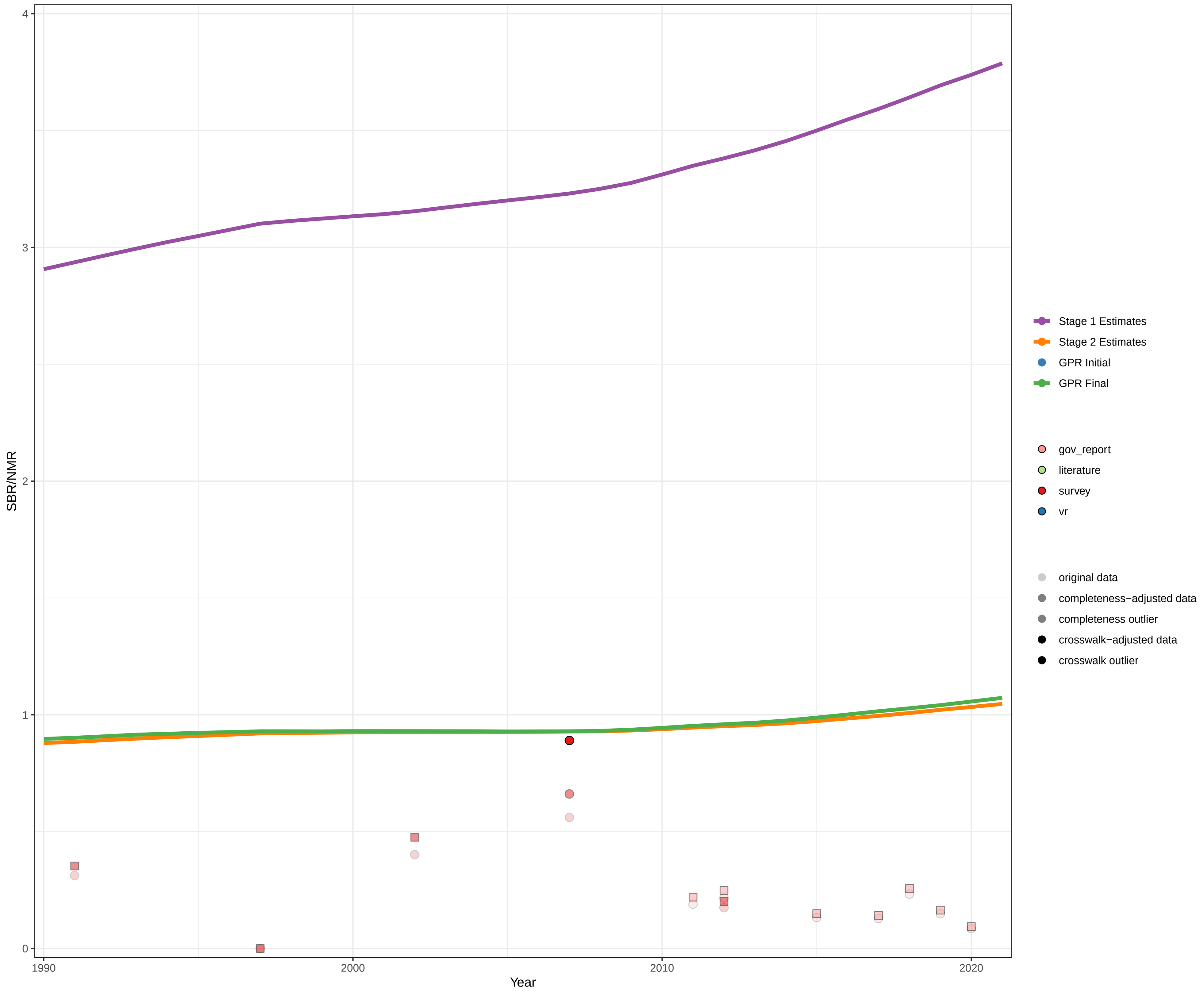

Banten IDN_4725 Southeast Asia Stillbirth Rate / Neonatal Mortality Rate (SBR/NMR) − 28 weeks
lambda = 0.8 ; zeta = 0.7 ; scale = 15 ; amp = 0.028

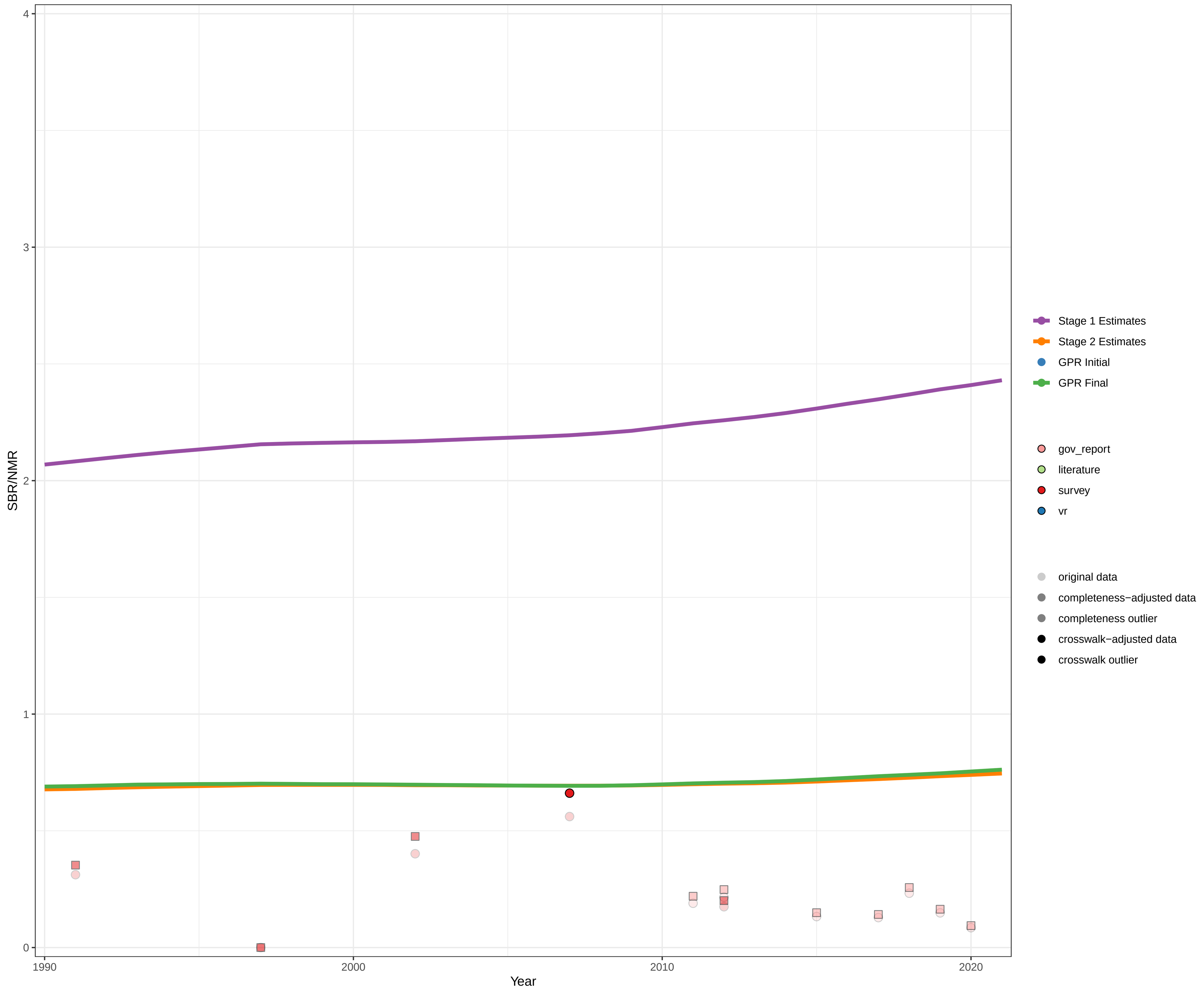

Definition	Years	Outlier	Source.Type	Title
28_weeks	2007	0	Survey	Indonesia Demographic and Health Survey 2007
28_weeks	1991	1	Survey	Indonesia Demographic and Health Survey 1991
28_weeks	1997	1	Survey	Indonesia Demographic and Health Survey 1997
28_weeks	2002	1	Survey	Indonesia Demographic and Health Survey 2002−2003
28_weeks	2011, 2012, 2015, 2017−2020	1	Statistical Report	Indonesia − Banten Province Health Profile
28_weeks	2012	1	Survey	Indonesia Demographic and Health Survey 2012

Banten IDN_4725 Southeast Asia Stillbirth Rate (SBR) − 20 weeks
lambda = 0.8 ; zeta = 0.7 ; scale = 15 ; amp = 0.028

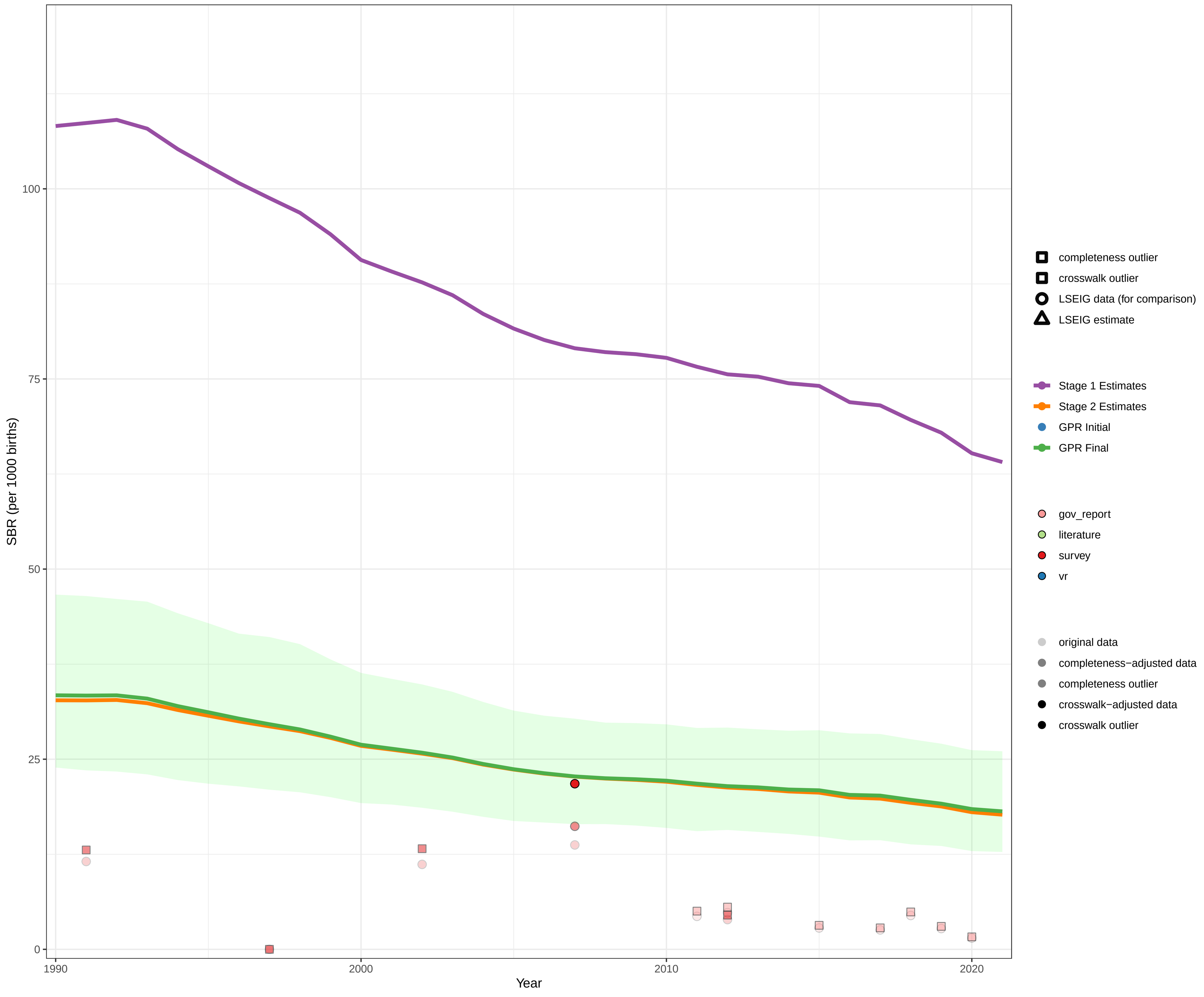

Banten IDN_4725 Southeast Asia Stillbirth Rate (SBR) − 28 weeks
lambda = 0.8 ; zeta = 0.7 ; scale = 15 ; amp = 0.028

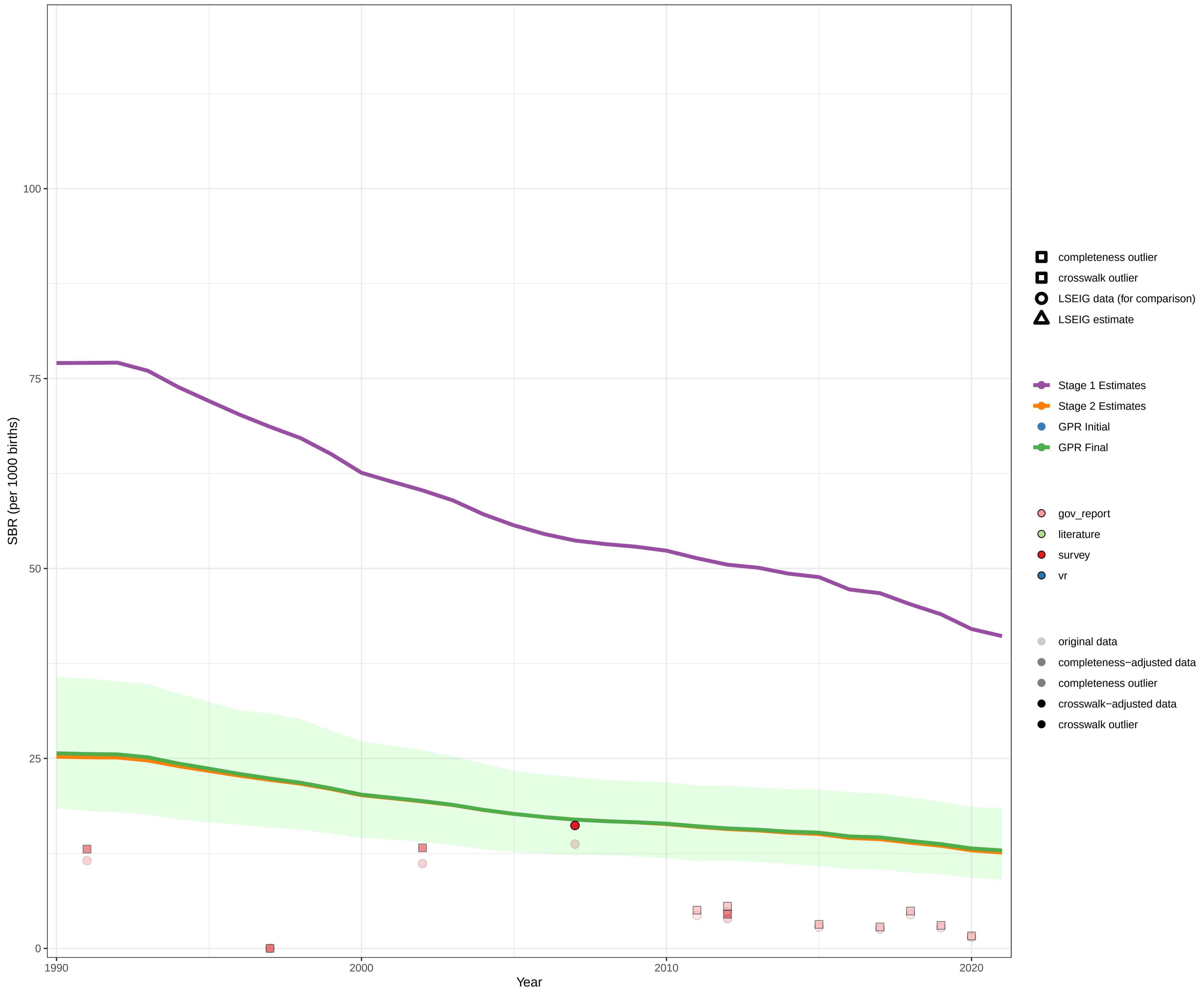

Year	Total Stillbirths (in thousands)	Total Stillbirths (in thousands)	SBR (per 1000 births)	SBR (per 1000 births)	SBR/NMR	SBR/NMR
	20 weeks	28 weeks	20 weeks	28 weeks	20 weeks	28 weeks
1990	8.2 (5.8 to 11.7)	6.3 (4.5 to 8.8)	33.4 (23.9 to 46.6)	25.7 (18.4 to 35.8)	0.90 (0.64 to 1.25)	0.69 (0.50 to 0.96)
1995	8.3 (5.7 to 11.5)	6.2 (4.3 to 8.6)	31.2 (21.8 to 42.9)	23.7 (16.6 to 32.5)	0.92 (0.64 to 1.27)	0.70 (0.49 to 0.96)
2000	7.1 (5.0 to 9.6)	5.3 (3.8 to 7.2)	26.9 (19.2 to 36.4)	20.2 (14.5 to 27.3)	0.93 (0.66 to 1.26)	0.70 (0.50 to 0.94)
2005	6.2 (4.4 to 8.2)	4.6 (3.2 to 6.1)	23.7 (16.9 to 31.4)	17.7 (12.6 to 23.4)	0.93 (0.66 to 1.23)	0.69 (0.50 to 0.92)
2010	5.5 (3.9 to 7.4)	4.0 (2.9 to 5.4)	22.2 (16.0 to 29.6)	16.4 (11.9 to 21.8)	0.94 (0.68 to 1.26)	0.70 (0.50 to 0.93)
2015	4.8 (3.4 to 6.7)	3.5 (2.5 to 4.8)	20.9 (14.8 to 28.8)	15.2 (10.8 to 20.9)	0.99 (0.70 to 1.36)	0.72 (0.51 to 0.99)
2020	4.0 (2.8 to 5.7)	2.8 (2.0 to 4.0)	18.4 (12.9 to 26.2)	13.2 (9.2 to 18.6)	1.06 (0.74 to 1.50)	0.75 (0.53 to 1.07)
2021	3.9 (2.7 to 5.6)	2.7 (1.9 to 3.9)	18.1 (12.8 to 26.0)	12.9 (9.1 to 18.4)	1.07 (0.76 to 1.54)	0.76 (0.54 to 1.09)

Bengkulu IDN_4715 Southeast Asia Stillbirth Rate / Neonatal Mortality Rate (SBR/NMR) − 20 weeks
lambda = 0.6 ; zeta = 0.8 ; scale = 15 ; amp = 0.028

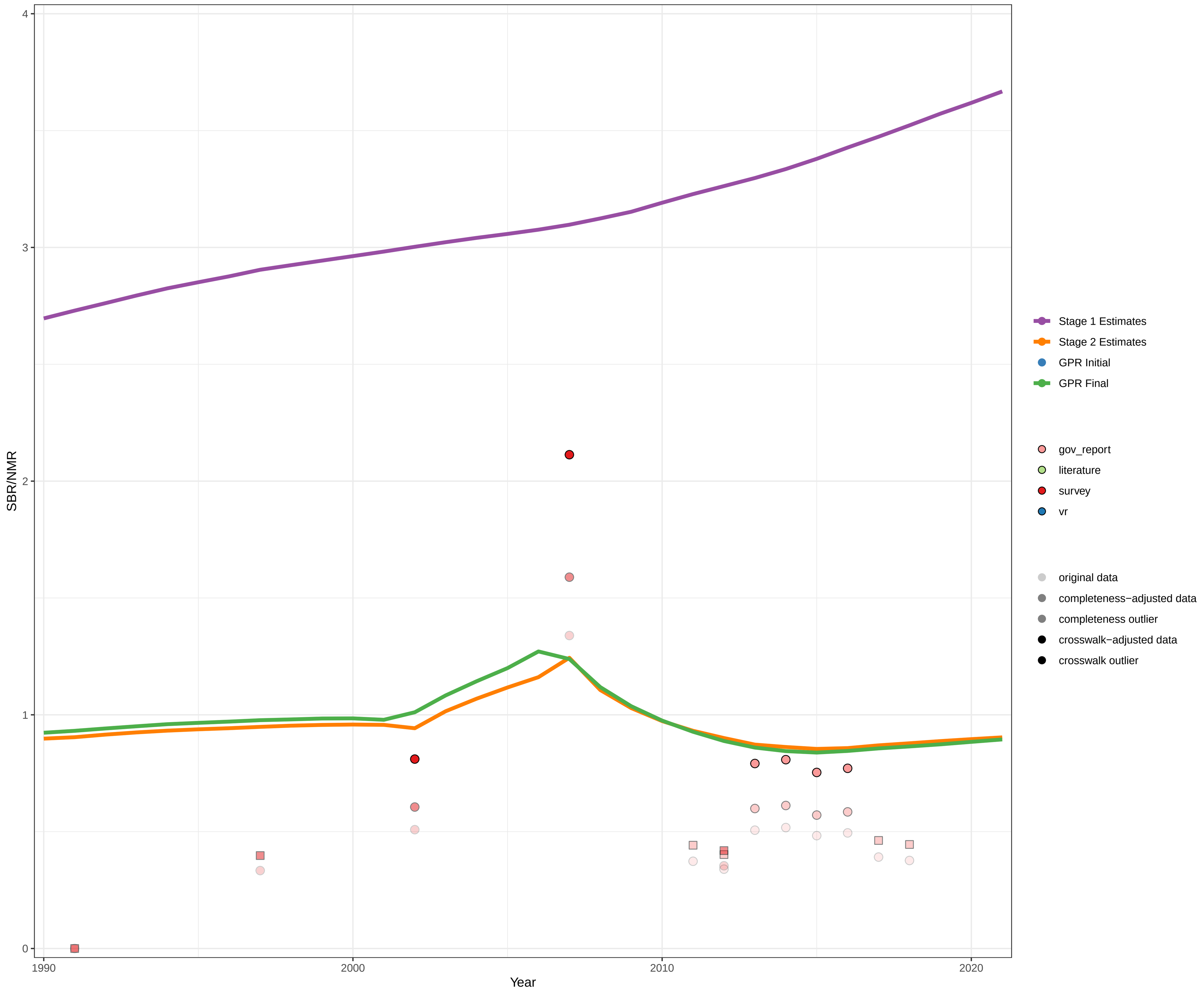

Bengkulu IDN_4715 Southeast Asia Stillbirth Rate / Neonatal Mortality Rate (SBR/NMR) − 28 weeks
lambda = 0.6 ; zeta = 0.8 ; scale = 15 ; amp = 0.028

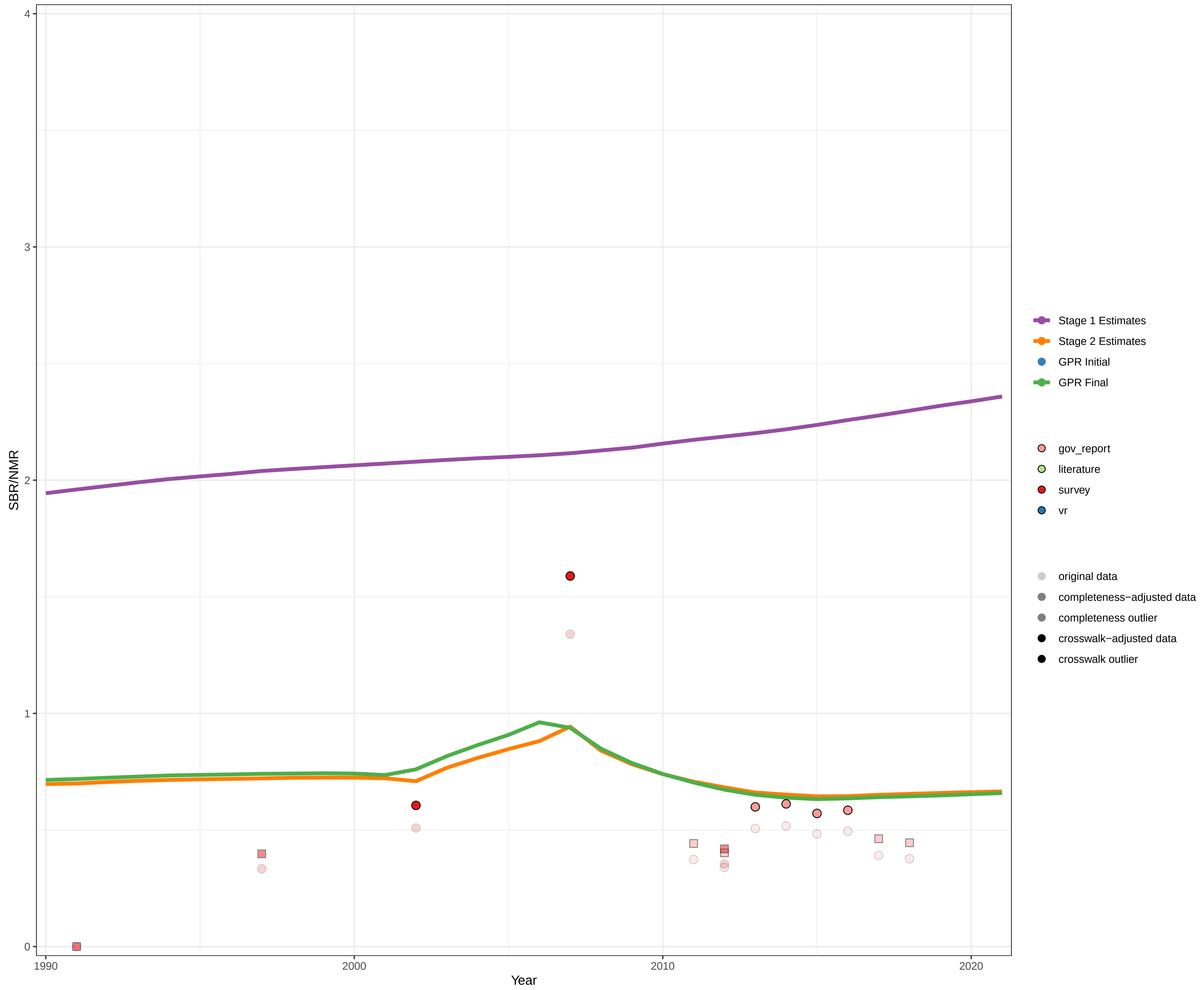

Definition	Years	Outlier	Source.Type	Title
28_weeks	2002	0	Survey	Indonesia Demographic and Health Survey 2002−2003
28_weeks	2007	0	Survey	Indonesia Demographic and Health Survey 2007
28_weeks	2013−2016	0	Statistical Report	Indonesia − Bengkulu Province Health Profile
28_weeks	1991	1	Survey	Indonesia Demographic and Health Survey 1991
28_weeks	1997	1	Survey	Indonesia Demographic and Health Survey 1997
28_weeks	2011, 2012, 2017, 2018	1	Statistical Report	Indonesia − Bengkulu Province Health Profile
28_weeks	2012	1	Survey	Indonesia Demographic and Health Survey 2012

Bengkulu IDN_4715 Southeast Asia Stillbirth Rate (SBR) − 20 weeks
lambda = 0.6 ; zeta = 0.8 ; scale = 15 ; amp = 0.028

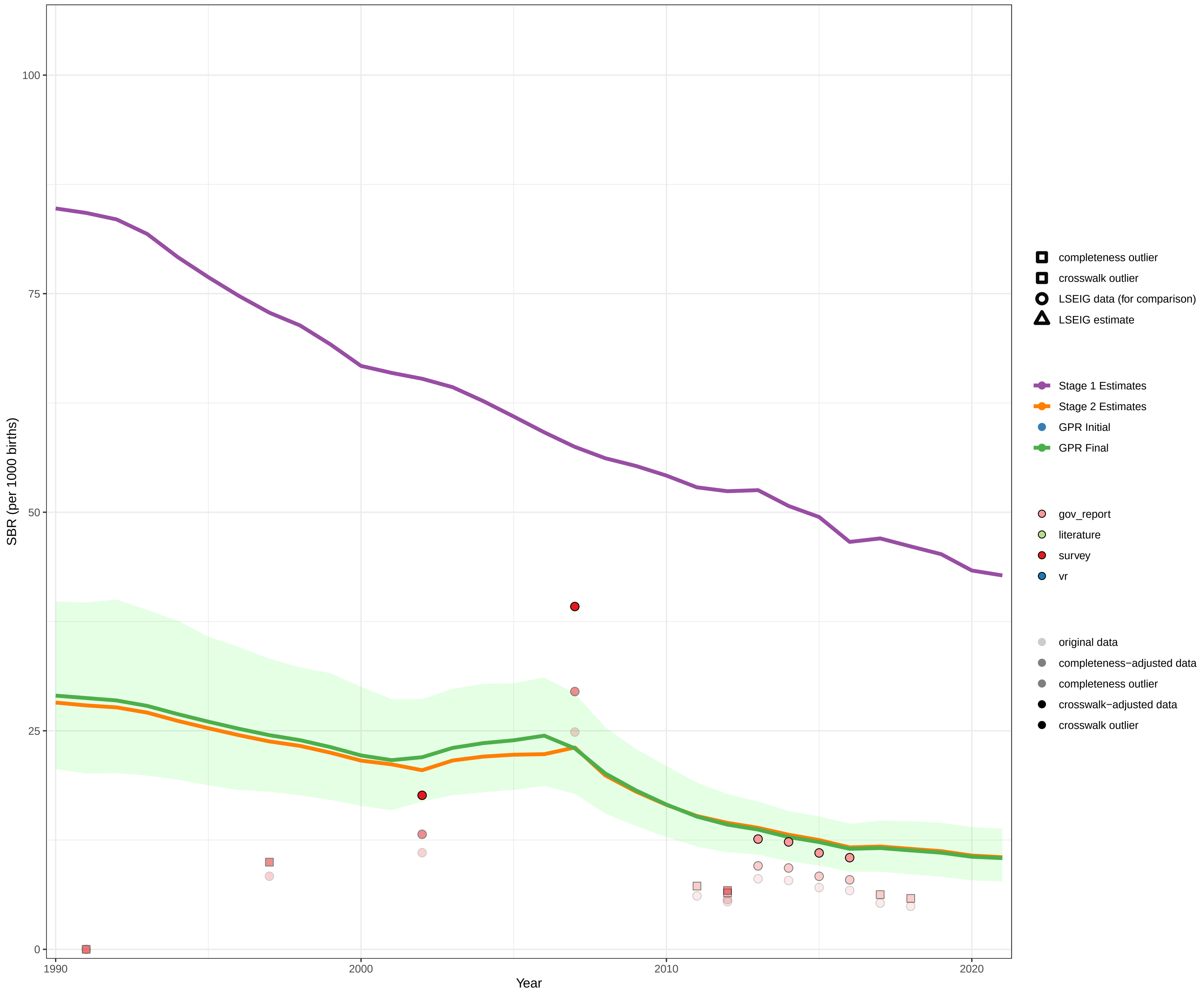

Bengkulu IDN_4715 Southeast Asia Stillbirth Rate (SBR) − 28 weeks
lambda = 0.6 ; zeta = 0.8 ; scale = 15 ; amp = 0.028

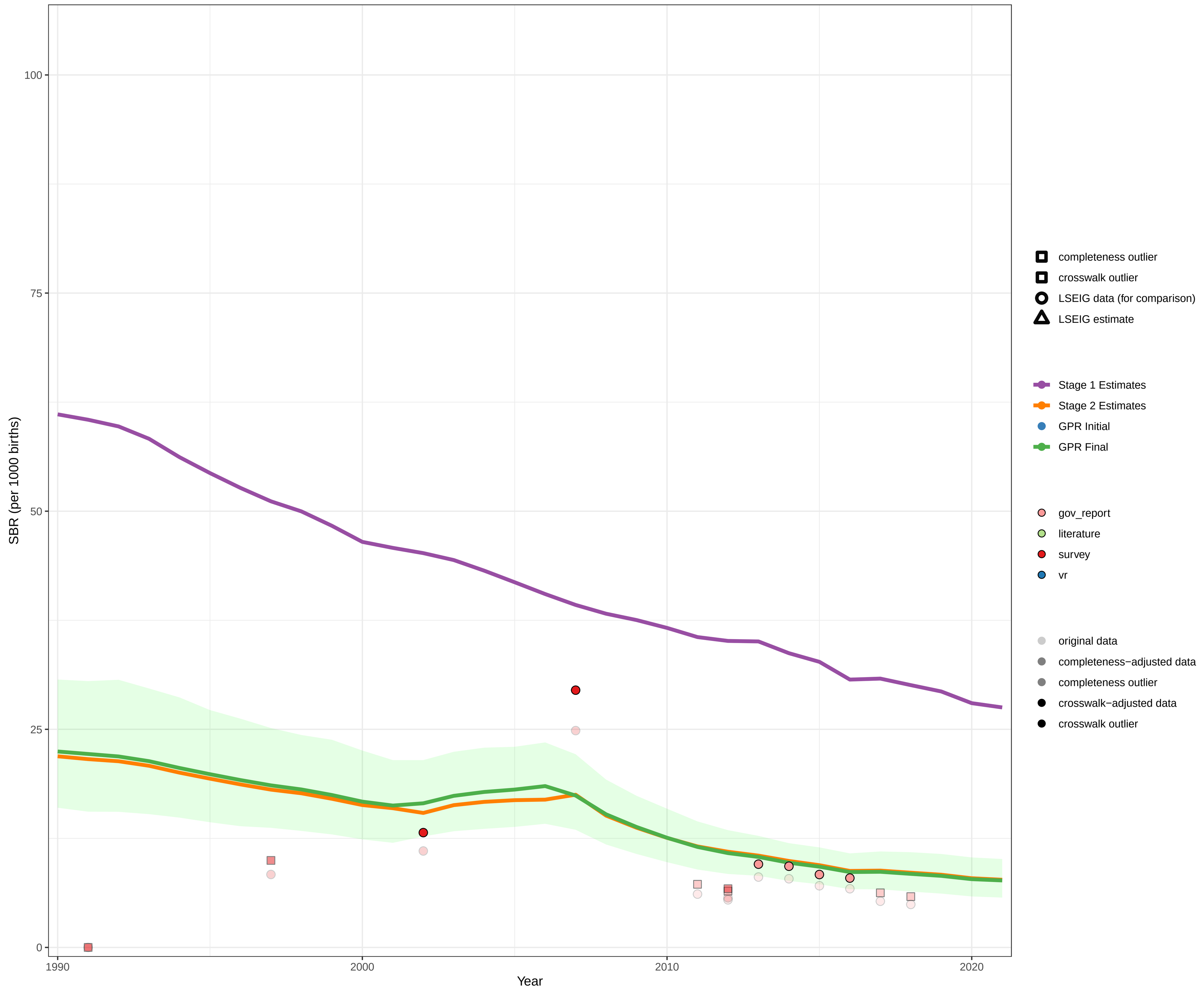

Year	Total Stillbirths (in thousands)	Total Stillbirths (in thousands)	SBR (per 1000 births)	SBR (per 1000 births)	SBR/NMR	SBR/NMR
	20 weeks	28 weeks	20 weeks	28 weeks	20 weeks	28 weeks
1990	1.1<br>(0.8 to 1.5)	0.8<br>(0.6 to 1.2)	29.0<br>(20.6 to 39.8)	22.5<br>(16.0 to 30.7)	0.92<br>(0.66 to 1.27)	0.71<br>(0.51 to 0.98)
1995	1.0<br>(0.7 to 1.4)	0.8<br>(0.5 to 1.0)	26.0<br>(18.8 to 35.8)	19.9<br>(14.3 to 27.2)	0.97<br>(0.70 to 1.33)	0.74<br>(0.53 to 1.01)
2000	0.9<br>(0.6 to 1.2)	0.6<br>(0.5 to 0.9)	22.2<br>(16.4 to 30.0)	16.7<br>(12.4 to 22.6)	0.98<br>(0.73 to 1.33)	0.74<br>(0.55 to 1.00)
2005	1.0<br>(0.7 to 1.2)	0.7<br>(0.5 to 0.9)	23.9<br>(18.2 to 30.4)	18.1<br>(13.8 to 23.0)	1.20<br>(0.91 to 1.53)	0.91<br>(0.69 to 1.15)
2010	0.7<br>(0.5 to 0.8)	0.5<br>(0.4 to 0.6)	16.6<br>(12.8 to 21.0)	12.6<br>(9.8 to 15.9)	0.98<br>(0.76 to 1.24)	0.74<br>(0.58 to 0.94)
2015	0.5<br>(0.4 to 0.6)	0.3<br>(0.3 to 0.4)	12.3<br>(9.6 to 15.2)	9.3<br>(7.2 to 11.5)	0.84<br>(0.66 to 1.04)	0.63<br>(0.49 to 0.78)
2020	0.4<br>(0.3 to 0.5)	0.3<br>(0.2 to 0.4)	10.6<br>(7.9 to 14.0)	7.8<br>(5.8 to 10.3)	0.88<br>(0.66 to 1.17)	0.65<br>(0.49 to 0.86)
2021	0.4<br>(0.3 to 0.5)	0.3<br>(0.2 to 0.4)	10.4<br>(7.7 to 13.8)	7.7<br>(5.7 to 10.1)	0.89<br>(0.66 to 1.18)	0.66<br>(0.49 to 0.87)

Gorontalo IDN_4737 Southeast Asia Stillbirth Rate / Neonatal Mortality Rate (SBR/NMR) − 20 weeks
lambda = 0.6 ; zeta = 0.8 ; scale = 15 ; amp = 0.028

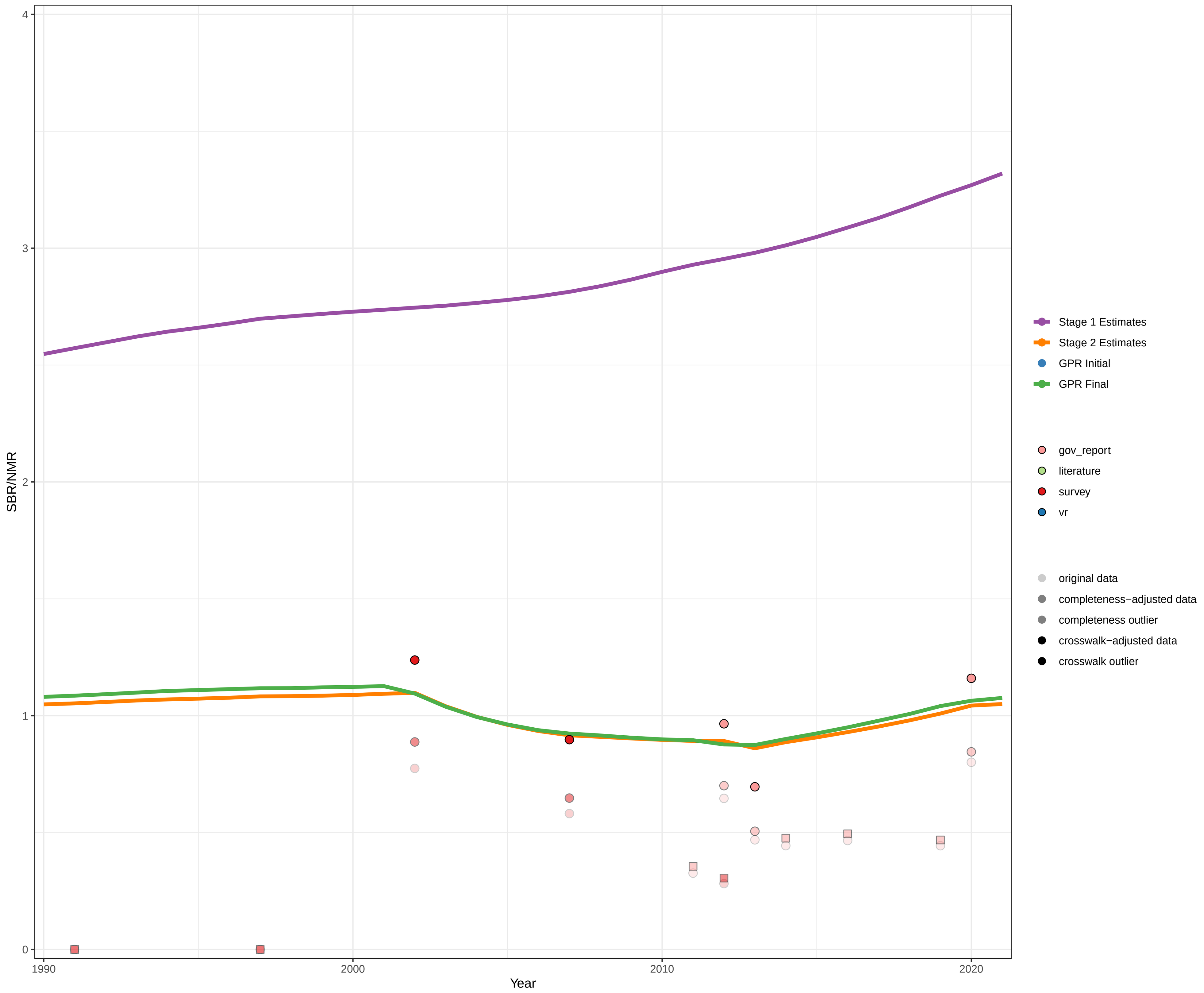

Gorontalo IDN_4737 Southeast Asia Stillbirth Rate / Neonatal Mortality Rate (SBR/NMR) − 28 weeks
lambda = 0.6 ; zeta = 0.8 ; scale = 15 ; amp = 0.028

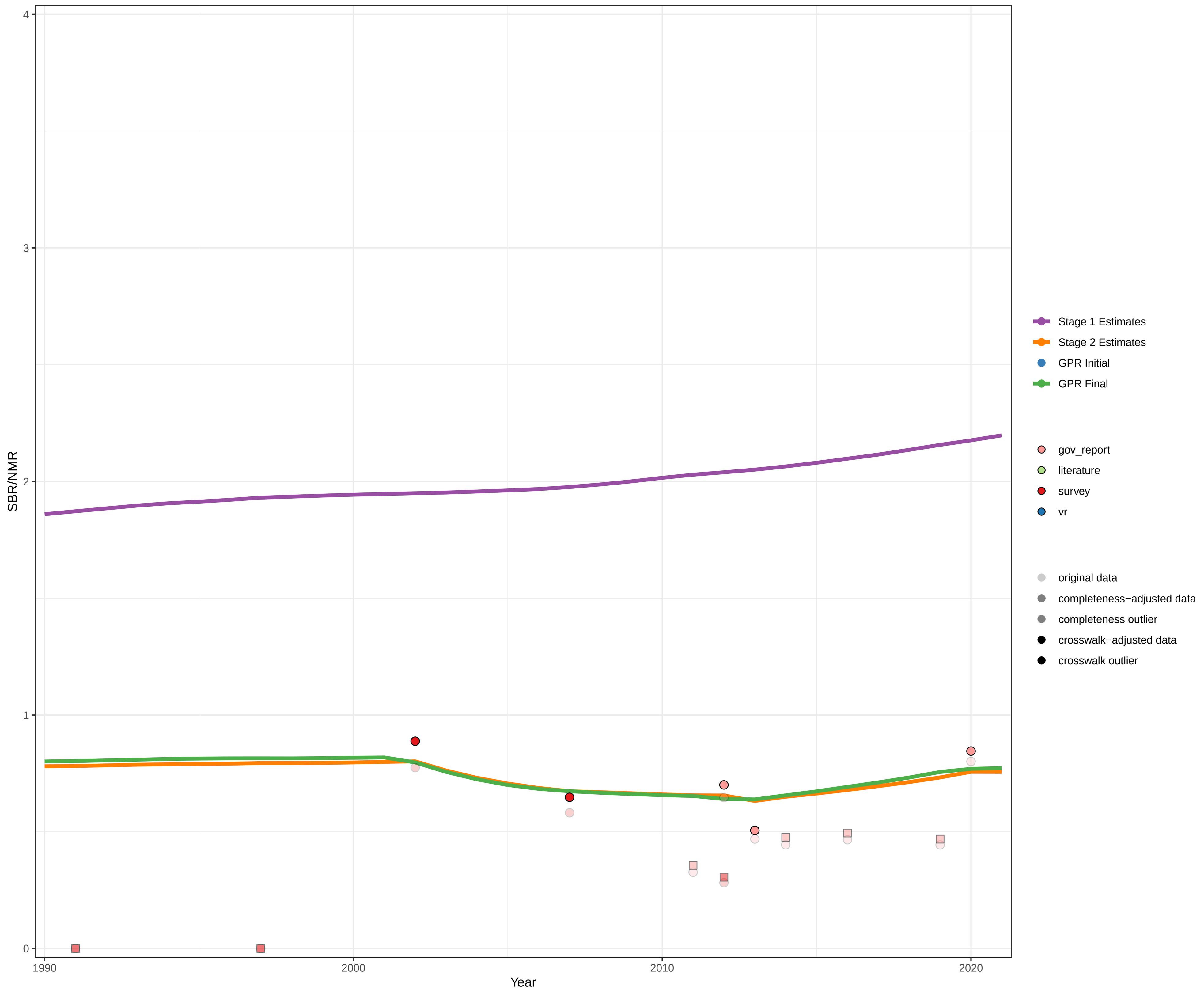

Definition	Years	Outlier	Source.Type	Title
28_weeks	2002	0	Survey	Indonesia Demographic and Health Survey 2002−2003
28_weeks	2007	0	Survey	Indonesia Demographic and Health Survey 2007
28_weeks	2012, 2013, 2020	0	Statistical Report	Indonesia − Gorontalo Province Health Profile
28_weeks	1991	1	Survey	Indonesia Demographic and Health Survey 1991
28_weeks	1997	1	Survey	Indonesia Demographic and Health Survey 1997
28_weeks	2011, 2014, 2016, 2019	1	Statistical Report	Indonesia − Gorontalo Province Health Profile
28_weeks	2012	1	Survey	Indonesia Demographic and Health Survey 2012

Gorontalo IDN_4737 Southeast Asia Stillbirth Rate (SBR) − 20 weeks
lambda = 0.6 ; zeta = 0.8 ; scale = 15 ; amp = 0.028

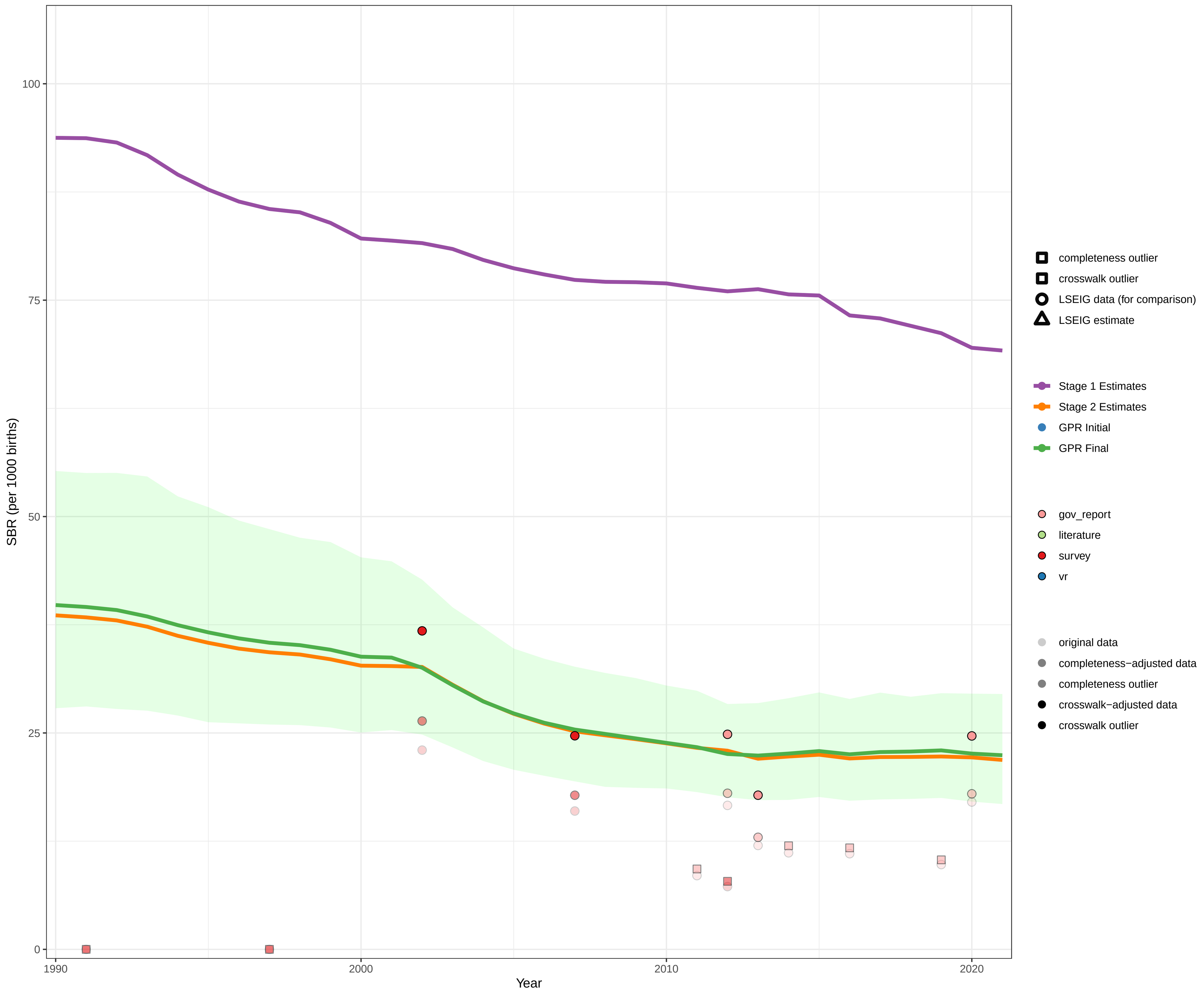

Gorontalo IDN_4737 Southeast Asia Stillbirth Rate (SBR) − 28 weeks
lambda = 0.6 ; zeta = 0.8 ; scale = 15 ; amp = 0.028

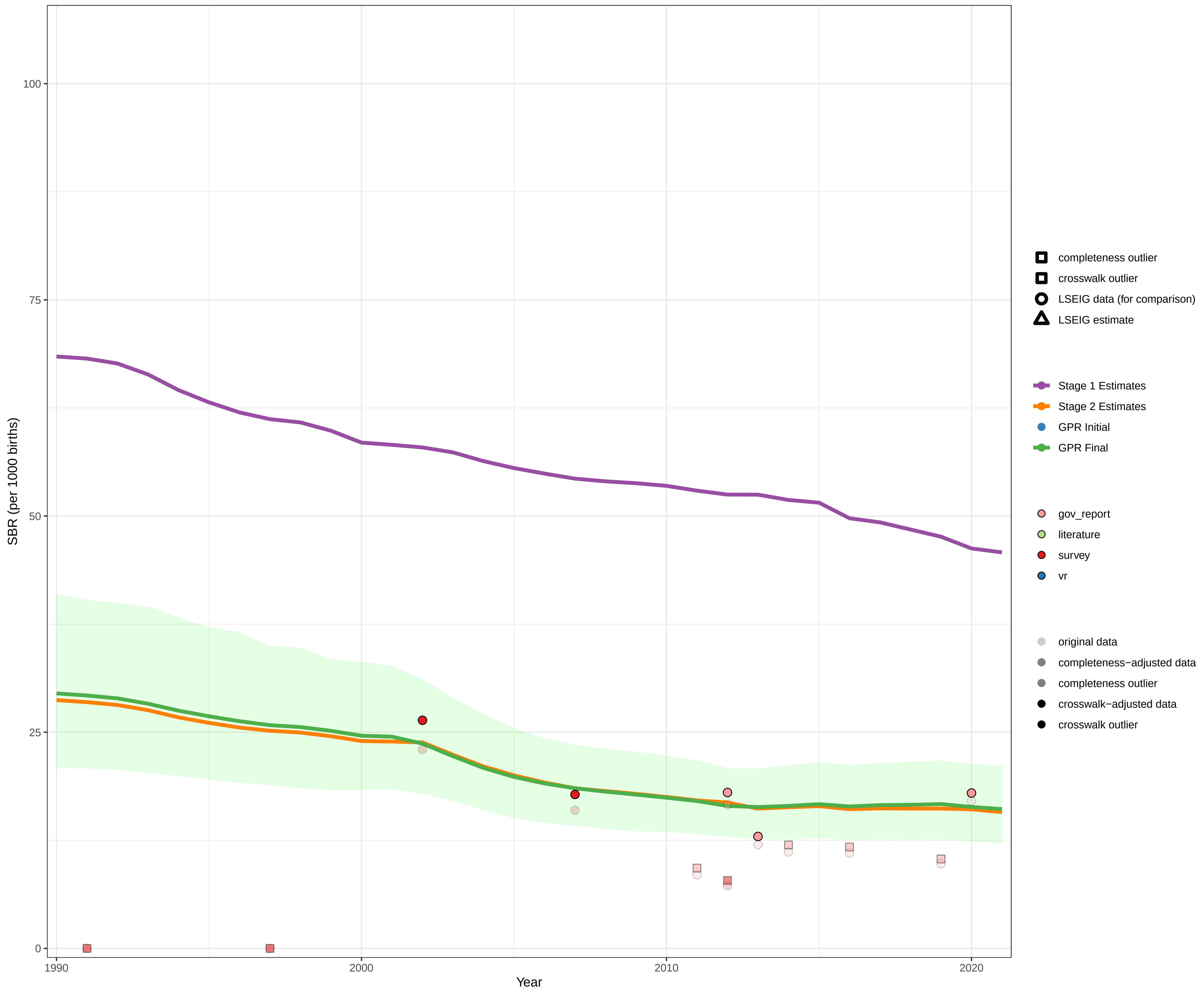

Year	Total Stillbirths (in thousands)	Total Stillbirths (in thousands)	SBR (per 1000 births)	SBR (per 1000 births)	SBR/NMR	SBR/NMR
	20 weeks	28 weeks	20 weeks	28 weeks	20 weeks	28 weeks
1990	0.7 (0.5 to 1.0)	0.5 (0.4 to 0.7)	39.8 (27.9 to 55.3)	29.5 (20.8 to 40.9)	1.08 (0.76 to 1.50)	0.80 (0.57 to 1.11)
1995	0.7 (0.5 to 1.0)	0.5 (0.4 to 0.7)	36.6 (26.2 to 51.1)	26.8 (19.6 to 37.1)	1.11 (0.80 to 1.55)	0.81 (0.59 to 1.12)
2000	0.7 (0.5 to 0.9)	0.5 (0.4 to 0.7)	33.8 (25.0 to 45.3)	24.6 (18.3 to 33.1)	1.12 (0.83 to 1.50)	0.82 (0.61 to 1.10)
2005	0.6 (0.5 to 0.8)	0.4 (0.3 to 0.6)	27.3 (20.7 to 34.8)	19.8 (15.1 to 25.5)	0.96 (0.73 to 1.23)	0.70 (0.53 to 0.90)
2010	0.6 (0.4 to 0.7)	0.4 (0.3 to 0.5)	23.9 (18.6 to 30.5)	17.4 (13.4 to 22.3)	0.90 (0.70 to 1.15)	0.66 (0.51 to 0.84)
2015	0.5 (0.4 to 0.7)	0.4 (0.3 to 0.5)	22.9 (17.6 to 29.7)	16.7 (12.7 to 21.5)	0.92 (0.71 to 1.20)	0.67 (0.51 to 0.87)
2020	0.5 (0.4 to 0.7)	0.4 (0.3 to 0.5)	22.6 (17.1 to 29.5)	16.4 (12.4 to 21.3)	1.06 (0.80 to 1.39)	0.77 (0.58 to 1.00)
2021	0.5 (0.4 to 0.7)	0.4 (0.3 to 0.5)	22.4 (16.8 to 29.5)	16.1 (12.1 to 21.1)	1.08 (0.81 to 1.42)	0.77 (0.58 to 1.01)

Jakarta IDN_4720 Southeast Asia Stillbirth Rate / Neonatal Mortality Rate (SBR/NMR) − 20 weeks
lambda = 0.8 ; zeta = 0.7 ; scale = 15 ; amp = 0.028

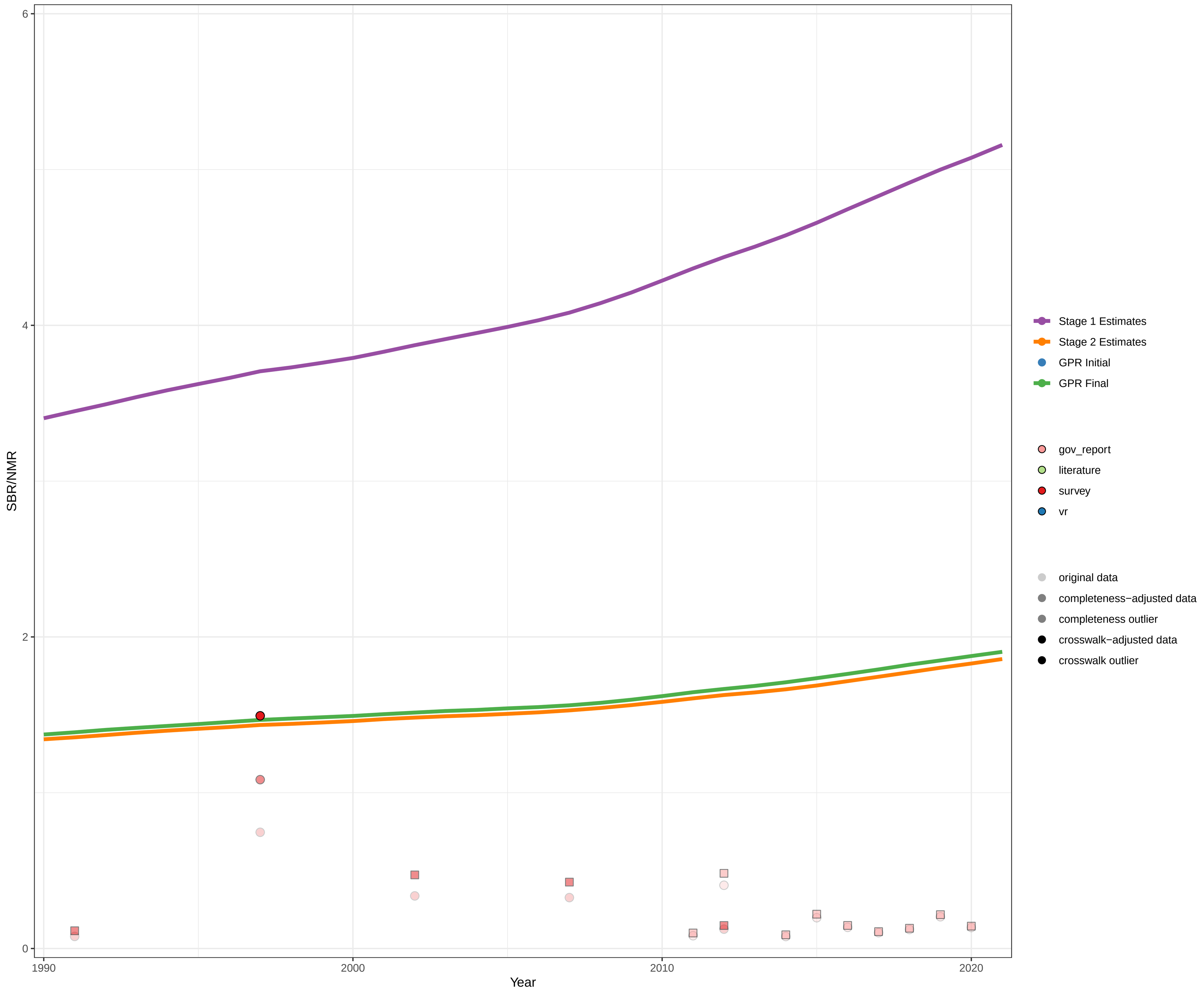

Jakarta IDN_4720 Southeast Asia Stillbirth Rate / Neonatal Mortality Rate (SBR/NMR) − 28 weeks
lambda = 0.8 ; zeta = 0.7 ; scale = 15 ; amp = 0.028

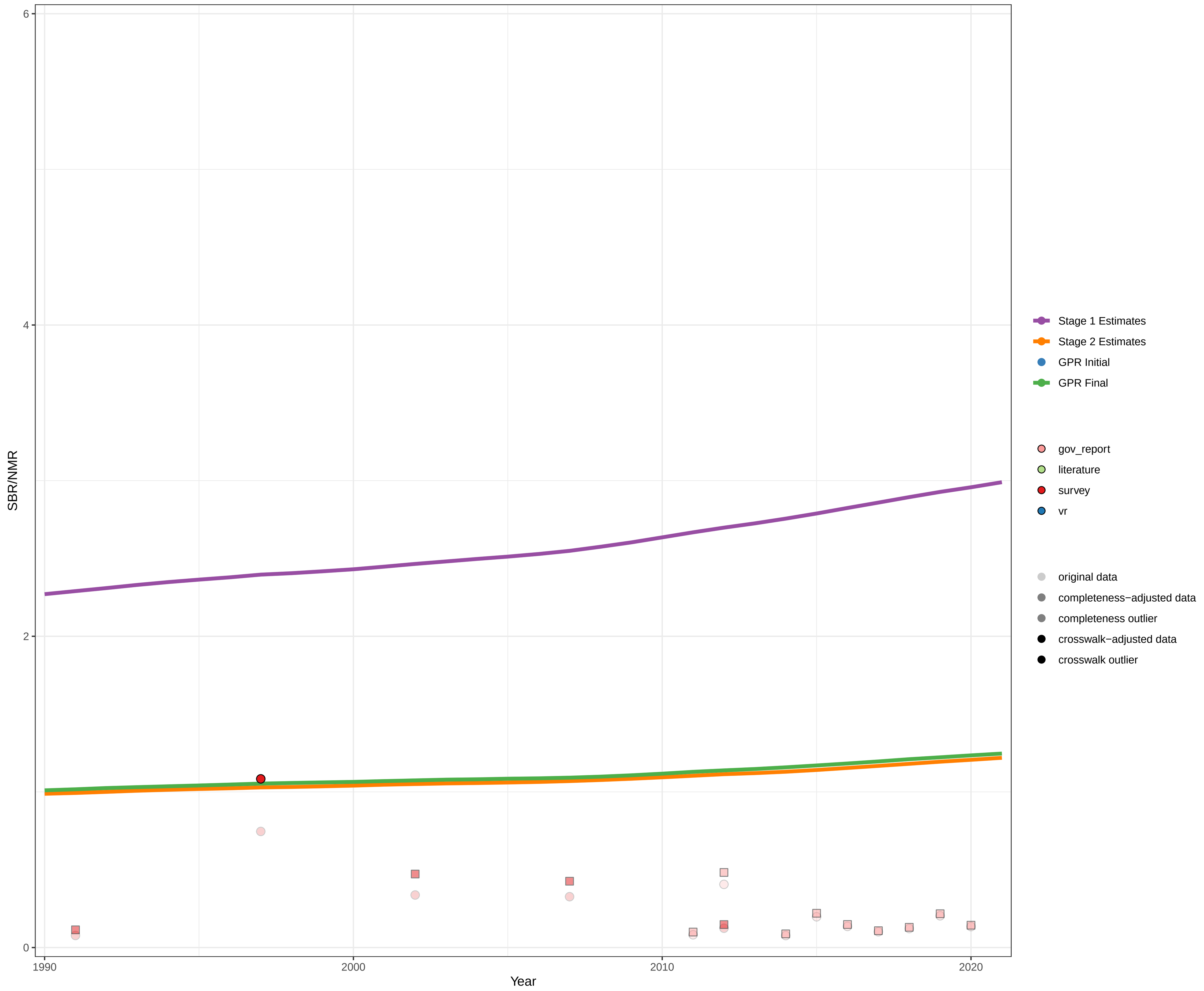

Definition	Years	Outlier	Source.Type	Title
28_weeks	1997	0	Survey	Indonesia Demographic and Health Survey 1997
28_weeks	1991	1	Survey	Indonesia Demographic and Health Survey 1991
28_weeks	2002	1	Survey	Indonesia Demographic and Health Survey 2002−2003
28_weeks	2007	1	Survey	Indonesia Demographic and Health Survey 2007
28_weeks	2011, 2012, 2014−2020	1	Statistical Report	Indonesia − Jakarta Health Profile
28_weeks	2012	1	Survey	Indonesia Demographic and Health Survey 2012

Jakarta IDN_4720 Southeast Asia Stillbirth Rate (SBR) − 20 weeks
lambda = 0.8 ; zeta = 0.7 ; scale = 15 ; amp = 0.028

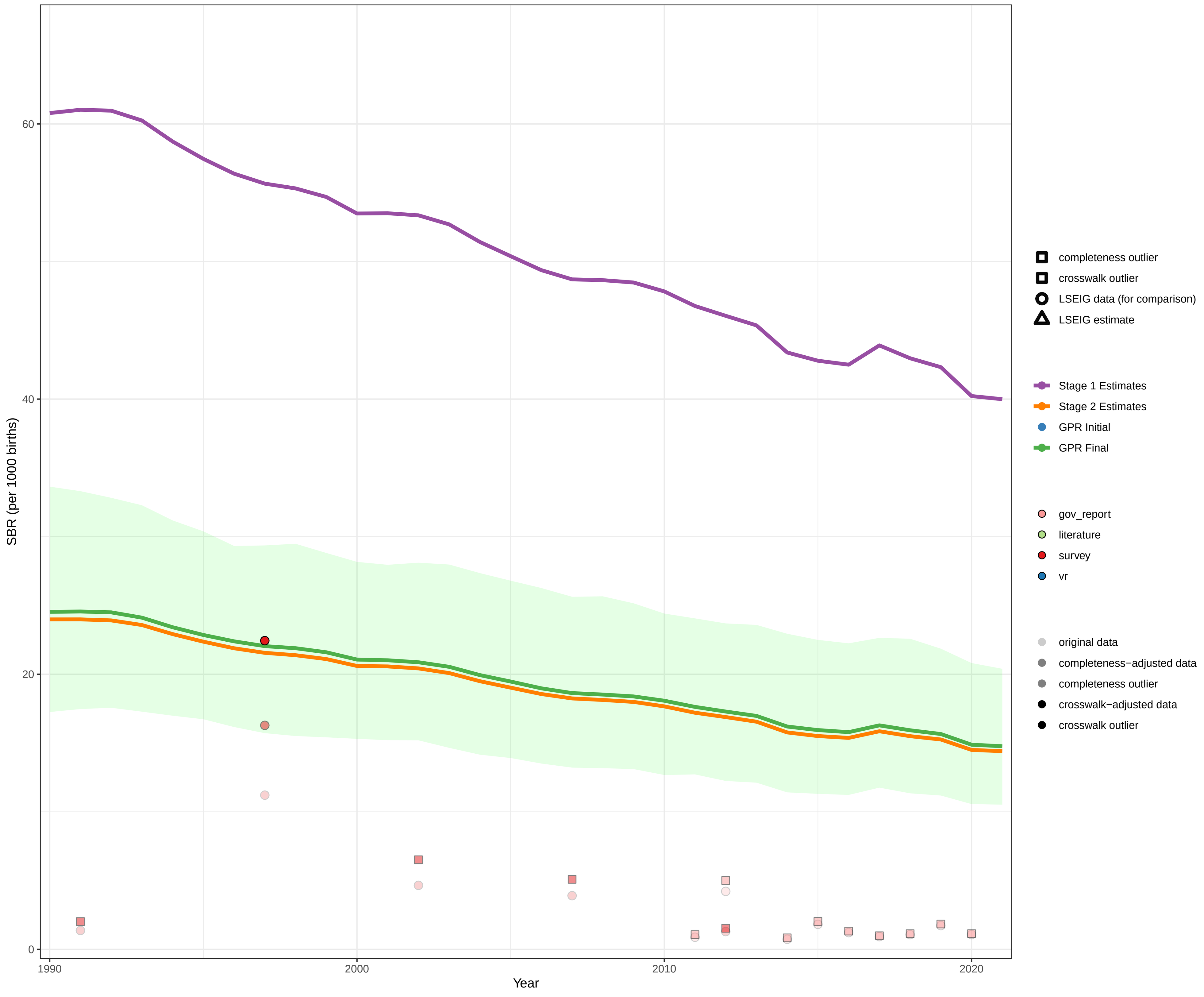

Jakarta IDN_4720 Southeast Asia Stillbirth Rate (SBR) − 28 weeks
lambda = 0.8 ; zeta = 0.7 ; scale = 15 ; amp = 0.028

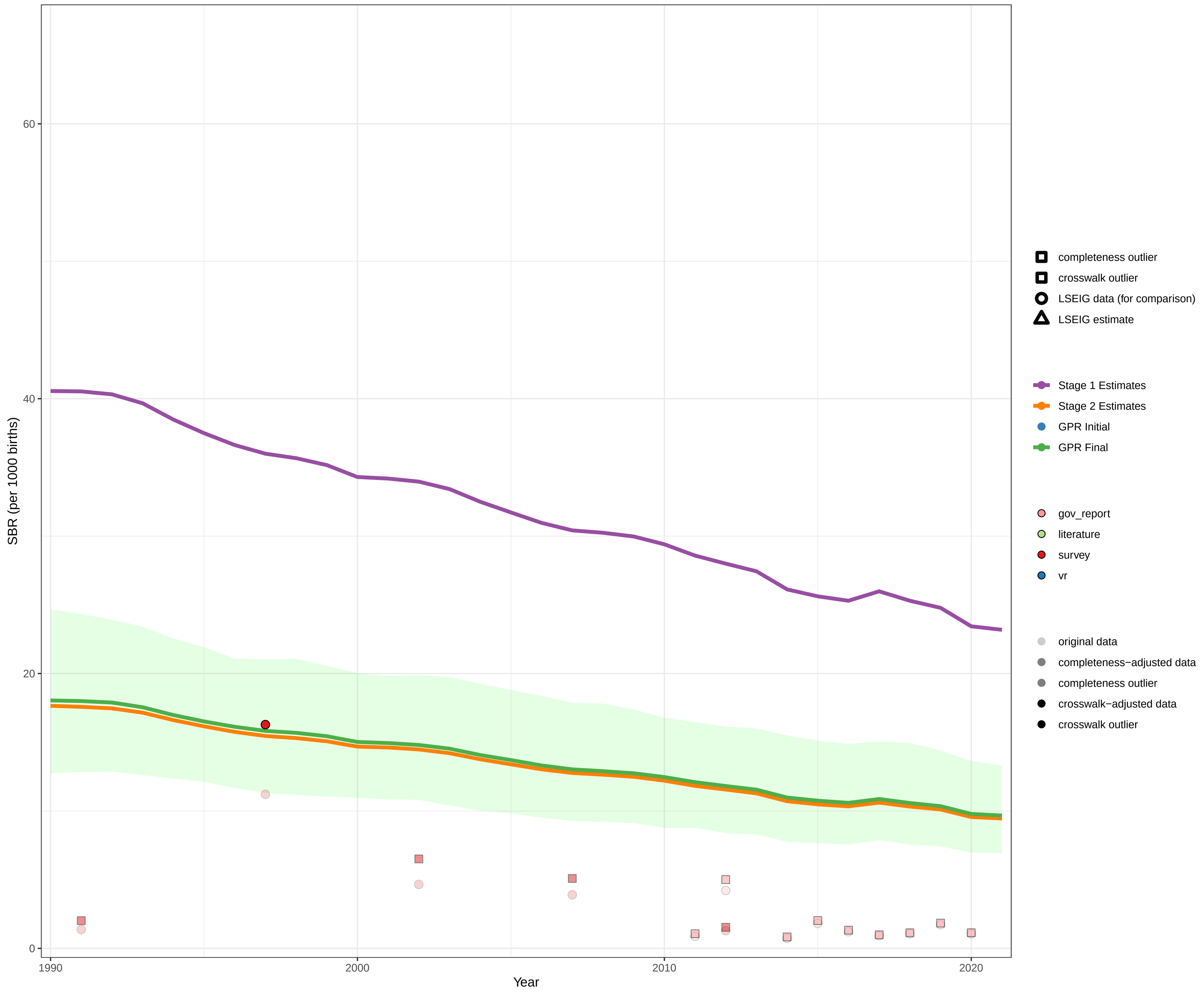

Year	Total Stillbirths (in thousands)	Total Stillbirths (in thousands)	SBR (per 1000 births)	SBR (per 1000 births)	SBR/NMR	SBR/NMR
	20 weeks	28 weeks	20 weeks	28 weeks	20 weeks	28 weeks
1990	5.4 (3.8 to 7.5)	4.0 (2.8 to 5.5)	24.5 (17.3 to 33.6)	18.0 (12.7 to 24.7)	1.37 (0.97 to 1.88)	1.01 (0.71 to 1.38)
1995	4.8 (3.5 to 6.4)	3.4 (2.5 to 4.6)	22.9 (16.7 to 30.4)	16.5 (12.1 to 21.9)	1.44 (1.05 to 1.92)	1.04 (0.76 to 1.38)
2000	4.3 (3.1 to 5.7)	3.0 (2.2 to 4.1)	21.1 (15.3 to 28.2)	15.0 (10.9 to 20.0)	1.49 (1.08 to 2.00)	1.06 (0.78 to 1.42)
2005	3.9 (2.8 to 5.4)	2.7 (2.0 to 3.8)	19.5 (13.9 to 26.8)	13.7 (9.8 to 18.8)	1.54 (1.10 to 2.12)	1.08 (0.78 to 1.49)
2010	3.5 (2.4 to 4.7)	2.4 (1.7 to 3.2)	18.1 (12.7 to 24.4)	12.5 (8.8 to 16.8)	1.62 (1.14 to 2.19)	1.12 (0.79 to 1.50)
2015	2.6 (1.9 to 3.7)	1.8 (1.3 to 2.5)	15.9 (11.3 to 22.5)	10.7 (7.6 to 15.1)	1.74 (1.23 to 2.45)	1.17 (0.83 to 1.65)
2020	2.1 (1.5 to 2.9)	1.4 (1.0 to 1.9)	14.9 (10.6 to 20.8)	9.8 (7.0 to 13.6)	1.88 (1.33 to 2.63)	1.23 (0.88 to 1.72)
2021	2.0 (1.4 to 2.8)	1.3 (0.9 to 1.8)	14.8 (10.5 to 20.4)	9.7 (6.9 to 13.3)	1.90 (1.36 to 2.63)	1.25 (0.89 to 1.72)

Jambi IDN_4713 Southeast Asia Stillbirth Rate / Neonatal Mortality Rate (SBR/NMR) − 20 weeks
lambda = 0.8 ; zeta = 0.7 ; scale = 15 ; amp = 0.028

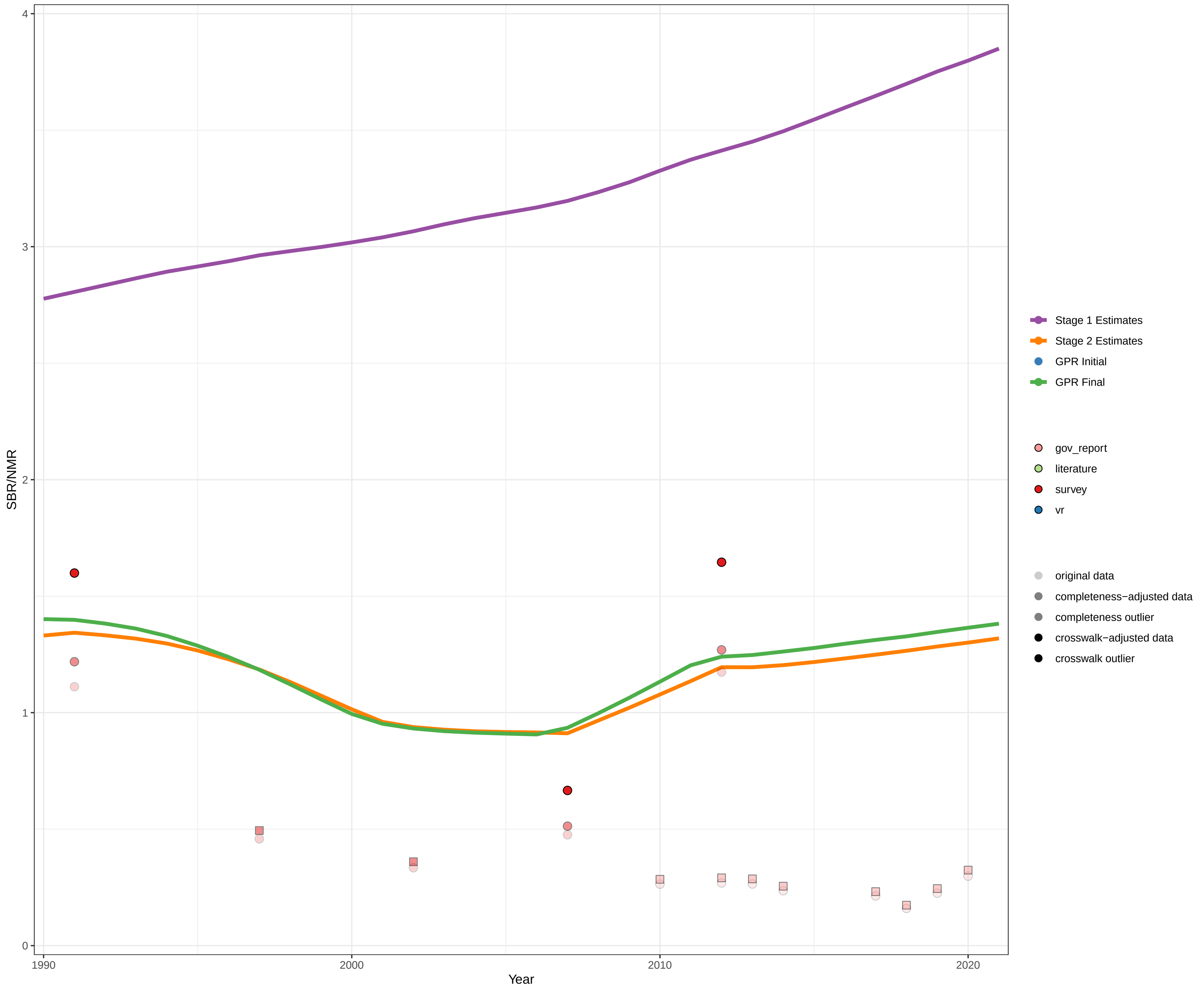

Jambi IDN_4713 Southeast Asia Stillbirth Rate / Neonatal Mortality Rate (SBR/NMR) − 28 weeks
lambda = 0.8 ; zeta = 0.7 ; scale = 15 ; amp = 0.028

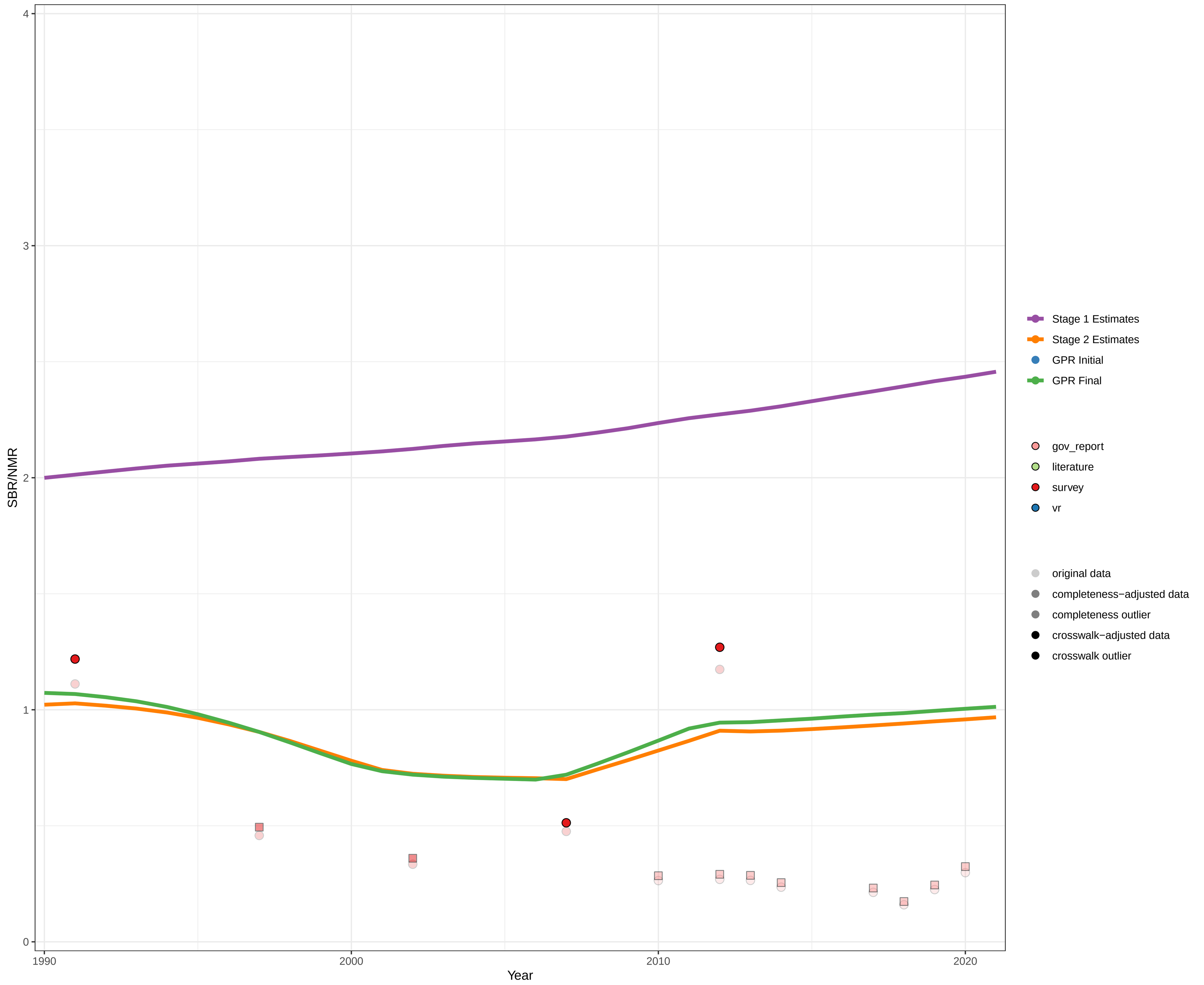

Definition	Years	Outlier	Source.Type	Title
28_weeks	1991	0	Survey	Indonesia Demographic and Health Survey 1991
28_weeks	2007	0	Survey	Indonesia Demographic and Health Survey 2007
28_weeks	2012	0	Survey	Indonesia Demographic and Health Survey 2012
28_weeks	1997	1	Survey	Indonesia Demographic and Health Survey 1997
28_weeks	2002	1	Survey	Indonesia Demographic and Health Survey 2002−2003
28_weeks	2010, 2012−2014, 2017−2020	1	Statistical Report	Indonesia − Jambi Province Health Profile

Jambi IDN_4713 Southeast Asia Stillbirth Rate (SBR) − 20 weeks
lambda = 0.8 ; zeta = 0.7 ; scale = 15 ; amp = 0.028

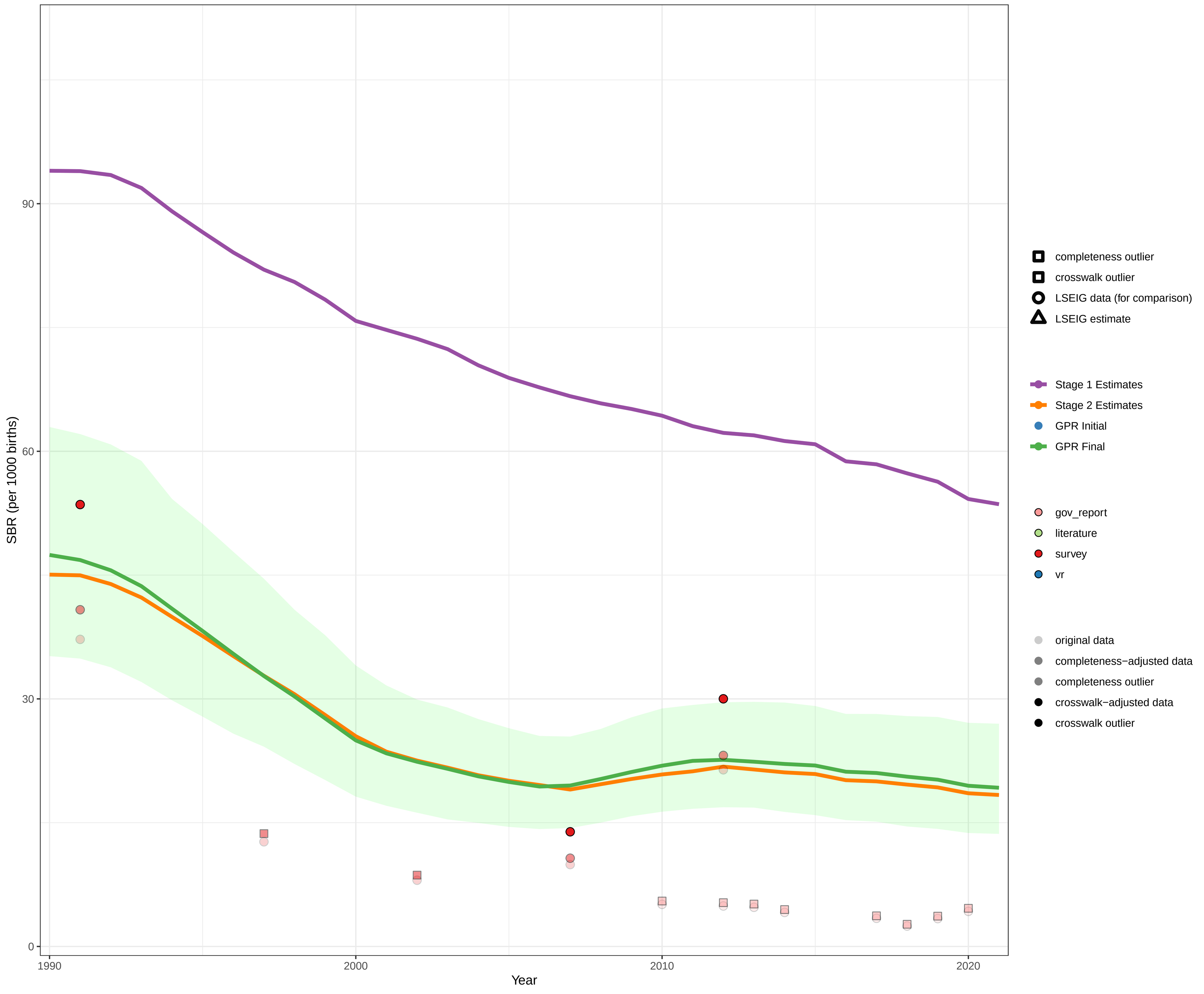

Jambi IDN_4713 Southeast Asia Stillbirth Rate (SBR) − 28 weeks
lambda = 0.8 ; zeta = 0.7 ; scale = 15 ; amp = 0.028

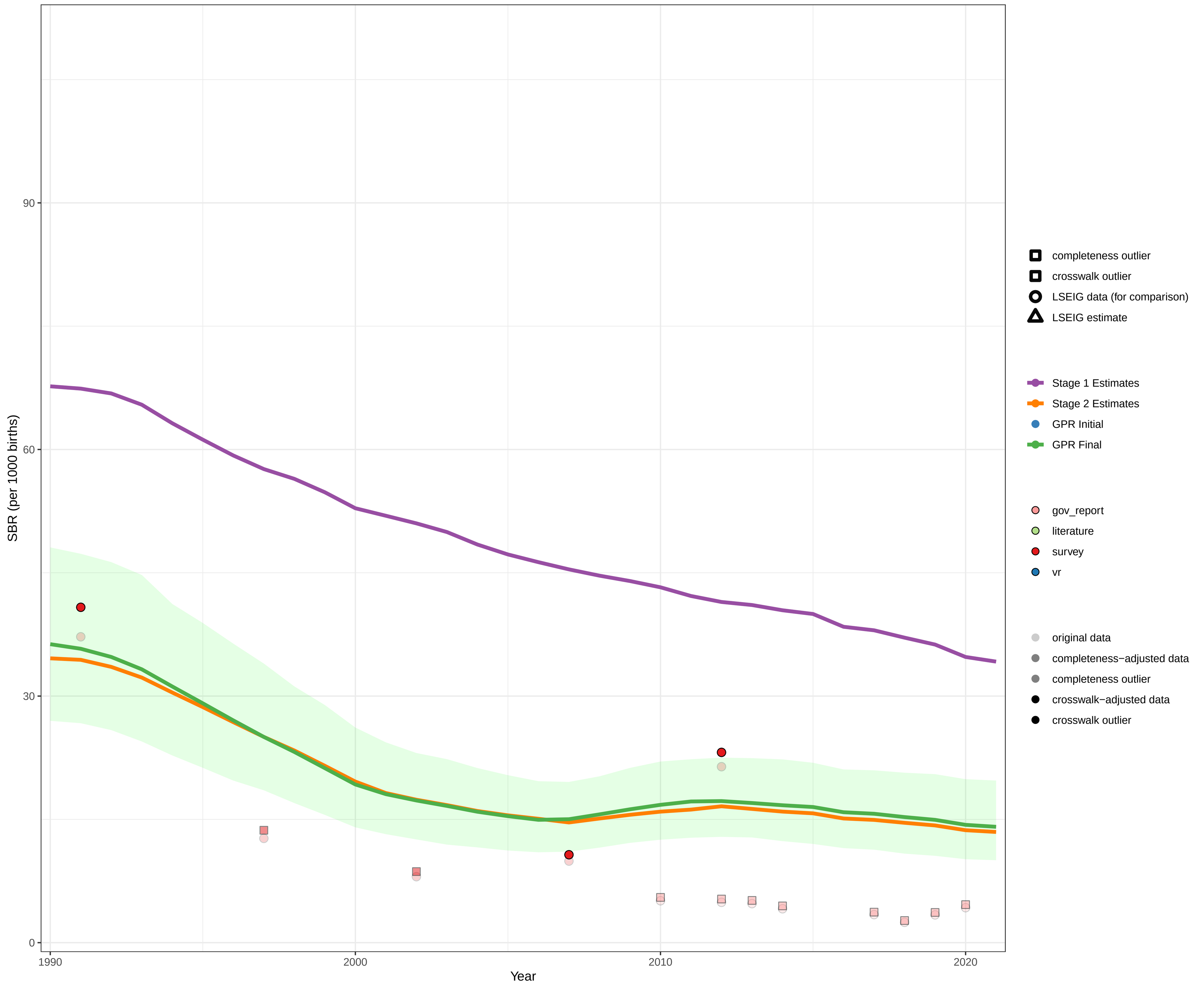

Year	Total Stillbirths (in thousands)	Total Stillbirths (in thousands)	SBR (per 1000 births)	SBR (per 1000 births)	SBR/NMR	SBR/NMR
	20 weeks	28 weeks	20 weeks	28 weeks	20 weeks	28 weeks
1990	2.8 (2.0 to 3.7)	2.1 (1.5 to 2.8)	47.4 (35.2 to 62.9)	36.3 (27.0 to 48.1)	1.40 (1.04 to 1.86)	1.07 (0.80 to 1.42)
1995	2.3 (1.7 to 3.1)	1.7 (1.3 to 2.3)	38.2 (27.8 to 51.2)	29.1 (21.3 to 38.9)	1.29 (0.94 to 1.72)	0.98 (0.72 to 1.31)
2000	1.6 (1.1 to 2.1)	1.2 (0.9 to 1.6)	25.0 (18.2 to 34.0)	19.2 (14.0 to 26.2)	0.99 (0.72 to 1.36)	0.77 (0.56 to 1.04)
2005	1.3 (1.0 to 1.8)	1.0 (0.7 to 1.4)	19.9 (14.5 to 26.4)	15.4 (11.2 to 20.4)	0.91 (0.66 to 1.21)	0.70 (0.51 to 0.93)
2010	1.5 (1.1 to 2.0)	1.1 (0.8 to 1.5)	21.9 (16.3 to 28.8)	16.8 (12.5 to 22.0)	1.13 (0.84 to 1.49)	0.87 (0.65 to 1.14)
2015	1.4 (1.0 to 1.9)	1.0 (0.8 to 1.4)	21.9 (15.9 to 29.1)	16.5 (12.0 to 21.9)	1.28 (0.93 to 1.70)	0.96 (0.70 to 1.28)
2020	1.2 (0.8 to 1.6)	0.9 (0.6 to 1.2)	19.5 (13.7 to 27.1)	14.3 (10.2 to 19.9)	1.36 (0.96 to 1.90)	1.00 (0.71 to 1.39)
2021	1.1 (0.8 to 1.6)	0.8 (0.6 to 1.2)	19.2 (13.6 to 27.0)	14.1 (10.0 to 19.7)	1.38 (0.98 to 1.94)	1.01 (0.72 to 1.42)

West Java IDN_4721 Southeast Asia Stillbirth Rate / Neonatal Mortality Rate (SBR/NMR) − 20 weeks
lambda = 0.8 ; zeta = 0.7 ; scale = 15 ; amp = 0.028

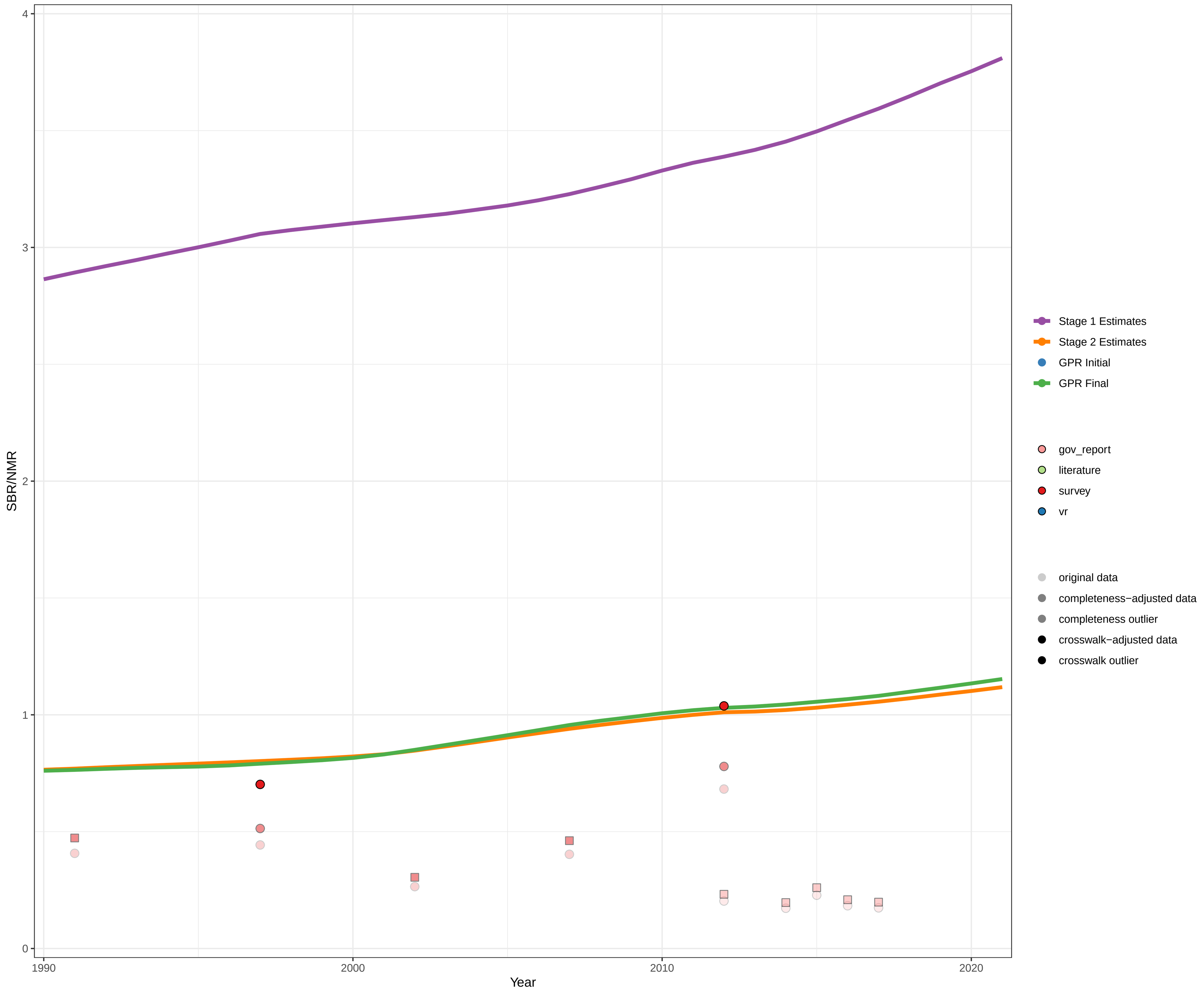

West Java IDN_4721 Southeast Asia Stillbirth Rate / Neonatal Mortality Rate (SBR/NMR) − 28 weeks
lambda = 0.8 ; zeta = 0.7 ; scale = 15 ; amp = 0.028

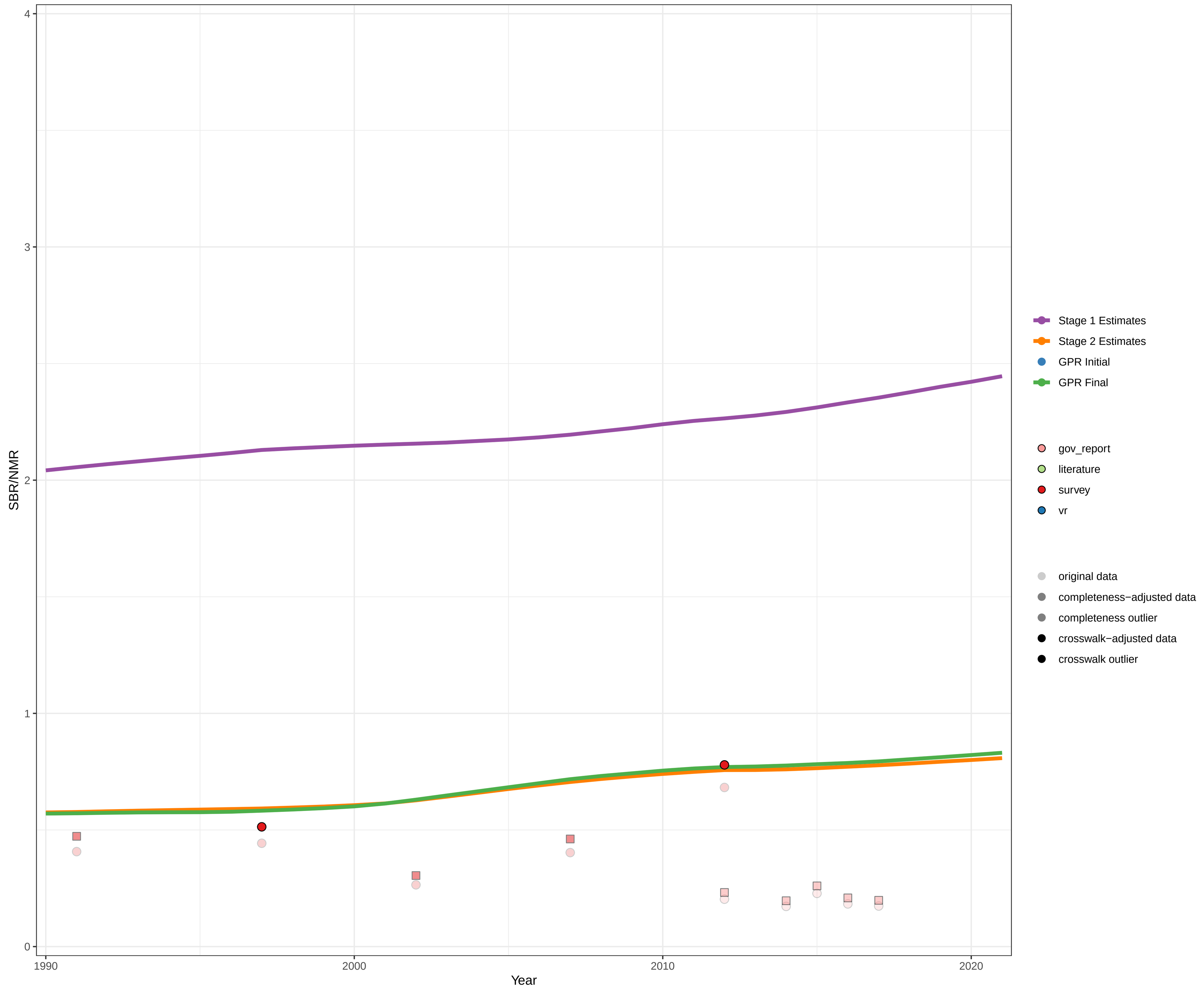

Definition	Years	Outlier	Source.Type	Title
28_weeks	1997	0	Survey	Indonesia Demographic and Health Survey 1997
28_weeks	2012	0	Survey	Indonesia Demographic and Health Survey 2012
28_weeks	1991	1	Survey	Indonesia Demographic and Health Survey 1991
28_weeks	2002	1	Survey	Indonesia Demographic and Health Survey 2002−2003
28_weeks	2007	1	Survey	Indonesia Demographic and Health Survey 2007
28_weeks	2012, 2014−2017	1	Statistical Report	Indonesia − Jawa Barat Province Health Profile

West Java IDN_4721 Southeast Asia Stillbirth Rate (SBR) − 20 weeks
lambda = 0.8 ; zeta = 0.7 ; scale = 15 ; amp = 0.028

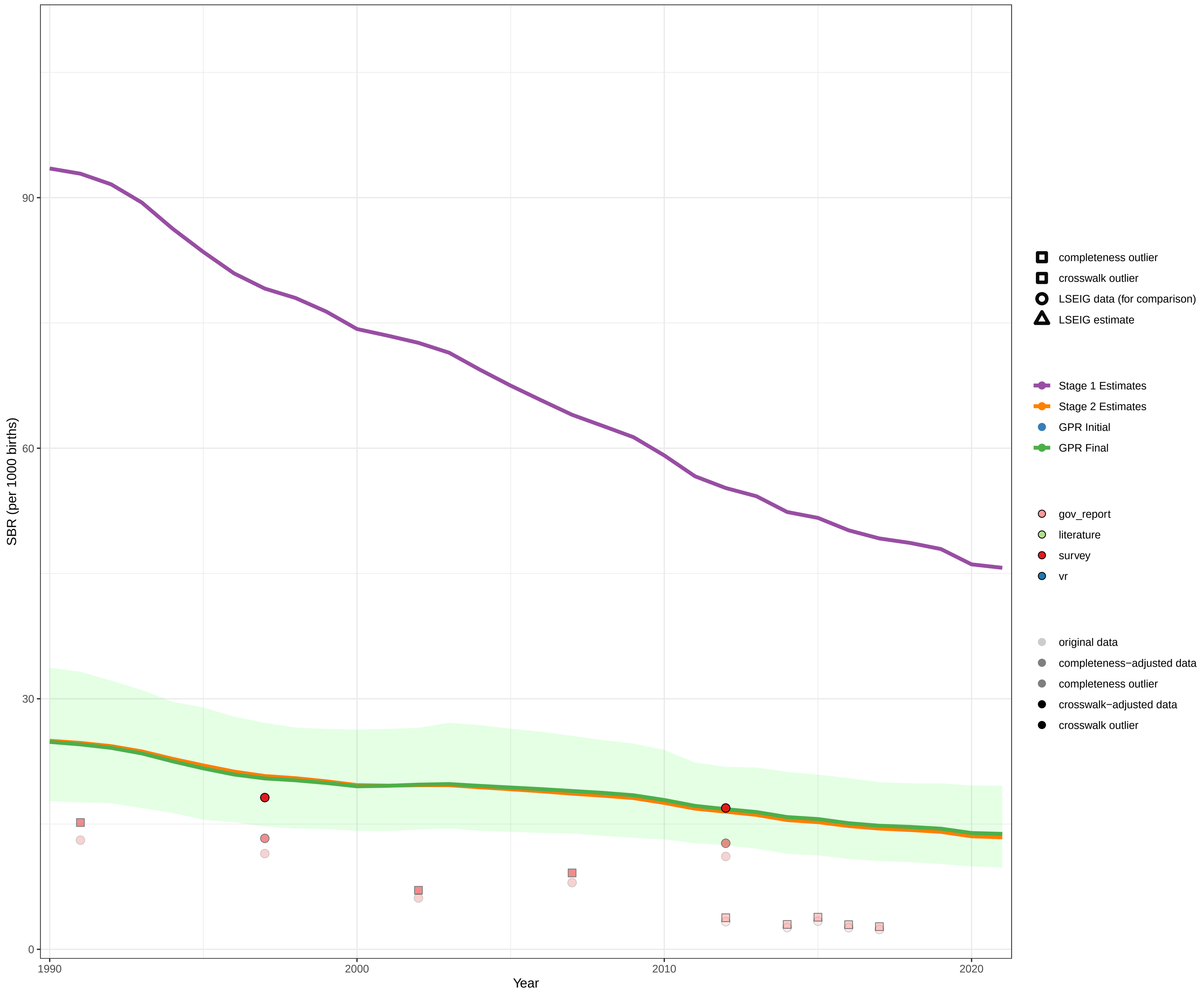

West Java IDN_4721 Southeast Asia Stillbirth Rate (SBR) − 28 weeks
lambda = 0.8 ; zeta = 0.7 ; scale = 15 ; amp = 0.028

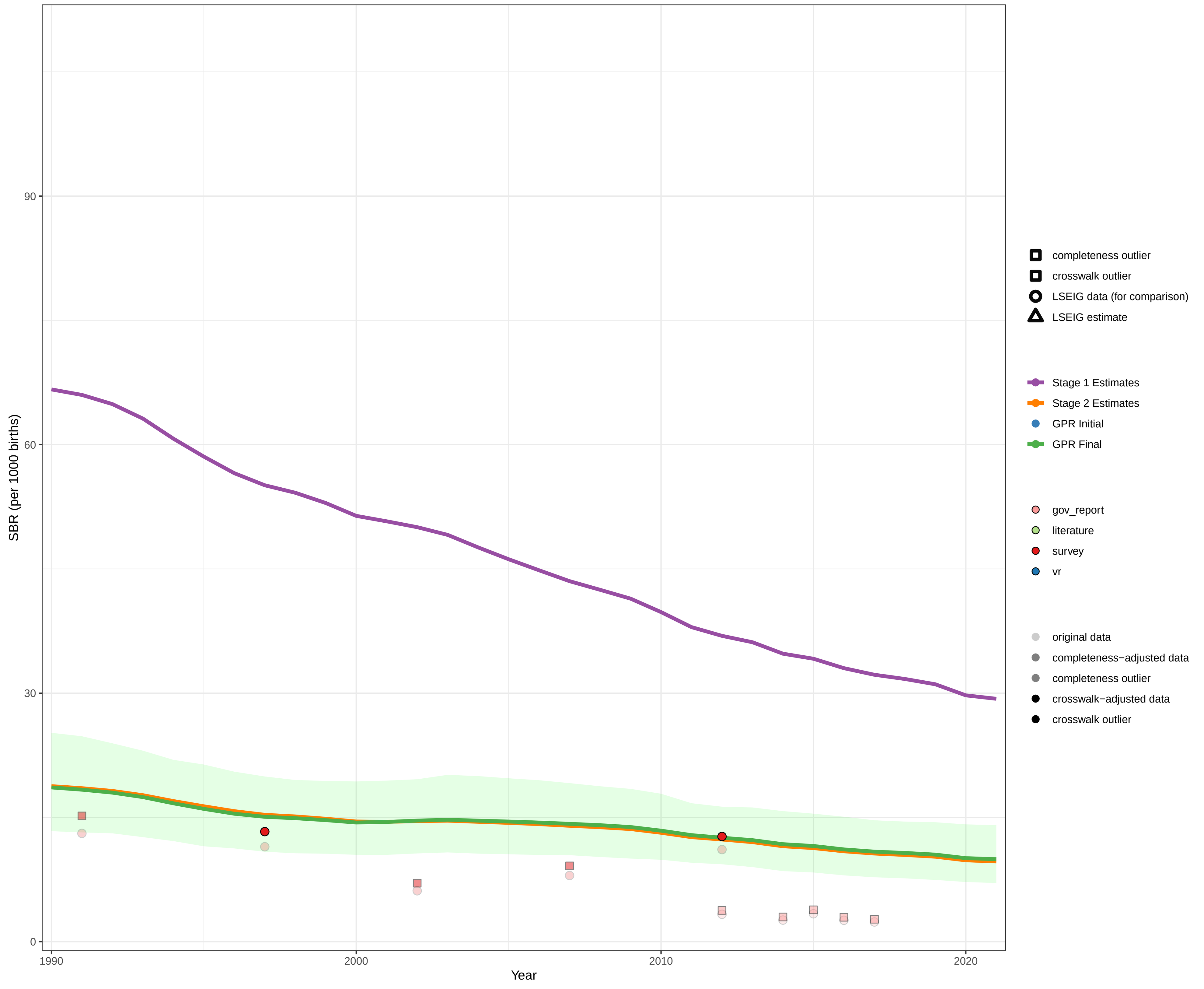

Year	Total Stillbirths (in thousands)	Total Stillbirths (in thousands)	SBR (per 1000 births)	SBR (per 1000 births)	SBR/NMR	SBR/NMR
	20 weeks	28 weeks	20 weeks	28 weeks	20 weeks	28 weeks
1990	18.7 (13.3 to 25.7)	14.0 (9.9 to 19.0)	24.8 (17.7 to 33.7)	18.6 (13.3 to 25.2)	0.76 (0.54 to 1.03)	0.57 (0.41 to 0.77)
1995	16.8 (11.9 to 22.6)	12.3 (8.8 to 16.6)	21.7 (15.5 to 29.0)	16.0 (11.5 to 21.4)	0.78 (0.56 to 1.04)	0.58 (0.41 to 0.77)
2000	15.9 (11.5 to 21.6)	11.7 (8.5 to 15.8)	19.5 (14.2 to 26.3)	14.4 (10.5 to 19.3)	0.82 (0.59 to 1.10)	0.60 (0.44 to 0.81)
2005	16.9 (12.2 to 23.3)	12.6 (9.1 to 17.2)	19.4 (14.1 to 26.4)	14.5 (10.6 to 19.7)	0.91 (0.66 to 1.24)	0.68 (0.50 to 0.93)
2010	16.1 (11.8 to 21.6)	12.0 (8.8 to 16.0)	17.9 (13.2 to 23.9)	13.4 (9.9 to 17.9)	1.01 (0.74 to 1.34)	0.75 (0.56 to 1.01)
2015	13.5 (9.7 to 18.2)	9.9 (7.2 to 13.4)	15.6 (11.3 to 20.9)	11.6 (8.4 to 15.5)	1.06 (0.76 to 1.42)	0.78 (0.57 to 1.05)
2020	11.5 (8.1 to 16.3)	8.3 (5.9 to 11.7)	13.9 (9.9 to 19.6)	10.1 (7.2 to 14.2)	1.13 (0.81 to 1.60)	0.82 (0.59 to 1.15)
2021	11.3 (8.0 to 16.1)	8.1 (5.8 to 11.5)	13.8 (9.8 to 19.6)	10.0 (7.1 to 14.1)	1.15 (0.82 to 1.63)	0.83 (0.59 to 1.17)

Central Java IDN_4722 Southeast Asia Stillbirth Rate / Neonatal Mortality Rate (SBR/NMR) − 20 weeks
lambda = 0.8 ; zeta = 0.7 ; scale = 15 ; amp = 0.028

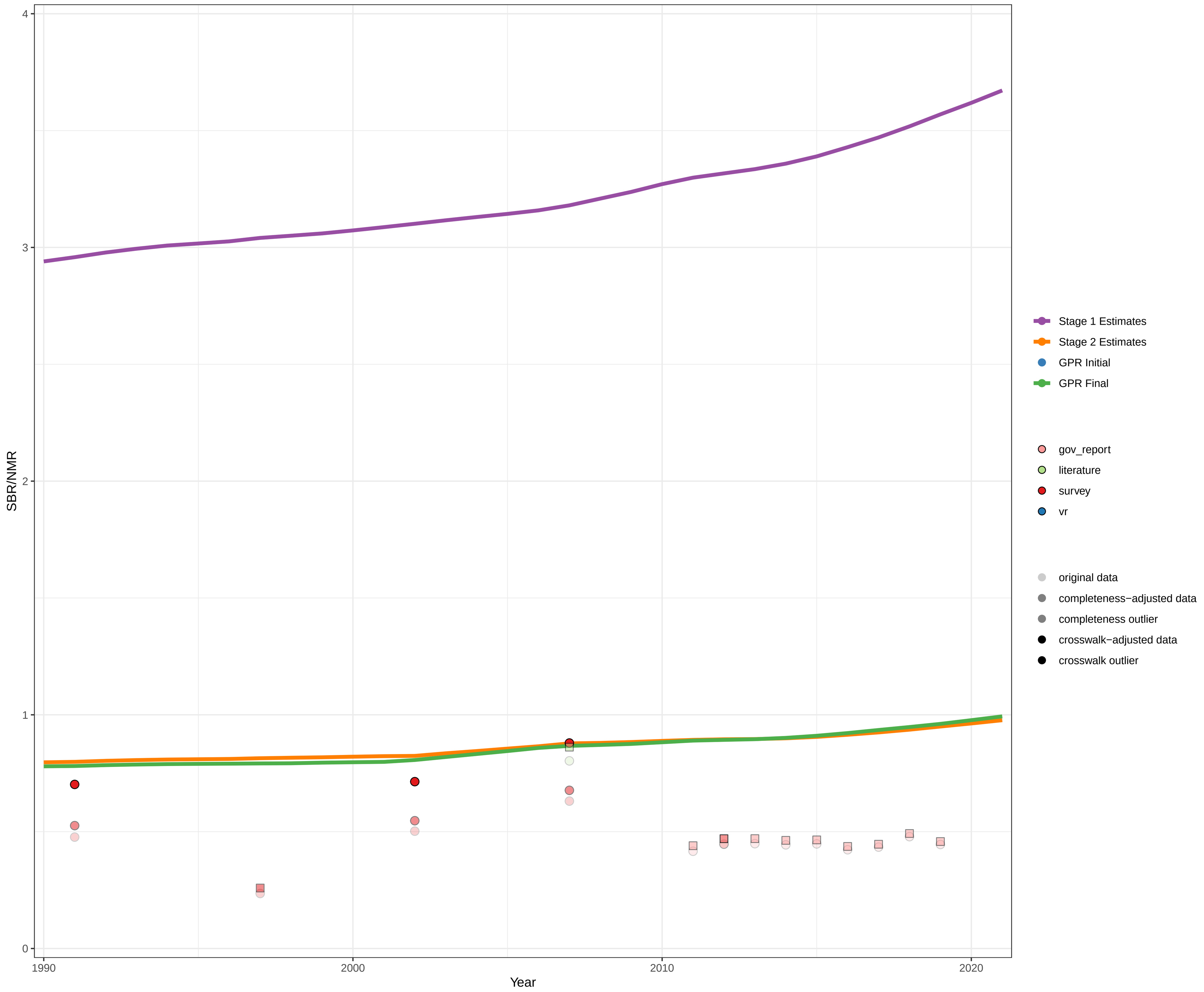

Central Java IDN_4722 Southeast Asia Stillbirth Rate / Neonatal Mortality Rate (SBR/NMR) − 28 weeks
lambda = 0.8 ; zeta = 0.7 ; scale = 15 ; amp = 0.028

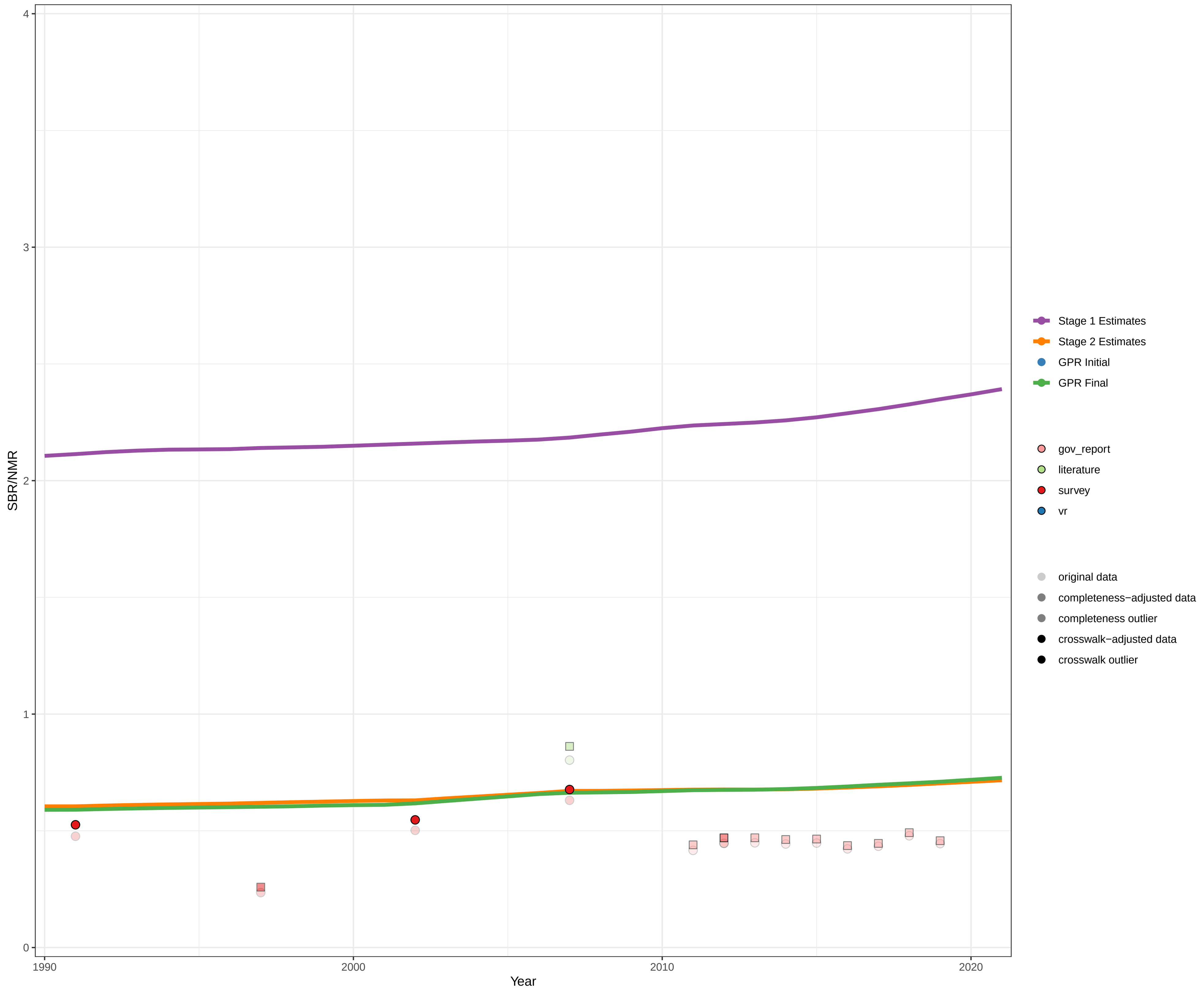

Definition	Years	Outlier	Source.Type	Title
28_weeks	1991	0	Survey	Indonesia Demographic and Health Survey 1991
28_weeks	2002	0	Survey	Indonesia Demographic and Health Survey 2002−2003
28_weeks	2007	0	Survey	Indonesia Demographic and Health Survey 2007
28_weeks	1997	1	Survey	Indonesia Demographic and Health Survey 1997
28_weeks	2007	1	Sci Lit	Utility of local health registers in measuring perinatal mortality: a case study in rural Indonesia
28_weeks	2011−2019	1	Statistical Report	Indonesia − Jawa Tengah Province Health Profile
28_weeks	2012	1	Survey	Indonesia Demographic and Health Survey 2012

Central Java IDN_4722 Southeast Asia Stillbirth Rate (SBR) − 20 weeks
lambda = 0.8 ; zeta = 0.7 ; scale = 15 ; amp = 0.028

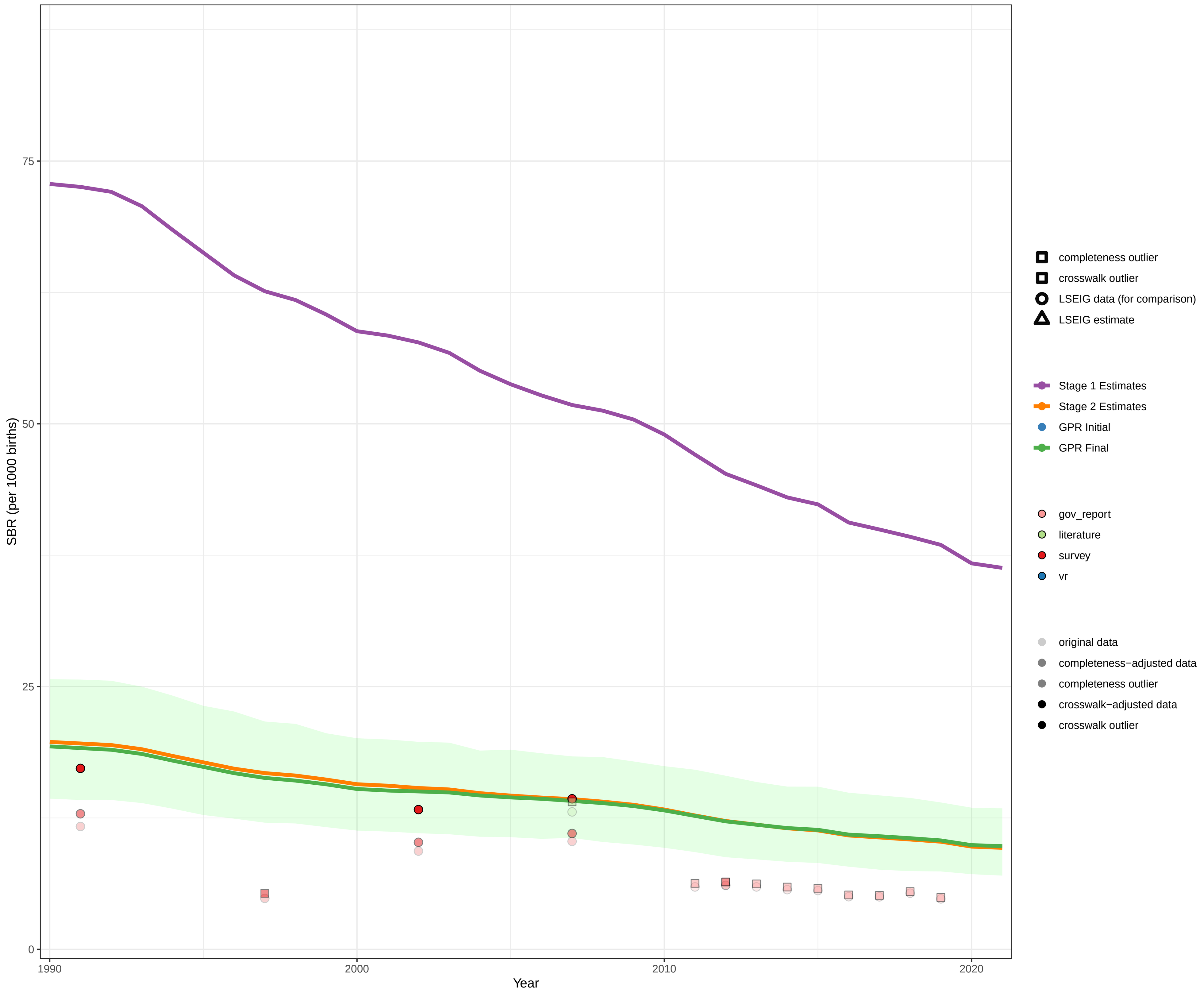

Central Java IDN_4722 Southeast Asia Stillbirth Rate (SBR) − 28 weeks
lambda = 0.8 ; zeta = 0.7 ; scale = 15 ; amp = 0.028

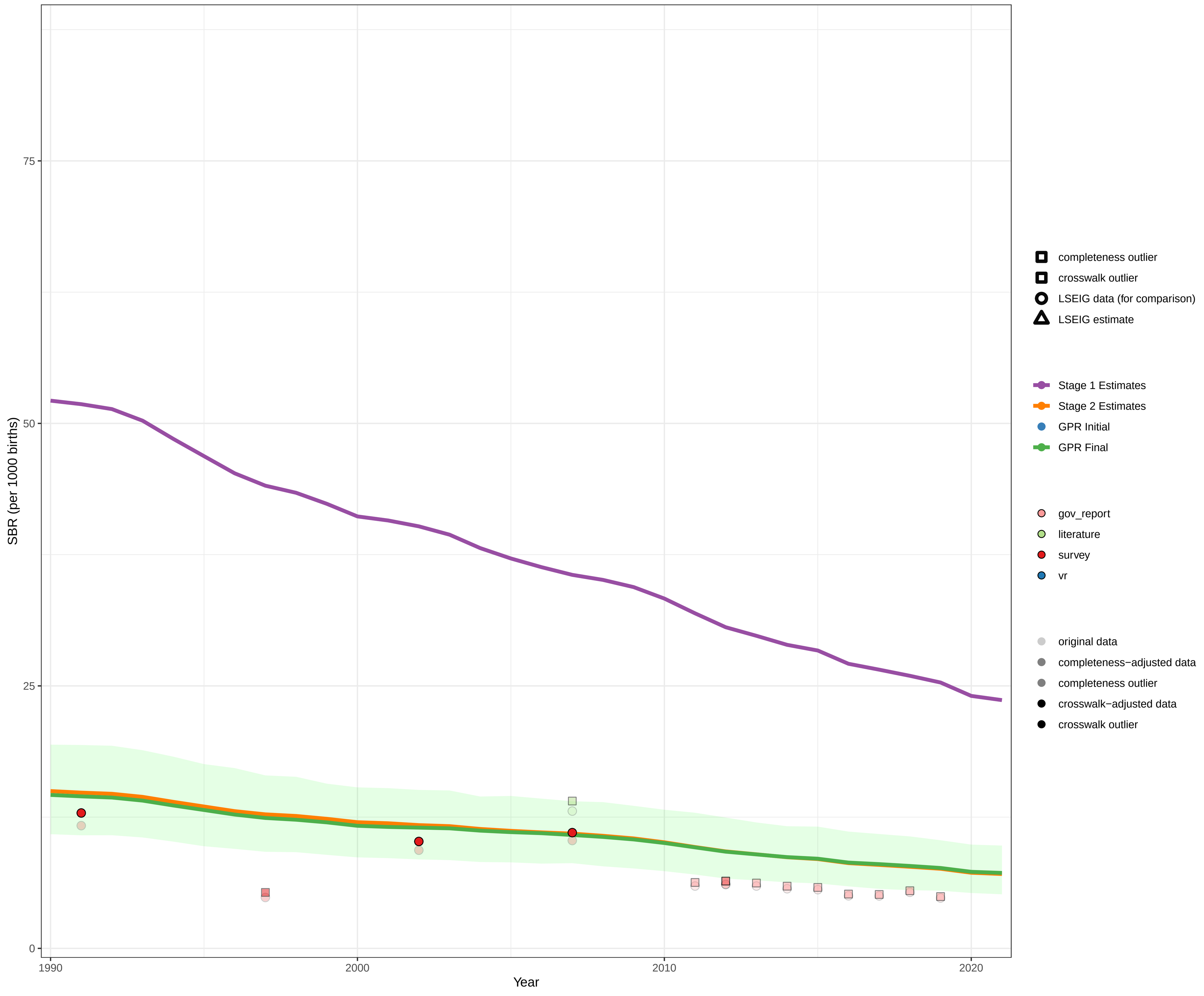

Year	Total Stillbirths (in thousands)	Total Stillbirths (in thousands)	SBR (per 1000 births)	SBR (per 1000 births)	SBR/NMR	SBR/NMR
	20 weeks	28 weeks	20 weeks	28 weeks	20 weeks	28 weeks
1990	13.1 (9.7 to 17.6)	9.9 (7.3 to 13.2)	19.3 (14.3 to 25.7)	14.6 (10.9 to 19.4)	0.78 (0.58 to 1.04)	0.59 (0.44 to 0.78)
1995	10.9 (8.0 to 14.6)	8.2 (6.0 to 11.0)	17.4 (12.8 to 23.2)	13.2 (9.7 to 17.6)	0.79 (0.58 to 1.05)	0.60 (0.44 to 0.80)
2000	8.9 (6.6 to 11.8)	6.8 (5.0 to 9.0)	15.3 (11.3 to 20.1)	11.7 (8.7 to 15.3)	0.80 (0.59 to 1.05)	0.61 (0.45 to 0.80)
2005	8.2 (6.0 to 10.8)	6.3 (4.6 to 8.2)	14.5 (10.7 to 19.0)	11.1 (8.2 to 14.5)	0.84 (0.62 to 1.11)	0.65 (0.48 to 0.85)
2010	7.4 (5.4 to 9.8)	5.6 (4.1 to 7.4)	13.2 (9.7 to 17.4)	10.0 (7.4 to 13.2)	0.88 (0.65 to 1.16)	0.67 (0.49 to 0.88)
2015	6.1 (4.4 to 8.3)	4.5 (3.3 to 6.2)	11.4 (8.2 to 15.5)	8.5 (6.2 to 11.6)	0.91 (0.66 to 1.24)	0.68 (0.50 to 0.93)
2020	5.0 (3.6 to 6.9)	3.7 (2.7 to 5.0)	9.9 (7.1 to 13.5)	7.3 (5.3 to 9.9)	0.98 (0.70 to 1.33)	0.72 (0.52 to 0.97)
2021	5.0 (3.5 to 6.8)	3.6 (2.6 to 4.9)	9.8 (7.0 to 13.4)	7.2 (5.2 to 9.8)	0.99 (0.71 to 1.36)	0.73 (0.52 to 0.99)

East Java IDN_4724 Southeast Asia Stillbirth Rate / Neonatal Mortality Rate (SBR/NMR) − 20 weeks
lambda = 0.8 ; zeta = 0.7 ; scale = 15 ; amp = 0.028

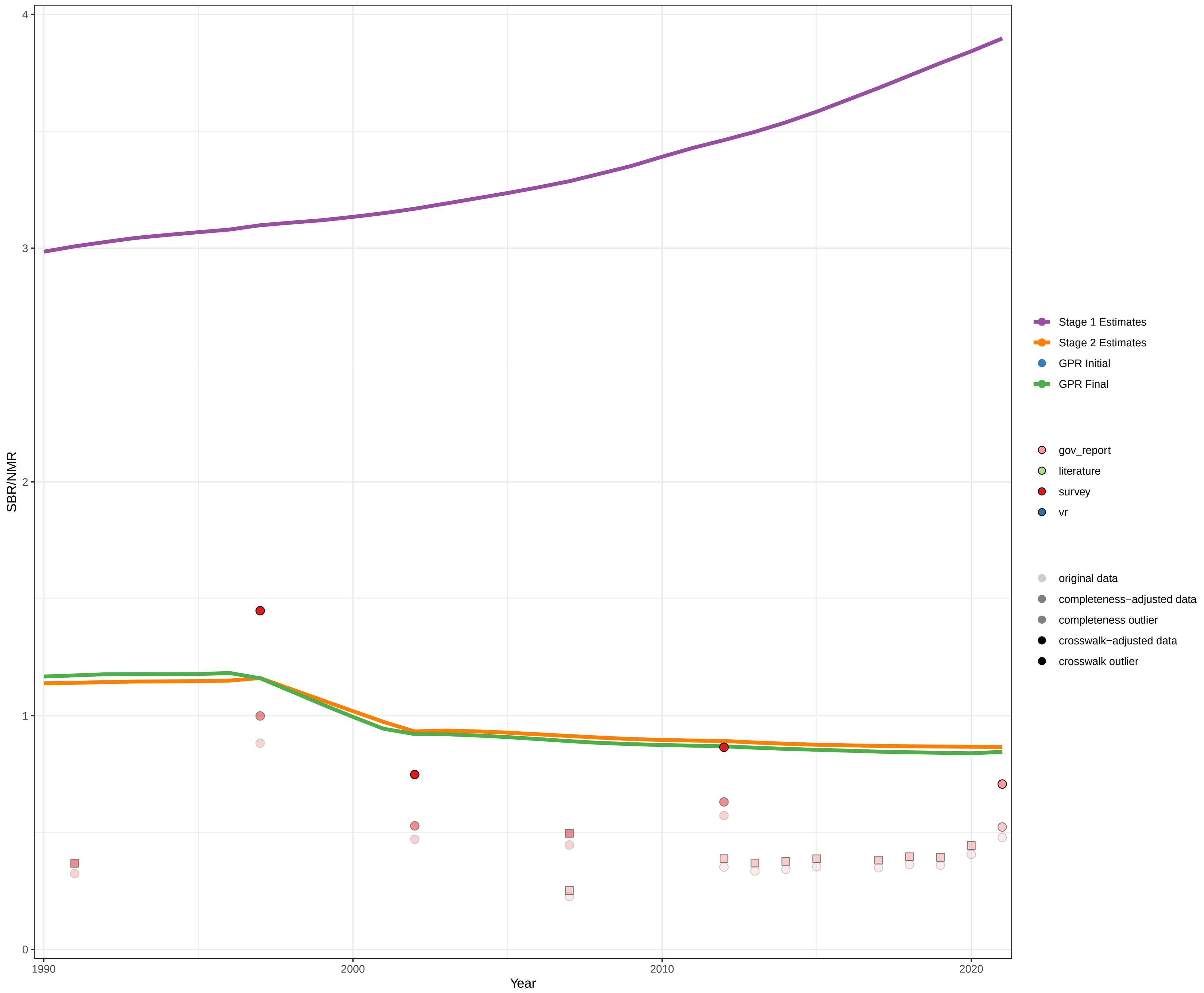

East Java IDN_4724 Southeast Asia Stillbirth Rate / Neonatal Mortality Rate (SBR/NMR) − 28 weeks
lambda = 0.8 ; zeta = 0.7 ; scale = 15 ; amp = 0.028

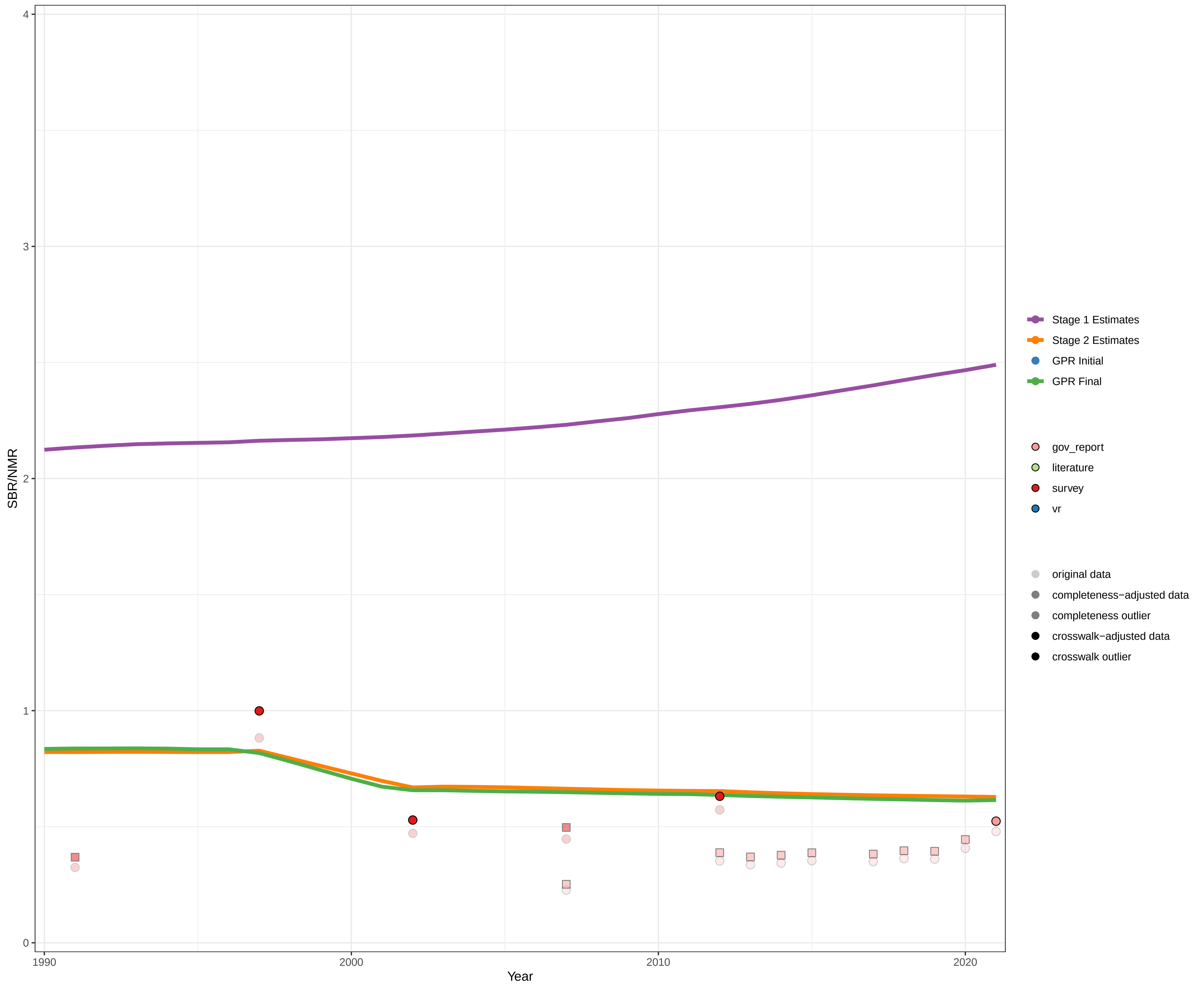

Definition	Years	Outlier	Source.Type	Title
28_weeks	1997	0	Survey	Indonesia Demographic and Health Survey 1997
28_weeks	2002	0	Survey	Indonesia Demographic and Health Survey 2002−2003
28_weeks	2012	0	Survey	Indonesia Demographic and Health Survey 2012
28_weeks	2021	0	Statistical Report	Indonesia − Jawa Timur Province Health Profile
28_weeks	1991	1	Survey	Indonesia Demographic and Health Survey 1991
28_weeks	2007	1	Survey	Indonesia Demographic and Health Survey 2007
28_weeks	2007, 2012−2015, 2017−2020	1	Statistical Report	Indonesia − Jawa Timur Province Health Profile

East Java IDN_4724 Southeast Asia Stillbirth Rate (SBR) − 20 weeks
lambda = 0.8 ; zeta = 0.7 ; scale = 15 ; amp = 0.028

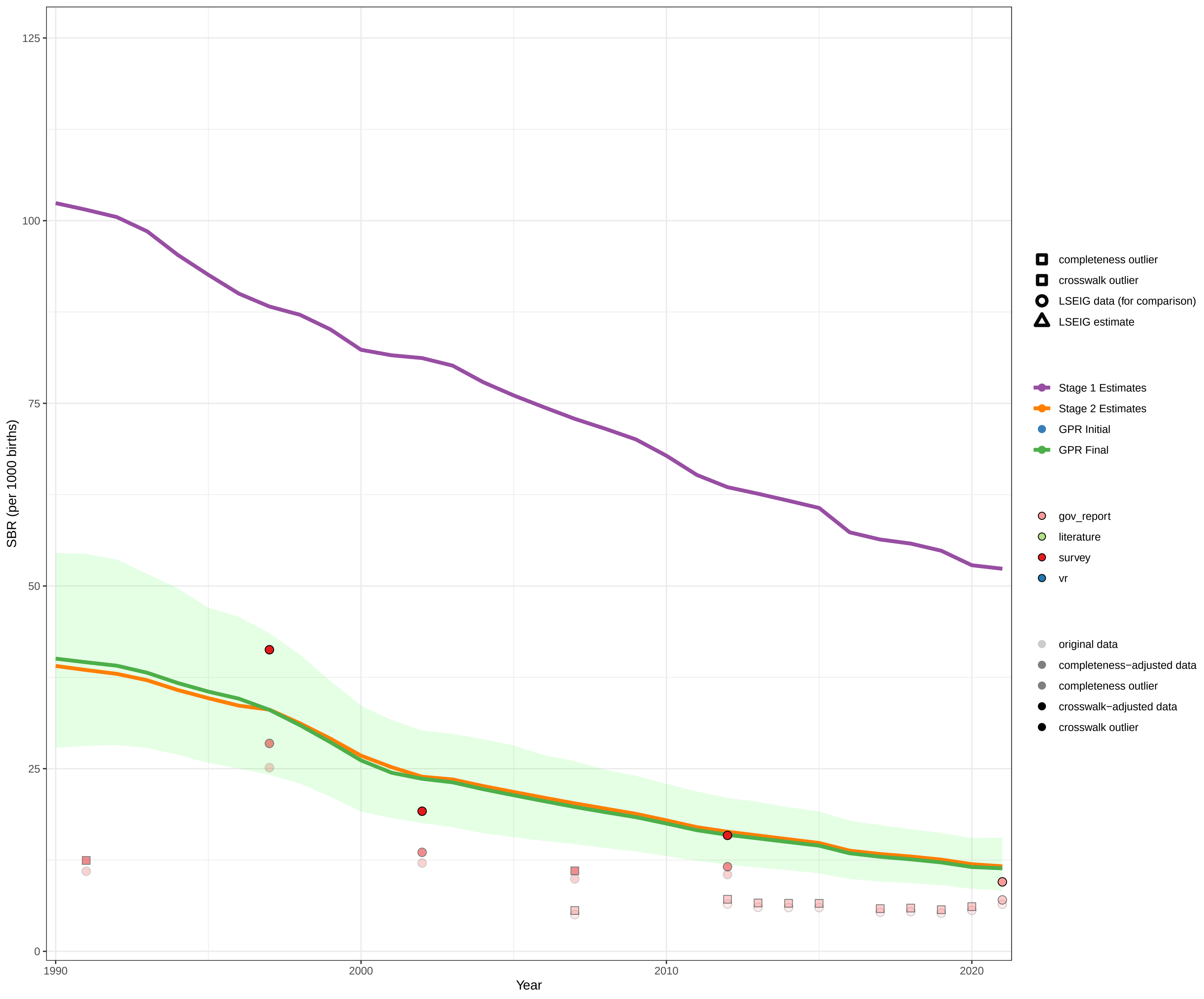

East Java IDN_4724 Southeast Asia Stillbirth Rate (SBR) − 28 weeks
lambda = 0.8 ; zeta = 0.7 ; scale = 15 ; amp = 0.028

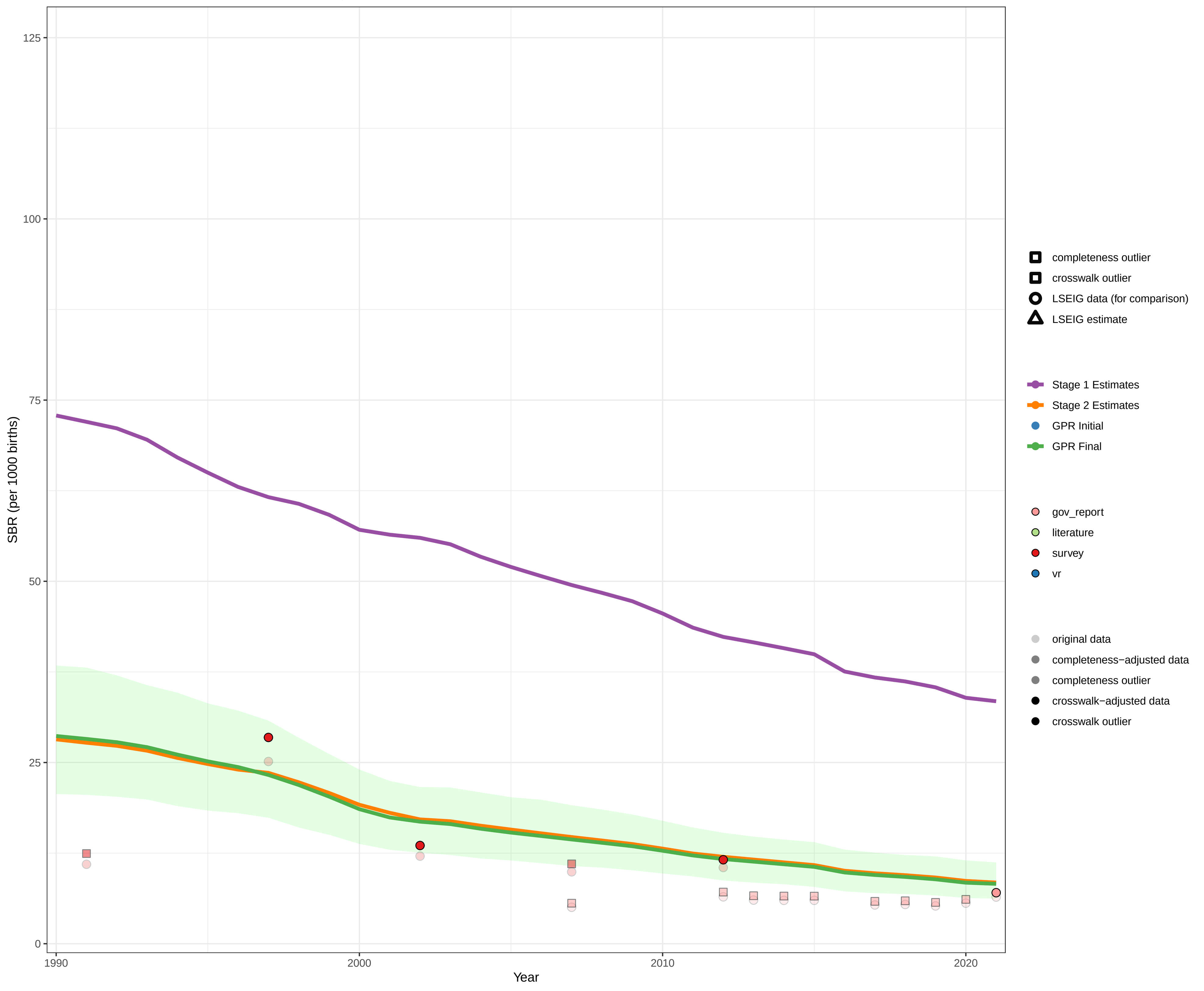

Year	Total Stillbirths (in thousands)	Total Stillbirths (in thousands)	SBR (per 1000 births)	SBR (per 1000 births)	SBR/NMR	SBR/NMR
	20 weeks	28 weeks	20 weeks	28 weeks	20 weeks	28 weeks
1990	27.5 (18.8 to 37.9)	19.4 (13.9 to 26.2)	40.1 (27.9 to 54.5)	28.7 (20.6 to 38.4)	1.17 (0.81 to 1.59)	0.84 (0.60 to 1.12)
1995	23.2 (16.7 to 31.1)	16.3 (11.8 to 21.6)	35.5 (25.8 to 47.0)	25.2 (18.4 to 33.2)	1.18 (0.85 to 1.56)	0.83 (0.61 to 1.10)
2000	16.1 (11.7 to 20.9)	11.4 (8.4 to 14.8)	26.1 (19.1 to 33.6)	18.6 (13.8 to 24.0)	0.99 (0.73 to 1.28)	0.71 (0.52 to 0.91)
2005	12.9 (9.3 to 17.1)	9.2 (6.8 to 12.1)	21.4 (15.6 to 28.2)	15.3 (11.5 to 20.2)	0.91 (0.66 to 1.20)	0.65 (0.49 to 0.86)
2010	10.1 (7.5 to 13.3)	7.4 (5.6 to 9.8)	17.5 (13.0 to 22.9)	12.8 (9.7 to 17.0)	0.87 (0.65 to 1.15)	0.64 (0.48 to 0.85)
2015	7.5 (5.5 to 10.0)	5.5 (4.0 to 7.3)	14.5 (10.7 to 19.2)	10.6 (7.8 to 14.0)	0.85 (0.63 to 1.13)	0.63 (0.46 to 0.83)
2020	5.4 (4.0 to 7.3)	3.9 (2.9 to 5.4)	11.5 (8.6 to 15.5)	8.4 (6.3 to 11.5)	0.84 (0.62 to 1.13)	0.61 (0.46 to 0.84)
2021	5.2 (3.8 to 7.2)	3.8 (2.8 to 5.2)	11.4 (8.3 to 15.6)	8.3 (6.2 to 11.2)	0.85 (0.62 to 1.16)	0.61 (0.46 to 0.83)

West Kalimantan IDN_4729 Southeast Asia Stillbirth Rate / Neonatal Mortality Rate (SBR/NMR) − 20 weeks
lambda = 0.4 ; zeta = 0.9 ; scale = 10 ; amp = 0.028

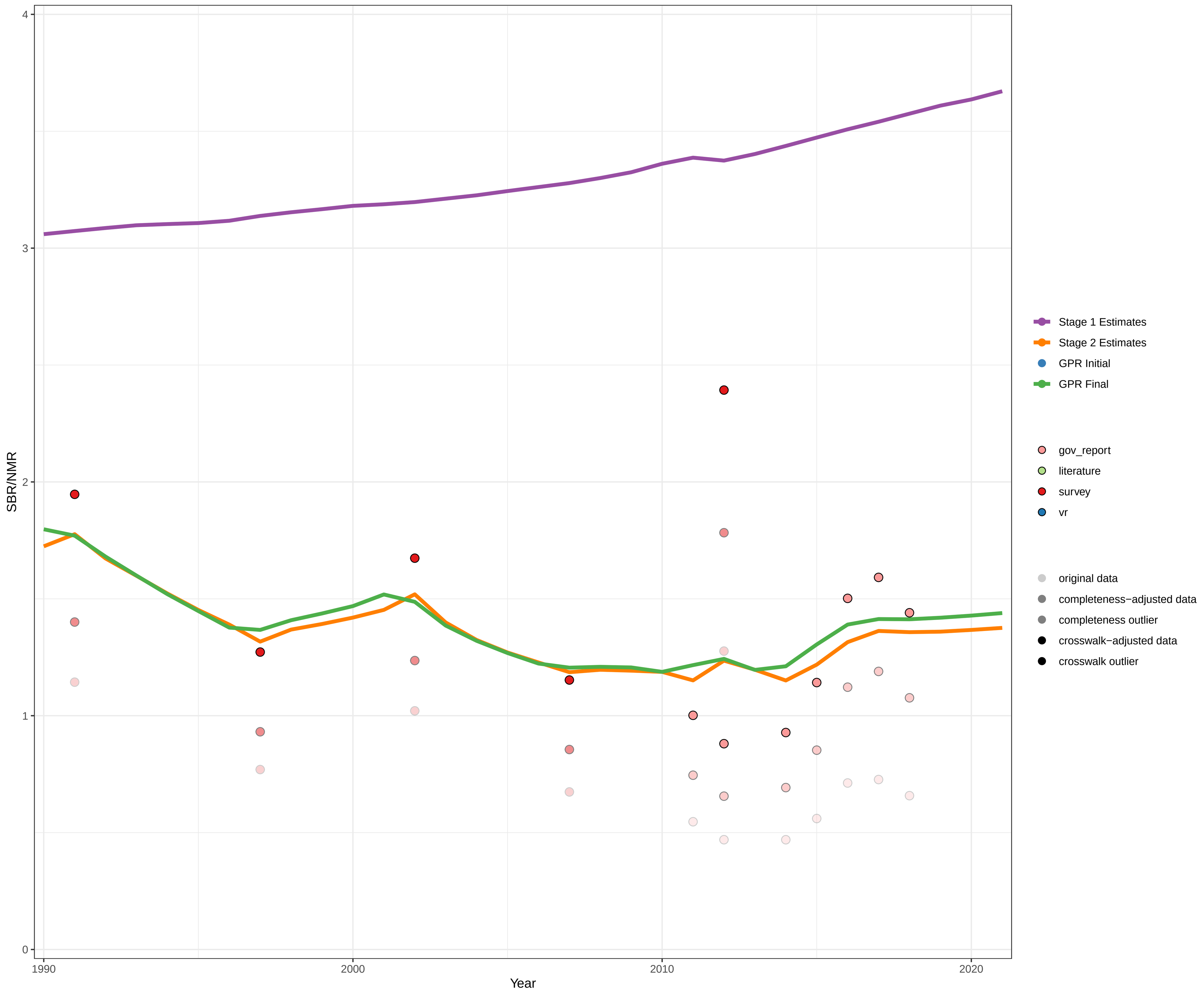

West Kalimantan IDN_4729 Southeast Asia Stillbirth Rate / Neonatal Mortality Rate (SBR/NMR) − 28 weeks
lambda = 0.4 ; zeta = 0.9 ; scale = 10 ; amp = 0.028

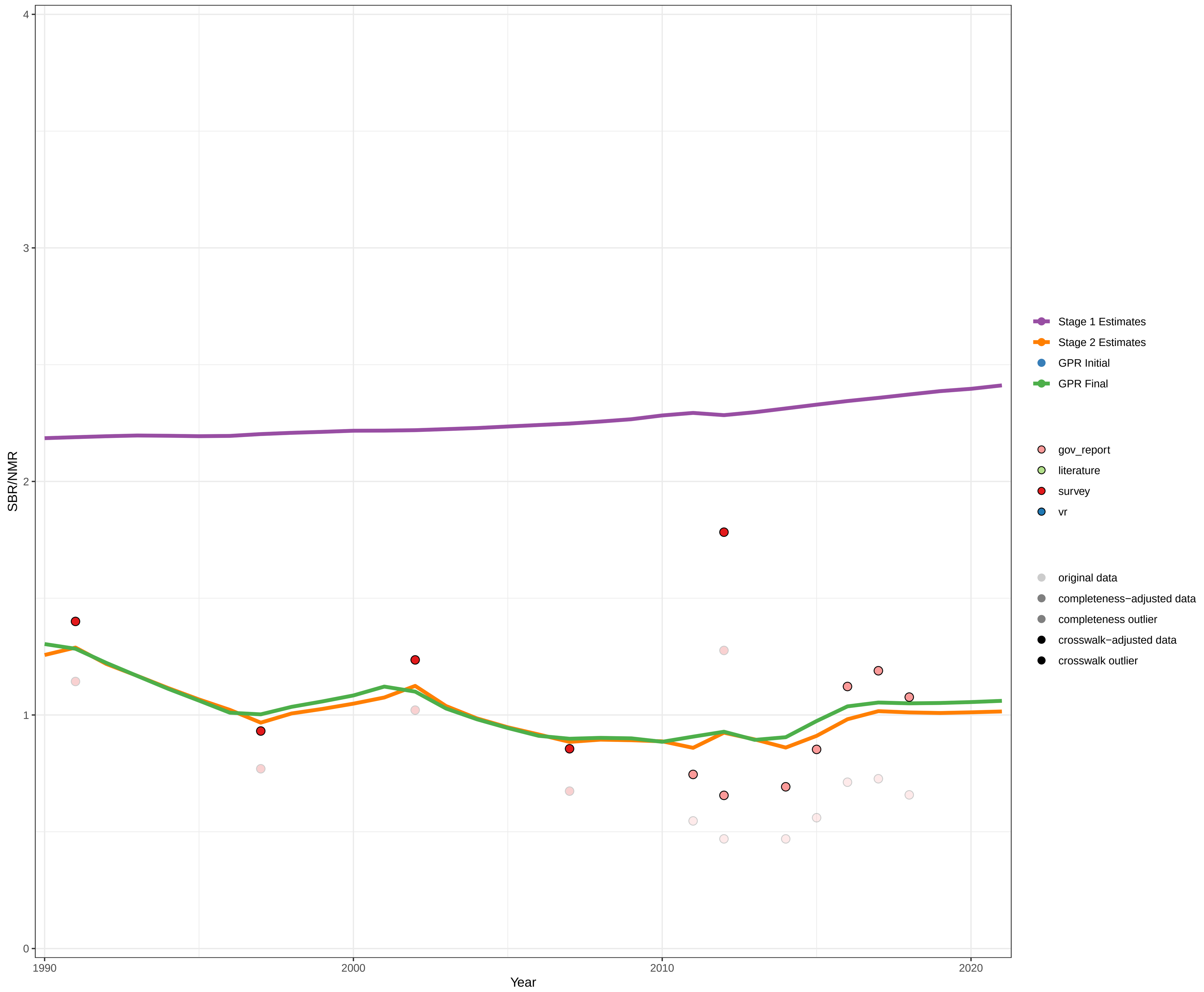

Definition	Years	Outlier	Source.Type	Title
28_weeks	1991	0	Survey	Indonesia Demographic and Health Survey 1991
28_weeks	1997	0	Survey	Indonesia Demographic and Health Survey 1997
28_weeks	2002	0	Survey	Indonesia Demographic and Health Survey 2002−2003
28_weeks	2007	0	Survey	Indonesia Demographic and Health Survey 2007
28_weeks	2011, 2012, 2014−2018	0	Statistical Report	Indonesia − Kalimantan Barat Province Health Profile
28_weeks	2012	0	Survey	Indonesia Demographic and Health Survey 2012

West Kalimantan IDN_4729 Southeast Asia Stillbirth Rate (SBR) − 20 weeks
lambda = 0.4 ; zeta = 0.9 ; scale = 10 ; amp = 0.028

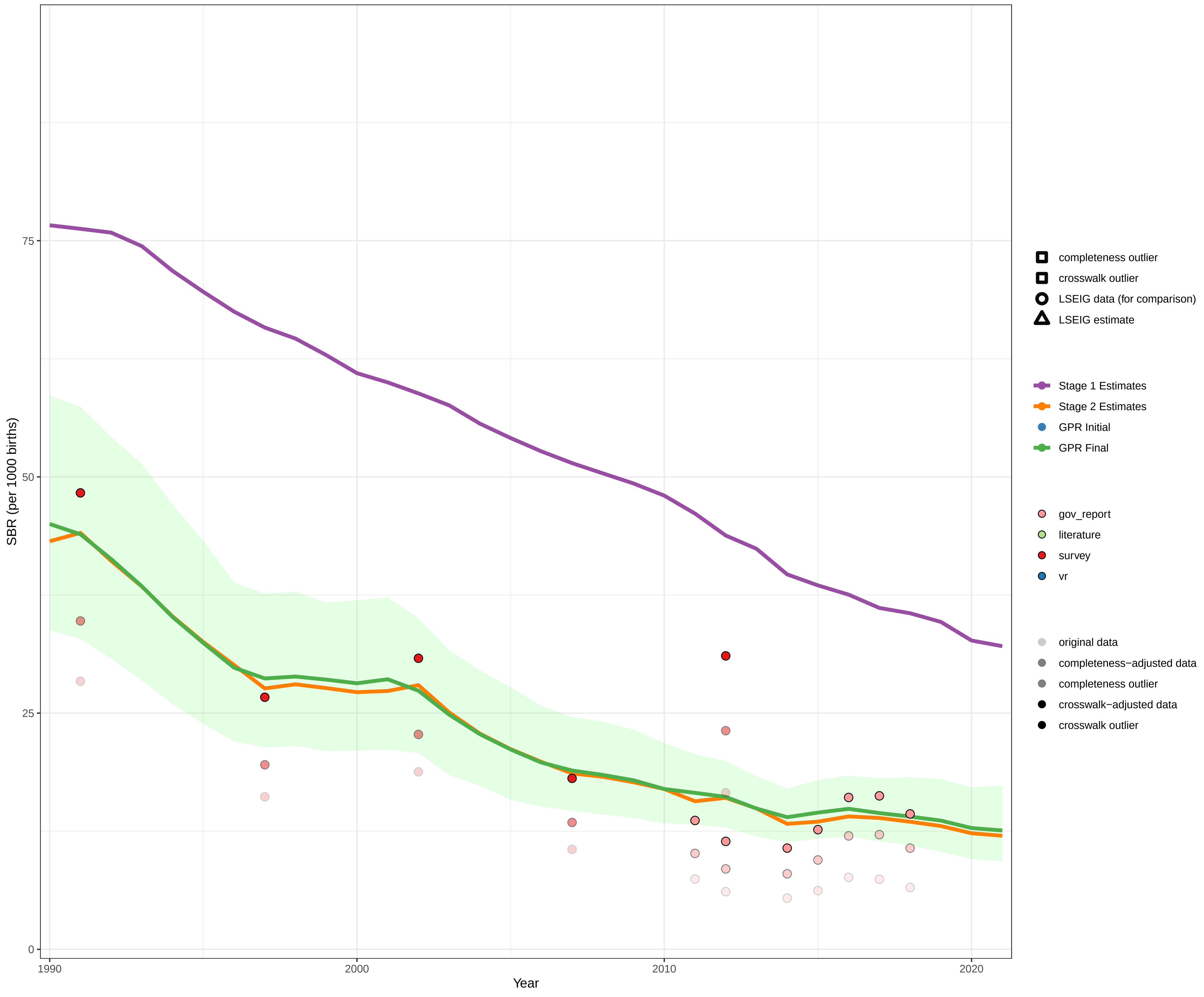

West Kalimantan IDN_4729 Southeast Asia Stillbirth Rate (SBR) − 28 weeks
lambda = 0.4 ; zeta = 0.9 ; scale = 10 ; amp = 0.028

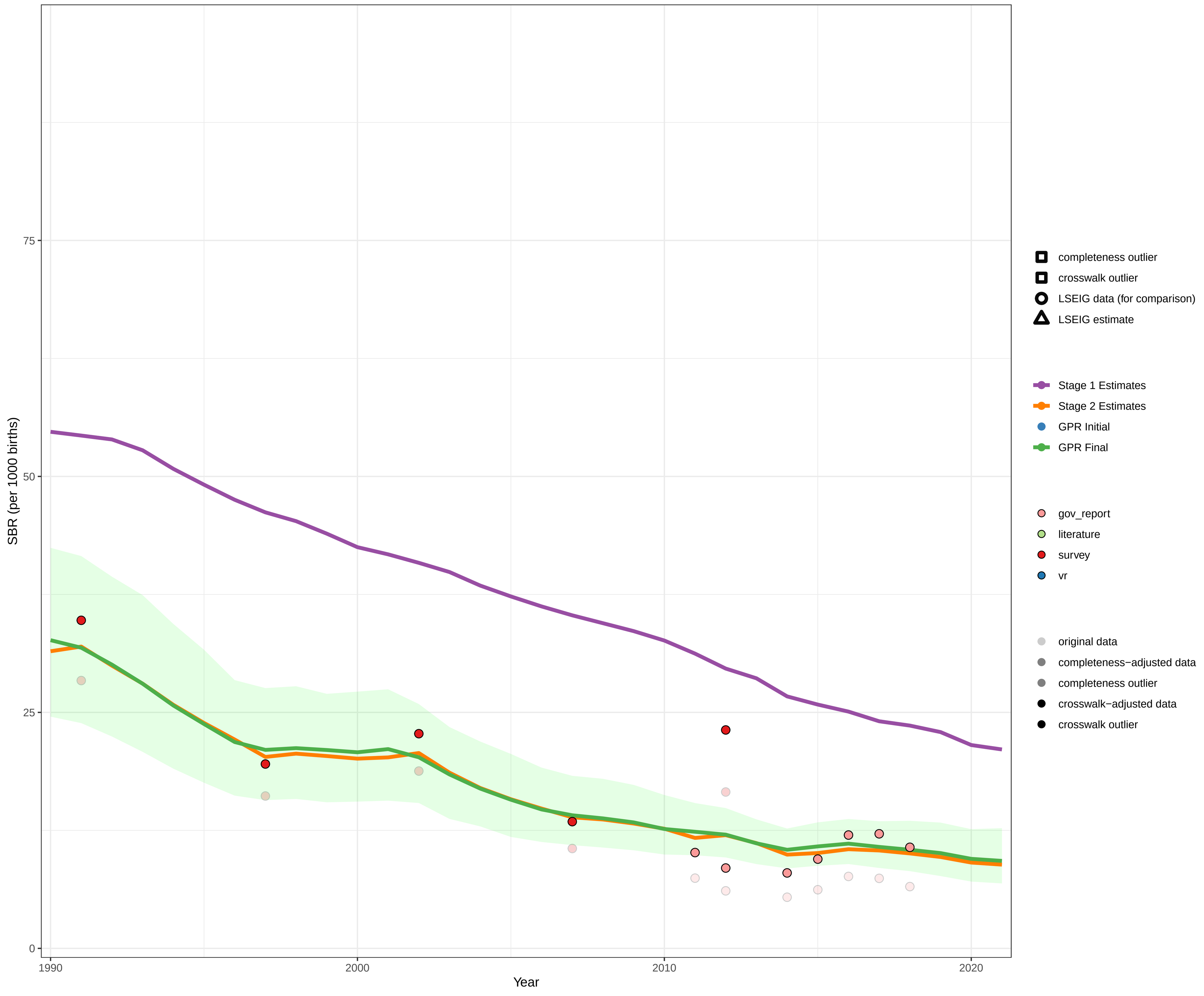

Year	Total Stillbirths (in thousands)	Total Stillbirths (in thousands)	SBR (per 1000 births)	SBR (per 1000 births)	SBR/NMR	SBR/NMR
	20 weeks	28 weeks	20 weeks	28 weeks	20 weeks	28 weeks
1990	4.6 (3.4 to 6.0)	3.3 (2.4 to 4.3)	45.0 (33.8 to 58.6)	32.7 (24.5 to 42.4)	1.80 (1.35 to 2.34)	1.30 (0.98 to 1.69)
1995	3.2 (2.3 to 4.3)	2.3 (1.7 to 3.1)	32.4 (23.9 to 43.2)	23.8 (17.5 to 31.6)	1.45 (1.07 to 1.93)	1.06 (0.78 to 1.41)
2000	2.9 (2.1 to 3.8)	2.1 (1.6 to 2.8)	28.2 (21.0 to 36.9)	20.8 (15.5 to 27.2)	1.47 (1.10 to 1.93)	1.08 (0.81 to 1.42)
2005	2.3 (1.7 to 3.0)	1.7 (1.2 to 2.2)	21.2 (15.8 to 27.7)	15.7 (11.8 to 20.6)	1.27 (0.95 to 1.66)	0.94 (0.71 to 1.24)
2010	1.9 (1.5 to 2.4)	1.4 (1.1 to 1.8)	17.0 (13.3 to 21.8)	12.6 (9.9 to 16.3)	1.19 (0.93 to 1.53)	0.89 (0.70 to 1.14)
2015	1.6 (1.3 to 2.0)	1.2 (1.0 to 1.5)	14.5 (11.7 to 17.9)	10.8 (8.8 to 13.4)	1.30 (1.06 to 1.62)	0.97 (0.79 to 1.20)
2020	1.5 (1.1 to 2.0)	1.1 (0.8 to 1.4)	12.8 (9.6 to 17.1)	9.5 (7.1 to 12.6)	1.43 (1.06 to 1.91)	1.06 (0.79 to 1.41)
2021	1.5 (1.1 to 2.0)	1.1 (0.8 to 1.5)	12.6 (9.3 to 17.3)	9.3 (6.9 to 12.7)	1.44 (1.07 to 1.98)	1.06 (0.79 to 1.46)

South Kalimantan IDN_4731 Southeast Asia Stillbirth Rate / Neonatal Mortality Rate (SBR/NMR) − 20 weeks
lambda = 0.8 ; zeta = 0.7 ; scale = 15 ; amp = 0.028

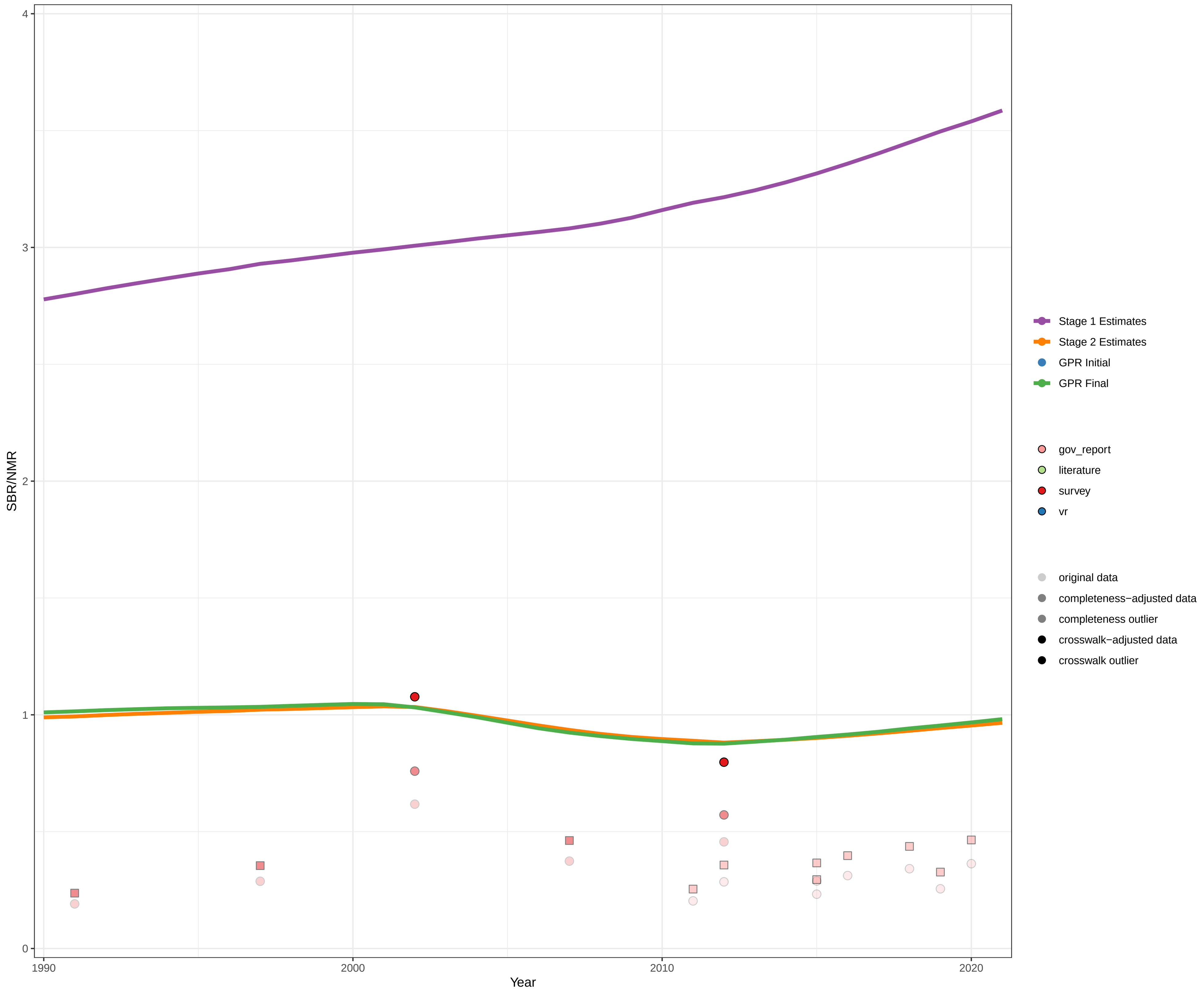

South Kalimantan IDN_4731 Southeast Asia Stillbirth Rate / Neonatal Mortality Rate (SBR/NMR) − 28 weeks
lambda = 0.8 ; zeta = 0.7 ; scale = 15 ; amp = 0.028

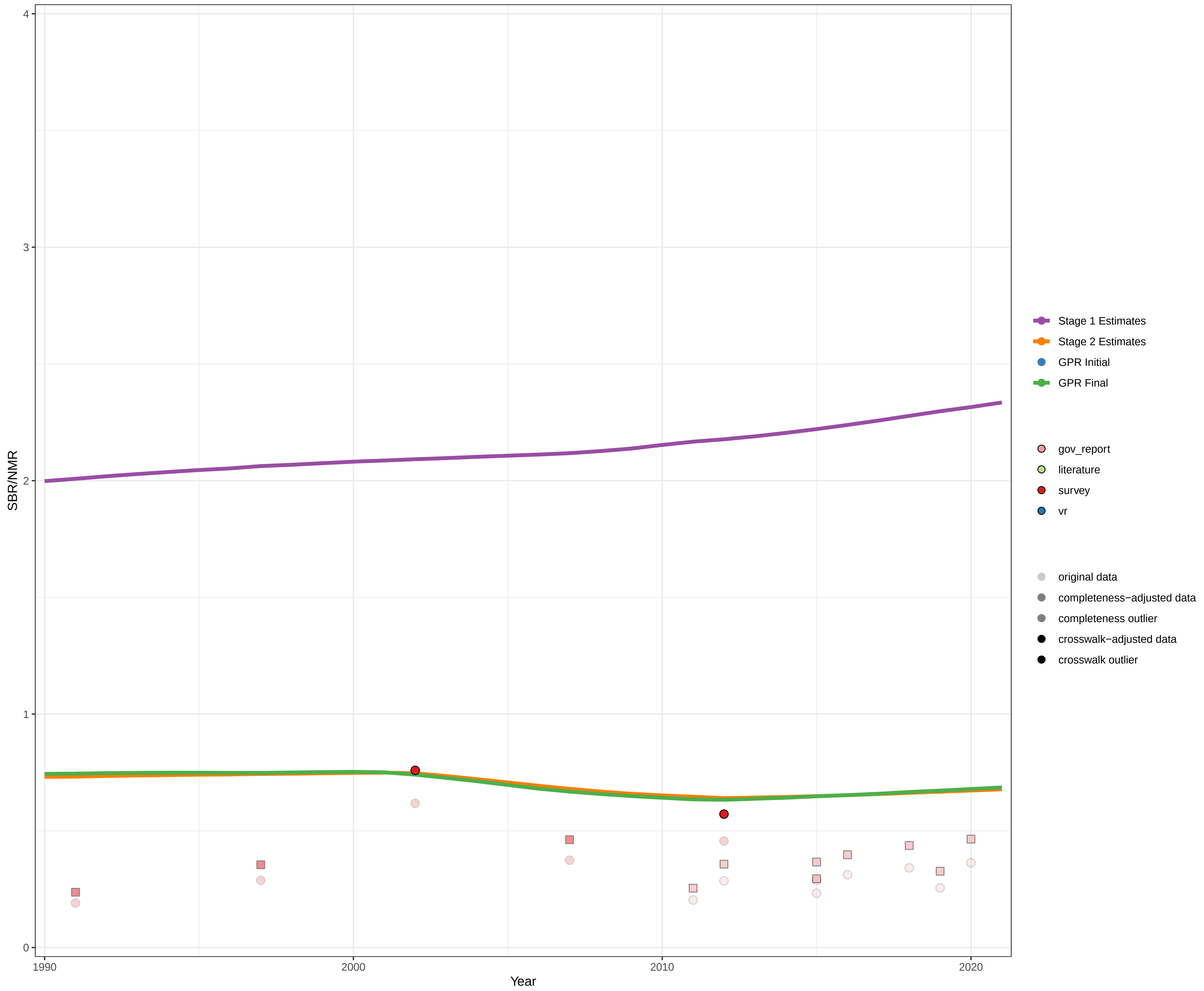

Definition	Years	Outlier	Source.Type	Title
28_weeks	2002	0	Survey	Indonesia Demographic and Health Survey 2002−2003
28_weeks	2012	0	Survey	Indonesia Demographic and Health Survey 2012
28_weeks	1991	1	Survey	Indonesia Demographic and Health Survey 1991
28_weeks	1997	1	Survey	Indonesia Demographic and Health Survey 1997
28_weeks	2007	1	Survey	Indonesia Demographic and Health Survey 2007
28_weeks	2011, 2012, 2015, 2016, 2018−2020	1	Statistical Report	Indonesia − Kalimantan Selatan Province Health Profile

South Kalimantan IDN_4731 Southeast Asia Stillbirth Rate (SBR) − 20 weeks
lambda = 0.8 ; zeta = 0.7 ; scale = 15 ; amp = 0.028

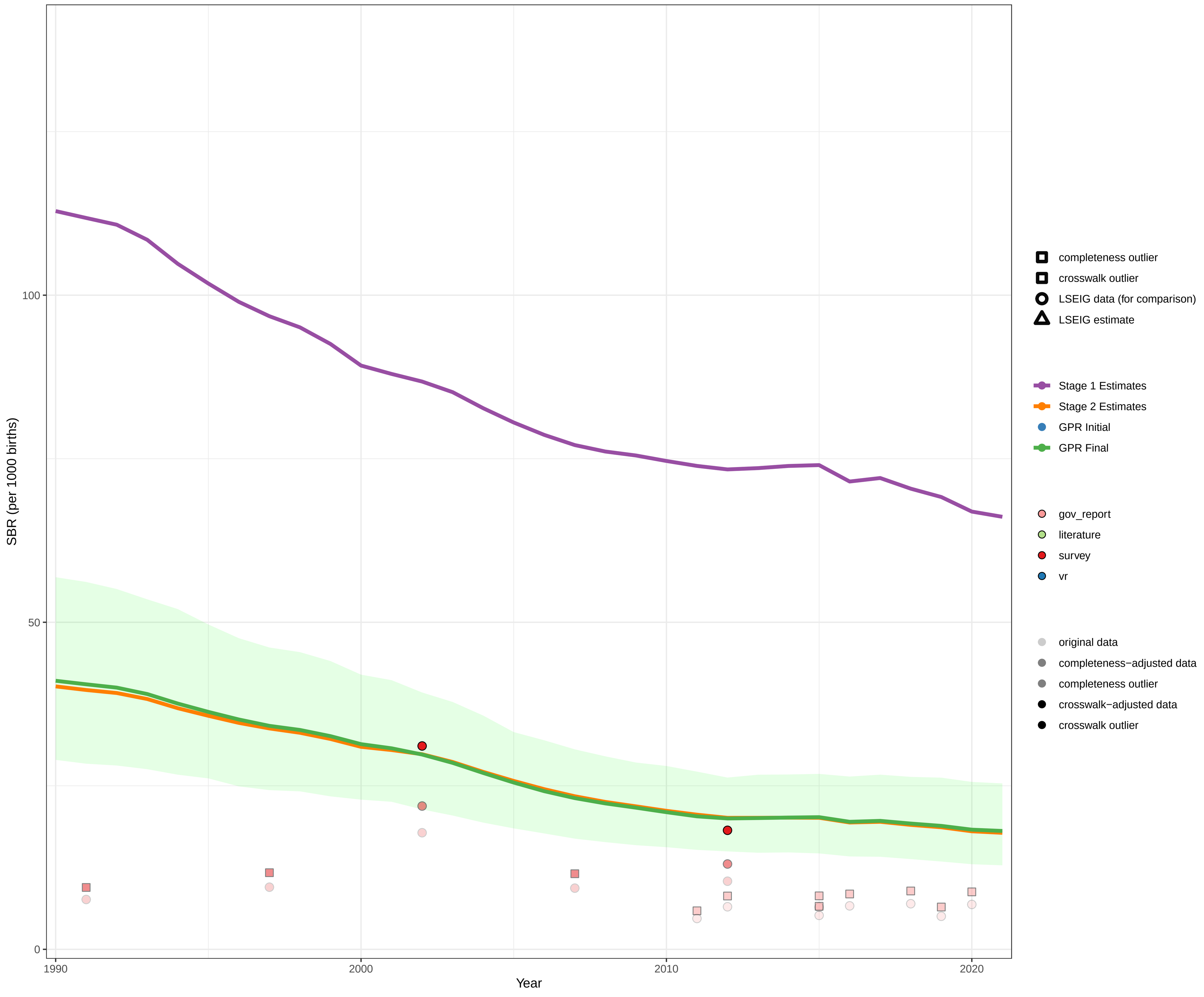

South Kalimantan IDN_4731 Southeast Asia Stillbirth Rate (SBR) − 28 weeks
lambda = 0.8 ; zeta = 0.7 ; scale = 15 ; amp = 0.028

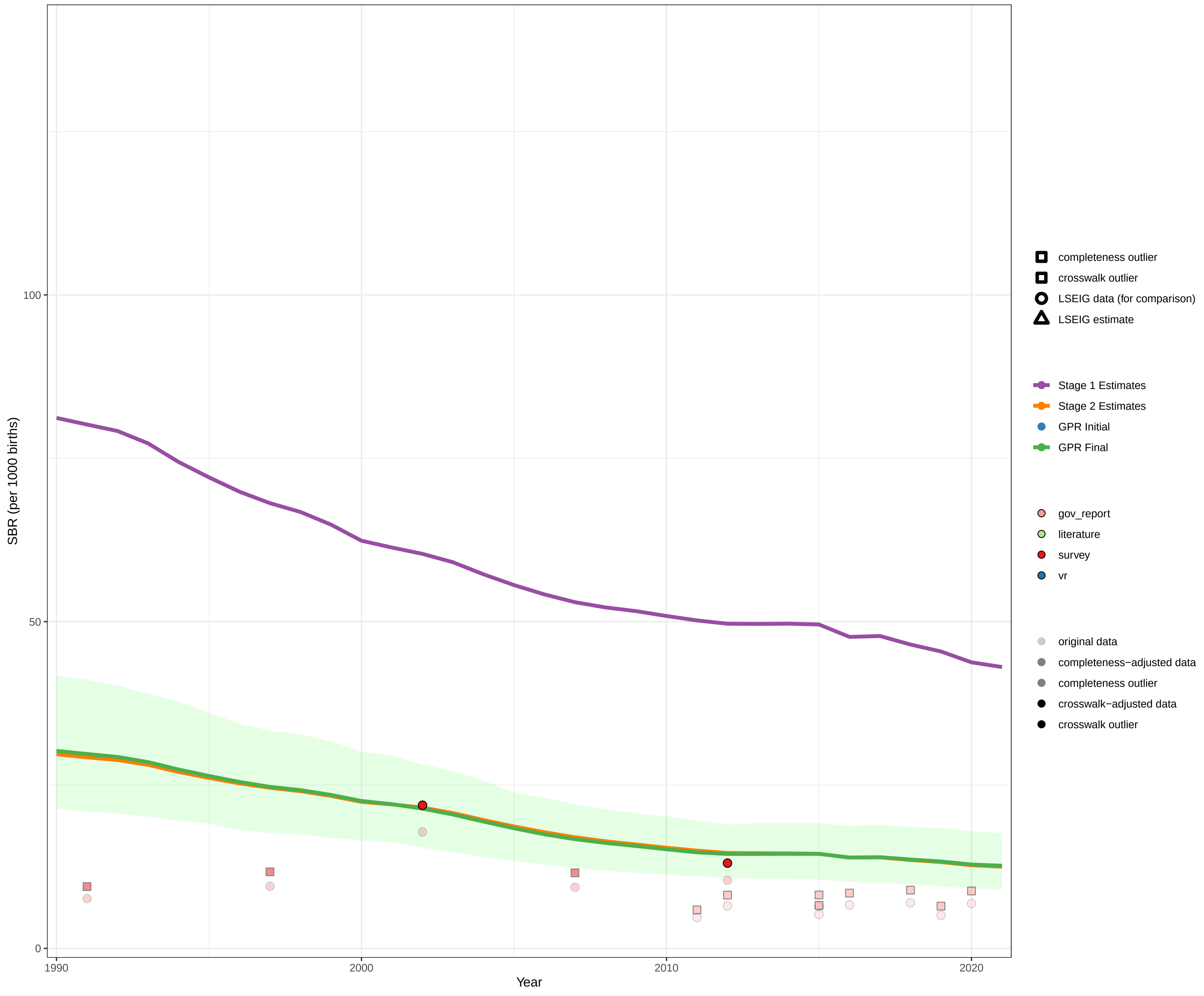

Year	Total Stillbirths (in thousands)	Total Stillbirths (in thousands)	SBR (per 1000 births)	SBR (per 1000 births)	SBR/NMR	SBR/NMR
	20 weeks	28 weeks	20 weeks	28 weeks	20 weeks	28 weeks
1990	3.0 (2.1 to 4.2)	2.2 (1.5 to 3.0)	41.1 (29.0 to 56.9)	30.2 (21.4 to 41.8)	1.01 (0.71 to 1.40)	0.74 (0.53 to 1.03)
1995	2.7 (1.9 to 3.8)	2.0 (1.4 to 2.7)	36.3 (26.1 to 49.7)	26.4 (19.1 to 36.0)	1.03 (0.74 to 1.41)	0.75 (0.54 to 1.02)
2000	2.5 (1.8 to 3.4)	1.8 (1.3 to 2.4)	31.4 (22.9 to 42.0)	22.6 (16.5 to 30.1)	1.05 (0.76 to 1.40)	0.75 (0.55 to 1.01)
2005	2.1 (1.5 to 2.8)	1.5 (1.1 to 2.0)	25.5 (18.5 to 33.2)	18.4 (13.4 to 23.9)	0.97 (0.70 to 1.26)	0.70 (0.51 to 0.91)
2010	1.7 (1.3 to 2.3)	1.2 (0.9 to 1.7)	21.0 (15.6 to 28.0)	15.2 (11.3 to 20.2)	0.89 (0.66 to 1.19)	0.64 (0.48 to 0.86)
2015	1.5 (1.1 to 2.0)	1.1 (0.8 to 1.4)	20.2 (14.7 to 26.8)	14.5 (10.5 to 19.1)	0.91 (0.66 to 1.20)	0.65 (0.47 to 0.86)
2020	1.2 (0.9 to 1.8)	0.9 (0.6 to 1.2)	18.3 (13.0 to 25.6)	12.8 (9.2 to 17.9)	0.97 (0.69 to 1.35)	0.68 (0.48 to 0.95)
2021	1.2 (0.9 to 1.7)	0.8 (0.6 to 1.2)	18.1 (12.8 to 25.4)	12.6 (9.0 to 17.7)	0.98 (0.70 to 1.38)	0.69 (0.49 to 0.96)

Central Kalimantan IDN_4730 Southeast Asia Stillbirth Rate / Neonatal Mortality Rate (SBR/NMR) − 20 weeks
lambda = 0.6 ; zeta = 0.8 ; scale = 15 ; amp = 0.028

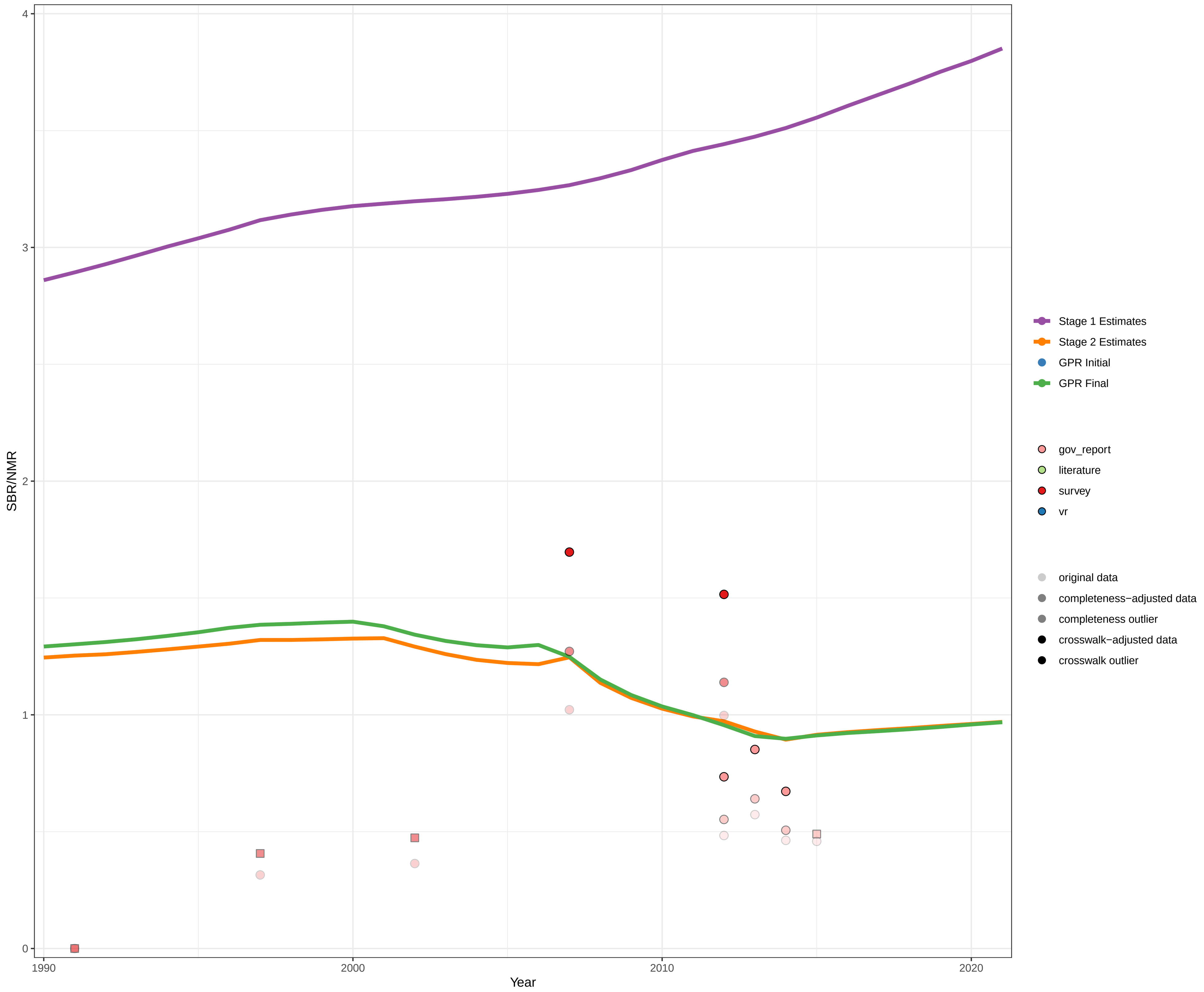

Central Kalimantan IDN_4730 Southeast Asia Stillbirth Rate / Neonatal Mortality Rate (SBR/NMR) − 28 weeks
lambda = 0.6 ; zeta = 0.8 ; scale = 15 ; amp = 0.028

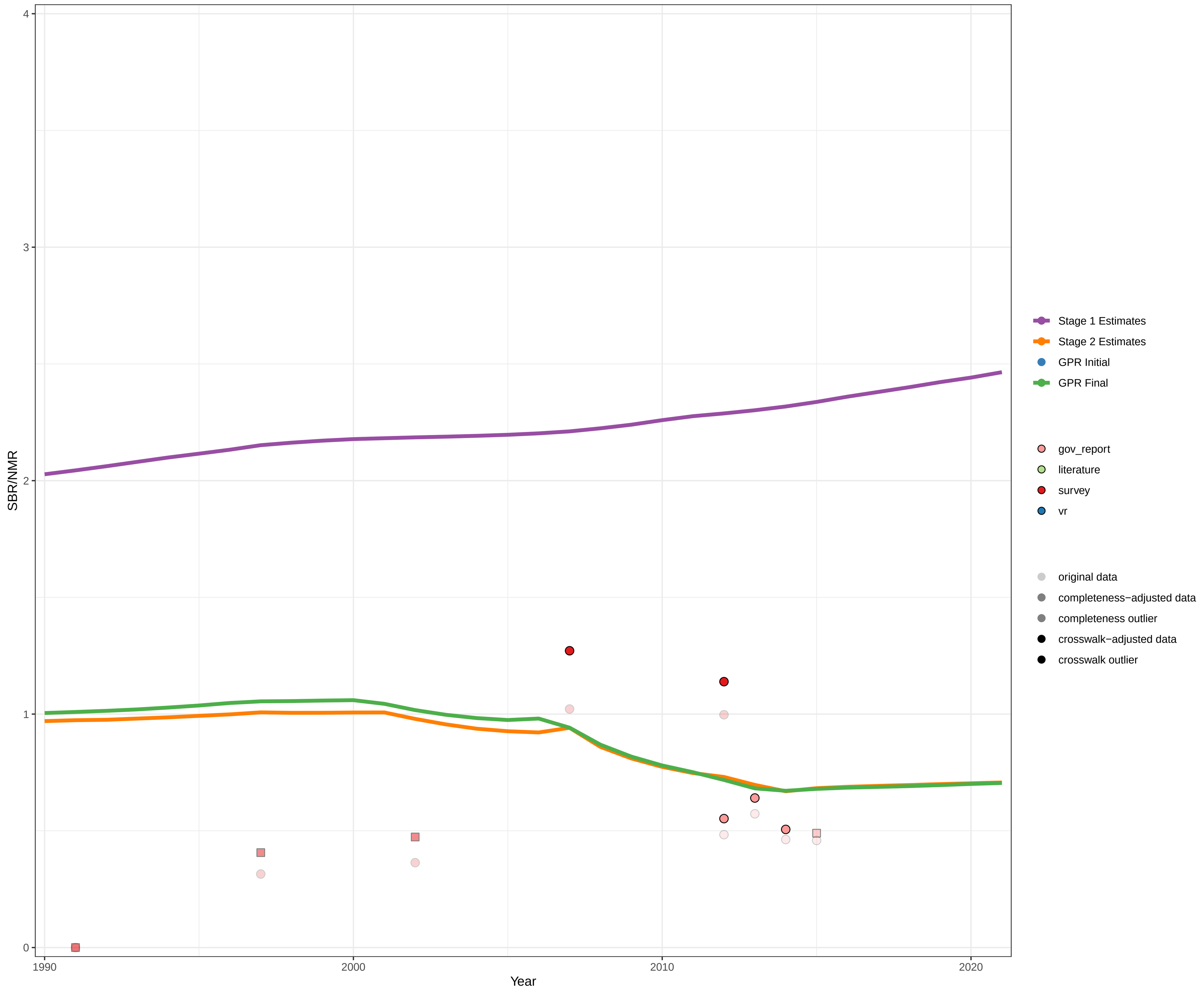

Definition	Years	Outlier	Source.Type	Title
28_weeks	2007	0	Survey	Indonesia Demographic and Health Survey 2007
28_weeks	2012	0	Survey	Indonesia Demographic and Health Survey 2012
28_weeks	2012−2014	0	Statistical Report	Indonesia − Kalimantan Tengah Province Health Profile
28_weeks	1991	1	Survey	Indonesia Demographic and Health Survey 1991
28_weeks	1997	1	Survey	Indonesia Demographic and Health Survey 1997
28_weeks	2002	1	Survey	Indonesia Demographic and Health Survey 2002−2003
28_weeks	2015	1	Statistical Report	Indonesia − Kalimantan Tengah Province Health Profile

Central Kalimantan IDN_4730 Southeast Asia Stillbirth Rate (SBR) − 20 weeks
lambda = 0.6 ; zeta = 0.8 ; scale = 15 ; amp = 0.028

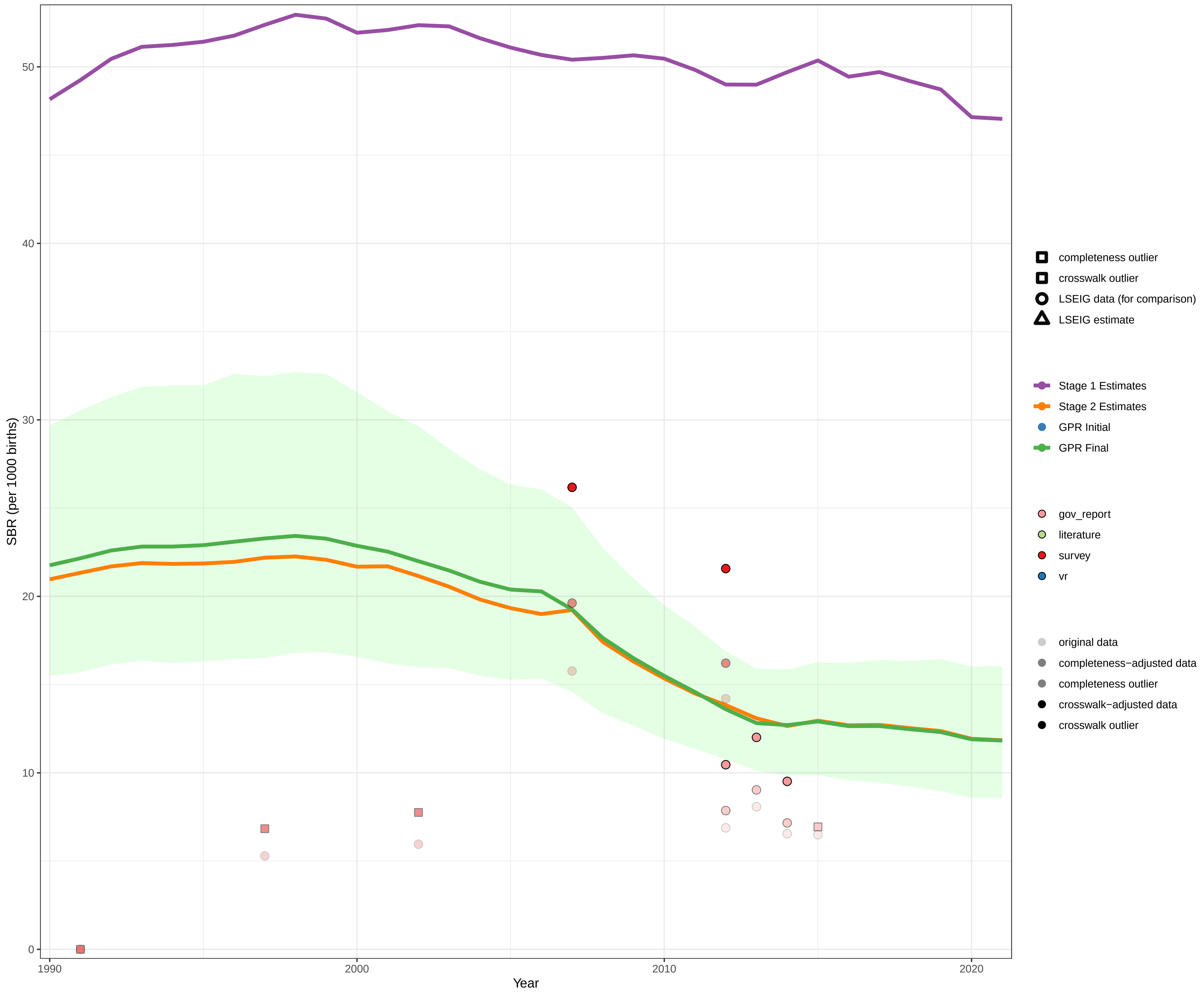

Central Kalimantan IDN_4730 Southeast Asia Stillbirth Rate (SBR) − 28 weeks
lambda = 0.6 ; zeta = 0.8 ; scale = 15 ; amp = 0.028

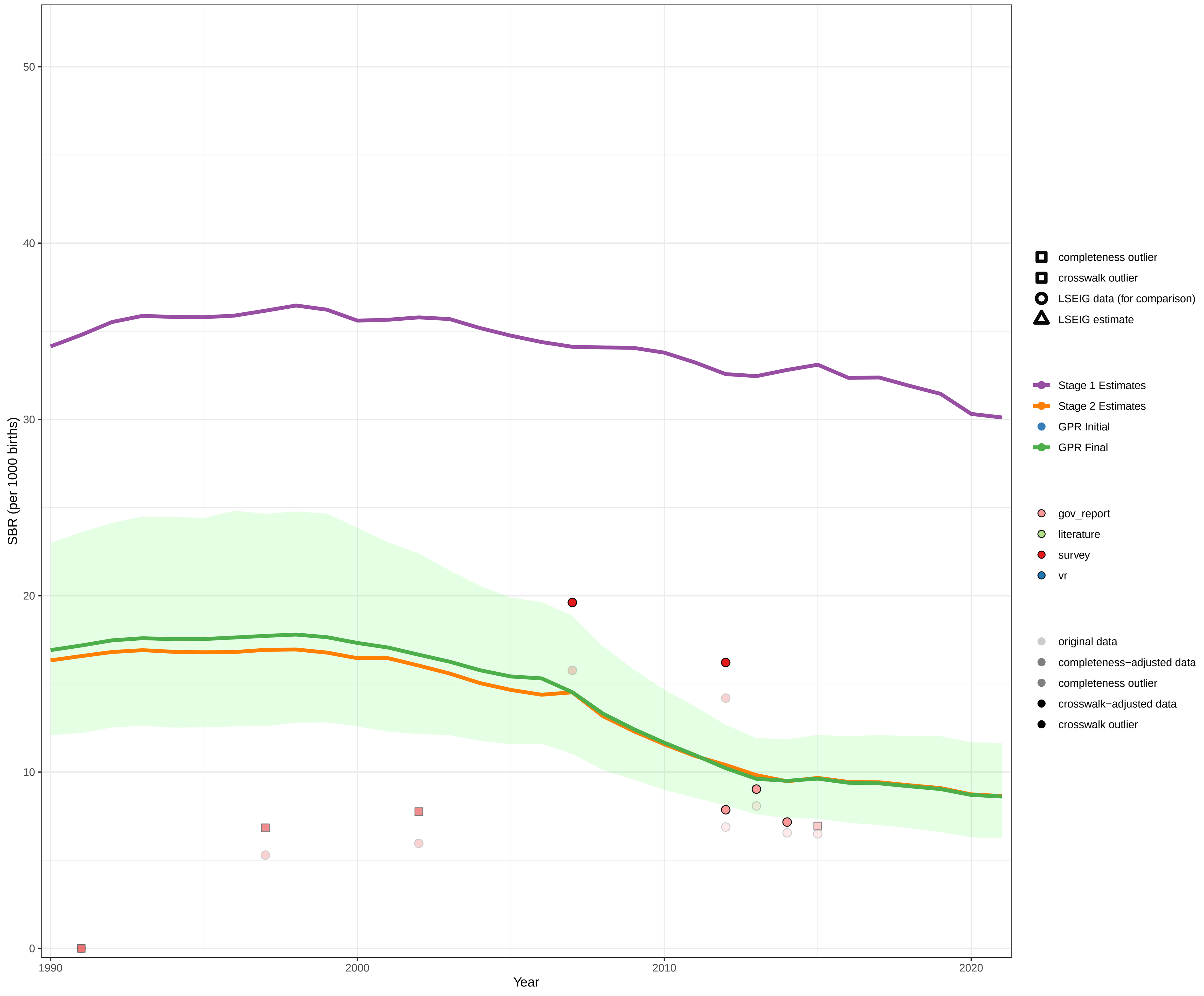

Year	Total Stillbirths (in thousands)	Total Stillbirths (in thousands)	SBR (per 1000 births)	SBR (per 1000 births)	SBR/NMR	SBR/NMR
	20 weeks	28 weeks	20 weeks	28 weeks	20 weeks	28 weeks
1990	0.8<br>(0.6 to 1.2)	0.6<br>(0.5 to 0.9)	21.8<br>(15.5 to 29.7)	16.9<br>(12.1 to 23.0)	1.29<br>(0.92 to 1.76)	1.00<br>(0.72 to 1.37)
1995	0.9<br>(0.7 to 1.3)	0.7<br>(0.5 to 1.0)	22.9<br>(16.3 to 32.0)	17.5<br>(12.5 to 24.4)	1.35<br>(0.96 to 1.89)	1.04<br>(0.74 to 1.44)
2000	1.0<br>(0.7 to 1.4)	0.8<br>(0.6 to 1.1)	22.9<br>(16.6 to 31.6)	17.3<br>(12.6 to 23.9)	1.40<br>(1.01 to 1.93)	1.06<br>(0.77 to 1.46)
2005	1.0<br>(0.7 to 1.3)	0.8<br>(0.6 to 1.0)	20.4<br>(15.3 to 26.3)	15.4<br>(11.6 to 19.9)	1.29<br>(0.96 to 1.66)	0.97<br>(0.73 to 1.26)
2010	0.8<br>(0.6 to 1.0)	0.6<br>(0.5 to 0.8)	15.5<br>(11.9 to 19.5)	11.7<br>(9.0 to 14.7)	1.04<br>(0.80 to 1.30)	0.78<br>(0.60 to 0.98)
2015	0.7<br>(0.5 to 0.8)	0.5<br>(0.4 to 0.6)	12.9<br>(9.9 to 16.3)	9.6<br>(7.4 to 12.1)	0.91<br>(0.70 to 1.15)	0.68<br>(0.52 to 0.86)
2020	0.6<br>(0.4 to 0.8)	0.4<br>(0.3 to 0.6)	11.9<br>(8.6 to 16.0)	8.7<br>(6.3 to 11.7)	0.96<br>(0.69 to 1.29)	0.70<br>(0.51 to 0.94)
2021	0.6<br>(0.4 to 0.8)	0.4<br>(0.3 to 0.6)	11.8<br>(8.6 to 16.1)	8.6<br>(6.3 to 11.7)	0.97<br>(0.70 to 1.31)	0.70<br>(0.51 to 0.95)

East Kalimantan IDN_4732 Southeast Asia Stillbirth Rate / Neonatal Mortality Rate (SBR/NMR) − 20 weeks
lambda = 0.4 ; zeta = 0.9 ; scale = 10 ; amp = 0.028

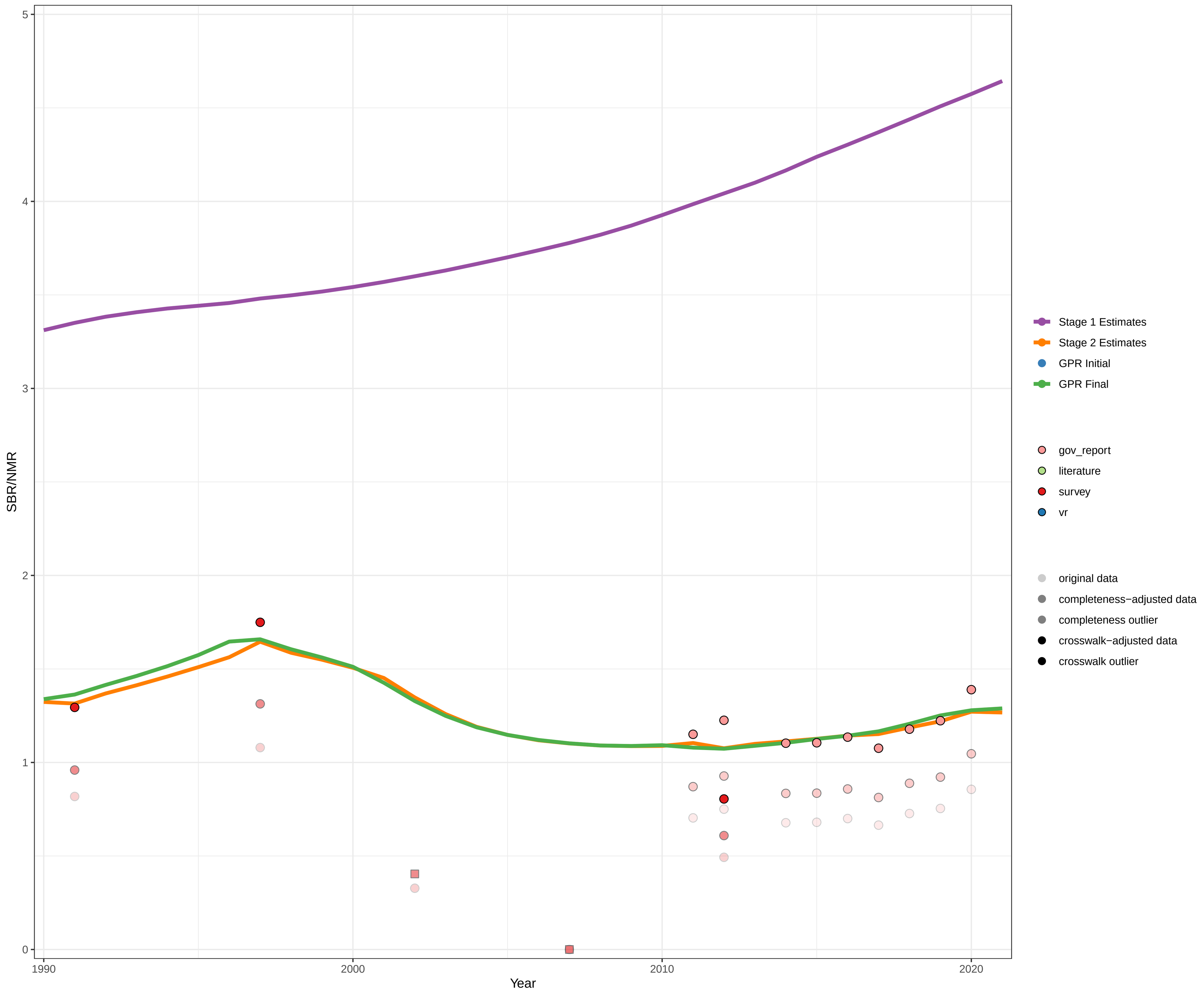

East Kalimantan IDN_4732 Southeast Asia Stillbirth Rate / Neonatal Mortality Rate (SBR/NMR) − 28 weeks
lambda = 0.4 ; zeta = 0.9 ; scale = 10 ; amp = 0.028

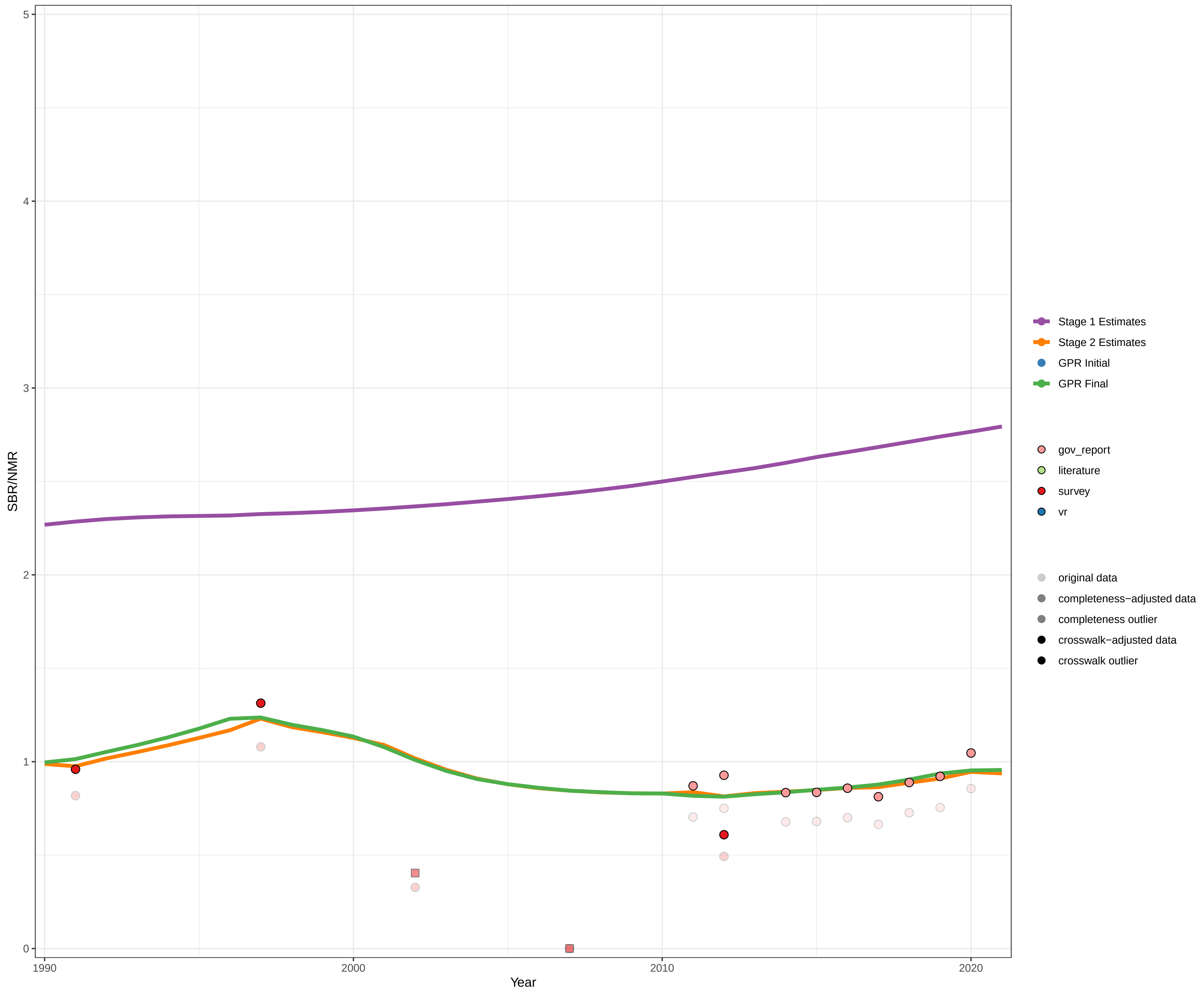

Definition	Years	Outlier	Source.Type	Title
28_weeks	1991	0	Survey	Indonesia Demographic and Health Survey 1991
28_weeks	1997	0	Survey	Indonesia Demographic and Health Survey 1997
28_weeks	2011, 2012, 2014−2020	0	Statistical Report	Indonesia − Kalimantan Timur Province Health Profile
28_weeks	2012	0	Survey	Indonesia Demographic and Health Survey 2012
28_weeks	2002	1	Survey	Indonesia Demographic and Health Survey 2002−2003
28_weeks	2007	1	Survey	Indonesia Demographic and Health Survey 2007

East Kalimantan IDN_4732 Southeast Asia Stillbirth Rate (SBR) − 20 weeks
lambda = 0.4 ; zeta = 0.9 ; scale = 10 ; amp = 0.028

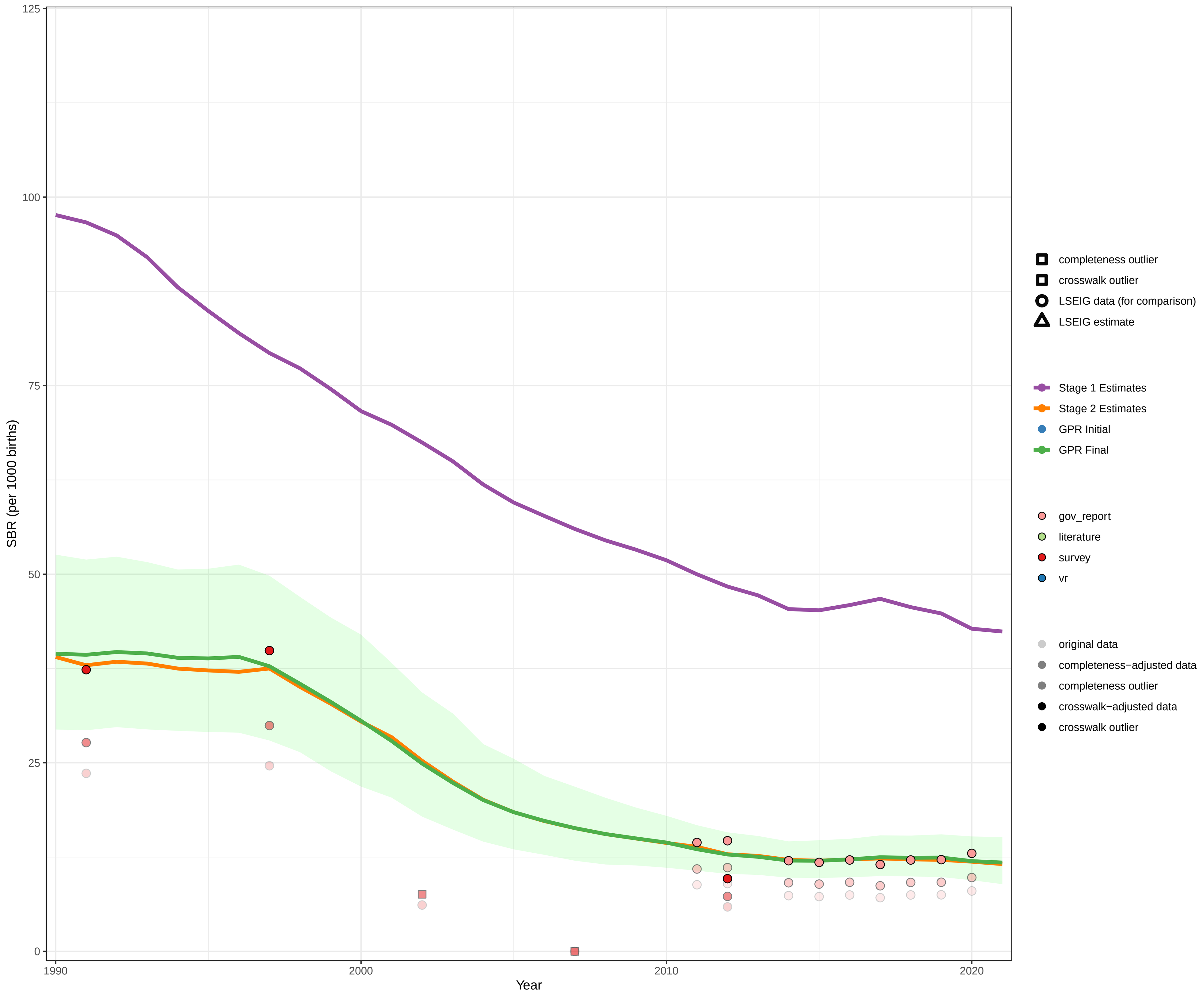

East Kalimantan IDN_4732 Southeast Asia Stillbirth Rate (SBR) − 28 weeks
lambda = 0.4 ; zeta = 0.9 ; scale = 10 ; amp = 0.028

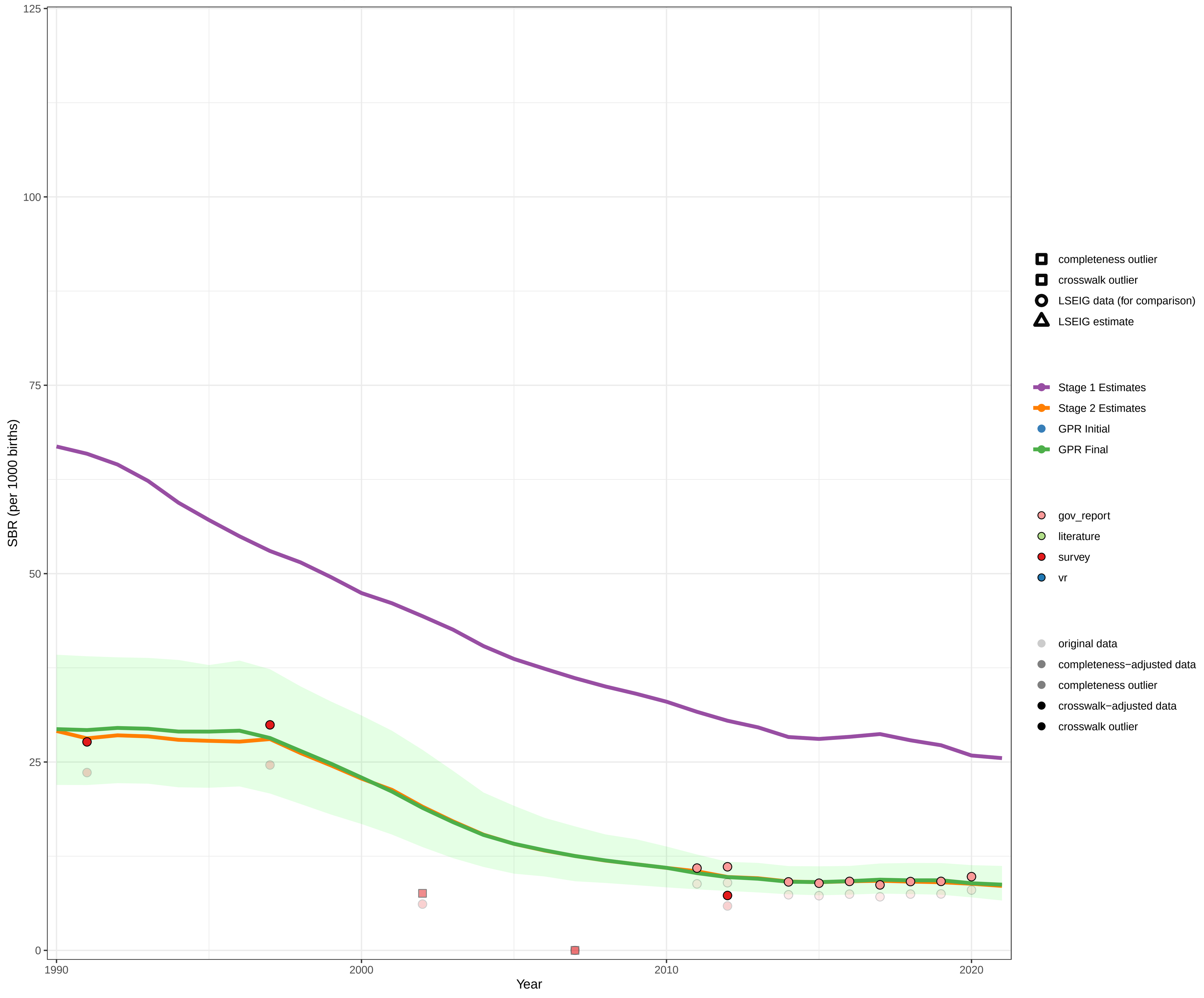

Year	Total Stillbirths (in thousands)	Total Stillbirths (in thousands)	SBR (per 1000 births)	SBR (per 1000 births)	SBR/NMR	SBR/NMR
	20 weeks	28 weeks	20 weeks	28 weeks	20 weeks	28 weeks
1990	1.8 (1.3 to 2.5)	1.3 (1.0 to 1.8)	39.5 (29.4 to 52.6)	29.4 (22.0 to 39.2)	1.34 (1.00 to 1.78)	1.00 (0.74 to 1.33)
1995	2.1 (1.5 to 2.7)	1.5 (1.1 to 2.0)	38.8 (29.1 to 50.7)	29.0 (21.6 to 37.9)	1.57 (1.18 to 2.06)	1.18 (0.87 to 1.54)
2000	1.8 (1.3 to 2.5)	1.4 (1.0 to 1.9)	30.6 (21.8 to 42.0)	22.9 (16.8 to 31.2)	1.51 (1.08 to 2.08)	1.13 (0.83 to 1.54)
2005	1.2 (0.9 to 1.7)	0.9 (0.7 to 1.3)	18.4 (13.5 to 25.5)	14.1 (10.2 to 19.2)	1.15 (0.84 to 1.59)	0.88 (0.63 to 1.19)
2010	1.0 (0.8 to 1.3)	0.8 (0.6 to 1.0)	14.4 (11.1 to 18.0)	11.0 (8.4 to 13.8)	1.09 (0.84 to 1.36)	0.83 (0.63 to 1.04)
2015	0.8 (0.7 to 1.0)	0.6 (0.5 to 0.7)	12.0 (9.7 to 14.7)	9.1 (7.3 to 11.2)	1.13 (0.91 to 1.38)	0.85 (0.69 to 1.05)
2020	0.8 (0.6 to 1.0)	0.6 (0.5 to 0.8)	12.0 (9.4 to 15.2)	8.9 (7.0 to 11.3)	1.28 (1.01 to 1.63)	0.95 (0.75 to 1.21)
2021	0.8 (0.6 to 1.0)	0.6 (0.4 to 0.7)	11.8 (8.9 to 15.2)	8.7 (6.6 to 11.2)	1.29 (0.98 to 1.66)	0.96 (0.72 to 1.23)

North Kalimantan   IDN_4719   Southeast Asia   Stillbirth Rate / Neonatal Mortality Rate (SBR/NMR) − 20 weeks
lambda = 0.8 ; zeta = 0.7 ; scale = 15 ; amp = 0.028

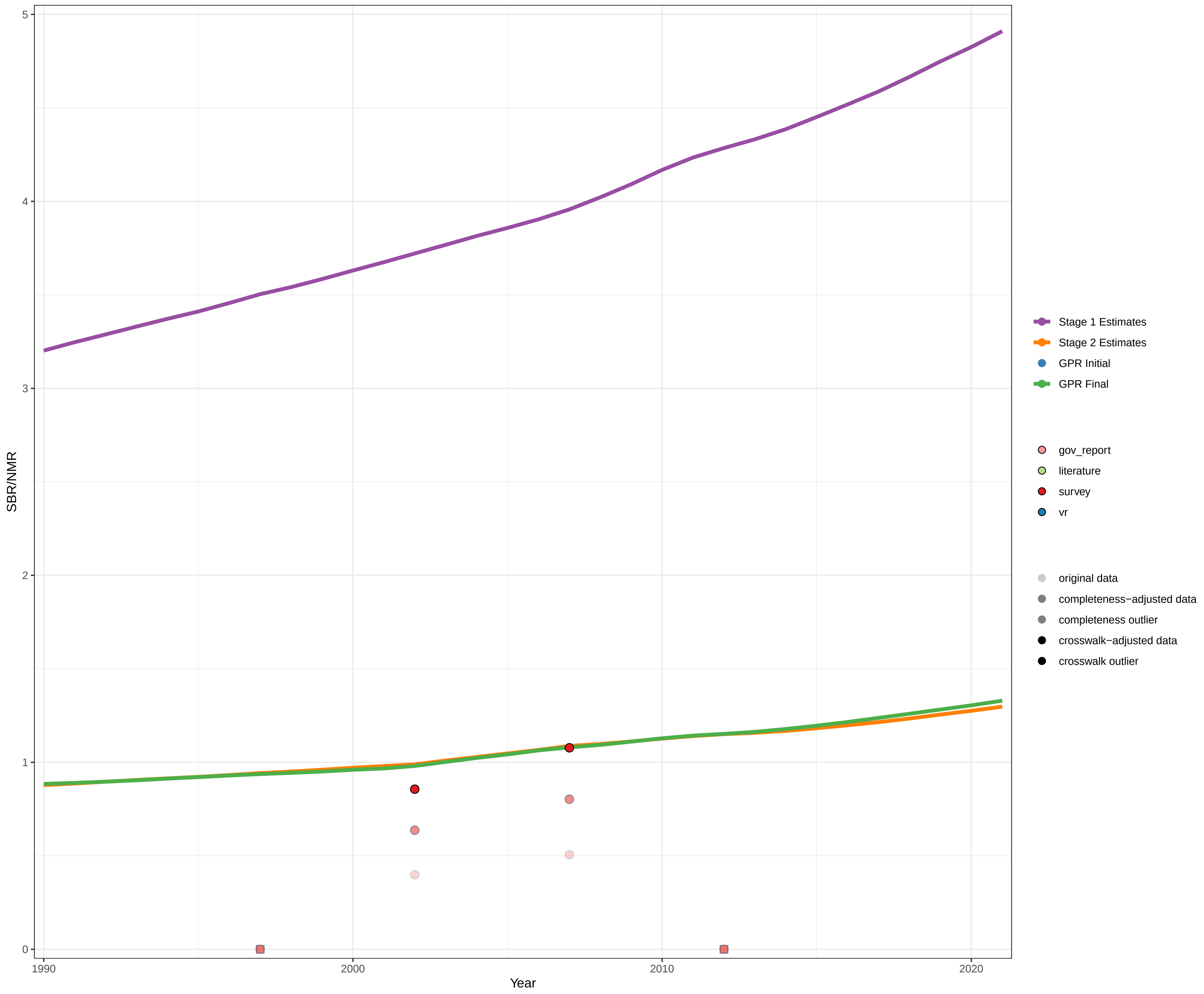

North Kalimantan   IDN_4719   Southeast Asia   Stillbirth Rate / Neonatal Mortality Rate (SBR/NMR) − 28 weeks
lambda = 0.8 ; zeta = 0.7 ; scale = 15 ; amp = 0.028

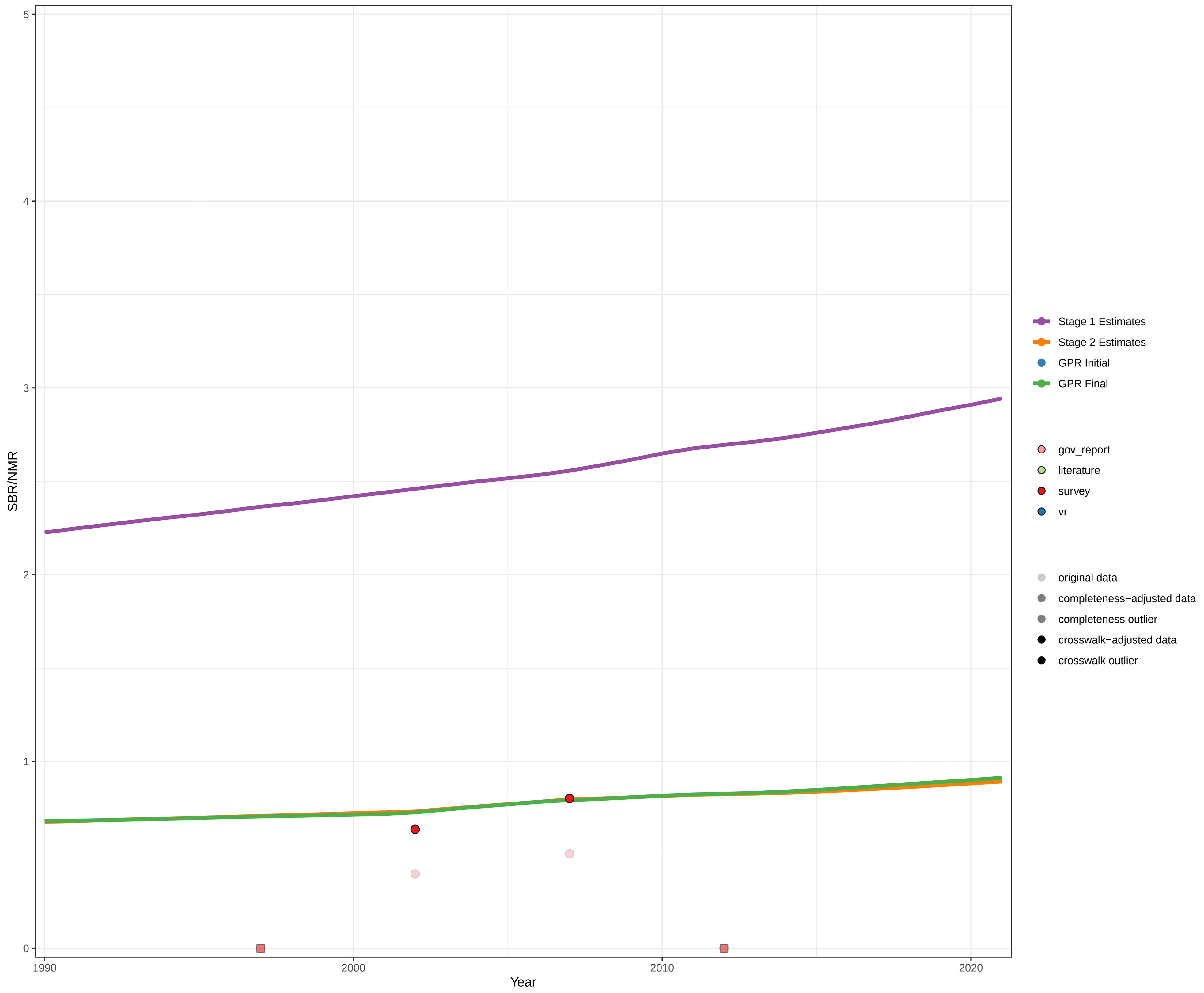

Definition	Years	Outlier	Source.Type	Title
28_weeks	2002	0	Survey	Indonesia Demographic and Health Survey 2002−2003
28_weeks	2007	0	Survey	Indonesia Demographic and Health Survey 2007
28_weeks	1997	1	Survey	Indonesia Demographic and Health Survey 1997
28_weeks	2012	1	Survey	Indonesia Demographic and Health Survey 2012

North Kalimantan   IDN_4719   Southeast Asia   Stillbirth Rate (SBR) − 20 weeks
lambda = 0.8 ; zeta = 0.7 ; scale = 15 ; amp = 0.028

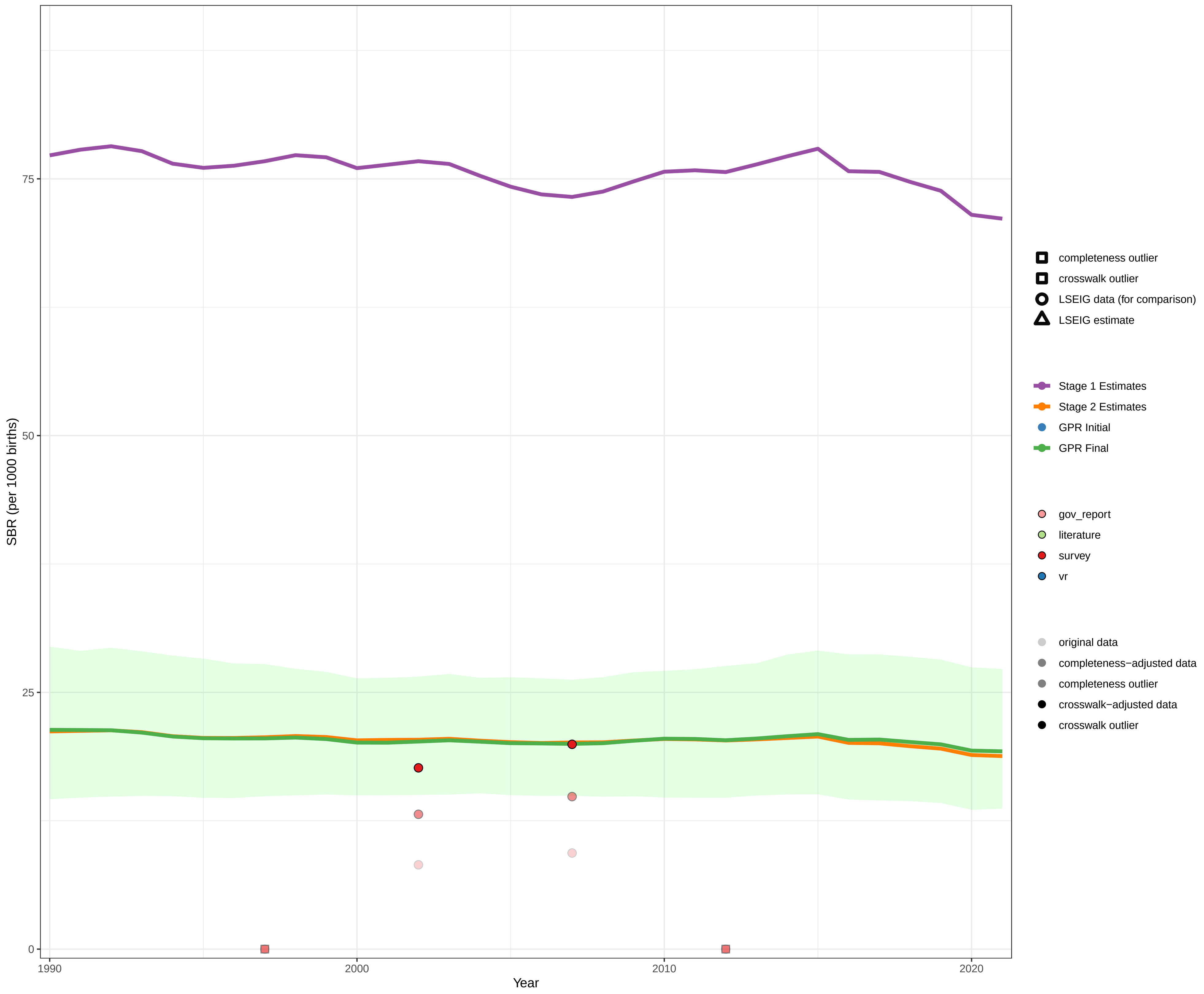

North Kalimantan   IDN_4719   Southeast Asia   Stillbirth Rate (SBR) − 28 weeks
lambda = 0.8 ; zeta = 0.7 ; scale = 15 ; amp = 0.028

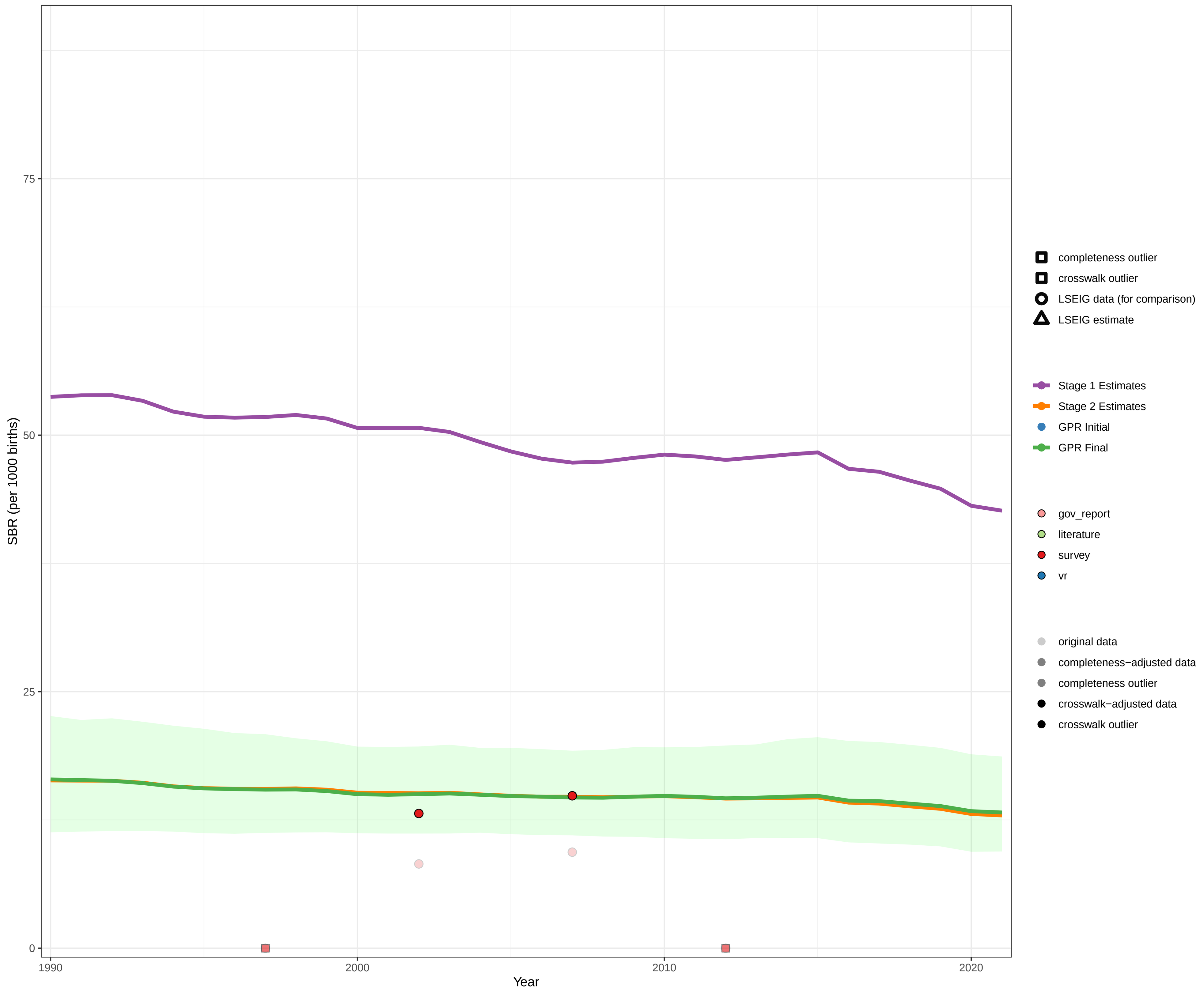

Year	Total Stillbirths (in thousands)	Total Stillbirths (in thousands)	SBR (per 1000 births)	SBR (per 1000 births)	SBR/NMR	SBR/NMR
	20 weeks	28 weeks	20 weeks	28 weeks	20 weeks	28 weeks
1990	0.2 (0.1 to 0.2)	0.1 (0.1 to 0.2)	21.3 (14.6 to 29.4)	16.4 (11.3 to 22.6)	0.88 (0.60 to 1.22)	0.68 (0.47 to 0.94)
1995	0.2 (0.1 to 0.2)	0.1 (0.1 to 0.2)	20.5 (14.7 to 28.3)	15.6 (11.2 to 21.4)	0.92 (0.66 to 1.27)	0.70 (0.50 to 0.96)
2000	0.2 (0.1 to 0.3)	0.1 (0.1 to 0.2)	20.1 (15.0 to 26.4)	15.0 (11.2 to 19.6)	0.96 (0.71 to 1.26)	0.72 (0.53 to 0.94)
2005	0.2 (0.2 to 0.3)	0.2 (0.1 to 0.2)	20.1 (15.0 to 26.5)	14.8 (11.1 to 19.5)	1.04 (0.78 to 1.38)	0.77 (0.58 to 1.01)
2010	0.3 (0.2 to 0.3)	0.2 (0.1 to 0.2)	20.5 (14.8 to 27.1)	14.8 (10.7 to 19.6)	1.13 (0.81 to 1.49)	0.82 (0.59 to 1.08)
2015	0.3 (0.2 to 0.4)	0.2 (0.1 to 0.3)	20.9 (15.1 to 29.1)	14.8 (10.7 to 20.6)	1.20 (0.86 to 1.66)	0.85 (0.61 to 1.17)
2020	0.3 (0.2 to 0.4)	0.2 (0.1 to 0.3)	19.3 (13.6 to 27.4)	13.4 (9.4 to 18.9)	1.30 (0.92 to 1.85)	0.90 (0.63 to 1.28)
2021	0.3 (0.2 to 0.4)	0.2 (0.1 to 0.3)	19.3 (13.7 to 27.3)	13.2 (9.4 to 18.7)	1.33 (0.94 to 1.88)	0.91 (0.65 to 1.29)

Riau Islands   IDN_4718   Southeast Asia   Stillbirth Rate / Neonatal Mortality Rate (SBR/NMR) − 20 weeks
lambda = 0.4 ; zeta = 0.9 ; scale = 10 ; amp = 0.028

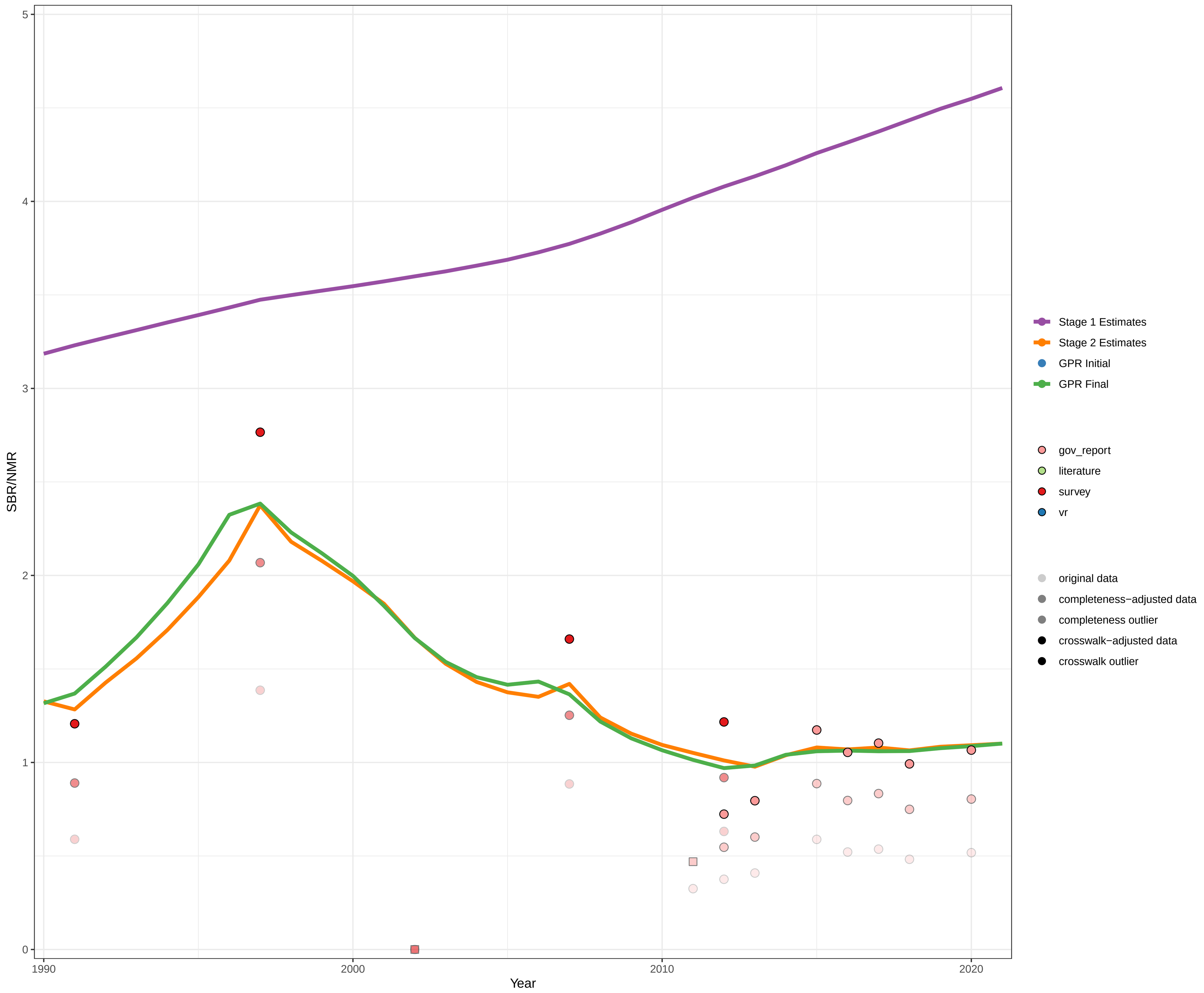

Riau Islands   IDN_4718   Southeast Asia   Stillbirth Rate / Neonatal Mortality Rate (SBR/NMR) − 28 weeks
lambda = 0.4 ; zeta = 0.9 ; scale = 10 ; amp = 0.028

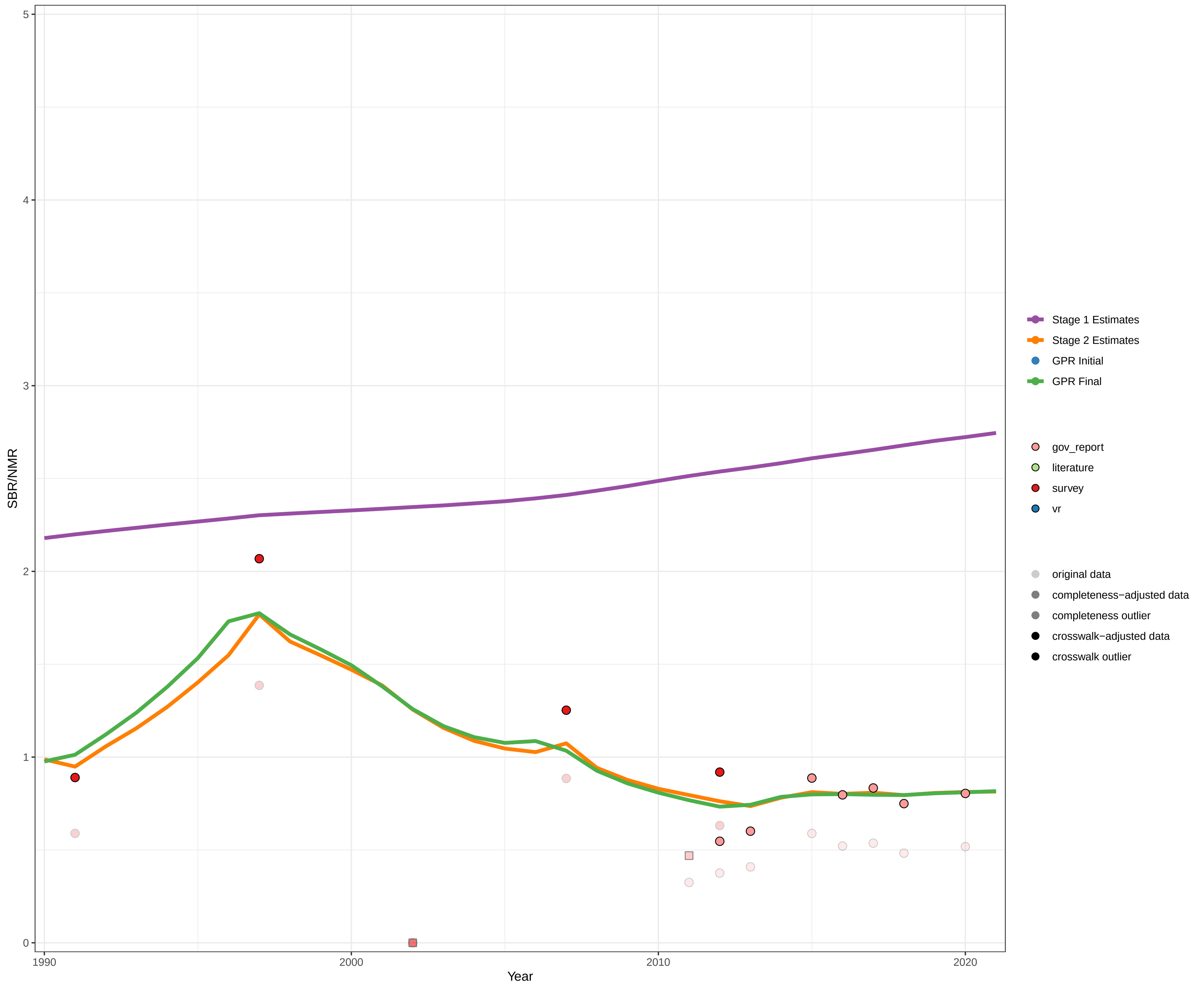

Definition	Years	Outlier	Source.Type	Title
28_weeks	1991	0	Survey	Indonesia Demographic and Health Survey 1991
28_weeks	1997	0	Survey	Indonesia Demographic and Health Survey 1997
28_weeks	2007	0	Survey	Indonesia Demographic and Health Survey 2007
28_weeks	2012	0	Survey	Indonesia Demographic and Health Survey 2012
28_weeks	2012, 2013, 2015−2018, 2020	0	Statistical Report	Indonesia − Kepulauan Riau Province Health Profile
28_weeks	2002	1	Survey	Indonesia Demographic and Health Survey 2002−2003
28_weeks	2011	1	Statistical Report	Indonesia − Kepulauan Riau Province Health Profile

Riau Islands   IDN_4718   Southeast Asia   Stillbirth Rate (SBR) − 20 weeks
lambda = 0.4 ; zeta = 0.9 ; scale = 10 ; amp = 0.028

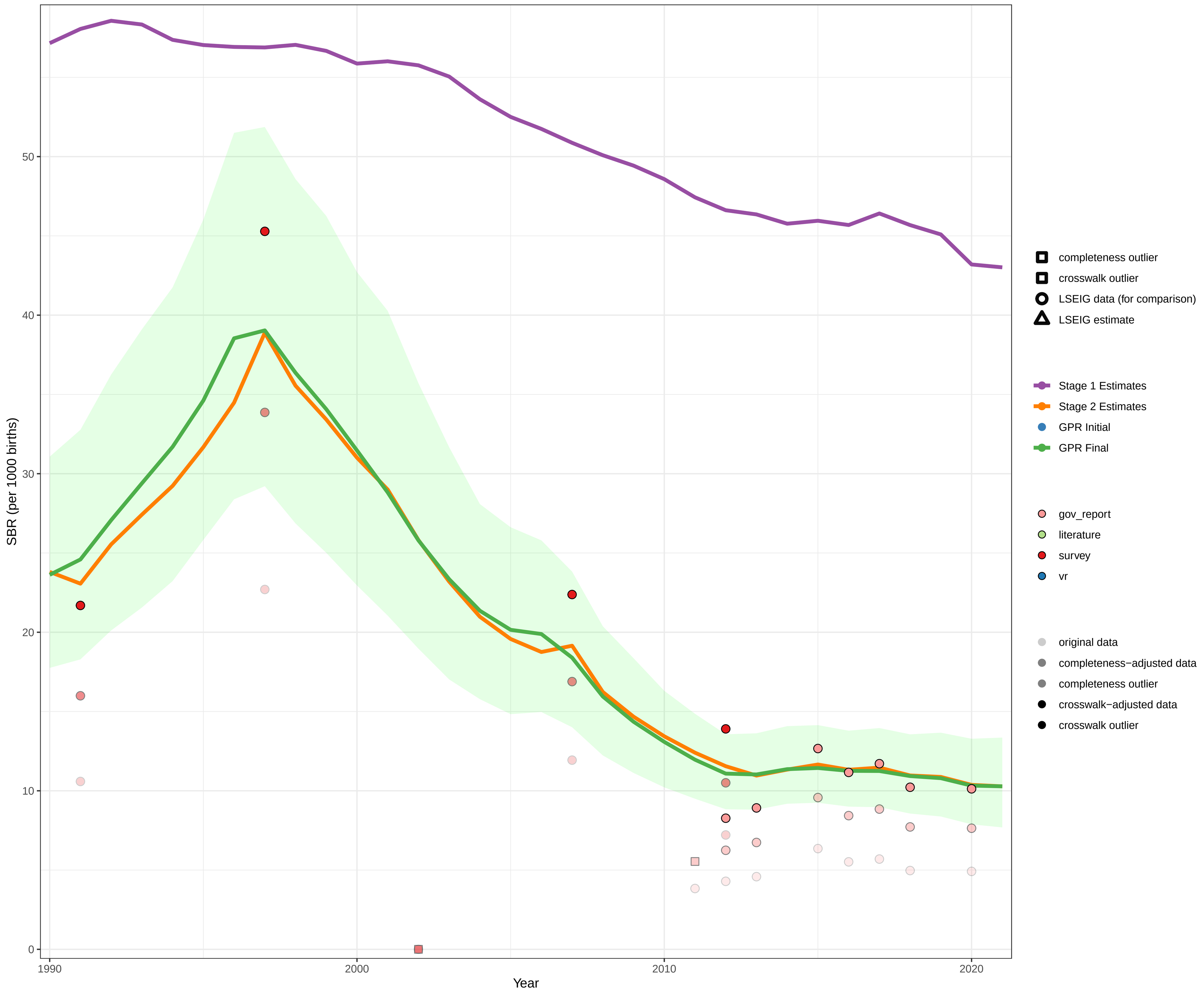

Riau Islands   IDN_4718   Southeast Asia   Stillbirth Rate (SBR) − 28 weeks
lambda = 0.4 ; zeta = 0.9 ; scale = 10 ; amp = 0.028

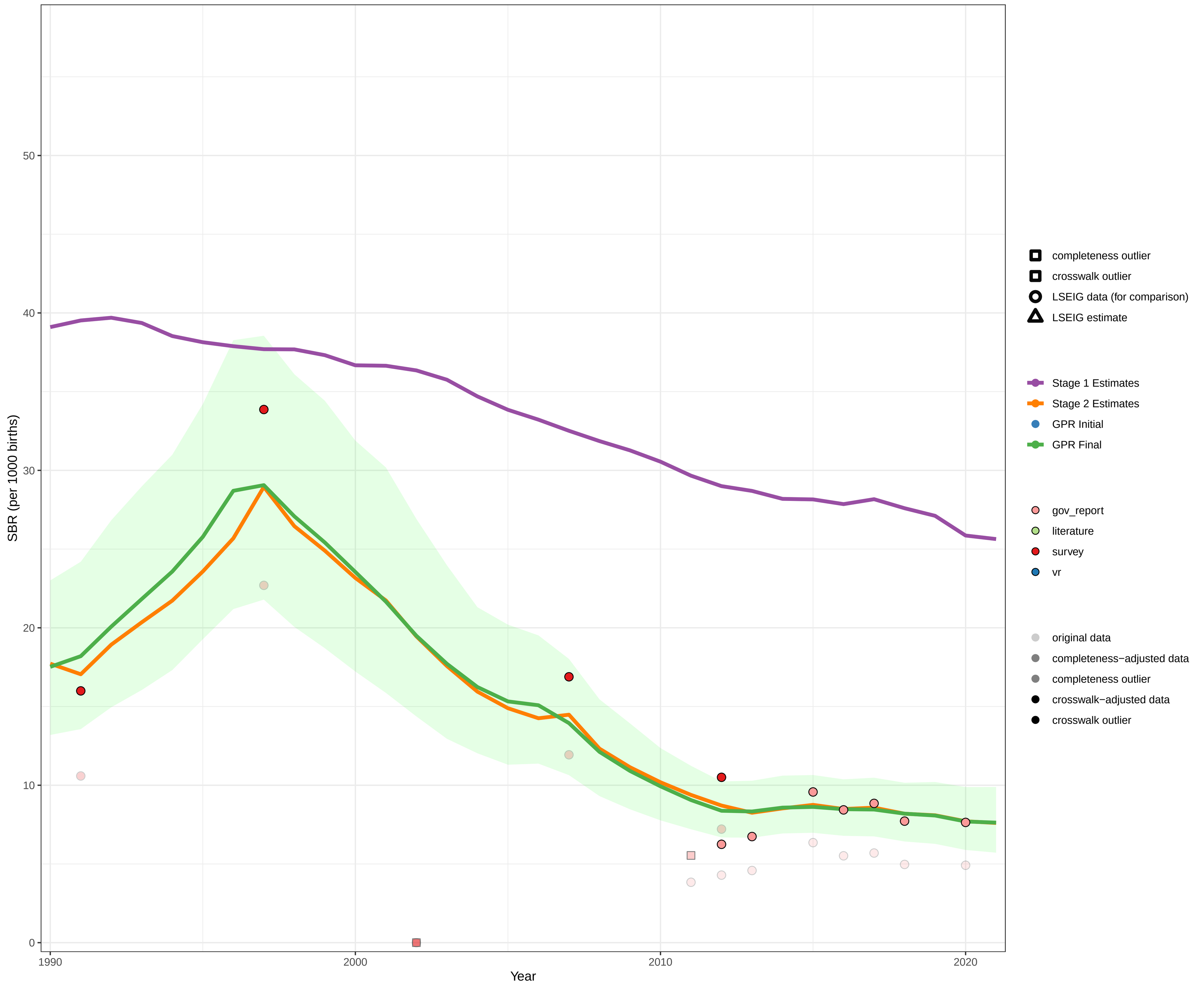

Year	Total Stillbirths (in thousands)	Total Stillbirths (in thousands)	SBR (per 1000 births)	SBR (per 1000 births)	SBR/NMR	SBR/NMR
	20 weeks	28 weeks	20 weeks	28 weeks	20 weeks	28 weeks
1990	0.6 (0.5 to 0.8)	0.5 (0.4 to 0.6)	23.6 (17.8 to 31.1)	17.5 (13.2 to 23.0)	1.32 (0.99 to 1.73)	0.98 (0.74 to 1.28)
1995	1.1 (0.8 to 1.4)	0.8 (0.6 to 1.1)	34.6 (25.8 to 46.0)	25.8 (19.3 to 34.2)	2.06 (1.54 to 2.74)	1.53 (1.15 to 2.03)
2000	1.1 (0.8 to 1.5)	0.8 (0.6 to 1.1)	31.5 (23.0 to 42.7)	23.5 (17.2 to 31.9)	2.00 (1.46 to 2.71)	1.49 (1.09 to 2.02)
2005	0.8 (0.6 to 1.1)	0.6 (0.5 to 0.8)	20.2 (14.8 to 26.6)	15.3 (11.3 to 20.2)	1.42 (1.04 to 1.87)	1.08 (0.79 to 1.42)
2010	0.6 (0.4 to 0.7)	0.4 (0.3 to 0.5)	13.1 (10.2 to 16.3)	9.9 (7.8 to 12.4)	1.07 (0.83 to 1.33)	0.81 (0.63 to 1.01)
2015	0.5 (0.4 to 0.6)	0.4 (0.3 to 0.4)	11.4 (9.2 to 14.1)	8.6 (7.0 to 10.6)	1.06 (0.86 to 1.31)	0.80 (0.65 to 0.99)
2020	0.4 (0.3 to 0.5)	0.3 (0.2 to 0.4)	10.3 (7.9 to 13.3)	7.7 (5.9 to 9.9)	1.09 (0.83 to 1.40)	0.81 (0.62 to 1.04)
2021	0.4 (0.3 to 0.5)	0.3 (0.2 to 0.4)	10.3 (7.7 to 13.4)	7.6 (5.7 to 9.9)	1.10 (0.82 to 1.43)	0.82 (0.61 to 1.06)

Lampung IDN_4716 Southeast Asia Stillbirth Rate / Neonatal Mortality Rate (SBR/NMR) − 20 weeks
lambda = 0.8 ; zeta = 0.7 ; scale = 15 ; amp = 0.028

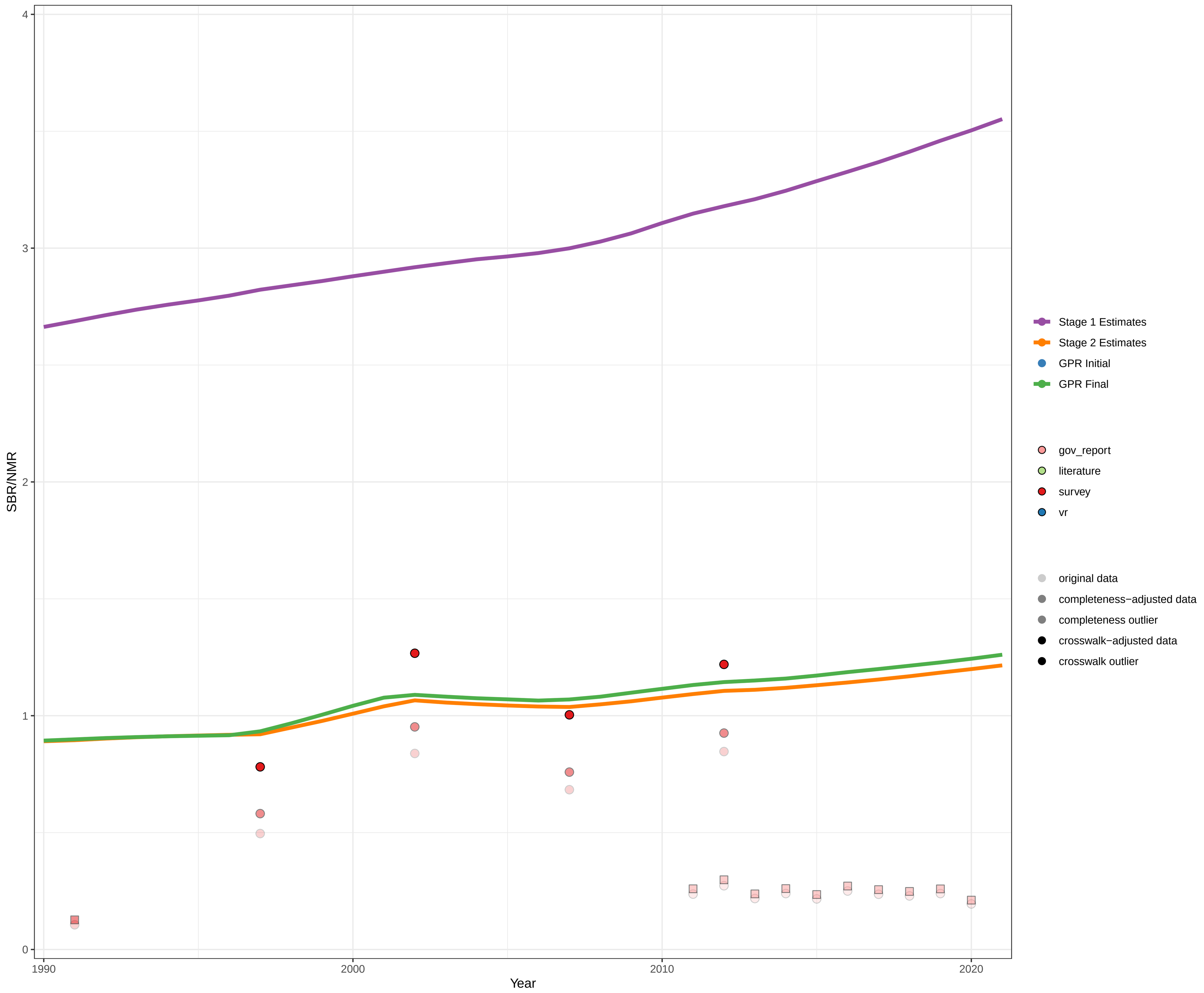

Lampung IDN_4716 Southeast Asia Stillbirth Rate / Neonatal Mortality Rate (SBR/NMR) − 28 weeks
lambda = 0.8 ; zeta = 0.7 ; scale = 15 ; amp = 0.028

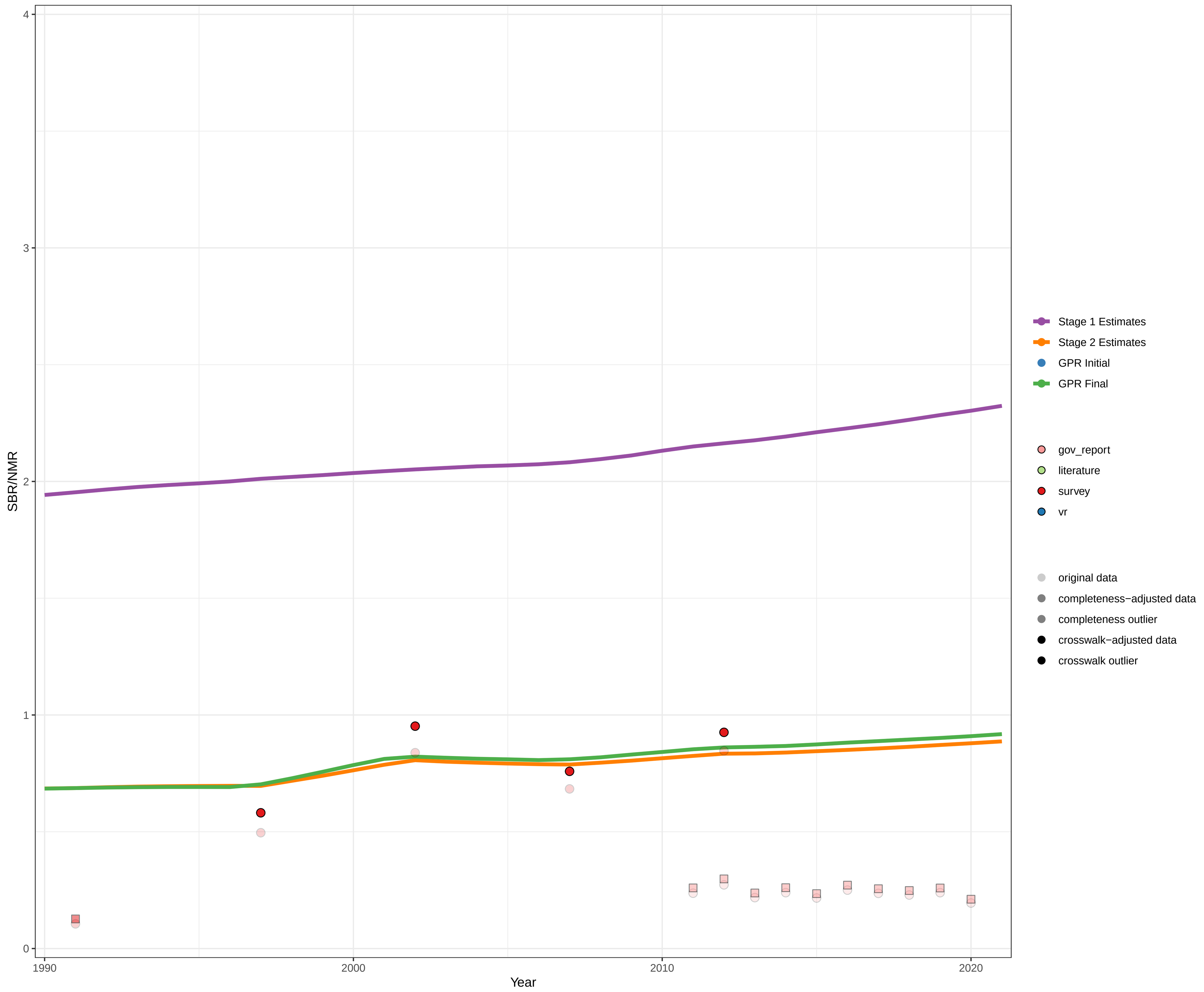

Definition	Years	Outlier	Source.Type	Title
28_weeks	1997	0	Survey	Indonesia Demographic and Health Survey 1997
28_weeks	2002	0	Survey	Indonesia Demographic and Health Survey 2002−2003
28_weeks	2007	0	Survey	Indonesia Demographic and Health Survey 2007
28_weeks	2012	0	Survey	Indonesia Demographic and Health Survey 2012
28_weeks	1991	1	Survey	Indonesia Demographic and Health Survey 1991
28_weeks	2011−2020	1	Statistical Report	Indonesia − Lampung Province Health Profile

Lampung IDN_4716 Southeast Asia Stillbirth Rate (SBR) − 20 weeks
lambda = 0.8 ; zeta = 0.7 ; scale = 15 ; amp = 0.028

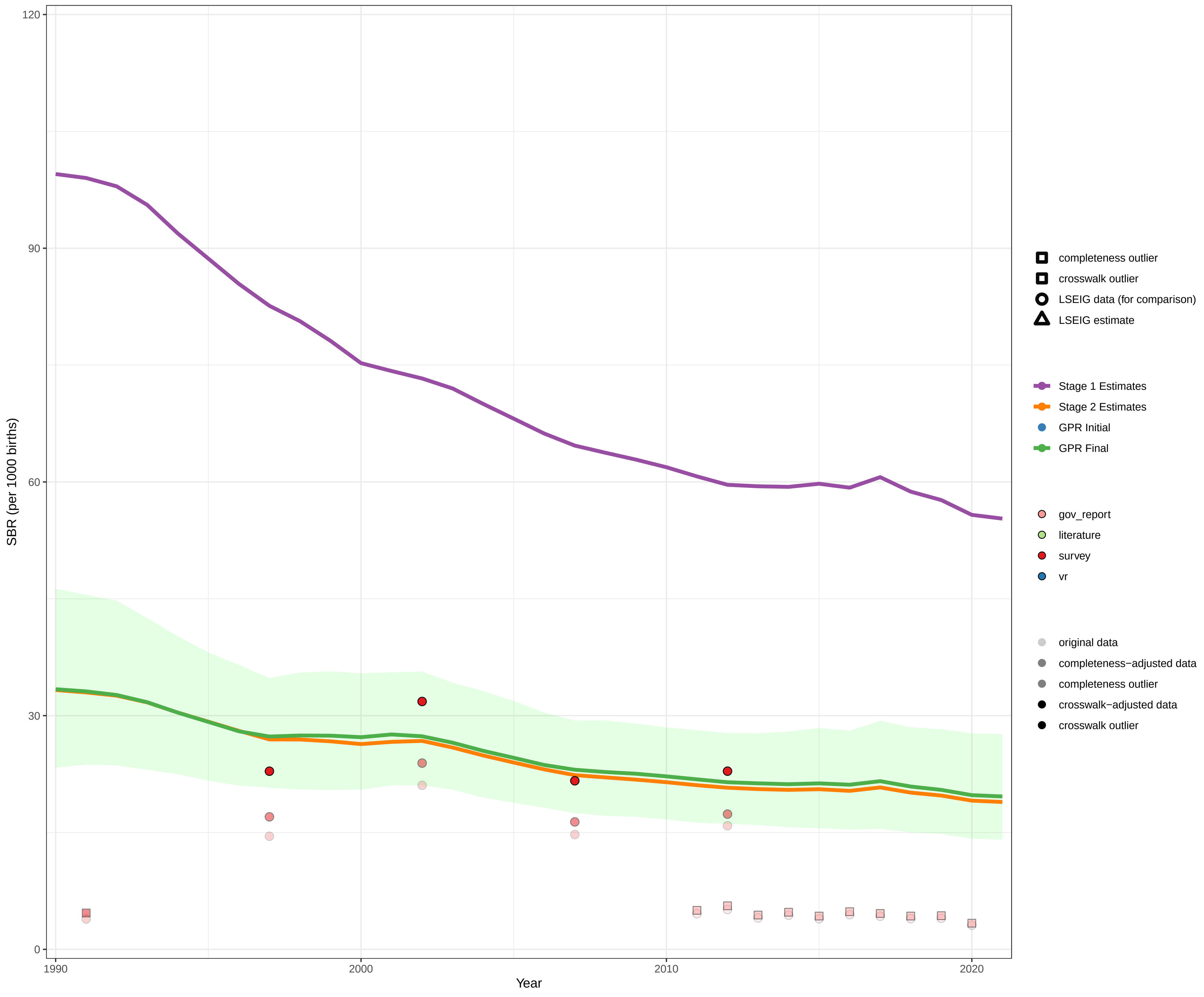

Lampung IDN_4716 Southeast Asia Stillbirth Rate (SBR) − 28 weeks
lambda = 0.8 ; zeta = 0.7 ; scale = 15 ; amp = 0.028

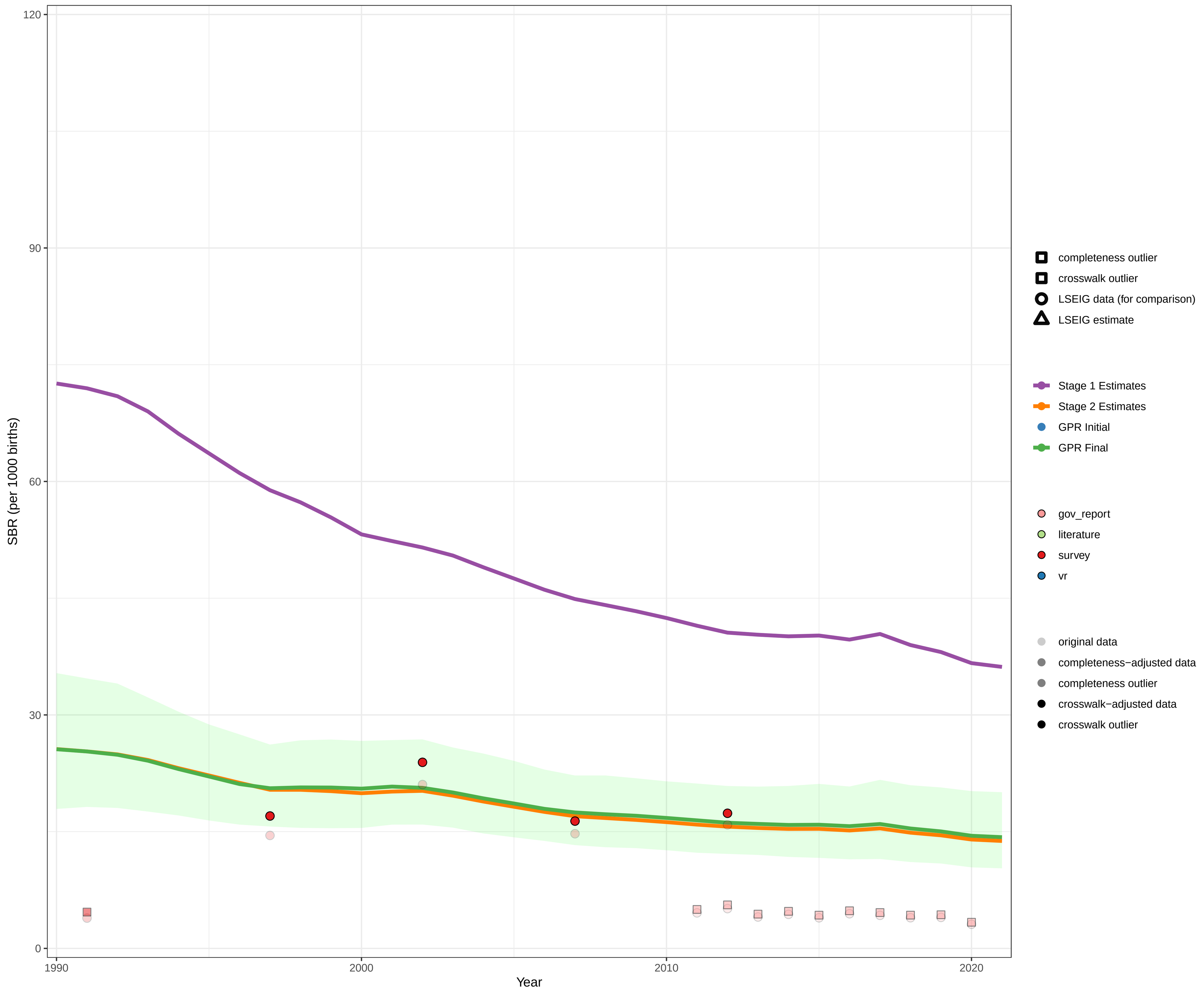

Year	Total Stillbirths (in thousands)	Total Stillbirths (in thousands)	SBR (per 1000 births)	SBR (per 1000 births)	SBR/NMR	SBR/NMR
	20 weeks	28 weeks	20 weeks	28 weeks	20 weeks	28 weeks
1990	5.8 (4.0 to 8.1)	4.4 (3.0 to 6.1)	33.4 (23.3 to 46.3)	25.6 (17.9 to 35.4)	0.89 (0.62 to 1.24)	0.68 (0.48 to 0.95)
1995	4.9 (3.6 to 6.4)	3.7 (2.7 to 4.8)	29.2 (21.7 to 38.1)	22.1 (16.4 to 28.8)	0.91 (0.68 to 1.19)	0.69 (0.51 to 0.90)
2000	4.5 (3.3 to 5.9)	3.3 (2.5 to 4.4)	27.2 (20.5 to 35.4)	20.5 (15.5 to 26.7)	1.04 (0.78 to 1.36)	0.79 (0.59 to 1.02)
2005	4.1 (3.1 to 5.3)	3.0 (2.3 to 4.0)	24.6 (18.8 to 31.9)	18.6 (14.3 to 24.1)	1.07 (0.82 to 1.39)	0.81 (0.62 to 1.05)
2010	3.6 (2.7 to 4.7)	2.7 (2.0 to 3.5)	22.2 (16.7 to 28.5)	16.8 (12.6 to 21.5)	1.12 (0.84 to 1.43)	0.84 (0.63 to 1.08)
2015	3.4 (2.5 to 4.6)	2.5 (1.8 to 3.4)	21.3 (15.6 to 28.4)	15.9 (11.6 to 21.1)	1.17 (0.86 to 1.56)	0.87 (0.64 to 1.16)
2020	3.1 (2.2 to 4.4)	2.3 (1.6 to 3.2)	19.8 (14.2 to 27.7)	14.5 (10.4 to 20.2)	1.24 (0.89 to 1.74)	0.91 (0.65 to 1.27)
2021	3.1 (2.2 to 4.4)	2.2 (1.6 to 3.2)	19.6 (14.1 to 27.6)	14.3 (10.3 to 20.1)	1.26 (0.90 to 1.78)	0.92 (0.66 to 1.29)

Maluku IDN_4739 Southeast Asia Stillbirth Rate / Neonatal Mortality Rate (SBR/NMR) − 20 weeks
lambda = 0.6 ; zeta = 0.8 ; scale = 15 ; amp = 0.028

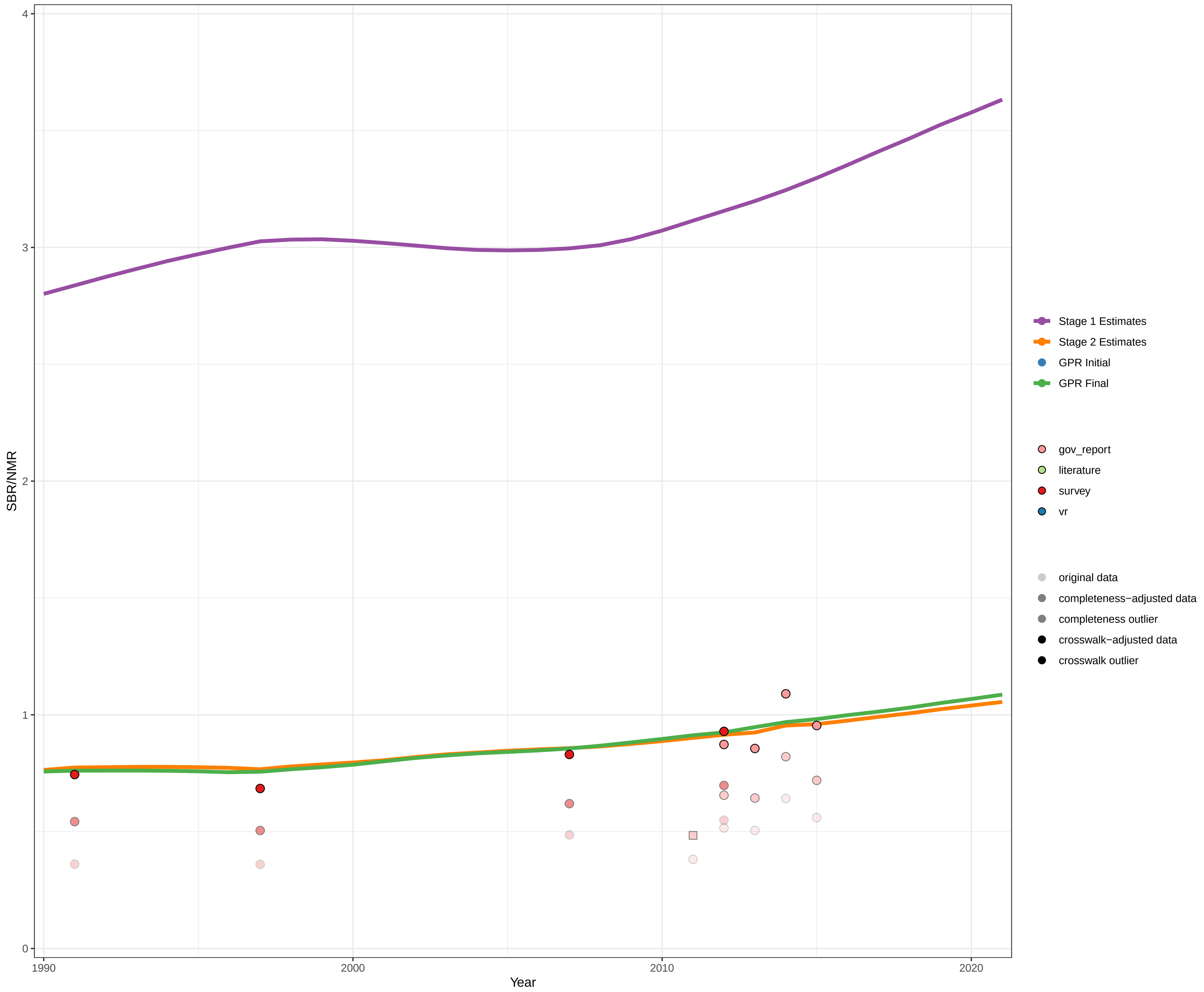

Maluku IDN_4739 Southeast Asia Stillbirth Rate / Neonatal Mortality Rate (SBR/NMR) − 28 weeks
lambda = 0.6 ; zeta = 0.8 ; scale = 15 ; amp = 0.028

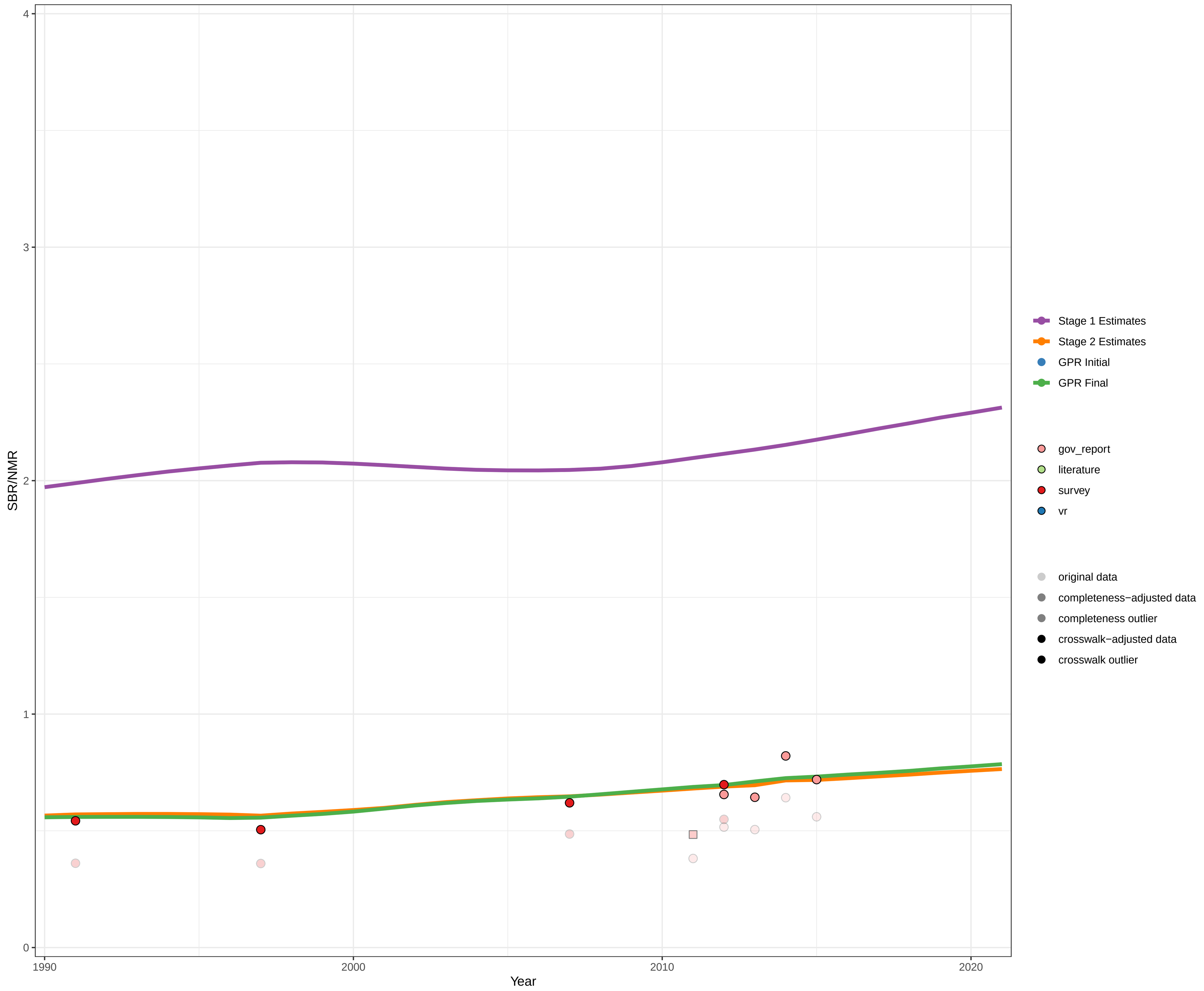

Definition	Years	Outlier	Source.Type	Title
28_weeks	1991	0	Survey	Indonesia Demographic and Health Survey 1991
28_weeks	1997	0	Survey	Indonesia Demographic and Health Survey 1997
28_weeks	2007	0	Survey	Indonesia Demographic and Health Survey 2007
28_weeks	2012	0	Survey	Indonesia Demographic and Health Survey 2012
28_weeks	2012−2015	0	Statistical Report	Indonesia − Maluku Province Health Profile
28_weeks	2011	1	Statistical Report	Indonesia − Maluku Province Health Profile

Maluku IDN_4739 Southeast Asia Stillbirth Rate (SBR) − 20 weeks
lambda = 0.6 ; zeta = 0.8 ; scale = 15 ; amp = 0.028

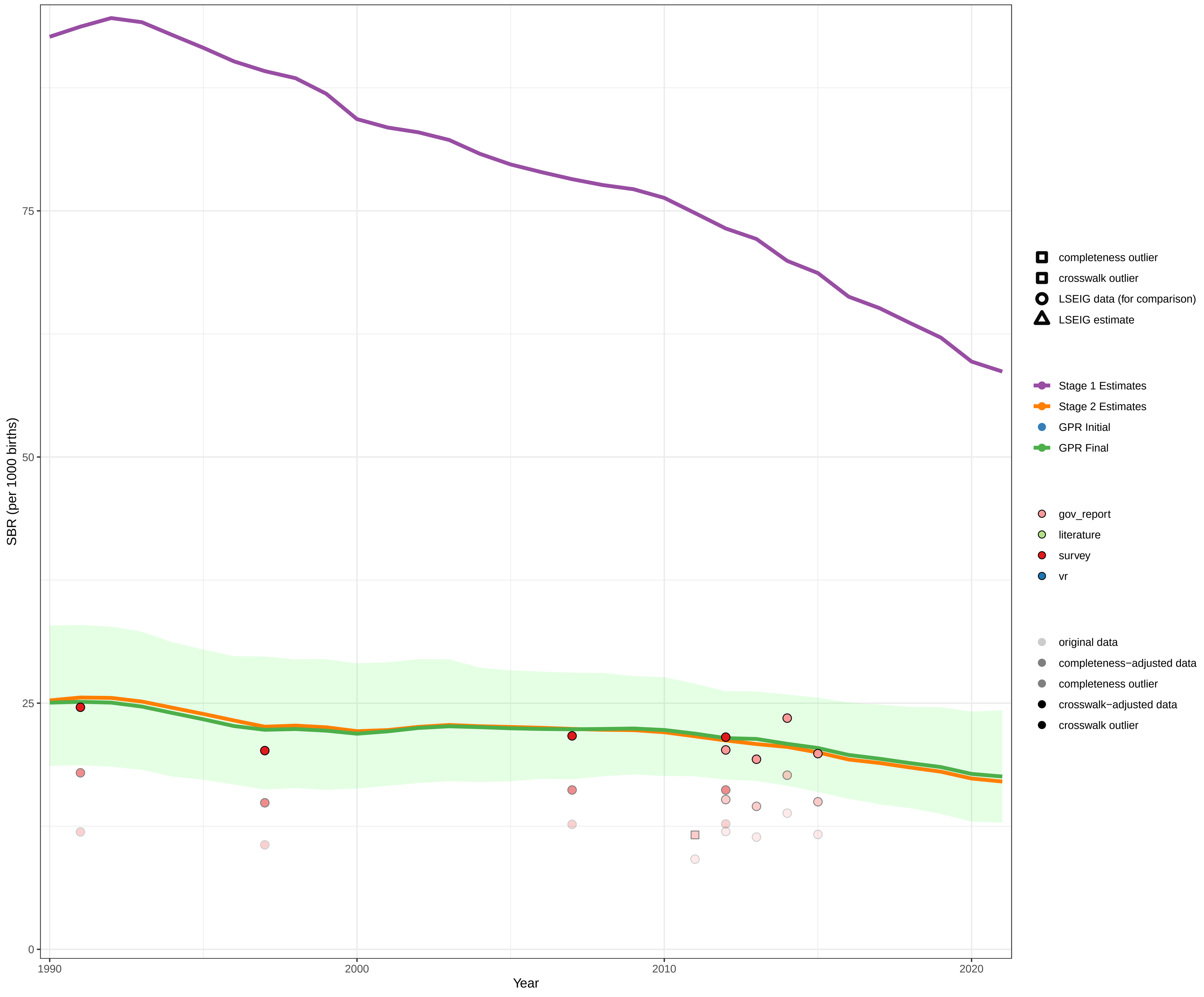

Maluku IDN_4739 Southeast Asia Stillbirth Rate (SBR) − 28 weeks
lambda = 0.6 ; zeta = 0.8 ; scale = 15 ; amp = 0.028

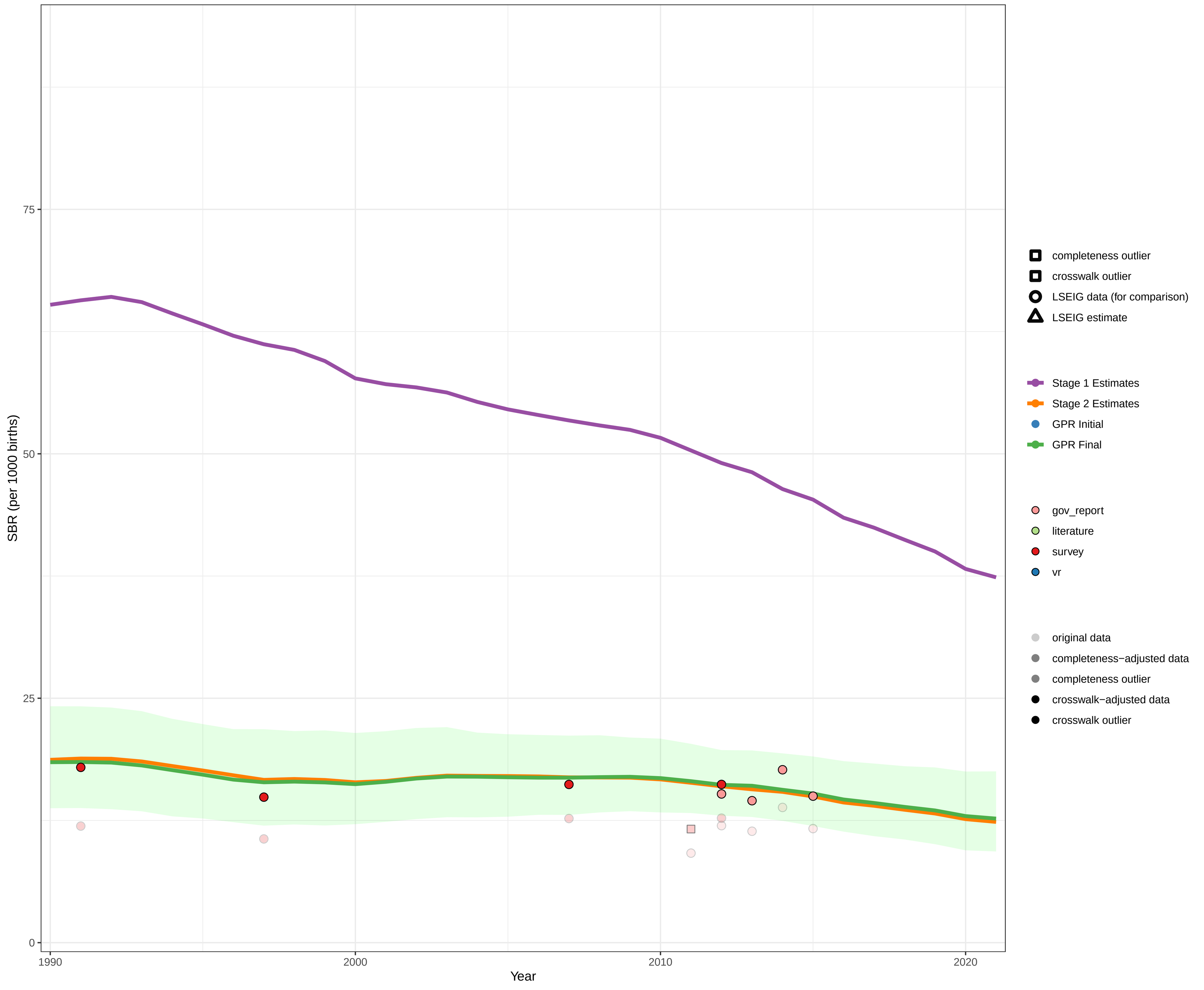

Year	Total Stillbirths (in thousands)	Total Stillbirths (in thousands)	SBR (per 1000 births)	SBR (per 1000 births)	SBR/NMR	SBR/NMR
	20 weeks	28 weeks	20 weeks	28 weeks	20 weeks	28 weeks
1990	1.0 (0.7 to 1.3)	0.7 (0.5 to 1.0)	25.1 (18.6 to 32.9)	18.5 (13.8 to 24.2)	0.76 (0.56 to 0.99)	0.56 (0.42 to 0.73)
1995	0.9 (0.7 to 1.2)	0.7 (0.5 to 0.9)	23.4 (17.2 to 30.5)	17.2 (12.7 to 22.4)	0.76 (0.56 to 0.99)	0.56 (0.41 to 0.73)
2000	0.9 (0.7 to 1.2)	0.7 (0.5 to 0.9)	21.9 (16.3 to 29.0)	16.2 (12.1 to 21.4)	0.79 (0.59 to 1.04)	0.58 (0.44 to 0.77)
2005	1.0 (0.7 to 1.2)	0.7 (0.6 to 0.9)	22.4 (17.1 to 28.3)	16.9 (12.9 to 21.3)	0.84 (0.64 to 1.06)	0.63 (0.48 to 0.80)
2010	0.9 (0.7 to 1.2)	0.7 (0.6 to 0.9)	22.3 (17.6 to 27.6)	16.8 (13.3 to 20.9)	0.90 (0.71 to 1.11)	0.68 (0.54 to 0.84)
2015	0.9 (0.7 to 1.1)	0.6 (0.5 to 0.8)	20.4 (16.0 to 25.6)	15.2 (11.9 to 19.0)	0.98 (0.77 to 1.23)	0.73 (0.57 to 0.91)
2020	0.8 (0.6 to 1.1)	0.6 (0.4 to 0.8)	17.8 (13.0 to 24.1)	12.9 (9.4 to 17.5)	1.07 (0.78 to 1.45)	0.78 (0.57 to 1.05)
2021	0.8 (0.6 to 1.1)	0.6 (0.4 to 0.8)	17.5 (12.9 to 24.3)	12.7 (9.3 to 17.5)	1.09 (0.80 to 1.50)	0.79 (0.58 to 1.08)

North Maluku IDN_4740 Southeast Asia Stillbirth Rate / Neonatal Mortality Rate (SBR/NMR) − 20 weeks
lambda = 0.6 ; zeta = 0.8 ; scale = 15 ; amp = 0.028

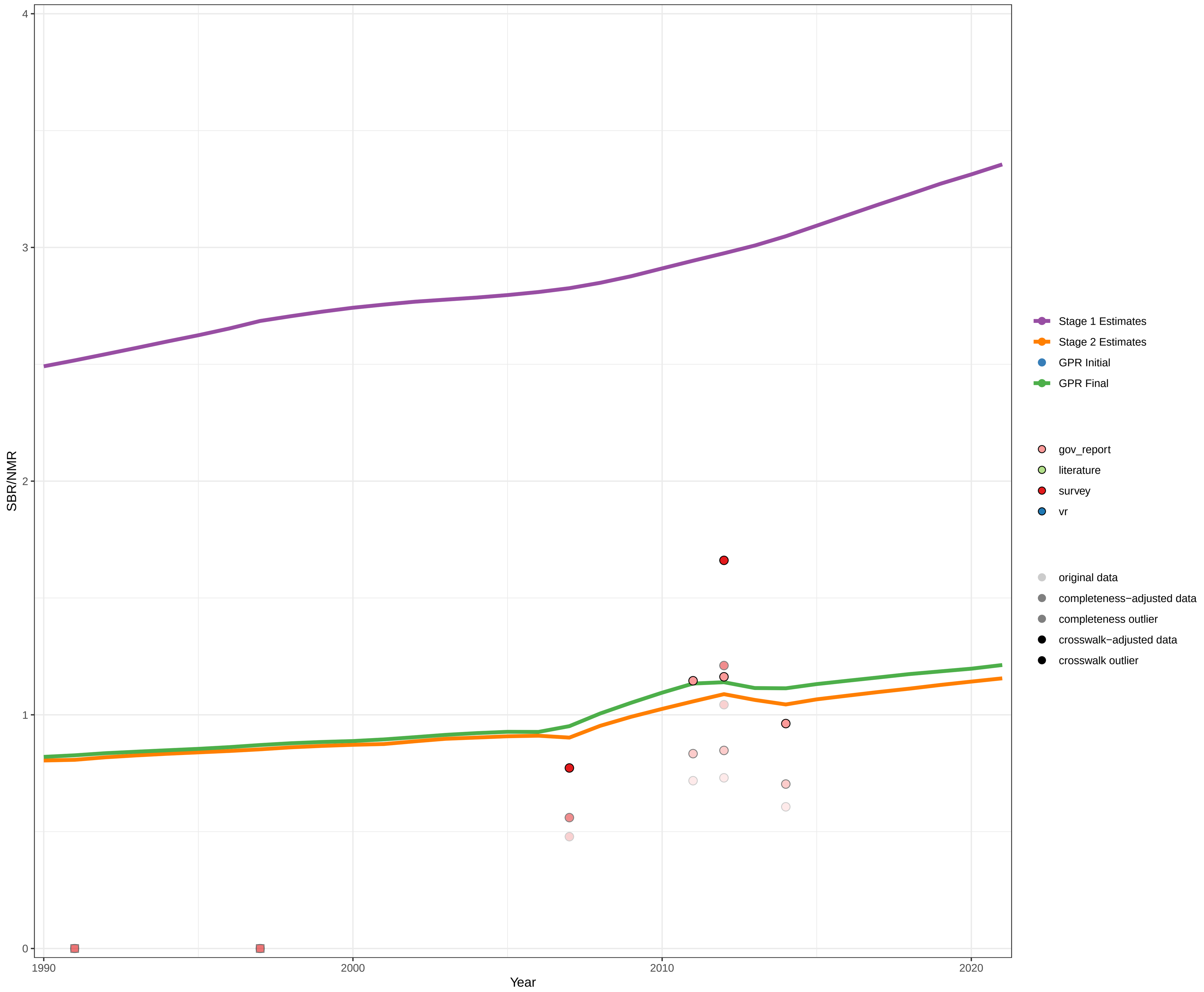

North Maluku IDN_4740 Southeast Asia Stillbirth Rate / Neonatal Mortality Rate (SBR/NMR) − 28 weeks
lambda = 0.6 ; zeta = 0.8 ; scale = 15 ; amp = 0.028

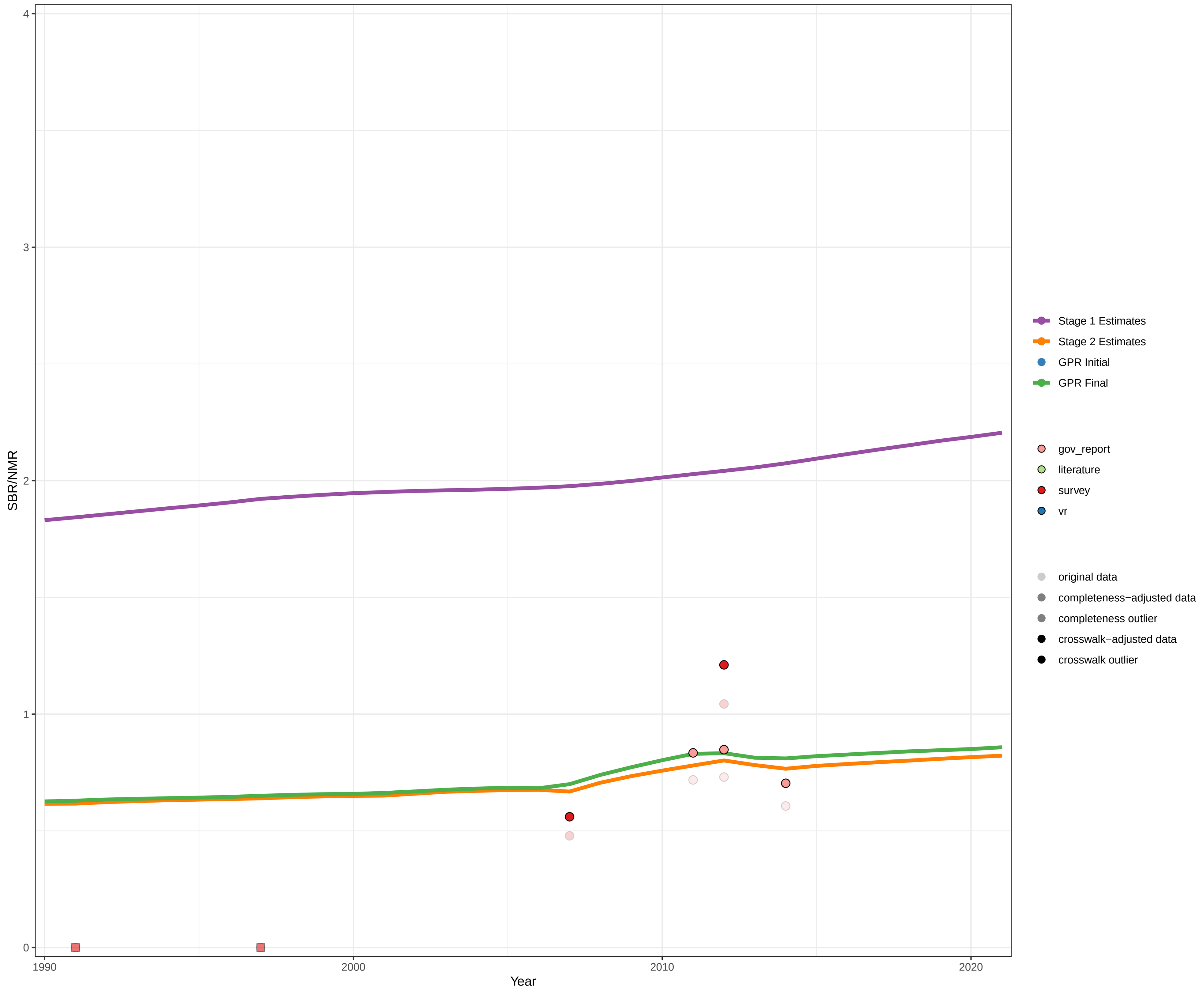

Definition	Years	Outlier	Source.Type	Title
28_weeks	2007	0	Survey	Indonesia Demographic and Health Survey 2007
28_weeks	2011, 2012, 2014	0	Statistical Report	Indonesia − Maluku Utara Province Health Profile
28_weeks	2012	0	Survey	Indonesia Demographic and Health Survey 2012
28_weeks	1991	1	Survey	Indonesia Demographic and Health Survey 1991
28_weeks	1997	1	Survey	Indonesia Demographic and Health Survey 1997

North Maluku IDN_4740 Southeast Asia Stillbirth Rate (SBR) − 20 weeks
lambda = 0.6 ; zeta = 0.8 ; scale = 15 ; amp = 0.028

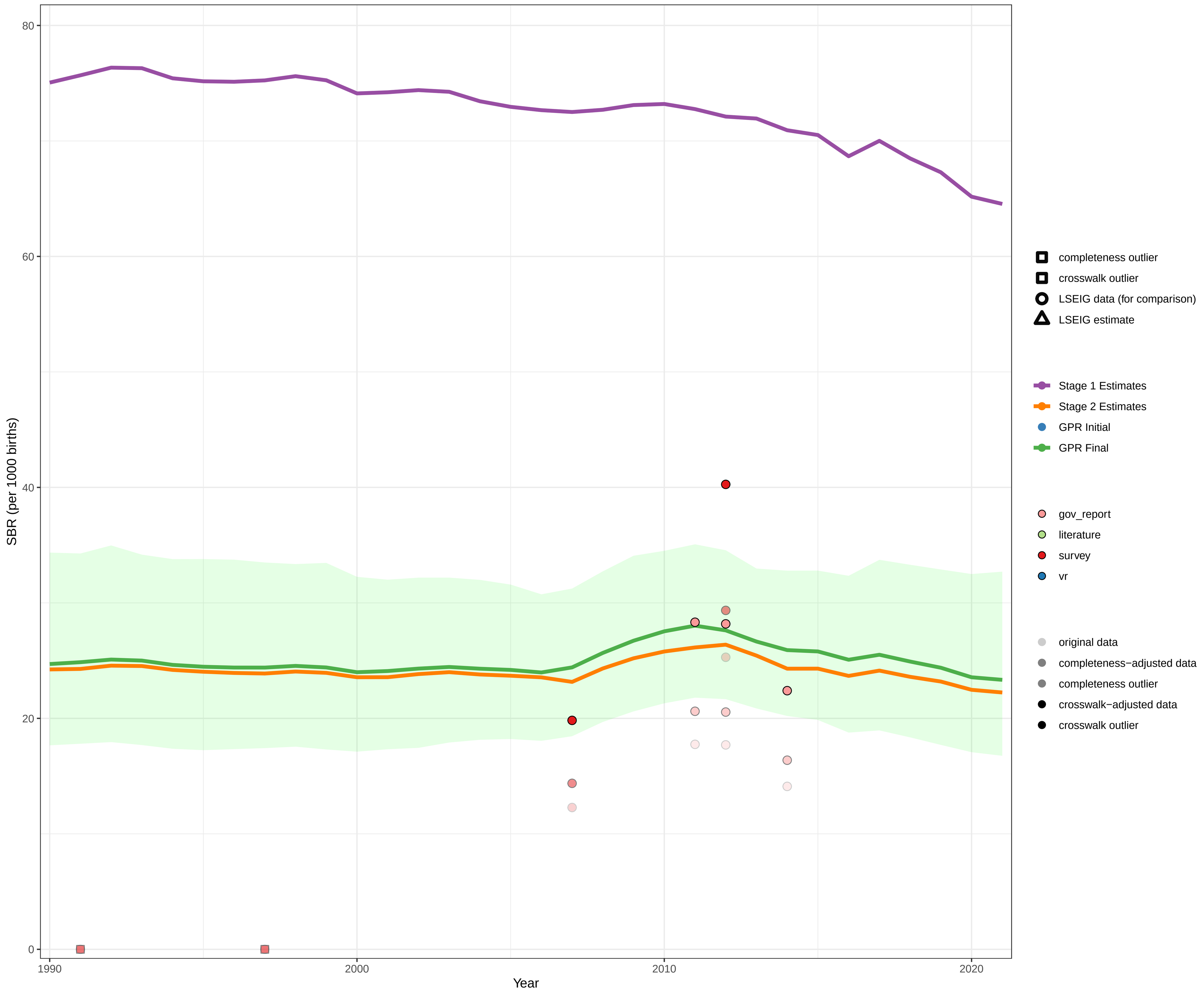

North Maluku IDN_4740 Southeast Asia Stillbirth Rate (SBR) − 28 weeks
lambda = 0.6 ; zeta = 0.8 ; scale = 15 ; amp = 0.028

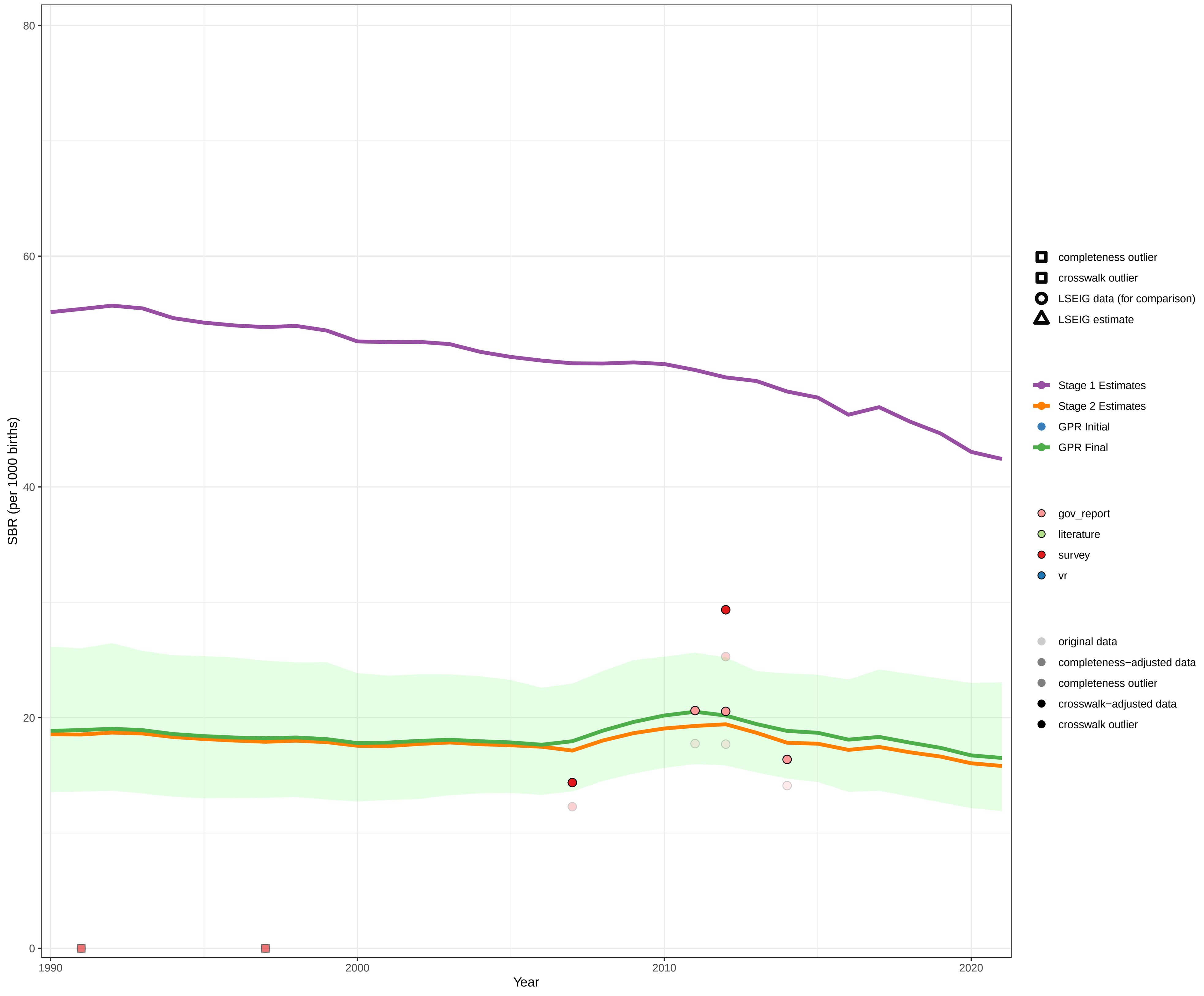

Year	Total Stillbirths (in thousands)	Total Stillbirths (in thousands)	SBR (per 1000 births)	SBR (per 1000 births)	SBR/NMR	SBR/NMR
	20 weeks	28 weeks	20 weeks	28 weeks	20 weeks	28 weeks
1990	0.6<br>(0.4 to 0.8)	0.4<br>(0.3 to 0.6)	24.7<br>(17.7 to 34.4)	18.9<br>(13.5 to 26.1)	0.82<br>(0.59 to 1.14)	0.63<br>(0.45 to 0.87)
1995	0.6<br>(0.4 to 0.9)	0.5<br>(0.3 to 0.6)	24.5<br>(17.2 to 33.8)	18.4<br>(13.0 to 25.3)	0.85<br>(0.60 to 1.18)	0.64<br>(0.45 to 0.88)
2000	0.6<br>(0.4 to 0.9)	0.5<br>(0.3 to 0.6)	24.0<br>(17.1 to 32.3)	17.8<br>(12.7 to 23.9)	0.89<br>(0.63 to 1.19)	0.66<br>(0.47 to 0.88)
2005	0.7<br>(0.5 to 0.9)	0.5<br>(0.4 to 0.6)	24.2<br>(18.2 to 31.6)	17.9<br>(13.5 to 23.3)	0.93<br>(0.70 to 1.21)	0.68<br>(0.52 to 0.89)
2010	0.7<br>(0.6 to 0.9)	0.5<br>(0.4 to 0.7)	27.5<br>(21.3 to 34.5)	20.2<br>(15.6 to 25.3)	1.09<br>(0.85 to 1.37)	0.80<br>(0.62 to 1.01)
2015	0.7<br>(0.5 to 0.9)	0.5<br>(0.4 to 0.6)	25.8<br>(19.9 to 32.8)	18.7<br>(14.4 to 23.7)	1.13<br>(0.87 to 1.44)	0.82<br>(0.63 to 1.04)
2020	0.7<br>(0.5 to 0.9)	0.5<br>(0.3 to 0.6)	23.6<br>(17.1 to 32.5)	16.7<br>(12.2 to 23.0)	1.20<br>(0.87 to 1.65)	0.85<br>(0.62 to 1.17)
2021	0.7<br>(0.5 to 0.9)	0.5<br>(0.3 to 0.7)	23.3<br>(16.8 to 32.7)	16.5<br>(11.9 to 23.1)	1.21<br>(0.87 to 1.70)	0.86<br>(0.62 to 1.20)

West Nusa Tenggara IDN_4727 Southeast Asia Stillbirth Rate / Neonatal Mortality Rate (SBR/NMR) − 20 weeks
lambda = 0.6 ; zeta = 0.8 ; scale = 15 ; amp = 0.028

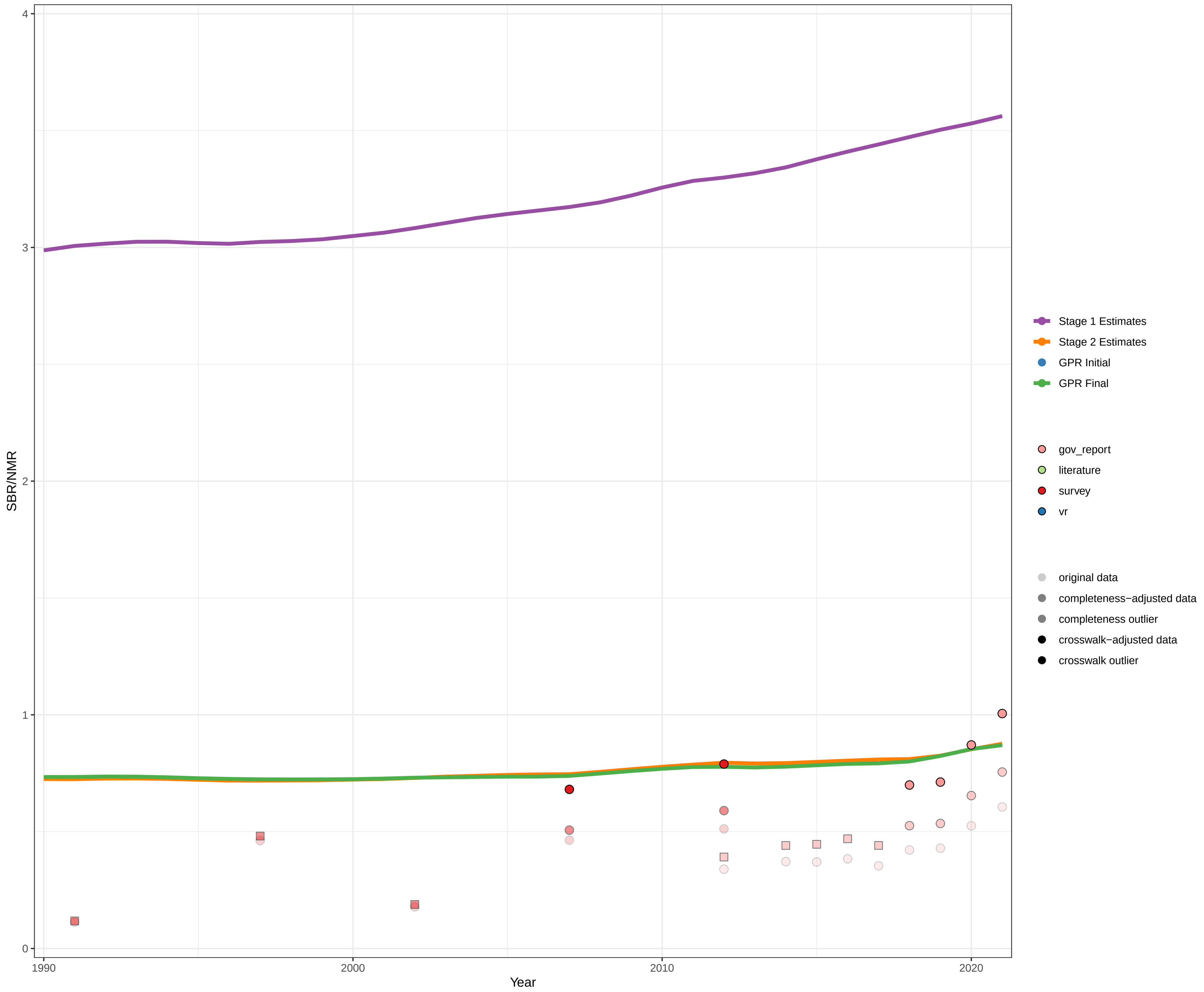

West Nusa Tenggara IDN_4727 Southeast Asia Stillbirth Rate / Neonatal Mortality Rate (SBR/NMR) − 28 weeks
lambda = 0.6 ; zeta = 0.8 ; scale = 15 ; amp = 0.028

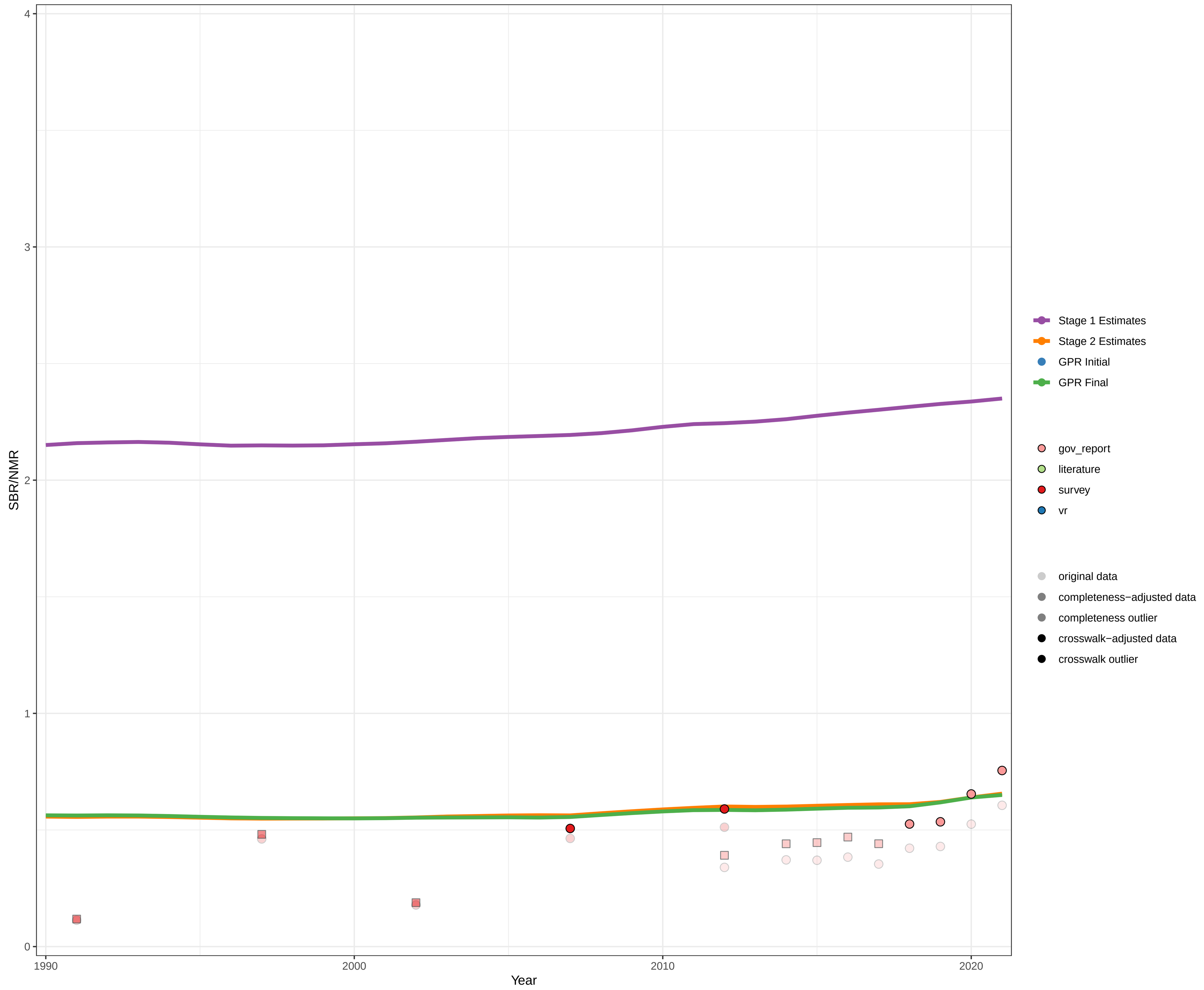

Definition	Years	Outlier	Source.Type	Title
28_weeks	2007	0	Survey	Indonesia Demographic and Health Survey 2007
28_weeks	2012	0	Survey	Indonesia Demographic and Health Survey 2012
28_weeks	2018, 2019	0	Statistical Report	Indonesia − Nusa Tenggara Barat Province Health Profile
28_weeks	2020, 2021	0	Statistical Report	Indonesia − Nusa Tenggara Timur Province Health Profile
28_weeks	1991	1	Survey	Indonesia Demographic and Health Survey 1991
28_weeks	1997	1	Survey	Indonesia Demographic and Health Survey 1997
28_weeks	2002	1	Survey	Indonesia Demographic and Health Survey 2002−2003
28_weeks	2012, 2014−2017	1	Statistical Report	Indonesia − Nusa Tenggara Barat Province Health Profile

West Nusa Tenggara IDN_4727 Southeast Asia Stillbirth Rate (SBR) − 20 weeks
lambda = 0.6 ; zeta = 0.8 ; scale = 15 ; amp = 0.028

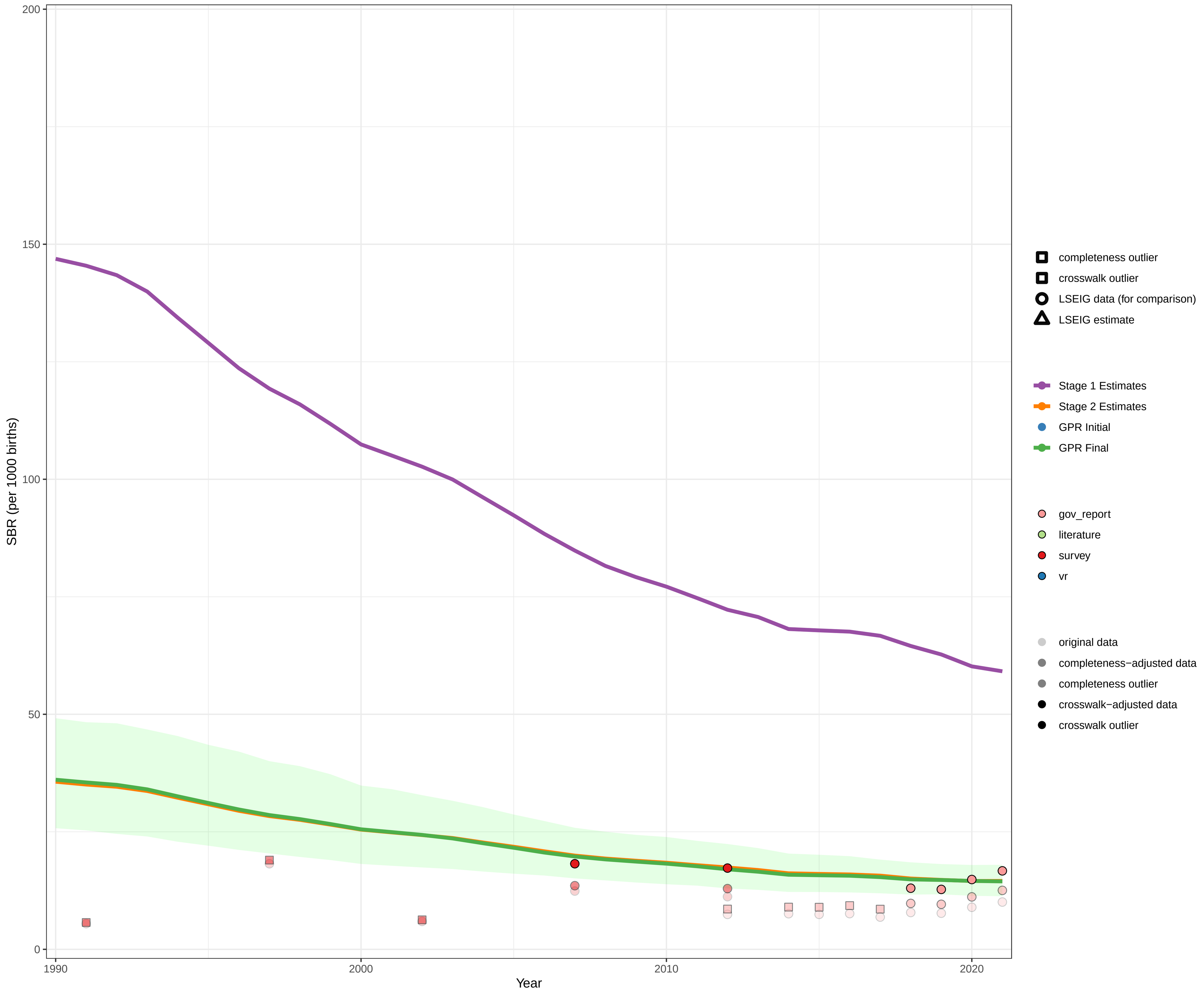

West Nusa Tenggara IDN_4727 Southeast Asia Stillbirth Rate (SBR) − 28 weeks
lambda = 0.6 ; zeta = 0.8 ; scale = 15 ; amp = 0.028

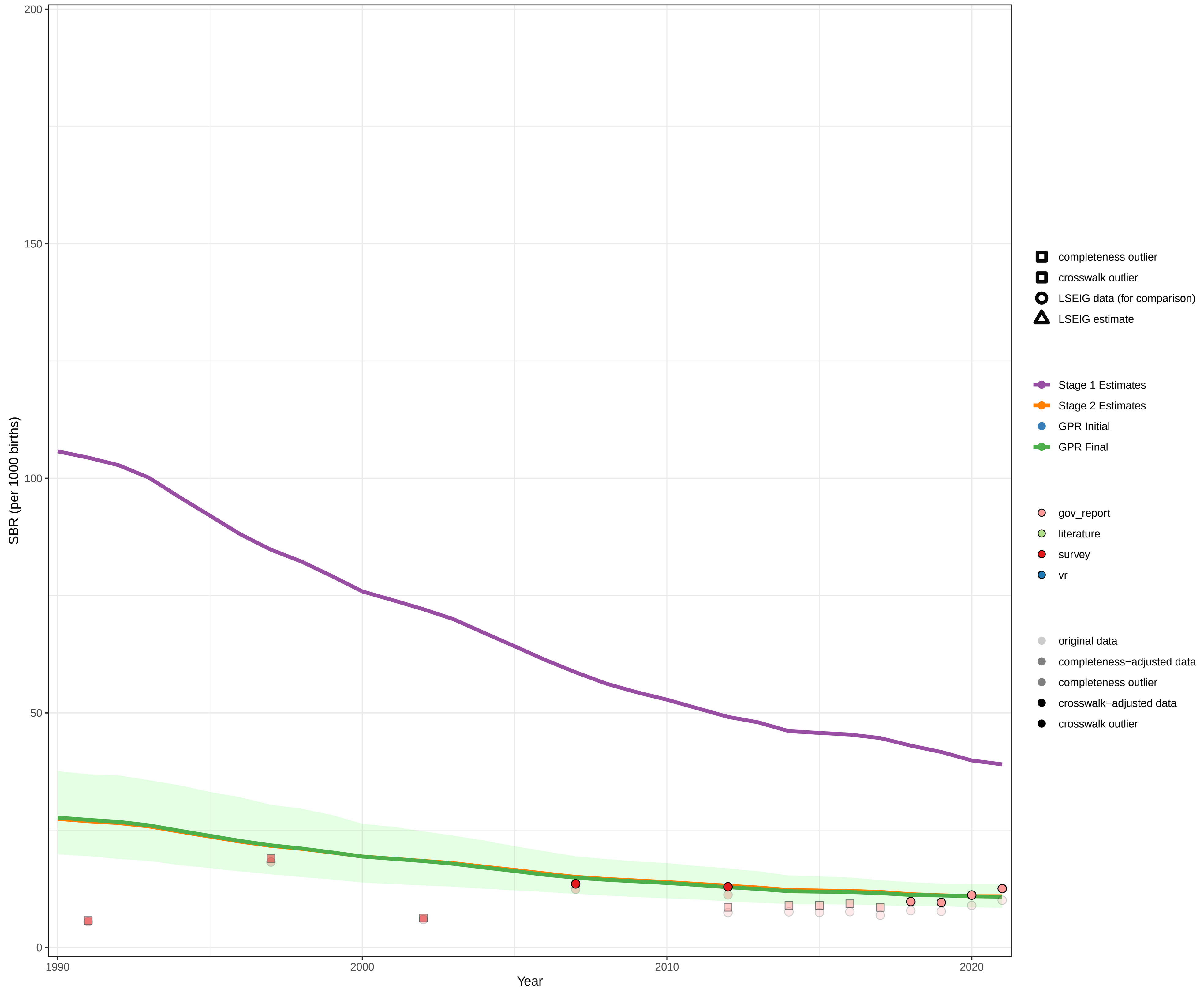

Year	Total Stillbirths (in thousands)	Total Stillbirths (in thousands)	SBR (per 1000 births)	SBR (per 1000 births)	SBR/NMR	SBR/NMR
	20 weeks	28 weeks	20 weeks	28 weeks	20 weeks	28 weeks
1990	3.8 (2.7 to 5.3)	2.9 (2.1 to 4.0)	36.1 (25.8 to 49.2)	27.7 (19.8 to 37.6)	0.73 (0.52 to 1.00)	0.56 (0.40 to 0.76)
1995	3.1 (2.2 to 4.4)	2.4 (1.7 to 3.3)	31.1 (22.0 to 43.5)	23.8 (16.9 to 33.1)	0.73 (0.52 to 1.02)	0.56 (0.40 to 0.78)
2000	2.6 (1.8 to 3.6)	2.0 (1.4 to 2.7)	25.5 (18.2 to 34.8)	19.4 (13.8 to 26.4)	0.72 (0.52 to 0.99)	0.55 (0.39 to 0.75)
2005	2.3 (1.7 to 3.1)	1.7 (1.3 to 2.3)	21.6 (16.1 to 28.7)	16.3 (12.2 to 21.6)	0.74 (0.55 to 0.98)	0.55 (0.41 to 0.73)
2010	1.9 (1.4 to 2.5)	1.4 (1.1 to 1.9)	18.2 (13.8 to 23.9)	13.7 (10.5 to 18.0)	0.77 (0.58 to 1.01)	0.58 (0.44 to 0.76)
2015	1.6 (1.2 to 2.0)	1.2 (0.9 to 1.5)	15.8 (12.2 to 20.1)	11.9 (9.2 to 15.1)	0.78 (0.61 to 1.00)	0.59 (0.46 to 0.75)
2020	1.4 (1.1 to 1.7)	1.0 (0.8 to 1.3)	14.6 (11.4 to 17.9)	10.9 (8.5 to 13.4)	0.85 (0.67 to 1.05)	0.64 (0.50 to 0.79)
2021	1.4 (1.1 to 1.7)	1.0 (0.8 to 1.3)	14.5 (11.3 to 18.0)	10.8 (8.4 to 13.4)	0.87 (0.68 to 1.08)	0.65 (0.51 to 0.81)

East Nusa Tenggara IDN_4728 Southeast Asia Stillbirth Rate / Neonatal Mortality Rate (SBR/NMR) − 20 weeks
lambda = 0.6 ; zeta = 0.8 ; scale = 15 ; amp = 0.028

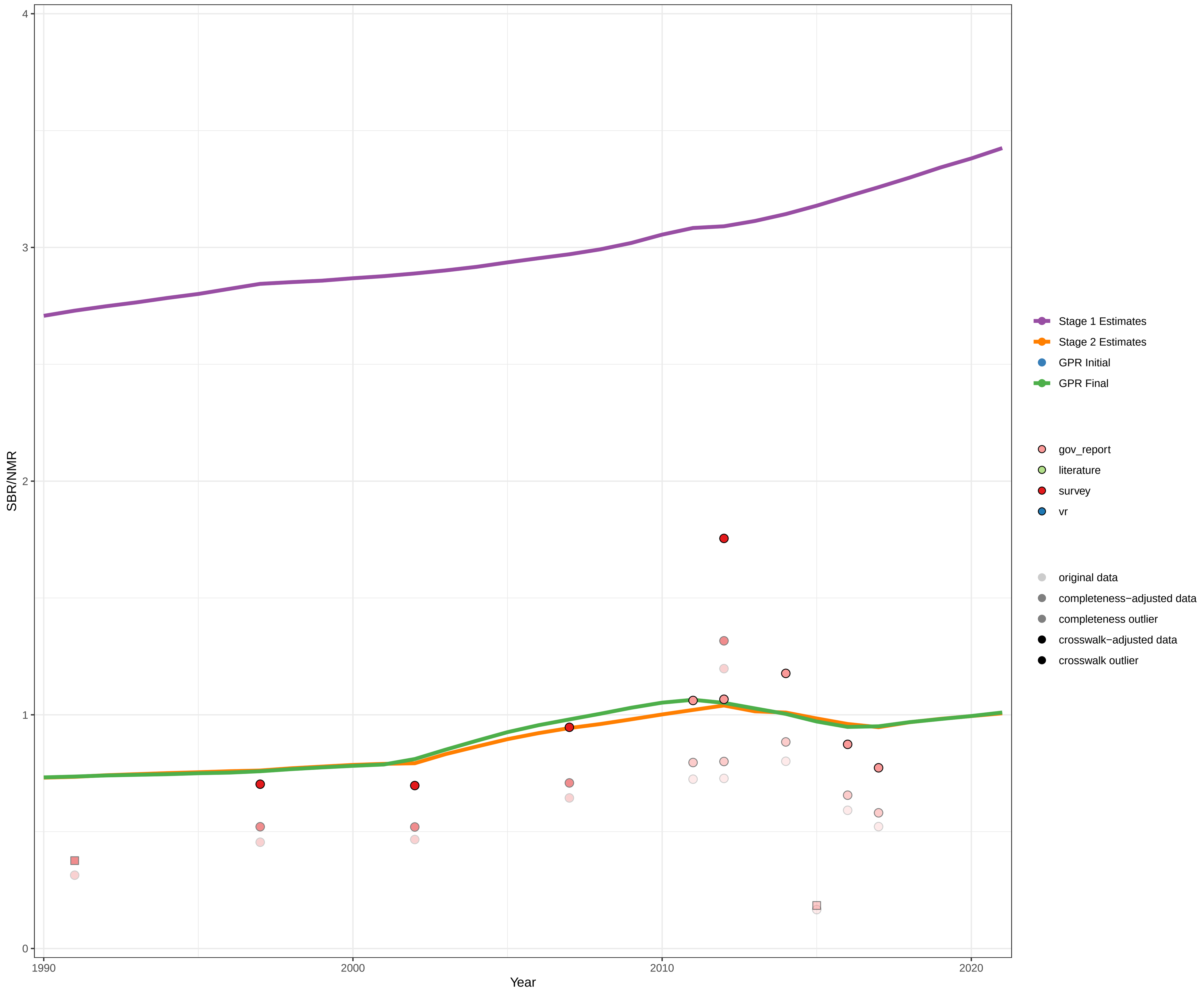

East Nusa Tenggara IDN_4728 Southeast Asia Stillbirth Rate / Neonatal Mortality Rate (SBR/NMR) − 28 weeks
lambda = 0.6 ; zeta = 0.8 ; scale = 15 ; amp = 0.028

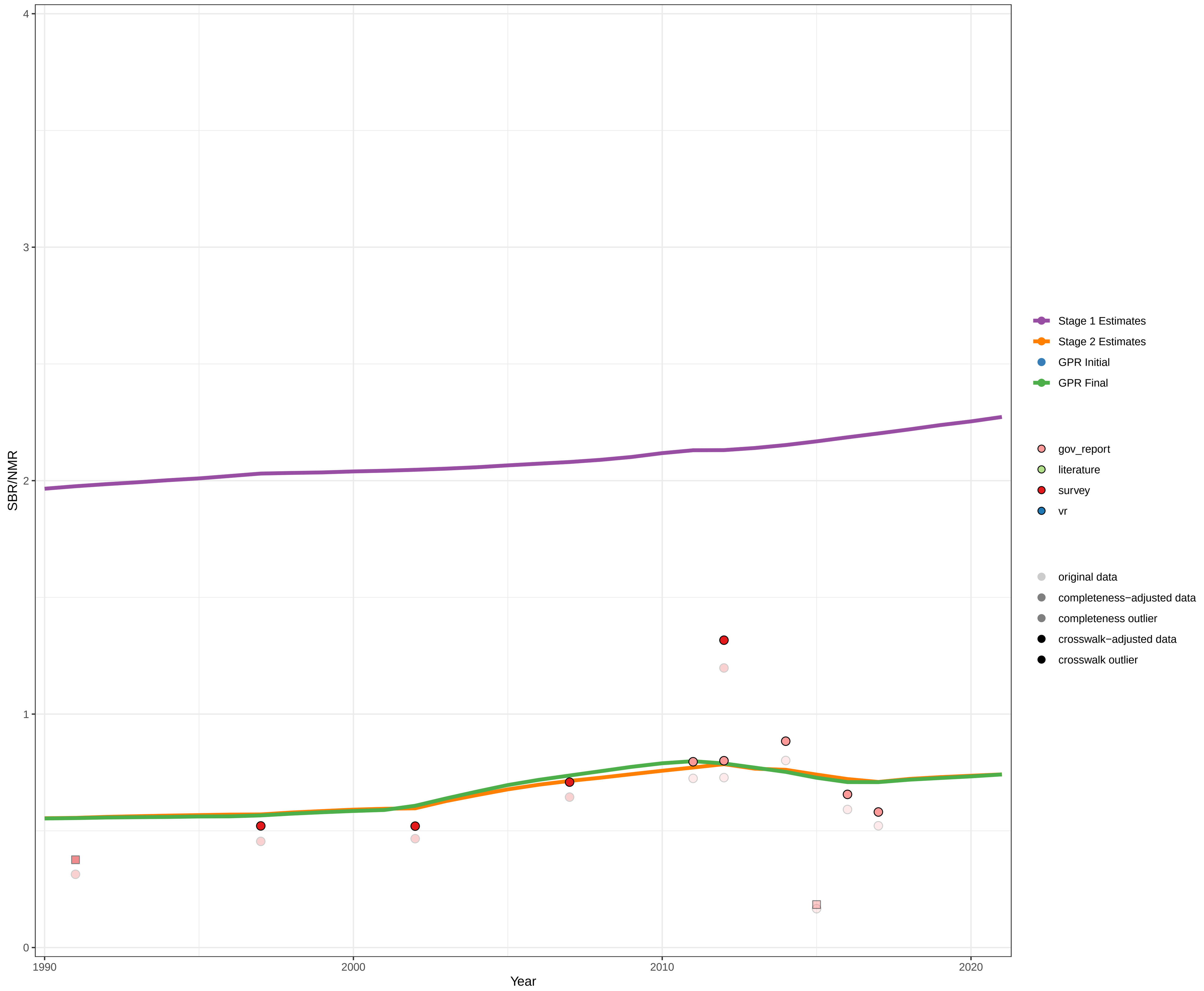

Definition	Years	Outlier	Source.Type	Title
28_weeks	1997	0	Survey	Indonesia Demographic and Health Survey 1997
28_weeks	2002	0	Survey	Indonesia Demographic and Health Survey 2002−2003
28_weeks	2007	0	Survey	Indonesia Demographic and Health Survey 2007
28_weeks	2011, 2012, 2014, 2017	0	Statistical Report	Indonesia − Nusa Tenggara Timur Province Health Profile
28_weeks	2012	0	Survey	Indonesia Demographic and Health Survey 2012
28_weeks	2016	0	Statistical Report	Angola Nodule Palpation Mapping Survey
28_weeks	1991	1	Survey	Indonesia Demographic and Health Survey 1991
28_weeks	2015	1	Statistical Report	Indonesia − Nusa Tenggara Timur Province Health Profile

East Nusa Tenggara IDN_4728 Southeast Asia Stillbirth Rate (SBR) − 20 weeks
lambda = 0.6 ; zeta = 0.8 ; scale = 15 ; amp = 0.028

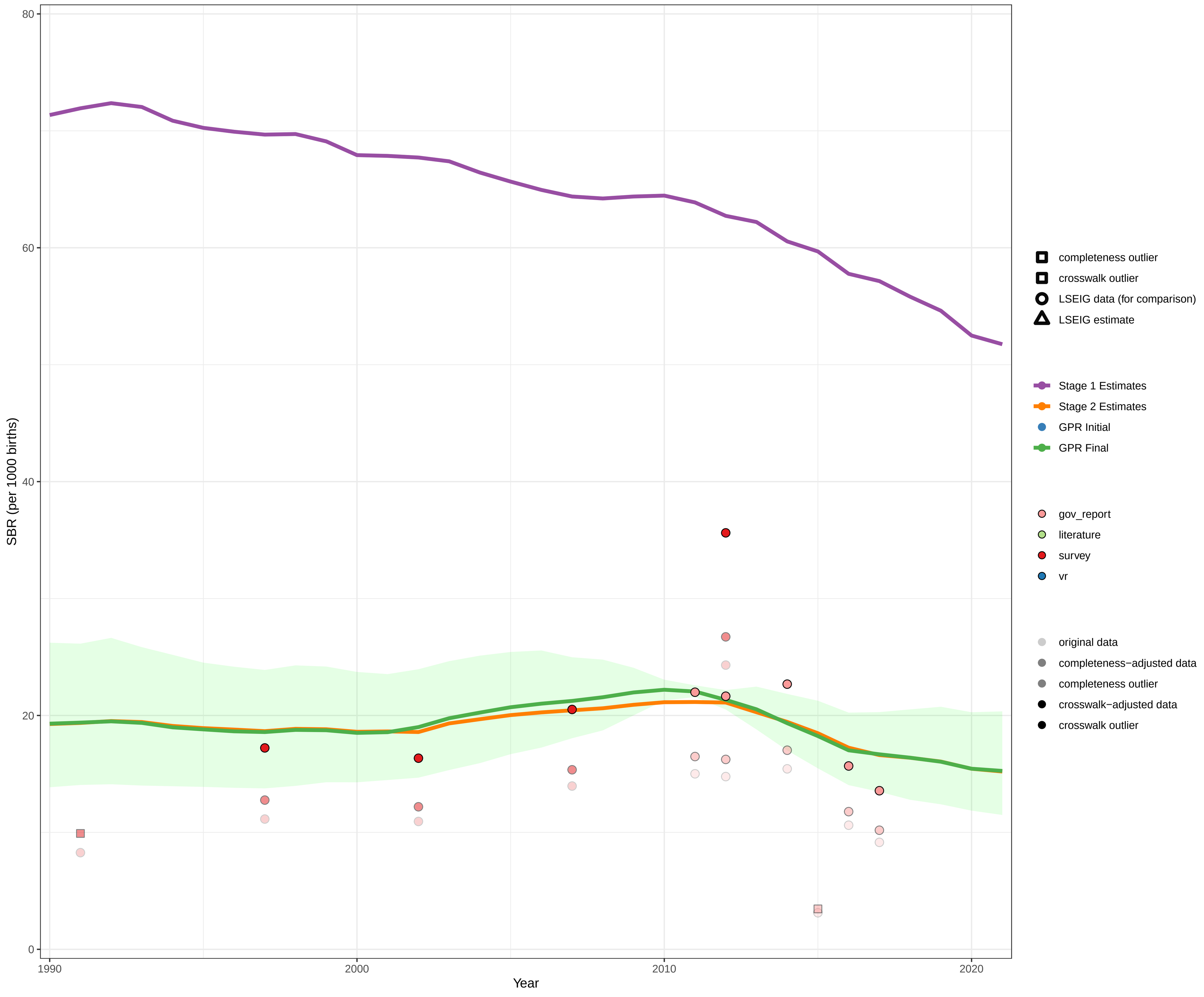

East Nusa Tenggara IDN_4728 Southeast Asia Stillbirth Rate (SBR) − 28 weeks
lambda = 0.6 ; zeta = 0.8 ; scale = 15 ; amp = 0.028

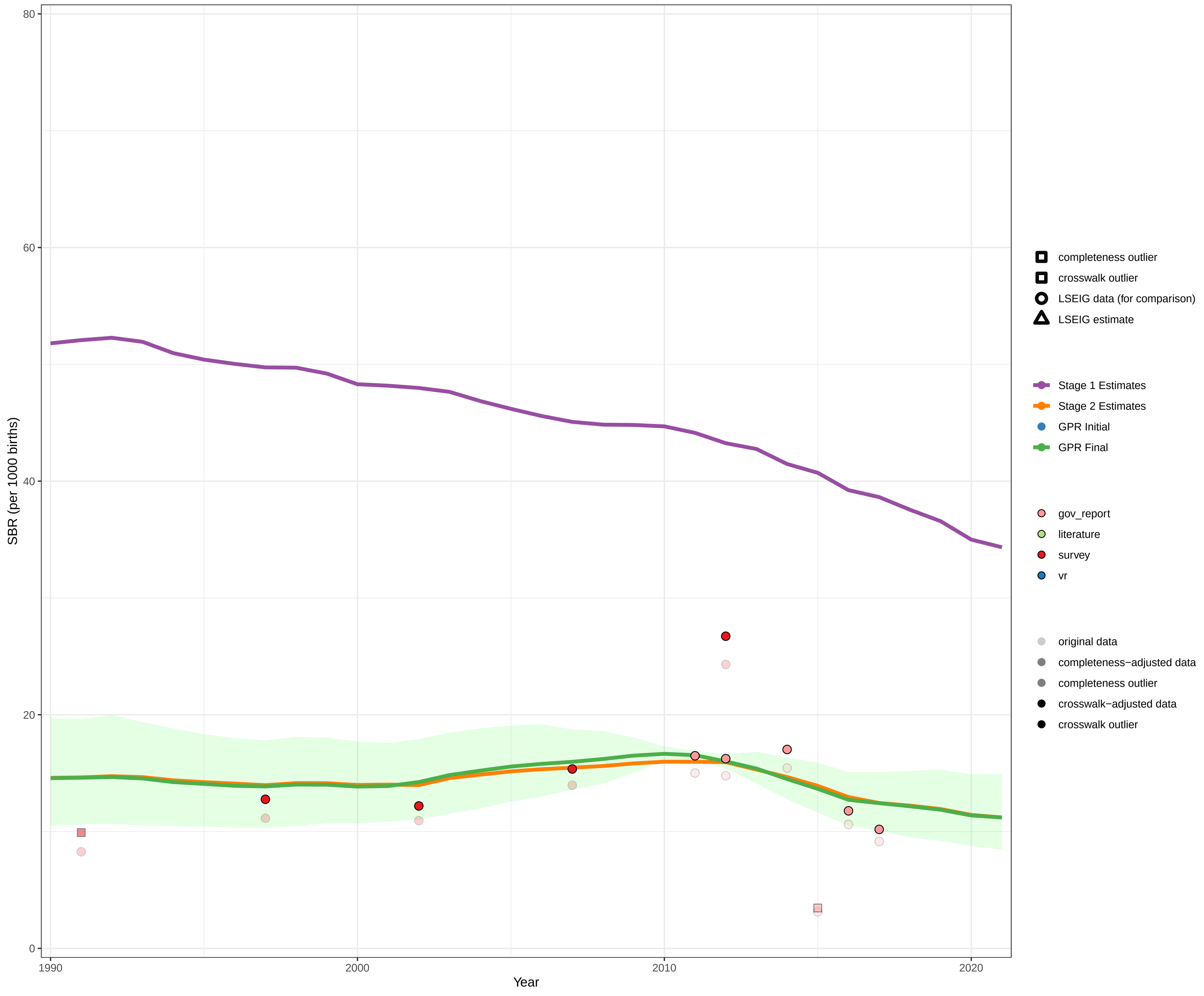

Year	Total Stillbirths (in thousands)	Total Stillbirths (in thousands)	SBR (per 1000 births)	SBR (per 1000 births)	SBR/NMR	SBR/NMR
	20 weeks	28 weeks	20 weeks	28 weeks	20 weeks	28 weeks
1990	2.0 (1.4 to 2.8)	1.5 (1.1 to 2.1)	19.3 (13.9 to 26.2)	14.6 (10.5 to 19.7)	0.73 (0.53 to 0.99)	0.55 (0.40 to 0.75)
1995	2.1 (1.5 to 2.7)	1.6 (1.1 to 2.0)	18.8 (13.9 to 24.5)	14.1 (10.4 to 18.3)	0.75 (0.55 to 0.98)	0.56 (0.42 to 0.73)
2000	2.2 (1.7 to 2.9)	1.7 (1.3 to 2.1)	18.5 (14.3 to 23.7)	13.9 (10.7 to 17.7)	0.78 (0.60 to 1.00)	0.58 (0.45 to 0.75)
2005	2.6 (2.1 to 3.3)	2.0 (1.6 to 2.4)	20.7 (16.7 to 25.4)	15.6 (12.6 to 19.1)	0.93 (0.75 to 1.14)	0.70 (0.56 to 0.85)
2010	2.8 (2.6 to 2.9)	2.1 (2.0 to 2.1)	22.2 (21.3 to 23.1)	16.7 (16.0 to 17.3)	1.05 (1.01 to 1.09)	0.79 (0.76 to 0.82)
2015	2.2 (1.9 to 2.6)	1.7 (1.4 to 1.9)	18.2 (15.5 to 21.3)	13.7 (11.6 to 15.9)	0.97 (0.82 to 1.13)	0.73 (0.62 to 0.85)
2020	2.0 (1.5 to 2.6)	1.4 (1.1 to 1.9)	15.4 (11.9 to 20.3)	11.4 (8.8 to 14.9)	0.99 (0.76 to 1.31)	0.73 (0.56 to 0.96)
2021	2.0 (1.5 to 2.6)	1.4 (1.1 to 1.9)	15.3 (11.5 to 20.4)	11.2 (8.5 to 14.9)	1.01 (0.76 to 1.35)	0.74 (0.56 to 0.99)

Papua IDN_4742 Southeast Asia Stillbirth Rate / Neonatal Mortality Rate (SBR/NMR) − 20 weeks
lambda = 0.8 ; zeta = 0.7 ; scale = 15 ; amp = 0.028

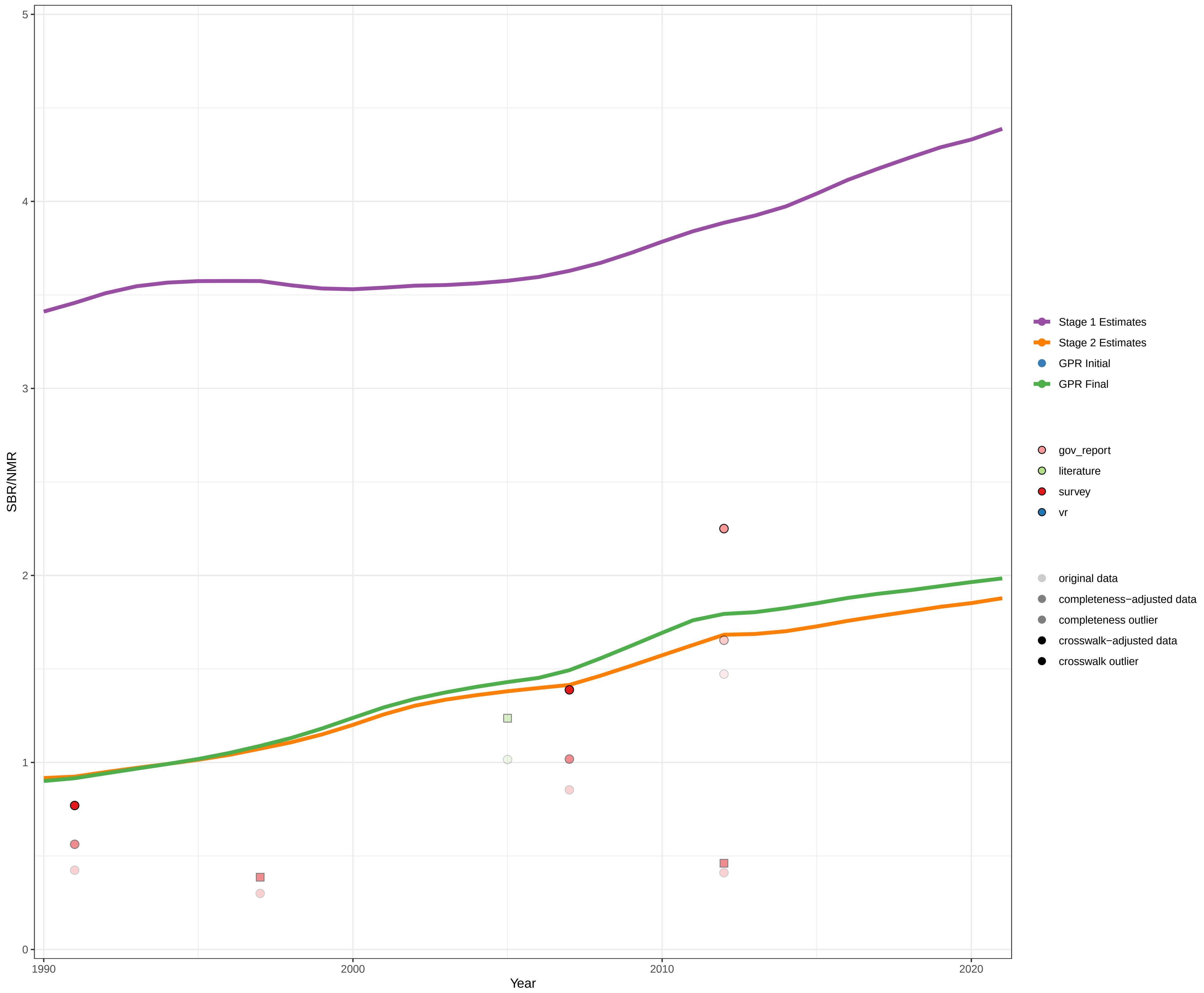

Papua IDN_4742 Southeast Asia Stillbirth Rate / Neonatal Mortality Rate (SBR/NMR) − 28 weeks
lambda = 0.8 ; zeta = 0.7 ; scale = 15 ; amp = 0.028

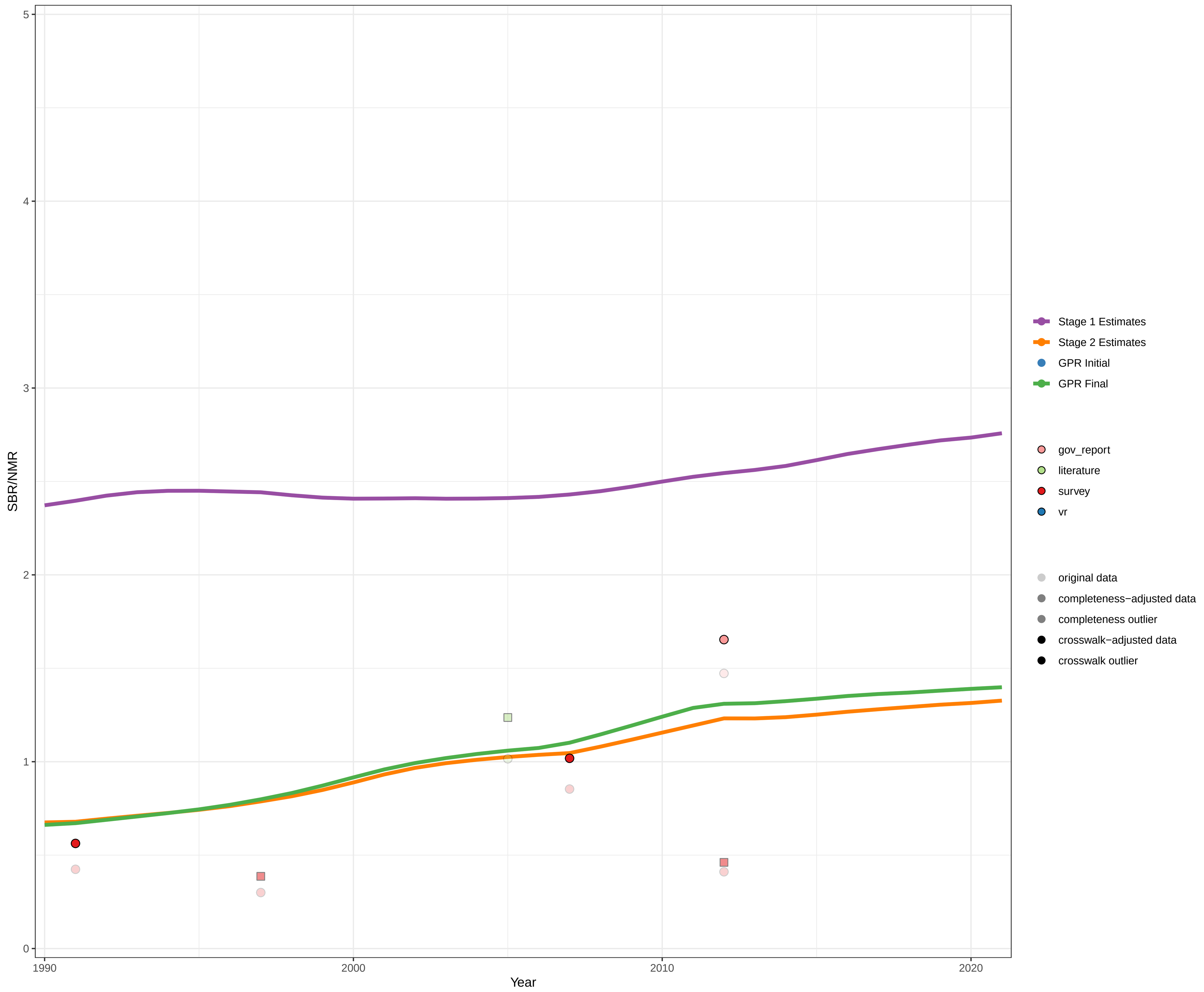

Definition	Years	Outlier	Source.Type	Title
28_weeks	1991	0	Survey	Indonesia Demographic and Health Survey 1991
28_weeks	2007	0	Survey	Indonesia Demographic and Health Survey 2007
28_weeks	2012	0	Statistical Report	Indonesia − Papua Province Health Profile
28_weeks	1997	1	Survey	Indonesia Demographic and Health Survey 1997
28_weeks	2005	1	Sci Lit	Adverse pregnancy outcomes in an area where multidrug−resistant plasmodium vivax and Plasmodium falciparum infections are endemic
28_weeks	2012	1	Survey	Indonesia Demographic and Health Survey 2012

Papua IDN_4742 Southeast Asia Stillbirth Rate (SBR) − 20 weeks
lambda = 0.8 ; zeta = 0.7 ; scale = 15 ; amp = 0.028

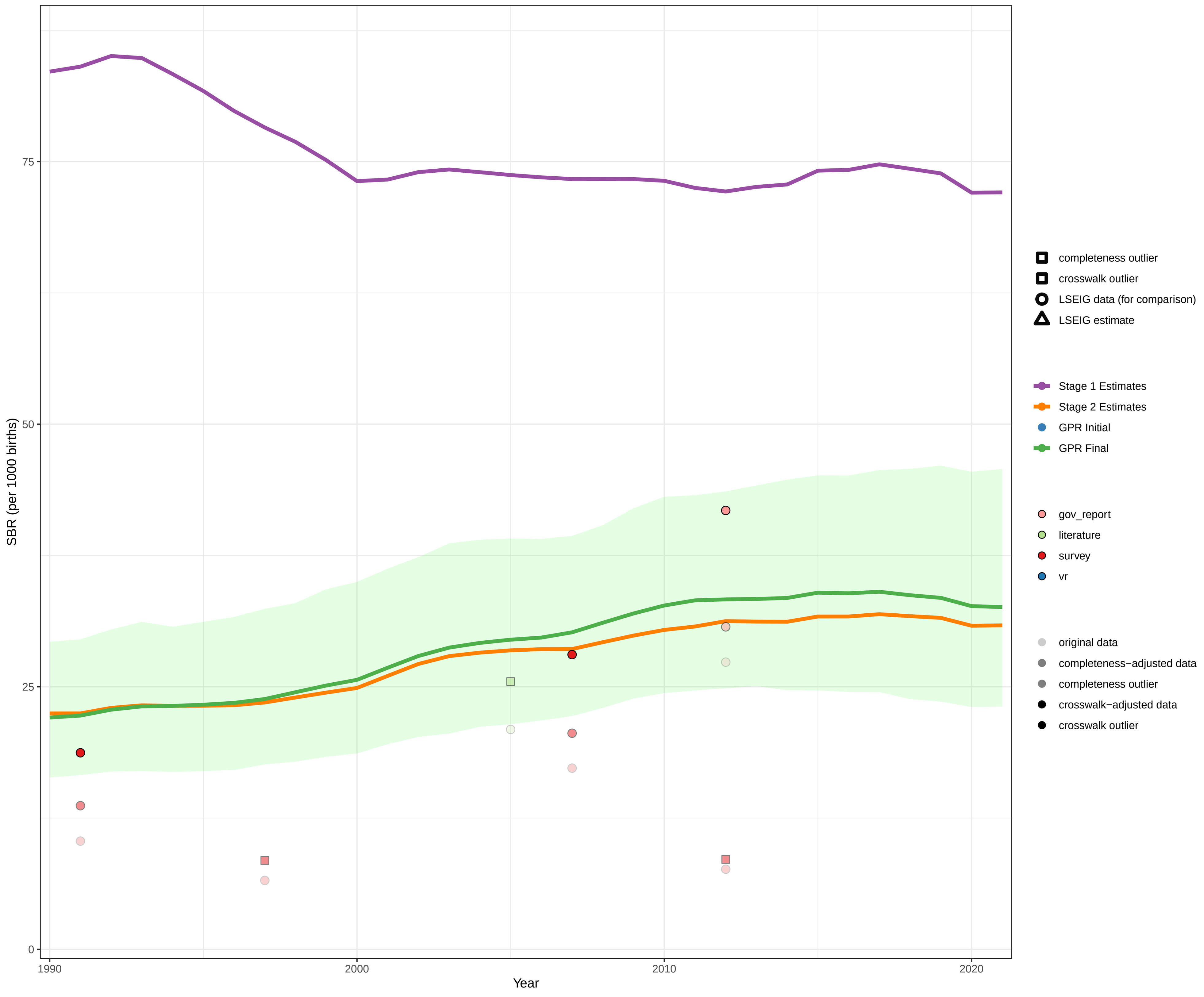

Papua IDN_4742 Southeast Asia Stillbirth Rate (SBR) − 28 weeks
lambda = 0.8 ; zeta = 0.7 ; scale = 15 ; amp = 0.028

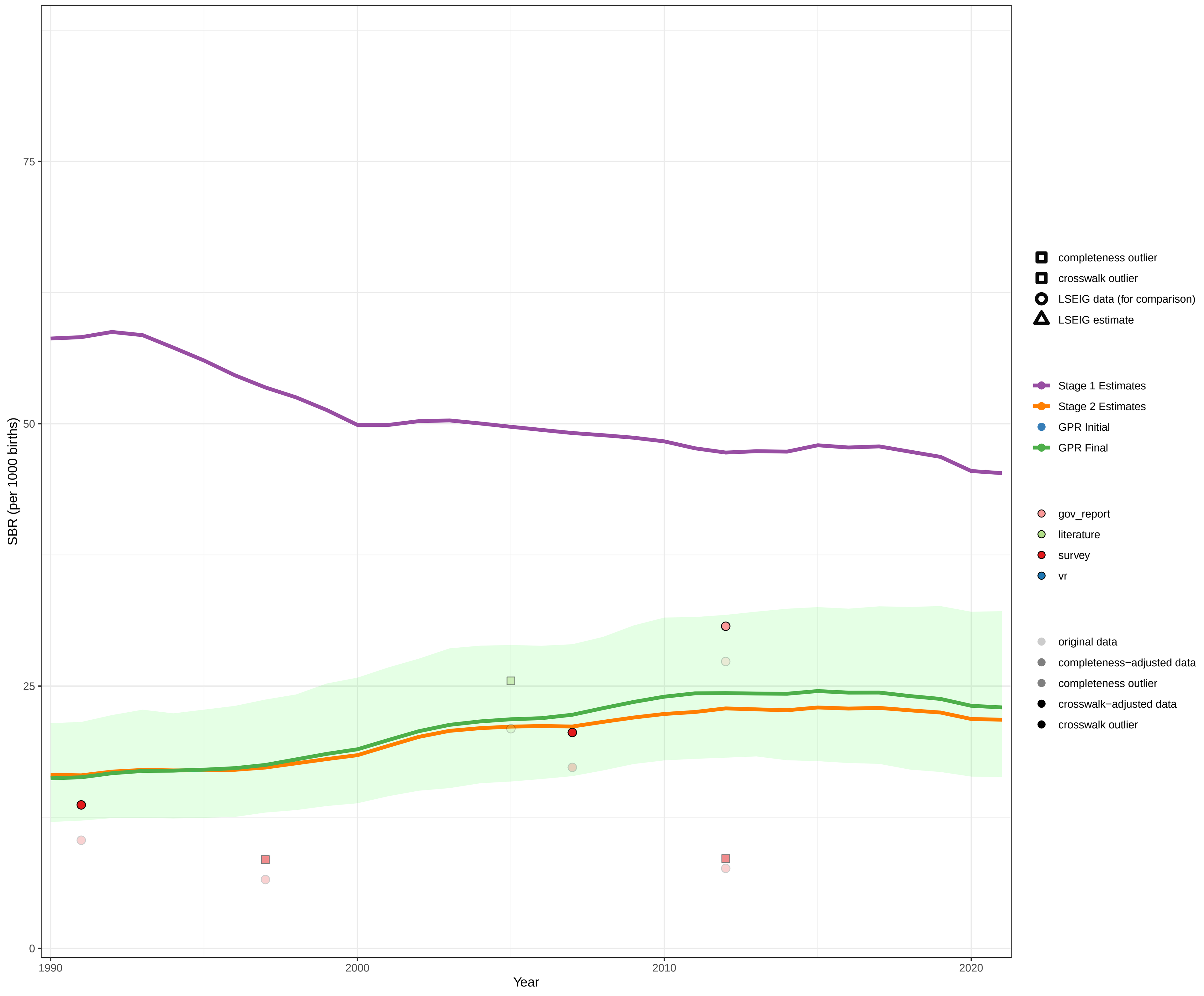

Year	Total Stillbirths (in thousands)	Total Stillbirths (in thousands)	SBR (per 1000 births)	SBR (per 1000 births)	SBR/NMR	SBR/NMR
	20 weeks	28 weeks	20 weeks	28 weeks	20 weeks	28 weeks
1990	0.7 (0.5 to 0.9)	0.5 (0.4 to 0.7)	22.1 (16.4 to 29.3)	16.2 (12.0 to 21.5)	0.90 (0.67 to 1.20)	0.66 (0.49 to 0.88)
1995	0.9 (0.7 to 1.3)	0.7 (0.5 to 0.9)	23.3 (17.0 to 31.2)	17.0 (12.4 to 22.8)	1.02 (0.74 to 1.36)	0.74 (0.54 to 1.00)
2000	1.3 (1.0 to 1.8)	1.0 (0.7 to 1.3)	25.7 (18.7 to 35.0)	19.0 (13.8 to 25.8)	1.24 (0.90 to 1.69)	0.92 (0.67 to 1.25)
2005	2.0 (1.4 to 2.6)	1.4 (1.0 to 1.9)	29.5 (21.4 to 39.1)	21.8 (15.9 to 28.9)	1.43 (1.04 to 1.90)	1.06 (0.77 to 1.40)
2010	2.7 (2.0 to 3.7)	2.0 (1.5 to 2.6)	32.7 (24.4 to 43.1)	24.0 (17.9 to 31.5)	1.69 (1.26 to 2.23)	1.24 (0.93 to 1.63)
2015	3.2 (2.3 to 4.3)	2.3 (1.6 to 3.0)	34.0 (24.6 to 45.1)	24.5 (17.8 to 32.5)	1.85 (1.34 to 2.46)	1.34 (0.97 to 1.77)
2020	3.2 (2.2 to 4.4)	2.2 (1.6 to 3.1)	32.7 (23.1 to 45.5)	23.1 (16.4 to 32.1)	1.96 (1.39 to 2.73)	1.39 (0.98 to 1.93)
2021	3.2 (2.2 to 4.5)	2.2 (1.6 to 3.1)	32.6 (23.1 to 45.7)	23.0 (16.3 to 32.1)	1.98 (1.41 to 2.78)	1.40 (1.00 to 1.96)

West Papua IDN_4741 Southeast Asia Stillbirth Rate / Neonatal Mortality Rate (SBR/NMR) − 20 weeks
lambda = 0.8 ; zeta = 0.7 ; scale = 15 ; amp = 0.028

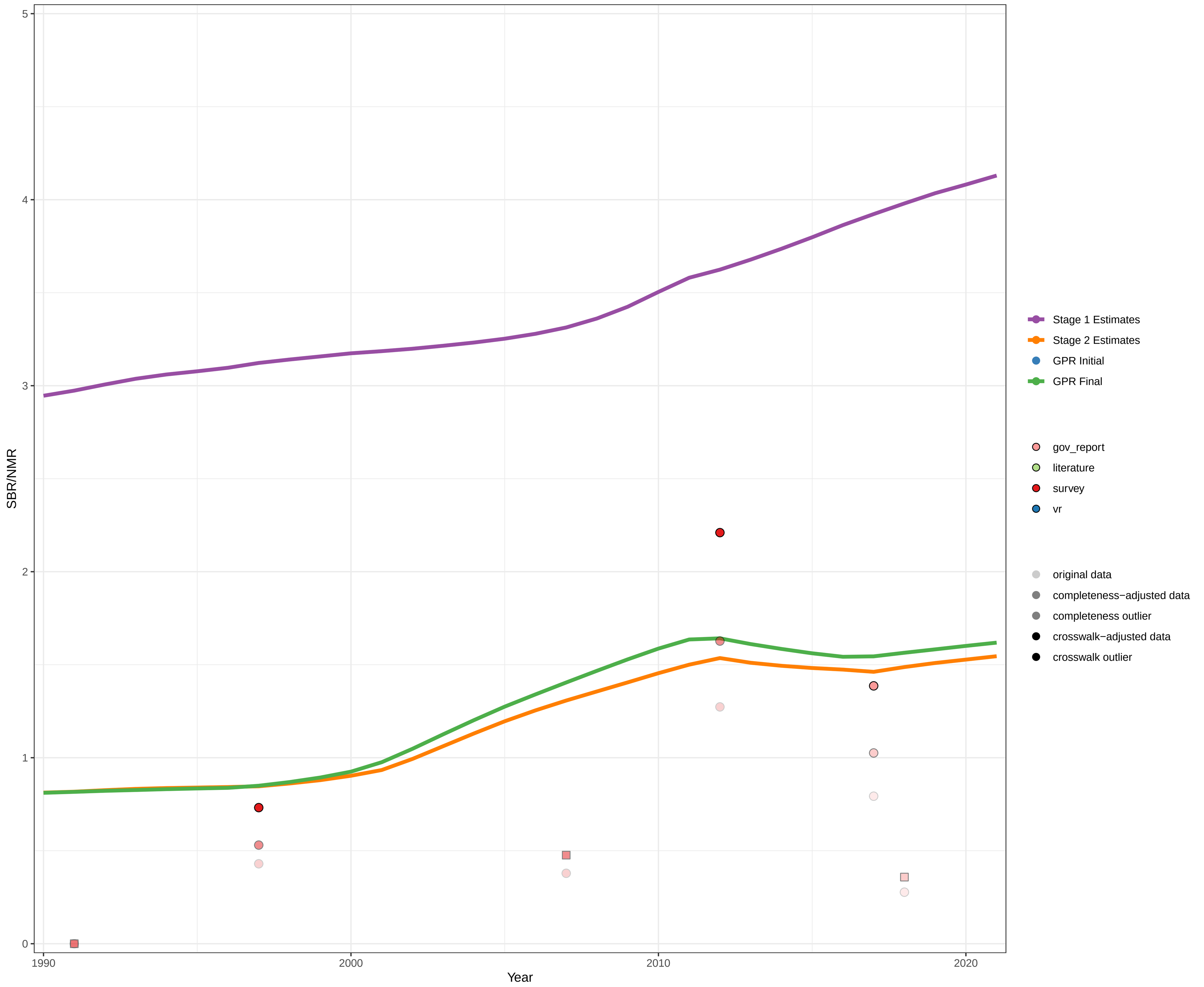

West Papua IDN_4741 Southeast Asia Stillbirth Rate / Neonatal Mortality Rate (SBR/NMR) − 28 weeks
lambda = 0.8 ; zeta = 0.7 ; scale = 15 ; amp = 0.028

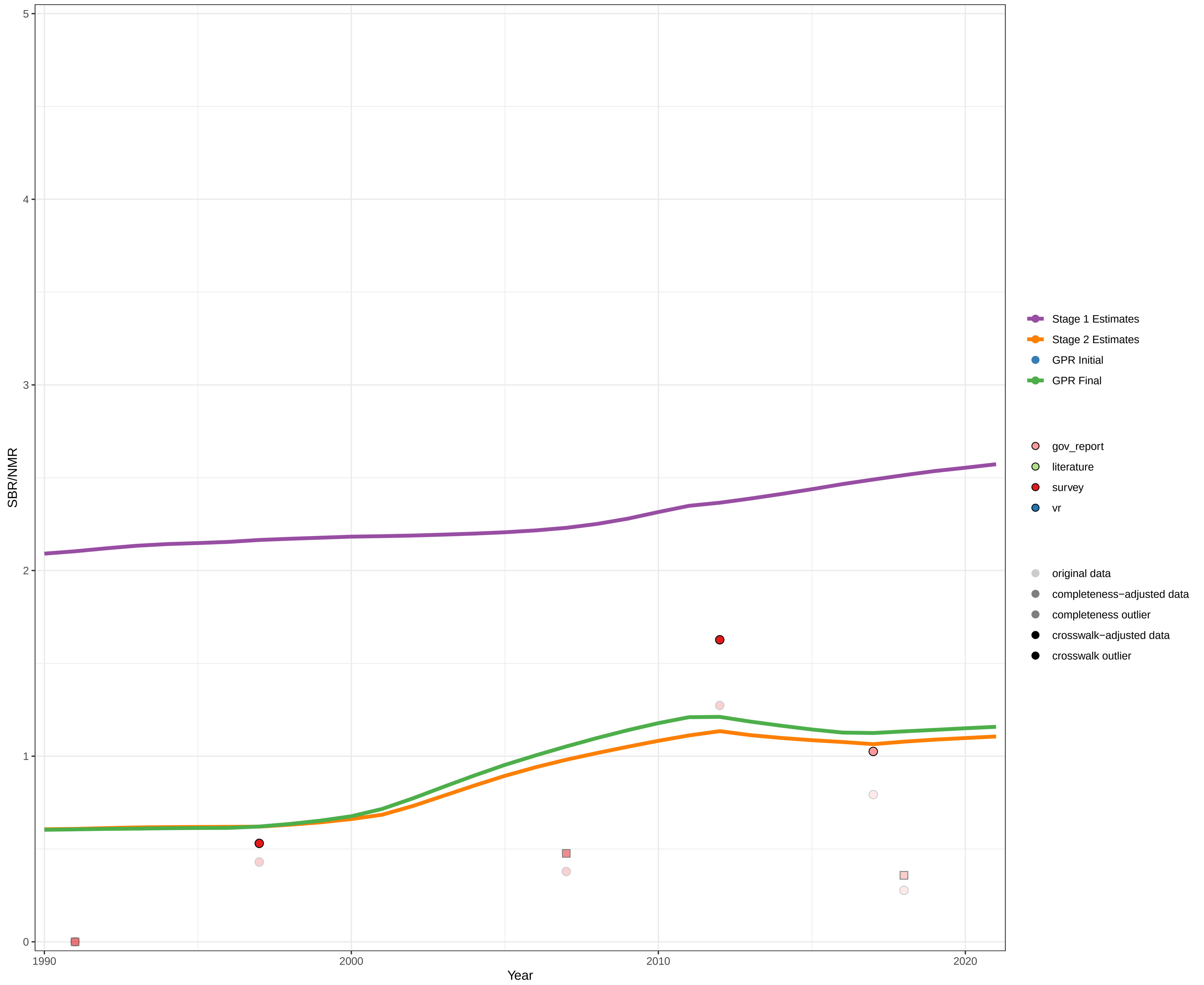

Definition	Years	Outlier	Source.Type	Title
28_weeks	1997	0	Survey	Indonesia Demographic and Health Survey 1997
28_weeks	2012	0	Survey	Indonesia Demographic and Health Survey 2012
28_weeks	2017	0	Statistical Report	Indonesia − Papua Barat Province Health Profile
28_weeks	1991	1	Survey	Indonesia Demographic and Health Survey 1991
28_weeks	2007	1	Survey	Indonesia Demographic and Health Survey 2007
28_weeks	2018	1	Statistical Report	Indonesia − Papua Barat Province Health Profile

West Papua IDN_4741 Southeast Asia Stillbirth Rate (SBR) − 20 weeks
lambda = 0.8 ; zeta = 0.7 ; scale = 15 ; amp = 0.028

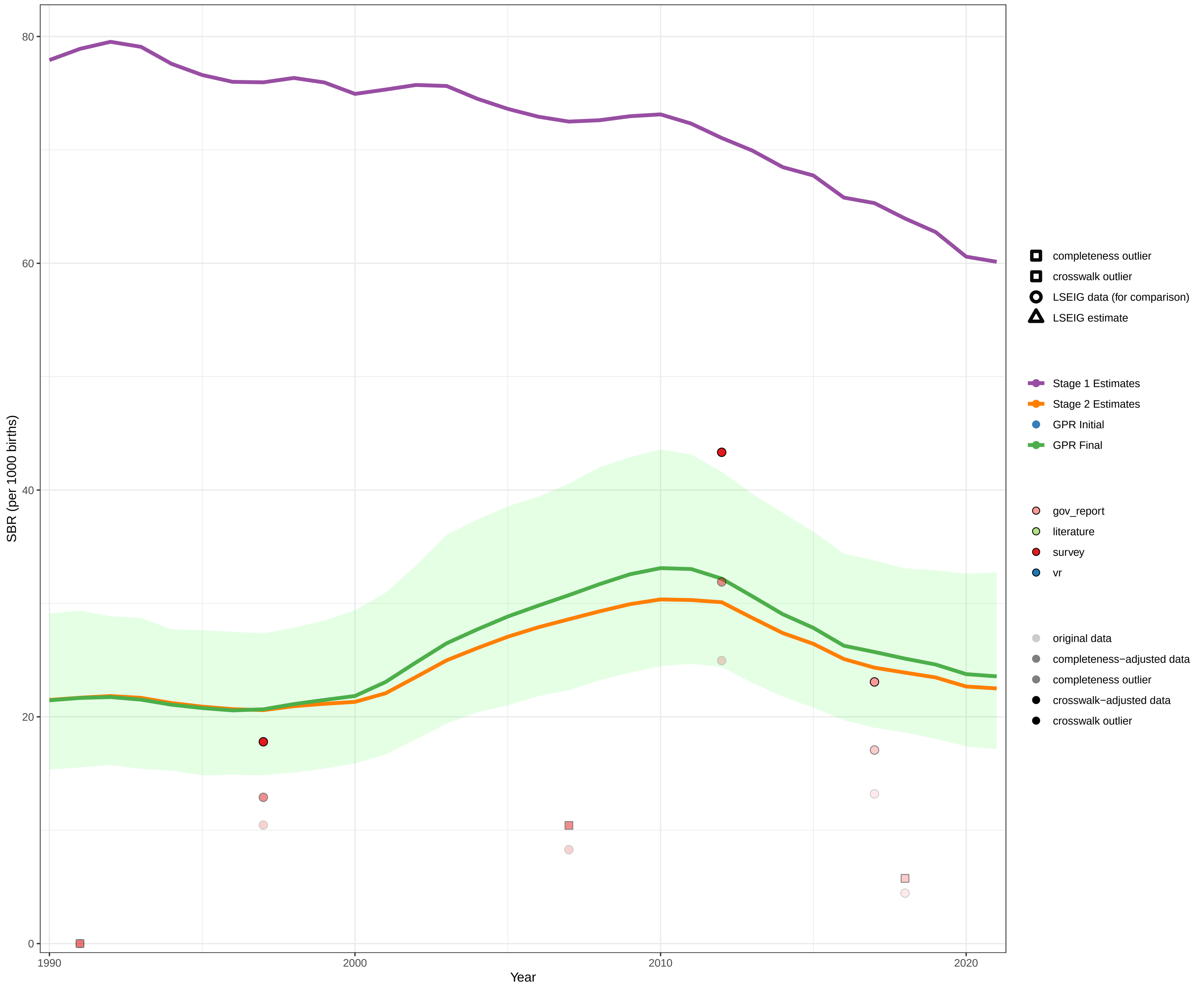

West Papua IDN_4741 Southeast Asia Stillbirth Rate (SBR) − 28 weeks
lambda = 0.8 ; zeta = 0.7 ; scale = 15 ; amp = 0.028

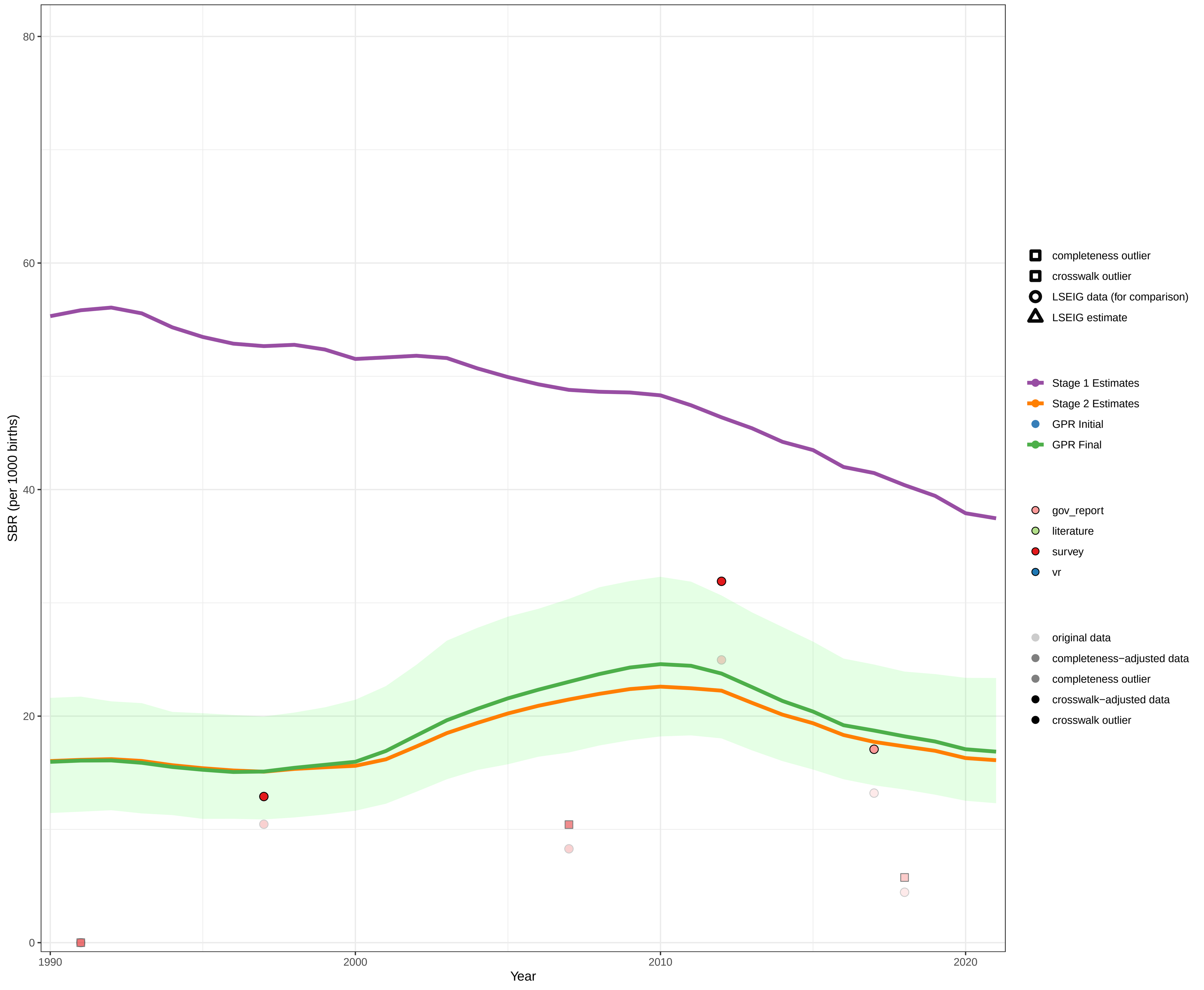

Year	Total Stillbirths (in thousands)	Total Stillbirths (in thousands)	SBR (per 1000 births)	SBR (per 1000 births)	SBR/NMR	SBR/NMR
	20 weeks	28 weeks	20 weeks	28 weeks	20 weeks	28 weeks
1990	0.5 (0.3 to 0.7)	0.4 (0.3 to 0.5)	21.5 (15.3 to 29.1)	16.0 (11.4 to 21.6)	0.81 (0.58 to 1.10)	0.60 (0.43 to 0.82)
1995	0.4 (0.3 to 0.6)	0.3 (0.2 to 0.4)	20.8 (14.8 to 27.6)	15.3 (10.9 to 20.3)	0.83 (0.60 to 1.11)	0.61 (0.44 to 0.81)
2000	0.4 (0.3 to 0.6)	0.3 (0.2 to 0.4)	21.8 (15.9 to 29.4)	16.0 (11.6 to 21.4)	0.93 (0.67 to 1.25)	0.68 (0.49 to 0.91)
2005	0.6 (0.4 to 0.8)	0.4 (0.3 to 0.6)	28.8 (21.0 to 38.6)	21.6 (15.7 to 28.8)	1.27 (0.93 to 1.70)	0.95 (0.70 to 1.27)
2010	0.7 (0.5 to 1.0)	0.5 (0.4 to 0.7)	33.1 (24.5 to 43.6)	24.6 (18.2 to 32.3)	1.59 (1.17 to 2.09)	1.18 (0.87 to 1.55)
2015	0.6 (0.5 to 0.9)	0.5 (0.4 to 0.6)	27.8 (20.8 to 36.3)	20.4 (15.3 to 26.6)	1.56 (1.17 to 2.04)	1.14 (0.86 to 1.49)
2020	0.6 (0.5 to 0.9)	0.5 (0.3 to 0.6)	23.8 (17.4 to 32.6)	17.1 (12.5 to 23.4)	1.60 (1.17 to 2.20)	1.15 (0.84 to 1.57)
2021	0.6 (0.5 to 0.9)	0.5 (0.3 to 0.6)	23.6 (17.2 to 32.7)	16.9 (12.3 to 23.4)	1.62 (1.18 to 2.25)	1.16 (0.85 to 1.60)

Riau IDN_4712 Southeast Asia Stillbirth Rate / Neonatal Mortality Rate (SBR/NMR) − 20 weeks
lambda = 0.8 ; zeta = 0.7 ; scale = 15 ; amp = 0.028

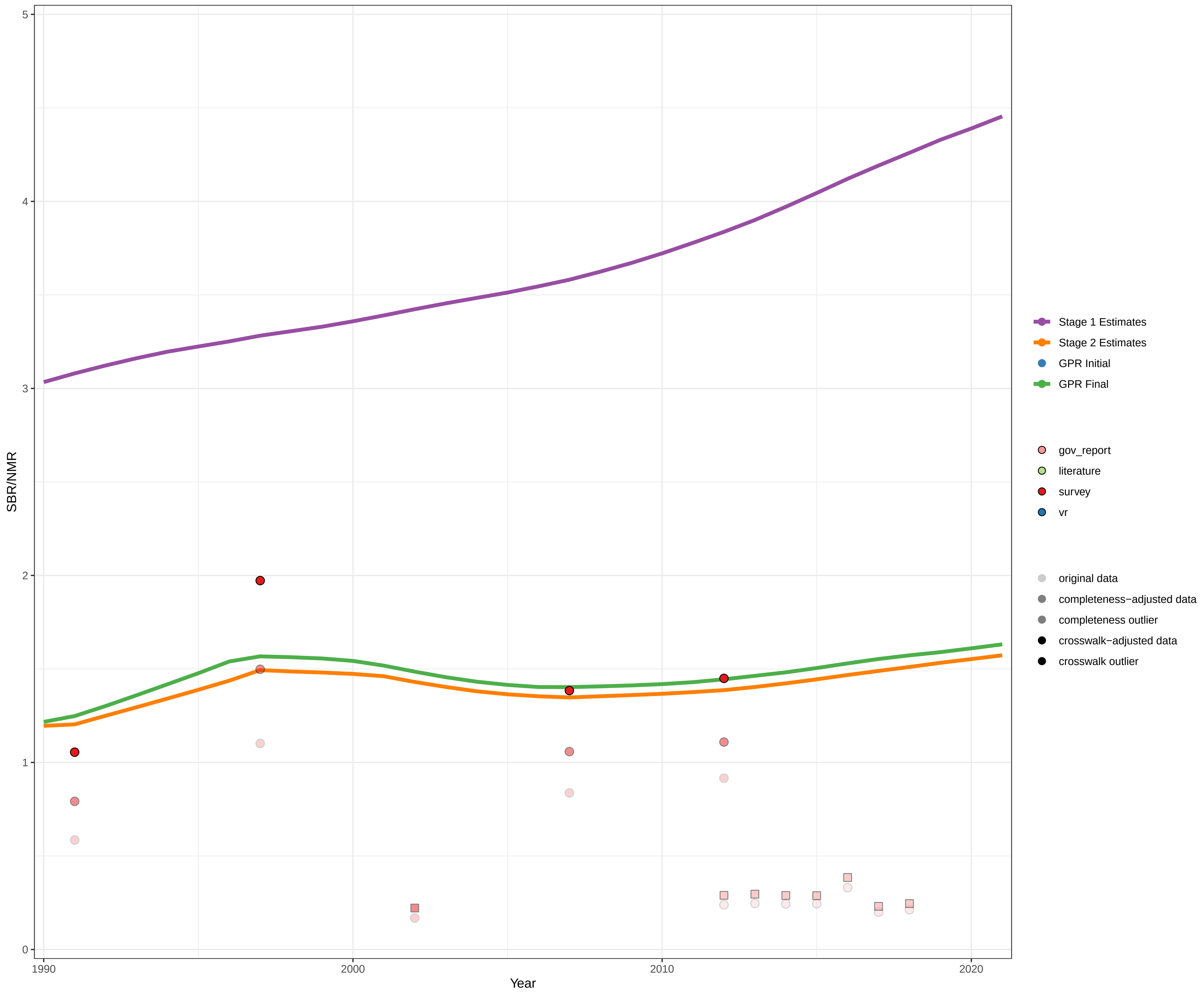

Riau IDN_4712 Southeast Asia Stillbirth Rate / Neonatal Mortality Rate (SBR/NMR) − 28 weeks
lambda = 0.8 ; zeta = 0.7 ; scale = 15 ; amp = 0.028

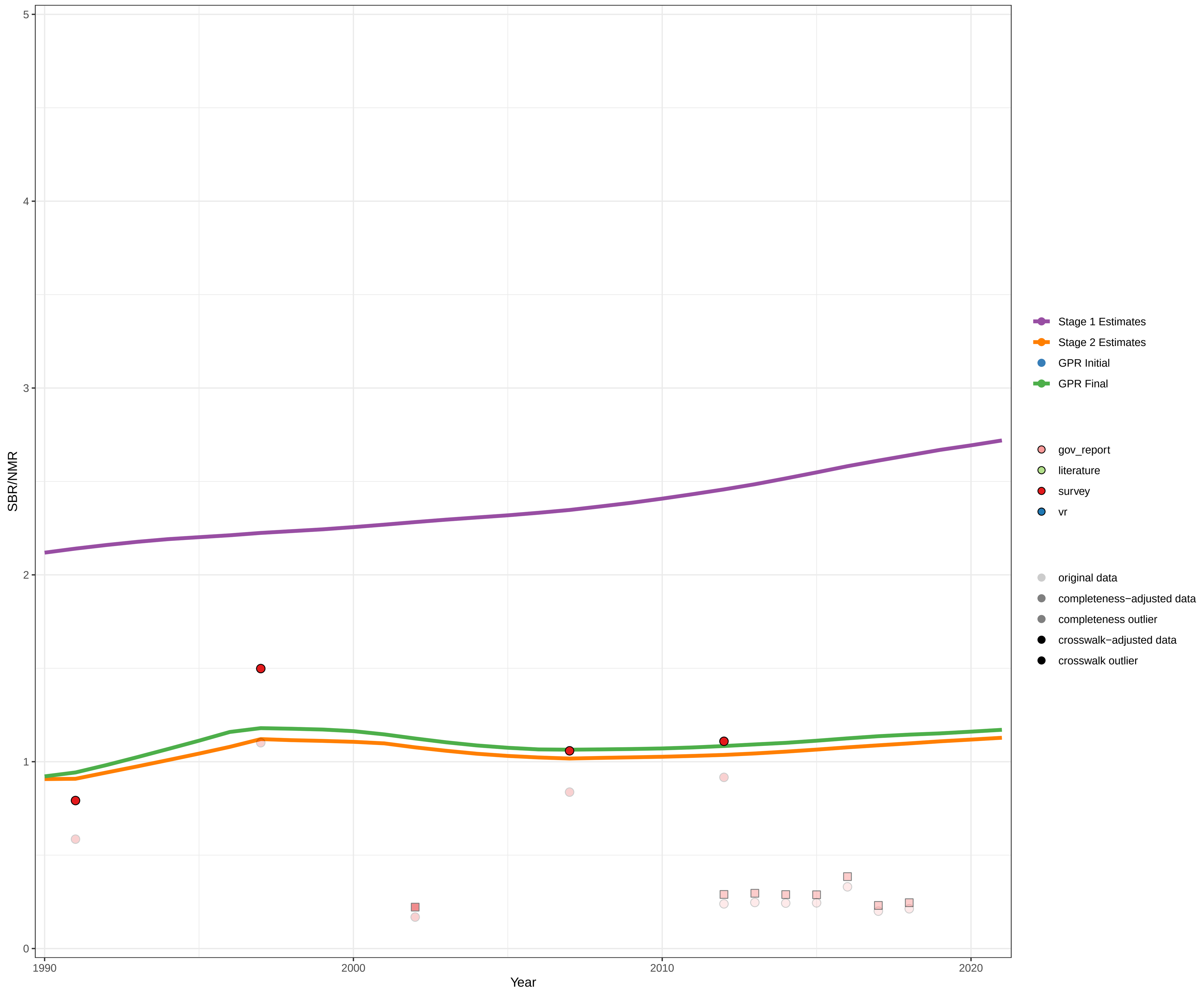

Definition	Years	Outlier	Source.Type	Title
28_weeks	1991	0	Survey	Indonesia Demographic and Health Survey 1991
28_weeks	1997	0	Survey	Indonesia Demographic and Health Survey 1997
28_weeks	2007	0	Survey	Indonesia Demographic and Health Survey 2007
28_weeks	2012	0	Survey	Indonesia Demographic and Health Survey 2012
28_weeks	2002	1	Survey	Indonesia Demographic and Health Survey 2002−2003
28_weeks	2012−2018	1	Statistical Report	Indonesia − Riau Province Health Profile

Riau IDN_4712 Southeast Asia Stillbirth Rate (SBR) − 20 weeks
lambda = 0.8 ; zeta = 0.7 ; scale = 15 ; amp = 0.028

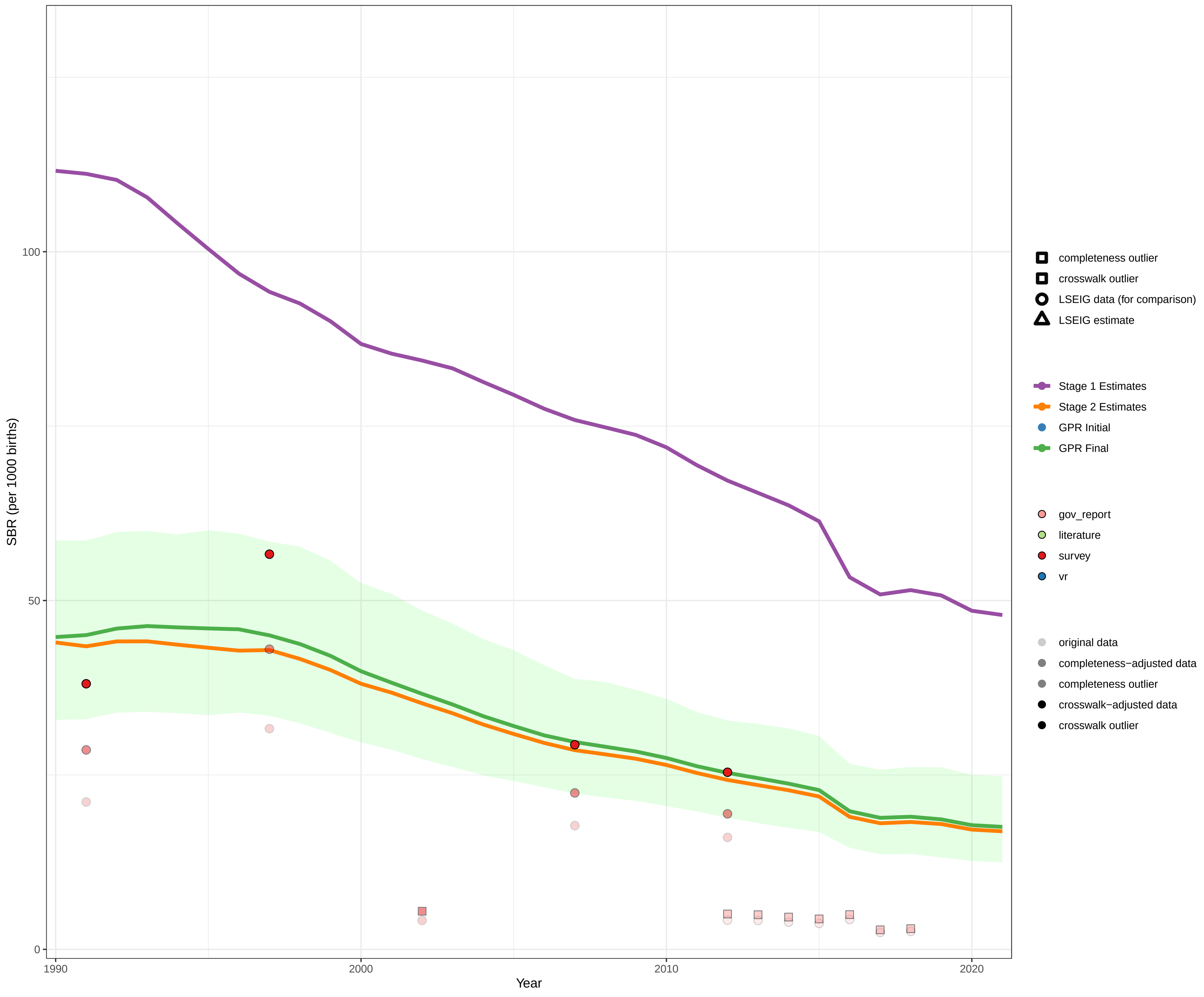

Riau IDN_4712 Southeast Asia Stillbirth Rate (SBR) − 28 weeks
lambda = 0.8 ; zeta = 0.7 ; scale = 15 ; amp = 0.028

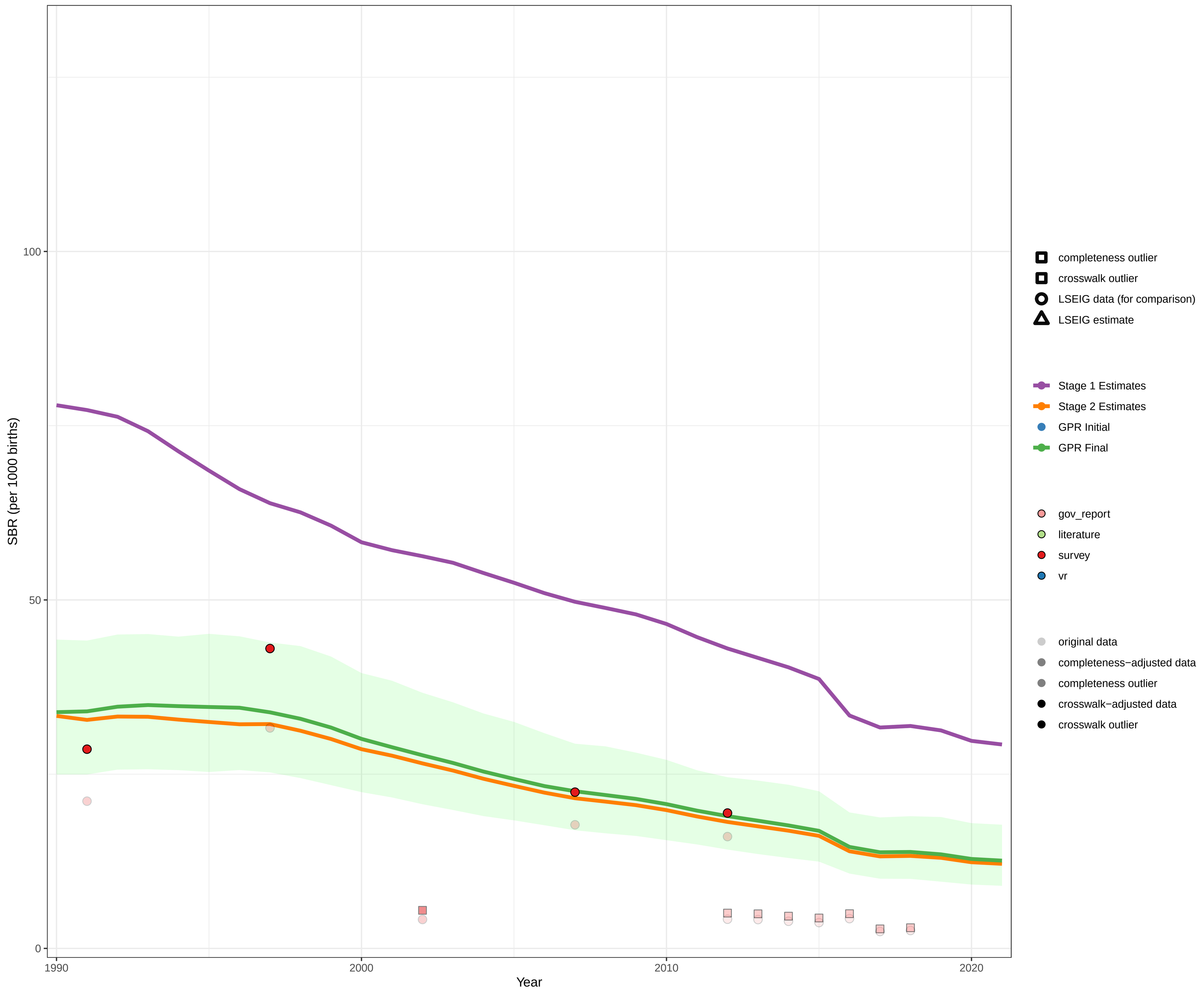

Year	Total Stillbirths (in thousands)	Total Stillbirths (in thousands)	SBR (per 1000 births)	SBR (per 1000 births)	SBR/NMR	SBR/NMR
	20 weeks	28 weeks	20 weeks	28 weeks	20 weeks	28 weeks
1990	3.3 (2.4 to 4.4)	2.5 (1.8 to 3.3)	44.8 (32.9 to 58.6)	33.9 (25.0 to 44.3)	1.22 (0.89 to 1.59)	0.92 (0.68 to 1.20)
1995	3.9 (2.8 to 5.1)	2.9 (2.1 to 3.8)	46.0 (33.5 to 60.1)	34.6 (25.3 to 45.1)	1.48 (1.08 to 1.93)	1.11 (0.81 to 1.45)
2000	4.2 (3.1 to 5.5)	3.1 (2.3 to 4.1)	39.9 (29.7 to 52.5)	30.1 (22.4 to 39.5)	1.54 (1.15 to 2.03)	1.16 (0.87 to 1.53)
2005	4.1 (3.1 to 5.6)	3.1 (2.3 to 4.2)	32.0 (24.1 to 42.9)	24.3 (18.4 to 32.5)	1.41 (1.07 to 1.89)	1.07 (0.81 to 1.44)
2010	3.9 (2.9 to 5.1)	2.9 (2.2 to 3.8)	27.4 (20.5 to 35.9)	20.7 (15.5 to 27.1)	1.42 (1.06 to 1.86)	1.07 (0.80 to 1.40)
2015	3.0 (2.2 to 4.1)	2.2 (1.6 to 3.0)	22.8 (16.8 to 30.6)	16.9 (12.4 to 22.6)	1.50 (1.11 to 2.02)	1.11 (0.82 to 1.49)
2020	2.3 (1.6 to 3.2)	1.6 (1.1 to 2.3)	17.8 (12.7 to 25.0)	12.8 (9.2 to 18.0)	1.61 (1.15 to 2.26)	1.16 (0.83 to 1.63)
2021	2.2 (1.6 to 3.1)	1.6 (1.1 to 2.2)	17.6 (12.5 to 24.8)	12.6 (9.0 to 17.7)	1.63 (1.16 to 2.31)	1.17 (0.83 to 1.65)

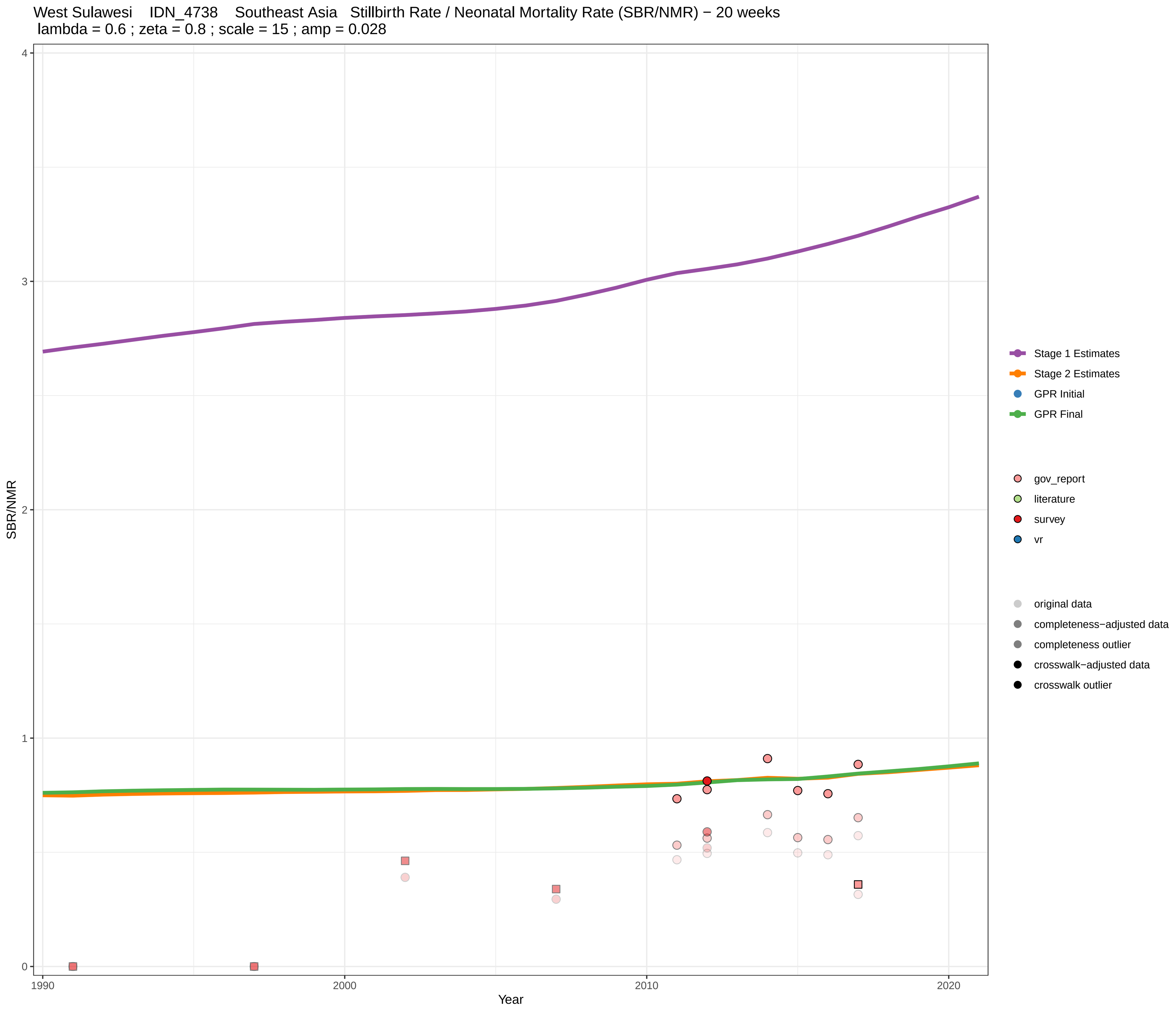

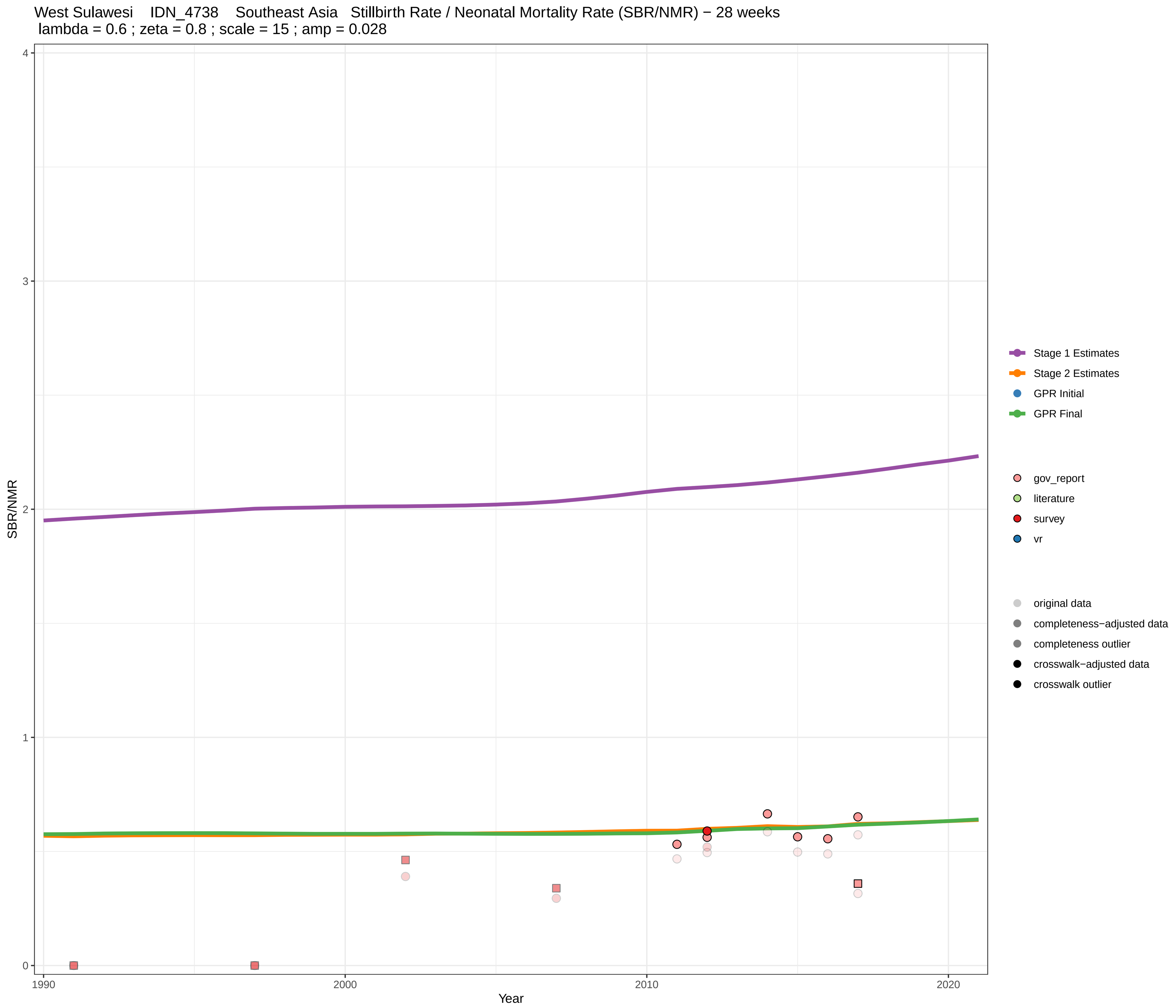

Definition	Years	Outlier	Source.Type	Title
28_weeks	2011, 2012, 2014−2017	0	Statistical Report	Indonesia − Sulawesi Barat Province Health Profile
28_weeks	2012	0	Survey	Indonesia Demographic and Health Survey 2012
28_weeks	1991	1	Survey	Indonesia Demographic and Health Survey 1991
28_weeks	1997	1	Survey	Indonesia Demographic and Health Survey 1997
28_weeks	2002	1	Survey	Indonesia Demographic and Health Survey 2002−2003
28_weeks	2007	1	Survey	Indonesia Demographic and Health Survey 2007
28_weeks	2017	2	Statistical Report	Indonesia − Sulawesi Barat Province Health Profile

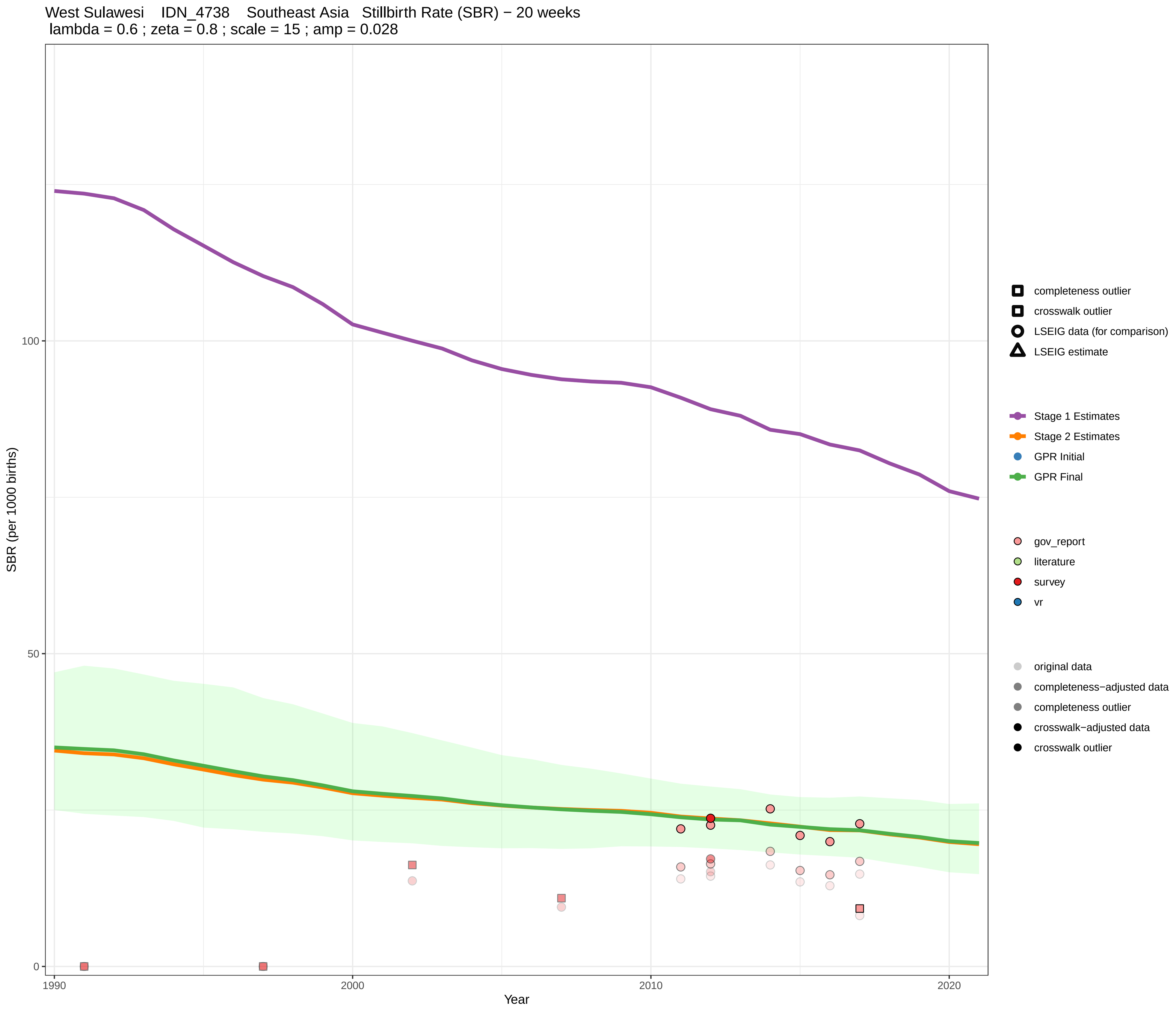

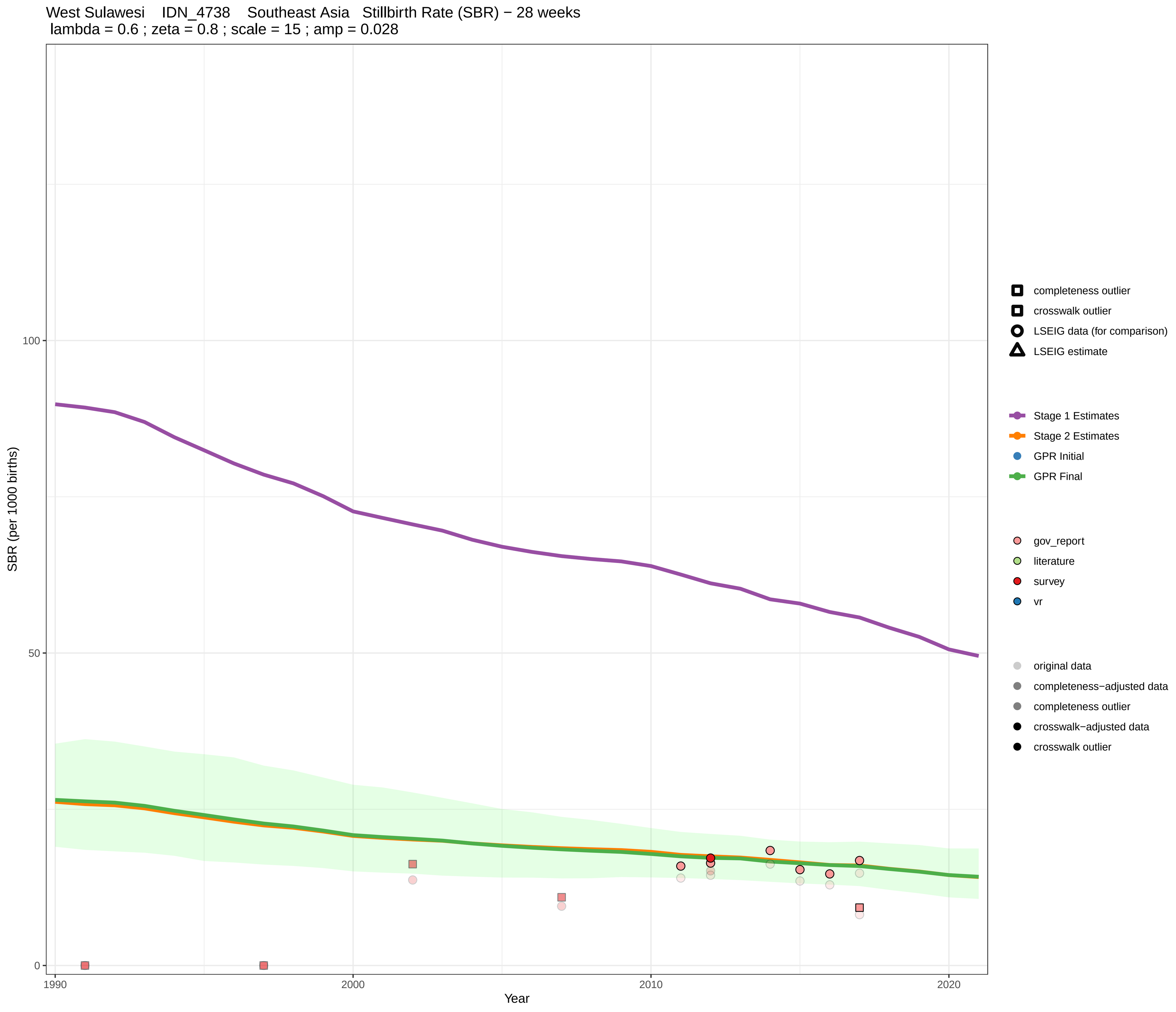

Year	Total Stillbirths (in thousands)	Total Stillbirths (in thousands)	SBR (per 1000 births)	SBR (per 1000 births)	SBR/NMR	SBR/NMR
	20 weeks	28 weeks	20 weeks	28 weeks	20 weeks	28 weeks
1990	0.9 (0.6 to 1.2)	0.7 (0.5 to 0.9)	35.0 (25.0 to 47.0)	26.5 (19.0 to 35.5)	0.76 (0.54 to 1.02)	0.58 (0.41 to 0.77)
1995	0.8 (0.6 to 1.2)	0.6 (0.4 to 0.9)	32.1 (22.2 to 45.2)	24.1 (16.7 to 33.8)	0.77 (0.54 to 1.09)	0.58 (0.40 to 0.82)
2000	0.8 (0.6 to 1.1)	0.6 (0.4 to 0.8)	28.0 (20.1 to 38.9)	20.9 (15.1 to 28.9)	0.77 (0.56 to 1.08)	0.58 (0.42 to 0.80)
2005	0.8 (0.6 to 1.1)	0.6 (0.4 to 0.8)	25.8 (18.9 to 33.8)	19.1 (14.1 to 25.0)	0.78 (0.57 to 1.02)	0.58 (0.42 to 0.76)
2010	0.8 (0.6 to 1.0)	0.6 (0.5 to 0.7)	24.3 (19.2 to 30.0)	17.8 (14.1 to 22.0)	0.79 (0.62 to 0.98)	0.58 (0.46 to 0.71)
2015	0.7 (0.6 to 0.9)	0.5 (0.4 to 0.7)	22.3 (17.9 to 27.1)	16.4 (13.1 to 19.8)	0.82 (0.66 to 1.00)	0.60 (0.48 to 0.73)
2020	0.7 (0.5 to 0.9)	0.5 (0.4 to 0.6)	20.0 (15.1 to 26.0)	14.5 (10.9 to 18.7)	0.88 (0.66 to 1.14)	0.63 (0.48 to 0.82)
2021	0.7 (0.5 to 0.9)	0.5 (0.4 to 0.6)	19.7 (14.8 to 26.1)	14.2 (10.7 to 18.7)	0.89 (0.67 to 1.18)	0.64 (0.48 to 0.84)

South Sulawesi IDN_4735 Southeast Asia Stillbirth Rate / Neonatal Mortality Rate (SBR/NMR) − 20 weeks
lambda = 0.4 ; zeta = 0.9 ; scale = 10 ; amp = 0.028

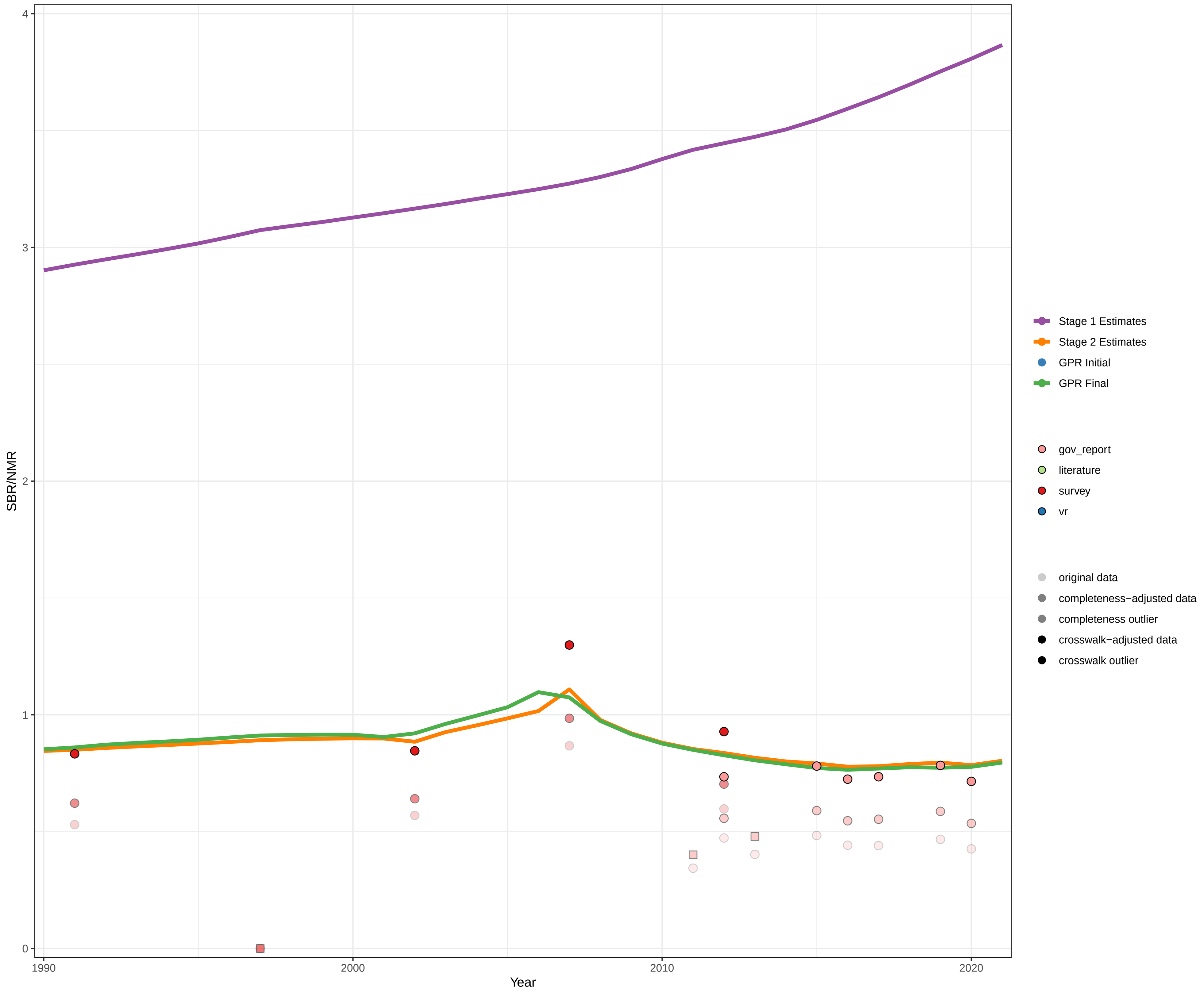

South Sulawesi IDN_4735 Southeast Asia Stillbirth Rate / Neonatal Mortality Rate (SBR/NMR) − 28 weeks
lambda = 0.4 ; zeta = 0.9 ; scale = 10 ; amp = 0.028

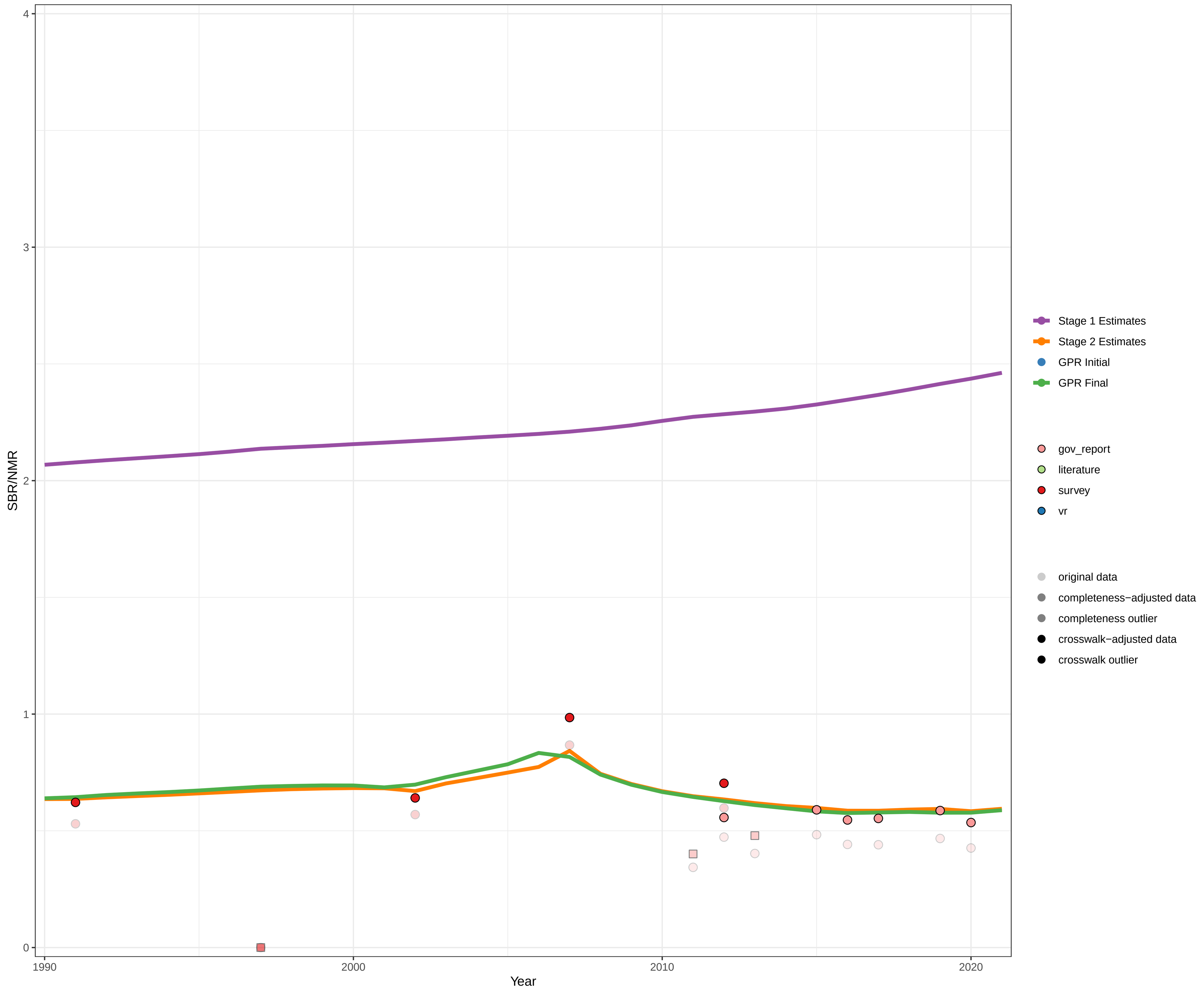

Definition	Years	Outlier	Source.Type	Title
28_weeks	1991	0	Survey	Indonesia Demographic and Health Survey 1991
28_weeks	2002	0	Survey	Indonesia Demographic and Health Survey 2002−2003
28_weeks	2007	0	Survey	Indonesia Demographic and Health Survey 2007
28_weeks	2012	0	Survey	Indonesia Demographic and Health Survey 2012
28_weeks	2012, 2015−2017, 2019, 2020	0	Statistical Report	Indonesia − Sulawesi Selatan Province Health Profile
28_weeks	1997	1	Survey	Indonesia Demographic and Health Survey 1997
28_weeks	2011, 2013	1	Statistical Report	Indonesia − Sulawesi Selatan Province Health Profile

South Sulawesi IDN_4735 Southeast Asia Stillbirth Rate (SBR) − 20 weeks
lambda = 0.4 ; zeta = 0.9 ; scale = 10 ; amp = 0.028

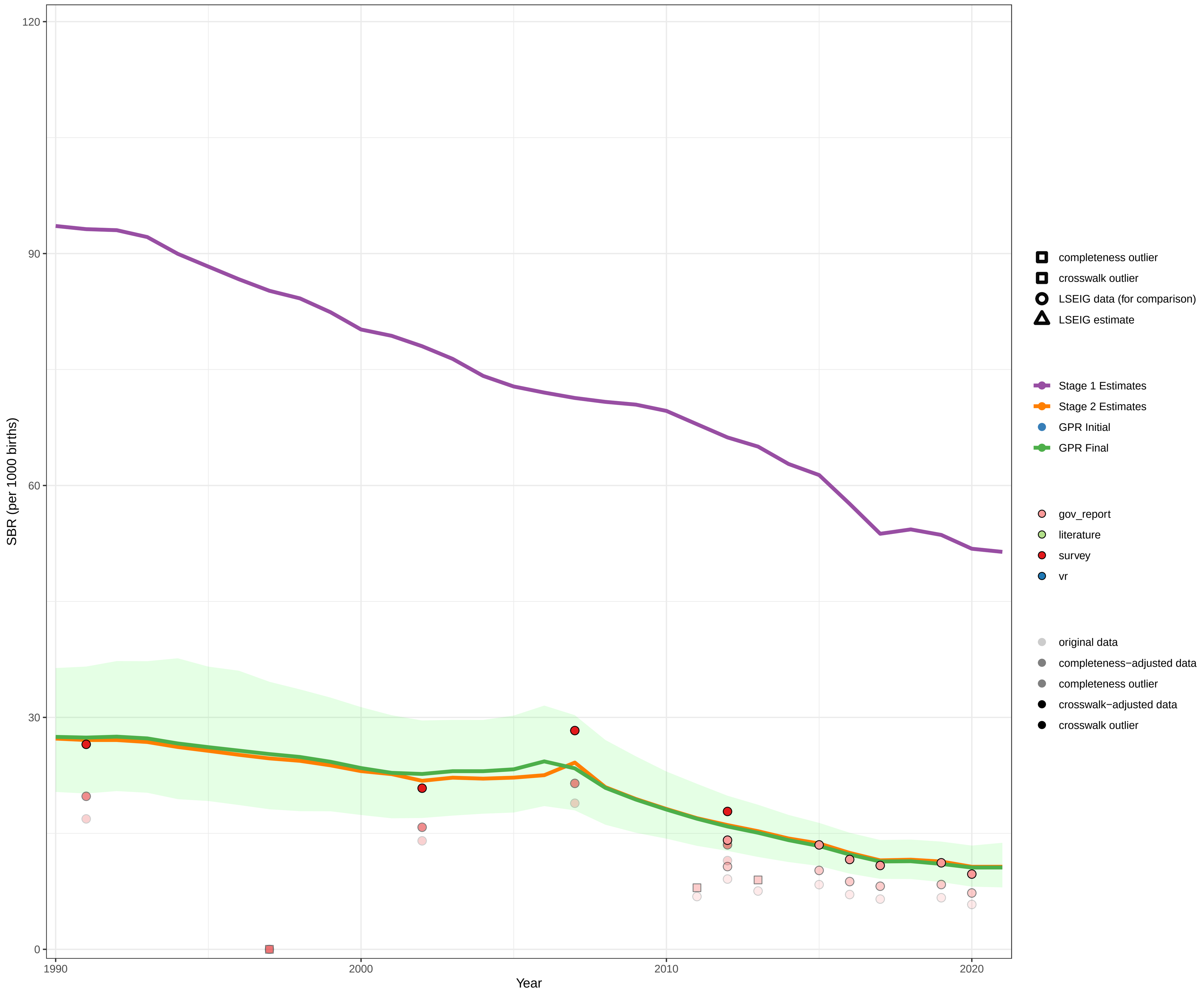

South Sulawesi IDN_4735 Southeast Asia Stillbirth Rate (SBR) − 28 weeks
lambda = 0.4 ; zeta = 0.9 ; scale = 10 ; amp = 0.028

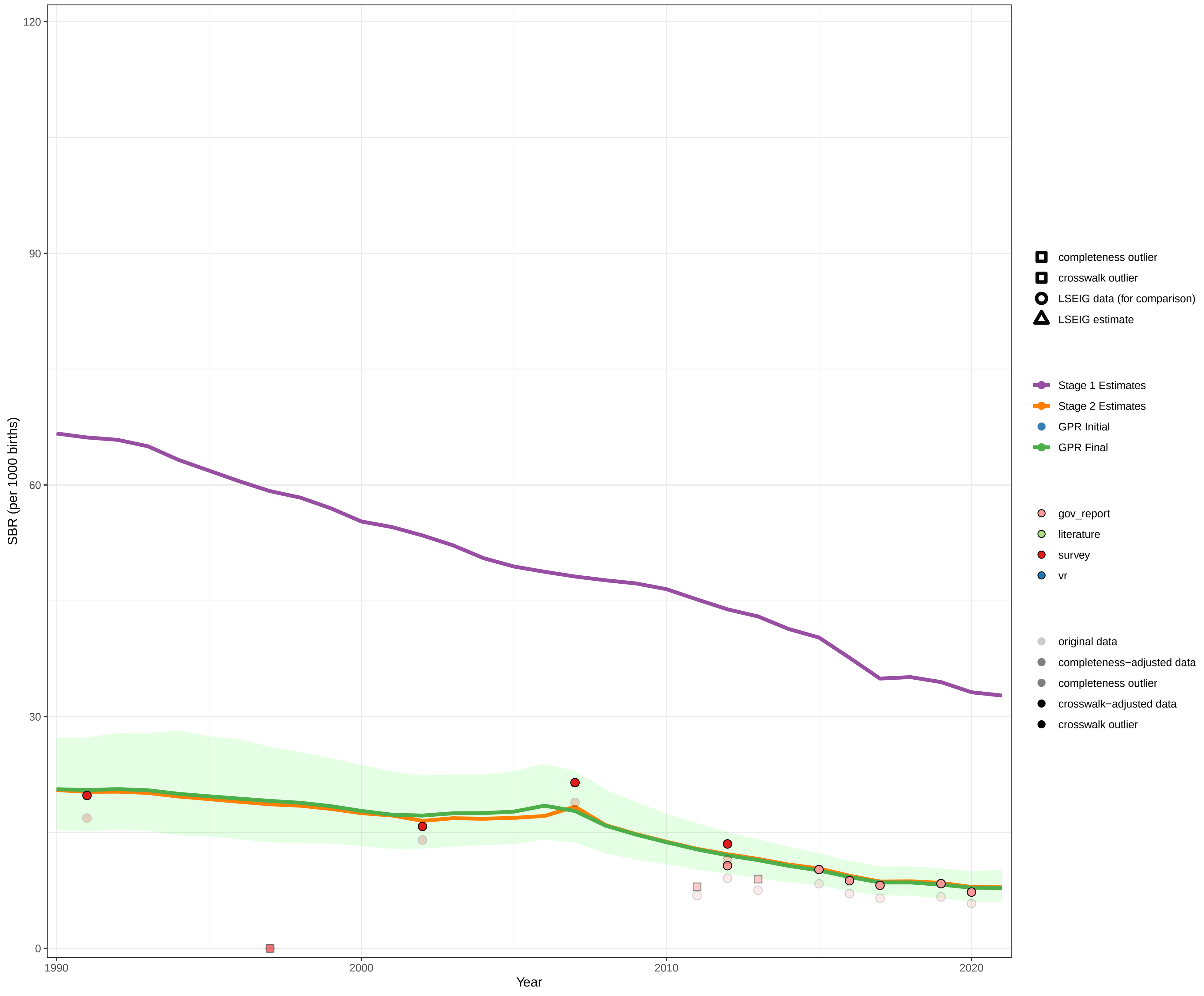

Year	Total Stillbirths (in thousands)	Total Stillbirths (in thousands)	SBR (per 1000 births)	SBR (per 1000 births)	SBR/NMR	SBR/NMR
	20 weeks	28 weeks	20 weeks	28 weeks	20 weeks	28 weeks
1990	5.2 (3.8 to 7.0)	3.9 (2.9 to 5.2)	27.5 (20.4 to 36.4)	20.6 (15.3 to 27.2)	0.85 (0.63 to 1.13)	0.64 (0.47 to 0.84)
1995	5.0 (3.6 to 7.0)	3.7 (2.7 to 5.2)	26.2 (19.2 to 36.6)	19.7 (14.5 to 27.5)	0.89 (0.66 to 1.25)	0.67 (0.49 to 0.94)
2000	4.4 (3.2 to 5.9)	3.3 (2.5 to 4.5)	23.4 (17.4 to 31.3)	17.8 (13.2 to 23.7)	0.91 (0.68 to 1.22)	0.69 (0.52 to 0.93)
2005	4.3 (3.3 to 5.7)	3.3 (2.5 to 4.3)	23.3 (17.7 to 30.2)	17.7 (13.5 to 23.0)	1.03 (0.78 to 1.34)	0.79 (0.60 to 1.02)
2010	3.2 (2.5 to 4.1)	2.4 (1.9 to 3.1)	18.1 (14.3 to 23.0)	13.7 (10.9 to 17.5)	0.88 (0.69 to 1.12)	0.67 (0.53 to 0.85)
2015	2.3 (1.8 to 2.8)	1.7 (1.4 to 2.1)	13.4 (10.8 to 16.4)	10.1 (8.2 to 12.3)	0.77 (0.62 to 0.95)	0.58 (0.47 to 0.71)
2020	1.8 (1.4 to 2.3)	1.3 (1.0 to 1.7)	10.6 (8.1 to 13.4)	7.9 (6.0 to 10.0)	0.78 (0.60 to 0.99)	0.58 (0.44 to 0.73)
2021	1.8 (1.3 to 2.3)	1.3 (1.0 to 1.7)	10.6 (8.0 to 13.8)	7.8 (5.9 to 10.2)	0.80 (0.60 to 1.04)	0.59 (0.45 to 0.76)

Central Sulawesi IDN_4734 Southeast Asia Stillbirth Rate / Neonatal Mortality Rate (SBR/NMR) − 20 weeks
lambda = 0.4 ; zeta = 0.9 ; scale = 10 ; amp = 0.028

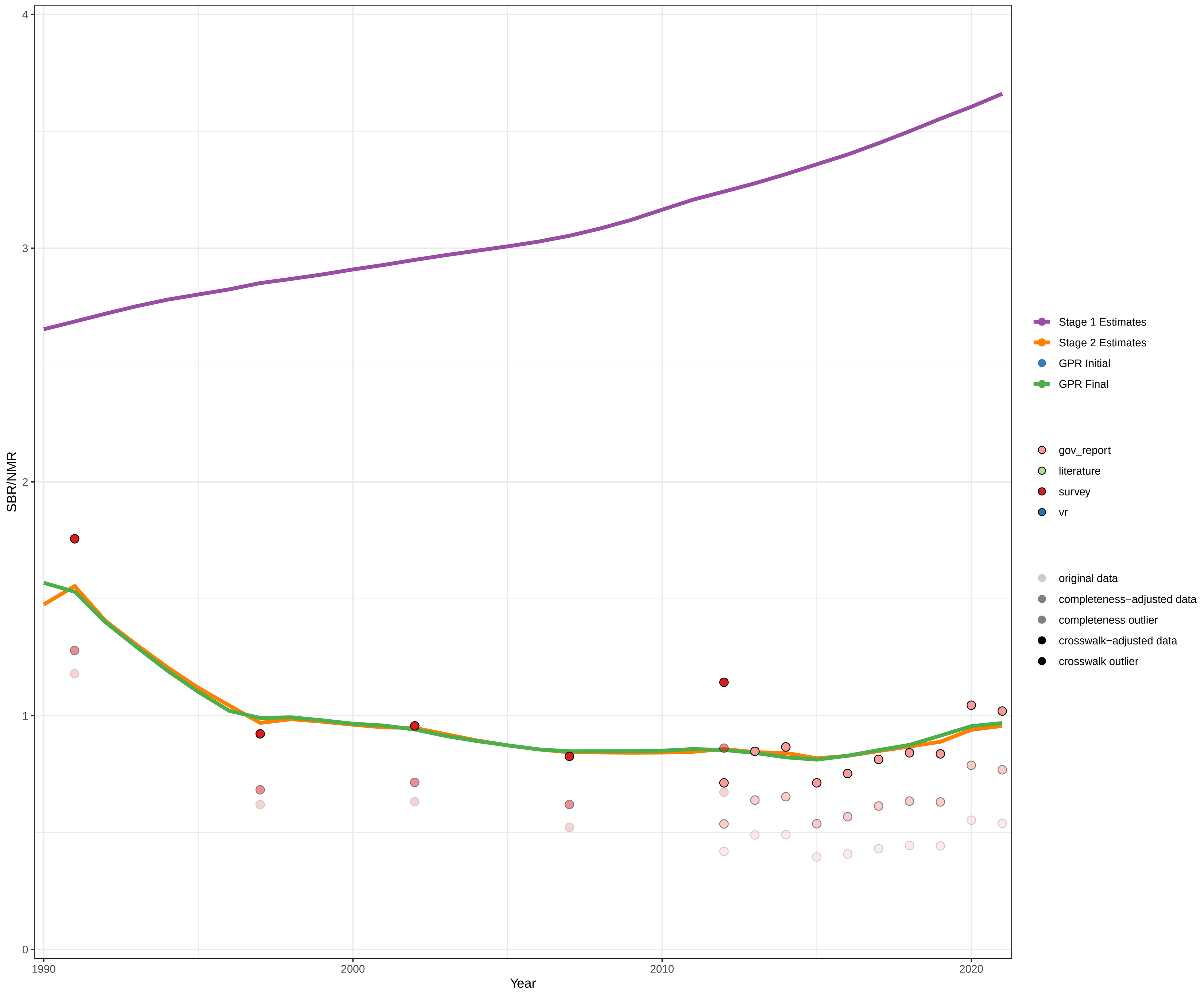

Central Sulawesi IDN_4734 Southeast Asia Stillbirth Rate / Neonatal Mortality Rate (SBR/NMR) − 28 weeks
lambda = 0.4 ; zeta = 0.9 ; scale = 10 ; amp = 0.028

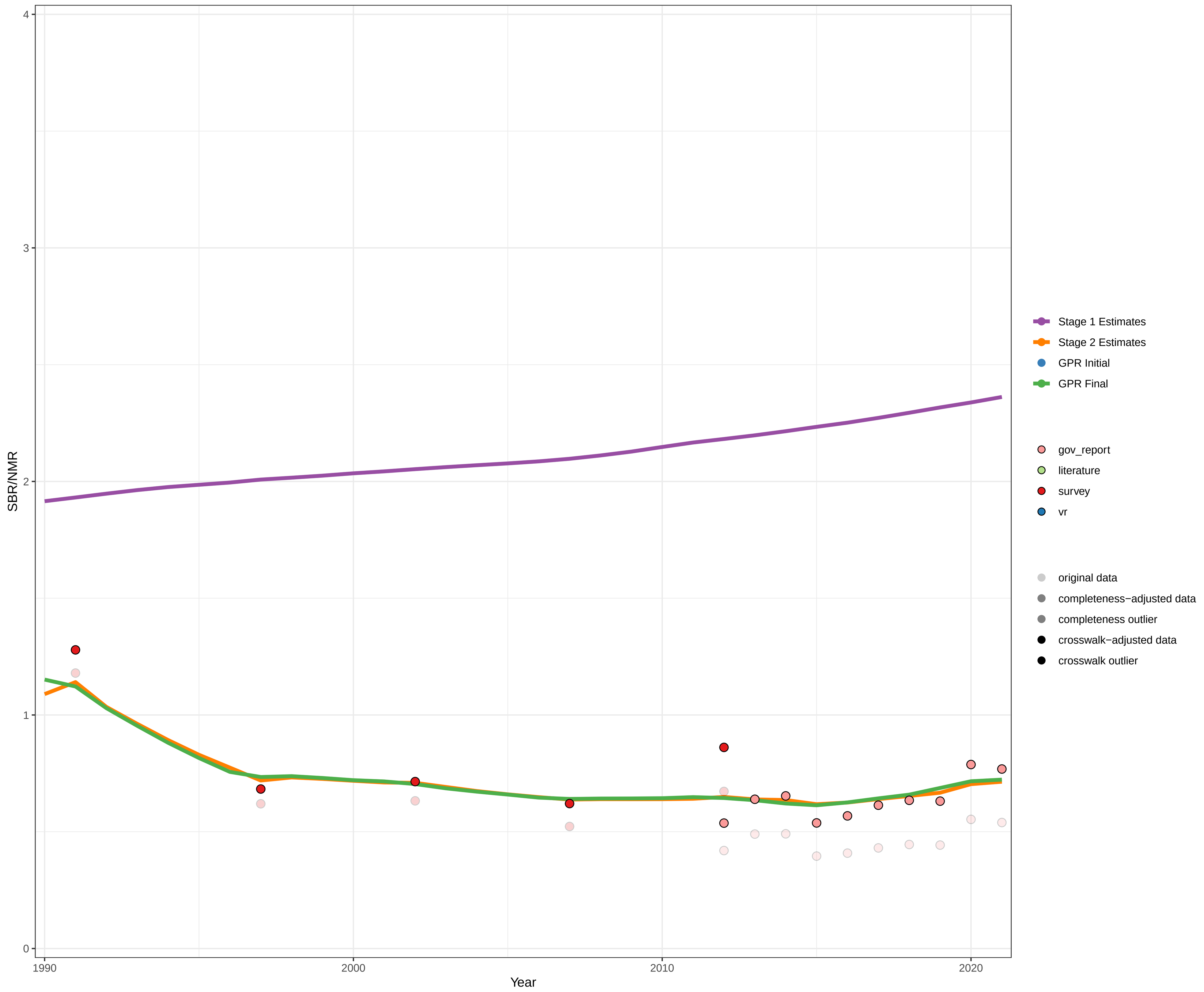

Definition	Years	Outlier	Source.Type	Title
28_weeks	1991	0	Survey	Indonesia Demographic and Health Survey 1991
28_weeks	1997	0	Survey	Indonesia Demographic and Health Survey 1997
28_weeks	2002	0	Survey	Indonesia Demographic and Health Survey 2002−2003
28_weeks	2007	0	Survey	Indonesia Demographic and Health Survey 2007
28_weeks	2012	0	Survey	Indonesia Demographic and Health Survey 2012
28_weeks	2012−2021	0	Statistical Report	Indonesia − Sulawesi Tengah Province Health Profile

Central Sulawesi IDN_4734 Southeast Asia Stillbirth Rate (SBR) − 20 weeks
lambda = 0.4 ; zeta = 0.9 ; scale = 10 ; amp = 0.028

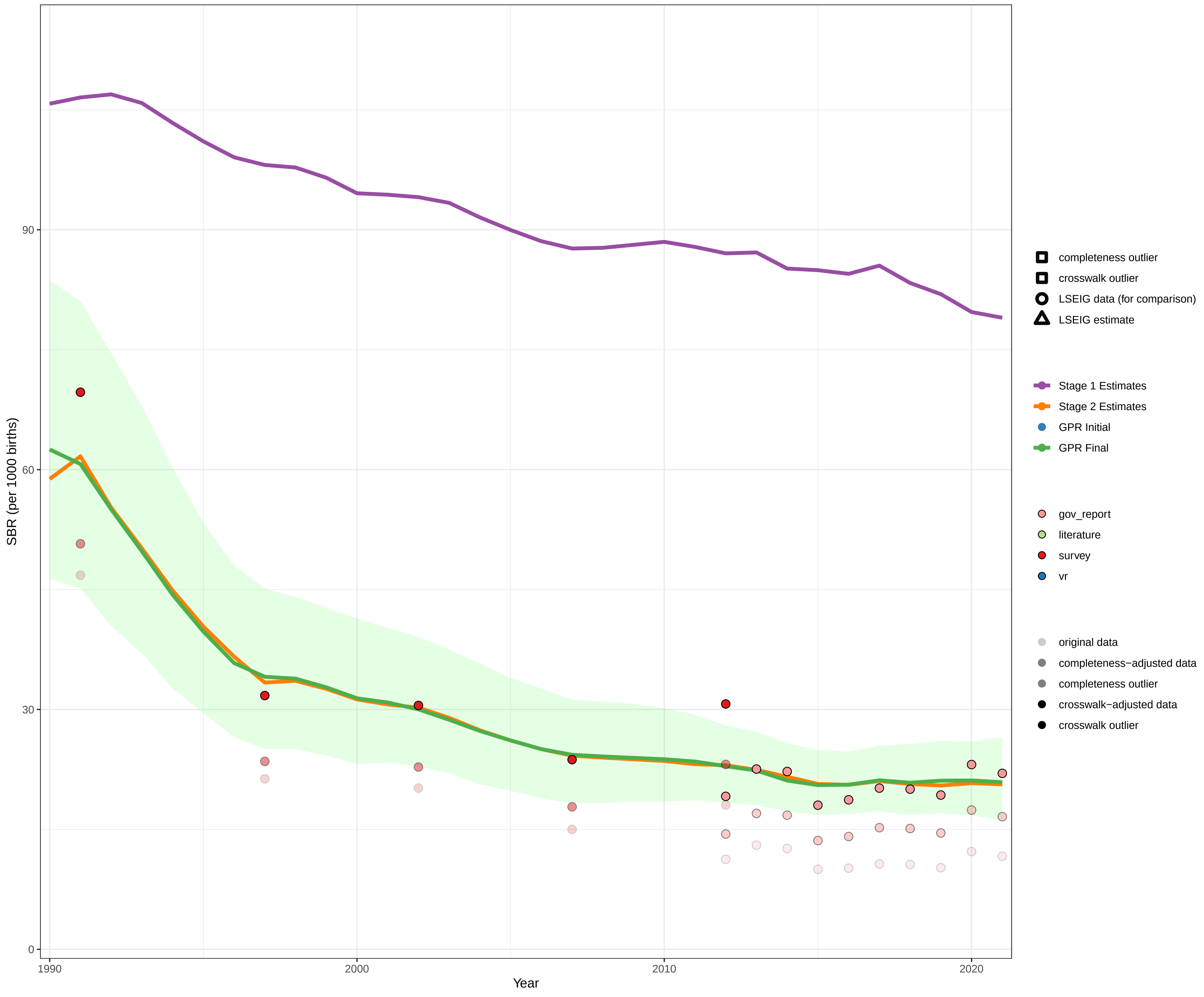

Central Sulawesi IDN_4734 Southeast Asia Stillbirth Rate (SBR) − 28 weeks
lambda = 0.4 ; zeta = 0.9 ; scale = 10 ; amp = 0.028

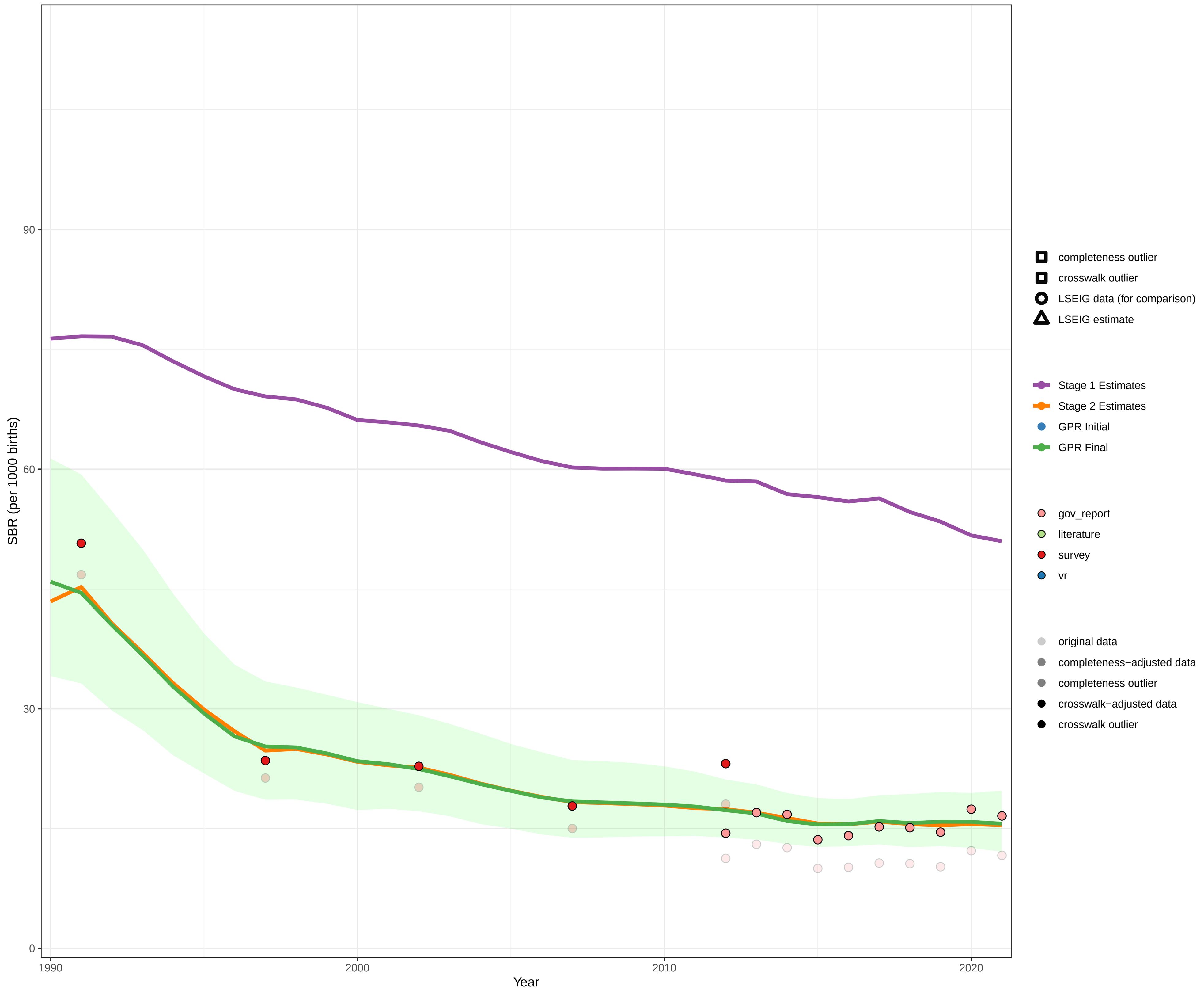

Year	Total Stillbirths (in thousands)	Total Stillbirths (in thousands)	SBR (per 1000 births)	SBR (per 1000 births)	SBR/NMR	SBR/NMR
	20 weeks	28 weeks	20 weeks	28 weeks	20 weeks	28 weeks
1990	3.3 (2.4 to 4.5)	2.4 (1.7 to 3.2)	62.5 (46.3 to 83.7)	45.9 (34.1 to 61.3)	1.57 (1.16 to 2.10)	1.15 (0.86 to 1.54)
1995	2.2 (1.6 to 3.0)	1.6 (1.2 to 2.2)	39.7 (29.5 to 53.4)	29.4 (21.9 to 39.4)	1.10 (0.82 to 1.48)	0.82 (0.61 to 1.09)
2000	2.0 (1.4 to 2.6)	1.5 (1.1 to 1.9)	31.4 (23.2 to 41.4)	23.4 (17.3 to 30.8)	0.97 (0.71 to 1.27)	0.72 (0.53 to 0.95)
2005	1.8 (1.3 to 2.3)	1.3 (1.0 to 1.7)	26.1 (19.8 to 34.0)	19.7 (15.0 to 25.6)	0.87 (0.66 to 1.14)	0.66 (0.50 to 0.86)
2010	1.6 (1.2 to 2.0)	1.2 (0.9 to 1.5)	23.8 (18.5 to 30.2)	18.0 (14.0 to 22.8)	0.85 (0.66 to 1.08)	0.64 (0.50 to 0.82)
2015	1.3 (1.0 to 1.6)	1.0 (0.8 to 1.2)	20.5 (16.8 to 25.0)	15.5 (12.7 to 18.8)	0.81 (0.66 to 0.99)	0.61 (0.50 to 0.74)
2020	1.3 (1.0 to 1.6)	1.0 (0.8 to 1.2)	21.1 (16.8 to 26.0)	15.8 (12.6 to 19.5)	0.96 (0.76 to 1.18)	0.72 (0.57 to 0.88)
2021	1.3 (1.0 to 1.6)	0.9 (0.7 to 1.2)	20.9 (16.2 to 26.5)	15.6 (12.1 to 19.8)	0.97 (0.75 to 1.23)	0.72 (0.56 to 0.92)

Southeast Sulawesi IDN_4736 Southeast Asia Stillbirth Rate / Neonatal Mortality Rate (SBR/NMR) − 20 weeks
lambda = 0.6 ; zeta = 0.8 ; scale = 15 ; amp = 0.028

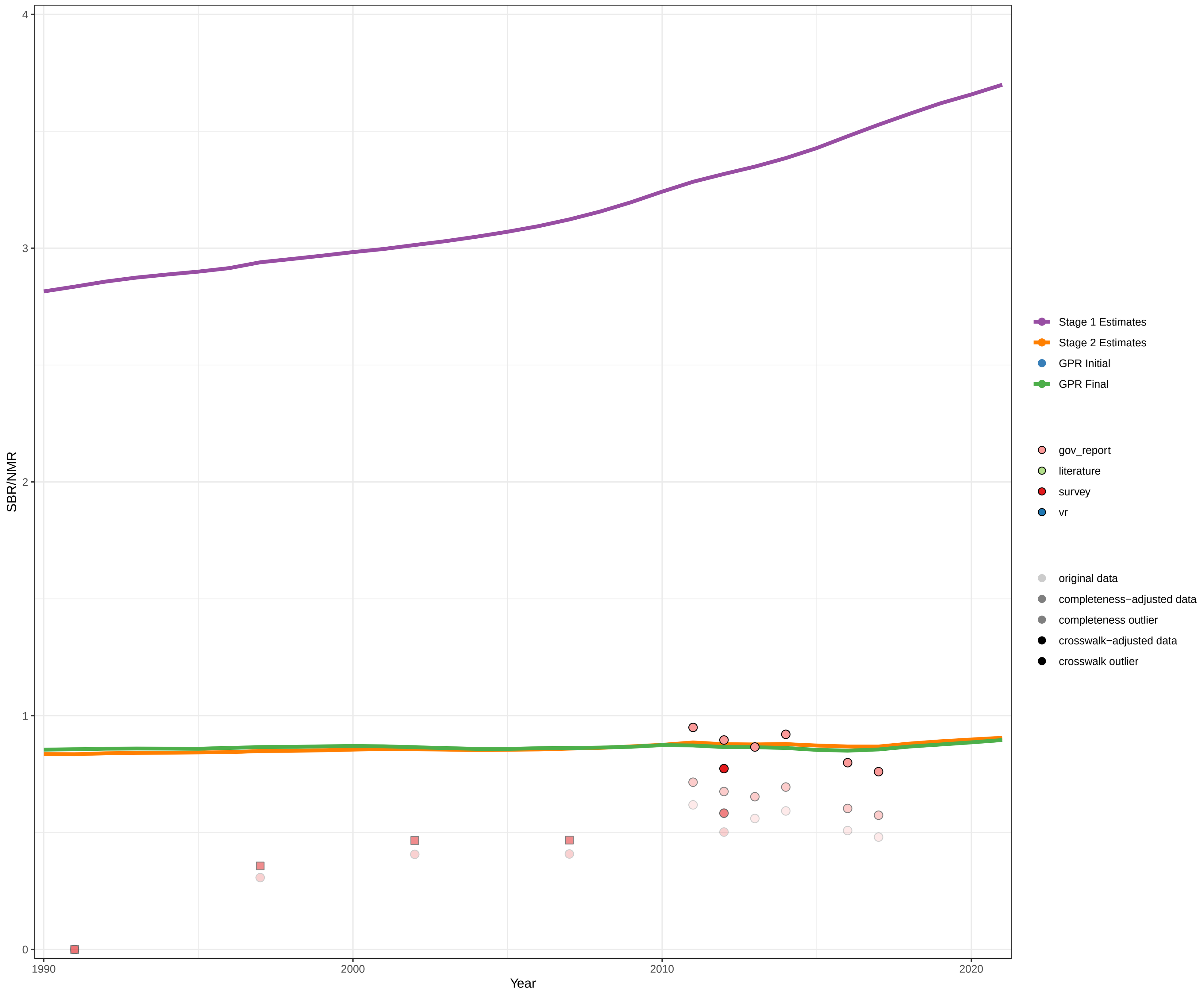

Southeast Sulawesi IDN_4736 Southeast Asia Stillbirth Rate / Neonatal Mortality Rate (SBR/NMR) − 28 weeks
lambda = 0.6 ; zeta = 0.8 ; scale = 15 ; amp = 0.028

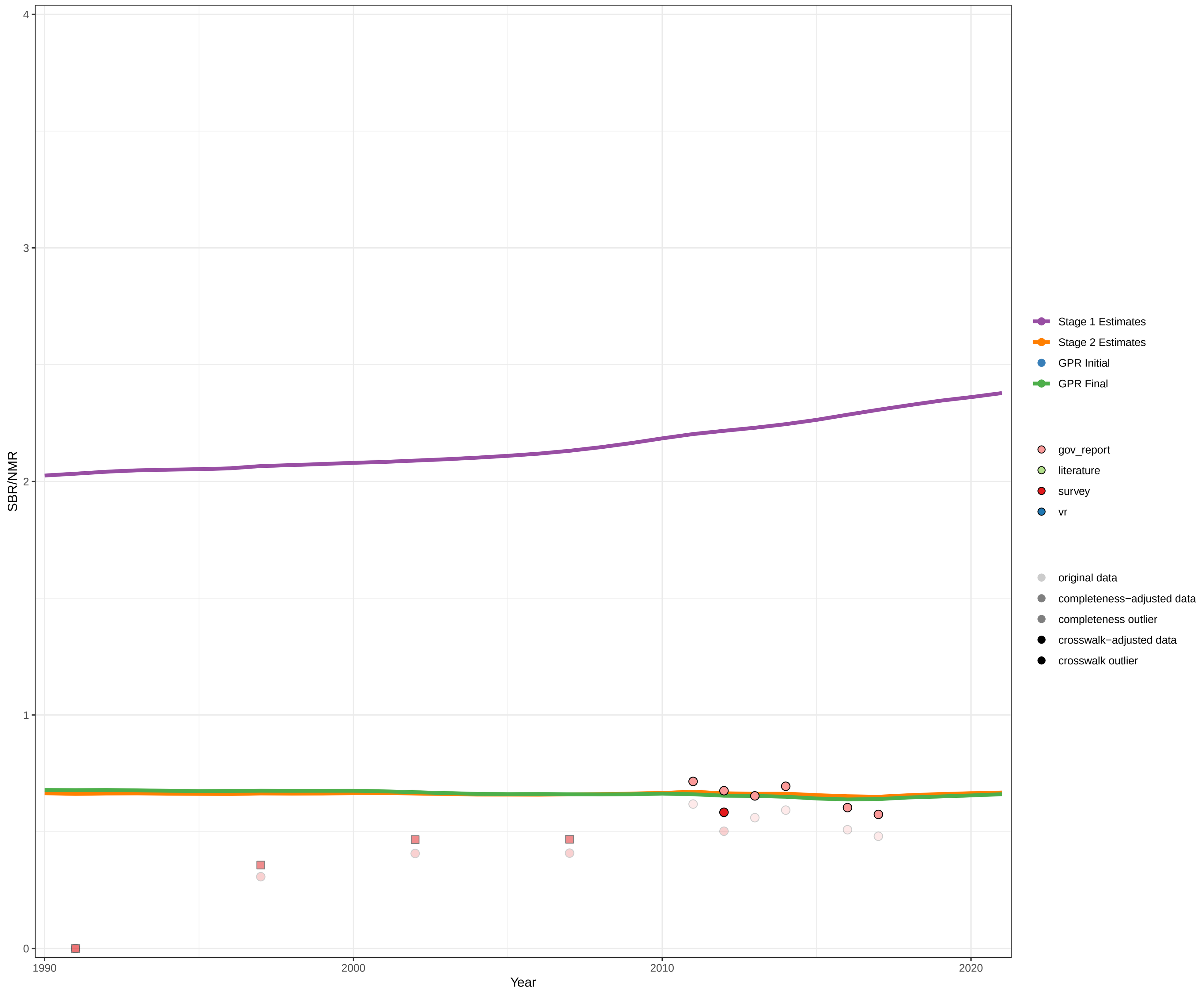

Definition	Years	Outlier	Source.Type	Title
28_weeks	2011−2014, 2016, 2017	0	Statistical Report	Indonesia − Sulawesi Tenggara Province Health Profile
28_weeks	2012	0	Survey	Indonesia Demographic and Health Survey 2012
28_weeks	1991	1	Survey	Indonesia Demographic and Health Survey 1991
28_weeks	1997	1	Survey	Indonesia Demographic and Health Survey 1997
28_weeks	2002	1	Survey	Indonesia Demographic and Health Survey 2002−2003
28_weeks	2007	1	Survey	Indonesia Demographic and Health Survey 2007

Southeast Sulawesi IDN_4736 Southeast Asia Stillbirth Rate (SBR) − 20 weeks
lambda = 0.6 ; zeta = 0.8 ; scale = 15 ; amp = 0.028

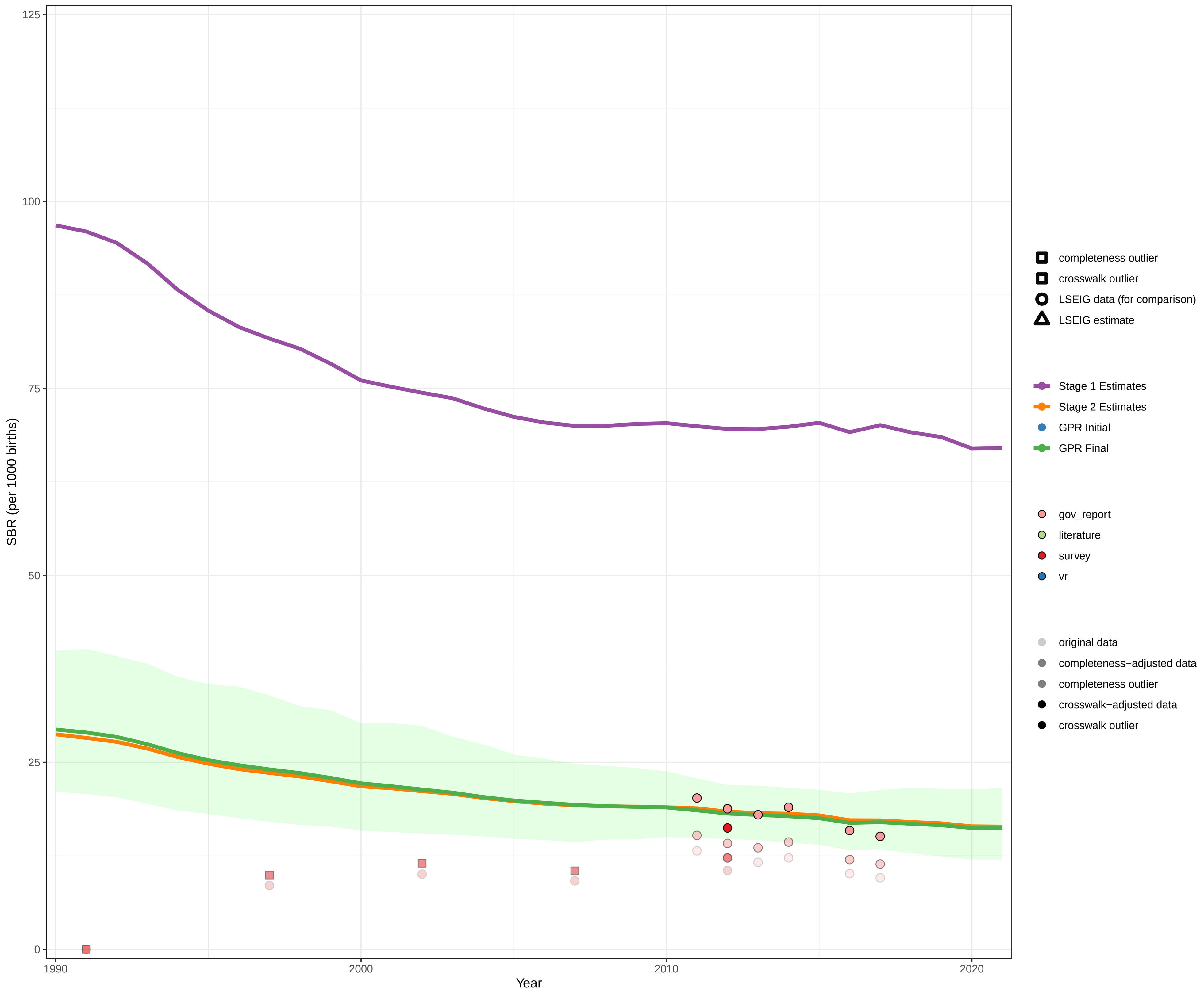

Southeast Sulawesi IDN_4736 Southeast Asia Stillbirth Rate (SBR) − 28 weeks
lambda = 0.6 ; zeta = 0.8 ; scale = 15 ; amp = 0.028

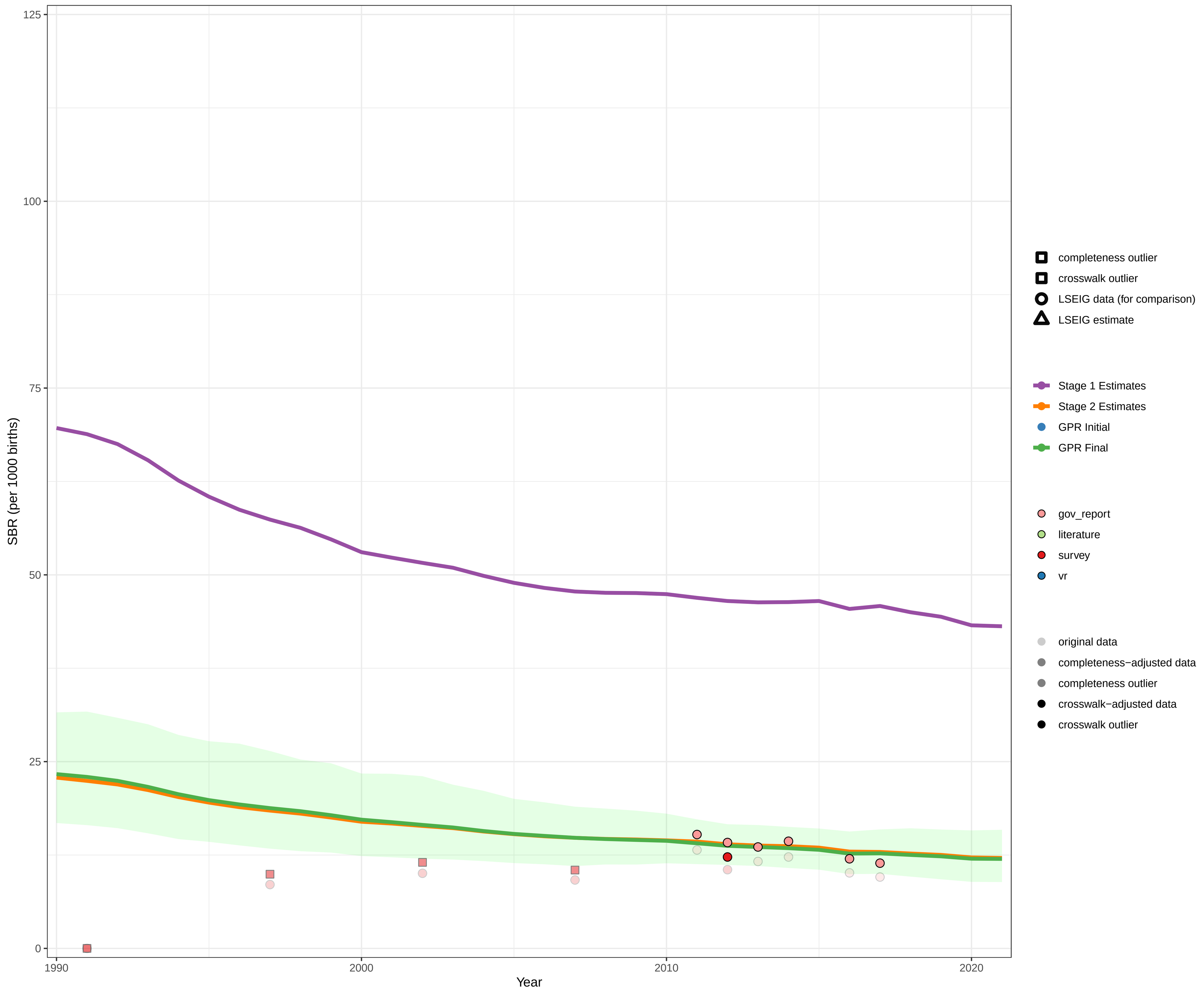

Year	Total Stillbirths (in thousands)	Total Stillbirths (in thousands)	SBR (per 1000 births)	SBR (per 1000 births)	SBR/NMR	SBR/NMR
	20 weeks	28 weeks	20 weeks	28 weeks	20 weeks	28 weeks
1990	1.3 (0.9 to 1.8)	1.0 (0.7 to 1.4)	29.4 (21.1 to 39.9)	23.3 (16.8 to 31.6)	0.85 (0.61 to 1.16)	0.68 (0.49 to 0.92)
1995	1.2 (0.8 to 1.7)	0.9 (0.7 to 1.3)	25.3 (18.1 to 35.4)	19.8 (14.3 to 27.7)	0.86 (0.62 to 1.20)	0.67 (0.48 to 0.94)
2000	1.2 (0.8 to 1.6)	0.9 (0.6 to 1.2)	22.2 (15.9 to 30.2)	17.2 (12.4 to 23.4)	0.87 (0.62 to 1.19)	0.68 (0.48 to 0.92)
2005	1.1 (0.8 to 1.5)	0.9 (0.6 to 1.1)	19.9 (14.8 to 26.1)	15.3 (11.4 to 20.0)	0.86 (0.64 to 1.12)	0.66 (0.49 to 0.86)
2010	1.1 (0.8 to 1.4)	0.8 (0.6 to 1.0)	19.0 (15.0 to 23.8)	14.4 (11.4 to 18.0)	0.87 (0.69 to 1.10)	0.66 (0.52 to 0.83)
2015	1.0 (0.8 to 1.2)	0.7 (0.6 to 0.9)	17.5 (14.0 to 21.3)	13.2 (10.5 to 16.0)	0.85 (0.68 to 1.04)	0.64 (0.51 to 0.78)
2020	0.9 (0.7 to 1.2)	0.7 (0.5 to 0.9)	16.2 (12.0 to 21.4)	12.0 (8.9 to 15.8)	0.89 (0.66 to 1.17)	0.66 (0.49 to 0.86)
2021	0.9 (0.7 to 1.2)	0.7 (0.5 to 0.9)	16.2 (12.0 to 21.6)	12.0 (8.9 to 15.9)	0.90 (0.66 to 1.19)	0.66 (0.49 to 0.88)

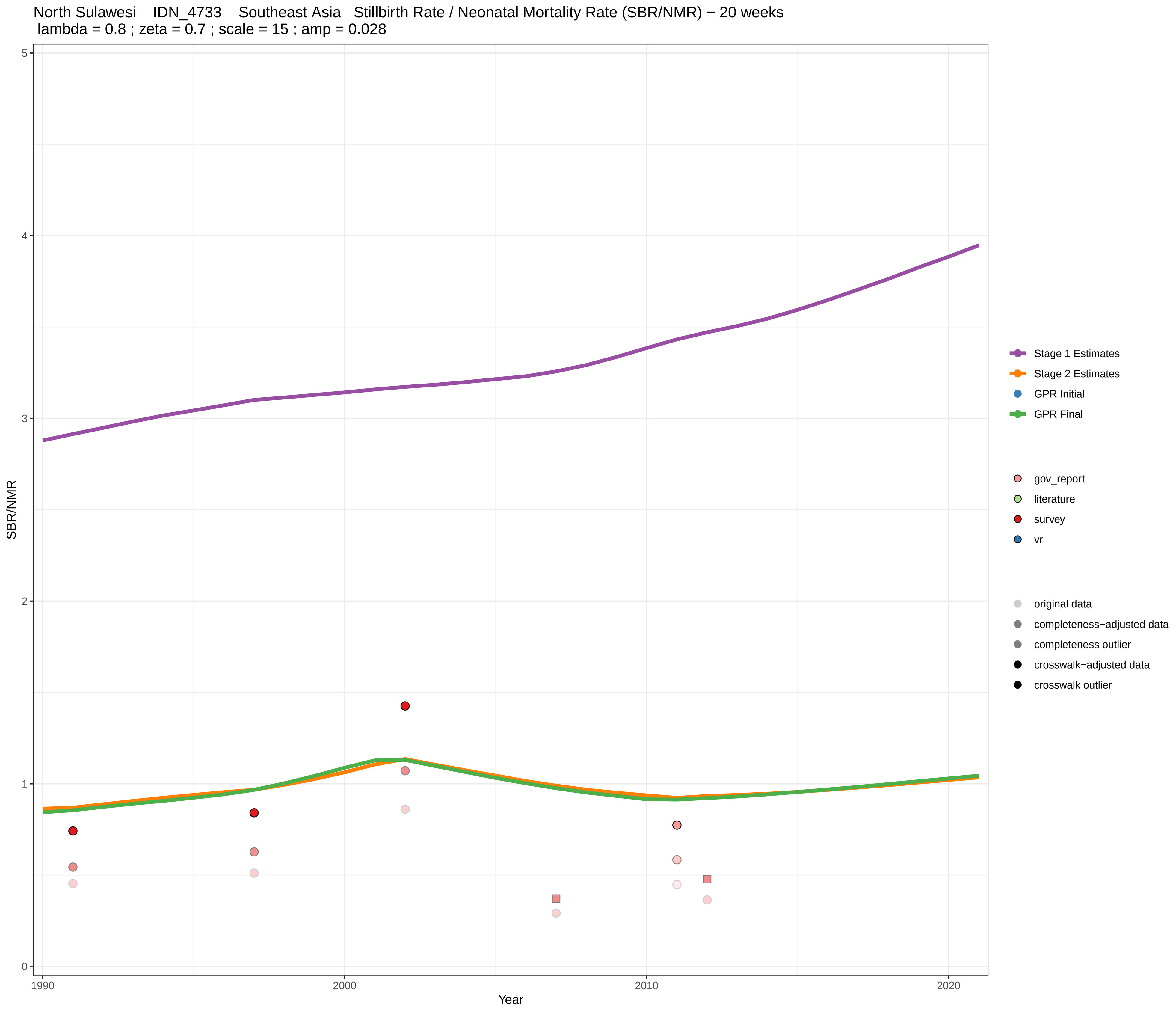

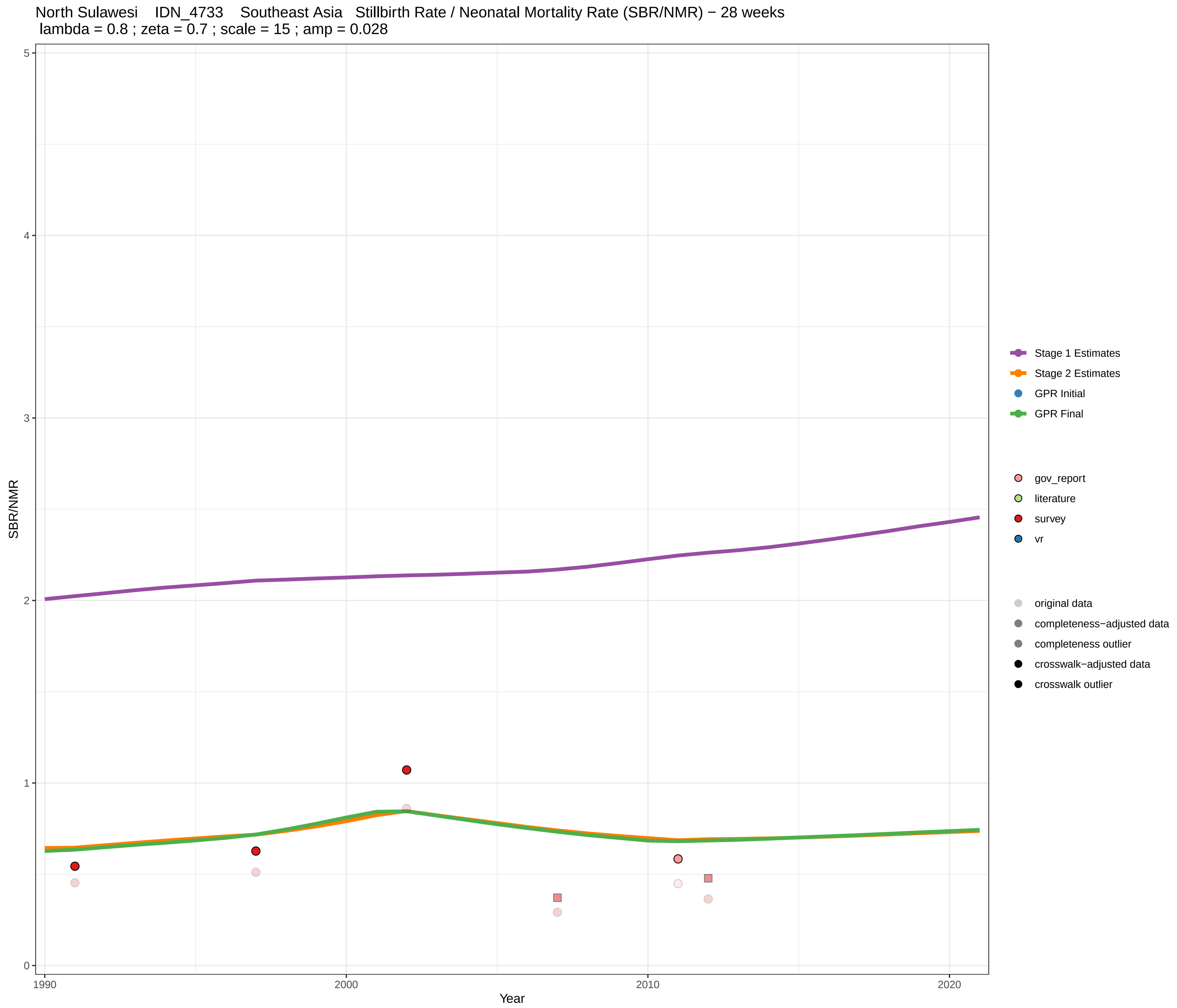

Definition	Years	Outlier	Source.Type	Title
28_weeks	1991	0	Survey	Indonesia Demographic and Health Survey 1991
28_weeks	1997	0	Survey	Indonesia Demographic and Health Survey 1997
28_weeks	2002	0	Survey	Indonesia Demographic and Health Survey 2002−2003
28_weeks	2011	0	Statistical Report	Indonesia − Sulawesi Utara Province Health Profile
28_weeks	2007	1	Survey	Indonesia Demographic and Health Survey 2007
28_weeks	2012	1	Survey	Indonesia Demographic and Health Survey 2012

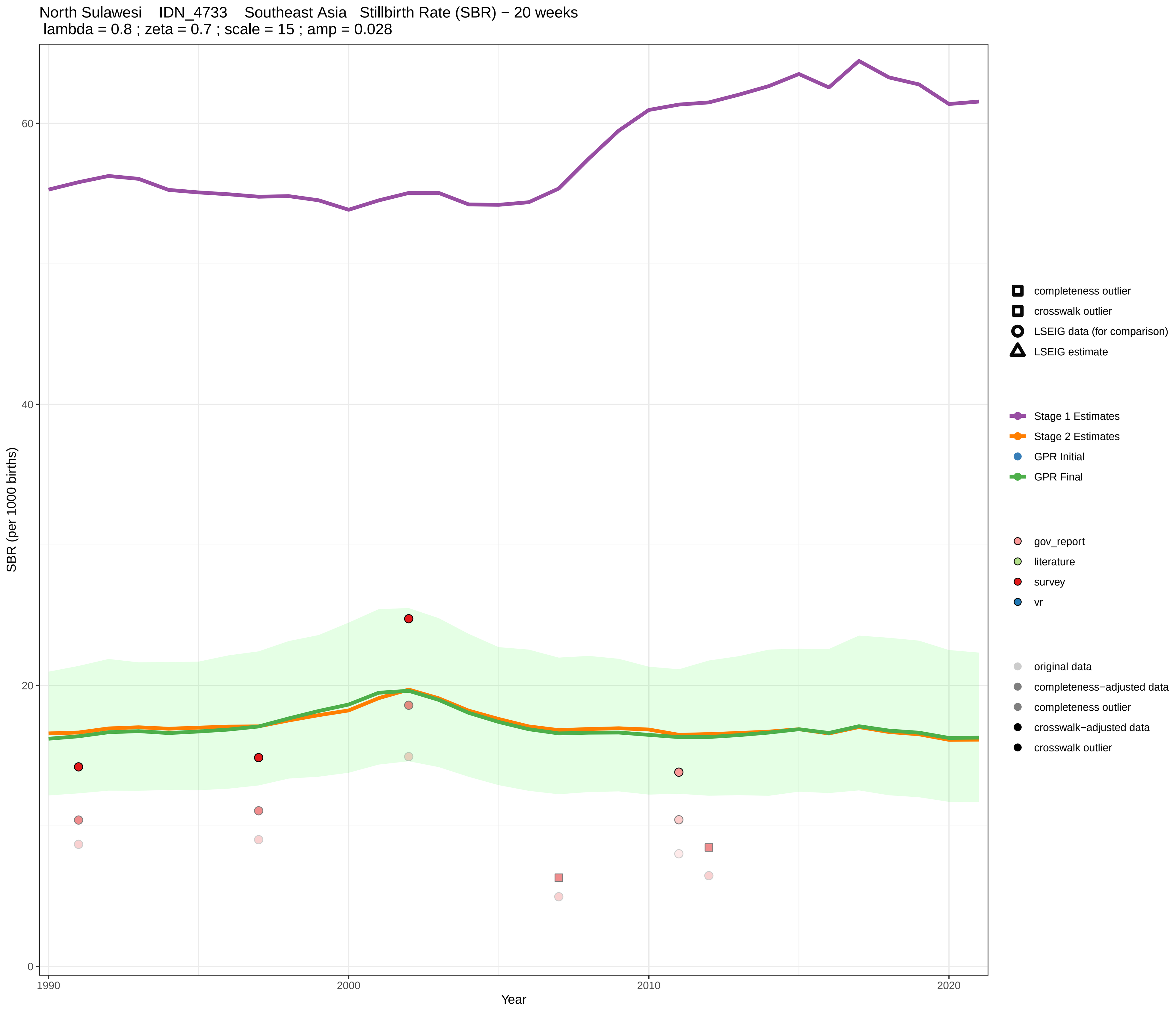

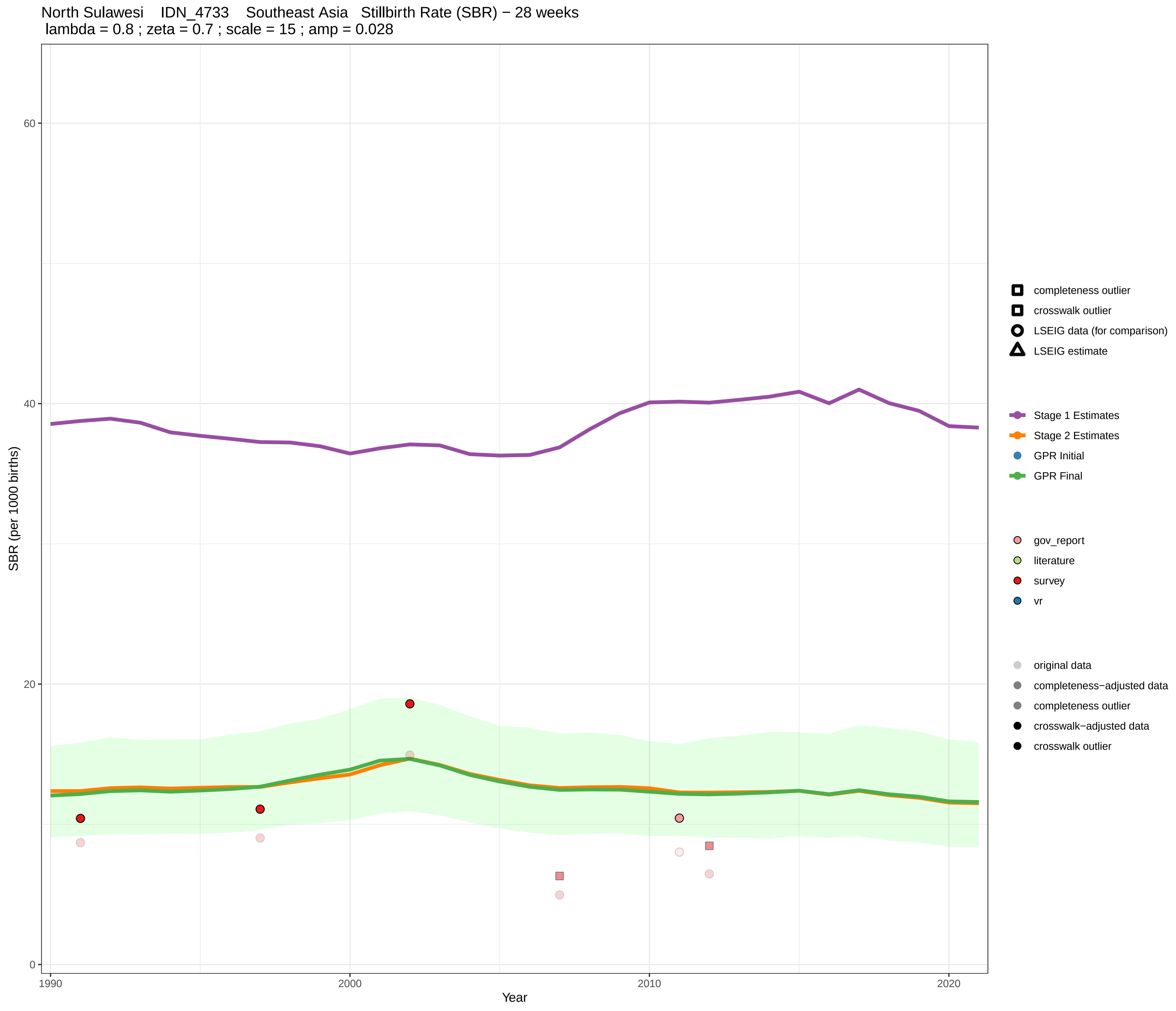

Year	Total Stillbirths (in thousands)	Total Stillbirths (in thousands)	SBR (per 1000 births)	SBR (per 1000 births)	SBR/NMR	SBR/NMR
	20 weeks	28 weeks	20 weeks	28 weeks	20 weeks	28 weeks
1990	0.7 (0.6 to 1.0)	0.6 (0.4 to 0.7)	16.2 (12.2 to 21.0)	12.1 (9.1 to 15.6)	0.84 (0.63 to 1.09)	0.63 (0.47 to 0.81)
1995	0.8 (0.6 to 1.0)	0.6 (0.4 to 0.7)	16.7 (12.5 to 21.7)	12.4 (9.3 to 16.1)	0.92 (0.69 to 1.20)	0.69 (0.52 to 0.89)
2000	0.9 (0.6 to 1.1)	0.6 (0.5 to 0.8)	18.6 (13.8 to 24.5)	13.9 (10.3 to 18.2)	1.09 (0.80 to 1.43)	0.81 (0.60 to 1.06)
2005	0.8 (0.6 to 1.0)	0.6 (0.4 to 0.8)	17.4 (12.9 to 22.7)	13.1 (9.7 to 17.0)	1.03 (0.77 to 1.35)	0.77 (0.58 to 1.01)
2010	0.7 (0.5 to 0.9)	0.5 (0.4 to 0.7)	16.5 (12.2 to 21.3)	12.3 (9.2 to 15.9)	0.92 (0.68 to 1.18)	0.68 (0.51 to 0.88)
2015	0.7 (0.5 to 1.0)	0.5 (0.4 to 0.7)	16.9 (12.4 to 22.6)	12.4 (9.2 to 16.6)	0.96 (0.70 to 1.28)	0.70 (0.52 to 0.94)
2020	0.7 (0.5 to 1.0)	0.5 (0.4 to 0.7)	16.3 (11.7 to 22.5)	11.6 (8.4 to 16.1)	1.03 (0.74 to 1.43)	0.74 (0.53 to 1.02)
2021	0.7 (0.5 to 0.9)	0.5 (0.3 to 0.7)	16.3 (11.7 to 22.3)	11.6 (8.4 to 15.9)	1.04 (0.75 to 1.43)	0.74 (0.54 to 1.02)

West Sumatra IDN_4711 Southeast Asia Stillbirth Rate / Neonatal Mortality Rate (SBR/NMR) − 20 weeks
lambda = 0.6 ; zeta = 0.8 ; scale = 15 ; amp = 0.028

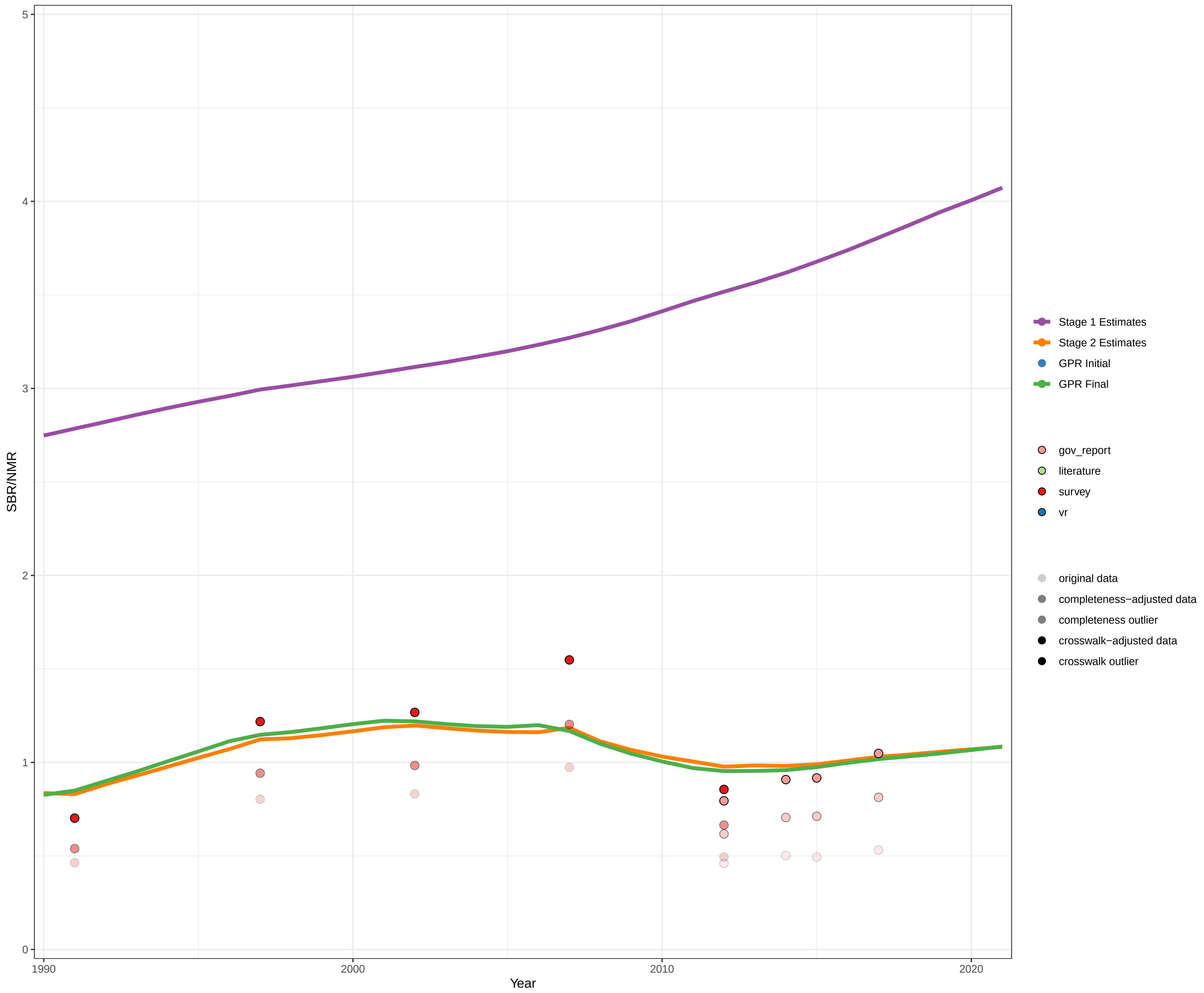

West Sumatra IDN_4711 Southeast Asia Stillbirth Rate / Neonatal Mortality Rate (SBR/NMR) − 28 weeks
lambda = 0.6 ; zeta = 0.8 ; scale = 15 ; amp = 0.028

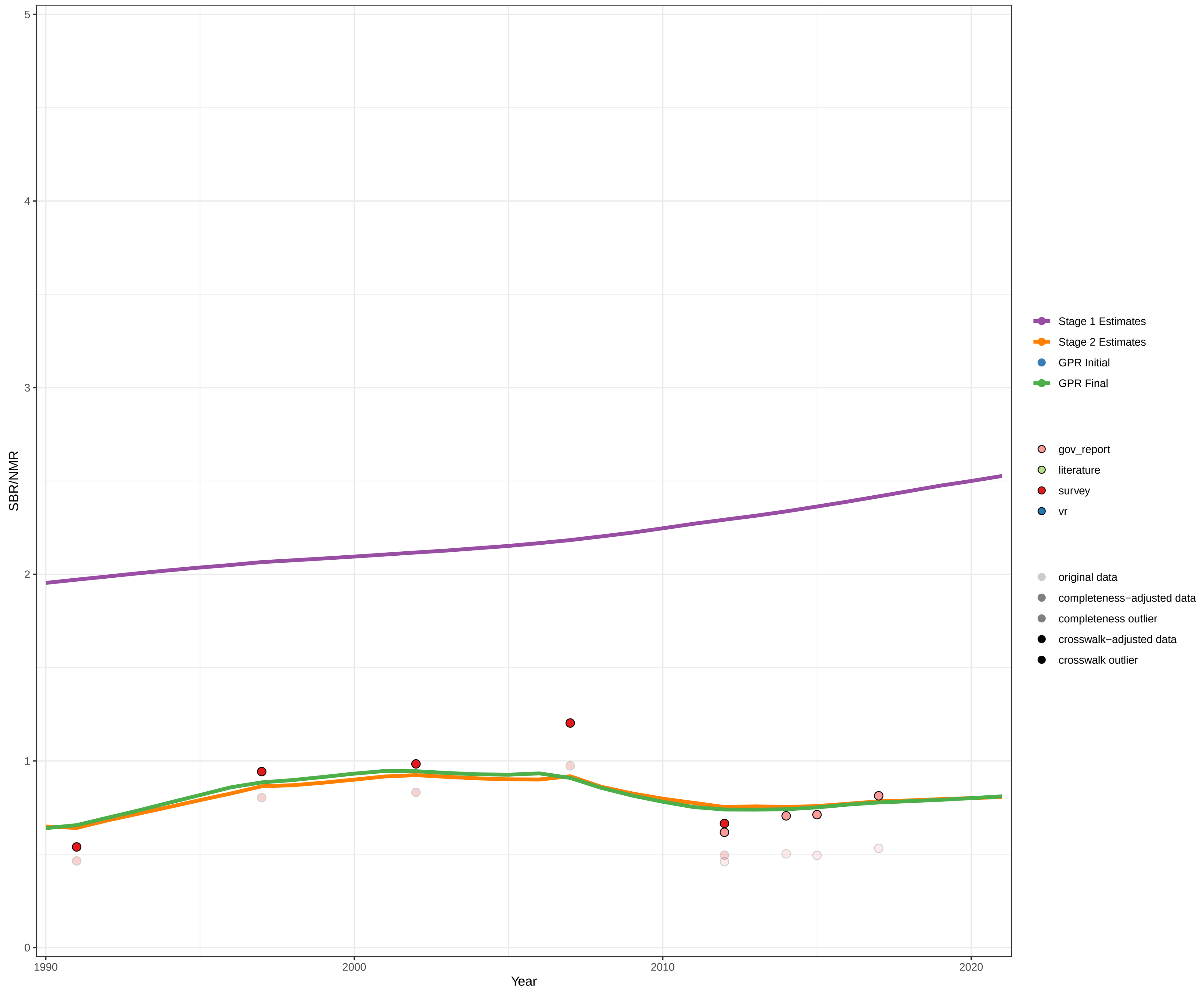

Definition	Years	Outlier	Source.Type	Title
28_weeks	1991	0	Survey	Indonesia Demographic and Health Survey 1991
28_weeks	1997	0	Survey	Indonesia Demographic and Health Survey 1997
28_weeks	2002	0	Survey	Indonesia Demographic and Health Survey 2002−2003
28_weeks	2007	0	Survey	Indonesia Demographic and Health Survey 2007
28_weeks	2012	0	Survey	Indonesia Demographic and Health Survey 2012
28_weeks	2012, 2014, 2015, 2017	0	Statistical Report	Indonesia − Sumatera Barat Province Health Profile

West Sumatra IDN_4711 Southeast Asia Stillbirth Rate (SBR) − 20 weeks
lambda = 0.6 ; zeta = 0.8 ; scale = 15 ; amp = 0.028

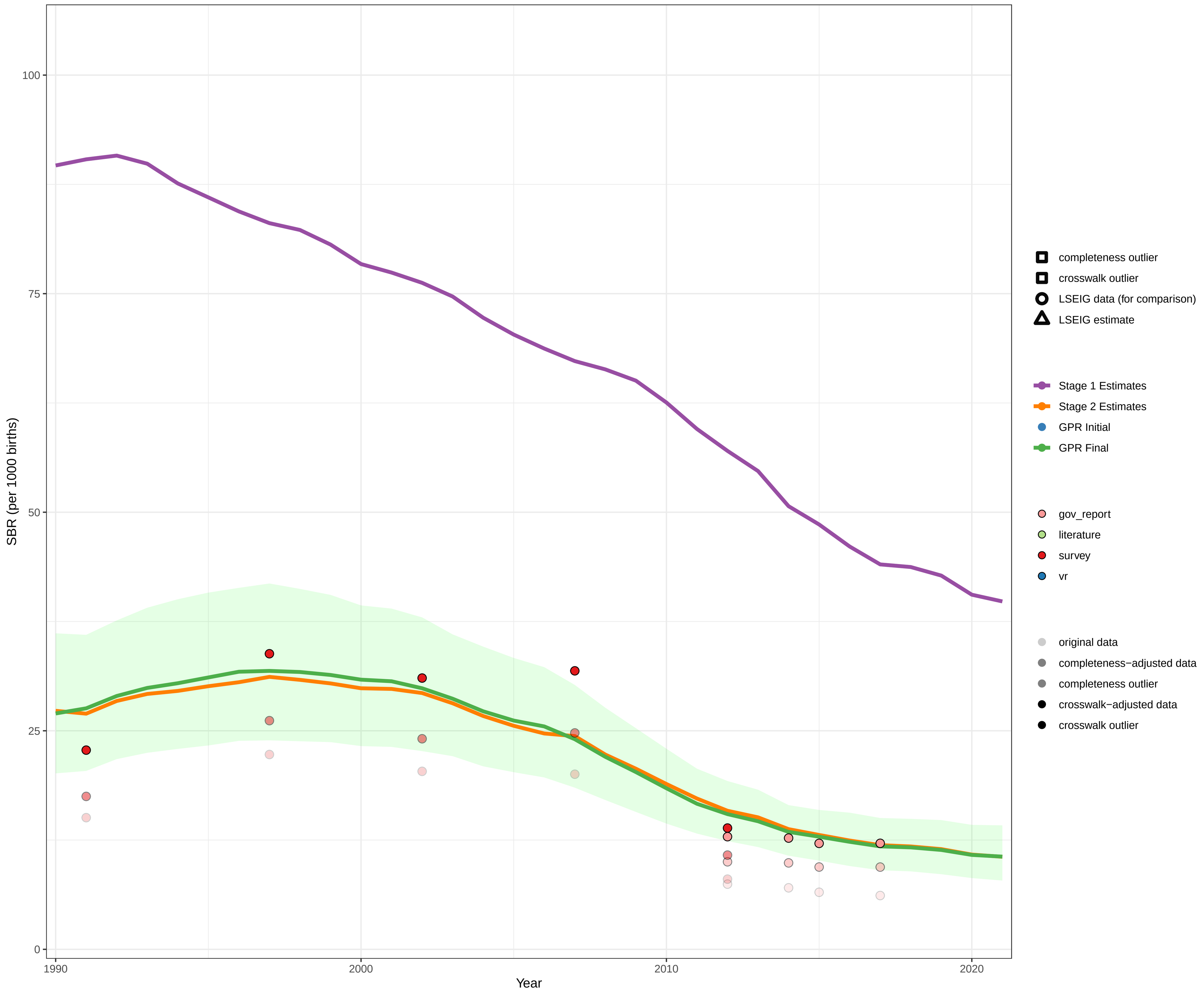

West Sumatra IDN_4711 Southeast Asia Stillbirth Rate (SBR) − 28 weeks
lambda = 0.6 ; zeta = 0.8 ; scale = 15 ; amp = 0.028

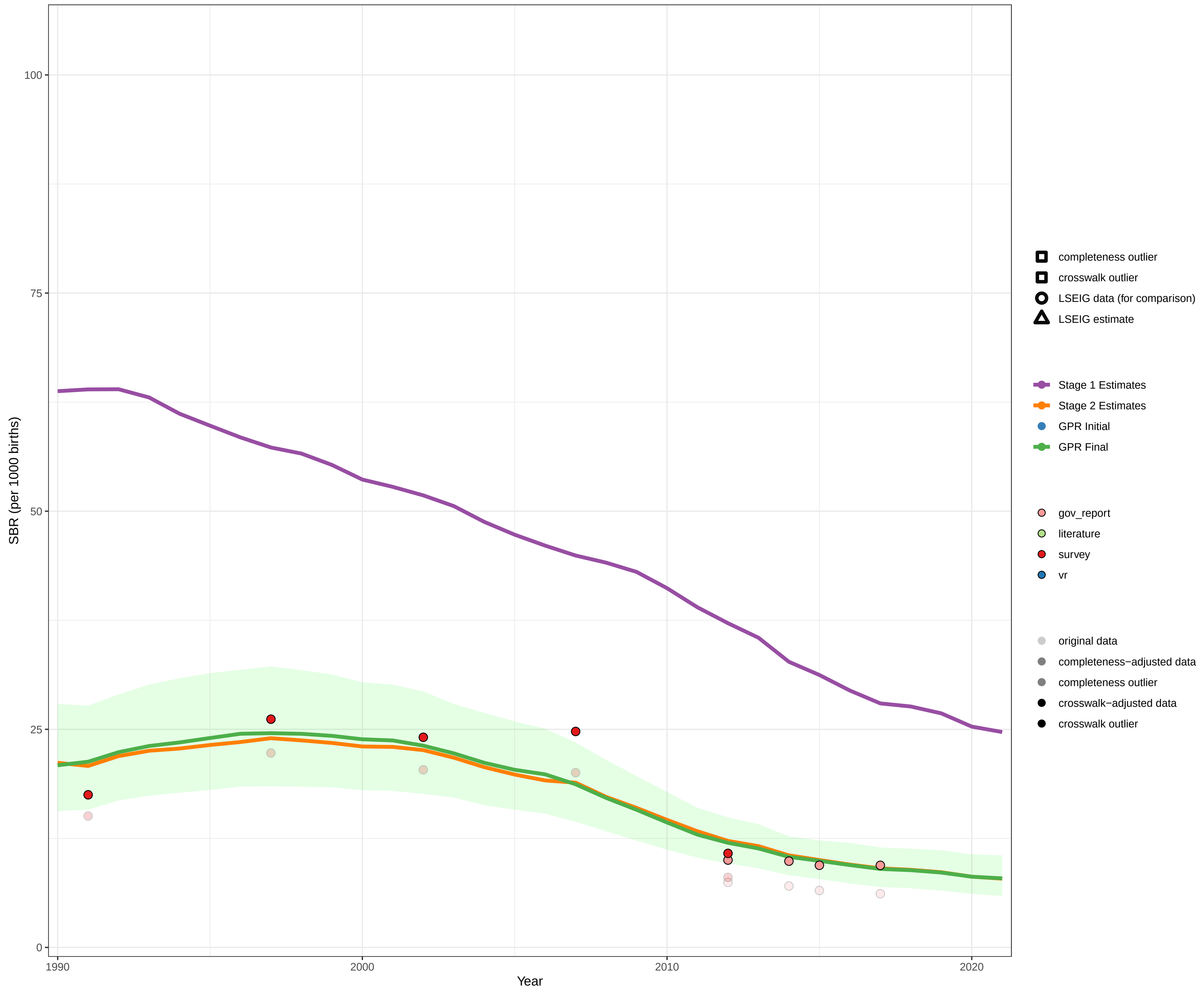

Year	Total Stillbirths (in thousands)	Total Stillbirths (in thousands)	SBR (per 1000 births)	SBR (per 1000 births)	SBR/NMR	SBR/NMR
	20 weeks	28 weeks	20 weeks	28 weeks	20 weeks	28 weeks
1990	3.2 (2.3 to 4.3)	2.4 (1.8 to 3.3)	27.0 (20.1 to 36.2)	20.9 (15.6 to 27.9)	0.83 (0.62 to 1.11)	0.64 (0.48 to 0.86)
1995	3.6 (2.7 to 4.8)	2.8 (2.1 to 3.7)	31.1 (23.3 to 40.8)	24.0 (18.0 to 31.4)	1.06 (0.79 to 1.39)	0.82 (0.61 to 1.07)
2000	3.6 (2.7 to 4.6)	2.8 (2.1 to 3.5)	30.8 (23.2 to 39.3)	23.9 (18.0 to 30.4)	1.21 (0.91 to 1.54)	0.93 (0.70 to 1.19)
2005	3.0 (2.3 to 3.9)	2.3 (1.8 to 3.0)	26.2 (20.3 to 33.3)	20.4 (15.8 to 25.9)	1.19 (0.92 to 1.52)	0.93 (0.72 to 1.18)
2010	2.0 (1.6 to 2.5)	1.6 (1.2 to 2.0)	18.4 (14.4 to 22.9)	14.3 (11.2 to 17.8)	1.01 (0.78 to 1.25)	0.78 (0.61 to 0.97)
2015	1.4 (1.1 to 1.7)	1.0 (0.8 to 1.3)	12.9 (10.1 to 15.9)	9.9 (7.8 to 12.3)	0.97 (0.77 to 1.21)	0.75 (0.59 to 0.93)
2020	1.1 (0.9 to 1.5)	0.9 (0.6 to 1.1)	10.8 (8.2 to 14.2)	8.1 (6.1 to 10.7)	1.07 (0.81 to 1.41)	0.80 (0.61 to 1.05)
2021	1.1 (0.8 to 1.5)	0.8 (0.6 to 1.1)	10.6 (7.9 to 14.2)	7.9 (5.9 to 10.6)	1.09 (0.81 to 1.45)	0.81 (0.60 to 1.08)

South Sumatra IDN_4714 Southeast Asia Stillbirth Rate / Neonatal Mortality Rate (SBR/NMR) − 20 weeks
lambda = 0.8 ; zeta = 0.7 ; scale = 15 ; amp = 0.028

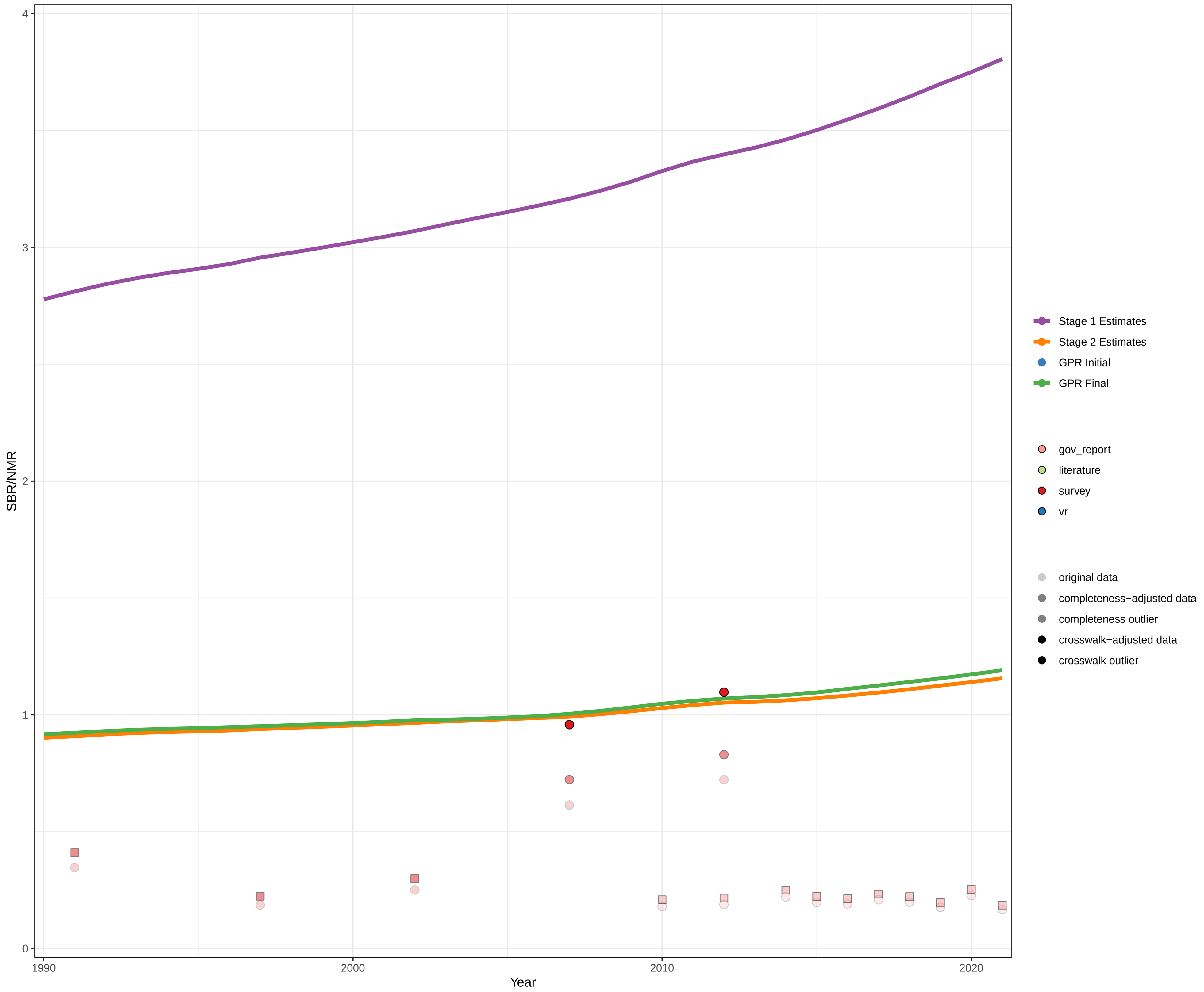

South Sumatra IDN_4714 Southeast Asia Stillbirth Rate / Neonatal Mortality Rate (SBR/NMR) − 28 weeks
lambda = 0.8 ; zeta = 0.7 ; scale = 15 ; amp = 0.028

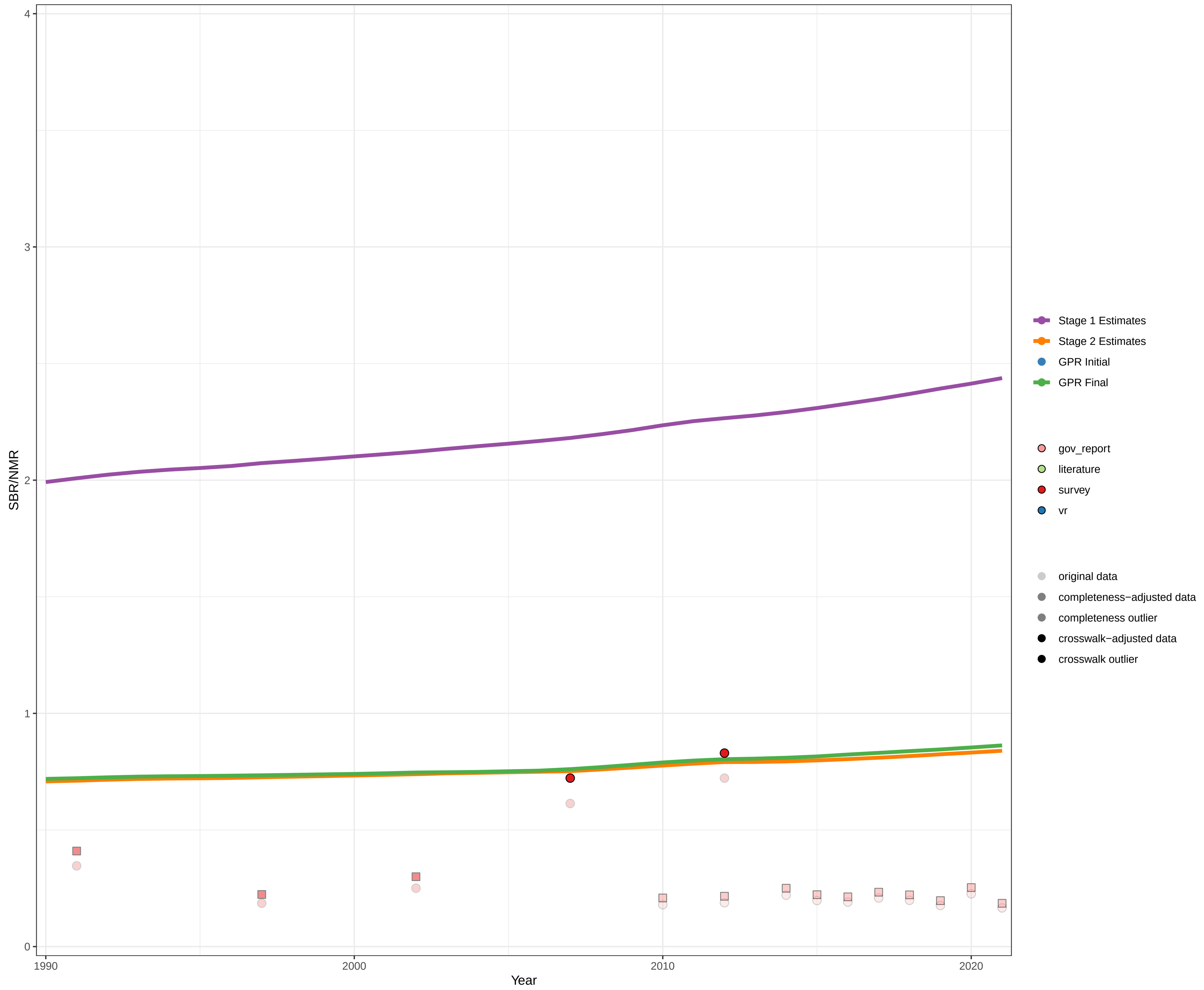

Definition	Years	Outlier	Source.Type	Title
28_weeks	2007	0	Survey	Indonesia Demographic and Health Survey 2007
28_weeks	2012	0	Survey	Indonesia Demographic and Health Survey 2012
28_weeks	1991	1	Survey	Indonesia Demographic and Health Survey 1991
28_weeks	1997	1	Survey	Indonesia Demographic and Health Survey 1997
28_weeks	2002	1	Survey	Indonesia Demographic and Health Survey 2002−2003
28_weeks	2010, 2012, 2014−2021	1	Statistical Report	Indonesia − Sumatera Selatan Province Health Profile

South Sumatra IDN_4714 Southeast Asia Stillbirth Rate (SBR) − 20 weeks
lambda = 0.8 ; zeta = 0.7 ; scale = 15 ; amp = 0.028

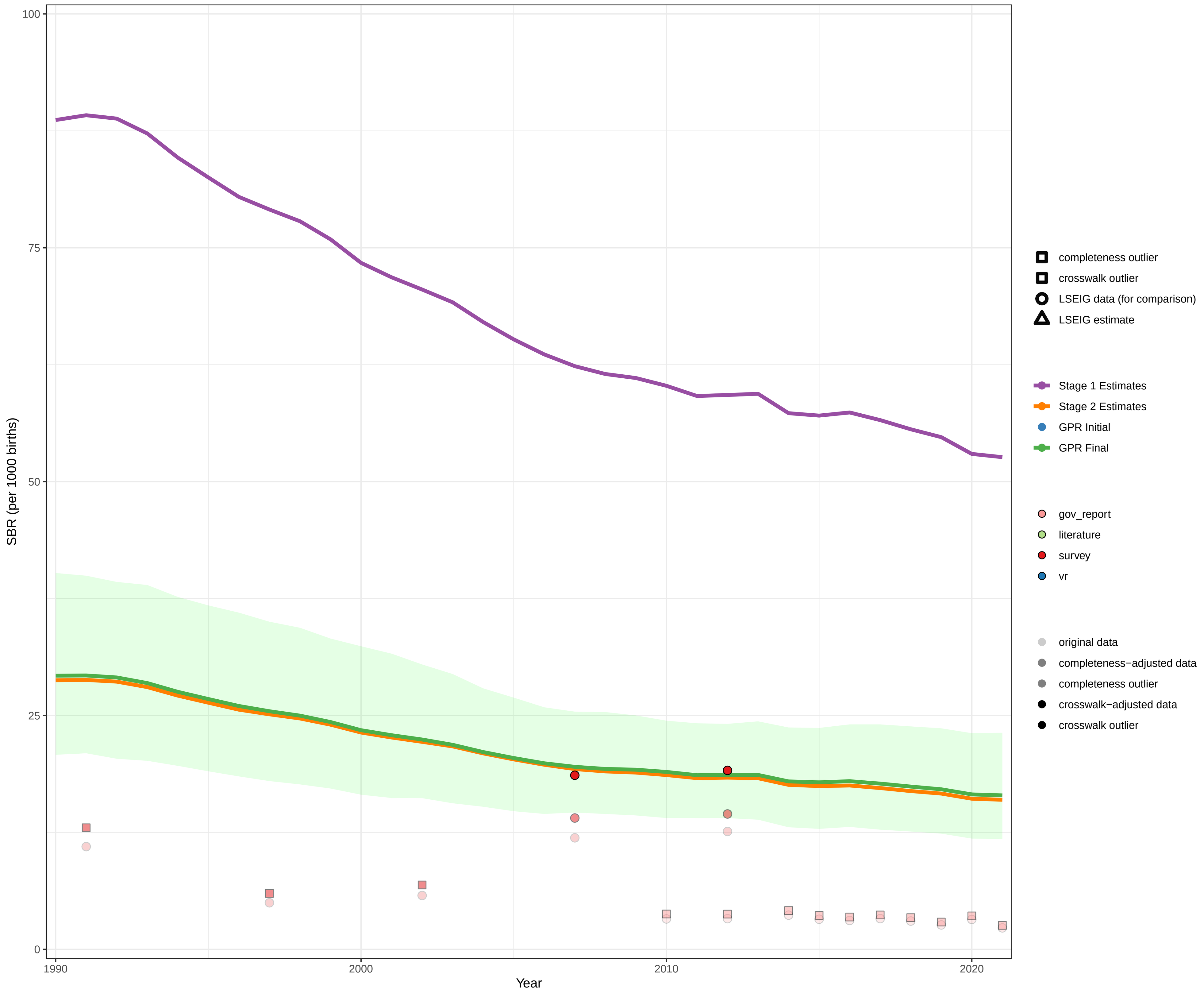

South Sumatra IDN_4714 Southeast Asia Stillbirth Rate (SBR) − 28 weeks
lambda = 0.8 ; zeta = 0.7 ; scale = 15 ; amp = 0.028

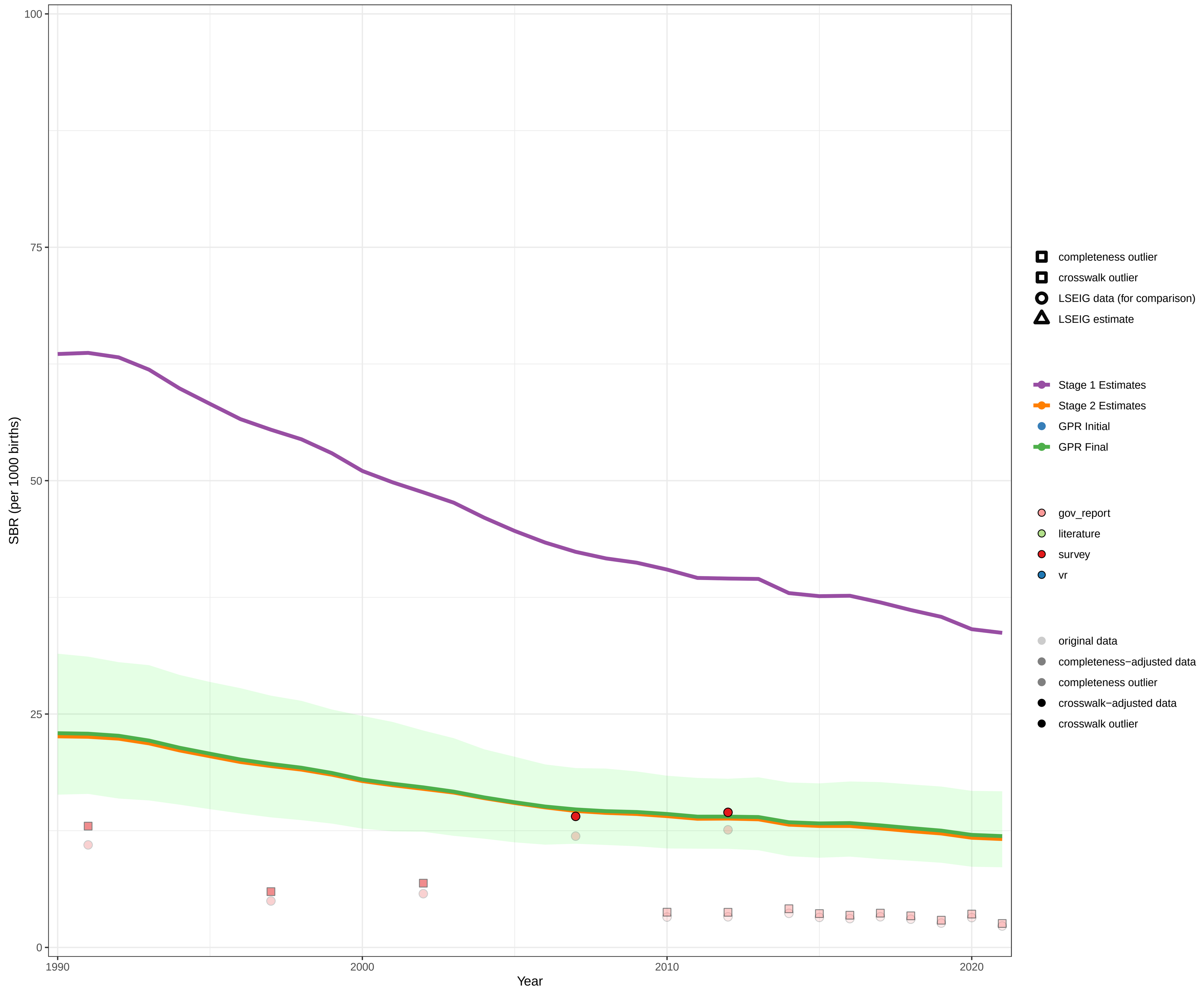

Year	Total Stillbirths (in thousands)	Total Stillbirths (in thousands)	SBR (per 1000 births)	SBR (per 1000 births)	SBR/NMR	SBR/NMR
	20 weeks	28 weeks	20 weeks	28 weeks	20 weeks	28 weeks
1990	4.8 (3.4 to 6.6)	3.7 (2.6 to 5.1)	29.3 (20.8 to 40.2)	22.9 (16.4 to 31.5)	0.92 (0.65 to 1.26)	0.72 (0.51 to 0.99)
1995	4.3 (3.1 to 6.0)	3.3 (2.4 to 4.6)	26.8 (19.0 to 36.8)	20.8 (14.8 to 28.4)	0.94 (0.67 to 1.30)	0.73 (0.52 to 1.00)
2000	3.9 (2.7 to 5.5)	3.0 (2.1 to 4.1)	23.4 (16.5 to 32.4)	18.0 (12.7 to 24.8)	0.96 (0.68 to 1.33)	0.74 (0.52 to 1.02)
2005	3.6 (2.6 to 4.7)	2.7 (1.9 to 3.6)	20.5 (14.8 to 26.9)	15.6 (11.3 to 20.4)	0.99 (0.71 to 1.30)	0.75 (0.54 to 0.99)
2010	3.3 (2.4 to 4.2)	2.5 (1.8 to 3.2)	19.0 (14.0 to 24.4)	14.3 (10.6 to 18.4)	1.05 (0.78 to 1.35)	0.79 (0.59 to 1.02)
2015	2.9 (2.1 to 3.8)	2.1 (1.5 to 2.8)	17.9 (12.9 to 23.7)	13.3 (9.6 to 17.6)	1.10 (0.79 to 1.45)	0.82 (0.59 to 1.08)
2020	2.5 (1.8 to 3.5)	1.8 (1.3 to 2.5)	16.6 (11.8 to 23.1)	12.1 (8.6 to 16.8)	1.17 (0.84 to 1.64)	0.85 (0.61 to 1.19)
2021	2.4 (1.7 to 3.5)	1.8 (1.3 to 2.5)	16.5 (11.8 to 23.2)	11.9 (8.6 to 16.7)	1.19 (0.85 to 1.68)	0.86 (0.62 to 1.21)

North Sumatra IDN_4710 Southeast Asia Stillbirth Rate / Neonatal Mortality Rate (SBR/NMR) − 20 weeks
lambda = 0.8 ; zeta = 0.7 ; scale = 15 ; amp = 0.028

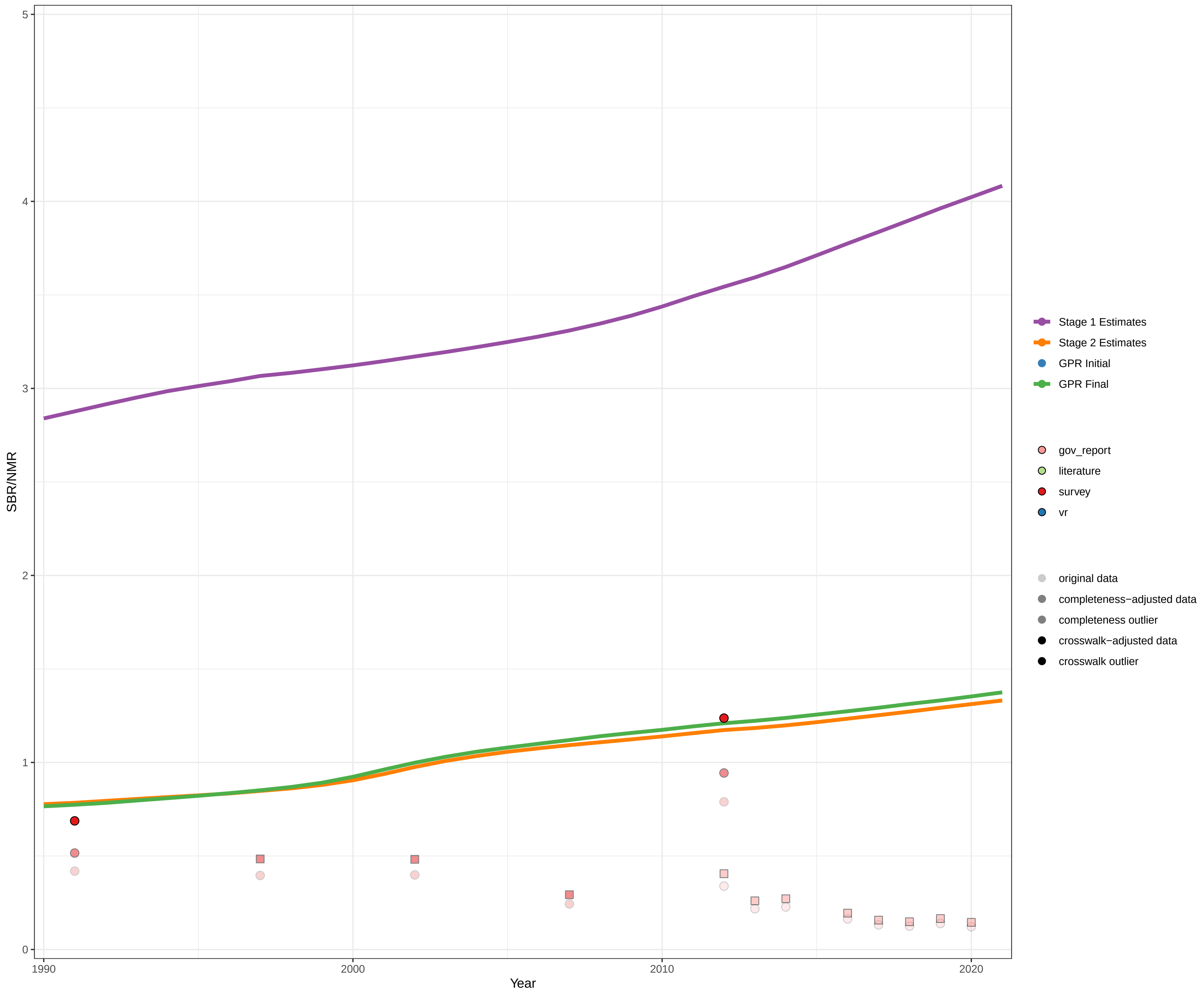

North Sumatra IDN_4710 Southeast Asia Stillbirth Rate / Neonatal Mortality Rate (SBR/NMR) − 28 weeks
lambda = 0.8 ; zeta = 0.7 ; scale = 15 ; amp = 0.028

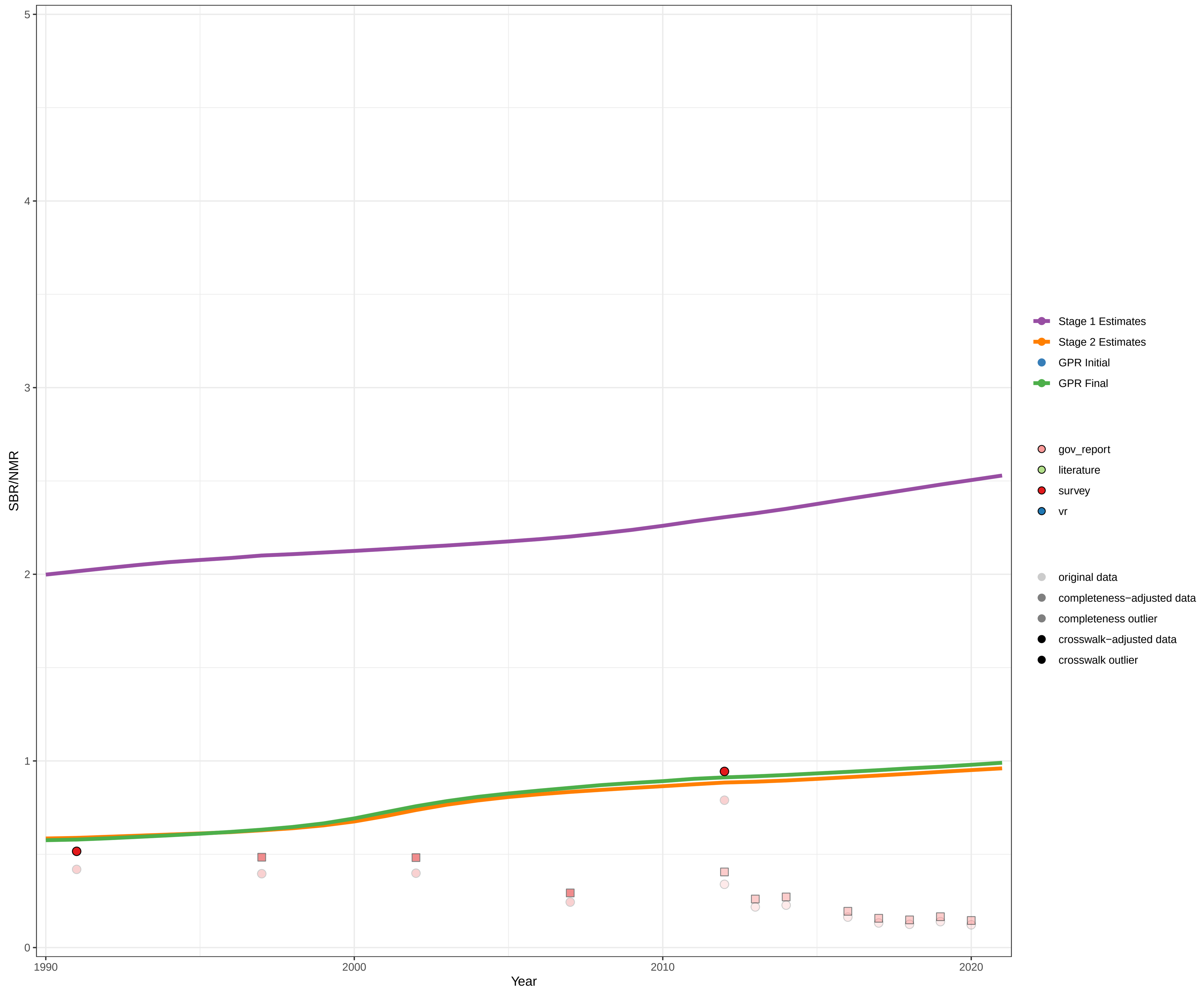

Definition	Years	Outlier	Source.Type	Title
28_weeks	1991	0	Survey	Indonesia Demographic and Health Survey 1991
28_weeks	2012	0	Survey	Indonesia Demographic and Health Survey 2012
28_weeks	1997	1	Survey	Indonesia Demographic and Health Survey 1997
28_weeks	2002	1	Survey	Indonesia Demographic and Health Survey 2002−2003
28_weeks	2007	1	Survey	Indonesia Demographic and Health Survey 2007
28_weeks	2012−2014, 2016−2020	1	Statistical Report	Indonesia − Sumatera Utara Province Health Profile

North Sumatra IDN_4710 Southeast Asia Stillbirth Rate (SBR) − 20 weeks
lambda = 0.8 ; zeta = 0.7 ; scale = 15 ; amp = 0.028

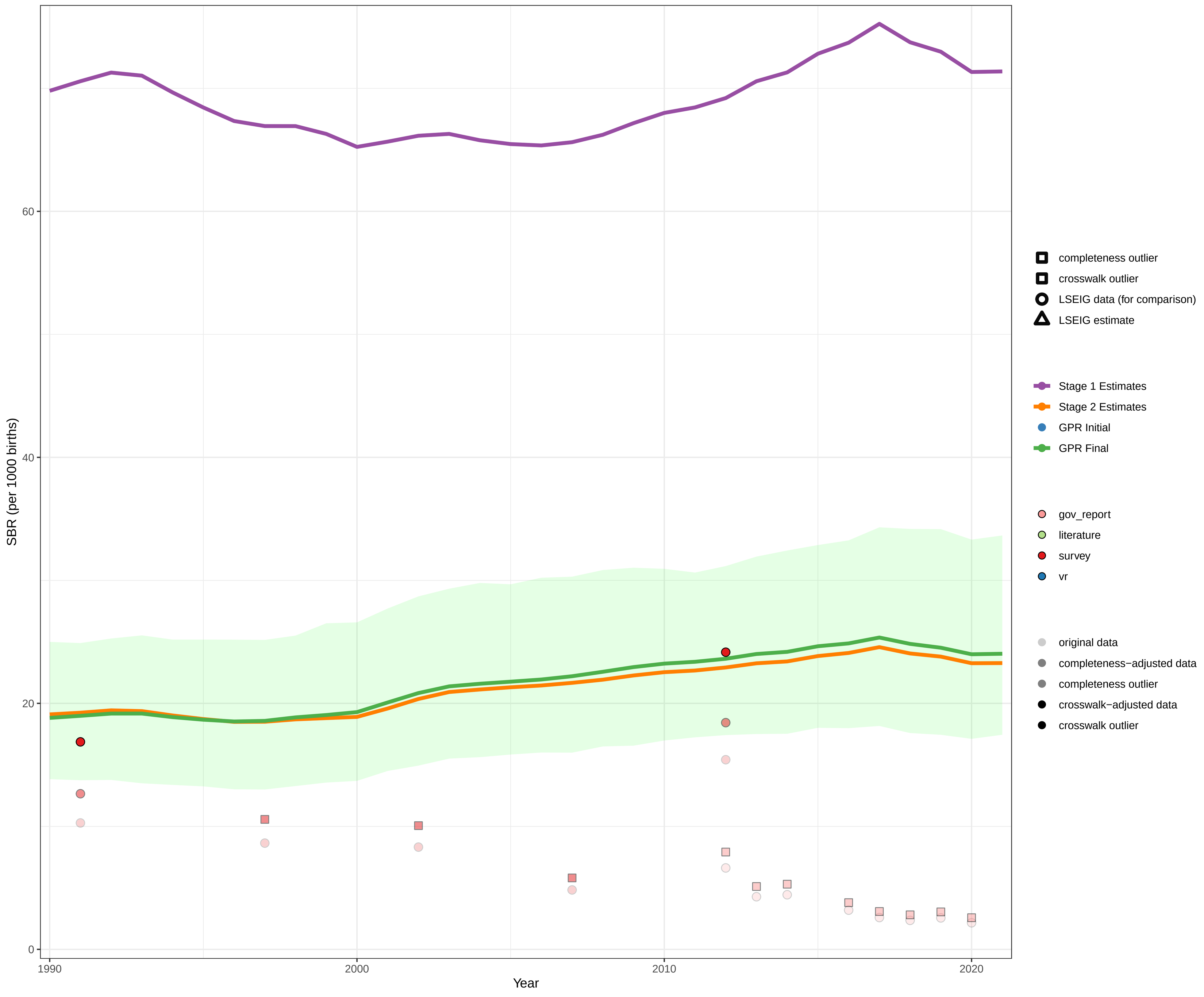

North Sumatra IDN_4710 Southeast Asia Stillbirth Rate (SBR) − 28 weeks
lambda = 0.8 ; zeta = 0.7 ; scale = 15 ; amp = 0.028

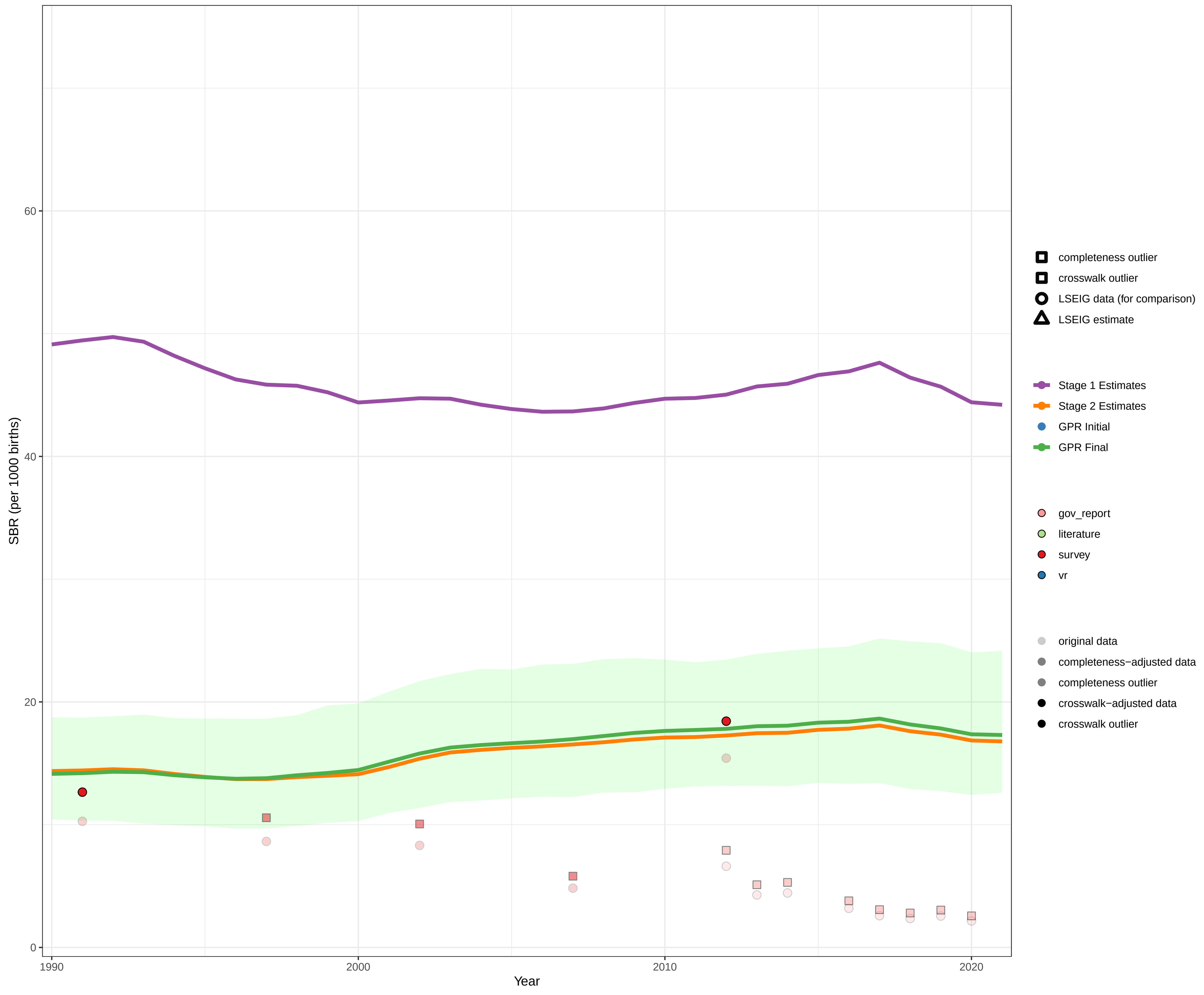

Year	Total Stillbirths (in thousands)	Total Stillbirths (in thousands)	SBR (per 1000 births)	SBR (per 1000 births)	SBR/NMR	SBR/NMR
	20 weeks	28 weeks	20 weeks	28 weeks	20 weeks	28 weeks
1990	6.4 (4.7 to 8.5)	4.8 (3.5 to 6.3)	18.8 (13.8 to 25.0)	14.1 (10.4 to 18.7)	0.77 (0.56 to 1.02)	0.58 (0.42 to 0.76)
1995	6.2 (4.4 to 8.4)	4.6 (3.3 to 6.2)	18.7 (13.3 to 25.2)	13.9 (9.9 to 18.6)	0.82 (0.58 to 1.11)	0.61 (0.43 to 0.82)
2000	6.6 (4.6 to 9.1)	4.9 (3.5 to 6.8)	19.3 (13.7 to 26.6)	14.5 (10.3 to 19.9)	0.92 (0.66 to 1.27)	0.69 (0.49 to 0.95)
2005	7.6 (5.5 to 10.5)	5.8 (4.2 to 7.9)	21.8 (15.8 to 29.7)	16.6 (12.1 to 22.6)	1.08 (0.79 to 1.47)	0.83 (0.60 to 1.12)
2010	8.0 (5.8 to 10.7)	6.0 (4.4 to 8.1)	23.2 (17.0 to 30.9)	17.6 (12.9 to 23.4)	1.17 (0.86 to 1.56)	0.89 (0.65 to 1.19)
2015	8.2 (5.9 to 11.0)	6.0 (4.4 to 8.1)	24.6 (18.0 to 32.9)	18.3 (13.4 to 24.4)	1.26 (0.92 to 1.68)	0.93 (0.68 to 1.24)
2020	8.0 (5.6 to 11.2)	5.7 (4.1 to 8.0)	24.0 (17.1 to 33.3)	17.4 (12.4 to 24.0)	1.35 (0.97 to 1.88)	0.98 (0.70 to 1.36)
2021	8.0 (5.7 to 11.3)	5.7 (4.1 to 8.0)	24.0 (17.4 to 33.6)	17.3 (12.6 to 24.2)	1.37 (1.00 to 1.93)	0.99 (0.72 to 1.38)

Yogyakarta IDN_4723 Southeast Asia Stillbirth Rate / Neonatal Mortality Rate (SBR/NMR) − 20 weeks
lambda = 0.4 ; zeta = 0.9 ; scale = 10 ; amp = 0.028

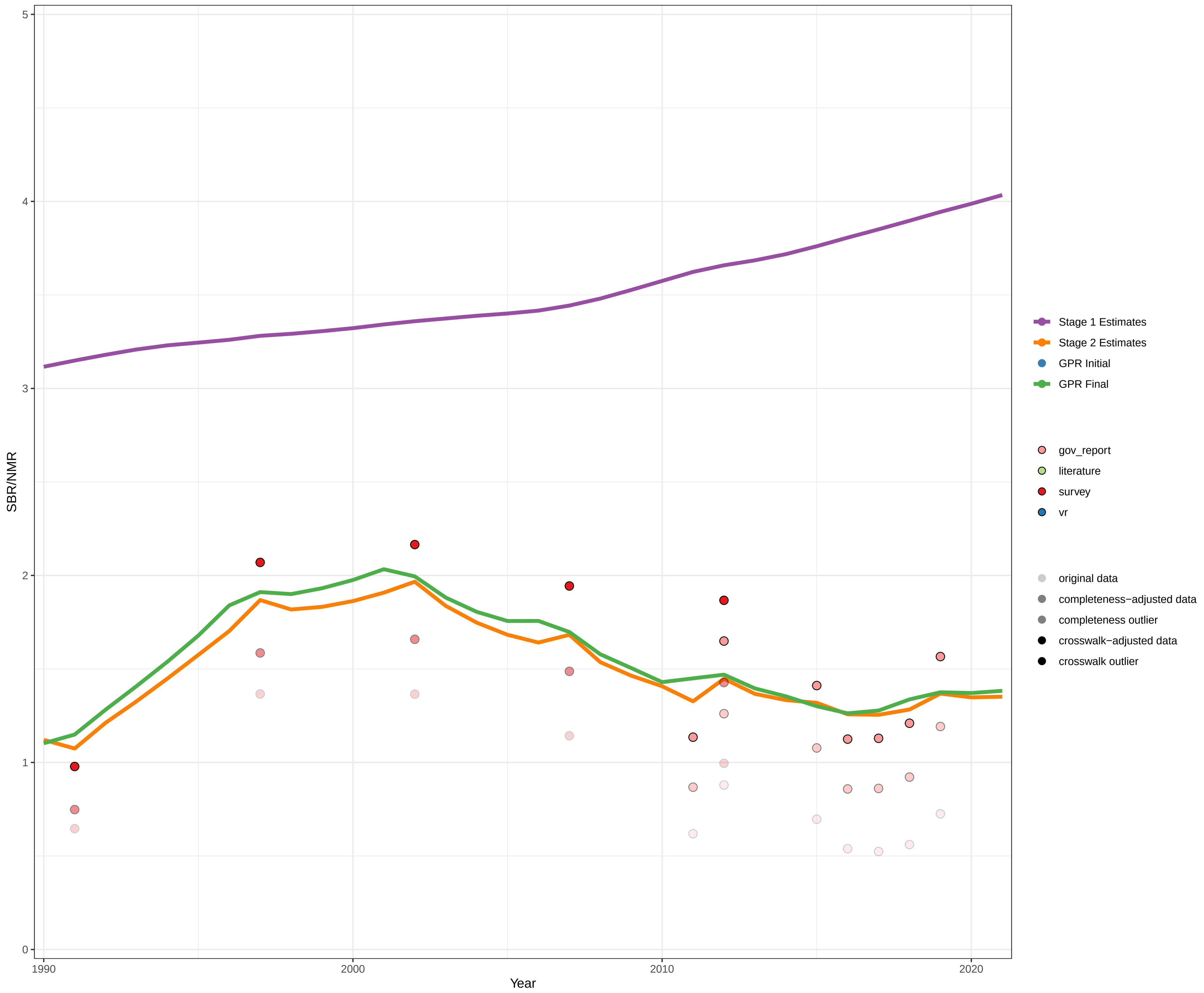

Yogyakarta IDN_4723 Southeast Asia Stillbirth Rate / Neonatal Mortality Rate (SBR/NMR) − 28 weeks
lambda = 0.4 ; zeta = 0.9 ; scale = 10 ; amp = 0.028

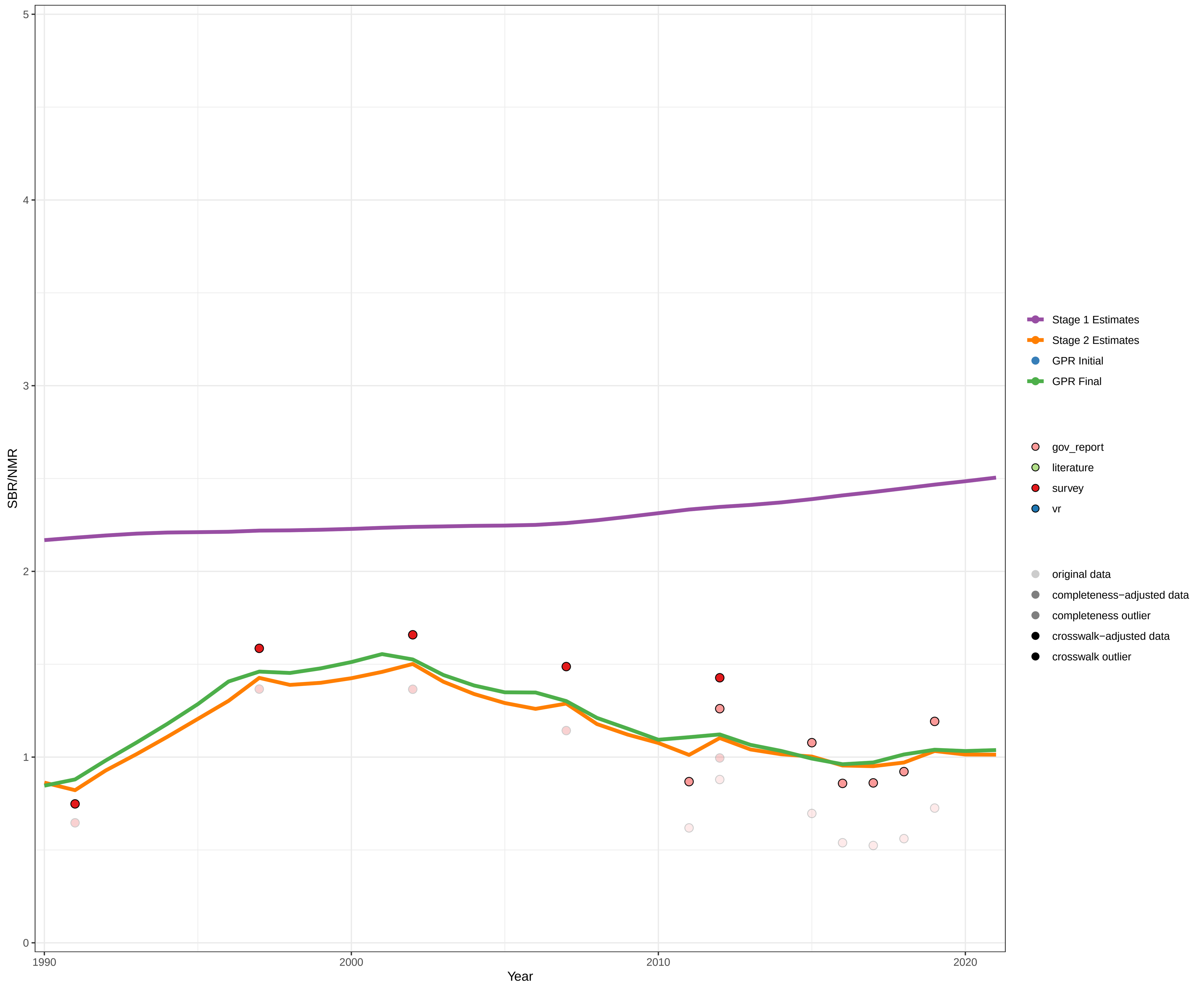

Definition	Years	Outlier	Source.Type	Title
28_weeks	1991	0	Survey	Indonesia Demographic and Health Survey 1991
28_weeks	1997	0	Survey	Indonesia Demographic and Health Survey 1997
28_weeks	2002	0	Survey	Indonesia Demographic and Health Survey 2002−2003
28_weeks	2007	0	Survey	Indonesia Demographic and Health Survey 2007
28_weeks	2011, 2012, 2015−2017, 2019	0	Statistical Report	Indonesia − Yogyakarta Health Profile
28_weeks	2012	0	Survey	Indonesia Demographic and Health Survey 2012
28_weeks	2018	0	Statistical Report	Indonesia Health Profile Province of Yogyakarta in

Yogyakarta IDN_4723 Southeast Asia Stillbirth Rate (SBR) − 20 weeks
lambda = 0.4 ; zeta = 0.9 ; scale = 10 ; amp = 0.028

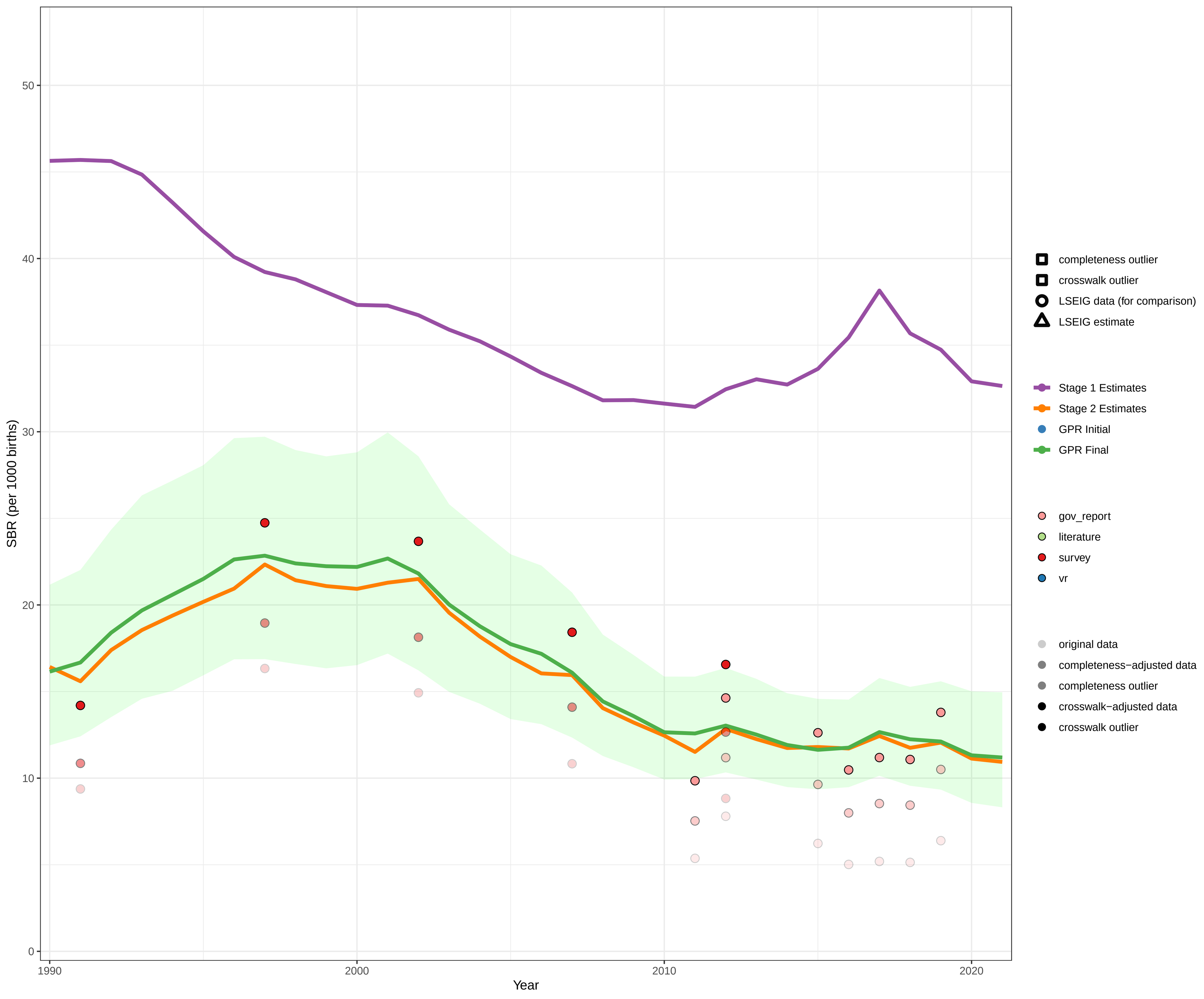

Yogyakarta IDN_4723 Southeast Asia Stillbirth Rate (SBR) − 28 weeks
lambda = 0.4 ; zeta = 0.9 ; scale = 10 ; amp = 0.028

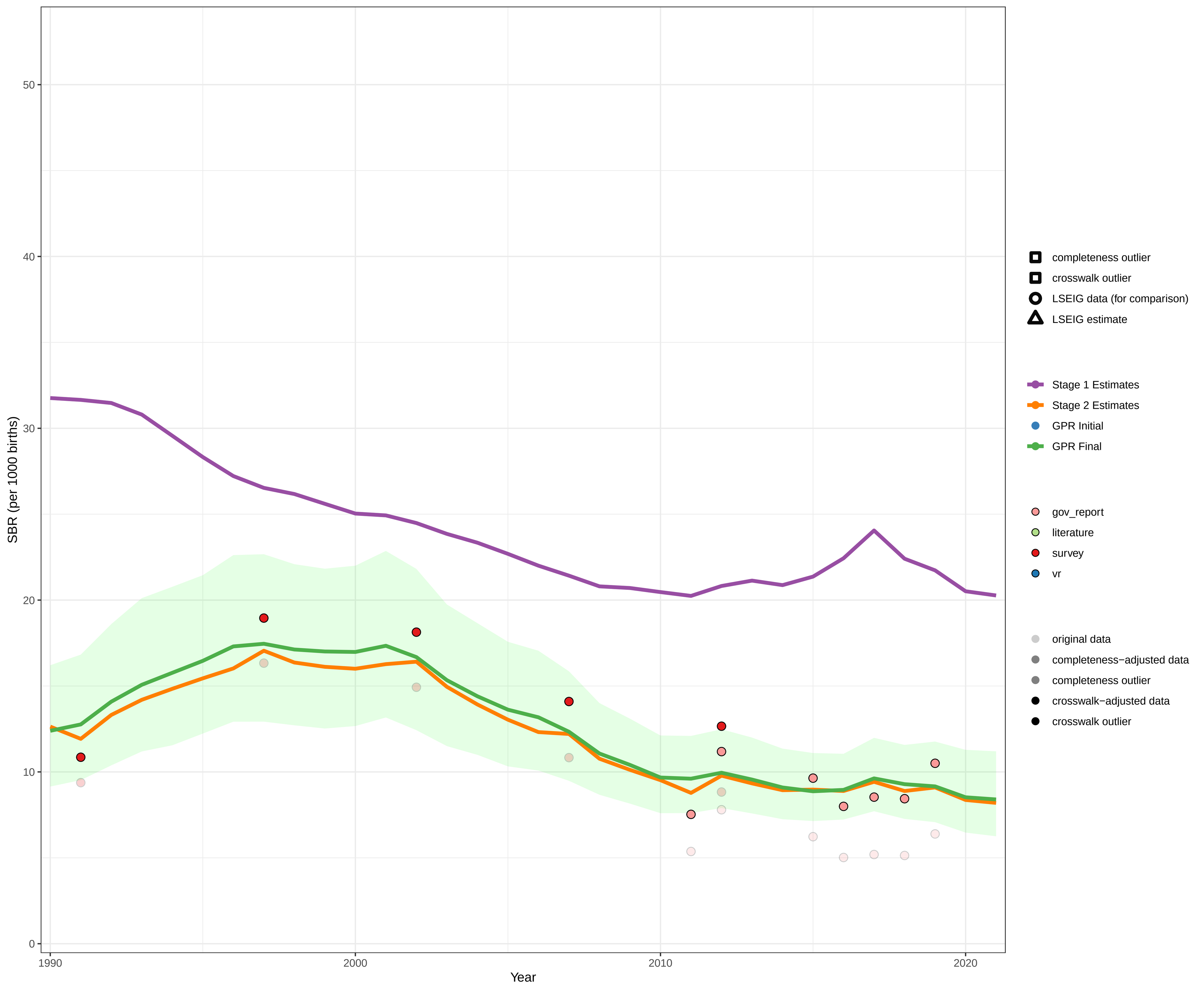

Year	Total Stillbirths (in thousands)	Total Stillbirths (in thousands)	SBR (per 1000 births)	SBR (per 1000 births)	SBR/NMR	SBR/NMR
	20 weeks	28 weeks	20 weeks	28 weeks	20 weeks	28 weeks
1990	0.9 (0.7 to 1.2)	0.7 (0.5 to 0.9)	16.1 (11.9 to 21.2)	12.4 (9.1 to 16.2)	1.10 (0.81 to 1.45)	0.85 (0.62 to 1.11)
1995	1.1 (0.8 to 1.5)	0.9 (0.6 to 1.1)	21.5 (15.9 to 28.1)	16.5 (12.2 to 21.4)	1.68 (1.24 to 2.19)	1.29 (0.95 to 1.67)
2000	1.1 (0.8 to 1.4)	0.8 (0.6 to 1.1)	22.2 (16.5 to 28.8)	17.0 (12.7 to 22.0)	1.98 (1.47 to 2.56)	1.51 (1.13 to 1.96)
2005	0.9 (0.6 to 1.1)	0.7 (0.5 to 0.9)	17.7 (13.4 to 22.9)	13.6 (10.3 to 17.6)	1.76 (1.33 to 2.27)	1.35 (1.02 to 1.74)
2010	0.6 (0.5 to 0.7)	0.5 (0.4 to 0.6)	12.6 (9.9 to 15.9)	9.7 (7.6 to 12.1)	1.43 (1.12 to 1.79)	1.09 (0.86 to 1.37)
2015	0.5 (0.4 to 0.6)	0.4 (0.3 to 0.5)	11.6 (9.4 to 14.6)	8.9 (7.1 to 11.1)	1.30 (1.05 to 1.63)	0.99 (0.80 to 1.24)
2020	0.4 (0.3 to 0.6)	0.3 (0.2 to 0.4)	11.3 (8.6 to 15.0)	8.5 (6.5 to 11.3)	1.37 (1.04 to 1.82)	1.03 (0.78 to 1.37)
2021	0.4 (0.3 to 0.5)	0.3 (0.2 to 0.4)	11.2 (8.3 to 15.0)	8.4 (6.3 to 11.2)	1.38 (1.03 to 1.85)	1.04 (0.77 to 1.38)

Lao People's Democratic Republic   LAO   Southeast Asia   Stillbirth Rate / Neonatal Mortality Rate (SBR/NMR) − 20 weeks
lambda = 1 ; zeta = 0.6 ; scale = 15 ; amp = 0.028

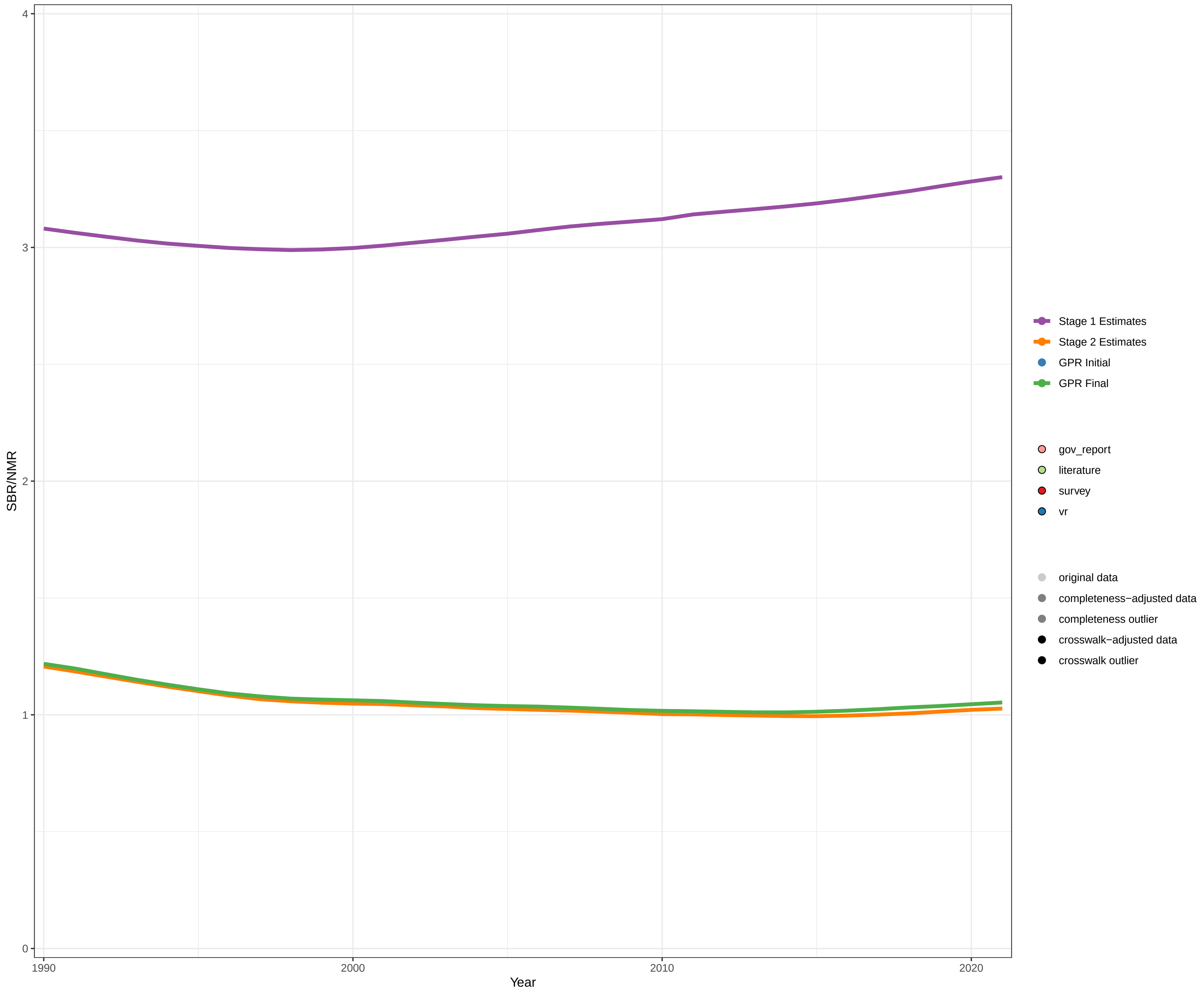

Lao People's Democratic Republic   LAO   Southeast Asia   Stillbirth Rate / Neonatal Mortality Rate (SBR/NMR) − 28 weeks
lambda = 1 ; zeta = 0.6 ; scale = 15 ; amp = 0.028

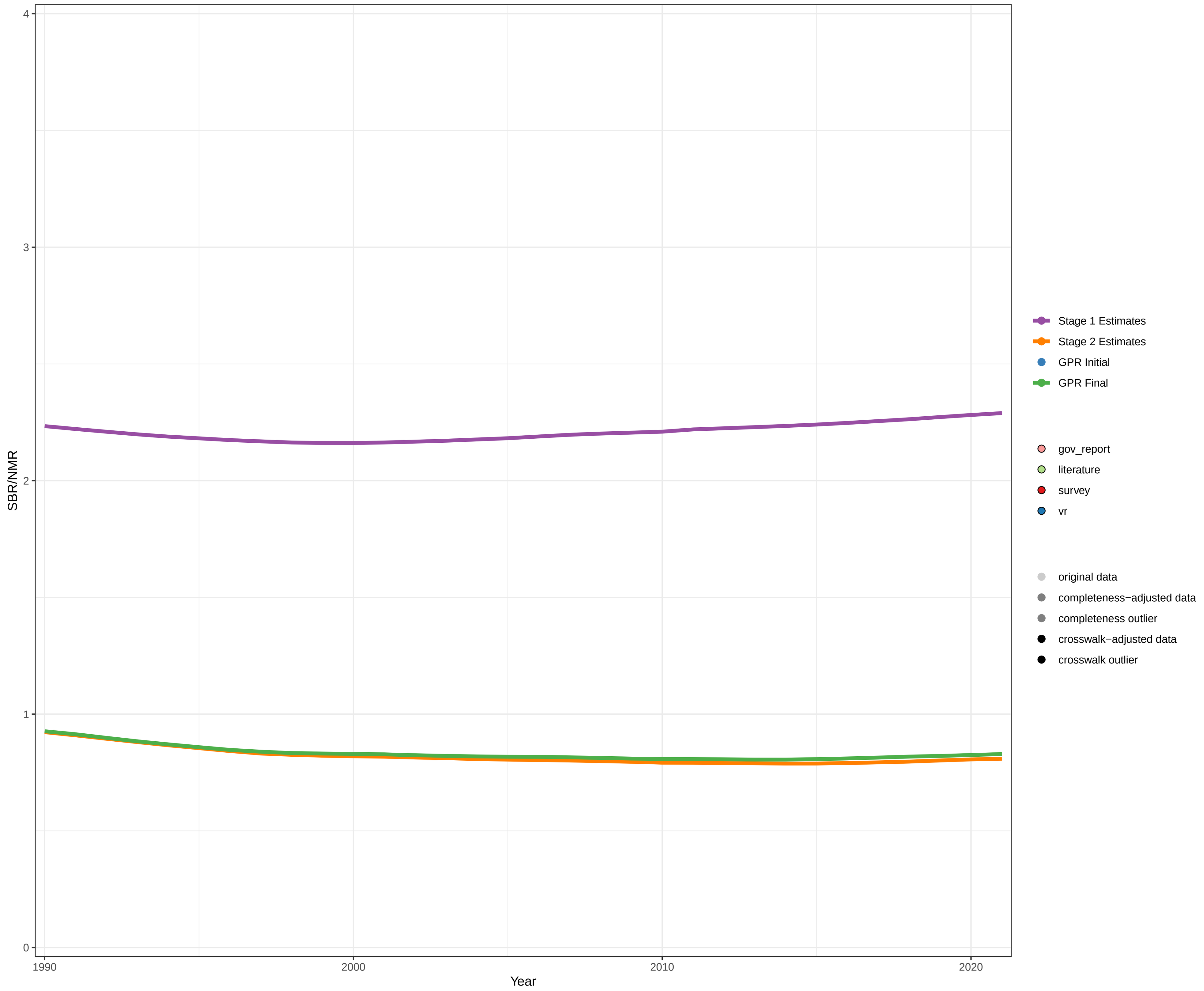

Definition	Years	Outlier	Source.Type	Title

Lao People's Democratic Republic   LAO   Southeast Asia   Stillbirth Rate (SBR) − 20 weeks
lambda = 1 ; zeta = 0.6 ; scale = 15 ; amp = 0.028

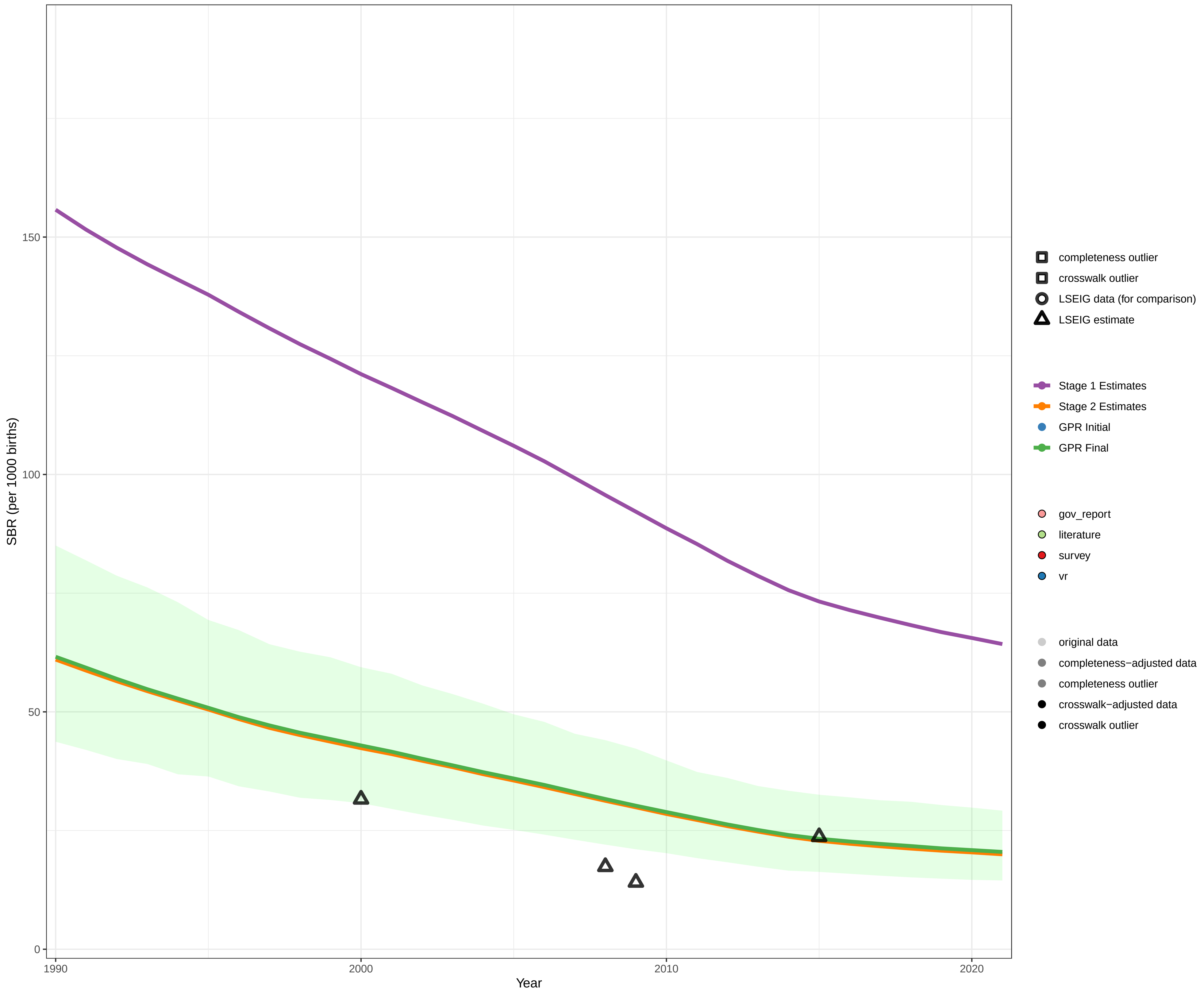

Lao People's Democratic Republic   LAO   Southeast Asia   Stillbirth Rate (SBR) − 28 weeks
lambda = 1 ; zeta = 0.6 ; scale = 15 ; amp = 0.028

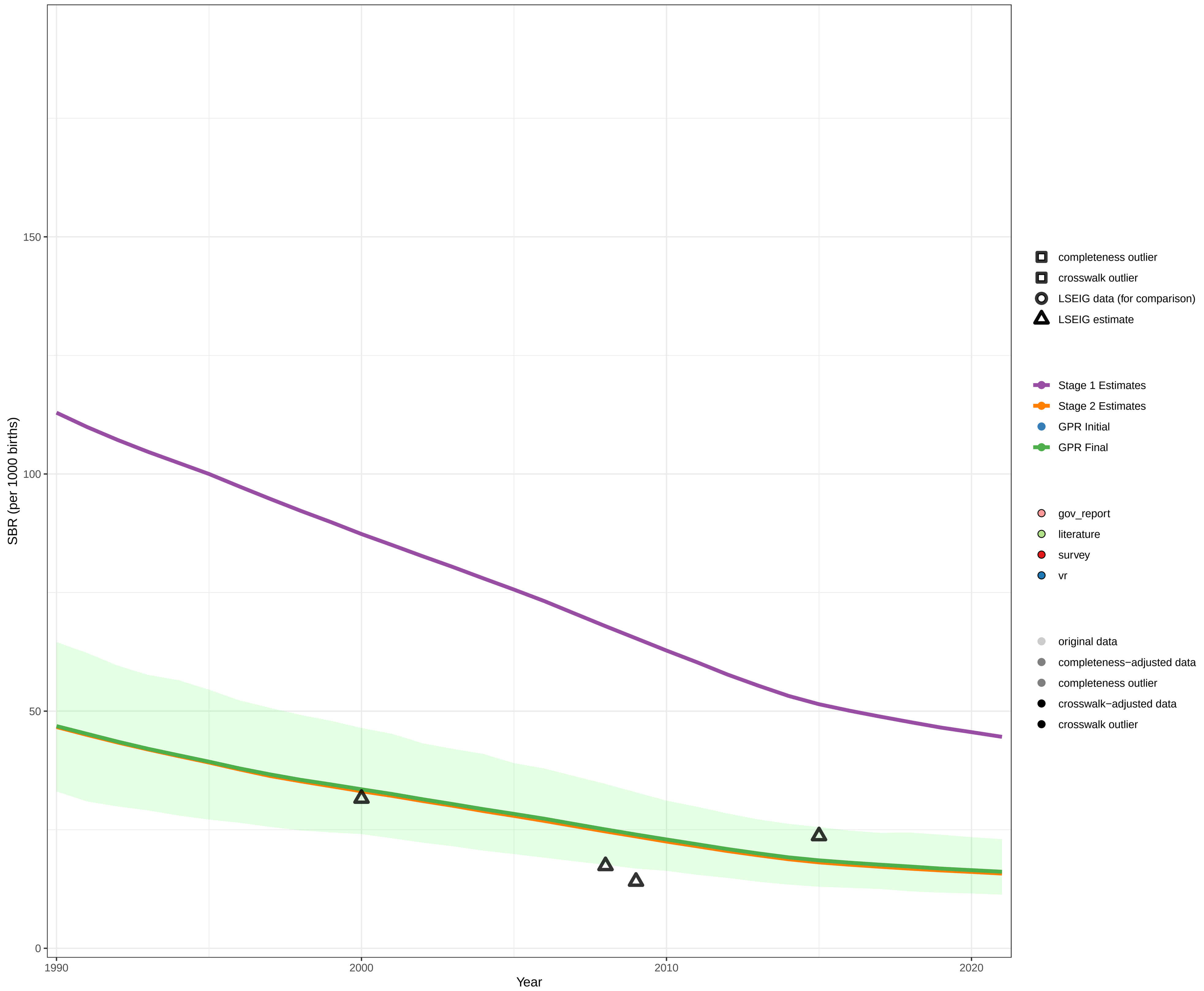

Year	Total Stillbirths (in thousands)	Total Stillbirths (in thousands)	SBR (per 1000 births)	SBR (per 1000 births)	SBR/NMR	SBR/NMR
	20 weeks	28 weeks	20 weeks	28 weeks	20 weeks	28 weeks
1990	11.4 (7.9 to 16.1)	8.5 (5.9 to 11.9)	61.6 (43.7 to 85.0)	46.8 (33.0 to 64.6)	1.22 (0.86 to 1.68)	0.93 (0.65 to 1.28)
1995	10.2 (7.2 to 14.1)	7.8 (5.3 to 11.0)	50.9 (36.4 to 69.4)	39.3 (27.1 to 54.5)	1.11 (0.79 to 1.51)	0.86 (0.59 to 1.19)
2000	8.7 (6.1 to 12.2)	6.7 (4.8 to 9.4)	42.9 (30.8 to 59.4)	33.5 (24.1 to 46.4)	1.06 (0.76 to 1.47)	0.83 (0.60 to 1.15)
2005	6.7 (4.6 to 9.4)	5.3 (3.6 to 7.3)	36.0 (25.1 to 49.5)	28.3 (19.9 to 39.0)	1.04 (0.73 to 1.43)	0.82 (0.57 to 1.13)
2010	5.2 (3.6 to 7.2)	4.1 (2.9 to 5.6)	28.9 (20.2 to 39.8)	22.9 (16.3 to 31.1)	1.02 (0.71 to 1.40)	0.81 (0.57 to 1.10)
2015	4.2 (2.9 to 6.0)	3.4 (2.3 to 4.7)	23.3 (16.3 to 32.5)	18.5 (13.0 to 25.6)	1.01 (0.71 to 1.42)	0.81 (0.56 to 1.11)
2020	3.8 (2.6 to 5.5)	3.0 (2.1 to 4.3)	20.9 (14.6 to 29.8)	16.5 (11.6 to 23.4)	1.05 (0.73 to 1.49)	0.82 (0.58 to 1.17)
2021	3.7 (2.6 to 5.3)	2.9 (2.0 to 4.2)	20.5 (14.5 to 29.2)	16.1 (11.3 to 23.0)	1.05 (0.74 to 1.50)	0.83 (0.58 to 1.18)

Malaysia MYS Southeast Asia Stillbirth Rate / Neonatal Mortality Rate (SBR/NMR) − 20 weeks
lambda = 0.2 ; zeta = 0.99 ; scale = 5 ; amp = 0.028

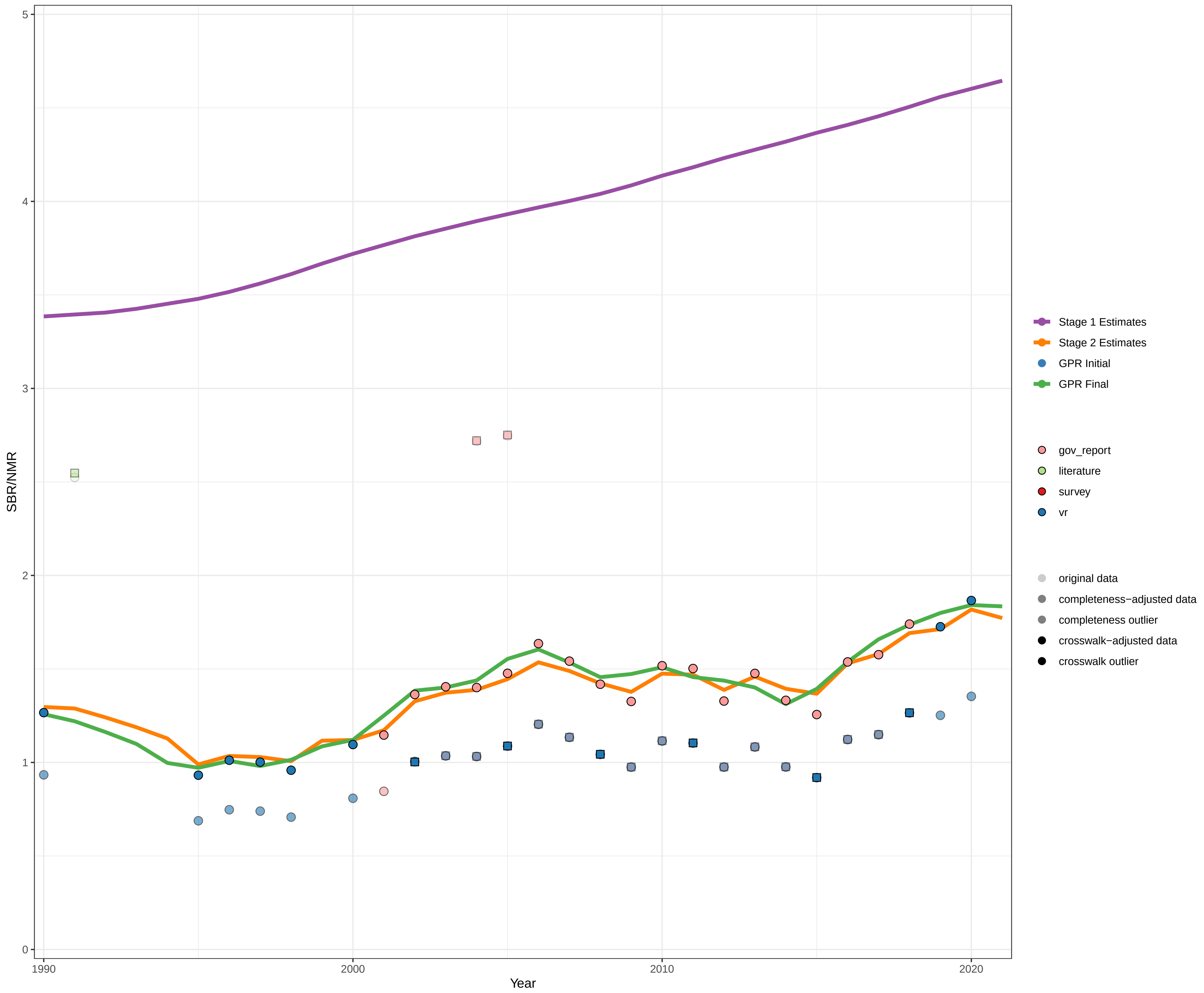

Malaysia MYS Southeast Asia Stillbirth Rate / Neonatal Mortality Rate (SBR/NMR) − 28 weeks
lambda = 0.2 ; zeta = 0.99 ; scale = 5 ; amp = 0.028

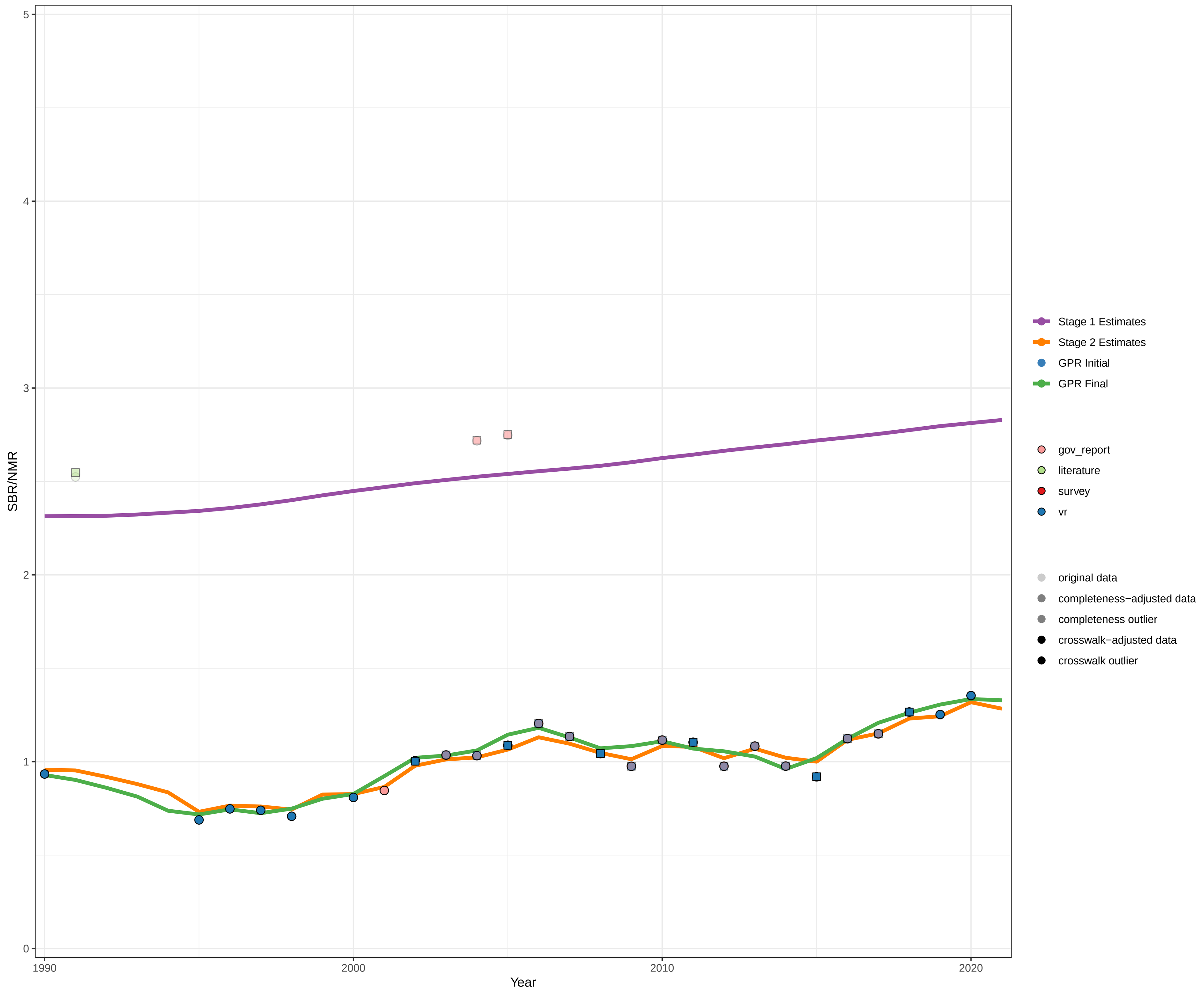

Definition	Years	Outlier	Source.Type	Title
28_weeks	1990	0	VR	Malaysia − Peninsular Vital Statistics
28_weeks	1995−1998, 2000, 2019, 2020	0	VR	United Nations Demographic Yearbook
28_weeks	2001−2018	0	Statistical Report	Malaysia Health Indicators
28_weeks	1991	1	Sci Lit	Changes of perinatal statistics in a semiurban setup between two time periods in Malaysia
28_weeks	2003, 2004, 2006, 2007, 2009, 2010, 2012−2014, 2016, 2017	1	VR	United Nations Demographic Yearbook
28_weeks	2004, 2005	1	Statistical Report	Malaysia Ministry of Health Annual Report
28_weeks	2002, 2005, 2008, 2011, 2015, 2018	2	VR	United Nations Demographic Yearbook

Malaysia MYS Southeast Asia Stillbirth Rate (SBR) − 20 weeks
lambda = 0.2 ; zeta = 0.99 ; scale = 5 ; amp = 0.028

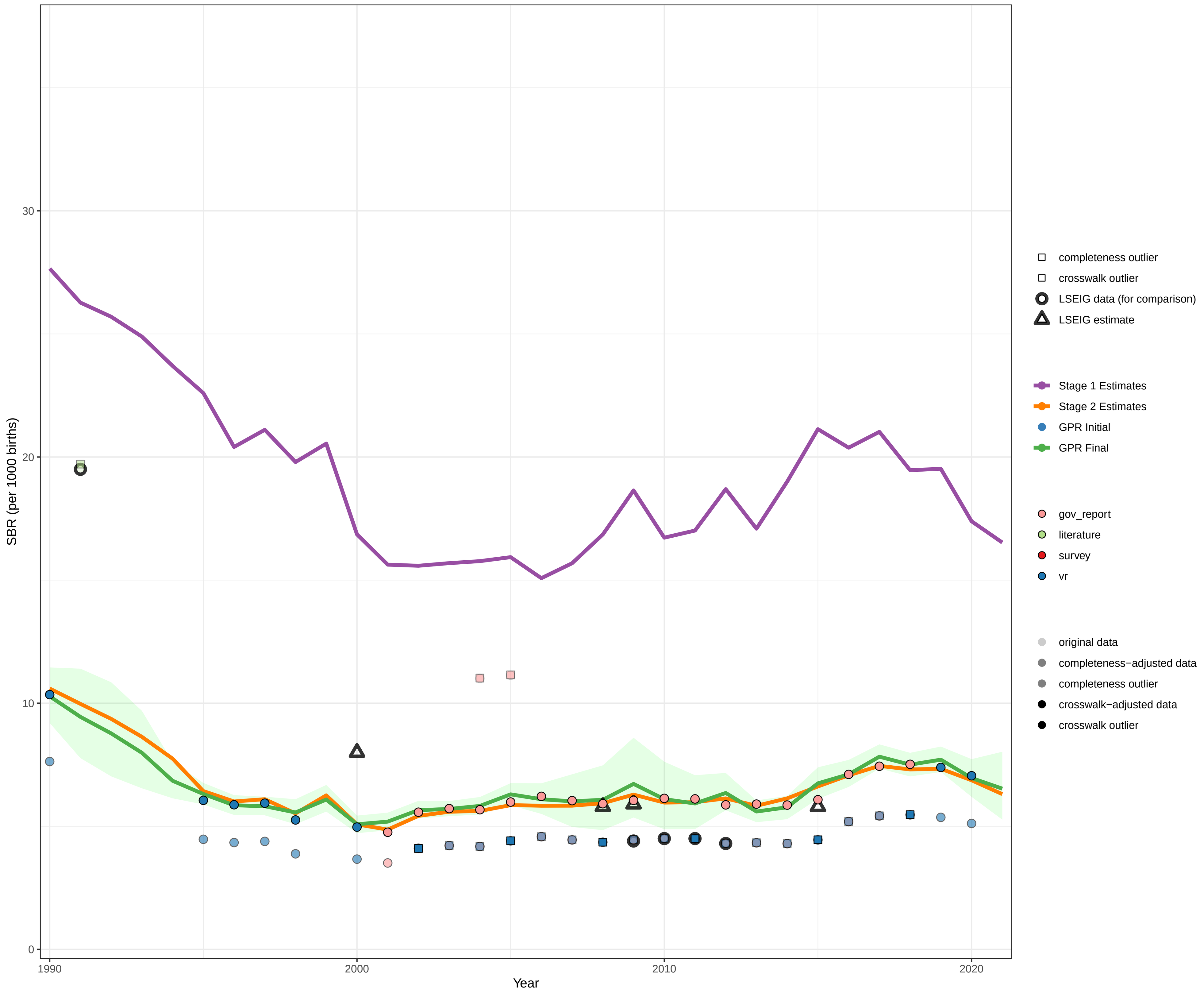

Malaysia MYS Southeast Asia Stillbirth Rate (SBR) − 28 weeks
lambda = 0.2 ; zeta = 0.99 ; scale = 5 ; amp = 0.028

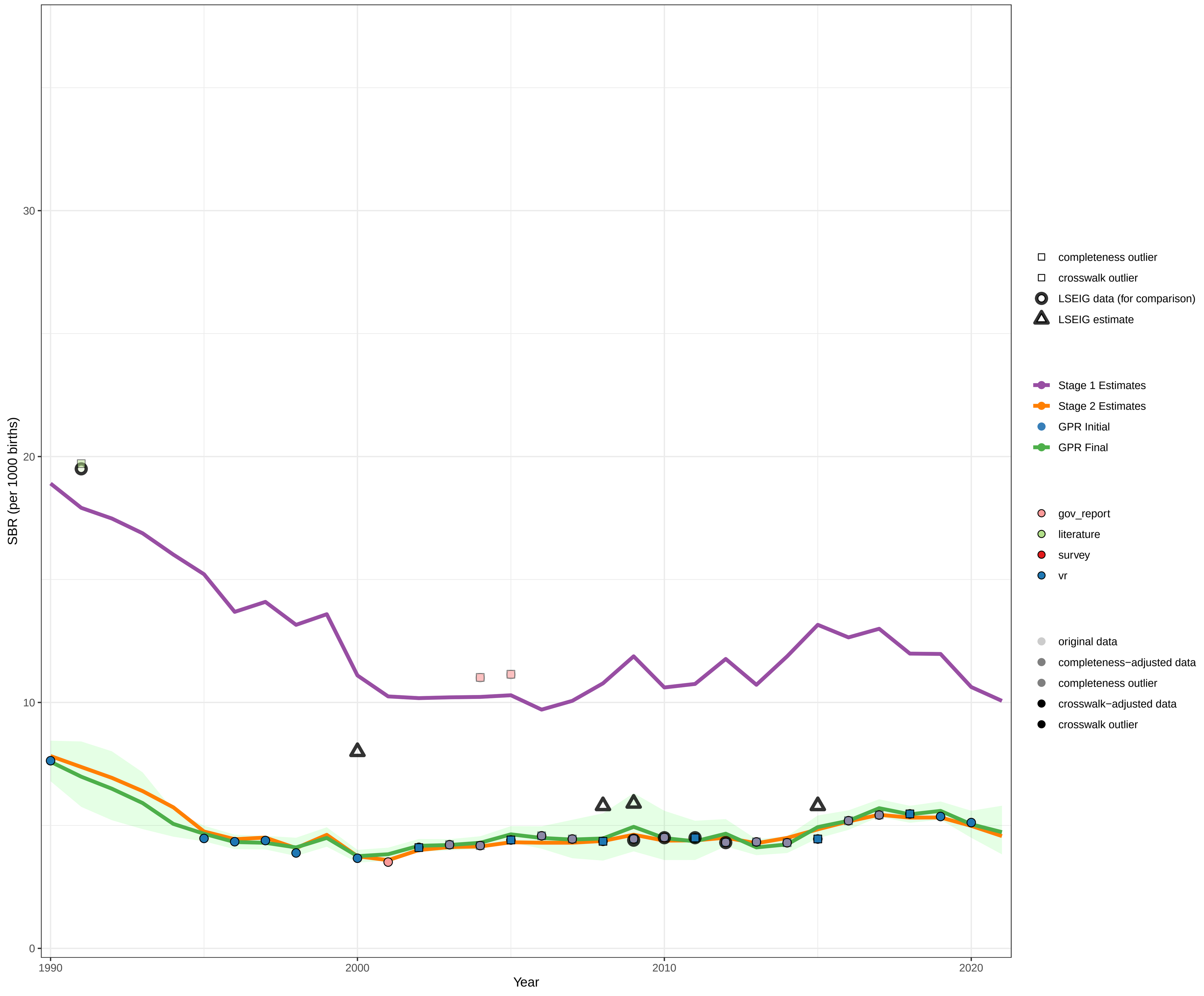

Year	Total Stillbirths (in thousands)	Total Stillbirths (in thousands)	SBR (per 1000 births)	SBR (per 1000 births)	SBR/NMR	SBR/NMR
	20 weeks	28 weeks	20 weeks	28 weeks	20 weeks	28 weeks
1990	5.1 (4.6 to 5.7)	3.8 (3.4 to 4.2)	10.3 (9.2 to 11.5)	7.6 (6.8 to 8.4)	1.26 (1.13 to 1.40)	0.93 (0.83 to 1.03)
1995	3.4 (3.2 to 3.7)	2.5 (2.4 to 2.7)	6.3 (5.9 to 6.8)	4.7 (4.4 to 5.0)	0.97 (0.91 to 1.04)	0.72 (0.67 to 0.77)
2000	2.7 (2.5 to 2.9)	2.0 (1.8 to 2.1)	5.1 (4.7 to 5.4)	3.7 (3.5 to 4.0)	1.12 (1.04 to 1.20)	0.83 (0.77 to 0.88)
2005	3.1 (2.9 to 3.3)	2.3 (2.1 to 2.4)	6.3 (5.9 to 6.8)	4.6 (4.3 to 5.0)	1.55 (1.44 to 1.67)	1.14 (1.06 to 1.23)
2010	3.0 (2.4 to 3.8)	2.2 (1.8 to 2.8)	6.1 (4.9 to 7.6)	4.5 (3.6 to 5.6)	1.51 (1.21 to 1.89)	1.11 (0.89 to 1.38)
2015	3.4 (3.0 to 3.7)	2.5 (2.2 to 2.7)	6.7 (6.1 to 7.4)	4.9 (4.5 to 5.4)	1.39 (1.26 to 1.53)	1.02 (0.92 to 1.12)
2020	3.3 (3.0 to 3.7)	2.4 (2.2 to 2.7)	7.0 (6.2 to 7.7)	5.0 (4.5 to 5.6)	1.84 (1.64 to 2.04)	1.34 (1.19 to 1.48)
2021	3.1 (2.5 to 3.8)	2.3 (1.8 to 2.8)	6.5 (5.3 to 8.0)	4.7 (3.8 to 5.8)	1.83 (1.48 to 2.26)	1.33 (1.07 to 1.63)

Maldives MDV Southeast Asia Stillbirth Rate / Neonatal Mortality Rate (SBR/NMR) − 20 weeks
lambda = 0.6 ; zeta = 0.8 ; scale = 15 ; amp = 0.028

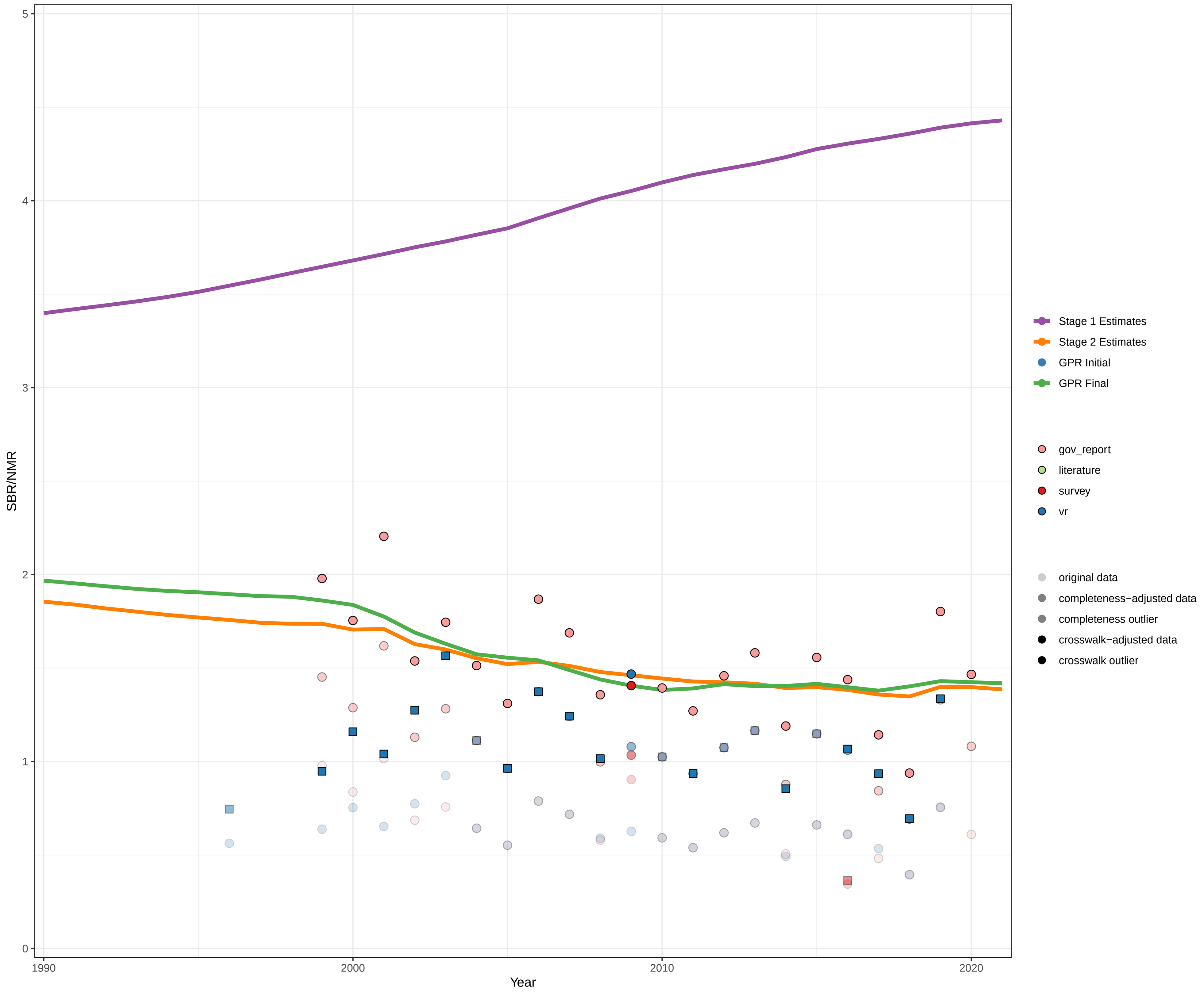

Maldives MDV Southeast Asia Stillbirth Rate / Neonatal Mortality Rate (SBR/NMR) − 28 weeks
lambda = 0.6 ; zeta = 0.8 ; scale = 15 ; amp = 0.028

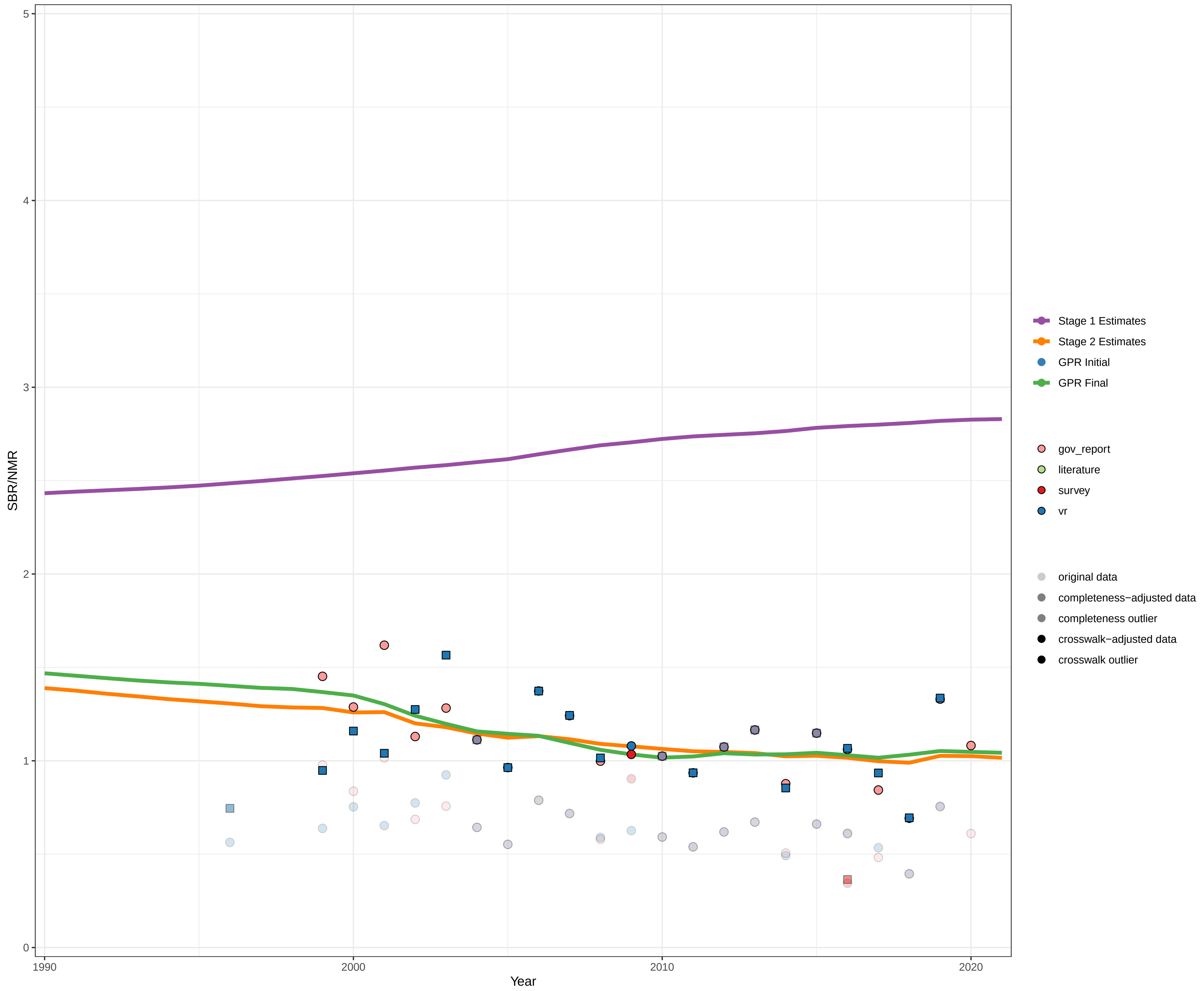

Definition	Years	Outlier	Source.Type	Title
28_weeks	1999−2008, 2010, 2011, 2016−2020	0	Statistical Report	Maldives Health Statistics
28_weeks	2009	0	Survey	Maldives Demographic and Health Survey 2009
28_weeks	2009	0	VR	United Nations Demographic Yearbook
28_weeks	2012−2015	0	Statistical Report	Maldives Health Statistics
28_weeks	1996, 2004, 2010, 2012, 2013, 2015	1	VR	United Nations Demographic Yearbook
28_weeks	2016	1	Survey	Maldives Demographic and Health Survey 2016−2017
28_weeks	1999−2003, 2005−2008, 2011, 2014, 2016−2019	2	VR	United Nations Demographic Yearbook

Maldives MDV Southeast Asia Stillbirth Rate (SBR) − 20 weeks
lambda = 0.6 ; zeta = 0.8 ; scale = 15 ; amp = 0.028

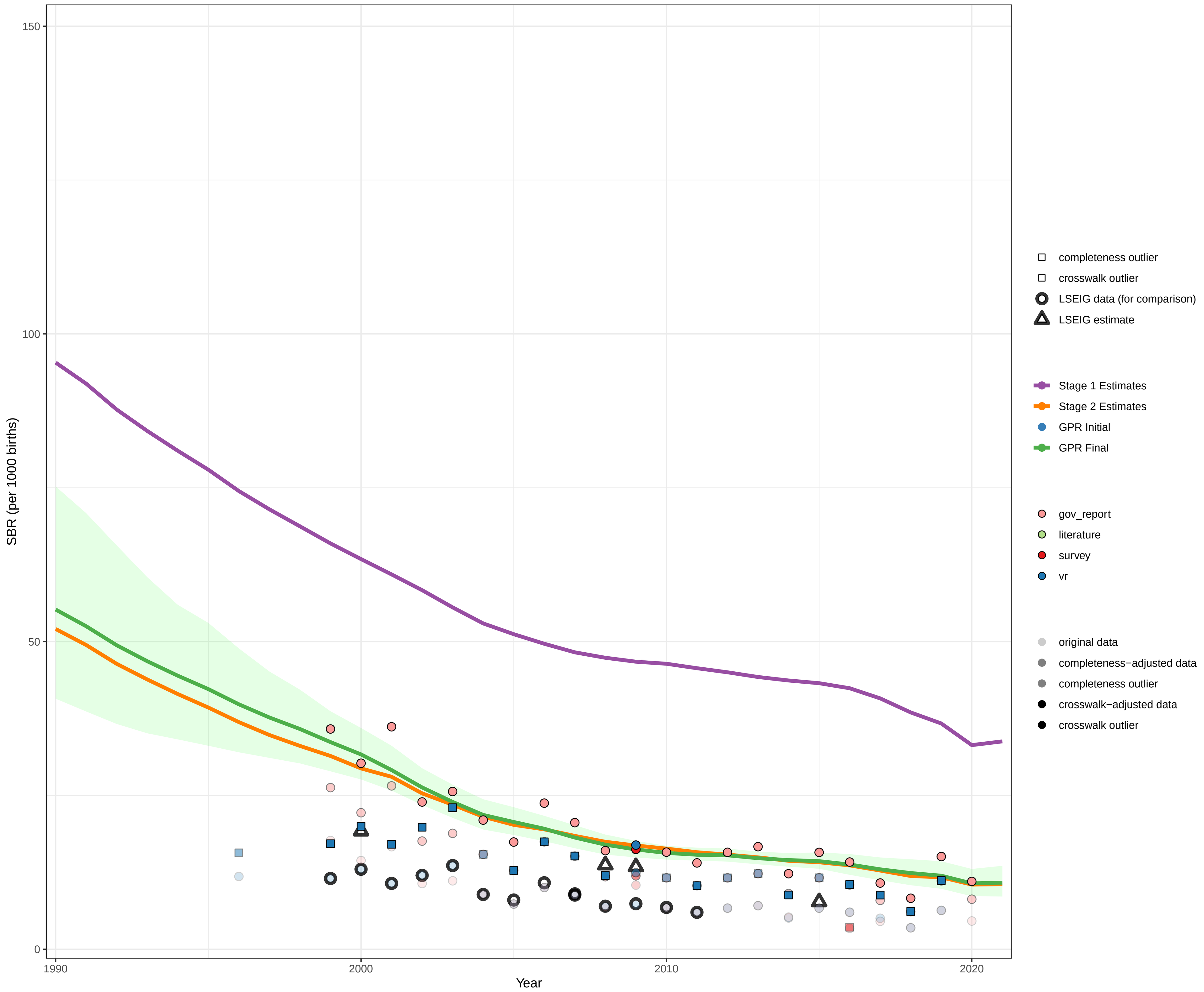

Maldives MDV Southeast Asia Stillbirth Rate (SBR) − 28 weeks
lambda = 0.6 ; zeta = 0.8 ; scale = 15 ; amp = 0.028

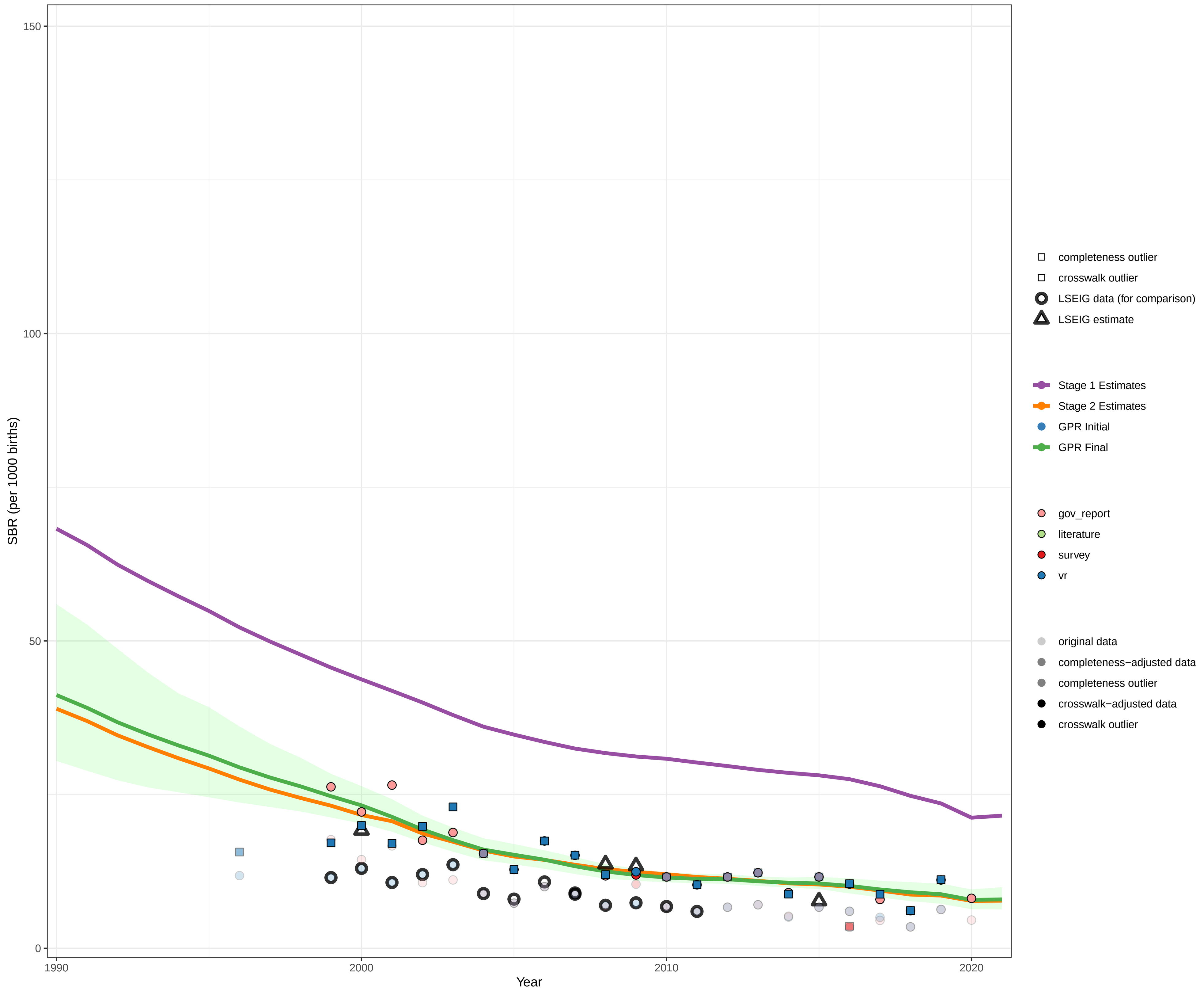

Year	Total Stillbirths (in thousands)	Total Stillbirths (in thousands)	SBR (per 1000 births)	SBR (per 1000 births)	SBR/NMR	SBR/NMR
	20 weeks	28 weeks	20 weeks	28 weeks	20 weeks	28 weeks
1990	0.5 (0.4 to 0.7)	0.4 (0.3 to 0.5)	55.2 (40.7 to 75.2)	41.2 (30.5 to 56.0)	1.97 (1.45 to 2.68)	1.47 (1.09 to 2.00)
1995	0.3 (0.3 to 0.4)	0.2 (0.2 to 0.3)	42.3 (33.1 to 53.0)	31.3 (24.6 to 39.2)	1.91 (1.49 to 2.39)	1.41 (1.11 to 1.77)
2000	0.2 (0.2 to 0.2)	0.1 (0.1 to 0.2)	31.7 (27.6 to 36.0)	23.2 (20.3 to 26.4)	1.84 (1.60 to 2.09)	1.35 (1.18 to 1.53)
2005	0.1 (0.1 to 0.1)	0.1 (0.1 to 0.1)	20.7 (18.6 to 23.1)	15.2 (13.7 to 17.0)	1.56 (1.40 to 1.74)	1.14 (1.03 to 1.28)
2010	0.1 (0.1 to 0.1)	0.1 (0.1 to 0.1)	15.7 (14.6 to 16.8)	11.5 (10.7 to 12.3)	1.38 (1.29 to 1.48)	1.02 (0.95 to 1.09)
2015	0.1 (0.1 to 0.1)	0.1 (0.1 to 0.1)	14.3 (13.1 to 15.7)	10.5 (9.6 to 11.6)	1.42 (1.29 to 1.56)	1.04 (0.95 to 1.15)
2020	0.1 (0.1 to 0.1)	0.0 (0.0 to 0.1)	10.7 (8.6 to 13.0)	7.9 (6.3 to 9.6)	1.42 (1.15 to 1.74)	1.05 (0.84 to 1.28)
2021	0.1 (0.1 to 0.1)	0.0 (0.0 to 0.1)	10.8 (8.6 to 13.6)	8.0 (6.3 to 10.0)	1.42 (1.12 to 1.78)	1.04 (0.83 to 1.30)

Mauritius MUS Southeast Asia Stillbirth Rate / Neonatal Mortality Rate (SBR/NMR) − 20 weeks
lambda = 0.2 ; zeta = 0.99 ; scale = 5 ; amp = 0.028

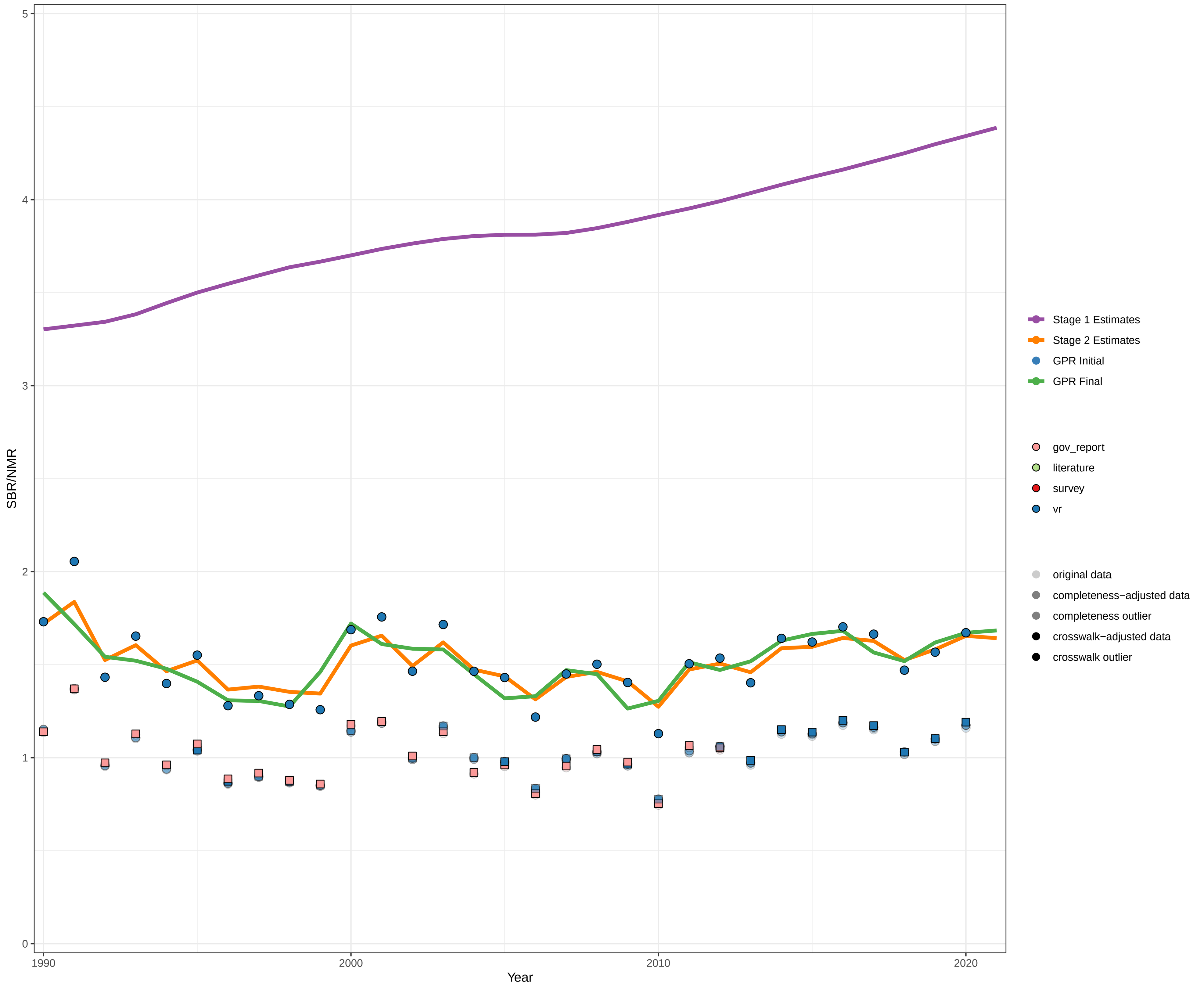

Mauritius MUS Southeast Asia Stillbirth Rate / Neonatal Mortality Rate (SBR/NMR) − 28 weeks
lambda = 0.2 ; zeta = 0.99 ; scale = 5 ; amp = 0.028

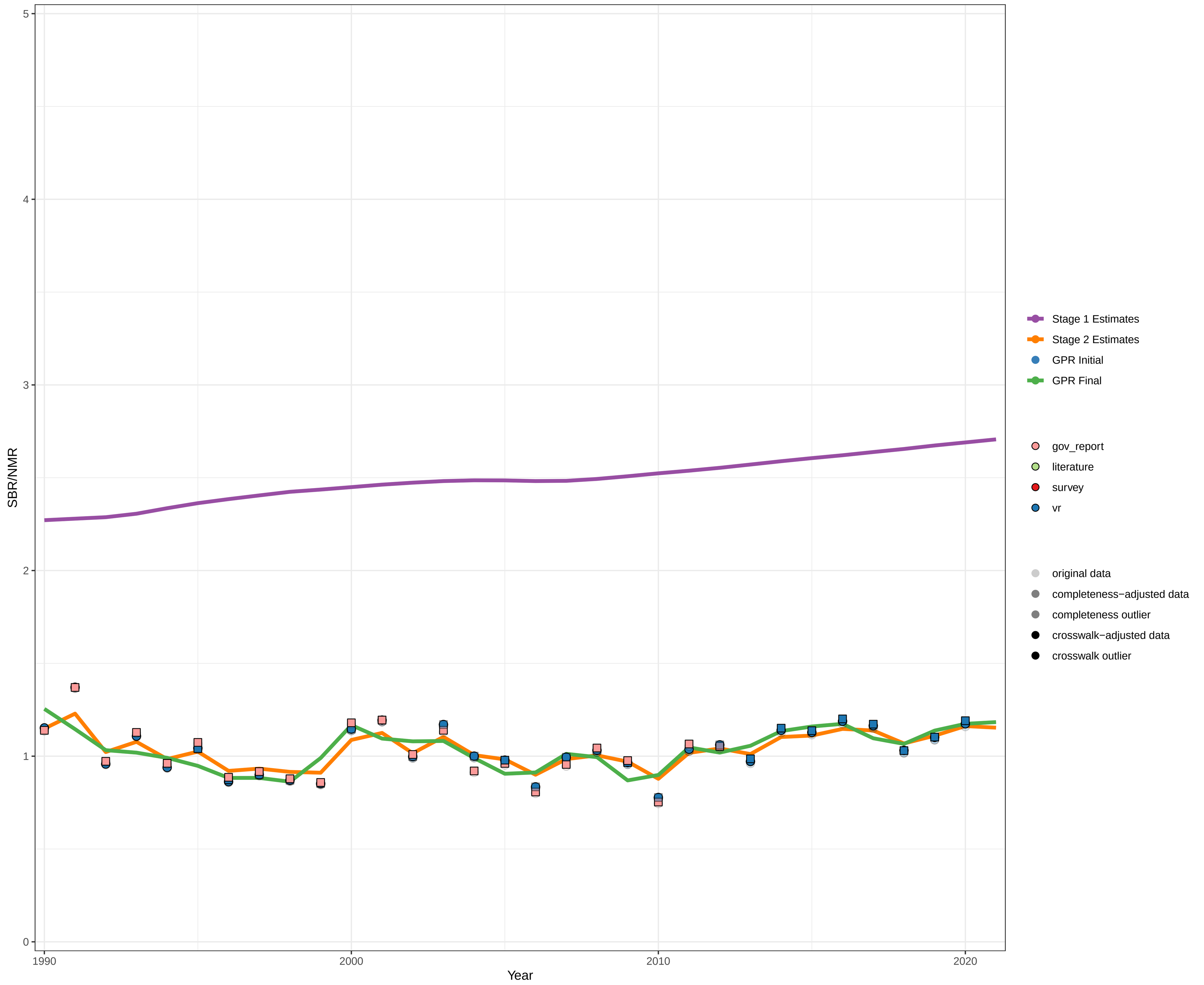

Definition	Years	Outlier	Source.Type	Title
28_weeks	1990−2020	0	VR	Mauritius Digest of Demographic Statistics
28_weeks	2011	0	VR	United Nations Demographic Yearbook
28_weeks	1997−2000, 2002−2004, 2006−2008, 2010, 2012	1	VR	United Nations Demographic Yearbook
28_weeks	1990−2012	2	Statistical Report	Mauritius Family Planning and Demographic Yearbook
28_weeks	1995, 1996, 2001, 2005, 2009, 2013−2020	2	VR	United Nations Demographic Yearbook

Mauritius MUS Southeast Asia Stillbirth Rate (SBR) − 20 weeks
lambda = 0.2 ; zeta = 0.99 ; scale = 5 ; amp = 0.028

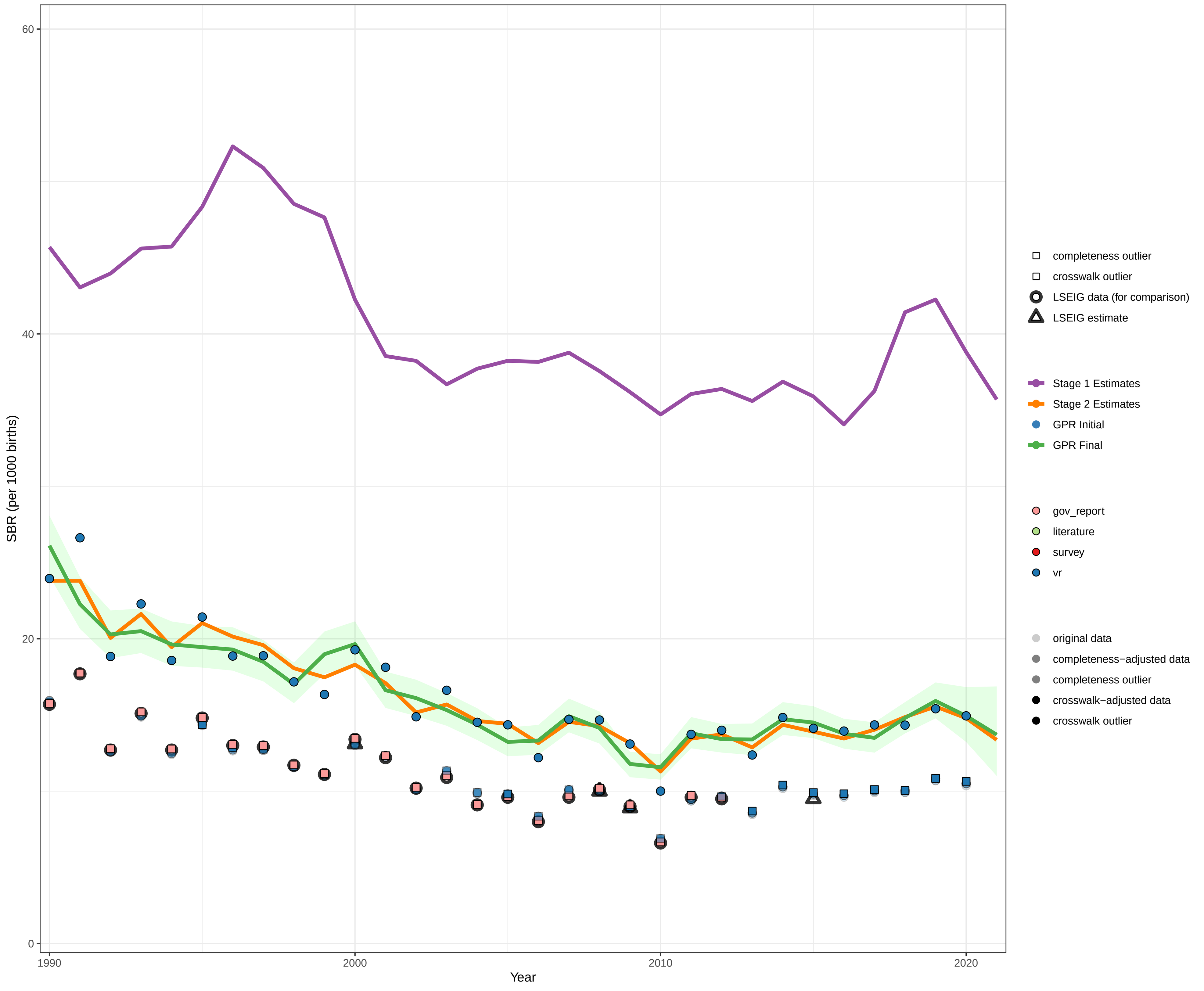

Mauritius MUS Southeast Asia Stillbirth Rate (SBR) − 28 weeks
lambda = 0.2 ; zeta = 0.99 ; scale = 5 ; amp = 0.028

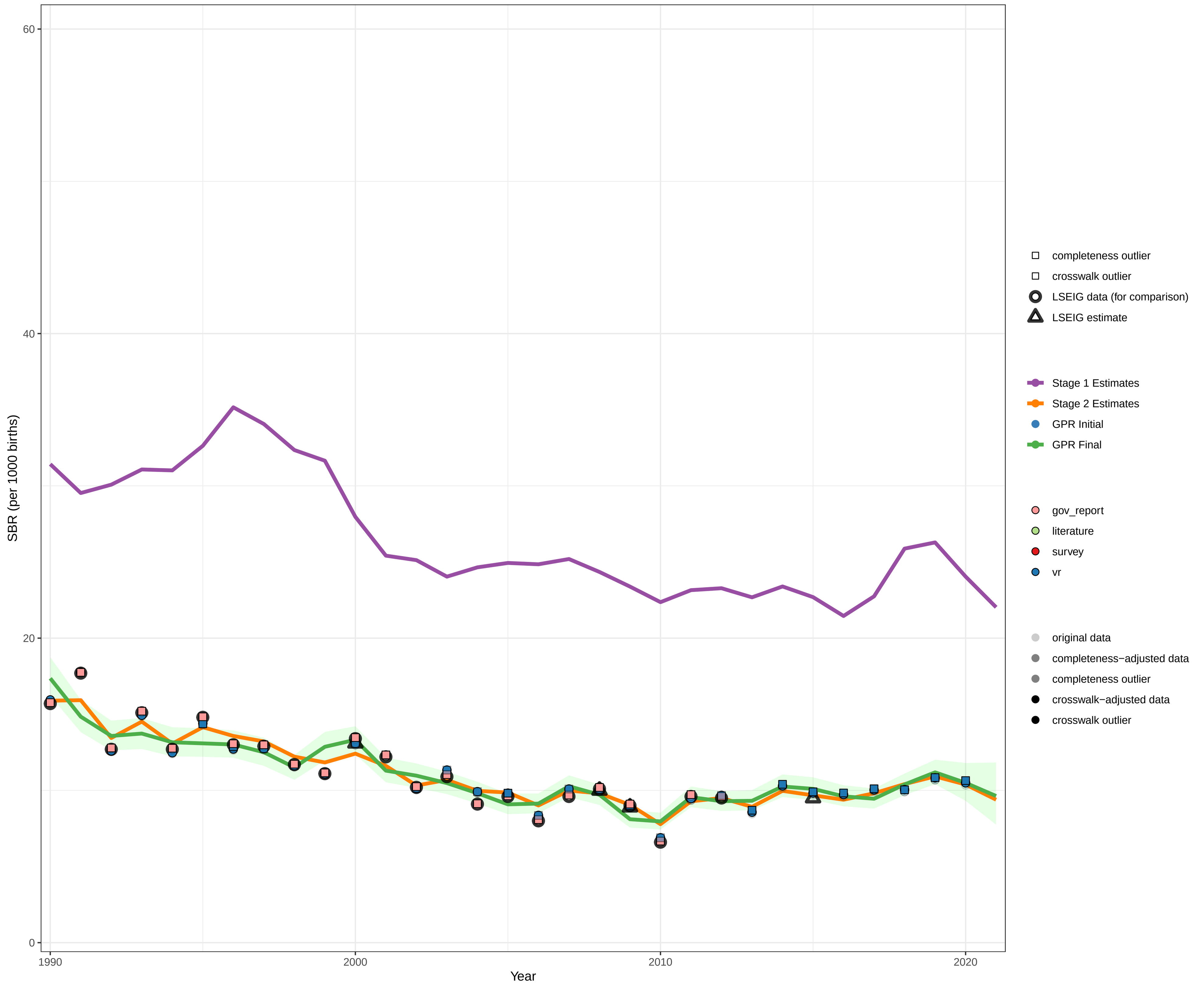

Year	Total Stillbirths (in thousands)	Total Stillbirths (in thousands)	SBR (per 1000 births)	SBR (per 1000 births)	SBR/NMR	SBR/NMR
	20 weeks	28 weeks	20 weeks	28 weeks	20 weeks	28 weeks
1990	0.6 (0.6 to 0.7)	0.4 (0.4 to 0.4)	26.1 (24.1 to 28.1)	17.4 (16.2 to 18.8)	1.89 (1.74 to 2.03)	1.25 (1.17 to 1.36)
1995	0.4 (0.4 to 0.5)	0.3 (0.3 to 0.3)	19.5 (18.1 to 20.8)	13.1 (12.2 to 14.1)	1.41 (1.31 to 1.51)	0.95 (0.88 to 1.02)
2000	0.4 (0.4 to 0.4)	0.3 (0.3 to 0.3)	19.7 (18.3 to 21.1)	13.3 (12.4 to 14.2)	1.72 (1.60 to 1.85)	1.17 (1.08 to 1.25)
2005	0.2 (0.2 to 0.2)	0.2 (0.1 to 0.2)	13.2 (12.3 to 14.2)	9.1 (8.4 to 9.8)	1.32 (1.23 to 1.41)	0.91 (0.84 to 0.98)
2010	0.2 (0.2 to 0.2)	0.1 (0.1 to 0.1)	11.6 (10.8 to 12.4)	8.0 (7.4 to 8.5)	1.31 (1.21 to 1.40)	0.90 (0.84 to 0.96)
2015	0.2 (0.2 to 0.2)	0.1 (0.1 to 0.1)	14.5 (13.5 to 15.6)	10.1 (9.4 to 10.9)	1.67 (1.55 to 1.79)	1.16 (1.08 to 1.25)
2020	0.2 (0.2 to 0.2)	0.1 (0.1 to 0.2)	14.9 (13.2 to 16.8)	10.5 (9.3 to 11.8)	1.67 (1.48 to 1.88)	1.17 (1.04 to 1.32)
2021	0.2 (0.1 to 0.2)	0.1 (0.1 to 0.2)	13.7 (11.0 to 16.9)	9.6 (7.8 to 11.8)	1.68 (1.35 to 2.07)	1.18 (0.95 to 1.45)

Myanmar MMR Southeast Asia Stillbirth Rate / Neonatal Mortality Rate (SBR/NMR) − 20 weeks
lambda = 0.8 ; zeta = 0.7 ; scale = 15 ; amp = 0.028

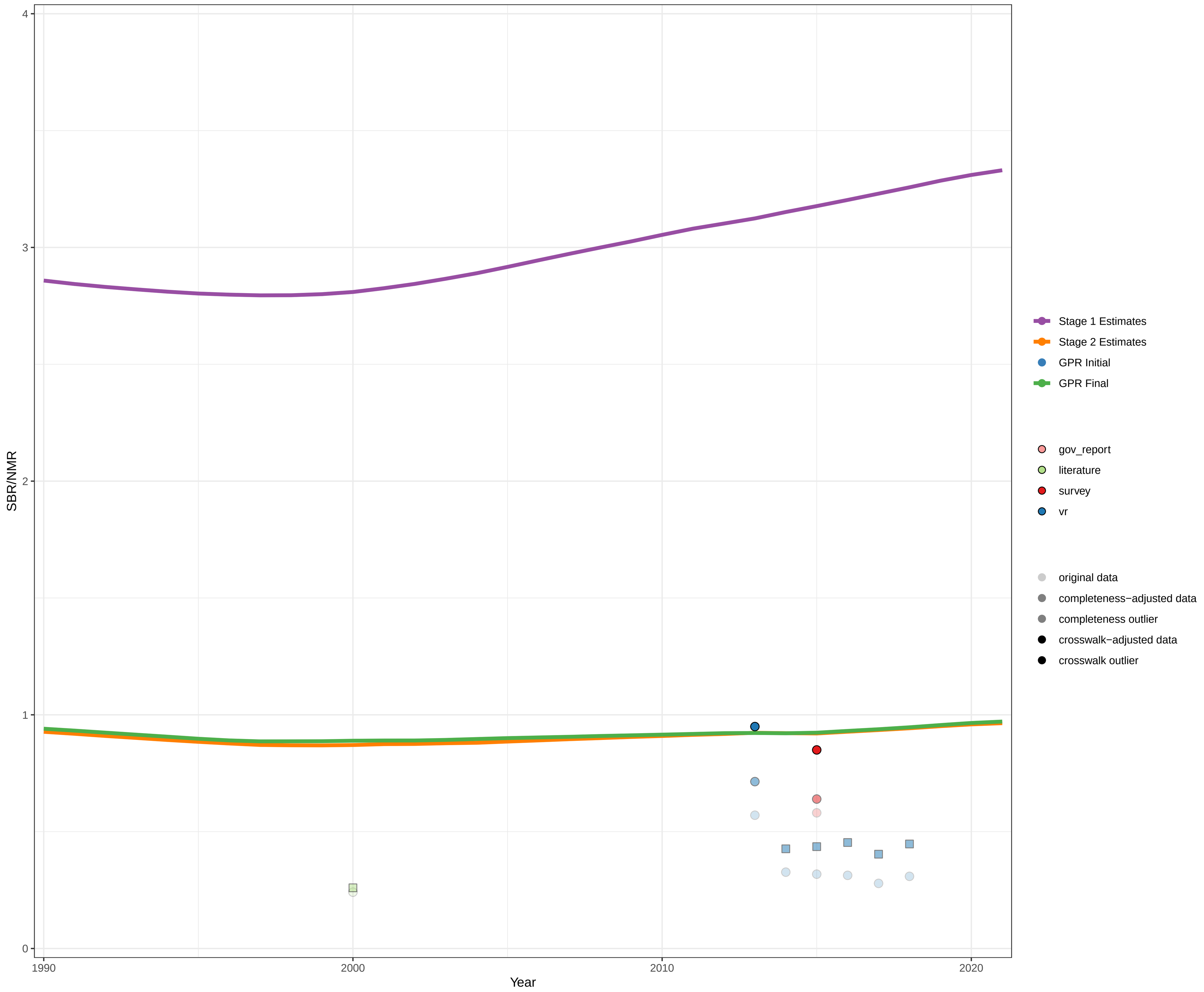

Myanmar MMR Southeast Asia Stillbirth Rate / Neonatal Mortality Rate (SBR/NMR) − 28 weeks
lambda = 0.8 ; zeta = 0.7 ; scale = 15 ; amp = 0.028

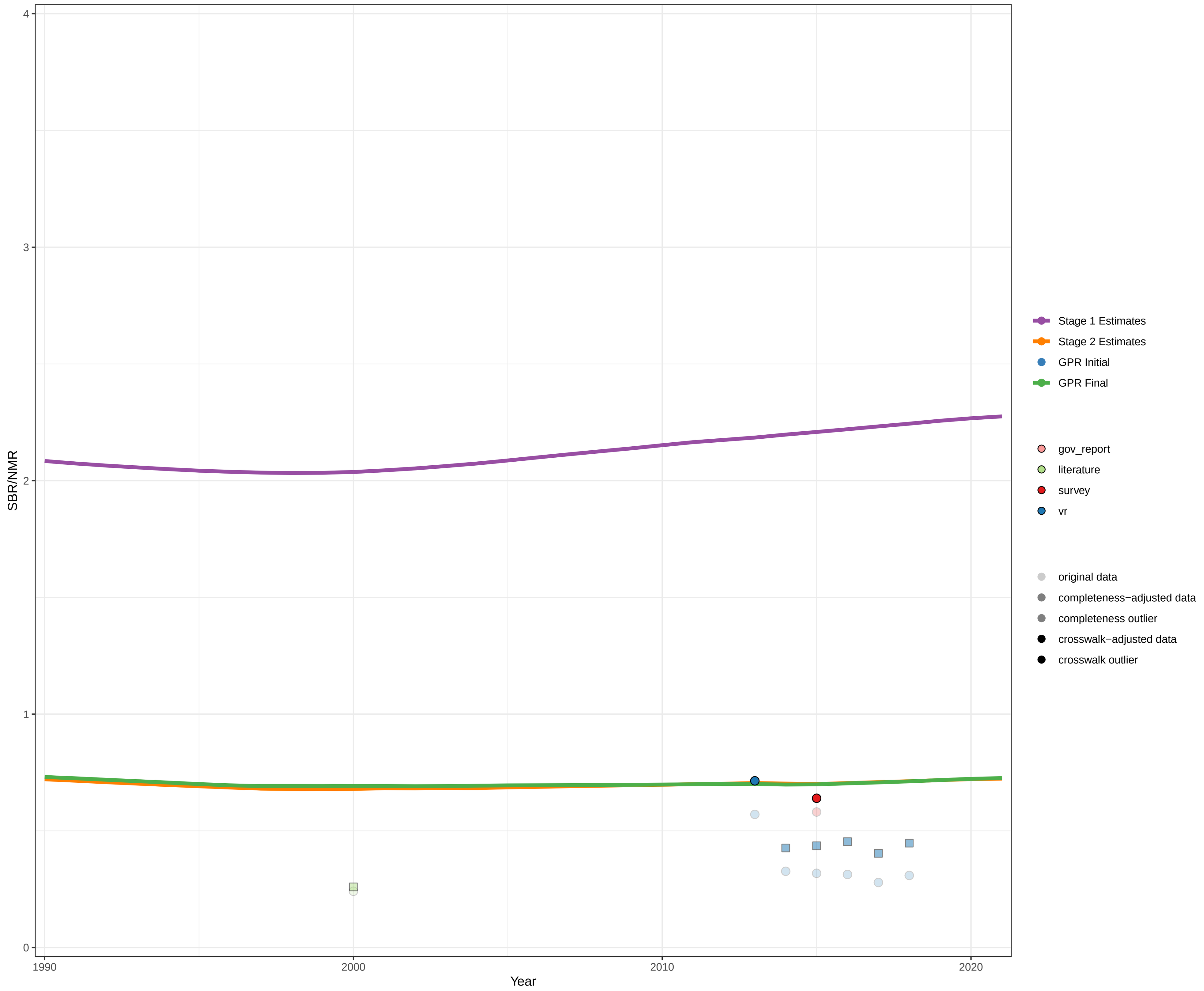

Definition	Years	Outlier	Source.Type	Title
28_weeks	2013	0	VR	United Nations Demographic Yearbook
28_weeks	2015	0	Survey	Myanmar Demographic and Health Survey 2015−2016
28_weeks	2000	1	Sci Lit	Mediation of the effect of malaria in pregnancy on stillbirth and neonatal death in an area of low transmission: observational data analysis
28_weeks	2014−2018	1	VR	United Nations Demographic Yearbook

Myanmar MMR Southeast Asia Stillbirth Rate (SBR) − 20 weeks
lambda = 0.8 ; zeta = 0.7 ; scale = 15 ; amp = 0.028

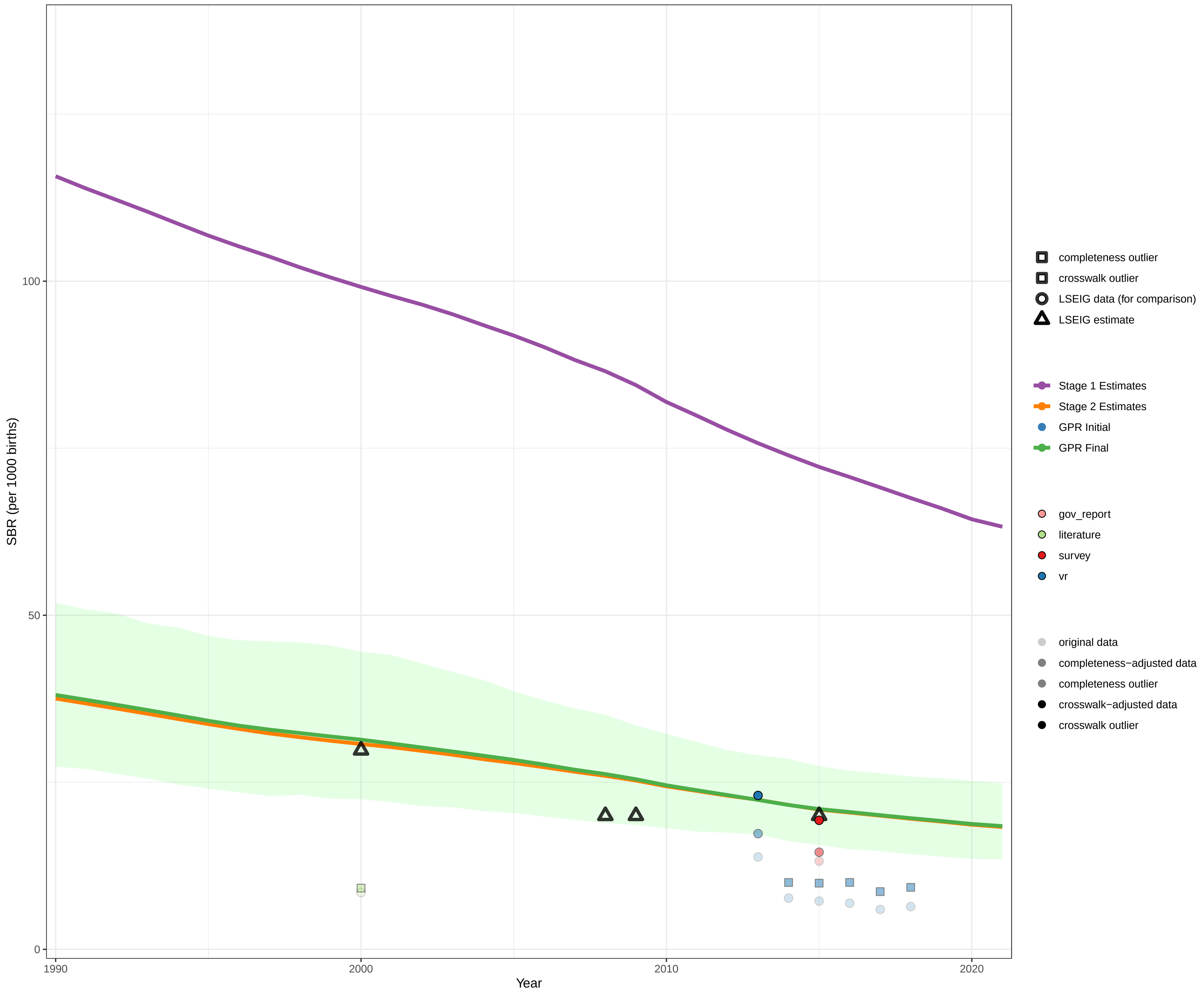

Myanmar MMR Southeast Asia Stillbirth Rate (SBR) − 28 weeks
lambda = 0.8 ; zeta = 0.7 ; scale = 15 ; amp = 0.028

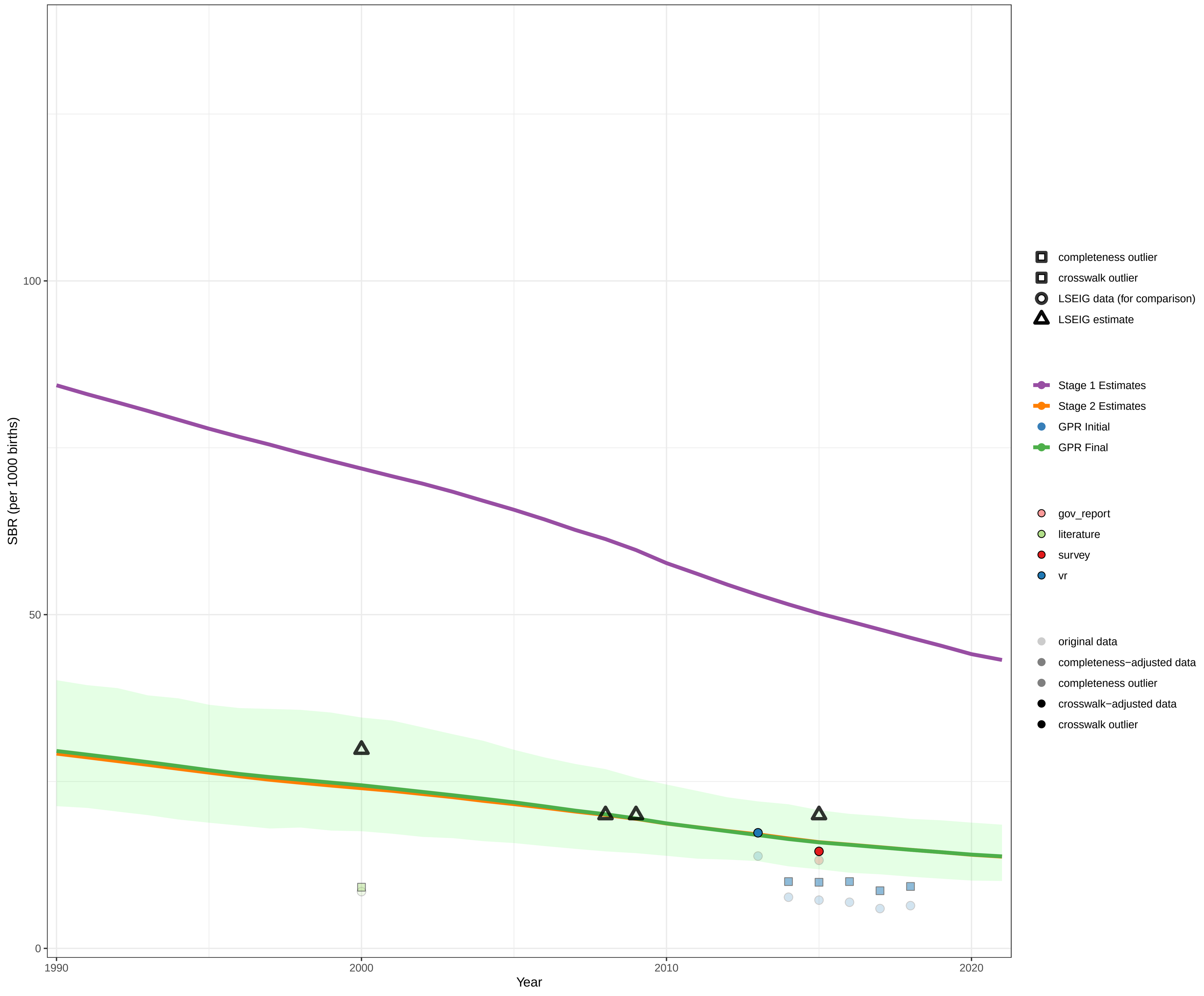

Year	Total Stillbirths (in thousands)	Total Stillbirths (in thousands)	SBR (per 1000 births)	SBR (per 1000 births)	SBR/NMR	SBR/NMR
	20 weeks	28 weeks	20 weeks	28 weeks	20 weeks	28 weeks
1990	44.3 (31.4 to 61.1)	34.1 (24.3 to 46.8)	38.1 (27.3 to 51.9)	29.6 (21.3 to 40.2)	0.94 (0.68 to 1.28)	0.73 (0.53 to 0.99)
1995	37.7 (26.2 to 52.3)	29.2 (20.4 to 40.3)	34.2 (24.0 to 46.9)	26.7 (18.8 to 36.5)	0.90 (0.63 to 1.23)	0.70 (0.49 to 0.96)
2000	34.2 (24.3 to 49.2)	26.5 (18.9 to 37.8)	31.4 (22.5 to 44.5)	24.4 (17.6 to 34.6)	0.89 (0.64 to 1.26)	0.69 (0.50 to 0.98)
2005	30.6 (21.8 to 42.2)	23.5 (16.8 to 32.2)	28.4 (20.4 to 38.7)	21.9 (15.8 to 29.8)	0.90 (0.65 to 1.23)	0.69 (0.50 to 0.94)
2010	26.6 (19.5 to 35.1)	20.1 (14.8 to 26.6)	24.5 (18.1 to 32.2)	18.7 (13.9 to 24.6)	0.92 (0.68 to 1.20)	0.70 (0.52 to 0.92)
2015	23.1 (17.1 to 30.3)	17.4 (12.9 to 22.8)	21.0 (15.6 to 27.4)	15.9 (11.8 to 20.7)	0.92 (0.69 to 1.21)	0.70 (0.52 to 0.91)
2020	20.6 (14.8 to 27.8)	15.3 (11.0 to 20.7)	18.8 (13.5 to 25.2)	14.1 (10.2 to 18.8)	0.97 (0.70 to 1.30)	0.72 (0.52 to 0.97)
2021	20.2 (14.7 to 27.4)	15.0 (10.9 to 20.3)	18.4 (13.5 to 24.9)	13.8 (10.1 to 18.5)	0.97 (0.71 to 1.31)	0.73 (0.53 to 0.98)

Philippines PHL Southeast Asia Stillbirth Rate / Neonatal Mortality Rate (SBR/NMR) − 20 weeks
lambda = 0.6 ; zeta = 0.8 ; scale = 15 ; amp = 0.028

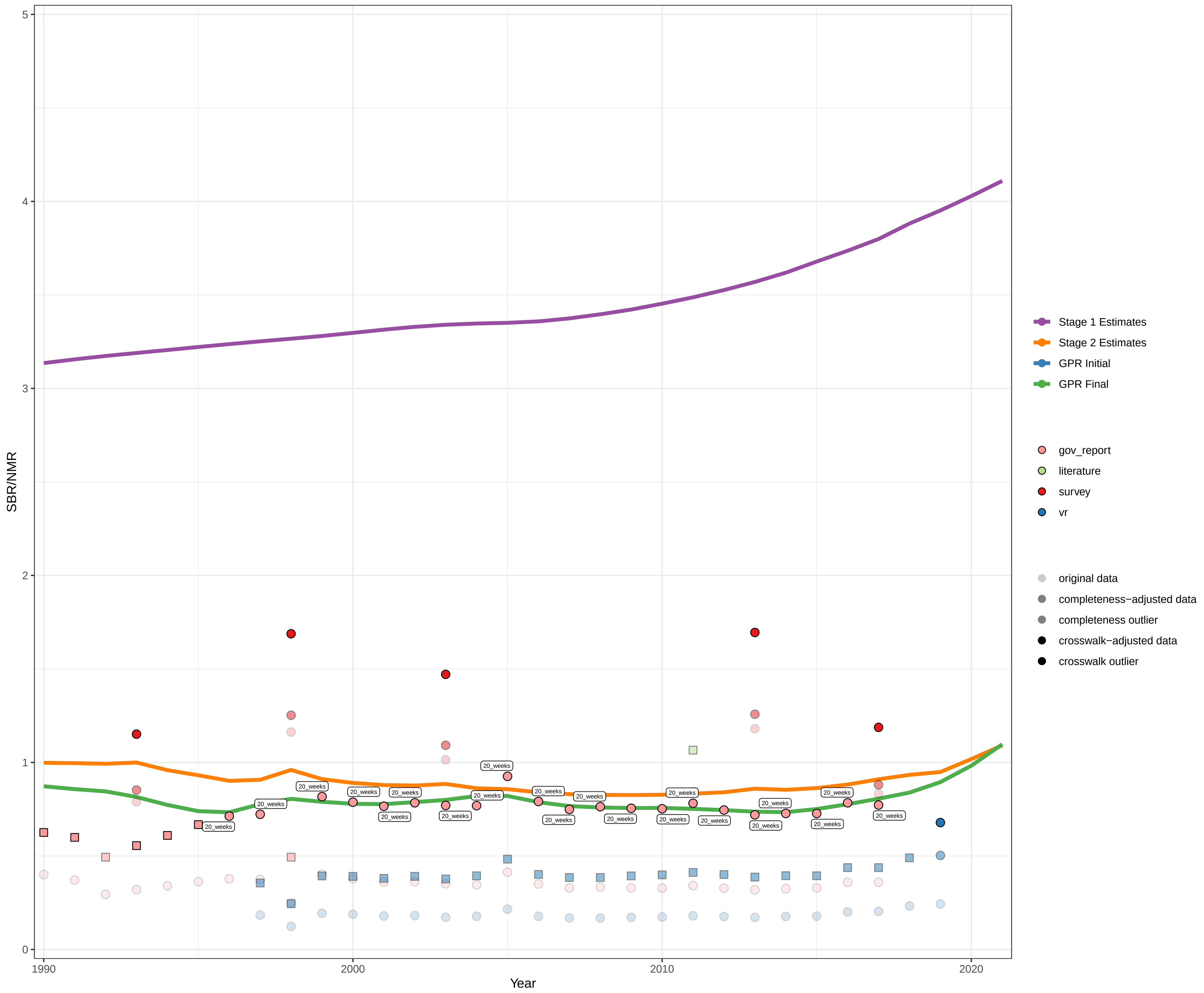

Philippines PHL Southeast Asia Stillbirth Rate / Neonatal Mortality Rate (SBR/NMR) − 28 weeks
lambda = 0.6 ; zeta = 0.8 ; scale = 15 ; amp = 0.028

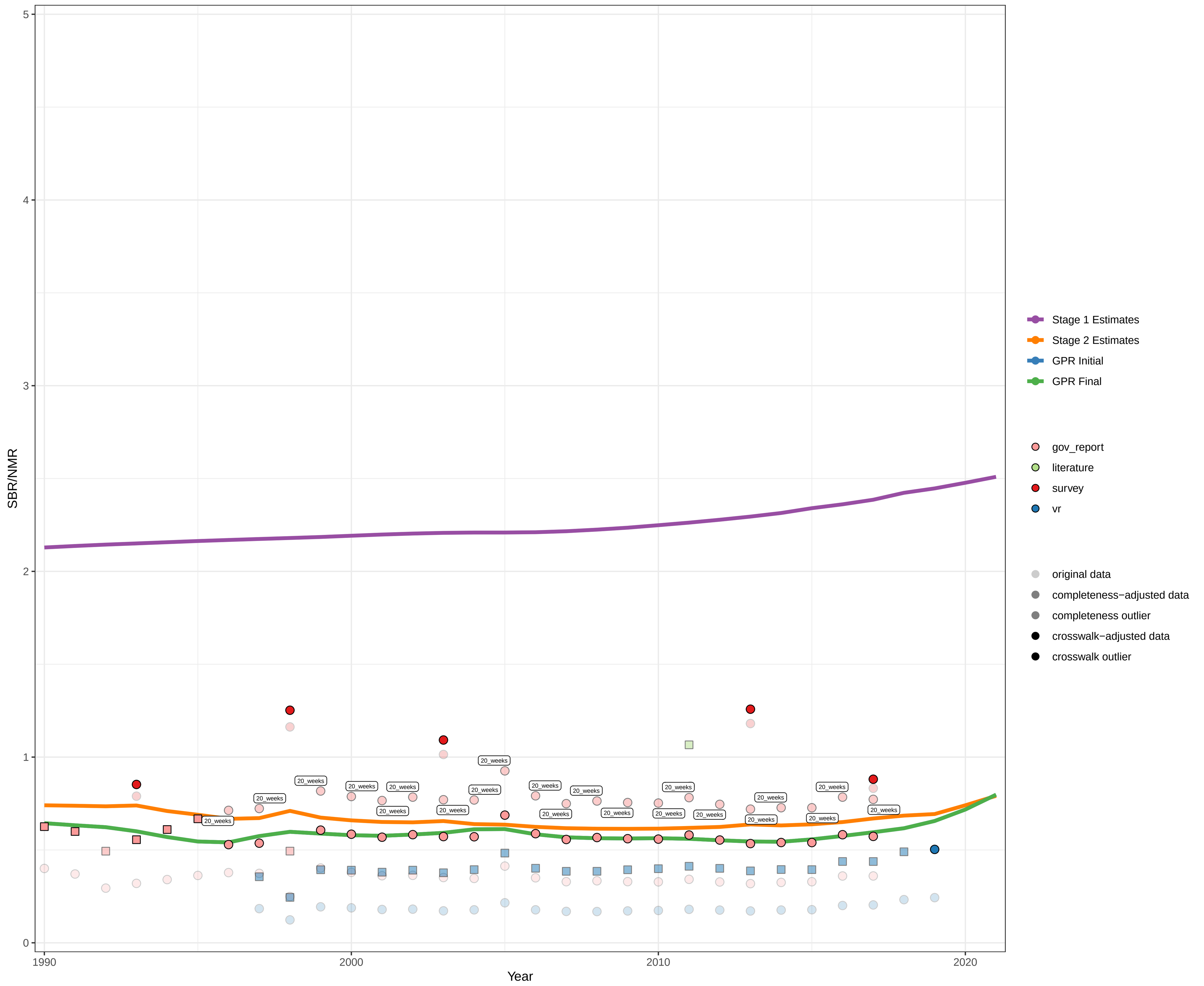

Definition	Years	Outlier	Source.Type	Title
20_weeks	1996, 1997, 1999−2017	0	Statistical Report	Philippines Statistical Yearbook
28_weeks	1993	0	Survey	Philippines Demographic and Health Survey 1993
28_weeks	1998	0	Survey	Philippines Demographic and Health Survey 1998
28_weeks	2003	0	Survey	Philippines Demographic and Health Survey 2003
28_weeks	2013	0	Survey	Philippines Demographic and Health Survey 2013
28_weeks	2017	0	Survey	Philippines Demographic and Health Survey 2017
28_weeks	2019	0	VR	United Nations Demographic Yearbook
28_weeks	2022	0	Survey	Philippines Demographic and Health Survey 2022
20_weeks	1992, 1998	1	Statistical Report	Philippines Statistical Yearbook
28_weeks	1997−2018	1	VR	United Nations Demographic Yearbook
28_weeks_OR_1000_grams	2011	1	Sci Lit	Maternal complications and perinatal mortality: findings of the World Health Organization Multicountry Survey on Maternal and Newborn Health
20_weeks	1990, 1991, 1993−1995	2	Statistical Report	Philippines Statistical Yearbook

Philippines PHL Southeast Asia Stillbirth Rate (SBR) − 20 weeks
lambda = 0.6 ; zeta = 0.8 ; scale = 15 ; amp = 0.028

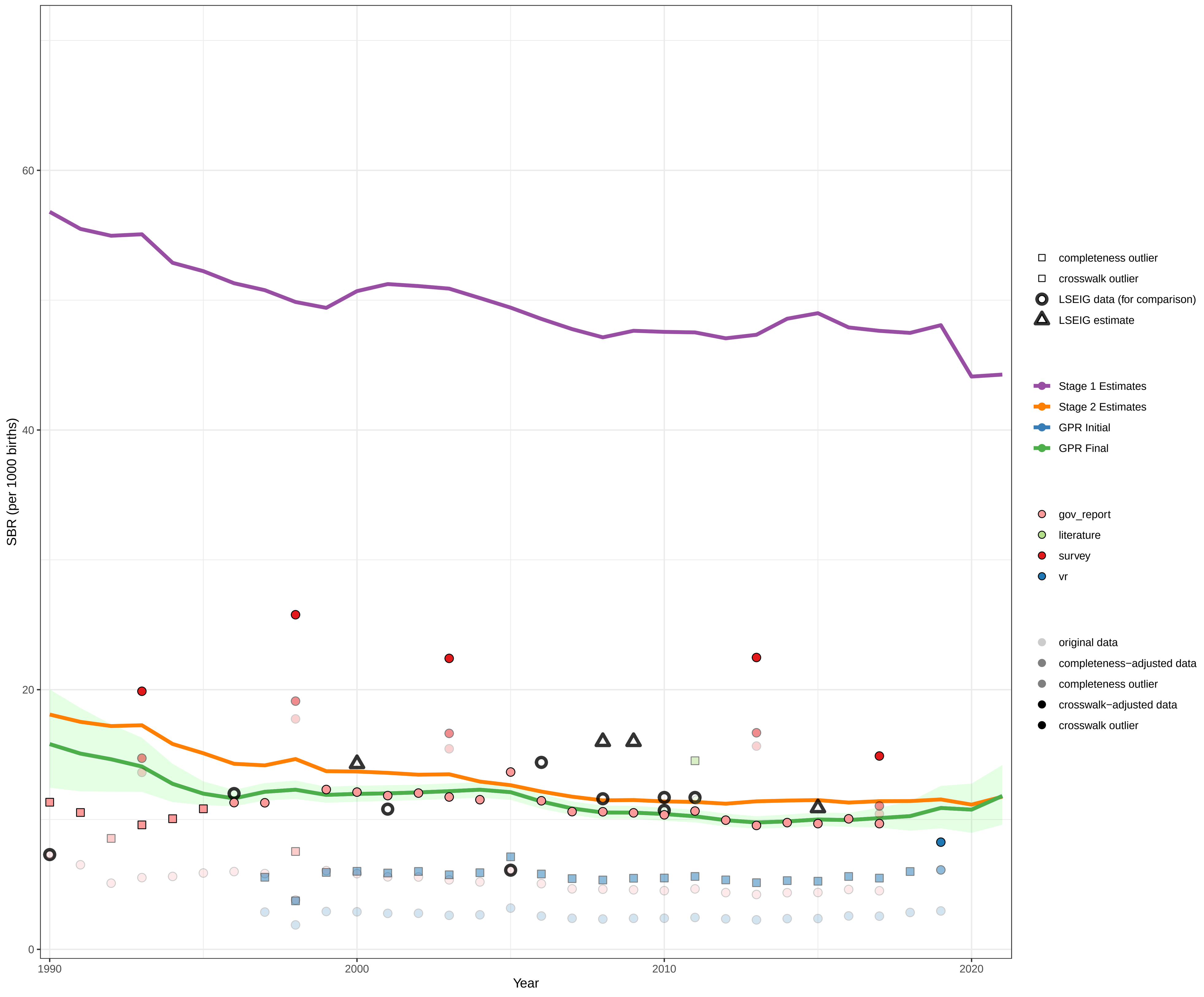

Philippines PHL Southeast Asia Stillbirth Rate (SBR) − 28 weeks
lambda = 0.6 ; zeta = 0.8 ; scale = 15 ; amp = 0.028

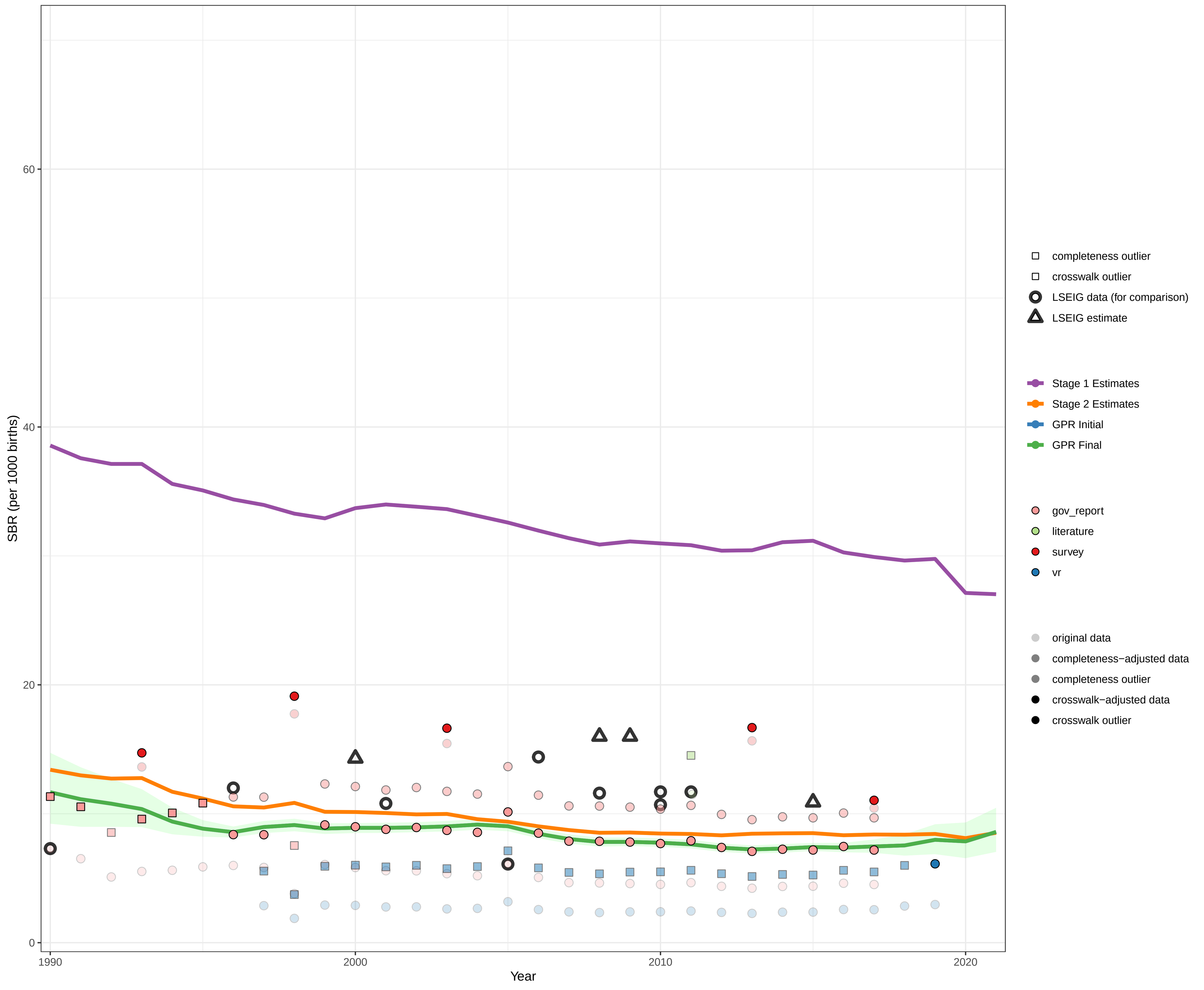

Year	Total Stillbirths (in thousands)	Total Stillbirths (in thousands)	SBR (per 1000 births)	SBR (per 1000 births)	SBR/NMR	SBR/NMR
	20 weeks	28 weeks	20 weeks	28 weeks	20 weeks	28 weeks
1990	32.4 (25.5 to 41.3)	23.8 (18.8 to 30.2)	15.8 (12.4 to 20.0)	11.7 (9.2 to 14.7)	0.87 (0.69 to 1.11)	0.64 (0.51 to 0.81)
1995	25.8 (23.8 to 27.8)	18.9 (17.6 to 20.4)	12.0 (11.1 to 12.9)	8.8 (8.2 to 9.5)	0.74 (0.69 to 0.80)	0.55 (0.51 to 0.59)
2000	27.0 (25.6 to 28.4)	20.0 (19.1 to 20.8)	12.0 (11.4 to 12.6)	8.9 (8.5 to 9.3)	0.78 (0.74 to 0.82)	0.58 (0.55 to 0.60)
2005	28.8 (27.4 to 30.2)	21.4 (20.5 to 22.4)	12.1 (11.5 to 12.7)	9.0 (8.6 to 9.4)	0.82 (0.78 to 0.86)	0.61 (0.59 to 0.64)
2010	26.1 (24.8 to 27.3)	19.4 (18.6 to 20.2)	10.4 (9.9 to 10.9)	7.8 (7.4 to 8.1)	0.76 (0.72 to 0.79)	0.56 (0.54 to 0.59)
2015	23.9 (22.7 to 25.2)	17.7 (17.0 to 18.5)	10.0 (9.5 to 10.5)	7.4 (7.1 to 7.7)	0.75 (0.71 to 0.79)	0.56 (0.53 to 0.58)
2020	23.9 (19.9 to 28.4)	17.4 (14.5 to 20.7)	10.8 (9.0 to 12.8)	7.9 (6.6 to 9.3)	0.98 (0.82 to 1.17)	0.72 (0.60 to 0.85)
2021	26.1 (21.2 to 31.5)	18.9 (15.5 to 23.1)	11.8 (9.6 to 14.2)	8.6 (7.0 to 10.5)	1.10 (0.89 to 1.32)	0.80 (0.65 to 0.97)

Seychelles SYC Southeast Asia Stillbirth Rate / Neonatal Mortality Rate (SBR/NMR) − 20 weeks
lambda = 0.4 ; zeta = 0.9 ; scale = 10 ; amp = 0.028

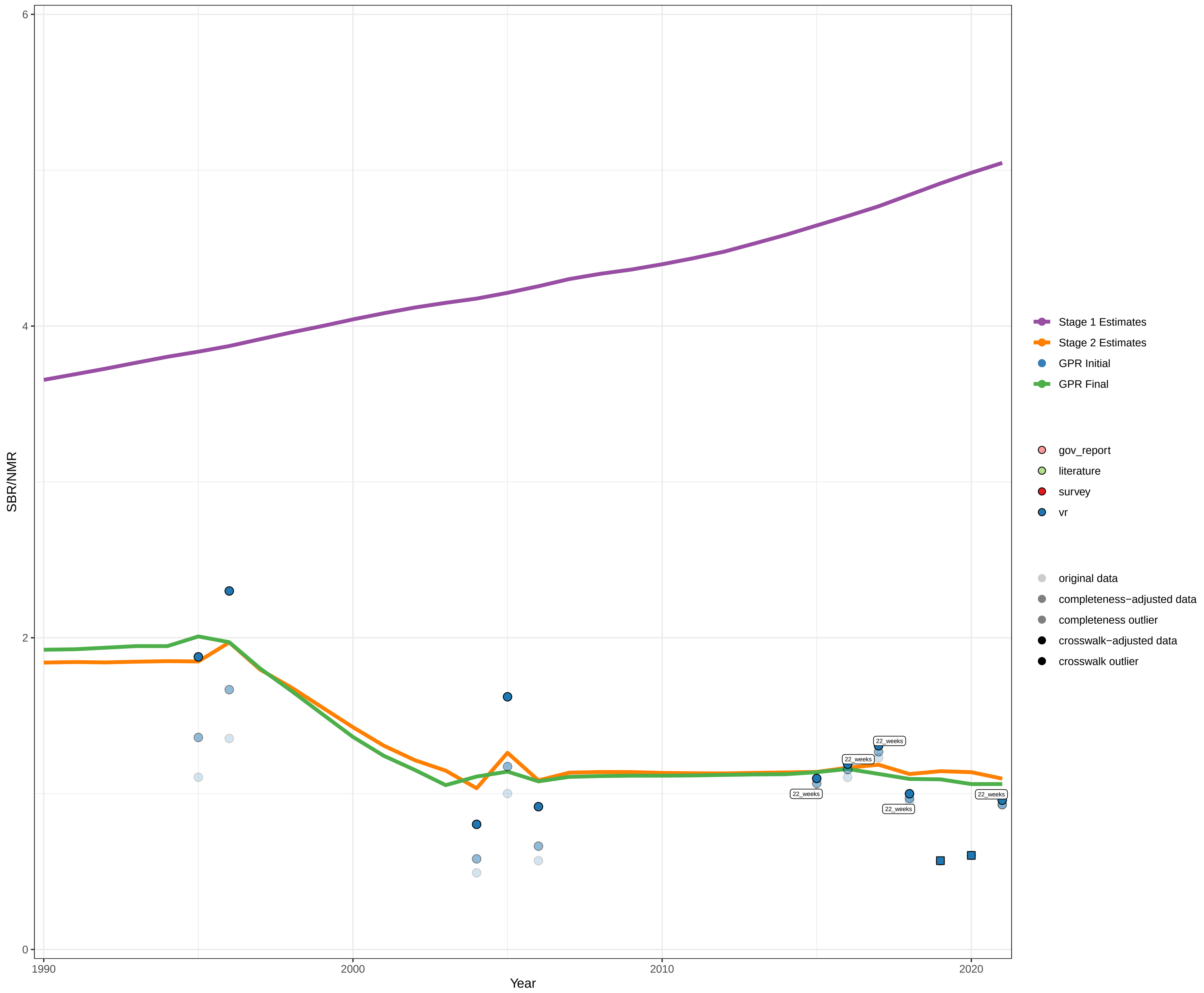

Seychelles SYC Southeast Asia Stillbirth Rate / Neonatal Mortality Rate (SBR/NMR) − 28 weeks
lambda = 0.4 ; zeta = 0.9 ; scale = 10 ; amp = 0.028

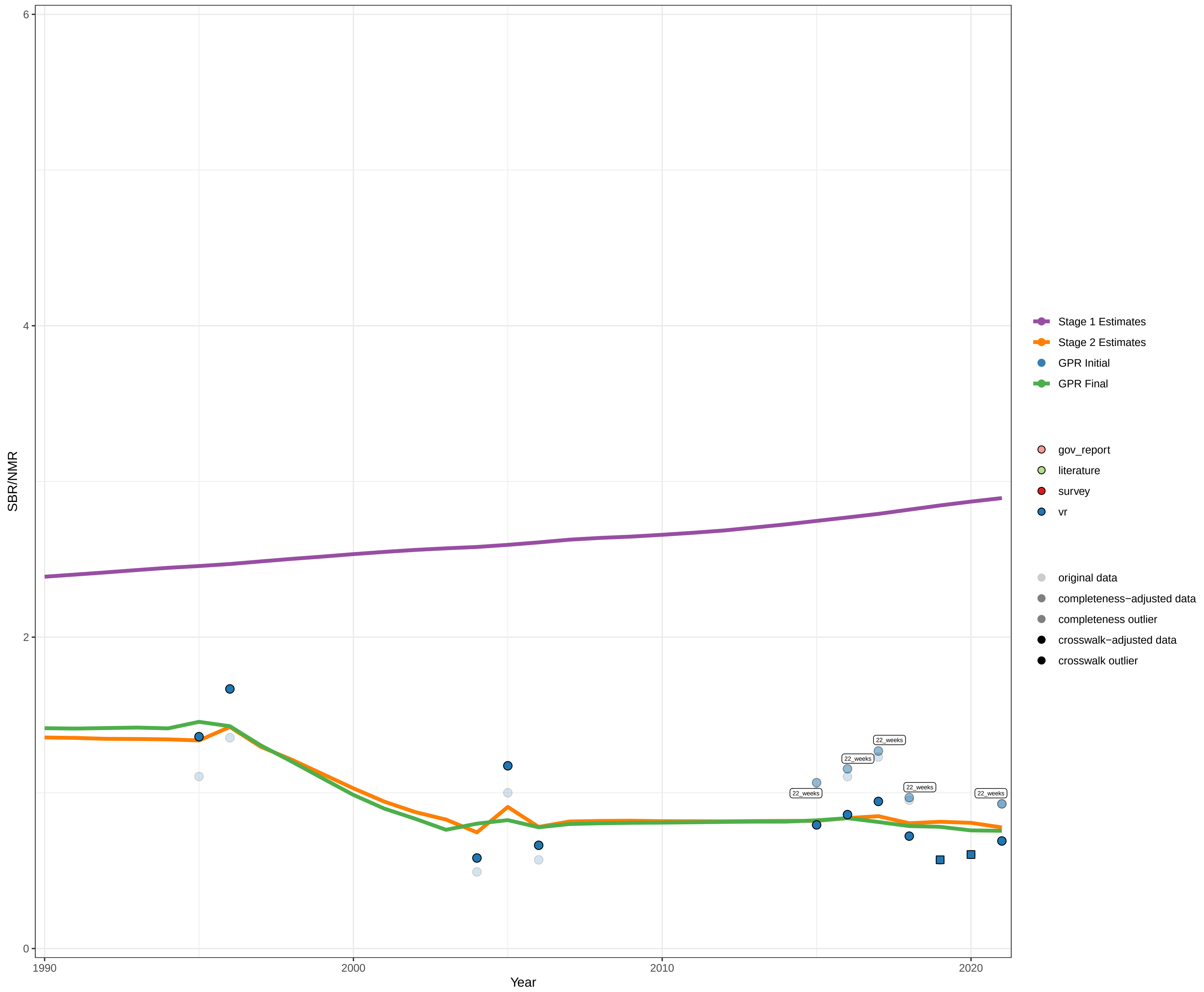

Definition	Years	Outlier	Source.Type	Title
22_weeks	2015−2021	0	VR	Seychelles Statistical Bulletin: Population and Vital Statistics
28_weeks	1995, 1996, 2004−2006	0	VR	United Nations Demographic Yearbook

Seychelles SYC Southeast Asia Stillbirth Rate (SBR) − 20 weeks
lambda = 0.4 ; zeta = 0.9 ; scale = 10 ; amp = 0.028

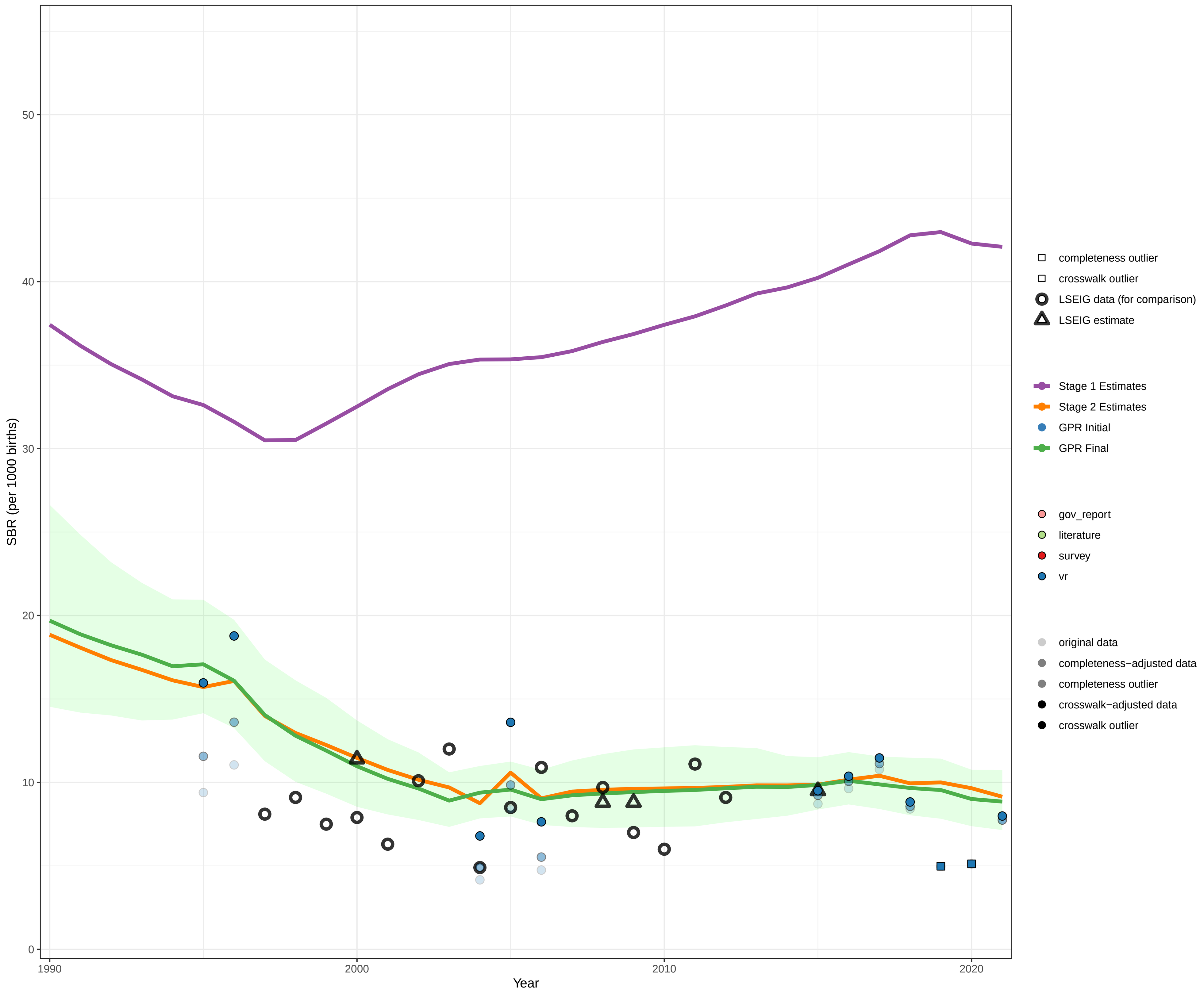

Seychelles SYC Southeast Asia Stillbirth Rate (SBR) − 28 weeks
lambda = 0.4 ; zeta = 0.9 ; scale = 10 ; amp = 0.028

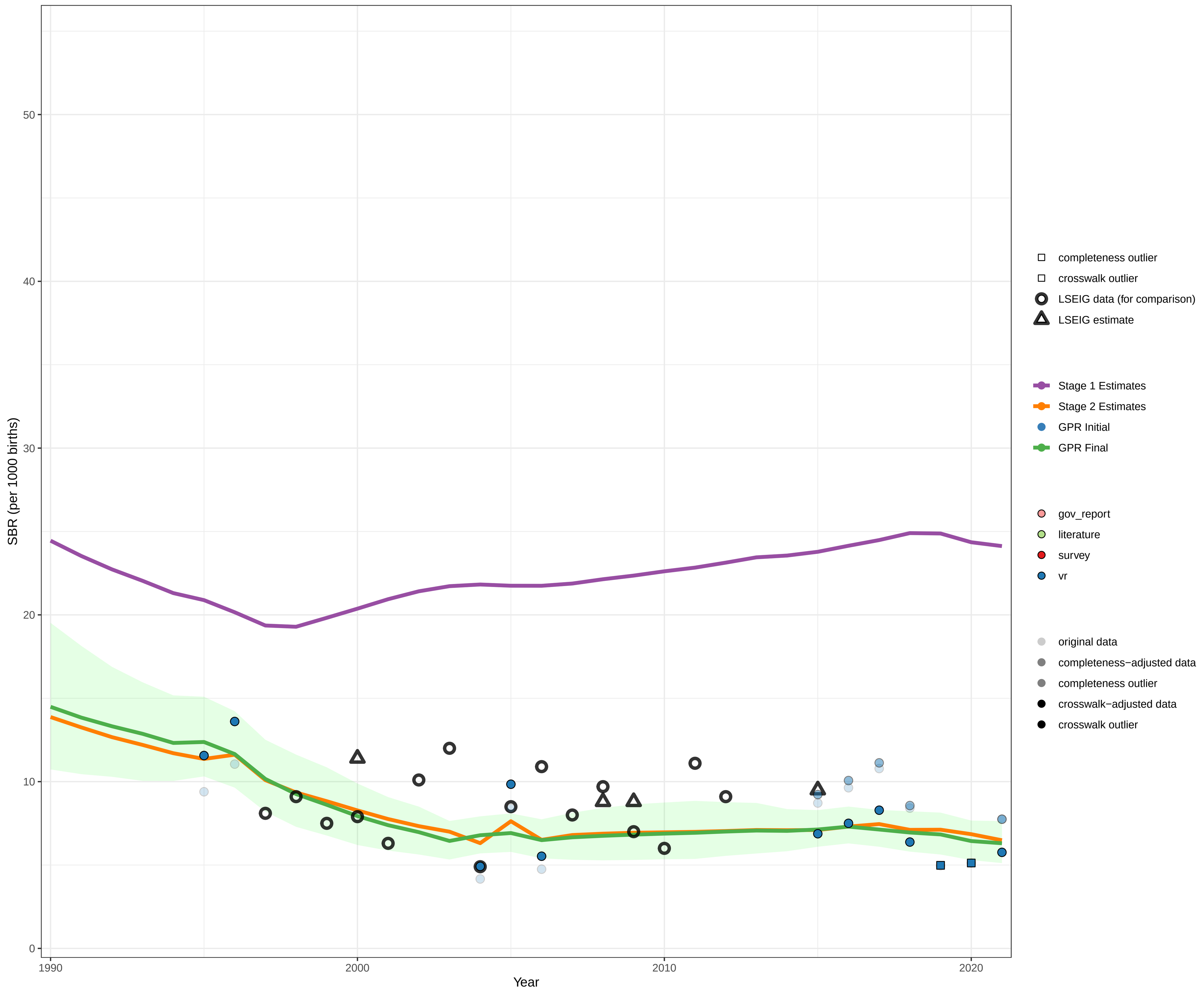

Year	Total Stillbirths (in thousands)	Total Stillbirths (in thousands)	SBR (per 1000 births)	SBR (per 1000 births)	SBR/NMR	SBR/NMR
	20 weeks	28 weeks	20 weeks	28 weeks	20 weeks	28 weeks
1990	0.0 (0.0 to 0.0)	0.0 (0.0 to 0.0)	19.7 (14.5 to 26.6)	14.5 (10.7 to 19.5)	1.92 (1.42 to 2.60)	1.42 (1.05 to 1.91)
1995	0.0 (0.0 to 0.0)	0.0 (0.0 to 0.0)	17.1 (14.1 to 20.9)	12.4 (10.3 to 15.1)	2.01 (1.66 to 2.46)	1.46 (1.21 to 1.78)
2000	0.0 (0.0 to 0.0)	0.0 (0.0 to 0.0)	11.0 (8.5 to 13.7)	7.9 (6.2 to 9.9)	1.36 (1.06 to 1.71)	0.99 (0.77 to 1.23)
2005	0.0 (0.0 to 0.0)	0.0 (0.0 to 0.0)	9.6 (8.0 to 11.2)	6.9 (5.8 to 8.1)	1.14 (0.95 to 1.34)	0.82 (0.69 to 0.97)
2010	0.0 (0.0 to 0.0)	0.0 (0.0 to 0.0)	9.5 (7.3 to 12.1)	6.9 (5.3 to 8.7)	1.12 (0.86 to 1.42)	0.81 (0.63 to 1.03)
2015	0.0 (0.0 to 0.0)	0.0 (0.0 to 0.0)	9.9 (8.4 to 11.5)	7.1 (6.1 to 8.3)	1.14 (0.97 to 1.33)	0.82 (0.70 to 0.96)
2020	0.0 (0.0 to 0.0)	0.0 (0.0 to 0.0)	9.0 (7.4 to 10.8)	6.4 (5.3 to 7.7)	1.06 (0.87 to 1.27)	0.76 (0.62 to 0.90)
2021	0.0 (0.0 to 0.0)	0.0 (0.0 to 0.0)	8.9 (7.1 to 10.8)	6.3 (5.1 to 7.6)	1.06 (0.86 to 1.29)	0.76 (0.61 to 0.92)

Sri Lanka LKA Southeast Asia Stillbirth Rate / Neonatal Mortality Rate (SBR/NMR) − 20 weeks
lambda = 0.2 ; zeta = 0.99 ; scale = 5 ; amp = 0.028

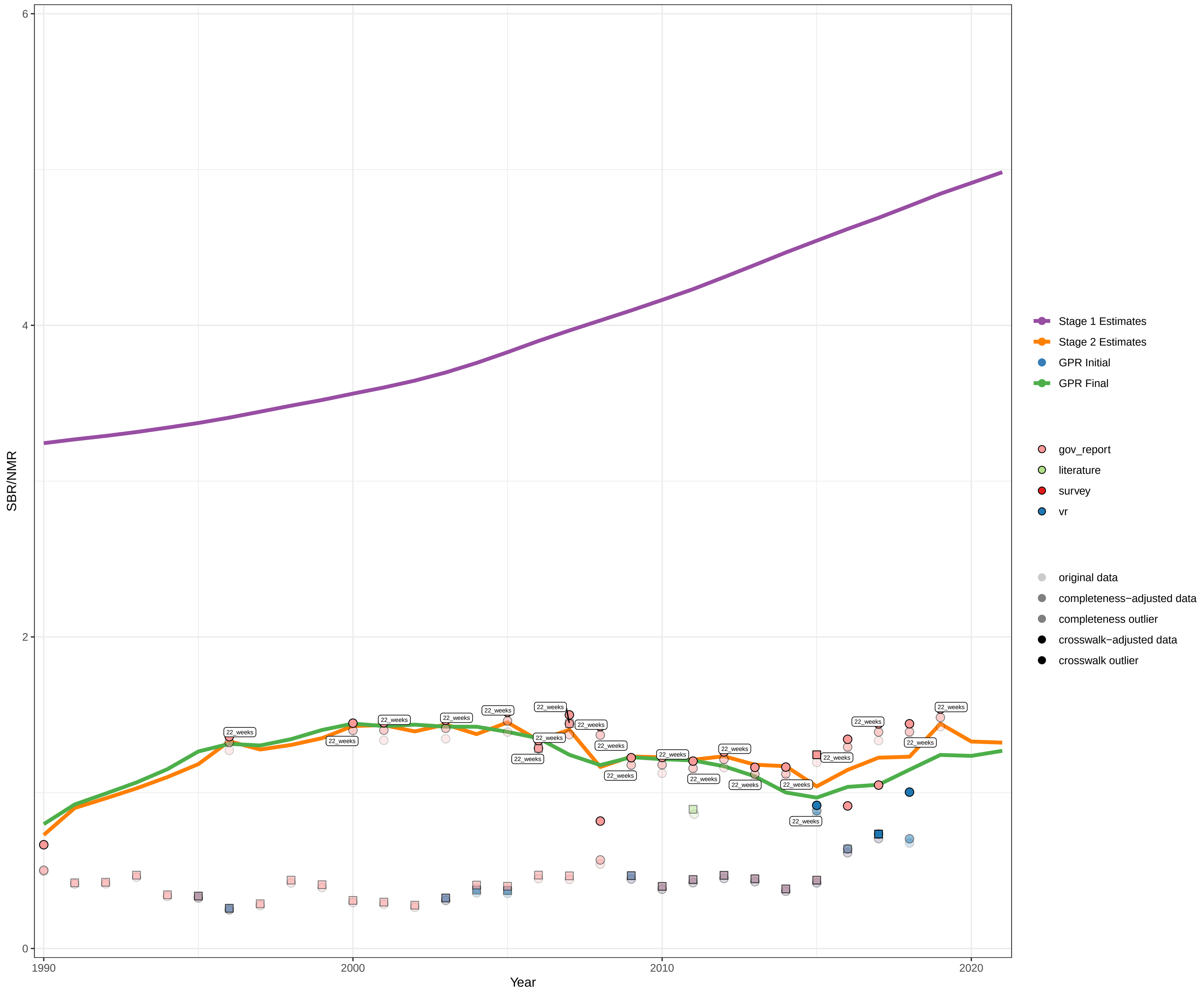

Sri Lanka LKA Southeast Asia Stillbirth Rate / Neonatal Mortality Rate (SBR/NMR) − 28 weeks
lambda = 0.2 ; zeta = 0.99 ; scale = 5 ; amp = 0.028

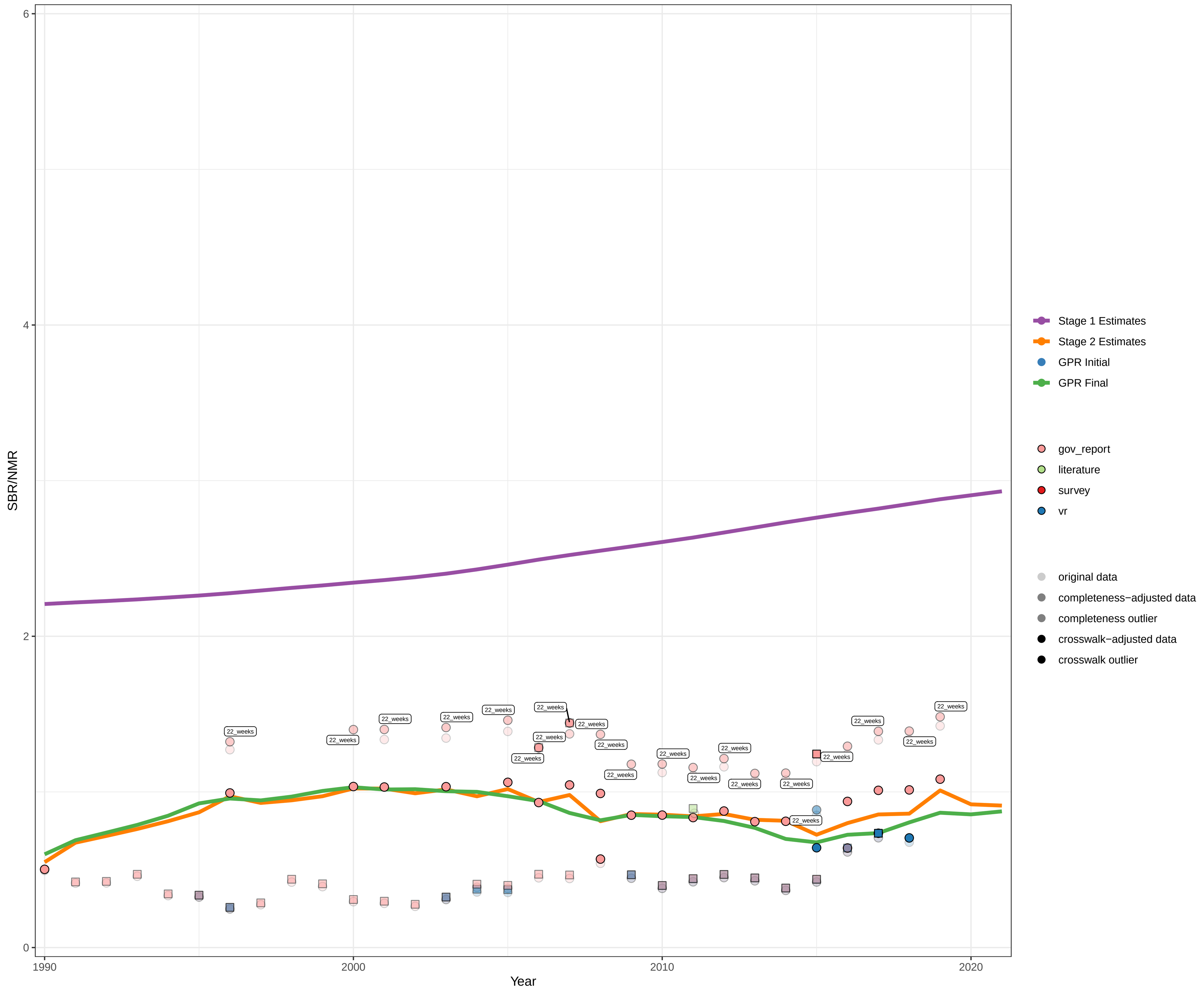

Definition	Years	Outlier	Source.Type	Title
22_weeks	1996, 2000, 2001, 2003, 2006, 2008, 2012−2014	0	Statistical Report	Sri Lanka Annual Health Bulletin
22_weeks	2005	0	Statistical Report	Sri Lanka Health at a Glance
22_weeks	2007, 2011, 2015−2019	0	Statistical Report	Sri Lanka Annual Health Statistics
22_weeks	2009, 2010	0	Statistical Report	Sri Lanka Annual Health Statistics
22_weeks	2015	0	VR	Sri Lanka Perinatal Death Surveillance Review
28_weeks	1990, 2008, 2016, 2017	0	Statistical Report	Sri Lanka Statistical Abstract
28_weeks	2018	0	VR	United Nations Demographic Yearbook
22_weeks	2006, 2007	1	Statistical Report	Sri Lanka Health at a Glance
28_weeks	1991−2007, 2009−2015	1	Statistical Report	Sri Lanka Statistical Abstract
28_weeks	1995, 1996, 2003−2005, 2009−2016	1	VR	United Nations Demographic Yearbook
28_weeks_OR_1000_grams	2011	1	Sci Lit	Maternal complications and perinatal mortality: findings of the World Health Organization Multicountry Survey on Maternal and Newborn Health
28_weeks	2017	2	VR	United Nations Demographic Yearbook

Sri Lanka LKA Southeast Asia Stillbirth Rate (SBR) − 20 weeks
lambda = 0.2 ; zeta = 0.99 ; scale = 5 ; amp = 0.028

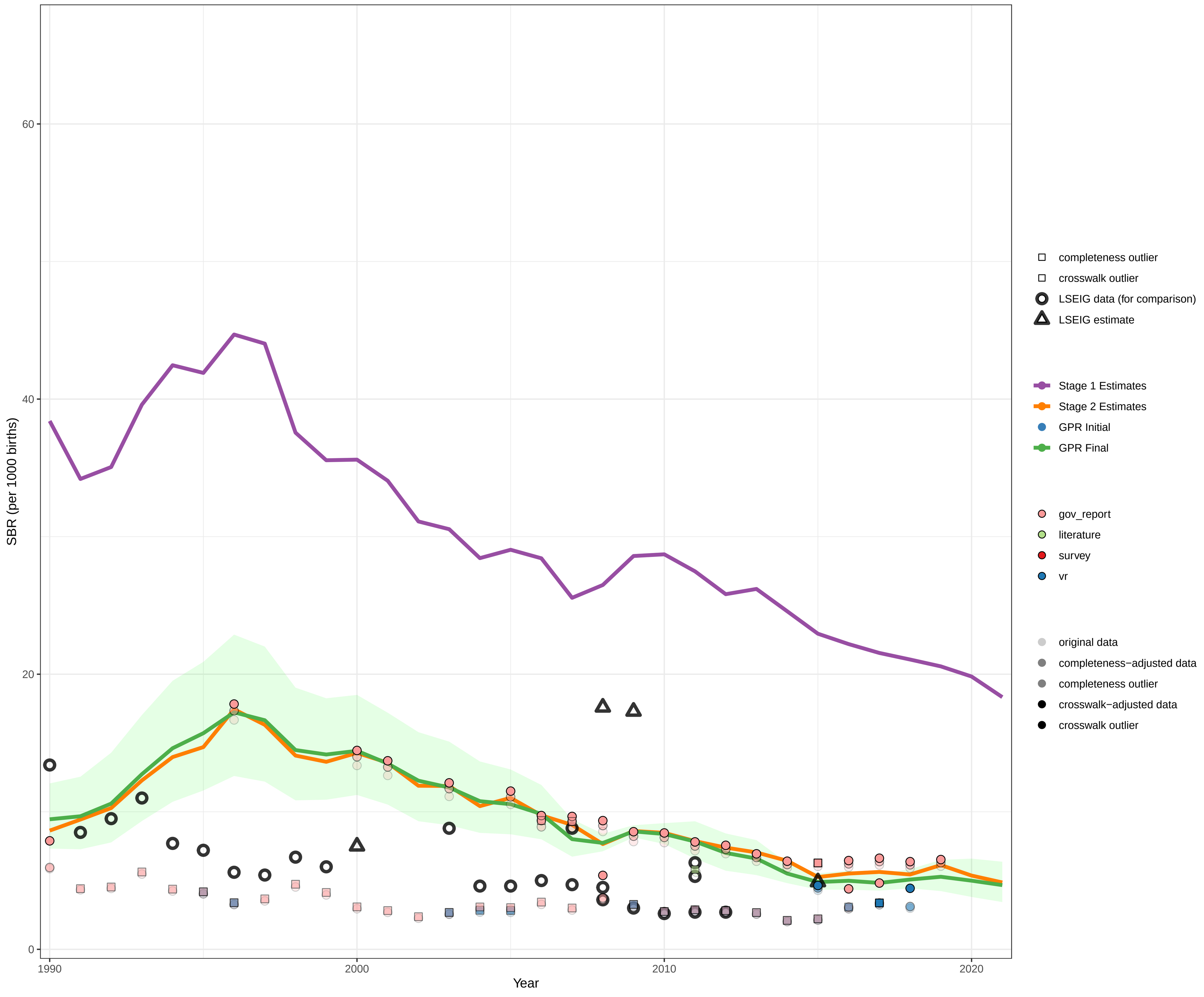

Sri Lanka LKA Southeast Asia Stillbirth Rate (SBR) − 28 weeks
lambda = 0.2 ; zeta = 0.99 ; scale = 5 ; amp = 0.028

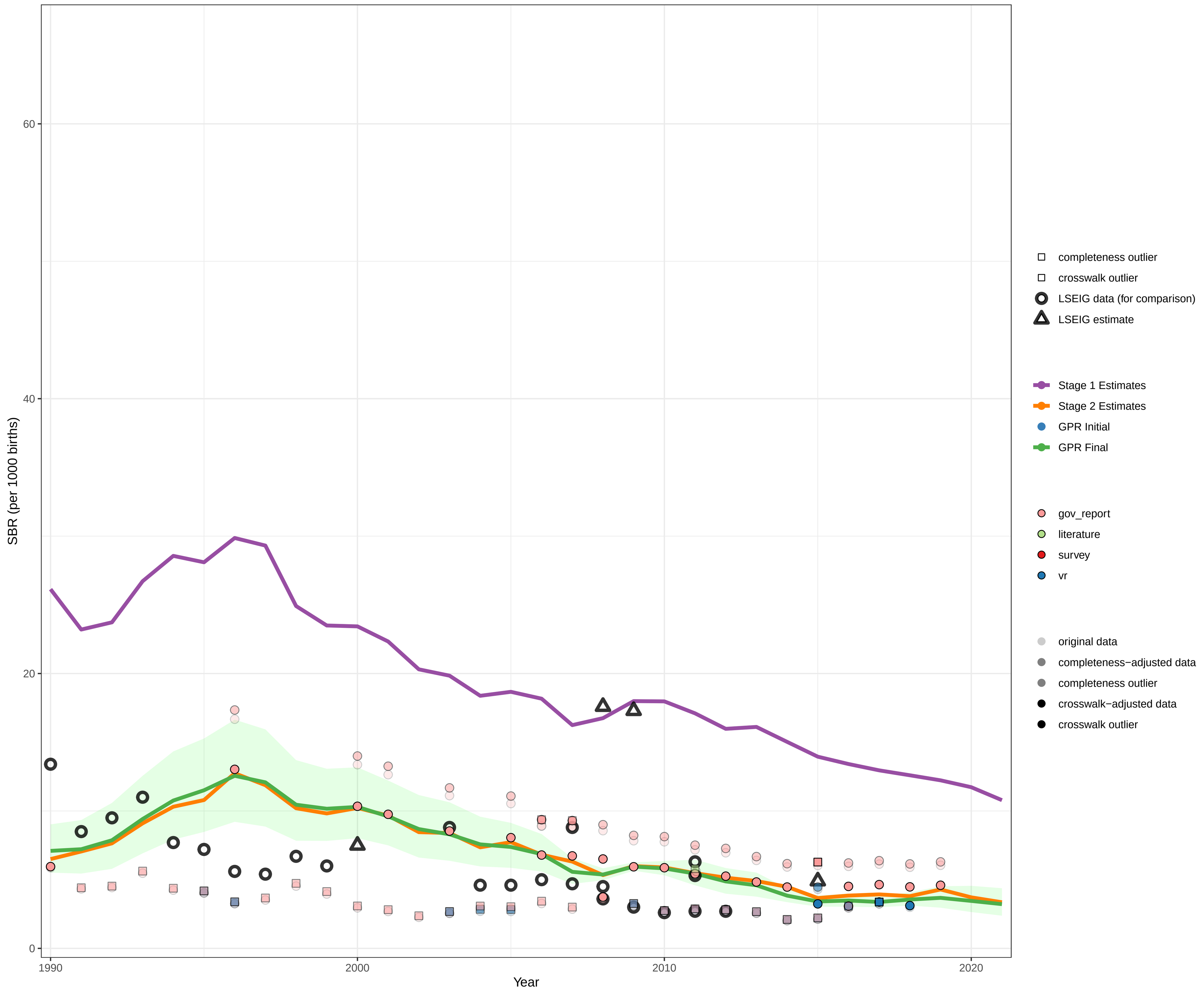

Year	Total Stillbirths (in thousands)	Total Stillbirths (in thousands)	SBR (per 1000 births)	SBR (per 1000 births)	SBR/NMR	SBR/NMR
	20 weeks	28 weeks	20 weeks	28 weeks	20 weeks	28 weeks
1990	3.4 (2.7 to 4.4)	2.6 (2.0 to 3.3)	9.5 (7.3 to 12.1)	7.1 (5.5 to 9.0)	0.80 (0.62 to 1.02)	0.60 (0.47 to 0.76)
1995	5.5 (4.0 to 7.3)	4.0 (2.9 to 5.3)	15.7 (11.5 to 20.9)	11.5 (8.5 to 15.3)	1.26 (0.93 to 1.68)	0.93 (0.68 to 1.23)
2000	5.1 (3.9 to 6.5)	3.6 (2.8 to 4.6)	14.4 (11.2 to 18.5)	10.3 (8.0 to 13.2)	1.44 (1.12 to 1.85)	1.03 (0.80 to 1.32)
2005	4.0 (3.2 to 5.0)	2.8 (2.2 to 3.5)	10.6 (8.4 to 13.1)	7.4 (5.9 to 9.1)	1.39 (1.10 to 1.72)	0.97 (0.77 to 1.20)
2010	3.1 (2.8 to 3.4)	2.2 (2.0 to 2.4)	8.4 (7.7 to 9.2)	5.8 (5.3 to 6.4)	1.22 (1.11 to 1.33)	0.84 (0.77 to 0.92)
2015	1.7 (1.5 to 1.9)	1.2 (1.0 to 1.3)	4.9 (4.3 to 5.5)	3.4 (3.0 to 3.8)	0.97 (0.86 to 1.09)	0.68 (0.60 to 0.76)
2020	1.5 (1.2 to 2.0)	1.1 (0.8 to 1.4)	5.0 (3.8 to 6.6)	3.5 (2.6 to 4.6)	1.24 (0.94 to 1.63)	0.86 (0.65 to 1.13)
2021	1.4 (1.0 to 1.9)	1.0 (0.7 to 1.3)	4.7 (3.4 to 6.4)	3.2 (2.4 to 4.4)	1.27 (0.93 to 1.73)	0.87 (0.65 to 1.19)

Thailand THA Southeast Asia Stillbirth Rate / Neonatal Mortality Rate (SBR/NMR) − 20 weeks
lambda = 1 ; zeta = 0.6 ; scale = 15 ; amp = 0.028

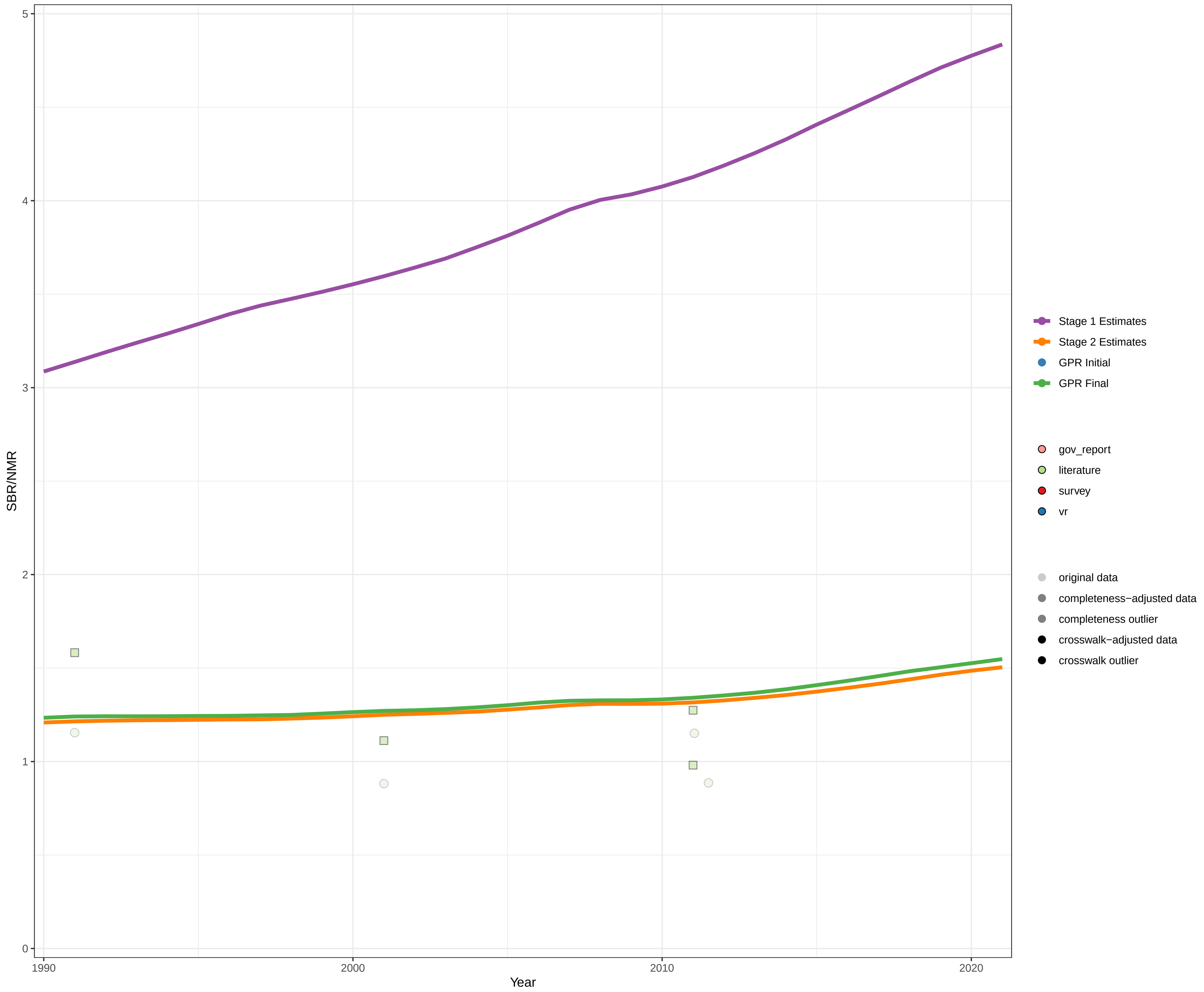

Thailand THA Southeast Asia Stillbirth Rate / Neonatal Mortality Rate (SBR/NMR) − 28 weeks
lambda = 1 ; zeta = 0.6 ; scale = 15 ; amp = 0.028

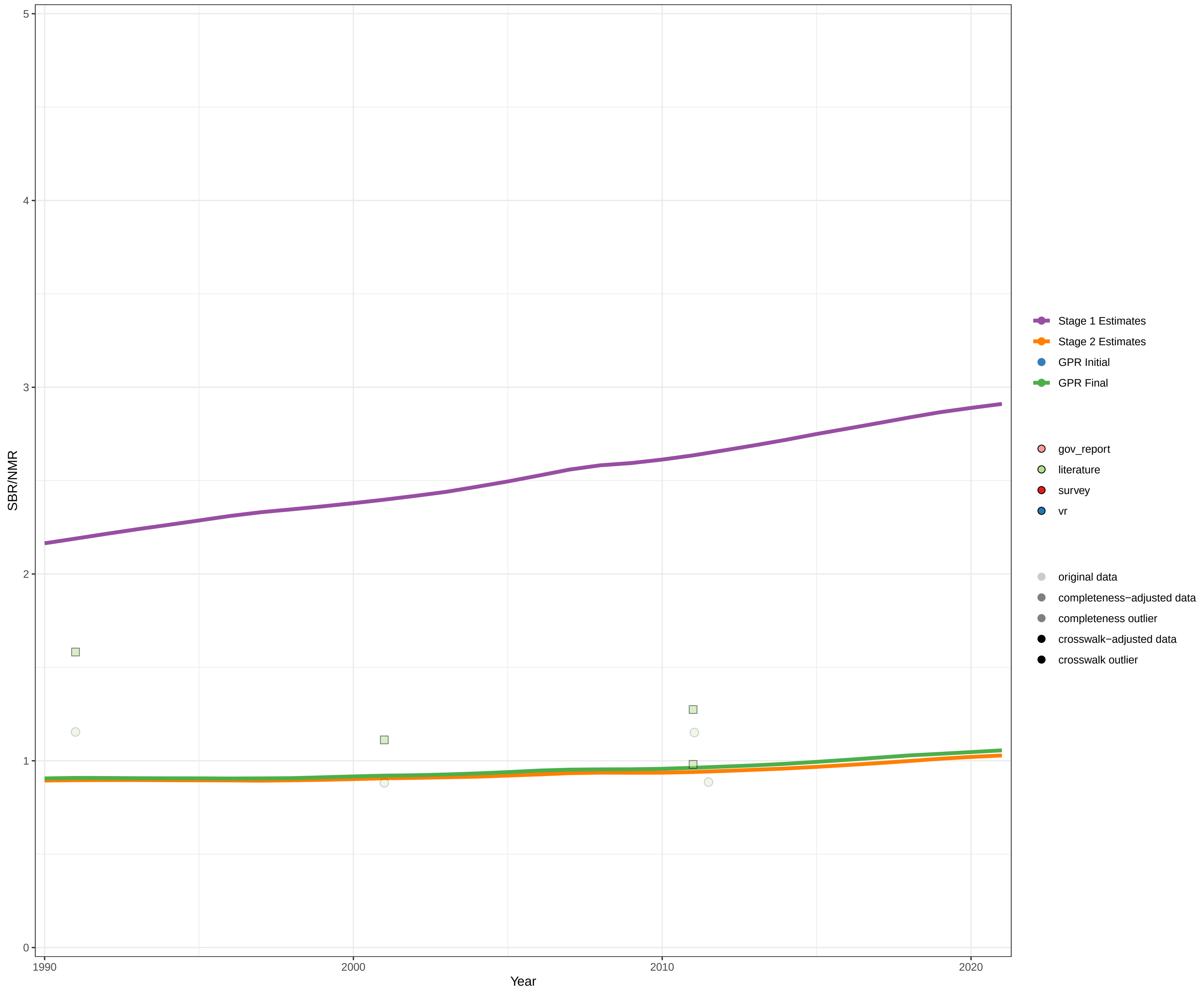

Definition	Years	Outlier	Source.Type	Title
28_weeks	1991	1	Sci Lit	A survey of neonatal tetanus and perinatal mortality in southern Muslim communities in Thailand
28_weeks	2001	1	Sci Lit	Perinatal death pattern in the four districts of Thailand: findings from the Prospective Cohort Study of Thai Children (PCTC)
28_weeks	2011	1	Sci Lit	Associations between pregnancy outcomes and unexplained high and low maternal serum alpha−fetoprotein levels
28_weeks_OR_1000_grams	2011	1	Sci Lit	Maternal complications and perinatal mortality: findings of the World Health Organization Multicountry Survey on Maternal and Newborn Health

Thailand THA Southeast Asia Stillbirth Rate (SBR) − 20 weeks
lambda = 1 ; zeta = 0.6 ; scale = 15 ; amp = 0.028

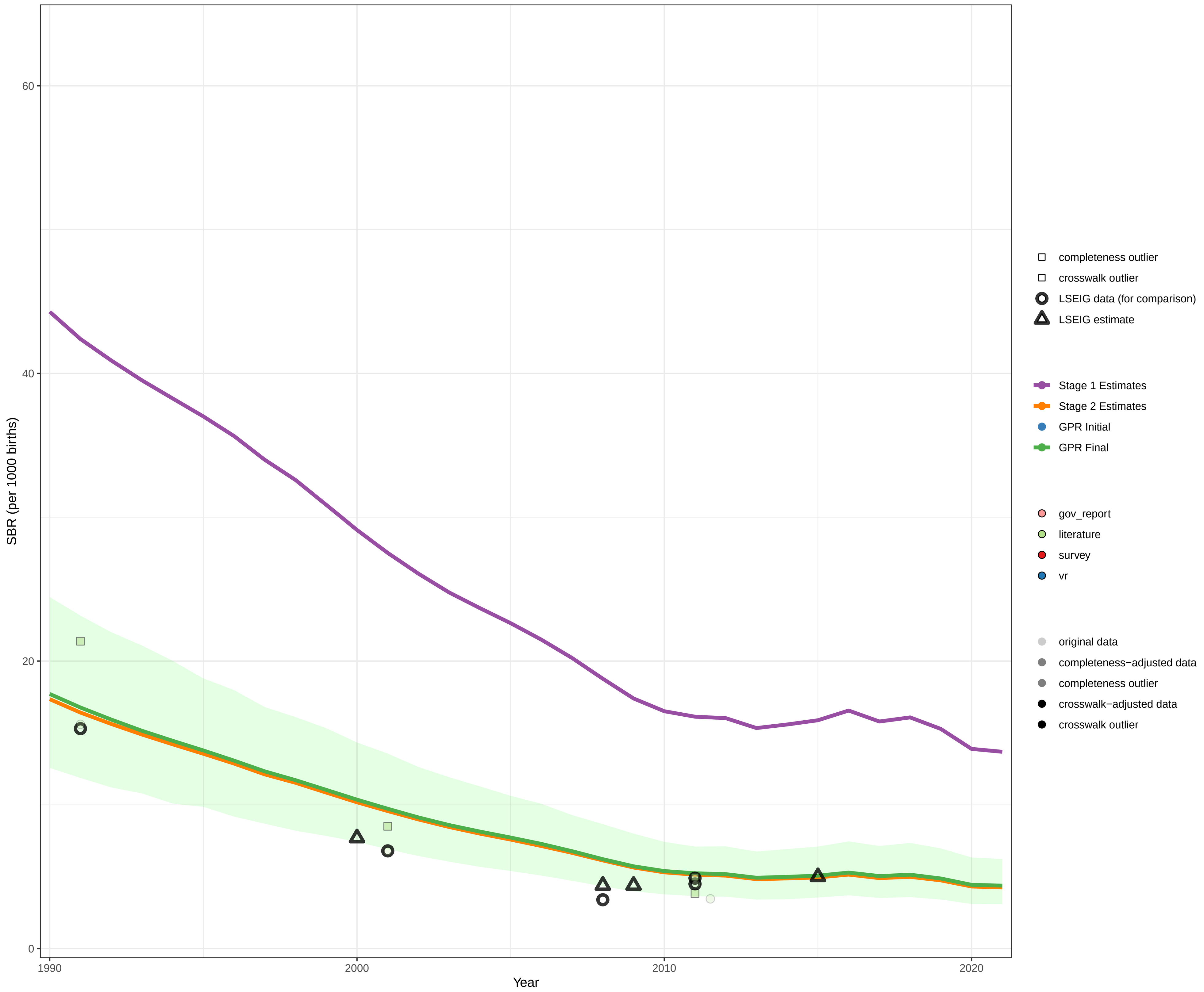

Thailand THA Southeast Asia Stillbirth Rate (SBR) − 28 weeks
lambda = 1 ; zeta = 0.6 ; scale = 15 ; amp = 0.028

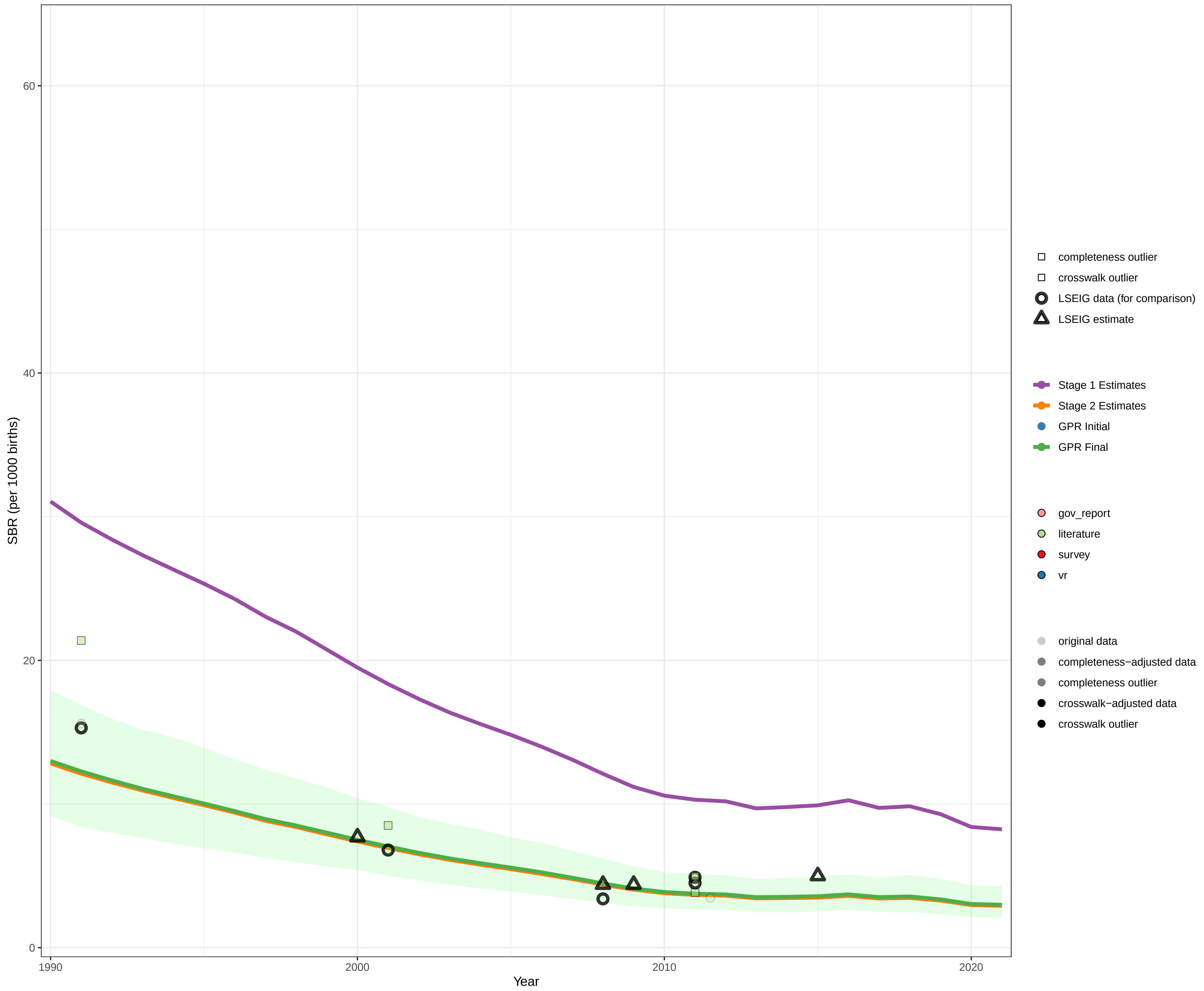

Year	Total Stillbirths (in thousands)	Total Stillbirths (in thousands)	SBR (per 1000 births)	SBR (per 1000 births)	SBR/NMR	SBR/NMR
	20 weeks	28 weeks	20 weeks	28 weeks	20 weeks	28 weeks
1990	18.6 (13.1 to 25.8)	13.6 (9.5 to 18.8)	17.7 (12.6 to 24.5)	13.0 (9.2 to 17.9)	1.23 (0.88 to 1.70)	0.91 (0.64 to 1.25)
1995	13.6 (9.7 to 18.6)	9.8 (6.8 to 13.7)	13.8 (9.9 to 18.8)	10.0 (6.9 to 13.9)	1.24 (0.89 to 1.70)	0.91 (0.62 to 1.26)
2000	9.1 (6.5 to 12.6)	6.6 (4.7 to 9.1)	10.4 (7.4 to 14.3)	7.5 (5.4 to 10.4)	1.26 (0.91 to 1.75)	0.92 (0.66 to 1.27)
2005	6.3 (4.4 to 8.7)	4.5 (3.2 to 6.3)	7.7 (5.4 to 10.6)	5.6 (3.9 to 7.7)	1.30 (0.91 to 1.79)	0.94 (0.66 to 1.29)
2010	4.3 (3.0 to 5.9)	3.1 (2.2 to 4.2)	5.4 (3.8 to 7.4)	3.9 (2.8 to 5.3)	1.33 (0.93 to 1.83)	0.96 (0.68 to 1.30)
2015	3.4 (2.4 to 4.8)	2.4 (1.7 to 3.3)	5.1 (3.6 to 7.1)	3.6 (2.5 to 4.9)	1.41 (0.99 to 1.97)	0.99 (0.70 to 1.37)
2020	2.6 (1.8 to 3.7)	1.8 (1.3 to 2.5)	4.4 (3.1 to 6.3)	3.0 (2.1 to 4.3)	1.53 (1.07 to 2.18)	1.05 (0.73 to 1.49)
2021	2.5 (1.8 to 3.6)	1.7 (1.2 to 2.5)	4.4 (3.1 to 6.2)	3.0 (2.1 to 4.3)	1.55 (1.09 to 2.20)	1.06 (0.74 to 1.51)

Timor−Leste TLS Southeast Asia Stillbirth Rate / Neonatal Mortality Rate (SBR/NMR) − 20 weeks
lambda = 1 ; zeta = 0.6 ; scale = 15 ; amp = 0.028

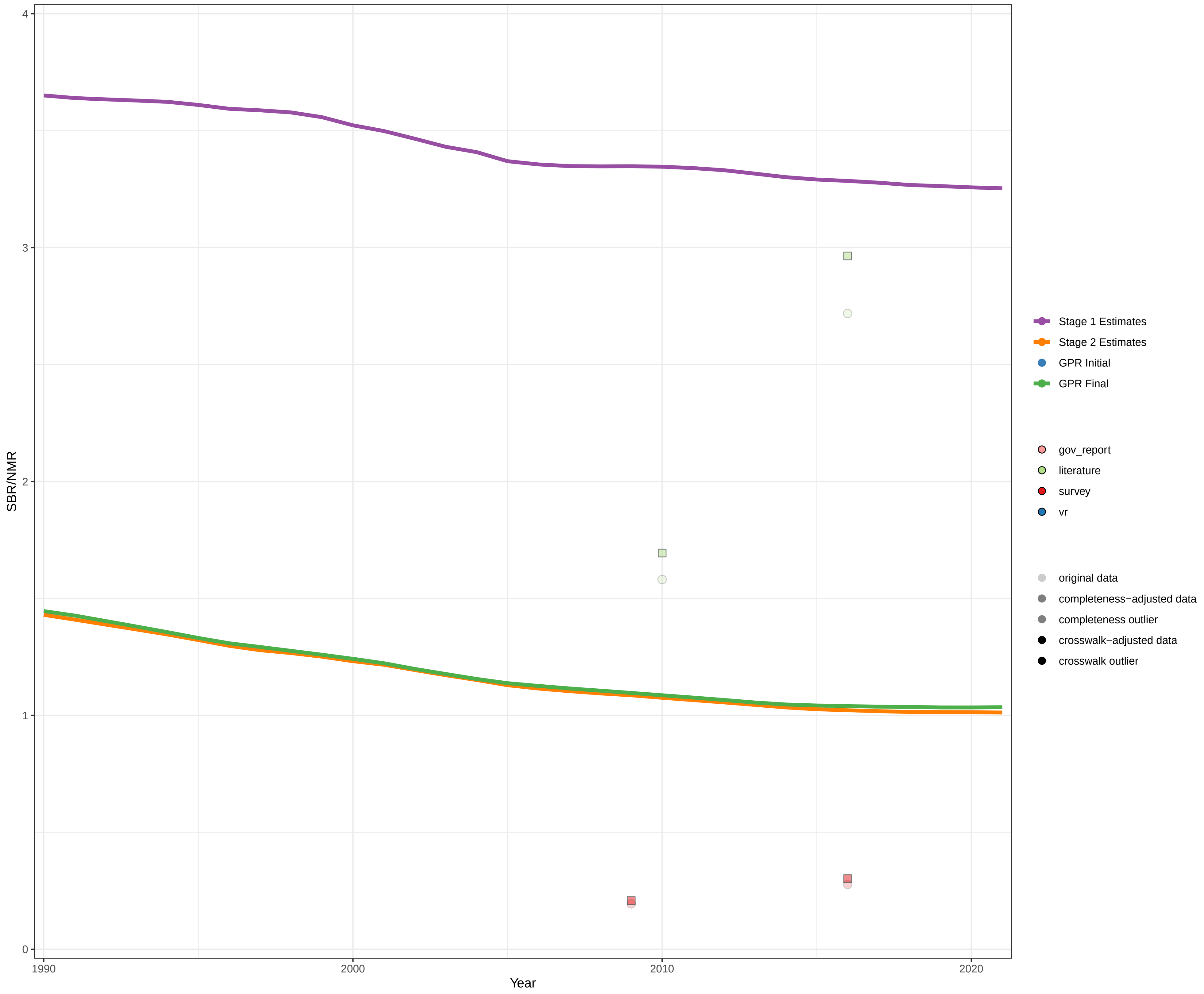

Timor−Leste TLS Southeast Asia Stillbirth Rate / Neonatal Mortality Rate (SBR/NMR) − 28 weeks
lambda = 1 ; zeta = 0.6 ; scale = 15 ; amp = 0.028

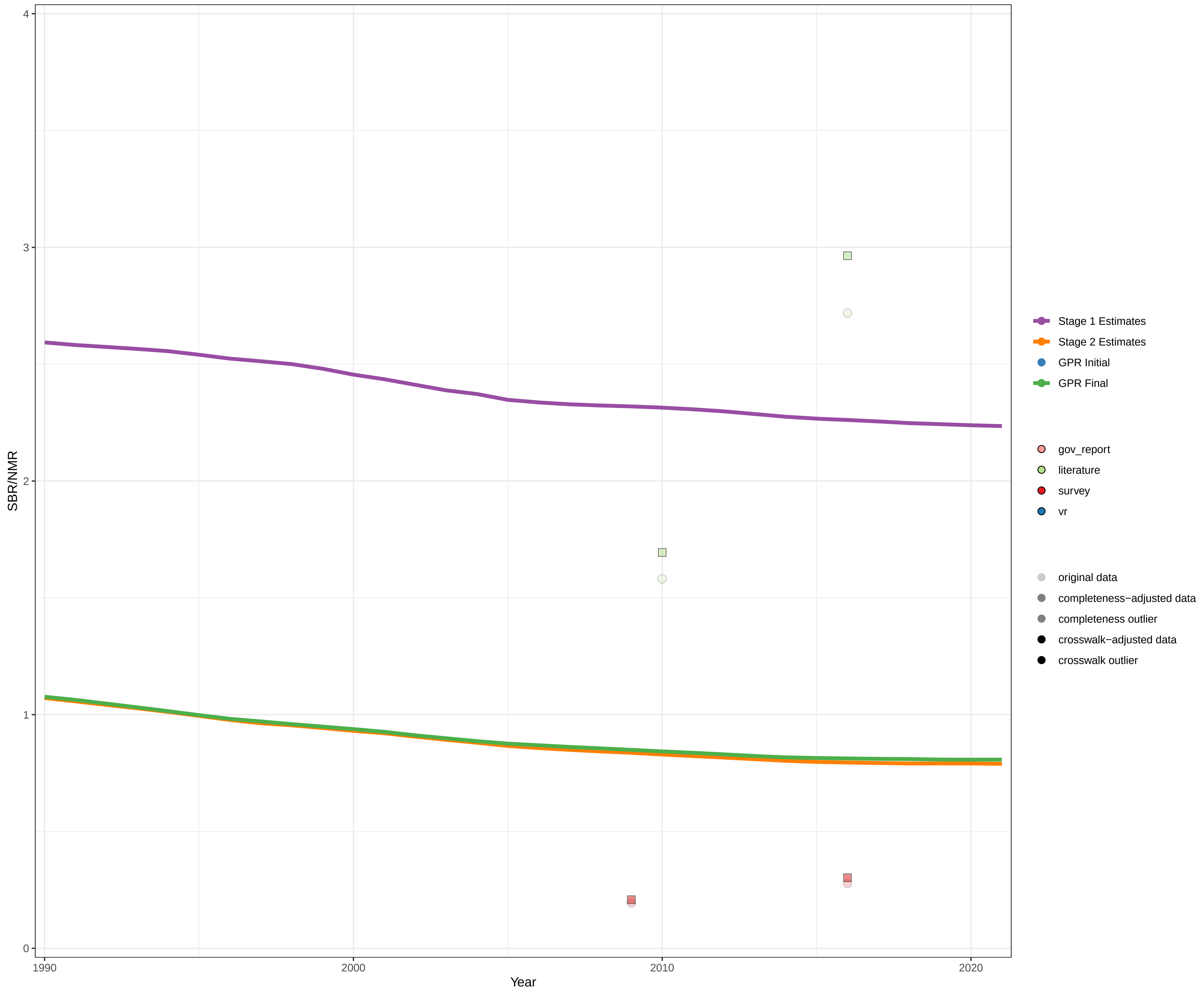

Definition	Years	Outlier	Source.Type	Title
22_weeks_OR_500_grams	2010	1	Sci Lit	A retrospective review of stillbirths at the national hospital in Timor−Leste
22_weeks_OR_500_grams	2016	1	Sci Lit	A prospective review of perinatal mortality at Hospital Nacional Guido Valadares (HNGV)
28_weeks	2009	1	Survey	Timor−Leste Demographic and Health Survey 2009−2010
28_weeks	2016	1	Survey	Timor−Leste Demographic and Health Survey 2016

Timor−Leste TLS Southeast Asia Stillbirth Rate (SBR) − 20 weeks
lambda = 1 ; zeta = 0.6 ; scale = 15 ; amp = 0.028

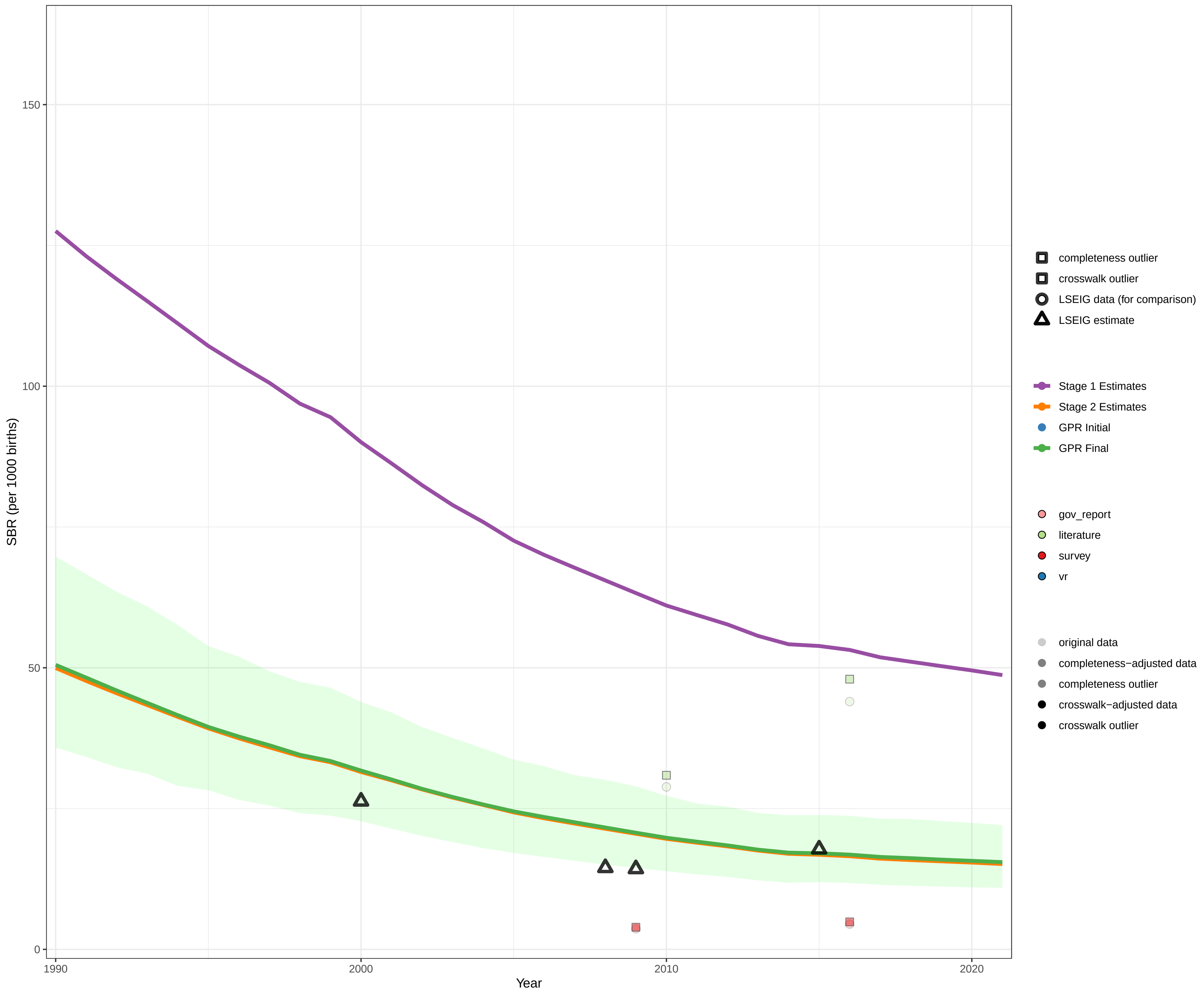

Timor−Leste TLS Southeast Asia Stillbirth Rate (SBR) − 28 weeks
lambda = 1 ; zeta = 0.6 ; scale = 15 ; amp = 0.028

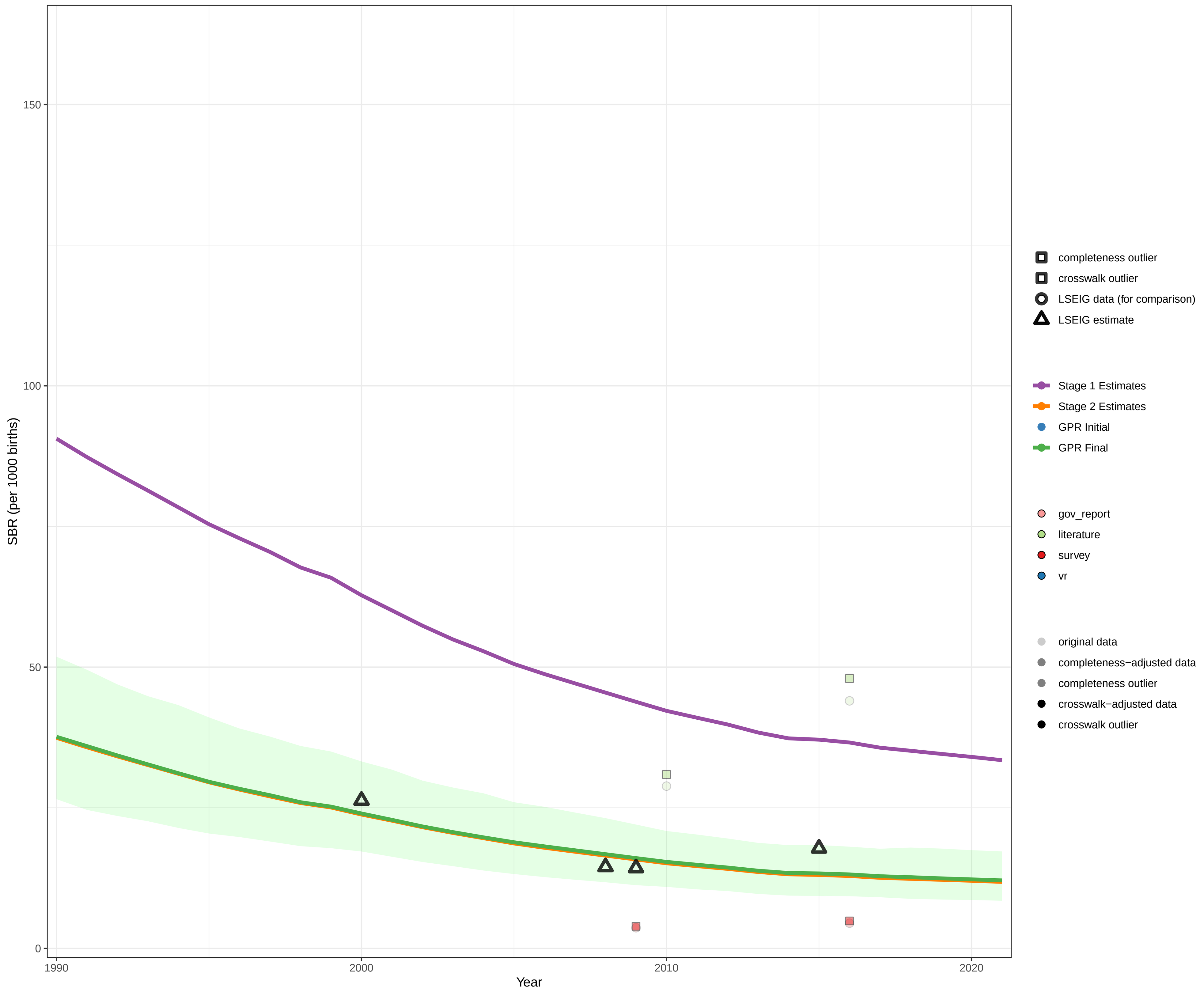

Year	Total Stillbirths (in thousands)	Total Stillbirths (in thousands)	SBR (per 1000 births)	SBR (per 1000 births)	SBR/NMR	SBR/NMR
	20 weeks	28 weeks	20 weeks	28 weeks	20 weeks	28 weeks
1990	2.0 (1.4 to 2.8)	1.5 (1.0 to 2.0)	50.5 (35.8 to 69.7)	37.6 (26.5 to 51.8)	1.45 (1.03 to 2.00)	1.08 (0.76 to 1.48)
1995	1.5 (1.1 to 2.1)	1.1 (0.8 to 1.6)	39.5 (28.3 to 53.9)	29.6 (20.4 to 41.1)	1.33 (0.95 to 1.81)	1.00 (0.69 to 1.38)
2000	1.2 (0.9 to 1.7)	0.9 (0.6 to 1.3)	31.7 (22.8 to 43.9)	24.0 (17.2 to 33.2)	1.24 (0.89 to 1.72)	0.94 (0.67 to 1.30)
2005	0.9 (0.6 to 1.2)	0.7 (0.5 to 0.9)	24.5 (17.1 to 33.7)	18.9 (13.2 to 26.0)	1.14 (0.80 to 1.57)	0.88 (0.61 to 1.21)
2010	0.7 (0.5 to 1.0)	0.5 (0.4 to 0.7)	19.8 (13.9 to 27.3)	15.4 (10.9 to 20.9)	1.09 (0.76 to 1.49)	0.84 (0.60 to 1.14)
2015	0.6 (0.4 to 0.9)	0.5 (0.3 to 0.7)	17.1 (11.9 to 23.9)	13.3 (9.3 to 18.4)	1.04 (0.73 to 1.46)	0.81 (0.57 to 1.12)
2020	0.6 (0.4 to 0.9)	0.5 (0.3 to 0.7)	15.7 (11.0 to 22.5)	12.3 (8.6 to 17.5)	1.03 (0.72 to 1.48)	0.81 (0.57 to 1.15)
2021	0.6 (0.5 to 0.9)	0.5 (0.4 to 0.7)	15.5 (10.9 to 22.1)	12.1 (8.5 to 17.2)	1.03 (0.73 to 1.47)	0.81 (0.57 to 1.15)

Viet Nam VNM Southeast Asia Stillbirth Rate / Neonatal Mortality Rate (SBR/NMR) − 20 weeks
lambda = 0.8 ; zeta = 0.7 ; scale = 15 ; amp = 0.028

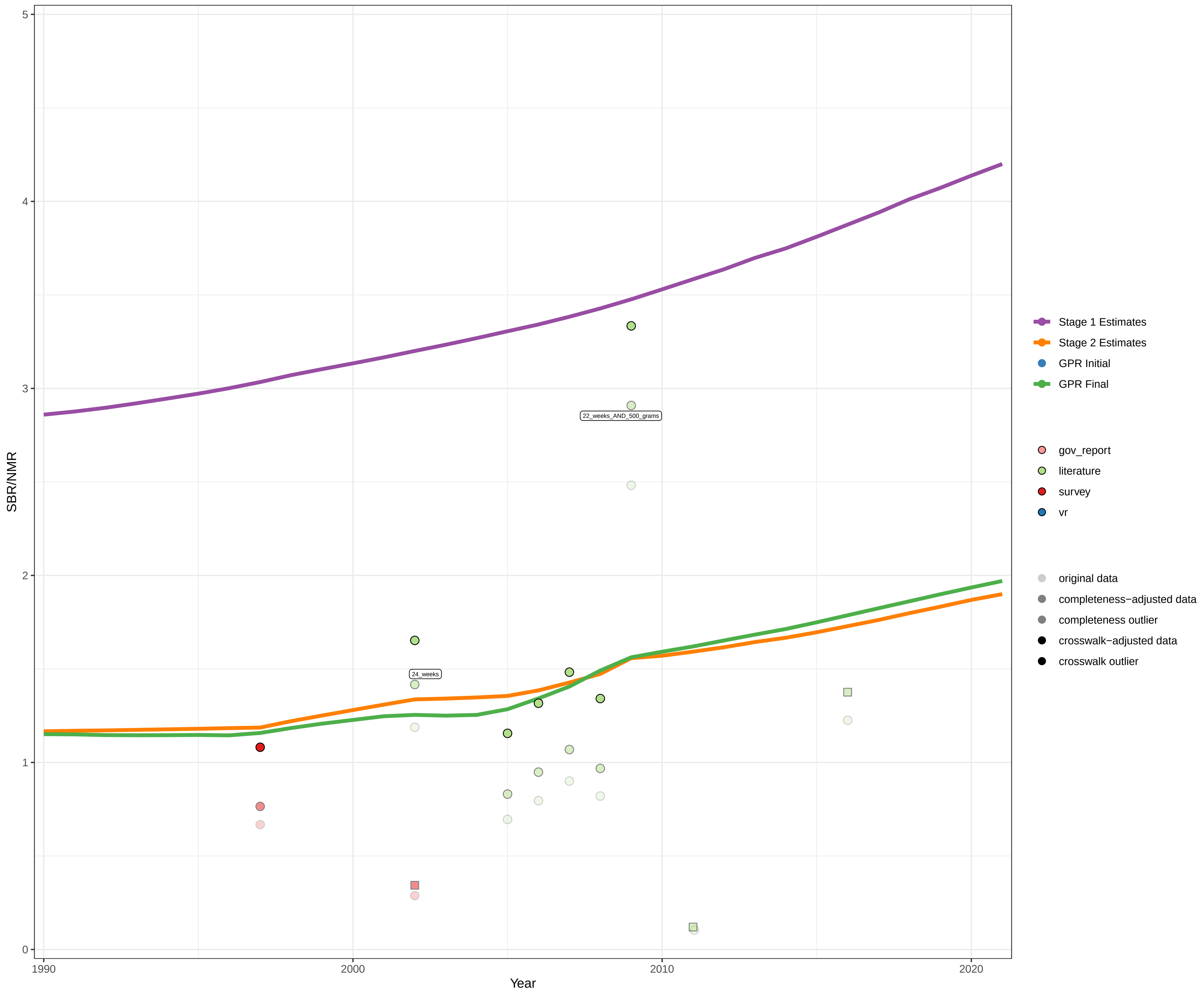

Viet Nam VNM Southeast Asia Stillbirth Rate / Neonatal Mortality Rate (SBR/NMR) − 28 weeks
lambda = 0.8 ; zeta = 0.7 ; scale = 15 ; amp = 0.028

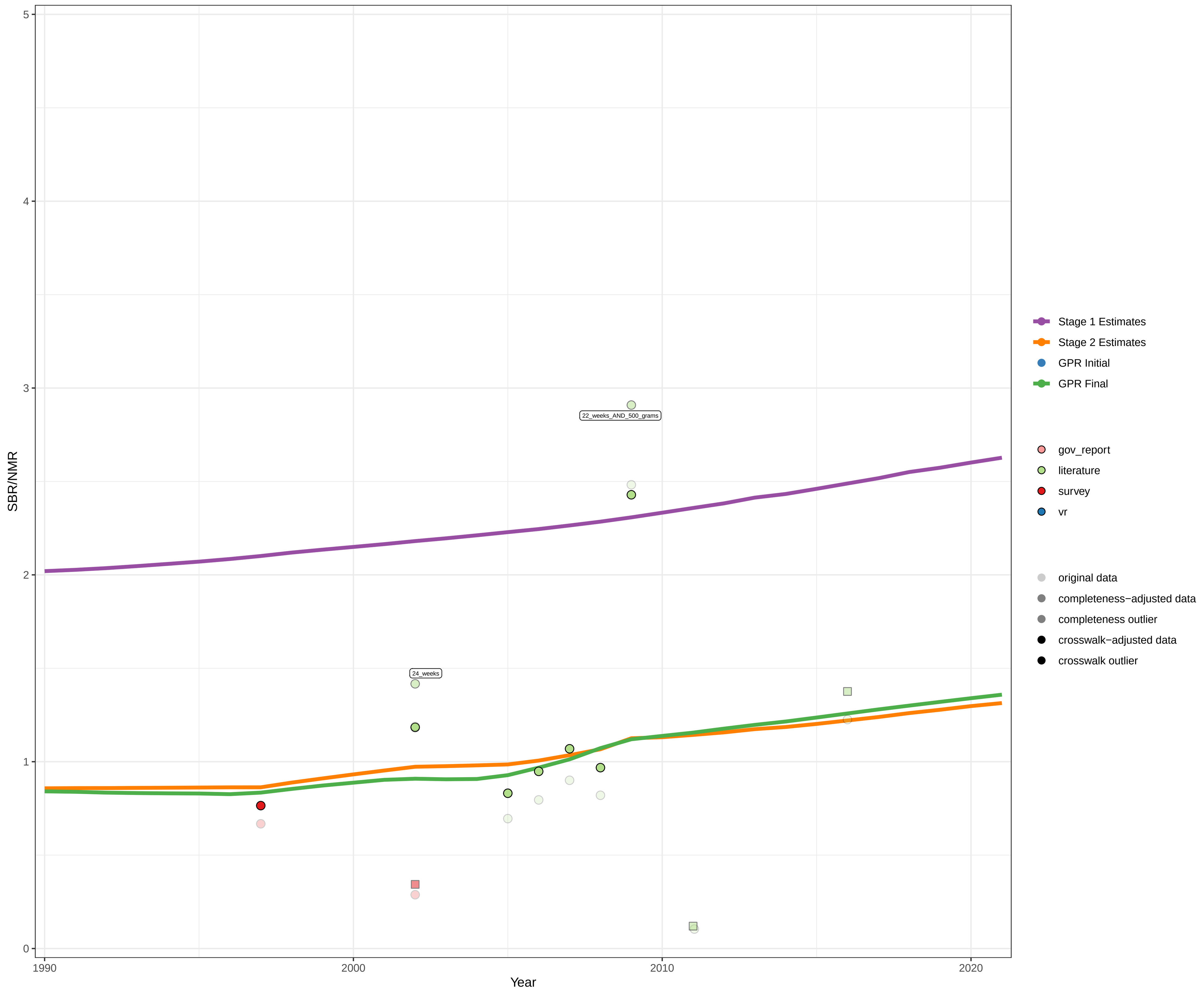

Definition	Years	Outlier	Source.Type	Title
22_weeks_AND_500_grams	2009	0	Sci Lit	Epidemiology of stillbirth and strategies for its prevention in Vietnam
24_weeks	2002	0	Sci Lit	Adverse perinatal and neonatal outcomes and their determinants in rural Vietnam 1999−2005
28_weeks	1997	0	Survey	Vietnam Demographic and Health Survey 1997
28_weeks	2005−2008	0	Sci Lit	Effect of Facilitation of Local Maternal−and−Newborn Stakeholder Groups on Neonatal Mortality: Cluster−Randomized Controlled Trial
22_weeks_OR_500_grams	2016	1	Sci Lit	Stillbirth and preterm birth and associated factors in one of the largest cities in central Vietnam
28_weeks	2002	1	Survey	Vietnam Demographic and Health Survey 2002
28_weeks_OR_1000_grams	2011	1	Sci Lit	Maternal complications and perinatal mortality: findings of the World Health Organization Multicountry Survey on Maternal and Newborn Health

Viet Nam VNM Southeast Asia Stillbirth Rate (SBR) − 20 weeks
lambda = 0.8 ; zeta = 0.7 ; scale = 15 ; amp = 0.028

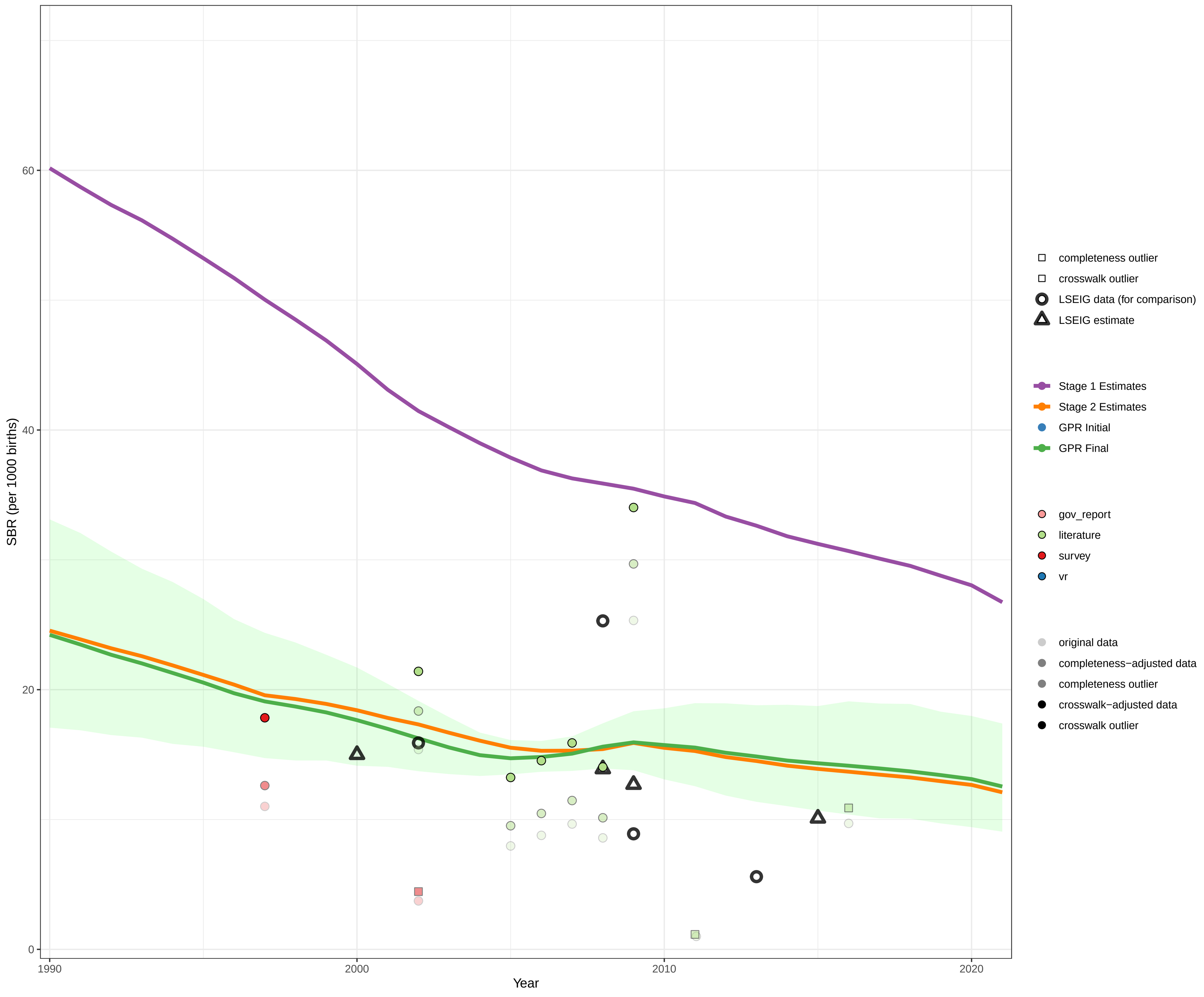

Viet Nam VNM Southeast Asia Stillbirth Rate (SBR) − 28 weeks
lambda = 0.8 ; zeta = 0.7 ; scale = 15 ; amp = 0.028

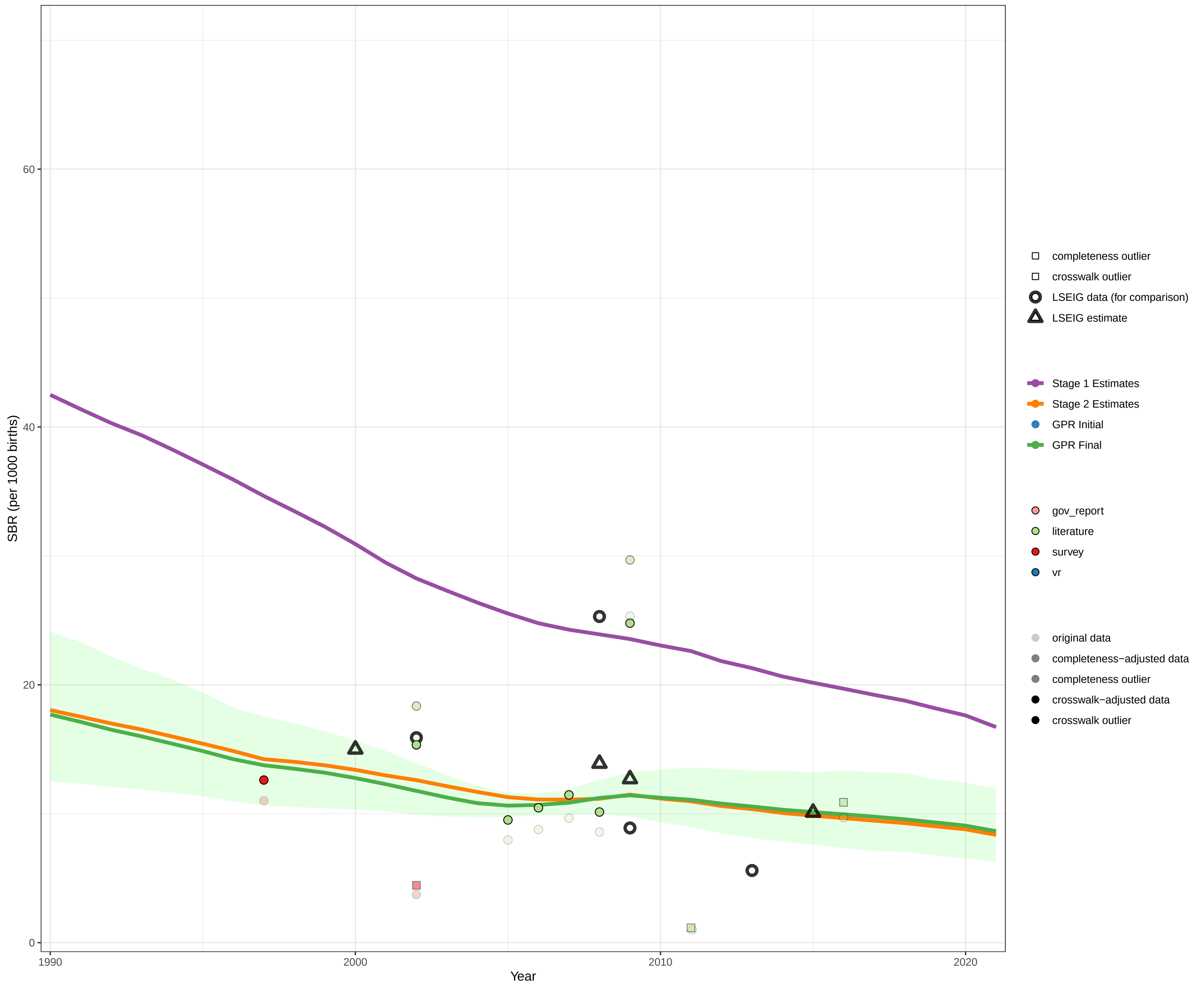

Year	Total Stillbirths (in thousands)	Total Stillbirths (in thousands)	SBR (per 1000 births)	SBR (per 1000 births)	SBR/NMR	SBR/NMR
	20 weeks	28 weeks	20 weeks	28 weeks	20 weeks	28 weeks
1990	47.7 (33.4 to 65.8)	34.6 (24.3 to 47.5)	24.2 (17.1 to 33.1)	17.7 (12.5 to 24.1)	1.15 (0.81 to 1.57)	0.84 (0.59 to 1.15)
1995	35.7 (26.9 to 47.1)	25.6 (19.5 to 33.7)	20.5 (15.6 to 27.0)	14.9 (11.4 to 19.4)	1.15 (0.87 to 1.51)	0.83 (0.63 to 1.08)
2000	27.4 (21.9 to 33.9)	19.7 (15.9 to 24.3)	17.7 (14.1 to 21.7)	12.8 (10.3 to 15.7)	1.23 (0.98 to 1.51)	0.89 (0.72 to 1.09)
2005	22.8 (20.8 to 25.0)	16.4 (15.0 to 17.9)	14.7 (13.5 to 16.1)	10.6 (9.7 to 11.6)	1.28 (1.18 to 1.41)	0.93 (0.85 to 1.01)
2010	26.0 (21.6 to 30.8)	18.5 (15.4 to 22.2)	15.7 (13.1 to 18.6)	11.2 (9.3 to 13.4)	1.59 (1.32 to 1.88)	1.14 (0.94 to 1.36)
2015	24.5 (18.2 to 32.2)	17.3 (12.9 to 22.6)	14.3 (10.7 to 18.7)	10.1 (7.6 to 13.2)	1.75 (1.30 to 2.29)	1.24 (0.93 to 1.61)
2020	21.0 (15.0 to 28.9)	14.5 (10.4 to 19.8)	13.1 (9.4 to 18.0)	9.1 (6.5 to 12.4)	1.94 (1.39 to 2.65)	1.34 (0.96 to 1.83)
2021	19.6 (14.1 to 27.4)	13.5 (9.8 to 18.7)	12.5 (9.1 to 17.4)	8.7 (6.3 to 12.0)	1.97 (1.42 to 2.73)	1.36 (0.99 to 1.88)

Sub−Saharan Africa S3 NA Stillbirth Rate / Neonatal Mortality Rate (SBR/NMR) − 20 weeks
lambda = 1 ; zeta = 0.6 ; scale = 15 ; amp = 0.028

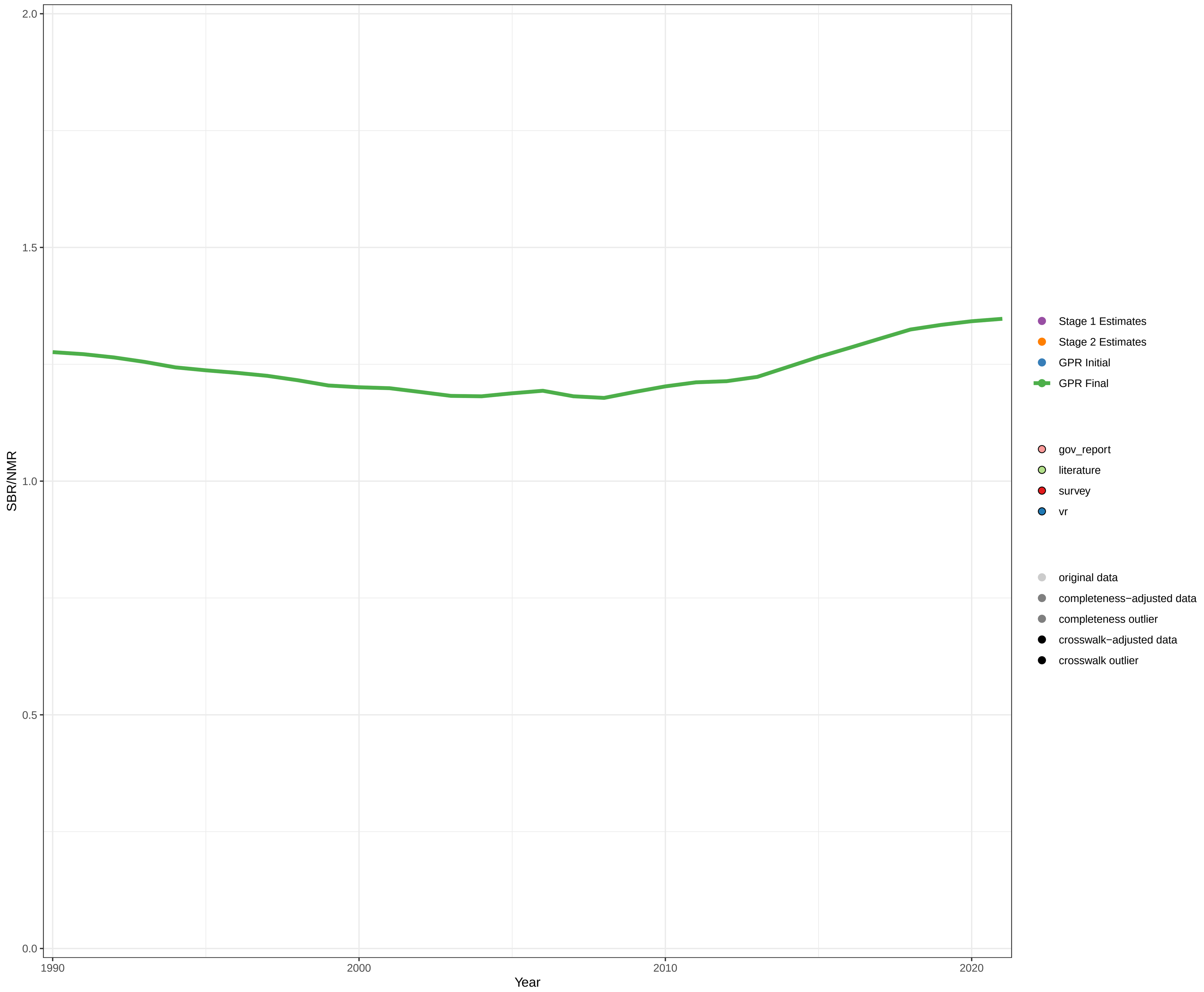

Sub−Saharan Africa S3 NA Stillbirth Rate / Neonatal Mortality Rate (SBR/NMR) − 28 weeks
lambda = 1 ; zeta = 0.6 ; scale = 15 ; amp = 0.028

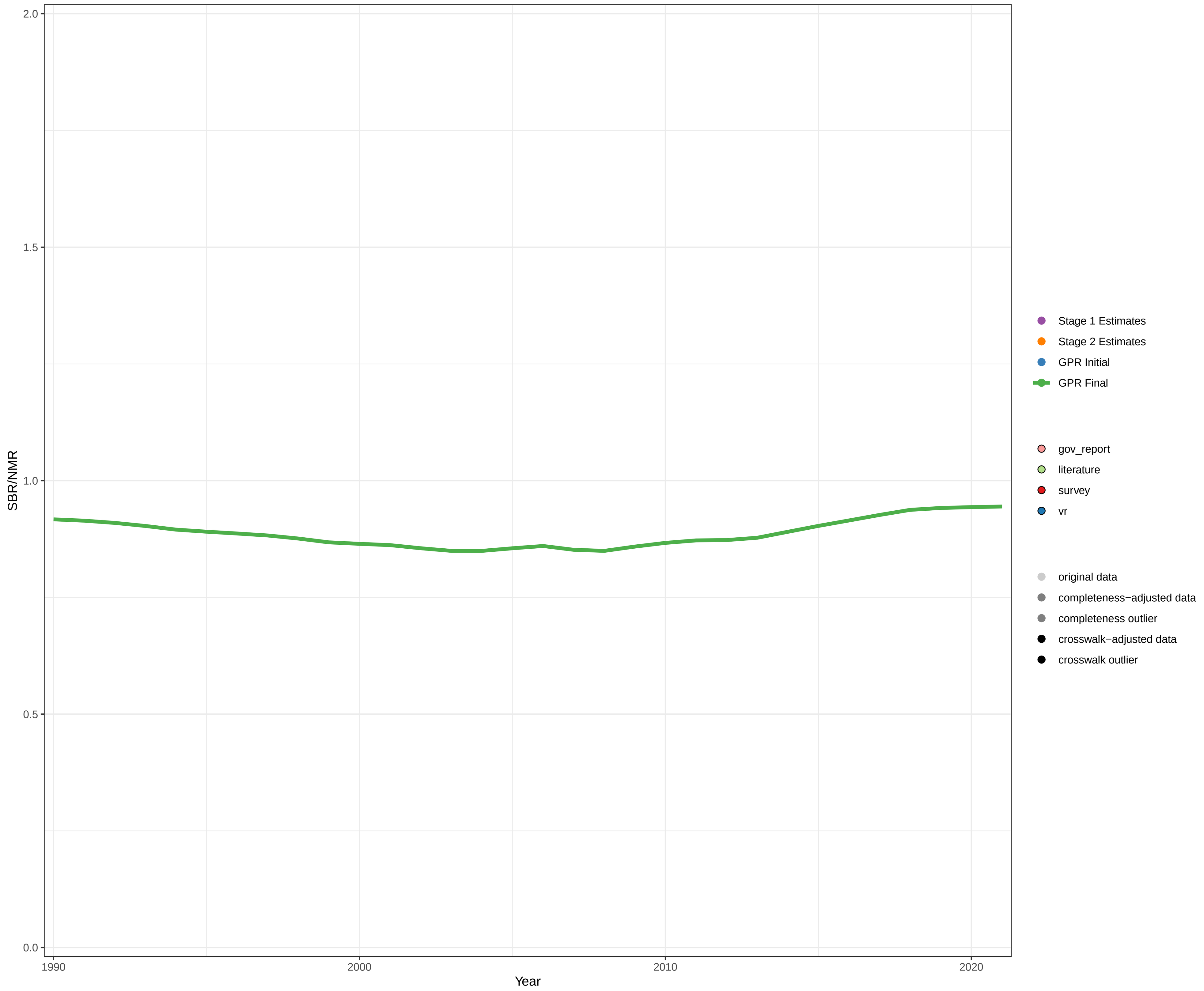

Definition	Years	Outlier	Source.Type	Title

Sub−Saharan Africa S3 NA Stillbirth Rate (SBR) − 20 weeks
lambda = 1 ; zeta = 0.6 ; scale = 15 ; amp = 0.028

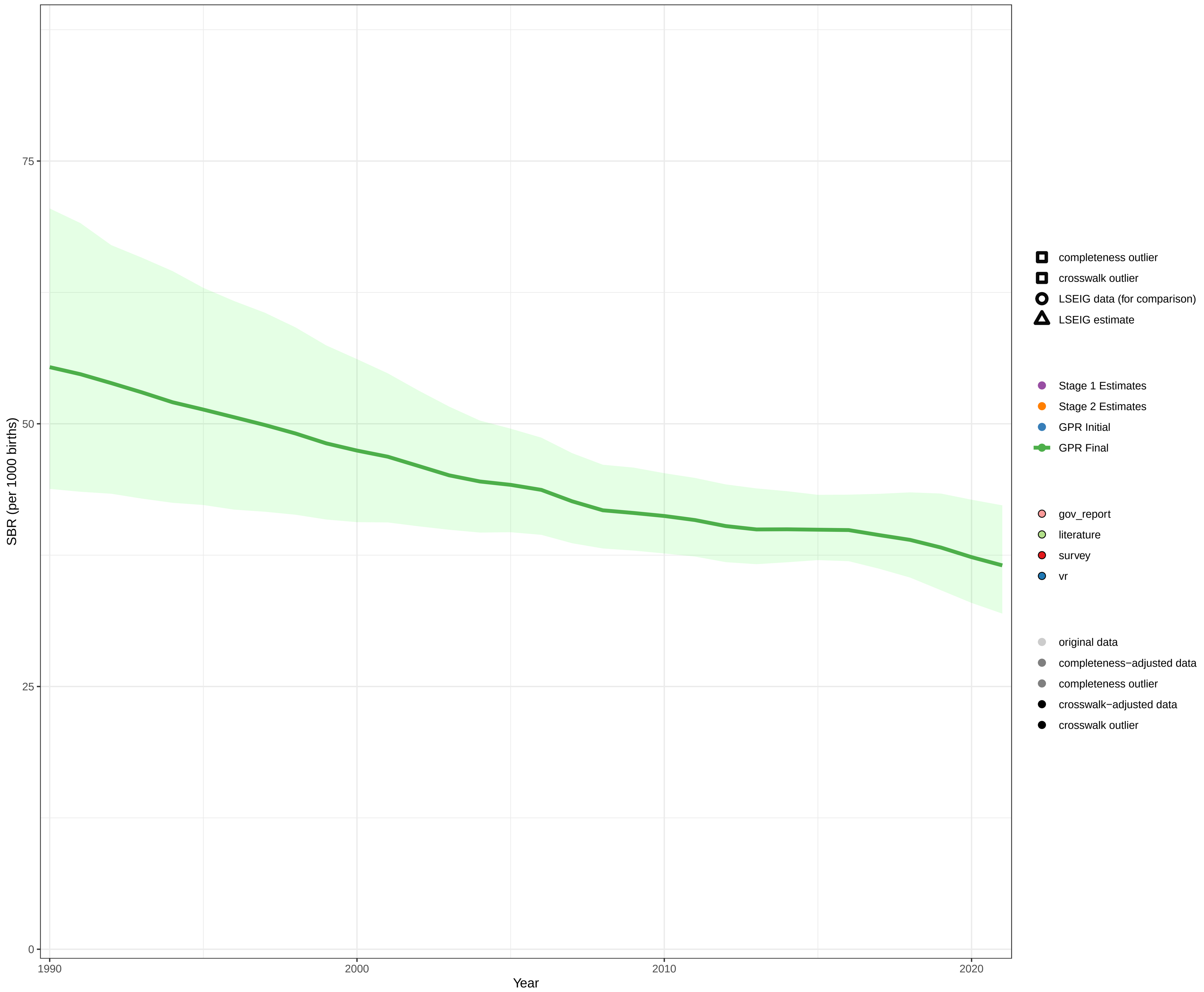

Sub−Saharan Africa S3 NA Stillbirth Rate (SBR) − 28 weeks
lambda = 1 ; zeta = 0.6 ; scale = 15 ; amp = 0.028

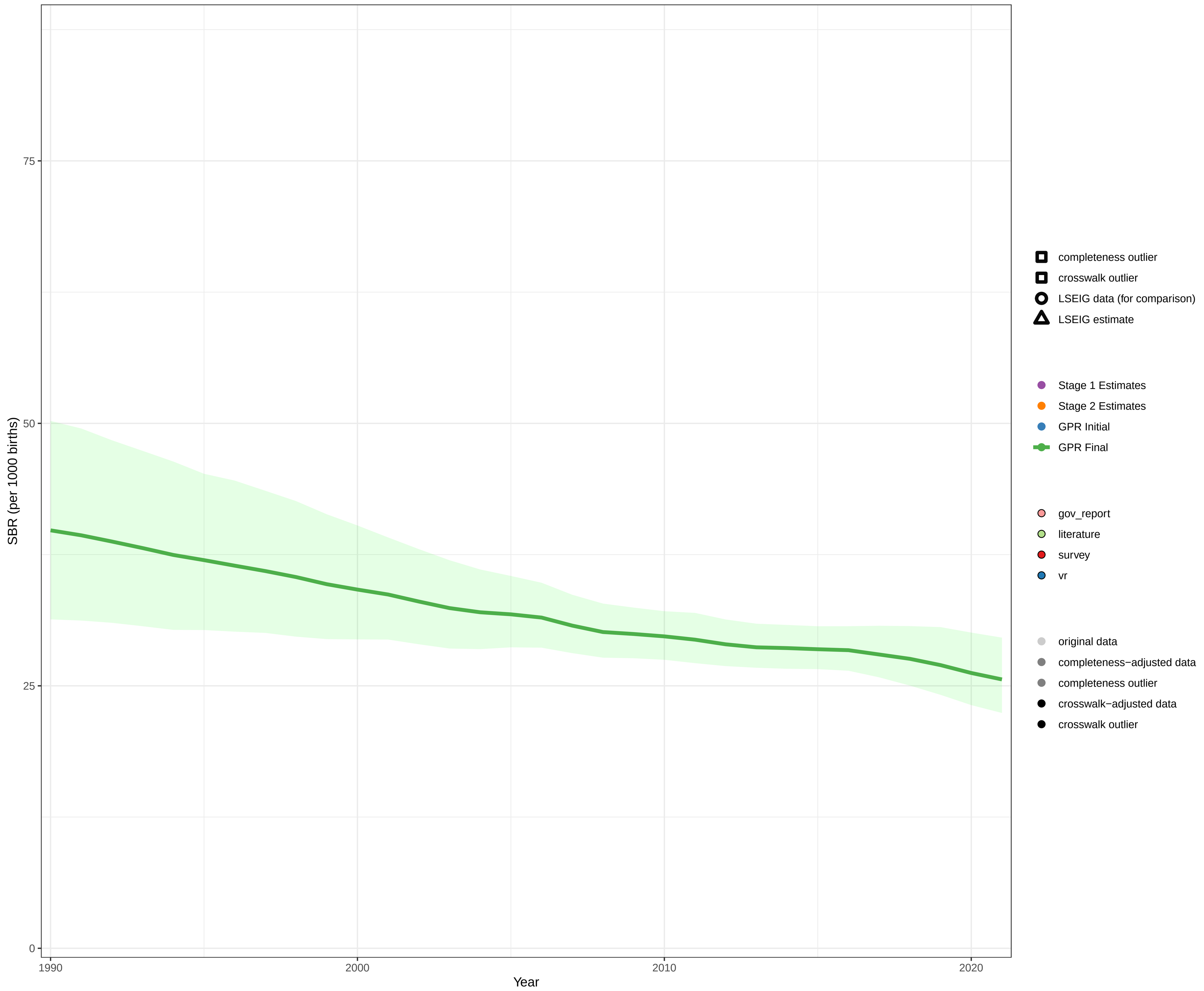

Year	Total Stillbirths (in thousands)	Total Stillbirths (in thousands)	SBR (per 1000 births)	SBR (per 1000 births)	SBR/NMR	SBR/NMR
	20 weeks	28 weeks	20 weeks	28 weeks	20 weeks	28 weeks
1990	1308.0 (1020.9 to 1690.0)	924.6 (720.8 to 1178.6)	55.4 (43.8 to 70.5)	39.8 (31.3 to 50.2)	1.28 (1.01 to 1.62)	0.92 (0.72 to 1.16)
1995	1344.1 (1095.2 to 1666.7)	953.1 (775.7 to 1174.7)	51.4 (42.3 to 62.9)	37.0 (30.3 to 45.2)	1.24 (1.02 to 1.52)	0.89 (0.73 to 1.09)
2000	1394.4 (1185.3 to 1664.7)	990.1 (848.3 to 1174.3)	47.5 (40.6 to 56.2)	34.2 (29.4 to 40.3)	1.20 (1.03 to 1.42)	0.86 (0.74 to 1.02)
2005	1447.7 (1293.7 to 1631.8)	1028.7 (924.0 to 1151.2)	44.2 (39.7 to 49.5)	31.8 (28.7 to 35.5)	1.19 (1.07 to 1.33)	0.86 (0.77 to 0.95)
2010	1473.0 (1340.5 to 1625.3)	1048.9 (967.9 to 1136.3)	41.2 (37.7 to 45.3)	29.7 (27.5 to 32.1)	1.20 (1.10 to 1.32)	0.87 (0.80 to 0.94)
2015	1513.7 (1399.6 to 1645.0)	1067.3 (994.4 to 1151.9)	39.9 (37.0 to 43.2)	28.5 (26.6 to 30.7)	1.27 (1.17 to 1.37)	0.90 (0.84 to 0.97)
2020	1450.5 (1275.2 to 1672.2)	1007.8 (887.3 to 1160.1)	37.3 (33.0 to 42.8)	26.2 (23.2 to 30.1)	1.34 (1.19 to 1.54)	0.94 (0.83 to 1.08)
2021	1430.1 (1244.2 to 1663.3)	991.4 (864.6 to 1150.0)	36.5 (31.9 to 42.2)	25.6 (22.4 to 29.6)	1.35 (1.18 to 1.56)	0.94 (0.83 to 1.09)

Central Sub−Saharan Africa R18 Central Sub−Saharan Africa Stillbirth Rate / Neonatal Mortality Rate (SBR/NMR) − 20 weeks
lambda = 1 ; zeta = 0.6 ; scale = 15 ; amp = 0.028

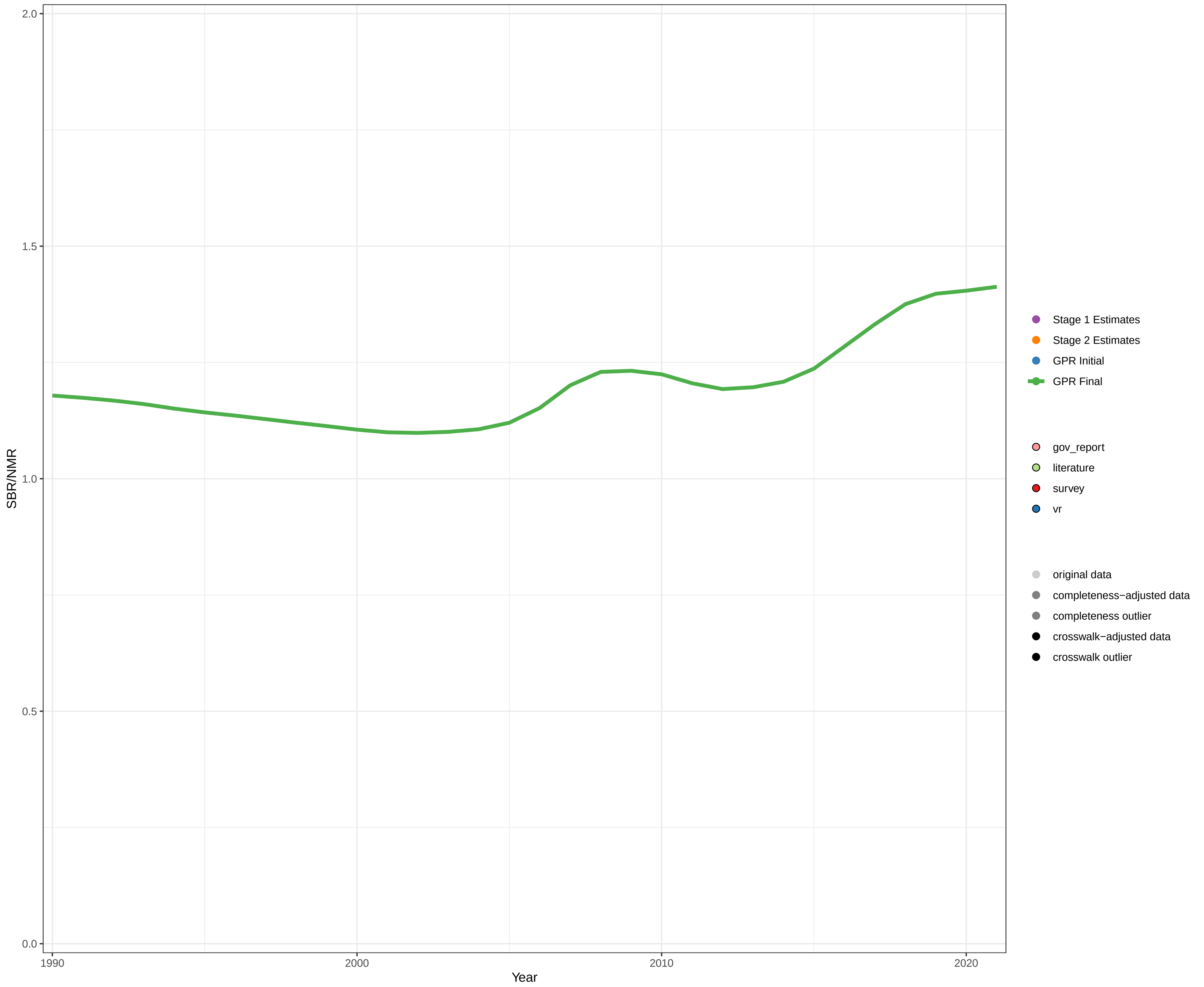

Central Sub−Saharan Africa R18 Central Sub−Saharan Africa Stillbirth Rate / Neonatal Mortality Rate (SBR/NMR) − 28 weeks
lambda = 1 ; zeta = 0.6 ; scale = 15 ; amp = 0.028

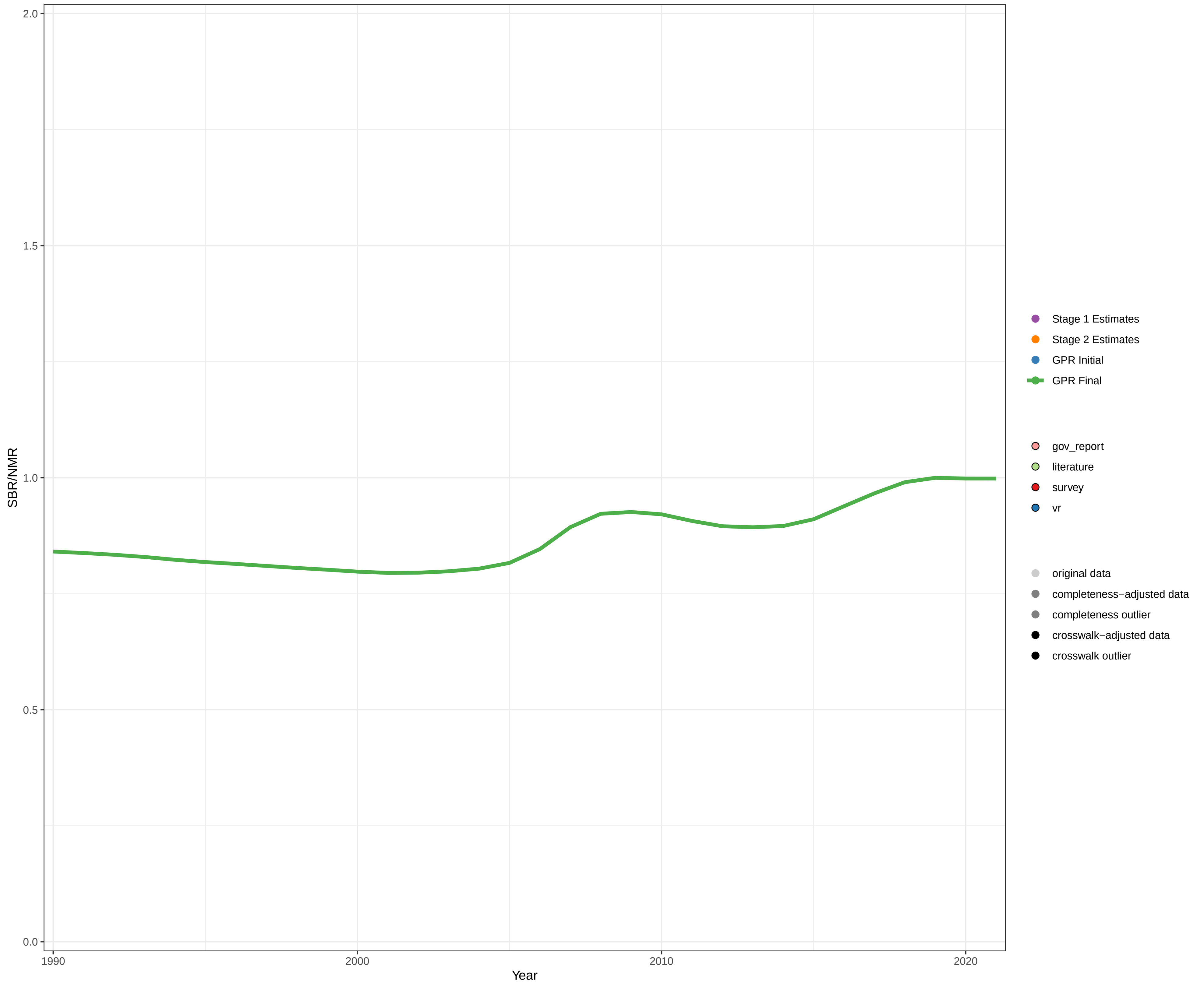

Definition	Years	Outlier	Source.Type	Title

Central Sub−Saharan Africa R18 Central Sub−Saharan Africa Stillbirth Rate (SBR) − 20 weeks
lambda = 1 ; zeta = 0.6 ; scale = 15 ; amp = 0.028

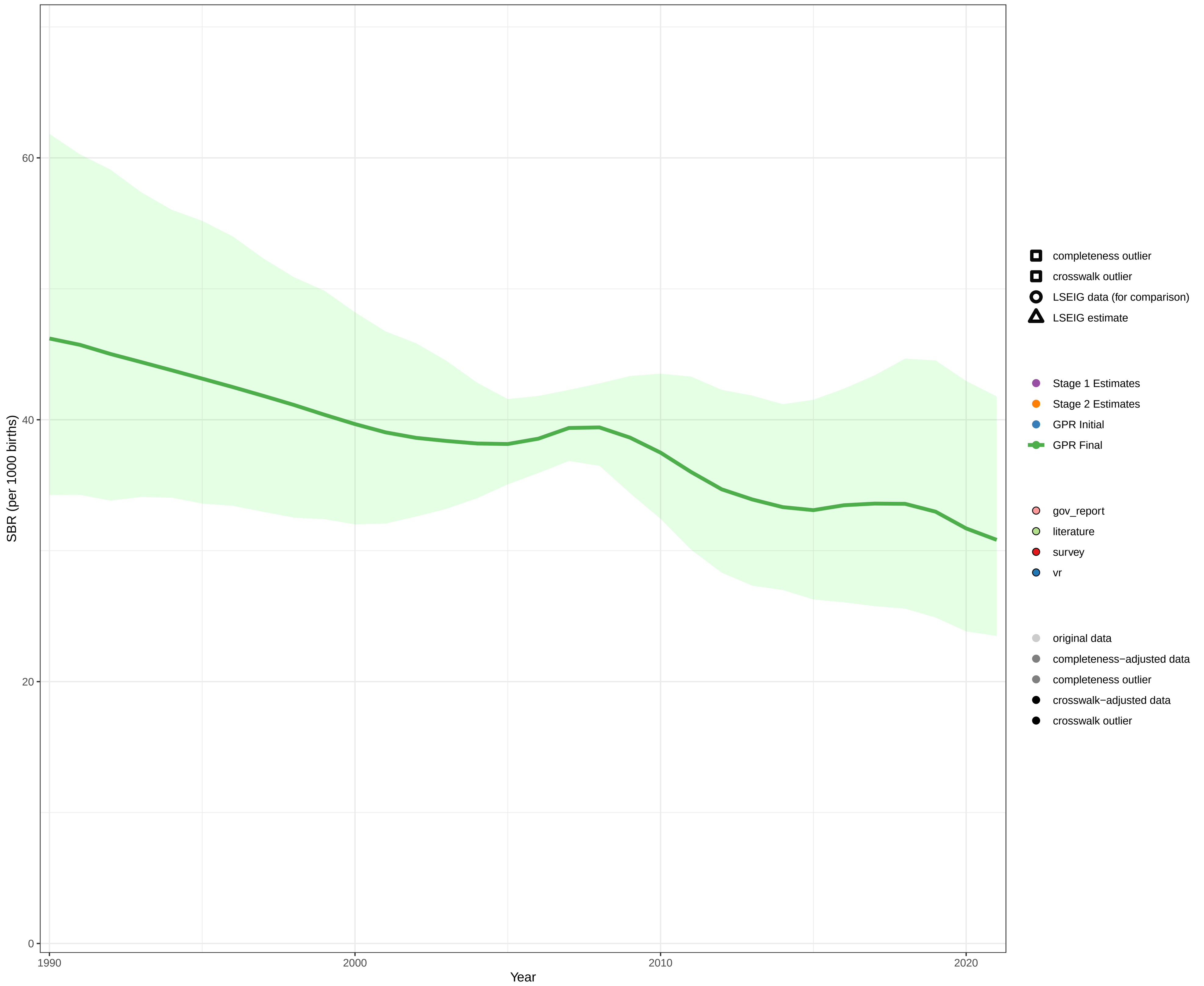

Central Sub−Saharan Africa R18 Central Sub−Saharan Africa Stillbirth Rate (SBR) − 28 weeks
lambda = 1 ; zeta = 0.6 ; scale = 15 ; amp = 0.028

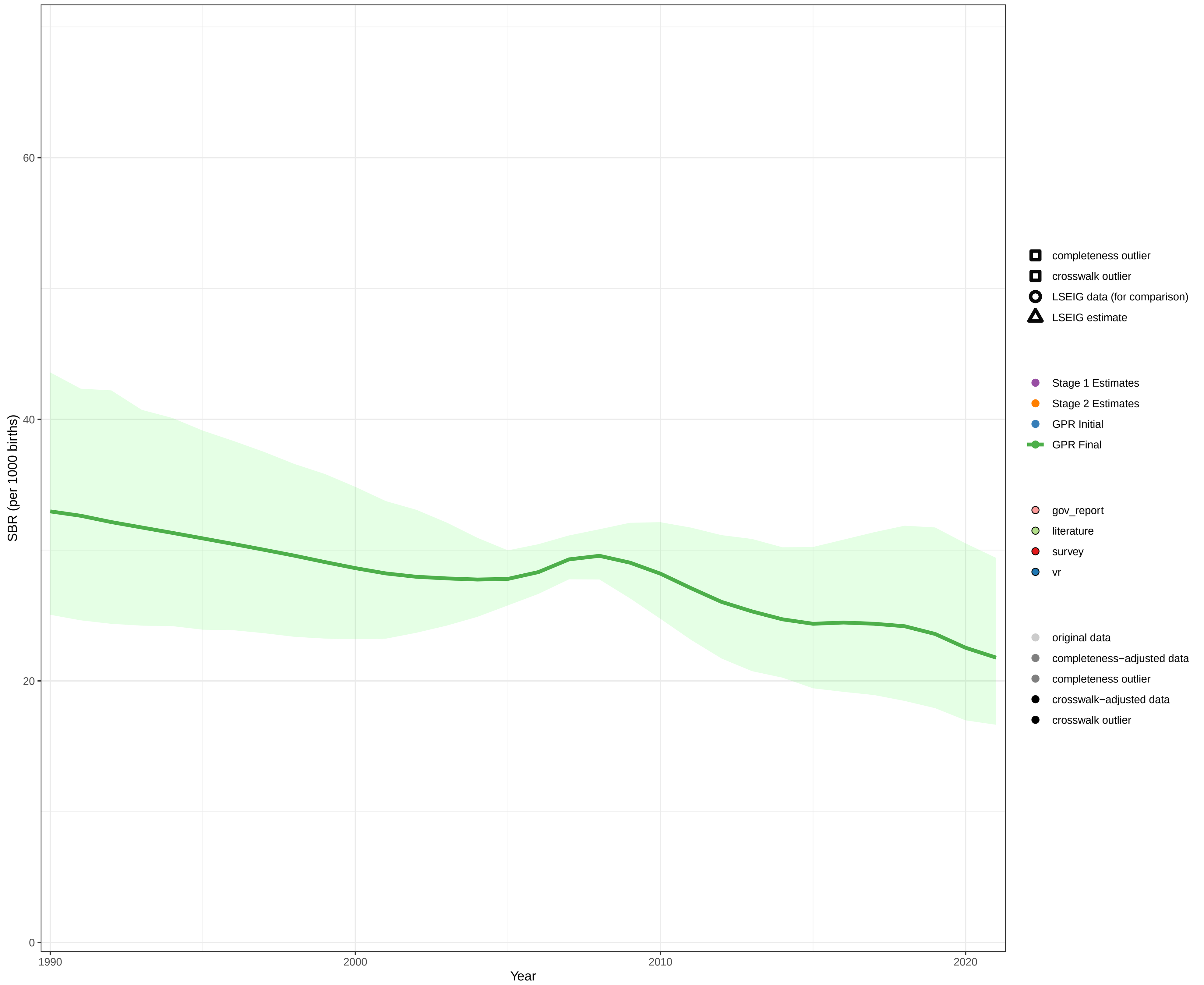

Year	Total Stillbirths (in thousands)	Total Stillbirths (in thousands)	SBR (per 1000 births)	SBR (per 1000 births)	SBR/NMR	SBR/NMR
	20 weeks	28 weeks	20 weeks	28 weeks	20 weeks	28 weeks
1990	125.6 (91.9 to 170.7)	88.4 (66.6 to 118.1)	46.2 (34.3 to 61.8)	33.0 (25.1 to 43.6)	1.18 (0.87 to 1.58)	0.84 (0.64 to 1.11)
1995	131.8 (101.6 to 170.7)	93.2 (71.6 to 119.0)	43.1 (33.6 to 55.2)	30.9 (23.9 to 39.1)	1.14 (0.89 to 1.46)	0.82 (0.63 to 1.04)
2000	137.2 (109.8 to 168.2)	97.9 (78.8 to 119.8)	39.7 (32.0 to 48.2)	28.6 (23.2 to 34.8)	1.11 (0.89 to 1.34)	0.80 (0.65 to 0.97)
2005	150.1 (137.6 to 164.2)	108.2 (100.1 to 117.0)	38.1 (35.1 to 41.6)	27.8 (25.8 to 30.0)	1.12 (1.03 to 1.22)	0.82 (0.76 to 0.88)
2010	164.1 (141.1 to 191.6)	122.2 (106.9 to 139.8)	37.5 (32.4 to 43.5)	28.2 (24.7 to 32.1)	1.22 (1.06 to 1.42)	0.92 (0.81 to 1.05)
2015	152.2 (119.9 to 192.6)	111.0 (88.1 to 138.6)	33.1 (26.3 to 41.5)	24.4 (19.4 to 30.2)	1.24 (0.98 to 1.55)	0.91 (0.73 to 1.13)
2020	145.8 (108.7 to 199.8)	102.6 (76.9 to 140.1)	31.7 (23.8 to 43.0)	22.5 (17.0 to 30.5)	1.40 (1.06 to 1.90)	1.00 (0.75 to 1.35)
2021	141.9 (107.3 to 194.4)	99.3 (75.5 to 135.1)	30.8 (23.5 to 41.8)	21.8 (16.6 to 29.4)	1.41 (1.08 to 1.91)	1.00 (0.76 to 1.35)

Angola AGO Central Sub−Saharan Africa Stillbirth Rate / Neonatal Mortality Rate (SBR/NMR) − 20 weeks
lambda = 0.8 ; zeta = 0.7 ; scale = 15 ; amp = 0.028

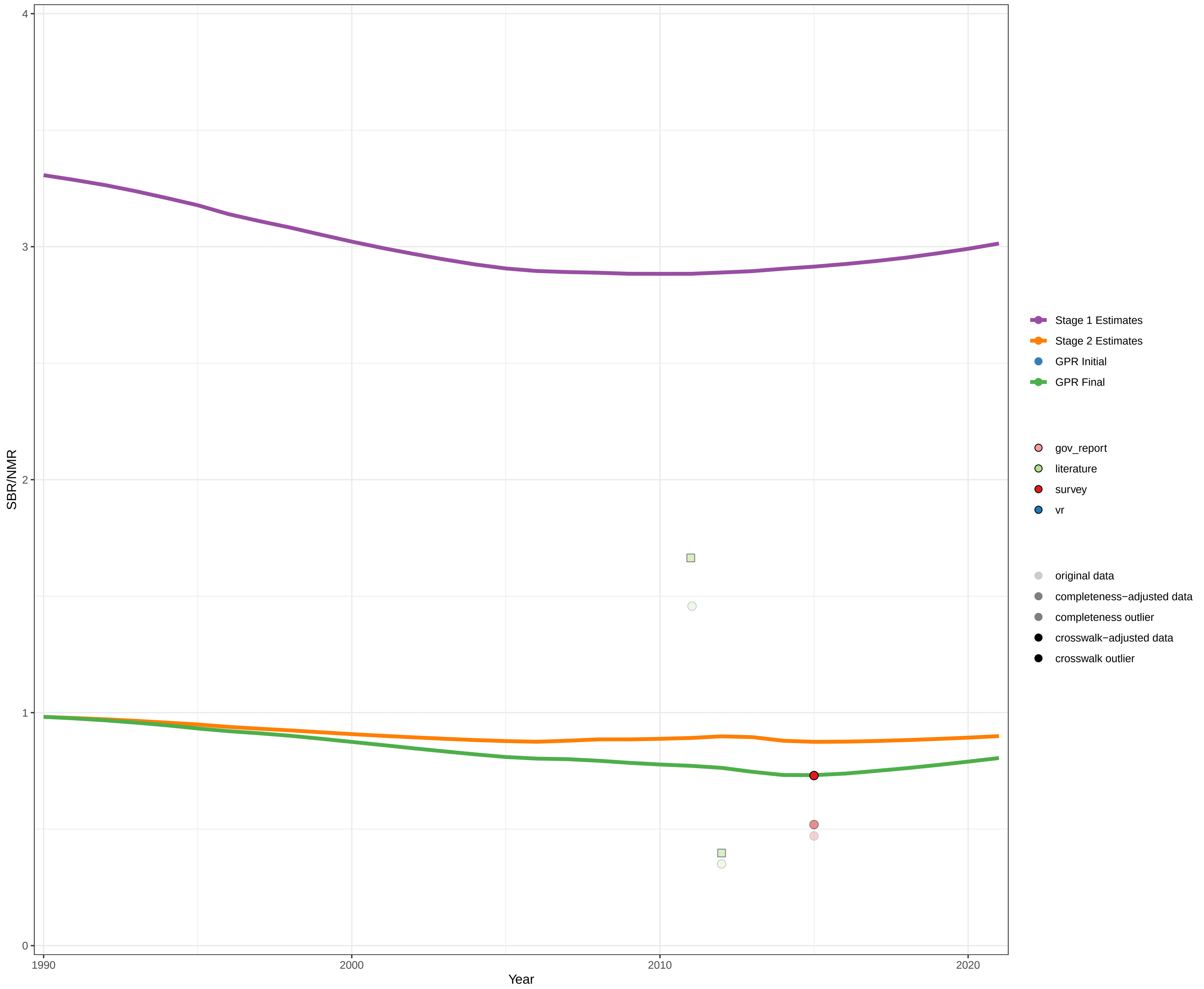

Angola AGO Central Sub−Saharan Africa Stillbirth Rate / Neonatal Mortality Rate (SBR/NMR) − 28 weeks
lambda = 0.8 ; zeta = 0.7 ; scale = 15 ; amp = 0.028

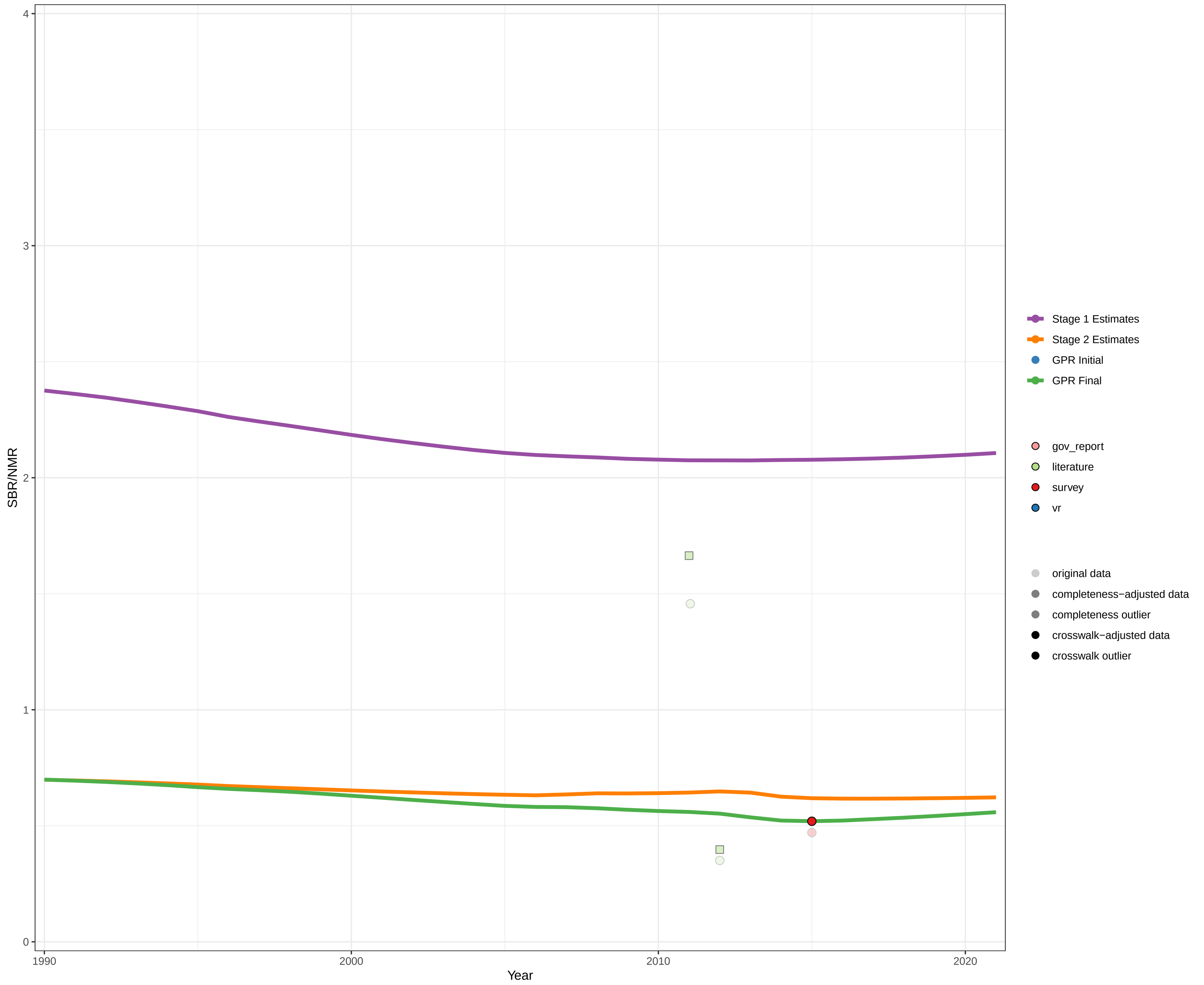

Definition	Years	Outlier	Source.Type	Title
28_weeks	2015	0	Survey	Angola Demographic and Health Survey 2015−2016
28_weeks	2012	1	Sci Lit	Determinants of maternal health care and birth outcome in the Dande Health and Demographic Surveillance System area, Angola
28_weeks_OR_1000_grams	2011	1	Sci Lit	Maternal complications and perinatal mortality: findings of the World Health Organization Multicountry Survey on Maternal and Newborn Health

Angola AGO Central Sub−Saharan Africa Stillbirth Rate (SBR) − 20 weeks
lambda = 0.8 ; zeta = 0.7 ; scale = 15 ; amp = 0.028

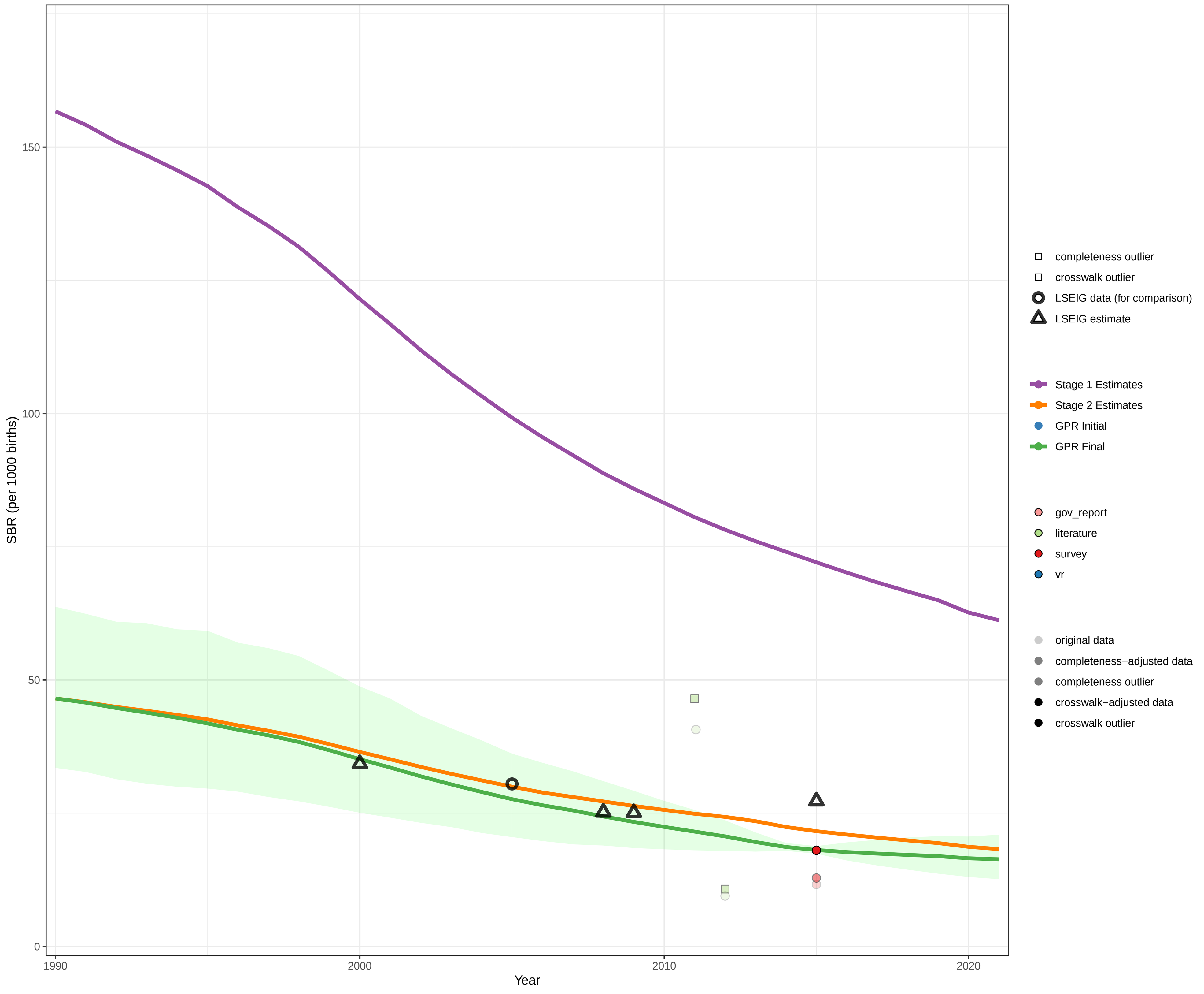

Angola AGO Central Sub−Saharan Africa Stillbirth Rate (SBR) − 28 weeks
lambda = 0.8 ; zeta = 0.7 ; scale = 15 ; amp = 0.028

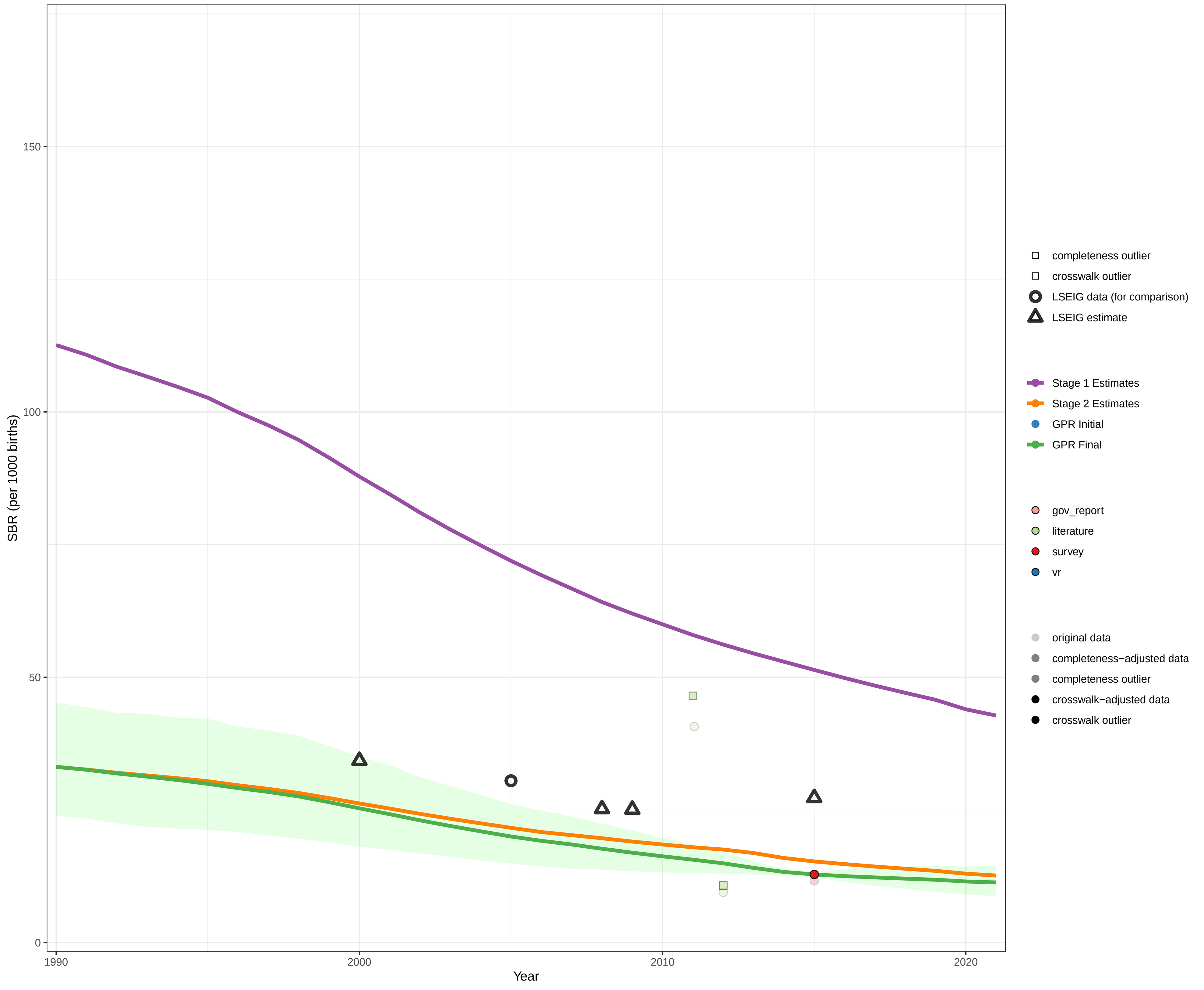

Year	Total Stillbirths (in thousands)	Total Stillbirths (in thousands)	SBR (per 1000 births)	SBR (per 1000 births)	SBR/NMR	SBR/NMR
	20 weeks	28 weeks	20 weeks	28 weeks	20 weeks	28 weeks
1990	24.7 (17.5 to 34.4)	17.3 (12.4 to 23.9)	46.5 (33.5 to 63.7)	33.1 (23.9 to 45.2)	0.98 (0.71 to 1.34)	0.70 (0.50 to 0.95)
1995	26.2 (18.3 to 37.7)	18.5 (13.0 to 26.4)	41.9 (29.6 to 59.2)	29.9 (21.3 to 42.2)	0.93 (0.66 to 1.32)	0.67 (0.47 to 0.94)
2000	25.9 (18.3 to 36.5)	18.5 (13.1 to 25.8)	35.2 (25.1 to 48.8)	25.3 (18.1 to 35.0)	0.87 (0.62 to 1.21)	0.63 (0.45 to 0.87)
2005	24.1 (17.8 to 31.9)	17.3 (12.8 to 22.8)	27.6 (20.5 to 36.2)	20.0 (14.9 to 26.1)	0.81 (0.60 to 1.06)	0.59 (0.44 to 0.77)
2010	22.8 (18.5 to 27.9)	16.4 (13.3 to 20.0)	22.4 (18.2 to 27.3)	16.3 (13.2 to 19.8)	0.78 (0.63 to 0.95)	0.56 (0.46 to 0.69)
2015	20.5 (19.7 to 21.4)	14.5 (13.9 to 15.1)	18.1 (17.4 to 18.8)	12.9 (12.4 to 13.4)	0.73 (0.70 to 0.76)	0.52 (0.50 to 0.54)
2020	19.9 (15.6 to 25.0)	13.8 (10.9 to 17.2)	16.5 (13.0 to 20.6)	11.5 (9.1 to 14.3)	0.79 (0.62 to 0.99)	0.55 (0.43 to 0.68)
2021	20.0 (15.4 to 25.8)	13.8 (10.7 to 17.7)	16.4 (12.6 to 21.0)	11.3 (8.8 to 14.5)	0.81 (0.62 to 1.03)	0.56 (0.43 to 0.71)

Central African Republic CAF Central Sub−Saharan Africa Stillbirth Rate / Neonatal Mortality Rate (SBR/NMR) − 20 weeks
lambda = 1 ; zeta = 0.6 ; scale = 15 ; amp = 0.028

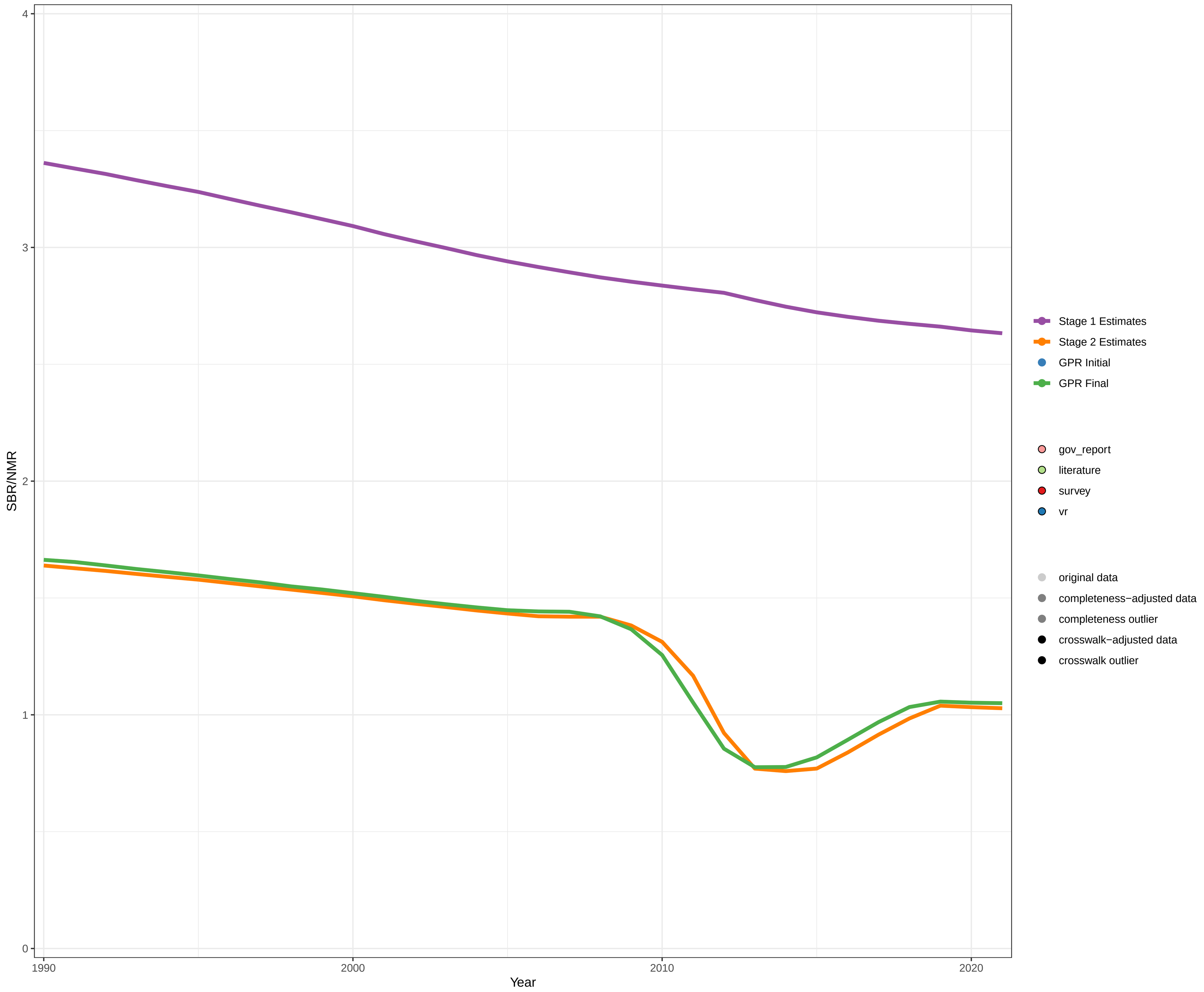

Central African Republic CAF Central Sub−Saharan Africa Stillbirth Rate / Neonatal Mortality Rate (SBR/NMR) − 28 weeks
lambda = 1 ; zeta = 0.6 ; scale = 15 ; amp = 0.028

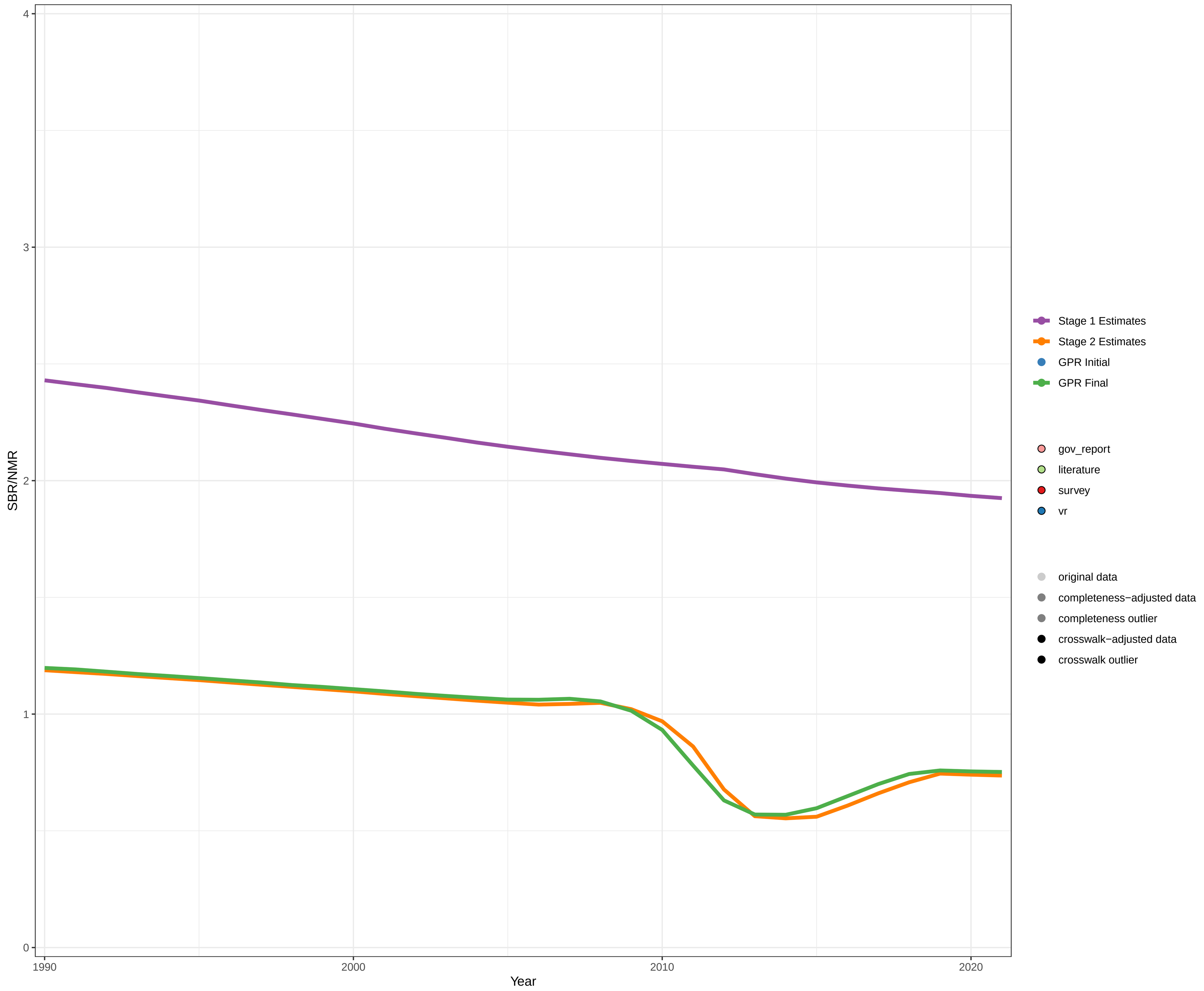

Definition	Years	Outlier	Source.Type	Title

Central African Republic CAF Central Sub−Saharan Africa Stillbirth Rate (SBR) − 20 weeks
lambda = 1 ; zeta = 0.6 ; scale = 15 ; amp = 0.028

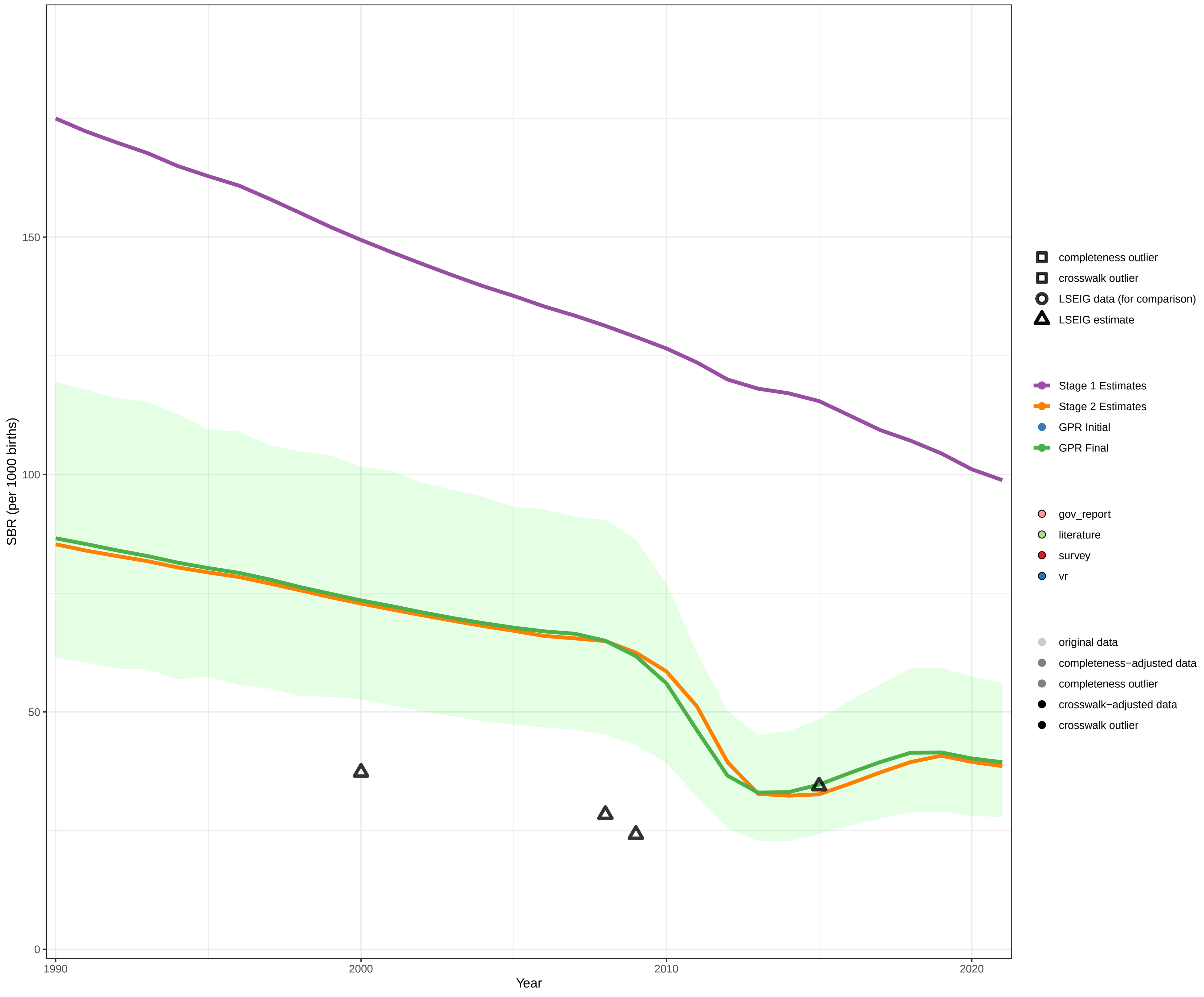

Central African Republic CAF Central Sub−Saharan Africa Stillbirth Rate (SBR) − 28 weeks
lambda = 1 ; zeta = 0.6 ; scale = 15 ; amp = 0.028

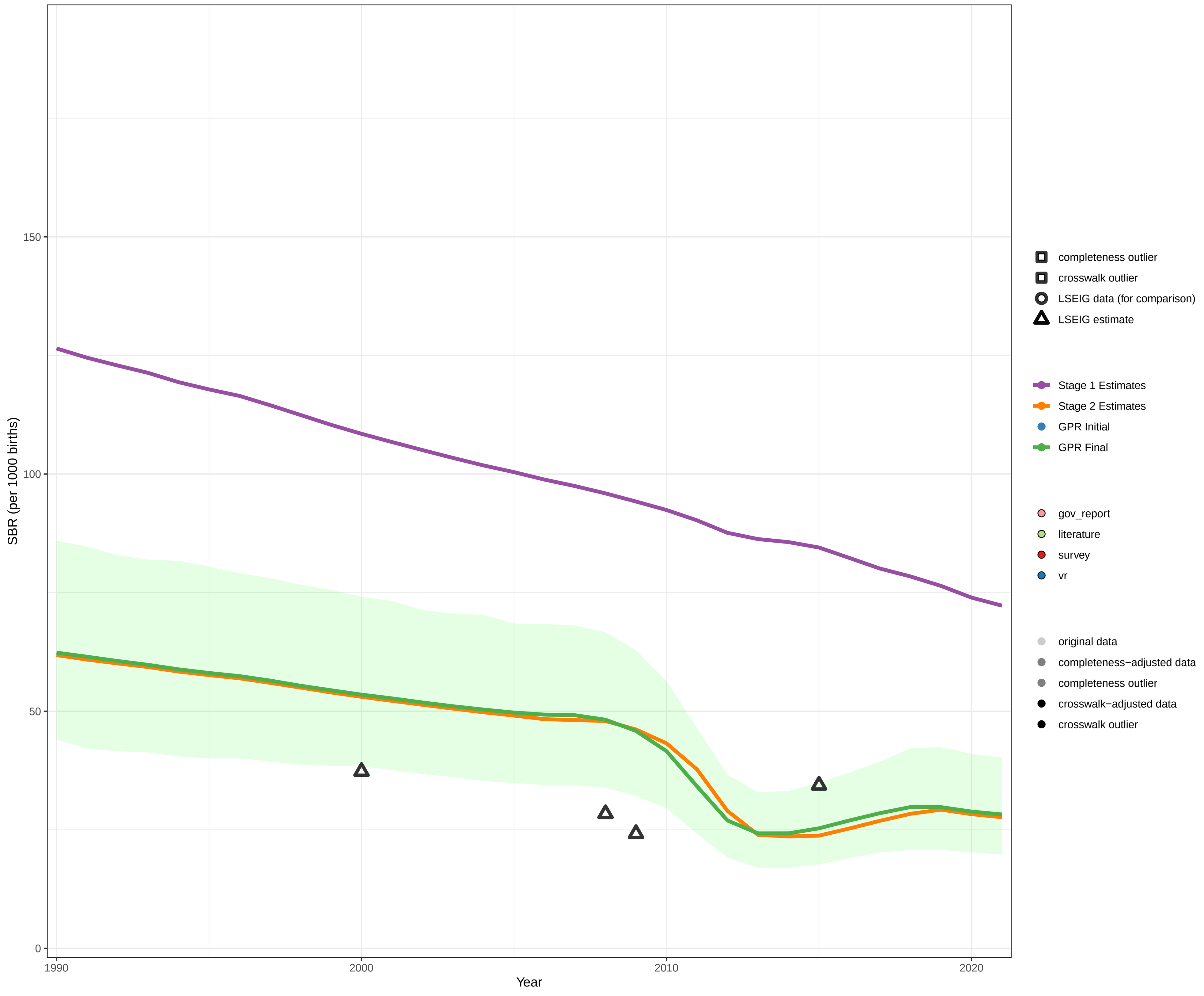

Year	Total Stillbirths (in thousands)	Total Stillbirths (in thousands)	SBR (per 1000 births)	SBR (per 1000 births)	SBR/NMR	SBR/NMR
	20 weeks	28 weeks	20 weeks	28 weeks	20 weeks	28 weeks
1990	12.2 (8.4 to 17.4)	8.5 (5.9 to 12.0)	86.6 (61.4 to 119.5)	62.3 (44.0 to 85.9)	1.66 (1.18 to 2.30)	1.20 (0.84 to 1.65)
1995	12.6 (8.7 to 17.6)	8.9 (6.0 to 12.6)	80.3 (57.4 to 109.5)	58.1 (40.0 to 80.5)	1.60 (1.14 to 2.18)	1.15 (0.80 to 1.60)
2000	12.7 (8.9 to 18.1)	9.1 (6.4 to 12.8)	73.5 (52.7 to 101.7)	53.5 (38.4 to 74.1)	1.52 (1.09 to 2.10)	1.11 (0.80 to 1.53)
2005	12.9 (8.8 to 18.2)	9.3 (6.4 to 13.0)	67.7 (47.4 to 93.2)	49.7 (34.8 to 68.5)	1.45 (1.01 to 1.99)	1.06 (0.74 to 1.46)
2010	11.4 (7.8 to 16.0)	8.3 (5.8 to 11.5)	56.0 (39.2 to 77.1)	41.6 (29.6 to 56.4)	1.26 (0.88 to 1.73)	0.93 (0.66 to 1.26)
2015	7.0 (4.8 to 9.9)	5.0 (3.5 to 7.0)	34.7 (24.3 to 48.5)	25.3 (17.7 to 34.9)	0.82 (0.57 to 1.14)	0.60 (0.42 to 0.82)
2020	8.0 (5.5 to 11.6)	5.7 (3.9 to 8.2)	40.2 (28.1 to 57.4)	28.8 (20.2 to 41.0)	1.05 (0.74 to 1.50)	0.75 (0.53 to 1.07)
2021	7.9 (5.5 to 11.4)	5.6 (3.9 to 8.0)	39.4 (27.8 to 56.1)	28.2 (19.8 to 40.3)	1.05 (0.74 to 1.49)	0.75 (0.53 to 1.07)

Congo COG Central Sub−Saharan Africa Stillbirth Rate / Neonatal Mortality Rate (SBR/NMR) − 20 weeks
lambda = 1 ; zeta = 0.6 ; scale = 15 ; amp = 0.028

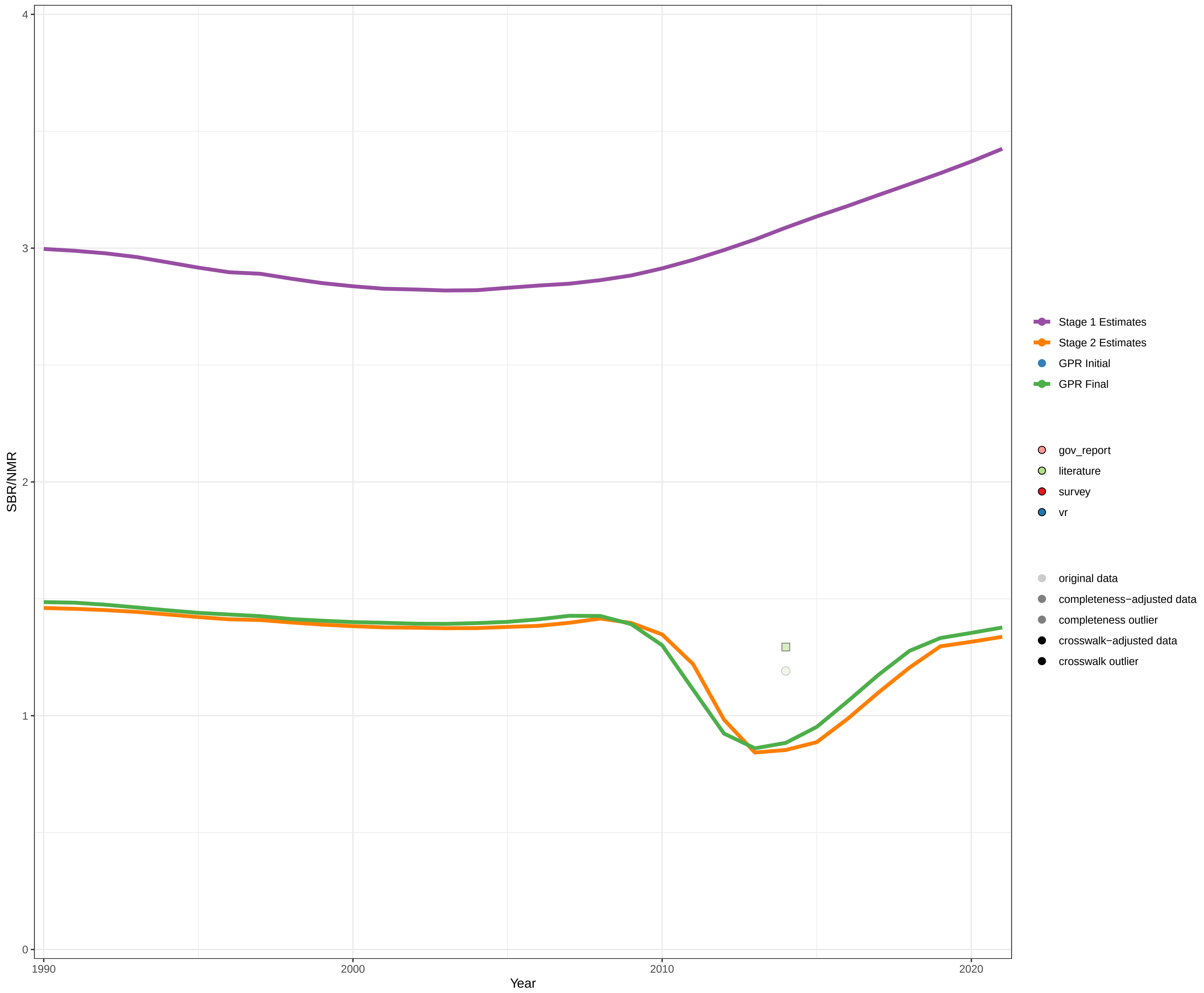

Congo COG Central Sub−Saharan Africa Stillbirth Rate / Neonatal Mortality Rate (SBR/NMR) − 28 weeks
lambda = 1 ; zeta = 0.6 ; scale = 15 ; amp = 0.028

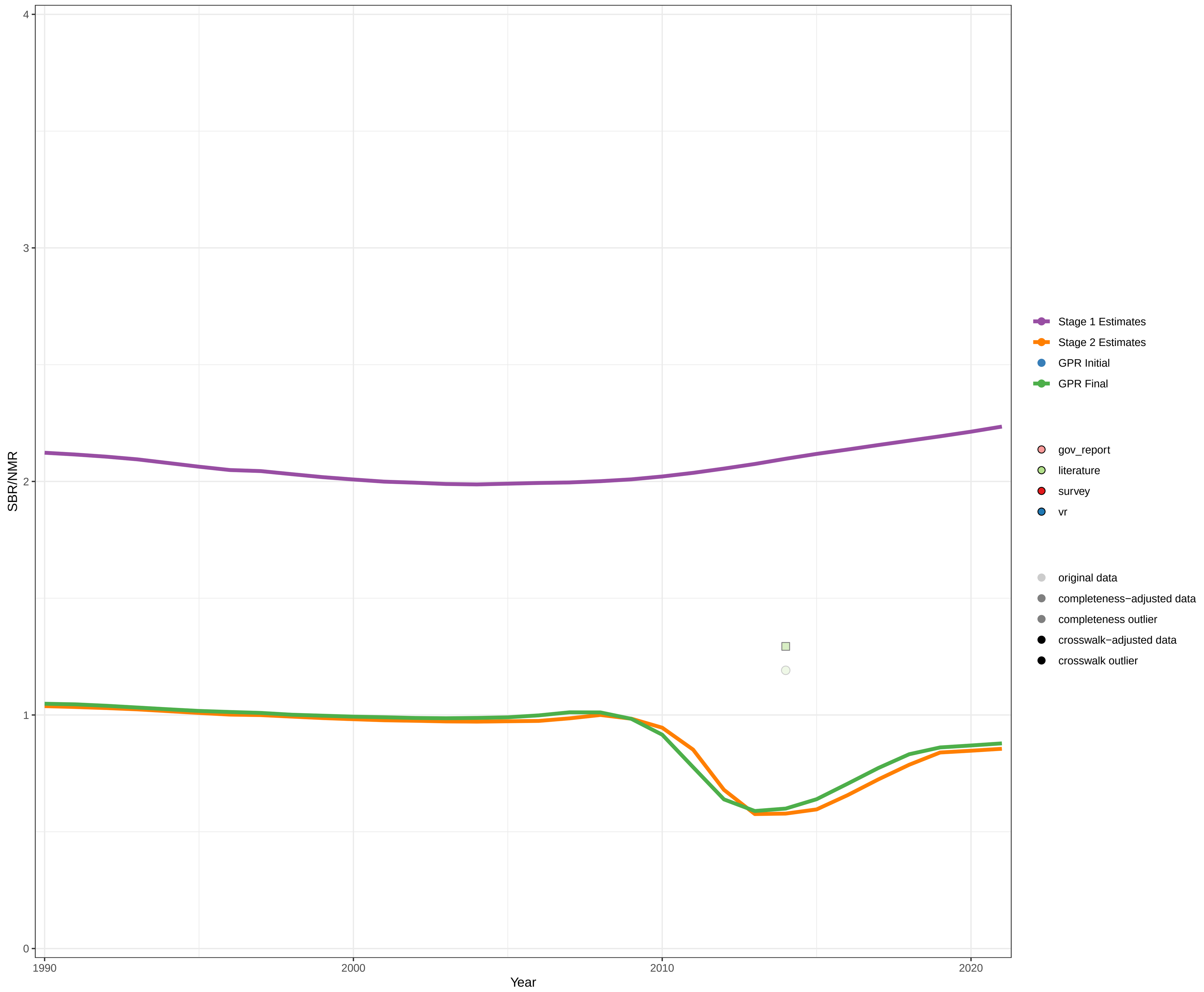

Definition	Years	Outlier	Source.Type	Title
28_weeks	2014	1	Sci Lit	Population−based rates, timing, and causes of maternal deaths, stillbirths, and neonatal deaths in south Asia and sub−Saharan Africa: a multi−country prospective cohort study

Congo COG Central Sub−Saharan Africa Stillbirth Rate (SBR) − 20 weeks
lambda = 1 ; zeta = 0.6 ; scale = 15 ; amp = 0.028

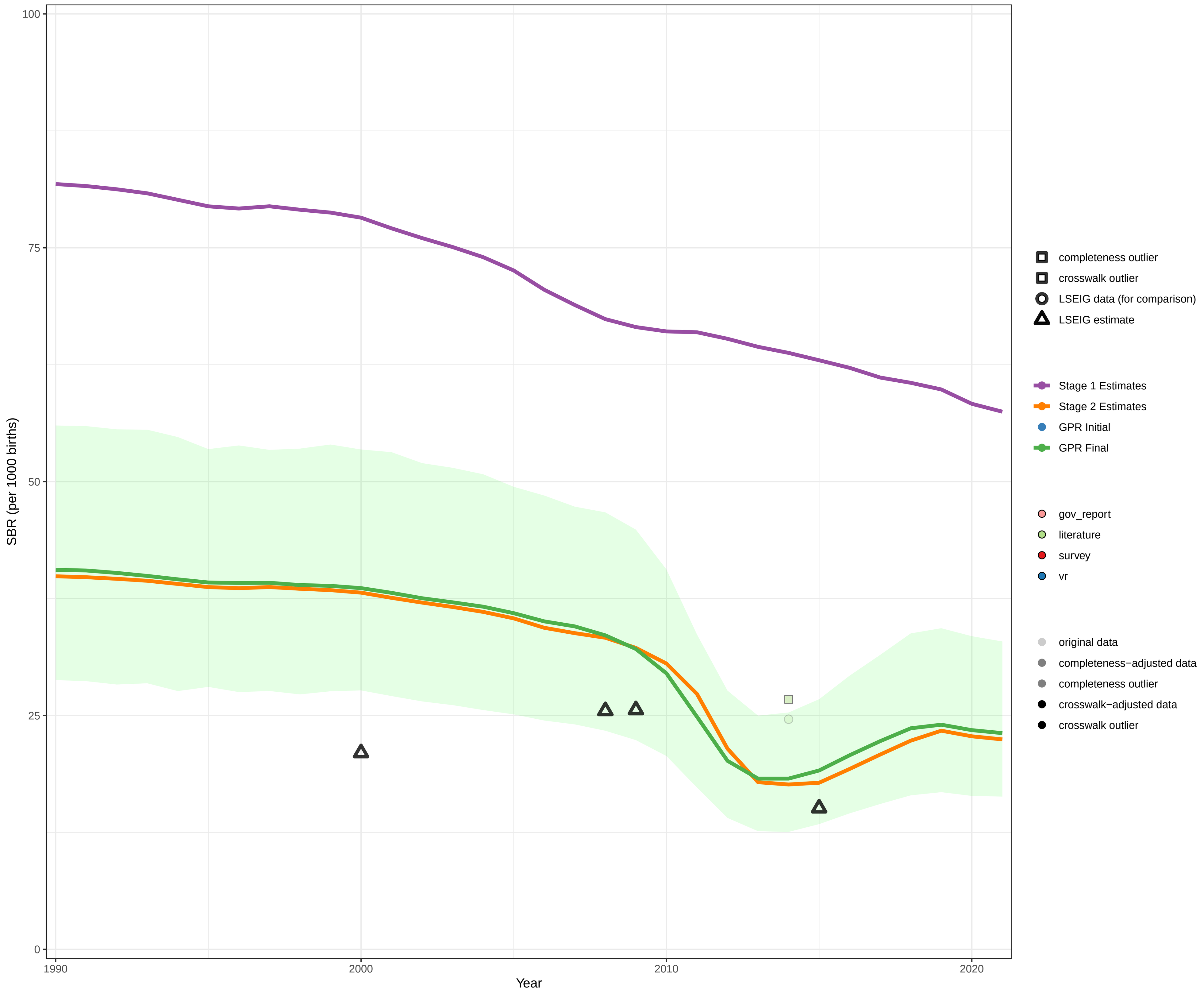

Congo COG Central Sub−Saharan Africa Stillbirth Rate (SBR) − 28 weeks
lambda = 1 ; zeta = 0.6 ; scale = 15 ; amp = 0.028

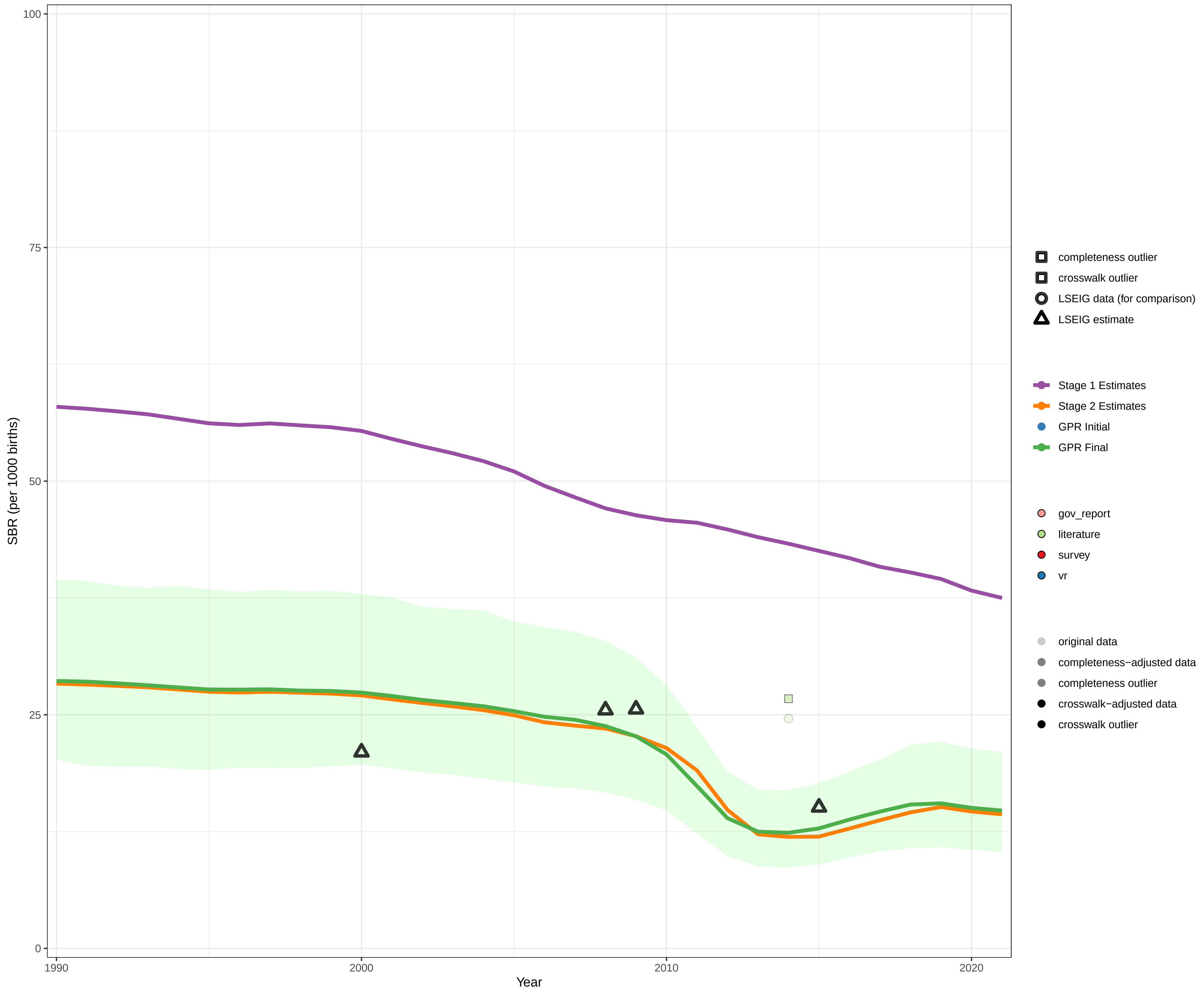

Year	Total Stillbirths (in thousands)	Total Stillbirths (in thousands)	SBR (per 1000 births)	SBR (per 1000 births)	SBR/NMR	SBR/NMR
	20 weeks	28 weeks	20 weeks	28 weeks	20 weeks	28 weeks
1990	3.9 (2.7 to 5.5)	2.7 (1.9 to 3.8)	40.6 (28.8 to 56.0)	28.6 (20.2 to 39.4)	1.49 (1.05 to 2.05)	1.05 (0.74 to 1.44)
1995	4.3 (3.0 to 5.9)	3.0 (2.0 to 4.2)	39.2 (28.1 to 53.5)	27.7 (19.1 to 38.4)	1.44 (1.03 to 1.96)	1.02 (0.70 to 1.41)
2000	4.8 (3.4 to 6.7)	3.4 (2.4 to 4.7)	38.6 (27.7 to 53.4)	27.4 (19.7 to 37.9)	1.40 (1.00 to 1.94)	0.99 (0.71 to 1.38)
2005	5.1 (3.5 to 7.2)	3.6 (2.5 to 5.0)	35.9 (25.1 to 49.4)	25.4 (17.8 to 35.0)	1.40 (0.98 to 1.93)	0.99 (0.69 to 1.36)
2010	4.6 (3.2 to 6.4)	3.2 (2.3 to 4.4)	29.5 (20.7 to 40.6)	20.8 (14.8 to 28.2)	1.30 (0.91 to 1.79)	0.92 (0.65 to 1.24)
2015	2.8 (2.0 to 4.0)	1.9 (1.3 to 2.6)	19.1 (13.4 to 26.7)	12.8 (9.0 to 17.7)	0.95 (0.67 to 1.33)	0.64 (0.45 to 0.88)
2020	3.1 (2.2 to 4.5)	2.0 (1.4 to 2.8)	23.4 (16.4 to 33.5)	15.0 (10.6 to 21.4)	1.35 (0.95 to 1.93)	0.87 (0.61 to 1.24)
2021	3.0 (2.1 to 4.4)	1.9 (1.3 to 2.8)	23.1 (16.3 to 32.9)	14.7 (10.3 to 21.0)	1.38 (0.97 to 1.96)	0.88 (0.62 to 1.25)

Democratic Republic of the Congo   COD   Central Sub−Saharan Africa   Stillbirth Rate / Neonatal Mortality Rate (SBR/NMR) − 20 weeks
lambda = 0.8 ; zeta = 0.7 ; scale = 15 ; amp = 0.028

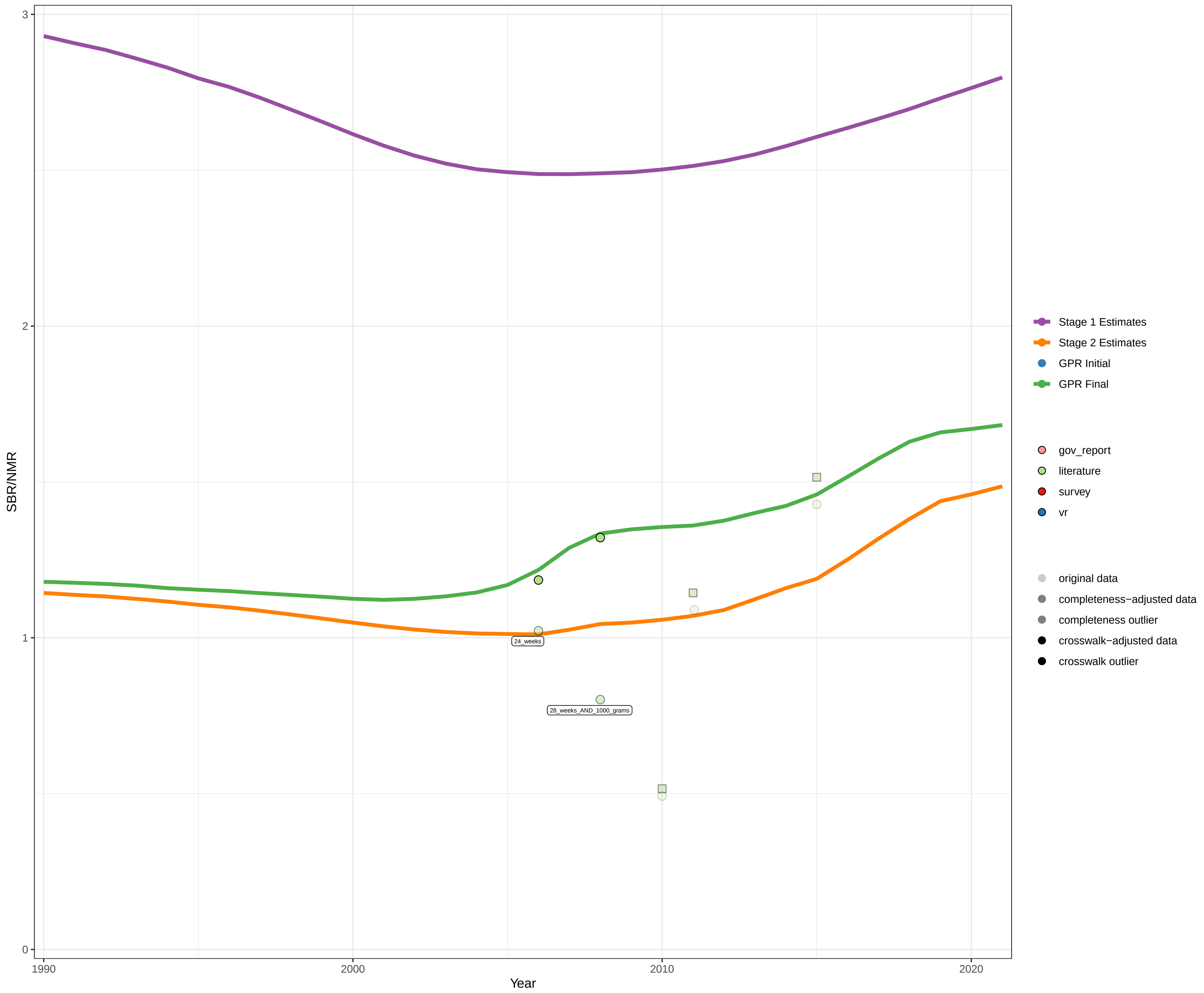

Democratic Republic of the Congo   COD   Central Sub−Saharan Africa   Stillbirth Rate / Neonatal Mortality Rate (SBR/NMR) − 28 weeks
lambda = 0.8 ; zeta = 0.7 ; scale = 15 ; amp = 0.028

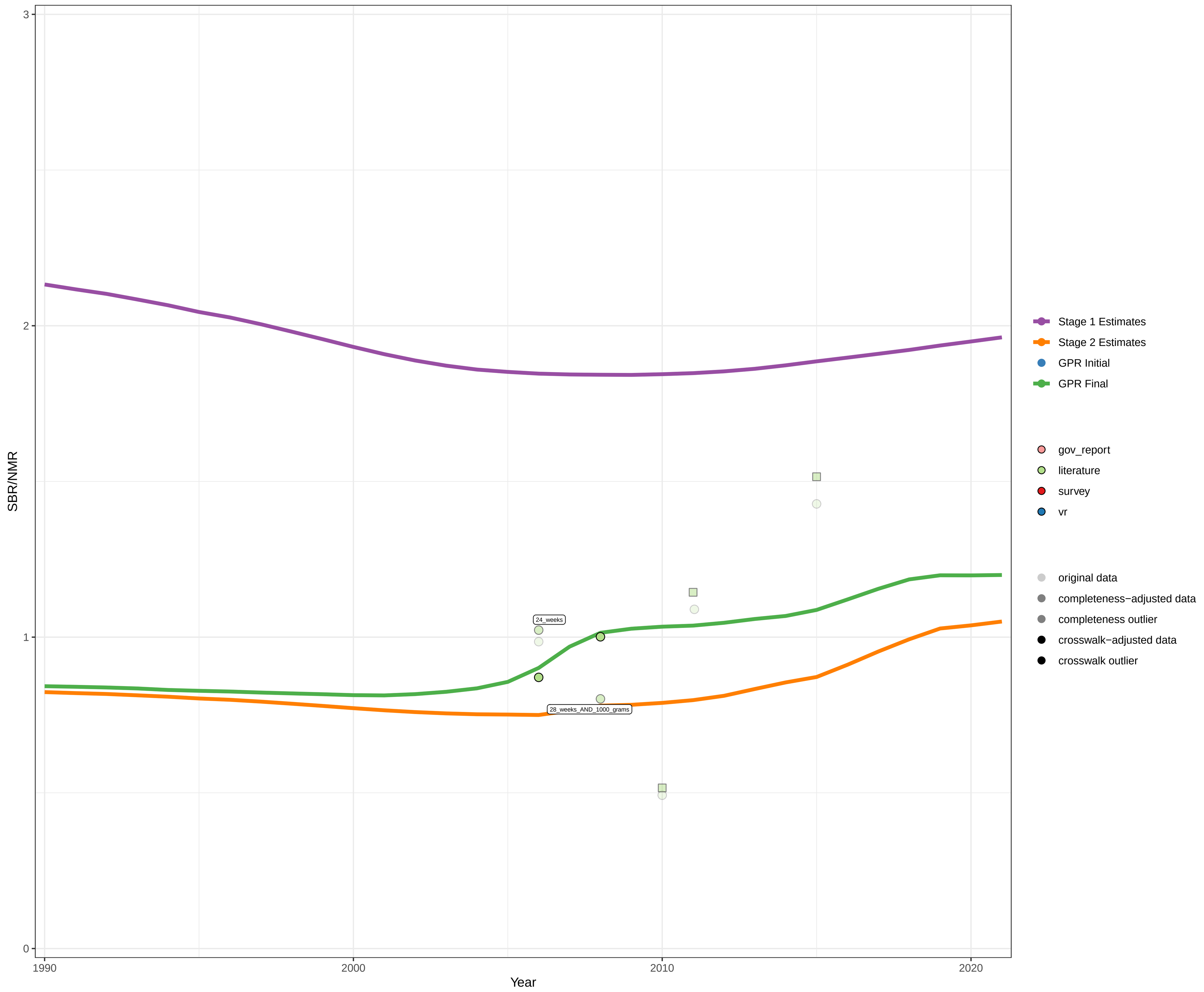

Definition	Years	Outlier	Source.Type	Title
24_weeks	2006	0	Sci Lit	Stillbirth and early neonatal mortality in rural Central Africa
28_weeks_AND_1000_grams	2008	0	Sci Lit	Epidemiology of stillbirth in low−middle income countries: A Global Network Study
20_weeks	2015	1	Sci Lit	Global Network for Women's and Children's Health Research: probable causes of stillbirth in low− and middle−income countries using a prospectively defined classification system
28_weeks	2010	1	Sci Lit	[Perinatal mortality: extent and causes in Lubumbashi, Democratic Republic of Congo]
28_weeks_OR_1000_grams	2011	1	Sci Lit	Maternal complications and perinatal mortality: findings of the World Health Organization Multicountry Survey on Maternal and Newborn Health

Democratic Republic of the Congo   COD   Central Sub−Saharan Africa   Stillbirth Rate (SBR) − 20 weeks
lambda = 0.8 ; zeta = 0.7 ; scale = 15 ; amp = 0.028

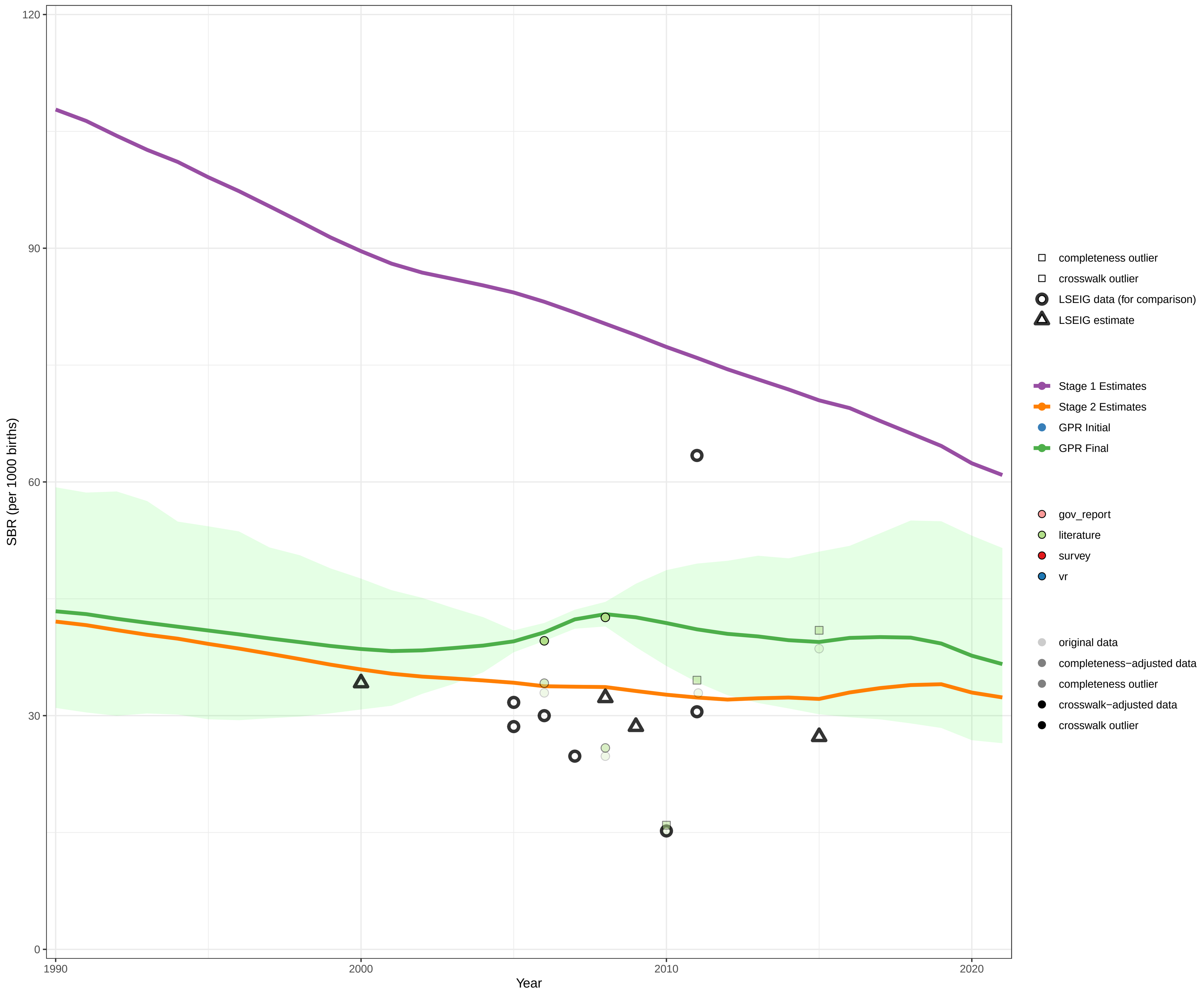

Democratic Republic of the Congo   COD   Central Sub−Saharan Africa   Stillbirth Rate (SBR) − 28 weeks
lambda = 0.8 ; zeta = 0.7 ; scale = 15 ; amp = 0.028

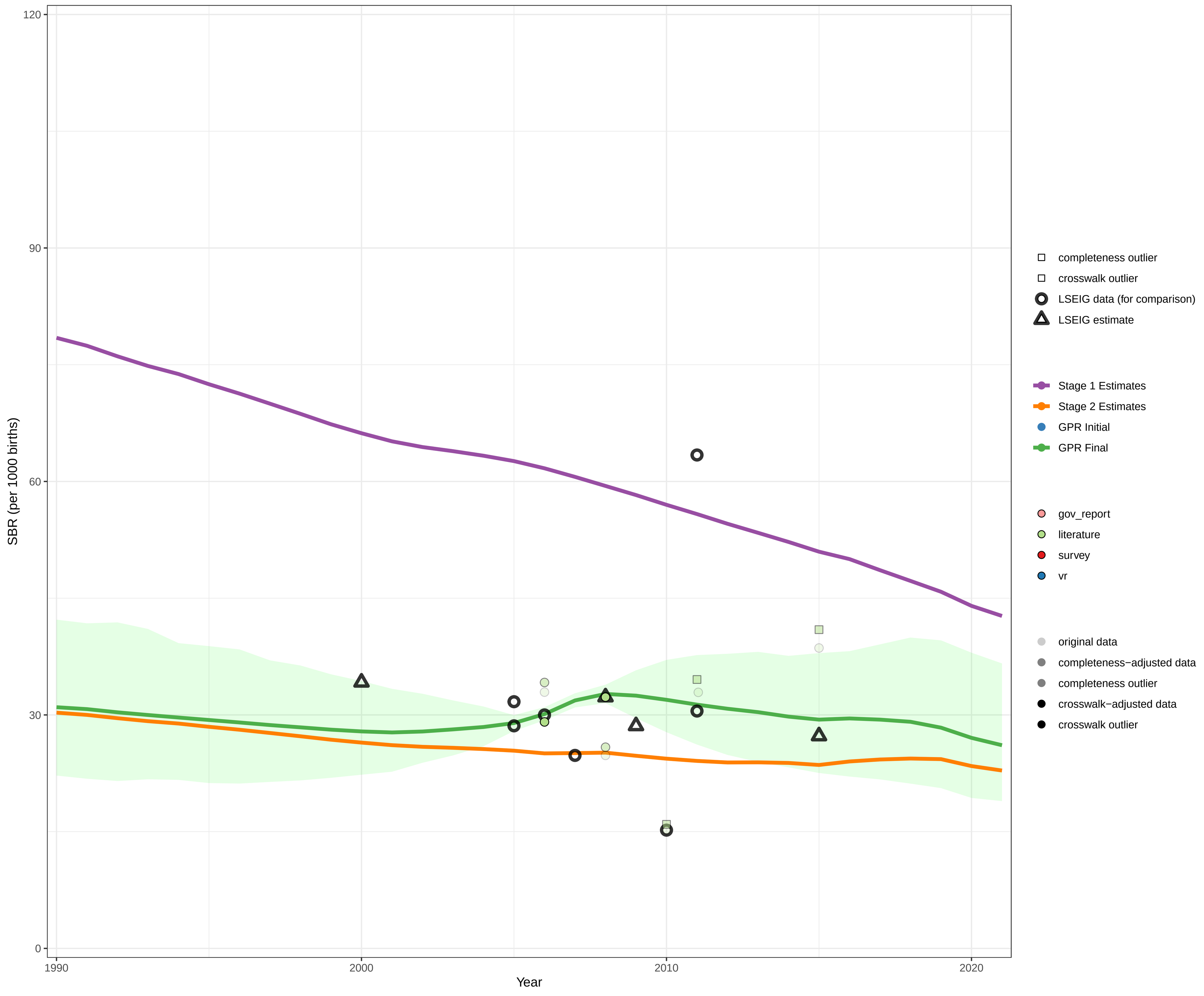

Year	Total Stillbirths (in thousands)	Total Stillbirths (in thousands)	SBR (per 1000 births)	SBR (per 1000 births)	SBR/NMR	SBR/NMR
	20 weeks	28 weeks	20 weeks	28 weeks	20 weeks	28 weeks
1990	82.2 (57.9 to 114.1)	57.9 (41.1 to 79.8)	43.4 (31.0 to 59.3)	31.0 (22.2 to 42.2)	1.18 (0.84 to 1.61)	0.84 (0.60 to 1.15)
1995	85.9 (61.2 to 115.5)	60.9 (43.7 to 81.3)	40.9 (29.5 to 54.3)	29.3 (21.2 to 38.8)	1.15 (0.83 to 1.53)	0.83 (0.60 to 1.10)
2000	90.6 (71.7 to 112.9)	64.8 (51.6 to 80.4)	38.6 (30.8 to 47.6)	27.9 (22.3 to 34.4)	1.13 (0.90 to 1.39)	0.81 (0.65 to 1.00)
2005	104.7 (100.8 to 108.6)	75.8 (73.1 to 78.6)	39.5 (38.1 to 40.9)	29.0 (27.9 to 30.0)	1.17 (1.13 to 1.21)	0.86 (0.83 to 0.89)
2010	122.0 (105.3 to 142.8)	92.1 (79.7 to 107.4)	41.9 (36.4 to 48.7)	31.9 (27.8 to 37.1)	1.36 (1.18 to 1.58)	1.03 (0.90 to 1.20)
2015	119.5 (90.4 to 156.4)	88.1 (67.0 to 114.7)	39.5 (30.2 to 51.1)	29.4 (22.5 to 37.9)	1.46 (1.12 to 1.89)	1.09 (0.83 to 1.40)
2020	112.3 (79.0 to 160.6)	79.7 (56.4 to 113.1)	37.7 (26.8 to 53.1)	27.1 (19.3 to 38.0)	1.67 (1.19 to 2.35)	1.20 (0.86 to 1.68)
2021	108.7 (77.6 to 155.1)	76.6 (55.1 to 108.6)	36.6 (26.5 to 51.5)	26.1 (18.9 to 36.6)	1.68 (1.22 to 2.37)	1.20 (0.87 to 1.68)

Equatorial Guinea GNQ Central Sub−Saharan Africa Stillbirth Rate / Neonatal Mortality Rate (SBR/NMR) − 20 weeks
lambda = 1 ; zeta = 0.6 ; scale = 15 ; amp = 0.028

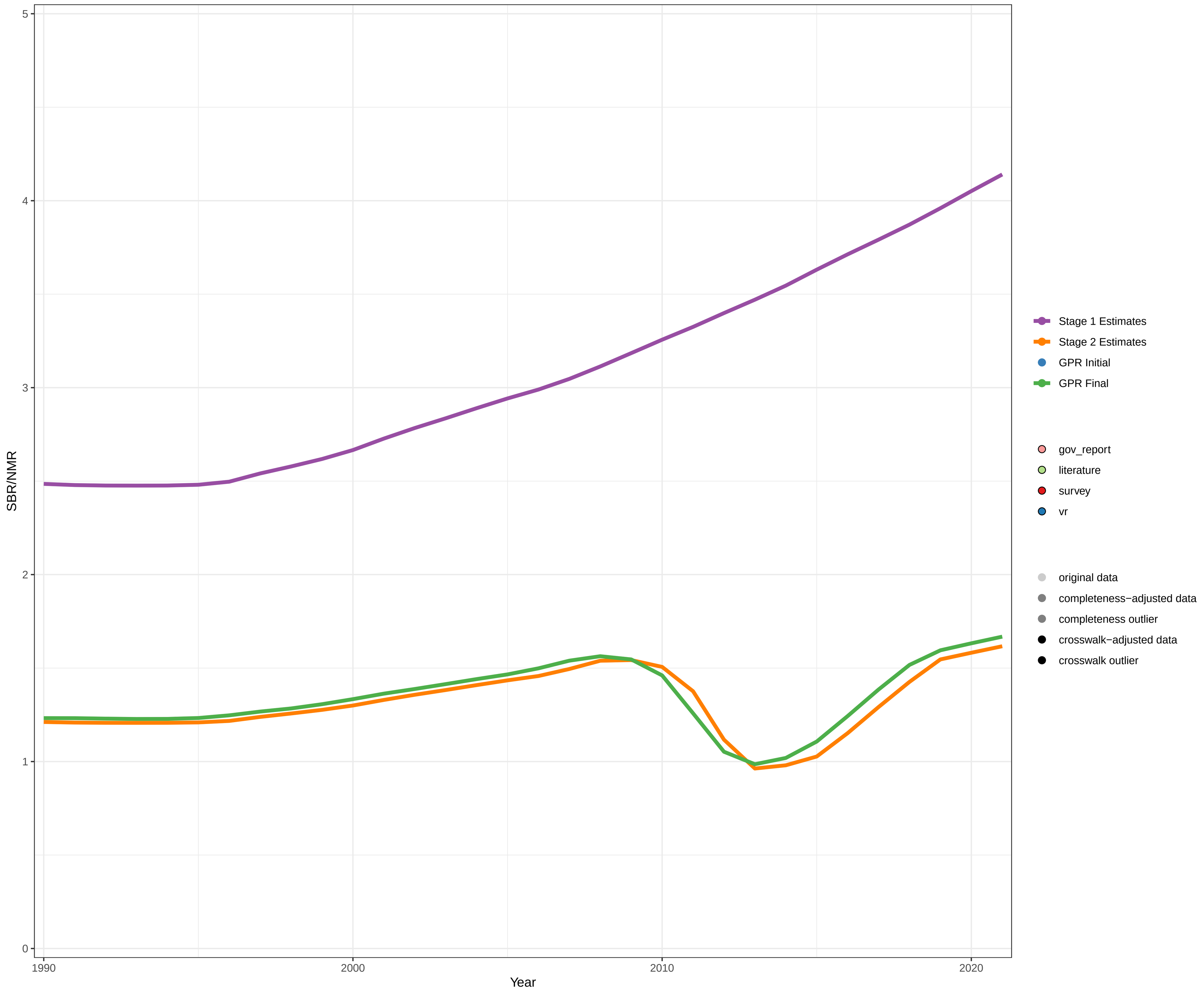

Equatorial Guinea GNQ Central Sub−Saharan Africa Stillbirth Rate / Neonatal Mortality Rate (SBR/NMR) − 28 weeks
lambda = 1 ; zeta = 0.6 ; scale = 15 ; amp = 0.028

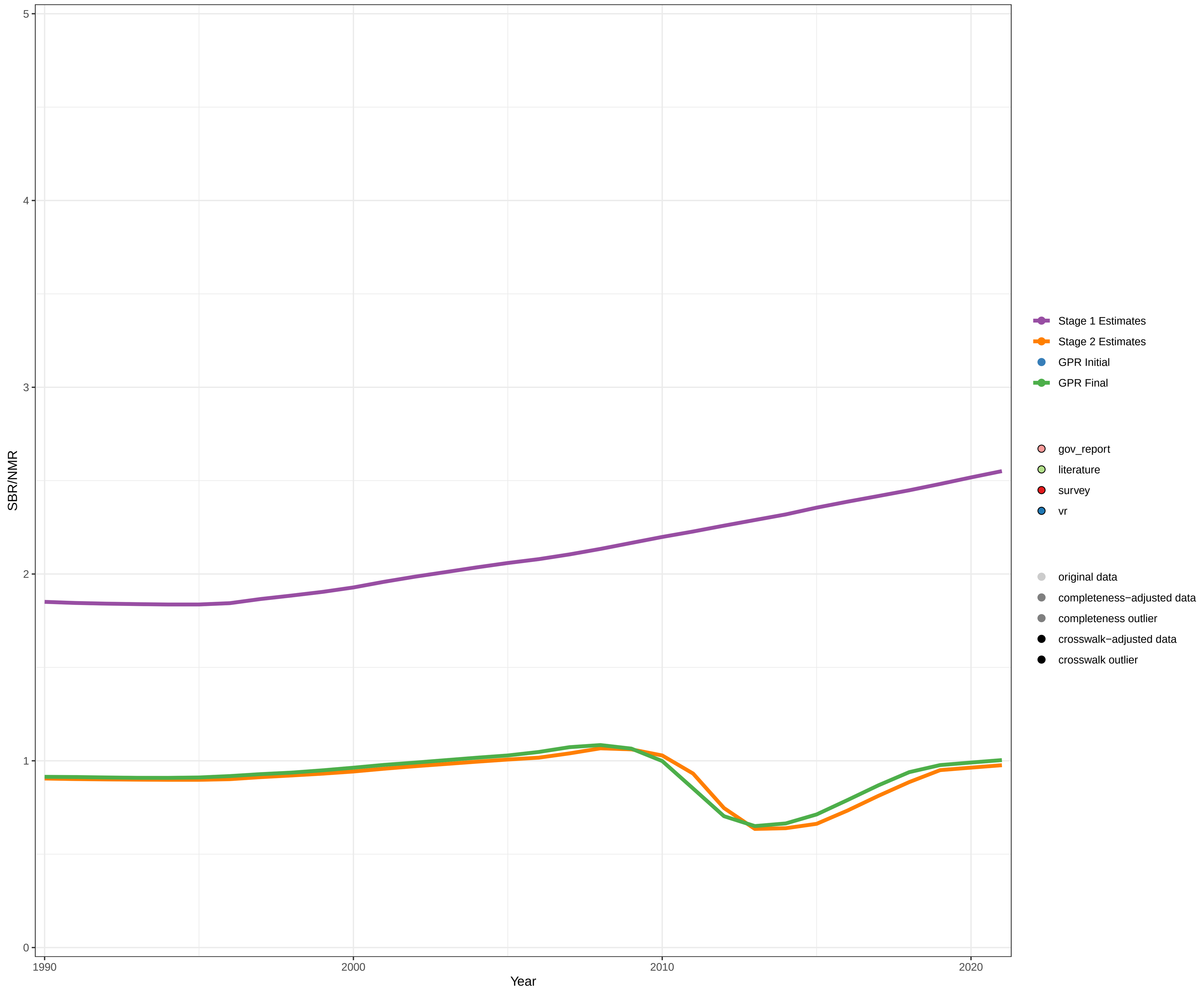

Definition	Years	Outlier	Source.Type	Title

Equatorial Guinea GNQ Central Sub−Saharan Africa Stillbirth Rate (SBR) − 20 weeks
lambda = 1 ; zeta = 0.6 ; scale = 15 ; amp = 0.028

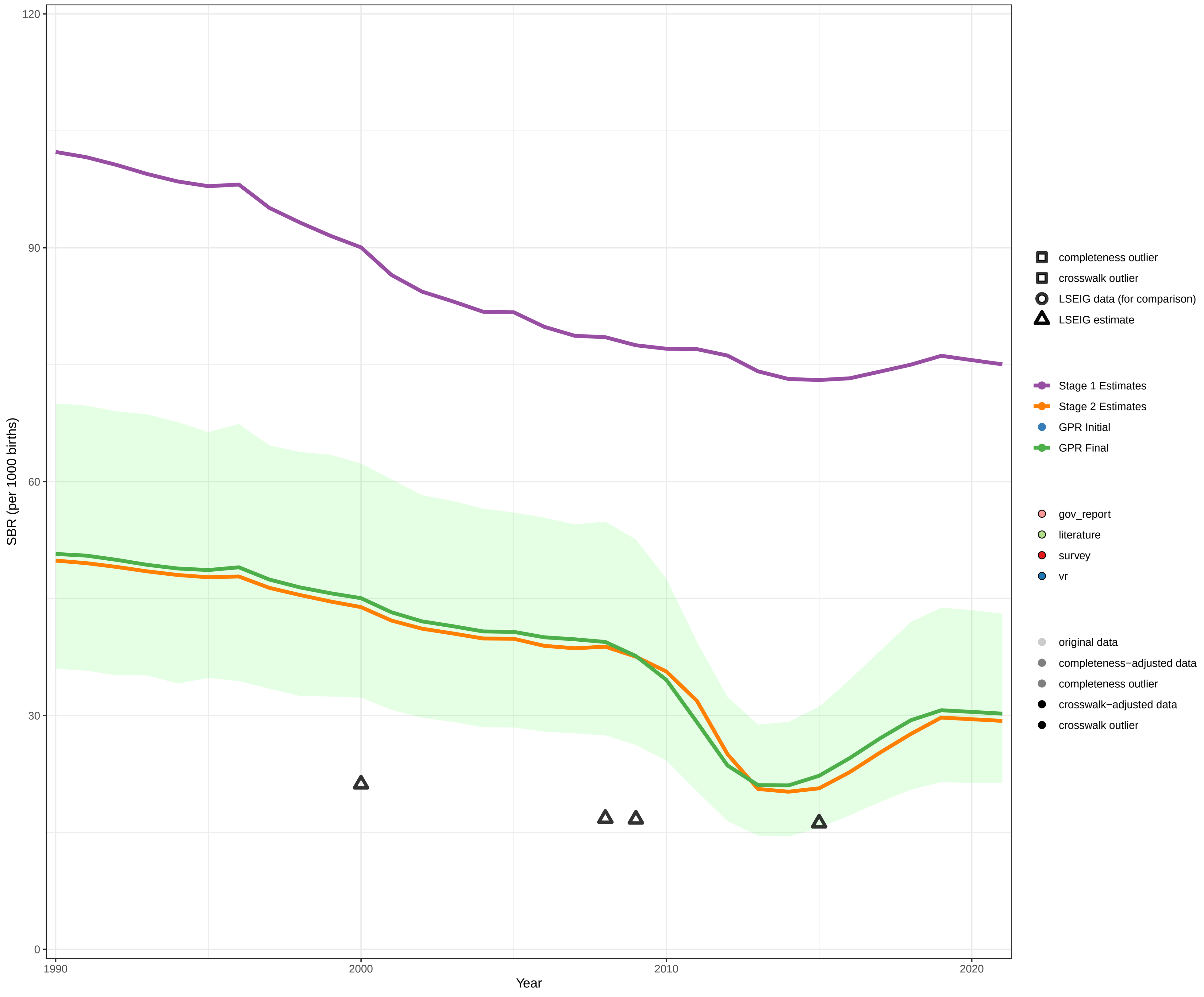

Equatorial Guinea GNQ Central Sub−Saharan Africa Stillbirth Rate (SBR) − 28 weeks
lambda = 1 ; zeta = 0.6 ; scale = 15 ; amp = 0.028

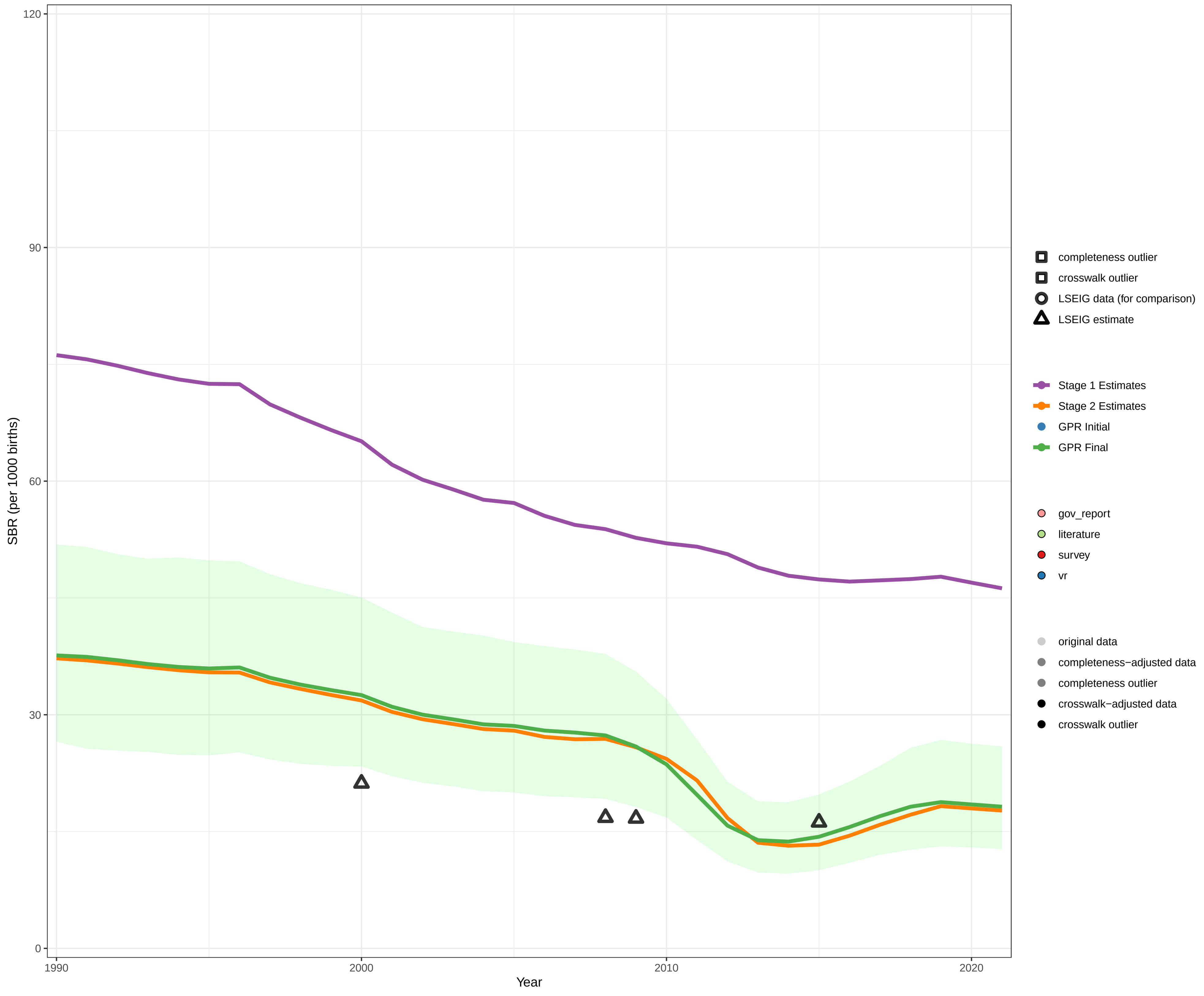

Year	Total Stillbirths (in thousands)	Total Stillbirths (in thousands)	SBR (per 1000 births)	SBR (per 1000 births)	SBR/NMR	SBR/NMR
	20 weeks	28 weeks	20 weeks	28 weeks	20 weeks	28 weeks
1990	1.1 (0.8 to 1.5)	0.8 (0.6 to 1.1)	50.7 (36.0 to 70.0)	37.6 (26.5 to 51.9)	1.23 (0.87 to 1.70)	0.91 (0.64 to 1.26)
1995	1.2 (0.9 to 1.7)	0.9 (0.6 to 1.3)	48.7 (34.8 to 66.4)	35.9 (24.8 to 49.8)	1.23 (0.88 to 1.68)	0.91 (0.63 to 1.26)
2000	1.3 (0.9 to 1.9)	0.9 (0.7 to 1.3)	45.0 (32.3 to 62.3)	32.5 (23.4 to 45.1)	1.33 (0.96 to 1.85)	0.96 (0.69 to 1.33)
2005	1.4 (1.0 to 1.9)	1.0 (0.7 to 1.3)	40.7 (28.5 to 56.0)	28.6 (20.0 to 39.4)	1.47 (1.02 to 2.02)	1.03 (0.72 to 1.42)
2010	1.3 (0.9 to 1.9)	0.9 (0.6 to 1.2)	34.6 (24.2 to 47.6)	23.6 (16.8 to 32.1)	1.46 (1.02 to 2.01)	1.00 (0.71 to 1.36)
2015	0.9 (0.6 to 1.2)	0.6 (0.4 to 0.8)	22.3 (15.6 to 31.1)	14.3 (10.0 to 19.8)	1.11 (0.77 to 1.55)	0.71 (0.50 to 0.98)
2020	1.2 (0.8 to 1.7)	0.7 (0.5 to 1.0)	30.5 (21.3 to 43.5)	18.5 (13.0 to 26.3)	1.63 (1.14 to 2.33)	0.99 (0.69 to 1.41)
2021	1.2 (0.8 to 1.7)	0.7 (0.5 to 1.0)	30.2 (21.4 to 43.1)	18.2 (12.7 to 25.9)	1.67 (1.18 to 2.38)	1.00 (0.70 to 1.43)

Gabon GAB Central Sub−Saharan Africa Stillbirth Rate / Neonatal Mortality Rate (SBR/NMR) − 20 weeks
lambda = 0.8 ; zeta = 0.7 ; scale = 15 ; amp = 0.028

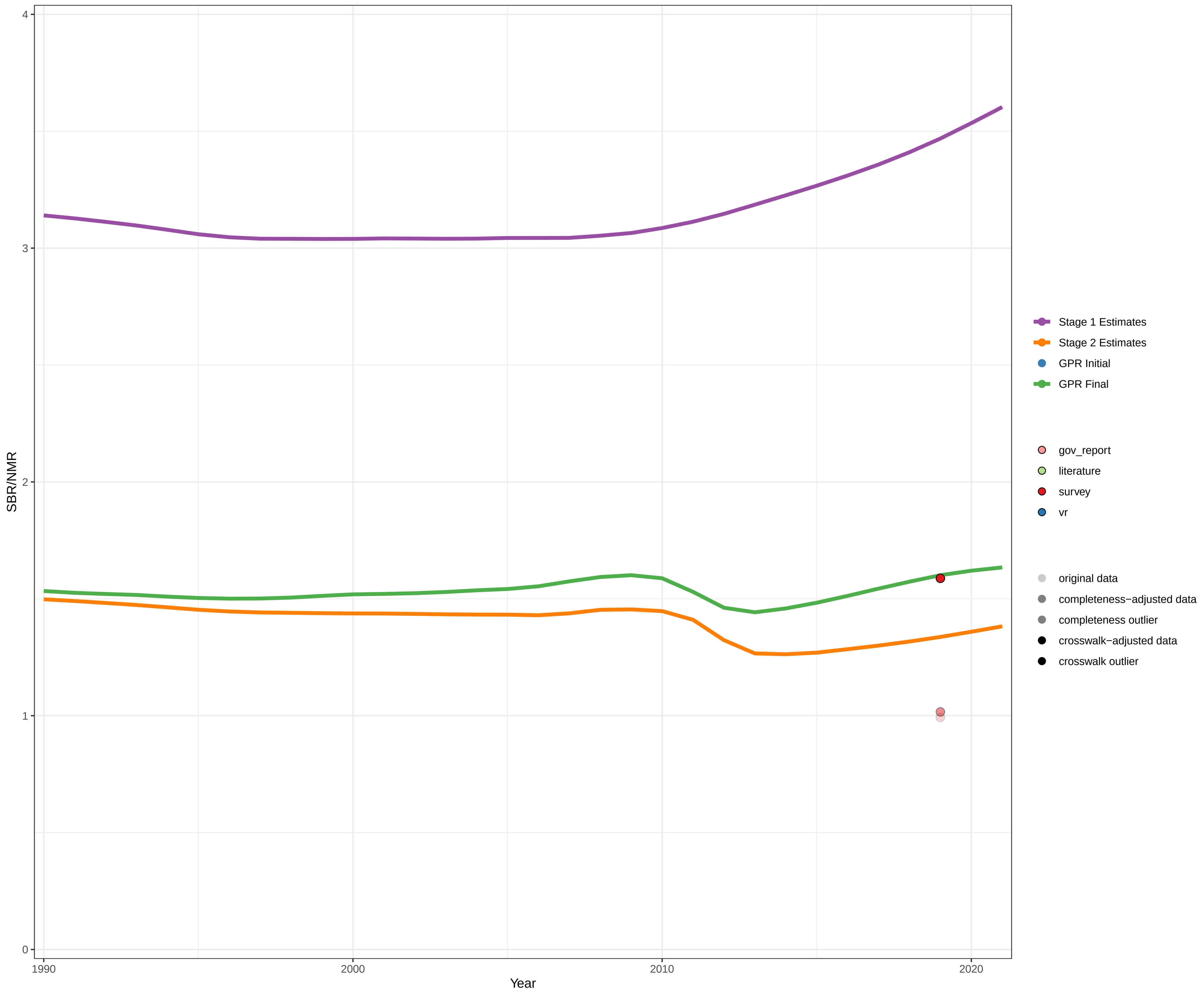

Gabon GAB Central Sub−Saharan Africa Stillbirth Rate / Neonatal Mortality Rate (SBR/NMR) − 28 weeks
lambda = 0.8 ; zeta = 0.7 ; scale = 15 ; amp = 0.028

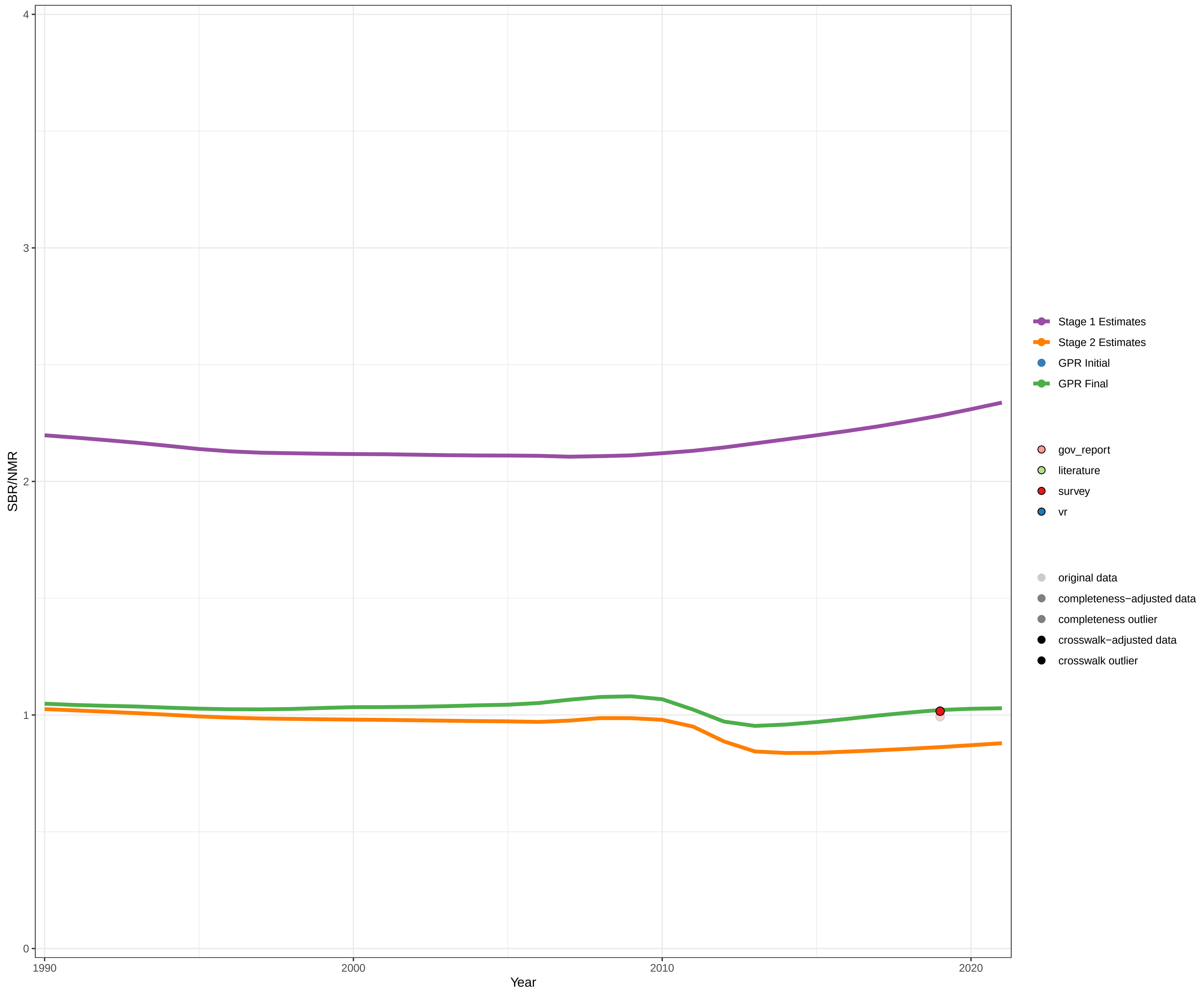

Definition	Years	Outlier	Source.Type	Title
28_weeks	2019	0	Survey	Gabon Demographic and Health Survey 2019−2021

Gabon GAB Central Sub−Saharan Africa Stillbirth Rate (SBR) − 20 weeks
lambda = 0.8 ; zeta = 0.7 ; scale = 15 ; amp = 0.028

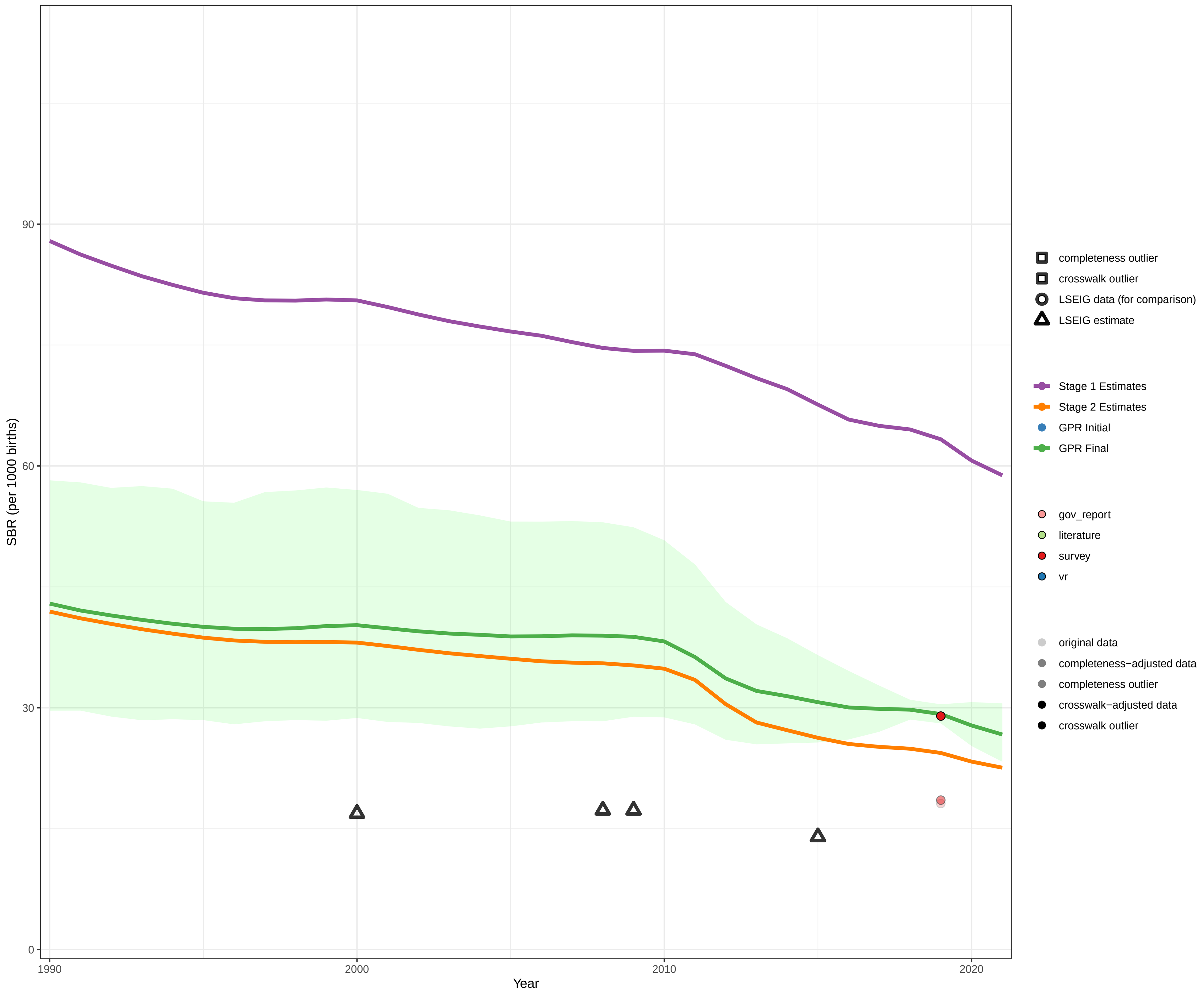

Gabon GAB Central Sub−Saharan Africa Stillbirth Rate (SBR) − 28 weeks
lambda = 0.8 ; zeta = 0.7 ; scale = 15 ; amp = 0.028

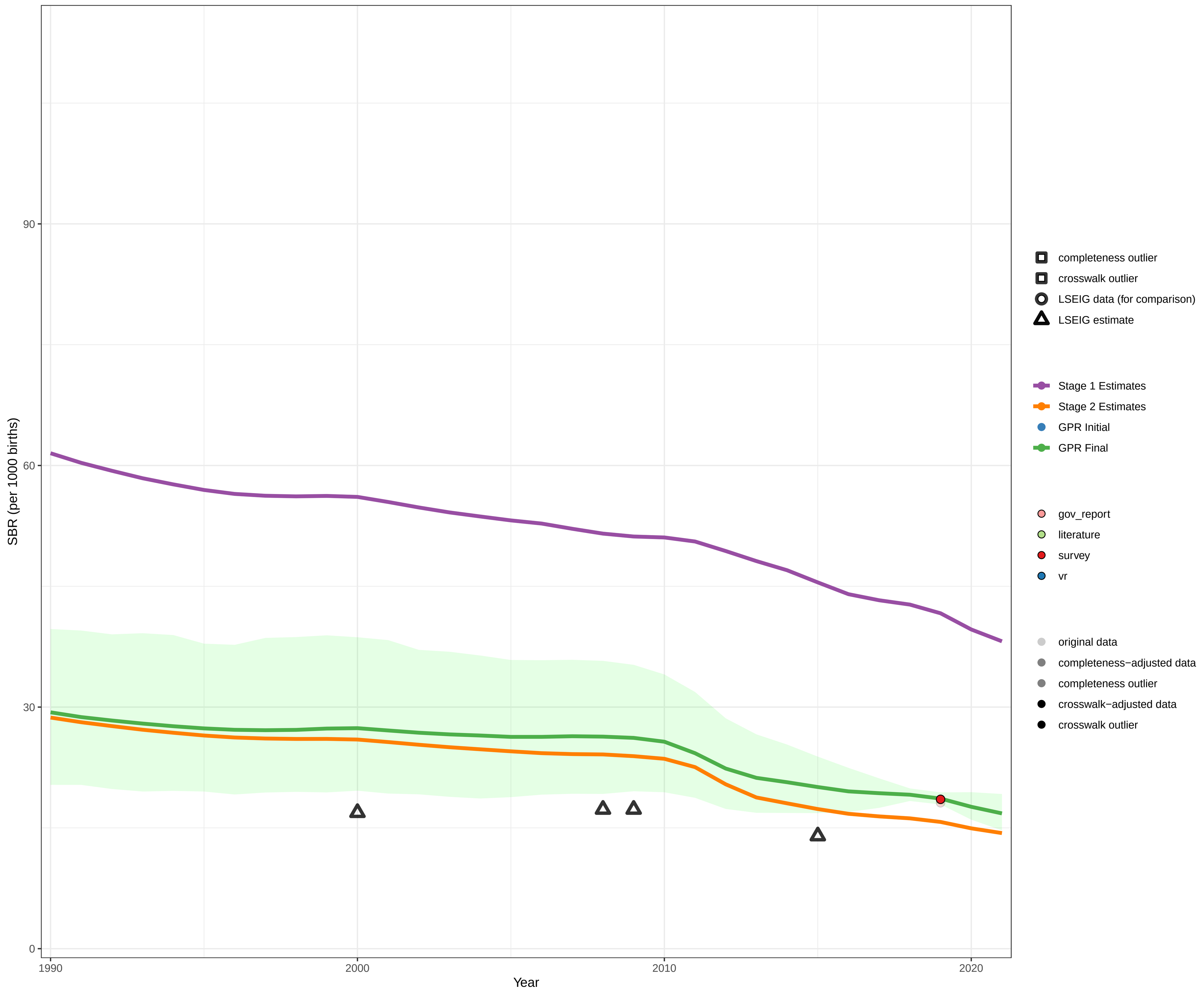

Year	Total Stillbirths (in thousands)	Total Stillbirths (in thousands)	SBR (per 1000 births)	SBR (per 1000 births)	SBR/NMR	SBR/NMR
	20 weeks	28 weeks	20 weeks	28 weeks	20 weeks	28 weeks
1990	1.6 (1.1 to 2.2)	1.1 (0.7 to 1.5)	42.9 (29.6 to 58.2)	29.3 (20.3 to 39.7)	1.53 (1.06 to 2.08)	1.05 (0.73 to 1.42)
1995	1.6 (1.2 to 2.3)	1.1 (0.8 to 1.6)	40.0 (28.5 to 55.6)	27.4 (19.5 to 37.9)	1.50 (1.07 to 2.09)	1.03 (0.73 to 1.42)
2000	1.8 (1.3 to 2.6)	1.2 (0.8 to 1.7)	40.2 (28.7 to 57.0)	27.4 (19.6 to 38.7)	1.52 (1.08 to 2.15)	1.03 (0.74 to 1.46)
2005	1.8 (1.3 to 2.5)	1.2 (0.9 to 1.7)	38.8 (27.7 to 53.1)	26.3 (18.8 to 35.9)	1.54 (1.10 to 2.11)	1.04 (0.75 to 1.42)
2010	1.9 (1.4 to 2.5)	1.3 (0.9 to 1.7)	38.2 (28.8 to 50.8)	25.7 (19.4 to 34.1)	1.59 (1.20 to 2.11)	1.07 (0.81 to 1.41)
2015	1.5 (1.2 to 1.8)	1.0 (0.8 to 1.2)	30.7 (25.7 to 36.5)	20.1 (16.8 to 23.9)	1.48 (1.24 to 1.77)	0.97 (0.81 to 1.15)
2020	1.3 (1.1 to 1.4)	0.8 (0.7 to 0.9)	27.8 (25.3 to 30.7)	17.6 (16.0 to 19.4)	1.62 (1.47 to 1.79)	1.03 (0.93 to 1.13)
2021	1.2 (1.0 to 1.4)	0.7 (0.6 to 0.9)	26.7 (23.3 to 30.6)	16.8 (14.7 to 19.2)	1.63 (1.43 to 1.87)	1.03 (0.90 to 1.18)

Eastern Sub−Saharan Africa R19 Eastern Sub−Saharan Africa Stillbirth Rate / Neonatal Mortality Rate (SBR/NMR) − 20 weeks
lambda = 1 ; zeta = 0.6 ; scale = 15 ; amp = 0.028

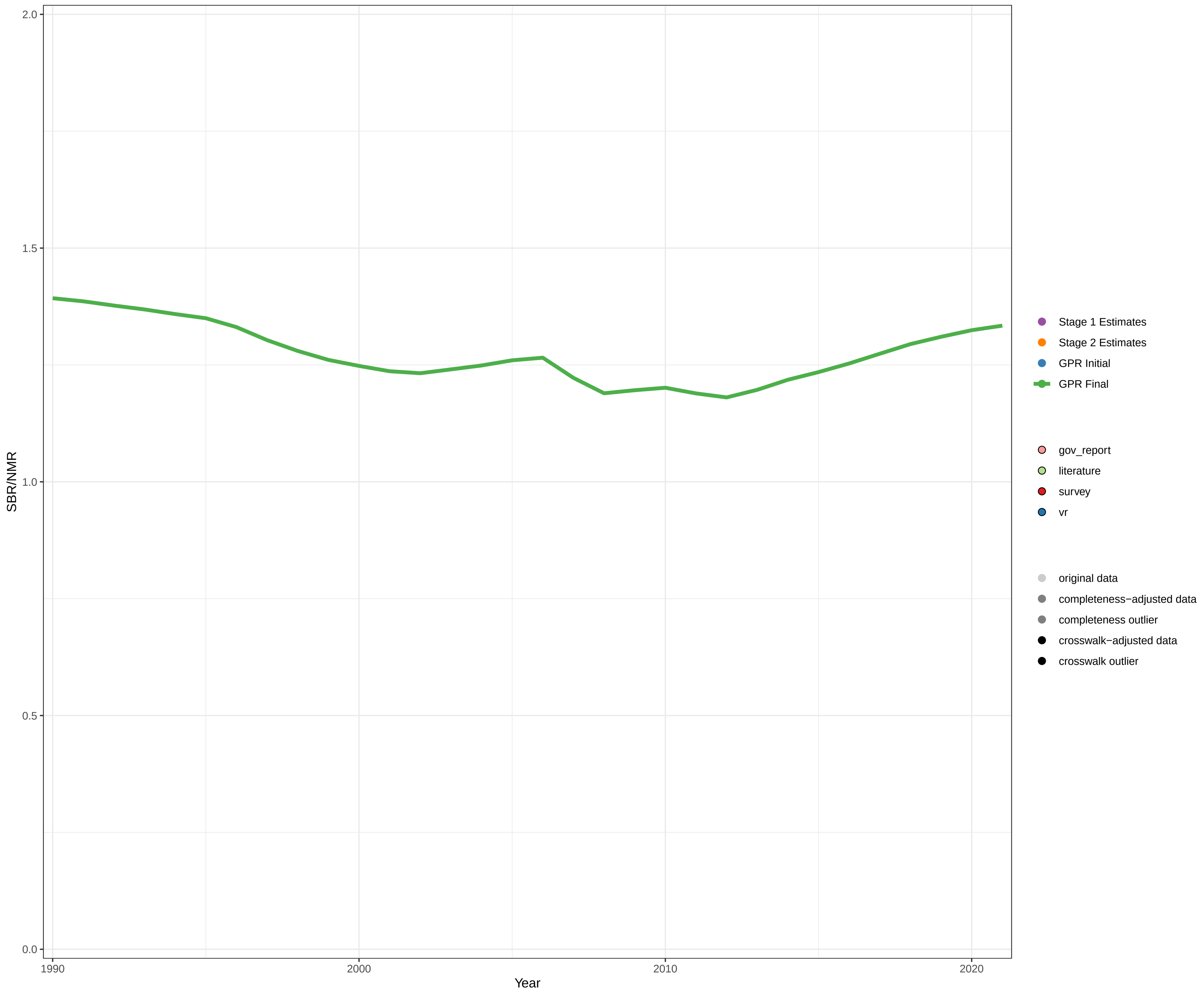

Eastern Sub−Saharan Africa R19 Eastern Sub−Saharan Africa Stillbirth Rate / Neonatal Mortality Rate (SBR/NMR) − 28 weeks
lambda = 1 ; zeta = 0.6 ; scale = 15 ; amp = 0.028

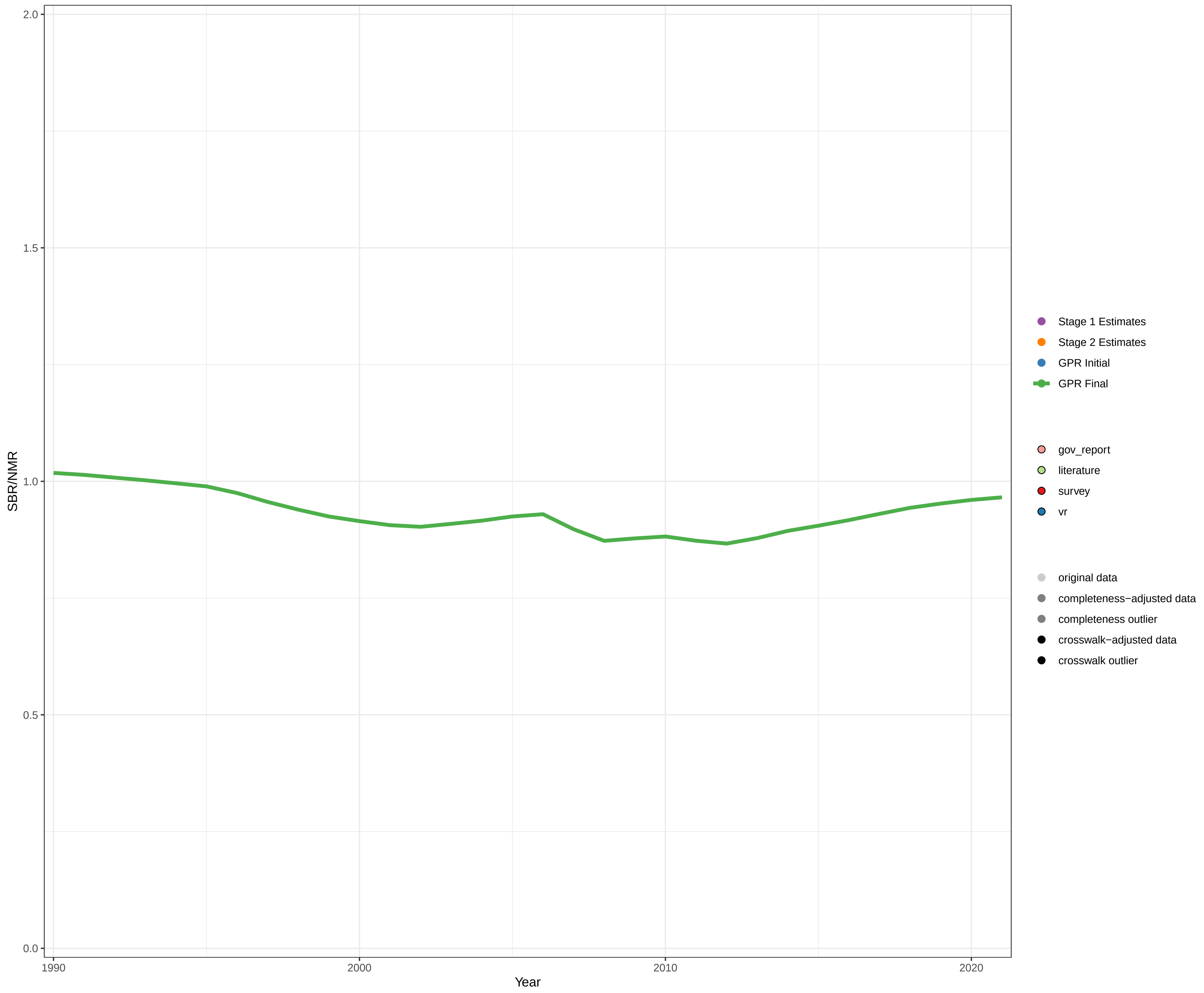

Definition	Years	Outlier	Source.Type	Title

Eastern Sub−Saharan Africa R19 Eastern Sub−Saharan Africa Stillbirth Rate (SBR) − 20 weeks
lambda = 1 ; zeta = 0.6 ; scale = 15 ; amp = 0.028

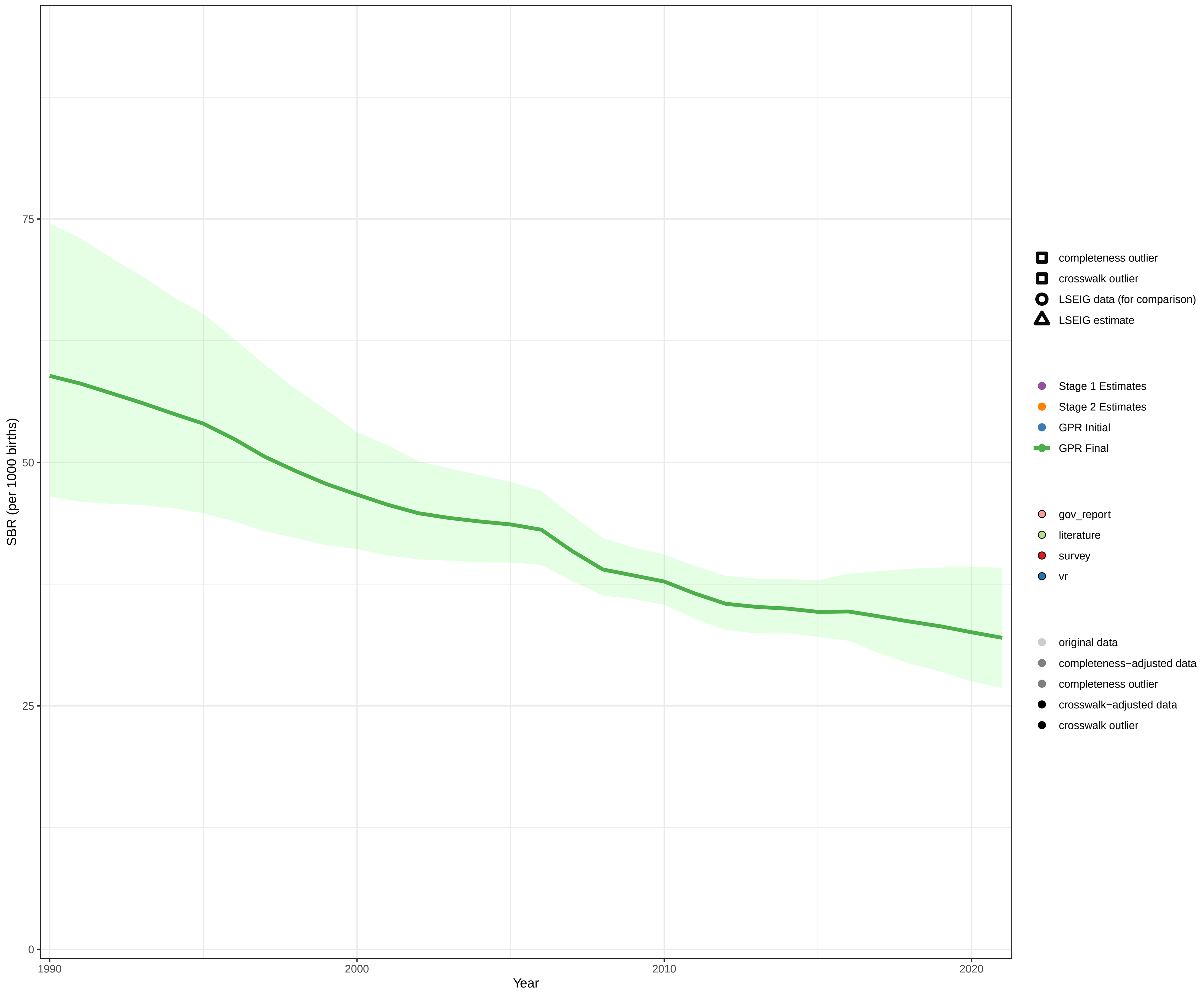

Eastern Sub−Saharan Africa R19 Eastern Sub−Saharan Africa Stillbirth Rate (SBR) − 28 weeks
lambda = 1 ; zeta = 0.6 ; scale = 15 ; amp = 0.028

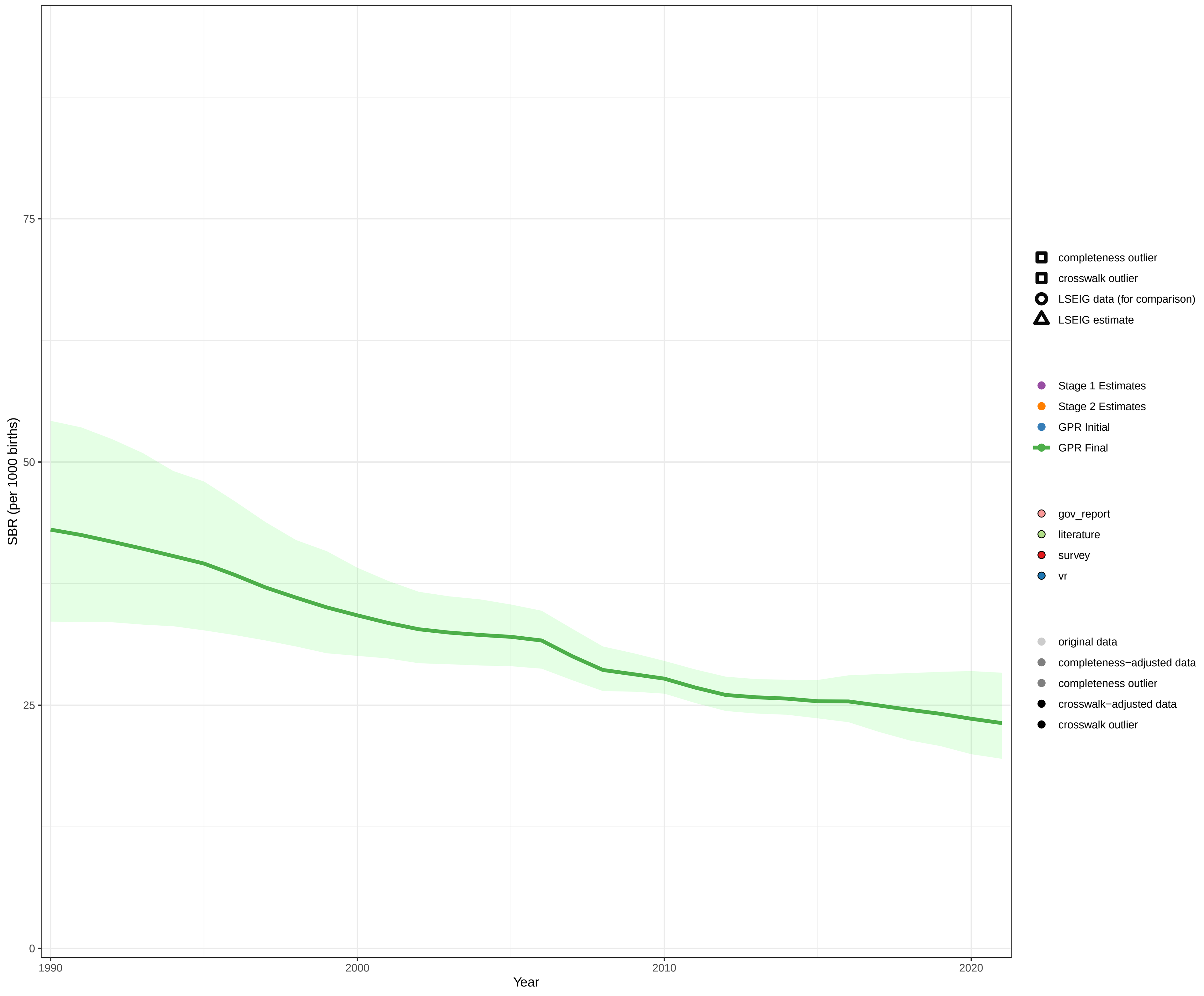

Year	Total Stillbirths (in thousands)	Total Stillbirths (in thousands)	SBR (per 1000 births)	SBR (per 1000 births)	SBR/NMR	SBR/NMR
	20 weeks	28 weeks	20 weeks	28 weeks	20 weeks	28 weeks
1990	566.3 (440.7 to 728.2)	406.9 (314.2 to 518.4)	58.9 (46.5 to 74.5)	43.0 (33.6 to 54.2)	1.39 (1.10 to 1.76)	1.02 (0.79 to 1.28)
1995	574.9 (472.1 to 703.3)	414.9 (340.3 to 507.6)	54.0 (44.8 to 65.3)	39.6 (32.7 to 48.0)	1.35 (1.12 to 1.63)	0.99 (0.82 to 1.20)
2000	551.8 (482.9 to 631.9)	399.3 (349.2 to 458.6)	46.7 (41.1 to 53.1)	34.2 (30.1 to 39.1)	1.25 (1.10 to 1.42)	0.91 (0.80 to 1.05)
2005	560.0 (507.7 to 618.8)	406.1 (366.6 to 449.7)	43.6 (39.7 to 48.0)	32.0 (29.0 to 35.4)	1.26 (1.15 to 1.39)	0.92 (0.84 to 1.02)
2010	508.9 (475.5 to 547.9)	369.7 (348.6 to 394.7)	37.8 (35.4 to 40.6)	27.7 (26.2 to 29.6)	1.20 (1.13 to 1.29)	0.88 (0.83 to 0.94)
2015	483.6 (446.3 to 530.8)	351.1 (326.2 to 382.3)	34.7 (32.1 to 37.9)	25.4 (23.6 to 27.6)	1.23 (1.14 to 1.35)	0.91 (0.84 to 0.98)
2020	460.6 (387.1 to 559.4)	330.9 (278.7 to 401.5)	32.6 (27.5 to 39.3)	23.6 (20.0 to 28.5)	1.32 (1.12 to 1.60)	0.96 (0.81 to 1.16)
2021	455.6 (379.9 to 561.9)	326.8 (273.9 to 401.8)	32.0 (26.8 to 39.2)	23.2 (19.5 to 28.3)	1.33 (1.12 to 1.63)	0.97 (0.81 to 1.18)

Burundi BDI Eastern Sub−Saharan Africa Stillbirth Rate / Neonatal Mortality Rate (SBR/NMR) − 20 weeks
lambda = 0.8 ; zeta = 0.7 ; scale = 15 ; amp = 0.028

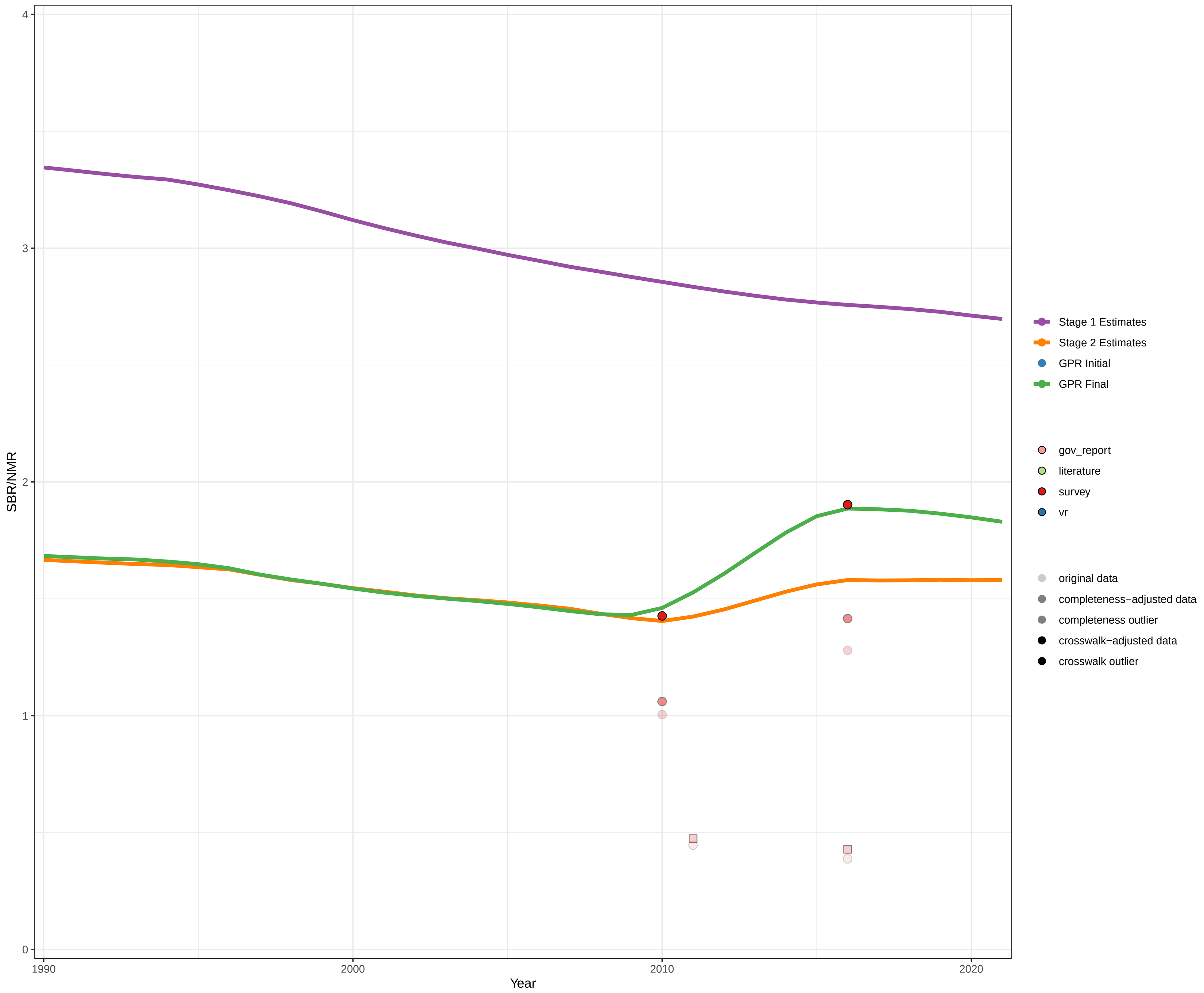

Burundi BDI Eastern Sub−Saharan Africa Stillbirth Rate / Neonatal Mortality Rate (SBR/NMR) − 28 weeks
lambda = 0.8 ; zeta = 0.7 ; scale = 15 ; amp = 0.028

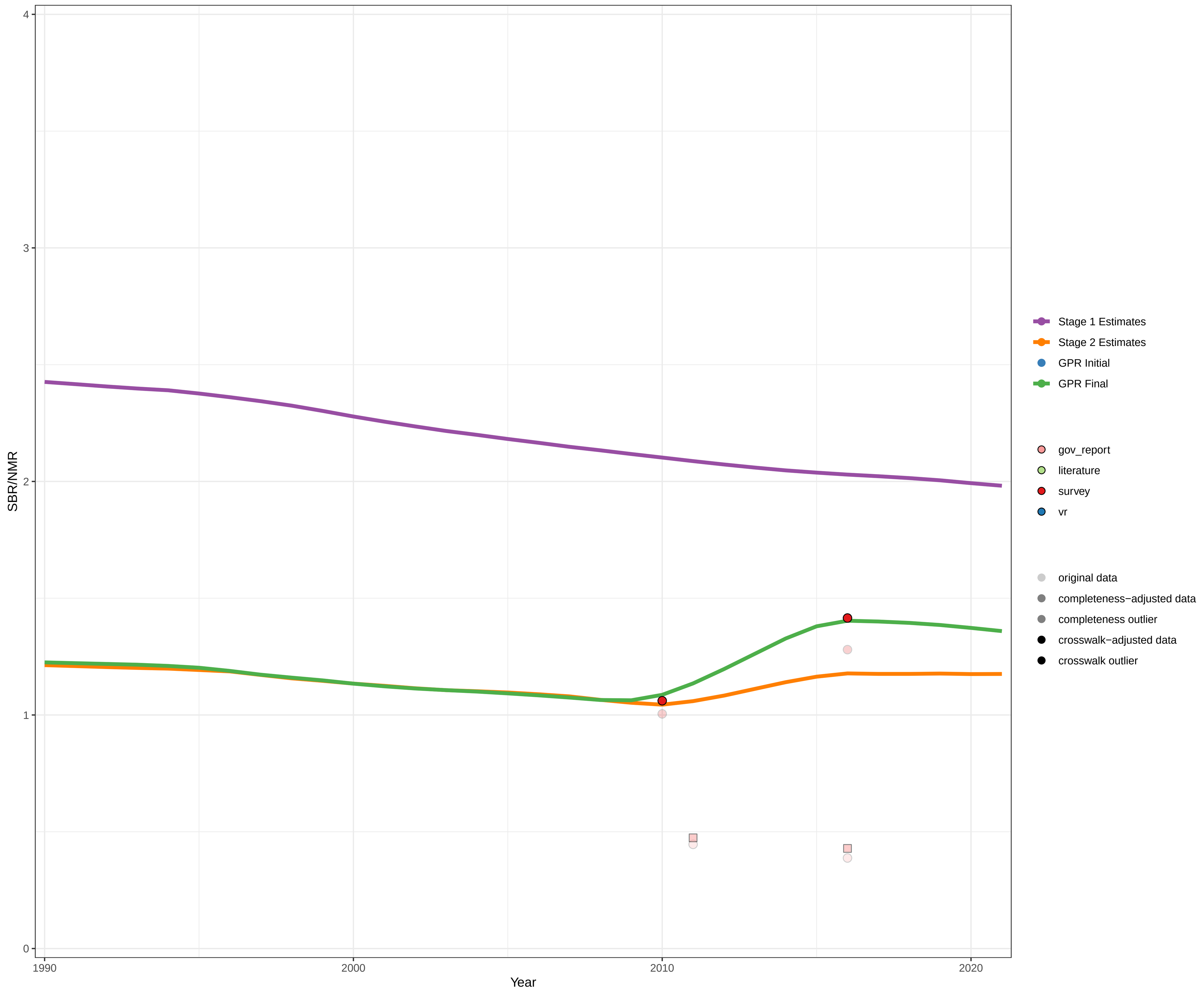

Definition	Years	Outlier	Source.Type	Title
28_weeks	2010	0	Survey	Burundi Demographic and Health Survey 2010−2011
28_weeks	2016	0	Survey	Burundi Demographic and Health Survey 2016−2017
28_weeks	2011	1	Statistical Report	Burundi Statistical Yearbook
28_weeks	2016	1	Statistical Report	Burundi Health Statistical Yearbook

Burundi BDI Eastern Sub−Saharan Africa Stillbirth Rate (SBR) − 20 weeks
lambda = 0.8 ; zeta = 0.7 ; scale = 15 ; amp = 0.028

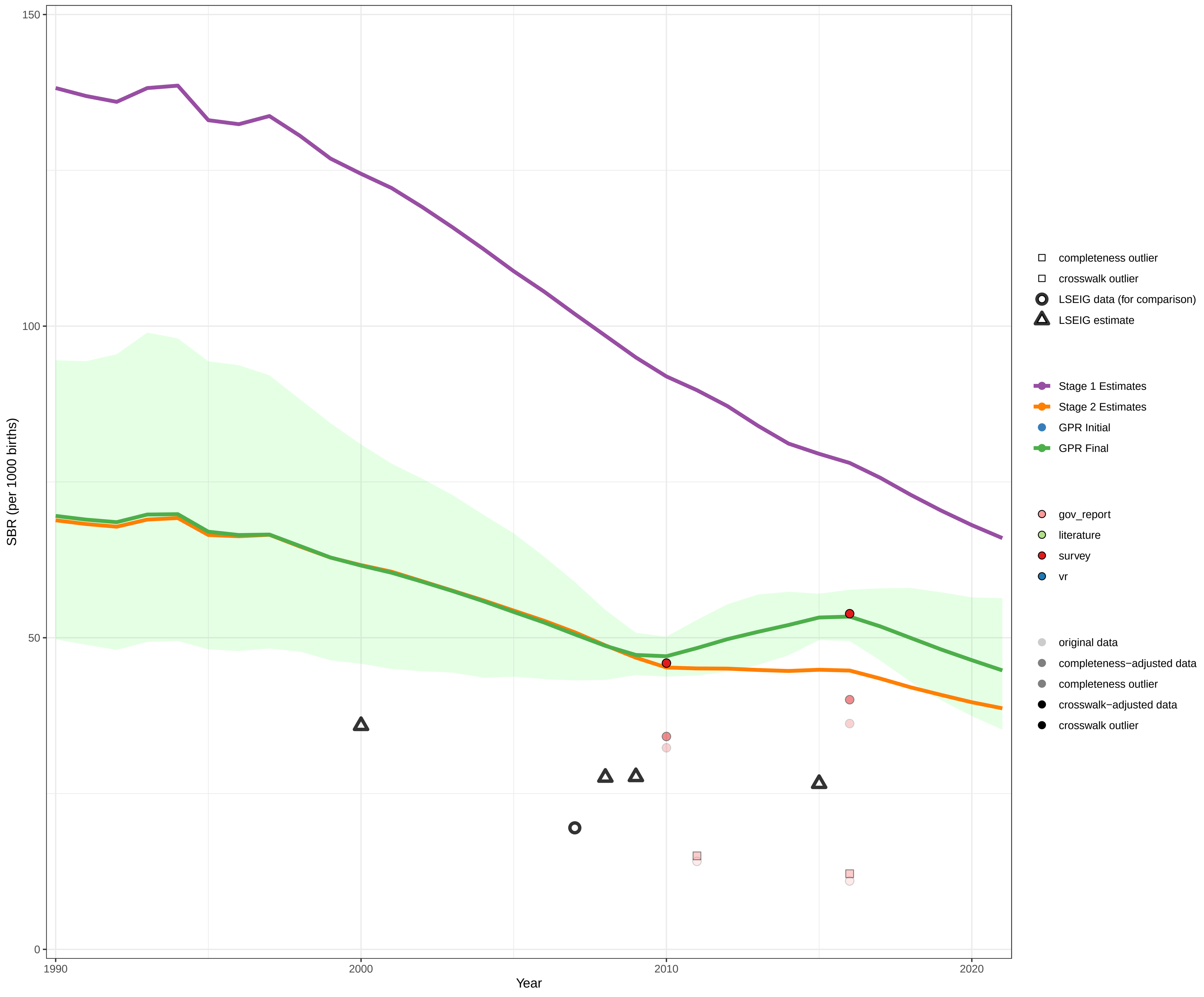

Burundi BDI Eastern Sub−Saharan Africa Stillbirth Rate (SBR) − 28 weeks
lambda = 0.8 ; zeta = 0.7 ; scale = 15 ; amp = 0.028

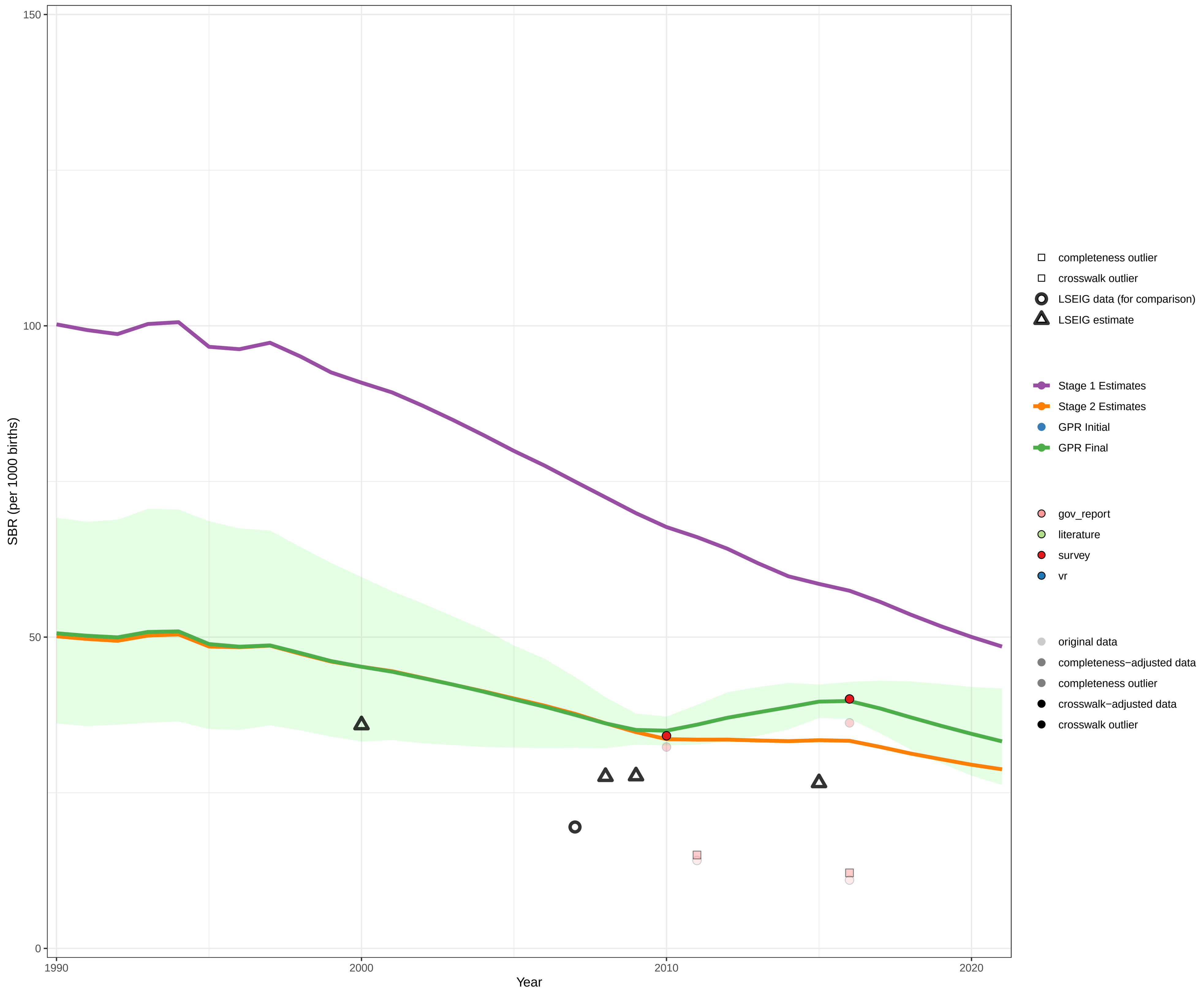

Year	Total Stillbirths (in thousands)	Total Stillbirths (in thousands)	SBR (per 1000 births)	SBR (per 1000 births)	SBR/NMR	SBR/NMR
	20 weeks	28 weeks	20 weeks	28 weeks	20 weeks	28 weeks
1990	19.4 (13.6 to 27.0)	13.8 (9.7 to 19.3)	69.5 (49.7 to 94.5)	50.6 (36.1 to 69.2)	1.68 (1.20 to 2.29)	1.23 (0.87 to 1.67)
1995	18.7 (13.1 to 27.0)	13.3 (9.5 to 19.1)	67.0 (48.2 to 94.4)	48.9 (35.2 to 68.6)	1.65 (1.18 to 2.32)	1.20 (0.87 to 1.69)
2000	17.7 (13.0 to 23.8)	12.8 (9.3 to 17.1)	61.6 (45.8 to 81.0)	45.2 (33.2 to 59.7)	1.54 (1.15 to 2.03)	1.13 (0.83 to 1.50)
2005	18.4 (14.7 to 22.9)	13.4 (10.7 to 16.4)	54.1 (43.8 to 66.8)	40.0 (32.2 to 48.7)	1.48 (1.19 to 1.82)	1.09 (0.88 to 1.33)
2010	19.7 (18.3 to 21.1)	14.5 (13.5 to 15.5)	47.0 (43.8 to 50.2)	35.0 (32.6 to 37.2)	1.46 (1.36 to 1.56)	1.09 (1.01 to 1.16)
2015	24.7 (22.9 to 26.6)	18.1 (16.9 to 19.4)	53.2 (49.6 to 57.1)	39.6 (37.0 to 42.4)	1.85 (1.73 to 1.99)	1.38 (1.29 to 1.48)
2020	22.5 (18.0 to 27.7)	16.5 (13.2 to 20.3)	46.4 (37.4 to 56.5)	34.5 (27.8 to 42.0)	1.85 (1.49 to 2.25)	1.37 (1.11 to 1.67)
2021	22.0 (17.1 to 28.0)	16.1 (12.6 to 20.4)	44.8 (35.2 to 56.3)	33.3 (26.3 to 41.8)	1.83 (1.44 to 2.30)	1.36 (1.07 to 1.71)

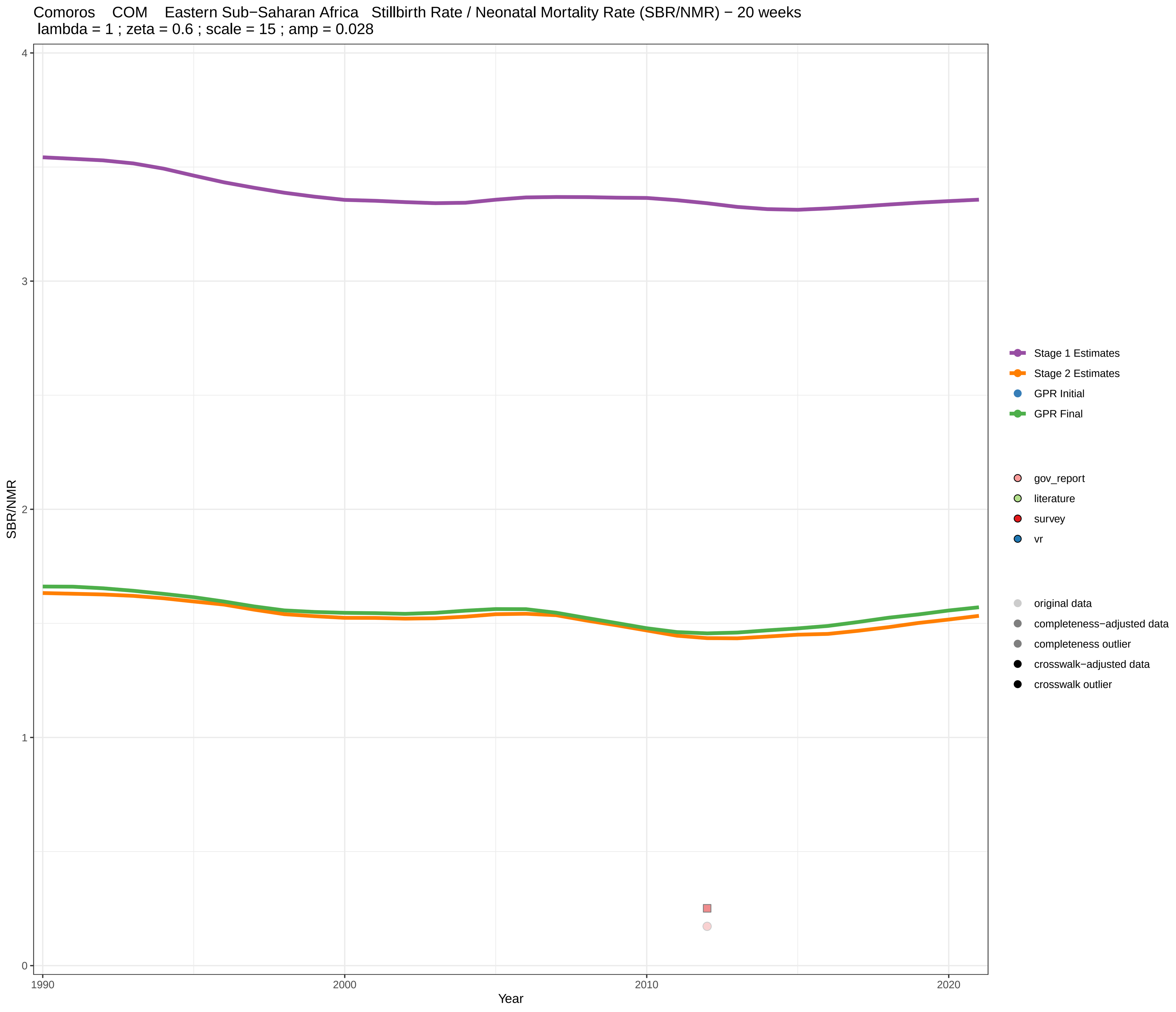

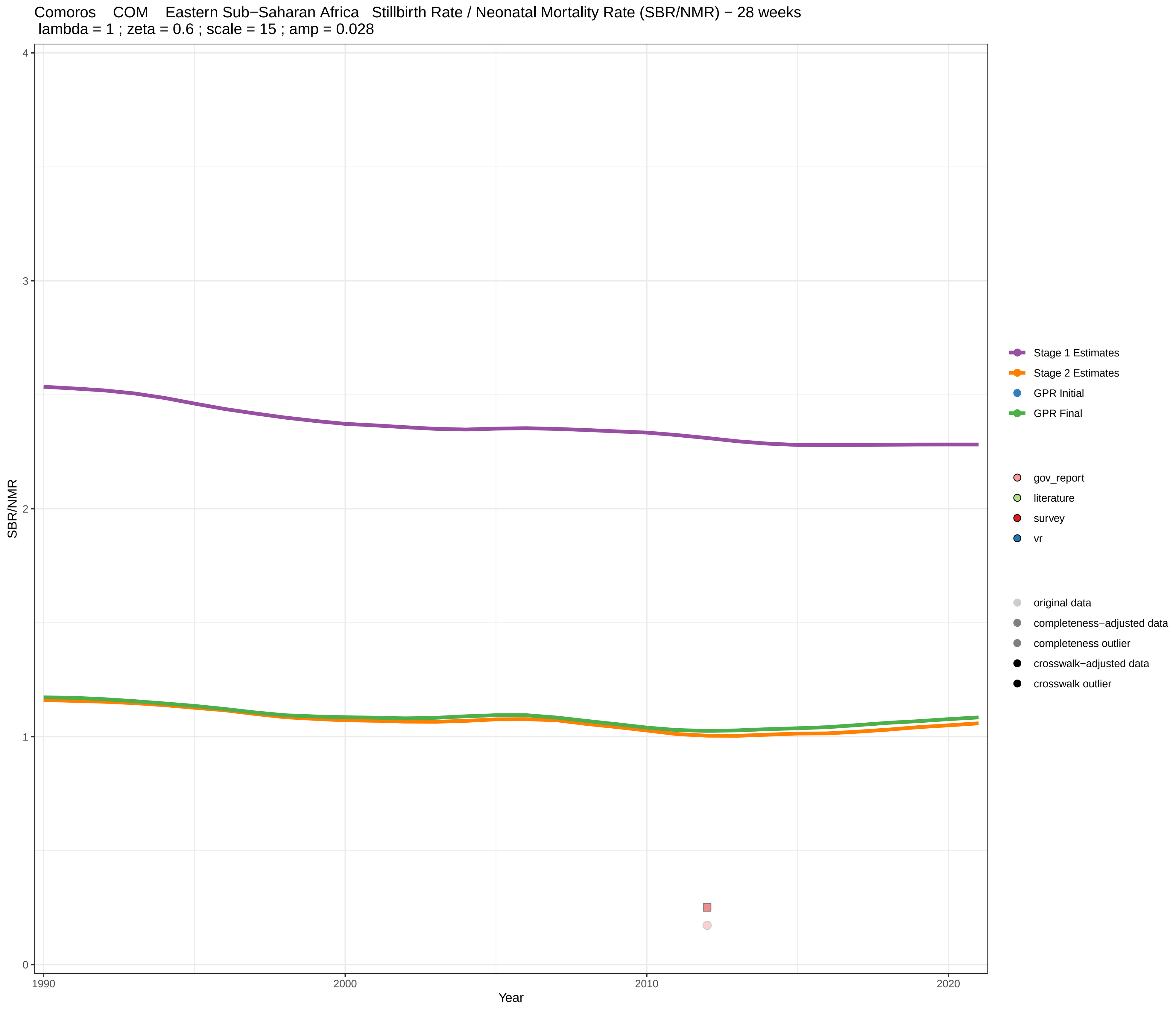

Definition	Years	Outlier	Source.Type	Title
28_weeks	2012	1	Survey	Comoros Demographic and Health Survey 2012−2013

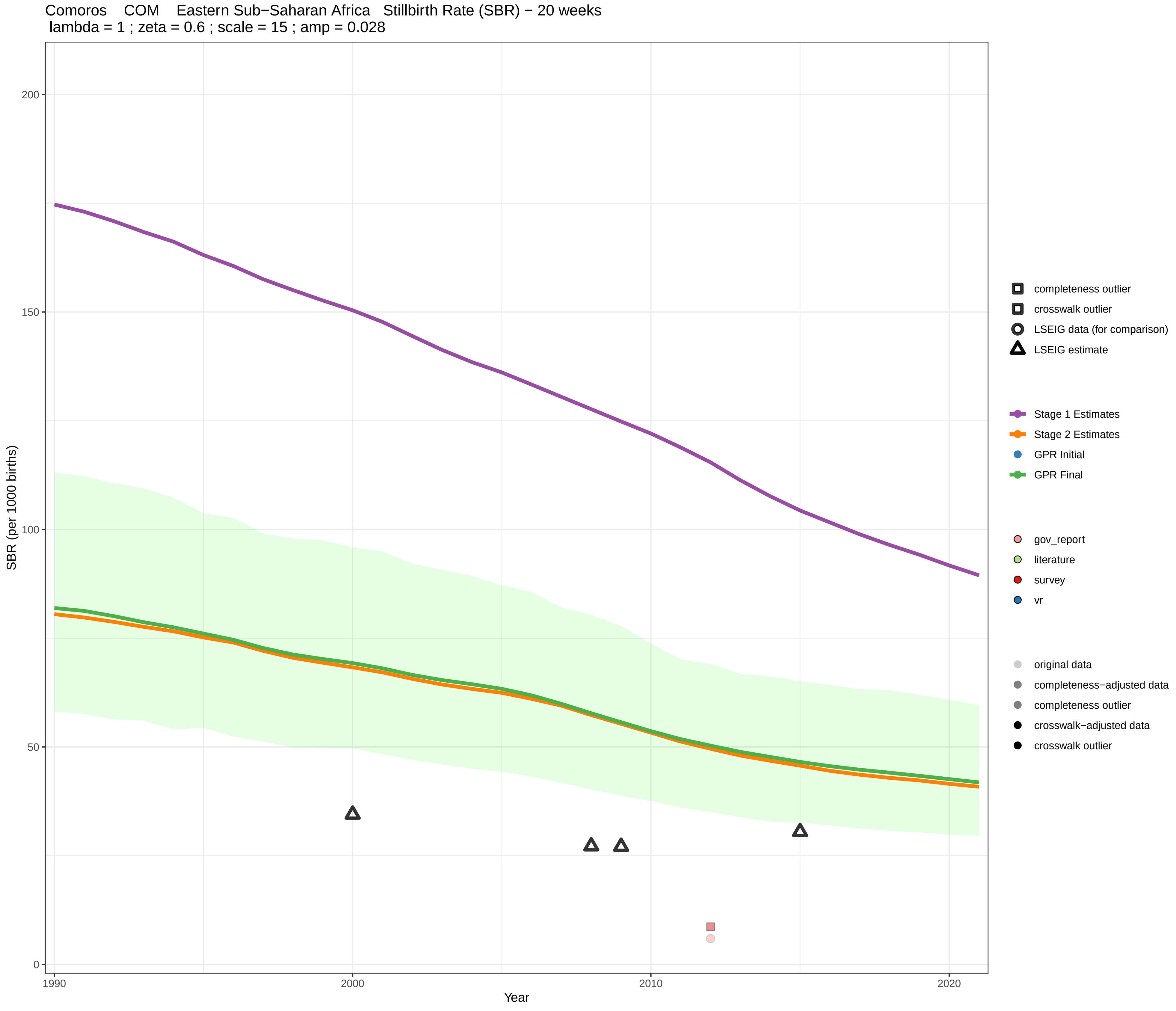

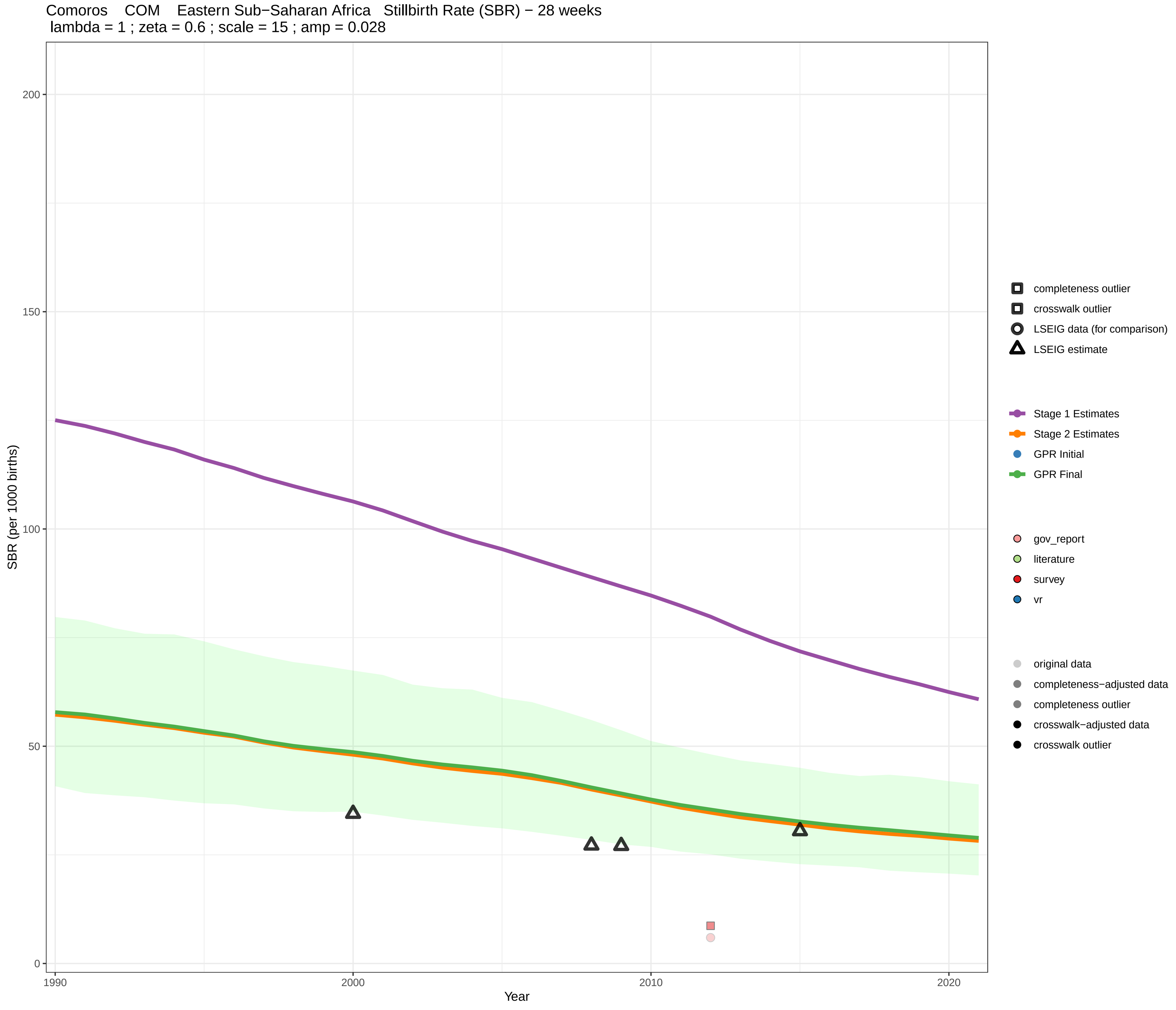

Year	Total Stillbirths (in thousands)	Total Stillbirths (in thousands)	SBR (per 1000 births)	SBR (per 1000 births)	SBR/NMR	SBR/NMR
	20 weeks	28 weeks	20 weeks	28 weeks	20 weeks	28 weeks
1990	1.8 (1.3 to 2.6)	1.2 (0.9 to 1.8)	81.9 (58.1 to 113.1)	57.8 (40.8 to 79.7)	1.66 (1.18 to 2.29)	1.17 (0.83 to 1.62)
1995	1.7 (1.2 to 2.4)	1.2 (0.8 to 1.7)	76.1 (54.4 to 103.7)	53.5 (36.9 to 74.1)	1.61 (1.16 to 2.20)	1.14 (0.78 to 1.57)
2000	1.5 (1.1 to 2.2)	1.0 (0.7 to 1.5)	69.3 (49.7 to 95.9)	48.6 (34.9 to 67.4)	1.55 (1.11 to 2.14)	1.09 (0.78 to 1.50)
2005	1.3 (0.9 to 1.9)	0.9 (0.6 to 1.3)	63.4 (44.3 to 87.2)	44.4 (31.1 to 61.1)	1.56 (1.09 to 2.15)	1.09 (0.77 to 1.51)
2010	1.1 (0.7 to 1.5)	0.8 (0.5 to 1.0)	53.6 (37.6 to 73.8)	37.7 (26.8 to 51.2)	1.48 (1.04 to 2.04)	1.04 (0.74 to 1.41)
2015	0.9 (0.6 to 1.3)	0.6 (0.4 to 0.9)	46.6 (32.6 to 65.1)	32.7 (22.9 to 45.0)	1.48 (1.03 to 2.07)	1.04 (0.73 to 1.43)
2020	0.8 (0.5 to 1.1)	0.5 (0.4 to 0.8)	42.6 (29.8 to 60.9)	29.5 (20.7 to 41.9)	1.56 (1.09 to 2.22)	1.08 (0.76 to 1.53)
2021	0.8 (0.5 to 1.1)	0.5 (0.4 to 0.7)	41.9 (29.6 to 59.6)	28.9 (20.3 to 41.2)	1.57 (1.11 to 2.24)	1.08 (0.76 to 1.55)

Djibouti   DJI   Eastern Sub−Saharan Africa   Stillbirth Rate / Neonatal Mortality Rate (SBR/NMR) − 20 weeks
lambda = 1 ; zeta = 0.6 ; scale = 15 ; amp = 0.028

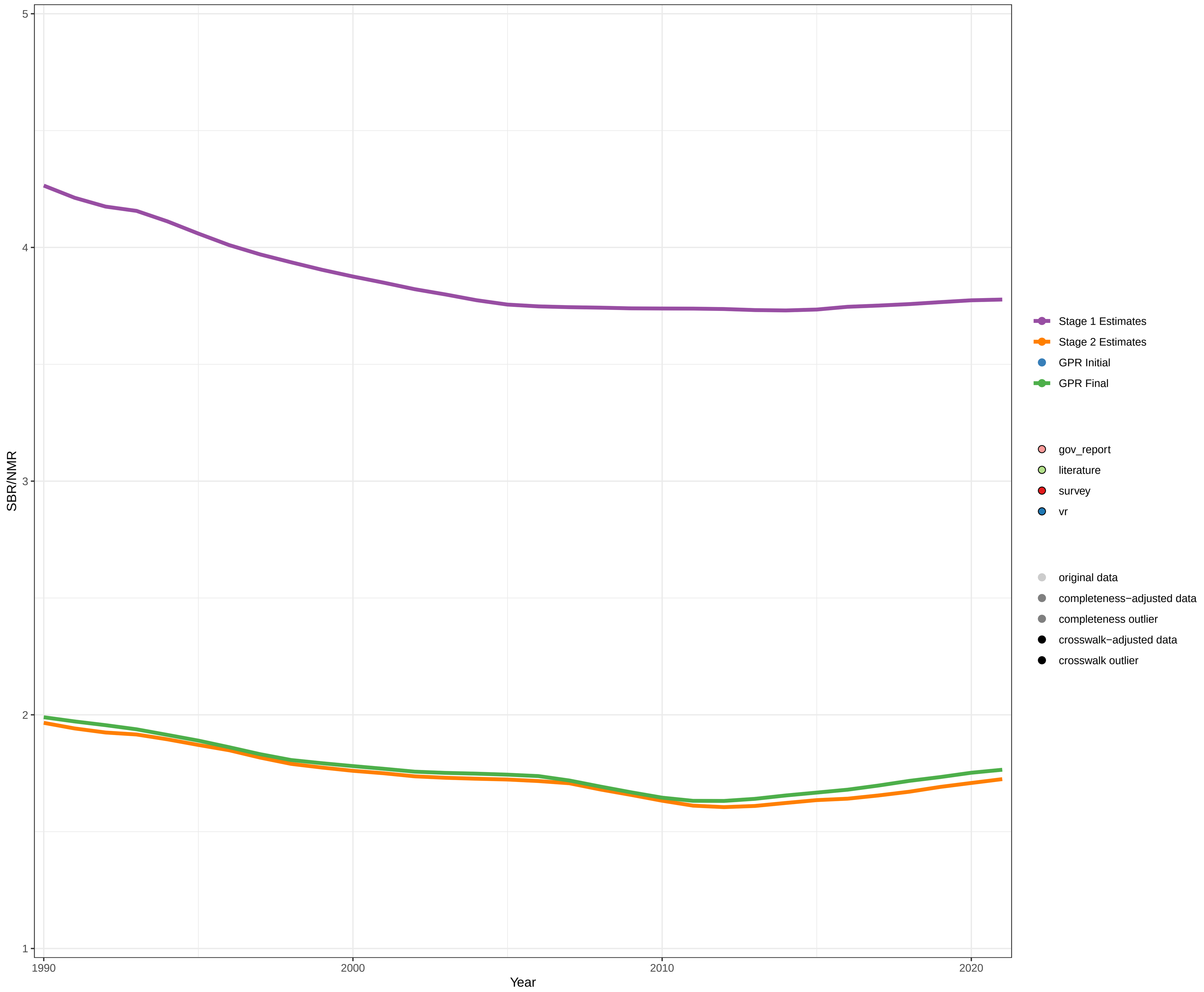

Djibouti   DJI   Eastern Sub−Saharan Africa   Stillbirth Rate / Neonatal Mortality Rate (SBR/NMR) − 28 weeks
lambda = 1 ; zeta = 0.6 ; scale = 15 ; amp = 0.028

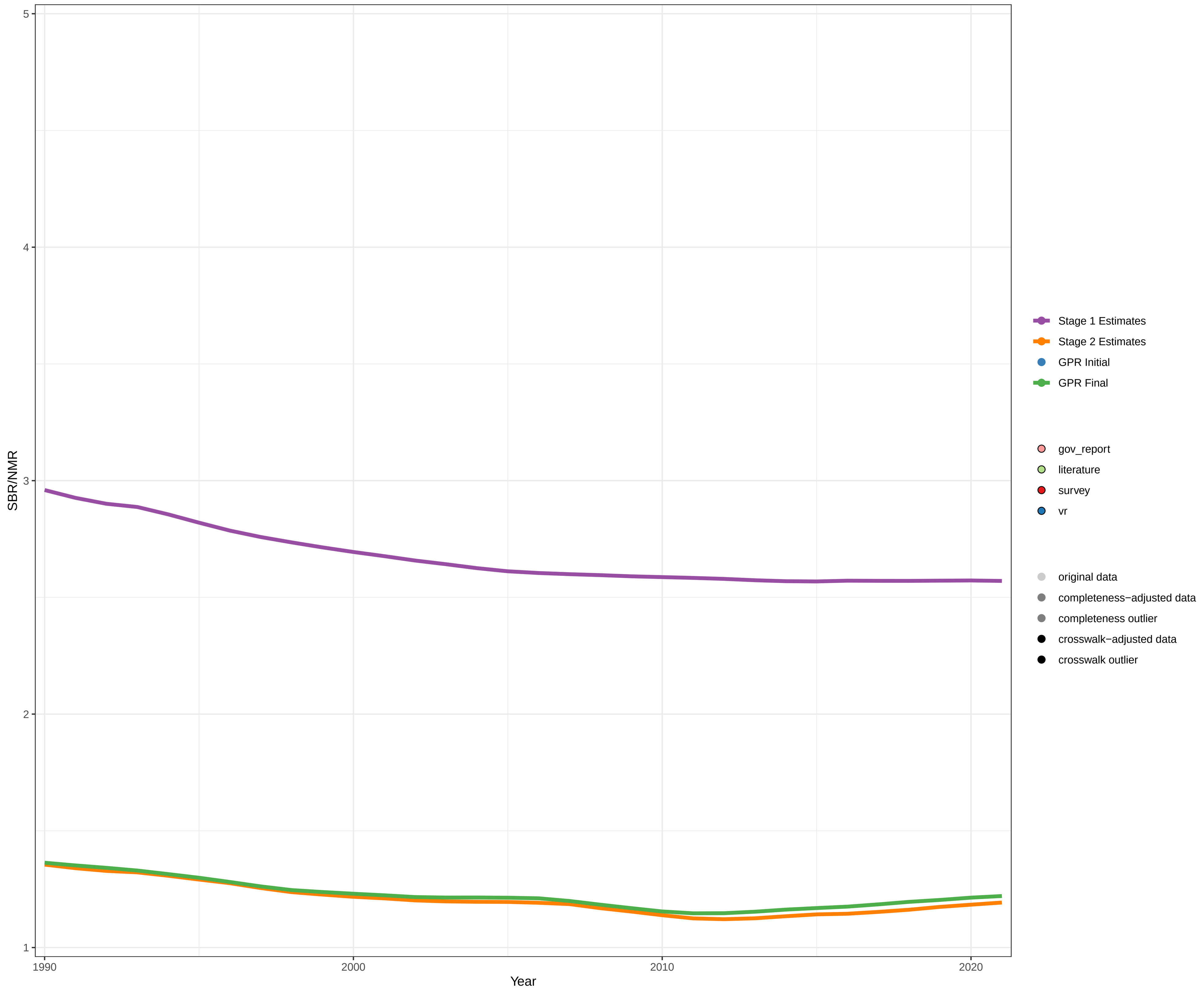

Definition	Years	Outlier	Source.Type	Title

Djibouti   DJI   Eastern Sub−Saharan Africa   Stillbirth Rate (SBR) − 20 weeks
lambda = 1 ; zeta = 0.6 ; scale = 15 ; amp = 0.028

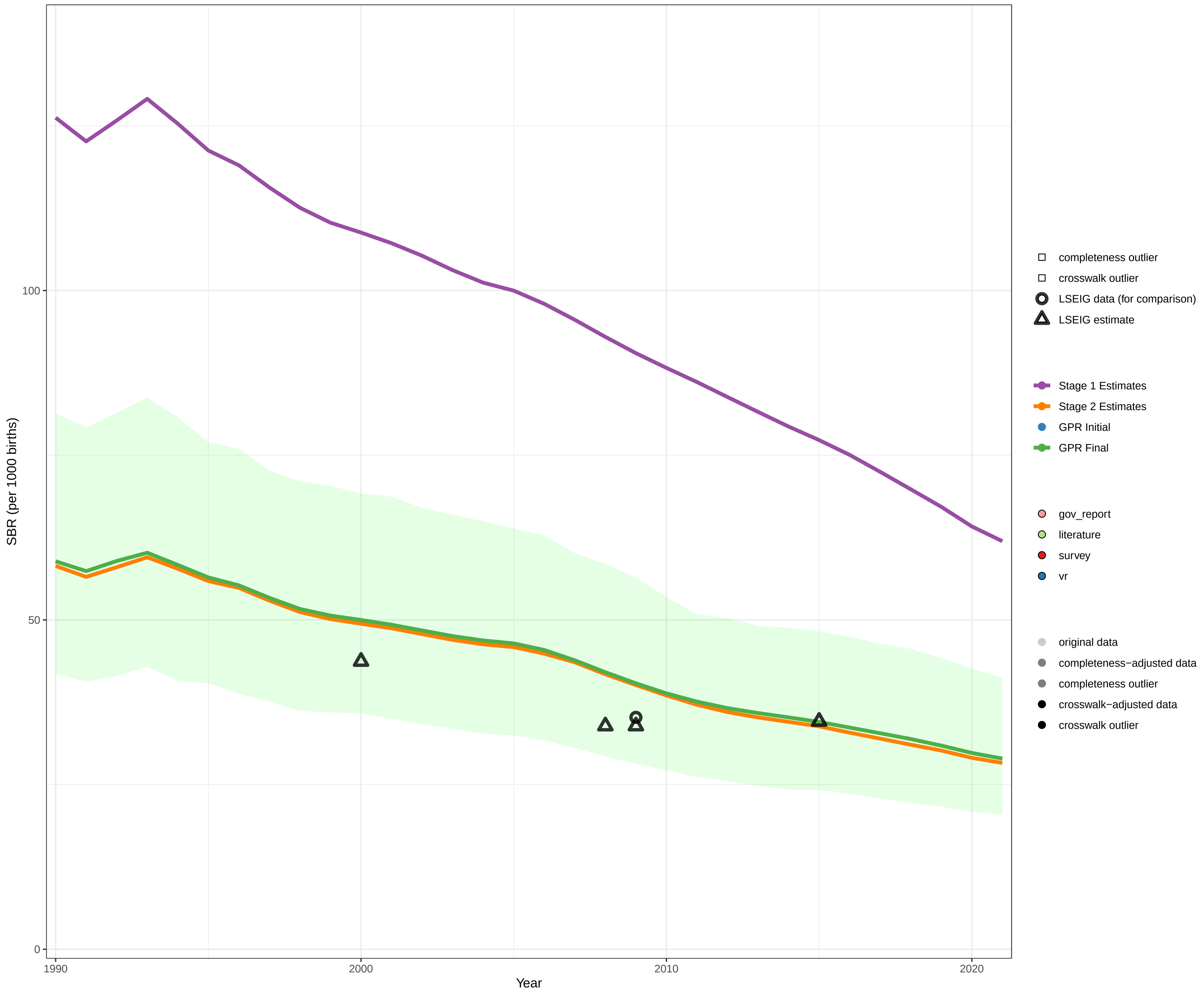

Djibouti   DJI   Eastern Sub−Saharan Africa   Stillbirth Rate (SBR) − 28 weeks
lambda = 1 ; zeta = 0.6 ; scale = 15 ; amp = 0.028

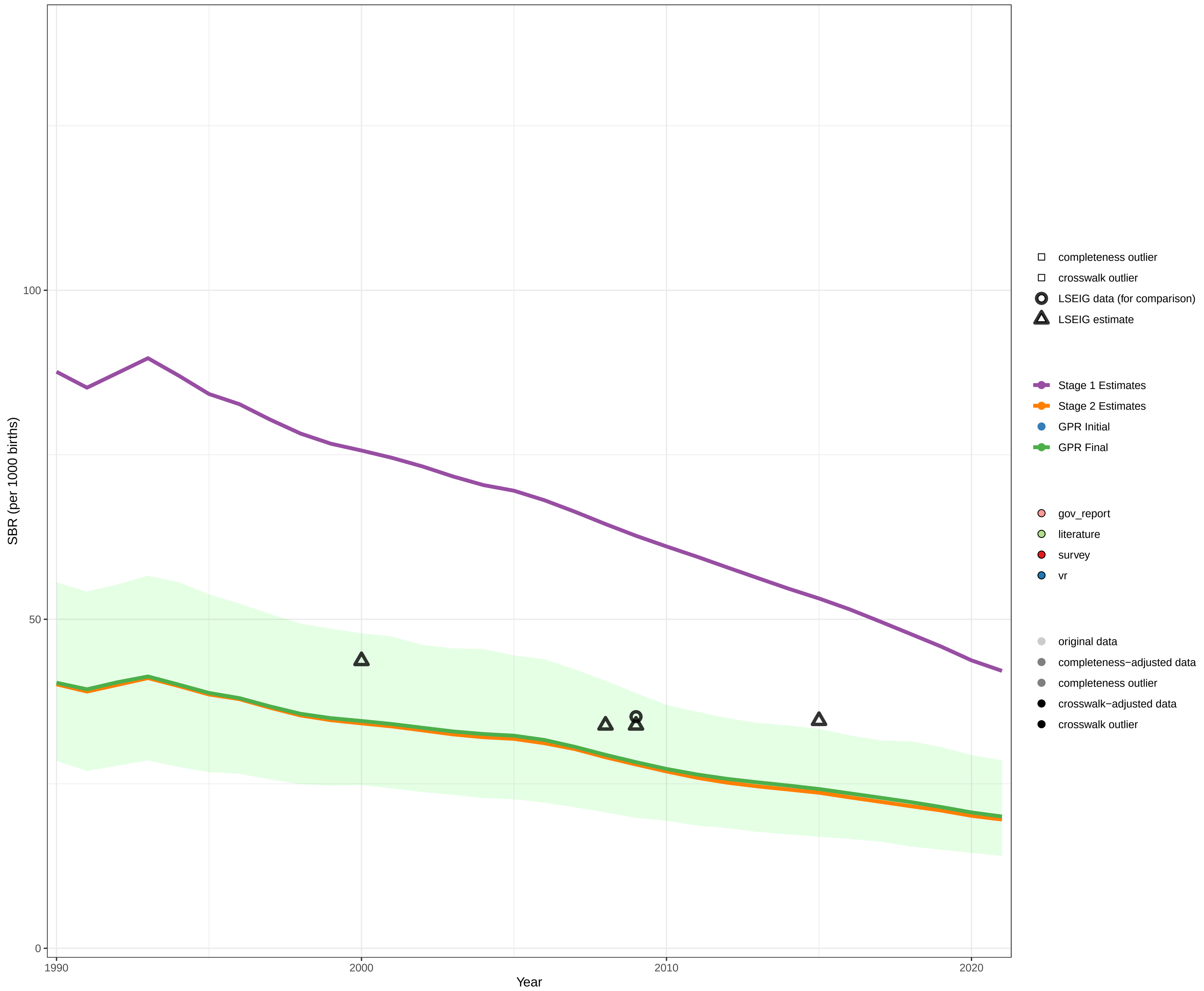

Year	Total Stillbirths (in thousands)	Total Stillbirths (in thousands)	SBR (per 1000 births)	SBR (per 1000 births)	SBR/NMR	SBR/NMR
	20 weeks	28 weeks	20 weeks	28 weeks	20 weeks	28 weeks
1990	0.9 (0.6 to 1.2)	0.6 (0.4 to 0.8)	58.9 (41.8 to 81.3)	40.4 (28.5 to 55.6)	1.99 (1.41 to 2.75)	1.36 (0.96 to 1.88)
1995	0.9 (0.7 to 1.3)	0.6 (0.4 to 0.9)	56.4 (40.4 to 77.0)	38.8 (26.8 to 53.8)	1.89 (1.35 to 2.58)	1.30 (0.90 to 1.80)
2000	1.0 (0.7 to 1.4)	0.7 (0.5 to 0.9)	50.0 (35.8 to 69.2)	34.5 (24.8 to 47.9)	1.78 (1.28 to 2.46)	1.23 (0.88 to 1.71)
2005	1.1 (0.7 to 1.5)	0.7 (0.5 to 1.0)	46.4 (32.5 to 63.9)	32.3 (22.6 to 44.5)	1.74 (1.22 to 2.40)	1.21 (0.85 to 1.67)
2010	1.0 (0.7 to 1.4)	0.7 (0.5 to 1.0)	38.8 (27.2 to 53.5)	27.3 (19.4 to 37.0)	1.65 (1.15 to 2.26)	1.15 (0.82 to 1.57)
2015	0.9 (0.7 to 1.3)	0.7 (0.5 to 0.9)	34.5 (24.2 to 48.3)	24.2 (16.9 to 33.4)	1.67 (1.17 to 2.33)	1.17 (0.82 to 1.61)
2020	0.8 (0.5 to 1.1)	0.5 (0.4 to 0.8)	29.8 (20.9 to 42.6)	20.6 (14.5 to 29.4)	1.75 (1.23 to 2.50)	1.21 (0.85 to 1.73)
2021	0.8 (0.5 to 1.1)	0.5 (0.4 to 0.7)	28.9 (20.4 to 41.2)	20.0 (14.0 to 28.6)	1.76 (1.25 to 2.51)	1.22 (0.86 to 1.74)

Eritrea ERI Eastern Sub−Saharan Africa Stillbirth Rate / Neonatal Mortality Rate (SBR/NMR) − 20 weeks
lambda = 1 ; zeta = 0.6 ; scale = 15 ; amp = 0.028

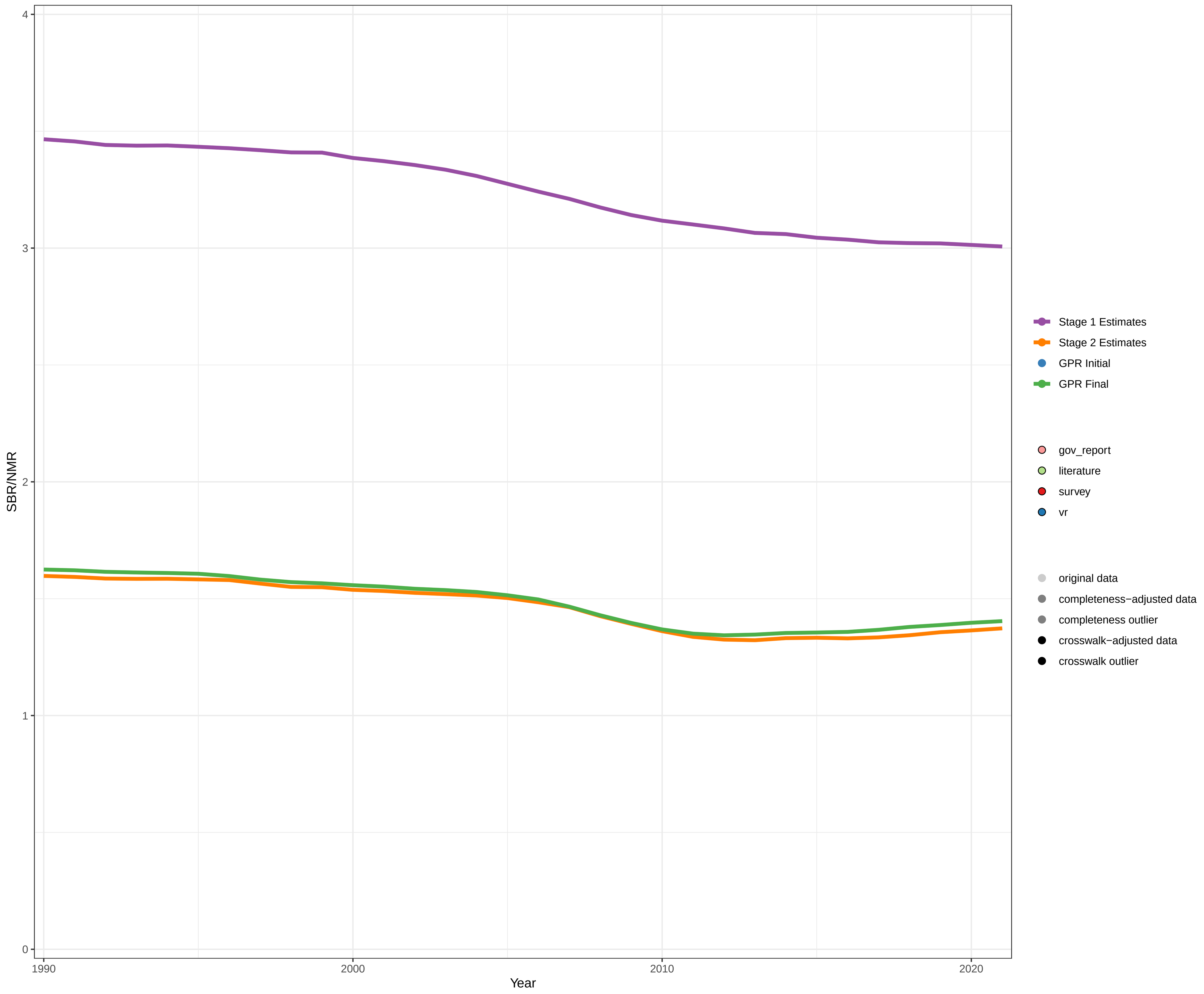

Eritrea ERI Eastern Sub−Saharan Africa Stillbirth Rate / Neonatal Mortality Rate (SBR/NMR) − 28 weeks
lambda = 1 ; zeta = 0.6 ; scale = 15 ; amp = 0.028

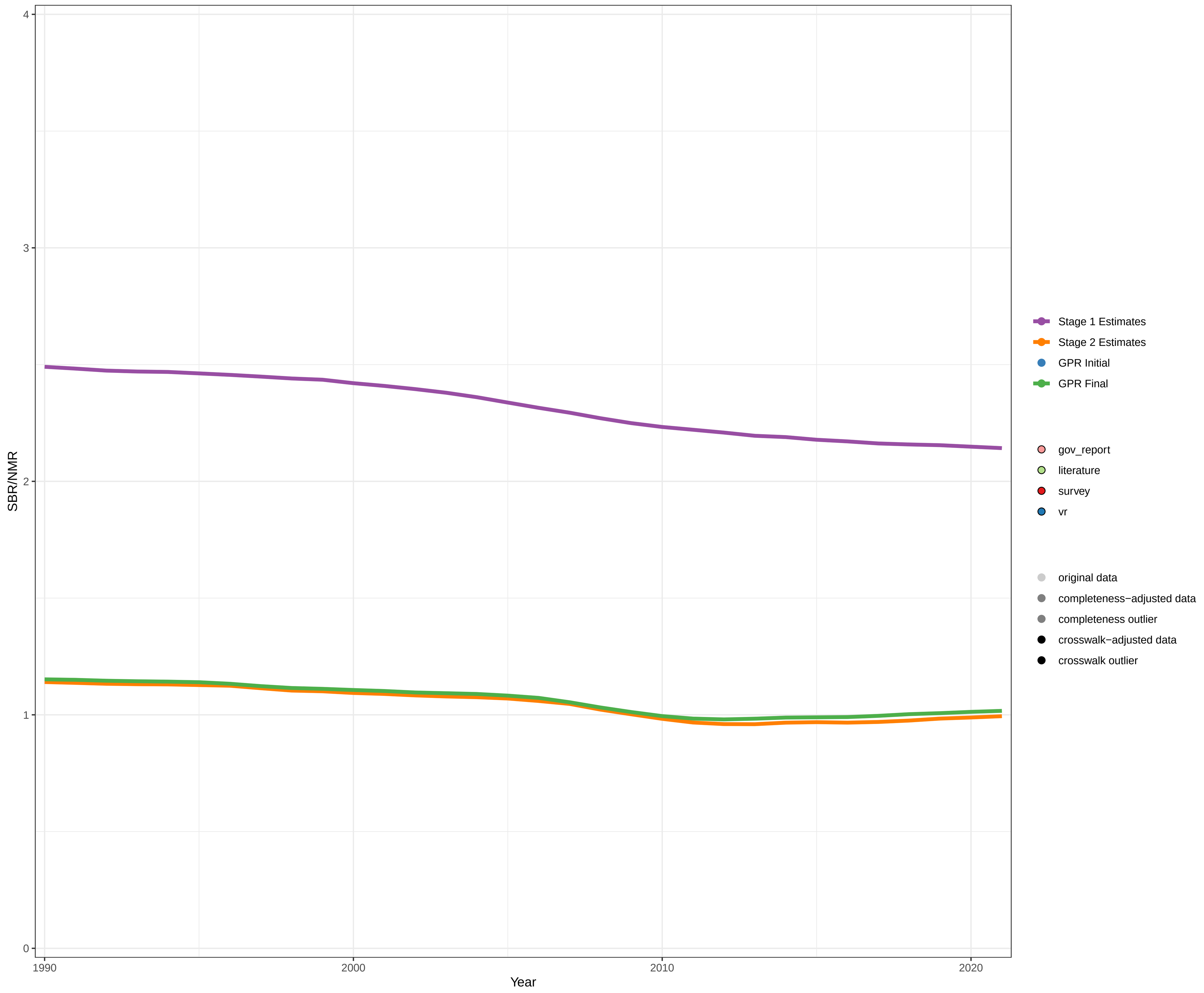

Definition	Years	Outlier	Source.Type	Title

Eritrea ERI Eastern Sub−Saharan Africa Stillbirth Rate (SBR) − 20 weeks
lambda = 1 ; zeta = 0.6 ; scale = 15 ; amp = 0.028

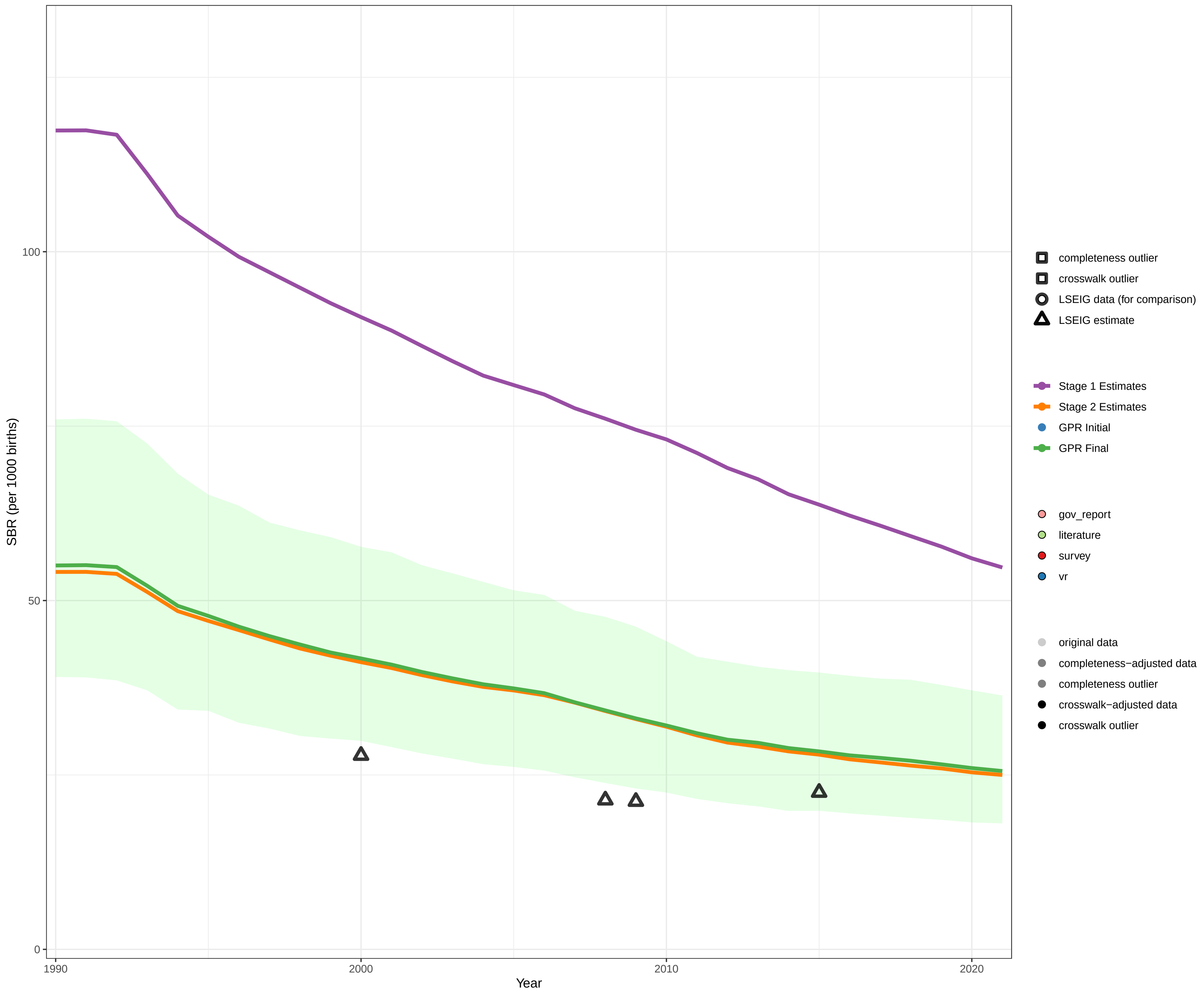

Eritrea ERI Eastern Sub−Saharan Africa Stillbirth Rate (SBR) − 28 weeks
lambda = 1 ; zeta = 0.6 ; scale = 15 ; amp = 0.028

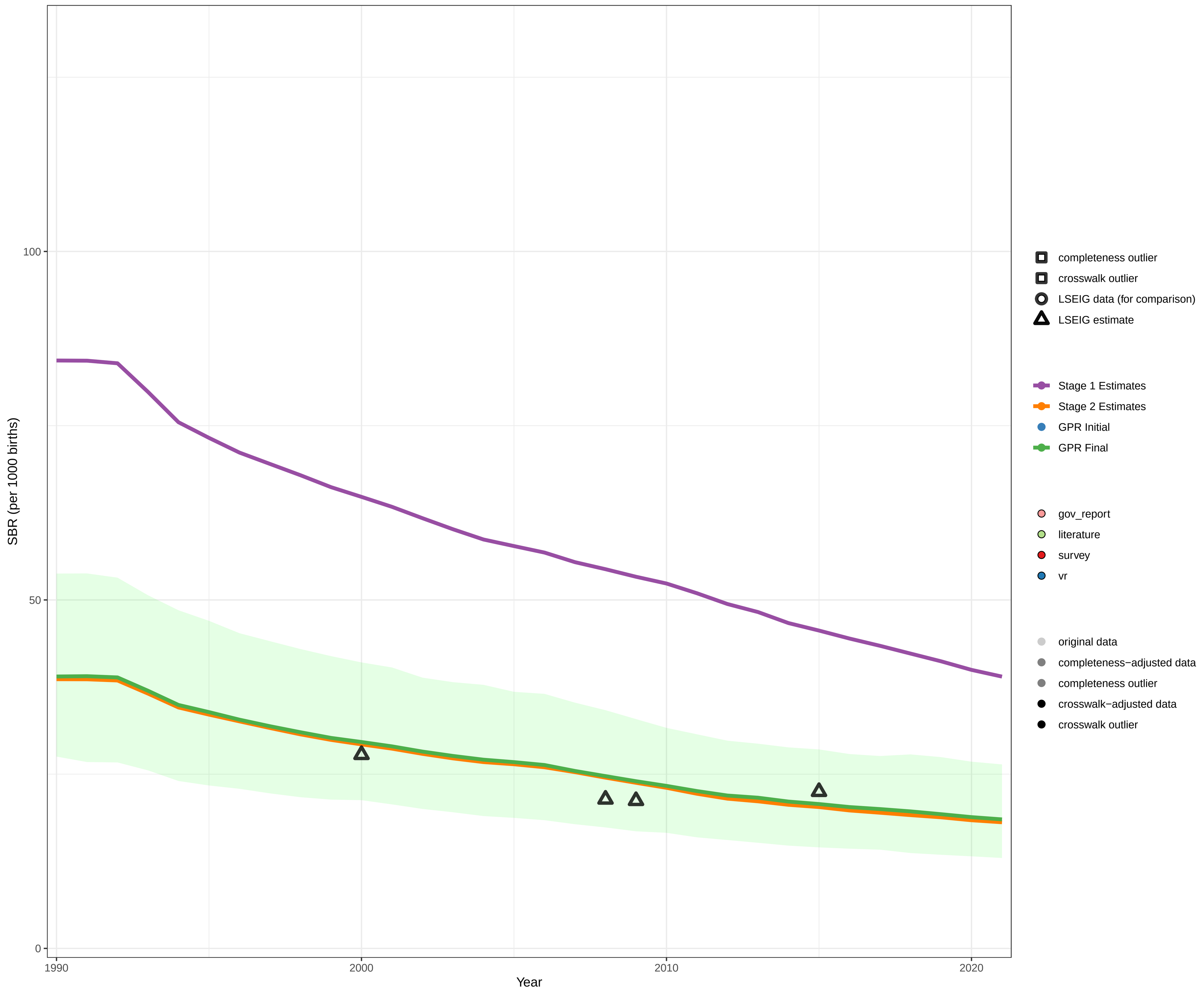

Year	Total Stillbirths (in thousands)	Total Stillbirths (in thousands)	SBR (per 1000 births)	SBR (per 1000 births)	SBR/NMR	SBR/NMR
	20 weeks	28 weeks	20 weeks	28 weeks	20 weeks	28 weeks
1990	8.6 (6.0 to 12.1)	6.0 (4.2 to 8.4)	55.0 (39.0 to 76.0)	39.0 (27.5 to 53.8)	1.62 (1.15 to 2.24)	1.15 (0.81 to 1.59)
1995	7.0 (4.9 to 9.7)	4.9 (3.3 to 6.9)	47.8 (34.2 to 65.2)	33.9 (23.4 to 47.0)	1.61 (1.15 to 2.19)	1.14 (0.79 to 1.58)
2000	6.5 (4.6 to 9.1)	4.6 (3.2 to 6.4)	41.7 (29.9 to 57.7)	29.6 (21.3 to 41.0)	1.56 (1.12 to 2.16)	1.11 (0.79 to 1.53)
2005	6.4 (4.4 to 9.0)	4.6 (3.2 to 6.3)	37.4 (26.1 to 51.5)	26.7 (18.7 to 36.8)	1.51 (1.06 to 2.08)	1.08 (0.76 to 1.49)
2010	6.0 (4.1 to 8.3)	4.3 (3.0 to 5.9)	32.1 (22.5 to 44.2)	23.3 (16.6 to 31.6)	1.37 (0.96 to 1.88)	0.99 (0.71 to 1.35)
2015	5.6 (3.9 to 7.9)	4.0 (2.8 to 5.6)	28.4 (19.9 to 39.7)	20.7 (14.5 to 28.6)	1.36 (0.95 to 1.90)	0.99 (0.69 to 1.36)
2020	5.2 (3.6 to 7.5)	3.7 (2.6 to 5.3)	26.0 (18.2 to 37.1)	18.8 (13.2 to 26.8)	1.40 (0.98 to 2.00)	1.01 (0.71 to 1.44)
2021	5.1 (3.6 to 7.4)	3.7 (2.6 to 5.3)	25.6 (18.1 to 36.4)	18.5 (13.0 to 26.4)	1.40 (0.99 to 2.00)	1.02 (0.71 to 1.45)

Ethiopia ETH Eastern Sub−Saharan Africa Stillbirth Rate / Neonatal Mortality Rate (SBR/NMR) − 20 weeks
lambda = 0.8 ; zeta = 0.7 ; scale = 15 ; amp = 0.028

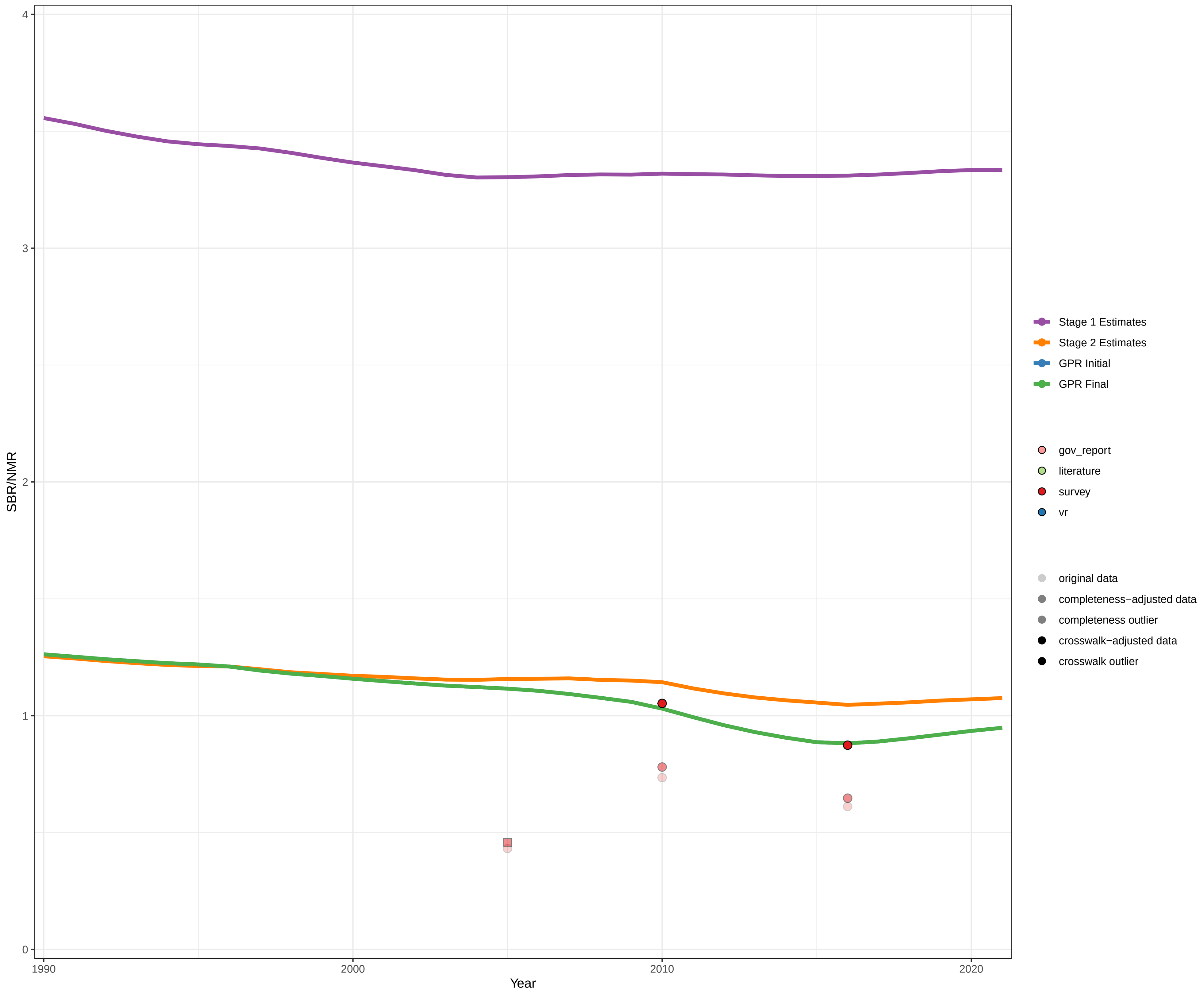

Ethiopia ETH Eastern Sub−Saharan Africa Stillbirth Rate / Neonatal Mortality Rate (SBR/NMR) − 28 weeks
lambda = 0.8 ; zeta = 0.7 ; scale = 15 ; amp = 0.028

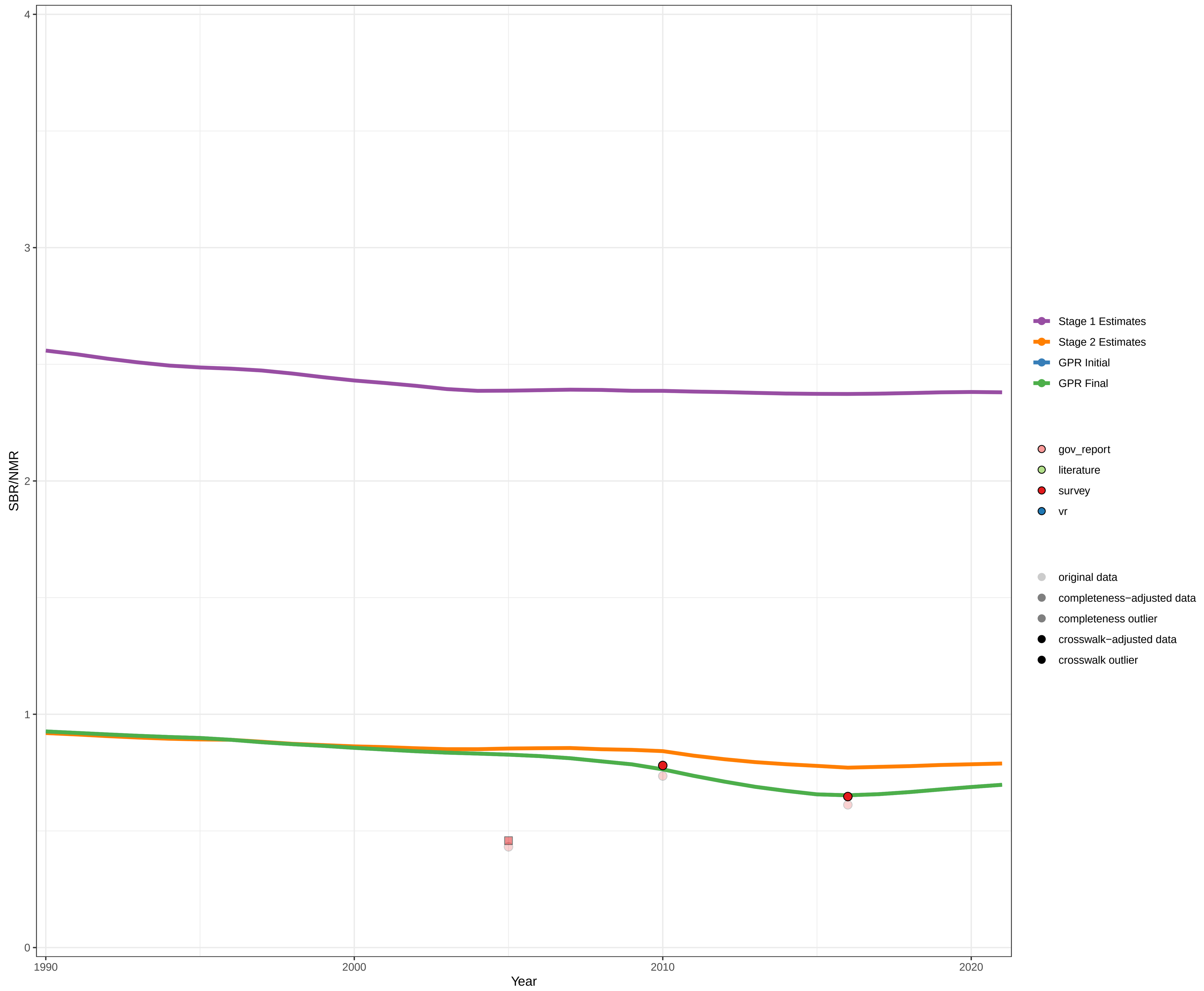

Definition	Years	Outlier	Source.Type	Title
28_weeks	2010	0	Survey	Ethiopia Demographic and Health Survey 2010−2011
28_weeks	2016	0	Survey	Ethiopia Demographic and Health Survey 2016
28_weeks	2005	1	Survey	Ethiopia Demographic and Health Survey 2005

Ethiopia ETH Eastern Sub−Saharan Africa Stillbirth Rate (SBR) − 20 weeks
lambda = 0.8 ; zeta = 0.7 ; scale = 15 ; amp = 0.028

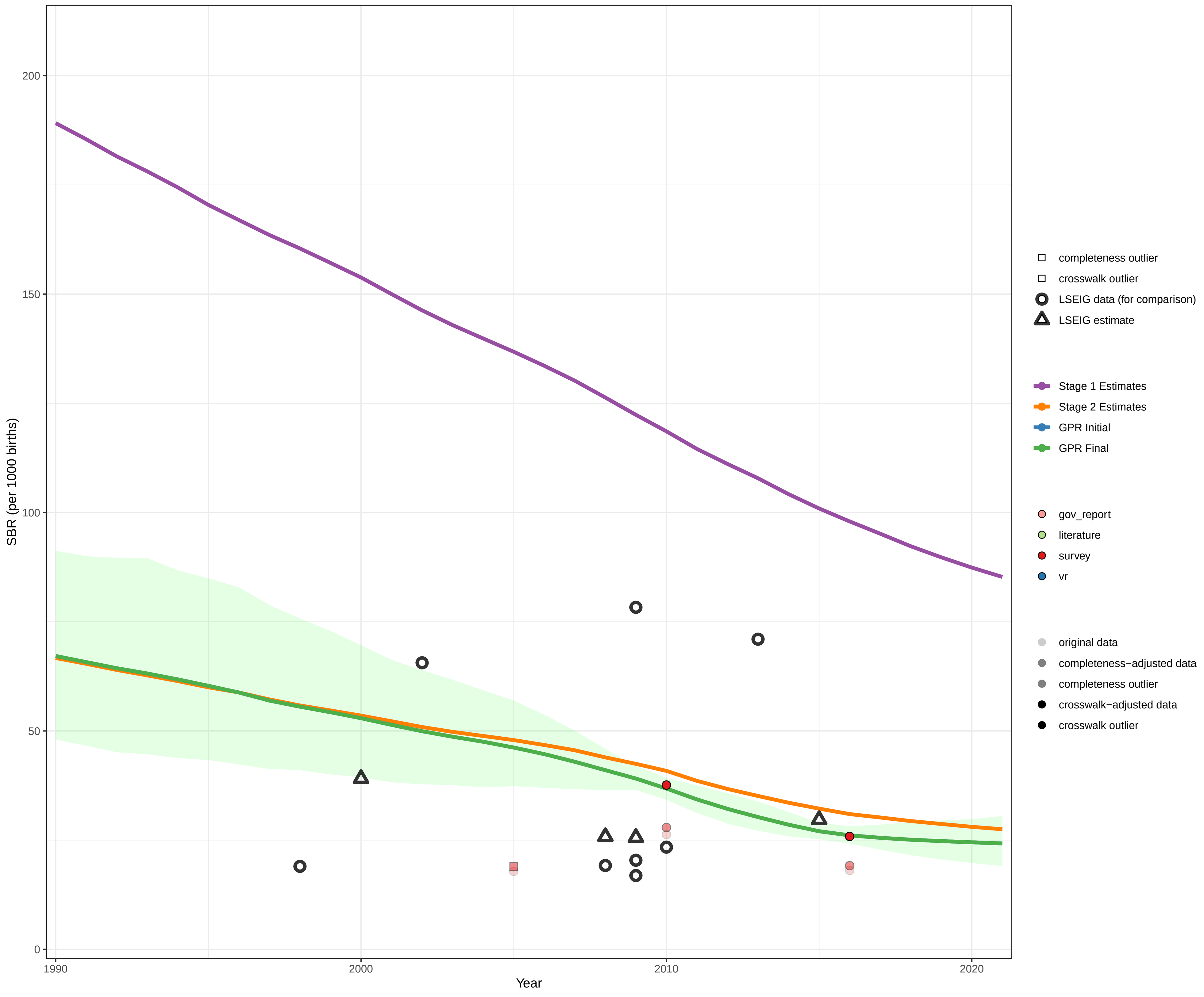

Ethiopia ETH Eastern Sub−Saharan Africa Stillbirth Rate (SBR) − 28 weeks
lambda = 0.8 ; zeta = 0.7 ; scale = 15 ; amp = 0.028

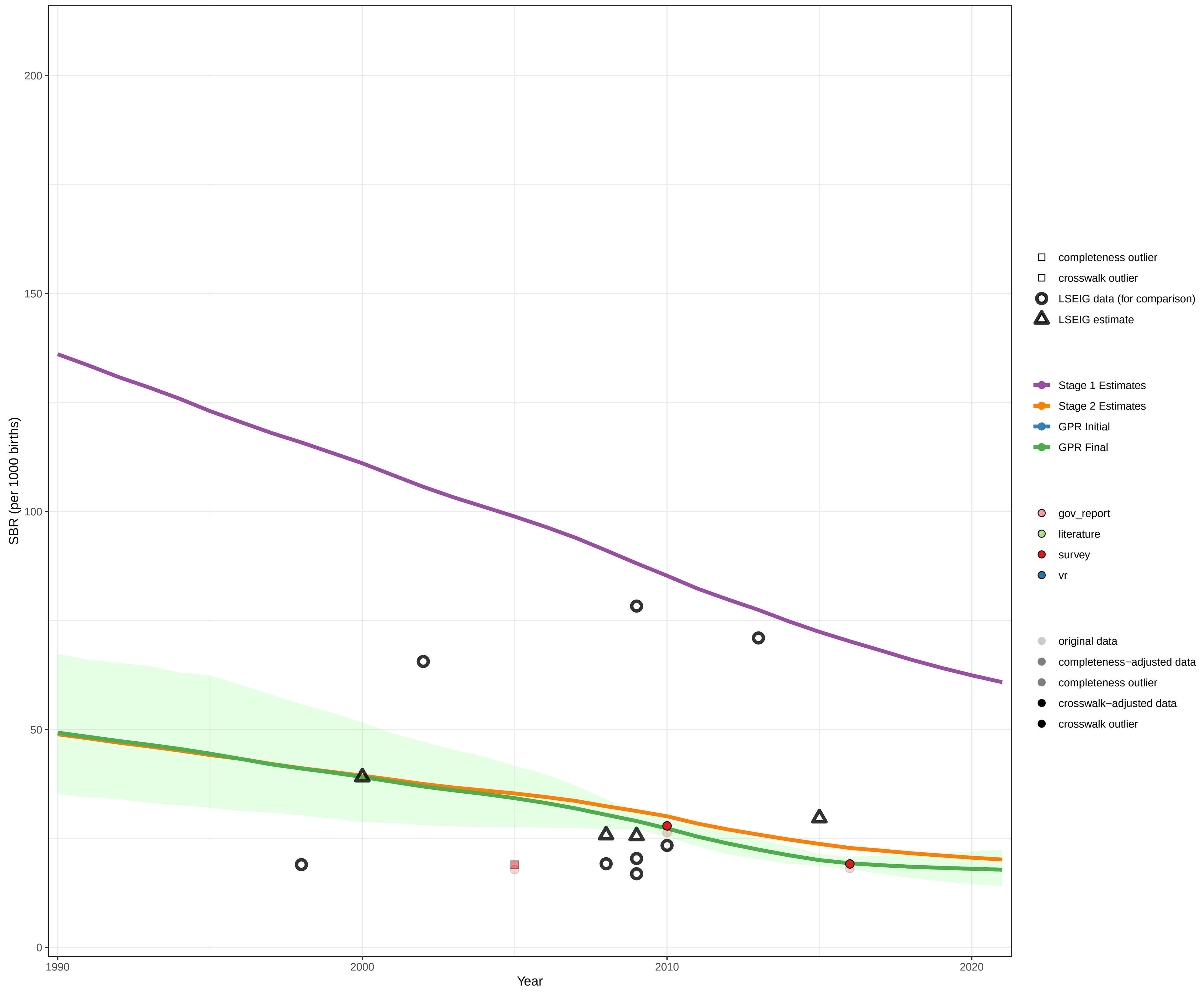

Year	Total Stillbirths (in thousands)	Total Stillbirths (in thousands)	SBR (per 1000 births)	SBR (per 1000 births)	SBR/NMR	SBR/NMR
	20 weeks	28 weeks	20 weeks	28 weeks	20 weeks	28 weeks
1990	175.3 (122.6 to 244.0)	126.1 (88.5 to 175.4)	67.2 (48.0 to 91.3)	49.3 (35.1 to 67.3)	1.26 (0.90 to 1.72)	0.93 (0.66 to 1.27)
1995	180.6 (127.2 to 260.6)	130.8 (92.9 to 186.8)	60.3 (43.3 to 84.9)	44.5 (32.0 to 62.4)	1.22 (0.88 to 1.72)	0.90 (0.65 to 1.26)
2000	170.6 (125.0 to 228.2)	124.3 (90.2 to 165.9)	52.9 (39.4 to 69.6)	39.1 (28.7 to 51.6)	1.16 (0.86 to 1.52)	0.86 (0.63 to 1.13)
2005	153.5 (122.8 to 191.3)	112.3 (89.9 to 137.6)	46.2 (37.3 to 57.0)	34.2 (27.6 to 41.6)	1.12 (0.90 to 1.38)	0.83 (0.67 to 1.01)
2010	120.4 (111.7 to 128.7)	88.4 (82.2 to 94.3)	36.8 (34.3 to 39.3)	27.3 (25.4 to 29.1)	1.03 (0.96 to 1.10)	0.76 (0.71 to 0.81)
2015	90.6 (84.3 to 97.3)	66.6 (62.1 to 71.4)	27.0 (25.2 to 29.0)	20.0 (18.7 to 21.4)	0.89 (0.83 to 0.95)	0.66 (0.61 to 0.70)
2020	86.7 (69.6 to 106.1)	63.4 (50.9 to 77.6)	24.5 (19.8 to 29.8)	18.0 (14.5 to 22.0)	0.93 (0.75 to 1.14)	0.69 (0.55 to 0.84)
2021	87.0 (68.1 to 110.1)	63.6 (50.0 to 80.2)	24.2 (19.1 to 30.5)	17.8 (14.1 to 22.4)	0.95 (0.75 to 1.19)	0.70 (0.55 to 0.88)

Addis Ababa ETH_44861 Eastern Sub−Saharan Africa Stillbirth Rate / Neonatal Mortality Rate (SBR/NMR) − 20 weeks
lambda = 0.8 ; zeta = 0.7 ; scale = 15 ; amp = 0.028

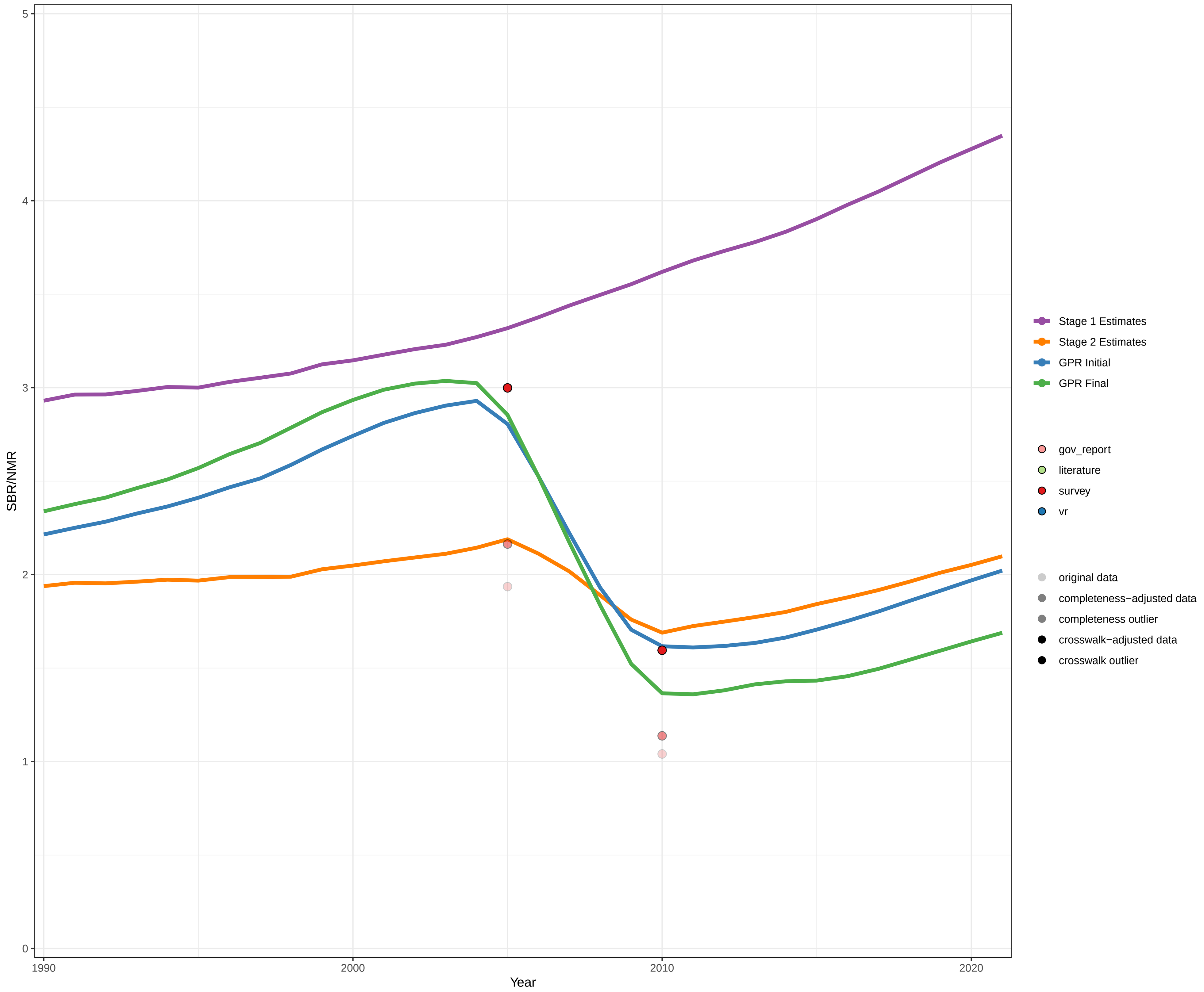

Addis Ababa ETH_44861 Eastern Sub−Saharan Africa Stillbirth Rate / Neonatal Mortality Rate (SBR/NMR) − 28 weeks
lambda = 0.8 ; zeta = 0.7 ; scale = 15 ; amp = 0.028

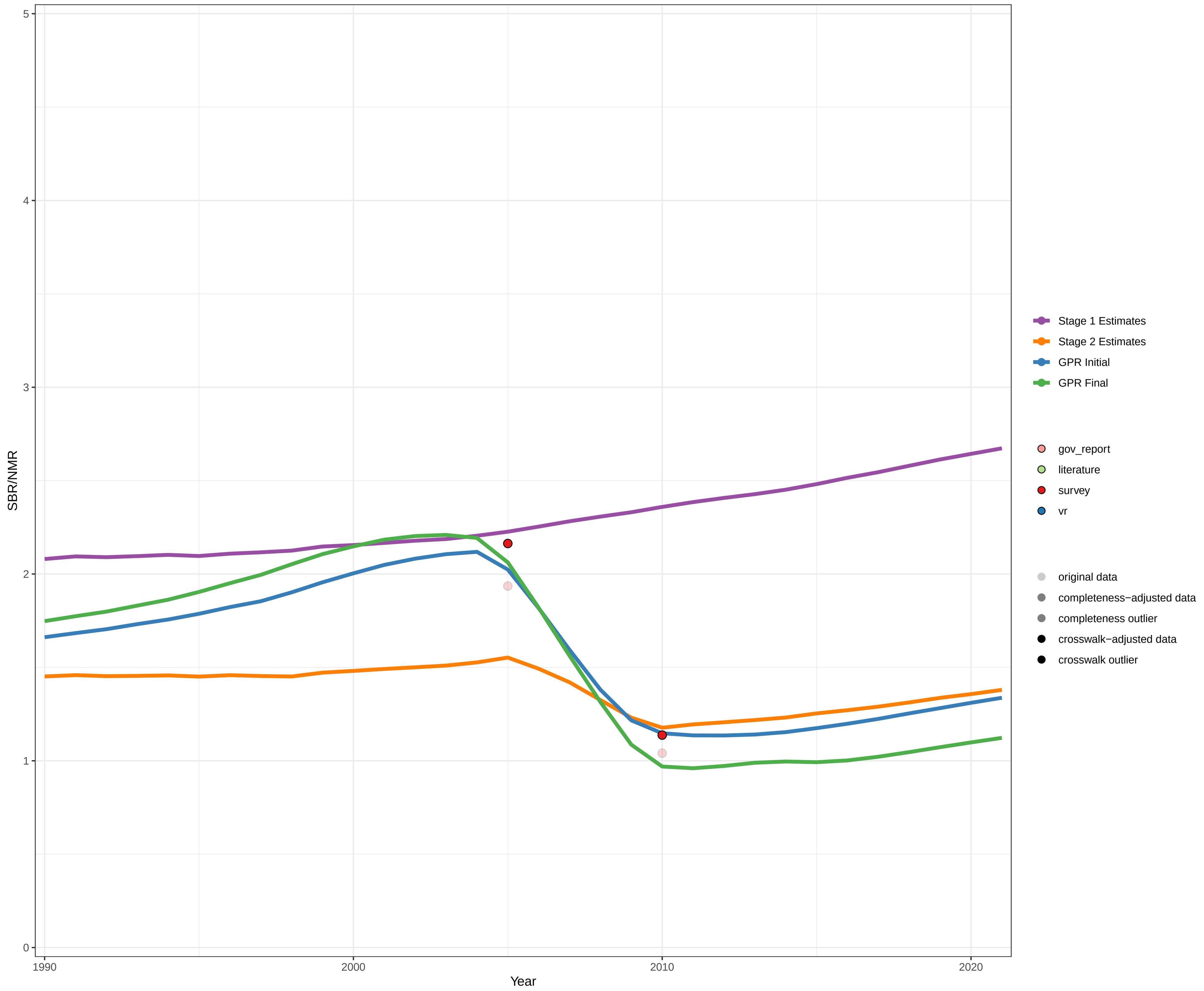

Definition	Years	Outlier	Source.Type	Title
28_weeks	2005	0	Survey	Ethiopia Demographic and Health Survey 2005
28_weeks	2010	0	Survey	Ethiopia Demographic and Health Survey 2010−2011

Addis Ababa ETH_44861 Eastern Sub−Saharan Africa Stillbirth Rate (SBR) − 20 weeks
lambda = 0.8 ; zeta = 0.7 ; scale = 15 ; amp = 0.028

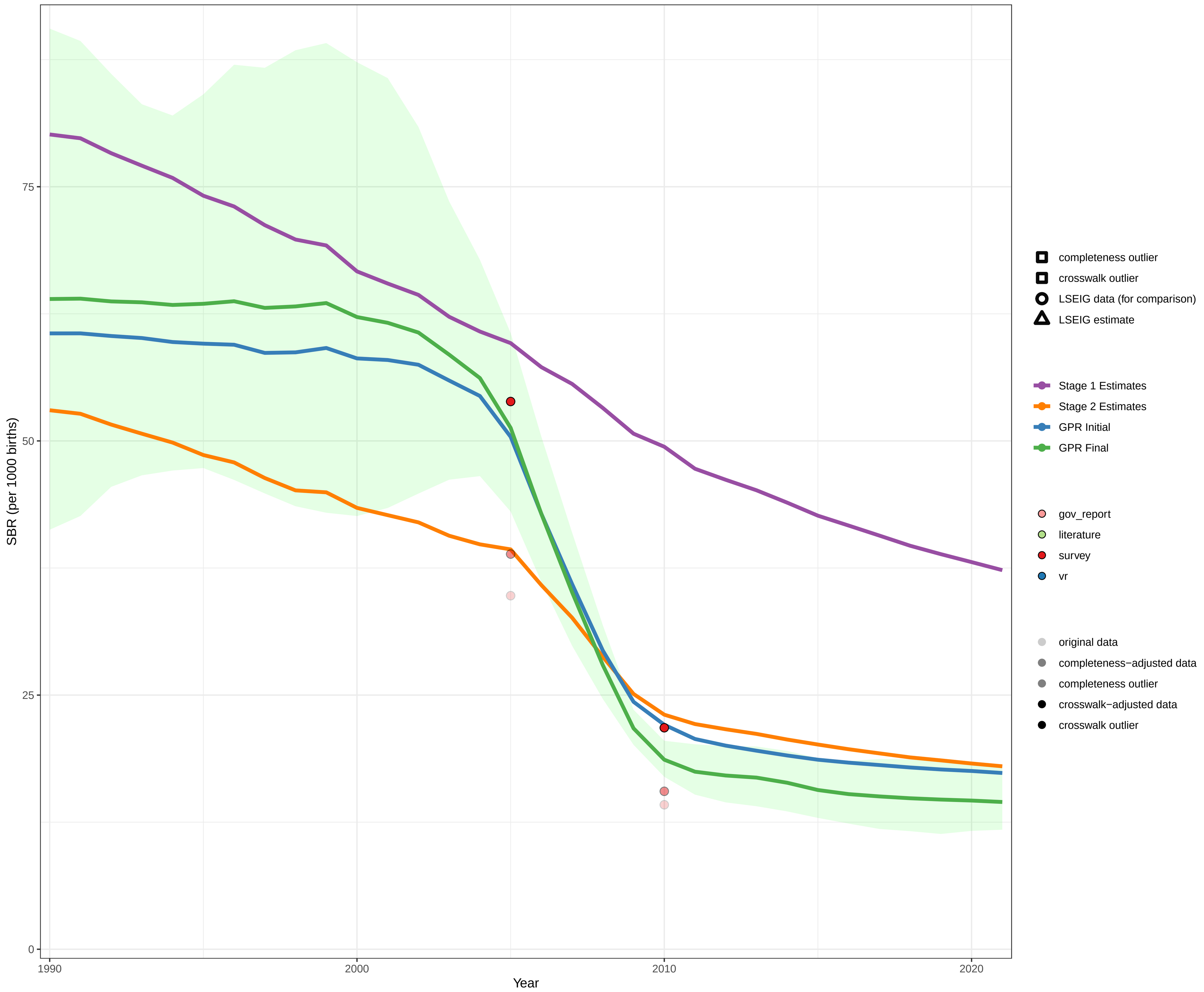

Addis Ababa ETH_44861 Eastern Sub−Saharan Africa Stillbirth Rate (SBR) − 28 weeks
lambda = 0.8 ; zeta = 0.7 ; scale = 15 ; amp = 0.028

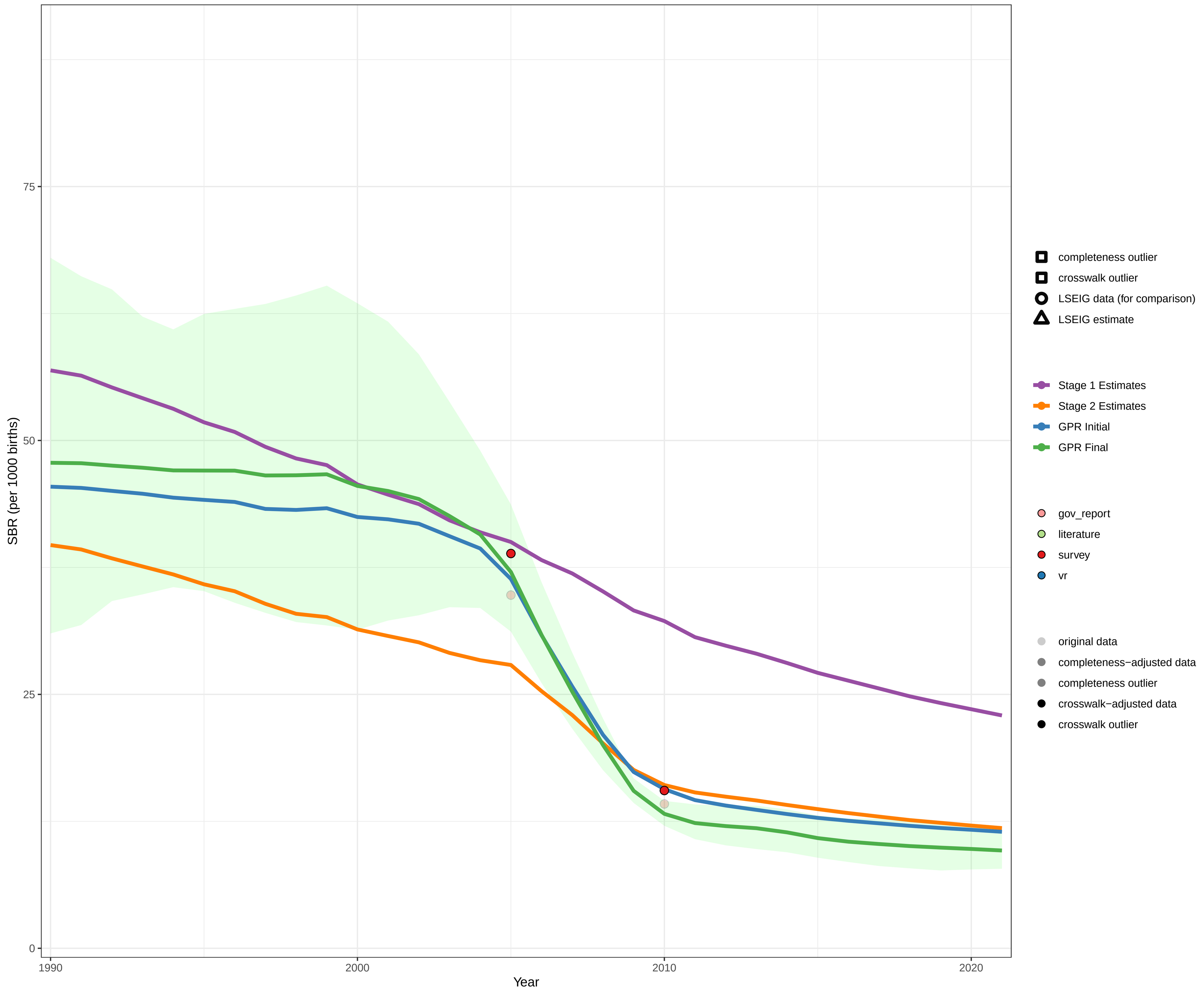

Year	Total Stillbirths (in thousands)	Total Stillbirths (in thousands)	SBR (per 1000 births)	SBR (per 1000 births)	SBR/NMR	SBR/NMR
	20 weeks	28 weeks	20 weeks	28 weeks	20 weeks	28 weeks
1990	4.2 (2.7 to 6.1)	3.1 (2.0 to 4.5)	64.0 (41.3 to 90.6)	47.8 (31.0 to 68.0)	2.34 (1.51 to 3.31)	1.75 (1.13 to 2.49)
1995	3.9 (2.8 to 5.3)	2.8 (2.1 to 3.8)	63.5 (47.3 to 84.1)	47.0 (35.2 to 62.5)	2.57 (1.92 to 3.40)	1.90 (1.42 to 2.53)
2000	3.7 (2.5 to 5.3)	2.6 (1.8 to 3.7)	62.2 (42.6 to 87.3)	45.5 (31.4 to 63.5)	2.93 (2.01 to 4.12)	2.15 (1.48 to 3.00)
2005	3.0 (2.5 to 3.6)	2.1 (1.8 to 2.5)	51.3 (43.1 to 60.6)	37.0 (31.2 to 43.7)	2.85 (2.40 to 3.37)	2.06 (1.74 to 2.43)
2010	1.1 (1.0 to 1.2)	0.7 (0.7 to 0.8)	18.6 (17.0 to 20.5)	13.2 (12.1 to 14.5)	1.37 (1.24 to 1.50)	0.97 (0.88 to 1.06)
2015	0.9 (0.7 to 1.1)	0.6 (0.5 to 0.7)	15.7 (12.9 to 18.8)	10.8 (8.9 to 13.1)	1.43 (1.18 to 1.72)	0.99 (0.82 to 1.20)
2020	0.7 (0.6 to 0.9)	0.5 (0.4 to 0.6)	14.6 (11.6 to 18.2)	9.8 (7.8 to 12.1)	1.64 (1.31 to 2.04)	1.10 (0.87 to 1.36)
2021	0.7 (0.6 to 0.9)	0.5 (0.4 to 0.6)	14.5 (11.8 to 17.9)	9.6 (7.8 to 11.9)	1.69 (1.37 to 2.09)	1.12 (0.91 to 1.39)

Afar ETH_44853 Eastern Sub−Saharan Africa Stillbirth Rate / Neonatal Mortality Rate (SBR/NMR) − 20 weeks
lambda = 1 ; zeta = 0.6 ; scale = 15 ; amp = 0.028

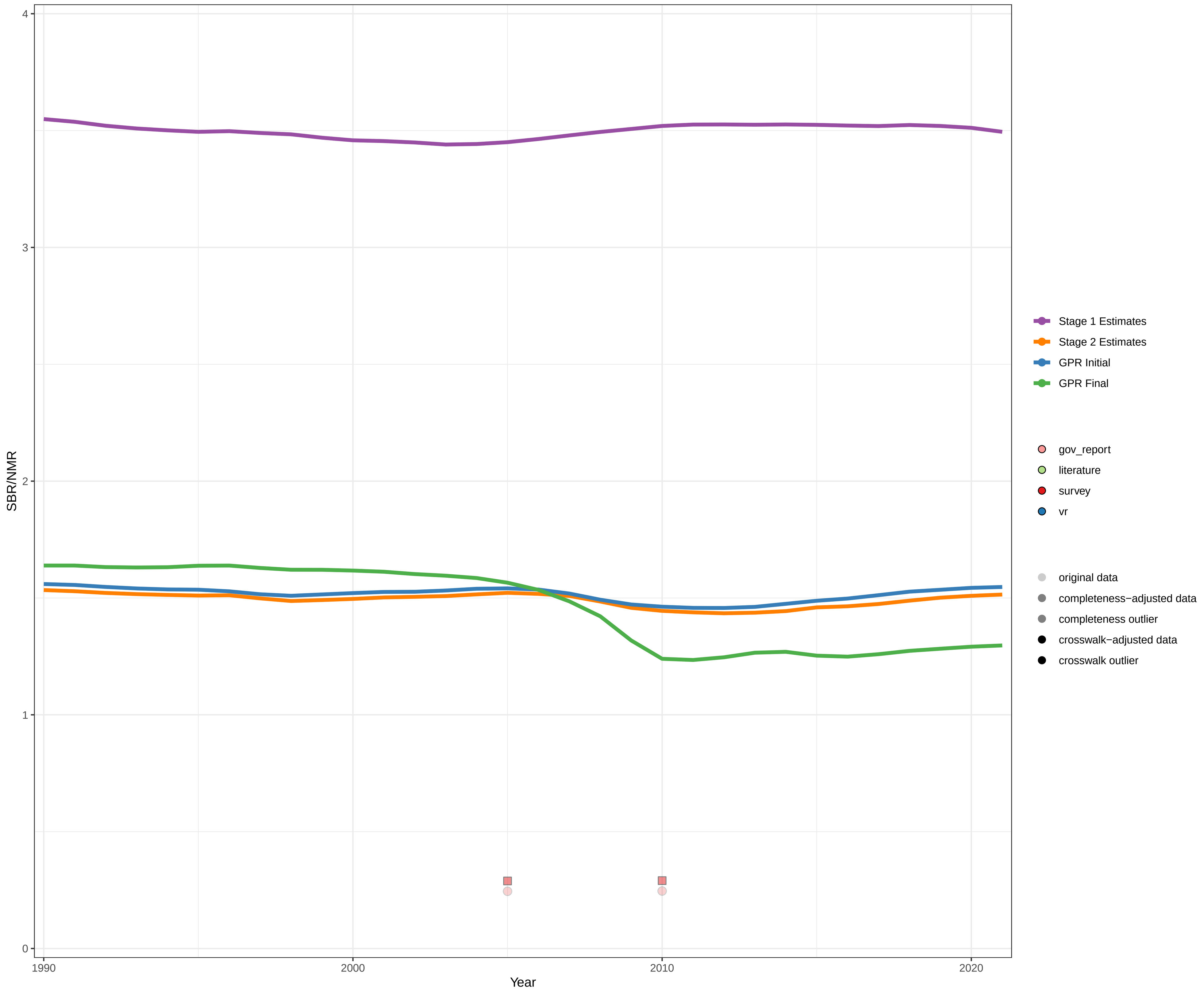

Afar ETH_44853 Eastern Sub−Saharan Africa Stillbirth Rate / Neonatal Mortality Rate (SBR/NMR) − 28 weeks
lambda = 1 ; zeta = 0.6 ; scale = 15 ; amp = 0.028

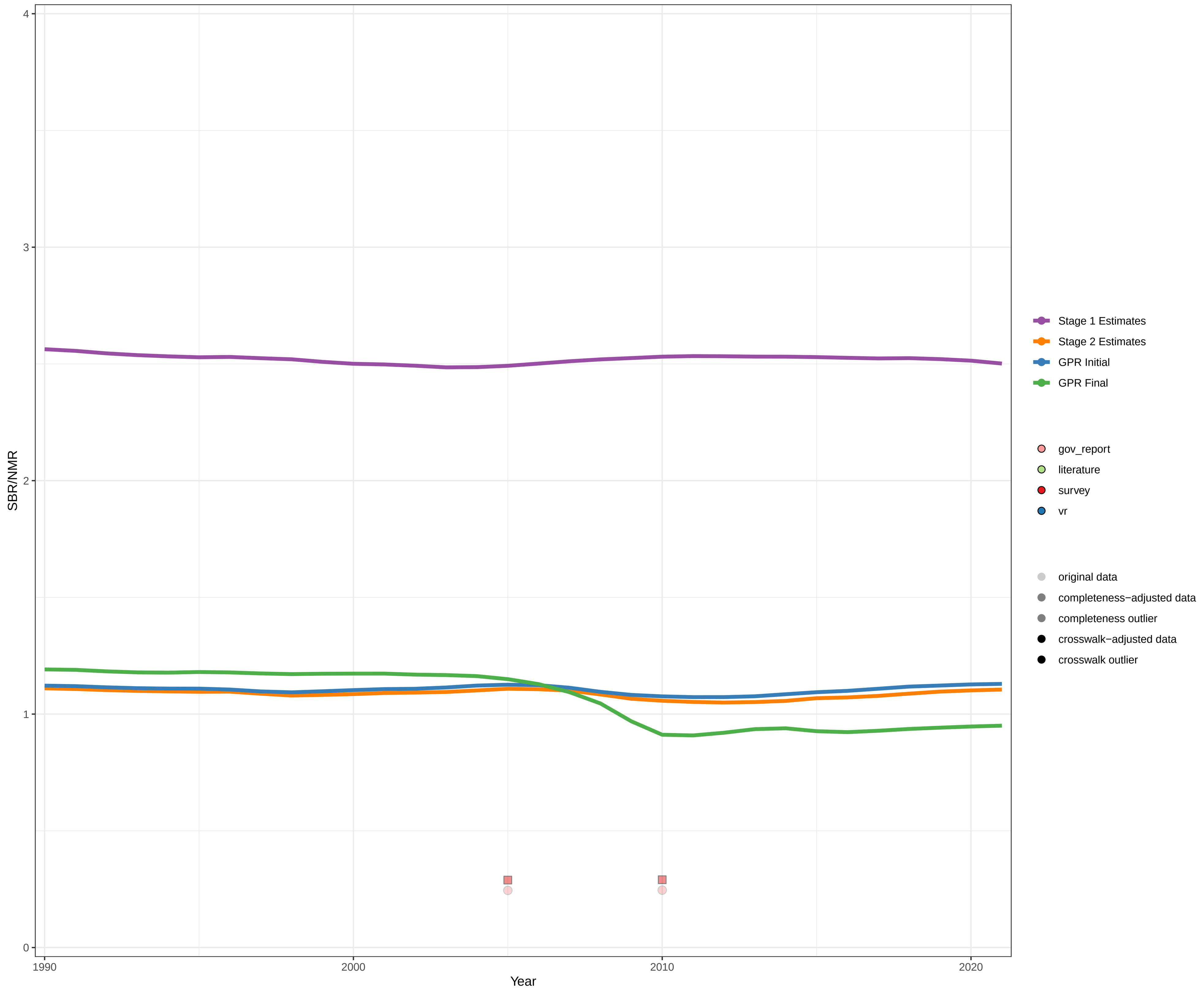

Definition	Years	Outlier	Source.Type	Title
28_weeks	2005	1	Survey	Ethiopia Demographic and Health Survey 2005
28_weeks	2010	1	Survey	Ethiopia Demographic and Health Survey 2010−2011

Afar ETH_44853 Eastern Sub−Saharan Africa Stillbirth Rate (SBR) − 20 weeks
lambda = 1 ; zeta = 0.6 ; scale = 15 ; amp = 0.028

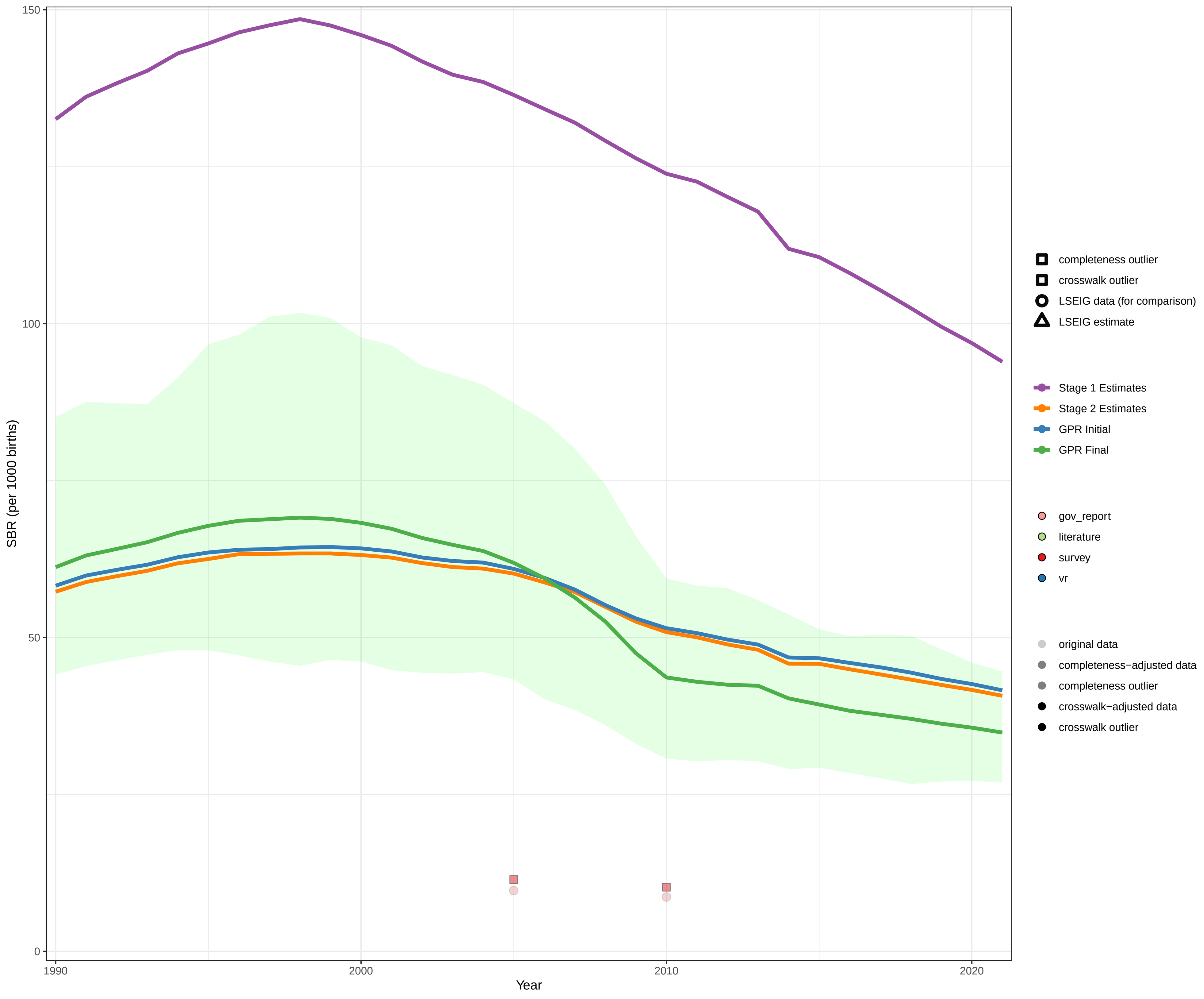

Afar ETH_44853 Eastern Sub−Saharan Africa Stillbirth Rate (SBR) − 28 weeks
lambda = 1 ; zeta = 0.6 ; scale = 15 ; amp = 0.028

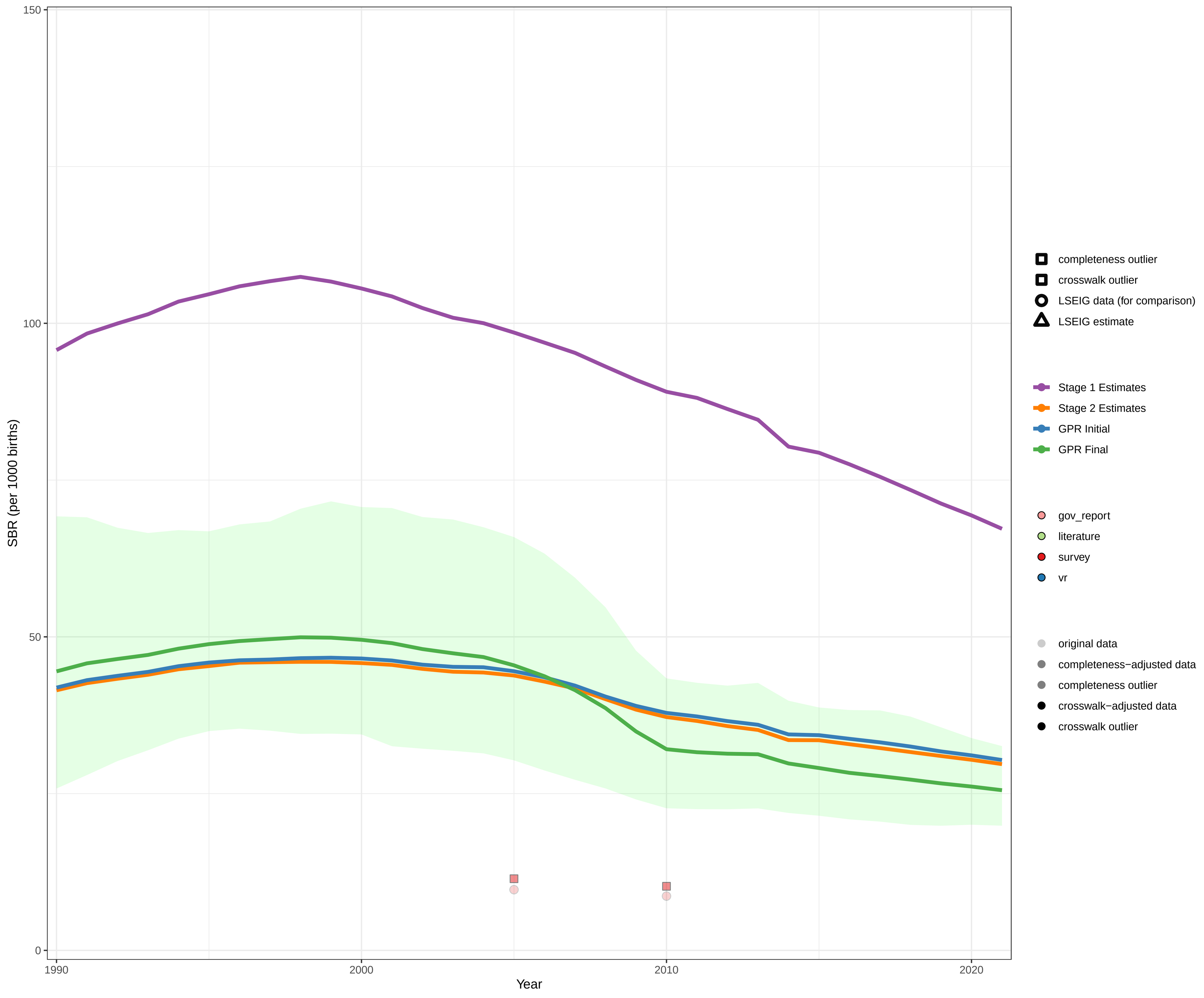

Year	Total Stillbirths (in thousands)	Total Stillbirths (in thousands)	SBR (per 1000 births)	SBR (per 1000 births)	SBR/NMR	SBR/NMR
	20 weeks	28 weeks	20 weeks	28 weeks	20 weeks	28 weeks
1990	2.4 (1.7 to 3.4)	1.7 (1.0 to 2.8)	61.2 (44.1 to 85.1)	44.5 (25.8 to 69.2)	1.64 (1.18 to 2.28)	1.19 (0.69 to 1.85)
1995	4.2 (2.9 to 6.2)	3.0 (2.1 to 4.1)	67.8 (47.9 to 96.7)	48.8 (35.0 to 66.8)	1.64 (1.16 to 2.34)	1.18 (0.84 to 1.61)
2000	4.9 (3.2 to 7.2)	3.5 (2.4 to 5.1)	68.3 (46.2 to 97.8)	49.5 (34.4 to 70.7)	1.62 (1.09 to 2.32)	1.17 (0.82 to 1.68)
2005	4.4 (3.0 to 6.3)	3.2 (2.1 to 4.7)	61.9 (43.3 to 87.3)	45.5 (30.3 to 65.9)	1.57 (1.10 to 2.21)	1.15 (0.77 to 1.67)
2010	2.9 (2.0 to 4.0)	2.1 (1.5 to 2.9)	43.6 (30.7 to 59.4)	32.1 (22.7 to 43.4)	1.24 (0.87 to 1.69)	0.91 (0.64 to 1.23)
2015	2.6 (1.9 to 3.4)	1.9 (1.4 to 2.6)	39.3 (29.3 to 51.3)	29.1 (21.5 to 38.7)	1.25 (0.93 to 1.64)	0.93 (0.68 to 1.23)
2020	2.3 (1.8 to 3.0)	1.7 (1.3 to 2.2)	35.6 (27.2 to 46.0)	26.1 (20.0 to 33.9)	1.29 (0.98 to 1.67)	0.95 (0.73 to 1.23)
2021	2.3 (1.7 to 2.9)	1.6 (1.3 to 2.1)	34.9 (26.9 to 44.6)	25.5 (19.9 to 32.6)	1.30 (1.00 to 1.66)	0.95 (0.74 to 1.21)

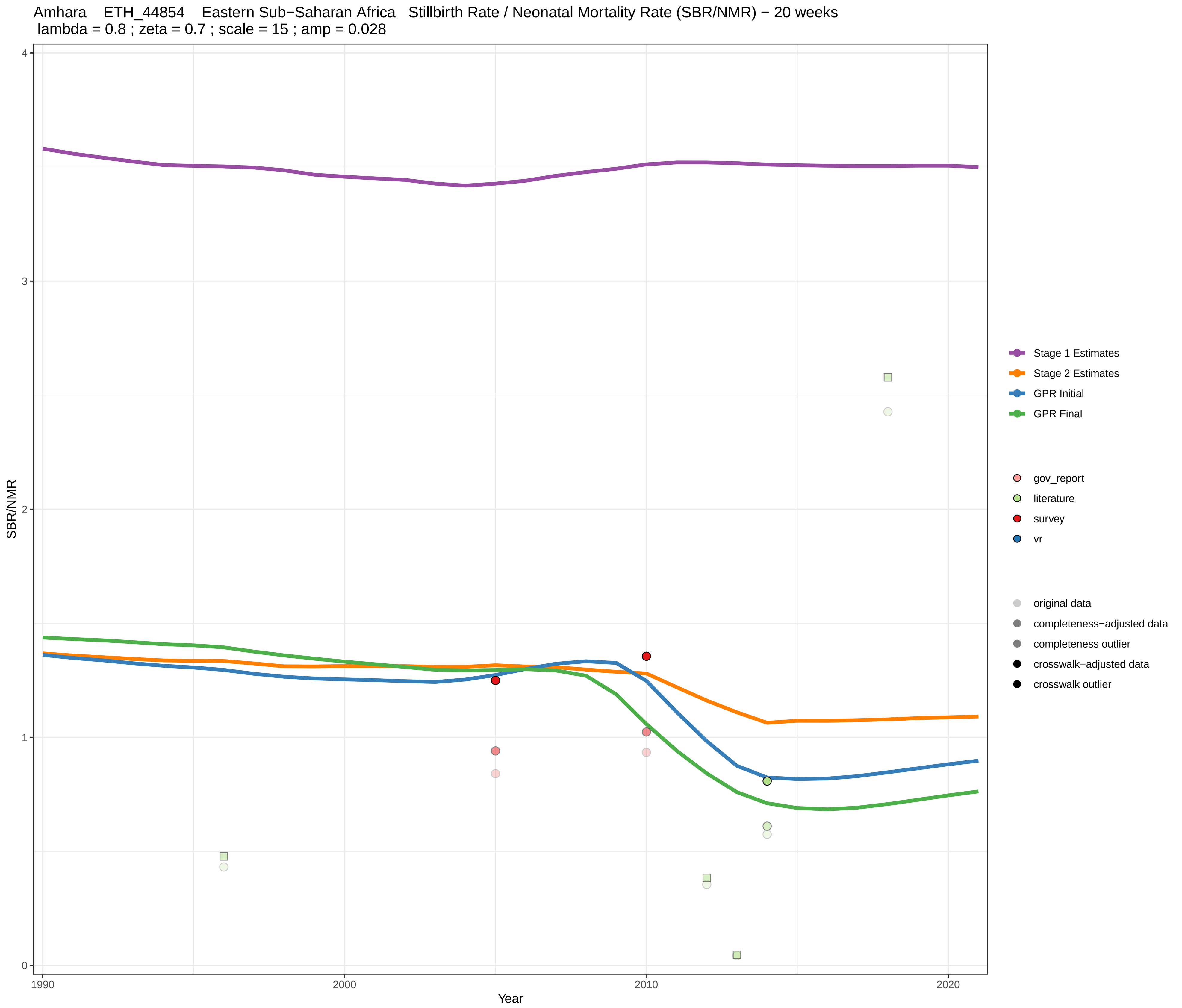

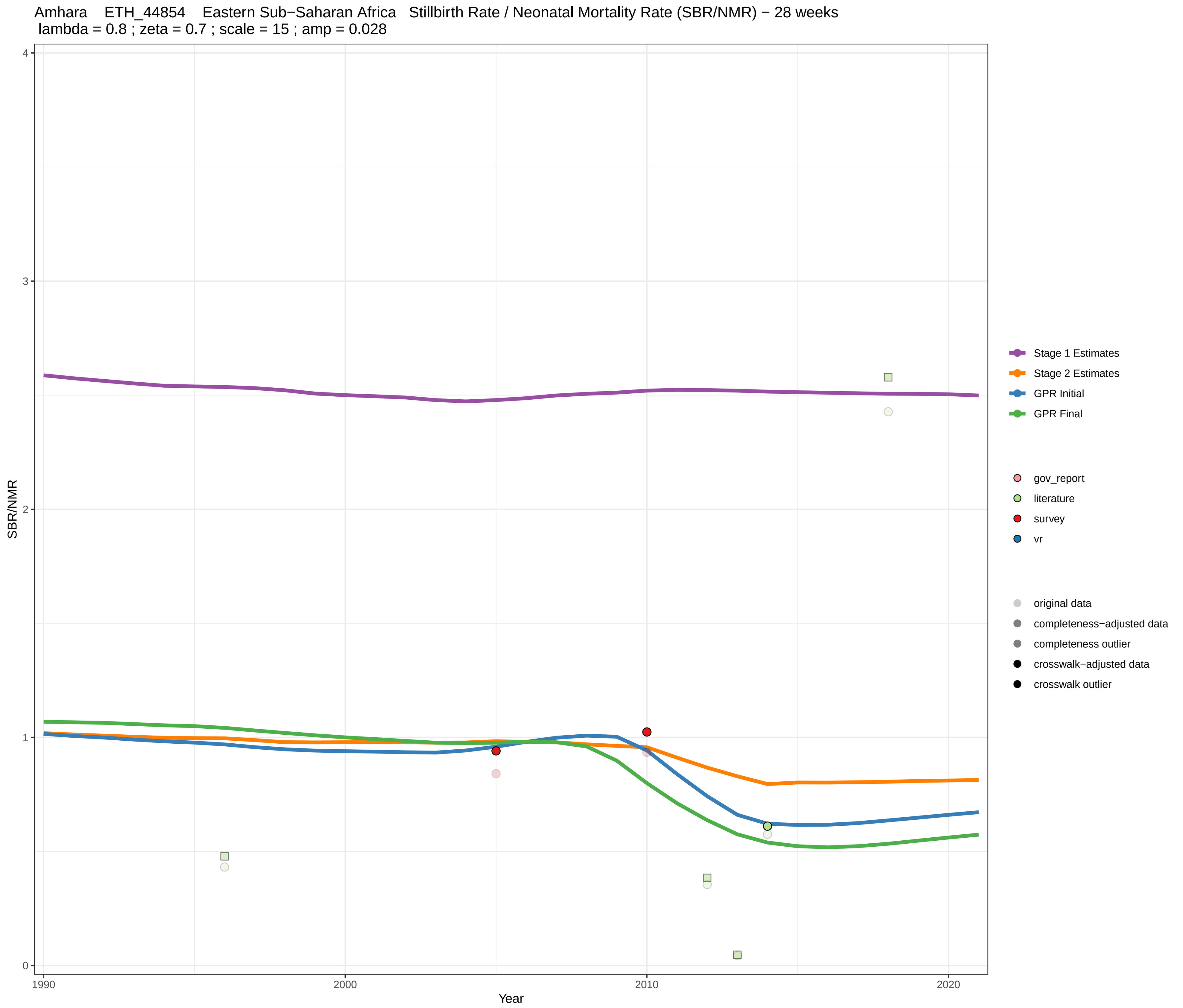

Definition	Years	Outlier	Source.Type	Title
28_weeks	2005	0	Survey	Ethiopia Demographic and Health Survey 2005
28_weeks	2010	0	Survey	Ethiopia Demographic and Health Survey 2010−2011
28_weeks	2014	0	Sci Lit	Effect of prenatal and intrapartum care on the stillbirth rate among women in rural Ethiopia
28_weeks	1996	1	Sci Lit	Predictors of perinatal mortality in rural population of Northwest Ethiopia: a prospective longitudinal study
28_weeks	2012	1	Sci Lit	Perinatal Mortality Magnitude, Determinants and Causes in West Gojam: Population−Based Nested Case−Control Study
28_weeks	2013	1	Sci Lit	Adverse birth outcomes among deliveries at Gondar University Hospital, Northwest Ethiopia
28_weeks	2018	1	Sci Lit	Adverse birth outcomes and its associated factors among women who delivered in North Wollo zone, northeast Ethiopia: a facility based cross−sectional study

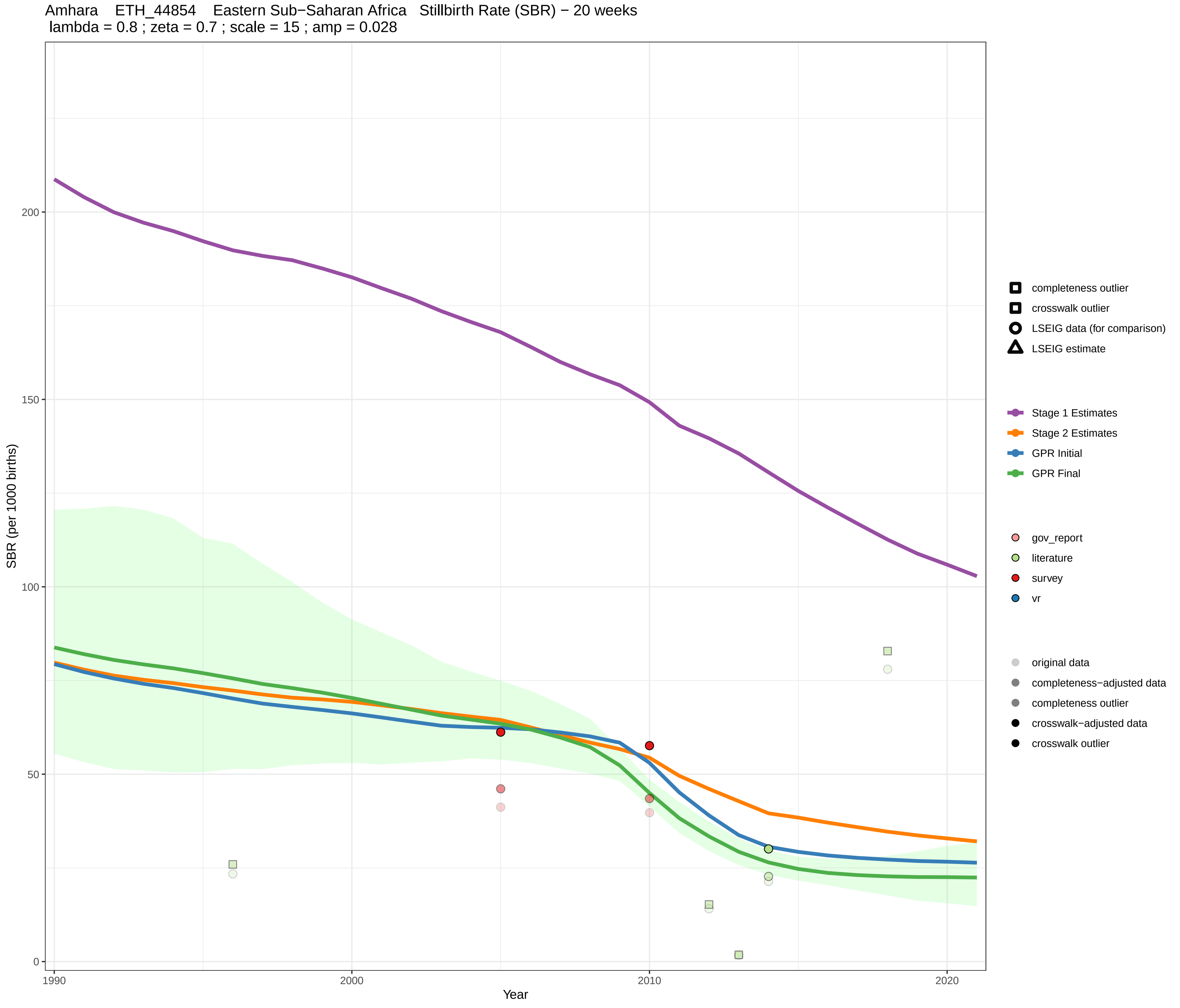

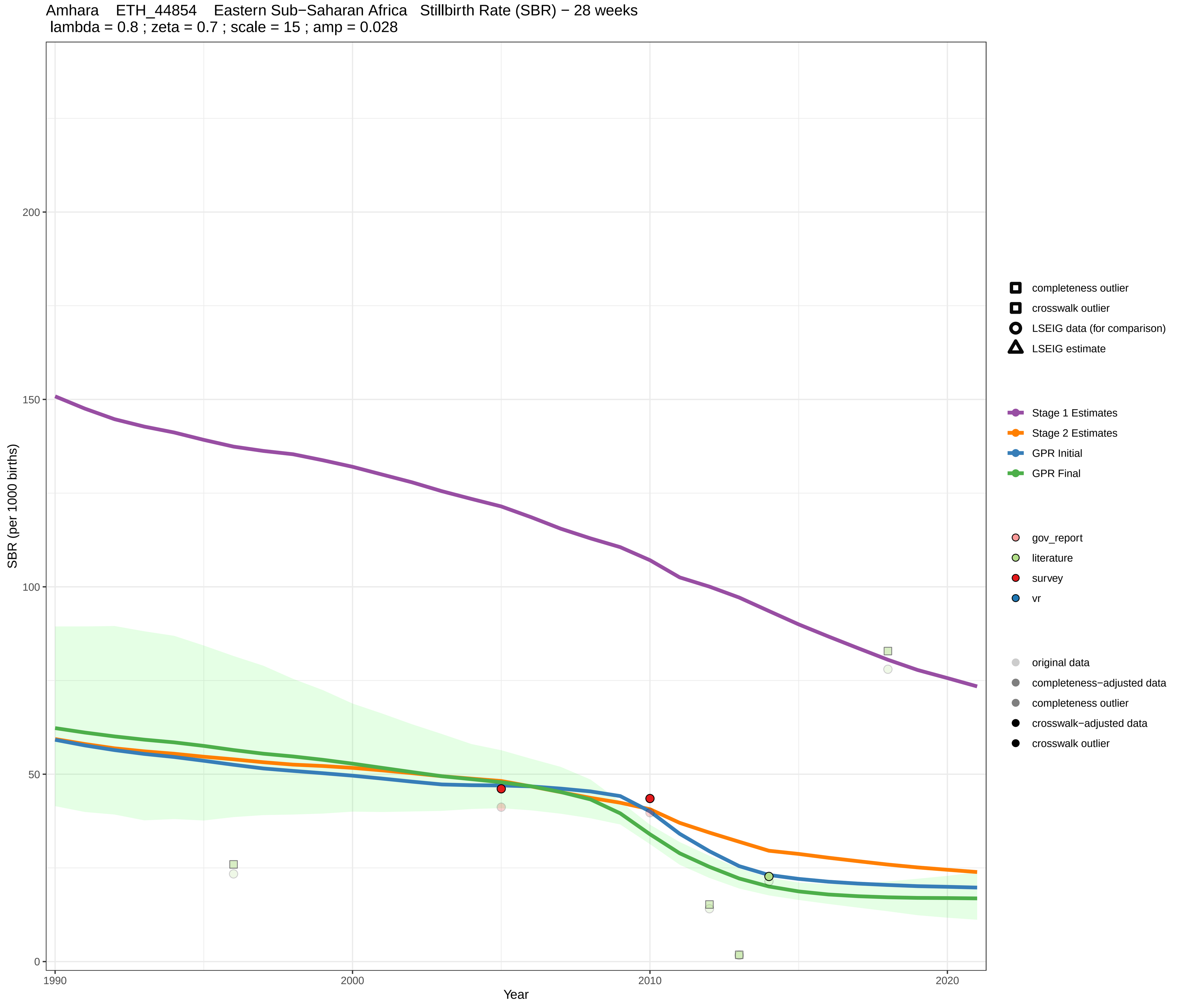

Year	Total Stillbirths (in thousands)	Total Stillbirths (in thousands)	SBR (per 1000 births)	SBR (per 1000 births)	SBR/NMR	SBR/NMR
	20 weeks	28 weeks	20 weeks	28 weeks	20 weeks	28 weeks
1990	59.9 (38.2 to 89.4)	43.4 (28.2 to 64.0)	83.8 (55.4 to 120.6)	62.3 (41.5 to 89.4)	1.44 (0.95 to 2.07)	1.07 (0.71 to 1.53)
1995	59.5 (37.8 to 90.6)	43.5 (27.8 to 65.5)	77.0 (50.5 to 113.1)	57.5 (37.6 to 84.3)	1.40 (0.92 to 2.06)	1.05 (0.69 to 1.54)
2000	54.3 (40.1 to 72.1)	40.0 (29.9 to 53.0)	70.3 (53.0 to 91.3)	52.8 (40.0 to 68.9)	1.33 (1.00 to 1.73)	1.00 (0.76 to 1.30)
2005	46.2 (38.8 to 55.2)	34.3 (29.1 to 40.7)	63.5 (53.9 to 74.9)	47.9 (40.9 to 56.4)	1.30 (1.10 to 1.53)	0.98 (0.84 to 1.15)
2010	29.1 (26.6 to 31.5)	21.7 (20.0 to 23.4)	45.0 (41.3 to 48.5)	34.0 (31.3 to 36.5)	1.06 (0.97 to 1.14)	0.80 (0.74 to 0.86)
2015	15.3 (13.3 to 17.4)	11.5 (10.1 to 13.0)	24.7 (21.6 to 28.0)	18.7 (16.4 to 21.1)	0.69 (0.60 to 0.78)	0.52 (0.46 to 0.59)
2020	14.4 (9.9 to 19.9)	10.8 (7.4 to 14.6)	22.5 (15.6 to 30.9)	16.9 (11.7 to 22.9)	0.75 (0.52 to 1.02)	0.56 (0.39 to 0.76)
2021	14.5 (9.5 to 20.8)	10.8 (7.1 to 15.4)	22.4 (14.8 to 31.9)	16.9 (11.2 to 23.9)	0.76 (0.50 to 1.08)	0.57 (0.38 to 0.81)

Benishangul−Gumuz ETH_44857 Eastern Sub−Saharan Africa Stillbirth Rate / Neonatal Mortality Rate (SBR/NMR) − 20 weeks
lambda = 0.8 ; zeta = 0.7 ; scale = 15 ; amp = 0.028

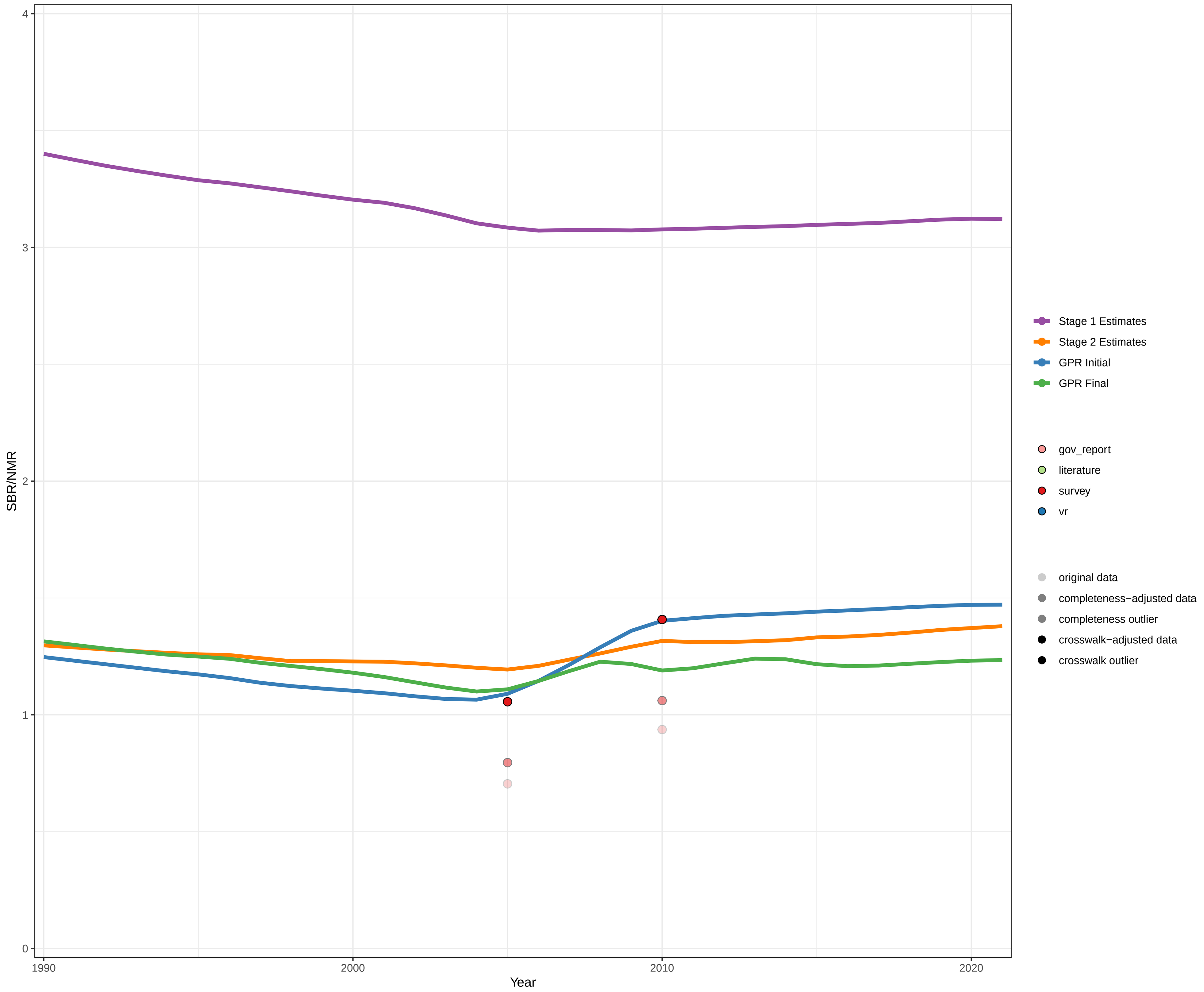

Benishangul−Gumuz ETH_44857 Eastern Sub−Saharan Africa Stillbirth Rate / Neonatal Mortality Rate (SBR/NMR) − 28 weeks
lambda = 0.8 ; zeta = 0.7 ; scale = 15 ; amp = 0.028

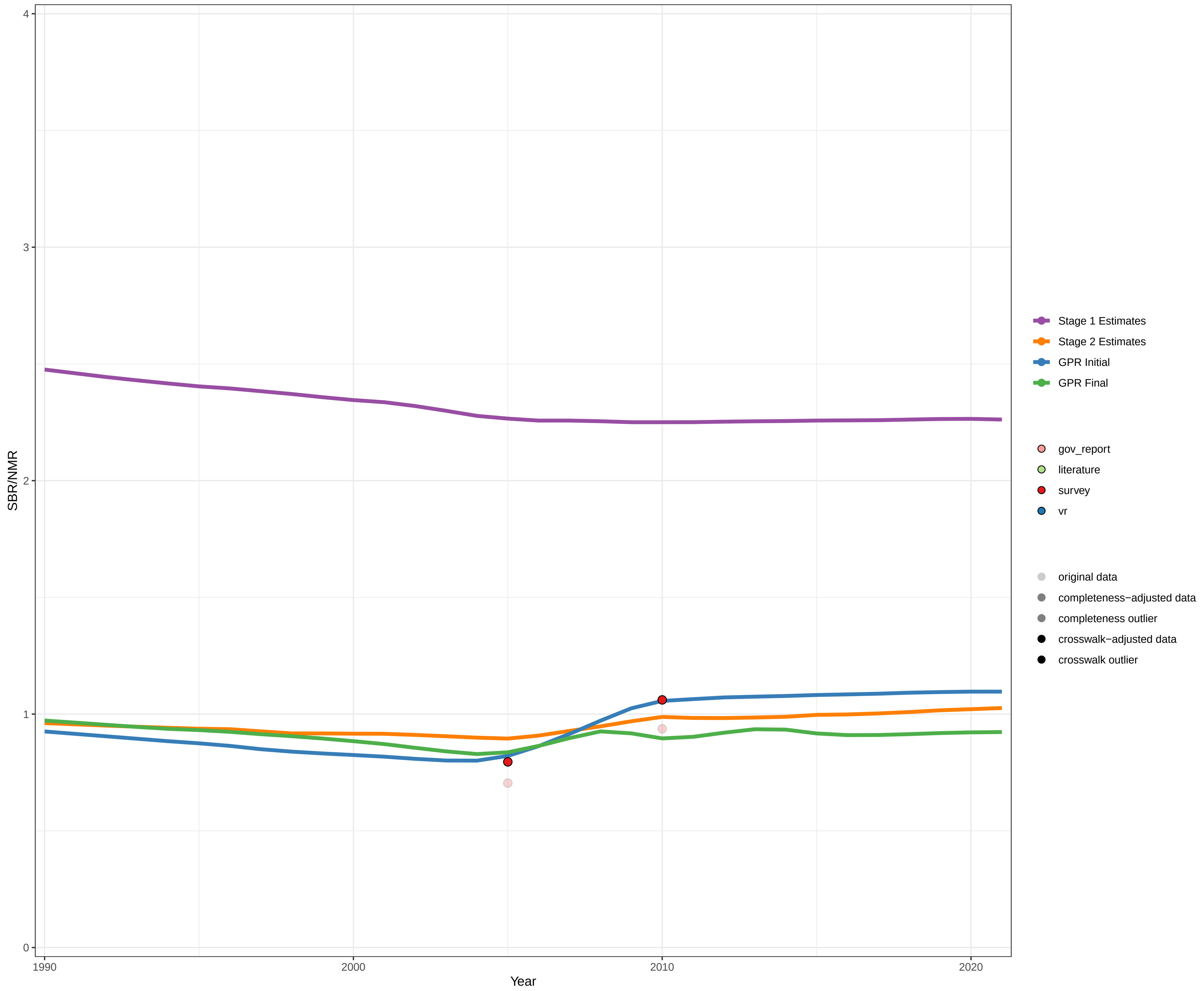

Definition	Years	Outlier	Source.Type	Title
28_weeks	2005	0	Survey	Ethiopia Demographic and Health Survey 2005
28_weeks	2010	0	Survey	Ethiopia Demographic and Health Survey 2010−2011

Benishangul−Gumuz ETH_44857 Eastern Sub−Saharan Africa Stillbirth Rate (SBR) − 20 weeks
lambda = 0.8 ; zeta = 0.7 ; scale = 15 ; amp = 0.028

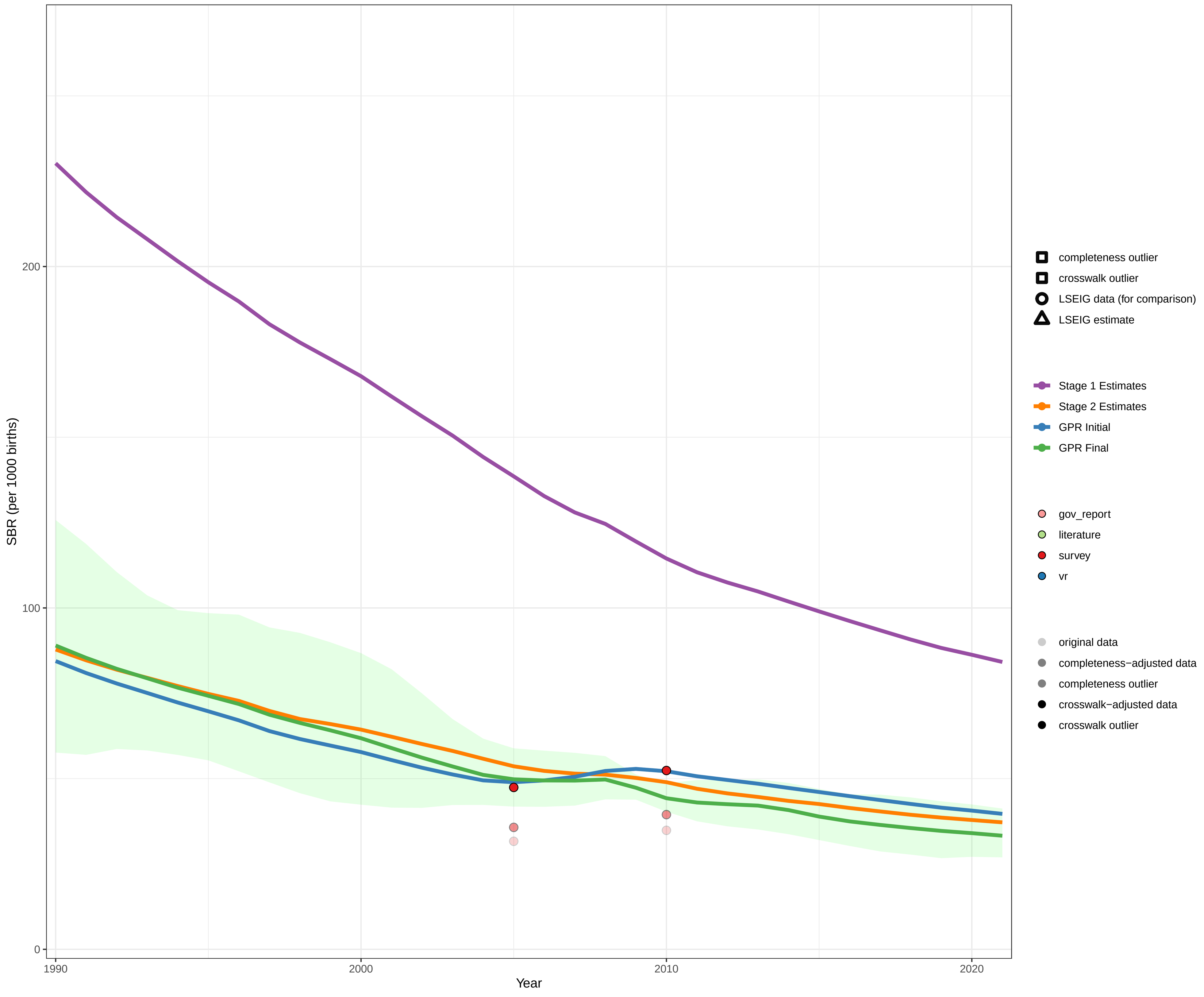

Benishangul−Gumuz ETH_44857 Eastern Sub−Saharan Africa Stillbirth Rate (SBR) − 28 weeks
lambda = 0.8 ; zeta = 0.7 ; scale = 15 ; amp = 0.028

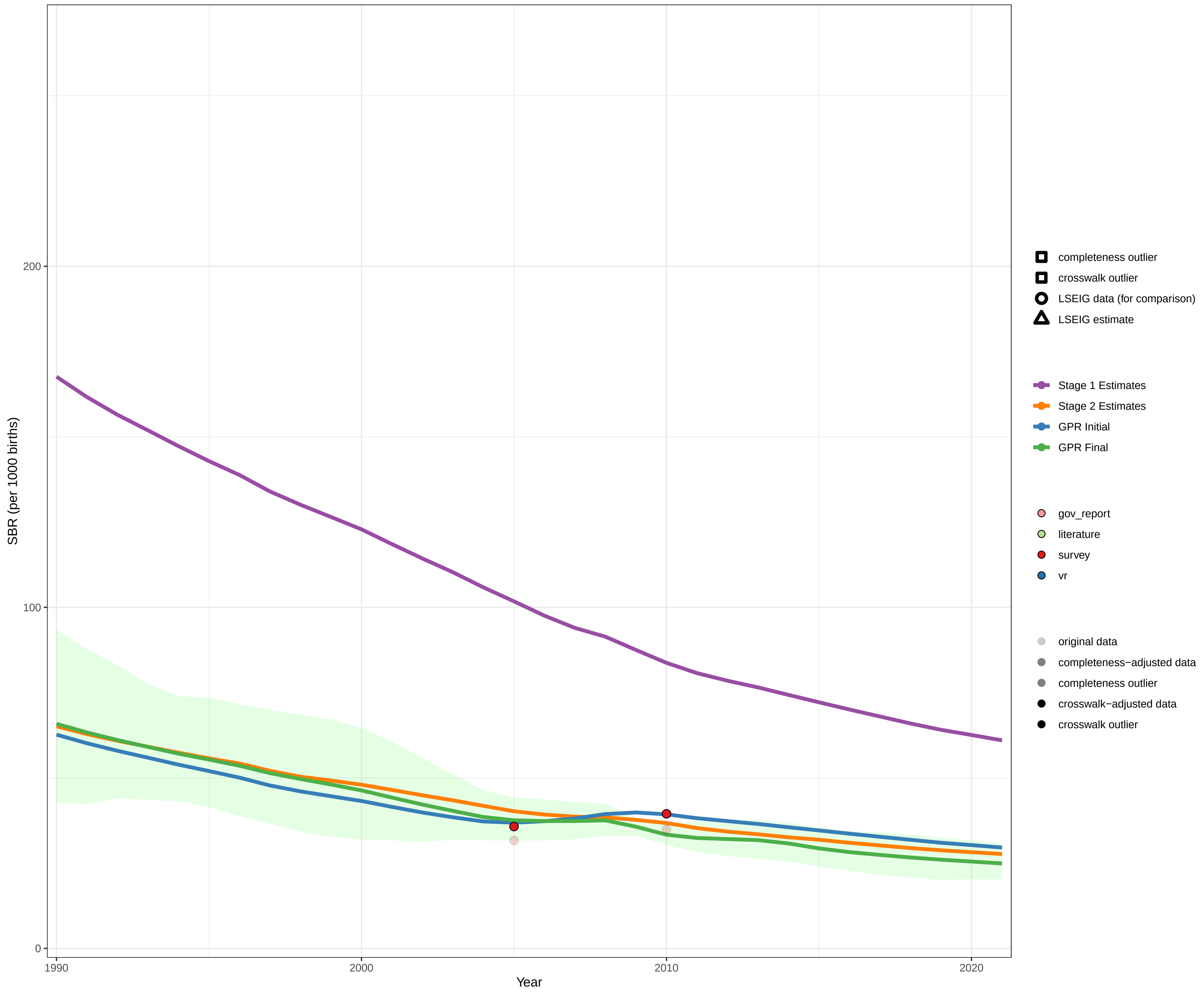

Year	Total Stillbirths (in thousands)	Total Stillbirths (in thousands)	SBR (per 1000 births)	SBR (per 1000 births)	SBR/NMR	SBR/NMR
	20 weeks	28 weeks	20 weeks	28 weeks	20 weeks	28 weeks
1990	2.4 (1.5 to 3.6)	1.8 (1.1 to 2.6)	89.0 (57.6 to 125.8)	65.8 (42.9 to 93.4)	1.31 (0.85 to 1.86)	0.97 (0.63 to 1.38)
1995	2.1 (1.5 to 2.8)	1.5 (1.1 to 2.1)	74.3 (55.4 to 98.5)	55.4 (41.4 to 73.6)	1.25 (0.93 to 1.66)	0.93 (0.70 to 1.24)
2000	1.8 (1.2 to 2.7)	1.4 (0.9 to 1.9)	61.8 (42.4 to 86.8)	46.3 (31.9 to 64.6)	1.18 (0.81 to 1.66)	0.88 (0.61 to 1.23)
2005	1.6 (1.4 to 2.0)	1.2 (1.0 to 1.5)	49.8 (41.8 to 58.9)	37.6 (31.6 to 44.3)	1.11 (0.93 to 1.31)	0.84 (0.70 to 0.99)
2010	1.6 (1.4 to 1.7)	1.2 (1.1 to 1.3)	44.3 (40.3 to 48.6)	33.3 (30.4 to 36.5)	1.19 (1.08 to 1.31)	0.90 (0.82 to 0.98)
2015	1.5 (1.2 to 1.8)	1.1 (0.9 to 1.3)	38.9 (32.0 to 47.0)	29.3 (24.1 to 35.5)	1.22 (1.00 to 1.47)	0.92 (0.75 to 1.11)
2020	1.4 (1.1 to 1.7)	1.0 (0.8 to 1.3)	34.0 (27.1 to 42.4)	25.5 (20.2 to 31.6)	1.23 (0.98 to 1.54)	0.92 (0.73 to 1.14)
2021	1.3 (1.1 to 1.7)	1.0 (0.8 to 1.2)	33.3 (26.9 to 41.2)	24.9 (20.2 to 30.8)	1.23 (1.00 to 1.53)	0.92 (0.75 to 1.14)

Dire Dawa   ETH_44862   Eastern Sub−Saharan Africa   Stillbirth Rate / Neonatal Mortality Rate (SBR/NMR) − 20 weeks
lambda = 0.8 ; zeta = 0.7 ; scale = 15 ; amp = 0.028

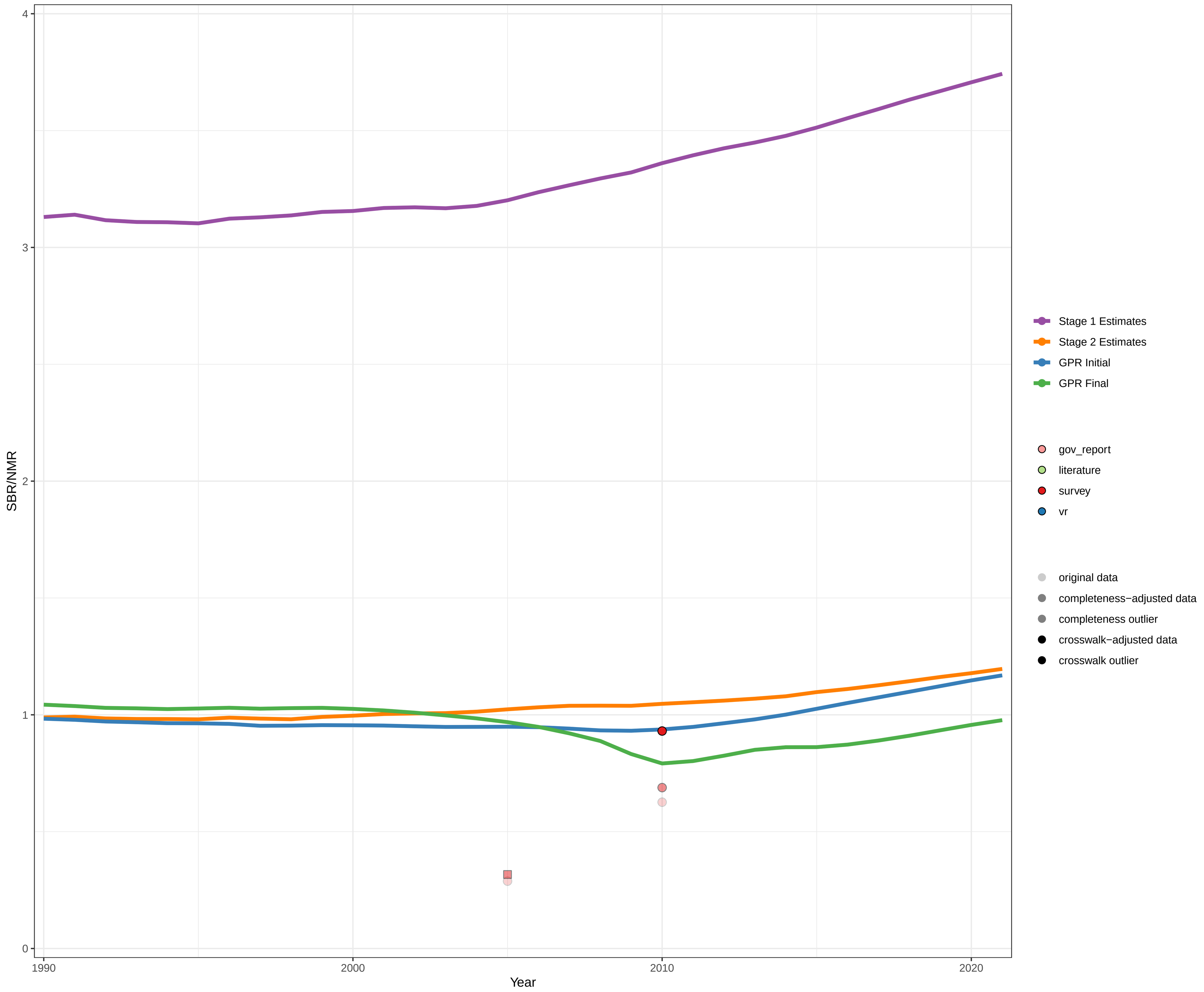

Dire Dawa   ETH_44862   Eastern Sub−Saharan Africa   Stillbirth Rate / Neonatal Mortality Rate (SBR/NMR) − 28 weeks
lambda = 0.8 ; zeta = 0.7 ; scale = 15 ; amp = 0.028

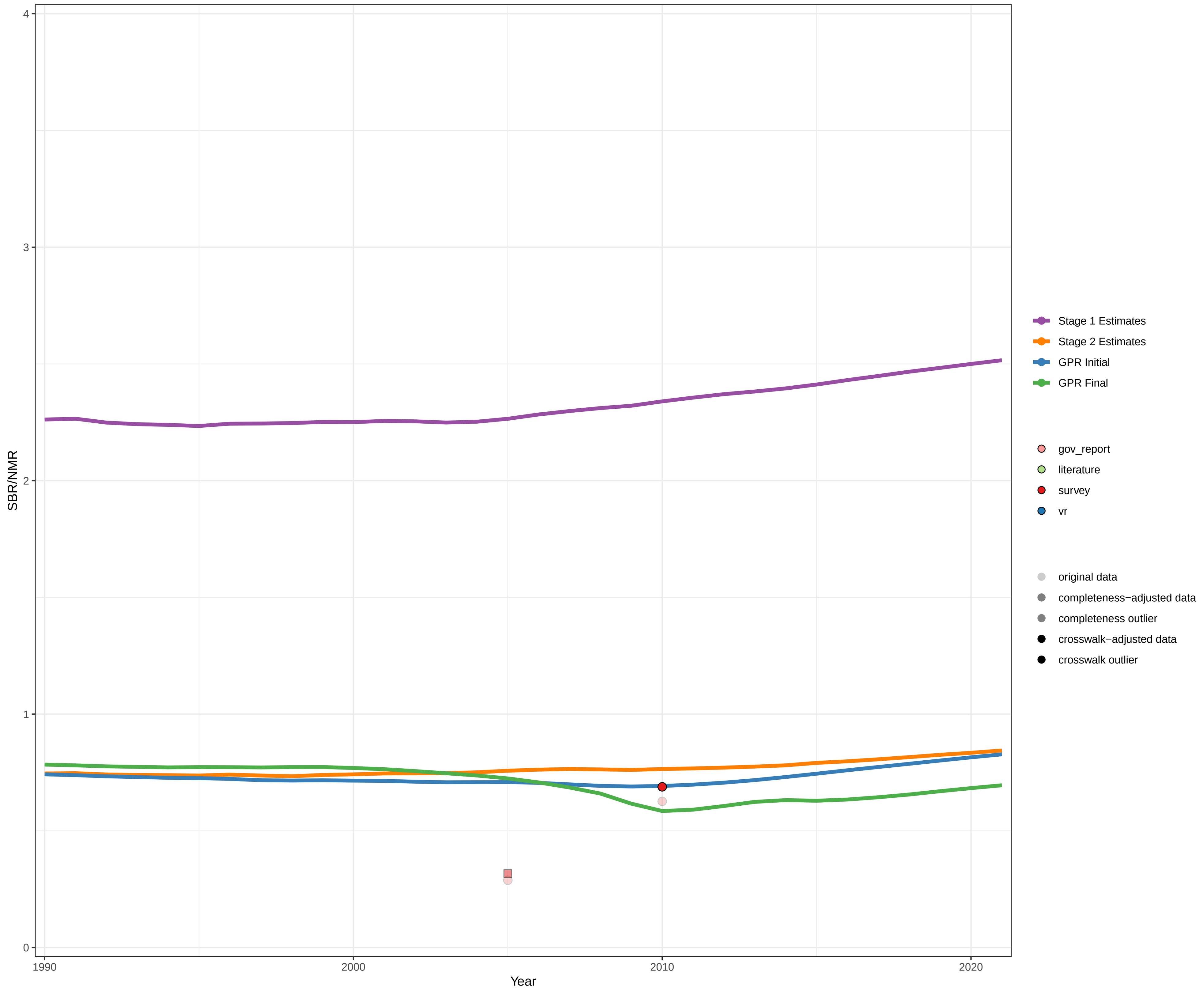

Definition	Years	Outlier	Source.Type	Title
28_weeks	2010	0	Survey	Ethiopia Demographic and Health Survey 2010−2011
28_weeks	2005	1	Survey	Ethiopia Demographic and Health Survey 2005

Dire Dawa   ETH_44862   Eastern Sub−Saharan Africa   Stillbirth Rate (SBR) − 20 weeks
lambda = 0.8 ; zeta = 0.7 ; scale = 15 ; amp = 0.028

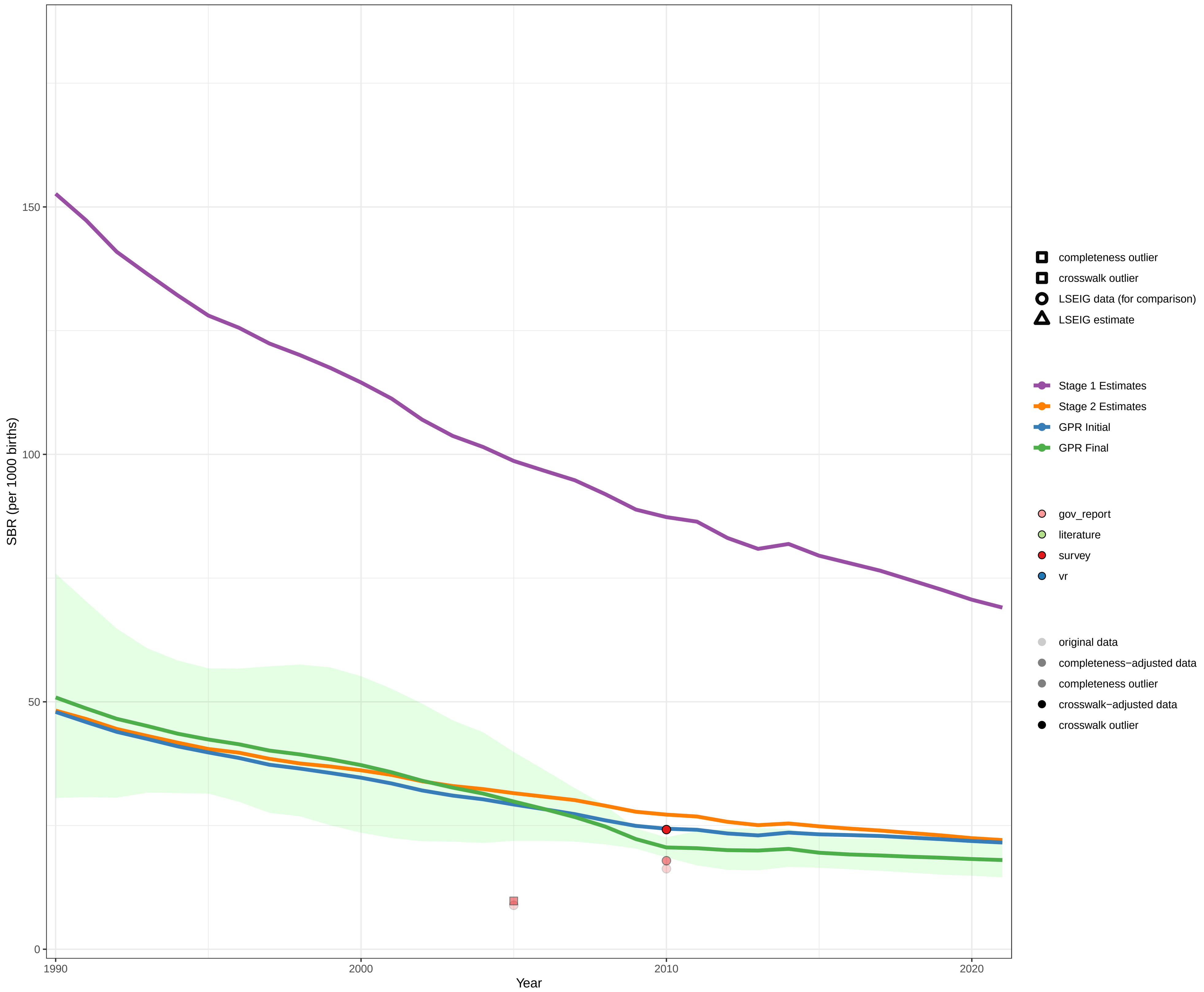

Dire Dawa   ETH_44862   Eastern Sub−Saharan Africa   Stillbirth Rate (SBR) − 28 weeks
lambda = 0.8 ; zeta = 0.7 ; scale = 15 ; amp = 0.028

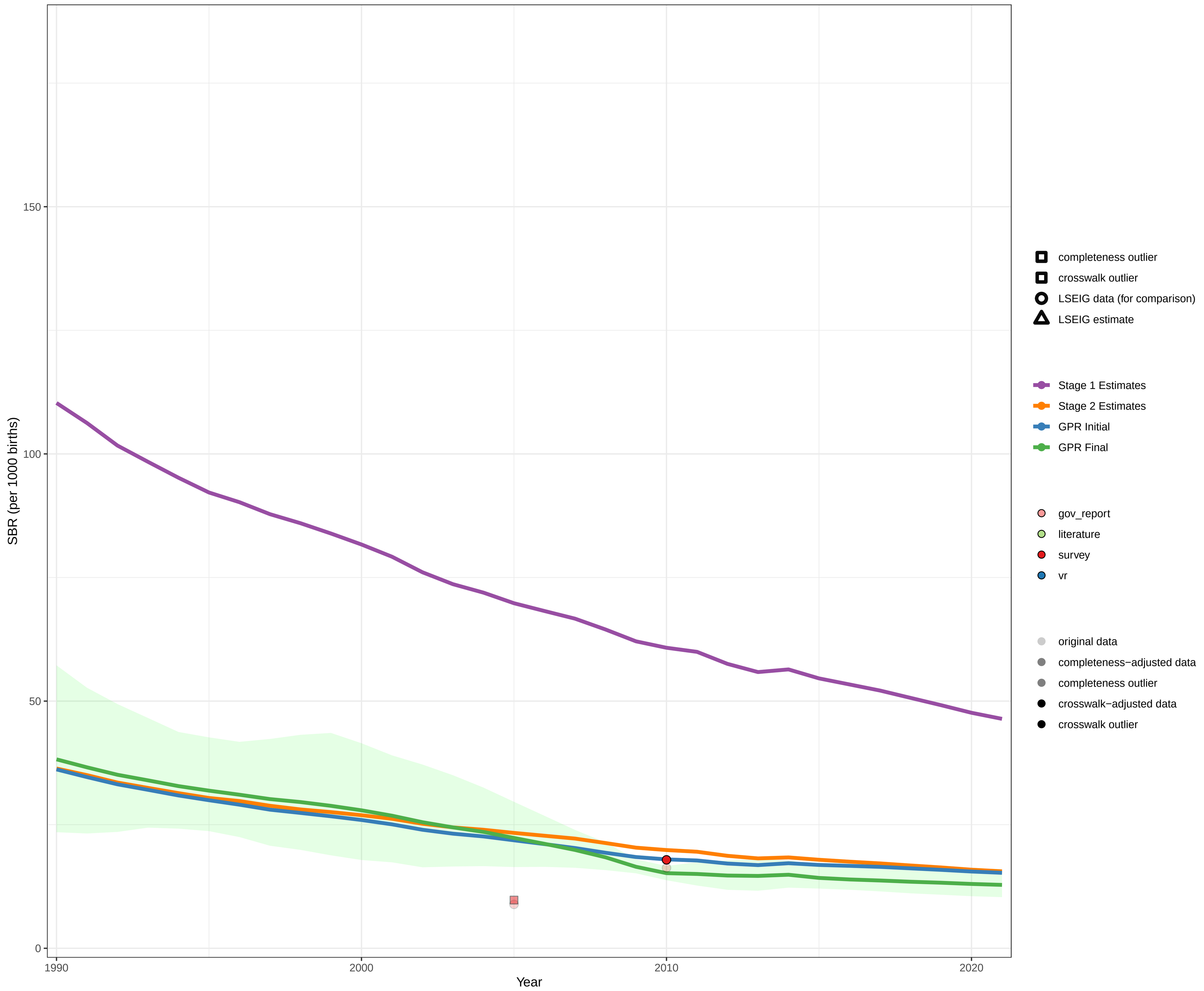

Year	Total Stillbirths (in thousands)	Total Stillbirths (in thousands)	SBR (per 1000 births)	SBR (per 1000 births)	SBR/NMR	SBR/NMR
	20 weeks	28 weeks	20 weeks	28 weeks	20 weeks	28 weeks
1990	0.6 (0.4 to 1.0)	0.5 (0.3 to 0.7)	50.9 (30.6 to 75.9)	38.2 (23.5 to 57.2)	1.04 (0.63 to 1.56)	0.78 (0.48 to 1.17)
1995	0.5 (0.4 to 0.7)	0.4 (0.3 to 0.5)	42.4 (31.4 to 56.8)	31.9 (23.7 to 42.7)	1.03 (0.76 to 1.38)	0.77 (0.57 to 1.03)
2000	0.4 (0.3 to 0.7)	0.3 (0.2 to 0.5)	37.2 (23.5 to 55.2)	27.9 (17.9 to 41.5)	1.03 (0.65 to 1.52)	0.77 (0.49 to 1.14)
2005	0.4 (0.3 to 0.5)	0.3 (0.2 to 0.4)	29.9 (21.9 to 39.9)	22.3 (16.4 to 29.6)	0.97 (0.71 to 1.29)	0.72 (0.53 to 0.96)
2010	0.3 (0.2 to 0.3)	0.2 (0.2 to 0.2)	20.6 (18.6 to 22.6)	15.2 (13.8 to 16.7)	0.79 (0.71 to 0.87)	0.58 (0.53 to 0.64)
2015	0.2 (0.2 to 0.3)	0.2 (0.1 to 0.2)	19.5 (16.5 to 22.8)	14.2 (12.1 to 16.7)	0.86 (0.73 to 1.01)	0.63 (0.53 to 0.74)
2020	0.2 (0.2 to 0.3)	0.1 (0.1 to 0.2)	18.2 (14.9 to 22.4)	13.0 (10.5 to 16.1)	0.96 (0.78 to 1.18)	0.68 (0.55 to 0.84)
2021	0.2 (0.2 to 0.2)	0.1 (0.1 to 0.2)	18.0 (14.5 to 22.1)	12.8 (10.3 to 15.7)	0.98 (0.79 to 1.20)	0.70 (0.56 to 0.85)

Gambella ETH_44860 Eastern Sub−Saharan Africa Stillbirth Rate / Neonatal Mortality Rate (SBR/NMR) − 20 weeks
lambda = 0.8 ; zeta = 0.7 ; scale = 15 ; amp = 0.028

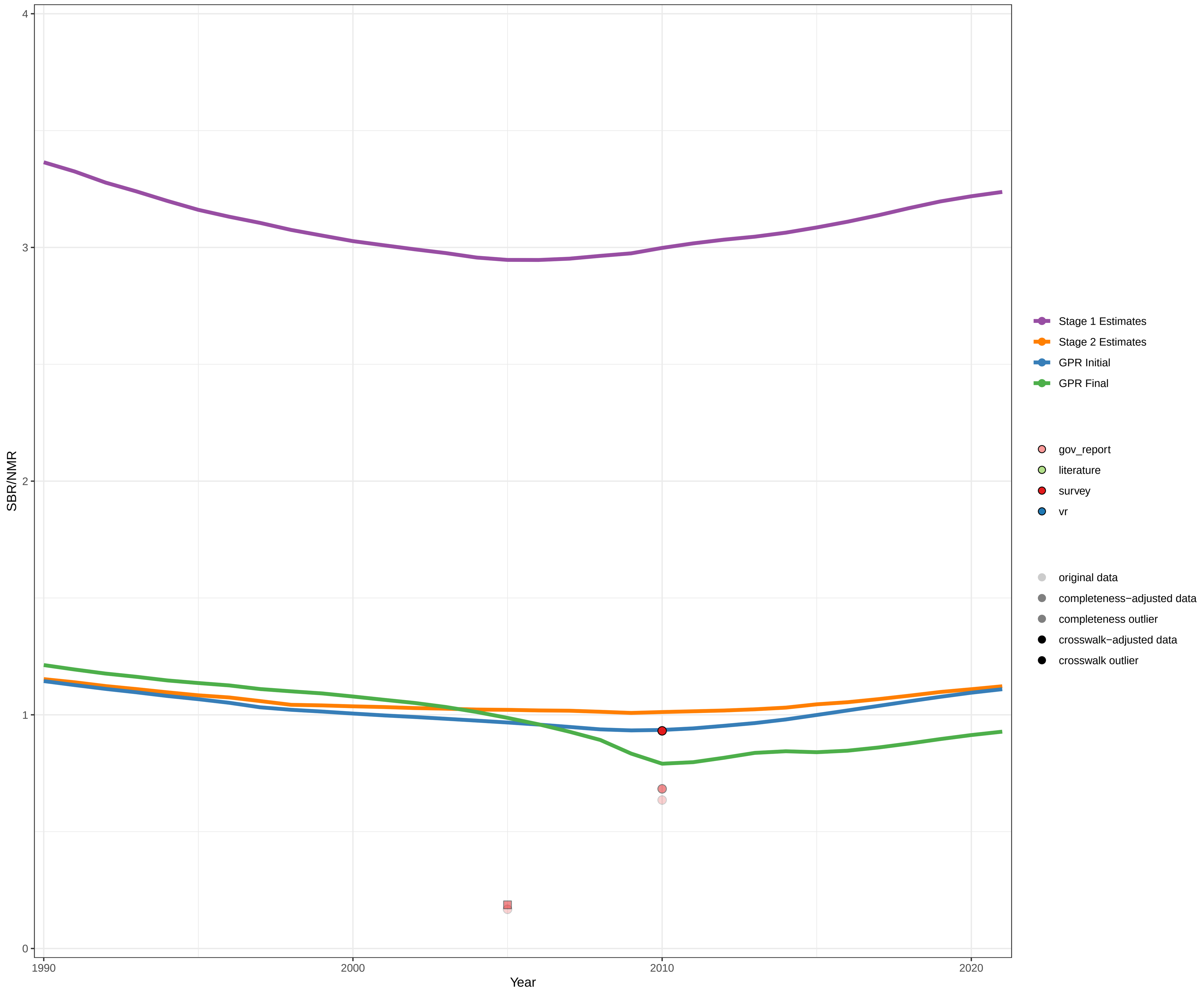

Gambella ETH_44860 Eastern Sub−Saharan Africa Stillbirth Rate / Neonatal Mortality Rate (SBR/NMR) − 28 weeks
lambda = 0.8 ; zeta = 0.7 ; scale = 15 ; amp = 0.028

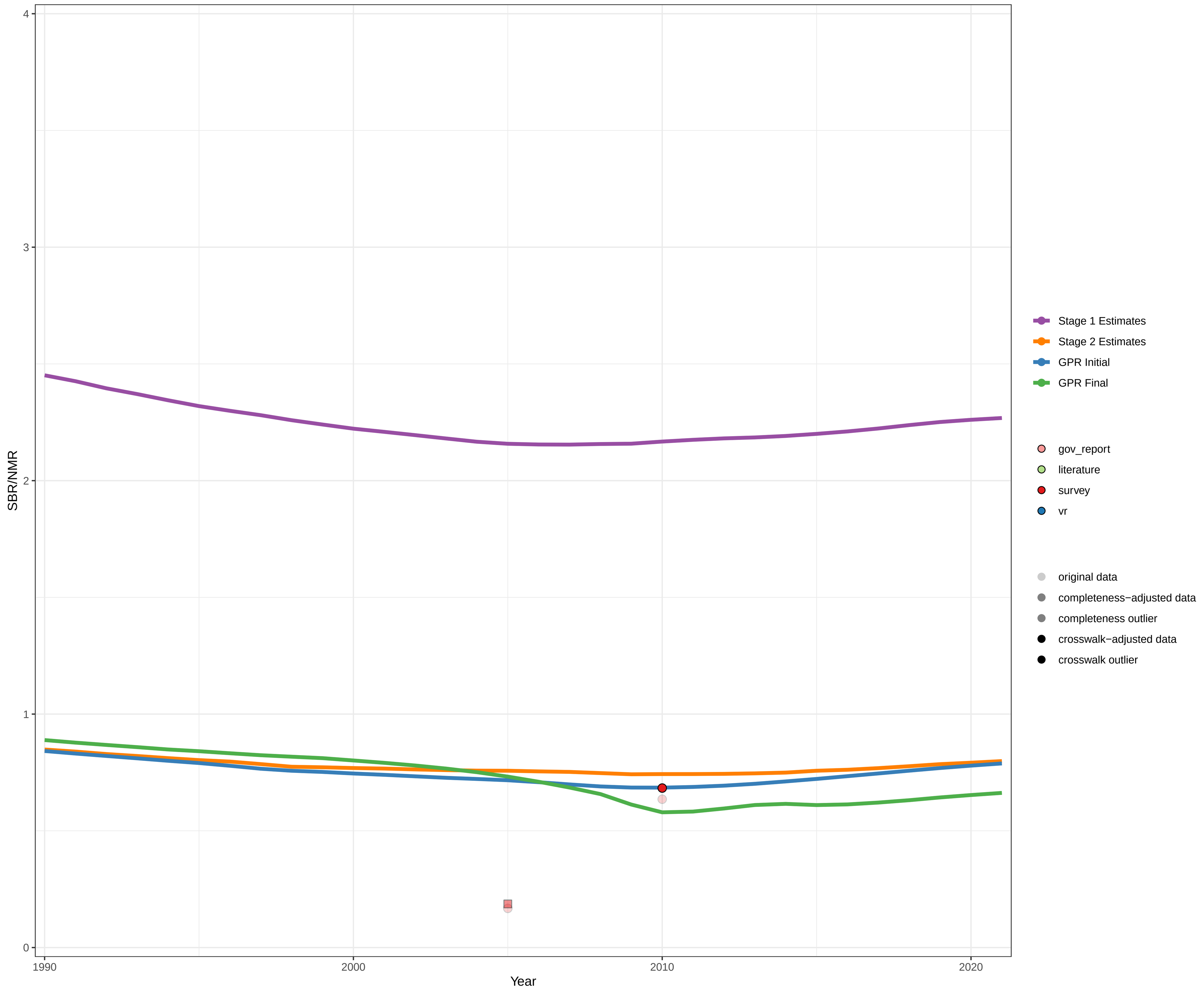

Definition	Years	Outlier	Source.Type	Title
28_weeks	2010	0	Survey	Ethiopia Demographic and Health Survey 2010−2011
28_weeks	2005	1	Survey	Ethiopia Demographic and Health Survey 2005

Gambella ETH_44860 Eastern Sub−Saharan Africa Stillbirth Rate (SBR) − 20 weeks
lambda = 0.8 ; zeta = 0.7 ; scale = 15 ; amp = 0.028

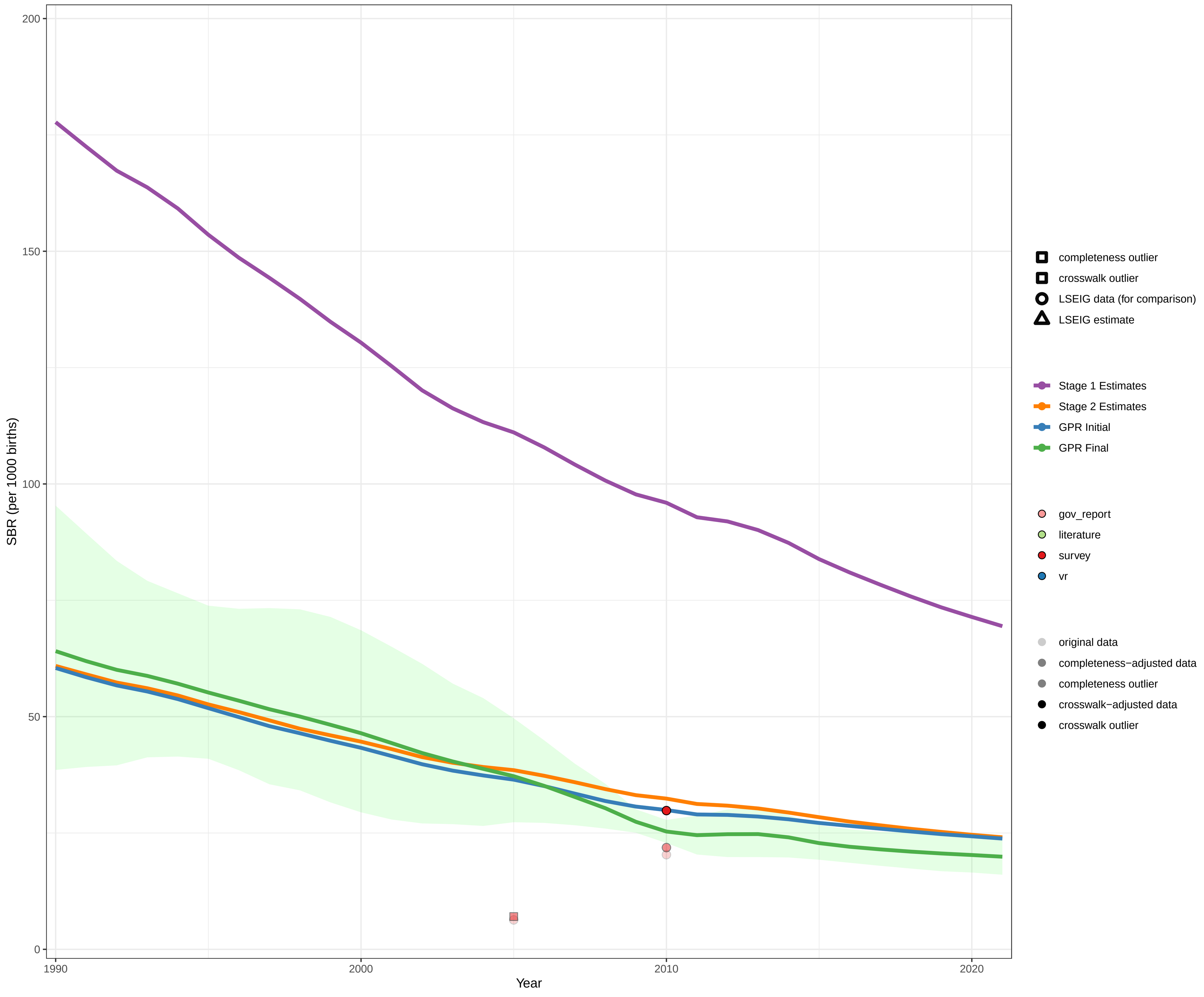

Gambella ETH_44860 Eastern Sub−Saharan Africa Stillbirth Rate (SBR) − 28 weeks
lambda = 0.8 ; zeta = 0.7 ; scale = 15 ; amp = 0.028

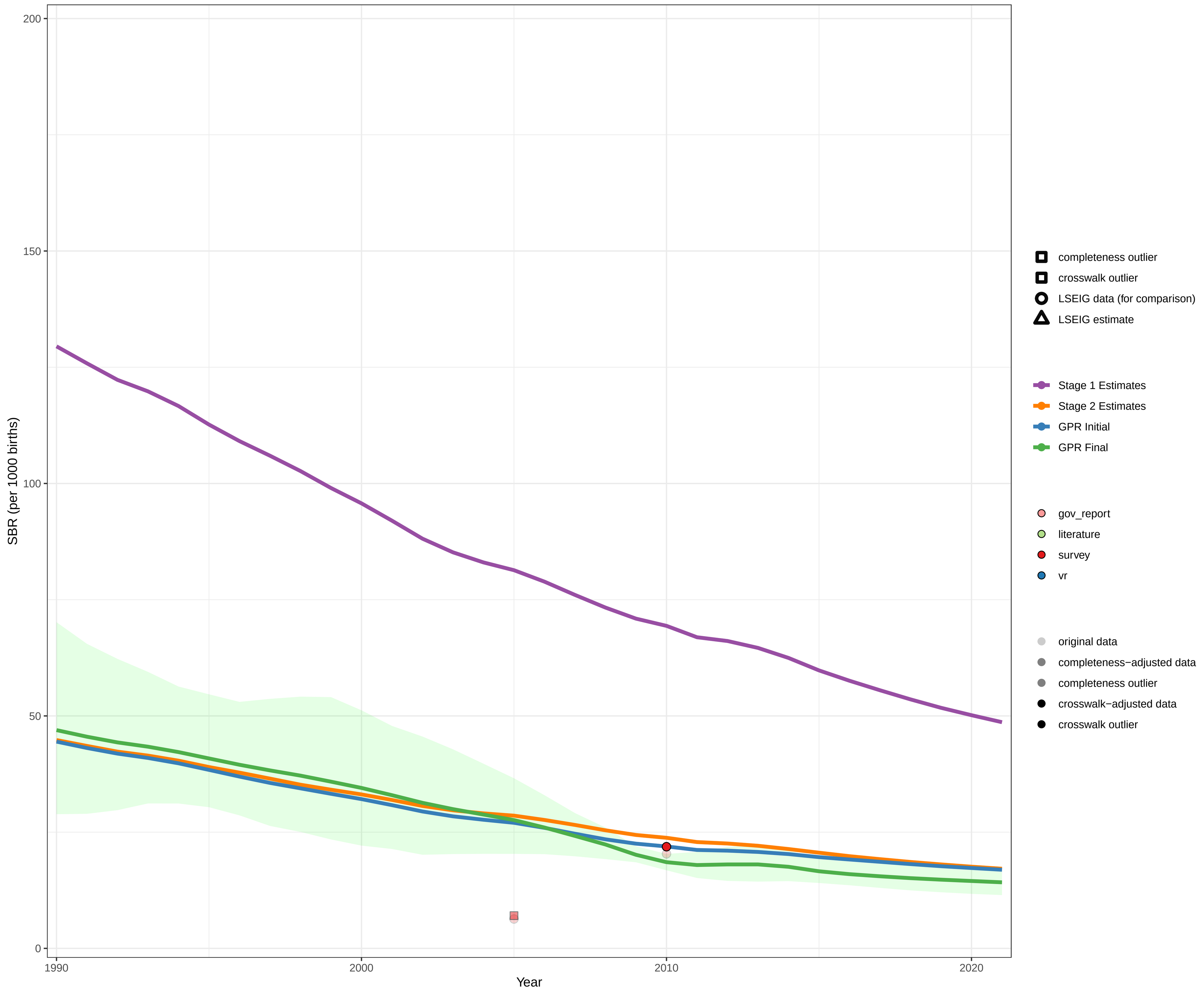

Year	Total Stillbirths (in thousands)	Total Stillbirths (in thousands)	SBR (per 1000 births)	SBR (per 1000 births)	SBR/NMR	SBR/NMR
	20 weeks	28 weeks	20 weeks	28 weeks	20 weeks	28 weeks
1990	0.6 (0.3 to 0.9)	0.4 (0.2 to 0.6)	64.1 (38.5 to 95.3)	46.9 (28.9 to 70.2)	1.21 (0.73 to 1.80)	0.89 (0.55 to 1.33)
1995	0.5 (0.4 to 0.7)	0.4 (0.3 to 0.5)	55.2 (40.9 to 73.8)	40.9 (30.4 to 54.7)	1.14 (0.84 to 1.52)	0.84 (0.63 to 1.13)
2000	0.5 (0.3 to 0.8)	0.4 (0.2 to 0.6)	46.4 (29.4 to 68.6)	34.5 (22.1 to 51.2)	1.08 (0.68 to 1.59)	0.80 (0.51 to 1.19)
2005	0.4 (0.3 to 0.6)	0.3 (0.2 to 0.4)	37.2 (27.3 to 49.7)	27.6 (20.3 to 36.6)	0.99 (0.72 to 1.32)	0.73 (0.54 to 0.97)
2010	0.3 (0.3 to 0.3)	0.2 (0.2 to 0.2)	25.3 (22.8 to 27.8)	18.5 (16.8 to 20.3)	0.79 (0.71 to 0.87)	0.58 (0.52 to 0.63)
2015	0.3 (0.2 to 0.3)	0.2 (0.2 to 0.2)	22.8 (19.3 to 26.7)	16.6 (14.1 to 19.4)	0.84 (0.71 to 0.98)	0.61 (0.52 to 0.71)
2020	0.2 (0.2 to 0.3)	0.2 (0.1 to 0.2)	20.3 (16.5 to 24.9)	14.5 (11.7 to 17.9)	0.91 (0.74 to 1.12)	0.65 (0.53 to 0.81)
2021	0.2 (0.2 to 0.3)	0.2 (0.1 to 0.2)	19.9 (16.0 to 24.4)	14.2 (11.5 to 17.4)	0.93 (0.75 to 1.14)	0.66 (0.53 to 0.81)

Harari ETH_44859 Eastern Sub−Saharan Africa Stillbirth Rate / Neonatal Mortality Rate (SBR/NMR) − 20 weeks
lambda = 1 ; zeta = 0.6 ; scale = 15 ; amp = 0.028

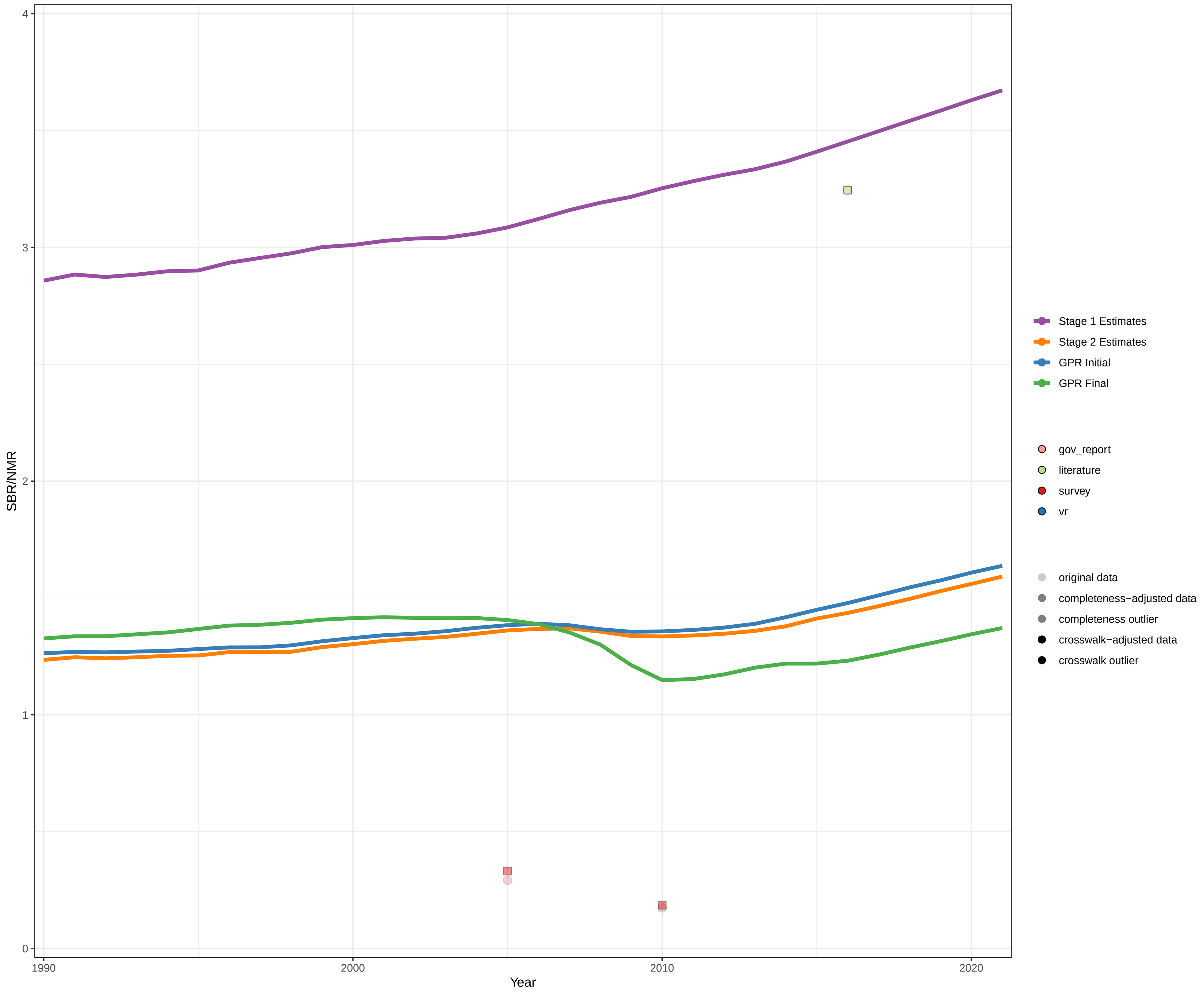

Harari ETH_44859 Eastern Sub−Saharan Africa Stillbirth Rate / Neonatal Mortality Rate (SBR/NMR) − 28 weeks
lambda = 1 ; zeta = 0.6 ; scale = 15 ; amp = 0.028

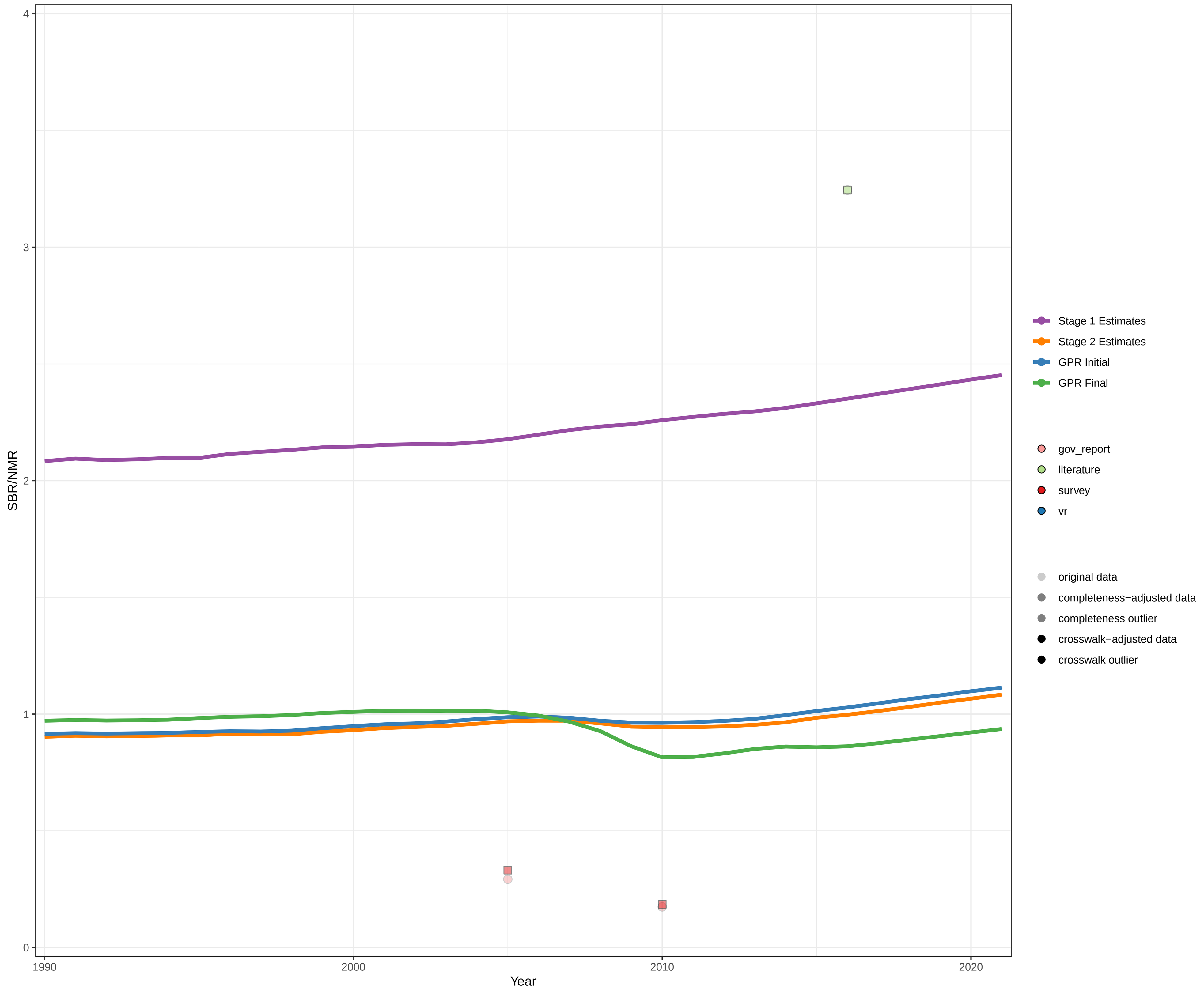

Definition	Years	Outlier	Source.Type	Title
28_weeks	2005	1	Survey	Ethiopia Demographic and Health Survey 2005
28_weeks	2010	1	Survey	Ethiopia Demographic and Health Survey 2010−2011
other	2016	1	Sci Lit	Maternal group B Streptococcus recto vaginal colonization increases the odds of stillbirth: evidence from Eastern Ethiopia

Harari ETH_44859 Eastern Sub−Saharan Africa Stillbirth Rate (SBR) − 20 weeks
lambda = 1 ; zeta = 0.6 ; scale = 15 ; amp = 0.028

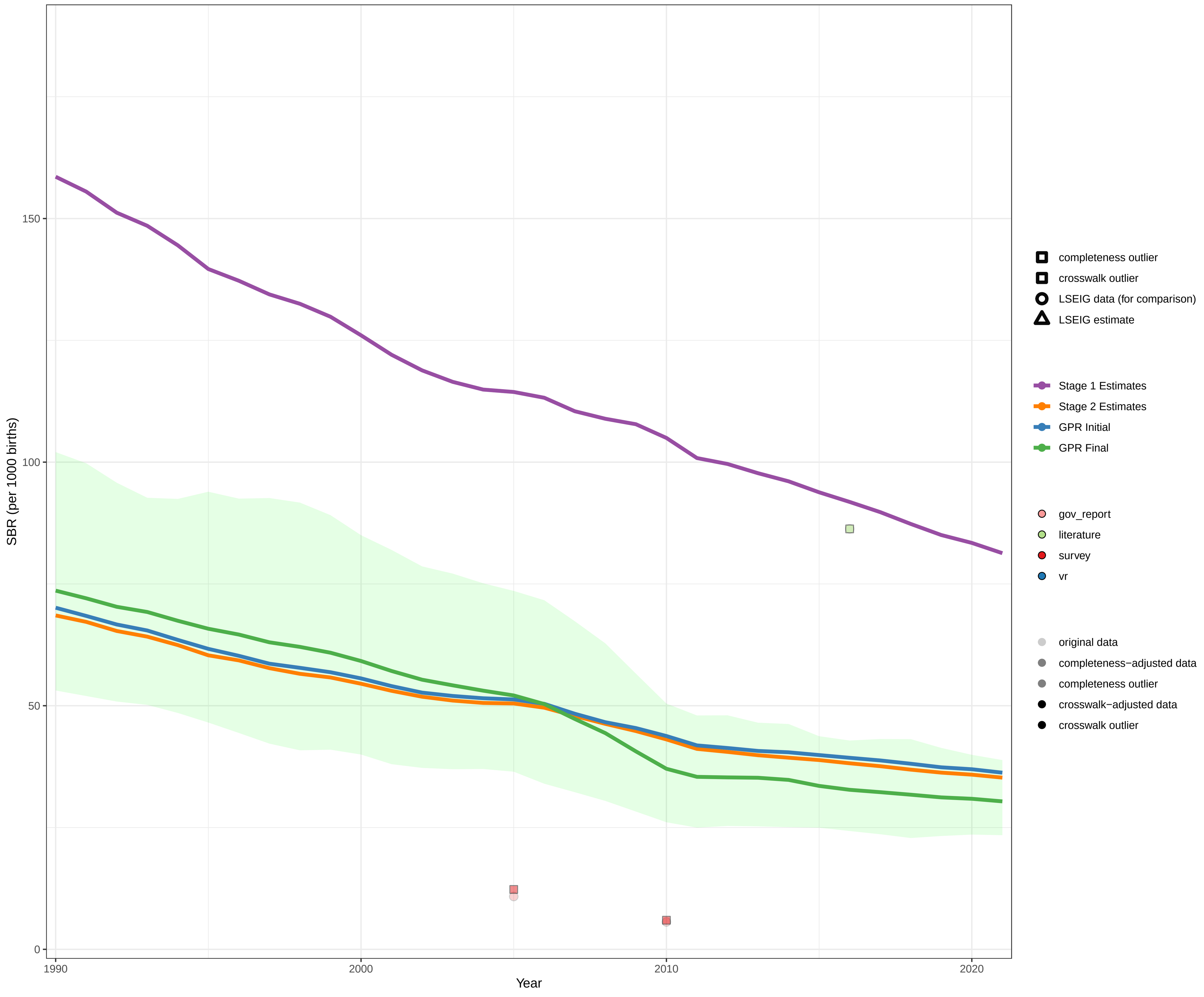

Harari ETH_44859 Eastern Sub−Saharan Africa Stillbirth Rate (SBR) − 28 weeks
lambda = 1 ; zeta = 0.6 ; scale = 15 ; amp = 0.028

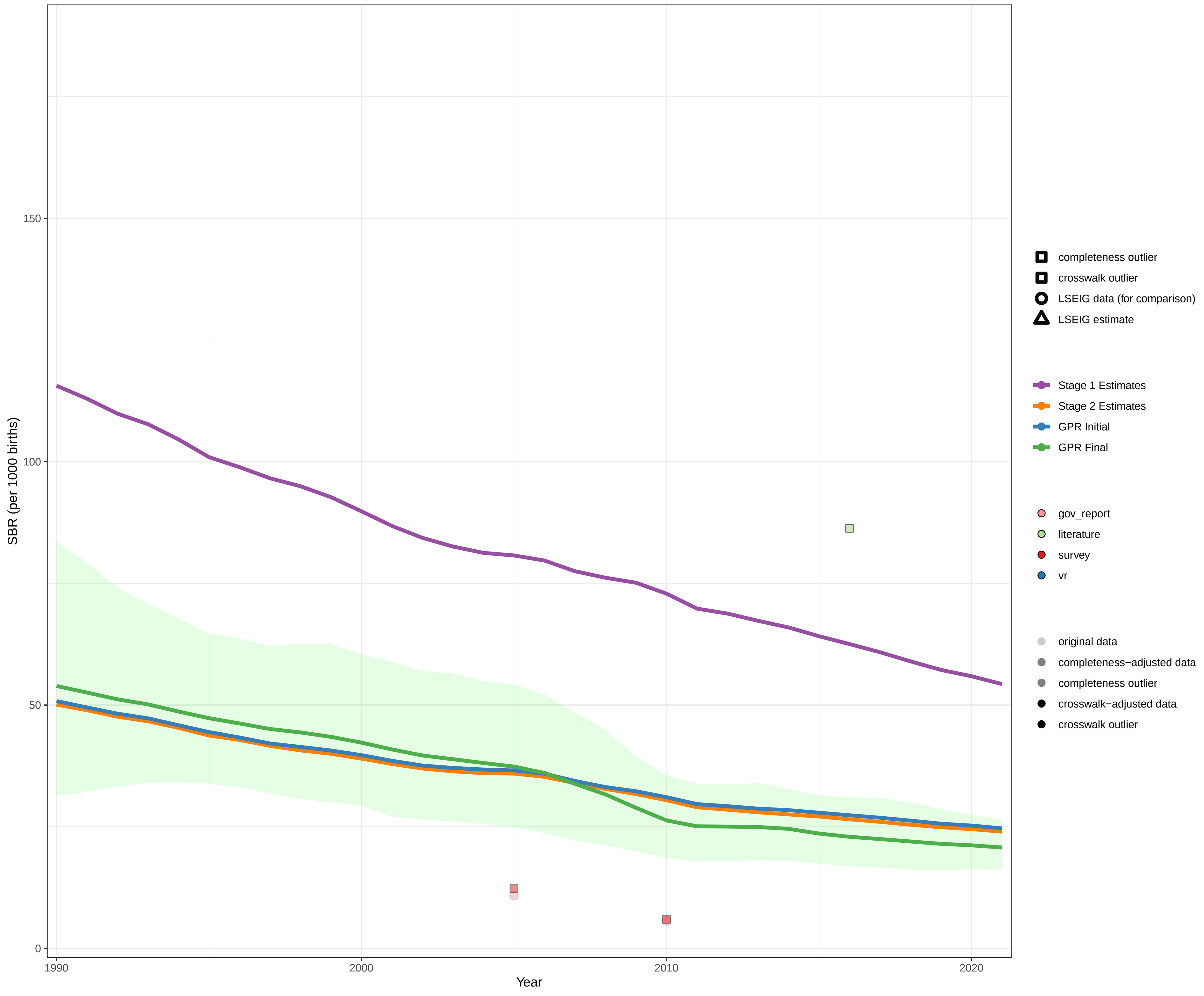

Year	Total Stillbirths (in thousands)	Total Stillbirths (in thousands)	SBR (per 1000 births)	SBR (per 1000 births)	SBR/NMR	SBR/NMR
	20 weeks	28 weeks	20 weeks	28 weeks	20 weeks	28 weeks
1990	0.4 (0.3 to 0.6)	0.3 (0.2 to 0.5)	73.6 (53.1 to 102.1)	53.9 (31.3 to 83.6)	1.33 (0.96 to 1.84)	0.97 (0.56 to 1.51)
1995	0.4 (0.3 to 0.6)	0.3 (0.2 to 0.4)	65.8 (46.5 to 93.9)	47.3 (33.9 to 64.7)	1.37 (0.97 to 1.95)	0.98 (0.70 to 1.34)
2000	0.4 (0.3 to 0.6)	0.3 (0.2 to 0.4)	59.2 (40.0 to 85.0)	42.3 (29.4 to 60.4)	1.41 (0.96 to 2.03)	1.01 (0.70 to 1.44)
2005	0.4 (0.2 to 0.5)	0.3 (0.2 to 0.4)	52.1 (36.5 to 73.6)	37.4 (24.9 to 54.2)	1.41 (0.98 to 1.98)	1.01 (0.67 to 1.46)
2010	0.3 (0.2 to 0.3)	0.2 (0.1 to 0.2)	37.1 (26.1 to 50.4)	26.3 (18.6 to 35.5)	1.15 (0.81 to 1.56)	0.81 (0.58 to 1.10)
2015	0.2 (0.2 to 0.3)	0.2 (0.1 to 0.2)	33.5 (25.0 to 43.7)	23.6 (17.4 to 31.4)	1.22 (0.91 to 1.59)	0.86 (0.63 to 1.14)
2020	0.2 (0.2 to 0.3)	0.1 (0.1 to 0.2)	30.9 (23.6 to 39.9)	21.2 (16.2 to 27.5)	1.34 (1.02 to 1.74)	0.92 (0.71 to 1.19)
2021	0.2 (0.1 to 0.3)	0.1 (0.1 to 0.2)	30.4 (23.4 to 38.8)	20.7 (16.1 to 26.4)	1.37 (1.06 to 1.75)	0.94 (0.73 to 1.19)

Oromia ETH_44855 Eastern Sub−Saharan Africa Stillbirth Rate / Neonatal Mortality Rate (SBR/NMR) − 20 weeks
lambda = 0.8 ; zeta = 0.7 ; scale = 15 ; amp = 0.028

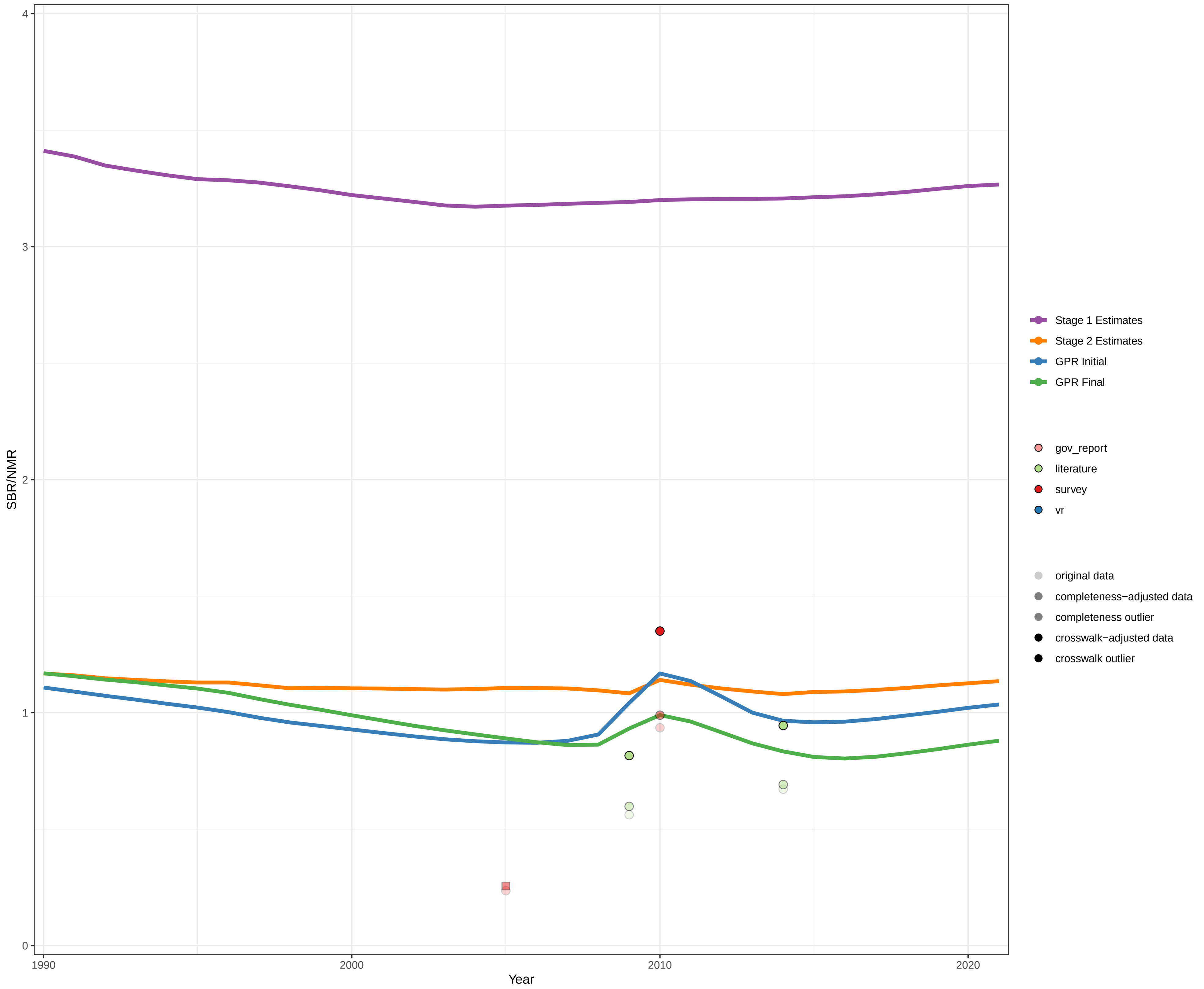

Oromia ETH_44855 Eastern Sub−Saharan Africa Stillbirth Rate / Neonatal Mortality Rate (SBR/NMR) − 28 weeks
lambda = 0.8 ; zeta = 0.7 ; scale = 15 ; amp = 0.028

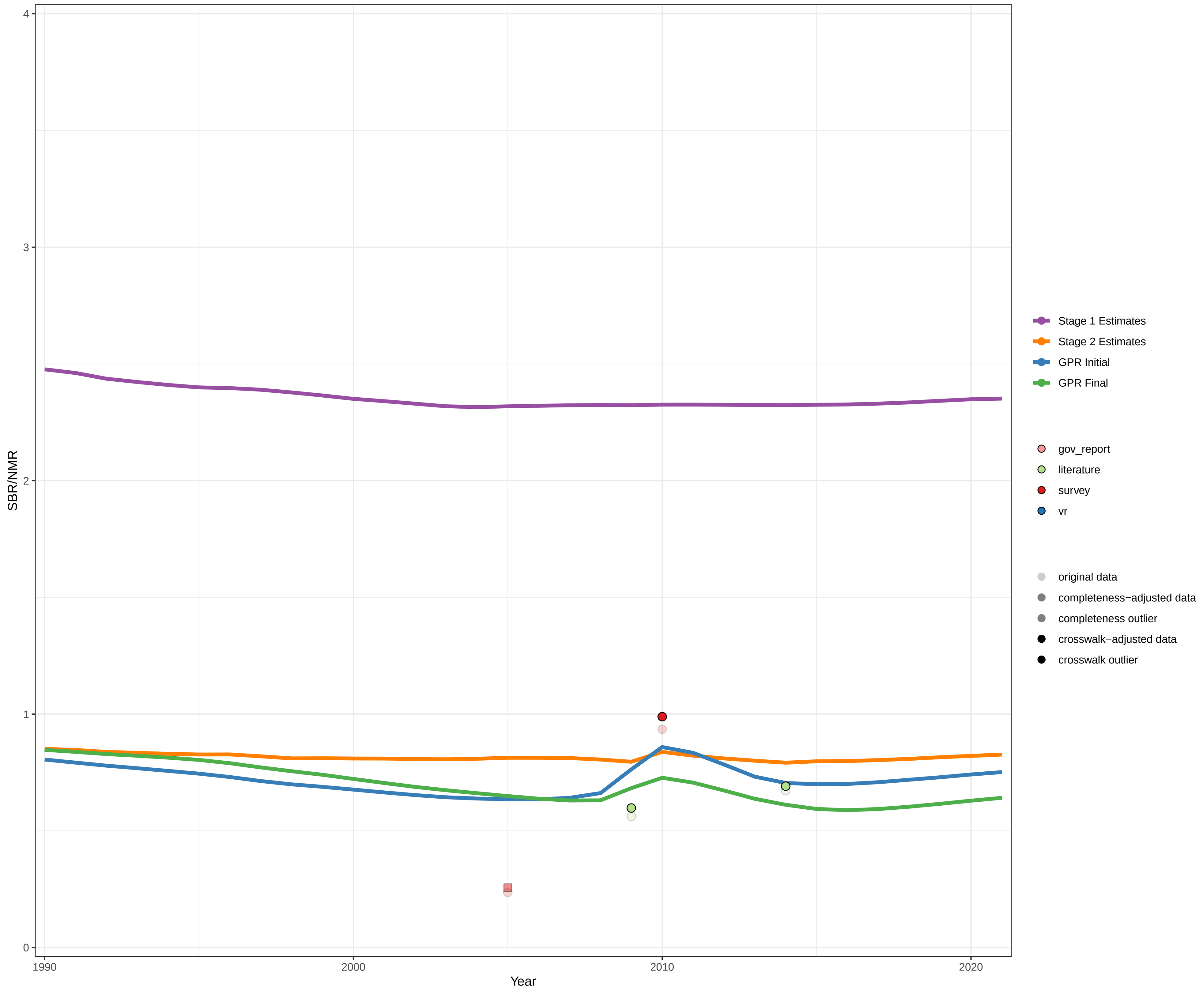

Definition	Years	Outlier	Source.Type	Title
28_weeks	2009	0	Sci Lit	Pregnancy rates and pregnancy loss in Eastern Ethiopia
28_weeks	2010	0	Survey	Ethiopia Demographic and Health Survey 2010−2011
28_weeks	2014	0	Sci Lit	Effect of prenatal and intrapartum care on the stillbirth rate among women in rural Ethiopia
28_weeks	2005	1	Survey	Ethiopia Demographic and Health Survey 2005

Oromia ETH_44855 Eastern Sub−Saharan Africa Stillbirth Rate (SBR) − 20 weeks
lambda = 0.8 ; zeta = 0.7 ; scale = 15 ; amp = 0.028

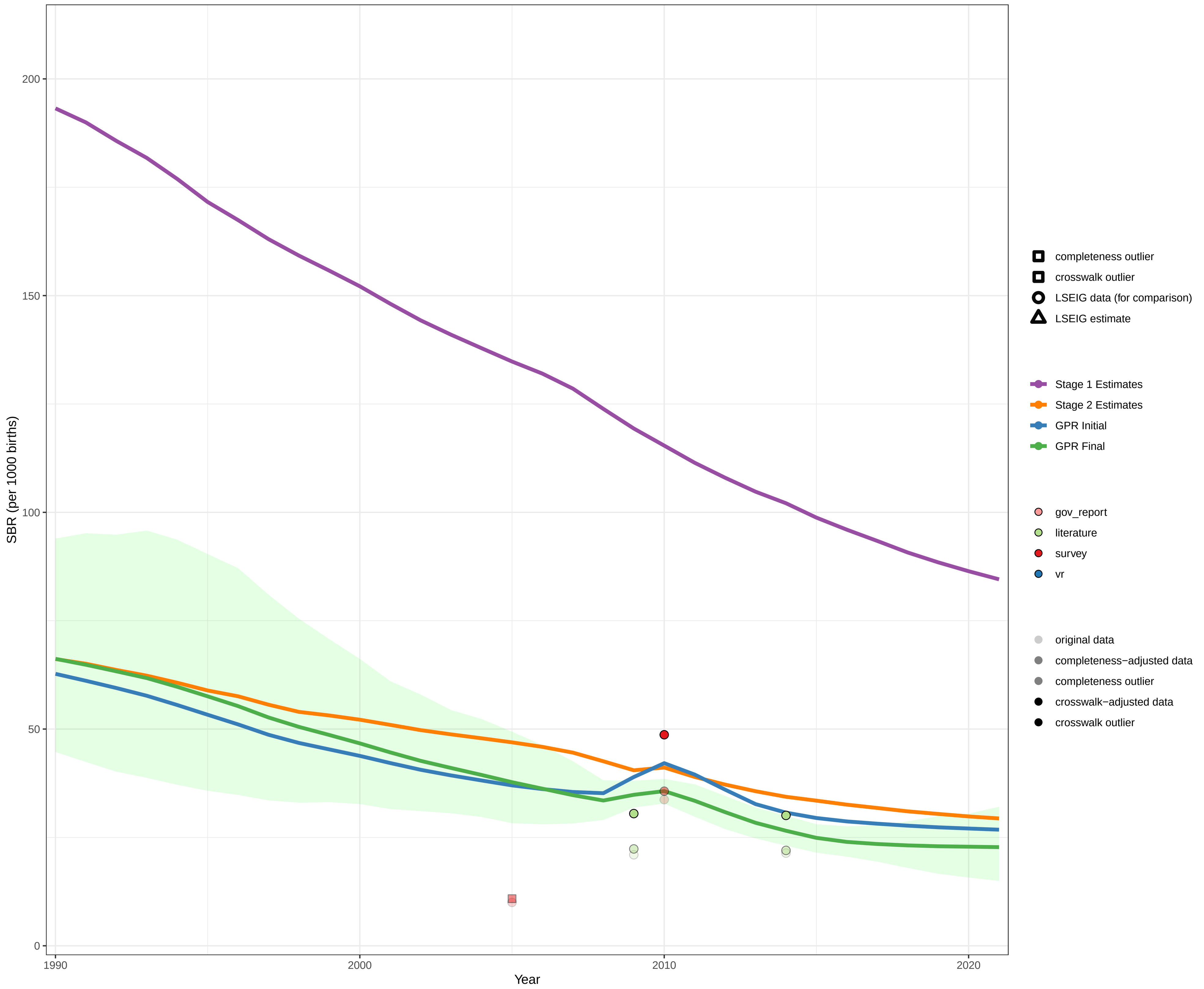

Oromia ETH_44855 Eastern Sub−Saharan Africa Stillbirth Rate (SBR) − 28 weeks
lambda = 0.8 ; zeta = 0.7 ; scale = 15 ; amp = 0.028

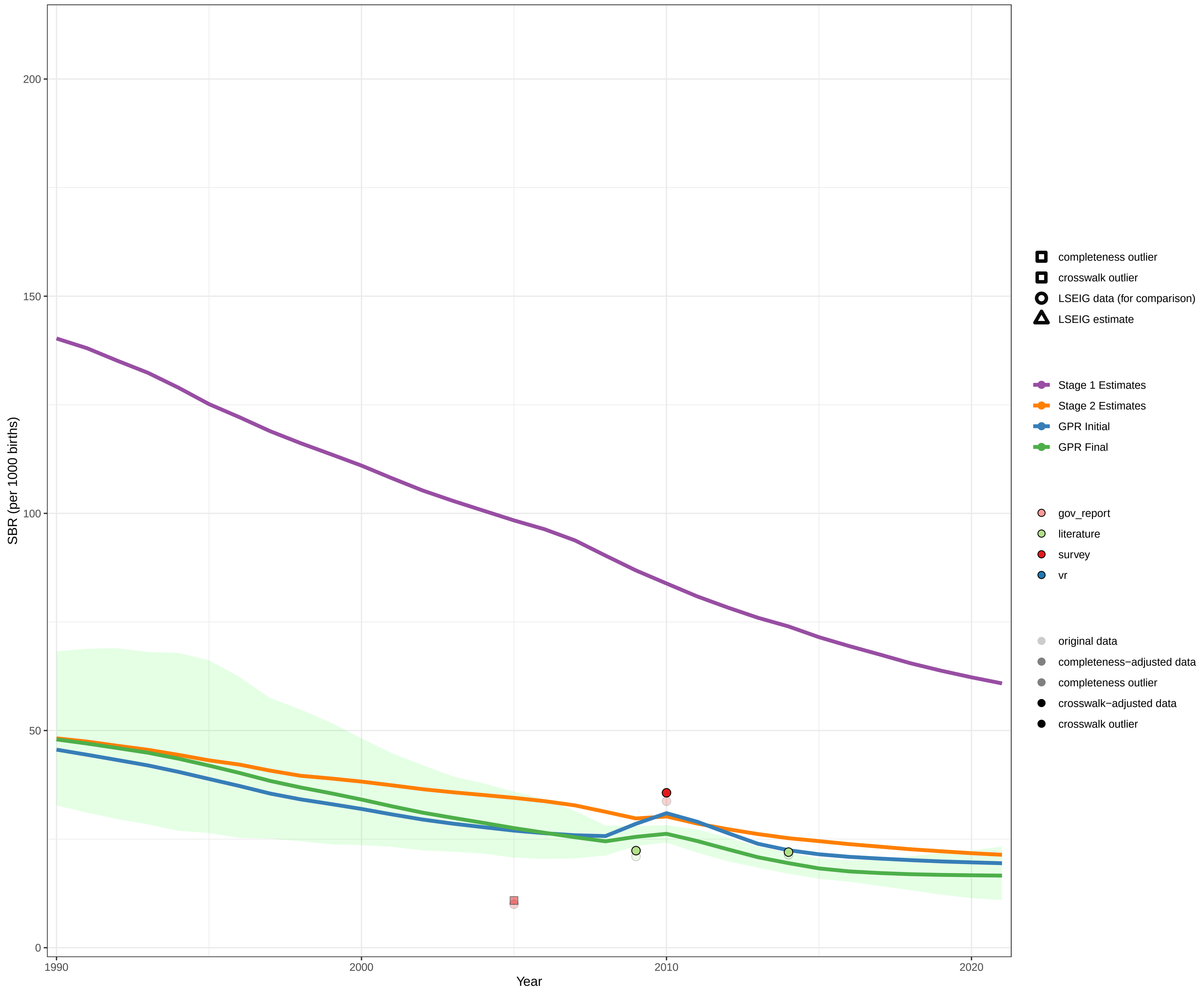

Year	Total Stillbirths (in thousands)	Total Stillbirths (in thousands)	SBR (per 1000 births)	SBR (per 1000 births)	SBR/NMR	SBR/NMR
	20 weeks	28 weeks	20 weeks	28 weeks	20 weeks	28 weeks
1990	57.8 (38.1 to 84.3)	41.1 (27.6 to 59.5)	66.2 (44.7 to 94.0)	48.0 (32.8 to 68.2)	1.17 (0.79 to 1.66)	0.85 (0.58 to 1.20)
1995	61.8 (37.4 to 100.2)	44.2 (27.3 to 71.5)	57.6 (35.8 to 90.4)	41.9 (26.4 to 66.2)	1.10 (0.69 to 1.73)	0.80 (0.51 to 1.27)
2000	56.3 (38.8 to 81.3)	40.6 (27.8 to 58.1)	46.7 (32.7 to 66.2)	34.1 (23.6 to 48.2)	0.99 (0.69 to 1.40)	0.72 (0.50 to 1.02)
2005	48.2 (35.7 to 63.8)	34.8 (26.0 to 45.8)	37.7 (28.3 to 49.4)	27.5 (20.7 to 35.9)	0.89 (0.67 to 1.16)	0.65 (0.49 to 0.85)
2010	46.5 (42.7 to 50.4)	33.9 (31.2 to 36.5)	35.7 (32.8 to 38.5)	26.2 (24.2 to 28.2)	0.99 (0.91 to 1.07)	0.73 (0.67 to 0.78)
2015	33.9 (29.2 to 38.4)	24.7 (21.4 to 27.9)	24.9 (21.5 to 28.1)	18.3 (15.9 to 20.5)	0.81 (0.70 to 0.91)	0.59 (0.52 to 0.67)
2020	33.7 (23.0 to 45.3)	24.4 (16.6 to 32.9)	22.9 (15.7 to 30.5)	16.7 (11.4 to 22.4)	0.86 (0.59 to 1.15)	0.63 (0.43 to 0.84)
2021	34.2 (22.3 to 48.6)	24.8 (16.3 to 35.0)	22.8 (15.0 to 32.1)	16.6 (11.0 to 23.3)	0.88 (0.58 to 1.24)	0.64 (0.42 to 0.90)

Somali ETH_44856 Eastern Sub−Saharan Africa Stillbirth Rate / Neonatal Mortality Rate (SBR/NMR) − 20 weeks
lambda = 0.8 ; zeta = 0.7 ; scale = 15 ; amp = 0.028

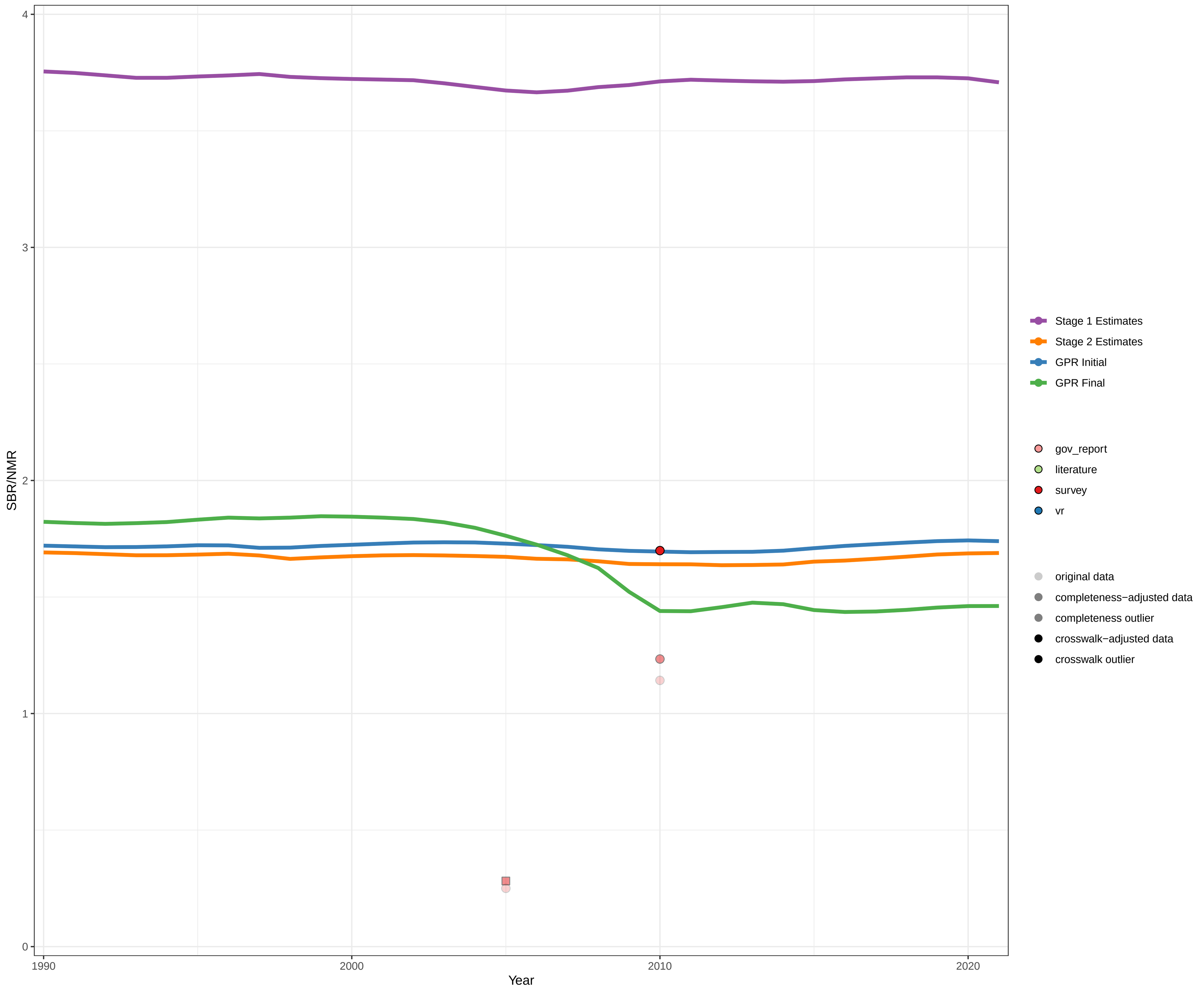

Somali ETH_44856 Eastern Sub−Saharan Africa Stillbirth Rate / Neonatal Mortality Rate (SBR/NMR) − 28 weeks
lambda = 0.8 ; zeta = 0.7 ; scale = 15 ; amp = 0.028

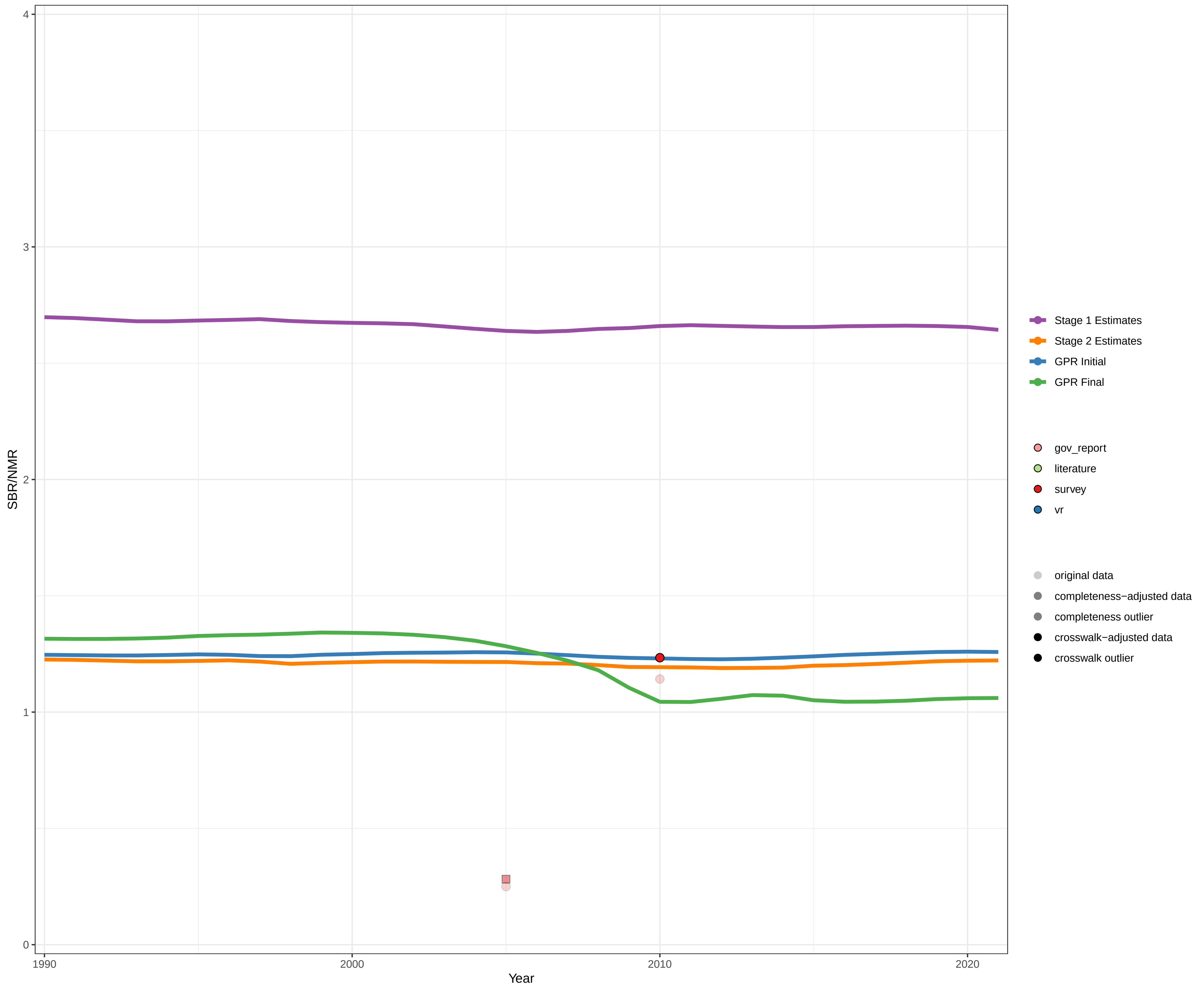

Definition	Years	Outlier	Source.Type	Title
28_weeks	2010	0	Survey	Ethiopia Demographic and Health Survey 2010−2011
28_weeks	2005	1	Survey	Ethiopia Demographic and Health Survey 2005

Somali ETH_44856 Eastern Sub−Saharan Africa Stillbirth Rate (SBR) − 20 weeks
lambda = 0.8 ; zeta = 0.7 ; scale = 15 ; amp = 0.028

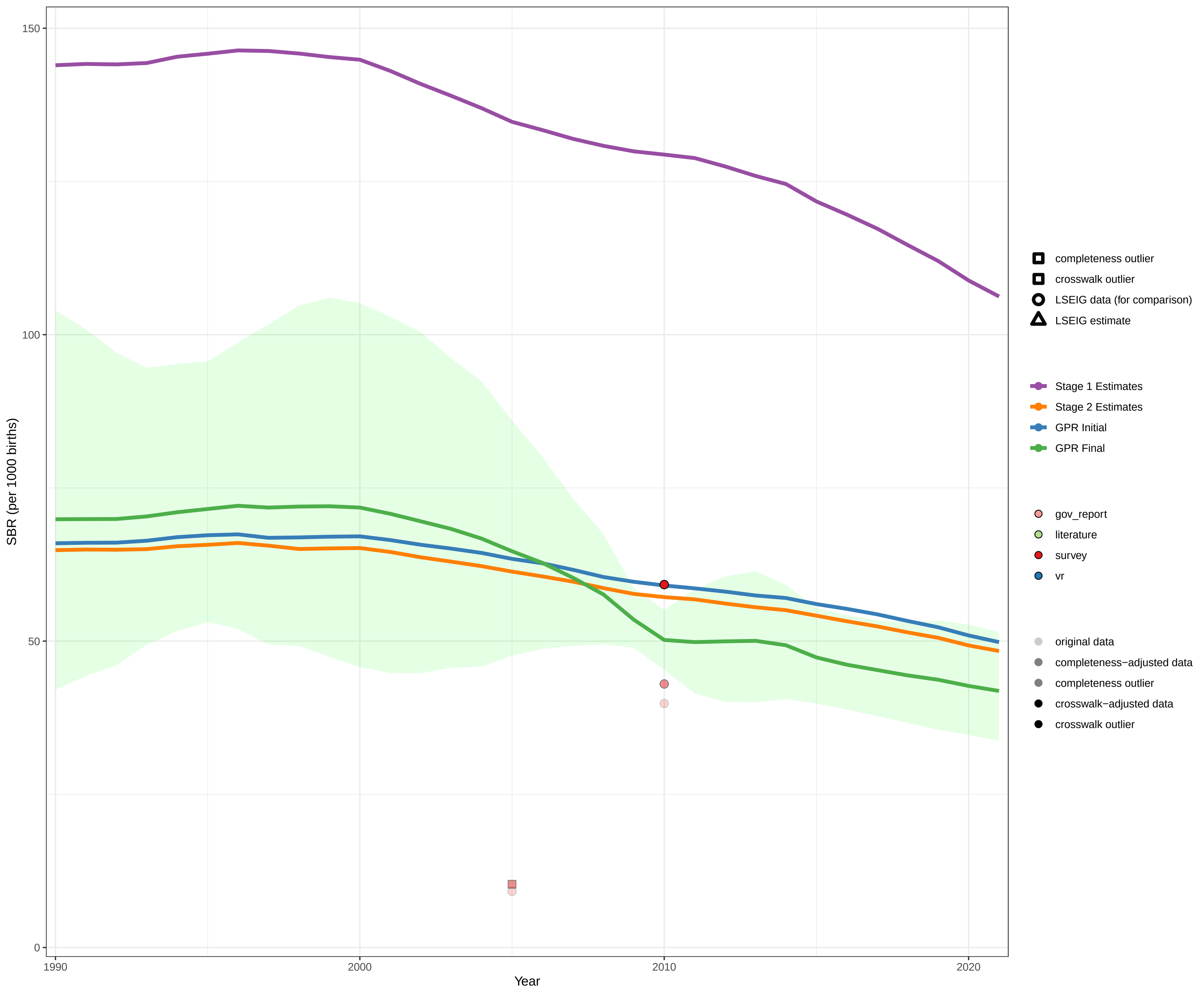

Somali ETH_44856 Eastern Sub−Saharan Africa Stillbirth Rate (SBR) − 28 weeks
lambda = 0.8 ; zeta = 0.7 ; scale = 15 ; amp = 0.028

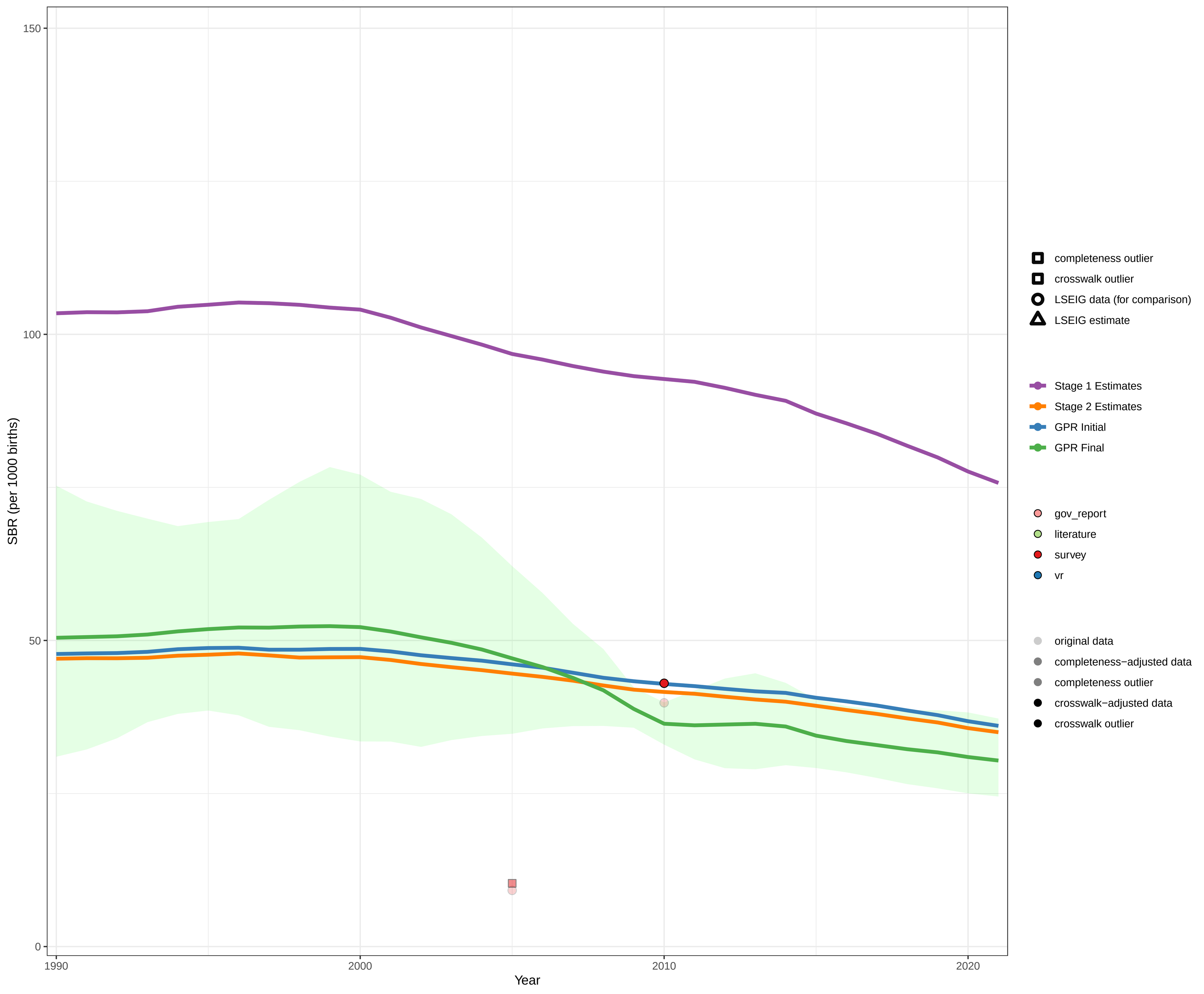

Year	Total Stillbirths (in thousands)	Total Stillbirths (in thousands)	SBR (per 1000 births)	SBR (per 1000 births)	SBR/NMR	SBR/NMR
	20 weeks	28 weeks	20 weeks	28 weeks	20 weeks	28 weeks
1990	10.6 (6.2 to 16.3)	7.5 (4.5 to 11.5)	69.9 (42.1 to 104.0)	50.4 (31.0 to 75.3)	1.82 (1.10 to 2.71)	1.32 (0.81 to 1.96)
1995	13.7 (10.0 to 18.8)	9.7 (7.1 to 13.2)	71.6 (53.1 to 95.7)	51.9 (38.6 to 69.3)	1.83 (1.36 to 2.45)	1.33 (0.99 to 1.78)
2000	16.0 (9.9 to 24.2)	11.4 (7.1 to 17.2)	71.8 (45.7 to 105.2)	52.2 (33.5 to 77.1)	1.84 (1.17 to 2.70)	1.34 (0.86 to 1.98)
2005	15.8 (11.4 to 21.4)	11.3 (8.2 to 15.1)	64.7 (47.6 to 86.0)	47.1 (34.8 to 62.1)	1.76 (1.30 to 2.34)	1.28 (0.95 to 1.69)
2010	12.8 (11.5 to 14.2)	9.2 (8.3 to 10.1)	50.2 (45.3 to 55.1)	36.4 (33.0 to 39.9)	1.44 (1.30 to 1.58)	1.04 (0.95 to 1.14)
2015	12.8 (10.6 to 15.1)	9.2 (7.7 to 10.8)	47.3 (39.8 to 55.4)	34.5 (29.2 to 40.4)	1.44 (1.21 to 1.69)	1.05 (0.89 to 1.23)
2020	11.9 (9.6 to 14.8)	8.5 (6.8 to 10.6)	42.7 (34.7 to 52.7)	31.0 (25.0 to 38.2)	1.46 (1.19 to 1.80)	1.06 (0.86 to 1.31)
2021	11.7 (9.4 to 14.6)	8.4 (6.7 to 10.4)	41.9 (33.7 to 51.5)	30.4 (24.5 to 37.2)	1.46 (1.18 to 1.80)	1.06 (0.86 to 1.30)

Southern Nations, Nationalities, and Peoples with Sidama and South West Regions   ETH_44858   Eastern Sub−Saharan Africa   Stillbirth Rate / Neonatal Mortality Rate
lambda = 0.8 ; zeta = 0.7 ; scale = 15 ; amp = 0.028

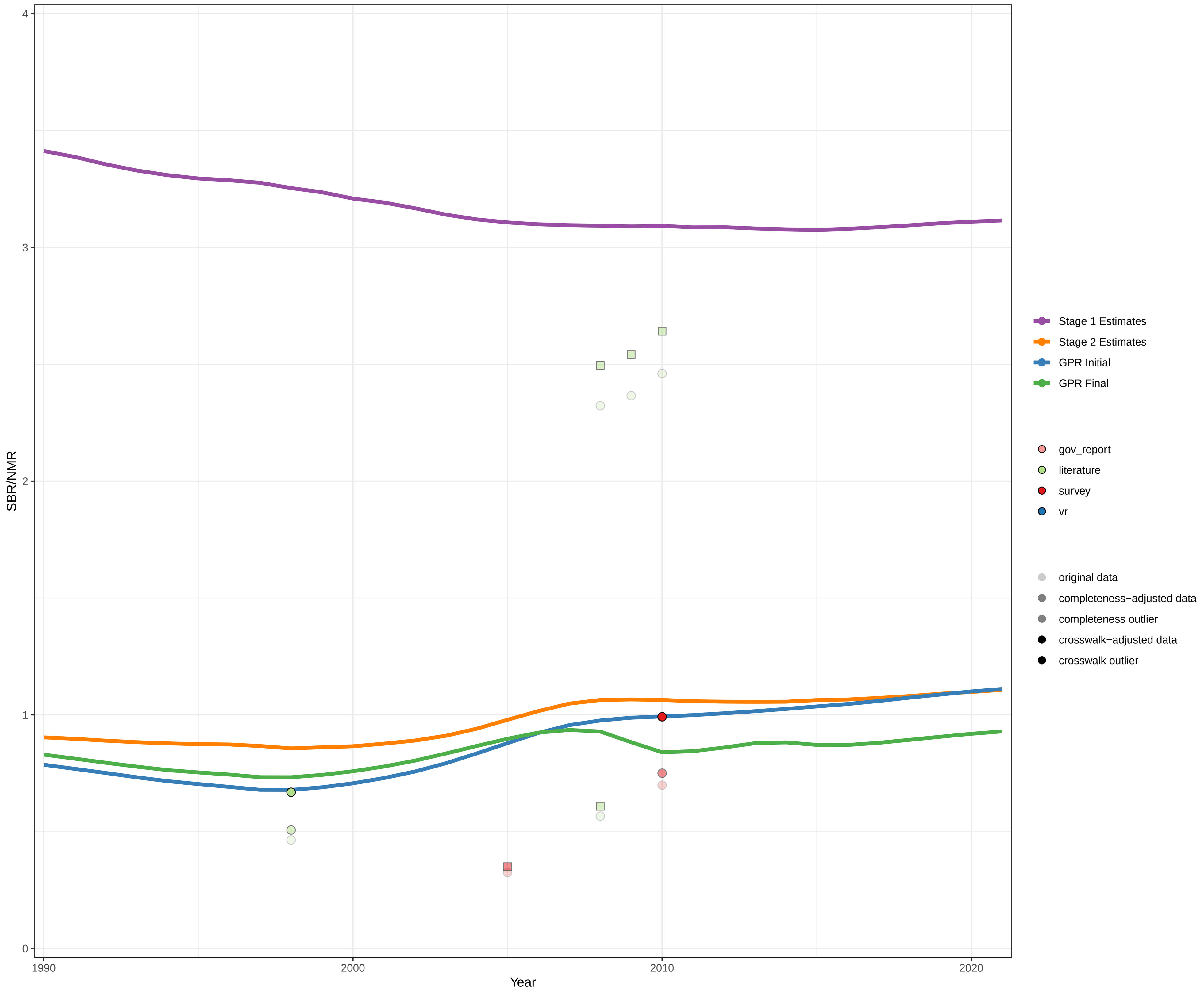

Southern Nations, Nationalities, and Peoples with Sidama and South West Regions   ETH_44858   Eastern Sub−Saharan Africa   Stillbirth Rate / Neonatal Mortality Rate (SBR/NMR) − 28 weeks
lambda = 0.8 ; zeta = 0.7 ; scale = 15 ; amp = 0.028

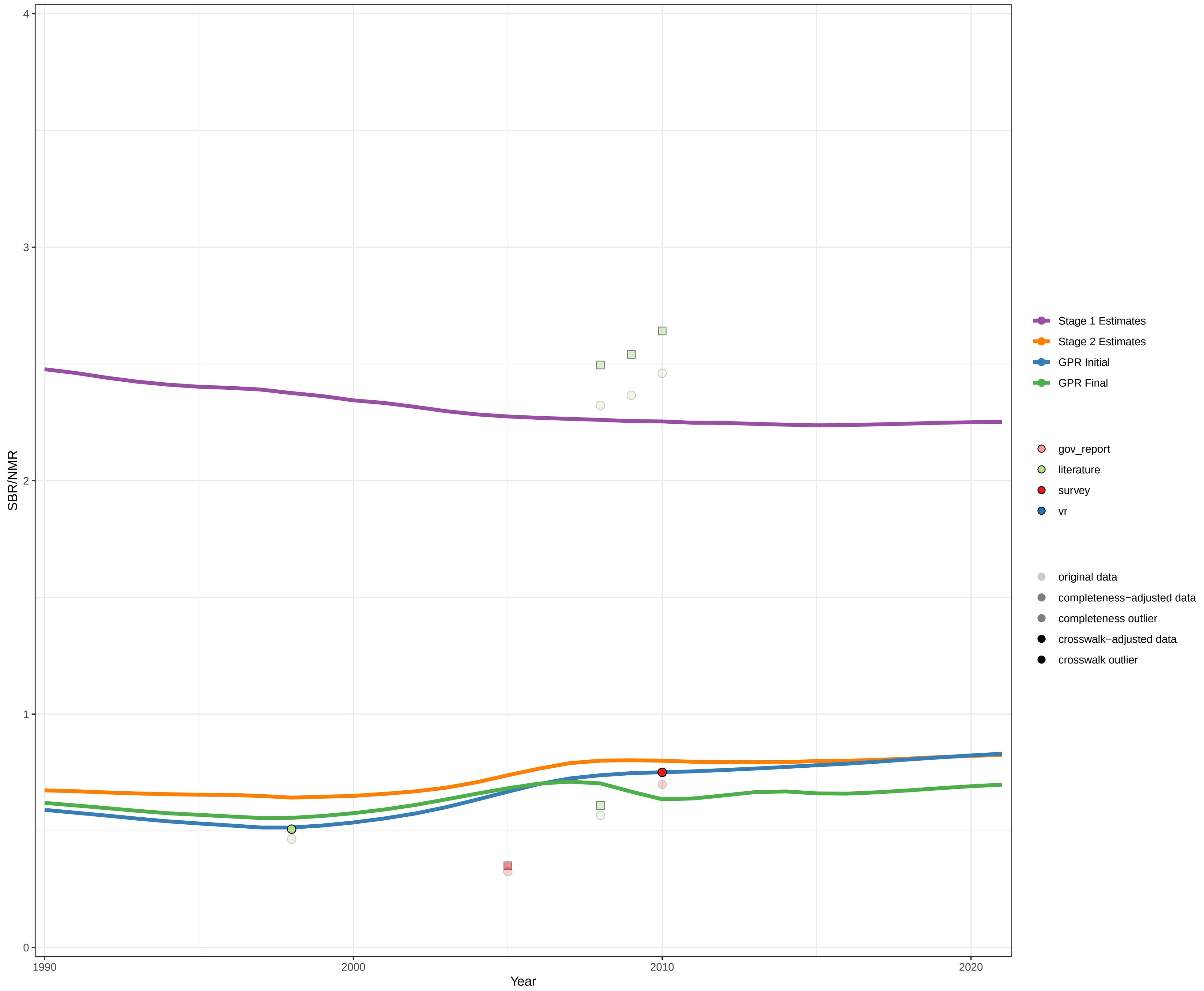

Definition	Years	Outlier	Source.Type	Title
28_weeks	1998	0	Sci Lit	Prolonged Labour in Rural Ethiopia: A Community Based Study
28_weeks	2010	0	Survey	Ethiopia Demographic and Health Survey 2010−2011
28_weeks	2005	1	Survey	Ethiopia Demographic and Health Survey 2005
28_weeks	2008	1	Sci Lit	Maternal and Neonatal Mortality in South−West Ethiopia: Estimates and Socio−Economic Inequality
28_weeks	2008−2010	1	Sci Lit	Perinatal mortality and associated risk factors: a case control study

Southern Nations, Nationalities, and Peoples with Sidama and South West Regions   ETH_44858   Eastern Sub−Saharan Africa   Stillbirth Rate (SBR) − 20 weeks
lambda = 0.8 ; zeta = 0.7 ; scale = 15 ; amp = 0.028

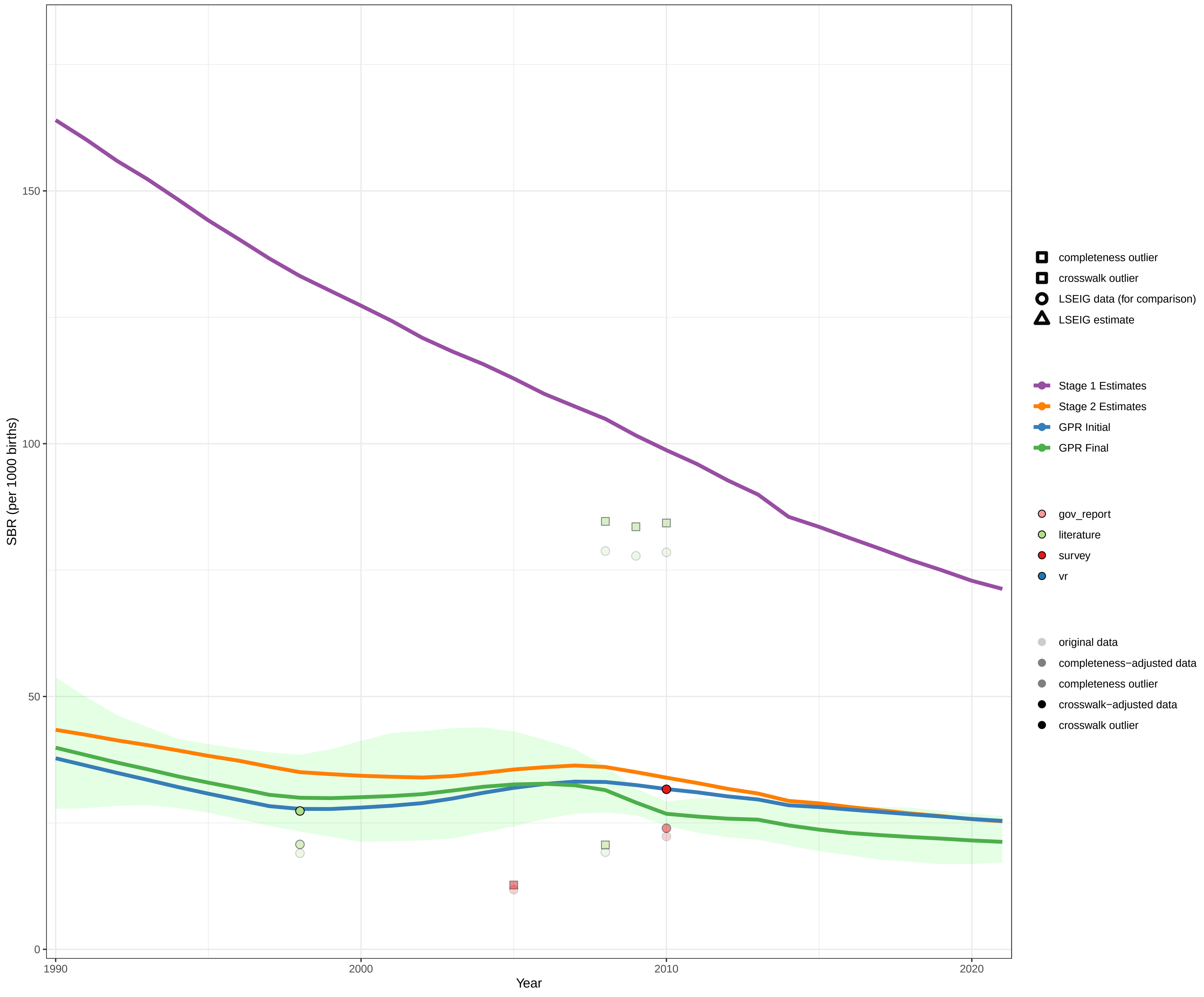

Southern Nations, Nationalities, and Peoples with Sidama and South West Regions   ETH_44858   Eastern Sub−Saharan Africa   Stillbirth Rate (SBR) − 28 weeks
lambda = 0.8 ; zeta = 0.7 ; scale = 15 ; amp = 0.028

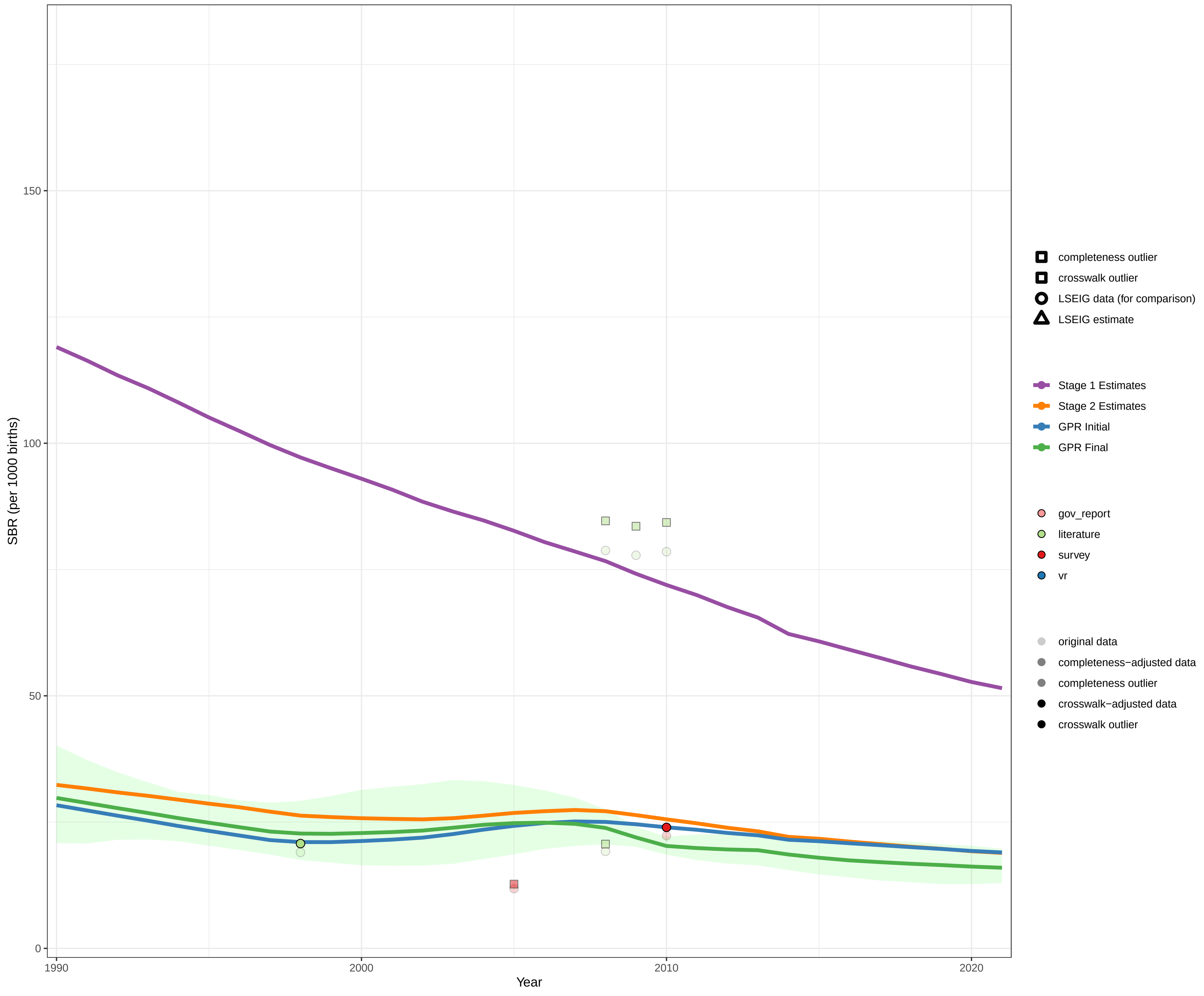

Year	Total Stillbirths (in thousands)	Total Stillbirths (in thousands)	SBR (per 1000 births)	SBR (per 1000 births)	SBR/NMR	SBR/NMR
	20 weeks	28 weeks	20 weeks	28 weeks	20 weeks	28 weeks
1990	22.2 (15.3 to 30.4)	16.4 (11.4 to 22.4)	39.9 (27.8 to 53.8)	29.8 (20.8 to 40.2)	0.83 (0.58 to 1.12)	0.62 (0.43 to 0.84)
1995	20.0 (16.3 to 24.9)	15.0 (12.2 to 18.4)	33.0 (27.0 to 40.6)	24.9 (20.3 to 30.4)	0.75 (0.62 to 0.93)	0.57 (0.46 to 0.69)
2000	19.7 (13.8 to 27.3)	14.9 (10.6 to 20.6)	30.1 (21.2 to 41.2)	22.8 (16.4 to 31.4)	0.76 (0.54 to 1.04)	0.58 (0.41 to 0.79)
2005	22.8 (16.8 to 30.5)	17.2 (12.8 to 22.6)	32.6 (24.3 to 43.1)	24.8 (18.6 to 32.3)	0.90 (0.67 to 1.19)	0.68 (0.51 to 0.89)
2010	18.9 (17.2 to 20.7)	14.2 (12.9 to 15.5)	26.8 (24.5 to 29.2)	20.3 (18.5 to 22.1)	0.84 (0.77 to 0.92)	0.64 (0.58 to 0.69)
2015	17.4 (14.2 to 20.9)	13.1 (10.7 to 15.7)	23.7 (19.4 to 28.3)	17.9 (14.6 to 21.4)	0.87 (0.71 to 1.04)	0.66 (0.54 to 0.79)
2020	16.9 (13.2 to 21.3)	12.7 (9.9 to 16.0)	21.5 (16.9 to 26.9)	16.2 (12.7 to 20.3)	0.92 (0.72 to 1.15)	0.69 (0.54 to 0.87)
2021	17.0 (13.7 to 21.2)	12.7 (10.2 to 15.7)	21.3 (17.2 to 26.4)	16.0 (12.9 to 19.7)	0.93 (0.75 to 1.15)	0.70 (0.56 to 0.86)

Tigray   ETH_44852   Eastern Sub−Saharan Africa   Stillbirth Rate / Neonatal Mortality Rate (SBR/NMR) − 20 weeks
lambda = 0.8 ; zeta = 0.7 ; scale = 15 ; amp = 0.028

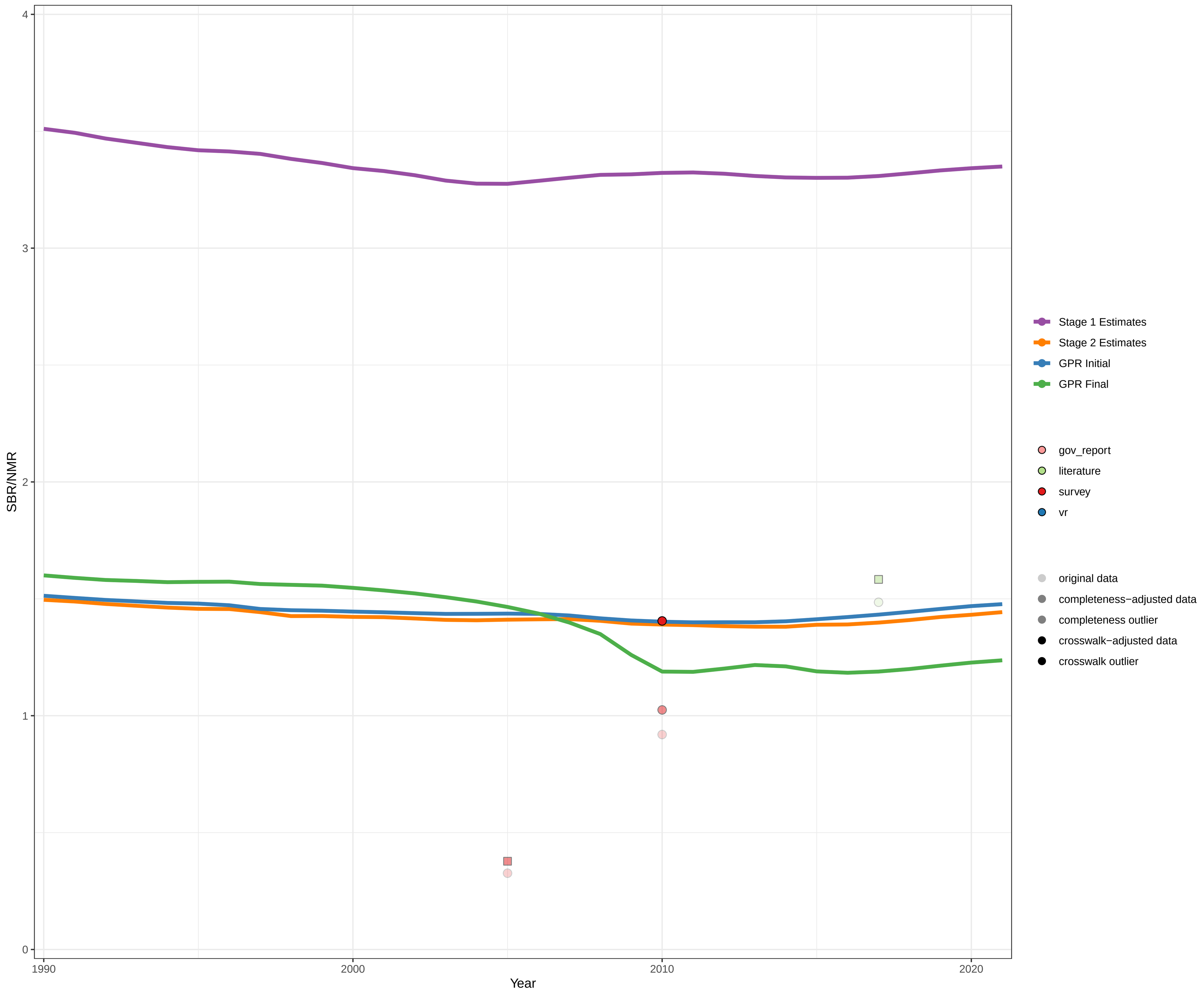

Tigray   ETH_44852   Eastern Sub−Saharan Africa   Stillbirth Rate / Neonatal Mortality Rate (SBR/NMR) − 28 weeks
lambda = 0.8 ; zeta = 0.7 ; scale = 15 ; amp = 0.028

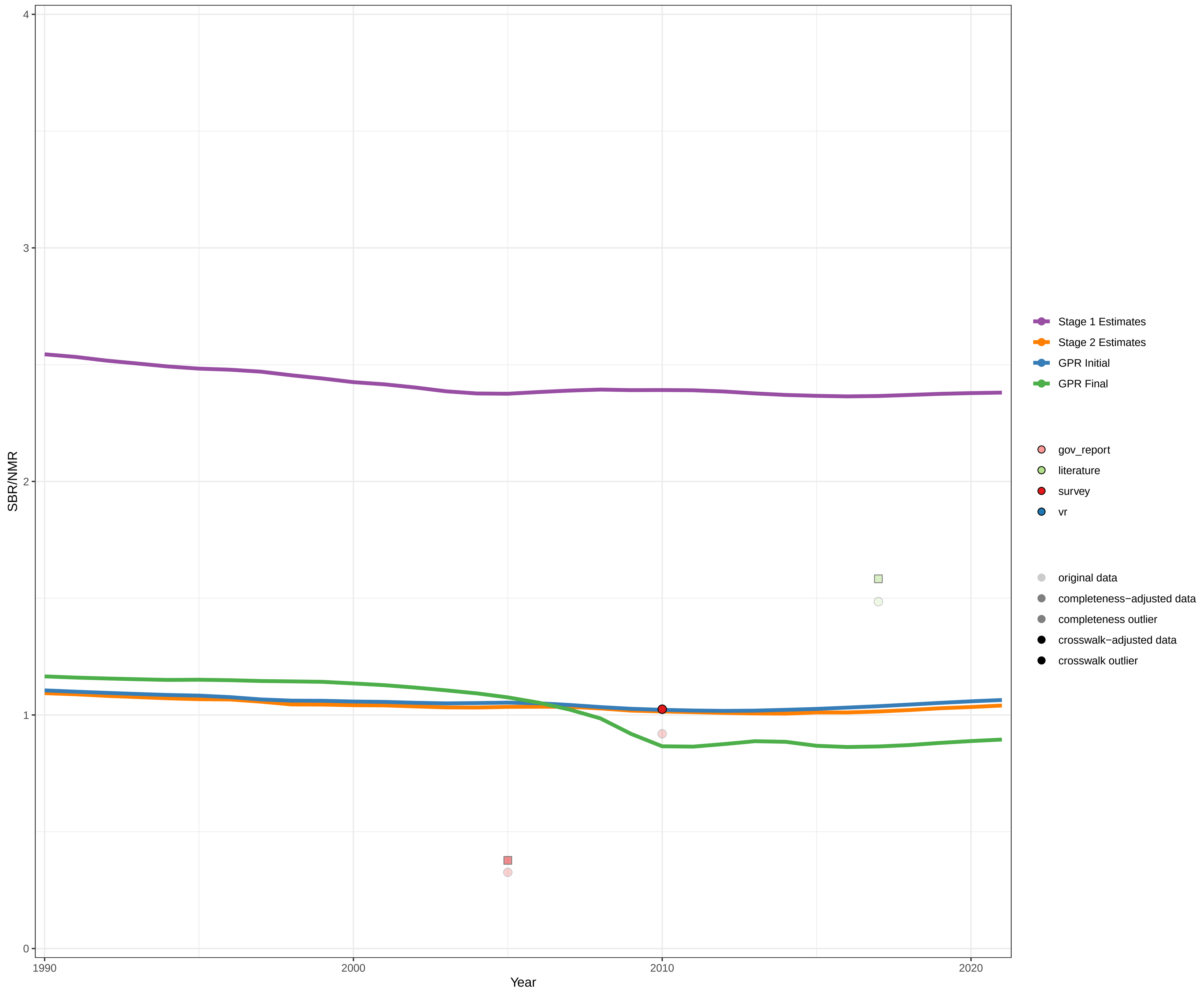

Definition	Years	Outlier	Source.Type	Title
28_weeks	2010	0	Survey	Ethiopia Demographic and Health Survey 2010−2011
28_weeks	2005	1	Survey	Ethiopia Demographic and Health Survey 2005
28_weeks	2017	1	Sci Lit	Prevalence and determinants of stillbirth among women attended deliveries in Aksum General Hospital: a facility based cross−sectional study

Tigray   ETH_44852   Eastern Sub−Saharan Africa   Stillbirth Rate (SBR) − 20 weeks
lambda = 0.8 ; zeta = 0.7 ; scale = 15 ; amp = 0.028

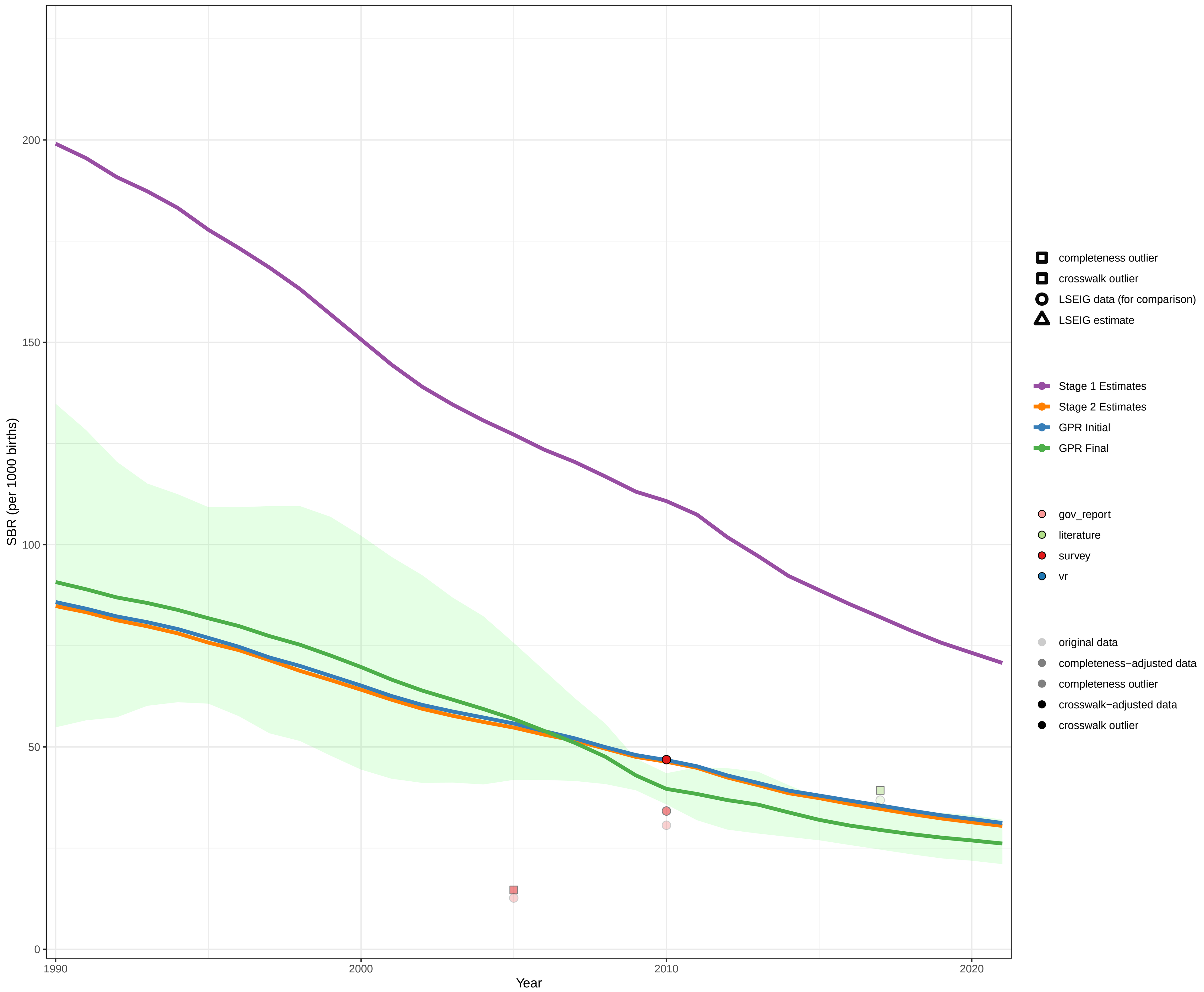

Tigray   ETH_44852   Eastern Sub−Saharan Africa   Stillbirth Rate (SBR) − 28 weeks
lambda = 0.8 ; zeta = 0.7 ; scale = 15 ; amp = 0.028

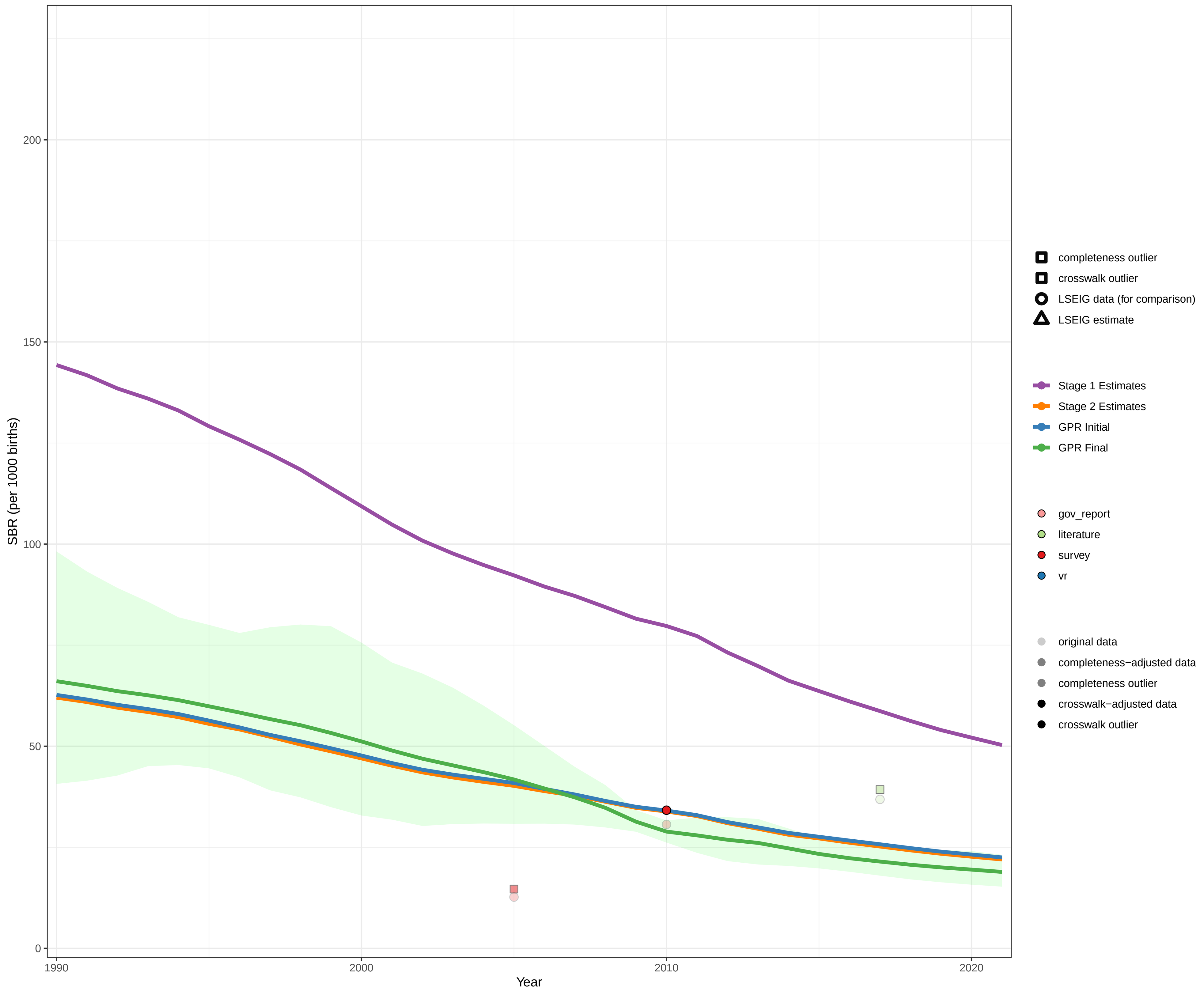

Year	Total Stillbirths (in thousands)	Total Stillbirths (in thousands)	SBR (per 1000 births)	SBR (per 1000 births)	SBR/NMR	SBR/NMR
	20 weeks	28 weeks	20 weeks	28 weeks	20 weeks	28 weeks
1990	14.1 (8.1 to 21.8)	9.9 (5.9 to 15.3)	90.7 (54.8 to 134.8)	66.1 (40.7 to 98.2)	1.60 (0.97 to 2.38)	1.17 (0.72 to 1.73)
1995	13.9 (10.0 to 19.1)	9.9 (7.2 to 13.5)	81.8 (60.7 to 109.3)	59.9 (44.5 to 80.0)	1.57 (1.17 to 2.10)	1.15 (0.86 to 1.54)
2000	12.5 (7.7 to 19.0)	9.0 (5.7 to 13.6)	69.7 (44.4 to 102.2)	51.2 (32.9 to 75.6)	1.55 (0.98 to 2.27)	1.14 (0.73 to 1.68)
2005	10.3 (7.4 to 14.0)	7.4 (5.4 to 9.9)	56.9 (41.9 to 75.7)	41.8 (30.8 to 55.2)	1.47 (1.08 to 1.95)	1.08 (0.79 to 1.42)
2010	6.7 (6.0 to 7.4)	4.8 (4.3 to 5.3)	39.6 (35.8 to 43.5)	28.9 (26.2 to 31.6)	1.19 (1.07 to 1.31)	0.87 (0.78 to 0.95)
2015	5.4 (4.6 to 6.4)	3.9 (3.3 to 4.6)	32.0 (26.9 to 37.4)	23.3 (19.8 to 27.3)	1.19 (1.00 to 1.39)	0.87 (0.74 to 1.02)
2020	4.7 (3.8 to 5.8)	3.4 (2.7 to 4.2)	26.9 (21.9 to 33.2)	19.5 (15.7 to 24.1)	1.23 (1.00 to 1.51)	0.89 (0.72 to 1.10)
2021	4.6 (3.7 to 5.7)	3.3 (2.7 to 4.1)	26.1 (21.0 to 32.0)	18.9 (15.3 to 23.1)	1.24 (1.00 to 1.52)	0.89 (0.72 to 1.09)

Kenya KEN Eastern Sub−Saharan Africa Stillbirth Rate / Neonatal Mortality Rate (SBR/NMR) − 20 weeks
lambda = 0.6 ; zeta = 0.8 ; scale = 15 ; amp = 0.028

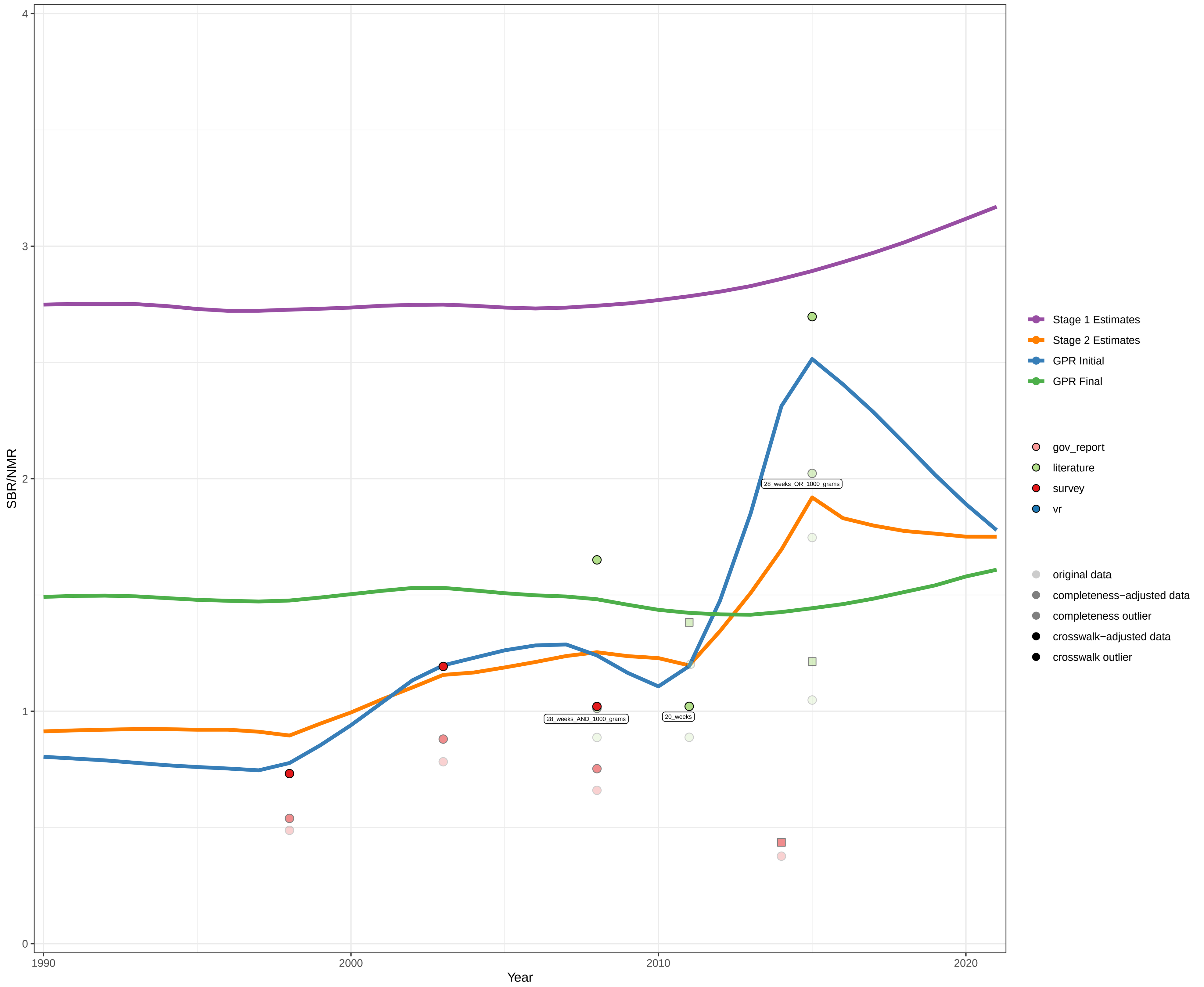

Kenya KEN Eastern Sub−Saharan Africa Stillbirth Rate / Neonatal Mortality Rate (SBR/NMR) − 28 weeks
lambda = 0.6 ; zeta = 0.8 ; scale = 15 ; amp = 0.028

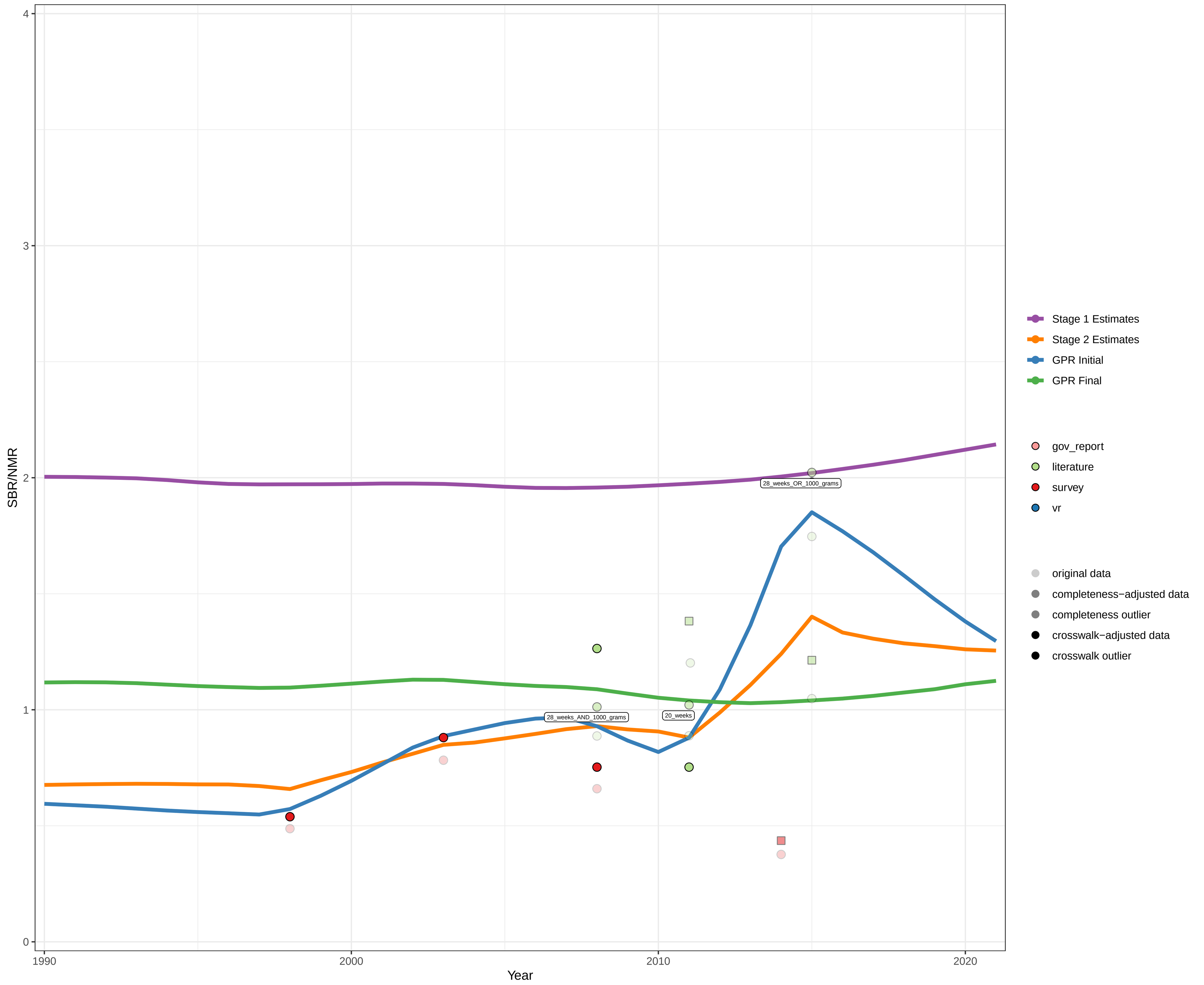

Definition	Years	Outlier	Source.Type	Title
20_weeks	2011	0	Sci Lit	A prospective study of maternal, fetal and neonatal deaths in low− and middle−income countries
28_weeks	1998	0	Survey	Kenya Demographic and Health Survey 1998
28_weeks	2003	0	Survey	Kenya Demographic and Health Survey 2003
28_weeks	2008	0	Survey	Kenya Demographic and Health Survey 2008−2009
28_weeks	2022	0	Survey	Kenya Demographic and Health Survey 2022
28_weeks_AND_1000_grams	2008	0	Sci Lit	Epidemiology of stillbirth in low−middle income countries: A Global Network Study
28_weeks_OR_1000_grams	2015	0	Sci Lit	Application of the ICD−PM classification system to stillbirth in four sub−Saharan African countries
20_weeks	2015	1	Sci Lit	Global Network for Women's and Children's Health Research: probable causes of stillbirth in low− and middle−income countries using a prospectively defined classification system
28_weeks	2014	1	Survey	Kenya Demographic and Health Survey 2014
28_weeks_OR_1000_grams	2011	1	Sci Lit	Maternal complications and perinatal mortality: findings of the World Health Organization Multicountry Survey on Maternal and Newborn Health

Kenya KEN Eastern Sub−Saharan Africa Stillbirth Rate (SBR) − 20 weeks
lambda = 0.6 ; zeta = 0.8 ; scale = 15 ; amp = 0.028

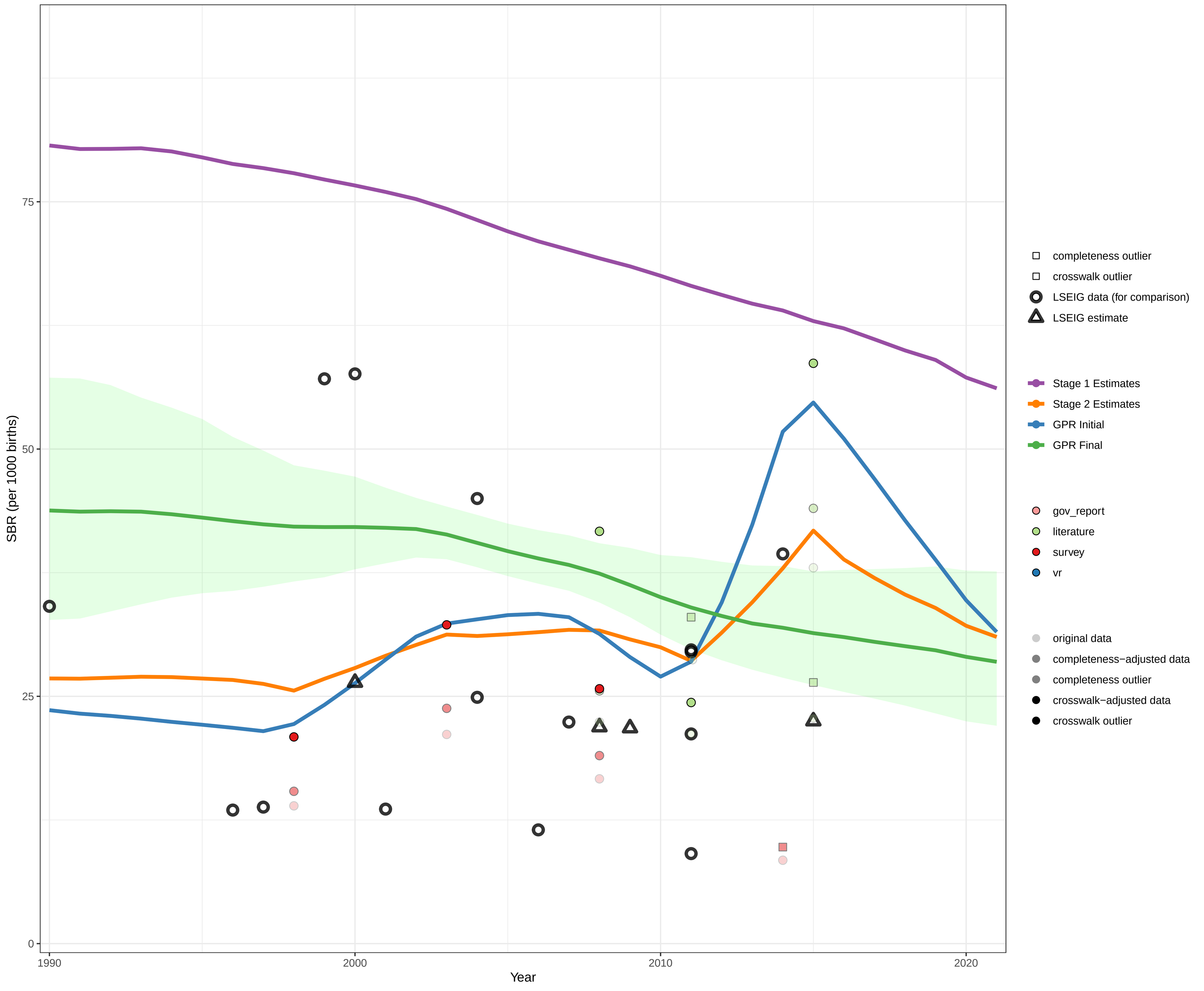

Kenya KEN Eastern Sub−Saharan Africa Stillbirth Rate (SBR) − 28 weeks
lambda = 0.6 ; zeta = 0.8 ; scale = 15 ; amp = 0.028

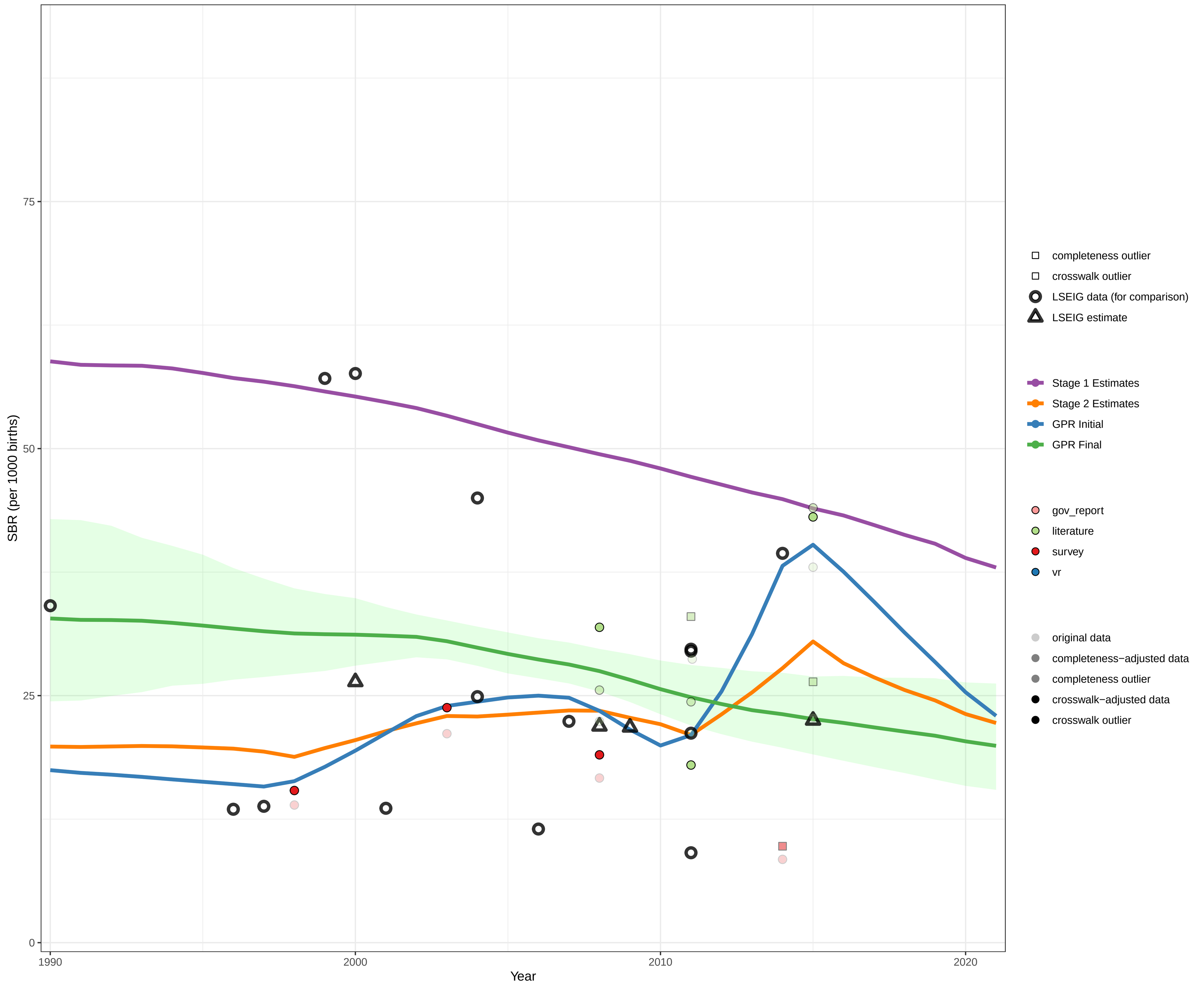

Year	Total Stillbirths (in thousands)	Total Stillbirths (in thousands)	SBR (per 1000 births)	SBR (per 1000 births)	SBR/NMR	SBR/NMR
	20 weeks	28 weeks	20 weeks	28 weeks	20 weeks	28 weeks
1990	45.5 (33.6 to 60.2)	33.7 (24.8 to 44.4)	43.8 (32.7 to 57.2)	32.8 (24.4 to 42.9)	1.49 (1.11 to 1.95)	1.12 (0.83 to 1.46)
1995	49.1 (40.0 to 61.1)	36.2 (29.3 to 44.6)	43.1 (35.4 to 53.0)	32.1 (26.2 to 39.3)	1.48 (1.22 to 1.82)	1.10 (0.90 to 1.35)
2000	53.3 (47.7 to 60.1)	39.0 (35.0 to 43.8)	42.1 (37.8 to 47.2)	31.2 (28.0 to 34.9)	1.50 (1.35 to 1.69)	1.11 (1.00 to 1.24)
2005	54.3 (50.7 to 58.3)	39.6 (36.8 to 42.6)	39.7 (37.2 to 42.5)	29.2 (27.2 to 31.4)	1.51 (1.41 to 1.61)	1.11 (1.04 to 1.19)
2010	49.3 (43.8 to 55.5)	35.7 (32.1 to 39.9)	35.0 (31.2 to 39.3)	25.7 (23.1 to 28.6)	1.44 (1.28 to 1.61)	1.05 (0.95 to 1.17)
2015	42.6 (35.3 to 51.4)	30.4 (25.5 to 36.4)	31.4 (26.1 to 37.6)	22.6 (19.1 to 26.9)	1.44 (1.20 to 1.73)	1.04 (0.88 to 1.24)
2020	35.9 (27.7 to 47.2)	25.0 (19.4 to 32.5)	29.0 (22.5 to 37.7)	20.4 (15.9 to 26.3)	1.58 (1.22 to 2.05)	1.11 (0.86 to 1.43)
2021	34.8 (26.7 to 46.4)	24.1 (18.6 to 32.0)	28.5 (22.0 to 37.6)	19.9 (15.5 to 26.2)	1.61 (1.24 to 2.12)	1.12 (0.87 to 1.48)

Central KEN_44793 Eastern Sub−Saharan Africa Stillbirth Rate / Neonatal Mortality Rate (SBR/NMR) − 20 weeks
lambda = 1 ; zeta = 0.6 ; scale = 15 ; amp = 0.028

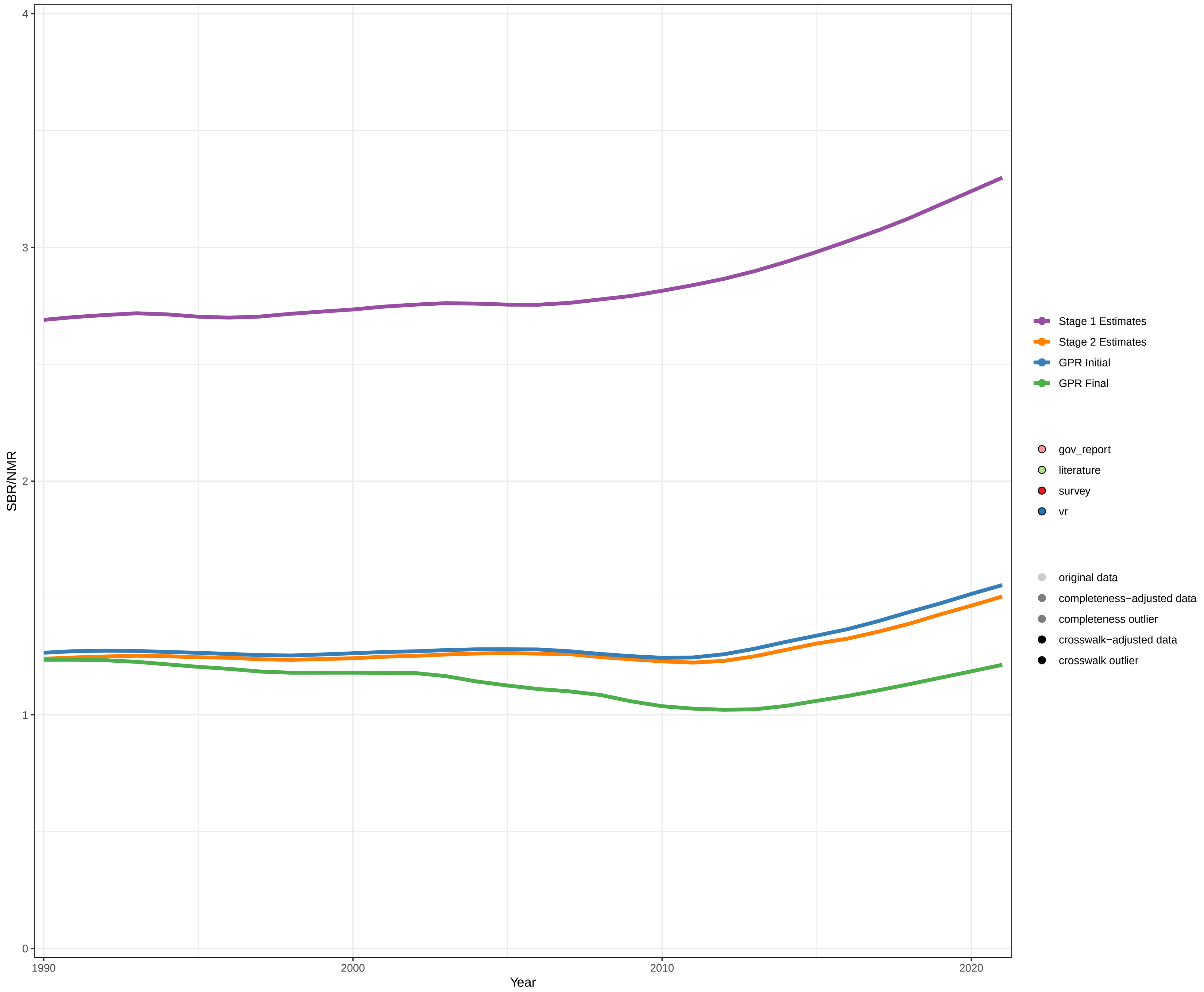

Central KEN_44793 Eastern Sub−Saharan Africa Stillbirth Rate / Neonatal Mortality Rate (SBR/NMR) − 28 weeks
lambda = 1 ; zeta = 0.6 ; scale = 15 ; amp = 0.028

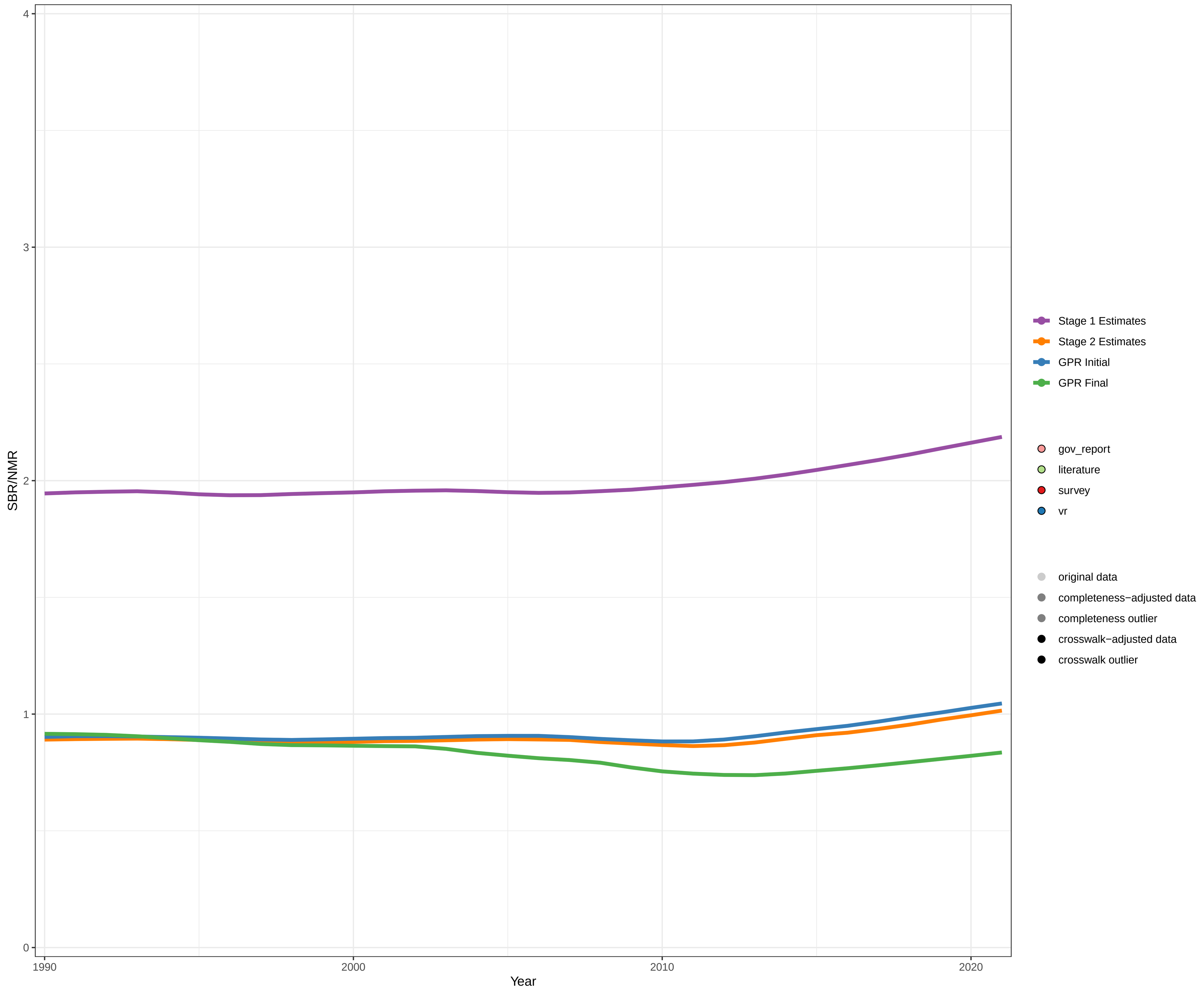

Definition	Years	Outlier	Source.Type	Title

Central KEN_44793 Eastern Sub−Saharan Africa Stillbirth Rate (SBR) − 20 weeks
lambda = 1 ; zeta = 0.6 ; scale = 15 ; amp = 0.028

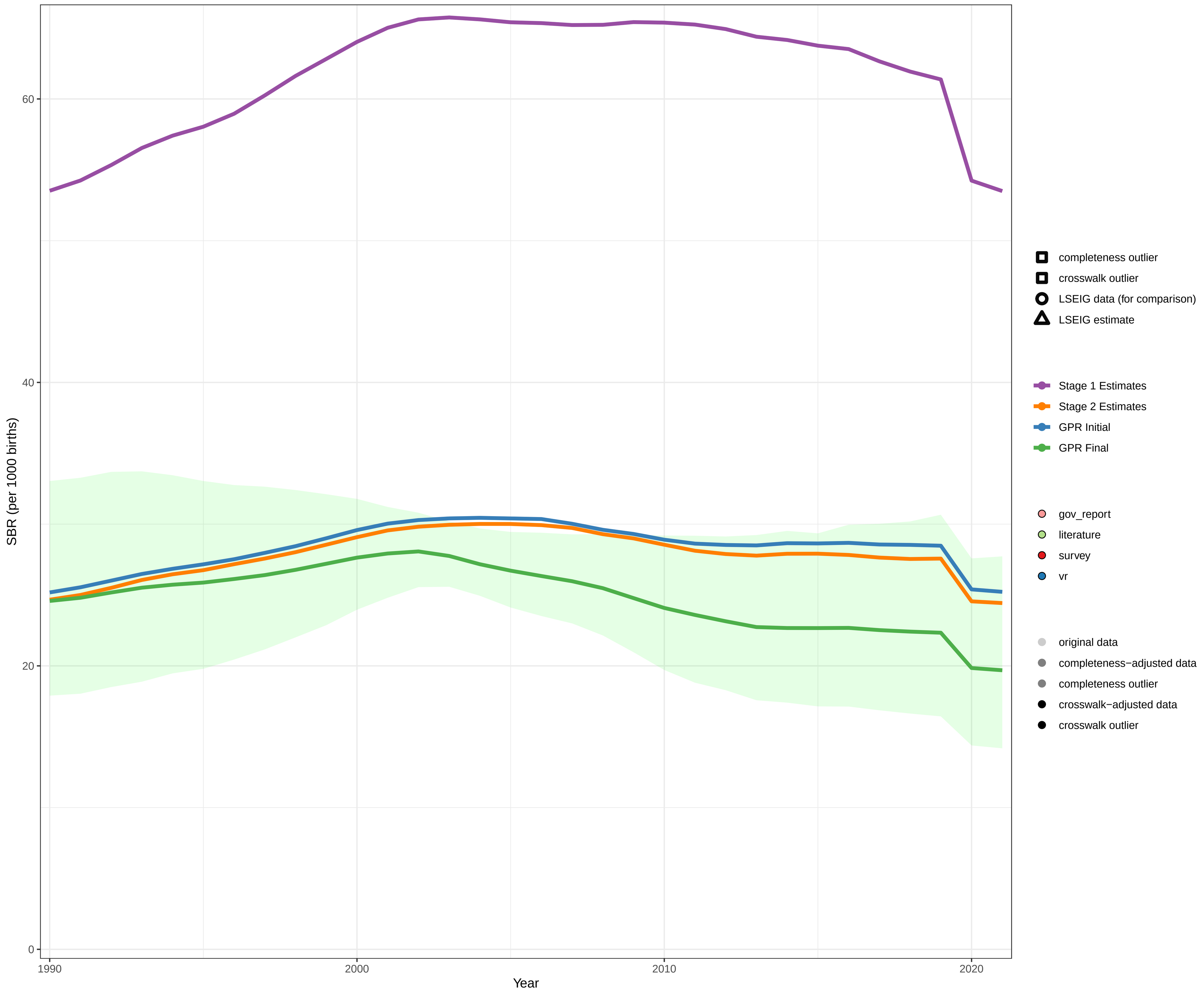

Central KEN_44793 Eastern Sub−Saharan Africa Stillbirth Rate (SBR) − 28 weeks
lambda = 1 ; zeta = 0.6 ; scale = 15 ; amp = 0.028

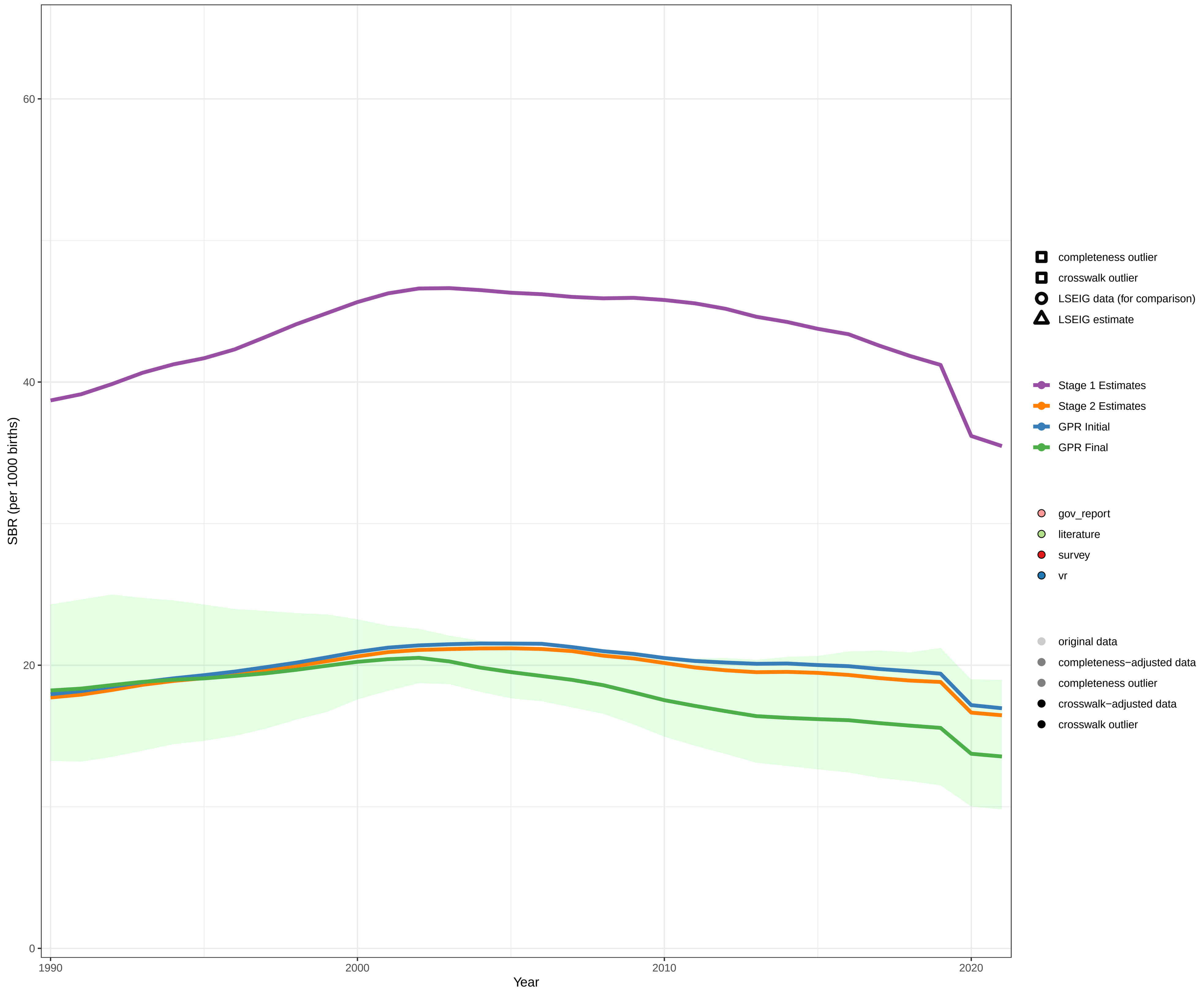

Year	Total Stillbirths (in thousands)	Total Stillbirths (in thousands)	SBR (per 1000 births)	SBR (per 1000 births)	SBR/NMR	SBR/NMR
	20 weeks	28 weeks	20 weeks	28 weeks	20 weeks	28 weeks
1990	3.0<br>(2.2 to 4.0)	2.2<br>(1.6 to 3.0)	24.6<br>(17.9 to 33.0)	18.2<br>(13.2 to 24.3)	1.24<br>(0.90 to 1.66)	0.92<br>(0.67 to 1.22)
1995	3.2<br>(2.5 to 4.2)	2.4<br>(1.8 to 3.0)	25.9<br>(19.8 to 33.0)	19.1<br>(14.7 to 24.3)	1.21<br>(0.92 to 1.54)	0.89<br>(0.68 to 1.13)
2000	3.6<br>(3.1 to 4.1)	2.6<br>(2.2 to 3.0)	27.6<br>(24.0 to 31.8)	20.2<br>(17.6 to 23.2)	1.18<br>(1.02 to 1.36)	0.86<br>(0.75 to 0.99)
2005	3.4<br>(3.0 to 3.7)	2.4<br>(2.2 to 2.7)	26.7<br>(24.1 to 29.5)	19.5<br>(17.7 to 21.4)	1.13<br>(1.02 to 1.24)	0.82<br>(0.74 to 0.90)
2010	2.8<br>(2.3 to 3.5)	2.1<br>(1.7 to 2.4)	24.1<br>(19.7 to 29.2)	17.5<br>(14.9 to 20.4)	1.04<br>(0.85 to 1.26)	0.75<br>(0.64 to 0.88)
2015	2.4<br>(1.8 to 3.1)	1.7<br>(1.3 to 2.2)	22.7<br>(17.1 to 29.4)	16.2<br>(12.6 to 20.7)	1.06<br>(0.80 to 1.37)	0.76<br>(0.59 to 0.97)
2020	1.9<br>(1.4 to 2.7)	1.3<br>(0.9 to 1.8)	19.8<br>(14.4 to 27.6)	13.7<br>(10.0 to 19.0)	1.19<br>(0.86 to 1.65)	0.82<br>(0.60 to 1.14)
2021	1.8<br>(1.3 to 2.6)	1.3<br>(0.9 to 1.8)	19.7<br>(14.2 to 27.7)	13.6<br>(9.8 to 19.0)	1.21<br>(0.87 to 1.71)	0.84<br>(0.60 to 1.17)

Coast KEN_44794 Eastern Sub−Saharan Africa Stillbirth Rate / Neonatal Mortality Rate (SBR/NMR) − 20 weeks
lambda = 1 ; zeta = 0.6 ; scale = 15 ; amp = 0.028

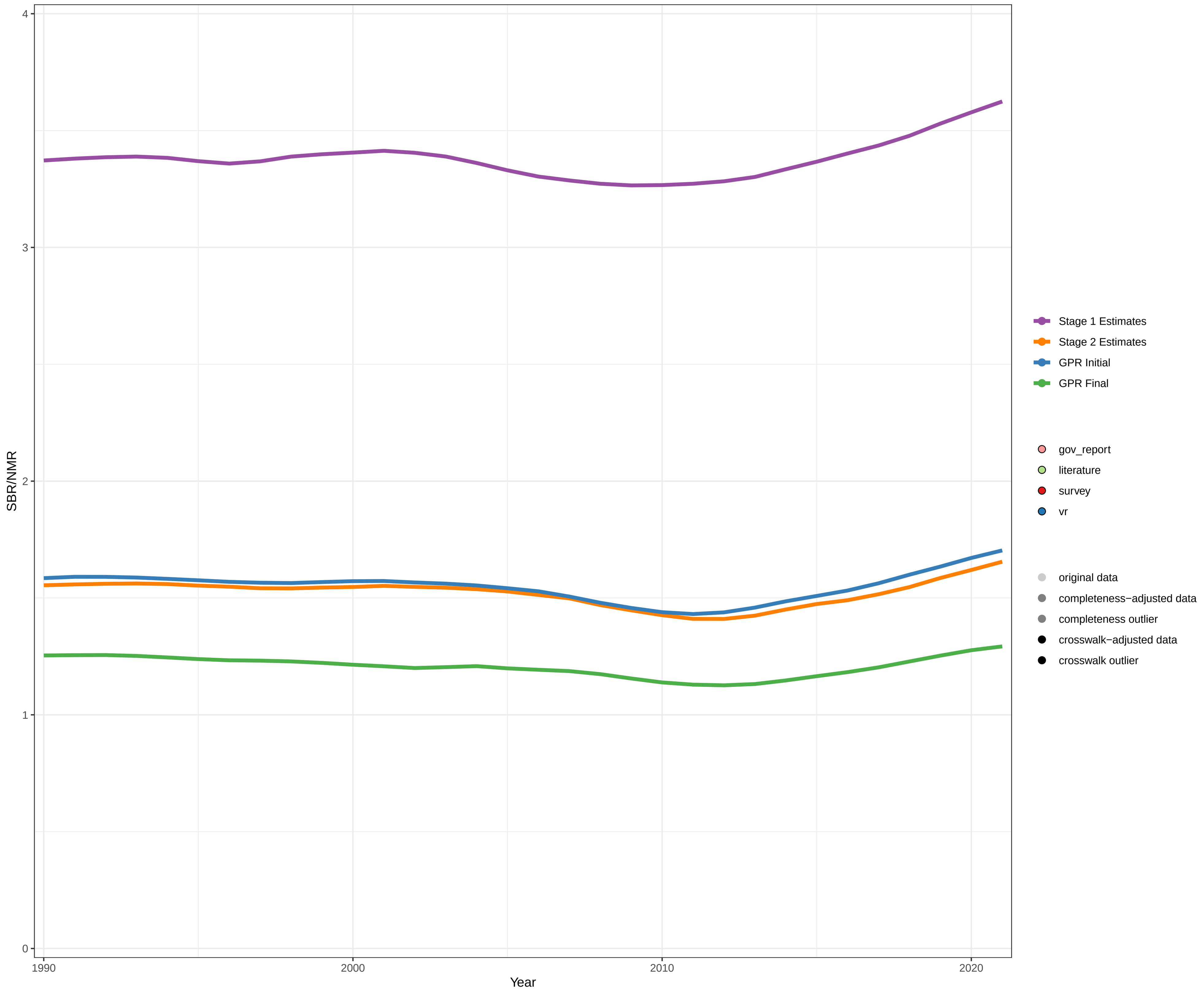

Coast KEN_44794 Eastern Sub−Saharan Africa Stillbirth Rate / Neonatal Mortality Rate (SBR/NMR) − 28 weeks
lambda = 1 ; zeta = 0.6 ; scale = 15 ; amp = 0.028

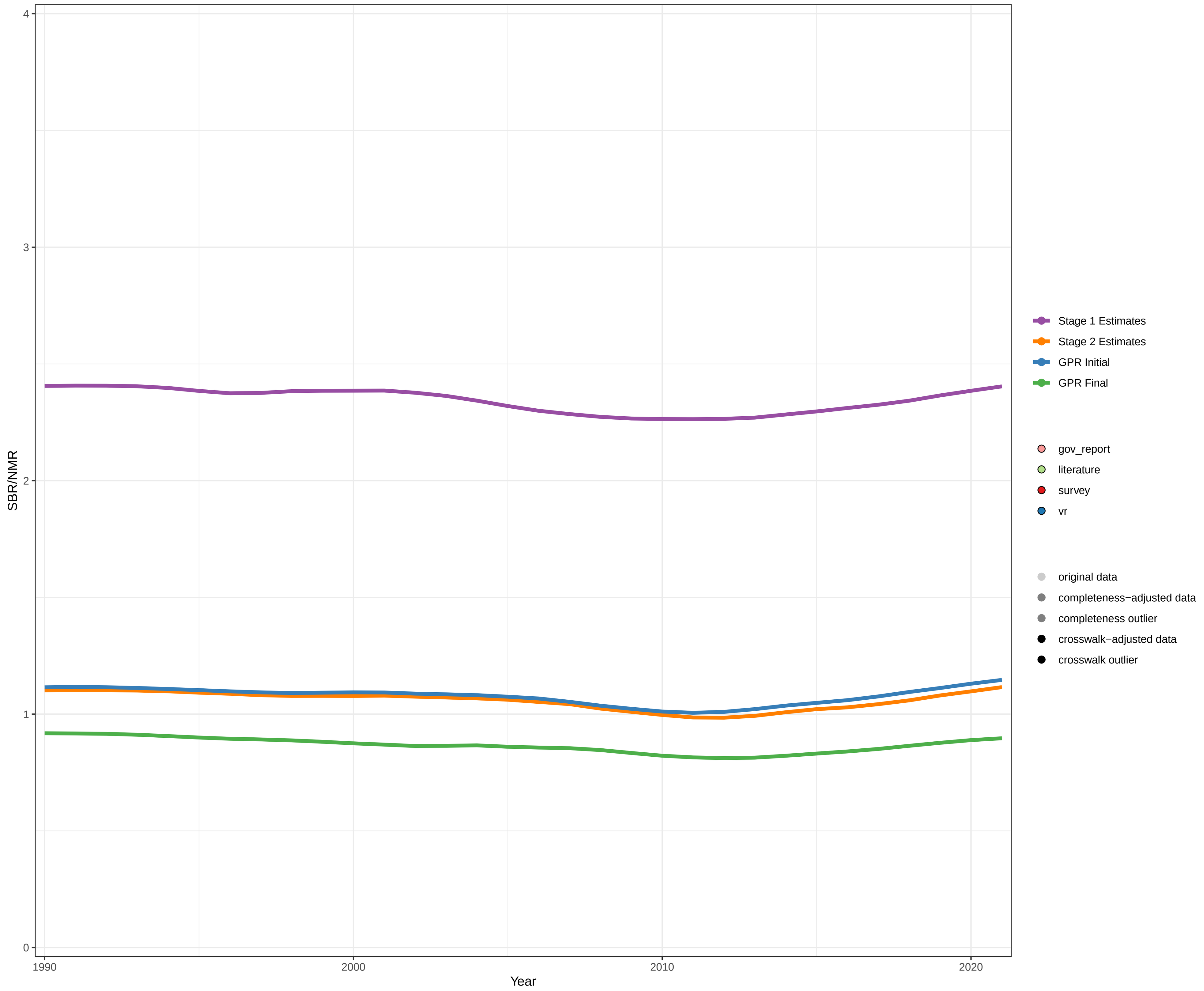

Definition	Years	Outlier	Source.Type	Title

Coast KEN_44794 Eastern Sub−Saharan Africa Stillbirth Rate (SBR) − 20 weeks
lambda = 1 ; zeta = 0.6 ; scale = 15 ; amp = 0.028

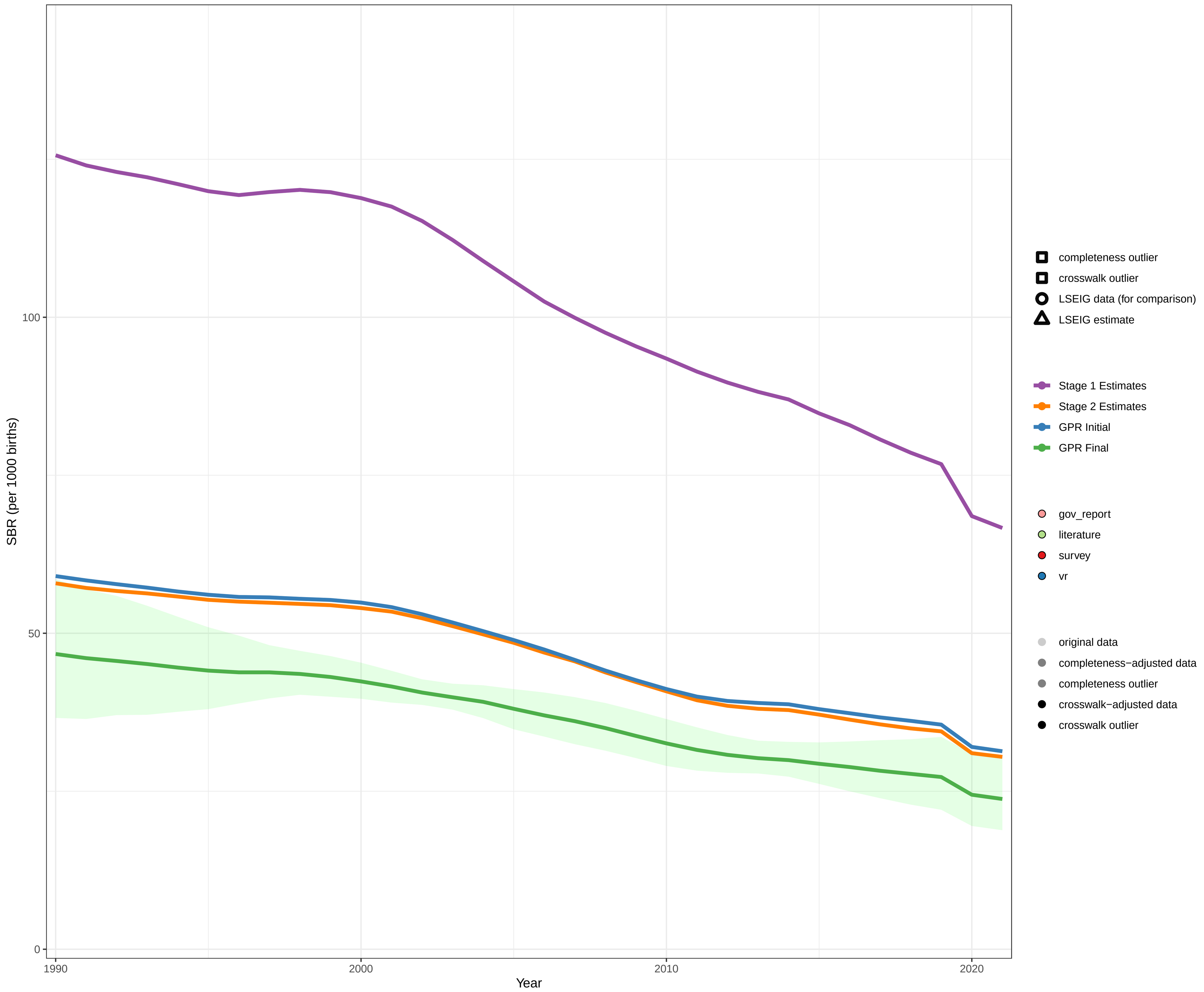

Coast KEN_44794 Eastern Sub−Saharan Africa Stillbirth Rate (SBR) − 28 weeks
lambda = 1 ; zeta = 0.6 ; scale = 15 ; amp = 0.028

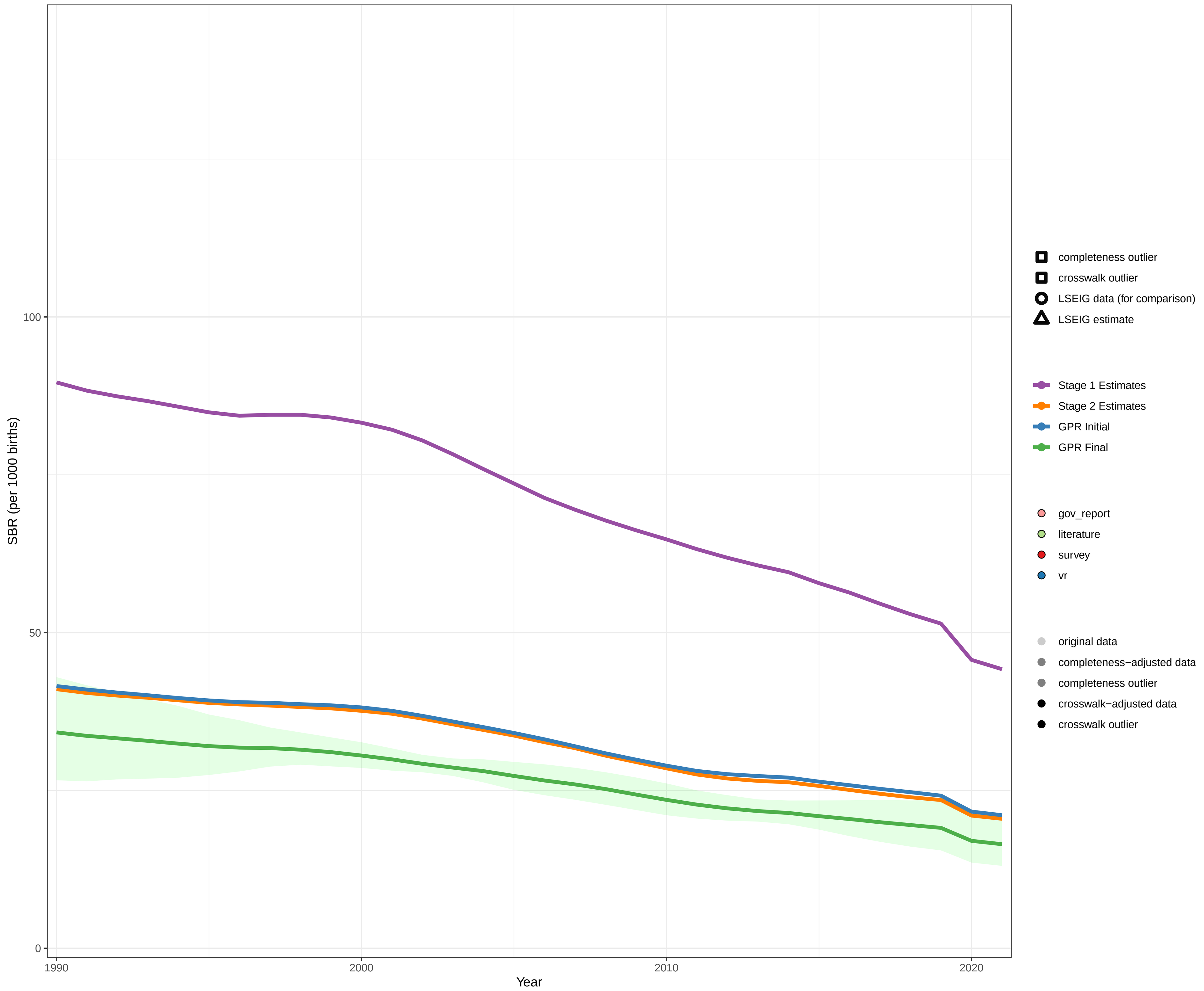

Year	Total Stillbirths (in thousands)	Total Stillbirths (in thousands)	SBR (per 1000 births)	SBR (per 1000 births)	SBR/NMR	SBR/NMR
	20 weeks	28 weeks	20 weeks	28 weeks	20 weeks	28 weeks
1990	4.1 (3.2 to 5.2)	3.0 (2.3 to 3.8)	46.7 (36.6 to 58.6)	34.2 (26.6 to 42.9)	1.25 (0.98 to 1.57)	0.92 (0.71 to 1.15)
1995	4.3 (3.6 to 5.0)	3.1 (2.6 to 3.5)	44.1 (38.0 to 50.9)	32.0 (27.5 to 37.0)	1.24 (1.07 to 1.43)	0.90 (0.77 to 1.04)
2000	4.6 (4.3 to 5.0)	3.3 (3.1 to 3.5)	42.4 (39.6 to 45.3)	30.5 (28.6 to 32.6)	1.21 (1.14 to 1.30)	0.87 (0.82 to 0.94)
2005	4.5 (4.1 to 4.9)	3.2 (3.0 to 3.5)	38.0 (34.8 to 41.2)	27.3 (25.1 to 29.5)	1.20 (1.10 to 1.30)	0.86 (0.79 to 0.93)
2010	4.1 (3.6 to 4.6)	2.9 (2.6 to 3.3)	32.6 (29.0 to 36.4)	23.5 (21.1 to 26.1)	1.14 (1.01 to 1.27)	0.82 (0.74 to 0.91)
2015	3.7 (3.3 to 4.1)	2.6 (2.3 to 2.9)	29.3 (26.2 to 32.7)	20.9 (18.8 to 23.4)	1.17 (1.04 to 1.30)	0.83 (0.75 to 0.93)
2020	2.8 (2.2 to 3.6)	1.9 (1.5 to 2.4)	24.4 (19.5 to 30.9)	17.0 (13.6 to 21.5)	1.28 (1.02 to 1.62)	0.89 (0.71 to 1.12)
2021	2.7 (2.1 to 3.5)	1.8 (1.5 to 2.4)	23.8 (18.8 to 30.5)	16.5 (13.1 to 21.1)	1.29 (1.02 to 1.66)	0.90 (0.71 to 1.15)

Eastern   KEN_44795   Eastern Sub−Saharan Africa   Stillbirth Rate / Neonatal Mortality Rate (SBR/NMR) − 20 weeks
lambda = 1 ; zeta = 0.6 ; scale = 15 ; amp = 0.028

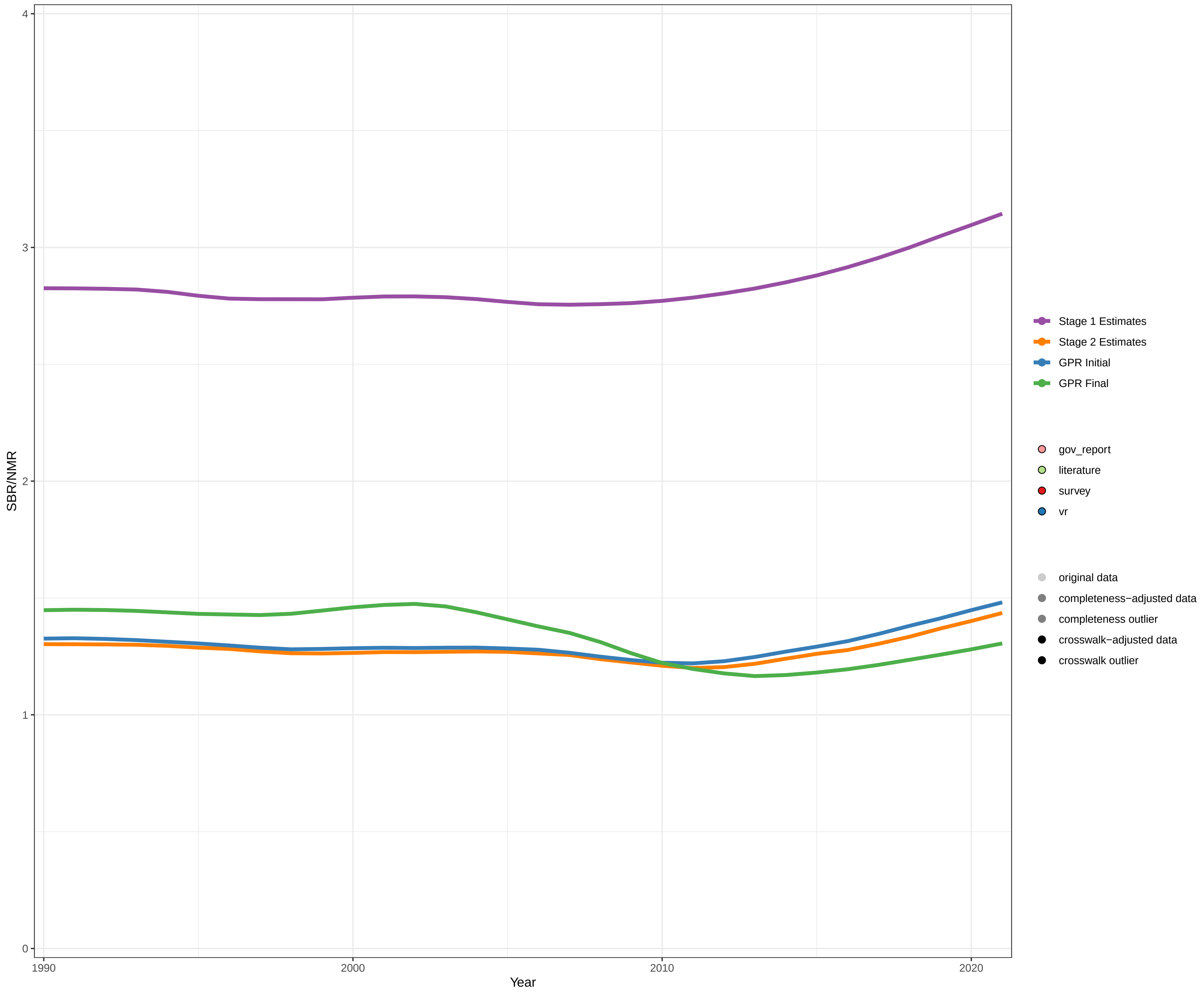

Eastern   KEN_44795   Eastern Sub−Saharan Africa   Stillbirth Rate / Neonatal Mortality Rate (SBR/NMR) − 28 weeks
lambda = 1 ; zeta = 0.6 ; scale = 15 ; amp = 0.028

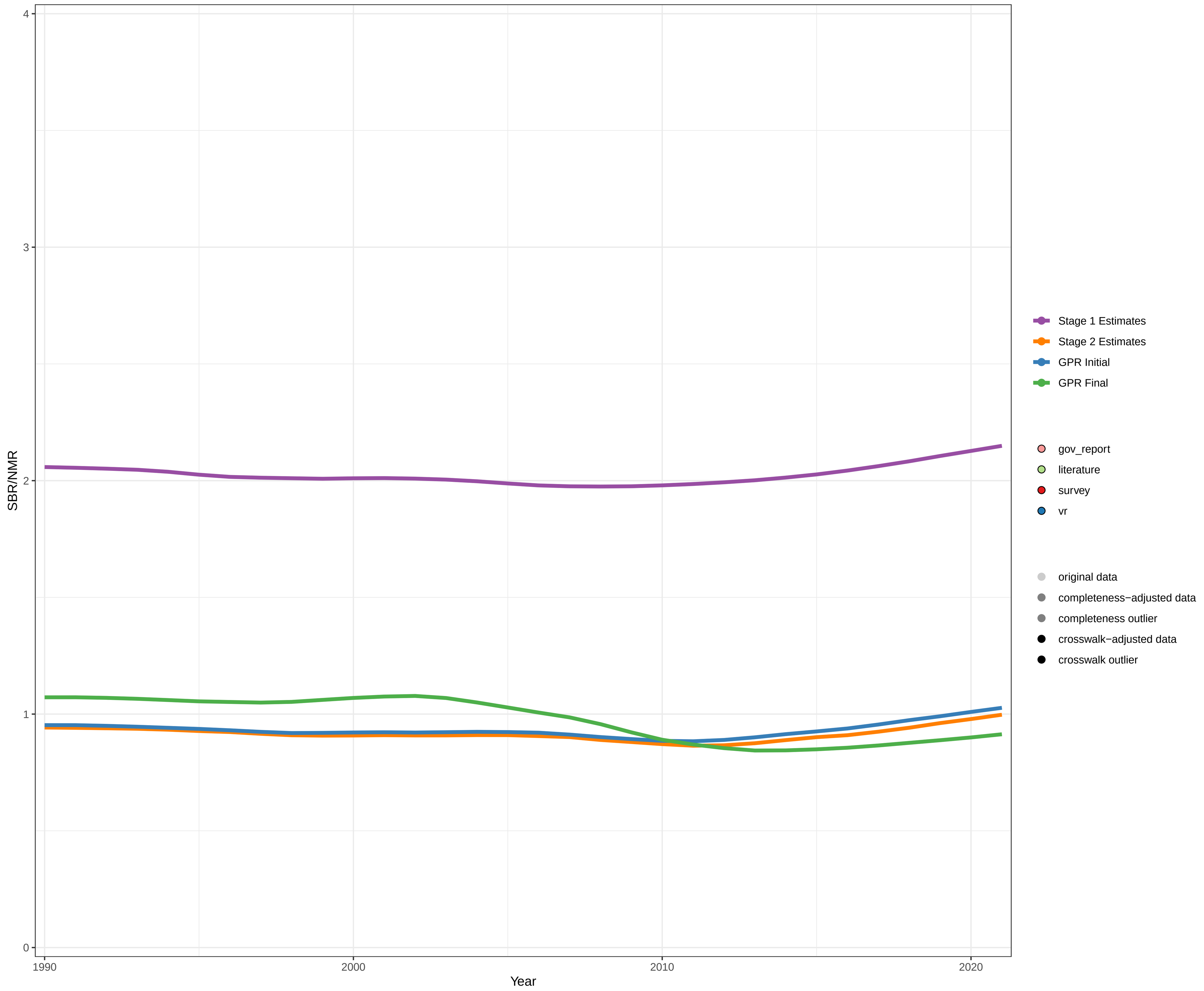

Definition	Years	Outlier	Source.Type	Title

Eastern   KEN_44795   Eastern Sub−Saharan Africa   Stillbirth Rate (SBR) − 20 weeks
lambda = 1 ; zeta = 0.6 ; scale = 15 ; amp = 0.028

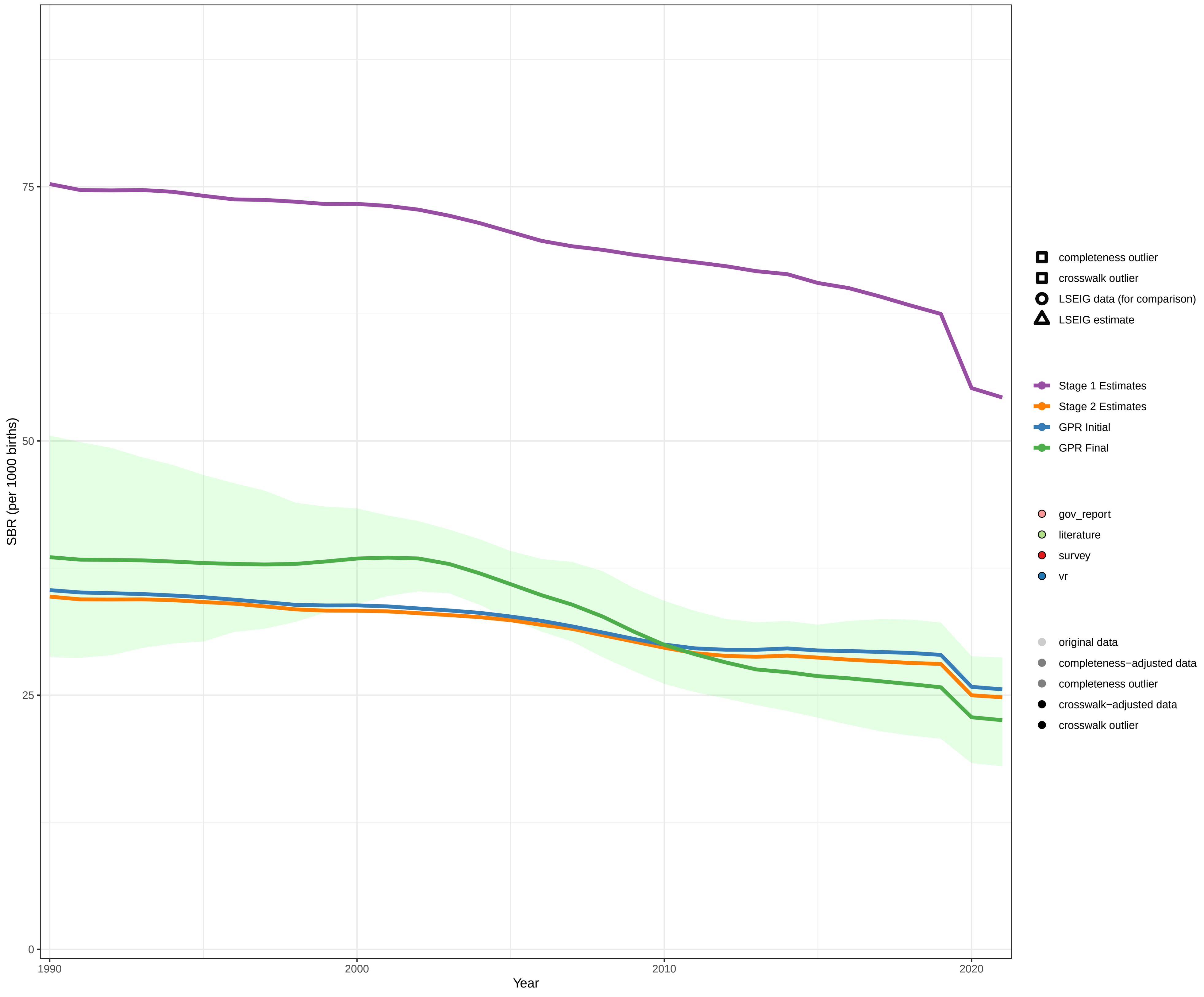

Eastern   KEN_44795   Eastern Sub−Saharan Africa   Stillbirth Rate (SBR) − 28 weeks
lambda = 1 ; zeta = 0.6 ; scale = 15 ; amp = 0.028

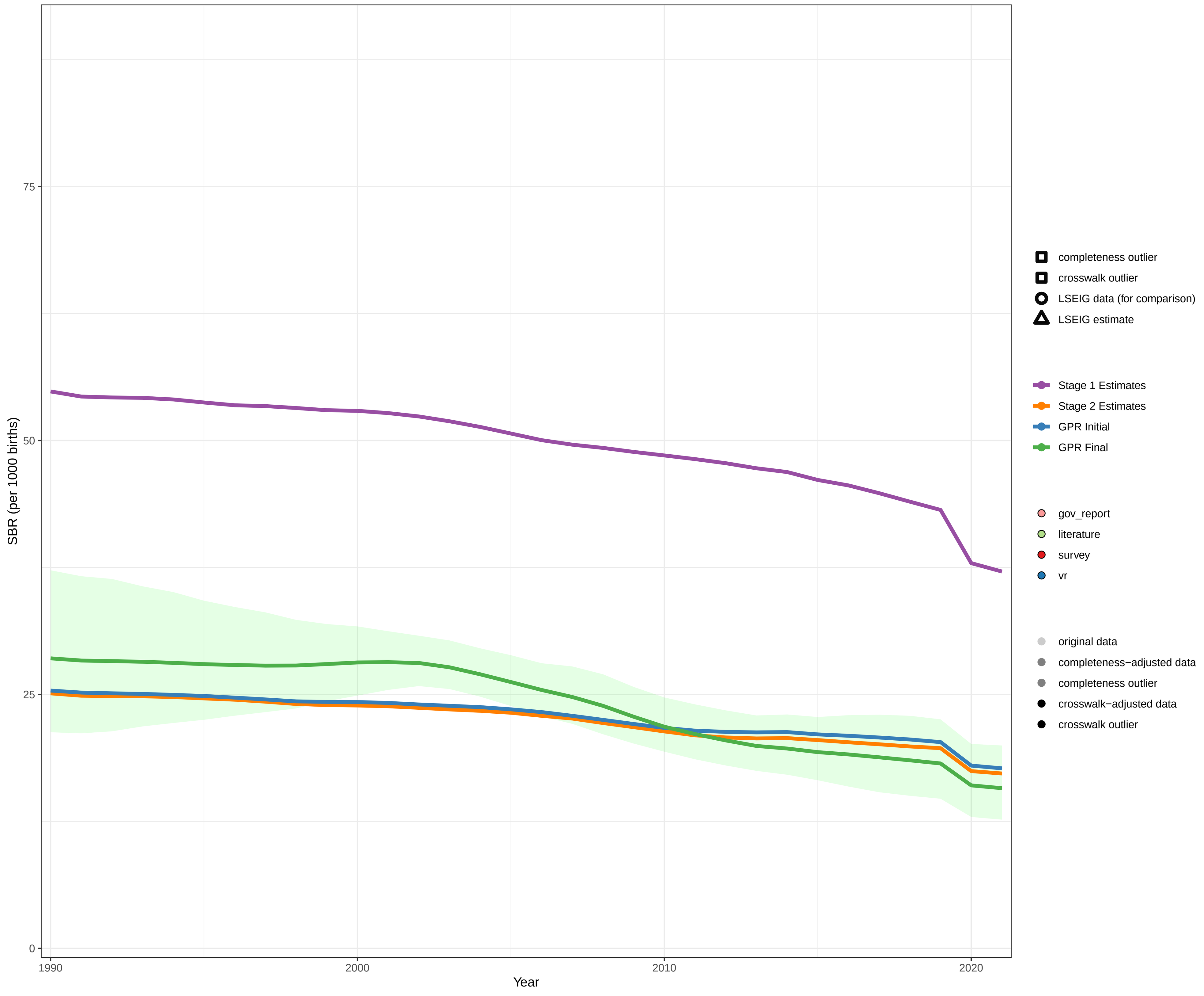

Year	Total Stillbirths (in thousands)	Total Stillbirths (in thousands)	SBR (per 1000 births)	SBR (per 1000 births)	SBR/NMR	SBR/NMR
	20 weeks	28 weeks	20 weeks	28 weeks	20 weeks	28 weeks
1990	6.7 (4.9 to 8.8)	4.9 (3.6 to 6.4)	38.6 (28.7 to 50.5)	28.6 (21.3 to 37.2)	1.45 (1.08 to 1.90)	1.07 (0.80 to 1.40)
1995	7.0 (5.5 to 8.6)	5.1 (4.1 to 6.2)	38.0 (30.3 to 46.7)	28.0 (22.5 to 34.2)	1.43 (1.14 to 1.76)	1.05 (0.85 to 1.29)
2000	7.4 (6.5 to 8.4)	5.4 (4.8 to 6.1)	38.4 (33.9 to 43.4)	28.1 (24.9 to 31.7)	1.46 (1.29 to 1.65)	1.07 (0.95 to 1.20)
2005	7.0 (6.3 to 7.7)	5.1 (4.6 to 5.6)	35.9 (32.5 to 39.2)	26.2 (23.9 to 28.9)	1.41 (1.27 to 1.54)	1.03 (0.94 to 1.13)
2010	5.6 (4.9 to 6.4)	4.1 (3.6 to 4.6)	29.9 (26.1 to 34.3)	21.8 (19.4 to 24.7)	1.22 (1.07 to 1.40)	0.89 (0.79 to 1.01)
2015	4.7 (4.0 to 5.6)	3.3 (2.9 to 4.0)	26.9 (22.8 to 31.9)	19.3 (16.6 to 22.8)	1.18 (1.00 to 1.40)	0.85 (0.73 to 1.00)
2020	3.5 (2.8 to 4.4)	2.4 (2.0 to 3.1)	22.8 (18.3 to 28.8)	16.0 (12.9 to 20.1)	1.28 (1.03 to 1.62)	0.90 (0.73 to 1.13)
2021	3.4 (2.7 to 4.3)	2.3 (1.9 to 3.0)	22.5 (18.0 to 28.7)	15.8 (12.7 to 20.0)	1.31 (1.04 to 1.66)	0.91 (0.73 to 1.16)

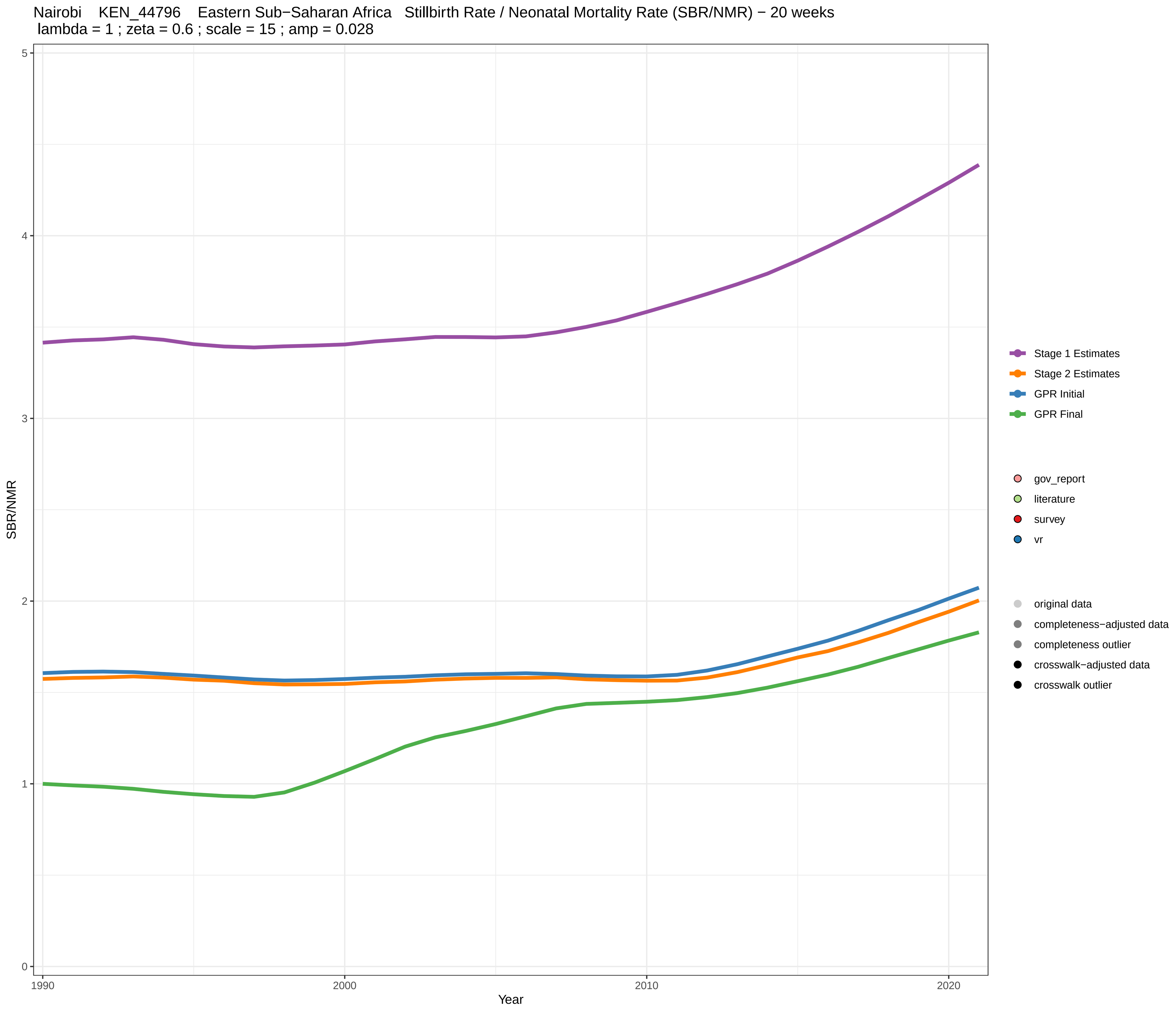

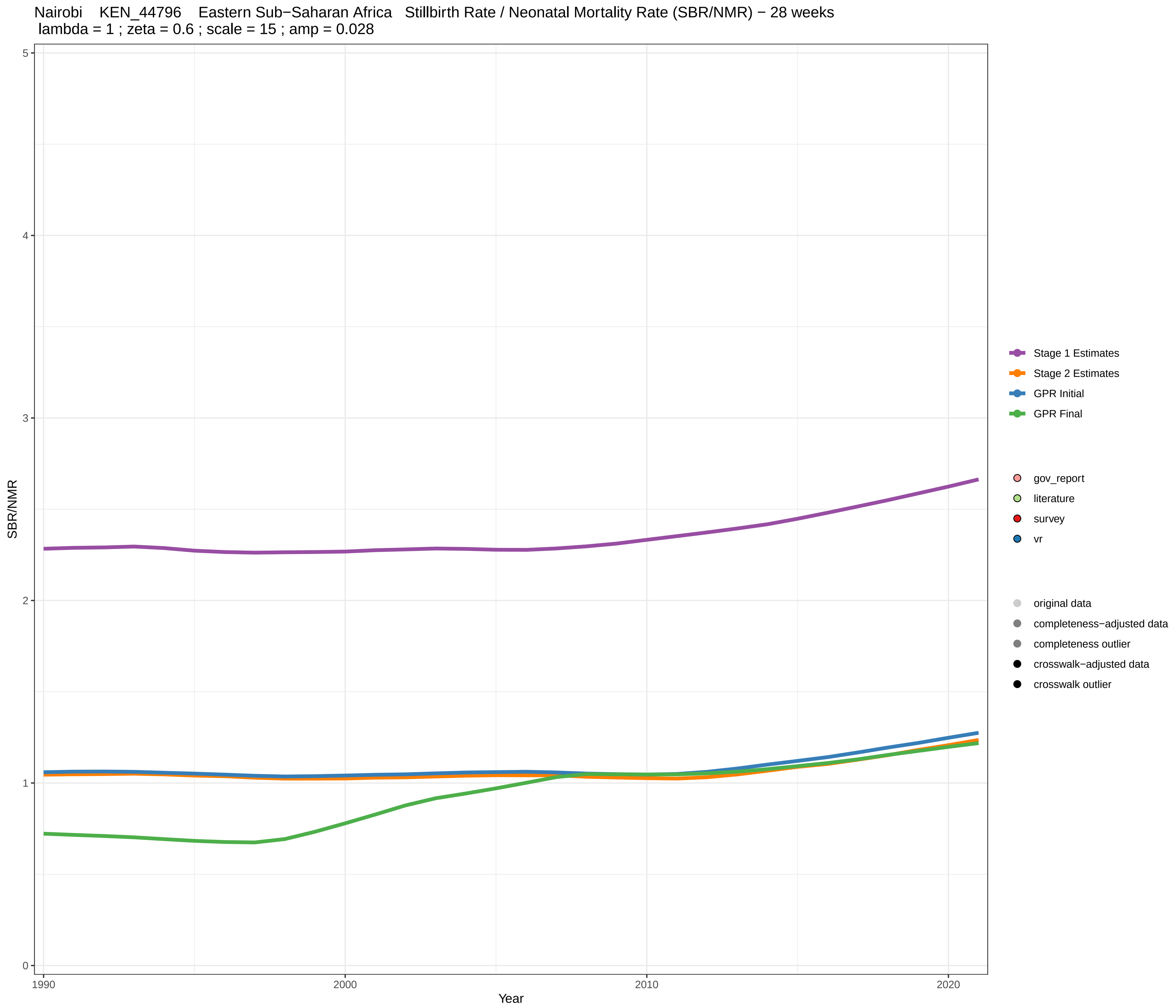

Definition	Years	Outlier	Source.Type	Title

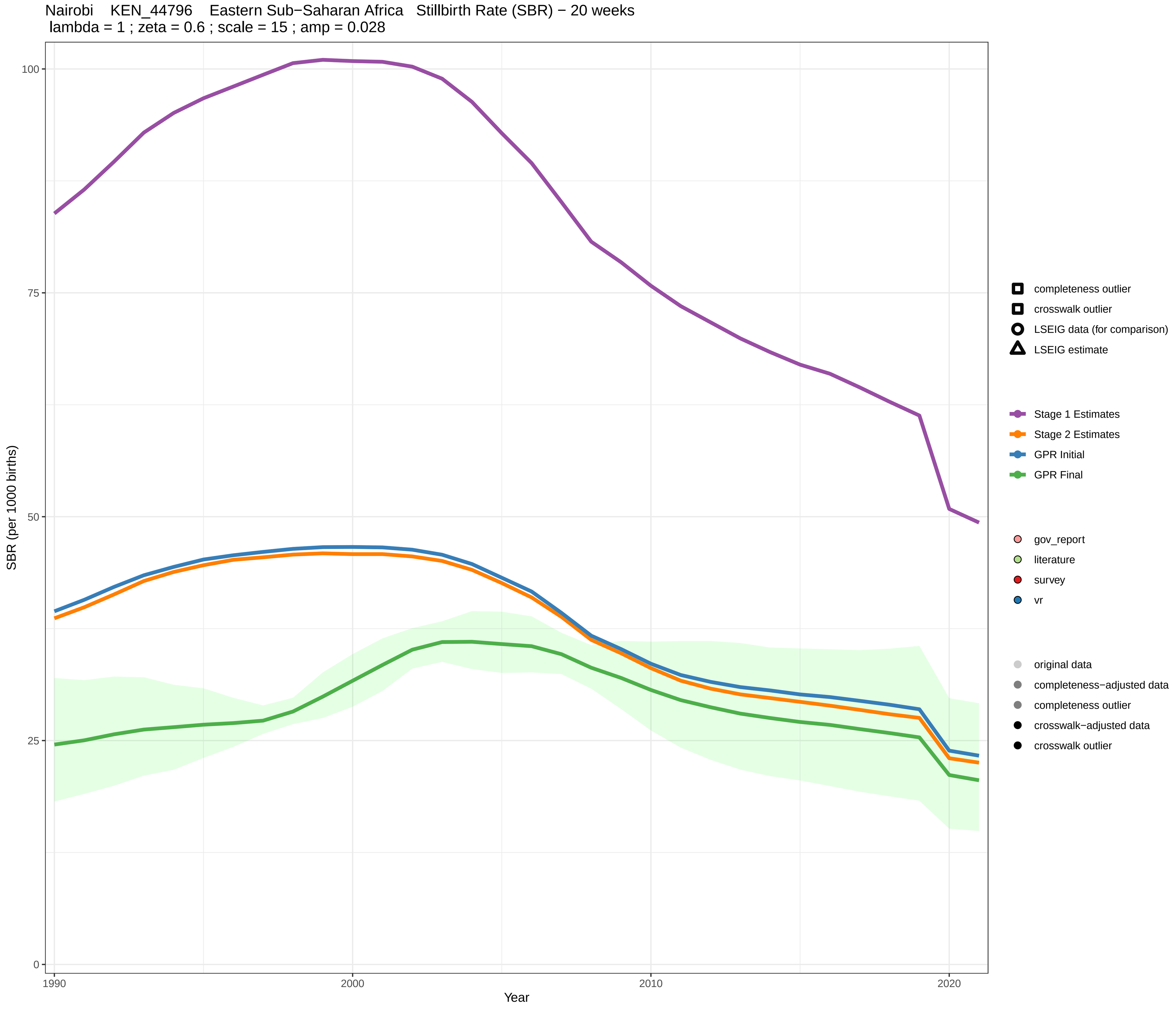

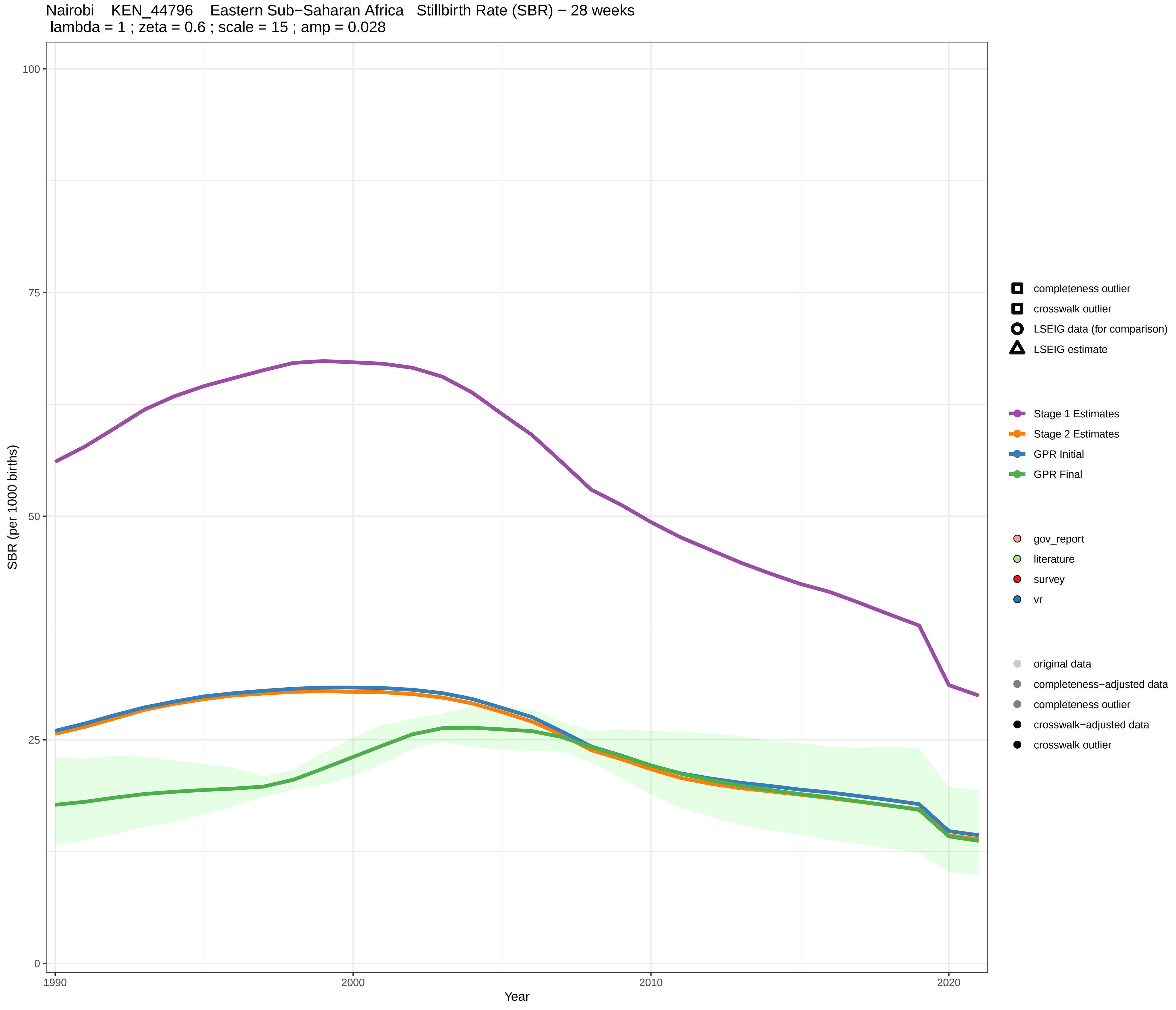

Year	Total Stillbirths (in thousands)	Total Stillbirths (in thousands)	SBR (per 1000 births)	SBR (per 1000 births)	SBR/NMR	SBR/NMR
	20 weeks	28 weeks	20 weeks	28 weeks	20 weeks	28 weeks
1990	1.1 (0.8 to 1.5)	0.8 (0.6 to 1.1)	24.6 (18.2 to 32.0)	17.7 (13.2 to 23.0)	1.00 (0.74 to 1.30)	0.72 (0.54 to 0.94)
1995	1.4 (1.2 to 1.7)	1.0 (0.9 to 1.2)	26.8 (23.1 to 30.8)	19.4 (16.7 to 22.3)	0.94 (0.81 to 1.09)	0.68 (0.59 to 0.79)
2000	2.1 (1.9 to 2.3)	1.5 (1.4 to 1.7)	31.7 (28.8 to 34.7)	23.1 (21.0 to 25.2)	1.07 (0.97 to 1.17)	0.78 (0.71 to 0.85)
2005	2.9 (2.6 to 3.2)	2.1 (1.9 to 2.3)	35.8 (32.6 to 39.4)	26.2 (23.9 to 28.8)	1.33 (1.21 to 1.46)	0.97 (0.88 to 1.07)
2010	2.6 (2.2 to 3.1)	1.9 (1.6 to 2.2)	30.7 (26.1 to 36.1)	22.1 (18.9 to 26.0)	1.45 (1.24 to 1.70)	1.05 (0.89 to 1.23)
2015	2.2 (1.6 to 2.8)	1.5 (1.1 to 2.0)	27.1 (20.5 to 35.3)	18.9 (14.4 to 24.6)	1.56 (1.19 to 2.04)	1.09 (0.83 to 1.42)
2020	1.4 (1.0 to 2.0)	0.9 (0.7 to 1.3)	21.2 (15.2 to 29.7)	14.2 (10.2 to 19.7)	1.78 (1.28 to 2.51)	1.20 (0.86 to 1.66)
2021	1.3 (1.0 to 1.9)	0.9 (0.6 to 1.2)	20.6 (14.9 to 29.2)	13.7 (10.0 to 19.4)	1.83 (1.33 to 2.59)	1.22 (0.89 to 1.72)

North Eastern KEN_44797 Eastern Sub−Saharan Africa Stillbirth Rate / Neonatal Mortality Rate (SBR/NMR) − 20 weeks
lambda = 1 ; zeta = 0.6 ; scale = 15 ; amp = 0.028

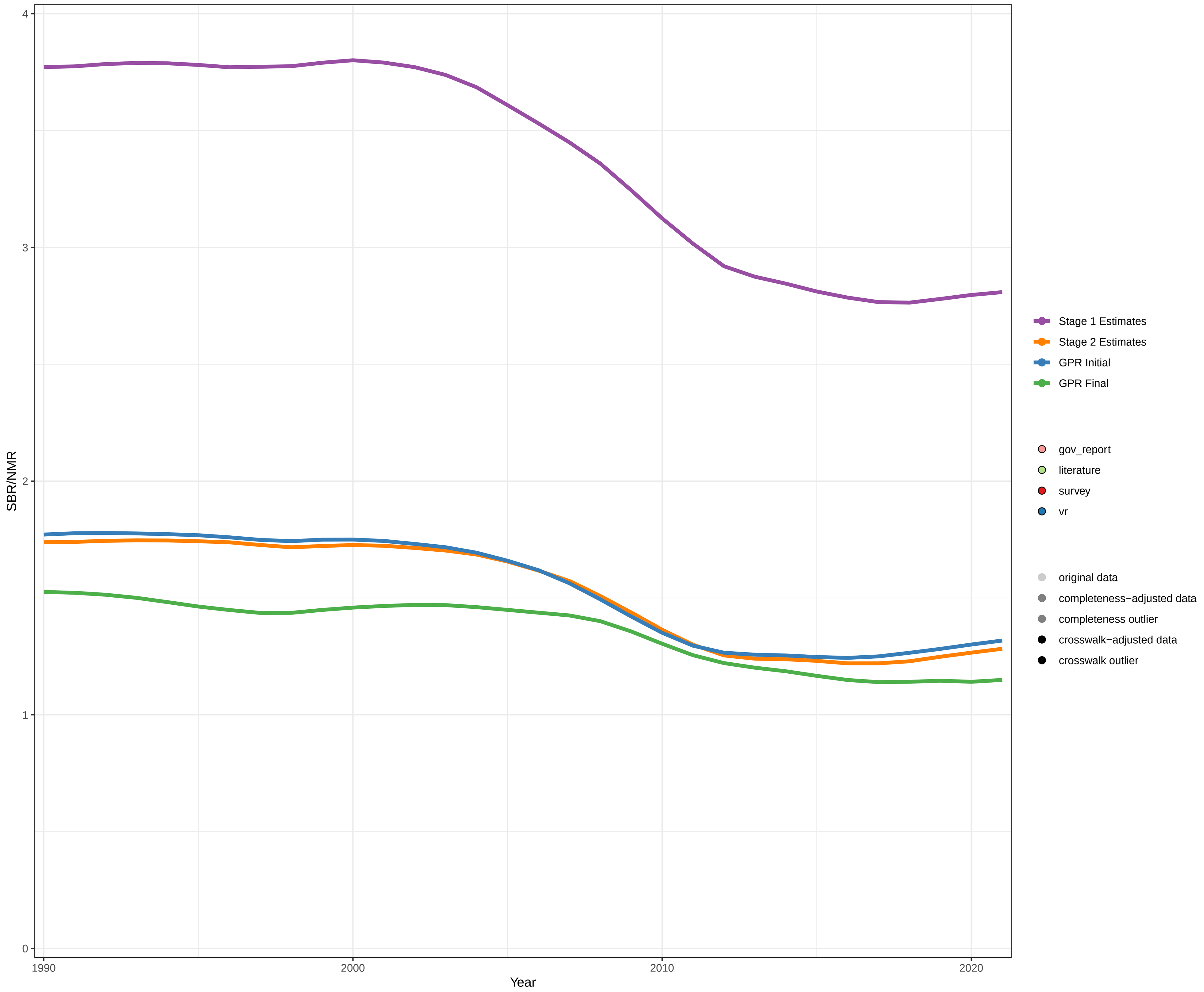

North Eastern KEN_44797 Eastern Sub−Saharan Africa Stillbirth Rate / Neonatal Mortality Rate (SBR/NMR) − 28 weeks
lambda = 1 ; zeta = 0.6 ; scale = 15 ; amp = 0.028

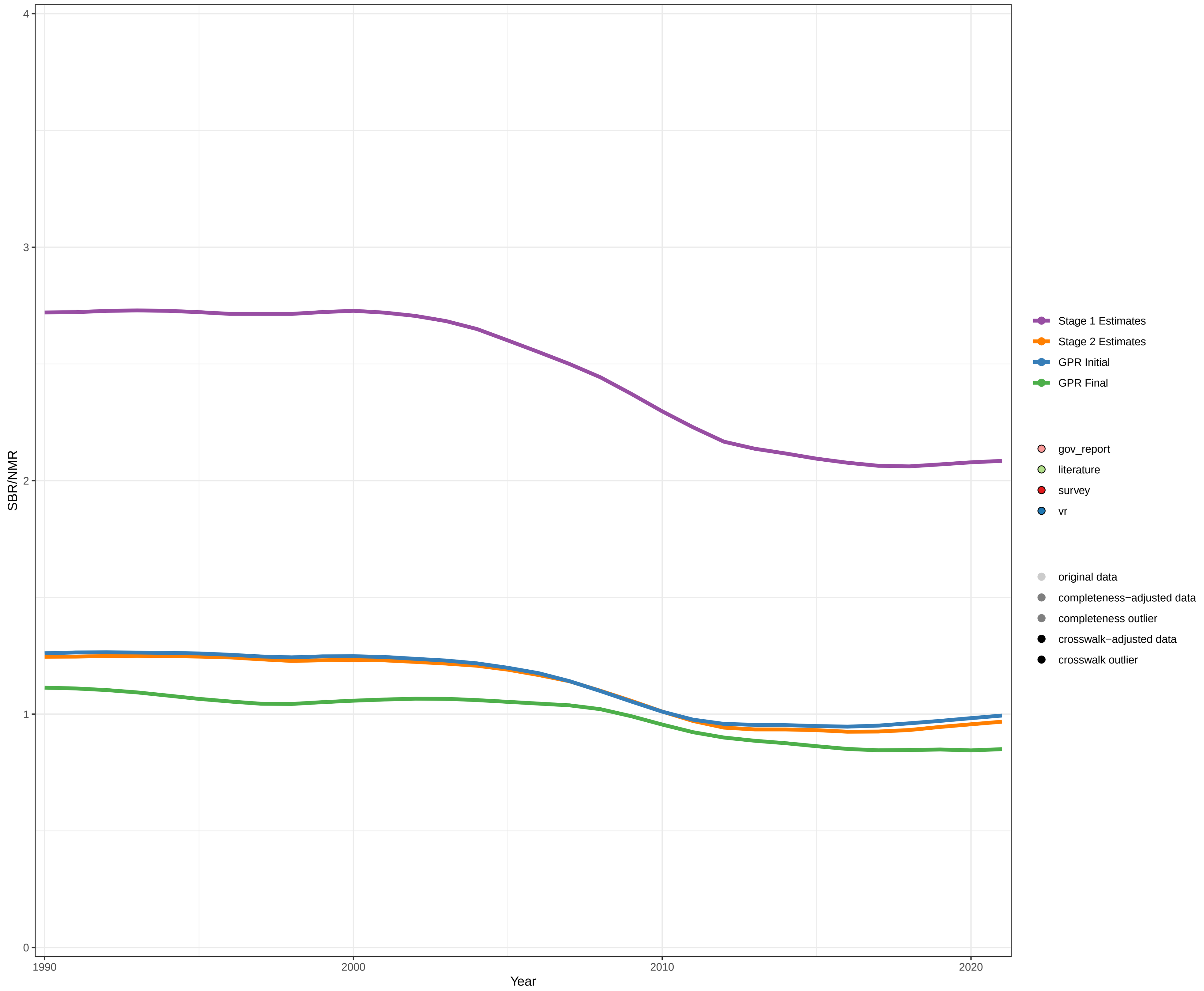

Definition	Years	Outlier	Source.Type	Title

North Eastern KEN_44797 Eastern Sub−Saharan Africa Stillbirth Rate (SBR) − 20 weeks
lambda = 1 ; zeta = 0.6 ; scale = 15 ; amp = 0.028

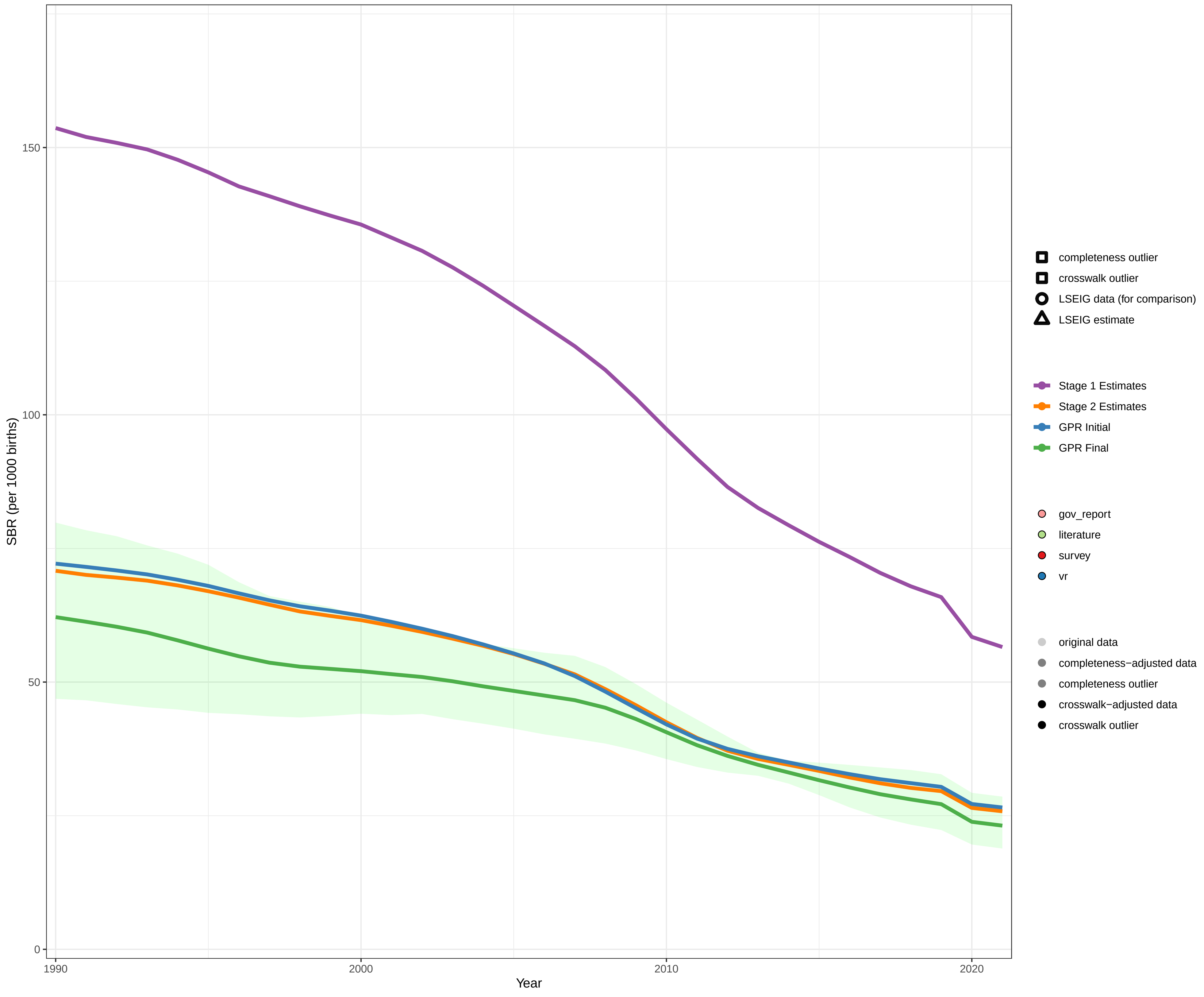

North Eastern KEN_44797 Eastern Sub−Saharan Africa Stillbirth Rate (SBR) − 28 weeks
lambda = 1 ; zeta = 0.6 ; scale = 15 ; amp = 0.028

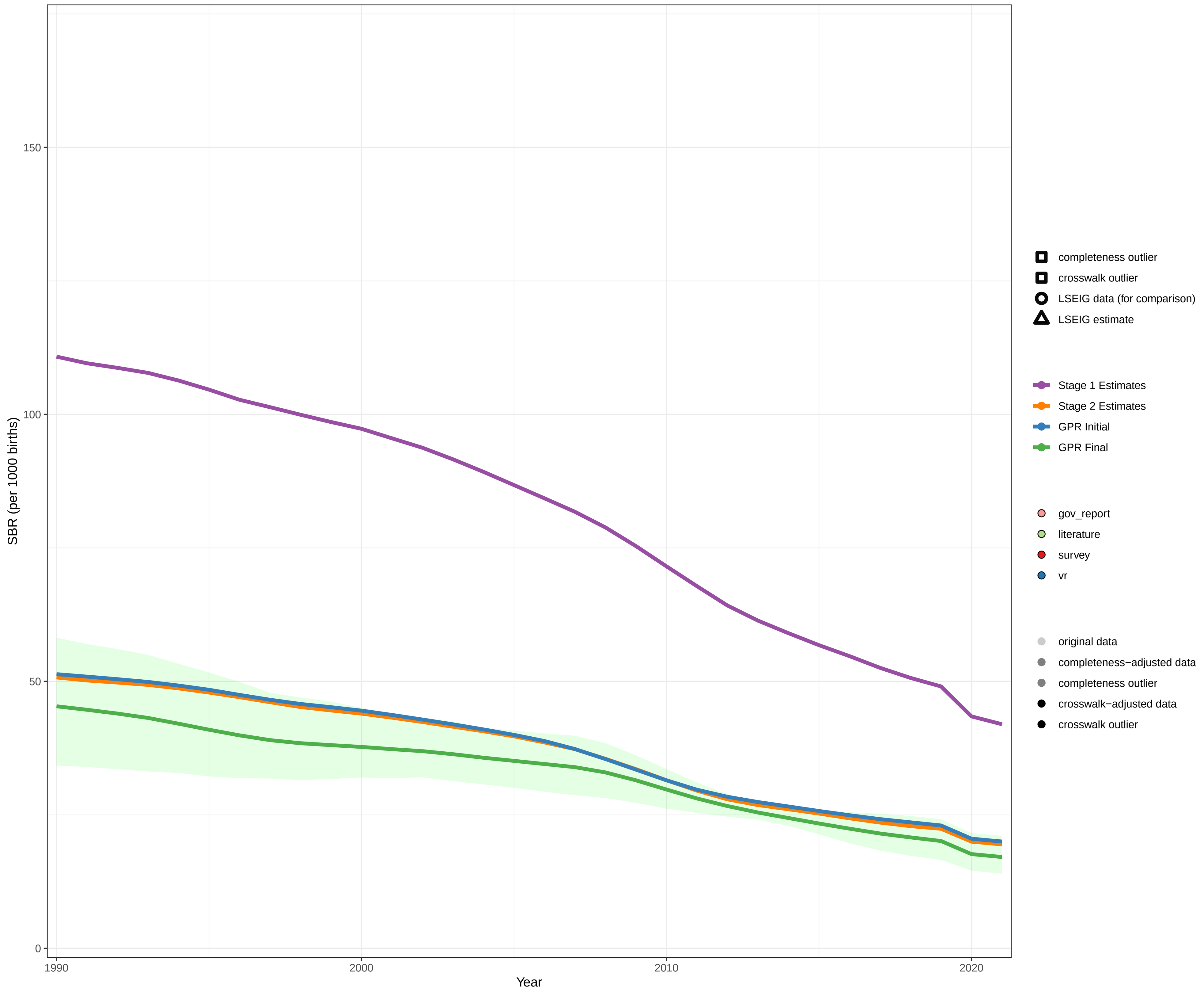

Year	Total Stillbirths (in thousands)	Total Stillbirths (in thousands)	SBR (per 1000 births)	SBR (per 1000 births)	SBR/NMR	SBR/NMR
	20 weeks	28 weeks	20 weeks	28 weeks	20 weeks	28 weeks
1990	1.3 (1.0 to 1.7)	1.0 (0.7 to 1.2)	62.2 (46.9 to 79.8)	45.3 (34.2 to 58.2)	1.53 (1.15 to 1.96)	1.11 (0.84 to 1.43)
1995	1.8 (1.4 to 2.3)	1.3 (1.0 to 1.6)	56.3 (44.2 to 71.9)	40.9 (32.2 to 51.7)	1.46 (1.15 to 1.87)	1.07 (0.84 to 1.34)
2000	2.7 (2.3 to 3.3)	2.0 (1.6 to 2.3)	52.0 (44.1 to 62.3)	37.7 (32.0 to 45.0)	1.46 (1.24 to 1.75)	1.06 (0.90 to 1.26)
2005	4.1 (3.5 to 4.9)	3.0 (2.5 to 3.5)	48.3 (41.3 to 56.3)	35.1 (30.1 to 40.8)	1.45 (1.24 to 1.69)	1.05 (0.90 to 1.22)
2010	4.7 (4.1 to 5.3)	3.4 (3.0 to 3.8)	40.6 (35.6 to 46.2)	29.8 (26.2 to 33.6)	1.30 (1.14 to 1.48)	0.96 (0.84 to 1.08)
2015	3.9 (3.6 to 4.3)	2.9 (2.6 to 3.2)	31.6 (28.9 to 34.9)	23.4 (21.4 to 25.8)	1.17 (1.06 to 1.29)	0.86 (0.79 to 0.95)
2020	2.9 (2.4 to 3.6)	2.2 (1.8 to 2.6)	23.9 (19.6 to 29.3)	17.7 (14.5 to 21.6)	1.14 (0.94 to 1.40)	0.84 (0.70 to 1.03)
2021	2.8 (2.3 to 3.5)	2.1 (1.7 to 2.6)	23.1 (18.9 to 28.6)	17.1 (14.0 to 21.1)	1.15 (0.94 to 1.42)	0.85 (0.69 to 1.05)

Nyanza KEN_44798 Eastern Sub−Saharan Africa Stillbirth Rate / Neonatal Mortality Rate (SBR/NMR) − 20 weeks
lambda = 1 ; zeta = 0.6 ; scale = 15 ; amp = 0.028

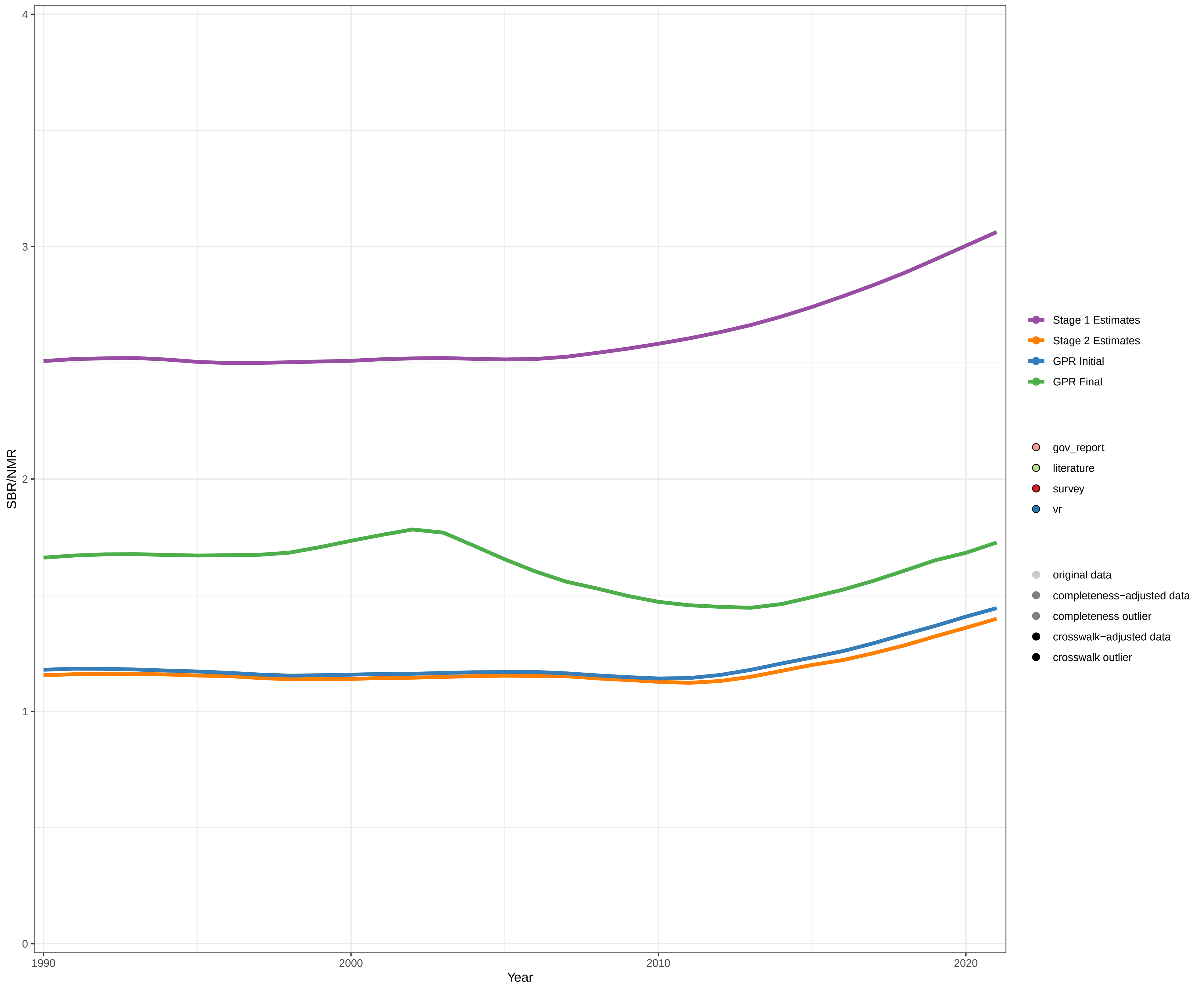

Nyanza KEN_44798 Eastern Sub−Saharan Africa Stillbirth Rate / Neonatal Mortality Rate (SBR/NMR) − 28 weeks
lambda = 1 ; zeta = 0.6 ; scale = 15 ; amp = 0.028

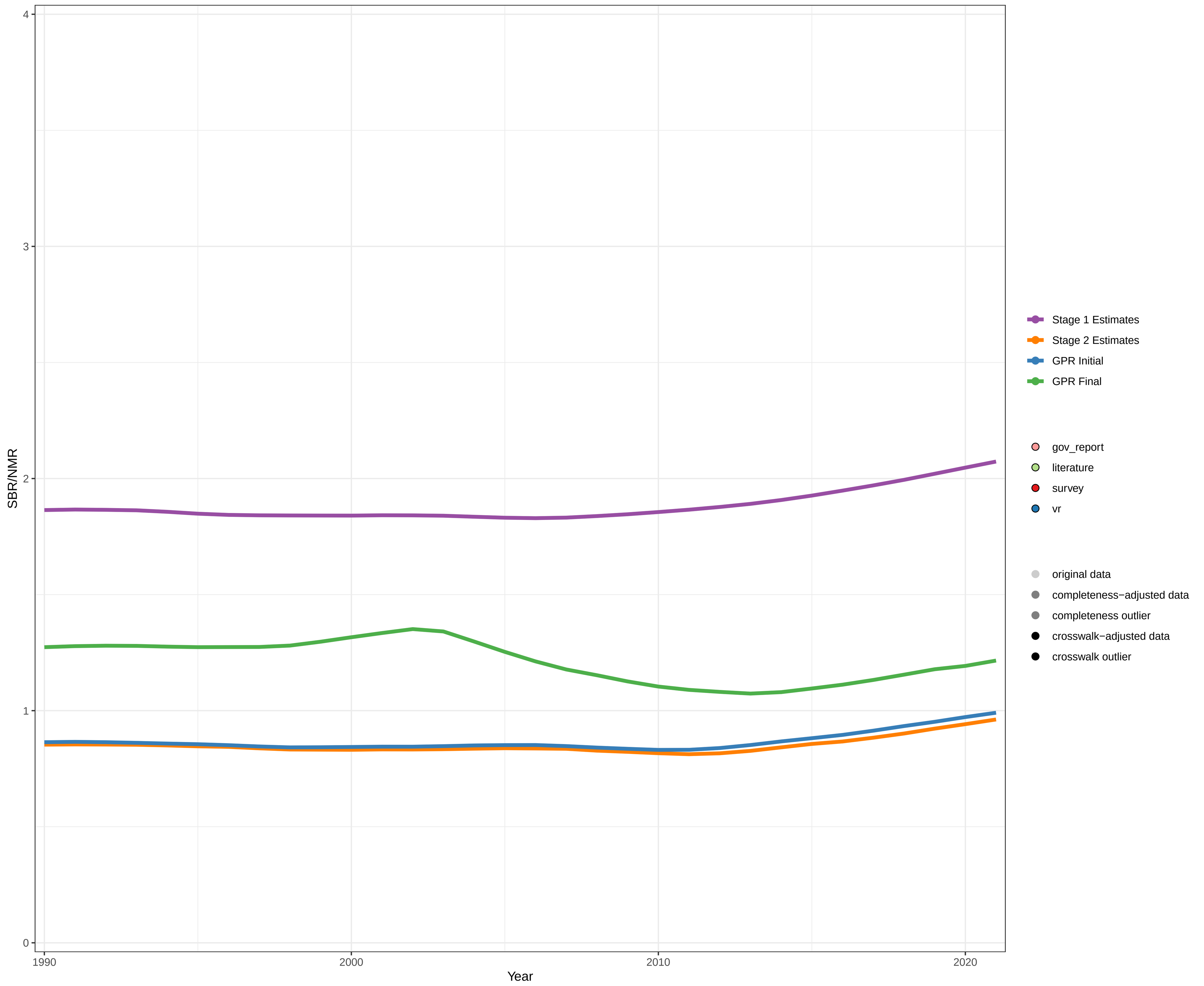

Definition	Years	Outlier	Source.Type	Title

Nyanza KEN_44798 Eastern Sub−Saharan Africa Stillbirth Rate (SBR) − 20 weeks
lambda = 1 ; zeta = 0.6 ; scale = 15 ; amp = 0.028

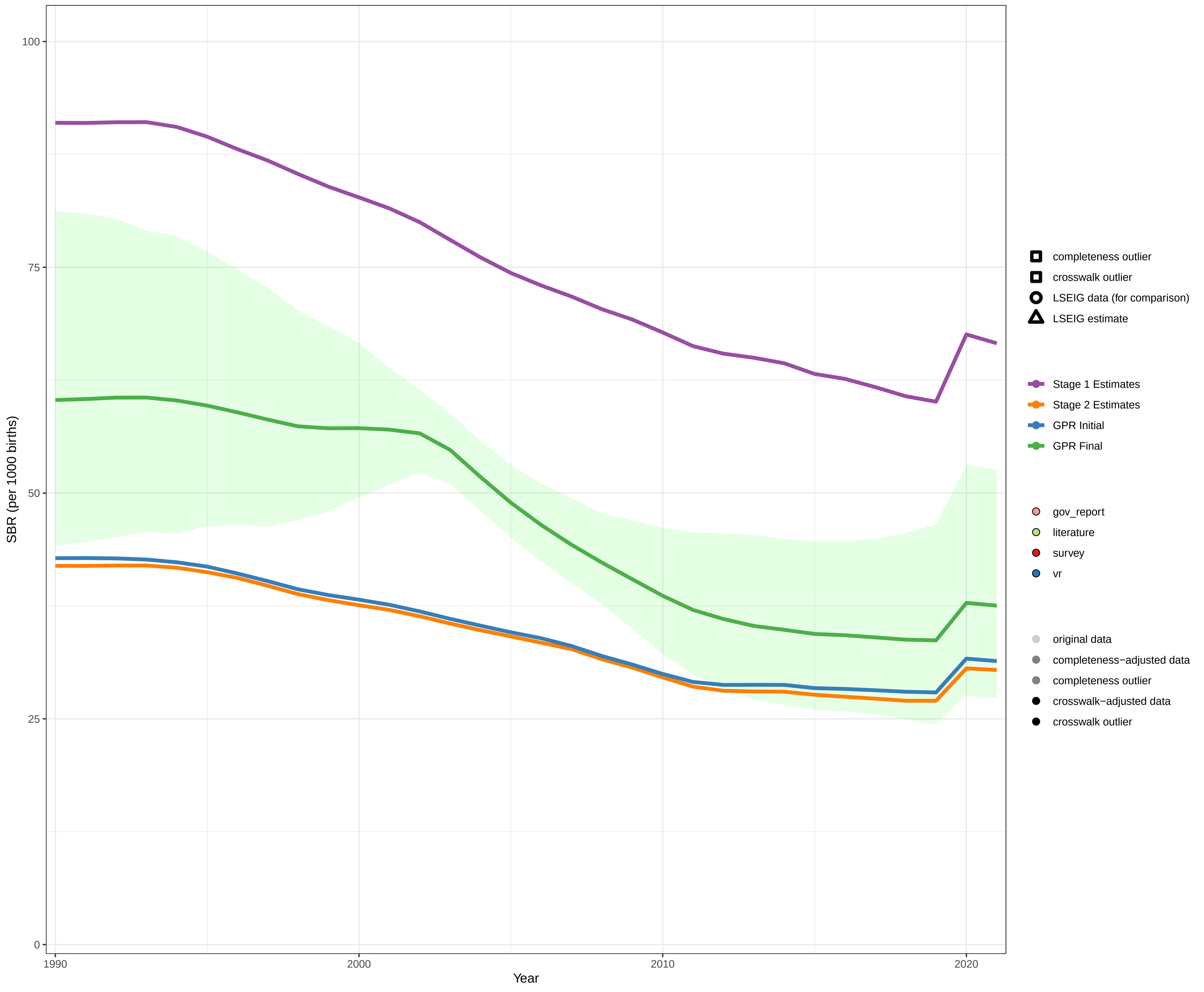

Nyanza KEN_44798 Eastern Sub−Saharan Africa Stillbirth Rate (SBR) − 28 weeks
lambda = 1 ; zeta = 0.6 ; scale = 15 ; amp = 0.028

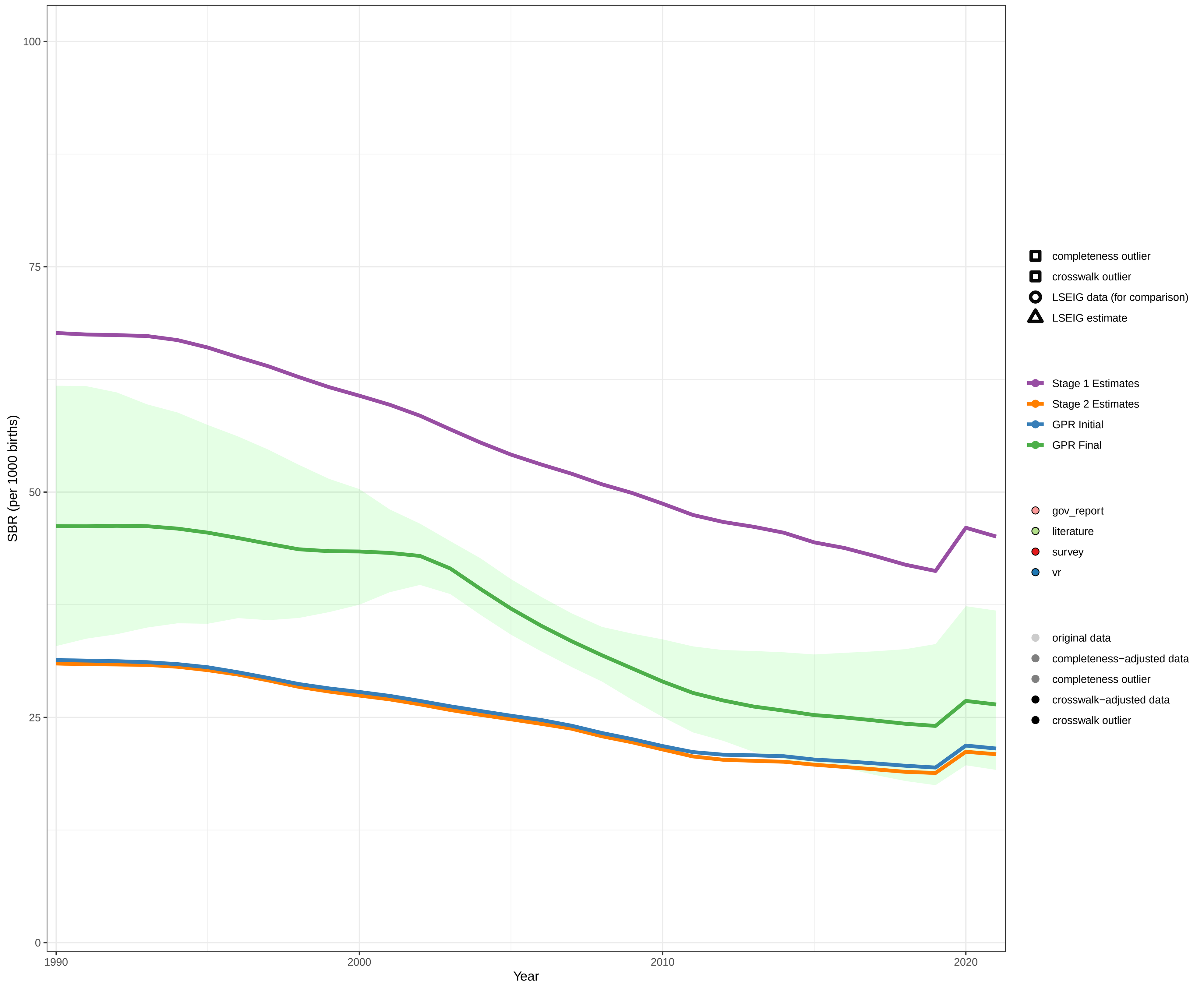

Year	Total Stillbirths (in thousands)	Total Stillbirths (in thousands)	SBR (per 1000 births)	SBR (per 1000 births)	SBR/NMR	SBR/NMR
	20 weeks	28 weeks	20 weeks	28 weeks	20 weeks	28 weeks
1990	11.7 (8.4 to 16.1)	8.8 (6.2 to 12.0)	60.3 (44.2 to 81.2)	46.2 (32.9 to 61.8)	1.66 (1.22 to 2.24)	1.27 (0.91 to 1.70)
1995	12.5 (9.6 to 16.4)	9.4 (7.2 to 12.0)	59.7 (46.3 to 76.8)	45.5 (35.4 to 57.4)	1.67 (1.30 to 2.15)	1.27 (0.99 to 1.61)
2000	12.8 (11.0 to 15.0)	9.6 (8.2 to 11.2)	57.2 (49.5 to 66.6)	43.4 (37.5 to 50.3)	1.73 (1.50 to 2.02)	1.32 (1.14 to 1.53)
2005	11.1 (10.2 to 12.1)	8.3 (7.6 to 9.1)	48.9 (45.1 to 53.1)	37.1 (34.2 to 40.3)	1.65 (1.52 to 1.80)	1.25 (1.16 to 1.36)
2010	8.5 (7.0 to 10.2)	6.3 (5.4 to 7.4)	38.6 (32.2 to 46.1)	29.0 (25.0 to 33.7)	1.47 (1.23 to 1.76)	1.10 (0.95 to 1.28)
2015	7.0 (5.3 to 9.2)	5.1 (4.0 to 6.5)	34.4 (26.0 to 44.7)	25.3 (19.8 to 32.0)	1.49 (1.13 to 1.94)	1.10 (0.86 to 1.39)
2020	6.8 (4.9 to 9.7)	4.8 (3.5 to 6.7)	37.8 (27.6 to 53.2)	26.8 (19.7 to 37.3)	1.68 (1.23 to 2.36)	1.19 (0.88 to 1.66)
2021	6.6 (4.7 to 9.4)	4.6 (3.3 to 6.4)	37.5 (27.2 to 52.6)	26.4 (19.2 to 36.8)	1.73 (1.25 to 2.42)	1.22 (0.88 to 1.70)

Rift Valley KEN_44799 Eastern Sub−Saharan Africa Stillbirth Rate / Neonatal Mortality Rate (SBR/NMR) − 20 weeks
lambda = 1 ; zeta = 0.6 ; scale = 15 ; amp = 0.028

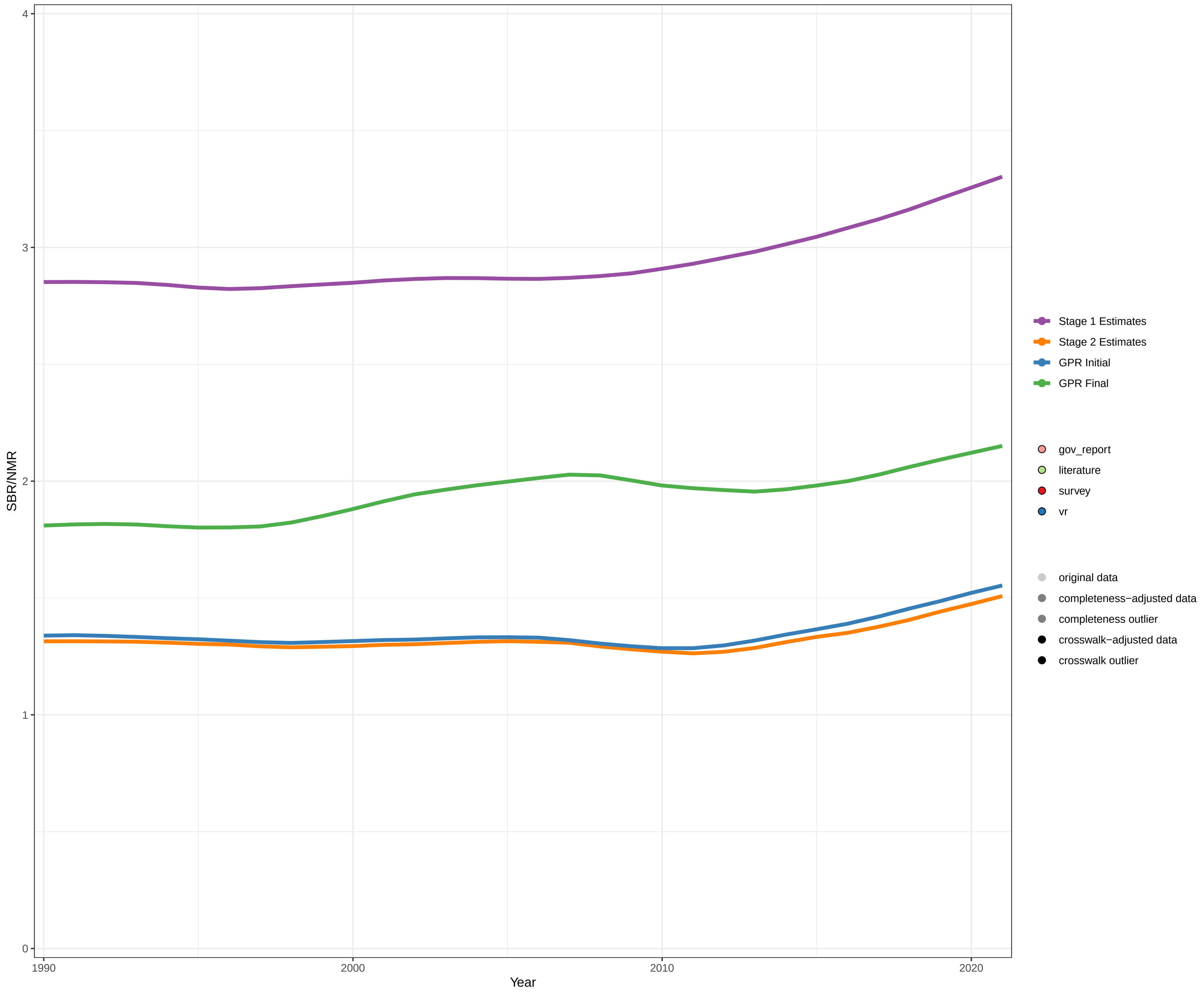

Rift Valley KEN_44799 Eastern Sub−Saharan Africa Stillbirth Rate / Neonatal Mortality Rate (SBR/NMR) − 28 weeks
lambda = 1 ; zeta = 0.6 ; scale = 15 ; amp = 0.028

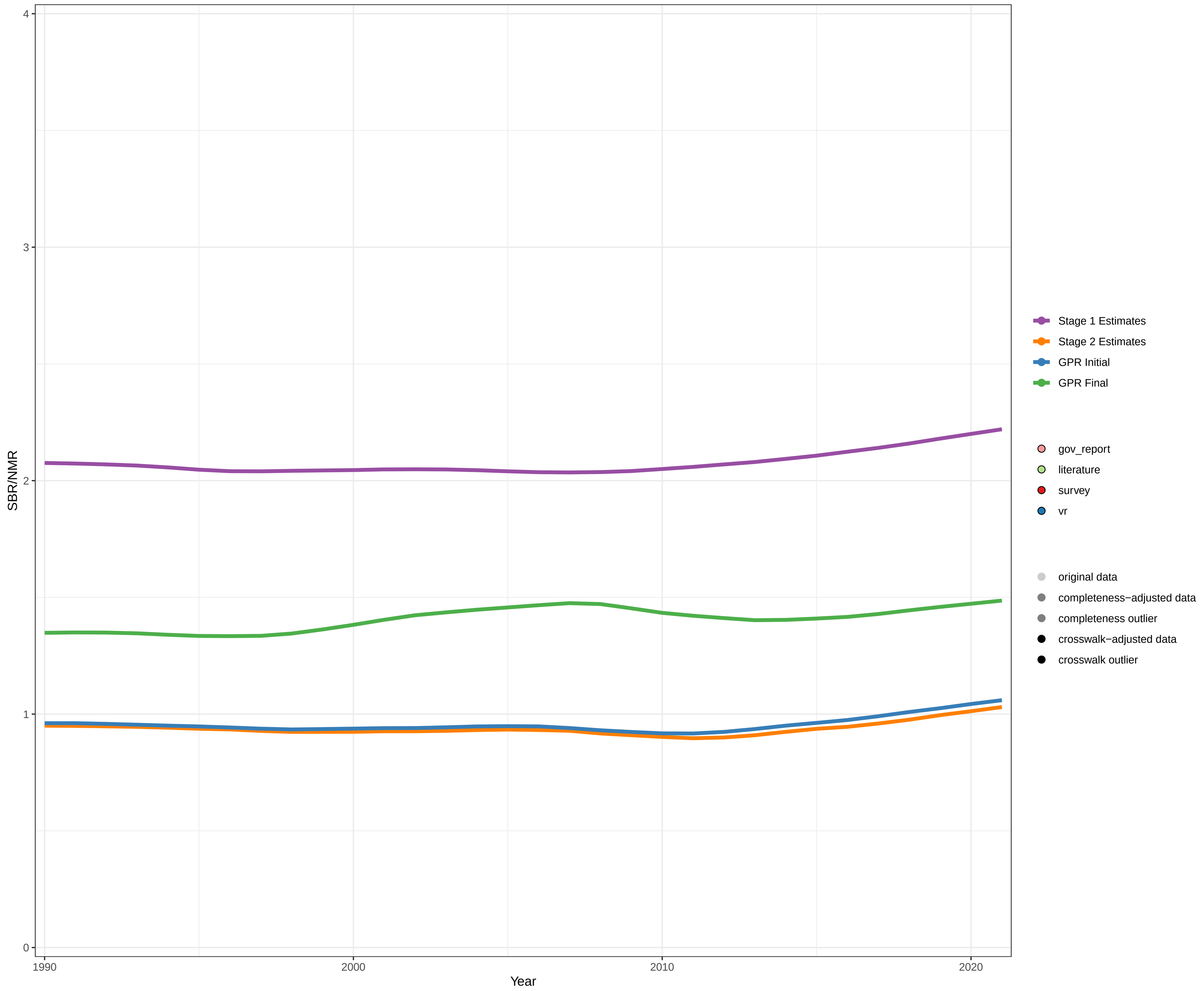

Definition	Years	Outlier	Source.Type	Title

Rift Valley KEN_44799 Eastern Sub−Saharan Africa Stillbirth Rate (SBR) − 20 weeks
lambda = 1 ; zeta = 0.6 ; scale = 15 ; amp = 0.028

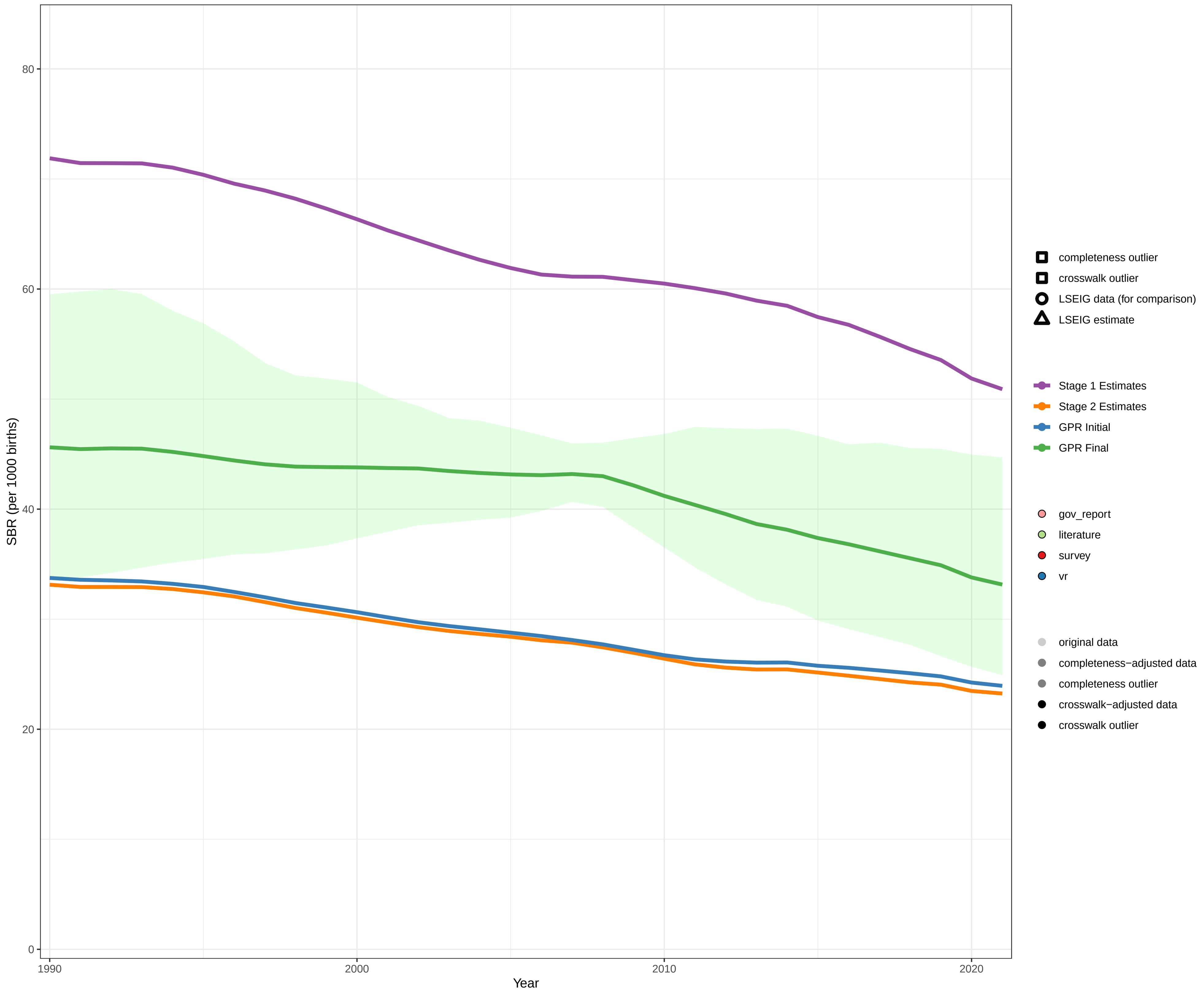

Rift Valley KEN_44799 Eastern Sub−Saharan Africa Stillbirth Rate (SBR) − 28 weeks
lambda = 1 ; zeta = 0.6 ; scale = 15 ; amp = 0.028

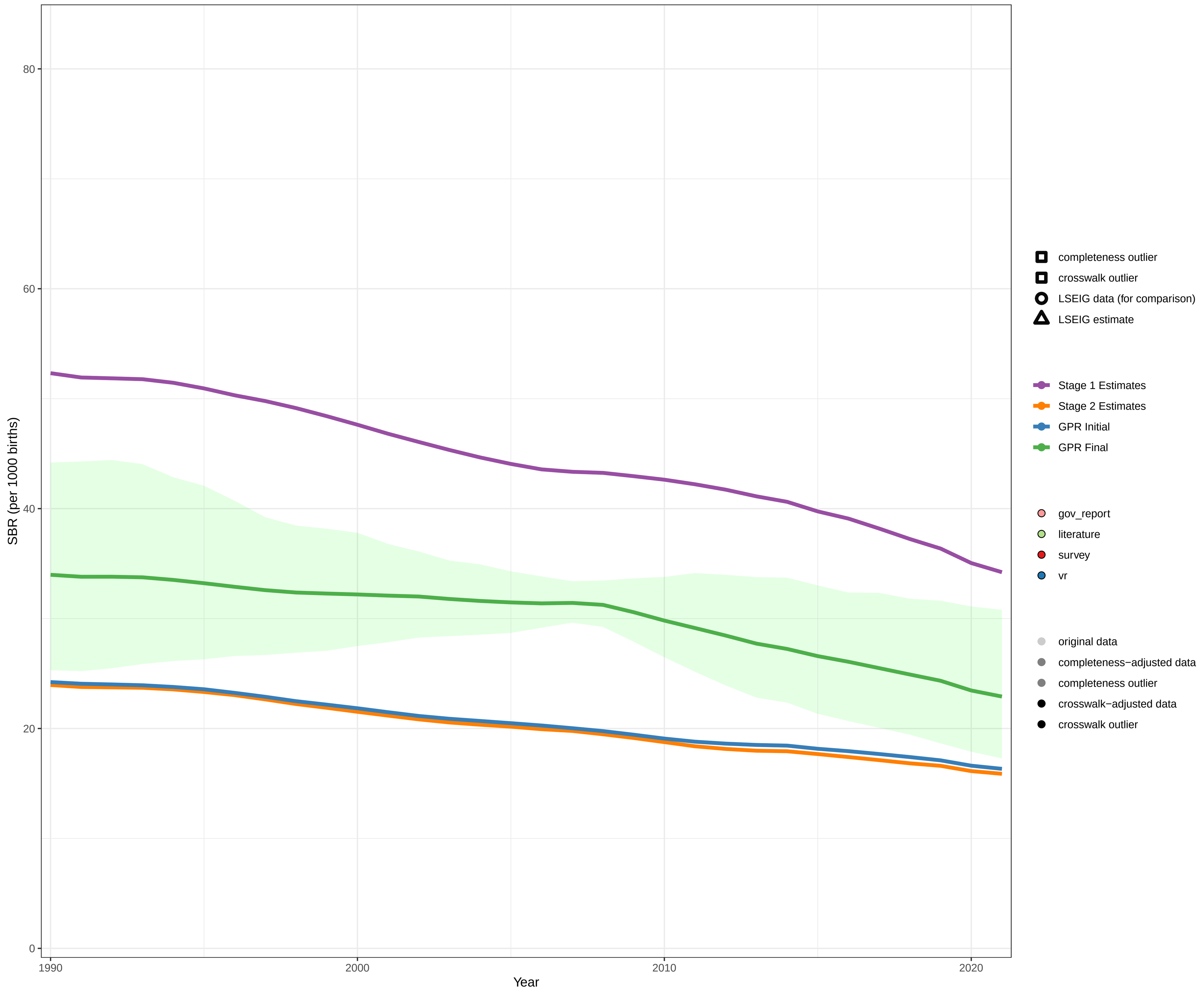

Year	Total Stillbirths (in thousands)	Total Stillbirths (in thousands)	SBR (per 1000 births)	SBR (per 1000 births)	SBR/NMR	SBR/NMR
	20 weeks	28 weeks	20 weeks	28 weeks	20 weeks	28 weeks
1990	11.6 (8.5 to 15.3)	8.5 (6.3 to 11.2)	45.6 (33.9 to 59.5)	34.0 (25.3 to 44.2)	1.81 (1.34 to 2.36)	1.35 (1.00 to 1.75)
1995	13.0 (10.1 to 16.6)	9.5 (7.5 to 12.1)	44.8 (35.5 to 56.9)	33.2 (26.3 to 42.1)	1.80 (1.43 to 2.29)	1.33 (1.06 to 1.69)
2000	14.4 (12.2 to 17.1)	10.5 (8.9 to 12.4)	43.8 (37.4 to 51.5)	32.2 (27.5 to 37.8)	1.88 (1.60 to 2.21)	1.38 (1.18 to 1.62)
2005	15.8 (14.3 to 17.5)	11.4 (10.4 to 12.5)	43.2 (39.2 to 47.4)	31.5 (28.7 to 34.3)	2.00 (1.82 to 2.19)	1.46 (1.33 to 1.59)
2010	16.0 (14.1 to 18.3)	11.4 (10.1 to 13.0)	41.2 (36.5 to 46.8)	29.8 (26.5 to 33.8)	1.98 (1.76 to 2.25)	1.43 (1.27 to 1.62)
2015	14.5 (11.5 to 18.3)	10.2 (8.1 to 12.8)	37.4 (29.9 to 46.7)	26.6 (21.3 to 33.0)	1.98 (1.58 to 2.47)	1.41 (1.13 to 1.75)
2020	12.4 (9.3 to 16.6)	8.5 (6.4 to 11.3)	33.8 (25.7 to 45.0)	23.5 (17.9 to 31.1)	2.12 (1.61 to 2.82)	1.47 (1.12 to 1.95)
2021	12.0 (8.9 to 16.4)	8.2 (6.2 to 11.1)	33.1 (24.9 to 44.7)	22.9 (17.3 to 30.8)	2.15 (1.62 to 2.90)	1.49 (1.12 to 2.00)

Western KEN_44800 Eastern Sub−Saharan Africa Stillbirth Rate / Neonatal Mortality Rate (SBR/NMR) − 20 weeks
lambda = 1 ; zeta = 0.6 ; scale = 15 ; amp = 0.028

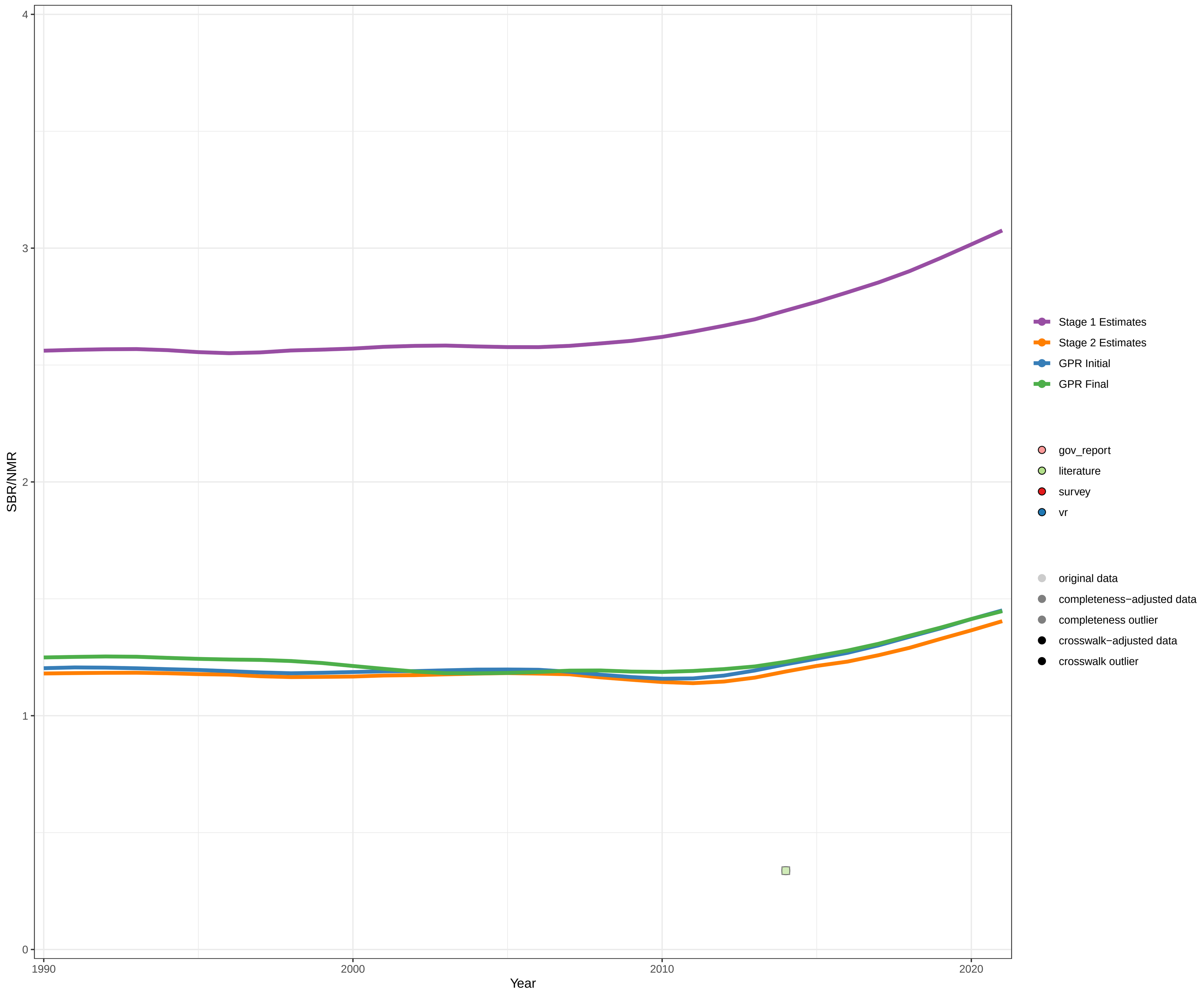

Western KEN_44800 Eastern Sub−Saharan Africa Stillbirth Rate / Neonatal Mortality Rate (SBR/NMR) − 28 weeks
lambda = 1 ; zeta = 0.6 ; scale = 15 ; amp = 0.028

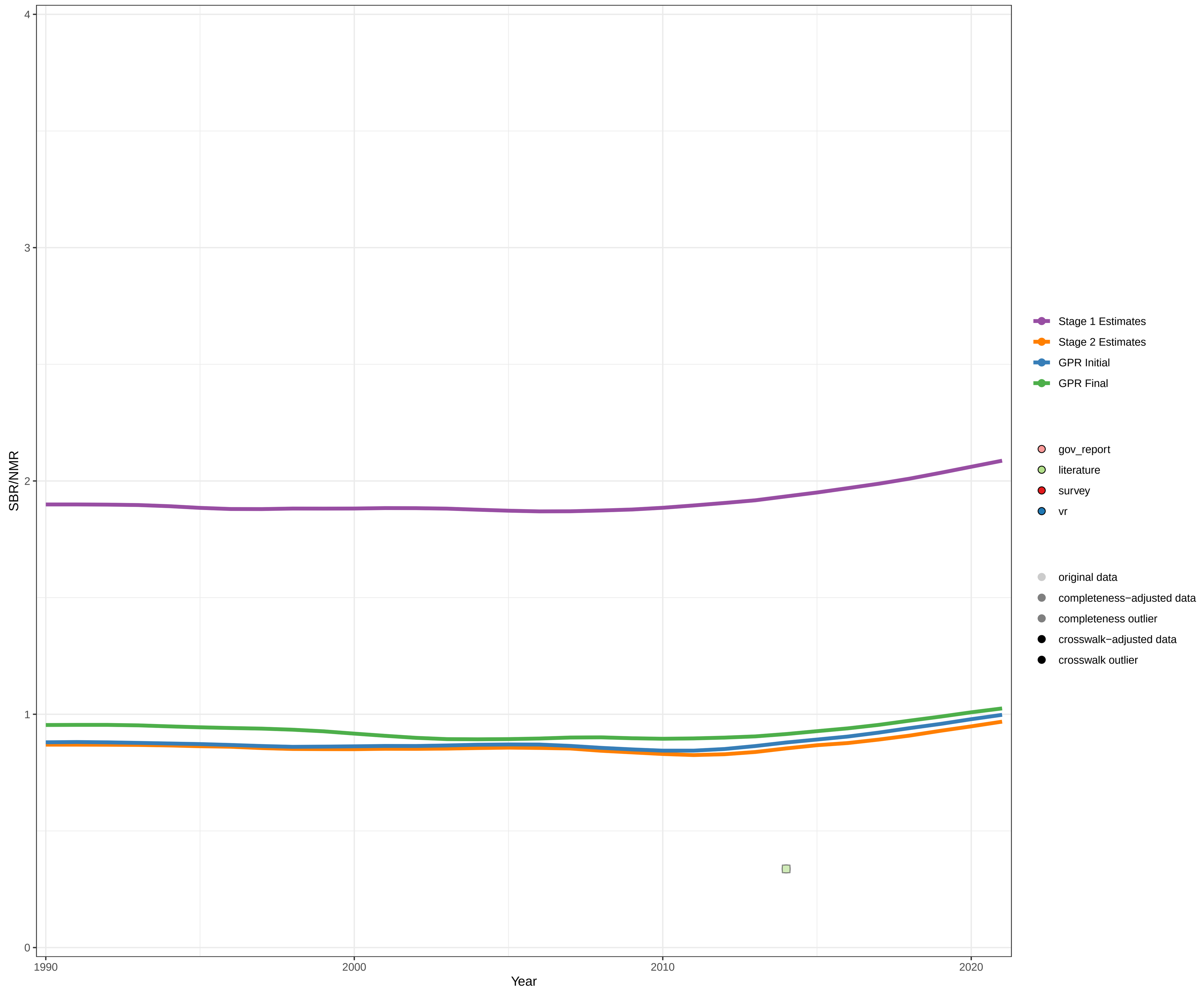

Definition	Years	Outlier	Source.Type	Title
28_weeks	2014	1	Sci Lit	Population−based rates, timing, and causes of maternal deaths, stillbirths, and neonatal deaths in south Asia and sub−Saharan Africa: a multi−country prospective cohort study

Western KEN_44800 Eastern Sub−Saharan Africa Stillbirth Rate (SBR) − 20 weeks
lambda = 1 ; zeta = 0.6 ; scale = 15 ; amp = 0.028

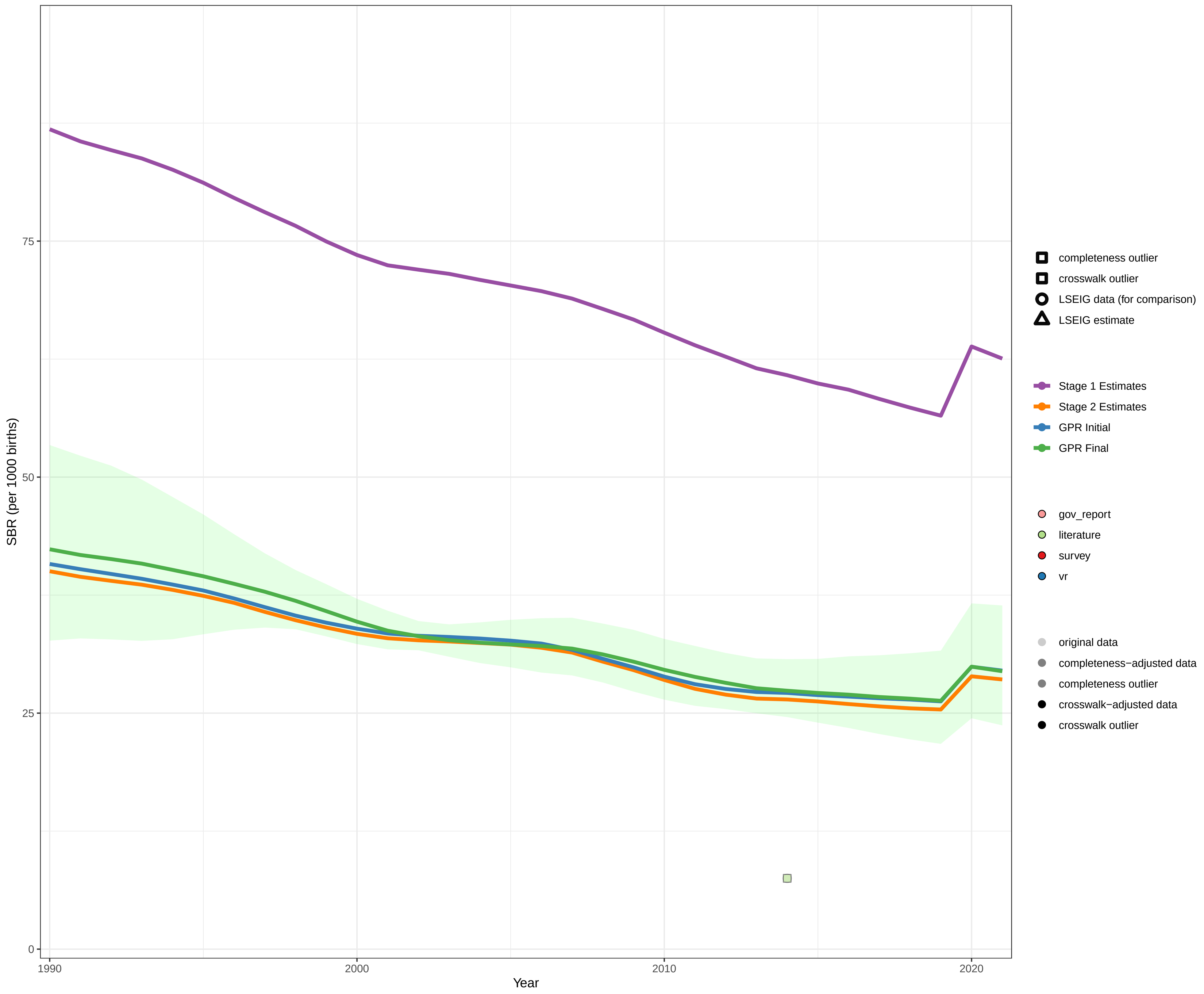

Western KEN_44800 Eastern Sub−Saharan Africa Stillbirth Rate (SBR) − 28 weeks
lambda = 1 ; zeta = 0.6 ; scale = 15 ; amp = 0.028

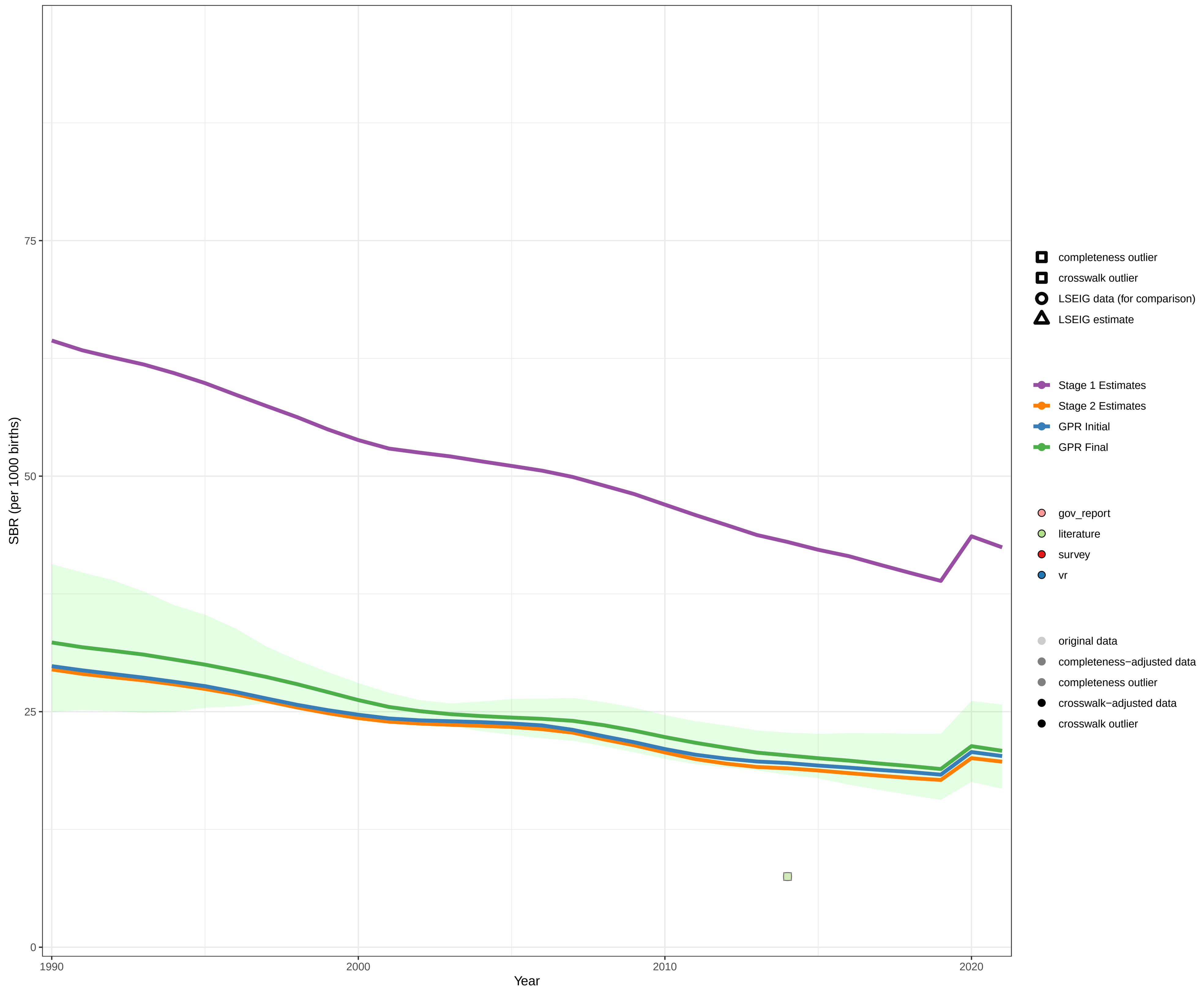

Year	Total Stillbirths (in thousands)	Total Stillbirths (in thousands)	SBR (per 1000 births)	SBR (per 1000 births)	SBR/NMR	SBR/NMR
	20 weeks	28 weeks	20 weeks	28 weeks	20 weeks	28 weeks
1990	6.0 (4.6 to 7.6)	4.5 (3.5 to 5.7)	42.4 (32.7 to 53.4)	32.3 (25.0 to 40.7)	1.25 (0.96 to 1.57)	0.95 (0.74 to 1.20)
1995	6.0 (5.0 to 7.0)	4.5 (3.8 to 5.3)	39.5 (33.4 to 46.0)	30.0 (25.4 to 35.3)	1.24 (1.05 to 1.45)	0.94 (0.80 to 1.11)
2000	5.6 (5.2 to 6.0)	4.2 (3.9 to 4.5)	34.7 (32.3 to 37.1)	26.2 (24.5 to 28.0)	1.21 (1.13 to 1.30)	0.92 (0.86 to 0.98)
2005	5.4 (5.0 to 5.9)	4.1 (3.8 to 4.4)	32.3 (29.9 to 34.9)	24.4 (22.6 to 26.4)	1.18 (1.09 to 1.28)	0.89 (0.83 to 0.97)
2010	4.9 (4.4 to 5.5)	3.7 (3.3 to 4.1)	29.6 (26.4 to 32.9)	22.3 (20.0 to 24.6)	1.19 (1.06 to 1.32)	0.90 (0.80 to 0.99)
2015	4.2 (3.7 to 4.8)	3.1 (2.8 to 3.5)	27.1 (24.0 to 30.7)	20.1 (17.9 to 22.7)	1.25 (1.11 to 1.42)	0.93 (0.83 to 1.05)
2020	4.3 (3.5 to 5.3)	3.0 (2.5 to 3.7)	29.9 (24.5 to 36.6)	21.3 (17.5 to 26.2)	1.41 (1.16 to 1.73)	1.01 (0.83 to 1.24)
2021	4.2 (3.3 to 5.2)	2.9 (2.4 to 3.6)	29.4 (23.7 to 36.4)	20.8 (16.8 to 25.8)	1.45 (1.17 to 1.79)	1.03 (0.83 to 1.27)

Baringo KEN_35617 Eastern Sub−Saharan Africa Stillbirth Rate / Neonatal Mortality Rate (SBR/NMR) − 20 weeks
lambda = 0.8 ; zeta = 0.7 ; scale = 15 ; amp = 0.028

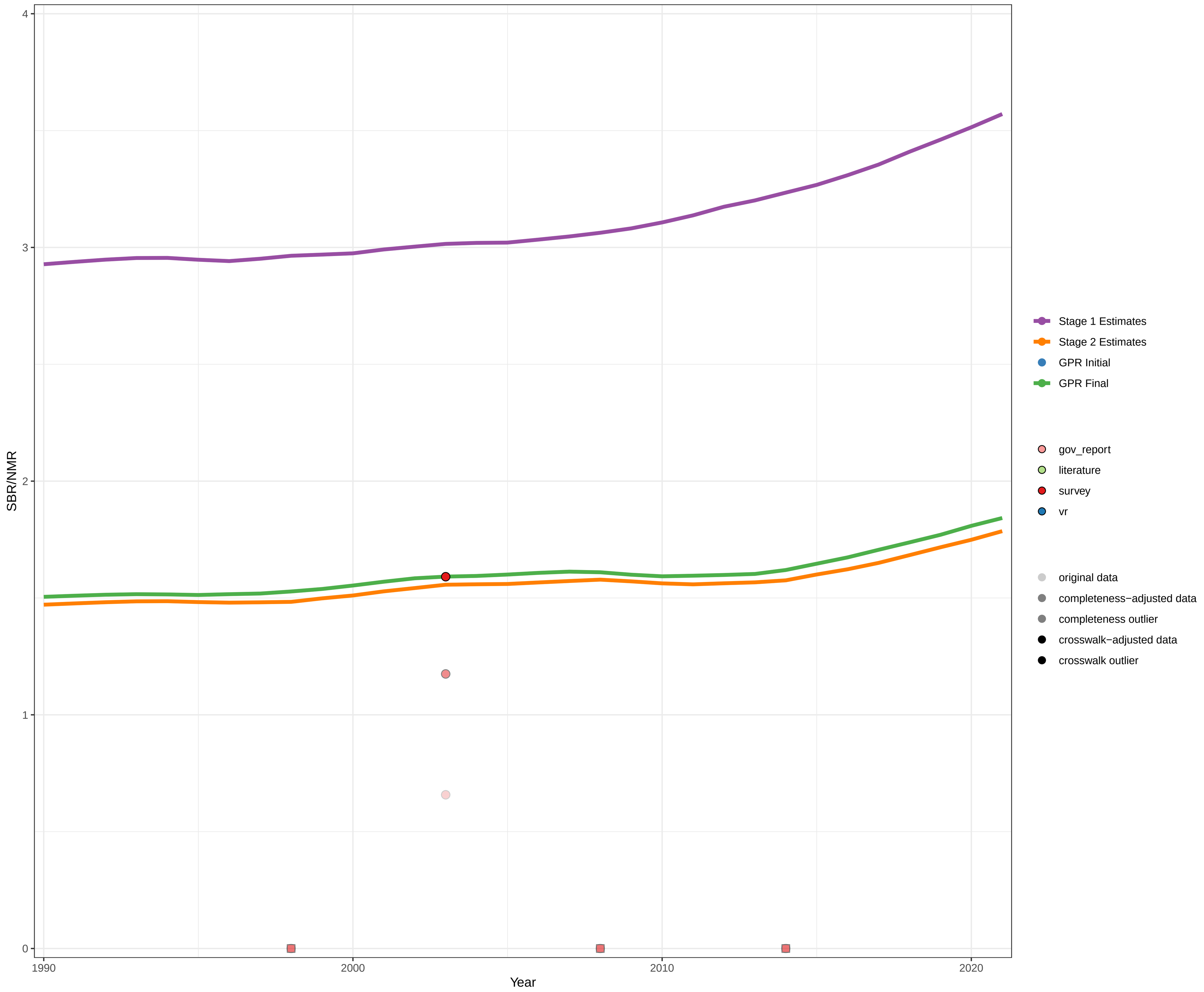

Baringo KEN_35617 Eastern Sub−Saharan Africa Stillbirth Rate / Neonatal Mortality Rate (SBR/NMR) − 28 weeks
lambda = 0.8 ; zeta = 0.7 ; scale = 15 ; amp = 0.028

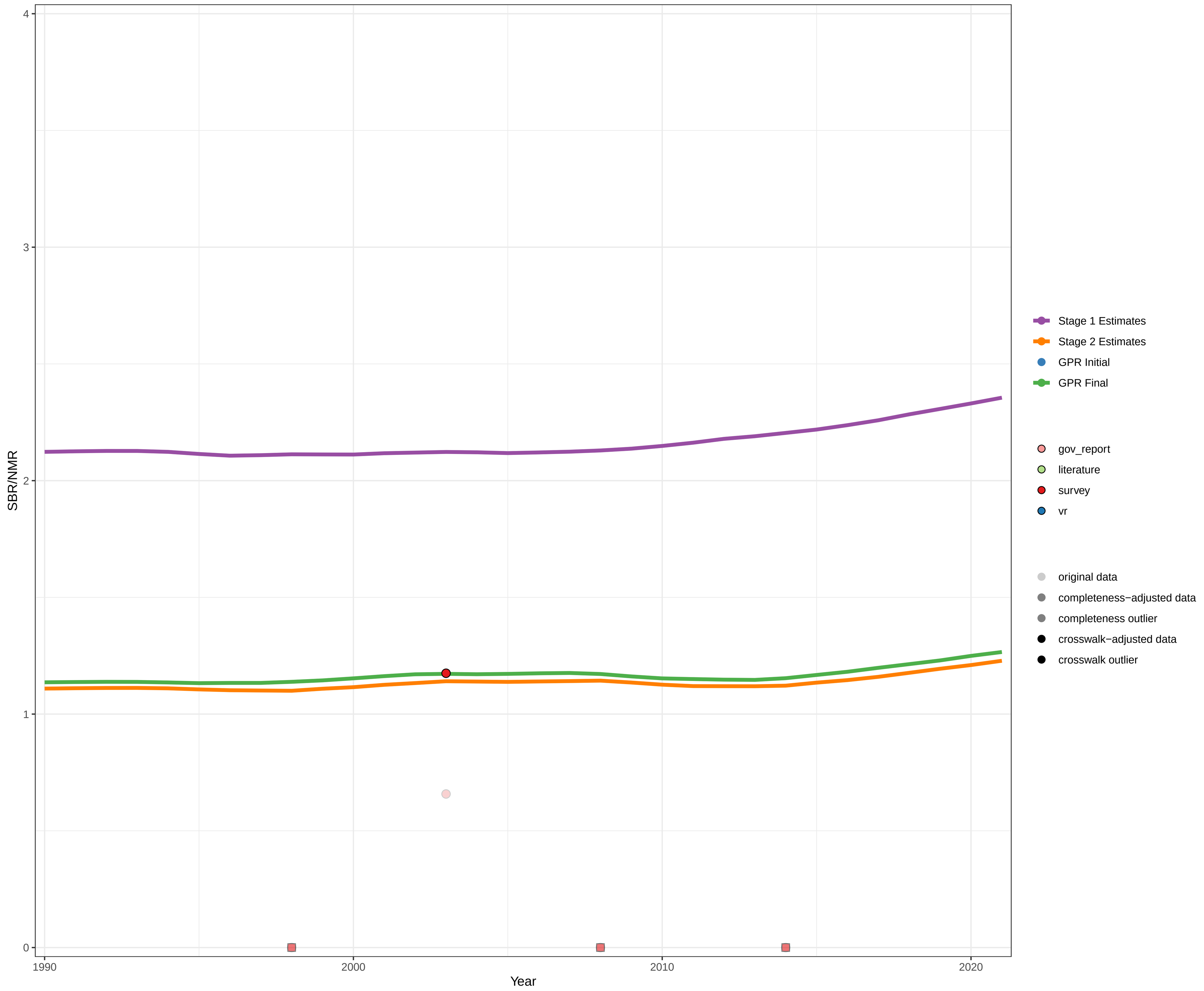

Definition	Years	Outlier	Source.Type	Title
28_weeks	2003	0	Survey	Kenya Demographic and Health Survey 2003
28_weeks	1998	1	Survey	Kenya Demographic and Health Survey 1998
28_weeks	2008	1	Survey	Kenya Demographic and Health Survey 2008−2009
28_weeks	2014	1	Survey	Kenya Demographic and Health Survey 2014

Baringo KEN_35617 Eastern Sub−Saharan Africa Stillbirth Rate (SBR) − 20 weeks
lambda = 0.8 ; zeta = 0.7 ; scale = 15 ; amp = 0.028

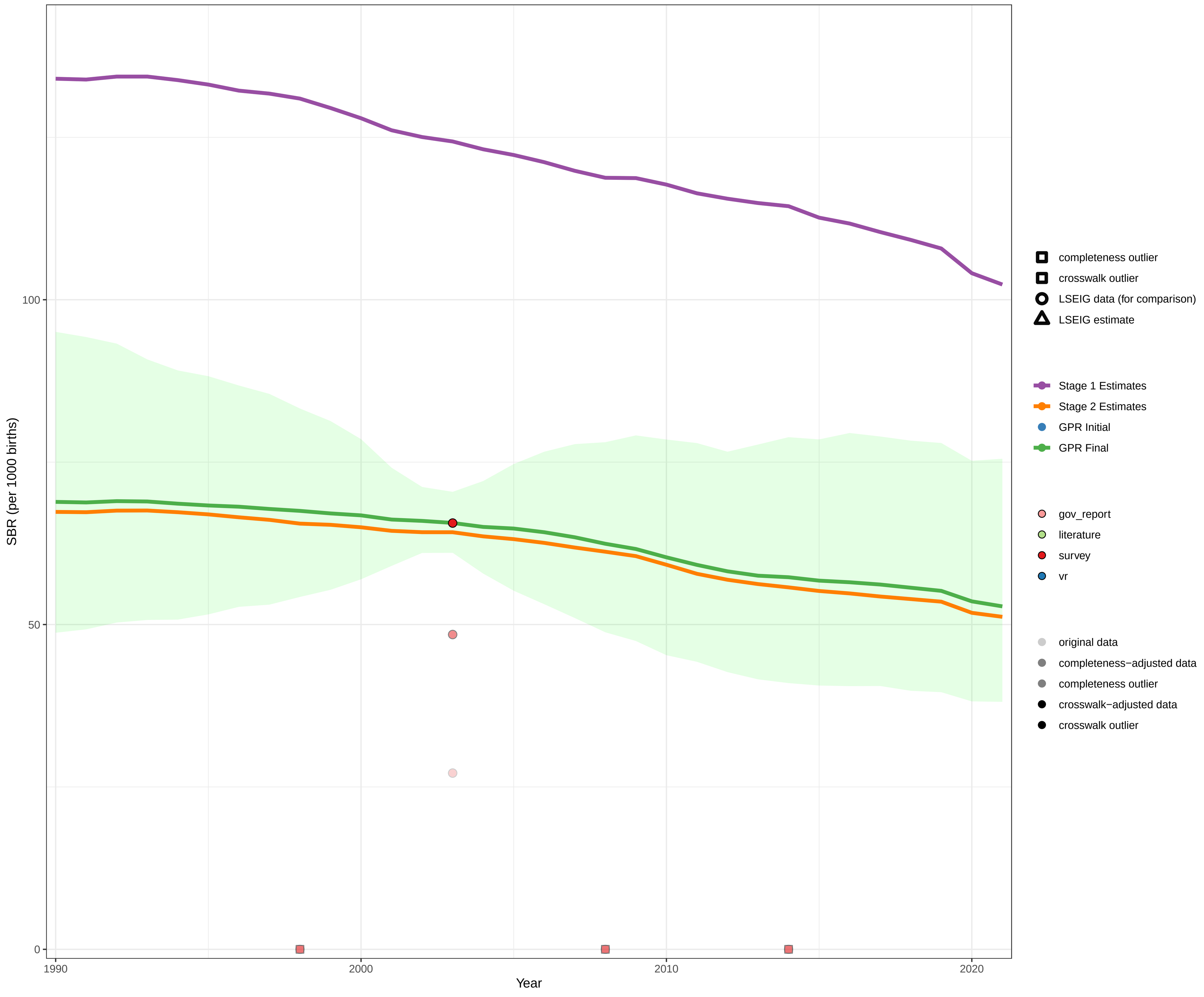

Baringo KEN_35617 Eastern Sub−Saharan Africa Stillbirth Rate (SBR) − 28 weeks
lambda = 0.8 ; zeta = 0.7 ; scale = 15 ; amp = 0.028

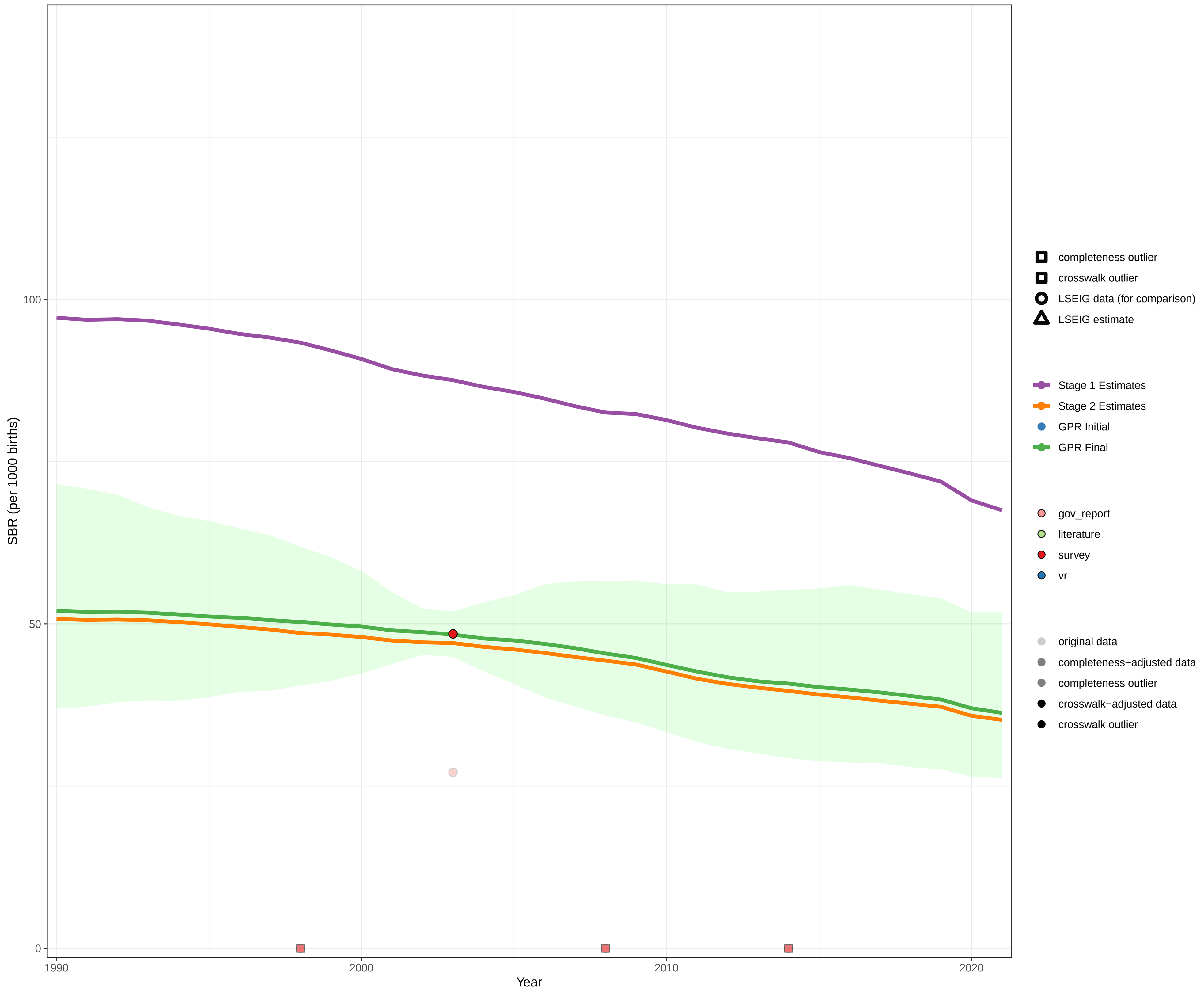

Year	Total Stillbirths (in thousands)	Total Stillbirths (in thousands)	SBR (per 1000 births)	SBR (per 1000 births)	SBR/NMR	SBR/NMR
	20 weeks	28 weeks	20 weeks	28 weeks	20 weeks	28 weeks
1990	1.1 (0.8 to 1.6)	0.8 (0.6 to 1.2)	68.9 (48.7 to 95.1)	52.0 (36.9 to 71.6)	1.50 (1.06 to 2.08)	1.14 (0.81 to 1.56)
1995	1.2 (0.9 to 1.6)	0.9 (0.7 to 1.2)	68.3 (51.6 to 88.2)	51.2 (38.7 to 65.9)	1.51 (1.14 to 1.95)	1.13 (0.86 to 1.46)
2000	1.2 (1.0 to 1.5)	0.9 (0.8 to 1.1)	66.8 (57.0 to 78.5)	49.6 (42.4 to 58.2)	1.55 (1.32 to 1.83)	1.15 (0.99 to 1.35)
2005	1.2 (1.0 to 1.4)	0.9 (0.8 to 1.0)	64.8 (55.2 to 74.7)	47.5 (40.8 to 54.4)	1.60 (1.36 to 1.85)	1.17 (1.01 to 1.34)
2010	1.1 (0.8 to 1.5)	0.8 (0.6 to 1.0)	60.3 (45.3 to 78.5)	43.7 (33.3 to 56.1)	1.59 (1.19 to 2.07)	1.15 (0.88 to 1.48)
2015	1.0 (0.7 to 1.4)	0.7 (0.5 to 1.0)	56.8 (40.6 to 78.5)	40.2 (28.8 to 55.5)	1.65 (1.18 to 2.28)	1.17 (0.84 to 1.61)
2020	0.9 (0.6 to 1.2)	0.6 (0.4 to 0.8)	53.6 (38.2 to 75.2)	37.0 (26.5 to 51.8)	1.81 (1.29 to 2.54)	1.25 (0.89 to 1.75)
2021	0.8 (0.6 to 1.2)	0.6 (0.4 to 0.8)	52.8 (38.1 to 75.5)	36.3 (26.3 to 51.7)	1.84 (1.33 to 2.63)	1.27 (0.92 to 1.81)

Bomet KEN_35618 Eastern Sub−Saharan Africa Stillbirth Rate / Neonatal Mortality Rate (SBR/NMR) − 20 weeks
lambda = 0.8 ; zeta = 0.7 ; scale = 15 ; amp = 0.028

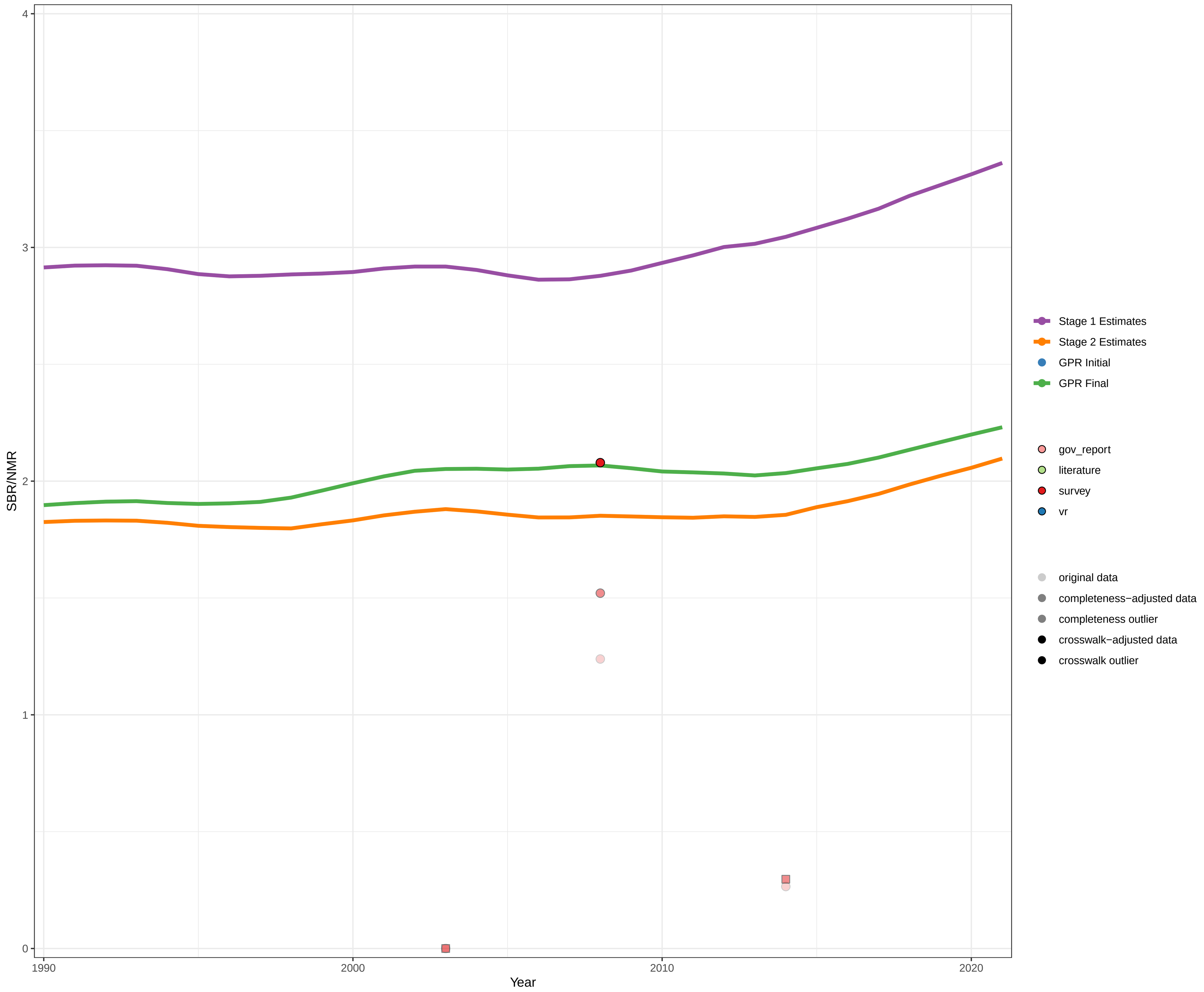

Bomet KEN_35618 Eastern Sub−Saharan Africa Stillbirth Rate / Neonatal Mortality Rate (SBR/NMR) − 28 weeks
lambda = 0.8 ; zeta = 0.7 ; scale = 15 ; amp = 0.028

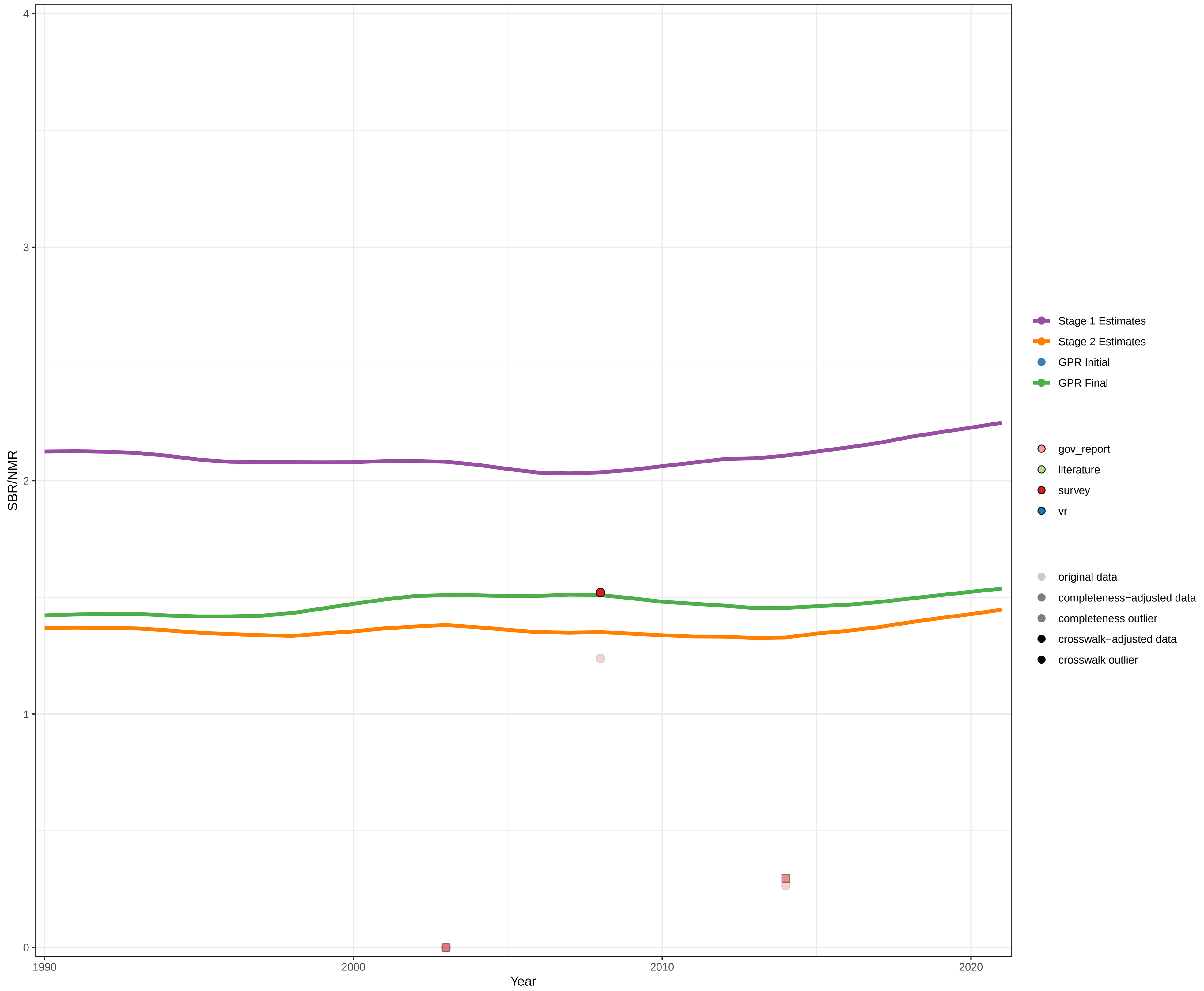

Definition	Years	Outlier	Source.Type	Title
28_weeks	2008	0	Survey	Kenya Demographic and Health Survey 2008−2009
28_weeks	2003	1	Survey	Kenya Demographic and Health Survey 2003
28_weeks	2014	1	Survey	Kenya Demographic and Health Survey 2014

Bomet KEN_35618 Eastern Sub−Saharan Africa Stillbirth Rate (SBR) − 20 weeks
lambda = 0.8 ; zeta = 0.7 ; scale = 15 ; amp = 0.028

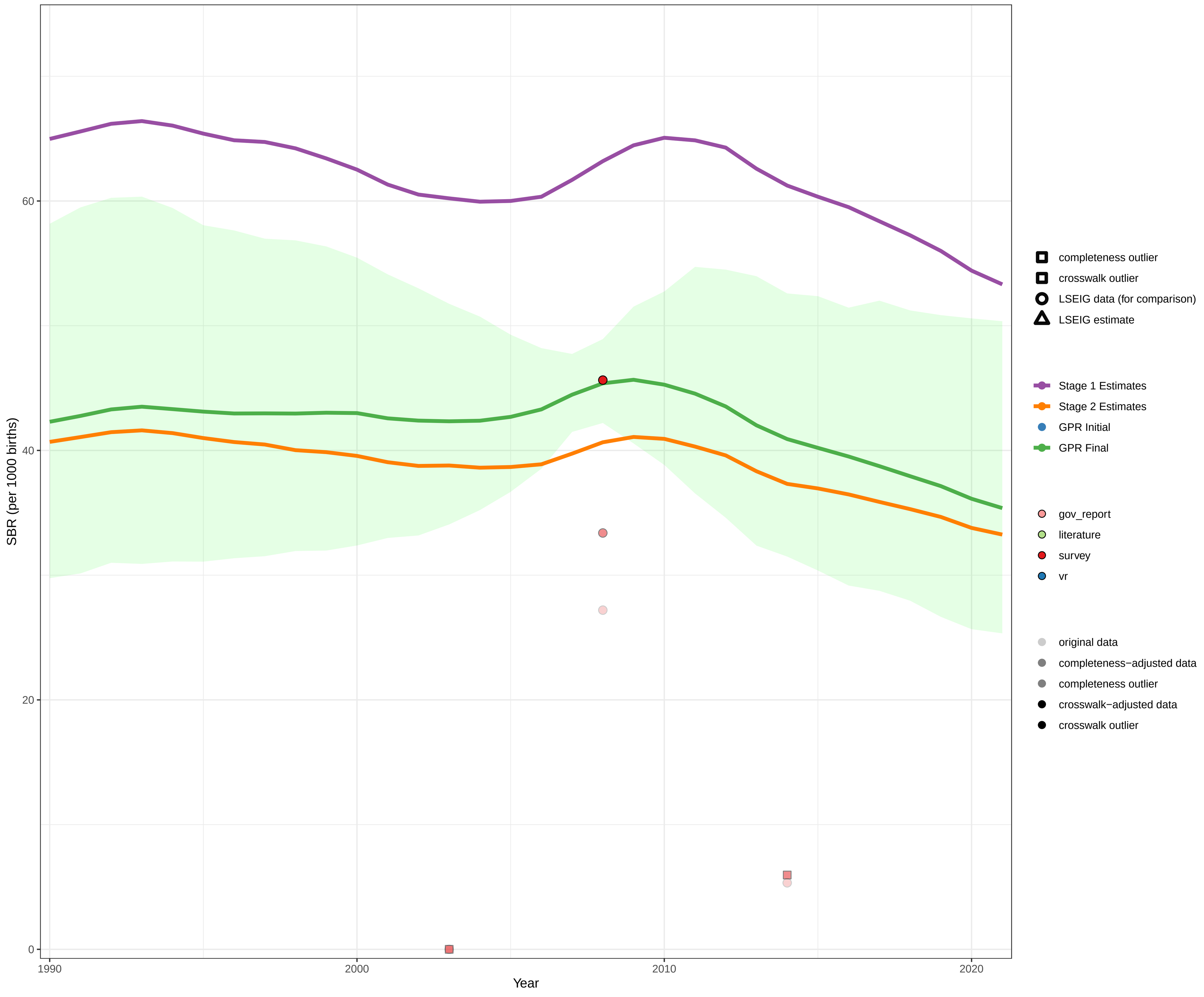

Bomet KEN_35618 Eastern Sub−Saharan Africa Stillbirth Rate (SBR) − 28 weeks
lambda = 0.8 ; zeta = 0.7 ; scale = 15 ; amp = 0.028

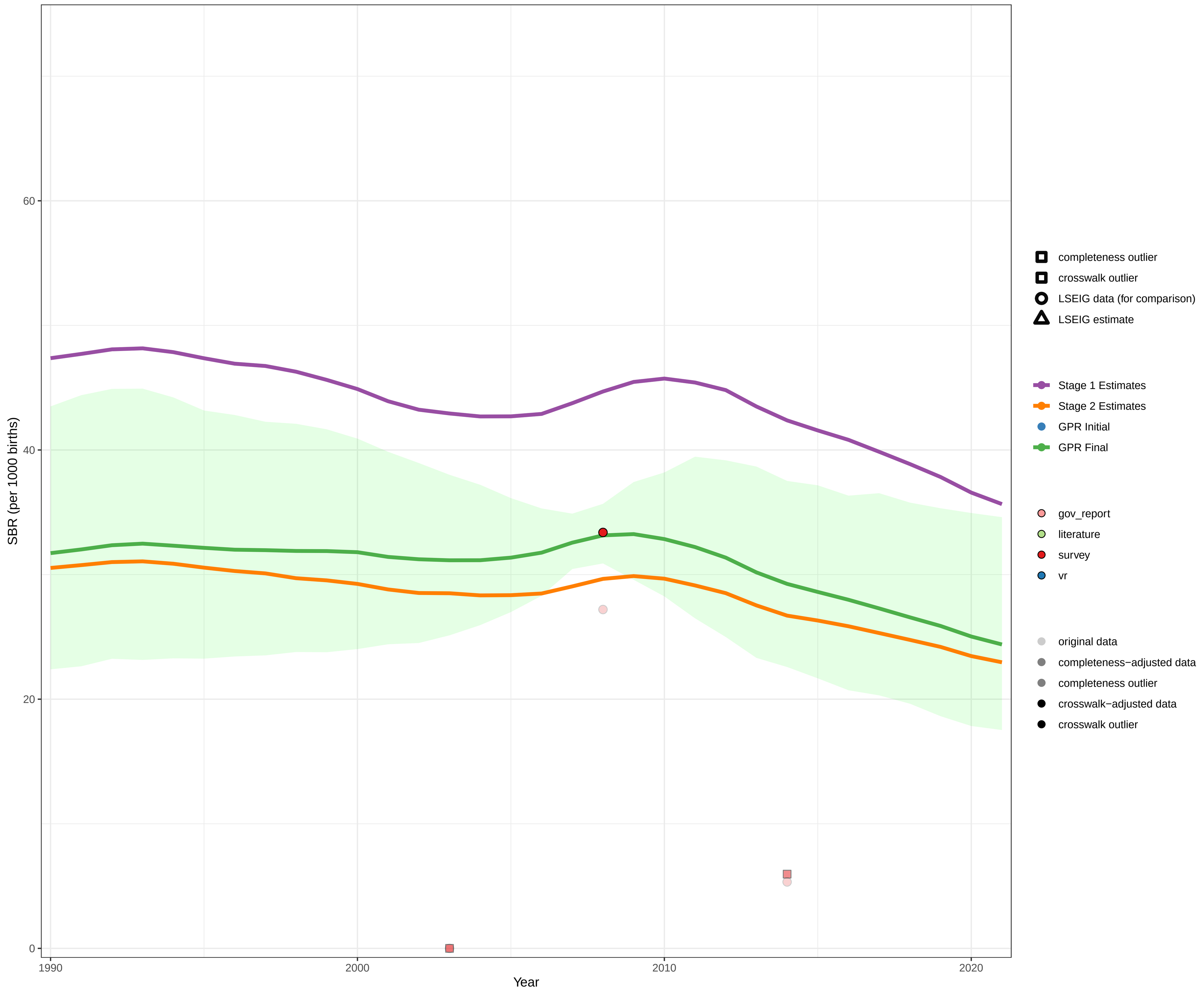

Year	Total Stillbirths (in thousands)	Total Stillbirths (in thousands)	SBR (per 1000 births)	SBR (per 1000 births)	SBR/NMR	SBR/NMR
	20 weeks	28 weeks	20 weeks	28 weeks	20 weeks	28 weeks
1990	0.7 (0.5 to 1.0)	0.5 (0.4 to 0.7)	42.3 (29.8 to 58.2)	31.7 (22.4 to 43.5)	1.90 (1.33 to 2.61)	1.42 (1.00 to 1.95)
1995	0.8 (0.6 to 1.1)	0.6 (0.4 to 0.8)	43.1 (31.1 to 58.1)	32.1 (23.3 to 43.2)	1.90 (1.37 to 2.56)	1.42 (1.03 to 1.90)
2000	1.0 (0.7 to 1.3)	0.7 (0.5 to 0.9)	43.0 (32.4 to 55.5)	31.8 (24.0 to 40.9)	1.99 (1.50 to 2.57)	1.47 (1.11 to 1.89)
2005	1.3 (1.1 to 1.5)	1.0 (0.8 to 1.1)	42.7 (36.7 to 49.3)	31.4 (27.0 to 36.1)	2.05 (1.76 to 2.37)	1.51 (1.30 to 1.73)
2010	1.7 (1.4 to 1.9)	1.2 (1.0 to 1.4)	45.3 (38.8 to 52.7)	32.8 (28.2 to 38.2)	2.04 (1.75 to 2.38)	1.48 (1.27 to 1.72)
2015	1.3 (1.0 to 1.7)	0.9 (0.7 to 1.2)	40.2 (30.4 to 52.4)	28.6 (21.7 to 37.2)	2.05 (1.55 to 2.68)	1.46 (1.11 to 1.90)
2020	0.9 (0.6 to 1.3)	0.6 (0.4 to 0.9)	36.1 (25.7 to 50.6)	25.0 (17.8 to 34.9)	2.20 (1.56 to 3.08)	1.52 (1.09 to 2.13)
2021	0.8 (0.6 to 1.2)	0.6 (0.4 to 0.8)	35.4 (25.3 to 50.4)	24.4 (17.5 to 34.6)	2.23 (1.60 to 3.18)	1.54 (1.10 to 2.18)

Bungoma KEN_35619 Eastern Sub−Saharan Africa Stillbirth Rate / Neonatal Mortality Rate (SBR/NMR) − 20 weeks
lambda = 0.8 ; zeta = 0.7 ; scale = 15 ; amp = 0.028

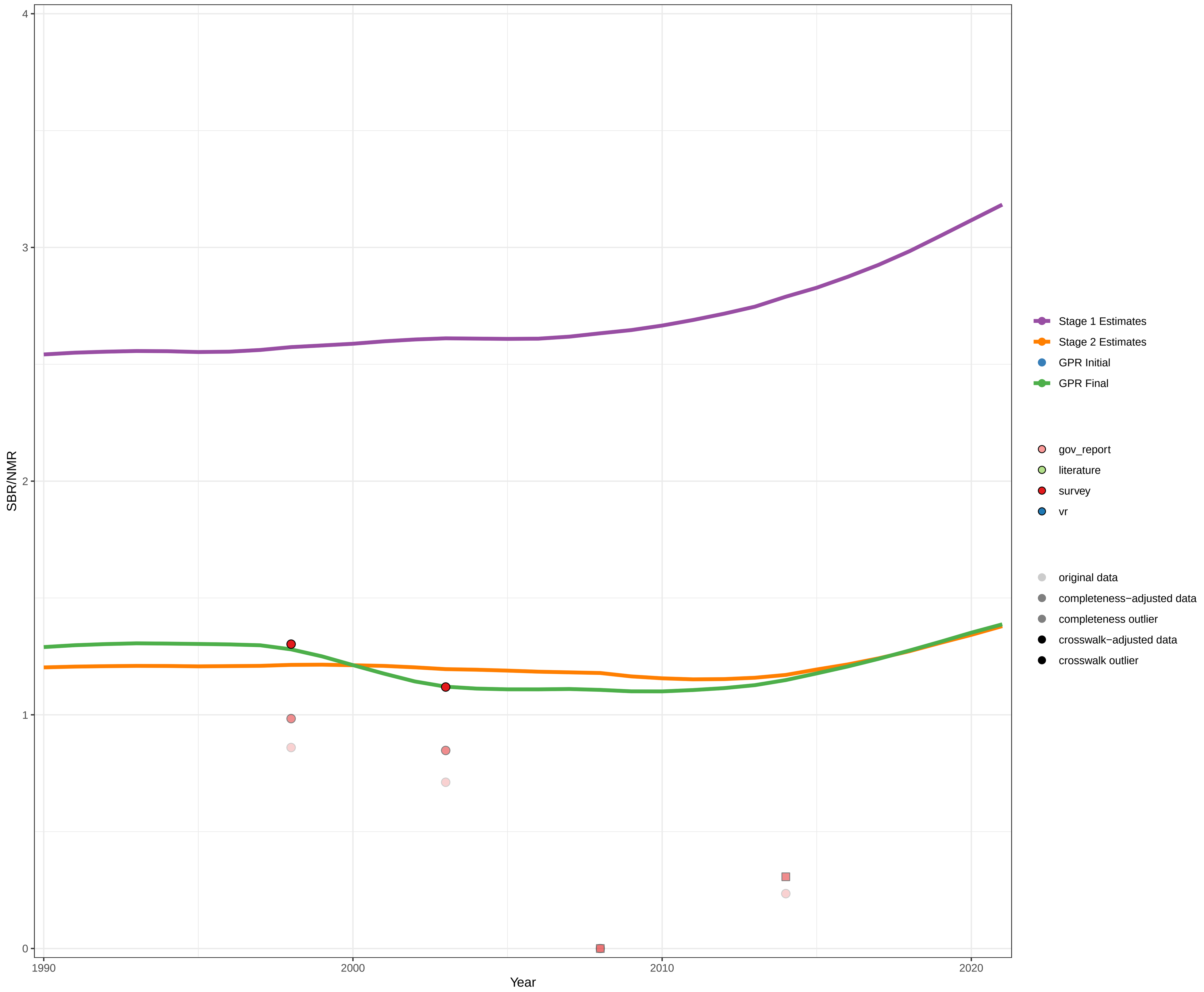

Bungoma KEN_35619 Eastern Sub−Saharan Africa Stillbirth Rate / Neonatal Mortality Rate (SBR/NMR) − 28 weeks
lambda = 0.8 ; zeta = 0.7 ; scale = 15 ; amp = 0.028

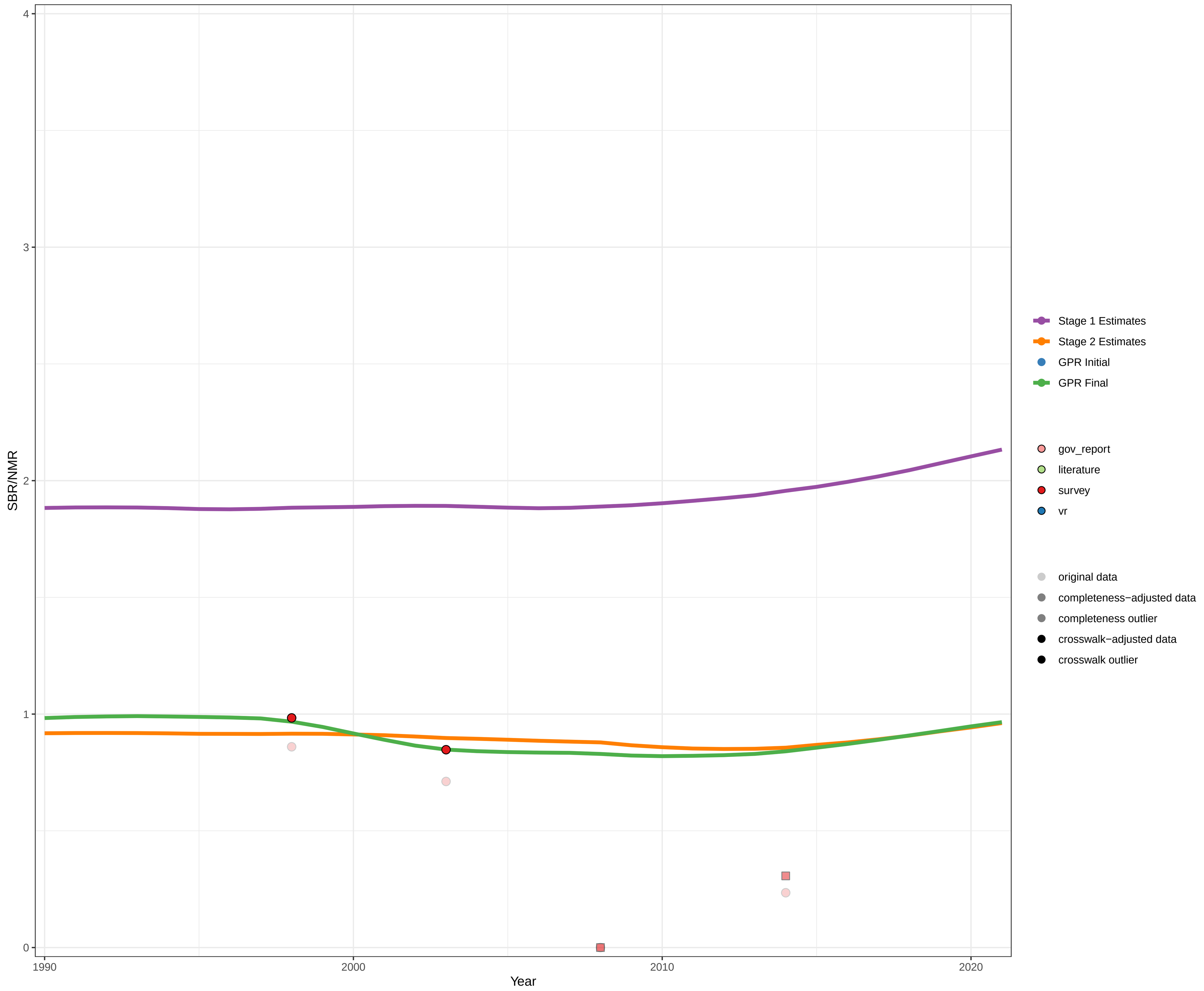

Definition	Years	Outlier	Source.Type	Title
28_weeks	1998	0	Survey	Kenya Demographic and Health Survey 1998
28_weeks	2003	0	Survey	Kenya Demographic and Health Survey 2003
28_weeks	2008	1	Survey	Kenya Demographic and Health Survey 2008−2009
28_weeks	2014	1	Survey	Kenya Demographic and Health Survey 2014

Bungoma KEN_35619 Eastern Sub−Saharan Africa Stillbirth Rate (SBR) − 20 weeks
lambda = 0.8 ; zeta = 0.7 ; scale = 15 ; amp = 0.028

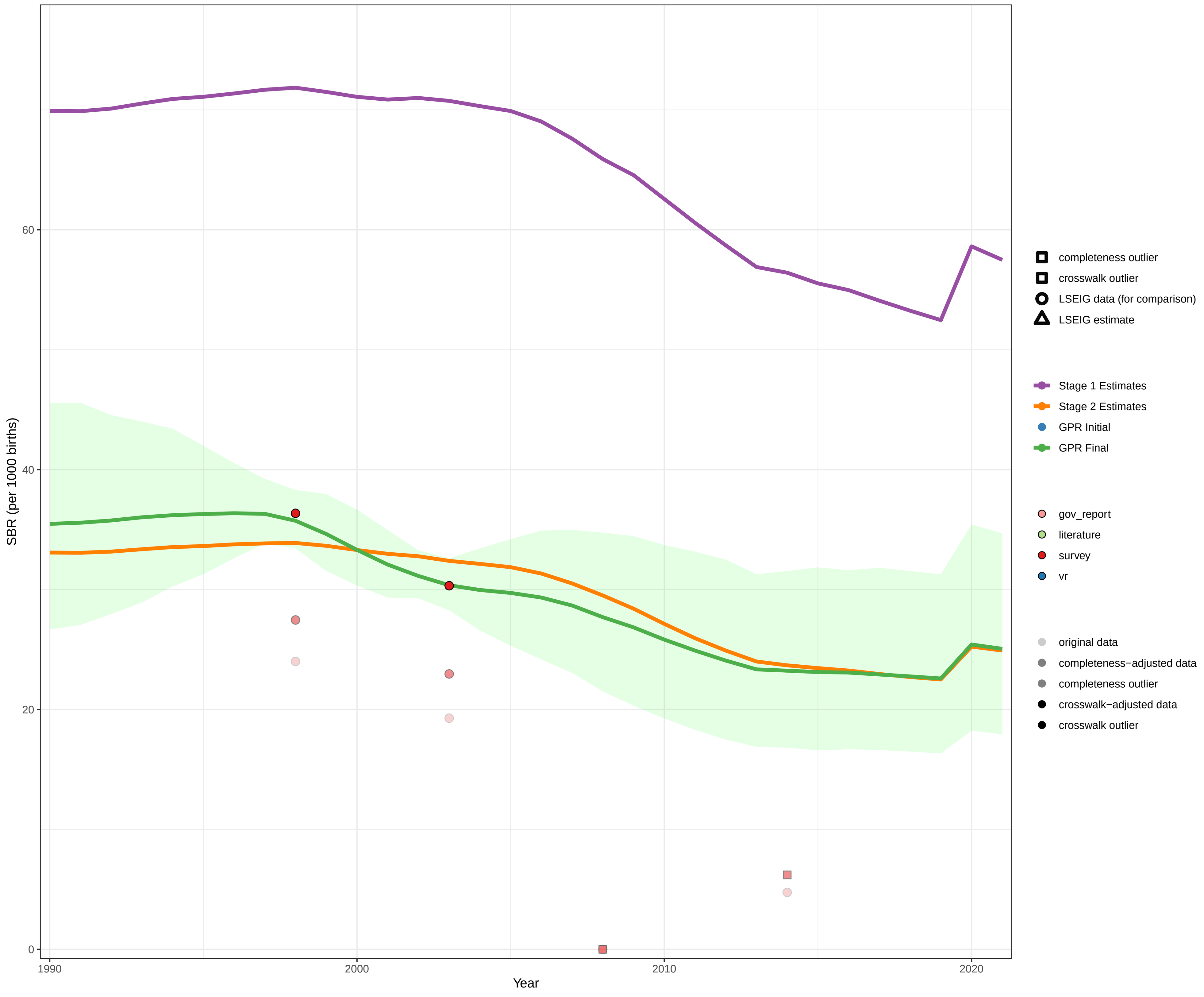

Bungoma KEN_35619 Eastern Sub−Saharan Africa Stillbirth Rate (SBR) − 28 weeks
lambda = 0.8 ; zeta = 0.7 ; scale = 15 ; amp = 0.028

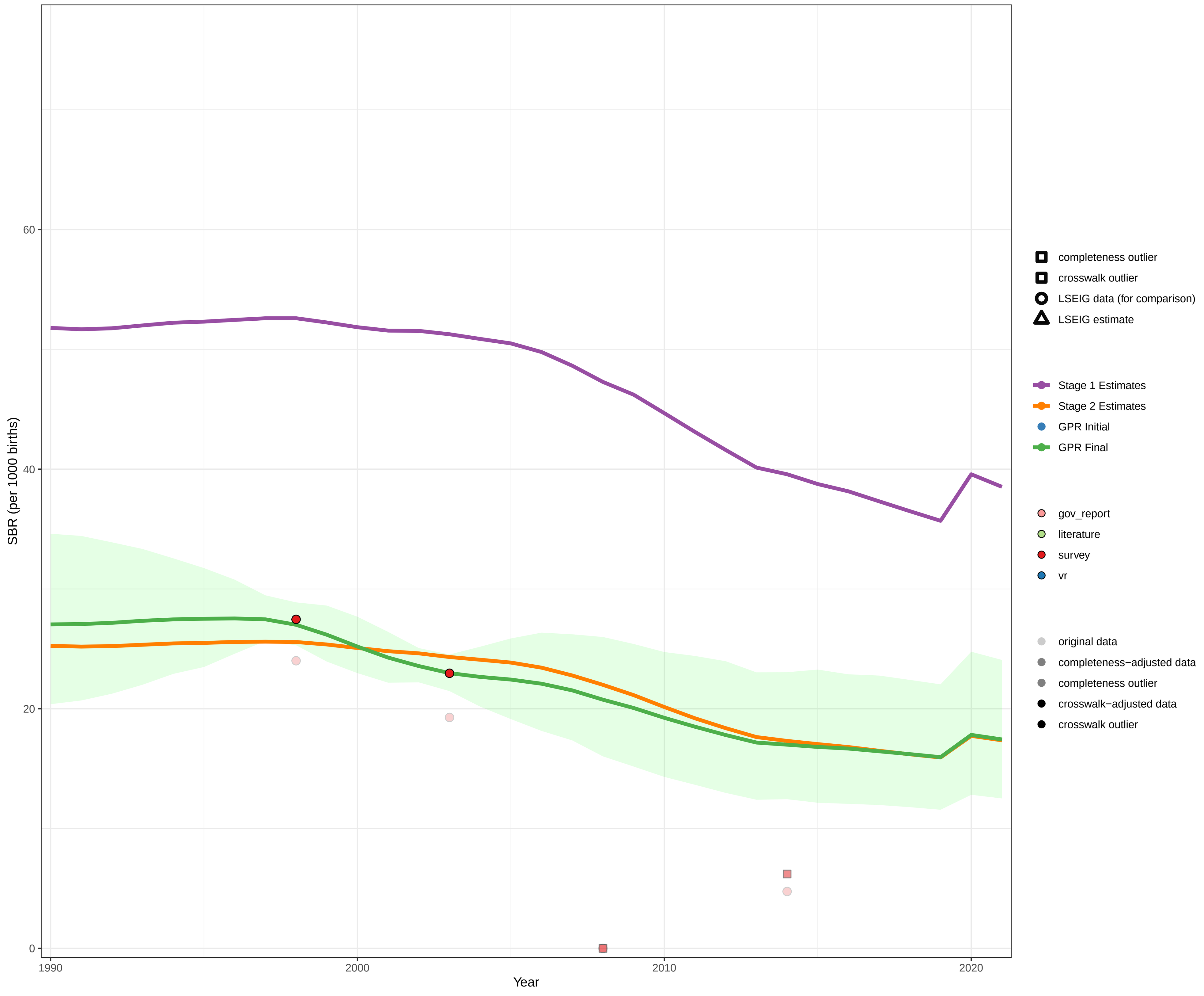

Year	Total Stillbirths (in thousands)	Total Stillbirths (in thousands)	SBR (per 1000 births)	SBR (per 1000 births)	SBR/NMR	SBR/NMR
	20 weeks	28 weeks	20 weeks	28 weeks	20 weeks	28 weeks
1990	1.5 (1.1 to 2.0)	1.2 (0.9 to 1.5)	35.5 (26.7 to 45.5)	27.0 (20.4 to 34.6)	1.29 (0.97 to 1.66)	0.98 (0.74 to 1.26)
1995	2.3 (2.0 to 2.7)	1.7 (1.5 to 2.0)	36.3 (31.3 to 42.0)	27.5 (23.5 to 31.7)	1.30 (1.12 to 1.51)	0.99 (0.84 to 1.14)
2000	2.6 (2.3 to 2.8)	1.9 (1.8 to 2.1)	33.3 (30.3 to 36.7)	25.2 (23.0 to 27.7)	1.21 (1.10 to 1.34)	0.92 (0.84 to 1.01)
2005	2.1 (1.8 to 2.4)	1.6 (1.3 to 1.8)	29.7 (25.3 to 34.2)	22.4 (19.1 to 25.9)	1.11 (0.94 to 1.28)	0.84 (0.71 to 0.97)
2010	1.6 (1.2 to 2.1)	1.2 (0.9 to 1.5)	25.8 (19.2 to 33.7)	19.2 (14.3 to 24.7)	1.10 (0.82 to 1.44)	0.82 (0.61 to 1.05)
2015	1.4 (1.0 to 1.9)	1.0 (0.7 to 1.4)	23.1 (16.6 to 31.9)	16.8 (12.2 to 23.3)	1.18 (0.85 to 1.62)	0.86 (0.62 to 1.18)
2020	1.6 (1.1 to 2.2)	1.1 (0.8 to 1.5)	25.4 (18.2 to 35.4)	17.8 (12.8 to 24.8)	1.35 (0.97 to 1.88)	0.95 (0.68 to 1.32)
2021	1.6 (1.1 to 2.2)	1.1 (0.8 to 1.5)	25.1 (17.9 to 34.7)	17.4 (12.5 to 24.1)	1.39 (0.99 to 1.92)	0.97 (0.69 to 1.33)

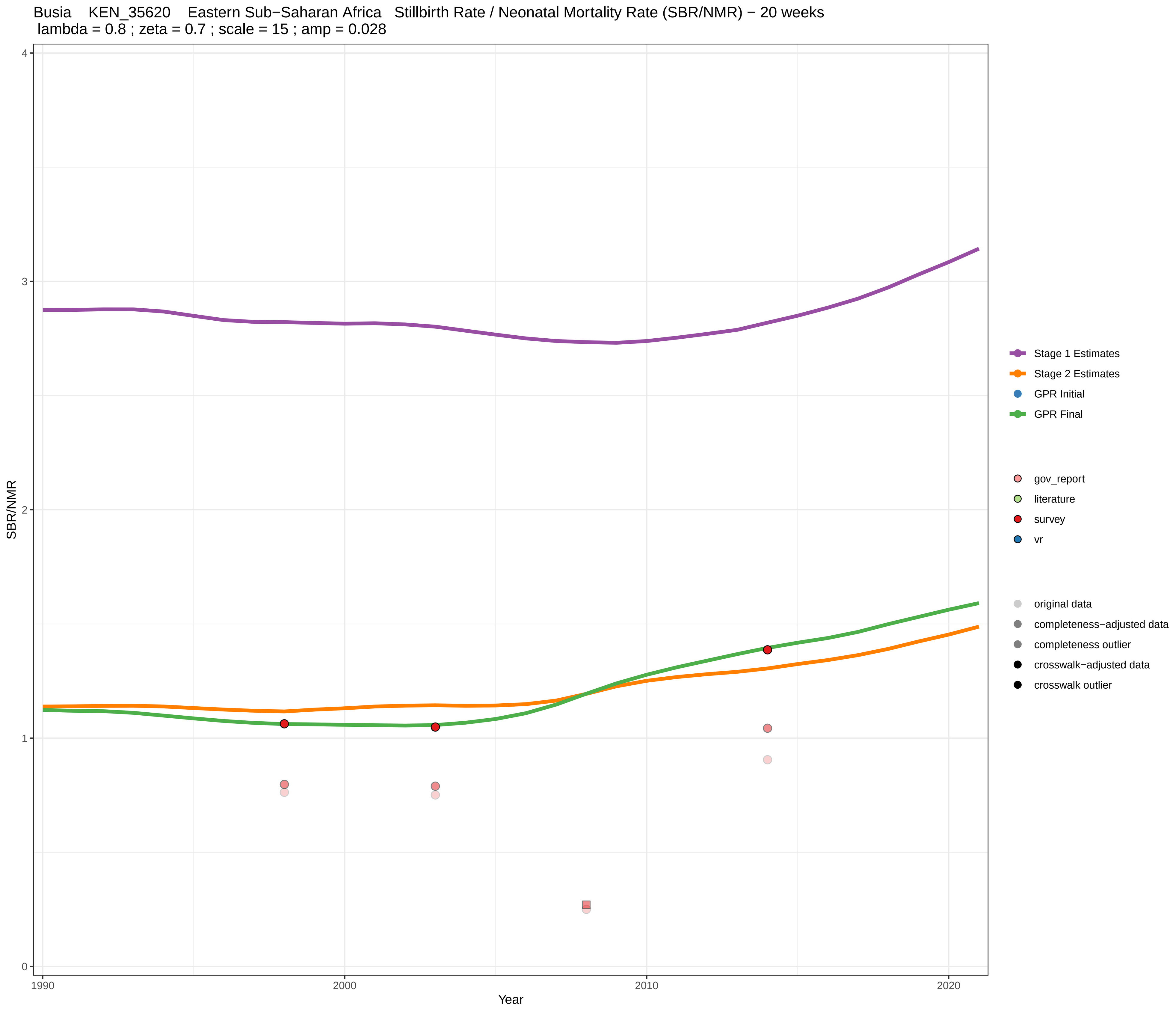

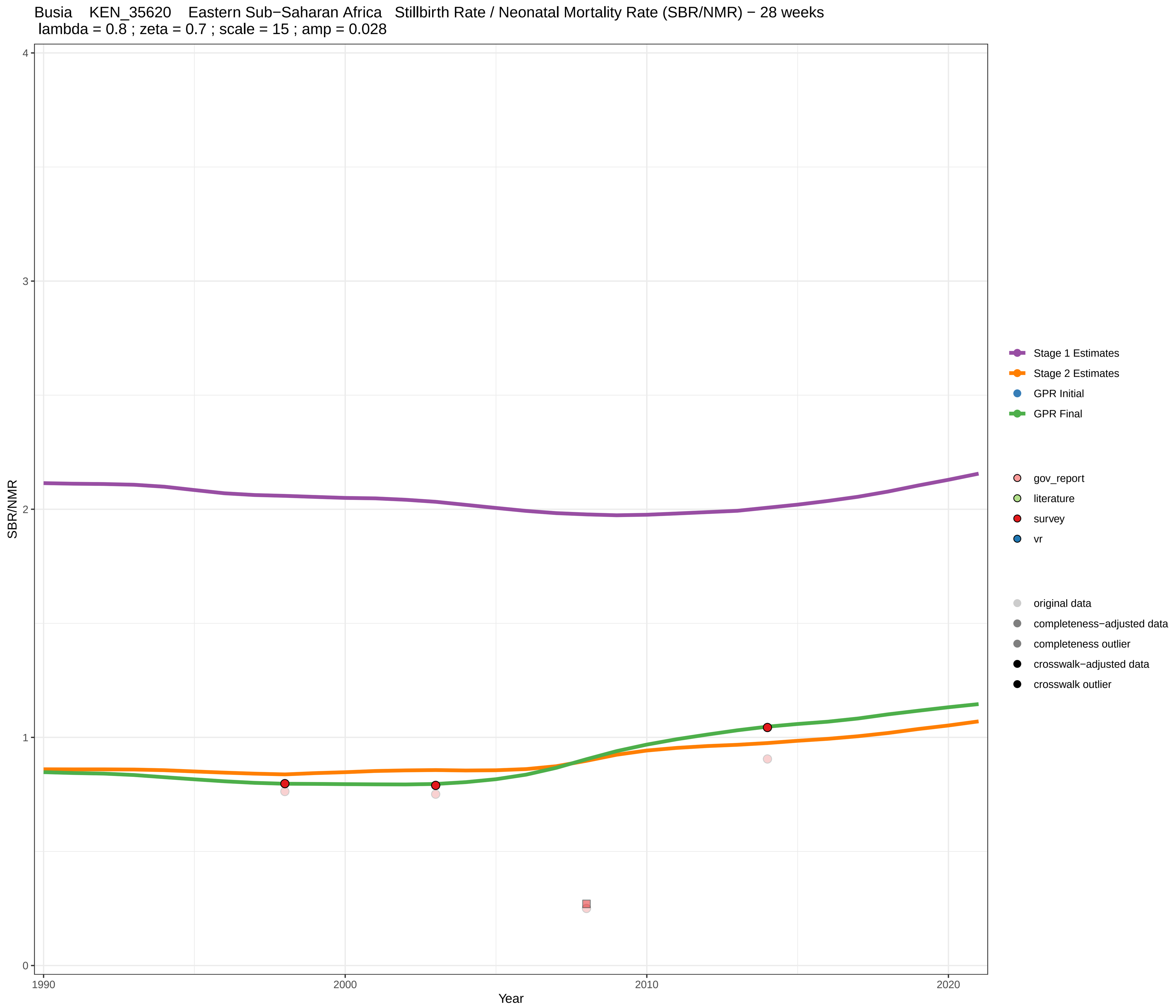

Definition	Years	Outlier	Source.Type	Title
28_weeks	1998	0	Survey	Kenya Demographic and Health Survey 1998
28_weeks	2003	0	Survey	Kenya Demographic and Health Survey 2003
28_weeks	2014	0	Survey	Kenya Demographic and Health Survey 2014
28_weeks	2008	1	Survey	Kenya Demographic and Health Survey 2008−2009

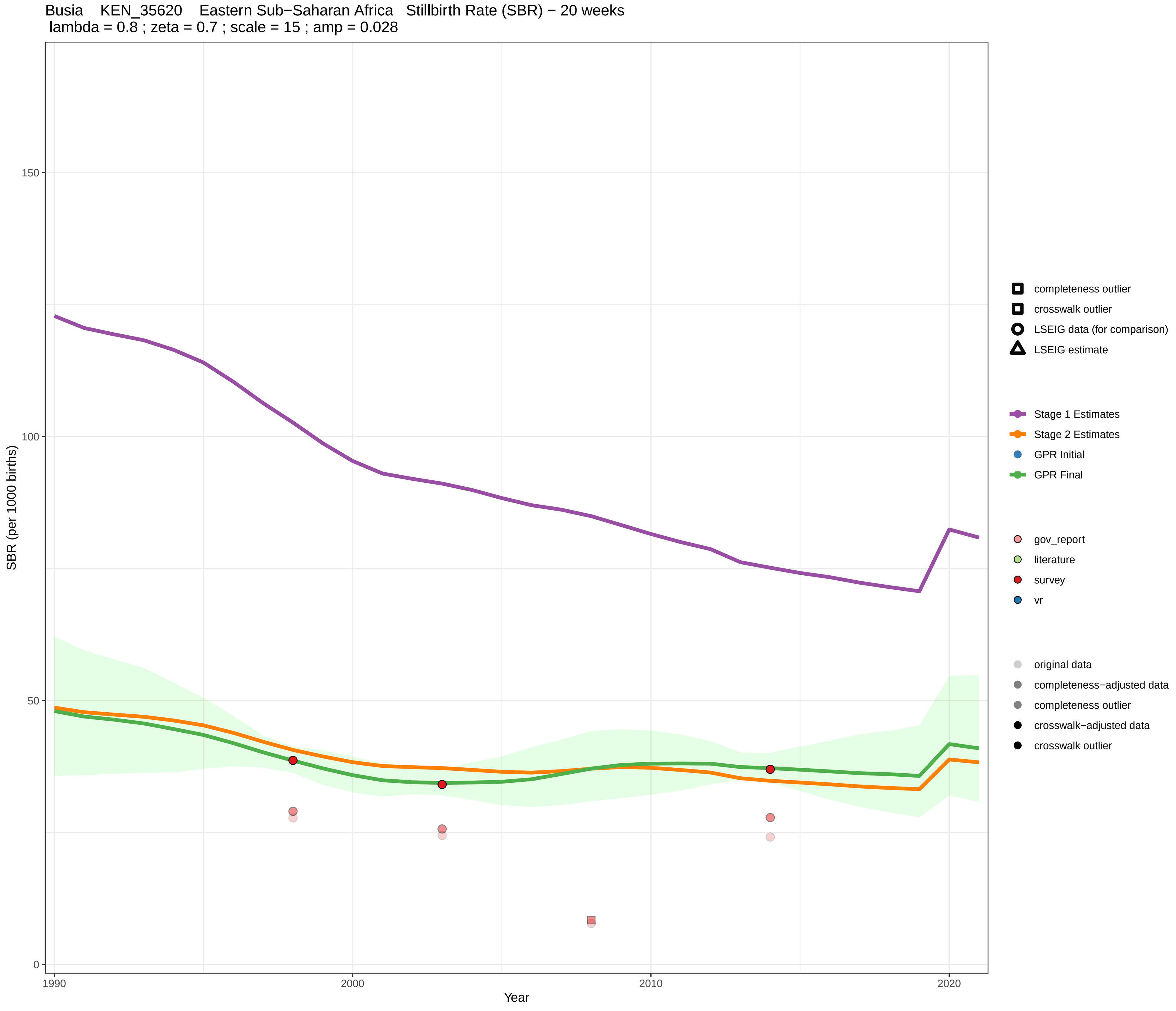

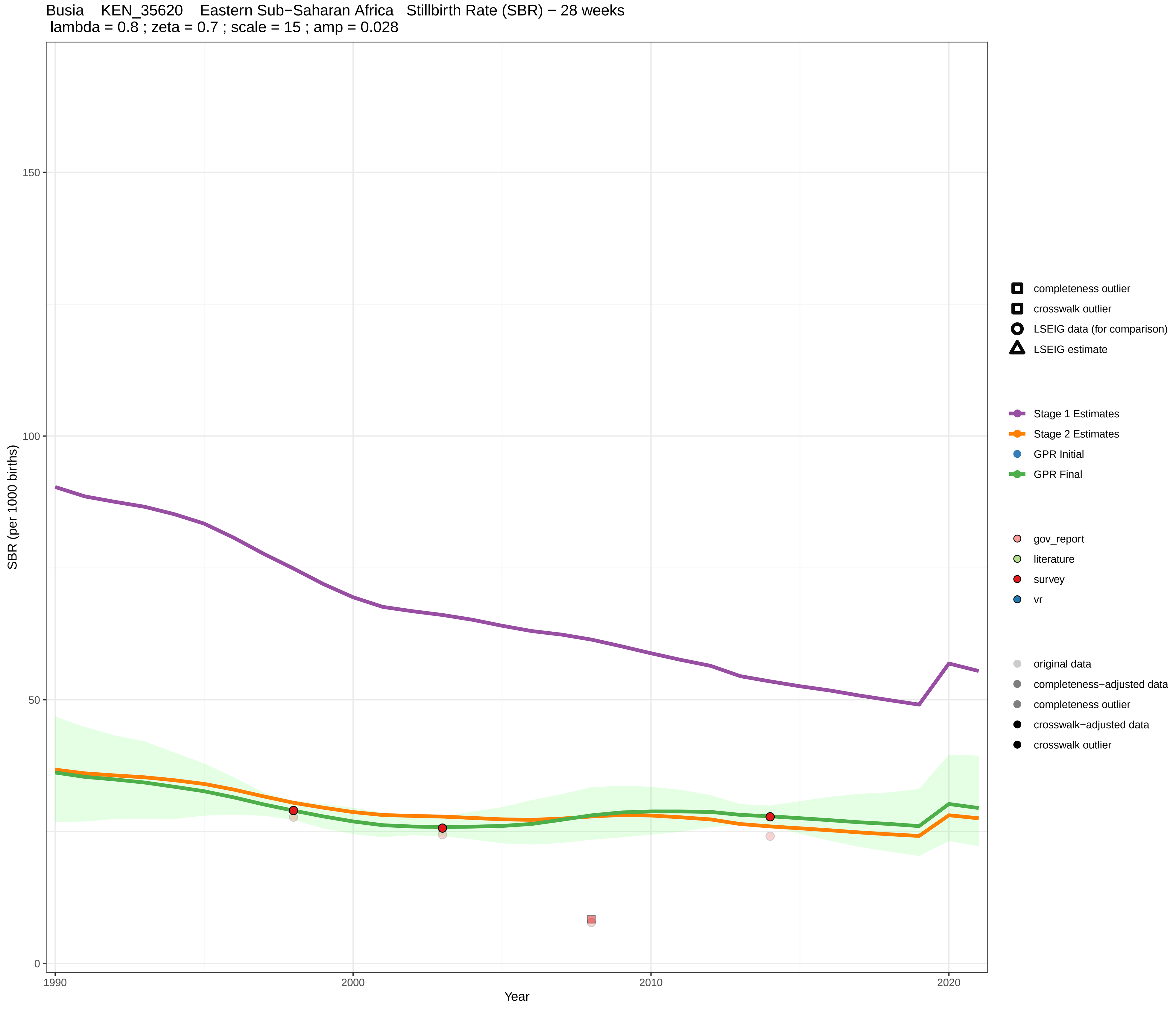

Year	Total Stillbirths (in thousands)	Total Stillbirths (in thousands)	SBR (per 1000 births)	SBR (per 1000 births)	SBR/NMR	SBR/NMR
	20 weeks	28 weeks	20 weeks	28 weeks	20 weeks	28 weeks
1990	1.2 (0.9 to 1.6)	0.9 (0.7 to 1.2)	48.0 (35.7 to 62.1)	36.2 (26.8 to 46.8)	1.12 (0.84 to 1.45)	0.85 (0.63 to 1.10)
1995	1.1 (1.0 to 1.3)	0.8 (0.7 to 1.0)	43.5 (37.1 to 50.5)	32.7 (28.0 to 37.9)	1.09 (0.93 to 1.26)	0.82 (0.70 to 0.95)
2000	1.0 (0.9 to 1.0)	0.7 (0.6 to 0.8)	35.9 (32.6 to 39.4)	26.9 (24.5 to 29.5)	1.06 (0.96 to 1.16)	0.80 (0.72 to 0.87)
2005	1.0 (0.9 to 1.1)	0.7 (0.6 to 0.8)	34.6 (30.2 to 39.4)	26.1 (22.8 to 29.6)	1.08 (0.94 to 1.23)	0.82 (0.71 to 0.93)
2010	1.1 (0.9 to 1.3)	0.8 (0.7 to 1.0)	38.0 (32.2 to 44.3)	28.8 (24.4 to 33.5)	1.28 (1.08 to 1.49)	0.97 (0.82 to 1.13)
2015	1.0 (0.9 to 1.2)	0.8 (0.7 to 0.8)	36.9 (32.9 to 41.3)	27.5 (24.6 to 30.8)	1.42 (1.26 to 1.59)	1.06 (0.95 to 1.18)
2020	1.1 (0.8 to 1.4)	0.8 (0.6 to 1.0)	41.7 (32.0 to 54.6)	30.2 (23.2 to 39.6)	1.56 (1.20 to 2.05)	1.13 (0.87 to 1.48)
2021	1.0 (0.8 to 1.4)	0.7 (0.6 to 1.0)	40.9 (30.8 to 54.8)	29.5 (22.2 to 39.4)	1.59 (1.20 to 2.13)	1.15 (0.86 to 1.53)

Elgeyo−Marakwet KEN_35621 Eastern Sub−Saharan Africa Stillbirth Rate / Neonatal Mortality Rate (SBR/NMR) − 20 weeks
lambda = 0.8 ; zeta = 0.7 ; scale = 15 ; amp = 0.028

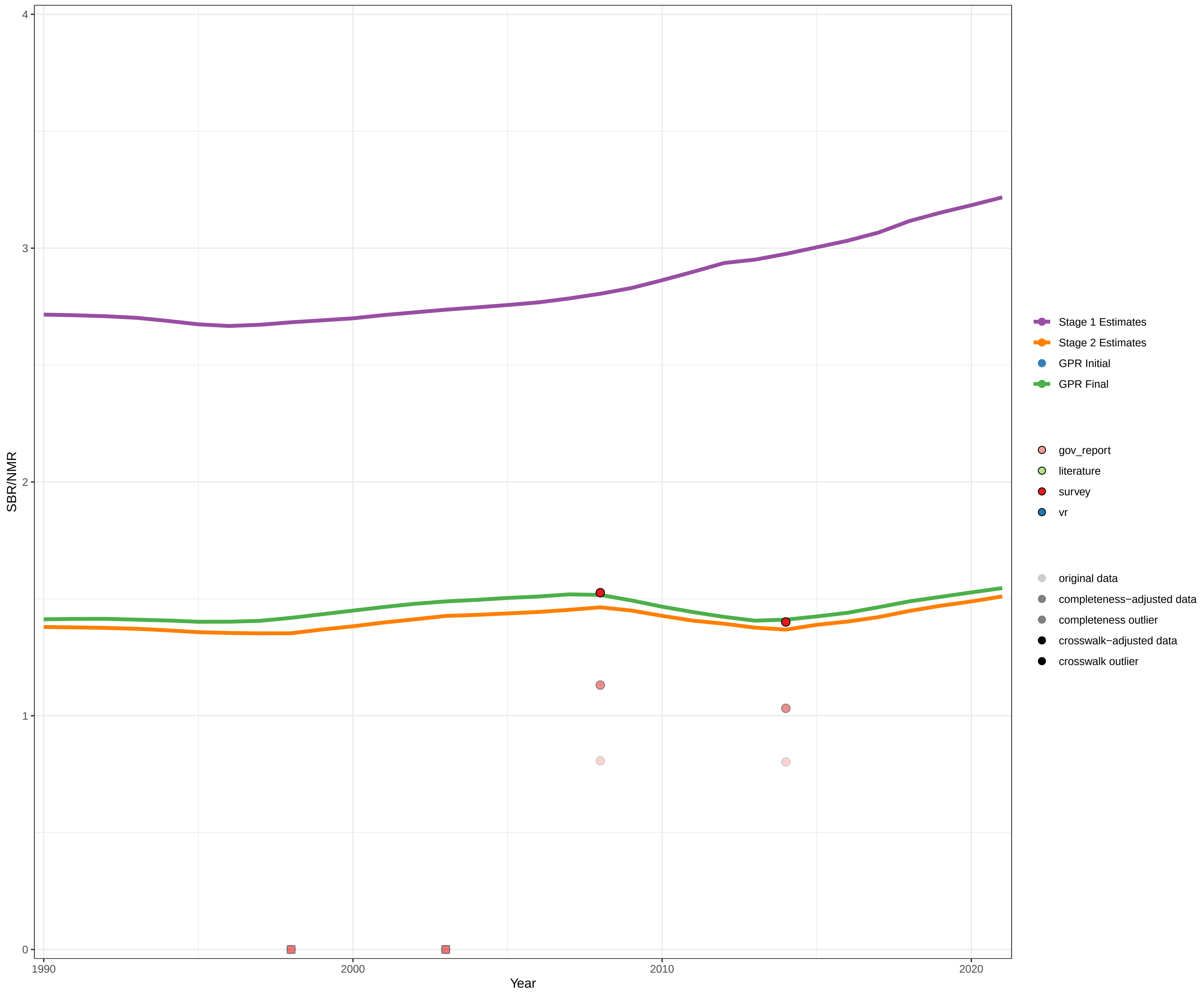

Elgeyo−Marakwet KEN_35621 Eastern Sub−Saharan Africa Stillbirth Rate / Neonatal Mortality Rate (SBR/NMR) − 28 weeks
lambda = 0.8 ; zeta = 0.7 ; scale = 15 ; amp = 0.028

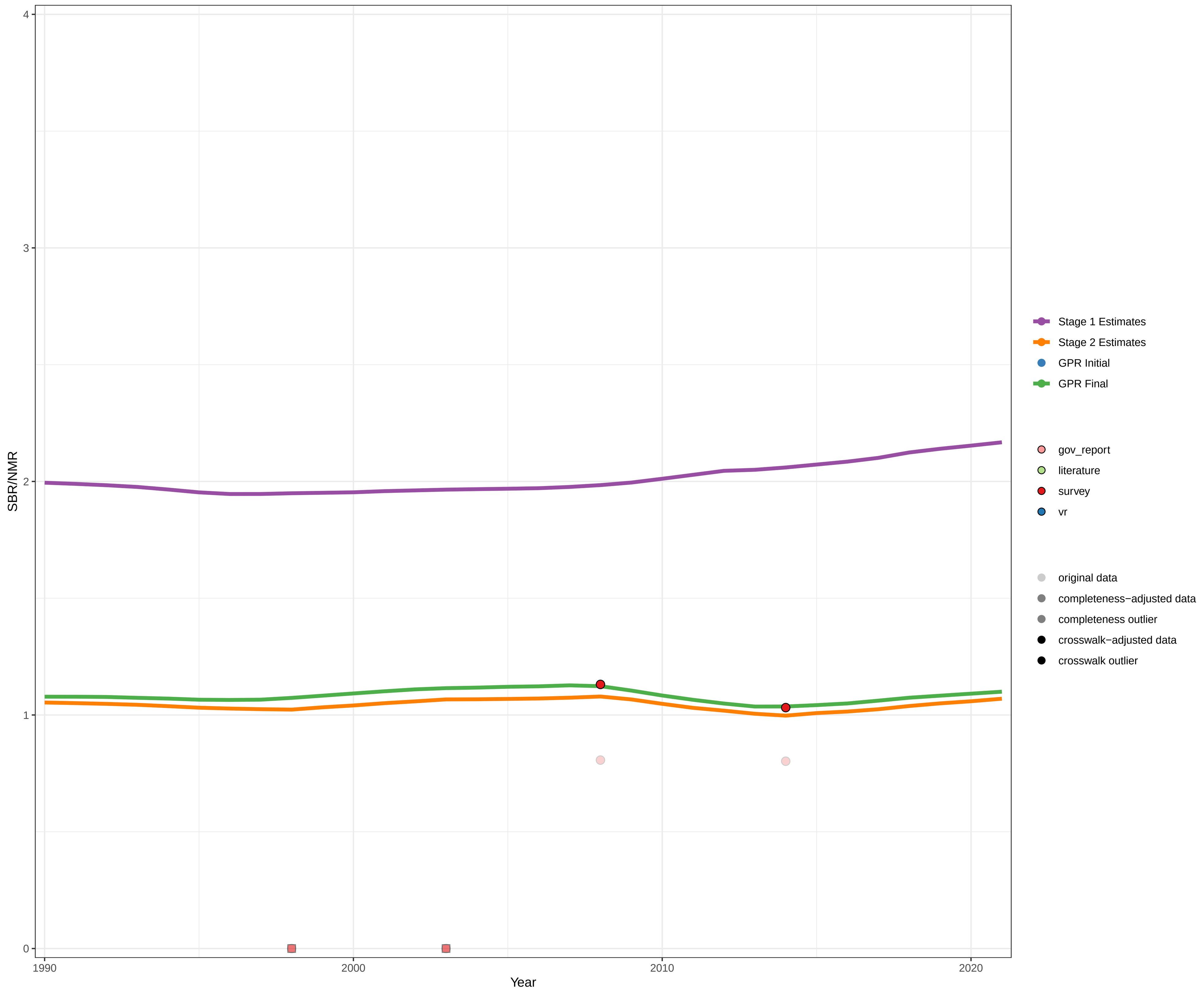

Definition	Years	Outlier	Source.Type	Title
28_weeks	2008	0	Survey	Kenya Demographic and Health Survey 2008−2009
28_weeks	2014	0	Survey	Kenya Demographic and Health Survey 2014
28_weeks	1998	1	Survey	Kenya Demographic and Health Survey 1998
28_weeks	2003	1	Survey	Kenya Demographic and Health Survey 2003

Elgeyo−Marakwet KEN_35621 Eastern Sub−Saharan Africa Stillbirth Rate (SBR) − 20 weeks
lambda = 0.8 ; zeta = 0.7 ; scale = 15 ; amp = 0.028

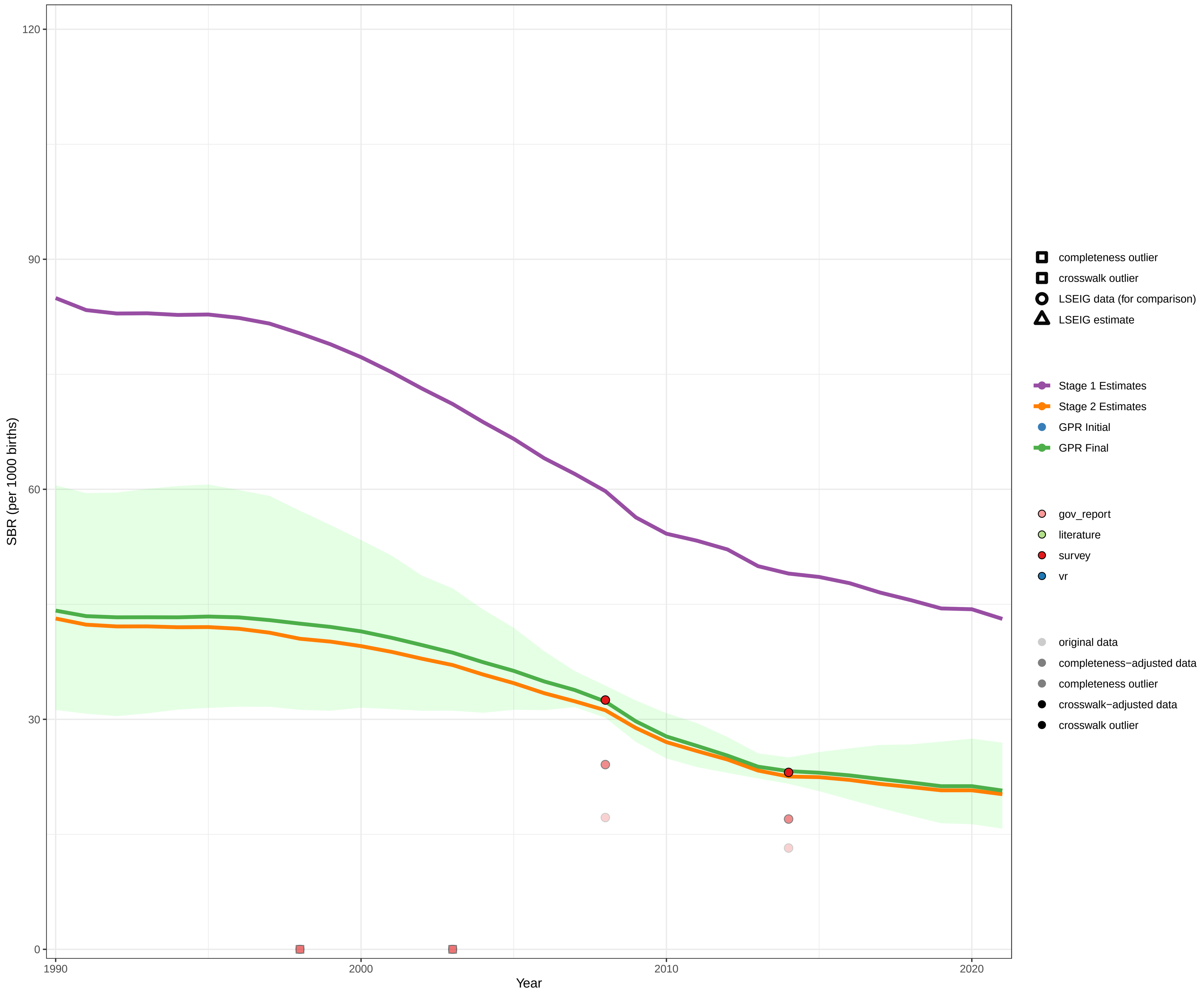

Elgeyo−Marakwet KEN_35621 Eastern Sub−Saharan Africa Stillbirth Rate (SBR) − 28 weeks
lambda = 0.8 ; zeta = 0.7 ; scale = 15 ; amp = 0.028

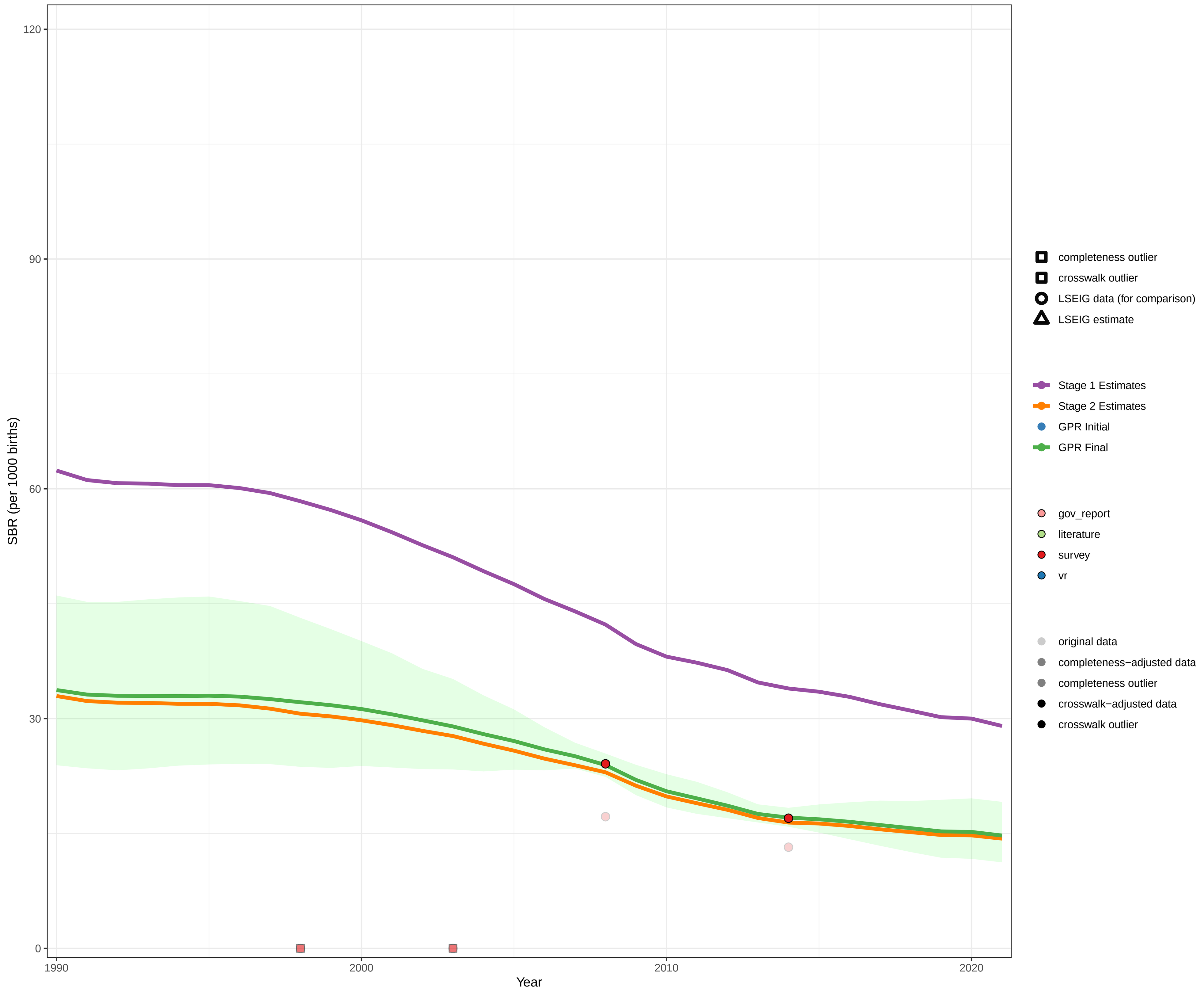

Year	Total Stillbirths (in thousands)	Total Stillbirths (in thousands)	SBR (per 1000 births)	SBR (per 1000 births)	SBR/NMR	SBR/NMR
	20 weeks	28 weeks	20 weeks	28 weeks	20 weeks	28 weeks
1990	0.5 (0.4 to 0.7)	0.4 (0.3 to 0.6)	44.2 (31.2 to 60.5)	33.7 (23.9 to 46.1)	1.41 (1.00 to 1.94)	1.08 (0.76 to 1.47)
1995	0.8 (0.5 to 1.1)	0.6 (0.4 to 0.8)	43.4 (31.5 to 60.6)	33.0 (24.0 to 45.9)	1.40 (1.02 to 1.96)	1.07 (0.78 to 1.48)
2000	0.8 (0.6 to 1.1)	0.6 (0.5 to 0.8)	41.5 (31.5 to 53.4)	31.2 (23.8 to 40.1)	1.45 (1.10 to 1.87)	1.09 (0.83 to 1.40)
2005	0.6 (0.5 to 0.6)	0.4 (0.4 to 0.5)	36.3 (31.2 to 41.9)	27.1 (23.3 to 31.2)	1.50 (1.29 to 1.74)	1.12 (0.97 to 1.29)
2010	0.3 (0.3 to 0.3)	0.2 (0.2 to 0.3)	27.8 (24.9 to 30.8)	20.5 (18.4 to 22.7)	1.47 (1.31 to 1.63)	1.08 (0.97 to 1.20)
2015	0.2 (0.2 to 0.3)	0.2 (0.2 to 0.2)	23.0 (20.6 to 25.7)	16.9 (15.1 to 18.8)	1.42 (1.28 to 1.59)	1.04 (0.93 to 1.16)
2020	0.2 (0.2 to 0.3)	0.2 (0.1 to 0.2)	21.3 (16.3 to 27.5)	15.2 (11.7 to 19.6)	1.53 (1.17 to 1.97)	1.09 (0.84 to 1.41)
2021	0.2 (0.2 to 0.3)	0.1 (0.1 to 0.2)	20.7 (15.7 to 27.0)	14.7 (11.2 to 19.1)	1.55 (1.18 to 2.01)	1.10 (0.84 to 1.43)

Embu KEN_35622 Eastern Sub−Saharan Africa Stillbirth Rate / Neonatal Mortality Rate (SBR/NMR) − 20 weeks
lambda = 0.8 ; zeta = 0.7 ; scale = 15 ; amp = 0.028

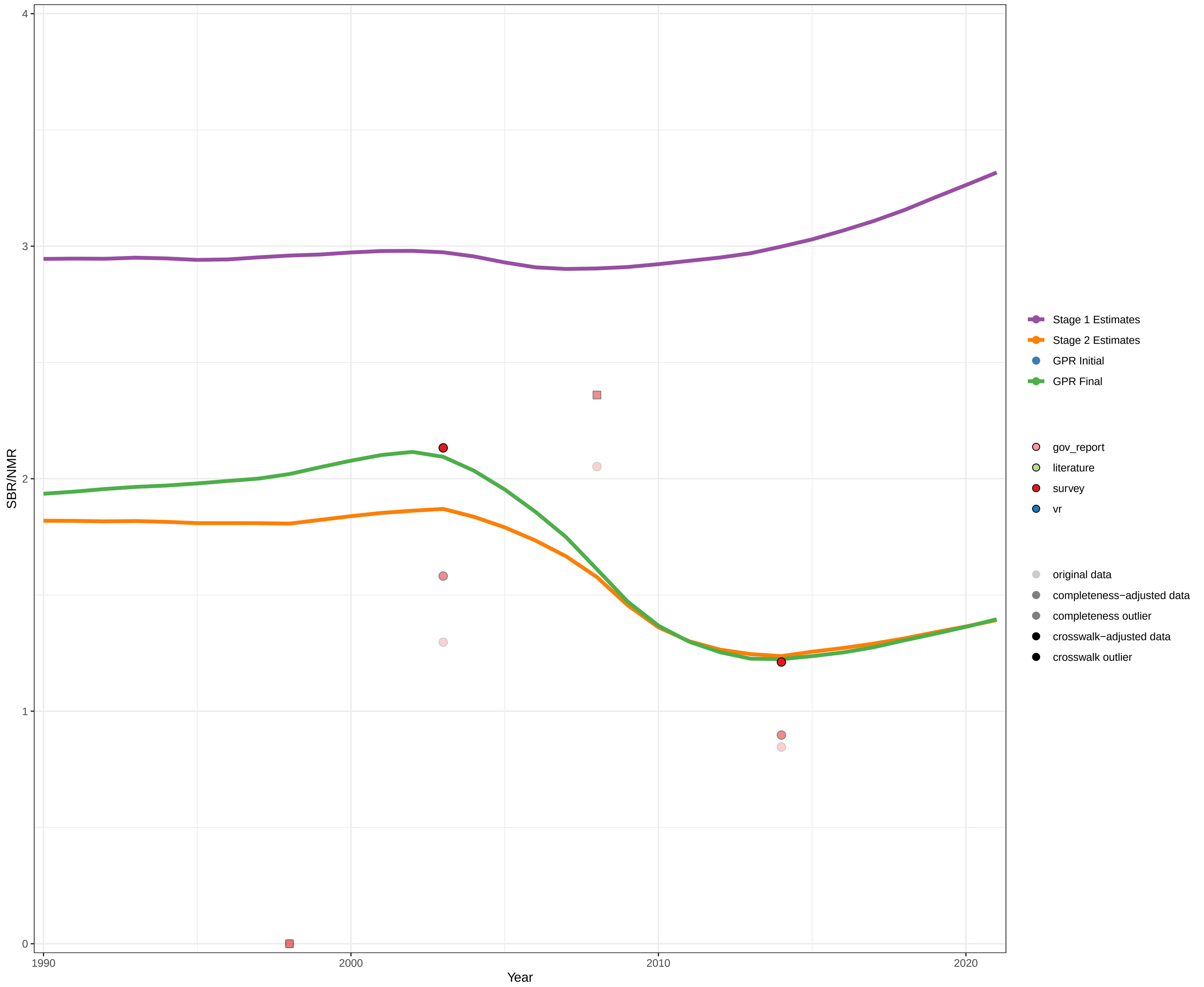

Embu KEN_35622 Eastern Sub−Saharan Africa Stillbirth Rate / Neonatal Mortality Rate (SBR/NMR) − 28 weeks
lambda = 0.8 ; zeta = 0.7 ; scale = 15 ; amp = 0.028

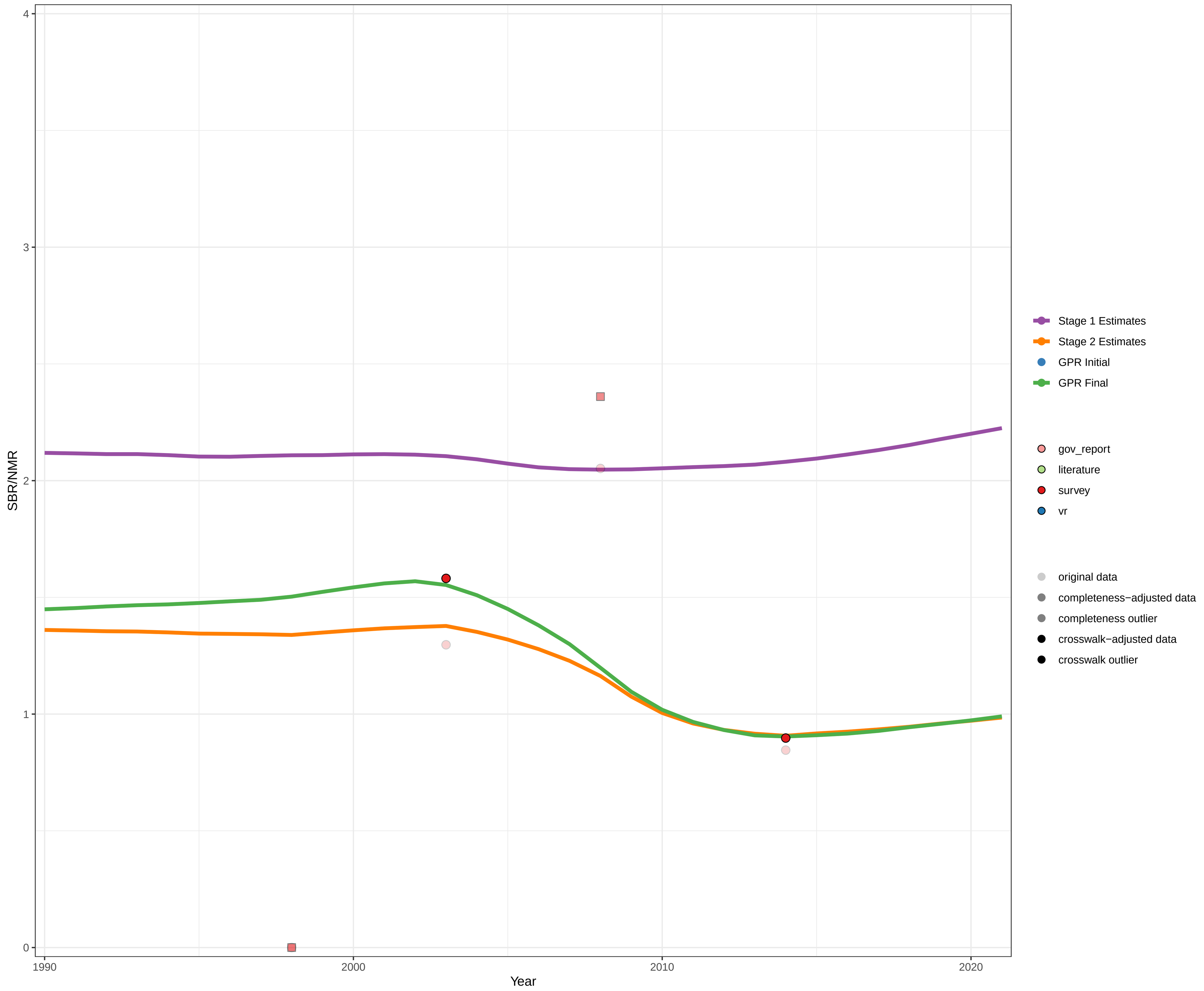

Definition	Years	Outlier	Source.Type	Title
28_weeks	2003	0	Survey	Kenya Demographic and Health Survey 2003
28_weeks	2014	0	Survey	Kenya Demographic and Health Survey 2014
28_weeks	1998	1	Survey	Kenya Demographic and Health Survey 1998
28_weeks	2008	1	Survey	Kenya Demographic and Health Survey 2008−2009

Embu KEN_35622 Eastern Sub−Saharan Africa Stillbirth Rate (SBR) − 20 weeks
lambda = 0.8 ; zeta = 0.7 ; scale = 15 ; amp = 0.028

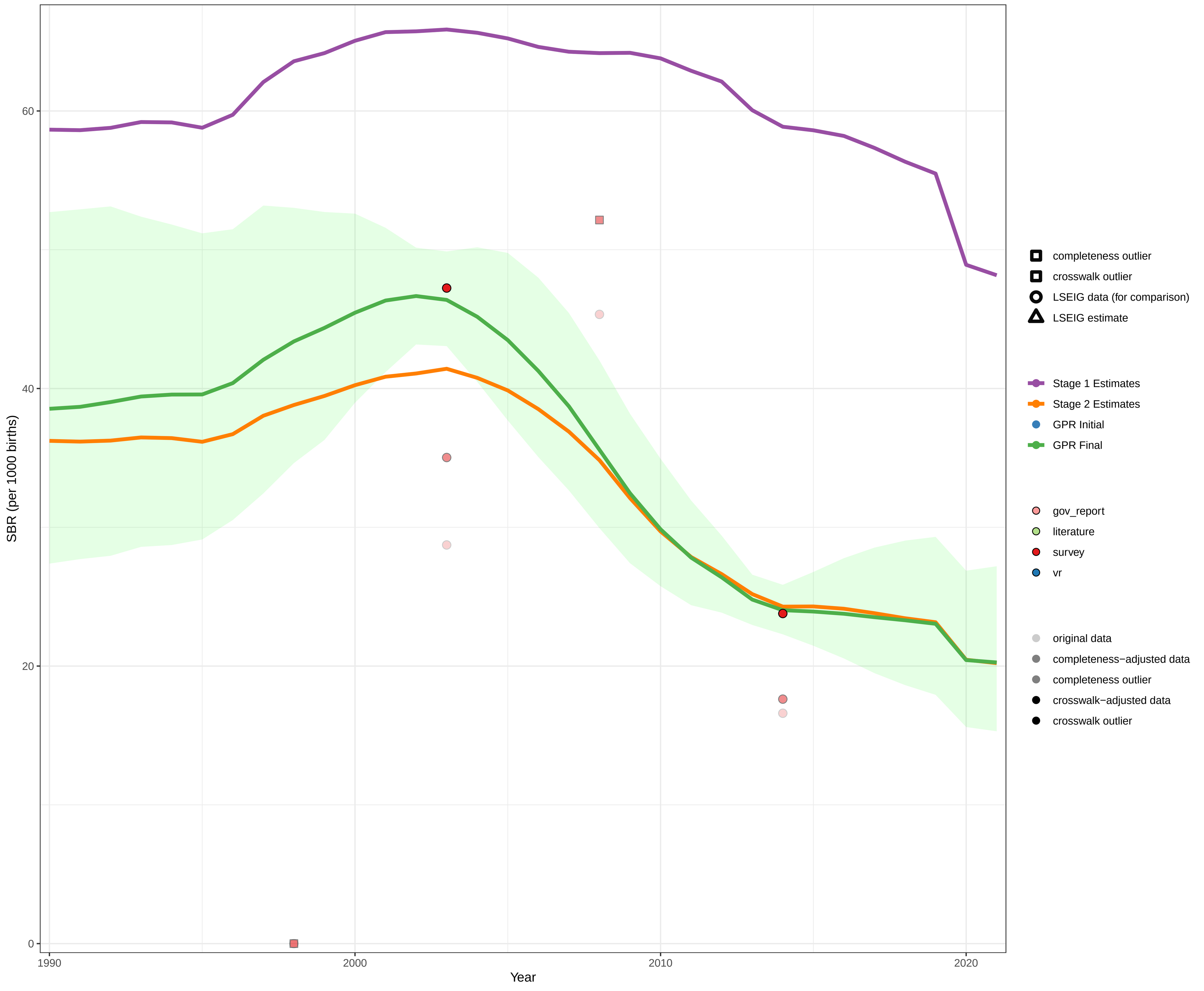

Embu KEN_35622 Eastern Sub−Saharan Africa Stillbirth Rate (SBR) − 28 weeks
lambda = 0.8 ; zeta = 0.7 ; scale = 15 ; amp = 0.028

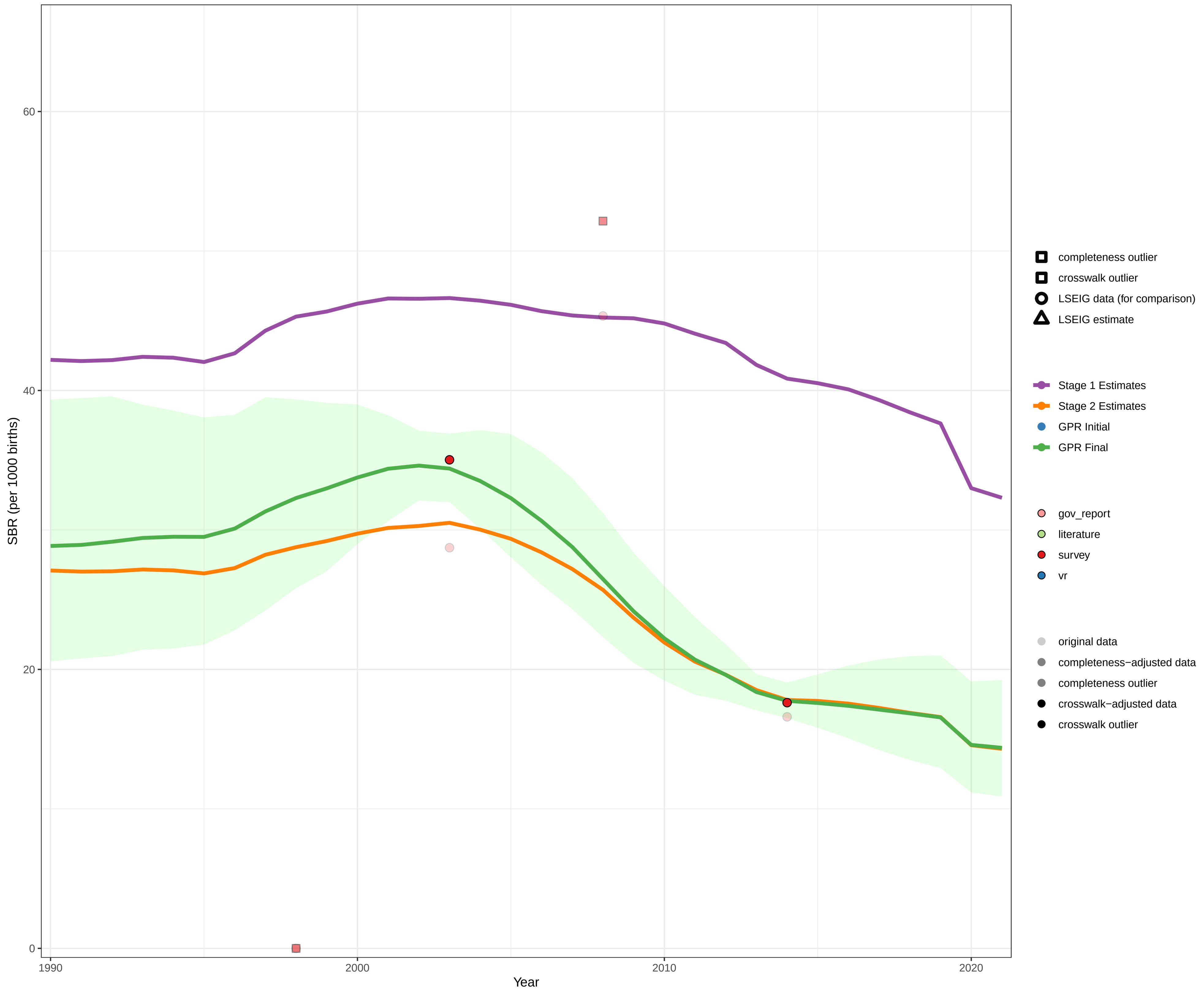

Year	Total Stillbirths (in thousands)	Total Stillbirths (in thousands)	SBR (per 1000 births)	SBR (per 1000 births)	SBR/NMR	SBR/NMR
	20 weeks	28 weeks	20 weeks	28 weeks	20 weeks	28 weeks
1990	0.6 (0.4 to 0.8)	0.5 (0.3 to 0.6)	38.5 (27.4 to 52.7)	28.9 (20.6 to 39.3)	1.94 (1.38 to 2.65)	1.45 (1.03 to 1.98)
1995	0.7 (0.5 to 0.9)	0.5 (0.4 to 0.6)	39.6 (29.1 to 51.2)	29.5 (21.8 to 38.1)	1.98 (1.46 to 2.56)	1.48 (1.09 to 1.90)
2000	0.8 (0.7 to 0.9)	0.6 (0.5 to 0.7)	45.5 (39.0 to 52.6)	33.8 (29.0 to 39.0)	2.08 (1.78 to 2.40)	1.54 (1.32 to 1.78)
2005	0.7 (0.6 to 0.8)	0.5 (0.5 to 0.6)	43.5 (37.7 to 49.8)	32.3 (28.0 to 36.9)	1.95 (1.69 to 2.24)	1.45 (1.26 to 1.66)
2010	0.4 (0.4 to 0.5)	0.3 (0.3 to 0.4)	29.9 (25.8 to 34.9)	22.2 (19.2 to 26.0)	1.37 (1.18 to 1.60)	1.02 (0.88 to 1.19)
2015	0.3 (0.3 to 0.4)	0.2 (0.2 to 0.3)	23.9 (21.5 to 26.8)	17.6 (15.8 to 19.6)	1.24 (1.11 to 1.38)	0.91 (0.82 to 1.02)
2020	0.2 (0.2 to 0.3)	0.2 (0.1 to 0.2)	20.4 (15.6 to 26.9)	14.6 (11.2 to 19.1)	1.36 (1.04 to 1.79)	0.97 (0.75 to 1.28)
2021	0.2 (0.2 to 0.3)	0.2 (0.1 to 0.2)	20.3 (15.3 to 27.2)	14.4 (10.9 to 19.2)	1.40 (1.05 to 1.87)	0.99 (0.75 to 1.33)

Garissa KEN_35623 Eastern Sub−Saharan Africa Stillbirth Rate / Neonatal Mortality Rate (SBR/NMR) − 20 weeks
lambda = 0.8 ; zeta = 0.7 ; scale = 15 ; amp = 0.028

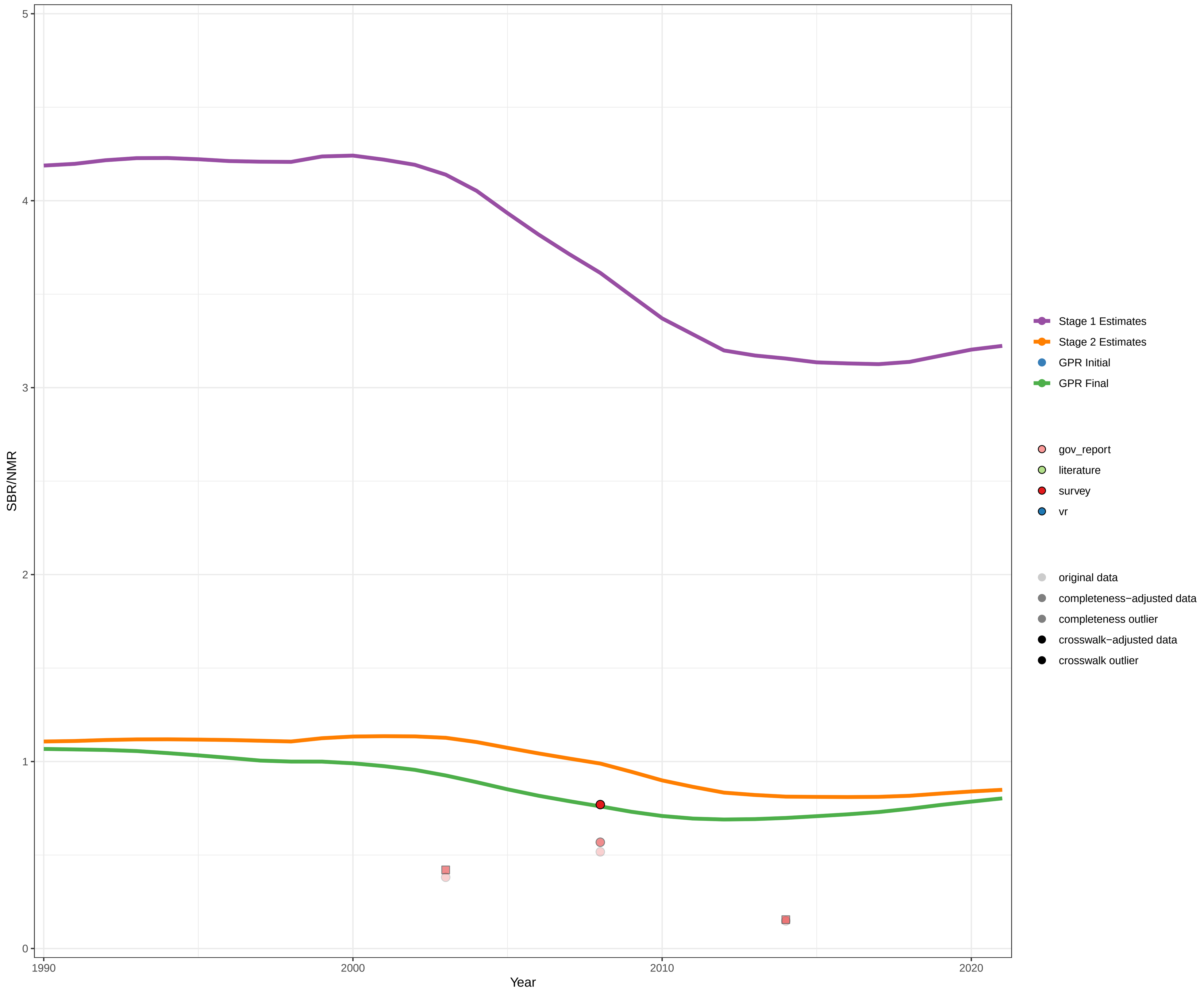

Garissa KEN_35623 Eastern Sub−Saharan Africa Stillbirth Rate / Neonatal Mortality Rate (SBR/NMR) − 28 weeks
lambda = 0.8 ; zeta = 0.7 ; scale = 15 ; amp = 0.028

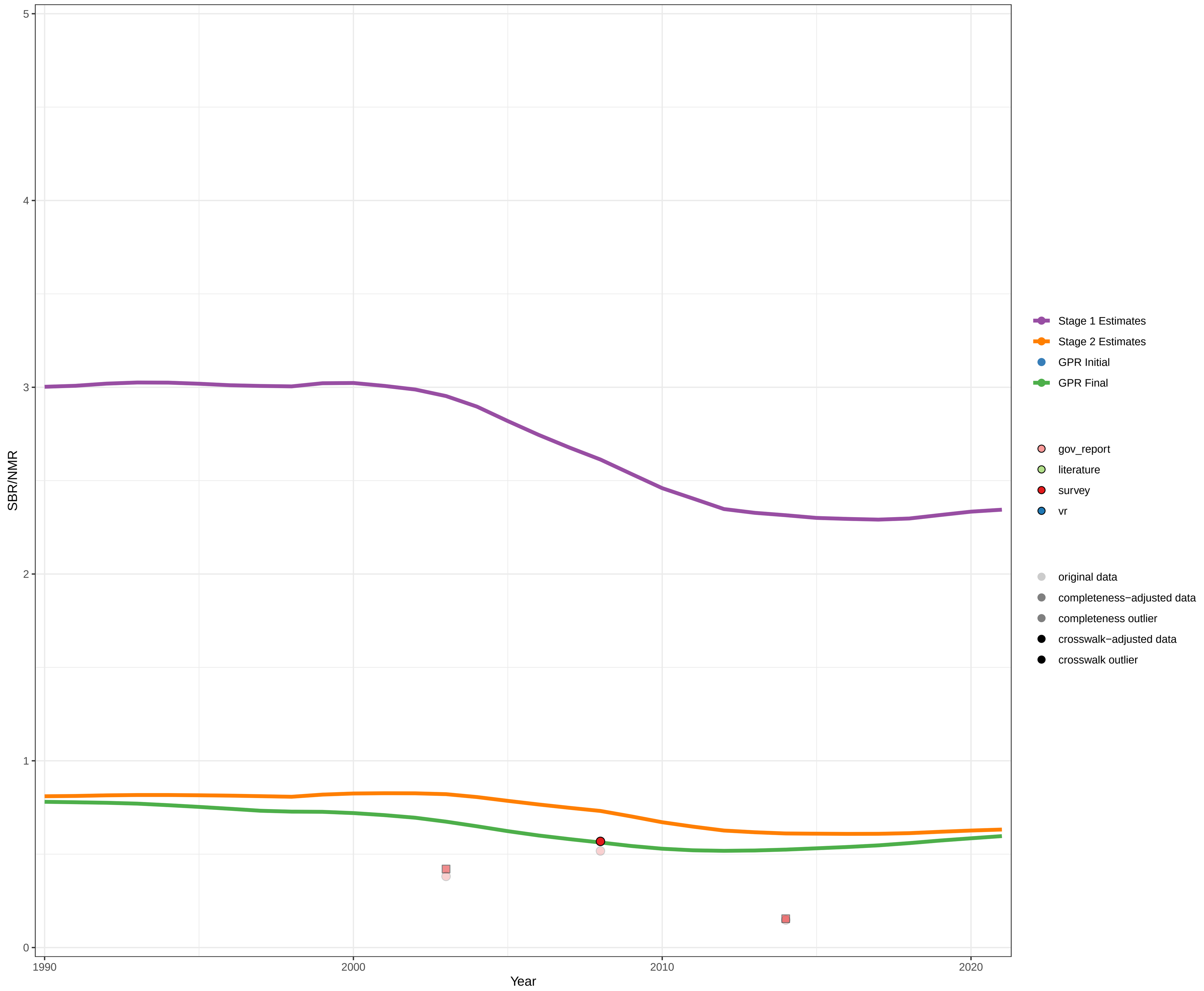

Definition	Years	Outlier	Source.Type	Title
28_weeks	2008	0	Survey	Kenya Demographic and Health Survey 2008−2009
28_weeks	2003	1	Survey	Kenya Demographic and Health Survey 2003
28_weeks	2014	1	Survey	Kenya Demographic and Health Survey 2014

Garissa KEN_35623 Eastern Sub−Saharan Africa Stillbirth Rate (SBR) − 20 weeks
lambda = 0.8 ; zeta = 0.7 ; scale = 15 ; amp = 0.028

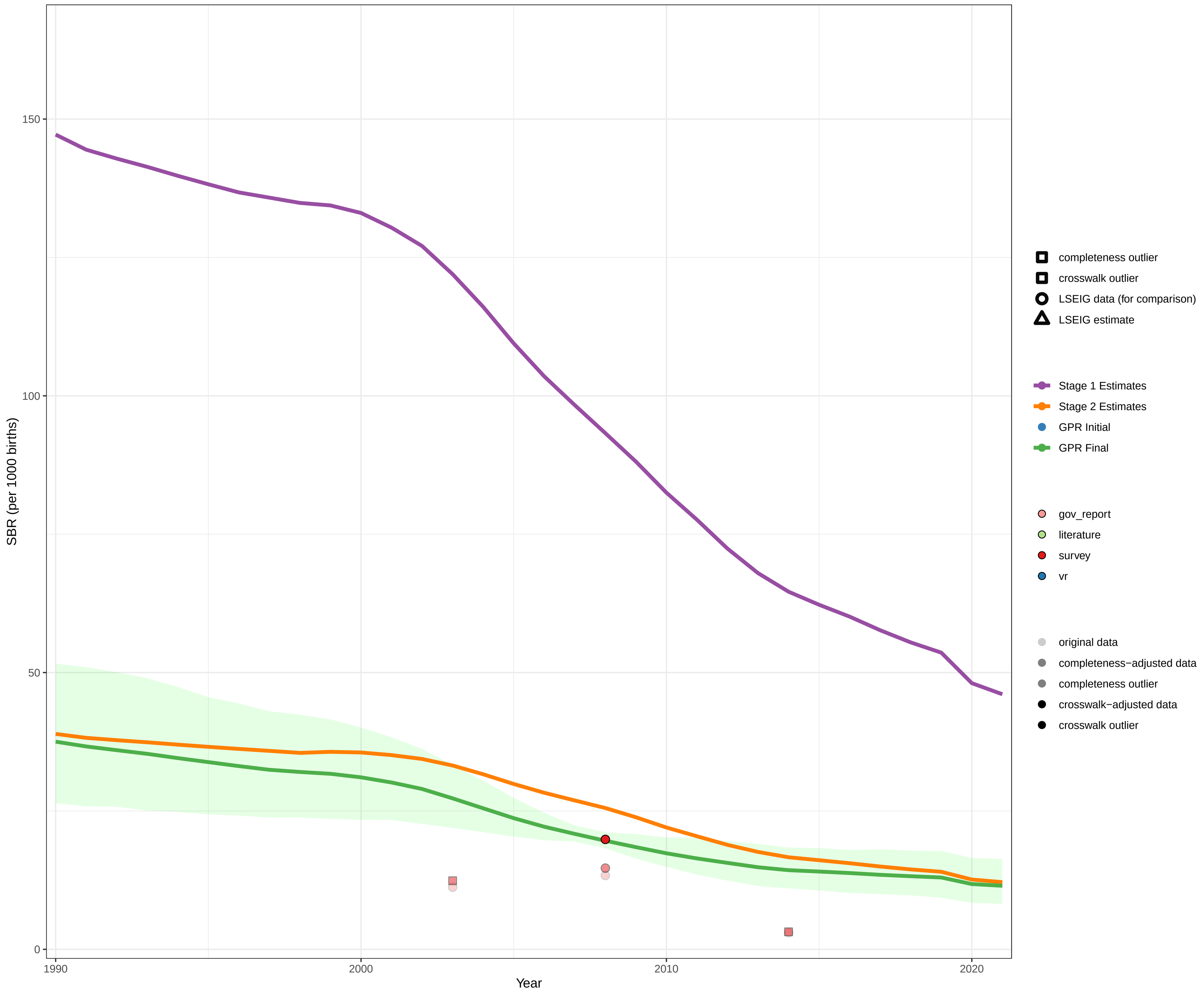

Garissa KEN_35623 Eastern Sub−Saharan Africa Stillbirth Rate (SBR) − 28 weeks
lambda = 0.8 ; zeta = 0.7 ; scale = 15 ; amp = 0.028

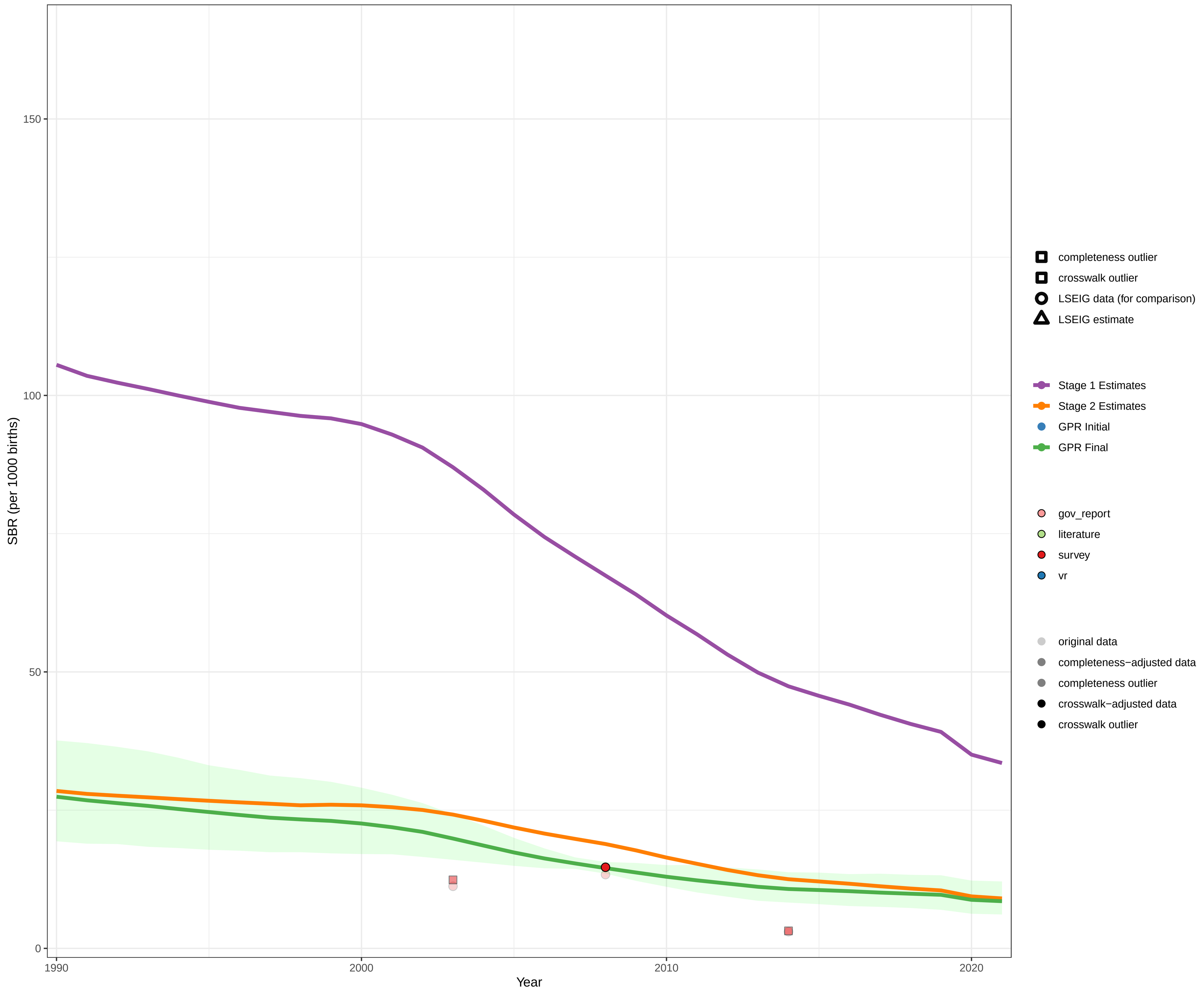

Year	Total Stillbirths (in thousands)	Total Stillbirths (in thousands)	SBR (per 1000 births)	SBR (per 1000 births)	SBR/NMR	SBR/NMR
	20 weeks	28 weeks	20 weeks	28 weeks	20 weeks	28 weeks
1990	0.3 (0.2 to 0.4)	0.2 (0.1 to 0.3)	37.5 (26.4 to 51.6)	27.4 (19.4 to 37.6)	1.07 (0.75 to 1.47)	0.78 (0.55 to 1.07)
1995	0.3 (0.2 to 0.5)	0.2 (0.2 to 0.3)	33.8 (24.4 to 45.6)	24.7 (17.8 to 33.1)	1.03 (0.74 to 1.39)	0.75 (0.54 to 1.01)
2000	0.5 (0.4 to 0.6)	0.4 (0.3 to 0.5)	31.1 (23.4 to 40.1)	22.6 (17.1 to 29.1)	0.99 (0.75 to 1.28)	0.72 (0.54 to 0.93)
2005	0.6 (0.5 to 0.7)	0.4 (0.4 to 0.5)	23.7 (20.4 to 27.4)	17.3 (14.9 to 20.0)	0.85 (0.73 to 0.98)	0.62 (0.54 to 0.72)
2010	0.5 (0.4 to 0.6)	0.4 (0.3 to 0.5)	17.4 (14.9 to 20.2)	13.0 (11.1 to 15.1)	0.71 (0.61 to 0.83)	0.53 (0.45 to 0.62)
2015	0.5 (0.4 to 0.6)	0.4 (0.3 to 0.5)	14.1 (10.6 to 18.3)	10.6 (8.0 to 13.7)	0.71 (0.53 to 0.92)	0.53 (0.40 to 0.69)
2020	0.4 (0.3 to 0.6)	0.3 (0.2 to 0.5)	11.8 (8.4 to 16.5)	8.8 (6.3 to 12.3)	0.79 (0.56 to 1.10)	0.58 (0.42 to 0.82)
2021	0.4 (0.3 to 0.6)	0.3 (0.2 to 0.5)	11.5 (8.2 to 16.3)	8.5 (6.1 to 12.1)	0.80 (0.58 to 1.14)	0.60 (0.43 to 0.85)

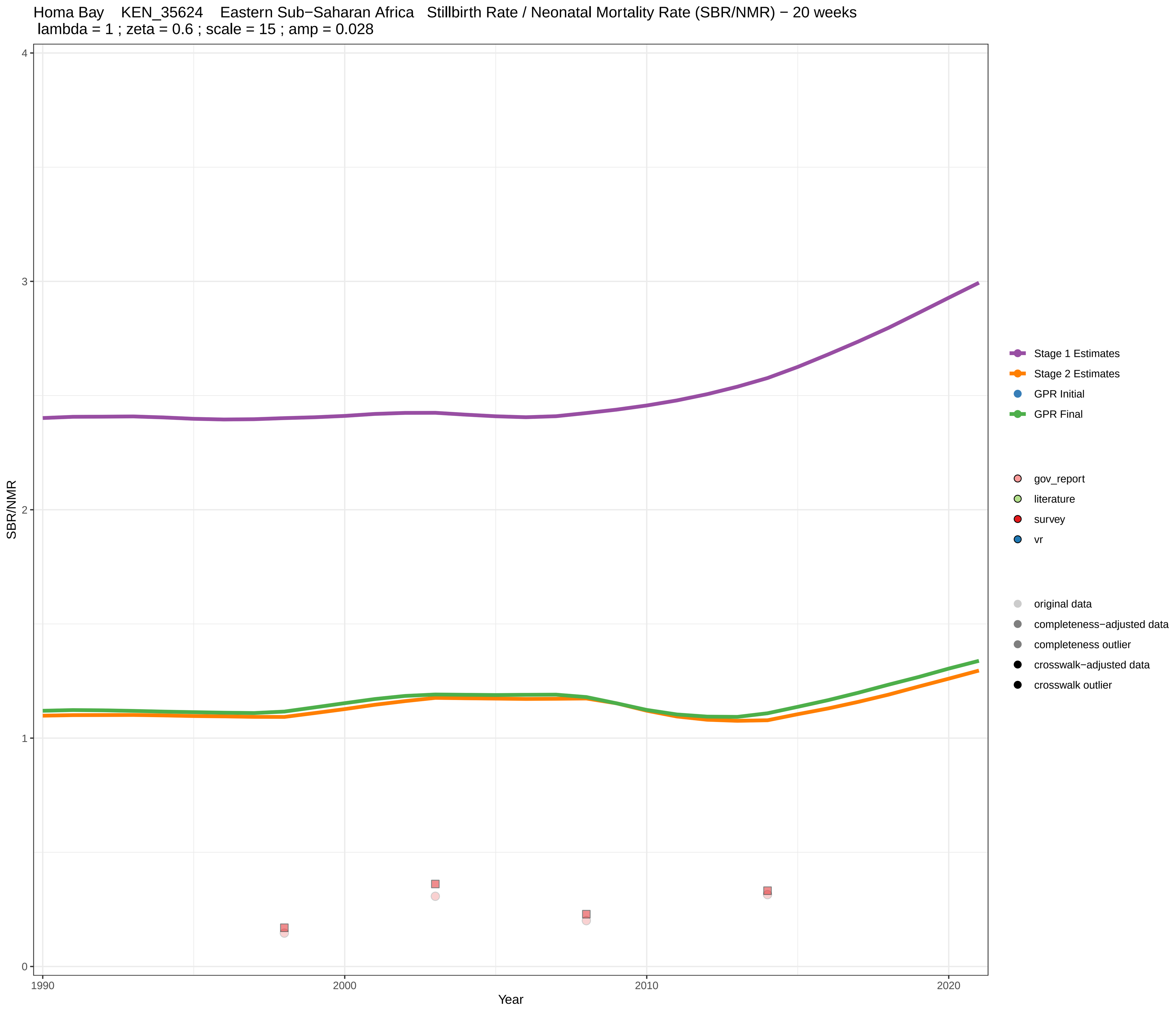

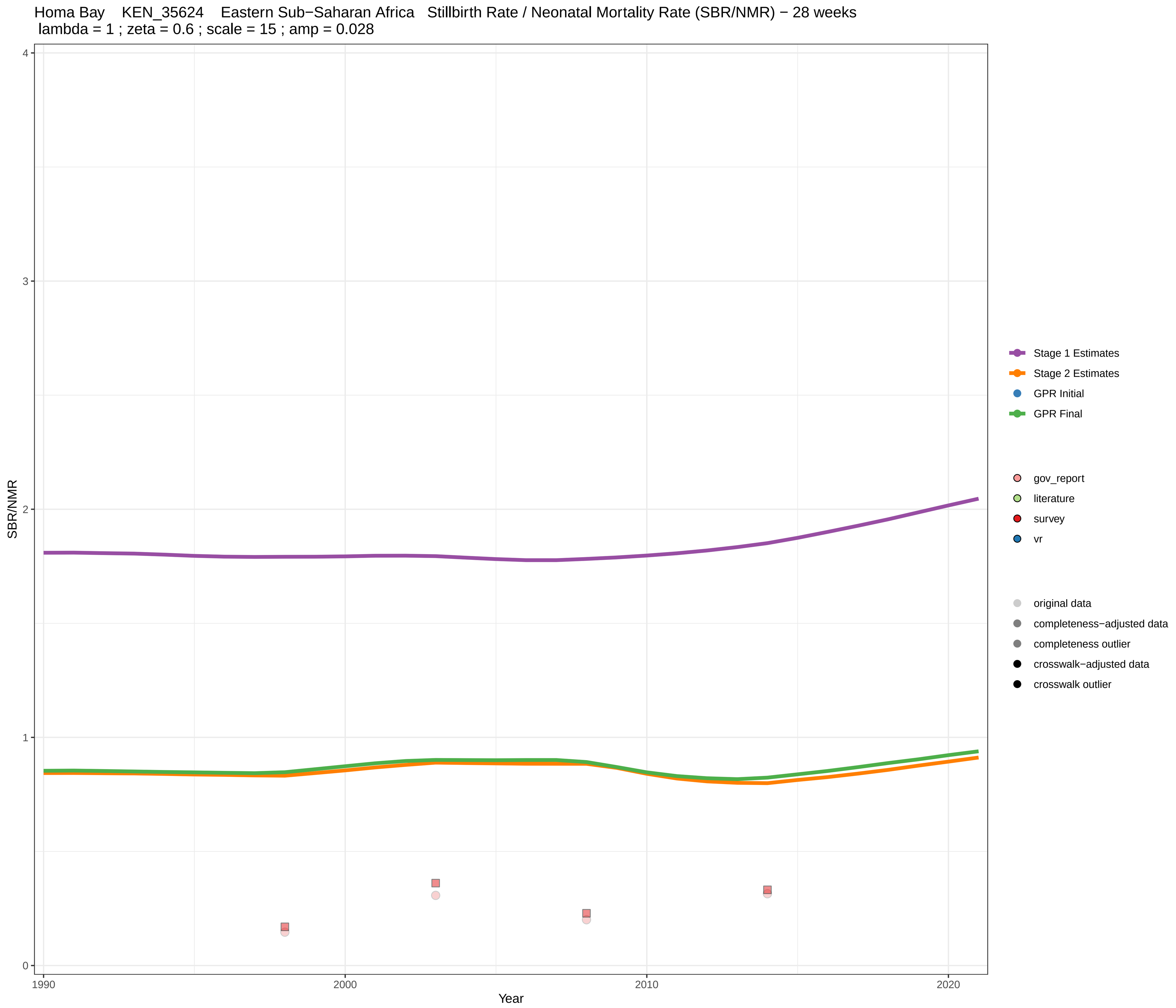

Definition	Years	Outlier	Source.Type	Title
28_weeks	1998	1	Survey	Kenya Demographic and Health Survey 1998
28_weeks	2003	1	Survey	Kenya Demographic and Health Survey 2003
28_weeks	2008	1	Survey	Kenya Demographic and Health Survey 2008−2009
28_weeks	2014	1	Survey	Kenya Demographic and Health Survey 2014

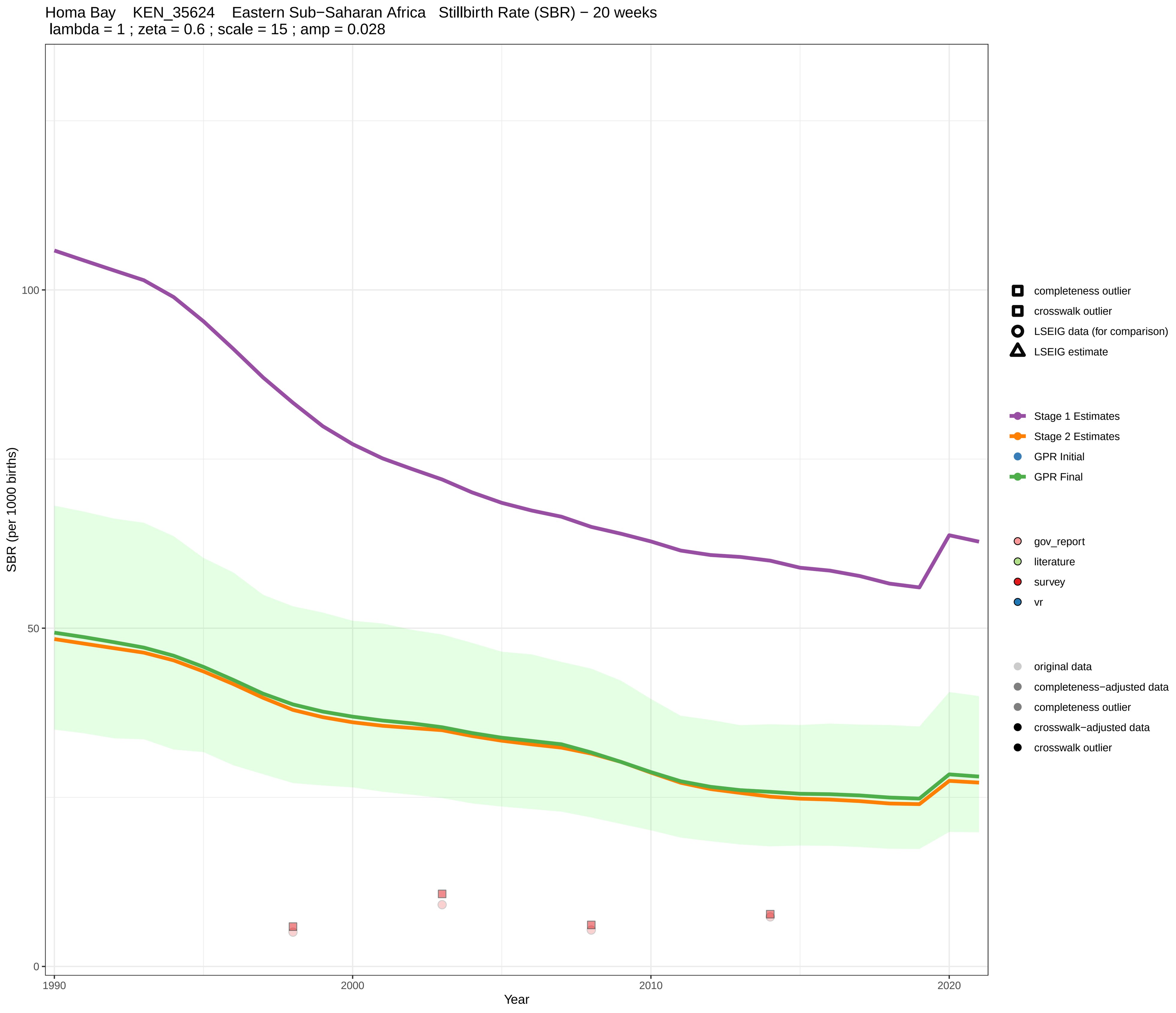

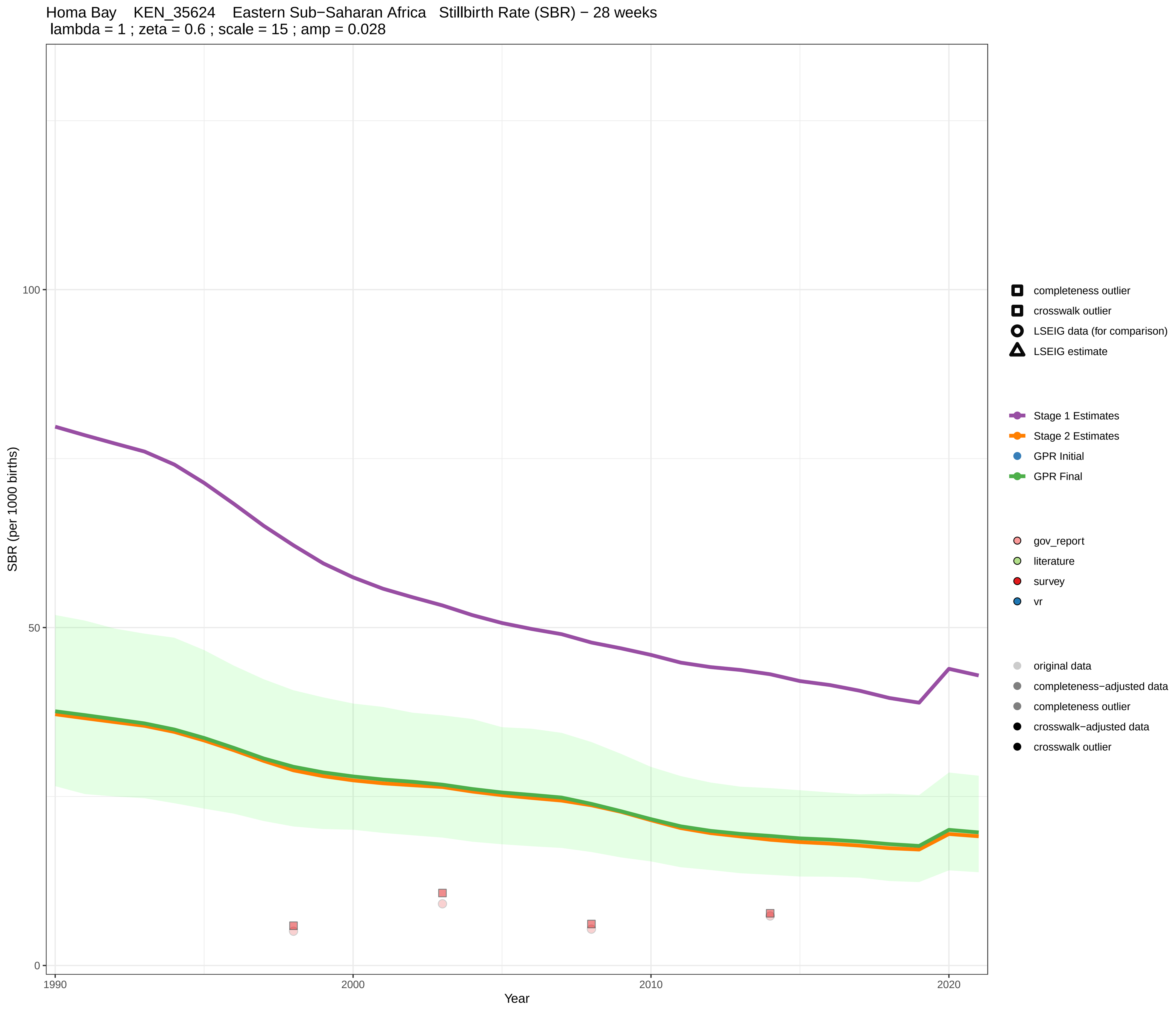

Year	Total Stillbirths (in thousands)	Total Stillbirths (in thousands)	SBR (per 1000 births)	SBR (per 1000 births)	SBR/NMR	SBR/NMR
	20 weeks	28 weeks	20 weeks	28 weeks	20 weeks	28 weeks
1990	1.8 (1.2 to 2.5)	1.3 (0.9 to 1.8)	49.3 (35.0 to 68.1)	37.6 (26.5 to 51.9)	1.12 (0.79 to 1.55)	0.85 (0.60 to 1.18)
1995	1.7 (1.2 to 2.3)	1.3 (0.9 to 1.8)	44.3 (31.7 to 60.4)	33.7 (23.2 to 46.7)	1.11 (0.80 to 1.52)	0.85 (0.58 to 1.17)
2000	1.5 (1.1 to 2.1)	1.1 (0.8 to 1.6)	36.9 (26.5 to 51.1)	28.0 (20.1 to 38.8)	1.15 (0.83 to 1.60)	0.87 (0.63 to 1.21)
2005	1.4 (1.0 to 2.0)	1.1 (0.7 to 1.5)	33.8 (23.6 to 46.5)	25.6 (17.9 to 35.3)	1.19 (0.83 to 1.64)	0.90 (0.63 to 1.24)
2010	1.2 (0.8 to 1.6)	0.9 (0.6 to 1.2)	28.7 (20.1 to 39.5)	21.7 (15.4 to 29.4)	1.12 (0.79 to 1.55)	0.85 (0.60 to 1.15)
2015	1.0 (0.7 to 1.4)	0.7 (0.5 to 1.0)	25.5 (17.9 to 35.7)	18.8 (13.2 to 25.9)	1.14 (0.80 to 1.59)	0.84 (0.59 to 1.16)
2020	0.9 (0.7 to 1.4)	0.7 (0.5 to 0.9)	28.4 (19.9 to 40.6)	20.1 (14.1 to 28.6)	1.30 (0.91 to 1.86)	0.92 (0.65 to 1.31)
2021	0.9 (0.6 to 1.3)	0.6 (0.4 to 0.9)	28.1 (19.8 to 40.0)	19.7 (13.8 to 28.1)	1.34 (0.95 to 1.91)	0.94 (0.66 to 1.34)

Isiolo KEN_35625 Eastern Sub−Saharan Africa Stillbirth Rate / Neonatal Mortality Rate (SBR/NMR) − 20 weeks
lambda = 0.8 ; zeta = 0.7 ; scale = 15 ; amp = 0.028

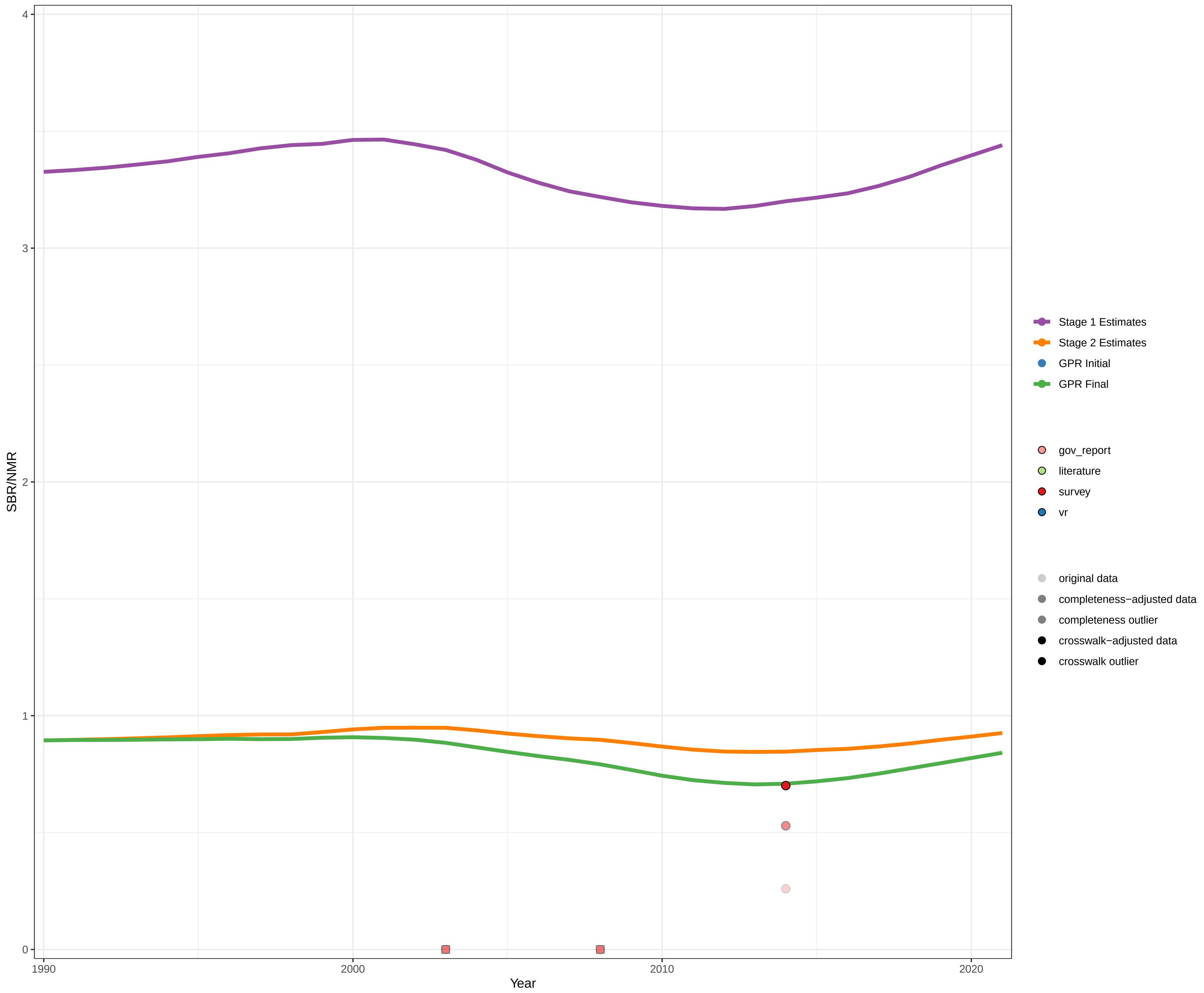

Isiolo KEN_35625 Eastern Sub−Saharan Africa Stillbirth Rate / Neonatal Mortality Rate (SBR/NMR) − 28 weeks
lambda = 0.8 ; zeta = 0.7 ; scale = 15 ; amp = 0.028

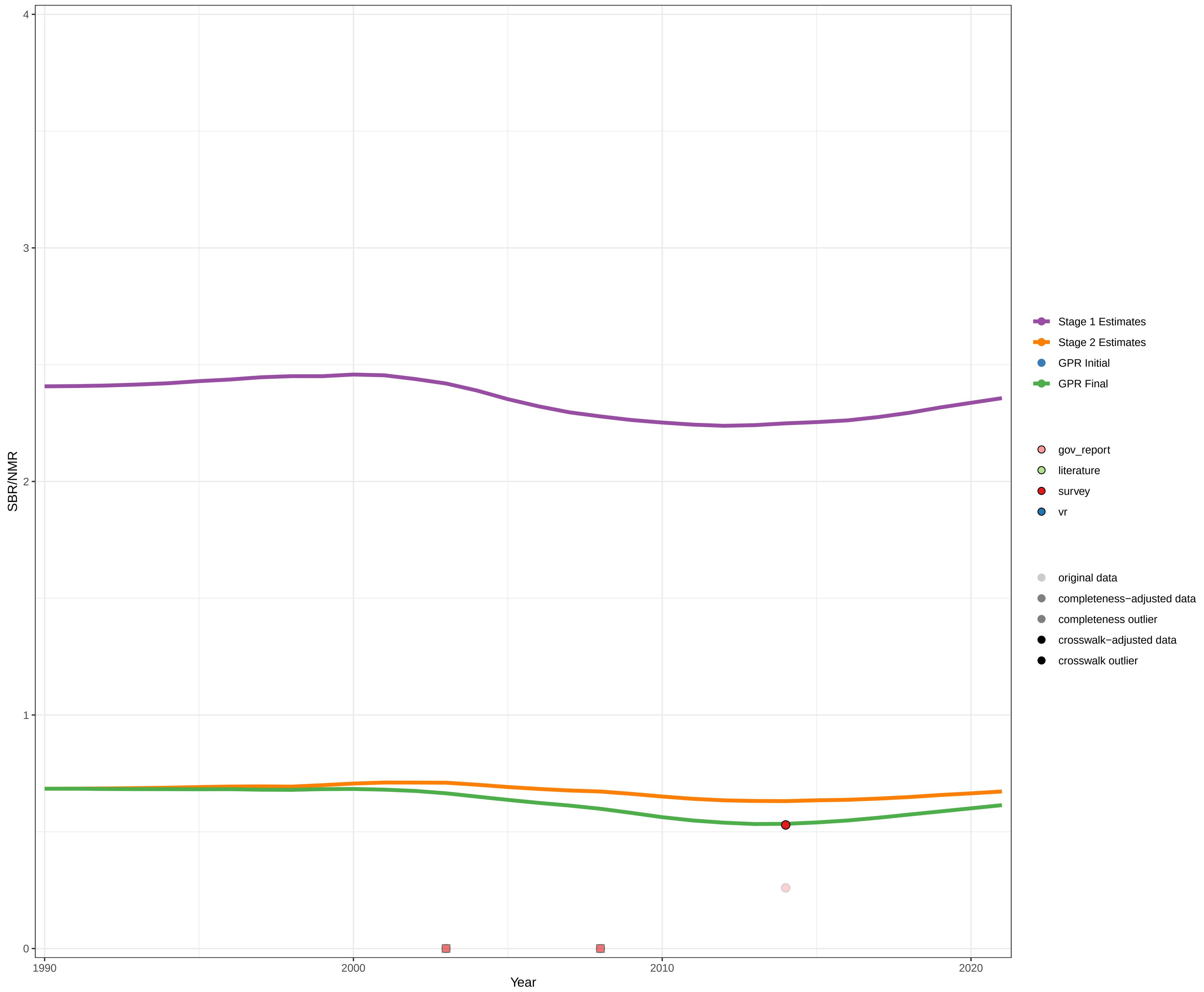

Definition	Years	Outlier	Source.Type	Title
28_weeks	2014	0	Survey	Kenya Demographic and Health Survey 2014
28_weeks	2003	1	Survey	Kenya Demographic and Health Survey 2003
28_weeks	2008	1	Survey	Kenya Demographic and Health Survey 2008−2009

Isiolo KEN_35625 Eastern Sub−Saharan Africa Stillbirth Rate (SBR) − 20 weeks
lambda = 0.8 ; zeta = 0.7 ; scale = 15 ; amp = 0.028

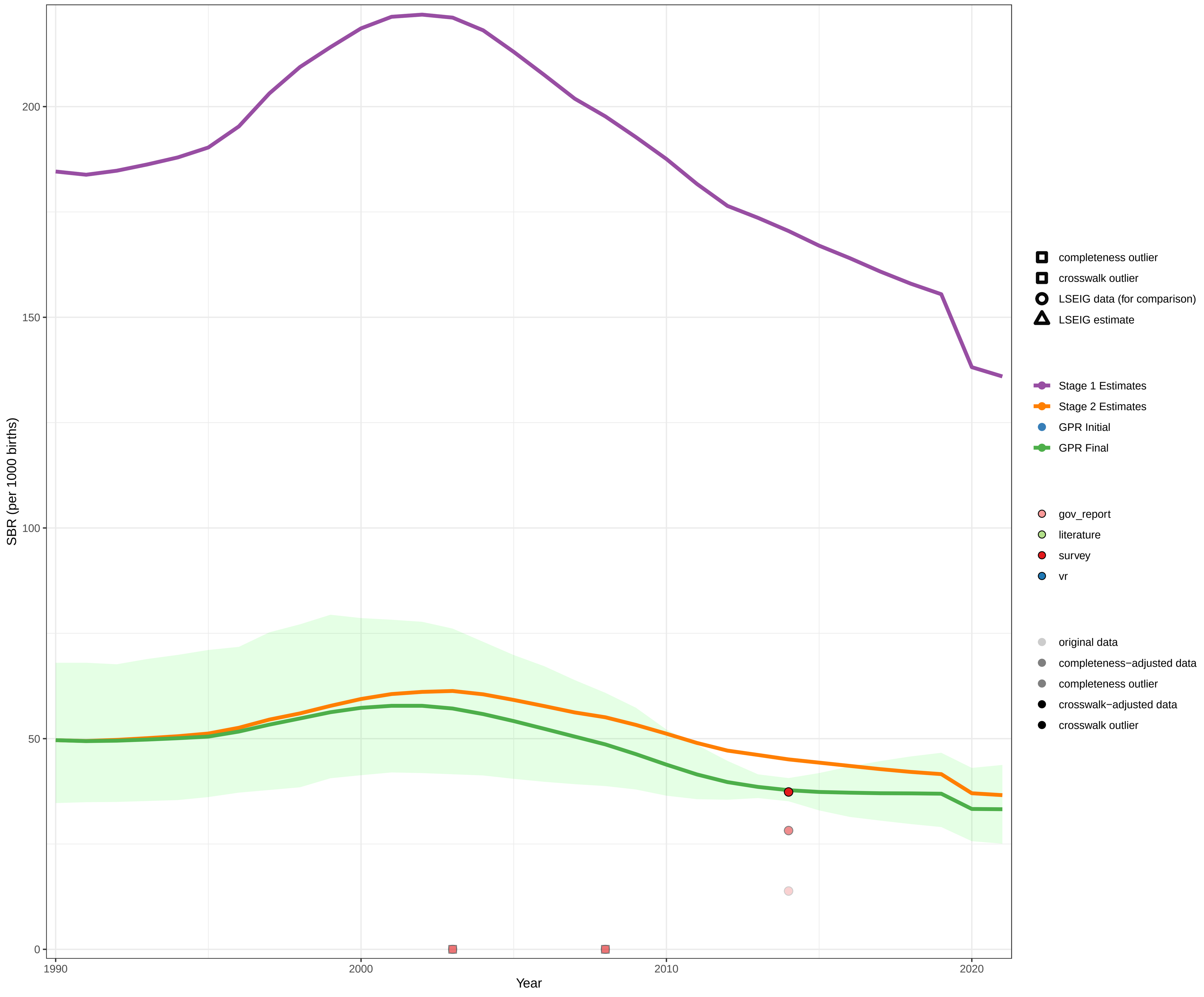

Isiolo KEN_35625 Eastern Sub−Saharan Africa Stillbirth Rate (SBR) − 28 weeks
lambda = 0.8 ; zeta = 0.7 ; scale = 15 ; amp = 0.028

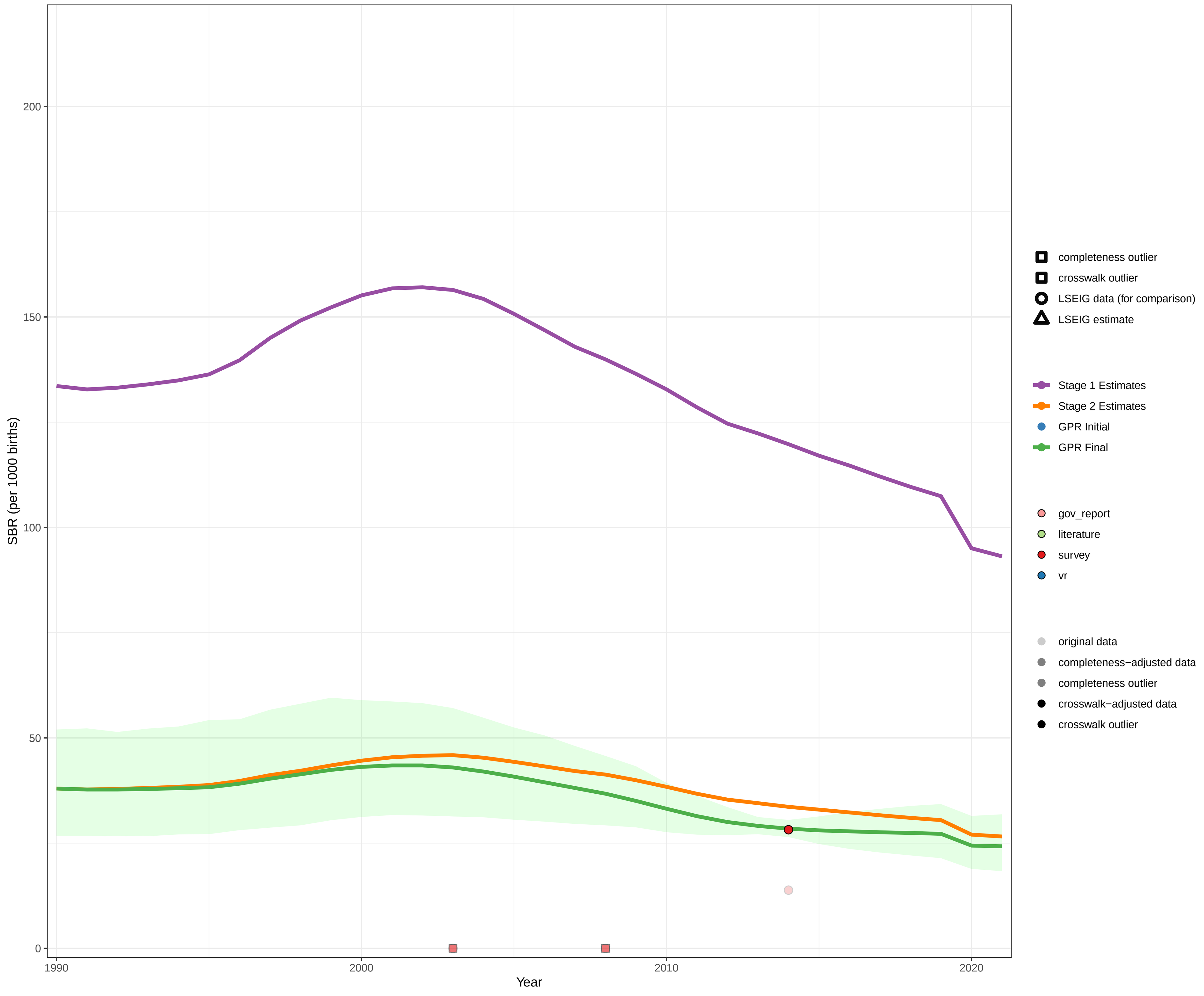

Year	Total Stillbirths (in thousands)	Total Stillbirths (in thousands)	SBR (per 1000 births)	SBR (per 1000 births)	SBR/NMR	SBR/NMR
	20 weeks	28 weeks	20 weeks	28 weeks	20 weeks	28 weeks
1990	0.2 (0.1 to 0.3)	0.1 (0.1 to 0.2)	49.6 (34.7 to 68.0)	38.0 (26.7 to 52.0)	0.89 (0.63 to 1.23)	0.68 (0.48 to 0.94)
1995	0.2 (0.2 to 0.3)	0.2 (0.1 to 0.2)	50.5 (36.1 to 71.0)	38.3 (27.1 to 54.2)	0.90 (0.64 to 1.27)	0.68 (0.48 to 0.97)
2000	0.3 (0.2 to 0.4)	0.2 (0.1 to 0.3)	57.3 (41.3 to 78.6)	43.1 (31.2 to 59.0)	0.91 (0.65 to 1.25)	0.68 (0.49 to 0.93)
2005	0.3 (0.2 to 0.4)	0.2 (0.2 to 0.3)	54.2 (40.5 to 69.8)	40.8 (30.6 to 52.5)	0.85 (0.63 to 1.09)	0.64 (0.48 to 0.82)
2010	0.2 (0.2 to 0.3)	0.2 (0.2 to 0.2)	43.8 (36.5 to 52.2)	33.2 (27.6 to 39.3)	0.74 (0.62 to 0.88)	0.56 (0.47 to 0.67)
2015	0.3 (0.2 to 0.3)	0.2 (0.2 to 0.2)	37.3 (33.0 to 41.8)	28.0 (24.8 to 31.3)	0.72 (0.63 to 0.81)	0.54 (0.48 to 0.60)
2020	0.3 (0.2 to 0.3)	0.2 (0.1 to 0.2)	33.3 (25.7 to 43.1)	24.4 (18.9 to 31.5)	0.82 (0.63 to 1.06)	0.60 (0.46 to 0.77)
2021	0.3 (0.2 to 0.3)	0.2 (0.1 to 0.2)	33.3 (25.1 to 43.8)	24.3 (18.3 to 31.9)	0.84 (0.63 to 1.11)	0.61 (0.46 to 0.81)

Kajiado KEN_35626 Eastern Sub−Saharan Africa Stillbirth Rate / Neonatal Mortality Rate (SBR/NMR) − 20 weeks
lambda = 0.8 ; zeta = 0.7 ; scale = 15 ; amp = 0.028

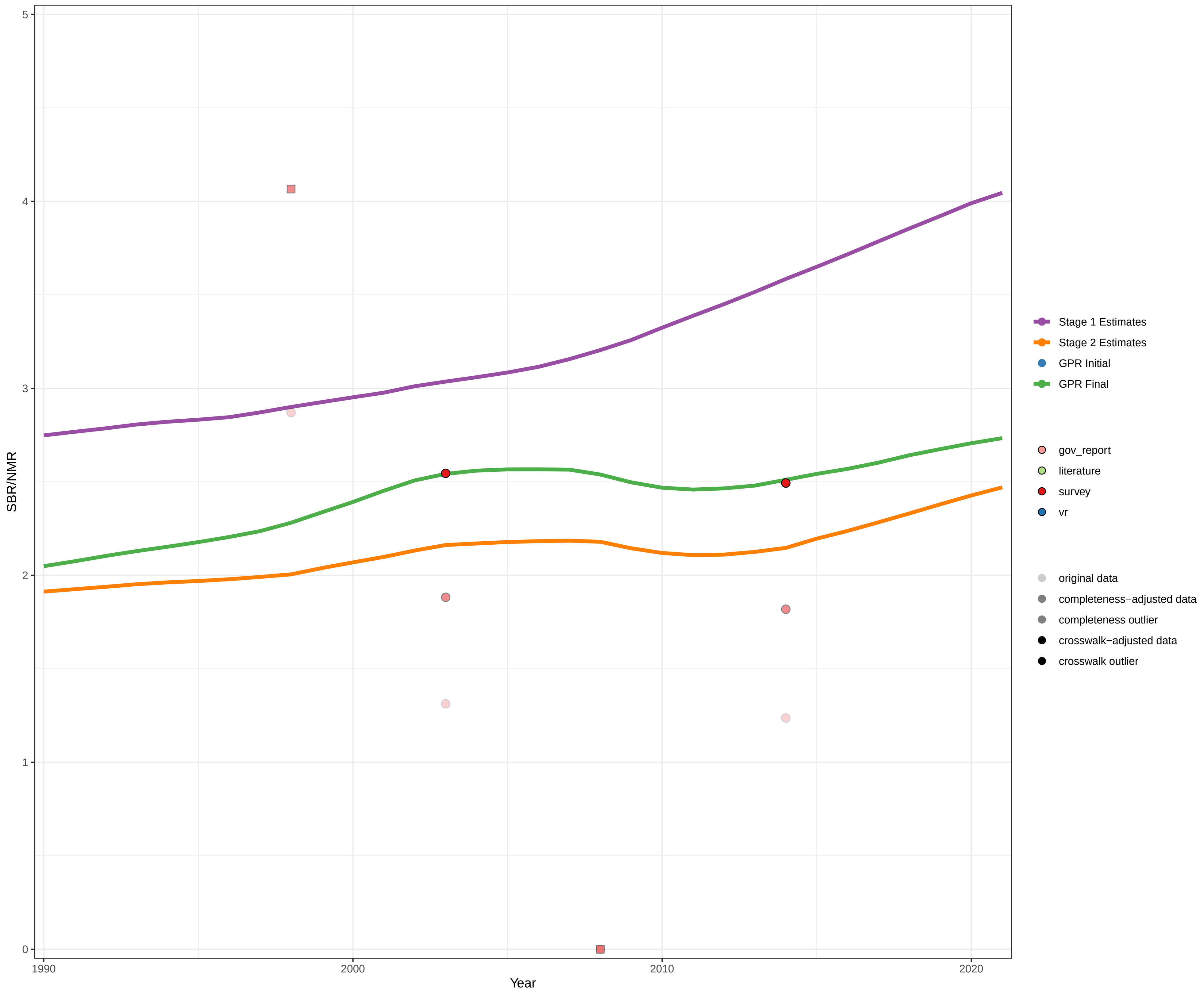

Kajiado KEN_35626 Eastern Sub−Saharan Africa Stillbirth Rate / Neonatal Mortality Rate (SBR/NMR) − 28 weeks
lambda = 0.8 ; zeta = 0.7 ; scale = 15 ; amp = 0.028

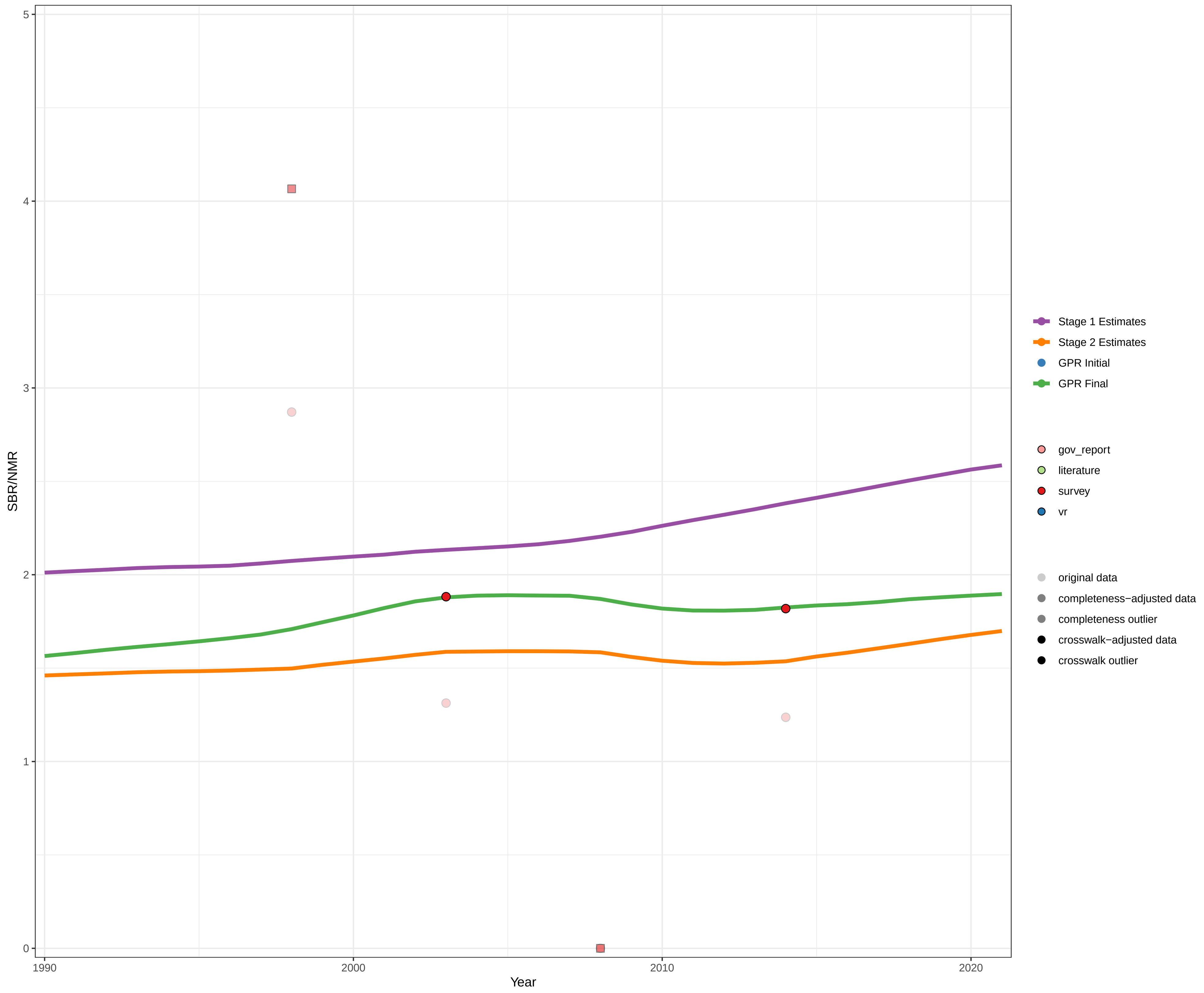

Definition	Years	Outlier	Source.Type	Title
28_weeks	2003	0	Survey	Kenya Demographic and Health Survey 2003
28_weeks	2014	0	Survey	Kenya Demographic and Health Survey 2014
28_weeks	1998	1	Survey	Kenya Demographic and Health Survey 1998
28_weeks	2008	1	Survey	Kenya Demographic and Health Survey 2008−2009

Kajiado KEN_35626 Eastern Sub−Saharan Africa Stillbirth Rate (SBR) − 20 weeks
lambda = 0.8 ; zeta = 0.7 ; scale = 15 ; amp = 0.028

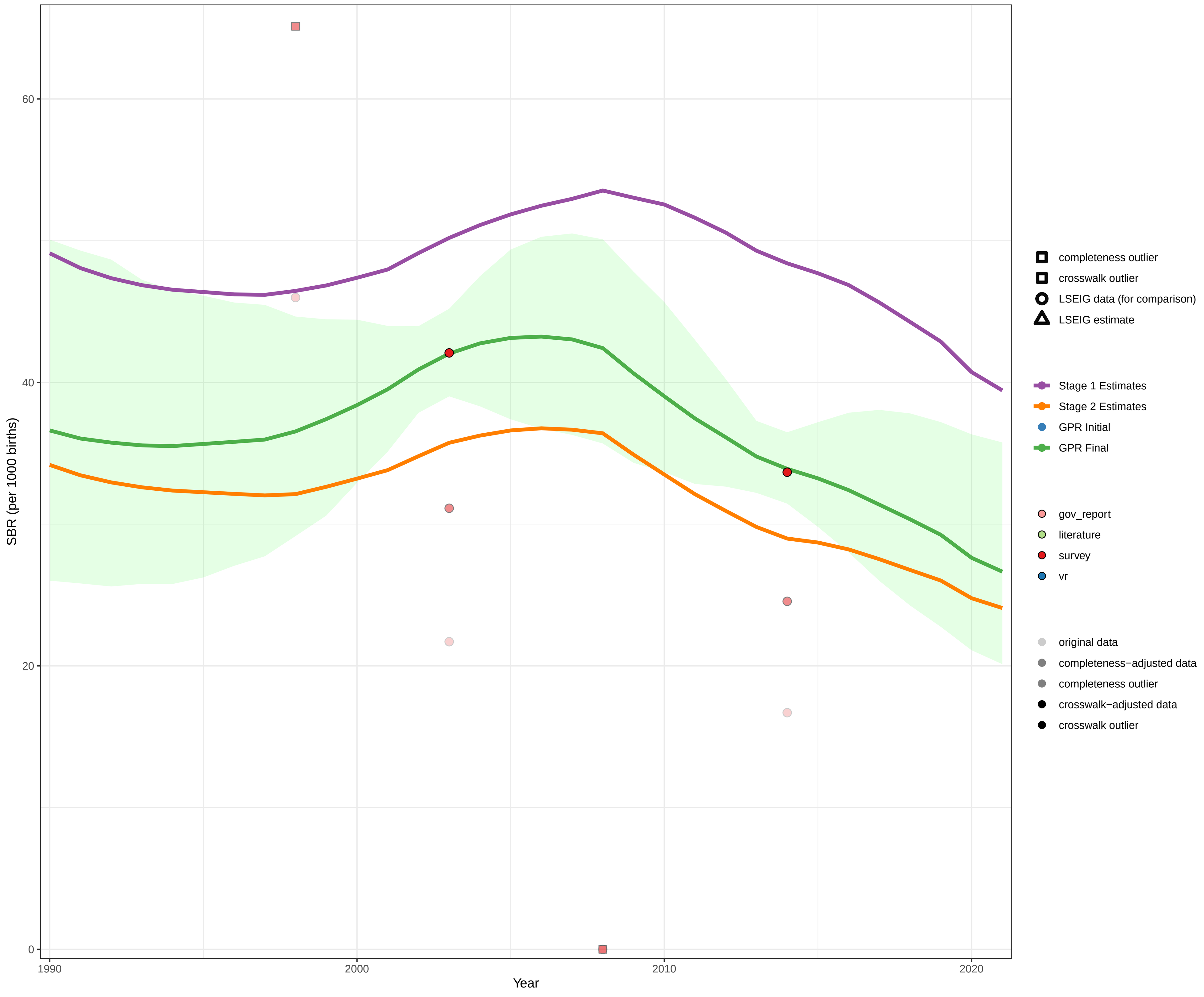

Kajiado KEN_35626 Eastern Sub−Saharan Africa Stillbirth Rate (SBR) − 28 weeks
lambda = 0.8 ; zeta = 0.7 ; scale = 15 ; amp = 0.028

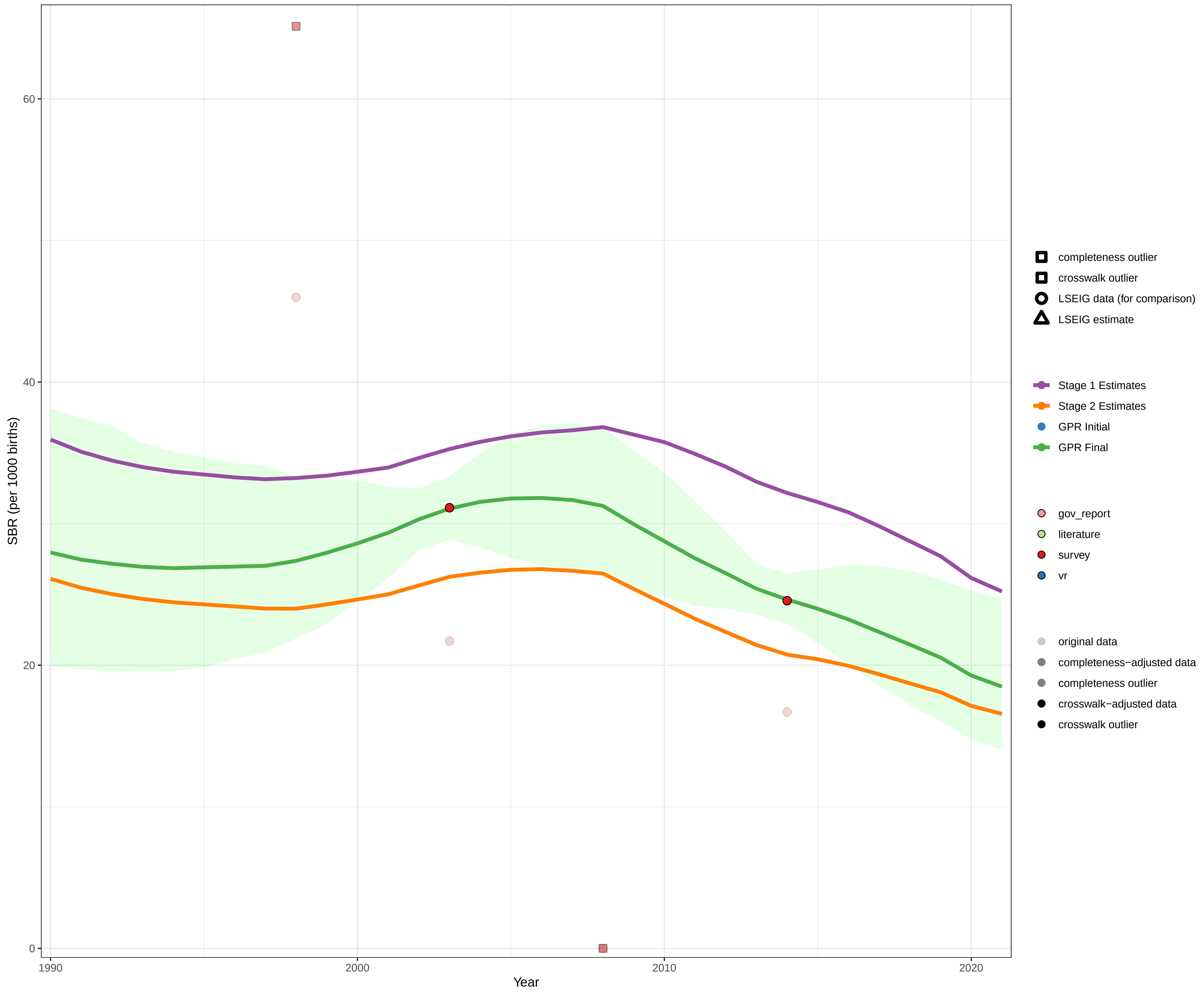

Year	Total Stillbirths (in thousands)	Total Stillbirths (in thousands)	SBR (per 1000 births)	SBR (per 1000 births)	SBR/NMR	SBR/NMR
	20 weeks	28 weeks	20 weeks	28 weeks	20 weeks	28 weeks
1990	0.5 (0.4 to 0.7)	0.4 (0.3 to 0.5)	36.6 (26.0 to 50.1)	28.0 (19.9 to 38.1)	2.05 (1.46 to 2.80)	1.56 (1.12 to 2.13)
1995	0.6 (0.5 to 0.8)	0.5 (0.3 to 0.6)	35.7 (26.2 to 46.1)	26.9 (19.9 to 34.7)	2.18 (1.60 to 2.82)	1.64 (1.21 to 2.12)
2000	0.9 (0.7 to 1.0)	0.6 (0.5 to 0.7)	38.4 (32.9 to 44.4)	28.6 (24.6 to 33.0)	2.39 (2.05 to 2.77)	1.78 (1.53 to 2.06)
2005	1.3 (1.1 to 1.4)	0.9 (0.8 to 1.1)	43.1 (37.4 to 49.4)	31.8 (27.6 to 36.3)	2.57 (2.22 to 2.94)	1.89 (1.64 to 2.16)
2010	1.4 (1.2 to 1.7)	1.0 (0.9 to 1.2)	39.0 (33.7 to 45.7)	28.8 (24.8 to 33.6)	2.47 (2.13 to 2.89)	1.82 (1.57 to 2.13)
2015	1.4 (1.3 to 1.6)	1.0 (0.9 to 1.1)	33.2 (29.8 to 37.2)	24.0 (21.5 to 26.8)	2.54 (2.28 to 2.85)	1.84 (1.65 to 2.05)
2020	1.3 (1.0 to 1.7)	0.9 (0.7 to 1.2)	27.6 (21.1 to 36.3)	19.3 (14.8 to 25.3)	2.71 (2.07 to 3.56)	1.89 (1.45 to 2.48)
2021	1.3 (1.0 to 1.7)	0.9 (0.7 to 1.2)	26.6 (20.1 to 35.8)	18.5 (14.0 to 24.7)	2.73 (2.06 to 3.67)	1.90 (1.44 to 2.54)

Kakamega KEN_35627 Eastern Sub−Saharan Africa Stillbirth Rate / Neonatal Mortality Rate (SBR/NMR) − 20 weeks
lambda = 0.8 ; zeta = 0.7 ; scale = 15 ; amp = 0.028

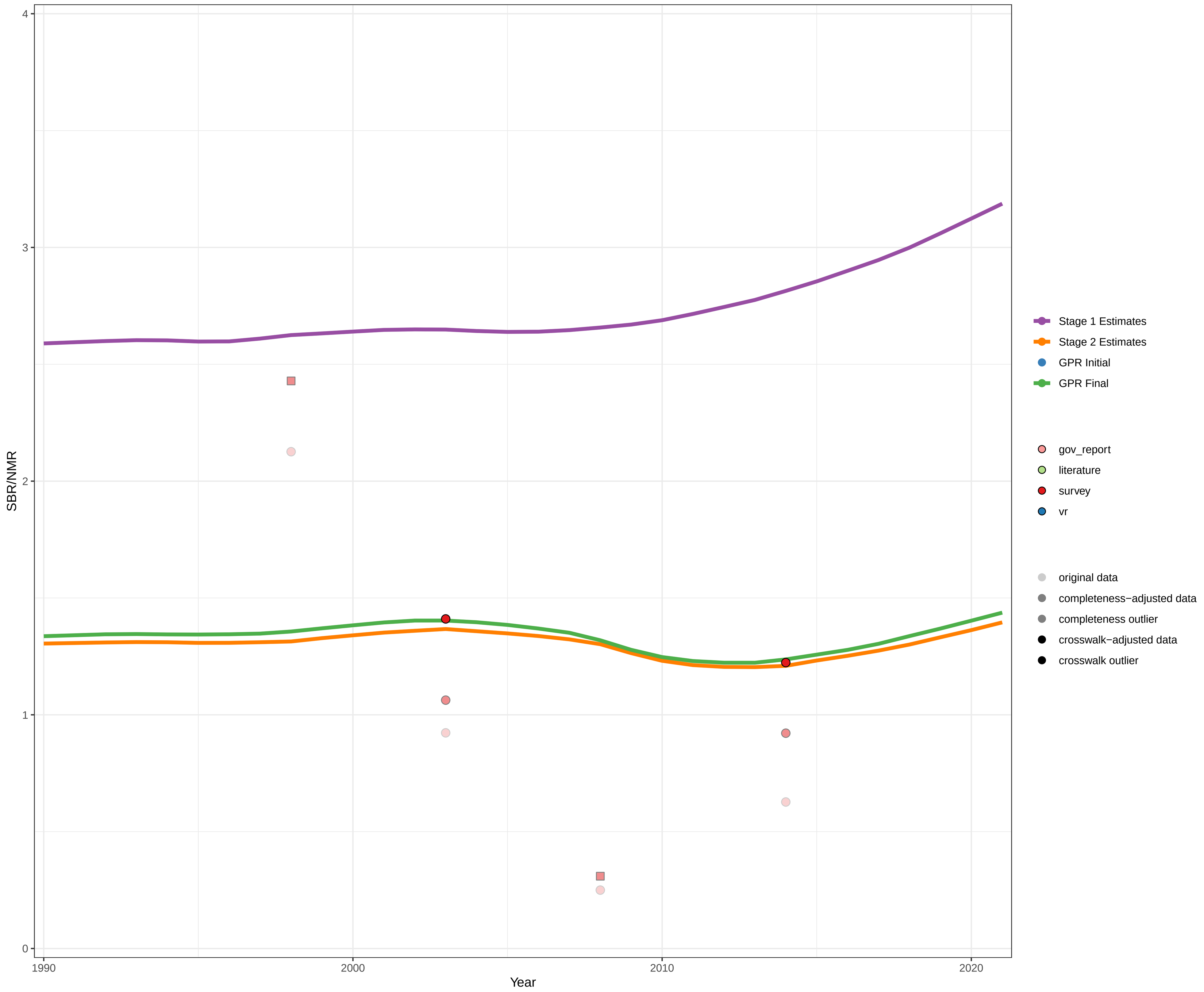

Kakamega KEN_35627 Eastern Sub−Saharan Africa Stillbirth Rate / Neonatal Mortality Rate (SBR/NMR) − 28 weeks
lambda = 0.8 ; zeta = 0.7 ; scale = 15 ; amp = 0.028

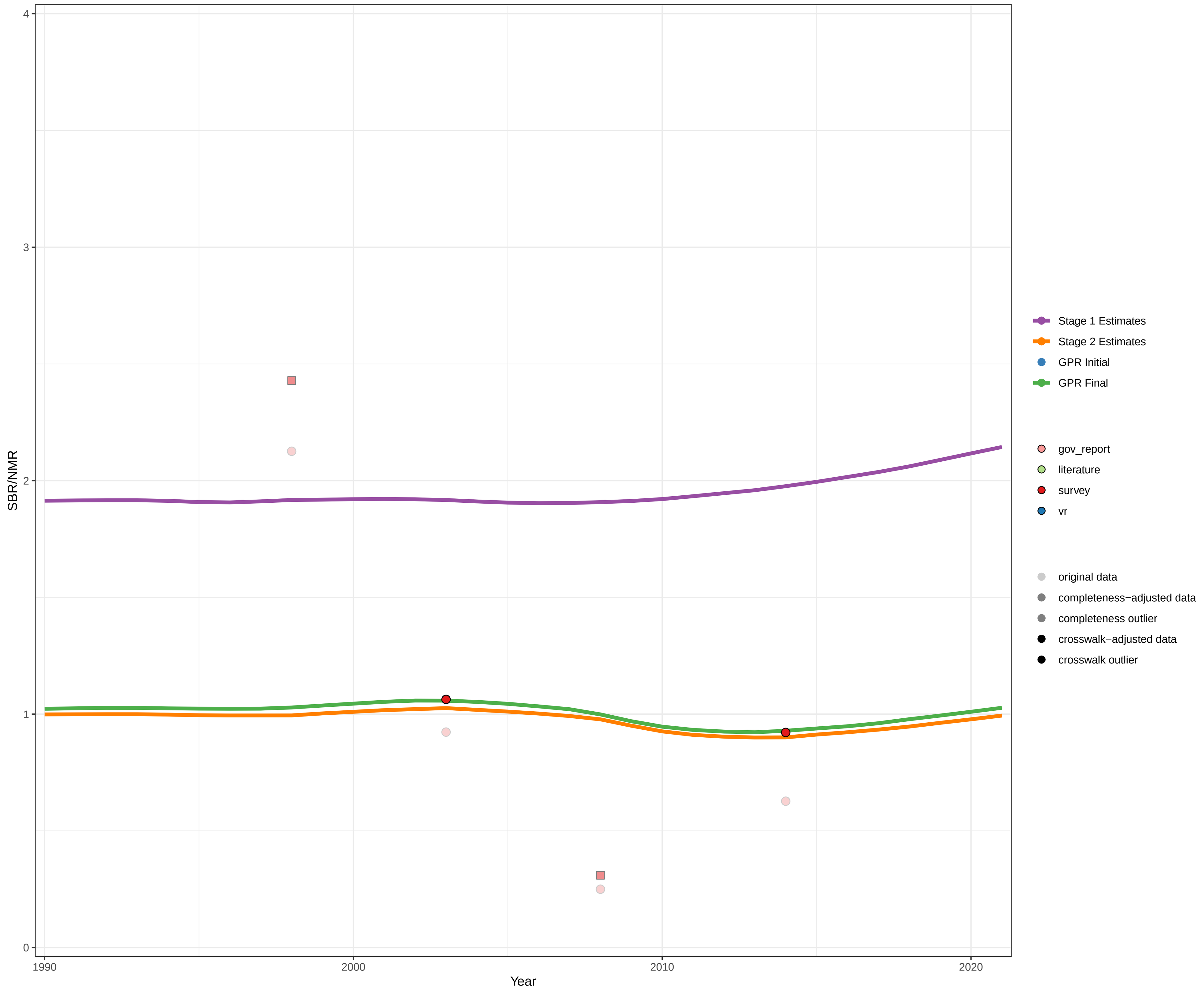

Definition	Years	Outlier	Source.Type	Title
28_weeks	2003	0	Survey	Kenya Demographic and Health Survey 2003
28_weeks	2014	0	Survey	Kenya Demographic and Health Survey 2014
28_weeks	1998	1	Survey	Kenya Demographic and Health Survey 1998
28_weeks	2008	1	Survey	Kenya Demographic and Health Survey 2008−2009

Kakamega KEN_35627 Eastern Sub−Saharan Africa Stillbirth Rate (SBR) − 20 weeks
lambda = 0.8 ; zeta = 0.7 ; scale = 15 ; amp = 0.028

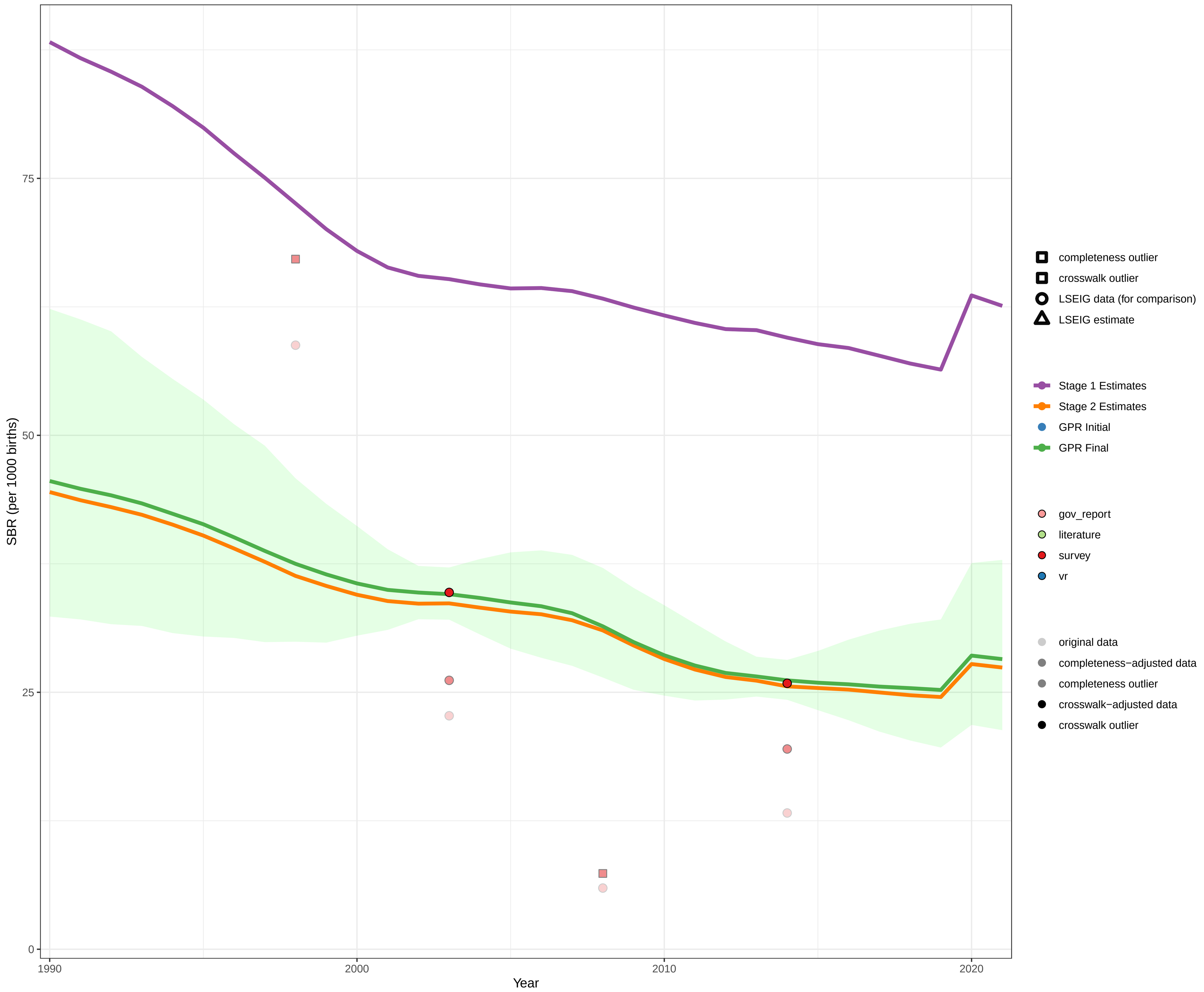

Kakamega KEN_35627 Eastern Sub−Saharan Africa Stillbirth Rate (SBR) − 28 weeks
lambda = 0.8 ; zeta = 0.7 ; scale = 15 ; amp = 0.028

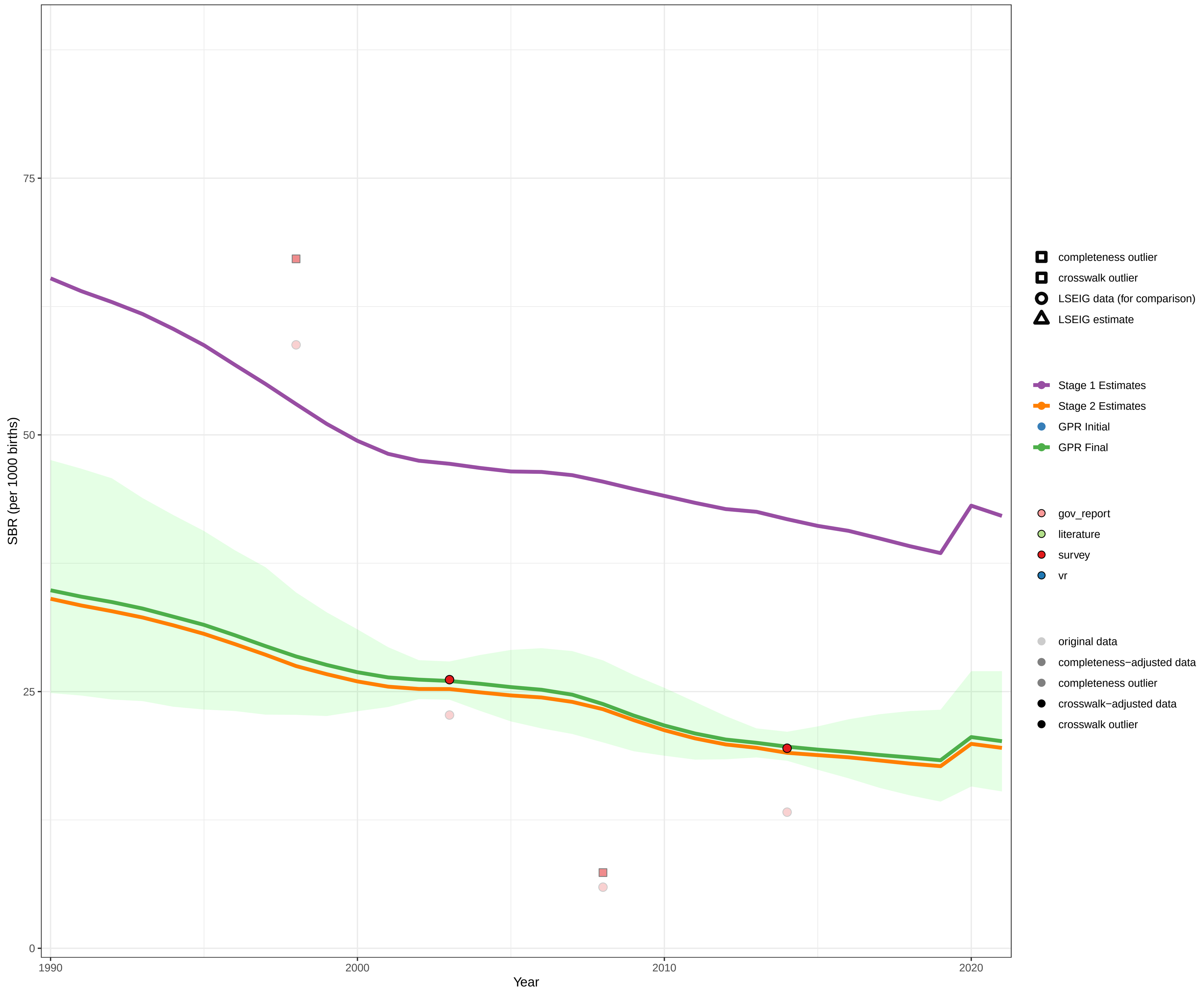

Year	Total Stillbirths (in thousands)	Total Stillbirths (in thousands)	SBR (per 1000 births)	SBR (per 1000 births)	SBR/NMR	SBR/NMR
	20 weeks	28 weeks	20 weeks	28 weeks	20 weeks	28 weeks
1990	2.5 (1.8 to 3.5)	1.9 (1.4 to 2.7)	45.6 (32.4 to 62.3)	34.9 (24.9 to 47.5)	1.34 (0.95 to 1.83)	1.02 (0.73 to 1.39)
1995	1.9 (1.4 to 2.5)	1.4 (1.0 to 1.9)	41.3 (30.4 to 53.5)	31.5 (23.3 to 40.6)	1.34 (0.99 to 1.74)	1.02 (0.76 to 1.32)
2000	1.5 (1.3 to 1.8)	1.1 (1.0 to 1.3)	35.6 (30.5 to 41.2)	26.9 (23.1 to 31.1)	1.38 (1.19 to 1.60)	1.04 (0.90 to 1.21)
2005	1.8 (1.6 to 2.1)	1.4 (1.2 to 1.6)	33.7 (29.2 to 38.6)	25.4 (22.1 to 29.1)	1.38 (1.20 to 1.58)	1.04 (0.91 to 1.19)
2010	1.8 (1.5 to 2.1)	1.3 (1.1 to 1.6)	28.6 (24.7 to 33.5)	21.7 (18.8 to 25.4)	1.25 (1.08 to 1.46)	0.95 (0.82 to 1.11)
2015	1.4 (1.3 to 1.6)	1.1 (1.0 to 1.2)	25.9 (23.3 to 29.0)	19.4 (17.4 to 21.6)	1.26 (1.13 to 1.41)	0.94 (0.84 to 1.05)
2020	1.3 (1.0 to 1.8)	0.9 (0.7 to 1.3)	28.6 (21.8 to 37.6)	20.6 (15.8 to 27.0)	1.40 (1.07 to 1.85)	1.01 (0.77 to 1.33)
2021	1.3 (1.0 to 1.7)	0.9 (0.7 to 1.2)	28.2 (21.3 to 37.9)	20.2 (15.3 to 27.0)	1.44 (1.09 to 1.93)	1.03 (0.78 to 1.37)

Kericho KEN_35628 Eastern Sub−Saharan Africa Stillbirth Rate / Neonatal Mortality Rate (SBR/NMR) − 20 weeks
lambda = 0.8 ; zeta = 0.7 ; scale = 15 ; amp = 0.028

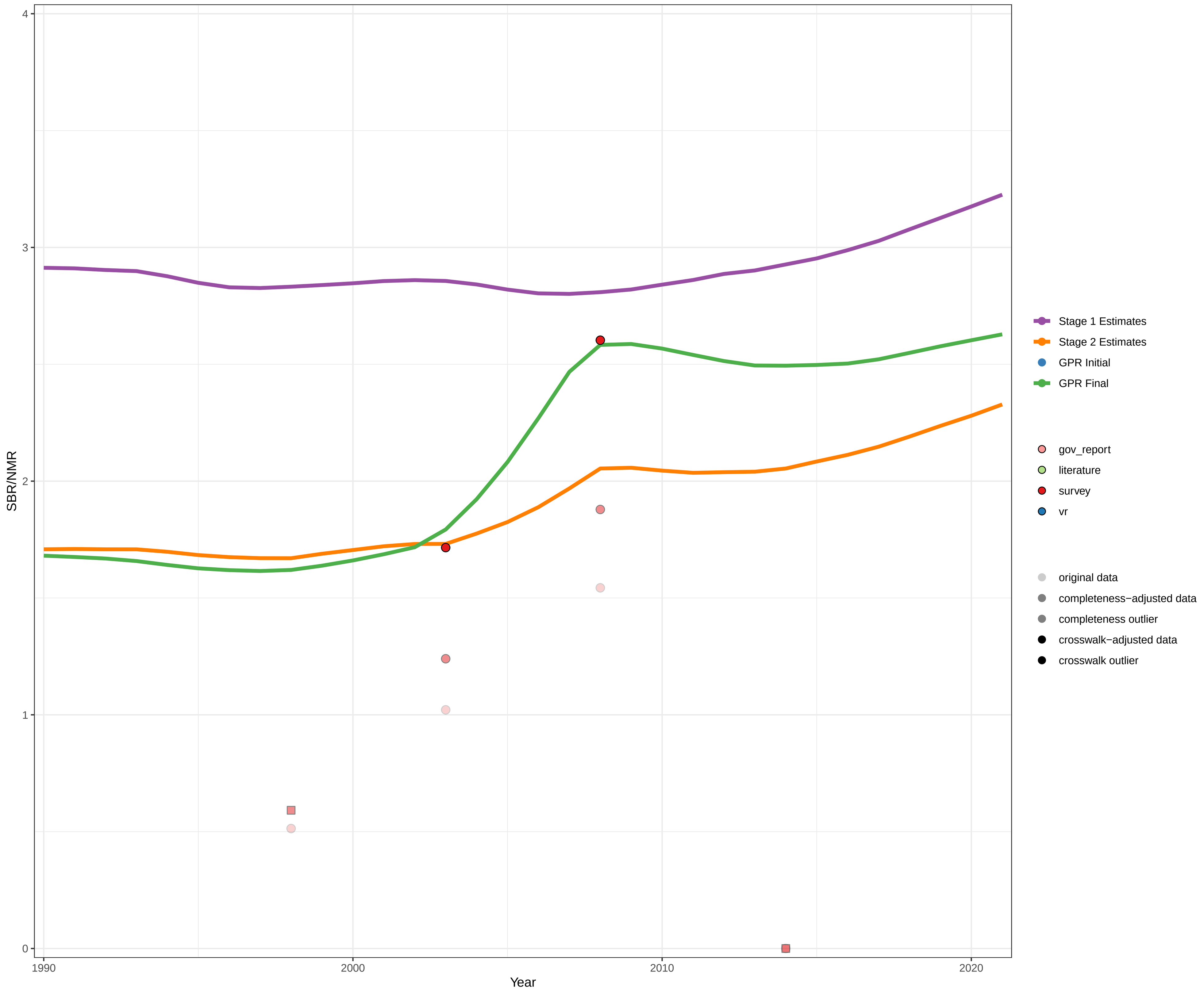

Kericho KEN_35628 Eastern Sub−Saharan Africa Stillbirth Rate / Neonatal Mortality Rate (SBR/NMR) − 28 weeks
lambda = 0.8 ; zeta = 0.7 ; scale = 15 ; amp = 0.028

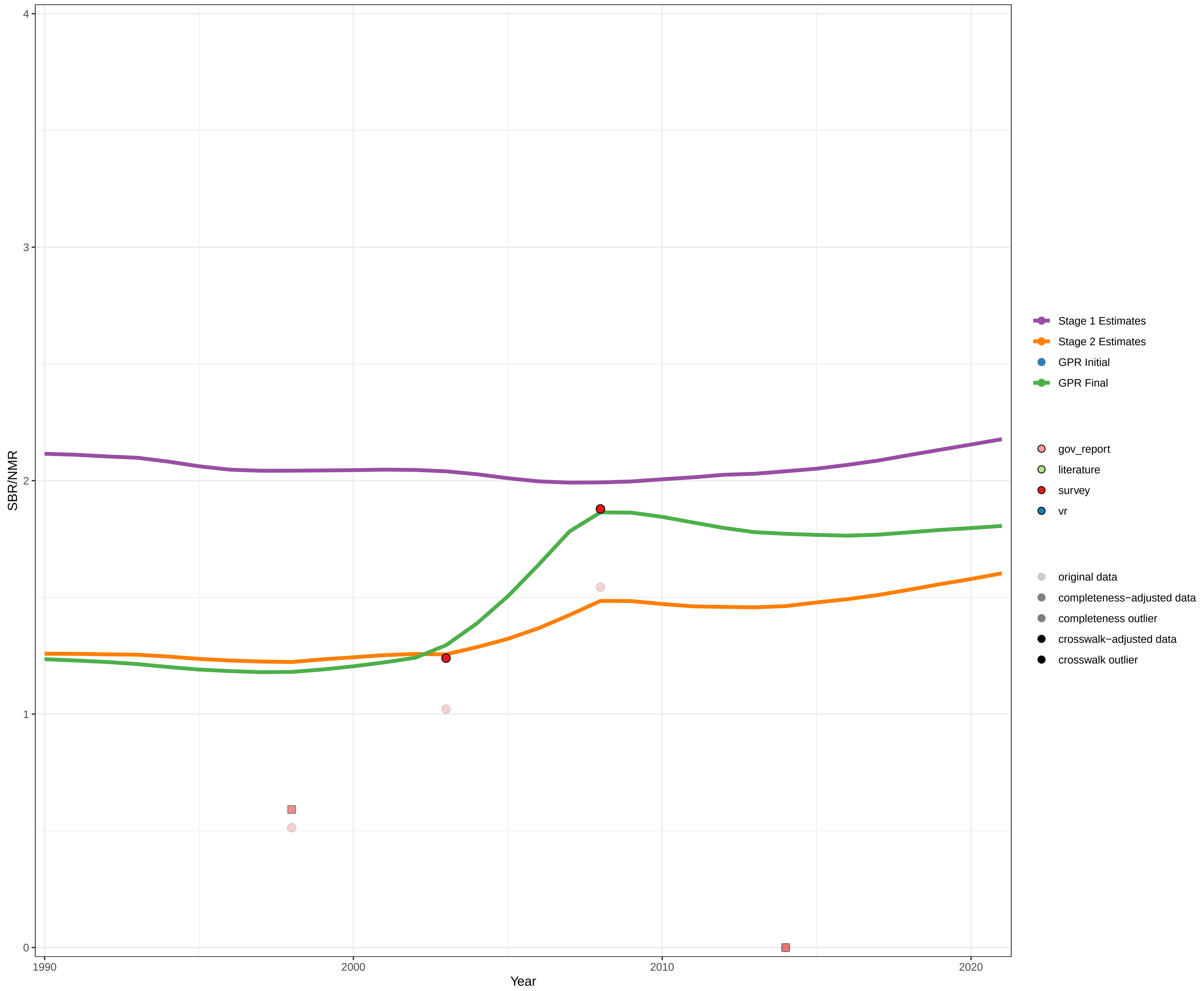

Definition	Years	Outlier	Source.Type	Title
28_weeks	2003	0	Survey	Kenya Demographic and Health Survey 2003
28_weeks	2008	0	Survey	Kenya Demographic and Health Survey 2008−2009
28_weeks	1998	1	Survey	Kenya Demographic and Health Survey 1998
28_weeks	2014	1	Survey	Kenya Demographic and Health Survey 2014

Kericho KEN_35628 Eastern Sub−Saharan Africa Stillbirth Rate (SBR) − 20 weeks
lambda = 0.8 ; zeta = 0.7 ; scale = 15 ; amp = 0.028

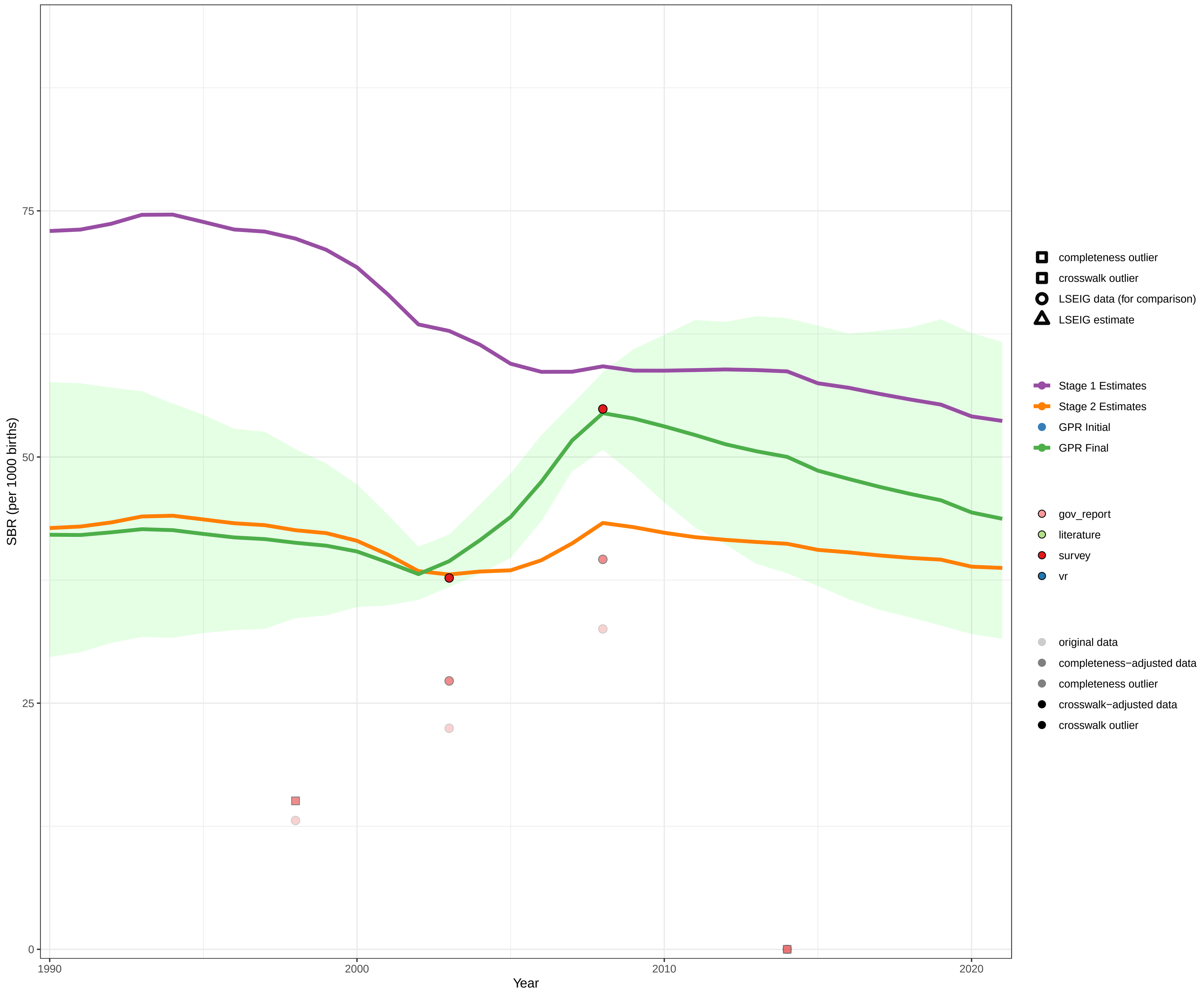

Kericho KEN_35628 Eastern Sub−Saharan Africa Stillbirth Rate (SBR) − 28 weeks
lambda = 0.8 ; zeta = 0.7 ; scale = 15 ; amp = 0.028

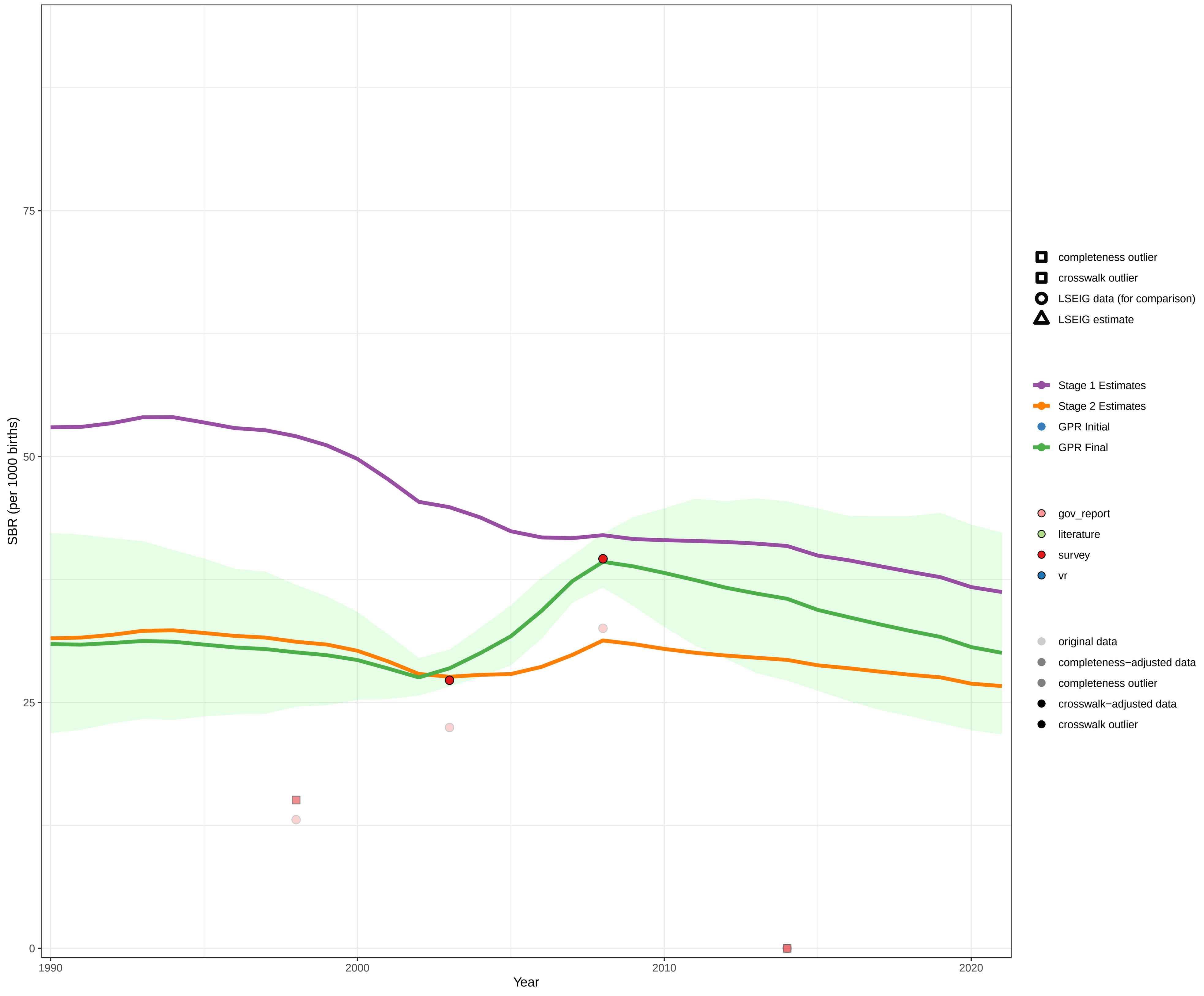

Year	Total Stillbirths (in thousands)	Total Stillbirths (in thousands)	SBR (per 1000 births)	SBR (per 1000 births)	SBR/NMR	SBR/NMR
	20 weeks	28 weeks	20 weeks	28 weeks	20 weeks	28 weeks
1990	1.3 (0.9 to 1.8)	0.9 (0.6 to 1.3)	42.1 (29.7 to 57.6)	30.9 (21.9 to 42.2)	1.68 (1.19 to 2.30)	1.24 (0.87 to 1.69)
1995	1.5 (1.1 to 1.9)	1.1 (0.8 to 1.4)	42.2 (32.1 to 54.3)	30.9 (23.6 to 39.6)	1.63 (1.24 to 2.09)	1.19 (0.91 to 1.53)
2000	1.4 (1.2 to 1.7)	1.0 (0.9 to 1.2)	40.4 (34.8 to 47.2)	29.3 (25.3 to 34.2)	1.66 (1.43 to 1.94)	1.20 (1.04 to 1.40)
2005	1.3 (1.2 to 1.4)	0.9 (0.8 to 1.0)	43.9 (39.7 to 48.3)	31.7 (28.8 to 34.9)	2.08 (1.88 to 2.29)	1.50 (1.36 to 1.65)
2010	1.4 (1.2 to 1.6)	1.0 (0.8 to 1.2)	53.1 (45.4 to 62.4)	38.2 (32.7 to 44.7)	2.57 (2.19 to 3.02)	1.84 (1.58 to 2.16)
2015	1.4 (1.0 to 1.8)	1.0 (0.7 to 1.3)	48.6 (36.9 to 63.3)	34.4 (26.2 to 44.7)	2.50 (1.89 to 3.25)	1.77 (1.35 to 2.30)
2020	1.4 (1.0 to 2.1)	1.0 (0.7 to 1.4)	44.4 (32.0 to 62.6)	30.6 (22.2 to 43.1)	2.60 (1.88 to 3.67)	1.80 (1.30 to 2.53)
2021	1.4 (1.0 to 2.1)	1.0 (0.7 to 1.4)	43.7 (31.5 to 61.7)	30.0 (21.7 to 42.2)	2.63 (1.90 to 3.71)	1.81 (1.31 to 2.54)

Kiambu KEN_35629 Eastern Sub−Saharan Africa Stillbirth Rate / Neonatal Mortality Rate (SBR/NMR) − 20 weeks
lambda = 0.8 ; zeta = 0.7 ; scale = 15 ; amp = 0.028

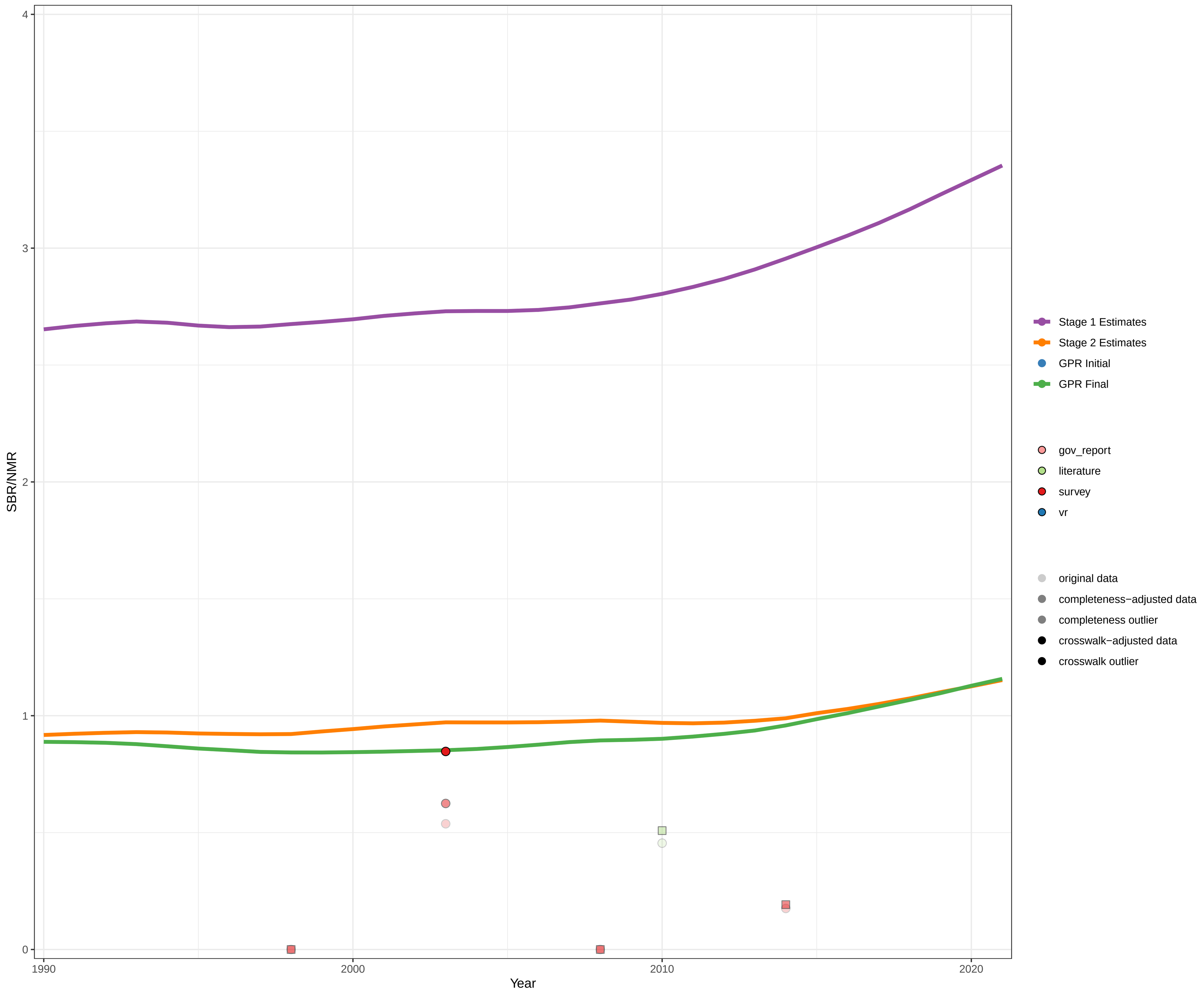

Kiambu KEN_35629 Eastern Sub−Saharan Africa Stillbirth Rate / Neonatal Mortality Rate (SBR/NMR) − 28 weeks
lambda = 0.8 ; zeta = 0.7 ; scale = 15 ; amp = 0.028

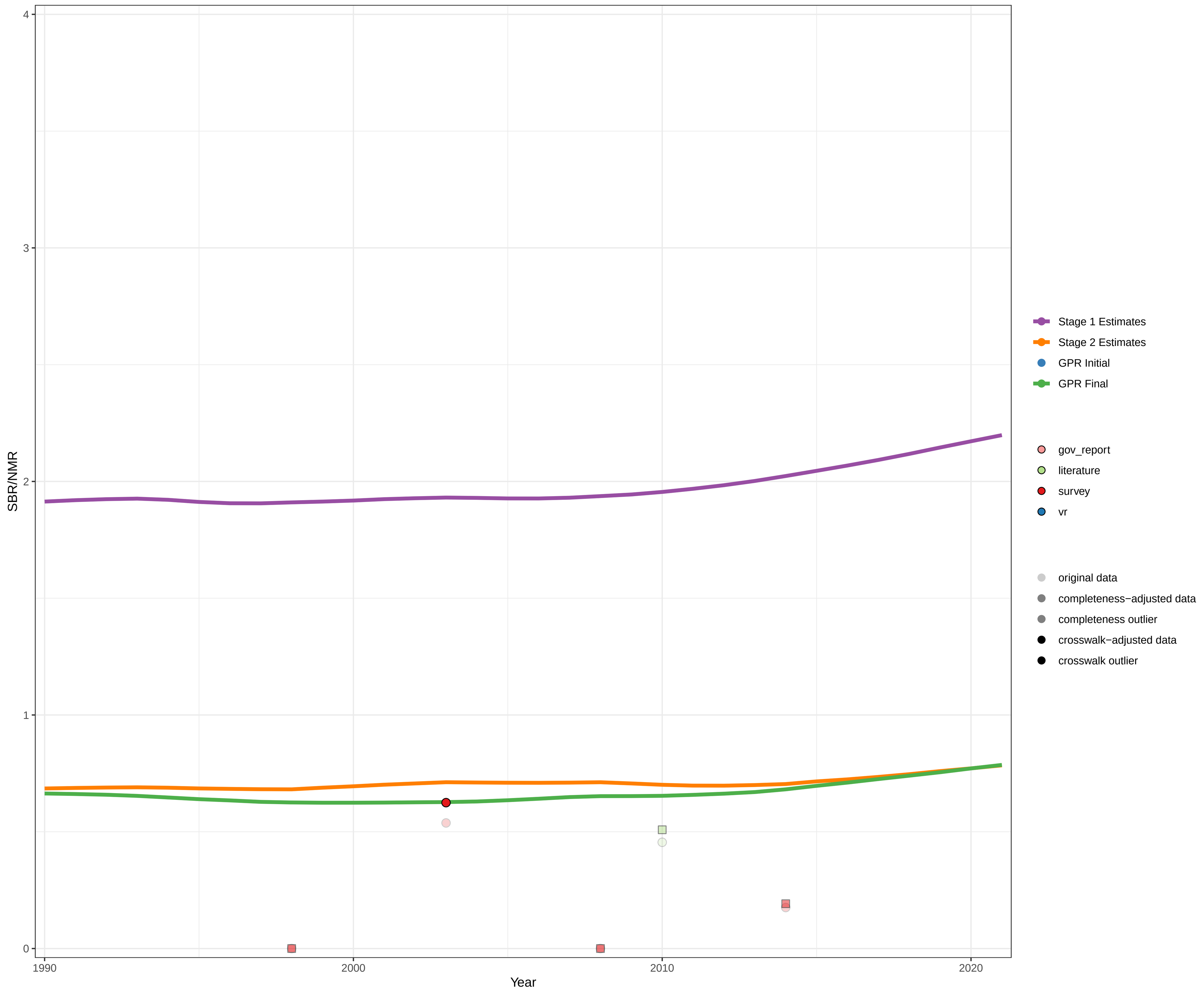

Definition	Years	Outlier	Source.Type	Title
28_weeks	2003	0	Survey	Kenya Demographic and Health Survey 2003
28_weeks	1998	1	Survey	Kenya Demographic and Health Survey 1998
28_weeks	2008	1	Survey	Kenya Demographic and Health Survey 2008−2009
28_weeks	2010	1	Sci Lit	Antenatal care and pregnancy outcomes in a safe motherhood health voucher system in rural Kenya, 2007−2013
28_weeks	2014	1	Survey	Kenya Demographic and Health Survey 2014

Kiambu KEN_35629 Eastern Sub−Saharan Africa Stillbirth Rate (SBR) − 20 weeks
lambda = 0.8 ; zeta = 0.7 ; scale = 15 ; amp = 0.028

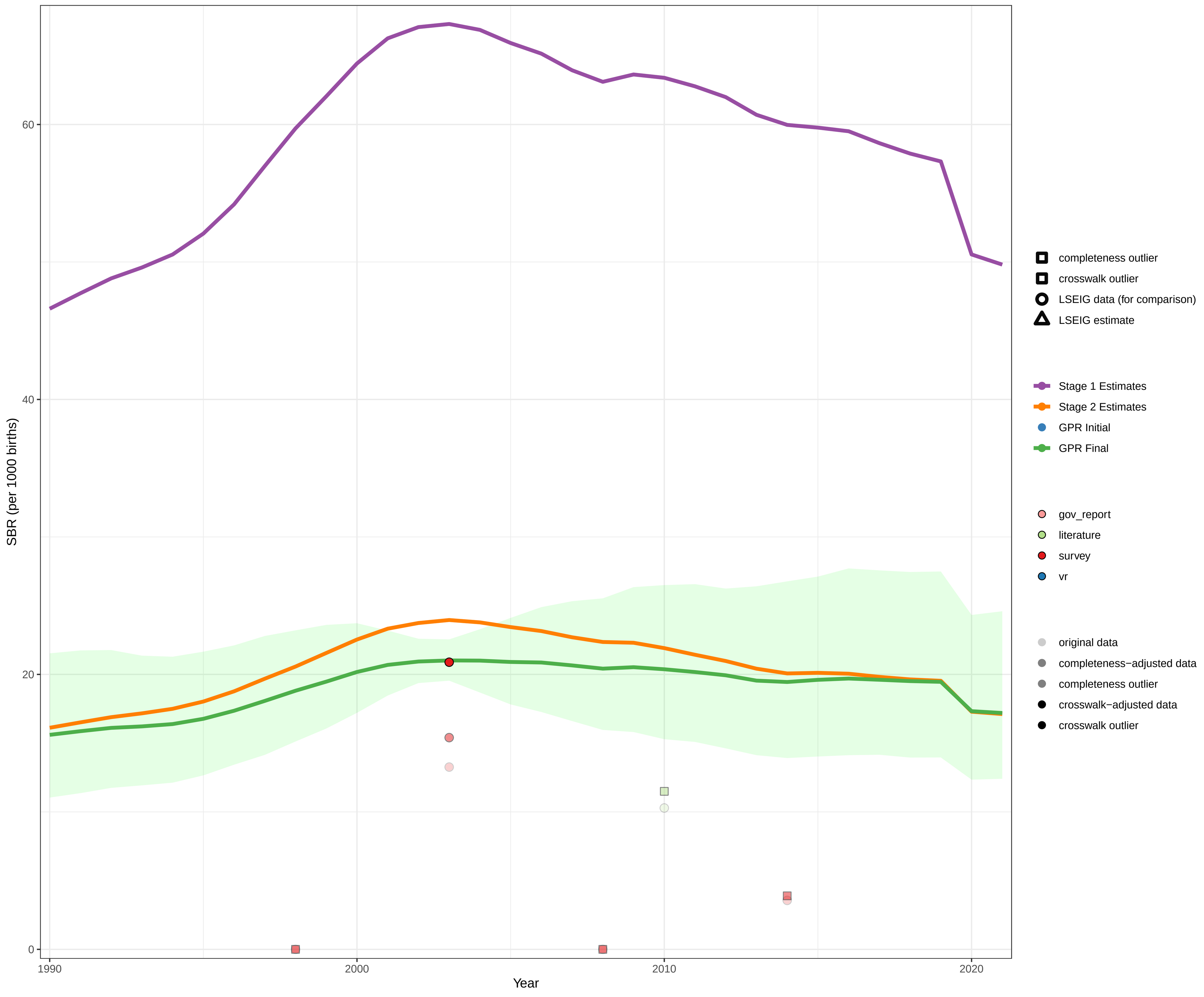

Kiambu KEN_35629 Eastern Sub−Saharan Africa Stillbirth Rate (SBR) − 28 weeks
lambda = 0.8 ; zeta = 0.7 ; scale = 15 ; amp = 0.028

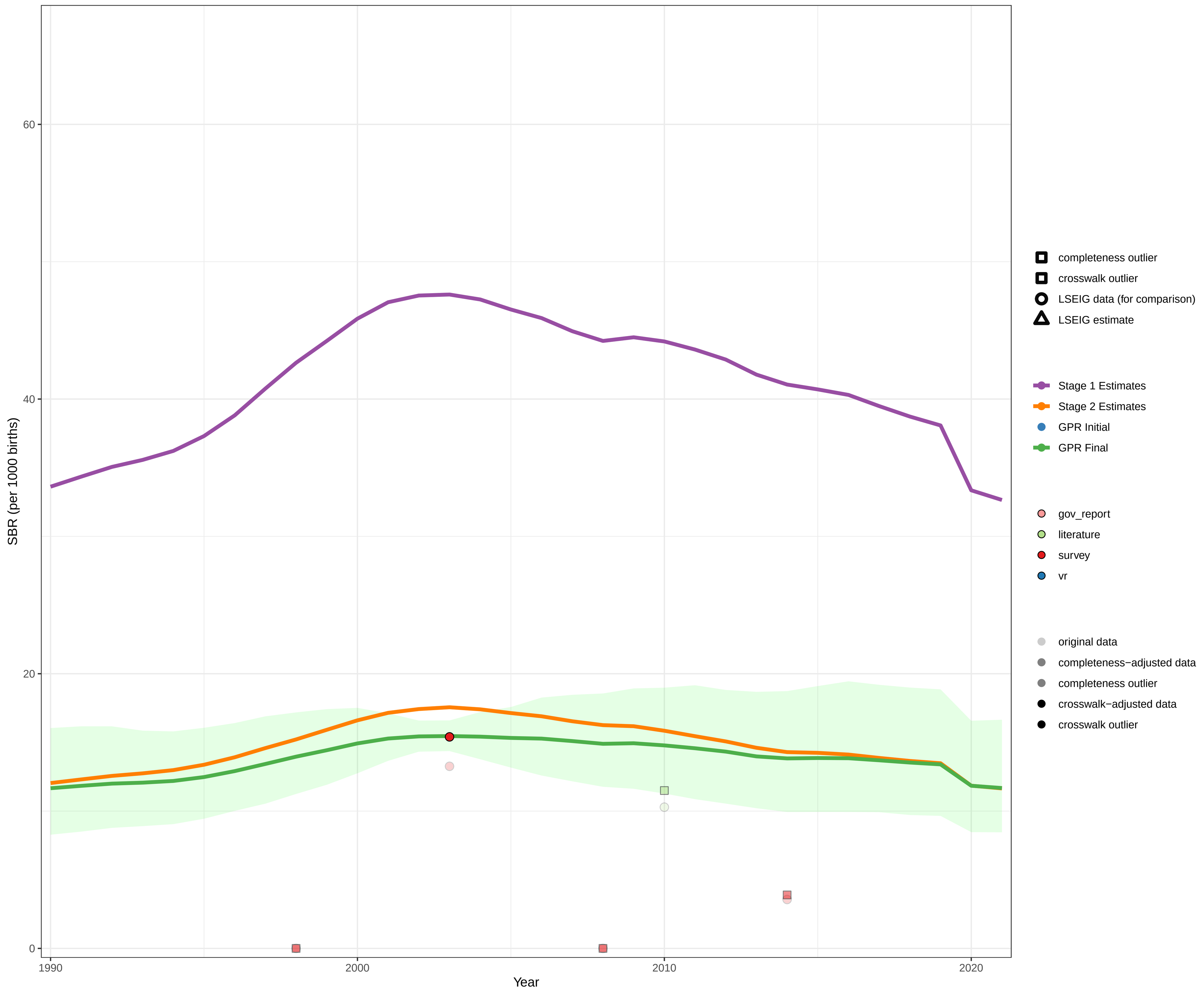

Year	Total Stillbirths (in thousands)	Total Stillbirths (in thousands)	SBR (per 1000 births)	SBR (per 1000 births)	SBR/NMR	SBR/NMR
	20 weeks	28 weeks	20 weeks	28 weeks	20 weeks	28 weeks
1990	0.6 (0.4 to 0.8)	0.4 (0.3 to 0.6)	15.6 (11.0 to 21.5)	11.7 (8.3 to 16.0)	0.89 (0.63 to 1.23)	0.66 (0.47 to 0.91)
1995	0.8 (0.6 to 1.0)	0.6 (0.4 to 0.7)	16.8 (12.7 to 21.7)	12.5 (9.4 to 16.1)	0.86 (0.65 to 1.11)	0.64 (0.48 to 0.82)
2000	1.1 (0.9 to 1.3)	0.8 (0.7 to 0.9)	20.2 (17.2 to 23.7)	14.9 (12.8 to 17.5)	0.84 (0.72 to 0.99)	0.62 (0.53 to 0.73)
2005	1.1 (0.9 to 1.3)	0.8 (0.7 to 0.9)	20.9 (17.8 to 24.1)	15.3 (13.2 to 17.6)	0.87 (0.74 to 1.00)	0.63 (0.55 to 0.73)
2010	1.1 (0.8 to 1.4)	0.8 (0.6 to 1.0)	20.4 (15.3 to 26.5)	14.8 (11.3 to 19.0)	0.90 (0.68 to 1.17)	0.65 (0.50 to 0.84)
2015	1.0 (0.7 to 1.4)	0.7 (0.5 to 1.0)	19.6 (14.0 to 27.1)	13.9 (9.9 to 19.1)	0.99 (0.70 to 1.36)	0.70 (0.50 to 0.96)
2020	0.9 (0.7 to 1.3)	0.6 (0.4 to 0.9)	17.3 (12.3 to 24.3)	11.8 (8.5 to 16.6)	1.13 (0.80 to 1.58)	0.77 (0.55 to 1.08)
2021	0.9 (0.7 to 1.3)	0.6 (0.4 to 0.9)	17.2 (12.4 to 24.6)	11.7 (8.5 to 16.7)	1.16 (0.84 to 1.66)	0.79 (0.57 to 1.12)

Kilifi KEN_35630 Eastern Sub−Saharan Africa Stillbirth Rate / Neonatal Mortality Rate (SBR/NMR) − 20 weeks
lambda = 0.8 ; zeta = 0.7 ; scale = 15 ; amp = 0.028

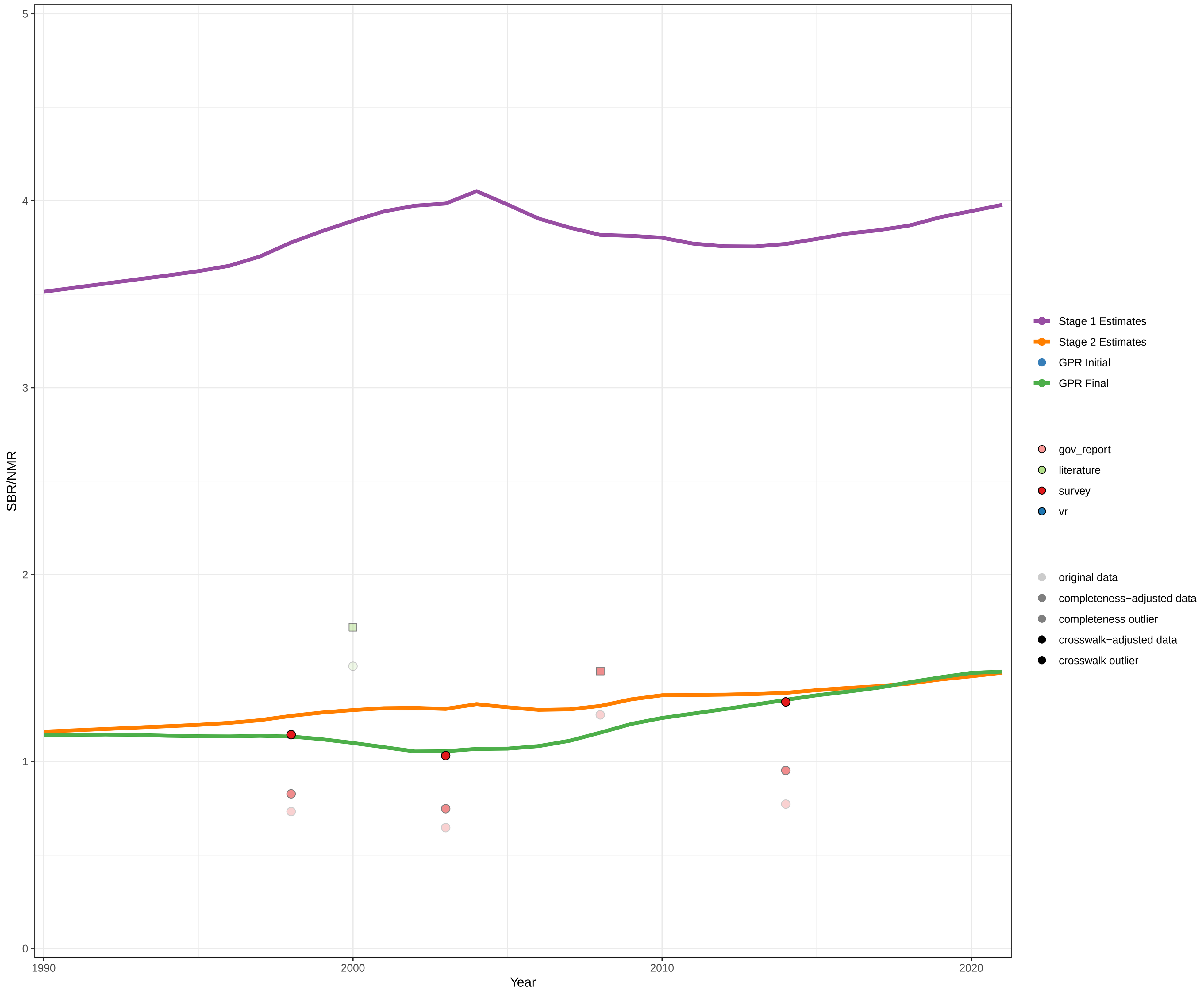

Kilifi KEN_35630 Eastern Sub−Saharan Africa Stillbirth Rate / Neonatal Mortality Rate (SBR/NMR) − 28 weeks
lambda = 0.8 ; zeta = 0.7 ; scale = 15 ; amp = 0.028

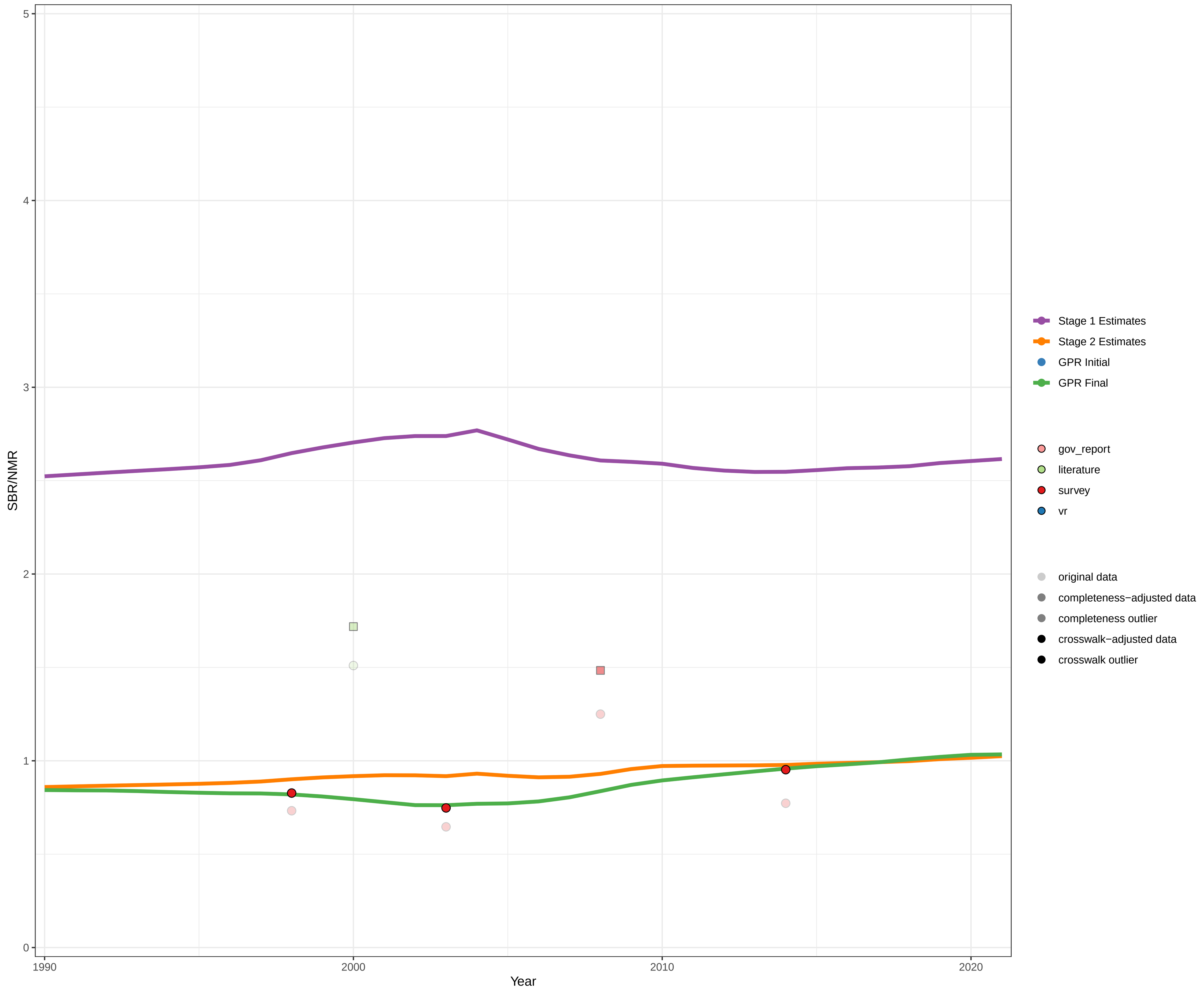

Definition	Years	Outlier	Source.Type	Title
28_weeks	1998	0	Survey	Kenya Demographic and Health Survey 1998
28_weeks	2003	0	Survey	Kenya Demographic and Health Survey 2003
28_weeks	2014	0	Survey	Kenya Demographic and Health Survey 2014
22_weeks	2000	1	Sci Lit	Outcome of Delivery and Cause−Specific Mortality and Severe Morbidity in Early Infancy: A Kenyan District Hospital Birth Cohort
28_weeks	2008	1	Survey	Kenya Demographic and Health Survey 2008−2009

Kilifi KEN_35630 Eastern Sub−Saharan Africa Stillbirth Rate (SBR) − 20 weeks
lambda = 0.8 ; zeta = 0.7 ; scale = 15 ; amp = 0.028

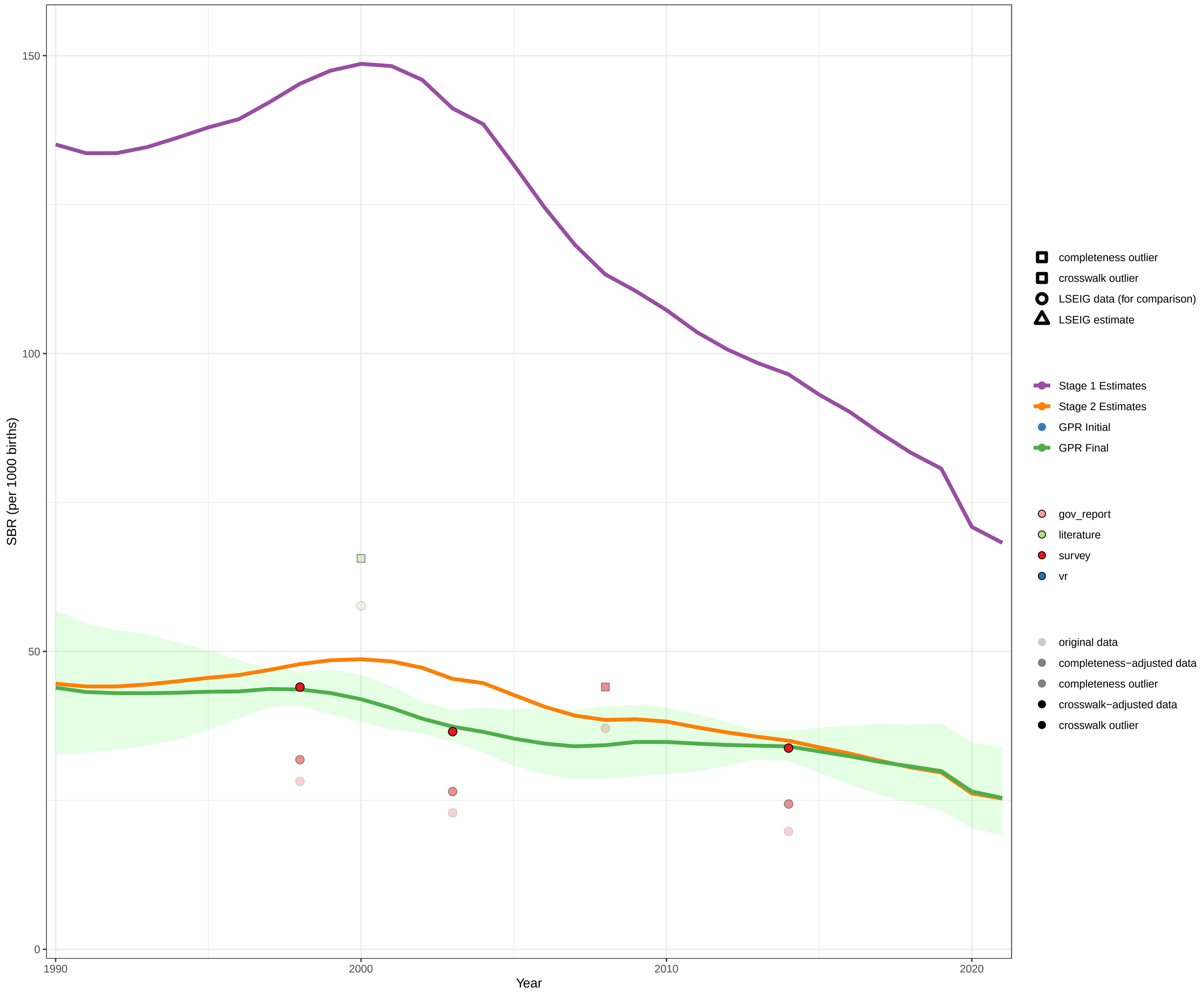

Kilifi KEN_35630 Eastern Sub−Saharan Africa Stillbirth Rate (SBR) − 28 weeks
lambda = 0.8 ; zeta = 0.7 ; scale = 15 ; amp = 0.028

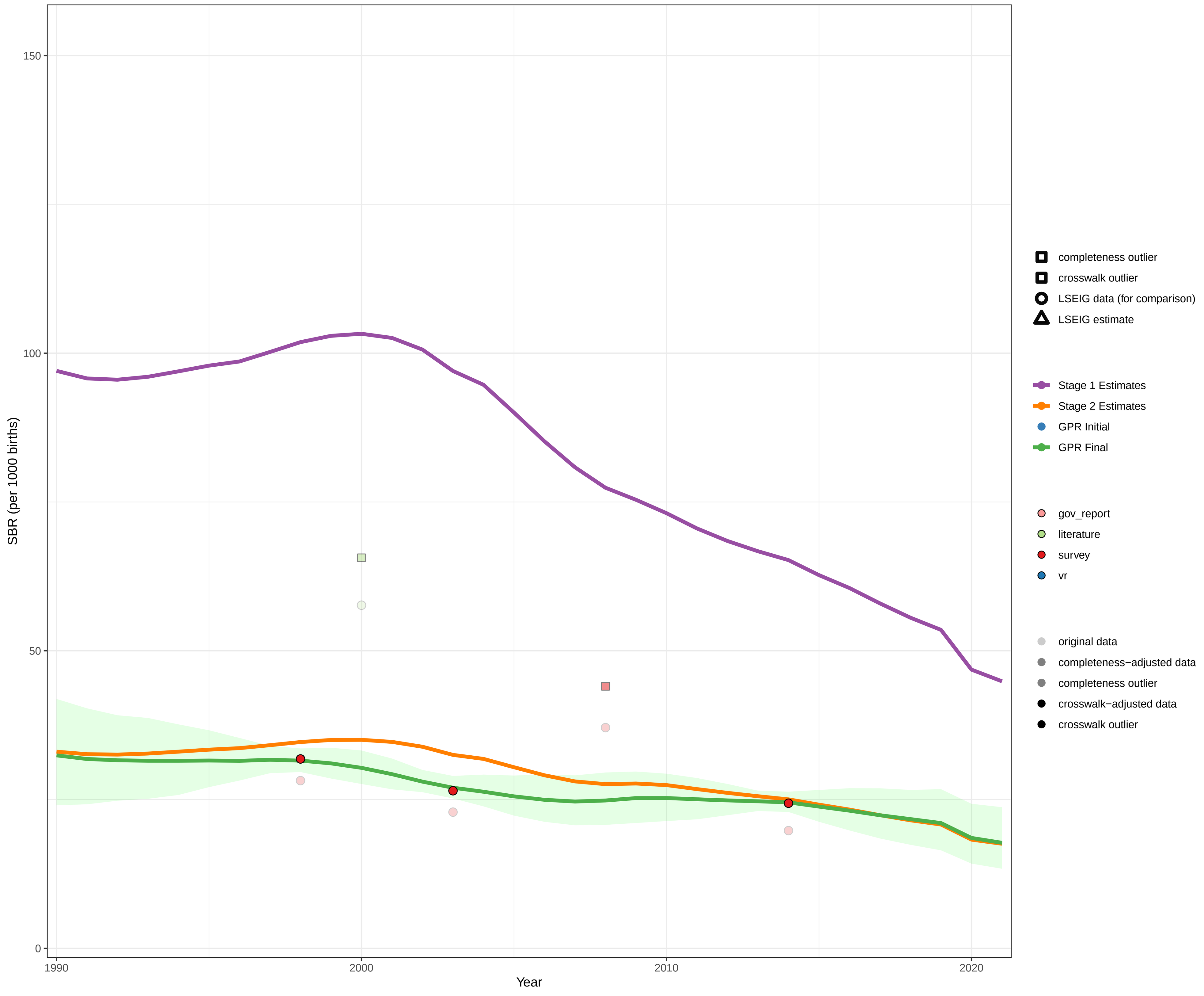

Year	Total Stillbirths (in thousands)	Total Stillbirths (in thousands)	SBR (per 1000 births)	SBR (per 1000 births)	SBR/NMR	SBR/NMR
	20 weeks	28 weeks	20 weeks	28 weeks	20 weeks	28 weeks
1990	1.5 (1.1 to 1.9)	1.1 (0.8 to 1.4)	43.9 (32.7 to 56.8)	32.4 (24.0 to 41.9)	1.14 (0.85 to 1.48)	0.84 (0.62 to 1.09)
1995	1.6 (1.4 to 1.9)	1.2 (1.0 to 1.4)	43.2 (36.9 to 50.2)	31.6 (27.1 to 36.6)	1.14 (0.97 to 1.32)	0.83 (0.71 to 0.96)
2000	1.8 (1.6 to 2.0)	1.3 (1.2 to 1.4)	42.0 (38.2 to 46.1)	30.3 (27.6 to 33.2)	1.10 (1.00 to 1.21)	0.79 (0.72 to 0.87)
2005	1.7 (1.4 to 1.9)	1.2 (1.0 to 1.3)	35.4 (30.8 to 40.3)	25.5 (22.3 to 29.0)	1.07 (0.93 to 1.22)	0.77 (0.67 to 0.88)
2010	1.7 (1.5 to 2.0)	1.2 (1.0 to 1.5)	34.8 (29.4 to 40.6)	25.3 (21.4 to 29.4)	1.23 (1.04 to 1.44)	0.89 (0.76 to 1.04)
2015	1.7 (1.5 to 1.9)	1.2 (1.1 to 1.3)	33.2 (29.6 to 37.2)	23.8 (21.3 to 26.6)	1.35 (1.21 to 1.52)	0.97 (0.87 to 1.08)
2020	1.3 (1.0 to 1.7)	0.9 (0.7 to 1.1)	26.5 (20.3 to 34.7)	18.6 (14.2 to 24.3)	1.47 (1.13 to 1.93)	1.03 (0.79 to 1.35)
2021	1.2 (0.9 to 1.6)	0.8 (0.6 to 1.1)	25.4 (19.1 to 34.0)	17.7 (13.4 to 23.7)	1.48 (1.11 to 1.98)	1.03 (0.78 to 1.38)

Kirinyaga KEN_35631 Eastern Sub−Saharan Africa Stillbirth Rate / Neonatal Mortality Rate (SBR/NMR) − 20 weeks
lambda = 1 ; zeta = 0.6 ; scale = 15 ; amp = 0.028

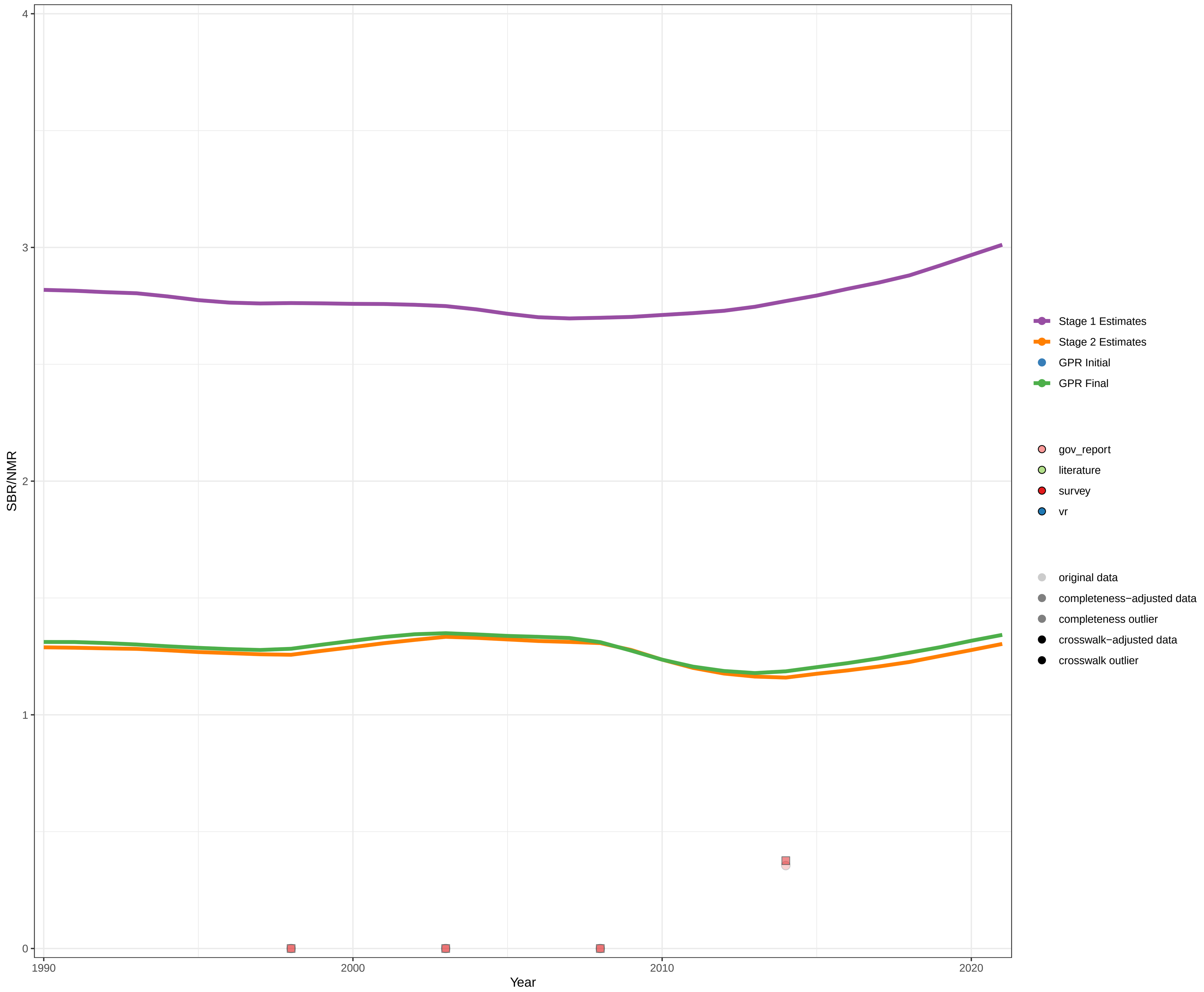

Kirinyaga KEN_35631 Eastern Sub−Saharan Africa Stillbirth Rate / Neonatal Mortality Rate (SBR/NMR) − 28 weeks
lambda = 1 ; zeta = 0.6 ; scale = 15 ; amp = 0.028

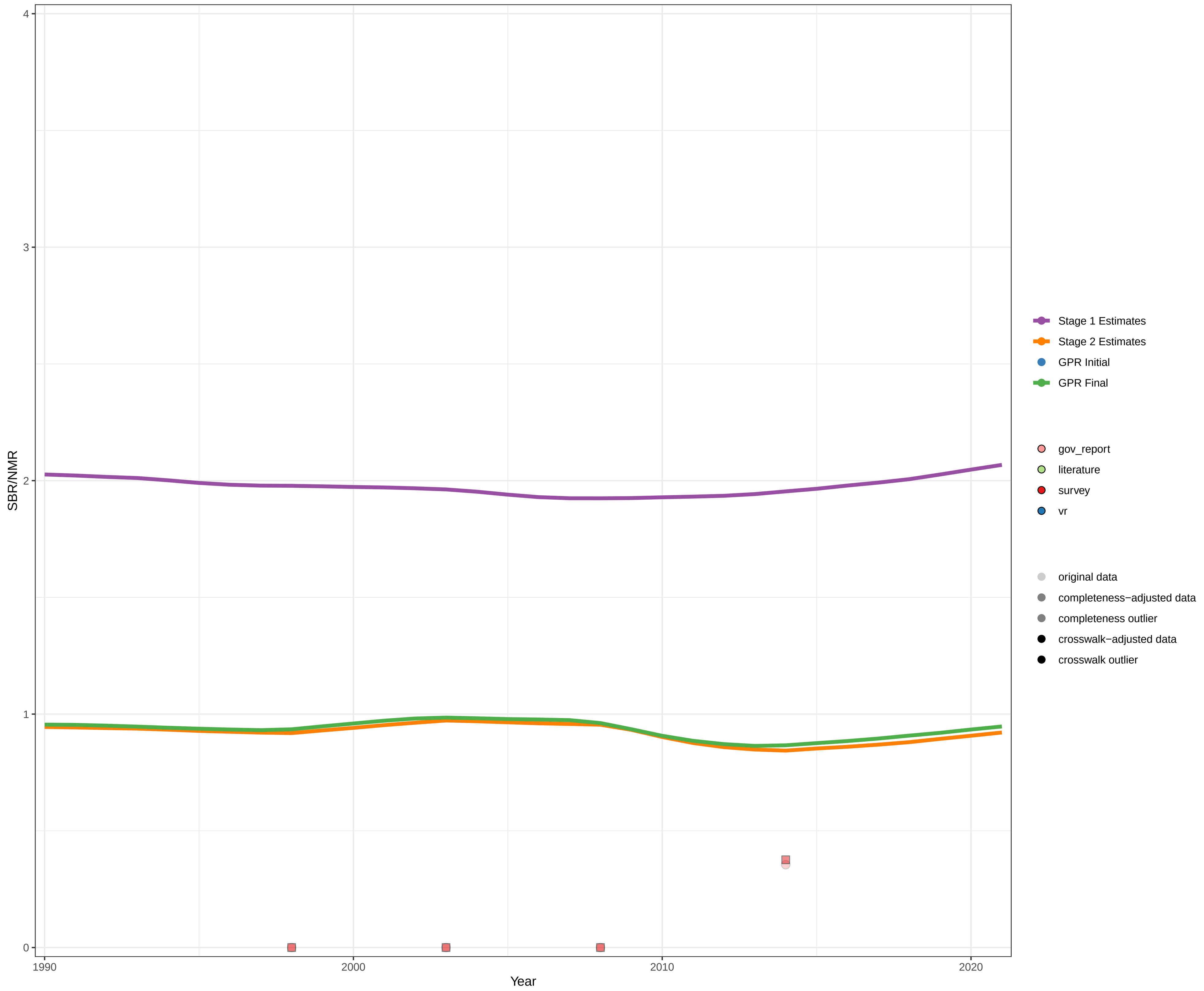

Definition	Years	Outlier	Source.Type	Title
28_weeks	1998	1	Survey	Kenya Demographic and Health Survey 1998
28_weeks	2003	1	Survey	Kenya Demographic and Health Survey 2003
28_weeks	2008	1	Survey	Kenya Demographic and Health Survey 2008−2009
28_weeks	2014	1	Survey	Kenya Demographic and Health Survey 2014

Kirinyaga KEN_35631 Eastern Sub−Saharan Africa Stillbirth Rate (SBR) − 20 weeks
lambda = 1 ; zeta = 0.6 ; scale = 15 ; amp = 0.028

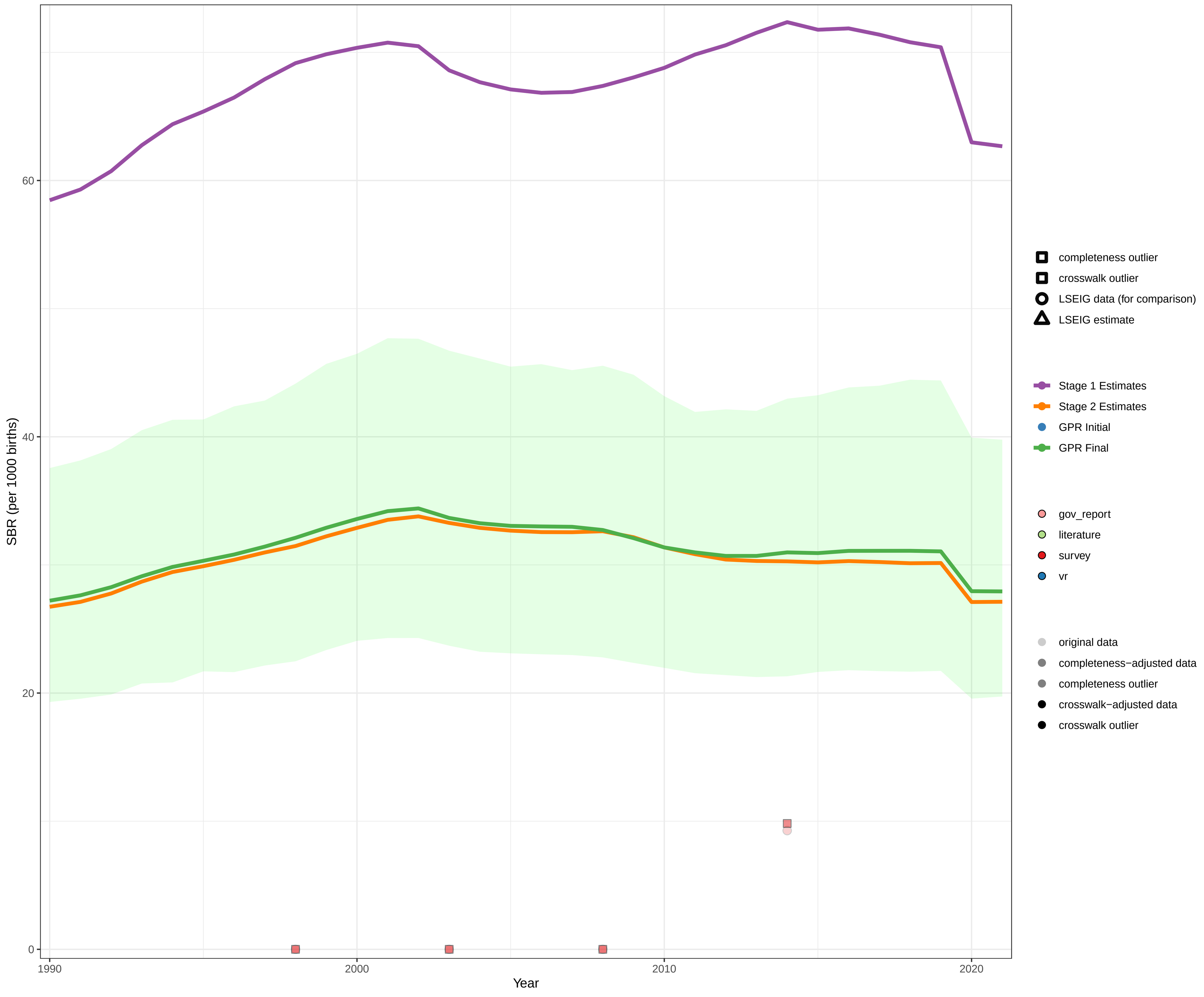

Kirinyaga KEN_35631 Eastern Sub−Saharan Africa Stillbirth Rate (SBR) − 28 weeks
lambda = 1 ; zeta = 0.6 ; scale = 15 ; amp = 0.028

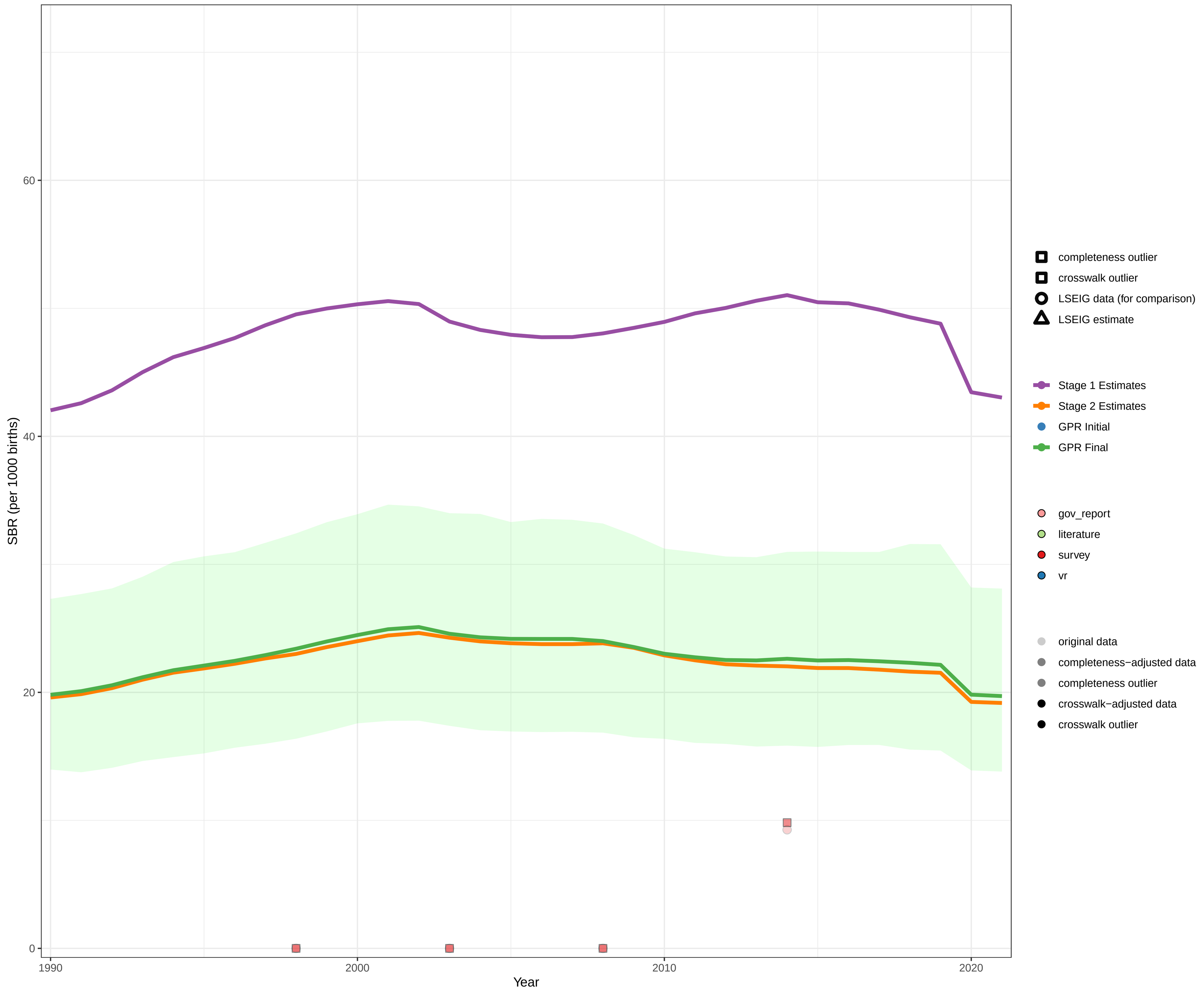

Year	Total Stillbirths (in thousands)	Total Stillbirths (in thousands)	SBR (per 1000 births)	SBR (per 1000 births)	SBR/NMR	SBR/NMR
	20 weeks	28 weeks	20 weeks	28 weeks	20 weeks	28 weeks
1990	0.4 (0.3 to 0.5)	0.3 (0.2 to 0.4)	27.2 (19.3 to 37.6)	19.8 (14.0 to 27.3)	1.31 (0.93 to 1.81)	0.95 (0.67 to 1.32)
1995	0.4 (0.3 to 0.6)	0.3 (0.2 to 0.4)	30.3 (21.7 to 41.4)	22.1 (15.2 to 30.6)	1.29 (0.92 to 1.75)	0.94 (0.65 to 1.30)
2000	0.5 (0.3 to 0.7)	0.4 (0.3 to 0.5)	33.6 (24.1 to 46.5)	24.5 (17.6 to 33.9)	1.32 (0.94 to 1.82)	0.96 (0.69 to 1.33)
2005	0.5 (0.3 to 0.7)	0.4 (0.3 to 0.5)	33.0 (23.1 to 45.5)	24.2 (16.9 to 33.3)	1.34 (0.93 to 1.84)	0.98 (0.69 to 1.35)
2010	0.4 (0.3 to 0.6)	0.3 (0.2 to 0.4)	31.4 (22.0 to 43.2)	23.0 (16.4 to 31.2)	1.24 (0.87 to 1.70)	0.91 (0.65 to 1.23)
2015	0.4 (0.3 to 0.5)	0.3 (0.2 to 0.4)	30.9 (21.6 to 43.2)	22.5 (15.7 to 31.0)	1.20 (0.84 to 1.68)	0.88 (0.61 to 1.21)
2020	0.3 (0.2 to 0.4)	0.2 (0.1 to 0.3)	27.9 (19.6 to 39.9)	19.8 (13.9 to 28.2)	1.32 (0.92 to 1.88)	0.93 (0.66 to 1.33)
2021	0.3 (0.2 to 0.4)	0.2 (0.1 to 0.3)	27.9 (19.7 to 39.8)	19.7 (13.8 to 28.1)	1.34 (0.95 to 1.91)	0.95 (0.66 to 1.35)

Kisii   KEN_35632   Eastern Sub−Saharan Africa   Stillbirth Rate / Neonatal Mortality Rate (SBR/NMR) − 20 weeks
lambda = 0.8 ; zeta = 0.7 ; scale = 15 ; amp = 0.028

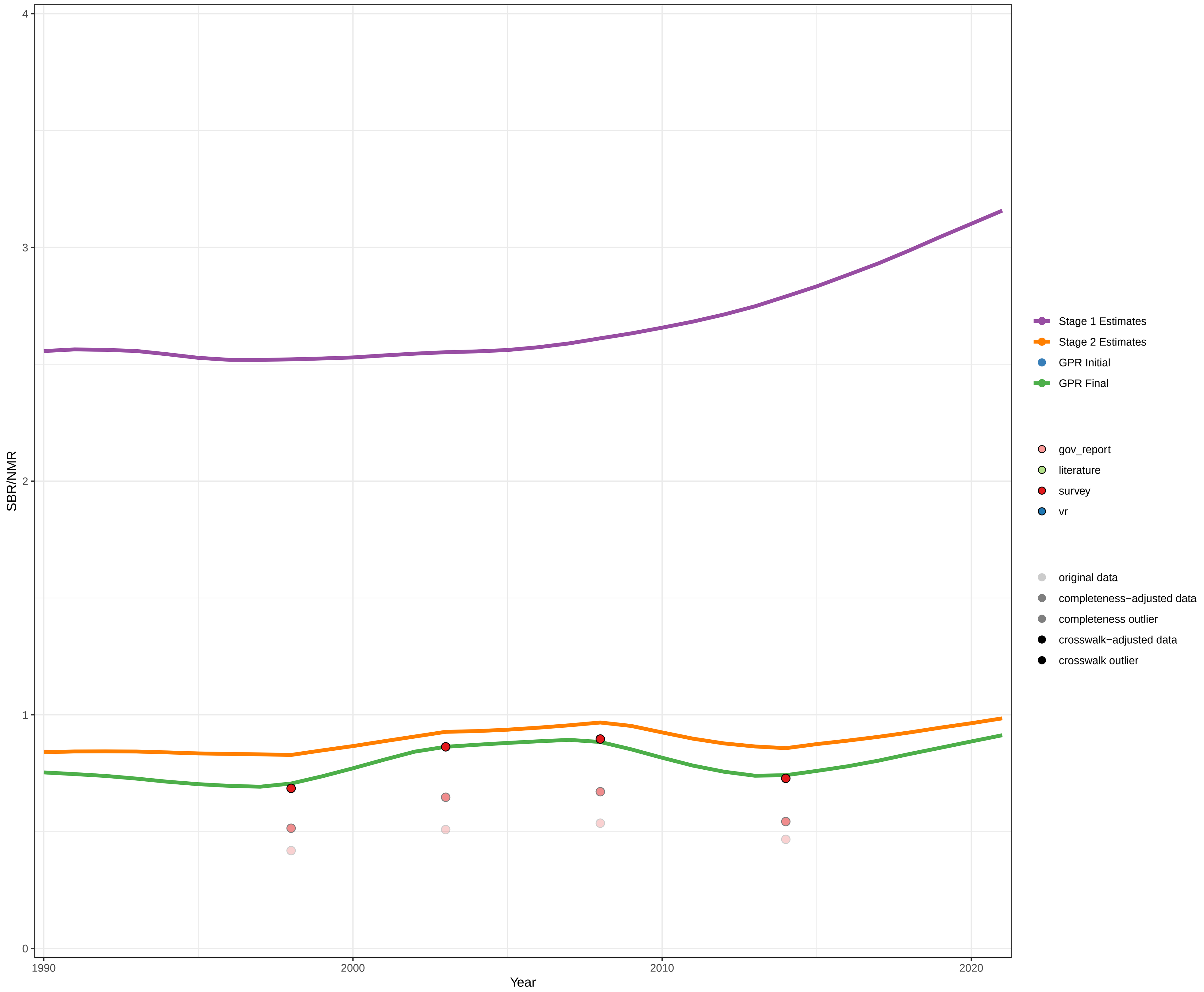

Kisii   KEN_35632   Eastern Sub−Saharan Africa   Stillbirth Rate / Neonatal Mortality Rate (SBR/NMR) − 28 weeks
lambda = 0.8 ; zeta = 0.7 ; scale = 15 ; amp = 0.028

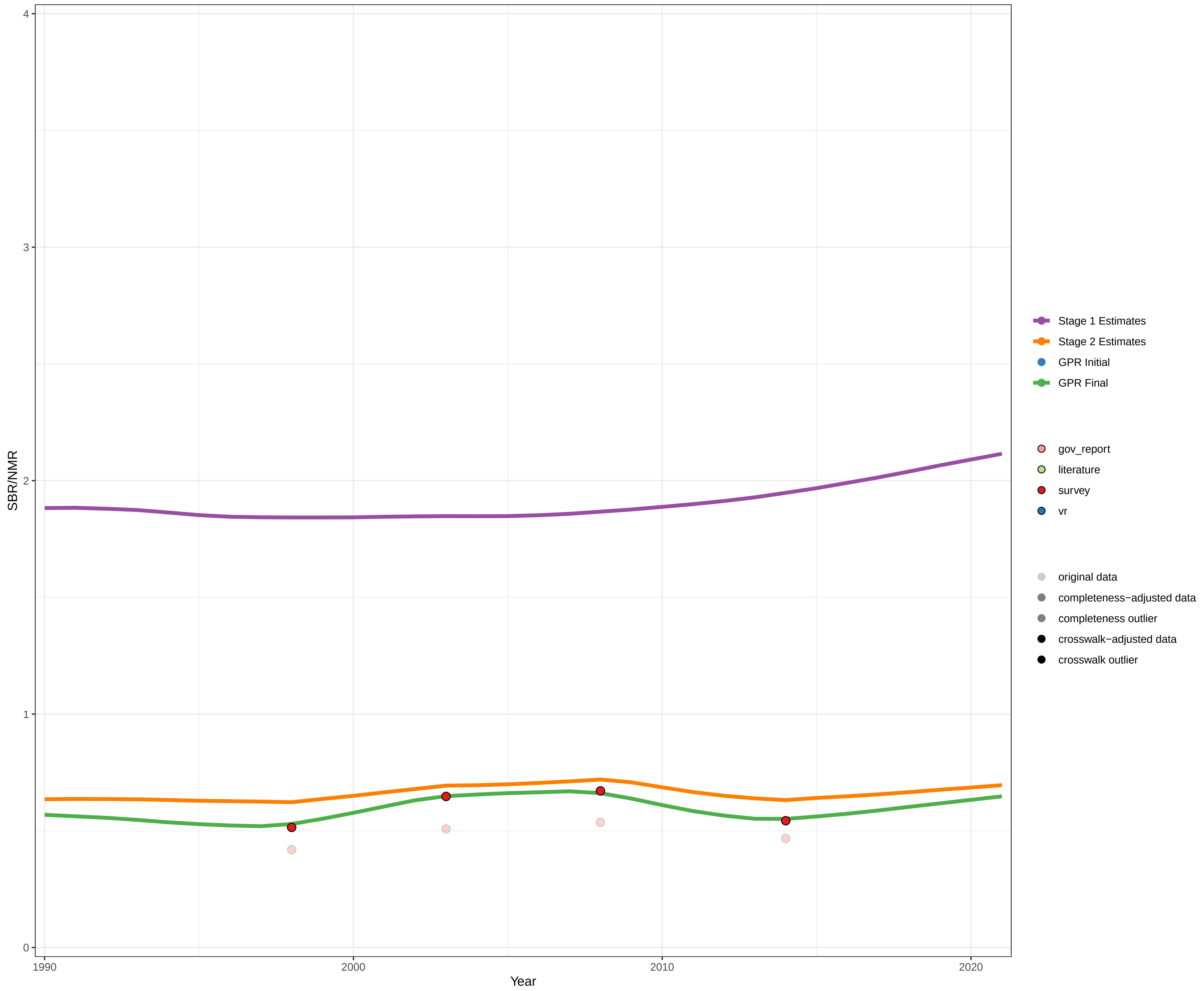

Definition	Years	Outlier	Source.Type	Title
28_weeks	1998	0	Survey	Kenya Demographic and Health Survey 1998
28_weeks	2003	0	Survey	Kenya Demographic and Health Survey 2003
28_weeks	2008	0	Survey	Kenya Demographic and Health Survey 2008−2009
28_weeks	2014	0	Survey	Kenya Demographic and Health Survey 2014

Kisii   KEN_35632   Eastern Sub−Saharan Africa   Stillbirth Rate (SBR) − 20 weeks
lambda = 0.8 ; zeta = 0.7 ; scale = 15 ; amp = 0.028

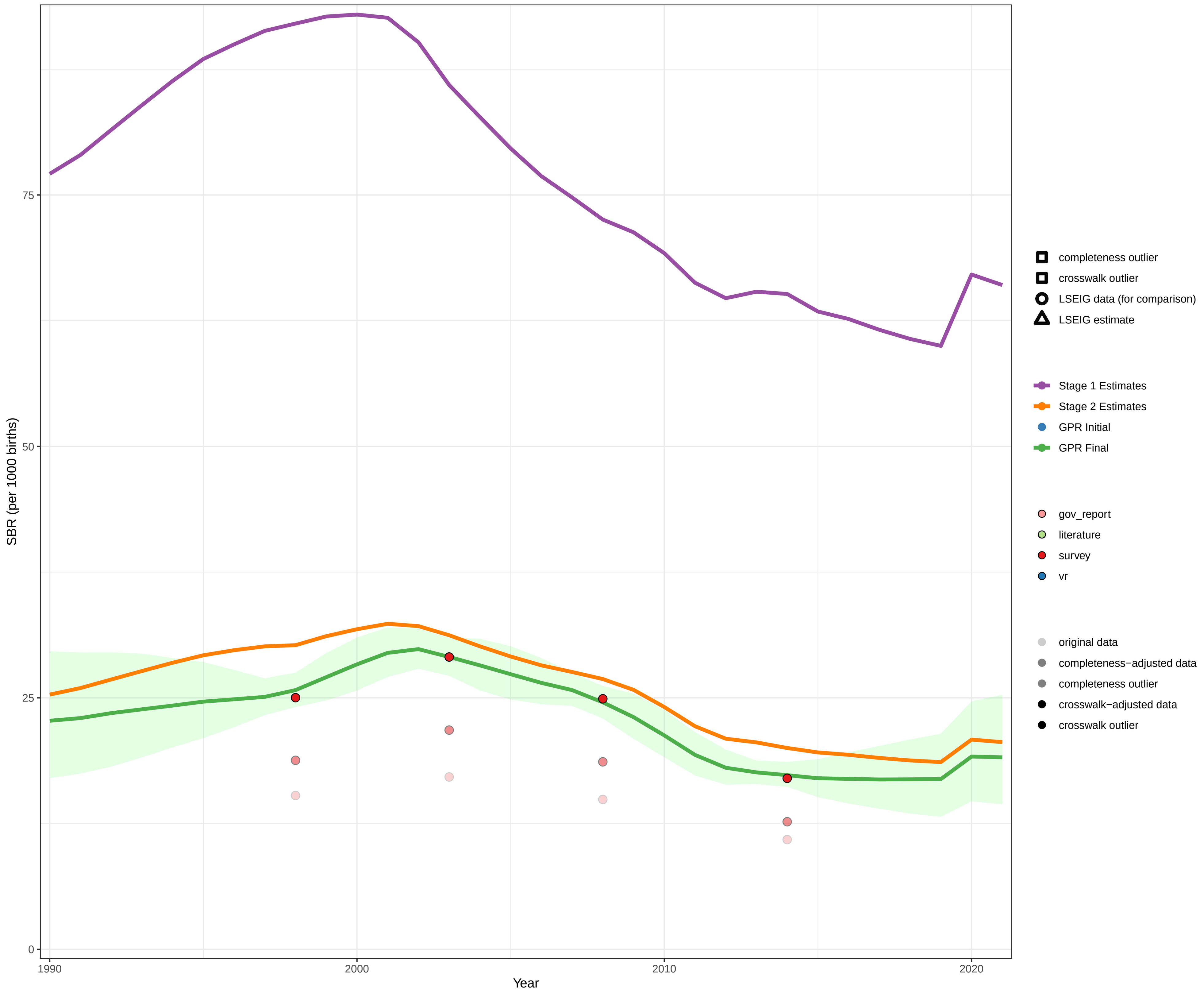

Kisii   KEN_35632   Eastern Sub−Saharan Africa   Stillbirth Rate (SBR) − 28 weeks
lambda = 0.8 ; zeta = 0.7 ; scale = 15 ; amp = 0.028

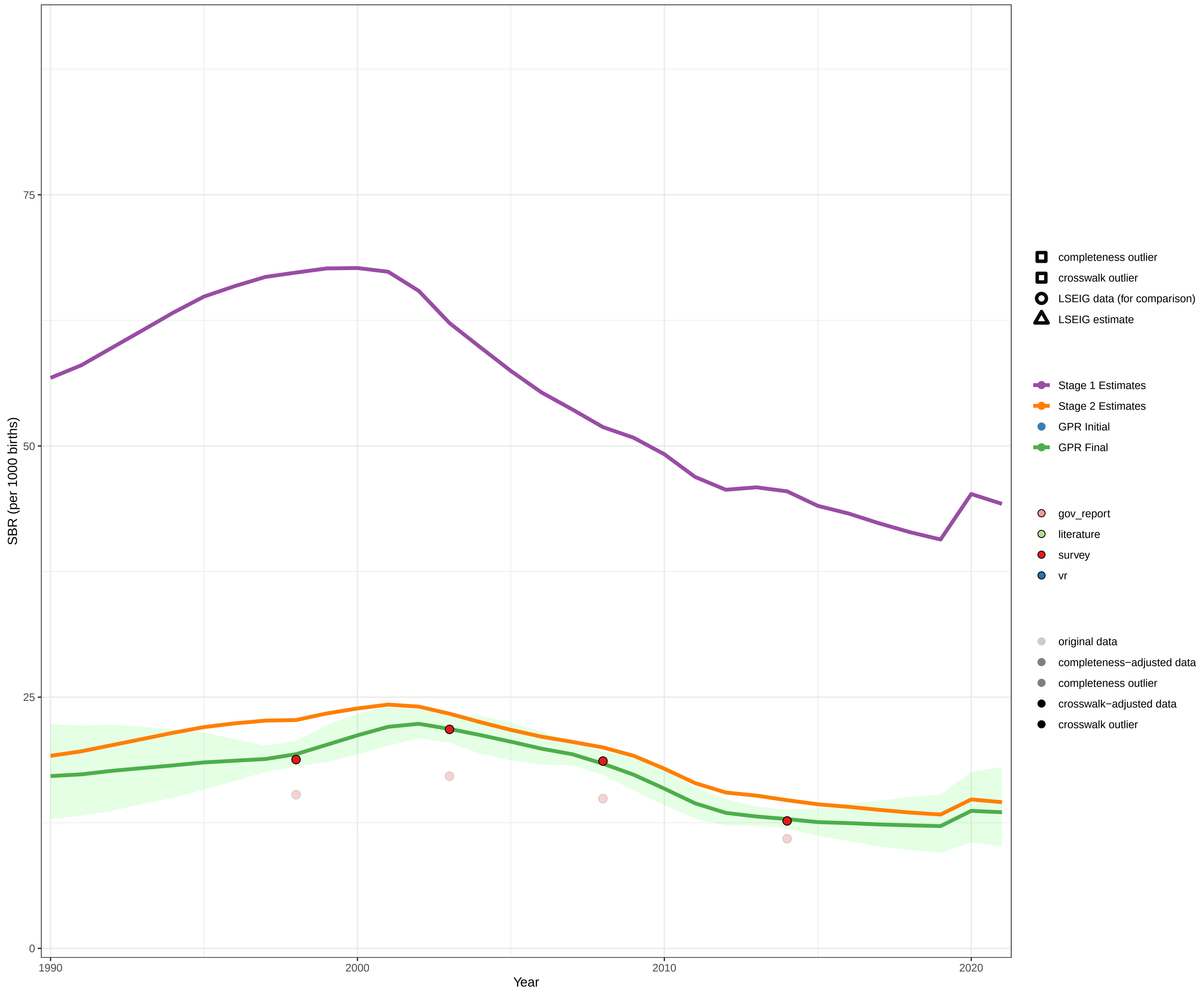

Year	Total Stillbirths (in thousands)	Total Stillbirths (in thousands)	SBR (per 1000 births)	SBR (per 1000 births)	SBR/NMR	SBR/NMR
	20 weeks	28 weeks	20 weeks	28 weeks	20 weeks	28 weeks
1990	1.0 (0.7 to 1.3)	0.7 (0.6 to 1.0)	22.7 (17.0 to 29.6)	17.1 (12.8 to 22.3)	0.75 (0.56 to 0.98)	0.57 (0.43 to 0.74)
1995	1.2 (1.0 to 1.4)	0.9 (0.8 to 1.0)	24.6 (21.0 to 28.6)	18.5 (15.8 to 21.5)	0.70 (0.60 to 0.82)	0.53 (0.45 to 0.61)
2000	1.5 (1.4 to 1.6)	1.1 (1.0 to 1.2)	28.3 (25.7 to 31.0)	21.2 (19.3 to 23.4)	0.77 (0.70 to 0.84)	0.58 (0.53 to 0.64)
2005	1.4 (1.3 to 1.5)	1.0 (0.9 to 1.1)	27.4 (24.8 to 30.1)	20.6 (18.7 to 22.5)	0.88 (0.80 to 0.97)	0.66 (0.60 to 0.72)
2010	0.9 (0.8 to 1.1)	0.7 (0.6 to 0.8)	21.3 (19.1 to 23.8)	15.9 (14.2 to 17.7)	0.82 (0.73 to 0.91)	0.61 (0.55 to 0.68)
2015	0.6 (0.6 to 0.7)	0.5 (0.4 to 0.5)	17.0 (15.1 to 18.9)	12.6 (11.2 to 13.9)	0.76 (0.68 to 0.84)	0.56 (0.50 to 0.62)
2020	0.6 (0.4 to 0.7)	0.4 (0.3 to 0.5)	19.2 (14.7 to 24.7)	13.7 (10.5 to 17.6)	0.89 (0.68 to 1.14)	0.63 (0.49 to 0.81)
2021	0.5 (0.4 to 0.7)	0.4 (0.3 to 0.5)	19.1 (14.4 to 25.3)	13.5 (10.1 to 18.0)	0.91 (0.69 to 1.21)	0.65 (0.49 to 0.86)

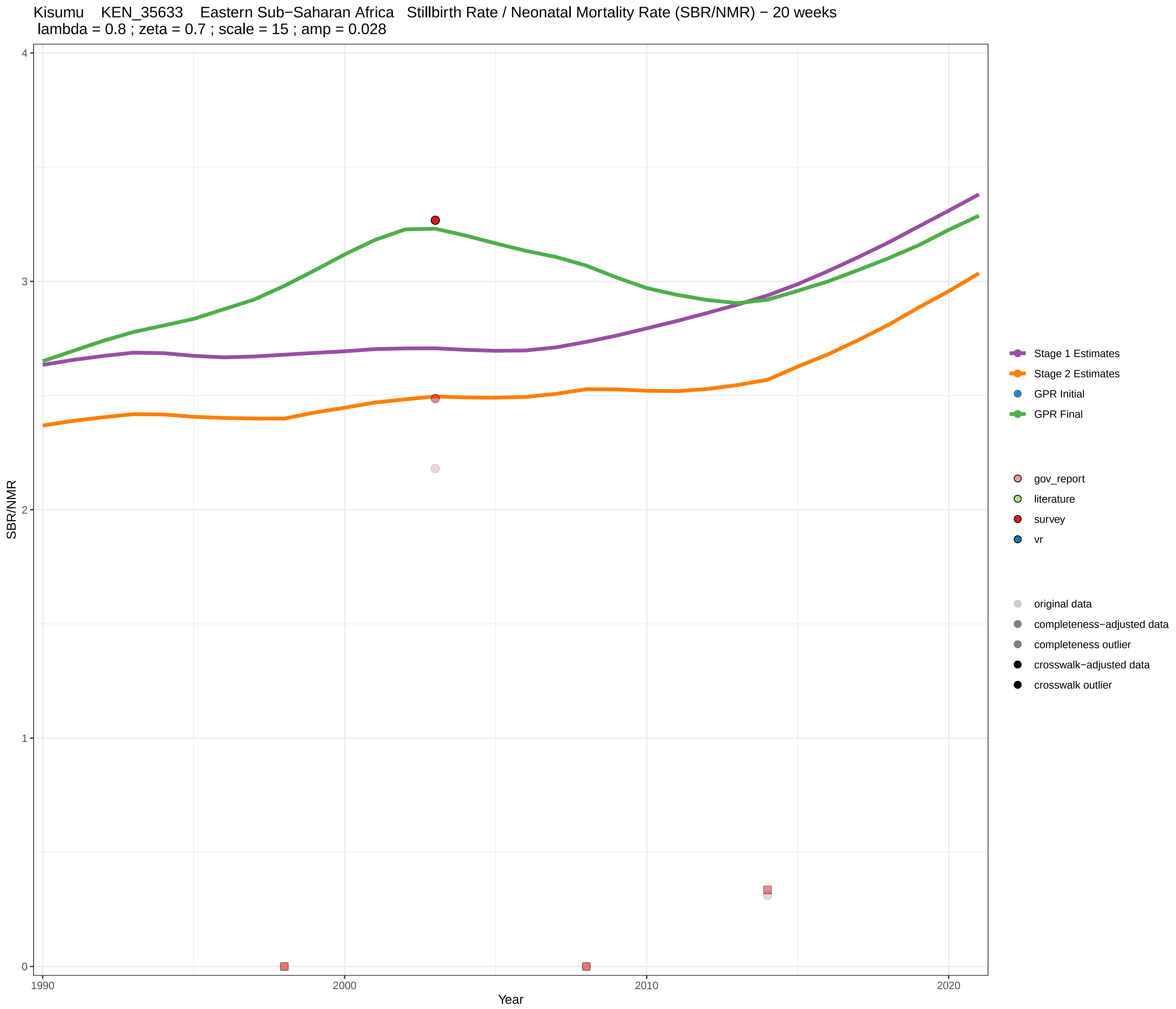

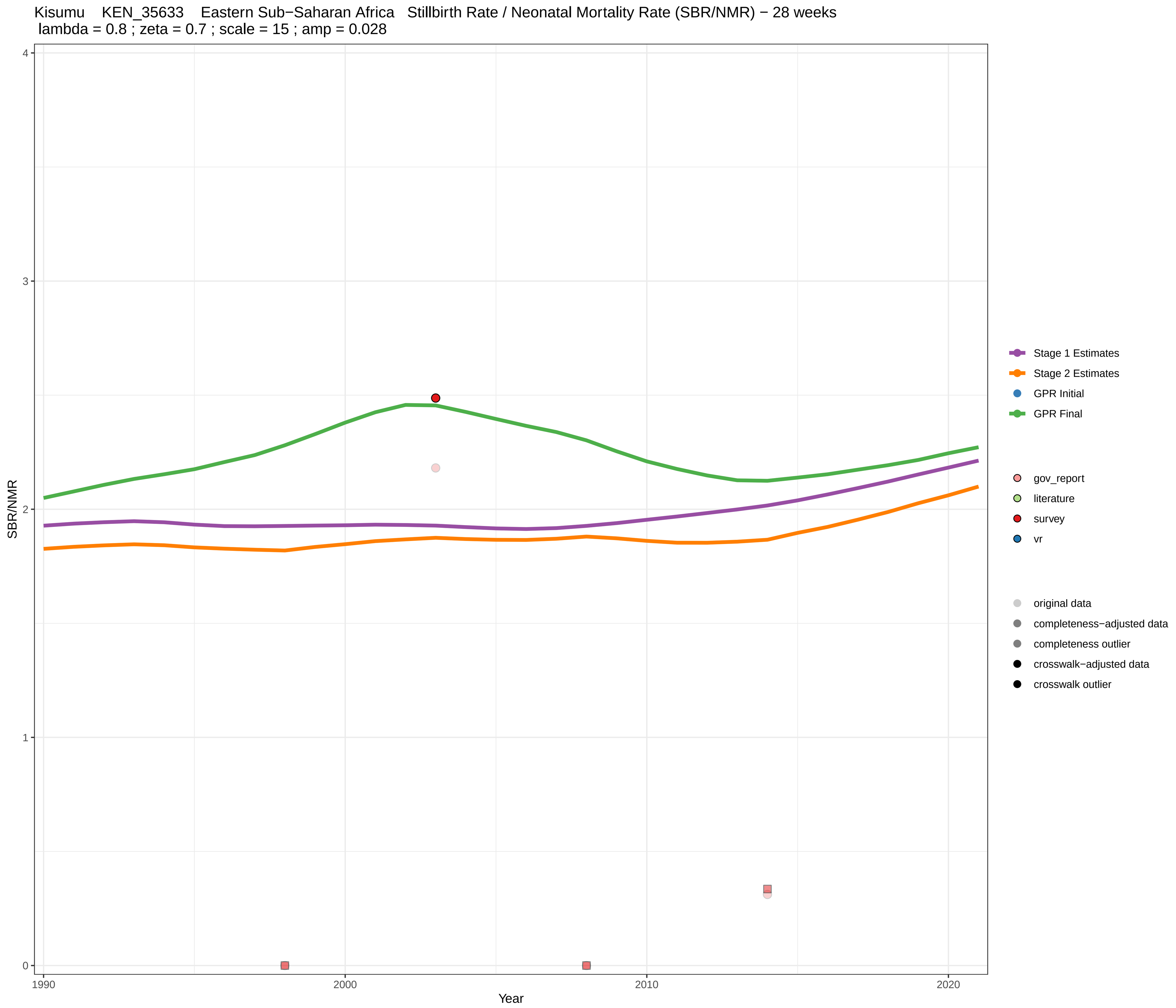

Definition	Years	Outlier	Source.Type	Title
28_weeks	2003	0	Survey	Kenya Demographic and Health Survey 2003
28_weeks	1998	1	Survey	Kenya Demographic and Health Survey 1998
28_weeks	2008	1	Survey	Kenya Demographic and Health Survey 2008−2009
28_weeks	2014	1	Survey	Kenya Demographic and Health Survey 2014

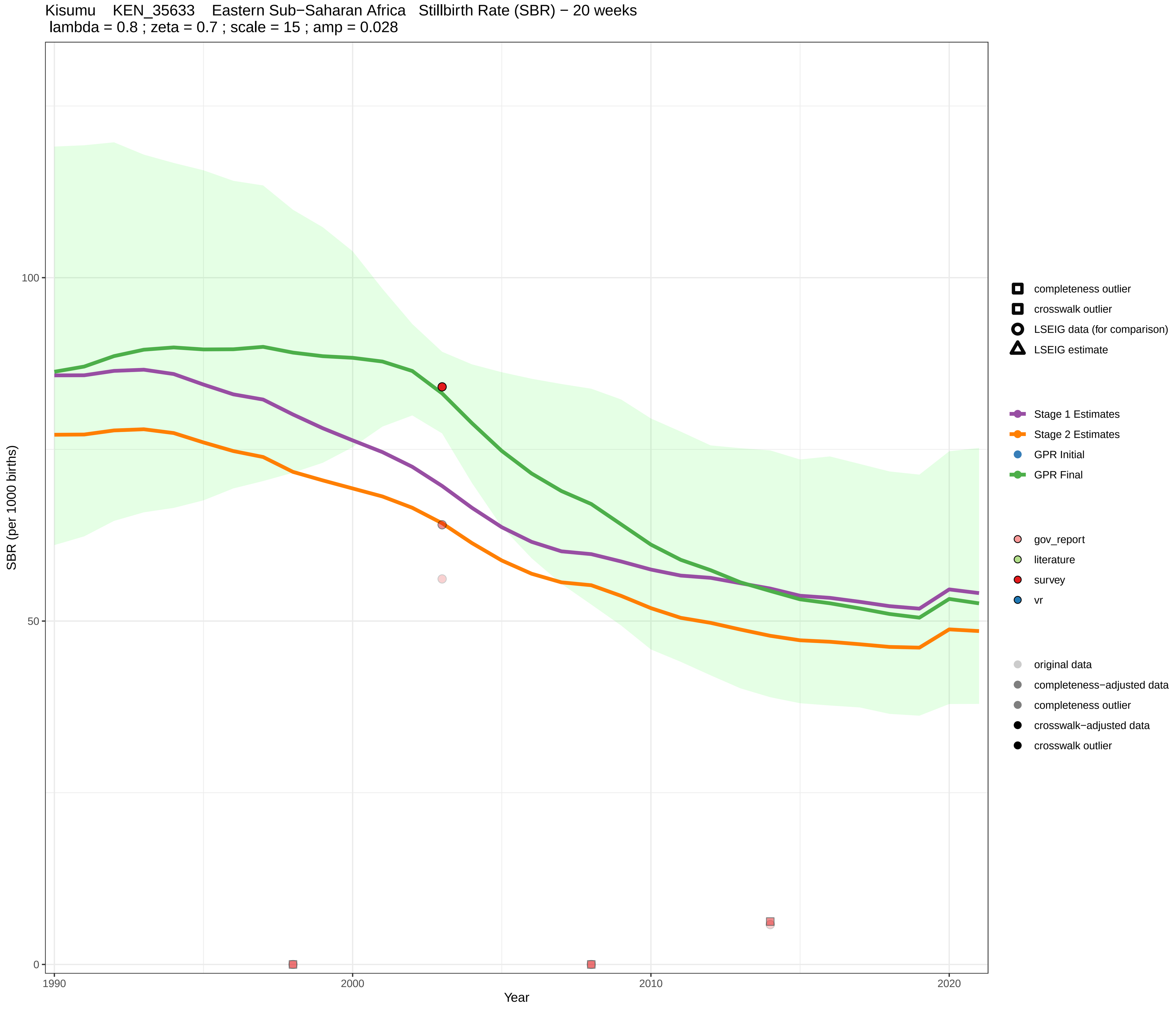

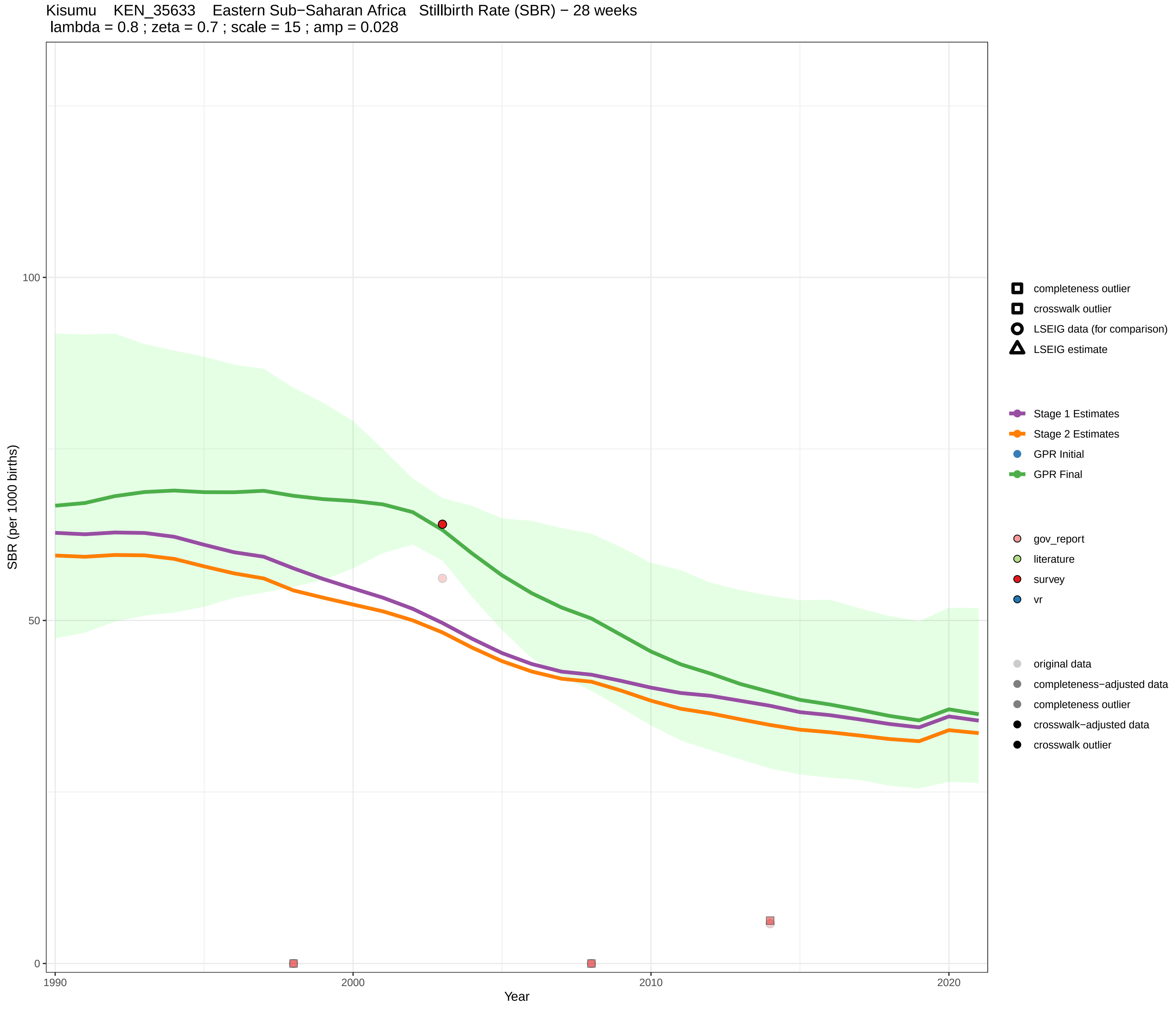

Year	Total Stillbirths (in thousands)	Total Stillbirths (in thousands)	SBR (per 1000 births)	SBR (per 1000 births)	SBR/NMR	SBR/NMR
	20 weeks	28 weeks	20 weeks	28 weeks	20 weeks	28 weeks
1990	3.2 (2.2 to 4.6)	2.5 (1.7 to 3.5)	86.3 (61.1 to 119.1)	66.7 (47.4 to 91.8)	2.65 (1.88 to 3.66)	2.05 (1.46 to 2.82)
1995	3.4 (2.5 to 4.5)	2.6 (1.9 to 3.4)	89.6 (67.6 to 115.6)	68.7 (52.0 to 88.5)	2.84 (2.14 to 3.66)	2.18 (1.65 to 2.80)
2000	3.4 (2.9 to 4.1)	2.5 (2.1 to 3.0)	88.3 (75.3 to 103.8)	67.4 (57.6 to 79.1)	3.12 (2.66 to 3.67)	2.38 (2.03 to 2.79)
2005	2.9 (2.4 to 3.4)	2.1 (1.8 to 2.5)	74.8 (63.7 to 86.2)	56.6 (48.6 to 64.9)	3.17 (2.70 to 3.65)	2.40 (2.06 to 2.75)
2010	2.3 (1.7 to 3.0)	1.7 (1.3 to 2.2)	61.1 (45.9 to 79.5)	45.5 (34.7 to 58.4)	2.97 (2.23 to 3.86)	2.21 (1.69 to 2.84)
2015	1.9 (1.3 to 2.7)	1.3 (0.9 to 1.9)	53.2 (38.0 to 73.5)	38.4 (27.5 to 53.0)	2.96 (2.12 to 4.09)	2.14 (1.53 to 2.95)
2020	1.7 (1.2 to 2.4)	1.2 (0.8 to 1.6)	53.2 (37.9 to 74.7)	37.0 (26.5 to 51.9)	3.23 (2.30 to 4.53)	2.25 (1.61 to 3.14)
2021	1.6 (1.2 to 2.4)	1.1 (0.8 to 1.6)	52.6 (37.9 to 75.2)	36.3 (26.3 to 51.8)	3.29 (2.37 to 4.70)	2.27 (1.64 to 3.24)

Kitui KEN_35634 Eastern Sub−Saharan Africa Stillbirth Rate / Neonatal Mortality Rate (SBR/NMR) − 20 weeks
lambda = 0.8 ; zeta = 0.7 ; scale = 15 ; amp = 0.028

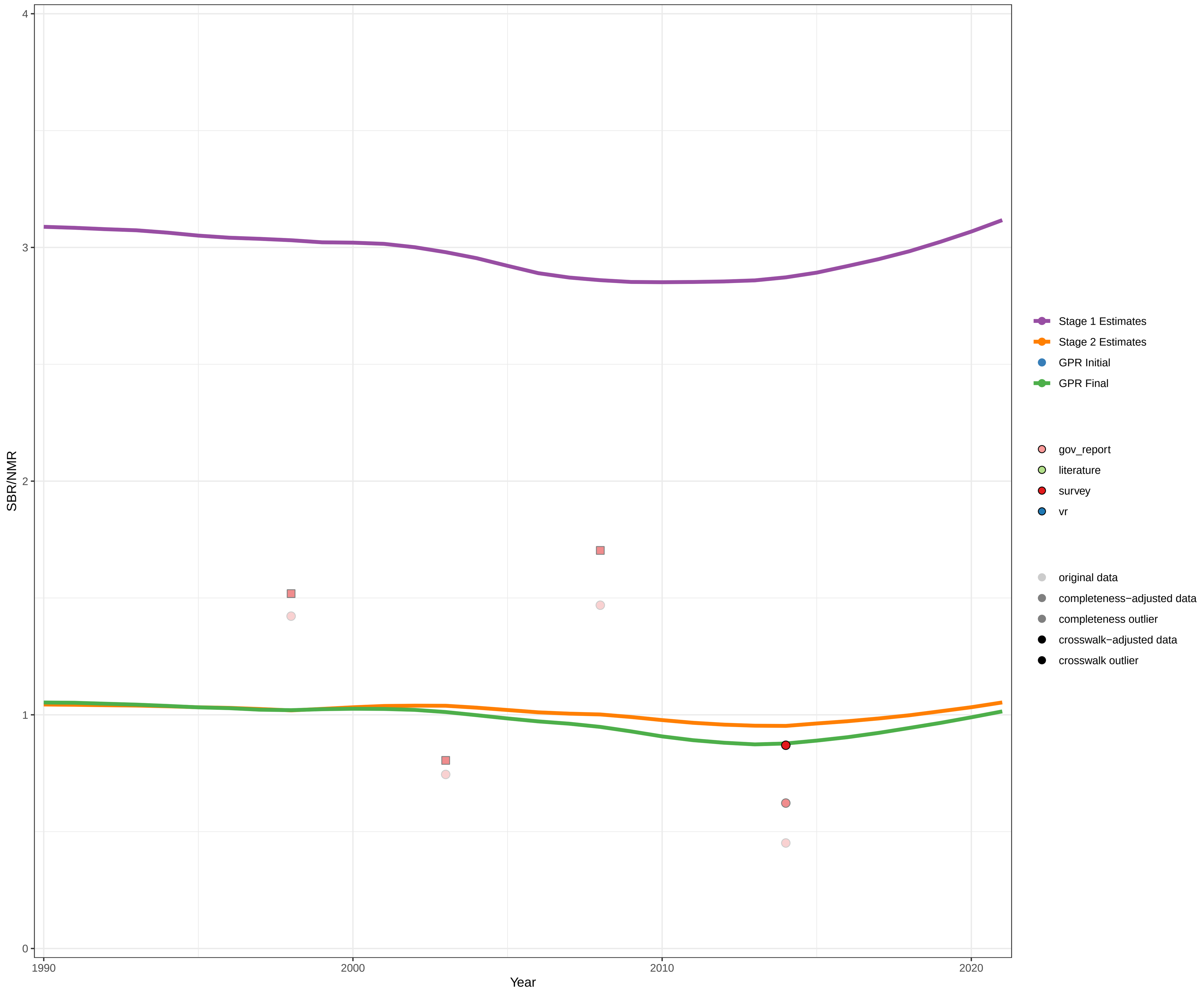

Kitui KEN_35634 Eastern Sub−Saharan Africa Stillbirth Rate / Neonatal Mortality Rate (SBR/NMR) − 28 weeks
lambda = 0.8 ; zeta = 0.7 ; scale = 15 ; amp = 0.028

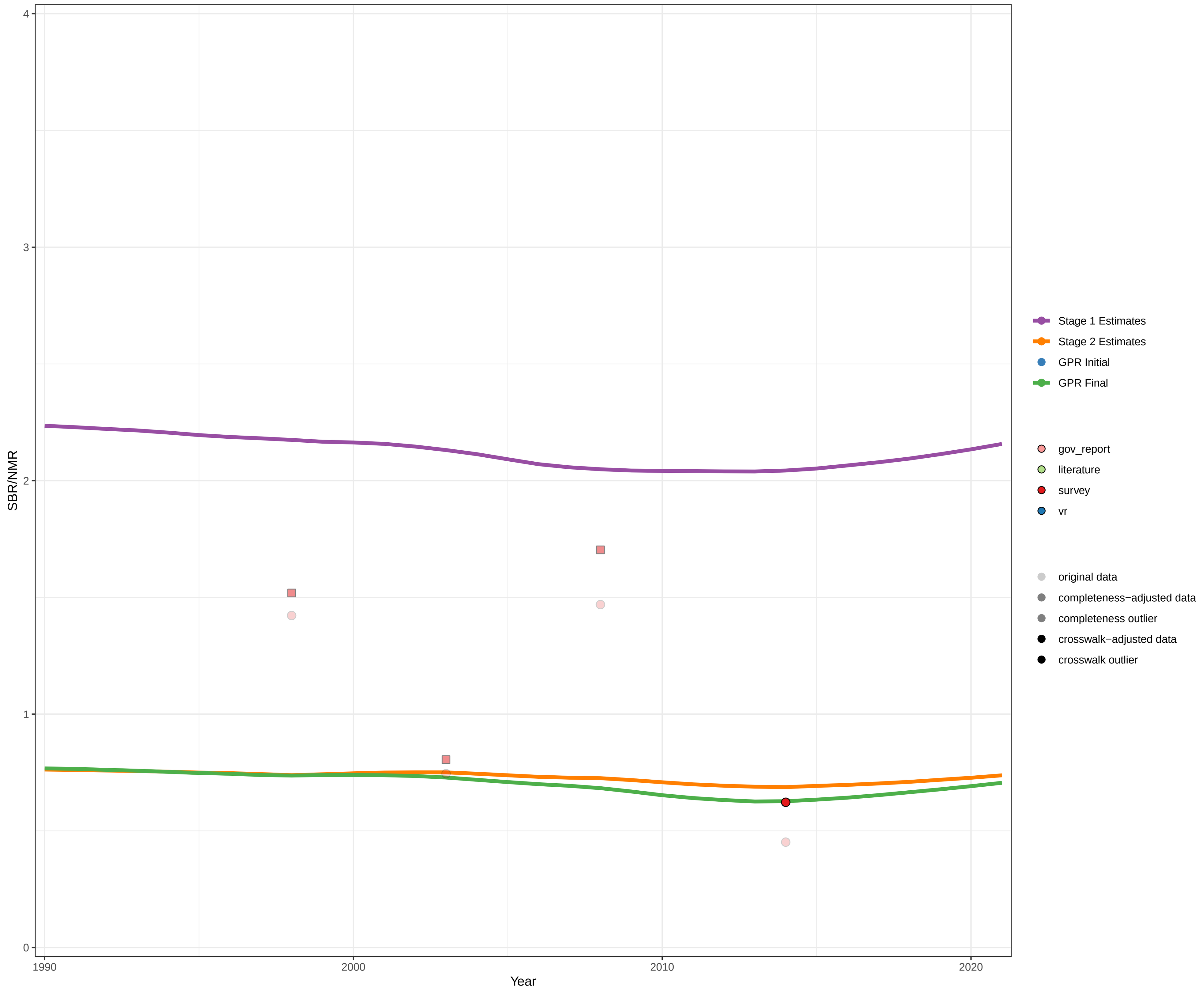

Definition	Years	Outlier	Source.Type	Title
28_weeks	2014	0	Survey	Kenya Demographic and Health Survey 2014
28_weeks	1998	1	Survey	Kenya Demographic and Health Survey 1998
28_weeks	2003	1	Survey	Kenya Demographic and Health Survey 2003
28_weeks	2008	1	Survey	Kenya Demographic and Health Survey 2008−2009

Kitui KEN_35634 Eastern Sub−Saharan Africa Stillbirth Rate (SBR) − 20 weeks
lambda = 0.8 ; zeta = 0.7 ; scale = 15 ; amp = 0.028

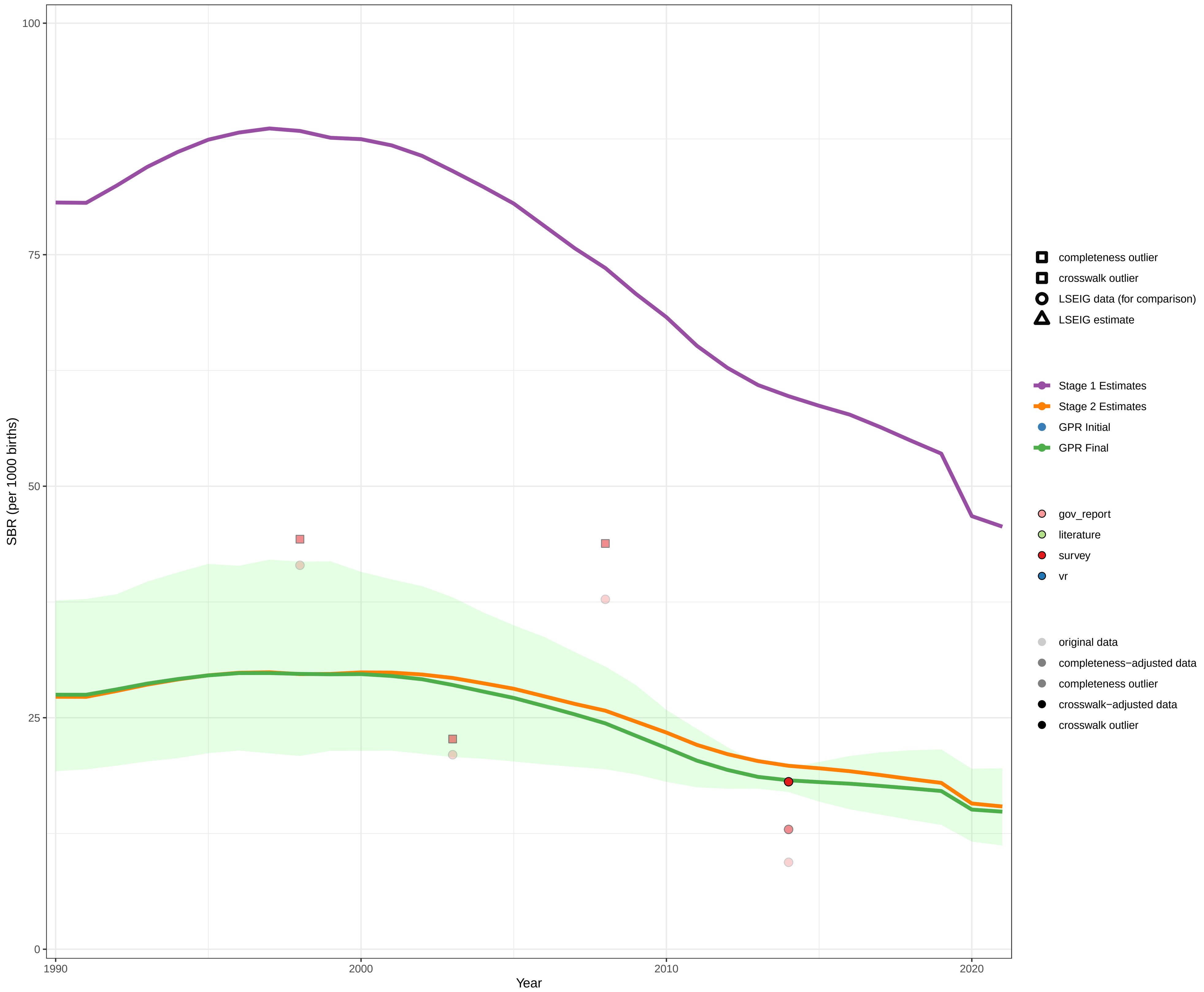

Kitui KEN_35634 Eastern Sub−Saharan Africa Stillbirth Rate (SBR) − 28 weeks
lambda = 0.8 ; zeta = 0.7 ; scale = 15 ; amp = 0.028

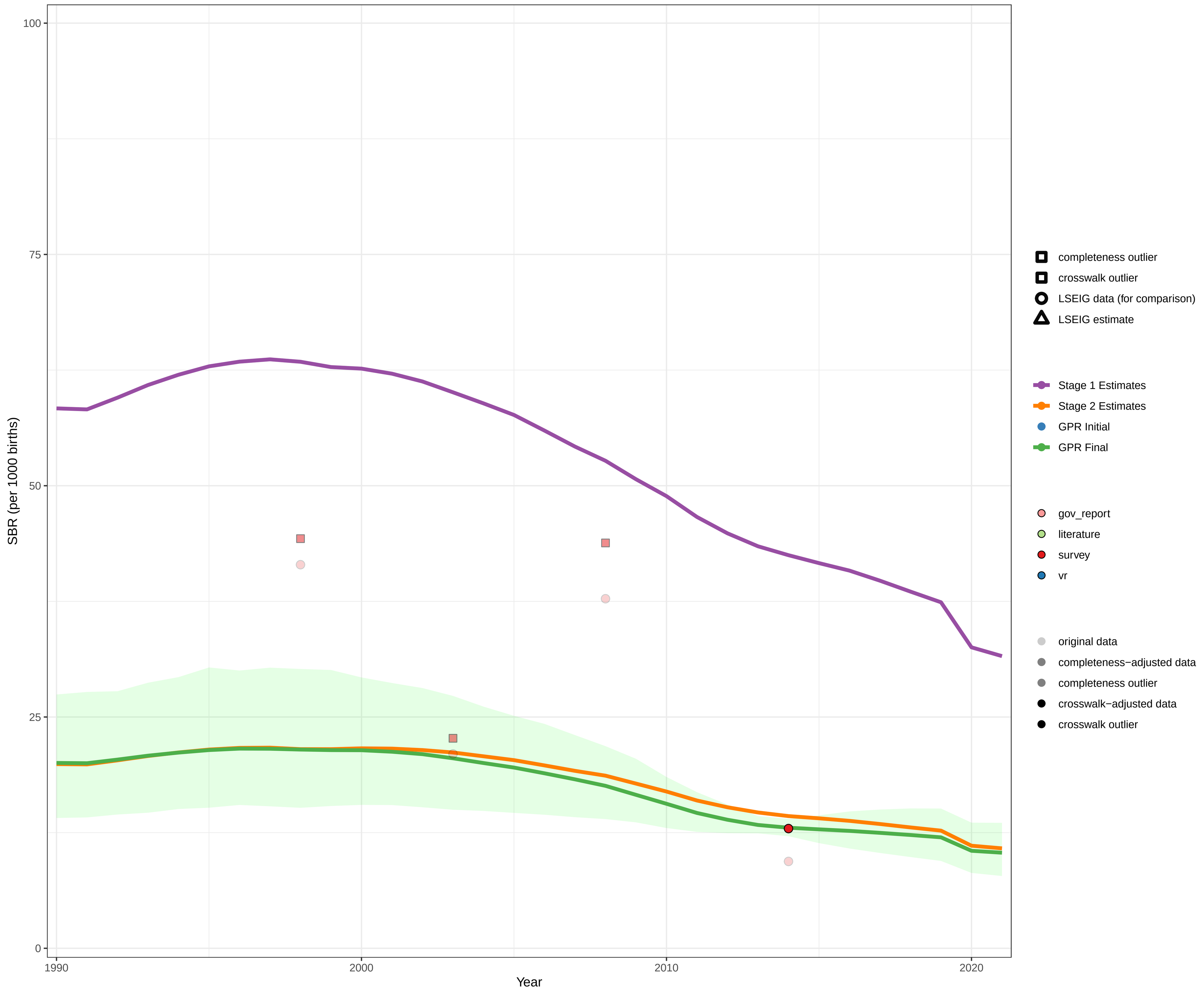

Year	Total Stillbirths (in thousands)	Total Stillbirths (in thousands)	SBR (per 1000 births)	SBR (per 1000 births)	SBR/NMR	SBR/NMR
	20 weeks	28 weeks	20 weeks	28 weeks	20 weeks	28 weeks
1990	0.9 (0.6 to 1.2)	0.6 (0.4 to 0.9)	27.5 (19.2 to 37.7)	20.0 (14.1 to 27.4)	1.05 (0.74 to 1.44)	0.77 (0.54 to 1.05)
1995	1.0 (0.7 to 1.4)	0.7 (0.5 to 1.0)	29.6 (21.2 to 41.6)	21.4 (15.2 to 30.4)	1.03 (0.74 to 1.45)	0.75 (0.53 to 1.06)
2000	1.1 (0.8 to 1.5)	0.8 (0.6 to 1.1)	29.7 (21.4 to 40.8)	21.4 (15.5 to 29.3)	1.03 (0.74 to 1.41)	0.74 (0.54 to 1.01)
2005	1.0 (0.7 to 1.3)	0.7 (0.5 to 0.9)	27.1 (20.3 to 35.0)	19.5 (14.6 to 25.1)	0.98 (0.74 to 1.27)	0.71 (0.53 to 0.91)
2010	0.8 (0.6 to 0.9)	0.5 (0.5 to 0.7)	21.7 (18.1 to 25.9)	15.6 (13.0 to 18.5)	0.91 (0.75 to 1.08)	0.65 (0.54 to 0.77)
2015	0.6 (0.5 to 0.7)	0.4 (0.4 to 0.5)	18.1 (15.9 to 20.2)	12.9 (11.4 to 14.4)	0.89 (0.79 to 1.00)	0.63 (0.56 to 0.71)
2020	0.4 (0.3 to 0.6)	0.3 (0.2 to 0.4)	15.1 (11.6 to 19.5)	10.5 (8.1 to 13.6)	0.99 (0.76 to 1.28)	0.69 (0.53 to 0.89)
2021	0.4 (0.3 to 0.6)	0.3 (0.2 to 0.4)	14.9 (11.2 to 19.5)	10.3 (7.8 to 13.6)	1.01 (0.77 to 1.34)	0.71 (0.53 to 0.93)

Kwale KEN_35635 Eastern Sub−Saharan Africa Stillbirth Rate / Neonatal Mortality Rate (SBR/NMR) − 20 weeks
lambda = 0.8 ; zeta = 0.7 ; scale = 15 ; amp = 0.028

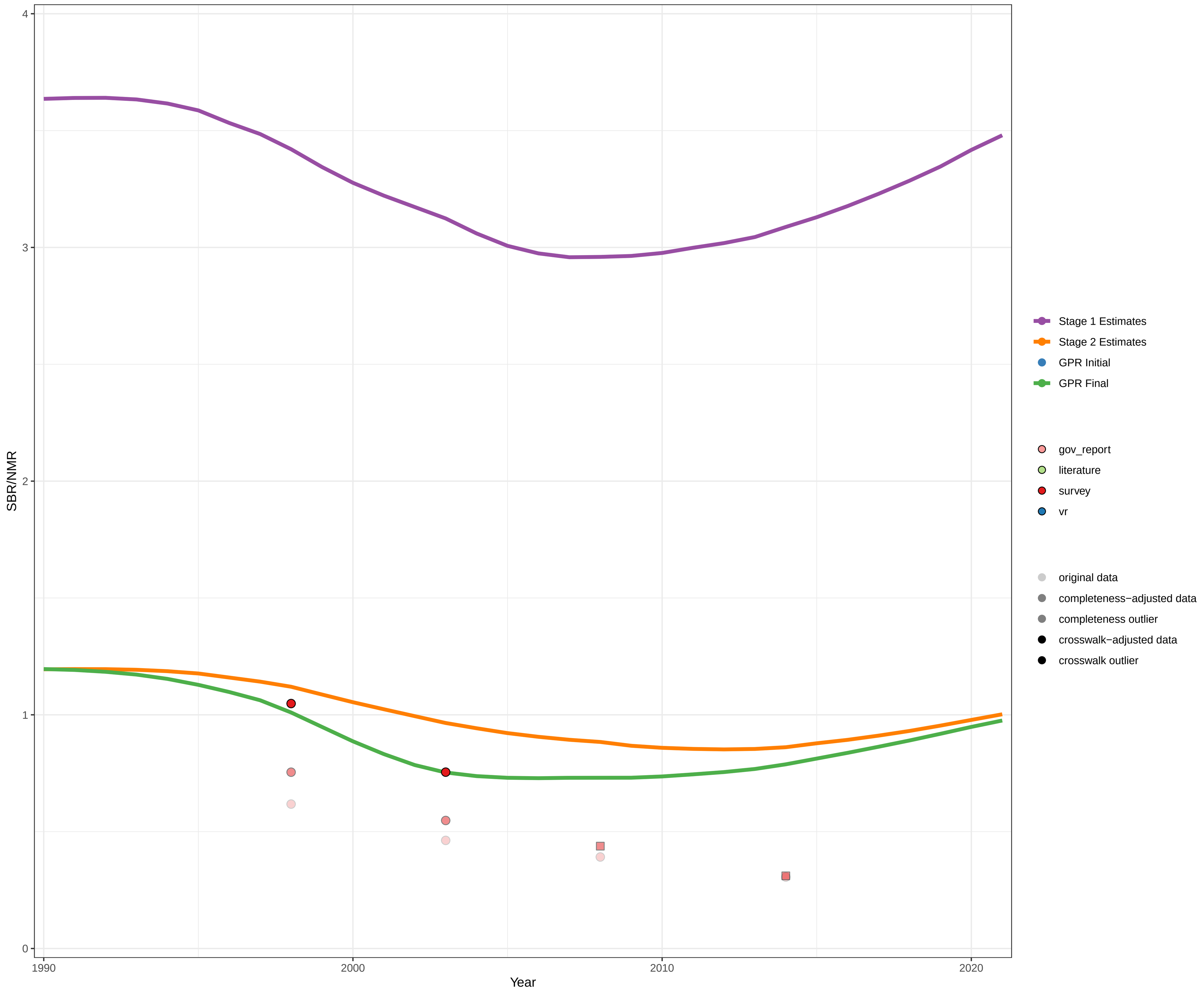

Kwale KEN_35635 Eastern Sub−Saharan Africa Stillbirth Rate / Neonatal Mortality Rate (SBR/NMR) − 28 weeks
lambda = 0.8 ; zeta = 0.7 ; scale = 15 ; amp = 0.028

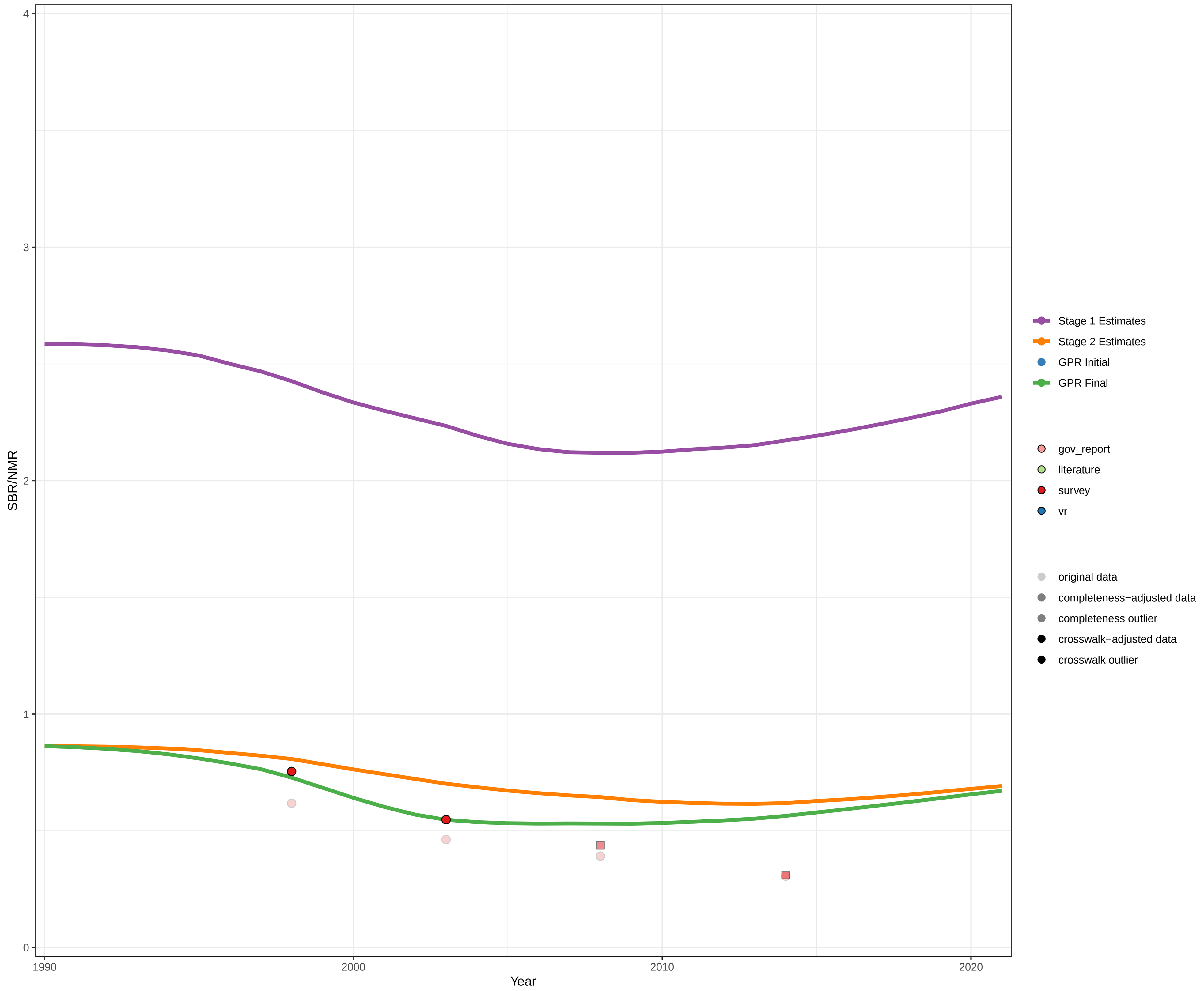

Definition	Years	Outlier	Source.Type	Title
28_weeks	1998	0	Survey	Kenya Demographic and Health Survey 1998
28_weeks	2003	0	Survey	Kenya Demographic and Health Survey 2003
28_weeks	2008	1	Survey	Kenya Demographic and Health Survey 2008−2009
28_weeks	2014	1	Survey	Kenya Demographic and Health Survey 2014

Kwale KEN_35635 Eastern Sub−Saharan Africa Stillbirth Rate (SBR) − 20 weeks
lambda = 0.8 ; zeta = 0.7 ; scale = 15 ; amp = 0.028

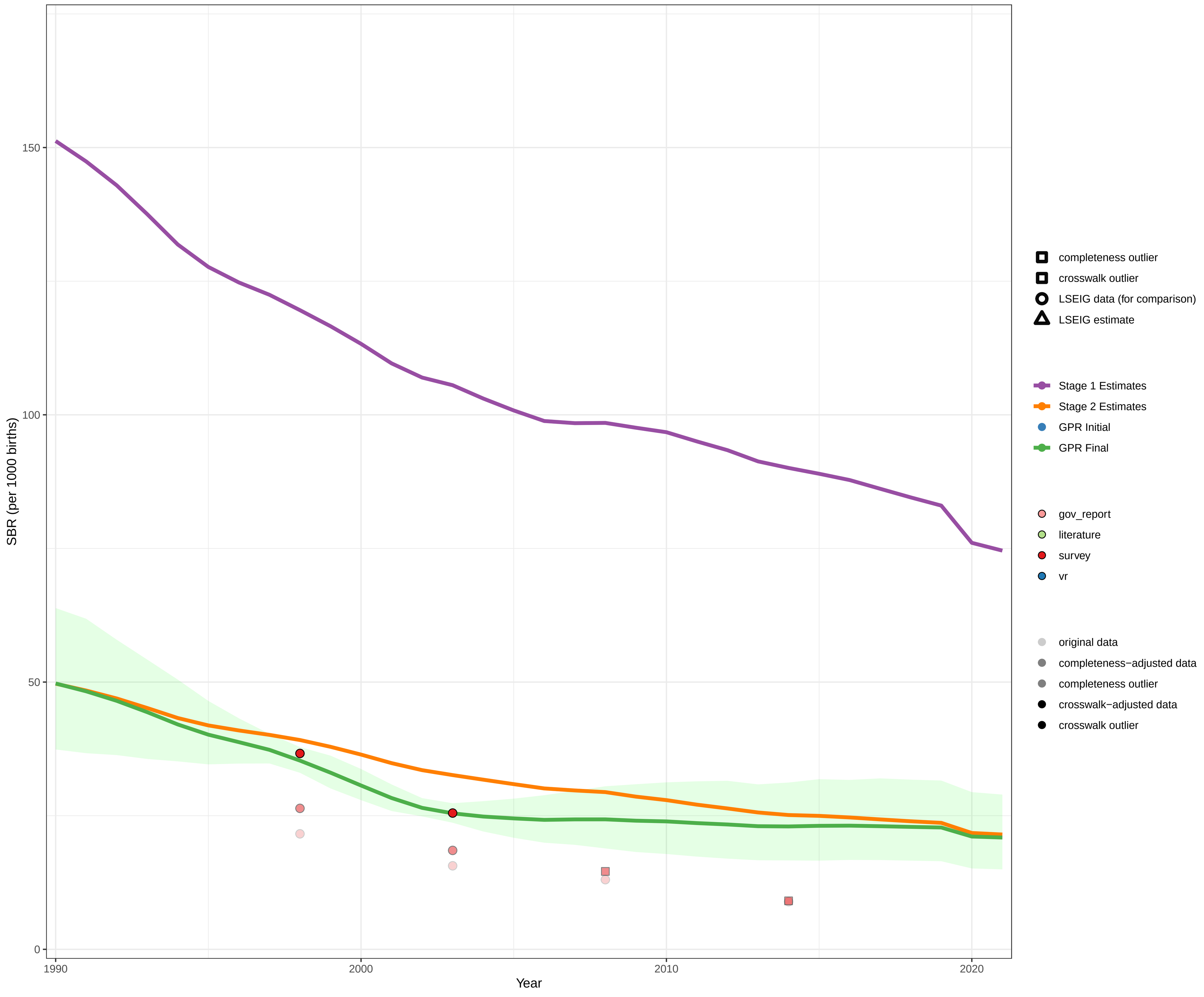

Kwale KEN_35635 Eastern Sub−Saharan Africa Stillbirth Rate (SBR) − 28 weeks
lambda = 0.8 ; zeta = 0.7 ; scale = 15 ; amp = 0.028

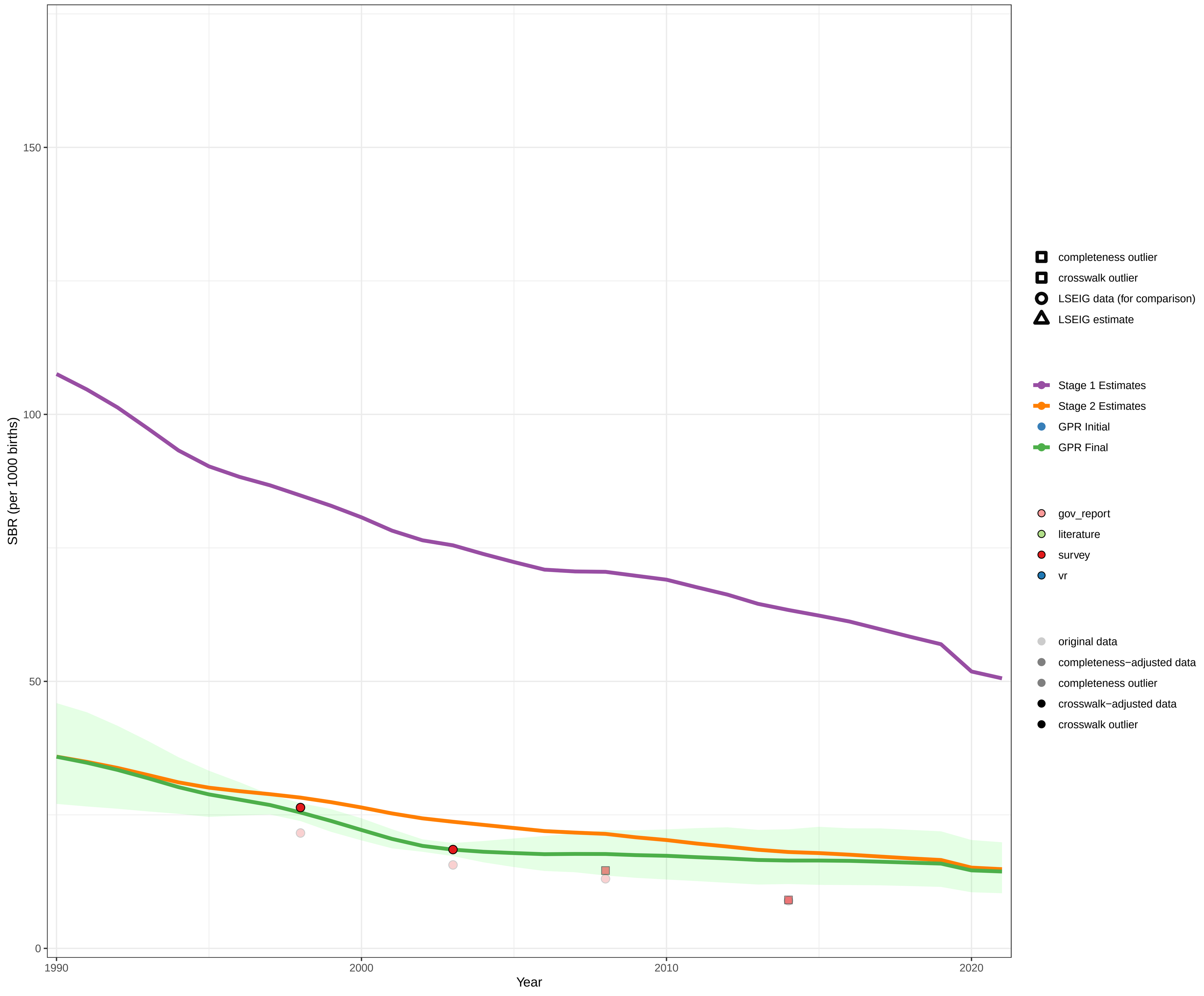

Year	Total Stillbirths (in thousands)	Total Stillbirths (in thousands)	SBR (per 1000 births)	SBR (per 1000 births)	SBR/NMR	SBR/NMR
	20 weeks	28 weeks	20 weeks	28 weeks	20 weeks	28 weeks
1990	1.0<br>(0.7 to 1.3)	0.7<br>(0.5 to 0.9)	49.7<br>(37.4 to 63.8)	35.9<br>(27.1 to 45.9)	1.20<br>(0.90 to 1.54)	0.86<br>(0.65 to 1.10)
1995	0.9<br>(0.7 to 1.0)	0.6<br>(0.5 to 0.7)	40.2<br>(34.6 to 46.5)	28.8<br>(24.6 to 33.3)	1.13<br>(0.97 to 1.31)	0.81<br>(0.69 to 0.93)
2000	0.7<br>(0.7 to 0.8)	0.5<br>(0.5 to 0.6)	30.7<br>(27.9 to 33.7)	22.2<br>(20.2 to 24.4)	0.89<br>(0.81 to 0.98)	0.64<br>(0.58 to 0.70)
2005	0.6<br>(0.5 to 0.7)	0.5<br>(0.4 to 0.5)	24.5<br>(20.9 to 28.2)	17.9<br>(15.2 to 20.6)	0.73<br>(0.62 to 0.84)	0.53<br>(0.45 to 0.61)
2010	0.7<br>(0.5 to 0.9)	0.5<br>(0.4 to 0.6)	23.9<br>(17.8 to 31.2)	17.3<br>(12.9 to 22.3)	0.74<br>(0.55 to 0.96)	0.53<br>(0.40 to 0.69)
2015	0.7<br>(0.5 to 0.9)	0.5<br>(0.3 to 0.7)	23.1<br>(16.6 to 31.8)	16.5<br>(11.9 to 22.8)	0.81<br>(0.58 to 1.12)	0.58<br>(0.42 to 0.80)
2020	0.6<br>(0.4 to 0.8)	0.4<br>(0.3 to 0.6)	21.1<br>(15.1 to 29.4)	14.6<br>(10.5 to 20.3)	0.95<br>(0.68 to 1.32)	0.66<br>(0.47 to 0.91)
2021	0.6<br>(0.4 to 0.8)	0.4<br>(0.3 to 0.6)	20.9<br>(15.0 to 29.0)	14.4<br>(10.3 to 19.9)	0.98<br>(0.70 to 1.35)	0.67<br>(0.48 to 0.93)

Laikipia KEN_35636 Eastern Sub−Saharan Africa Stillbirth Rate / Neonatal Mortality Rate (SBR/NMR) − 20 weeks
lambda = 0.8 ; zeta = 0.7 ; scale = 15 ; amp = 0.028

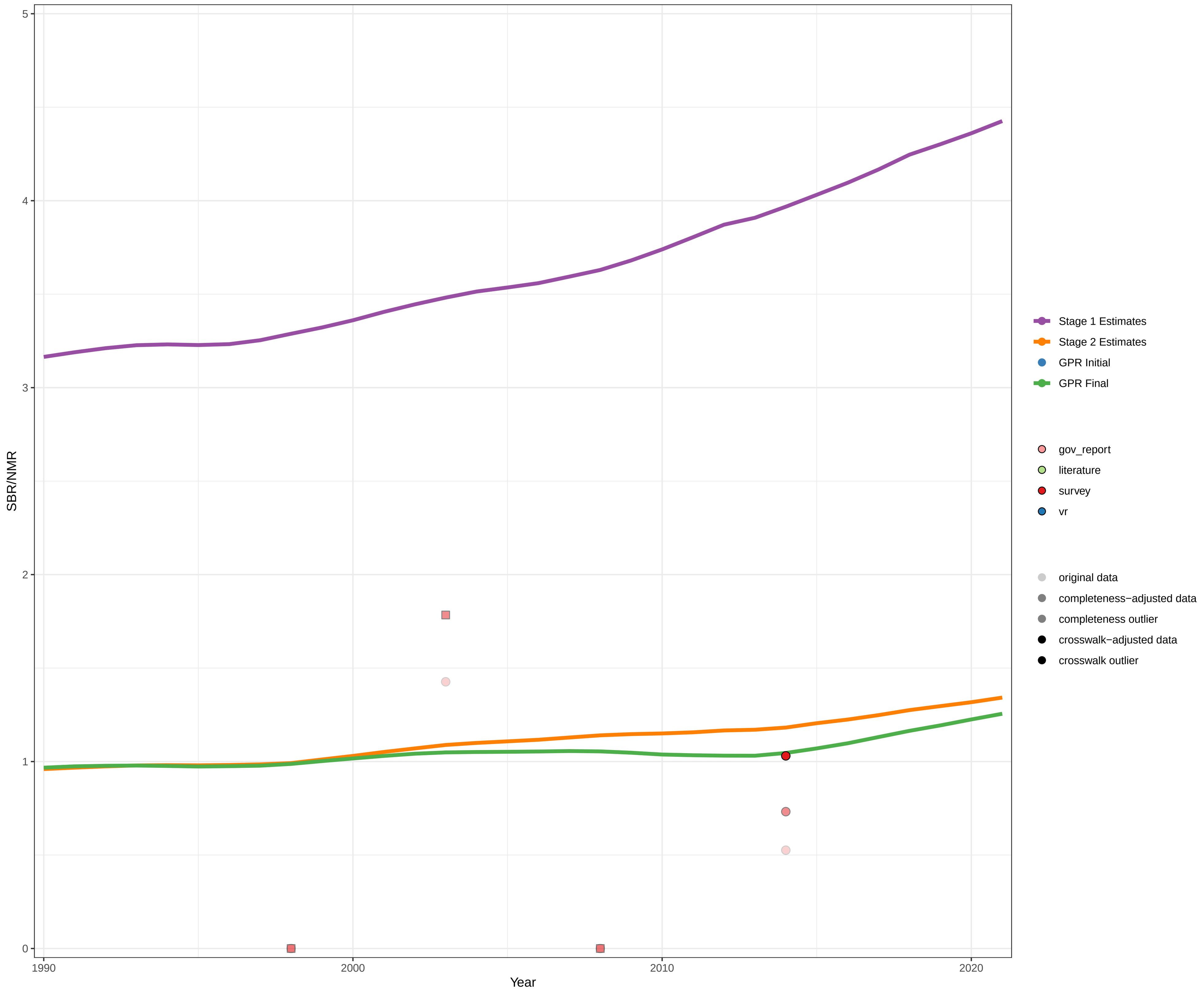

Laikipia KEN_35636 Eastern Sub−Saharan Africa Stillbirth Rate / Neonatal Mortality Rate (SBR/NMR) − 28 weeks
lambda = 0.8 ; zeta = 0.7 ; scale = 15 ; amp = 0.028

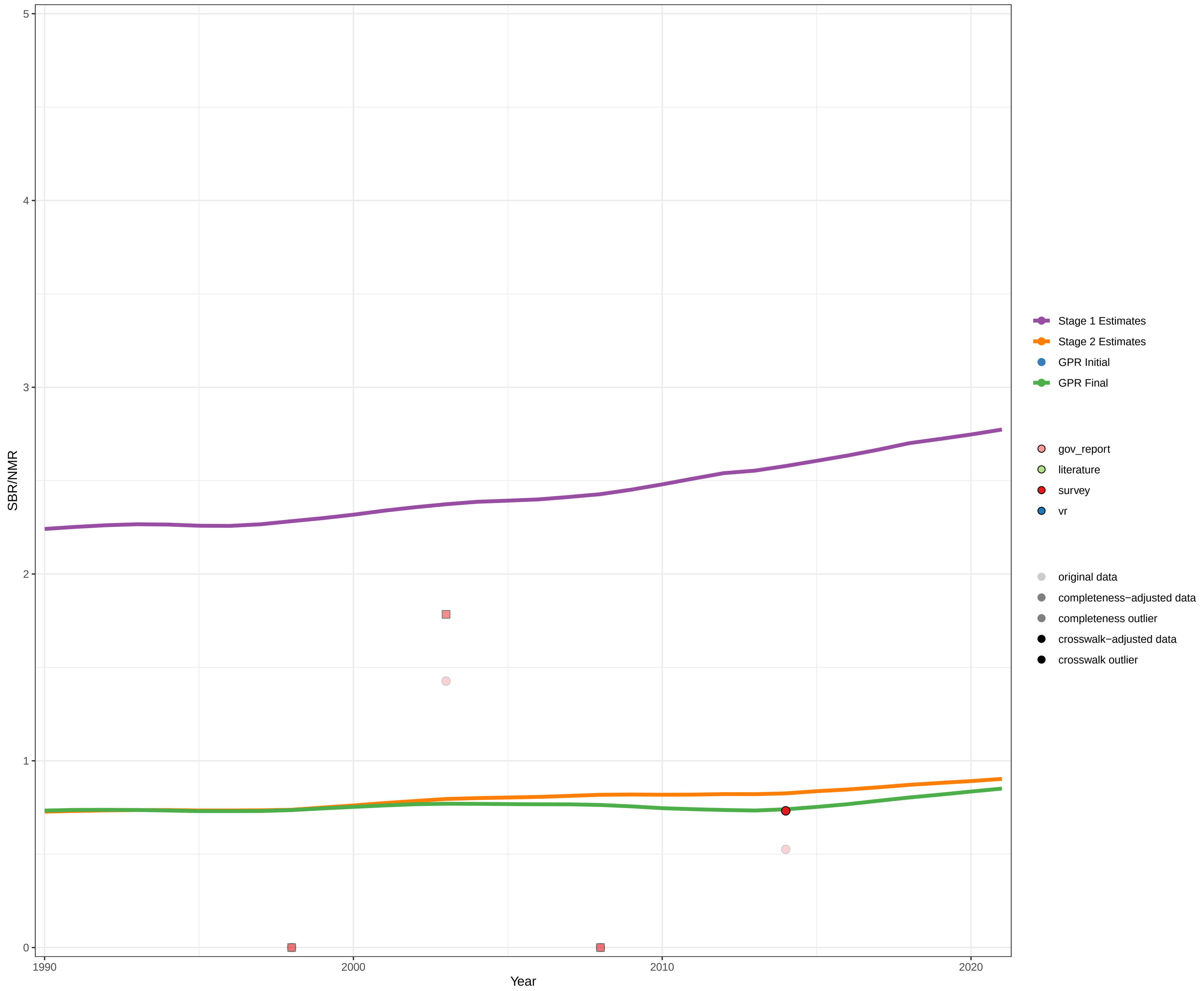

Definition	Years	Outlier	Source.Type	Title
28_weeks	2014	0	Survey	Kenya Demographic and Health Survey 2014
28_weeks	1998	1	Survey	Kenya Demographic and Health Survey 1998
28_weeks	2003	1	Survey	Kenya Demographic and Health Survey 2003
28_weeks	2008	1	Survey	Kenya Demographic and Health Survey 2008−2009

Laikipia KEN_35636 Eastern Sub−Saharan Africa Stillbirth Rate (SBR) − 20 weeks
lambda = 0.8 ; zeta = 0.7 ; scale = 15 ; amp = 0.028

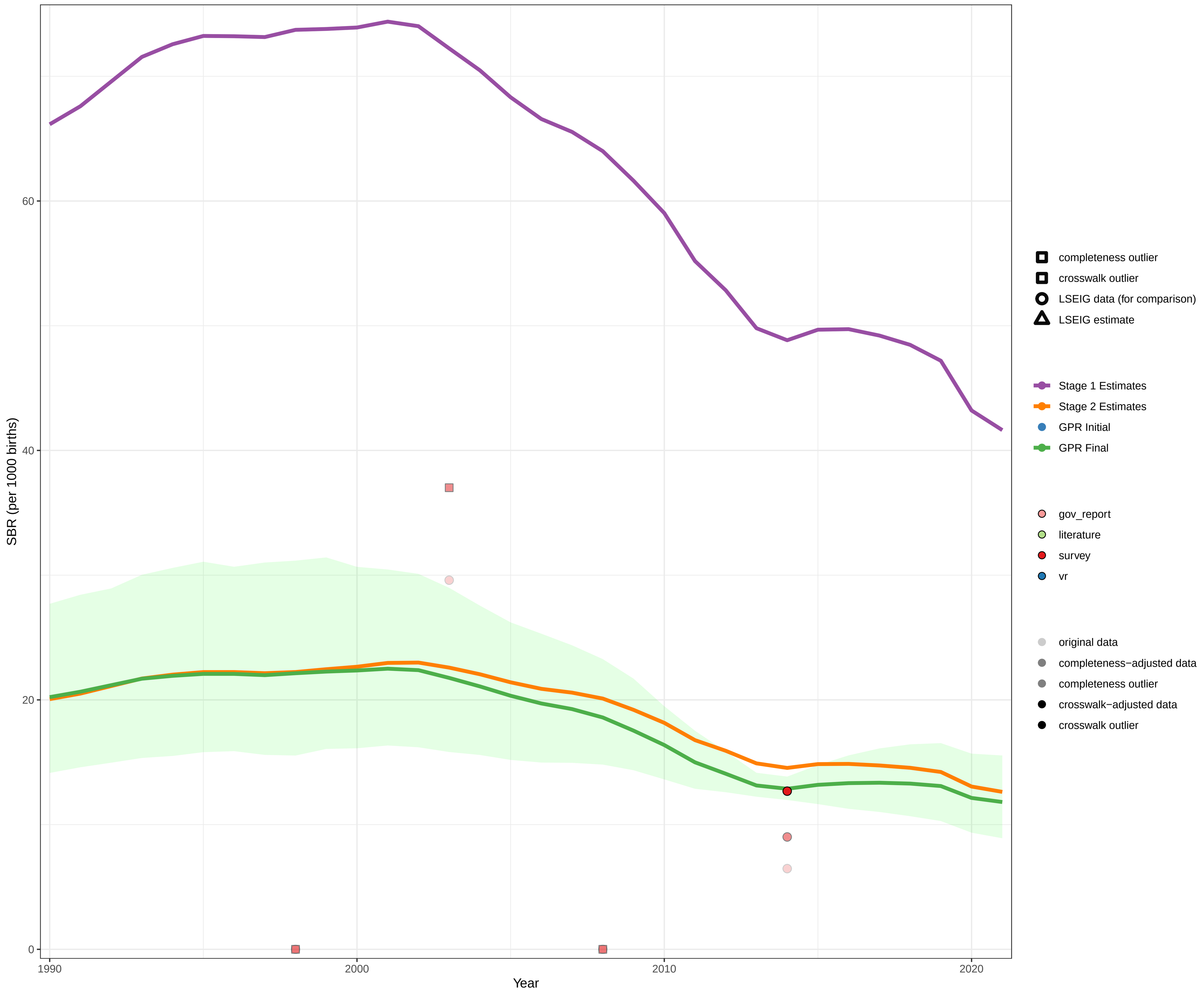

Laikipia KEN_35636 Eastern Sub−Saharan Africa Stillbirth Rate (SBR) − 28 weeks
lambda = 0.8 ; zeta = 0.7 ; scale = 15 ; amp = 0.028

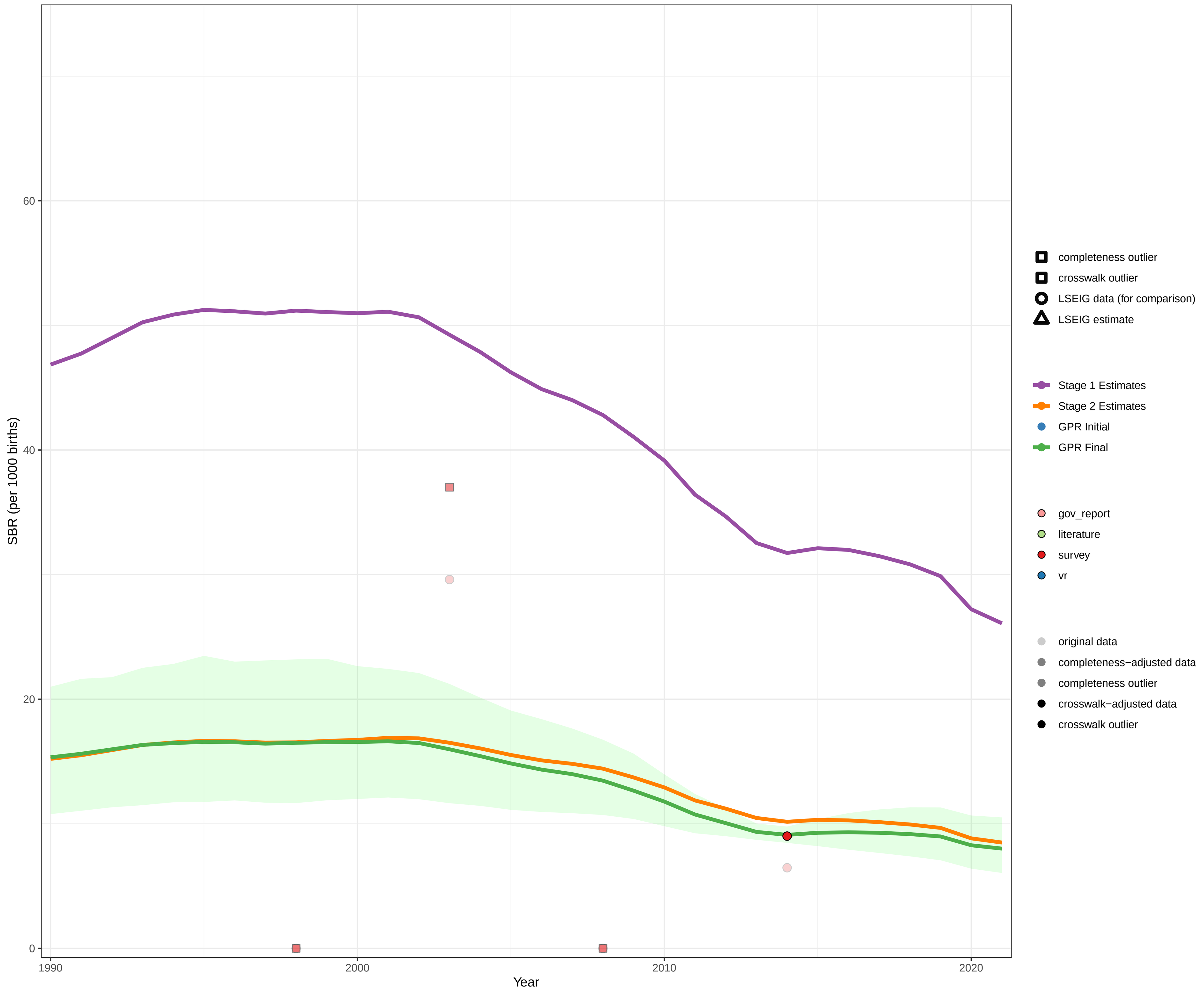

Year	Total Stillbirths (in thousands)	Total Stillbirths (in thousands)	SBR (per 1000 births)	SBR (per 1000 births)	SBR/NMR	SBR/NMR
	20 weeks	28 weeks	20 weeks	28 weeks	20 weeks	28 weeks
1990	0.2 (0.1 to 0.2)	0.1 (0.1 to 0.2)	20.2 (14.1 to 27.7)	15.3 (10.8 to 21.0)	0.97 (0.68 to 1.33)	0.73 (0.52 to 1.00)
1995	0.3 (0.2 to 0.4)	0.2 (0.1 to 0.3)	22.1 (15.8 to 31.1)	16.6 (11.8 to 23.5)	0.97 (0.70 to 1.37)	0.73 (0.52 to 1.03)
2000	0.3 (0.2 to 0.4)	0.2 (0.2 to 0.3)	22.4 (16.1 to 30.7)	16.6 (12.0 to 22.7)	1.02 (0.73 to 1.39)	0.75 (0.55 to 1.03)
2005	0.3 (0.2 to 0.4)	0.2 (0.2 to 0.3)	20.3 (15.2 to 26.2)	14.8 (11.1 to 19.1)	1.05 (0.79 to 1.36)	0.77 (0.58 to 0.99)
2010	0.2 (0.2 to 0.2)	0.1 (0.1 to 0.2)	16.4 (13.6 to 19.5)	11.8 (9.8 to 14.0)	1.04 (0.86 to 1.23)	0.75 (0.62 to 0.88)
2015	0.2 (0.1 to 0.2)	0.1 (0.1 to 0.1)	13.2 (11.6 to 14.8)	9.3 (8.2 to 10.4)	1.07 (0.94 to 1.20)	0.75 (0.67 to 0.84)
2020	0.1 (0.1 to 0.2)	0.1 (0.1 to 0.1)	12.1 (9.4 to 15.7)	8.3 (6.4 to 10.7)	1.23 (0.94 to 1.58)	0.84 (0.65 to 1.08)
2021	0.1 (0.1 to 0.2)	0.1 (0.1 to 0.1)	11.8 (8.9 to 15.5)	8.0 (6.1 to 10.5)	1.26 (0.95 to 1.65)	0.85 (0.64 to 1.12)

Lamu KEN_35637 Eastern Sub−Saharan Africa Stillbirth Rate / Neonatal Mortality Rate (SBR/NMR) − 20 weeks
lambda = 1 ; zeta = 0.6 ; scale = 15 ; amp = 0.028

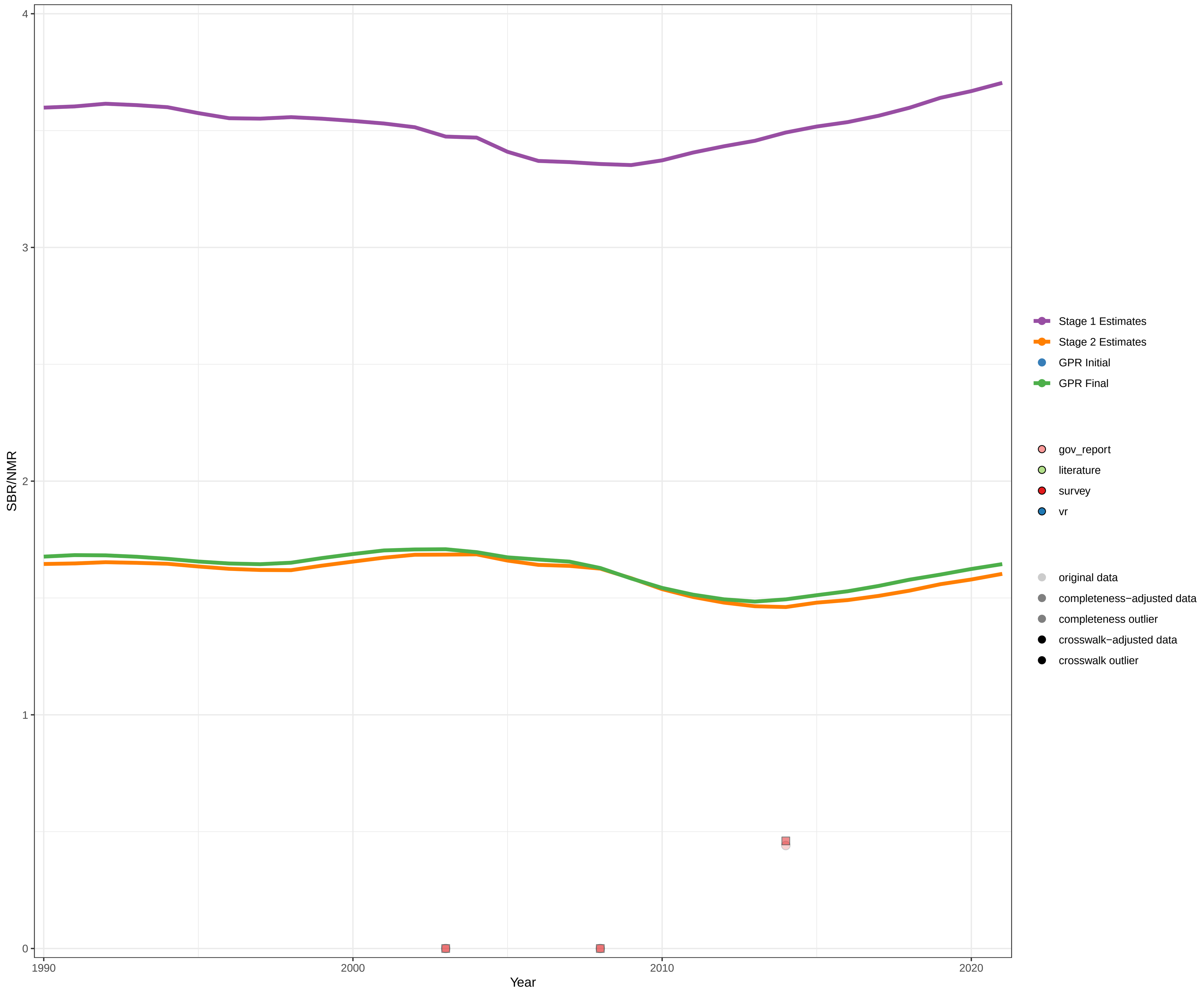

Lamu KEN_35637 Eastern Sub−Saharan Africa Stillbirth Rate / Neonatal Mortality Rate (SBR/NMR) − 28 weeks
lambda = 1 ; zeta = 0.6 ; scale = 15 ; amp = 0.028

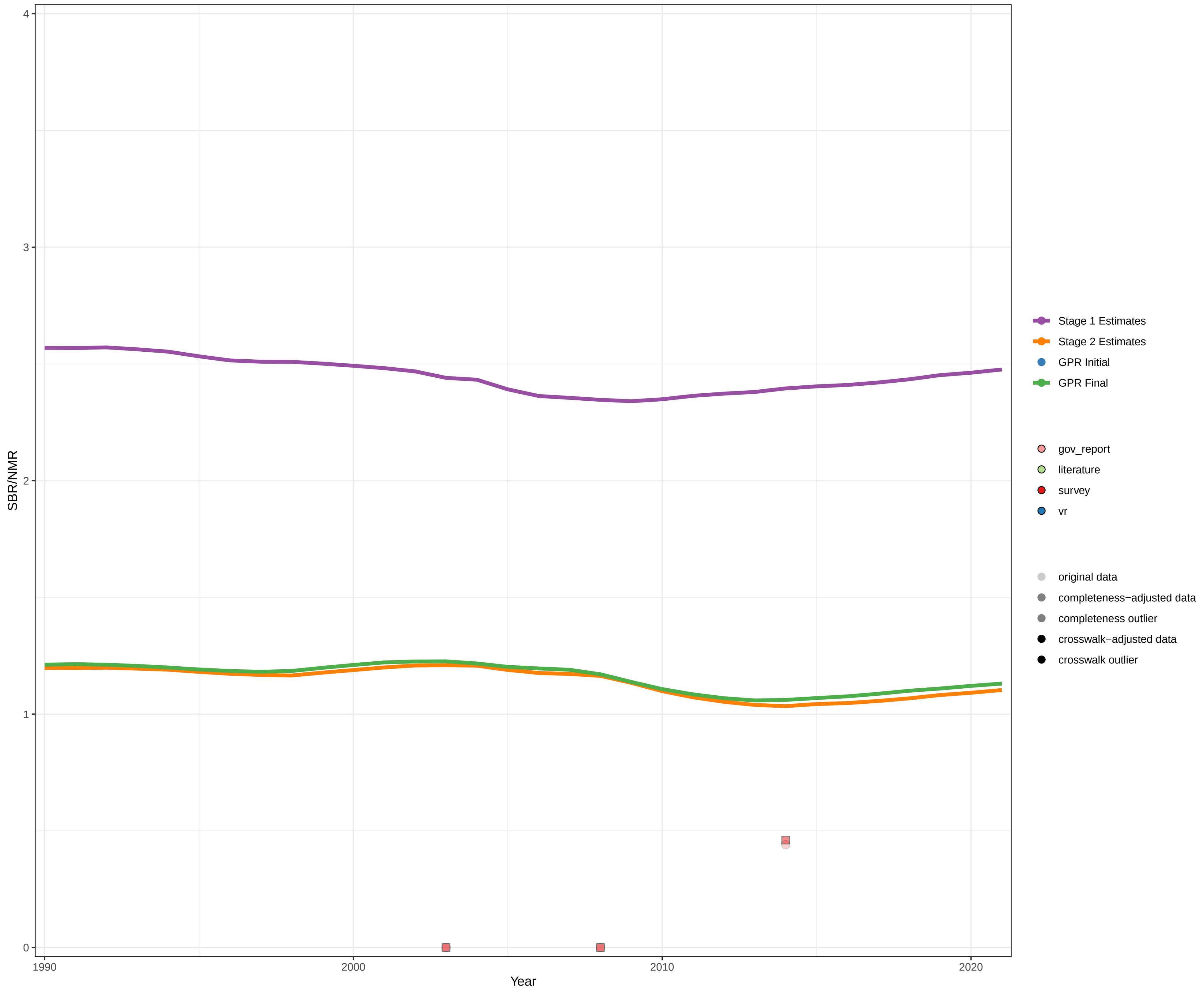

Definition	Years	Outlier	Source.Type	Title
28_weeks	2003	1	Survey	Kenya Demographic and Health Survey 2003
28_weeks	2008	1	Survey	Kenya Demographic and Health Survey 2008−2009
28_weeks	2014	1	Survey	Kenya Demographic and Health Survey 2014

Lamu KEN_35637 Eastern Sub−Saharan Africa Stillbirth Rate (SBR) − 20 weeks
lambda = 1 ; zeta = 0.6 ; scale = 15 ; amp = 0.028

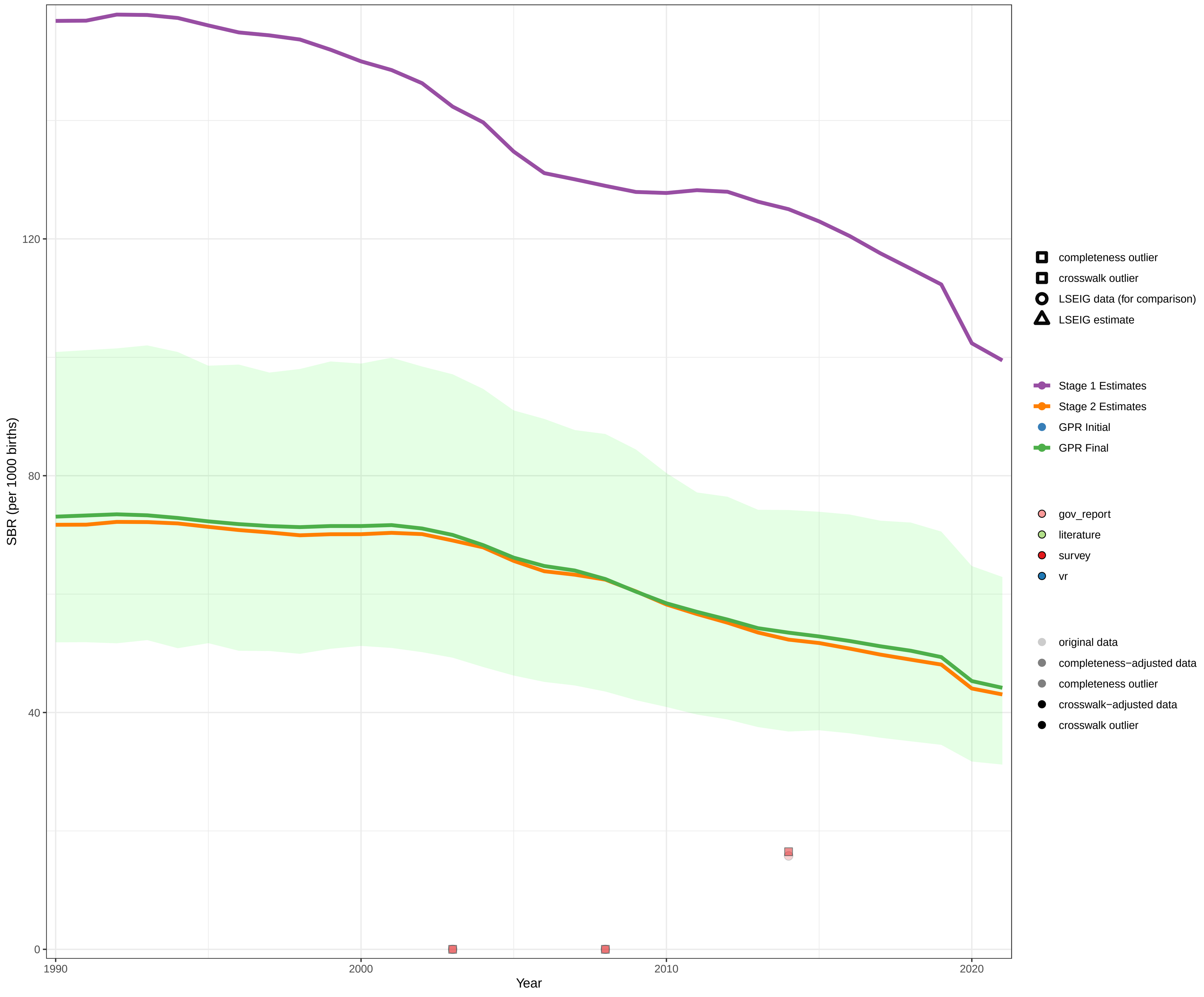

Lamu KEN_35637 Eastern Sub−Saharan Africa Stillbirth Rate (SBR) − 28 weeks
lambda = 1 ; zeta = 0.6 ; scale = 15 ; amp = 0.028

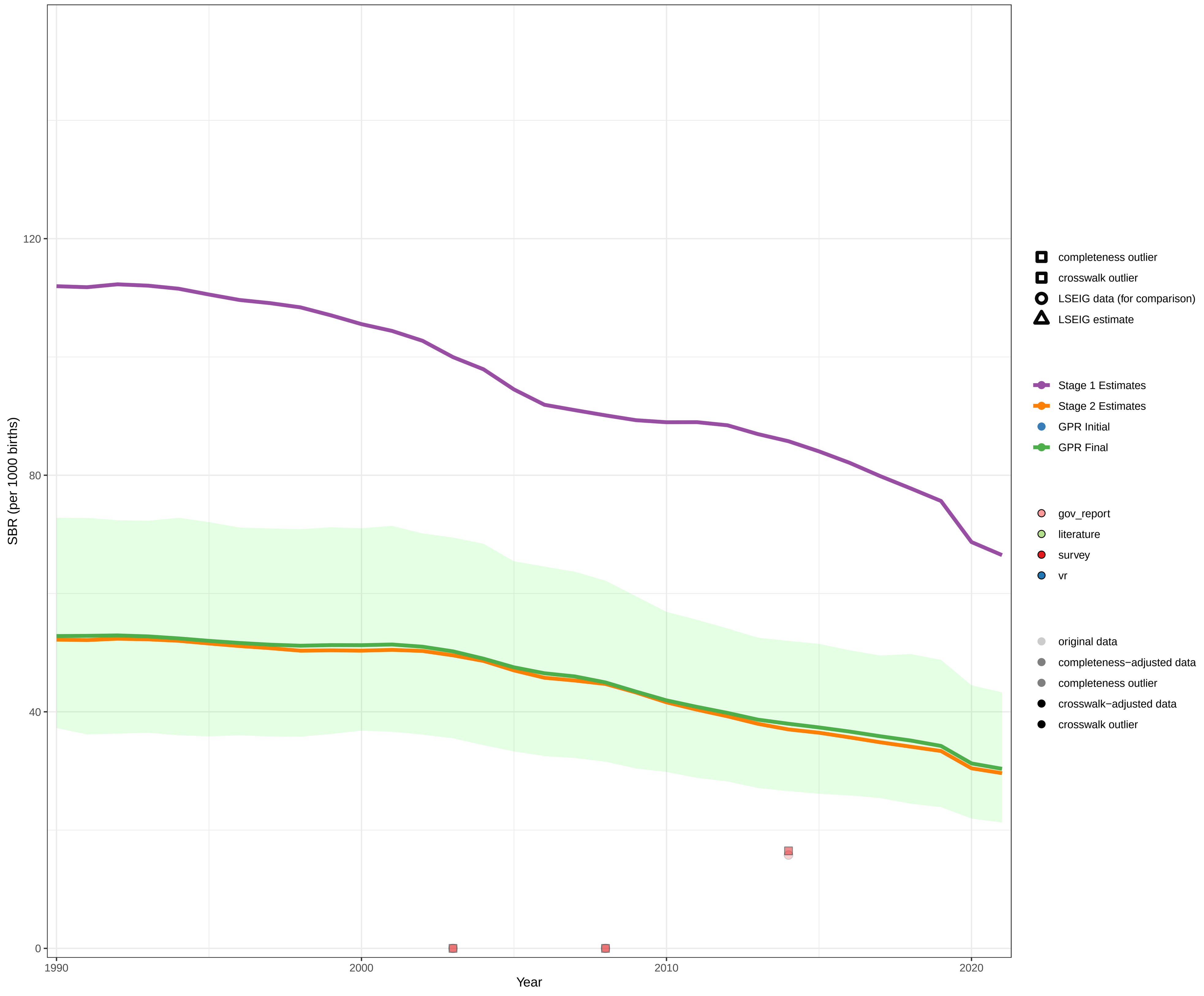

Year	Total Stillbirths (in thousands)	Total Stillbirths (in thousands)	SBR (per 1000 births)	SBR (per 1000 births)	SBR/NMR	SBR/NMR
	20 weeks	28 weeks	20 weeks	28 weeks	20 weeks	28 weeks
1990	0.2 (0.1 to 0.3)	0.1 (0.1 to 0.2)	73.1 (51.9 to 100.9)	52.8 (37.3 to 72.8)	1.68 (1.19 to 2.32)	1.21 (0.85 to 1.67)
1995	0.2 (0.1 to 0.3)	0.1 (0.1 to 0.2)	72.3 (51.7 to 98.6)	52.0 (35.9 to 72.1)	1.66 (1.18 to 2.26)	1.19 (0.82 to 1.65)
2000	0.2 (0.2 to 0.3)	0.2 (0.1 to 0.2)	71.5 (51.3 to 98.9)	51.3 (36.8 to 71.0)	1.69 (1.21 to 2.34)	1.21 (0.87 to 1.68)
2005	0.2 (0.1 to 0.3)	0.2 (0.1 to 0.2)	66.2 (46.2 to 91.0)	47.5 (33.3 to 65.5)	1.67 (1.17 to 2.30)	1.20 (0.84 to 1.66)
2010	0.2 (0.1 to 0.3)	0.1 (0.1 to 0.2)	58.4 (40.9 to 80.5)	41.9 (29.8 to 56.9)	1.54 (1.08 to 2.12)	1.11 (0.79 to 1.50)
2015	0.2 (0.1 to 0.2)	0.1 (0.1 to 0.2)	52.9 (37.0 to 73.9)	37.4 (26.1 to 51.5)	1.51 (1.06 to 2.11)	1.07 (0.75 to 1.47)
2020	0.1 (0.1 to 0.2)	0.1 (0.1 to 0.1)	45.3 (31.7 to 64.7)	31.3 (21.9 to 44.5)	1.62 (1.14 to 2.32)	1.12 (0.79 to 1.59)
2021	0.1 (0.1 to 0.2)	0.1 (0.1 to 0.1)	44.2 (31.2 to 62.9)	30.4 (21.3 to 43.3)	1.64 (1.16 to 2.34)	1.13 (0.79 to 1.61)

Machakos KEN_35638 Eastern Sub−Saharan Africa Stillbirth Rate / Neonatal Mortality Rate (SBR/NMR) − 20 weeks
lambda = 0.8 ; zeta = 0.7 ; scale = 15 ; amp = 0.028

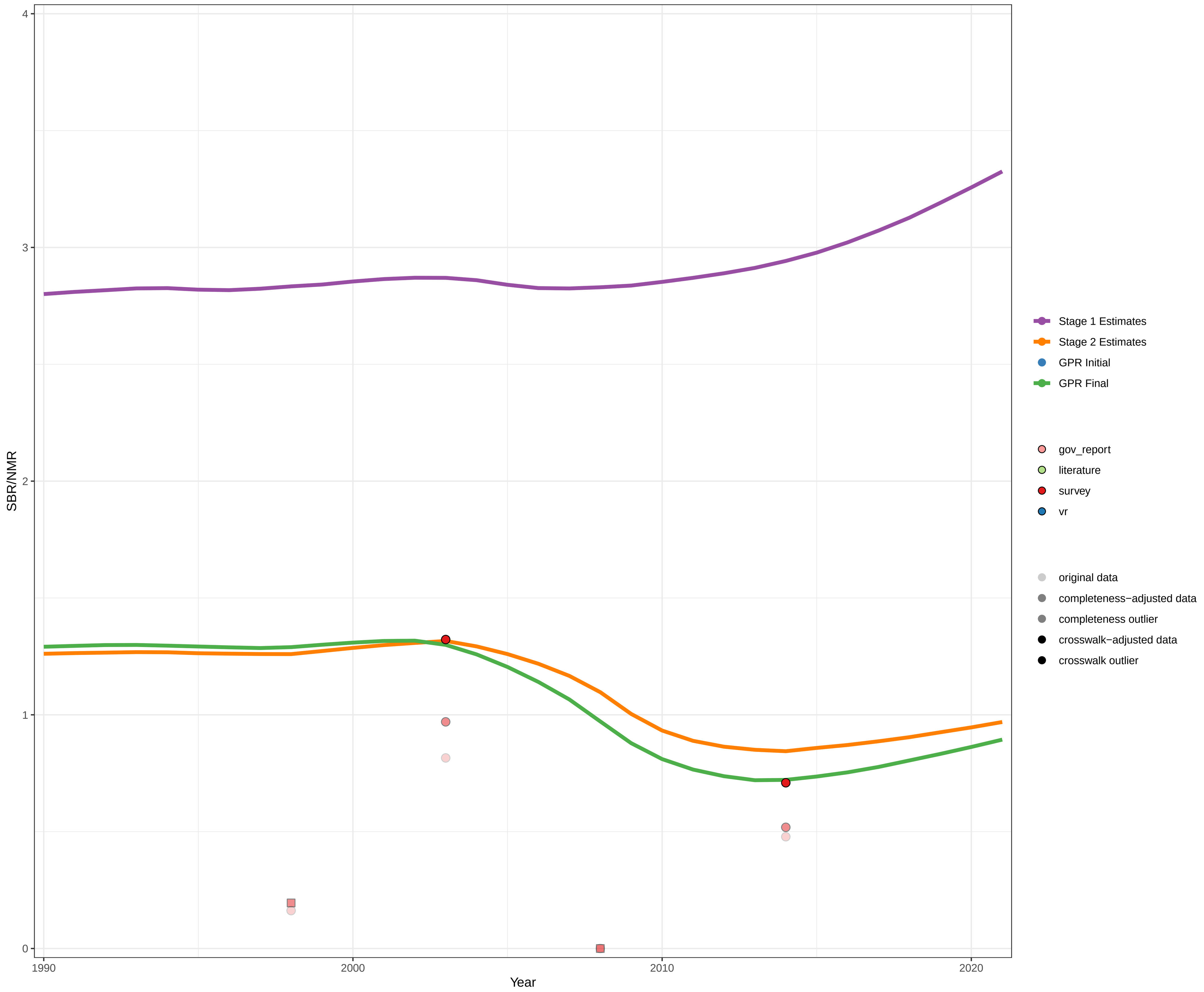

Machakos KEN_35638 Eastern Sub−Saharan Africa Stillbirth Rate / Neonatal Mortality Rate (SBR/NMR) − 28 weeks
lambda = 0.8 ; zeta = 0.7 ; scale = 15 ; amp = 0.028

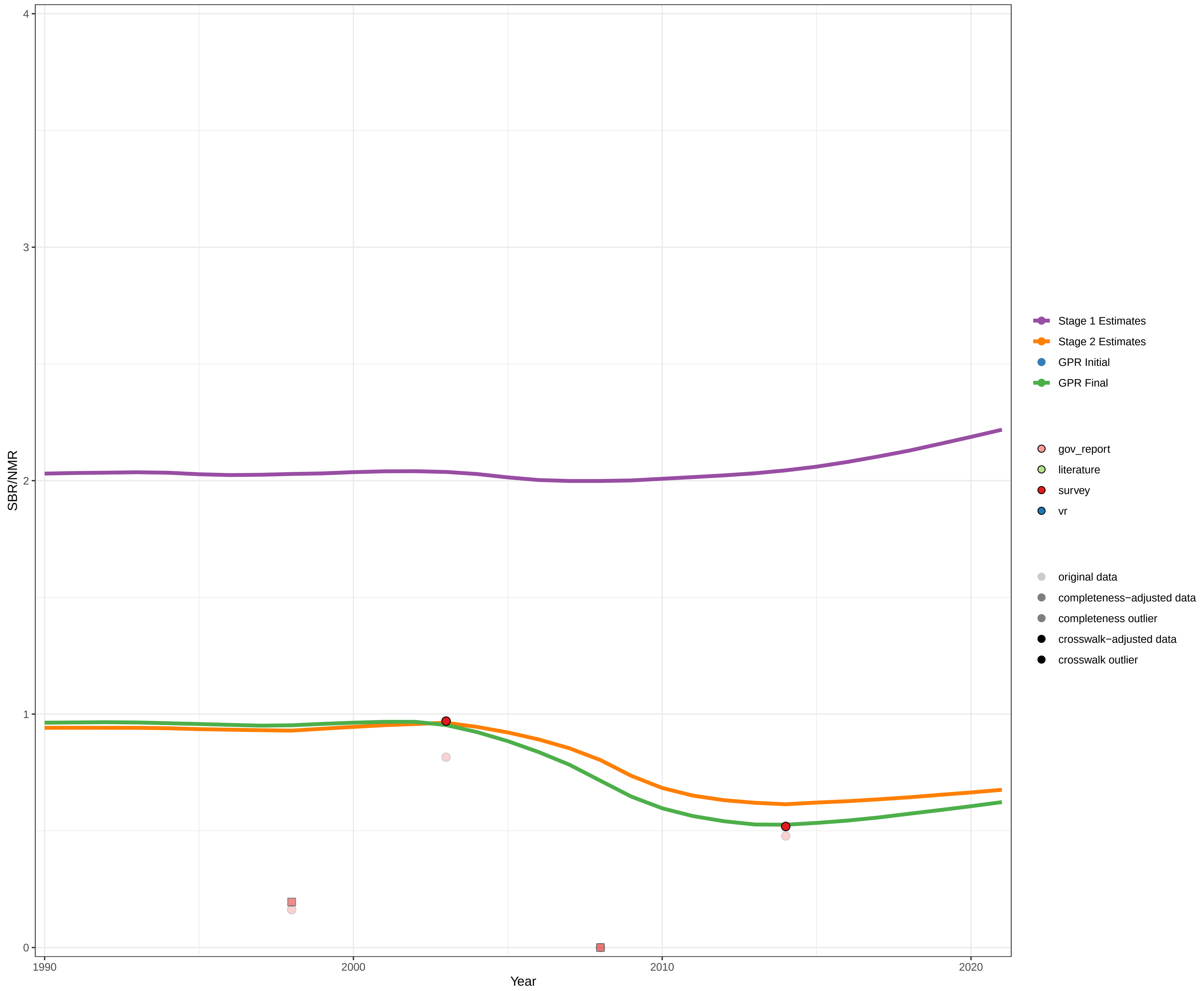

Definition	Years	Outlier	Source.Type	Title
28_weeks	2003	0	Survey	Kenya Demographic and Health Survey 2003
28_weeks	2014	0	Survey	Kenya Demographic and Health Survey 2014
28_weeks	1998	1	Survey	Kenya Demographic and Health Survey 1998
28_weeks	2008	1	Survey	Kenya Demographic and Health Survey 2008−2009

Machakos KEN_35638 Eastern Sub−Saharan Africa Stillbirth Rate (SBR) − 20 weeks
lambda = 0.8 ; zeta = 0.7 ; scale = 15 ; amp = 0.028

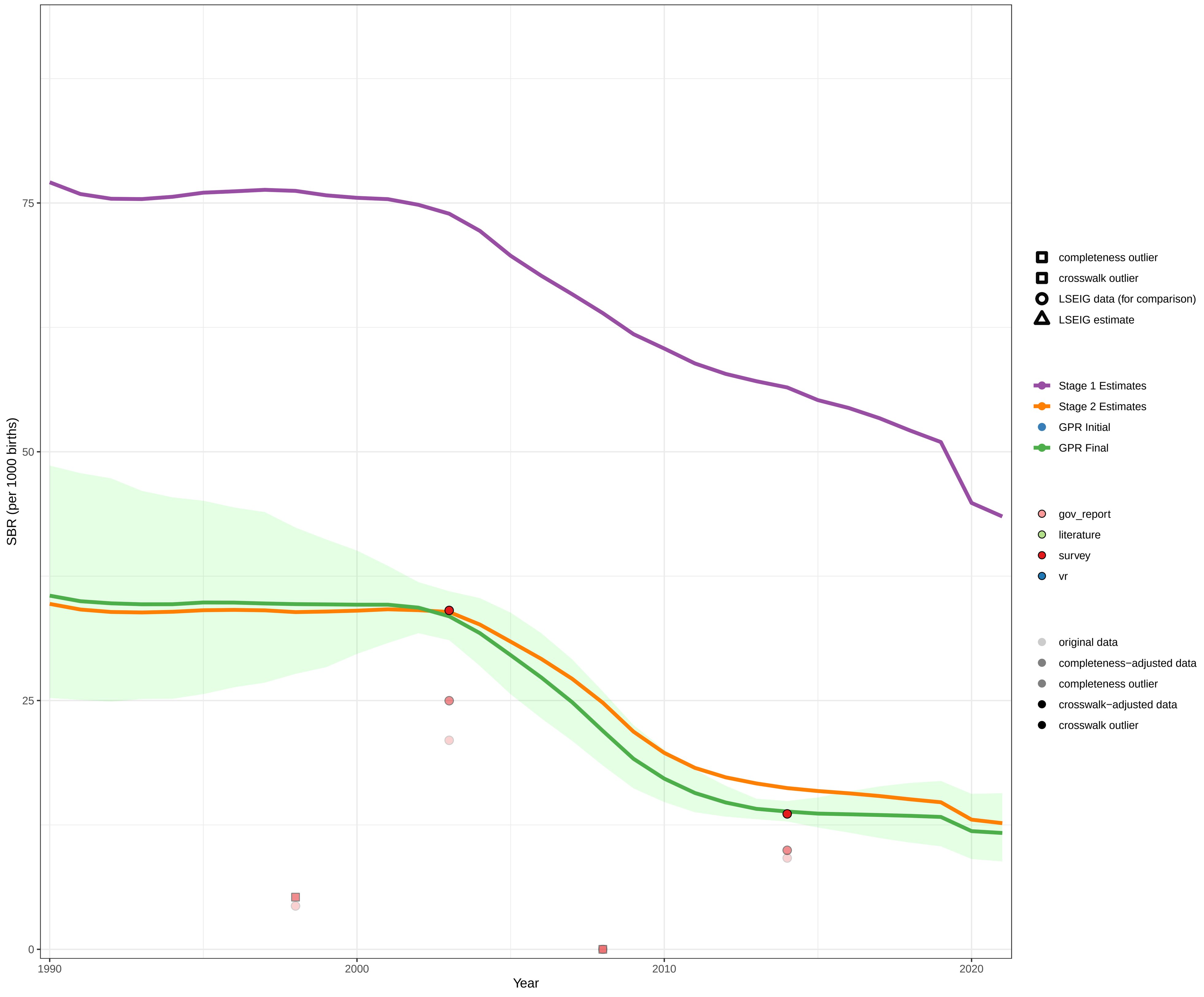

Machakos KEN_35638 Eastern Sub−Saharan Africa Stillbirth Rate (SBR) − 28 weeks
lambda = 0.8 ; zeta = 0.7 ; scale = 15 ; amp = 0.028

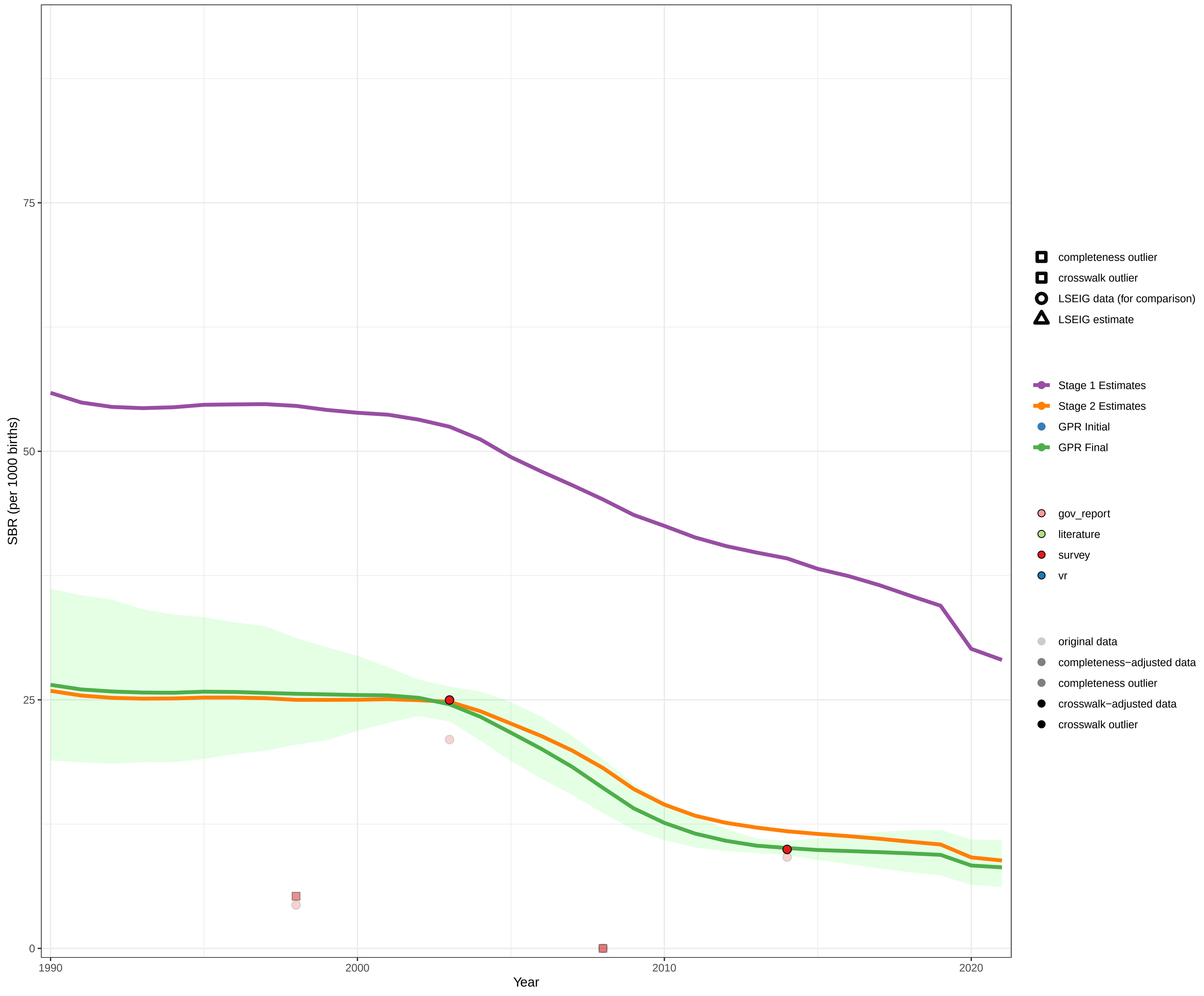

Year	Total Stillbirths (in thousands)	Total Stillbirths (in thousands)	SBR (per 1000 births)	SBR (per 1000 births)	SBR/NMR	SBR/NMR
	20 weeks	28 weeks	20 weeks	28 weeks	20 weeks	28 weeks
1990	1.4 (1.0 to 1.9)	1.0 (0.7 to 1.4)	35.5 (25.3 to 48.6)	26.5 (18.9 to 36.2)	1.29 (0.92 to 1.77)	0.96 (0.69 to 1.31)
1995	1.4 (1.0 to 1.9)	1.1 (0.8 to 1.4)	34.9 (25.6 to 45.1)	25.8 (19.1 to 33.3)	1.29 (0.95 to 1.67)	0.96 (0.71 to 1.24)
2000	1.4 (1.2 to 1.6)	1.0 (0.9 to 1.2)	34.6 (29.7 to 40.1)	25.5 (21.9 to 29.4)	1.31 (1.12 to 1.51)	0.96 (0.83 to 1.11)
2005	1.1 (0.9 to 1.2)	0.8 (0.7 to 0.9)	29.6 (25.6 to 33.8)	21.7 (18.8 to 24.8)	1.20 (1.04 to 1.38)	0.88 (0.77 to 1.01)
2010	0.5 (0.5 to 0.6)	0.4 (0.3 to 0.5)	17.2 (14.8 to 20.1)	12.6 (10.9 to 14.8)	0.81 (0.70 to 0.95)	0.60 (0.52 to 0.70)
2015	0.4 (0.4 to 0.5)	0.3 (0.3 to 0.3)	13.6 (12.2 to 15.3)	9.9 (8.9 to 11.1)	0.74 (0.66 to 0.82)	0.53 (0.48 to 0.60)
2020	0.3 (0.2 to 0.4)	0.2 (0.2 to 0.3)	11.9 (9.1 to 15.6)	8.3 (6.4 to 10.9)	0.86 (0.66 to 1.13)	0.61 (0.46 to 0.79)
2021	0.3 (0.2 to 0.4)	0.2 (0.2 to 0.3)	11.7 (8.8 to 15.7)	8.1 (6.2 to 10.9)	0.89 (0.67 to 1.20)	0.62 (0.47 to 0.83)

Makueni KEN_35639 Eastern Sub−Saharan Africa Stillbirth Rate / Neonatal Mortality Rate (SBR/NMR) − 20 weeks
lambda = 0.8 ; zeta = 0.7 ; scale = 15 ; amp = 0.028

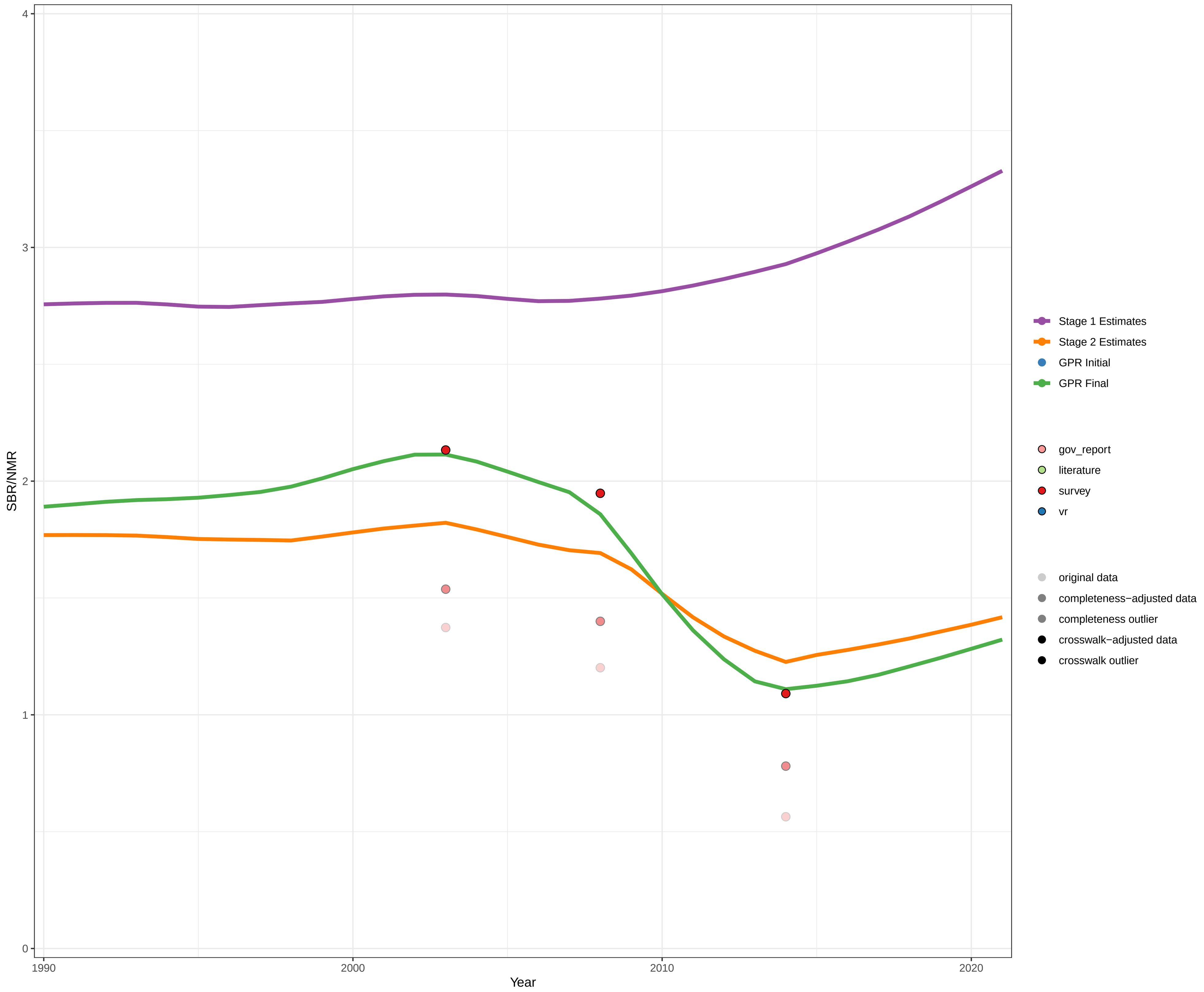

Makueni KEN_35639 Eastern Sub−Saharan Africa Stillbirth Rate / Neonatal Mortality Rate (SBR/NMR) − 28 weeks
lambda = 0.8 ; zeta = 0.7 ; scale = 15 ; amp = 0.028

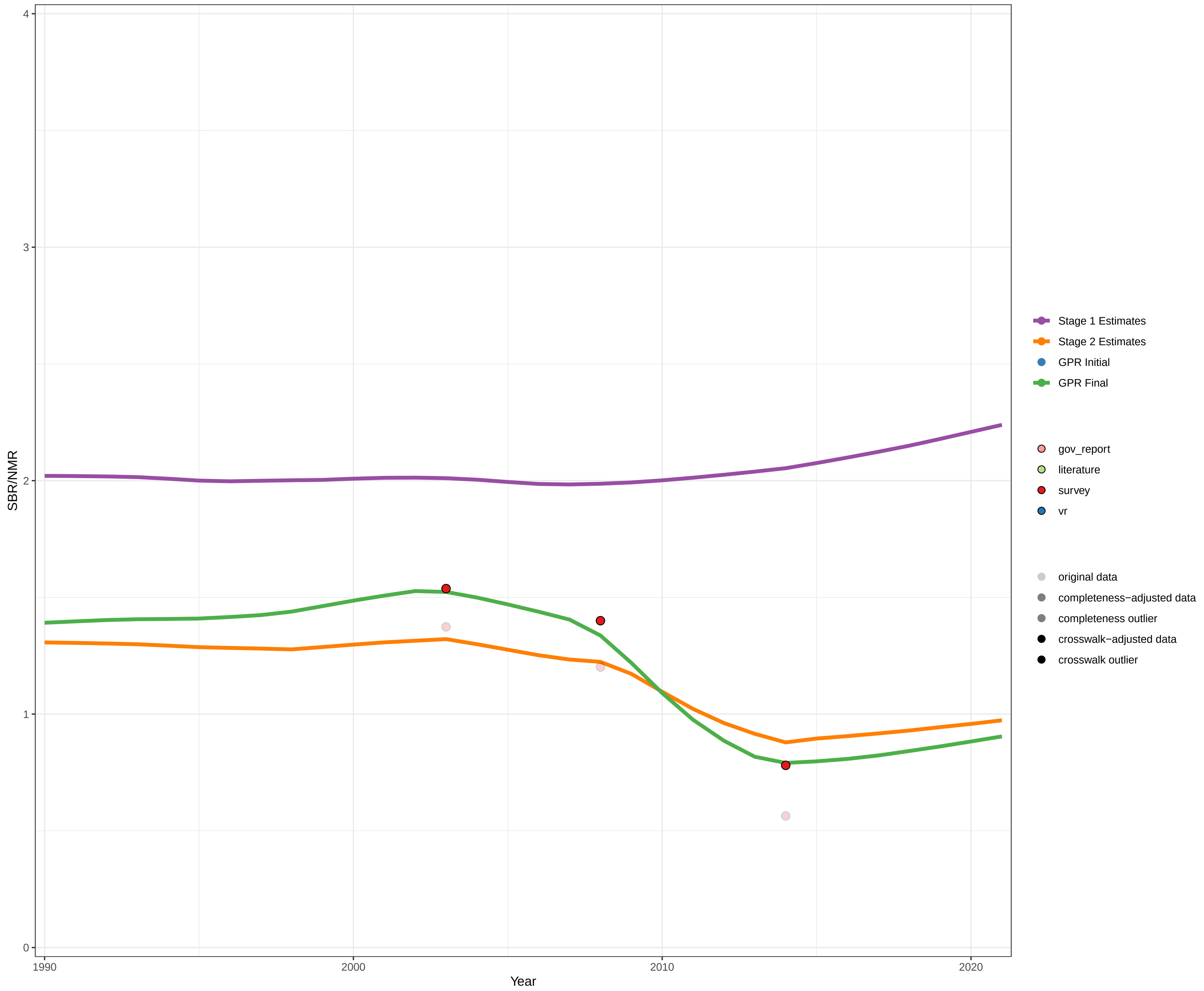

Definition	Years	Outlier	Source.Type	Title
28_weeks	2003	0	Survey	Kenya Demographic and Health Survey 2003
28_weeks	2008	0	Survey	Kenya Demographic and Health Survey 2008−2009
28_weeks	2014	0	Survey	Kenya Demographic and Health Survey 2014

Makueni KEN_35639 Eastern Sub−Saharan Africa Stillbirth Rate (SBR) − 20 weeks
lambda = 0.8 ; zeta = 0.7 ; scale = 15 ; amp = 0.028

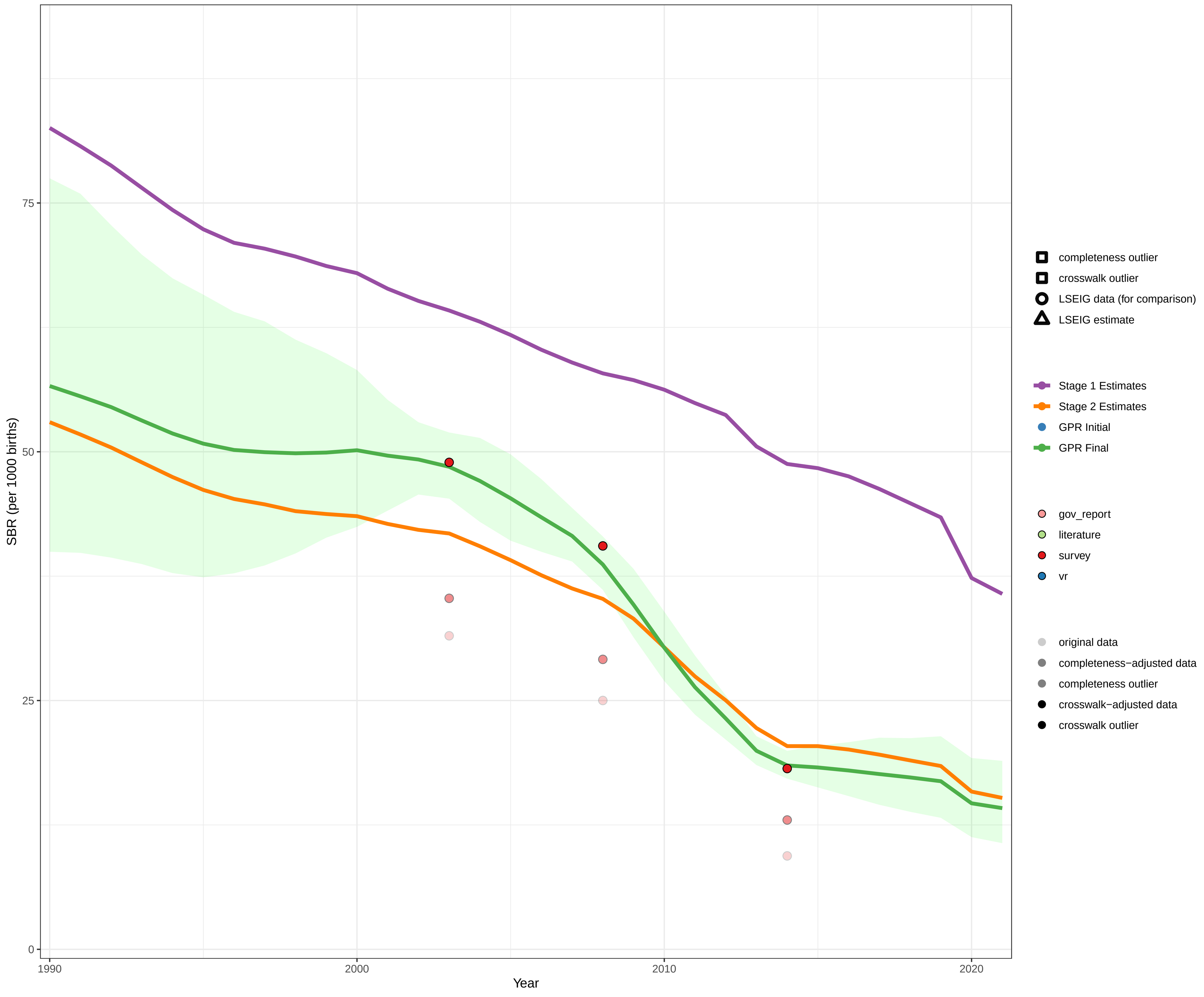

Makueni KEN_35639 Eastern Sub−Saharan Africa Stillbirth Rate (SBR) − 28 weeks
lambda = 0.8 ; zeta = 0.7 ; scale = 15 ; amp = 0.028

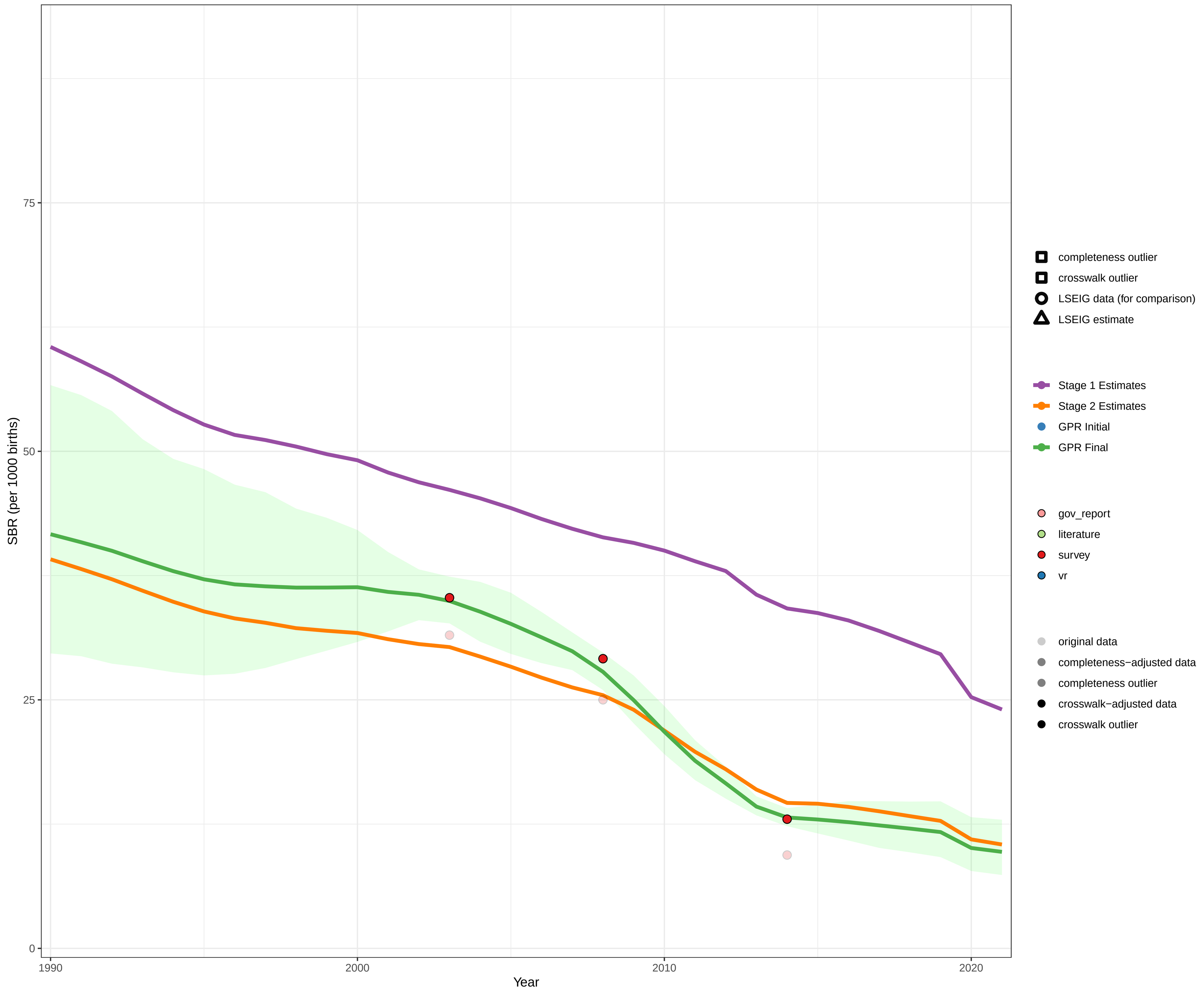

Year	Total Stillbirths (in thousands)	Total Stillbirths (in thousands)	SBR (per 1000 births)	SBR (per 1000 births)	SBR/NMR	SBR/NMR
	20 weeks	28 weeks	20 weeks	28 weeks	20 weeks	28 weeks
1990	1.2 (0.8 to 1.7)	0.9 (0.6 to 1.2)	56.6 (40.0 to 77.5)	41.7 (29.7 to 56.7)	1.89 (1.33 to 2.59)	1.39 (0.99 to 1.89)
1995	1.1 (0.8 to 1.4)	0.8 (0.6 to 1.0)	50.8 (37.4 to 65.8)	37.1 (27.5 to 48.2)	1.93 (1.42 to 2.50)	1.41 (1.04 to 1.83)
2000	1.1 (1.0 to 1.3)	0.8 (0.7 to 0.9)	50.2 (42.5 to 58.2)	36.3 (30.8 to 42.1)	2.05 (1.74 to 2.38)	1.49 (1.26 to 1.72)
2005	1.2 (1.0 to 1.3)	0.8 (0.7 to 0.9)	45.3 (41.1 to 49.7)	32.6 (29.6 to 35.8)	2.04 (1.85 to 2.24)	1.47 (1.33 to 1.61)
2010	0.8 (0.7 to 0.9)	0.6 (0.5 to 0.6)	30.3 (27.0 to 33.9)	21.8 (19.5 to 24.4)	1.52 (1.35 to 1.70)	1.09 (0.98 to 1.22)
2015	0.4 (0.4 to 0.5)	0.3 (0.3 to 0.3)	18.3 (16.3 to 20.5)	13.0 (11.6 to 14.5)	1.12 (1.00 to 1.26)	0.80 (0.71 to 0.89)
2020	0.3 (0.2 to 0.3)	0.2 (0.1 to 0.2)	14.7 (11.3 to 19.2)	10.1 (7.8 to 13.2)	1.28 (0.99 to 1.68)	0.88 (0.68 to 1.15)
2021	0.2 (0.2 to 0.3)	0.2 (0.1 to 0.2)	14.2 (10.7 to 18.9)	9.7 (7.4 to 12.9)	1.32 (0.99 to 1.76)	0.90 (0.69 to 1.21)

Mandera KEN_35640 Eastern Sub−Saharan Africa Stillbirth Rate / Neonatal Mortality Rate (SBR/NMR) − 20 weeks
lambda = 0.8 ; zeta = 0.7 ; scale = 15 ; amp = 0.028

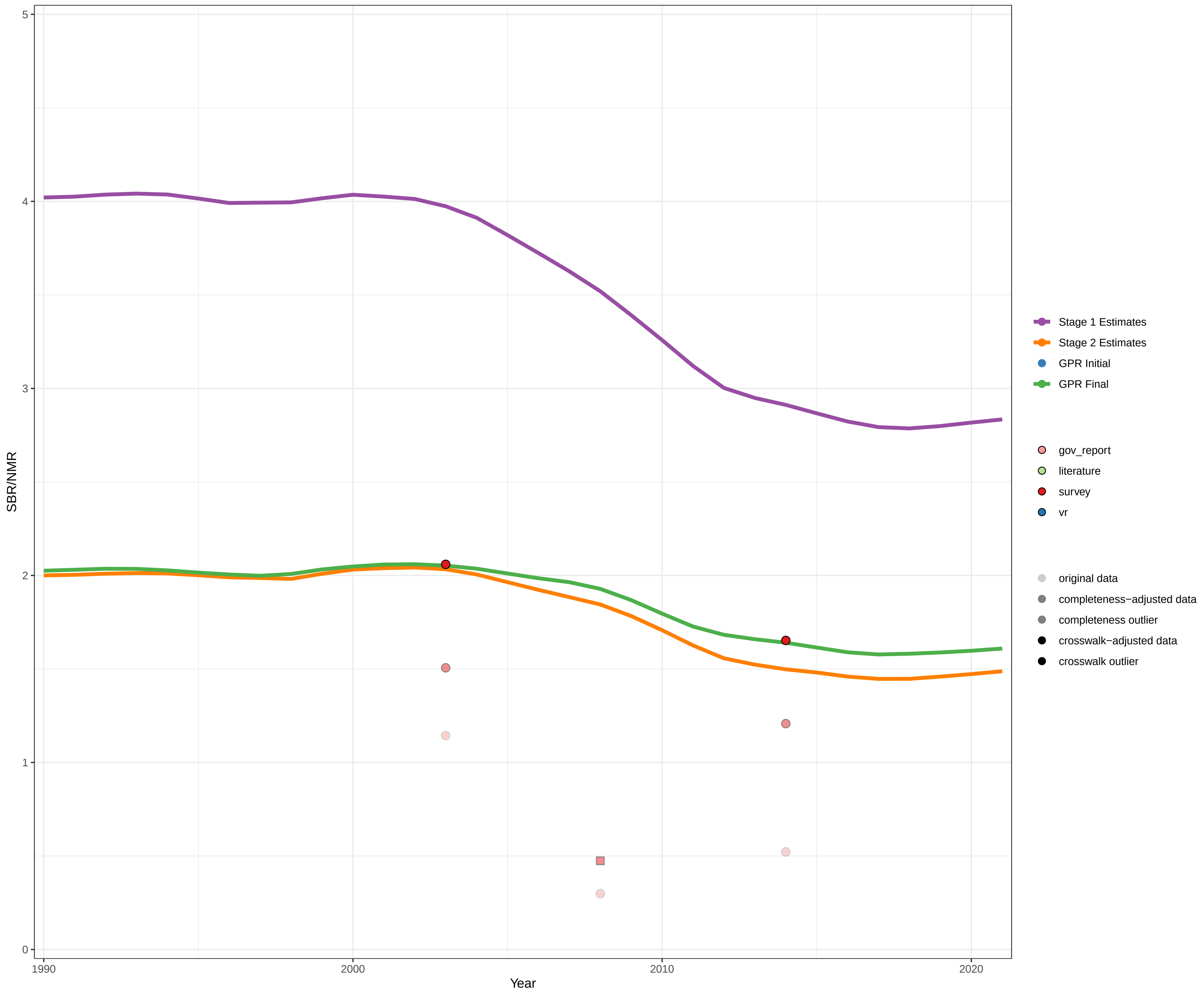

Mandera KEN_35640 Eastern Sub−Saharan Africa Stillbirth Rate / Neonatal Mortality Rate (SBR/NMR) − 28 weeks
lambda = 0.8 ; zeta = 0.7 ; scale = 15 ; amp = 0.028

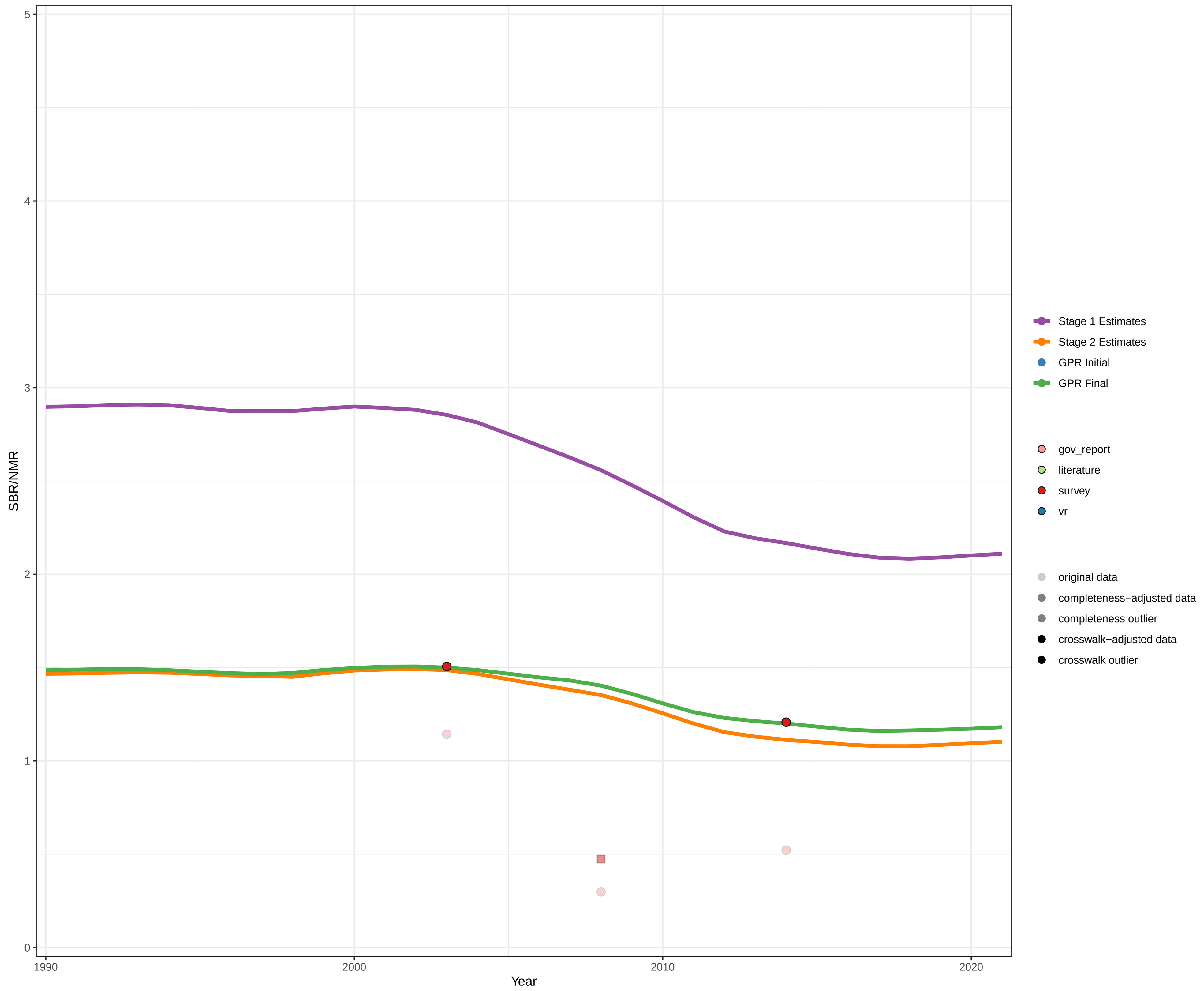

Definition	Years	Outlier	Source.Type	Title
28_weeks	2003	0	Survey	Kenya Demographic and Health Survey 2003
28_weeks	2014	0	Survey	Kenya Demographic and Health Survey 2014
28_weeks	2008	1	Survey	Kenya Demographic and Health Survey 2008−2009

Mandera KEN_35640 Eastern Sub−Saharan Africa Stillbirth Rate (SBR) − 20 weeks
lambda = 0.8 ; zeta = 0.7 ; scale = 15 ; amp = 0.028

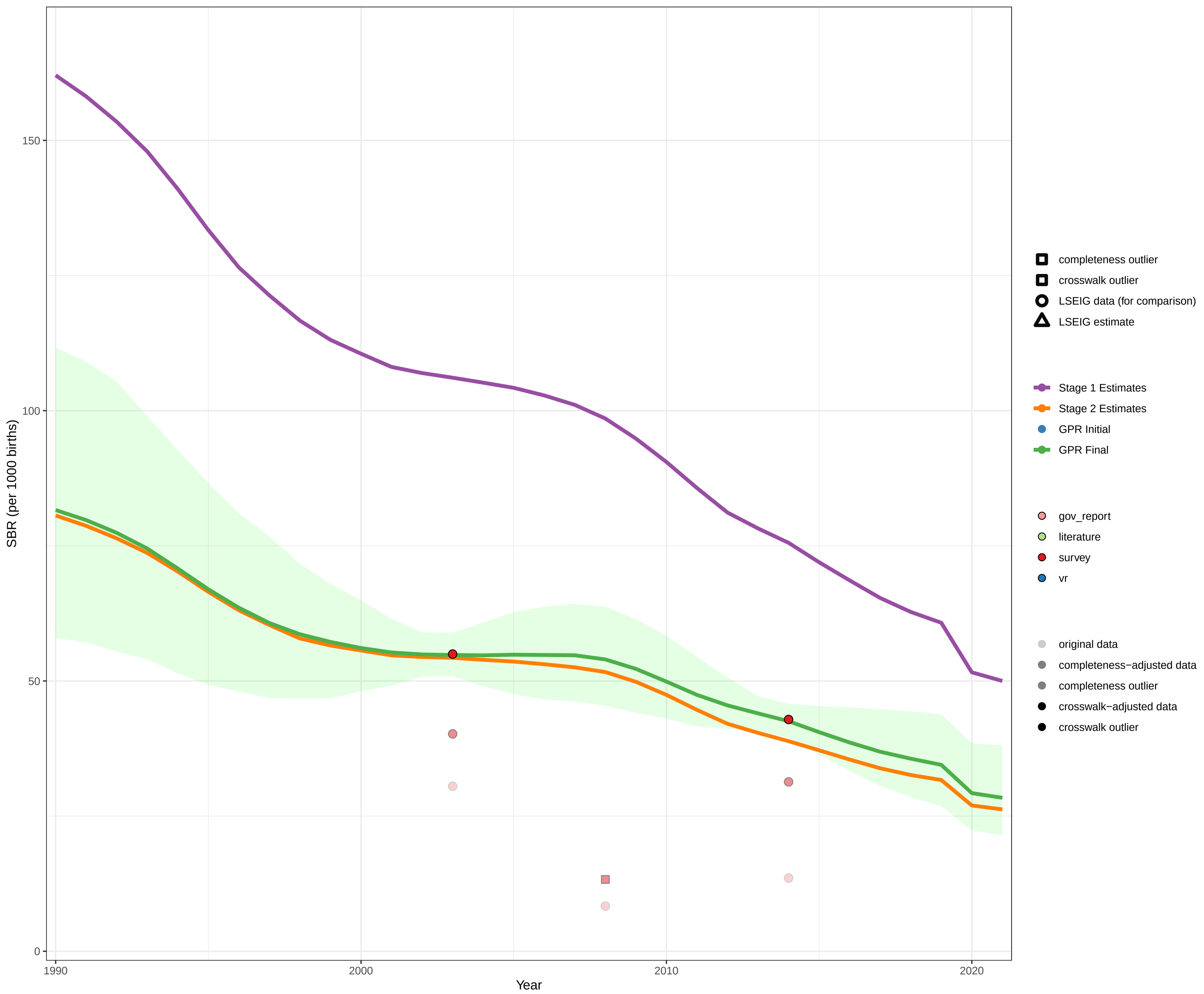

Mandera KEN_35640 Eastern Sub−Saharan Africa Stillbirth Rate (SBR) − 28 weeks
lambda = 0.8 ; zeta = 0.7 ; scale = 15 ; amp = 0.028

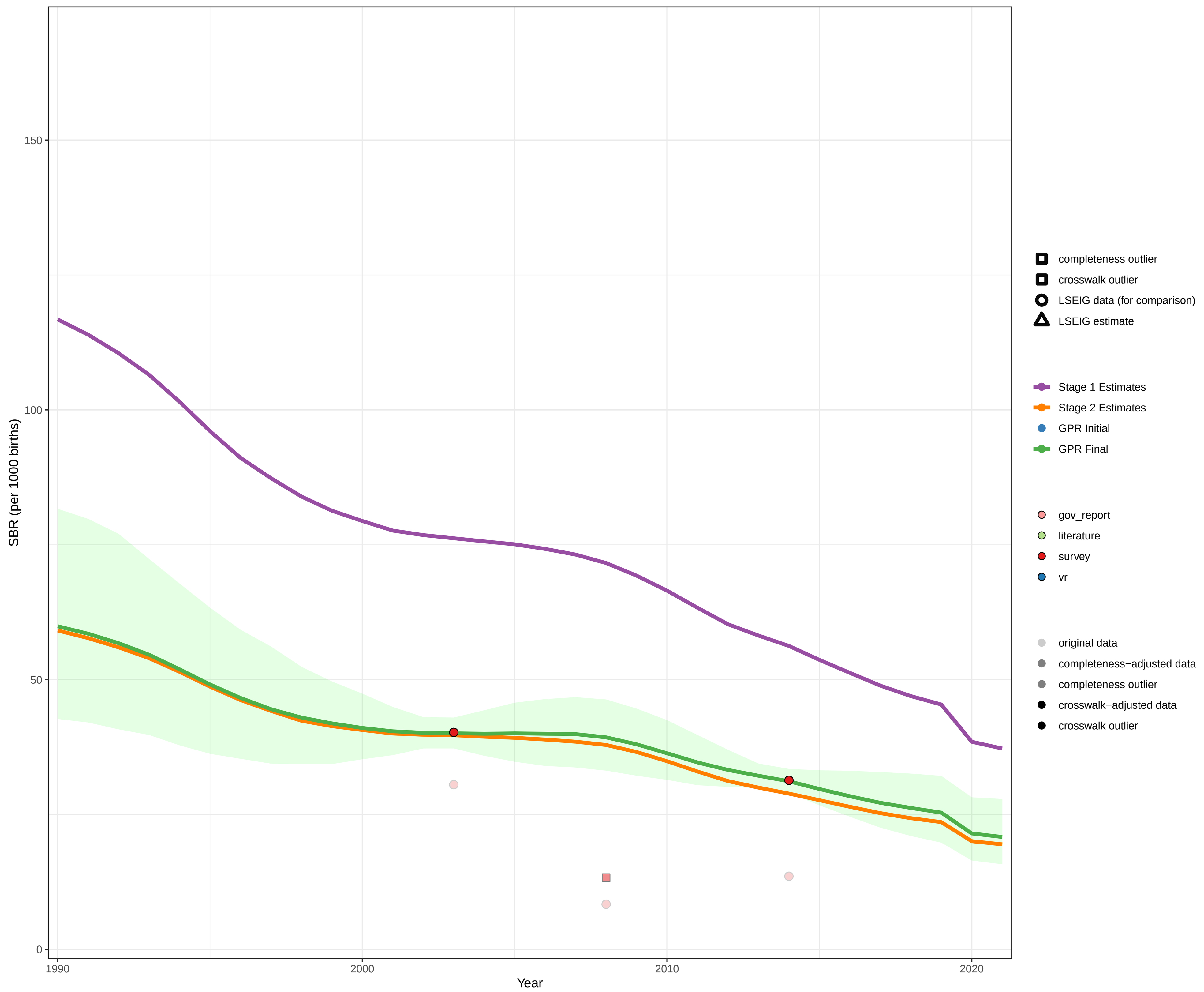

Year	Total Stillbirths (in thousands)	Total Stillbirths (in thousands)	SBR (per 1000 births)	SBR (per 1000 births)	SBR/NMR	SBR/NMR
	20 weeks	28 weeks	20 weeks	28 weeks	20 weeks	28 weeks
1990	0.6 (0.4 to 0.9)	0.4 (0.3 to 0.6)	81.6 (58.0 to 111.6)	59.9 (42.7 to 81.7)	2.03 (1.44 to 2.77)	1.49 (1.06 to 2.03)
1995	0.7 (0.5 to 0.9)	0.5 (0.3 to 0.6)	66.9 (49.3 to 86.6)	49.1 (36.2 to 63.3)	2.02 (1.48 to 2.61)	1.48 (1.09 to 1.91)
2000	0.9 (0.8 to 1.1)	0.7 (0.6 to 0.8)	56.1 (48.1 to 64.9)	41.0 (35.2 to 47.4)	2.05 (1.76 to 2.37)	1.50 (1.29 to 1.73)
2005	1.8 (1.6 to 2.1)	1.3 (1.1 to 1.5)	54.9 (47.6 to 62.8)	40.0 (34.8 to 45.7)	2.01 (1.74 to 2.30)	1.47 (1.27 to 1.68)
2010	2.6 (2.2 to 3.1)	1.9 (1.6 to 2.2)	49.9 (43.0 to 58.4)	36.3 (31.4 to 42.5)	1.80 (1.55 to 2.10)	1.31 (1.13 to 1.53)
2015	2.1 (1.9 to 2.4)	1.6 (1.4 to 1.7)	40.5 (36.4 to 45.4)	29.7 (26.7 to 33.2)	1.61 (1.45 to 1.81)	1.18 (1.06 to 1.32)
2020	1.3 (1.0 to 1.7)	0.9 (0.7 to 1.3)	29.3 (22.3 to 38.5)	21.5 (16.5 to 28.2)	1.60 (1.22 to 2.10)	1.17 (0.90 to 1.54)
2021	1.2 (0.9 to 1.7)	0.9 (0.7 to 1.2)	28.4 (21.4 to 38.1)	20.8 (15.8 to 27.9)	1.61 (1.22 to 2.16)	1.18 (0.89 to 1.58)

Marsabit KEN_35641 Eastern Sub−Saharan Africa Stillbirth Rate / Neonatal Mortality Rate (SBR/NMR) − 20 weeks
lambda = 0.8 ; zeta = 0.7 ; scale = 15 ; amp = 0.028

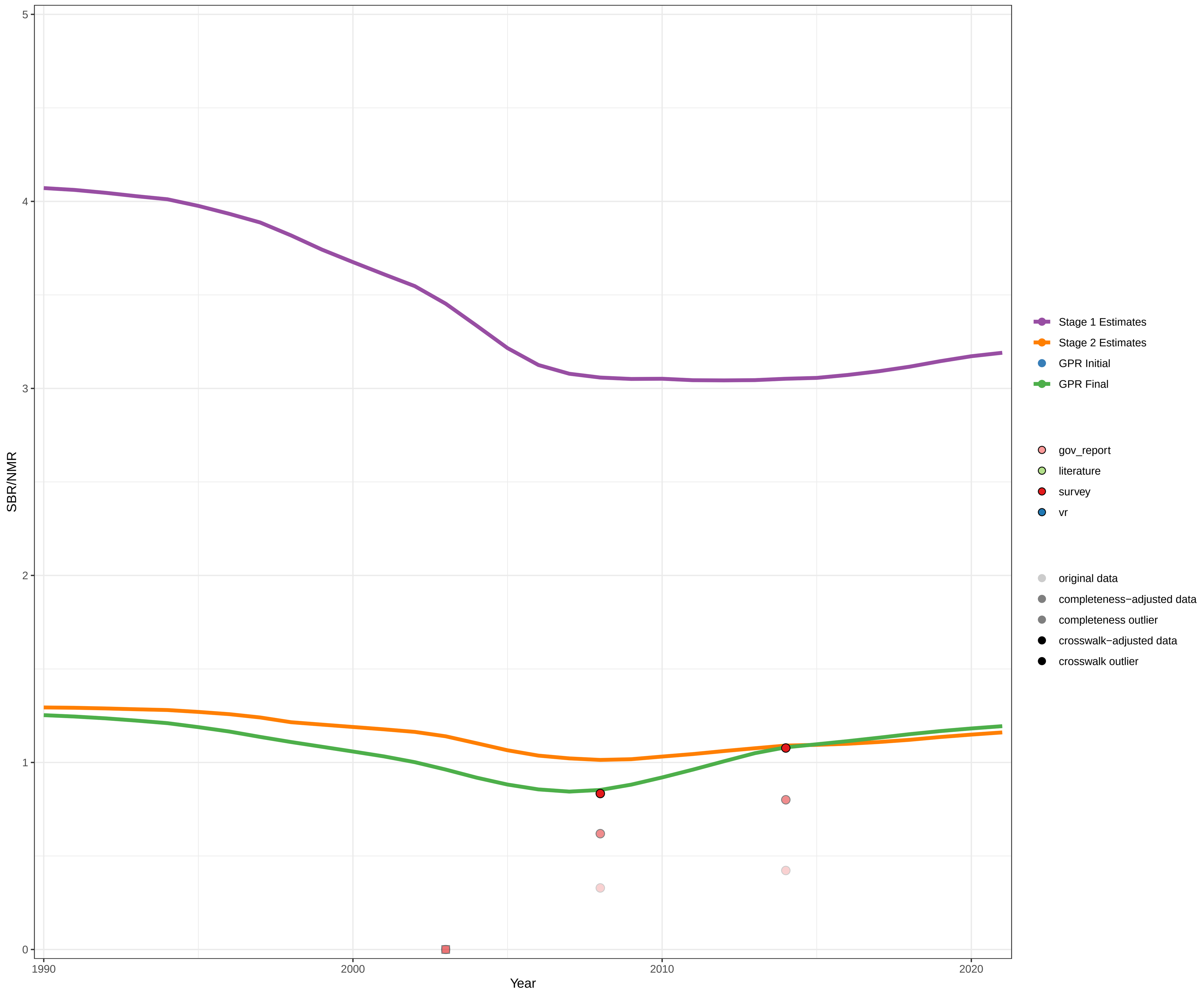

Marsabit KEN_35641 Eastern Sub−Saharan Africa Stillbirth Rate / Neonatal Mortality Rate (SBR/NMR) − 28 weeks
lambda = 0.8 ; zeta = 0.7 ; scale = 15 ; amp = 0.028

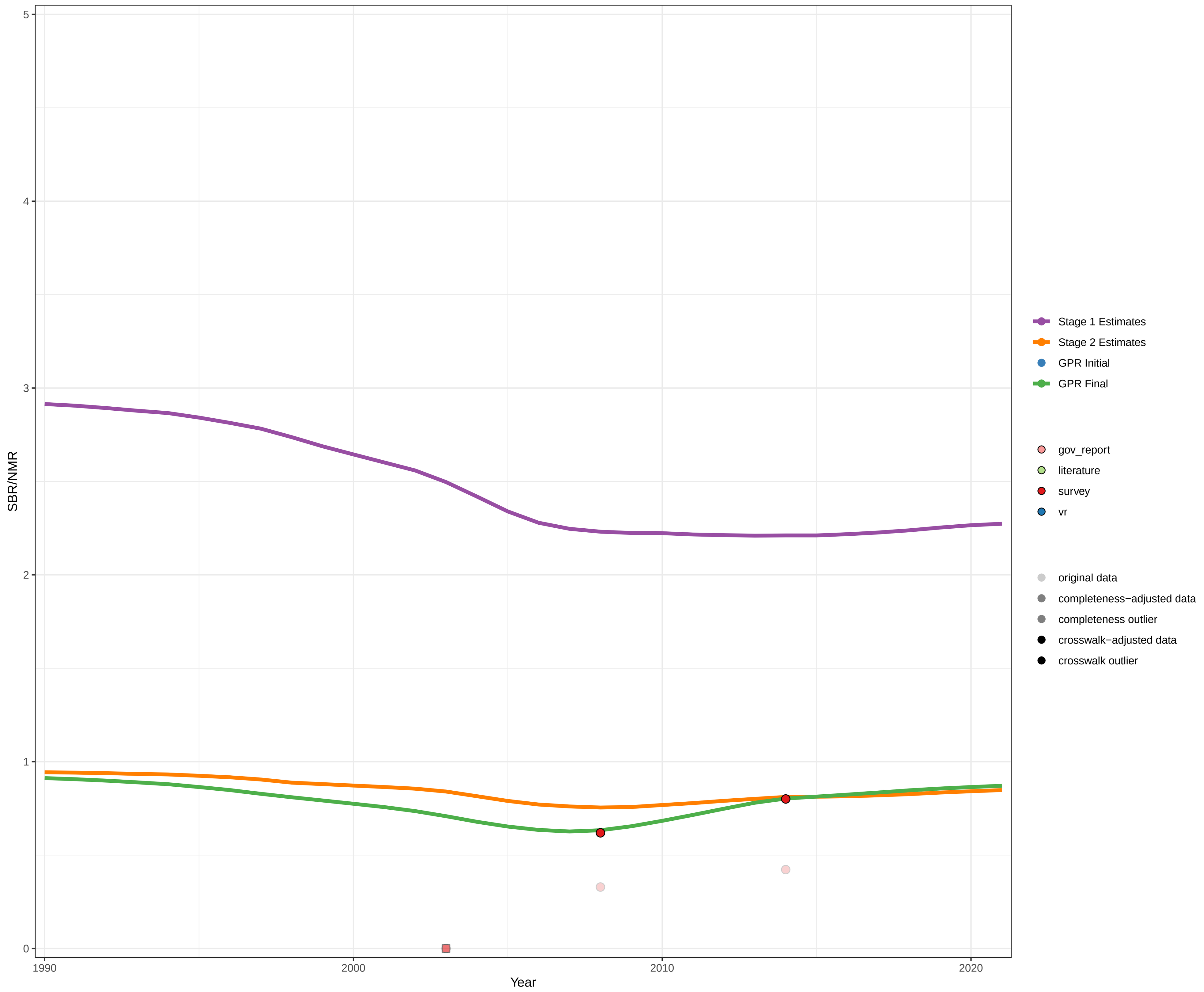

Definition	Years	Outlier	Source.Type	Title
28_weeks	2008	0	Survey	Kenya Demographic and Health Survey 2008−2009
28_weeks	2014	0	Survey	Kenya Demographic and Health Survey 2014
28_weeks	2003	1	Survey	Kenya Demographic and Health Survey 2003

Marsabit KEN_35641 Eastern Sub−Saharan Africa Stillbirth Rate (SBR) − 20 weeks
lambda = 0.8 ; zeta = 0.7 ; scale = 15 ; amp = 0.028

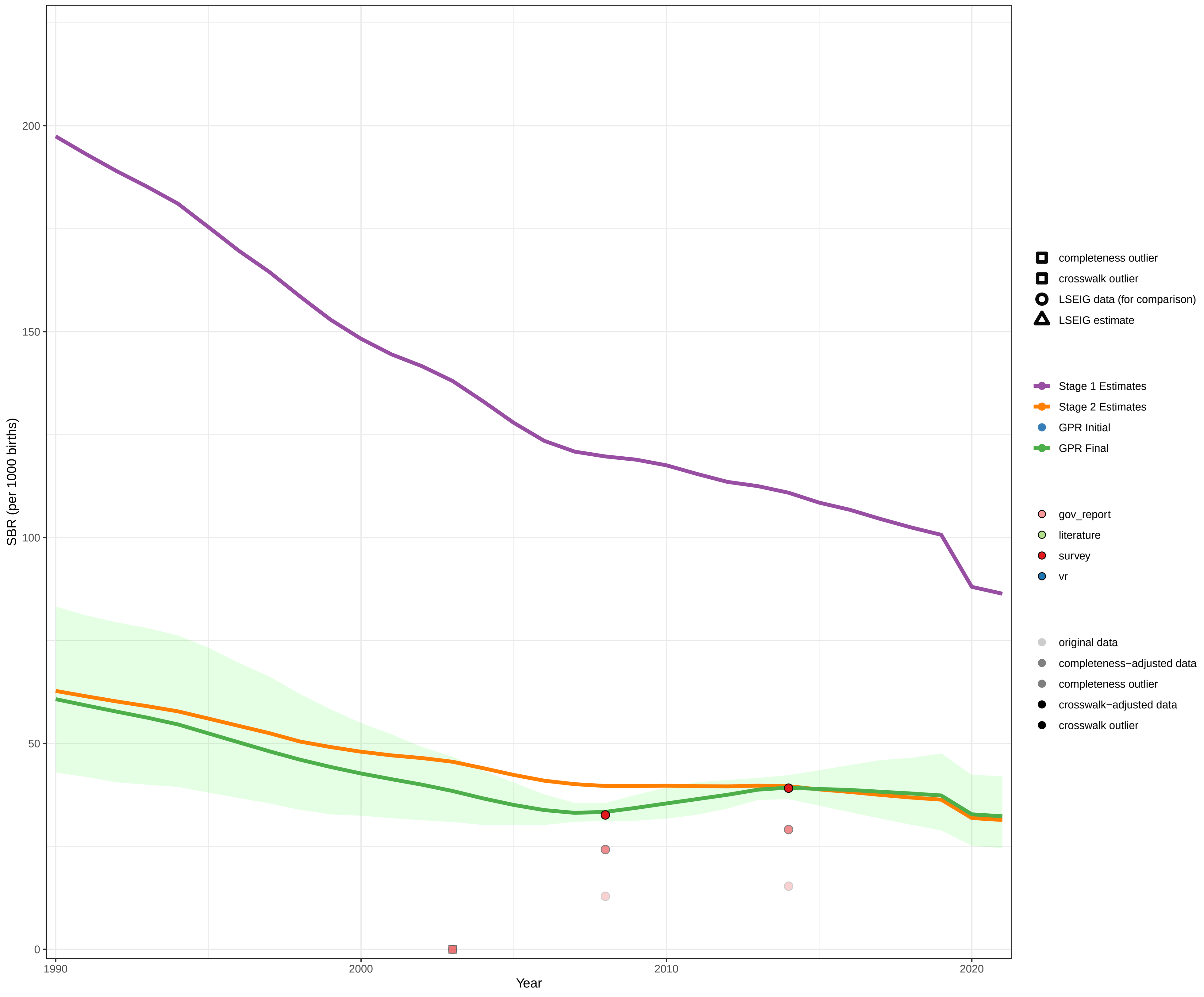

Marsabit KEN_35641 Eastern Sub−Saharan Africa Stillbirth Rate (SBR) − 28 weeks
lambda = 0.8 ; zeta = 0.7 ; scale = 15 ; amp = 0.028

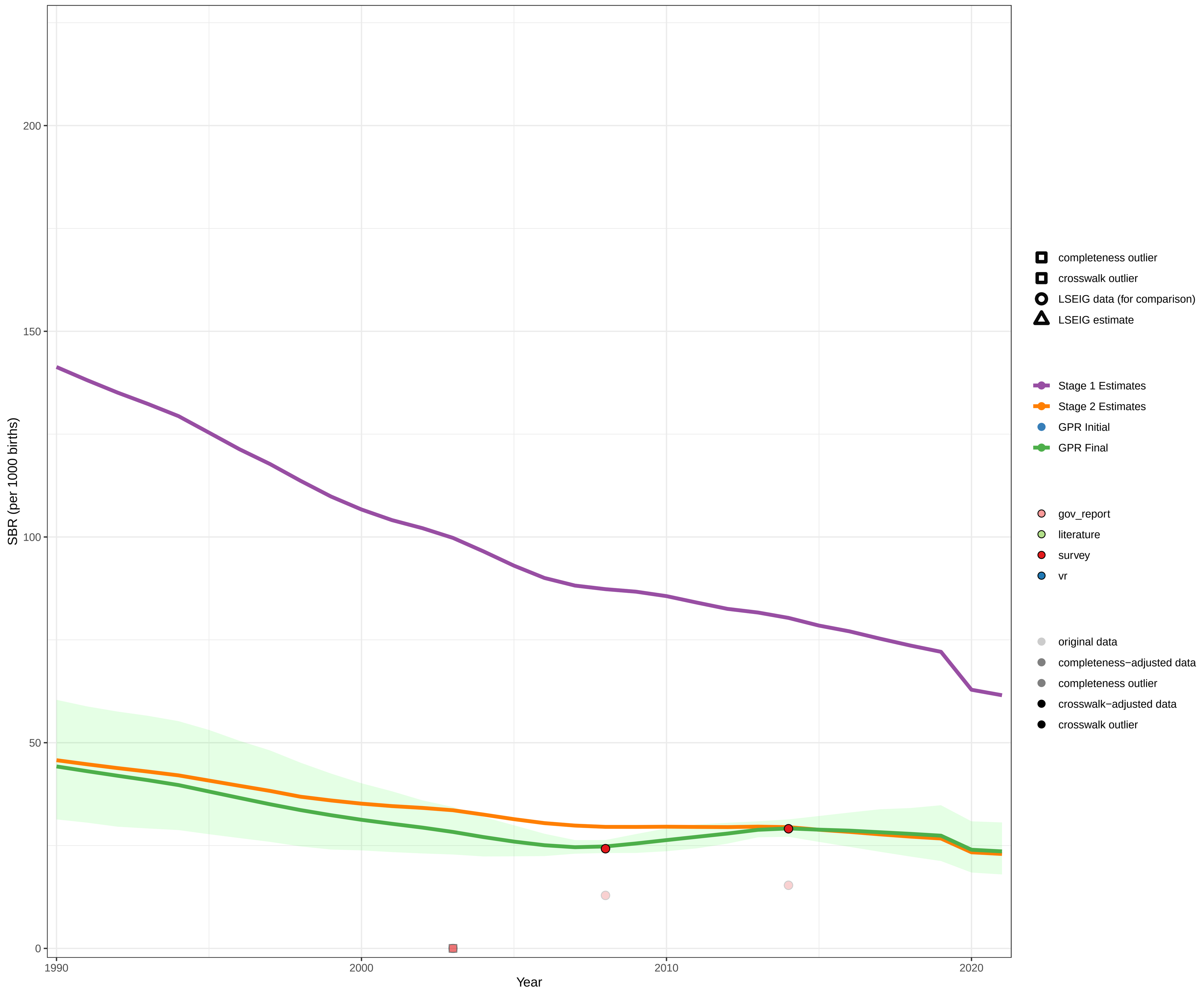

Year	Total Stillbirths (in thousands)	Total Stillbirths (in thousands)	SBR (per 1000 births)	SBR (per 1000 births)	SBR/NMR	SBR/NMR
	20 weeks	28 weeks	20 weeks	28 weeks	20 weeks	28 weeks
1990	0.4 (0.3 to 0.5)	0.3 (0.2 to 0.4)	60.8 (42.9 to 83.2)	44.2 (31.4 to 60.4)	1.25 (0.89 to 1.72)	0.91 (0.65 to 1.25)
1995	0.4 (0.3 to 0.5)	0.3 (0.2 to 0.4)	52.4 (38.1 to 73.3)	38.1 (27.8 to 53.1)	1.19 (0.86 to 1.66)	0.86 (0.63 to 1.20)
2000	0.3 (0.3 to 0.4)	0.2 (0.2 to 0.3)	42.7 (32.5 to 55.0)	31.3 (23.8 to 40.1)	1.06 (0.80 to 1.36)	0.77 (0.59 to 1.00)
2005	0.3 (0.3 to 0.4)	0.2 (0.2 to 0.3)	35.1 (30.2 to 40.5)	26.0 (22.4 to 29.9)	0.88 (0.76 to 1.02)	0.65 (0.56 to 0.75)
2010	0.4 (0.4 to 0.4)	0.3 (0.3 to 0.3)	35.4 (31.8 to 39.3)	26.3 (23.6 to 29.2)	0.92 (0.82 to 1.02)	0.68 (0.61 to 0.76)
2015	0.5 (0.5 to 0.6)	0.4 (0.3 to 0.4)	38.9 (34.9 to 43.5)	28.9 (25.9 to 32.2)	1.10 (0.98 to 1.23)	0.81 (0.73 to 0.91)
2020	0.5 (0.4 to 0.6)	0.4 (0.3 to 0.5)	32.8 (25.1 to 42.4)	24.0 (18.4 to 30.9)	1.18 (0.91 to 1.53)	0.86 (0.66 to 1.11)
2021	0.5 (0.4 to 0.6)	0.4 (0.3 to 0.5)	32.3 (24.6 to 42.1)	23.6 (18.0 to 30.6)	1.19 (0.91 to 1.55)	0.87 (0.66 to 1.13)

Meru KEN_35642 Eastern Sub−Saharan Africa Stillbirth Rate / Neonatal Mortality Rate (SBR/NMR) − 20 weeks
lambda = 0.8 ; zeta = 0.7 ; scale = 15 ; amp = 0.028

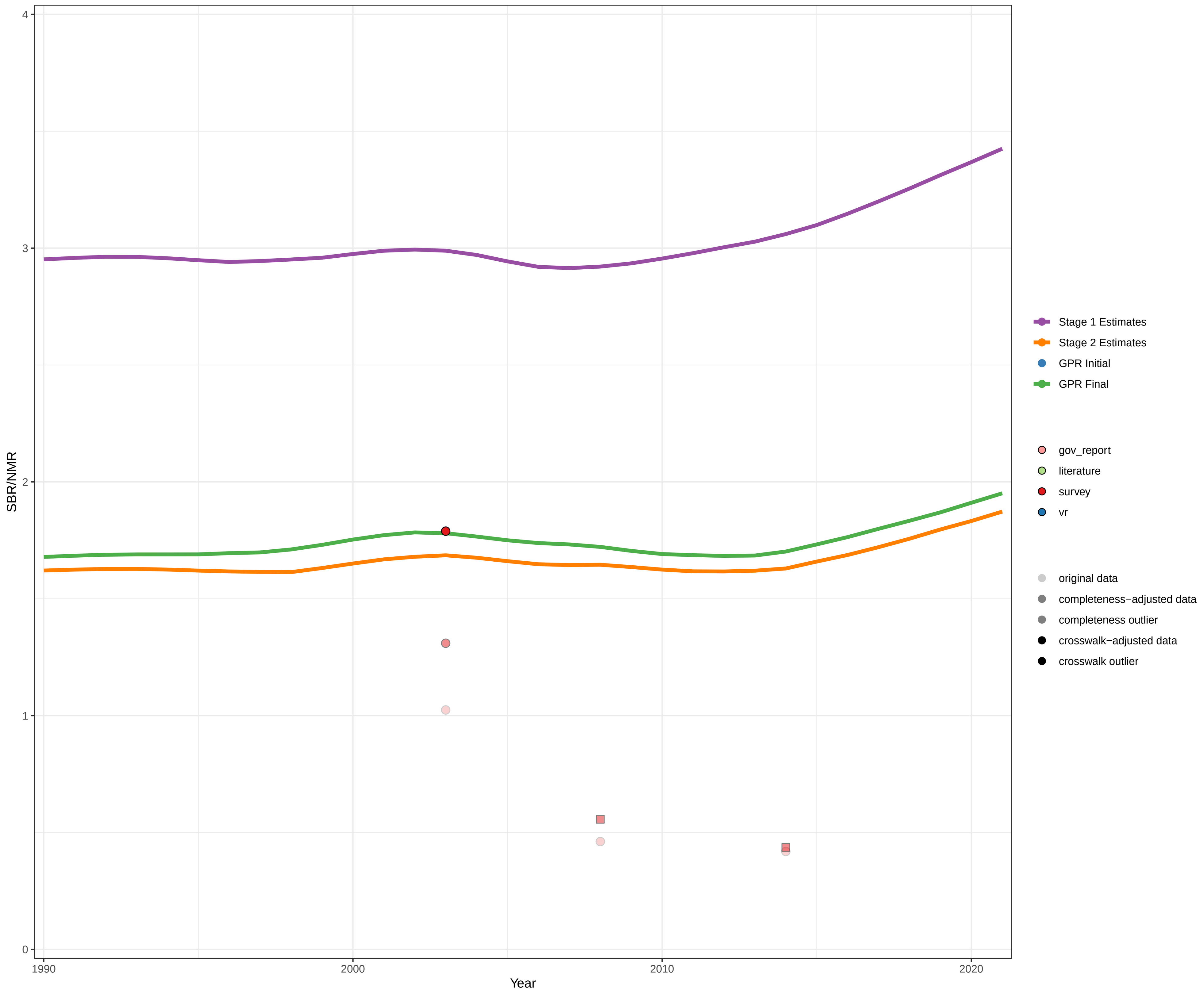

Meru KEN_35642 Eastern Sub−Saharan Africa Stillbirth Rate / Neonatal Mortality Rate (SBR/NMR) − 28 weeks
lambda = 0.8 ; zeta = 0.7 ; scale = 15 ; amp = 0.028

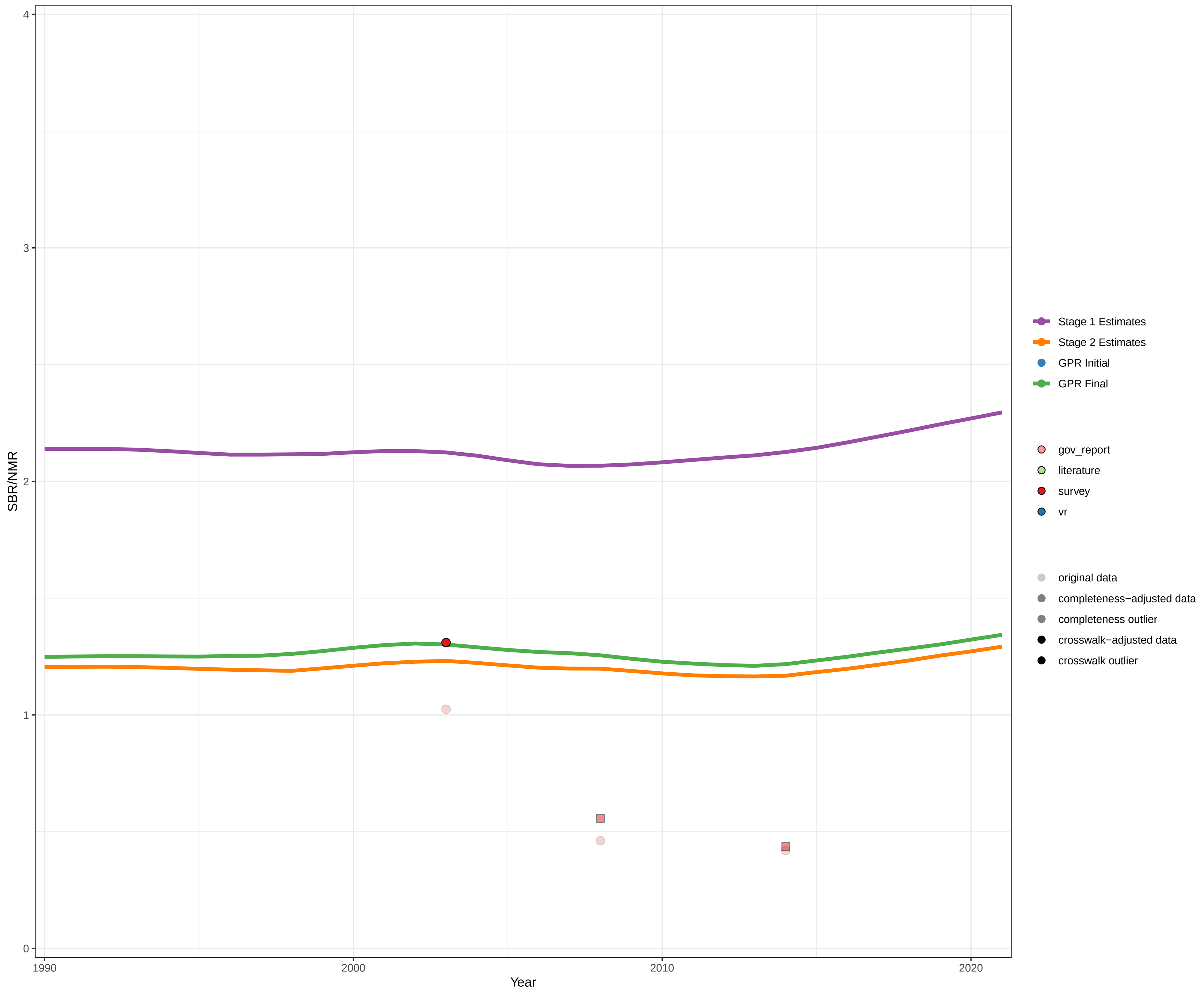

Definition	Years	Outlier	Source.Type	Title
28_weeks	2003	0	Survey	Kenya Demographic and Health Survey 2003
28_weeks	2008	1	Survey	Kenya Demographic and Health Survey 2008−2009
28_weeks	2014	1	Survey	Kenya Demographic and Health Survey 2014

Meru KEN_35642 Eastern Sub−Saharan Africa Stillbirth Rate (SBR) − 20 weeks
lambda = 0.8 ; zeta = 0.7 ; scale = 15 ; amp = 0.028

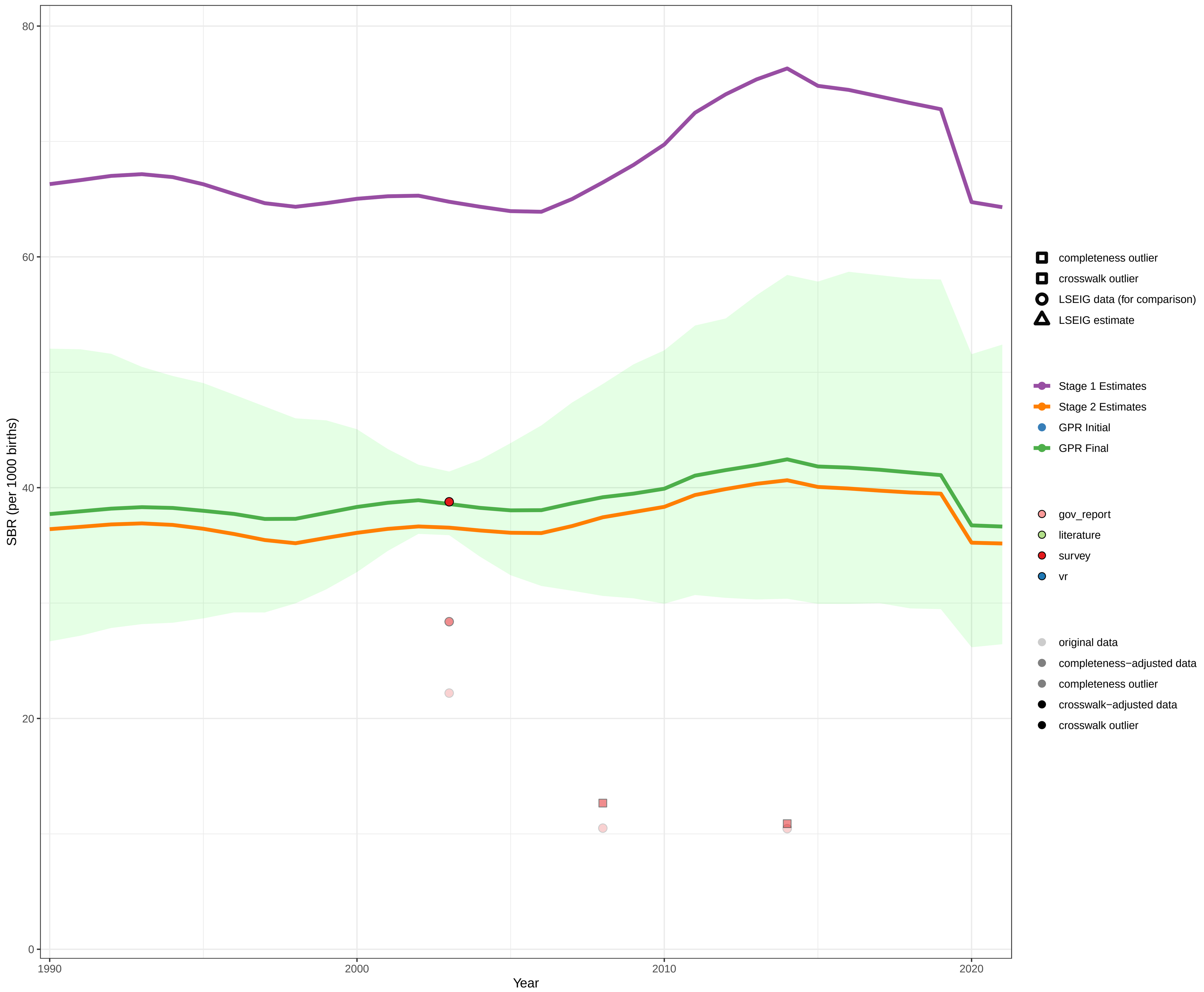

Meru KEN_35642 Eastern Sub−Saharan Africa Stillbirth Rate (SBR) − 28 weeks
lambda = 0.8 ; zeta = 0.7 ; scale = 15 ; amp = 0.028

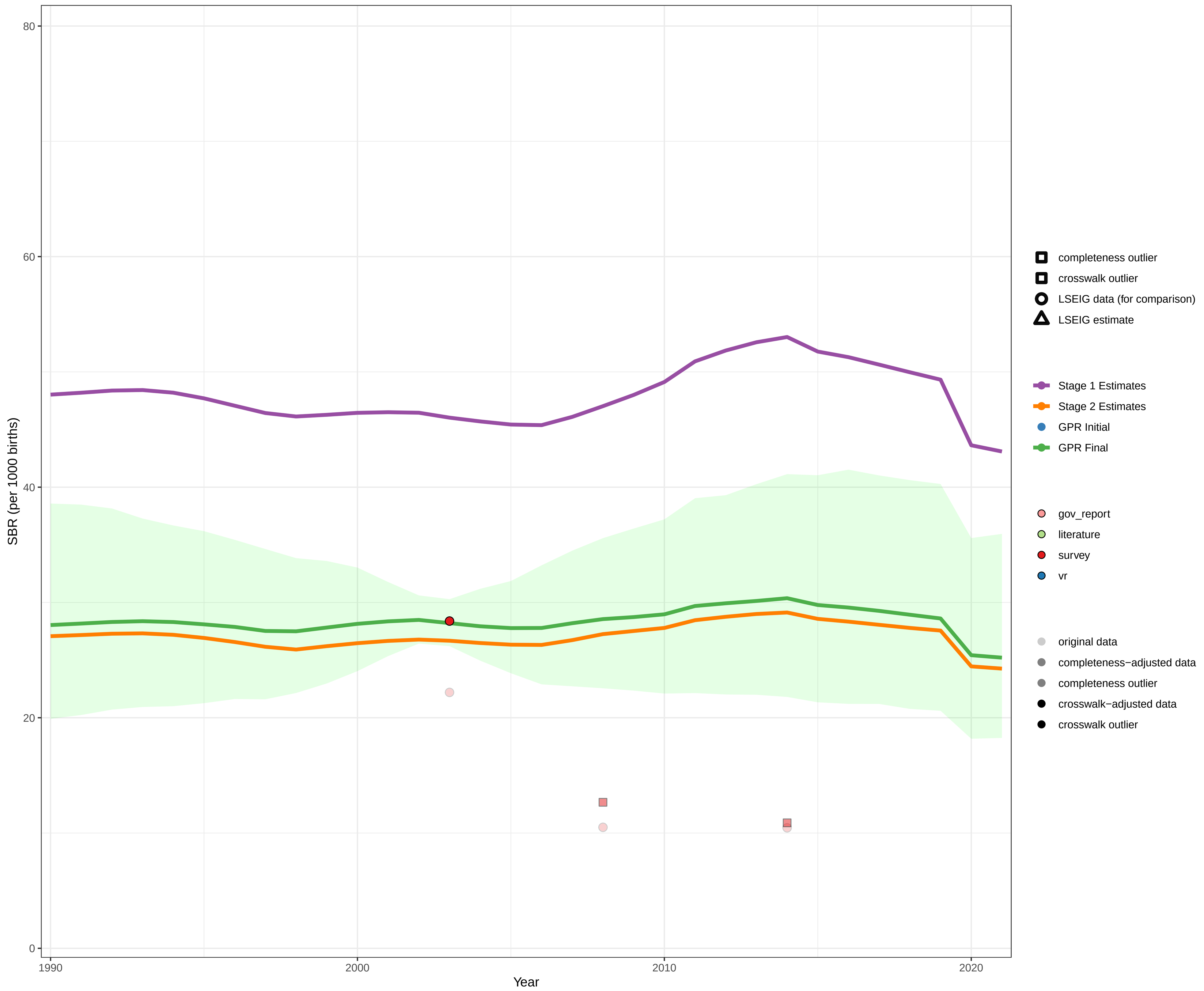

Year	Total Stillbirths (in thousands)	Total Stillbirths (in thousands)	SBR (per 1000 births)	SBR (per 1000 births)	SBR/NMR	SBR/NMR
	20 weeks	28 weeks	20 weeks	28 weeks	20 weeks	28 weeks
1990	1.9 (1.3 to 2.7)	1.4 (1.0 to 2.0)	37.7 (26.7 to 52.0)	28.0 (19.9 to 38.6)	1.68 (1.19 to 2.32)	1.25 (0.89 to 1.72)
1995	2.1 (1.6 to 2.7)	1.5 (1.1 to 2.0)	38.0 (28.7 to 49.1)	28.1 (21.3 to 36.2)	1.69 (1.28 to 2.18)	1.25 (0.95 to 1.61)
2000	2.3 (1.9 to 2.7)	1.7 (1.4 to 2.0)	38.3 (32.7 to 45.1)	28.1 (24.0 to 33.0)	1.75 (1.50 to 2.06)	1.29 (1.10 to 1.51)
2005	2.4 (2.0 to 2.7)	1.7 (1.5 to 2.0)	38.0 (32.4 to 43.9)	27.8 (23.9 to 31.9)	1.75 (1.49 to 2.02)	1.28 (1.10 to 1.47)
2010	2.3 (1.7 to 3.1)	1.7 (1.3 to 2.2)	39.9 (29.9 to 51.9)	29.0 (22.1 to 37.2)	1.69 (1.27 to 2.20)	1.23 (0.94 to 1.58)
2015	2.1 (1.4 to 2.9)	1.4 (1.0 to 2.0)	41.8 (29.9 to 57.9)	29.8 (21.3 to 41.0)	1.73 (1.24 to 2.40)	1.23 (0.88 to 1.70)
2020	1.4 (1.0 to 1.9)	0.9 (0.7 to 1.3)	36.7 (26.2 to 51.6)	25.4 (18.2 to 35.6)	1.91 (1.36 to 2.68)	1.32 (0.95 to 1.85)
2021	1.3 (0.9 to 1.9)	0.9 (0.6 to 1.3)	36.6 (26.4 to 52.4)	25.2 (18.3 to 35.9)	1.95 (1.41 to 2.79)	1.34 (0.97 to 1.91)

Migori KEN_35643 Eastern Sub−Saharan Africa Stillbirth Rate / Neonatal Mortality Rate (SBR/NMR) − 20 weeks
lambda = 0.8 ; zeta = 0.7 ; scale = 15 ; amp = 0.028

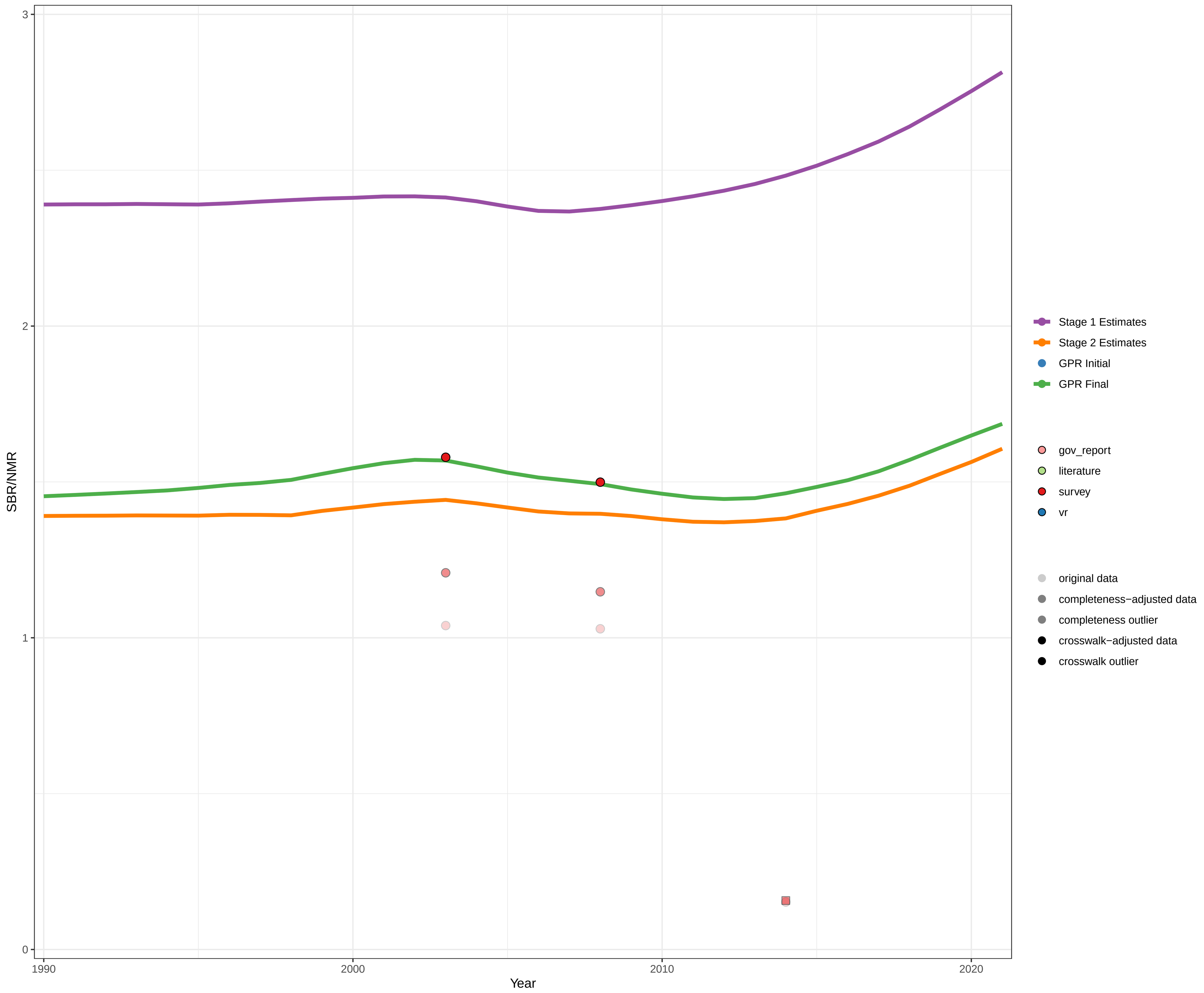

Migori KEN_35643 Eastern Sub−Saharan Africa Stillbirth Rate / Neonatal Mortality Rate (SBR/NMR) − 28 weeks
lambda = 0.8 ; zeta = 0.7 ; scale = 15 ; amp = 0.028

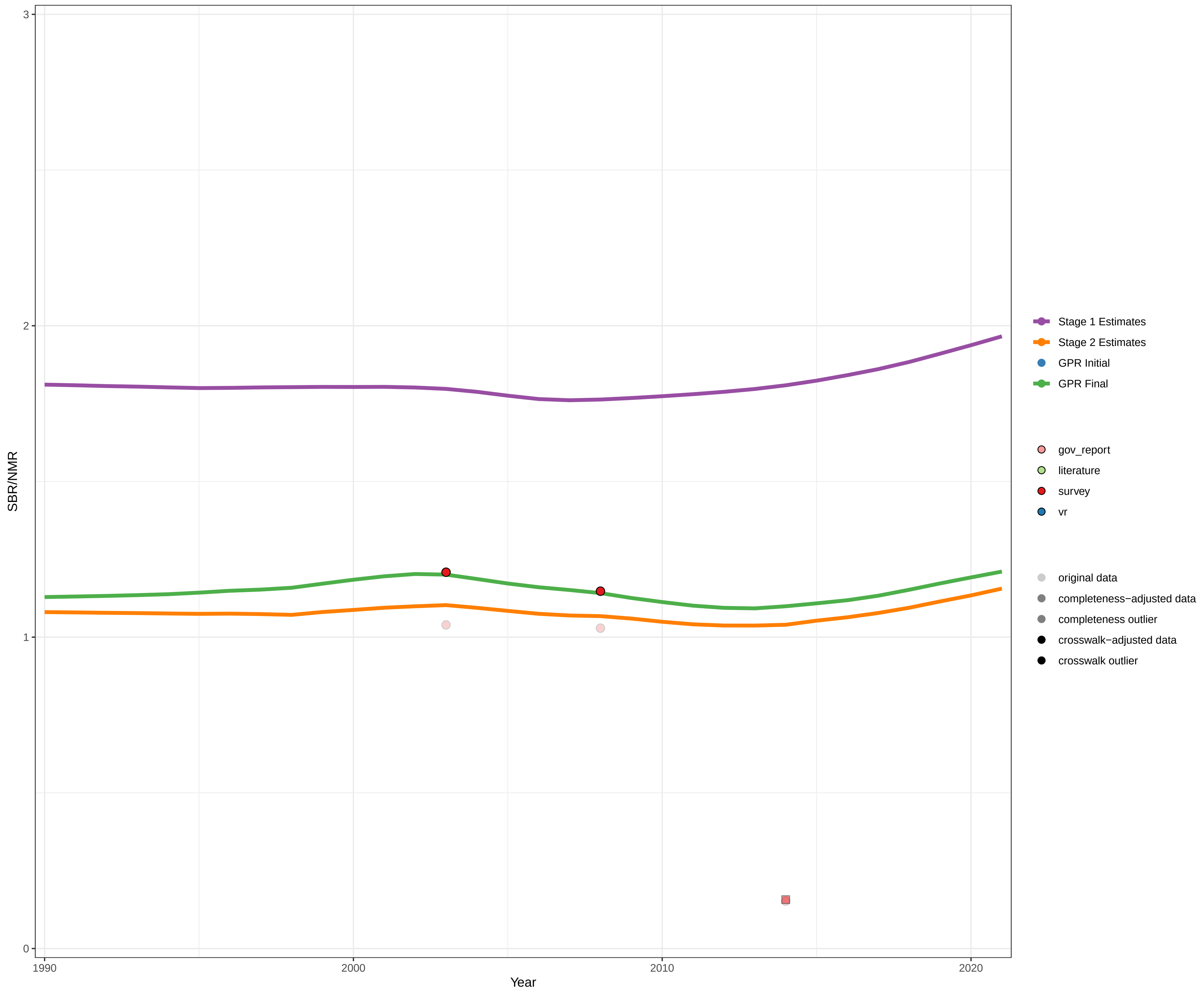

Definition	Years	Outlier	Source.Type	Title
28_weeks	2003	0	Survey	Kenya Demographic and Health Survey 2003
28_weeks	2008	0	Survey	Kenya Demographic and Health Survey 2008−2009
28_weeks	2014	1	Survey	Kenya Demographic and Health Survey 2014

Migori KEN_35643 Eastern Sub−Saharan Africa Stillbirth Rate (SBR) − 20 weeks
lambda = 0.8 ; zeta = 0.7 ; scale = 15 ; amp = 0.028

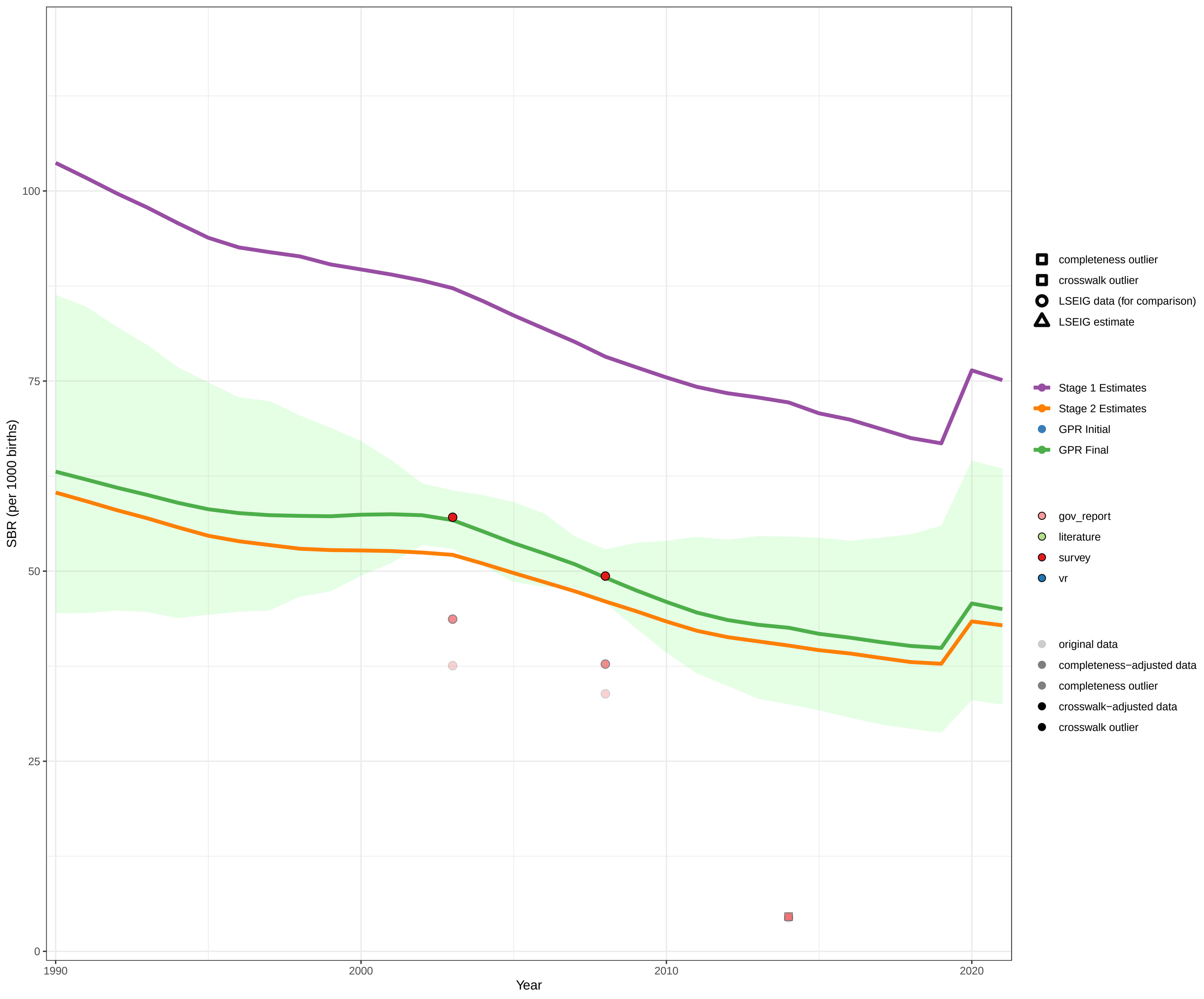

Migori KEN_35643 Eastern Sub−Saharan Africa Stillbirth Rate (SBR) − 28 weeks
lambda = 0.8 ; zeta = 0.7 ; scale = 15 ; amp = 0.028

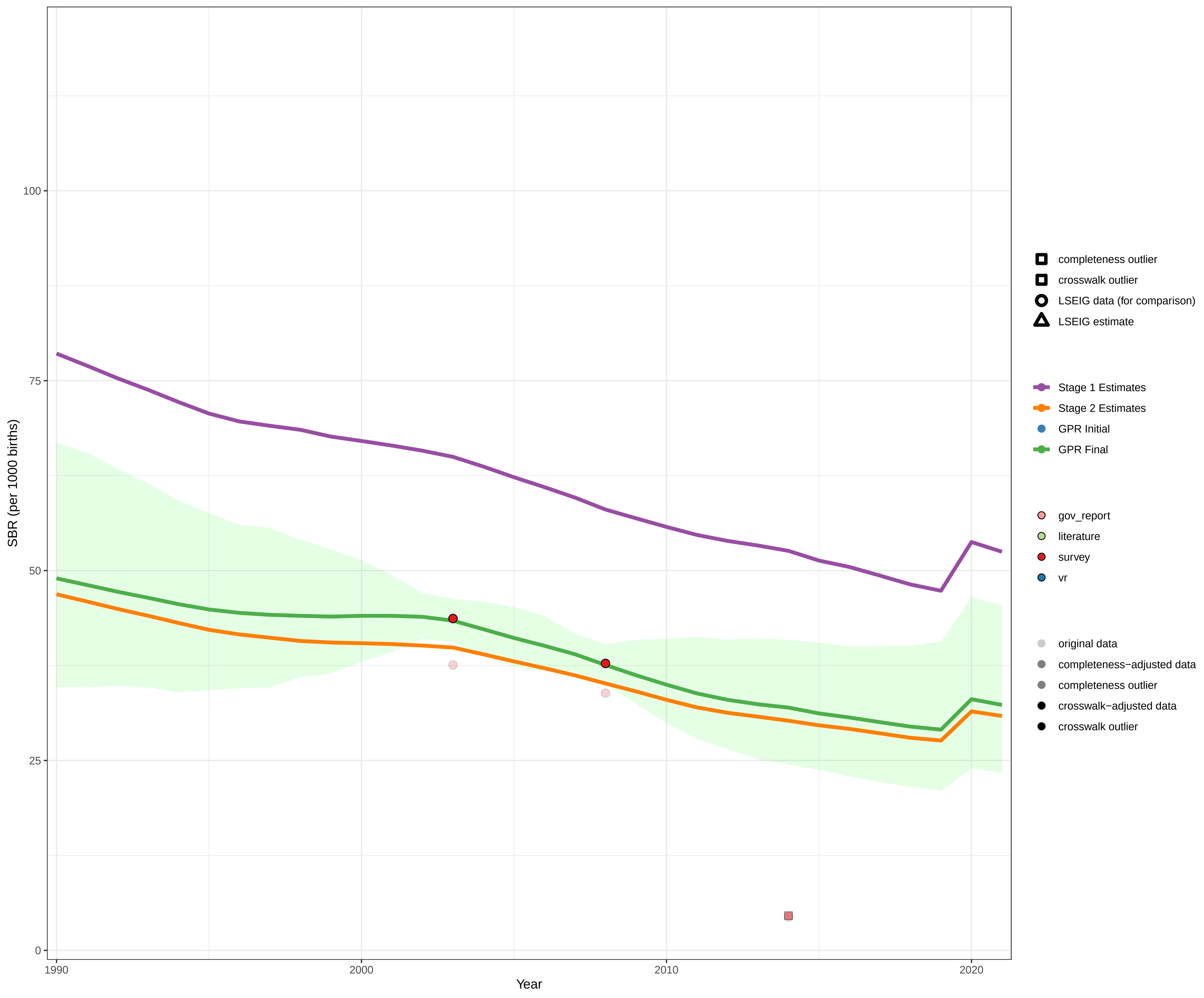

Year	Total Stillbirths (in thousands)	Total Stillbirths (in thousands)	SBR (per 1000 births)	SBR (per 1000 births)	SBR/NMR	SBR/NMR
	20 weeks	28 weeks	20 weeks	28 weeks	20 weeks	28 weeks
1990	2.0 (1.4 to 2.7)	1.5 (1.0 to 2.1)	63.1 (44.5 to 86.3)	49.0 (34.6 to 66.8)	1.45 (1.03 to 1.99)	1.13 (0.80 to 1.54)
1995	2.1 (1.6 to 2.7)	1.6 (1.2 to 2.0)	58.1 (44.3 to 74.8)	44.9 (34.3 to 57.6)	1.48 (1.13 to 1.91)	1.14 (0.87 to 1.47)
2000	2.3 (2.0 to 2.7)	1.7 (1.5 to 2.1)	57.4 (49.4 to 67.1)	44.0 (38.0 to 51.4)	1.54 (1.33 to 1.80)	1.18 (1.02 to 1.38)
2005	2.3 (2.1 to 2.6)	1.8 (1.6 to 2.0)	53.7 (48.6 to 59.1)	41.1 (37.3 to 45.2)	1.53 (1.38 to 1.68)	1.17 (1.06 to 1.29)
2010	2.1 (1.8 to 2.4)	1.6 (1.3 to 1.8)	46.0 (39.3 to 54.0)	35.0 (30.0 to 41.0)	1.46 (1.25 to 1.72)	1.11 (0.95 to 1.30)
2015	1.9 (1.4 to 2.4)	1.4 (1.0 to 1.8)	41.8 (31.7 to 54.4)	31.2 (23.7 to 40.5)	1.48 (1.13 to 1.93)	1.11 (0.84 to 1.44)
2020	1.9 (1.3 to 2.7)	1.3 (1.0 to 1.9)	45.7 (33.0 to 64.5)	33.1 (23.9 to 46.5)	1.65 (1.19 to 2.33)	1.19 (0.86 to 1.68)
2021	1.8 (1.3 to 2.6)	1.3 (0.9 to 1.8)	45.0 (32.5 to 63.5)	32.3 (23.4 to 45.4)	1.69 (1.22 to 2.38)	1.21 (0.88 to 1.70)

Mombasa KEN_35644 Eastern Sub−Saharan Africa Stillbirth Rate / Neonatal Mortality Rate (SBR/NMR) − 20 weeks
lambda = 0.8 ; zeta = 0.7 ; scale = 15 ; amp = 0.028

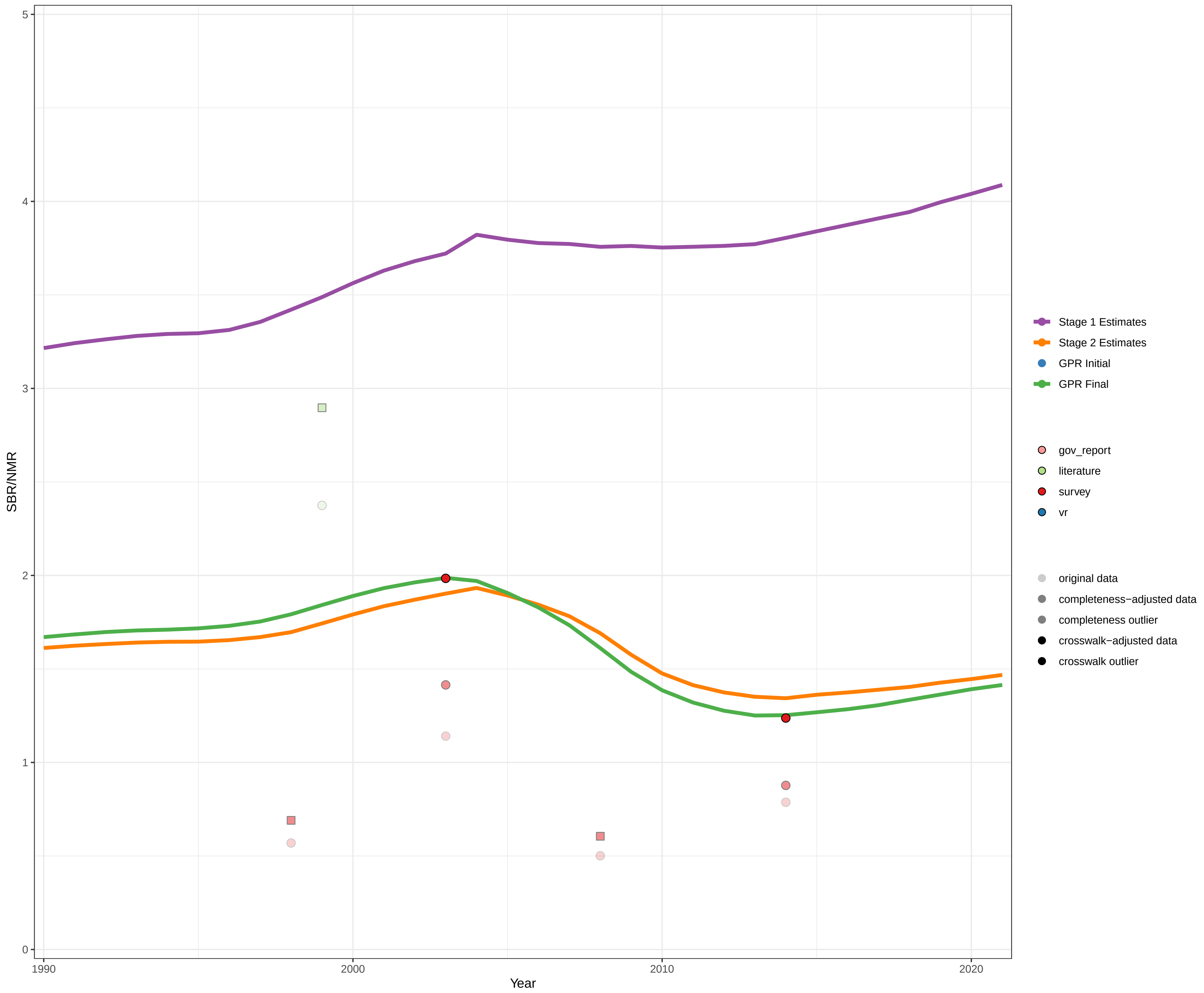

Mombasa KEN_35644 Eastern Sub−Saharan Africa Stillbirth Rate / Neonatal Mortality Rate (SBR/NMR) − 28 weeks
lambda = 0.8 ; zeta = 0.7 ; scale = 15 ; amp = 0.028

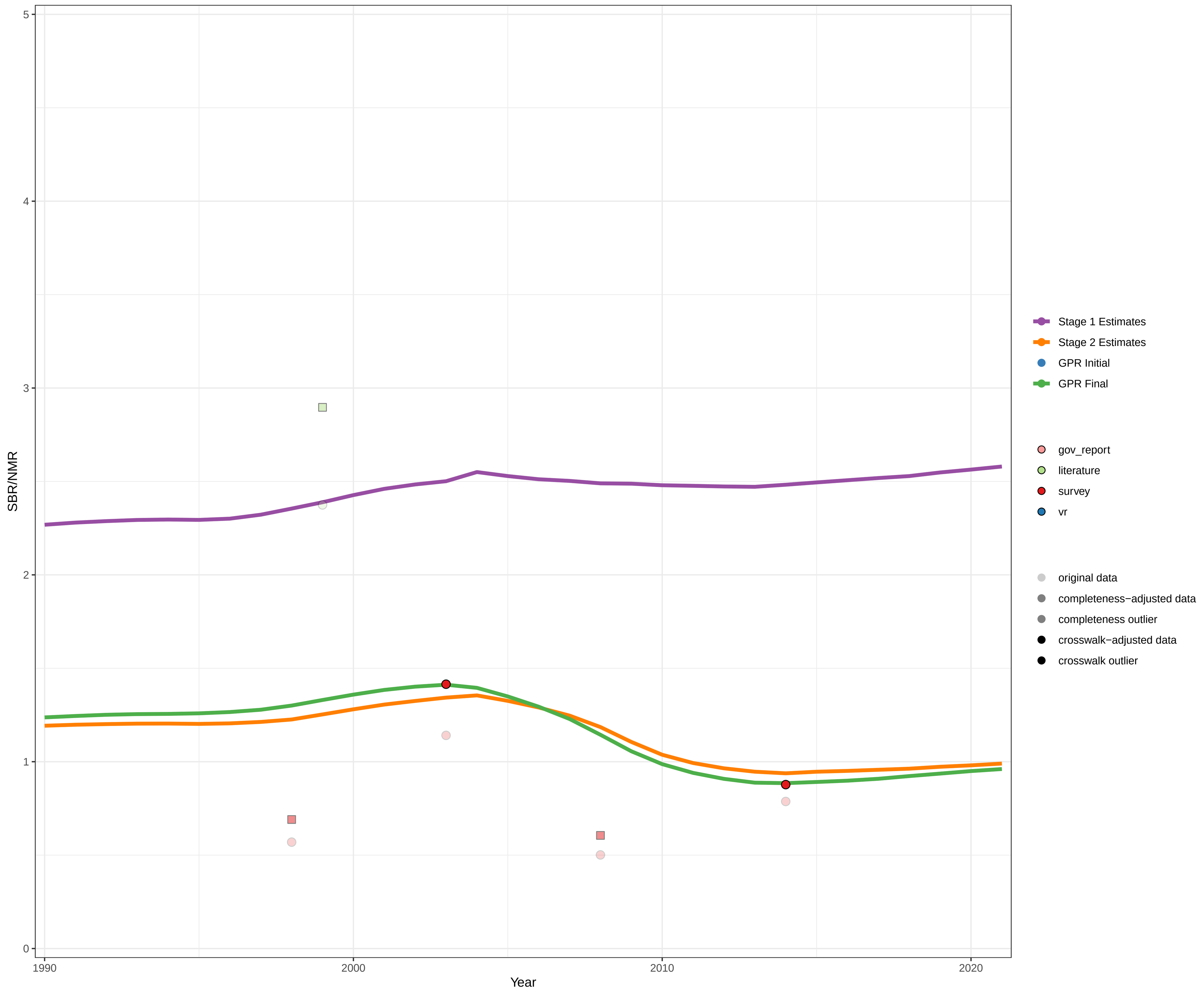

Definition	Years	Outlier	Source.Type	Title
28_weeks	2003	0	Survey	Kenya Demographic and Health Survey 2003
28_weeks	2014	0	Survey	Kenya Demographic and Health Survey 2014
22_weeks	1999	1	Sci Lit	Placental inflammation and perinatal outcome
28_weeks	1998	1	Survey	Kenya Demographic and Health Survey 1998
28_weeks	2008	1	Survey	Kenya Demographic and Health Survey 2008−2009

Mombasa KEN_35644 Eastern Sub−Saharan Africa Stillbirth Rate (SBR) − 20 weeks
lambda = 0.8 ; zeta = 0.7 ; scale = 15 ; amp = 0.028

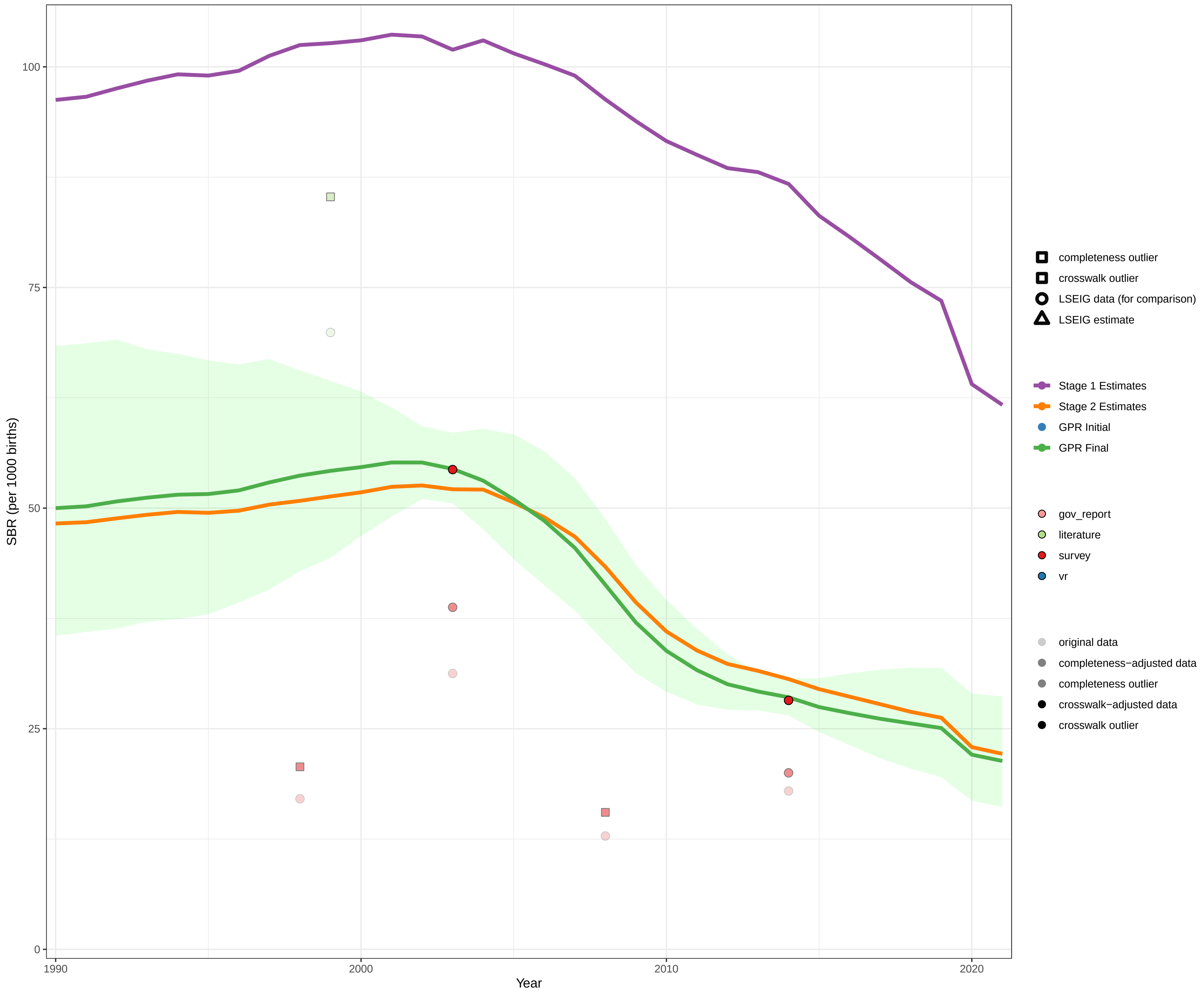

Mombasa KEN_35644 Eastern Sub−Saharan Africa Stillbirth Rate (SBR) − 28 weeks
lambda = 0.8 ; zeta = 0.7 ; scale = 15 ; amp = 0.028

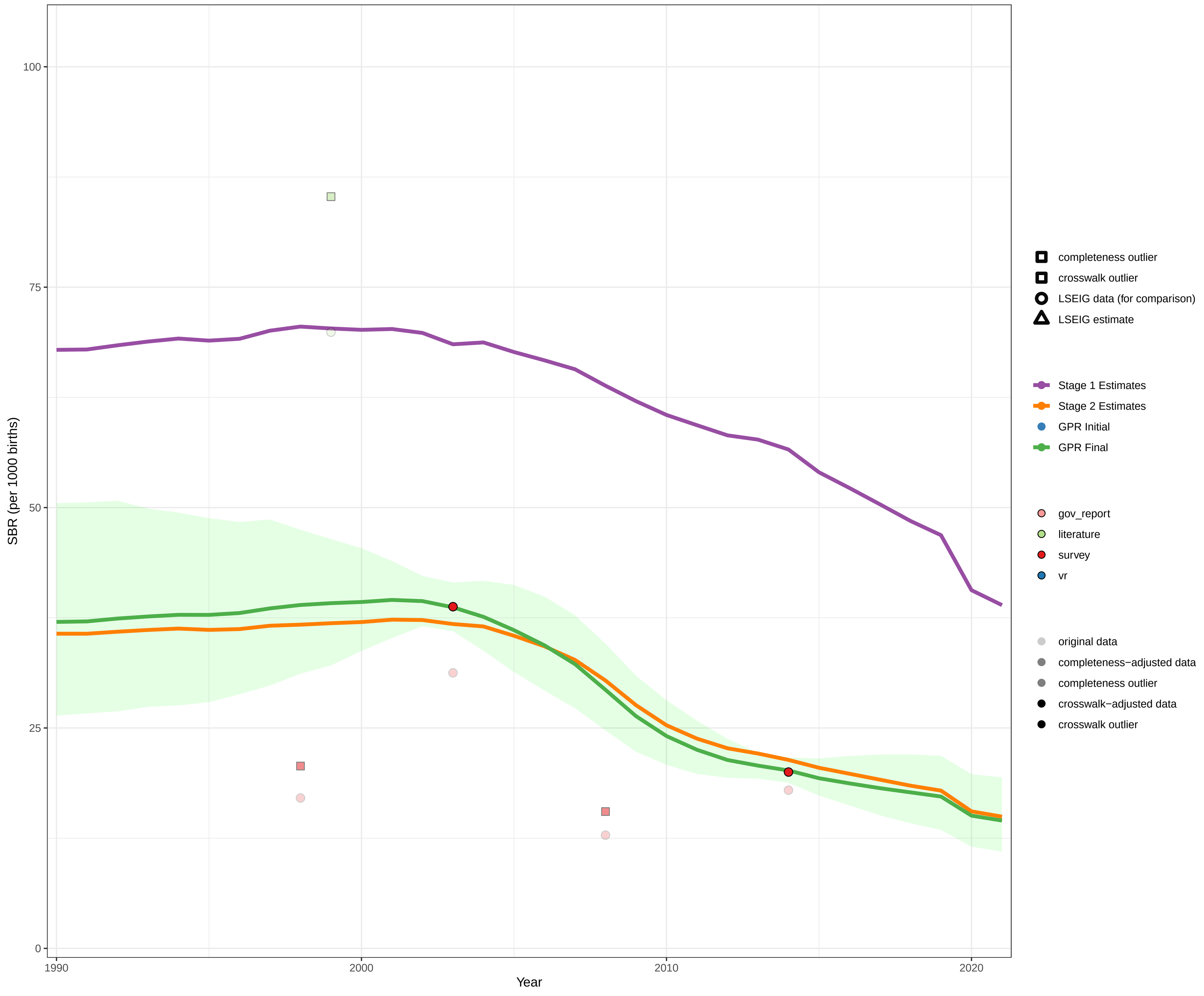

Year	Total Stillbirths (in thousands)	Total Stillbirths (in thousands)	SBR (per 1000 births)	SBR (per 1000 births)	SBR/NMR	SBR/NMR
	20 weeks	28 weeks	20 weeks	28 weeks	20 weeks	28 weeks
1990	0.9 (0.6 to 1.2)	0.6 (0.4 to 0.9)	50.0 (35.5 to 68.4)	37.0 (26.4 to 50.5)	1.67 (1.19 to 2.28)	1.24 (0.88 to 1.69)
1995	1.0 (0.7 to 1.3)	0.7 (0.5 to 0.9)	51.6 (38.0 to 66.7)	37.8 (27.9 to 48.8)	1.72 (1.26 to 2.22)	1.26 (0.93 to 1.62)
2000	1.2 (1.0 to 1.4)	0.8 (0.7 to 1.0)	54.6 (46.8 to 63.2)	39.3 (33.7 to 45.4)	1.89 (1.62 to 2.19)	1.36 (1.17 to 1.57)
2005	1.3 (1.1 to 1.4)	0.9 (0.8 to 1.0)	51.0 (44.2 to 58.4)	36.1 (31.3 to 41.2)	1.91 (1.65 to 2.18)	1.35 (1.17 to 1.54)
2010	0.9 (0.7 to 1.0)	0.6 (0.5 to 0.7)	33.8 (29.2 to 39.6)	24.1 (20.8 to 28.2)	1.39 (1.20 to 1.62)	0.99 (0.85 to 1.15)
2015	0.6 (0.6 to 0.7)	0.4 (0.4 to 0.5)	27.5 (24.6 to 30.7)	19.3 (17.3 to 21.6)	1.27 (1.14 to 1.42)	0.89 (0.80 to 1.00)
2020	0.4 (0.3 to 0.5)	0.3 (0.2 to 0.4)	22.1 (16.8 to 29.0)	15.1 (11.5 to 19.8)	1.39 (1.06 to 1.83)	0.95 (0.73 to 1.25)
2021	0.4 (0.3 to 0.5)	0.2 (0.2 to 0.3)	21.3 (16.1 to 28.6)	14.5 (11.0 to 19.4)	1.41 (1.07 to 1.90)	0.96 (0.73 to 1.29)

Murang'a KEN_35645 Eastern Sub−Saharan Africa Stillbirth Rate / Neonatal Mortality Rate (SBR/NMR) − 20 weeks
lambda = 0.8 ; zeta = 0.7 ; scale = 15 ; amp = 0.028

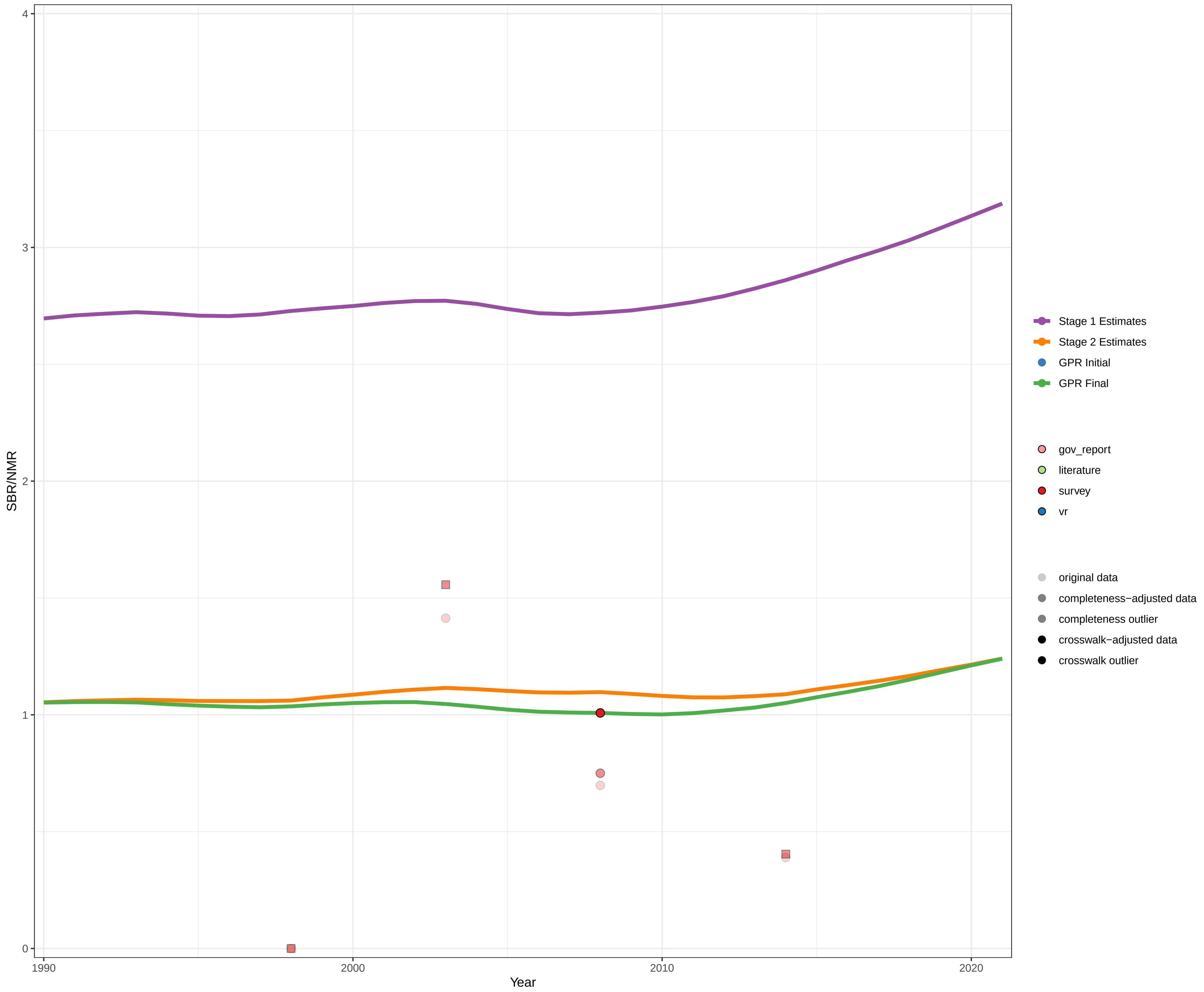

Murang'a KEN_35645 Eastern Sub−Saharan Africa Stillbirth Rate / Neonatal Mortality Rate (SBR/NMR) − 28 weeks
lambda = 0.8 ; zeta = 0.7 ; scale = 15 ; amp = 0.028

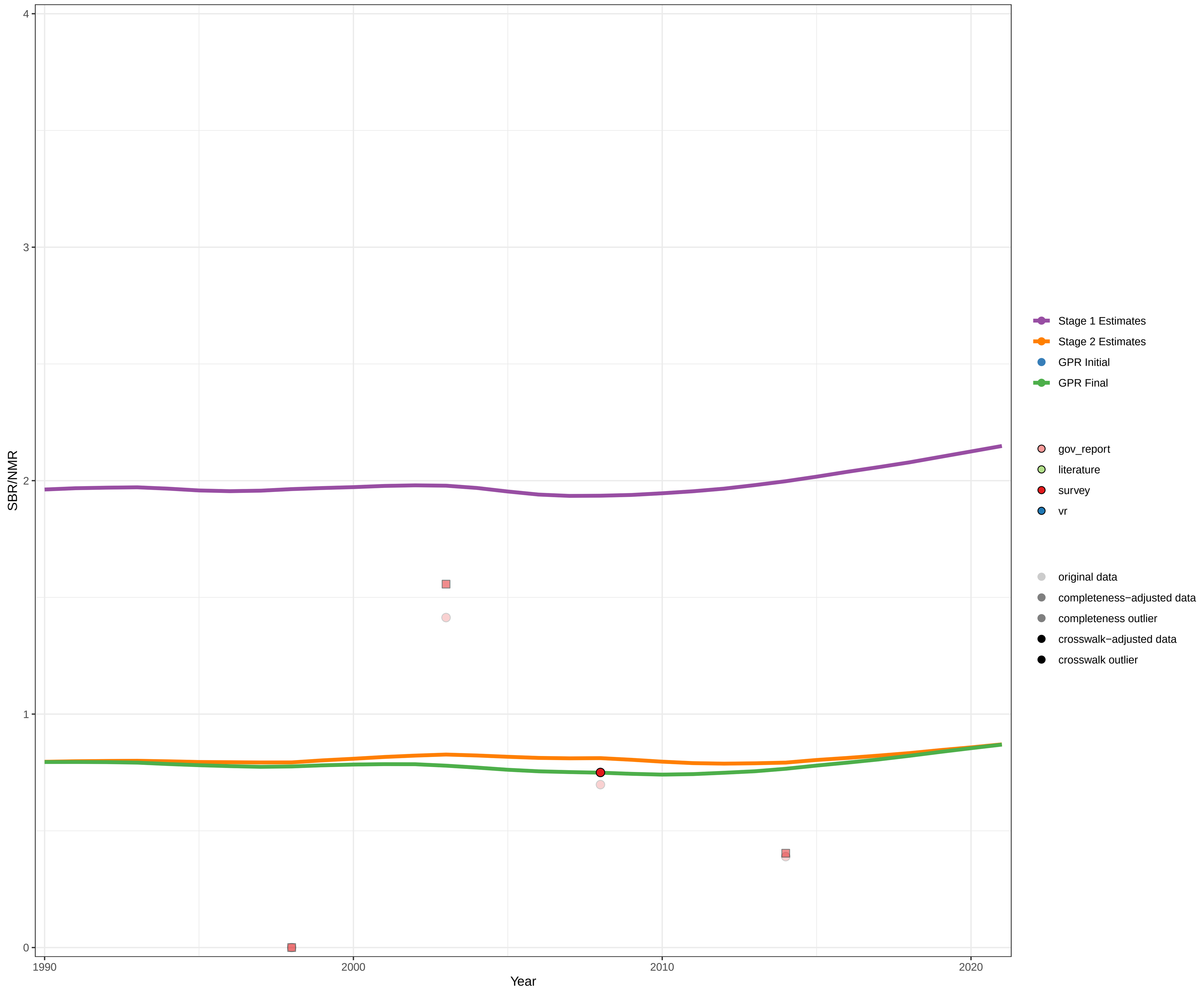

Definition	Years	Outlier	Source.Type	Title
28_weeks	2008	0	Survey	Kenya Demographic and Health Survey 2008−2009
28_weeks	1998	1	Survey	Kenya Demographic and Health Survey 1998
28_weeks	2003	1	Survey	Kenya Demographic and Health Survey 2003
28_weeks	2014	1	Survey	Kenya Demographic and Health Survey 2014

Murang'a KEN_35645 Eastern Sub−Saharan Africa Stillbirth Rate (SBR) − 20 weeks
lambda = 0.8 ; zeta = 0.7 ; scale = 15 ; amp = 0.028

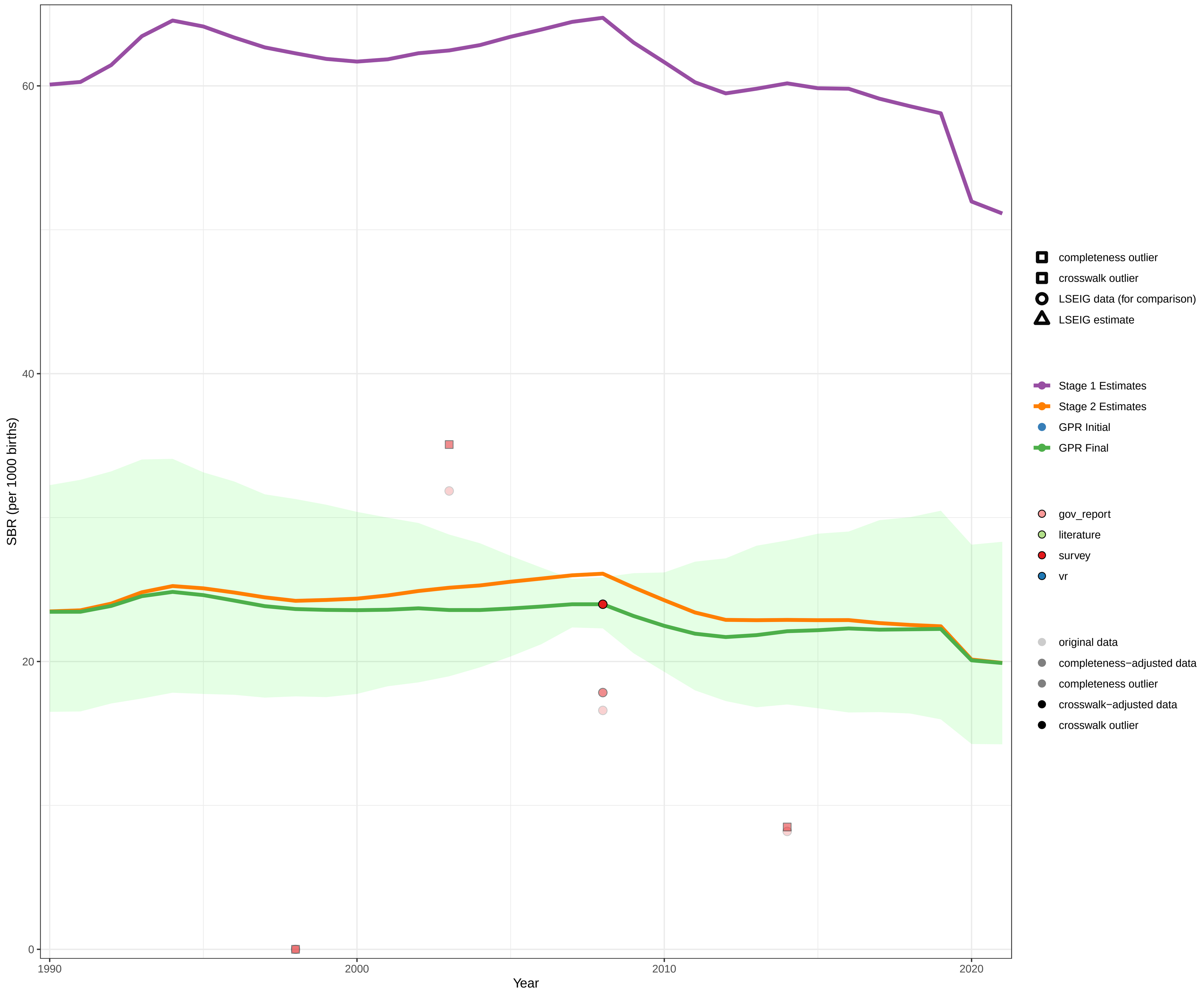

Murang'a KEN_35645 Eastern Sub−Saharan Africa Stillbirth Rate (SBR) − 28 weeks
lambda = 0.8 ; zeta = 0.7 ; scale = 15 ; amp = 0.028

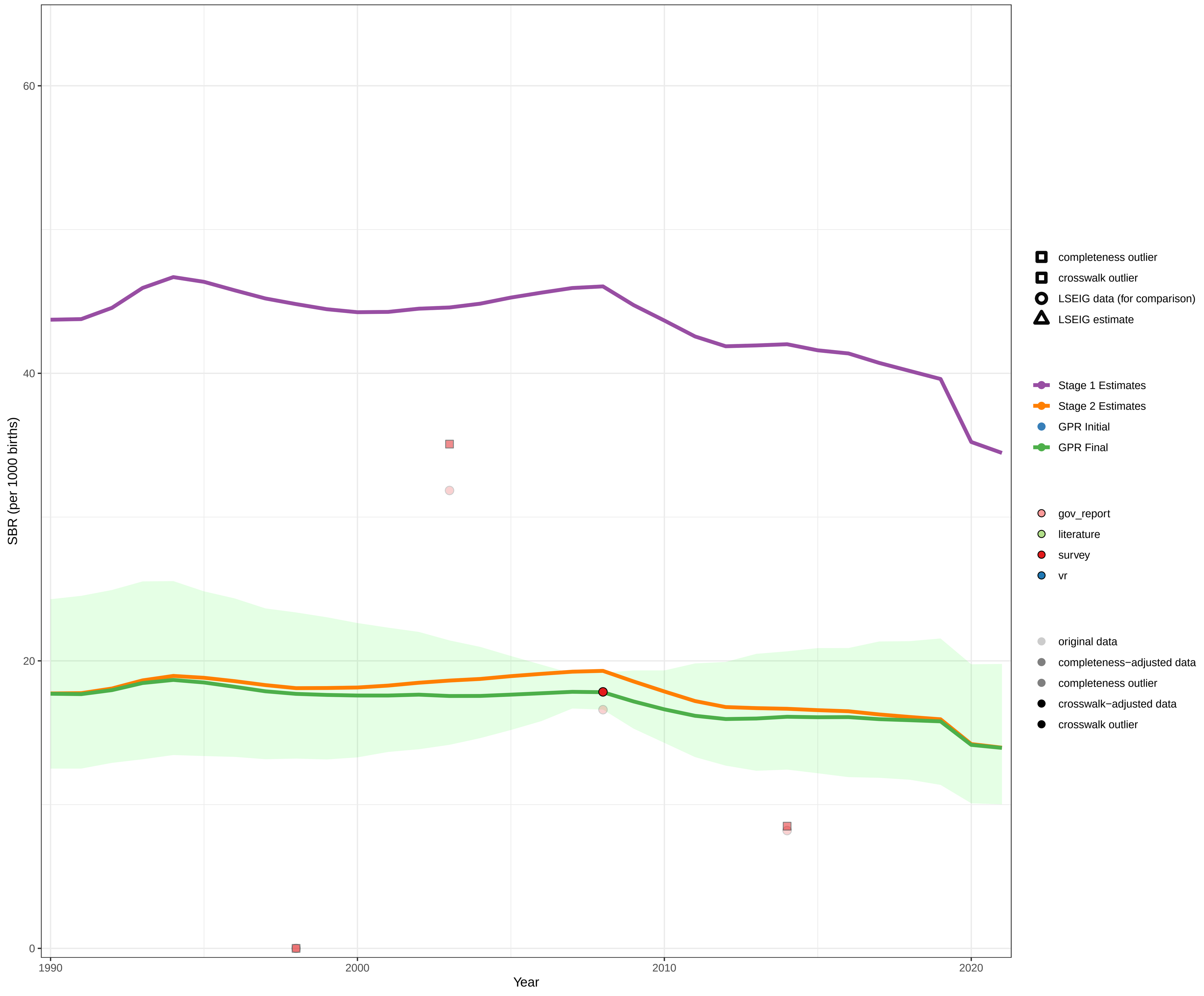

Year	Total Stillbirths (in thousands)	Total Stillbirths (in thousands)	SBR (per 1000 births)	SBR (per 1000 births)	SBR/NMR	SBR/NMR
	20 weeks	28 weeks	20 weeks	28 weeks	20 weeks	28 weeks
1990	0.8 (0.6 to 1.1)	0.6 (0.4 to 0.8)	23.5 (16.5 to 32.3)	17.7 (12.5 to 24.3)	1.05 (0.74 to 1.45)	0.79 (0.56 to 1.09)
1995	0.7 (0.5 to 1.0)	0.5 (0.4 to 0.7)	24.6 (17.7 to 33.1)	18.5 (13.4 to 24.8)	1.04 (0.75 to 1.40)	0.78 (0.56 to 1.05)
2000	0.6 (0.5 to 0.8)	0.4 (0.3 to 0.6)	23.6 (17.7 to 30.4)	17.6 (13.3 to 22.6)	1.05 (0.79 to 1.35)	0.78 (0.59 to 1.01)
2005	0.6 (0.5 to 0.7)	0.4 (0.4 to 0.5)	23.7 (20.3 to 27.3)	17.6 (15.2 to 20.3)	1.02 (0.88 to 1.18)	0.76 (0.66 to 0.88)
2010	0.5 (0.5 to 0.6)	0.4 (0.3 to 0.5)	22.5 (19.3 to 26.2)	16.6 (14.3 to 19.3)	1.00 (0.86 to 1.17)	0.74 (0.64 to 0.86)
2015	0.4 (0.3 to 0.6)	0.3 (0.2 to 0.4)	22.2 (16.8 to 28.9)	16.1 (12.2 to 20.9)	1.08 (0.81 to 1.40)	0.78 (0.59 to 1.01)
2020	0.3 (0.2 to 0.4)	0.2 (0.2 to 0.3)	20.1 (14.3 to 28.1)	14.2 (10.1 to 19.8)	1.21 (0.86 to 1.70)	0.85 (0.61 to 1.19)
2021	0.3 (0.2 to 0.4)	0.2 (0.2 to 0.3)	19.9 (14.2 to 28.3)	13.9 (10.0 to 19.8)	1.24 (0.89 to 1.77)	0.87 (0.62 to 1.23)

Nairobi KEN_35646 Eastern Sub−Saharan Africa Stillbirth Rate / Neonatal Mortality Rate (SBR/NMR) − 20 weeks
lambda = 0.8 ; zeta = 0.7 ; scale = 15 ; amp = 0.028

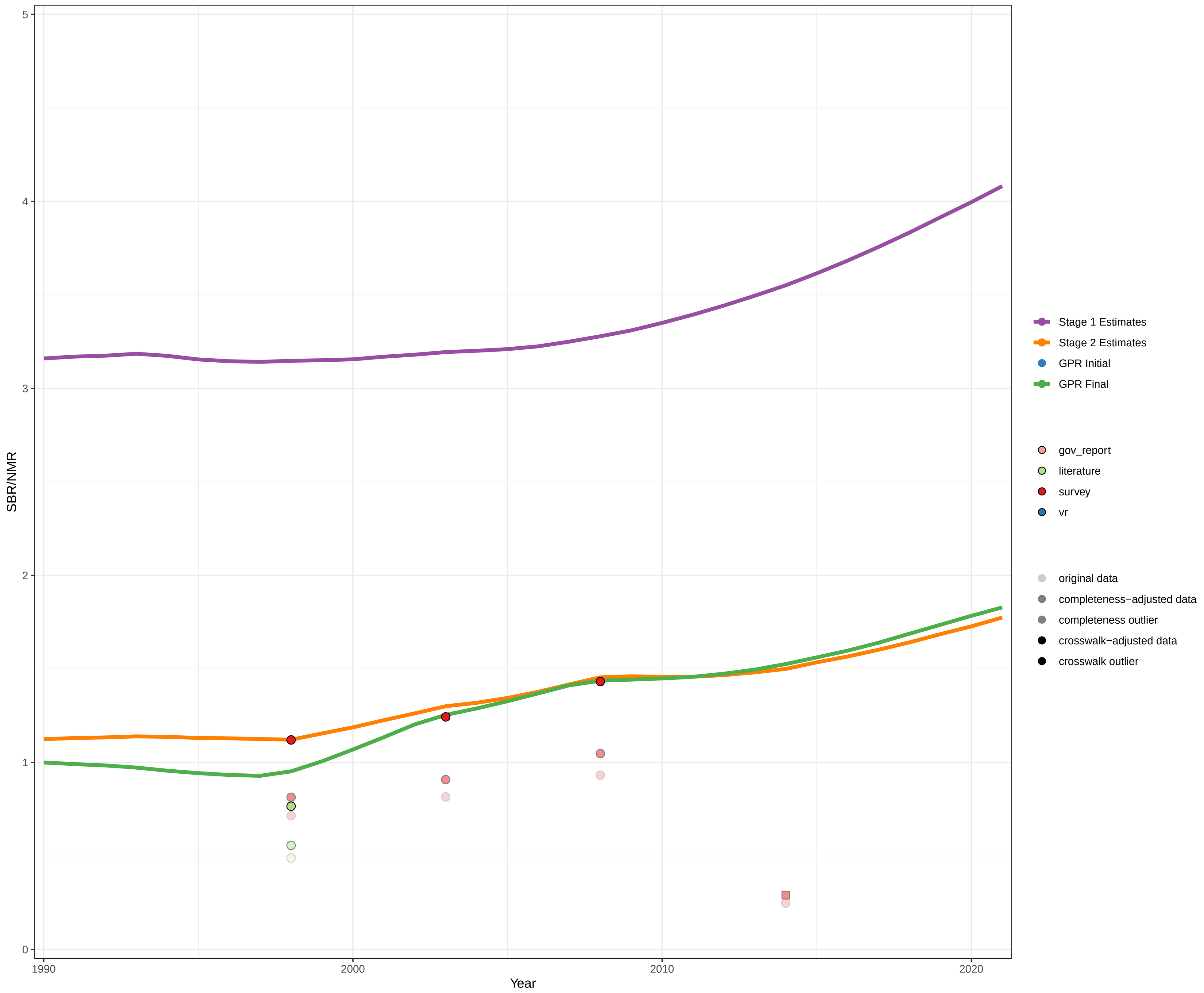

Nairobi KEN_35646 Eastern Sub−Saharan Africa Stillbirth Rate / Neonatal Mortality Rate (SBR/NMR) − 28 weeks
lambda = 0.8 ; zeta = 0.7 ; scale = 15 ; amp = 0.028

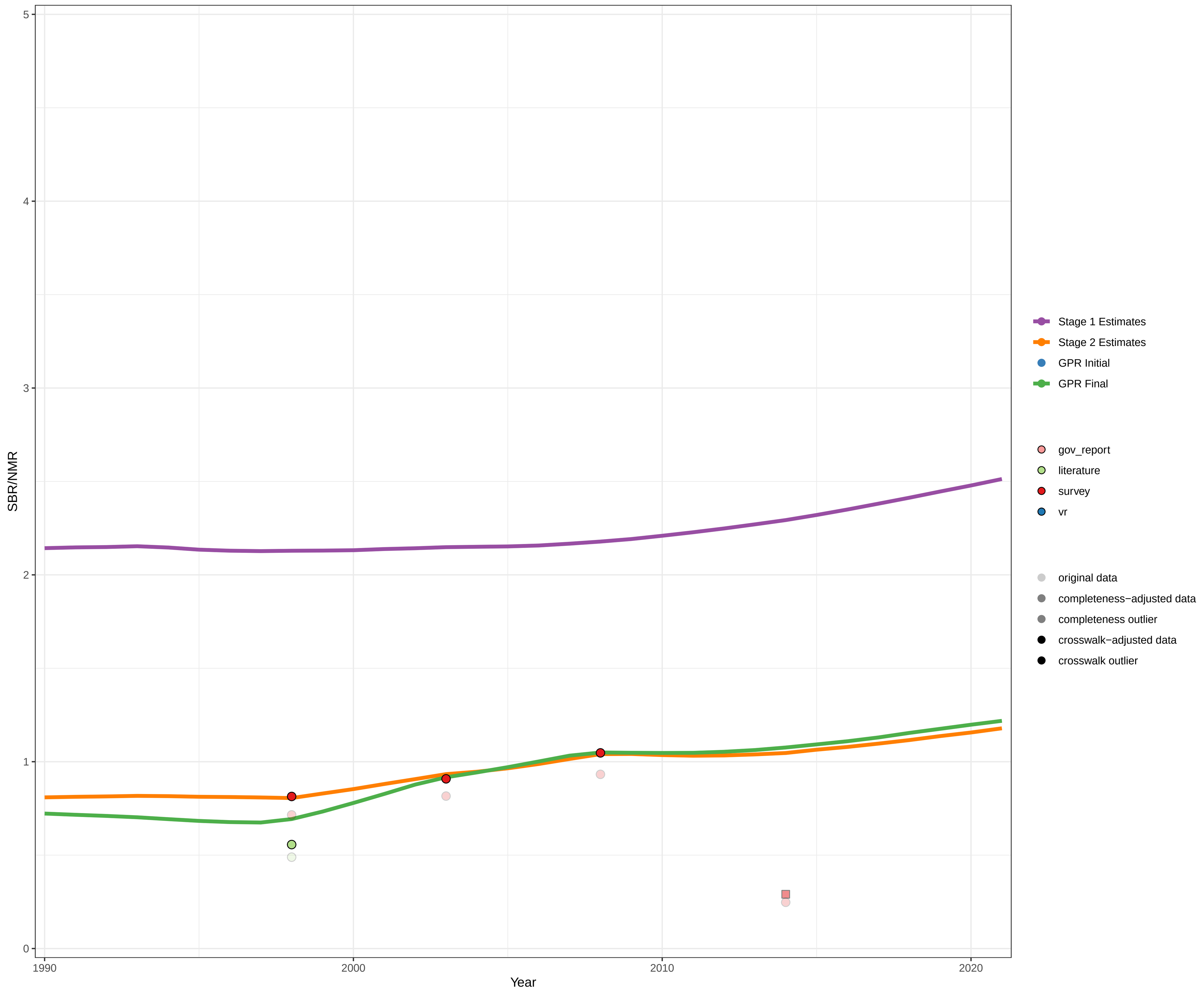

Definition	Years	Outlier	Source.Type	Title
28_weeks	1998	0	Sci Lit	Congenital syphilis in a Nairobi maternity hospital
28_weeks	1998	0	Survey	Kenya Demographic and Health Survey 1998
28_weeks	2003	0	Survey	Kenya Demographic and Health Survey 2003
28_weeks	2008	0	Survey	Kenya Demographic and Health Survey 2008−2009
28_weeks	2014	1	Survey	Kenya Demographic and Health Survey 2014

Nairobi KEN_35646 Eastern Sub−Saharan Africa Stillbirth Rate (SBR) − 20 weeks
lambda = 0.8 ; zeta = 0.7 ; scale = 15 ; amp = 0.028

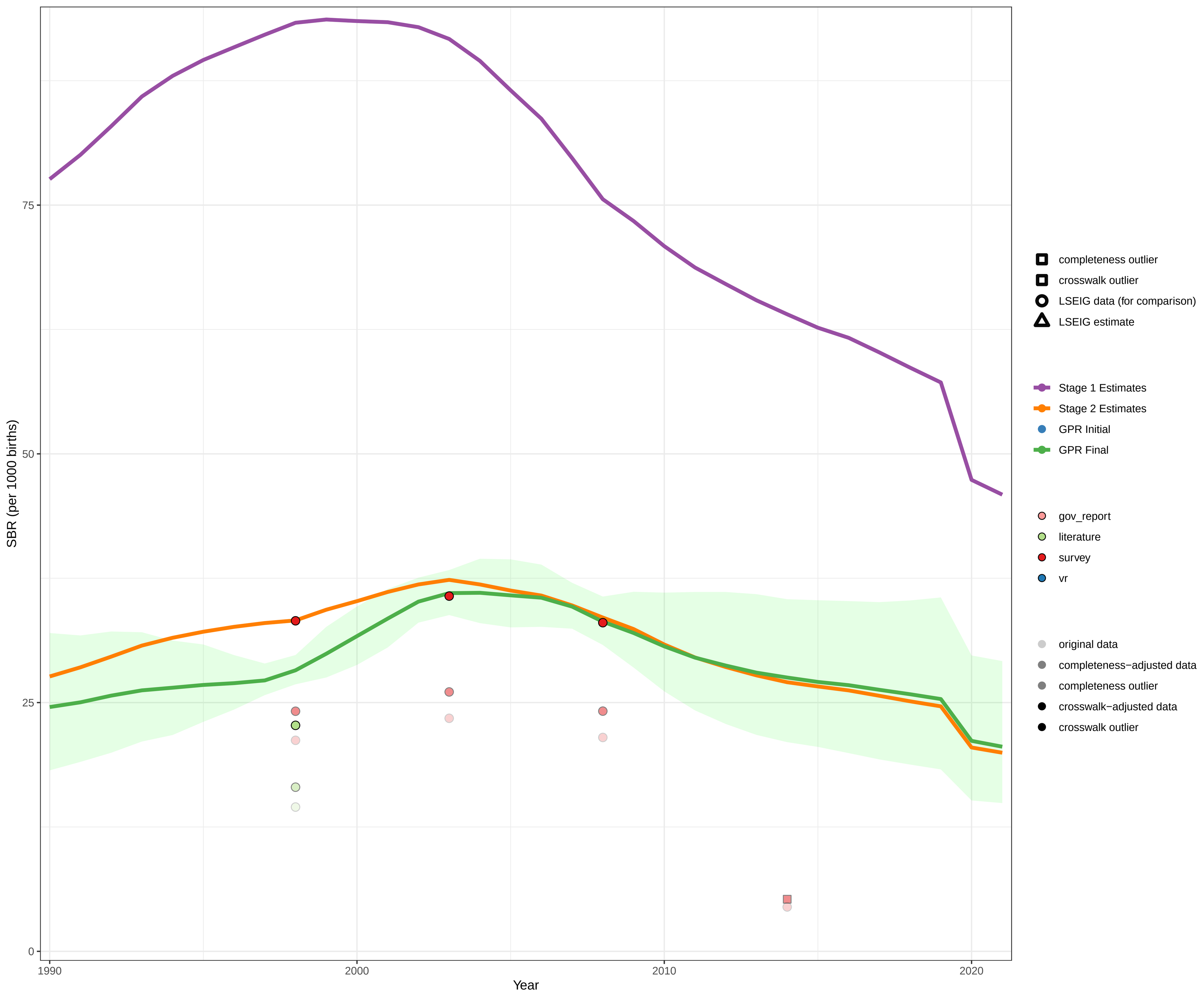

Nairobi KEN_35646 Eastern Sub−Saharan Africa Stillbirth Rate (SBR) − 28 weeks
lambda = 0.8 ; zeta = 0.7 ; scale = 15 ; amp = 0.028

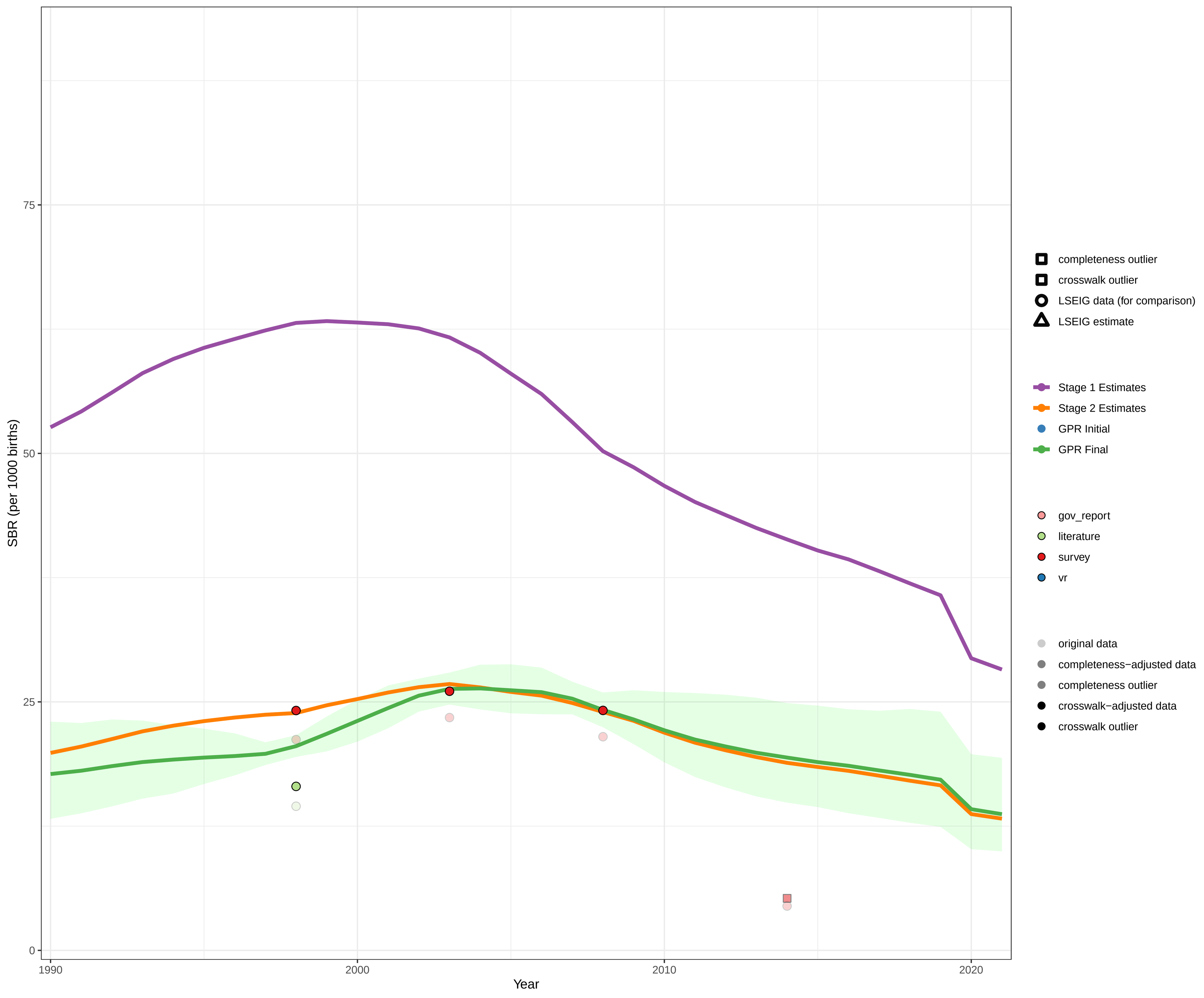

Year	Total Stillbirths (in thousands)	Total Stillbirths (in thousands)	SBR (per 1000 births)	SBR (per 1000 births)	SBR/NMR	SBR/NMR
	20 weeks	28 weeks	20 weeks	28 weeks	20 weeks	28 weeks
1990	1.1 (0.8 to 1.5)	0.8 (0.6 to 1.1)	24.6 (18.2 to 32.0)	17.7 (13.2 to 23.0)	1.00 (0.74 to 1.30)	0.72 (0.54 to 0.94)
1995	1.4 (1.2 to 1.7)	1.0 (0.9 to 1.2)	26.8 (23.1 to 30.8)	19.4 (16.7 to 22.3)	0.94 (0.81 to 1.09)	0.68 (0.59 to 0.79)
2000	2.1 (1.9 to 2.3)	1.5 (1.4 to 1.7)	31.7 (28.8 to 34.7)	23.1 (21.0 to 25.2)	1.07 (0.97 to 1.17)	0.78 (0.71 to 0.85)
2005	2.9 (2.6 to 3.2)	2.1 (1.9 to 2.3)	35.8 (32.6 to 39.4)	26.2 (23.9 to 28.8)	1.33 (1.21 to 1.46)	0.97 (0.88 to 1.07)
2010	2.6 (2.2 to 3.1)	1.9 (1.6 to 2.2)	30.7 (26.1 to 36.1)	22.1 (18.9 to 26.0)	1.45 (1.24 to 1.70)	1.05 (0.89 to 1.23)
2015	2.2 (1.6 to 2.8)	1.5 (1.1 to 2.0)	27.1 (20.5 to 35.3)	18.9 (14.4 to 24.6)	1.56 (1.19 to 2.04)	1.09 (0.83 to 1.42)
2020	1.4 (1.0 to 2.0)	0.9 (0.7 to 1.3)	21.2 (15.2 to 29.7)	14.2 (10.2 to 19.7)	1.78 (1.28 to 2.51)	1.20 (0.86 to 1.66)
2021	1.3 (1.0 to 1.9)	0.9 (0.6 to 1.2)	20.6 (14.9 to 29.2)	13.7 (10.0 to 19.4)	1.83 (1.33 to 2.59)	1.22 (0.89 to 1.72)

Nakuru KEN_35647 Eastern Sub−Saharan Africa Stillbirth Rate / Neonatal Mortality Rate (SBR/NMR) − 20 weeks
lambda = 0.8 ; zeta = 0.7 ; scale = 15 ; amp = 0.028

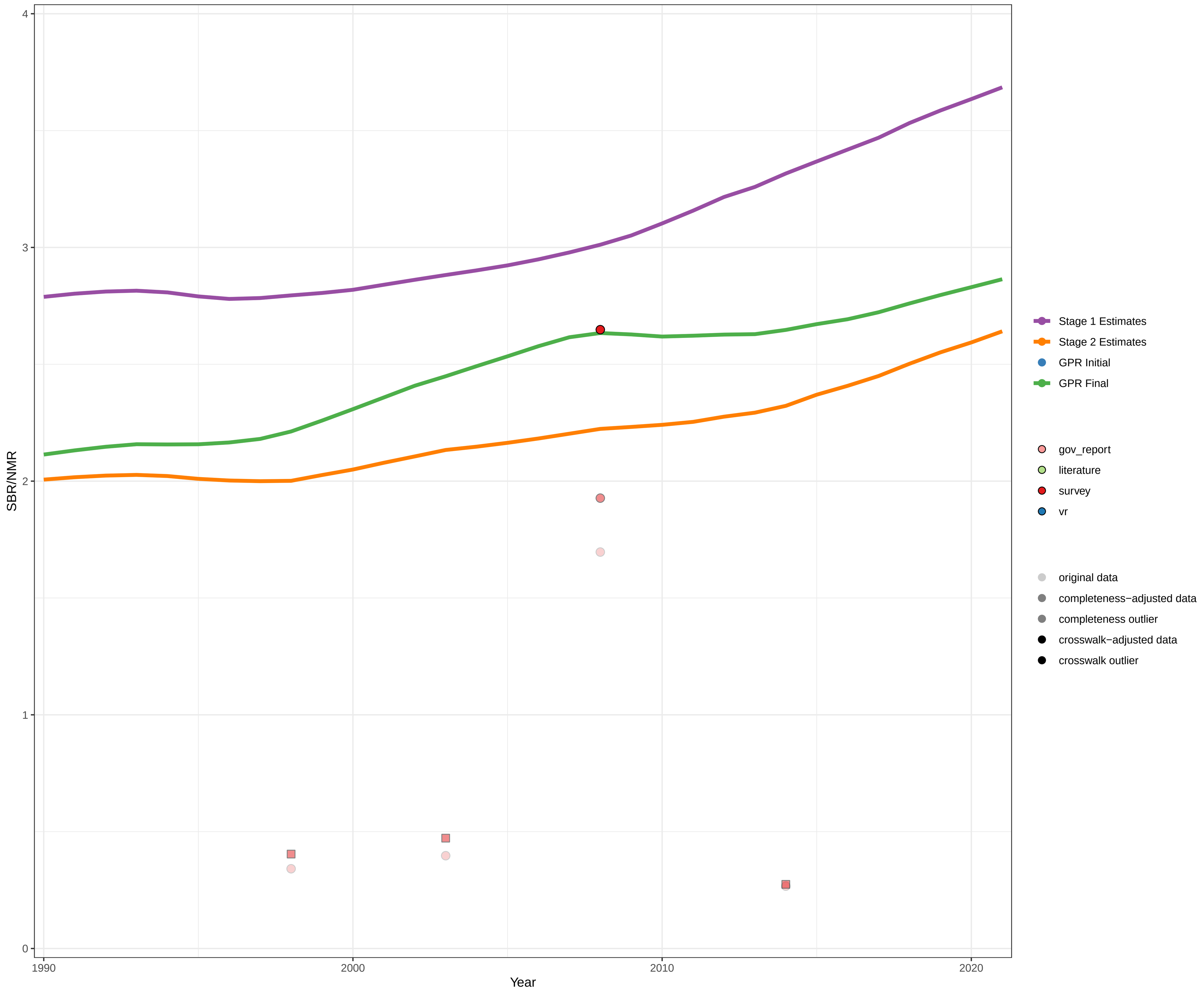

Nakuru KEN_35647 Eastern Sub−Saharan Africa Stillbirth Rate / Neonatal Mortality Rate (SBR/NMR) − 28 weeks
lambda = 0.8 ; zeta = 0.7 ; scale = 15 ; amp = 0.028

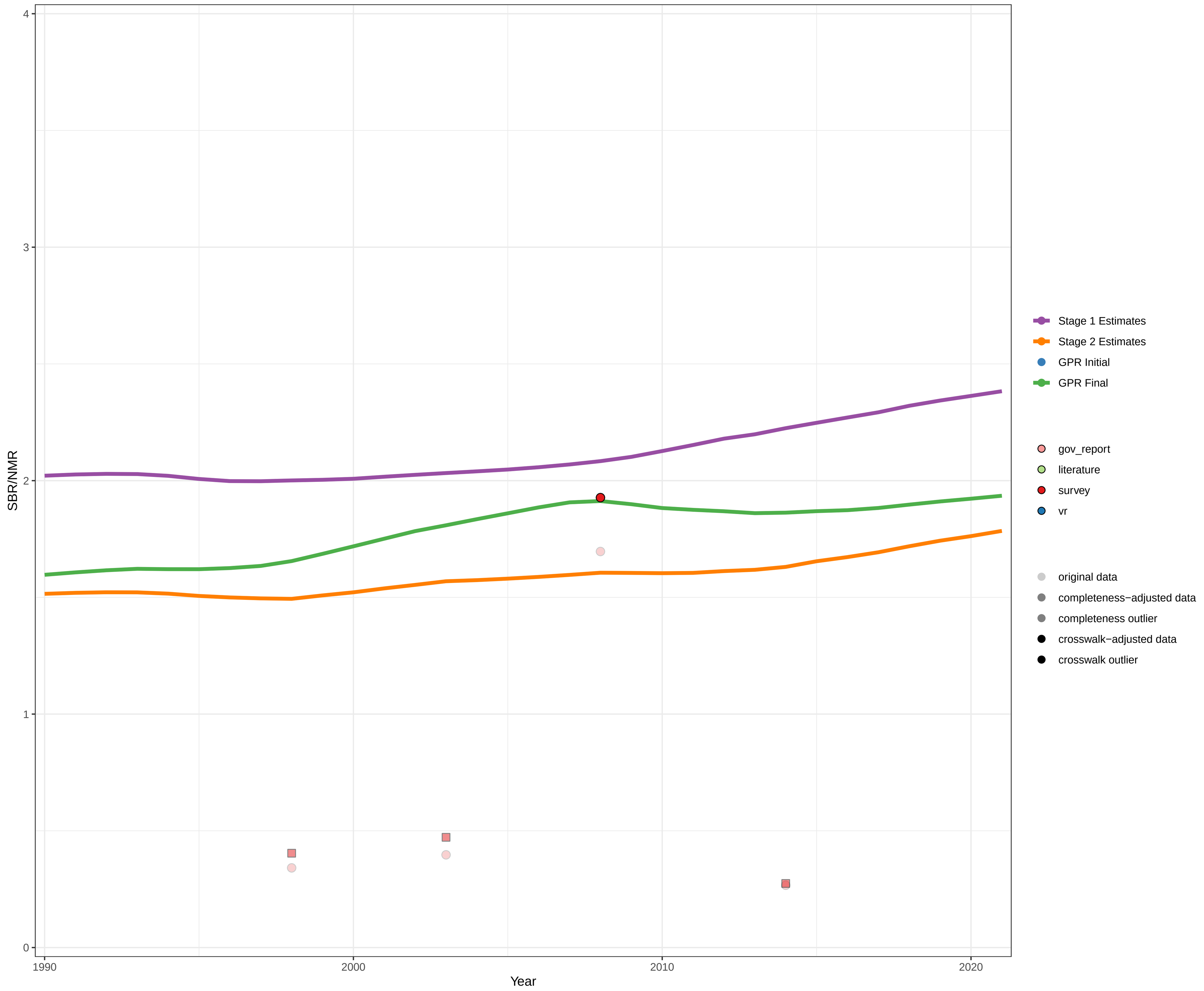

Definition	Years	Outlier	Source.Type	Title
28_weeks	2008	0	Survey	Kenya Demographic and Health Survey 2008−2009
28_weeks	1998	1	Survey	Kenya Demographic and Health Survey 1998
28_weeks	2003	1	Survey	Kenya Demographic and Health Survey 2003
28_weeks	2014	1	Survey	Kenya Demographic and Health Survey 2014

Nakuru KEN_35647 Eastern Sub−Saharan Africa Stillbirth Rate (SBR) − 20 weeks
lambda = 0.8 ; zeta = 0.7 ; scale = 15 ; amp = 0.028

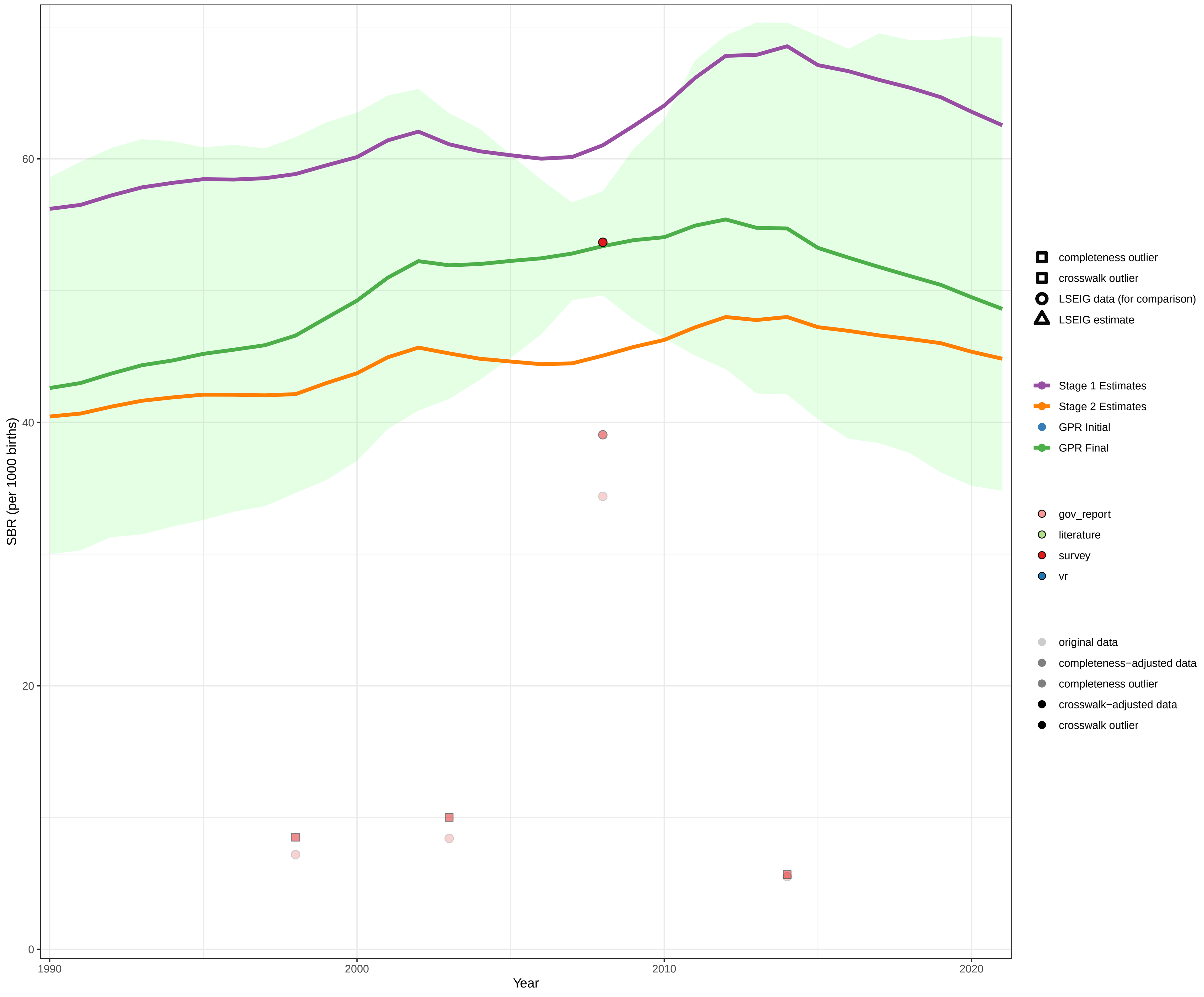

Nakuru KEN_35647 Eastern Sub−Saharan Africa Stillbirth Rate (SBR) − 28 weeks
lambda = 0.8 ; zeta = 0.7 ; scale = 15 ; amp = 0.028

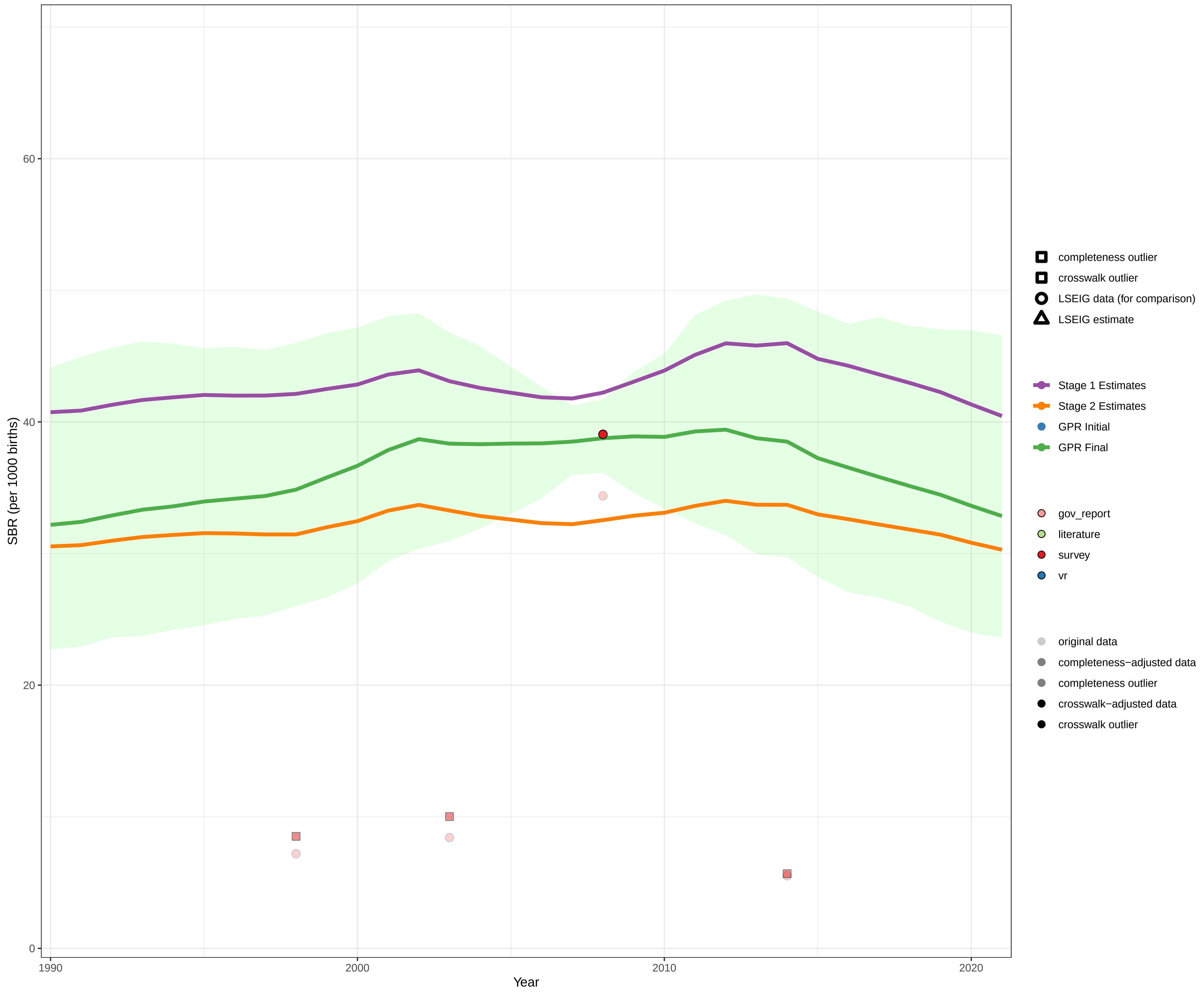

Year	Total Stillbirths (in thousands)	Total Stillbirths (in thousands)	SBR (per 1000 births)	SBR (per 1000 births)	SBR/NMR	SBR/NMR
	20 weeks	28 weeks	20 weeks	28 weeks	20 weeks	28 weeks
1990	1.6 (1.1 to 2.2)	1.2 (0.8 to 1.7)	42.6 (30.0 to 58.6)	32.2 (22.7 to 44.1)	2.11 (1.49 to 2.91)	1.60 (1.13 to 2.19)
1995	1.9 (1.3 to 2.6)	1.4 (1.0 to 1.9)	45.2 (32.6 to 60.9)	34.0 (24.6 to 45.6)	2.16 (1.56 to 2.91)	1.62 (1.17 to 2.18)
2000	2.3 (1.7 to 3.0)	1.7 (1.2 to 2.2)	49.2 (37.1 to 63.5)	36.7 (27.7 to 47.2)	2.31 (1.74 to 2.98)	1.72 (1.30 to 2.21)
2005	2.4 (2.1 to 2.8)	1.8 (1.5 to 2.0)	52.3 (44.9 to 60.3)	38.4 (33.0 to 44.2)	2.53 (2.18 to 2.93)	1.86 (1.60 to 2.14)
2010	2.4 (2.0 to 2.8)	1.7 (1.4 to 2.0)	54.1 (46.4 to 63.0)	38.9 (33.4 to 45.2)	2.62 (2.25 to 3.05)	1.88 (1.62 to 2.19)
2015	2.1 (1.6 to 2.8)	1.5 (1.1 to 1.9)	53.2 (40.2 to 69.3)	37.2 (28.2 to 48.4)	2.67 (2.02 to 3.48)	1.87 (1.42 to 2.43)
2020	1.8 (1.3 to 2.6)	1.2 (0.9 to 1.7)	49.5 (35.2 to 69.3)	33.6 (24.0 to 46.9)	2.83 (2.01 to 3.96)	1.92 (1.37 to 2.68)
2021	1.8 (1.3 to 2.6)	1.2 (0.8 to 1.7)	48.6 (34.8 to 69.2)	32.8 (23.6 to 46.6)	2.86 (2.05 to 4.08)	1.94 (1.39 to 2.75)

Nandi KEN_35648 Eastern Sub−Saharan Africa Stillbirth Rate / Neonatal Mortality Rate (SBR/NMR) − 20 weeks
lambda = 0.8 ; zeta = 0.7 ; scale = 15 ; amp = 0.028

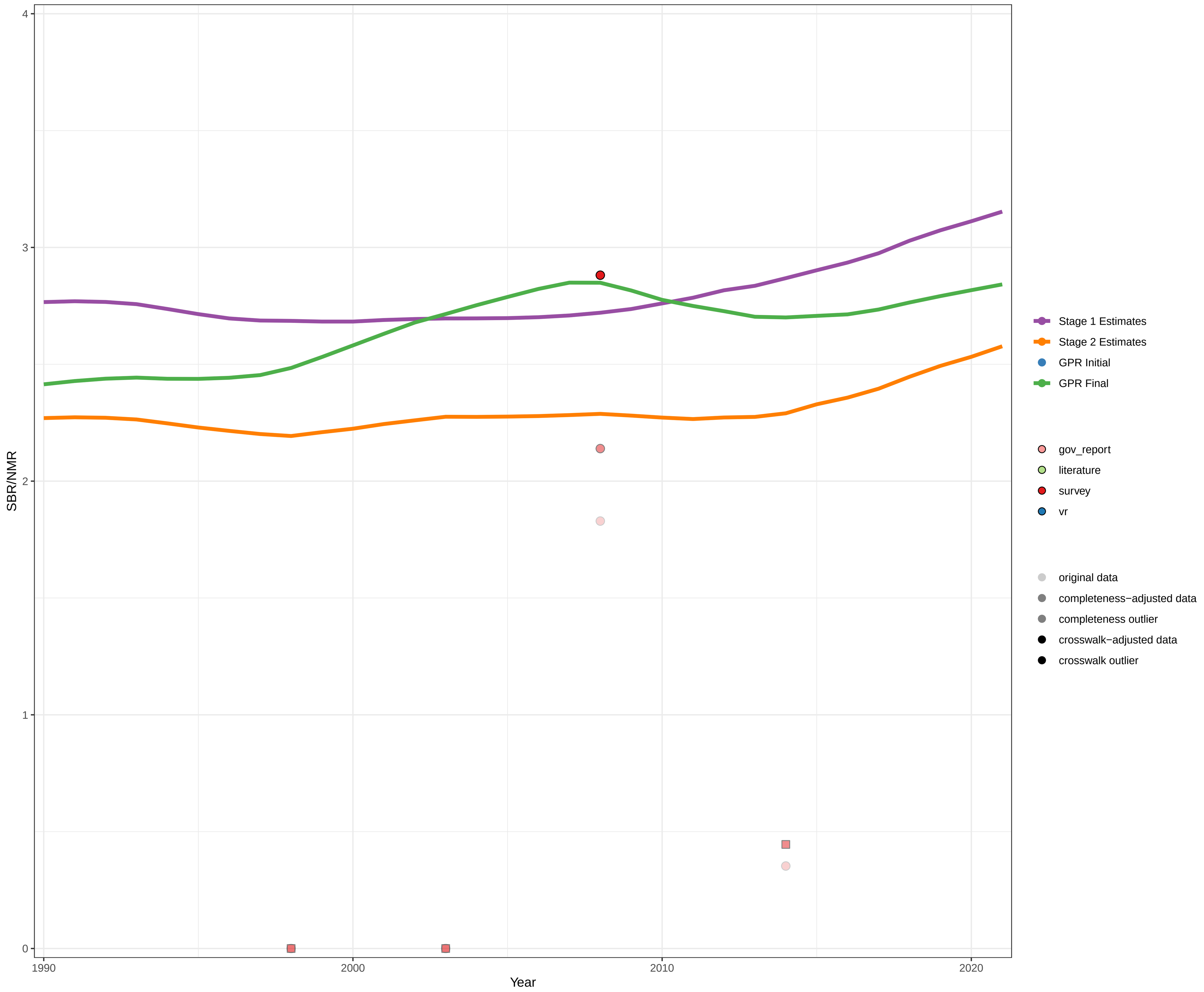

Nandi KEN_35648 Eastern Sub−Saharan Africa Stillbirth Rate / Neonatal Mortality Rate (SBR/NMR) − 28 weeks
lambda = 0.8 ; zeta = 0.7 ; scale = 15 ; amp = 0.028

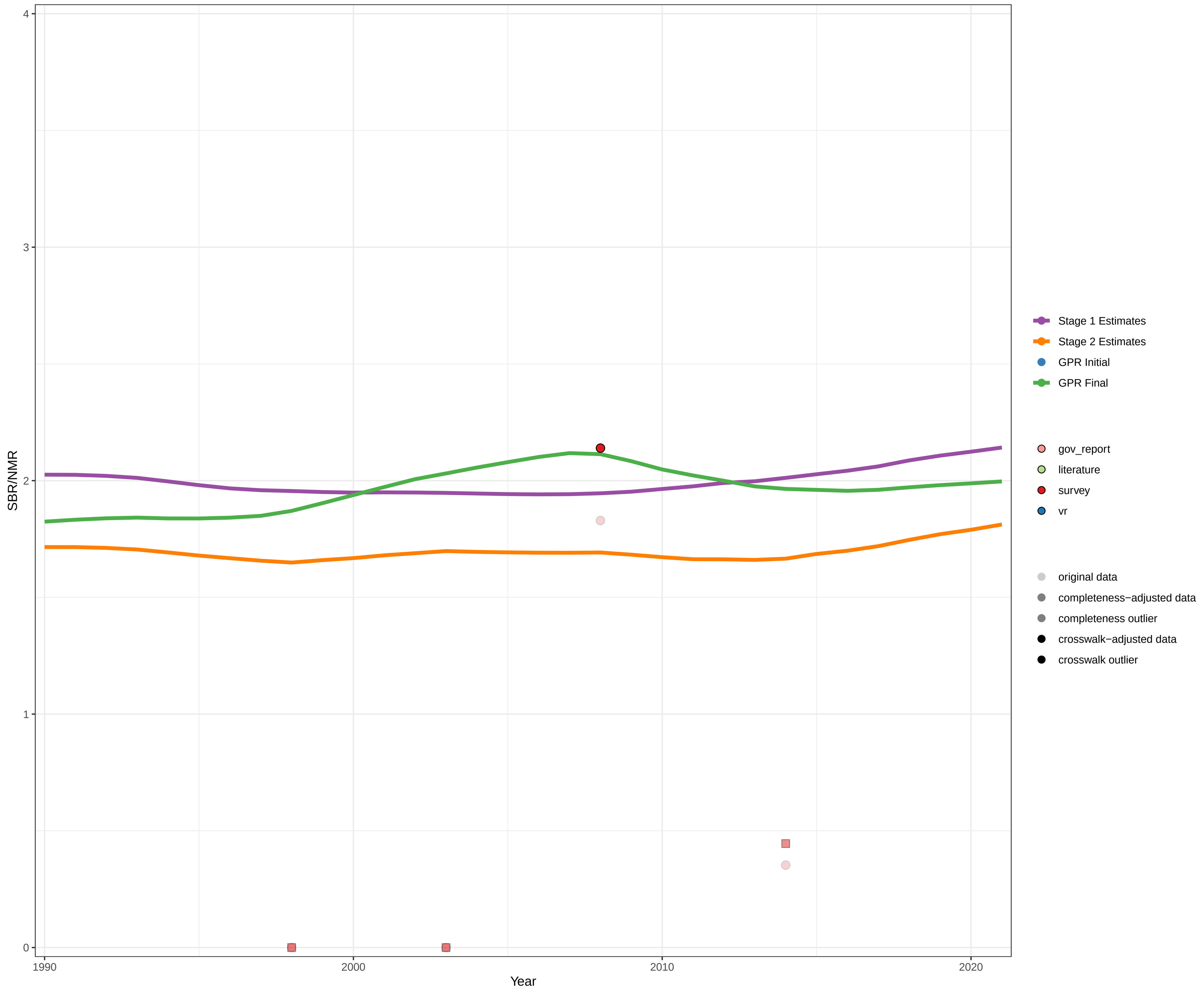

Definition	Years	Outlier	Source.Type	Title
28_weeks	2008	0	Survey	Kenya Demographic and Health Survey 2008−2009
28_weeks	1998	1	Survey	Kenya Demographic and Health Survey 1998
28_weeks	2003	1	Survey	Kenya Demographic and Health Survey 2003
28_weeks	2014	1	Survey	Kenya Demographic and Health Survey 2014

Nandi KEN_35648 Eastern Sub−Saharan Africa Stillbirth Rate (SBR) − 20 weeks
lambda = 0.8 ; zeta = 0.7 ; scale = 15 ; amp = 0.028

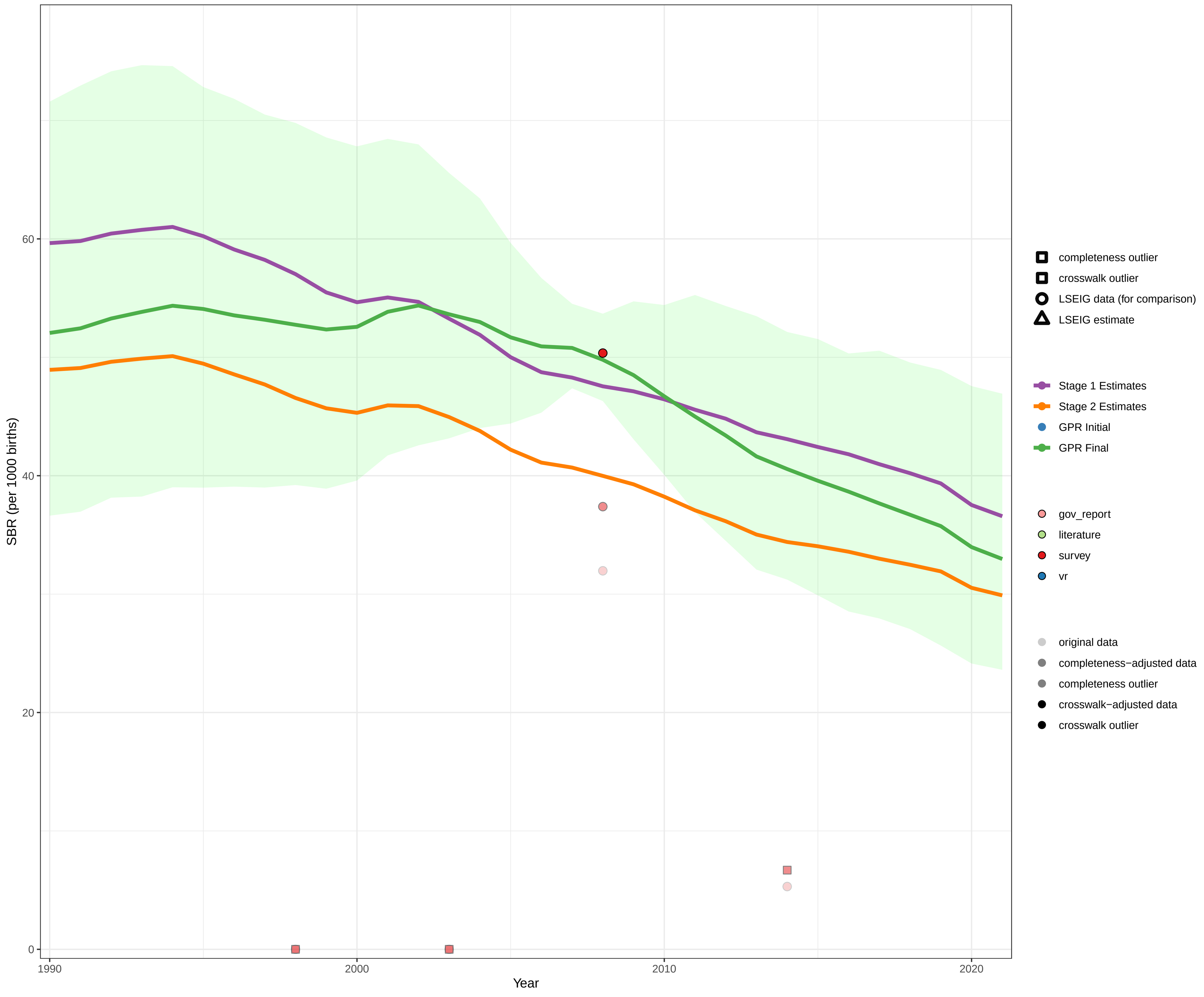

Nandi KEN_35648 Eastern Sub−Saharan Africa Stillbirth Rate (SBR) − 28 weeks
lambda = 0.8 ; zeta = 0.7 ; scale = 15 ; amp = 0.028

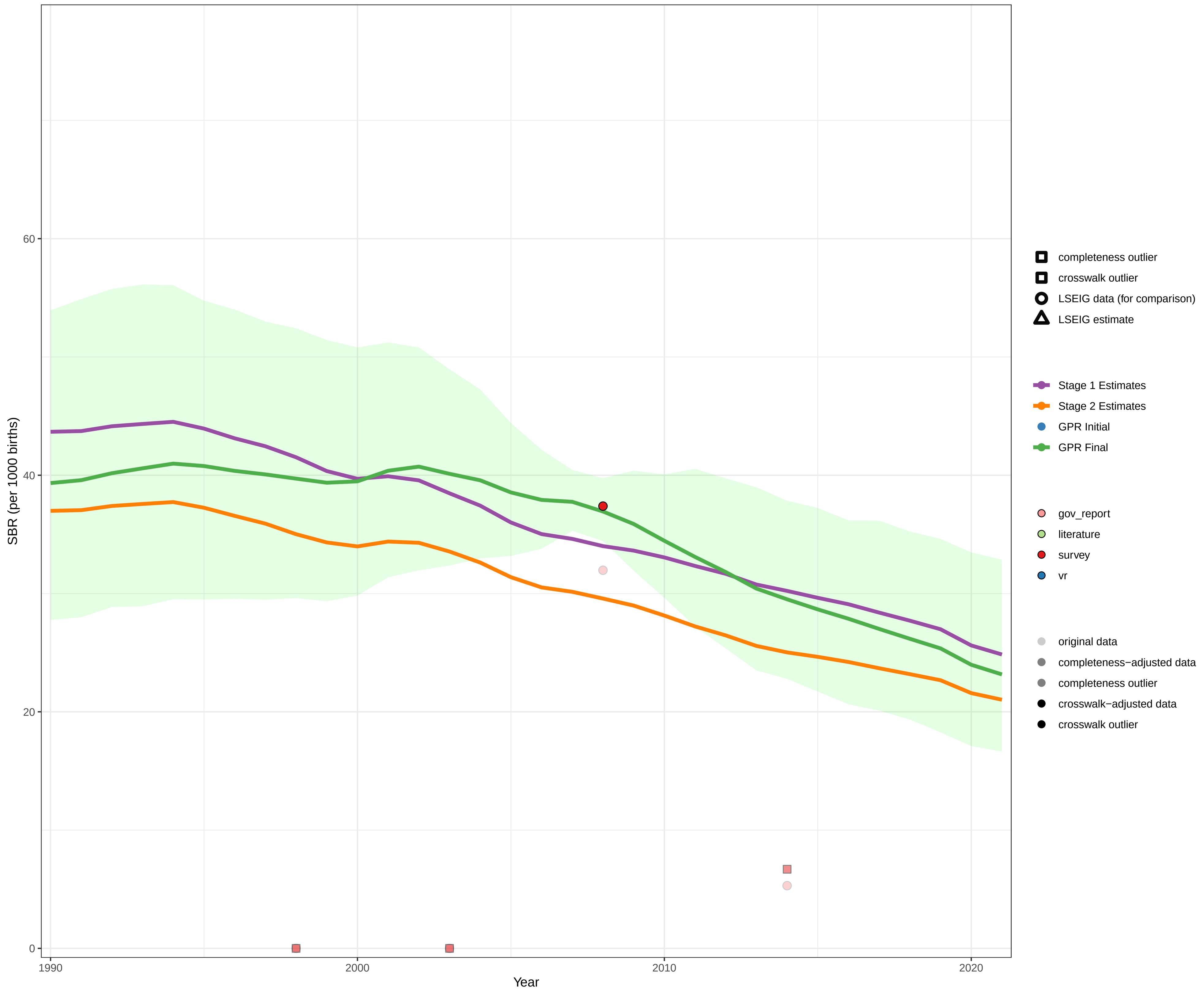

Year	Total Stillbirths (in thousands)	Total Stillbirths (in thousands)	SBR (per 1000 births)	SBR (per 1000 births)	SBR/NMR	SBR/NMR
	20 weeks	28 weeks	20 weeks	28 weeks	20 weeks	28 weeks
1990	1.1 (0.8 to 1.6)	0.8 (0.6 to 1.2)	52.1 (36.6 to 71.6)	39.3 (27.8 to 53.9)	2.41 (1.70 to 3.32)	1.82 (1.29 to 2.50)
1995	1.3 (0.9 to 1.8)	1.0 (0.7 to 1.3)	54.1 (39.0 to 72.8)	40.8 (29.5 to 54.8)	2.44 (1.76 to 3.28)	1.84 (1.33 to 2.47)
2000	1.4 (1.0 to 1.8)	1.0 (0.8 to 1.4)	52.6 (39.6 to 67.8)	39.5 (29.8 to 50.8)	2.58 (1.94 to 3.33)	1.94 (1.46 to 2.49)
2005	1.5 (1.3 to 1.7)	1.1 (0.9 to 1.3)	51.7 (44.4 to 59.7)	38.5 (33.2 to 44.4)	2.79 (2.40 to 3.22)	2.08 (1.79 to 2.40)
2010	1.3 (1.1 to 1.5)	0.9 (0.8 to 1.1)	46.7 (40.1 to 54.4)	34.5 (29.6 to 40.1)	2.78 (2.38 to 3.23)	2.05 (1.76 to 2.38)
2015	1.0 (0.8 to 1.4)	0.7 (0.6 to 1.0)	39.6 (29.9 to 51.5)	28.7 (21.7 to 37.2)	2.71 (2.05 to 3.53)	1.96 (1.49 to 2.55)
2020	0.8 (0.6 to 1.2)	0.6 (0.4 to 0.8)	34.0 (24.1 to 47.6)	24.0 (17.1 to 33.5)	2.82 (2.00 to 3.95)	1.99 (1.42 to 2.78)
2021	0.8 (0.6 to 1.1)	0.5 (0.4 to 0.8)	33.0 (23.6 to 46.9)	23.2 (16.6 to 32.9)	2.84 (2.04 to 4.05)	2.00 (1.43 to 2.83)

Narok KEN_35649 Eastern Sub−Saharan Africa Stillbirth Rate / Neonatal Mortality Rate (SBR/NMR) − 20 weeks
lambda = 0.8 ; zeta = 0.7 ; scale = 15 ; amp = 0.028

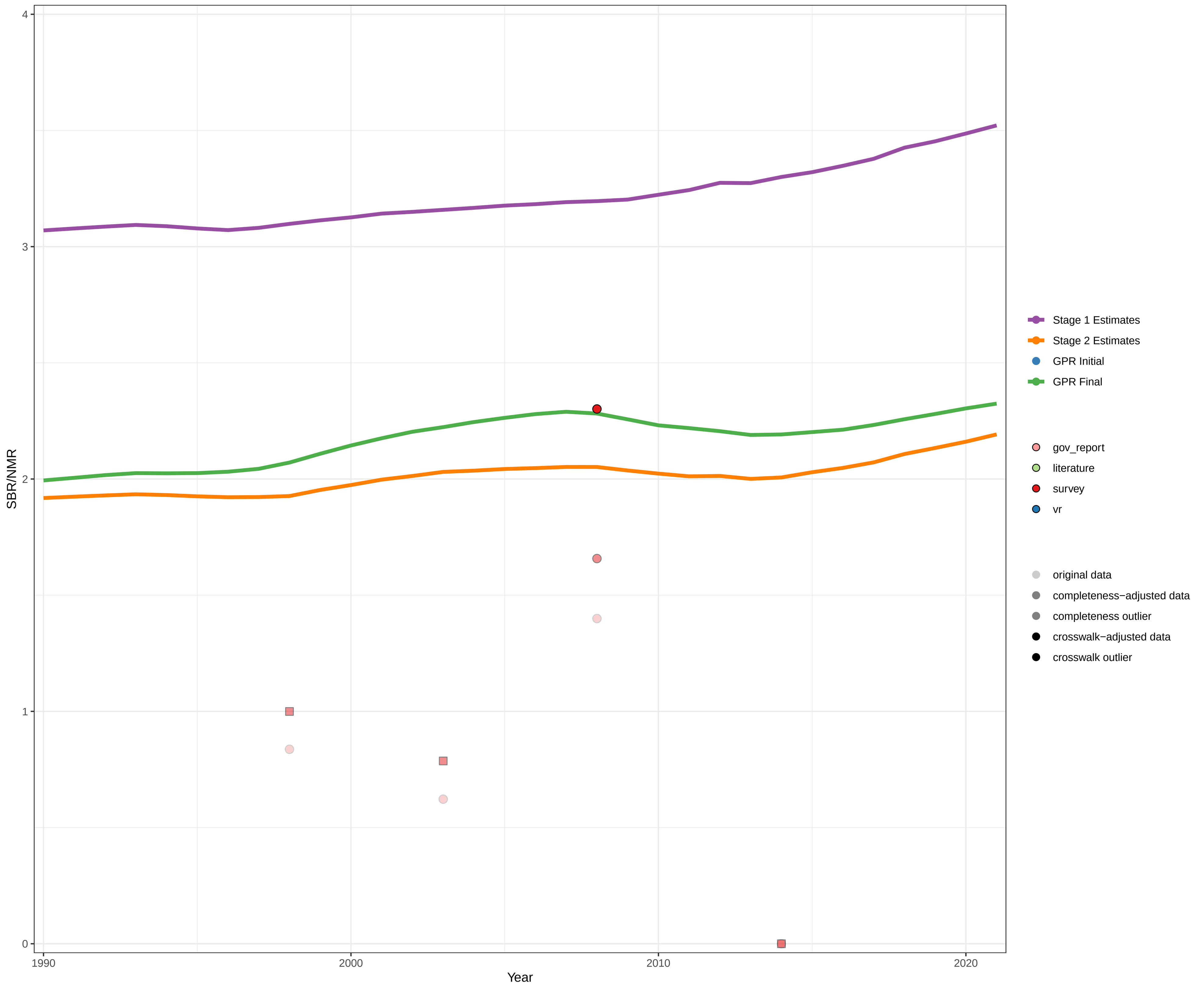

Narok KEN_35649 Eastern Sub−Saharan Africa Stillbirth Rate / Neonatal Mortality Rate (SBR/NMR) − 28 weeks
lambda = 0.8 ; zeta = 0.7 ; scale = 15 ; amp = 0.028

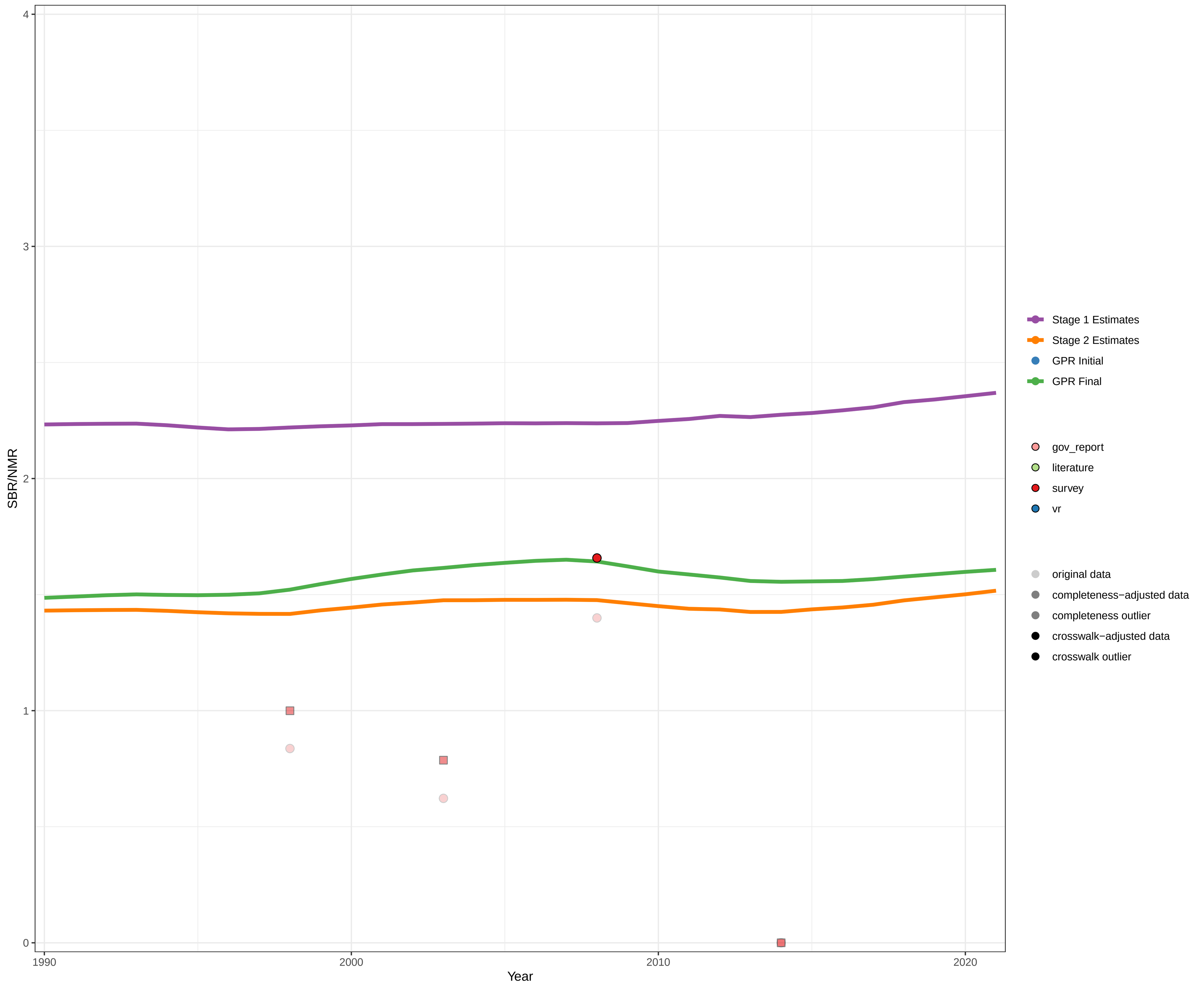

Definition	Years	Outlier	Source.Type	Title
28_weeks	2008	0	Survey	Kenya Demographic and Health Survey 2008−2009
28_weeks	1998	1	Survey	Kenya Demographic and Health Survey 1998
28_weeks	2003	1	Survey	Kenya Demographic and Health Survey 2003
28_weeks	2014	1	Survey	Kenya Demographic and Health Survey 2014

Narok KEN_35649 Eastern Sub−Saharan Africa Stillbirth Rate (SBR) − 20 weeks
lambda = 0.8 ; zeta = 0.7 ; scale = 15 ; amp = 0.028

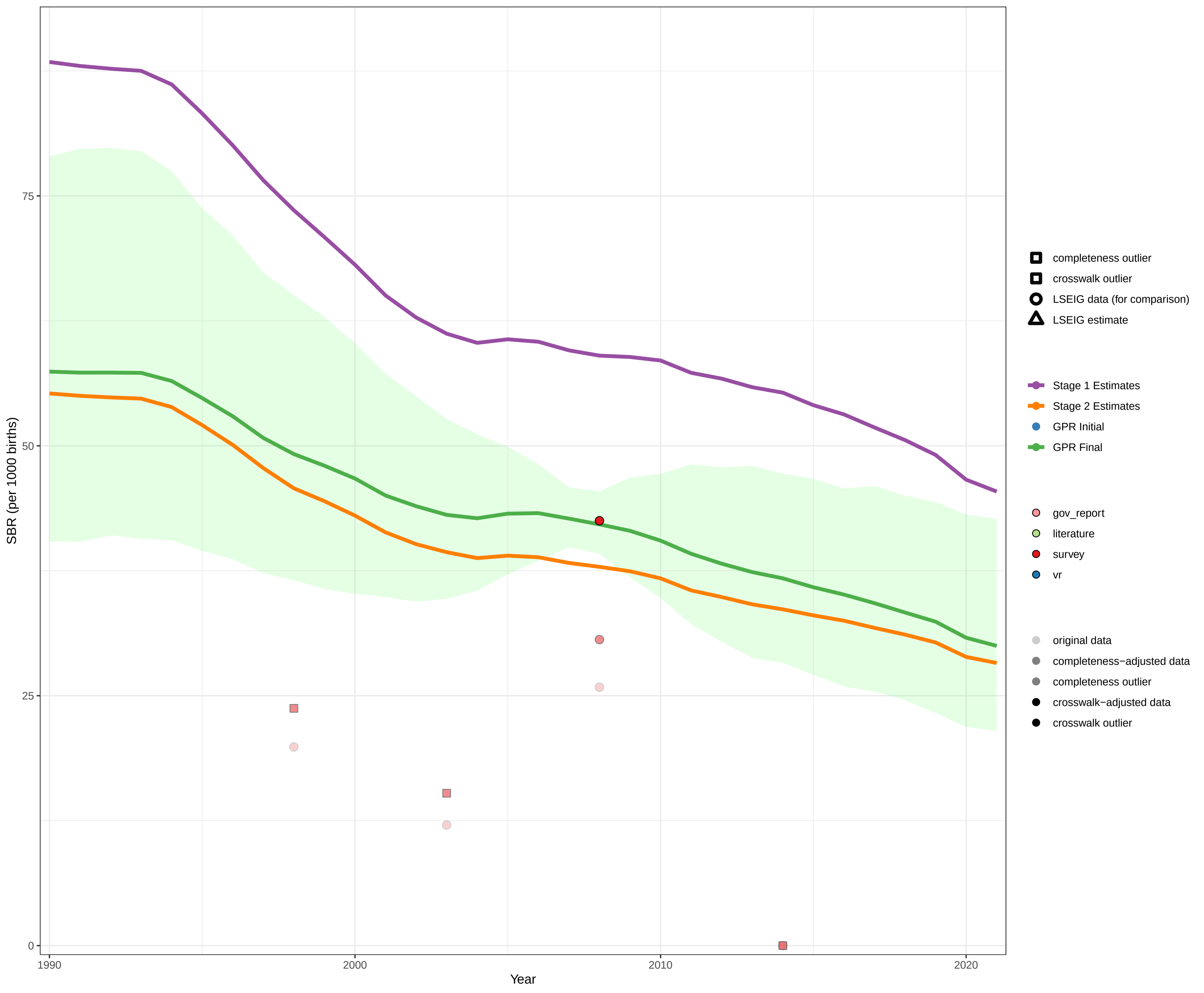

Narok KEN_35649 Eastern Sub−Saharan Africa Stillbirth Rate (SBR) − 28 weeks
lambda = 0.8 ; zeta = 0.7 ; scale = 15 ; amp = 0.028

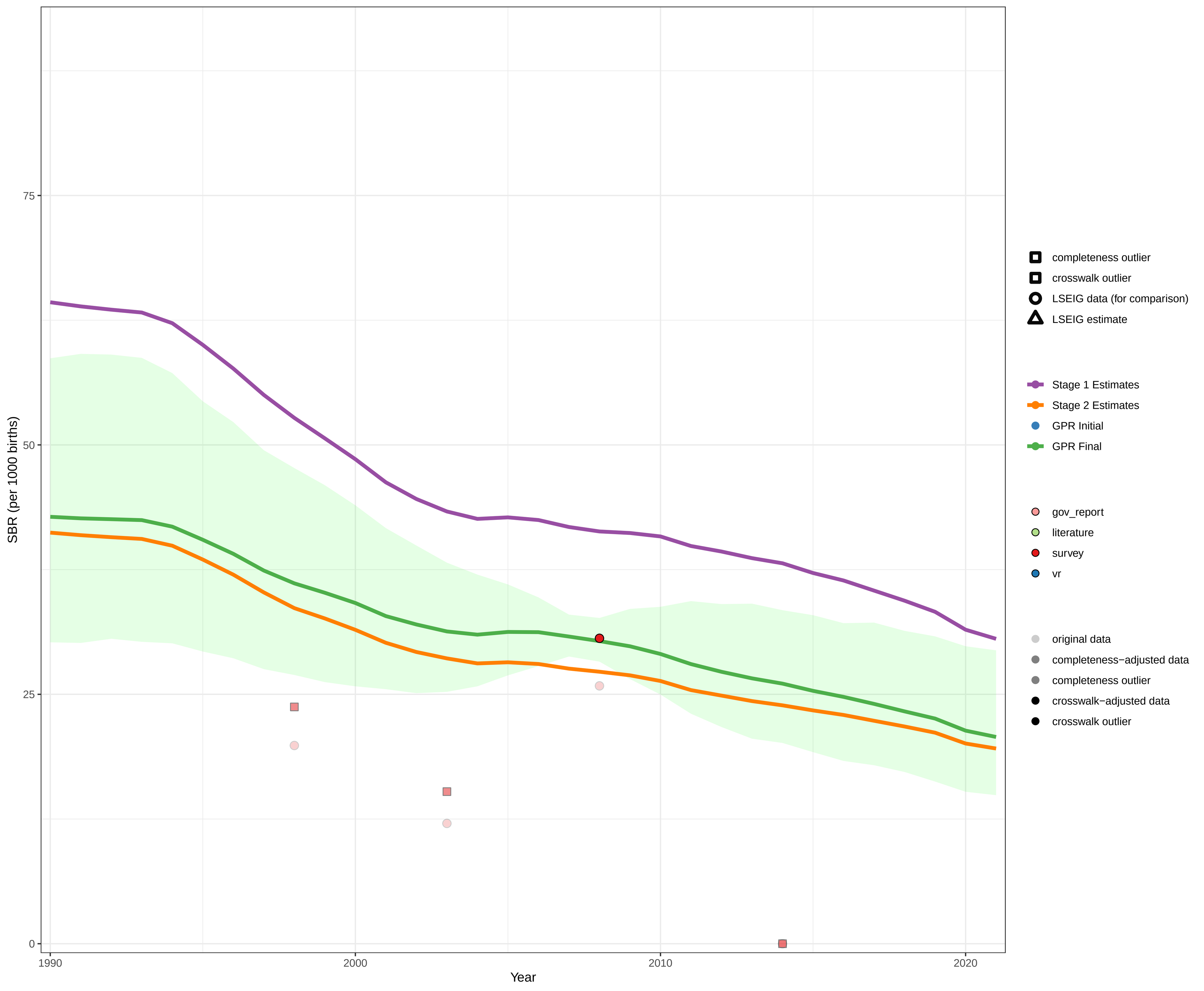

Year	Total Stillbirths (in thousands)	Total Stillbirths (in thousands)	SBR (per 1000 births)	SBR (per 1000 births)	SBR/NMR	SBR/NMR
	20 weeks	28 weeks	20 weeks	28 weeks	20 weeks	28 weeks
1990	1.3 (0.9 to 1.8)	1.0 (0.7 to 1.3)	57.4 (40.4 to 79.0)	42.8 (30.2 to 58.7)	1.99 (1.40 to 2.74)	1.49 (1.05 to 2.04)
1995	1.1 (0.8 to 1.5)	0.8 (0.6 to 1.1)	54.8 (39.5 to 73.8)	40.5 (29.3 to 54.4)	2.03 (1.46 to 2.73)	1.50 (1.08 to 2.01)
2000	1.0 (0.7 to 1.2)	0.7 (0.5 to 0.9)	46.7 (35.2 to 60.3)	34.2 (25.8 to 43.9)	2.14 (1.61 to 2.77)	1.57 (1.18 to 2.02)
2005	1.3 (1.1 to 1.5)	0.9 (0.8 to 1.1)	43.2 (37.1 to 49.9)	31.3 (26.9 to 36.0)	2.26 (1.94 to 2.61)	1.64 (1.41 to 1.89)
2010	1.7 (1.4 to 2.0)	1.2 (1.0 to 1.4)	40.5 (34.8 to 47.2)	29.0 (25.0 to 33.8)	2.23 (1.91 to 2.60)	1.60 (1.38 to 1.86)
2015	1.6 (1.2 to 2.1)	1.1 (0.9 to 1.5)	35.9 (27.1 to 46.7)	25.3 (19.2 to 32.9)	2.20 (1.66 to 2.87)	1.56 (1.18 to 2.02)
2020	1.3 (0.9 to 1.9)	0.9 (0.6 to 1.3)	30.8 (21.9 to 43.1)	21.4 (15.2 to 29.8)	2.30 (1.64 to 3.23)	1.60 (1.14 to 2.23)
2021	1.3 (0.9 to 1.8)	0.9 (0.6 to 1.2)	30.0 (21.5 to 42.7)	20.7 (14.9 to 29.4)	2.32 (1.66 to 3.31)	1.61 (1.15 to 2.28)

Nyamira KEN_35650 Eastern Sub−Saharan Africa Stillbirth Rate / Neonatal Mortality Rate (SBR/NMR) − 20 weeks
lambda = 1 ; zeta = 0.6 ; scale = 15 ; amp = 0.028

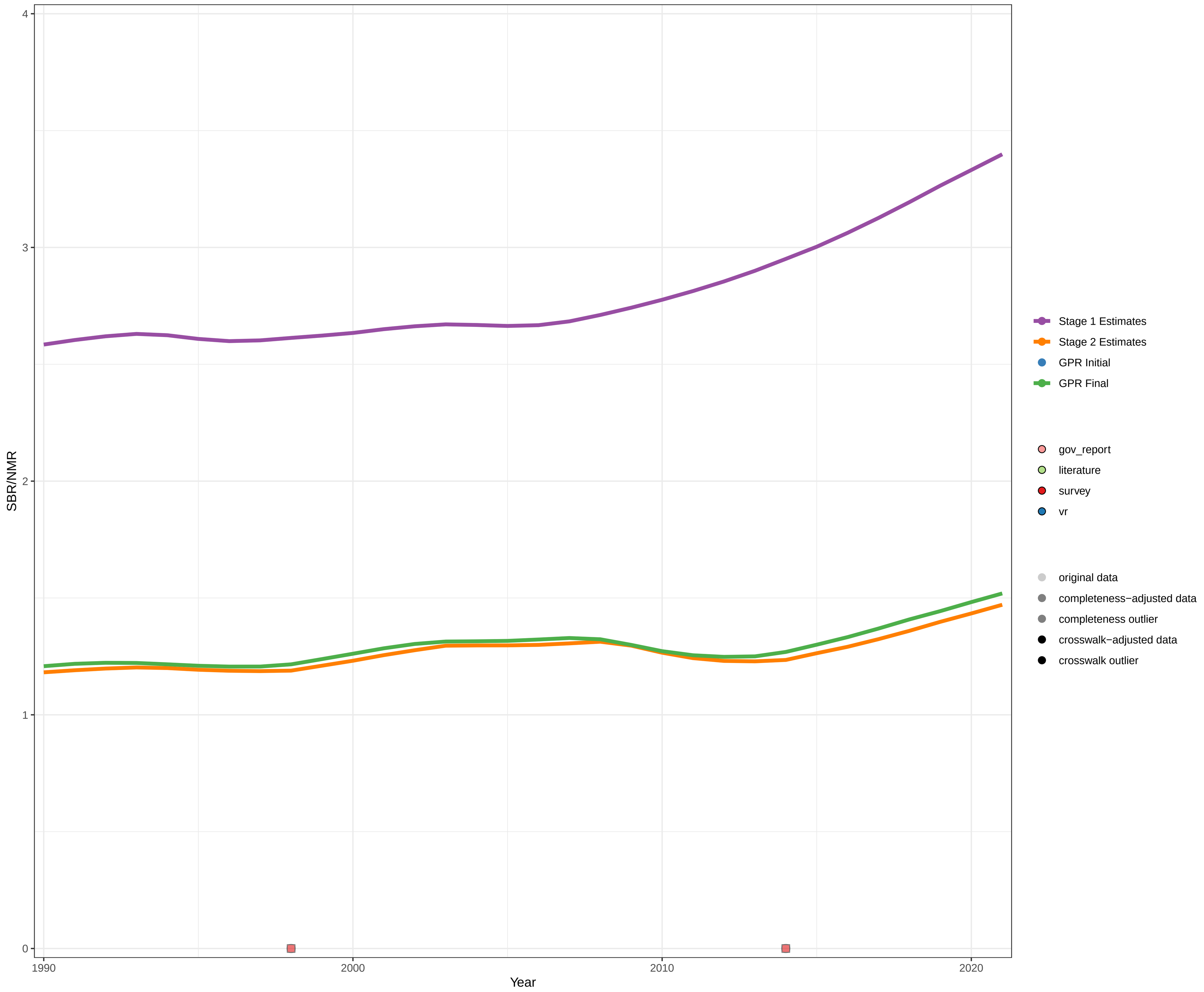

Nyamira KEN_35650 Eastern Sub−Saharan Africa Stillbirth Rate / Neonatal Mortality Rate (SBR/NMR) − 28 weeks
lambda = 1 ; zeta = 0.6 ; scale = 15 ; amp = 0.028

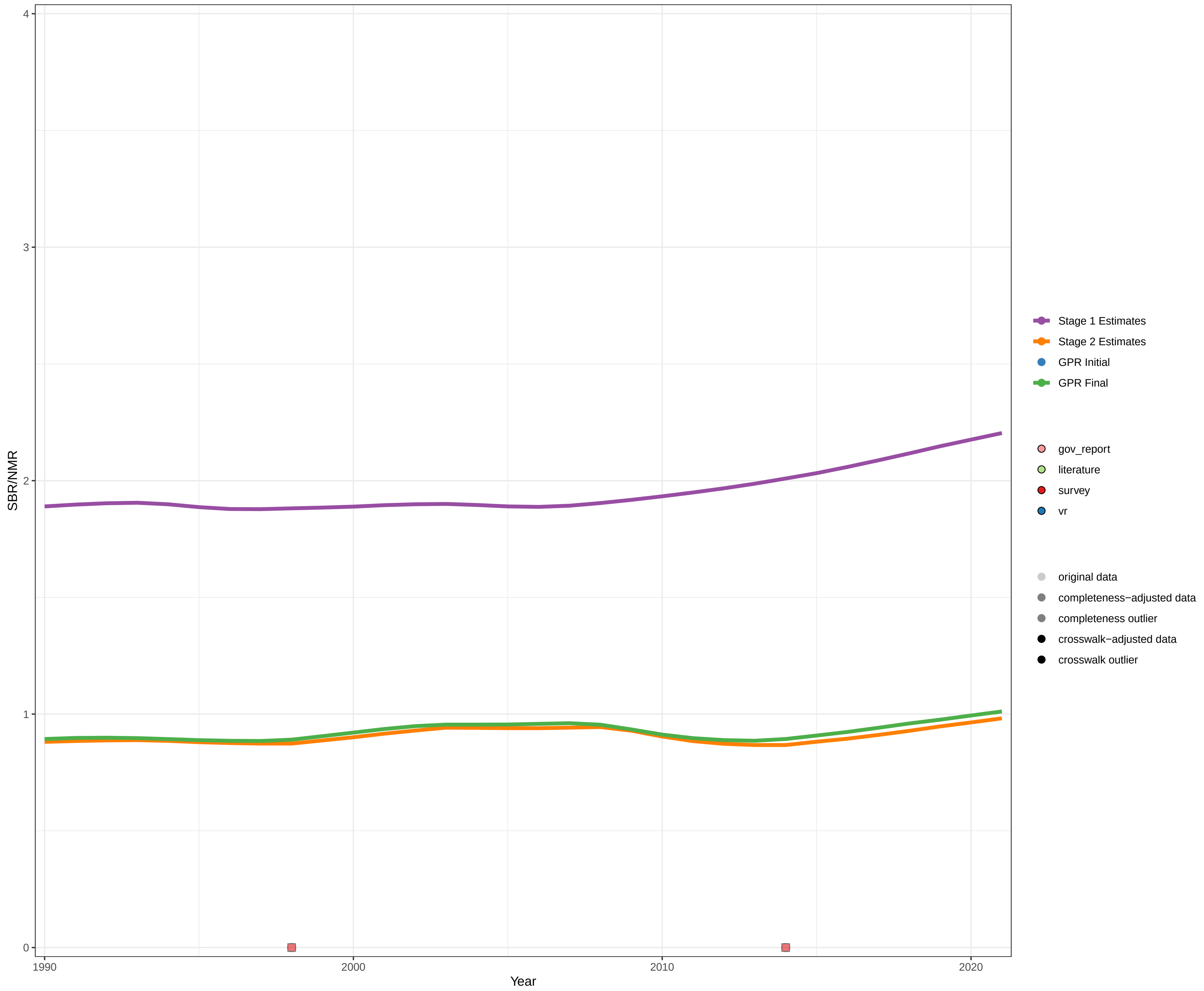

Definition	Years	Outlier	Source.Type	Title
28_weeks	1998	1	Survey	Kenya Demographic and Health Survey 1998
28_weeks	2014	1	Survey	Kenya Demographic and Health Survey 2014

Nyamira KEN_35650 Eastern Sub−Saharan Africa Stillbirth Rate (SBR) − 20 weeks
lambda = 1 ; zeta = 0.6 ; scale = 15 ; amp = 0.028

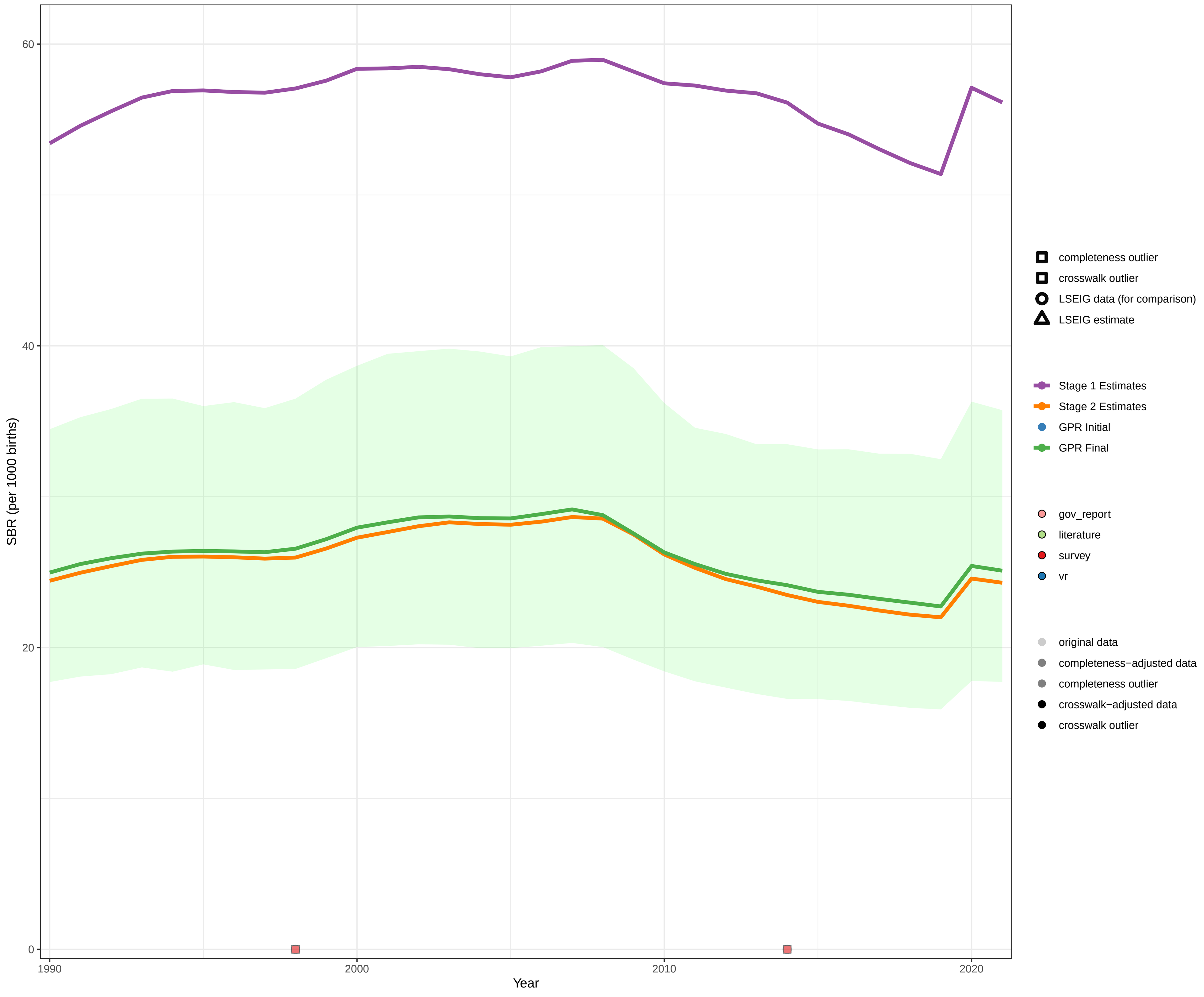

Nyamira KEN_35650 Eastern Sub−Saharan Africa Stillbirth Rate (SBR) − 28 weeks
lambda = 1 ; zeta = 0.6 ; scale = 15 ; amp = 0.028

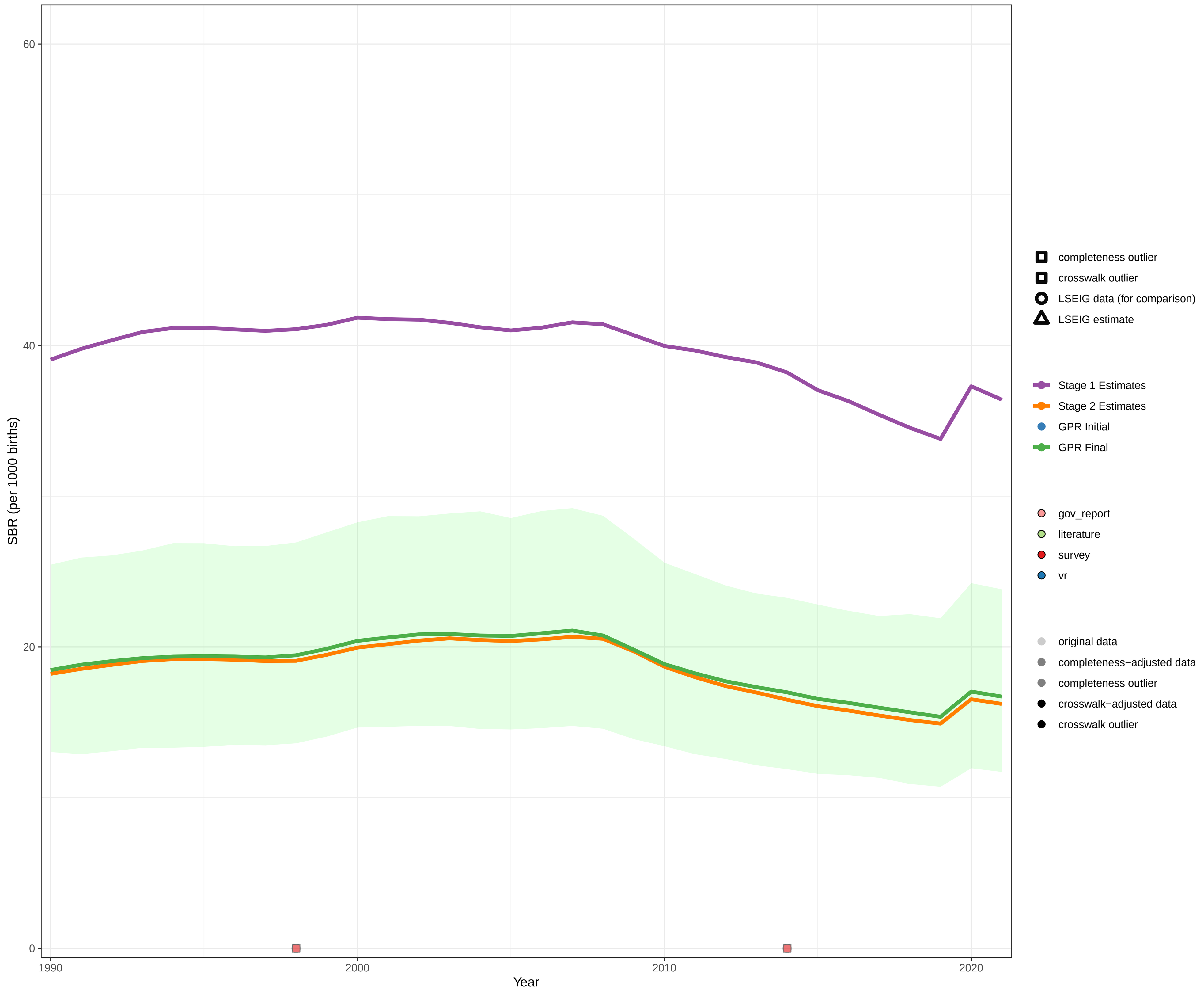

Year	Total Stillbirths (in thousands)	Total Stillbirths (in thousands)	SBR (per 1000 births)	SBR (per 1000 births)	SBR/NMR	SBR/NMR
	20 weeks	28 weeks	20 weeks	28 weeks	20 weeks	28 weeks
1990	0.3 (0.2 to 0.4)	0.2 (0.1 to 0.3)	25.0 (17.7 to 34.5)	18.5 (13.0 to 25.5)	1.21 (0.86 to 1.67)	0.89 (0.63 to 1.23)
1995	0.3 (0.2 to 0.4)	0.2 (0.2 to 0.3)	26.4 (18.9 to 36.0)	19.4 (13.4 to 26.9)	1.21 (0.87 to 1.65)	0.89 (0.61 to 1.23)
2000	0.4 (0.3 to 0.5)	0.3 (0.2 to 0.4)	27.9 (20.0 to 38.7)	20.4 (14.7 to 28.3)	1.26 (0.90 to 1.75)	0.92 (0.66 to 1.28)
2005	0.5 (0.3 to 0.6)	0.3 (0.2 to 0.5)	28.6 (20.0 to 39.3)	20.7 (14.5 to 28.6)	1.32 (0.92 to 1.81)	0.96 (0.67 to 1.32)
2010	0.4 (0.3 to 0.6)	0.3 (0.2 to 0.4)	26.3 (18.4 to 36.2)	18.9 (13.4 to 25.6)	1.27 (0.89 to 1.75)	0.91 (0.65 to 1.24)
2015	0.4 (0.3 to 0.5)	0.3 (0.2 to 0.3)	23.7 (16.6 to 33.1)	16.6 (11.6 to 22.8)	1.30 (0.91 to 1.82)	0.91 (0.64 to 1.25)
2020	0.3 (0.2 to 0.5)	0.2 (0.1 to 0.3)	25.4 (17.8 to 36.3)	17.0 (12.0 to 24.2)	1.48 (1.04 to 2.12)	0.99 (0.70 to 1.41)
2021	0.3 (0.2 to 0.4)	0.2 (0.1 to 0.3)	25.1 (17.7 to 35.7)	16.7 (11.7 to 23.8)	1.52 (1.07 to 2.16)	1.01 (0.71 to 1.44)

Nyandarua KEN_35651 Eastern Sub−Saharan Africa Stillbirth Rate / Neonatal Mortality Rate (SBR/NMR) − 20 weeks
lambda = 0.8 ; zeta = 0.7 ; scale = 15 ; amp = 0.028

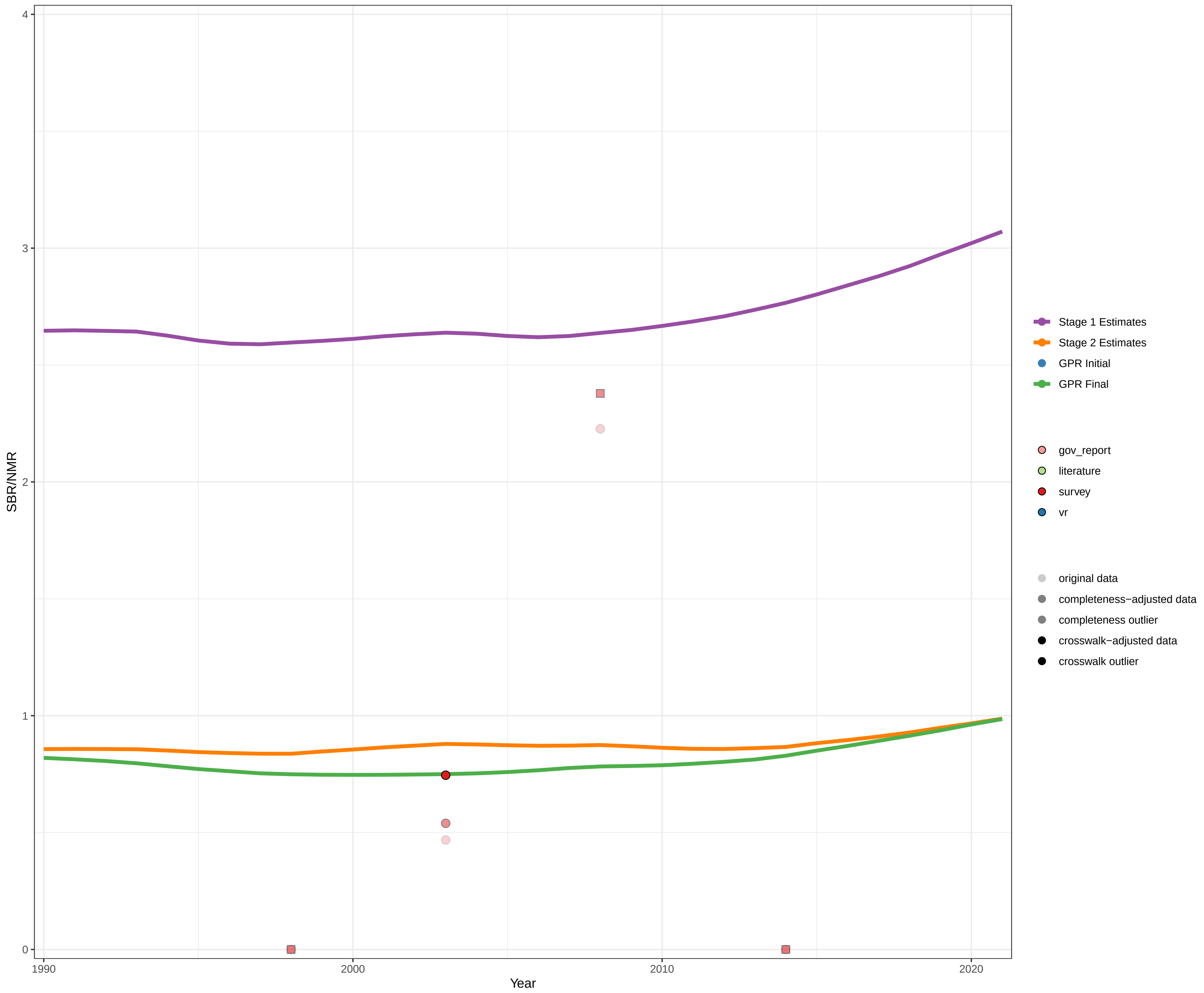

Nyandarua KEN_35651 Eastern Sub−Saharan Africa Stillbirth Rate / Neonatal Mortality Rate (SBR/NMR) − 28 weeks
lambda = 0.8 ; zeta = 0.7 ; scale = 15 ; amp = 0.028

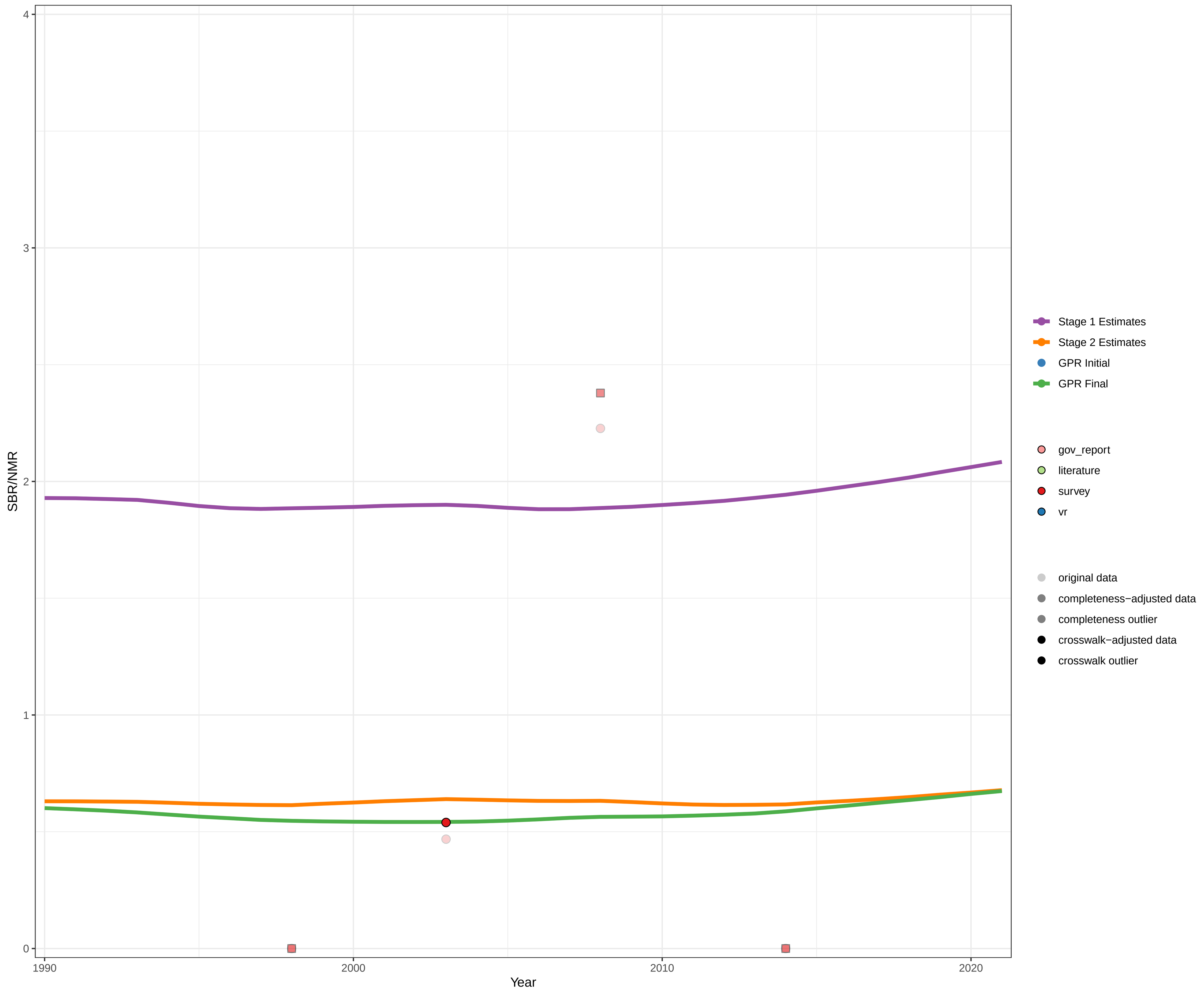

Definition	Years	Outlier	Source.Type	Title
28_weeks	2003	0	Survey	Kenya Demographic and Health Survey 2003
28_weeks	1998	1	Survey	Kenya Demographic and Health Survey 1998
28_weeks	2008	1	Survey	Kenya Demographic and Health Survey 2008−2009
28_weeks	2014	1	Survey	Kenya Demographic and Health Survey 2014

Nyandarua KEN_35651 Eastern Sub−Saharan Africa Stillbirth Rate (SBR) − 20 weeks
lambda = 0.8 ; zeta = 0.7 ; scale = 15 ; amp = 0.028

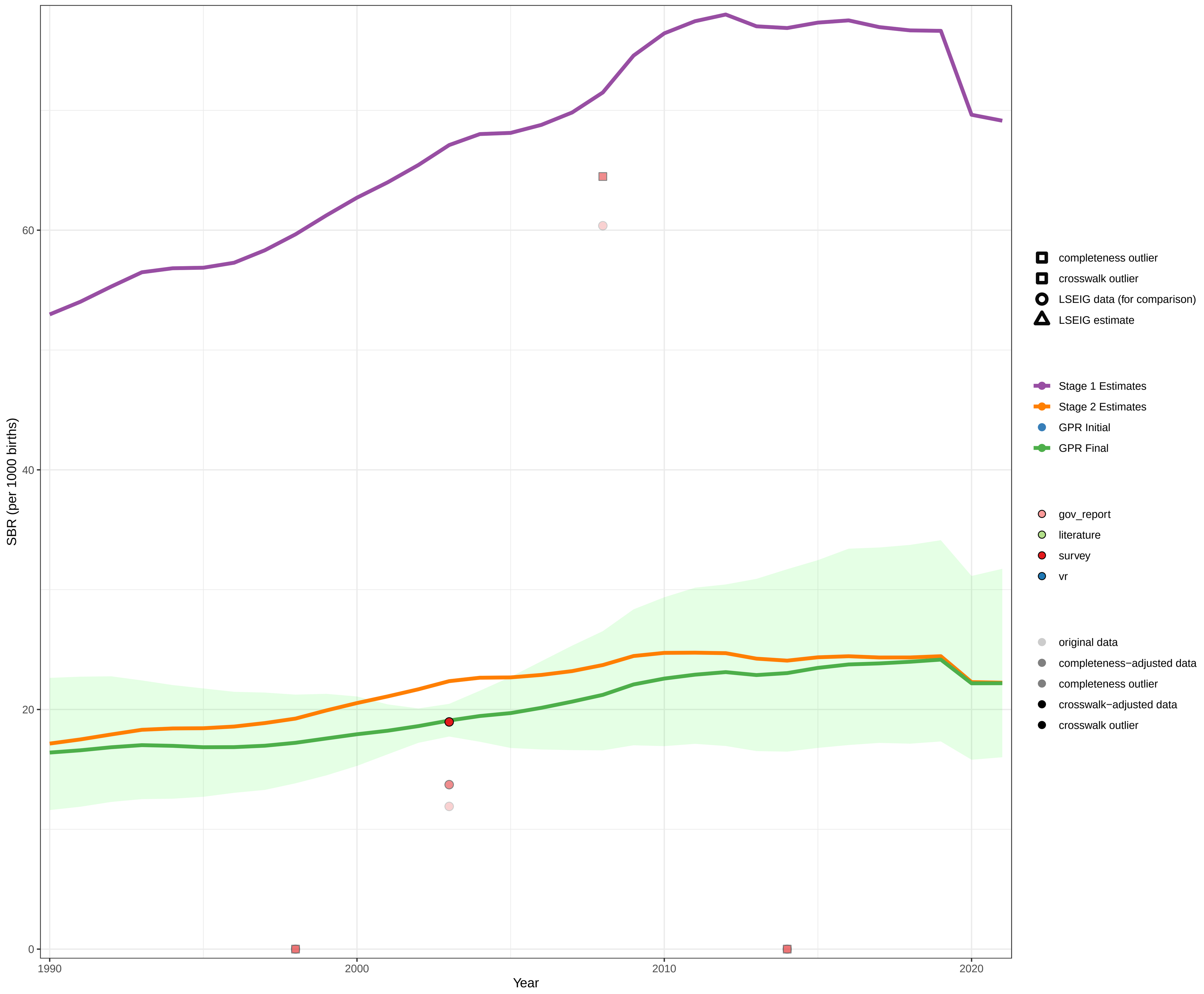

Nyandarua KEN_35651 Eastern Sub−Saharan Africa Stillbirth Rate (SBR) − 28 weeks
lambda = 0.8 ; zeta = 0.7 ; scale = 15 ; amp = 0.028

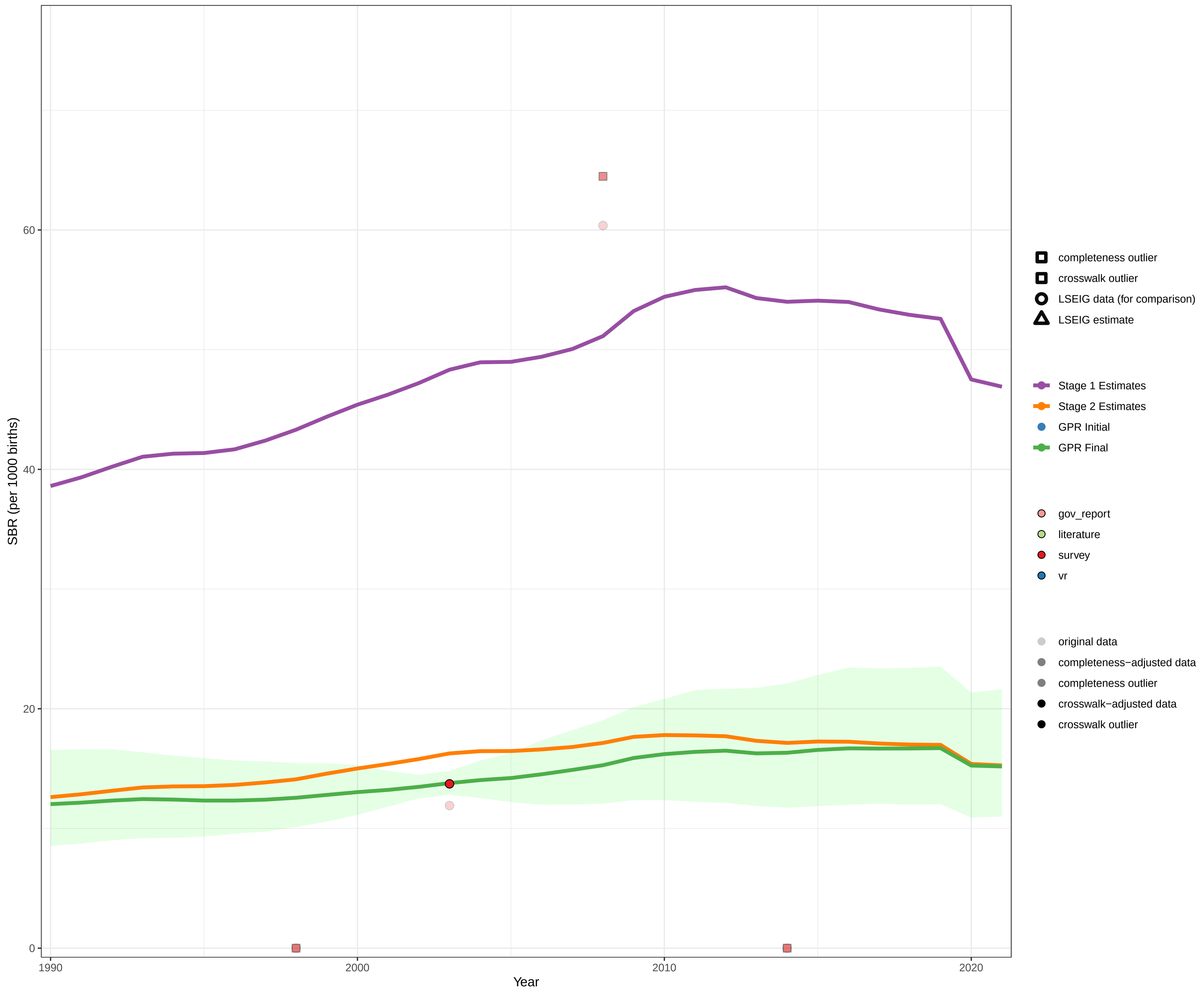

Year	Total Stillbirths (in thousands)	Total Stillbirths (in thousands)	SBR (per 1000 births)	SBR (per 1000 births)	SBR/NMR	SBR/NMR
	20 weeks	28 weeks	20 weeks	28 weeks	20 weeks	28 weeks
1990	0.2<br>(0.2 to 0.3)	0.2<br>(0.1 to 0.2)	16.4<br>(11.6 to 22.6)	12.0<br>(8.5 to 16.6)	0.82<br>(0.58 to 1.13)	0.60<br>(0.43 to 0.83)
1995	0.3<br>(0.2 to 0.4)	0.2<br>(0.2 to 0.3)	16.8<br>(12.7 to 21.8)	12.3<br>(9.3 to 15.9)	0.77<br>(0.58 to 1.00)	0.56<br>(0.43 to 0.73)
2000	0.3<br>(0.3 to 0.4)	0.2<br>(0.2 to 0.3)	17.9<br>(15.3 to 21.1)	13.0<br>(11.1 to 15.3)	0.75<br>(0.64 to 0.88)	0.54<br>(0.46 to 0.64)
2005	0.3<br>(0.3 to 0.4)	0.2<br>(0.2 to 0.3)	19.7<br>(16.8 to 22.7)	14.2<br>(12.2 to 16.3)	0.76<br>(0.65 to 0.88)	0.55<br>(0.47 to 0.63)
2010	0.3<br>(0.2 to 0.4)	0.2<br>(0.2 to 0.3)	22.6<br>(16.9 to 29.4)	16.2<br>(12.4 to 20.8)	0.79<br>(0.59 to 1.02)	0.57<br>(0.43 to 0.73)
2015	0.2<br>(0.2 to 0.3)	0.2<br>(0.1 to 0.2)	23.5<br>(16.8 to 32.5)	16.6<br>(11.9 to 22.8)	0.85<br>(0.61 to 1.18)	0.60<br>(0.43 to 0.83)
2020	0.2<br>(0.1 to 0.3)	0.1<br>(0.1 to 0.2)	22.2<br>(15.8 to 31.1)	15.3<br>(10.9 to 21.4)	0.96<br>(0.69 to 1.35)	0.66<br>(0.47 to 0.93)
2021	0.2<br>(0.1 to 0.2)	0.1<br>(0.1 to 0.2)	22.2<br>(16.0 to 31.7)	15.2<br>(11.0 to 21.6)	0.99<br>(0.71 to 1.41)	0.67<br>(0.49 to 0.96)

Nyeri KEN_35652 Eastern Sub−Saharan Africa Stillbirth Rate / Neonatal Mortality Rate (SBR/NMR) − 20 weeks
lambda = 0.8 ; zeta = 0.7 ; scale = 15 ; amp = 0.028

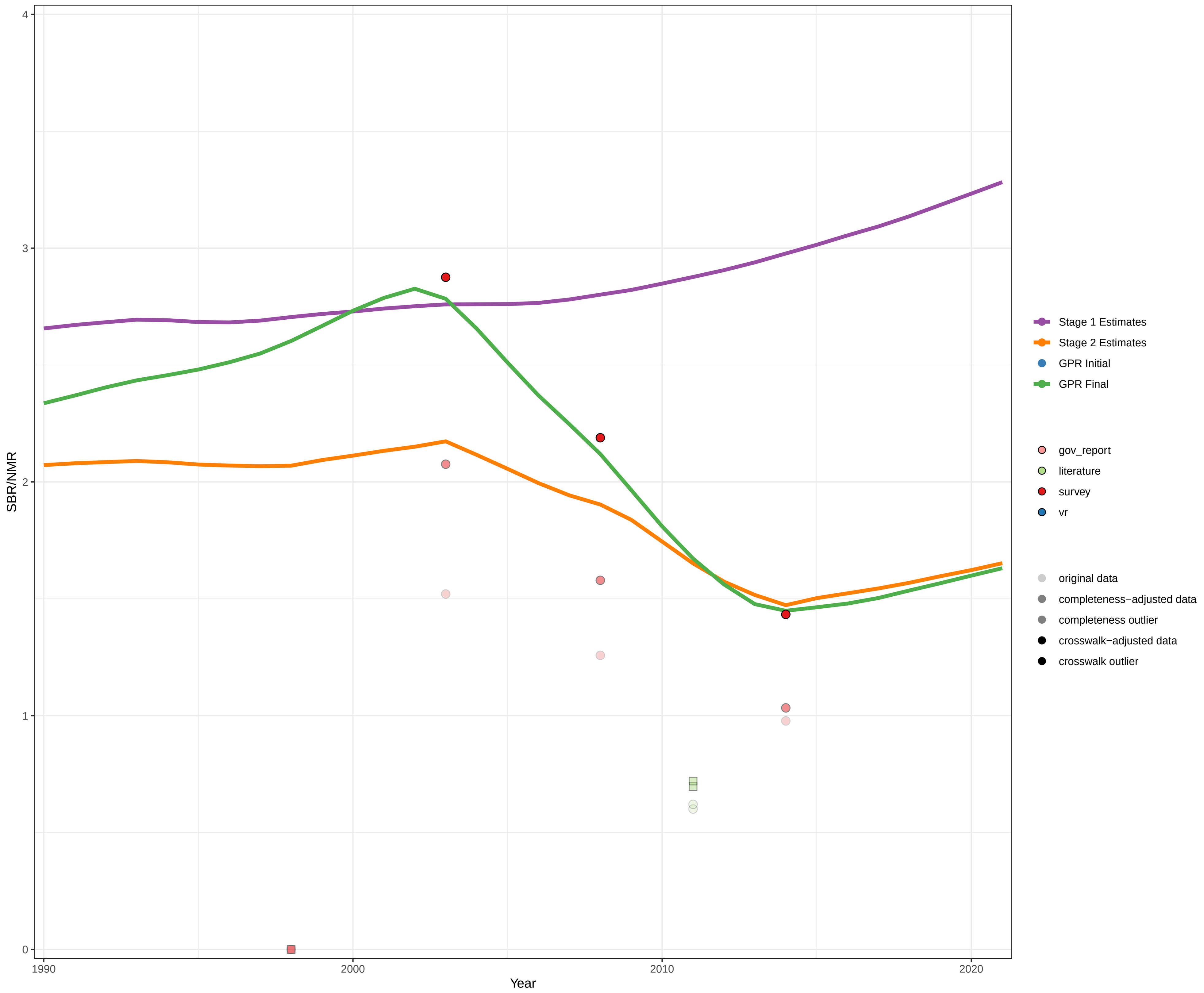

Nyeri KEN_35652 Eastern Sub−Saharan Africa Stillbirth Rate / Neonatal Mortality Rate (SBR/NMR) − 28 weeks
lambda = 0.8 ; zeta = 0.7 ; scale = 15 ; amp = 0.028

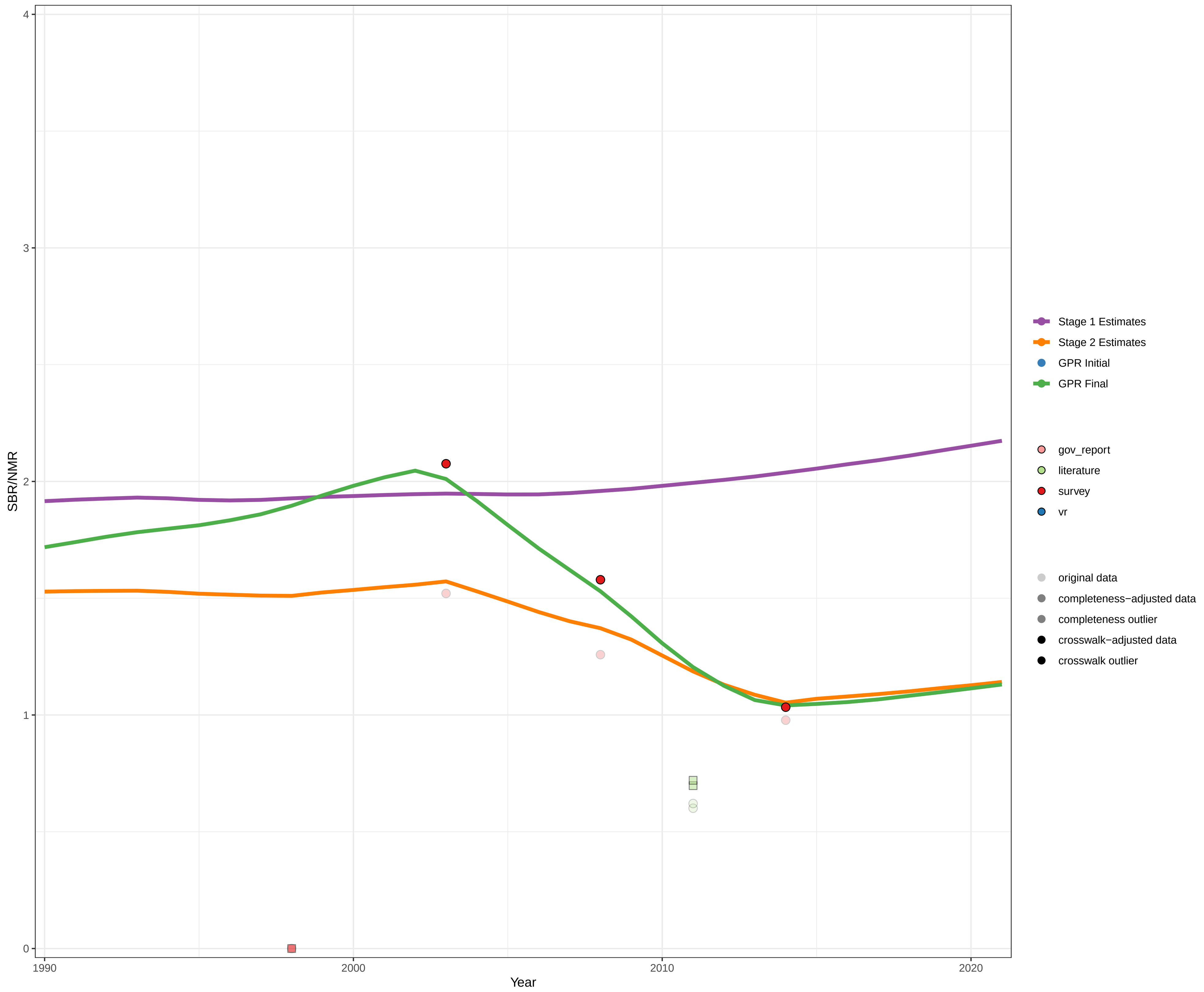

Definition	Years	Outlier	Source.Type	Title
28_weeks	2003	0	Survey	Kenya Demographic and Health Survey 2003
28_weeks	2008	0	Survey	Kenya Demographic and Health Survey 2008−2009
28_weeks	2014	0	Survey	Kenya Demographic and Health Survey 2014
24_weeks, 28_weeks	2011	1	Sci Lit	Correlates of Stillbirths at Nyeri Provincial General Hospital, Kenya, 2009−2013: A Retrospective Study
28_weeks	1998	1	Survey	Kenya Demographic and Health Survey 1998

Nyeri KEN_35652 Eastern Sub−Saharan Africa Stillbirth Rate (SBR) − 20 weeks
lambda = 0.8 ; zeta = 0.7 ; scale = 15 ; amp = 0.028

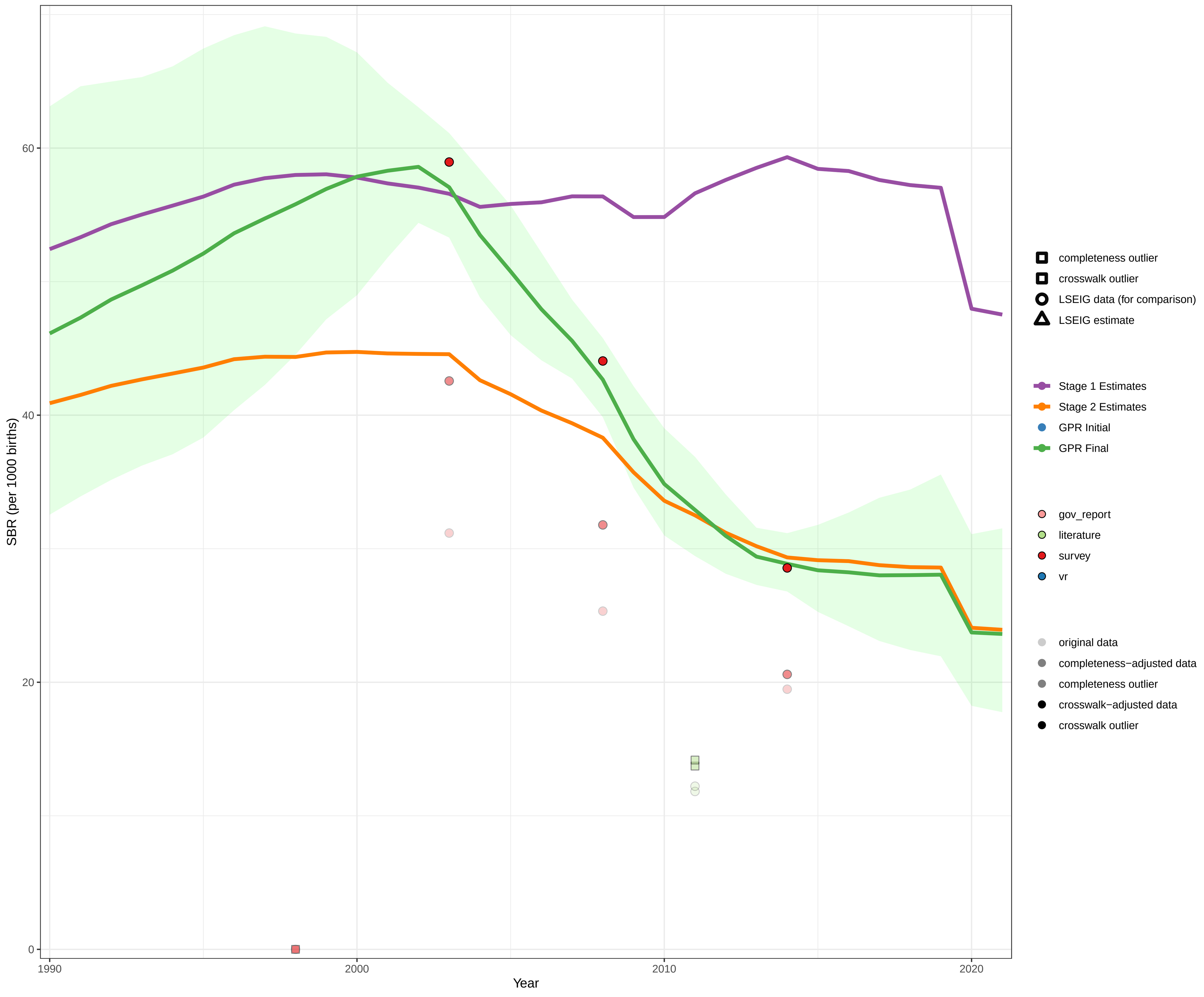

Nyeri KEN_35652 Eastern Sub−Saharan Africa Stillbirth Rate (SBR) − 28 weeks
lambda = 0.8 ; zeta = 0.7 ; scale = 15 ; amp = 0.028

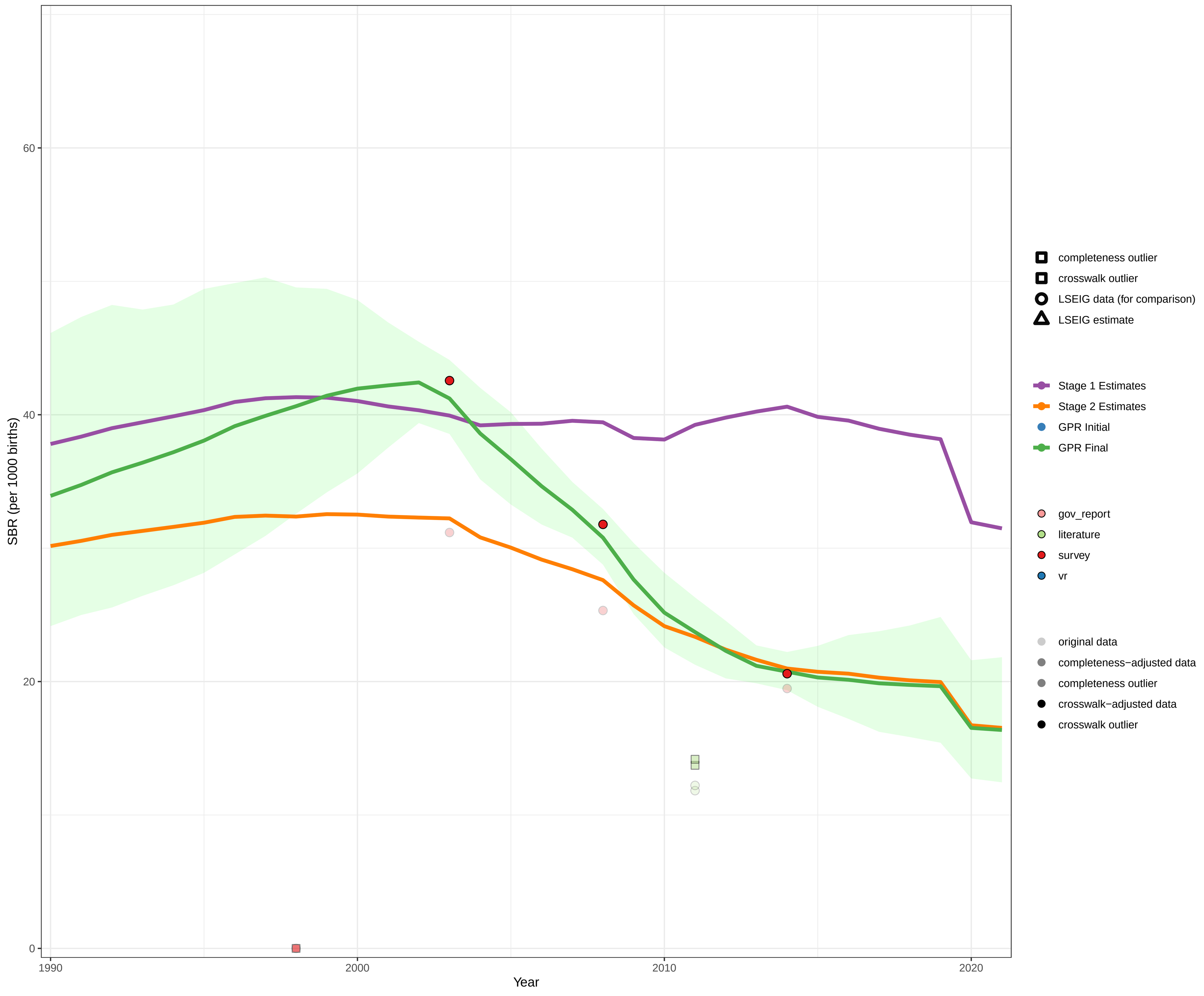

Year	Total Stillbirths (in thousands)	Total Stillbirths (in thousands)	SBR (per 1000 births)	SBR (per 1000 births)	SBR/NMR	SBR/NMR
	20 weeks	28 weeks	20 weeks	28 weeks	20 weeks	28 weeks
1990	1.0 (0.7 to 1.4)	0.7 (0.5 to 1.0)	46.1 (32.5 to 63.1)	33.9 (24.2 to 46.1)	2.34 (1.65 to 3.20)	1.72 (1.22 to 2.34)
1995	1.1 (0.8 to 1.4)	0.8 (0.6 to 1.0)	52.1 (38.3 to 67.5)	38.1 (28.2 to 49.4)	2.48 (1.82 to 3.21)	1.81 (1.34 to 2.35)
2000	1.1 (0.9 to 1.3)	0.8 (0.7 to 0.9)	57.9 (49.0 to 67.2)	42.0 (35.6 to 48.6)	2.73 (2.31 to 3.17)	1.98 (1.68 to 2.30)
2005	0.8 (0.8 to 0.9)	0.6 (0.5 to 0.7)	50.8 (46.0 to 55.7)	36.7 (33.3 to 40.2)	2.51 (2.27 to 2.76)	1.81 (1.65 to 1.99)
2010	0.5 (0.4 to 0.5)	0.3 (0.3 to 0.4)	34.8 (31.0 to 39.0)	25.2 (22.6 to 28.2)	1.81 (1.61 to 2.03)	1.31 (1.17 to 1.46)
2015	0.3 (0.3 to 0.4)	0.2 (0.2 to 0.3)	28.4 (25.3 to 31.8)	20.3 (18.1 to 22.7)	1.46 (1.30 to 1.64)	1.05 (0.93 to 1.17)
2020	0.2 (0.2 to 0.3)	0.1 (0.1 to 0.2)	23.7 (18.2 to 31.1)	16.5 (12.7 to 21.6)	1.60 (1.23 to 2.10)	1.11 (0.86 to 1.46)
2021	0.2 (0.1 to 0.3)	0.1 (0.1 to 0.2)	23.6 (17.8 to 31.5)	16.4 (12.4 to 21.8)	1.63 (1.23 to 2.18)	1.13 (0.86 to 1.51)

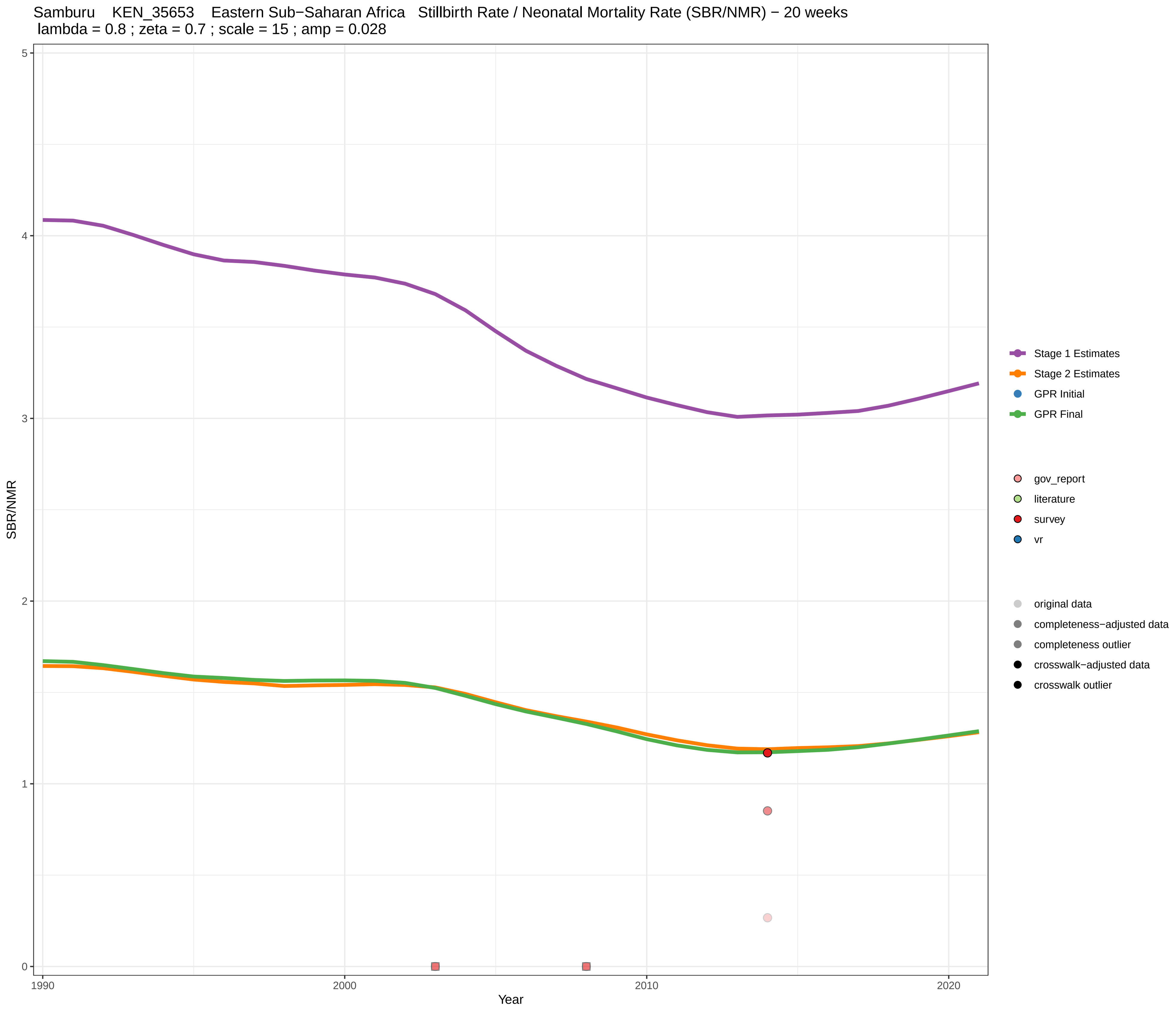

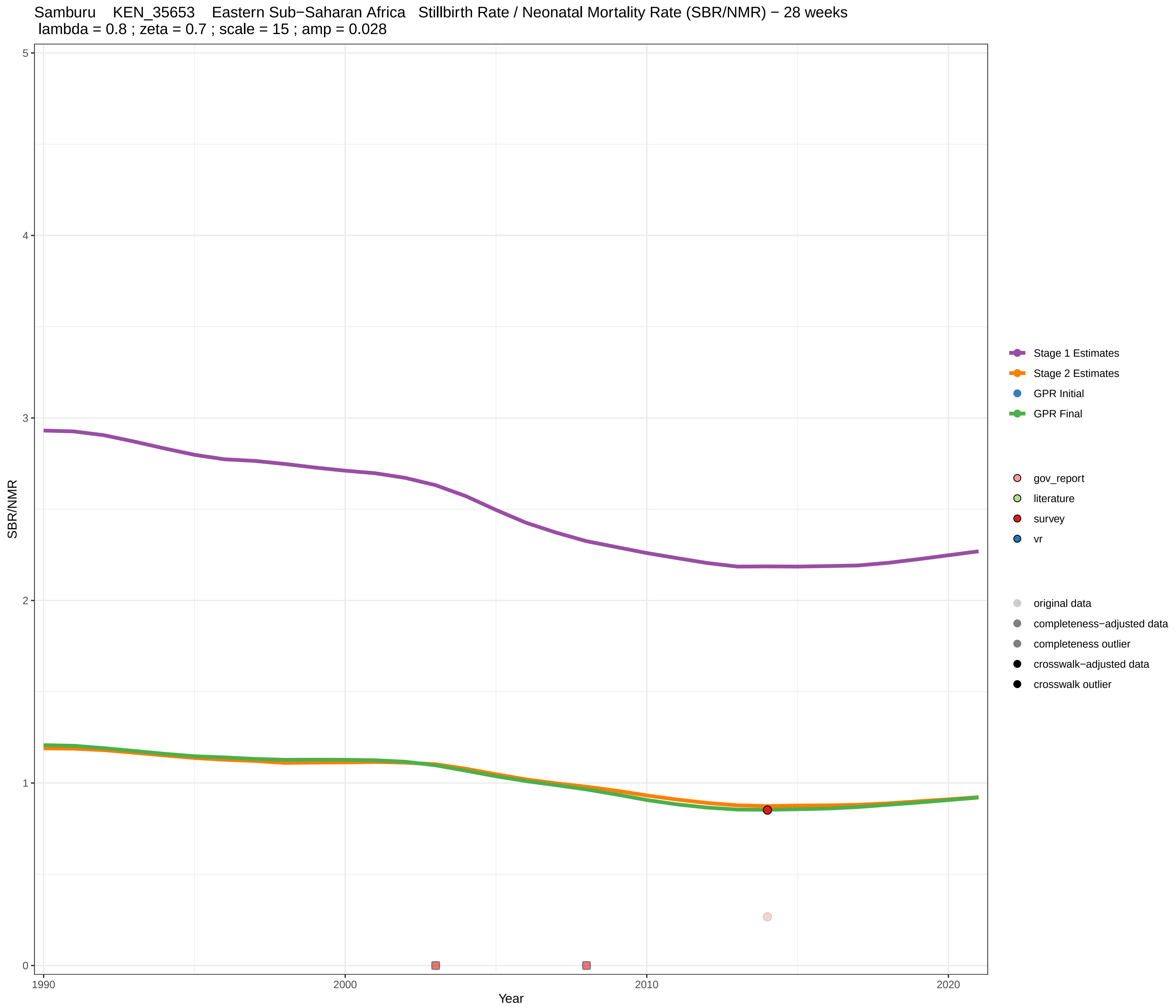

Definition	Years	Outlier	Source.Type	Title
28_weeks	2014	0	Survey	Kenya Demographic and Health Survey 2014
28_weeks	2003	1	Survey	Kenya Demographic and Health Survey 2003
28_weeks	2008	1	Survey	Kenya Demographic and Health Survey 2008−2009

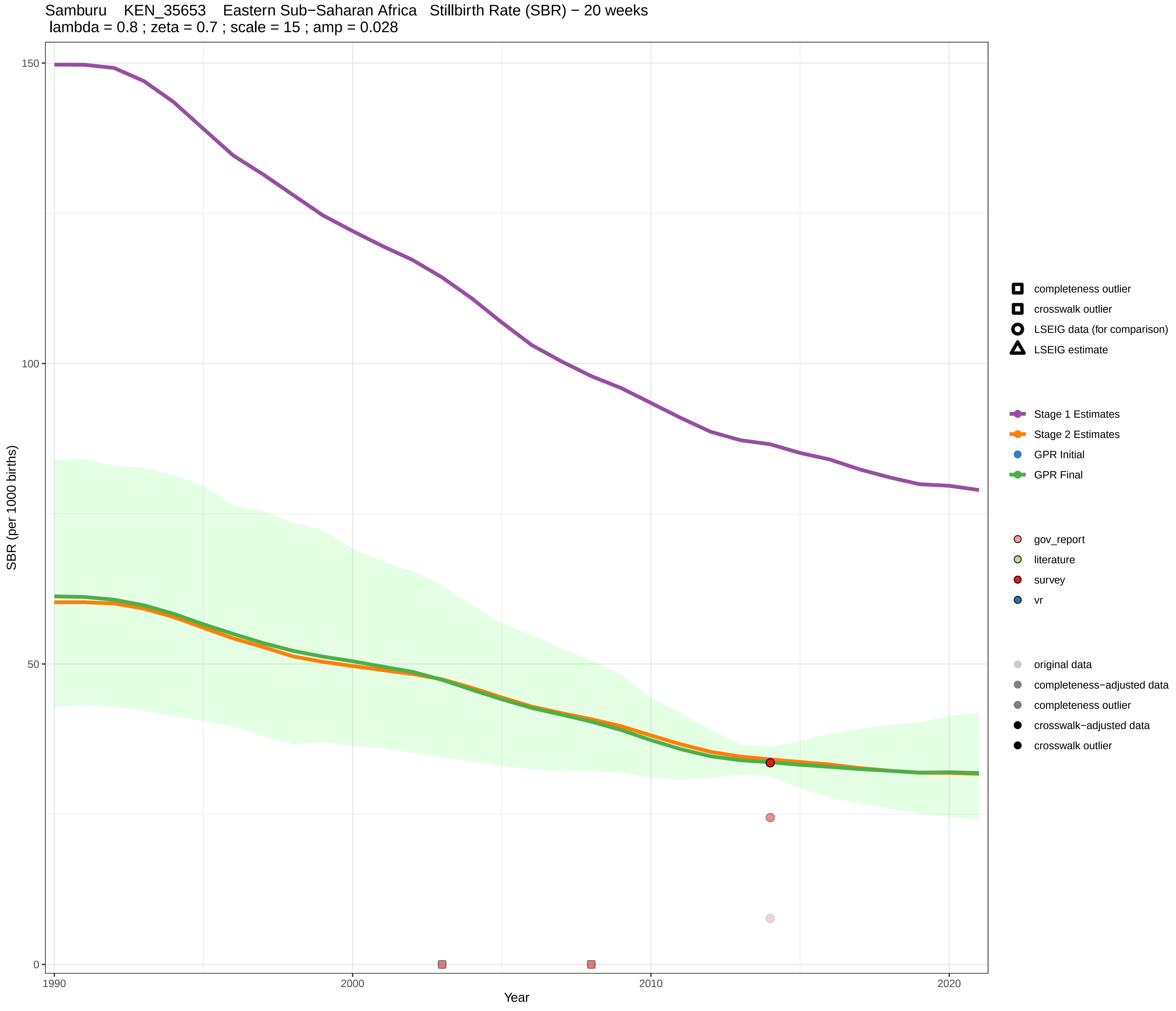

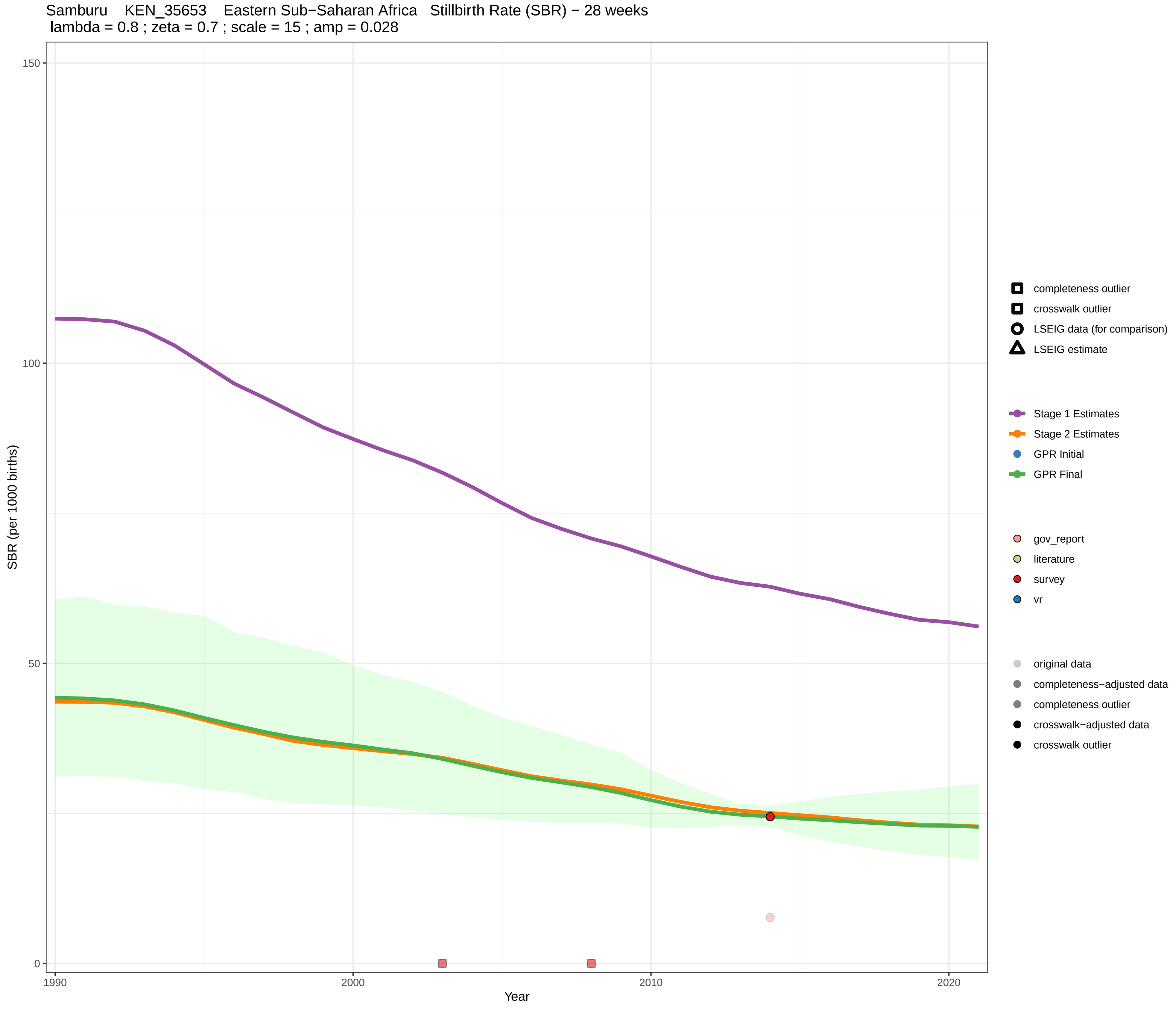

Year	Total Stillbirths (in thousands)	Total Stillbirths (in thousands)	SBR (per 1000 births)	SBR (per 1000 births)	SBR/NMR	SBR/NMR
	20 weeks	28 weeks	20 weeks	28 weeks	20 weeks	28 weeks
1990	0.4 (0.3 to 0.6)	0.3 (0.2 to 0.4)	61.3 (42.8 to 83.9)	44.3 (31.1 to 60.6)	1.67 (1.17 to 2.29)	1.21 (0.85 to 1.65)
1995	0.4 (0.3 to 0.6)	0.3 (0.2 to 0.4)	56.6 (40.5 to 79.7)	40.9 (29.0 to 58.0)	1.59 (1.14 to 2.23)	1.15 (0.81 to 1.62)
2000	0.4 (0.3 to 0.5)	0.3 (0.2 to 0.4)	50.5 (36.4 to 69.2)	36.3 (26.3 to 49.7)	1.57 (1.13 to 2.15)	1.13 (0.82 to 1.54)
2005	0.4 (0.3 to 0.5)	0.3 (0.2 to 0.4)	44.1 (33.0 to 56.9)	31.9 (23.9 to 41.0)	1.44 (1.07 to 1.85)	1.04 (0.78 to 1.33)
2010	0.4 (0.3 to 0.4)	0.3 (0.2 to 0.3)	37.3 (31.1 to 44.4)	27.2 (22.6 to 32.3)	1.24 (1.04 to 1.48)	0.91 (0.75 to 1.08)
2015	0.4 (0.3 to 0.4)	0.3 (0.2 to 0.3)	33.2 (29.3 to 37.2)	24.1 (21.3 to 27.0)	1.18 (1.04 to 1.32)	0.86 (0.76 to 0.96)
2020	0.4 (0.3 to 0.5)	0.3 (0.2 to 0.3)	32.0 (24.6 to 41.3)	22.9 (17.7 to 29.6)	1.26 (0.97 to 1.63)	0.91 (0.70 to 1.17)
2021	0.4 (0.3 to 0.5)	0.3 (0.2 to 0.4)	31.9 (24.0 to 41.9)	22.8 (17.2 to 29.9)	1.29 (0.97 to 1.69)	0.92 (0.70 to 1.21)

Siaya KEN_35654 Eastern Sub−Saharan Africa Stillbirth Rate / Neonatal Mortality Rate (SBR/NMR) − 20 weeks
lambda = 0.8 ; zeta = 0.7 ; scale = 15 ; amp = 0.028

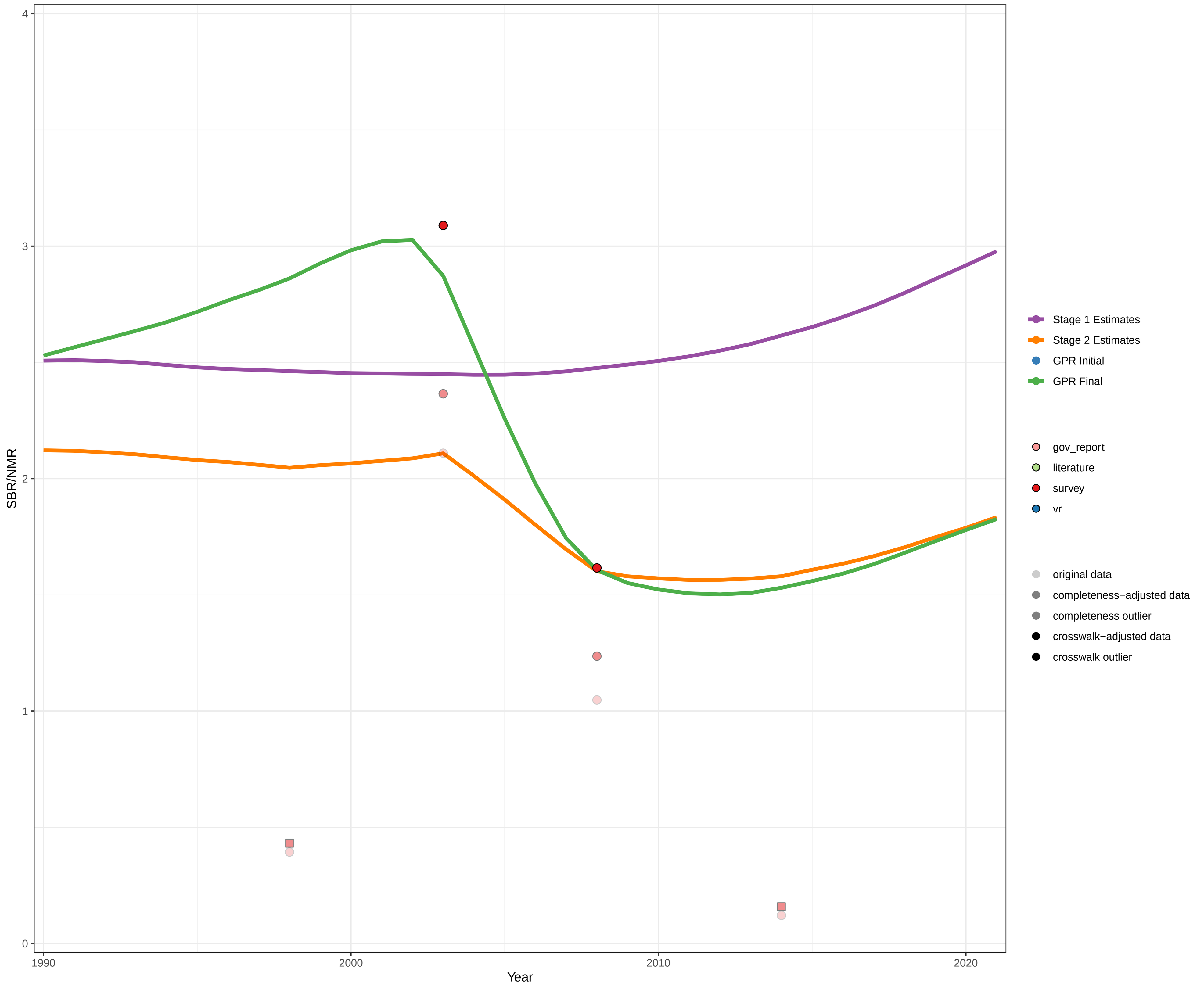

Siaya KEN_35654 Eastern Sub−Saharan Africa Stillbirth Rate / Neonatal Mortality Rate (SBR/NMR) − 28 weeks
lambda = 0.8 ; zeta = 0.7 ; scale = 15 ; amp = 0.028

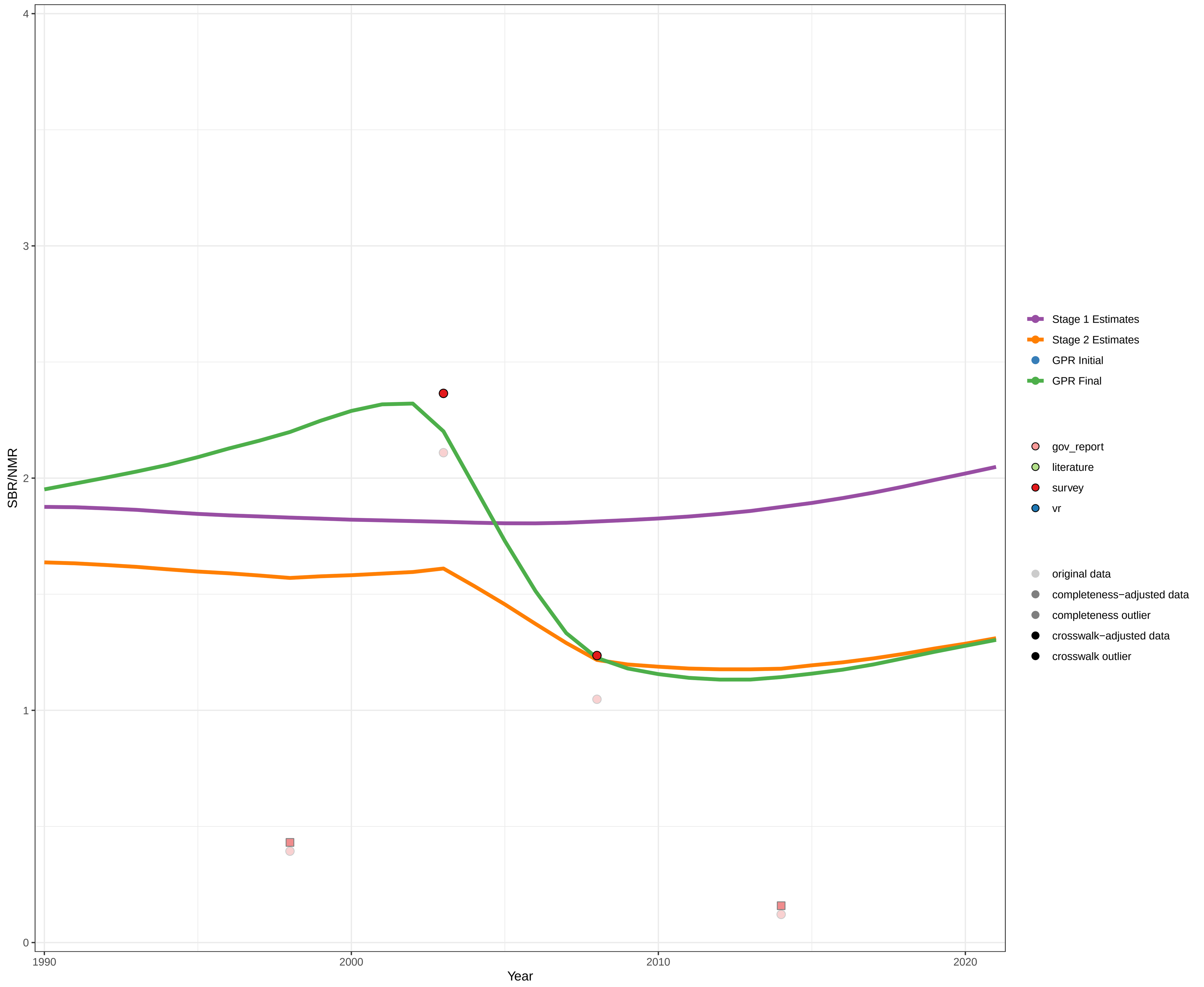

Definition	Years	Outlier	Source.Type	Title
28_weeks	2003	0	Survey	Kenya Demographic and Health Survey 2003
28_weeks	2008	0	Survey	Kenya Demographic and Health Survey 2008−2009
28_weeks	1998	1	Survey	Kenya Demographic and Health Survey 1998
28_weeks	2014	1	Survey	Kenya Demographic and Health Survey 2014

Siaya KEN_35654 Eastern Sub−Saharan Africa Stillbirth Rate (SBR) − 20 weeks
lambda = 0.8 ; zeta = 0.7 ; scale = 15 ; amp = 0.028

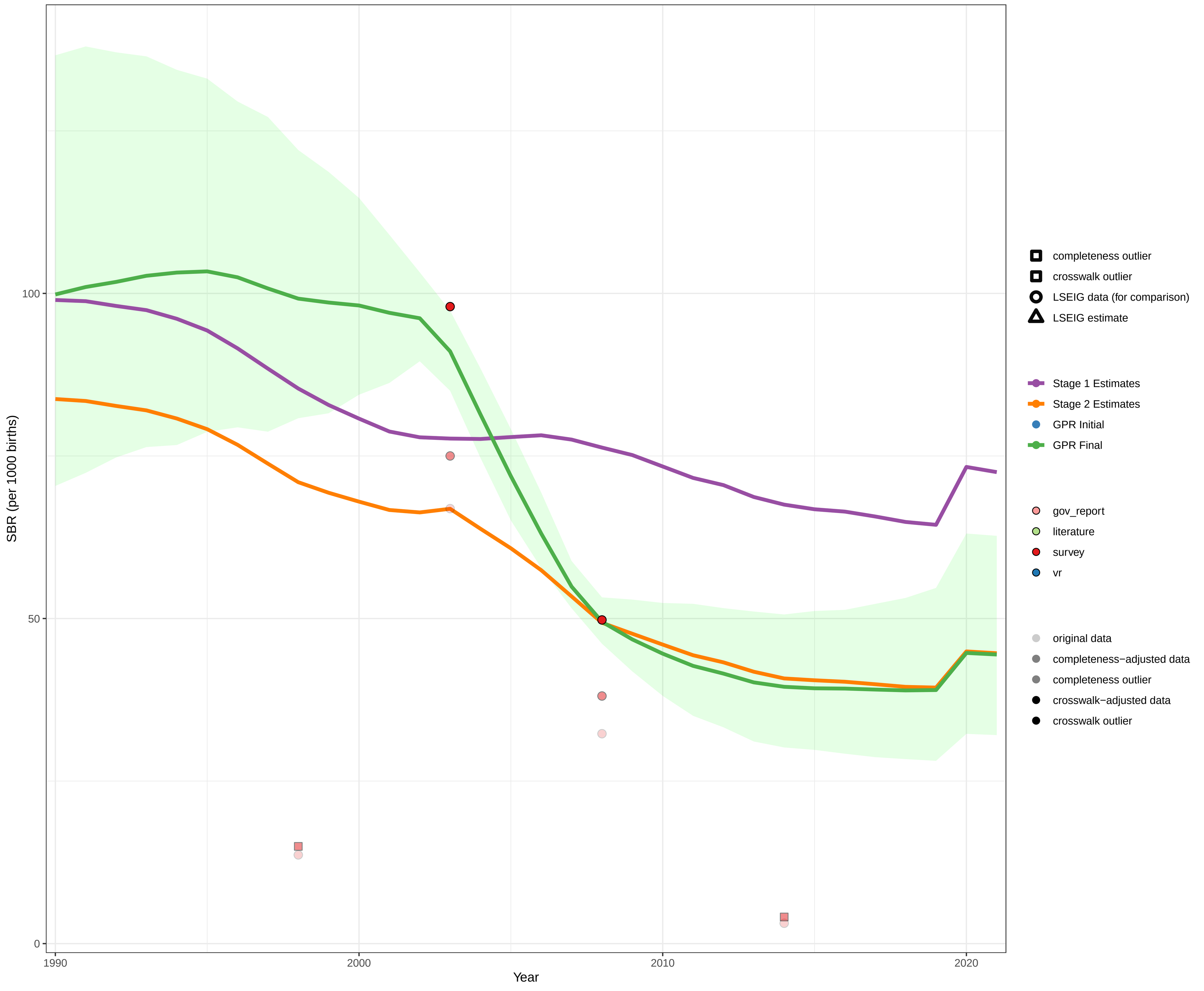

Siaya KEN_35654 Eastern Sub−Saharan Africa Stillbirth Rate (SBR) − 28 weeks
lambda = 0.8 ; zeta = 0.7 ; scale = 15 ; amp = 0.028

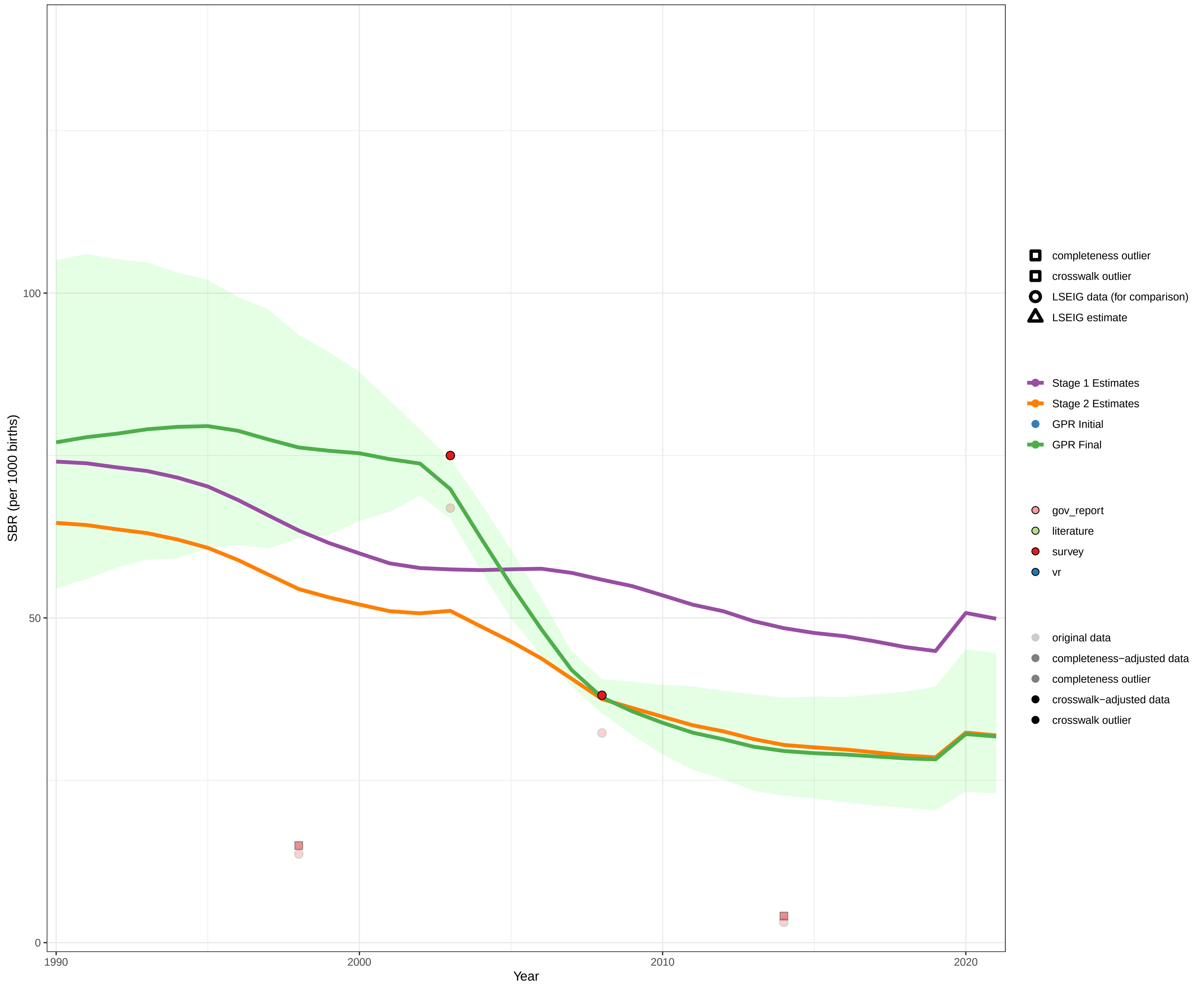

Year	Total Stillbirths (in thousands)	Total Stillbirths (in thousands)	SBR (per 1000 births)	SBR (per 1000 births)	SBR/NMR	SBR/NMR
	20 weeks	28 weeks	20 weeks	28 weeks	20 weeks	28 weeks
1990	3.5 (2.4 to 4.9)	2.6 (1.8 to 3.7)	99.8 (70.4 to 136.6)	77.0 (54.5 to 105.1)	2.53 (1.78 to 3.46)	1.95 (1.38 to 2.66)
1995	3.8 (2.8 to 5.0)	2.8 (2.1 to 3.7)	103.4 (78.7 to 133.0)	79.5 (60.7 to 102.1)	2.72 (2.07 to 3.50)	2.09 (1.60 to 2.68)
2000	3.7 (3.1 to 4.4)	2.8 (2.4 to 3.3)	98.1 (84.4 to 114.7)	75.3 (64.9 to 87.9)	2.98 (2.57 to 3.48)	2.29 (1.97 to 2.67)
2005	2.6 (2.4 to 2.9)	2.0 (1.8 to 2.2)	71.9 (65.1 to 79.1)	55.1 (49.9 to 60.5)	2.26 (2.04 to 2.49)	1.73 (1.57 to 1.90)
2010	1.6 (1.3 to 1.9)	1.2 (1.0 to 1.4)	44.6 (38.1 to 52.4)	33.8 (29.0 to 39.7)	1.52 (1.30 to 1.79)	1.16 (0.99 to 1.36)
2015	1.3 (1.0 to 1.7)	1.0 (0.7 to 1.3)	39.3 (29.8 to 51.2)	29.2 (22.2 to 37.9)	1.56 (1.18 to 2.03)	1.16 (0.88 to 1.50)
2020	1.4 (1.0 to 2.0)	1.0 (0.7 to 1.4)	44.7 (32.3 to 63.1)	32.1 (23.3 to 45.2)	1.78 (1.28 to 2.51)	1.28 (0.93 to 1.80)
2021	1.4 (1.0 to 2.0)	1.0 (0.7 to 1.4)	44.5 (32.1 to 62.7)	31.7 (23.0 to 44.6)	1.83 (1.32 to 2.58)	1.30 (0.94 to 1.83)

Taita Taveta KEN_35655 Eastern Sub−Saharan Africa Stillbirth Rate / Neonatal Mortality Rate (SBR/NMR) − 20 weeks
lambda = 0.8 ; zeta = 0.7 ; scale = 15 ; amp = 0.028

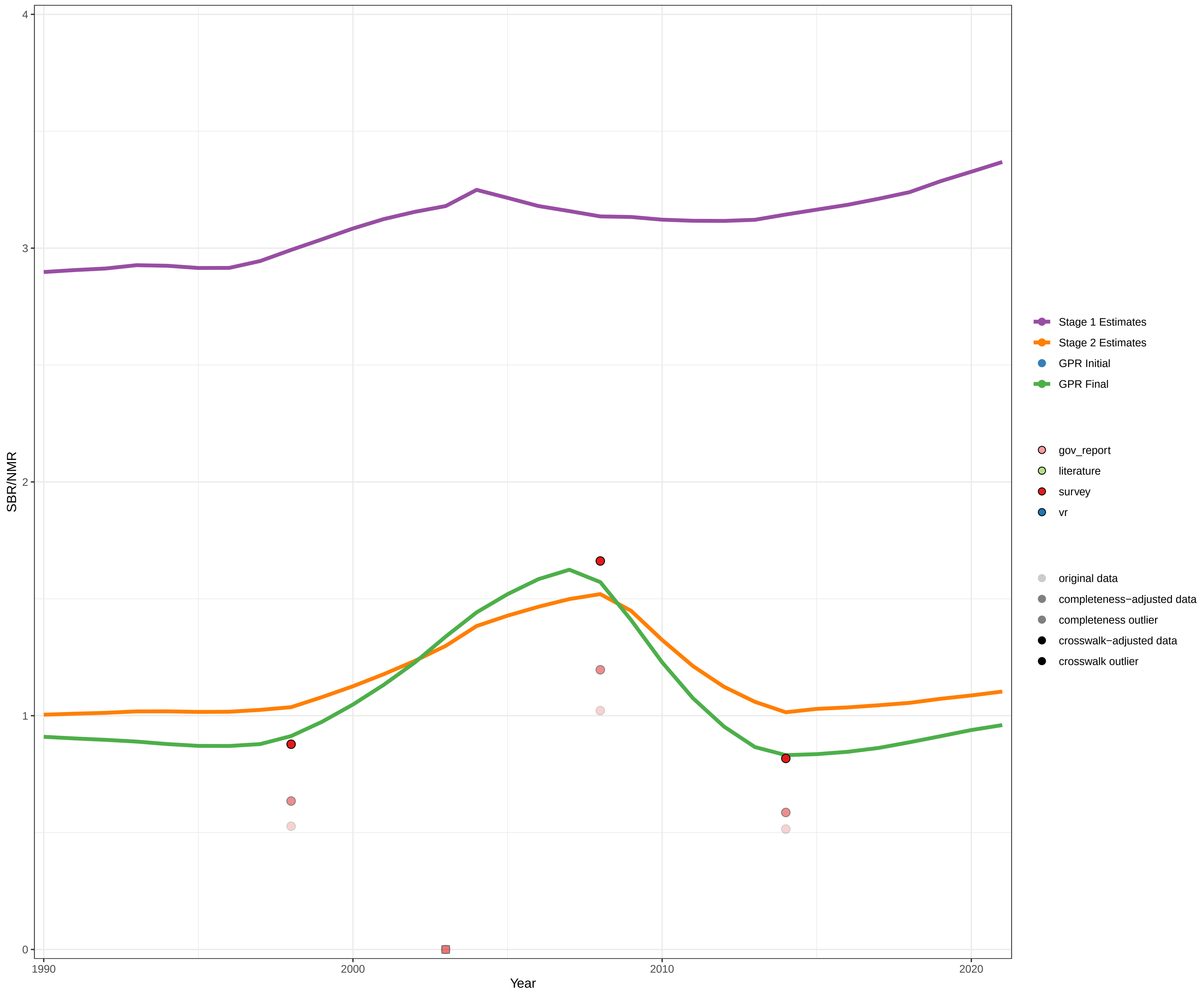

Taita Taveta KEN_35655 Eastern Sub−Saharan Africa Stillbirth Rate / Neonatal Mortality Rate (SBR/NMR) − 28 weeks
lambda = 0.8 ; zeta = 0.7 ; scale = 15 ; amp = 0.028

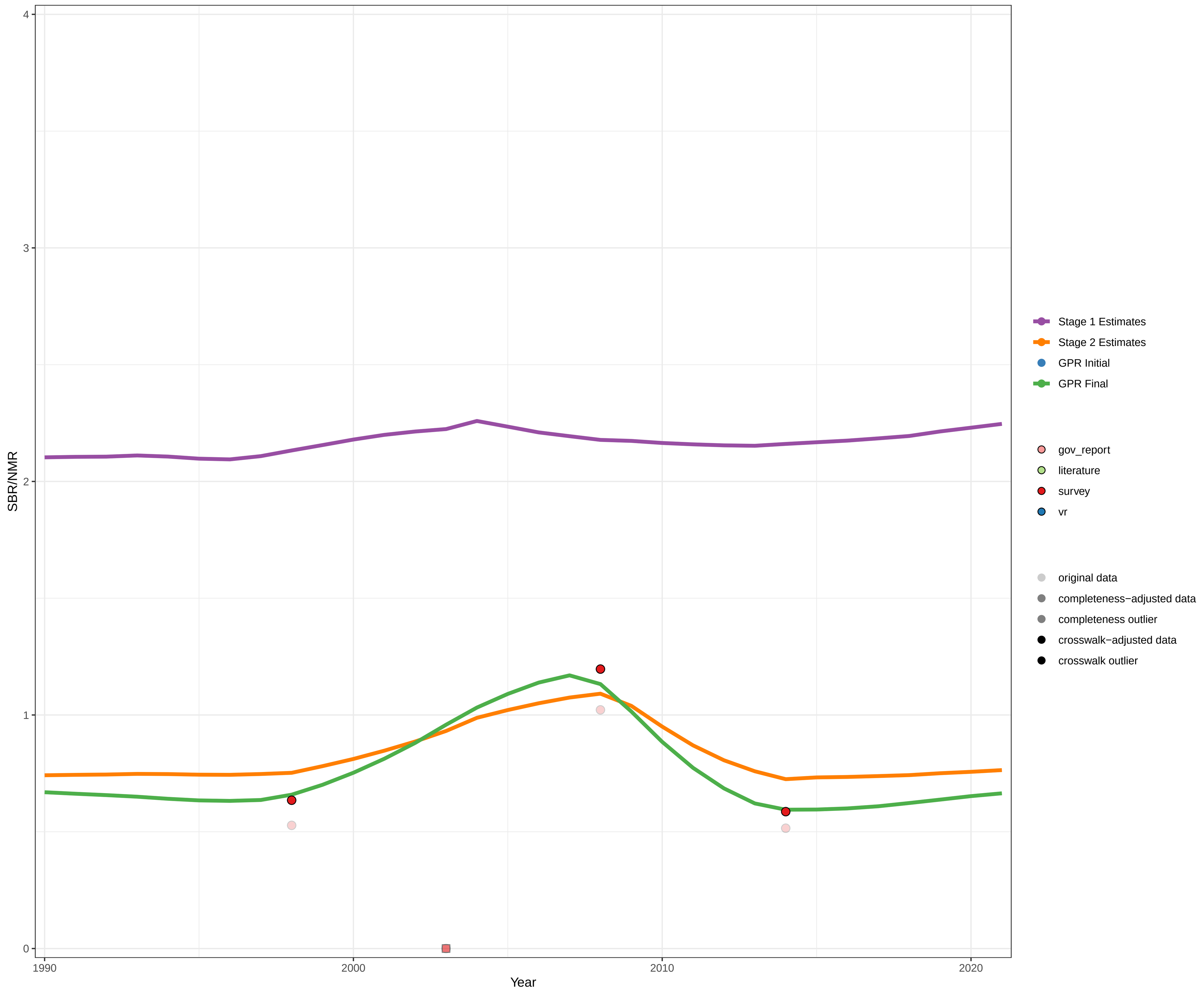

Definition	Years	Outlier	Source.Type	Title
28_weeks	1998	0	Survey	Kenya Demographic and Health Survey 1998
28_weeks	2008	0	Survey	Kenya Demographic and Health Survey 2008−2009
28_weeks	2014	0	Survey	Kenya Demographic and Health Survey 2014
28_weeks	2003	1	Survey	Kenya Demographic and Health Survey 2003

Taita Taveta KEN_35655 Eastern Sub−Saharan Africa Stillbirth Rate (SBR) − 20 weeks
lambda = 0.8 ; zeta = 0.7 ; scale = 15 ; amp = 0.028

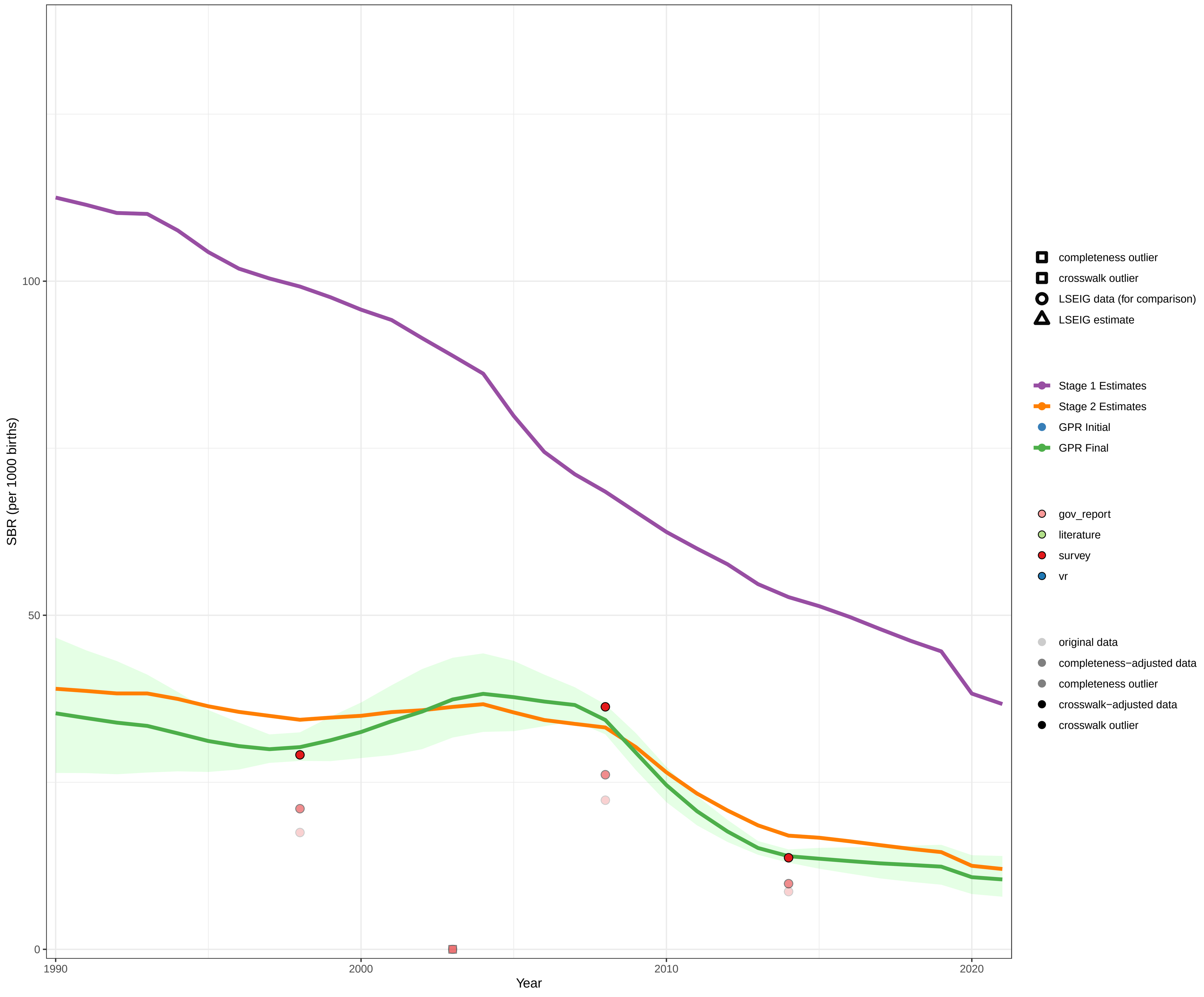

Taita Taveta KEN_35655 Eastern Sub−Saharan Africa Stillbirth Rate (SBR) − 28 weeks
lambda = 0.8 ; zeta = 0.7 ; scale = 15 ; amp = 0.028

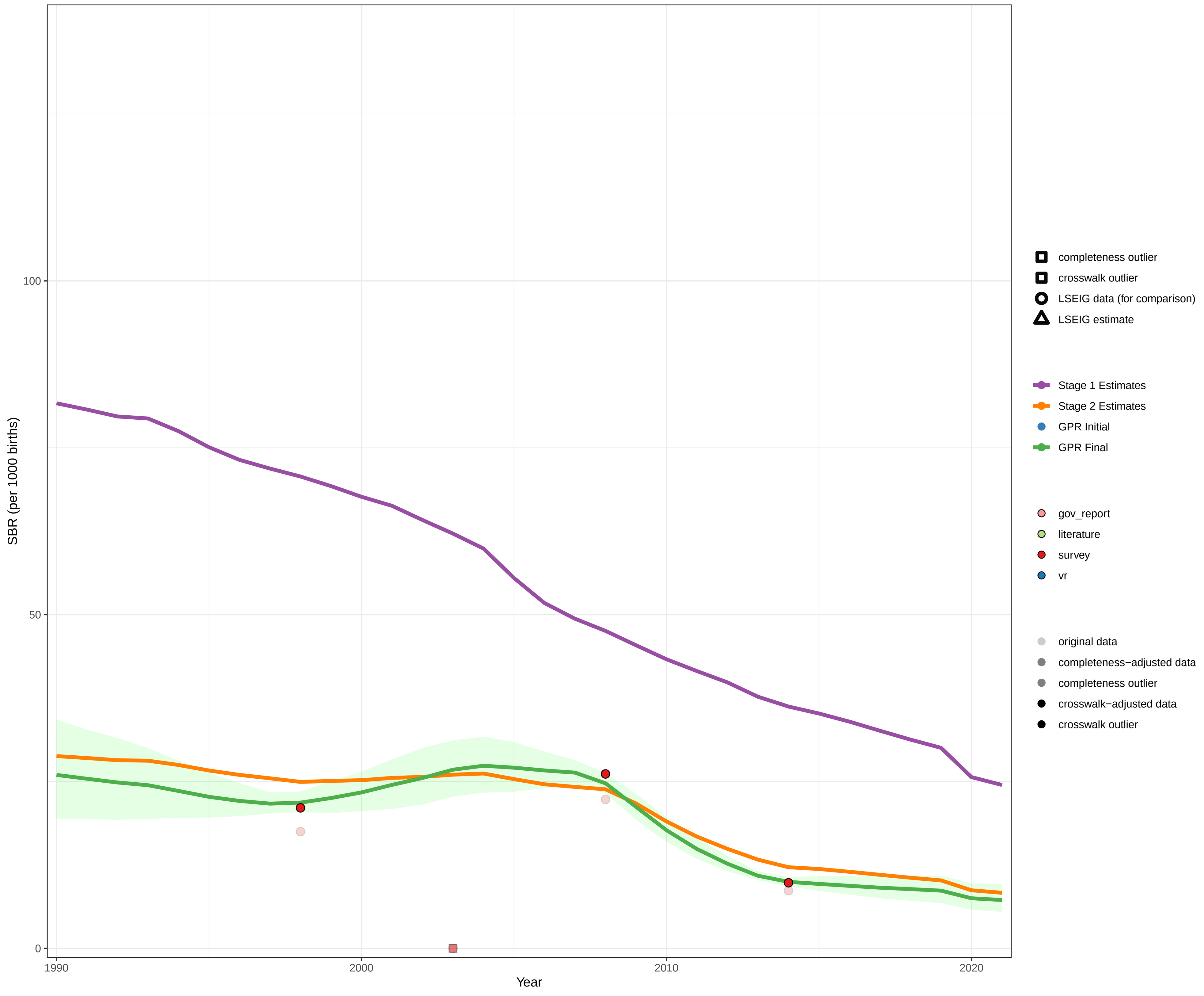

Year	Total Stillbirths (in thousands)	Total Stillbirths (in thousands)	SBR (per 1000 births)	SBR (per 1000 births)	SBR/NMR	SBR/NMR
	20 weeks	28 weeks	20 weeks	28 weeks	20 weeks	28 weeks
1990	0.3 (0.2 to 0.4)	0.2 (0.2 to 0.3)	35.3 (26.4 to 46.7)	26.0 (19.5 to 34.3)	0.91 (0.68 to 1.20)	0.67 (0.50 to 0.88)
1995	0.3 (0.2 to 0.3)	0.2 (0.2 to 0.2)	31.2 (26.5 to 35.8)	22.7 (19.6 to 26.3)	0.87 (0.74 to 1.00)	0.63 (0.55 to 0.73)
2000	0.3 (0.3 to 0.3)	0.2 (0.2 to 0.2)	32.5 (28.6 to 36.9)	23.4 (20.6 to 26.5)	1.05 (0.92 to 1.19)	0.75 (0.66 to 0.85)
2005	0.3 (0.3 to 0.4)	0.2 (0.2 to 0.3)	37.7 (32.7 to 43.2)	27.1 (23.5 to 30.9)	1.52 (1.32 to 1.74)	1.09 (0.95 to 1.25)
2010	0.2 (0.2 to 0.2)	0.1 (0.1 to 0.2)	24.6 (22.0 to 27.3)	17.7 (15.9 to 19.7)	1.23 (1.10 to 1.37)	0.88 (0.80 to 0.98)
2015	0.1 (0.1 to 0.1)	0.1 (0.1 to 0.1)	13.6 (12.1 to 15.2)	9.7 (8.6 to 10.8)	0.84 (0.74 to 0.94)	0.60 (0.53 to 0.66)
2020	0.1 (0.1 to 0.1)	0.0 (0.0 to 0.1)	10.8 (8.3 to 14.1)	7.5 (5.8 to 9.8)	0.94 (0.72 to 1.22)	0.65 (0.50 to 0.85)
2021	0.1 (0.1 to 0.1)	0.0 (0.0 to 0.1)	10.5 (7.9 to 14.0)	7.2 (5.5 to 9.6)	0.96 (0.72 to 1.28)	0.66 (0.50 to 0.89)

Tana River KEN_35656 Eastern Sub−Saharan Africa Stillbirth Rate / Neonatal Mortality Rate (SBR/NMR) − 20 weeks
lambda = 0.8 ; zeta = 0.7 ; scale = 15 ; amp = 0.028

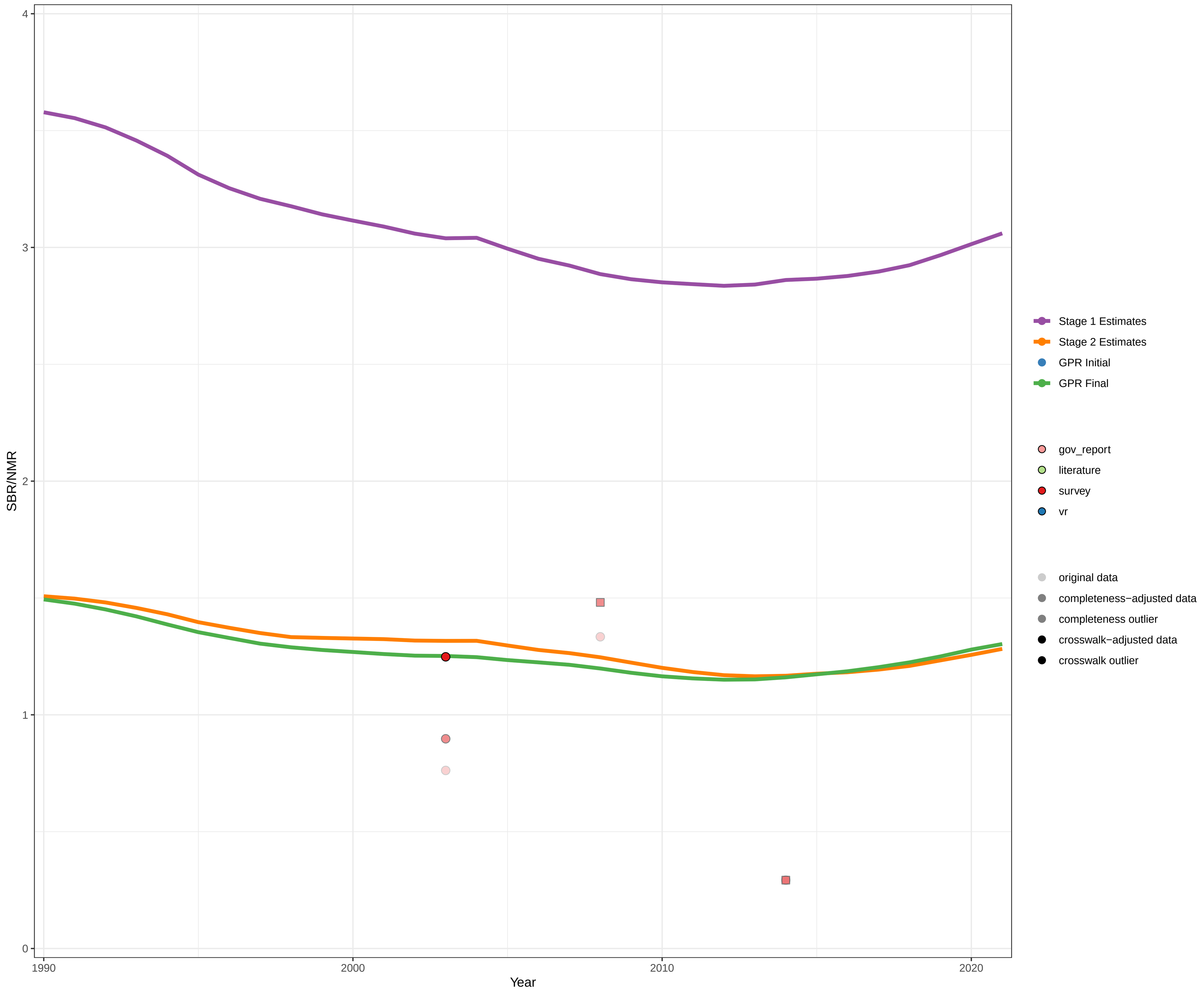

Tana River KEN_35656 Eastern Sub−Saharan Africa Stillbirth Rate / Neonatal Mortality Rate (SBR/NMR) − 28 weeks
lambda = 0.8 ; zeta = 0.7 ; scale = 15 ; amp = 0.028

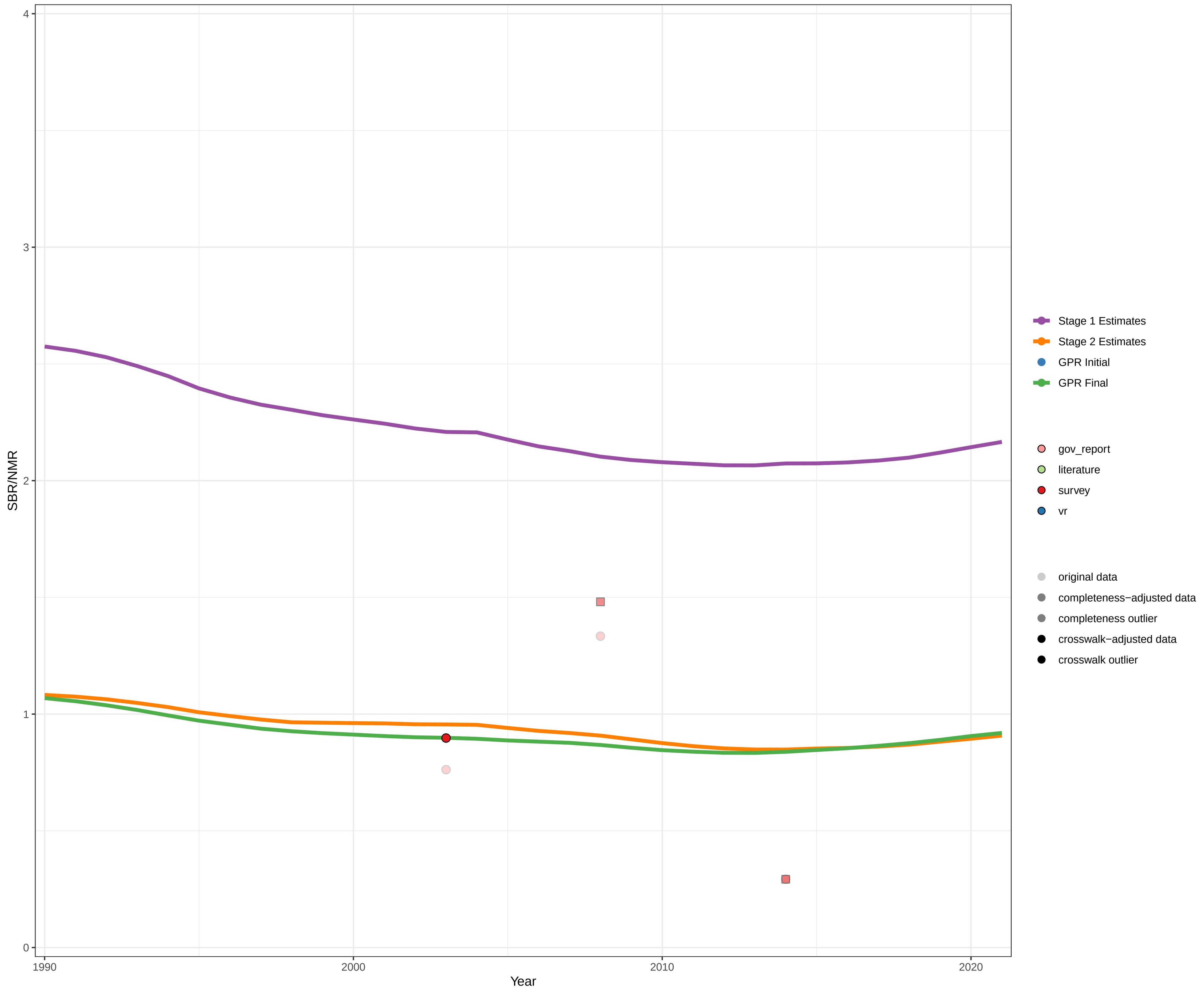

Definition	Years	Outlier	Source.Type	Title
28_weeks	2003	0	Survey	Kenya Demographic and Health Survey 2003
28_weeks	2008	1	Survey	Kenya Demographic and Health Survey 2008−2009
28_weeks	2014	1	Survey	Kenya Demographic and Health Survey 2014

Tana River KEN_35656 Eastern Sub−Saharan Africa Stillbirth Rate (SBR) − 20 weeks
lambda = 0.8 ; zeta = 0.7 ; scale = 15 ; amp = 0.028

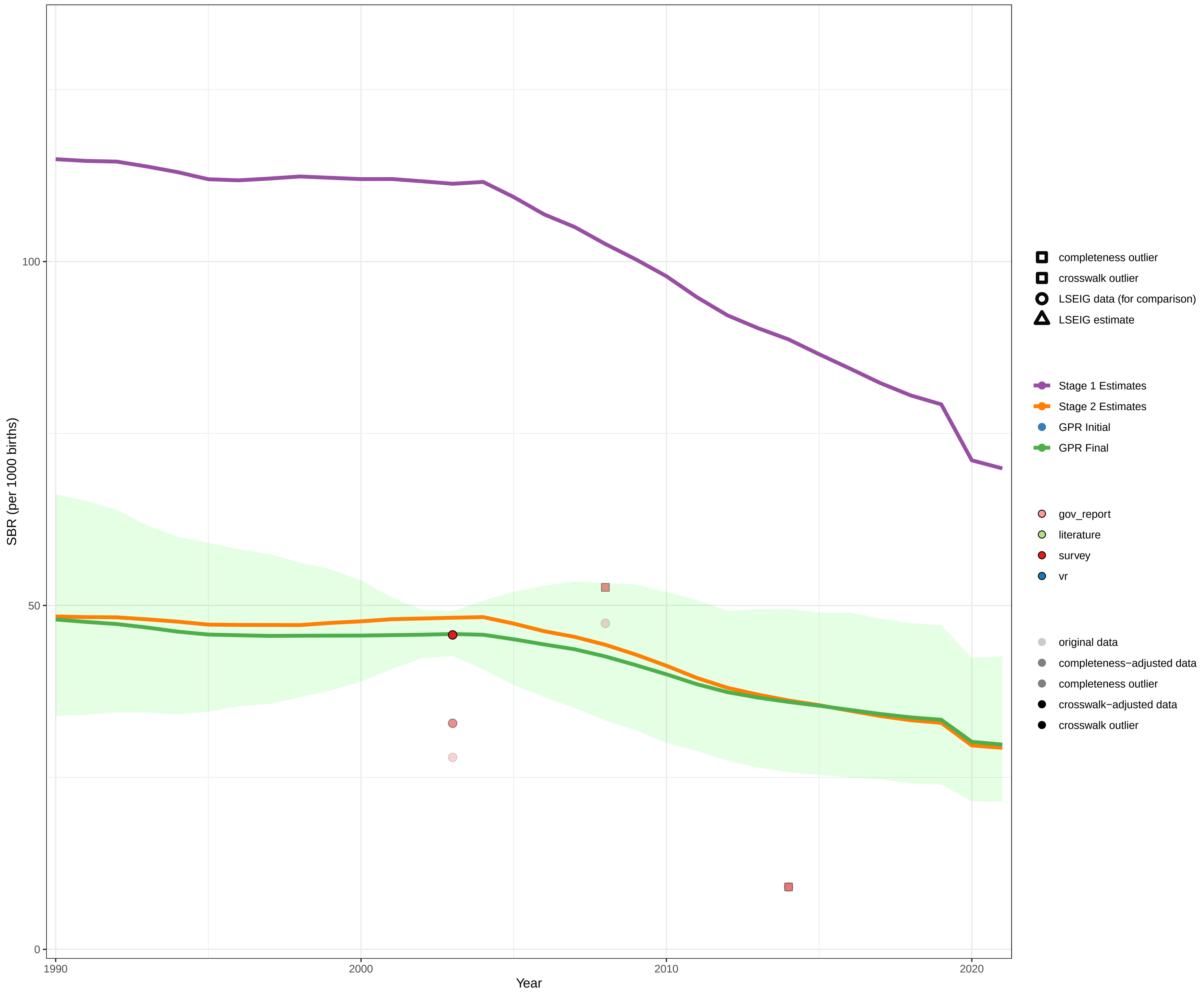

Tana River KEN_35656 Eastern Sub−Saharan Africa Stillbirth Rate (SBR) − 28 weeks
lambda = 0.8 ; zeta = 0.7 ; scale = 15 ; amp = 0.028

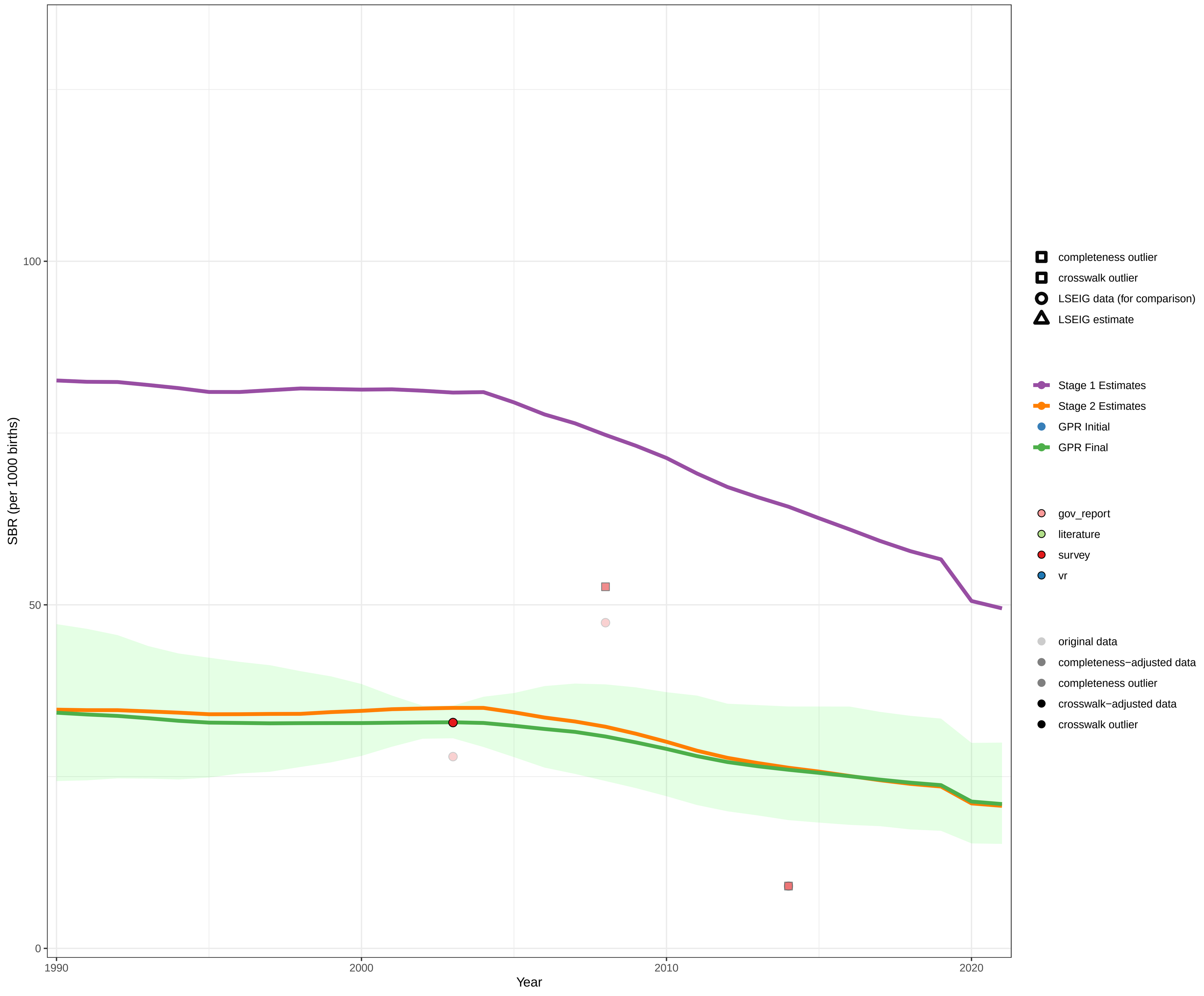

Year	Total Stillbirths (in thousands)	Total Stillbirths (in thousands)	SBR (per 1000 births)	SBR (per 1000 births)	SBR/NMR	SBR/NMR
	20 weeks	28 weeks	20 weeks	28 weeks	20 weeks	28 weeks
1990	0.3 (0.2 to 0.4)	0.2 (0.1 to 0.3)	48.0 (33.9 to 66.2)	34.3 (24.4 to 47.2)	1.49 (1.06 to 2.06)	1.07 (0.76 to 1.47)
1995	0.3 (0.3 to 0.4)	0.2 (0.2 to 0.3)	45.8 (34.5 to 59.1)	32.9 (24.9 to 42.3)	1.35 (1.02 to 1.75)	0.97 (0.74 to 1.25)
2000	0.4 (0.3 to 0.5)	0.3 (0.2 to 0.3)	45.6 (38.9 to 53.6)	32.8 (28.0 to 38.5)	1.27 (1.08 to 1.49)	0.91 (0.78 to 1.07)
2005	0.5 (0.4 to 0.5)	0.3 (0.3 to 0.4)	45.1 (38.4 to 52.0)	32.4 (27.8 to 37.2)	1.23 (1.05 to 1.42)	0.89 (0.76 to 1.02)
2010	0.4 (0.3 to 0.6)	0.3 (0.2 to 0.4)	40.0 (30.0 to 52.0)	29.0 (22.1 to 37.3)	1.16 (0.87 to 1.51)	0.85 (0.65 to 1.09)
2015	0.4 (0.3 to 0.6)	0.3 (0.2 to 0.4)	35.4 (25.3 to 49.0)	25.5 (18.3 to 35.2)	1.17 (0.84 to 1.62)	0.85 (0.61 to 1.17)
2020	0.4 (0.2 to 0.5)	0.2 (0.2 to 0.4)	30.2 (21.5 to 42.4)	21.4 (15.3 to 29.9)	1.28 (0.91 to 1.80)	0.91 (0.65 to 1.27)
2021	0.4 (0.3 to 0.5)	0.2 (0.2 to 0.4)	29.8 (21.5 to 42.6)	21.0 (15.2 to 29.9)	1.30 (0.94 to 1.86)	0.92 (0.67 to 1.31)

Tharaka Nithi KEN_35657 Eastern Sub−Saharan Africa Stillbirth Rate / Neonatal Mortality Rate (SBR/NMR) − 20 weeks
lambda = 1 ; zeta = 0.6 ; scale = 15 ; amp = 0.028

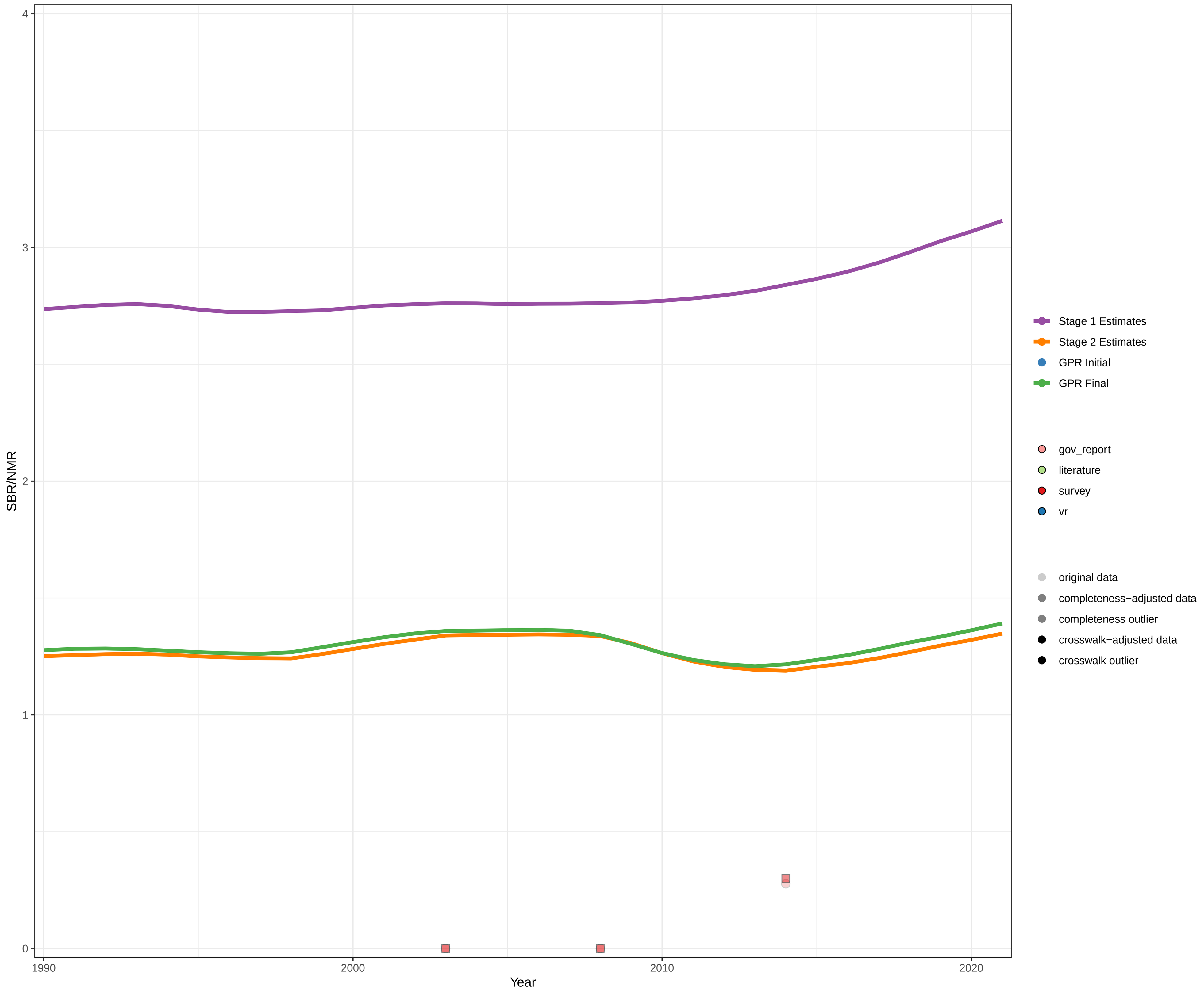

Tharaka Nithi KEN_35657 Eastern Sub−Saharan Africa Stillbirth Rate / Neonatal Mortality Rate (SBR/NMR) − 28 weeks
lambda = 1 ; zeta = 0.6 ; scale = 15 ; amp = 0.028

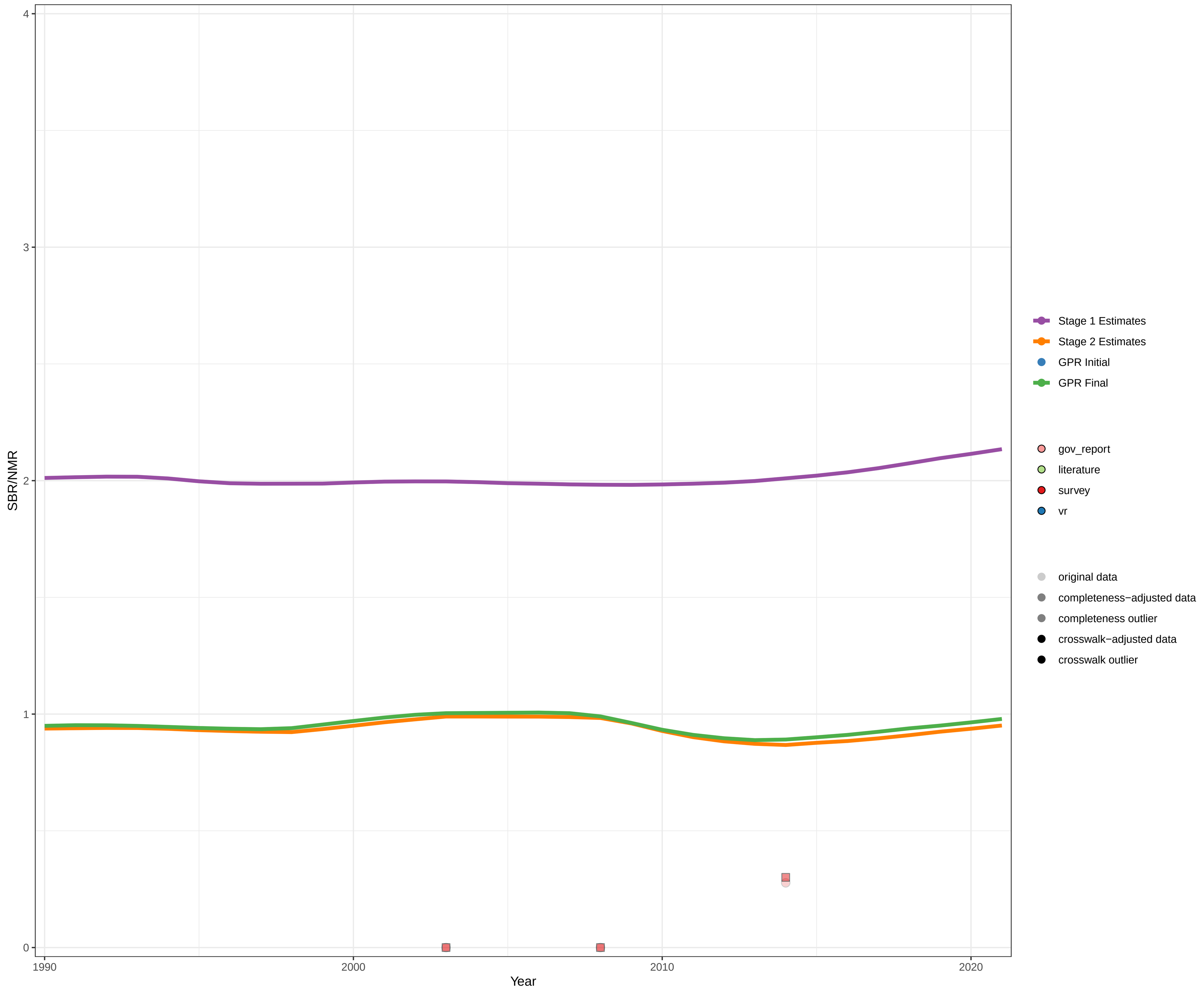

Definition	Years	Outlier	Source.Type	Title
28_weeks	2003	1	Survey	Kenya Demographic and Health Survey 2003
28_weeks	2008	1	Survey	Kenya Demographic and Health Survey 2008−2009
28_weeks	2014	1	Survey	Kenya Demographic and Health Survey 2014

Tharaka Nithi KEN_35657 Eastern Sub−Saharan Africa Stillbirth Rate (SBR) − 20 weeks
lambda = 1 ; zeta = 0.6 ; scale = 15 ; amp = 0.028

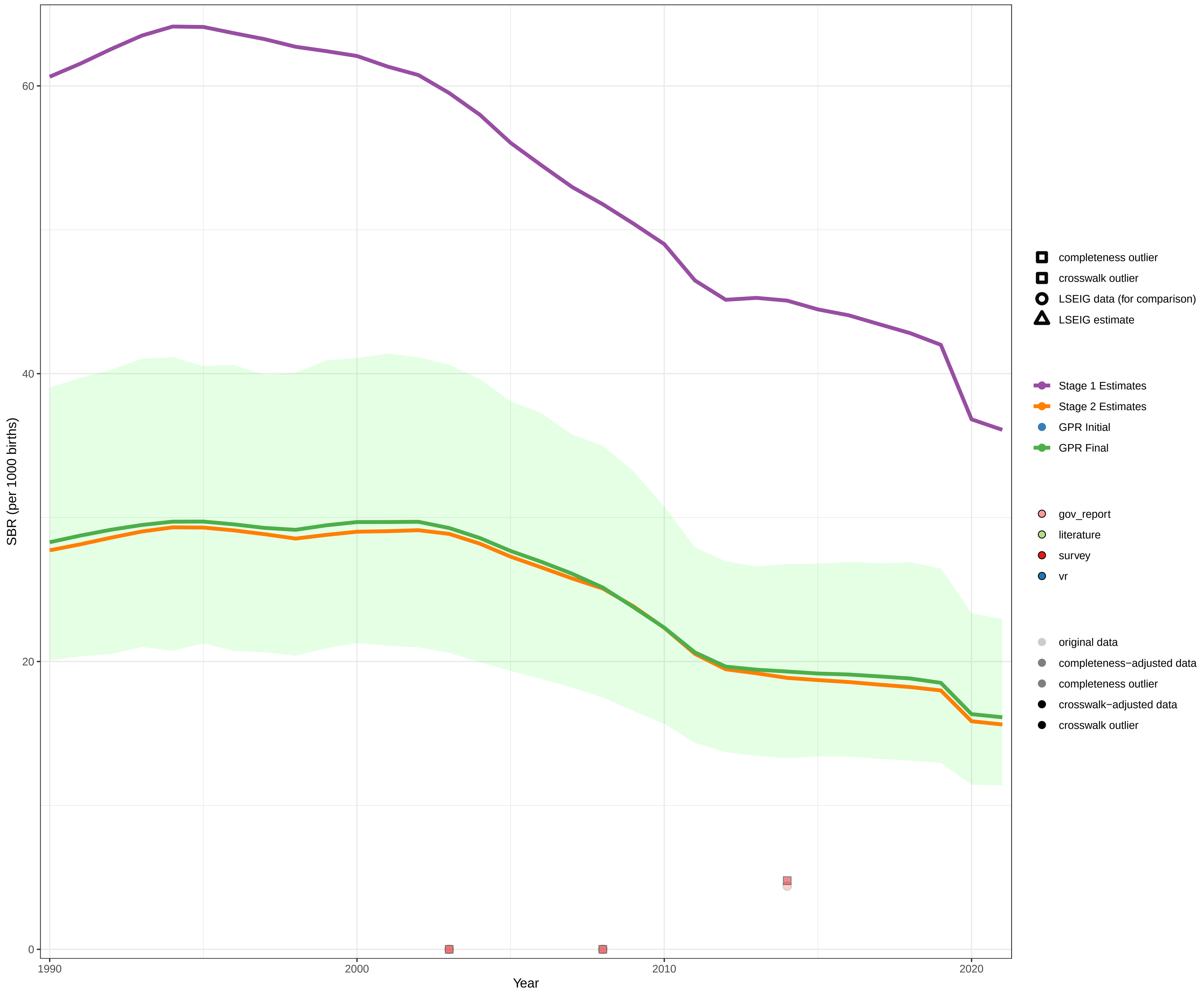

Tharaka Nithi KEN_35657 Eastern Sub−Saharan Africa Stillbirth Rate (SBR) − 28 weeks
lambda = 1 ; zeta = 0.6 ; scale = 15 ; amp = 0.028

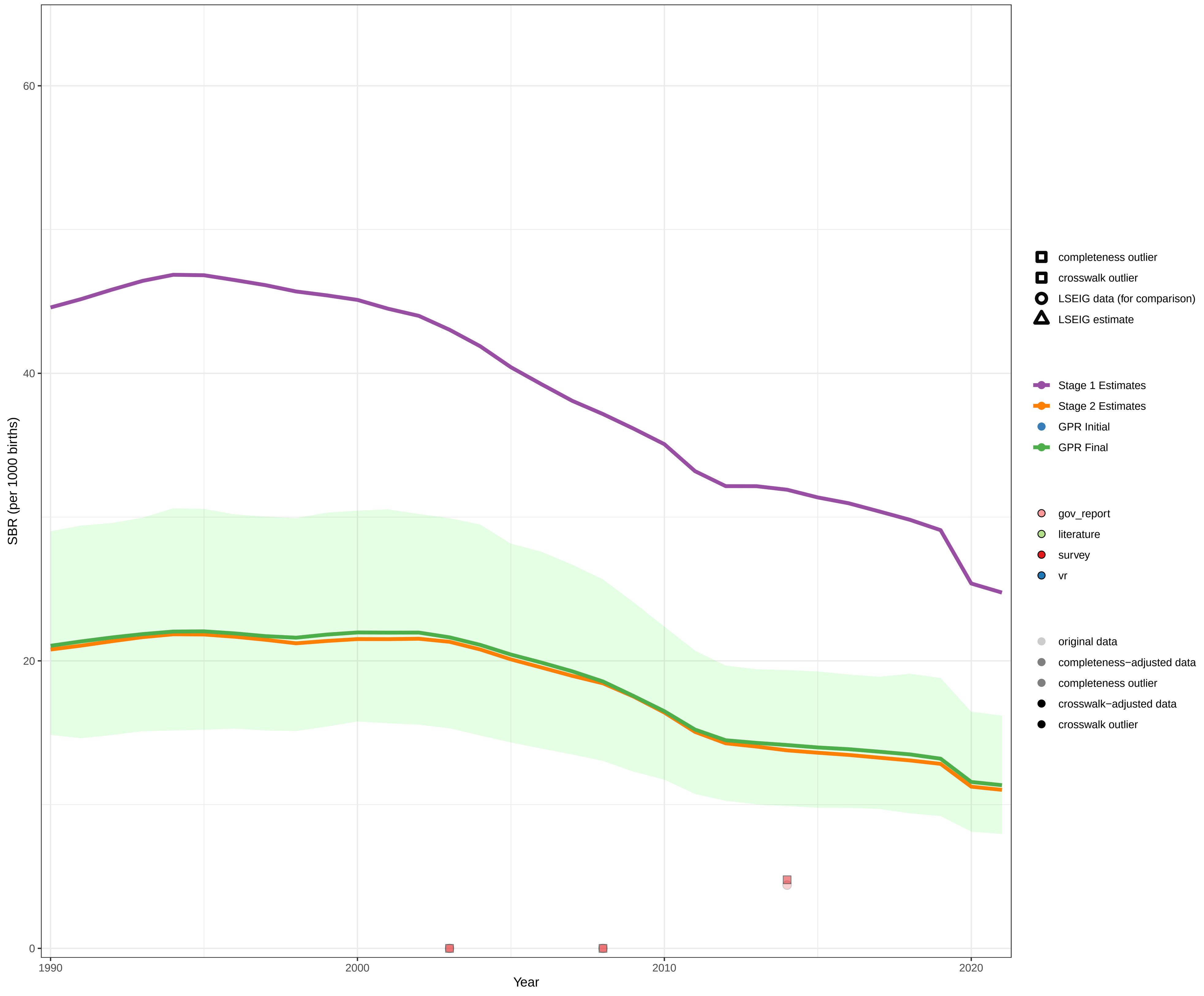

Year	Total Stillbirths (in thousands)	Total Stillbirths (in thousands)	SBR (per 1000 births)	SBR (per 1000 births)	SBR/NMR	SBR/NMR
	20 weeks	28 weeks	20 weeks	28 weeks	20 weeks	28 weeks
1990	0.1 (0.1 to 0.2)	0.1 (0.1 to 0.1)	28.3 (20.1 to 39.1)	21.0 (14.8 to 29.0)	1.28 (0.91 to 1.76)	0.95 (0.67 to 1.31)
1995	0.1 (0.1 to 0.2)	0.1 (0.1 to 0.1)	29.7 (21.3 to 40.5)	22.1 (15.2 to 30.6)	1.27 (0.91 to 1.73)	0.94 (0.65 to 1.30)
2000	0.1 (0.1 to 0.2)	0.1 (0.1 to 0.1)	29.7 (21.3 to 41.1)	22.0 (15.8 to 30.4)	1.31 (0.94 to 1.81)	0.97 (0.70 to 1.34)
2005	0.1 (0.1 to 0.2)	0.1 (0.1 to 0.1)	27.7 (19.3 to 38.1)	20.4 (14.3 to 28.2)	1.36 (0.95 to 1.87)	1.01 (0.70 to 1.39)
2010	0.1 (0.1 to 0.1)	0.1 (0.1 to 0.1)	22.4 (15.7 to 30.8)	16.5 (11.7 to 22.4)	1.26 (0.89 to 1.74)	0.93 (0.66 to 1.27)
2015	0.1 (0.1 to 0.2)	0.1 (0.1 to 0.1)	19.2 (13.4 to 26.8)	14.0 (9.8 to 19.3)	1.24 (0.86 to 1.73)	0.90 (0.63 to 1.24)
2020	0.1 (0.1 to 0.2)	0.1 (0.1 to 0.1)	16.3 (11.4 to 23.3)	11.6 (8.1 to 16.5)	1.36 (0.95 to 1.95)	0.96 (0.68 to 1.37)
2021	0.1 (0.1 to 0.2)	0.1 (0.1 to 0.1)	16.1 (11.4 to 23.0)	11.4 (8.0 to 16.2)	1.39 (0.98 to 1.98)	0.98 (0.69 to 1.40)

Trans Nzoia KEN_35658 Eastern Sub−Saharan Africa Stillbirth Rate / Neonatal Mortality Rate (SBR/NMR) − 20 weeks
lambda = 0.8 ; zeta = 0.7 ; scale = 15 ; amp = 0.028

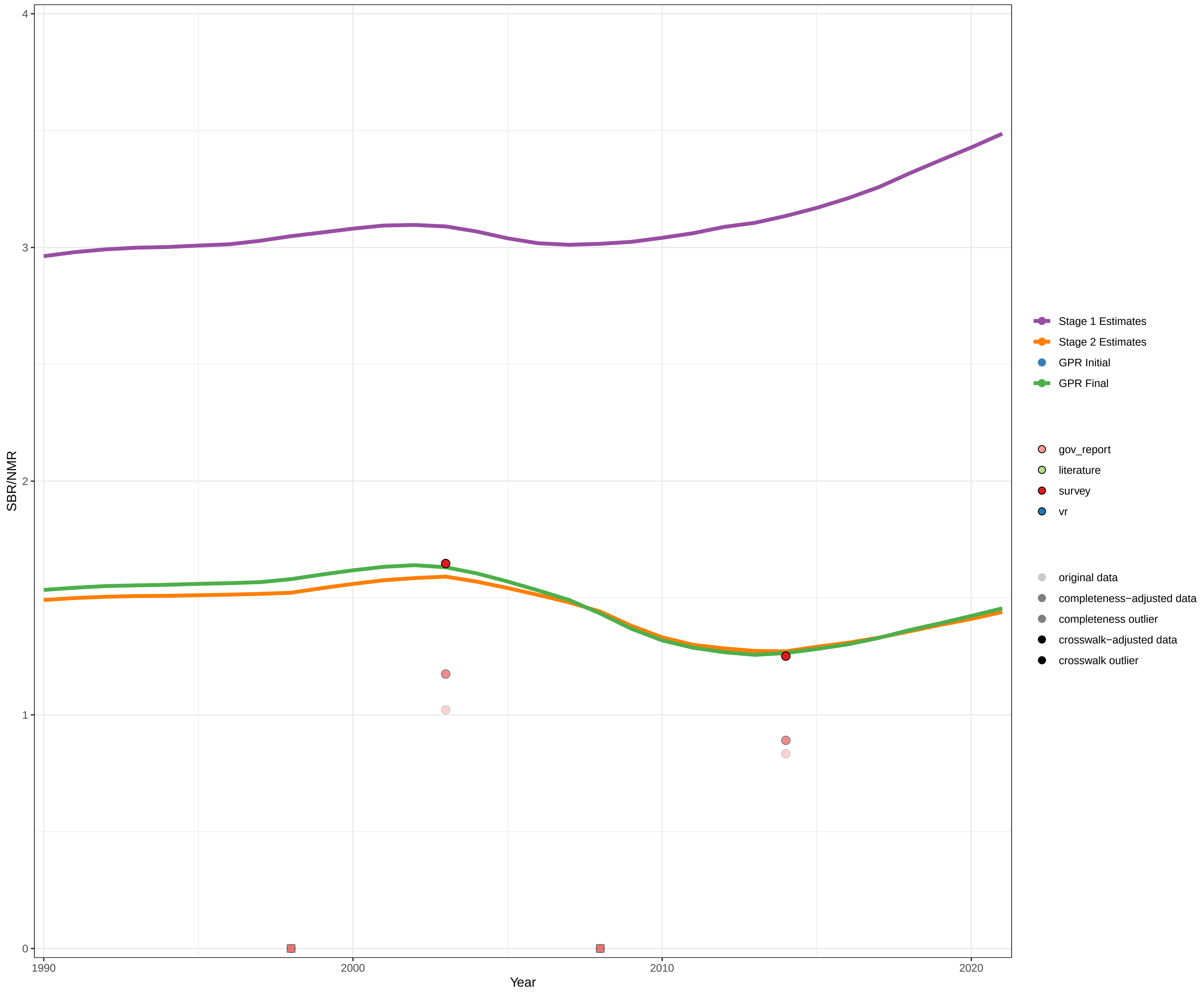

Trans Nzoia KEN_35658 Eastern Sub−Saharan Africa Stillbirth Rate / Neonatal Mortality Rate (SBR/NMR) − 28 weeks
lambda = 0.8 ; zeta = 0.7 ; scale = 15 ; amp = 0.028

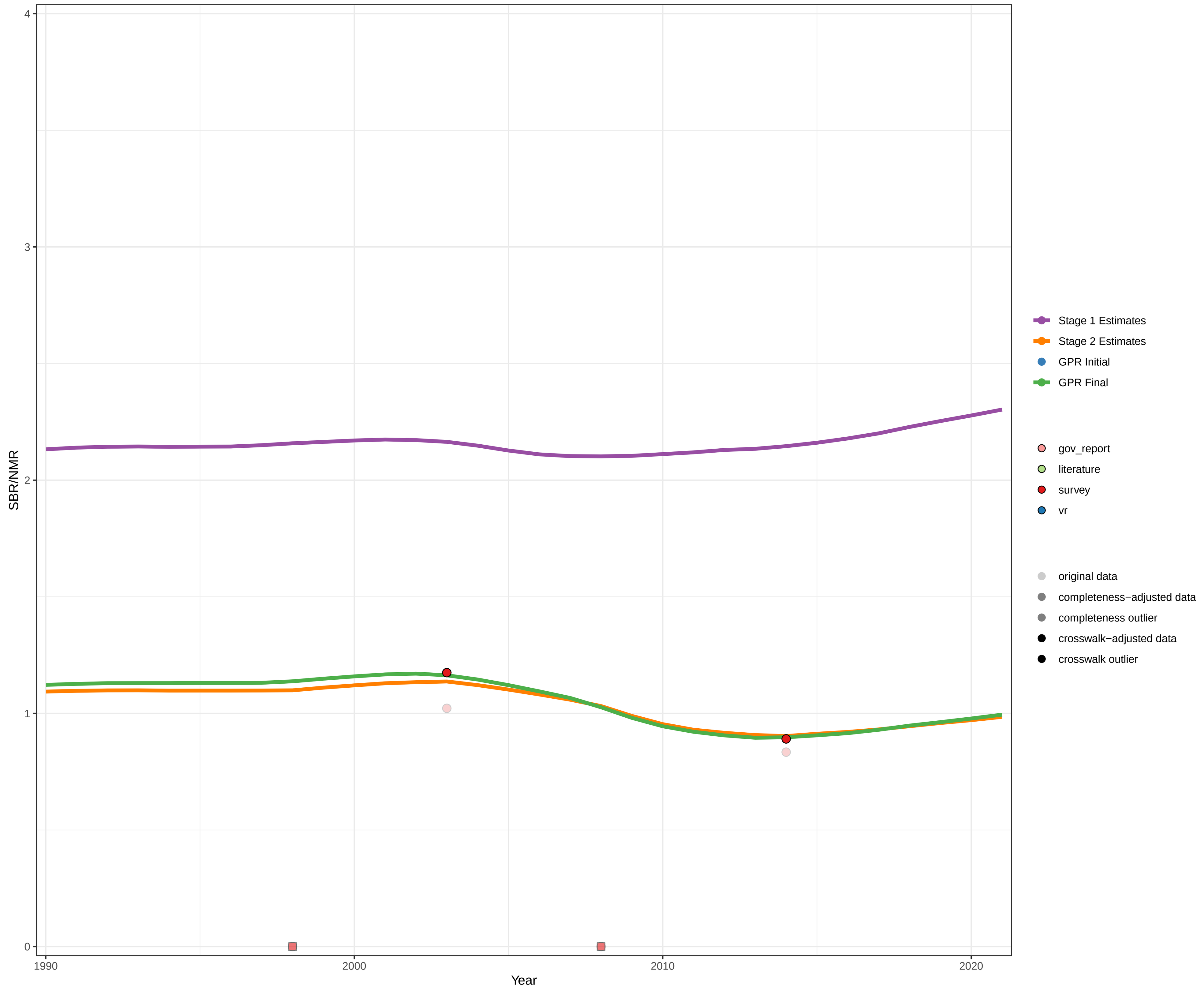

Definition	Years	Outlier	Source.Type	Title
28_weeks	2003	0	Survey	Kenya Demographic and Health Survey 2003
28_weeks	2014	0	Survey	Kenya Demographic and Health Survey 2014
28_weeks	1998	1	Survey	Kenya Demographic and Health Survey 1998
28_weeks	2008	1	Survey	Kenya Demographic and Health Survey 2008−2009

Trans Nzoia KEN_35658 Eastern Sub−Saharan Africa Stillbirth Rate (SBR) − 20 weeks
lambda = 0.8 ; zeta = 0.7 ; scale = 15 ; amp = 0.028

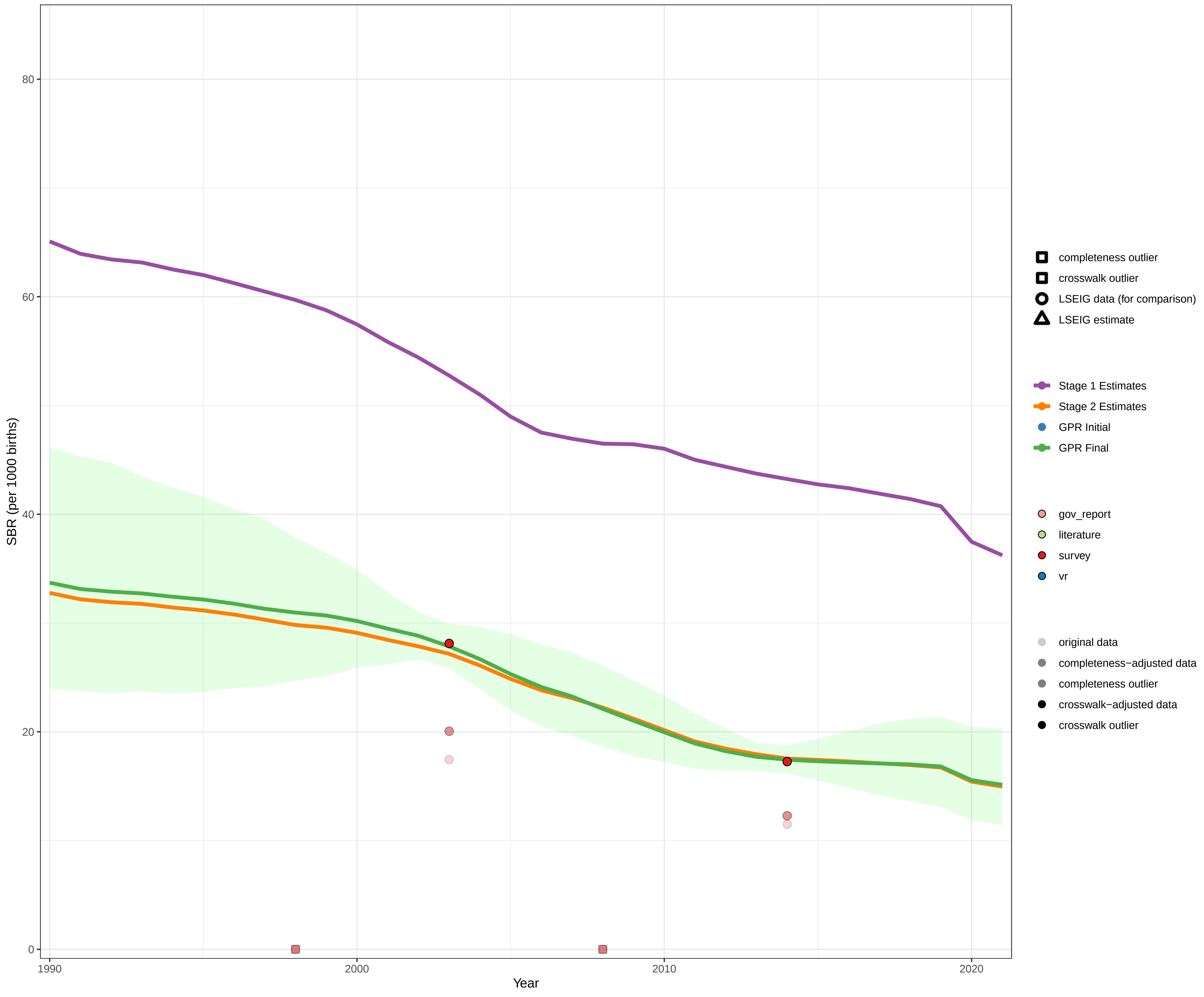

Trans Nzoia KEN_35658 Eastern Sub−Saharan Africa Stillbirth Rate (SBR) − 28 weeks
lambda = 0.8 ; zeta = 0.7 ; scale = 15 ; amp = 0.028

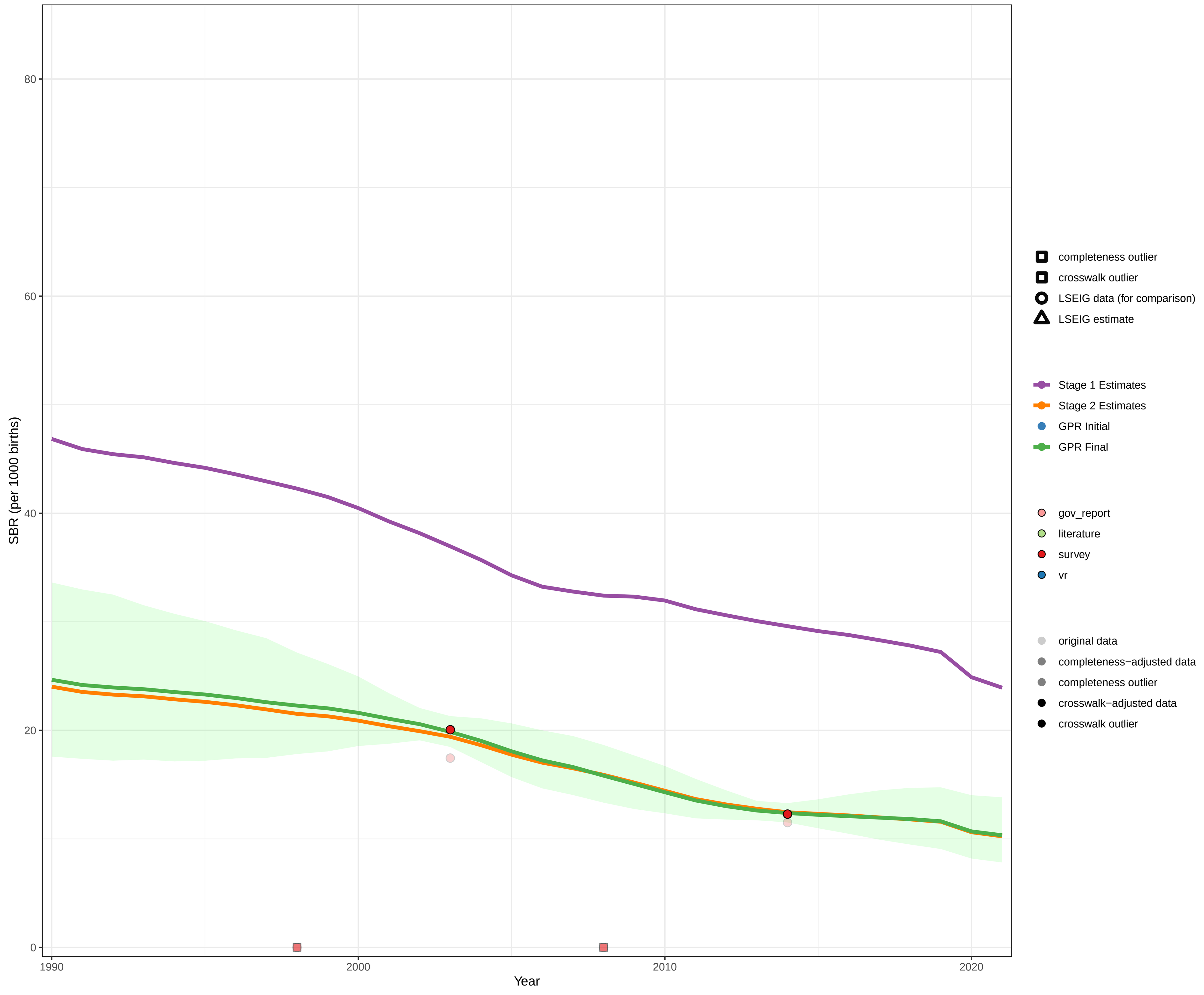

Year	Total Stillbirths (in thousands)	Total Stillbirths (in thousands)	SBR (per 1000 births)	SBR (per 1000 births)	SBR/NMR	SBR/NMR
	20 weeks	28 weeks	20 weeks	28 weeks	20 weeks	28 weeks
1990	0.7 (0.5 to 0.9)	0.5 (0.3 to 0.7)	33.7 (23.9 to 46.1)	24.7 (17.6 to 33.6)	1.53 (1.09 to 2.10)	1.12 (0.80 to 1.53)
1995	0.7 (0.5 to 0.9)	0.5 (0.4 to 0.7)	32.2 (23.7 to 41.6)	23.3 (17.2 to 30.1)	1.56 (1.15 to 2.02)	1.13 (0.83 to 1.46)
2000	0.8 (0.7 to 0.9)	0.6 (0.5 to 0.6)	30.2 (25.9 to 34.9)	21.6 (18.6 to 25.0)	1.62 (1.39 to 1.87)	1.16 (0.99 to 1.34)
2005	0.7 (0.6 to 0.8)	0.5 (0.4 to 0.6)	25.3 (21.9 to 29.0)	18.1 (15.7 to 20.6)	1.57 (1.36 to 1.80)	1.12 (0.97 to 1.28)
2010	0.6 (0.5 to 0.7)	0.4 (0.3 to 0.5)	20.0 (17.2 to 23.4)	14.3 (12.4 to 16.7)	1.32 (1.14 to 1.54)	0.94 (0.82 to 1.10)
2015	0.4 (0.4 to 0.5)	0.3 (0.3 to 0.4)	17.3 (15.5 to 19.3)	12.2 (11.0 to 13.6)	1.28 (1.15 to 1.43)	0.91 (0.81 to 1.01)
2020	0.3 (0.3 to 0.5)	0.2 (0.2 to 0.3)	15.6 (11.9 to 20.5)	10.7 (8.2 to 14.0)	1.42 (1.09 to 1.87)	0.98 (0.75 to 1.28)
2021	0.3 (0.2 to 0.4)	0.2 (0.2 to 0.3)	15.1 (11.4 to 20.3)	10.3 (7.8 to 13.8)	1.46 (1.10 to 1.95)	0.99 (0.75 to 1.33)

Turkana KEN_35659 Eastern Sub−Saharan Africa Stillbirth Rate / Neonatal Mortality Rate (SBR/NMR) − 20 weeks
lambda = 0.8 ; zeta = 0.7 ; scale = 15 ; amp = 0.028

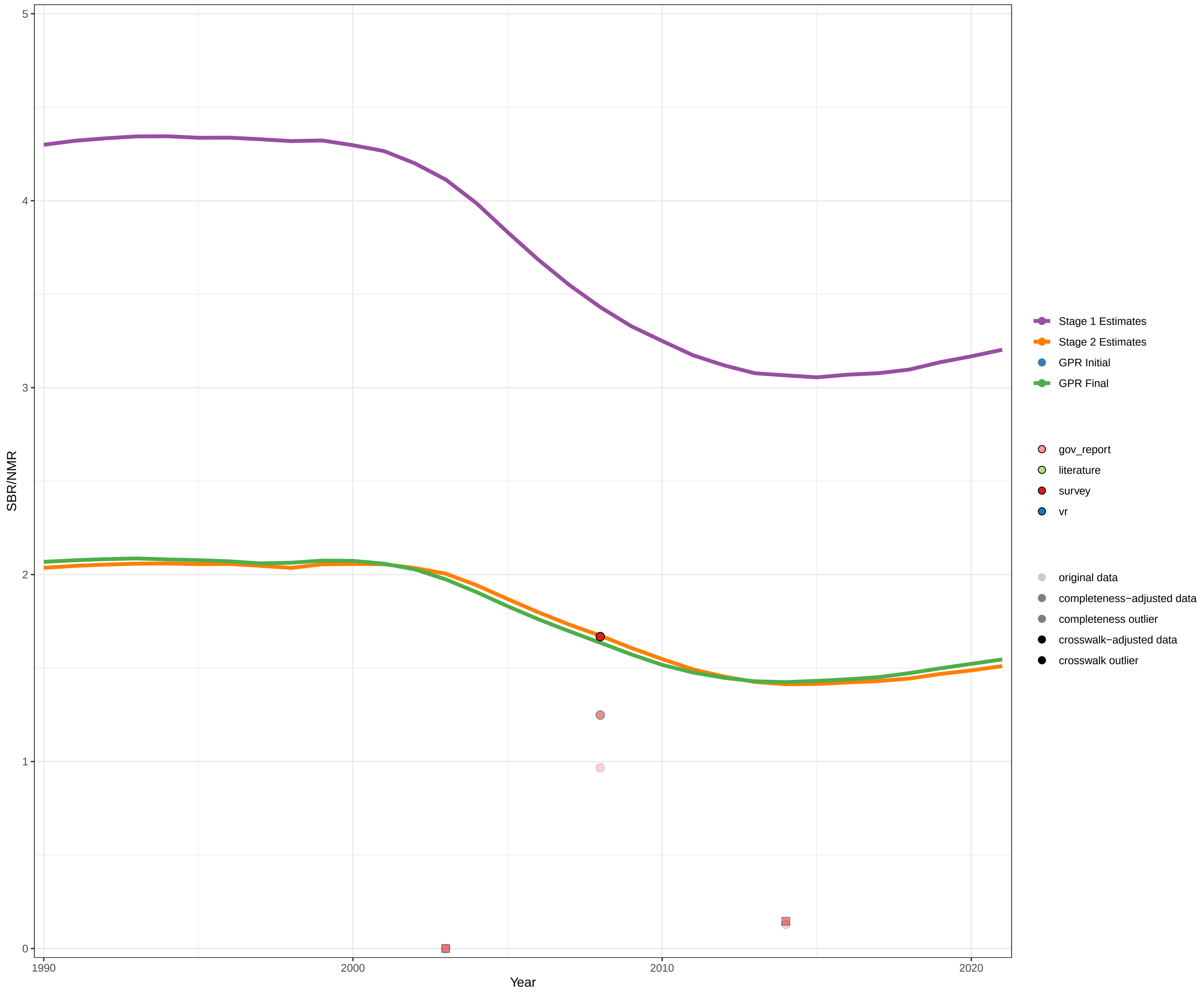

Turkana KEN_35659 Eastern Sub−Saharan Africa Stillbirth Rate / Neonatal Mortality Rate (SBR/NMR) − 28 weeks
lambda = 0.8 ; zeta = 0.7 ; scale = 15 ; amp = 0.028

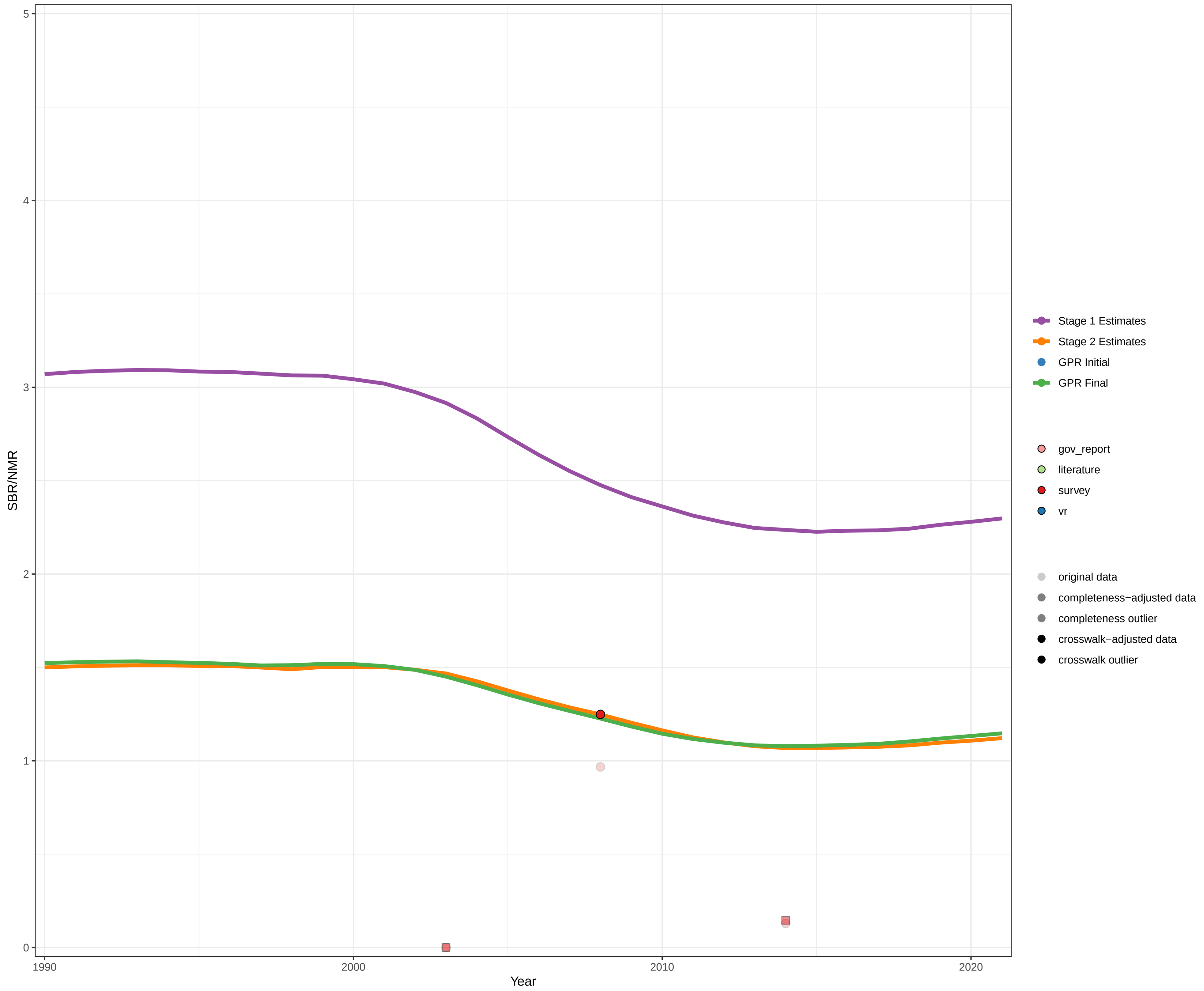

Definition	Years	Outlier	Source.Type	Title
28_weeks	2008	0	Survey	Kenya Demographic and Health Survey 2008−2009
28_weeks	2003	1	Survey	Kenya Demographic and Health Survey 2003
28_weeks	2014	1	Survey	Kenya Demographic and Health Survey 2014

Turkana KEN_35659 Eastern Sub−Saharan Africa Stillbirth Rate (SBR) − 20 weeks
lambda = 0.8 ; zeta = 0.7 ; scale = 15 ; amp = 0.028

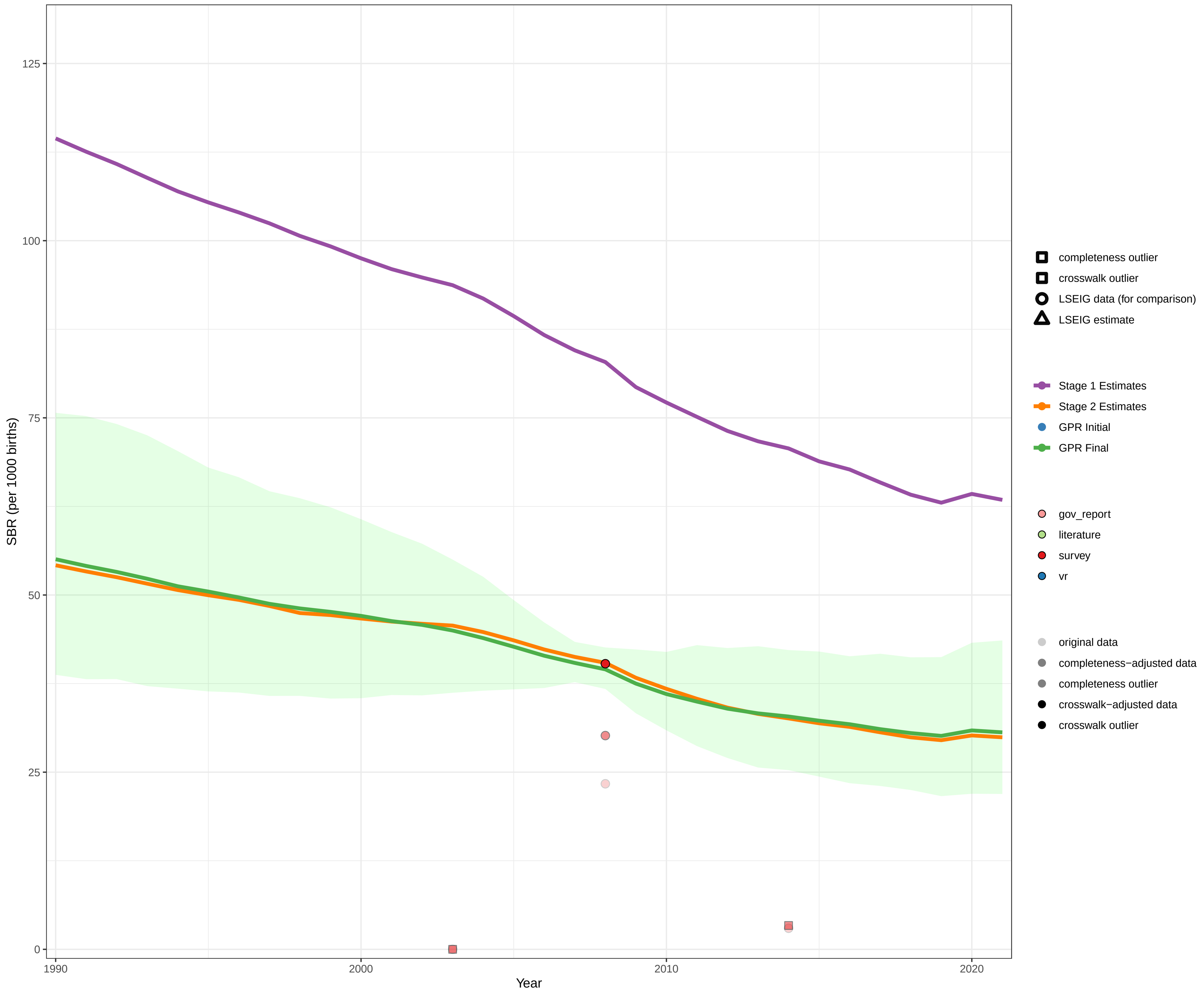

Turkana KEN_35659 Eastern Sub−Saharan Africa Stillbirth Rate (SBR) − 28 weeks
lambda = 0.8 ; zeta = 0.7 ; scale = 15 ; amp = 0.028

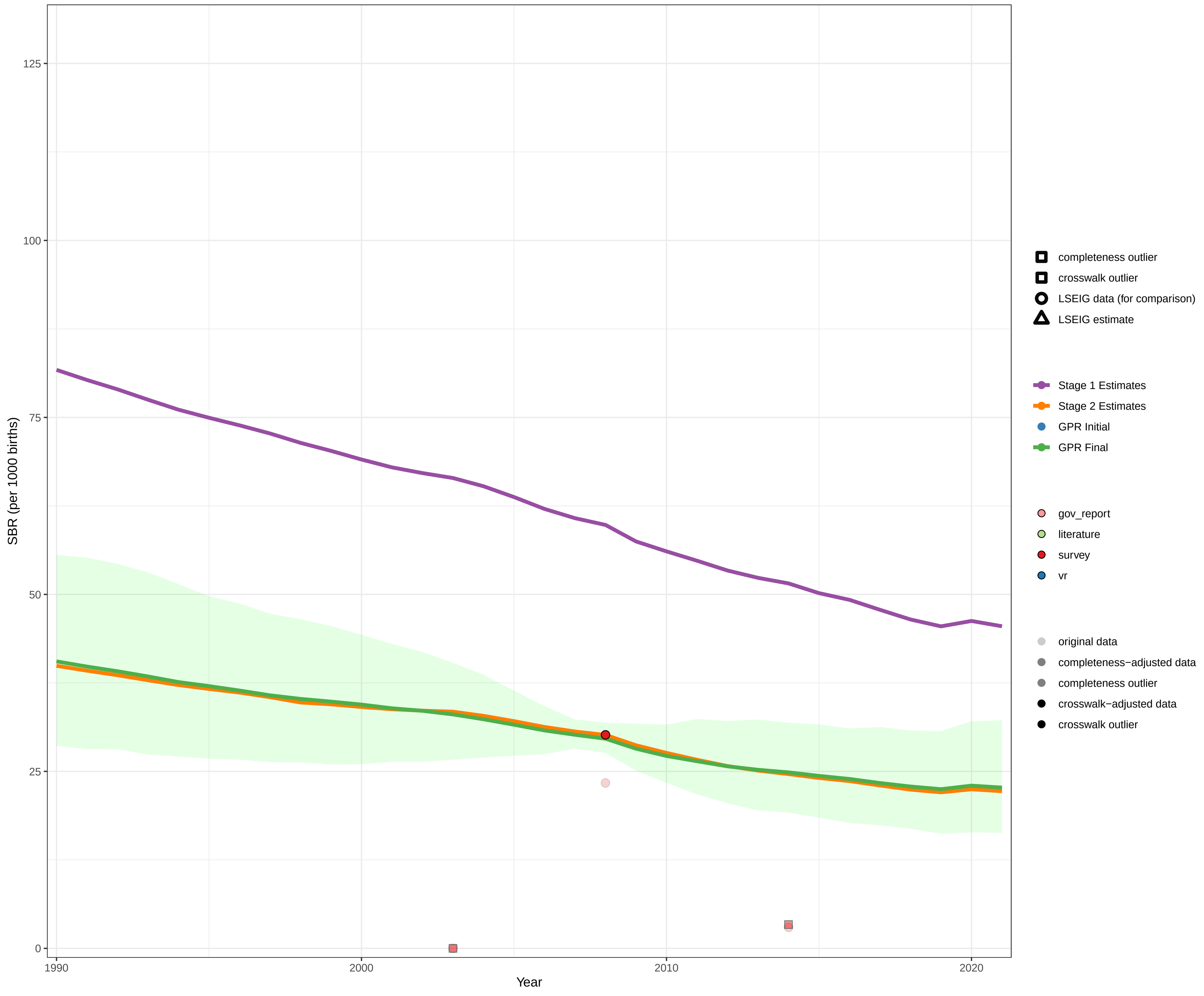

Year	Total Stillbirths (in thousands)	Total Stillbirths (in thousands)	SBR (per 1000 births)	SBR (per 1000 births)	SBR/NMR	SBR/NMR
	20 weeks	28 weeks	20 weeks	28 weeks	20 weeks	28 weeks
1990	0.7 (0.5 to 0.9)	0.5 (0.3 to 0.7)	55.0 (38.7 to 75.7)	40.5 (28.6 to 55.6)	2.07 (1.46 to 2.84)	1.52 (1.08 to 2.09)
1995	0.8 (0.6 to 1.1)	0.6 (0.4 to 0.8)	50.5 (36.4 to 68.0)	37.0 (26.8 to 49.7)	2.08 (1.50 to 2.80)	1.52 (1.10 to 2.05)
2000	1.1 (0.8 to 1.5)	0.8 (0.6 to 1.0)	47.1 (35.4 to 60.7)	34.4 (26.0 to 44.3)	2.07 (1.56 to 2.67)	1.52 (1.15 to 1.95)
2005	1.4 (1.2 to 1.6)	1.0 (0.9 to 1.2)	42.7 (36.7 to 49.3)	31.6 (27.2 to 36.4)	1.83 (1.57 to 2.11)	1.35 (1.17 to 1.56)
2010	1.4 (1.2 to 1.6)	1.0 (0.9 to 1.2)	36.0 (30.9 to 42.0)	27.2 (23.4 to 31.6)	1.52 (1.30 to 1.77)	1.14 (0.98 to 1.33)
2015	1.2 (0.9 to 1.6)	0.9 (0.7 to 1.2)	32.3 (24.4 to 42.0)	24.4 (18.5 to 31.7)	1.43 (1.08 to 1.86)	1.08 (0.82 to 1.40)
2020	1.1 (0.7 to 1.5)	0.8 (0.6 to 1.1)	30.9 (22.0 to 43.3)	23.0 (16.4 to 32.1)	1.52 (1.08 to 2.13)	1.13 (0.81 to 1.58)
2021	1.0 (0.7 to 1.5)	0.8 (0.5 to 1.1)	30.6 (21.9 to 43.6)	22.7 (16.3 to 32.2)	1.55 (1.11 to 2.20)	1.15 (0.82 to 1.63)

Uasin Gishu KEN_35660 Eastern Sub−Saharan Africa Stillbirth Rate / Neonatal Mortality Rate (SBR/NMR) − 20 weeks
lambda = 0.8 ; zeta = 0.7 ; scale = 15 ; amp = 0.028

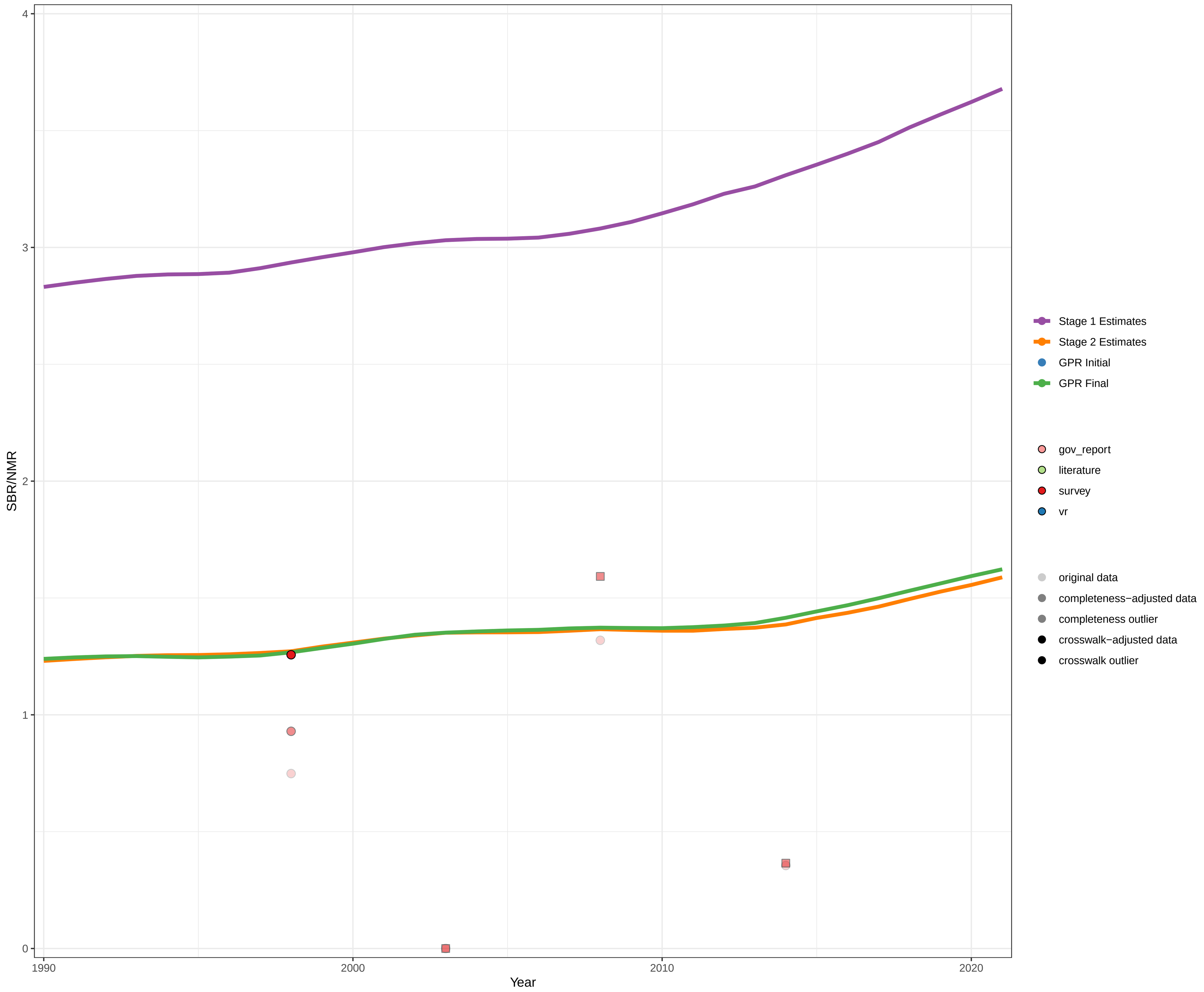

Uasin Gishu KEN_35660 Eastern Sub−Saharan Africa Stillbirth Rate / Neonatal Mortality Rate (SBR/NMR) − 28 weeks
lambda = 0.8 ; zeta = 0.7 ; scale = 15 ; amp = 0.028

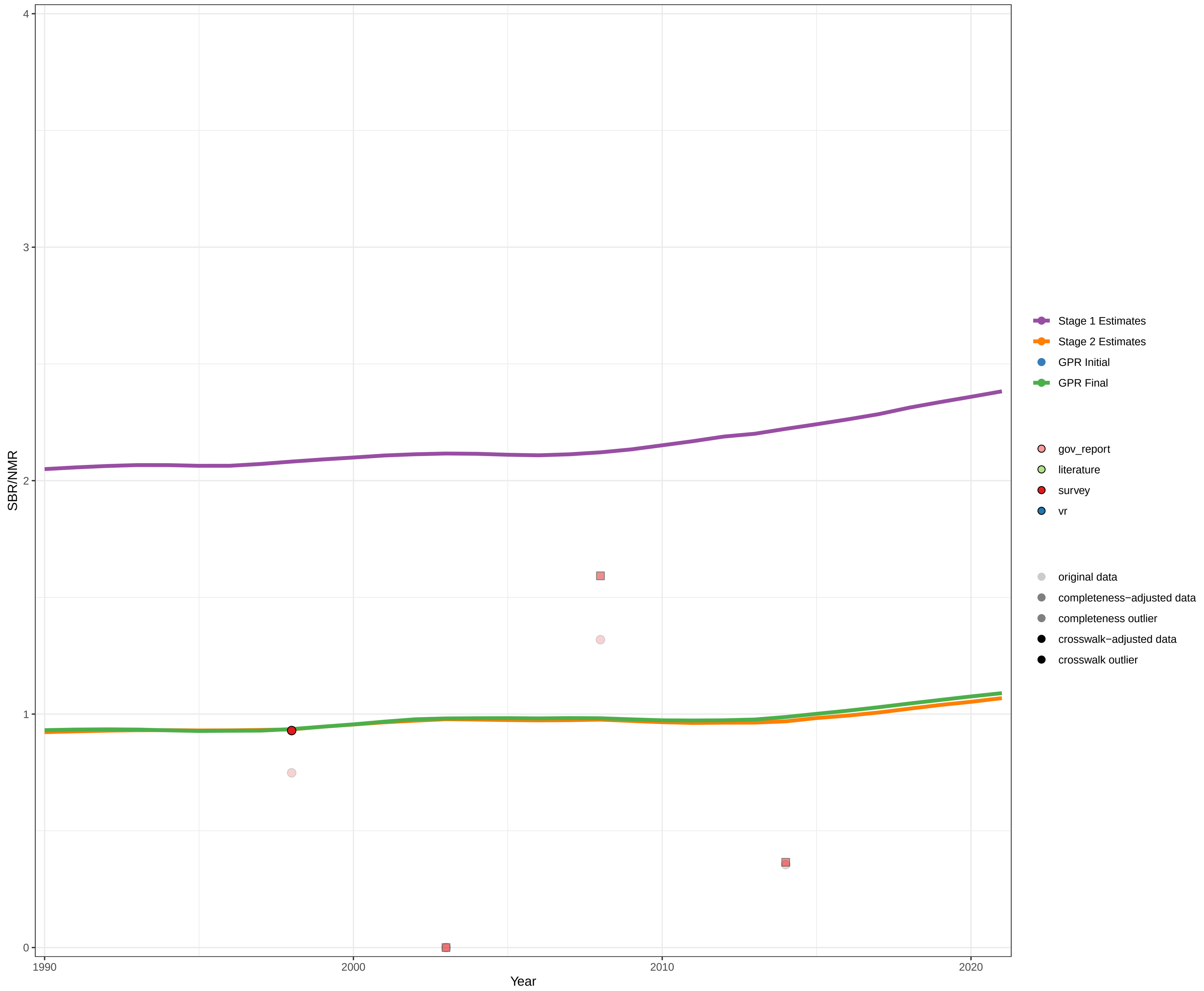

Definition	Years	Outlier	Source.Type	Title
28_weeks	1998	0	Survey	Kenya Demographic and Health Survey 1998
28_weeks	2003	1	Survey	Kenya Demographic and Health Survey 2003
28_weeks	2008	1	Survey	Kenya Demographic and Health Survey 2008−2009
28_weeks	2014	1	Survey	Kenya Demographic and Health Survey 2014

Uasin Gishu KEN_35660 Eastern Sub−Saharan Africa Stillbirth Rate (SBR) − 20 weeks
lambda = 0.8 ; zeta = 0.7 ; scale = 15 ; amp = 0.028

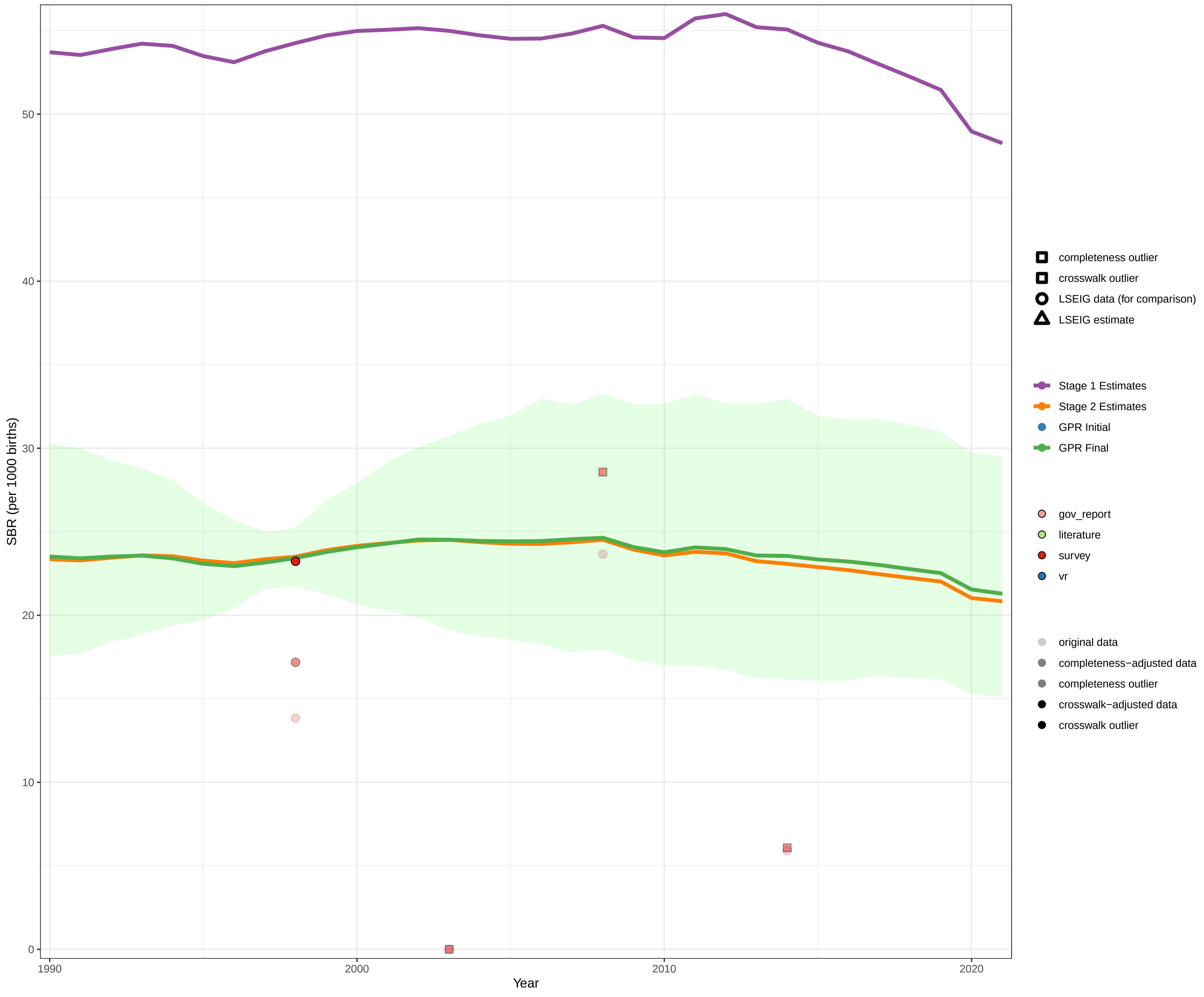

Uasin Gishu KEN_35660 Eastern Sub−Saharan Africa Stillbirth Rate (SBR) − 28 weeks
lambda = 0.8 ; zeta = 0.7 ; scale = 15 ; amp = 0.028

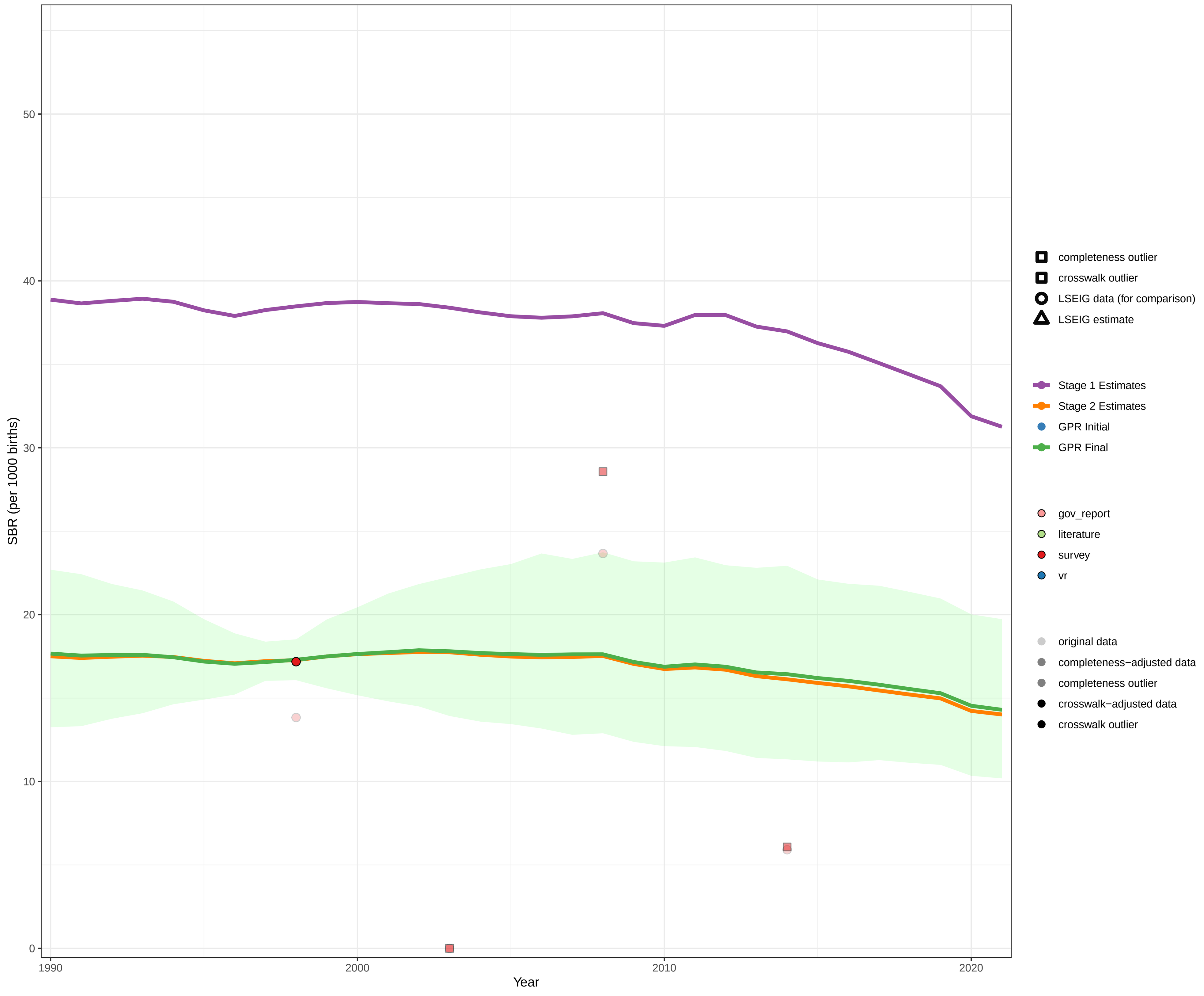

Year	Total Stillbirths (in thousands)	Total Stillbirths (in thousands)	SBR (per 1000 births)	SBR (per 1000 births)	SBR/NMR	SBR/NMR
	20 weeks	28 weeks	20 weeks	28 weeks	20 weeks	28 weeks
1990	0.5 (0.4 to 0.7)	0.4 (0.3 to 0.5)	23.5 (17.6 to 30.3)	17.7 (13.3 to 22.7)	1.24 (0.93 to 1.60)	0.93 (0.70 to 1.20)
1995	0.6 (0.5 to 0.7)	0.4 (0.4 to 0.5)	23.1 (19.7 to 26.7)	17.2 (14.9 to 19.7)	1.25 (1.06 to 1.44)	0.93 (0.80 to 1.06)
2000	0.7 (0.6 to 0.9)	0.5 (0.5 to 0.6)	24.1 (20.7 to 27.9)	17.6 (15.2 to 20.4)	1.30 (1.12 to 1.51)	0.96 (0.82 to 1.11)
2005	0.9 (0.6 to 1.1)	0.6 (0.5 to 0.8)	24.4 (18.6 to 32.0)	17.6 (13.4 to 23.0)	1.36 (1.03 to 1.78)	0.98 (0.75 to 1.28)
2010	0.9 (0.6 to 1.2)	0.6 (0.4 to 0.9)	23.8 (17.0 to 32.7)	16.9 (12.1 to 23.1)	1.37 (0.98 to 1.88)	0.97 (0.70 to 1.33)
2015	0.9 (0.6 to 1.2)	0.6 (0.4 to 0.8)	23.3 (16.1 to 31.9)	16.2 (11.2 to 22.1)	1.44 (0.99 to 1.97)	1.00 (0.69 to 1.37)
2020	0.8 (0.5 to 1.1)	0.5 (0.4 to 0.7)	21.5 (15.3 to 29.7)	14.5 (10.3 to 20.0)	1.59 (1.13 to 2.20)	1.08 (0.77 to 1.48)
2021	0.7 (0.5 to 1.0)	0.5 (0.3 to 0.7)	21.3 (15.1 to 29.5)	14.3 (10.2 to 19.7)	1.62 (1.15 to 2.25)	1.09 (0.78 to 1.50)

Vihiga KEN_35661 Eastern Sub−Saharan Africa Stillbirth Rate / Neonatal Mortality Rate (SBR/NMR) − 20 weeks
lambda = 0.8 ; zeta = 0.7 ; scale = 15 ; amp = 0.028

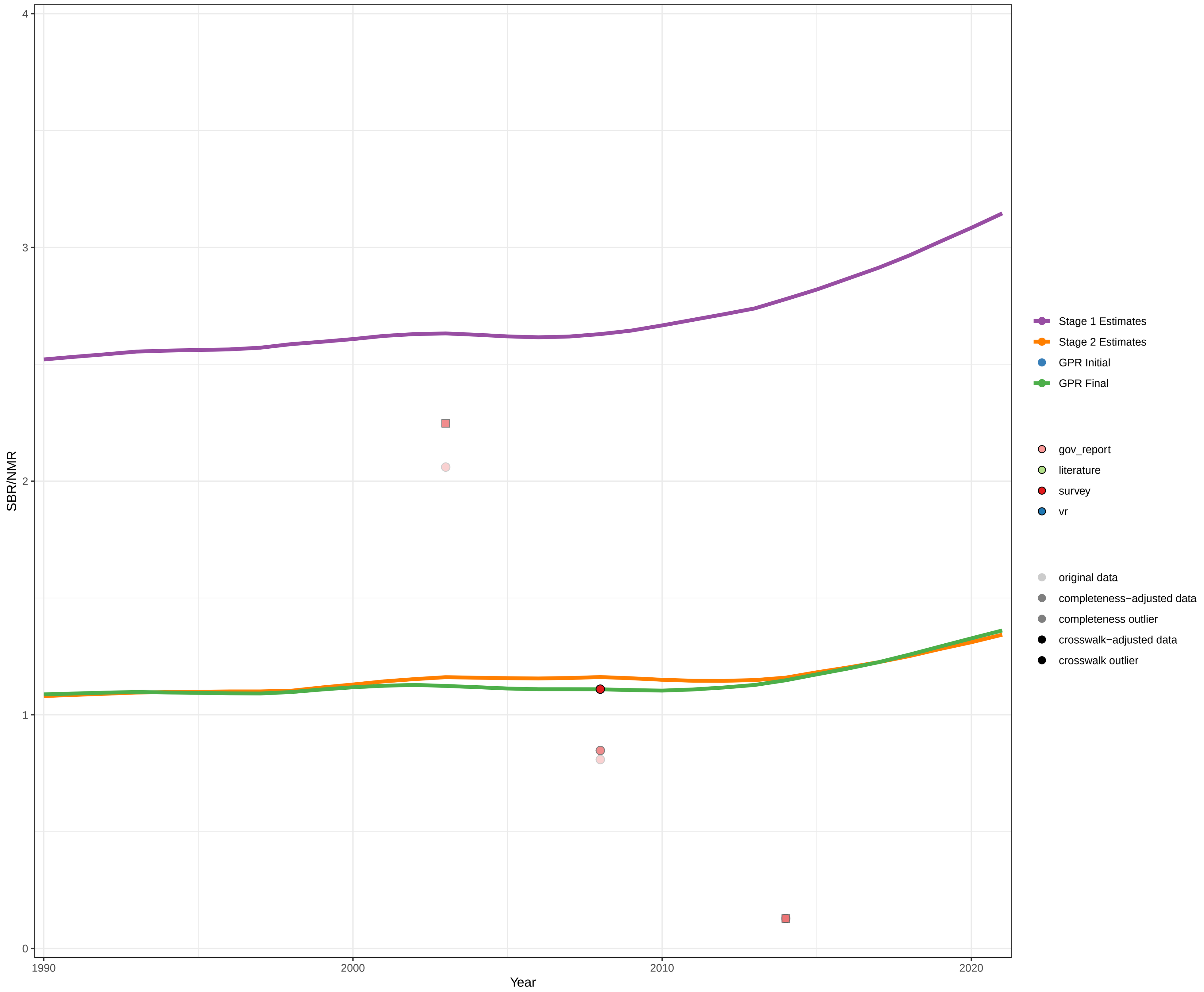

Vihiga KEN_35661 Eastern Sub−Saharan Africa Stillbirth Rate / Neonatal Mortality Rate (SBR/NMR) − 28 weeks
lambda = 0.8 ; zeta = 0.7 ; scale = 15 ; amp = 0.028

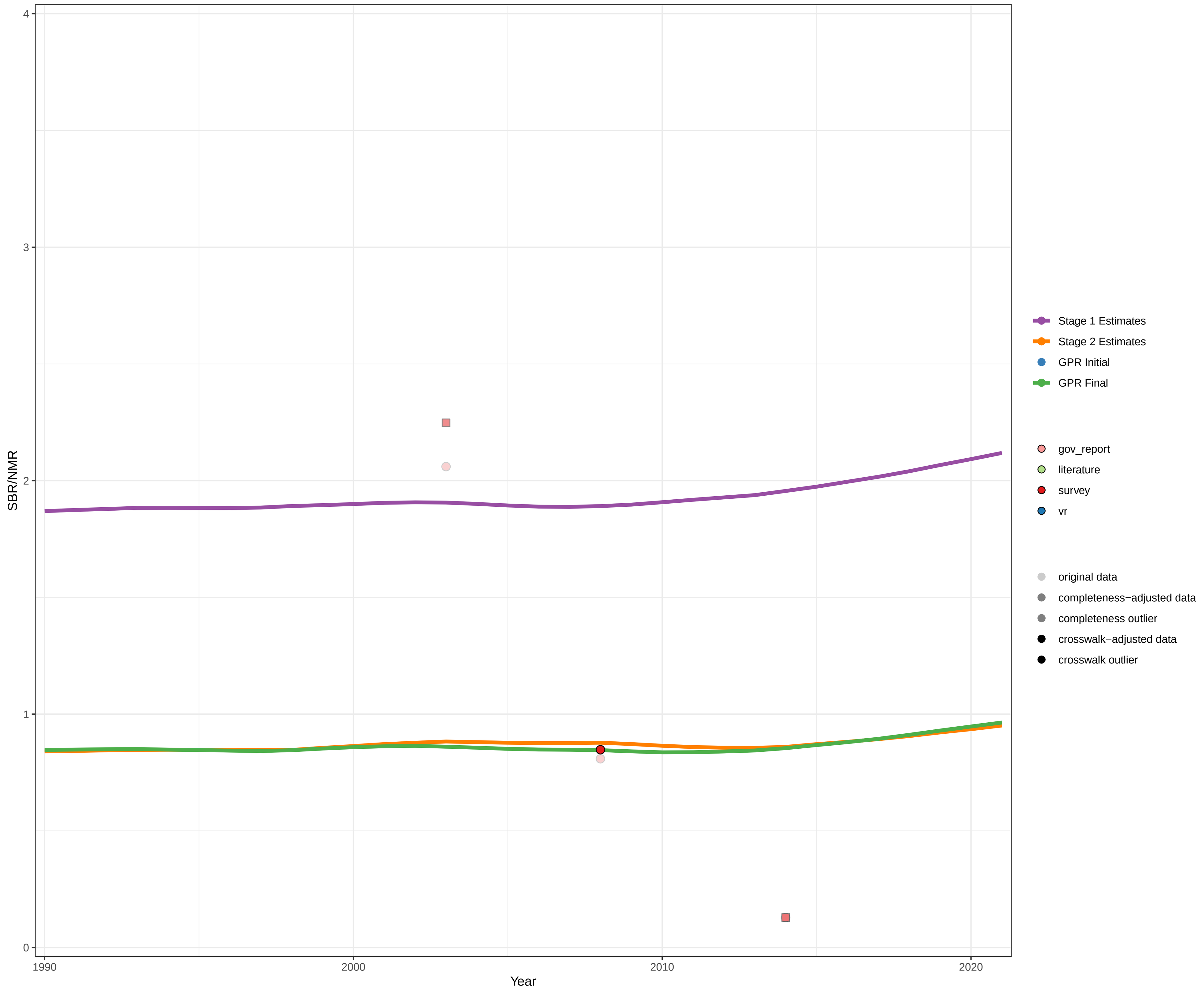

Definition	Years	Outlier	Source.Type	Title
28_weeks	2008	0	Survey	Kenya Demographic and Health Survey 2008−2009
28_weeks	2003	1	Survey	Kenya Demographic and Health Survey 2003
28_weeks	2014	1	Survey	Kenya Demographic and Health Survey 2014

Vihiga KEN_35661 Eastern Sub−Saharan Africa Stillbirth Rate (SBR) − 20 weeks
lambda = 0.8 ; zeta = 0.7 ; scale = 15 ; amp = 0.028

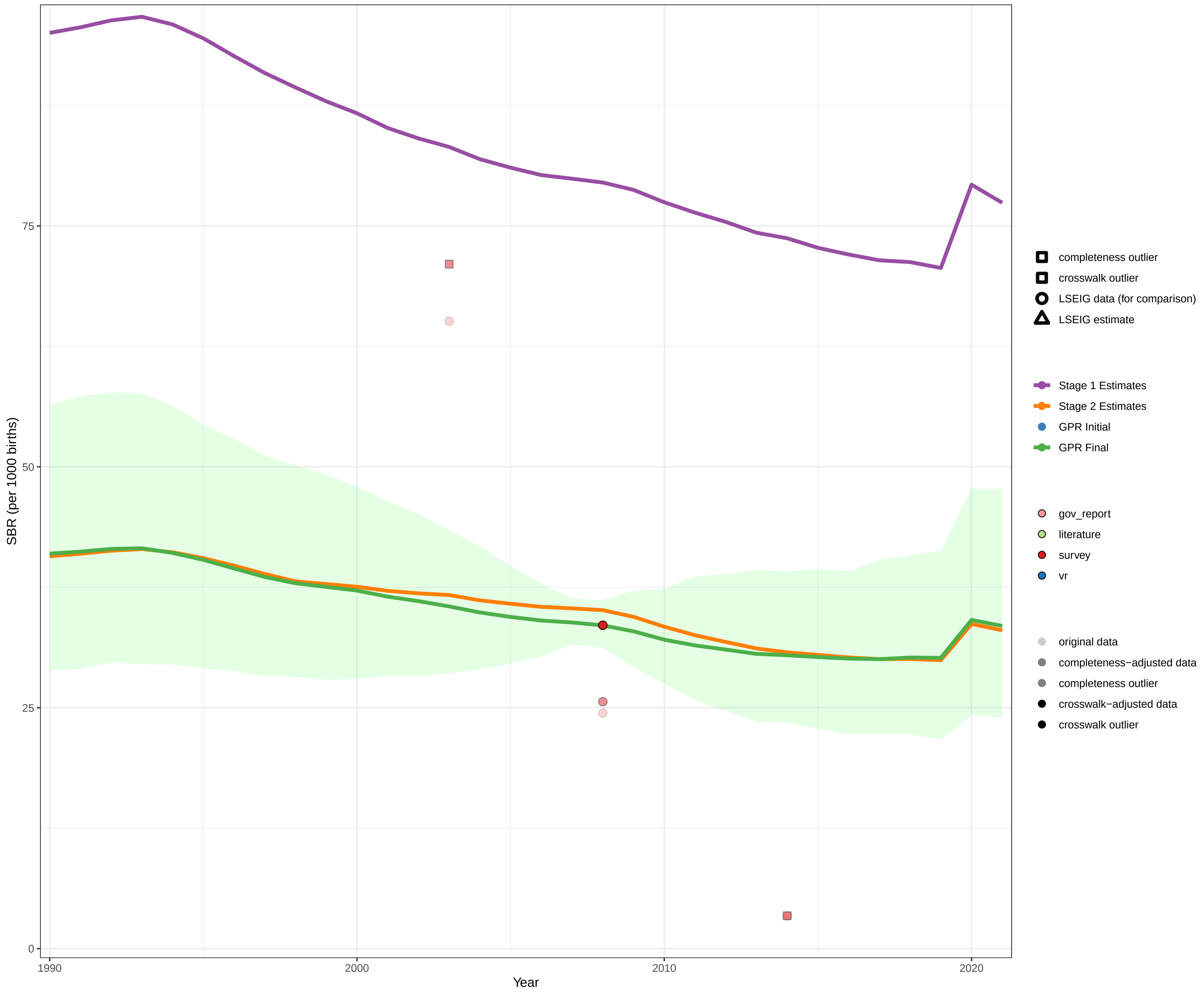

Vihiga KEN_35661 Eastern Sub−Saharan Africa Stillbirth Rate (SBR) − 28 weeks
lambda = 0.8 ; zeta = 0.7 ; scale = 15 ; amp = 0.028

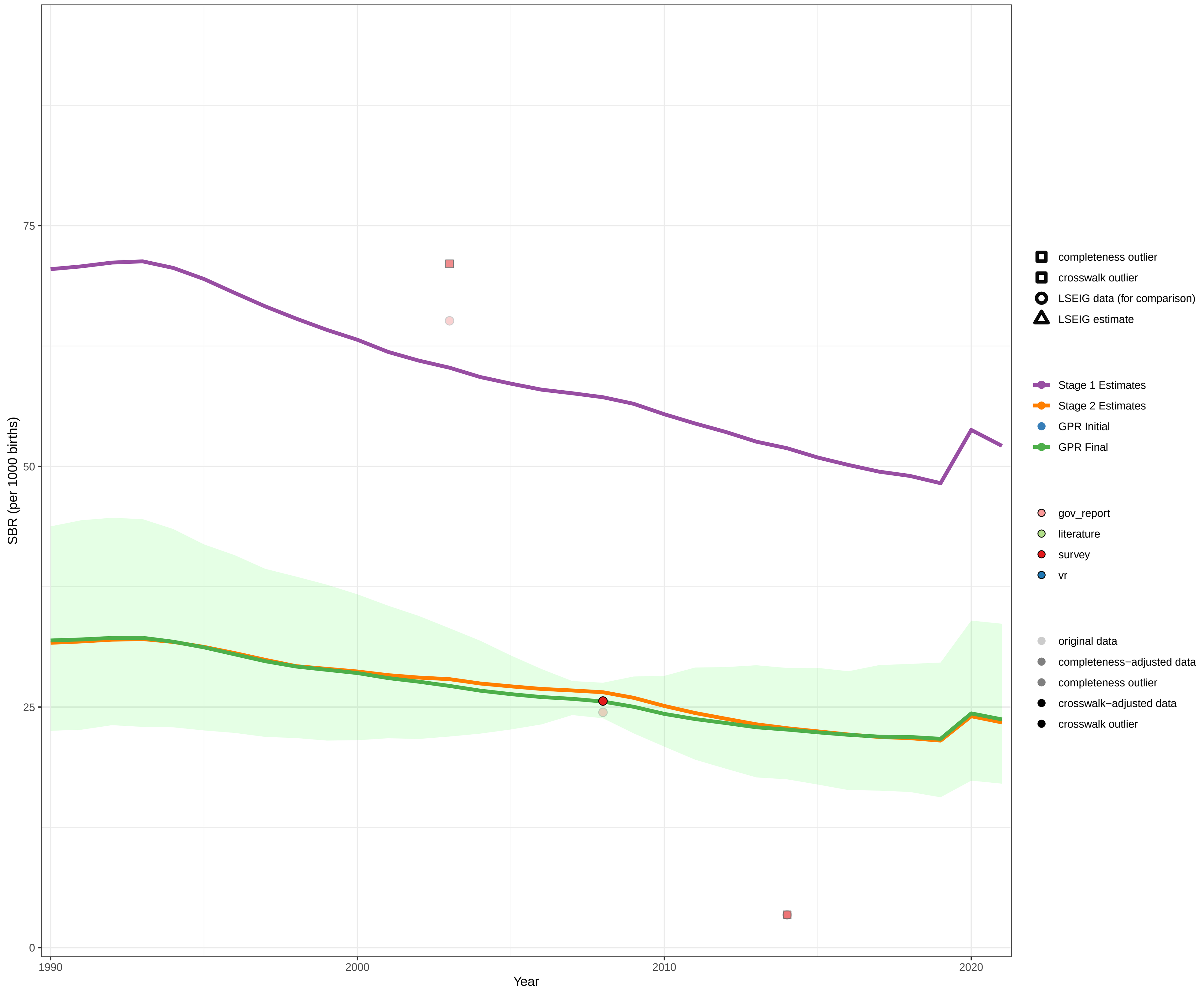

Year	Total Stillbirths (in thousands)	Total Stillbirths (in thousands)	SBR (per 1000 births)	SBR (per 1000 births)	SBR/NMR	SBR/NMR
	20 weeks	28 weeks	20 weeks	28 weeks	20 weeks	28 weeks
1990	0.7 (0.5 to 1.0)	0.5 (0.4 to 0.7)	41.0 (28.8 to 56.4)	31.9 (22.5 to 43.8)	1.09 (0.77 to 1.50)	0.85 (0.60 to 1.16)
1995	0.6 (0.5 to 0.9)	0.5 (0.3 to 0.7)	40.3 (29.1 to 54.3)	31.2 (22.6 to 41.9)	1.09 (0.79 to 1.47)	0.85 (0.61 to 1.14)
2000	0.5 (0.4 to 0.7)	0.4 (0.3 to 0.5)	37.2 (28.0 to 47.9)	28.5 (21.6 to 36.7)	1.12 (0.84 to 1.44)	0.86 (0.65 to 1.10)
2005	0.5 (0.5 to 0.6)	0.4 (0.4 to 0.5)	34.4 (29.6 to 39.7)	26.3 (22.7 to 30.4)	1.11 (0.96 to 1.28)	0.85 (0.73 to 0.98)
2010	0.5 (0.4 to 0.6)	0.4 (0.3 to 0.4)	32.1 (27.5 to 37.4)	24.3 (20.9 to 28.2)	1.10 (0.95 to 1.29)	0.84 (0.72 to 0.97)
2015	0.4 (0.3 to 0.5)	0.3 (0.2 to 0.4)	30.3 (22.9 to 39.4)	22.4 (16.9 to 29.1)	1.17 (0.89 to 1.53)	0.87 (0.66 to 1.13)
2020	0.3 (0.2 to 0.4)	0.2 (0.2 to 0.3)	34.1 (24.2 to 47.8)	24.3 (17.4 to 34.0)	1.33 (0.94 to 1.86)	0.95 (0.68 to 1.32)
2021	0.3 (0.2 to 0.4)	0.2 (0.1 to 0.3)	33.5 (24.0 to 47.7)	23.7 (17.0 to 33.7)	1.36 (0.97 to 1.94)	0.96 (0.69 to 1.37)

Wajir KEN_35662 Eastern Sub−Saharan Africa Stillbirth Rate / Neonatal Mortality Rate (SBR/NMR) − 20 weeks
lambda = 0.8 ; zeta = 0.7 ; scale = 15 ; amp = 0.028

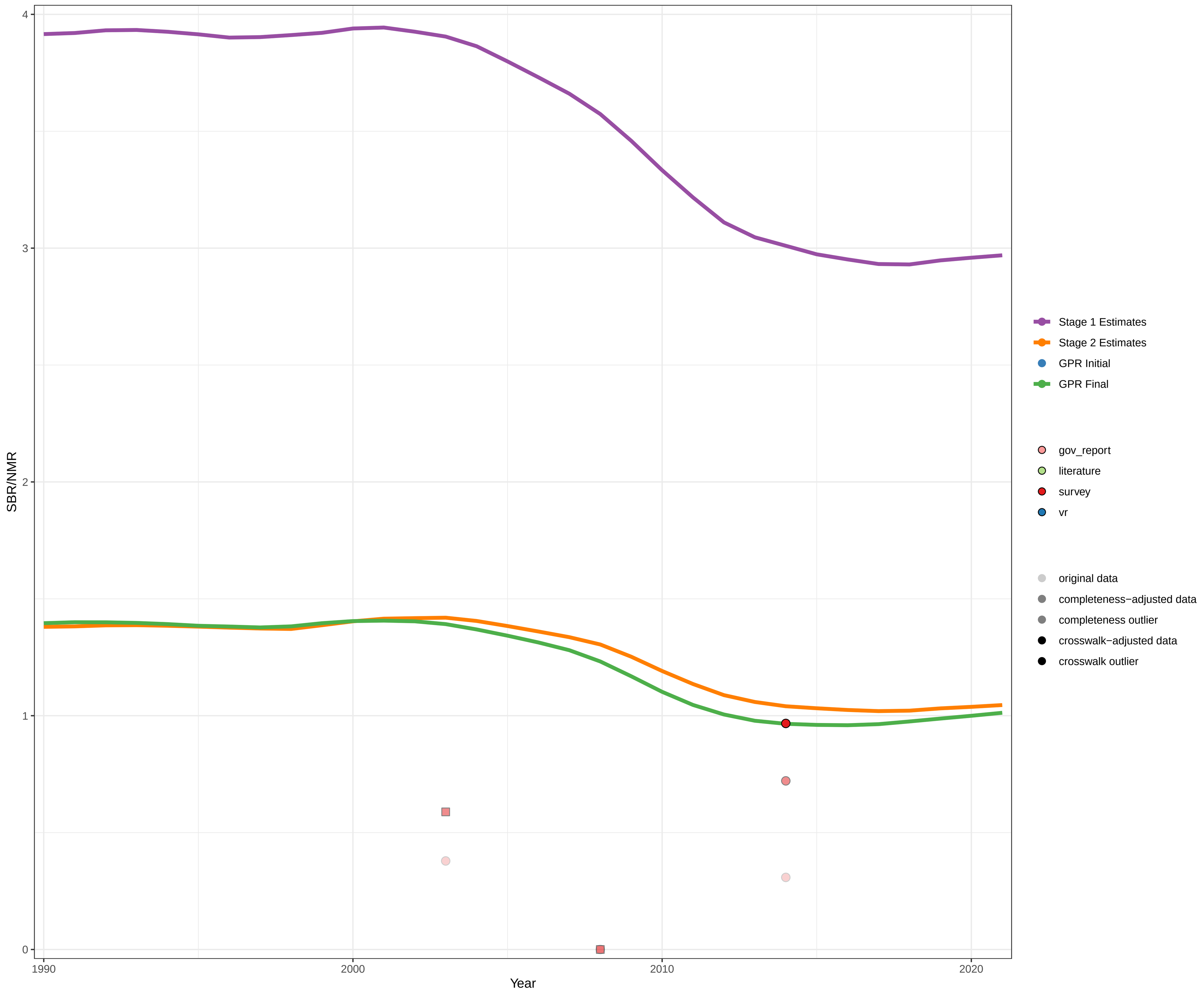

Wajir KEN_35662 Eastern Sub−Saharan Africa Stillbirth Rate / Neonatal Mortality Rate (SBR/NMR) − 28 weeks
lambda = 0.8 ; zeta = 0.7 ; scale = 15 ; amp = 0.028

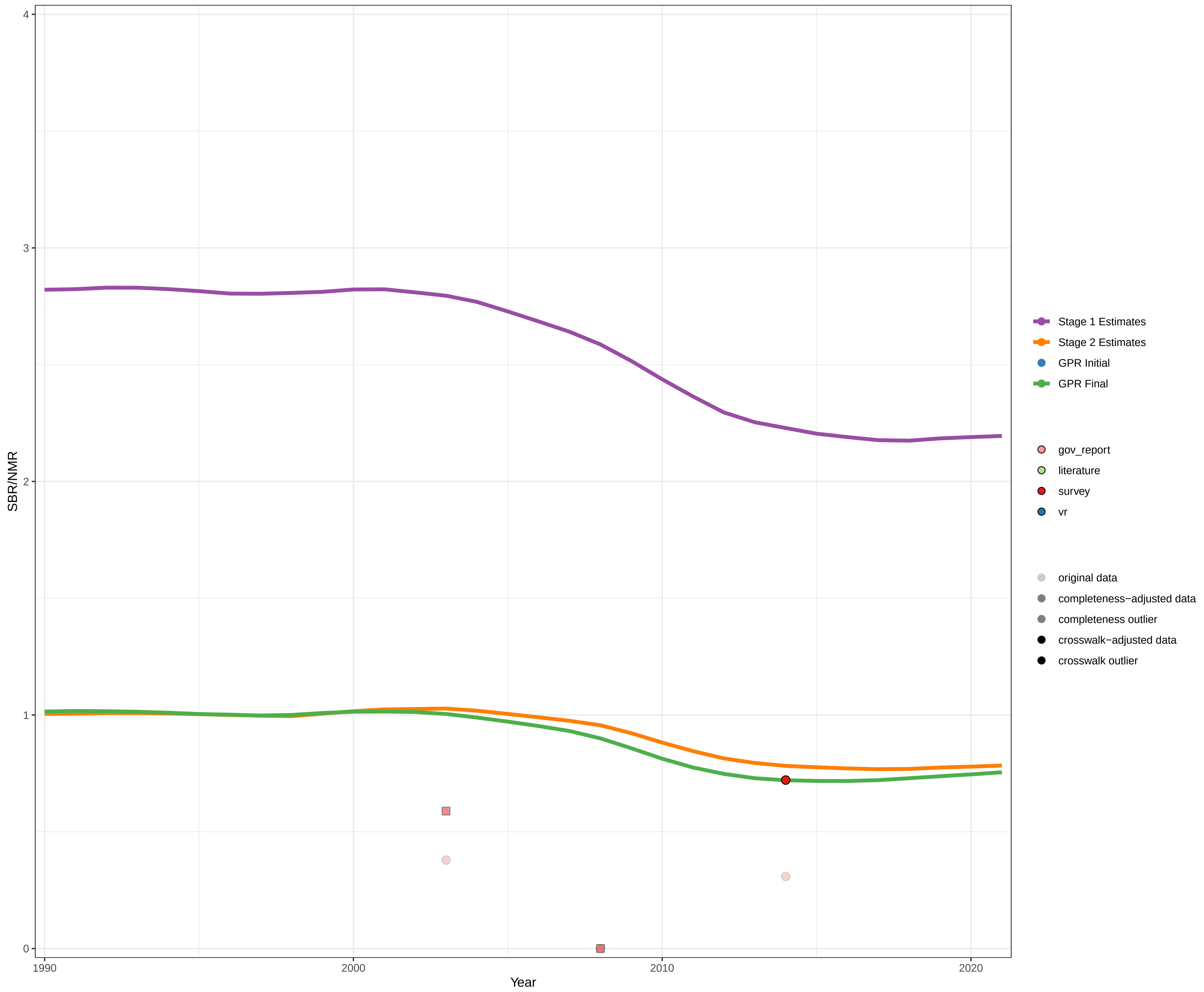

Definition	Years	Outlier	Source.Type	Title
28_weeks	2014	0	Survey	Kenya Demographic and Health Survey 2014
28_weeks	2003	1	Survey	Kenya Demographic and Health Survey 2003
28_weeks	2008	1	Survey	Kenya Demographic and Health Survey 2008−2009

Wajir KEN_35662 Eastern Sub−Saharan Africa Stillbirth Rate (SBR) − 20 weeks
lambda = 0.8 ; zeta = 0.7 ; scale = 15 ; amp = 0.028

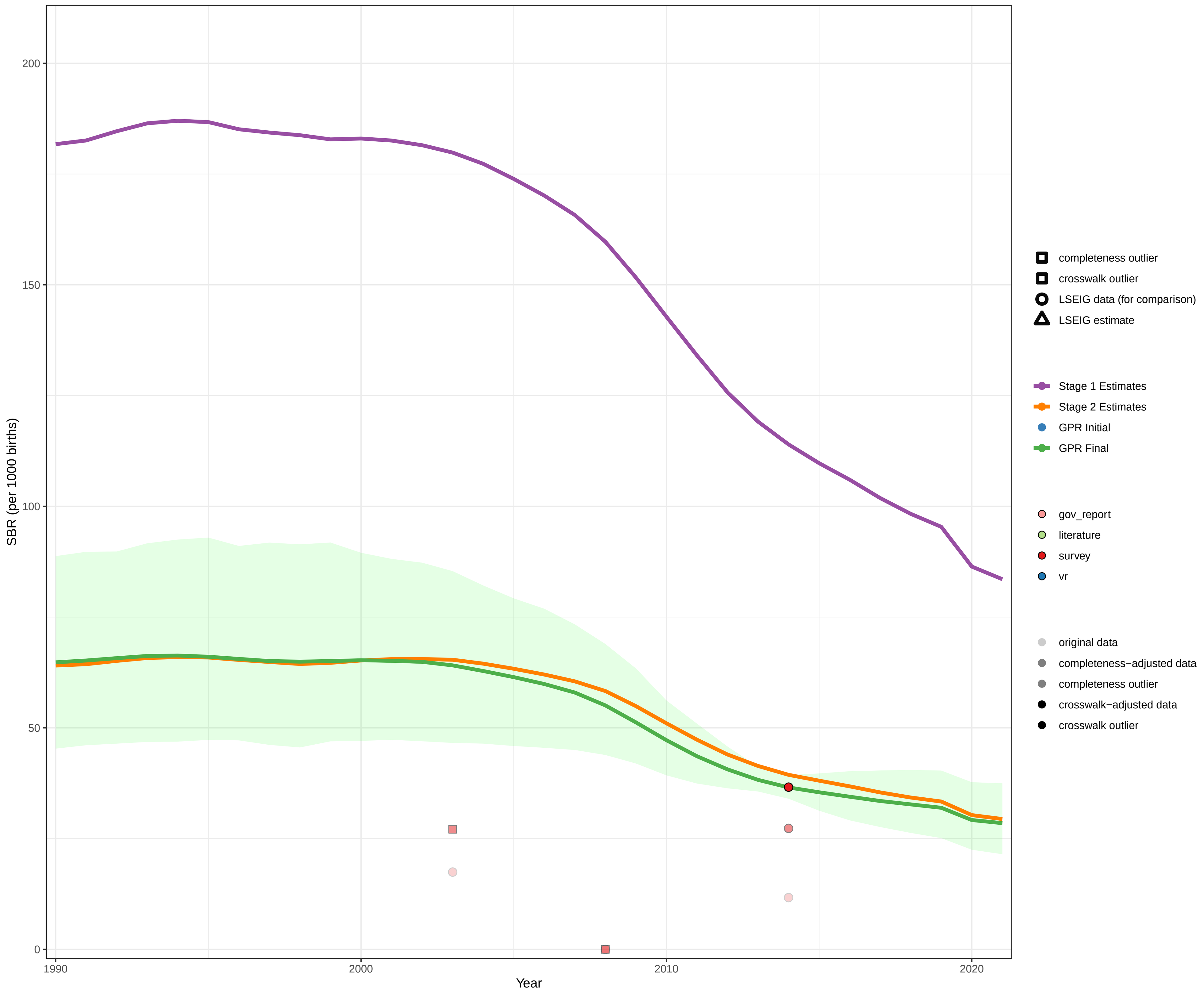

Wajir KEN_35662 Eastern Sub−Saharan Africa Stillbirth Rate (SBR) − 28 weeks
lambda = 0.8 ; zeta = 0.7 ; scale = 15 ; amp = 0.028

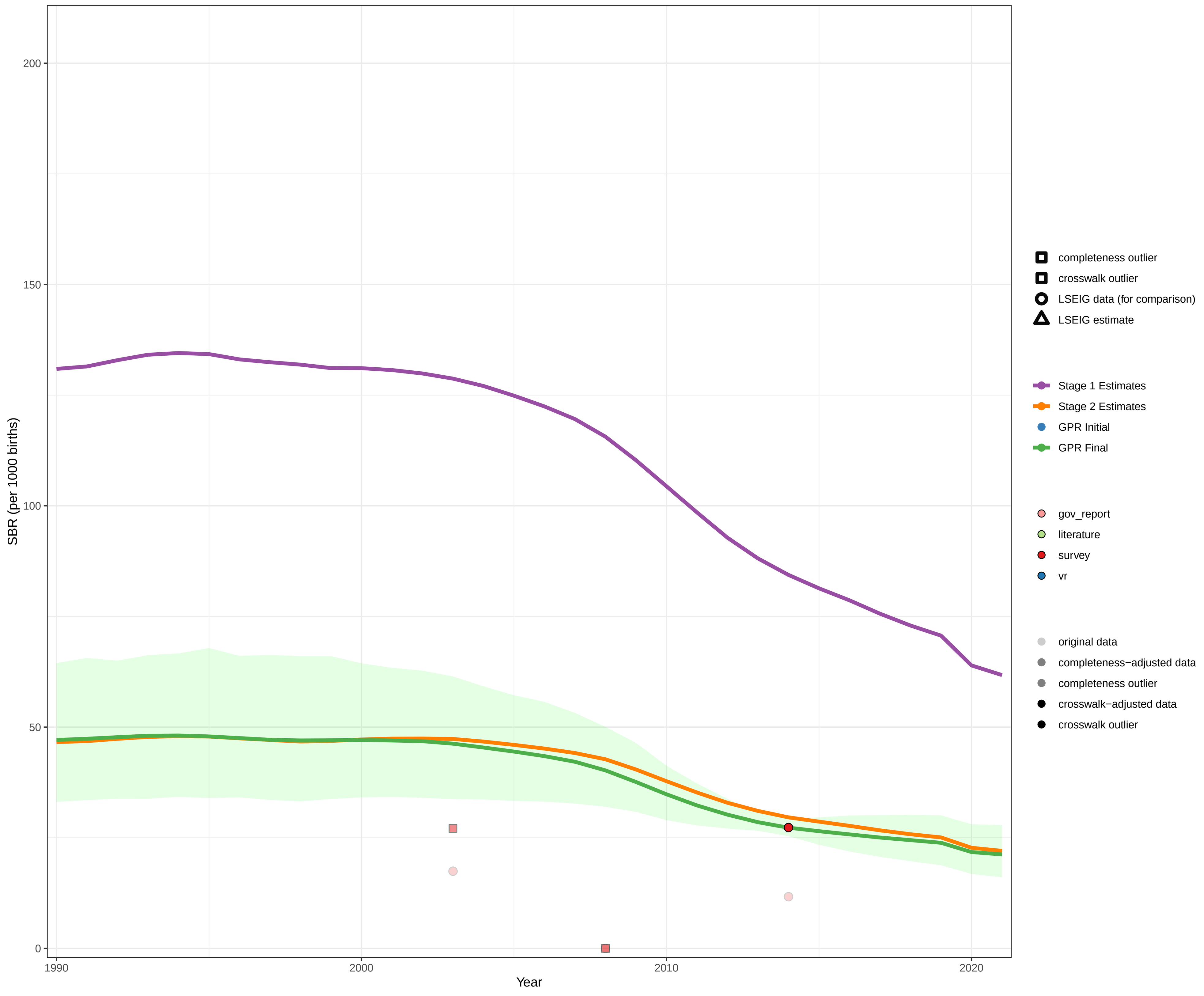

Year	Total Stillbirths (in thousands)	Total Stillbirths (in thousands)	SBR (per 1000 births)	SBR (per 1000 births)	SBR/NMR	SBR/NMR
	20 weeks	28 weeks	20 weeks	28 weeks	20 weeks	28 weeks
1990	0.5 (0.3 to 0.6)	0.3 (0.2 to 0.5)	64.8 (45.3 to 88.8)	47.1 (33.1 to 64.5)	1.40 (0.98 to 1.91)	1.01 (0.71 to 1.39)
1995	0.8 (0.5 to 1.1)	0.5 (0.4 to 0.8)	66.0 (47.3 to 92.9)	47.9 (34.0 to 67.9)	1.38 (0.99 to 1.95)	1.00 (0.71 to 1.42)
2000	1.3 (0.9 to 1.8)	0.9 (0.7 to 1.3)	65.3 (47.1 to 89.5)	47.1 (34.1 to 64.4)	1.40 (1.01 to 1.93)	1.01 (0.73 to 1.39)
2005	1.7 (1.3 to 2.3)	1.2 (0.9 to 1.6)	61.4 (45.9 to 79.2)	44.5 (33.3 to 57.2)	1.34 (1.00 to 1.73)	0.97 (0.73 to 1.25)
2010	1.5 (1.3 to 1.8)	1.1 (0.9 to 1.3)	47.2 (39.3 to 56.2)	34.8 (29.0 to 41.3)	1.10 (0.92 to 1.31)	0.81 (0.68 to 0.96)
2015	1.3 (1.1 to 1.5)	1.0 (0.8 to 1.1)	35.5 (31.3 to 39.7)	26.5 (23.4 to 29.6)	0.96 (0.85 to 1.08)	0.72 (0.63 to 0.80)
2020	1.2 (0.9 to 1.5)	0.9 (0.7 to 1.1)	29.2 (22.5 to 37.7)	21.8 (16.8 to 28.1)	1.00 (0.77 to 1.29)	0.75 (0.58 to 0.96)
2021	1.2 (0.9 to 1.6)	0.9 (0.7 to 1.1)	28.5 (21.5 to 37.5)	21.2 (16.1 to 27.9)	1.01 (0.76 to 1.33)	0.75 (0.57 to 0.99)

West Pokot   KEN_35663   Eastern Sub−Saharan Africa   Stillbirth Rate / Neonatal Mortality Rate (SBR/NMR) − 20 weeks
lambda = 0.8 ; zeta = 0.7 ; scale = 15 ; amp = 0.028

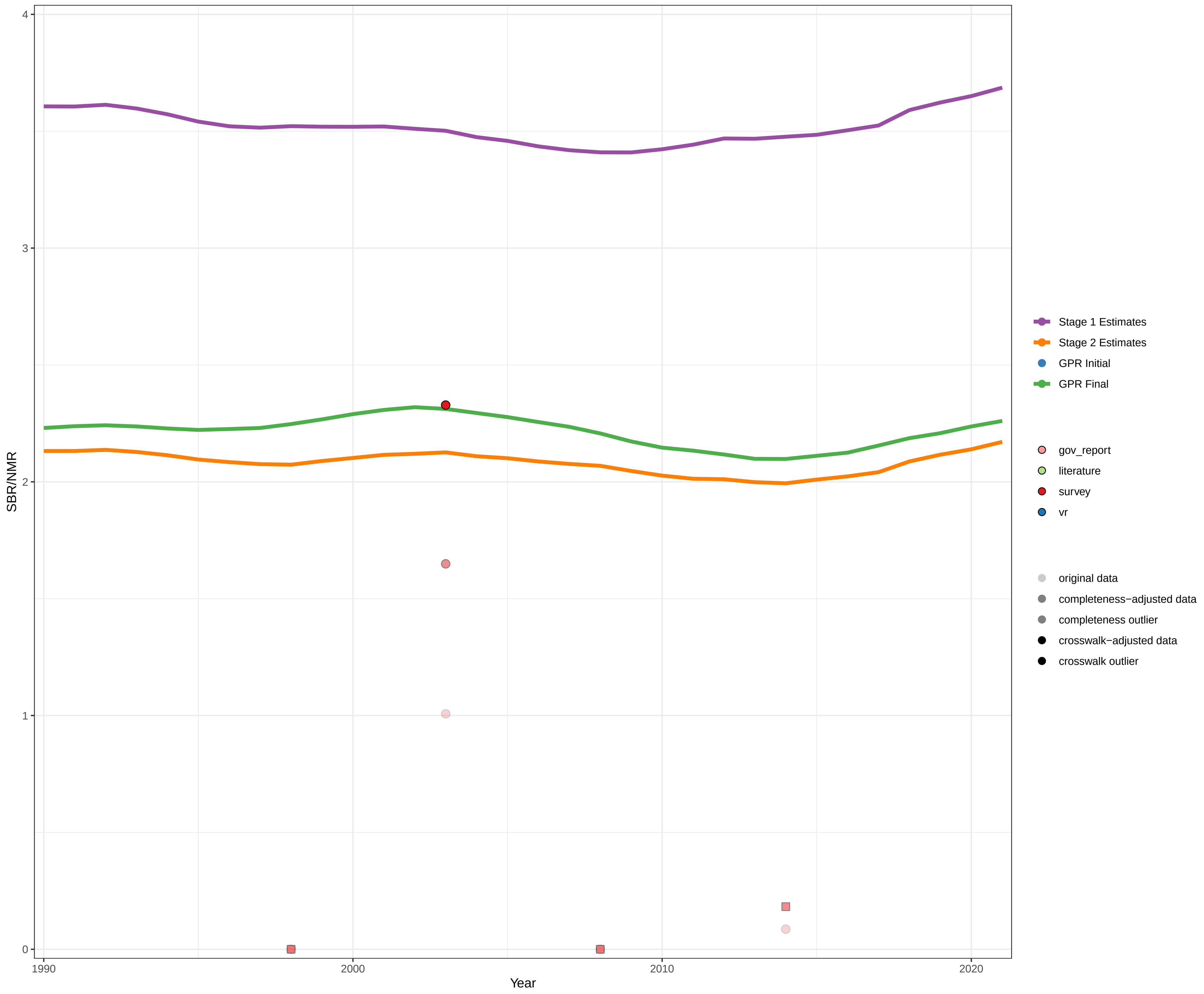

West Pokot   KEN_35663   Eastern Sub−Saharan Africa   Stillbirth Rate / Neonatal Mortality Rate (SBR/NMR) − 28 weeks
lambda = 0.8 ; zeta = 0.7 ; scale = 15 ; amp = 0.028

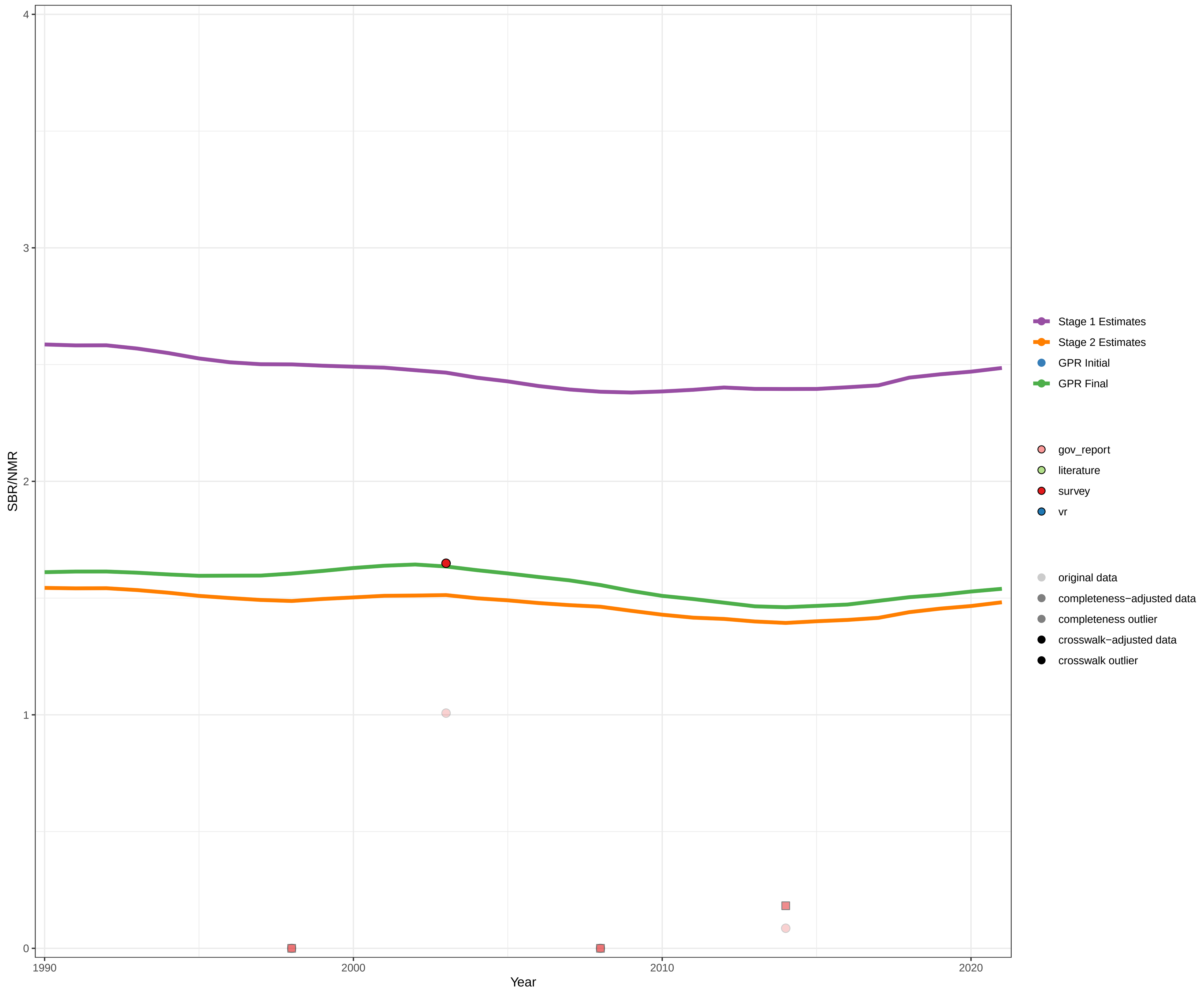

Definition	Years	Outlier	Source.Type	Title
28_weeks	2003	0	Survey	Kenya Demographic and Health Survey 2003
28_weeks	1998	1	Survey	Kenya Demographic and Health Survey 1998
28_weeks	2008	1	Survey	Kenya Demographic and Health Survey 2008−2009
28_weeks	2014	1	Survey	Kenya Demographic and Health Survey 2014

West Pokot   KEN_35663   Eastern Sub−Saharan Africa   Stillbirth Rate (SBR) − 20 weeks
lambda = 0.8 ; zeta = 0.7 ; scale = 15 ; amp = 0.028

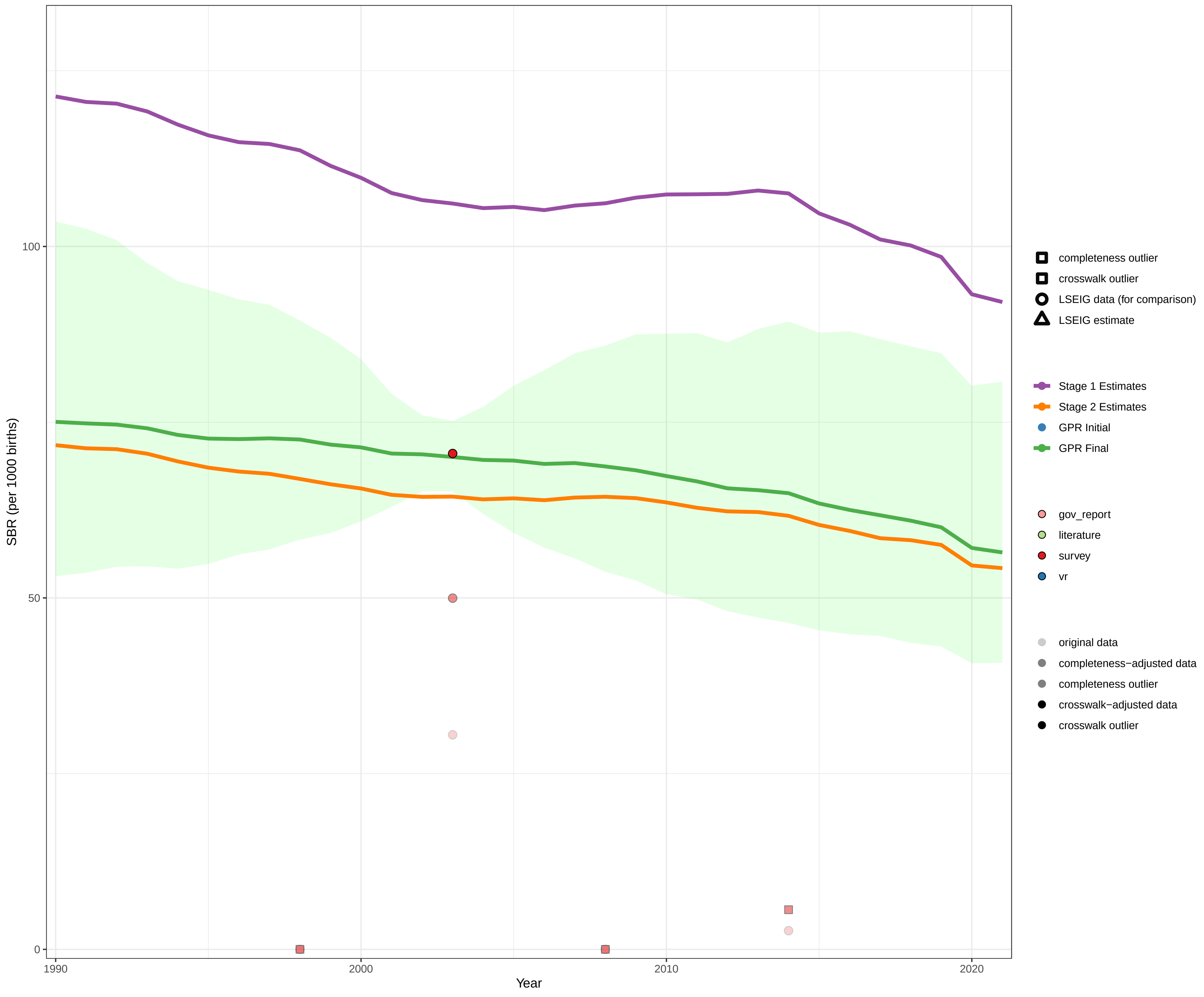

West Pokot   KEN_35663   Eastern Sub−Saharan Africa   Stillbirth Rate (SBR) − 28 weeks
lambda = 0.8 ; zeta = 0.7 ; scale = 15 ; amp = 0.028

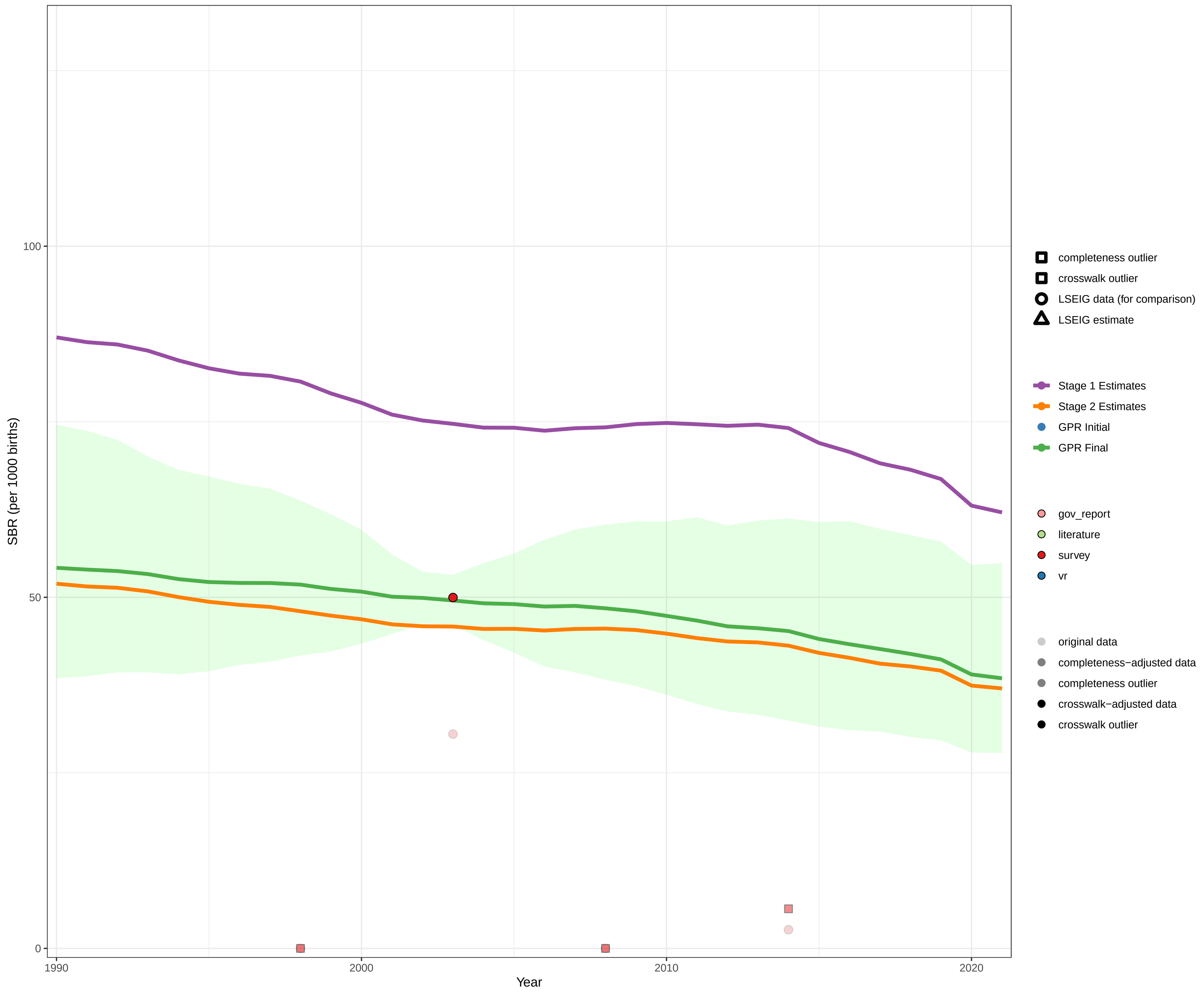

Year	Total Stillbirths (in thousands)	Total Stillbirths (in thousands)	SBR (per 1000 births)	SBR (per 1000 births)	SBR/NMR	SBR/NMR
	20 weeks	28 weeks	20 weeks	28 weeks	20 weeks	28 weeks
1990	1.0 (0.7 to 1.4)	0.7 (0.5 to 1.0)	75.0 (53.1 to 103.6)	54.2 (38.5 to 74.6)	2.23 (1.58 to 3.08)	1.61 (1.14 to 2.22)
1995	1.0 (0.8 to 1.3)	0.7 (0.5 to 0.9)	72.7 (54.8 to 93.8)	52.2 (39.5 to 67.2)	2.22 (1.68 to 2.87)	1.60 (1.21 to 2.05)
2000	1.2 (1.0 to 1.4)	0.8 (0.7 to 1.0)	71.4 (60.9 to 84.0)	50.8 (43.4 to 59.6)	2.29 (1.95 to 2.69)	1.63 (1.39 to 1.91)
2005	1.3 (1.1 to 1.5)	0.9 (0.8 to 1.0)	69.5 (59.3 to 80.2)	49.0 (42.1 to 56.2)	2.28 (1.94 to 2.63)	1.61 (1.38 to 1.84)
2010	1.4 (1.0 to 1.8)	0.9 (0.7 to 1.2)	67.3 (50.5 to 87.6)	47.4 (36.1 to 60.8)	2.15 (1.61 to 2.79)	1.51 (1.15 to 1.94)
2015	1.2 (0.9 to 1.8)	0.8 (0.6 to 1.2)	63.4 (45.4 to 87.7)	44.1 (31.6 to 60.7)	2.11 (1.51 to 2.92)	1.47 (1.05 to 2.02)
2020	1.0 (0.7 to 1.4)	0.7 (0.5 to 1.0)	57.1 (40.7 to 80.2)	39.0 (27.9 to 54.6)	2.24 (1.59 to 3.14)	1.53 (1.09 to 2.14)
2021	1.0 (0.7 to 1.4)	0.6 (0.5 to 0.9)	56.5 (40.8 to 80.8)	38.5 (27.8 to 54.8)	2.26 (1.63 to 3.23)	1.54 (1.11 to 2.20)

Madagascar MDG Eastern Sub−Saharan Africa Stillbirth Rate / Neonatal Mortality Rate (SBR/NMR) − 20 weeks
lambda = 0.8 ; zeta = 0.7 ; scale = 15 ; amp = 0.028

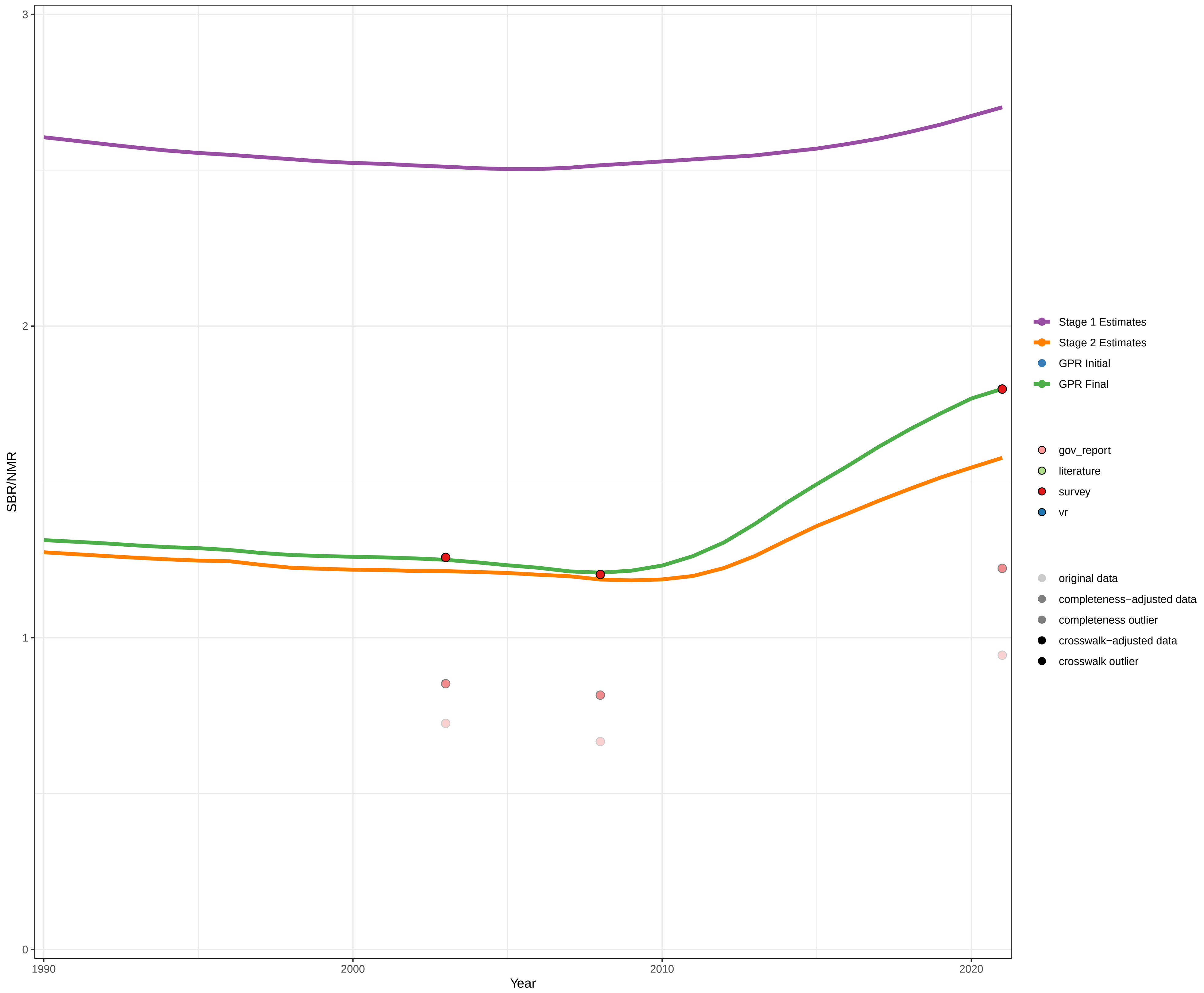

Madagascar MDG Eastern Sub−Saharan Africa Stillbirth Rate / Neonatal Mortality Rate (SBR/NMR) − 28 weeks
lambda = 0.8 ; zeta = 0.7 ; scale = 15 ; amp = 0.028

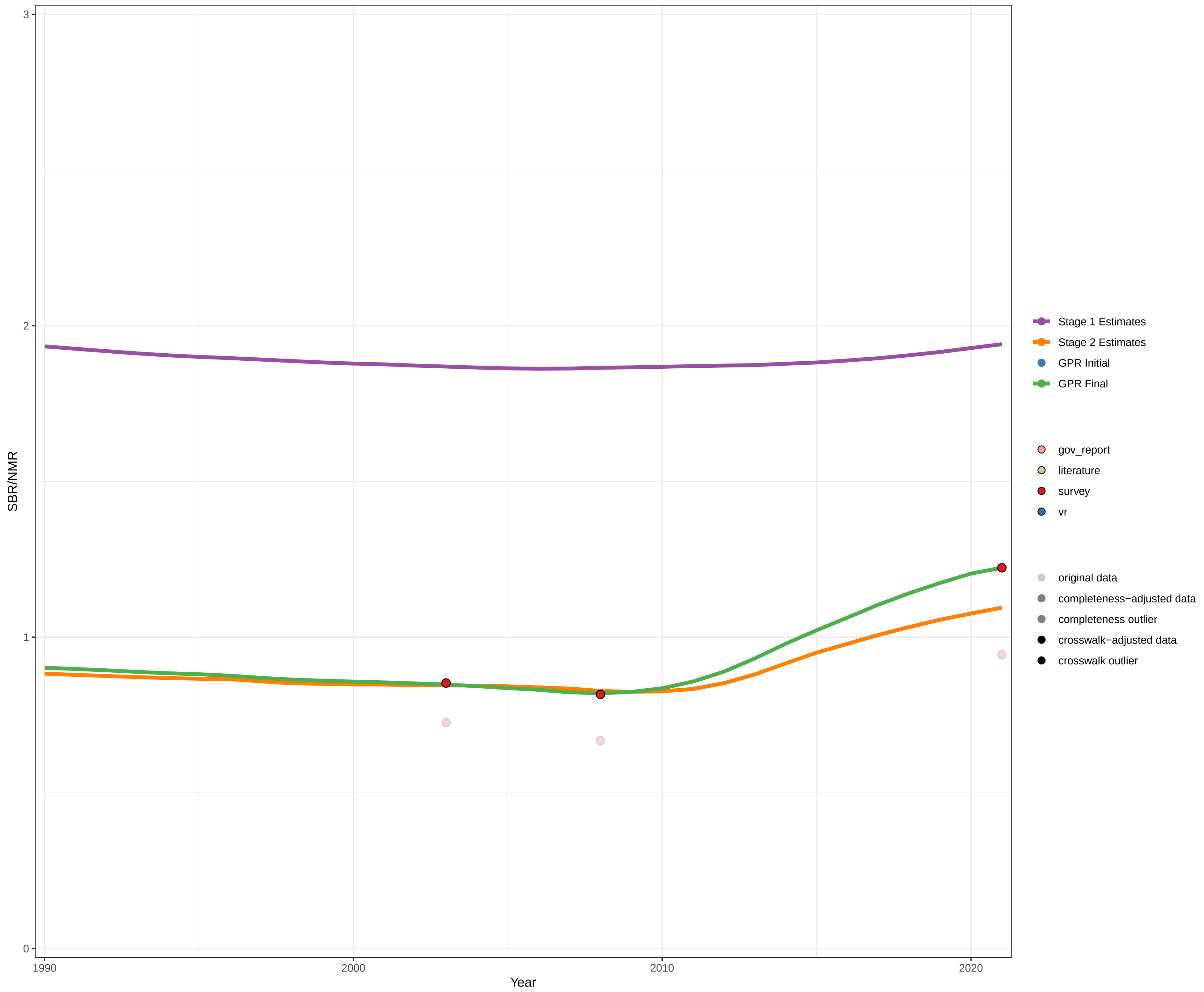

Definition	Years	Outlier	Source.Type	Title
28_weeks	2003	0	Survey	Madagascar Demographic and Health Survey 2003−2004
28_weeks	2008	0	Survey	Madagascar Demographic and Health Survey 2008−2009
28_weeks	2021	0	Survey	Madagascar Demographic and Health Survey 2021

Madagascar MDG Eastern Sub−Saharan Africa Stillbirth Rate (SBR) − 20 weeks
lambda = 0.8 ; zeta = 0.7 ; scale = 15 ; amp = 0.028

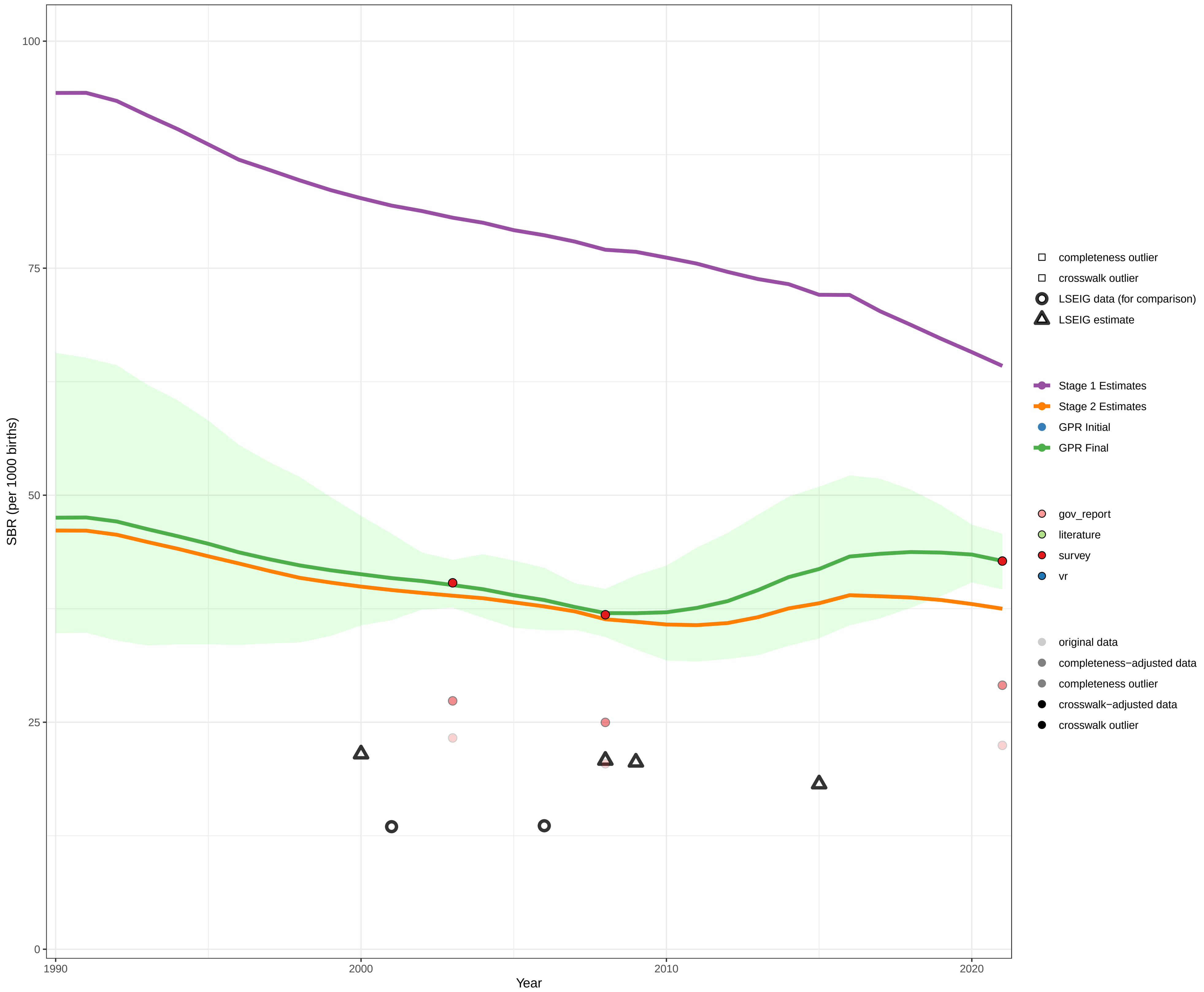

Madagascar MDG Eastern Sub−Saharan Africa Stillbirth Rate (SBR) − 28 weeks
lambda = 0.8 ; zeta = 0.7 ; scale = 15 ; amp = 0.028

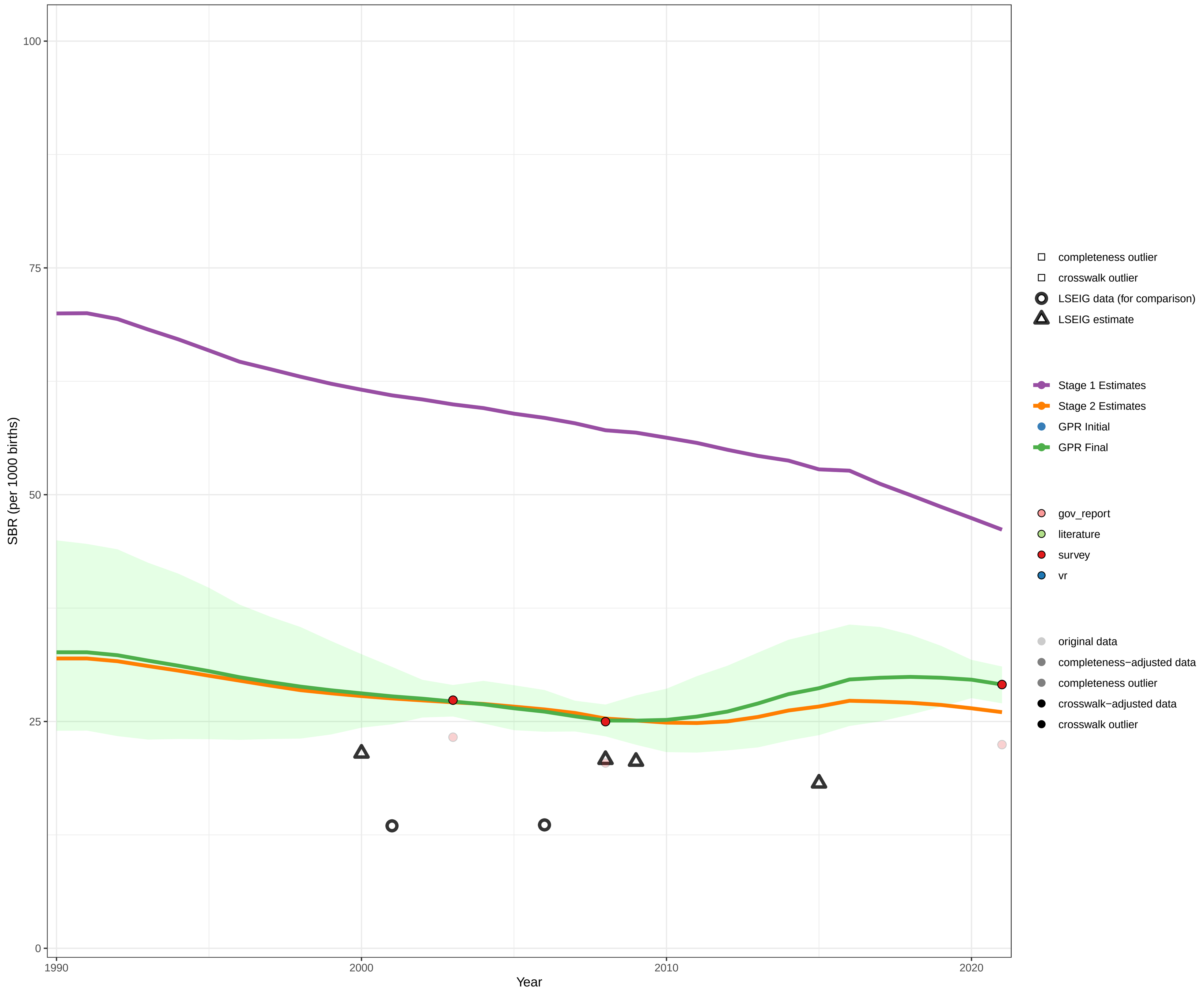

Year	Total Stillbirths (in thousands)	Total Stillbirths (in thousands)	SBR (per 1000 births)	SBR (per 1000 births)	SBR/NMR	SBR/NMR
	20 weeks	28 weeks	20 weeks	28 weeks	20 weeks	28 weeks
1990	26.3 (19.0 to 37.0)	17.8 (12.9 to 24.8)	47.5 (34.8 to 65.7)	32.6 (24.0 to 45.0)	1.31 (0.96 to 1.82)	0.90 (0.66 to 1.24)
1995	28.4 (21.1 to 37.5)	19.1 (14.3 to 25.1)	44.7 (33.6 to 58.2)	30.6 (23.0 to 39.7)	1.29 (0.97 to 1.68)	0.88 (0.66 to 1.15)
2000	29.8 (25.6 to 34.7)	20.0 (17.2 to 23.2)	41.3 (35.7 to 47.7)	28.1 (24.3 to 32.4)	1.26 (1.09 to 1.46)	0.86 (0.74 to 0.99)
2005	31.4 (28.4 to 34.7)	21.1 (19.1 to 23.1)	39.0 (35.4 to 42.8)	26.5 (24.0 to 29.0)	1.23 (1.12 to 1.35)	0.84 (0.76 to 0.92)
2010	33.0 (28.1 to 37.8)	22.1 (18.9 to 25.2)	37.1 (31.8 to 42.2)	25.2 (21.6 to 28.6)	1.23 (1.06 to 1.40)	0.84 (0.72 to 0.95)
2015	39.5 (32.0 to 48.5)	26.7 (21.7 to 32.6)	41.9 (34.2 to 50.9)	28.7 (23.5 to 34.8)	1.49 (1.22 to 1.82)	1.02 (0.84 to 1.24)
2020	40.1 (37.2 to 43.3)	26.9 (25.0 to 29.0)	43.5 (40.4 to 46.8)	29.6 (27.6 to 31.8)	1.77 (1.64 to 1.90)	1.20 (1.12 to 1.29)
2021	39.2 (36.2 to 42.1)	26.3 (24.3 to 28.1)	42.8 (39.6 to 45.8)	29.1 (27.0 to 31.1)	1.80 (1.67 to 1.92)	1.22 (1.14 to 1.31)

Malawi MWI Eastern Sub−Saharan Africa Stillbirth Rate / Neonatal Mortality Rate (SBR/NMR) − 20 weeks
lambda = 0.6 ; zeta = 0.8 ; scale = 15 ; amp = 0.028

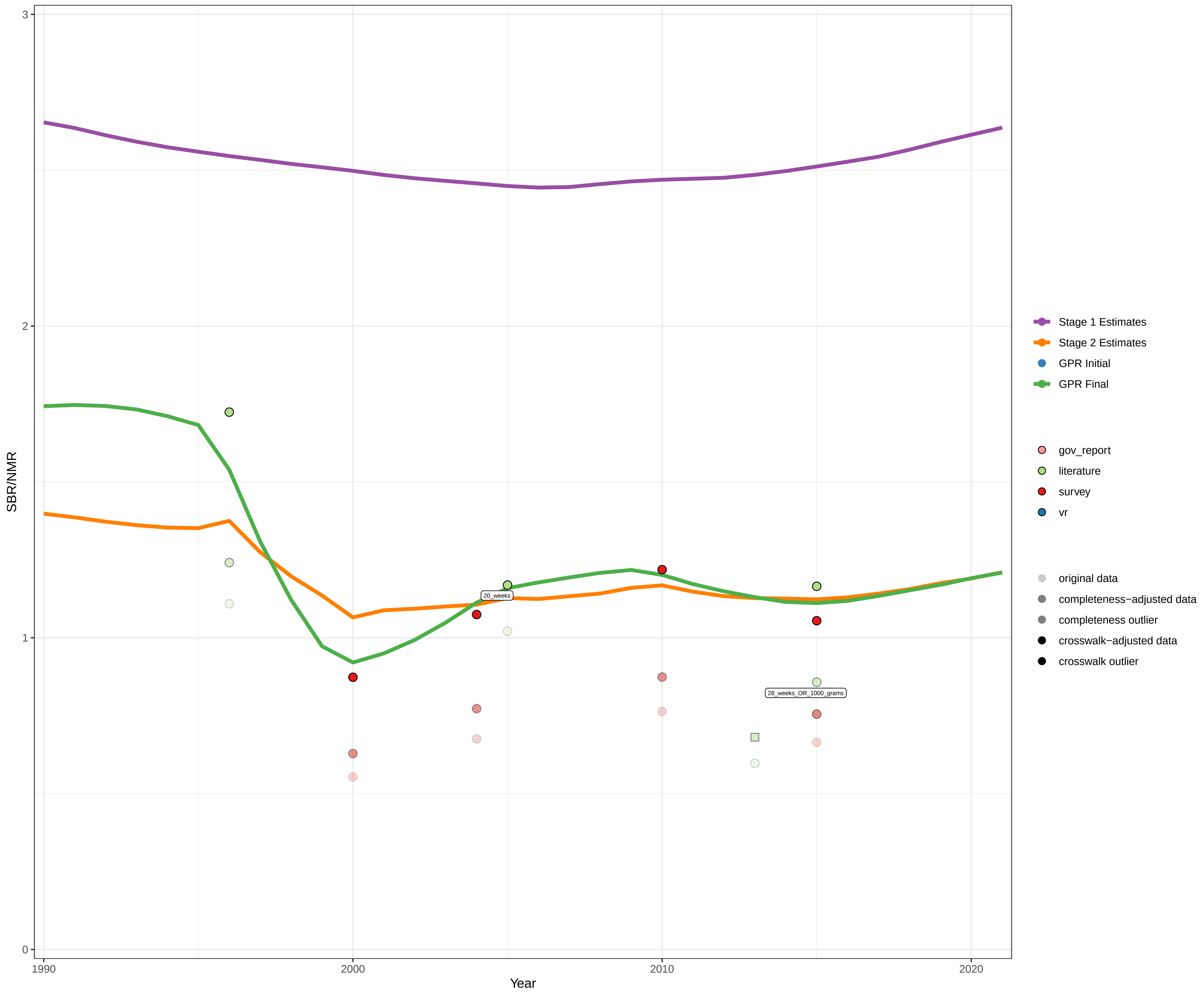

Malawi MWI Eastern Sub−Saharan Africa Stillbirth Rate / Neonatal Mortality Rate (SBR/NMR) − 28 weeks
lambda = 0.6 ; zeta = 0.8 ; scale = 15 ; amp = 0.028

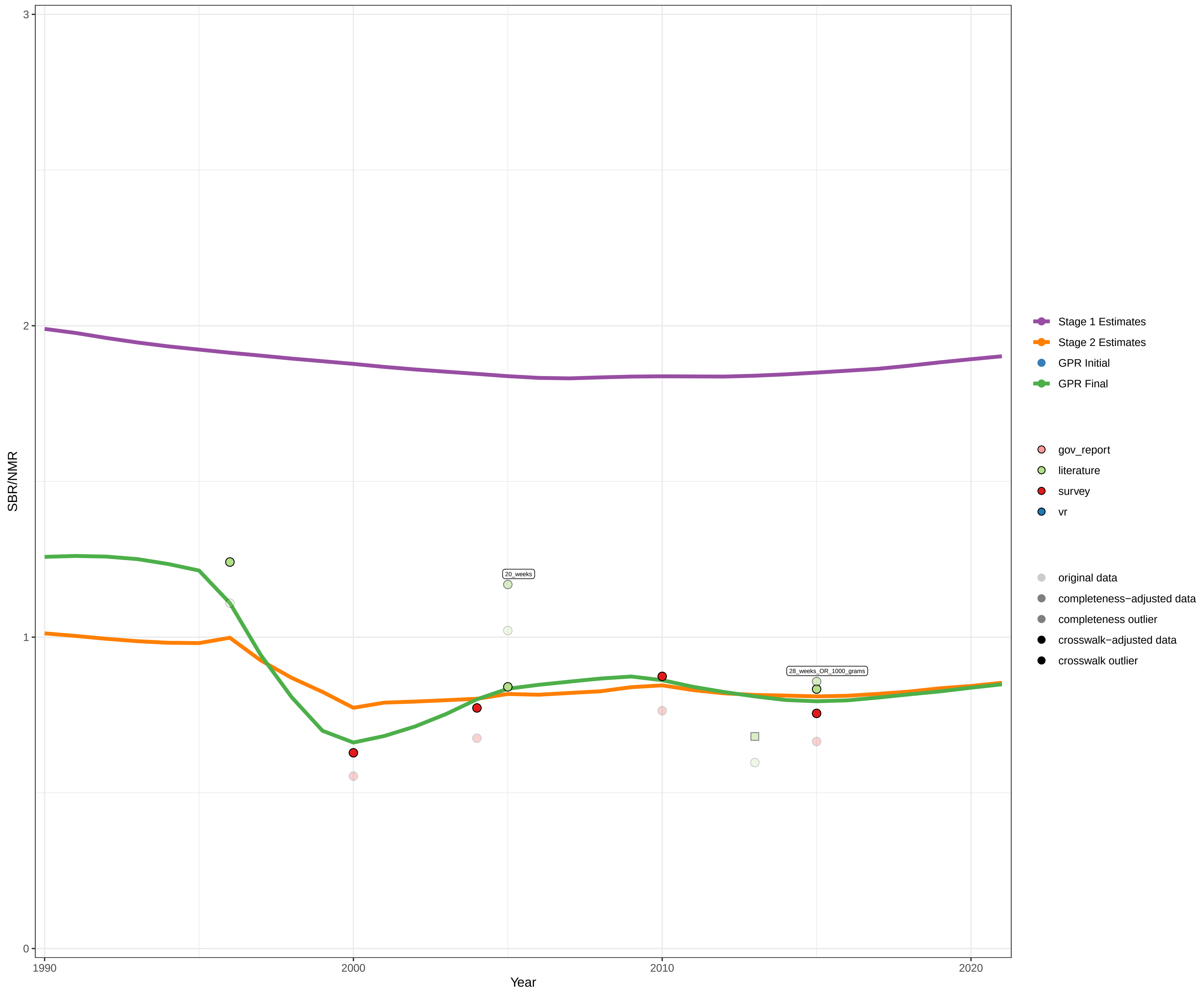

Definition	Years	Outlier	Source.Type	Title
20_weeks	2005	0	Sci Lit	Stillbirths and hospital early neonatal deaths at Queen Elizabeth Central Hospital, Blantyre−Malawi
28_weeks	1996	0	Sci Lit	The importance of preterm births for peri− and neonatal mortality in rural Malawi
28_weeks	2000	0	Survey	Malawi Demographic and Health Survey 2000
28_weeks	2004	0	Survey	Malawi Demographic and Health Survey 2004−2005
28_weeks	2010	0	Survey	Malawi Demographic and Health Survey 2010
28_weeks	2015	0	Survey	Malawi Demographic and Health Survey 2015−2016
28_weeks_OR_1000_grams	2015	0	Sci Lit	Application of the ICD−PM classification system to stillbirth in four sub−Saharan African countries
28_weeks	2013	1	Sci Lit	Reassessing pregnancy intention and its relation to maternal, perinatal and neonatal outcomes in a low−income setting: A cohort study

Malawi MWI Eastern Sub−Saharan Africa Stillbirth Rate (SBR) − 20 weeks
lambda = 0.6 ; zeta = 0.8 ; scale = 15 ; amp = 0.028

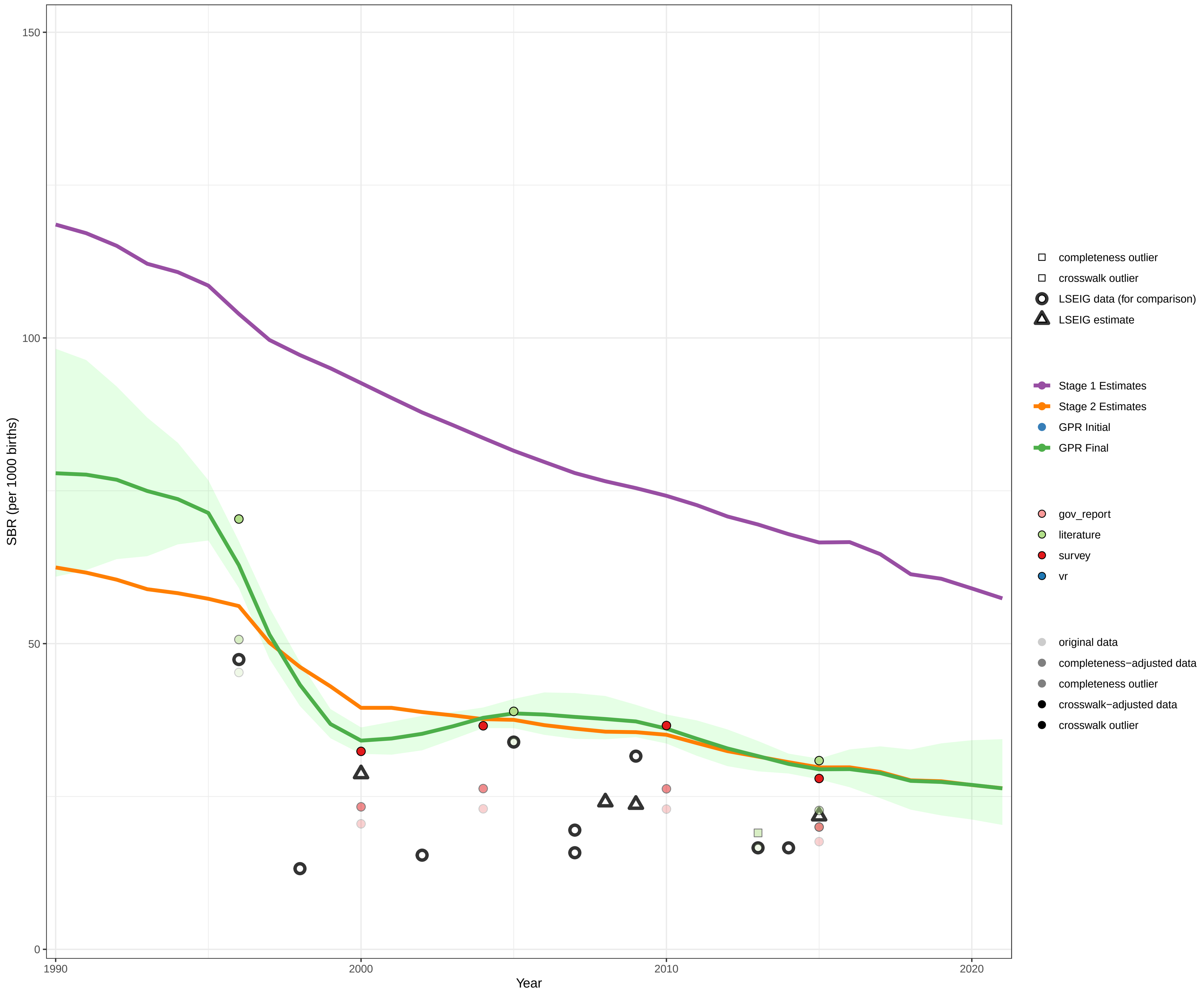

Malawi MWI Eastern Sub−Saharan Africa Stillbirth Rate (SBR) − 28 weeks
lambda = 0.6 ; zeta = 0.8 ; scale = 15 ; amp = 0.028

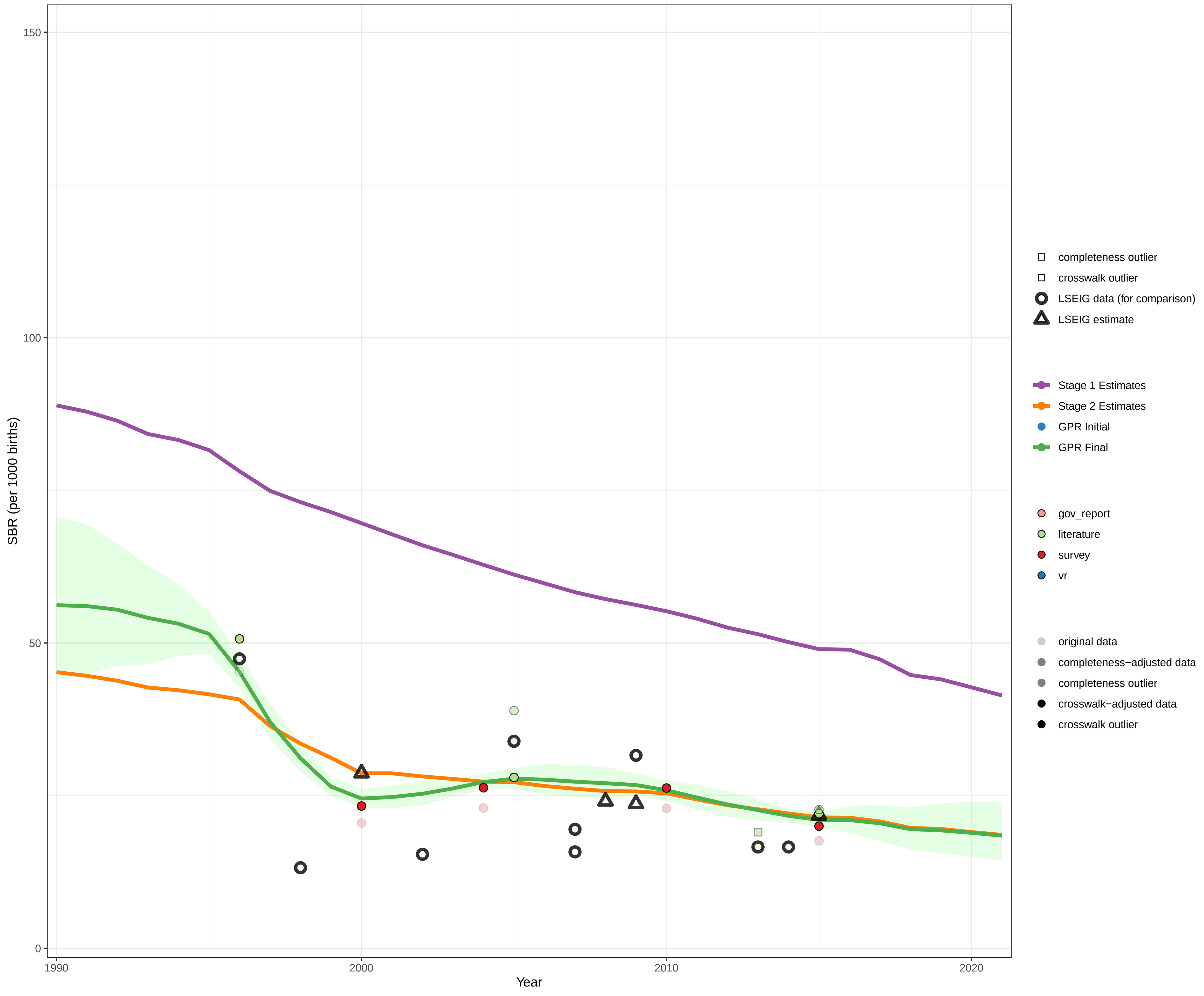

Year	Total Stillbirths (in thousands)	Total Stillbirths (in thousands)	SBR (per 1000 births)	SBR (per 1000 births)	SBR/NMR	SBR/NMR
	20 weeks	28 weeks	20 weeks	28 weeks	20 weeks	28 weeks
1990	43.0 (33.0 to 55.4)	30.3 (23.5 to 38.7)	77.9 (60.9 to 98.2)	56.2 (44.1 to 70.7)	1.74 (1.36 to 2.20)	1.26 (0.99 to 1.58)
1995	40.2 (37.5 to 43.5)	28.4 (26.5 to 30.6)	71.4 (66.9 to 76.7)	51.5 (48.3 to 55.2)	1.68 (1.58 to 1.81)	1.21 (1.14 to 1.30)
2000	19.6 (18.3 to 20.9)	14.0 (13.1 to 14.9)	34.1 (32.0 to 36.3)	24.5 (23.1 to 26.1)	0.92 (0.86 to 0.98)	0.66 (0.62 to 0.70)
2005	23.8 (22.2 to 25.3)	16.9 (15.8 to 18.0)	38.6 (36.2 to 41.0)	27.8 (26.0 to 29.6)	1.16 (1.09 to 1.23)	0.83 (0.78 to 0.89)
2010	22.5 (20.9 to 24.0)	16.0 (14.9 to 17.0)	36.1 (33.7 to 38.4)	25.9 (24.2 to 27.5)	1.20 (1.12 to 1.28)	0.86 (0.81 to 0.92)
2015	17.8 (16.8 to 18.9)	12.6 (11.9 to 13.3)	29.4 (27.8 to 31.2)	21.0 (19.9 to 22.2)	1.11 (1.05 to 1.18)	0.79 (0.75 to 0.84)
2020	15.9 (12.4 to 20.3)	11.1 (8.7 to 14.1)	26.9 (21.2 to 34.2)	18.9 (15.0 to 24.0)	1.19 (0.94 to 1.51)	0.84 (0.66 to 1.06)
2021	15.5 (11.9 to 20.4)	10.8 (8.3 to 14.2)	26.3 (20.3 to 34.4)	18.5 (14.3 to 24.1)	1.21 (0.93 to 1.58)	0.85 (0.66 to 1.10)

Mozambique MOZ Eastern Sub−Saharan Africa Stillbirth Rate / Neonatal Mortality Rate (SBR/NMR) − 20 weeks
lambda = 0.8 ; zeta = 0.7 ; scale = 15 ; amp = 0.028

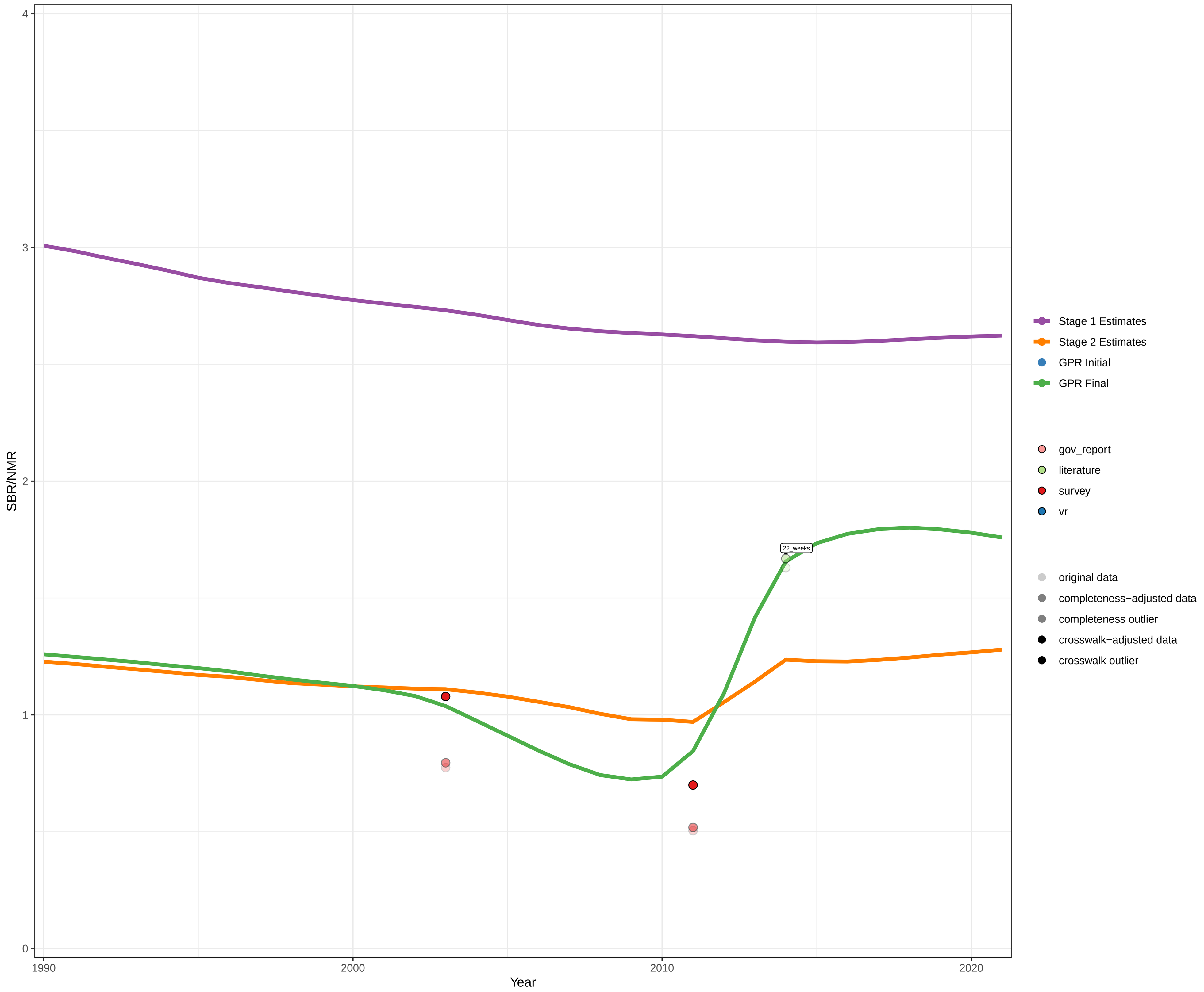

Mozambique MOZ Eastern Sub−Saharan Africa Stillbirth Rate / Neonatal Mortality Rate (SBR/NMR) − 28 weeks
lambda = 0.8 ; zeta = 0.7 ; scale = 15 ; amp = 0.028

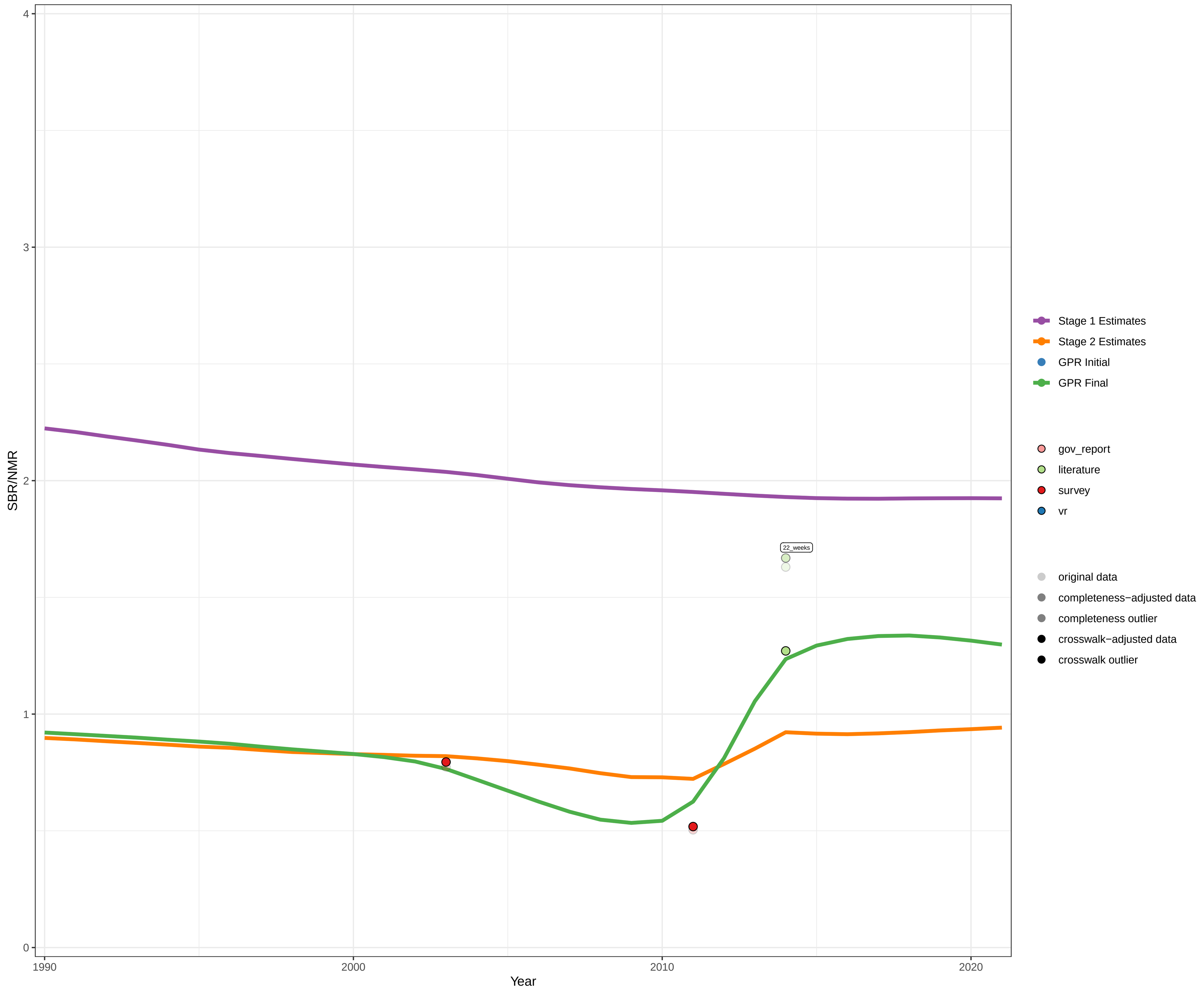

Definition	Years	Outlier	Source.Type	Title
22_weeks	2014	0	Sci Lit	Place−specific factors associated with adverse maternal and perinatal outcomes in Southern Mozambique: a retrospective cohort study
28_weeks	2003	0	Survey	Mozambique Demographic and Health Survey 2003−2004
28_weeks	2011	0	Survey	Mozambique Demographic and Health Survey 2011

Mozambique MOZ Eastern Sub−Saharan Africa Stillbirth Rate (SBR) − 20 weeks
lambda = 0.8 ; zeta = 0.7 ; scale = 15 ; amp = 0.028

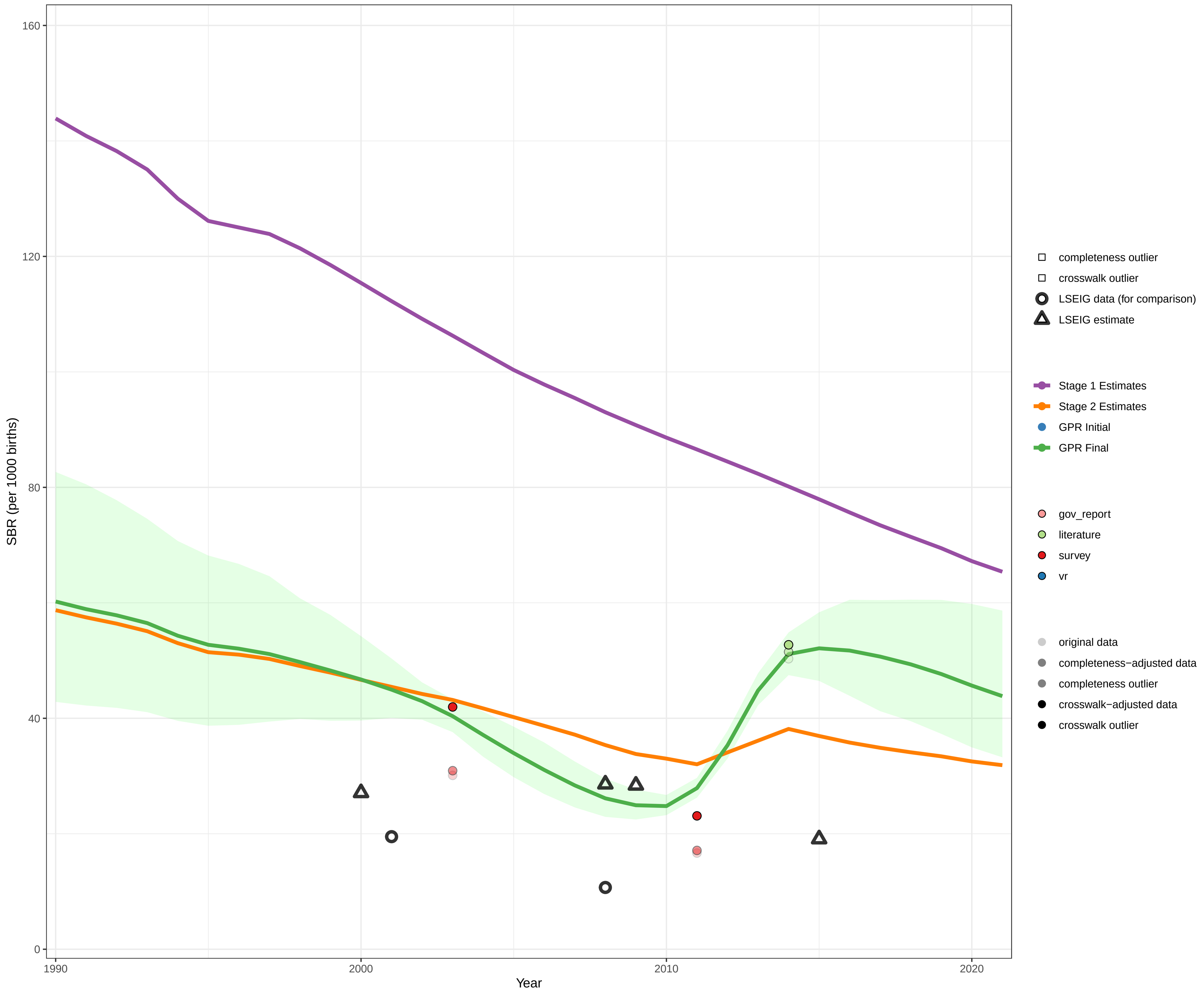

Mozambique MOZ Eastern Sub−Saharan Africa Stillbirth Rate (SBR) − 28 weeks
lambda = 0.8 ; zeta = 0.7 ; scale = 15 ; amp = 0.028

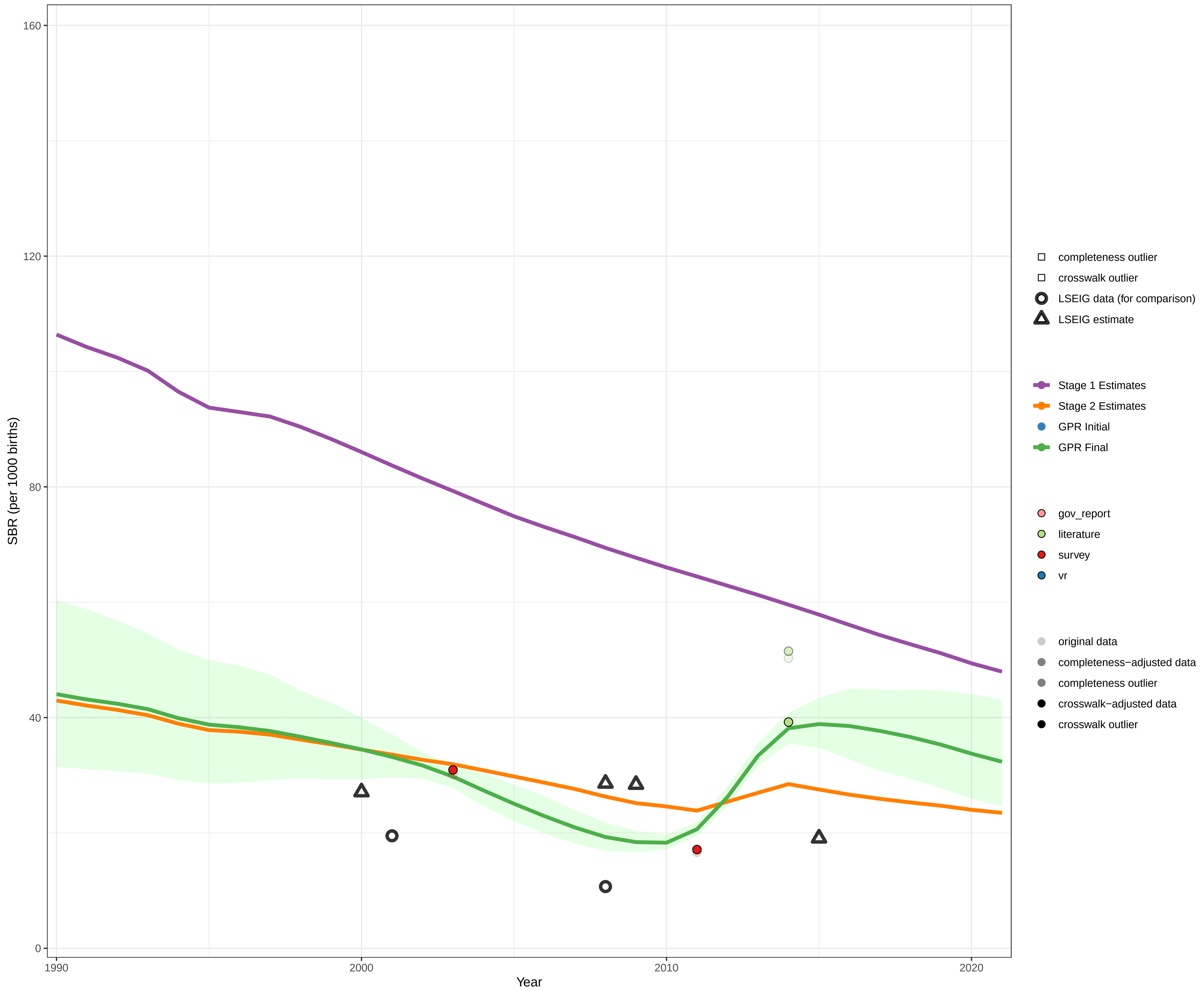

Year	Total Stillbirths (in thousands)	Total Stillbirths (in thousands)	SBR (per 1000 births)	SBR (per 1000 births)	SBR/NMR	SBR/NMR
	20 weeks	28 weeks	20 weeks	28 weeks	20 weeks	28 weeks
1990	40.0 (27.9 to 56.2)	28.8 (20.2 to 40.0)	60.2 (42.8 to 82.7)	44.1 (31.5 to 60.3)	1.26 (0.90 to 1.73)	0.92 (0.66 to 1.26)
1995	40.4 (29.2 to 53.0)	29.2 (21.3 to 38.1)	52.7 (38.7 to 68.2)	38.8 (28.6 to 50.0)	1.20 (0.88 to 1.55)	0.88 (0.65 to 1.14)
2000	40.4 (34.0 to 47.2)	29.4 (24.9 to 34.3)	46.7 (39.6 to 54.2)	34.5 (29.3 to 39.9)	1.12 (0.95 to 1.30)	0.83 (0.70 to 0.96)
2005	31.7 (27.7 to 36.2)	23.2 (20.3 to 26.4)	34.0 (29.8 to 38.6)	25.1 (22.0 to 28.4)	0.91 (0.80 to 1.03)	0.67 (0.59 to 0.76)
2010	24.8 (23.2 to 26.8)	18.2 (17.1 to 19.6)	24.8 (23.2 to 26.7)	18.3 (17.2 to 19.7)	0.74 (0.69 to 0.79)	0.54 (0.51 to 0.58)
2015	57.5 (50.9 to 64.8)	42.3 (37.6 to 47.5)	52.1 (46.5 to 58.4)	38.9 (34.7 to 43.4)	1.73 (1.55 to 1.94)	1.29 (1.16 to 1.45)
2020	52.3 (39.6 to 69.5)	38.2 (29.1 to 50.4)	45.7 (35.0 to 59.8)	33.7 (25.9 to 44.1)	1.78 (1.36 to 2.33)	1.31 (1.01 to 1.72)
2021	50.7 (38.0 to 68.8)	37.0 (27.9 to 49.8)	43.8 (33.2 to 58.7)	32.4 (24.6 to 43.2)	1.76 (1.33 to 2.35)	1.30 (0.99 to 1.73)

Rwanda   RWA   Eastern Sub−Saharan Africa   Stillbirth Rate / Neonatal Mortality Rate (SBR/NMR) − 20 weeks
lambda = 0.4 ; zeta = 0.9 ; scale = 10 ; amp = 0.028

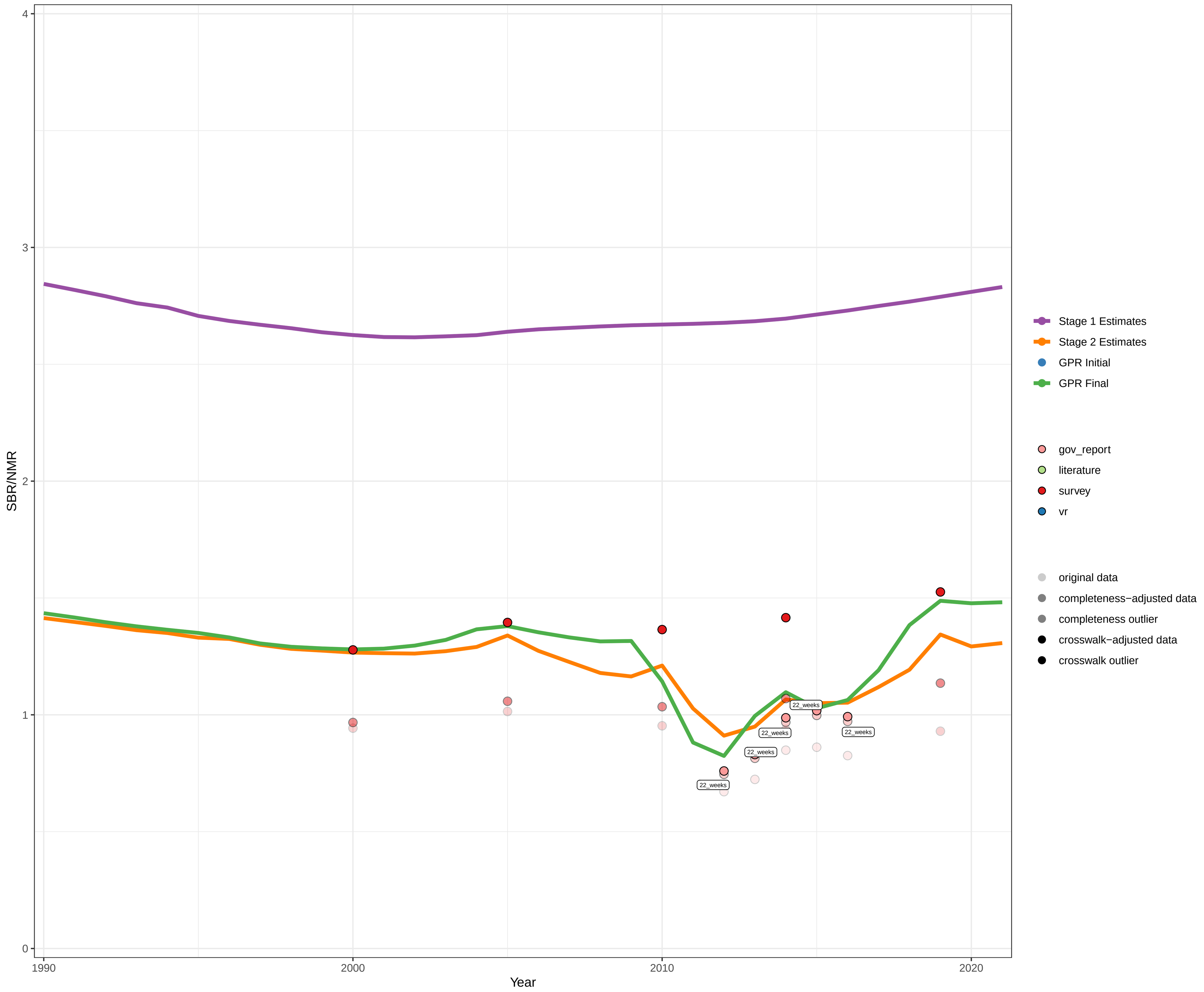

Rwanda   RWA   Eastern Sub−Saharan Africa   Stillbirth Rate / Neonatal Mortality Rate (SBR/NMR) − 28 weeks
lambda = 0.4 ; zeta = 0.9 ; scale = 10 ; amp = 0.028

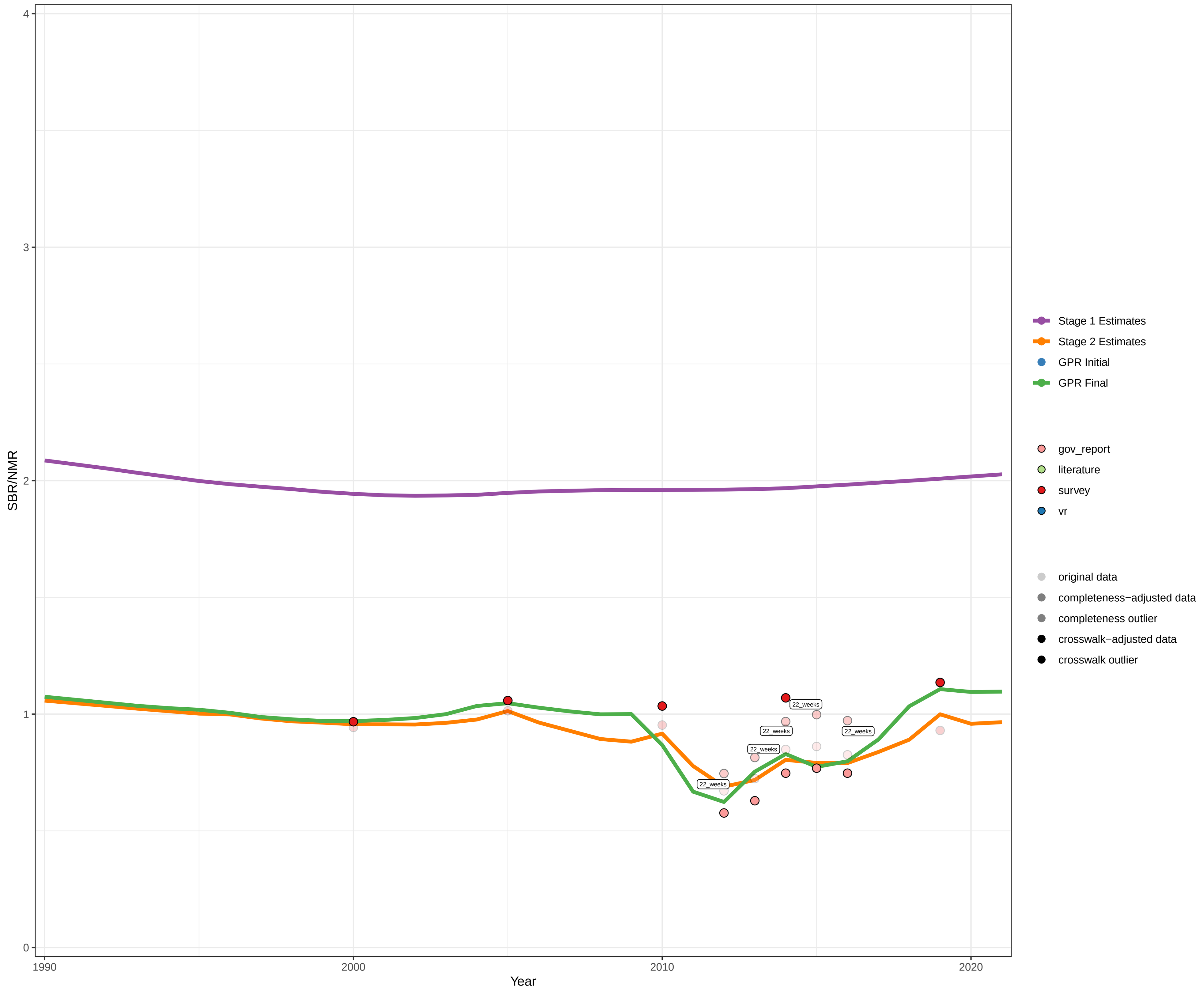

Definition	Years	Outlier	Source.Type	Title
22_weeks	2012	0	Statistical Report	Rwanda Health Statistics Booklet
22_weeks	2013−2016	0	Statistical Report	Rwanda Annual Health Statistics Booklet
28_weeks	2000	0	Survey	Rwanda Demographic and Health Survey 2000
28_weeks	2005	0	Survey	Rwanda Demographic and Health Survey 2005
28_weeks	2010	0	Survey	Rwanda Demographic and Health Survey 2010−2011
28_weeks	2014	0	Survey	Rwanda Demographic and Health Survey 2014−2015
28_weeks	2019	0	Survey	Rwanda Demographic and Health Survey 2019−2020

Rwanda   RWA   Eastern Sub−Saharan Africa   Stillbirth Rate (SBR) − 20 weeks
lambda = 0.4 ; zeta = 0.9 ; scale = 10 ; amp = 0.028

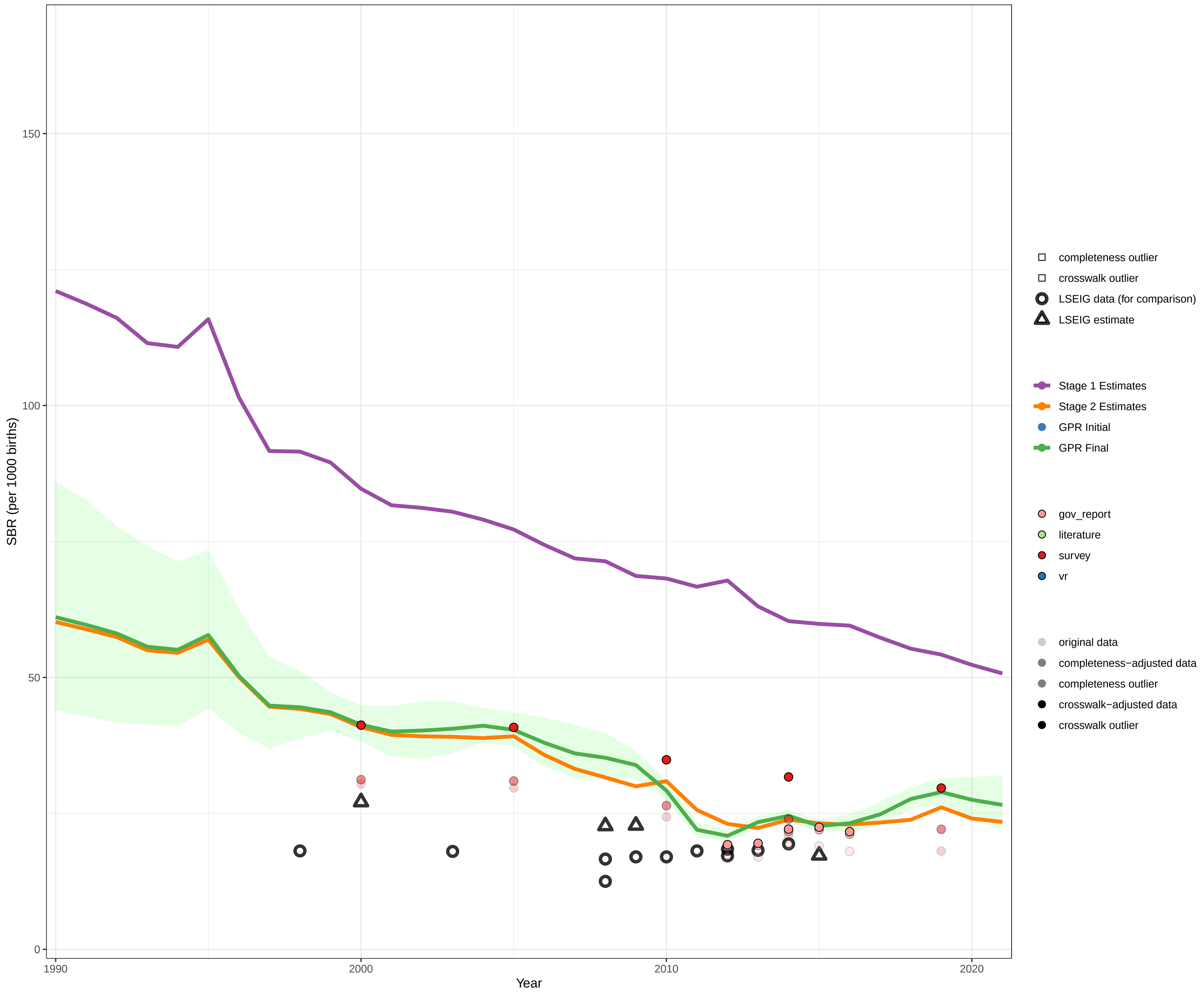

Rwanda   RWA   Eastern Sub−Saharan Africa   Stillbirth Rate (SBR) − 28 weeks
lambda = 0.4 ; zeta = 0.9 ; scale = 10 ; amp = 0.028

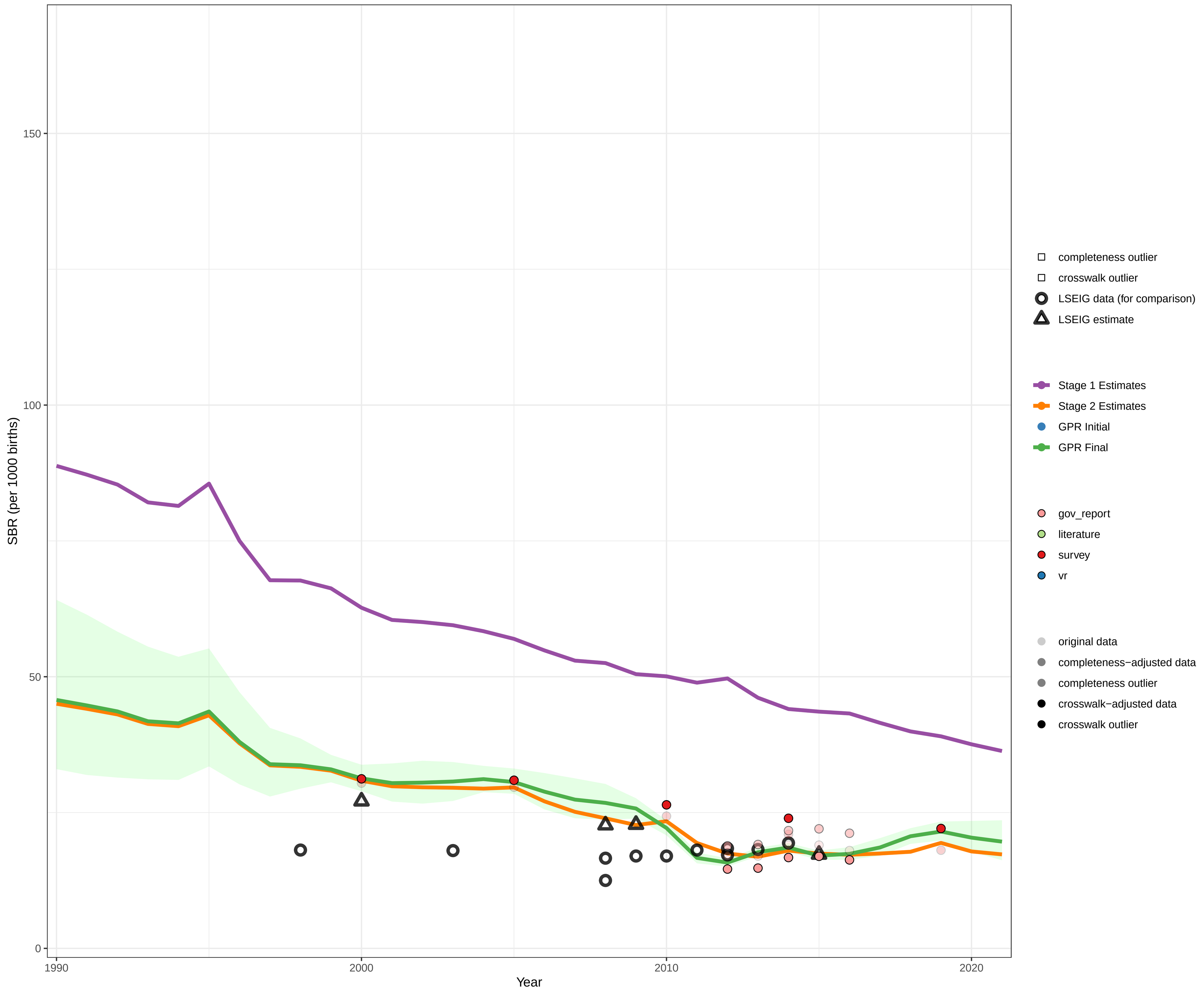

Year	Total Stillbirths (in thousands)	Total Stillbirths (in thousands)	SBR (per 1000 births)	SBR (per 1000 births)	SBR/NMR	SBR/NMR
	20 weeks	28 weeks	20 weeks	28 weeks	20 weeks	28 weeks
1990	20.7 (14.6 to 29.9)	15.3 (10.9 to 21.8)	61.1 (43.9 to 85.9)	45.7 (33.0 to 64.1)	1.43 (1.03 to 2.02)	1.07 (0.78 to 1.51)
1995	16.1 (12.1 to 20.7)	11.9 (9.1 to 15.3)	57.8 (44.3 to 73.4)	43.6 (33.5 to 55.2)	1.35 (1.03 to 1.71)	1.02 (0.78 to 1.29)
2000	15.0 (13.8 to 16.3)	11.2 (10.4 to 12.2)	41.3 (38.3 to 44.8)	31.3 (28.9 to 33.8)	1.28 (1.19 to 1.39)	0.97 (0.90 to 1.05)
2005	15.5 (14.3 to 16.8)	11.6 (10.8 to 12.6)	40.4 (37.2 to 43.6)	30.6 (28.5 to 33.1)	1.38 (1.27 to 1.49)	1.05 (0.97 to 1.13)
2010	10.8 (10.0 to 11.5)	8.1 (7.6 to 8.6)	29.2 (27.3 to 31.1)	22.2 (20.8 to 23.6)	1.14 (1.07 to 1.22)	0.87 (0.81 to 0.92)
2015	8.5 (8.1 to 9.0)	6.4 (6.1 to 6.7)	22.7 (21.6 to 23.9)	17.1 (16.3 to 18.0)	1.03 (0.98 to 1.08)	0.77 (0.74 to 0.82)
2020	10.5 (9.1 to 12.1)	7.7 (6.7 to 8.9)	27.5 (24.0 to 31.7)	20.4 (17.8 to 23.5)	1.48 (1.29 to 1.70)	1.09 (0.96 to 1.26)
2021	10.2 (8.4 to 12.3)	7.5 (6.2 to 9.0)	26.6 (21.9 to 31.9)	19.6 (16.2 to 23.6)	1.48 (1.22 to 1.78)	1.10 (0.90 to 1.32)

Somalia SOM Eastern Sub−Saharan Africa Stillbirth Rate / Neonatal Mortality Rate (SBR/NMR) − 20 weeks
lambda = 1 ; zeta = 0.6 ; scale = 15 ; amp = 0.028

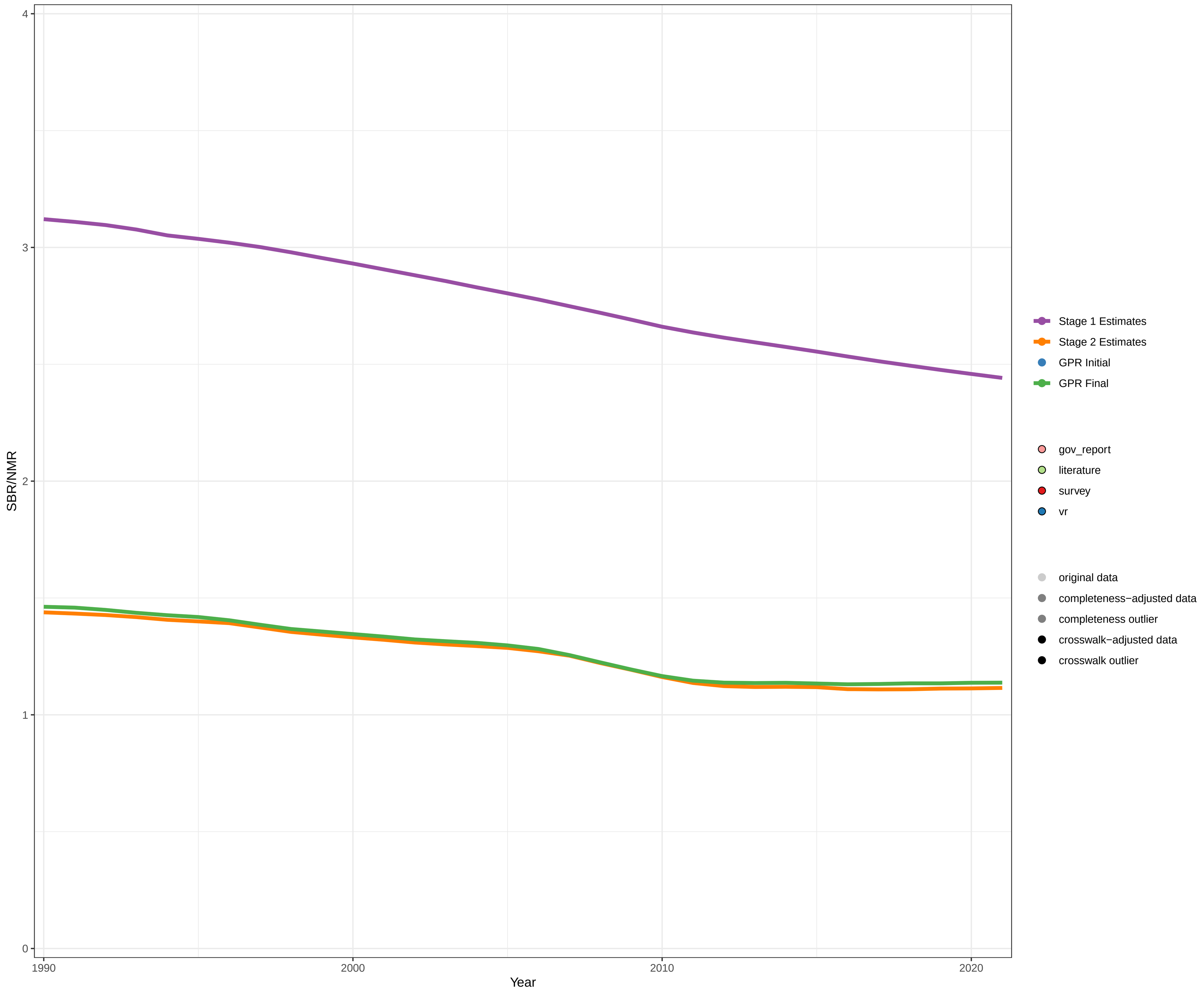

Somalia SOM Eastern Sub−Saharan Africa Stillbirth Rate / Neonatal Mortality Rate (SBR/NMR) − 28 weeks
lambda = 1 ; zeta = 0.6 ; scale = 15 ; amp = 0.028

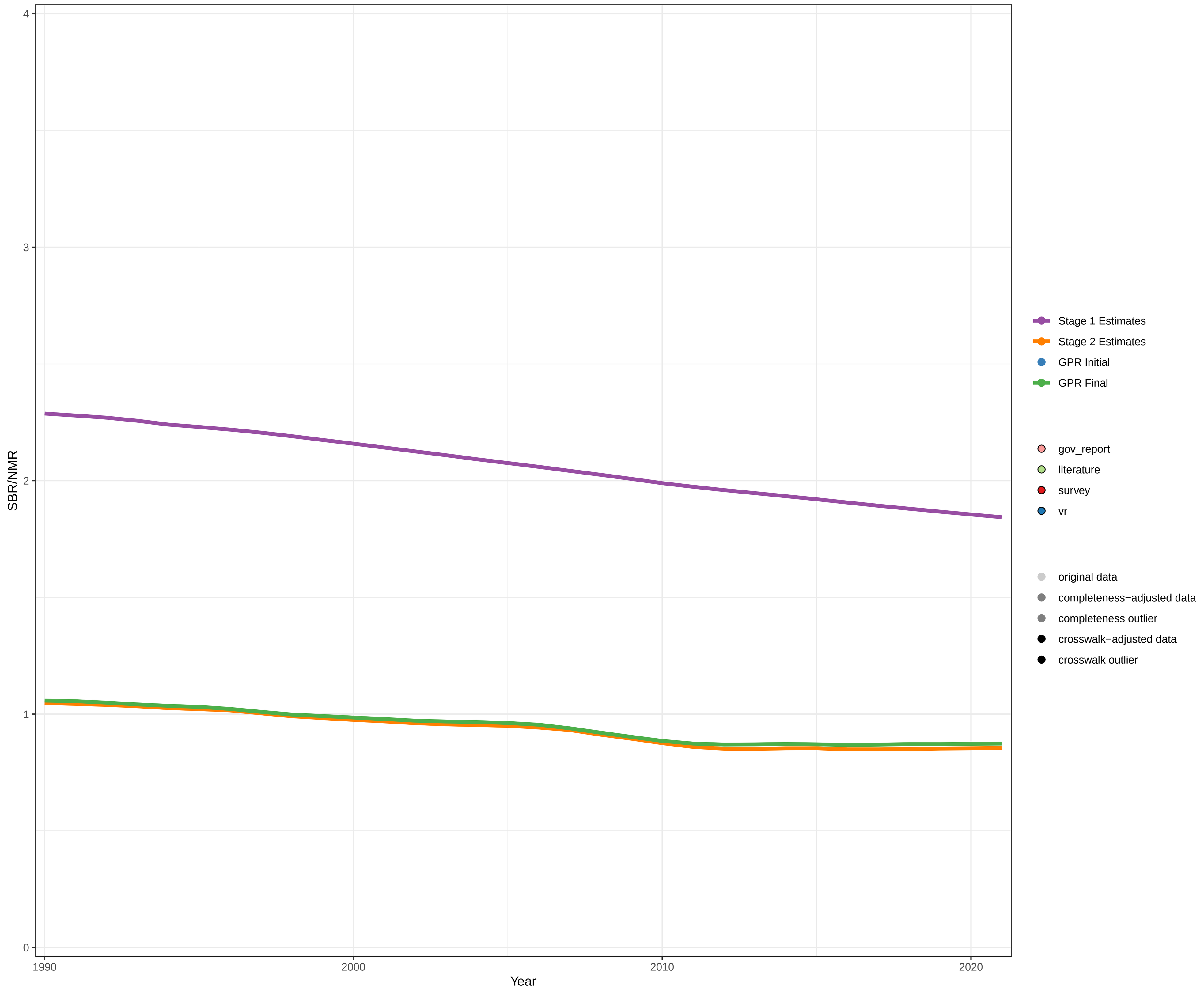

Definition	Years	Outlier	Source.Type	Title

Somalia SOM Eastern Sub−Saharan Africa Stillbirth Rate (SBR) − 20 weeks
lambda = 1 ; zeta = 0.6 ; scale = 15 ; amp = 0.028

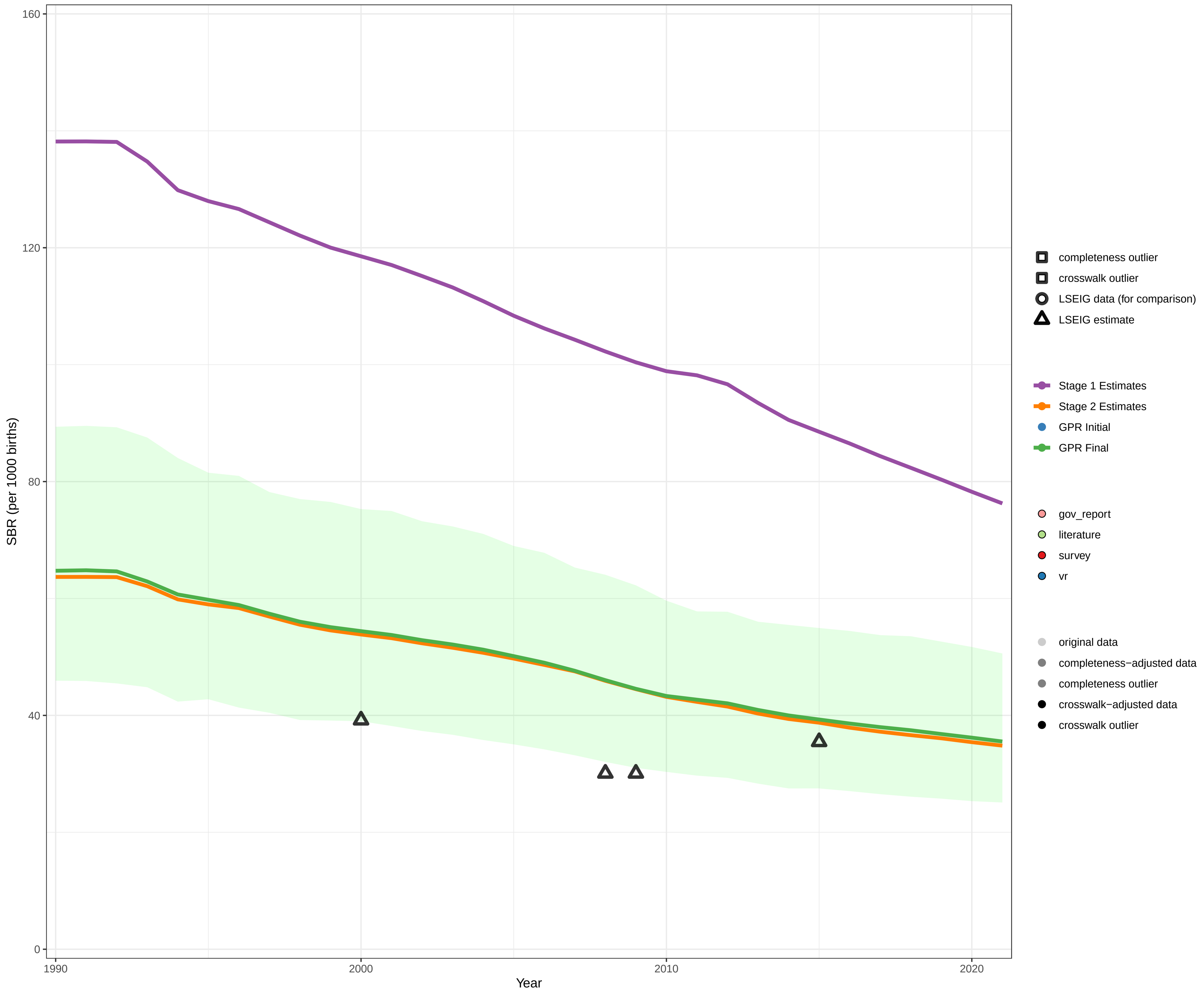

Somalia SOM Eastern Sub−Saharan Africa Stillbirth Rate (SBR) − 28 weeks
lambda = 1 ; zeta = 0.6 ; scale = 15 ; amp = 0.028

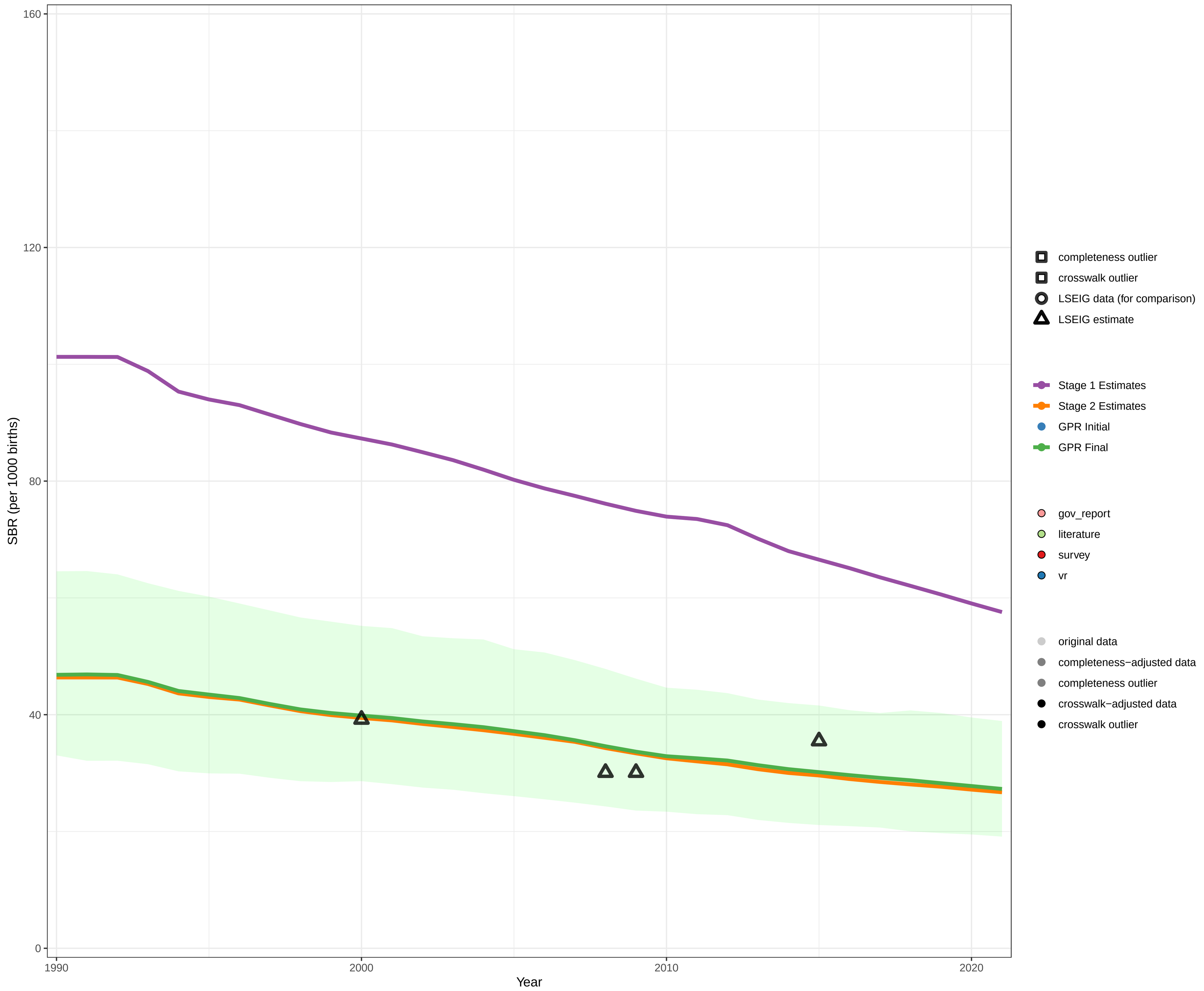

Year	Total Stillbirths (in thousands)	Total Stillbirths (in thousands)	SBR (per 1000 births)	SBR (per 1000 births)	SBR/NMR	SBR/NMR
	20 weeks	28 weeks	20 weeks	28 weeks	20 weeks	28 weeks
1990	27.2 (18.8 to 38.4)	19.3 (13.4 to 27.0)	64.7 (45.9 to 89.4)	46.8 (33.0 to 64.6)	1.46 (1.04 to 2.02)	1.06 (0.75 to 1.46)
1995	25.7 (18.0 to 35.8)	18.3 (12.4 to 25.8)	59.8 (42.8 to 81.5)	43.4 (30.0 to 60.2)	1.42 (1.01 to 1.93)	1.03 (0.71 to 1.43)
2000	29.2 (20.5 to 41.2)	21.0 (14.9 to 29.6)	54.4 (39.0 to 75.3)	39.8 (28.6 to 55.2)	1.35 (0.96 to 1.86)	0.99 (0.71 to 1.37)
2005	32.9 (22.6 to 46.1)	24.1 (16.6 to 33.6)	50.1 (35.0 to 69.0)	37.2 (26.1 to 51.2)	1.30 (0.91 to 1.78)	0.96 (0.67 to 1.32)
2010	32.5 (22.4 to 45.4)	24.4 (17.1 to 33.4)	43.3 (30.3 to 59.6)	32.9 (23.4 to 44.6)	1.17 (0.82 to 1.60)	0.88 (0.63 to 1.20)
2015	32.5 (22.4 to 46.1)	24.7 (17.1 to 34.4)	39.3 (27.5 to 55.0)	30.2 (21.1 to 41.6)	1.13 (0.79 to 1.59)	0.87 (0.61 to 1.20)
2020	34.9 (24.2 to 50.7)	26.6 (18.5 to 38.2)	36.2 (25.3 to 51.7)	27.8 (19.5 to 39.5)	1.14 (0.80 to 1.62)	0.87 (0.61 to 1.24)
2021	35.4 (24.7 to 51.2)	27.0 (18.7 to 38.9)	35.5 (25.1 to 50.6)	27.3 (19.1 to 38.9)	1.14 (0.80 to 1.62)	0.87 (0.61 to 1.25)

South Sudan SSD Eastern Sub−Saharan Africa Stillbirth Rate / Neonatal Mortality Rate (SBR/NMR) − 20 weeks
lambda = 1 ; zeta = 0.6 ; scale = 15 ; amp = 0.028

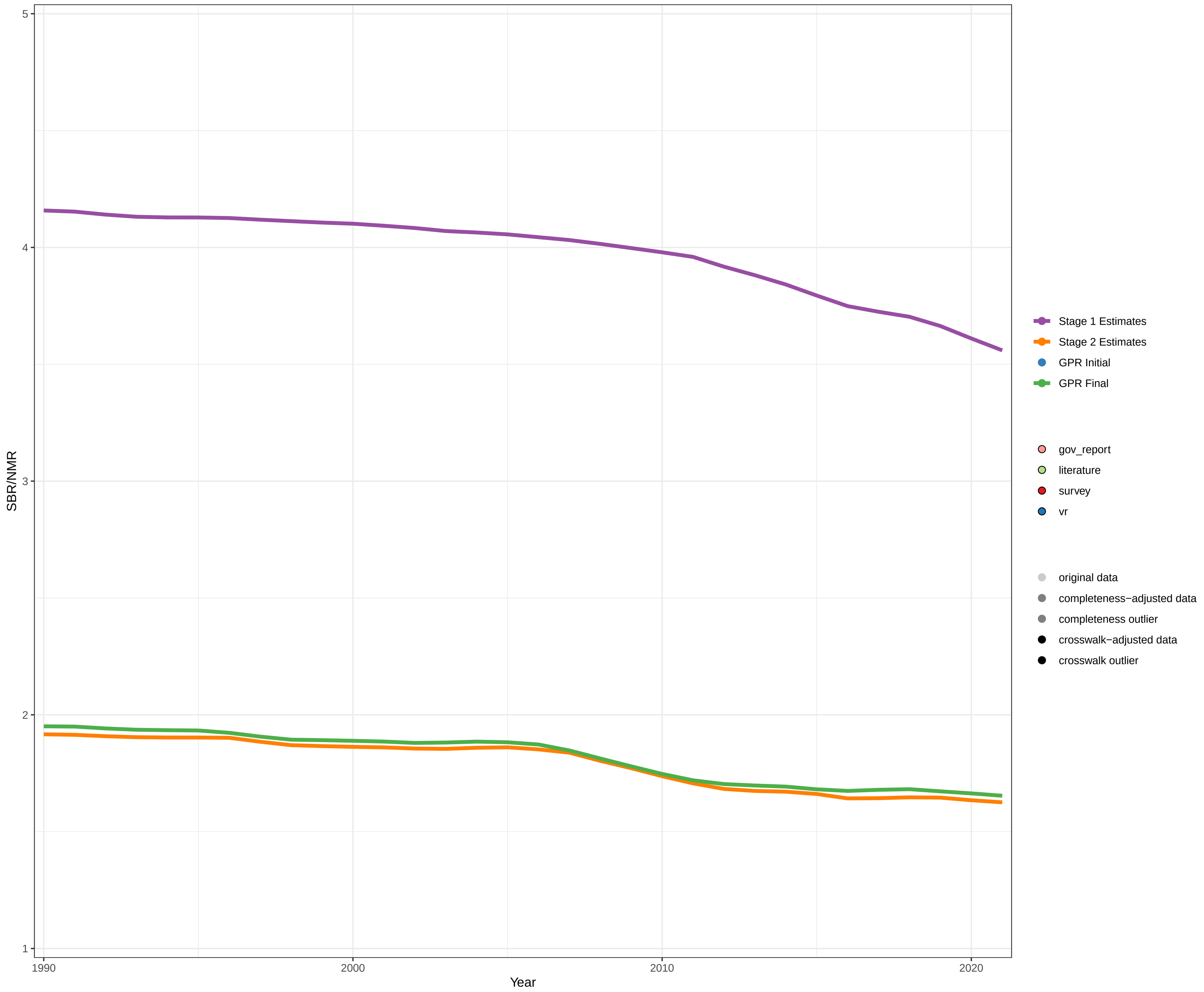

South Sudan SSD Eastern Sub−Saharan Africa Stillbirth Rate / Neonatal Mortality Rate (SBR/NMR) − 28 weeks
lambda = 1 ; zeta = 0.6 ; scale = 15 ; amp = 0.028

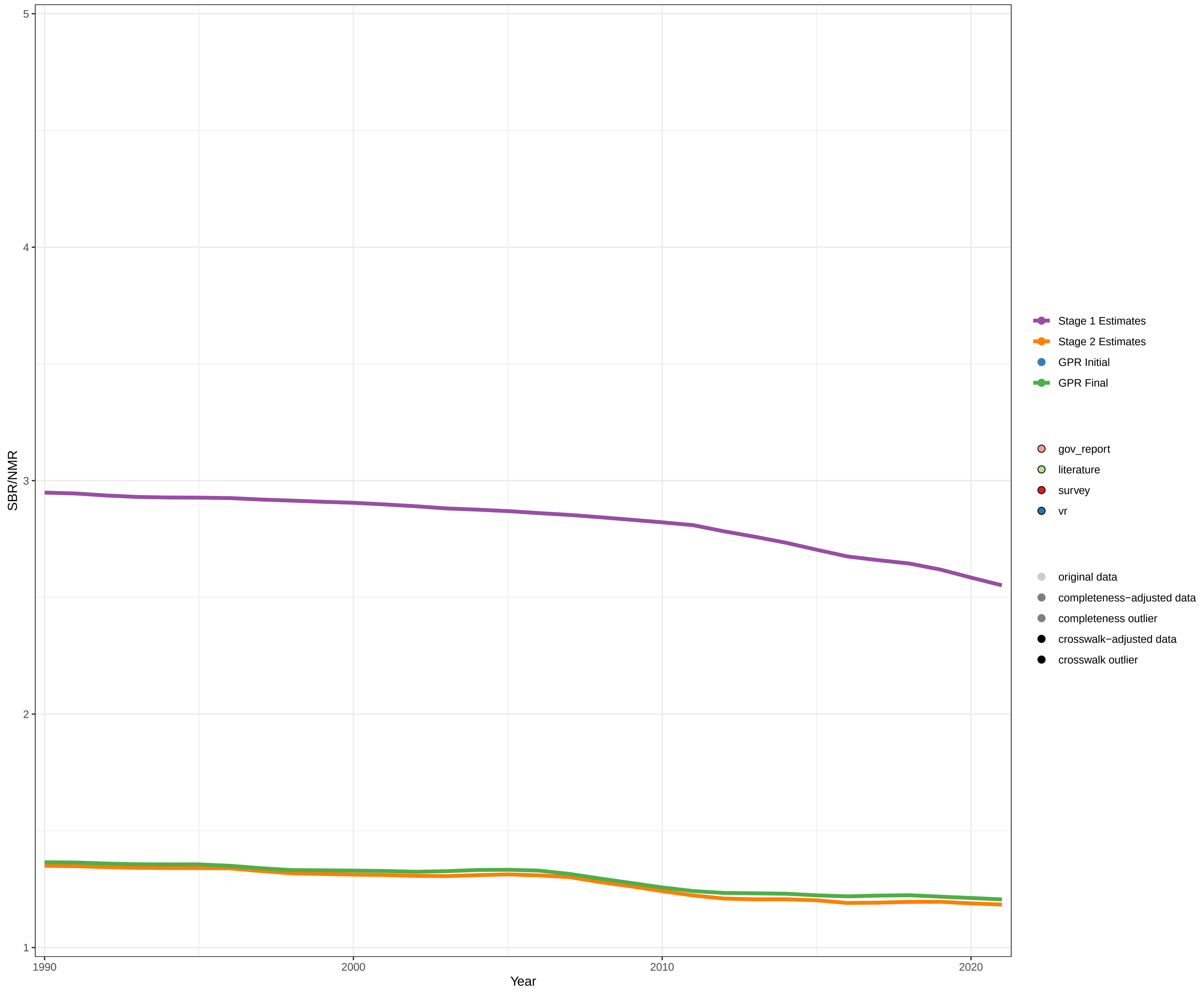

Definition	Years	Outlier	Source.Type	Title

South Sudan SSD Eastern Sub−Saharan Africa Stillbirth Rate (SBR) − 20 weeks
lambda = 1 ; zeta = 0.6 ; scale = 15 ; amp = 0.028

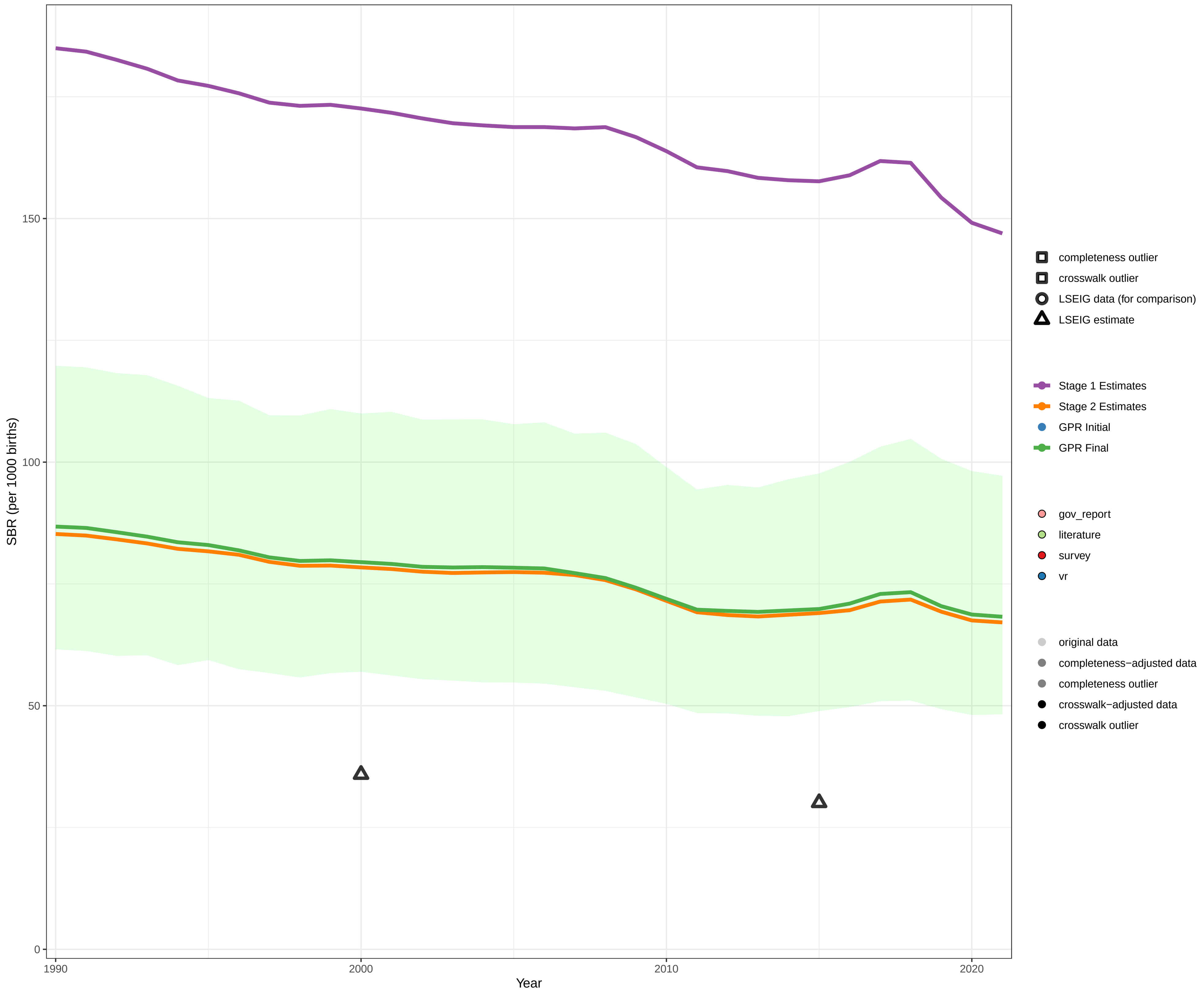

South Sudan SSD Eastern Sub−Saharan Africa Stillbirth Rate (SBR) − 28 weeks
lambda = 1 ; zeta = 0.6 ; scale = 15 ; amp = 0.028

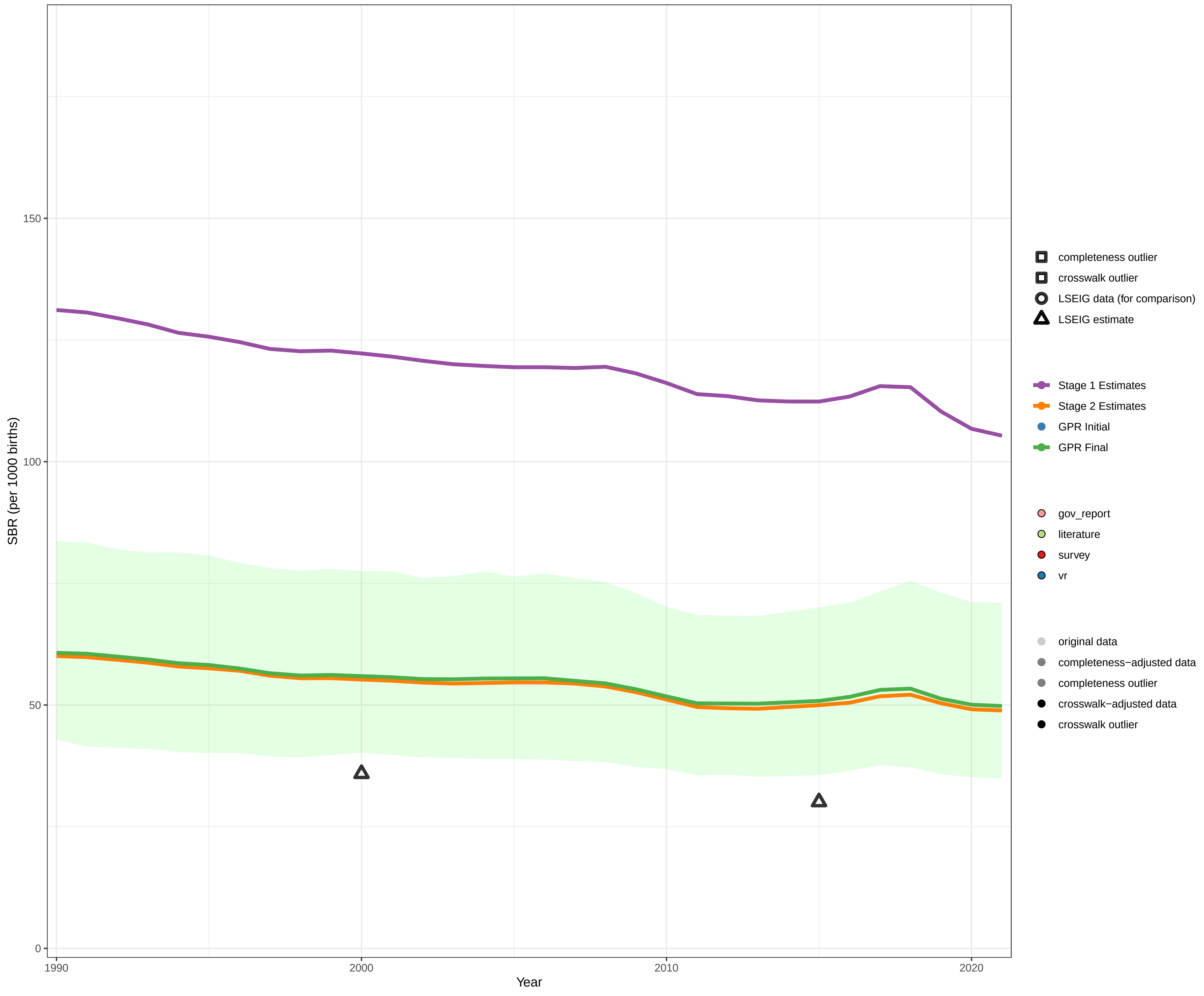

Year	Total Stillbirths (in thousands)	Total Stillbirths (in thousands)	SBR (per 1000 births)	SBR (per 1000 births)	SBR/NMR	SBR/NMR
	20 weeks	28 weeks	20 weeks	28 weeks	20 weeks	28 weeks
1990	24.5 (16.9 to 35.0)	16.7 (11.5 to 23.5)	86.8 (61.6 to 119.8)	60.7 (42.8 to 83.7)	1.95 (1.38 to 2.69)	1.37 (0.96 to 1.88)
1995	26.3 (18.3 to 37.0)	18.0 (12.1 to 25.5)	83.0 (59.4 to 113.2)	58.2 (40.1 to 80.7)	1.93 (1.38 to 2.64)	1.36 (0.94 to 1.88)
2000	28.4 (19.8 to 40.5)	19.5 (13.7 to 27.5)	79.5 (57.0 to 110.0)	55.9 (40.2 to 77.5)	1.89 (1.35 to 2.61)	1.33 (0.95 to 1.84)
2005	31.5 (21.4 to 44.6)	21.7 (14.9 to 30.6)	78.3 (54.8 to 107.8)	55.5 (38.9 to 76.4)	1.88 (1.32 to 2.59)	1.33 (0.93 to 1.84)
2010	32.1 (21.9 to 45.3)	22.6 (15.8 to 31.2)	71.9 (50.4 to 99.0)	51.8 (36.8 to 70.2)	1.75 (1.22 to 2.40)	1.26 (0.89 to 1.71)
2015	32.5 (22.2 to 46.7)	23.1 (15.9 to 32.5)	69.8 (48.9 to 97.7)	50.8 (35.6 to 70.1)	1.68 (1.18 to 2.35)	1.22 (0.86 to 1.69)
2020	27.5 (18.8 to 40.4)	19.6 (13.5 to 28.5)	68.7 (48.1 to 98.2)	50.1 (35.1 to 71.2)	1.66 (1.16 to 2.38)	1.21 (0.85 to 1.72)
2021	28.2 (19.5 to 41.4)	20.2 (13.9 to 29.4)	68.3 (48.2 to 97.2)	49.8 (34.9 to 71.0)	1.65 (1.17 to 2.35)	1.21 (0.85 to 1.72)

Uganda UGA Eastern Sub−Saharan Africa Stillbirth Rate / Neonatal Mortality Rate (SBR/NMR) − 20 weeks
lambda = 0.6 ; zeta = 0.8 ; scale = 15 ; amp = 0.028

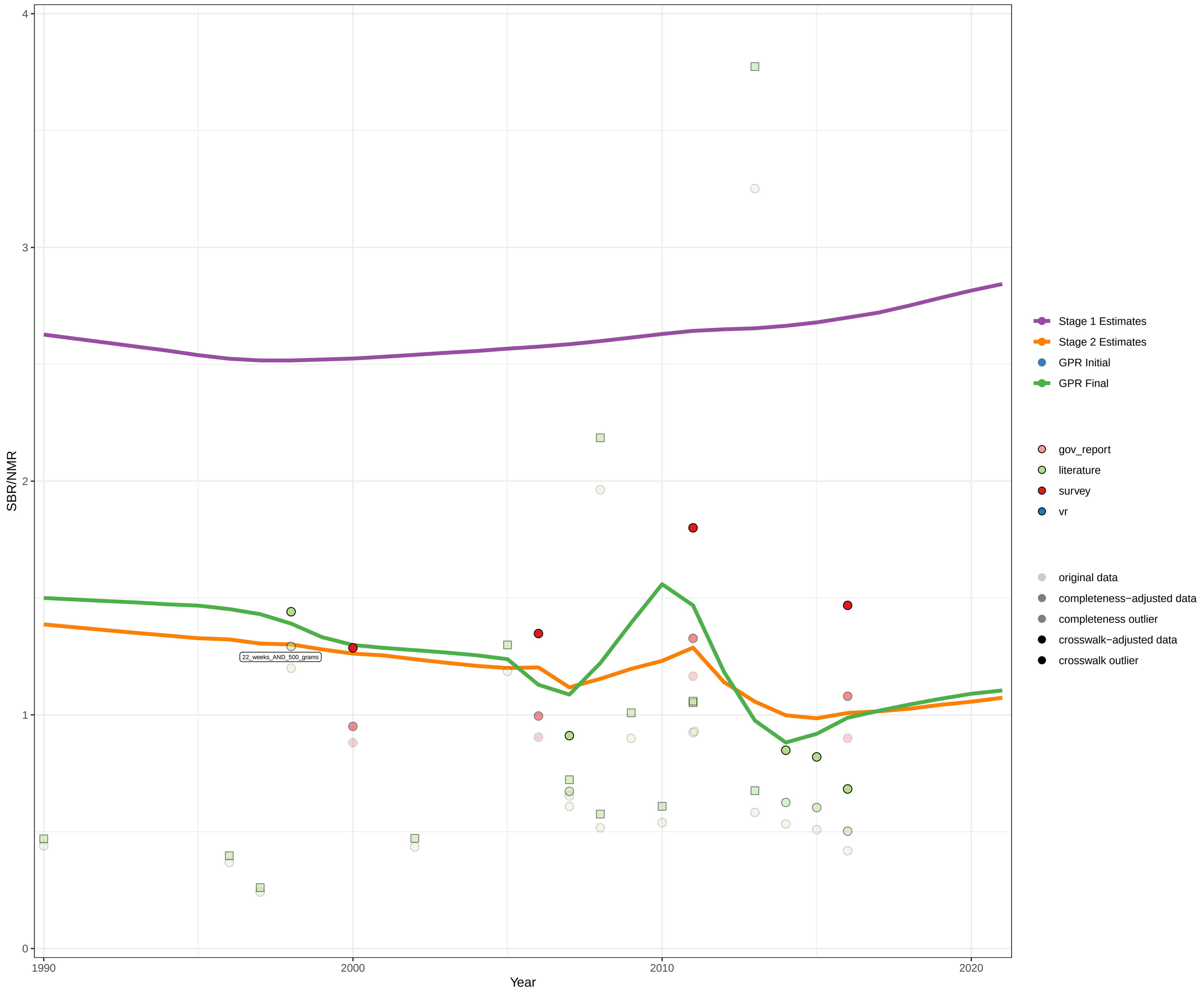

Uganda UGA Eastern Sub−Saharan Africa Stillbirth Rate / Neonatal Mortality Rate (SBR/NMR) − 28 weeks
lambda = 0.6 ; zeta = 0.8 ; scale = 15 ; amp = 0.028

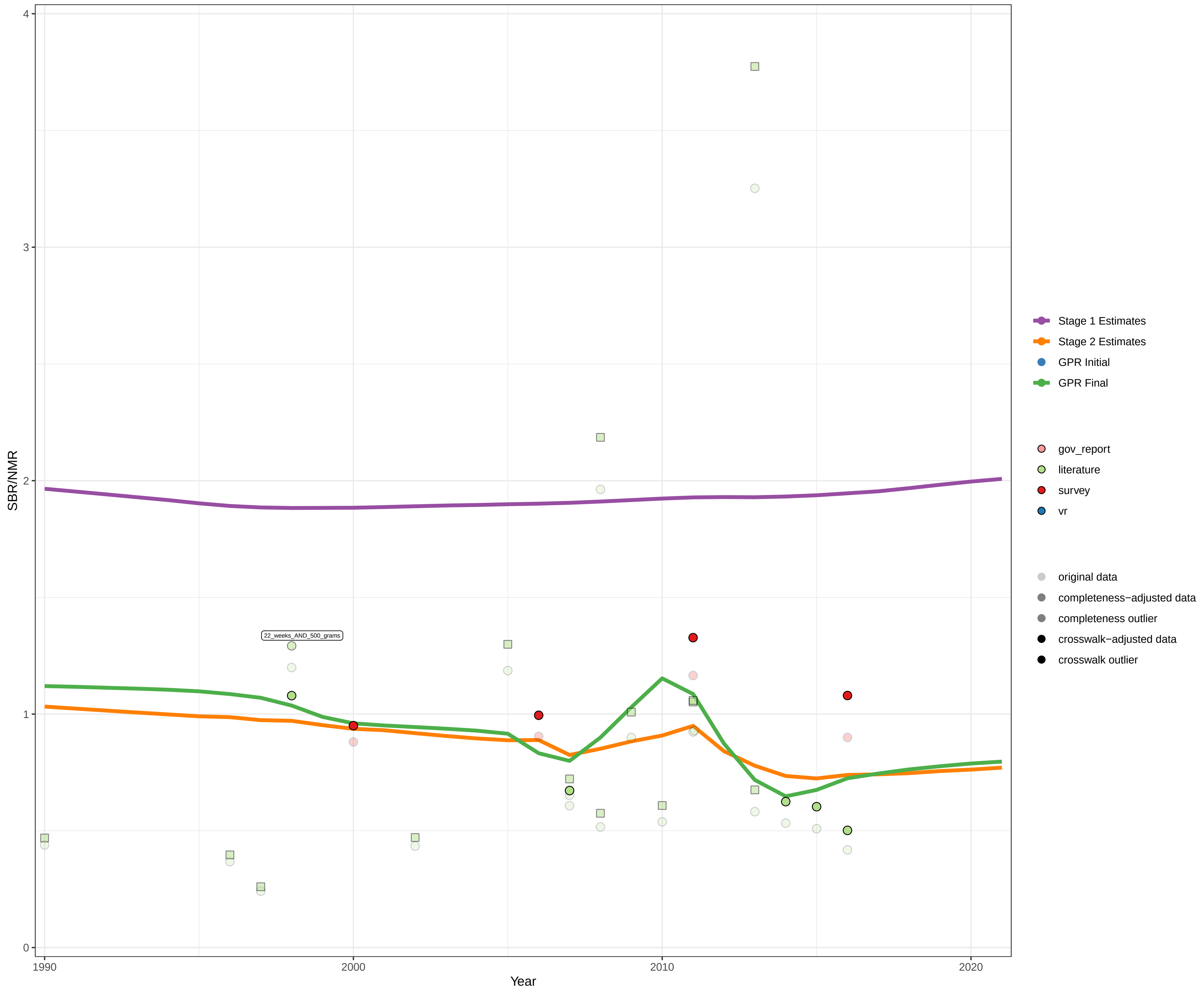

Definition	Years	Outlier	Source.Type	Title
22_weeks_AND_500_grams	1998	0	Sci Lit	Improving healthcare by perinatal mortality audit and feedback
28_weeks	2000	0	Survey	Uganda Demographic and Health Survey 2000−2001
28_weeks	2006	0	Survey	Uganda Demographic and Health Survey 2006
28_weeks	2007	0	Sci Lit	Perinatal Mortality in Eastern Uganda: A Community Based Prospective Cohort Study
28_weeks	2011	0	Survey	Uganda Demographic and Health Survey 2011
28_weeks	2014−2016	0	Sci Lit	Saving Mothers, Giving Life Approach for Strengthening Health Systems to Reduce Maternal and Newborn Deaths in 7 Scale−up Districts in Northern Uganda
28_weeks	2016	0	Survey	Uganda Demographic and Health Survey 2016
28_weeks	1990, 1996, 2002, 2008	1	Sci Lit	Trends and risk factors of stillbirths and neonatal deaths in Eastern Uganda (1982−2011): a cross−sectional, population−based study
28_weeks	1997, 2005, 2007−2011, 2013	1	Sci Lit	Adverse pregnancy outcomes in rural Uganda (1996−2013): trends and associated factors from serial cross sectional surveys
28_weeks	2013	1	Sci Lit	Predictors of stillbirths and neonatal deaths in rural western Uganda
28_weeks_OR_1000_grams	2011	1	Sci Lit	Maternal complications and perinatal mortality: findings of the World Health Organization Multicountry Survey on Maternal and Newborn Health

Uganda UGA Eastern Sub−Saharan Africa Stillbirth Rate (SBR) − 20 weeks
lambda = 0.6 ; zeta = 0.8 ; scale = 15 ; amp = 0.028

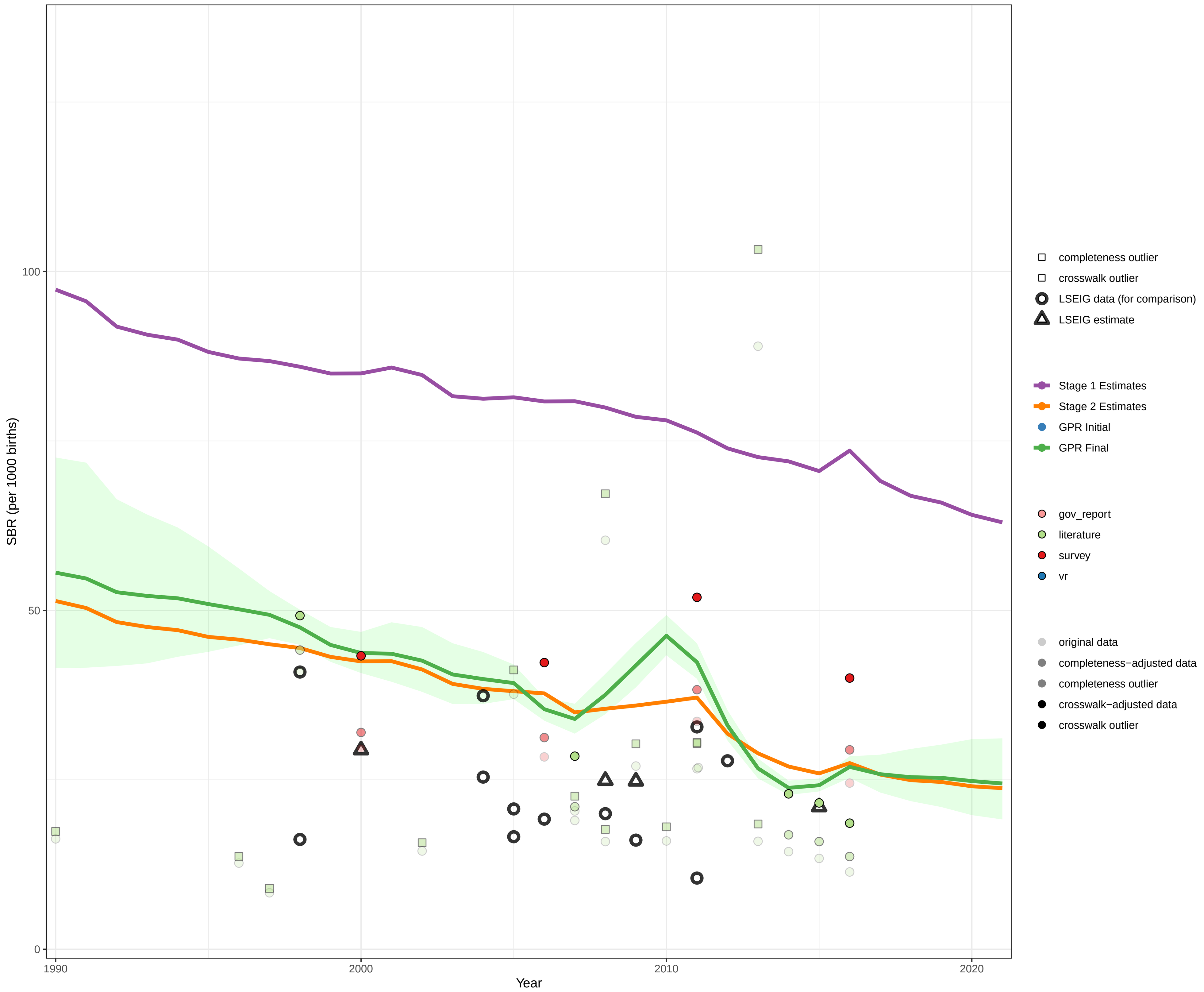

Uganda UGA Eastern Sub−Saharan Africa Stillbirth Rate (SBR) − 28 weeks
lambda = 0.6 ; zeta = 0.8 ; scale = 15 ; amp = 0.028

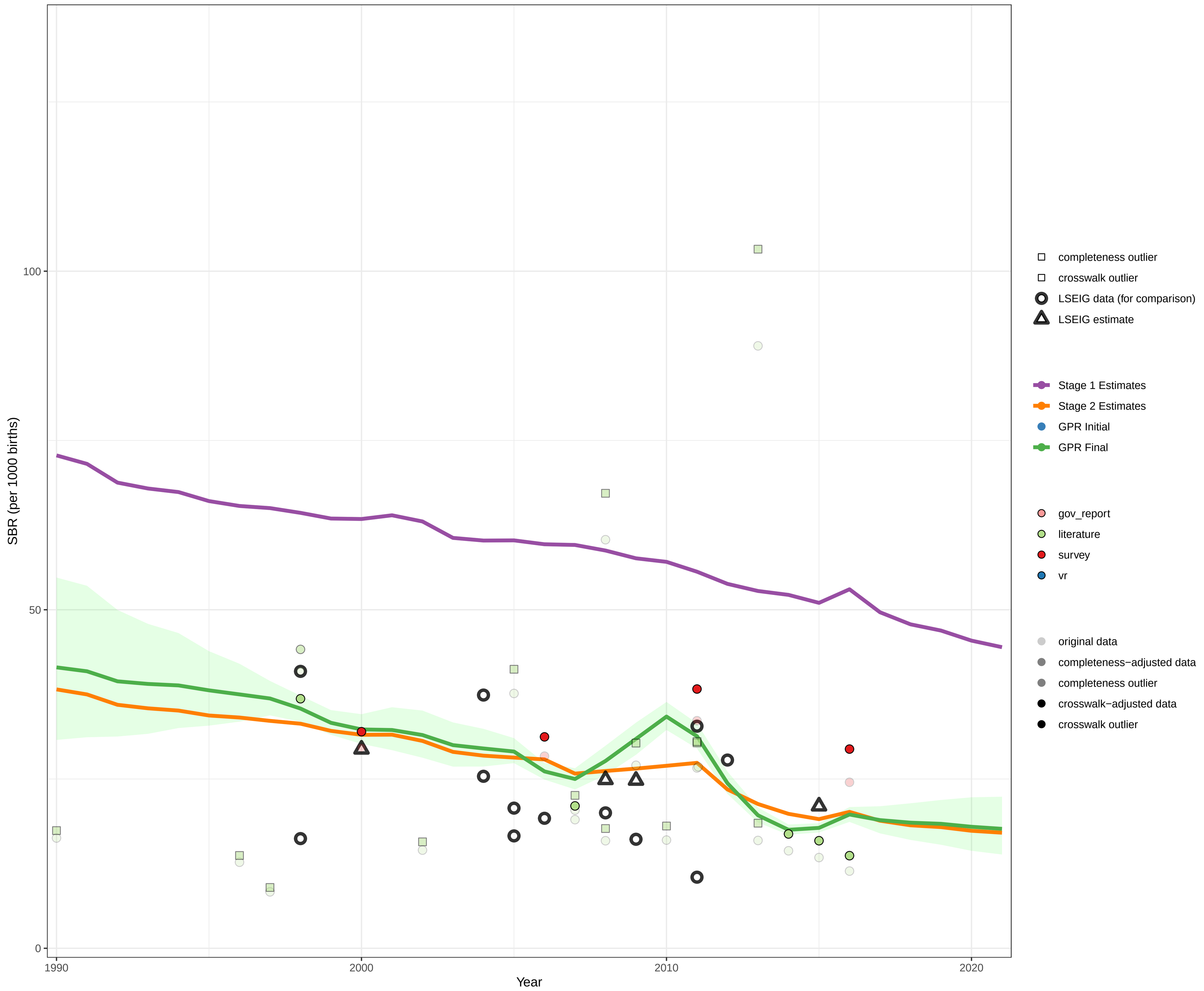

Year	Total Stillbirths (in thousands)	Total Stillbirths (in thousands)	SBR (per 1000 births)	SBR (per 1000 births)	SBR/NMR	SBR/NMR
	20 weeks	28 weeks	20 weeks	28 weeks	20 weeks	28 weeks
1990	55.1 (40.5 to 73.2)	40.5 (29.7 to 54.2)	55.6 (41.5 to 72.6)	41.5 (30.8 to 54.8)	1.50 (1.12 to 1.96)	1.12 (0.83 to 1.48)
1995	58.7 (50.1 to 69.1)	43.3 (37.2 to 50.2)	50.9 (43.9 to 59.4)	38.1 (32.9 to 43.9)	1.47 (1.26 to 1.71)	1.10 (0.95 to 1.26)
2000	57.6 (53.5 to 61.9)	42.1 (39.2 to 45.1)	43.7 (40.7 to 46.8)	32.3 (30.2 to 34.6)	1.30 (1.21 to 1.39)	0.96 (0.90 to 1.03)
2005	57.2 (53.6 to 61.3)	41.9 (39.3 to 44.8)	39.3 (36.9 to 42.0)	29.1 (27.3 to 31.0)	1.24 (1.16 to 1.32)	0.92 (0.86 to 0.98)
2010	72.3 (67.5 to 77.3)	52.8 (49.6 to 56.3)	46.3 (43.4 to 49.3)	34.2 (32.2 to 36.4)	1.56 (1.46 to 1.66)	1.15 (1.09 to 1.23)
2015	38.7 (37.2 to 40.3)	28.2 (27.1 to 29.4)	24.2 (23.3 to 25.2)	17.8 (17.1 to 18.5)	0.92 (0.88 to 0.96)	0.68 (0.65 to 0.70)
2020	40.4 (32.0 to 50.7)	29.0 (23.2 to 36.2)	24.8 (19.8 to 31.0)	17.9 (14.4 to 22.3)	1.09 (0.87 to 1.36)	0.79 (0.63 to 0.98)
2021	39.9 (31.1 to 51.1)	28.6 (22.4 to 36.4)	24.5 (19.2 to 31.1)	17.6 (13.9 to 22.4)	1.10 (0.86 to 1.40)	0.80 (0.63 to 1.01)

United Republic of Tanzania TZA Eastern Sub−Saharan Africa Stillbirth Rate / Neonatal Mortality Rate (SBR/NMR) − 20 weeks
lambda = 0.6 ; zeta = 0.8 ; scale = 15 ; amp = 0.028

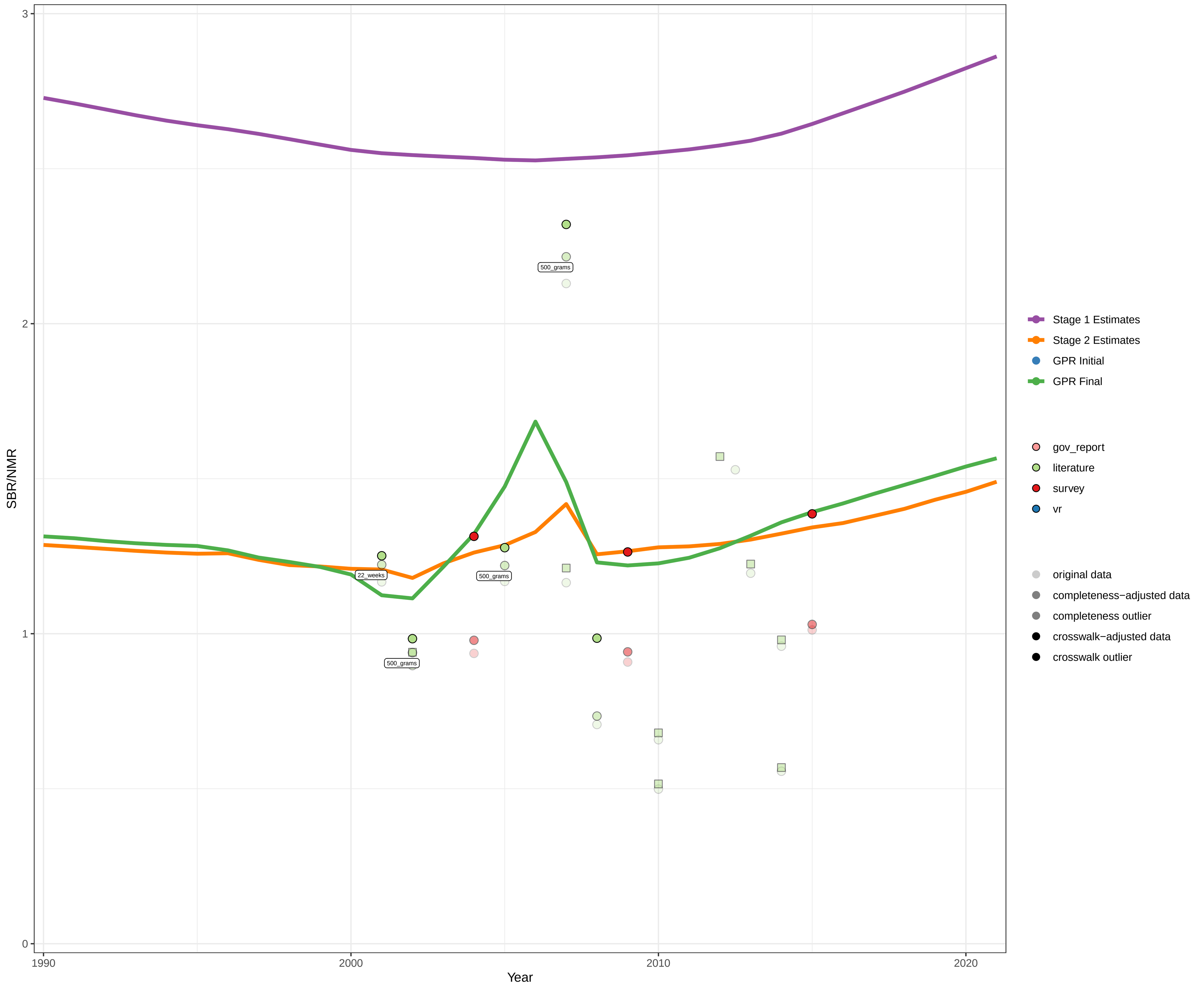

United Republic of Tanzania TZA Eastern Sub−Saharan Africa Stillbirth Rate / Neonatal Mortality Rate (SBR/NMR) − 28 weeks
lambda = 0.6 ; zeta = 0.8 ; scale = 15 ; amp = 0.028

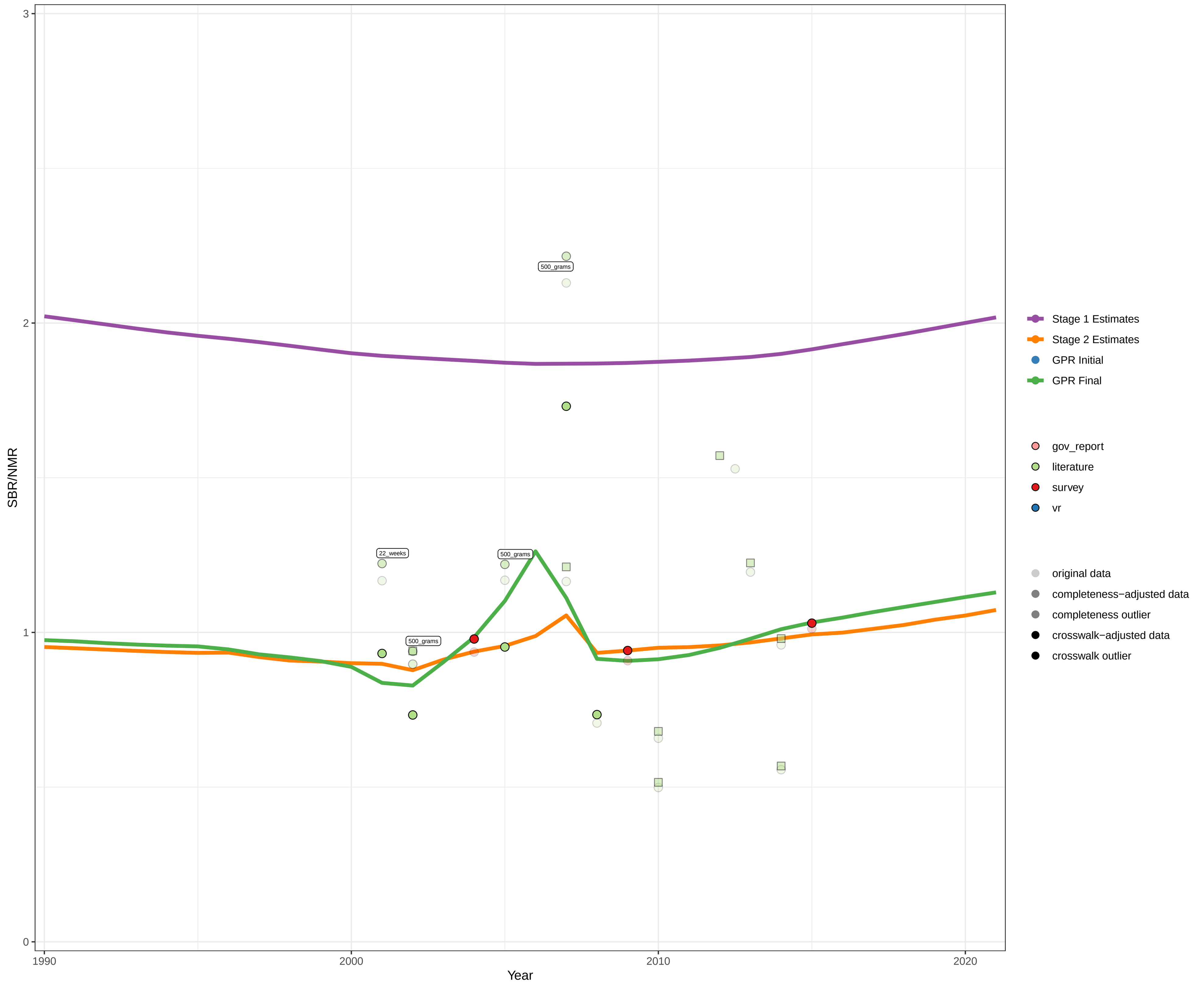

Definition	Years	Outlier	Source.Type	Title
22_weeks	2001	0	Sci Lit	Improving Surveillance for Maternal and Perinatal Health in 2 Districts of Rural Tanzania
28_weeks	2004	0	Survey	Tanzania Demographic and Health Survey 2004−2005
28_weeks	2008	0	Sci Lit	Impact of ALSO training on the management of prolonged labor and neonatal care at Kagera Regional Hospital, Tanzania
28_weeks	2009	0	Survey	Tanzania Demographic and Health Survey 2009−2010
28_weeks	2015	0	Survey	Tanzania Demographic and Health Survey 2015−2016
500_grams	2002	0	Sci Lit	Sociodemographic characteristics and perinatal mortality among singletons in North East Tanzania: a registry−based study
500_grams	2005	0	Sci Lit	Causes of perinatal death at a tertiary care hospital in Northern Tanzania 2000−2010: a registry based study
500_grams	2007	0	Sci Lit	Introduction of a qualitative perinatal audit at Muhimbili National Hospital, Dar es Salaam, Tanzania
22_weeks	2012	1	Sci Lit	Medication exposure during pregnancy: a pilot pharmacovigilance system using health and demographic surveillance platform
24_weeks	2007	1	Sci Lit	Maternal and fetal risk factors for stillbirth in Northern Tanzania: A registry−based retrospective cohort study
28_weeks	2010	1	Sci Lit	Newborn Mortality and Fresh Stillbirth Rates in Tanzania After Helping Babies Breathe Training
28_weeks	2014	1	Sci Lit	Population−based rates, timing, and causes of maternal deaths, stillbirths, and neonatal deaths in south Asia and sub−Saharan Africa: a multi−country prospective cohort study
28_weeks_AND_1000_grams	2013	1	Sci Lit	Predisposing factors associated with stillbirth in Tanzania
500_grams	2002	1	Sci Lit	Birthweight, preterm birth and perinatal mortality: A comparison of black babies in Tanzania and the USA

United Republic of Tanzania TZA Eastern Sub−Saharan Africa Stillbirth Rate (SBR) − 20 weeks
lambda = 0.6 ; zeta = 0.8 ; scale = 15 ; amp = 0.028

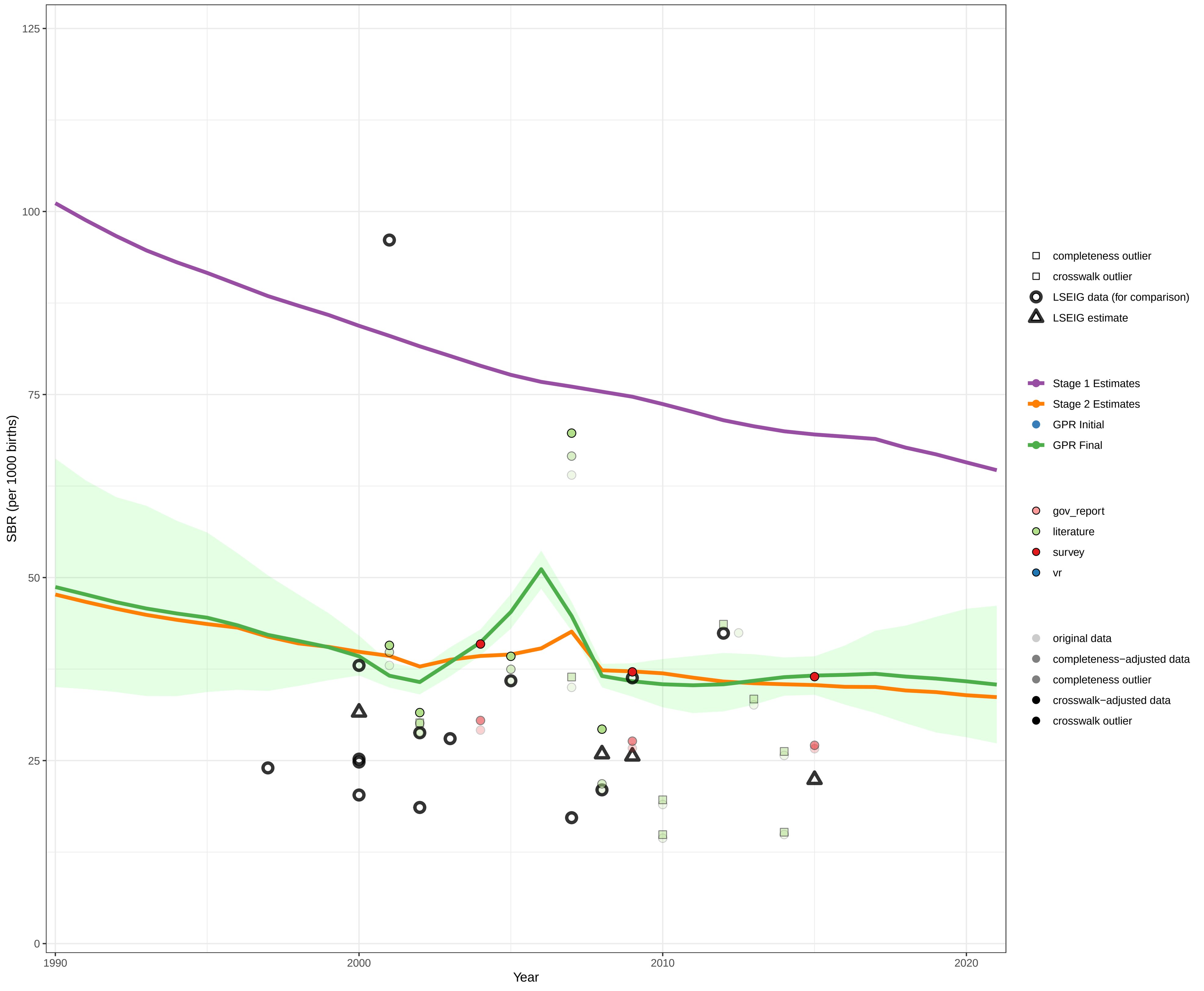

United Republic of Tanzania TZA Eastern Sub−Saharan Africa Stillbirth Rate (SBR) − 28 weeks
lambda = 0.6 ; zeta = 0.8 ; scale = 15 ; amp = 0.028

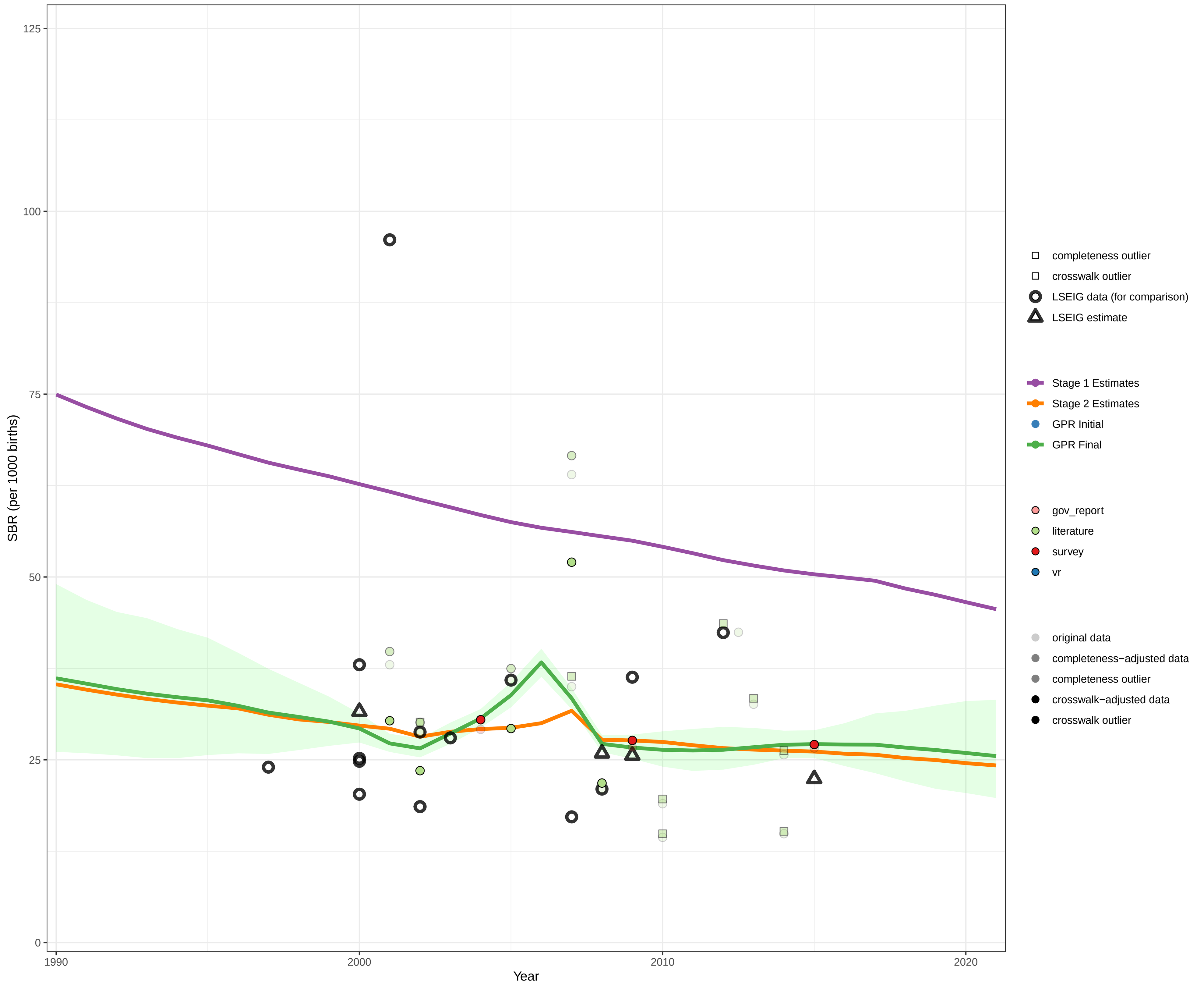

Year	Total Stillbirths (in thousands)	Total Stillbirths (in thousands)	SBR (per 1000 births)	SBR (per 1000 births)	SBR/NMR	SBR/NMR
	20 weeks	28 weeks	20 weeks	28 weeks	20 weeks	28 weeks
1990	62.5 (44.3 to 86.5)	45.8 (32.7 to 62.9)	48.7 (35.0 to 66.3)	36.1 (26.1 to 49.0)	1.31 (0.95 to 1.79)	0.98 (0.70 to 1.32)
1995	65.0 (49.6 to 82.9)	47.8 (36.7 to 60.6)	44.5 (34.4 to 56.2)	33.1 (25.7 to 41.7)	1.28 (0.99 to 1.62)	0.95 (0.74 to 1.20)
2000	63.3 (58.9 to 68.0)	46.7 (43.6 to 50.1)	39.3 (36.6 to 42.1)	29.3 (27.4 to 31.4)	1.19 (1.11 to 1.28)	0.89 (0.83 to 0.95)
2005	80.4 (76.2 to 85.0)	59.4 (56.3 to 62.6)	45.3 (43.0 to 47.7)	33.8 (32.2 to 35.6)	1.47 (1.40 to 1.55)	1.10 (1.05 to 1.16)
2010	66.7 (60.6 to 73.4)	49.2 (44.8 to 54.0)	35.4 (32.3 to 38.9)	26.4 (24.1 to 28.9)	1.23 (1.12 to 1.35)	0.91 (0.83 to 1.00)
2015	72.0 (66.7 to 77.4)	52.9 (49.1 to 56.7)	36.6 (34.0 to 39.2)	27.1 (25.2 to 29.0)	1.39 (1.29 to 1.49)	1.03 (0.96 to 1.10)
2020	70.7 (55.1 to 91.1)	50.6 (39.7 to 64.9)	35.8 (28.2 to 45.7)	25.9 (20.5 to 33.0)	1.54 (1.21 to 1.97)	1.11 (0.88 to 1.42)
2021	69.8 (53.5 to 92.0)	49.8 (38.4 to 65.3)	35.4 (27.3 to 46.2)	25.5 (19.8 to 33.2)	1.57 (1.21 to 2.04)	1.13 (0.88 to 1.47)

Zambia ZMB Eastern Sub−Saharan Africa Stillbirth Rate / Neonatal Mortality Rate (SBR/NMR) − 20 weeks
lambda = 0.6 ; zeta = 0.8 ; scale = 15 ; amp = 0.028

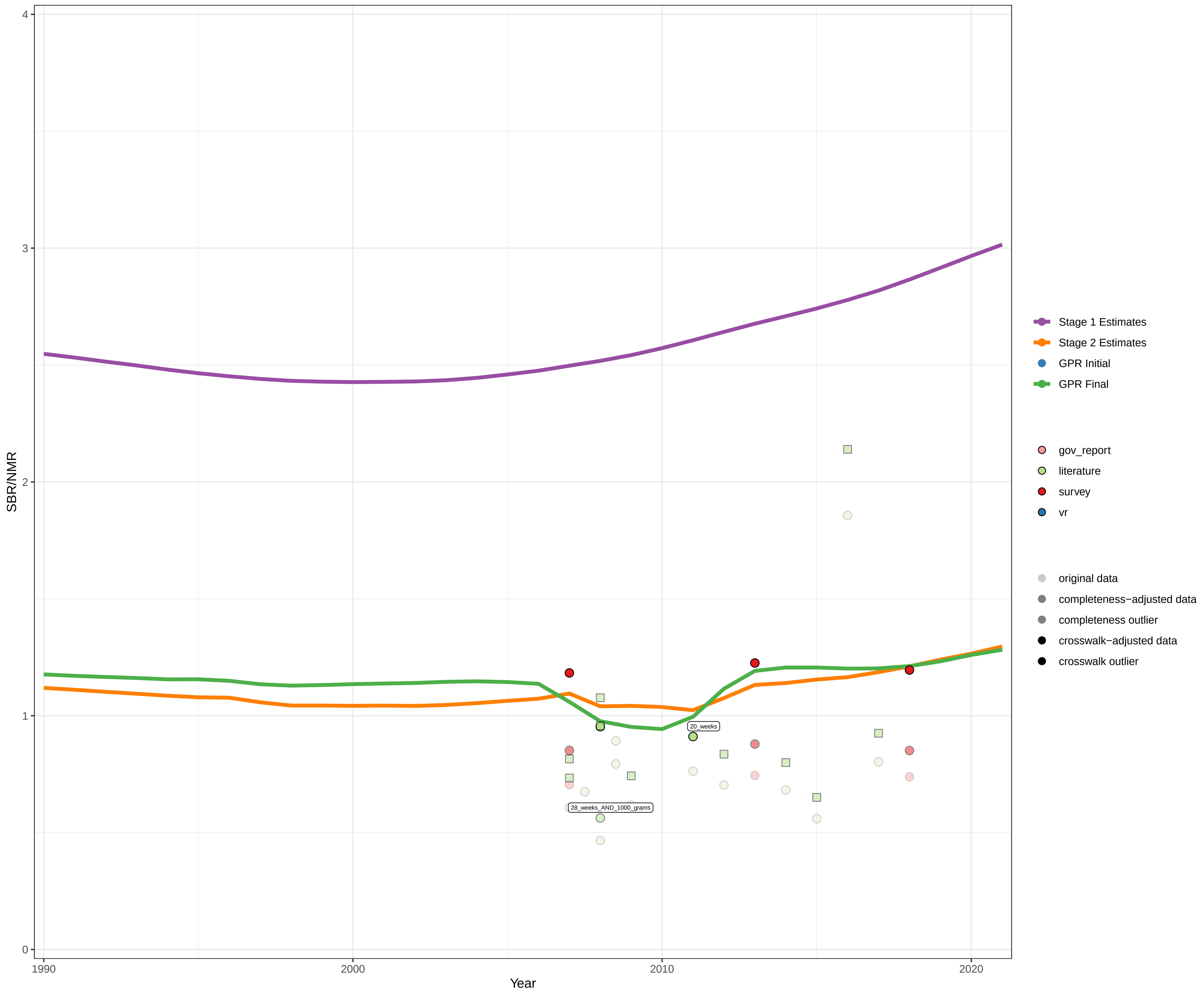

Zambia ZMB Eastern Sub−Saharan Africa Stillbirth Rate / Neonatal Mortality Rate (SBR/NMR) − 28 weeks
lambda = 0.6 ; zeta = 0.8 ; scale = 15 ; amp = 0.028

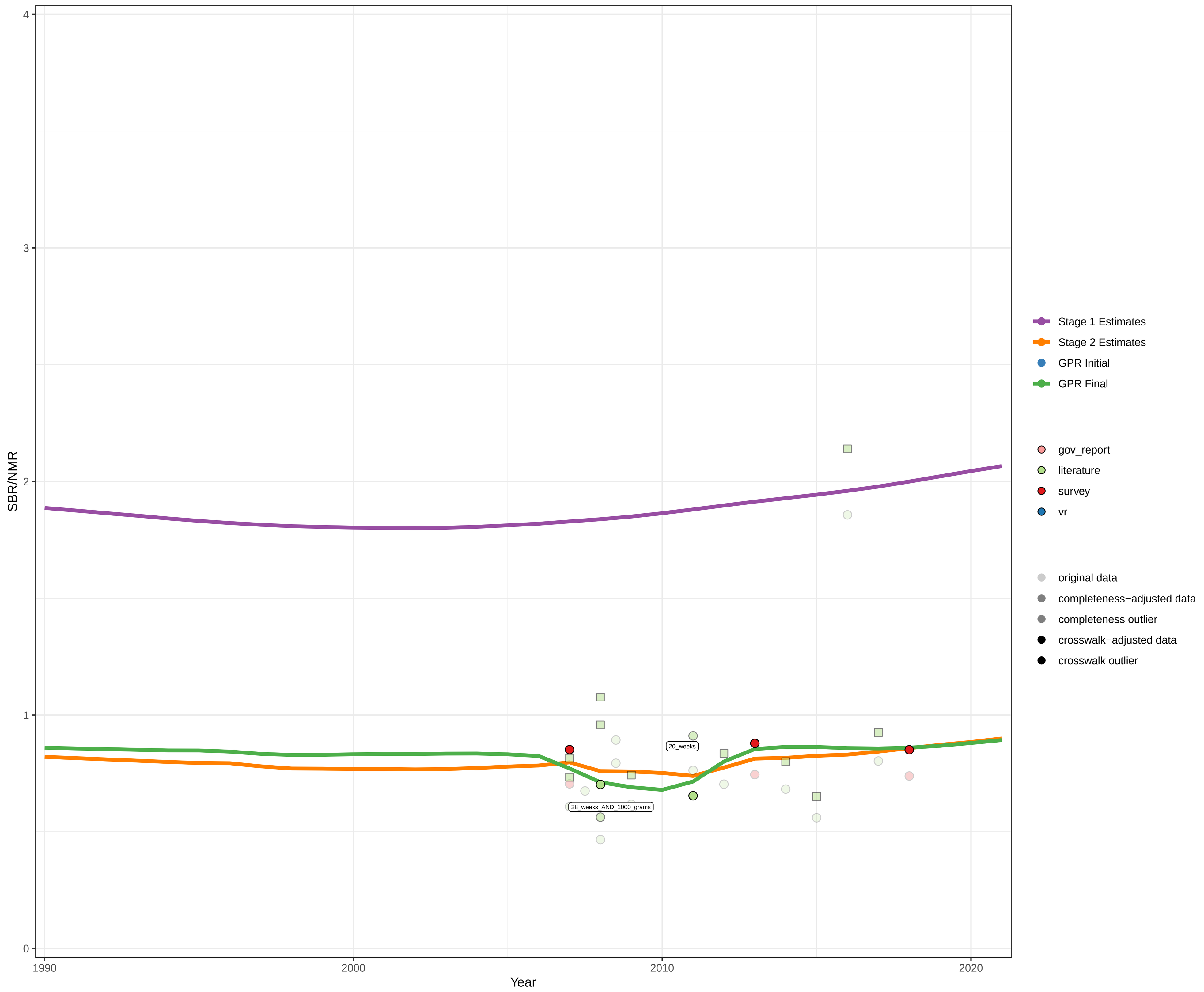

Definition	Years	Outlier	Source.Type	Title
20_weeks	2011	0	Sci Lit	A prospective study of maternal, fetal and neonatal deaths in low− and middle−income countries
28_weeks	2007	0	Survey	Zambia Demographic and Health Survey 2007
28_weeks	2013	0	Survey	Zambia Demographic and Health Survey 2013−2014
28_weeks	2018	0	Survey	Zambia Demographic and Health Survey 2018−2019
28_weeks_AND_1000_grams	2008	0	Sci Lit	Epidemiology of stillbirth in low−middle income countries: A Global Network Study
20_weeks	2015	1	Sci Lit	Global Network for Women's and Children's Health Research: probable causes of stillbirth in low− and middle−income countries using a prospectively defined classification system
22_weeks	2007	1	Sci Lit	Determinants of stillbirth in Zambia
22_weeks	2012	1	Sci Lit	Effectiveness of 4% chlorhexidine umbilical cord care on neonatal mortality in Southern Province, Zambia (ZamCAT): a cluster−randomised controlled trial
26_weeks	2007	1	Sci Lit	Effect of training traditional birth attendants on neonatal mortality (Lufwanyama Neonatal Survival Project): randomised controlled study
28_weeks	2008	1	Sci Lit	Causes of stillbirth, neonatal death and early childhood death in rural Zambia by verbal autopsy assessments
28_weeks	2008	1	Sci Lit	Implementation of the Zambia Electronic Perinatal Record System for comprehensive prenatal and delivery care
28_weeks	2014	1	Sci Lit	Population−based rates, timing, and causes of maternal deaths, stillbirths, and neonatal deaths in south Asia and sub−Saharan Africa: a multi−country prospective cohort study
28_weeks	2016	1	Sci Lit	Adverse birth outcomes and their clinical phenotypes in an urban Zambian cohort
28_weeks_OR_1000_grams	2009	1	Sci Lit	Predictors and outcomes of low birth weight in Lusaka, Zambia
28_weeks_OR_1000_grams	2017	1	Sci Lit	Baby survival in Zambia: stillbirth and neonatal death in a local hospital setting

Zambia ZMB Eastern Sub−Saharan Africa Stillbirth Rate (SBR) − 20 weeks
lambda = 0.6 ; zeta = 0.8 ; scale = 15 ; amp = 0.028

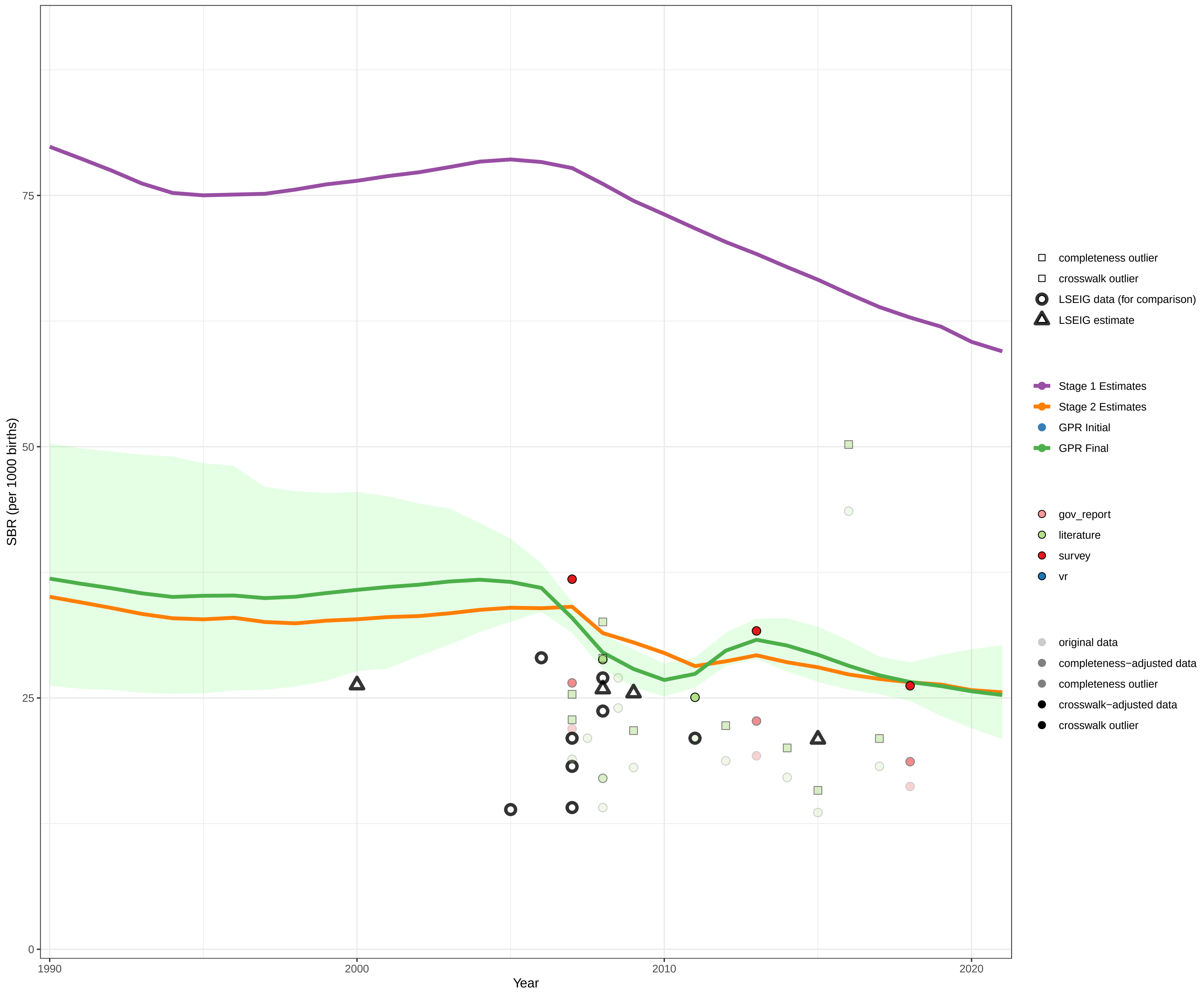

Zambia ZMB Eastern Sub−Saharan Africa Stillbirth Rate (SBR) − 28 weeks
lambda = 0.6 ; zeta = 0.8 ; scale = 15 ; amp = 0.028

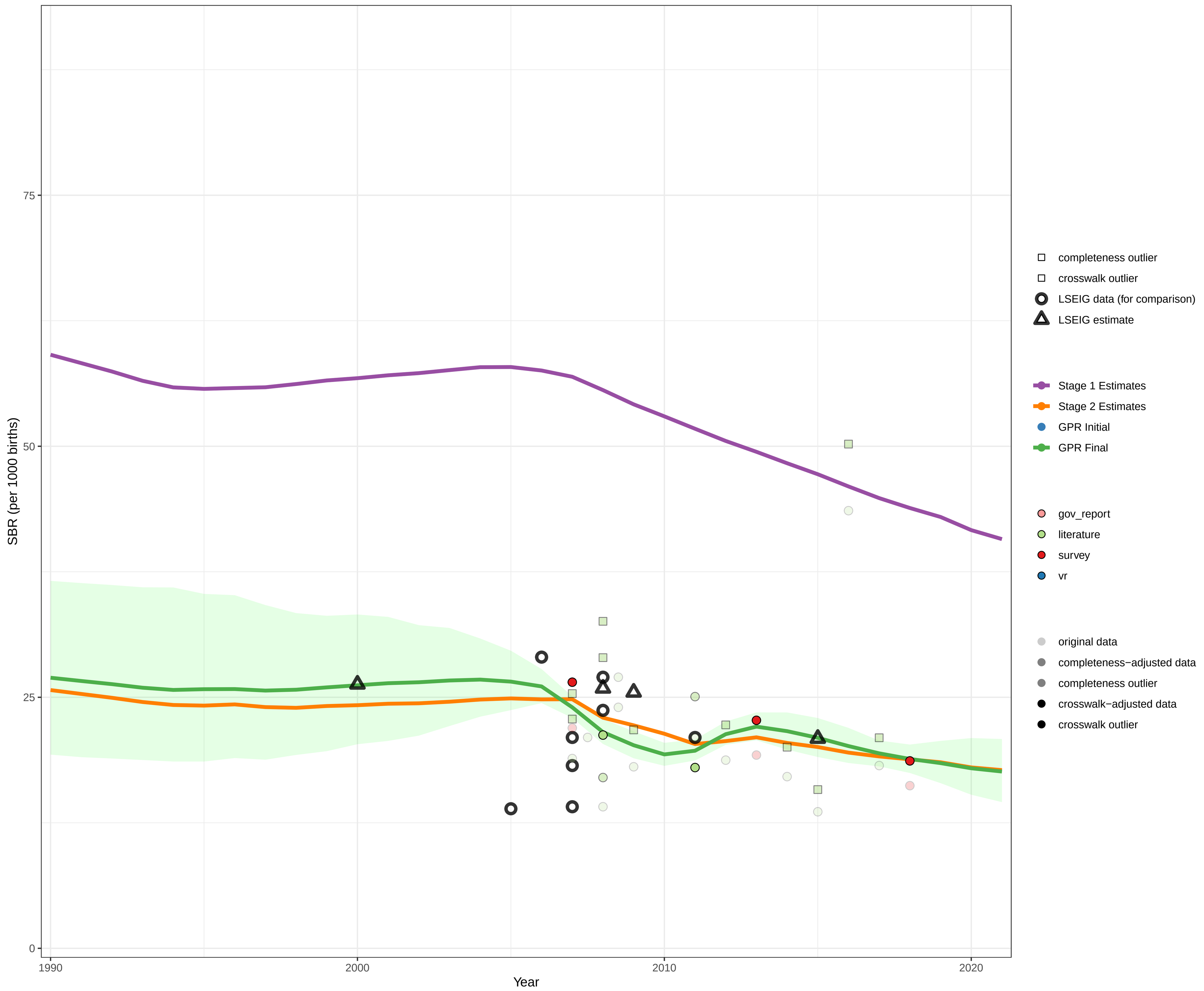

Year	Total Stillbirths (in thousands)	Total Stillbirths (in thousands)	SBR (per 1000 births)	SBR (per 1000 births)	SBR/NMR	SBR/NMR
	20 weeks	28 weeks	20 weeks	28 weeks	20 weeks	28 weeks
1990	15.0 (10.5 to 20.7)	10.8 (7.7 to 14.8)	36.9 (26.2 to 50.3)	26.9 (19.3 to 36.6)	1.18 (0.84 to 1.61)	0.86 (0.62 to 1.17)
1995	15.8 (11.3 to 22.0)	11.5 (8.2 to 15.8)	35.2 (25.5 to 48.4)	25.8 (18.6 to 35.3)	1.16 (0.84 to 1.59)	0.85 (0.61 to 1.16)
2000	17.5 (13.4 to 22.5)	12.7 (9.8 to 16.2)	35.8 (27.7 to 45.5)	26.2 (20.3 to 33.2)	1.14 (0.88 to 1.44)	0.83 (0.65 to 1.06)
2005	20.1 (17.8 to 22.6)	14.4 (12.9 to 16.2)	36.5 (32.6 to 40.9)	26.6 (23.7 to 29.6)	1.14 (1.02 to 1.28)	0.83 (0.74 to 0.93)
2010	16.4 (15.4 to 17.5)	11.7 (11.0 to 12.5)	26.8 (25.2 to 28.4)	19.3 (18.2 to 20.5)	0.94 (0.88 to 1.00)	0.68 (0.64 to 0.72)
2015	19.0 (17.2 to 20.9)	13.5 (12.2 to 14.8)	29.3 (26.6 to 32.1)	21.0 (19.1 to 22.9)	1.21 (1.09 to 1.32)	0.86 (0.78 to 0.94)
2020	16.1 (13.7 to 18.8)	11.2 (9.5 to 13.1)	25.7 (22.0 to 29.9)	17.9 (15.3 to 20.9)	1.26 (1.08 to 1.47)	0.88 (0.75 to 1.03)
2021	15.8 (13.0 to 18.9)	10.9 (9.0 to 12.9)	25.3 (20.9 to 30.3)	17.6 (14.6 to 20.8)	1.28 (1.06 to 1.53)	0.89 (0.74 to 1.06)

Southern Sub−Saharan Africa R20 Southern Sub−Saharan Africa Stillbirth Rate / Neonatal Mortality Rate (SBR/NMR) − 20 weeks
lambda = 1 ; zeta = 0.6 ; scale = 15 ; amp = 0.028

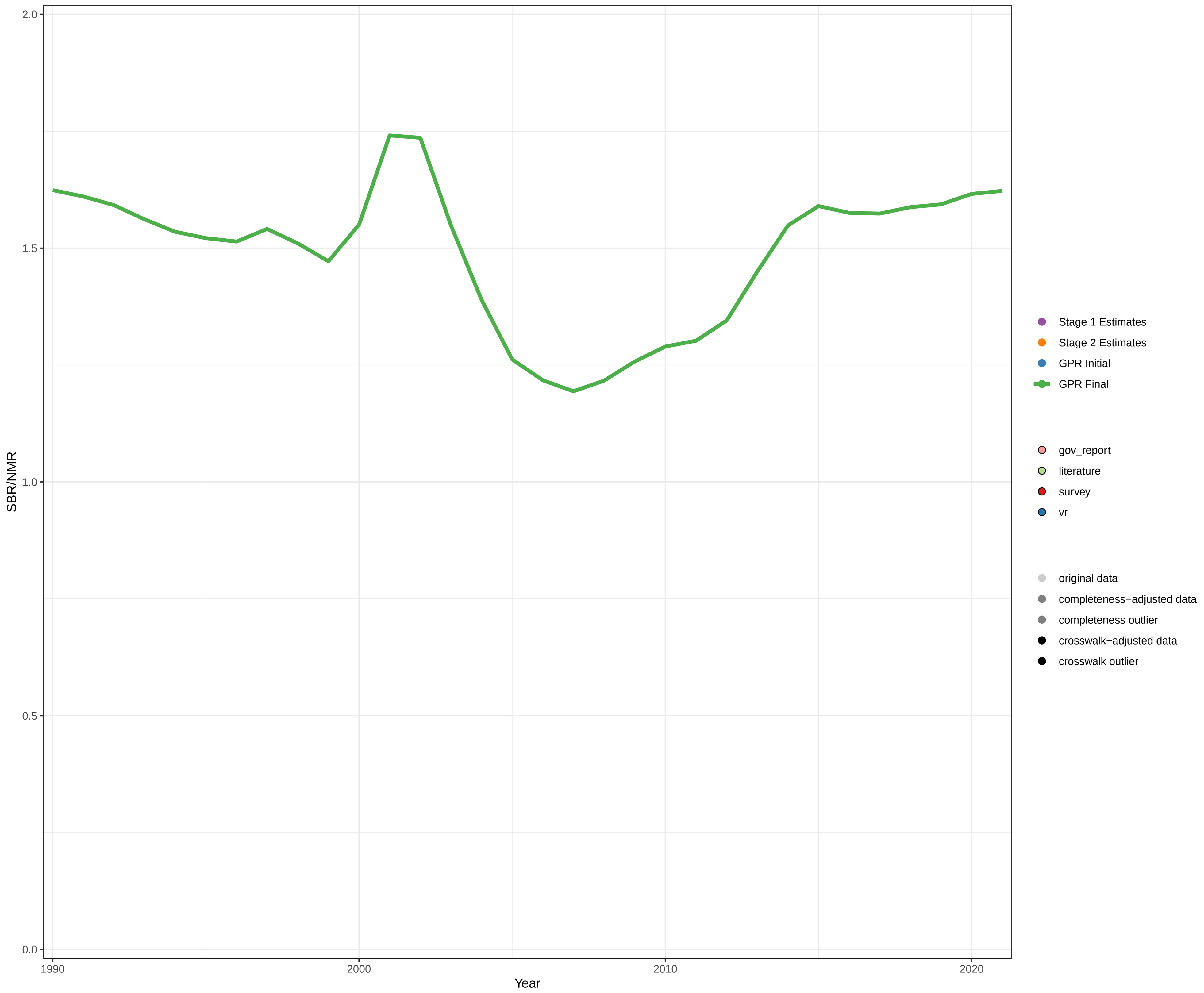

Southern Sub−Saharan Africa R20 Southern Sub−Saharan Africa Stillbirth Rate / Neonatal Mortality Rate (SBR/NMR) − 28 weeks
lambda = 1 ; zeta = 0.6 ; scale = 15 ; amp = 0.028

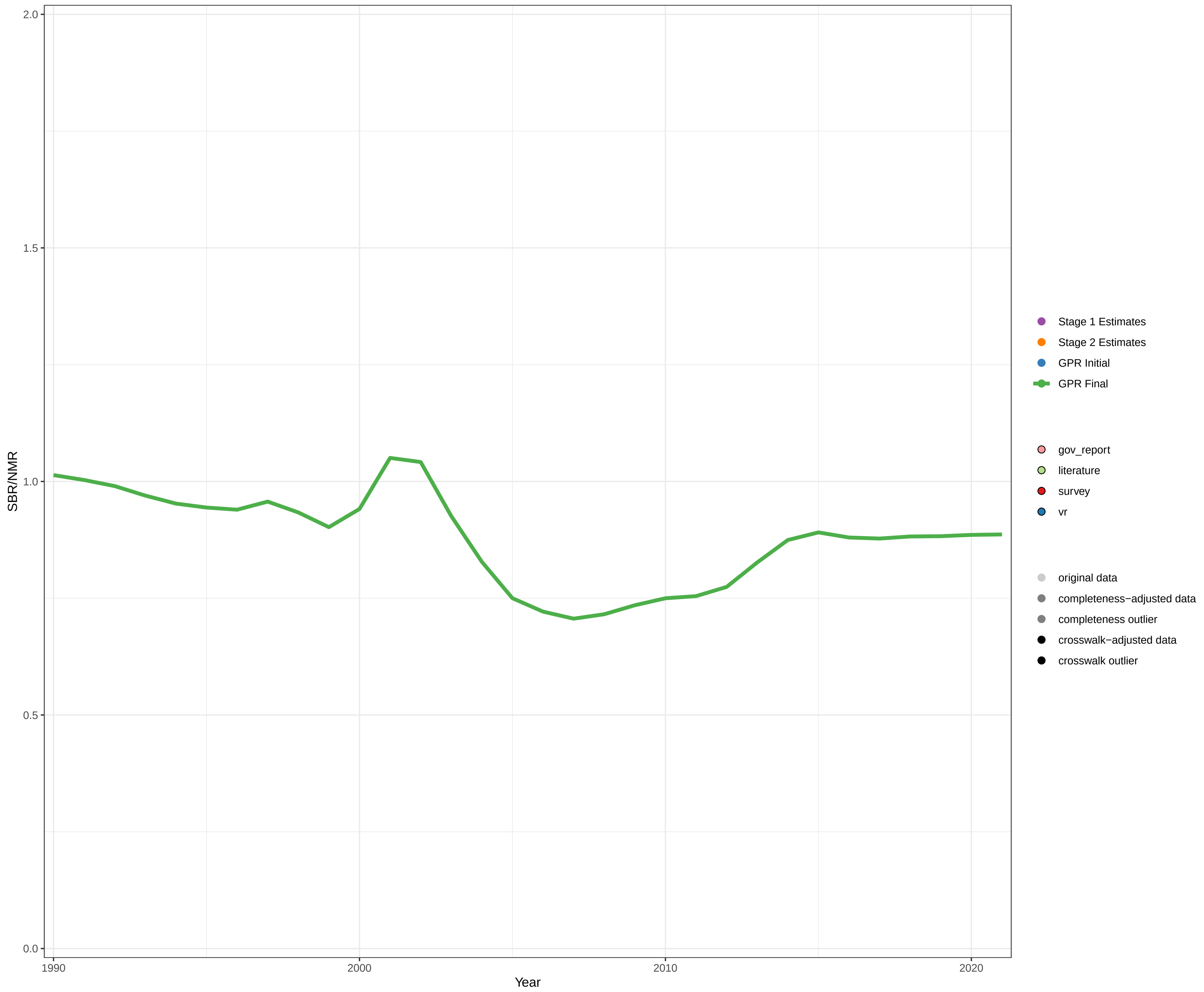

Definition	Years	Outlier	Source.Type	Title

Southern Sub−Saharan Africa R20 Southern Sub−Saharan Africa Stillbirth Rate (SBR) − 20 weeks
lambda = 1 ; zeta = 0.6 ; scale = 15 ; amp = 0.028

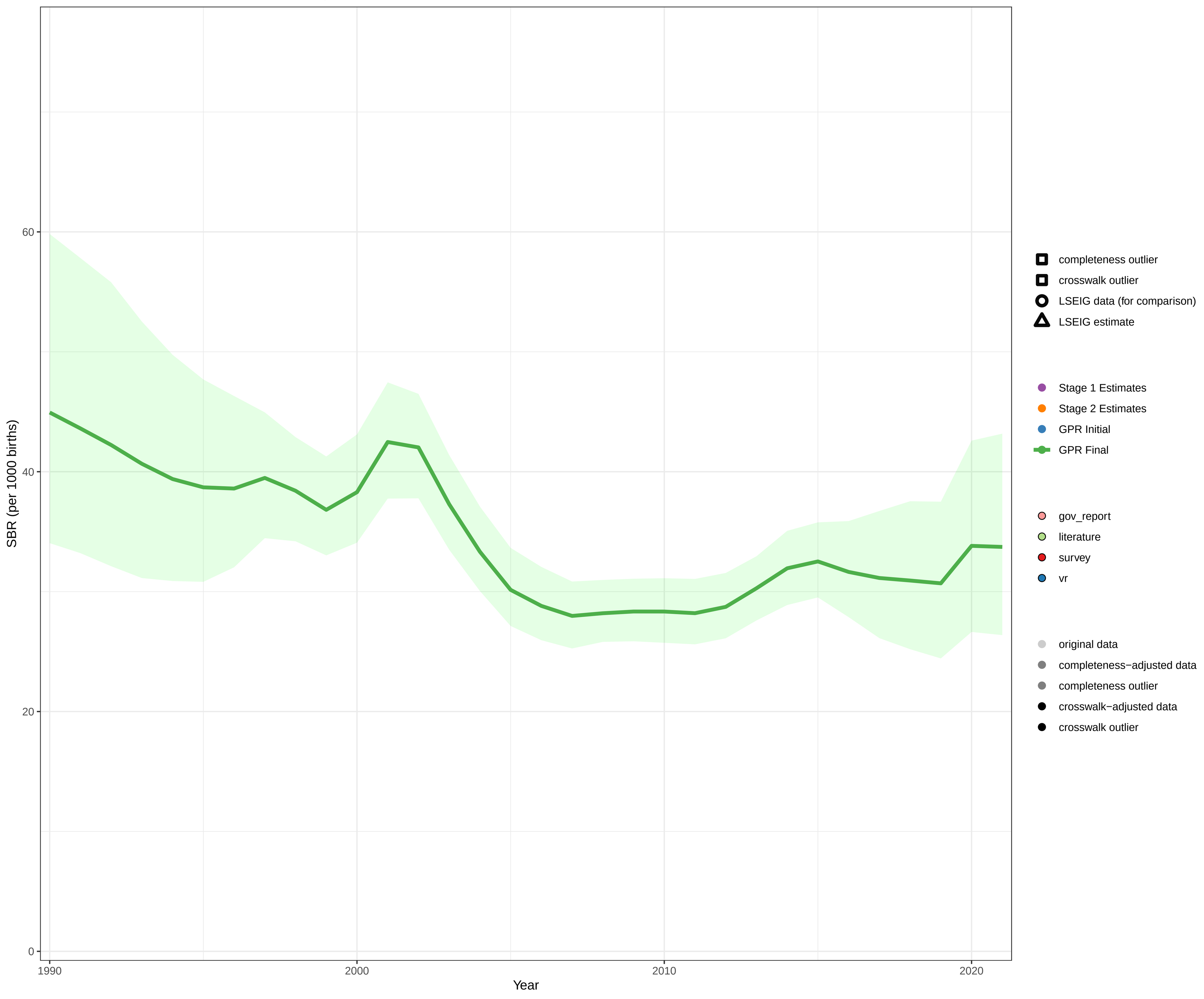

Southern Sub−Saharan Africa R20 Southern Sub−Saharan Africa Stillbirth Rate (SBR) − 28 weeks
lambda = 1 ; zeta = 0.6 ; scale = 15 ; amp = 0.028

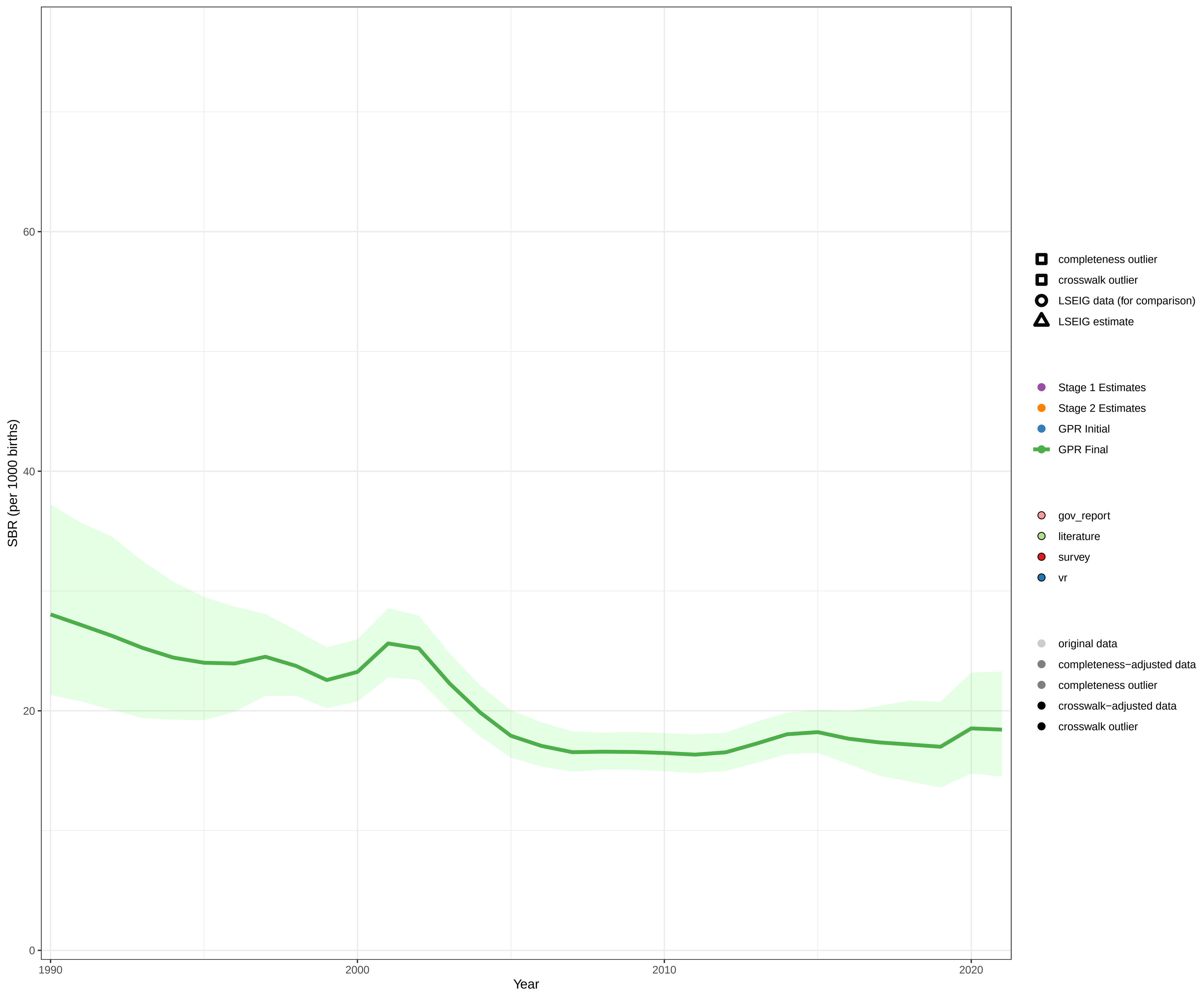

Year	Total Stillbirths (in thousands)	Total Stillbirths (in thousands)	SBR (per 1000 births)	SBR (per 1000 births)	SBR/NMR	SBR/NMR
	20 weeks	28 weeks	20 weeks	28 weeks	20 weeks	28 weeks
1990	76.8 (57.5 to 103.7)	47.1 (35.5 to 63.0)	44.9 (34.0 to 59.8)	28.0 (21.3 to 37.2)	1.62 (1.23 to 2.16)	1.01 (0.77 to 1.34)
1995	65.8 (51.9 to 81.8)	40.2 (32.0 to 49.6)	38.7 (30.8 to 47.7)	24.0 (19.2 to 29.5)	1.52 (1.21 to 1.88)	0.94 (0.76 to 1.16)
2000	65.6 (58.1 to 74.2)	39.2 (35.0 to 43.9)	38.3 (34.1 to 43.1)	23.2 (20.8 to 26.0)	1.55 (1.38 to 1.75)	0.94 (0.84 to 1.05)
2005	54.2 (48.7 to 60.8)	31.8 (28.5 to 35.7)	30.1 (27.1 to 33.7)	17.9 (16.1 to 20.0)	1.26 (1.14 to 1.41)	0.75 (0.67 to 0.84)
2010	52.9 (47.9 to 58.3)	30.4 (27.6 to 33.5)	28.3 (25.7 to 31.1)	16.5 (15.0 to 18.1)	1.29 (1.17 to 1.42)	0.75 (0.68 to 0.83)
2015	59.4 (53.7 to 65.6)	32.8 (29.6 to 36.2)	32.5 (29.5 to 35.8)	18.2 (16.5 to 20.1)	1.59 (1.44 to 1.75)	0.89 (0.81 to 0.98)
2020	58.1 (45.4 to 73.8)	31.3 (24.9 to 39.4)	33.8 (26.6 to 42.6)	18.5 (14.8 to 23.2)	1.62 (1.27 to 2.04)	0.89 (0.71 to 1.11)
2021	57.2 (44.3 to 73.9)	30.7 (24.1 to 39.0)	33.7 (26.4 to 43.2)	18.4 (14.5 to 23.3)	1.62 (1.27 to 2.08)	0.89 (0.70 to 1.12)

Botswana BWA Southern Sub−Saharan Africa Stillbirth Rate / Neonatal Mortality Rate (SBR/NMR) − 20 weeks
lambda = 0.4 ; zeta = 0.9 ; scale = 10 ; amp = 0.028

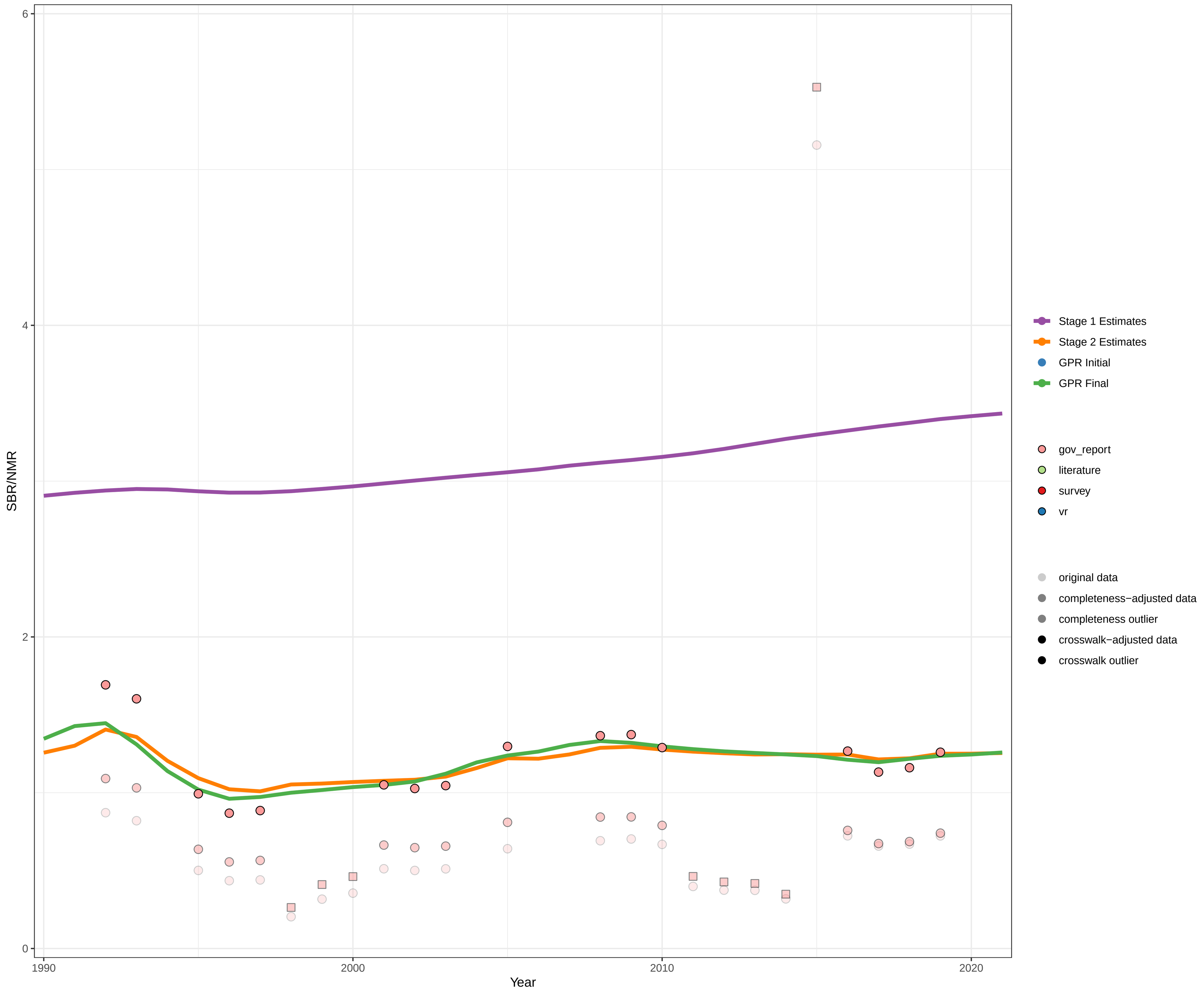

Botswana BWA Southern Sub−Saharan Africa Stillbirth Rate / Neonatal Mortality Rate (SBR/NMR) − 28 weeks
lambda = 0.4 ; zeta = 0.9 ; scale = 10 ; amp = 0.028

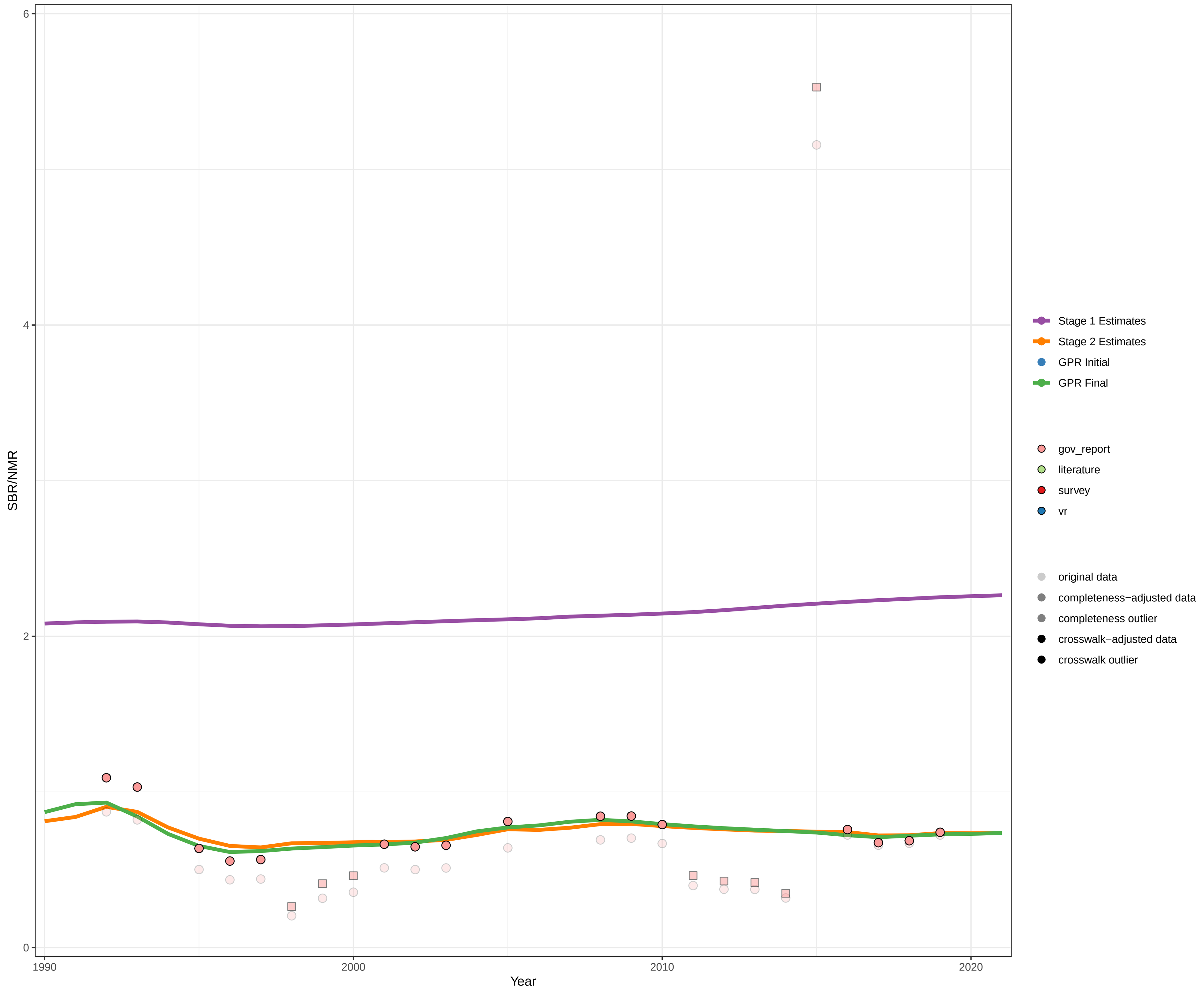

Definition	Years	Outlier	Source.Type	Title
28_weeks	1992, 1993, 1995−1997, 2001−2003, 2005, 2008−2010	0	Statistical Report	Botswana Health Statistics Report
28_weeks	2016−2019	0	Statistical Report	Botswana Vital Statistics Report
28_weeks	1998−2000	1	Statistical Report	Botswana Health Statistics Report
28_weeks	2011−2015	1	Statistical Report	Botswana Vital Statistics Report

Botswana BWA Southern Sub−Saharan Africa Stillbirth Rate (SBR) − 20 weeks
lambda = 0.4 ; zeta = 0.9 ; scale = 10 ; amp = 0.028

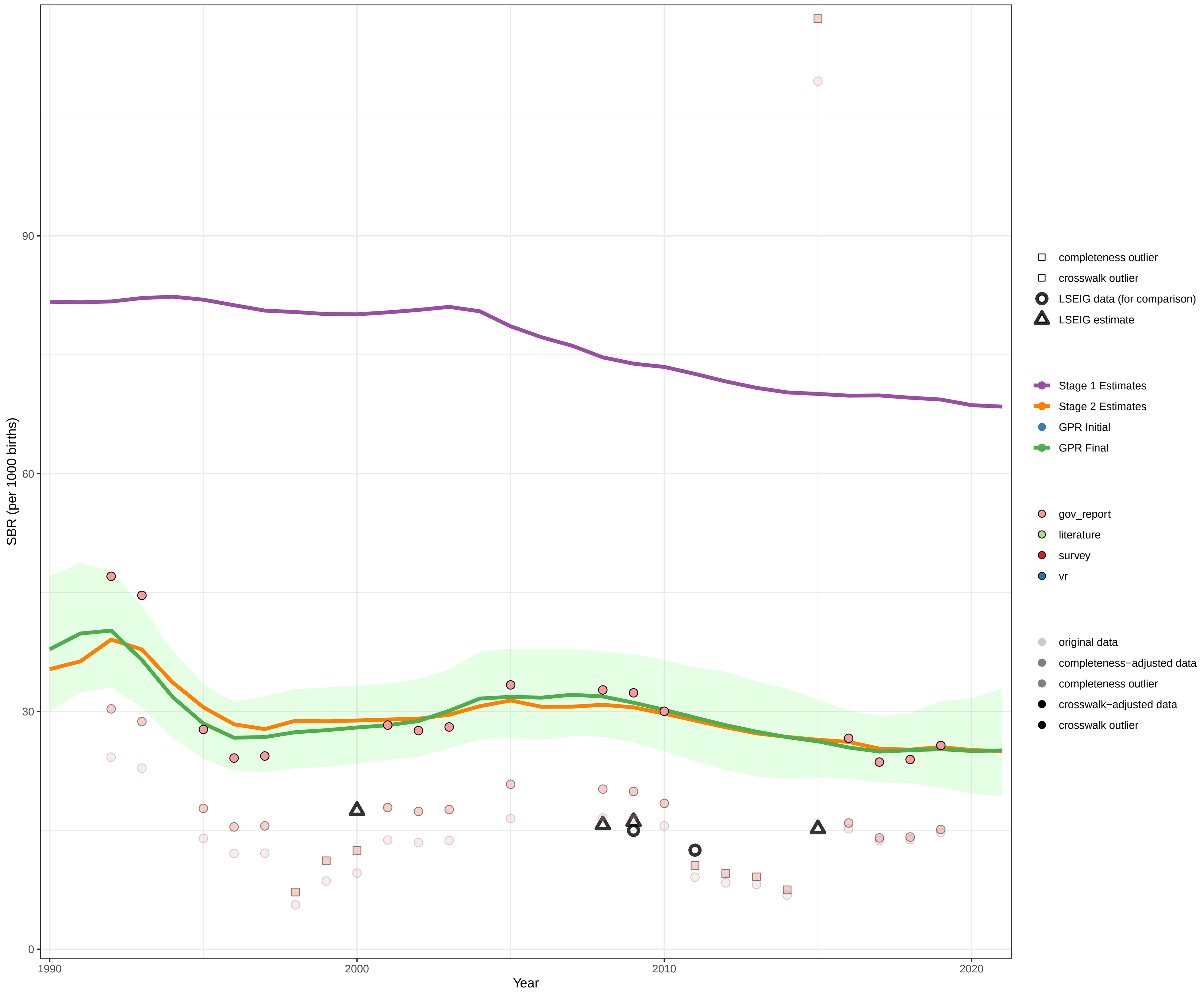

Botswana BWA Southern Sub−Saharan Africa Stillbirth Rate (SBR) − 28 weeks
lambda = 0.4 ; zeta = 0.9 ; scale = 10 ; amp = 0.028

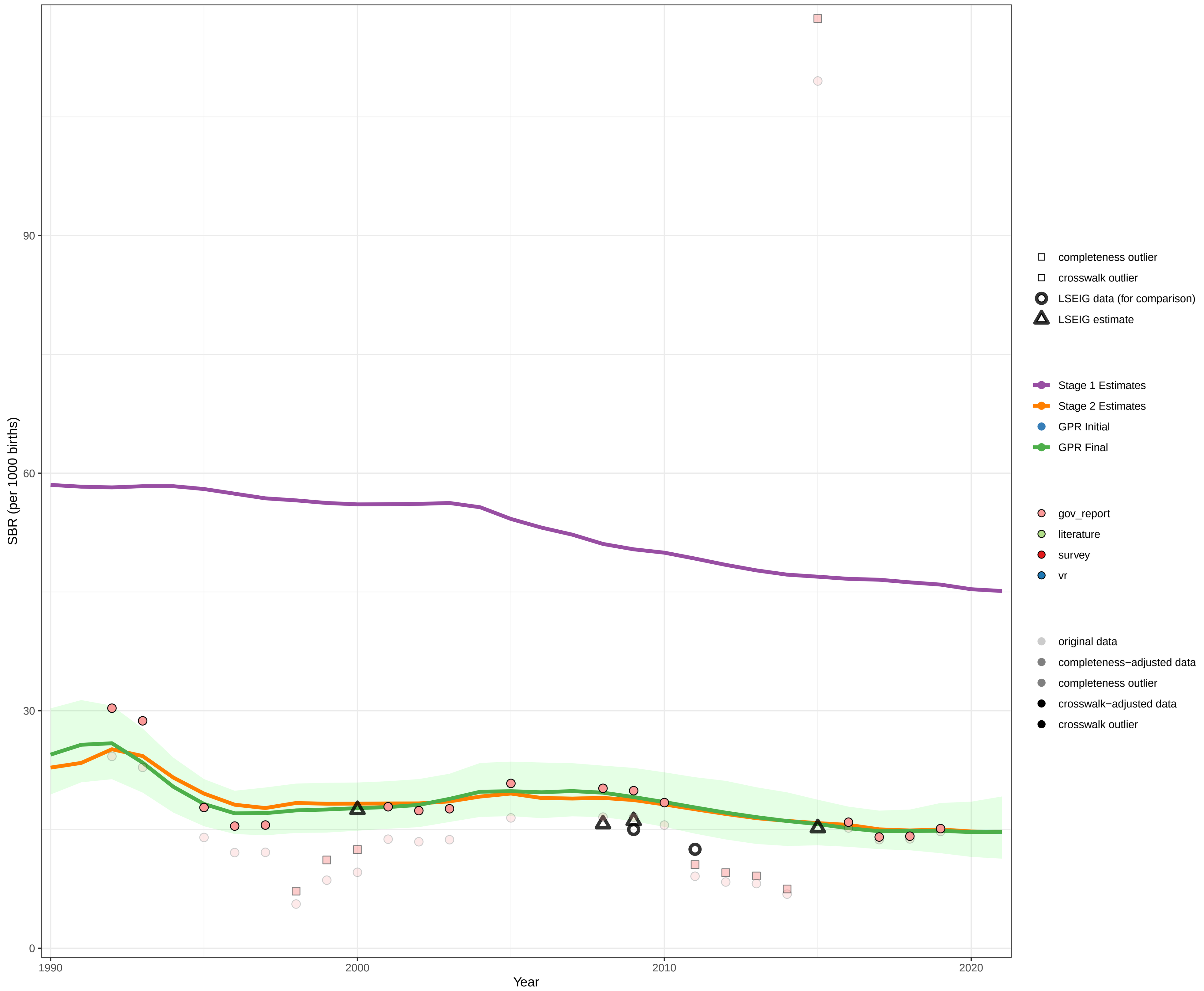

Year	Total Stillbirths (in thousands)	Total Stillbirths (in thousands)	SBR (per 1000 births)	SBR (per 1000 births)	SBR/NMR	SBR/NMR
	20 weeks	28 weeks	20 weeks	28 weeks	20 weeks	28 weeks
1990	1.8 (1.4 to 2.3)	1.2 (0.9 to 1.5)	37.8 (29.9 to 47.0)	24.5 (19.4 to 30.3)	1.35 (1.06 to 1.67)	0.87 (0.69 to 1.08)
1995	1.4 (1.2 to 1.7)	0.9 (0.8 to 1.1)	28.5 (24.0 to 33.5)	18.2 (15.4 to 21.4)	1.02 (0.86 to 1.20)	0.65 (0.55 to 0.77)
2000	1.4 (1.2 to 1.7)	0.9 (0.7 to 1.0)	28.0 (23.4 to 33.2)	17.7 (14.8 to 20.9)	1.04 (0.87 to 1.23)	0.66 (0.55 to 0.78)
2005	1.6 (1.3 to 1.9)	1.0 (0.8 to 1.2)	31.8 (26.7 to 37.9)	19.8 (16.7 to 23.6)	1.24 (1.04 to 1.47)	0.77 (0.65 to 0.92)
2010	1.5 (1.2 to 1.8)	0.9 (0.8 to 1.1)	30.2 (25.0 to 36.5)	18.5 (15.3 to 22.2)	1.30 (1.07 to 1.57)	0.79 (0.66 to 0.95)
2015	1.3 (1.1 to 1.6)	0.8 (0.6 to 0.9)	26.2 (21.7 to 31.5)	15.7 (13.0 to 18.8)	1.24 (1.02 to 1.48)	0.74 (0.61 to 0.88)
2020	1.3 (1.0 to 1.6)	0.7 (0.6 to 0.9)	25.0 (19.6 to 31.7)	14.7 (11.5 to 18.5)	1.25 (0.98 to 1.58)	0.73 (0.57 to 0.92)
2021	1.3 (1.0 to 1.7)	0.7 (0.6 to 1.0)	25.1 (19.3 to 32.9)	14.7 (11.3 to 19.2)	1.26 (0.97 to 1.65)	0.74 (0.57 to 0.96)

Eswatini SWZ Southern Sub−Saharan Africa Stillbirth Rate / Neonatal Mortality Rate (SBR/NMR) − 20 weeks
lambda = 0.8 ; zeta = 0.7 ; scale = 15 ; amp = 0.028

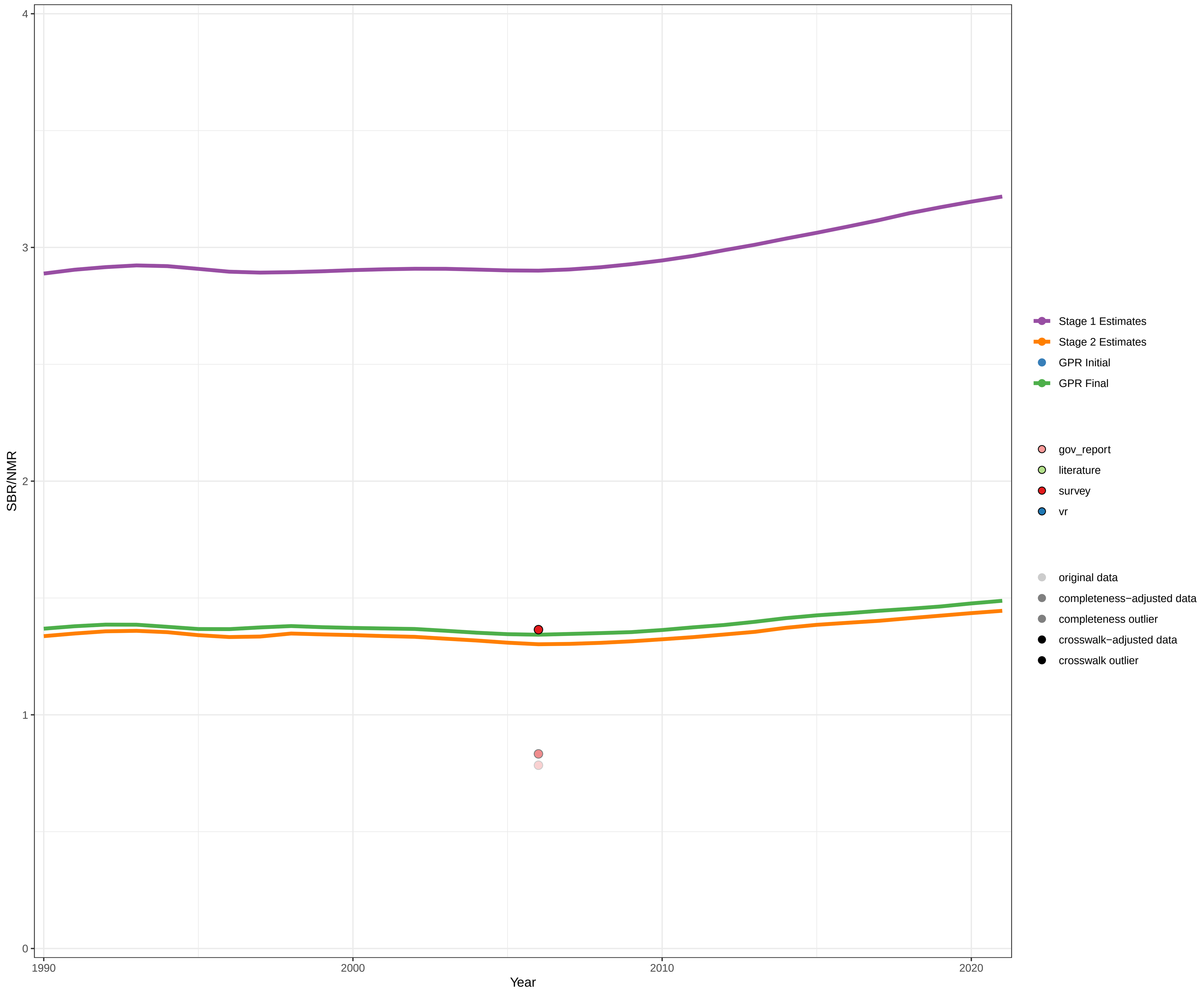

Eswatini SWZ Southern Sub−Saharan Africa Stillbirth Rate / Neonatal Mortality Rate (SBR/NMR) − 28 weeks
lambda = 0.8 ; zeta = 0.7 ; scale = 15 ; amp = 0.028

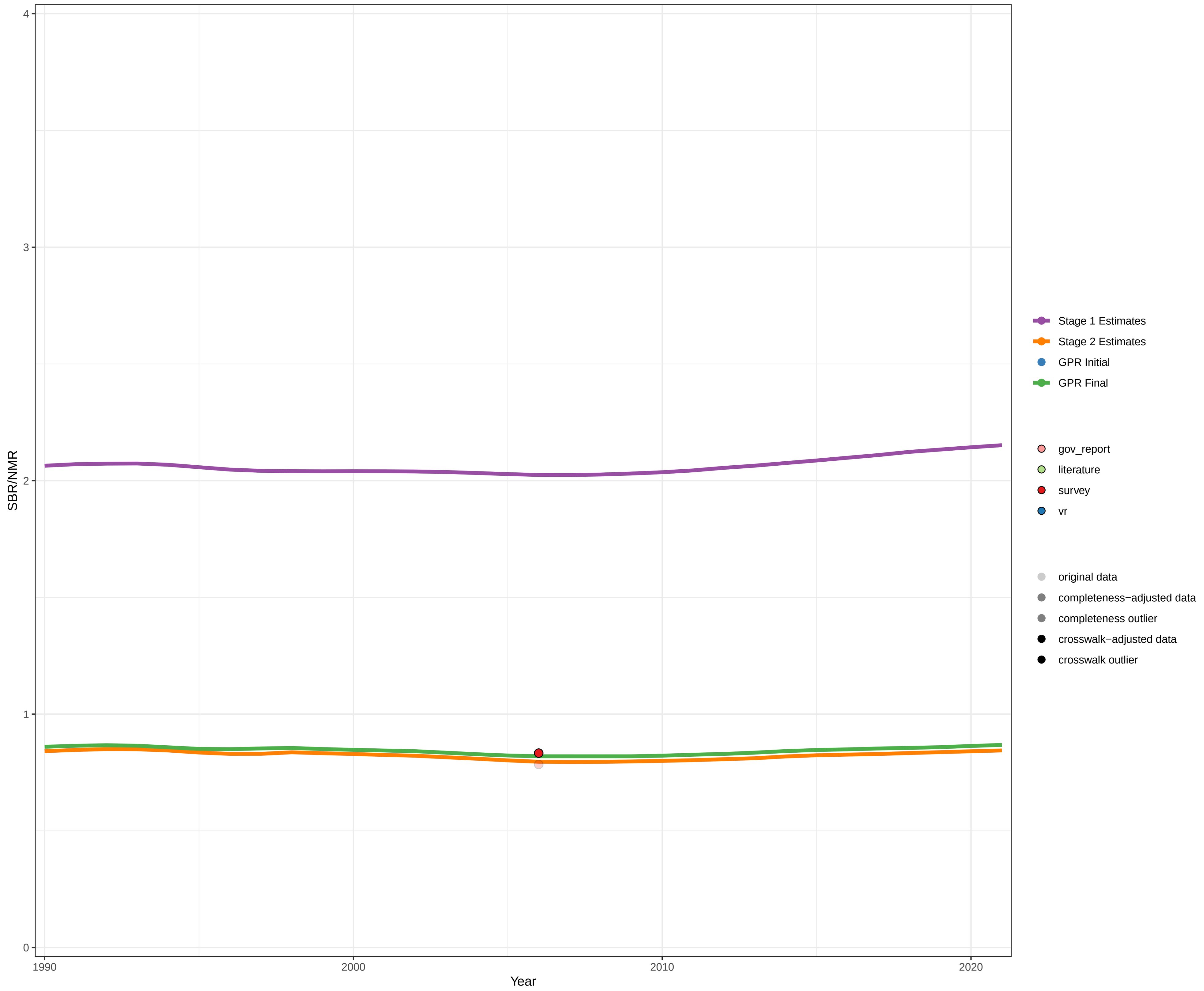

Definition	Years	Outlier	Source.Type	Title
28_weeks	2006	0	Survey	Swaziland Demographic and Health Survey 2006−2007

Eswatini SWZ Southern Sub−Saharan Africa Stillbirth Rate (SBR) − 20 weeks
lambda = 0.8 ; zeta = 0.7 ; scale = 15 ; amp = 0.028

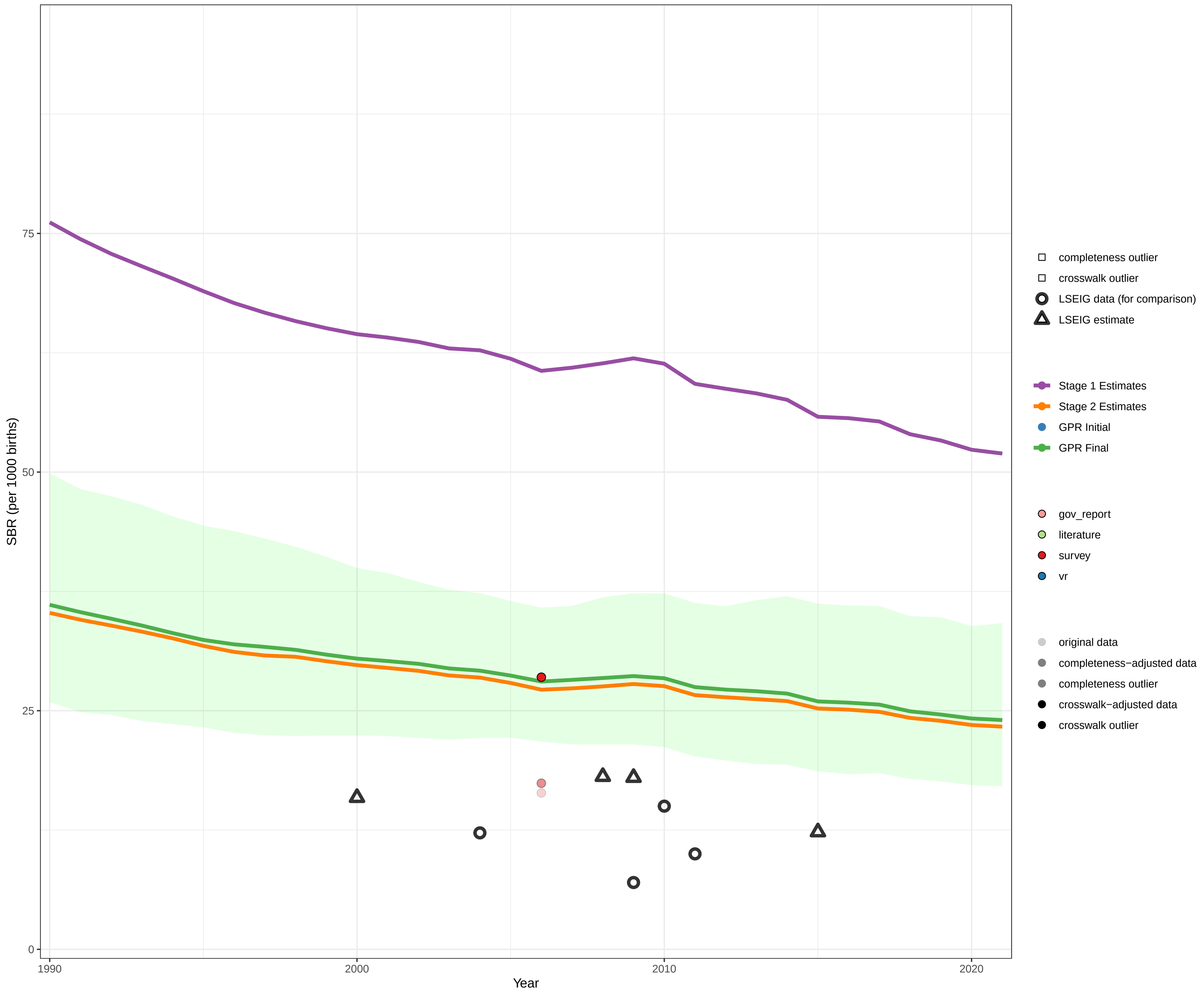

Eswatini SWZ Southern Sub−Saharan Africa Stillbirth Rate (SBR) − 28 weeks
lambda = 0.8 ; zeta = 0.7 ; scale = 15 ; amp = 0.028

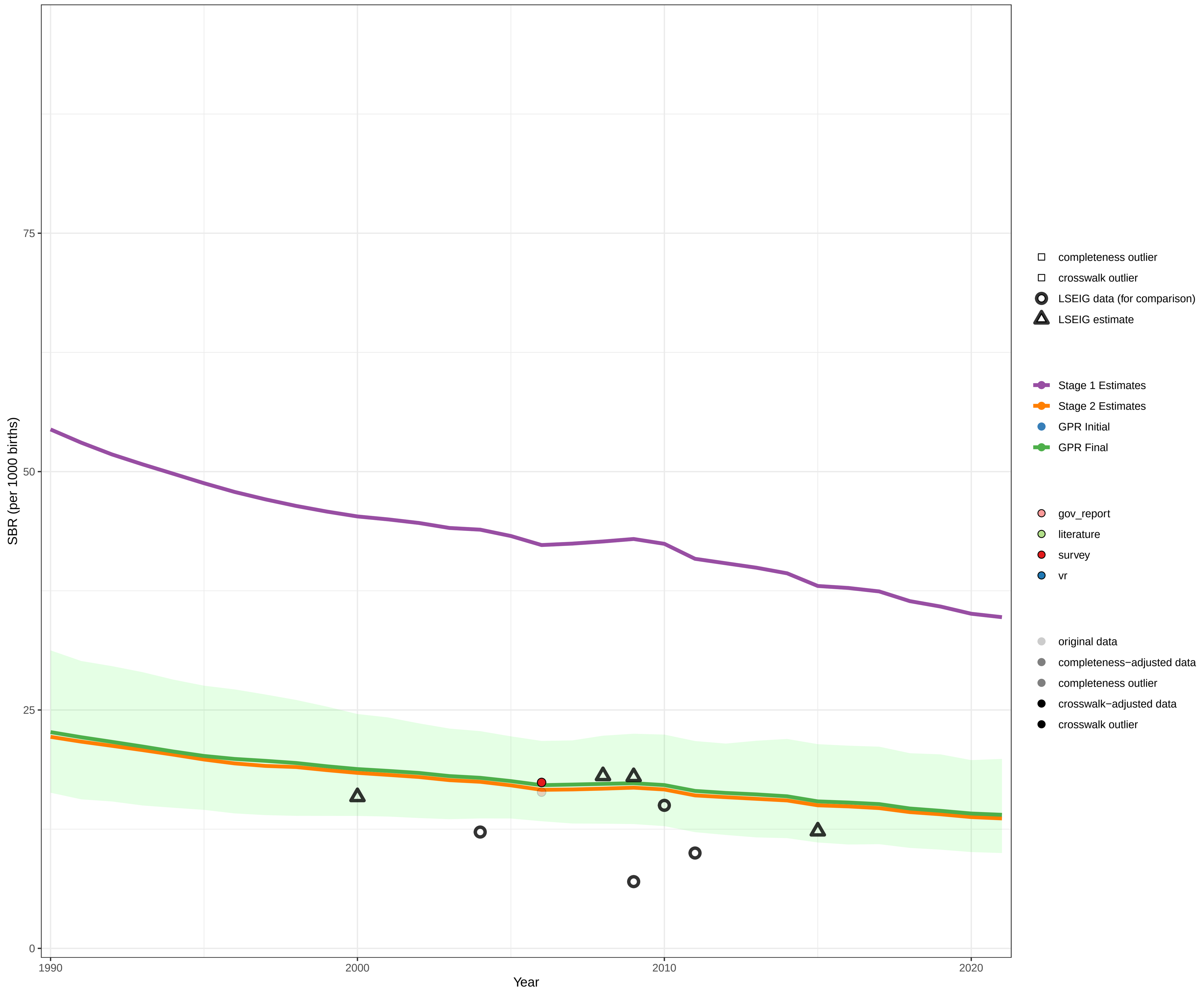

Year	Total Stillbirths (in thousands)	Total Stillbirths (in thousands)	SBR (per 1000 births)	SBR (per 1000 births)	SBR/NMR	SBR/NMR
	20 weeks	28 weeks	20 weeks	28 weeks	20 weeks	28 weeks
1990	1.2 (0.9 to 1.7)	0.8 (0.5 to 1.1)	36.1 (25.9 to 49.9)	22.7 (16.3 to 31.3)	1.37 (0.98 to 1.89)	0.86 (0.62 to 1.19)
1995	1.2 (0.8 to 1.6)	0.7 (0.5 to 1.0)	32.4 (23.3 to 44.4)	20.2 (14.5 to 27.5)	1.37 (0.98 to 1.87)	0.85 (0.61 to 1.16)
2000	1.1 (0.8 to 1.5)	0.7 (0.5 to 0.9)	30.5 (22.4 to 40.0)	18.8 (13.9 to 24.6)	1.37 (1.01 to 1.80)	0.85 (0.63 to 1.11)
2005	1.1 (0.8 to 1.4)	0.6 (0.5 to 0.8)	28.7 (22.2 to 36.5)	17.5 (13.6 to 22.2)	1.35 (1.04 to 1.71)	0.82 (0.64 to 1.04)
2010	1.0 (0.8 to 1.3)	0.6 (0.5 to 0.8)	28.4 (21.2 to 37.3)	17.1 (12.8 to 22.4)	1.36 (1.02 to 1.79)	0.82 (0.62 to 1.08)
2015	0.9 (0.6 to 1.2)	0.5 (0.4 to 0.7)	26.0 (18.6 to 36.2)	15.4 (11.1 to 21.4)	1.43 (1.02 to 1.99)	0.85 (0.61 to 1.18)
2020	0.7 (0.5 to 1.0)	0.4 (0.3 to 0.6)	24.2 (17.2 to 33.9)	14.1 (10.1 to 19.7)	1.48 (1.05 to 2.07)	0.86 (0.62 to 1.21)
2021	0.7 (0.5 to 1.0)	0.4 (0.3 to 0.6)	24.0 (17.1 to 34.2)	14.0 (10.0 to 19.9)	1.49 (1.06 to 2.12)	0.87 (0.62 to 1.23)

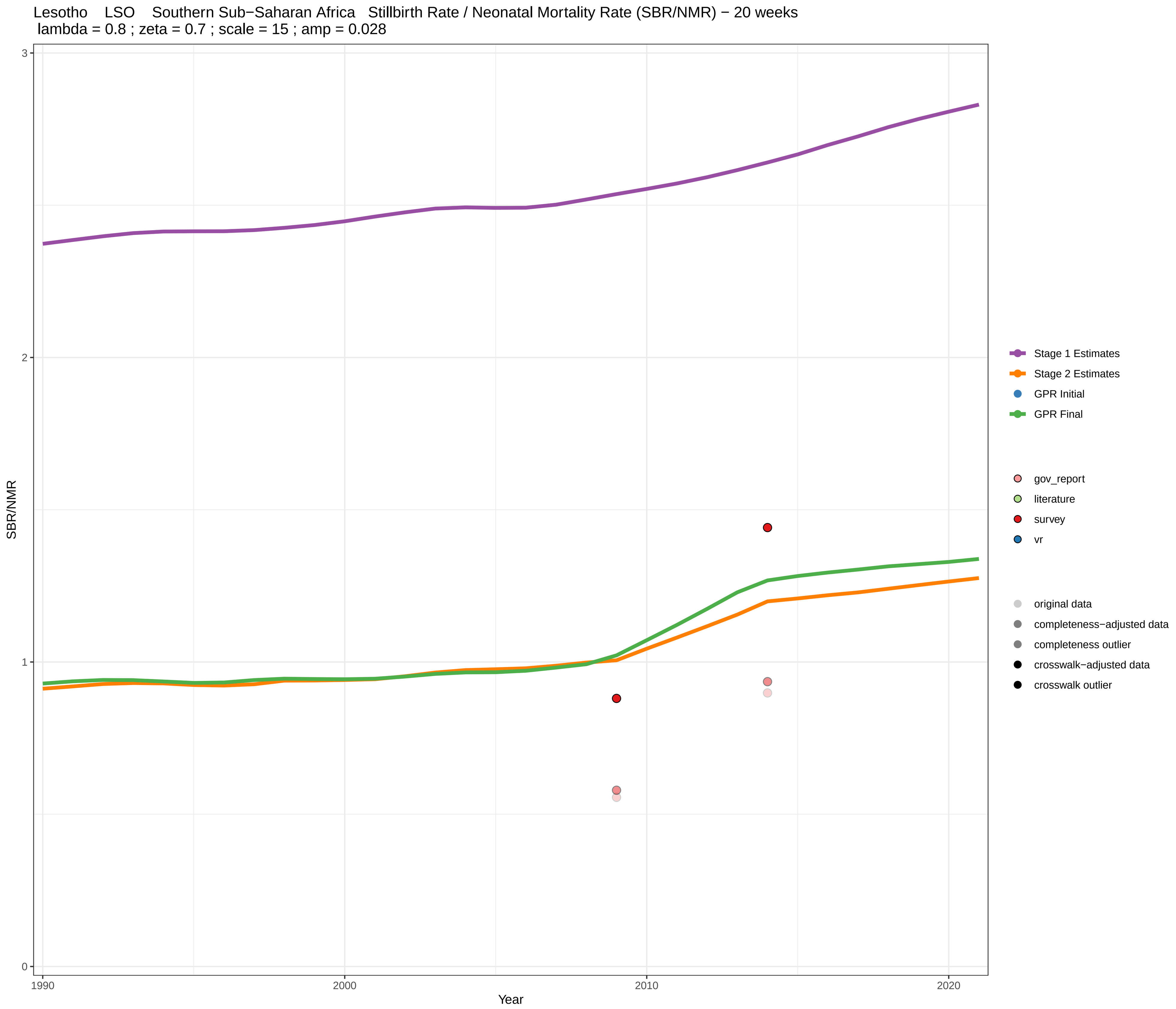

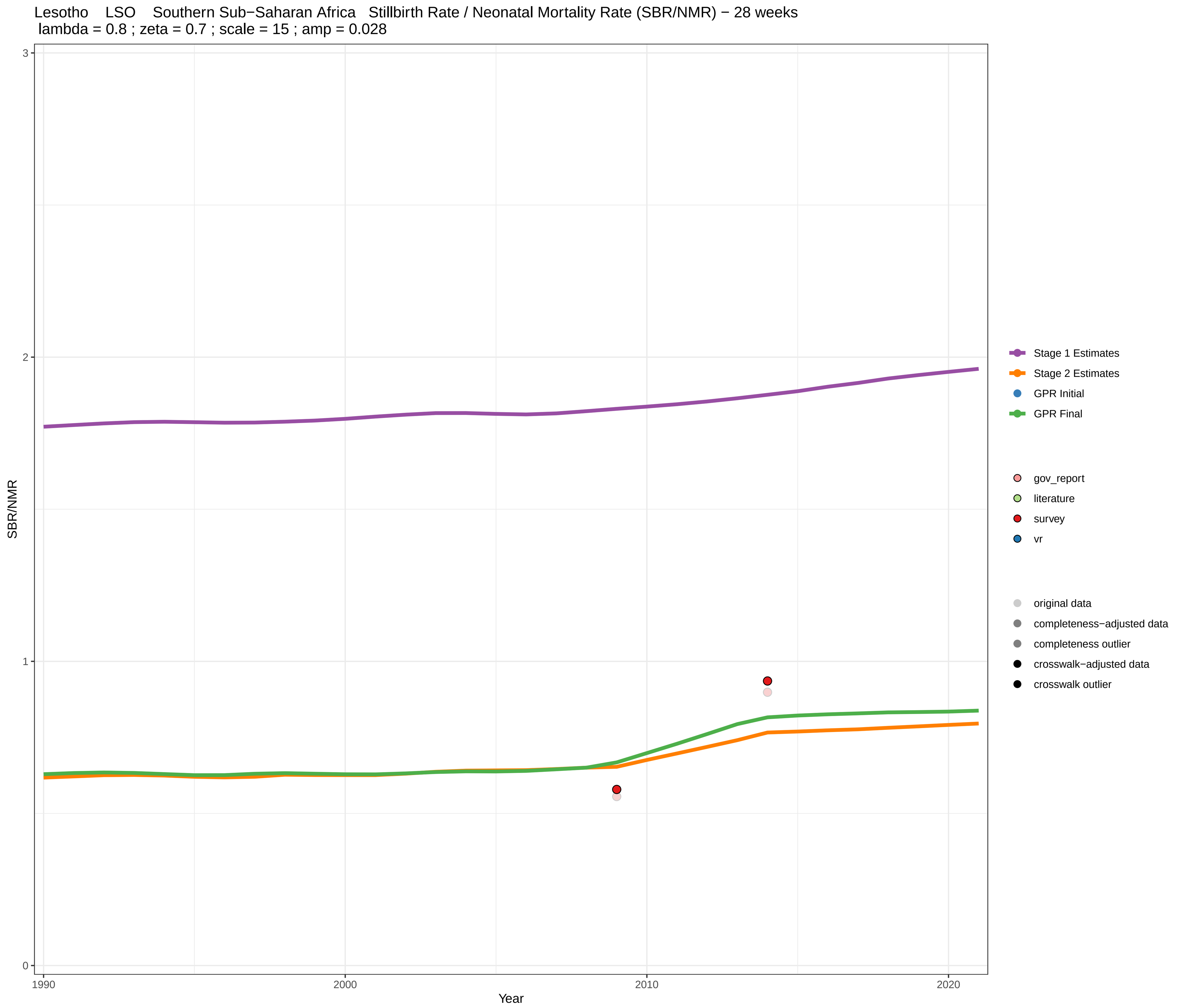

Definition	Years	Outlier	Source.Type	Title
28_weeks	2009	0	Survey	Lesotho Demographic and Health Survey 2009−2010
28_weeks	2014	0	Survey	Lesotho Demographic and Health Survey 2014

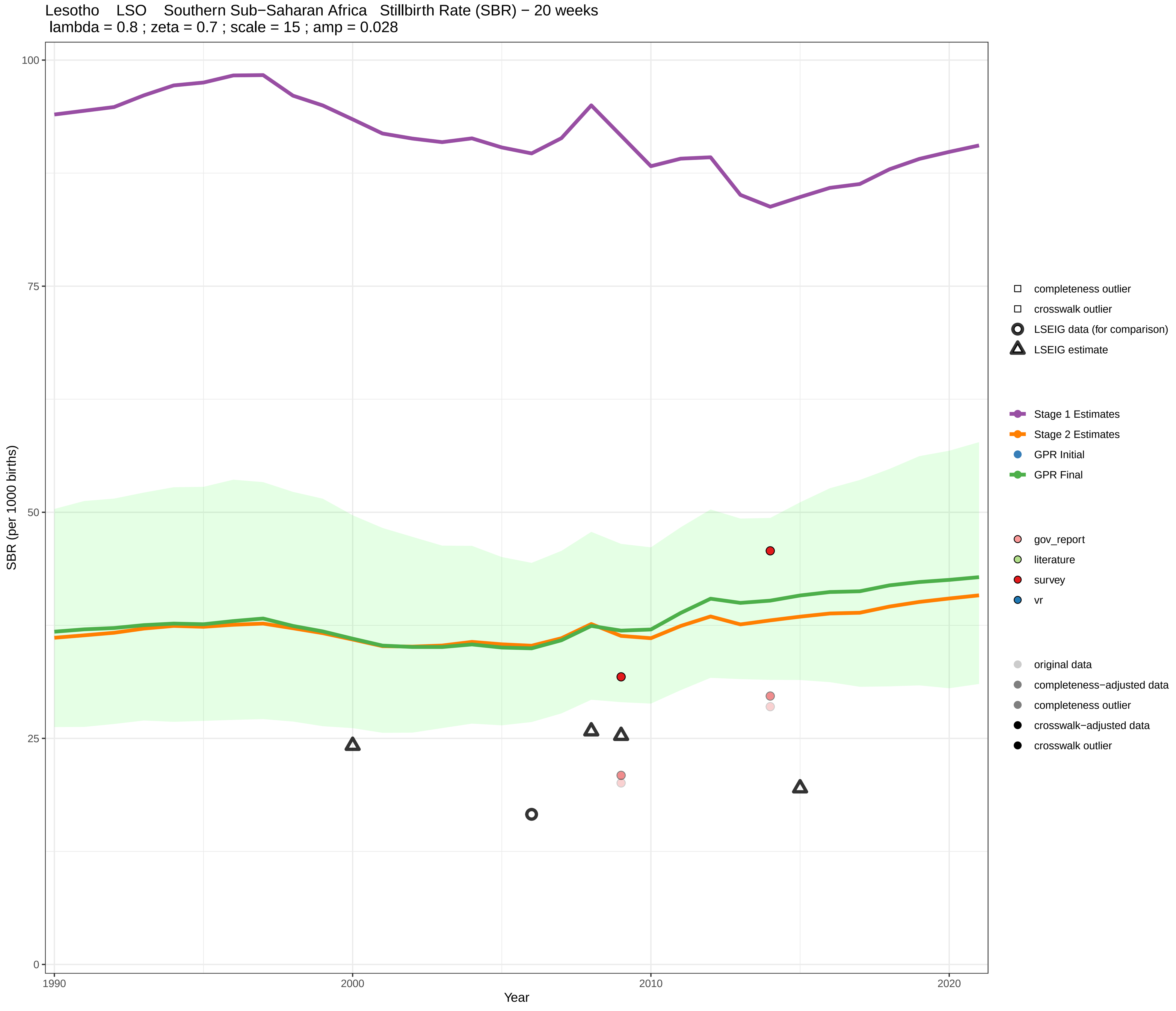

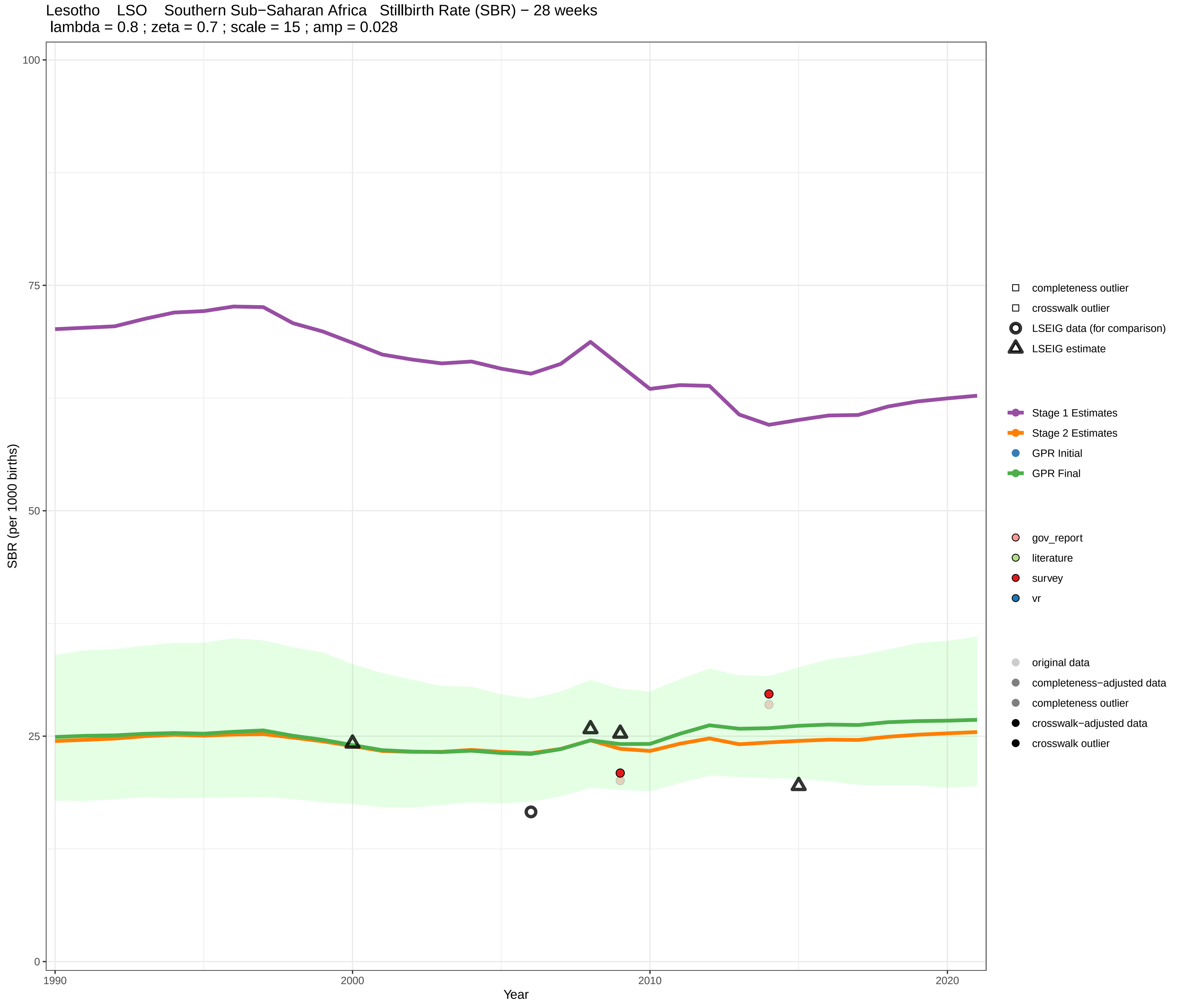

Year	Total Stillbirths (in thousands)	Total Stillbirths (in thousands)	SBR (per 1000 births)	SBR (per 1000 births)	SBR/NMR	SBR/NMR
	20 weeks	28 weeks	20 weeks	28 weeks	20 weeks	28 weeks
1990	2.1 (1.5 to 2.9)	1.4 (1.0 to 1.9)	36.8 (26.2 to 50.4)	24.9 (17.8 to 34.0)	0.93 (0.66 to 1.27)	0.63 (0.45 to 0.86)
1995	2.1 (1.5 to 2.9)	1.4 (1.0 to 1.9)	37.6 (26.9 to 52.8)	25.3 (18.2 to 35.4)	0.93 (0.67 to 1.31)	0.63 (0.45 to 0.88)
2000	1.9 (1.4 to 2.7)	1.3 (0.9 to 1.8)	36.0 (26.1 to 49.7)	24.0 (17.5 to 33.0)	0.94 (0.68 to 1.30)	0.63 (0.46 to 0.86)
2005	1.8 (1.4 to 2.4)	1.2 (0.9 to 1.5)	35.0 (26.4 to 45.1)	23.1 (17.5 to 29.6)	0.97 (0.73 to 1.24)	0.64 (0.48 to 0.82)
2010	1.9 (1.4 to 2.4)	1.2 (0.9 to 1.5)	37.0 (28.8 to 46.1)	24.1 (18.9 to 30.0)	1.07 (0.83 to 1.33)	0.70 (0.55 to 0.87)
2015	2.0 (1.5 to 2.5)	1.2 (1.0 to 1.6)	40.8 (31.5 to 51.1)	26.1 (20.2 to 32.6)	1.28 (0.99 to 1.61)	0.82 (0.64 to 1.03)
2020	1.9 (1.4 to 2.6)	1.2 (0.8 to 1.6)	42.5 (30.5 to 56.8)	26.7 (19.3 to 35.6)	1.33 (0.95 to 1.77)	0.83 (0.60 to 1.11)
2021	1.9 (1.4 to 2.6)	1.2 (0.8 to 1.6)	42.8 (31.0 to 57.8)	26.8 (19.5 to 36.1)	1.34 (0.97 to 1.81)	0.84 (0.61 to 1.13)

Namibia NAM Southern Sub−Saharan Africa Stillbirth Rate / Neonatal Mortality Rate (SBR/NMR) − 20 weeks
lambda = 0.8 ; zeta = 0.7 ; scale = 15 ; amp = 0.028

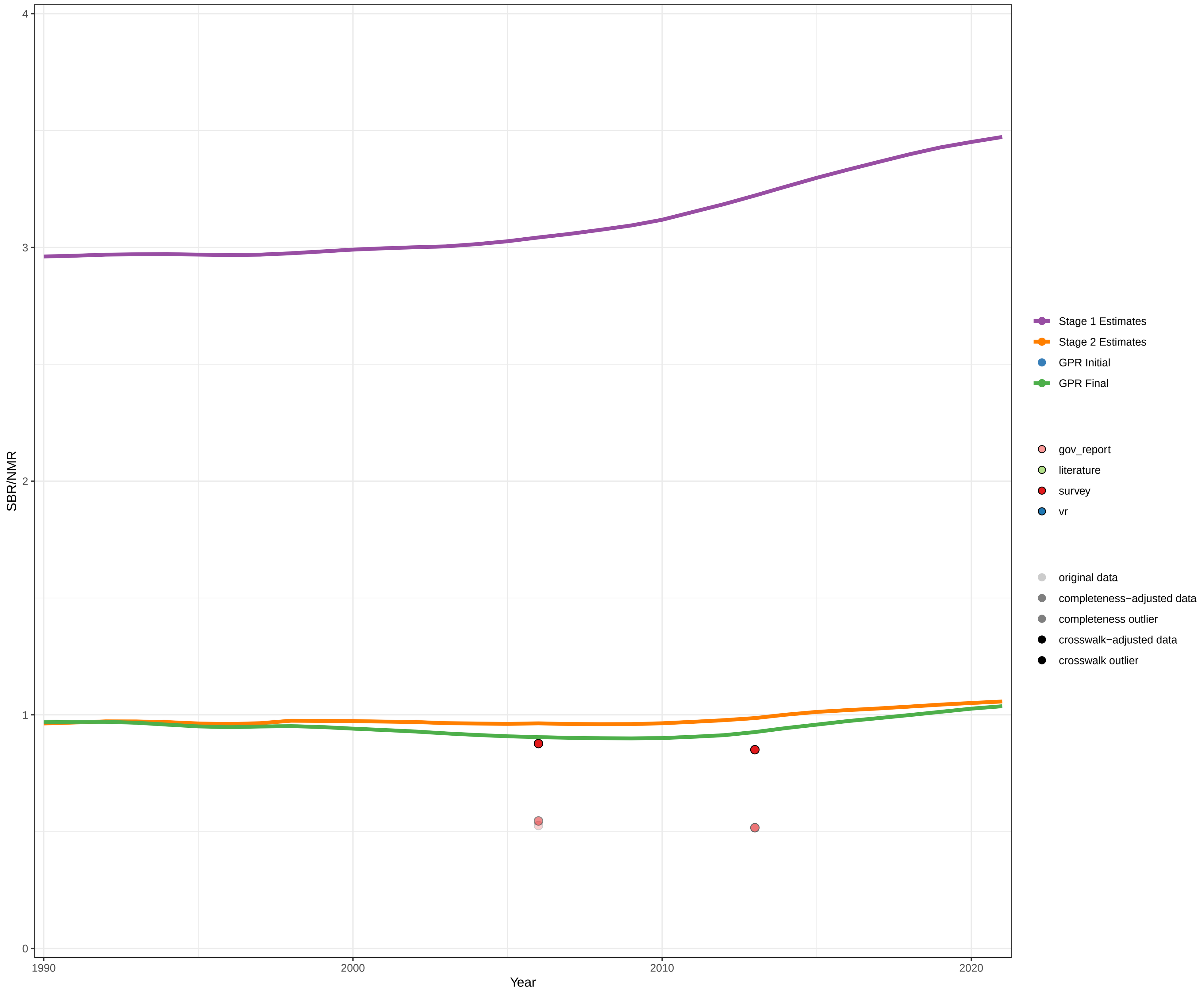

Namibia NAM Southern Sub−Saharan Africa Stillbirth Rate / Neonatal Mortality Rate (SBR/NMR) − 28 weeks
lambda = 0.8 ; zeta = 0.7 ; scale = 15 ; amp = 0.028

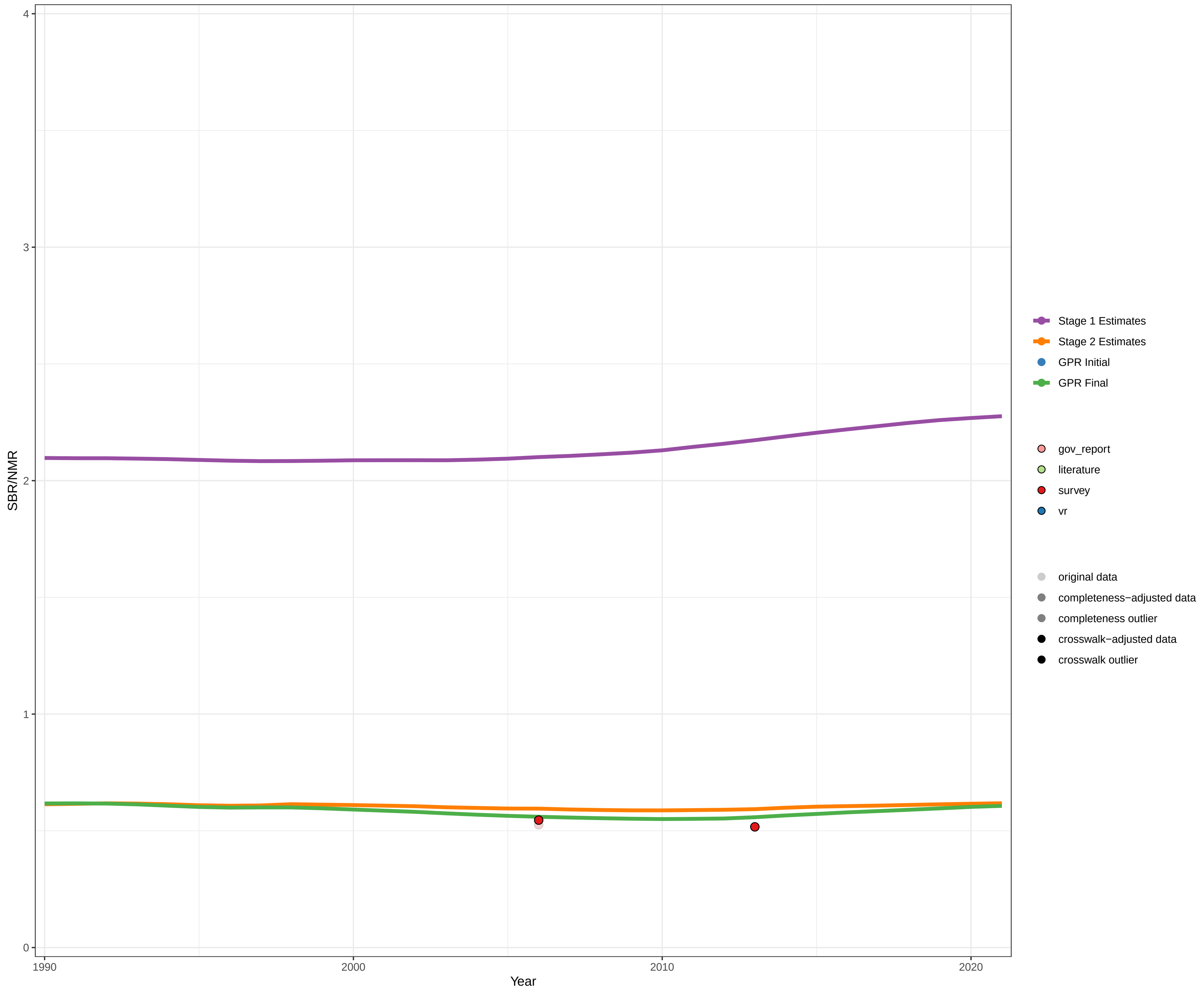

Definition	Years	Outlier	Source.Type	Title
28_weeks	2006	0	Survey	Namibia Demographic and Health Survey 2006−2007
28_weeks	2013	0	Survey	Namibia Demographic and Health Survey 2013

Namibia NAM Southern Sub−Saharan Africa Stillbirth Rate (SBR) − 20 weeks
lambda = 0.8 ; zeta = 0.7 ; scale = 15 ; amp = 0.028

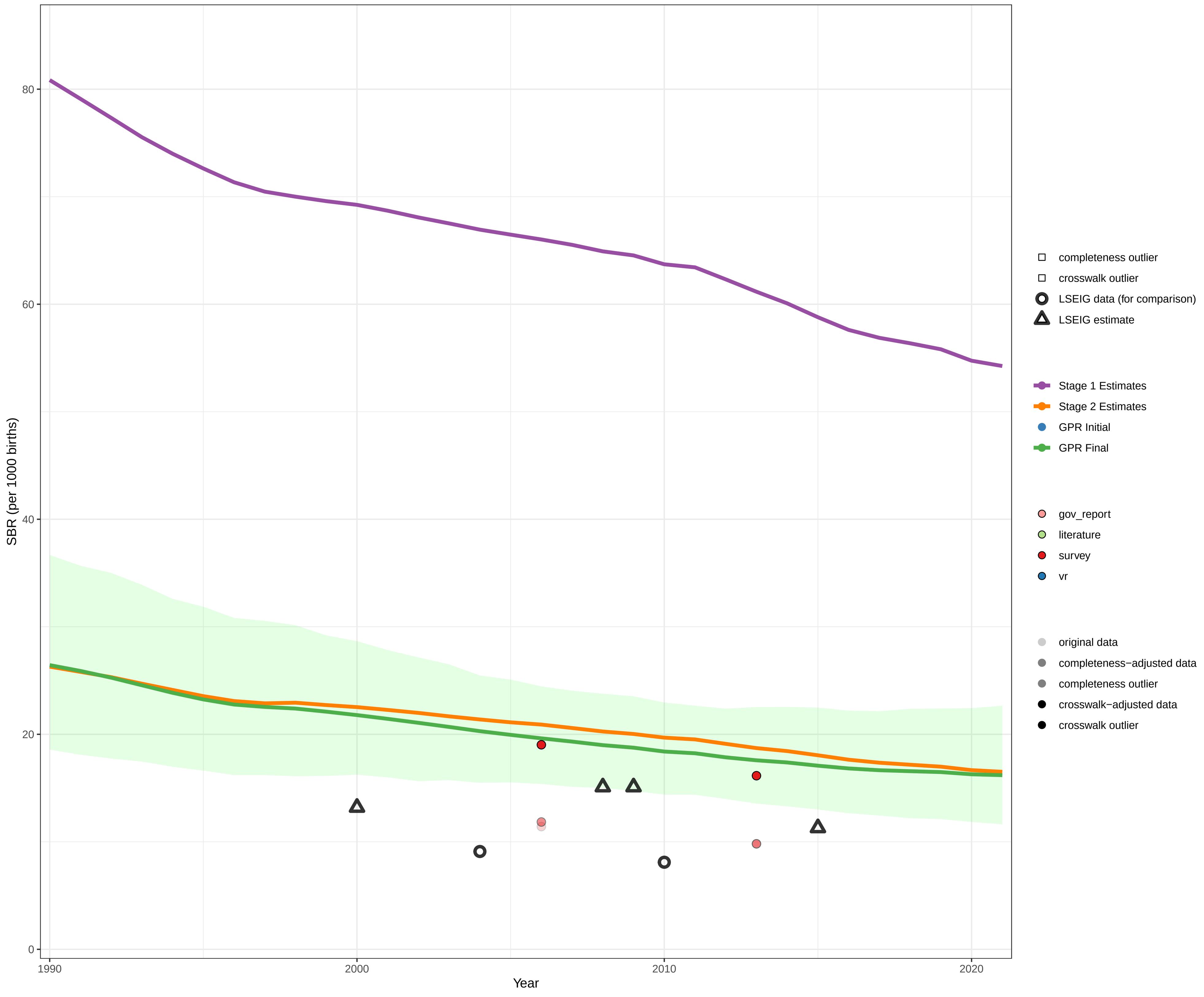

Namibia NAM Southern Sub−Saharan Africa Stillbirth Rate (SBR) − 28 weeks
lambda = 0.8 ; zeta = 0.7 ; scale = 15 ; amp = 0.028

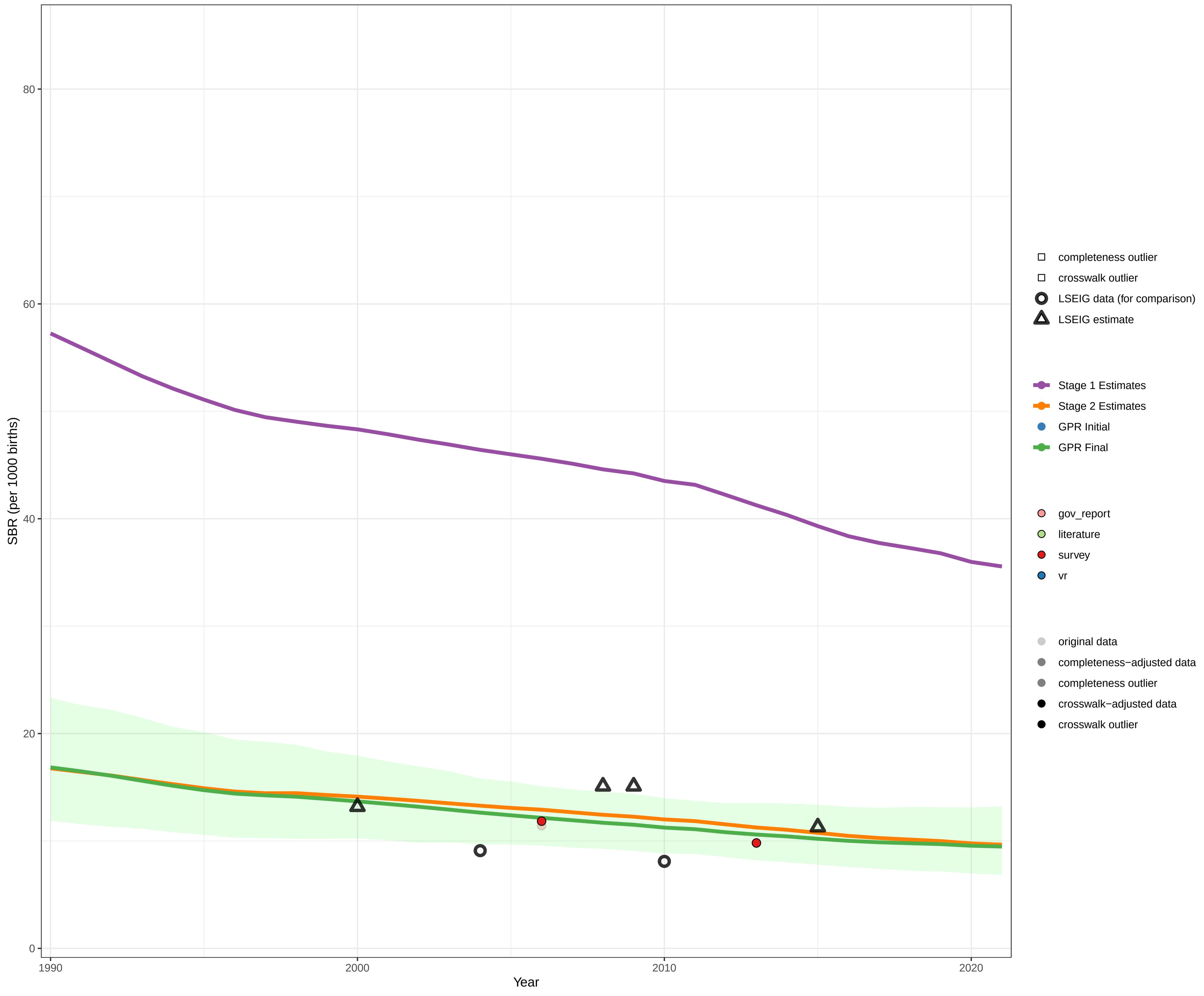

Year	Total Stillbirths (in thousands)	Total Stillbirths (in thousands)	SBR (per 1000 births)	SBR (per 1000 births)	SBR/NMR	SBR/NMR
	20 weeks	28 weeks	20 weeks	28 weeks	20 weeks	28 weeks
1990	1.4 (1.0 to 2.0)	0.9 (0.6 to 1.2)	26.4 (18.6 to 36.7)	16.9 (11.9 to 23.3)	0.97 (0.68 to 1.34)	0.62 (0.44 to 0.85)
1995	1.3 (0.9 to 1.8)	0.8 (0.6 to 1.1)	23.2 (16.6 to 31.9)	14.7 (10.6 to 20.1)	0.95 (0.68 to 1.30)	0.60 (0.43 to 0.82)
2000	1.3 (0.9 to 1.7)	0.8 (0.6 to 1.0)	21.8 (16.2 to 28.7)	13.7 (10.2 to 17.9)	0.94 (0.70 to 1.24)	0.59 (0.44 to 0.77)
2005	1.2 (1.0 to 1.6)	0.8 (0.6 to 1.0)	19.9 (15.5 to 25.1)	12.4 (9.7 to 15.5)	0.91 (0.71 to 1.14)	0.56 (0.44 to 0.71)
2010	1.2 (0.9 to 1.5)	0.7 (0.6 to 0.9)	18.4 (14.4 to 23.0)	11.2 (8.8 to 14.0)	0.90 (0.70 to 1.12)	0.55 (0.43 to 0.68)
2015	1.1 (0.8 to 1.4)	0.6 (0.5 to 0.8)	17.1 (13.0 to 22.5)	10.2 (7.8 to 13.4)	0.96 (0.73 to 1.26)	0.57 (0.44 to 0.75)
2020	1.0 (0.7 to 1.3)	0.6 (0.4 to 0.8)	16.3 (11.8 to 22.4)	9.6 (7.0 to 13.1)	1.03 (0.75 to 1.41)	0.60 (0.44 to 0.83)
2021	1.0 (0.7 to 1.3)	0.6 (0.4 to 0.8)	16.2 (11.6 to 22.7)	9.5 (6.8 to 13.2)	1.04 (0.74 to 1.45)	0.61 (0.44 to 0.85)

South Africa   ZAF   Southern Sub−Saharan Africa   Stillbirth Rate / Neonatal Mortality Rate (SBR/NMR) − 20 weeks
lambda = 0.2 ; zeta = 0.99 ; scale = 5 ; amp = 0.028

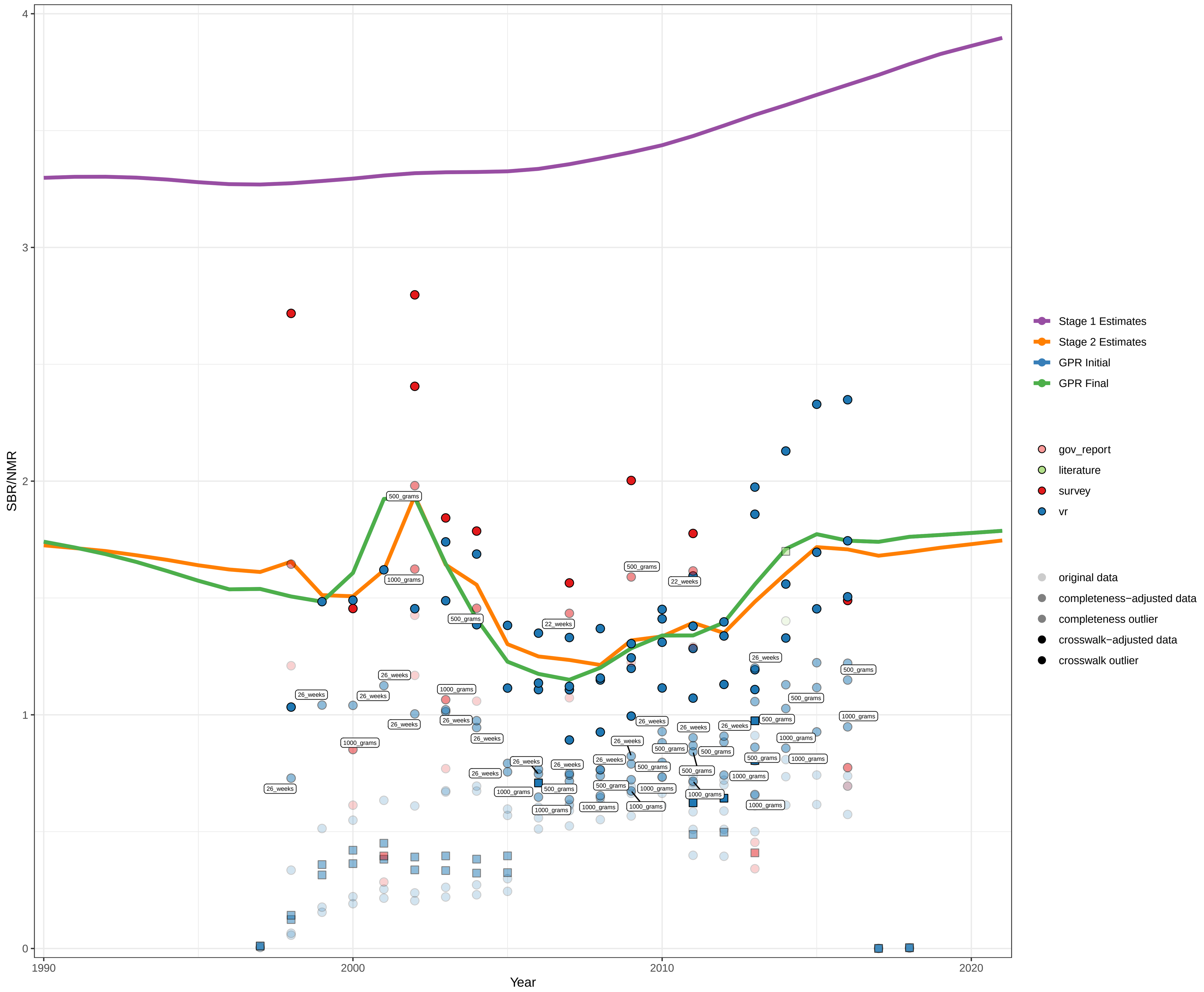

South Africa   ZAF   Southern Sub−Saharan Africa   Stillbirth Rate / Neonatal Mortality Rate (SBR/NMR) − 28 weeks
lambda = 0.2 ; zeta = 0.99 ; scale = 5 ; amp = 0.028

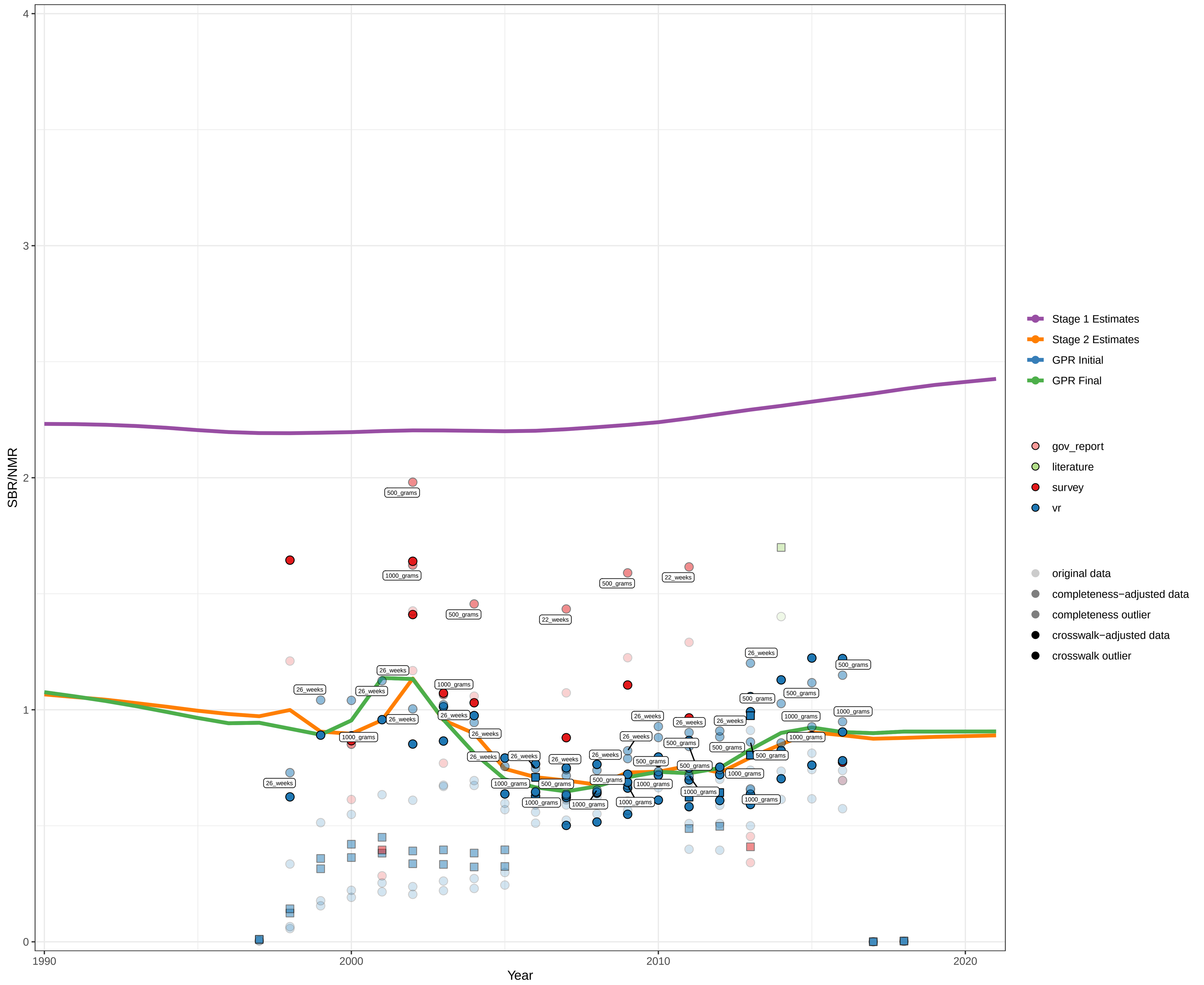

Definition	Years	Outlier	Source.Type	Title
1000_grams	2000	0	Survey	South Africa − Saving Babies: A Perinatal Care Survey 2000
1000_grams	2003	0	Survey	South Africa − Saving Babies: A Perinatal Care Survey 2003
1000_grams	2013	0	VR	South Africa Perinatal Deaths
1000_grams, 500_grams	2002	0	Survey	South Africa − Saving Babies: A Perinatal Care Survey 2002
1000_grams, 500_grams	2006−2016	0	VR	South Africa Vital Registration − Causes of Death
22_weeks	2007	0	Survey	South Africa − Saving Babies: A Perinatal Care Survey 2006−2007
22_weeks	2011	0	Survey	South Africa − Saving Babies: A Perinatal Care Survey 2010−2011
26_weeks	1998−2013	0	VR	South Africa Perinatal Deaths
28_weeks	1998	0	Survey	South Africa Demographic and Health Survey 1998
28_weeks	2003−2011, 2013−2016	0	VR	United Nations Demographic Yearbook
28_weeks	2016	0	Survey	South Africa Demographic and Health Survey 2016
500_grams	2004	0	Survey	South Africa − Saving Babies: A Perinatal Care Survey 2003−2005
500_grams	2009	0	Survey	South Africa − Saving Babies: A Perinatal Care Survey 2008−2009
500_grams	2011−2013	0	VR	South Africa Perinatal Deaths
500_grams	2013	0	Survey	South Africa − Saving Babies: A Perinatal Care Survey 2012−2013
1000_grams	2001	1	Survey	South Africa − Saving Babies: A Perinatal Care Survey 2001
1000_grams	2011, 2012	1	VR	South Africa Perinatal Deaths
1000_grams	2013	1	Survey	South Africa − Saving Babies: A Perinatal Care Survey 2012−2013
1000_grams, 500_grams	1997−2005	1	VR	South Africa Vital Registration − Causes of Death
1000_grams, 500_grams	2017, 2018	1	VR	South Africa Vital Registration − Causes of Death
22_weeks	2014	1	Sci Lit	Effects of emergency obstetric care training on maternal and perinatal outcomes: a stepped wedge cluster randomised trial in South Africa

South Africa   ZAF   Southern Sub−Saharan Africa   Stillbirth Rate (SBR) − 20 weeks
lambda = 0.2 ; zeta = 0.99 ; scale = 5 ; amp = 0.028

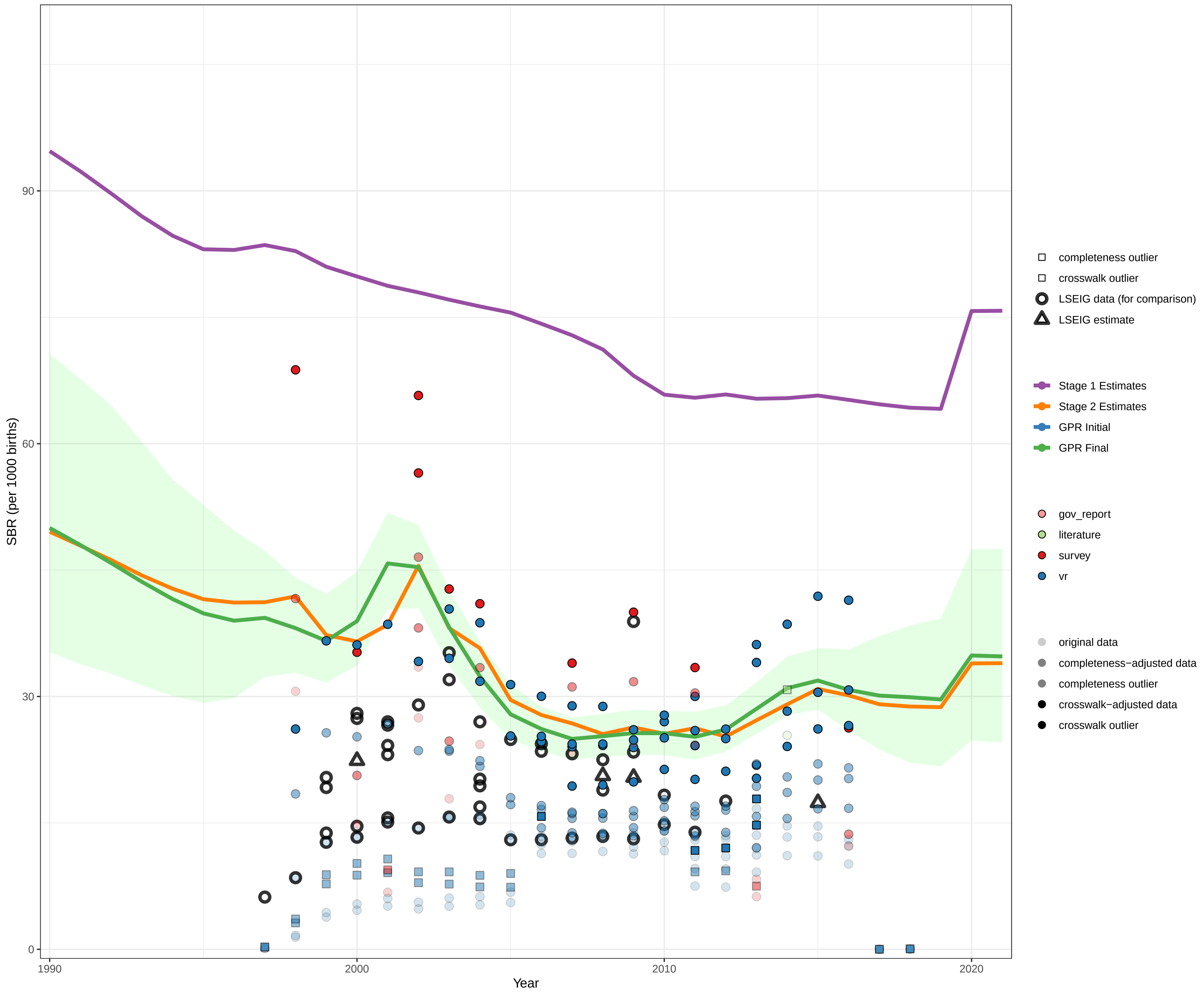

South Africa   ZAF   Southern Sub−Saharan Africa   Stillbirth Rate (SBR) − 28 weeks
lambda = 0.2 ; zeta = 0.99 ; scale = 5 ; amp = 0.028

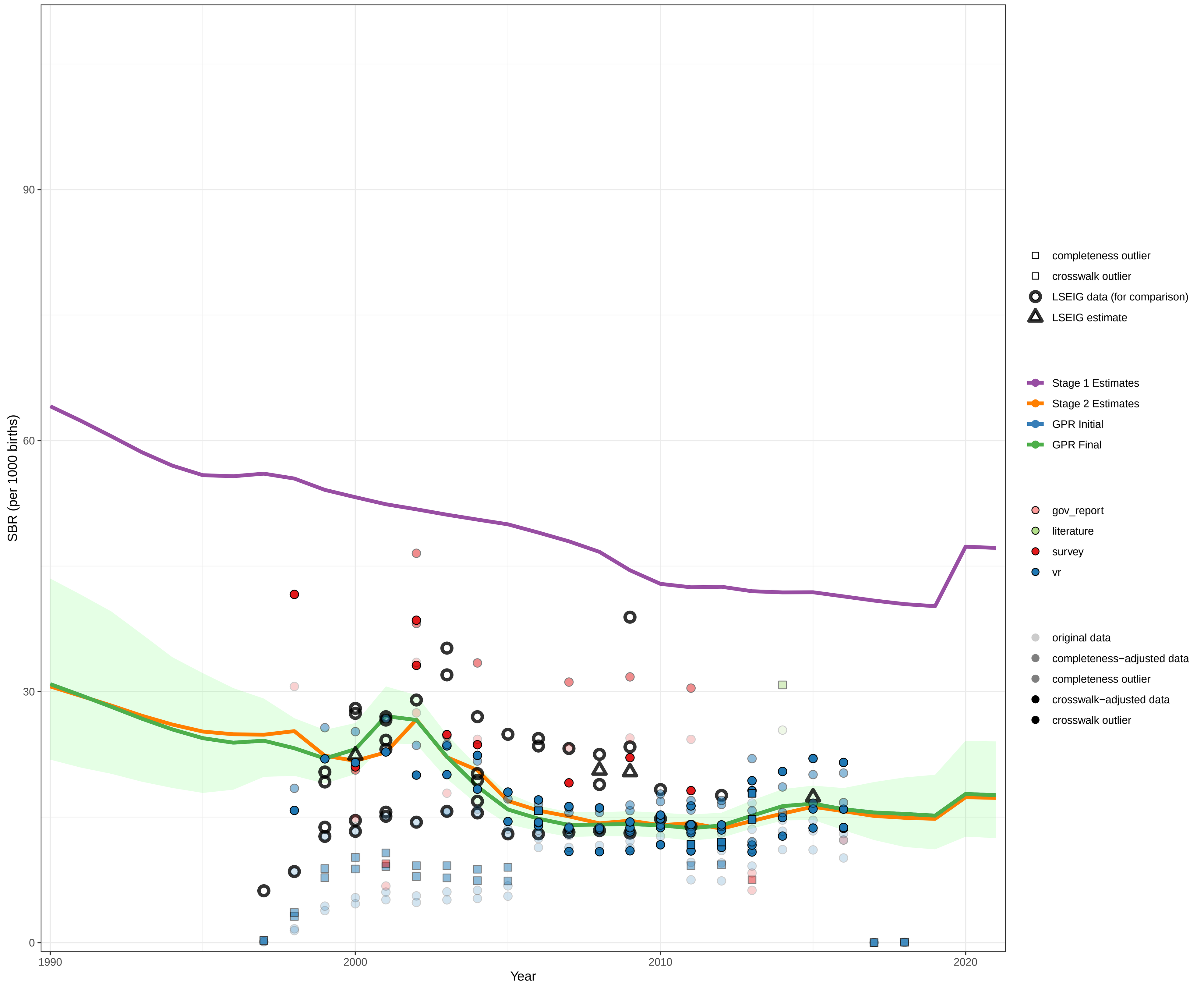

Year	Total Stillbirths (in thousands)	Total Stillbirths (in thousands)	SBR (per 1000 births)	SBR (per 1000 births)	SBR/NMR	SBR/NMR
	20 weeks	28 weeks	20 weeks	28 weeks	20 weeks	28 weeks
1990	55.4 (38.4 to 79.8)	33.5 (23.5 to 47.8)	50.0 (35.3 to 70.6)	30.9 (21.9 to 43.5)	1.74 (1.23 to 2.46)	1.08 (0.76 to 1.52)
1995	43.0 (31.2 to 57.6)	25.9 (18.8 to 34.4)	39.9 (29.2 to 52.8)	24.4 (17.9 to 32.2)	1.57 (1.15 to 2.08)	0.96 (0.71 to 1.27)
2000	41.7 (35.8 to 48.3)	24.4 (21.1 to 27.7)	38.9 (33.7 to 44.8)	23.1 (20.1 to 26.3)	1.61 (1.39 to 1.85)	0.95 (0.83 to 1.08)
2005	32.0 (28.5 to 36.2)	18.1 (16.0 to 20.3)	27.9 (24.9 to 31.4)	15.9 (14.1 to 17.9)	1.23 (1.09 to 1.38)	0.70 (0.62 to 0.79)
2010	30.0 (26.9 to 33.2)	16.2 (14.6 to 18.0)	25.6 (23.0 to 28.3)	14.0 (12.6 to 15.5)	1.34 (1.20 to 1.48)	0.73 (0.66 to 0.81)
2015	36.2 (32.2 to 40.8)	18.6 (16.4 to 21.0)	31.9 (28.4 to 35.8)	16.6 (14.7 to 18.8)	1.77 (1.58 to 1.99)	0.92 (0.82 to 1.04)
2020	36.5 (25.6 to 50.3)	18.3 (12.9 to 24.9)	34.9 (24.8 to 47.5)	17.8 (12.7 to 24.1)	1.78 (1.26 to 2.42)	0.91 (0.65 to 1.23)
2021	35.6 (24.9 to 49.3)	17.7 (12.5 to 24.3)	34.8 (24.6 to 47.5)	17.6 (12.5 to 24.0)	1.79 (1.26 to 2.44)	0.91 (0.64 to 1.24)

Eastern Cape ZAF_482 Southern Sub−Saharan Africa Stillbirth Rate / Neonatal Mortality Rate (SBR/NMR) − 20 weeks
lambda = 0.6 ; zeta = 0.8 ; scale = 15 ; amp = 0.028

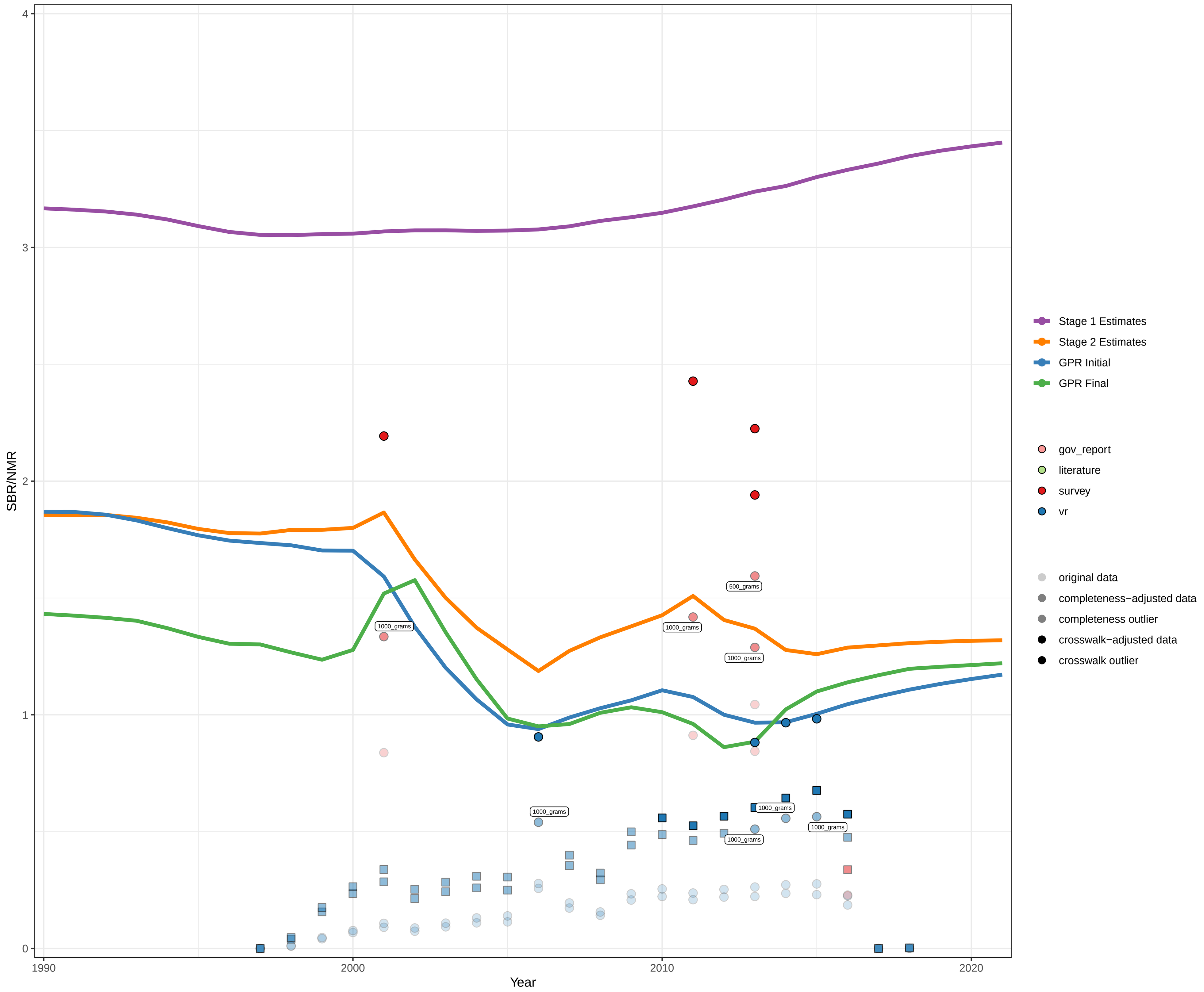

Eastern Cape ZAF_482 Southern Sub−Saharan Africa Stillbirth Rate / Neonatal Mortality Rate (SBR/NMR) − 28 weeks
lambda = 0.6 ; zeta = 0.8 ; scale = 15 ; amp = 0.028

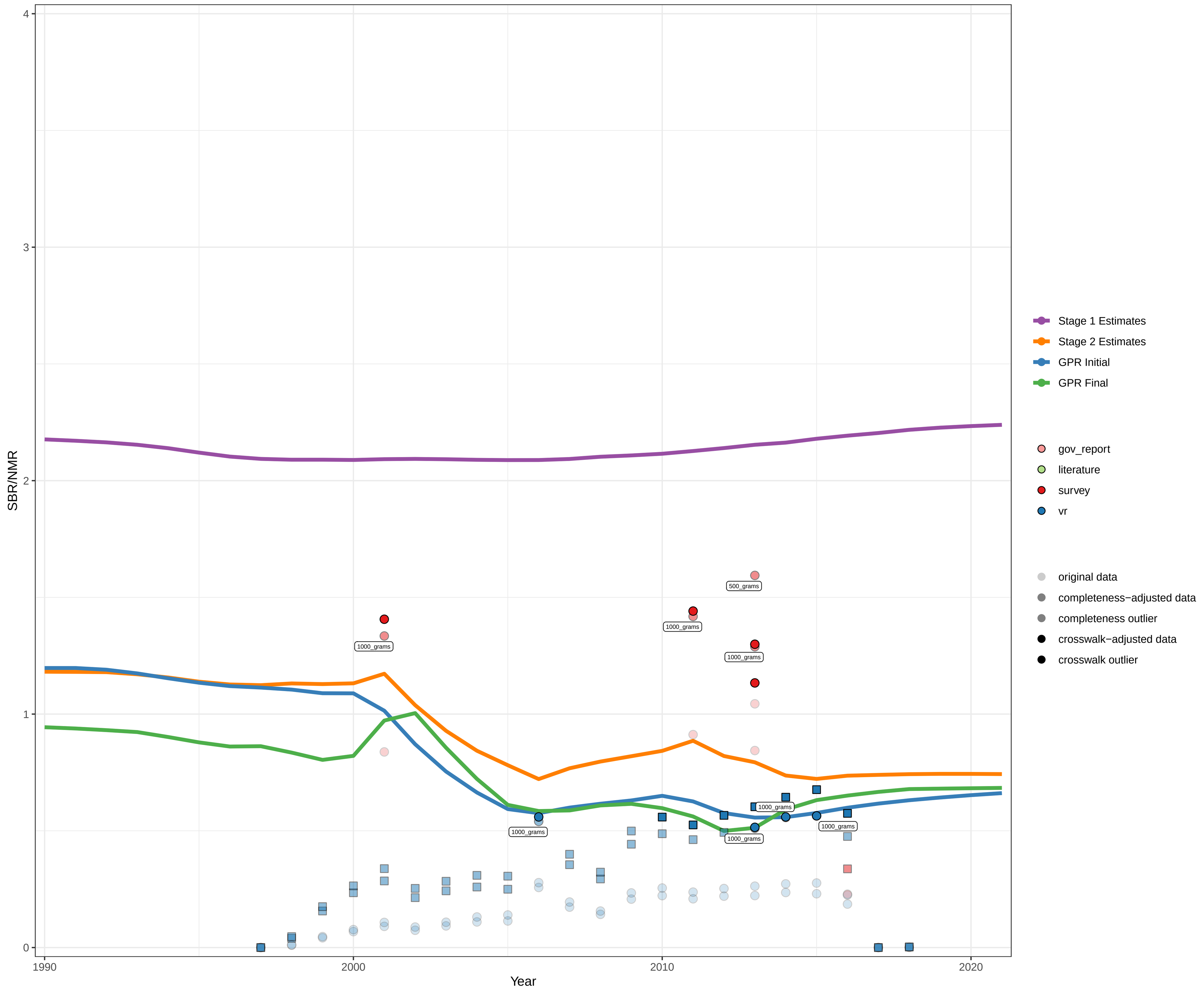

Definition	Years	Outlier	Source.Type	Title
1000_grams	2001	0	Survey	South Africa − Saving Babies: A Perinatal Care Survey 2001
1000_grams	2006, 2013−2015	0	VR	South Africa Vital Registration − Causes of Death
1000_grams	2011	0	Survey	South Africa − Saving Babies: A Perinatal Care Survey 2010−2011
1000_grams, 500_grams	2013	0	Survey	South Africa − Saving Babies: A Perinatal Care Survey 2012−2013
500_grams	2006, 2010−2016	0	VR	South Africa Vital Registration − Causes of Death
1000_grams	2007−2012, 2016−2018	1	VR	South Africa Vital Registration − Causes of Death
1000_grams, 500_grams	1997−2005	1	VR	South Africa Vital Registration − Causes of Death
28_weeks	2016	1	Survey	South Africa Demographic and Health Survey 2016
500_grams	2007−2009, 2017, 2018	1	VR	South Africa Vital Registration − Causes of Death

Eastern Cape ZAF_482 Southern Sub−Saharan Africa Stillbirth Rate (SBR) − 20 weeks
lambda = 0.6 ; zeta = 0.8 ; scale = 15 ; amp = 0.028

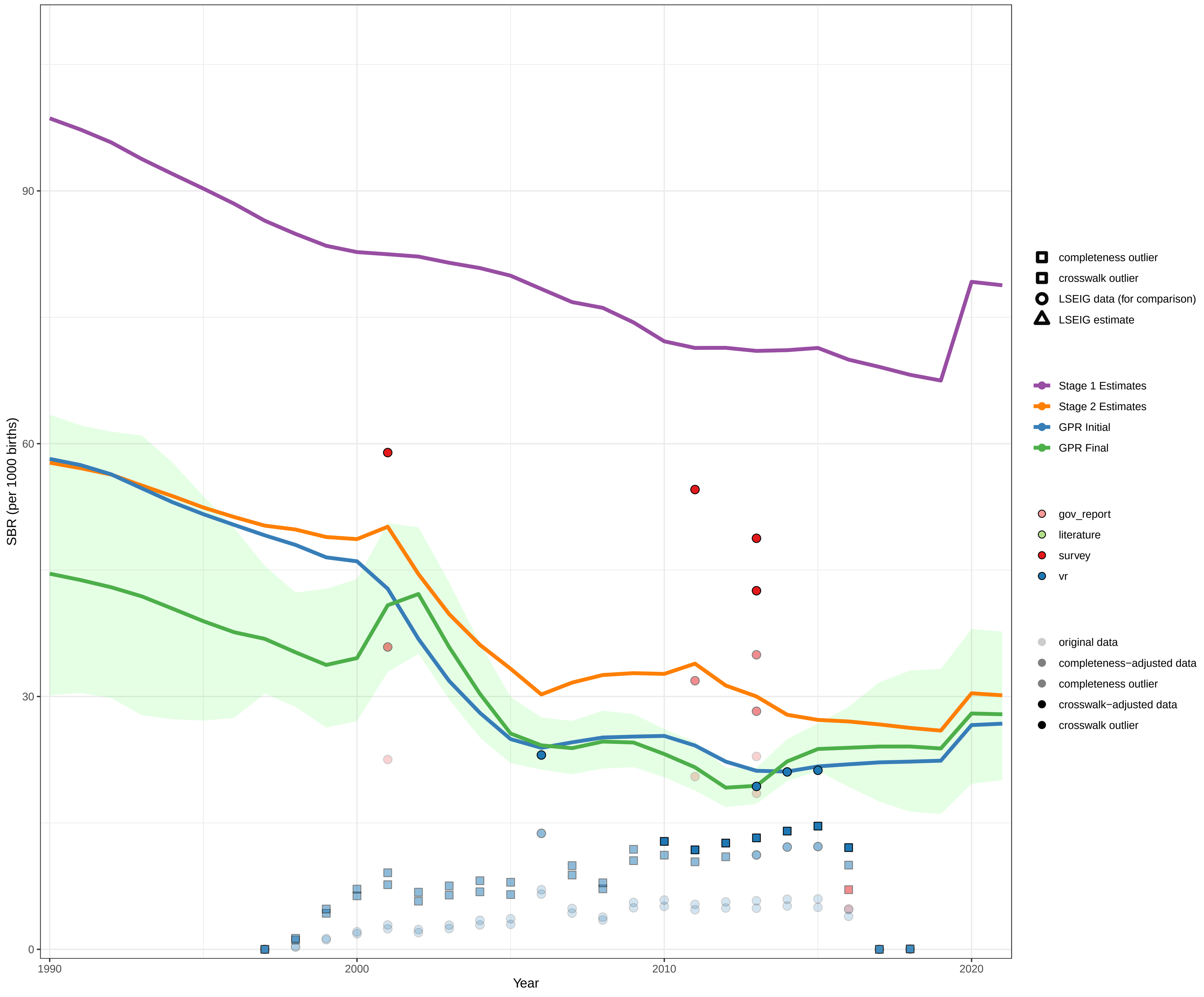

Eastern Cape ZAF_482 Southern Sub−Saharan Africa Stillbirth Rate (SBR) − 28 weeks
lambda = 0.6 ; zeta = 0.8 ; scale = 15 ; amp = 0.028

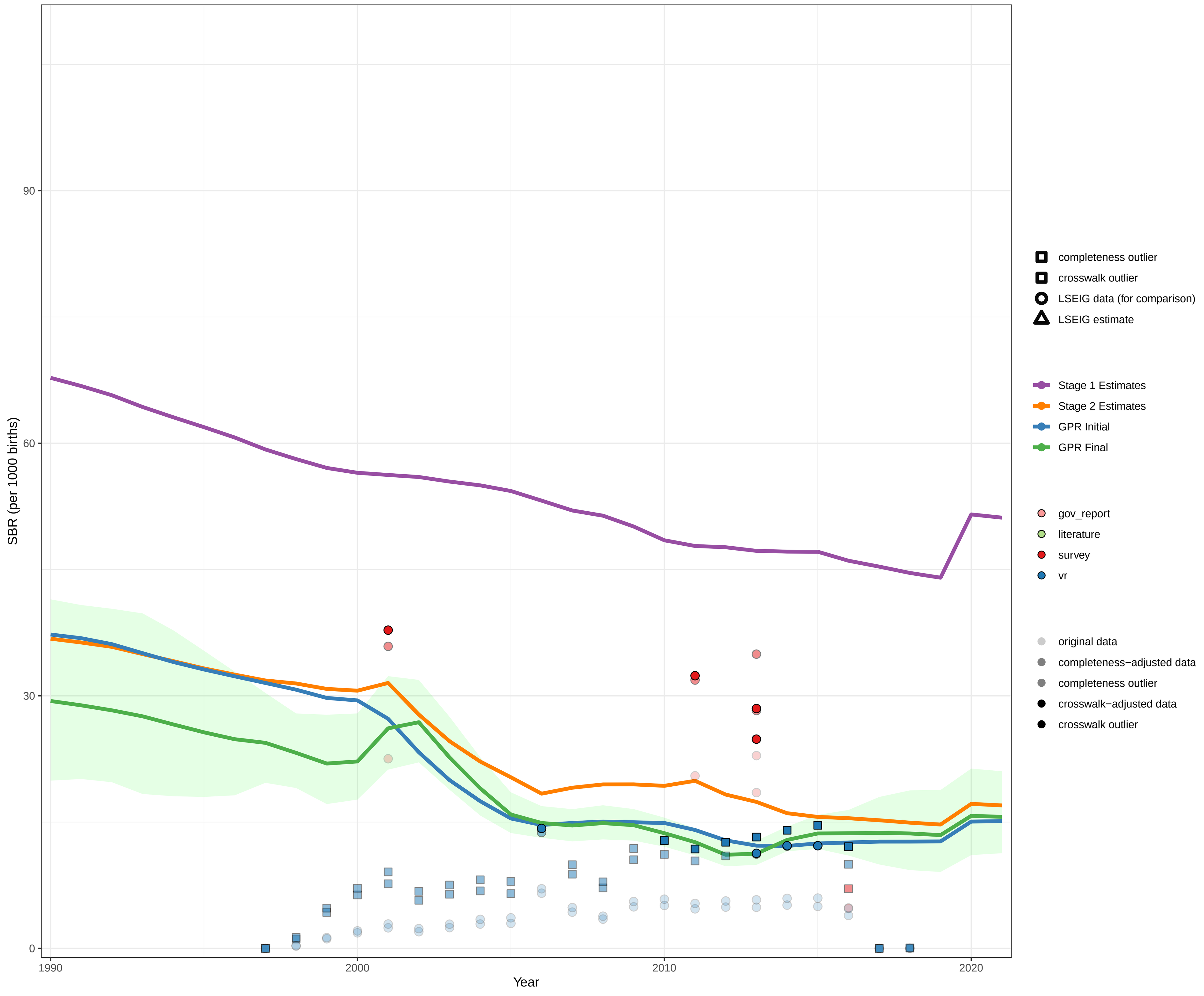

Year	Total Stillbirths (in thousands)	Total Stillbirths (in thousands)	SBR (per 1000 births)	SBR (per 1000 births)	SBR/NMR	SBR/NMR
	20 weeks	28 weeks	20 weeks	28 weeks	20 weeks	28 weeks
1990	8.3 (5.5 to 12.1)	5.4 (3.6 to 7.7)	44.6 (30.1 to 63.4)	29.4 (19.9 to 41.5)	1.43 (0.97 to 2.04)	0.94 (0.64 to 1.33)
1995	6.7 (4.6 to 9.4)	4.4 (3.0 to 6.1)	38.9 (27.1 to 53.8)	25.7 (18.0 to 35.4)	1.33 (0.93 to 1.84)	0.88 (0.62 to 1.21)
2000	5.4 (4.2 to 6.9)	3.4 (2.7 to 4.3)	34.6 (27.1 to 43.9)	22.2 (17.7 to 27.9)	1.28 (1.00 to 1.62)	0.82 (0.65 to 1.03)
2005	3.9 (3.3 to 4.6)	2.4 (2.1 to 2.8)	25.6 (22.1 to 30.0)	15.9 (13.7 to 18.5)	0.98 (0.85 to 1.15)	0.61 (0.53 to 0.71)
2010	3.4 (3.0 to 3.8)	2.0 (1.7 to 2.2)	23.2 (20.4 to 26.1)	13.7 (12.1 to 15.5)	1.01 (0.89 to 1.14)	0.60 (0.53 to 0.68)
2015	3.2 (2.9 to 3.7)	1.8 (1.6 to 2.1)	23.8 (21.2 to 26.8)	13.7 (11.8 to 15.9)	1.10 (0.98 to 1.24)	0.63 (0.55 to 0.73)
2020	3.5 (2.4 to 4.7)	1.9 (1.3 to 2.6)	28.0 (19.6 to 38.0)	15.7 (11.1 to 21.4)	1.21 (0.85 to 1.65)	0.68 (0.48 to 0.93)
2021	3.4 (2.4 to 4.6)	1.9 (1.3 to 2.5)	27.9 (20.1 to 37.7)	15.6 (11.3 to 21.0)	1.22 (0.88 to 1.65)	0.68 (0.49 to 0.92)

Free State ZAF_483 Southern Sub−Saharan Africa Stillbirth Rate / Neonatal Mortality Rate (SBR/NMR) − 20 weeks
lambda = 0.2 ; zeta = 0.99 ; scale = 5 ; amp = 0.028

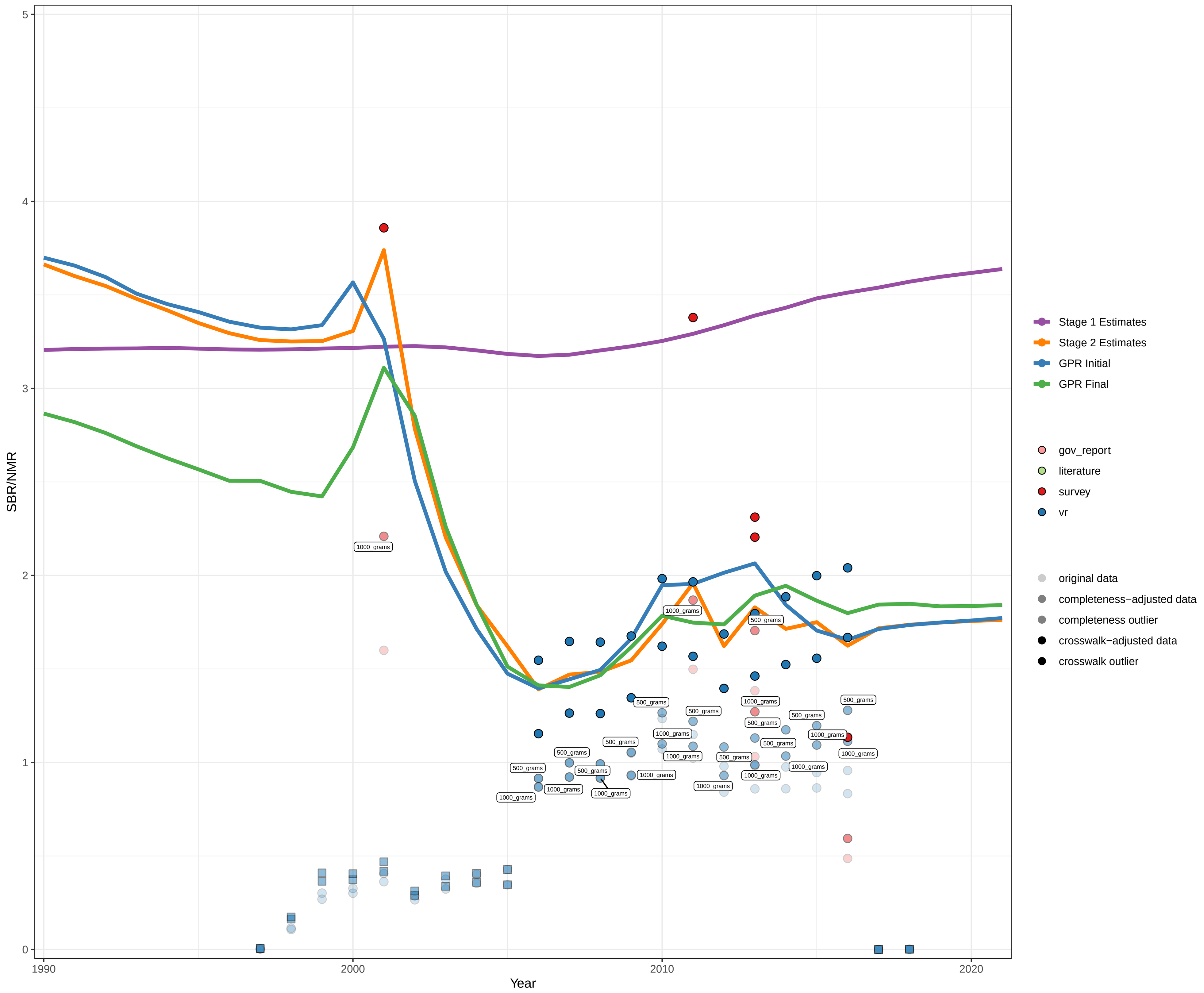

Free State ZAF_483 Southern Sub−Saharan Africa Stillbirth Rate / Neonatal Mortality Rate (SBR/NMR) − 28 weeks
lambda = 0.2 ; zeta = 0.99 ; scale = 5 ; amp = 0.028

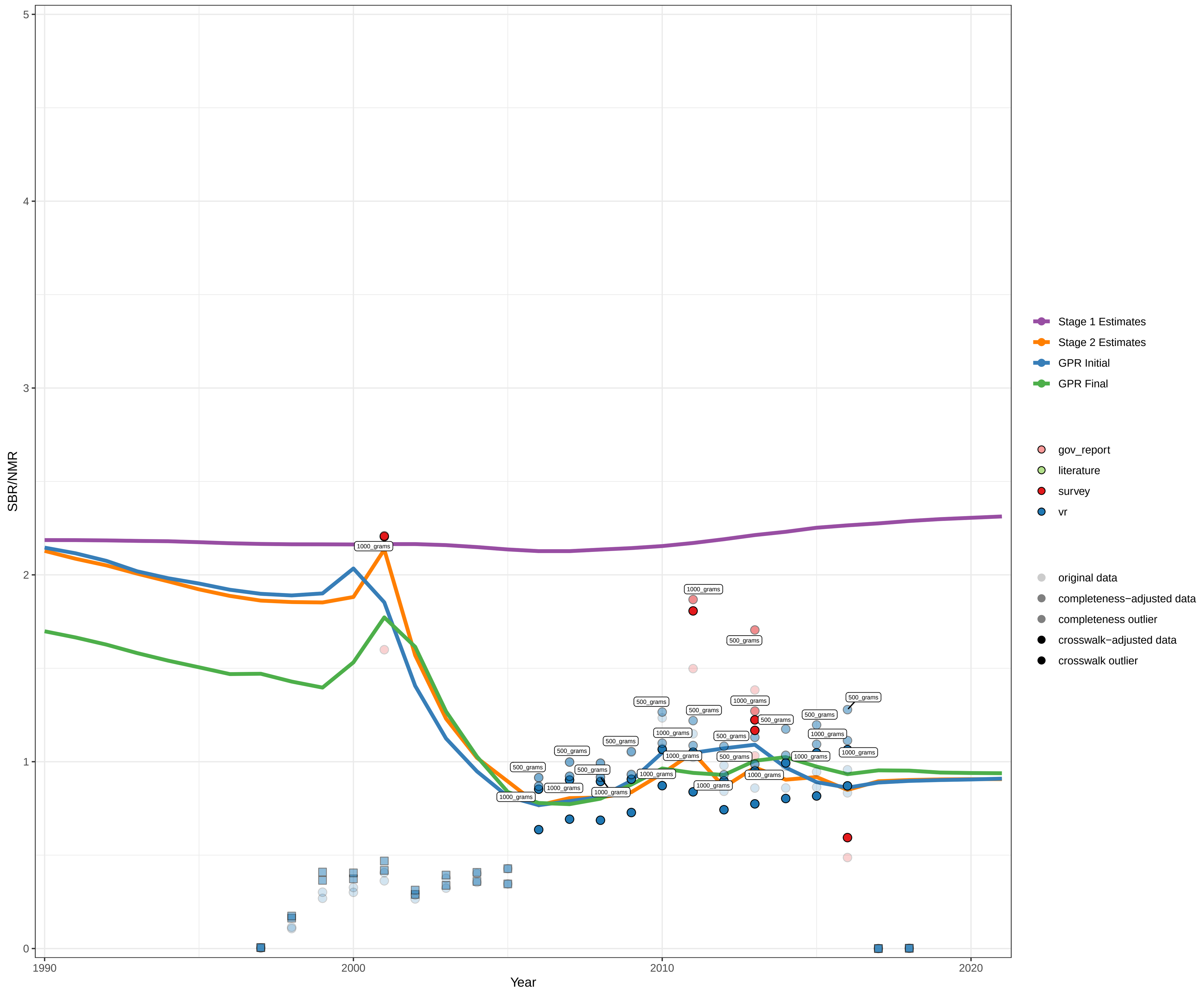

Definition	Years	Outlier	Source.Type	Title
1000_grams	2001	0	Survey	South Africa − Saving Babies: A Perinatal Care Survey 2001
1000_grams	2011	0	Survey	South Africa − Saving Babies: A Perinatal Care Survey 2010−2011
1000_grams, 500_grams	2006−2016	0	VR	South Africa Vital Registration − Causes of Death
1000_grams, 500_grams	2013	0	Survey	South Africa − Saving Babies: A Perinatal Care Survey 2012−2013
28_weeks	2016	0	Survey	South Africa Demographic and Health Survey 2016
1000_grams, 500_grams	1997−2005	1	VR	South Africa Vital Registration − Causes of Death
1000_grams, 500_grams	2017, 2018	1	VR	South Africa Vital Registration − Causes of Death

Free State ZAF_483 Southern Sub−Saharan Africa Stillbirth Rate (SBR) − 20 weeks
lambda = 0.2 ; zeta = 0.99 ; scale = 5 ; amp = 0.028

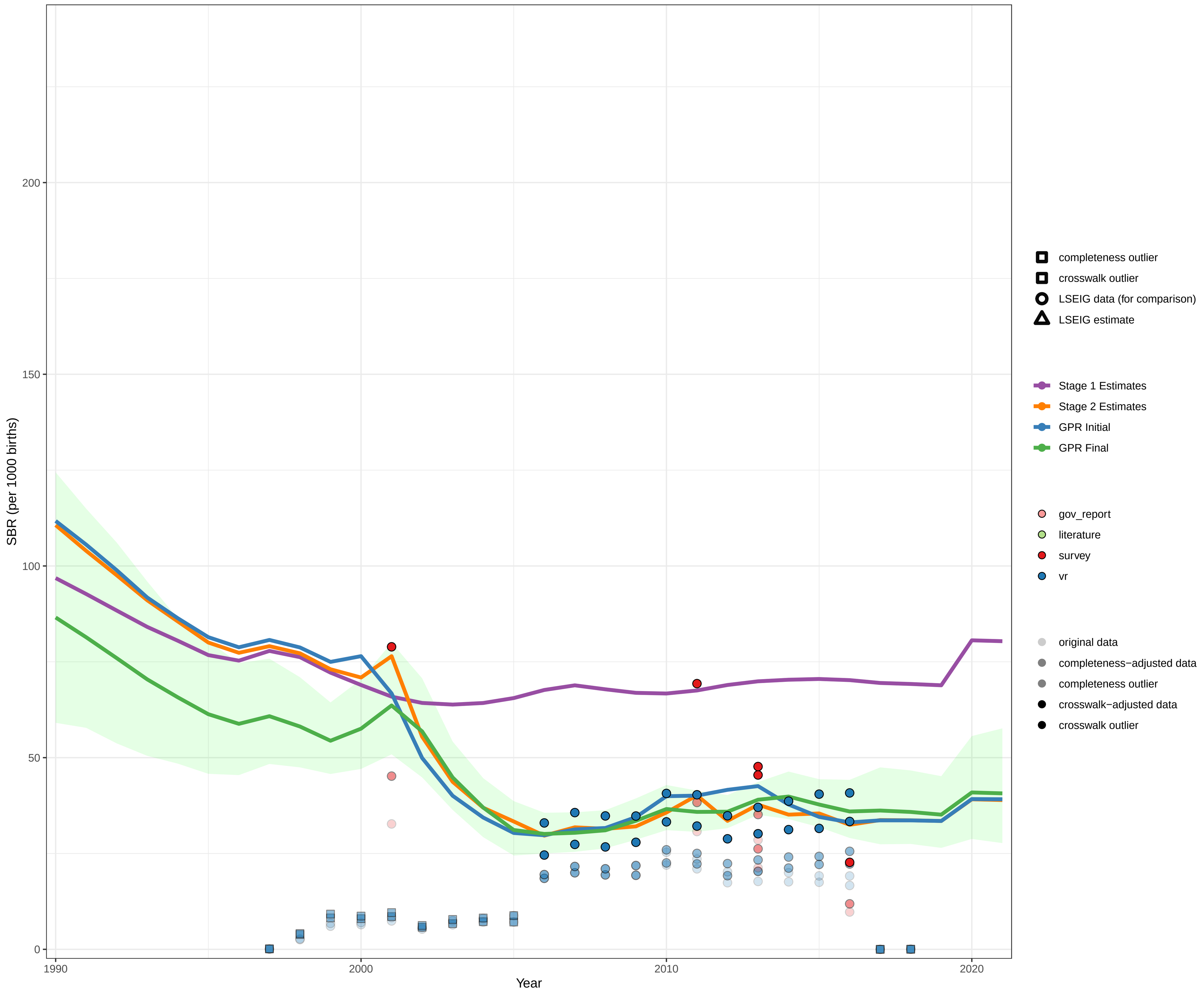

Free State ZAF_483 Southern Sub−Saharan Africa Stillbirth Rate (SBR) − 28 weeks
lambda = 0.2 ; zeta = 0.99 ; scale = 5 ; amp = 0.028

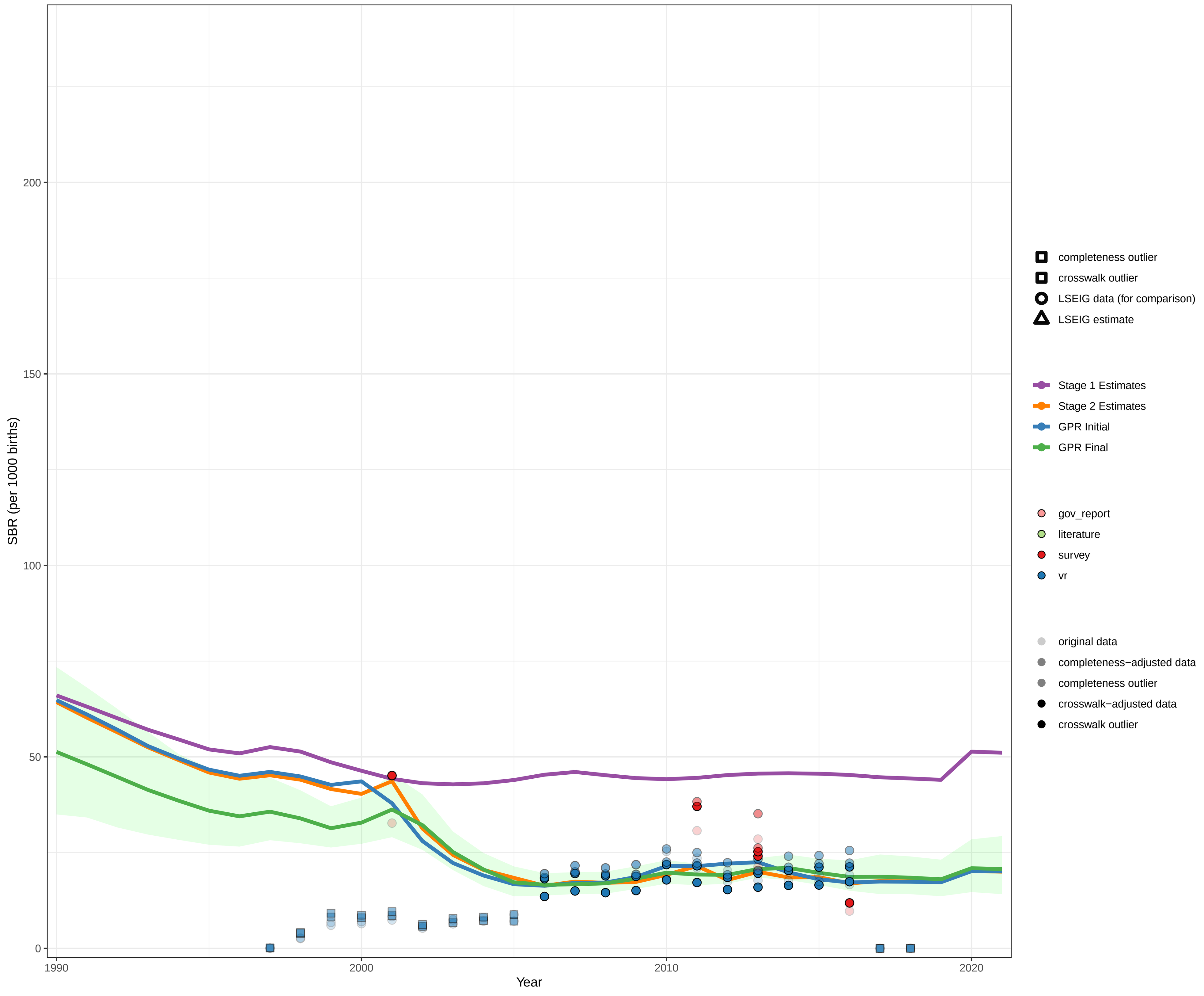

Year	Total Stillbirths (in thousands)	Total Stillbirths (in thousands)	SBR (per 1000 births)	SBR (per 1000 births)	SBR/NMR	SBR/NMR
	20 weeks	28 weeks	20 weeks	28 weeks	20 weeks	28 weeks
1990	5.9 (3.9 to 8.9)	3.4 (2.3 to 4.9)	86.6 (59.1 to 124.4)	51.3 (35.0 to 73.5)	2.87 (1.96 to 4.12)	1.70 (1.16 to 2.43)
1995	3.9 (2.8 to 5.2)	2.2 (1.6 to 2.9)	61.3 (45.8 to 80.4)	36.0 (27.1 to 47.2)	2.57 (1.92 to 3.37)	1.51 (1.13 to 1.97)
2000	3.5 (2.8 to 4.3)	1.9 (1.6 to 2.3)	57.6 (47.1 to 70.4)	32.8 (27.3 to 39.4)	2.68 (2.19 to 3.28)	1.53 (1.27 to 1.84)
2005	1.9 (1.5 to 2.4)	1.1 (0.8 to 1.3)	31.1 (24.5 to 38.7)	17.2 (13.6 to 21.4)	1.51 (1.19 to 1.88)	0.84 (0.66 to 1.04)
2010	2.2 (1.8 to 2.6)	1.1 (1.0 to 1.3)	36.6 (31.1 to 42.9)	19.8 (16.9 to 23.0)	1.78 (1.52 to 2.09)	0.96 (0.82 to 1.12)
2015	2.0 (1.7 to 2.3)	1.0 (0.8 to 1.2)	37.8 (31.9 to 44.4)	19.7 (16.6 to 23.4)	1.86 (1.57 to 2.19)	0.97 (0.82 to 1.16)
2020	1.9 (1.3 to 2.6)	0.9 (0.7 to 1.3)	40.9 (28.8 to 55.6)	20.9 (14.7 to 28.5)	1.84 (1.29 to 2.50)	0.94 (0.66 to 1.28)
2021	1.8 (1.2 to 2.6)	0.9 (0.6 to 1.3)	40.7 (27.7 to 57.6)	20.7 (14.2 to 29.3)	1.84 (1.25 to 2.61)	0.94 (0.64 to 1.33)

Gauteng ZAF_484 Southern Sub−Saharan Africa Stillbirth Rate / Neonatal Mortality Rate (SBR/NMR) − 20 weeks
lambda = 0.2 ; zeta = 0.99 ; scale = 5 ; amp = 0.028

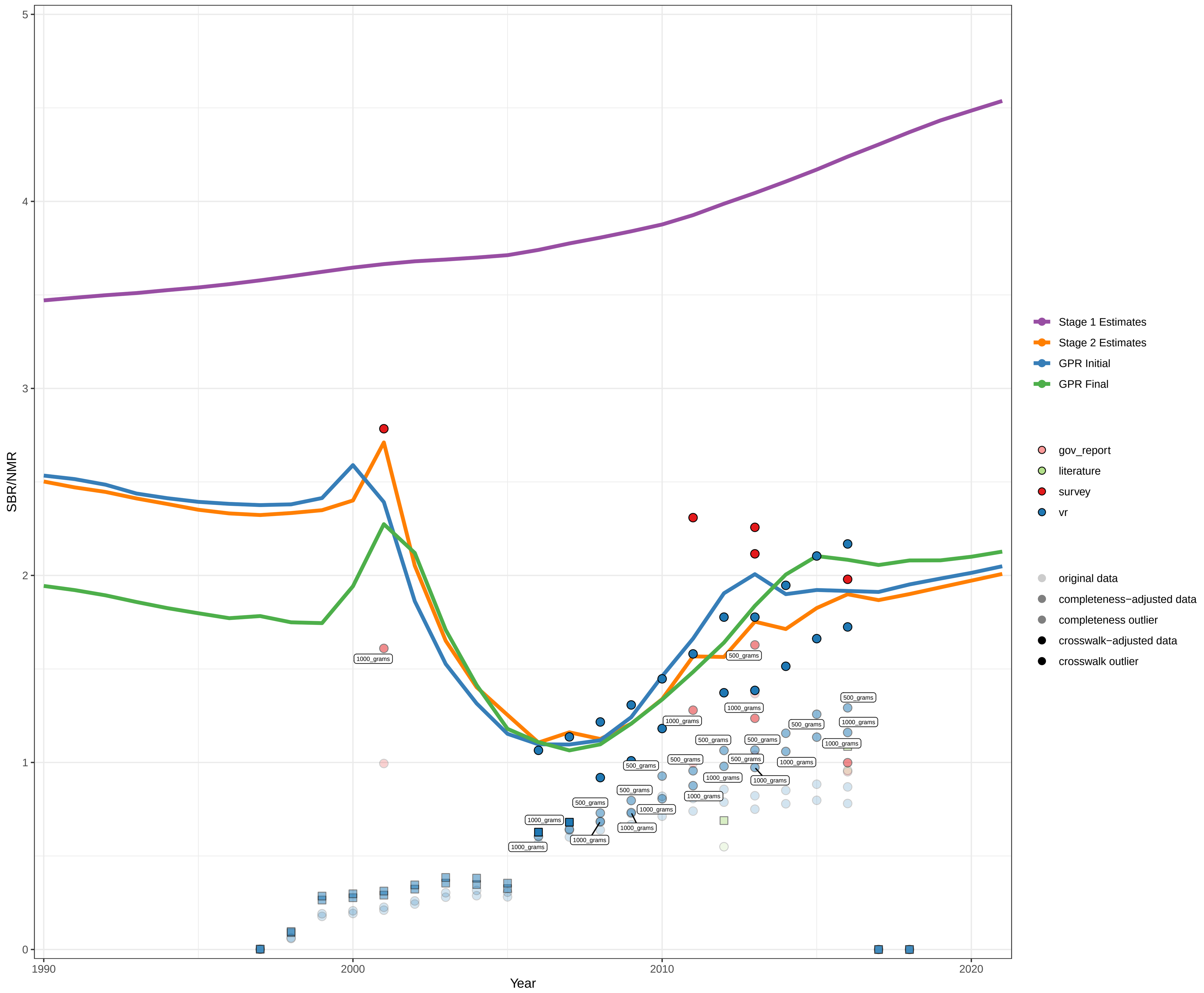

Gauteng ZAF_484 Southern Sub−Saharan Africa Stillbirth Rate / Neonatal Mortality Rate (SBR/NMR) − 28 weeks
lambda = 0.2 ; zeta = 0.99 ; scale = 5 ; amp = 0.028

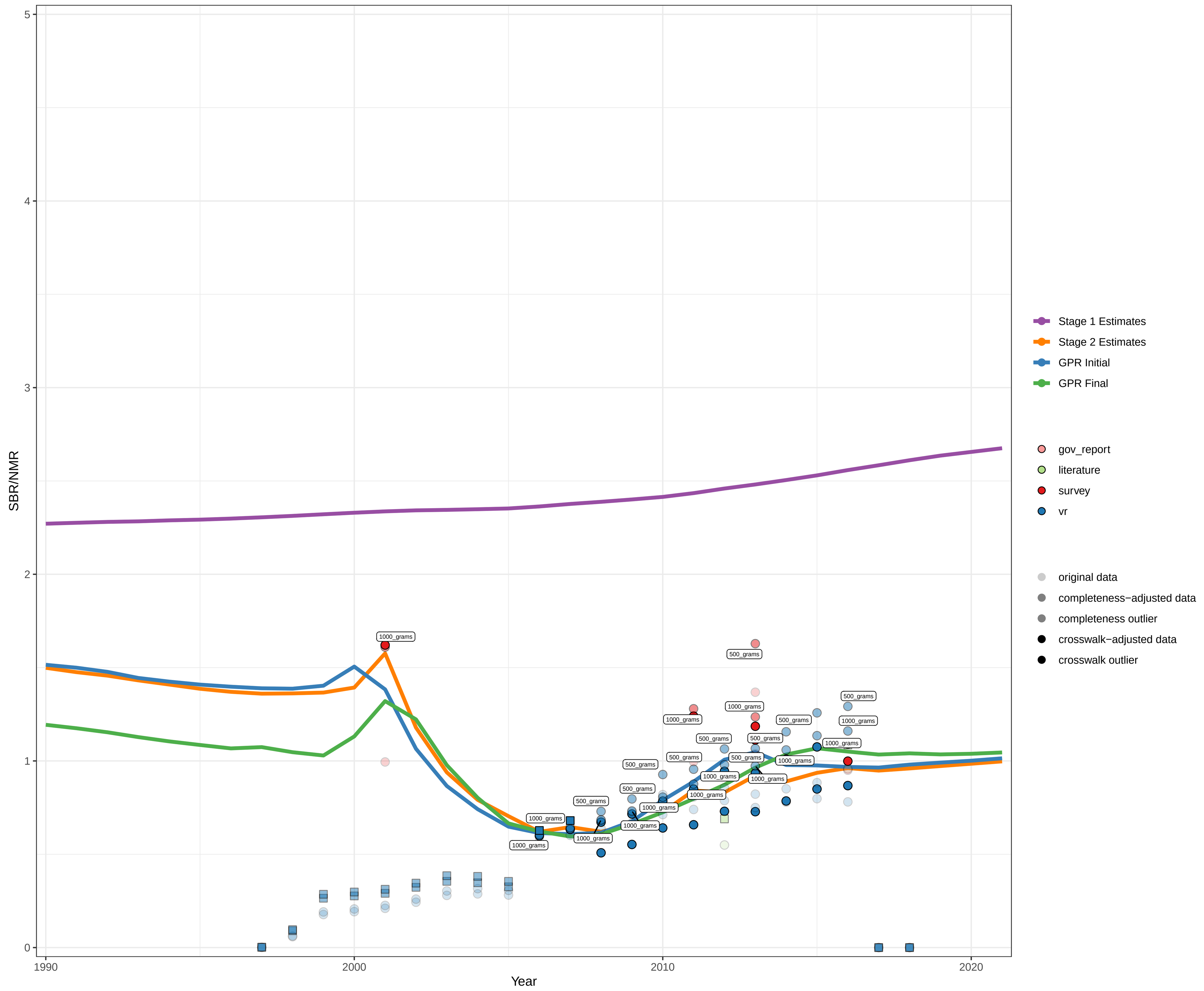

Definition	Years	Outlier	Source.Type	Title
1000_grams	2001	0	Survey	South Africa − Saving Babies: A Perinatal Care Survey 2001
1000_grams	2011	0	Survey	South Africa − Saving Babies: A Perinatal Care Survey 2010−2011
1000_grams, 500_grams	2006−2016	0	VR	South Africa Vital Registration − Causes of Death
1000_grams, 500_grams	2013	0	Survey	South Africa − Saving Babies: A Perinatal Care Survey 2012−2013
28_weeks	2016	0	Survey	South Africa Demographic and Health Survey 2016
1000_grams, 500_grams	1997−2005	1	VR	South Africa Vital Registration − Causes of Death
1000_grams, 500_grams	2017, 2018	1	VR	South Africa Vital Registration − Causes of Death
28_weeks	2012	1	Sci Lit	Trivalent influenza vaccination randomized control trial of pregnant women and adverse fetal outcomes
28_weeks	2016	1	Sci Lit	Screening and managing a low−risk pregnant population using continuous−wave Doppler ultrasound in a low−income population: A cohort analytical study

Gauteng ZAF_484 Southern Sub−Saharan Africa Stillbirth Rate (SBR) − 20 weeks
lambda = 0.2 ; zeta = 0.99 ; scale = 5 ; amp = 0.028

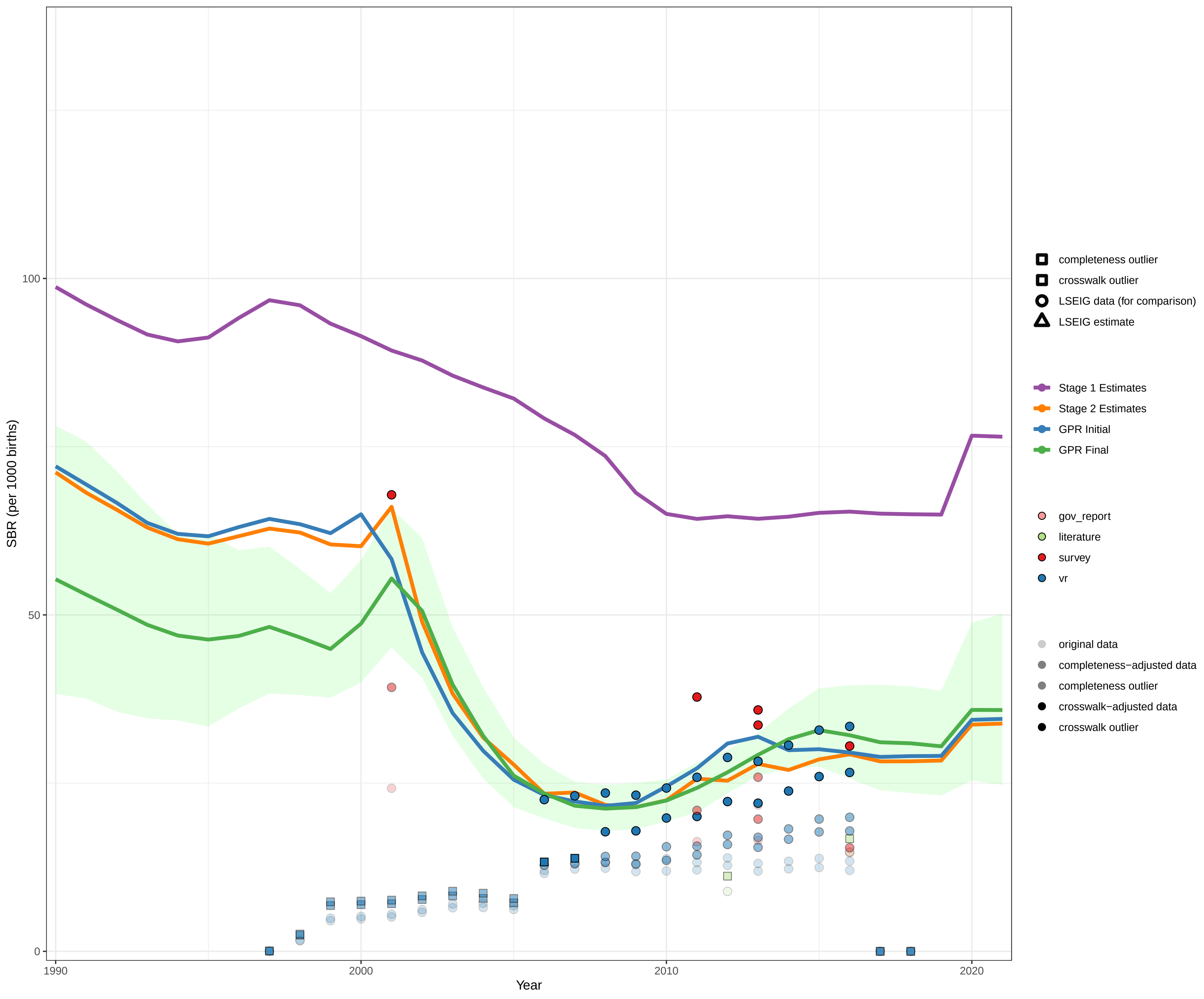

Gauteng ZAF_484 Southern Sub−Saharan Africa Stillbirth Rate (SBR) − 28 weeks
lambda = 0.2 ; zeta = 0.99 ; scale = 5 ; amp = 0.028

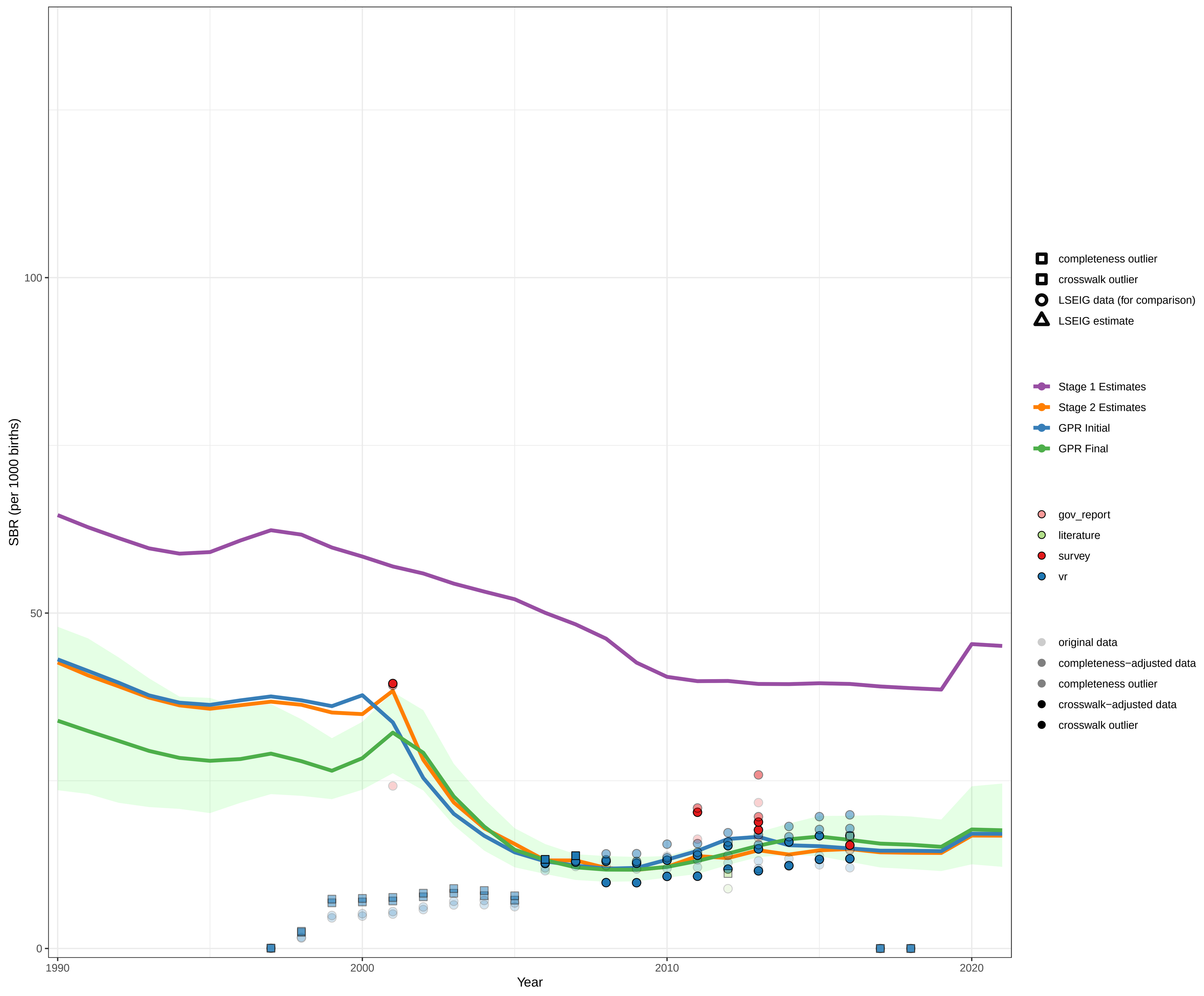

Year	Total Stillbirths (in thousands)	Total Stillbirths (in thousands)	SBR (per 1000 births)	SBR (per 1000 births)	SBR/NMR	SBR/NMR
	20 weeks	28 weeks	20 weeks	28 weeks	20 weeks	28 weeks
1990	9.3 (6.3 to 13.5)	5.6 (3.8 to 8.0)	55.3 (38.2 to 78.1)	34.0 (23.6 to 48.0)	1.94 (1.34 to 2.75)	1.19 (0.83 to 1.69)
1995	8.4 (5.9 to 11.4)	5.0 (3.5 to 6.7)	46.3 (33.4 to 62.0)	28.0 (20.2 to 37.4)	1.80 (1.30 to 2.41)	1.09 (0.78 to 1.45)
2000	10.0 (8.1 to 12.1)	5.7 (4.7 to 6.8)	48.7 (40.0 to 58.1)	28.4 (23.7 to 33.8)	1.94 (1.59 to 2.32)	1.13 (0.94 to 1.35)
2005	6.5 (5.3 to 7.9)	3.6 (3.0 to 4.4)	26.1 (21.4 to 31.8)	14.7 (12.1 to 17.9)	1.18 (0.97 to 1.44)	0.67 (0.55 to 0.81)
2010	6.2 (5.4 to 7.1)	3.3 (2.9 to 3.8)	22.4 (19.3 to 25.5)	12.2 (10.6 to 13.8)	1.34 (1.15 to 1.52)	0.72 (0.63 to 0.82)
2015	9.5 (7.9 to 11.4)	4.7 (3.9 to 5.6)	32.9 (27.4 to 39.1)	16.7 (13.8 to 19.8)	2.10 (1.76 to 2.50)	1.07 (0.88 to 1.26)
2020	9.5 (6.7 to 13.2)	4.6 (3.3 to 6.3)	35.9 (25.4 to 48.9)	17.7 (12.5 to 24.2)	2.10 (1.49 to 2.86)	1.04 (0.73 to 1.42)
2021	9.3 (6.3 to 13.2)	4.5 (3.1 to 6.3)	35.9 (24.7 to 50.2)	17.6 (12.2 to 24.6)	2.13 (1.47 to 2.98)	1.05 (0.72 to 1.46)

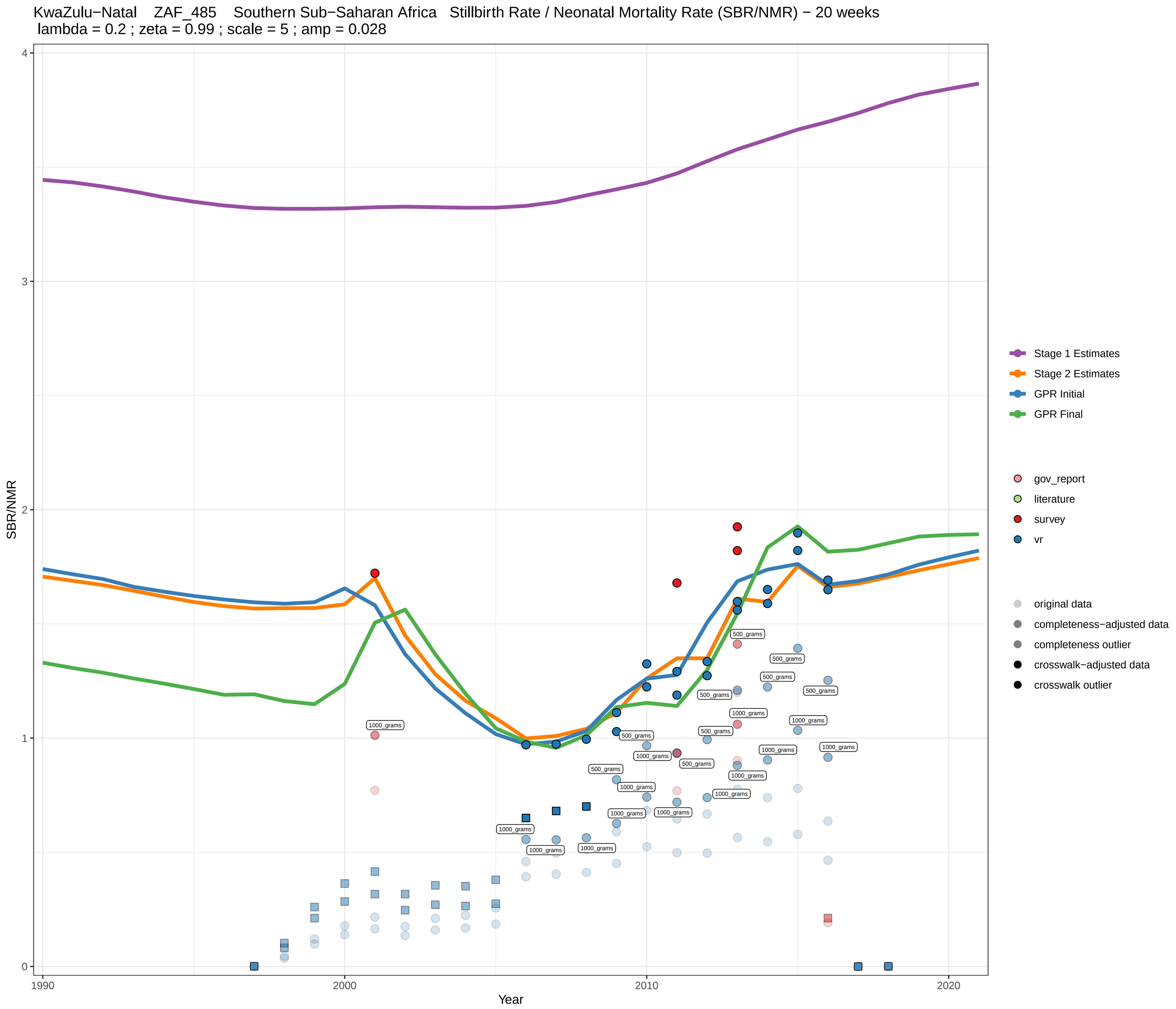

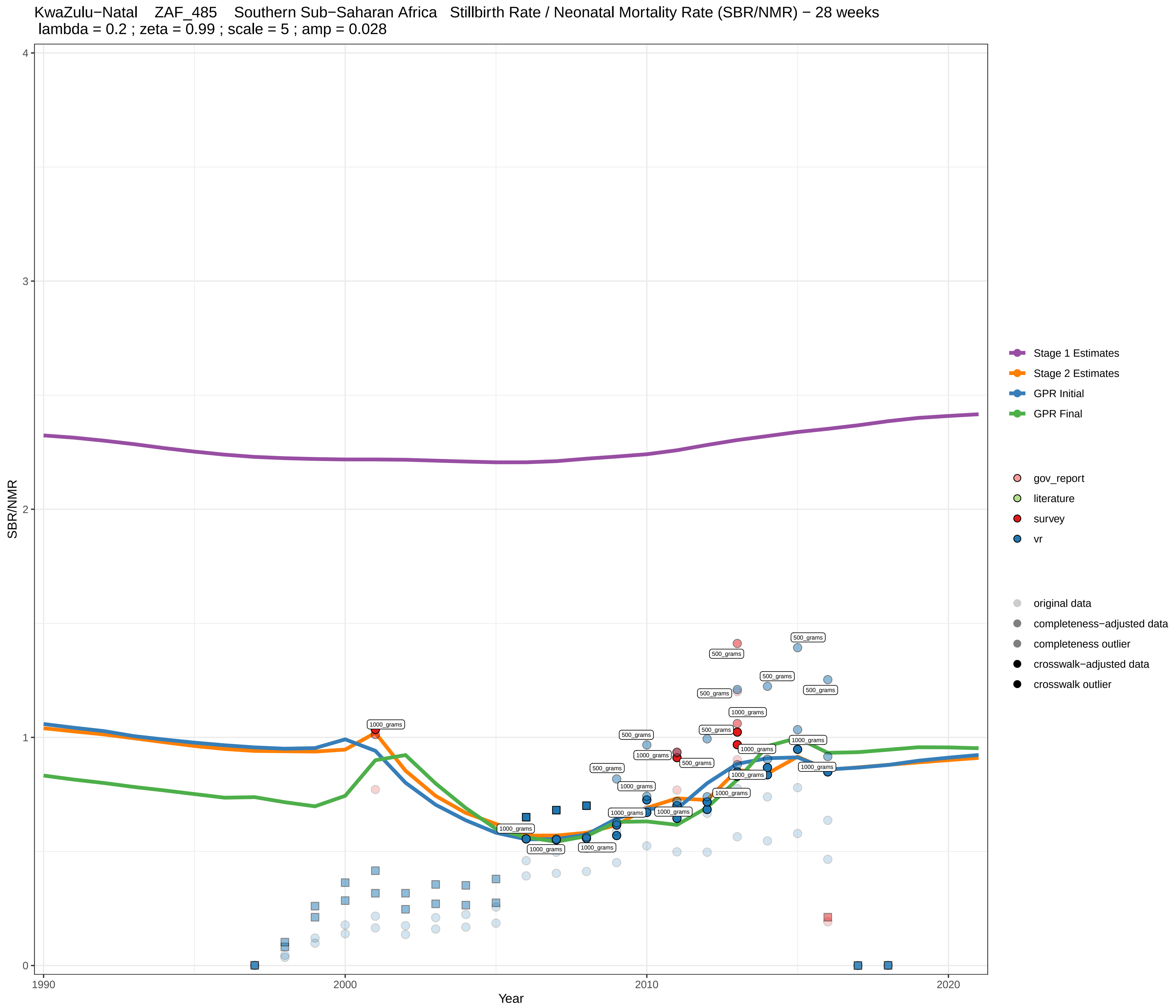

Definition	Years	Outlier	Source.Type	Title
1000_grams	2001	0	Survey	South Africa − Saving Babies: A Perinatal Care Survey 2001
1000_grams	2011	0	Survey	South Africa − Saving Babies: A Perinatal Care Survey 2010−2011
1000_grams, 500_grams	2006−2016	0	VR	South Africa Vital Registration − Causes of Death
1000_grams, 500_grams	2013	0	Survey	South Africa − Saving Babies: A Perinatal Care Survey 2012−2013
1000_grams, 500_grams	1997−2005	1	VR	South Africa Vital Registration − Causes of Death
1000_grams, 500_grams	2017, 2018	1	VR	South Africa Vital Registration − Causes of Death
28_weeks	2016	1	Survey	South Africa Demographic and Health Survey 2016

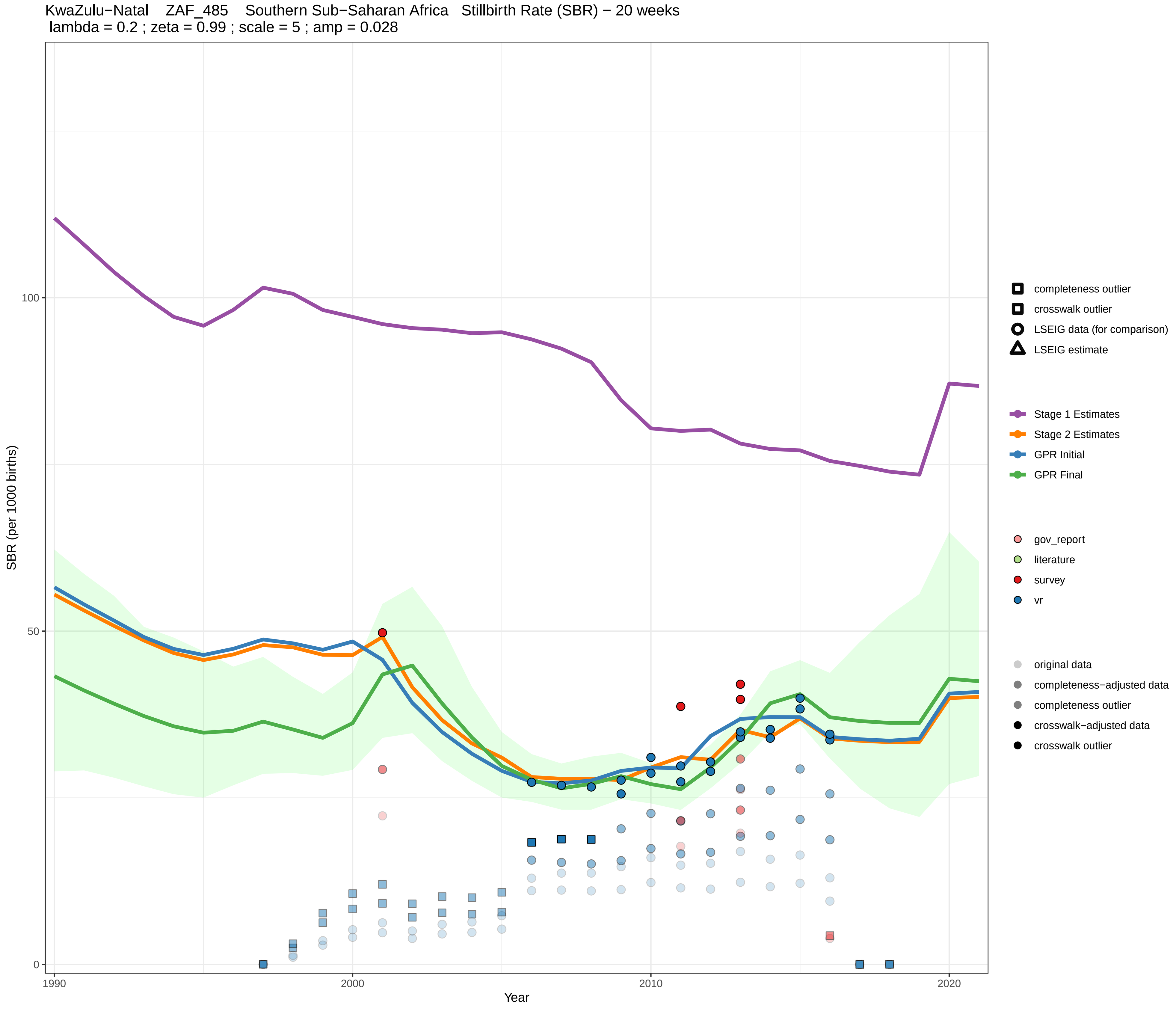

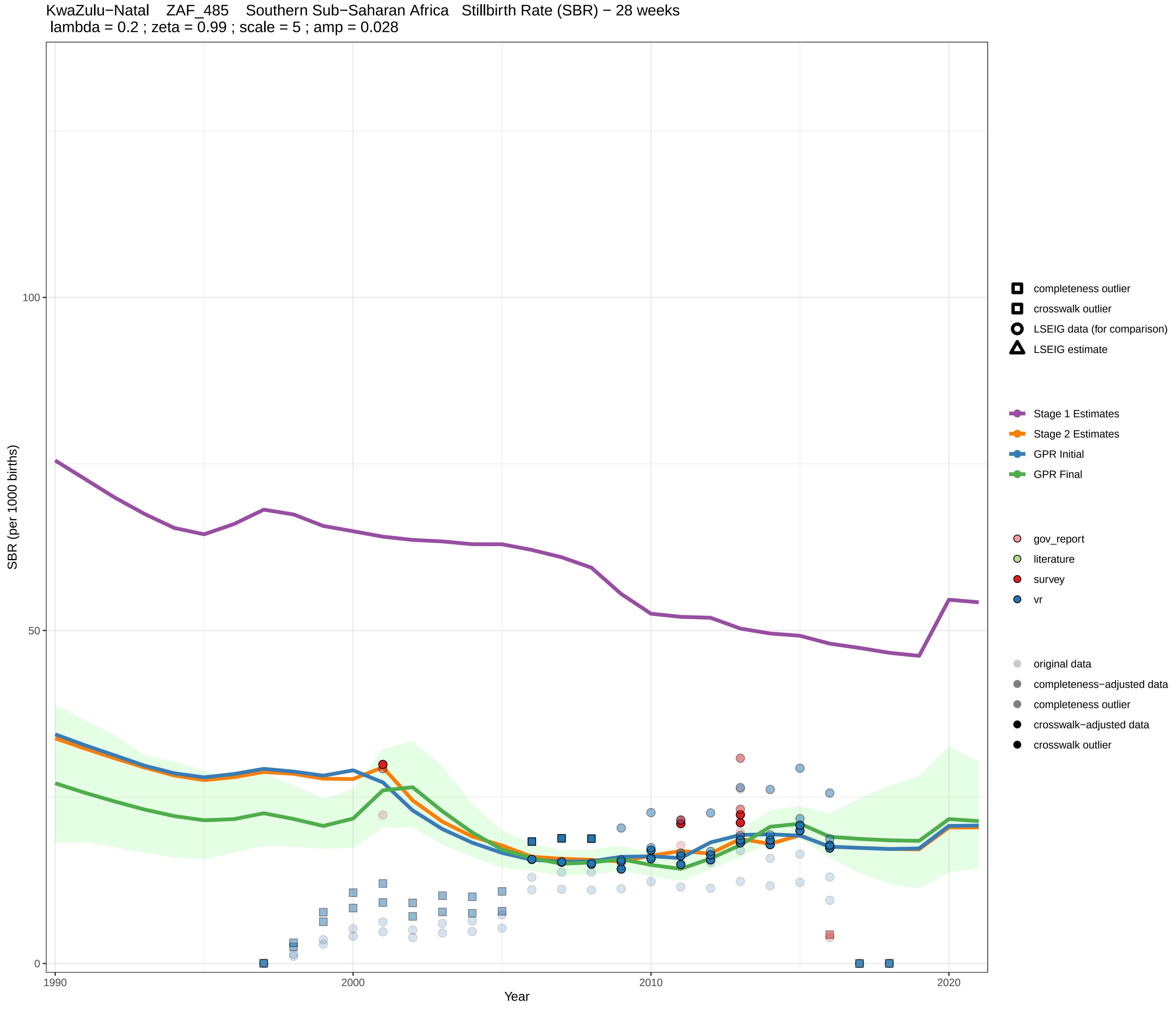

Year	Total Stillbirths (in thousands)	Total Stillbirths (in thousands)	SBR (per 1000 births)	SBR (per 1000 births)	SBR/NMR	SBR/NMR
	20 weeks	28 weeks	20 weeks	28 weeks	20 weeks	28 weeks
1990	10.6 (7.0 to 15.5)	6.5 (4.3 to 9.4)	43.2 (29.0 to 62.2)	27.1 (18.1 to 38.9)	1.33 (0.89 to 1.91)	0.83 (0.56 to 1.20)
1995	8.2 (5.8 to 11.2)	5.0 (3.6 to 6.8)	34.8 (25.0 to 47.0)	21.5 (15.6 to 29.0)	1.22 (0.87 to 1.64)	0.75 (0.55 to 1.01)
2000	8.4 (6.7 to 10.3)	5.0 (4.0 to 6.0)	36.2 (29.2 to 43.8)	21.8 (17.4 to 26.2)	1.24 (1.00 to 1.50)	0.74 (0.59 to 0.89)
2005	7.2 (6.0 to 8.5)	4.1 (3.4 to 4.8)	29.8 (25.0 to 34.9)	17.1 (14.4 to 20.0)	1.04 (0.88 to 1.22)	0.60 (0.50 to 0.70)
2010	6.5 (5.7 to 7.2)	3.5 (3.1 to 3.9)	27.1 (24.1 to 30.2)	14.8 (13.1 to 16.6)	1.15 (1.03 to 1.29)	0.63 (0.56 to 0.71)
2015	9.3 (8.2 to 10.5)	4.7 (4.2 to 5.3)	40.5 (36.1 to 45.6)	21.0 (18.8 to 23.6)	1.93 (1.71 to 2.17)	1.00 (0.89 to 1.12)
2020	9.0 (5.6 to 14.0)	4.5 (2.8 to 6.8)	42.8 (27.1 to 64.8)	21.7 (13.7 to 32.6)	1.89 (1.19 to 2.86)	0.96 (0.60 to 1.44)
2021	8.8 (5.7 to 12.7)	4.3 (2.9 to 6.2)	42.5 (28.3 to 60.4)	21.4 (14.3 to 30.3)	1.89 (1.26 to 2.69)	0.95 (0.64 to 1.35)

Limpopo ZAF_486 Southern Sub−Saharan Africa Stillbirth Rate / Neonatal Mortality Rate (SBR/NMR) − 20 weeks
lambda = 0.4 ; zeta = 0.9 ; scale = 10 ; amp = 0.028

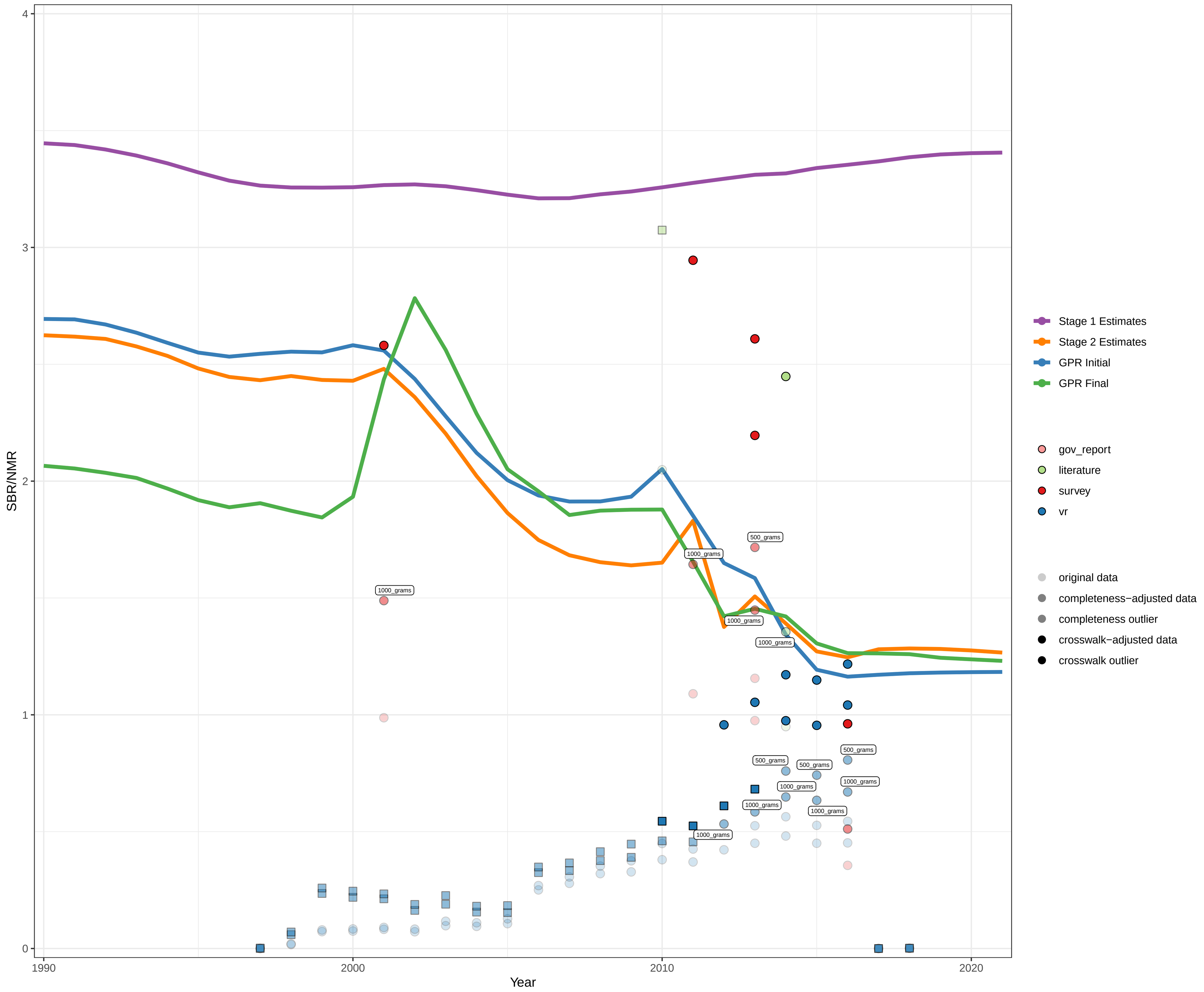

Limpopo ZAF_486 Southern Sub−Saharan Africa Stillbirth Rate / Neonatal Mortality Rate (SBR/NMR) − 28 weeks
lambda = 0.4 ; zeta = 0.9 ; scale = 10 ; amp = 0.028

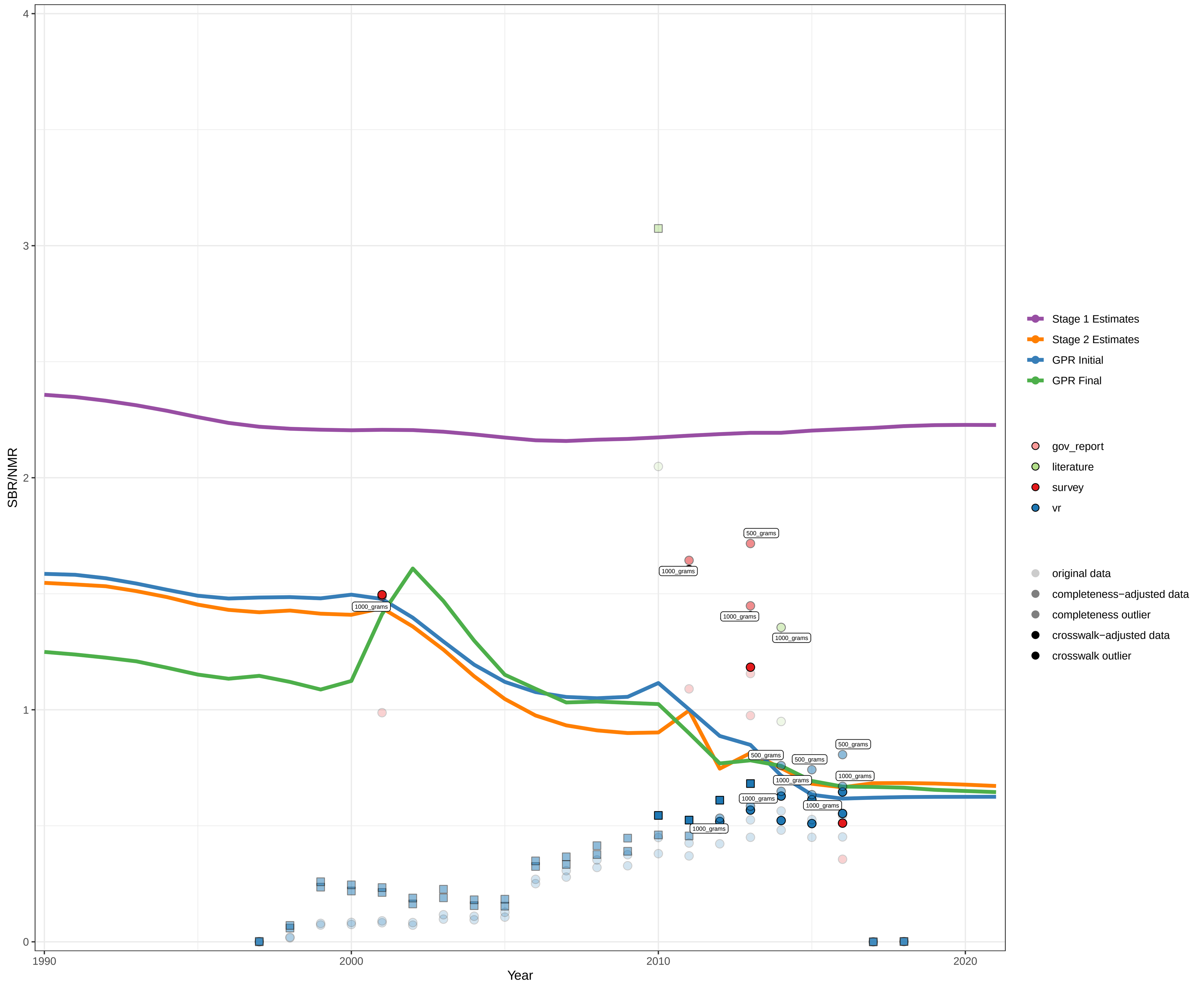

Definition	Years	Outlier	Source.Type	Title
1000_grams	2001	0	Survey	South Africa − Saving Babies: A Perinatal Care Survey 2001
1000_grams	2011	0	Survey	South Africa − Saving Babies: A Perinatal Care Survey 2010−2011
1000_grams	2012−2016	0	VR	South Africa Vital Registration − Causes of Death
1000_grams	2014	0	Sci Lit	Timing and cause of perinatal mortality for small−for−gestational−age babies in South Africa: critical periods and challenges with detection
1000_grams, 500_grams	2013	0	Survey	South Africa − Saving Babies: A Perinatal Care Survey 2012−2013
28_weeks	2016	0	Survey	South Africa Demographic and Health Survey 2016
500_grams	2010−2016	0	VR	South Africa Vital Registration − Causes of Death
1000_grams	2006−2011, 2017, 2018	1	VR	South Africa Vital Registration − Causes of Death
1000_grams, 500_grams	1997−2005	1	VR	South Africa Vital Registration − Causes of Death
20_weeks	2010	1	Sci Lit	An Investigation of the Stillbirths at a Tertiary Hospital in Limpopo Province of South Africa
500_grams	2006−2009, 2017, 2018	1	VR	South Africa Vital Registration − Causes of Death

Limpopo ZAF_486 Southern Sub−Saharan Africa Stillbirth Rate (SBR) − 20 weeks
lambda = 0.4 ; zeta = 0.9 ; scale = 10 ; amp = 0.028

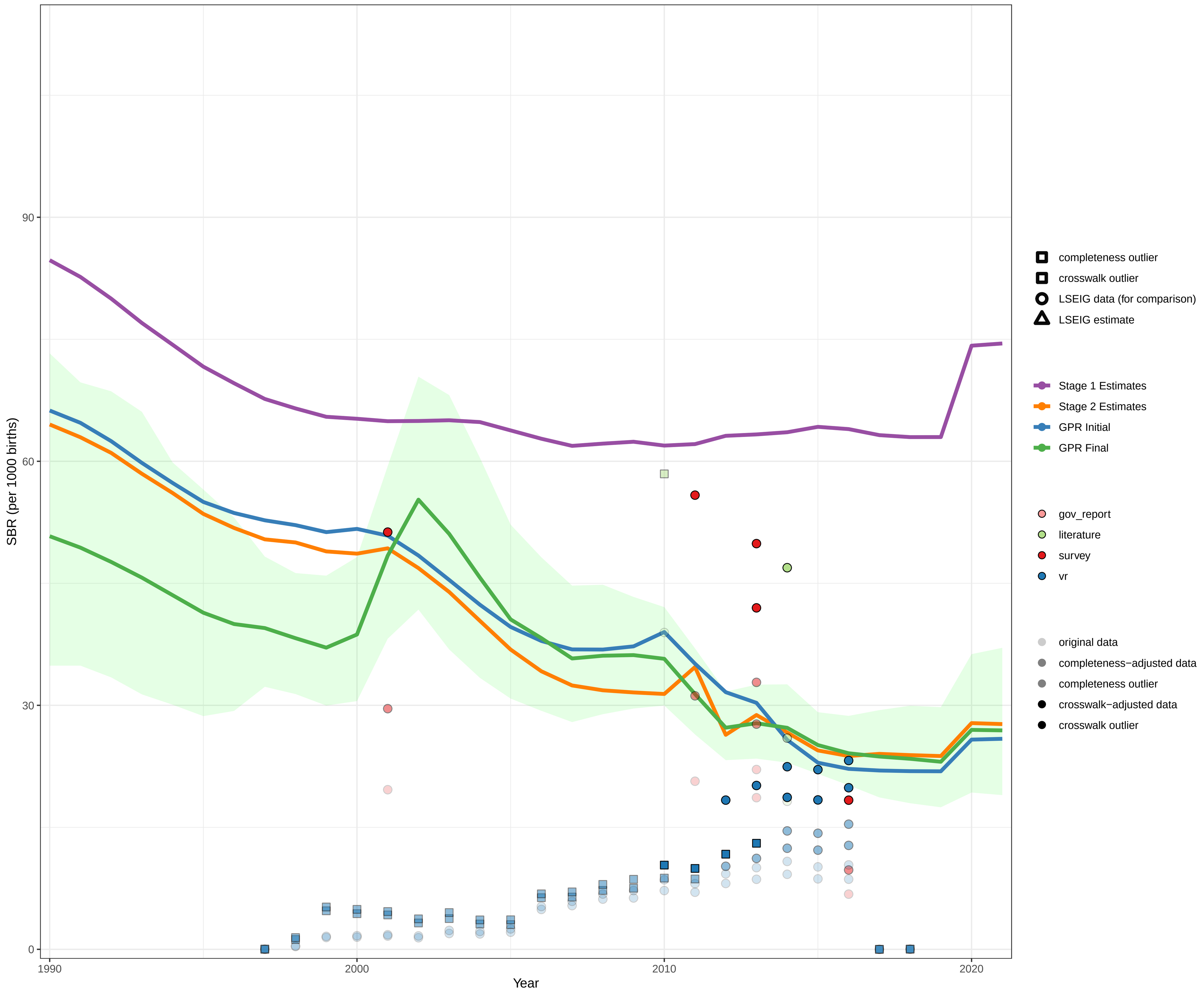

Limpopo ZAF_486 Southern Sub−Saharan Africa Stillbirth Rate (SBR) − 28 weeks
lambda = 0.4 ; zeta = 0.9 ; scale = 10 ; amp = 0.028

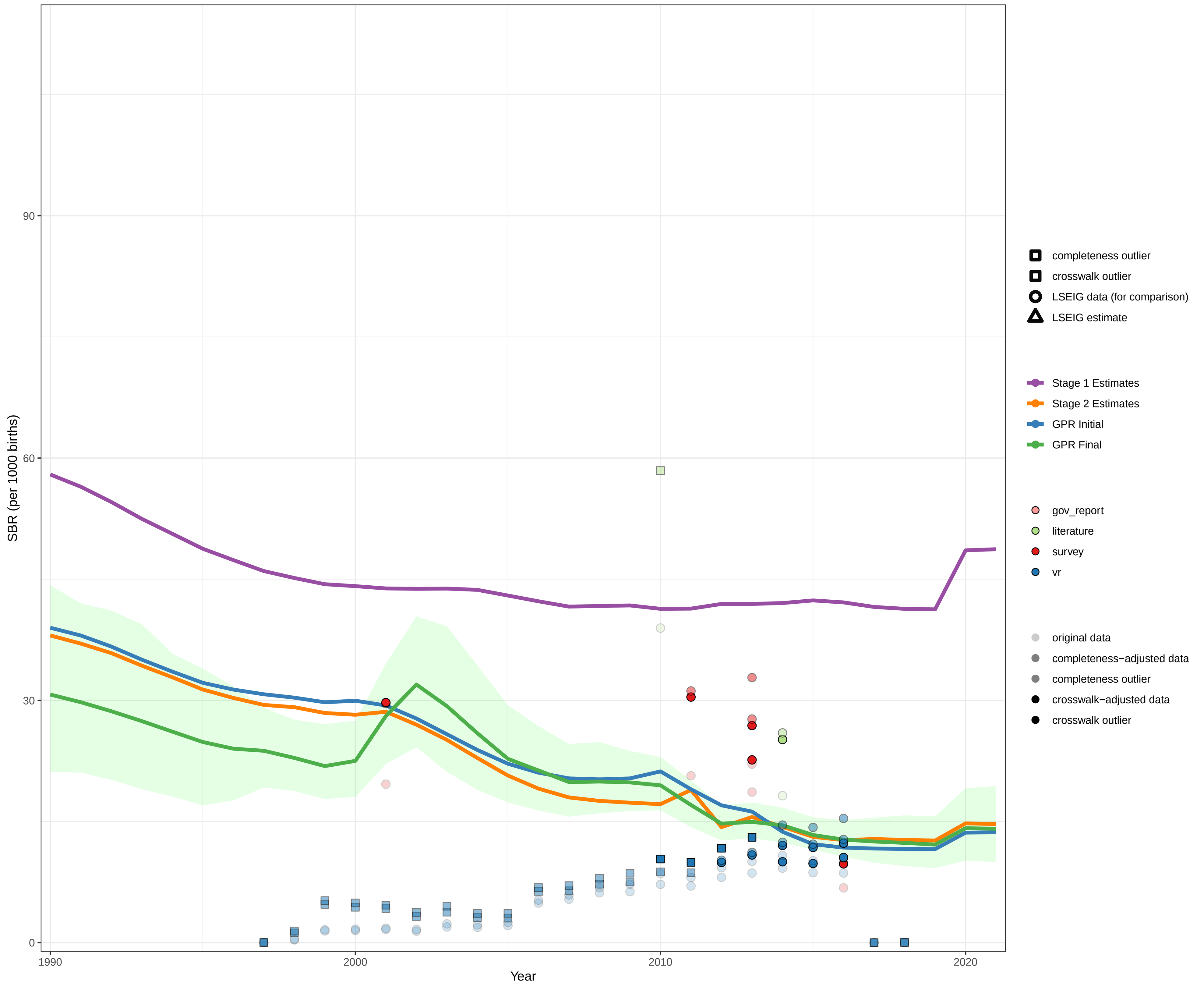

Year	Total Stillbirths (in thousands)	Total Stillbirths (in thousands)	SBR (per 1000 births)	SBR (per 1000 births)	SBR/NMR	SBR/NMR
	20 weeks	28 weeks	20 weeks	28 weeks	20 weeks	28 weeks
1990	8.4 (5.6 to 12.3)	4.9 (3.4 to 7.2)	50.8 (34.9 to 73.3)	30.7 (21.1 to 44.3)	2.07 (1.42 to 2.98)	1.25 (0.86 to 1.80)
1995	6.1 (4.2 to 8.5)	3.6 (2.5 to 5.0)	41.4 (28.7 to 56.5)	24.8 (17.0 to 33.9)	1.92 (1.33 to 2.62)	1.15 (0.79 to 1.57)
2000	5.2 (4.1 to 6.5)	3.0 (2.4 to 3.6)	38.7 (30.5 to 48.2)	22.5 (18.0 to 27.5)	1.93 (1.52 to 2.41)	1.12 (0.90 to 1.37)
2005	5.6 (4.2 to 7.3)	3.1 (2.3 to 4.0)	40.6 (30.8 to 52.2)	22.8 (17.4 to 29.4)	2.05 (1.56 to 2.64)	1.15 (0.88 to 1.49)
2010	4.8 (4.0 to 5.6)	2.5 (2.1 to 3.0)	35.7 (30.0 to 42.1)	19.5 (16.4 to 23.0)	1.88 (1.58 to 2.21)	1.02 (0.86 to 1.21)
2015	3.1 (2.7 to 3.7)	1.6 (1.4 to 1.9)	25.1 (21.6 to 29.2)	13.3 (11.5 to 15.5)	1.31 (1.12 to 1.52)	0.69 (0.60 to 0.81)
2020	3.2 (2.3 to 4.4)	1.7 (1.2 to 2.3)	27.0 (19.3 to 36.3)	14.2 (10.2 to 19.1)	1.24 (0.88 to 1.66)	0.65 (0.47 to 0.88)
2021	3.2 (2.2 to 4.4)	1.6 (1.1 to 2.3)	26.9 (19.0 to 37.1)	14.1 (10.0 to 19.4)	1.23 (0.87 to 1.69)	0.65 (0.46 to 0.89)

Mpumalanga   ZAF_487   Southern Sub−Saharan Africa   Stillbirth Rate / Neonatal Mortality Rate (SBR/NMR) − 20 weeks
lambda = 0.2 ; zeta = 0.99 ; scale = 5 ; amp = 0.028

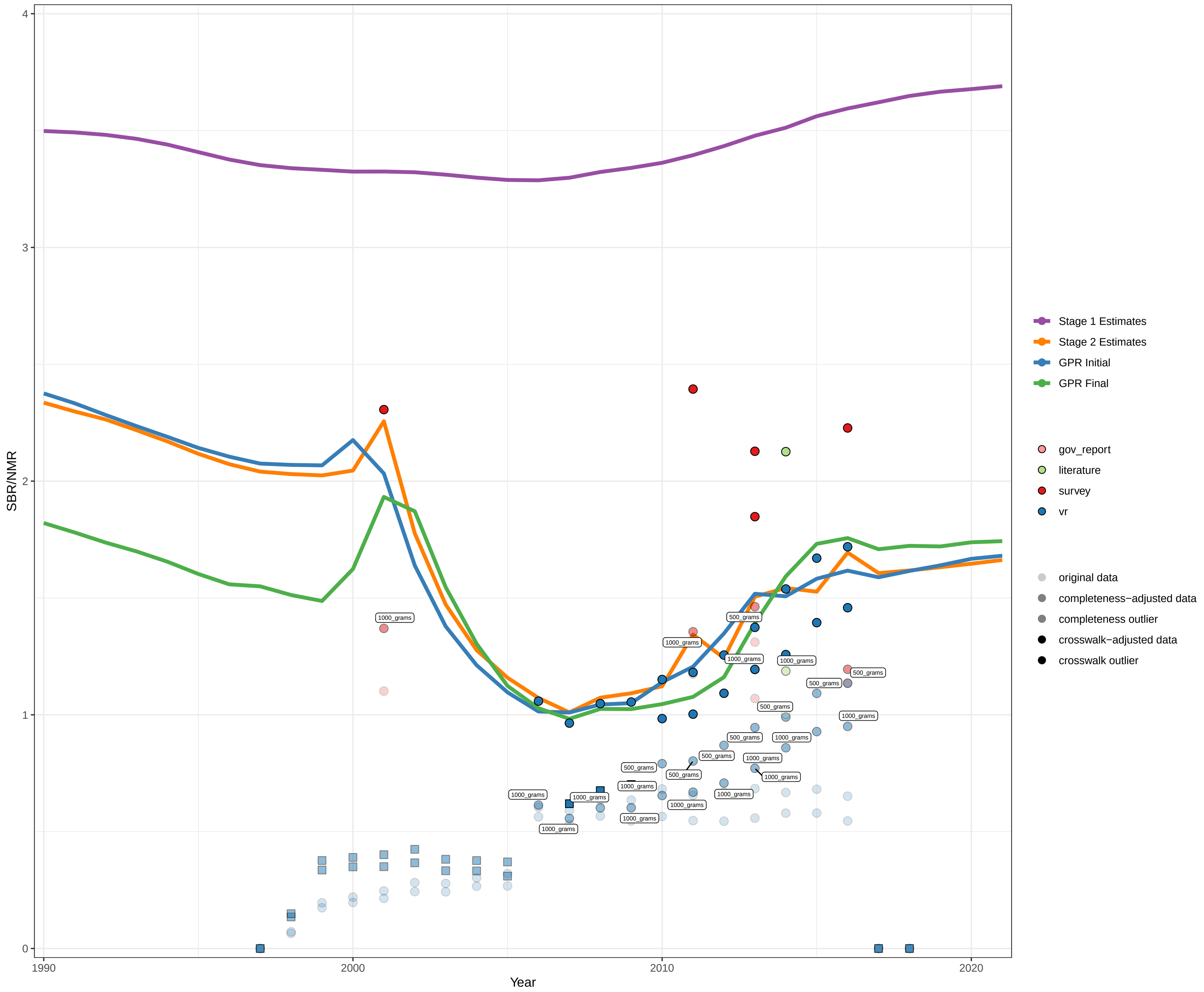

Mpumalanga   ZAF_487   Southern Sub−Saharan Africa   Stillbirth Rate / Neonatal Mortality Rate (SBR/NMR) − 28 weeks
lambda = 0.2 ; zeta = 0.99 ; scale = 5 ; amp = 0.028

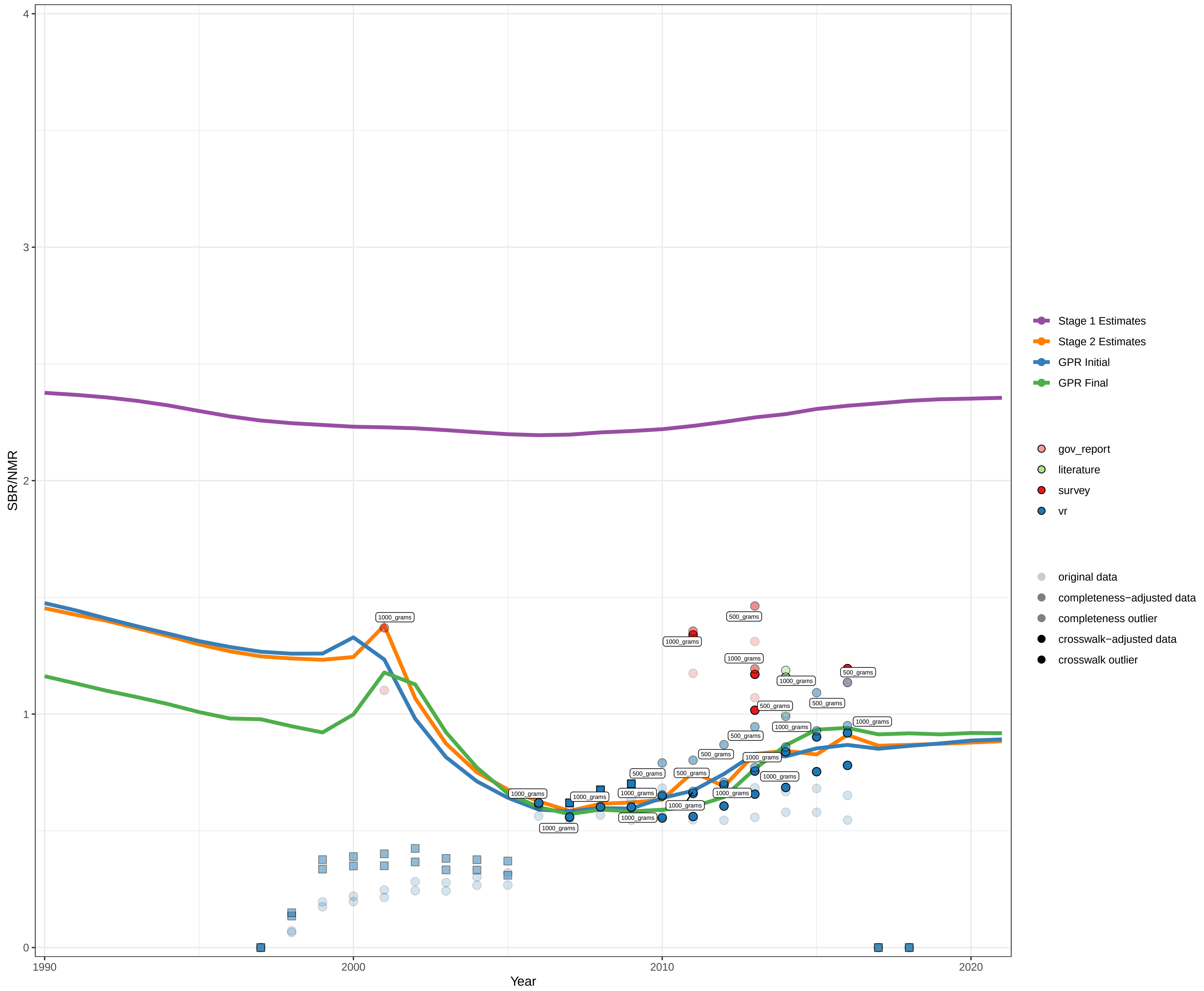

Definition	Years	Outlier	Source.Type	Title
1000_grams	2001	0	Survey	South Africa − Saving Babies: A Perinatal Care Survey 2001
1000_grams	2011	0	Survey	South Africa − Saving Babies: A Perinatal Care Survey 2010−2011
1000_grams	2014	0	Sci Lit	Timing and cause of perinatal mortality for small−for−gestational−age babies in South Africa: critical periods and challenges with detection
1000_grams, 500_grams	2006−2016	0	VR	South Africa Vital Registration − Causes of Death
1000_grams, 500_grams	2013	0	Survey	South Africa − Saving Babies: A Perinatal Care Survey 2012−2013
28_weeks	2016	0	Survey	South Africa Demographic and Health Survey 2016
1000_grams, 500_grams	1997−2005	1	VR	South Africa Vital Registration − Causes of Death
1000_grams, 500_grams	2017, 2018	1	VR	South Africa Vital Registration − Causes of Death

Mpumalanga   ZAF_487   Southern Sub−Saharan Africa   Stillbirth Rate (SBR) − 20 weeks
lambda = 0.2 ; zeta = 0.99 ; scale = 5 ; amp = 0.028

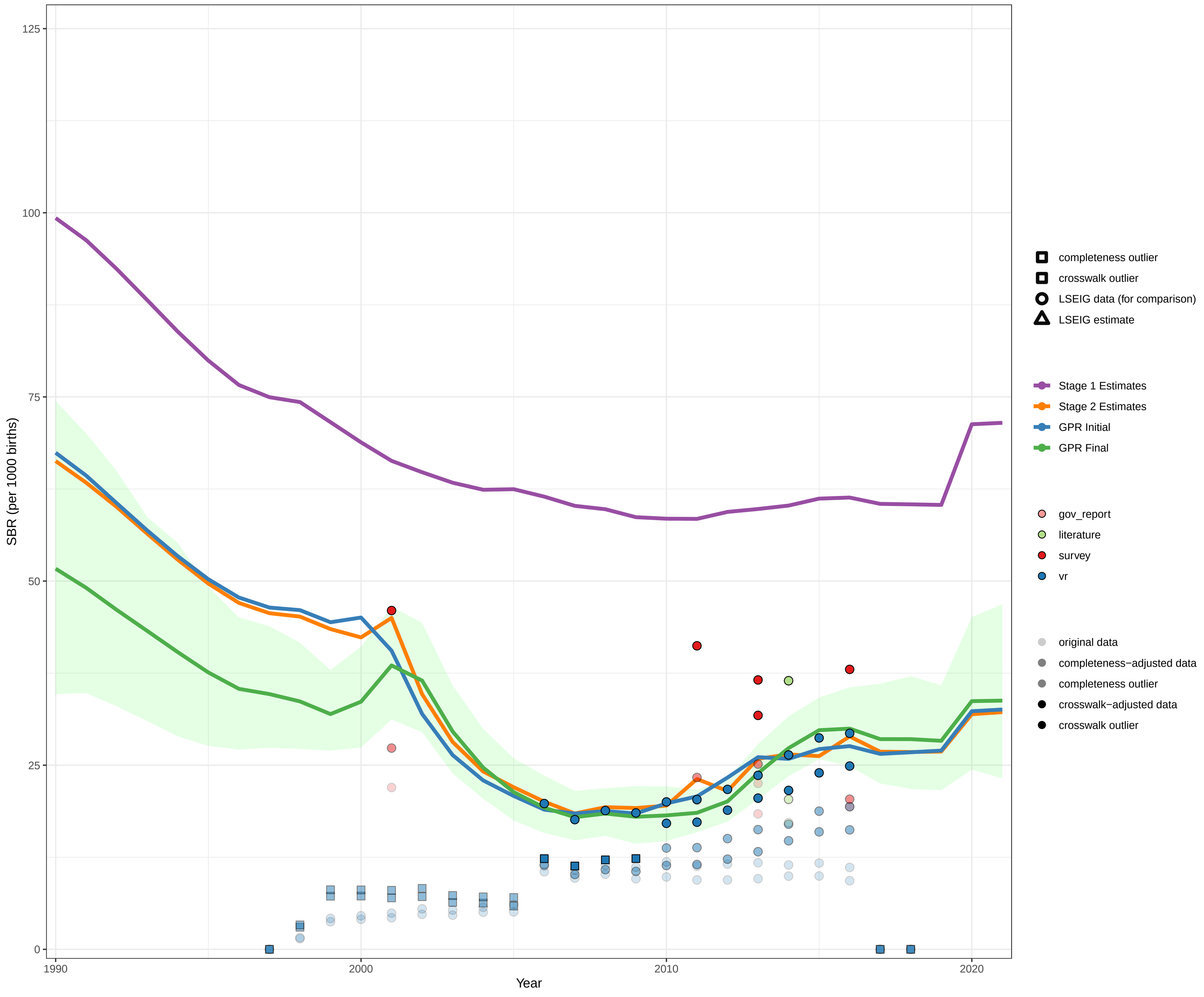

Mpumalanga   ZAF_487   Southern Sub−Saharan Africa   Stillbirth Rate (SBR) − 28 weeks
lambda = 0.2 ; zeta = 0.99 ; scale = 5 ; amp = 0.028

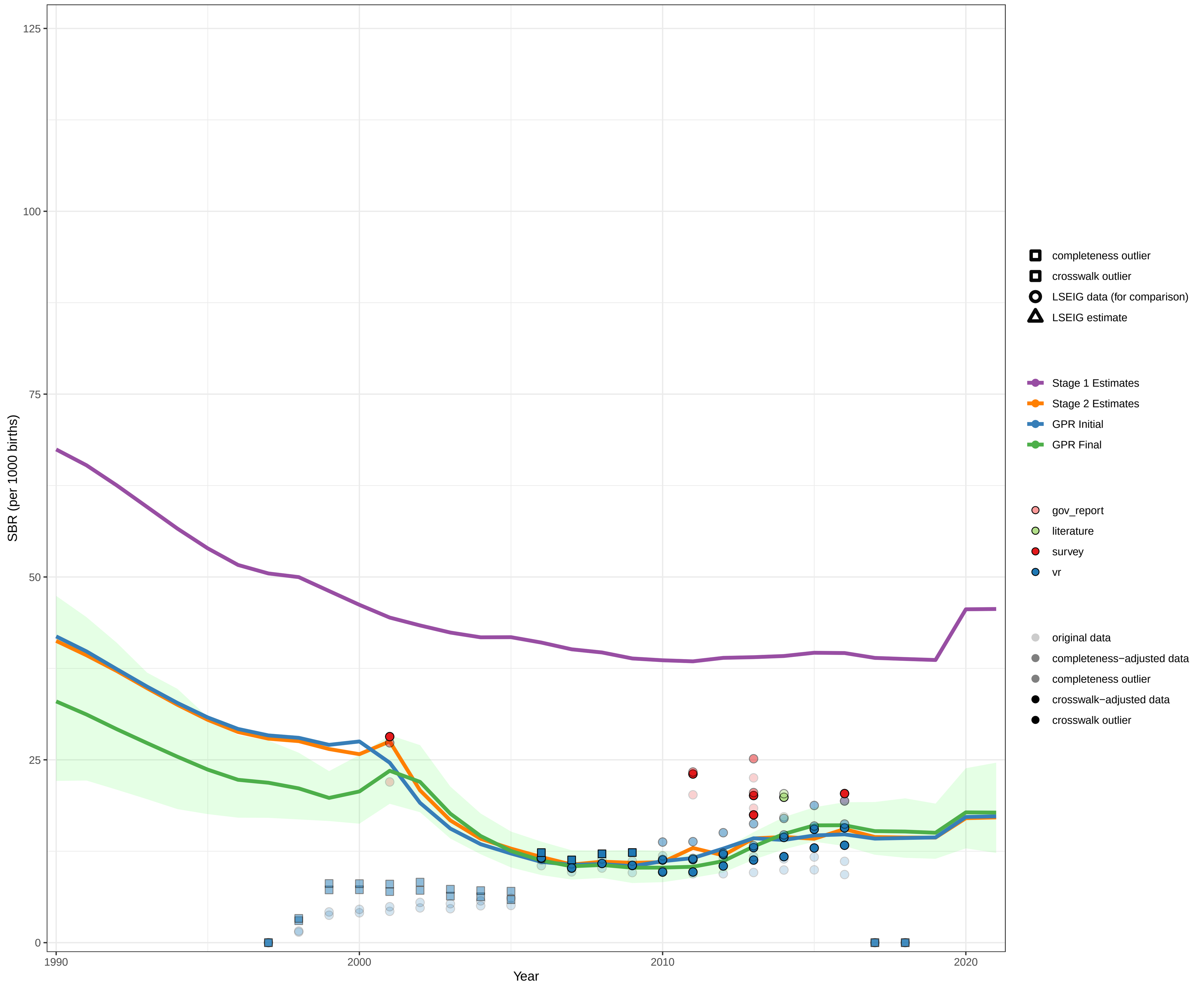

Year	Total Stillbirths (in thousands)	Total Stillbirths (in thousands)	SBR (per 1000 births)	SBR (per 1000 births)	SBR/NMR	SBR/NMR
	20 weeks	28 weeks	20 weeks	28 weeks	20 weeks	28 weeks
1990	4.2 (2.8 to 6.2)	2.6 (1.7 to 3.9)	51.7 (34.6 to 74.4)	33.0 (22.1 to 47.4)	1.82 (1.22 to 2.62)	1.16 (0.78 to 1.67)
1995	3.0 (2.2 to 4.0)	1.9 (1.4 to 2.5)	37.6 (27.6 to 49.2)	23.7 (17.6 to 30.9)	1.60 (1.18 to 2.10)	1.01 (0.75 to 1.32)
2000	2.7 (2.2 to 3.3)	1.6 (1.3 to 2.0)	33.6 (27.4 to 41.1)	20.7 (16.3 to 25.7)	1.62 (1.32 to 1.99)	1.00 (0.79 to 1.24)
2005	1.9 (1.6 to 2.3)	1.1 (0.9 to 1.3)	21.3 (17.5 to 25.9)	12.5 (10.3 to 15.2)	1.12 (0.92 to 1.37)	0.66 (0.54 to 0.80)
2010	1.7 (1.4 to 2.1)	1.0 (0.8 to 1.2)	18.2 (14.7 to 22.1)	10.3 (8.3 to 12.6)	1.05 (0.84 to 1.27)	0.59 (0.48 to 0.72)
2015	2.9 (2.5 to 3.4)	1.6 (1.3 to 1.8)	29.8 (25.8 to 34.2)	16.0 (13.8 to 18.5)	1.73 (1.50 to 1.99)	0.93 (0.80 to 1.08)
2020	3.3 (2.4 to 4.5)	1.7 (1.2 to 2.3)	33.7 (24.4 to 45.1)	17.8 (12.9 to 23.9)	1.74 (1.26 to 2.33)	0.92 (0.67 to 1.23)
2021	3.3 (2.2 to 4.6)	1.7 (1.2 to 2.4)	33.8 (23.2 to 46.8)	17.8 (12.3 to 24.6)	1.74 (1.20 to 2.42)	0.92 (0.63 to 1.27)

North−West ZAF_488 Southern Sub−Saharan Africa Stillbirth Rate / Neonatal Mortality Rate (SBR/NMR) − 20 weeks
lambda = 0.2 ; zeta = 0.99 ; scale = 5 ; amp = 0.028

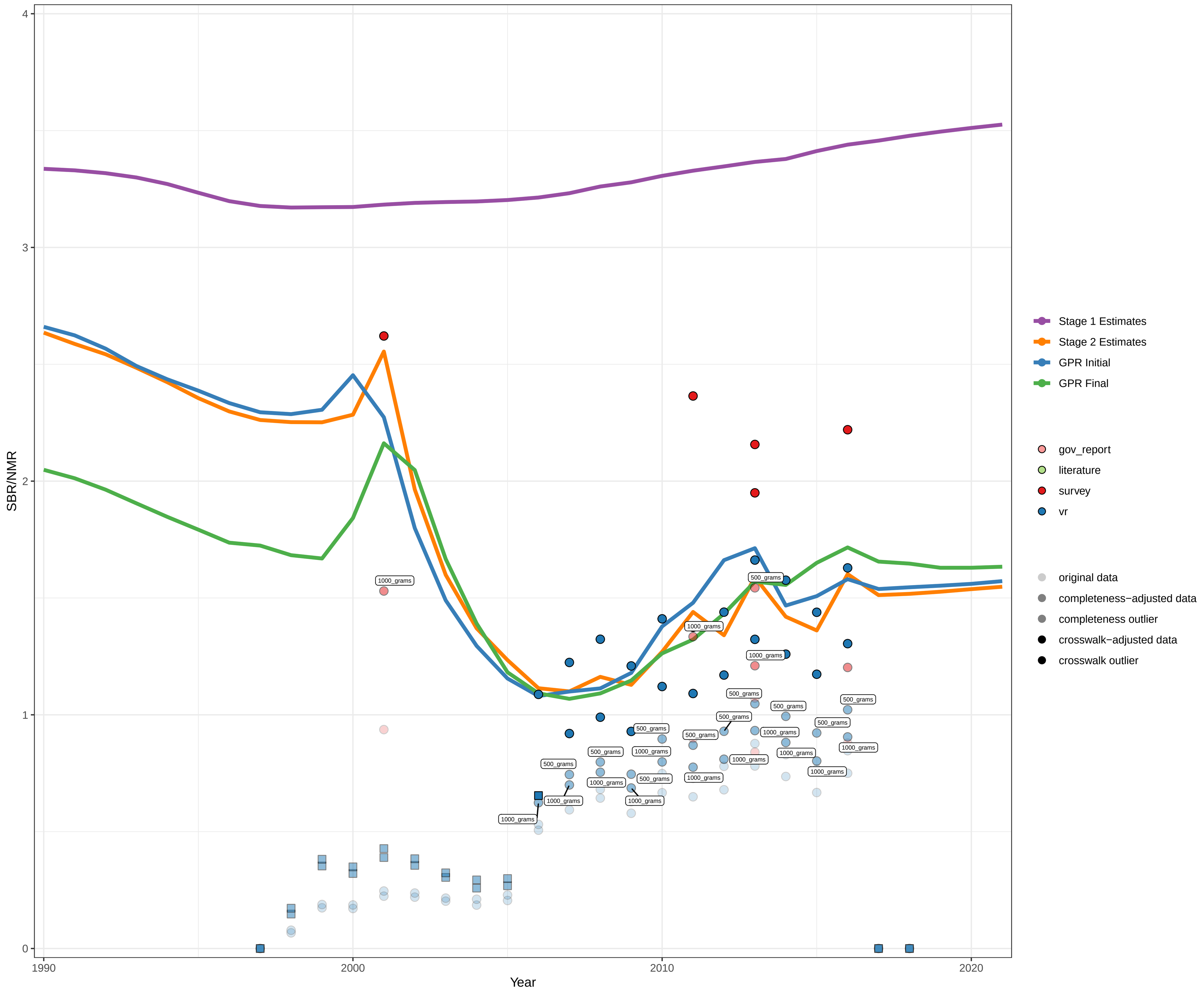

North−West ZAF_488 Southern Sub−Saharan Africa Stillbirth Rate / Neonatal Mortality Rate (SBR/NMR) − 28 weeks
lambda = 0.2 ; zeta = 0.99 ; scale = 5 ; amp = 0.028

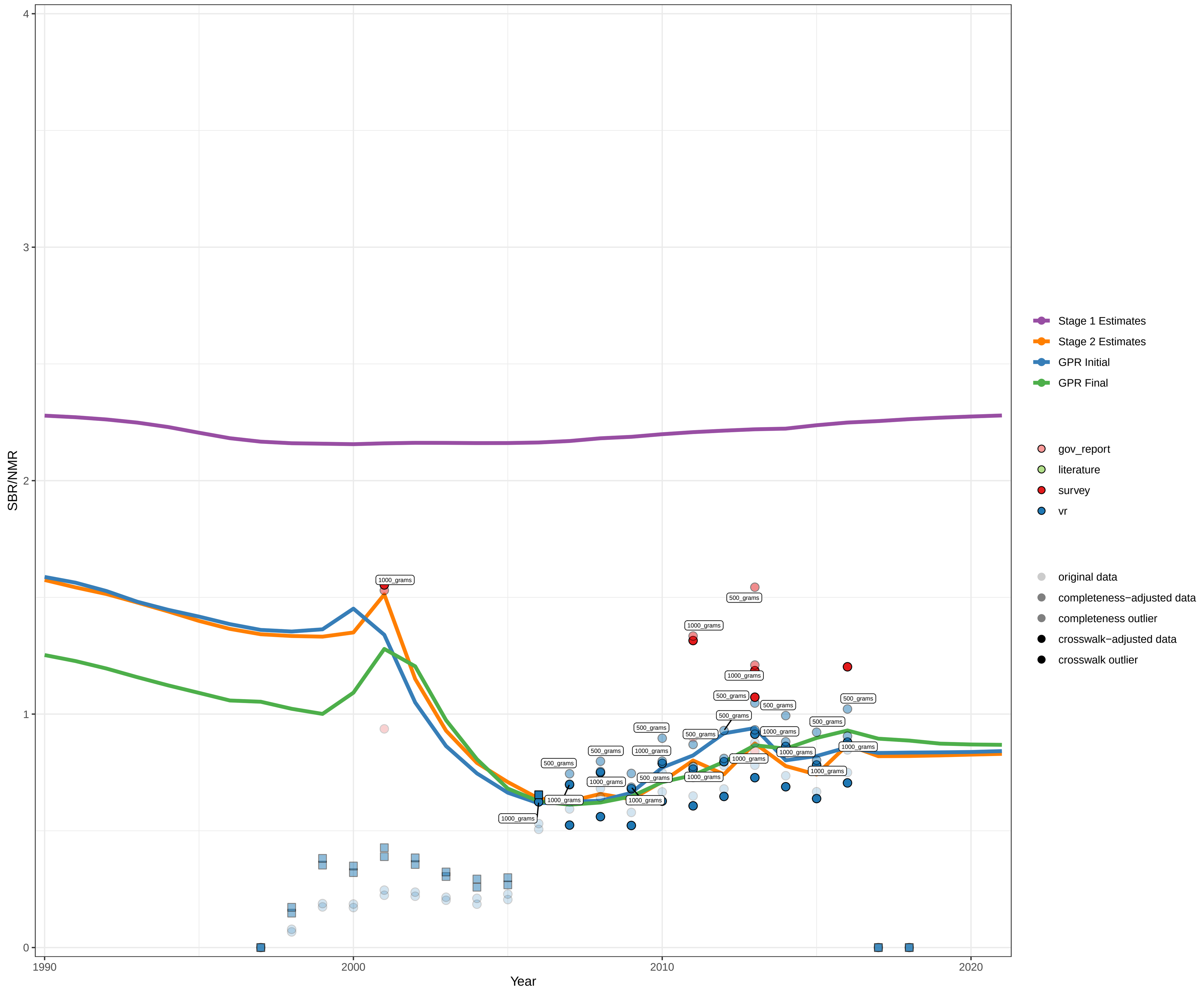

Definition	Years	Outlier	Source.Type	Title
1000_grams	2001	0	Survey	South Africa − Saving Babies: A Perinatal Care Survey 2001
1000_grams	2011	0	Survey	South Africa − Saving Babies: A Perinatal Care Survey 2010−2011
1000_grams, 500_grams	2006−2016	0	VR	South Africa Vital Registration − Causes of Death
1000_grams, 500_grams	2013	0	Survey	South Africa − Saving Babies: A Perinatal Care Survey 2012−2013
28_weeks	2016	0	Survey	South Africa Demographic and Health Survey 2016
1000_grams, 500_grams	1997−2005	1	VR	South Africa Vital Registration − Causes of Death
1000_grams, 500_grams	2017, 2018	1	VR	South Africa Vital Registration − Causes of Death

North−West ZAF_488 Southern Sub−Saharan Africa Stillbirth Rate (SBR) − 20 weeks
lambda = 0.2 ; zeta = 0.99 ; scale = 5 ; amp = 0.028

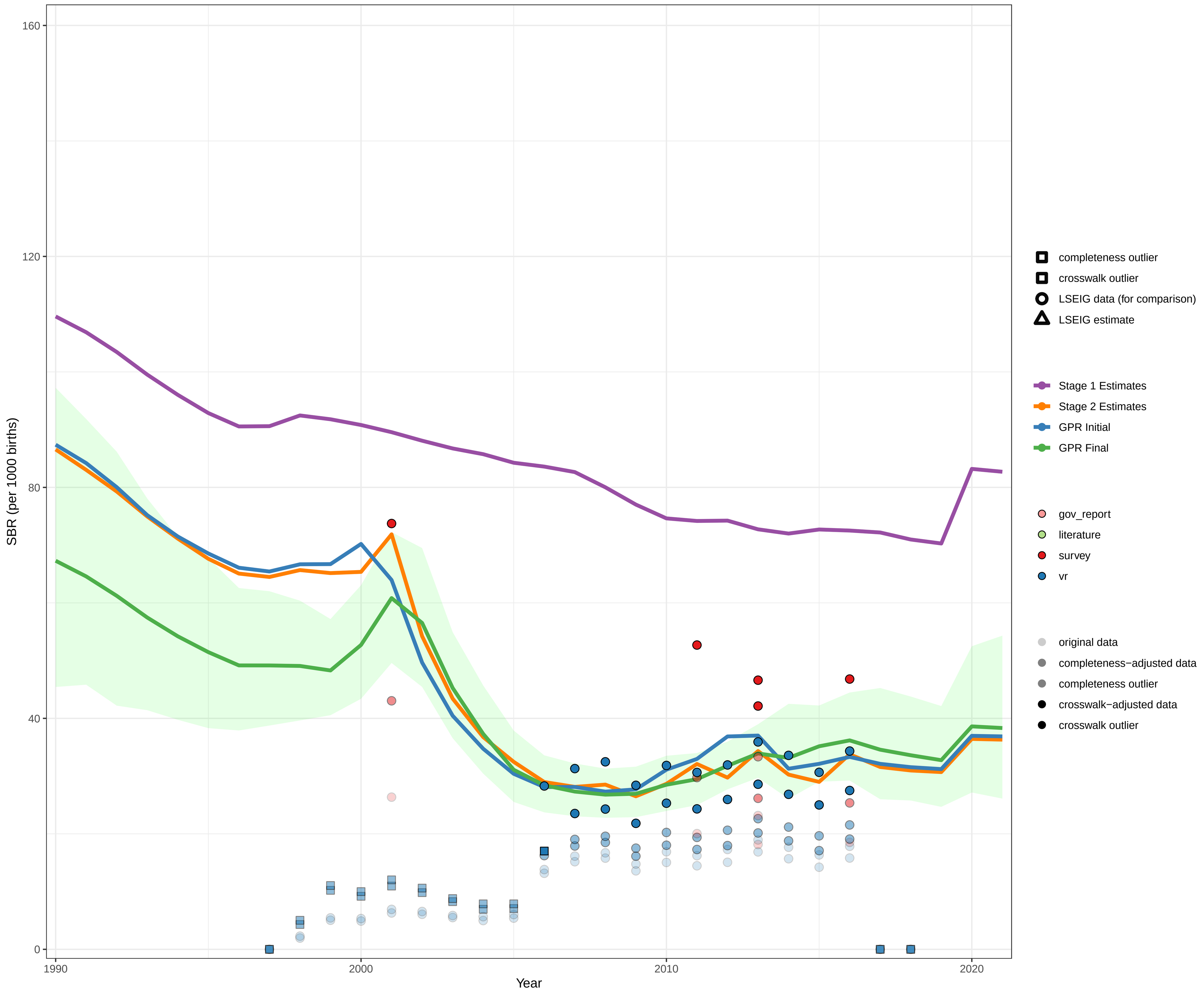

North−West ZAF_488 Southern Sub−Saharan Africa Stillbirth Rate (SBR) − 28 weeks
lambda = 0.2 ; zeta = 0.99 ; scale = 5 ; amp = 0.028

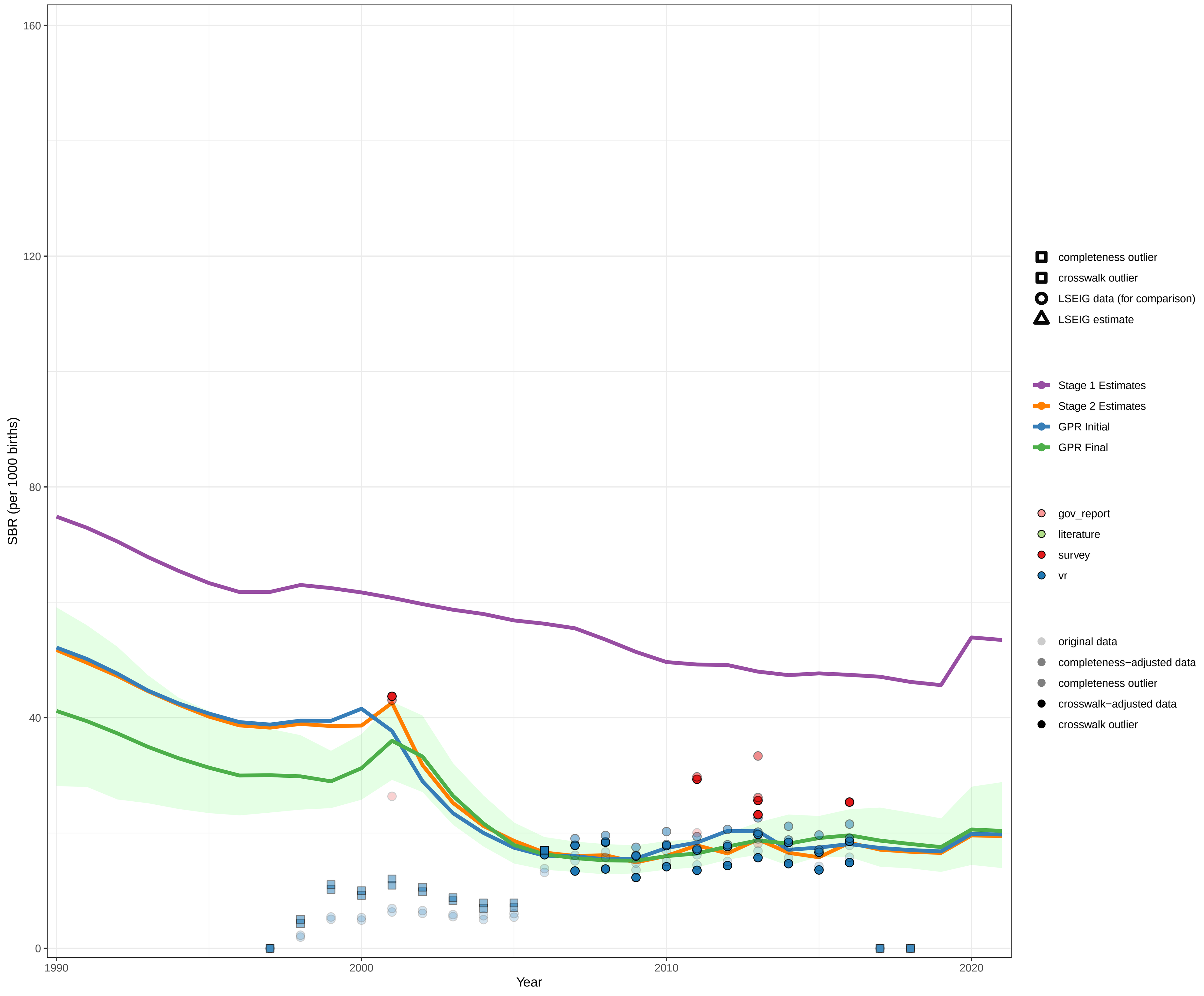

Year	Total Stillbirths (in thousands)	Total Stillbirths (in thousands)	SBR (per 1000 births)	SBR (per 1000 births)	SBR/NMR	SBR/NMR
	20 weeks	28 weeks	20 weeks	28 weeks	20 weeks	28 weeks
1990	6.0 (3.9 to 8.9)	3.6 (2.4 to 5.2)	67.3 (45.4 to 97.3)	41.2 (28.1 to 59.1)	2.05 (1.38 to 2.96)	1.25 (0.86 to 1.80)
1995	4.5 (3.3 to 6.1)	2.7 (2.0 to 3.6)	51.5 (38.3 to 67.8)	31.3 (23.4 to 41.2)	1.79 (1.33 to 2.36)	1.09 (0.82 to 1.43)
2000	4.7 (3.8 to 5.7)	2.7 (2.2 to 3.3)	52.7 (43.4 to 63.1)	31.2 (25.8 to 37.1)	1.84 (1.52 to 2.20)	1.09 (0.90 to 1.30)
2005	2.8 (2.3 to 3.4)	1.6 (1.3 to 1.9)	31.1 (25.5 to 37.9)	17.9 (14.7 to 21.8)	1.18 (0.97 to 1.44)	0.68 (0.56 to 0.83)
2010	2.3 (1.9 to 2.7)	1.3 (1.1 to 1.5)	28.5 (24.0 to 33.5)	16.0 (13.7 to 18.6)	1.26 (1.06 to 1.49)	0.71 (0.61 to 0.82)
2015	2.4 (2.0 to 2.9)	1.3 (1.1 to 1.6)	35.2 (29.0 to 42.2)	19.1 (15.9 to 23.0)	1.65 (1.36 to 1.98)	0.90 (0.75 to 1.08)
2020	2.2 (1.5 to 3.0)	1.2 (0.8 to 1.6)	38.6 (27.2 to 52.5)	20.6 (14.5 to 28.0)	1.63 (1.15 to 2.22)	0.87 (0.61 to 1.18)
2021	2.1 (1.4 to 3.0)	1.1 (0.7 to 1.6)	38.3 (26.1 to 54.3)	20.4 (13.9 to 28.8)	1.63 (1.11 to 2.32)	0.87 (0.59 to 1.23)

Northern Cape ZAF_489 Southern Sub−Saharan Africa Stillbirth Rate / Neonatal Mortality Rate (SBR/NMR) − 20 weeks
lambda = 0.2 ; zeta = 0.99 ; scale = 5 ; amp = 0.028

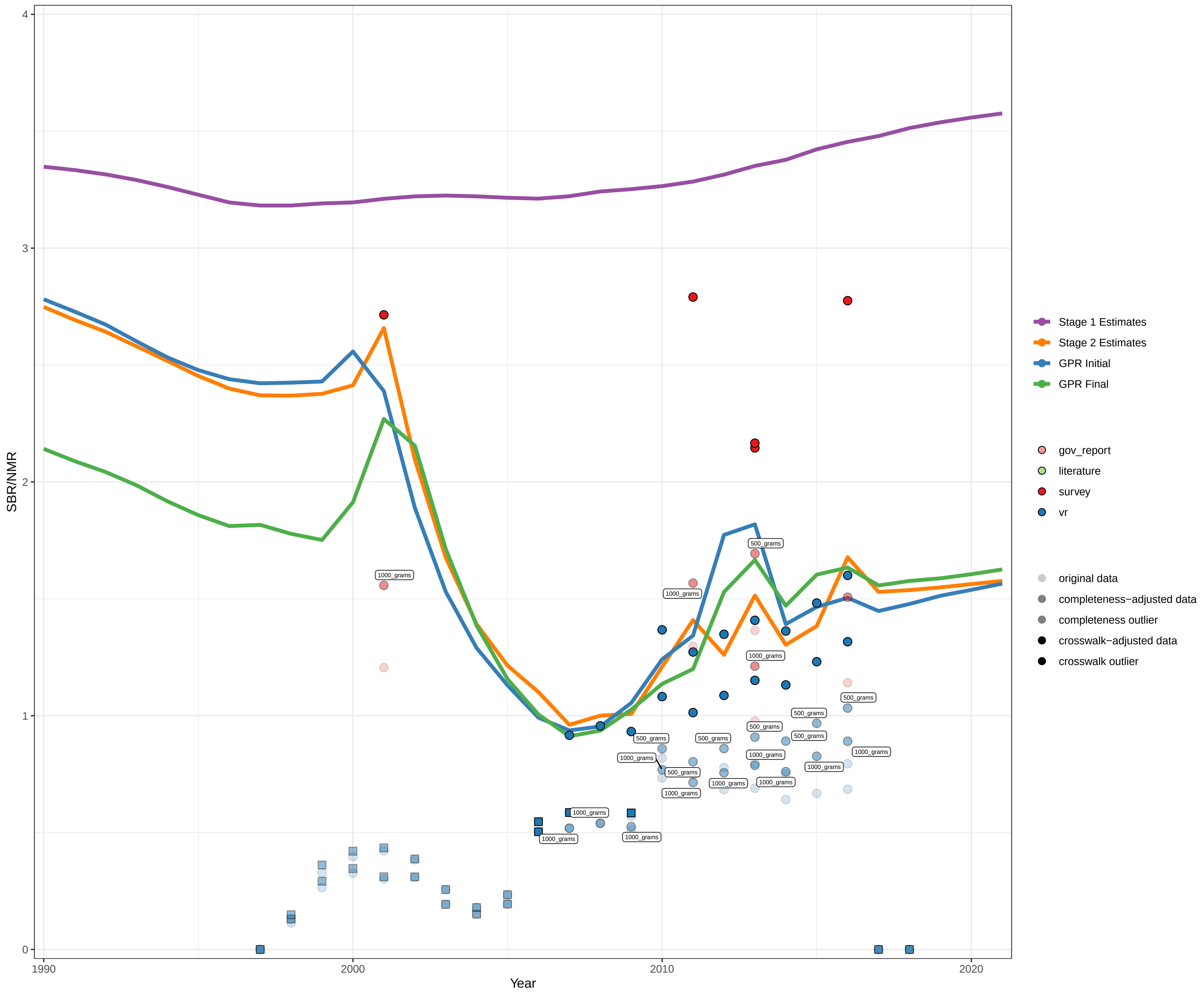

Northern Cape ZAF_489 Southern Sub−Saharan Africa Stillbirth Rate / Neonatal Mortality Rate (SBR/NMR) − 28 weeks
lambda = 0.2 ; zeta = 0.99 ; scale = 5 ; amp = 0.028

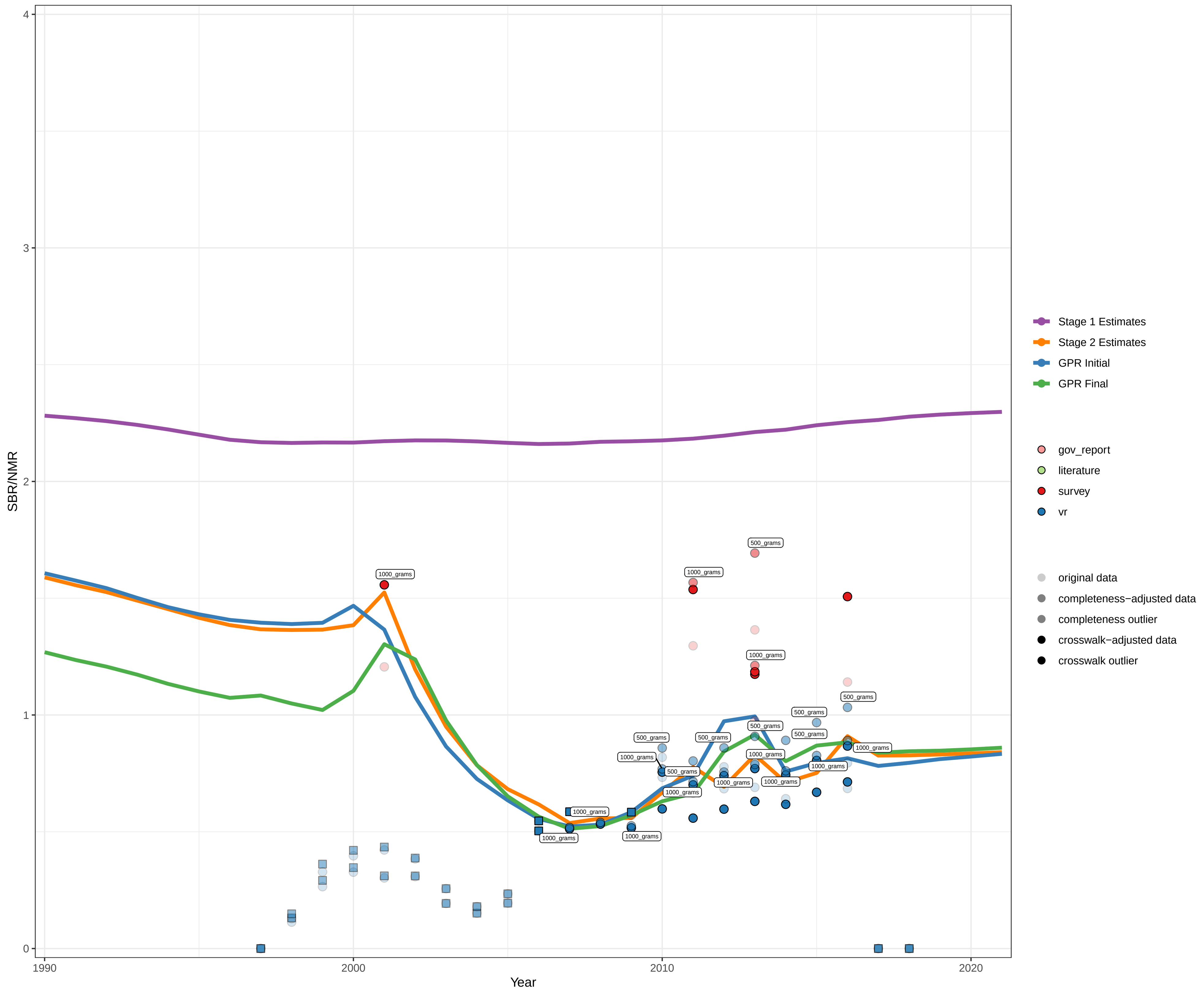

Definition	Years	Outlier	Source.Type	Title
1000_grams	2001	0	Survey	South Africa − Saving Babies: A Perinatal Care Survey 2001
1000_grams	2011	0	Survey	South Africa − Saving Babies: A Perinatal Care Survey 2010−2011
1000_grams, 500_grams	2006−2016	0	VR	South Africa Vital Registration − Causes of Death
1000_grams, 500_grams	2013	0	Survey	South Africa − Saving Babies: A Perinatal Care Survey 2012−2013
28_weeks	2016	0	Survey	South Africa Demographic and Health Survey 2016
1000_grams, 500_grams	1997−2005	1	VR	South Africa Vital Registration − Causes of Death
1000_grams, 500_grams	2017, 2018	1	VR	South Africa Vital Registration − Causes of Death

Northern Cape ZAF_489 Southern Sub−Saharan Africa Stillbirth Rate (SBR) − 20 weeks
lambda = 0.2 ; zeta = 0.99 ; scale = 5 ; amp = 0.028

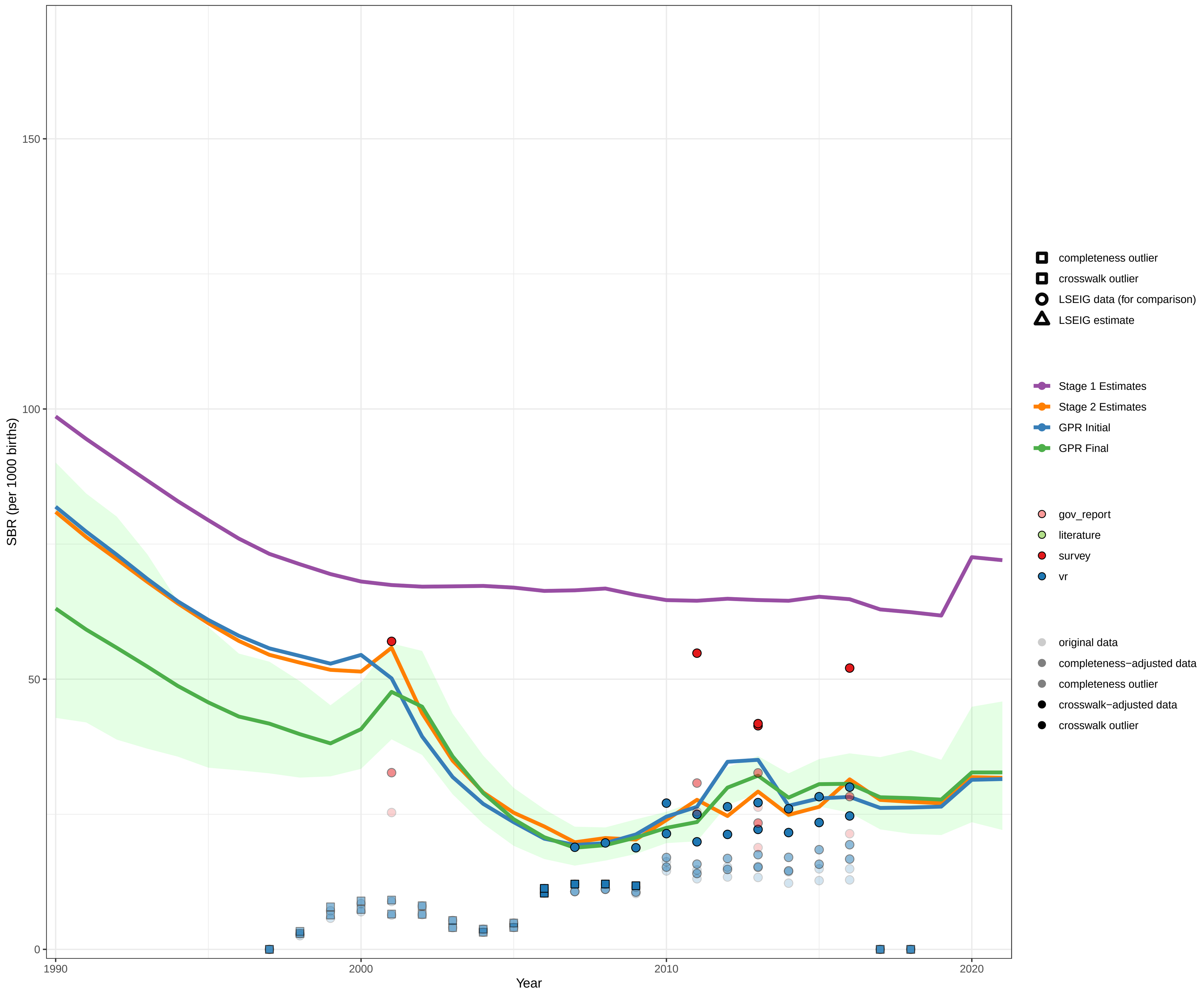

Northern Cape ZAF_489 Southern Sub−Saharan Africa Stillbirth Rate (SBR) − 28 weeks
lambda = 0.2 ; zeta = 0.99 ; scale = 5 ; amp = 0.028

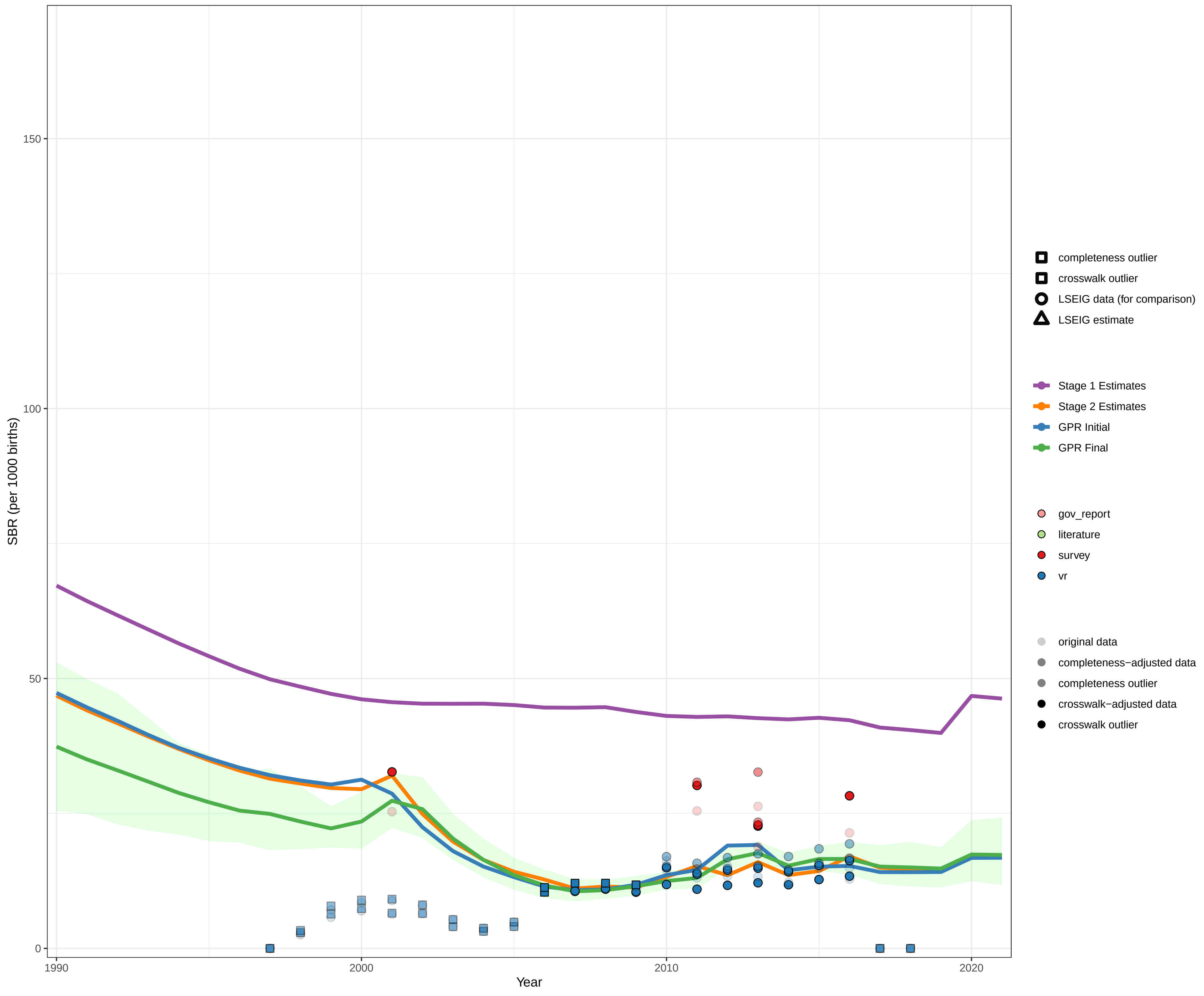

Year	Total Stillbirths (in thousands)	Total Stillbirths (in thousands)	SBR (per 1000 births)	SBR (per 1000 births)	SBR/NMR	SBR/NMR
	20 weeks	28 weeks	20 weeks	28 weeks	20 weeks	28 weeks
1990	1.4<br>(0.9 to 2.1)	0.8<br>(0.6 to 1.2)	63.1<br>(42.8 to 90.1)	37.4<br>(25.5 to 53.0)	2.14<br>(1.45 to 3.06)	1.27<br>(0.86 to 1.80)
1995	0.9<br>(0.7 to 1.2)	0.5<br>(0.4 to 0.7)	45.7<br>(33.6 to 59.6)	27.1<br>(19.9 to 35.9)	1.86<br>(1.37 to 2.42)	1.10<br>(0.81 to 1.46)
2000	0.7<br>(0.6 to 0.9)	0.4<br>(0.3 to 0.5)	40.8<br>(33.4 to 49.4)	23.5<br>(18.5 to 29.0)	1.91<br>(1.57 to 2.32)	1.10<br>(0.87 to 1.36)
2005	0.5<br>(0.4 to 0.6)	0.3<br>(0.2 to 0.3)	24.1<br>(19.2 to 29.9)	13.6<br>(10.9 to 16.8)	1.16<br>(0.92 to 1.44)	0.65<br>(0.52 to 0.81)
2010	0.5<br>(0.5 to 0.6)	0.3<br>(0.3 to 0.3)	22.5<br>(19.7 to 25.5)	12.5<br>(10.9 to 14.1)	1.14<br>(0.99 to 1.29)	0.63<br>(0.55 to 0.71)
2015	0.8<br>(0.7 to 0.9)	0.4<br>(0.4 to 0.5)	30.6<br>(26.4 to 35.2)	16.6<br>(14.4 to 19.0)	1.60<br>(1.39 to 1.85)	0.87<br>(0.75 to 1.00)
2020	0.8<br>(0.6 to 1.1)	0.4<br>(0.3 to 0.6)	32.7<br>(23.5 to 44.9)	17.4<br>(12.5 to 23.7)	1.61<br>(1.15 to 2.20)	0.85<br>(0.61 to 1.16)
2021	0.8<br>(0.5 to 1.2)	0.4<br>(0.3 to 0.6)	32.7<br>(22.1 to 45.9)	17.3<br>(11.7 to 24.2)	1.63<br>(1.10 to 2.28)	0.86<br>(0.58 to 1.20)

Western Cape ZAF_490 Southern Sub−Saharan Africa Stillbirth Rate / Neonatal Mortality Rate (SBR/NMR) − 20 weeks
lambda = 0.2 ; zeta = 0.99 ; scale = 5 ; amp = 0.028

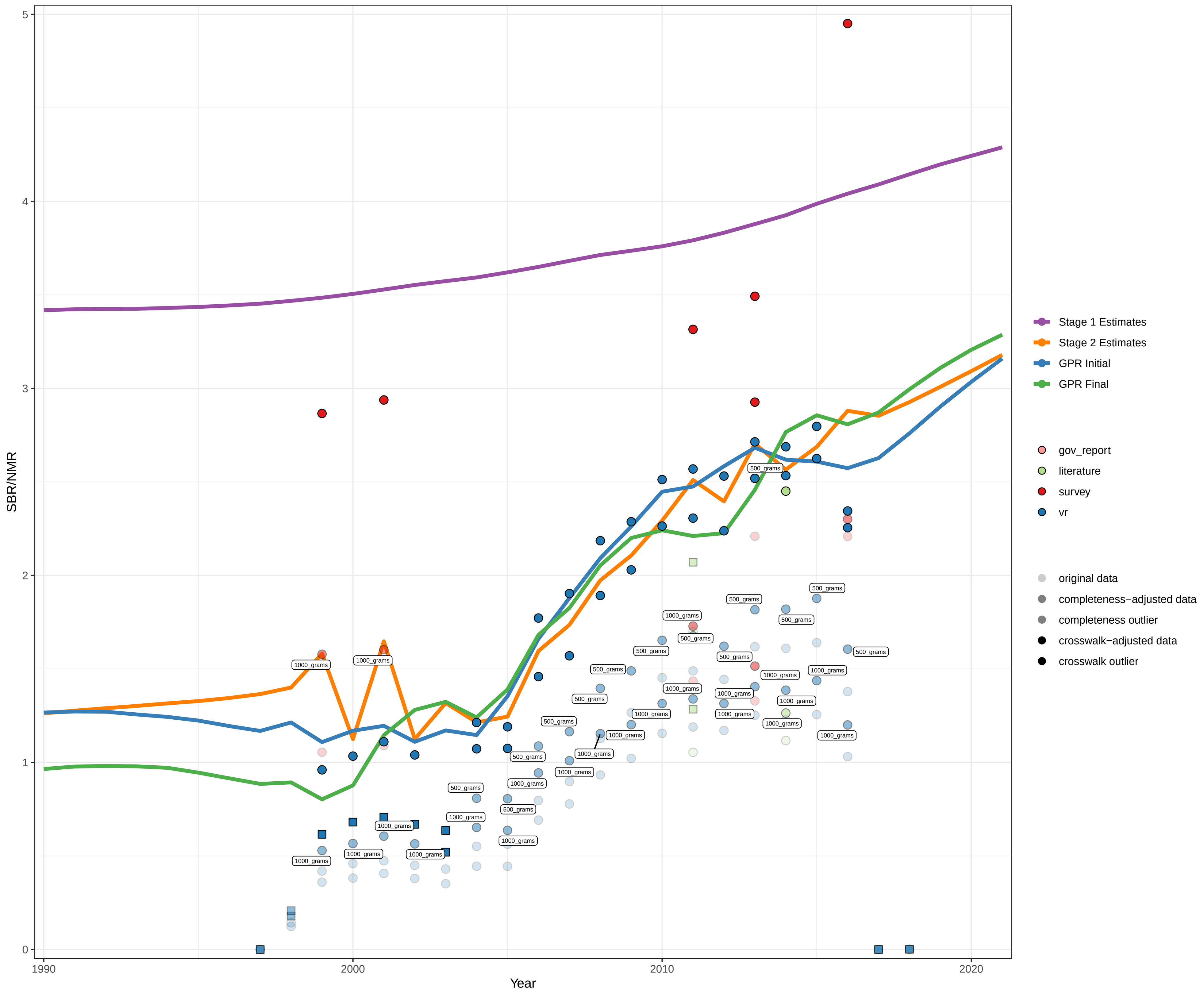

Western Cape ZAF_490 Southern Sub−Saharan Africa Stillbirth Rate / Neonatal Mortality Rate (SBR/NMR) − 28 weeks
lambda = 0.2 ; zeta = 0.99 ; scale = 5 ; amp = 0.028

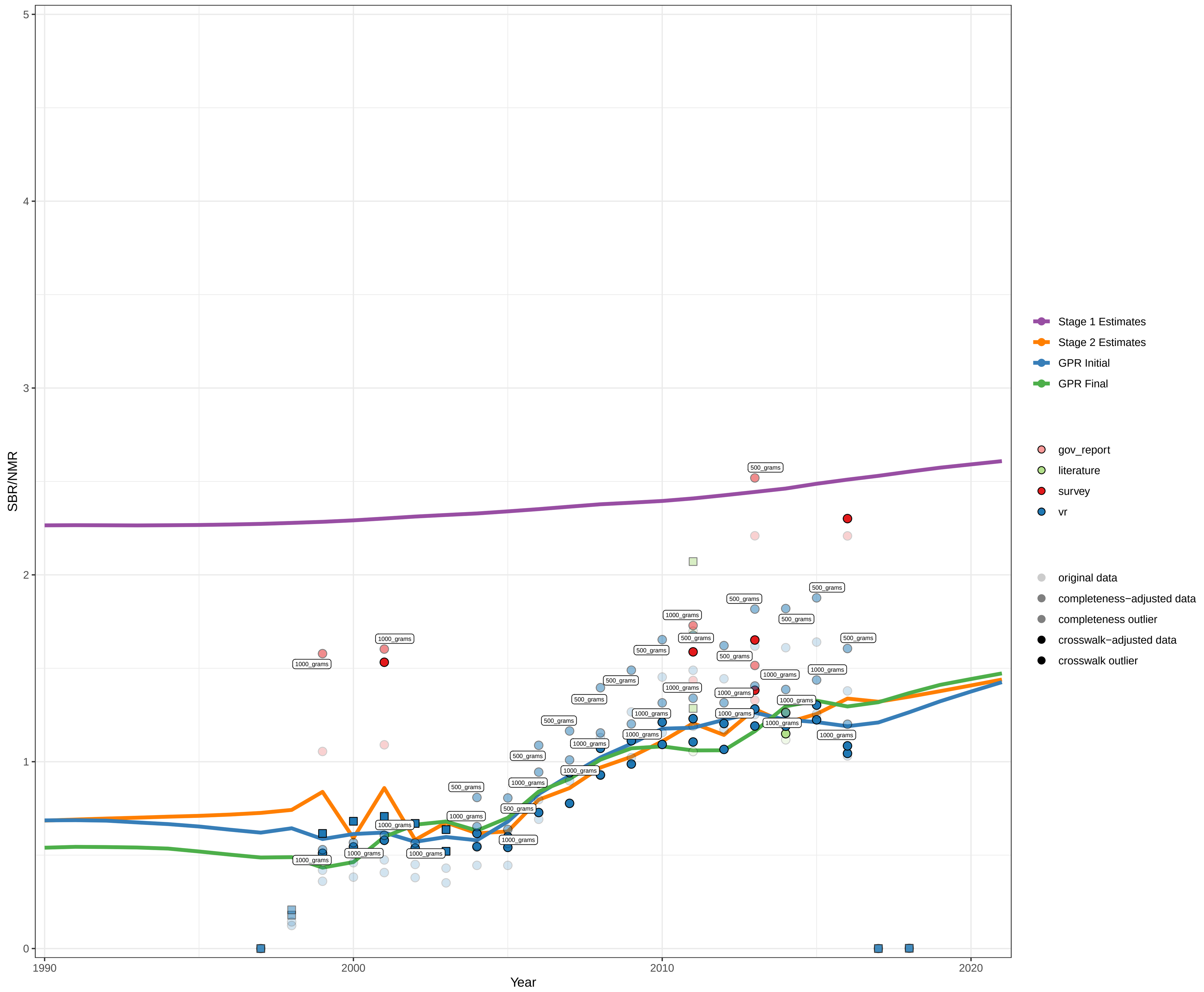

Definition	Years	Outlier	Source.Type	Title
1000_grams	1999	0	Survey	South Africa − Saving Babies: A Perinatal Care Survey 2000
1000_grams	2001	0	Survey	South Africa − Saving Babies: A Perinatal Care Survey 2001
1000_grams	2011	0	Survey	South Africa − Saving Babies: A Perinatal Care Survey 2010−2011
1000_grams	2014	0	Sci Lit	Timing and cause of perinatal mortality for small−for−gestational−age babies in South Africa: critical periods and challenges with detection
1000_grams, 500_grams	1999−2005	0	VR	South Africa Vital Registration − Causes of Death
1000_grams, 500_grams	2006−2016	0	VR	South Africa Vital Registration − Causes of Death
1000_grams, 500_grams	2013	0	Survey	South Africa − Saving Babies: A Perinatal Care Survey 2012−2013
28_weeks	2016	0	Survey	South Africa Demographic and Health Survey 2016
1000_grams, 500_grams	1997, 1998	1	VR	South Africa Vital Registration − Causes of Death
1000_grams, 500_grams	2017, 2018	1	VR	South Africa Vital Registration − Causes of Death
22_weeks, 28_weeks	2011	1	Sci Lit	Association between preterm labour, perinatal mortality and infant death (during the first year) in Bishop Lavis, Cape Town, South Africa

Western Cape ZAF_490 Southern Sub−Saharan Africa Stillbirth Rate (SBR) − 20 weeks
lambda = 0.2 ; zeta = 0.99 ; scale = 5 ; amp = 0.028

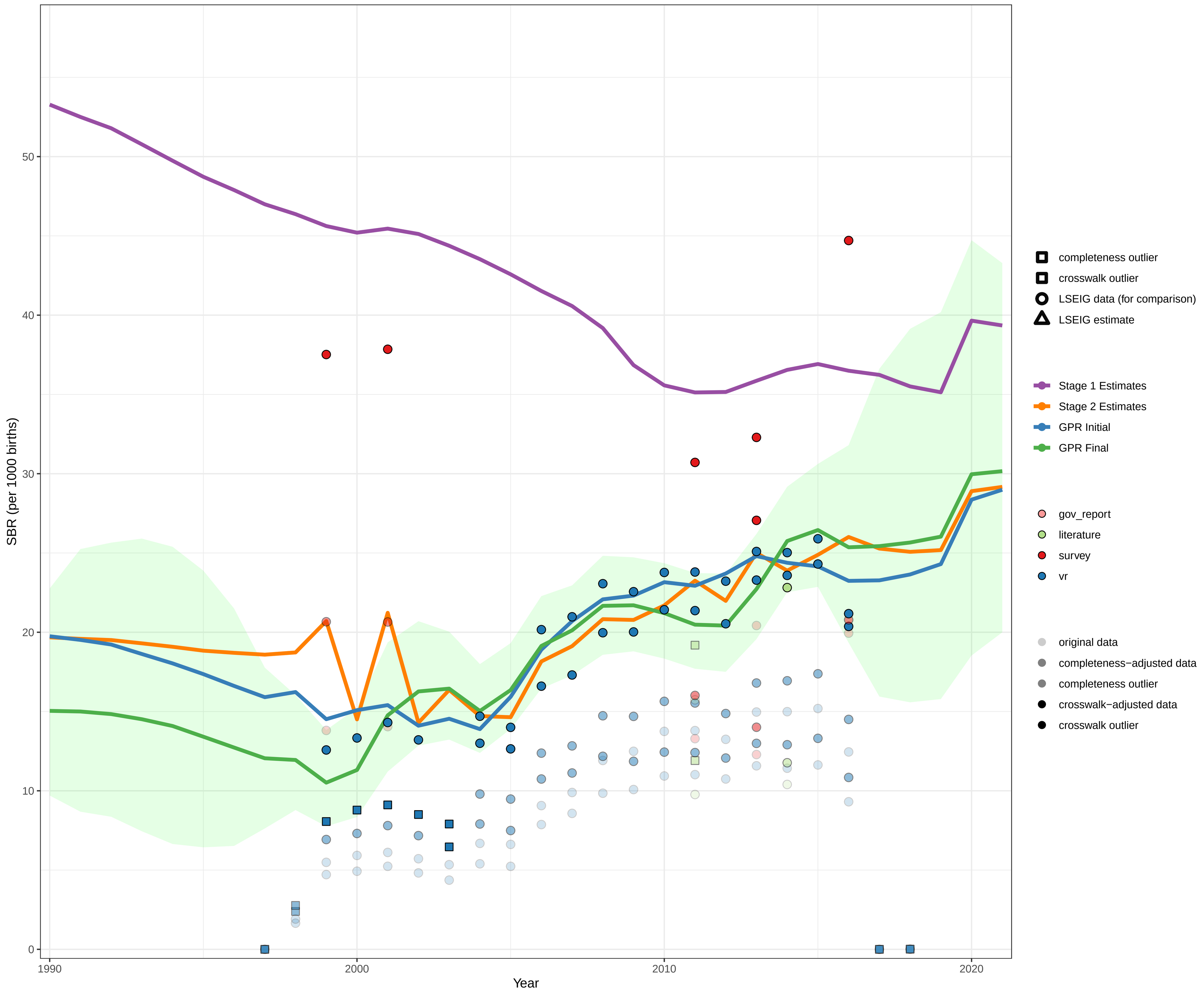

Western Cape ZAF_490 Southern Sub−Saharan Africa Stillbirth Rate (SBR) − 28 weeks
lambda = 0.2 ; zeta = 0.99 ; scale = 5 ; amp = 0.028

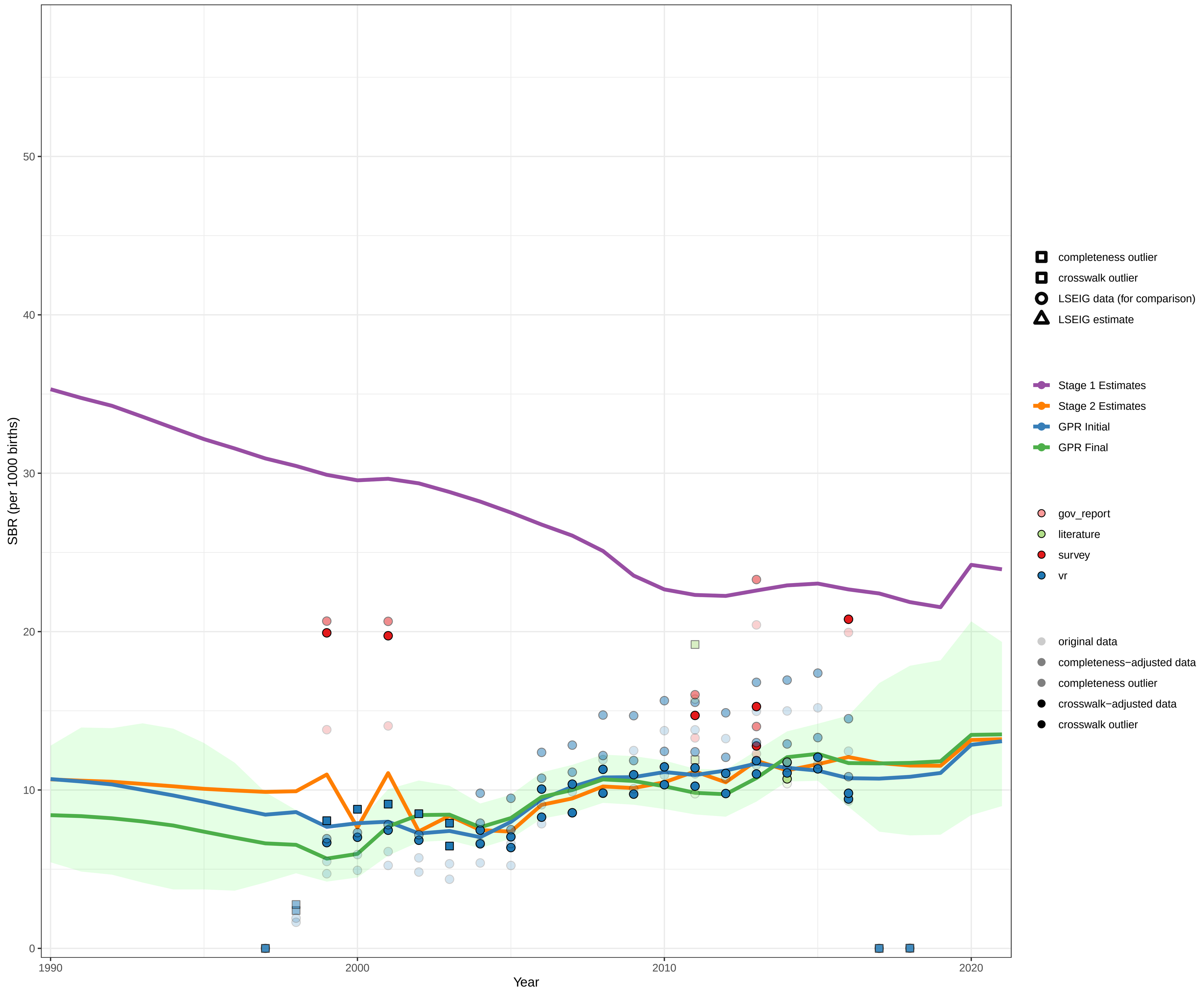

Year	Total Stillbirths (in thousands)	Total Stillbirths (in thousands)	SBR (per 1000 births)	SBR (per 1000 births)	SBR/NMR	SBR/NMR
	20 weeks	28 weeks	20 weeks	28 weeks	20 weeks	28 weeks
1990	1.2 (0.8 to 1.9)	0.7 (0.4 to 1.0)	15.0 (9.7 to 22.8)	8.4 (5.4 to 12.8)	0.97 (0.62 to 1.46)	0.54 (0.35 to 0.82)
1995	1.2 (0.6 to 2.1)	0.7 (0.3 to 1.2)	13.4 (6.4 to 23.9)	7.4 (3.7 to 13.0)	0.95 (0.45 to 1.68)	0.52 (0.26 to 0.91)
2000	1.1 (0.8 to 1.5)	0.6 (0.4 to 0.8)	11.3 (8.3 to 15.2)	6.0 (4.5 to 7.9)	0.88 (0.65 to 1.18)	0.46 (0.35 to 0.61)
2005	1.8 (1.5 to 2.1)	0.9 (0.7 to 1.0)	16.4 (13.9 to 19.3)	8.2 (6.9 to 9.7)	1.39 (1.18 to 1.64)	0.70 (0.59 to 0.82)
2010	2.4 (2.1 to 2.8)	1.2 (1.0 to 1.3)	21.2 (18.3 to 24.4)	10.2 (8.8 to 11.9)	2.24 (1.94 to 2.57)	1.08 (0.93 to 1.25)
2015	3.0 (2.5 to 3.4)	1.4 (1.2 to 1.6)	26.4 (22.9 to 30.6)	12.3 (10.6 to 14.2)	2.86 (2.47 to 3.31)	1.33 (1.14 to 1.53)
2020	3.0 (1.9 to 4.6)	1.3 (0.8 to 2.1)	30.0 (18.5 to 44.7)	13.5 (8.4 to 20.6)	3.21 (1.98 to 4.79)	1.44 (0.90 to 2.21)
2021	3.0 (2.0 to 4.3)	1.3 (0.9 to 1.9)	30.2 (20.0 to 43.3)	13.5 (9.0 to 19.3)	3.29 (2.18 to 4.72)	1.47 (0.98 to 2.11)

Zimbabwe ZWE Southern Sub−Saharan Africa Stillbirth Rate / Neonatal Mortality Rate (SBR/NMR) − 20 weeks
lambda = 0.6 ; zeta = 0.8 ; scale = 15 ; amp = 0.028

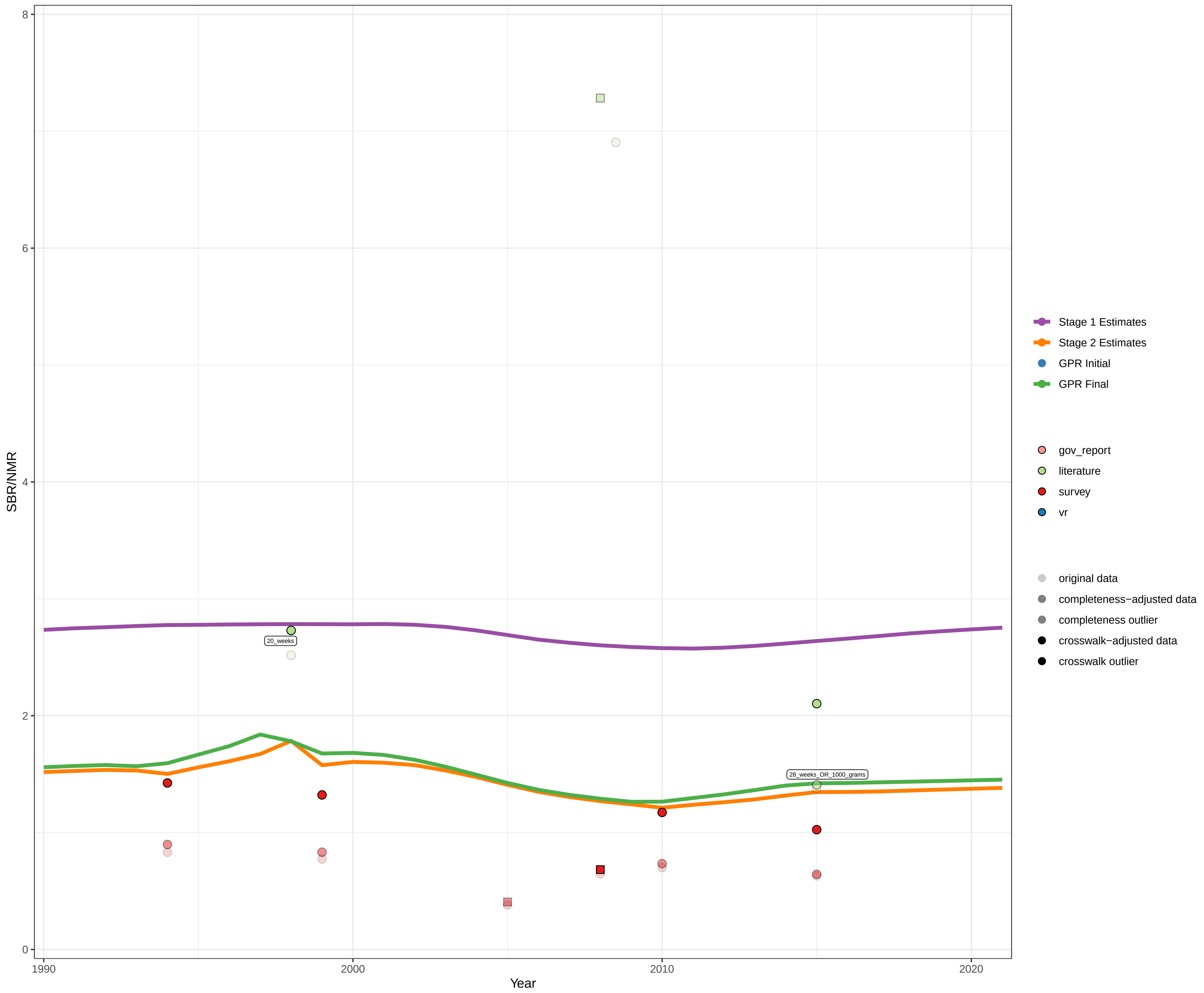

Zimbabwe ZWE Southern Sub−Saharan Africa Stillbirth Rate / Neonatal Mortality Rate (SBR/NMR) − 28 weeks
lambda = 0.6 ; zeta = 0.8 ; scale = 15 ; amp = 0.028

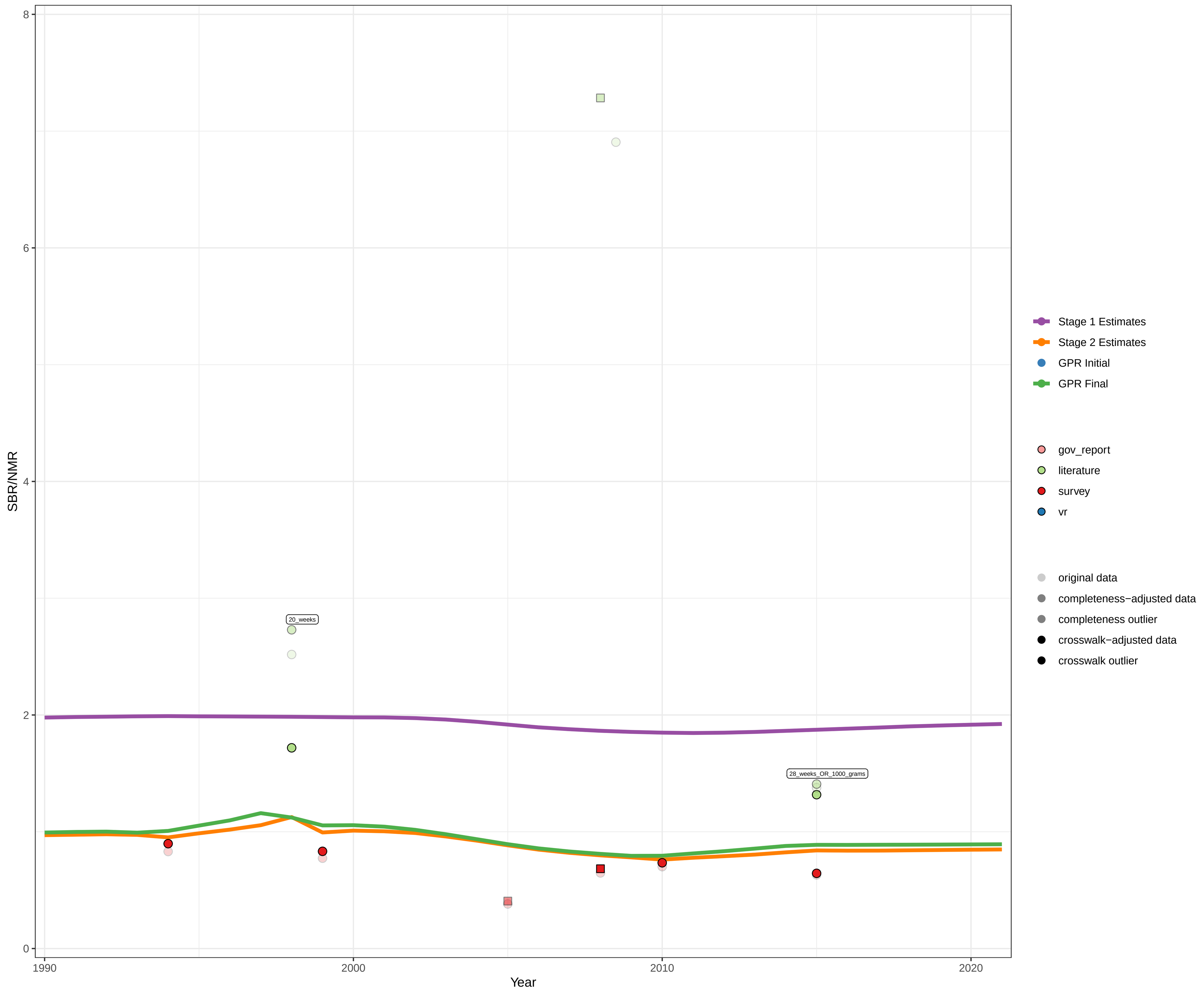

Definition	Years	Outlier	Source.Type	Title
20_weeks	1998	0	Sci Lit	Incidence of and socio−demographic risk factors for stillbirth, preterm birth and low birthweight among Zimbabwean women
22_weeks_OR_500_grams	2008	0	Survey	Zimbabwe Maternal and Perinatal Mortality Study 2007−2008
28_weeks	1994	0	Survey	Zimbabwe Demographic and Health Survey 1994
28_weeks	1999	0	Survey	Zimbabwe Demographic and Health Survey 1999
28_weeks	2010	0	Survey	Zimbabwe Demographic and Health Survey 2010−2011
28_weeks	2015	0	Survey	Zimbabwe Demographic and Health Survey 2015
28_weeks_OR_1000_grams	2015	0	Sci Lit	Application of the ICD−PM classification system to stillbirth in four sub−Saharan African countries
22_weeks	2008	1	Sci Lit	Determinants of perinatal mortality in Marondera district, Mashonaland East Province of Zimbabwe, 2009: a case control study
28_weeks	2005	1	Survey	Zimbabwe Demographic and Health Survey 2005−2006

Zimbabwe ZWE Southern Sub−Saharan Africa Stillbirth Rate (SBR) − 20 weeks
lambda = 0.6 ; zeta = 0.8 ; scale = 15 ; amp = 0.028

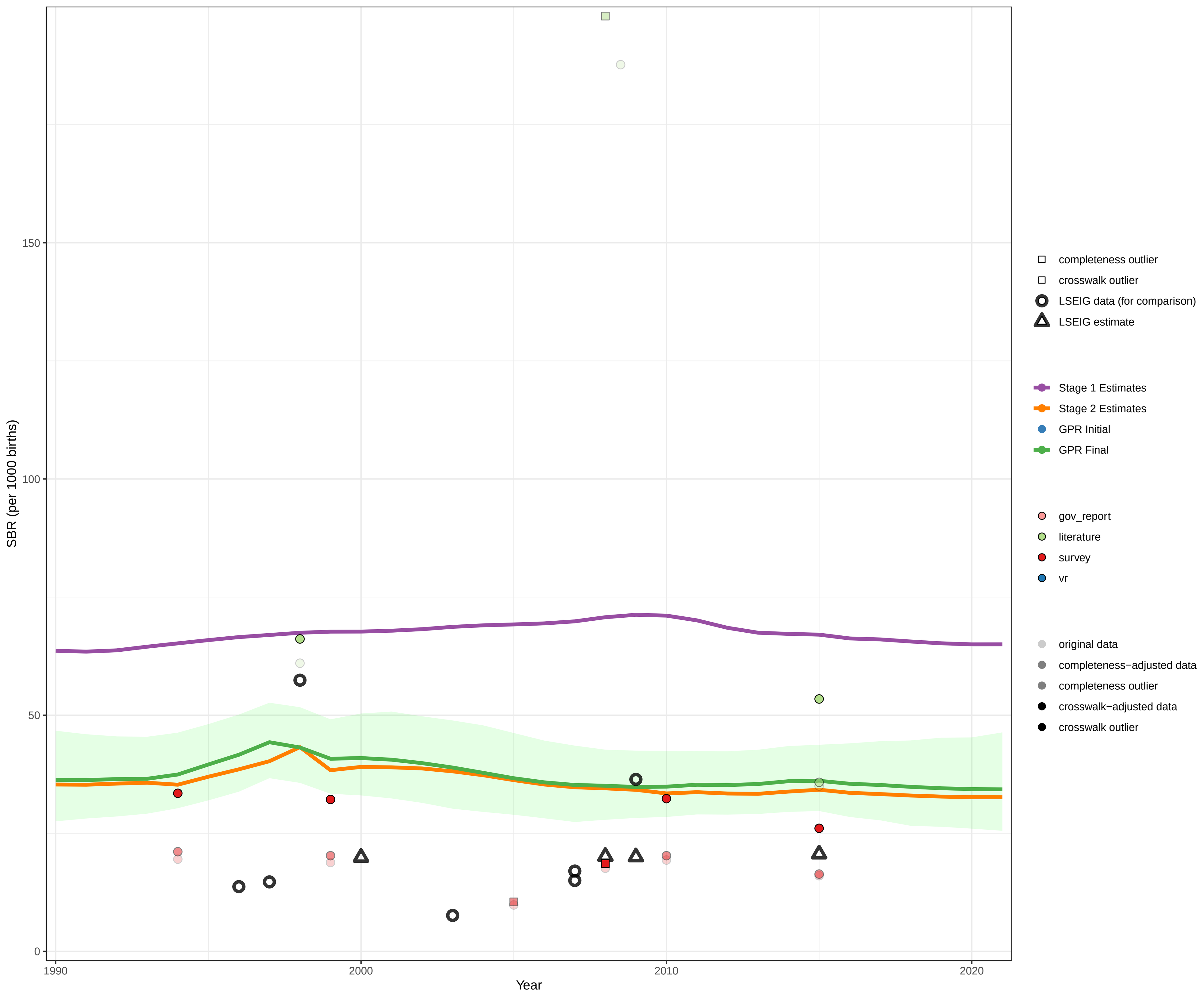

Zimbabwe ZWE Southern Sub−Saharan Africa Stillbirth Rate (SBR) − 28 weeks
lambda = 0.6 ; zeta = 0.8 ; scale = 15 ; amp = 0.028

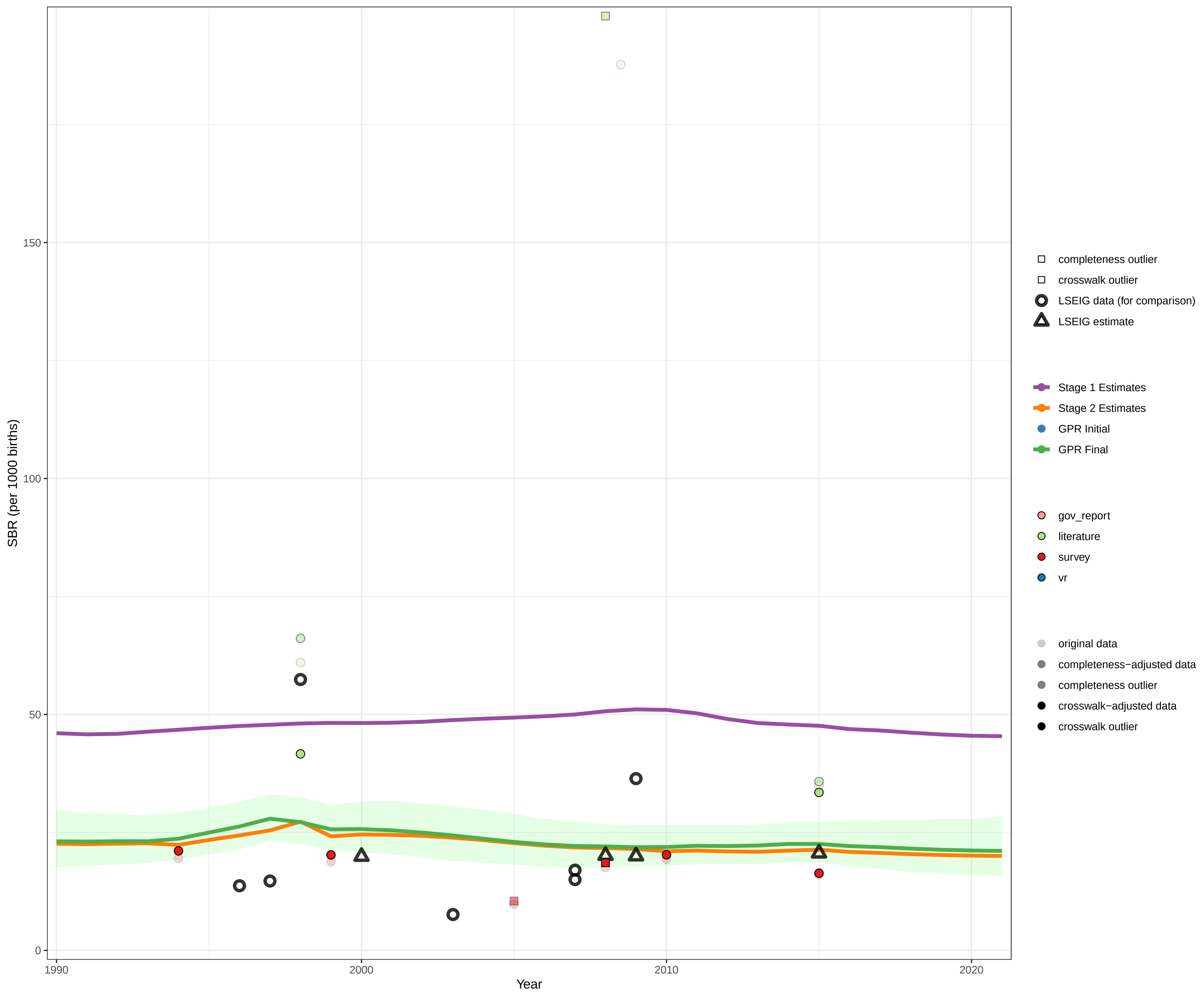

Year	Total Stillbirths (in thousands)	Total Stillbirths (in thousands)	SBR (per 1000 births)	SBR (per 1000 births)	SBR/NMR	SBR/NMR
	20 weeks	28 weeks	20 weeks	28 weeks	20 weeks	28 weeks
1990	14.9 (11.2 to 19.4)	9.4 (7.1 to 12.1)	36.3 (27.5 to 46.7)	23.1 (17.6 to 29.7)	1.56 (1.18 to 2.01)	0.99 (0.76 to 1.28)
1995	16.9 (13.5 to 20.7)	10.5 (8.5 to 12.8)	39.6 (32.0 to 48.1)	25.0 (20.3 to 30.3)	1.67 (1.35 to 2.03)	1.05 (0.85 to 1.28)
2000	18.2 (14.6 to 22.6)	11.3 (9.1 to 13.9)	40.9 (33.0 to 50.3)	25.7 (20.8 to 31.5)	1.68 (1.36 to 2.07)	1.06 (0.85 to 1.30)
2005	16.5 (12.9 to 21.0)	10.2 (8.0 to 12.9)	36.6 (28.9 to 46.2)	23.0 (18.2 to 28.9)	1.42 (1.12 to 1.80)	0.89 (0.71 to 1.12)
2010	17.3 (14.1 to 21.3)	10.8 (8.8 to 13.1)	34.9 (28.4 to 42.5)	21.9 (17.9 to 26.6)	1.26 (1.03 to 1.54)	0.79 (0.65 to 0.96)
2015	17.9 (14.7 to 21.9)	11.1 (9.1 to 13.4)	36.1 (29.7 to 43.7)	22.6 (18.6 to 27.2)	1.42 (1.17 to 1.72)	0.89 (0.73 to 1.07)
2020	16.7 (12.5 to 22.3)	10.2 (7.7 to 13.5)	34.3 (26.0 to 45.3)	21.2 (16.1 to 27.8)	1.45 (1.09 to 1.91)	0.89 (0.68 to 1.17)
2021	16.7 (12.3 to 22.8)	10.1 (7.5 to 13.7)	34.3 (25.5 to 46.4)	21.1 (15.7 to 28.4)	1.45 (1.08 to 1.96)	0.89 (0.67 to 1.20)

Western Sub−Saharan Africa R21 Western Sub−Saharan Africa Stillbirth Rate / Neonatal Mortality Rate (SBR/NMR) − 20 weeks
lambda = 1 ; zeta = 0.6 ; scale = 15 ; amp = 0.028

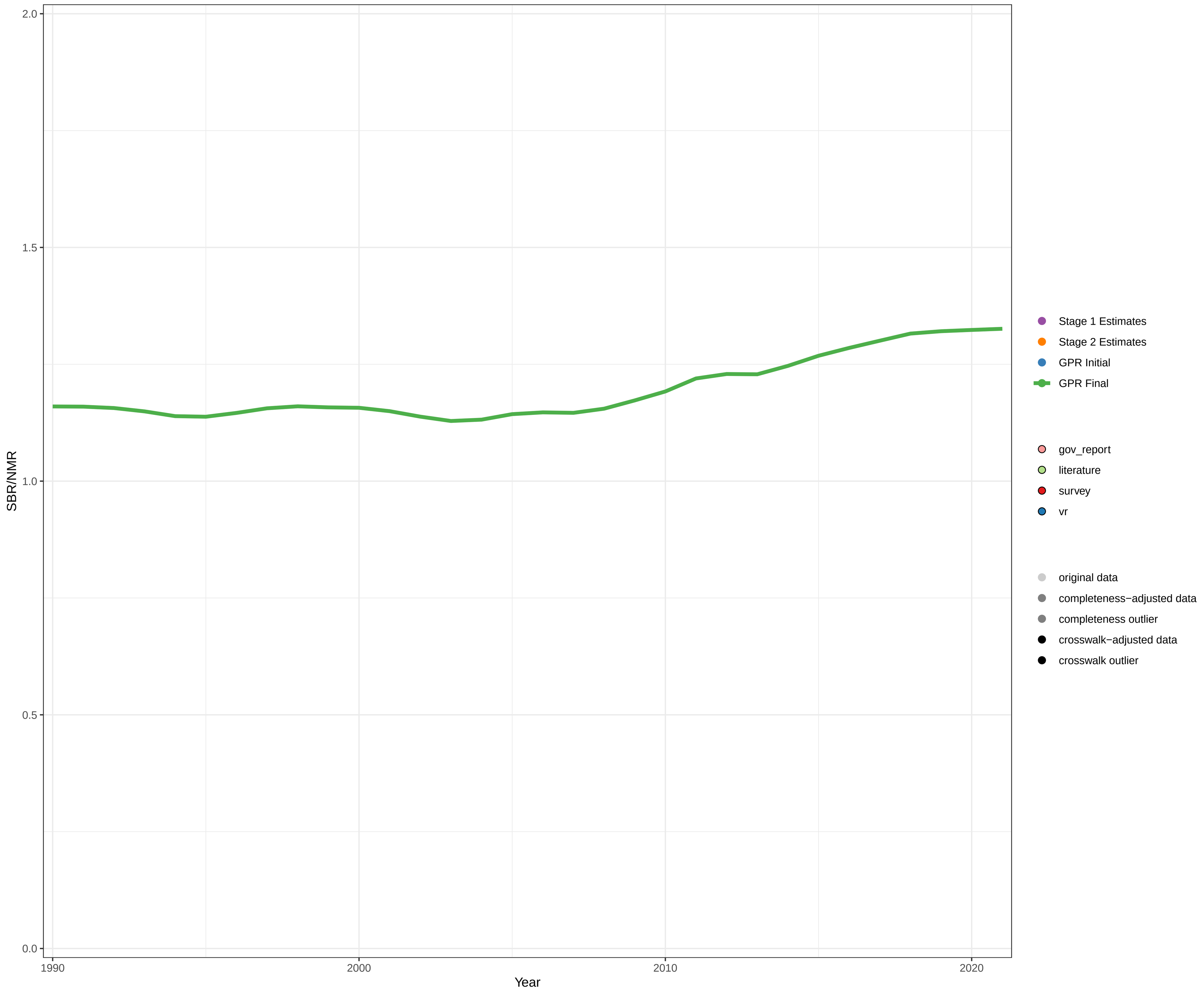

Western Sub−Saharan Africa R21 Western Sub−Saharan Africa Stillbirth Rate / Neonatal Mortality Rate (SBR/NMR) − 28 weeks
lambda = 1 ; zeta = 0.6 ; scale = 15 ; amp = 0.028

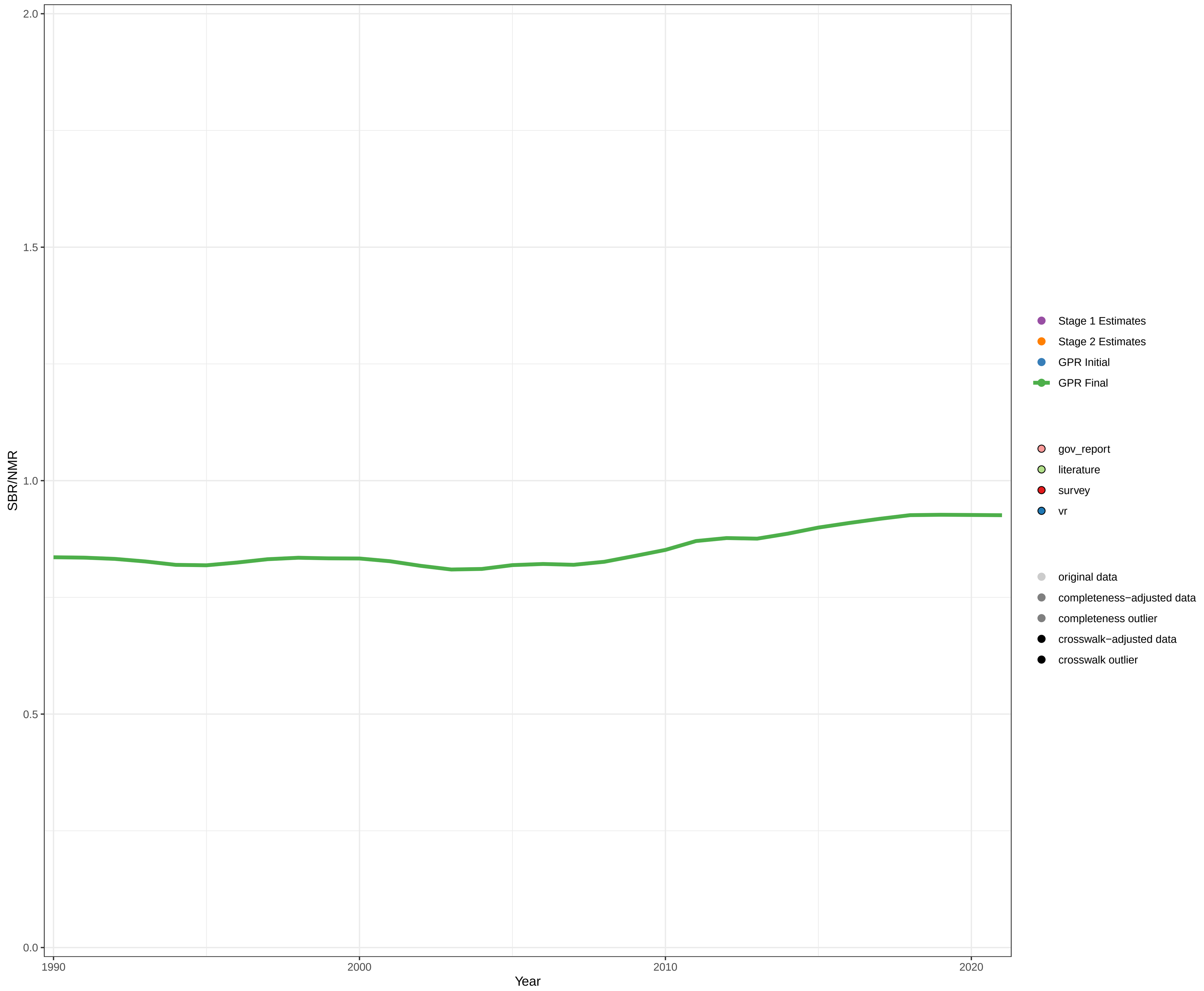

Definition	Years	Outlier	Source.Type	Title

Western Sub−Saharan Africa R21 Western Sub−Saharan Africa Stillbirth Rate (SBR) − 20 weeks
lambda = 1 ; zeta = 0.6 ; scale = 15 ; amp = 0.028

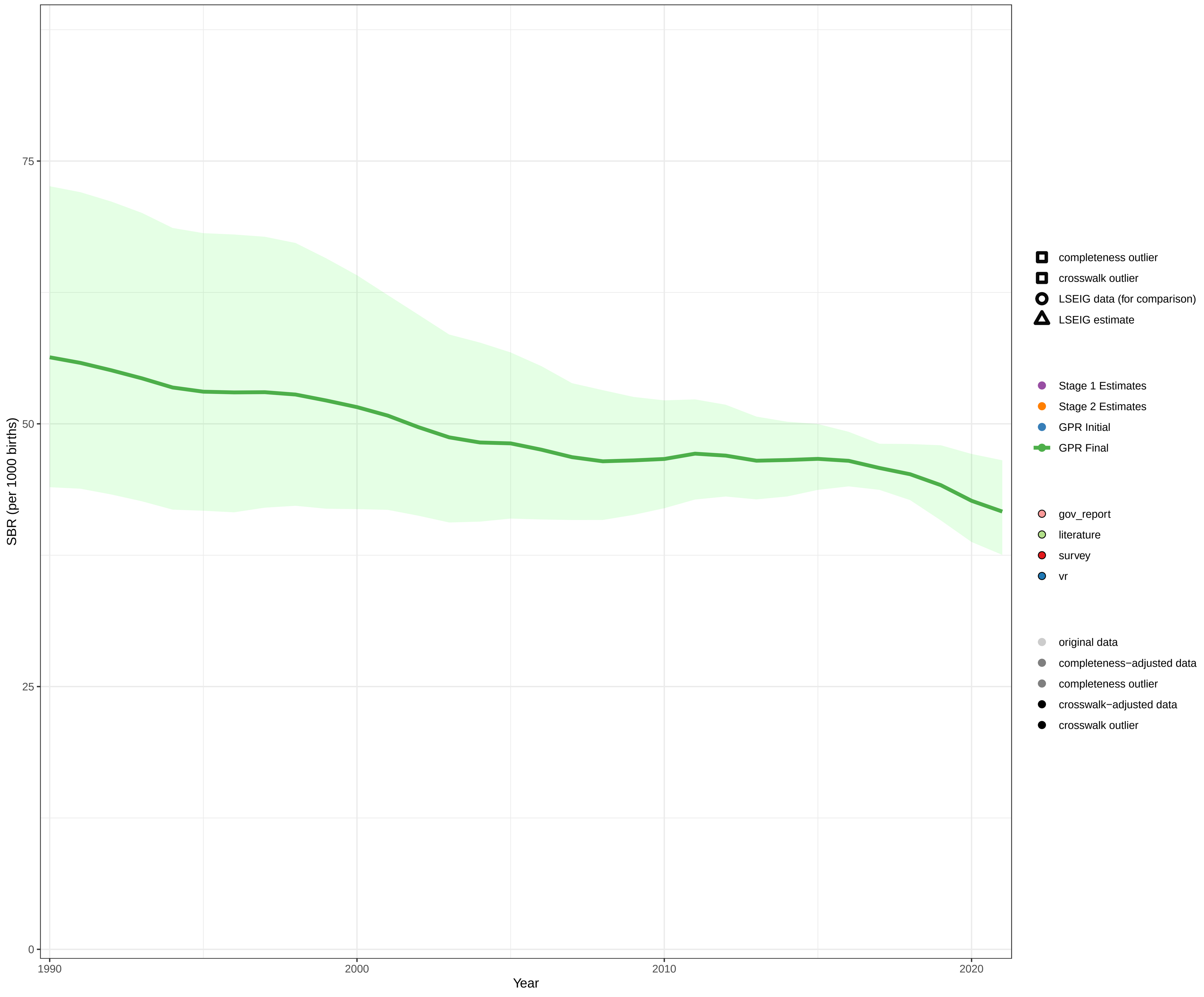

Western Sub−Saharan Africa R21 Western Sub−Saharan Africa Stillbirth Rate (SBR) − 28 weeks
lambda = 1 ; zeta = 0.6 ; scale = 15 ; amp = 0.028

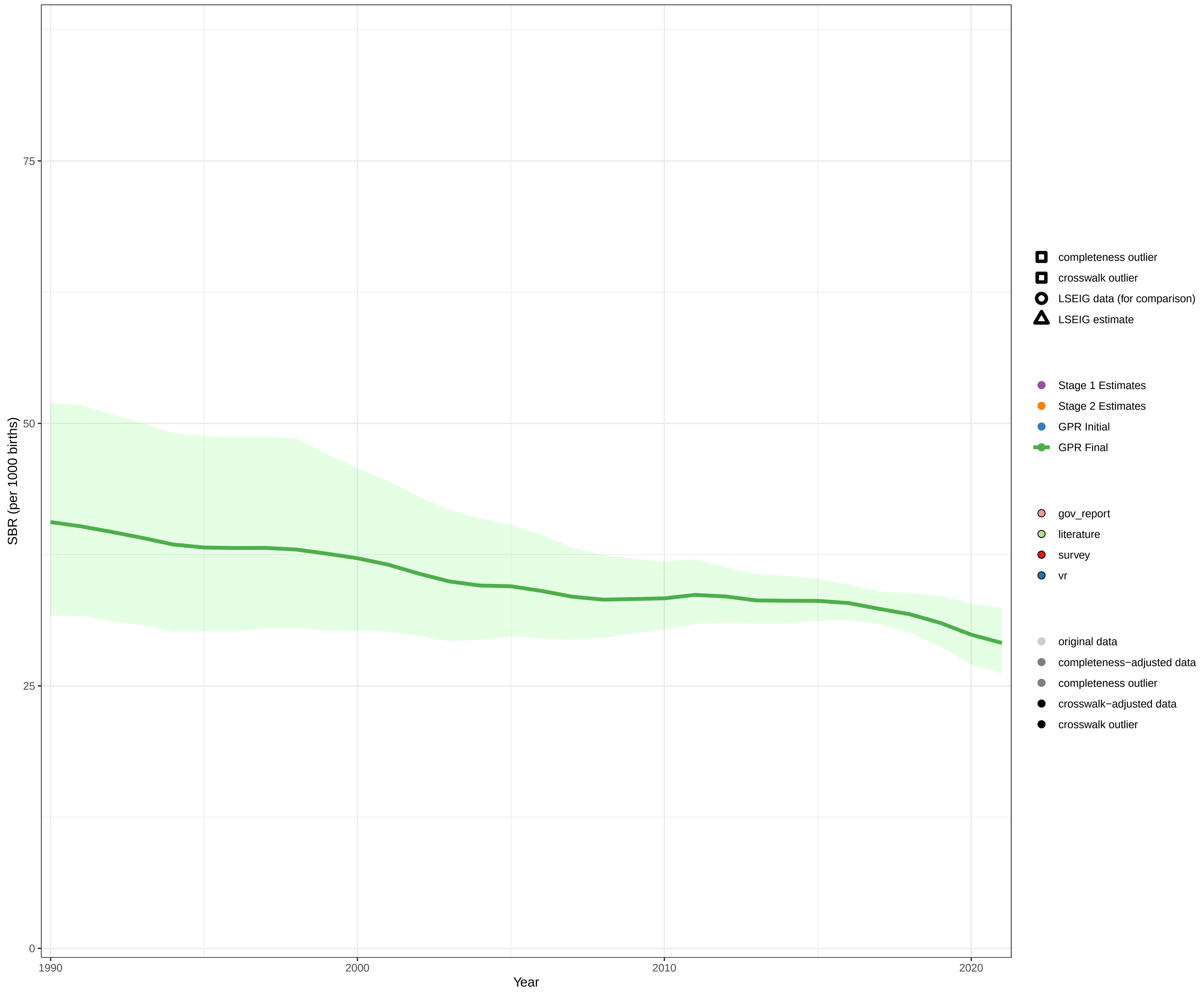

Year	Total Stillbirths (in thousands)	Total Stillbirths (in thousands)	SBR (per 1000 births)	SBR (per 1000 births)	SBR/NMR	SBR/NMR
	20 weeks	28 weeks	20 weeks	28 weeks	20 weeks	28 weeks
1990	539.3<br>(415.1 to 706.6)	382.2<br>(295.5 to 494.4)	56.3<br>(44.0 to 72.6)	40.6<br>(31.7 to 51.9)	1.16<br>(0.91 to 1.49)	0.84<br>(0.65 to 1.07)
1995	571.6<br>(443.8 to 745.2)	404.8<br>(317.2 to 522.5)	53.1<br>(41.7 to 68.1)	38.2<br>(30.2 to 48.8)	1.14<br>(0.89 to 1.46)	0.82<br>(0.65 to 1.05)
2000	639.7<br>(513.8 to 805.4)	453.7<br>(367.3 to 563.2)	51.6<br>(41.9 to 64.1)	37.2<br>(30.3 to 45.7)	1.16<br>(0.94 to 1.44)	0.83<br>(0.68 to 1.03)
2005	683.4<br>(577.2 to 813.3)	482.6<br>(413.6 to 567.9)	48.1<br>(41.0 to 56.8)	34.5<br>(29.7 to 40.3)	1.14<br>(0.97 to 1.35)	0.82<br>(0.71 to 0.96)
2010	747.2<br>(668.8 to 841.3)	526.5<br>(477.7 to 583.9)	46.7<br>(42.0 to 52.2)	33.3<br>(30.3 to 36.8)	1.19<br>(1.07 to 1.33)	0.85<br>(0.78 to 0.94)
2015	818.5<br>(764.3 to 879.6)	572.3<br>(538.7 to 610.3)	46.7<br>(43.7 to 50.0)	33.1<br>(31.2 to 35.2)	1.27<br>(1.19 to 1.36)	0.90<br>(0.85 to 0.96)
2020	786.0<br>(710.8 to 872.0)	542.9<br>(490.2 to 598.5)	42.7<br>(38.8 to 47.1)	29.9<br>(27.1 to 32.8)	1.32<br>(1.20 to 1.46)	0.93<br>(0.84 to 1.02)
2021	775.4<br>(695.6 to 870.6)	534.5<br>(479.2 to 597.3)	41.7<br>(37.5 to 46.5)	29.1<br>(26.2 to 32.4)	1.33<br>(1.19 to 1.48)	0.93<br>(0.83 to 1.03)

Benin BEN Western Sub−Saharan Africa Stillbirth Rate / Neonatal Mortality Rate (SBR/NMR) − 20 weeks
lambda = 0.4 ; zeta = 0.9 ; scale = 10 ; amp = 0.028

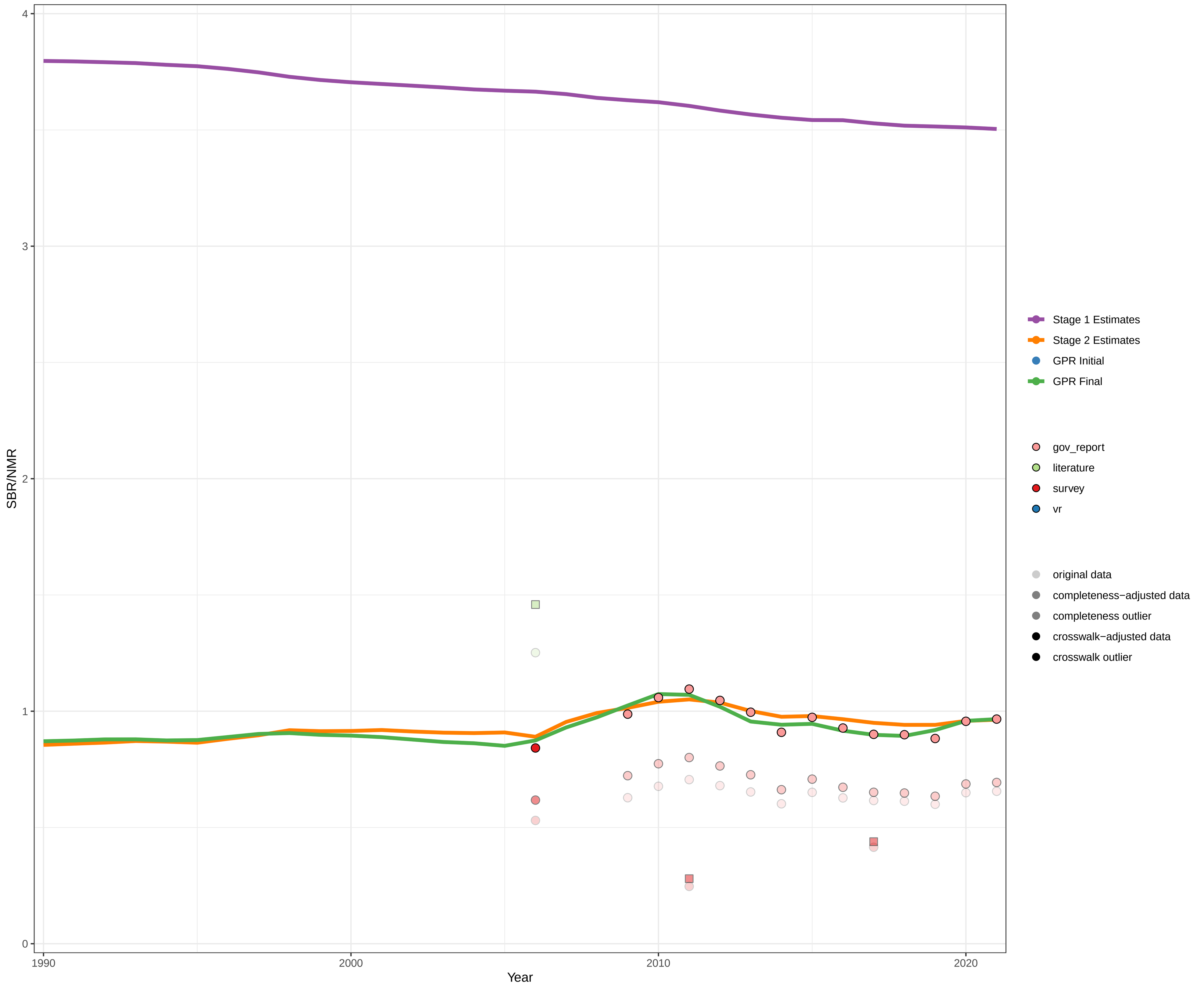

Benin BEN Western Sub−Saharan Africa Stillbirth Rate / Neonatal Mortality Rate (SBR/NMR) − 28 weeks
lambda = 0.4 ; zeta = 0.9 ; scale = 10 ; amp = 0.028

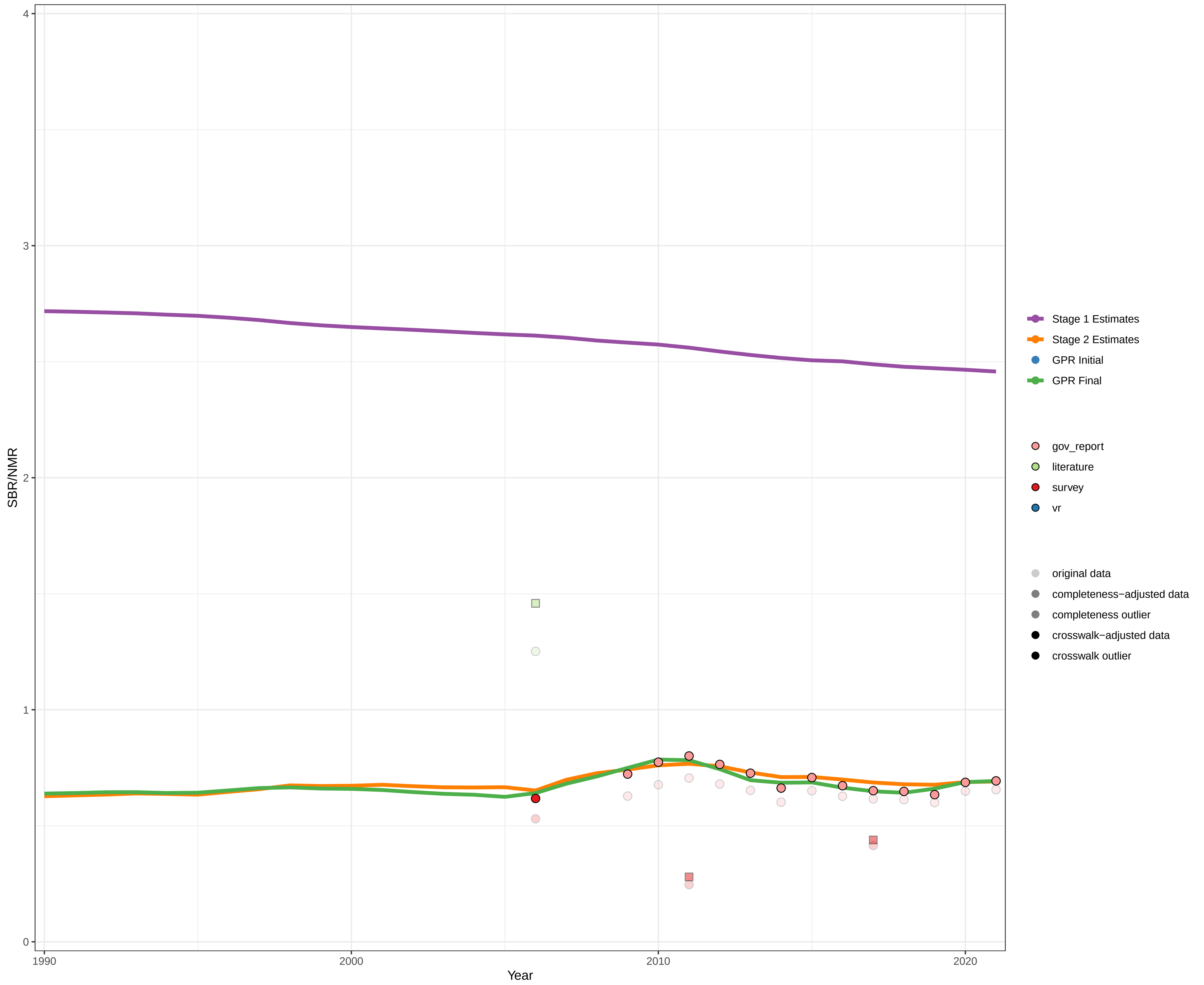

Definition	Years	Outlier	Source.Type	Title
28_weeks	2006	0	Survey	Benin Demographic and Health Survey 2006
28_weeks	2009−2021	0	Statistical Report	Benin Health Statistical Yearbook
22_weeks	2006	1	Sci Lit	Is chloroquine chemoprophylaxis still effective to prevent low birth weight? Results of a study in Benin
28_weeks	2011	1	Survey	Benin Demographic and Health Survey 2011−2012
28_weeks	2017	1	Survey	Benin Demographic and Health Survey 2017−2018

Benin BEN Western Sub−Saharan Africa Stillbirth Rate (SBR) − 20 weeks
lambda = 0.4 ; zeta = 0.9 ; scale = 10 ; amp = 0.028

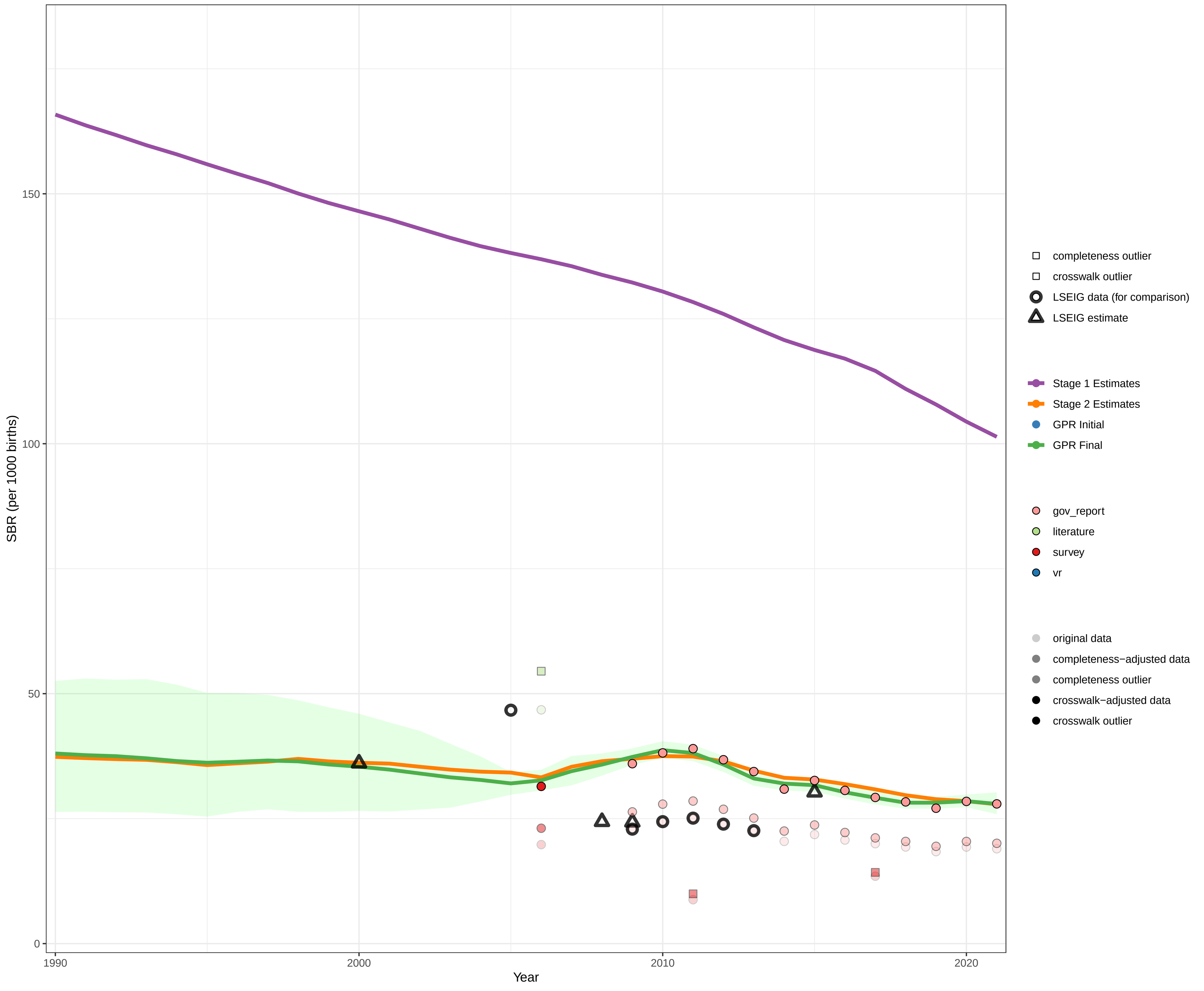

Benin BEN Western Sub−Saharan Africa Stillbirth Rate (SBR) − 28 weeks
lambda = 0.4 ; zeta = 0.9 ; scale = 10 ; amp = 0.028

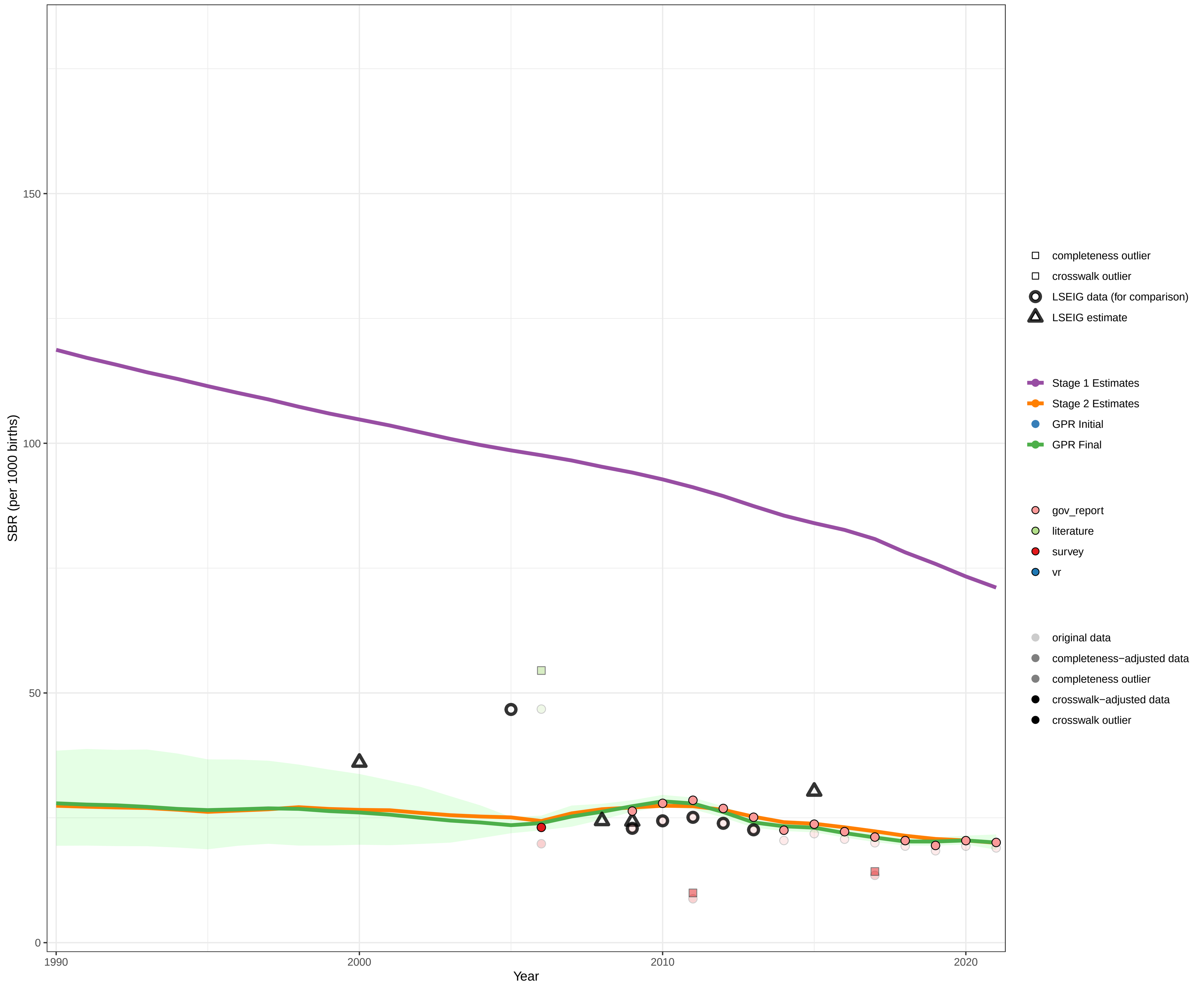

Year	Total Stillbirths (in thousands)	Total Stillbirths (in thousands)	SBR (per 1000 births)	SBR (per 1000 births)	SBR/NMR	SBR/NMR
	20 weeks	28 weeks	20 weeks	28 weeks	20 weeks	28 weeks
1990	9.4 (6.4 to 13.1)	6.8 (4.7 to 9.4)	38.0 (26.3 to 52.6)	27.9 (19.4 to 38.4)	0.87 (0.60 to 1.20)	0.64 (0.44 to 0.88)
1995	10.1 (7.0 to 14.2)	7.3 (5.1 to 10.2)	36.2 (25.4 to 50.2)	26.5 (18.7 to 36.7)	0.88 (0.61 to 1.21)	0.64 (0.45 to 0.89)
2000	11.3 (8.4 to 14.9)	8.3 (6.2 to 10.8)	35.4 (26.5 to 46.0)	26.1 (19.6 to 33.8)	0.90 (0.67 to 1.16)	0.66 (0.50 to 0.85)
2005	12.0 (11.1 to 12.9)	8.7 (8.1 to 9.3)	32.0 (29.8 to 34.3)	23.5 (21.9 to 25.2)	0.85 (0.79 to 0.91)	0.62 (0.58 to 0.67)
2010	16.7 (16.0 to 17.5)	12.1 (11.6 to 12.7)	38.7 (37.1 to 40.5)	28.3 (27.2 to 29.6)	1.07 (1.03 to 1.12)	0.79 (0.75 to 0.82)
2015	15.3 (14.6 to 16.0)	11.0 (10.5 to 11.5)	31.7 (30.4 to 33.1)	23.0 (22.1 to 24.0)	0.95 (0.91 to 0.99)	0.69 (0.66 to 0.72)
2020	15.0 (14.4 to 15.8)	10.7 (10.3 to 11.2)	28.5 (27.3 to 29.8)	20.5 (19.6 to 21.4)	0.96 (0.92 to 1.00)	0.69 (0.66 to 0.72)
2021	15.0 (13.9 to 16.3)	10.7 (9.9 to 11.6)	27.9 (25.9 to 30.3)	20.1 (18.6 to 21.7)	0.97 (0.90 to 1.05)	0.69 (0.64 to 0.75)

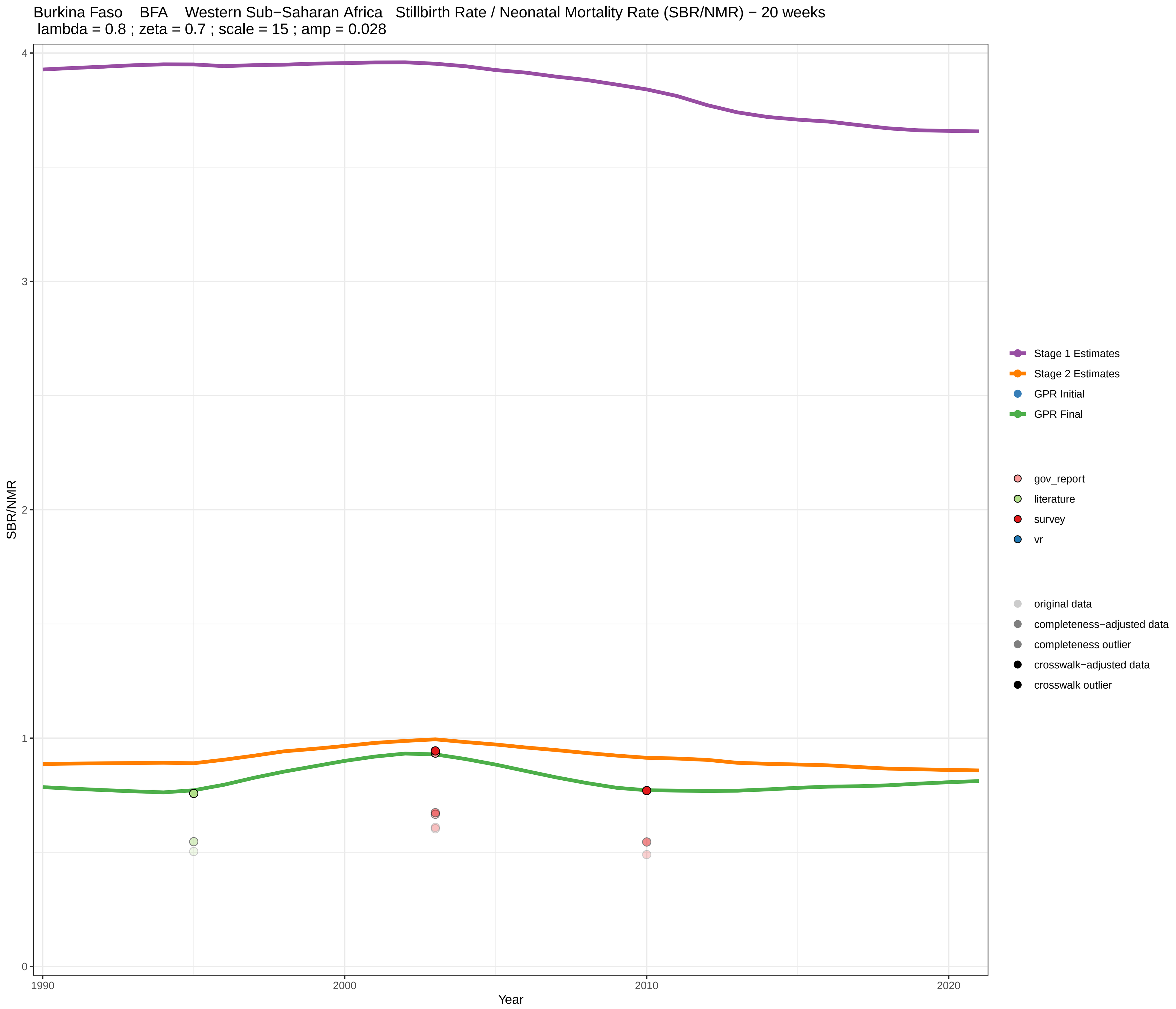

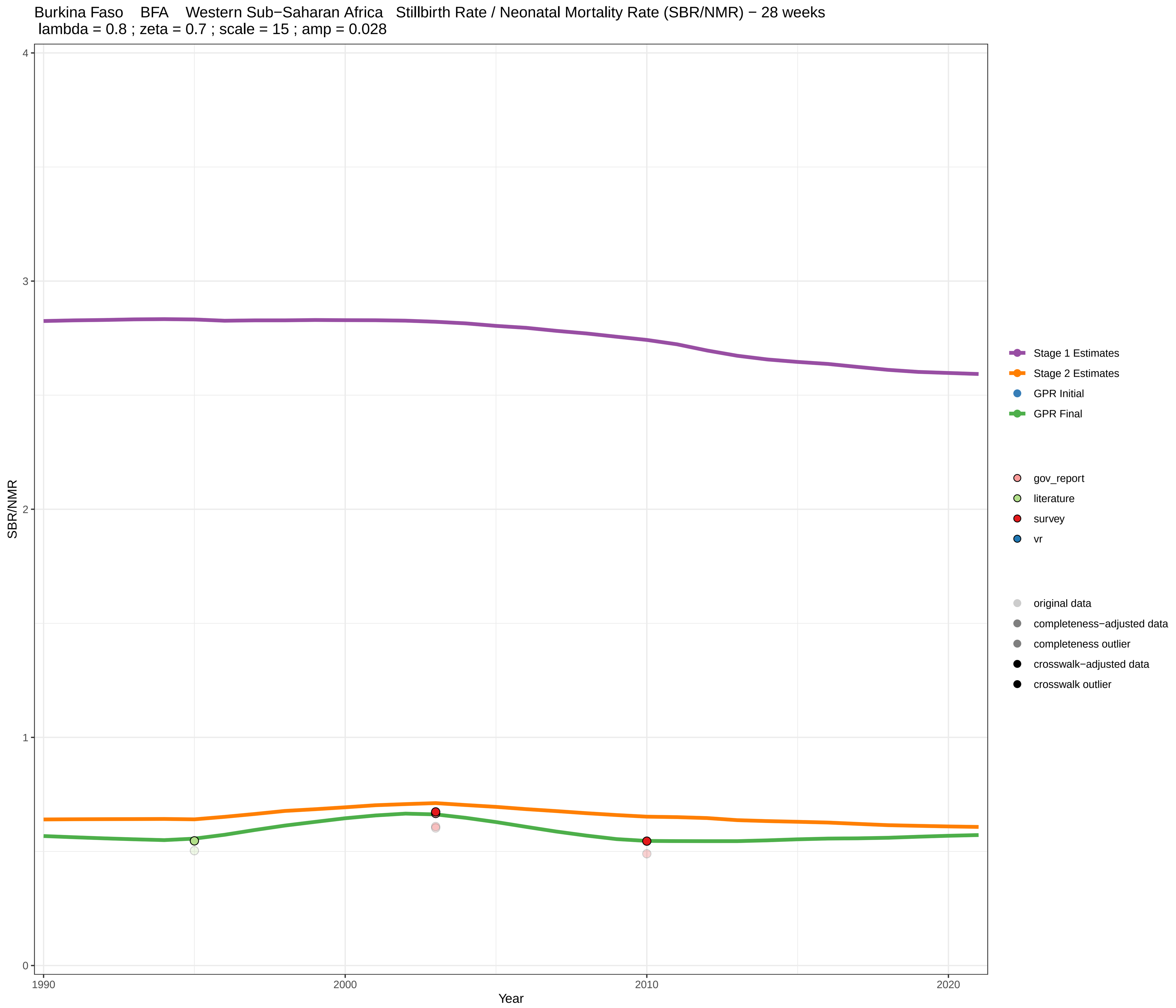

Definition	Years	Outlier	Source.Type	Title
28_weeks	1995	0	Sci Lit	Risk factors for perinatal mortality in West Africa: a population−based study of 20326 pregnancies. MOMA group
28_weeks	2003	0	Survey	Burkina Faso Demographic and Health Survey 2003
28_weeks	2003	0	Statistical Report	Burkina Faso Health Statistical Yearbook
28_weeks	2010	0	Survey	Burkina Faso Demographic and Health Survey 2010−2011

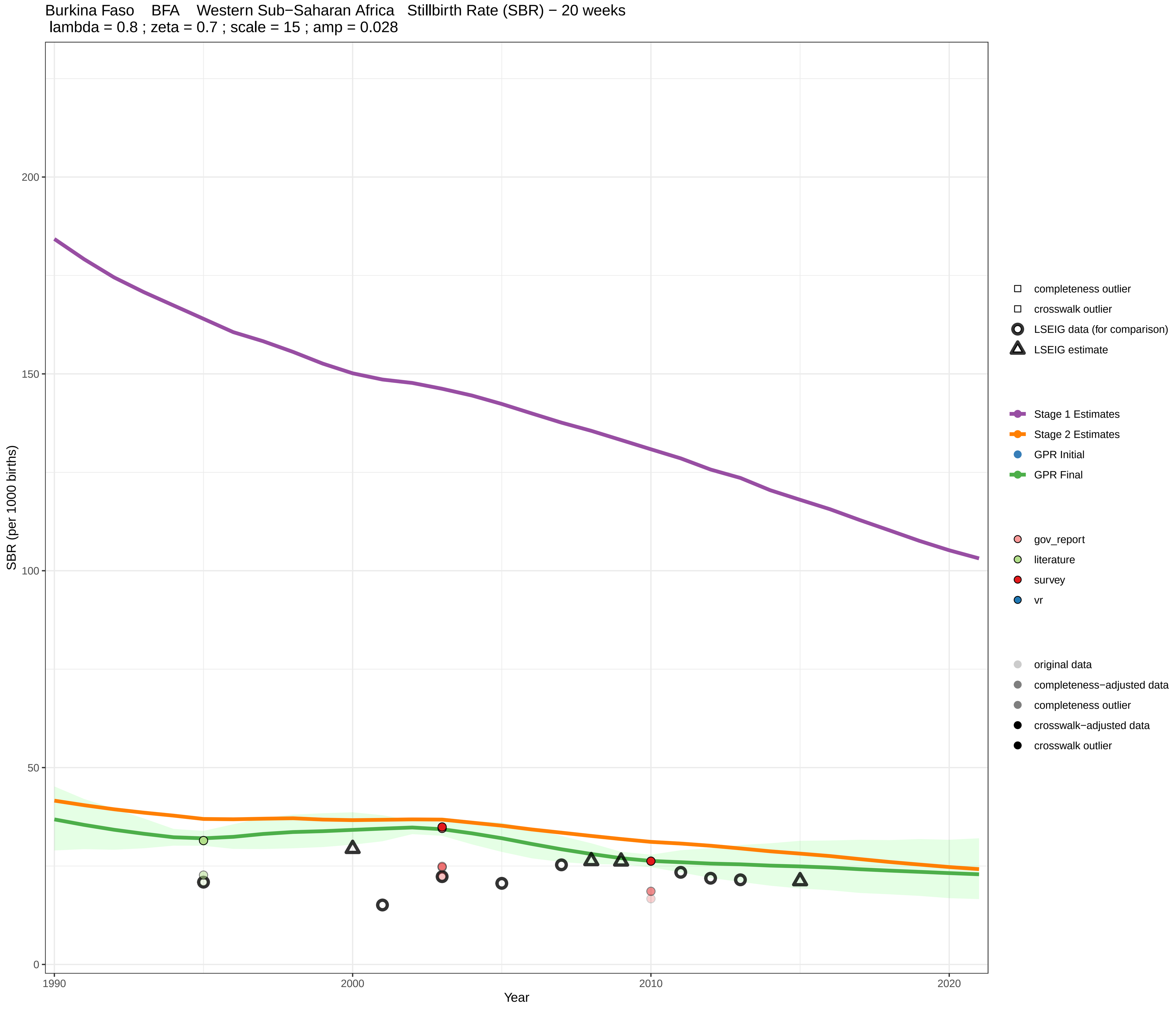

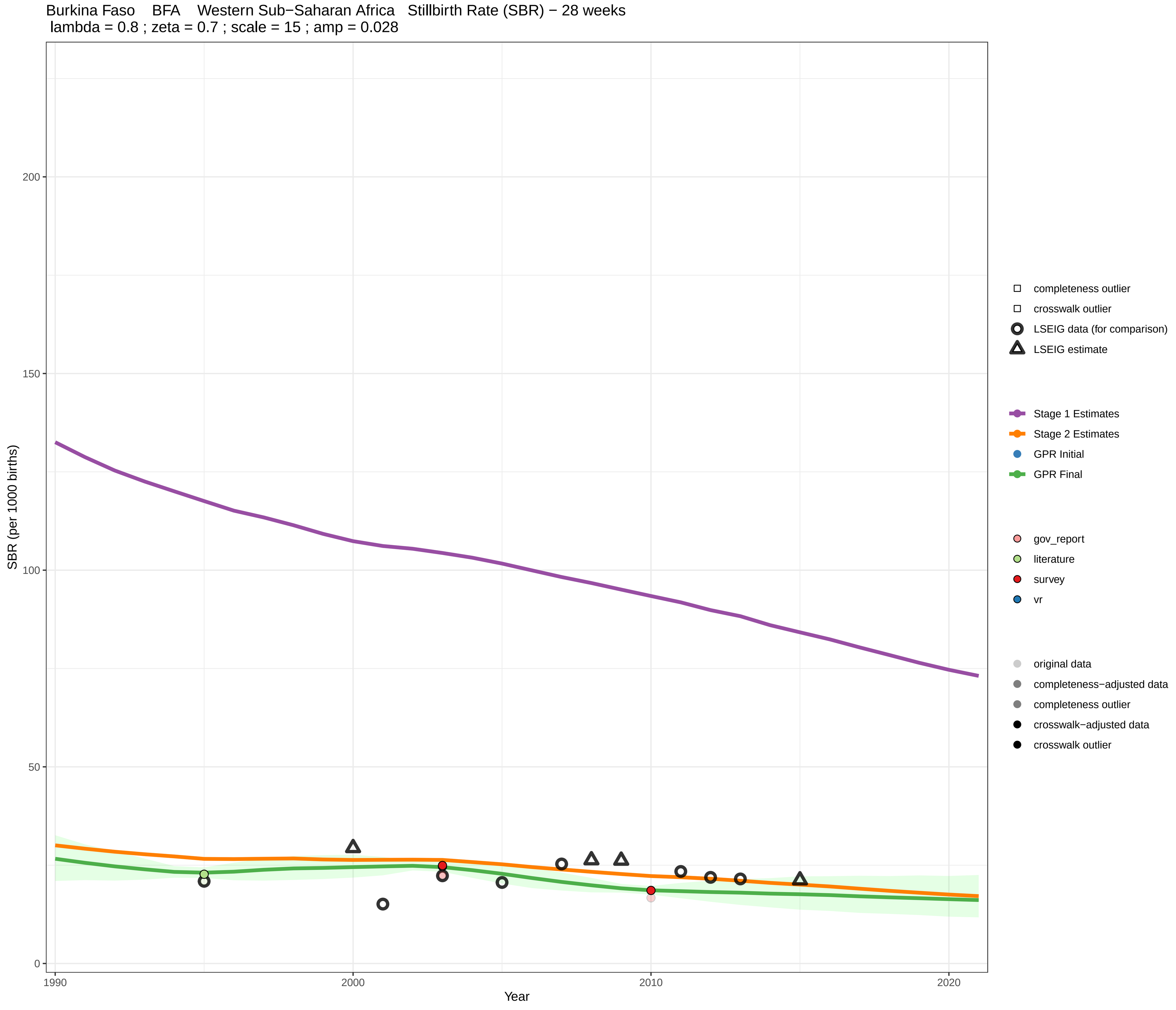

Year	Total Stillbirths (in thousands)	Total Stillbirths (in thousands)	SBR (per 1000 births)	SBR (per 1000 births)	SBR/NMR	SBR/NMR
	20 weeks	28 weeks	20 weeks	28 weeks	20 weeks	28 weeks
1990	17.6 (13.7 to 21.8)	12.6 (9.9 to 15.5)	36.8 (29.0 to 45.2)	26.6 (21.0 to 32.6)	0.79 (0.62 to 0.96)	0.57 (0.45 to 0.70)
1995	17.2 (16.1 to 18.2)	12.3 (11.5 to 13.0)	32.0 (30.1 to 33.9)	23.1 (21.7 to 24.4)	0.77 (0.73 to 0.82)	0.56 (0.52 to 0.59)
2000	21.1 (18.7 to 23.9)	14.9 (13.3 to 16.9)	34.2 (30.4 to 38.6)	24.5 (21.8 to 27.7)	0.90 (0.80 to 1.02)	0.65 (0.58 to 0.73)
2005	22.6 (20.1 to 25.5)	15.9 (14.2 to 17.8)	32.1 (28.6 to 36.0)	22.8 (20.3 to 25.5)	0.88 (0.79 to 0.99)	0.63 (0.56 to 0.70)
2010	20.7 (19.4 to 22.0)	14.5 (13.6 to 15.4)	26.3 (24.7 to 27.9)	18.6 (17.5 to 19.7)	0.77 (0.72 to 0.82)	0.55 (0.51 to 0.58)
2015	21.6 (16.6 to 27.4)	15.2 (11.7 to 19.2)	24.9 (19.3 to 31.4)	17.6 (13.7 to 22.2)	0.78 (0.61 to 0.99)	0.55 (0.43 to 0.70)
2020	22.1 (16.0 to 30.5)	15.5 (11.2 to 21.2)	23.2 (16.8 to 31.7)	16.4 (11.9 to 22.3)	0.81 (0.59 to 1.10)	0.57 (0.41 to 0.77)
2021	22.3 (16.1 to 31.5)	15.6 (11.3 to 21.9)	22.9 (16.6 to 32.0)	16.1 (11.7 to 22.5)	0.81 (0.59 to 1.14)	0.57 (0.42 to 0.80)

Cabo Verde   CPV   Western Sub−Saharan Africa   Stillbirth Rate / Neonatal Mortality Rate (SBR/NMR) − 20 weeks
lambda = 0.6 ; zeta = 0.8 ; scale = 15 ; amp = 0.028

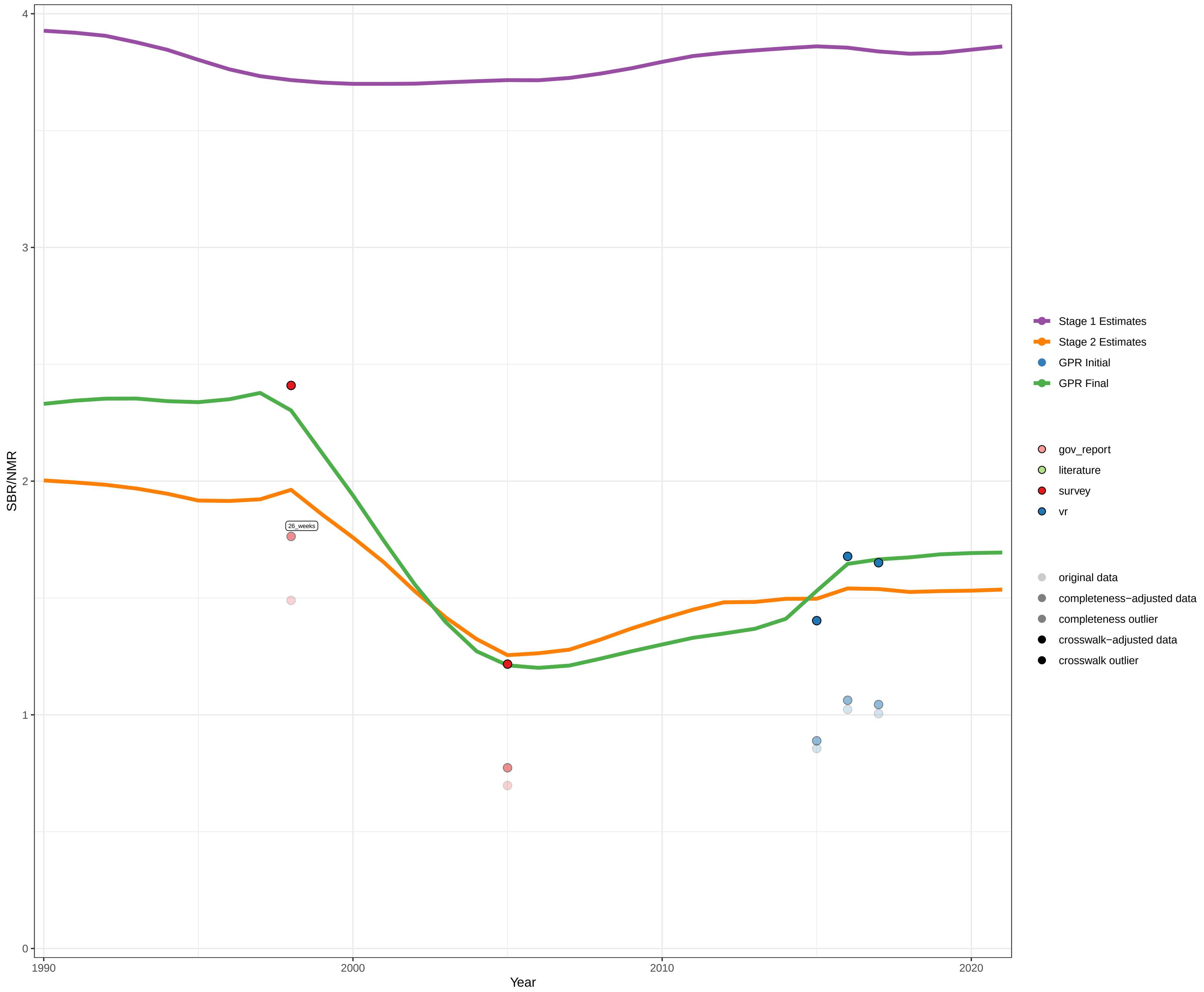

Cabo Verde   CPV   Western Sub−Saharan Africa   Stillbirth Rate / Neonatal Mortality Rate (SBR/NMR) − 28 weeks
lambda = 0.6 ; zeta = 0.8 ; scale = 15 ; amp = 0.028

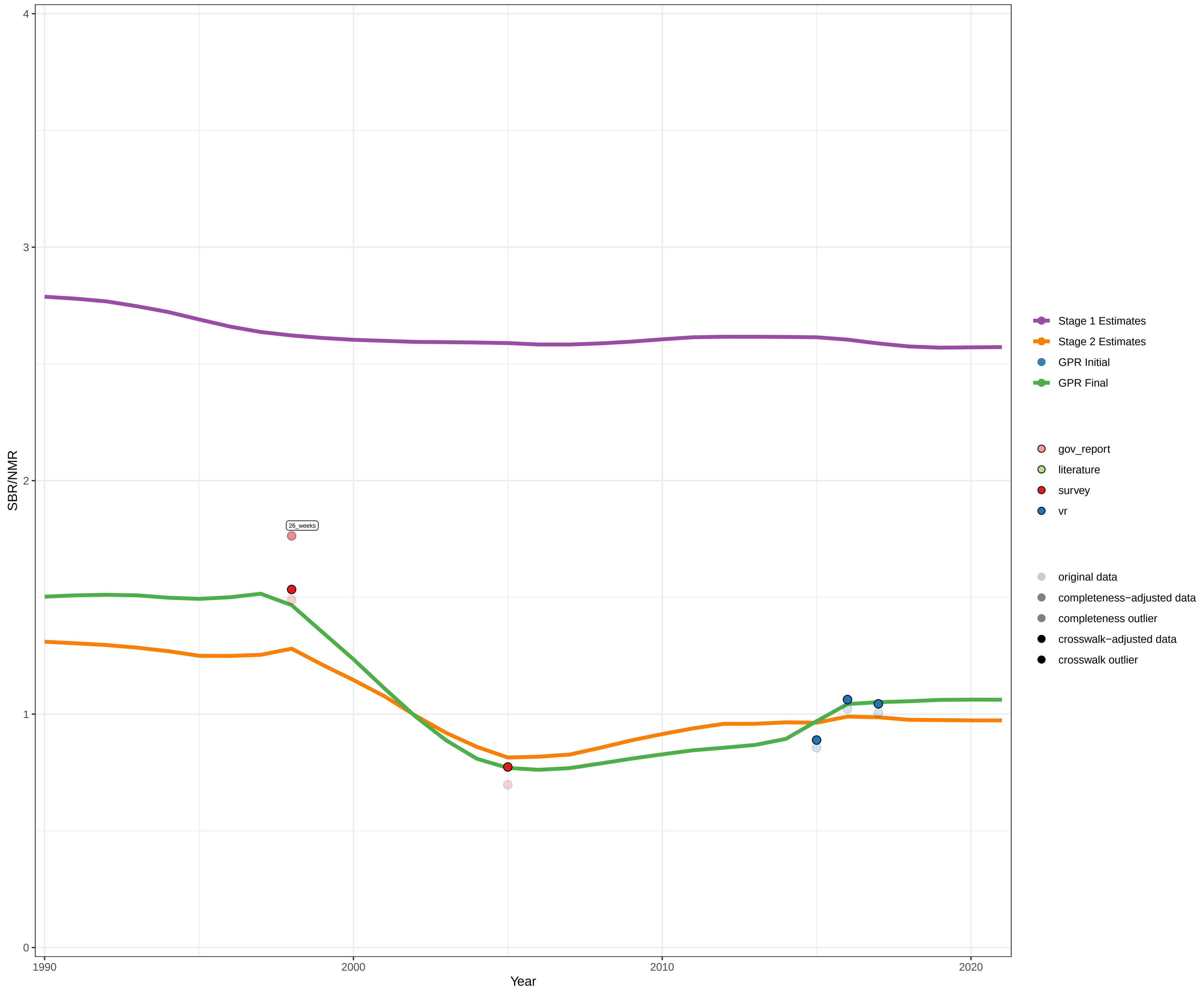

Definition	Years	Outlier	Source.Type	Title
26_weeks	1998	0	Survey	Cape Verde Reproductive Health Survey 1998
28_weeks	2005	0	Survey	Cape Verde Demographic and Health Survey 2005
28_weeks	2015−2017	0	VR	United Nations Demographic Yearbook

Cabo Verde   CPV   Western Sub−Saharan Africa   Stillbirth Rate (SBR) − 20 weeks
lambda = 0.6 ; zeta = 0.8 ; scale = 15 ; amp = 0.028

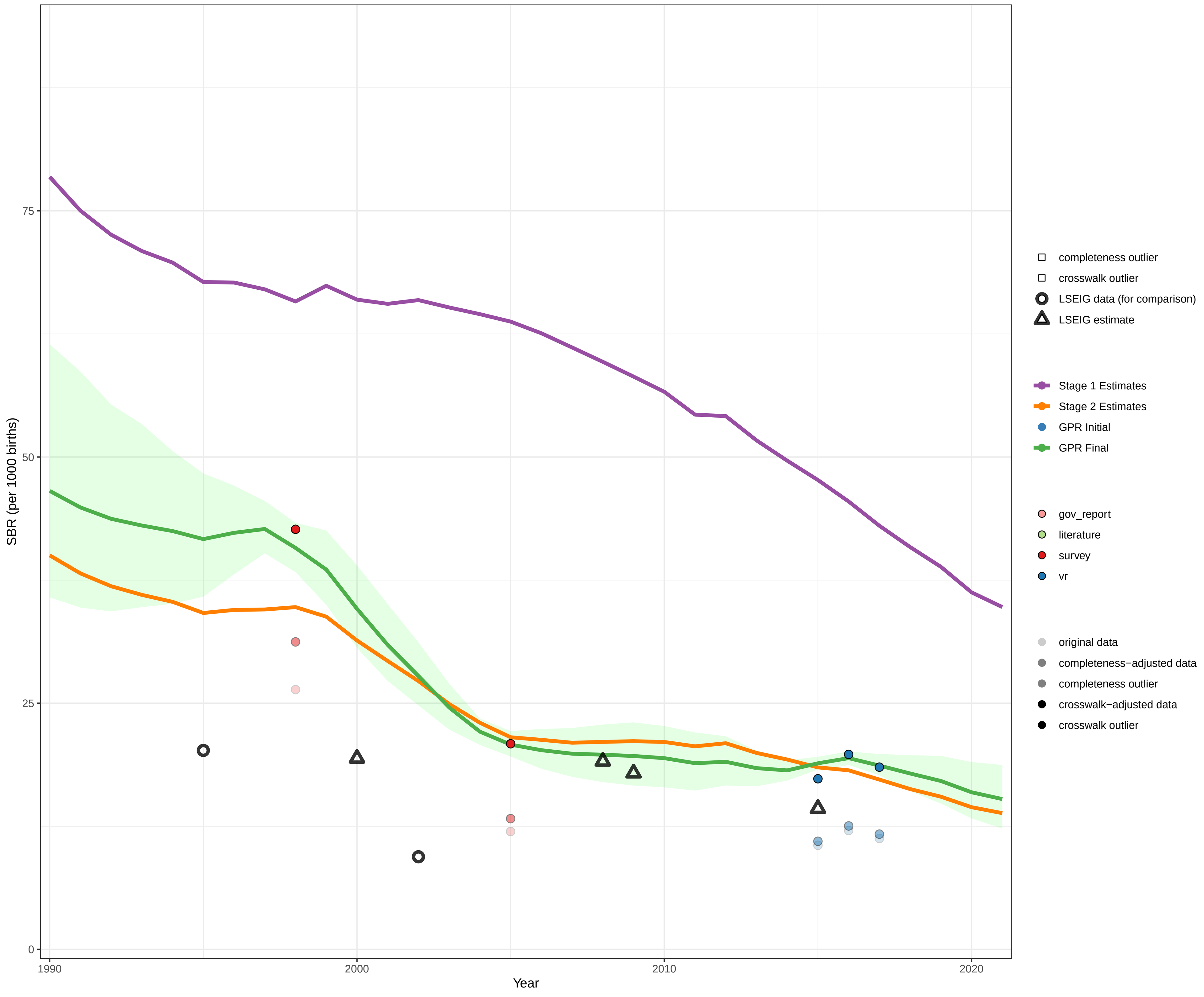

Cabo Verde   CPV   Western Sub−Saharan Africa   Stillbirth Rate (SBR) − 28 weeks
lambda = 0.6 ; zeta = 0.8 ; scale = 15 ; amp = 0.028

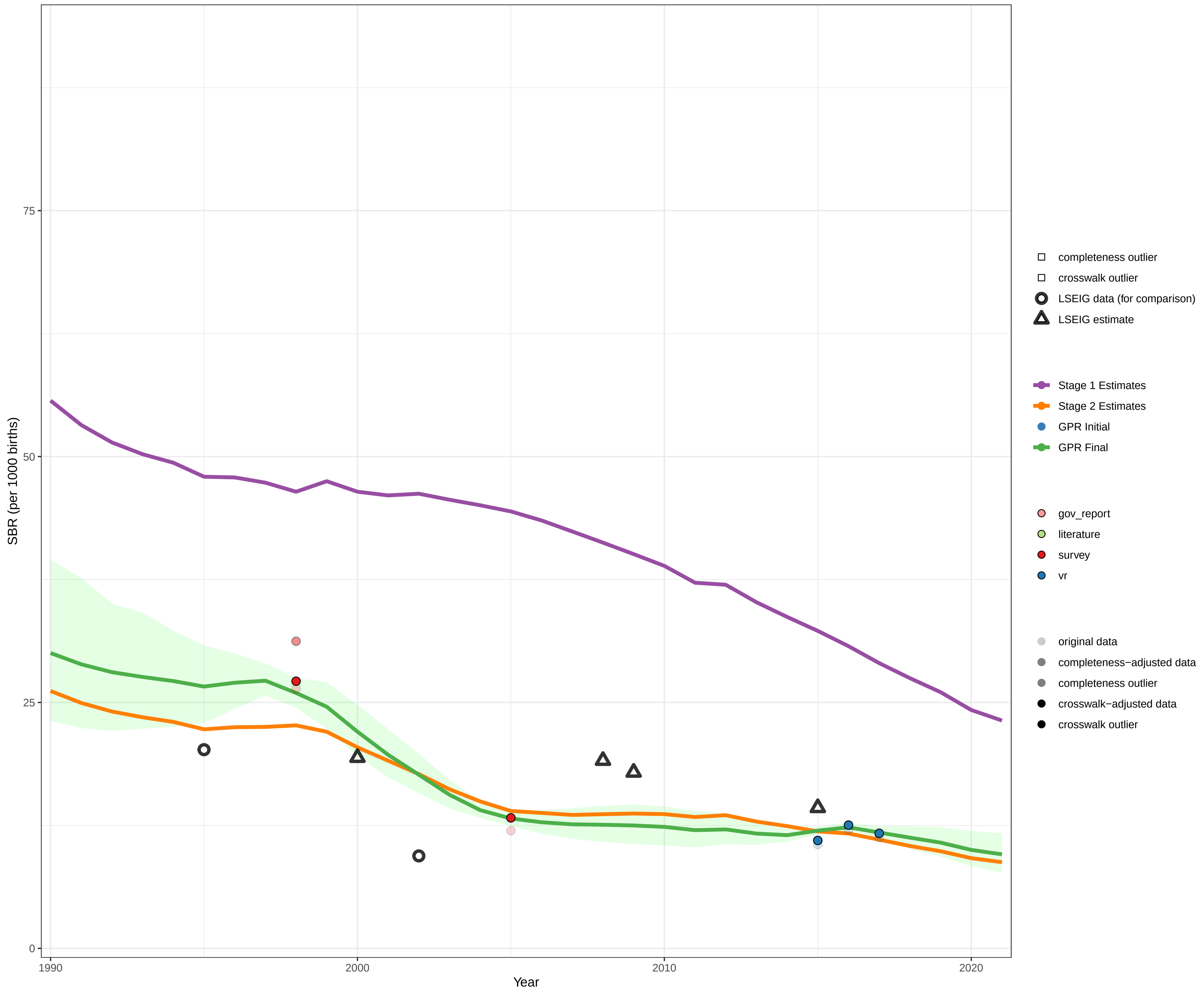

Year	Total Stillbirths (in thousands)	Total Stillbirths (in thousands)	SBR (per 1000 births)	SBR (per 1000 births)	SBR/NMR	SBR/NMR
	20 weeks	28 weeks	20 weeks	28 weeks	20 weeks	28 weeks
1990	0.6 (0.5 to 0.8)	0.4 (0.3 to 0.5)	46.6 (35.7 to 61.4)	30.0 (23.1 to 39.5)	2.33 (1.79 to 3.08)	1.50 (1.16 to 1.98)
1995	0.6 (0.5 to 0.7)	0.4 (0.3 to 0.4)	41.7 (35.8 to 48.3)	26.6 (22.9 to 30.8)	2.34 (2.01 to 2.71)	1.49 (1.29 to 1.73)
2000	0.5 (0.4 to 0.5)	0.3 (0.3 to 0.3)	34.6 (30.6 to 39.0)	22.0 (19.5 to 24.8)	1.94 (1.72 to 2.19)	1.24 (1.10 to 1.39)
2005	0.3 (0.2 to 0.3)	0.2 (0.2 to 0.2)	20.8 (19.6 to 22.2)	13.2 (12.5 to 14.0)	1.21 (1.14 to 1.29)	0.77 (0.73 to 0.82)
2010	0.2 (0.2 to 0.3)	0.1 (0.1 to 0.2)	19.4 (16.4 to 22.7)	12.4 (10.5 to 14.4)	1.30 (1.10 to 1.52)	0.83 (0.70 to 0.97)
2015	0.2 (0.2 to 0.2)	0.1 (0.1 to 0.1)	18.9 (18.3 to 19.6)	12.0 (11.6 to 12.4)	1.53 (1.48 to 1.58)	0.97 (0.94 to 1.00)
2020	0.1 (0.1 to 0.2)	0.1 (0.1 to 0.1)	16.0 (13.3 to 19.0)	10.0 (8.4 to 11.9)	1.69 (1.41 to 2.02)	1.06 (0.89 to 1.27)
2021	0.1 (0.1 to 0.2)	0.1 (0.1 to 0.1)	15.3 (12.3 to 18.7)	9.6 (7.7 to 11.7)	1.69 (1.36 to 2.08)	1.06 (0.86 to 1.30)

Cameroon CMR Western Sub−Saharan Africa Stillbirth Rate / Neonatal Mortality Rate (SBR/NMR) − 20 weeks
lambda = 0.8 ; zeta = 0.7 ; scale = 15 ; amp = 0.028

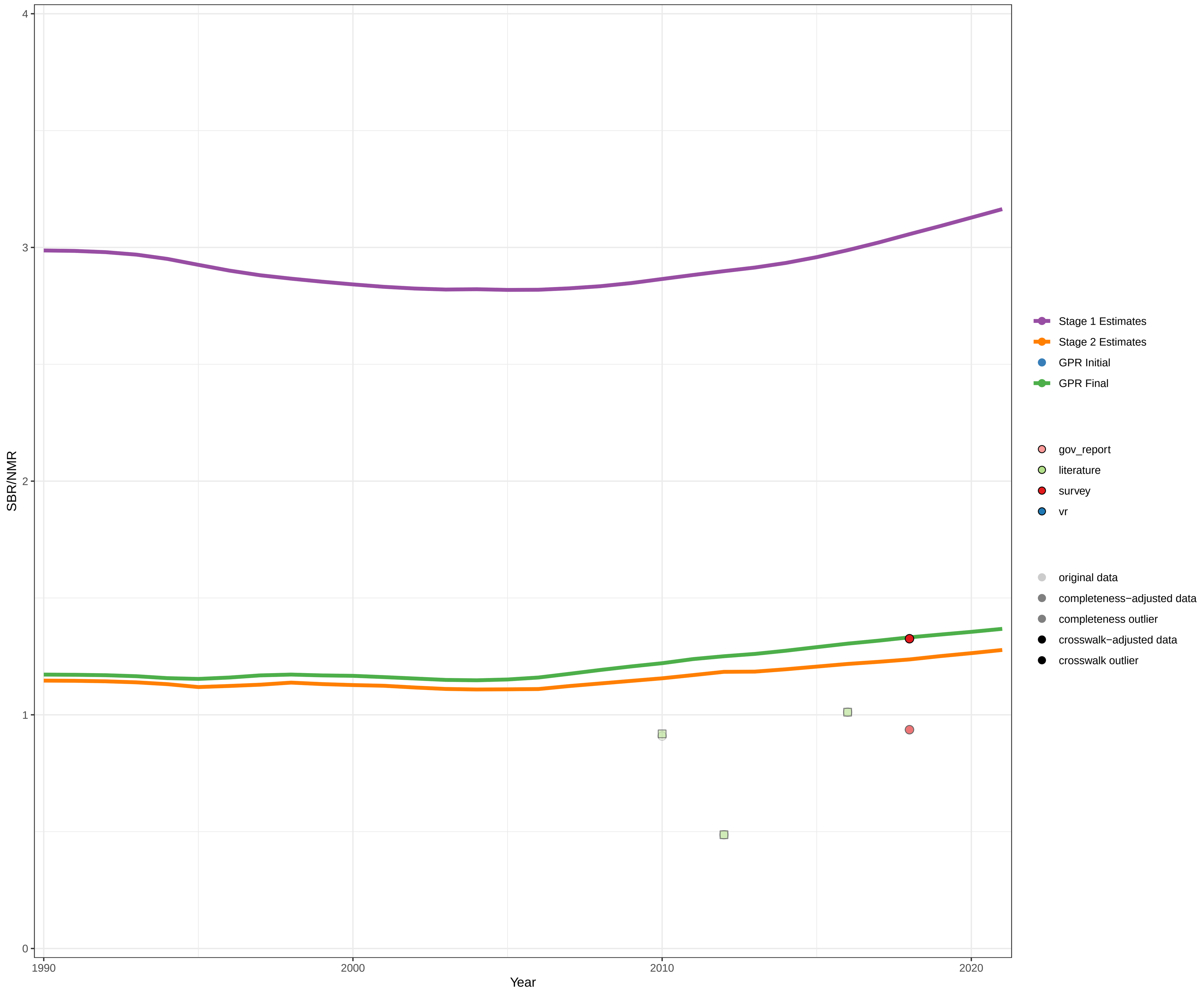

Cameroon CMR Western Sub−Saharan Africa Stillbirth Rate / Neonatal Mortality Rate (SBR/NMR) − 28 weeks
lambda = 0.8 ; zeta = 0.7 ; scale = 15 ; amp = 0.028

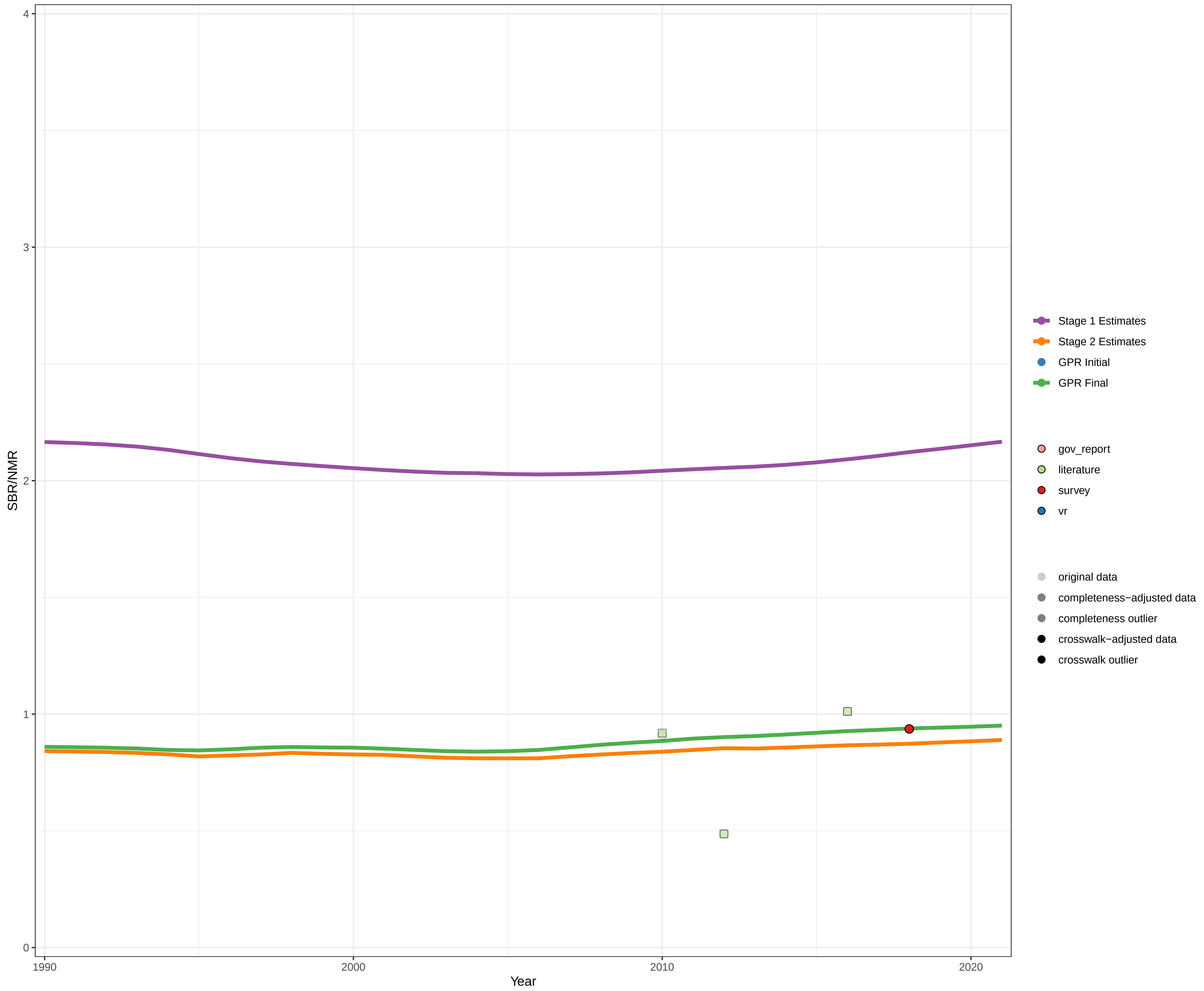

Definition	Years	Outlier	Source.Type	Title
28_weeks	2018	0	Survey	Cameroon Demographic and Health Survey 2018−2019
22_weeks	2012	1	Sci Lit	Low birthweight in rural Cameroon: an analysis of a cut−off value
22_weeks_AND_500_grams	2010	1	Sci Lit	Ten years analysis of stillbirth in a tertiary hospital in sub−Sahara Africa: a case control study
500_grams	2016	1	Sci Lit	Prevalence of stillbirth at the Buea Regional Hospital, Fako Division south−west region, Cameroon

Cameroon CMR Western Sub−Saharan Africa Stillbirth Rate (SBR) − 20 weeks
lambda = 0.8 ; zeta = 0.7 ; scale = 15 ; amp = 0.028

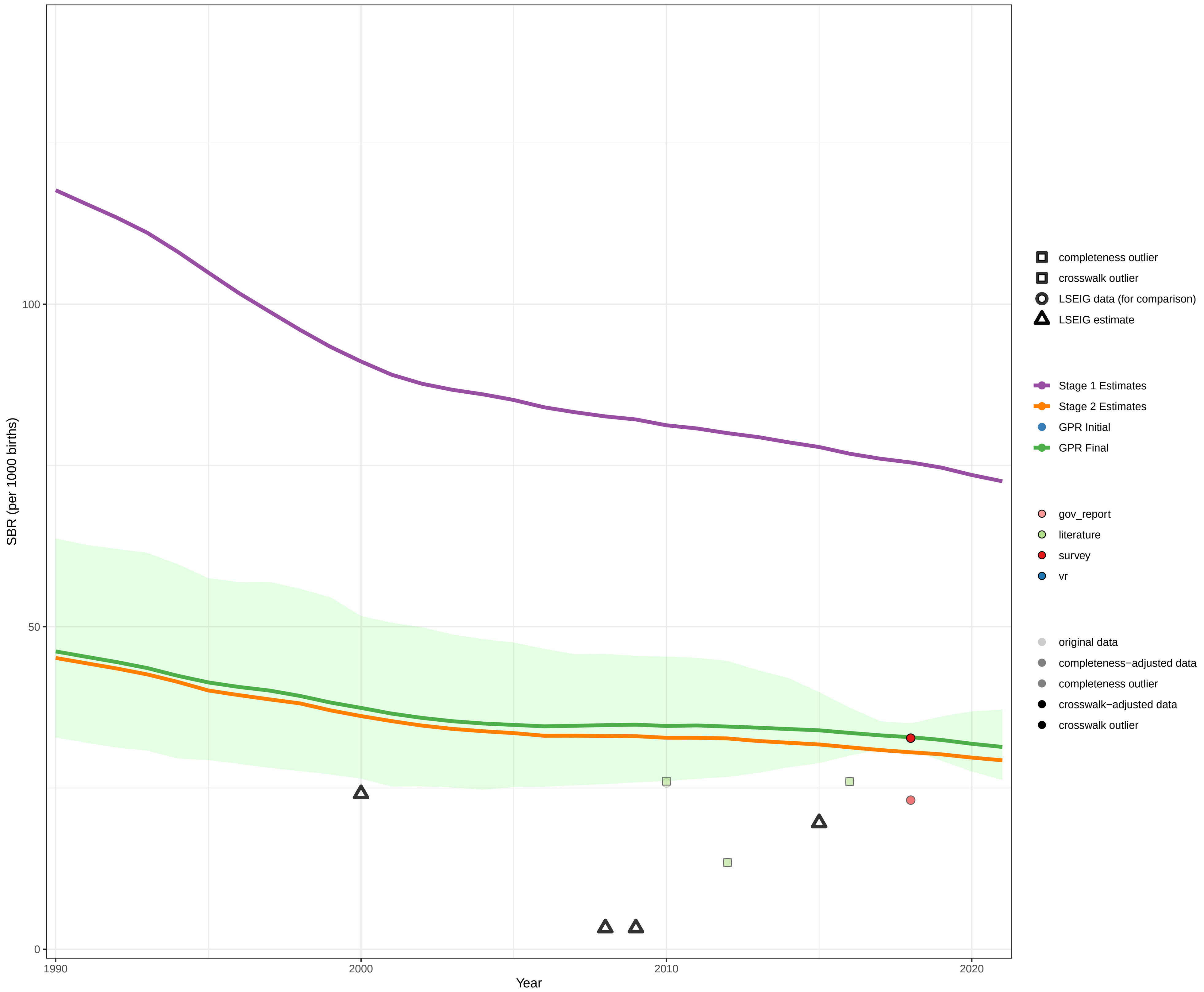

Cameroon CMR Western Sub−Saharan Africa Stillbirth Rate (SBR) − 28 weeks
lambda = 0.8 ; zeta = 0.7 ; scale = 15 ; amp = 0.028

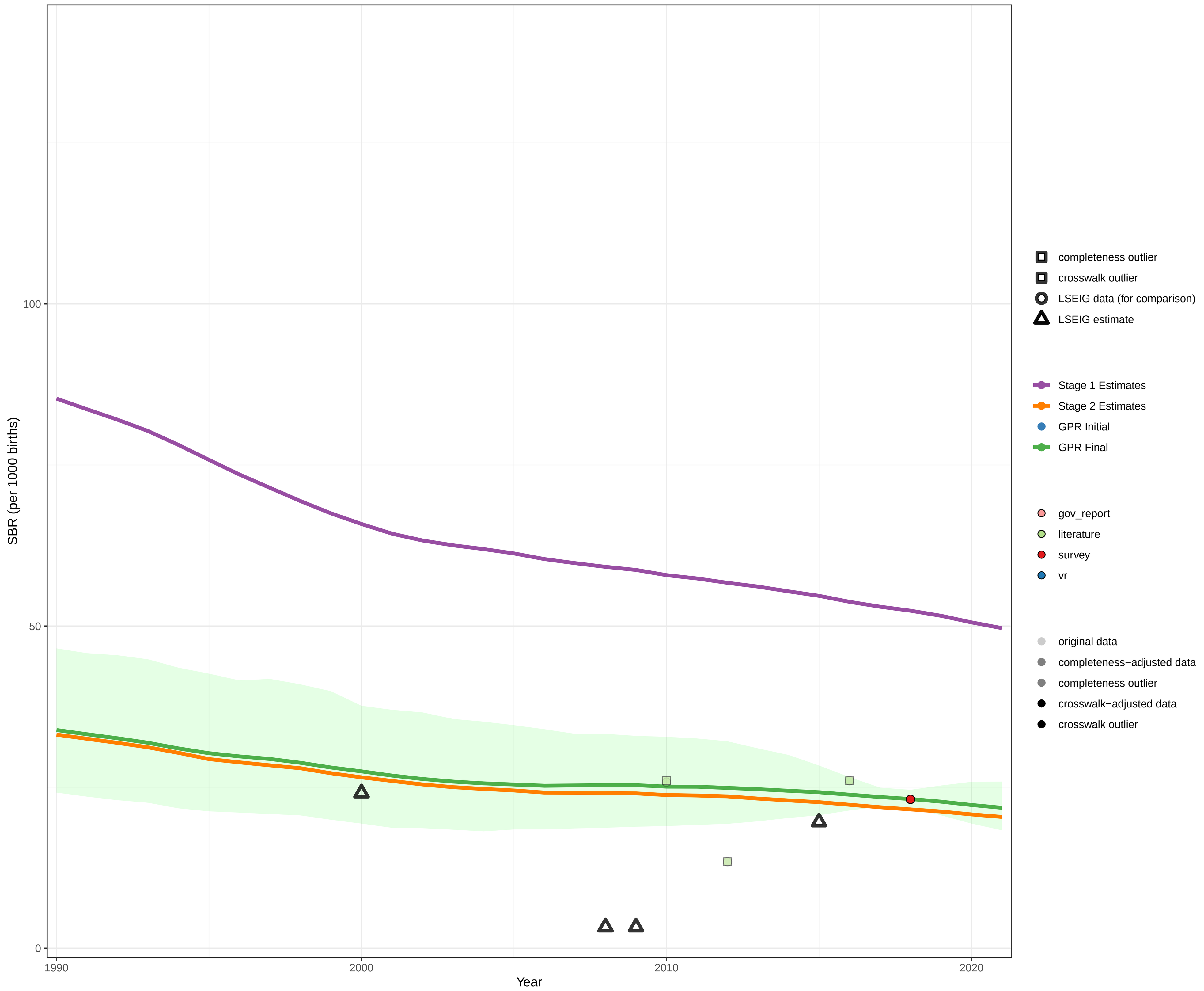

Year	Total Stillbirths (in thousands)	Total Stillbirths (in thousands)	SBR (per 1000 births)	SBR (per 1000 births)	SBR/NMR	SBR/NMR
	20 weeks	28 weeks	20 weeks	28 weeks	20 weeks	28 weeks
1990	23.1 (16.2 to 32.5)	16.7 (11.8 to 23.3)	46.2 (32.8 to 63.7)	33.9 (24.2 to 46.5)	1.17 (0.83 to 1.62)	0.86 (0.61 to 1.18)
1995	23.0 (16.1 to 32.5)	16.7 (11.6 to 23.7)	41.4 (29.3 to 57.5)	30.3 (21.2 to 42.6)	1.15 (0.82 to 1.60)	0.84 (0.59 to 1.19)
2000	24.7 (17.2 to 34.5)	17.9 (12.5 to 24.8)	37.4 (26.5 to 51.7)	27.5 (19.3 to 37.6)	1.17 (0.82 to 1.61)	0.86 (0.60 to 1.17)
2005	27.9 (19.9 to 38.6)	20.2 (14.5 to 27.7)	34.8 (25.1 to 47.6)	25.4 (18.4 to 34.6)	1.15 (0.83 to 1.57)	0.84 (0.61 to 1.15)
2010	32.5 (24.3 to 43.1)	23.3 (17.5 to 30.7)	34.6 (26.1 to 45.4)	25.1 (19.0 to 32.8)	1.22 (0.92 to 1.60)	0.88 (0.67 to 1.16)
2015	35.0 (29.6 to 41.4)	24.7 (21.0 to 29.1)	33.9 (28.9 to 39.9)	24.2 (20.6 to 28.4)	1.29 (1.10 to 1.51)	0.92 (0.78 to 1.08)
2020	33.8 (29.1 to 39.3)	23.4 (20.3 to 27.2)	31.9 (27.6 to 36.9)	22.2 (19.4 to 25.8)	1.36 (1.17 to 1.57)	0.95 (0.82 to 1.10)
2021	33.4 (27.8 to 39.8)	23.0 (19.3 to 27.4)	31.4 (26.2 to 37.2)	21.8 (18.3 to 25.9)	1.37 (1.14 to 1.62)	0.95 (0.80 to 1.13)

Chad TCD Western Sub−Saharan Africa Stillbirth Rate / Neonatal Mortality Rate (SBR/NMR) − 20 weeks
lambda = 1 ; zeta = 0.6 ; scale = 15 ; amp = 0.028

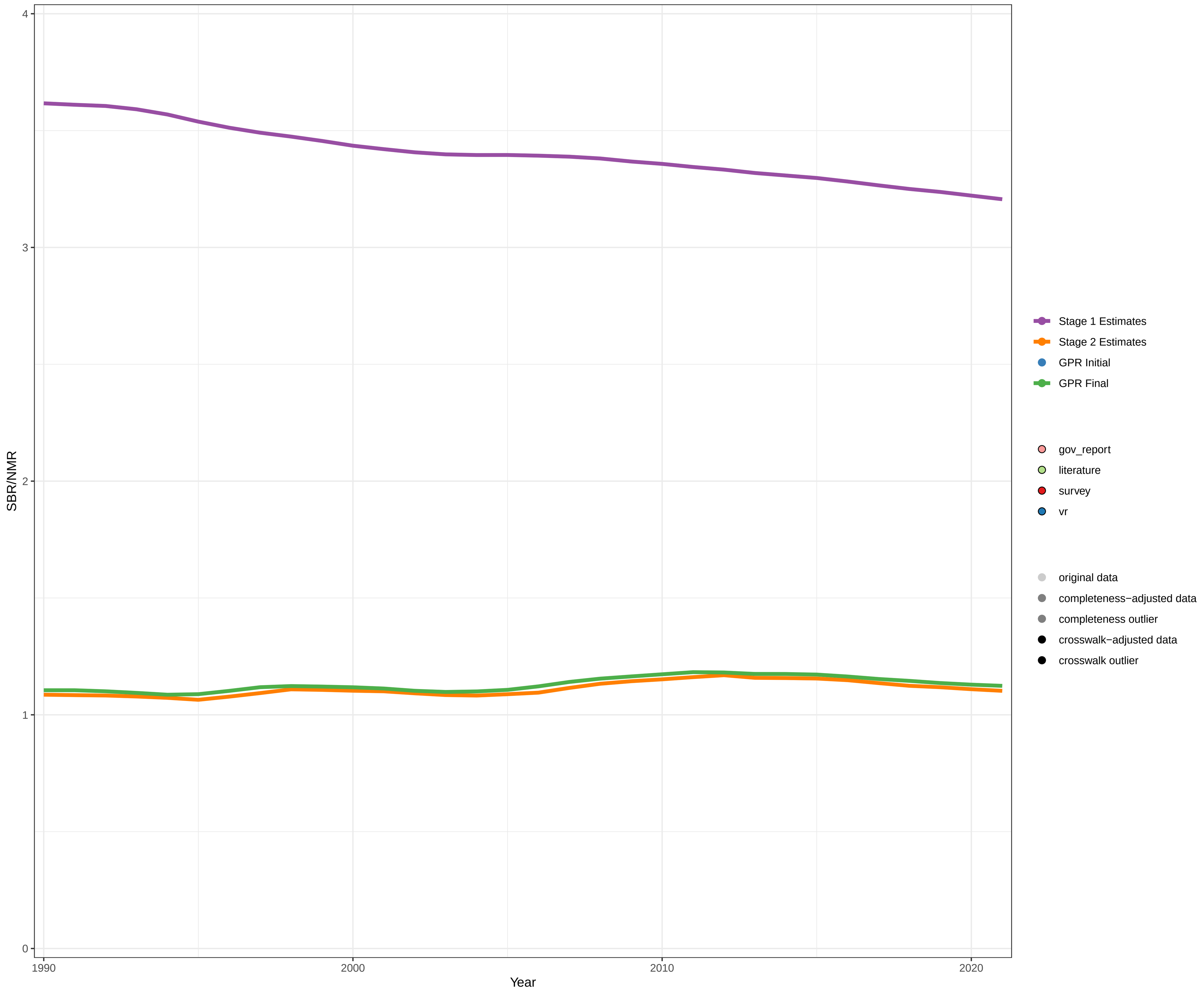

Chad TCD Western Sub−Saharan Africa Stillbirth Rate / Neonatal Mortality Rate (SBR/NMR) − 28 weeks
lambda = 1 ; zeta = 0.6 ; scale = 15 ; amp = 0.028

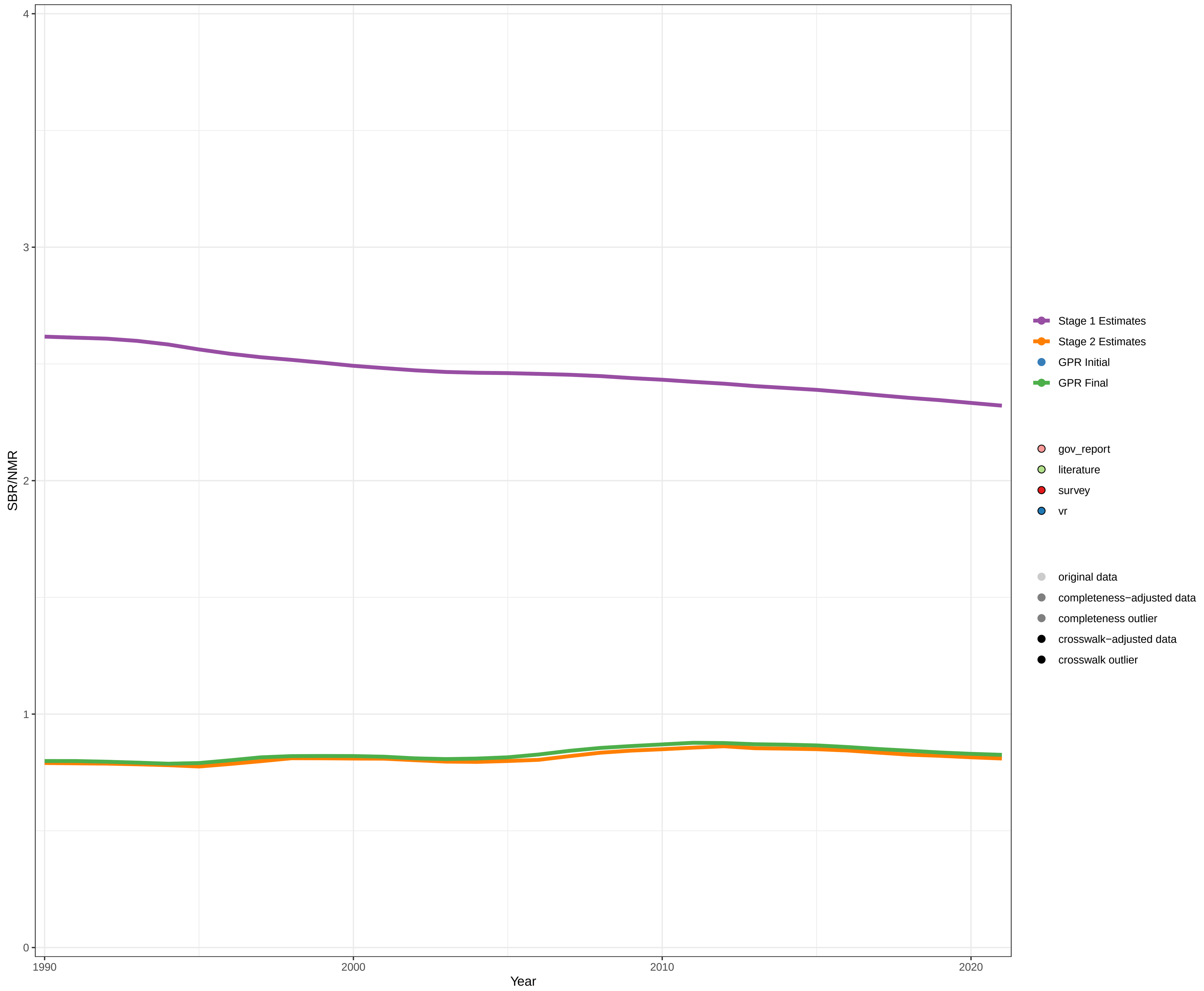

Definition	Years	Outlier	Source.Type	Title

Chad TCD Western Sub−Saharan Africa Stillbirth Rate (SBR) − 20 weeks
lambda = 1 ; zeta = 0.6 ; scale = 15 ; amp = 0.028

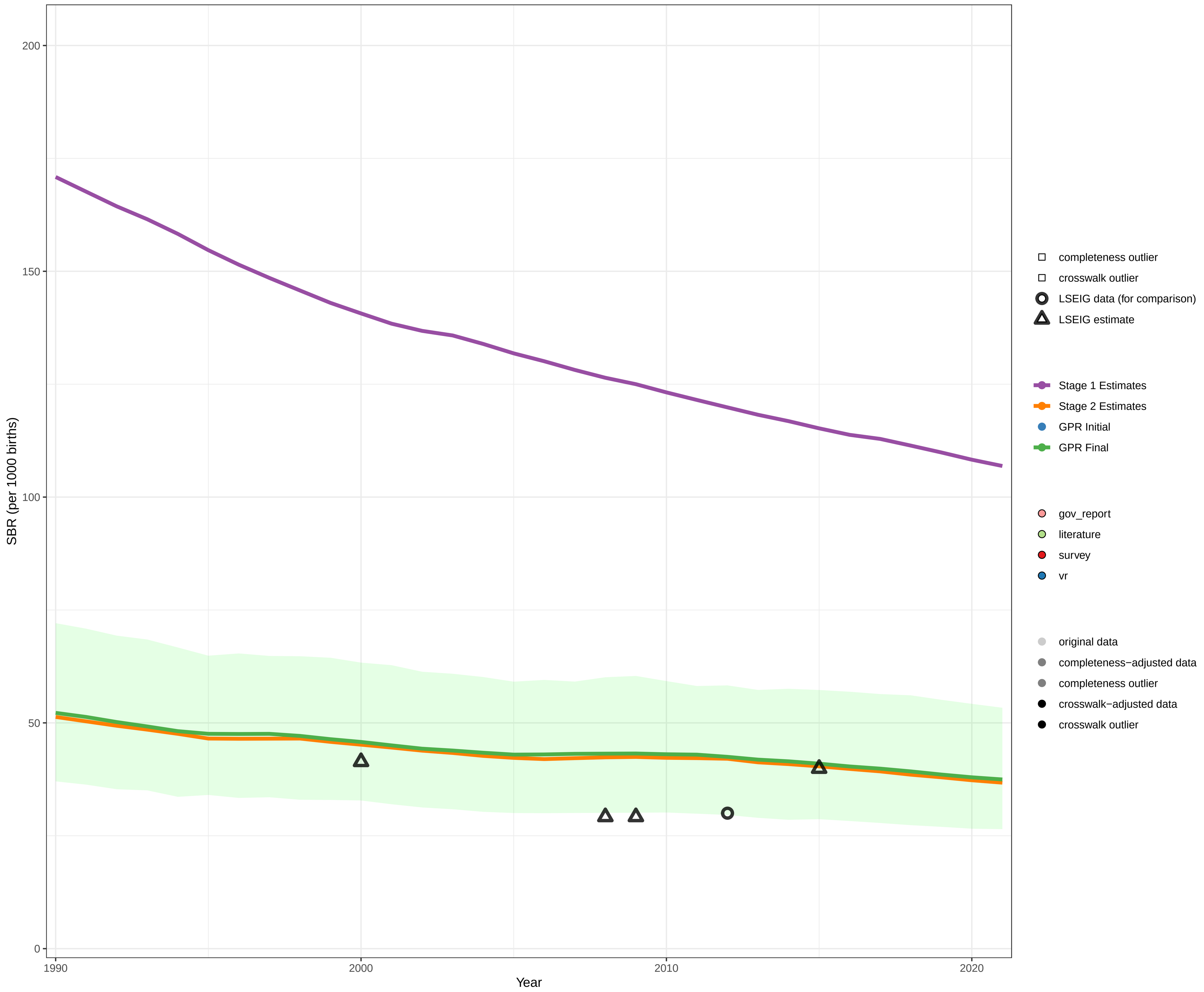

Chad TCD Western Sub−Saharan Africa Stillbirth Rate (SBR) − 28 weeks
lambda = 1 ; zeta = 0.6 ; scale = 15 ; amp = 0.028

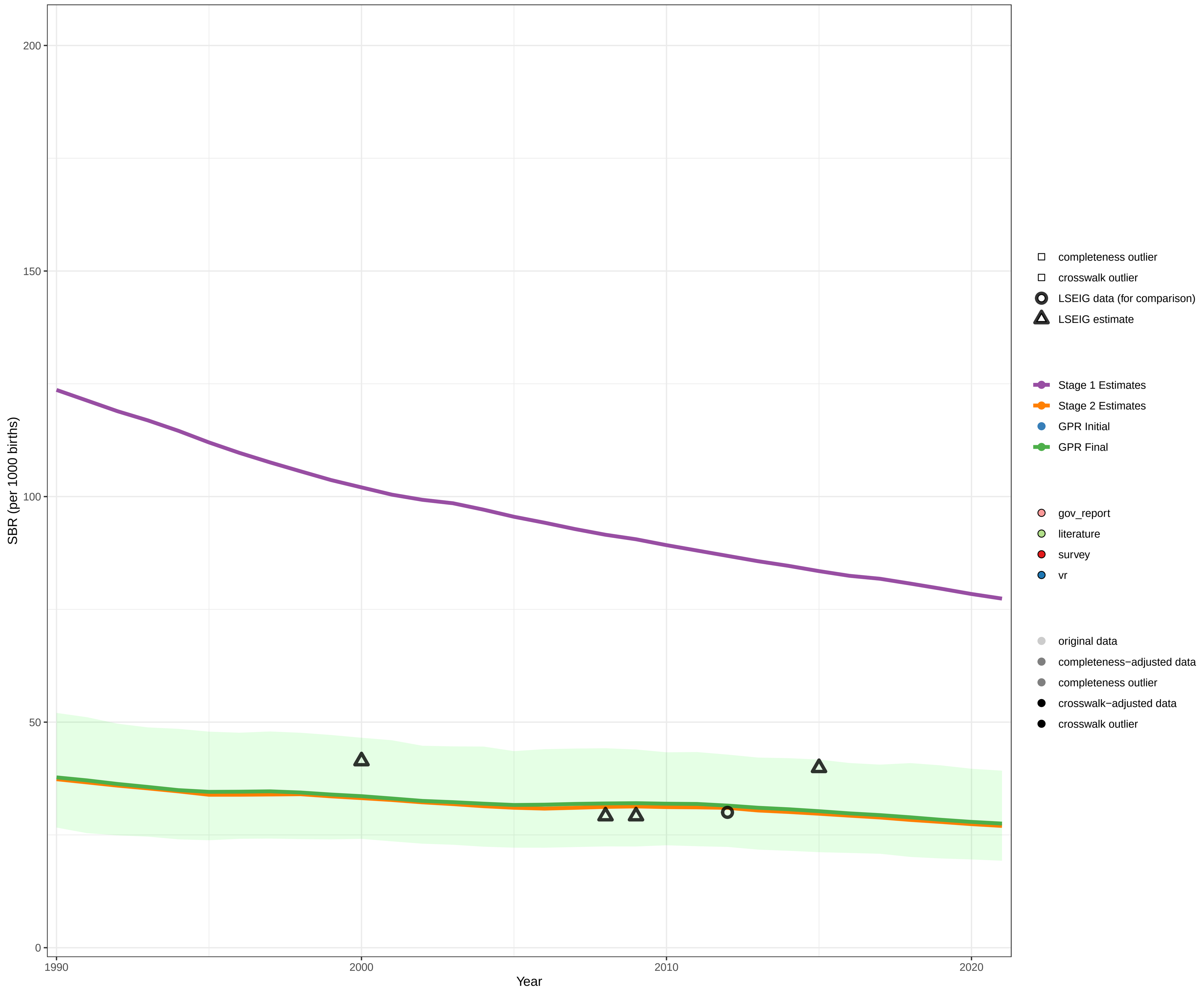

Year	Total Stillbirths (in thousands)	Total Stillbirths (in thousands)	SBR (per 1000 births)	SBR (per 1000 births)	SBR/NMR	SBR/NMR
	20 weeks	28 weeks	20 weeks	28 weeks	20 weeks	28 weeks
1990	17.8 (12.4 to 25.0)	12.6 (8.8 to 17.7)	52.2 (37.0 to 72.1)	37.8 (26.6 to 52.0)	1.11 (0.78 to 1.53)	0.80 (0.56 to 1.10)
1995	18.7 (13.2 to 25.9)	13.4 (9.1 to 18.8)	47.6 (34.0 to 64.9)	34.6 (23.8 to 47.9)	1.09 (0.78 to 1.48)	0.79 (0.54 to 1.10)
2000	21.1 (14.9 to 29.6)	15.3 (10.8 to 21.4)	45.8 (32.8 to 63.3)	33.6 (24.1 to 46.5)	1.12 (0.80 to 1.55)	0.82 (0.59 to 1.14)
2005	23.1 (15.9 to 32.2)	16.8 (11.6 to 23.4)	43.0 (30.0 to 59.1)	31.6 (22.2 to 43.6)	1.11 (0.77 to 1.52)	0.81 (0.57 to 1.12)
2010	27.1 (18.7 to 37.8)	19.8 (14.0 to 27.2)	43.1 (30.1 to 59.3)	31.9 (22.7 to 43.3)	1.17 (0.82 to 1.62)	0.87 (0.62 to 1.18)
2015	30.1 (20.8 to 42.8)	22.0 (15.2 to 30.6)	41.0 (28.7 to 57.3)	30.3 (21.2 to 41.7)	1.17 (0.82 to 1.64)	0.87 (0.61 to 1.19)
2020	32.8 (22.7 to 47.6)	23.9 (16.6 to 34.3)	37.9 (26.6 to 54.2)	27.9 (19.6 to 39.7)	1.13 (0.79 to 1.61)	0.83 (0.58 to 1.18)
2021	33.5 (23.4 to 48.5)	24.4 (16.9 to 35.1)	37.5 (26.5 to 53.4)	27.5 (19.3 to 39.2)	1.12 (0.79 to 1.60)	0.83 (0.58 to 1.18)

Cote d'Ivoire CIV Western Sub−Saharan Africa Stillbirth Rate / Neonatal Mortality Rate (SBR/NMR) − 20 weeks
lambda = 0.8 ; zeta = 0.7 ; scale = 15 ; amp = 0.028

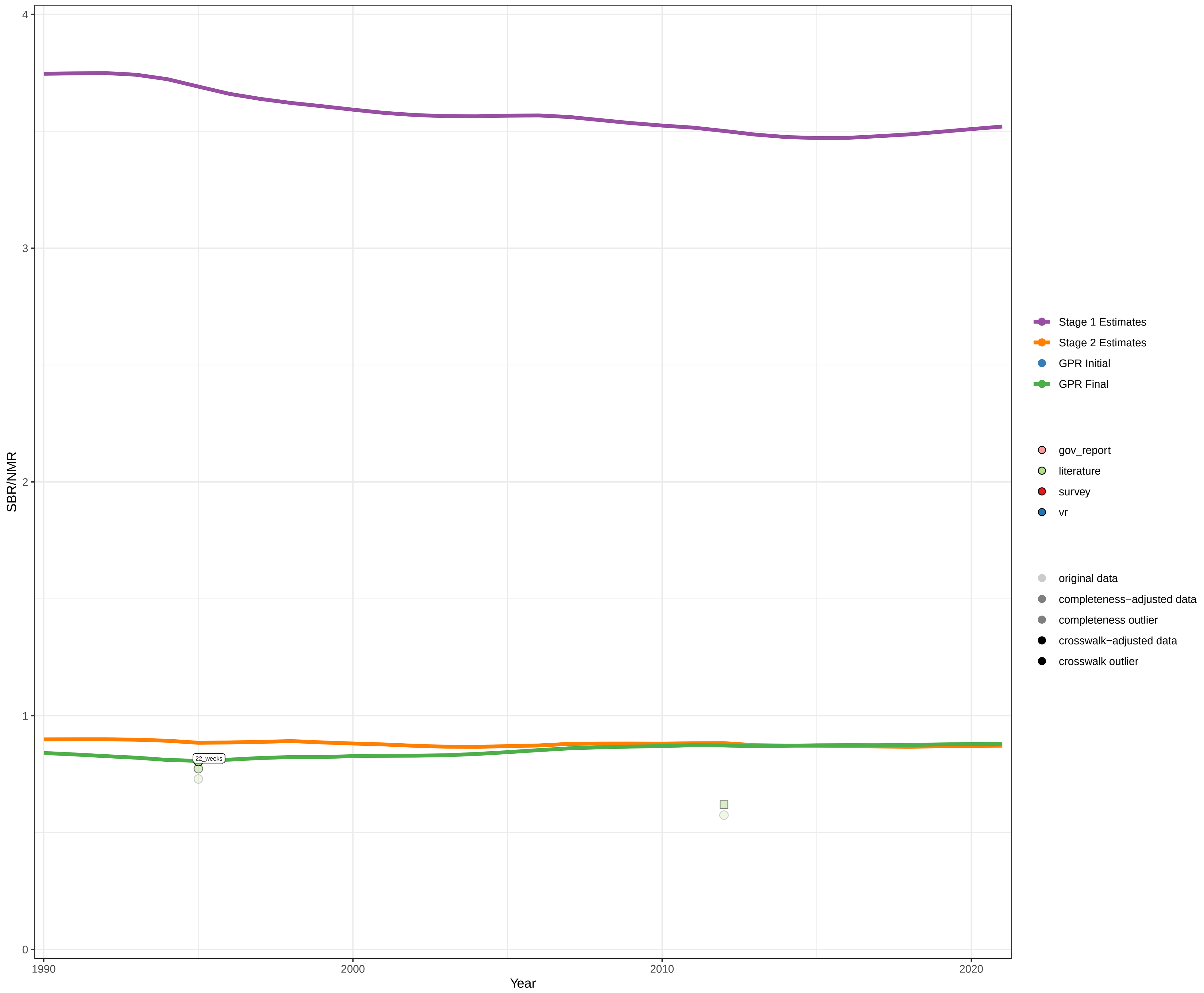

Cote d'Ivoire CIV Western Sub−Saharan Africa Stillbirth Rate / Neonatal Mortality Rate (SBR/NMR) − 28 weeks
lambda = 0.8 ; zeta = 0.7 ; scale = 15 ; amp = 0.028

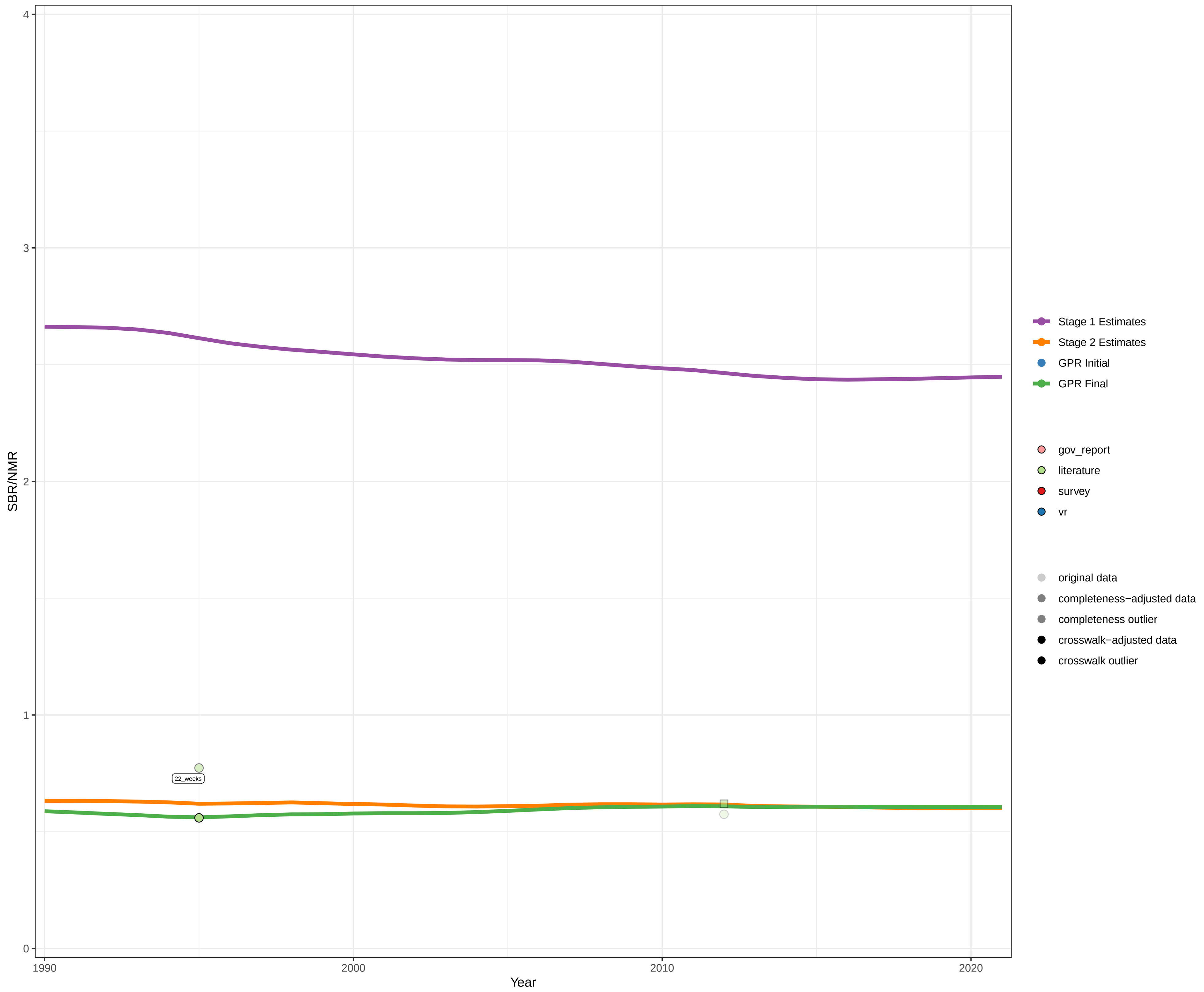

Definition	Years	Outlier	Source.Type	Title
22_weeks	1995	0	Sci Lit	Risk factors for perinatal mortality in West Africa: a population−based study of 20326 pregnancies. MOMA group
28_weeks	2012	1	Sci Lit	Cote d'Ivoire

Cote d'Ivoire CIV Western Sub−Saharan Africa Stillbirth Rate (SBR) − 20 weeks
lambda = 0.8 ; zeta = 0.7 ; scale = 15 ; amp = 0.028

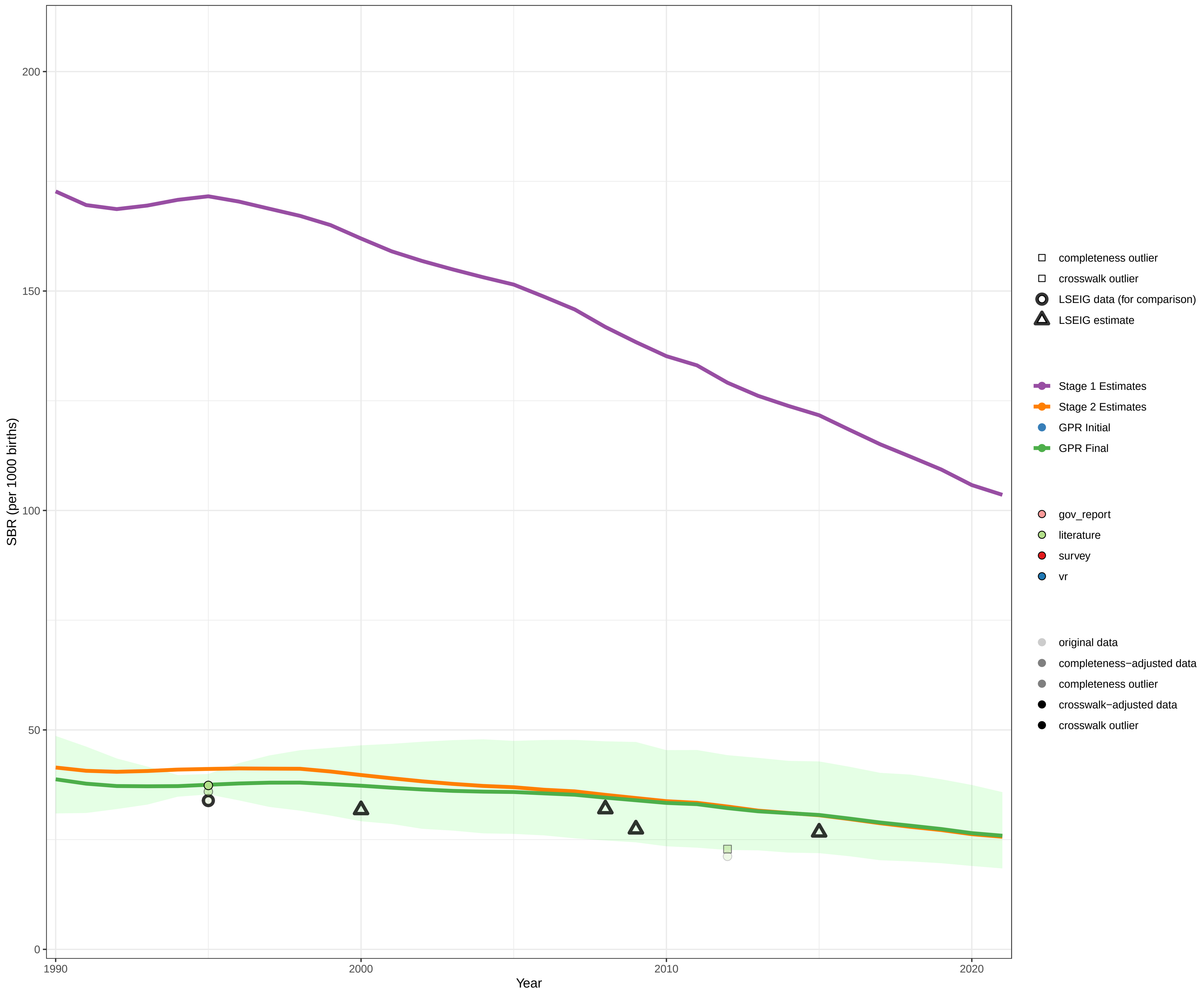

Cote d'Ivoire CIV Western Sub−Saharan Africa Stillbirth Rate (SBR) − 28 weeks
lambda = 0.8 ; zeta = 0.7 ; scale = 15 ; amp = 0.028

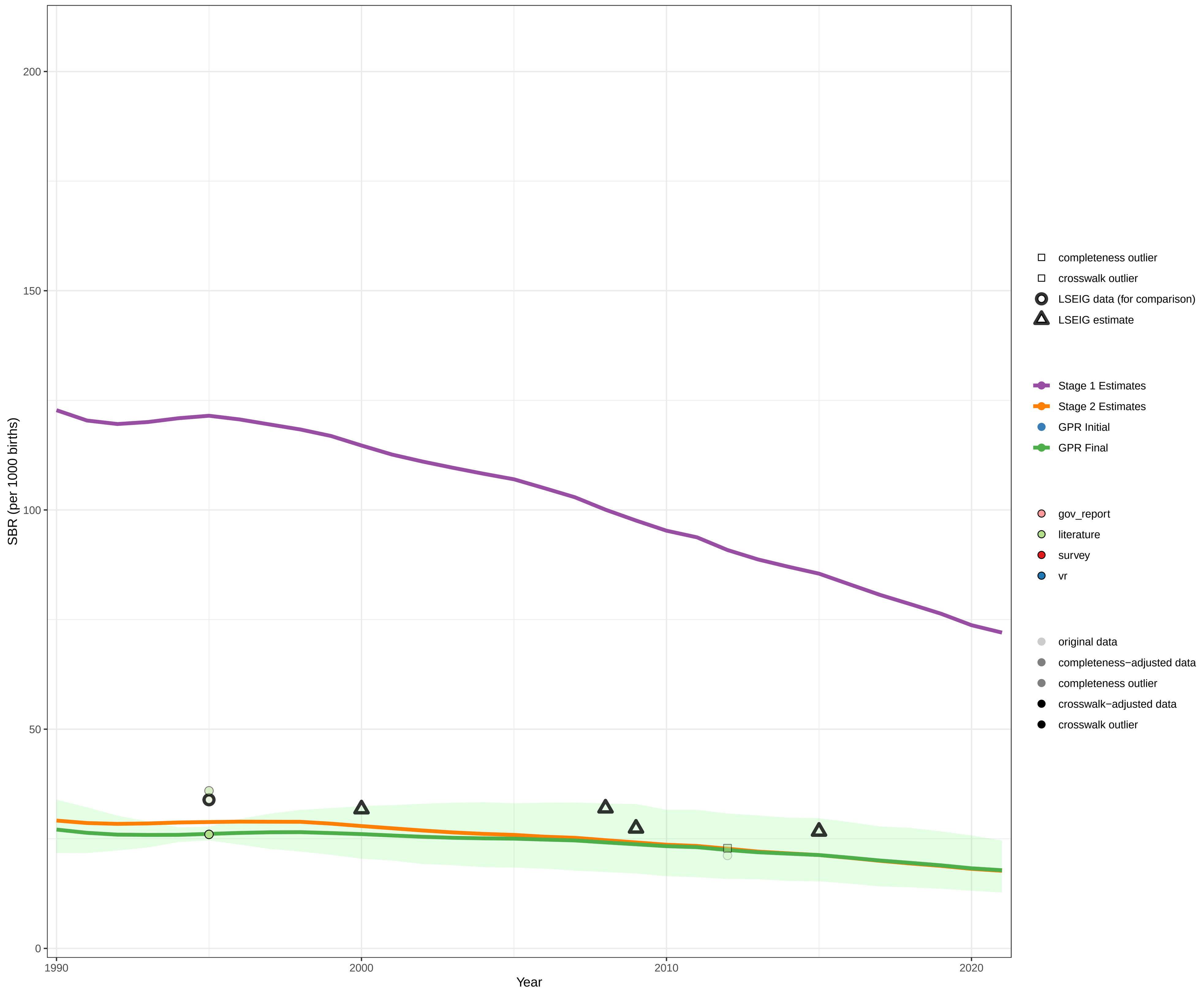

Year	Total Stillbirths (in thousands)	Total Stillbirths (in thousands)	SBR (per 1000 births)	SBR (per 1000 births)	SBR/NMR	SBR/NMR
	20 weeks	28 weeks	20 weeks	28 weeks	20 weeks	28 weeks
1990	22.3 (17.7 to 28.3)	15.4 (12.3 to 19.4)	38.8 (31.0 to 48.6)	27.1 (21.7 to 33.9)	0.84 (0.67 to 1.05)	0.59 (0.47 to 0.74)
1995	23.9 (22.4 to 25.5)	16.4 (15.5 to 17.5)	37.5 (35.3 to 40.0)	26.1 (24.6 to 27.8)	0.81 (0.76 to 0.86)	0.56 (0.53 to 0.60)
2000	26.7 (20.7 to 33.6)	18.4 (14.4 to 23.1)	37.3 (29.2 to 46.5)	26.1 (20.5 to 32.4)	0.83 (0.65 to 1.03)	0.58 (0.45 to 0.72)
2005	28.4 (20.6 to 38.1)	19.6 (14.3 to 26.1)	35.8 (26.3 to 47.5)	25.0 (18.4 to 33.1)	0.84 (0.62 to 1.12)	0.59 (0.43 to 0.78)
2010	29.4 (20.4 to 40.4)	20.3 (14.2 to 27.8)	33.4 (23.5 to 45.4)	23.3 (16.5 to 31.6)	0.87 (0.61 to 1.18)	0.61 (0.43 to 0.82)
2015	28.9 (20.5 to 40.9)	19.9 (14.2 to 28.0)	30.6 (21.9 to 42.8)	21.3 (15.3 to 29.7)	0.87 (0.63 to 1.22)	0.61 (0.44 to 0.85)
2020	25.7 (18.3 to 36.8)	17.6 (12.6 to 25.0)	26.5 (19.0 to 37.5)	18.3 (13.2 to 25.8)	0.88 (0.63 to 1.24)	0.61 (0.44 to 0.85)
2021	25.3 (17.8 to 35.3)	17.3 (12.3 to 24.0)	25.9 (18.4 to 35.8)	17.8 (12.7 to 24.6)	0.88 (0.63 to 1.22)	0.61 (0.43 to 0.84)

Gambia GMB Western Sub−Saharan Africa Stillbirth Rate / Neonatal Mortality Rate (SBR/NMR) − 20 weeks
lambda = 0.6 ; zeta = 0.8 ; scale = 15 ; amp = 0.028

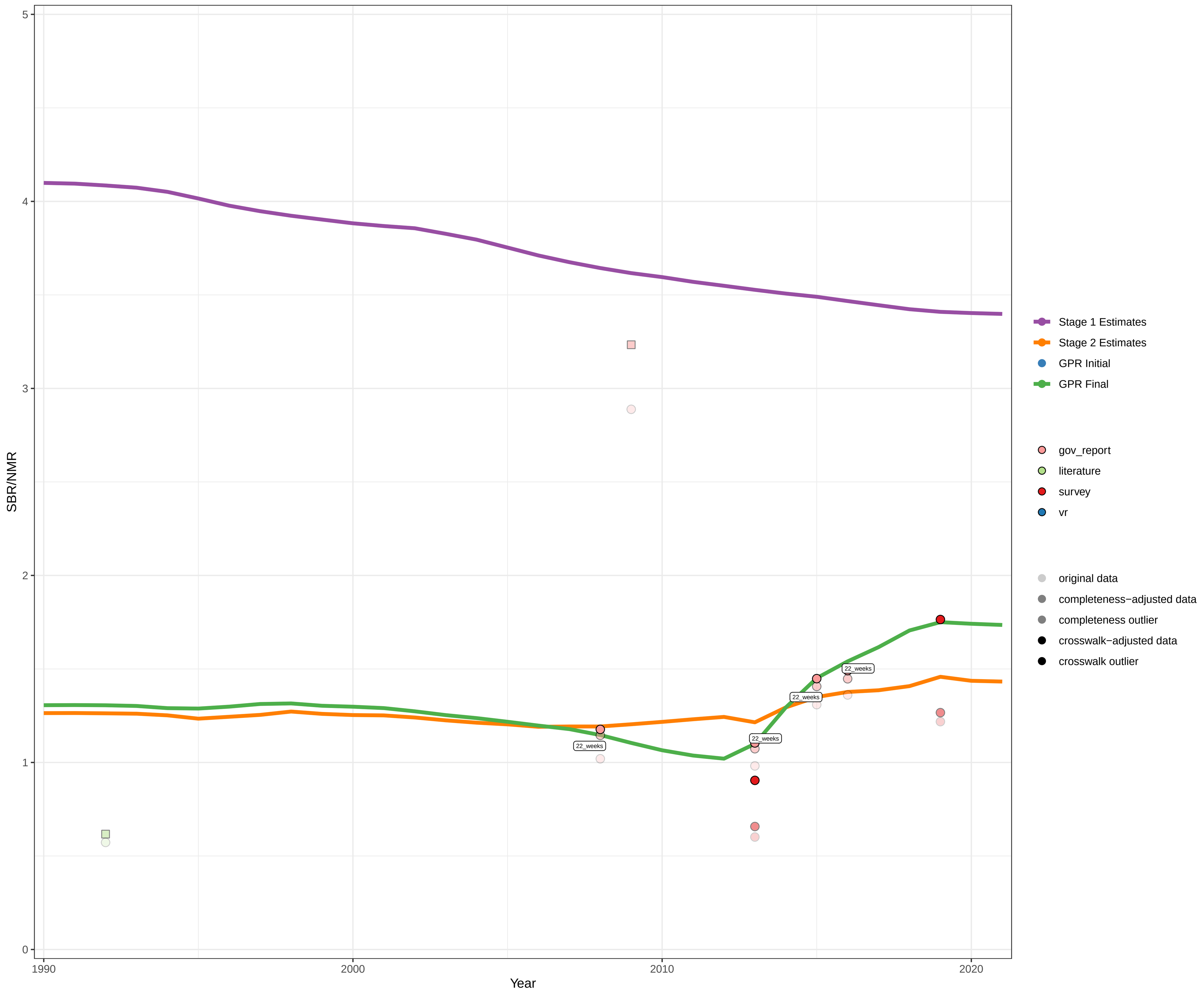

Gambia GMB Western Sub−Saharan Africa Stillbirth Rate / Neonatal Mortality Rate (SBR/NMR) − 28 weeks
lambda = 0.6 ; zeta = 0.8 ; scale = 15 ; amp = 0.028

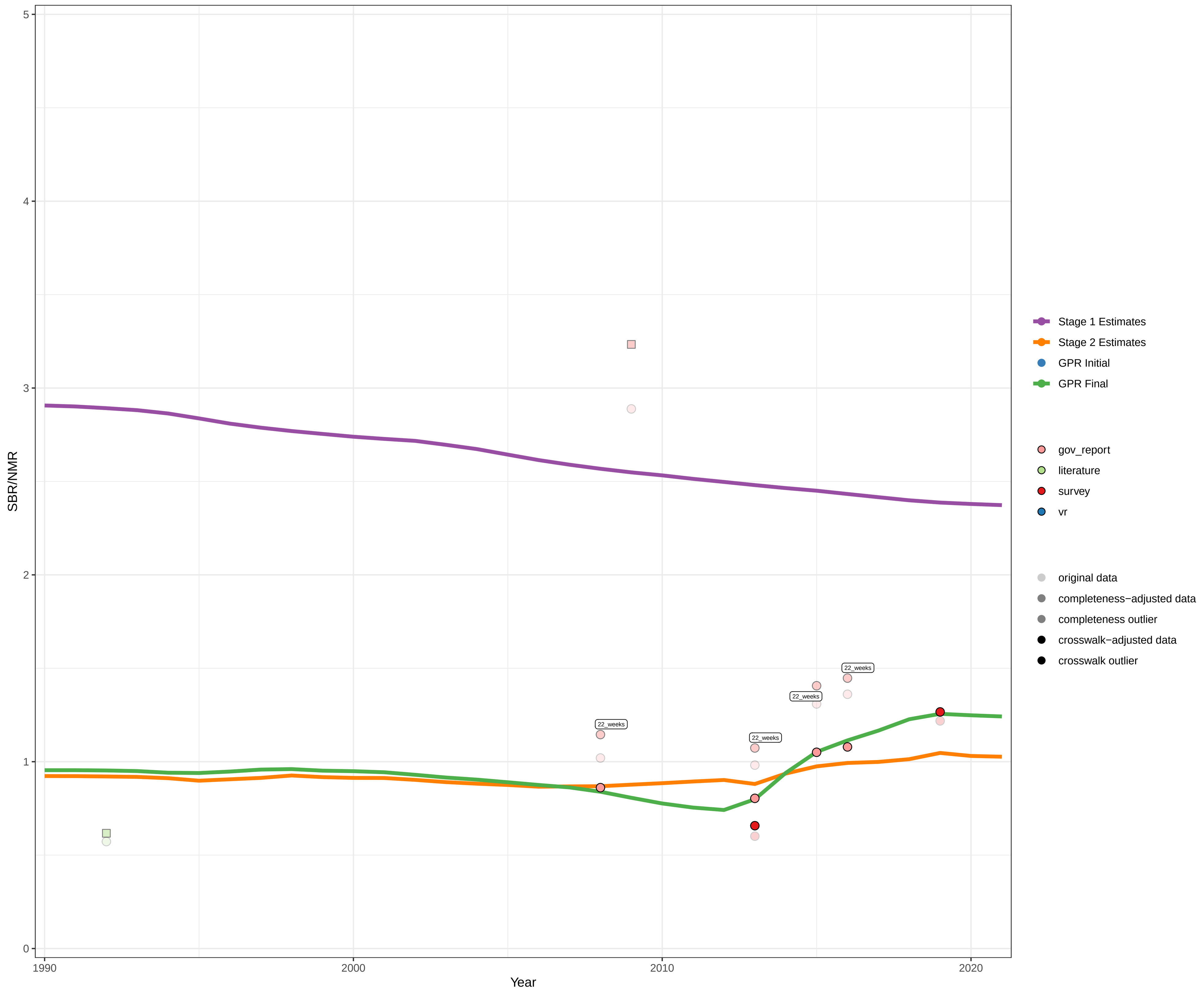

Definition	Years	Outlier	Source.Type	Title
22_weeks	2008, 2013	0	Statistical Report	Gambia Service Statistics Report
22_weeks	2015, 2016	0	Statistical Report	Gambia Statistical Abstract
28_weeks	2013	0	Survey	Gambia Demographic and Health Survey 2013
28_weeks	2019	0	Survey	Gambia Demographic and Health Survey 2019−2020
22_weeks	2009	1	Statistical Report	Gambia Service Statistics Report
28_weeks	1992	1	Sci Lit	Effects on birth weight and perinatal mortality of maternal dietary supplements in rural Gambia: 5 year randomised controlled trial

Gambia GMB Western Sub−Saharan Africa Stillbirth Rate (SBR) − 20 weeks
lambda = 0.6 ; zeta = 0.8 ; scale = 15 ; amp = 0.028

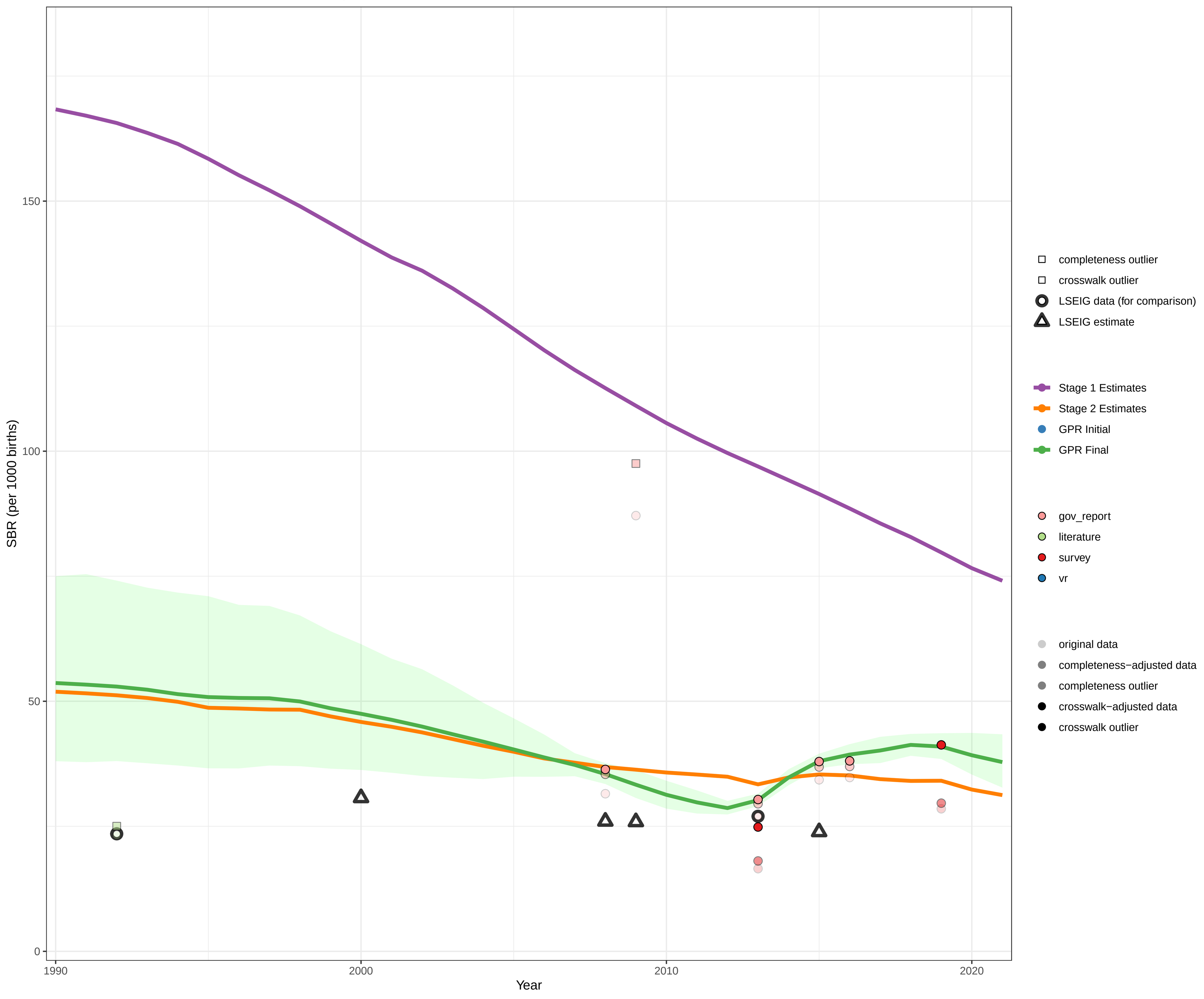

Gambia GMB Western Sub−Saharan Africa Stillbirth Rate (SBR) − 28 weeks
lambda = 0.6 ; zeta = 0.8 ; scale = 15 ; amp = 0.028

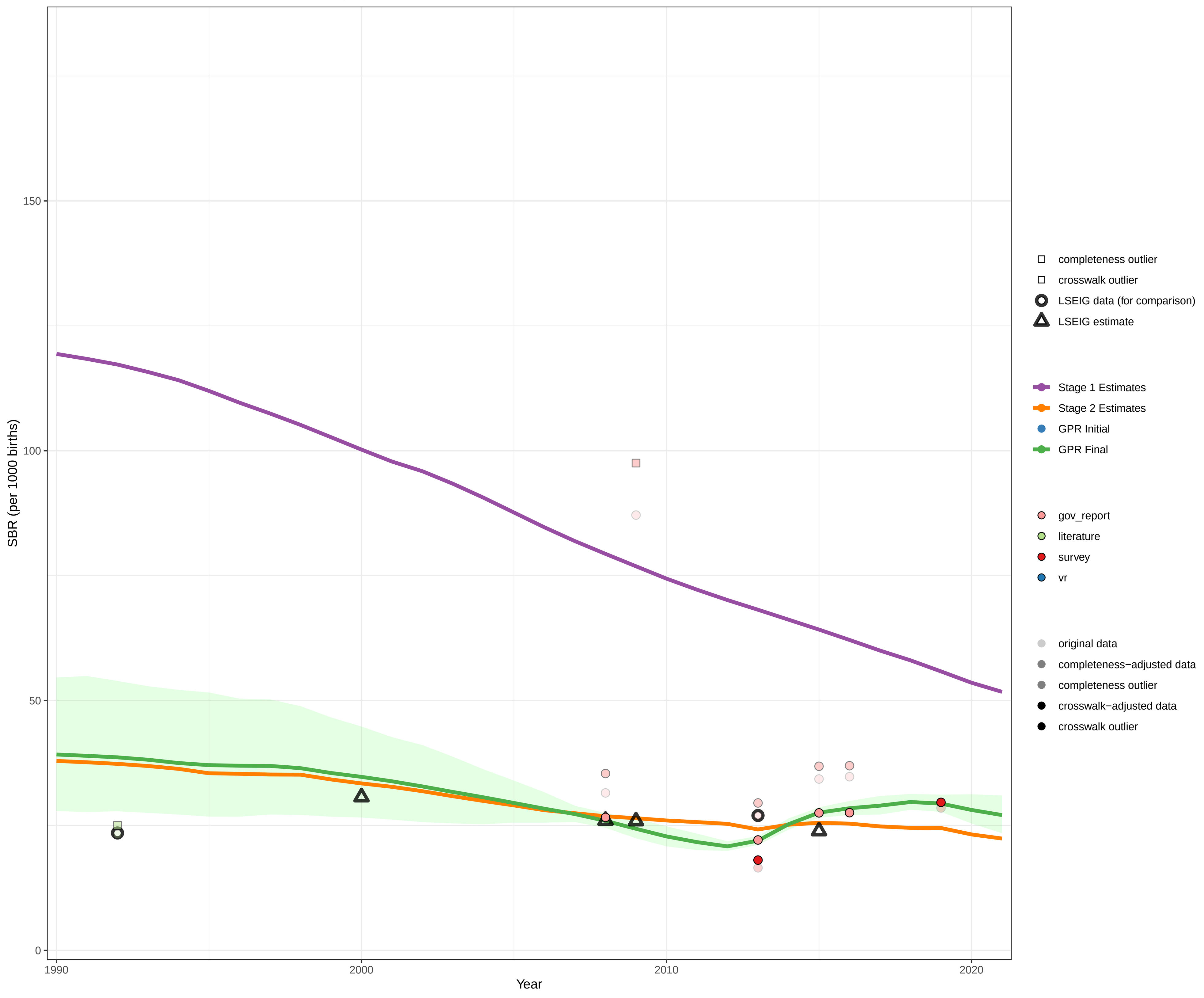

Year	Total Stillbirths (in thousands)	Total Stillbirths (in thousands)	SBR (per 1000 births)	SBR (per 1000 births)	SBR/NMR	SBR/NMR
	20 weeks	28 weeks	20 weeks	28 weeks	20 weeks	28 weeks
1990	2.6 (1.8 to 3.8)	1.9 (1.3 to 2.7)	53.6 (38.0 to 75.0)	39.2 (27.9 to 54.6)	1.31 (0.93 to 1.83)	0.95 (0.68 to 1.33)
1995	2.9 (2.1 to 4.2)	2.1 (1.5 to 3.0)	50.8 (36.6 to 71.0)	37.1 (26.8 to 51.6)	1.29 (0.93 to 1.80)	0.94 (0.68 to 1.31)
2000	3.1 (2.3 to 4.0)	2.2 (1.7 to 2.9)	47.5 (36.3 to 61.4)	34.7 (26.6 to 44.8)	1.30 (0.99 to 1.68)	0.95 (0.73 to 1.22)
2005	2.8 (2.4 to 3.3)	2.1 (1.8 to 2.4)	40.4 (34.9 to 46.6)	29.5 (25.6 to 34.0)	1.22 (1.05 to 1.41)	0.89 (0.77 to 1.03)
2010	2.4 (2.2 to 2.6)	1.7 (1.6 to 1.9)	31.3 (28.5 to 34.1)	22.8 (20.8 to 24.8)	1.07 (0.97 to 1.16)	0.78 (0.71 to 0.85)
2015	3.0 (2.9 to 3.1)	2.2 (2.1 to 2.2)	38.0 (36.6 to 39.5)	27.6 (26.6 to 28.6)	1.45 (1.40 to 1.51)	1.05 (1.01 to 1.09)
2020	3.1 (2.8 to 3.5)	2.2 (2.0 to 2.5)	39.2 (35.3 to 43.7)	28.1 (25.4 to 31.2)	1.74 (1.57 to 1.94)	1.25 (1.13 to 1.39)
2021	3.1 (2.6 to 3.5)	2.2 (1.9 to 2.5)	37.8 (32.7 to 43.4)	27.1 (23.5 to 31.0)	1.74 (1.50 to 1.99)	1.24 (1.08 to 1.42)

Ghana GHA Western Sub−Saharan Africa Stillbirth Rate / Neonatal Mortality Rate (SBR/NMR) − 20 weeks
lambda = 0.4 ; zeta = 0.9 ; scale = 10 ; amp = 0.028

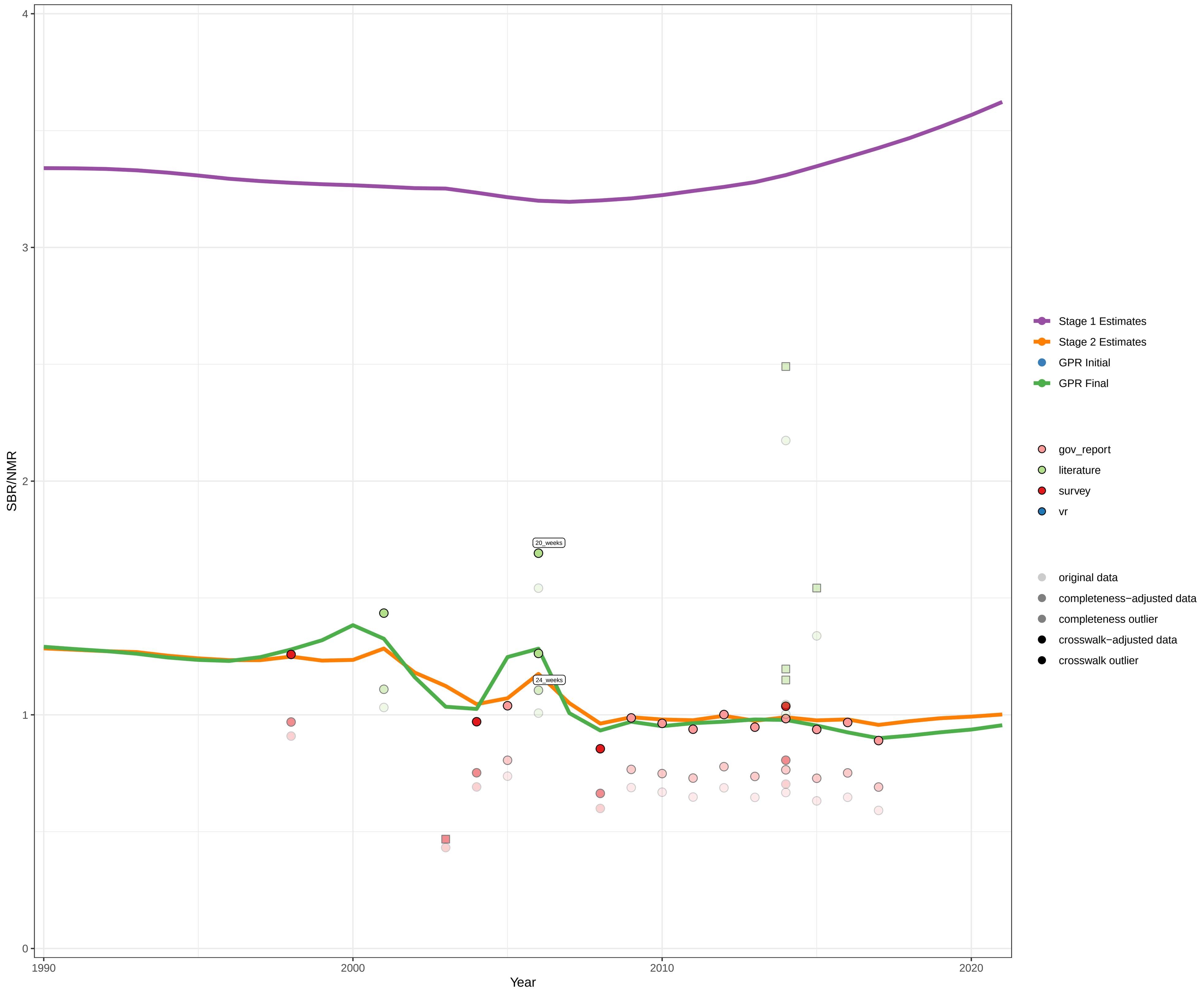

Ghana GHA Western Sub−Saharan Africa Stillbirth Rate / Neonatal Mortality Rate (SBR/NMR) − 28 weeks
lambda = 0.4 ; zeta = 0.9 ; scale = 10 ; amp = 0.028

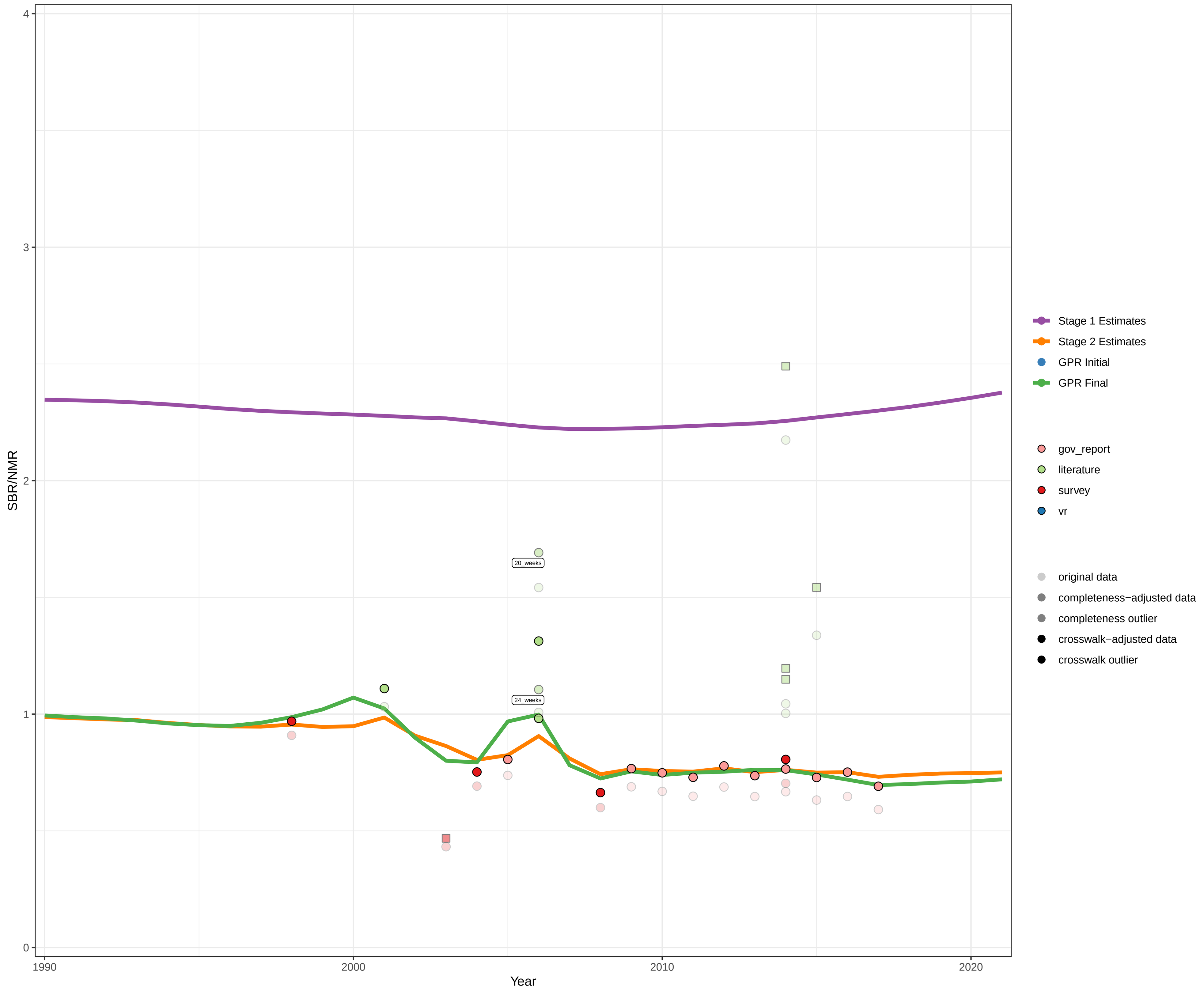

Definition	Years	Outlier	Source.Type	Title
20_weeks	2006	0	Sci Lit	Malaria, intestinal helminths and other risk factors for stillbirth in Ghana
24_weeks	2006	0	Sci Lit	Effect of Socioeconomic Deprivation and Health Service Utilisation on Antepartum and Intrapartum Stillbirth: Population Cohort Study from Rural Ghana
28_weeks	1998	0	Survey	Ghana Demographic and Health Survey 1998−1999
28_weeks	2001	0	Sci Lit	Aetiology of stillbirths and neonatal deaths in rural Ghana: implications for health programming in developing countries
28_weeks	2004	0	Survey	Ghana Special Demographic and Health Survey 2007−2008
28_weeks	2005, 2010, 2011	0	Statistical Report	Ghana Health Service Annual Report
28_weeks	2008	0	Survey	Ghana Demographic and Health Survey 2008
28_weeks	2009, 2013	0	Statistical Report	Ghana Annual Reproductive and Child Health Report
28_weeks	2012, 2014−2017	0	Statistical Report	Ghana Ministry of Health Holistic Assessment of the Health Sector Programme of Work
28_weeks	2014	0	Survey	Ghana Demographic and Health Survey 2014
28_weeks	2003	1	Survey	Ghana Demographic and Health Survey 2003
28_weeks	2014	1	Sci Lit	A review of Intra−uterine foetal deaths at the Cape Coast Teaching Hospital
28_weeks	2014	1	Sci Lit	Population−based rates, timing, and causes of maternal deaths, stillbirths, and neonatal deaths in south Asia and sub−Saharan Africa: a multi−country prospective cohort study
28_weeks	2014	1	Sci Lit	Prevalence of low birth weight, macrosomia and stillbirth and their relationship to associated maternal risk factors in Hohoe Municipality, Ghana
28_weeks	2015	1	Sci Lit	Determinants and outcomes of preterm births at a tertiary hospital in Ghana

Ghana GHA Western Sub−Saharan Africa Stillbirth Rate (SBR) − 20 weeks
lambda = 0.4 ; zeta = 0.9 ; scale = 10 ; amp = 0.028

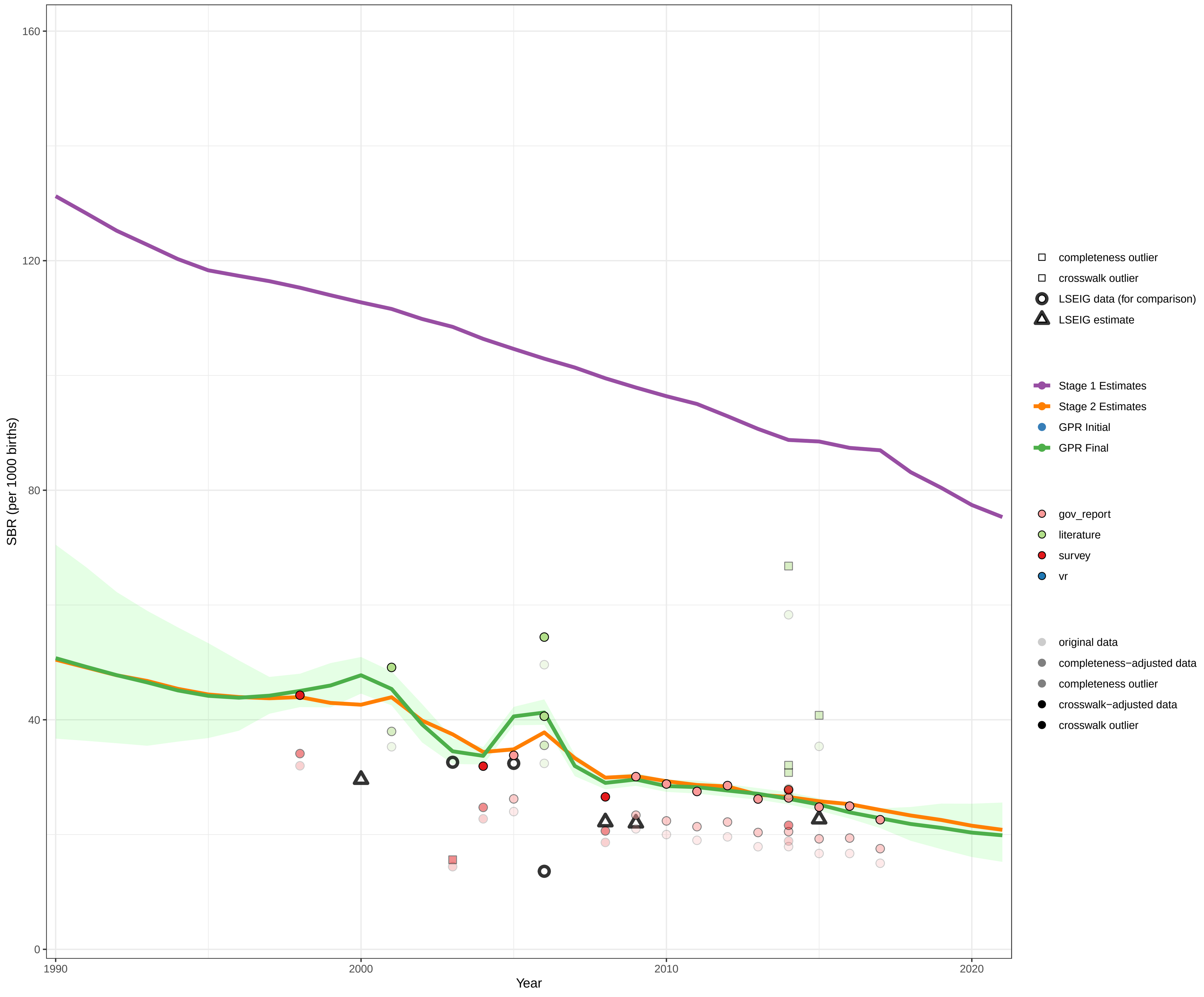

Ghana GHA Western Sub−Saharan Africa Stillbirth Rate (SBR) − 28 weeks
lambda = 0.4 ; zeta = 0.9 ; scale = 10 ; amp = 0.028

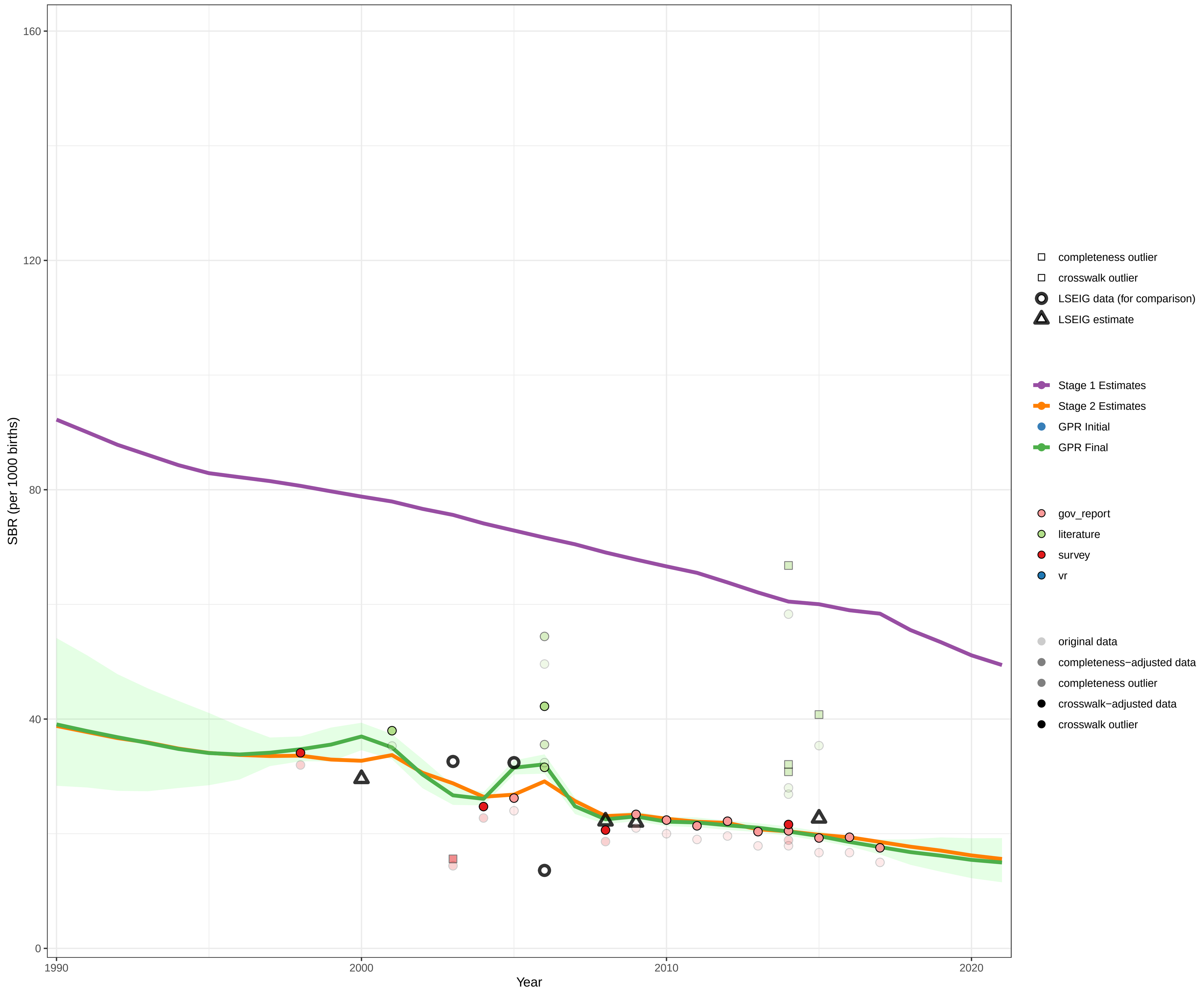

Year	Total Stillbirths (in thousands)	Total Stillbirths (in thousands)	SBR (per 1000 births)	SBR (per 1000 births)	SBR/NMR	SBR/NMR
	20 weeks	28 weeks	20 weeks	28 weeks	20 weeks	28 weeks
1990	32.4 (23.1 to 46.0)	24.7 (17.7 to 34.7)	50.7 (36.7 to 70.5)	39.1 (28.4 to 54.1)	1.29 (0.93 to 1.79)	0.99 (0.72 to 1.38)
1995	29.2 (24.1 to 35.5)	22.2 (18.5 to 27.0)	44.2 (36.8 to 53.4)	34.1 (28.5 to 41.1)	1.23 (1.03 to 1.49)	0.95 (0.80 to 1.15)
2000	33.6 (31.3 to 36.0)	25.7 (24.0 to 27.5)	47.8 (44.6 to 50.9)	37.0 (34.6 to 39.4)	1.38 (1.29 to 1.48)	1.07 (1.00 to 1.14)
2005	31.6 (30.3 to 32.9)	24.3 (23.3 to 25.3)	40.6 (39.0 to 42.3)	31.5 (30.3 to 32.8)	1.25 (1.20 to 1.30)	0.97 (0.93 to 1.01)
2010	25.0 (24.0 to 26.0)	19.3 (18.6 to 20.0)	28.4 (27.4 to 29.6)	22.1 (21.3 to 22.9)	0.95 (0.92 to 0.99)	0.74 (0.71 to 0.77)
2015	24.4 (23.3 to 25.5)	18.9 (18.1 to 19.7)	25.2 (24.1 to 26.3)	19.6 (18.8 to 20.4)	0.95 (0.91 to 1.00)	0.74 (0.71 to 0.77)
2020	20.0 (15.8 to 25.1)	15.1 (11.9 to 18.9)	20.3 (16.1 to 25.4)	15.4 (12.2 to 19.2)	0.94 (0.74 to 1.17)	0.71 (0.56 to 0.89)
2021	19.6 (15.0 to 25.4)	14.7 (11.3 to 19.0)	19.9 (15.2 to 25.6)	15.0 (11.5 to 19.2)	0.96 (0.73 to 1.23)	0.72 (0.55 to 0.93)

Guinea GIN Western Sub−Saharan Africa Stillbirth Rate / Neonatal Mortality Rate (SBR/NMR) − 20 weeks
lambda = 0.8 ; zeta = 0.7 ; scale = 15 ; amp = 0.028

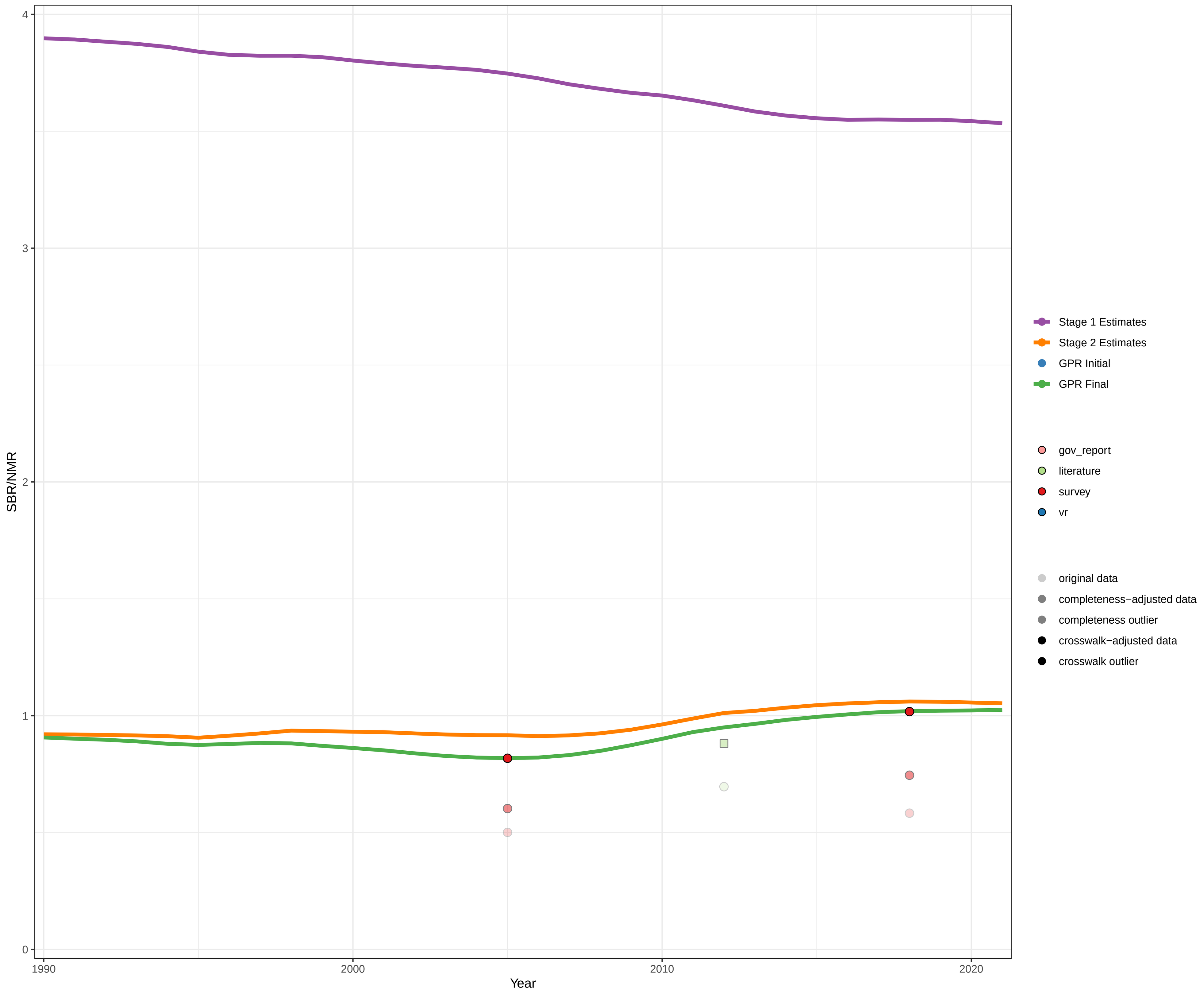

Guinea GIN Western Sub−Saharan Africa Stillbirth Rate / Neonatal Mortality Rate (SBR/NMR) − 28 weeks
lambda = 0.8 ; zeta = 0.7 ; scale = 15 ; amp = 0.028

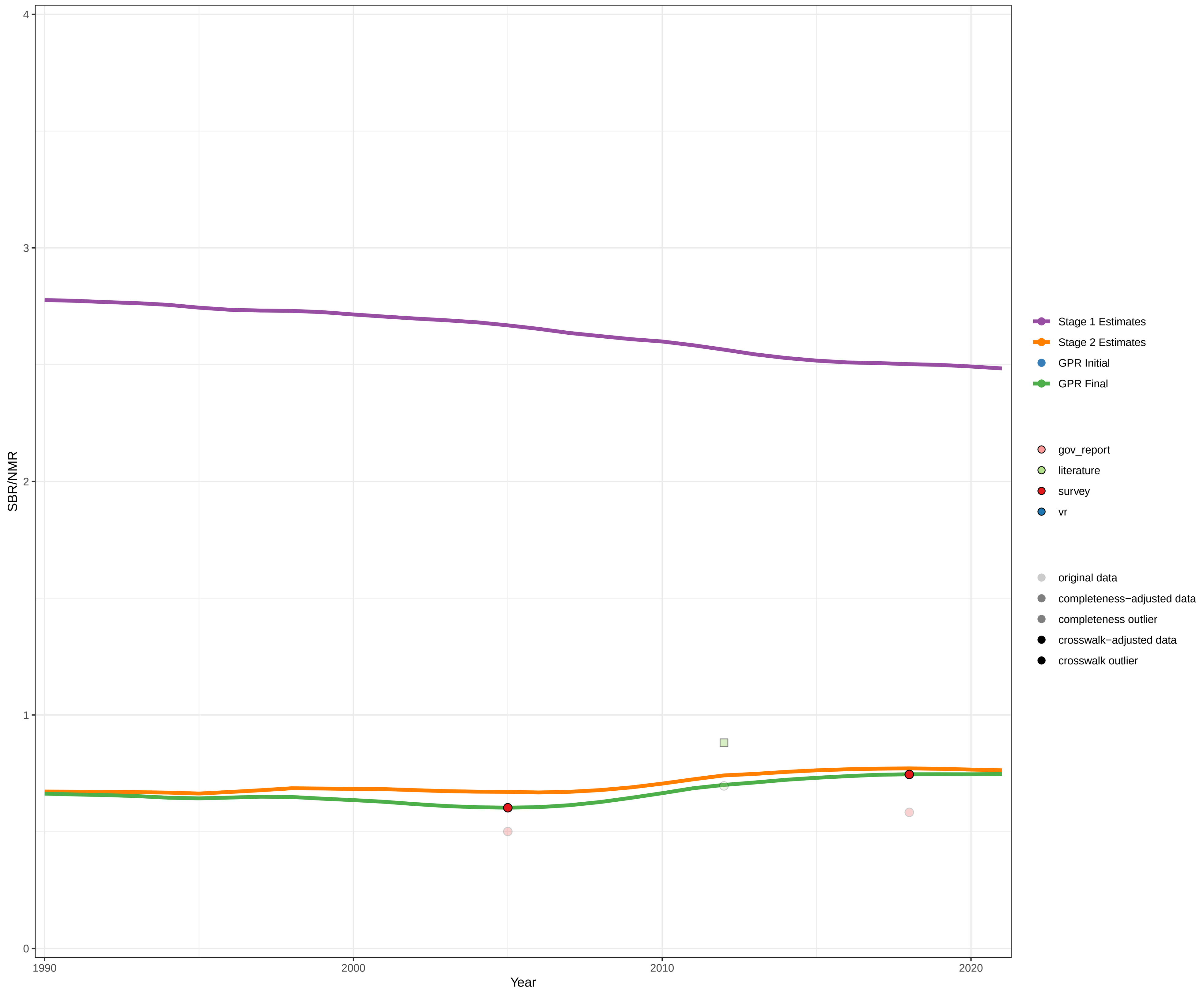

Definition	Years	Outlier	Source.Type	Title
28_weeks	2005	0	Survey	Guinea Demographic and Health Survey 2005
28_weeks	2018	0	Survey	Guinea Demographic and Health Survey 2018
20_weeks	2012	1	Sci Lit	Pregnancy Outcomes after a Mass Vaccination Campaign with an Oral Cholera Vaccine in Guinea: A Retrospective Cohort Study

Guinea GIN Western Sub−Saharan Africa Stillbirth Rate (SBR) − 20 weeks
lambda = 0.8 ; zeta = 0.7 ; scale = 15 ; amp = 0.028

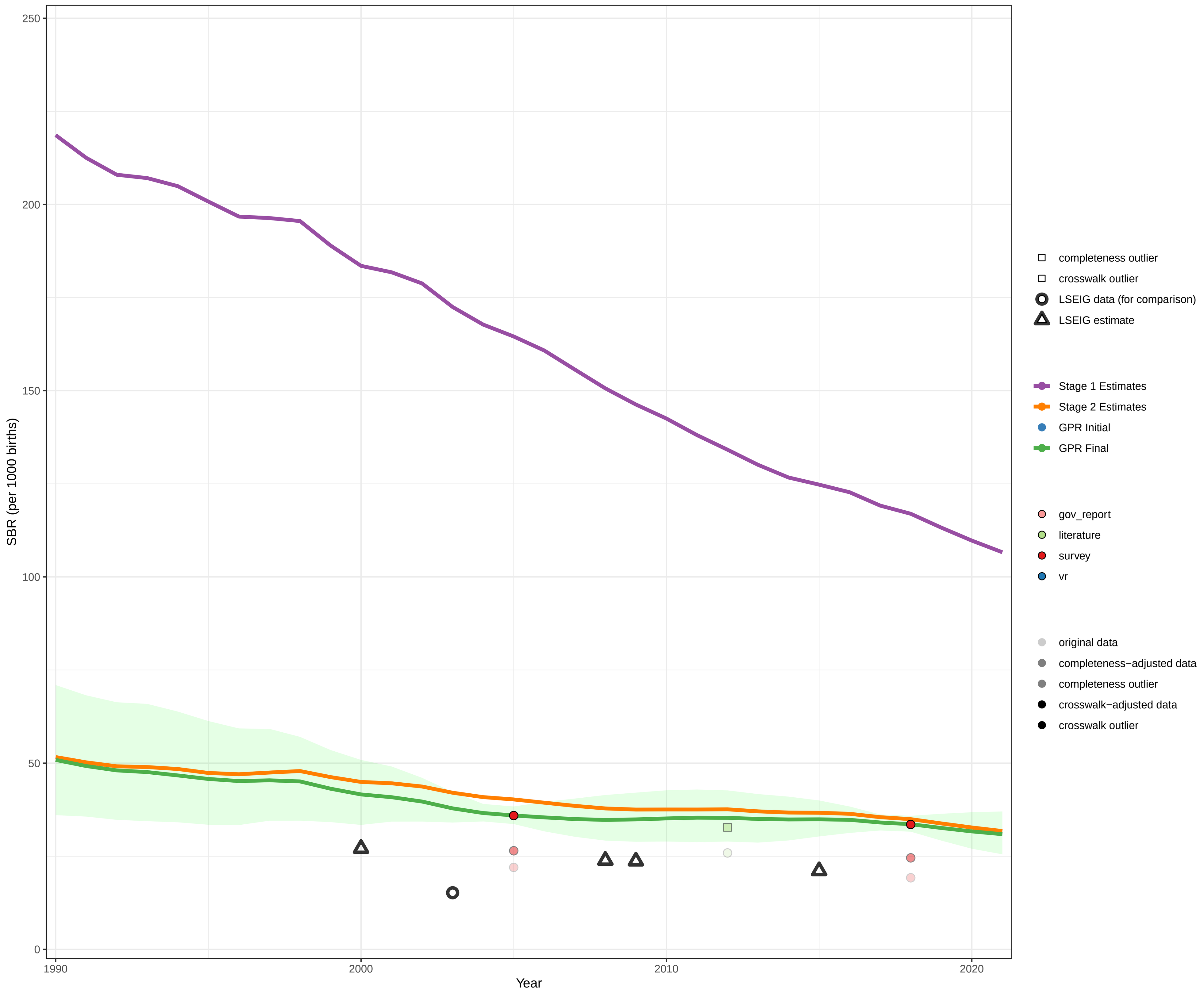

Guinea GIN Western Sub−Saharan Africa Stillbirth Rate (SBR) − 28 weeks
lambda = 0.8 ; zeta = 0.7 ; scale = 15 ; amp = 0.028

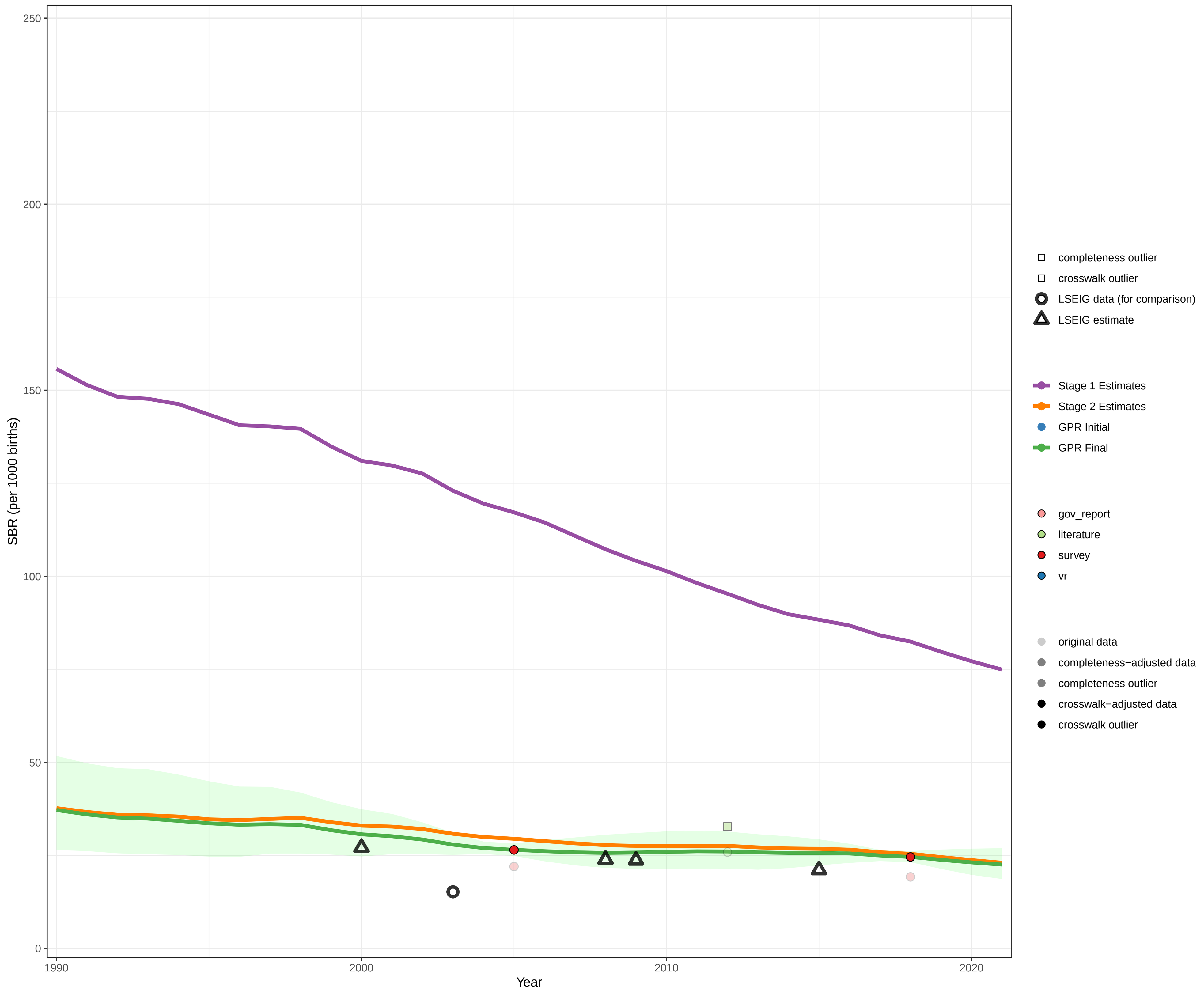

Year	Total Stillbirths (in thousands)	Total Stillbirths (in thousands)	SBR (per 1000 births)	SBR (per 1000 births)	SBR/NMR	SBR/NMR
	20 weeks	28 weeks	20 weeks	28 weeks	20 weeks	28 weeks
1990	15.5 (10.8 to 22.0)	11.2 (7.8 to 15.7)	50.9 (36.0 to 71.0)	37.2 (26.4 to 51.7)	0.91 (0.64 to 1.27)	0.66 (0.47 to 0.92)
1995	15.9 (11.4 to 21.6)	11.5 (8.4 to 15.6)	45.8 (33.5 to 61.3)	33.6 (24.7 to 44.9)	0.88 (0.64 to 1.17)	0.64 (0.47 to 0.86)
2000	15.4 (12.3 to 19.0)	11.2 (9.0 to 13.8)	41.6 (33.4 to 50.9)	30.7 (24.7 to 37.4)	0.86 (0.69 to 1.05)	0.64 (0.51 to 0.78)
2005	14.0 (13.1 to 15.0)	10.2 (9.6 to 10.9)	36.0 (33.6 to 38.4)	26.5 (24.9 to 28.2)	0.82 (0.77 to 0.87)	0.60 (0.57 to 0.64)
2010	15.4 (12.6 to 18.8)	11.2 (9.2 to 13.7)	35.2 (29.0 to 42.7)	26.0 (21.4 to 31.5)	0.90 (0.74 to 1.09)	0.67 (0.55 to 0.81)
2015	16.7 (14.4 to 19.2)	12.2 (10.5 to 13.9)	34.9 (30.3 to 40.0)	25.7 (22.3 to 29.3)	0.99 (0.86 to 1.14)	0.73 (0.64 to 0.84)
2020	16.0 (13.5 to 18.7)	11.5 (9.8 to 13.4)	31.7 (27.0 to 36.8)	23.1 (19.8 to 26.8)	1.02 (0.87 to 1.19)	0.75 (0.64 to 0.87)
2021	15.8 (13.0 to 19.0)	11.4 (9.4 to 13.7)	30.9 (25.5 to 37.0)	22.5 (18.6 to 26.9)	1.03 (0.85 to 1.23)	0.75 (0.62 to 0.89)

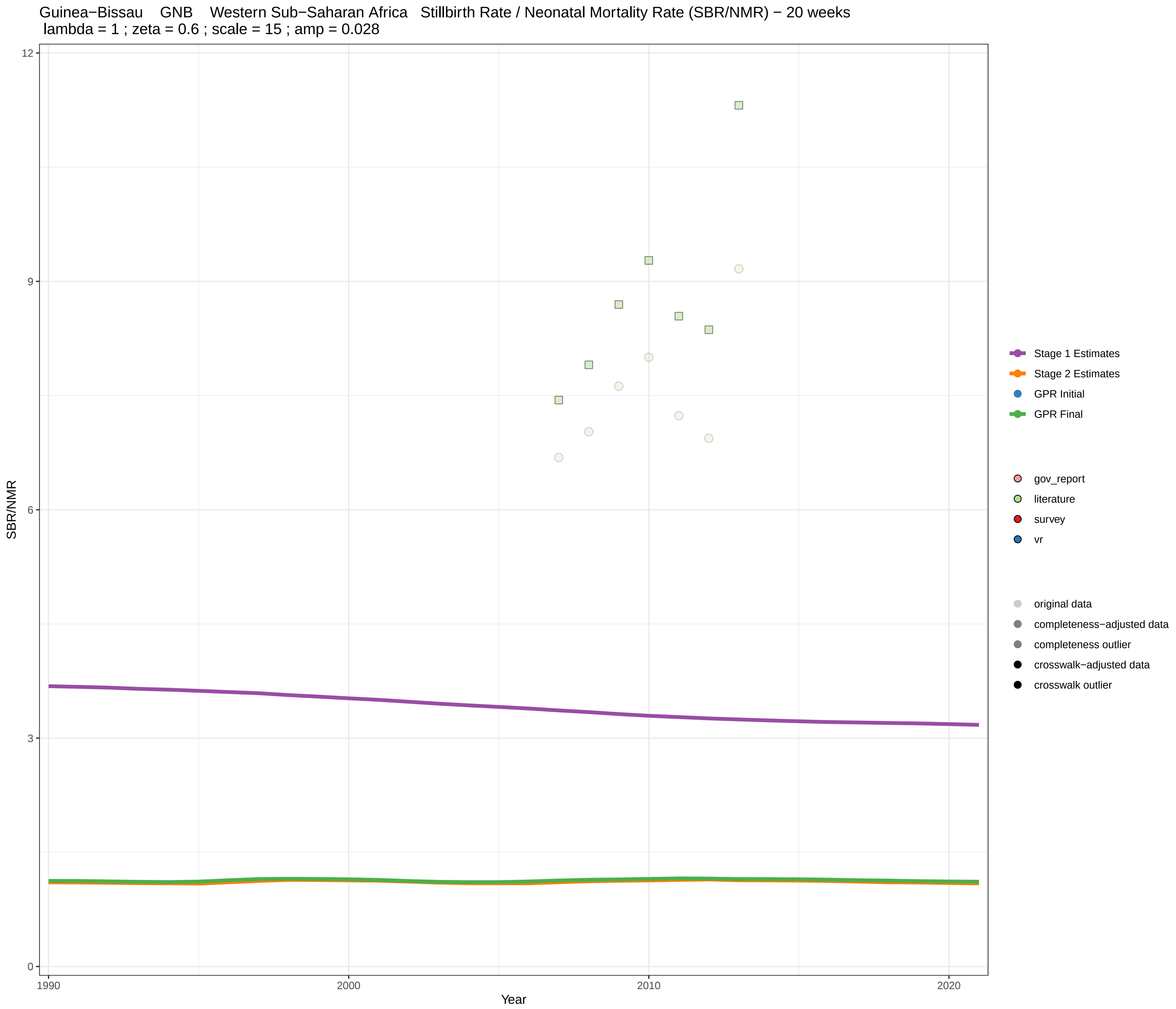

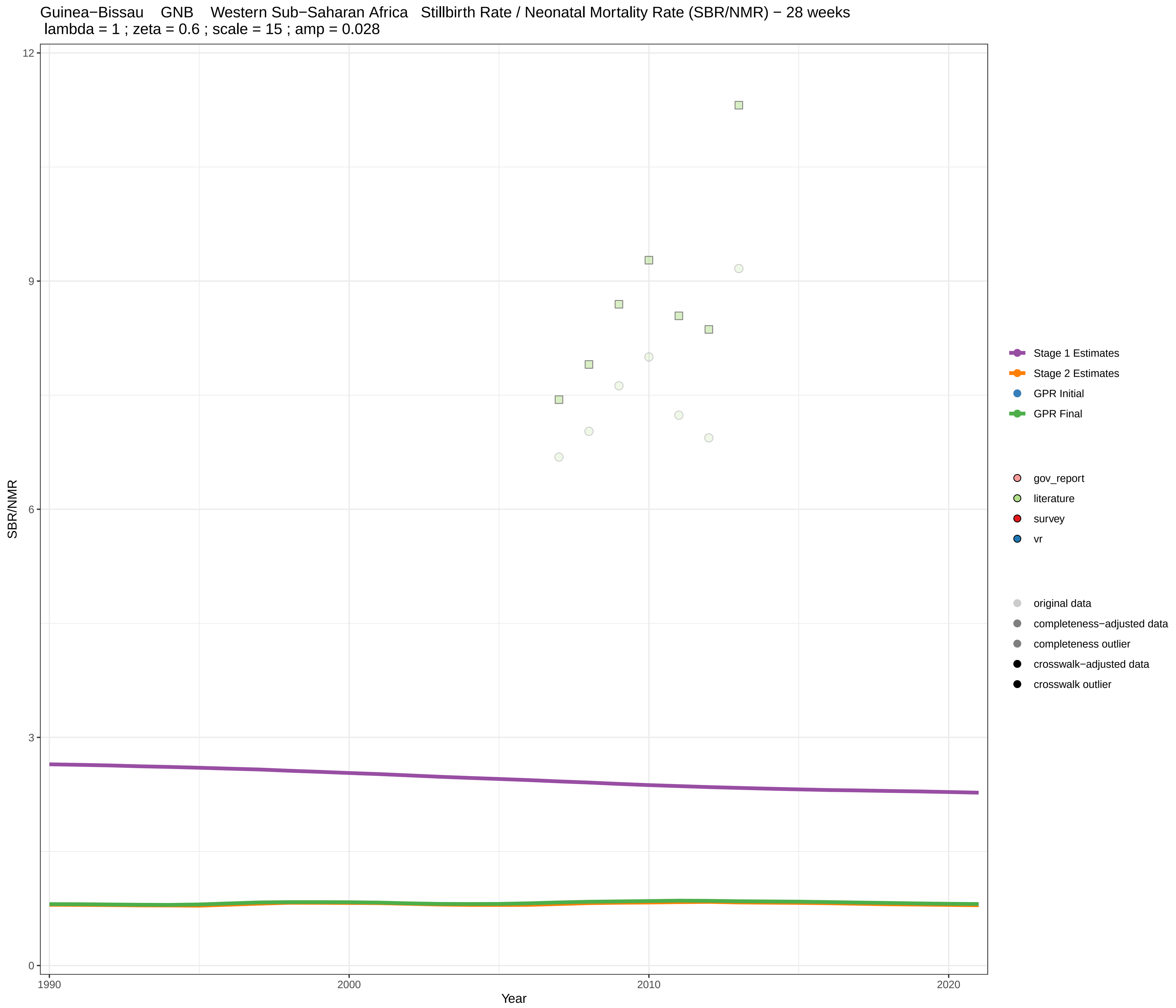

Definition	Years	Outlier	Source.Type	Title
1000_grams	2007−2013	1	Sci Lit	Stillbirths in urban Guinea−Bissau: A hospital− and community−based study

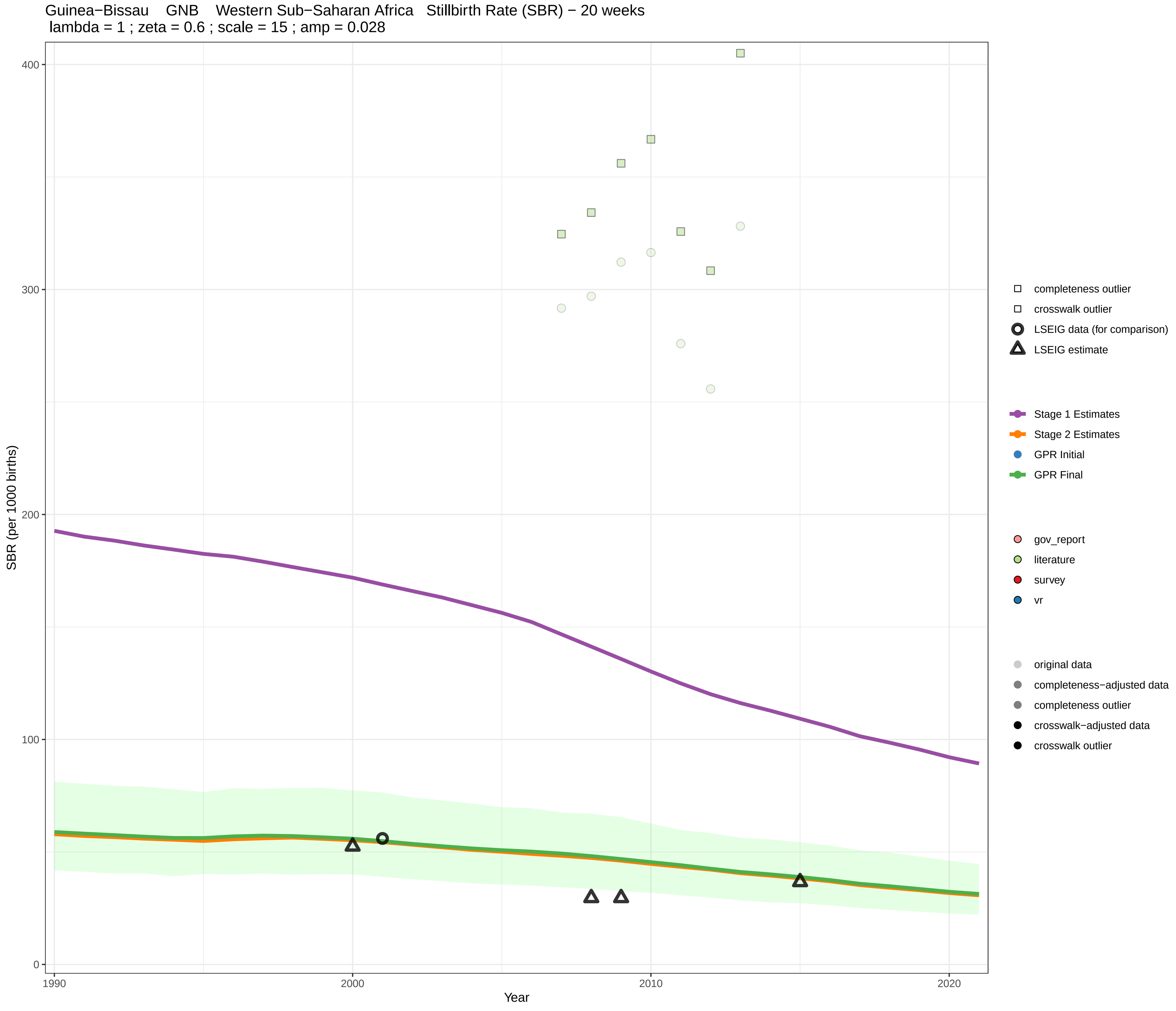

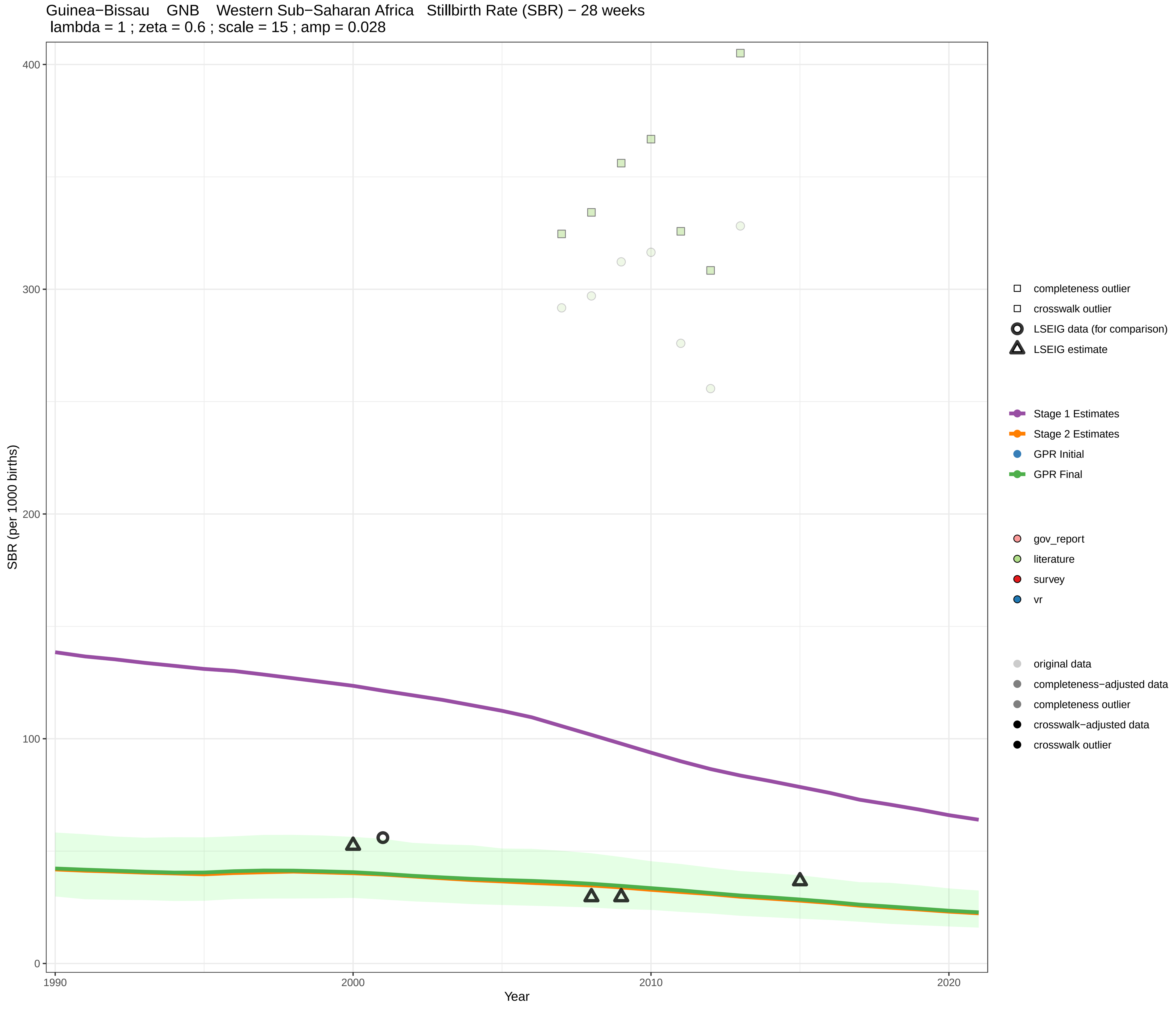

Year	Total Stillbirths (in thousands)	Total Stillbirths (in thousands)	SBR (per 1000 births)	SBR (per 1000 births)	SBR/NMR	SBR/NMR
	20 weeks	28 weeks	20 weeks	28 weeks	20 weeks	28 weeks
1990	2.9 (2.0 to 4.1)	2.0 (1.4 to 2.8)	58.9 (41.8 to 81.3)	42.3 (29.8 to 58.3)	1.12 (0.80 to 1.55)	0.81 (0.57 to 1.11)
1995	3.0 (2.1 to 4.1)	2.1 (1.4 to 2.9)	56.2 (40.2 to 76.7)	40.5 (27.9 to 56.1)	1.12 (0.80 to 1.52)	0.80 (0.55 to 1.11)
2000	3.2 (2.3 to 4.5)	2.3 (1.6 to 3.2)	55.9 (40.1 to 77.4)	40.6 (29.2 to 56.3)	1.15 (0.82 to 1.59)	0.83 (0.60 to 1.15)
2005	3.2 (2.2 to 4.5)	2.3 (1.6 to 3.2)	50.8 (35.5 to 69.9)	37.1 (26.0 to 51.2)	1.11 (0.77 to 1.53)	0.81 (0.57 to 1.12)
2010	3.2 (2.2 to 4.4)	2.3 (1.6 to 3.2)	45.5 (31.9 to 62.6)	33.5 (23.8 to 45.5)	1.15 (0.81 to 1.58)	0.85 (0.60 to 1.15)
2015	2.8 (2.0 to 4.0)	2.1 (1.4 to 2.9)	38.9 (27.2 to 54.3)	28.5 (19.9 to 39.3)	1.15 (0.80 to 1.60)	0.84 (0.59 to 1.16)
2020	2.4 (1.7 to 3.5)	1.7 (1.2 to 2.5)	32.3 (22.6 to 46.2)	23.5 (16.5 to 33.4)	1.12 (0.78 to 1.60)	0.81 (0.57 to 1.16)
2021	2.3 (1.6 to 3.4)	1.7 (1.2 to 2.4)	31.4 (22.1 to 44.6)	22.8 (16.0 to 32.5)	1.11 (0.79 to 1.59)	0.81 (0.57 to 1.15)

Liberia LBR Western Sub−Saharan Africa Stillbirth Rate / Neonatal Mortality Rate (SBR/NMR) − 20 weeks
lambda = 0.8 ; zeta = 0.7 ; scale = 15 ; amp = 0.028

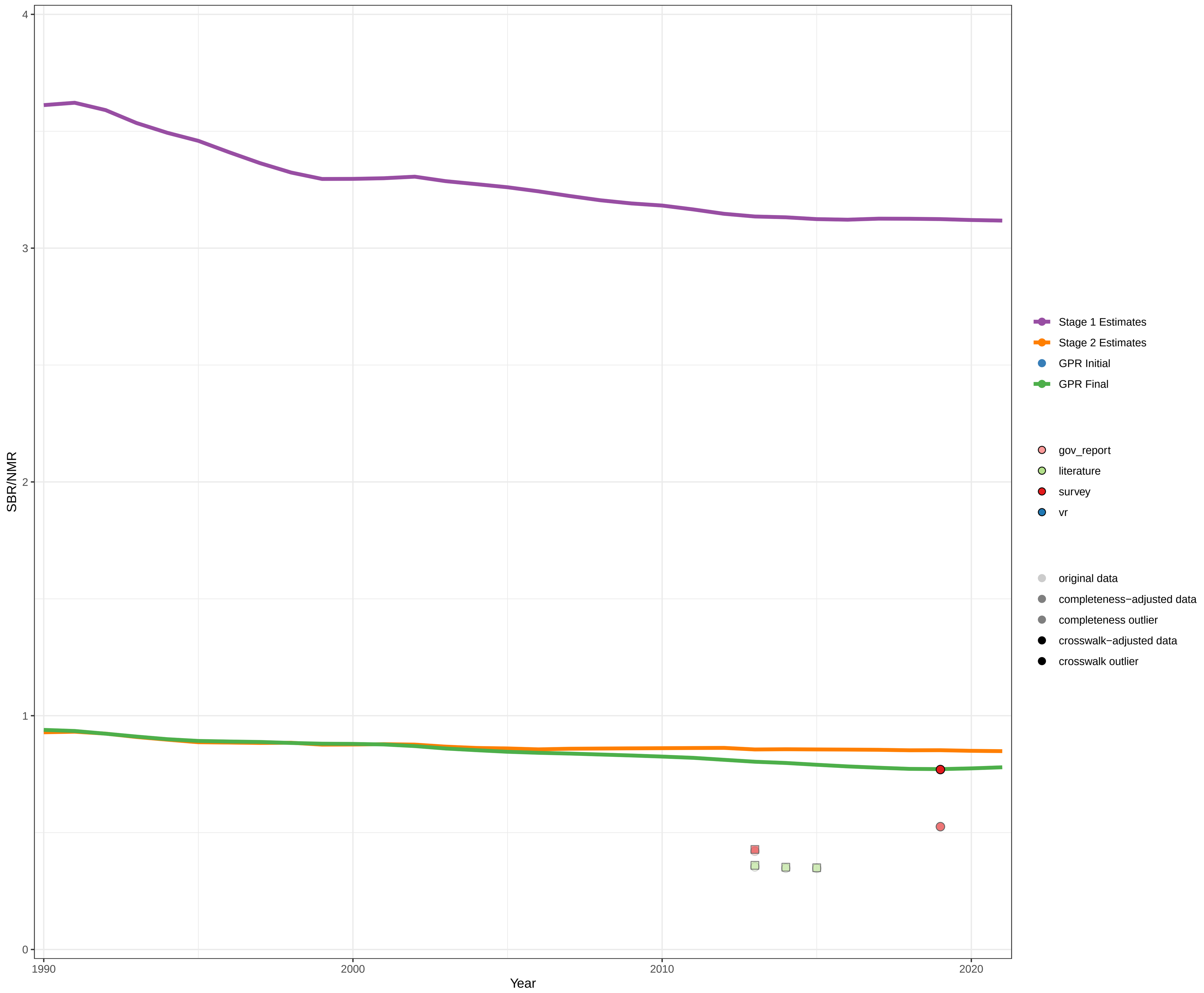

Liberia LBR Western Sub−Saharan Africa Stillbirth Rate / Neonatal Mortality Rate (SBR/NMR) − 28 weeks
lambda = 0.8 ; zeta = 0.7 ; scale = 15 ; amp = 0.028

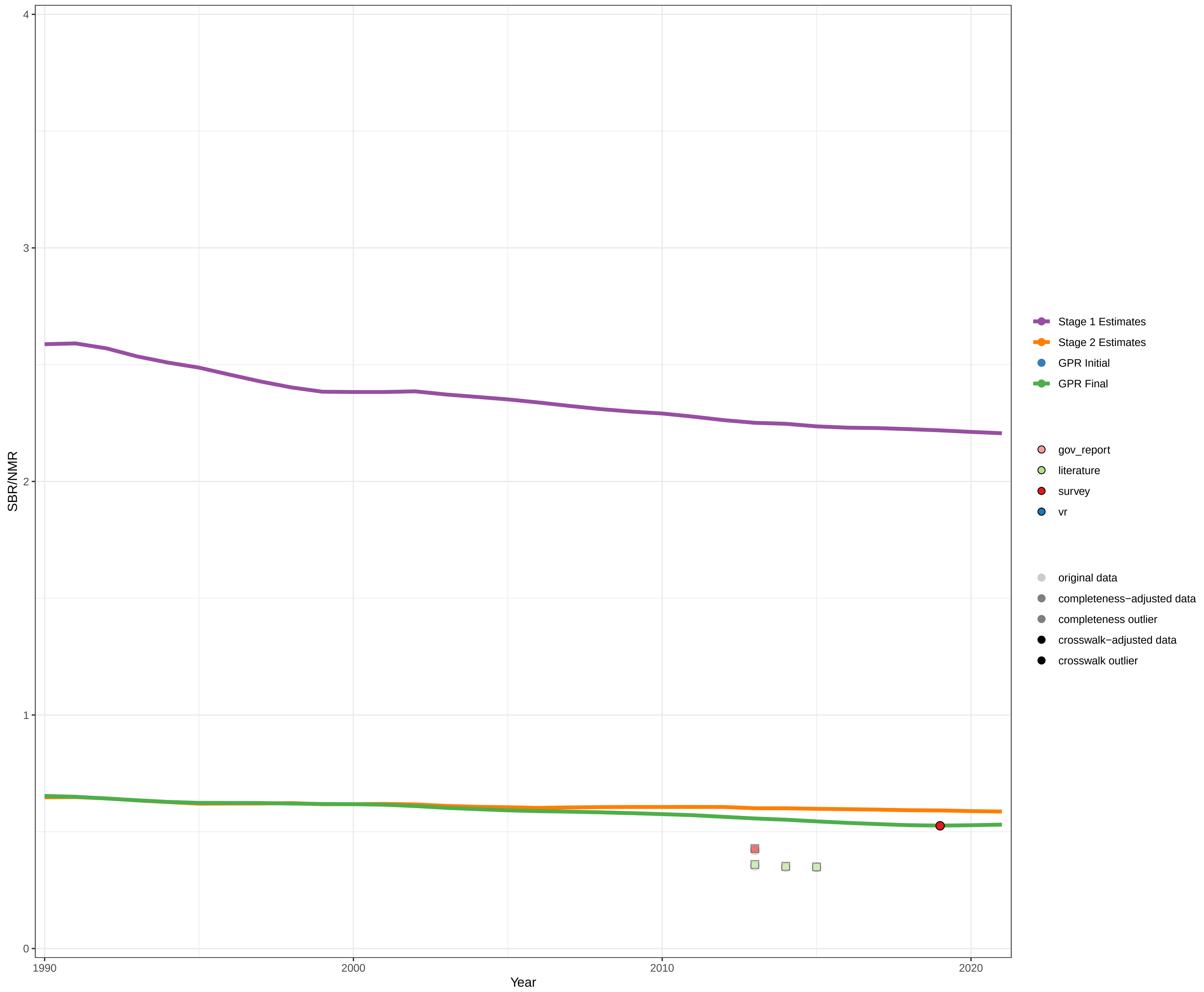

Definition	Years	Outlier	Source.Type	Title
28_weeks	2019	0	Survey	Liberia Demographic and Health Survey 2019−2020
28_weeks	2013	1	Survey	Liberia Demographic and Health Survey 2013
28_weeks	2013−2015	1	Sci Lit	Effects of the 2014 Ebola outbreak on antenatal care and delivery outcomes in Liberia: a nationwide analysis

Liberia LBR Western Sub−Saharan Africa Stillbirth Rate (SBR) − 20 weeks
lambda = 0.8 ; zeta = 0.7 ; scale = 15 ; amp = 0.028

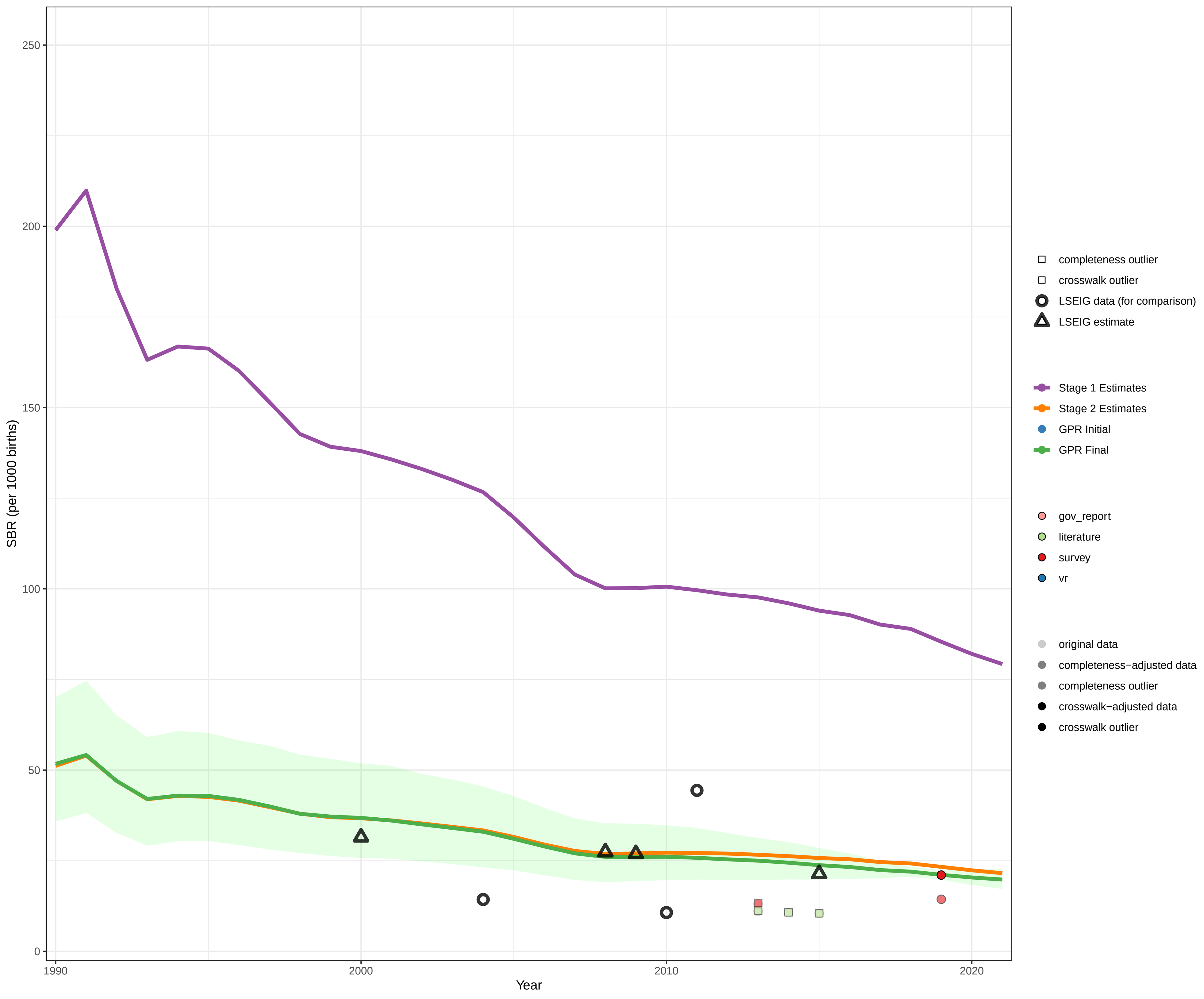

Liberia LBR Western Sub−Saharan Africa Stillbirth Rate (SBR) − 28 weeks
lambda = 0.8 ; zeta = 0.7 ; scale = 15 ; amp = 0.028

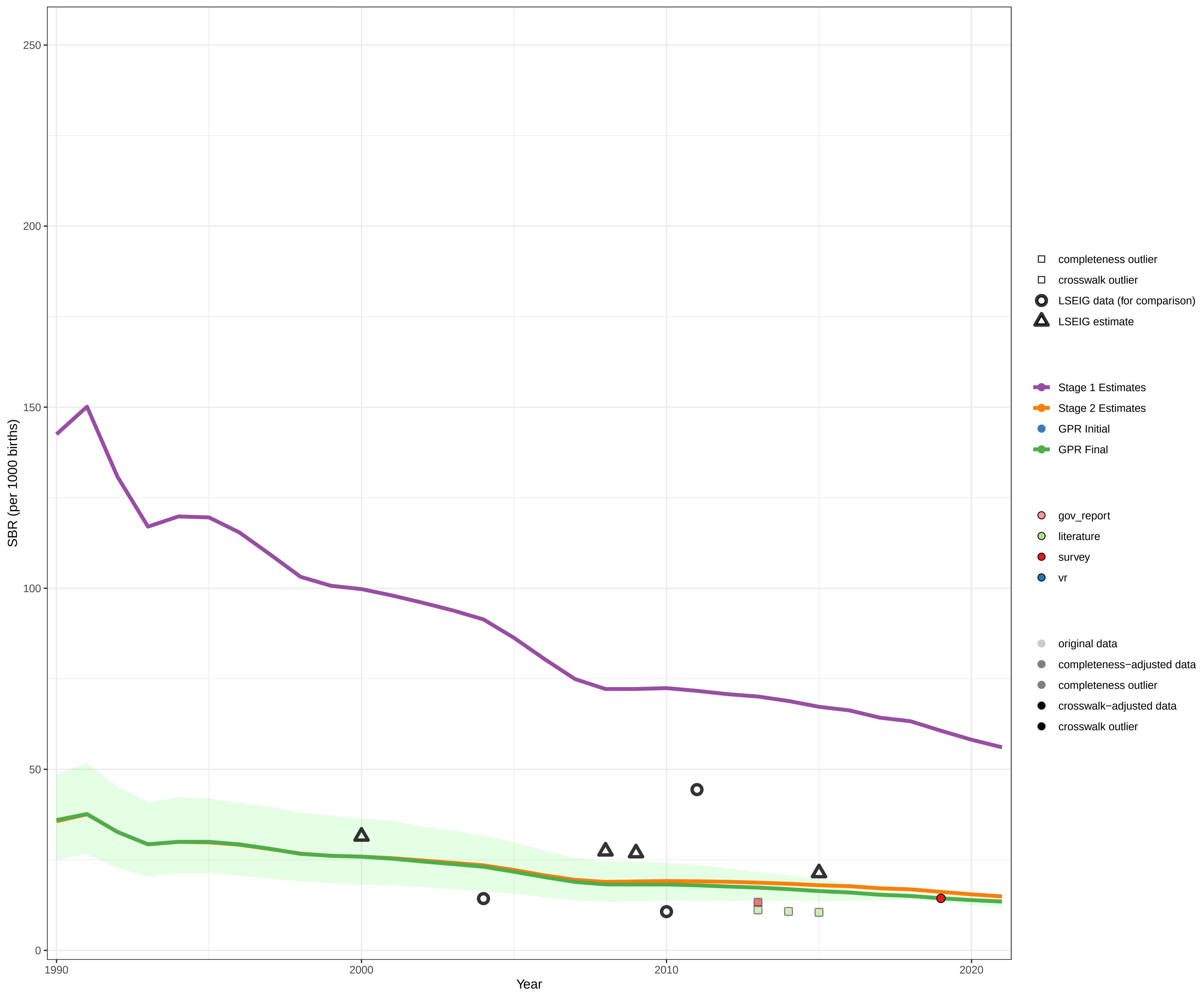

Year	Total Stillbirths (in thousands)	Total Stillbirths (in thousands)	SBR (per 1000 births)	SBR (per 1000 births)	SBR/NMR	SBR/NMR
	20 weeks	28 weeks	20 weeks	28 weeks	20 weeks	28 weeks
1990	6.6 (4.5 to 9.1)	4.5 (3.1 to 6.2)	51.7 (35.8 to 70.1)	36.0 (25.0 to 48.7)	0.94 (0.65 to 1.27)	0.65 (0.45 to 0.88)
1995	4.3 (3.0 to 6.1)	2.9 (2.1 to 4.2)	42.9 (30.4 to 60.3)	30.0 (21.3 to 42.0)	0.89 (0.63 to 1.25)	0.62 (0.44 to 0.87)
2000	5.0 (3.5 to 7.2)	3.5 (2.4 to 5.0)	36.8 (25.8 to 51.9)	25.9 (18.2 to 36.3)	0.88 (0.62 to 1.24)	0.62 (0.43 to 0.87)
2005	4.5 (3.2 to 6.3)	3.1 (2.2 to 4.3)	31.0 (22.3 to 42.8)	21.7 (15.7 to 29.9)	0.85 (0.61 to 1.17)	0.59 (0.43 to 0.81)
2010	4.4 (3.3 to 5.9)	3.0 (2.3 to 4.0)	26.1 (19.6 to 34.7)	18.2 (13.7 to 24.2)	0.83 (0.62 to 1.10)	0.58 (0.43 to 0.76)
2015	4.1 (3.4 to 5.0)	2.8 (2.3 to 3.4)	23.8 (19.8 to 28.5)	16.4 (13.7 to 19.6)	0.79 (0.66 to 0.95)	0.54 (0.45 to 0.65)
2020	3.4 (3.0 to 3.8)	2.3 (2.1 to 2.6)	20.4 (18.3 to 22.6)	13.9 (12.5 to 15.4)	0.77 (0.69 to 0.86)	0.53 (0.47 to 0.59)
2021	3.3 (2.9 to 3.8)	2.2 (1.9 to 2.6)	19.8 (17.2 to 22.8)	13.5 (11.7 to 15.5)	0.78 (0.68 to 0.90)	0.53 (0.46 to 0.61)

Mali MLI Western Sub−Saharan Africa Stillbirth Rate / Neonatal Mortality Rate (SBR/NMR) − 20 weeks
lambda = 0.8 ; zeta = 0.7 ; scale = 15 ; amp = 0.028

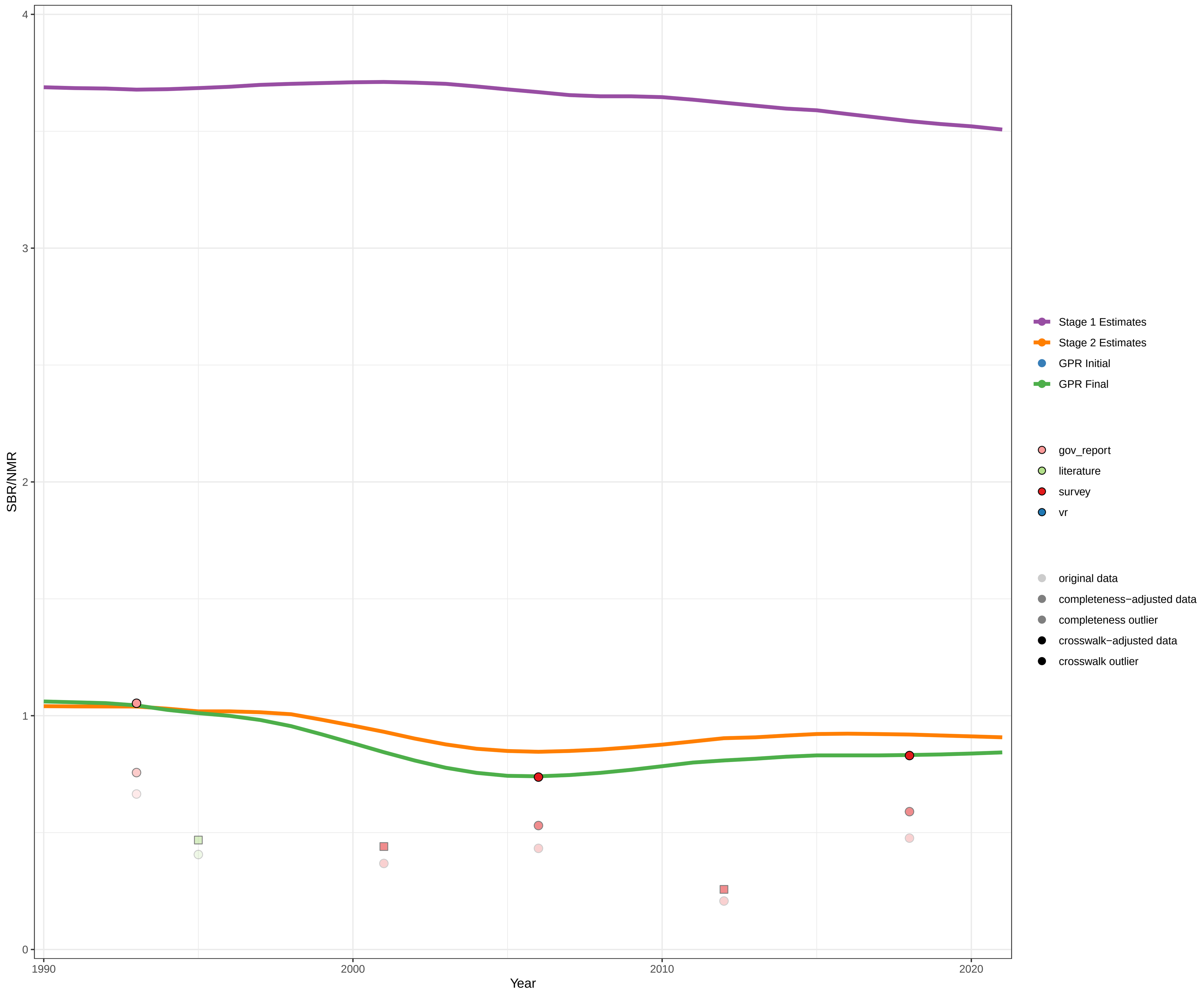

Mali MLI Western Sub−Saharan Africa Stillbirth Rate / Neonatal Mortality Rate (SBR/NMR) − 28 weeks
lambda = 0.8 ; zeta = 0.7 ; scale = 15 ; amp = 0.028

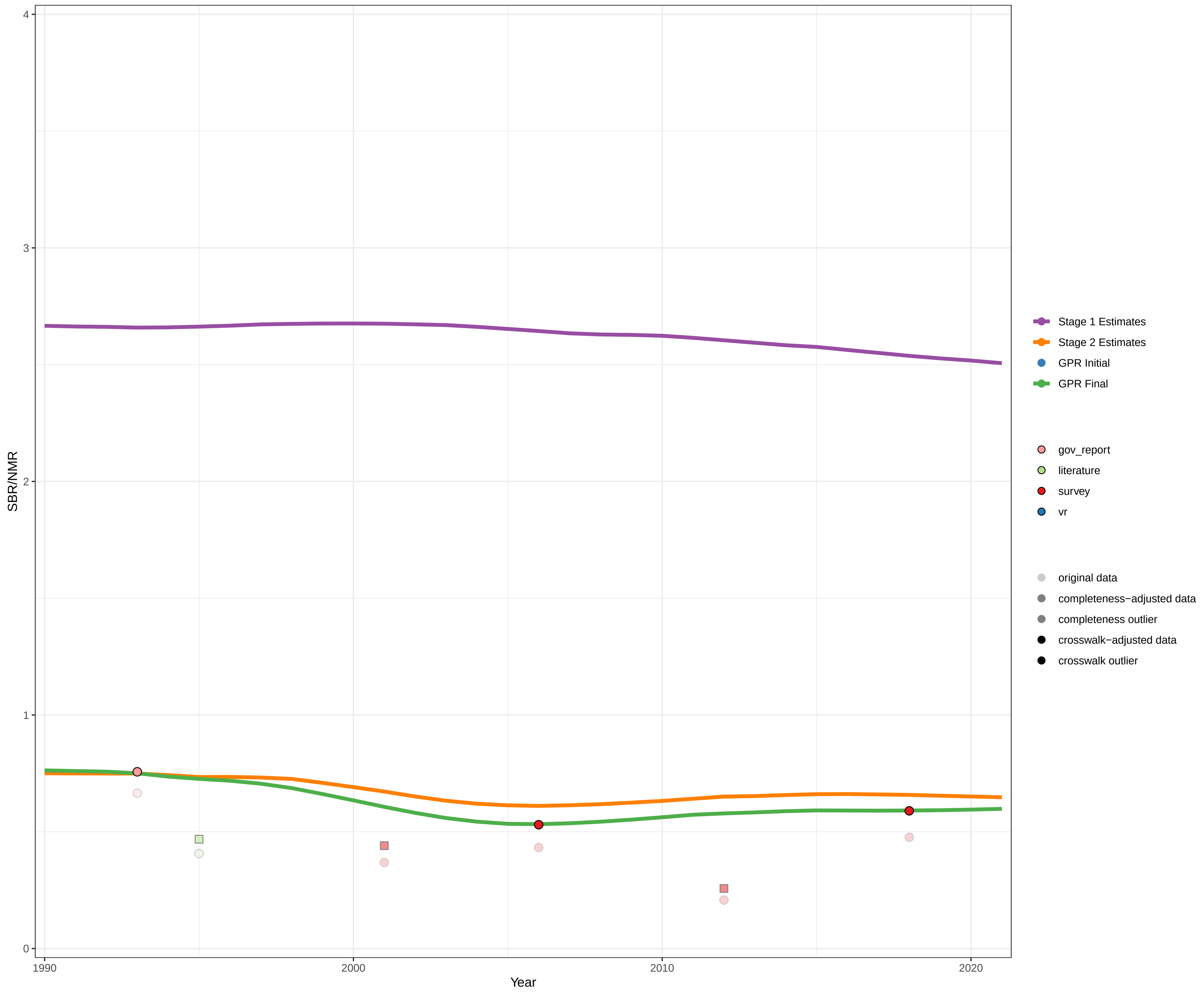

Definition	Years	Outlier	Source.Type	Title
28_weeks	1993	0	Statistical Report	Mali Health Services Statistical Yearbook
28_weeks	2006	0	Survey	Mali Demographic and Health Survey 2006
28_weeks	2018	0	Survey	Mali Demographic and Health Survey 2018
28_weeks	1995	1	Sci Lit	Risk factors for perinatal mortality in West Africa: a population−based study of 20326 pregnancies. MOMA group
28_weeks	2001	1	Survey	Mali Demographic and Health Survey 2001
28_weeks	2012	1	Survey	Mali Demographic and Health Survey 2012−2013

Mali MLI Western Sub−Saharan Africa Stillbirth Rate (SBR) − 20 weeks
lambda = 0.8 ; zeta = 0.7 ; scale = 15 ; amp = 0.028

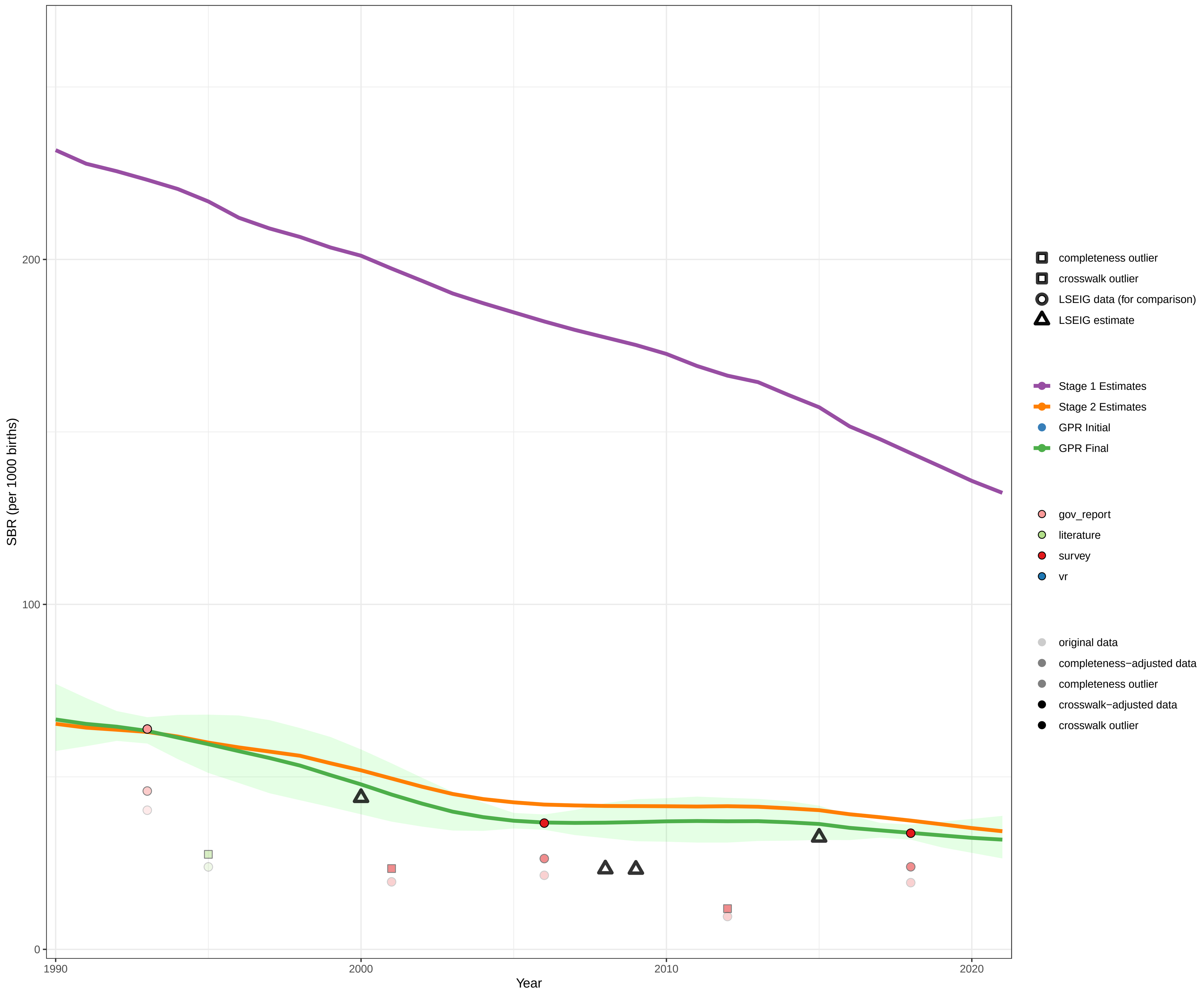

Mali MLI Western Sub−Saharan Africa Stillbirth Rate (SBR) − 28 weeks
lambda = 0.8 ; zeta = 0.7 ; scale = 15 ; amp = 0.028

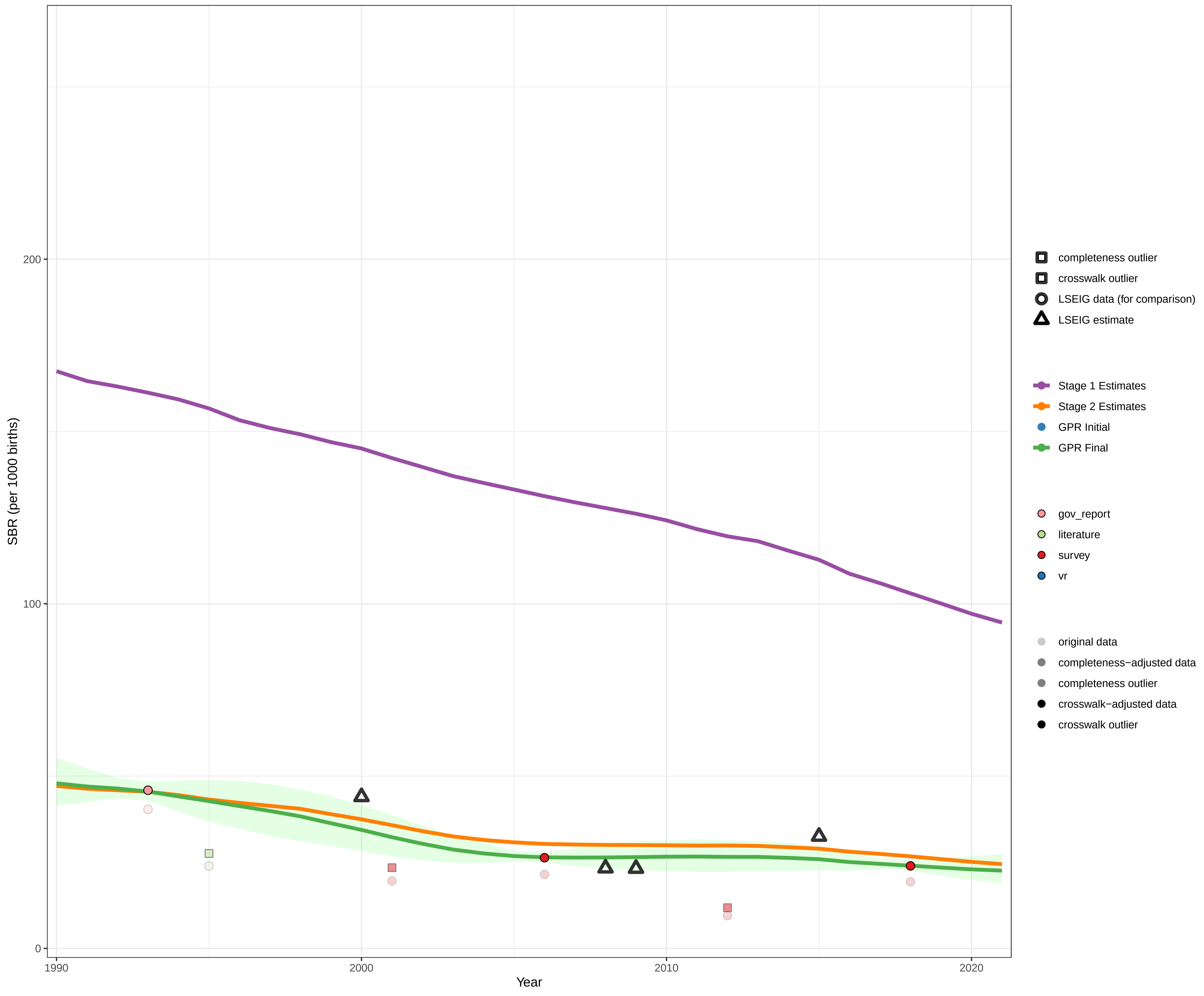

Year	Total Stillbirths (in thousands)	Total Stillbirths (in thousands)	SBR (per 1000 births)	SBR (per 1000 births)	SBR/NMR	SBR/NMR
	20 weeks	28 weeks	20 weeks	28 weeks	20 weeks	28 weeks
1990	32.4 (27.6 to 37.8)	22.8 (19.6 to 26.5)	66.6 (57.5 to 77.0)	47.9 (41.4 to 55.2)	1.06 (0.92 to 1.23)	0.76 (0.66 to 0.88)
1995	30.7 (26.2 to 35.5)	21.7 (18.6 to 24.9)	59.5 (51.1 to 68.0)	42.7 (36.8 to 48.8)	1.01 (0.87 to 1.16)	0.73 (0.63 to 0.83)
2000	28.0 (22.7 to 34.2)	19.8 (16.2 to 24.1)	47.8 (39.1 to 58.0)	34.4 (28.3 to 41.5)	0.88 (0.72 to 1.07)	0.63 (0.52 to 0.77)
2005	25.2 (23.7 to 26.8)	17.9 (16.9 to 19.0)	37.3 (35.0 to 39.6)	26.8 (25.2 to 28.4)	0.74 (0.70 to 0.79)	0.53 (0.50 to 0.57)
2010	29.8 (24.9 to 35.4)	21.1 (17.7 to 24.9)	37.1 (31.2 to 43.8)	26.6 (22.5 to 31.3)	0.78 (0.66 to 0.93)	0.56 (0.47 to 0.66)
2015	33.8 (29.3 to 38.9)	23.8 (20.7 to 27.3)	36.3 (31.7 to 41.7)	25.9 (22.6 to 29.6)	0.83 (0.72 to 0.95)	0.59 (0.52 to 0.68)
2020	34.5 (29.8 to 40.6)	24.3 (21.0 to 28.4)	32.3 (28.0 to 37.8)	22.9 (19.9 to 26.8)	0.84 (0.73 to 0.98)	0.59 (0.52 to 0.69)
2021	35.0 (28.8 to 42.8)	24.6 (20.3 to 30.0)	31.8 (26.4 to 38.7)	22.6 (18.7 to 27.4)	0.84 (0.70 to 1.03)	0.60 (0.50 to 0.73)

Mauritania MRT Western Sub−Saharan Africa Stillbirth Rate / Neonatal Mortality Rate (SBR/NMR) − 20 weeks
lambda = 0.8 ; zeta = 0.7 ; scale = 15 ; amp = 0.028

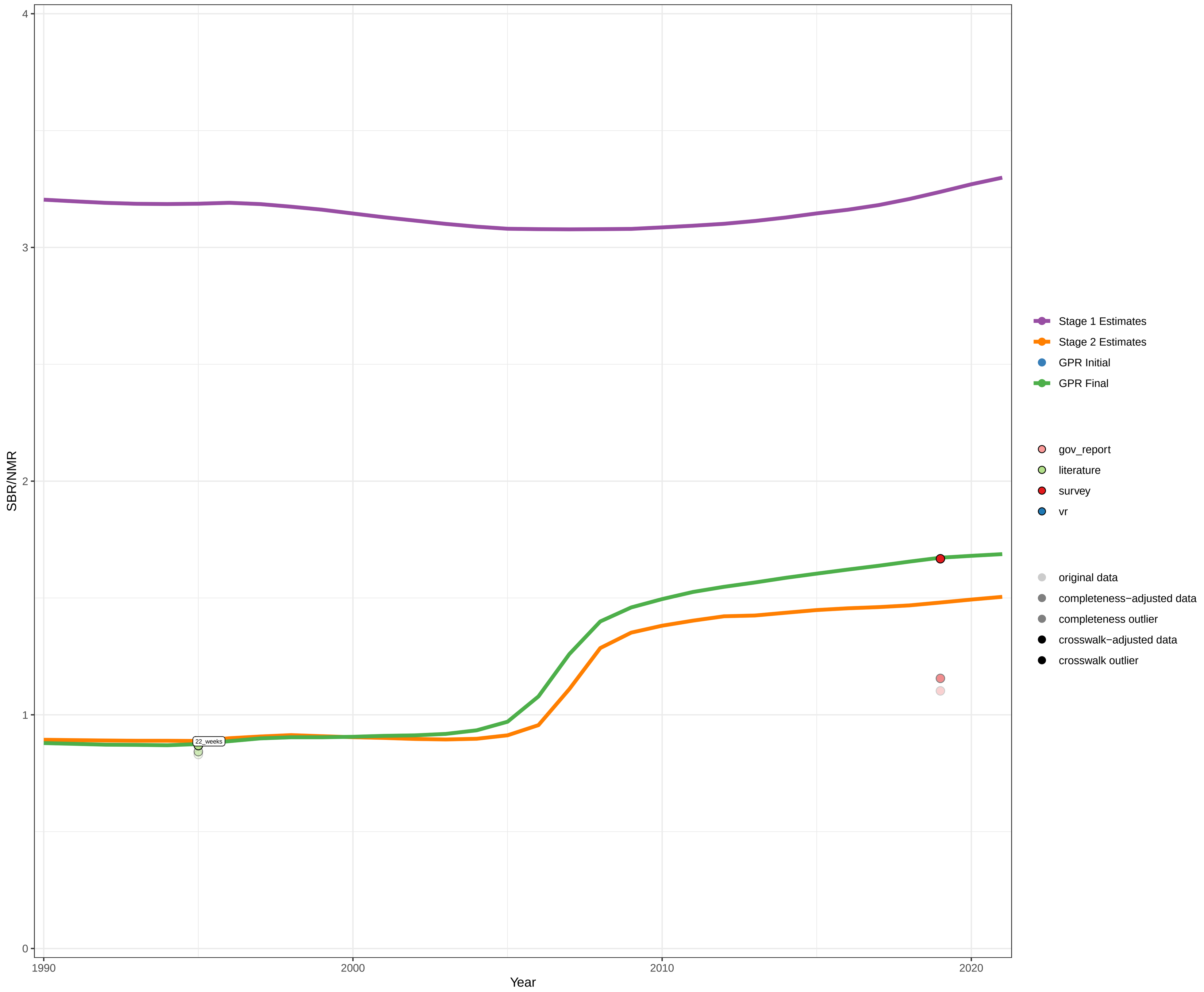

Mauritania MRT Western Sub−Saharan Africa Stillbirth Rate / Neonatal Mortality Rate (SBR/NMR) − 28 weeks
lambda = 0.8 ; zeta = 0.7 ; scale = 15 ; amp = 0.028

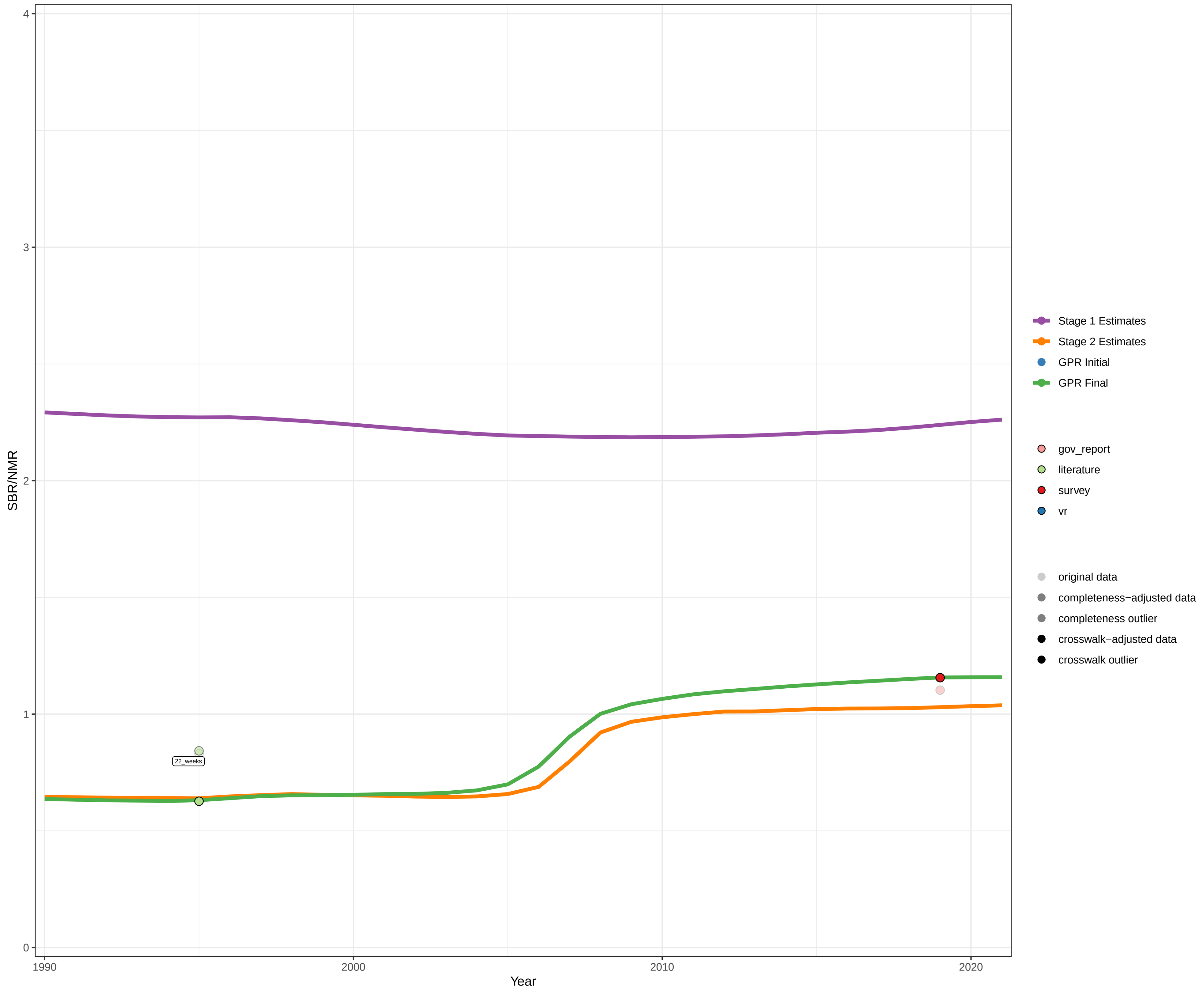

Definition	Years	Outlier	Source.Type	Title
22_weeks	1995	0	Sci Lit	Risk factors for perinatal mortality in West Africa: a population−based study of 20326 pregnancies. MOMA group
28_weeks	2019	0	Survey	Mauritania Demographic and Health Survey 2019−2020

Mauritania MRT Western Sub−Saharan Africa Stillbirth Rate (SBR) − 20 weeks
lambda = 0.8 ; zeta = 0.7 ; scale = 15 ; amp = 0.028

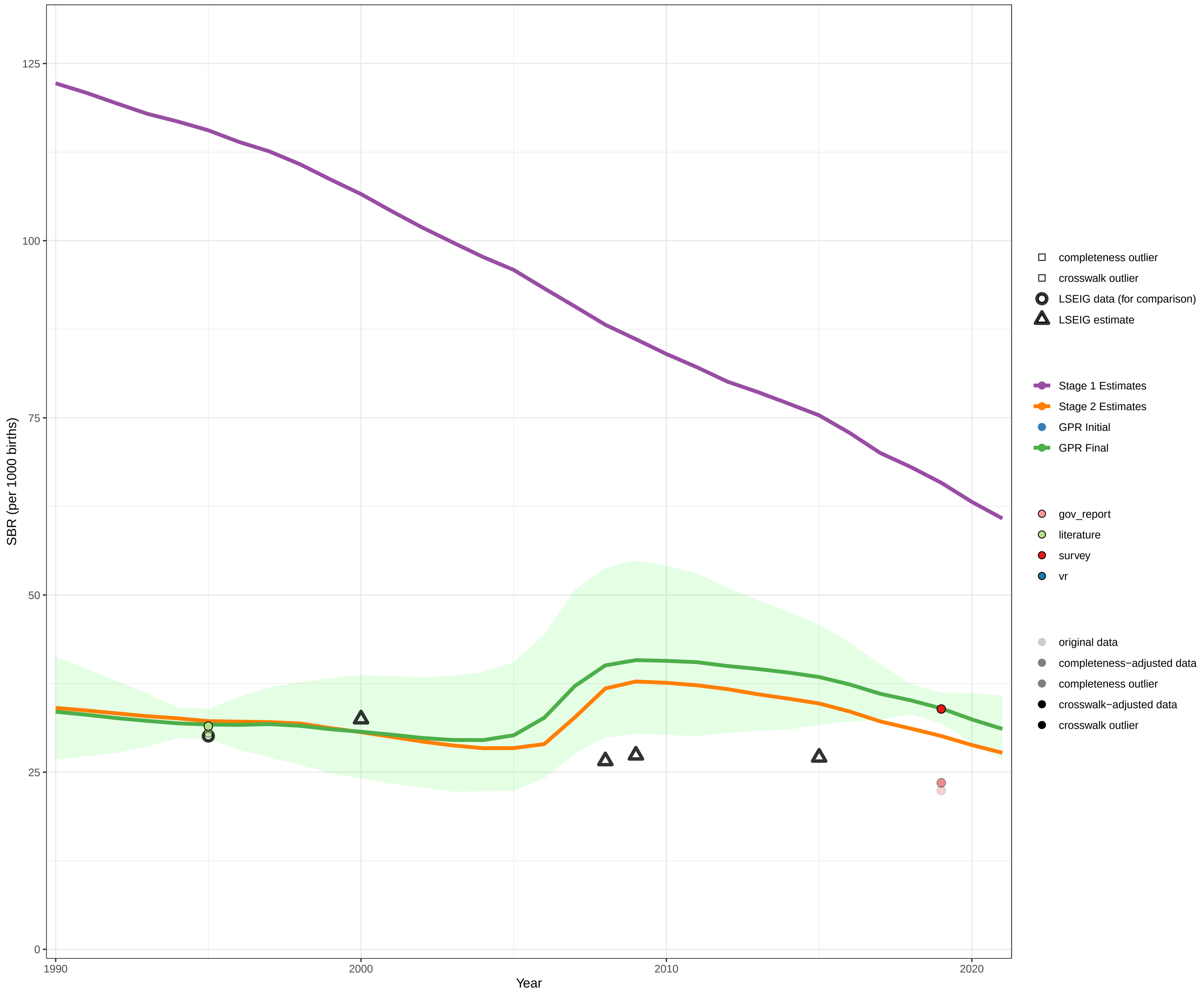

Mauritania MRT Western Sub−Saharan Africa Stillbirth Rate (SBR) − 28 weeks
lambda = 0.8 ; zeta = 0.7 ; scale = 15 ; amp = 0.028

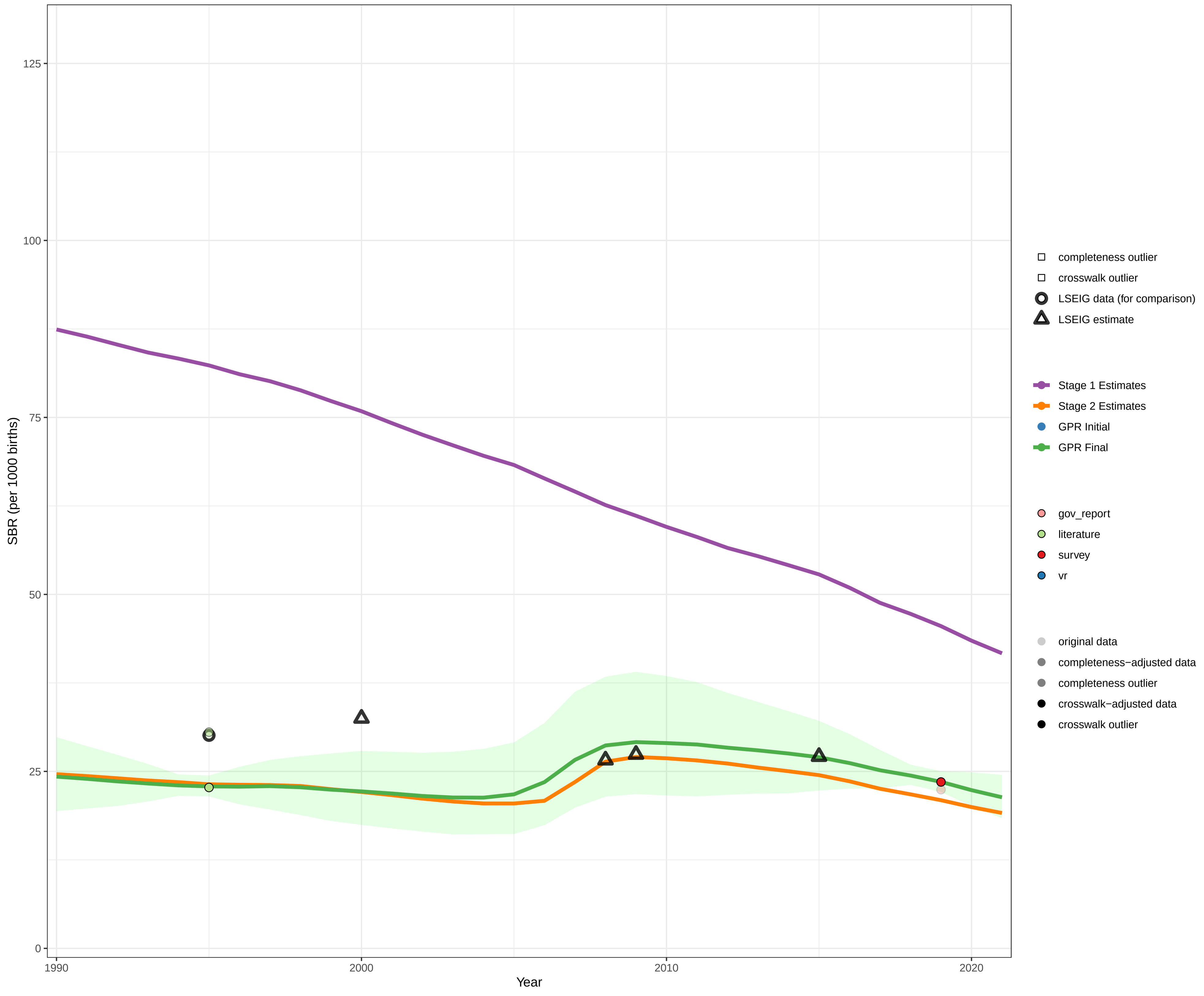

Year	Total Stillbirths (in thousands)	Total Stillbirths (in thousands)	SBR (per 1000 births)	SBR (per 1000 births)	SBR/NMR	SBR/NMR
	20 weeks	28 weeks	20 weeks	28 weeks	20 weeks	28 weeks
1990	3.0 (2.4 to 3.8)	2.2 (1.7 to 2.7)	33.5 (26.7 to 41.3)	24.3 (19.4 to 29.8)	0.88 (0.70 to 1.08)	0.64 (0.51 to 0.78)
1995	3.1 (2.9 to 3.3)	2.2 (2.1 to 2.4)	31.7 (29.7 to 33.9)	22.9 (21.5 to 24.4)	0.87 (0.82 to 0.94)	0.63 (0.59 to 0.67)
2000	3.3 (2.6 to 4.2)	2.4 (1.9 to 3.0)	30.7 (24.1 to 38.7)	22.2 (17.4 to 27.9)	0.91 (0.71 to 1.14)	0.65 (0.51 to 0.82)
2005	3.7 (2.7 to 5.0)	2.6 (1.9 to 3.5)	30.2 (22.4 to 40.5)	21.8 (16.1 to 29.1)	0.97 (0.72 to 1.30)	0.70 (0.52 to 0.93)
2010	5.6 (4.1 to 7.5)	3.9 (2.9 to 5.2)	40.7 (30.2 to 54.1)	29.0 (21.6 to 38.5)	1.50 (1.11 to 1.99)	1.07 (0.79 to 1.41)
2015	5.5 (4.5 to 6.6)	3.8 (3.1 to 4.6)	38.4 (31.7 to 45.9)	27.0 (22.3 to 32.1)	1.60 (1.32 to 1.91)	1.13 (0.93 to 1.34)
2020	4.5 (4.1 to 5.1)	3.1 (2.8 to 3.5)	32.4 (29.1 to 36.2)	22.4 (20.1 to 24.9)	1.68 (1.51 to 1.87)	1.16 (1.04 to 1.29)
2021	4.3 (3.7 to 5.0)	3.0 (2.5 to 3.4)	31.1 (26.8 to 35.8)	21.3 (18.4 to 24.5)	1.69 (1.45 to 1.94)	1.16 (1.00 to 1.33)

Niger NER Western Sub−Saharan Africa Stillbirth Rate / Neonatal Mortality Rate (SBR/NMR) − 20 weeks
lambda = 0.6 ; zeta = 0.8 ; scale = 15 ; amp = 0.028

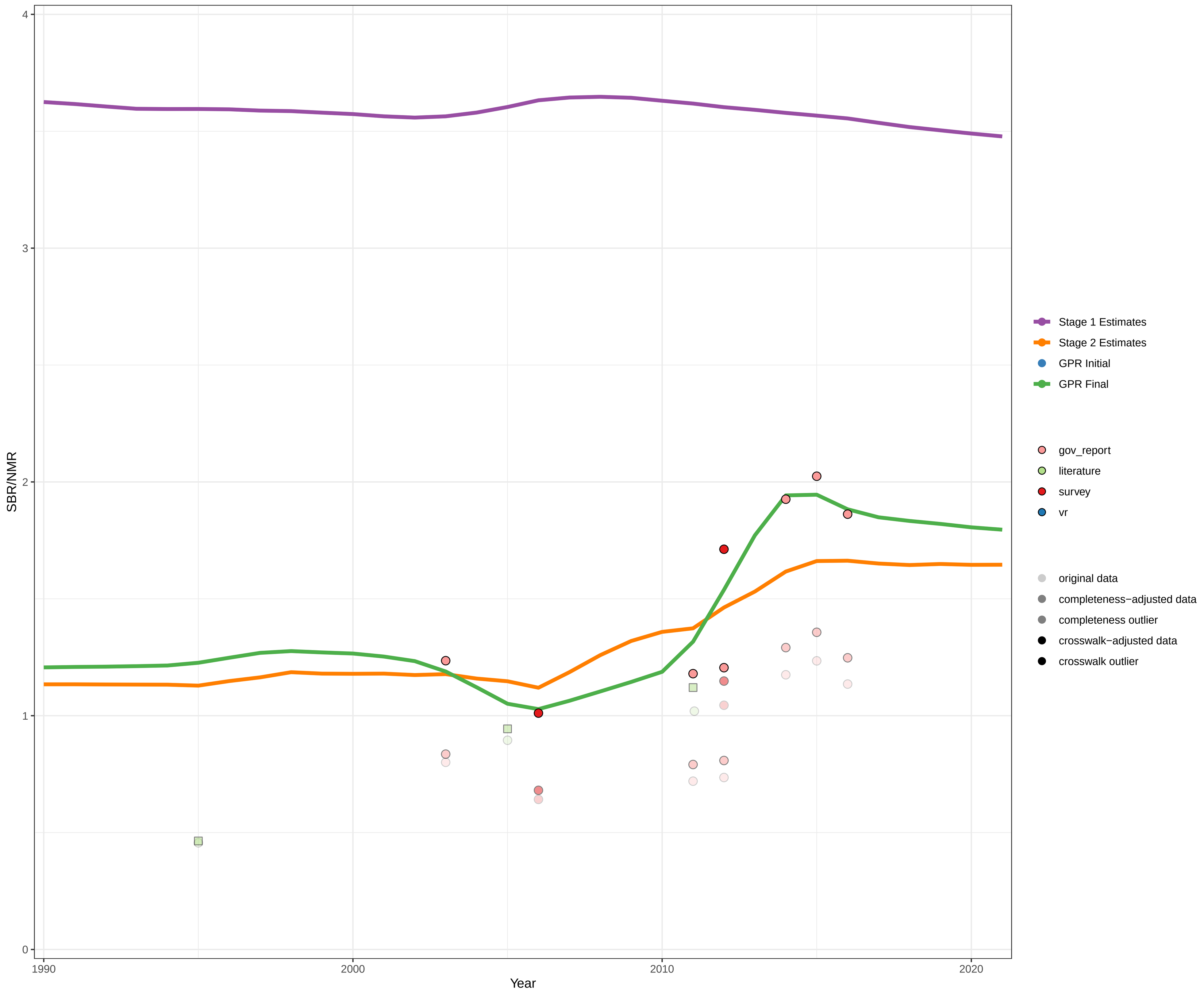

Niger NER Western Sub−Saharan Africa Stillbirth Rate / Neonatal Mortality Rate (SBR/NMR) − 28 weeks
lambda = 0.6 ; zeta = 0.8 ; scale = 15 ; amp = 0.028

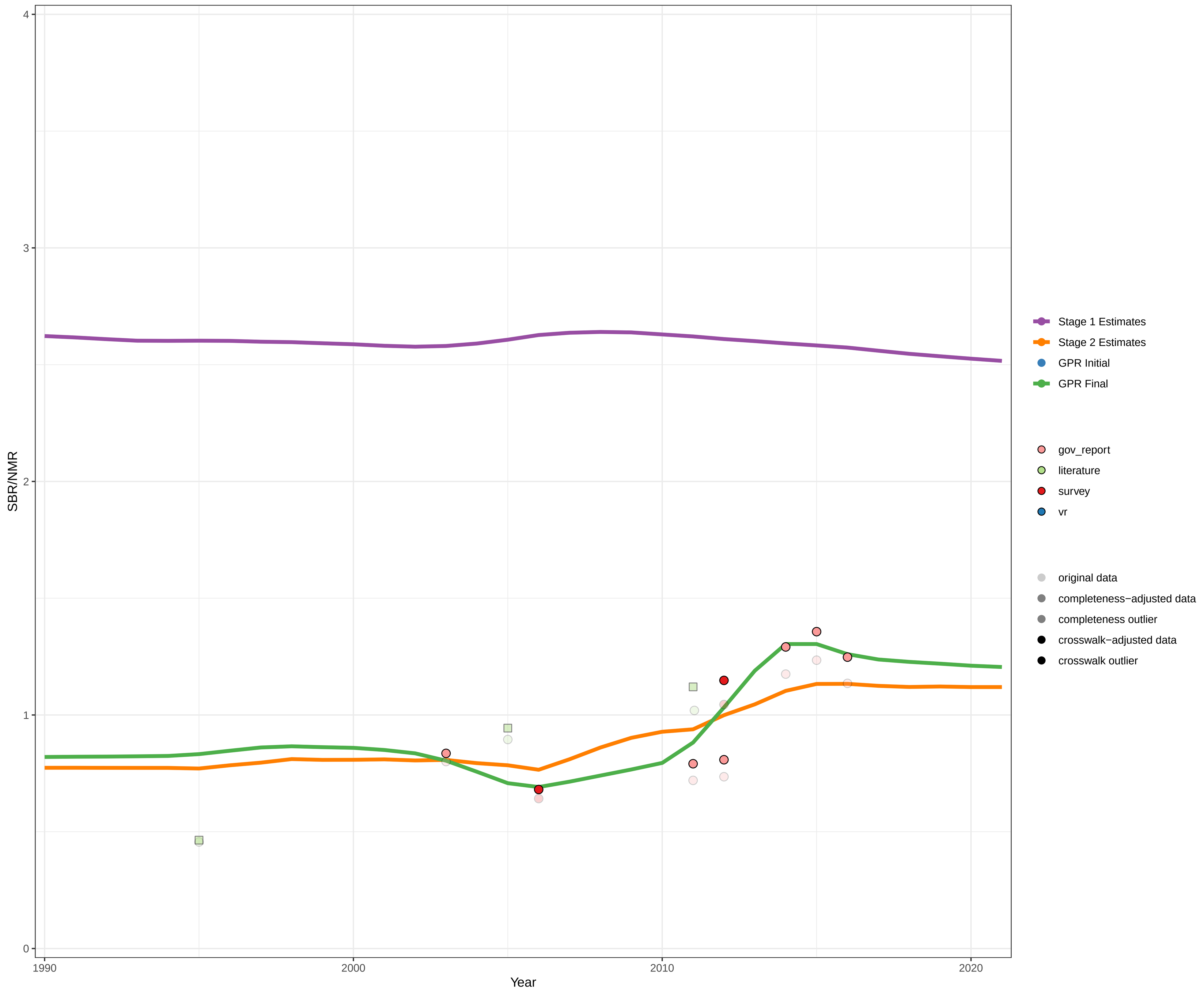

Definition	Years	Outlier	Source.Type	Title
28_weeks	2003, 2011, 2012, 2014−2016	0	Statistical Report	Niger Health Statistical Yearbook
28_weeks	2006	0	Survey	Niger Demographic and Health Survey 2006
28_weeks	2012	0	Survey	Niger Demographic and Health Survey 2012
22_weeks	1995	1	Sci Lit	Risk factors for perinatal mortality in West Africa: a population−based study of 20326 pregnancies. MOMA group
24_weeks	2005	1	Sci Lit	Prenatal Multiple Micronutrient Supplementation has Greater Impact on Birthweight than Supplementation with Iron and Folic Acid: A Cluster−Randomized, Double−Blind, Controlled Programmatic Study in Rural Niger
28_weeks_OR_1000_grams	2011	1	Sci Lit	Maternal complications and perinatal mortality: findings of the World Health Organization Multicountry Survey on Maternal and Newborn Health

Niger NER Western Sub−Saharan Africa Stillbirth Rate (SBR) − 20 weeks
lambda = 0.6 ; zeta = 0.8 ; scale = 15 ; amp = 0.028

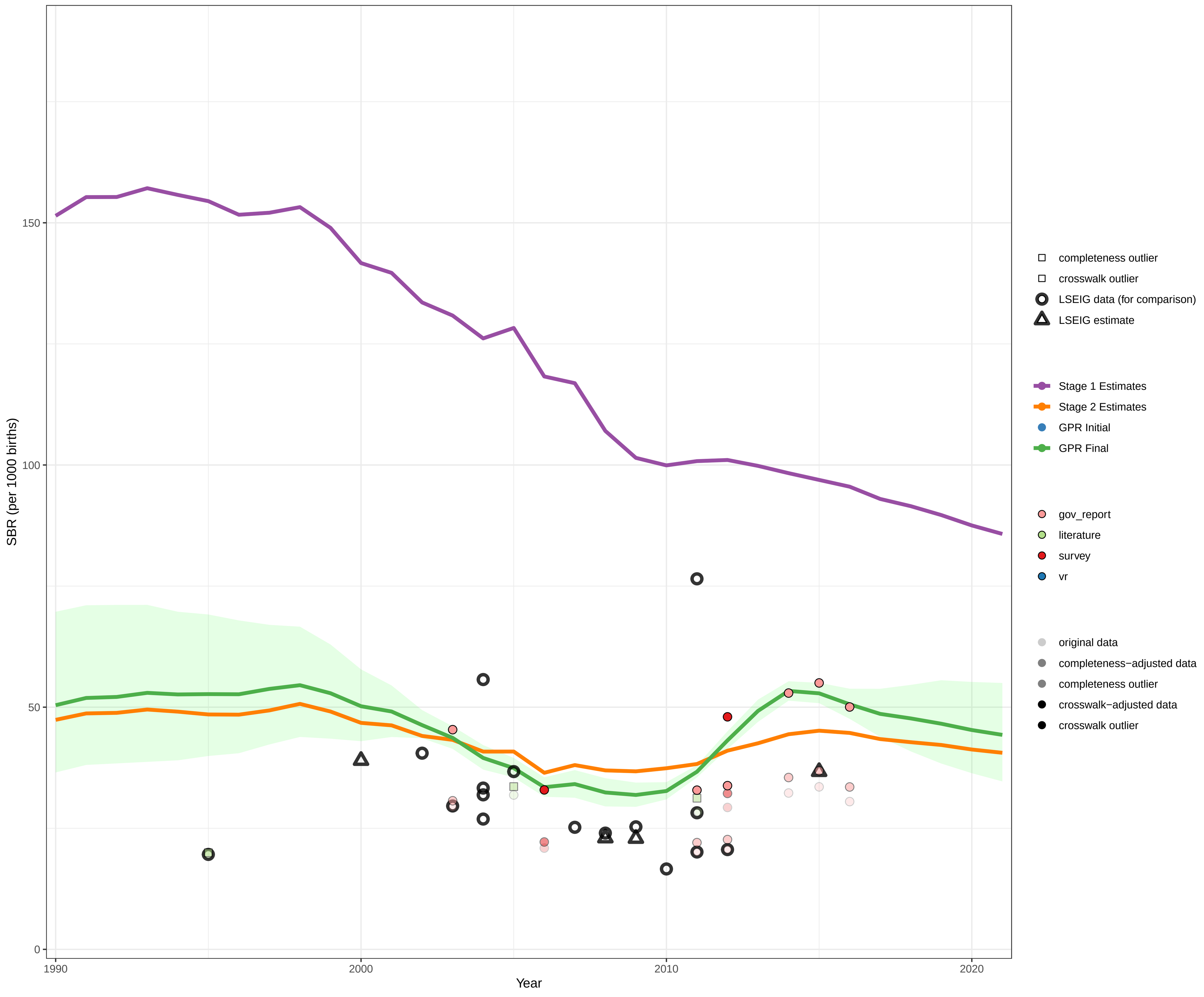

Niger NER Western Sub−Saharan Africa Stillbirth Rate (SBR) − 28 weeks
lambda = 0.6 ; zeta = 0.8 ; scale = 15 ; amp = 0.028

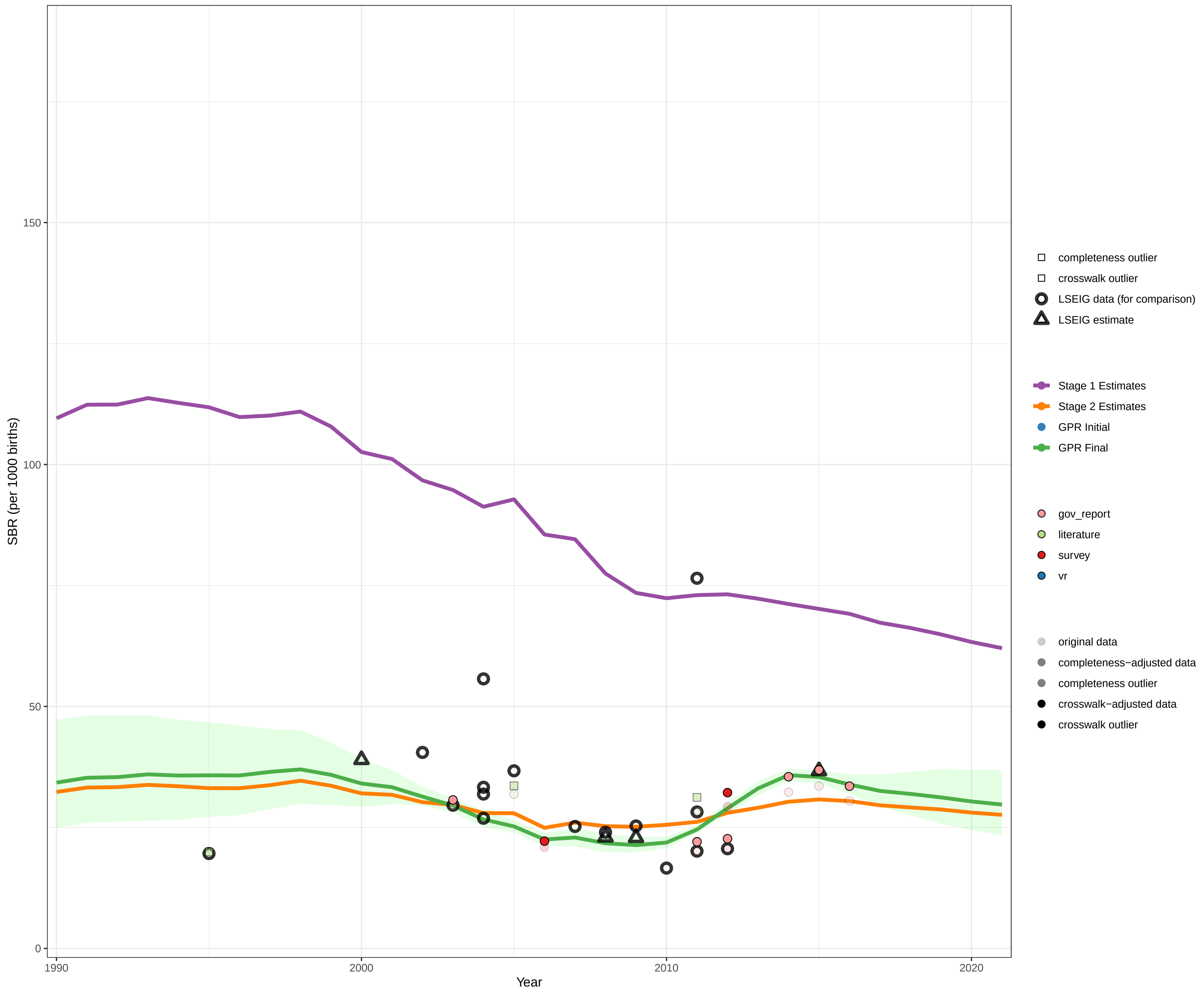

Year	Total Stillbirths (in thousands)	Total Stillbirths (in thousands)	SBR (per 1000 births)	SBR (per 1000 births)	SBR/NMR	SBR/NMR
	20 weeks	28 weeks	20 weeks	28 weeks	20 weeks	28 weeks
1990	23.6 (16.8 to 33.3)	15.8 (11.4 to 22.0)	50.4 (36.5 to 69.7)	34.3 (24.9 to 47.2)	1.21 (0.87 to 1.67)	0.82 (0.60 to 1.13)
1995	29.4 (21.9 to 39.2)	19.6 (14.7 to 25.9)	52.7 (39.9 to 69.1)	35.8 (27.2 to 46.8)	1.23 (0.93 to 1.61)	0.83 (0.63 to 1.09)
2000	33.0 (28.0 to 38.3)	22.0 (18.8 to 25.5)	50.2 (43.0 to 57.8)	34.1 (29.2 to 39.2)	1.27 (1.08 to 1.46)	0.86 (0.74 to 0.99)
2005	27.8 (26.3 to 29.4)	18.5 (17.5 to 19.5)	37.4 (35.5 to 39.5)	25.2 (24.0 to 26.6)	1.05 (1.00 to 1.11)	0.71 (0.67 to 0.75)
2010	27.6 (26.1 to 29.2)	18.2 (17.2 to 19.3)	32.7 (31.0 to 34.5)	21.9 (20.7 to 23.1)	1.19 (1.13 to 1.25)	0.79 (0.75 to 0.84)
2015	53.1 (50.9 to 55.4)	34.9 (33.6 to 36.3)	52.9 (50.8 to 55.0)	35.4 (34.1 to 36.8)	1.95 (1.87 to 2.03)	1.30 (1.26 to 1.35)
2020	53.7 (42.7 to 66.1)	35.5 (28.4 to 43.4)	45.3 (36.4 to 55.2)	30.4 (24.5 to 36.9)	1.81 (1.45 to 2.20)	1.21 (0.98 to 1.47)
2021	54.5 (42.2 to 68.4)	36.0 (28.1 to 44.9)	44.3 (34.7 to 55.0)	29.7 (23.3 to 36.8)	1.80 (1.41 to 2.23)	1.21 (0.95 to 1.49)

Nigeria NGA Western Sub−Saharan Africa Stillbirth Rate / Neonatal Mortality Rate (SBR/NMR) − 20 weeks
lambda = 0.8 ; zeta = 0.7 ; scale = 15 ; amp = 0.028

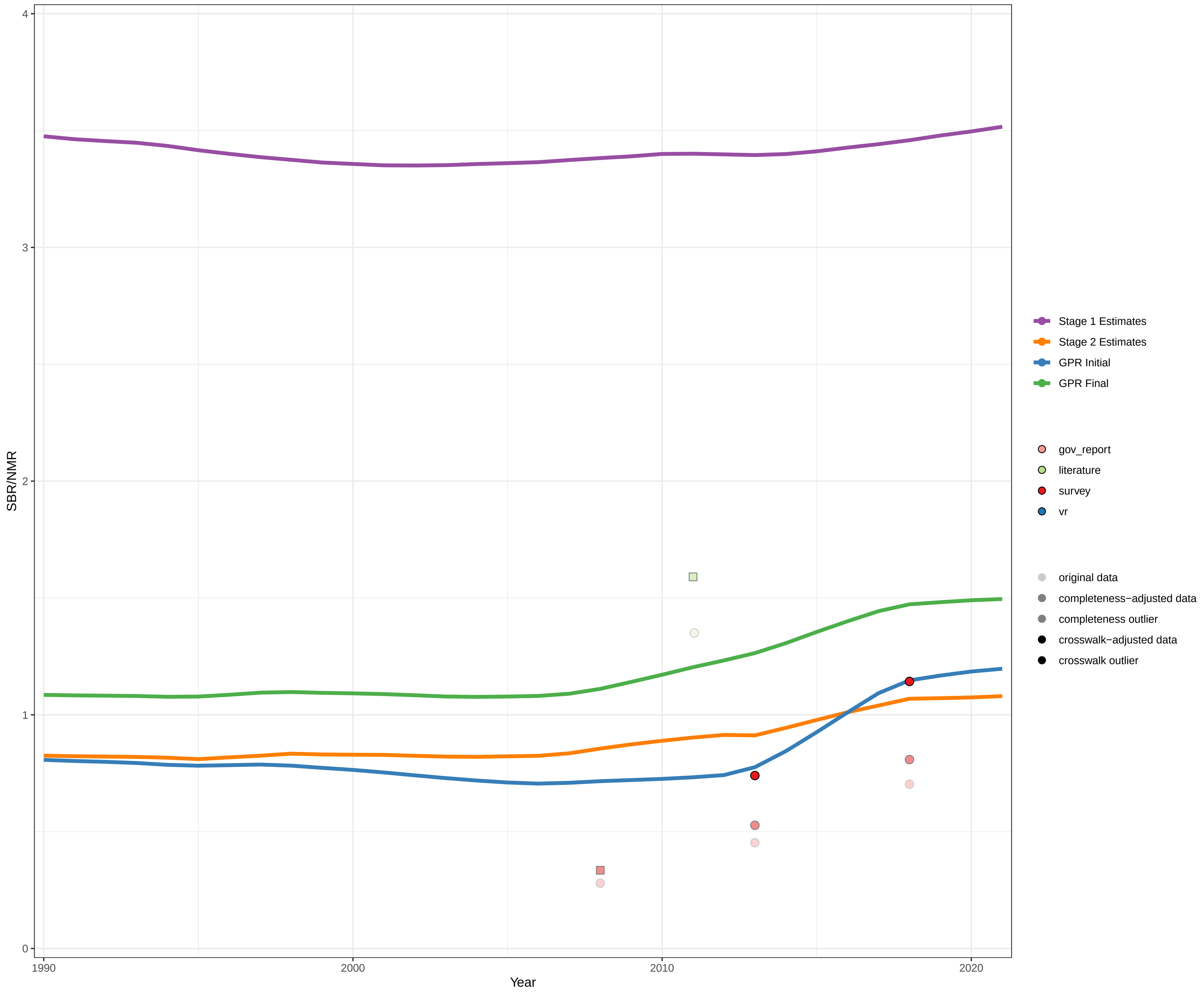

Nigeria NGA Western Sub−Saharan Africa Stillbirth Rate / Neonatal Mortality Rate (SBR/NMR) − 28 weeks
lambda = 0.8 ; zeta = 0.7 ; scale = 15 ; amp = 0.028

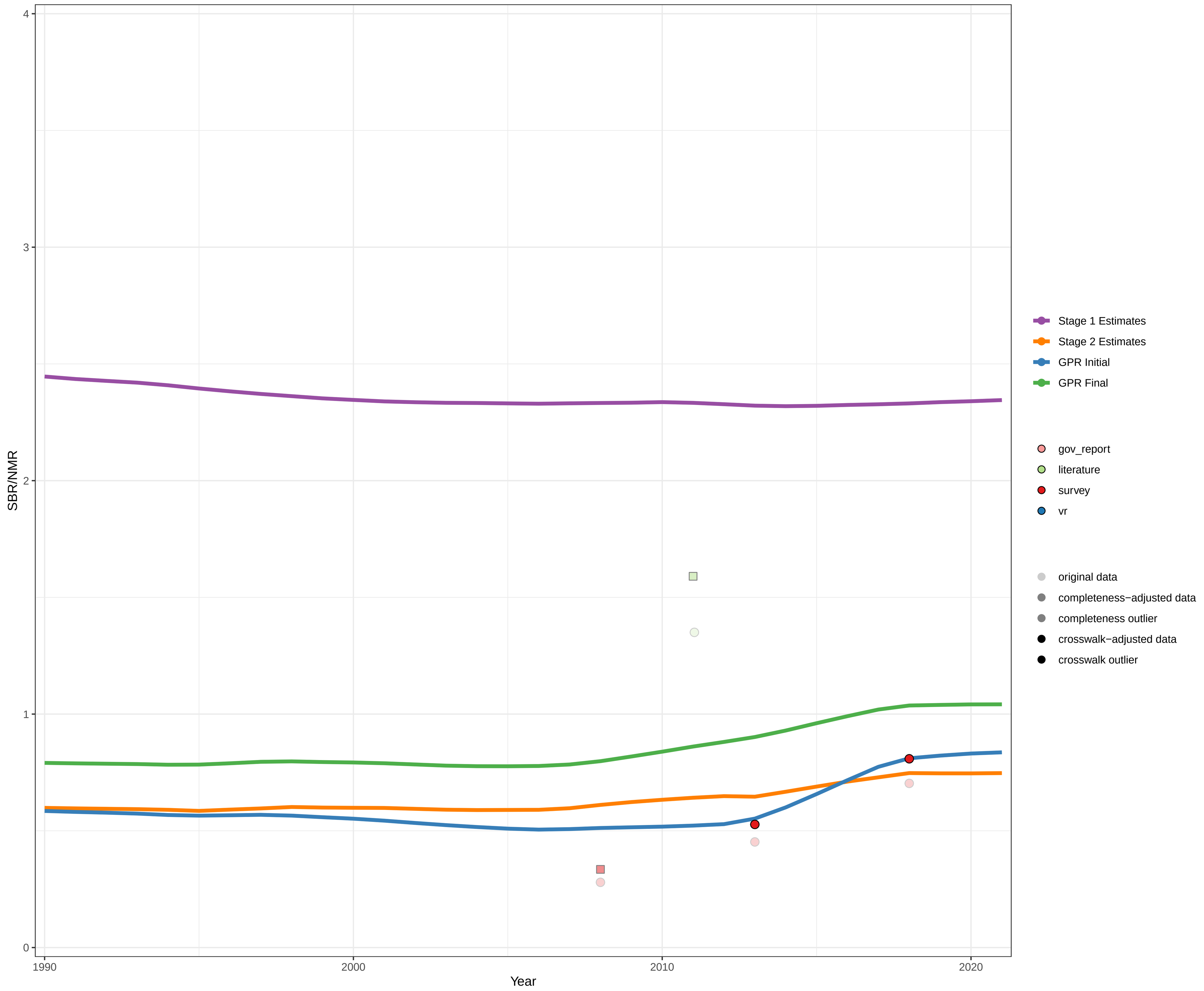

Definition	Years	Outlier	Source.Type	Title
28_weeks	2013	0	Survey	Nigeria Demographic and Health Survey 2013
28_weeks	2018	0	Survey	Nigeria Demographic and Health Survey 2018
28_weeks	2008	1	Survey	Nigeria Demographic and Health Survey 2008
28_weeks_OR_1000_grams	2011	1	Sci Lit	Maternal complications and perinatal mortality: findings of the World Health Organization Multicountry Survey on Maternal and Newborn Health

Nigeria NGA Western Sub−Saharan Africa Stillbirth Rate (SBR) − 20 weeks
lambda = 0.8 ; zeta = 0.7 ; scale = 15 ; amp = 0.028

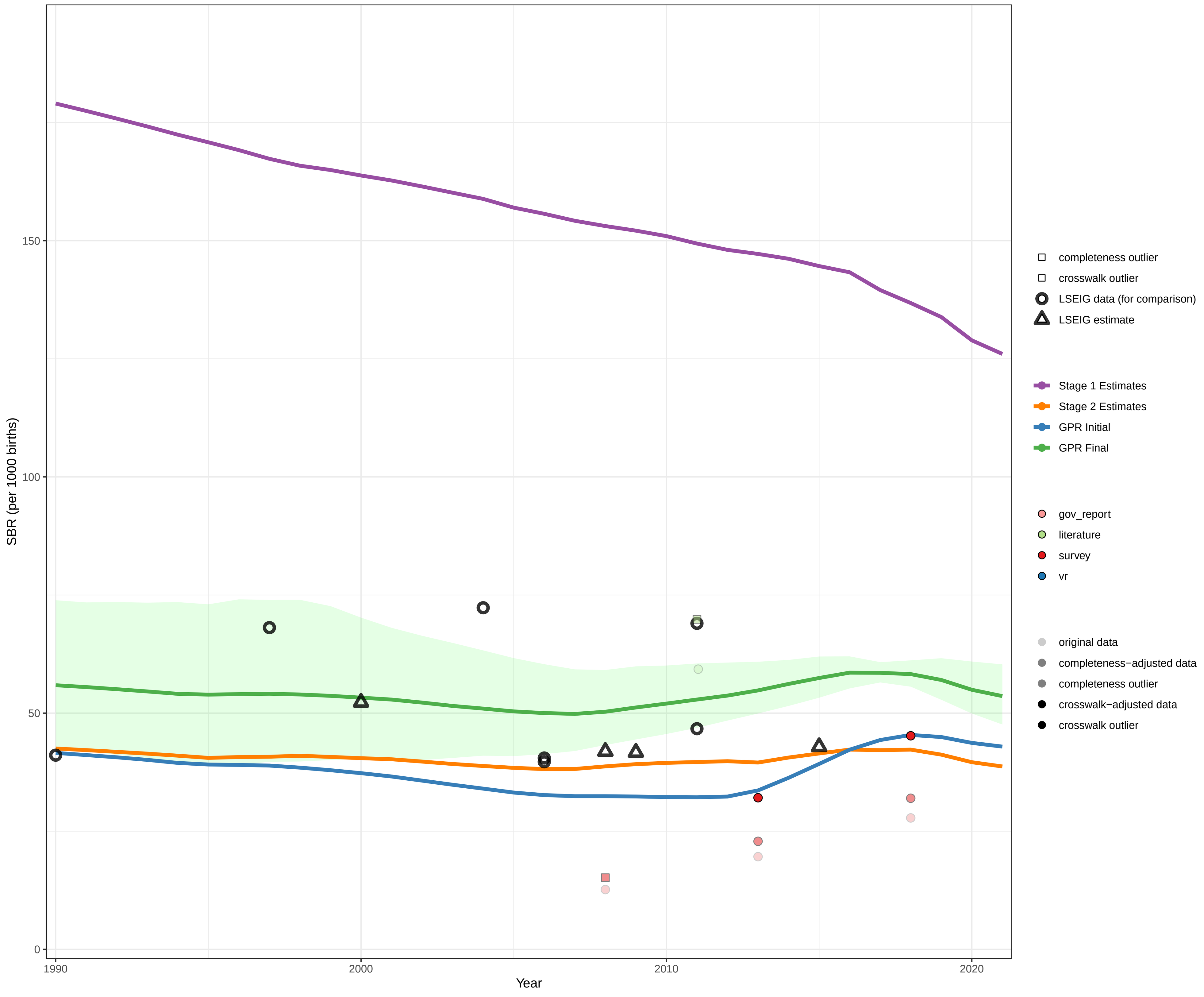

Nigeria NGA Western Sub−Saharan Africa Stillbirth Rate (SBR) − 28 weeks
lambda = 0.8 ; zeta = 0.7 ; scale = 15 ; amp = 0.028

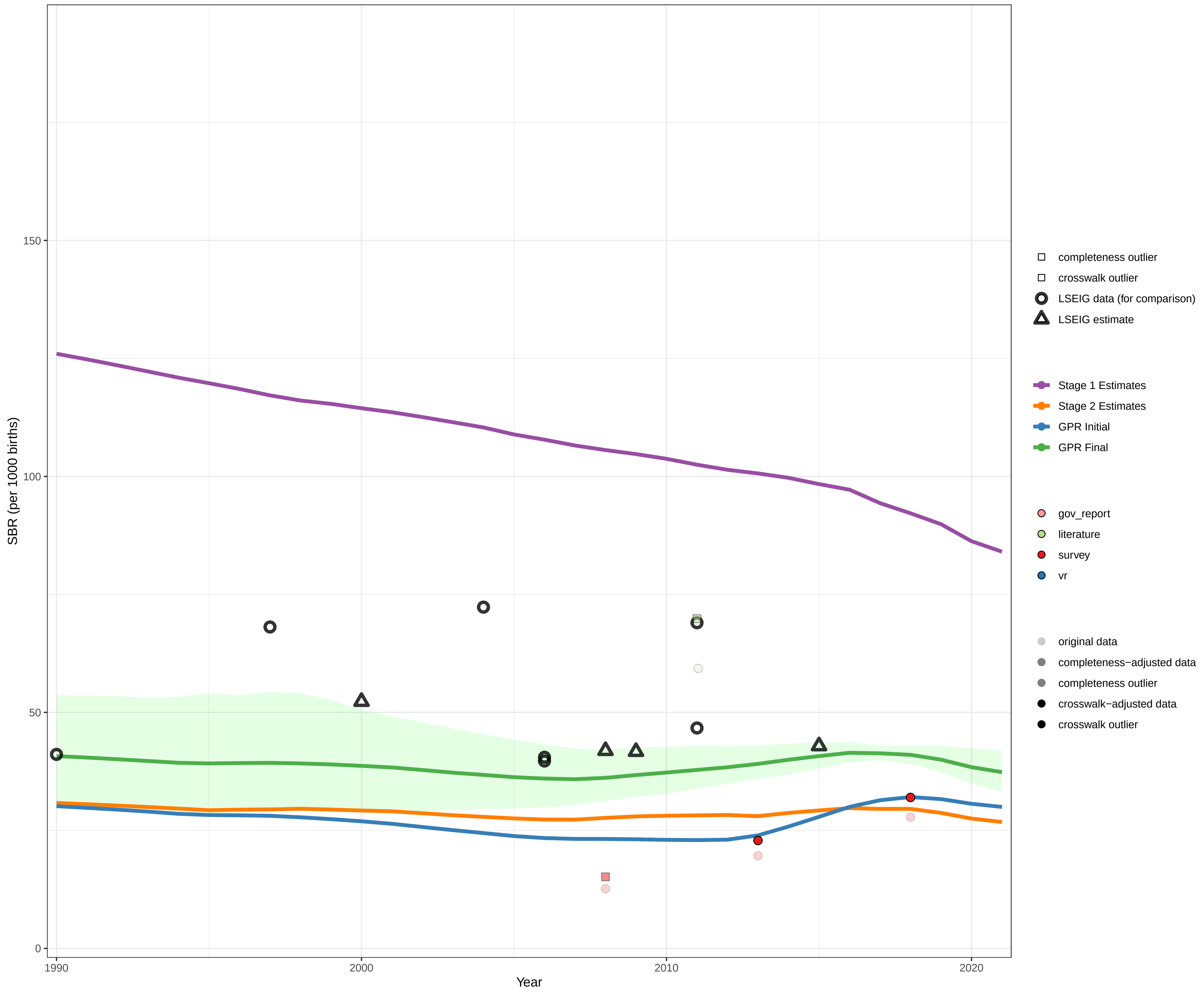

Year	Total Stillbirths (in thousands)	Total Stillbirths (in thousands)	SBR (per 1000 births)	SBR (per 1000 births)	SBR/NMR	SBR/NMR
	20 weeks	28 weeks	20 weeks	28 weeks	20 weeks	28 weeks
1990	246.3 (182.9 to 331.4)	176.6 (132.0 to 235.2)	55.9 (42.2 to 73.9)	40.7 (30.8 to 53.6)	1.09 (0.82 to 1.43)	0.79 (0.60 to 1.04)
1995	277.0 (198.2 to 382.3)	198.1 (143.2 to 277.0)	53.9 (39.2 to 73.0)	39.2 (28.7 to 54.0)	1.08 (0.78 to 1.46)	0.78 (0.57 to 1.08)
2000	320.9 (239.4 to 430.2)	229.4 (172.0 to 304.0)	53.3 (40.3 to 70.2)	38.7 (29.3 to 50.7)	1.09 (0.83 to 1.44)	0.79 (0.60 to 1.04)
2005	349.7 (280.9 to 432.9)	248.1 (201.1 to 304.8)	50.4 (40.9 to 61.6)	36.3 (29.6 to 44.2)	1.08 (0.88 to 1.32)	0.78 (0.63 to 0.95)
2010	407.0 (354.2 to 474.0)	287.0 (251.2 to 330.3)	52.0 (45.6 to 60.1)	37.3 (32.8 to 42.6)	1.17 (1.03 to 1.35)	0.84 (0.74 to 0.96)
2015	490.4 (452.7 to 531.8)	341.8 (318.2 to 368.1)	57.4 (53.3 to 62.0)	40.7 (38.0 to 43.7)	1.35 (1.26 to 1.46)	0.96 (0.90 to 1.03)
2020	481.2 (434.9 to 536.9)	330.5 (299.3 to 366.6)	54.9 (49.9 to 60.9)	38.4 (34.9 to 42.4)	1.49 (1.35 to 1.65)	1.04 (0.95 to 1.15)
2021	472.1 (416.4 to 535.0)	323.3 (285.9 to 364.5)	53.6 (47.6 to 60.3)	37.3 (33.2 to 41.9)	1.50 (1.33 to 1.68)	1.04 (0.93 to 1.17)

Sao Tome and Principe   STP   Western Sub−Saharan Africa   Stillbirth Rate / Neonatal Mortality Rate (SBR/NMR) − 20 weeks
lambda = 1 ; zeta = 0.6 ; scale = 15 ; amp = 0.028

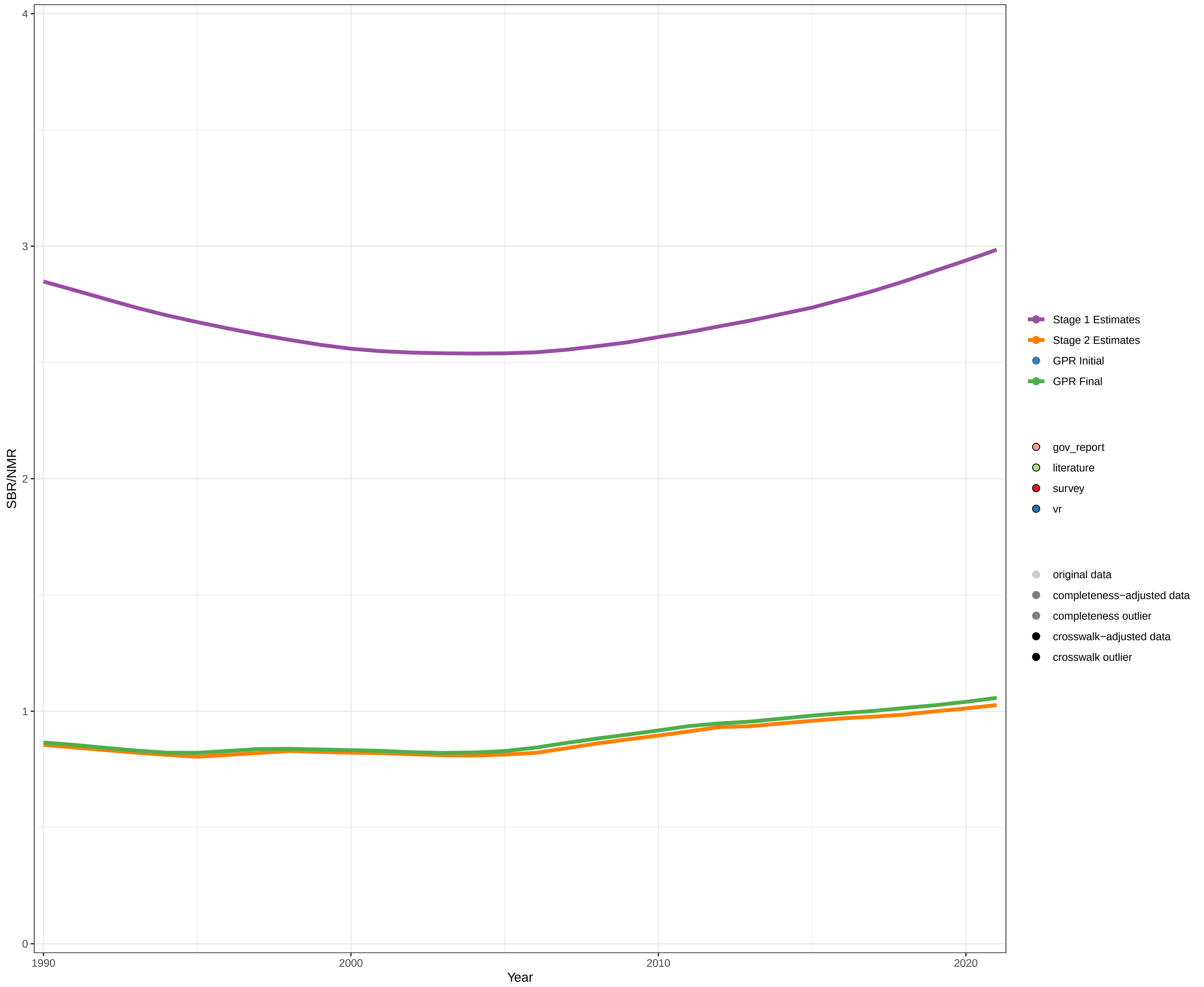

Sao Tome and Principe   STP   Western Sub−Saharan Africa   Stillbirth Rate / Neonatal Mortality Rate (SBR/NMR) − 28 weeks
lambda = 1 ; zeta = 0.6 ; scale = 15 ; amp = 0.028

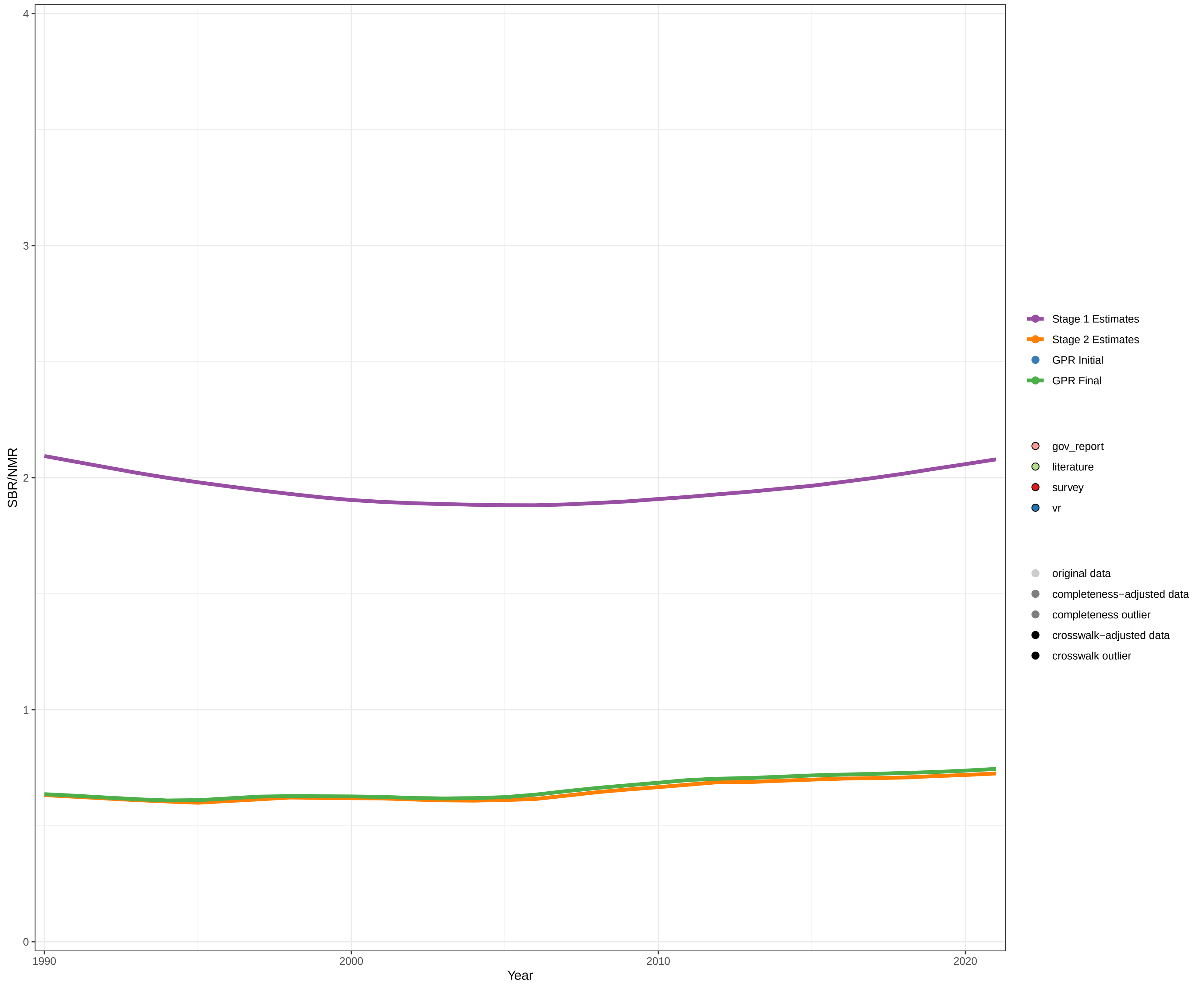

Definition	Years	Outlier	Source.Type	Title

Sao Tome and Principe   STP   Western Sub−Saharan Africa   Stillbirth Rate (SBR) − 20 weeks
lambda = 1 ; zeta = 0.6 ; scale = 15 ; amp = 0.028

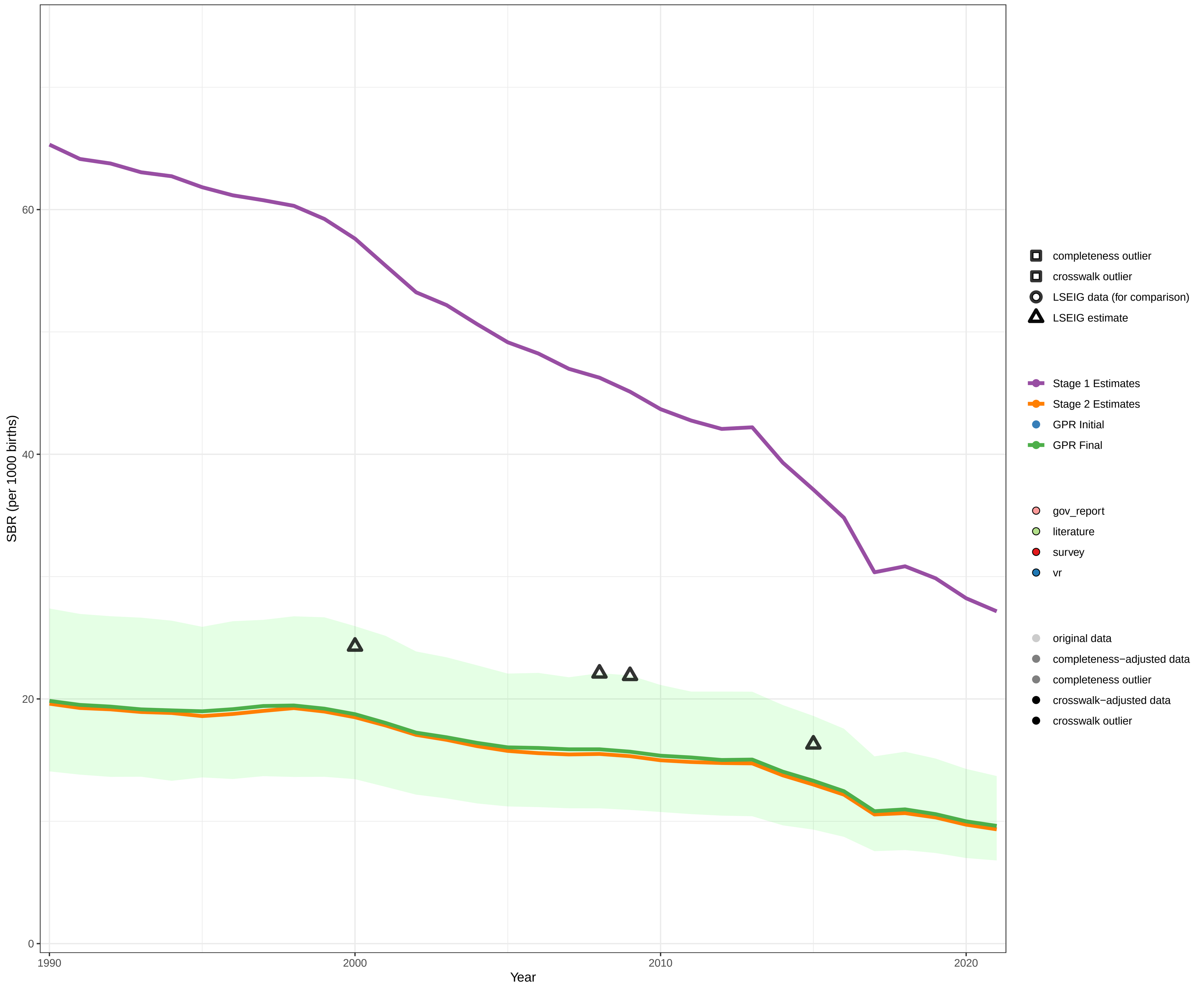

Sao Tome and Principe   STP   Western Sub−Saharan Africa   Stillbirth Rate (SBR) − 28 weeks
lambda = 1 ; zeta = 0.6 ; scale = 15 ; amp = 0.028

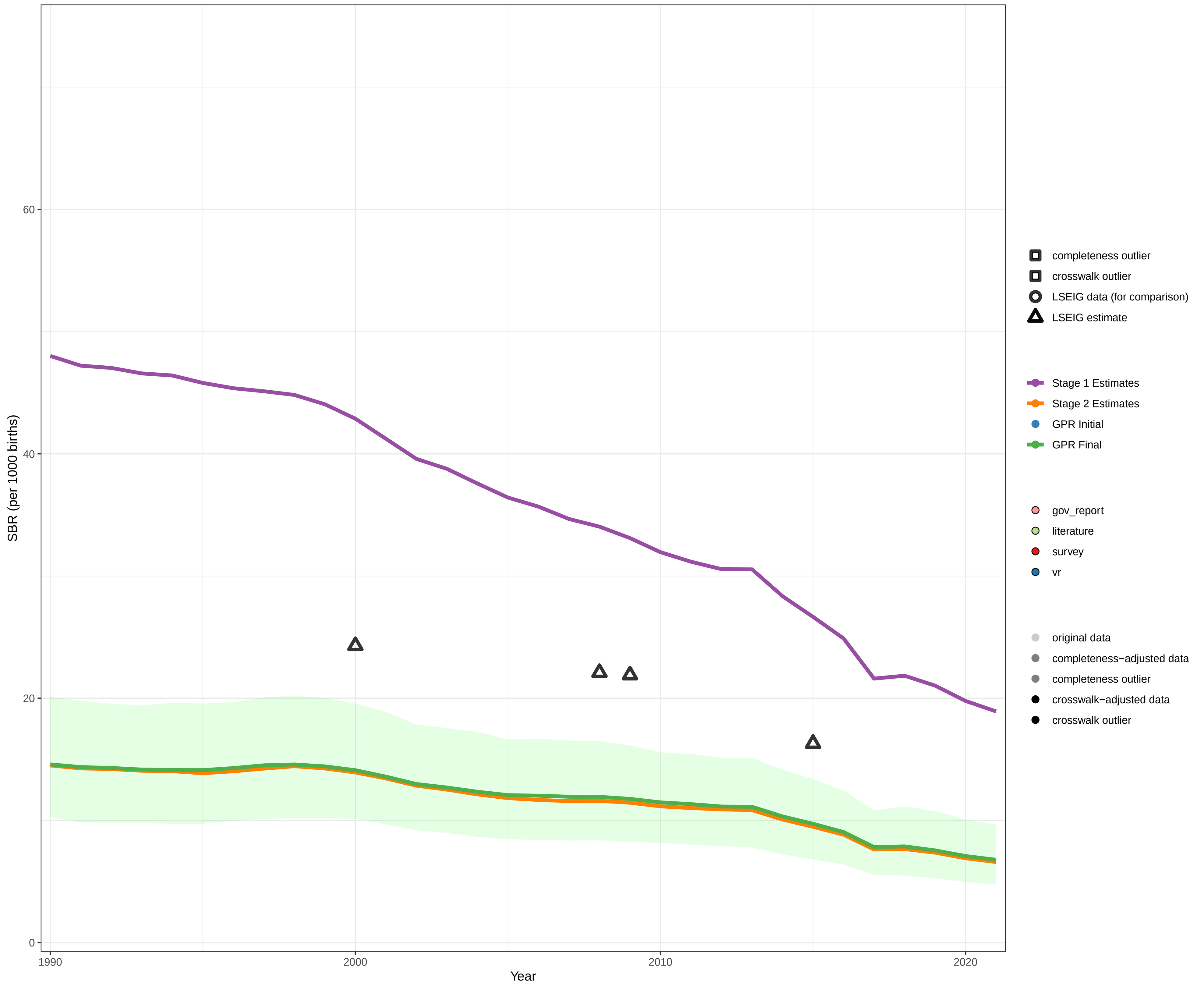

Year	Total Stillbirths (in thousands)	Total Stillbirths (in thousands)	SBR (per 1000 births)	SBR (per 1000 births)	SBR/NMR	SBR/NMR
	20 weeks	28 weeks	20 weeks	28 weeks	20 weeks	28 weeks
1990	0.1<br>(0.1 to 0.1)	0.1<br>(0.0 to 0.1)	19.8<br>(14.1 to 27.4)	14.6<br>(10.3 to 20.1)	0.87<br>(0.61 to 1.19)	0.64<br>(0.45 to 0.88)
1995	0.1<br>(0.1 to 0.1)	0.1<br>(0.1 to 0.1)	19.0<br>(13.6 to 25.9)	14.1<br>(9.7 to 19.6)	0.82<br>(0.59 to 1.12)	0.61<br>(0.42 to 0.85)
2000	0.1<br>(0.1 to 0.2)	0.1<br>(0.1 to 0.1)	18.8<br>(13.4 to 26.0)	14.1<br>(10.1 to 19.6)	0.83<br>(0.60 to 1.15)	0.63<br>(0.45 to 0.87)
2005	0.1<br>(0.1 to 0.1)	0.1<br>(0.1 to 0.1)	16.0<br>(11.2 to 22.1)	12.1<br>(8.5 to 16.6)	0.83<br>(0.58 to 1.14)	0.62<br>(0.44 to 0.86)
2010	0.1<br>(0.1 to 0.1)	0.1<br>(0.0 to 0.1)	15.4<br>(10.8 to 21.1)	11.5<br>(8.2 to 15.6)	0.92<br>(0.64 to 1.26)	0.69<br>(0.49 to 0.93)
2015	0.1<br>(0.1 to 0.1)	0.1<br>(0.0 to 0.1)	13.3<br>(9.3 to 18.6)	9.7<br>(6.8 to 13.4)	0.98<br>(0.69 to 1.37)	0.72<br>(0.50 to 0.99)
2020	0.1<br>(0.0 to 0.1)	0.0<br>(0.0 to 0.1)	10.0<br>(7.0 to 14.3)	7.1<br>(5.0 to 10.1)	1.04<br>(0.73 to 1.49)	0.74<br>(0.52 to 1.05)
2021	0.0<br>(0.0 to 0.1)	0.0<br>(0.0 to 0.0)	9.6<br>(6.8 to 13.7)	6.8<br>(4.8 to 9.7)	1.06<br>(0.75 to 1.51)	0.75<br>(0.52 to 1.06)

Senegal SEN Western Sub−Saharan Africa Stillbirth Rate / Neonatal Mortality Rate (SBR/NMR) − 20 weeks
lambda = 0.4 ; zeta = 0.9 ; scale = 10 ; amp = 0.028

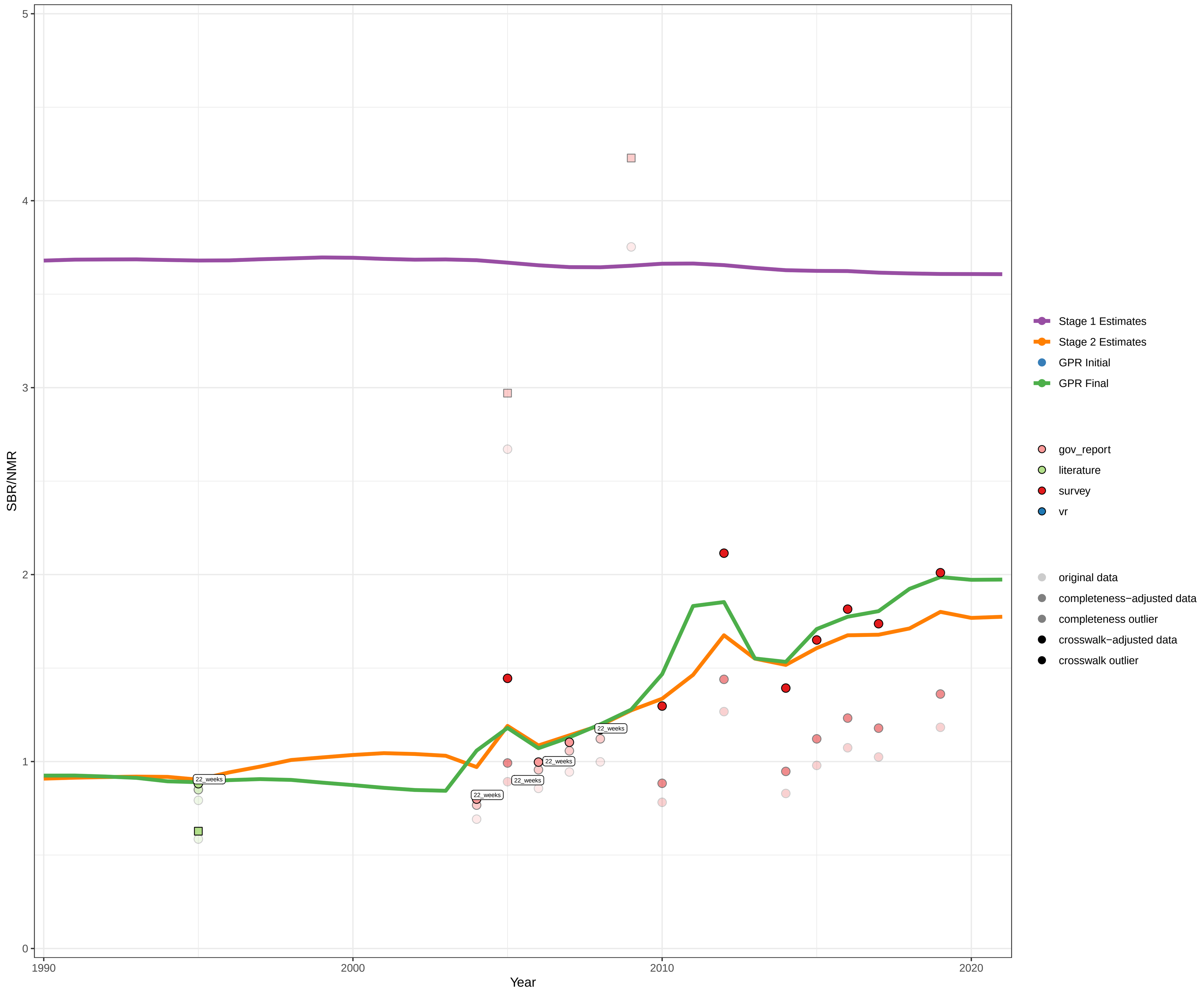

Senegal SEN Western Sub−Saharan Africa Stillbirth Rate / Neonatal Mortality Rate (SBR/NMR) − 28 weeks
lambda = 0.4 ; zeta = 0.9 ; scale = 10 ; amp = 0.028

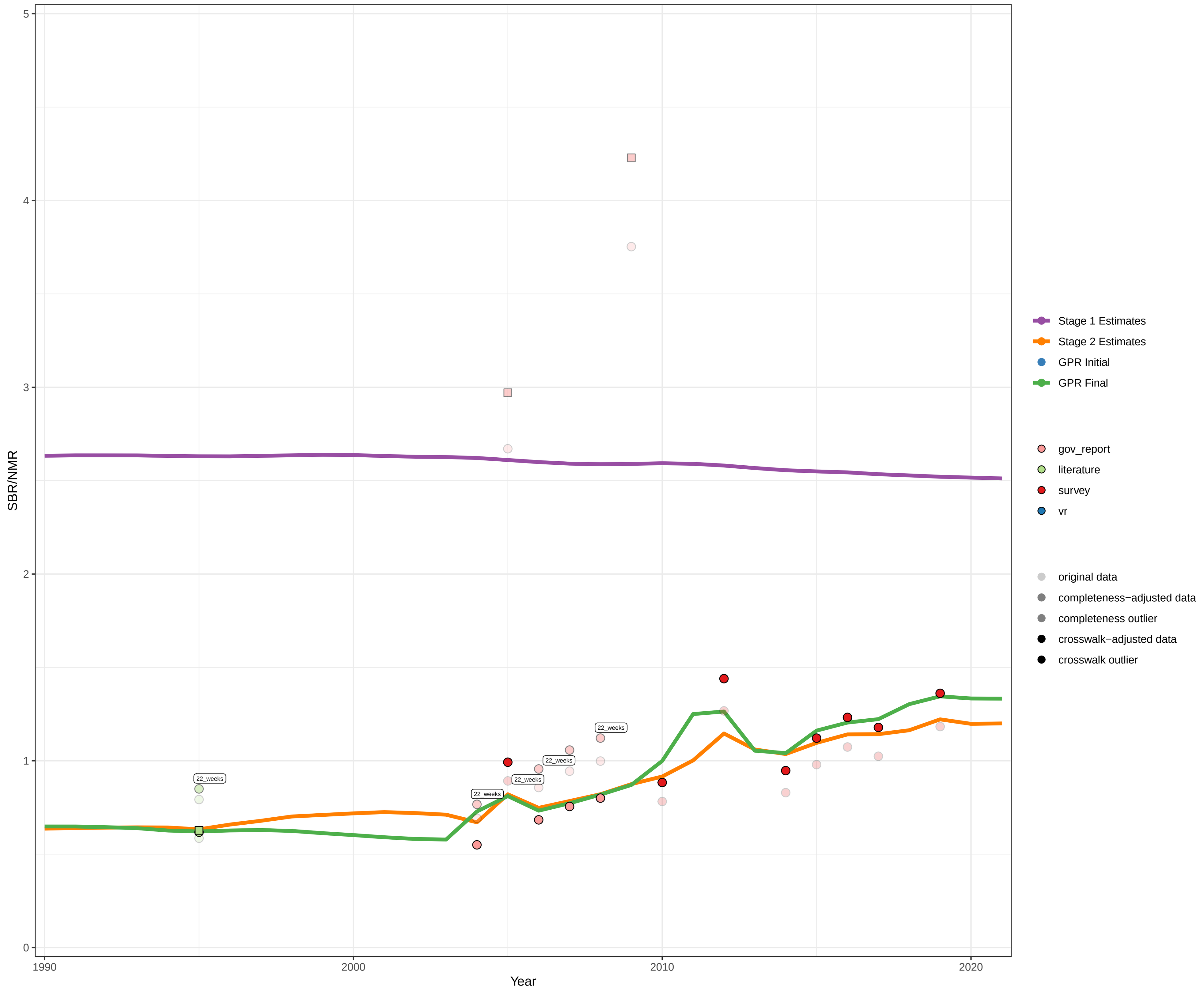

Definition	Years	Outlier	Source.Type	Title
22_weeks	1995	0	Sci Lit	Risk factors for perinatal mortality in West Africa: a population−based study of 20326 pregnancies. MOMA group
22_weeks	2004, 2006−2008	0	Statistical Report	Senegal Health Statistical Yearbook
28_weeks	2005	0	Survey	Senegal Demographic and Health Survey 2005
28_weeks	2010	0	Survey	Senegal Demographic and Health Survey 2010−2011
28_weeks	2012	0	Survey	Senegal Continuous Demographic and Health Survey 2012−2013
28_weeks	2014	0	Survey	Senegal Continuous Demographic and Health Survey 2014
28_weeks	2015	0	Survey	Senegal Continuous Demographic and Health Survey 2015
28_weeks	2016	0	Survey	Senegal Continuous Demographic and Health Survey 2016
28_weeks	2017	0	Survey	Senegal Continuous Demographic and Health Survey 2017
28_weeks	2019	0	Survey	Senegal Continuous Demographic and Health Survey 2019
28_weeks	2005, 2009	1	Statistical Report	Senegal Health Statistical Yearbook

Senegal SEN Western Sub−Saharan Africa Stillbirth Rate (SBR) − 20 weeks
lambda = 0.4 ; zeta = 0.9 ; scale = 10 ; amp = 0.028

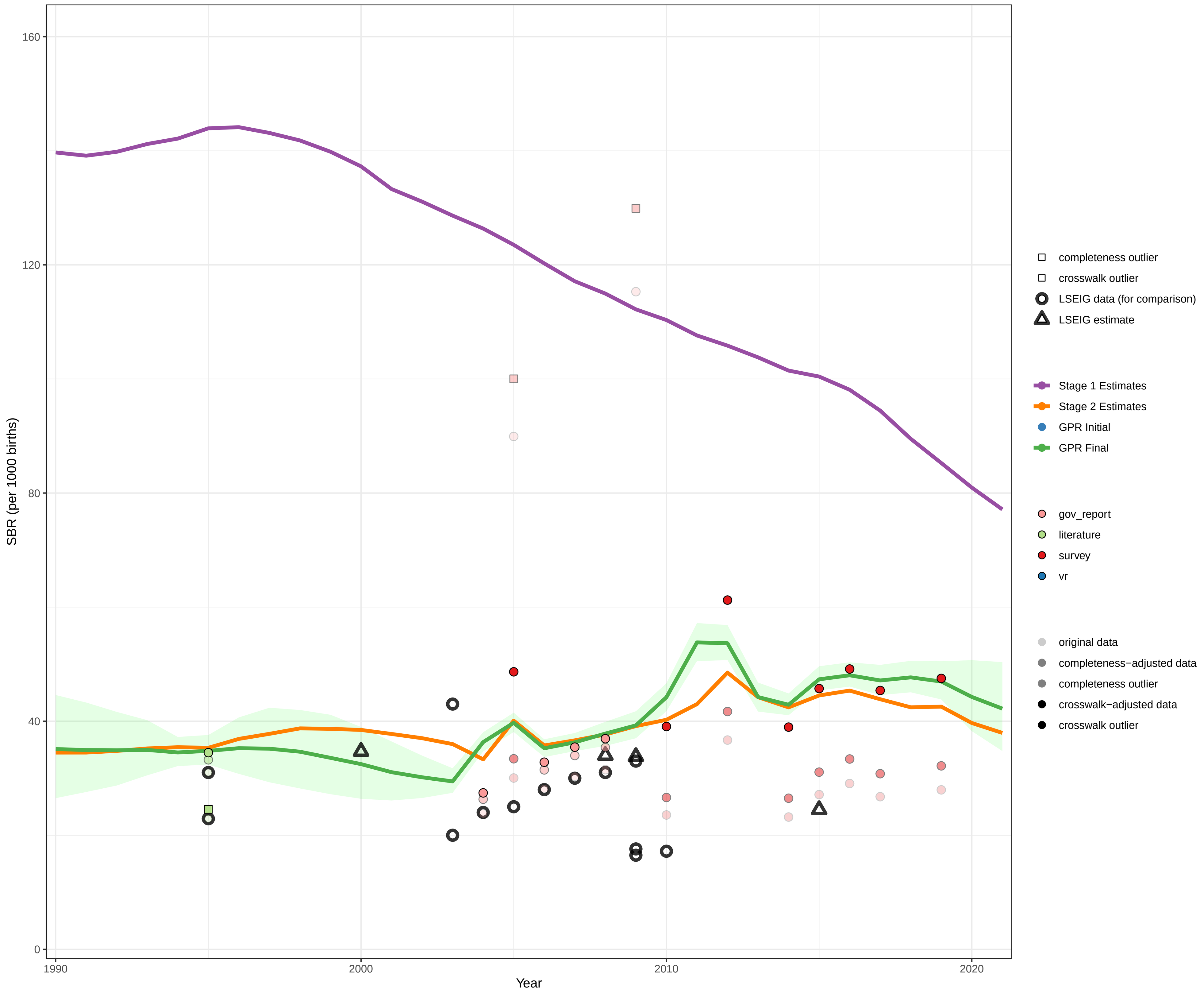

Senegal SEN Western Sub−Saharan Africa Stillbirth Rate (SBR) − 28 weeks
lambda = 0.4 ; zeta = 0.9 ; scale = 10 ; amp = 0.028

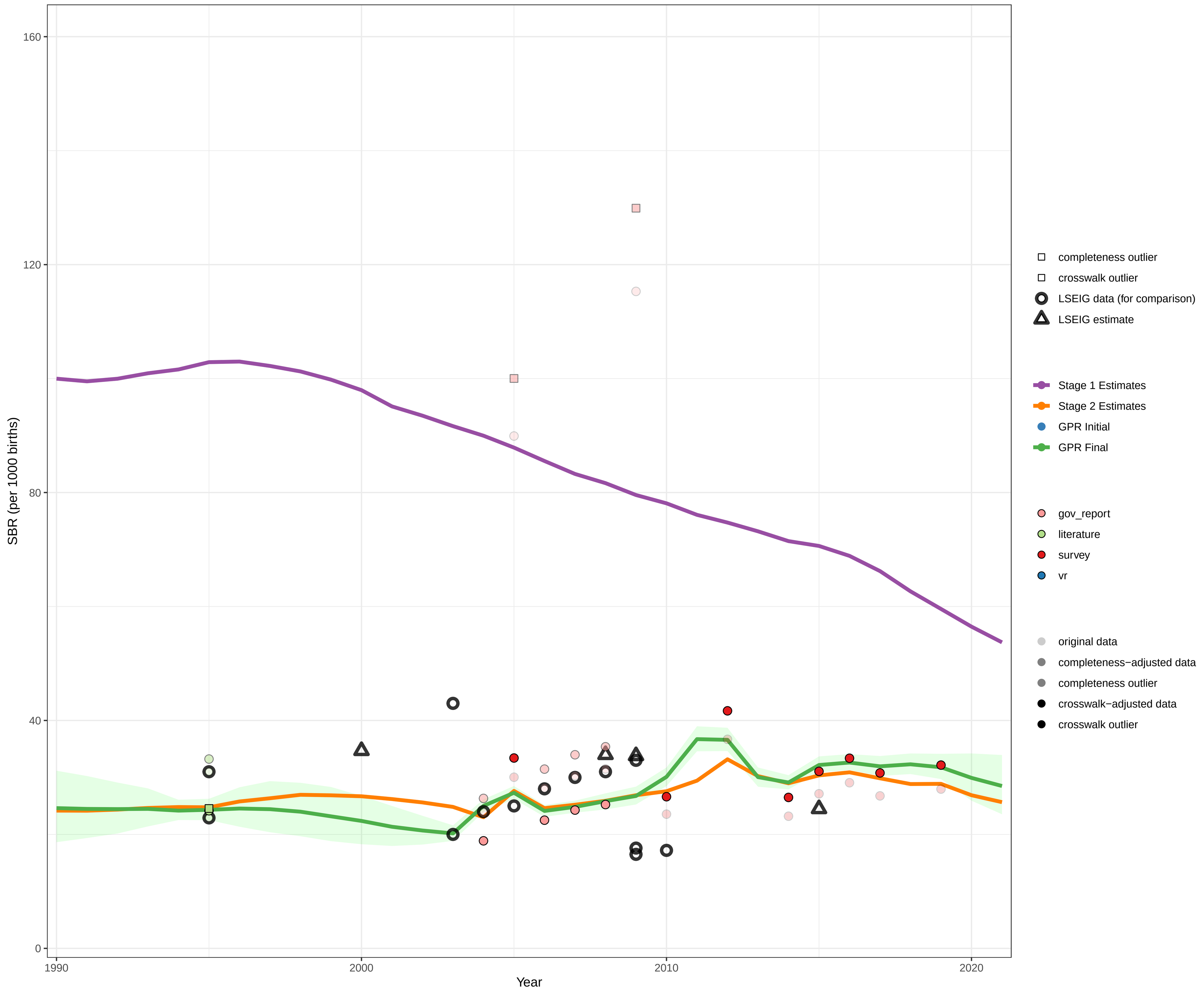

Year	Total Stillbirths (in thousands)	Total Stillbirths (in thousands)	SBR (per 1000 births)	SBR (per 1000 births)	SBR/NMR	SBR/NMR
	20 weeks	28 weeks	20 weeks	28 weeks	20 weeks	28 weeks
1990	12.7 (9.5 to 16.2)	8.8 (6.6 to 11.2)	35.1 (26.5 to 44.6)	24.6 (18.6 to 31.2)	0.92 (0.70 to 1.17)	0.65 (0.49 to 0.82)
1995	13.5 (12.5 to 14.6)	9.3 (8.6 to 10.1)	34.8 (32.4 to 37.6)	24.3 (22.5 to 26.3)	0.89 (0.83 to 0.96)	0.62 (0.58 to 0.67)
2000	13.9 (11.2 to 16.8)	9.5 (7.7 to 11.4)	32.5 (26.4 to 39.0)	22.4 (18.3 to 26.8)	0.87 (0.71 to 1.05)	0.60 (0.49 to 0.72)
2005	19.1 (18.3 to 20.0)	13.0 (12.4 to 13.5)	39.7 (38.1 to 41.5)	27.3 (26.2 to 28.5)	1.18 (1.13 to 1.23)	0.81 (0.78 to 0.85)
2010	22.8 (21.5 to 24.1)	15.3 (14.5 to 16.2)	44.2 (41.8 to 46.7)	30.1 (28.5 to 31.7)	1.47 (1.39 to 1.55)	1.00 (0.95 to 1.05)
2015	24.2 (23.2 to 25.5)	16.2 (15.6 to 17.0)	47.3 (45.4 to 49.6)	32.2 (30.9 to 33.7)	1.71 (1.64 to 1.79)	1.16 (1.12 to 1.22)
2020	22.3 (19.1 to 25.7)	14.8 (12.8 to 17.1)	44.3 (38.2 to 50.7)	29.9 (25.9 to 34.2)	1.97 (1.70 to 2.26)	1.33 (1.16 to 1.52)
2021	21.1 (17.3 to 25.4)	14.1 (11.6 to 16.8)	42.2 (34.8 to 50.4)	28.5 (23.5 to 33.9)	1.97 (1.63 to 2.36)	1.33 (1.10 to 1.59)

Sierra Leone   SLE   Western Sub−Saharan Africa   Stillbirth Rate / Neonatal Mortality Rate (SBR/NMR) − 20 weeks
lambda = 0.8 ; zeta = 0.7 ; scale = 15 ; amp = 0.028

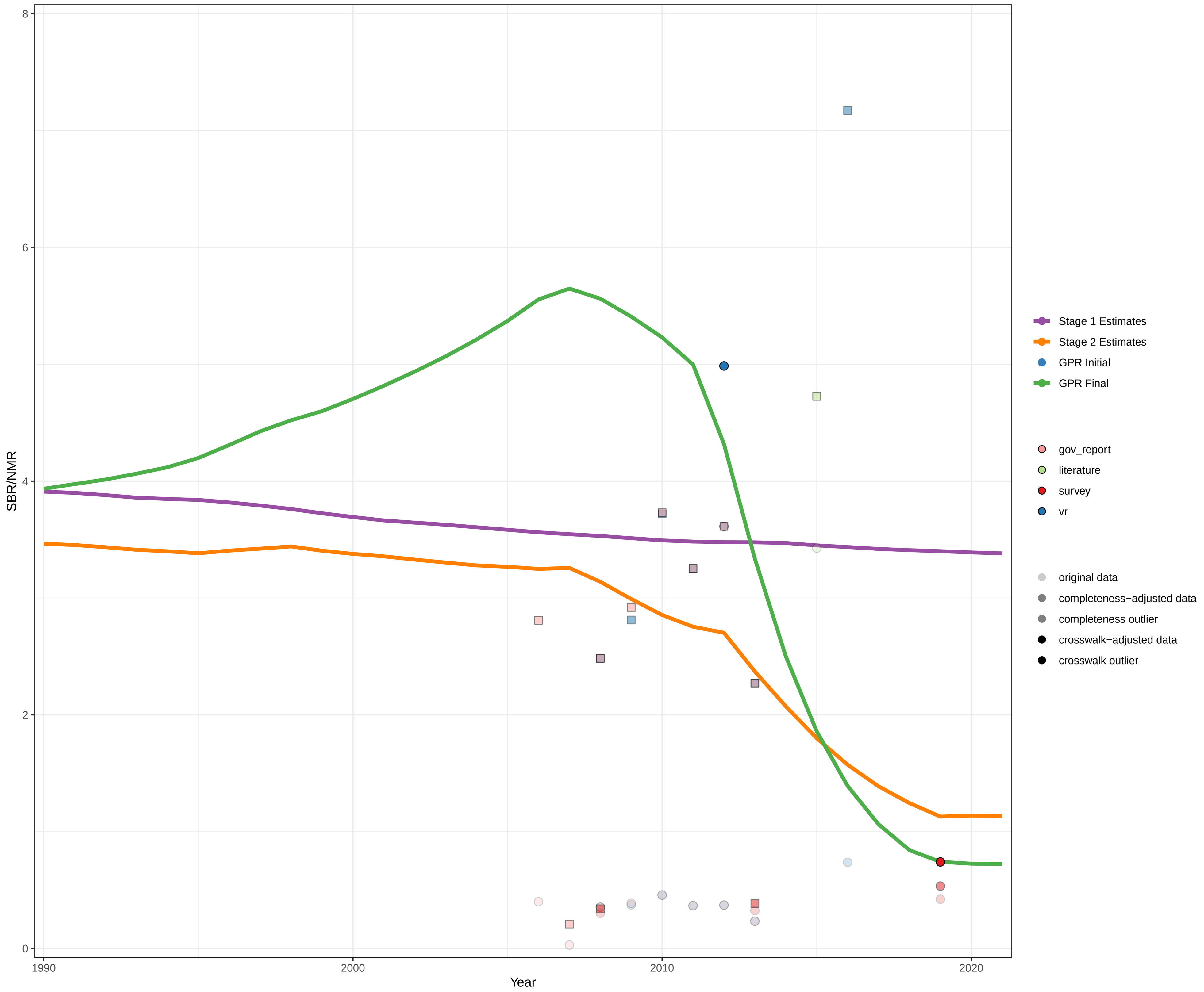

Sierra Leone   SLE   Western Sub−Saharan Africa   Stillbirth Rate / Neonatal Mortality Rate (SBR/NMR) − 28 weeks
lambda = 0.8 ; zeta = 0.7 ; scale = 15 ; amp = 0.028

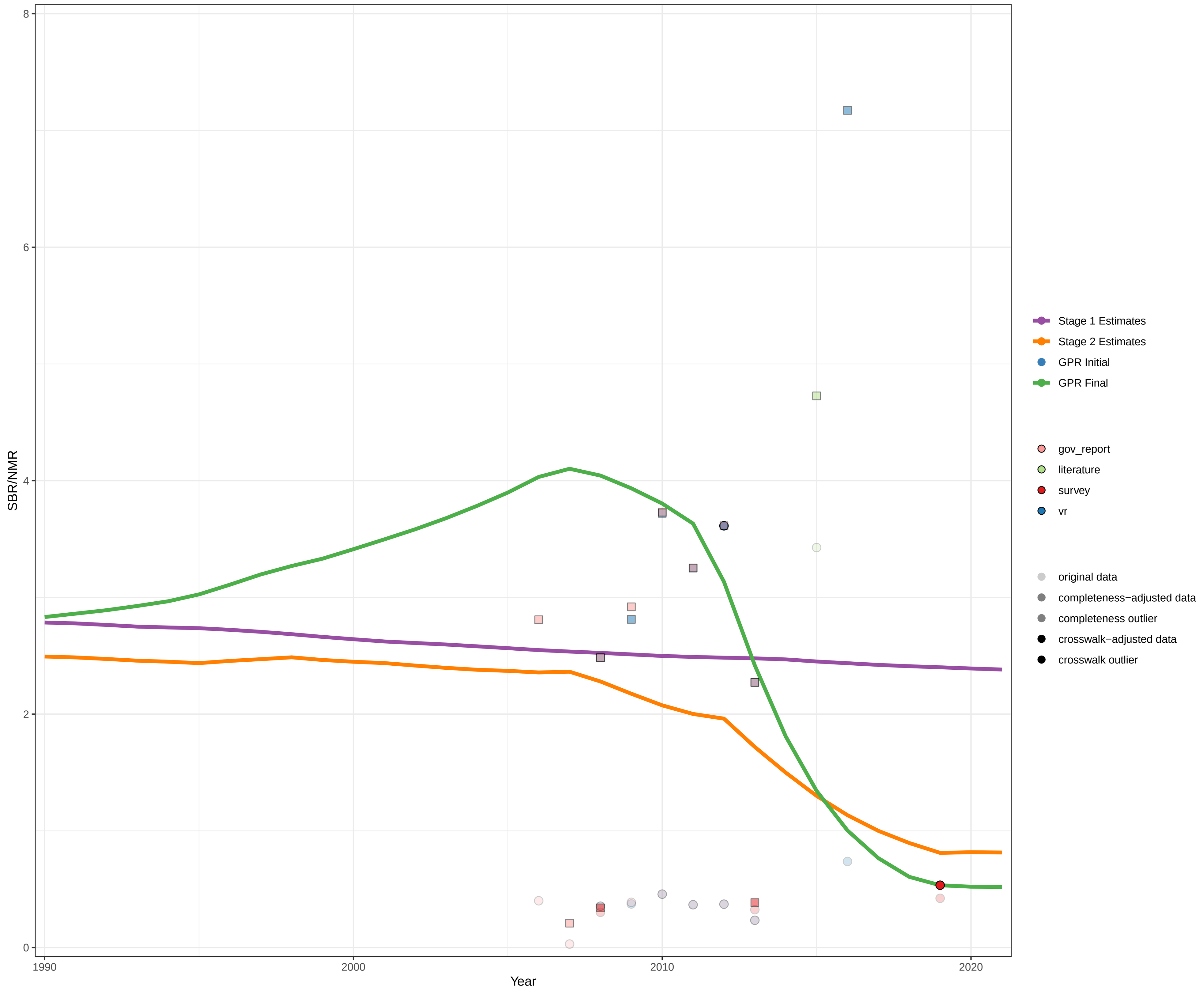

Definition	Years	Outlier	Source.Type	Title
28_weeks	2012	0	VR	United Nations Demographic Yearbook
28_weeks	2019	0	Survey	Sierra Leone Demographic and Health Survey 2019
28_weeks	2006−2013	1	Statistical Report	Sierra Leone Statistical Digest
28_weeks	2008	1	Survey	Sierra Leone Demographic and Health Survey 2008
28_weeks	2008−2011, 2013	1	VR	United Nations Demographic Yearbook
28_weeks	2013	1	Survey	Sierra Leone Demographic and Health Survey 2013
28_weeks	2016	1	VR	Sierra Leone Maternal Death Surveillance and Response Annual Report
28_weeks_OR_1000_grams	2015	1	Sci Lit	Application of the ICD−PM classification system to stillbirth in four sub−Saharan African countries

Sierra Leone   SLE   Western Sub−Saharan Africa   Stillbirth Rate (SBR) − 20 weeks
lambda = 0.8 ; zeta = 0.7 ; scale = 15 ; amp = 0.028

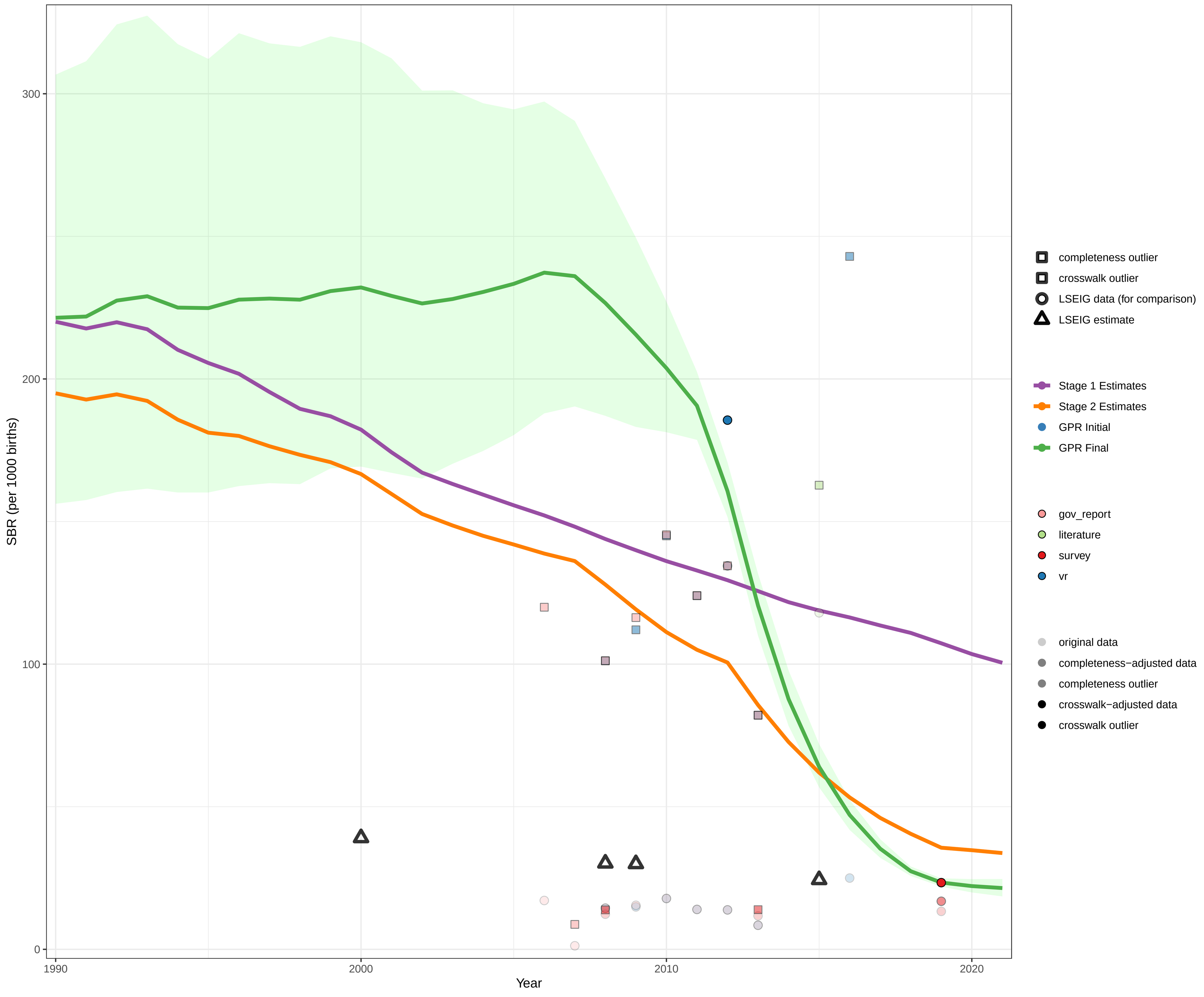

Sierra Leone   SLE   Western Sub−Saharan Africa   Stillbirth Rate (SBR) − 28 weeks
lambda = 0.8 ; zeta = 0.7 ; scale = 15 ; amp = 0.028

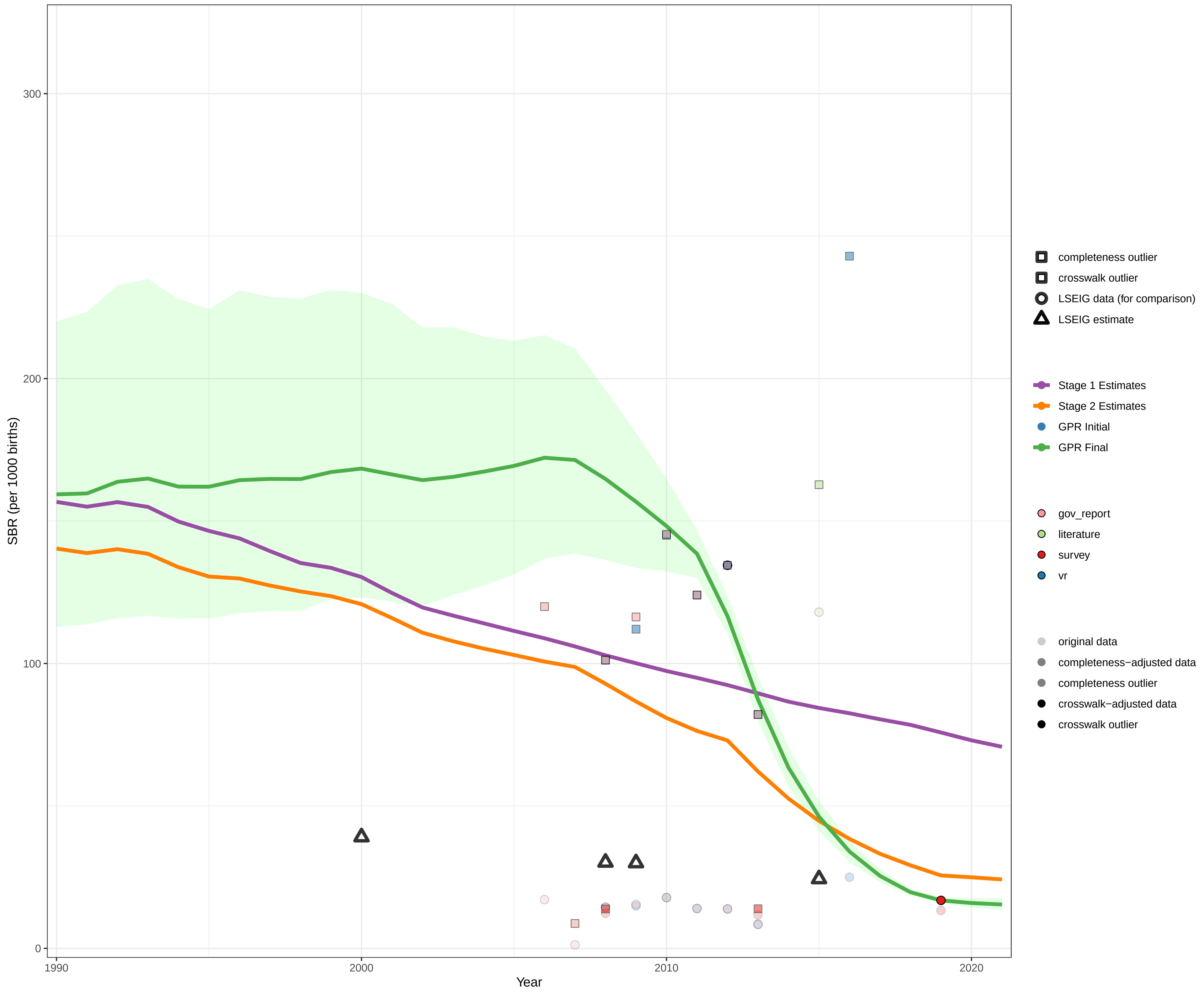

Year	Total Stillbirths (in thousands)	Total Stillbirths (in thousands)	SBR (per 1000 births)	SBR (per 1000 births)	SBR/NMR	SBR/NMR
	20 weeks	28 weeks	20 weeks	28 weeks	20 weeks	28 weeks
1990	59.1 (38.0 to 90.7)	39.2 (26.1 to 57.9)	221.5 (156.2 to 306.7)	159.4 (112.8 to 220.1)	3.93 (2.78 to 5.45)	2.83 (2.00 to 3.91)
1995	57.9 (37.6 to 89.6)	38.4 (25.8 to 57.1)	224.8 (160.2 to 312.2)	162.0 (115.8 to 224.3)	4.20 (2.99 to 5.83)	3.03 (2.16 to 4.19)
2000	62.4 (41.6 to 95.2)	41.6 (28.7 to 61.0)	232.1 (169.3 to 318.1)	168.4 (123.2 to 230.1)	4.70 (3.43 to 6.45)	3.41 (2.50 to 4.66)
2005	74.7 (53.6 to 101.8)	49.9 (36.8 to 66.1)	233.4 (180.3 to 294.5)	169.4 (131.2 to 213.3)	5.37 (4.15 to 6.78)	3.90 (3.02 to 4.91)
2010	66.6 (57.5 to 76.4)	45.2 (39.6 to 51.3)	203.8 (181.3 to 227.1)	148.2 (132.2 to 165.0)	5.23 (4.65 to 5.83)	3.80 (3.39 to 4.23)
2015	19.3 (17.0 to 21.9)	13.7 (12.1 to 15.4)	64.0 (56.8 to 72.0)	46.2 (41.1 to 51.9)	1.86 (1.65 to 2.09)	1.34 (1.19 to 1.51)
2020	6.8 (6.1 to 7.5)	4.8 (4.3 to 5.4)	22.2 (19.9 to 24.6)	15.9 (14.3 to 17.6)	0.73 (0.65 to 0.81)	0.52 (0.47 to 0.58)
2021	6.6 (5.7 to 7.7)	4.7 (4.1 to 5.4)	21.5 (18.6 to 24.7)	15.4 (13.3 to 17.6)	0.72 (0.62 to 0.83)	0.52 (0.45 to 0.59)

Togo TGO Western Sub−Saharan Africa Stillbirth Rate / Neonatal Mortality Rate (SBR/NMR) − 20 weeks
lambda = 0.6 ; zeta = 0.8 ; scale = 15 ; amp = 0.028

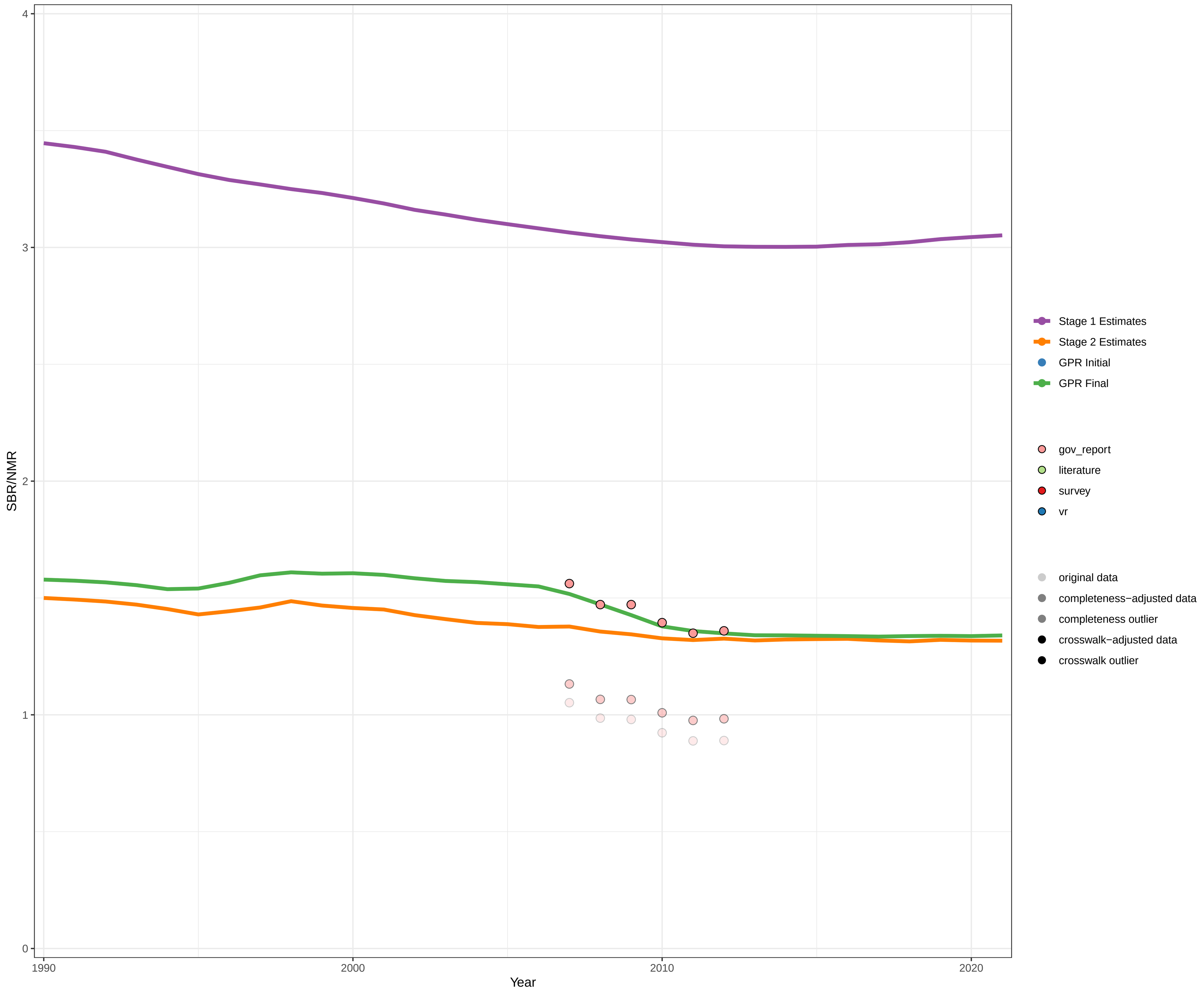

Togo TGO Western Sub−Saharan Africa Stillbirth Rate / Neonatal Mortality Rate (SBR/NMR) − 28 weeks
lambda = 0.6 ; zeta = 0.8 ; scale = 15 ; amp = 0.028

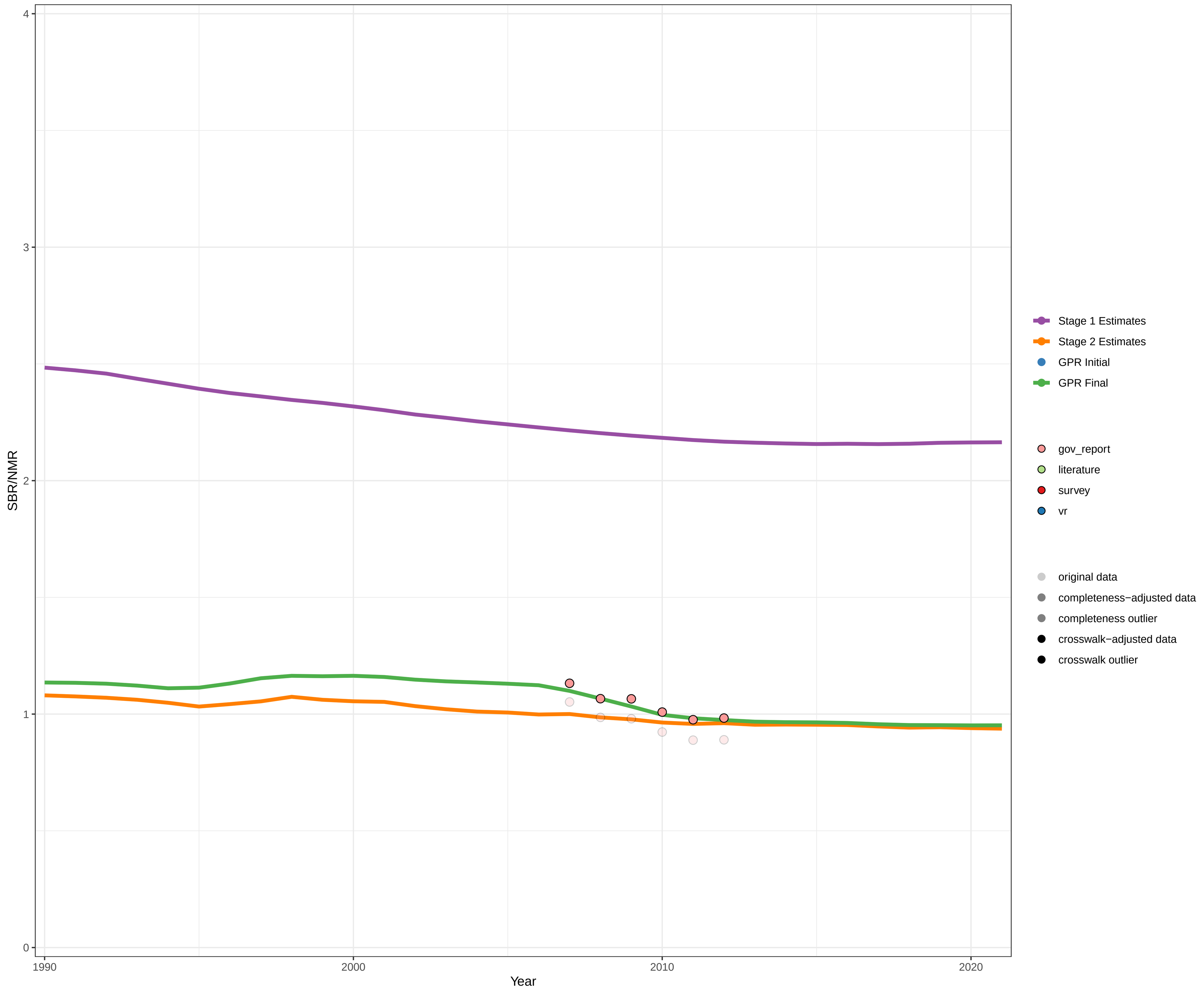

Definition	Years	Outlier	Source.Type	Title
28_weeks	2007	0	Statistical Report	Togo Principal Health Indicators
28_weeks	2008−2012	0	Statistical Report	Togo Key Health Indicators

Togo TGO Western Sub−Saharan Africa Stillbirth Rate (SBR) − 20 weeks
lambda = 0.6 ; zeta = 0.8 ; scale = 15 ; amp = 0.028

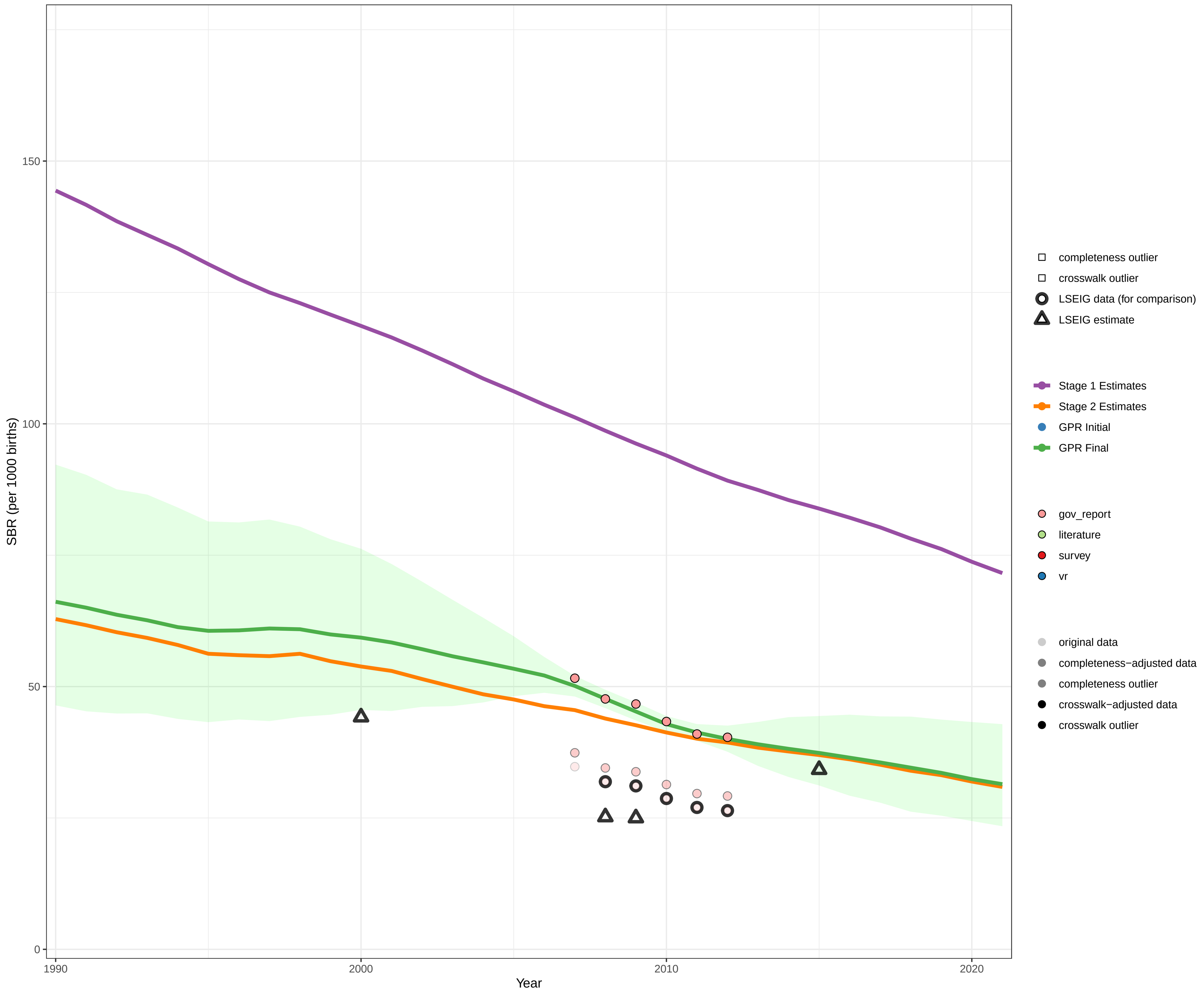

Togo TGO Western Sub−Saharan Africa Stillbirth Rate (SBR) − 28 weeks
lambda = 0.6 ; zeta = 0.8 ; scale = 15 ; amp = 0.028

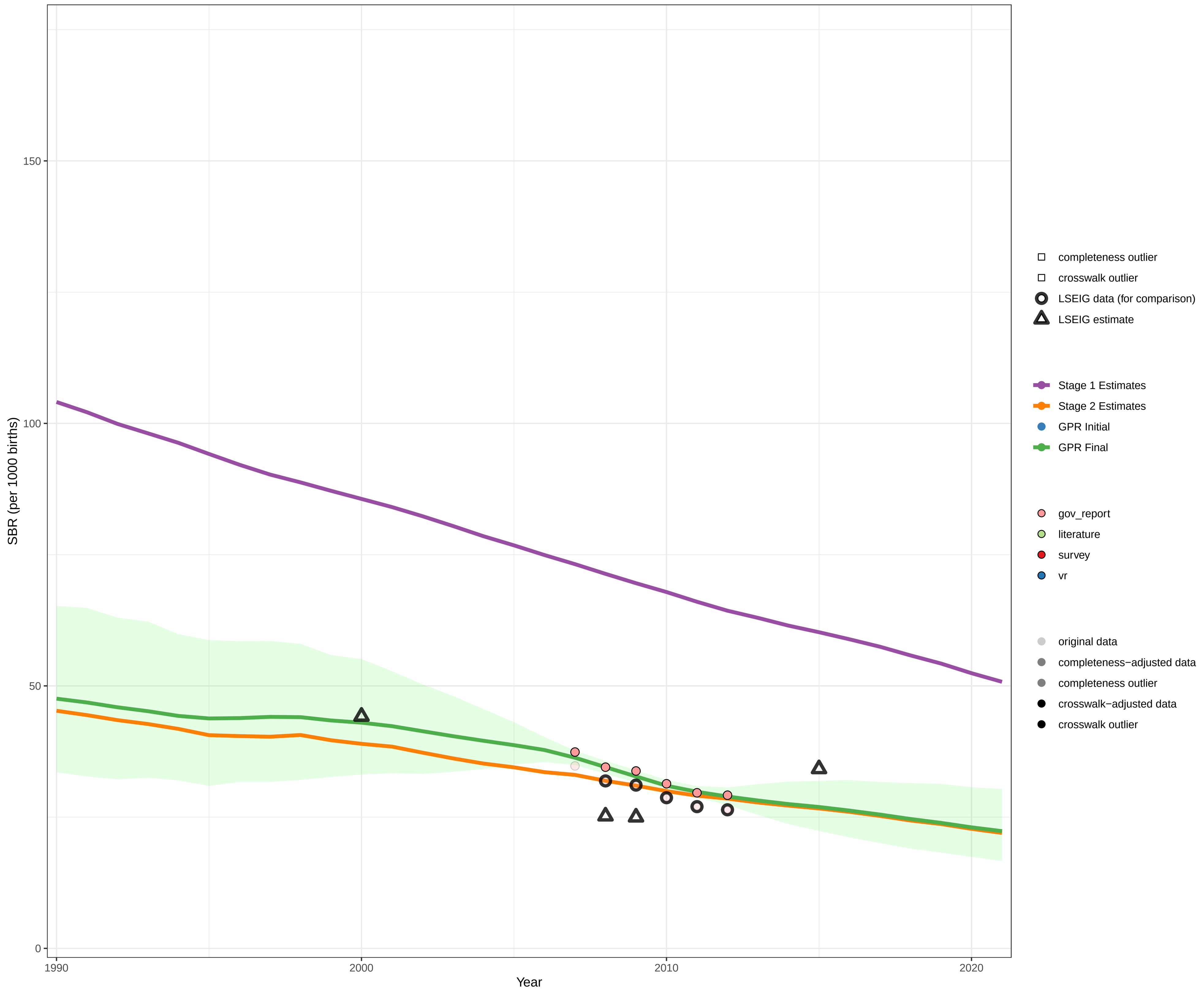

Year	Total Stillbirths (in thousands)	Total Stillbirths (in thousands)	SBR (per 1000 births)	SBR (per 1000 births)	SBR/NMR	SBR/NMR
	20 weeks	28 weeks	20 weeks	28 weeks	20 weeks	28 weeks
1990	11.3 (7.8 to 16.2)	8.0 (5.5 to 11.2)	66.1 (46.4 to 92.2)	47.6 (33.5 to 65.2)	1.58 (1.11 to 2.20)	1.14 (0.80 to 1.56)
1995	11.4 (7.9 to 15.6)	8.1 (5.6 to 11.0)	60.6 (43.2 to 81.4)	43.8 (30.9 to 58.7)	1.54 (1.10 to 2.07)	1.11 (0.79 to 1.49)
2000	12.5 (9.5 to 16.4)	8.9 (6.8 to 11.6)	59.3 (45.6 to 76.2)	43.0 (33.1 to 55.1)	1.61 (1.23 to 2.06)	1.16 (0.90 to 1.49)
2005	12.7 (11.4 to 14.2)	9.1 (8.2 to 10.1)	53.4 (48.2 to 59.6)	38.7 (35.0 to 43.1)	1.56 (1.41 to 1.74)	1.13 (1.02 to 1.26)
2010	11.0 (10.6 to 11.4)	7.8 (7.5 to 8.1)	42.9 (41.4 to 44.4)	31.0 (29.9 to 32.1)	1.38 (1.33 to 1.43)	1.00 (0.96 to 1.03)
2015	9.9 (8.2 to 11.8)	7.1 (5.8 to 8.4)	37.4 (31.2 to 44.4)	26.9 (22.4 to 31.9)	1.34 (1.12 to 1.59)	0.96 (0.80 to 1.14)
2020	8.2 (6.1 to 11.1)	5.8 (4.4 to 7.8)	32.4 (24.4 to 43.3)	23.1 (17.4 to 30.7)	1.34 (1.01 to 1.79)	0.95 (0.72 to 1.27)
2021	7.9 (5.8 to 10.9)	5.6 (4.1 to 7.6)	31.4 (23.4 to 42.9)	22.3 (16.6 to 30.4)	1.34 (1.00 to 1.83)	0.95 (0.71 to 1.29)

Appendix 2 Table S1A: Annual estimates of stillbirth (in thousands) for ≥20 weeks, by location

	1990	1991	1992	1993	1994
Global	5080 (4070 to 6350)	5020 (4050 to 6240)	4960 (4020 to 6180)	4930 (4000 to 6070)	4890 (4020 to 5970)
Central Europe, eastern Europe, and central Asia	93·4 (85·2 to 103)	87·6 (80·7 to 95·6)	79·6 (73·8 to 86·5)	74·8 (69·8 to 80·5)	71·6 (67·5 to 75·8)
Central Asia	38·8 (33·7 to 45·5)	37·5 (33 to 43·2)	35·4 (31·5 to 40·3)	34 (30·5 to 38·2)	33·1 (30 to 36·6)
Armenia	1·65 (1·51 to 1·81)	1·7 (1·41 to 2·01)	1·53 (1·24 to 1·88)	1·39 (1·13 to 1·7)	1·21 (0·987 to 1·46)
Azerbaijan	5·36 (4·65 to 6·2)	5·14 (4·64 to 5·69)	4·61 (4·16 to 5·04)	4·33 (3·94 to 4·8)	4·16 (3·77 to 4·58)
Georgia	1·67 (1·39 to 1·97)	1·59 (1·36 to 1·85)	1·44 (1·25 to 1·64)	1·58 (1·38 to 1·79)	1·78 (1·58 to 2)
Kazakhstan	6·58 (5·84 to 7·4)	6·05 (5·37 to 6·76)	5·71 (5·08 to 6·42)	5·25 (4·65 to 5·92)	4·97 (4·46 to 5·49)
Kyrgyzstan	3·55 (2·48 to 5·17)	3·43 (2·36 to 4·86)	3·23 (2·25 to 4·62)	3·05 (2·14 to 4·23)	2·86 (2·06 to 3·91)
Mongolia	1·78 (1·25 to 2·64)	1·68 (1·18 to 2·45)	1·59 (1·11 to 2·31)	1·51 (1·06 to 2·11)	1·43 (0·992 to 1·97)
Tajikistan	6·35 (5·18 to 7·7)	6·28 (5·27 to 7·54)	5·87 (4·93 to 7)	5·71 (4·75 to 6·83)	5·79 (4·84 to 6·91)
Turkmenistan	2·98 (2·72 to 3·27)	2·88 (2·69 to 3·08)	2·74 (2·59 to 2·91)	2·61 (2·47 to 2·76)	2·51 (2·38 to 2·64)
Uzbekistan	8·89 (6·33 to 12·7)	8·79 (6·16 to 12)	8·72 (6·15 to 12)	8·56 (6·11 to 11·7)	8·38 (6·06 to 11·3)
Central Europe	16·5 (14 to 19·5)	15·6 (13·4 to 18·3)	14·4 (12·7 to 16·2)	13·4 (12·2 to 14·6)	13·7 (12·8 to 14·5)
Albania	0·711 (0·507 to 0·966)	0·692 (0·488 to 0·947)	0·676 (0·474 to 0·922)	0·666 (0·468 to 0·919)	0·657 (0·461 to 0·902)
Bosnia and Herzegovina	0·86 (0·803 to 0·92)	0·819 (0·732 to 0·905)	0·835 (0·752 to 0·923)	0·813 (0·761 to 0·872)	0·743 (0·689 to 0·799)
Bulgaria	0·926 (0·681 to 1·29)	0·985 (0·721 to 1·33)	0·943 (0·711 to 1·24)	0·854 (0·684 to 1·05)	0·85 (0·754 to 0·956)
Croatia	0·348 (0·323 to 0·377)	0·36 (0·331 to 0·388)	0·326 (0·301 to 0·354)	0·292 (0·27 to 0·317)	0·313 (0·29 to 0·337)
Czechia	0·873 (0·797 to 0·95)	0·821 (0·71 to 0·965)	0·758 (0·689 to 0·836)	0·648 (0·595 to 0·709)	0·545 (0·479 to 0·619)
Hungary	1·04 (0·738 to 1·49)	1·09 (0·767 to 1·49)	0·961 (0·694 to 1·31)	0·849 (0·626 to 1·14)	0·776 (0·596 to 0·989)
Montenegro	0·083 (0·06 to 0·114)	0·0755 (0·0542 to 0·104)	0·072 (0·0519 to 0·0989)	0·0717 (0·0521 to 0·0966)	0·0723 (0·0529 to 0·0968)
North Macedonia	0·559 (0·422 to 0·718)	0·489 (0·397 to 0·602)	0·5 (0·448 to 0·553)	0·486 (0·454 to 0·519)	0·485 (0·452 to 0·518)
Poland	5·43 (3·96 to 7·18)	5·07 (3·81 to 6·52)	4·65 (3·69 to 5·81)	4·13 (3·58 to 4·77)	4·54 (4·16 to 4·97)
Romania	2·89 (2·69 to 3·09)	2·45 (2·27 to 2·63)	2·23 (2·07 to 2·4)	2·21 (2·06 to 2·37)	2·31 (2·17 to 2·46)
Serbia	1·74 (1·24 to 2·46)	1·69 (1·2 to 2·32)	1·43 (1·03 to 1·98)	1·47 (1·05 to 1·99)	1·57 (1·16 to 2·1)
Slovakia	0·632 (0·479 to 0·82)	0·651 (0·516 to 0·814)	0·604 (0·525 to 0·698)	0·512 (0·467 to 0·563)	0·451 (0·414 to 0·491)
Slovenia	0·166 (0·139 to 0·195)	0·152 (0·136 to 0·172)	0·141 (0·126 to 0·158)	0·132 (0·118 to 0·148)	0·125 (0·112 to 0·138)
Eastern Europe	38·1 (35·7 to 40·4)	34·5 (32·2 to 36·8)	29·8 (28 to 31·8)	27·5 (25·8 to 29·3)	24·8 (23·4 to 26·3)
Belarus	1·46 (1·28 to 1·66)	1·42 (1·2 to 1·68)	1·28 (1·12 to 1·44)	1·23 (1·13 to 1·34)	1·27 (1·17 to 1·38)
Estonia	0·239 (0·204 to 0·278)	0·202 (0·176 to 0·233)	0·173 (0·153 to 0·198)	0·147 (0·13 to 0·165)	0·127 (0·112 to 0·143)
Latvia	0·301 (0·266 to 0·338)	0·331 (0·304 to 0·36)	0·327 (0·301 to 0·354)	0·281 (0·26 to 0·307)	0·23 (0·211 to 0·25)
Lithuania	0·407 (0·371 to 0·446)	0·458 (0·418 to 0·496)	0·466 (0·424 to 0·507)	0·403 (0·368 to 0·44)	0·356 (0·327 to 0·387)
Moldova	1·12 (1·05 to 1·2)	1·03 (0·96 to 1·1)	0·937 (0·878 to 1)	0·841 (0·788 to 0·899)	0·828 (0·773 to 0·884)
Russia	25·7 (23·6 to 27·8)	23 (20·9 to 25·1)	19·8 (18·2 to 21·5)	18·6 (17 to 20·3)	16·7 (15·3 to 18·1)
Ukraine	8·88 (8·21 to 9·58)	8·05 (7·49 to 8·67)	6·88 (6·33 to 7·42)	5·99 (5·56 to 6·48)	5·34 (4·98 to 5·76)
High income	96·5 (88·6 to 107)	93·1 (85·7 to 102)	88·4 (82 to 95·6)	84·6 (79·7 to 90)	82·2 (78·4 to 86·3)
Australasia	3·15 (2·65 to 3·76)	2·87 (2·46 to 3·36)	2·65 (2·34 to 3)	2·43 (2·27 to 2·58)	2·4 (2·3 to 2·5)
Australia	2·08 (1·59 to 2·71)	1·86 (1·45 to 2·35)	1·77 (1·46 to 2·12)	1·6 (1·45 to 1·74)	1·58 (1·5 to 1·68)
New Zealand	1·07 (1·02 to 1·12)	1·01 (0·962 to 1·06)	0·876 (0·834 to 0·92)	0·834 (0·792 to 0·876)	0·813 (0·769 to 0·858)
High-income Asia Pacific	10·3 (7·56 to 13·6)	9·85 (7·48 to 12·8)	9·78 (7·71 to 12·4)	9·45 (7·71 to 11·6)	9·27 (8·03 to 11)
Brunei	0·0628 (0·0448 to 0·0891)	0·0648 (0·0463 to 0·0891)	0·0636 (0·0454 to 0·088)	0·0629 (0·0448 to 0·0861)	0·0618 (0·0452 to 0·0834)
Japan	5·69 (4 to 7·67)	5·42 (3·98 to 7·15)	5·51 (4·22 to 7·17)	5·37 (4·34 to 6·56)	5·36 (4·86 to 5·89)
Aichi	0·3 (0·211 to 0·405)	0·291 (0·214 to 0·384)	0·296 (0·226 to 0·384)	0·291 (0·235 to 0·355)	0·296 (0·268 to 0·325)
Akita	0·0554 (0·039 to 0·0748)	0·0519 (0·0381 to 0·0684)	0·0508 (0·0389 to 0·0661)	0·0478 (0·0387 to 0·0584)	0·046 (0·0417 to 0·0505)
Aomori	0·0758 (0·0533 to 0·102)	0·0712 (0·0523 to 0·0939)	0·0704 (0·0538 to 0·0915)	0·0675 (0·0546 to 0·0824)	0·0662 (0·06 to 0·0727)
Chiba	0·219 (0·154 to 0·295)	0·214 (0·157 to 0·283)	0·222 (0·17 to 0·289)	0·221 (0·179 to 0·27)	0·221 (0·2 to 0·243)
Ehime	0·069 (0·0485 to 0·0931)	0·0654 (0·048 to 0·0862)	0·0655 (0·0501 to 0·0852)	0·0632 (0·0511 to 0·0771)	0·0622 (0·0564 to 0·0684)
Fukui	0·0433 (0·0304 to 0·0584)	0·0415 (0·0305 to 0·0547)	0·0426 (0·0326 to 0·0554)	0·0417 (0·0337 to 0·0509)	0·0417 (0·0378 to 0·0458)
Fukuoka	0·227 (0·159 to 0·306)	0·223 (0·164 to 0·294)	0·228 (0·174 to 0·297)	0·222 (0·179 to 0·271)	0·223 (0·202 to 0·245)
Fukushima	0·107 (0·0751 to 0·144)	0·0999 (0·0734 to 0·132)	0·0985 (0·0754 to 0·128)	0·0927 (0·0749 to 0·113)	0·0893 (0·081 to 0·0982)
Gifu	0·0858 (0·0603 to 0·116)	0·0824 (0·0606 to 0·109)	0·0838 (0·0641 to 0·109)	0·0822 (0·0665 to 0·1)	0·0832 (0·0754 to 0·0914)
Gunma	0·0916 (0·0644 to 0·124)	0·0872 (0·0641 to 0·115)	0·0881 (0·0674 to 0·115)	0·0855 (0·0691 to 0·104)	0·0852 (0·0772 to 0·0936)
Hiroshima	0·129 (0·0907 to 0·174)	0·121 (0·0892 to 0·16)	0·122 (0·0931 to 0·158)	0·118 (0·0951 to 0·143)	0·117 (0·106 to 0·128)
Hokkaidō	0·219 (0·154 to 0·295)	0·205 (0·15 to 0·27)	0·202 (0·155 to 0·263)	0·191 (0·154 to 0·233)	0·191 (0·173 to 0·21)
Hyōgo	0·258 (0·182 to 0·348)	0·244 (0·179 to 0·321)	0·245 (0·187 to 0·319)	0·24 (0·194 to 0·293)	0·24 (0·217 to 0·264)
Ibaraki	0·124 (0·0871 to 0·167)	0·117 (0·0858 to 0·154)	0·116 (0·0888 to 0·151)	0·113 (0·0914 to 0·138)	0·113 (0·102 to 0·124)
Ishikawa	0·0529 (0·0372 to 0·0713)	0·0516 (0·0379 to 0·0681)	0·0529 (0·0405 to 0·0688)	0·052 (0·042 to 0·0634)	0·0522 (0·0473 to 0·0574)
Iwate	0·0528 (0·0371 to 0·0712)	0·0494 (0·0363 to 0·0652)	0·0485 (0·0371 to 0·063)	0·0455 (0·0368 to 0·0555)	0·0441 (0·04 to 0·0485)
Kagawa	0·0474 (0·0333 to 0·0639)	0·0442 (0·0325 to 0·0583)	0·0434 (0·0332 to 0·0564)	0·0409 (0·0331 to 0·0499)	0·0399 (0·0362 to 0·0438)
Kagoshima	0·0754 (0·053 to 0·102)	0·0684 (0·0503 to 0·0902)	0·0652 (0·0499 to 0·0848)	0·0605 (0·0489 to 0·0738)	0·0577 (0·0523 to 0·0634)
Kanagawa	0·406 (0·285 to 0·547)	0·398 (0·293 to 0·525)	0·428 (0·328 to 0·557)	0·426 (0·345 to 0·521)	0·441 (0·4 to 0·485)
Kōchi	0·0394 (0·0277 to 0·0532)	0·0363 (0·0267 to 0·0479)	0·0354 (0·0271 to 0·046)	0·0333 (0·027 to 0·0407)	0·0324 (0·0293 to 0·0356)
Kumamoto	0·0862 (0·0606 to 0·116)	0·0813 (0·0597 to 0·107)	0·0808 (0·0618 to 0·105)	0·0776 (0·0627 to 0·0947)	0·0768 (0·0696 to 0·0844)
Kyōto	0·13 (0·0911 to 0·175)	0·127 (0·093 to 0·167)	0·13 (0·0997 to 0·17)	0·127 (0·103 to 0·155)	0·126 (0·114 to 0·138)
Mie	0·09 (0·0632 to 0·121)	0·0862 (0·0633 to 0·114)	0·0867 (0·0663 to 0·113)	0·0841 (0·0681 to 0·103)	0·0834 (0·0756 to 0·0916)
Miyagi	0·104 (0·0729 to 0·14)	0·0951 (0·0699 to 0·125)	0·092 (0·0704 to 0·12)	0·0865 (0·07 to 0·106)	0·0847 (0·0767 to 0·093)
Miyazaki	0·0613 (0·0431 to 0·0826)	0·058 (0·0426 to 0·0765)	0·0578 (0·0442 to 0·0752)	0·0555 (0·0449 to 0·0678)	0·0544 (0·0493 to 0·0598)
Nagano	0·1 (0·0705 to 0·135)	0·0934 (0·0686 to 0·123)	0·0913 (0·0699 to 0·119)	0·0859 (0·0695 to 0·105)	0·0833 (0·0755 to 0·0915)
Nagasaki	0·0637 (0·0448 to 0·086)	0·061 (0·0448 to 0·0804)	0·0612 (0·0468 to 0·0795)	0·059 (0·0477 to 0·0719)	0·0582 (0·0528 to 0·064)
Nara	0·0633 (0·0445 to 0·0854)	0·0611 (0·0449 to 0·0806)	0·0627 (0·048 to 0·0816)	0·0613 (0·0496 to 0·0748)	0·0608 (0·0551 to 0·0668)
Niigata	0·0961 (0·0676 to 0·13)	0·0917 (0·0674 to 0·121)	0·0939 (0·0719 to 0·122)	0·0917 (0·0741 to 0·112)	0·0907 (0·0822 to 0·0996)
Ōita	0·0559 (0·0393 to 0·0754)	0·0525 (0·0386 to 0·0693)	0·0525 (0·0402 to 0·0683)	0·0498 (0·0403 to 0·0608)	0·0486 (0·0441 to 0·0534)
Okayama	0·0789 (0·0555 to 0·106)	0·0751 (0·0552 to 0·0991)	0·0755 (0·0578 to 0·0982)	0·0737 (0·0596 to 0·0899)	0·0736 (0·0667 to 0·0808)
Okinawa	0·0902 (0·0634 to 0·122)	0·0866 (0·0636 to 0·114)	0·0869 (0·0665 to 0·113)	0·0841 (0·068 to 0·103)	0·0835 (0·0757 to 0·0918)
Ōsaka	0·405 (0·285 to 0·547)	0·371 (0·273 to 0·489)	0·369 (0·282 to 0·48)	0·352 (0·285 to 0·43)	0·35 (0·317 to 0·385)
Saga	0·0433 (0·0304 to 0·0584)	0·0408 (0·03 to 0·0538)	0·0404 (0·0309 to 0·0526)	0·0385 (0·0312 to 0·0471)	0·0382 (0·0346 to 0·042)
Saitama	0·285 (0·201 to 0·385)	0·283 (0·208 to 0·373)	0·293 (0·224 to 0·381)	0·287 (0·232 to 0·35)	0·291 (0·264 to 0·32)
Shiga	0·0691 (0·0485 to 0·0932)	0·065 (0·0478 to 0·0858)	0·0661 (0·0505 to 0·0859)	0·0653 (0·0528 to 0·0798)	0·0671 (0·0608 to 0·0737)
Shimane	0·035 (0·0246 to 0·0472)	0·0325 (0·0239 to 0·0429)	0·0321 (0·0246 to 0·0418)	0·0307 (0·0248 to 0·0374)	0·0304 (0·0276 to 0·0334)
Shizuoka	0·155 (0·109 to 0·209)	0·15 (0·11 to 0·197)	0·15 (0·115 to 0·196)	0·146 (0·118 to 0·178)	0·144 (0·131 to 0·159)
Tochigi	0·1 (0·0704 to 0·135)	0·0969 (0·0712 to 0·128)	0·0993 (0·0759 to 0·129)	0·0958 (0·0775 to 0·117)	0·0947 (0·0858 to 0·104)
Tokushima	0·0377 (0·0265 to 0·0509)	0·0354 (0·026 to 0·0467)	0·0354 (0·0271 to 0·046)	0·0343 (0·0278 to 0·0419)	0·0346 (0·0313 to 0·038)
Tōkyō	0·558 (0·392 to 0·753)	0·53 (0·39 to 0·7)	0·56 (0·428 to 0·728)	0·57 (0·461 to 0·696)	0·568 (0·515 to 0·624)
Tottori	0·0289 (0·0203 to 0·0389)	0·0273 (0·02 to 0·036)	0·027 (0·0207 to 0·0352)	0·0259 (0·021 to 0·0316)	0·0256 (0·0232 to 0·0281)
Toyama	0·0581 (0·0408 to 0·0784)	0·0552 (0·0405 to 0·0728)	0·0559 (0·0427 to 0·0727)	0·0543 (0·044 to 0·0664)	0·0546 (0·0495 to 0·06)
Wakayama	0·0485 (0·0341 to 0·0655)	0·0454 (0·0334 to 0·0599)	0·0454 (0·0347 to 0·059)	0·0441 (0·0356 to 0·0538)	0·0444 (0·0402 to 0·0488)
Yamagata	0·0523 (0·0368 to 0·0705)	0·0491 (0·0361 to 0·0648)	0·0487 (0·0373 to 0·0634)	0·0467 (0·0378 to 0·0571)	0·0463 (0·0419 to 0·0508)
Yamaguchi	0·0724 (0·0509 to 0·0977)	0·0683 (0·0502 to 0·0901)	0·0683 (0·0523 to 0·0889)	0·0655 (0·053 to 0·08)	0·0644 (0·0583 to 0·0707)
Yamanashi	0·0423 (0·0297 to 0·0571)	0·0419 (0·0308 to 0·0553)	0·0441 (0·0337 to 0·0573)	0·0441 (0·0356 to 0·0538)	0·0445 (0·0403 to 0·0489)
Singapore	0·461 (0·331 to 0·632)	0·374 (0·274 to 0·505)	0·34 (0·259 to 0·451)	0·319 (0·255 to 0·392)	0·299 (0·265 to 0·337)
South Korea	4·09 (2·88 to 5·85)	3·99 (2·78 to 5·69)	3·87 (2·72 to 5·48)	3·7 (2·61 to 5·3)	3·55 (2·5 to 5·1)
High-income North America	34·3 (32·7 to 35·9)	33·5 (32 to 35·1)	31·7 (30·2 to 33·1)	30·7 (29·2 to 32·1)	29·8 (28·6 to 31·1)
Canada	2·5 (1·97 to 3·15)	2·4 (1·98 to 2·93)	2·3 (1·9 to 2·78)	2·25 (1·87 to 2·71)	2·25 (1·88 to 2·69)
Greenland	0·0236 (0·017 to 0·0323)	0·0223 (0·0161 to 0·0302)	0·0211 (0·0154 to 0·0288)	0·0194 (0·0142 to 0·0259)	0·018 (0·0133 to 0·024)
USA	31·7 (30·3 to 33·2)	31·1 (29·8 to 32·4)	29·4 (28·1 to 30·6)	28·4 (27·2 to 29·7)	27·5 (26·5 to 28·7)
Alabama	0·655 (0·611 to 0·701)	0·658 (0·614 to 0·705)	0·634 (0·592 to 0·681)	0·61 (0·569 to 0·656)	0·581 (0·543 to 0·624)
Alaska	0·0622 (0·0565 to 0·0689)	0·061 (0·0552 to 0·0669)	0·0533 (0·0481 to 0·0593)	0·0467 (0·0419 to 0·0521)	0·0431 (0·039 to 0·0474)
Arizona	0·405 (0·36 to 0·452)	0·423 (0·378 to 0·47)	0·431 (0·385 to 0·481)	0·419 (0·374 to 0·469)	0·412 (0·365 to 0·462)
Arkansas	0·273 (0·25 to 0·297)	0·289 (0·263 to 0·314)	0·283 (0·258 to 0·311)	0·249 (0·228 to 0·272)	0·241 (0·221 to 0·262)
California	3·97 (3·41 to 4·55)	3·88 (3·4 to 4·42)	3·58 (3·12 to 4·1)	3·47 (3·01 to 3·98)	3·43 (2·97 to 3·96)
Colorado	0·375 (0·339 to 0·416)	0·37 (0·335 to 0·407)	0·331 (0·299 to 0·365)	0·327 (0·297 to 0·361)	0·341 (0·309 to 0·377)
Connecticut	0·362 (0·308 to 0·422)	0·339 (0·29 to 0·399)	0·288 (0·249 to 0·338)	0·263 (0·228 to 0·301)	0·28 (0·241 to 0·325)
Delaware	0·103 (0·0954 to 0·112)	0·0922 (0·0849 to 0·1)	0·0813 (0·0724 to 0·0922)	0·0782 (0·0666 to 0·0913)	0·0747 (0·0671 to 0·083)
Florida	1·69 (1·48 to 1·92)	1·59 (1·4 to 1·82)	1·49 (1·3 to 1·7)	1·49 (1·31 to 1·7)	1·53 (1·35 to 1·72)
Georgia	1·34 (1·15 to 1·55)	1·3 (1·13 to 1·48)	1·13 (0·978 to 1·3)	1·09 (0·942 to 1·26)	1·14 (0·98 to 1·32)
Hawaii	0·143 (0·12 to 0·17)	0·132 (0·112 to 0·155)	0·117 (0·0983 to 0·136)	0·119 (0·1 to 0·139)	0·112 (0·095 to 0·134)
Idaho	0·106 (0·0948 to 0·119)	0·0958 (0·0855 to 0·107)	0·0929 (0·0833 to 0·103)	0·0897 (0·0802 to 0·1)	0·0925 (0·0825 to 0·103)
Illinois	1·56 (1·42 to 1·71)	1·6 (1·46 to 1·77)	1·55 (1·41 to 1·7)	1·51 (1·37 to 1·66)	1·38 (1·25 to 1·52)
Indiana	0·646 (0·598 to 0·698)	0·635 (0·588 to 0·684)	0·616 (0·574 to 0·666)	0·569 (0·529 to 0·616)	0·565 (0·525 to 0·609)
Iowa	0·245 (0·219 to 0·278)	0·233 (0·208 to 0·261)	0·218 (0·193 to 0·244)	0·201 (0·179 to 0·226)	0·198 (0·176 to 0·223)
Kansas	0·203 (0·183 to 0·224)	0·211 (0·192 to 0·231)	0·221 (0·201 to 0·244)	0·23 (0·209 to 0·253)	0·205 (0·186 to 0·226)
Kentucky	0·38 (0·346 to 0·417)	0·379 (0·345 to 0·417)	0·371 (0·338 to 0·407)	0·367 (0·333 to 0·398)	0·364 (0·333 to 0·398)
Louisiana	0·646 (0·602 to 0·691)	0·613 (0·572 to 0·656)	0·553 (0·516 to 0·592)	0·563 (0·522 to 0·605)	0·549 (0·512 to 0·591)
Maine	0·0805 (0·0712 to 0·0919)	0·0835 (0·0733 to 0·0942)	0·0816 (0·0715 to 0·0923)	0·0693 (0·0608 to 0·0792)	0·0643 (0·0566 to 0·073)
Maryland	0·608 (0·516 to 0·707)	0·646 (0·552 to 0·747)	0·66 (0·564 to 0·767)	0·648 (0·561 to 0·747)	0·631 (0·547 to 0·727)
Massachusetts	0·561 (0·52 to 0·6)	0·503 (0·468 to 0·541)	0·498 (0·459 to 0·537)	0·493 (0·456 to 0·531)	0·468 (0·436 to 0·505)
Michigan	1·04 (0·782 to 1·38)	0·997 (0·765 to 1·3)	0·907 (0·731 to 1·11)	0·833 (0·732 to 0·943)	0·776 (0·713 to 0·843)
Minnesota	0·378 (0·338 to 0·421)	0·38 (0·339 to 0·424)	0·353 (0·316 to 0·394)	0·355 (0·316 to 0·395)	0·359 (0·319 to 0·399)
Mississippi	0·507 (0·465 to 0·552)	0·495 (0·45 to 0·541)	0·504 (0·463 to 0·552)	0·491 (0·45 to 0·536)	0·479 (0·439 to 0·52)
Missouri	0·583 (0·534 to 0·632)	0·584 (0·54 to 0·631)	0·538 (0·495 to 0·587)	0·515 (0·475 to 0·559)	0·467 (0·431 to 0·508)
Montana	0·0804 (0·073 to 0·088)	0·0778 (0·0711 to 0·0855)	0·0736 (0·0671 to 0·0807)	0·0613 (0·056 to 0·0668)	0·0568 (0·0519 to 0·062)
Nebraska	0·17 (0·156 to 0·185)	0·17 (0·156 to 0·185)	0·154 (0·141 to 0·168)	0·149 (0·137 to 0·161)	0·134 (0·124 to 0·147)
Nevada	0·159 (0·138 to 0·182)	0·145 (0·127 to 0·166)	0·137 (0·12 to 0·156)	0·139 (0·121 to 0·158)	0·144 (0·126 to 0·168)
New Hampshire	0·104 (0·0938 to 0·114)	0·094 (0·0849 to 0·104)	0·0908 (0·0823 to 0·1)	0·0864 (0·0781 to 0·0962)	0·0707 (0·0639 to 0·0785)
New Jersey	0·906 (0·791 to 1·04)	0·868 (0·757 to 0·994)	0·826 (0·725 to 0·94)	0·783 (0·689 to 0·895)	0·744 (0·657 to 0·85)
New Mexico	0·135 (0·123 to 0·148)	0·135 (0·121 to 0·149)	0·139 (0·125 to 0·155)	0·133 (0·12 to 0·146)	0·129 (0·118 to 0·142)
New York	2·8 (2·39 to 3·32)	2·87 (2·39 to 3·39)	2·79 (2·34 to 3·24)	2·66 (2·24 to 3·1)	2·38 (2·01 to 2·79)
North Carolina	0·918 (0·846 to 0·992)	0·933 (0·863 to 1·01)	0·887 (0·818 to 0·958)	0·828 (0·766 to 0·893)	0·845 (0·781 to 0·916)
North Dakota	0·0825 (0·0736 to 0·0928)	0·0748 (0·0664 to 0·0845)	0·0643 (0·0573 to 0·0718)	0·0627 (0·0561 to 0·0696)	0·0683 (0·0608 to 0·0767)
Ohio	1·27 (1·16 to 1·41)	1·23 (1·12 to 1·36)	1·18 (1·07 to 1·3)	1·17 (1·05 to 1·28)	1·12 (1·02 to 1·24)
Oklahoma	0·342 (0·311 to 0·373)	0·324 (0·297 to 0·353)	0·311 (0·285 to 0·338)	0·347 (0·317 to 0·381)	0·338 (0·308 to 0·372)
Oregon	0·255 (0·229 to 0·283)	0·245 (0·22 to 0·271)	0·218 (0·197 to 0·24)	0·217 (0·195 to 0·239)	0·239 (0·216 to 0·265)
Pennsylvania	1·46 (1·3 to 1·66)	1·39 (1·23 to 1·56)	1·28 (1·13 to 1·44)	1·23 (1·09 to 1·39)	1·19 (1·05 to 1·35)
Rhode Island	0·0915 (0·0823 to 0·101)	0·0889 (0·0807 to 0·0979)	0·0942 (0·085 to 0·103)	0·0903 (0·0813 to 0·0995)	0·0745 (0·0679 to 0·0822)
South Carolina	0·611 (0·558 to 0·665)	0·599 (0·547 to 0·655)	0·574 (0·526 to 0·628)	0·557 (0·51 to 0·608)	0·506 (0·463 to 0·554)
South Dakota	0·0793 (0·0698 to 0·09)	0·0767 (0·0677 to 0·0869)	0·0647 (0·0566 to 0·074)	0·0546 (0·0479 to 0·0621)	0·0527 (0·0459 to 0·0602)
Tennessee	0·577 (0·498 to 0·667)	0·539 (0·467 to 0·615)	0·504 (0·44 to 0·572)	0·494 (0·434 to 0·561)	0·492 (0·429 to 0·56)
Texas	2·21 (1·98 to 2·45)	2·13 (1·92 to 2·37)	2·05 (1·83 to 2·28)	2·01 (1·8 to 2·22)	2·01 (1·79 to 2·24)
Utah	0·206 (0·182 to 0·236)	0·212 (0·186 to 0·239)	0·212 (0·187 to 0·24)	0·2 (0·176 to 0·227)	0·204 (0·179 to 0·232)
Vermont	0·0438 (0·0386 to 0·0494)	0·0447 (0·0397 to 0·0506)	0·0385 (0·034 to 0·0435)	0·0313 (0·0275 to 0·0355)	0·0303 (0·0265 to 0·0345)
Virginia	0·786 (0·678 to 0·911)	0·759 (0·641 to 0·884)	0·747 (0·633 to 0·876)	0·702 (0·596 to 0·822)	0·673 (0·586 to 0·774)
Washington	0·429 (0·359 to 0·503)	0·425 (0·362 to 0·498)	0·408 (0·347 to 0·477)	0·383 (0·326 to 0·45)	0·383 (0·327 to 0·45)
Washington, DC	0·4 (0·353 to 0·451)	0·364 (0·312 to 0·422)	0·313 (0·27 to 0·362)	0·284 (0·249 to 0·324)	0·26 (0·226 to 0·294)
West Virginia	0·195 (0·179 to 0·212)	0·176 (0·163 to 0·193)	0·175 (0·161 to 0·19)	0·173 (0·158 to 0·188)	0·159 (0·146 to 0·171)

Wisconsin	0·439 (0·405 to 0·474)	0·467 (0·433 to 0·504)	0·422 (0·39 to 0·456)	0·416 (0·386 to 0·449)	0·408 (0·378 to 0·442)	[illegible]	[illegible]	[illegible]
Wyoming	0·0494 (0·0446 to 0·0548)	0·0474 (0·0426 to 0·0524)	0·0395 (0·0355 to 0·0436)	0·0344 (0·0308 to 0·038)	0·031 (0·0279 to 0·0345)	[illegible] (0·0307 to 0·0376)	[illegible] (0·0281 to 0·0344)	[illegible] (0·021 to 0·026)
Southern Latin America	15·2 (12·7 to 18·6)	14·7 (12·2 to 18)	14·1 (11·9 to 16·9)	13·4 (11·7 to 15·4)	12·7 (11·5 to 14)	12·4 (11·7 to 13·4)	11·8 (11 to 12·7)	11 (10·3 to 11·7)
Argentina	8·59 (6·16 to 11·8)	8·61 (6·28 to 11·7)	8·26 (6·27 to 11)	8·04 (6·37 to 9·98)	7·8 (6·79 to 8·94)	7·71 (7·01 to 8·39)	7·5 (6·84 to 8·21)	7·11 (6·47 to 7·84)
Chile	5·85 (5·31 to 6·45)	5·3 (4·8 to 5·85)	5·1 (4·61 to 5·66)	4·55 (4·09 to 5·04)	4·07 (3·66 to 4·46)	3·8 (3·45 to 4·19)	3·44 (3·1 to 3·78)	3·04 (2·77 to 3·37)
Uruguay	0·784 (0·596 to 1·03)	0·767 (0·606 to 0·974)	0·708 (0·622 to 0·808)	0·764 (0·708 to 0·829)	0·855 (0·79 to 0·921)	0·937 (0·865 to 1·01)	0·879 (0·81 to 0·949)	0·816 (0·753 to 0·884)
Western Europe	33·6 (30·6 to 37·5)	32·2 (29·6 to 35·4)	30·2 (27·9 to 32·7)	28·7 (26·7 to 30·7)	28 (26·5 to 30)	27·4 (26 to 29·1)	27 (25·8 to 28·4)	26·6 (25·6 to 27·7)
Andorra	0·00469 (0·00335 to 0·00658)	0·00501 (0·00356 to 0·00688)	0·00568 (0·00407 to 0·00763)	0·00507 (0·00367 to 0·00691)	0·00496 (0·0037 to 0·00661)	0·00513 (0·00401 to 0·0066)	0·00468 (0·00372 to 0·00582)	0·00305 (0·00246 to 0·00376)
Austria	0·539 (0·503 to 0·578)	0·549 (0·511 to 0·588)	0·572 (0·533 to 0·615)	0·529 (0·493 to 0·566)	0·506 (0·474 to 0·541)	0·458 (0·431 to 0·487)	0·443 (0·415 to 0·472)	0·407 (0·382 to 0·434)
Belgium	0·999 (0·71 to 1·37)	1·01 (0·715 to 1·36)	0·991 (0·73 to 1·31)	0·965 (0·734 to 1·24)	0·91 (0·72 to 1·15)	0·796 (0·648 to 0·964)	0·708 (0·59 to 0·848)	0·712 (0·61 to 0·82)
Cyprus	0·181 (0·128 to 0·247)	0·175 (0·123 to 0·244)	0·164 (0·117 to 0·234)	0·152 (0·107 to 0·219)	0·139 (0·0983 to 0·2)	0·128 (0·0918 to 0·182)	0·118 (0·0843 to 0·163)	0·109 (0·0771 to 0·148)
Denmark	0·454 (0·396 to 0·52)	0·48 (0·415 to 0·553)	0·5 (0·432 to 0·576)	0·472 (0·407 to 0·535)	0·496 (0·438 to 0·564)	0·502 (0·444 to 0·568)	0·548 (0·476 to 0·629)	0·55 (0·475 to 0·635)
Finland	0·315 (0·293 to 0·34)	0·311 (0·288 to 0·335)	0·286 (0·265 to 0·306)	0·266 (0·246 to 0·288)	0·281 (0·262 to 0·301)	0·271 (0·254 to 0·292)	0·244 (0·228 to 0·262)	0·239 (0·224 to 0·255)
France	5·59 (5·23 to 5·96)	5·39 (5·05 to 5·75)	4·99 (4·68 to 5·32)	4·75 (4·44 to 5·08)	4·83 (4·56 to 5·17)	4·69 (4·42 to 4·97)	4·57 (4·31 to 4·85)	4·74 (4·47 to 5·04)
Germany	5·51 (5·18 to 5·85)	5·16 (4·85 to 5·46)	4·69 (4·42 to 4·97)	4·36 (4·1 to 4·62)	4·4 (4·16 to 4·66)	4·59 (4·34 to 4·84)	4·49 (4·24 to 4·74)	4·31 (4·08 to 4·55)
Greece	0·895 (0·751 to 1·05)	0·826 (0·7 to 0·972)	0·796 (0·675 to 0·935)	0·8 (0·679 to 0·941)	0·829 (0·712 to 0·962)	0·891 (0·774 to 1·03)	0·877 (0·76 to 1)	0·871 (0·756 to 1·01)
Iceland	0·0243 (0·0212 to 0·0277)	0·0257 (0·0227 to 0·0291)	0·0232 (0·0203 to 0·0264)	0·022 (0·0192 to 0·025)	0·0204 (0·0179 to 0·0233)	0·0229 (0·0201 to 0·0261)	0·0271 (0·0237 to 0·0305)	0·0208 (0·0184 to 0·0235)
Ireland	0·406 (0·373 to 0·44)	0·403 (0·373 to 0·433)	0·378 (0·349 to 0·408)	0·372 (0·334 to 0·41)	0·382 (0·34 to 0·428)	0·468 (0·427 to 0·514)	0·549 (0·499 to 0·605)	0·508 (0·451 to 0·571)
Israel	0·691 (0·631 to 0·751)	0·727 (0·666 to 0·793)	0·785 (0·719 to 0·858)	0·804 (0·741 to 0·879)	0·829 (0·764 to 0·9)	0·787 (0·725 to 0·852)	0·713 (0·657 to 0·771)	0·777 (0·713 to 0·85)
Italy	4·36 (3·85 to 4·89)	4·2 (3·73 to 4·71)	3·91 (3·46 to 4·39)	3·56 (3·15 to 3·98)	3·33 (2·97 to 3·67)	3·22 (2·92 to 3·56)	3·15 (2·84 to 3·48)	2·95 (2·62 to 3·29)
Luxembourg	0·033 (0·0287 to 0·0378)	0·0366 (0·0321 to 0·0421)	0·0325 (0·0284 to 0·0371)	0·0279 (0·0244 to 0·0318)	0·0301 (0·0261 to 0·0346)	0·0277 (0·024 to 0·0314)	0·0333 (0·0291 to 0·0376)	0·0391 (0·0344 to 0·0444)
Malta	0·0494 (0·0371 to 0·0648)	0·0475 (0·0373 to 0·0607)	0·0447 (0·036 to 0·0546)	0·0454 (0·0377 to 0·0548)	0·0462 (0·0378 to 0·0558)	0·043 (0·0353 to 0·0514)	0·04 (0·0337 to 0·0474)	0·0359 (0·0308 to 0·0414)
Monaco	0·00164 (0·00115 to 0·00222)	0·00158 (0·00111 to 0·00218)	0·00154 (0·00109 to 0·00212)	0·00151 (0·00106 to 0·00208)	0·00148 (0·00103 to 0·00204)	0·00145 (0·000999 to 0·00203)	0·00142 (0·000993 to 0·00202)	0·00139 (0·000982 to 0·00198)
Netherlands	1·81 (1·3 to 2·47)	1·71 (1·25 to 2·31)	1·63 (1·23 to 2·16)	1·62 (1·28 to 2·01)	1·57 (1·36 to 1·8)	1·42 (1·29 to 1·56)	1·36 (1·24 to 1·49)	1·41 (1·29 to 1·54)
Norway	0·448 (0·421 to 0·475)	0·438 (0·414 to 0·465)	0·424 (0·399 to 0·451)	0·419 (0·397 to 0·443)	0·426 (0·405 to 0·447)	0·391 (0·372 to 0·412)	0·37 (0·352 to 0·389)	0·373 (0·355 to 0·392)
Agder	0·0248 (0·0218 to 0·0282)	0·0262 (0·0227 to 0·0302)	0·0249 (0·0217 to 0·0281)	0·0205 (0·018 to 0·0231)	0·019 (0·0166 to 0·0216)	0·0176 (0·0155 to 0·0201)	0·0155 (0·0136 to 0·0177)	0·0172 (0·0151 to 0·0194)
Innlandet	0·0305 (0·0268 to 0·0346)	0·0304 (0·027 to 0·0344)	0·034 (0·0298 to 0·0383)	0·0358 (0·0316 to 0·0404)	0·0342 (0·0302 to 0·0389)	0·0286 (0·0252 to 0·0322)	0·0187 (0·0165 to 0·0213)	0·0187 (0·0165 to 0·0212)
Møre og Romsdal	0·0224 (0·0191 to 0·026)	0·0187 (0·016 to 0·0219)	0·02 (0·0171 to 0·0229)	0·0238 (0·0205 to 0·0274)	0·018 (0·0154 to 0·0209)	0·014 (0·012 to 0·0163)	0·0172 (0·0147 to 0·02)	0·0163 (0·014 to 0·0188)
Nordland	0·022 (0·0189 to 0·0254)	0·0278 (0·0241 to 0·0321)	0·0273 (0·0235 to 0·0315)	0·026 (0·0223 to 0·0299)	0·0272 (0·0233 to 0·0312)	0·0271 (0·0232 to 0·0315)	0·0228 (0·0196 to 0·0266)	0·0196 (0·017 to 0·0229)
Oslo	0·058 (0·0519 to 0·0644)	0·0555 (0·0498 to 0·0615)	0·0472 (0·042 to 0·0527)	0·0477 (0·0428 to 0·0531)	0·0561 (0·0501 to 0·0623)	0·0476 (0·0428 to 0·0528)	0·047 (0·0421 to 0·0525)	0·0532 (0·0475 to 0·0593)
Rogaland	0·0426 (0·0378 to 0·0478)	0·0404 (0·0357 to 0·0454)	0·0365 (0·0323 to 0·041)	0·037 (0·0329 to 0·0419)	0·0367 (0·0324 to 0·041)	0·0405 (0·0356 to 0·0461)	0·0403 (0·0357 to 0·0455)	0·035 (0·0309 to 0·0398)
Troms og Finnmark	0·0267 (0·0229 to 0·0308)	0·0227 (0·0194 to 0·0262)	0·0212 (0·0182 to 0·0248)	0·0218 (0·0188 to 0·0254)	0·0251 (0·0216 to 0·029)	0·0226 (0·0192 to 0·0261)	0·0239 (0·0204 to 0·028)	0·0289 (0·0249 to 0·0336)
Trøndelag	0·0283 (0·0248 to 0·0323)	0·0317 (0·0275 to 0·0363)	0·0371 (0·0325 to 0·0424)	0·0403 (0·0356 to 0·0458)	0·0375 (0·033 to 0·0426)	0·0356 (0·0315 to 0·0406)	0·0331 (0·0289 to 0·0375)	0·0333 (0·0292 to 0·038)
Vestfold og Telemark	0·0452 (0·0388 to 0·0521)	0·0423 (0·0367 to 0·0488)	0·0376 (0·0323 to 0·0434)	0·0295 (0·0252 to 0·0339)	0·0291 (0·0249 to 0·0335)	0·0331 (0·0283 to 0·0384)	0·0343 (0·0294 to 0·0399)	0·0313 (0·0271 to 0·0365)
Vestland	0·0594 (0·053 to 0·0663)	0·0557 (0·0496 to 0·0627)	0·0518 (0·0463 to 0·0578)	0·0511 (0·0454 to 0·0568)	0·0525 (0·0463 to 0·0585)	0·0428 (0·0384 to 0·0477)	0·0461 (0·041 to 0·0514)	0·0507 (0·0451 to 0·0569)
Viken	0·0883 (0·078 to 0·1)	0·0869 (0·0768 to 0·0979)	0·0868 (0·0771 to 0·0973)	0·0858 (0·0762 to 0·0965)	0·0907 (0·0804 to 0·102)	0·0815 (0·0724 to 0·0918)	0·0714 (0·0639 to 0·0804)	0·0691 (0·0615 to 0·0779)
Portugal	1·25 (1·17 to 1·35)	1·25 (1·16 to 1·34)	1·12 (1·04 to 1·2)	1·04 (0·975 to 1·12)	0·918 (0·859 to 0·98)	0·851 (0·796 to 0·908)	0·797 (0·748 to 0·849)	0·737 (0·691 to 0·786)
San Marino	0·00288 (0·00205 to 0·00409)	0·00284 (0·00203 to 0·00405)	0·00283 (0·00203 to 0·00392)	0·00282 (0·00203 to 0·0038)	0·00292 (0·0021 to 0·00397)	0·003 (0·00219 to 0·004)	0·00317 (0·00239 to 0·00419)	0·00293 (0·00224 to 0·00379)
Spain	2·63 (1·92 to 3·67)	2·46 (1·79 to 3·33)	2·4 (1·79 to 3·2)	2·19 (1·71 to 2·76)	1·95 (1·64 to 2·27)	1·71 (1·53 to 1·91)	1·75 (1·58 to 1·93)	1·78 (1·6 to 1·96)
Sweden	0·598 (0·551 to 0·649)	0·592 (0·546 to 0·643)	0·546 (0·503 to 0·589)	0·524 (0·483 to 0·568)	0·502 (0·464 to 0·542)	0·471 (0·439 to 0·504)	0·468 (0·435 to 0·501)	0·449 (0·418 to 0·484)
Stockholm	0·124 (0·114 to 0·135)	0·121 (0·111 to 0·131)	0·114 (0·105 to 0·123)	0·113 (0·104 to 0·122)	0·111 (0·103 to 0·12)	0·107 (0·0997 to 0·115)	0·108 (0·1 to 0·116)	0·105 (0·0979 to 0·113)
Sweden except Stockholm	0·474 (0·437 to 0·514)	0·472 (0·435 to 0·512)	0·432 (0·398 to 0·466)	0·412 (0·379 to 0·446)	0·391 (0·362 to 0·422)	0·364 (0·339 to 0·39)	0·36 (0·335 to 0·385)	0·344 (0·32 to 0·37)
Switzerland	0·462 (0·4 to 0·534)	0·435 (0·373 to 0·503)	0·437 (0·377 to 0·504)	0·401 (0·346 to 0·459)	0·405 (0·354 to 0·464)	0·421 (0·368 to 0·474)	0·415 (0·365 to 0·472)	0·415 (0·362 to 0·474)
UK	6·31 (4·7 to 8·63)	5·97 (4·41 to 7·98)	5·42 (4·02 to 7·16)	5·31 (4·01 to 6·91)	5·21 (3·94 to 6·74)	5·23 (4·1 to 6·73)	5·3 (4·4 to 6·43)	5·14 (4·66 to 5·71)
England	5·24 (3·73 to 7·43)	4·95 (3·47 to 6·86)	4·49 (3·17 to 6·15)	4·38 (3·14 to 5·91)	4·3 (3·09 to 5·73)	4·3 (3·22 to 5·72)	4·42 (3·56 to 5·48)	4·33 (3·86 to 4·85)
East Midlands	0·402 (0·287 to 0·571)	0·384 (0·269 to 0·532)	0·351 (0·248 to 0·481)	0·343 (0·246 to 0·463)	0·338 (0·243 to 0·451)	0·33 (0·247 to 0·439)	0·336 (0·27 to 0·417)	0·328 (0·291 to 0·369)
Derby	0·0284 (0·0189 to 0·042)	0·0276 (0·0172 to 0·0412)	0·0254 (0·0158 to 0·0382)	0·0246 (0·0161 to 0·0355)	0·0242 (0·0162 to 0·0339)	0·0236 (0·0172 to 0·0324)	0·0241 (0·0187 to 0·031)	0·0238 (0·0205 to 0·0276)
Derbyshire	0·067 (0·0465 to 0·096)	0·063 (0·0425 to 0·091)	0·0573 (0·0376 to 0·0842)	0·0559 (0·036 to 0·0832)	0·0552 (0·0362 to 0·0813)	0·0541 (0·0373 to 0·0774)	0·0556 (0·0417 to 0·0753)	0·0549 (0·0443 to 0·0668)
Leicester	0·046 (0·0312 to 0·0661)	0·0438 (0·0315 to 0·06)	0·0404 (0·0303 to 0·0539)	0·0395 (0·0302 to 0·0509)	0·039 (0·0293 to 0·0498)	0·0383 (0·0291 to 0·0489)	0·039 (0·0313 to 0·0475)	0·0382 (0·0336 to 0·0438)
Leicestershire	0·0463 (0·0315 to 0·0666)	0·0439 (0·0317 to 0·0602)	0·0402 (0·0302 to 0·0536)	0·0395 (0·0302 to 0·0509)	0·0389 (0·0293 to 0·0497)	0·0379 (0·0288 to 0·0484)	0·0387 (0·0311 to 0·047)	0·0374 (0·0329 to 0·0429)
Lincolnshire	0·054 (0·0359 to 0·0789)	0·0523 (0·0325 to 0·0776)	0·0485 (0·0307 to 0·0725)	0·0477 (0·0312 to 0·0688)	0·0473 (0·0318 to 0·0668)	0·0466 (0·0335 to 0·0633)	0·0477 (0·0373 to 0·0616)	0·0469 (0·0406 to 0·0546)
Northamptonshire	0·071 (0·0484 to 0·104)	0·0666 (0·0444 to 0·0972)	0·0602 (0·0404 to 0·0872)	0·0583 (0·039 to 0·0849)	0·0571 (0·0385 to 0·0847)	0·0549 (0·0384 to 0·0783)	0·0557 (0·0429 to 0·0722)	0·0545 (0·0477 to 0·0627)
Nottingham	0·0369 (0·0251 to 0·0531)	0·0362 (0·026 to 0·0496)	0·0335 (0·0252 to 0·0447)	0·0331 (0·0252 to 0·0426)	0·0323 (0·0243 to 0·0413)	0·0313 (0·0238 to 0·0399)	0·0315 (0·0253 to 0·0382)	0·0305 (0·0268 to 0·0349)
Nottinghamshire	0·0493 (0·0326 to 0·0722)	0·0467 (0·0293 to 0·0697)	0·0426 (0·027 to 0·0636)	0·0414 (0·0271 to 0·0597)	0·041 (0·0275 to 0·0579)	0·0398 (0·0289 to 0·0544)	0·0406 (0·0316 to 0·0521)	0·0395 (0·0341 to 0·0457)
Rutland	0·00359 (0·00246 to 0·0052)	0·00343 (0·00224 to 0·00498)	0·00324 (0·00203 to 0·0048)	0·00312 (0·00194 to 0·00469)	0·00309 (0·00197 to 0·00458)	0·00298 (0·00207 to 0·0042)	0·00296 (0·0022 to 0·00393)	0·00286 (0·00234 to 0·00344)
East of England	0·479 (0·341 to 0·679)	0·453 (0·317 to 0·627)	0·421 (0·298 to 0·577)	0·425 (0·304 to 0·573)	0·423 (0·303 to 0·564)	0·422 (0·315 to 0·562)	0·433 (0·348 to 0·538)	0·416 (0·368 to 0·469)
Bedford	0·016 (0·0112 to 0·0226)	0·0151 (0·0108 to 0·0204)	0·0141 (0·0104 to 0·0188)	0·0142 (0·0105 to 0·0187)	0·0143 (0·0102 to 0·0193)	0·0146 (0·0106 to 0·0199)	0·0151 (0·0118 to 0·0194)	0·0144 (0·0126 to 0·0167)
Cambridgeshire	0·0389 (0·0274 to 0·0549)	0·0373 (0·0266 to 0·0503)	0·0352 (0·0262 to 0·047)	0·0362 (0·0267 to 0·0474)	0·037 (0·0264 to 0·0499)	0·0379 (0·0274 to 0·0516)	0·04 (0·0312 to 0·0512)	0·0394 (0·0344 to 0·0454)
Central Bedfordshire	0·0261 (0·0178 to 0·0387)	0·0247 (0·0163 to 0·0359)	0·023 (0·0156 to 0·0328)	0·0232 (0·0167 to 0·0321)	0·0233 (0·0174 to 0·0301)	0·0231 (0·0183 to 0·0288)	0·0235 (0·0188 to 0·0293)	0·0225 (0·0198 to 0·0259)
Essex	0·112 (0·0785 to 0·157)	0·104 (0·0747 to 0·14)	0·0962 (0·0691 to 0·131)	0·0963 (0·0704 to 0·129)	0·0943 (0·0694 to 0·121)	0·0924 (0·0718 to 0·115)	0·0944 (0·0783 to 0·111)	0·0908 (0·0803 to 0·103)
Hertfordshire	0·0845 (0·054 to 0·127)	0·0797 (0·0502 to 0·121)	0·0742 (0·0456 to 0·112)	0·0751 (0·0459 to 0·114)	0·0754 (0·0474 to 0·114)	0·0756 (0·0512 to 0·109)	0·0783 (0·0572 to 0·109)	0·0757 (0·0628 to 0·0921)
Luton	0·0283 (0·02 to 0·0405)	0·0268 (0·0192 to 0·0368)	0·025 (0·0185 to 0·0339)	0·0252 (0·0186 to 0·0334)	0·0251 (0·018 to 0·034)	0·025 (0·0178 to 0·0342)	0·0254 (0·0196 to 0·0328)	0·0244 (0·0206 to 0·0283)
Norfolk	0·0601 (0·0425 to 0·0892)	0·0571 (0·0399 to 0·0807)	0·0534 (0·0377 to 0·0743)	0·0541 (0·0391 to 0·0729)	0·0546 (0·0404 to 0·071)	0·0549 (0·0439 to 0·0681)	0·0563 (0·0471 to 0·0674)	0·0543 (0·0485 to 0·061)
Peterborough	0·0161 (0·0104 to 0·0241)	0·0159 (0·00987 to 0·0242)	0·0151 (0·00936 to 0·023)	0·0154 (0·00937 to 0·0234)	0·0155 (0·00971 to 0·0234)	0·0153 (0·0102 to 0·0226)	0·0155 (0·0116 to 0·0206)	0·0148 (0·0123 to 0·0174)
Southend-on-Sea	0·0187 (0·012 to 0·028)	0·018 (0·0111 to 0·0275)	0·0167 (0·0102 to 0·0252)	0·0165 (0·00989 to 0·0253)	0·0159 (0·00992 to 0·024)	0·0154 (0·0102 to 0·0226)	0·0152 (0·0113 to 0·0203)	0·014 (0·0116 to 0·0166)
Suffolk	0·0602 (0·0385 to 0·0908)	0·0566 (0·0357 to 0·0862)	0·052 (0·0319 to 0·0783)	0·0519 (0·0317 to 0·0788)	0·0512 (0·0322 to 0·0777)	0·0511 (0·0346 to 0·0739)	0·0521 (0·0381 to 0·0729)	0·0495 (0·0411 to 0·0603)
Thurrock	0·0184 (0·0128 to 0·0269)	0·0174 (0·0123 to 0·0242)	0·0163 (0·012 to 0·0219)	0·0165 (0·0122 to 0·0218)	0·0164 (0·012 to 0·022)	0·0163 (0·0121 to 0·0221)	0·0168 (0·0129 to 0·0212)	0·0161 (0·014 to 0·0185)
Greater London	0·917 (0·653 to 1·3)	0·883 (0·618 to 1·22)	0·839 (0·594 to 1·15)	0·829 (0·595 to 1·12)	0·832 (0·599 to 1·11)	0·844 (0·634 to 1·12)	0·877 (0·712 to 1·08)	0·857 (0·773 to 0·95)
Barking and Dagenham	0·0226 (0·0153 to 0·0338)	0·0213 (0·0149 to 0·0303)	0·0196 (0·014 to 0·0271)	0·0184 (0·0135 to 0·0243)	0·0175 (0·0132 to 0·0227)	0·0172 (0·0136 to 0·0215)	0·0175 (0·0141 to 0·0217)	0·0167 (0·0148 to 0·0189)
Barnet	0·0347 (0·0228 to 0·051)	0·034 (0·0213 to 0·0504)	0·0332 (0·021 to 0·0502)	0·0337 (0·0219 to 0·0489)	0·0345 (0·0237 to 0·0475)	0·0356 (0·0269 to 0·0467)	0·0374 (0·0306 to 0·0463)	0·0368 (0·0329 to 0·0412)
Bexley	0·0188 (0·0128 to 0·0281)	0·0173 (0·012 to 0·0247)	0·0159 (0·0115 to 0·0218)	0·0153 (0·0112 to 0·0202)	0·015 (0·0113 to 0·0193)	0·0153 (0·0122 to 0·0192)	0·0163 (0·013 to 0·0202)	0·016 (0·0143 to 0·0181)
Brent	0·0497 (0·0339 to 0·0719)	0·0485 (0·032 to 0·0704)	0·0464 (0·0304 to 0·0673)	0·0462 (0·0301 to 0·0684)	0·0465 (0·0297 to 0·0686)	0·0472 (0·0313 to 0·0687)	0·0488 (0·0366 to 0·065)	0·0473 (0·0401 to 0·055)
Bromley	0·0215 (0·0146 to 0·0313)	0·0207 (0·0137 to 0·0303)	0·0196 (0·0129 to 0·0284)	0·0194 (0·0126 to 0·0282)	0·0194 (0·0124 to 0·0285)	0·0191 (0·0129 to 0·028)	0·0194 (0·0141 to 0·0271)	0·0185 (0·0156 to 0·0224)
Camden	0·0226 (0·0149 to 0·0332)	0·0217 (0·0136 to 0·0322)	0·0203 (0·0129 to 0·0308)	0·0198 (0·0129 to 0·0288)	0·0198 (0·0136 to 0·0273)	0·02 (0·0151 to 0·0262)	0·0206 (0·0168 to 0·0255)	0·0203 (0·0182 to 0·0228)
Croydon	0·0355 (0·0242 to 0·051)	0·0336 (0·024 to 0·046)	0·0315 (0·0229 to 0·0423)	0·0311 (0·0228 to 0·041)	0·0312 (0·023 to 0·0404)	0·0317 (0·024 to 0·0406)	0·0333 (0·0268 to 0·0403)	0·0326 (0·0285 to 0·0376)
Ealing	0·0377 (0·0255 to 0·0565)	0·0363 (0·0252 to 0·0519)	0·0347 (0·0249 to 0·0475)	0·0338 (0·0249 to 0·0448)	0·0339 (0·0254 to 0·044)	0·0343 (0·0272 to 0·0429)	0·0356 (0·028 to 0·0441)	0·0344 (0·0305 to 0·0388)
Enfield	0·0368 (0·0249 to 0·0535)	0·0357 (0·0236 to 0·0523)	0·034 (0·0223 to 0·0493)	0·0336 (0·0219 to 0·0488)	0·0339 (0·0216 to 0·0498)	0·0347 (0·0234 to 0·0508)	0·0362 (0·0262 to 0·0505)	0·0355 (0·0297 to 0·0429)
Greenwich	0·0212 (0·0145 to 0·0306)	0·0199 (0·0131 to 0·0289)	0·0184 (0·012 to 0·0266)	0·0178 (0·0115 to 0·0262)	0·0177 (0·0113 to 0·0261)	0·0179 (0·0121 to 0·0261)	0·0185 (0·0138 to 0·0247)	0·0183 (0·0155 to 0·0213)
Hackney	0·0314 (0·0209 to 0·0457)	0·0305 (0·0196 to 0·0452)	0·0292 (0·0187 to 0·0435)	0·0293 (0·0191 to 0·0426)	0·0302 (0·0205 to 0·0421)	0·0313 (0·0232 to 0·0416)	0·0332 (0·0268 to 0·0413)	0·0331 (0·0295 to 0·0371)
Hammersmith and Fulham	0·021 (0·0144 to 0·0304)	0·0205 (0·0135 to 0·0297)	0·0194 (0·0127 to 0·028)	0·0193 (0·0126 to 0·0284)	0·0194 (0·0124 to 0·0286)	0·0193 (0·0128 to 0·028)	0·0197 (0·0148 to 0·0262)	0·0194 (0·0165 to 0·0226)
Haringey	0·039 (0·0259 to 0·057)	0·038 (0·0239 to 0·0568)	0·0366 (0·0232 to 0·0552)	0·0364 (0·0237 to 0·0529)	0·0369 (0·0255 to 0·0505)	0·0378 (0·0287 to 0·0493)	0·0397 (0·0311 to 0·0504)	0·0395 (0·035 to 0·0453)
Harrow	0·0179 (0·0122 to 0·0258)	0·0173 (0·0115 to 0·0252)	0·0168 (0·011 to 0·0242)	0·017 (0·011 to 0·025)	0·0176 (0·0113 to 0·026)	0·0182 (0·0123 to 0·0266)	0·019 (0·0142 to 0·0254)	0·0187 (0·0159 to 0·0218)
Havering	0·0303 (0·0206 to 0·0435)	0·0284 (0·0203 to 0·0388)	0·0263 (0·0191 to 0·0354)	0·0255 (0·0187 to 0·0337)	0·0248 (0·0183 to 0·0322)	0·0242 (0·0183 to 0·031)	0·0247 (0·0198 to 0·0298)	0·0234 (0·0205 to 0·027)
Hillingdon	0·0325 (0·0216 to 0·0475)	0·031 (0·0192 to 0·046)	0·029 (0·0183 to 0·0438)	0·028 (0·0182 to 0·0406)	0·0275 (0·019 to 0·0381)	0·0275 (0·0207 to 0·0362)	0·0283 (0·0232 to 0·0343)	0·0275 (0·0246 to 0·031)
Hounslow	0·0253 (0·0168 to 0·0376)	0·0235 (0·0157 to 0·0338)	0·0217 (0·0149 to 0·0298)	0·021 (0·0153 to 0·0285)	0·021 (0·0154 to 0·0277)	0·0213 (0·0166 to 0·0269)	0·0224 (0·0181 to 0·0279)	0·0223 (0·019 to 0·0262)
Islington	0·0241 (0·0162 to 0·0356)	0·0229 (0·0151 to 0·0336)	0·0215 (0·0142 to 0·031)	0·0207 (0·014 to 0·029)	0·02 (0·014 to 0·0272)	0·0199 (0·0152 to 0·026)	0·0203 (0·0171 to 0·0241)	0·0199 (0·0182 to 0·022)
Kensington and Chelsea	0·0166 (0·011 to 0·0241)	0·0157 (0·01 to 0·0232)	0·0148 (0·0095 to 0·0221)	0·0148 (0·00962 to 0·0215)	0·0148 (0·01 to 0·0206)	0·0152 (0·0113 to 0·0203)	0·0158 (0·0127 to 0·0196)	0·0152 (0·0135 to 0·017)
Kingston upon Thames	0·0071 (0·00486 to 0·0104)	0·00664 (0·00481 to 0·00924)	0·00619 (0·00468 to 0·00828)	0·00608 (0·00449 to 0·0081)	0·00618 (0·00428 to 0·00861)	0·00628 (0·00436 to 0·00874)	0·00642 (0·00488 to 0·00839)	0·00617 (0·00526 to 0·00715)
Lambeth	0·0404 (0·0269 to 0·0596)	0·0389 (0·0255 to 0·0564)	0·0372 (0·0258 to 0·052)	0·0372 (0·0281 to 0·0492)	0·0377 (0·0293 to 0·0473)	0·039 (0·0314 to 0·0482)	0·0414 (0·0327 to 0·052)	0·0408 (0·0362 to 0·0461)
Lewisham	0·0342 (0·0232 to 0·0495)	0·0334 (0·0238 to 0·0455)	0·0322 (0·0239 to 0·0428)	0·0322 (0·0236 to 0·0428)	0·0332 (0·023 to 0·0466)	0·0343 (0·0236 to 0·0483)	0·036 (0·0272 to 0·0469)	0·0352 (0·0302 to 0·0411)
Merton	0·0213 (0·0145 to 0·0311)	0·0199 (0·0144 to 0·0278)	0·0187 (0·0141 to 0·025)	0·0183 (0·0135 to 0·0244)	0·018 (0·0125 to 0·0252)	0·0181 (0·0126 to 0·0253)	0·0184 (0·014 to 0·0241)	0·0177 (0·0151 to 0·0205)
Newham	0·052 (0·0355 to 0·0753)	0·0516 (0·0341 to 0·0753)	0·05 (0·0326 to 0·0722)	0·0505 (0·0327 to 0·0744)	0·0515 (0·0331 to 0·0763)	0·0527 (0·0355 to 0·0771)	0·0555 (0·0415 to 0·0742)	0·055 (0·0467 to 0·0641)
Redbridge	0·0181 (0·0124 to 0·026)	0·0172 (0·0123 to 0·0235)	0·016 (0·0117 to 0·0216)	0·0156 (0·0114 to 0·0205)	0·0153 (0·0113 to 0·0198)	0·0153 (0·0116 to 0·0196)	0·0158 (0·0127 to 0·0191)	0·0155 (0·0135 to 0·0179)
Richmond upon Thames	0·0145 (0·00994 to 0·021)	0·0138 (0·00913 to 0·0201)	0·013 (0·00849 to 0·0188)	0·0128 (0·00829 to 0·0188)	0·0128 (0·00825 to 0·019)	0·0131 (0·00885 to 0·0192)	0·0136 (0·0102 to 0·0182)	0·0134 (0·0114 to 0·0156)
Southwark	0·0404 (0·0268 to 0·0587)	0·0403 (0·0258 to 0·0597)	0·0399 (0·0255 to 0·0594)	0·0407 (0·0265 to 0·0592)	0·0423 (0·0287 to 0·059)	0·0435 (0·0322 to 0·0579)	0·0456 (0·0368 to 0·0568)	0·0451 (0·0402 to 0·0506)
Sutton	0·0138 (0·00945 to 0·0199)	0·0129 (0·0084 to 0·0189)	0·0122 (0·00761 to 0·018)	0·0118 (0·00737 to 0·0176)	0·0115 (0·00744 to 0·0167)	0·0113 (0·0081 to 0·0154)	0·0115 (0·00902 to 0·0145)	0·011 (0·00921 to 0·013)
Tower Hamlets	0·0311 (0·0212 to 0·0449)	0·0297 (0·0196 to 0·0433)	0·0286 (0·0186 to 0·0412)	0·0285 (0·0184 to 0·0419)	0·0292 (0·0188 to 0·0432)	0·0303 (0·0204 to 0·0443)	0·0316 (0·0236 to 0·0423)	0·0311 (0·0265 to 0·0363)
Waltham Forest	0·043 (0·0289 to 0·0637)	0·0419 (0·0275 to 0·0617)	0·04 (0·0262 to 0·0577)	0·0389 (0·0264 to 0·0546)	0·0379 (0·0264 to 0·0516)	0·037 (0·0282 to 0·0485)	0·0377 (0·0317 to 0·0448)	0·0362 (0·033 to 0·0399)
Wandsworth	0·031 (0·0207 to 0·0452)	0·0303 (0·019 to 0·0449)	0·0289 (0·0183 to 0·0436)	0·0284 (0·0185 to 0·0412)	0·0281 (0·0193 to 0·0386)	0·0282 (0·0213 to 0·037)	0·0289 (0·0236 to 0·0357)	0·0273 (0·0244 to 0·0305)
Westminster	0·0306 (0·0208 to 0·044)	0·0296 (0·0211 to 0·0405)	0·0278 (0·0202 to 0·0374)	0·0273 (0·02 to 0·0361)	0·027 (0·0198 to 0·0351)	0·0269 (0·0204 to 0·0345)	0·0279 (0·0224 to 0·0337)	0·0271 (0·0236 to 0·0312)
North East England	0·293 (0·209 to 0·416)	0·291 (0·203 to 0·403)	0·267 (0·189 to 0·367)	0·258 (0·185 to 0·349)	0·247 (0·177 to 0·33)	0·239 (0·179 to 0·319)	0·236 (0·19 to 0·294)	0·226 (0·2 to 0·255)
County Durham	0·039 (0·0274 to 0·0546)	0·039 (0·0264 to 0·055)	0·0362 (0·0242 to 0·0505)	0·0353 (0·0233 to 0·0506)	0·0342 (0·023 to 0·0482)	0·0339 (0·0248 to 0·0459)	0·0345 (0·0275 to 0·0431)	0·0341 (0·0288 to 0·0396)
Darlington	0·0139 (0·00989 to 0·0196)	0·0139 (0·00956 to 0·0191)	0·0126 (0·00876 to 0·0175)	0·012 (0·00801 to 0·0175)	0·0116 (0·00752 to 0·017)	0·0114 (0·00748 to 0·0169)	0·0114 (0·00831 to 0·0155)	0·011 (0·00912 to 0·0131)
Gateshead	0·0288 (0·0199 to 0·0418)	0·0283 (0·0191 to 0·0404)	0·0257 (0·0174 to 0·0366)	0·0248 (0·0172 to 0·0342)	0·0232 (0·0165 to 0·0313)	0·0219 (0·0168 to 0·0288)	0·0211 (0·017 to 0·0263)	0·0199 (0·0175 to 0·0225)
Hartlepool	0·0136 (0·00965 to 0·0189)	0·0132 (0·00929 to 0·0186)	0·012 (0·00815 to 0·017)	0·0115 (0·00745 to 0·0164)	0·0111 (0·00741 to 0·016)	0·0108 (0·0073 to 0·0153)	0·0105 (0·00776 to 0·0139)	0·0101 (0·00813 to 0·0124)
Middlesbrough	0·0224 (0·0155 to 0·0325)	0·022 (0·0149 to 0·0315)	0·0203 (0·0137 to 0·0288)	0·0195 (0·0135 to 0·0268)	0·0186 (0·0133 to 0·0251)	0·0181 (0·0139 to 0·0238)	0·0179 (0·0144 to 0·0222)	0·0169 (0·0149 to 0·0191)
Newcastle upon Tyne	0·0256 (0·0182 to 0·036)	0·0255 (0·0176 to 0·0351)	0·0232 (0·0162 to 0·0323)	0·0224 (0·0149 to 0·0327)	0·0214 (0·0139 to 0·0315)	0·0207 (0·0137 to 0·0307)	0·0201 (0·0146 to 0·0273)	0·0189 (0·0156 to 0·0225)
North Tyneside	0·0218 (0·0151 to 0·0315)	0·0213 (0·0144 to 0·0304)	0·0196 (0·0133 to 0·0279)	0·0191 (0·0133 to 0·0263)	0·0183 (0·013 to 0·0247)	0·0175 (0·0134 to 0·0231)	0·0172 (0·0138 to 0·0213)	0·0164 (0·0145 to 0·0186)
Northumberland	0·0342 (0·0236 to 0·0498)	0·0349 (0·0231 to 0·0501)	0·0331 (0·0221 to 0·0479)	0·0326 (0·0224 to 0·045)	0·0314 (0·0226 to 0·0421)	0·0304 (0·0232 to 0·0396)	0·0302 (0·0247 to 0·037)	0·0287 (0·0254 to 0·0325)
Redcar and Cleveland	0·0205 (0·0135 to 0·0305)	0·0204 (0·0138 to 0·0287)	0·0189 (0·0136 to 0·0255)	0·018 (0·0137 to 0·0234)	0·017 (0·0128 to 0·0219)	0·016 (0·0128 to 0·0199)	0·0154 (0·0125 to 0·0188)	0·0144 (0·0123 to 0·017)
South Tyneside	0·0158 (0·0109 to 0·0231)	0·0157 (0·0104 to 0·0225)	0·0146 (0·00973 to 0·021)	0·014 (0·00965 to 0·0194)	0·0135 (0·0097 to 0·018)	0·0129 (0·00987 to 0·0168)	0·0128 (0·0105 to 0·0157)	0·0123 (0·0109 to 0·0139)
Stockton-on-Tees	0·0239 (0·0152 to 0·037)	0·0231 (0·0153 to 0·0343)	0·0208 (0·0145 to 0·0291)	0·0197 (0·015 to 0·0258)	0·0189 (0·0144 to 0·0237)	0·0187 (0·0147 to 0·0234)	0·0191 (0·0158 to 0·023)	0·019 (0·0169 to 0·0213)
Sunderland	0·0338 (0·0237 to 0·0481)	0·0334 (0·0229 to 0·0472)	0·0304 (0·0212 to 0·0424)	0·0291 (0·0193 to 0·0415)	0·0278 (0·0181 to 0·0408)	0·0268 (0·0178 to 0·0404)	0·0261 (0·0185 to 0·0365)	0·0246 (0·0199 to 0·0301)
North West England	0·775 (0·552 to 1·1)	0·729 (0·51 to 1·01)	0·642 (0·454 to 0·879)	0·611 (0·438 to 0·825)	0·591 (0·424 to 0·788)	0·6 (0·449 to 0·8)	0·616 (0·496 to 0·765)	0·62 (0·55 to 0·697)
Blackburn with Darwen	0·0314 (0·02 to 0·0471)	0·0308 (0·0186 to 0·0476)	0·028 (0·0168 to 0·0437)	0·0271 (0·0167 to 0·0406)	0·0264 (0·0174 to 0·0373)	0·0267 (0·0194 to 0·0359)	0·0274 (0·0219 to 0·0346)	0·0278 (0·0245 to 0·0318)
Blackpool	0·0143 (0·0091 to 0·0215)	0·0136 (0·00821 to 0·0209)	0·0122 (0·00742 to 0·0189)	0·0119 (0·00738 to 0·0178)	0·0119 (0·00786 to 0·0171)	0·0124 (0·00911 to 0·0169)	0·013 (0·0104 to 0·0161)	0·013 (0·0114 to 0·0149)
Bolton	0·0418 (0·0274 to 0·0617)	0·04 (0·0246 to 0·0604)	0·0357 (0·0214 to 0·0549)	0·0335 (0·02 to 0·0516)	0·0321 (0·0202 to 0·0475)	0·0322 (0·0225 to 0·0449)	0·0328 (0·0252 to 0·0424)	0·033 (0·0271 to 0·0393)
Bury	0·019 (0·0121 to 0·0285)	0·0176 (0·0106 to 0·027)	0·0151 (0·00907 to 0·0237)	0·014 (0·00869 to 0·021)	0·0133 (0·00883 to 0·019)	0·0132 (0·00966 to 0·0179)	0·0133 (0·0107 to 0·0166)	0·0131 (0·0115 to 0·0151)
Cheshire East	0·0384 (0·0272 to 0·0542)	0·0363 (0·0266 to 0·0487)	0·0321 (0·0235 to 0·0433)	0·0309 (0·0225 to 0·0409)	0·0304 (0·022 to 0·0394)	0·0306 (0·0229 to 0·0398)	0·0309 (0·0247 to 0·0378)	0·0309 (0·0268 to 0·0355)
Cheshire West and Chester	0·0278 (0·0183 to 0·0415)	0·0259 (0·0168 to 0·0379)	0·0229 (0·0154 to 0·032)	0·0219 (0·0151 to 0·03)	0·0213 (0·0152 to 0·0289)	0·0217 (0·0164 to 0·0279)	0·0224 (0·0179 to 0·028)	0·0226 (0·0192 to 0·0268)
Cumbria	0·0421 (0·03 to 0·0597)	0·0396 (0·0282 to 0·0543)	0·0345 (0·025 to 0·0468)	0·0322 (0·0238 to 0·0428)	0·0304 (0·0221 to 0·0395)	0·0303 (0·0234 to 0·0386)	0·0304 (0·0245 to 0·0374)	0·0302 (0·0256 to 0·0355)
Halton	0·021 (0·0133 to 0·0315)	0·0199 (0·012 to 0·0306)	0·0176 (0·0107 to 0·0272)	0·0167 (0·0103 to 0·025)	0·0159 (0·0105 to 0·0228)	0·016 (0·0117 to 0·0216)	0·0163 (0·0132 to 0·0204)	0·0165 (0·0144 to 0·0189)
Knowsley	0·0262 (0·0172 to 0·0391)	0·024 (0·0152 to 0·0363)	0·0211 (0·0131 to 0·0318)	0·0202 (0·0125 to 0·031)	0·0197 (0·0123 to 0·0305)	0·02 (0·0129 to 0·0297)	0·02 (0·0145 to 0·0274)	0·0199 (0·0163 to 0·0239)
Lancashire	0·113 (0·0778 to 0·165)	0·103 (0·073 to 0·144)	0·0897 (0·066 to 0·12)	0·0843 (0·062 to 0·114)	0·0809 (0·0577 to 0·112)	0·0827 (0·0591 to 0·116)	0·0863 (0·0637 to 0·113)	0·0881 (0·0758 to 0·103)
Liverpool	0·038 (0·0241 to 0·057)	0·0359 (0·0217 to 0·0553)	0·0317 (0·0193 to 0·0491)	0·0303 (0·0188 to 0·0454)	0·0301 (0·02 to 0·0425)	0·0314 (0·0229 to 0·0422)	0·033 (0·0263 to 0·0417)	0·0335 (0·0295 to 0·0383)
Manchester	0·0516 (0·0362 to 0·0729)	0·0492 (0·0356 to 0·0659)	0·0436 (0·0325 to 0·0575)	0·0417 (0·0306 to 0·0554)	0·0405 (0·028 to 0·0566)	0·0412 (0·0283 to 0·0589)	0·0423 (0·0318 to 0·056)	0·043 (0·0364 to 0·0509)
Oldham	0·0253 (0·0162 to 0·0378)	0·024 (0·0147 to 0·0367)	0·0212 (0·013 to 0·0325)	0·0202 (0·0126 to 0·0299)	0·0196 (0·0129 to 0·0281)	0·0203 (0·0146 to 0·0275)	0·0211 (0·0165 to 0·0268)	0·0214 (0·0187 to 0·0246)
Rochdale	0·0262 (0·0172 to 0·0387)	0·0248 (0·0153 to 0·0374)	0·0218 (0·0131 to 0·0336)	0·0208 (0·0125 to 0·0321)	0·0199 (0·0125 to 0·0295)	0·02 (0·0139 to 0·0278)	0·0202 (0·0155 to 0·026)	0·02 (0·0165 to 0·0238)
Salford	0·0302 (0·0213 to 0·0426)	0·0287 (0·021 to 0·0385)	0·0256 (0·0188 to 0·0345)	0·0245 (0·0179 to 0·0324)	0·0237 (0·0172 to 0·0308)	0·0239 (0·0179 to 0·0311)	0·0244 (0·0195 to 0·0298)	0·0245 (0·0213 to 0·0282)
Sefton	0·025 (0·0171 to 0·0368)	0·0232 (0·0155 to 0·0333)	0·0204 (0·0144 to 0·0285)	0·0192 (0·0142 to 0·0254)	0·0185 (0·0141 to 0·0235)	0·019 (0·0151 to 0·0238)	0·0199 (0·0156 to 0·0251)	0·0203 (0·0176 to 0·0233)
St Helens	0·0201 (0·0132 to 0·0301)	0·0193 (0·0122 to 0·0291)	0·0173 (0·0108 to 0·0262)	0·0167 (0·0103 to 0·0255)	0·0162 (0·0101 to 0·0251)	0·0167 (0·0108 to 0·0248)	0·0173 (0·0126 to 0·0237)	0·0177 (0·0145 to 0·0213)
Stockport	0·032 (0·0223 to 0·047)	0·03 (0·021 to 0·0419)	0·0263 (0·0189 to 0·036)	0·025 (0·0181 to 0·0333)	0·0241 (0·0178 to 0·0322)	0·0246 (0·0188 to 0·0318)	0·025 (0·0194 to 0·0317)	0·0249 (0·0211 to 0·0291)
Tameside	0·0291 (0·0186 to 0·0435)	0·027 (0·0165 to 0·0412)	0·0232 (0·0142 to 0·0356)	0·0215 (0·0134 to 0·0318)	0·0203 (0·0133 to 0·0291)	0·0203 (0·0146 to 0·0275)	0·0204 (0·016 to 0·0259)	0·02 (0·0174 to 0·023)
Trafford	0·0271 (0·019 to 0·0382)	0·0252 (0·0182 to 0·0338)	0·0218 (0·0162 to 0·0287)	0·0204 (0·015 to 0·0272)	0·0198 (0·0137 to 0·0277)	0·0199 (0·0137 to 0·0284)	0·0203 (0·0153 to 0·0269)	0·0203 (0·0172 to 0·024)
Warrington	0·0263 (0·0187 to 0·0374)	0·0252 (0·0181 to 0·0345)	0·0226 (0·0164 to 0·0307)	0·022 (0·0159 to 0·0296)	0·0215 (0·0156 to 0·0282)	0·022 (0·0163 to 0·0287)	0·0226 (0·0175 to 0·0284)	0·0225 (0·0188 to 0·0268)
Wigan	0·0332 (0·0235 to 0·0468)	0·0318 (0·0233 to 0·0426)	0·0284 (0·0208 to 0·0384)	0·0279 (0·0203 to 0·0369)	0·0275 (0·0199 to 0·0357)	0·0282 (0·0211 to 0·0367)	0·0293 (0·0234 to 0·0358)	0·0295 (0·0255 to 0·0338)
Wirral	0·0361 (0·0234 to 0·053)	0·0334 (0·0212 to 0·0505)	0·0291 (0·018 to 0·0439)	0·0277 (0·017 to 0·0422)	0·0266 (0·0166 to 0·0408)	0·0271 (0·0177 to 0·0406)	0·0276 (0·0201 to 0·0378)	0·0275 (0·0225 to 0·033)
South East England	0·718 (0·512 to 1·02)	0·661 (0·463 to 0·916)	0·58 (0·41 to 0·795)	0·563 (0·404 to 0·759)	0·543 (0·39 to 0·723)	0·565 (0·424 to 0·751)	0·595 (0·481 to 0·737)	0·58 (0·518 to 0·648)
Bracknell Forest	0·0137 (0·00905 to 0·0204)	0·0126 (0·0085 to 0·0181)	0·0112 (0·00803 to 0·0152)	0·0112 (0·00811 to 0·0151)	0·0112 (0·00795 to 0·0156)	0·012 (0·0084 to 0·0169)	0·0128 (0·00928 to 0·0173)	0·0125 (0·0103 to 0·0151)
Brighton and Hove	0·0254 (0·0164 to 0·038)	0·0234 (0·0144 to 0·0354)	0·0207 (0·0128 to 0·0318)	0·0199 (0·0125 to 0·0291)	0·0191 (0·0128 to 0·0264)	0·0198 (0·0148 to 0·0263)	0·021 (0·0168 to 0·0263)	0·0207 (0·0182 to 0·0236)
Buckinghamshire	0·0405 (0·0263 to 0·0599)	0·0383 (0·024 to 0·0577)	0·034 (0·0212 to 0·0513)	0·0332 (0·0212 to 0·0485)	0·0318 (0·0213 to 0·045)	0·0332 (0·0242 to 0·0449)	0·0348 (0·0277 to 0·0439)	0·0339 (0·0298 to 0·0388)
East Sussex	0·0351 (0·0242 to 0·0503)	0·0324 (0·0233 to 0·0441)	0·0285 (0·0213 to 0·0377)	0·0277 (0·0204 to 0·0369)	0·0269 (0·0186 to 0·0374)	0·0278 (0·0194 to 0·039)	0·0293 (0·0224 to 0·0383)	0·0288 (0·0249 to 0·0334)
Hampshire	0·0824 (0·0571 to 0·117)	0·0739 (0·0533 to 0·101)	0·0643 (0·0475 to 0·0868)	0·0622 (0·0459 to 0·082)	0·06 (0·0444 to 0·0768)	0·0628 (0·0479 to 0·0796)	0·066 (0·0539 to 0·0795)	0·0643 (0·0568 to 0·0729)
Isle of Wight	0·01 (0·00677 to 0·0147)	0·00887 (0·00559 to 0·0132)	0·0075 (0·00454 to 0·0113)	0·0071 (0·00432 to 0·0108)	0·00656 (0·00423 to 0·00967)	0·00661 (0·00465 to 0·00911)	0·00655 (0·00506 to 0·00841)	0·00596 (0·00491 to 0·00712)
Kent	0·133 (0·0914 to 0·19)	0·121 (0·087 to 0·165)	0·105 (0·0784 to 0·138)	0·1 (0·0735 to 0·133)	0·0954 (0·066 to 0·133)	0·0989 (0·0689 to 0·138)	0·104 (0·0798 to 0·136)	0·101 (0·0877 to 0·118)

Medway	0·0314 (0·0209 to 0·0461)	0·0285 (0·0183 to 0·042)	0·0247 (0·0156 to 0·0365)	0·0238 (0·0151 to 0·0357)	0·0224 (0·0143 to 0·0337)	0·0227 (0·0151 to 0·0338)	0·0233 (0·0171 to 0·0316)	0·0222 (0·0183 to 0·0263)	0·0212 (0·0189 to 0·0238)	0·0207 (0·0193 to 0·0222)	0·0204 (0·0182 to 0·023)	0·0212 (0·0184 to 0·0242)	0·022 (0·0185 to 0·0259)	0·0239 (0·0194 to 0·0284)	0·0232 (0·0189 to 0·0279)	0·0224 (0·0177 to 0·0274)	0·0227 (0·0181 to 0·0278)	0·022 (0·0174 to 0·0266)	0·0223 (0·0182 to 0·027)	0·023 (0·0187 to 0·0276)	0·0236 (0·0191 to 0·0285)	0·0231 (0·0182 to 0·0282)	0·0209 (0·0165 to 0·0262)	0·0202 (0·0162 to 0·0255)	0·02 (0·0161 to 0·0246)	0·0192 (0·0157 to 0·0234)	0·0188 (0·0159 to 0·0221)	0·0187 (0·0164 to 0·0213)	0·0165 (0·0148 to 0·0183)	0·0155 (0·0139 to 0·0171)	0·0159 (0·014 to 0·0181)	0·017 (0·0135 to 0·0217)
Milton Keynes	0·0232 (0·015 to 0·0343)	0·022 (0·0134 to 0·0333)	0·0197 (0·0123 to 0·0304)	0·0195 (0·0122 to 0·0285)	0·0191 (0·0129 to 0·0269)	0·0202 (0·0151 to 0·027)	0·0218 (0·0177 to 0·0269)	0·0217 (0·0192 to 0·0246)	0·0214 (0·0198 to 0·023)	0·0216 (0·0199 to 0·0236)	0·0219 (0·0194 to 0·0245)	0·023 (0·0198 to 0·0265)	0·024 (0·0203 to 0·028)	0·0266 (0·022 to 0·0315)	0·0263 (0·0215 to 0·0313)	0·0261 (0·0211 to 0·0317)	0·0271 (0·022 to 0·0328)	0·027 (0·0219 to 0·0321)	0·0279 (0·0225 to 0·0334)	0·0292 (0·0237 to 0·0352)	0·03 (0·0244 to 0·0362)	0·029 (0·0239 to 0·0342)	0·0265 (0·0223 to 0·0309)	0·0255 (0·0221 to 0·0292)	0·0244 (0·0212 to 0·0279)	0·0224 (0·0198 to 0·0253)	0·0212 (0·019 to 0·0236)	0·0202 (0·0183 to 0·0223)	0·0174 (0·0156 to 0·0193)	0·0164 (0·0146 to 0·0183)	0·0164 (0·0137 to 0·0193)	0·0184 (0·0147 to 0·0231)
Oxfordshire	0·0513 (0·0355 to 0·0728)	0·0478 (0·0345 to 0·0652)	0·0422 (0·0312 to 0·057)	0·0408 (0·0301 to 0·0538)	0·0392 (0·029 to 0·0501)	0·0405 (0·0309 to 0·0514)	0·0431 (0·0352 to 0·052)	0·0428 (0·0378 to 0·0486)	0·0422 (0·0387 to 0·0455)	0·0424 (0·0396 to 0·0456)	0·0422 (0·0381 to 0·0461)	0·0442 (0·0388 to 0·05)	0·0466 (0·0404 to 0·0542)	0·0527 (0·0443 to 0·0619)	0·0539 (0·0443 to 0·0639)	0·0548 (0·0449 to 0·065)	0·0583 (0·0469 to 0·0701)	0·0586 (0·0472 to 0·0711)	0·0604 (0·049 to 0·0727)	0·0632 (0·0516 to 0·0756)	0·0658 (0·0533 to 0·0792)	0·0636 (0·0522 to 0·0763)	0·0571 (0·047 to 0·0687)	0·0538 (0·0441 to 0·065)	0·0514 (0·0435 to 0·0615)	0·0473 (0·0407 to 0·0547)	0·0437 (0·0387 to 0·0492)	0·043 (0·0387 to 0·0477)	0·0388 (0·0355 to 0·0425)	0·0364 (0·0333 to 0·0398)	0·0324 (0·0288 to 0·0364)	0·0386 (0·0308 to 0·0481)
Portsmouth	0·0233 (0·0152 to 0·0346)	0·0216 (0·0135 to 0·0326)	0·019 (0·0118 to 0·0287)	0·0182 (0·0116 to 0·0266)	0·0172 (0·0115 to 0·0243)	0·0177 (0·013 to 0·024)	0·0184 (0·0146 to 0·0232)	0·0176 (0·0155 to 0·0201)	0·0166 (0·0154 to 0·0179)	0·0161 (0·0147 to 0·0177)	0·0157 (0·0137 to 0·0178)	0·0158 (0·0133 to 0·0187)	0·0158 (0·0129 to 0·0192)	0·0169 (0·0135 to 0·0207)	0·0164 (0·0133 to 0·0199)	0·0159 (0·0126 to 0·0196)	0·0161 (0·0128 to 0·0197)	0·0155 (0·0126 to 0·0187)	0·0153 (0·0123 to 0·0183)	0·0154 (0·0125 to 0·0186)	0·0159 (0·013 to 0·0192)	0·0157 (0·0126 to 0·019)	0·0146 (0·012 to 0·0176)	0·0142 (0·0118 to 0·0169)	0·0138 (0·0117 to 0·0163)	0·0128 (0·0111 to 0·0147)	0·012 (0·0107 to 0·0134)	0·0118 (0·0106 to 0·013)	0·011 (0·0101 to 0·012)	0·0116 (0·0106 to 0·0125)	0·0107 (0·0096 to 0·0119)	0·0121 (0·00978 to 0·0151)
Reading	0·0223 (0·015 to 0·0326)	0·0216 (0·0136 to 0·0323)	0·0199 (0·012 to 0·03)	0·0202 (0·0122 to 0·0308)	0·0202 (0·013 to 0·0299)	0·0217 (0·0153 to 0·03)	0·0236 (0·0182 to 0·0304)	0·0237 (0·0195 to 0·0283)	0·0234 (0·0205 to 0·0267)	0·0235 (0·0214 to 0·0258)	0·0234 (0·0216 to 0·0252)	0·0236 (0·0212 to 0·0261)	0·0237 (0·0203 to 0·0274)	0·0257 (0·0212 to 0·0303)	0·0253 (0·0202 to 0·0311)	0·025 (0·0196 to 0·0316)	0·0258 (0·0198 to 0·0338)	0·0254 (0·0192 to 0·0331)	0·0256 (0·019 to 0·0337)	0·026 (0·0192 to 0·0343)	0·0267 (0·0199 to 0·0358)	0·0257 (0·0195 to 0·0337)	0·0232 (0·0176 to 0·0298)	0·0217 (0·017 to 0·0277)	0·0205 (0·0164 to 0·0252)	0·0186 (0·015 to 0·0225)	0·0167 (0·0136 to 0·0202)	0·0147 (0·0118 to 0·0179)	0·0124 (0·00996 to 0·0152)	0·0115 (0·00886 to 0·0143)	0·0112 (0·0084 to 0·0142)	0·0125 (0·00931 to 0·0165)
Slough	0·0177 (0·0115 to 0·0262)	0·0165 (0·0103 to 0·0248)	0·0146 (0·00913 to 0·0221)	0·0144 (0·0092 to 0·0211)	0·014 (0·00937 to 0·0198)	0·0147 (0·0108 to 0·02)	0·0158 (0·0126 to 0·02)	0·0157 (0·0138 to 0·018)	0·0155 (0·0143 to 0·0167)	0·0157 (0·0143 to 0·0173)	0·0159 (0·0139 to 0·0181)	0·0167 (0·014 to 0·0198)	0·0174 (0·0142 to 0·0212)	0·0195 (0·0155 to 0·0238)	0·0198 (0·0161 to 0·024)	0·0206 (0·0164 to 0·0254)	0·0224 (0·0178 to 0·0274)	0·0232 (0·0189 to 0·028)	0·0247 (0·0199 to 0·0296)	0·0262 (0·0212 to 0·0315)	0·0273 (0·0223 to 0·0329)	0·0271 (0·0218 to 0·0328)	0·0247 (0·0204 to 0·0298)	0·0236 (0·0196 to 0·0281)	0·0227 (0·0192 to 0·0266)	0·021 (0·0182 to 0·024)	0·0193 (0·0173 to 0·0215)	0·0173 (0·0157 to 0·019)	0·0135 (0·0124 to 0·0147)	0·0109 (0·01 to 0·0118)	0·0108 (0·00967 to 0·012)	0·0121 (0·00981 to 0·0149)
Southampton	0·0308 (0·0204 to 0·0457)	0·0276 (0·0182 to 0·0398)	0·0233 (0·0159 to 0·0323)	0·0222 (0·0158 to 0·0303)	0·0213 (0·0154 to 0·0284)	0·022 (0·017 to 0·0277)	0·0228 (0·0183 to 0·0284)	0·0219 (0·0187 to 0·026)	0·021 (0·0185 to 0·0239)	0·0206 (0·0187 to 0·0226)	0·0204 (0·0188 to 0·022)	0·0209 (0·0189 to 0·0231)	0·0213 (0·0186 to 0·0243)	0·0231 (0·0197 to 0·0266)	0·023 (0·0193 to 0·0269)	0·0232 (0·0196 to 0·0275)	0·0243 (0·0199 to 0·029)	0·0241 (0·0198 to 0·0288)	0·0245 (0·0204 to 0·0291)	0·0255 (0·021 to 0·0305)	0·0272 (0·0222 to 0·033)	0·0271 (0·022 to 0·0326)	0·0249 (0·0201 to 0·0301)	0·024 (0·0196 to 0·029)	0·0234 (0·0192 to 0·0283)	0·0221 (0·0179 to 0·027)	0·0207 (0·0164 to 0·0256)	0·0202 (0·0157 to 0·0251)	0·018 (0·0139 to 0·023)	0·0165 (0·0124 to 0·0212)	0·016 (0·0119 to 0·0209)	0·018 (0·0133 to 0·0241)
Surrey	0·0808 (0·0524 to 0·119)	0·0737 (0·0453 to 0·112)	0·0647 (0·0402 to 0·0989)	0·0626 (0·0393 to 0·0916)	0·0608 (0·0414 to 0·0849)	0·0638 (0·0475 to 0·0849)	0·0675 (0·0529 to 0·0865)	0·0657 (0·0572 to 0·0762)	0·0636 (0·0588 to 0·0683)	0·0625 (0·0574 to 0·0682)	0·0613 (0·0543 to 0·0688)	0·0623 (0·0539 to 0·0715)	0·0634 (0·0538 to 0·074)	0·0684 (0·057 to 0·0804)	0·0677 (0·0556 to 0·0809)	0·0677 (0·055 to 0·0815)	0·0712 (0·0575 to 0·0855)	0·071 (0·058 to 0·0839)	0·0723 (0·0593 to 0·0858)	0·0757 (0·0616 to 0·0909)	0·0798 (0·0662 to 0·095)	0·0789 (0·0662 to 0·0919)	0·0727 (0·0615 to 0·0841)	0·0698 (0·0605 to 0·0799)	0·0674 (0·0588 to 0·0781)	0·0628 (0·0545 to 0·072)	0·0585 (0·0507 to 0·0665)	0·0554 (0·0481 to 0·0627)	0·0463 (0·0404 to 0·0527)	0·0378 (0·0331 to 0·043)	0·0328 (0·0282 to 0·0378)	0·038 (0·0313 to 0·0461)
West Berkshire	0·0161 (0·0109 to 0·0235)	0·0151 (0·00977 to 0·0223)	0·0132 (0·00829 to 0·02)	0·0127 (0·00777 to 0·0192)	0·0122 (0·00793 to 0·0183)	0·0125 (0·00849 to 0·0176)	0·013 (0·00976 to 0·0173)	0·0125 (0·00995 to 0·0152)	0·0121 (0·0101 to 0·0141)	0·0119 (0·0104 to 0·0137)	0·0115 (0·0104 to 0·0128)	0·0114 (0·0104 to 0·0125)	0·011 (0·0097 to 0·0125)	0·0117 (0·00989 to 0·0137)	0·0114 (0·00937 to 0·0137)	0·0112 (0·00905 to 0·0137)	0·0115 (0·00908 to 0·0145)	0·0112 (0·00851 to 0·0145)	0·0111 (0·00842 to 0·0147)	0·0112 (0·00848 to 0·0148)	0·0114 (0·00858 to 0·015)	0·0108 (0·0081 to 0·0142)	0·00953 (0·00715 to 0·0125)	0·00894 (0·00687 to 0·0116)	0·00845 (0·00659 to 0·011)	0·00781 (0·00608 to 0·01)	0·00723 (0·00565 to 0·00918)	0·00652 (0·00511 to 0·00821)	0·00513 (0·00406 to 0·00643)	0·00439 (0·00345 to 0·00554)	0·00418 (0·00322 to 0·00533)	0·00495 (0·00372 to 0·00647)
West Sussex	0·0589 (0·0388 to 0·0876)	0·0548 (0·0354 to 0·0825)	0·0487 (0·0312 to 0·0729)	0·0481 (0·0303 to 0·0716)	0·047 (0·03 to 0·0705)	0·0491 (0·0332 to 0·0735)	0·0514 (0·037 to 0·0719)	0·0498 (0·041 to 0·0608)	0·0477 (0·0432 to 0·0527)	0·0468 (0·0437 to 0·0503)	0·0458 (0·0417 to 0·0501)	0·0464 (0·0413 to 0·0527)	0·0475 (0·0408 to 0·0549)	0·052 (0·0444 to 0·0607)	0·0517 (0·043 to 0·0611)	0·0513 (0·0417 to 0·0618)	0·0536 (0·0436 to 0·0654)	0·0532 (0·043 to 0·0647)	0·0543 (0·0438 to 0·0653)	0·0566 (0·0457 to 0·0683)	0·0587 (0·0469 to 0·0709)	0·0571 (0·0455 to 0·0692)	0·0518 (0·0413 to 0·0625)	0·0496 (0·0407 to 0·0592)	0·0485 (0·0402 to 0·0578)	0·0459 (0·038 to 0·0539)	0·0431 (0·0367 to 0·0501)	0·0409 (0·0351 to 0·0476)	0·0357 (0·0313 to 0·0404)	0·0332 (0·0295 to 0·0371)	0·033 (0·028 to 0·0389)	0·0388 (0·0301 to 0·0499)
Windsor and Maidenhead	0·0101 (0·0066 to 0·015)	0·00973 (0·00595 to 0·0147)	0·00892 (0·00546 to 0·0136)	0·00881 (0·00553 to 0·0129)	0·00857 (0·0058 to 0·012)	[illegible]	[illegible]	[illegible]	[illegible]	[illegible]	[illegible]	[illegible]	[illegible]	[illegible]	[illegible]	[illegible]	[illegible]	[illegible]	[illegible]	[illegible]	[illegible]	[illegible]	[illegible]	[illegible]	[illegible]	[illegible]	[illegible]	[illegible]	[illegible]	[illegible]	[illegible]	[illegible]
Wokingham	0·0125 (0·00834 to 0·0184)	0·0118 (0·00756 to 0·0173)	0·0105 (0·00663 to 0·0155)	0·0101 (0·00637 to 0·0151)	0·00976 (0·00622 to 0·0147)	[illegible]	[illegible]	[illegible]	[illegible]	[illegible]	[illegible]	[illegible]	[illegible]	[illegible]	[illegible]	[illegible]	[illegible]	[illegible]	[illegible]	[illegible]	[illegible]	[illegible]	[illegible]	[illegible]	[illegible]	[illegible]	[illegible]	[illegible]	[illegible]	[illegible]	[illegible]	[illegible]
South West England	0·422 (0·301 to 0·599)	0·392 (0·274 to 0·543)	0·361 (0·255 to 0·494)	0·357 (0·256 to 0·482)	0·352 (0·252 to 0·469)	[illegible]	[illegible]	[illegible]	[illegible]	[illegible]	[illegible]	[illegible]	[illegible]	[illegible]	[illegible]	[illegible]	[illegible]	[illegible]	[illegible]	[illegible]	[illegible]	[illegible]	[illegible]	[illegible]	[illegible]	[illegible]	[illegible]	[illegible]	[illegible]	[illegible]	[illegible]	[illegible]
Bath and North East Somerset	0·0138 (0·00953 to 0·0195)	0·0127 (0·00821 to 0·0185)	0·0116 (0·00707 to 0·0178)	0·0114 (0·00671 to 0·0174)	0·011 (0·00691 to 0·0165)	[illegible]	[illegible]	[illegible]	[illegible]	[illegible]	[illegible]	[illegible]	[illegible]	[illegible]	[illegible]	[illegible]	[illegible]	[illegible]	[illegible]	[illegible]	[illegible]	[illegible]	[illegible]	[illegible]	[illegible]	[illegible]	[illegible]	[illegible]	[illegible]	[illegible]	[illegible]	[illegible]
Bournemouth	0·0125 (0·0082 to 0·019)	0·0117 (0·00771 to 0·0173)	0·0107 (0·00742 to 0·0151)	0·0108 (0·00804 to 0·0145)	0·011 (0·00833 to 0·014)	[illegible]	[illegible]	[illegible]	[illegible]	[illegible]	[illegible]	[illegible]	[illegible]	[illegible]	[illegible]	[illegible]	[illegible]	[illegible]	[illegible]	[illegible]	[illegible]	[illegible]	[illegible]	[illegible]	[illegible]	[illegible]	[illegible]	[illegible]	[illegible]	[illegible]	[illegible]	[illegible]
Bristol, City of	0·0447 (0·03 to 0·0656)	0·0414 (0·0269 to 0·0597)	0·0381 (0·0248 to 0·0553)	0·0377 (0·0244 to 0·0558)	0·0364 (0·0238 to 0·054)	[illegible]	[illegible]	[illegible]	[illegible]	[illegible]	[illegible]	[illegible]	[illegible]	[illegible]	[illegible]	[illegible]	[illegible]	[illegible]	[illegible]	[illegible]	[illegible]	[illegible]	[illegible]	[illegible]	[illegible]	[illegible]	[illegible]	[illegible]	[illegible]	[illegible]	[illegible]	[illegible]
Cornwall	0·0442 (0·0312 to 0·061)	[illegible]	[illegible]	[illegible]	[illegible]	[illegible]	[illegible]	[illegible]	[illegible]	[illegible]	[illegible]	[illegible]	[illegible]	[illegible]	[illegible]	[illegible]	[illegible]	[illegible]	[illegible]	[illegible]	[illegible]	[illegible]	[illegible]	[illegible]	[illegible]	[illegible]	[illegible]	[illegible]	[illegible]	[illegible]	[illegible]	[illegible]
Devon	0·0493 (0·0335 to 0·0734)	[illegible]	[illegible]	[illegible]	[illegible]	[illegible]	[illegible]	[illegible]	[illegible]	[illegible]	[illegible]	[illegible]	[illegible]	[illegible]	[illegible]	[illegible]	[illegible]	[illegible]	[illegible]	[illegible]	[illegible]	[illegible]	[illegible]	[illegible]	[illegible]	[illegible]	[illegible]	[illegible]	[illegible]	[illegible]	[illegible]	[illegible]
Dorset	0·0231 (0·0151 to 0·0345)	[illegible]	[illegible]	[illegible]	[illegible]	[illegible]	[illegible]	[illegible]	[illegible]	[illegible]	[illegible]	[illegible]	[illegible]	[illegible]	[illegible]	[illegible]	[illegible]	[illegible]	[illegible]	[illegible]	[illegible]	[illegible]	[illegible]	[illegible]	[illegible]	[illegible]	[illegible]	[illegible]	[illegible]	[illegible]	[illegible]	[illegible]
Gloucestershire	0·0527 (0·0341 to 0·0783)	[illegible]	[illegible]	[illegible]	[illegible]	[illegible]	[illegible]	[illegible]	[illegible]	[illegible]	[illegible]	[illegible]	[illegible]	[illegible]	[illegible]	[illegible]	[illegible]	[illegible]	[illegible]	[illegible]	[illegible]	[illegible]	[illegible]	[illegible]	[illegible]	[illegible]	[illegible]	[illegible]	[illegible]	[illegible]	[illegible]	[illegible]
North Somerset	0·0129 (0·00868 to 0·0189)	[illegible]	[illegible]	[illegible]	[illegible]	[illegible]	[illegible]	[illegible]	[illegible]	[illegible]	[illegible]	[illegible]	[illegible]	[illegible]	[illegible]	[illegible]	[illegible]	[illegible]	[illegible]	[illegible]	[illegible]	[illegible]	[illegible]	[illegible]	[illegible]	[illegible]	[illegible]	[illegible]	[illegible]	[illegible]	[illegible]	[illegible]
Plymouth	0·0226 (0·0148 to 0·0332)	[illegible]	[illegible]	[illegible]	[illegible]	[illegible]	[illegible]	[illegible]	[illegible]	[illegible]	[illegible]	[illegible]	[illegible]	[illegible]	[illegible]	[illegible]	[illegible]	[illegible]	[illegible]	[illegible]	[illegible]	[illegible]	[illegible]	[illegible]	[illegible]	[illegible]	[illegible]	[illegible]	[illegible]	[illegible]	[illegible]	[illegible]
Poole	0·0129 (0·00877 to 0·0183)	[illegible]	[illegible]	[illegible]	[illegible]	[illegible]	[illegible]	[illegible]	[illegible]	[illegible]	[illegible]	[illegible]	[illegible]	[illegible]	[illegible]	[illegible]	[illegible]	[illegible]	[illegible]	[illegible]	[illegible]	[illegible]	[illegible]	[illegible]	[illegible]	[illegible]	[illegible]	[illegible]	[illegible]	[illegible]	[illegible]	[illegible]
Somerset	0·0294 (0·0199 to 0·0437)	[illegible]	[illegible]	[illegible]	[illegible]	[illegible]	[illegible]	[illegible]	[illegible]	[illegible]	[illegible]	[illegible]	[illegible]	[illegible]	[illegible]	[illegible]	[illegible]	[illegible]	[illegible]	[illegible]	[illegible]	[illegible]	[illegible]	[illegible]	[illegible]	[illegible]	[illegible]	[illegible]	[illegible]	[illegible]	[illegible]	[illegible]
South Gloucestershire	0·0241 (0·0162 to 0·0353)	[illegible]	[illegible]	[illegible]	[illegible]	[illegible]	[illegible]	[illegible]	[illegible]	[illegible]	[illegible]	[illegible]	[illegible]	[illegible]	[illegible]	[illegible]	[illegible]	[illegible]	[illegible]	[illegible]	[illegible]	[illegible]	[illegible]	[illegible]	[illegible]	[illegible]	[illegible]	[illegible]	[illegible]	[illegible]	[illegible]	[illegible]
Swindon	0·0198 (0·0133 to 0·0289)	[illegible]	[illegible]	[illegible]	[illegible]	[illegible]	[illegible]	[illegible]	[illegible]	[illegible]	[illegible]	[illegible]	[illegible]	[illegible]	[illegible]	[illegible]	[illegible]	[illegible]	[illegible]	[illegible]	[illegible]	[illegible]	[illegible]	[illegible]	[illegible]	[illegible]	[illegible]	[illegible]	[illegible]	[illegible]	[illegible]	[illegible]
Torbay	0·0161 (0·0103 to 0·0242)	[illegible]	[illegible]	[illegible]	[illegible]	[illegible]	[illegible]	[illegible]	[illegible]	[illegible]	[illegible]	[illegible]	[illegible]	[illegible]	[illegible]	[illegible]	[illegible]	[illegible]	[illegible]	[illegible]	[illegible]	[illegible]	[illegible]	[illegible]	[illegible]	[illegible]	[illegible]	[illegible]	[illegible]	[illegible]	[illegible]	[illegible]
Wiltshire	0·0442 (0·0296 to 0·0649)	[illegible]	[illegible]	[illegible]	[illegible]	[illegible]	[illegible]	[illegible]	[illegible]	[illegible]	[illegible]	[illegible]	[illegible]	[illegible]	[illegible]	[illegible]	[illegible]	[illegible]	[illegible]	[illegible]	[illegible]	[illegible]	[illegible]	[illegible]	[illegible]	[illegible]	[illegible]	[illegible]	[illegible]	[illegible]	[illegible]	[illegible]
West Midlands	0·706 (0·502 to 1)	[illegible]	[illegible]	[illegible]	[illegible]	[illegible]	[illegible]	[illegible]	[illegible]	[illegible]	[illegible]	[illegible]	[illegible]	[illegible]	[illegible]	[illegible]	[illegible]	[illegible]	[illegible]	[illegible]	[illegible]	[illegible]	[illegible]	[illegible]	[illegible]	[illegible]	[illegible]	[illegible]	[illegible]	[illegible]	[illegible]	[illegible]
Birmingham	0·158 (0·108 to 0·232)	[illegible]	[illegible]	[illegible]	[illegible]	[illegible]	[illegible]	[illegible]	[illegible]	[illegible]	[illegible]	[illegible]	[illegible]	[illegible]	[illegible]	[illegible]	[illegible]	[illegible]	[illegible]	[illegible]	[illegible]	[illegible]	[illegible]	[illegible]	[illegible]	[illegible]	[illegible]	[illegible]	[illegible]	[illegible]	[illegible]	[illegible]
Coventry	0·054 (0·0379 to 0·0766)	[illegible]	[illegible]	[illegible]	[illegible]	[illegible]	[illegible]	[illegible]	[illegible]	[illegible]	[illegible]	[illegible]	[illegible]	[illegible]	[illegible]	[illegible]	[illegible]	[illegible]	[illegible]	[illegible]	[illegible]	[illegible]	[illegible]	[illegible]	[illegible]	[illegible]	[illegible]	[illegible]	[illegible]	[illegible]	[illegible]	[illegible]
Dudley	0·0405 (0·0258 to 0·0611)	[illegible]	[illegible]	[illegible]	[illegible]	[illegible]	[illegible]	[illegible]	[illegible]	[illegible]	[illegible]	[illegible]	[illegible]	[illegible]	[illegible]	[illegible]	[illegible]	[illegible]	[illegible]	[illegible]	[illegible]	[illegible]	[illegible]	[illegible]	[illegible]	[illegible]	[illegible]	[illegible]	[illegible]	[illegible]	[illegible]	[illegible]
Herefordshire, County of	0·0121 (0·00854 to 0·0174)	[illegible]	[illegible]	[illegible]	[illegible]	[illegible]	[illegible]	[illegible]	[illegible]	[illegible]	[illegible]	[illegible]	[illegible]	[illegible]	[illegible]	[illegible]	[illegible]	[illegible]	[illegible]	[illegible]	[illegible]	[illegible]	[illegible]	[illegible]	[illegible]	[illegible]	[illegible]	[illegible]	[illegible]	[illegible]	[illegible]	[illegible]
Sandwell	0·0544 (0·0344 to 0·0823)	[illegible]	[illegible]	[illegible]	[illegible]	[illegible]	[illegible]	[illegible]	[illegible]	[illegible]	[illegible]	[illegible]	[illegible]	[illegible]	[illegible]	[illegible]	[illegible]	[illegible]	[illegible]	[illegible]	[illegible]	[illegible]	[illegible]	[illegible]	[illegible]	[illegible]	[illegible]	[illegible]	[illegible]	[illegible]	[illegible]	[illegible]
Shropshire	0·0295 (0·0202 to 0·0432)	[illegible]	[illegible]	[illegible]	[illegible]	[illegible]	[illegible]	[illegible]	[illegible]	[illegible]	[illegible]	[illegible]	[illegible]	[illegible]	[illegible]	[illegible]	[illegible]	[illegible]	[illegible]	[illegible]	[illegible]	[illegible]	[illegible]	[illegible]	[illegible]	[illegible]	[illegible]	[illegible]	[illegible]	[illegible]	[illegible]	[illegible]
Solihull	0·0231 (0·016 to 0·0327)	[illegible]	[illegible]	[illegible]	[illegible]	[illegible]	[illegible]	[illegible]	[illegible]	[illegible]	[illegible]	[illegible]	[illegible]	[illegible]	[illegible]	[illegible]	[illegible]	[illegible]	[illegible]	[illegible]	[illegible]	[illegible]	[illegible]	[illegible]	[illegible]	[illegible]	[illegible]	[illegible]	[illegible]	[illegible]	[illegible]	[illegible]
Staffordshire	0·0924 (0·0625 to 0·136)	[illegible]	[illegible]	[illegible]	[illegible]	[illegible]	[illegible]	[illegible]	[illegible]	[illegible]	[illegible]	[illegible]	[illegible]	[illegible]	[illegible]	[illegible]	[illegible]	[illegible]	[illegible]	[illegible]	[illegible]	[illegible]	[illegible]	[illegible]	[illegible]	[illegible]	[illegible]	[illegible]	[illegible]	[illegible]	[illegible]	[illegible]
Stoke-on-Trent	0·0307 (0·0216 to 0·0454)	[illegible]	[illegible]	[illegible]	[illegible]	[illegible]	[illegible]	[illegible]	[illegible]	[illegible]	[illegible]	[illegible]	[illegible]	[illegible]	[illegible]	[illegible]	[illegible]	[illegible]	[illegible]	[illegible]	[illegible]	[illegible]	[illegible]	[illegible]	[illegible]	[illegible]	[illegible]	[illegible]	[illegible]	[illegible]	[illegible]	[illegible]
Telford and Wrekin	0·0302 (0·0199 to 0·0452)	[illegible]	[illegible]	[illegible]	[illegible]	[illegible]	[illegible]	[illegible]	[illegible]	[illegible]	[illegible]	[illegible]	[illegible]	[illegible]	[illegible]	[illegible]	[illegible]	[illegible]	[illegible]	[illegible]	[illegible]	[illegible]	[illegible]	[illegible]	[illegible]	[illegible]	[illegible]	[illegible]	[illegible]	[illegible]	[illegible]	[illegible]
Walsall	0·0347 (0·0228 to 0·0519)	[illegible]	[illegible]	[illegible]	[illegible]	[illegible]	[illegible]	[illegible]	[illegible]	[illegible]	[illegible]	[illegible]	[illegible]	[illegible]	[illegible]	[illegible]	[illegible]	[illegible]	[illegible]	[illegible]	[illegible]	[illegible]	[illegible]	[illegible]	[illegible]	[illegible]	[illegible]	[illegible]	[illegible]	[illegible]	[illegible]	[illegible]
Warwickshire	0·0454 (0·0315 to 0·0643)	[illegible]	[illegible]	[illegible]	[illegible]	[illegible]	[illegible]	[illegible]	[illegible]	[illegible]	[illegible]	[illegible]	[illegible]	[illegible]	[illegible]	[illegible]	[illegible]	[illegible]	[illegible]	[illegible]	[illegible]	[illegible]	[illegible]	[illegible]	[illegible]	[illegible]	[illegible]	[illegible]	[illegible]	[illegible]	[illegible]	[illegible]
Wolverhampton	0·0482 (0·0316 to 0·0721)	[illegible]	[illegible]	[illegible]	[illegible]	[illegible]	[illegible]	[illegible]	[illegible]	[illegible]	[illegible]	[illegible]	[illegible]	[illegible]	[illegible]	[illegible]	[illegible]	[illegible]	[illegible]	[illegible]	[illegible]	[illegible]	[illegible]	[illegible]	[illegible]	[illegible]	[illegible]	[illegible]	[illegible]	[illegible]	[illegible]	[illegible]
Worcestershire	0·0524 (0·0359 to 0·0771)	[illegible]	[illegible]	[illegible]	[illegible]	[illegible]	[illegible]	[illegible]	[illegible]	[illegible]	[illegible]	[illegible]	[illegible]	[illegible]	[illegible]	[illegible]	[illegible]	[illegible]	[illegible]	[illegible]	[illegible]	[illegible]	[illegible]	[illegible]	[illegible]	[illegible]	[illegible]	[illegible]	[illegible]	[illegible]	[illegible]	[illegible]
Yorkshire and the Humber	0·525 (0·374 to 0·745)	[illegible]	[illegible]	[illegible]	[illegible]	[illegible]	[illegible]	[illegible]	[illegible]	[illegible]	[illegible]	[illegible]	[illegible]	[illegible]	[illegible]	[illegible]	[illegible]	[illegible]	[illegible]	[illegible]	[illegible]	[illegible]	[illegible]	[illegible]	[illegible]	[illegible]	[illegible]	[illegible]	[illegible]	[illegible]	[illegible]	[illegible]
Barnsley	0·0357 (0·0238 to 0·0524)	[illegible]	[illegible]	[illegible]	[illegible]	[illegible]	[illegible]	[illegible]	[illegible]	[illegible]	[illegible]	[illegible]	[illegible]	[illegible]	[illegible]	[illegible]	[illegible]	[illegible]	[illegible]	[illegible]	[illegible]	[illegible]	[illegible]	[illegible]	[illegible]	[illegible]	[illegible]	[illegible]	[illegible]	[illegible]	[illegible]	[illegible]
Bradford	0·066 (0·0454 to 0·0932)	[illegible]	[illegible]	[illegible]	[illegible]	[illegible]	[illegible]	[illegible]	[illegible]	[illegible]	[illegible]	[illegible]	[illegible]	[illegible]	[illegible]	[illegible]	[illegible]	[illegible]	[illegible]	[illegible]	[illegible]	[illegible]	[illegible]	[illegible]	[illegible]	[illegible]	[illegible]	[illegible]	[illegible]	[illegible]	[illegible]	[illegible]
Calderdale	0·0244 (0·0162 to 0·0367)	[illegible]	[illegible]	[illegible]	[illegible]	[illegible]	[illegible]	[illegible]	[illegible]	[illegible]	[illegible]	[illegible]	[illegible]	[illegible]	[illegible]	[illegible]	[illegible]	[illegible]	[illegible]	[illegible]	[illegible]	[illegible]	[illegible]	[illegible]	[illegible]	[illegible]	[illegible]	[illegible]	[illegible]	[illegible]	[illegible]	[illegible]
Doncaster	0·0264 (0·0184 to 0·0382)	[illegible]	[illegible]	[illegible]	[illegible]	[illegible]	[illegible]	[illegible]	[illegible]	[illegible]	[illegible]	[illegible]	[illegible]	[illegible]	[illegible]	[illegible]	[illegible]	[illegible]	[illegible]	[illegible]	[illegible]	[illegible]	[illegible]	[illegible]	[illegible]	[illegible]	[illegible]	[illegible]	[illegible]	[illegible]	[illegible]	[illegible]
East Riding of Yorkshire	0·0222 (0·0146 to 0·0331)	[illegible]	[illegible]	[illegible]	[illegible]	[illegible]	[illegible]	[illegible]	[illegible]	[illegible]	[illegible]	[illegible]	[illegible]	[illegible]	[illegible]	[illegible]	[illegible]	[illegible]	[illegible]	[illegible]	[illegible]	[illegible]	[illegible]	[illegible]	[illegible]	[illegible]	[illegible]	[illegible]	[illegible]	[illegible]	[illegible]	[illegible]
Kingston upon Hull, City of	0·043 (0·0277 to 0·0647)	[illegible]	[illegible]	[illegible]	[illegible]	[illegible]	[illegible]	[illegible]	[illegible]	[illegible]	[illegible]	[illegible]	[illegible]	[illegible]	[illegible]	[illegible]	[illegible]	[illegible]	[illegible]	[illegible]	[illegible]	[illegible]	[illegible]	[illegible]	[illegible]	[illegible]	[illegible]	[illegible]	[illegible]	[illegible]	[illegible]	[illegible]
Kirklees	0·0409 (0·027 to 0·0604)	[illegible]	[illegible]	[illegible]	[illegible]	[illegible]	[illegible]	[illegible]	[illegible]	[illegible]	[illegible]	[illegible]	[illegible]	[illegible]	[illegible]	[illegible]	[illegible]	[illegible]	[illegible]	[illegible]	[illegible]	[illegible]	[illegible]	[illegible]	[illegible]	[illegible]	[illegible]	[illegible]	[illegible]	[illegible]	[illegible]	[illegible]
Leeds	0·0645 (0·0449 to 0·0932)	[illegible]	[illegible]	[illegible]	[illegible]	[illegible]	[illegible]	[illegible]	[illegible]	[illegible]	[illegible]	[illegible]	[illegible]	[illegible]	[illegible]	[illegible]	[illegible]	[illegible]	[illegible]	[illegible]	[illegible]	[illegible]	[illegible]	[illegible]	[illegible]	[illegible]	[illegible]	[illegible]	[illegible]	[illegible]	[illegible]	[illegible]
North East Lincolnshire	0·0107 (0·00736 to 0·0151)	[illegible]	[illegible]	[illegible]	[illegible]	[illegible]	[illegible]	[illegible]	[illegible]	[illegible]	[illegible]	[illegible]	[illegible]	[illegible]	[illegible]	[illegible]	[illegible]	[illegible]	[illegible]	[illegible]	[illegible]	[illegible]	[illegible]	[illegible]	[illegible]	[illegible]	[illegible]	[illegible]	[illegible]	[illegible]	[illegible]	[illegible]
North Lincolnshire	0·0182 (0·0122 to 0·0258)	[illegible]	[illegible]	[illegible]	[illegible]	[illegible]	[illegible]	[illegible]	[illegible]	[illegible]	[illegible]	[illegible]	[illegible]	[illegible]	[illegible]	[illegible]	[illegible]	[illegible]	[illegible]	[illegible]	[illegible]	[illegible]	[illegible]	[illegible]	[illegible]	[illegible]	[illegible]	[illegible]	[illegible]	[illegible]	[illegible]	[illegible]
North Yorkshire	0·0357 (0·0236 to 0·0528)	[illegible]	[illegible]	[illegible]	[illegible]	[illegible]	[illegible]	[illegible]	[illegible]	[illegible]	[illegible]	[illegible]	[illegible]	[illegible]	[illegible]	[illegible]	[illegible]	[illegible]	[illegible]	[illegible]	[illegible]	[illegible]	[illegible]	[illegible]	[illegible]	[illegible]	[illegible]	[illegible]	[illegible]	[illegible]	[illegible]	[illegible]
Rotherham	0·0356 (0·0248 to 0·0515)	[illegible]	[illegible]	[illegible]	[illegible]	[illegible]	[illegible]	[illegible]	[illegible]	[illegible]	[illegible]	[illegible]	[illegible]	[illegible]	[illegible]	[illegible]	[illegible]	[illegible]	[illegible]	[illegible]	[illegible]	[illegible]	[illegible]	[illegible]	[illegible]	[illegible]	[illegible]	[illegible]	[illegible]	[illegible]	[illegible]	[illegible]
Sheffield	0·0582 (0·0376 to 0·0868)	[illegible]	[illegible]	[illegible]	[illegible]	[illegible]	[illegible]	[illegible]	[illegible]	[illegible]	[illegible]	[illegible]	[illegible]	[illegible]	[illegible]	[illegible]	[illegible]	[illegible]	[illegible]	[illegible]	[illegible]	[illegible]	[illegible]	[illegible]	[illegible]	[illegible]	[illegible]	[illegible]	[illegible]	[illegible]	[illegible]	[illegible]
Wakefield	0·0323 (0·0225 to 0·0466)	[illegible]	[illegible]	[illegible]	[illegible]	[illegible]	[illegible]	[illegible]	[illegible]	[illegible]	[illegible]	[illegible]	[illegible]	[illegible]	[illegible]	[illegible]	[illegible]	[illegible]	[illegible]	[illegible]	[illegible]	[illegible]	[illegible]	[illegible]	[illegible]	[illegible]	[illegible]	[illegible]	[illegible]	[illegible]	[illegible]	[illegible]
York	0·0116 (0·00746 to 0·0175)	[illegible]	[illegible]	[illegible]	[illegible]	[illegible]	[illegible]	[illegible]	[illegible]	[illegible]	[illegible]	[illegible]	[illegible]	[illegible]	[illegible]	[illegible]	[illegible]	[illegible]	[illegible]	[illegible]	[illegible]	[illegible]	[illegible]	[illegible]	[illegible]	[illegible]	[illegible]	[illegible]	[illegible]	[illegible]	[illegible]	[illegible]
Northern Ireland	0·216 (0·195 to 0·237)	[illegible]	[illegible]	[illegible]	[illegible]	[illegible]	[illegible]	[illegible]	[illegible]	[illegible]	[illegible]	[illegible]	[illegible]	[illegible]	[illegible]	[illegible]	[illegible]	[illegible]	[illegible]	[illegible]	[illegible]	[illegible]	[illegible]	[illegible]	[illegible]	[illegible]	[illegible]	[illegible]	[illegible]	[illegible]	[illegible]	[illegible]
Scotland	0·574 (0·532 to 0·616)	[illegible]	[illegible]	[illegible]	[illegible]	[illegible]	[illegible]	[illegible]	[illegible]	[illegible]	[illegible]	[illegible]	[illegible]	[illegible]	[illegible]	[illegible]	[illegible]	[illegible]	[illegible]	[illegible]	[illegible]	[illegible]	[illegible]	[illegible]	[illegible]	[illegible]	[illegible]	[illegible]	[illegible]	[illegible]	[illegible]	[illegible]
Wales	0·281 (0·2 to 0·398)	[illegible]	[illegible]	[illegible]	[illegible]	[illegible]	[illegible]	[illegible]	[illegible]	[illegible]	[illegible]	[illegible]	[illegible]	[illegible]	[illegible]	[illegible]	[illegible]	[illegible]	[illegible]	[illegible]	[illegible]	[illegible]	[illegible]	[illegible]	[illegible]	[illegible]	[illegible]	[illegible]	[illegible]	[illegible]	[illegible]	[illegible]
Latin America and Caribbean	302 (246 to 369)	[illegible]	[illegible]	[illegible]	[illegible]	[illegible]	[illegible]	[illegible]	[illegible]	[illegible]	[illegible]	[illegible]	[illegible]	[illegible]	[illegible]	[illegible]	[illegible]	[illegible]	[illegible]	[illegible]	[illegible]	[illegible]	[illegible]	[illegible]	[illegible]	[illegible]	[illegible]	[illegible]	[illegible]	[illegible]	[illegible]	[illegible]
Andean Latin America	23·8 (21·4 to 26·8)	[illegible]	[illegible]	[illegible]	[illegible]	[illegible]	[illegible]	[illegible]	[illegible]	[illegible]	[illegible]	[illegible]	[illegible]	[illegible]	[illegible]	[illegible]	[illegible]	[illegible]	[illegible]	[illegible]	[illegible]	[illegible]	[illegible]	[illegible]	[illegible]	[illegible]	[illegible]	[illegible]	[illegible]	[illegible]	[illegible]	[illegible]
Bolivia	6·78 (5·71 to 8·01)	[illegible]	[illegible]	[illegible]	[illegible]	[illegible]	[illegible]	[illegible]	[illegible]	[illegible]	[illegible]	[illegible]	[illegible]	[illegible]	[illegible]	[illegible]	[illegible]	[illegible]	[illegible]	[illegible]	[illegible]	[illegible]	[illegible]	[illegible]	[illegible]	[illegible]	[illegible]	[illegible]	[illegible]	[illegible]	[illegible]	[illegible]
Ecuador	4·04 (2·85 to 5·81)	[illegible]	[illegible]	[illegible]	[illegible]	[illegible]	[illegible]	[illegible]	[illegible]	[illegible]	[illegible]	[illegible]	[illegible]	[illegible]	[illegible]	[illegible]	[illegible]	[illegible]	[illegible]	[illegible]	[illegible]	[illegible]	[illegible]	[illegible]	[illegible]	[illegible]	[illegible]	[illegible]	[illegible]	[illegible]	[illegible]	[illegible]
Peru	13 (11·7 to 14·4)	[illegible]	[illegible]	[illegible]	[illegible]	[illegible]	[illegible]	[illegible]	[illegible]	[illegible]	[illegible]	[illegible]	[illegible]	[illegible]	[illegible]	[illegible]	[illegible]	[illegible]	[illegible]	[illegible]	[illegible]	[illegible]	[illegible]	[illegible]	[illegible]	[illegible]	[illegible]	[illegible]	[illegible]	[illegible]	[illegible]	[illegible]
Caribbean	37·8 (29·7 to 48)	[illegible]	[illegible]	[illegible]	[illegible]	[illegible]	[illegible]	[illegible]	[illegible]	[illegible]	[illegible]	[illegible]	[illegible]	[illegible]	[illegible]	[illegible]	[illegible]	[illegible]	[illegible]	[illegible]	[illegible]	[illegible]	[illegible]	[illegible]	[illegible]	[illegible]	[illegible]	[illegible]	[illegible]	[illegible]	[illegible]	[illegible]
Antigua and Barbuda	0·0236 (0·0165 to 0·0327)	[illegible]	[illegible]	[illegible]	[illegible]	[illegible]	[illegible]	[illegible]	[illegible]	[illegible]	[illegible]	[illegible]	[illegible]	[illegible]	[illegible]	[illegible]	[illegible]	[illegible]	[illegible]	[illegible]	[illegible]	[illegible]	[illegible]	[illegible]	[illegible]	[illegible]	[illegible]	[illegible]	[illegible]	[illegible]	[illegible]	[illegible]
Barbados	0·0608 (0·0446 to 0·081)	[illegible]	[illegible]	[illegible]	[illegible]	[illegible]	[illegible]	[illegible]	[illegible]	[illegible]	[illegible]	[illegible]	[illegible]	[illegible]	[illegible]	[illegible]	[illegible]	[illegible]	[illegible]	[illegible]	[illegible]	[illegible]	[illegible]	[illegible]	[illegible]	[illegible]	[illegible]	[illegible]	[illegible]	[illegible]	[illegible]	[illegible]
Belize	0·198 (0·144 to 0·265)	[illegible]	[illegible]	[illegible]	[illegible]	[illegible]	[illegible]	[illegible]	[illegible]	[illegible]	[illegible]	[illegible]	[illegible]	[illegible]	[illegible]	[illegible]	[illegible]	[illegible]	[illegible]	[illegible]	[illegible]	[illegible]	[illegible]	[illegible]	[illegible]	[illegible]	[illegible]	[illegible]	[illegible]	[illegible]	[illegible]	[illegible]
Bermuda	0·0147 (0·0106 to 0·0201)	[illegible]	[illegible]	[illegible]	[illegible]	[illegible]	[illegible]	[illegible]	[illegible]	[illegible]	[illegible]	[illegible]	[illegible]	[illegible]	[illegible]	[illegible]	[illegible]	[illegible]	[illegible]	[illegible]	[illegible]	[illegible]	[illegible]	[illegible]	[illegible]	[illegible]	[illegible]	[illegible]	[illegible]	[illegible]	[illegible]	[illegible]
Cuba	2·91 (2·75 to 3·08)	[illegible]	[illegible]	[illegible]	[illegible]	[illegible]	[illegible]	[illegible]	[illegible]	[illegible]	[illegible]	[illegible]	[illegible]	[illegible]	[illegible]	[illegible]	[illegible]	[illegible]	[illegible]	[illegible]	[illegible]	[illegible]	[illegible]	[illegible]	[illegible]	[illegible]	[illegible]	[illegible]	[illegible]	[illegible]	[illegible]	[illegible]
Dominica	0·039 (0·0275 to 0·0542)	[illegible]	[illegible]	[illegible]	[illegible]	[illegible]	[illegible]	[illegible]	[illegible]	[illegible]	[illegible]	[illegible]	[illegible]	[illegible]	[illegible]	[illegible]	[illegible]	[illegible]	[illegible]	[illegible]	[illegible]	[illegible]	[illegible]	[illegible]	[illegible]	[illegible]	[illegible]	[illegible]	[illegible]	[illegible]	[illegible]	[illegible]
Dominican Republic	10·4 (7·27 to 14·8)	[illegible]	[illegible]	[illegible]	[illegible]	[illegible]	[illegible]	[illegible]	[illegible]	[illegible]	[illegible]	[illegible]	[illegible]	[illegible]	[illegible]	[illegible]	[illegible]	[illegible]	[illegible]	[illegible]	[illegible]	[illegible]	[illegible]	[illegible]	[illegible]	[illegible]	[illegible]	[illegible]	[illegible]	[illegible]	[illegible]	[illegible]
Grenada	0·0706 (0·0514 to 0·0934)	[illegible]	[illegible]	[illegible]	[illegible]	[illegible]	[illegible]	[illegible]	[illegible]	[illegible]	[illegible]	[illegible]	[illegible]	[illegible]	[illegible]	[illegible]	[illegible]	[illegible]	[illegible]	[illegible]	[illegible]	[illegible]	[illegible]	[illegible]	[illegible]	[illegible]	[illegible]	[illegible]	[illegible]	[illegible]	[illegible]	[illegible]
Guyana	1·12 (0·783 to 1·59)	[illegible]	[illegible]	[illegible]	[illegible]	[illegible]	[illegible]	[illegible]	[illegible]	[illegible]	[illegible]	[illegible]	[illegible]	[illegible]	[illegible]	[illegible]	[illegible]	[illegible]	[illegible]	[illegible]	[illegible]	[illegible]	[illegible]	[illegible]	[illegible]	[illegible]	[illegible]	[illegible]	[illegible]	[illegible]	[illegible]	[illegible]
Haiti	17·3 (12 to 24·4)	[illegible]	[illegible]	[illegible]	[illegible]	[illegible]	[illegible]	[illegible]	[illegible]	[illegible]	[illegible]	[illegible]	[illegible]	[illegible]	[illegible]	[illegible]	[illegible]	[illegible]	[illegible]	[illegible]	[illegible]	[illegible]	[illegible]	[illegible]	[illegible]	[illegible]	[illegible]	[illegible]	[illegible]	[illegible]	[illegible]	[illegible]
Jamaica	1·19 (0·901 to 1·54)	[illegible]	[illegible]	[illegible]	[illegible]	[illegible]	[illegible]	[illegible]	[illegible]	[illegible]	[illegible]	[illegible]	[illegible]	[illegible]	[illegible]	[illegible]	[illegible]	[illegible]	[illegible]	[illegible]	[illegible]	[illegible]	[illegible]	[illegible]	[illegible]	[illegible]	[illegible]	[illegible]	[illegible]	[illegible]	[illegible]	[illegible]
Puerto Rico	1·69 (1·17 to 2·39)	[illegible]	[illegible]	[illegible]	[illegible]	[illegible]	[illegible]	[illegible]	[illegible]	[illegible]	[illegible]	[illegible]	[illegible]	[illegible]	[illegible]	[illegible]	[illegible]	[illegible]	[illegible]	[illegible]	[illegible]	[illegible]	[illegible]	[illegible]	[illegible]	[illegible]	[illegible]	[illegible]	[illegible]	[illegible]	[illegible]	[illegible]
Saint Kitts and Nevis	0·031 (0·0215 to 0·0431)	[illegible]	[illegible]	[illegible]	[illegible]	[illegible]	[illegible]	[illegible]	[illegible]	[illegible]	[illegible]	[illegible]	[illegible]	[illegible]	[illegible]	[illegible]	[illegible]	[illegible]	[illegible]	[illegible]	[illegible]	[illegible]	[illegible]	[illegible]	[illegible]	[illegible]	[illegible]	[illegible]	[illegible]	[illegible]	[illegible]	[illegible]
Saint Lucia	0·0882 (0·0755 to 0·102)	[illegible]	[illegible]	[illegible]	[illegible]	[illegible]	[illegible]	[illegible]	[illegible]	[illegible]	[illegible]	[illegible]	[illegible]	[illegible]	[illegible]	[illegible]	[illegible]	[illegible]	[illegible]	[illegible]	[illegible]	[illegible]	[illegible]	[illegible]	[illegible]	[illegible]	[illegible]	[illegible]	[illegible]	[illegible]	[illegible]	[illegible]
Saint Vincent and the Grenadines	0·0404 (0·0285 to 0·0562)	[illegible]	[illegible]	[illegible]	[illegible]	[illegible]	[illegible]	[illegible]	[illegible]	[illegible]	[illegible]	[illegible]	[illegible]	[illegible]	[illegible]	[illegible]	[illegible]	[illegible]	[illegible]	[illegible]	[illegible]	[illegible]	[illegible]	[illegible]	[illegible]	[illegible]	[illegible]	[illegible]	[illegible]	[illegible]	[illegible]	[illegible]
Suriname	0·554 (0·385 to 0·764)	[illegible]	[illegible]	[illegible]	[illegible]	[illegible]	[illegible]	[illegible]	[illegible]	[illegible]	[illegible]	[illegible]	[illegible]	[illegible]	[illegible]	[illegible]	[illegible]	[illegible]	[illegible]	[illegible]	[illegible]	[illegible]	[illegible]	[illegible]	[illegible]	[illegible]	[illegible]	[illegible]	[illegible]	[illegible]	[illegible]	[illegible]
The Bahamas	0·082 (0·0608 to 0·108)	[illegible]	[illegible]	[illegible]	[illegible]	[illegible]	[illegible]	[illegible]	[illegible]	[illegible]	[illegible]	[illegible]	[illegible]	[illegible]	[illegible]	[illegible]	[illegible]	[illegible]	[illegible]	[illegible]	[illegible]	[illegible]	[illegible]	[illegible]	[illegible]	[illegible]	[illegible]	[illegible]	[illegible]	[illegible]	[illegible]	[illegible]
Trinidad and Tobago	0·723 (0·652 to 0·802)	[illegible]	[illegible]	[illegible]	[illegible]	[illegible]	[illegible]	[illegible]	[illegible]	[illegible]	[illegible]	[illegible]	[illegible]	[illegible]	[illegible]	[illegible]	[illegible]	[illegible]	[illegible]	[illegible]	[illegible]	[illegible]	[illegible]	[illegible]	[illegible]	[illegible]	[illegible]	[illegible]	[illegible]	[illegible]	[illegible]	[illegible]
Virgin Islands	0·0688 (0·0477 to 0·0949)	[illegible]	[illegible]	[illegible]	[illegible]	[illegible]	[illegible]	[illegible]	[illegible]	[illegible]	[illegible]	[illegible]	[illegible]	[illegible]	[illegible]	[illegible]	[illegible]	[illegible]	[illegible]	[illegible]	[illegible]	[illegible]	[illegible]	[illegible]	[illegible]	[illegible]	[illegible]	[illegible]	[illegible]	[illegible]	[illegible]	[illegible]
Central Latin America	144 (119 to 173)	[illegible]	[illegible]	[illegible]	[illegible]	[illegible]	[illegible]	[illegible]	[illegible]	[illegible]	[illegible]	[illegible]	[illegible]	[illegible]	[illegible]	[illegible]	[illegible]	[illegible]	[illegible]	[illegible]	[illegible]	[illegible]	[illegible]	[illegible]	[illegible]	[illegible]	[illegible]	[illegible]	[illegible]	[illegible]	[illegible]	[illegible]
Colombia	31·2 (21·5 to 44·4)	[illegible]	[illegible]	[illegible]	[illegible]	[illegible]	[illegible]	[illegible]	[illegible]	[illegible]	[illegible]	[illegible]	[illegible]	[illegible]	[illegible]	[illegible]	[illegible]	[illegible]	[illegible]	[illegible]	[illegible]	[illegible]	[illegible]	[illegible]	[illegible]	[illegible]	[illegible]	[illegible]	[illegible]	[illegible]	[illegible]	[illegible]
Costa Rica	0·74 (0·685 to 0·802)	[illegible]	[illegible]	[illegible]	[illegible]	[illegible]	[illegible]	[illegible]	[illegible]	[illegible]	[illegible]	[illegible]	[illegible]	[illegible]	[illegible]	[illegible]	[illegible]	[illegible]	[illegible]	[illegible]	[illegible]	[illegible]	[illegible]	[illegible]	[illegible]	[illegible]	[illegible]	[illegible]	[illegible]	[illegible]	[illegible]	[illegible]
El Salvador	3·39 (2·48 to 4·54)	[illegible]	[illegible]	[illegible]	[illegible]	[illegible]	[illegible]	[illegible]	[illegible]	[illegible]	[illegible]	[illegible]	[illegible]	[illegible]	[illegible]	[illegible]	[illegible]	[illegible]	[illegible]	[illegible]	[illegible]	[illegible]	[illegible]	[illegible]	[illegible]	[illegible]	[illegible]	[illegible]	[illegible]	[illegible]	[illegible]	[illegible]
Guatemala	44·3 (31·4 to 60·1)	[illegible]	[illegible]	[illegible]	[illegible]	[illegible]	[illegible]	[illegible]	[illegible]	[illegible]	[illegible]	[illegible]	[illegible]	[illegible]	[illegible]	[illegible]	[illegible]	[illegible]	[illegible]	[illegible]	[illegible]	[illegible]	[illegible]	[illegible]	[illegible]	[illegible]	[illegible]	[illegible]	[illegible]	[illegible]	[illegible]	[illegible]
Honduras	6·13 (4·53 to 8·28)	[illegible]	[illegible]	[illegible]	[illegible]	[illegible]	[illegible]	[illegible]	[illegible]	[illegible]	[illegible]	[illegible]	[illegible]	[illegible]	[illegible]	[illegible]	[illegible]	[illegible]	[illegible]	[illegible]	[illegible]	[illegible]	[illegible]	[illegible]	[illegible]	[illegible]	[illegible]	[illegible]	[illegible]	[illegible]	[illegible]	[illegible]
Mexico	44·9 (43 to 47)	[illegible]	[illegible]	[illegible]	[illegible]	[illegible]	[illegible]	[illegible]	[illegible]	[illegible]	[illegible]	[illegible]	[illegible]	[illegible]	[illegible]	[illegible]	[illegible]	[illegible]	[illegible]	[illegible]	[illegible]	[illegible]	[illegible]	[illegible]	[illegible]	[illegible]	[illegible]	[illegible]	[illegible]	[illegible]	[illegible]	[illegible]
Aguascalientes	0·453 (0·418 to 0·491)	[illegible]	[illegible]	[illegible]	[illegible]	[illegible]	[illegible]	[illegible]	[illegible]	[illegible]	[illegible]	[illegible]	[illegible]	[illegible]	[illegible]	[illegible]	[illegible]	[illegible]	[illegible]	[illegible]	[illegible]	[illegible]	[illegible]	[illegible]	[illegible]	[illegible]	[illegible]	[illegible]	[illegible]	[illegible]	[illegible]	[illegible]
Baja California	0·722 (0·668 to 0·785)	[illegible]	[illegible]	[illegible]	[illegible]	[illegible]	[illegible]	[illegible]	[illegible]	[illegible]	[illegible]	[illegible]	[illegible]	[illegible]	[illegible]	[illegible]	[illegible]	[illegible]	[illegible]	[illegible]	[illegible]	[illegible]	[illegible]	[illegible]	[illegible]	[illegible]	[illegible]	[illegible]	[illegible]	[illegible]	[illegible]	[illegible]
Baja California Sur	0·115 (0·104 to 0·126)	[illegible]	[illegible]	[illegible]	[illegible]	[illegible]	[illegible]	[illegible]	[illegible]	[illegible]	[illegible]	[illegible]	[illegible]	[illegible]	[illegible]	[illegible]	[illegible]	[illegible]	[illegible]	[illegible]	[illegible]	[illegible]	[illegible]	[illegible]	[illegible]	[illegible]	[illegible]	[illegible]	[illegible]	[illegible]	[illegible]	[illegible]
Campeche	0·333 (0·307 to 0·359)	[illegible]	[illegible]	[illegible]	[illegible]	[illegible]	[illegible]	[illegible]	[illegible]	[illegible]	[illegible]	[illegible]	[illegible]	[illegible]	[illegible]	[illegible]	[illegible]	[illegible]	[illegible]	[illegible]	[illegible]	[illegible]	[illegible]	[illegible]	[illegible]	[illegible]	[illegible]	[illegible]	[illegible]	[illegible]	[illegible]	[illegible]
Chiapas	2·14 (1·96 to 2·33)	[illegible]	[illegible]	[illegible]	[illegible]	[illegible]	[illegible]	[illegible]	[illegible]	[illegible]	[illegible]	[illegible]	[illegible]	[illegible]	[illegible]	[illegible]	[illegible]	[illegible]	[illegible]	[illegible]	[illegible]	[illegible]	[illegible]	[illegible]	[illegible]	[illegible]	[illegible]	[illegible]	[illegible]	[illegible]	[illegible]	[illegible]
Chihuahua	1·89 (1·75 to 2·03)	[illegible]	[illegible]	[illegible]	[illegible]	[illegible]	[illegible]	[illegible]	[illegible]	[illegible]	[illegible]	[illegible]	[illegible]	[illegible]	[illegible]	[illegible]	[illegible]	[illegible]	[illegible]	[illegible]	[illegible]	[illegible]	[illegible]	[illegible]	[illegible]	[illegible]	[illegible]	[illegible]	[illegible]	[illegible]	[illegible]	[illegible]
Coahuila	1·25 (1·16 to 1·33)	[illegible]	[illegible]	[illegible]	[illegible]	[illegible]	[illegible]	[illegible]	[illegible]	[illegible]	[illegible]	[illegible]	[illegible]	[illegible]	[illegible]	[illegible]	[illegible]	[illegible]	[illegible]	[illegible]	[illegible]	[illegible]	[illegible]	[illegible]	[illegible]	[illegible]	[illegible]	[illegible]	[illegible]	[illegible]	[illegible]	[illegible]
Colima	0·206 (0·192 to 0·221)	[illegible]	[illegible]	[illegible]	[illegible]	[illegible]	[illegible]	[illegible]	[illegible]	[illegible]	[illegible]	[illegible]	[illegible]	[illegible]	[illegible]	[illegible]	[illegible]	[illegible]	[illegible]	[illegible]	[illegible]	[illegible]	[illegible]	[illegible]	[illegible]	[illegible]	[illegible]	[illegible]	[illegible]	[illegible]	[illegible]	[illegible]
Durango	0·974 (0·847 to 1·1)	[illegible]	[illegible]	[illegible]	[illegible]	[illegible]	[illegible]	[illegible]	[illegible]	[illegible]	[illegible]	[illegible]	[illegible]	[illegible]	[illegible]	[illegible]	[illegible]	[illegible]	[illegible]	[illegible]	[illegible]	[illegible]	[illegible]	[illegible]	[illegible]	[illegible]	[illegible]	[illegible]	[illegible]	[illegible]	[illegible]	[illegible]
Guanajuato	2·47 (2·29 to 2·67)	[illegible]	[illegible]	[illegible]	[illegible]	[illegible]	[illegible]	[illegible]	[illegible]	[illegible]	[illegible]	[illegible]	[illegible]	[illegible]	[illegible]	[illegible]	[illegible]	[illegible]	[illegible]	[illegible]	[illegible]	[illegible]	[illegible]	[illegible]	[illegible]	[illegible]	[illegible]	[illegible]	[illegible]	[illegible]	[illegible]	[illegible]
Guerrero	1·87 (1·4 to 2·47)	[illegible]	[illegible]	[illegible]	[illegible]	[illegible]	[illegible]	[illegible]	[illegible]	[illegible]	[illegible]	[illegible]	[illegible]	[illegible]	[illegible]	[illegible]	[illegible]	[illegible]	[illegible]	[illegible]	[illegible]	[illegible]	[illegible]	[illegible]	[illegible]	[illegible]	[illegible]	[illegible]	[illegible]	[illegible]	[illegible]	[illegible]
Hidalgo	0·93 (0·864 to 0·995)	[illegible]	[illegible]	[illegible]	[illegible]	[illegible]	[illegible]	[illegible]	[illegible]	[illegible]	[illegible]	[illegible]	[illegible]	[illegible]	[illegible]	[illegible]	[illegible]	[illegible]	[illegible]	[illegible]	[illegible]	[illegible]	[illegible]	[illegible]	[illegible]	[illegible]	[illegible]	[illegible]	[illegible]	[illegible]	[illegible]	[illegible]
Jalisco	2·42 (2·22 to 2·63)	[illegible]	[illegible]	[illegible]	[illegible]	[illegible]	[illegible]	[illegible]	[illegible]	[illegible]	[illegible]	[illegible]	[illegible]	[illegible]	[illegible]	[illegible]	[illegible]	[illegible]	[illegible]	[illegible]	[illegible]	[illegible]	[illegible]	[illegible]	[illegible]	[illegible]	[illegible]	[illegible]	[illegible]	[illegible]	[illegible]	[illegible]
México	4·58 (4·15 to 5·05)	[illegible]	[illegible]	[illegible]	[illegible]	[illegible]	[illegible]	[illegible]	[illegible]	[illegible]	[illegible]	[illegible]	[illegible]	[illegible]	[illegible]	[illegible]	[illegible]	[illegible]	[illegible]	[illegible]	[illegible]	[illegible]	[illegible]	[illegible]	[illegible]	[illegible]	[illegible]	[illegible]	[illegible]	[illegible]	[illegible]	[illegible]
Mexico City	3·99 (2·81 to 5·55)	[illegible]	[illegible]	[illegible]	[illegible]	[illegible]	[illegible]	[illegible]	[illegible]	[illegible]	[illegible]	[illegible]	[illegible]	[illegible]	[illegible]	[illegible]	[illegible]	[illegible]	[illegible]	[illegible]	[illegible]	[illegible]	[illegible]	[illegible]	[illegible]	[illegible]	[illegible]	[illegible]	[illegible]	[illegible]	[illegible]	[illegible]
Michoacán de Ocampo	2·34 (2·17 to 2·52)	[illegible]	[illegible]	[illegible]	[illegible]	[illegible]	[illegible]	[illegible]	[illegible]	[illegible]	[illegible]	[illegible]	[illegible]	[illegible]	[illegible]	[illegible]	[illegible]	[illegible]	[illegible]	[illegible]	[illegible]	[illegible]	[illegible]	[illegible]	[illegible]	[illegible]	[illegible]	[illegible]	[illegible]	[illegible]	[illegible]	[illegible]
Morelos	0·585 (0·539 to 0·635)	[illegible]	[illegible]	[illegible]	[illegible]	[illegible]	[illegible]	[illegible]	[illegible]	[illegible]	[illegible]	[illegible]	[illegible]	[illegible]	[illegible]	[illegible]	[illegible]	[illegible]	[illegible]	[illegible]	[illegible]	[illegible]	[illegible]	[illegible]	[illegible]	[illegible]	[illegible]	[illegible]	[illegible]	[illegible]	[illegible]	[illegible]
Nayarit	0·299 (0·271 to 0·33)	[illegible]	[illegible]	[illegible]	[illegible]	[illegible]	[illegible]	[illegible]	[illegible]	[illegible]	[illegible]	[illegible]	[illegible]	[illegible]	[illegible]	[illegible]	[illegible]	[illegible]	[illegible]	[illegible]	[illegible]	[illegible]	[illegible]	[illegible]	[illegible]	[illegible]	[illegible]	[illegible]	[illegible]	[illegible]	[illegible]	[illegible]
Nuevo León	1·25 (1·15 to 1·36)	[illegible]	[illegible]	[illegible]	[illegible]	[illegible]	[illegible]	[illegible]	[illegible]	[illegible]	[illegible]	[illegible]	[illegible]	[illegible]	[illegible]	[illegible]	[illegible]	[illegible]	[illegible]	[illegible]	[illegible]	[illegible]	[illegible]	[illegible]	[illegible]	[illegible]	[illegible]	[illegible]	[illegible]	[illegible]	[illegible]	[illegible]
Oaxaca	1·33 (1·22 to 1·44)	[illegible]	[illegible]	[illegible]	[illegible]	[illegible]	[illegible]	[illegible]	[illegible]	[illegible]	[illegible]	[illegible]	[illegible]	[illegible]	[illegible]	[illegible]	[illegible]	[illegible]	[illegible]	[illegible]	[illegible]	[illegible]	[illegible]	[illegible]	[illegible]	[illegible]	[illegible]	[illegible]	[illegible]	[illegible]	[illegible]	[illegible]
Puebla	2·61 (2·43 to 2·8)	[illegible]	[illegible]	[illegible]	[illegible]	[illegible]	[illegible]	[illegible]	[illegible]	[illegible]	[illegible]	[illegible]	[illegible]	[illegible]	[illegible]	[illegible]	[illegible]	[illegible]	[illegible]	[illegible]	[illegible]	[illegible]	[illegible]	[illegible]	[illegible]	[illegible]	[illegible]	[illegible]	[illegible]	[illegible]	[illegible]	[illegible]
Querétaro	0·627 (0·583 to 0·671)	[illegible]	[illegible]	[illegible]	[illegible]	[illegible]	[illegible]	[illegible]	[illegible]	[illegible]	[illegible]	[illegible]	[illegible]	[illegible]	[illegible]	[illegible]	[illegible]	[illegible]	[illegible]	[illegible]	[illegible]	[illegible]	[illegible]	[illegible]	[illegible]	[illegible]	[illegible]	[illegible]	[illegible]	[illegible]	[illegible]	[illegible]
Quintana Roo	0·253 (0·236 to 0·27)	[illegible]	[illegible]	[illegible]	[illegible]	[illegible]	[illegible]	[illegible]	[illegible]	[illegible]	[illegible]	[illegible]	[illegible]	[illegible]	[illegible]	[illegible]	[illegible]	[illegible]	[illegible]	[illegible]	[illegible]	[illegible]	[illegible]	[illegible]	[illegible]	[illegible]	[illegible]	[illegible]	[illegible]	[illegible]	[illegible]	[illegible]
San Luis Potosí	1·64 (1·51 to 1·77)	[illegible]	[illegible]	[illegible]	[illegible]	[illegible]	[illegible]	[illegible]	[illegible]	[illegible]	[illegible]	[illegible]	[illegible]	[illegible]	[illegible]	[illegible]	[illegible]	[illegible]	[illegible]	[illegible]	[illegible]	[illegible]	[illegible]	[illegible]	[illegible]	[illegible]	[illegible]	[illegible]	[illegible]	[illegible]	[illegible]	[illegible]
Sinaloa	0·627 (0·595 to 0·662)	[illegible]	[illegible]	[illegible]	[illegible]	[illegible]	[illegible]	[illegible]	[illegible]	[illegible]	[illegible]	[illegible]	[illegible]	[illegible]	[illegible]	[illegible]	[illegible]	[illegible]	[illegible]	[illegible]	[illegible]	[illegible]	[illegible]	[illegible]	[illegible]	[illegible]	[illegible]	[illegible]	[illegible]	[illegible]	[illegible]	[illegible]
Sonora	0·621 (0·58 to 0·663)	[illegible]	[illegible]	[illegible]	[illegible]	[illegible]	[illegible]	[illegible]	[illegible]	[illegible]	[illegible]	[illegible]	[illegible]	[illegible]	[illegible]	[illegible]	[illegible]	[illegible]	[illegible]	[illegible]	[illegible]	[illegible]	[illegible]	[illegible]	[illegible]	[illegible]	[illegible]	[illegible]	[illegible]	[illegible]	[illegible]	[illegible]
Tabasco	0·982 (0·902 to 1·07)	[illegible]	[illegible]	[illegible]	[illegible]	[illegible]	[illegible]	[illegible]	[illegible]	[illegible]	[illegible]	[illegible]	[illegible]	[illegible]	[illegible]	[illegible]	[illegible]	[illegible]	[illegible]	[illegible]	[illegible]	[illegible]	[illegible]	[illegible]	[illegible]	[illegible]	[illegible]	[illegible]	[illegible]	[illegible]	[illegible]	[illegible]
Tamaulipas	1·21 (1·13 to 1·31)	[illegible]	[illegible]	[illegible]	[illegible]	[illegible]	[illegible]	[illegible]	[illegible]	[illegible]	[illegible]	[illegible]	[illegible]	[illegible]	[illegible]	[illegible]	[illegible]	[illegible]	[illegible]	[illegible]	[illegible]	[illegible]	[illegible]	[illegible]	[illegible]	[illegible]	[illegible]	[illegible]	[illegible]	[illegible]	[illegible]	[illegible]
Tlaxcala	0·348 (0·318 to 0·381)	[illegible]	[illegible]	[illegible]	[illegible]	[illegible]	[illegible]	[illegible]	[illegible]	[illegible]	[illegible]	[illegible]	[illegible]	[illegible]	[illegible]	[illegible]	[illegible]	[illegible]	[illegible]	[illegible]	[illegible]	[illegible]	[illegible]	[illegible]	[illegible]	[illegible]	[illegible]	[illegible]	[illegible]	[illegible]	[illegible]	[illegible]
Veracruz de Ignacio de la Llave	4·32 (4·03 to 4·62)	[illegible]	[illegible]	[illegible]	[illegible]	[illegible]	[illegible]	[illegible]	[illegible]	[illegible]	[illegible]	[illegible]	[illegible]	[illegible]	[illegible]	[illegible]	[illegible]	[illegible]	[illegible]	[illegible]	[illegible]	[illegible]	[illegible]	[illegible]	[illegible]	[illegible]	[illegible]	[illegible]	[illegible]	[illegible]	[illegible]	[illegible]
Yucatán	0·794 (0·722 to 0·873)	[illegible]	[illegible]	[illegible]	[illegible]	[illegible]	[illegible]	[illegible]	[illegible]	[illegible]	[illegible]	[illegible]	[illegible]	[illegible]	[illegible]	[illegible]	[illegible]	[illegible]	[illegible]	[illegible]	[illegible]	[illegible]	[illegible]	[illegible]	[illegible]	[illegible]	[illegible]	[illegible]	[illegible]	[illegible]	[illegible]	[illegible]
Zacatecas	0·703 (0·65 to 0·76)	[illegible]	[illegible]	[illegible]	[illegible]	[illegible]	[illegible]	[illegible]	[illegible]	[illegible]	[illegible]	[illegible]	[illegible]	[illegible]	[illegible]	[illegible]	[illegible]	[illegible]	[illegible]	[illegible]	[illegible]	[illegible]	[illegible]	[illegible]	[illegible]	[illegible]	[illegible]	[illegible]	[illegible]	[illegible]	[illegible]	[illegible]
Nicaragua	2·84 (2·05 to 3·87)	[illegible]	[illegible]	[illegible]	[illegible]	[illegible]	[illegible]	[illegible]	[illegible]	[illegible]	[illegible]	[illegible]	[illegible]	[illegible]	[illegible]	[illegible]	[illegible]	[illegible]	[illegible]	[illegible]	[illegible]	[illegible]	[illegible]	[illegible]	[illegible]	[illegible]	[illegible]	[illegible]	[illegible]	[illegible]	[illegible]	[illegible]
Panama	0·578 (0·415 to 0·793)	[illegible]	[illegible]	[illegible]	[illegible]	[illegible]	[illegible]	[illegible]	[illegible]	[illegible]	[illegible]	[illegible]	[illegible]	[illegible]	[illegible]	[illegible]	[illegible]	[illegible]	[illegible]	[illegible]	[illegible]	[illegible]	[illegible]	[illegible]	[illegible]	[illegible]	[illegible]	[illegible]	[illegible]	[illegible]	[illegible]	[illegible]
Venezuela	9·92 (7·68 to 12·4)	[illegible]	[illegible]	[illegible]	[illegible]	[illegible]	[illegible]	[illegible]	[illegible]	[illegible]	[illegible]	[illegible]	[illegible]	[illegible]	[illegible]	[illegible]	[illegible]	[illegible]	[illegible]	[illegible]	[illegible]	[illegible]	[illegible]	[illegible]	[illegible]	[illegible]	[illegible]	[illegible]	[illegible]	[illegible]	[illegible]	[illegible]
Tropical Latin America	96·4 (70·4 to 130)	[illegible]	[illegible]	[illegible]	[illegible]	[illegible]	[illegible]	[illegible]	[illegible]	[illegible]	[illegible]	[illegible]	[illegible]	[illegible]	[illegible]	[illegible]	[illegible]	[illegible]	[illegible]	[illegible]	[illegible]	[illegible]	[illegible]	[illegible]	[illegible]	[illegible]	[illegible]	[illegible]	[illegible]	[illegible]	[illegible]	[illegible]
Brazil	91·4 (65·7 to 125)	[illegible]	[illegible]	[illegible]	[illegible]	[illegible]	[illegible]	[illegible]	[illegible]	[illegible]	[illegible]	[illegible]	[illegible]	[illegible]	[illegible]	[illegible]	[illegible]	[illegible]	[illegible]	[illegible]	[illegible]	[illegible]	[illegible]	[illegible]	[illegible]	[illegible]	[illegible]	[illegible]	[illegible]	[illegible]	[illegible]	[illegible]
Acre	0·307 (0·215 to 0·447)	[illegible]	[illegible]	[illegible]	[illegible]	[illegible]	[illegible]	[illegible]	[illegible]	[illegible]	[illegible]	[illegible]	[illegible]	[illegible]	[illegible]	[illegible]	[illegible]	[illegible]	[illegible]	[illegible]	[illegible]	[illegible]	[illegible]	[illegible]	[illegible]	[illegible]	[illegible]	[illegible]	[illegible]	[illegible]	[illegible]	[illegible]
Alagoas	3·74 (2·59 to 5·5)	[illegible]	[illegible]	[illegible]	[illegible]	[illegible]	[illegible]	[illegible]	[illegible]	[illegible]	[illegible]	[illegible]	[illegible]	[illegible]	[illegible]	[illegible]	[illegible]	[illegible]	[illegible]	[illegible]	[illegible]	[illegible]	[illegible]	[illegible]	[illegible]	[illegible]	[illegible]	[illegible]	[illegible]	[illegible]	[illegible]	[illegible]
Amapá	0·0897 (0·0637 to 0·124)	[illegible]	[illegible]	[illegible]	[illegible]	[illegible]	[illegible]	[illegible]	[illegible]	[illegible]	[illegible]	[illegible]	[illegible]	[illegible]	[illegible]	[illegible]	[illegible]	[illegible]	[illegible]	[illegible]	[illegible]	[illegible]	[illegible]	[illegible]	[illegible]	[illegible]	[illegible]	[illegible]	[illegible]	[illegible]	[illegible]	[illegible]
Amazonas	1·11 (0·775 to 1·55)	[illegible]	[illegible]	[illegible]	[illegible]	[illegible]	[illegible]	[illegible]	[illegible]	[illegible]	[illegible]	[illegible]	[illegible]	[illegible]	[illegible]	[illegible]	[illegible]	[illegible]	[illegible]	[illegible]	[illegible]	[illegible]	[illegible]	[illegible]	[illegible]	[illegible]	[illegible]	[illegible]	[illegible]	[illegible]	[illegible]	[illegible]
Bahia	5·97 (4·16 to 8·31)	[illegible]	[illegible]	[illegible]	[illegible]	[illegible]	[illegible]	[illegible]	[illegible]	[illegible]	[illegible]	[illegible]	[illegible]	[illegible]	[illegible]	[illegible]	[illegible]	[illegible]	[illegible]	[illegible]	[illegible]	[illegible]	[illegible]	[illegible]	[illegible]	[illegible]	[illegible]	[illegible]	[illegible]	[illegible]	[illegible]	[illegible]
Ceará	4·37 (3·06 to 6·38)	[illegible]	[illegible]	[illegible]	[illegible]	[illegible]	[illegible]	[illegible]	[illegible]	[illegible]	[illegible]	[illegible]	[illegible]	[illegible]	[illegible]	[illegible]	[illegible]	[illegible]	[illegible]	[illegible]	[illegible]	[illegible]	[illegible]	[illegible]	[illegible]	[illegible]	[illegible]	[illegible]	[illegible]	[illegible]	[illegible]	[illegible]
Distrito Federal	0·651 (0·454 to 0·906)	[illegible]	[illegible]	[illegible]	[illegible]	[illegible]	[illegible]	[illegible]	[illegible]	[illegible]	[illegible]	[illegible]	[illegible]	[illegible]	[illegible]	[illegible]	[illegible]	[illegible]	[illegible]	[illegible]	[illegible]	[illegible]	[illegible]	[illegible]	[illegible]	[illegible]	[illegible]	[illegible]	[illegible]	[illegible]	[illegible]	[illegible]
Espírito Santo	1·2 (0·845 to 1·75)	[illegible]	[illegible]	[illegible]	[illegible]	[illegible]	[illegible]	[illegible]	[illegible]	[illegible]	[illegible]	[illegible]	[illegible]	[illegible]	[illegible]	[illegible]	[illegible]	[illegible]	[illegible]	[illegible]	[illegible]	[illegible]	[illegible]	[illegible]	[illegible]	[illegible]	[illegible]	[illegible]	[illegible]	[illegible]	[illegible]	[illegible]
Goiás	1·75 (1·22 to 2·43)	[illegible]	[illegible]	[illegible]	[illegible]	[illegible]	[illegible]	[illegible]	[illegible]	[illegible]	[illegible]	[illegible]	[illegible]	[illegible]	[illegible]	[illegible]	[illegible]	[illegible]	[illegible]	[illegible]	[illegible]	[illegible]	[illegible]	[illegible]	[illegible]	[illegible]	[illegible]	[illegible]	[illegible]	[illegible]	[illegible]	[illegible]
Maranhão	13·9 (9·51 to 20·7)	[illegible]	[illegible]	[illegible]	[illegible]	[illegible]	[illegible]	[illegible]	[illegible]	[illegible]	[illegible]	[illegible]	[illegible]	[illegible]	[illegible]	[illegible]	[illegible]	[illegible]	[illegible]	[illegible]	[illegible]	[illegible]	[illegible]	[illegible]	[illegible]	[illegible]	[illegible]	[illegible]	[illegible]	[illegible]	[illegible]	[illegible]
Mato Grosso	1·32 (0·918 to 1·85)	[illegible]	[illegible]	[illegible]	[illegible]	[illegible]	[illegible]	[illegible]	[illegible]	[illegible]	[illegible]	[illegible]	[illegible]	[illegible]	[illegible]	[illegible]	[illegible]	[illegible]	[illegible]	[illegible]	[illegible]	[illegible]	[illegible]	[illegible]	[illegible]	[illegible]	[illegible]	[illegible]	[illegible]	[illegible]	[illegible]	[illegible]
Mato Grosso do Sul	0·678 (0·476 to 0·984)	[illegible]	[illegible]	[illegible]	[illegible]	[illegible]	[illegible]	[illegible]	[illegible]	[illegible]	[illegible]	[illegible]	[illegible]	[illegible]	[illegible]	[illegible]	[illegible]	[illegible]	[illegible]	[illegible]	[illegible]	[illegible]	[illegible]	[illegible]	[illegible]	[illegible]	[illegible]	[illegible]	[illegible]	[illegible]	[illegible]	[illegible]
Minas Gerais	7·62 (5·25 to 10·8)	[illegible]	[illegible]	[illegible]	[illegible]	[illegible]	[illegible]	[illegible]	[illegible]	[illegible]	[illegible]	[illegible]	[illegible]	[illegible]	[illegible]	[illegible]	[illegible]	[illegible]	[illegible]	[illegible]	[illegible]	[illegible]	[illegible]	[illegible]	[illegible]	[illegible]	[illegible]	[illegible]	[illegible]	[illegible]	[illegible]	[illegible]
Pará	3·44 (2·43 to 4·76)	[illegible]	[illegible]	[illegible]	[illegible]	[illegible]	[illegible]	[illegible]	[illegible]	[illegible]	[illegible]	[illegible]	[illegible]	[illegible]	[illegible]	[illegible]	[illegible]	[illegible]	[illegible]	[illegible]	[illegible]	[illegible]	[illegible]	[illegible]	[illegible]	[illegible]	[illegible]	[illegible]	[illegible]	[illegible]	[illegible]	[illegible]

Paraíba	2·1 (1·47 to 3·07)	2·06 (1·44 to 2·94)	2·04 (1·43 to 2·92)
Paraná	6·86 (4·75 to 9·62)	6·57 (4·61 to 9·01)	5·99 (4·3 to 8·08)
Pernambuco	3·73 (2·6 to 5·18)	3·72 (2·61 to 5·07)	3·63 (2·62 to 4·88)
Piauí	1·71 (1·19 to 2·38)	1·7 (1·19 to 2·32)	1·67 (1·2 to 2·25)
Rio de Janeiro	7·52 (5·22 to 10·5)	7·11 (5 to 9·74)	7·23 (5·2 to 9·75)
Rio Grande do Norte	2·9 (2·02 to 4·27)	2·68 (1·86 to 3·85)	2·49 (1·75 to 3·58)
Rio Grande do Sul	2·98 (2·08 to 4·16)	3·13 (2·21 to 4·28)	2·84 (2·05 to 3·81)
Rondônia	1·05 (0·729 to 1·48)	1·03 (0·716 to 1·41)	0·988 (0·709 to 1·33)
Roraima	0·1 (0·0703 to 0·146)	0·106 (0·0713 to 0·153)	0·11 (0·0775 to 0·157)
Santa Catarina	1·06 (0·742 to 1·53)	1·09 (0·768 to 1·55)	1·09 (0·768 to 1·55)
São Paulo	13·6 (9·55 to 19·8)	12·4 (8·69 to 17·6)	11·6 (8·22 to 16·6)
Sergipe	1·03 (0·723 to 1·51)	1·05 (0·708 to 1·52)	1·08 (0·761 to 1·55)
Tocantins	0·621 (0·435 to 0·905)	0·625 (0·438 to 0·889)	0·635 (0·447 to 0·908)
Paraguay	5·03 (4·26 to 5·89)	4·69 (3·89 to 5·55)	4·59 (3·75 to 5·59)
North Africa and Middle East	557 (454 to 684)	544 (448 to 662)	532 (440 to 642)
Afghanistan	38·7 (26·5 to 55·7)	38·6 (26·2 to 54·9)	39·5 (27·3 to 56·5)
Algeria	30·9 (27·9 to 34·4)	31·5 (25·1 to 38·5)	31·8 (24 to 41·6)
Bahrain	0·712 (0·499 to 0·999)	0·711 (0·495 to 0·978)	0·682 (0·479 to 0·949)
Egypt	82·4 (59·9 to 113)	76·9 (56·1 to 105)	73·4 (53·2 to 98·2)
Iran	41·2 (28·7 to 56·2)	38·1 (26·5 to 52·9)	35·9 (25 to 49·9)
Alborz	0·539 (0·377 to 0·735)	0·517 (0·36 to 0·717)	0·501 (0·351 to 0·696)
Ardebil	1·24 (0·866 to 1·7)	1·13 (0·785 to 1·57)	1·05 (0·732 to 1·46)
Bushehr	0·564 (0·394 to 0·769)	0·527 (0·367 to 0·734)	0·5 (0·349 to 0·695)
Chahar Mahaal and Bakhtiari	0·628 (0·438 to 0·856)	0·571 (0·398 to 0·795)	0·528 (0·369 to 0·735)
East Azarbayejan	3·03 (2·11 to 4·13)	2·72 (1·89 to 3·79)	2·51 (1·75 to 3·5)
Fars	2·37 (1·66 to 3·23)	2·26 (1·57 to 3·14)	2·15 (1·5 to 2·99)
Gilan	1·25 (0·876 to 1·71)	1·14 (0·792 to 1·58)	1·05 (0·732 to 1·46)
Golestan	1·18 (0·825 to 1·61)	1·08 (0·753 to 1·51)	1·01 (0·708 to 1·41)
Hamadan	1·46 (1·02 to 1·99)	1·36 (0·945 to 1·89)	1·28 (0·894 to 1·78)
Hormozgan	0·814 (0·568 to 1·11)	0·763 (0·531 to 1·06)	0·734 (0·513 to 1·02)
Ilam	0·388 (0·271 to 0·529)	0·365 (0·254 to 0·508)	0·35 (0·244 to 0·486)
Isfahan	2·25 (1·57 to 3·07)	2·09 (1·45 to 2·9)	1·94 (1·36 to 2·7)
Kerman	0·835 (0·573 to 1·16)	0·764 (0·518 to 1·09)	0·713 (0·465 to 1·03)
Kermanshah	1·6 (1·12 to 2·19)	1·48 (1·03 to 2·06)	1·4 (0·977 to 1·95)
Khorasan-e-Razavi	4·65 (3·24 to 6·36)	4·2 (2·93 to 5·85)	3·88 (2·71 to 5·41)
Khuzestan	2·13 (1·49 to 2·9)	2·01 (1·4 to 2·79)	1·94 (1·36 to 2·7)
Kohgiluyeh and Boyer-Ahmad	0·458 (0·32 to 0·625)	0·433 (0·301 to 0·602)	0·42 (0·293 to 0·584)
Kurdistan	1·44 (1 to 1·98)	1·32 (0·921 to 1·84)	1·24 (0·862 to 1·72)
Lorestan	1·35 (0·944 to 1·84)	1·24 (0·865 to 1·73)	1·16 (0·813 to 1·62)
Markazi	0·818 (0·571 to 1·11)	0·745 (0·518 to 1·04)	0·691 (0·482 to 0·962)
Mazandaran	0·599 (0·376 to 0·895)	0·54 (0·346 to 0·798)	0·498 (0·325 to 0·731)
North Khorasan	0·697 (0·486 to 0·951)	0·641 (0·446 to 0·892)	0·608 (0·424 to 0·845)
Qazvin	0·652 (0·456 to 0·889)	0·608 (0·423 to 0·845)	0·576 (0·402 to 0·802)
Qom	0·424 (0·297 to 0·578)	0·397 (0·277 to 0·552)	0·376 (0·263 to 0·523)
Semnan	0·265 (0·185 to 0·361)	0·246 (0·172 to 0·343)	0·233 (0·163 to 0·324)
Sistan and Baluchistan	1·61 (1·12 to 2·2)	1·51 (1·05 to 2·1)	1·45 (1·01 to 2·01)
South Khorasan	0·713 (0·497 to 0·974)	0·633 (0·441 to 0·882)	0·579 (0·404 to 0·807)
Tehran	3·87 (2·71 to 5·27)	3·65 (2·54 to 5·07)	3·53 (2·47 to 4·9)
West Azarbayejan	2·18 (1·52 to 2·97)	2 (1·39 to 2·79)	1·88 (1·31 to 2·62)
Yazd	0·413 (0·289 to 0·563)	0·391 (0·273 to 0·543)	0·374 (0·261 to 0·52)
Zanjan	0·832 (0·58 to 1·13)	0·759 (0·529 to 1·06)	0·712 (0·496 to 0·991)
Iraq	23·7 (16·9 to 32·1)	23·9 (16·8 to 32·7)	23·6 (16·5 to 32·7)
Jordan	4·75 (3·43 to 6·42)	4·68 (3·4 to 6·32)	4·57 (3·32 to 6·21)
Kuwait	0·931 (0·666 to 1·29)	0·645 (0·469 to 0·877)	0·778 (0·58 to 1·03)
Lebanon	1·04 (0·727 to 1·44)	1·02 (0·715 to 1·39)	1·01 (0·701 to 1·38)
Libya	5·07 (3·55 to 7·09)	5·01 (3·51 to 7·02)	4·82 (3·35 to 6·75)
Morocco	106 (76·1 to 144)	105 (78·5 to 137)	99·1 (76·4 to 126)
Oman	1·2 (1·13 to 1·28)	1·21 (1·16 to 1·26)	1·19 (1·14 to 1·24)
Palestine	2·33 (1·65 to 3·2)	2·34 (1·63 to 3·34)	2·33 (1·65 to 3·34)
Qatar	0·374 (0·286 to 0·479)	0·384 (0·292 to 0·5)	0·386 (0·295 to 0·5)
Saudi Arabia	11 (10·4 to 11·7)	10·7 (9·7 to 11·7)	11·2 (9·28 to 13·5)
Sudan	51 (36 to 71·1)	52·1 (37·3 to 72·7)	53·7 (37·7 to 75·6)
Syria	16·4 (11·5 to 23)	15·9 (11·2 to 22·3)	15·4 (10·7 to 21·5)
Tunisia	6·41 (5·93 to 6·91)	6·02 (5·59 to 6·48)	6·24 (5·77 to 6·72)
Türkiye	79·5 (56·5 to 114)	75·7 (54·4 to 108)	72·2 (51·8 to 99·1)
United Arab Emirates	0·916 (0·647 to 1·31)	0·876 (0·614 to 1·25)	0·881 (0·622 to 1·27)
Yemen	52·2 (43 to 63·1)	52·5 (44·3 to 62·3)	53·3 (45·2 to 63·3)
South Asia	1760 (1380 to 2240)	1740 (1370 to 2220)	1730 (1370 to 2200)
Bangladesh	313 (225 to 419)	296 (220 to 385)	277 (217 to 351)
Bhutan	2·23 (1·53 to 3·19)	2·2 (1·51 to 3·14)	2·1 (1·43 to 3)
India	923 (721 to 1170)	926 (724 to 1170)	932 (735 to 1180)
Nepal	76·3 (54·8 to 108)	73·3 (52·2 to 101)	70·3 (51·4 to 93·9)
Pakistan	444 (304 to 633)	447 (307 to 640)	452 (310 to 660)
Azad Jammu & Kashmir	9·28 (5·14 to 15·4)	9·28 (5·36 to 15·1)	9·27 (5·48 to 14·7)
Balochistan	26·8 (14·6 to 46)	27·3 (15·2 to 46)	28 (15·8 to 46·5)
Gilgit-Baltistan	3·07 (1·5 to 5·7)	3·1 (1·53 to 5·8)	3·13 (1·53 to 6·13)
Islamabad Capital Territory	2·76 (1·33 to 5·2)	2·82 (1·37 to 5·32)	2·87 (1·38 to 5·7)
Khyber Pakhtunkhwa	41·7 (28·7 to 59·2)	42·1 (29·2 to 60·1)	42·5 (29·4 to 61·8)
Punjab	206 (141 to 292)	206 (144 to 292)	206 (143 to 301)
Sindh	154 (106 to 219)	157 (108 to 223)	160 (108 to 236)
Southeast Asia, east Asia, and Oceania	966 (709 to 1300)	943 (700 to 1280)	913 (677 to 1230)
East Asia	634 (449 to 881)	620 (438 to 860)	597 (418 to 829)
China	621 (438 to 863)	606 (428 to 842)	584 (408 to 812)
North Korea	10·7 (7·56 to 14·9)	10·7 (7·56 to 14·9)	10·7 (7·51 to 14·9)
Taiwan (province of China)	1·81 (1·26 to 2·5)	1·78 (1·24 to 2·49)	1·88 (1·32 to 2·67)
Oceania	8·97 (6·38 to 12·5)	9·05 (6·4 to 12·5)	9·05 (6·34 to 12·5)
American Samoa	0·0343 (0·0298 to 0·0394)	0·0406 (0·0352 to 0·047)	0·0311 (0·0269 to 0·0362)
Cook Islands	0·00602 (0·00476 to 0·00755)	0·00603 (0·00476 to 0·00751)	0·00594 (0·00472 to 0·00731)
Federated States of Micronesia	0·0803 (0·0565 to 0·112)	0·0794 (0·0558 to 0·111)	0·0775 (0·0541 to 0·108)
Fiji	0·19 (0·135 to 0·259)	0·19 (0·134 to 0·263)	0·182 (0·127 to 0·251)
Guam	0·0879 (0·0615 to 0·125)	0·088 (0·0618 to 0·128)	0·0876 (0·0611 to 0·125)
Kiribati	0·0845 (0·0594 to 0·118)	0·0839 (0·0588 to 0·117)	0·082 (0·0571 to 0·115)
Marshall Islands	0·0302 (0·0214 to 0·0424)	0·0305 (0·022 to 0·0432)	0·0308 (0·0217 to 0·0429)
Nauru	0·00768 (0·00541 to 0·0107)	0·00771 (0·00542 to 0·0107)	0·00764 (0·00534 to 0·0106)
Niue	0·00109 (0·000765 to 0·00151)	0·00106 (0·000747 to 0·00148)	0·00106 (0·00074 to 0·00148)
Northern Mariana Islands	0·0474 (0·0333 to 0·0682)	0·0475 (0·0332 to 0·0685)	0·0443 (0·0311 to 0·0626)
Palau	0·00545 (0·0039 to 0·00765)	0·00569 (0·00408 to 0·00805)	0·00585 (0·00419 to 0·00836)
Papua New Guinea	7·1 (4·96 to 9·96)	7·17 (5 to 10·1)	7·2 (4·99 to 10·1)
Samoa	0·0623 (0·0442 to 0·087)	0·0605 (0·0428 to 0·0845)	0·0588 (0·041 to 0·0818)
Solomon Islands	0·431 (0·303 to 0·601)	0·438 (0·307 to 0·612)	0·44 (0·307 to 0·614)
Tokelau	0·000691 (0·000487 to 0·000961)	0·000635 (0·000447 to 0·000882)	0·000603 (0·000422 to 0·000838)
Tonga	0·0706 (0·0487 to 0·0977)	0·0696 (0·0482 to 0·0963)	0·0678 (0·0479 to 0·0943)
Tuvalu	0·0136 (0·00954 to 0·019)	0·0127 (0·00891 to 0·0178)	0·0119 (0·00825 to 0·0166)
Vanuatu	0·145 (0·102 to 0·201)	0·144 (0·101 to 0·201)	0·144 (0·1 to 0·2)
Southeast Asia	323 (257 to 404)	314 (249 to 394)	306 (244 to 384)
Cambodia	18·9 (12·8 to 26·2)	18·2 (12·3 to 25·1)	17·4 (11·9 to 24·2)
Indonesia	138 (108 to 171)	136 (107 to 170)	135 (106 to 169)
Aceh	2·42 (1·71 to 3·33)	2·44 (1·71 to 3·39)	2·46 (1·75 to 3·44)
Bali	1·11 (0·801 to 1·55)	1·09 (0·781 to 1·51)	1·07 (0·775 to 1·5)
Bangka-Belitung Islands	1·19 (0·829 to 1·65)	1·16 (0·82 to 1·6)	1·13 (0·806 to 1·57)
Banten	8·24 (5·83 to 11·7)	8·46 (5·9 to 11·9)	8·63 (5·97 to 12·1)
Bengkulu	1·1 (0·775 to 1·53)	1·09 (0·755 to 1·52)	1·08 (0·756 to 1·53)
Gorontalo	0·713 (0·493 to 1·01)	0·713 (0·499 to 1·01)	0·711 (0·497 to 1·01)
Jakarta	5·44 (3·8 to 7·53)	5·39 (3·8 to 7·37)	5·31 (3·78 to 7·17)
Jambi	2·76 (2·02 to 3·72)	2·73 (2·01 to 3·67)	2·67 (1·95 to 3·61)
West Java	18·7 (13·3 to 25·7)	18·5 (13·2 to 25·3)	18·2 (13·1 to 24·5)
Central Java	13·1 (9·68 to 17·6)	12·8 (9·43 to 17·2)	12·5 (9·27 to 16·9)
East Java	27·5 (18·8 to 37·9)	26·8 (18·8 to 37·4)	26·2 (18·7 to 36·4)
West Kalimantan	4·58 (3·39 to 6·05)	4·42 (3·26 to 5·86)	4·12 (3·03 to 5·48)
South Kalimantan	2·99 (2·08 to 4·21)	2·96 (2·05 to 4·17)	2·94 (2·03 to 4·1)
Central Kalimantan	0·838 (0·593 to 1·15)	0·861 (0·605 to 1·2)	0·886 (0·629 to 1·24)
East Kalimantan	1·82 (1·34 to 2·46)	1·86 (1·37 to 2·49)	1·93 (1·43 to 2·58)
North Kalimantan	0·152 (0·103 to 0·211)	0·155 (0·107 to 0·213)	0·16 (0·11 to 0·221)
Riau Islands	0·636 (0·475 to 0·843)	0·684 (0·505 to 0·918)	0·775 (0·571 to 1·05)
Lampung	5·77 (3·98 to 8·1)	5·68 (4·03 to 7·91)	5·56 (3·98 to 7·71)
Maluku	0·993 (0·733 to 1·31)	0·996 (0·735 to 1·32)	0·991 (0·728 to 1·31)
North Maluku	0·589 (0·418 to 0·826)	0·599 (0·425 to 0·833)	0·609 (0·432 to 0·857)
West Nusa Tenggara	3·82 (2·7 to 5·27)	3·7 (2·6 to 5·09)	3·6 (2·5 to 5·02)
East Nusa Tenggara	2·02 (1·44 to 2·76)	2·05 (1·48 to 2·78)	2·08 (1·5 to 2·86)
Papua	0·709 (0·523 to 0·948)	0·745 (0·552 to 0·995)	0·797 (0·588 to 1·07)
West Papua	0·491 (0·349 to 0·671)	0·488 (0·348 to 0·666)	0·48 (0·346 to 0·642)
Riau	3·3 (2·39 to 4·38)	3·36 (2·43 to 4·44)	3·51 (2·56 to 4·63)
West Sulawesi	0·869 (0·614 to 1·18)	0·869 (0·603 to 1·22)	0·871 (0·601 to 1·22)
South Sulawesi	5·24 (3·85 to 6·99)	5·2 (3·8 to 7·01)	5·21 (3·85 to 7·13)
Central Sulawesi	3·3 (2·4 to 4·51)	3·23 (2·36 to 4·4)	2·94 (2·12 to 4·07)
Southeast Sulawesi	1·3 (0·926 to 1·79)	1·29 (0·916 to 1·81)	1·27 (0·902 to 1·78)
North Sulawesi	0·745 (0·557 to 0·968)	0·756 (0·566 to 0·992)	0·771 (0·576 to 1·02)
West Sumatra	3·15 (2·33 to 4·26)	3·2 (2·35 to 4·21)	3·35 (2·5 to 4·39)
South Sumatra	4·76 (3·35 to 6·62)	4·73 (3·36 to 6·52)	4·69 (3·25 to 6·39)
North Sumatra	6·37 (4·66 to 8·52)	6·4 (4·6 to 8·44)	6·42 (4·58 to 8·51)
Yogyakarta	0·923 (0·677 to 1·22)	0·93 (0·689 to 1·23)	1 (0·734 to 1·34)
Laos	11·4 (7·9 to 16·1)	11·1 (7·71 to 15·7)	10·9 (7·49 to 15·3)
Malaysia	5·11 (4·56 to 5·7)	4·81 (3·95 to 5·82)	4·59 (3·67 to 5·69)
Maldives	0·512 (0·371 to 0·712)	0·483 (0·35 to 0·664)	0·446 (0·326 to 0·603)
Mauritius	0·615 (0·567 to 0·663)	0·53 (0·491 to 0·574)	0·481 (0·443 to 0·519)

Myanmar	44·3 (31·4 to 61·1)	42·8 (30·5 to 59)	41·3 (29·3 to 57·5)	40 (28·2 to 55·2)	38·8 (27 to 54·1)	37·7 (26·2 to 52·3)	36·7 (25·4 to 51·3)	36 (24·8 to 51·1)
Philippines	32·4 (25·5 to 41·3)	31·3 (25·2 to 38·8)	30·8 (25·5 to 36·5)	29·9 (25·6 to 34·6)	27·2 (24·1 to 30·5)	25·8 (23·8 to 27·8)	25·2 (23·9 to 26·7)	26·6 (25·1 to 28·1)
Seychelles	0·0333 (0·0245 to 0·0454)	0·0319 (0·0238 to 0·0422)	0·0306 (0·0234 to 0·0391)	0·0293 (0·0227 to 0·0367)	0·0277 (0·0224 to 0·0344)	0·0272 (0·0225 to 0·0335)	0·025 (0·0205 to 0·0308)	0·0212 (0·017 to 0·0263)
Sri Lanka	3·43 (2·65 to 4·39)	3·53 (2·65 to 4·6)	3·85 (2·82 to 5·22)	4·59 (3·35 to 6·16)	5·18 (3·79 to 6·95)	5·48 (4·01 to 7·32)	5·92 (4·31 to 7·9)	5·65 (4·11 to 7·5)
Thailand	18·6 (13·1 to 25·8)	17·4 (12·3 to 24·2)	16·4 (11·5 to 22·8)	15·4 (10·9 to 21·5)	14·5 (10·1 to 20·2)	13·6 (9·67 to 18·6)	12·6 (8·8 to 17·4)	11·6 (8·13 to 15·9)
Timor-Leste	1·98 (1·38 to 2·78)	1·9 (1·32 to 2·67)	1·81 (1·25 to 2·54)	1·72 (1·21 to 2·44)	1·64 (1·13 to 2·3)	1·54 (1·09 to 2·13)	1·47 (1·02 to 2·05)	1·41 (0·979 to 1·94)
Viet Nam	47·7 (33·4 to 65·8)	45·5 (32·5 to 62·7)	43 (31·1 to 58·6)	40·6 (29·8 to 54·4)	38 (28·1 to 50·8)	35·7 (26·9 to 47·1)	33·3 (25·5 to 43·2)	31·4 (24·1 to 40·3)
Sub-Saharan Africa	1310 (1020 to 1690)	1320 (1040 to 1690)	1330 (1050 to 1670)	1330 (1060 to 1670)	1330 (1080 to 1670)	1340 (1100 to 1670)	1360 (1110 to 1670)	1370 (1130 to 1680)
Central sub-Saharan Africa	126 (91·9 to 171)	127 (94 to 170)	128 (95 to 170)	129 (98·1 to 169)	131 (100 to 169)	132 (102 to 171)	133 (104 to 171)	134 (105 to 169)
Angola	24·7 (17·5 to 34·4)	25·1 (17·7 to 34·8)	25·3 (17·5 to 35)	25·7 (17·6 to 36·1)	26 (17·9 to 36·6)	26·2 (18·3 to 37·7)	26·3 (18·5 to 37·4)	26·5 (18·5 to 38)
Central African Republic	12·2 (8·37 to 17·4)	12·2 (8·39 to 17·4)	12·3 (8·38 to 17·5)	12·4 (8·58 to 17·8)	12·5 (8·46 to 17·8)	12·6 (8·74 to 17·6)	12·6 (8·61 to 17·9)	12·6 (8·66 to 17·7)
Congo (Brazzaville)	3·9 (2·73 to 5·47)	3·99 (2·78 to 5·59)	4·06 (2·82 to 5·69)	4·13 (2·9 to 5·83)	4·2 (2·89 to 5·9)	4·28 (3·02 to 5·91)	4·38 (3·04 to 6·11)	4·49 (3·13 to 6·21)
DR Congo	82·2 (57·9 to 114)	83·1 (57·9 to 115)	83·6 (58·3 to 118)	84·3 (60·1 to 117)	85·1 (61·1 to 114)	85·9 (61·2 to 116)	86·8 (62·3 to 117)	87·5 (64·3 to 114)
Equatorial Guinea	1·09 (0·763 to 1·54)	1·12 (0·783 to 1·58)	1·15 (0·794 to 1·62)	1·17 (0·82 to 1·66)	1·2 (0·821 to 1·69)	1·23 (0·867 to 1·71)	1·28 (0·883 to 1·79)	1·27 (0·884 to 1·77)
Gabon	1·62 (1·1 to 2·22)	1·61 (1·12 to 2·25)	1·62 (1·11 to 2·27)	1·62 (1·11 to 2·32)	1·63 (1·14 to 2·35)	1·65 (1·16 to 2·32)	1·66 (1·15 to 2·35)	1·69 (1·19 to 2·45)
Eastern sub-Saharan Africa	566 (441 to 728)	571 (446 to 729)	574 (454 to 724)	576 (463 to 719)	575 (468 to 709)	575 (472 to 703)	571 (474 to 690)	563 (474 to 675)
Burundi	19·4 (13·6 to 27)	19·5 (13·5 to 27·3)	19·6 (13·4 to 28·1)	19·6 (13·5 to 28·6)	19 (13·1 to 27·4)	18·7 (13·1 to 27)	18·9 (13·3 to 27·4)	18·5 (13·2 to 26·3)
Comoros	1·82 (1·25 to 2·59)	1·81 (1·25 to 2·59)	1·79 (1·23 to 2·56)	1·76 (1·22 to 2·53)	1·74 (1·18 to 2·48)	1·71 (1·19 to 2·39)	1·67 (1·14 to 2·36)	1·62 (1·11 to 2·26)
Djibouti	0·861 (0·599 to 1·22)	0·951 (0·66 to 1·34)	1·03 (0·71 to 1·45)	1·02 (0·709 to 1·45)	0·971 (0·664 to 1·37)	0·948 (0·665 to 1·32)	0·941 (0·648 to 1·32)	0·937 (0·648 to 1·3)
Eritrea	8·61 (6 to 12·1)	8·39 (5·83 to 11·8)	7·78 (5·37 to 11)	7·24 (5·07 to 10·3)	7·01 (4·81 to 9·89)	7 (4·93 to 9·71)	6·96 (4·81 to 9·74)	6·86 (4·76 to 9·49)
Ethiopia	175 (123 to 244)	177 (123 to 248)	179 (123 to 256)	181 (125 to 263)	180 (125 to 260)	181 (127 to 261)	180 (127 to 260)	177 (126 to 251)
Addis Ababa	4·23 (2·65 to 6·14)	4·15 (2·7 to 5·95)	4·09 (2·85 to 5·65)	4·01 (2·88 to 5·34)	3·93 (2·86 to 5·18)	3·89 (2·85 to 5·26)	3·87 (2·75 to 5·41)	3·79 (2·63 to 5·33)
Afar	2·42 (1·71 to 3·45)	2·79 (1·97 to 3·96)	3·15 (2·23 to 4·39)	3·51 (2·49 to 4·79)	3·87 (2·72 to 5·44)	4·21 (2·91 to 6·19)	4·49 (3·01 to 6·63)	4·69 (3·06 to 7·1)
Amhara	59·9 (38·2 to 89·4)	59·8 (37·4 to 91·5)	60 (37 to 94·4)	59·9 (37·2 to 95)	59·7 (37·2 to 94)	59·5 (37·8 to 90·6)	58·9 (38·9 to 90·1)	57·9 (39·1 to 85·7)
Benishangul-Gumuz	2·43 (1·52 to 3·57)	2·34 (1·51 to 3·36)	2·27 (1·57 to 3·14)	2·2 (1·57 to 2·94)	2·12 (1·54 to 2·82)	2·08 (1·51 to 2·82)	2·03 (1·44 to 2·85)	1·97 (1·37 to 2·77)
Dire Dawa	0·646 (0·378 to 0·987)	0·607 (0·375 to 0·895)	0·574 (0·371 to 0·813)	0·547 (0·378 to 0·748)	0·518 (0·37 to 0·704)	0·5 (0·367 to 0·68)	0·488 (0·346 to 0·678)	0·472 (0·319 to 0·684)
Gambella	0·563 (0·329 to 0·864)	0·549 (0·338 to 0·814)	0·54 (0·347 to 0·768)	0·534 (0·367 to 0·734)	0·525 (0·374 to 0·717)	0·518 (0·378 to 0·707)	0·516 (0·365 to 0·72)	0·512 (0·346 to 0·744)
Harari	0·44 (0·31 to 0·628)	0·434 (0·306 to 0·618)	0·428 (0·303 to 0·599)	0·425 (0·301 to 0·582)	0·415 (0·292 to 0·583)	0·409 (0·283 to 0·601)	0·406 (0·273 to 0·598)	0·401 (0·262 to 0·606)
Oromia	57·8 (38·1 to 84·3)	59·4 (37·8 to 89·9)	61 (37·6 to 94·2)	61·9 (37·8 to 99·2)	61·9 (37·4 to 100)	61·8 (37·4 to 100)	61·3 (37·6 to 99·6)	59·9 (37·3 to 94·7)
Somali	10·6 (6·19 to 16·3)	11·2 (6·88 to 16·7)	11·8 (7·57 to 16·9)	12·4 (8·52 to 17·1)	13·1 (9·28 to 17·9)	13·7 (9·96 to 18·8)	14·4 (10·1 to 20·2)	14·8 (9·92 to 21·6)
Southern Nations, Nationalities, and Peoples	22·2 (15·3 to 30·4)	21·9 (15·7 to 28·7)	21·5 (16·4 to 27·2)	21 (16·7 to 26·2)	20·4 (16·6 to 25·1)	20 (16·3 to 24·9)	19·7 (15·8 to 24·7)	19·2 (15·2 to 24·7)
Tigray	14·1 (8·13 to 21·8)	14·1 (8·6 to 21·1)	14 (8·93 to 20·1)	14 (9·57 to 19·5)	13·9 (9·88 to 19·3)	13·9 (10 to 19·1)	13·8 (9·68 to 19·4)	13·5 (9·05 to 19·7)
Kenya	45·5 (33·6 to 60·2)	46·1 (34·3 to 61·1)	47 (35·7 to 61·5)	47·8 (37·2 to 61·2)	48·5 (38·7 to 61·2)	49·1 (40 to 61·1)	49·7 (41·2 to 60·1)	50·4 (42·6 to 59·7)
Baringo	1·11 (0·766 to 1·57)	1·13 (0·79 to 1·59)	1·15 (0·824 to 1·6)	1·17 (0·845 to 1·58)	1·19 (0·86 to 1·57)	1·2 (0·887 to 1·58)	1·21 (0·92 to 1·57)	1·22 (0·938 to 1·57)
Bomet	0·688 (0·477 to 0·961)	0·709 (0·492 to 1)	0·732 (0·517 to 1·04)	0·751 (0·526 to 1·06)	0·764 (0·541 to 1·07)	0·781 (0·555 to 1·07)	0·801 (0·577 to 1·09)	0·83 (0·601 to 1·12)
Bungoma	1·54 (1·15 to 2)	1·67 (1·26 to 2·16)	1·82 (1·41 to 2·29)	1·99 (1·58 to 2·45)	2·15 (1·79 to 2·6)	2·31 (1·98 to 2·69)	2·47 (2·2 to 2·76)	2·59 (2·41 to 2·81)
Busia	1·2 (0·879 to 1·57)	1·19 (0·897 to 1·53)	1·19 (0·918 to 1·5)	1·18 (0·932 to 1·47)	1·16 (0·94 to 1·4)	1·14 (0·963 to 1·33)	1·1 (0·976 to 1·24)	1·05 (0·972 to 1·13)
Elgeyo Marakwet	0·532 (0·37 to 0·74)	0·563 (0·392 to 0·783)	0·605 (0·419 to 0·845)	0·652 (0·457 to 0·92)	0·701 (0·499 to 0·994)	0·751 (0·537 to 1·07)	0·793 (0·572 to 1·12)	0·824 (0·599 to 1·15)
Embu	0·608 (0·426 to 0·843)	0·619 (0·438 to 0·858)	0·633 (0·447 to 0·873)	0·647 (0·464 to 0·871)	0·657 (0·471 to 0·871)	0·664 (0·483 to 0·869)	0·685 (0·512 to 0·882)	0·72 (0·549 to 0·921)
Garissa	0·254 (0·177 to 0·354)	0·262 (0·182 to 0·37)	0·275 (0·194 to 0·387)	0·291 (0·204 to 0·409)	0·309 (0·219 to 0·429)	0·331 (0·236 to 0·45)	0·356 (0·257 to 0·482)	0·384 (0·279 to 0·514)
Homa Bay	1·75 (1·22 to 2·46)	1·75 (1·22 to 2·47)	1·75 (1·21 to 2·47)	1·75 (1·23 to 2·48)	1·73 (1·19 to 2·44)	1·69 (1·19 to 2·35)	1·64 (1·13 to 2·29)	1·58 (1·1 to 2·18)
Isiolo	0·182 (0·125 to 0·253)	0·186 (0·13 to 0·261)	0·193 (0·134 to 0·268)	0·2 (0·139 to 0·281)	0·207 (0·144 to 0·294)	0·214 (0·151 to 0·307)	0·225 (0·159 to 0·319)	0·238 (0·166 to 0·344)
Kajiado	0·517 (0·363 to 0·716)	0·531 (0·376 to 0·736)	0·55 (0·39 to 0·758)	0·572 (0·41 to 0·768)	0·597 (0·428 to 0·79)	0·627 (0·456 to 0·819)	0·658 (0·492 to 0·846)	0·692 (0·529 to 0·883)
Kakamega	2·54 (1·78 to 3·53)	2·43 (1·71 to 3·37)	2·31 (1·63 to 3·19)	2·17 (1·56 to 2·93)	2·03 (1·46 to 2·7)	1·9 (1·38 to 2·49)	1·77 (1·32 to 2·28)	1·66 (1·27 to 2·12)
Kericho	1·27 (0·885 to 1·77)	1·32 (0·932 to 1·83)	1·37 (0·995 to 1·87)	1·43 (1·05 to 1·92)	1·47 (1·08 to 1·93)	1·49 (1·12 to 1·94)	1·5 (1·15 to 1·92)	1·51 (1·17 to 1·93)
Kiambu	0·591 (0·416 to 0·82)	0·618 (0·44 to 0·851)	0·648 (0·47 to 0·881)	0·677 (0·496 to 0·897)	0·713 (0·525 to 0·93)	0·759 (0·57 to 0·985)	0·817 (0·63 to 1·05)	0·883 (0·688 to 1·12)
Kilifi	1·46 (1·08 to 1·92)	1·47 (1·11 to 1·88)	1·49 (1·15 to 1·88)	1·53 (1·2 to 1·9)	1·57 (1·27 to 1·89)	1·61 (1·37 to 1·89)	1·66 (1·48 to 1·87)	1·72 (1·59 to 1·86)
Kirinyaga	0·358 (0·252 to 0·499)	0·364 (0·256 to 0·508)	0·375 (0·261 to 0·523)	0·39 (0·275 to 0·549)	0·405 (0·28 to 0·567)	0·417 (0·295 to 0·574)	0·429 (0·298 to 0·597)	0·444 (0·31 to 0·612)
Kisii	0·994 (0·74 to 1·3)	1·02 (0·773 to 1·32)	1·07 (0·82 to 1·35)	1·11 (0·88 to 1·37)	1·15 (0·947 to 1·38)	1·19 (1·01 to 1·39)	1·23 (1·09 to 1·38)	1·27 (1·17 to 1·36)
Kisumu	3·25 (2·23 to 4·64)	3·3 (2·29 to 4·67)	3·37 (2·39 to 4·71)	3·42 (2·44 to 4·64)	3·44 (2·47 to 4·59)	3·43 (2·52 to 4·55)	3·43 (2·59 to 4·48)	3·45 (2·64 to 4·46)
Kitui	0·877 (0·608 to 1·21)	0·887 (0·621 to 1·23)	0·918 (0·642 to 1·27)	0·95 (0·665 to 1·33)	0·979 (0·685 to 1·38)	1·01 (0·713 to 1·43)	1·03 (0·732 to 1·44)	1·04 (0·73 to 1·48)
Kwale	0·969 (0·718 to 1·26)	0·954 (0·716 to 1·24)	0·934 (0·722 to 1·18)	0·907 (0·721 to 1·12)	0·875 (0·726 to 1·06)	0·851 (0·729 to 0·991)	0·839 (0·749 to 0·94)	0·826 (0·768 to 0·895)
Laikipia	0·18 (0·125 to 0·249)	0·193 (0·136 to 0·268)	0·208 (0·146 to 0·287)	0·225 (0·158 to 0·313)	0·239 (0·168 to 0·336)	0·252 (0·179 to 0·358)	0·264 (0·188 to 0·369)	0·274 (0·193 to 0·389)
Lamu	0·201 (0·139 to 0·285)	0·202 (0·139 to 0·287)	0·203 (0·139 to 0·288)	0·203 (0·141 to 0·291)	0·203 (0·138 to 0·29)	0·203 (0·142 to 0·285)	0·204 (0·14 to 0·289)	0·206 (0·142 to 0·288)
Machakos	1·41 (0·988 to 1·95)	1·39 (0·988 to 1·93)	1·4 (0·989 to 1·93)	1·4 (1·01 to 1·89)	1·42 (1·02 to 1·87)	1·43 (1·04 to 1·87)	1·44 (1·08 to 1·85)	1·44 (1·1 to 1·84)
Makueni	1·19 (0·825 to 1·66)	1·17 (0·822 to 1·63)	1·14 (0·811 to 1·55)	1·11 (0·798 to 1·49)	1·09 (0·781 to 1·44)	1·07 (0·776 to 1·41)	1·06 (0·789 to 1·38)	1·07 (0·815 to 1·37)
Mandera	0·617 (0·426 to 0·87)	0·626 (0·437 to 0·883)	0·635 (0·443 to 0·889)	0·643 (0·455 to 0·875)	0·648 (0·46 to 0·867)	0·656 (0·473 to 0·866)	0·675 (0·501 to 0·875)	0·704 (0·535 to 0·905)
Marsabit	0·378 (0·262 to 0·529)	0·373 (0·258 to 0·521)	0·369 (0·254 to 0·518)	0·366 (0·255 to 0·519)	0·363 (0·258 to 0·518)	0·357 (0·255 to 0·509)	0·352 (0·253 to 0·496)	0·347 (0·252 to 0·486)
Meru	1·91 (1·33 to 2·67)	1·95 (1·38 to 2·7)	1·99 (1·43 to 2·72)	2·03 (1·47 to 2·7)	2·06 (1·51 to 2·7)	2·08 (1·55 to 2·72)	2·1 (1·61 to 2·7)	2·12 (1·64 to 2·69)
Migori	1·96 (1·35 to 2·74)	1·98 (1·39 to 2·77)	2 (1·44 to 2·75)	2·02 (1·48 to 2·74)	2·04 (1·49 to 2·71)	2·07 (1·55 to 2·71)	2·11 (1·61 to 2·7)	2·15 (1·66 to 2·75)
Mombasa	0·853 (0·596 to 1·19)	0·869 (0·612 to 1·21)	0·892 (0·628 to 1·24)	0·916 (0·653 to 1·24)	0·942 (0·673 to 1·25)	0·964 (0·699 to 1·27)	0·998 (0·743 to 1·29)	1·04 (0·795 to 1·34)
Murang'a	0·805 (0·562 to 1·12)	0·782 (0·547 to 1·1)	0·77 (0·547 to 1·08)	0·766 (0·54 to 1·07)	0·751 (0·535 to 1·04)	0·72 (0·515 to 0·978)	0·685 (0·497 to 0·927)	0·653 (0·476 to 0·873)
Nairobi	1·13 (0·831 to 1·48)	1·17 (0·885 to 1·5)	1·23 (0·952 to 1·55)	1·3 (1·04 to 1·6)	1·37 (1·12 to 1·62)	1·44 (1·24 to 1·67)	1·52 (1·37 to 1·69)	1·61 (1·52 to 1·71)
Nakuru	1·6 (1·11 to 2·24)	1·65 (1·15 to 2·33)	1·72 (1·21 to 2·43)	1·78 (1·25 to 2·51)	1·83 (1·3 to 2·56)	1·89 (1·35 to 2·59)	1·95 (1·4 to 2·65)	2 (1·45 to 2·69)
Nandi	1·13 (0·784 to 1·59)	1·17 (0·809 to 1·66)	1·21 (0·853 to 1·72)	1·25 (0·873 to 1·77)	1·29 (0·909 to 1·8)	1·31 (0·927 to 1·79)	1·32 (0·947 to 1·8)	1·34 (0·964 to 1·8)
Narok	1·3 (0·896 to 1·83)	1·28 (0·882 to 1·82)	1·25 (0·875 to 1·77)	1·21 (0·842 to 1·71)	1·15 (0·814 to 1·62)	1·09 (0·77 to 1·49)	1·02 (0·736 to 1·4)	0·969 (0·7 to 1·31)
Nyamira	0·291 (0·204 to 0·405)	0·299 (0·21 to 0·417)	0·306 (0·213 to 0·426)	0·313 (0·221 to 0·44)	0·319 (0·221 to 0·446)	0·326 (0·231 to 0·449)	0·332 (0·231 to 0·461)	0·34 (0·237 to 0·467)
Nyandarua	0·241 (0·169 to 0·334)	0·25 (0·178 to 0·345)	0·261 (0·189 to 0·355)	0·27 (0·198 to 0·358)	0·275 (0·203 to 0·359)	0·278 (0·209 to 0·361)	0·282 (0·218 to 0·361)	0·287 (0·224 to 0·364)
Nyeri	0·993 (0·69 to 1·38)	1·01 (0·711 to 1·4)	1·03 (0·73 to 1·39)	1·04 (0·744 to 1·38)	1·05 (0·755 to 1·39)	1·07 (0·773 to 1·4)	1·08 (0·805 to 1·41)	1·09 (0·831 to 1·4)
Samburu	0·397 (0·272 to 0·556)	0·406 (0·281 to 0·571)	0·411 (0·284 to 0·574)	0·411 (0·285 to 0·582)	0·408 (0·282 to 0·582)	0·401 (0·282 to 0·577)	0·395 (0·279 to 0·56)	0·389 (0·271 to 0·561)
Siaya	3·48 (2·37 to 4·94)	3·57 (2·47 to 5·06)	3·64 (2·59 to 5·08)	3·71 (2·67 to 5·11)	3·76 (2·71 to 5·06)	3·8 (2·81 to 5·05)	3·79 (2·86 to 4·93)	3·74 (2·85 to 4·86)
Taita Taveta	0·32 (0·237 to 0·427)	0·316 (0·238 to 0·412)	0·311 (0·238 to 0·399)	0·307 (0·241 to 0·38)	0·297 (0·243 to 0·356)	0·285 (0·242 to 0·329)	0·278 (0·245 to 0·311)	0·273 (0·254 to 0·294)
Tana River	0·301 (0·209 to 0·422)	0·307 (0·216 to 0·428)	0·315 (0·226 to 0·432)	0·323 (0·234 to 0·431)	0·331 (0·242 to 0·436)	0·341 (0·254 to 0·446)	0·354 (0·271 to 0·457)	0·368 (0·285 to 0·469)
Tharaka Nithi	0·119 (0·0837 to 0·166)	0·121 (0·0851 to 0·169)	0·123 (0·086 to 0·172)	0·125 (0·0885 to 0·176)	0·127 (0·0877 to 0·178)	0·127 (0·0902 to 0·175)	0·126 (0·0879 to 0·176)	0·125 (0·0874 to 0·172)
Trans Nzoia	0·666 (0·468 to 0·921)	0·671 (0·476 to 0·929)	0·684 (0·485 to 0·942)	0·7 (0·502 to 0·939)	0·712 (0·512 to 0·942)	0·725 (0·528 to 0·946)	0·736 (0·552 to 0·946)	0·746 (0·571 to 0·951)
Turkana	0·667 (0·46 to 0·936)	0·69 (0·477 to 0·98)	0·718 (0·505 to 1·02)	0·748 (0·522 to 1·06)	0·78 (0·551 to 1·09)	0·821 (0·583 to 1·13)	0·866 (0·623 to 1·18)	0·915 (0·661 to 1·23)
Uasin Gishu	0·532 (0·394 to 0·69)	0·545 (0·409 to 0·702)	0·563 (0·438 to 0·705)	0·581 (0·462 to 0·713)	0·594 (0·49 to 0·716)	0·602 (0·513 to 0·699)	0·616 (0·548 to 0·693)	0·642 (0·596 to 0·694)
Vihiga	0·695 (0·482 to 0·97)	0·694 (0·482 to 0·98)	0·691 (0·488 to 0·978)	0·682 (0·478 to 0·961)	0·661 (0·468 to 0·92)	0·635 (0·452 to 0·867)	0·606 (0·437 to 0·823)	0·579 (0·42 to 0·777)
Wajir	0·462 (0·316 to 0·648)	0·5 (0·346 to 0·705)	0·551 (0·38 to 0·771)	0·613 (0·424 to 0·871)	0·685 (0·473 to 0·98)	0·764 (0·535 to 1·1)	0·852 (0·6 to 1·21)	0·95 (0·659 to 1·38)
West Pokot	0·964 (0·664 to 1·37)	0·975 (0·681 to 1·37)	0·99 (0·705 to 1·37)	1 (0·719 to 1·35)	1·01 (0·729 to 1·34)	1·02 (0·756 to 1·35)	1·05 (0·794 to 1·36)	1·07 (0·826 to 1·38)
Madagascar	26·3 (19 to 37)	27 (19·5 to 37·7)	27·5 (19·6 to 38·2)	27·8 (19·8 to 38)	28·1 (20·5 to 37·9)	28·4 (21·1 to 37·5)	28·5 (21·6 to 36·7)	28·7 (22·3 to 36·3)
Malawi	43 (33 to 55·4)	44·6 (35 to 56·5)	45·6 (37·3 to 55·5)	45·2 (38·3 to 53·1)	43·3 (38·7 to 49·2)	40·2 (37·5 to 43·5)	34·8 (32·6 to 37·1)	28·7 (26·3 to 31·2)
Mozambique	40 (27·9 to 56·2)	39·3 (27·6 to 54·8)	38·8 (27·6 to 53·3)	38·5 (27·5 to 51·8)	38·9 (27·8 to 51·4)	40·4 (29·2 to 53)	41·7 (30·6 to 54·2)	41·9 (31·9 to 53·6)
Rwanda	20·7 (14·6 to 29·9)	20·6 (14·5 to 29·2)	20·4 (14·4 to 27·9)	20·1 (14·7 to 27·3)	18·3 (13·4 to 24)	16·1 (12·1 to 20·7)	14·1 (11 to 17·7)	14·2 (11·6 to 17·2)
Somalia	27·2 (18·8 to 38·4)	26·7 (18·5 to 37·8)	25·9 (17·8 to 36·6)	25 (17·4 to 35·6)	24·9 (17 to 35·3)	25·7 (18 to 35·8)	26·3 (18·1 to 37)	26·7 (18·5 to 37·1)
South Sudan	24·5 (16·9 to 35)	25 (17·2 to 35·7)	25·3 (17·3 to 36·2)	25·6 (17·7 to 36·9)	25·9 (17·5 to 37)	26·3 (18·3 to 37)	26·6 (18·1 to 37·7)	26·7 (18·3 to 37·5)
Uganda	55·1 (40·5 to 73·2)	55·8 (41·8 to 74·5)	55·3 (43·3 to 70·6)	56·4 (45·1 to 70·2)	57·8 (47·7 to 70·2)	58·7 (50·1 to 69·1)	59·5 (52·9 to 67)	60·2 (55·8 to 64·7)
Tanzania	62·5 (44·3 to 86·5)	62·8 (45·1 to 84·7)	63·1 (45·8 to 83·7)	63·6 (46·3 to 84·2)	64·3 (47·6 to 83·3)	65 (49·6 to 82·9)	64·8 (51·1 to 80·2)	64·1 (52 to 77)
Zambia	15 (10·5 to 20·7)	15·1 (10·6 to 21)	15·2 (10·8 to 21·3)	15·3 (10·9 to 21·6)	15·5 (11·1 to 21·9)	15·8 (11·3 to 22)	16·1 (11·6 to 22·2)	16·2 (11·9 to 21·6)
Southern sub-Saharan Africa	76·8 (57·5 to 104)	74·4 (56 to 100)	71·9 (54 to 96·2)	69·1 (52·3 to 90·3)	66·9 (52 to 85·4)	65·8 (51·9 to 81·8)	65·6 (54·1 to 79·4)	67·3 (58·4 to 77)
Botswana	1·84 (1·44 to 2·3)	1·96 (1·58 to 2·42)	1·99 (1·63 to 2·38)	1·81 (1·51 to 2·17)	1·58 (1·32 to 1·88)	1·42 (1·19 to 1·68)	1·33 (1·12 to 1·56)	1·33 (1·11 to 1·6)
Eswatini	1·22 (0·865 to 1·71)	1·21 (0·841 to 1·67)	1·2 (0·841 to 1·67)	1·19 (0·831 to 1·65)	1·18 (0·83 to 1·63)	1·17 (0·828 to 1·62)	1·16 (0·817 to 1·61)	1·17 (0·817 to 1·6)
Lesotho	2·08 (1·46 to 2·88)	2·08 (1·45 to 2·91)	2·07 (1·46 to 2·91)	2·07 (1·47 to 2·92)	2·06 (1·45 to 2·93)	2·05 (1·45 to 2·92)	2·06 (1·45 to 2·96)	2·08 (1·45 to 2·94)
Namibia	1·39 (0·97 to 1·95)	1·39 (0·964 to 1·94)	1·38 (0·959 to 1·93)	1·35 (0·951 to 1·88)	1·31 (0·927 to 1·81)	1·28 (0·911 to 1·77)	1·27 (0·896 to 1·73)	1·27 (0·907 to 1·74)
South Africa	55·4 (38·4 to 79·8)	52·8 (36·6 to 75·9)	50 (35·2 to 71·8)	47·4 (33·5 to 66·4)	45 (32·1 to 61·2)	43 (31·2 to 57·6)	41·9 (31·7 to 53·9)	42·1 (34·4 to 51·1)
Eastern Cape	8·32 (5·53 to 12·1)	8·08 (5·52 to 11·7)	7·79 (5·33 to 11·3)	7·48 (4·88 to 11·1)	7·1 (4·72 to 10·3)	6·73 (4·63 to 9·42)	6·39 (4·61 to 8·59)	6·11 (5 to 7·62)
Free State	5·93 (3·92 to 8·87)	5·48 (3·78 to 8)	5·02 (3·46 to 7·23)	4·58 (3·21 to 6·41)	4·21 (3·04 to 5·65)	3·87 (2·84 to 5·18)	3·67 (2·8 to 4·74)	3·78 (2·96 to 4·78)
Gauteng	9·34 (6·33 to 13·5)	9·05 (6·3 to 13·2)	8·76 (6·03 to 12·6)	8·49 (5·96 to 11·8)	8·34 (6·01 to 11·2)	8·37 (5·95 to 11·4)	8·64 (6·58 to 11·1)	9·11 (7·16 to 11·5)
KwaZulu-Natal	10·6 (6·95 to 15·5)	9·92 (6·93 to 14·4)	9·34 (6·6 to 13·4)	8·82 (6·25 to 12·2)	8·43 (5·96 to 11·7)	8·2 (5·84 to 11·2)	8·25 (6·27 to 10·6)	8·55 (6·66 to 10·9)
Limpopo	8·36 (5·63 to 12·3)	8 (5·56 to 11·5)	7·58 (5·23 to 11·1)	7·13 (4·81 to 10·5)	6·64 (4·52 to 9·27)	6·14 (4·2 to 8·52)	5·76 (4·17 to 7·75)	5·54 (4·5 to 6·84)
Mpumalanga	4·22 (2·78 to 6·22)	4 (2·79 to 5·82)	3·72 (2·62 to 5·34)	3·47 (2·45 to 4·78)	3·23 (2·29 to 4·49)	3·01 (2·19 to 3·98)	2·82 (2·15 to 3·64)	2·76 (2·17 to 3·53)
North West	6 (3·95 to 8·94)	5·72 (3·97 to 8·35)	5·37 (3·62 to 7·75)	5·03 (3·56 to 6·97)	4·76 (3·43 to 6·38)	4·53 (3·32 to 6·07)	4·34 (3·31 to 5·6)	4·36 (3·4 to 5·57)
Northern Cape	1·42 (0·943 to 2·09)	1·3 (0·907 to 1·91)	1·21 (0·824 to 1·78)	1·12 (0·779 to 1·59)	1·03 (0·739 to 1·38)	0·942 (0·684 to 1·25)	0·867 (0·659 to 1·11)	0·816 (0·63 to 1·05)
Western Cape	1·23 (0·789 to 1·88)	1·24 (0·713 to 2·11)	1·25 (0·696 to 2·17)	1·24 (0·632 to 2·24)	1·23 (0·576 to 2·24)	1·19 (0·567 to 2·14)	1·15 (0·585 to 1·96)	1·1 (0·694 to 1·63)
Zimbabwe	14·9 (11·2 to 19·4)	15 (11·5 to 19·2)	15·2 (11·8 to 19·1)	15·3 (12·1 to 19·2)	15·8 (12·7 to 19·7)	16·9 (13·5 to 20·7)	17·9 (14·4 to 21·8)	19·3 (15·8 to 23·1)
Western sub-Saharan Africa	539 (415 to 707)	546 (423 to 716)	551 (427 to 723)	557 (431 to 729)	561 (434 to 732)	572 (444 to 745)	587 (455 to 765)	604 (473 to 785)
Benin	9·35 (6·39 to 13·1)	9·52 (6·58 to 13·6)	9·72 (6·72 to 13·9)	9·83 (6·87 to 14·2)	9·91 (6·93 to 14·3)	10·1 (6·98 to 14·2)	10·4 (7·44 to 14·5)	10·8 (7·81 to 14·8)
Burkina Faso	17·6 (13·7 to 21·8)	17·3 (14·2 to 20·7)	17·1 (14·5 to 19·9)	16·9 (15 to 19)	16·9 (15·7 to 18)	17·2 (16·1 to 18·2)	17·8 (16·1 to 19·6)	18·7 (16·5 to 21·1)
Cabo Verde	0·615 (0·466 to 0·823)	0·594 (0·454 to 0·787)	0·583 (0·453 to 0·746)	0·578 (0·462 to 0·724)	0·572 (0·468 to 0·686)	0·561 (0·479 to 0·655)	0·569 (0·51 to 0·637)	0·573 (0·538 to 0·613)
Cameroon	23·1 (16·2 to 32·5)	23·2 (16·1 to 32·6)	23·2 (16·1 to 32·9)	23·2 (16·1 to 33·3)	23 (15·8 to 33)	23 (16·1 to 32·5)	23·3 (16·2 to 33·1)	23·7 (16·4 to 34·2)
Chad	17·8 (12·4 to 25)	17·9 (12·5 to 25·2)	18 (12·5 to 25·4)	18·2 (12·7 to 25·8)	18·3 (12·6 to 25·9)	18·7 (13·2 to 25·9)	19·3 (13·3 to 26·9)	19·9 (13·8 to 27·6)
Côte d'Ivoire	22·3 (17·7 to 28·3)	22·2 (18·1 to 27·4)	22·3 (19 to 26·2)	22·6 (20 to 25·5)	23·1 (21·6 to 24·8)	23·9 (22·4 to 25·5)	24·7 (22·1 to 27·9)	25·4 (21·6 to 29·8)
Ghana	32·4 (23·1 to 46)	31·7 (23 to 43·6)	30·9 (22·9 to 40·8)	30·3 (22·8 to 38·9)	29·6 (23·5 to 37·2)	29·2 (24·1 to 35·5)	29·2 (25·2 to 33·8)	29·8 (27·6 to 32·1)
Guinea	15·5 (10·8 to 22)	15·4 (11 to 21·7)	15·5 (11·1 to 21·8)	15·8 (11·2 to 22·2)	15·8 (11·4 to 22)	15·9 (11·4 to 21·6)	16·1 (11·7 to 21·4)	16·3 (12·3 to 21·6)
Guinea-Bissau	2·87 (2 to 4·05)	2·87 (1·99 to 4·06)	2·88 (1·99 to 4·07)	2·89 (2·02 to 4·11)	2·9 (1·99 to 4·11)	2·95 (2·07 to 4·11)	3·04 (2·09 to 4·27)	3·11 (2·15 to 4·33)
Liberia	6·58 (4·48 to 9·09)	5·32 (3·68 to 7·48)	4·41 (3·01 to 6·22)	4·28 (2·92 to 6·11)	4·39 (3·06 to 6·31)	4·26 (2·98 to 6·08)	4·21 (2·91 to 5·96)	4·27 (2·96 to 6·16)
Mali	32·4 (27·6 to 37·8)	32·2 (28·8 to 36·2)	32·2 (30 to 34·6)	31·9 (30 to 34·1)	31·2 (27·9 to 34·8)	30·7 (26·2 to 35·5)	30·4 (25·3 to 36·3)	30·1 (24·3 to 36·5)
Mauritania	3·04 (2·41 to 3·78)	3·05 (2·5 to 3·67)	3·05 (2·58 to 3·56)	3·06 (2·71 to 3·44)	3·07 (2·86 to 3·29)	3·1 (2·89 to 3·32)	3·14 (2·79 to 3·55)	3·21 (2·73 to 3·76)
Niger	23·6 (16·8 to 33·3)	25·2 (18·2 to 35·1)	26·1 (19 to 36·3)	27·6 (19·8 to 37·7)	28·4 (20·7 to 38·2)	29·4 (21·9 to 39·2)	30·5 (23·1 to 39·9)	32·2 (25 to 40·7)
Nigeria	246 (183 to 331)	252 (187 to 339)	258 (190 to 350)	263 (192 to 361)	269 (193 to 373)	277 (198 to 382)	287 (204 to 401)	297 (214 to 414)
São Tomé and Príncipe	0·0943 (0·0665 to 0·131)	0·095 (0·0668 to 0·132)	0·0967 (0·0676 to 0·134)	0·0977 (0·0692 to 0·137)	0·0995 (0·069 to 0·139)	0·101 (0·072 to 0·139)	0·104 (0·0727 to 0·144)	0·108 (0·0753 to 0·148)
Senegal	12·7 (9·47 to 16·2)	12·7 (9·96 to 15·9)	12·9 (10·5 to 15·5)	13·1 (11·4 to 15·1)	13·1 (12·2 to 14·2)	13·5 (12·5 to 14·6)	13·9 (12·1 to 16·1)	14·1 (11·7 to 17·1)
Sierra Leone	59·1 (38 to 90·7)	60·3 (39·1 to 94·5)	60·5 (38·7 to 97·3)	58·8 (37·7 to 95·2)	57·5 (37·3 to 90·9)	57·9 (37·6 to 89·6)	59 (38·3 to 93·5)	59·9 (39·2 to 93·3)
The Gambia	2·63 (1·83 to 3·76)	2·71 (1·89 to 3·92)	2·79 (1·97 to 3·98)	2·85 (2·01 to 4·04)	2·89 (2·05 to 4·11)	2·94 (2·08 to 4·19)	3·01 (2·14 to 4·2)	3·09 (2·23 to 4·29)
Togo	11·3 (7·78 to 16·2)	11·4 (7·73 to 16·2)	11·3 (7·8 to 15·9)	11·3 (7·95 to 16)	11·3 (7·9 to 15·8)	11·4 (7·94 to 15·6)	11·6 (8·21 to 15·9)	12 (8·33 to 16·3)

Appendix 2 Table S1B: Annual estimates of stillbirth (in thousands) for ≥22 weeks, by location

Location	1990	1991	1992	1993	1994	1995	1996	1997	1998
Global	4890 (3890 to 6070)	4830 (3880 to 5970)	4770 (3860 to 5920)	4740 (3850 to 5860)	4710 (3830 to 5800)	4660 (3830 to 5730)	4610 (3830 to 5630)	4540 (3800 to 5460)	4480 (3780 to 5330)
Central Europe, eastern Europe, and central Asia	91·1 (83·1 to 100)	85·4 (78·6 to 93·3)	77·6 (71·9 to 84·3)	72·9 (68 to 78·6)	69·7 (65·8 to 73·9)	65·9 (62·3 to 69·8)	63·7 (60·4 to 67·4)	62·4 (58·8 to 66·4)	59·6 (56·1 to 63·5)
Central Asia	38 (33 to 44·6)	36·8 (32·3 to 42·4)	34·7 (30·9 to 39·5)	33·3 (29·8 to 37·3)	32·4 (29·4 to 35·9)	31·2 (28·2 to 34·5)	30·5 (27·5 to 34·1)	30·5 (27·5 to 34·1)	29·7 (26·6 to 33·3)
Armenia	1·59 (1·45 to 1·75)	1·64 (1·36 to 1·93)	1·48 (1·19 to 1·81)	1·34 (1·09 to 1·64)	1·17 (0·953 to 1·41)	1·04 (0·872 to 1·26)	1 (0·837 to 1·2)	0·955 (0·791 to 1·15)	0·945 (0·779 to 1·14)
Azerbaijan	5·19 (4·51 to 6·01)	4·98 (4·5 to 5·51)	4·46 (4·03 to 4·88)	4·2 (3·82 to 4·65)	4·04 (3·65 to 4·43)	3·82 (3·46 to 4·23)	3·7 (3·33 to 4·07)	3·85 (3·47 to 4·26)	3·87 (3·52 to 4·23)
Georgia	1·65 (1·38 to 1·95)	1·57 (1·34 to 1·82)	1·42 (1·23 to 1·62)	1·56 (1·36 to 1·77)	1·76 (1·56 to 1·97)	1·94 (1·73 to 2·19)	2·27 (2 to 2·57)	2·35 (2·06 to 2·68)	2·09 (1·83 to 2·37)
Kazakhstan	6·3 (5·59 to 7·08)	5·79 (5·14 to 6·47)	5·47 (4·87 to 6·15)	5·03 (4·46 to 5·67)	4·76 (4·28 to 5·26)	4·58 (4·18 to 5·05)	4·41 (3·98 to 4·83)	4·46 (4·03 to 4·91)	4·28 (3·89 to 4·7)
Kyrgyzstan	3·47 (2·43 to 5·05)	3·36 (2·36 to 4·88)	3·16 (2·21 to 4·51)	2·97 (2·12 to 4·12)	2·8 (2·02 to 3·82)	2·68 (1·91 to 3·63)	2·57 (1·86 to 3·43)	2·34 (1·68 to 3·1)	2·06 (1·49 to 2·7)
Mongolia	1·76 (1·24 to 2·61)	1·66 (1·17 to 2·42)	1·57 (1·1 to 2·27)	1·49 (1·05 to 2·08)	1·41 (0·981 to 1·94)	1·31 (0·924 to 1·81)	1·22 (0·867 to 1·71)	1·15 (0·816 to 1·61)	1·07 (0·764 to 1·49)
Tajikistan	6·25 (5·1 to 7·57)	6·18 (5·19 to 7·41)	5·78 (4·85 to 6·88)	5·62 (4·68 to 6·72)	5·7 (4·77 to 6·8)	5·35 (4·48 to 6·39)	5·04 (4·21 to 5·95)	5·13 (4·3 to 6·11)	5·08 (4·28 to 5·98)
Turkmenistan	2·93 (2·67 to 3·21)	2·83 (2·65 to 3·02)	2·69 (2·55 to 2·85)	2·56 (2·42 to 2·71)	2·46 (2·33 to 2·59)	2·37 (2·24 to 2·51)	2·26 (2·13 to 2·42)	2·22 (2·03 to 2·44)	2·22 (1·97 to 2·53)
Uzbekistan	8·86 (6·32 to 12·6)	8·76 (6·15 to 12)	8·69 (6·14 to 11·9)	8·52 (6·1 to 11·6)	8·34 (6·04 to 11·2)	8·1 (5·89 to 11)	7·99 (5·72 to 10·9)	8·08 (5·87 to 11)	8·1 (5·79 to 10·9)
Central Europe	15·7 (13·4 to 18·6)	14·8 (12·7 to 17·4)	13·7 (12·1 to 15·4)	12·7 (11·6 to 13·9)	13 (12·2 to 13·8)	11·9 (11·2 to 12·7)	11·4 (10·9 to 12·1)	11 (10·5 to 11·5)	10·6 (10·2 to 11·1)
Albania	0·699 (0·499 to 0·947)	0·68 (0·48 to 0·928)	0·664 (0·466 to 0·903)	0·654 (0·46 to 0·9)	0·645 (0·453 to 0·902)	0·631 (0·436 to 0·891)	0·618 (0·438 to 0·874)	0·6 (0·425 to 0·848)	0·575 (0·413 to 0·812)
Bosnia and Herzegovina	0·8 (0·747 to 0·855)	0·761 (0·682 to 0·841)	0·776 (0·699 to 0·858)	0·756 (0·707 to 0·81)	0·69 (0·641 to 0·742)	0·614 (0·541 to 0·695)	0·542 (0·463 to 0·629)	0·468 (0·4 to 0·545)	0·426 (0·368 to 0·49)
Bulgaria	0·892 (0·657 to 1·24)	0·948 (0·695 to 1·28)	0·907 (0·685 to 1·19)	0·821 (0·658 to 1·01)	0·817 (0·724 to 0·919)	0·738 (0·674 to 0·803)	0·769 (0·707 to 0·836)	0·817 (0·753 to 0·89)	0·784 (0·716 to 0·851)
Croatia	0·34 (0·315 to 0·368)	0·351 (0·324 to 0·379)	0·318 (0·294 to 0·346)	0·285 (0·264 to 0·309)	0·305 (0·284 to 0·329)	0·302 (0·278 to 0·325)	0·304 (0·281 to 0·328)	0·318 (0·294 to 0·343)	0·292 (0·271 to 0·317)
Czechia	0·82 (0·748 to 0·892)	0·774 (0·67 to 0·909)	0·718 (0·653 to 0·791)	0·617 (0·566 to 0·674)	0·52 (0·457 to 0·59)	0·483 (0·43 to 0·546)	0·43 (0·383 to 0·485)	0·43 (0·382 to 0·482)	0·438 (0·388 to 0·491)
Hungary	0·993 (0·704 to 1·42)	1·03 (0·731 to 1·42)	0·914 (0·662 to 1·24)	0·808 (0·597 to 1·08)	0·738 (0·567 to 0·94)	0·649 (0·533 to 0·777)	0·655 (0·57 to 0·744)	0·688 (0·612 to 0·766)	0·671 (0·594 to 0·743)
Montenegro	0·0814 (0·0589 to 0·112)	0·074 (0·0532 to 0·101)	0·0705 (0·0509 to 0·0967)	0·0701 (0·0511 to 0·0943)	0·0706 (0·0517 to 0·0944)	0·0694 (0·0509 to 0·0907)	0·0687 (0·0509 to 0·0881)	0·0653 (0·0502 to 0·0818)	0·0618 (0·0488 to 0·0766)
North Macedonia	0·538 (0·407 to 0·691)	0·47 (0·382 to 0·578)	0·48 (0·431 to 0·531)	0·466 (0·436 to 0·497)	0·463 (0·432 to 0·495)	0·47 (0·441 to 0·503)	0·467 (0·422 to 0·518)	0·445 (0·373 to 0·54)	0·499 (0·397 to 0·623)
Poland	5·12 (3·74 to 6·75)	4·78 (3·6 to 6·13)	4·38 (3·47 to 5·46)	3·88 (3·37 to 4·48)	4·27 (3·91 to 4·67)	3·82 (3·52 to 4·12)	3·53 (3·27 to 3·82)	3·09 (2·85 to 3·34)	2·95 (2·72 to 3·18)
Romania	2·83 (2·63 to 3·02)	2·39 (2·22 to 2·57)	2·18 (2·02 to 2·34)	2·15 (2·01 to 2·32)	2·25 (2·12 to 2·4)	2 (1·87 to 2·14)	2·05 (1·92 to 2·19)	2·15 (2·02 to 2·3)	2·13 (1·99 to 2·28)
Serbia	1·65 (1·19 to 2·33)	1·6 (1·15 to 2·21)	1·36 (0·976 to 1·87)	1·39 (1 to 1·89)	1·49 (1·1 to 1·99)	1·42 (1·08 to 1·82)	1·28 (1 to 1·61)	1·14 (0·922 to 1·4)	1·03 (0·866 to 1·23)
Slovakia	0·582 (0·441 to 0·755)	0·599 (0·474 to 0·749)	0·555 (0·482 to 0·644)	0·47 (0·428 to 0·519)	0·414 (0·379 to 0·451)	0·4 (0·365 to 0·434)	0·416 (0·378 to 0·451)	0·437 (0·402 to 0·474)	0·462 (0·427 to 0·501)
Slovenia	0·155 (0·13 to 0·182)	0·142 (0·127 to 0·16)	0·132 (0·117 to 0·148)	0·123 (0·11 to 0·138)	0·116 (0·105 to 0·129)	0·11 (0·0997 to 0·121)	0·116 (0·105 to 0·129)	0·125 (0·112 to 0·139)	0·12 (0·108 to 0·132)
Eastern Europe	37·3 (35 to 39·6)	33·8 (31·5 to 36)	29·2 (27·5 to 31·1)	26·9 (25·3 to 28·7)	24·3 (23 to 25·7)	22·8 (21·6 to 24·1)	21·8 (20·6 to 23)	20·9 (19·7 to 22·1)	19·3 (18·3 to 20·4)
Belarus	1·44 (1·26 to 1·63)	1·4 (1·18 to 1·65)	1·25 (1·1 to 1·42)	1·21 (1·11 to 1·32)	1·24 (1·15 to 1·35)	1·11 (1·02 to 1·19)	0·987 (0·907 to 1·07)	0·945 (0·864 to 1·03)	0·872 (0·796 to 0·954)
Estonia	0·235 (0·201 to 0·274)	0·199 (0·174 to 0·229)	0·17 (0·15 to 0·194)	0·144 (0·127 to 0·162)	0·125 (0·11 to 0·14)	0·118 (0·105 to 0·131)	0·119 (0·107 to 0·133)	0·118 (0·105 to 0·133)	0·103 (0·0921 to 0·116)
Latvia	0·298 (0·264 to 0·335)	0·328 (0·303 to 0·357)	0·323 (0·297 to 0·351)	0·278 (0·256 to 0·303)	0·228 (0·211 to 0·249)	0·208 (0·19 to 0·226)	0·195 (0·179 to 0·212)	0·189 (0·174 to 0·206)	0·19 (0·174 to 0·207)
Lithuania	0·398 (0·362 to 0·435)	0·447 (0·408 to 0·484)	0·455 (0·414 to 0·495)	0·392 (0·359 to 0·431)	0·347 (0·319 to 0·378)	0·314 (0·289 to 0·341)	0·283 (0·261 to 0·308)	0·285 (0·262 to 0·31)	0·264 (0·244 to 0·286)
Moldova	1·11 (1·04 to 1·19)	1·02 (0·952 to 1·09)	0·928 (0·871 to 0·992)	0·833 (0·781 to 0·891)	0·82 (0·766 to 0·876)	0·755 (0·707 to 0·805)	0·698 (0·628 to 0·773)	0·692 (0·568 to 0·831)	0·667 (0·534 to 0·833)
Russia	25·1 (23·1 to 27·2)	22·5 (20·5 to 24·6)	19·3 (17·8 to 21)	18·2 (16·6 to 19·9)	16·3 (15 to 17·7)	15·6 (14·5 to 16·8)	15·2 (14·1 to 16·4)	14·8 (13·7 to 15·9)	13·7 (12·7 to 14·7)
Ukraine	8·71 (8·06 to 9·39)	7·9 (7·35 to 8·51)	6·75 (6·21 to 7·28)	5·87 (5·45 to 6·36)	5·24 (4·88 to 5·65)	4·68 (4·37 to 5)	4·29 (4·01 to 4·57)	3·86 (3·62 to 4·15)	3·49 (3·23 to 3·73)
High income	90·5 (82·9 to 100)	87·3 (80·4 to 95·5)	82·9 (77·1 to 89·7)	79·3 (74·8 to 84·5)	77·2 (73·7 to 80·9)	75 (72·3 to 77·8)	72·5 (70 to 75·2)	70·1 (68 to 72·3)	69 (67 to 70·9)
Australasia	2·98 (2·51 to 3·56)	2·71 (2·33 to 3·18)	2·51 (2·22 to 2·84)	2·31 (2·17 to 2·45)	2·28 (2·19 to 2·37)	2·18 (2·07 to 2·29)	2·08 (1·96 to 2·2)	2 (1·89 to 2·12)	1·87 (1·78 to 1·96)
Australia	1·97 (1·5 to 2·56)	1·76 (1·38 to 2·22)	1·68 (1·39 to 2·01)	1·52 (1·38 to 1·65)	1·51 (1·43 to 1·6)	1·48 (1·38 to 1·58)	1·43 (1·32 to 1·54)	1·39 (1·3 to 1·49)	1·32 (1·23 to 1·41)
New Zealand	1·01 (0·941 to 1·08)	0·956 (0·91 to 1·01)	0·828 (0·788 to 0·87)	0·788 (0·75 to 0·83)	0·769 (0·725 to 0·808)	0·697 (0·66 to 0·738)	0·648 (0·618 to 0·681)	0·611 (0·583 to 0·642)	0·55 (0·523 to 0·578)
High-income Asia Pacific	9·95 (7·31 to 13·1)	9·5 (7·23 to 12·4)	9·43 (7·44 to 12)	9·11 (7·45 to 11·2)	8·93 (7·74 to 10·6)	8·66 (7·67 to 10)	8·11 (7·14 to 9·3)	7·59 (6·74 to 8·67)	7·42 (6·67 to 8·36)
Brunei	0·0609 (0·0435 to 0·0862)	0·0627 (0·0449 to 0·0862)	0·0616 (0·044 to 0·0851)	0·0609 (0·0435 to 0·0832)	0·0598 (0·0438 to 0·0806)	0·0581 (0·0437 to 0·0756)	0·0569 (0·0427 to 0·0741)	0·0565 (0·0438 to 0·0716)	0·0558 (0·0448 to 0·0697)
Japan	5·53 (3·89 to 7·44)	5·27 (3·88 to 6·94)	5·35 (4·1 to 6·95)	5·21 (4·22 to 6·36)	5·2 (4·72 to 5·71)	5·13 (4·85 to 5·41)	4·77 (4·51 to 5·04)	4·47 (4·24 to 4·72)	4·51 (4·26 to 4·76)
Aichi	0·292 (0·205 to 0·393)	0·283 (0·208 to 0·373)	0·287 (0·22 to 0·373)	0·282 (0·229 to 0·344)	0·287 (0·26 to 0·315)	0·287 (0·271 to 0·302)	0·273 (0·259 to 0·289)	0·259 (0·245 to 0·273)	0·258 (0·244 to 0·273)
Akita	0·0543 (0·0383 to 0·0732)	0·0509 (0·0374 to 0·067)	0·0498 (0·0382 to 0·0647)	0·0469 (0·0379 to 0·0571)	0·045 (0·0408 to 0·0494)	0·0431 (0·0407 to 0·0454)	0·0388 (0·0368 to 0·041)	0·0352 (0·0334 to 0·0372)	0·0347 (0·0328 to 0·0366)
Aomori	0·0744 (0·0524 to 0·1)	0·0698 (0·0514 to 0·092)	0·0691 (0·0529 to 0·0897)	0·0663 (0·0537 to 0·0808)	0·0649 (0·0589 to 0·0713)	0·0636 (0·0601 to 0·067)	0·059 (0·0558 to 0·0623)	0·0549 (0·052 to 0·0579)	0·0552 (0·0523 to 0·0583)
Chiba	0·212 (0·149 to 0·286)	0·208 (0·153 to 0·274)	0·215 (0·165 to 0·28)	0·214 (0·173 to 0·261)	0·214 (0·194 to 0·235)	0·212 (0·2 to 0·224)	0·199 (0·189 to 0·211)	0·191 (0·181 to 0·202)	0·196 (0·186 to 0·207)
Ehime	0·0673 (0·0474 to 0·0907)	0·0637 (0·0469 to 0·0839)	0·0638 (0·0489 to 0·0829)	0·0616 (0·0498 to 0·0751)	0·0606 (0·0549 to 0·0665)	0·0589 (0·0557 to 0·0622)	0·054 (0·0511 to 0·057)	0·0494 (0·0469 to 0·0522)	0·0483 (0·0457 to 0·051)
Fukui	0·0423 (0·0298 to 0·057)	0·0406 (0·0299 to 0·0534)	0·0417 (0·0319 to 0·0541)	0·0408 (0·033 to 0·0497)	0·0408 (0·037 to 0·0448)	0·0407 (0·0384 to 0·0429)	0·0382 (0·0362 to 0·0404)	0·036 (0·0341 to 0·038)	0·0357 (0·0338 to 0·0377)
Fukuoka	0·22 (0·155 to 0·297)	0·216 (0·159 to 0·285)	0·221 (0·17 to 0·288)	0·215 (0·174 to 0·263)	0·217 (0·196 to 0·238)	0·216 (0·204 to 0·228)	0·199 (0·188 to 0·21)	0·188 (0·178 to 0·198)	0·189 (0·179 to 0·2)
Fukushima	0·105 (0·0737 to 0·141)	0·0978 (0·072 to 0·129)	0·0964 (0·0739 to 0·125)	0·0907 (0·0734 to 0·111)	0·0874 (0·0793 to 0·096)	0·0834 (0·0788 to 0·088)	0·0759 (0·0719 to 0·0802)	0·0695 (0·0659 to 0·0734)	0·0683 (0·0646 to 0·0721)
Gifu	0·0839 (0·0591 to 0·113)	0·0806 (0·0593 to 0·106)	0·0819 (0·0627 to 0·106)	0·0803 (0·065 to 0·0979)	0·0812 (0·0736 to 0·0891)	0·0808 (0·0764 to 0·0852)	0·0763 (0·0723 to 0·0806)	0·0725 (0·0687 to 0·0765)	0·0733 (0·0694 to 0·0774)
Gunma	0·0894 (0·063 to 0·12)	0·0851 (0·0626 to 0·112)	0·0859 (0·0658 to 0·112)	0·0833 (0·0675 to 0·102)	0·083 (0·0753 to 0·0912)	0·0819 (0·0774 to 0·0864)	0·0762 (0·0722 to 0·0806)	0·0716 (0·0679 to 0·0756)	0·0731 (0·0692 to 0·0772)
Hiroshima	0·125 (0·0882 to 0·169)	0·118 (0·0866 to 0·155)	0·118 (0·0903 to 0·153)	0·114 (0·0922 to 0·139)	0·113 (0·102 to 0·124)	0·109 (0·103 to 0·115)	0·0986 (0·0933 to 0·104)	0·0909 (0·0861 to 0·0959)	0·0904 (0·0855 to 0·0954)
Hokkaidō	0·214 (0·151 to 0·288)	0·2 (0·147 to 0·263)	0·197 (0·151 to 0·256)	0·187 (0·151 to 0·228)	0·187 (0·169 to 0·205)	0·183 (0·173 to 0·193)	0·167 (0·158 to 0·176)	0·154 (0·146 to 0·162)	0·154 (0·145 to 0·162)
Hyōgo	0·25 (0·176 to 0·337)	0·236 (0·174 to 0·311)	0·237 (0·182 to 0·308)	0·232 (0·188 to 0·283)	0·232 (0·21 to 0·255)	0·23 (0·217 to 0·242)	0·218 (0·207 to 0·23)	0·205 (0·194 to 0·216)	0·208 (0·197 to 0·22)
Ibaraki	0·121 (0·0852 to 0·163)	0·114 (0·0838 to 0·15)	0·113 (0·0867 to 0·147)	0·11 (0·0892 to 0·134)	0·11 (0·0994 to 0·12)	0·109 (0·103 to 0·114)	0·101 (0·0954 to 0·106)	0·0933 (0·0884 to 0·0985)	0·093 (0·0881 to 0·0982)
Ishikawa	0·0516 (0·0364 to 0·0695)	0·0504 (0·0371 to 0·0663)	0·0516 (0·0395 to 0·067)	0·0507 (0·041 to 0·0618)	0·0509 (0·0461 to 0·0559)	0·0506 (0·0478 to 0·0533)	0·0475 (0·045 to 0·0502)	0·0449 (0·0426 to 0·0474)	0·0457 (0·0433 to 0·0483)
Iwate	0·0518 (0·0365 to 0·0698)	0·0485 (0·0357 to 0·0638)	0·0476 (0·0365 to 0·0618)	0·0447 (0·0362 to 0·0544)	0·0433 (0·0392 to 0·0475)	0·0415 (0·0393 to 0·0438)	0·0381 (0·0361 to 0·0402)	0·0354 (0·0336 to 0·0374)	0·0358 (0·0338 to 0·0377)
Kagawa	0·046 (0·0324 to 0·062)	0·043 (0·0316 to 0·0566)	0·0422 (0·0323 to 0·0548)	0·0397 (0·0321 to 0·0484)	0·0387 (0·0351 to 0·0425)	0·0381 (0·036 to 0·0402)	0·0358 (0·0339 to 0·0378)	0·034 (0·0322 to 0·0359)	0·0342 (0·0324 to 0·0361)
Kagoshima	0·0736 (0·0519 to 0·0991)	0·0668 (0·0492 to 0·088)	0·0637 (0·0488 to 0·0827)	0·0591 (0·0478 to 0·072)	0·0563 (0·051 to 0·0618)	0·0537 (0·0508 to 0·0567)	0·0491 (0·0465 to 0·0519)	0·0462 (0·0438 to 0·0487)	0·0467 (0·0442 to 0·0493)
Kanagawa	0·391 (0·276 to 0·527)	0·384 (0·283 to 0·506)	0·413 (0·316 to 0·536)	0·411 (0·333 to 0·501)	0·425 (0·385 to 0·467)	0·424 (0·4 to 0·447)	0·385 (0·365 to 0·407)	0·359 (0·341 to 0·379)	0·374 (0·354 to 0·395)
Kōchi	0·0385 (0·0271 to 0·0519)	0·0354 (0·0261 to 0·0467)	0·0345 (0·0264 to 0·0448)	0·0325 (0·0263 to 0·0397)	0·0315 (0·0286 to 0·0346)	0·0307 (0·029 to 0·0324)	0·0283 (0·0268 to 0·0299)	0·0265 (0·0251 to 0·0279)	0·0265 (0·0251 to 0·028)
Kumamoto	0·0842 (0·0593 to 0·113)	0·0794 (0·0584 to 0·105)	0·0789 (0·0605 to 0·103)	0·0757 (0·0613 to 0·0924)	0·075 (0·068 to 0·0823)	0·0736 (0·0695 to 0·0776)	0·0682 (0·0646 to 0·0721)	0·0632 (0·0599 to 0·0667)	0·063 (0·0596 to 0·0665)
Kyōto	0·125 (0·0883 to 0·169)	0·122 (0·0901 to 0·161)	0·126 (0·0965 to 0·164)	0·123 (0·0994 to 0·15)	0·121 (0·11 to 0·133)	0·118 (0·111 to 0·124)	0·11 (0·104 to 0·116)	0·102 (0·097 to 0·108)	0·102 (0·0962 to 0·107)
Mie	0·088 (0·062 to 0·119)	0·0842 (0·062 to 0·111)	0·0847 (0·0649 to 0·11)	0·0822 (0·0666 to 0·1)	0·0814 (0·0738 to 0·0894)	0·0802 (0·0758 to 0·0845)	0·0746 (0·0706 to 0·0788)	0·0696 (0·0659 to 0·0734)	0·07 (0·0662 to 0·0739)
Miyagi	0·101 (0·0713 to 0·136)	0·0928 (0·0683 to 0·122)	0·0898 (0·0688 to 0·117)	0·0844 (0·0683 to 0·103)	0·0826 (0·0748 to 0·0907)	0·0803 (0·0758 to 0·0846)	0·0744 (0·0704 to 0·0786)	0·0701 (0·0664 to 0·074)	0·0707 (0·0669 to 0·0746)
Miyazaki	0·06 (0·0422 to 0·0808)	0·0568 (0·0418 to 0·0748)	0·0566 (0·0434 to 0·0735)	0·0544 (0·044 to 0·0663)	0·0533 (0·0483 to 0·0585)	0·0514 (0·0486 to 0·0542)	0·0468 (0·0443 to 0·0494)	0·0428 (0·0405 to 0·0451)	0·0415 (0·0392 to 0·0438)
Nagano	0·0979 (0·0689 to 0·132)	0·0911 (0·067 to 0·12)	0·0891 (0·0682 to 0·116)	0·0838 (0·0678 to 0·102)	0·0811 (0·0736 to 0·0891)	0·0778 (0·0736 to 0·0821)	0·0708 (0·0671 to 0·0748)	0·0653 (0·0619 to 0·0689)	0·065 (0·0615 to 0·0686)
Nagasaki	0·0624 (0·0439 to 0·084)	0·0596 (0·0439 to 0·0785)	0·0598 (0·0458 to 0·0777)	0·0577 (0·0467 to 0·0703)	0·0569 (0·0516 to 0·0625)	0·056 (0·053 to 0·0591)	0·0521 (0·0494 to 0·0551)	0·049 (0·0464 to 0·0517)	0·0493 (0·0466 to 0·052)
Nara	0·0613 (0·0432 to 0·0826)	0·0591 (0·0435 to 0·0779)	0·0607 (0·0465 to 0·0789)	0·0593 (0·048 to 0·0723)	0·0588 (0·0533 to 0·0646)	0·0579 (0·0547 to 0·0611)	0·0537 (0·0508 to 0·0567)	0·0495 (0·0469 to 0·0522)	0·0487 (0·0461 to 0·0514)
Niigata	0·0945 (0·0665 to 0·127)	0·0901 (0·0663 to 0·119)	0·0922 (0·0707 to 0·12)	0·09 (0·0729 to 0·11)	0·089 (0·0806 to 0·0977)	0·0872 (0·0824 to 0·092)	0·0824 (0·078 to 0·0871)	0·0777 (0·0736 to 0·082)	0·0787 (0·0744 to 0·083)
Ōita	0·0544 (0·0383 to 0·0733)	0·0511 (0·0376 to 0·0673)	0·0511 (0·0392 to 0·0664)	0·0485 (0·0393 to 0·0592)	0·0473 (0·0429 to 0·052)	0·0462 (0·0436 to 0·0487)	0·0429 (0·0407 to 0·0454)	0·0403 (0·0382 to 0·0425)	0·0406 (0·0384 to 0·0429)
Okayama	0·0766 (0·054 to 0·103)	0·0729 (0·0537 to 0·096)	0·0733 (0·0562 to 0·0952)	0·0715 (0·0579 to 0·0871)	0·0714 (0·0647 to 0·0784)	0·0709 (0·067 to 0·0747)	0·0665 (0·0629 to 0·0702)	0·0625 (0·0592 to 0·066)	0·0629 (0·0596 to 0·0664)
Okinawa	0·0882 (0·0621 to 0·119)	0·0846 (0·0622 to 0·111)	0·0849 (0·065 to 0·11)	0·0821 (0·0665 to 0·1)	0·0815 (0·0739 to 0·0895)	0·0803 (0·0759 to 0·0847)	0·0747 (0·0708 to 0·079)	0·0699 (0·0663 to 0·0738)	0·0694 (0·0657 to 0·0733)
Ōsaka	0·393 (0·276 to 0·529)	0·359 (0·264 to 0·473)	0·357 (0·274 to 0·464)	0·341 (0·276 to 0·415)	0·339 (0·307 to 0·372)	0·339 (0·321 to 0·358)	0·321 (0·304 to 0·34)	0·303 (0·287 to 0·319)	0·315 (0·299 to 0·333)
Saga	0·0423 (0·0298 to 0·0569)	0·0399 (0·0293 to 0·0525)	0·0395 (0·0303 to 0·0513)	0·0376 (0·0305 to 0·0459)	0·0373 (0·0338 to 0·041)	0·0367 (0·0347 to 0·0387)	0·0341 (0·0323 to 0·0361)	0·0317 (0·03 to 0·0334)	0·0313 (0·0296 to 0·033)
Saitama	0·277 (0·195 to 0·374)	0·275 (0·202 to 0·362)	0·284 (0·218 to 0·369)	0·279 (0·226 to 0·34)	0·282 (0·256 to 0·31)	0·279 (0·264 to 0·294)	0·261 (0·248 to 0·276)	0·246 (0·233 to 0·26)	0·24 (0·227 to 0·254)
Shiga	0·0671 (0·0473 to 0·0904)	0·0632 (0·0465 to 0·0832)	0·0642 (0·0492 to 0·0834)	0·0634 (0·0514 to 0·0774)	0·0651 (0·059 to 0·0715)	0·0663 (0·0627 to 0·07)	0·0634 (0·0601 to 0·067)	0·0608 (0·0576 to 0·0641)	0·0622 (0·0588 to 0·0657)
Shimane	0·0343 (0·0242 to 0·0462)	0·0318 (0·0234 to 0·0419)	0·0314 (0·0241 to 0·0408)	0·03 (0·0243 to 0·0366)	0·0297 (0·0269 to 0·0326)	0·0291 (0·0275 to 0·0307)	0·0268 (0·0254 to 0·0284)	0·0248 (0·0235 to 0·0261)	0·0246 (0·0233 to 0·026)
Shizuoka	0·151 (0·107 to 0·204)	0·146 (0·107 to 0·192)	0·147 (0·112 to 0·191)	0·142 (0·115 to 0·173)	0·141 (0·128 to 0·155)	0·139 (0·131 to 0·146)	0·127 (0·121 to 0·134)	0·119 (0·113 to 0·126)	0·119 (0·113 to 0·126)
Tochigi	0·0978 (0·0689 to 0·132)	0·0946 (0·0696 to 0·125)	0·0968 (0·0742 to 0·126)	0·0935 (0·0757 to 0·114)	0·0923 (0·0837 to 0·101)	0·0911 (0·0861 to 0·0961)	0·0845 (0·0801 to 0·0893)	0·0787 (0·0746 to 0·083)	0·0794 (0·0751 to 0·0839)
Tokushima	0·0367 (0·0259 to 0·0495)	0·0345 (0·0254 to 0·0454)	0·0345 (0·0264 to 0·0448)	0·0334 (0·027 to 0·0407)	0·0336 (0·0305 to 0·0369)	0·0336 (0·0318 to 0·0354)	0·0317 (0·0301 to 0·0335)	0·0303 (0·0287 to 0·032)	0·0307 (0·0291 to 0·0324)
Tōkyō	0·535 (0·377 to 0·721)	0·508 (0·374 to 0·67)	0·536 (0·411 to 0·697)	0·546 (0·442 to 0·666)	0·544 (0·493 to 0·597)	0·536 (0·506 to 0·565)	0·492 (0·466 to 0·52)	0·47 (0·446 to 0·496)	0·47 (0·444 to 0·496)
Tottori	0·0281 (0·0198 to 0·0379)	0·0266 (0·0196 to 0·035)	0·0263 (0·0202 to 0·0342)	0·0252 (0·0204 to 0·0308)	0·0249 (0·0226 to 0·0274)	0·0245 (0·0232 to 0·0259)	0·0227 (0·0215 to 0·024)	0·0212 (0·0201 to 0·0224)	0·0212 (0·0201 to 0·0224)
Toyama	0·0567 (0·04 to 0·0765)	0·0538 (0·0396 to 0·0709)	0·0545 (0·0418 to 0·0708)	0·053 (0·0429 to 0·0647)	0·0532 (0·0482 to 0·0584)	0·0532 (0·0502 to 0·0561)	0·0502 (0·0475 to 0·053)	0·0472 (0·0447 to 0·0498)	0·0472 (0·0447 to 0·0499)
Wakayama	0·0474 (0·0334 to 0·0638)	0·0443 (0·0326 to 0·0583)	0·0443 (0·0339 to 0·0575)	0·043 (0·0348 to 0·0524)	0·0433 (0·0392 to 0·0475)	0·0428 (0·0405 to 0·0452)	0·0399 (0·0378 to 0·0422)	0·0372 (0·0353 to 0·0392)	0·037 (0·035 to 0·039)
Yamagata	0·0513 (0·0361 to 0·0691)	0·0482 (0·0355 to 0·0634)	0·0478 (0·0366 to 0·0621)	0·0458 (0·0371 to 0·0559)	0·0453 (0·0411 to 0·0498)	0·0444 (0·042 to 0·0469)	0·0413 (0·0391 to 0·0437)	0·0386 (0·0366 to 0·0407)	0·039 (0·0369 to 0·0411)
Yamaguchi	0·0705 (0·0496 to 0·095)	0·0665 (0·0489 to 0·0876)	0·0665 (0·0509 to 0·0863)	0·0637 (0·0516 to 0·0777)	0·0626 (0·0567 to 0·0687)	0·0607 (0·0574 to 0·064)	0·0557 (0·0528 to 0·0589)	0·0516 (0·0489 to 0·0544)	0·0511 (0·0483 to 0·0539)
Yamanashi	0·0412 (0·029 to 0·0555)	0·0408 (0·03 to 0·0538)	0·0429 (0·0329 to 0·0557)	0·0429 (0·0347 to 0·0523)	0·0433 (0·0392 to 0·0475)	0·0429 (0·0406 to 0·0453)	0·0395 (0·0375 to 0·0418)	0·0363 (0·0345 to 0·0384)	0·0356 (0·0337 to 0·0376)
Singapore	0·435 (0·313 to 0·595)	0·353 (0·259 to 0·475)	0·32 (0·245 to 0·424)	0·3 (0·241 to 0·369)	0·282 (0·25 to 0·317)	0·277 (0·258 to 0·299)	0·279 (0·258 to 0·3)	0·278 (0·259 to 0·298)	0·292 (0·269 to 0·315)
South Korea	3·92 (2·77 to 5·59)	3·82 (2·66 to 5·43)	3·7 (2·6 to 5·23)	3·53 (2·5 to 5·05)	3·39 (2·38 to 4·86)	3·19 (2·26 to 4·53)	3 (2·12 to 4·17)	2·78 (1·96 to 3·84)	2·57 (1·86 to 3·48)
High-income North America	31·4 (29·9 to 32·8)	30·7 (29·3 to 32·1)	29·1 (27·8 to 30·4)	28·1 (26·8 to 29·5)	27·4 (26·2 to 28·6)	26·4 (25·4 to 27·6)	25·6 (24·6 to 26·6)	25 (24 to 26)	25 (24·1 to 26)
Canada	2·36 (1·86 to 2·97)	2·26 (1·86 to 2·76)	2·18 (1·79 to 2·63)	2·13 (1·77 to 2·56)	2·13 (1·77 to 2·54)	2·04 (1·68 to 2·45)	1·88 (1·58 to 2·25)	1·75 (1·47 to 2·08)	1·56 (1·38 to 1·74)
Greenland	0·0212 (0·0153 to 0·029)	0·02 (0·0145 to 0·0271)	0·0189 (0·0138 to 0·0258)	0·0174 (0·0128 to 0·0232)	0·0162 (0·0119 to 0·0215)	0·0154 (0·0112 to 0·0204)	0·0146 (0·0106 to 0·0196)	0·0138 (0·00988 to 0·0186)	0·0125 (0·00914 to 0·017)
USA	29 (27·8 to 30·3)	28·4 (27·2 to 29·6)	26·9 (25·8 to 28)	26 (24·8 to 27·2)	25·2 (24·3 to 26·3)	24·4 (23·5 to 25·3)	23·7 (22·8 to 24·6)	23·2 (22·3 to 24·1)	23·4 (22·6 to 24·4)
Alabama	0·583 (0·544 to 0·624)	0·584 (0·543 to 0·625)	0·562 (0·525 to 0·601)	0·542 (0·505 to 0·583)	0·516 (0·482 to 0·554)	0·504 (0·47 to 0·541)	0·501 (0·466 to 0·54)	0·486 (0·455 to 0·525)	0·541 (0·505 to 0·581)
Alaska	0·0589 (0·0536 to 0·0652)	0·0578 (0·0523 to 0·0633)	0·0505 (0·0456 to 0·0561)	0·0442 (0·0397 to 0·0492)	0·0408 (0·037 to 0·0448)	0·0389 (0·0353 to 0·0431)	0·0369 (0·0333 to 0·0409)	0·0385 (0·0348 to 0·0431)	0·0438 (0·0394 to 0·0481)
Arizona	0·38 (0·338 to 0·424)	0·397 (0·354 to 0·441)	0·404 (0·361 to 0·451)	0·393 (0·351 to 0·44)	0·387 (0·342 to 0·434)	0·366 (0·328 to 0·408)	0·393 (0·35 to 0·437)	0·418 (0·373 to 0·469)	0·458 (0·405 to 0·513)
Arkansas	0·252 (0·231 to 0·274)	0·266 (0·243 to 0·291)	0·262 (0·24 to 0·286)	0·23 (0·211 to 0·251)	0·223 (0·204 to 0·244)	0·229 (0·209 to 0·25)	0·243 (0·222 to 0·264)	0·221 (0·202 to 0·241)	0·214 (0·194 to 0·235)
California	3·7 (3·18 to 4·24)	3·62 (3·18 to 4·13)	3·35 (2·93 to 3·84)	3·26 (2·83 to 3·74)	3·23 (2·79 to 3·72)	3·04 (2·65 to 3·49)	2·87 (2·49 to 3·23)	2·78 (2·42 to 3·19)	2·75 (2·39 to 3·13)
Colorado	0·352 (0·318 to 0·39)	0·347 (0·315 to 0·381)	0·31 (0·28 to 0·342)	0·306 (0·278 to 0·338)	0·319 (0·29 to 0·353)	0·317 (0·288 to 0·349)	0·312 (0·282 to 0·343)	0·327 (0·295 to 0·357)	0·358 (0·325 to 0·395)
Connecticut	0·34 (0·289 to 0·395)	0·318 (0·272 to 0·374)	0·27 (0·233 to 0·316)	0·247 (0·214 to 0·281)	0·262 (0·226 to 0·304)	0·26 (0·226 to 0·299)	0·236 (0·205 to 0·27)	0·226 (0·198 to 0·257)	0·221 (0·195 to 0·251)
Delaware	0·0916 (0·0845 to 0·0995)	0·0819 (0·0753 to 0·0893)	0·0722 (0·0643 to 0·082)	0·0695 (0·0592 to 0·0811)	0·0663 (0·0596 to 0·0738)	0·0657 (0·0606 to 0·0711)	0·0606 (0·0557 to 0·0657)	0·0589 (0·0519 to 0·0669)	0·064 (0·0528 to 0·0789)
Florida	1·54 (1·35 to 1·75)	1·45 (1·28 to 1·66)	1·36 (1·18 to 1·55)	1·36 (1·19 to 1·55)	1·4 (1·23 to 1·57)	1·32 (1·15 to 1·49)	1·32 (1·16 to 1·51)	1·32 (1·15 to 1·5)	1·34 (1·17 to 1·54)
Georgia	1·19 (1·01 to 1·37)	1·15 (1 to 1·31)	1 (0·867 to 1·15)	0·97 (0·838 to 1·12)	1·02 (0·874 to 1·18)	0·983 (0·852 to 1·13)	0·957 (0·826 to 1·09)	0·935 (0·809 to 1·08)	0·955 (0·826 to 1·09)
Hawaii	0·133 (0·112 to 0·158)	0·123 (0·104 to 0·144)	0·109 (0·0917 to 0·127)	0·11 (0·0935 to 0·13)	0·105 (0·0885 to 0·125)	0·0968 (0·0812 to 0·114)	0·095 (0·0804 to 0·111)	0·0944 (0·0799 to 0·111)	0·101 (0·0857 to 0·118)
Idaho	0·101 (0·0905 to 0·113)	0·0914 (0·0821 to 0·102)	0·0888 (0·0794 to 0·0989)	0·0859 (0·0761 to 0·096)	0·0882 (0·078 to 0·0984)	0·101 (0·0908 to 0·113)	0·101 (0·0896 to 0·112)	0·0924 (0·0824 to 0·103)	0·0931 (0·0836 to 0·104)
Illinois	1·39 (1·27 to 1·52)	1·43 (1·3 to 1·58)	1·39 (1·26 to 1·52)	1·36 (1·23 to 1·49)	1·25 (1·13 to 1·37)	1·18 (1·07 to 1·28)	1·1 (1 to 1·2)	1·04 (0·943 to 1·14)	1·07 (0·976 to 1·18)
Indiana	0·599 (0·558 to 0·644)	0·589 (0·546 to 0·637)	0·569 (0·527 to 0·613)	0·525 (0·488 to 0·563)	0·522 (0·487 to 0·565)	0·557 (0·518 to 0·598)	0·563 (0·524 to 0·606)	0·513 (0·475 to 0·548)	0·462 (0·429 to 0·497)
Iowa	0·233 (0·208 to 0·264)	0·221 (0·197 to 0·248)	0·206 (0·183 to 0·231)	0·19 (0·169 to 0·214)	0·187 (0·167 to 0·211)	0·186 (0·165 to 0·209)	0·179 (0·16 to 0·201)	0·172 (0·152 to 0·195)	0·182 (0·161 to 0·203)
Kansas	0·19 (0·172 to 0·21)	0·197 (0·18 to 0·216)	0·207 (0·188 to 0·227)	0·215 (0·195 to 0·237)	0·191 (0·173 to 0·21)	0·172 (0·157 to 0·189)	0·177 (0·161 to 0·194)	0·179 (0·163 to 0·197)	0·181 (0·164 to 0·199)
Kentucky	0·352 (0·32 to 0·386)	0·352 (0·32 to 0·387)	0·343 (0·313 to 0·374)	0·341 (0·311 to 0·375)	0·34 (0·309 to 0·375)	0·338 (0·308 to 0·369)	0·321 (0·292 to 0·35)	0·304 (0·277 to 0·332)	0·308 (0·28 to 0·336)
Louisiana	0·573 (0·535 to 0·614)	0·543 (0·507 to 0·582)	0·49 (0·457 to 0·524)	0·497 (0·462 to 0·535)	0·485 (0·452 to 0·521)	0·481 (0·449 to 0·513)	0·493 (0·461 to 0·527)	0·464 (0·433 to 0·497)	0·455 (0·426 to 0·484)
Maine	0·0761 (0·0673 to 0·0868)	0·0789 (0·0693 to 0·0889)	0·077 (0·0675 to 0·0871)	0·0654 (0·0574 to 0·0746)	0·0606 (0·0534 to 0·0688)	0·0663 (0·0584 to 0·0748)	0·0619 (0·0546 to 0·0702)	0·0577 (0·0508 to 0·0649)	0·0566 (0·05 to 0·0635)
Maryland	0·549 (0·467 to 0·638)	0·583 (0·499 to 0·673)	0·596 (0·51 to 0·691)	0·584 (0·506 to 0·672)	0·569 (0·494 to 0·654)	0·549 (0·477 to 0·63)	0·495 (0·432 to 0·563)	0·535 (0·462 to 0·611)	0·575 (0·502 to 0·651)
Massachusetts	0·519 (0·484 to 0·559)	0·466 (0·433 to 0·5)	0·463 (0·429 to 0·498)	0·456 (0·425 to 0·488)	0·434 (0·401 to 0·468)	0·412 (0·382 to 0·442)	0·395 (0·368 to 0·425)	0·387 (0·359 to 0·419)	0·38 (0·353 to 0·411)
Michigan	0·932 (0·702 to 1·24)	0·894 (0·687 to 1·16)	0·814 (0·656 to 0·994)	0·747 (0·657 to 0·846)	0·696 (0·64 to 0·757)	0·674 (0·62 to 0·73)	0·67 (0·621 to 0·726)	0·663 (0·619 to 0·713)	0·691 (0·641 to 0·747)
Minnesota	0·354 (0·317 to 0·395)	0·356 (0·318 to 0·398)	0·331 (0·296 to 0·369)	0·332 (0·296 to 0·37)	0·336 (0·299 to 0·374)	0·328 (0·29 to 0·368)	0·315 (0·281 to 0·353)	0·324 (0·29 to 0·363)	0·328 (0·291 to 0·367)
Mississippi	0·455 (0·417 to 0·495)	0·443 (0·403 to 0·485)	0·451 (0·414 to 0·494)	0·439 (0·402 to 0·479)	0·428 (0·393 to 0·464)	0·403 (0·37 to 0·441)	0·411 (0·376 to 0·449)	0·403 (0·37 to 0·442)	0·377 (0·346 to 0·41)
Missouri	0·524 (0·481 to 0·563)	0·526 (0·486 to 0·572)	0·484 (0·446 to 0·525)	0·462 (0·425 to 0·502)	0·419 (0·388 to 0·453)	0·402 (0·371 to 0·436)	0·43 (0·395 to 0·467)	0·435 (0·4 to 0·469)	0·433 (0·4 to 0·471)
Montana	0·0767 (0·0696 to 0·0839)	0·0742 (0·0677 to 0·0814)	0·0701 (0·0639 to 0·0768)	0·0583 (0·0533 to 0·0636)	0·054 (0·0493 to 0·0589)	0·0553 (0·0505 to 0·0602)	0·0485 (0·0439 to 0·0534)	0·0555 (0·0507 to 0·0608)	0·0624 (0·057 to 0·0683)
Nebraska	0·16 (0·146 to 0·174)	0·16 (0·147 to 0·174)	0·144 (0·133 to 0·156)	0·139 (0·128 to 0·152)	0·126 (0·116 to 0·137)	0·129 (0·119 to 0·139)	0·143 (0·131 to 0·156)	0·136 (0·125 to 0·148)	0·133 (0·122 to 0·145)
Nevada	0·148 (0·129 to 0·17)	0·136 (0·119 to 0·155)	0·128 (0·112 to 0·145)	0·129 (0·113 to 0·148)	0·135 (0·118 to 0·157)	0·136 (0·119 to 0·156)	0·14 (0·122 to 0·16)	0·146 (0·128 to 0·165)	0·148 (0·128 to 0·169)
New Hampshire	0·0985 (0·0889 to 0·109)	0·0889 (0·0801 to 0·0986)	0·0858 (0·0772 to 0·0959)	0·0814 (0·0735 to 0·0906)	0·0669 (0·0604 to 0·0741)	0·0577 (0·0522 to 0·064)	0·0621 (0·056 to 0·0686)	0·0682 (0·0616 to 0·0755)	0·0661 (0·0596 to 0·0729)
New Jersey	0·821 (0·717 to 0·937)	0·787 (0·687 to 0·901)	0·75 (0·658 to 0·853)	0·712 (0·626 to 0·813)	0·676 (0·598 to 0·772)	0·632 (0·555 to 0·715)	0·624 (0·55 to 0·71)	0·601 (0·525 to 0·686)	0·621 (0·546 to 0·703)
New Mexico	0·124 (0·113 to 0·136)	0·124 (0·112 to 0·137)	0·128 (0·116 to 0·143)	0·122 (0·111 to 0·134)	0·119 (0·108 to 0·13)	0·113 (0·103 to 0·124)	0·11 (0·0999 to 0·121)	0·104 (0·0953 to 0·115)	0·104 (0·0945 to 0·114)
New York	2·58 (2·2 to 3·06)	2·65 (2·21 to 3·12)	2·58 (2·17 to 2·98)	2·45 (2·07 to 2·86)	2·19 (1·86 to 2·57)	2·16 (1·83 to 2·53)	2·07 (1·76 to 2·44)	1·99 (1·69 to 2·35)	1·93 (1·64 to 2·29)
North Carolina	0·817 (0·752 to 0·883)	0·831 (0·769 to 0·899)	0·791 (0·729 to 0·855)	0·74 (0·684 to 0·798)	0·756 (0·698 to 0·82)	0·784 (0·726 to 0·849)	0·79 (0·732 to 0·854)	0·764 (0·704 to 0·828)	0·761 (0·703 to 0·824)
North Dakota	0·0771 (0·0688 to 0·0867)	0·0699 (0·062 to 0·079)	0·0601 (0·0535 to 0·0671)	0·0586 (0·0525 to 0·065)	0·0638 (0·0568 to 0·0716)	0·0608 (0·054 to 0·0681)	0·0522 (0·0463 to 0·0584)	0·0501 (0·0447 to 0·0559)	0·046 (0·0411 to 0·0518)
Ohio	1·15 (1·05 to 1·27)	1·11 (1·01 to 1·23)	1·06 (0·966 to 1·18)	1·06 (0·954 to 1·16)	1·02 (0·927 to 1·12)	1·01 (0·915 to 1·11)	0·936 (0·847 to 1·04)	0·91 (0·823 to 1·01)	0·916 (0·83 to 1·01)
Oklahoma	0·322 (0·292 to 0·351)	0·304 (0·28 to 0·332)	0·292 (0·267 to 0·319)	0·325 (0·297 to 0·357)	0·317 (0·288 to 0·348)	0·302 (0·276 to 0·33)	0·294 (0·268 to 0·321)	0·292 (0·265 to 0·321)	0·289 (0·263 to 0·317)
Oregon	0·242 (0·218 to 0·268)	0·232 (0·209 to 0·257)	0·207 (0·187 to 0·228)	0·206 (0·186 to 0·227)	0·227 (0·205 to 0·252)	0·235 (0·211 to 0·259)	0·231 (0·209 to 0·256)	0·224 (0·202 to 0·249)	0·213 (0·192 to 0·236)
Pennsylvania	1·33 (1·18 to 1·51)	1·27 (1·12 to 1·42)	1·17 (1·03 to 1·31)	1·12 (0·993 to 1·27)	1·09 (0·961 to 1·23)	1 (0·882 to 1·13)	0·945 (0·842 to 1·07)	0·945 (0·827 to 1·08)	0·941 (0·831 to 1·06)
Rhode Island	0·0859 (0·0772 to 0·095)	0·0833 (0·0757 to 0·0918)	0·0881 (0·0794 to 0·0964)	0·0843 (0·0759 to 0·0929)	0·0695 (0·0634 to 0·0768)	0·0632 (0·0569 to 0·0695)	0·0615 (0·056 to 0·0679)	0·0683 (0·062 to 0·0751)	0·0741 (0·0668 to 0·0819)
South Carolina	0·55 (0·503 to 0·6)	0·539 (0·493 to 0·587)	0·515 (0·472 to 0·559)	0·501 (0·455 to 0·547)	0·456 (0·417 to 0·498)	0·439 (0·402 to 0·48)	0·425 (0·389 to 0·465)	0·407 (0·372 to 0·444)	0·403 (0·37 to 0·438)
South Dakota	0·0746 (0·0657 to 0·0847)	0·0721 (0·0636 to 0·0816)	0·0609 (0·0533 to 0·0696)	0·0513 (0·045 to 0·0584)	0·0496 (0·0432 to 0·0566)	0·052 (0·0453 to 0·0591)	0·0466 (0·0409 to 0·0532)	0·0402 (0·0352 to 0·0454)	0·0426 (0·0375 to 0·0483)
Tennessee	0·52 (0·45 to 0·602)	0·487 (0·421 to 0·556)	0·455 (0·397 to 0·517)	0·446 (0·392 to 0·506)	0·445 (0·388 to 0·506)	0·456 (0·399 to 0·522)	0·444 (0·388 to 0·508)	0·441 (0·387 to 0·51)	0·457 (0·396 to 0·525)
Texas	2·02 (1·81 to 2·24)	1·95 (1·75 to 2·17)	1·87 (1·68 to 2·08)	1·84 (1·65 to 2·04)	1·84 (1·64 to 2·06)	1·73 (1·55 to 1·91)	1·67 (1·5 to 1·86)	1·67 (1·51 to 1·86)	1·72 (1·54 to 1·92)
Utah	0·195 (0·173 to 0·224)	0·202 (0·177 to 0·227)	0·201 (0·177 to 0·228)	0·19 (0·167 to 0·215)	0·193 (0·17 to 0·22)	0·202 (0·178 to 0·23)	0·203 (0·18 to 0·231)	0·195 (0·17 to 0·224)	0·194 (0·171 to 0·219)
Vermont	0·0413 (0·0365 to 0·0466)	0·0422 (0·0375 to 0·0477)	0·0363 (0·0321 to 0·041)	0·0295 (0·0259 to 0·0334)	0·0286 (0·025 to 0·0325)	0·0294 (0·0259 to 0·0333)	0·0285 (0·0252 to 0·0322)	0·0257 (0·0226 to 0·029)	0·027 (0·0239 to 0·0303)
Virginia	0·714 (0·616 to 0·827)	0·689 (0·583 to 0·803)	0·678 (0·576 to 0·796)	0·638 (0·542 to 0·746)	0·611 (0·533 to 0·703)	0·584 (0·51 to 0·669)	0·563 (0·489 to 0·644)	0·563 (0·488 to 0·637)	0·606 (0·529 to 0·699)
Washington	0·404 (0·344 to 0·482)	0·401 (0·339 to 0·473)	0·386 (0·325 to 0·452)	0·362 (0·308 to 0·424)	0·362 (0·307 to 0·425)	0·363 (0·31 to 0·426)	0·371 (0·317 to 0·433)	0·374 (0·318 to 0·435)	0·394 (0·335 to 0·46)
Washington, DC	0·328 (0·289 to 0·374)	0·3 (0·258 to 0·347)	0·259 (0·223 to 0·3)	0·236 (0·208 to 0·268)	0·216 (0·192 to 0·244)	0·187 (0·167 to 0·207)	0·174 (0·157 to 0·193)	0·16 (0·145 to 0·176)	0·145 (0·131 to 0·159)
West Virginia	0·181 (0·166 to 0·198)	0·164 (0·15 to 0·178)	0·162 (0·149 to 0·176)	0·16 (0·146 to 0·174)	0·147 (0·136 to 0·159)	0·148 (0·136 to 0·16)	0·139 (0·128 to 0·15)	0·138 (0·127 to 0·15)	0·137 (0·126 to 0·148)

Location	1999	2000	2001	2002	2003	2004	2005	2006	2007	2008	2009	2010	2011	2012	2013	2014	2015	2016	2017	2018	2019	2020	2021
Global	[illegible]	[illegible]	[illegible]	[illegible]	[illegible]	[illegible]	4060 (3590 to 4620)	4020 (3570 to 4520)	3940 (3520 to 4420)	3880 (3480 to 4350)	3840 (3450 to 4310)	3760 (3390 to 4190)	3710 (3350 to 4120)	3670 (3320 to 4070)	3610 (3270 to 4010)	3540 (3190 to 3940)	3490 (3130 to 3880)	3400 (3050 to 3810)	3310 (2950 to 3750)	3220 (2840 to 3710)	3130 (2720 to 3650)	3030 (2610 to 3580)	2930 (2510 to 3480)
Central Europe, eastern Europe, and central Asia	[illegible]	[illegible]	[illegible]	[illegible]	[illegible]	[illegible]	57·8 (55·8 to 60)	59·4 (56·9 to 62)	61·5 (58·7 to 64·5)	63·1 (60·4 to 66·2)	64·4 (61·9 to 67·2)	63·9 (61·4 to 66·7)	64·3 (61·8 to 66·9)	66·4 (63·4 to 69·3)	67·2 (63·4 to 71·1)	68·5 (65·5 to 71·9)	66·3 (63·5 to 69·2)	64·6 (62 to 67·6)	61·7 (59·2 to 64·5)	56·9 (54·2 to 60)	56·9 (53·6 to 60·8)	55·5 (50·3 to 61·2)	54·5 (47·3 to 62·4)
Central Asia	[illegible]	[illegible]	[illegible]	[illegible]	[illegible]	[illegible]	32·6 (30·9 to 34·6)	33·8 (31·9 to 35·9)	35·2 (32·6 to 38·1)	37·2 (34·6 to 39·9)	38·4 (36·3 to 40·9)	39·2 (37·1 to 41·4)	39·5 (37·3 to 41·8)	39·6 (37·3 to 41·9)	40·5 (38 to 43)	42·5 (40·3 to 44·7)	42·8 (40·6 to 45)	42·6 (40·5 to 45·1)	41·2 (39 to 43·5)	37·5 (35·5 to 39·7)	37·6 (34·9 to 40·5)	38 (33·6 to 42·7)	37·8 (32·1 to 44·3)
Armenia	[illegible]	[illegible]	[illegible]	[illegible]	[illegible]	[illegible]	0·887 (0·746 to 1·06)	1·01 (0·828 to 1·22)	1·01 (0·816 to 1·22)	1 (0·825 to 1·21)	0·96 (0·785 to 1·17)	0·939 (0·77 to 1·14)	0·97 (0·788 to 1·17)	0·935 (0·766 to 1·13)	0·93 (0·761 to 1·12)	0·964 (0·812 to 1·14)	1·05 (0·905 to 1·24)	1·09 (0·955 to 1·26)	1·02 (0·905 to 1·15)	0·985 (0·871 to 1·1)	0·86 (0·761 to 0·966)	0·82 (0·717 to 0·93)	0·774 (0·645 to 0·925)
Azerbaijan	[illegible]	[illegible]	[illegible]	[illegible]	[illegible]	[illegible]	4·12 (3·75 to 4·52)	4·19 (3·79 to 4·63)	3·93 (3·6 to 4·27)	3·86 (3·55 to 4·19)	3·8 (3·49 to 4·11)	3·83 (3·5 to 4·19)	4·13 (3·75 to 4·56)	3·87 (3·56 to 4·18)	3·45 (3·19 to 3·74)	3·71 (3·44 to 4·05)	4·1 (3·78 to 4·44)	4·57 (4·21 to 4·97)	4·32 (3·96 to 4·72)	3·25 (2·94 to 3·59)	2·88 (2·49 to 3·32)	2·89 (2·29 to 3·61)	2·83 (2·12 to 3·66)
Georgia	[illegible]	[illegible]	[illegible]	[illegible]	[illegible]	[illegible]	0·892 (0·803 to 0·996)	0·811 (0·724 to 0·893)	0·717 (0·646 to 0·796)	0·609 (0·551 to 0·677)	0·559 (0·504 to 0·62)	0·557 (0·501 to 0·62)	0·565 (0·507 to 0·632)	0·573 (0·515 to 0·638)	0·563 (0·506 to 0·62)	0·561 (0·505 to 0·628)	0·54 (0·484 to 0·602)	0·542 (0·502 to 0·587)	0·515 (0·484 to 0·548)	0·496 (0·468 to 0·527)	0·495 (0·466 to 0·525)	0·468 (0·441 to 0·496)	0·42 (0·381 to 0·466)
Kazakhstan	[illegible]	[illegible]	[illegible]	[illegible]	[illegible]	[illegible]	3·91 (3·53 to 4·3)	4·18 (3·78 to 4·6)	4·9 (4·46 to 5·37)	5·61 (5·04 to 6·18)	5·34 (4·83 to 5·91)	5·6 (5·1 to 6·17)	5·41 (4·96 to 5·95)	5·03 (4·56 to 5·51)	4·59 (4·17 to 5·04)	4·39 (4 to 4·79)	4·28 (3·9 to 4·7)	4·17 (3·79 to 4·58)	3·86 (3·51 to 4·24)	3·6 (3·28 to 3·97)	3·74 (3·33 to 4·19)	3·84 (3·25 to 4·53)	3·61 (2·84 to 4·59)
Kyrgyzstan	[illegible]	[illegible]	[illegible]	[illegible]	[illegible]	[illegible]	2·41 (2·17 to 2·69)	2·49 (2·21 to 2·79)	2·62 (2·35 to 2·95)	2·59 (2·32 to 2·91)	2·54 (2·26 to 2·84)	2·47 (2·2 to 2·77)	2·33 (2·09 to 2·61)	2·2 (1·96 to 2·46)	2·17 (1·95 to 2·42)	2·1 (1·88 to 2·33)	2·04 (1·82 to 2·28)	1·98 (1·78 to 2·2)	1·8 (1·61 to 2·01)	1·75 (1·54 to 1·98)	1·81 (1·59 to 2·04)	1·74 (1·52 to 1·98)	1·67 (1·38 to 2)
Mongolia	[illegible]	[illegible]	[illegible]	[illegible]	[illegible]	[illegible]	[illegible]	[illegible]	[illegible]	[illegible]	[illegible]	[illegible]	[illegible]	[illegible]	[illegible]	[illegible]	[illegible]	[illegible]	[illegible]	[illegible]	[illegible]	[illegible]	[illegible]
Tajikistan	[illegible]	[illegible]	[illegible]	[illegible]	[illegible]	[illegible]	[illegible]	[illegible]	[illegible]	[illegible]	[illegible]	[illegible]	[illegible]	[illegible]	[illegible]	[illegible]	[illegible]	[illegible]	[illegible]	[illegible]	[illegible]	[illegible]	[illegible]
Turkmenistan	[illegible]	[illegible]	[illegible]	[illegible]	[illegible]	[illegible]	[illegible]	[illegible]	[illegible]	[illegible]	[illegible]	[illegible]	[illegible]	[illegible]	[illegible]	[illegible]	[illegible]	[illegible]	[illegible]	[illegible]	[illegible]	[illegible]	[illegible]
Uzbekistan	[illegible]	[illegible]	[illegible]	[illegible]	[illegible]	[illegible]	[illegible]	[illegible]	[illegible]	[illegible]	[illegible]	[illegible]	[illegible]	[illegible]	[illegible]	[illegible]	[illegible]	[illegible]	[illegible]	[illegible]	[illegible]	[illegible]	[illegible]
Central Europe	[illegible]	[illegible]	[illegible]	[illegible]	[illegible]	[illegible]	[illegible]	[illegible]	[illegible]	[illegible]	[illegible]	[illegible]	[illegible]	[illegible]	[illegible]	[illegible]	[illegible]	[illegible]	[illegible]	[illegible]	[illegible]	[illegible]	[illegible]
Albania	[illegible]	[illegible]	[illegible]	[illegible]	[illegible]	[illegible]	[illegible]	[illegible]	[illegible]	[illegible]	[illegible]	[illegible]	[illegible]	[illegible]	[illegible]	[illegible]	[illegible]	[illegible]	[illegible]	[illegible]	[illegible]	[illegible]	[illegible]
Bosnia and Herzegovina	[illegible]	[illegible]	[illegible]	[illegible]	[illegible]	[illegible]	[illegible]	[illegible]	[illegible]	[illegible]	[illegible]	[illegible]	[illegible]	[illegible]	[illegible]	[illegible]	[illegible]	[illegible]	[illegible]	[illegible]	[illegible]	[illegible]	[illegible]
Bulgaria	[illegible]	[illegible]	[illegible]	[illegible]	[illegible]	[illegible]	[illegible]	[illegible]	[illegible]	[illegible]	[illegible]	[illegible]	[illegible]	[illegible]	[illegible]	[illegible]	[illegible]	[illegible]	[illegible]	[illegible]	[illegible]	[illegible]	[illegible]
Croatia	[illegible]	[illegible]	[illegible]	[illegible]	[illegible]	[illegible]	[illegible]	[illegible]	[illegible]	[illegible]	[illegible]	[illegible]	[illegible]	[illegible]	[illegible]	[illegible]	[illegible]	[illegible]	[illegible]	[illegible]	[illegible]	[illegible]	[illegible]
Czechia	[illegible]	[illegible]	[illegible]	[illegible]	[illegible]	[illegible]	[illegible]	[illegible]	[illegible]	[illegible]	[illegible]	[illegible]	[illegible]	[illegible]	[illegible]	[illegible]	[illegible]	[illegible]	[illegible]	[illegible]	[illegible]	[illegible]	[illegible]
Hungary	[illegible]	[illegible]	[illegible]	[illegible]	[illegible]	[illegible]	[illegible]	[illegible]	[illegible]	[illegible]	[illegible]	[illegible]	[illegible]	[illegible]	[illegible]	[illegible]	[illegible]	[illegible]	[illegible]	[illegible]	[illegible]	[illegible]	[illegible]
Montenegro	[illegible]	[illegible]	[illegible]	[illegible]	[illegible]	[illegible]	[illegible]	[illegible]	[illegible]	[illegible]	[illegible]	[illegible]	[illegible]	[illegible]	[illegible]	[illegible]	[illegible]	[illegible]	[illegible]	[illegible]	[illegible]	[illegible]	[illegible]
North Macedonia	[illegible]	[illegible]	[illegible]	[illegible]	[illegible]	[illegible]	[illegible]	[illegible]	[illegible]	[illegible]	[illegible]	[illegible]	[illegible]	[illegible]	[illegible]	[illegible]	[illegible]	[illegible]	[illegible]	[illegible]	[illegible]	[illegible]	[illegible]
Poland	[illegible]	[illegible]	[illegible]	[illegible]	[illegible]	[illegible]	[illegible]	[illegible]	[illegible]	[illegible]	[illegible]	[illegible]	[illegible]	[illegible]	[illegible]	[illegible]	[illegible]	[illegible]	[illegible]	[illegible]	[illegible]	[illegible]	[illegible]
Romania	[illegible]	[illegible]	[illegible]	[illegible]	[illegible]	[illegible]	[illegible]	[illegible]	[illegible]	[illegible]	[illegible]	[illegible]	[illegible]	[illegible]	[illegible]	[illegible]	[illegible]	[illegible]	[illegible]	[illegible]	[illegible]	[illegible]	[illegible]
Serbia	[illegible]	[illegible]	[illegible]	[illegible]	[illegible]	[illegible]	[illegible]	[illegible]	[illegible]	[illegible]	[illegible]	[illegible]	[illegible]	[illegible]	[illegible]	[illegible]	[illegible]	[illegible]	[illegible]	[illegible]	[illegible]	[illegible]	[illegible]
Slovakia	[illegible]	[illegible]	[illegible]	[illegible]	[illegible]	[illegible]	[illegible]	[illegible]	[illegible]	[illegible]	[illegible]	[illegible]	[illegible]	[illegible]	[illegible]	[illegible]	[illegible]	[illegible]	[illegible]	[illegible]	[illegible]	[illegible]	[illegible]
Slovenia	[illegible]	[illegible]	[illegible]	[illegible]	[illegible]	[illegible]	[illegible]	[illegible]	[illegible]	[illegible]	[illegible]	[illegible]	[illegible]	[illegible]	[illegible]	[illegible]	[illegible]	[illegible]	[illegible]	[illegible]	[illegible]	[illegible]	[illegible]
Eastern Europe	[illegible]	[illegible]	[illegible]	[illegible]	[illegible]	[illegible]	[illegible]	[illegible]	[illegible]	[illegible]	[illegible]	[illegible]	[illegible]	[illegible]	[illegible]	[illegible]	[illegible]	[illegible]	[illegible]	[illegible]	[illegible]	[illegible]	[illegible]
Belarus	[illegible]	[illegible]	[illegible]	[illegible]	[illegible]	[illegible]	[illegible]	[illegible]	[illegible]	[illegible]	[illegible]	[illegible]	[illegible]	[illegible]	[illegible]	[illegible]	[illegible]	[illegible]	[illegible]	[illegible]	[illegible]	[illegible]	[illegible]
Estonia	[illegible]	[illegible]	[illegible]	[illegible]	[illegible]	[illegible]	[illegible]	[illegible]	[illegible]	[illegible]	[illegible]	[illegible]	[illegible]	[illegible]	[illegible]	[illegible]	[illegible]	[illegible]	[illegible]	[illegible]	[illegible]	[illegible]	[illegible]
Latvia	[illegible]	[illegible]	[illegible]	[illegible]	[illegible]	[illegible]	[illegible]	[illegible]	[illegible]	[illegible]	[illegible]	[illegible]	[illegible]	[illegible]	[illegible]	[illegible]	[illegible]	[illegible]	[illegible]	[illegible]	[illegible]	[illegible]	[illegible]
Lithuania	[illegible]	[illegible]	[illegible]	[illegible]	[illegible]	[illegible]	[illegible]	[illegible]	[illegible]	[illegible]	[illegible]	[illegible]	[illegible]	[illegible]	[illegible]	[illegible]	[illegible]	[illegible]	[illegible]	[illegible]	[illegible]	[illegible]	[illegible]
Moldova	[illegible]	[illegible]	[illegible]	[illegible]	[illegible]	[illegible]	[illegible]	[illegible]	[illegible]	[illegible]	[illegible]	[illegible]	[illegible]	[illegible]	[illegible]	[illegible]	[illegible]	[illegible]	[illegible]	[illegible]	[illegible]	[illegible]	[illegible]
Russia	[illegible]	[illegible]	[illegible]	[illegible]	[illegible]	[illegible]	[illegible]	[illegible]	[illegible]	[illegible]	[illegible]	[illegible]	[illegible]	[illegible]	[illegible]	[illegible]	[illegible]	[illegible]	[illegible]	[illegible]	[illegible]	[illegible]	[illegible]
Ukraine	[illegible]	[illegible]	[illegible]	[illegible]	[illegible]	[illegible]	[illegible]	[illegible]	[illegible]	[illegible]	[illegible]	[illegible]	[illegible]	[illegible]	[illegible]	[illegible]	[illegible]	[illegible]	[illegible]	[illegible]	[illegible]	[illegible]	[illegible]
High income	[illegible]	[illegible]	[illegible]	[illegible]	[illegible]	[illegible]	[illegible]	[illegible]	[illegible]	[illegible]	[illegible]	[illegible]	[illegible]	[illegible]	[illegible]	[illegible]	[illegible]	[illegible]	[illegible]	[illegible]	[illegible]	[illegible]	[illegible]
Australasia	[illegible]	[illegible]	[illegible]	[illegible]	[illegible]	[illegible]	[illegible]	[illegible]	[illegible]	[illegible]	[illegible]	[illegible]	[illegible]	[illegible]	[illegible]	[illegible]	[illegible]	[illegible]	[illegible]	[illegible]	[illegible]	[illegible]	[illegible]
Australia	[illegible]	[illegible]	[illegible]	[illegible]	[illegible]	[illegible]	[illegible]	[illegible]	[illegible]	[illegible]	[illegible]	[illegible]	[illegible]	[illegible]	[illegible]	[illegible]	[illegible]	[illegible]	[illegible]	[illegible]	[illegible]	[illegible]	[illegible]
New Zealand	[illegible]	[illegible]	[illegible]	[illegible]	[illegible]	[illegible]	[illegible]	[illegible]	[illegible]	[illegible]	[illegible]	[illegible]	[illegible]	[illegible]	[illegible]	[illegible]	[illegible]	[illegible]	[illegible]	[illegible]	[illegible]	[illegible]	[illegible]
High-income Asia Pacific	[illegible]	[illegible]	[illegible]	[illegible]	[illegible]	[illegible]	[illegible]	[illegible]	[illegible]	[illegible]	[illegible]	[illegible]	[illegible]	[illegible]	[illegible]	[illegible]	[illegible]	[illegible]	[illegible]	[illegible]	[illegible]	[illegible]	[illegible]
Brunei	[illegible]	[illegible]	[illegible]	[illegible]	[illegible]	[illegible]	[illegible]	[illegible]	[illegible]	[illegible]	[illegible]	[illegible]	[illegible]	[illegible]	[illegible]	[illegible]	[illegible]	[illegible]	[illegible]	[illegible]	[illegible]	[illegible]	[illegible]
Japan	[illegible]	[illegible]	[illegible]	[illegible]	[illegible]	[illegible]	[illegible]	[illegible]	[illegible]	[illegible]	[illegible]	[illegible]	[illegible]	[illegible]	[illegible]	[illegible]	[illegible]	[illegible]	[illegible]	[illegible]	[illegible]	[illegible]	[illegible]
Aichi	[illegible]	[illegible]	[illegible]	[illegible]	[illegible]	[illegible]	[illegible]	[illegible]	[illegible]	[illegible]	[illegible]	[illegible]	[illegible]	[illegible]	[illegible]	[illegible]	[illegible]	[illegible]	[illegible]	[illegible]	[illegible]	[illegible]	[illegible]
Akita	[illegible]	[illegible]	[illegible]	[illegible]	[illegible]	[illegible]	[illegible]	[illegible]	[illegible]	[illegible]	[illegible]	[illegible]	[illegible]	[illegible]	[illegible]	[illegible]	[illegible]	[illegible]	[illegible]	[illegible]	[illegible]	[illegible]	[illegible]
Aomori	[illegible]	[illegible]	[illegible]	[illegible]	[illegible]	[illegible]	[illegible]	[illegible]	[illegible]	[illegible]	[illegible]	[illegible]	[illegible]	[illegible]	[illegible]	[illegible]	[illegible]	[illegible]	[illegible]	[illegible]	[illegible]	[illegible]	[illegible]
Chiba	[illegible]	[illegible]	[illegible]	[illegible]	[illegible]	[illegible]	[illegible]	[illegible]	[illegible]	[illegible]	[illegible]	[illegible]	[illegible]	[illegible]	[illegible]	[illegible]	[illegible]	[illegible]	[illegible]	[illegible]	[illegible]	[illegible]	[illegible]
Ehime	[illegible]	[illegible]	[illegible]	[illegible]	[illegible]	[illegible]	[illegible]	[illegible]	[illegible]	[illegible]	[illegible]	[illegible]	[illegible]	[illegible]	[illegible]	[illegible]	[illegible]	[illegible]	[illegible]	[illegible]	[illegible]	[illegible]	[illegible]
Fukui	[illegible]	[illegible]	[illegible]	[illegible]	[illegible]	[illegible]	[illegible]	[illegible]	[illegible]	[illegible]	[illegible]	[illegible]	[illegible]	[illegible]	[illegible]	[illegible]	[illegible]	[illegible]	[illegible]	[illegible]	[illegible]	[illegible]	[illegible]
Fukuoka	[illegible]	[illegible]	[illegible]	[illegible]	[illegible]	[illegible]	[illegible]	[illegible]	[illegible]	[illegible]	[illegible]	[illegible]	[illegible]	[illegible]	[illegible]	[illegible]	[illegible]	[illegible]	[illegible]	[illegible]	[illegible]	[illegible]	[illegible]
Fukushima	[illegible]	[illegible]	[illegible]	[illegible]	[illegible]	[illegible]	[illegible]	[illegible]	[illegible]	[illegible]	[illegible]	[illegible]	[illegible]	[illegible]	[illegible]	[illegible]	[illegible]	[illegible]	[illegible]	[illegible]	[illegible]	[illegible]	[illegible]
Gifu	[illegible]	[illegible]	[illegible]	[illegible]	[illegible]	[illegible]	[illegible]	[illegible]	[illegible]	[illegible]	[illegible]	[illegible]	[illegible]	[illegible]	[illegible]	[illegible]	[illegible]	[illegible]	[illegible]	[illegible]	[illegible]	[illegible]	[illegible]
Gunma	[illegible]	[illegible]	[illegible]	[illegible]	[illegible]	[illegible]	[illegible]	[illegible]	[illegible]	[illegible]	[illegible]	[illegible]	[illegible]	[illegible]	[illegible]	[illegible]	[illegible]	[illegible]	[illegible]	[illegible]	[illegible]	[illegible]	[illegible]
Hiroshima	[illegible]	[illegible]	[illegible]	[illegible]	[illegible]	[illegible]	[illegible]	[illegible]	[illegible]	[illegible]	[illegible]	[illegible]	[illegible]	[illegible]	[illegible]	[illegible]	[illegible]	[illegible]	[illegible]	[illegible]	[illegible]	[illegible]	[illegible]
Hokkaidō	[illegible]	[illegible]	[illegible]	[illegible]	[illegible]	[illegible]	[illegible]	[illegible]	[illegible]	[illegible]	[illegible]	[illegible]	[illegible]	[illegible]	[illegible]	[illegible]	[illegible]	[illegible]	[illegible]	[illegible]	[illegible]	[illegible]	[illegible]
Hyōgo	[illegible]	[illegible]	[illegible]	[illegible]	[illegible]	[illegible]	[illegible]	[illegible]	[illegible]	[illegible]	[illegible]	[illegible]	[illegible]	[illegible]	[illegible]	[illegible]	[illegible]	[illegible]	[illegible]	[illegible]	[illegible]	[illegible]	[illegible]
Ibaraki	[illegible]	[illegible]	[illegible]	[illegible]	[illegible]	[illegible]	[illegible]	[illegible]	[illegible]	[illegible]	[illegible]	[illegible]	[illegible]	[illegible]	[illegible]	[illegible]	[illegible]	[illegible]	[illegible]	[illegible]	[illegible]	[illegible]	[illegible]
Ishikawa	[illegible]	[illegible]	[illegible]	[illegible]	[illegible]	[illegible]	[illegible]	[illegible]	[illegible]	[illegible]	[illegible]	[illegible]	[illegible]	[illegible]	[illegible]	[illegible]	[illegible]	[illegible]	[illegible]	[illegible]	[illegible]	[illegible]	[illegible]
Iwate	[illegible]	[illegible]	[illegible]	[illegible]	[illegible]	[illegible]	[illegible]	[illegible]	[illegible]	[illegible]	[illegible]	[illegible]	[illegible]	[illegible]	[illegible]	[illegible]	[illegible]	[illegible]	[illegible]	[illegible]	[illegible]	[illegible]	[illegible]
Kagawa	[illegible]	[illegible]	[illegible]	[illegible]	[illegible]	[illegible]	[illegible]	[illegible]	[illegible]	[illegible]	[illegible]	[illegible]	[illegible]	[illegible]	[illegible]	[illegible]	[illegible]	[illegible]	[illegible]	[illegible]	[illegible]	[illegible]	[illegible]
Kagoshima	[illegible]	[illegible]	[illegible]	[illegible]	[illegible]	[illegible]	[illegible]	[illegible]	[illegible]	[illegible]	[illegible]	[illegible]	[illegible]	[illegible]	[illegible]	[illegible]	[illegible]	[illegible]	[illegible]	[illegible]	[illegible]	[illegible]	[illegible]
Kanagawa	[illegible]	[illegible]	[illegible]	[illegible]	[illegible]	[illegible]	[illegible]	[illegible]	[illegible]	[illegible]	[illegible]	[illegible]	[illegible]	[illegible]	[illegible]	[illegible]	[illegible]	[illegible]	[illegible]	[illegible]	[illegible]	[illegible]	[illegible]
Kōchi	[illegible]	[illegible]	[illegible]	[illegible]	[illegible]	[illegible]	[illegible]	[illegible]	[illegible]	[illegible]	[illegible]	[illegible]	[illegible]	[illegible]	[illegible]	[illegible]	[illegible]	[illegible]	[illegible]	[illegible]	[illegible]	[illegible]	[illegible]
Kumamoto	[illegible]	[illegible]	[illegible]	[illegible]	[illegible]	[illegible]	[illegible]	[illegible]	[illegible]	[illegible]	[illegible]	[illegible]	[illegible]	[illegible]	[illegible]	[illegible]	[illegible]	[illegible]	[illegible]	[illegible]	[illegible]	[illegible]	[illegible]
Kyōto	[illegible]	[illegible]	[illegible]	[illegible]	[illegible]	[illegible]	[illegible]	[illegible]	[illegible]	[illegible]	[illegible]	[illegible]	[illegible]	[illegible]	[illegible]	[illegible]	[illegible]	[illegible]	[illegible]	[illegible]	[illegible]	[illegible]	[illegible]
Mie	[illegible]	[illegible]	[illegible]	[illegible]	[illegible]	[illegible]	[illegible]	[illegible]	[illegible]	[illegible]	[illegible]	[illegible]	[illegible]	[illegible]	[illegible]	[illegible]	[illegible]	[illegible]	[illegible]	[illegible]	[illegible]	[illegible]	[illegible]
Miyagi	[illegible]	[illegible]	[illegible]	[illegible]	[illegible]	[illegible]	[illegible]	[illegible]	[illegible]	[illegible]	[illegible]	[illegible]	[illegible]	[illegible]	[illegible]	[illegible]	[illegible]	[illegible]	[illegible]	[illegible]	[illegible]	[illegible]	[illegible]
Miyazaki	[illegible]	[illegible]	[illegible]	[illegible]	[illegible]	[illegible]	[illegible]	[illegible]	[illegible]	[illegible]	[illegible]	[illegible]	[illegible]	[illegible]	[illegible]	[illegible]	[illegible]	[illegible]	[illegible]	[illegible]	[illegible]	[illegible]	[illegible]
Nagano	[illegible]	[illegible]	[illegible]	[illegible]	[illegible]	[illegible]	[illegible]	[illegible]	[illegible]	[illegible]	[illegible]	[illegible]	[illegible]	[illegible]	[illegible]	[illegible]	[illegible]	[illegible]	[illegible]	[illegible]	[illegible]	[illegible]	[illegible]
Nagasaki	[illegible]	[illegible]	[illegible]	[illegible]	[illegible]	[illegible]	[illegible]	[illegible]	[illegible]	[illegible]	[illegible]	[illegible]	[illegible]	[illegible]	[illegible]	[illegible]	[illegible]	[illegible]	[illegible]	[illegible]	[illegible]	[illegible]	[illegible]
Nara	[illegible]	[illegible]	[illegible]	[illegible]	[illegible]	[illegible]	[illegible]	[illegible]	[illegible]	[illegible]	[illegible]	[illegible]	[illegible]	[illegible]	[illegible]	[illegible]	[illegible]	[illegible]	[illegible]	[illegible]	[illegible]	[illegible]	[illegible]
Niigata	[illegible]	[illegible]	[illegible]	[illegible]	[illegible]	[illegible]	[illegible]	[illegible]	[illegible]	[illegible]	[illegible]	[illegible]	[illegible]	[illegible]	[illegible]	[illegible]	[illegible]	[illegible]	[illegible]	[illegible]	[illegible]	[illegible]	[illegible]
Ōita	[illegible]	[illegible]	[illegible]	[illegible]	[illegible]	[illegible]	[illegible]	[illegible]	[illegible]	[illegible]	[illegible]	[illegible]	[illegible]	[illegible]	[illegible]	[illegible]	[illegible]	[illegible]	[illegible]	[illegible]	[illegible]	[illegible]	[illegible]
Okayama	[illegible]	[illegible]	[illegible]	[illegible]	[illegible]	[illegible]	[illegible]	[illegible]	[illegible]	[illegible]	[illegible]	[illegible]	[illegible]	[illegible]	[illegible]	[illegible]	[illegible]	[illegible]	[illegible]	[illegible]	[illegible]	[illegible]	[illegible]
Okinawa	[illegible]	[illegible]	[illegible]	[illegible]	[illegible]	[illegible]	[illegible]	[illegible]	[illegible]	[illegible]	[illegible]	[illegible]	[illegible]	[illegible]	[illegible]	[illegible]	[illegible]	[illegible]	[illegible]	[illegible]	[illegible]	[illegible]	[illegible]
Ōsaka	[illegible]	[illegible]	[illegible]	[illegible]	[illegible]	[illegible]	[illegible]	[illegible]	[illegible]	[illegible]	[illegible]	[illegible]	[illegible]	[illegible]	[illegible]	[illegible]	[illegible]	[illegible]	[illegible]	[illegible]	[illegible]	[illegible]	[illegible]
Saga	[illegible]	[illegible]	[illegible]	[illegible]	[illegible]	[illegible]	[illegible]	[illegible]	[illegible]	[illegible]	[illegible]	[illegible]	[illegible]	[illegible]	[illegible]	[illegible]	[illegible]	[illegible]	[illegible]	[illegible]	[illegible]	[illegible]	[illegible]
Saitama	[illegible]	[illegible]	[illegible]	[illegible]	[illegible]	[illegible]	[illegible]	[illegible]	[illegible]	[illegible]	[illegible]	[illegible]	[illegible]	[illegible]	[illegible]	[illegible]	[illegible]	[illegible]	[illegible]	[illegible]	[illegible]	[illegible]	[illegible]
Shiga	[illegible]	[illegible]	[illegible]	[illegible]	[illegible]	[illegible]	[illegible]	[illegible]	[illegible]	[illegible]	[illegible]	[illegible]	[illegible]	[illegible]	[illegible]	[illegible]	[illegible]	[illegible]	[illegible]	[illegible]	[illegible]	[illegible]	[illegible]
Shimane	[illegible]	[illegible]	[illegible]	[illegible]	[illegible]	[illegible]	[illegible]	[illegible]	[illegible]	[illegible]	[illegible]	[illegible]	[illegible]	[illegible]	[illegible]	[illegible]	[illegible]	[illegible]	[illegible]	[illegible]	[illegible]	[illegible]	[illegible]
Shizuoka	[illegible]	[illegible]	[illegible]	[illegible]	[illegible]	[illegible]	[illegible]	[illegible]	[illegible]	[illegible]	[illegible]	[illegible]	[illegible]	[illegible]	[illegible]	[illegible]	[illegible]	[illegible]	[illegible]	[illegible]	[illegible]	[illegible]	[illegible]
Tochigi	[illegible]	[illegible]	[illegible]	[illegible]	[illegible]	[illegible]	[illegible]	[illegible]	[illegible]	[illegible]	[illegible]	[illegible]	[illegible]	[illegible]	[illegible]	[illegible]	[illegible]	[illegible]	[illegible]	[illegible]	[illegible]	[illegible]	[illegible]
Tokushima	[illegible]	[illegible]	[illegible]	[illegible]	[illegible]	[illegible]	[illegible]	[illegible]	[illegible]	[illegible]	[illegible]	[illegible]	[illegible]	[illegible]	[illegible]	[illegible]	[illegible]	[illegible]	[illegible]	[illegible]	[illegible]	[illegible]	[illegible]
Tōkyō	[illegible]	[illegible]	[illegible]	[illegible]	[illegible]	[illegible]	[illegible]	[illegible]	[illegible]	[illegible]	[illegible]	[illegible]	[illegible]	[illegible]	[illegible]	[illegible]	[illegible]	[illegible]	[illegible]	[illegible]	[illegible]	[illegible]	[illegible]
Tottori	[illegible]	[illegible]	[illegible]	[illegible]	[illegible]	[illegible]	[illegible]	[illegible]	[illegible]	[illegible]	[illegible]	[illegible]	[illegible]	[illegible]	[illegible]	[illegible]	[illegible]	[illegible]	[illegible]	[illegible]	[illegible]	[illegible]	[illegible]
Toyama	[illegible]	[illegible]	[illegible]	[illegible]	[illegible]	[illegible]	[illegible]	[illegible]	[illegible]	[illegible]	[illegible]	[illegible]	[illegible]	[illegible]	[illegible]	[illegible]	[illegible]	[illegible]	[illegible]	[illegible]	[illegible]	[illegible]	[illegible]
Wakayama	[illegible]	[illegible]	[illegible]	[illegible]	[illegible]	[illegible]	[illegible]	[illegible]	[illegible]	[illegible]	[illegible]	[illegible]	[illegible]	[illegible]	[illegible]	[illegible]	[illegible]	[illegible]	[illegible]	[illegible]	[illegible]	[illegible]	[illegible]
Yamagata	[illegible]	[illegible]	[illegible]	[illegible]	[illegible]	[illegible]	[illegible]	[illegible]	[illegible]	[illegible]	[illegible]	[illegible]	[illegible]	[illegible]	[illegible]	[illegible]	[illegible]	[illegible]	[illegible]	[illegible]	[illegible]	[illegible]	[illegible]
Yamaguchi	[illegible]	[illegible]	[illegible]	[illegible]	[illegible]	[illegible]	[illegible]	[illegible]	[illegible]	[illegible]	[illegible]	[illegible]	[illegible]	[illegible]	[illegible]	[illegible]	[illegible]	[illegible]	[illegible]	[illegible]	[illegible]	[illegible]	[illegible]
Yamanashi	[illegible]	[illegible]	[illegible]	[illegible]	[illegible]	[illegible]	[illegible]	[illegible]	[illegible]	[illegible]	[illegible]	[illegible]	[illegible]	[illegible]	[illegible]	[illegible]	[illegible]	[illegible]	[illegible]	[illegible]	[illegible]	[illegible]	[illegible]
Singapore	[illegible]	[illegible]	[illegible]	[illegible]	[illegible]	[illegible]	[illegible]	[illegible]	[illegible]	[illegible]	[illegible]	[illegible]	[illegible]	[illegible]	[illegible]	[illegible]	[illegible]	[illegible]	[illegible]	[illegible]	[illegible]	[illegible]	[illegible]
South Korea	[illegible]	[illegible]	[illegible]	[illegible]	[illegible]	[illegible]	[illegible]	[illegible]	[illegible]	[illegible]	[illegible]	[illegible]	[illegible]	[illegible]	[illegible]	[illegible]	[illegible]	[illegible]	[illegible]	[illegible]	[illegible]	[illegible]	[illegible]
High-income North America	[illegible]	[illegible]	[illegible]	[illegible]	[illegible]	[illegible]	[illegible]	[illegible]	[illegible]	[illegible]	[illegible]	[illegible]	[illegible]	[illegible]	[illegible]	[illegible]	[illegible]	[illegible]	[illegible]	[illegible]	[illegible]	[illegible]	[illegible]
Canada	[illegible]	[illegible]	[illegible]	[illegible]	[illegible]	[illegible]	[illegible]	[illegible]	[illegible]	[illegible]	[illegible]	[illegible]	[illegible]	[illegible]	[illegible]	[illegible]	[illegible]	[illegible]	[illegible]	[illegible]	[illegible]	[illegible]	[illegible]
Greenland	[illegible]	[illegible]	[illegible]	[illegible]	[illegible]	[illegible]	[illegible]	[illegible]	[illegible]	[illegible]	[illegible]	[illegible]	[illegible]	[illegible]	[illegible]	[illegible]	[illegible]	[illegible]	[illegible]	[illegible]	[illegible]	[illegible]	[illegible]
USA	[illegible]	[illegible]	[illegible]	[illegible]	[illegible]	[illegible]	[illegible]	[illegible]	[illegible]	[illegible]	[illegible]	[illegible]	[illegible]	[illegible]	[illegible]	[illegible]	[illegible]	[illegible]	[illegible]	[illegible]	[illegible]	[illegible]	[illegible]
Alabama	[illegible]	[illegible]	[illegible]	[illegible]	[illegible]	[illegible]	[illegible]	[illegible]	[illegible]	[illegible]	[illegible]	[illegible]	[illegible]	[illegible]	[illegible]	[illegible]	[illegible]	[illegible]	[illegible]	[illegible]	[illegible]	[illegible]	[illegible]
Alaska	[illegible]	[illegible]	[illegible]	[illegible]	[illegible]	[illegible]	[illegible]	[illegible]	[illegible]	[illegible]	[illegible]	[illegible]	[illegible]	[illegible]	[illegible]	[illegible]	[illegible]	[illegible]	[illegible]	[illegible]	[illegible]	[illegible]	[illegible]
Arizona	[illegible]	[illegible]	[illegible]	[illegible]	[illegible]	[illegible]	[illegible]	[illegible]	[illegible]	[illegible]	[illegible]	[illegible]	[illegible]	[illegible]	[illegible]	[illegible]	[illegible]	[illegible]	[illegible]	[illegible]	[illegible]	[illegible]	[illegible]
Arkansas	[illegible]	[illegible]	[illegible]	[illegible]	[illegible]	[illegible]	[illegible]	[illegible]	[illegible]	[illegible]	[illegible]	[illegible]	[illegible]	[illegible]	[illegible]	[illegible]	[illegible]	[illegible]	[illegible]	[illegible]	[illegible]	[illegible]	[illegible]
California	[illegible]	[illegible]	[illegible]	[illegible]	[illegible]	[illegible]	[illegible]	[illegible]	[illegible]	[illegible]	[illegible]	[illegible]	[illegible]	[illegible]	[illegible]	[illegible]	[illegible]	[illegible]	[illegible]	[illegible]	[illegible]	[illegible]	[illegible]
Colorado	[illegible]	[illegible]	[illegible]	[illegible]	[illegible]	[illegible]	[illegible]	[illegible]	[illegible]	[illegible]	[illegible]	[illegible]	[illegible]	[illegible]	[illegible]	[illegible]	[illegible]	[illegible]	[illegible]	[illegible]	[illegible]	[illegible]	[illegible]
Connecticut	[illegible]	[illegible]	[illegible]	[illegible]	[illegible]	[illegible]	[illegible]	[illegible]	[illegible]	[illegible]	[illegible]	[illegible]	[illegible]	[illegible]	[illegible]	[illegible]	[illegible]	[illegible]	[illegible]	[illegible]	[illegible]	[illegible]	[illegible]
Delaware	[illegible]	[illegible]	[illegible]	[illegible]	[illegible]	[illegible]	[illegible]	[illegible]	[illegible]	[illegible]	[illegible]	[illegible]	[illegible]	[illegible]	[illegible]	[illegible]	[illegible]	[illegible]	[illegible]	[illegible]	[illegible]	[illegible]	[illegible]
Florida	[illegible]	[illegible]	[illegible]	[illegible]	[illegible]	[illegible]	[illegible]	[illegible]	[illegible]	[illegible]	[illegible]	[illegible]	[illegible]	[illegible]	[illegible]	[illegible]	[illegible]	[illegible]	[illegible]	[illegible]	[illegible]	[illegible]	[illegible]
Georgia	[illegible]	[illegible]	[illegible]	[illegible]	[illegible]	[illegible]	[illegible]	[illegible]	[illegible]	[illegible]	[illegible]	[illegible]	[illegible]	[illegible]	[illegible]	[illegible]	[illegible]	[illegible]	[illegible]	[illegible]	[illegible]	[illegible]	[illegible]
Hawaii	[illegible]	[illegible]	[illegible]	[illegible]	[illegible]	[illegible]	[illegible]	[illegible]	[illegible]	[illegible]	[illegible]	[illegible]	[illegible]	[illegible]	[illegible]	[illegible]	[illegible]	[illegible]	[illegible]	[illegible]	[illegible]	[illegible]	[illegible]
Idaho	[illegible]	[illegible]	[illegible]	[illegible]	[illegible]	[illegible]	[illegible]	[illegible]	[illegible]	[illegible]	[illegible]	[illegible]	[illegible]	[illegible]	[illegible]	[illegible]	[illegible]	[illegible]	[illegible]	[illegible]	[illegible]	[illegible]	[illegible]
Illinois	[illegible]	[illegible]	[illegible]	[illegible]	[illegible]	[illegible]	[illegible]	[illegible]	[illegible]	[illegible]	[illegible]	[illegible]	[illegible]	[illegible]	[illegible]	[illegible]	[illegible]	[illegible]	[illegible]	[illegible]	[illegible]	[illegible]	[illegible]
Indiana	[illegible]	[illegible]	[illegible]	[illegible]	[illegible]	[illegible]	[illegible]	[illegible]	[illegible]	[illegible]	[illegible]	[illegible]	[illegible]	[illegible]	[illegible]	[illegible]	[illegible]	[illegible]	[illegible]	[illegible]	[illegible]	[illegible]	[illegible]
Iowa	[illegible]	[illegible]	[illegible]	[illegible]	[illegible]	[illegible]	[illegible]	[illegible]	[illegible]	[illegible]	[illegible]	[illegible]	[illegible]	[illegible]	[illegible]	[illegible]	[illegible]	[illegible]	[illegible]	[illegible]	[illegible]	[illegible]	[illegible]
Kansas	[illegible]	[illegible]	[illegible]	[illegible]	[illegible]	[illegible]	[illegible]	[illegible]	[illegible]	[illegible]	[illegible]	[illegible]	[illegible]	[illegible]	[illegible]	[illegible]	[illegible]	[illegible]	[illegible]	[illegible]	[illegible]	[illegible]	[illegible]
Kentucky	[illegible]	[illegible]	[illegible]	[illegible]	[illegible]	[illegible]	[illegible]	[illegible]	[illegible]	[illegible]	[illegible]	[illegible]	[illegible]	[illegible]	[illegible]	[illegible]	[illegible]	[illegible]	[illegible]	[illegible]	[illegible]	[illegible]	[illegible]
Louisiana	[illegible]	[illegible]	[illegible]	[illegible]	[illegible]	[illegible]	[illegible]	[illegible]	[illegible]	[illegible]	[illegible]	[illegible]	[illegible]	[illegible]	[illegible]	[illegible]	[illegible]	[illegible]	[illegible]	[illegible]	[illegible]	[illegible]	[illegible]
Maine	[illegible]	[illegible]	[illegible]	[illegible]	[illegible]	[illegible]	[illegible]	[illegible]	[illegible]	[illegible]	[illegible]	[illegible]	[illegible]	[illegible]	[illegible]	[illegible]	[illegible]	[illegible]	[illegible]	[illegible]	[illegible]	[illegible]	[illegible]
Maryland	[illegible]	[illegible]	[illegible]	[illegible]	[illegible]	[illegible]	[illegible]	[illegible]	[illegible]	[illegible]	[illegible]	[illegible]	[illegible]	[illegible]	[illegible]	[illegible]	[illegible]	[illegible]	[illegible]	[illegible]	[illegible]	[illegible]	[illegible]
Massachusetts	[illegible]	[illegible]	[illegible]	[illegible]	[illegible]	[illegible]	[illegible]	[illegible]	[illegible]	[illegible]	[illegible]	[illegible]	[illegible]	[illegible]	[illegible]	[illegible]	[illegible]	[illegible]	[illegible]	[illegible]	[illegible]	[illegible]	[illegible]
Michigan	[illegible]	[illegible]	[illegible]	[illegible]	[illegible]	[illegible]	[illegible]	[illegible]	[illegible]	[illegible]	[illegible]	[illegible]	[illegible]	[illegible]	[illegible]	[illegible]	[illegible]	[illegible]	[illegible]	[illegible]	[illegible]	[illegible]	[illegible]
Minnesota	[illegible]	[illegible]	[illegible]	[illegible]	[illegible]	[illegible]	[illegible]	[illegible]	[illegible]	[illegible]	[illegible]	[illegible]	[illegible]	[illegible]	[illegible]	[illegible]	[illegible]	[illegible]	[illegible]	[illegible]	[illegible]	[illegible]	[illegible]
Mississippi	[illegible]	[illegible]	[illegible]	[illegible]	[illegible]	[illegible]	[illegible]	[illegible]	[illegible]	[illegible]	[illegible]	[illegible]	[illegible]	[illegible]	[illegible]	[illegible]	[illegible]	[illegible]	[illegible]	[illegible]	[illegible]	[illegible]	[illegible]
Missouri	[illegible]	[illegible]	[illegible]	[illegible]	[illegible]	[illegible]	[illegible]	[illegible]	[illegible]	[illegible]	[illegible]	[illegible]	[illegible]	[illegible]	[illegible]	[illegible]	[illegible]	[illegible]	[illegible]	[illegible]	[illegible]	[illegible]	[illegible]
Montana	[illegible]	[illegible]	[illegible]	[illegible]	[illegible]	[illegible]	[illegible]	[illegible]	[illegible]	[illegible]	[illegible]	[illegible]	[illegible]	[illegible]	[illegible]	[illegible]	[illegible]	[illegible]	[illegible]	[illegible]	[illegible]	[illegible]	[illegible]
Nebraska	[illegible]	[illegible]	[illegible]	[illegible]	[illegible]	[illegible]	[illegible]	[illegible]	[illegible]	[illegible]	[illegible]	[illegible]	[illegible]	[illegible]	[illegible]	[illegible]	[illegible]	[illegible]	[illegible]	[illegible]	[illegible]	[illegible]	[illegible]
Nevada	[illegible]	[illegible]	[illegible]	[illegible]	[illegible]	[illegible]	[illegible]	[illegible]	[illegible]	[illegible]	[illegible]	[illegible]	[illegible]	[illegible]	[illegible]	[illegible]	[illegible]	[illegible]	[illegible]	[illegible]	[illegible]	[illegible]	[illegible]
New Hampshire	[illegible]	[illegible]	[illegible]	[illegible]	[illegible]	[illegible]	[illegible]	[illegible]	[illegible]	[illegible]	[illegible]	[illegible]	[illegible]	[illegible]	[illegible]	[illegible]	[illegible]	[illegible]	[illegible]	[illegible]	[illegible]	[illegible]	[illegible]
New Jersey	[illegible]	[illegible]	[illegible]	[illegible]	[illegible]	[illegible]	[illegible]	[illegible]	[illegible]	[illegible]	[illegible]	[illegible]	[illegible]	[illegible]	[illegible]	[illegible]	[illegible]	[illegible]	[illegible]	[illegible]	[illegible]	[illegible]	[illegible]
New Mexico	[illegible]	[illegible]	[illegible]	[illegible]	[illegible]	[illegible]	[illegible]	[illegible]	[illegible]	[illegible]	[illegible]	[illegible]	[illegible]	[illegible]	[illegible]	[illegible]	[illegible]	[illegible]	[illegible]	[illegible]	[illegible]	[illegible]	[illegible]
New York	[illegible]	[illegible]	[illegible]	[illegible]	[illegible]	[illegible]	[illegible]	[illegible]	[illegible]	[illegible]	[illegible]	[illegible]	[illegible]	[illegible]	[illegible]	[illegible]	[illegible]	[illegible]	[illegible]	[illegible]	[illegible]	[illegible]	[illegible]
North Carolina	[illegible]	[illegible]	[illegible]	[illegible]	[illegible]	[illegible]	[illegible]	[illegible]	[illegible]	[illegible]	[illegible]	[illegible]	[illegible]	[illegible]	[illegible]	[illegible]	[illegible]	[illegible]	[illegible]	[illegible]	[illegible]	[illegible]	[illegible]
North Dakota	[illegible]	[illegible]	[illegible]	[illegible]	[illegible]	[illegible]	[illegible]	[illegible]	[illegible]	[illegible]	[illegible]	[illegible]	[illegible]	[illegible]	[illegible]	[illegible]	[illegible]	[illegible]	[illegible]	[illegible]	[illegible]	[illegible]	[illegible]
Ohio	[illegible]	[illegible]	[illegible]	[illegible]	[illegible]	[illegible]	[illegible]	[illegible]	[illegible]	[illegible]	[illegible]	[illegible]	[illegible]	[illegible]	[illegible]	[illegible]	[illegible]	[illegible]	[illegible]	[illegible]	[illegible]	[illegible]	[illegible]
Oklahoma	[illegible]	[illegible]	[illegible]	[illegible]	[illegible]	[illegible]	[illegible]	[illegible]	[illegible]	[illegible]	[illegible]	[illegible]	[illegible]	[illegible]	[illegible]	[illegible]	[illegible]	[illegible]	[illegible]	[illegible]	[illegible]	[illegible]	[illegible]
Oregon	[illegible]	[illegible]	[illegible]	[illegible]	[illegible]	[illegible]	[illegible]	[illegible]	[illegible]	[illegible]	[illegible]	[illegible]	[illegible]	[illegible]	[illegible]	[illegible]	[illegible]	[illegible]	[illegible]	[illegible]	[illegible]	[illegible]	[illegible]
Pennsylvania	[illegible]	[illegible]	[illegible]	[illegible]	[illegible]	[illegible]	[illegible]	[illegible]	[illegible]	[illegible]	[illegible]	[illegible]	[illegible]	[illegible]	[illegible]	[illegible]	[illegible]	[illegible]	[illegible]	[illegible]	[illegible]	[illegible]	[illegible]
Rhode Island	[illegible]	[illegible]	[illegible]	[illegible]	[illegible]	[illegible]	[illegible]	[illegible]	[illegible]	[illegible]	[illegible]	[illegible]	[illegible]	[illegible]	[illegible]	[illegible]	[illegible]	[illegible]	[illegible]	[illegible]	[illegible]	[illegible]	[illegible]
South Carolina	[illegible]	[illegible]	[illegible]	[illegible]	[illegible]	[illegible]	[illegible]	[illegible]	[illegible]	[illegible]	[illegible]	[illegible]	[illegible]	[illegible]	[illegible]	[illegible]	[illegible]	[illegible]	[illegible]	[illegible]	[illegible]	[illegible]	[illegible]
South Dakota	[illegible]	[illegible]	[illegible]	[illegible]	[illegible]	[illegible]	[illegible]	[illegible]	[illegible]	[illegible]	[illegible]	[illegible]	[illegible]	[illegible]	[illegible]	[illegible]	[illegible]	[illegible]	[illegible]	[illegible]	[illegible]	[illegible]	[illegible]
Tennessee	[illegible]	[illegible]	[illegible]	[illegible]	[illegible]	[illegible]	[illegible]	[illegible]	[illegible]	[illegible]	[illegible]	[illegible]	[illegible]	[illegible]	[illegible]	[illegible]	[illegible]	[illegible]	[illegible]	[illegible]	[illegible]	[illegible]	[illegible]
Texas	[illegible]	[illegible]	[illegible]	[illegible]	[illegible]	[illegible]	[illegible]	[illegible]	[illegible]	[illegible]	[illegible]	[illegible]	[illegible]	[illegible]	[illegible]	[illegible]	[illegible]	[illegible]	[illegible]	[illegible]	[illegible]	[illegible]	[illegible]
Utah	[illegible]	[illegible]	[illegible]	[illegible]	[illegible]	[illegible]	[illegible]	[illegible]	[illegible]	[illegible]	[illegible]	[illegible]	[illegible]	[illegible]	[illegible]	[illegible]	[illegible]	[illegible]	[illegible]	[illegible]	[illegible]	[illegible]	[illegible]
Vermont	[illegible]	[illegible]	[illegible]	[illegible]	[illegible]	[illegible]	[illegible]	[illegible]	[illegible]	[illegible]	[illegible]	[illegible]	[illegible]	[illegible]	[illegible]	[illegible]	[illegible]	[illegible]	[illegible]	[illegible]	[illegible]	[illegible]	[illegible]
Virginia	[illegible]	[illegible]	[illegible]	[illegible]	[illegible]	[illegible]	[illegible]	[illegible]	[illegible]	[illegible]	[illegible]	[illegible]	[illegible]	[illegible]	[illegible]	[illegible]	[illegible]	[illegible]	[illegible]	[illegible]	[illegible]	[illegible]	[illegible]
Washington	[illegible]	[illegible]	[illegible]	[illegible]	[illegible]	[illegible]	[illegible]	[illegible]	[illegible]	[illegible]	[illegible]	[illegible]	[illegible]	[illegible]	[illegible]	[illegible]	[illegible]	[illegible]	[illegible]	[illegible]	[illegible]	[illegible]	[illegible]
Washington, DC	[illegible]	[illegible]	[illegible]	[illegible]	[illegible]	[illegible]	[illegible]	[illegible]	[illegible]	[illegible]	[illegible]	[illegible]	[illegible]	[illegible]	[illegible]	[illegible]	[illegible]	[illegible]	[illegible]	[illegible]	[illegible]	[illegible]	[illegible]
West Virginia	[illegible]	[illegible]	[illegible]	[illegible]	[illegible]	[illegible]	[illegible]	[illegible]	[illegible]	[illegible]	[illegible]	[illegible]	[illegible]	[illegible]	[illegible]	[illegible]	[illegible]	[illegible]	[illegible]	[illegible]	[illegible]	[illegible]	[illegible]

Wisconsin	0·407 (0·38 to 0·438)	0·433 (0·402 to 0·468)	0·392 (0·363 to 0·422)	0·386 (0·358 to 0·416)
Wyoming	0·0474 (0·0424 to 0·0527)	0·0455 (0·0408 to 0·0503)	0·038 (0·0341 to 0·0419)	0·0331 (0·0297 to 0·0367)
Southern Latin America	14·6 (12·2 to 17·9)	14·1 (11·8 to 17·3)	13·5 (11·5 to 16·2)	12·8 (11·2 to 14·8)
Argentina	8·31 (5·97 to 11·4)	8·32 (6·08 to 11·2)	7·98 (6·07 to 10·6)	7·76 (6·15 to 9·62)
Chile	5·56 (5·06 to 6·13)	5·04 (4·56 to 5·56)	4·84 (4·38 to 5·37)	4·32 (3·88 to 4·78)
Uruguay	0·756 (0·575 to 0·995)	0·739 (0·585 to 0·937)	0·683 (0·6 to 0·779)	0·737 (0·683 to 0·798)
Western Europe	31·5 (28·8 to 35·1)	30·3 (27·9 to 33·2)	28·4 (26·4 to 30·7)	27 (25·2 to 28·9)
Andorra	0·00442 (0·00316 to 0·0062)	0·00473 (0·00336 to 0·00647)	0·00535 (0·00384 to 0·00717)	0·00478 (0·00346 to 0·0065)
Austria	0·512 (0·477 to 0·548)	0·52 (0·484 to 0·557)	0·541 (0·504 to 0·581)	0·5 (0·466 to 0·535)
Belgium	0·965 (0·688 to 1·32)	0·974 (0·692 to 1·31)	0·957 (0·706 to 1·26)	0·932 (0·709 to 1·2)
Cyprus	0·175 (0·125 to 0·24)	0·17 (0·12 to 0·237)	0·159 (0·114 to 0·227)	0·147 (0·104 to 0·212)
Denmark	0·427 (0·373 to 0·489)	0·451 (0·39 to 0·52)	0·469 (0·406 to 0·54)	0·442 (0·382 to 0·501)
Finland	0·303 (0·282 to 0·327)	0·299 (0·278 to 0·322)	0·276 (0·255 to 0·295)	0·256 (0·237 to 0·277)
France	5·42 (5·08 to 5·78)	5·24 (4·9 to 5·58)	4·86 (4·55 to 5·17)	4·63 (4·33 to 4·95)
Germany	4·99 (4·69 to 5·3)	4·68 (4·41 to 4·96)	4·27 (4·02 to 4·52)	3·97 (3·74 to 4·21)
Greece	0·822 (0·689 to 0·961)	0·755 (0·64 to 0·888)	0·724 (0·614 to 0·85)	0·724 (0·615 to 0·852)
Iceland	0·0224 (0·0196 to 0·0255)	0·0238 (0·021 to 0·0269)	0·0215 (0·0188 to 0·0245)	0·0204 (0·0179 to 0·0232)
Ireland	0·382 (0·351 to 0·413)	0·379 (0·352 to 0·408)	0·357 (0·33 to 0·385)	0·351 (0·316 to 0·387)
Israel	0·635 (0·581 to 0·69)	0·67 (0·615 to 0·731)	0·726 (0·665 to 0·792)	0·745 (0·687 to 0·814)
Italy	4·12 (3·65 to 4·61)	3·97 (3·53 to 4·45)	3·72 (3·31 to 4·22)	3·38 (2·99 to 3·78)
Luxembourg	0·032 (0·0278 to 0·0366)	0·0355 (0·0311 to 0·0407)	0·0315 (0·0276 to 0·0359)	0·027 (0·0237 to 0·0308)
Malta	0·0435 (0·0327 to 0·057)	0·0419 (0·0329 to 0·0535)	0·0394 (0·0318 to 0·0482)	0·0402 (0·0333 to 0·0485)
Monaco	0·0016 (0·00112 to 0·00216)	0·00153 (0·00108 to 0·00211)	0·00149 (0·00106 to 0·00206)	0·00146 (0·00103 to 0·00201)
Netherlands	1·74 (1·26 to 2·38)	1·65 (1·21 to 2·23)	1·57 (1·19 to 2·08)	1·56 (1·24 to 1·94)
Norway	0·436 (0·41 to 0·463)	0·427 (0·405 to 0·451)	0·414 (0·39 to 0·438)	0·409 (0·386 to 0·433)
Agder	0·0241 (0·0212 to 0·0274)	0·0255 (0·0221 to 0·0294)	0·0243 (0·0211 to 0·0274)	0·0199 (0·0175 to 0·0225)
Innlandet	0·0299 (0·0262 to 0·0341)	0·0297 (0·0264 to 0·0334)	0·033 (0·0291 to 0·0375)	0·0349 (0·0306 to 0·0398)
Møre og Romsdal	0·0218 (0·0186 to 0·0253)	0·0182 (0·0156 to 0·0213)	0·0194 (0·0167 to 0·0223)	0·0232 (0·02 to 0·0267)
Nordland	0·0214 (0·0184 to 0·0247)	0·0271 (0·0235 to 0·0312)	0·0266 (0·0229 to 0·0307)	0·0254 (0·0217 to 0·0292)
Oslo	0·0565 (0·0506 to 0·0627)	0·054 (0·0486 to 0·0599)	0·046 (0·041 to 0·0513)	0·0465 (0·0417 to 0·0517)
Rogaland	0·0415 (0·0369 to 0·0466)	0·0394 (0·0349 to 0·0443)	0·0356 (0·0316 to 0·04)	0·0362 (0·0322 to 0·0408)
Troms og Finnmark	0·026 (0·0223 to 0·03)	0·0221 (0·0189 to 0·0255)	0·0206 (0·0177 to 0·0242)	0·0213 (0·0183 to 0·0247)
Trøndelag	0·0276 (0·024 to 0·0315)	0·0308 (0·0272 to 0·0351)	0·0363 (0·0316 to 0·041)	0·0394 (0·0345 to 0·0446)
Vestfold og Telemark	0·0439 (0·0376 to 0·0511)	0·0412 (0·0352 to 0·0481)	0·0366 (0·0313 to 0·042)	0·0288 (0·0249 to 0·0332)
Vestland	0·0579 (0·0516 to 0·0646)	0·0543 (0·0484 to 0·0611)	0·0505 (0·0451 to 0·0563)	0·0498 (0·0443 to 0·0553)
Viken	0·086 (0·0761 to 0·0975)	0·0847 (0·0749 to 0·0953)	0·0845 (0·0751 to 0·0947)	0·0836 (0·0743 to 0·094)
Portugal	1·2 (1·13 to 1·29)	1·2 (1·12 to 1·29)	1·08 (1·01 to 1·15)	1·01 (0·942 to 1·08)
San Marino	0·00282 (0·00201 to 0·004)	0·00278 (0·00199 to 0·00396)	0·00277 (0·00199 to 0·00383)	0·00276 (0·00199 to 0·00372)
Spain	2·51 (1·84 to 3·49)	2·34 (1·71 to 3·17)	2·29 (1·72 to 3·05)	2·09 (1·64 to 2·63)
Sweden	0·579 (0·534 to 0·628)	0·574 (0·529 to 0·622)	0·529 (0·488 to 0·57)	0·508 (0·468 to 0·55)
Stockholm	0·119 (0·11 to 0·129)	0·116 (0·107 to 0·126)	0·109 (0·101 to 0·118)	0·108 (0·0999 to 0·117)
Sweden except Stockholm	0·46 (0·424 to 0·499)	0·458 (0·422 to 0·496)	0·419 (0·387 to 0·452)	0·4 (0·369 to 0·433)
Switzerland	0·438 (0·38 to 0·503)	0·412 (0·358 to 0·474)	0·415 (0·355 to 0·48)	0·381 (0·329 to 0·438)
[illegible]	[illegible]	[illegible]	[illegible]	[illegible]

Medway	0·0289 (0·0188 to 0·0435)	0·0263 (0·0165 to 0·04)
Milton Keynes	0·0211 (0·0134 to 0·0318)	0·02 (0·012 to 0·031)
Oxfordshire	0·0474 (0·0334 to 0·0667)	0·0441 (0·0322 to 0·0595)
Portsmouth	0·0213 (0·015 to 0·03)	0·0196 (0·0143 to 0·0264)
Reading	0·0205 (0·0134 to 0·0305)	0·0199 (0·0121 to 0·0304)
Slough	0·0164 (0·0104 to 0·0247)	0·0152 (0·00921 to 0·0233)
Southampton	0·0284 (0·0187 to 0·0421)	0·0254 (0·0163 to 0·0373)
Surrey	0·0745 (0·051 to 0·11)	0·0676 (0·0447 to 0·0972)
West Berkshire	0·0152 (0·00989 to 0·0225)	0·0141 (0·00888 to 0·0216)
West Sussex	0·054 (0·0351 to 0·0811)	0·0502 (0·0314 to 0·0764)
Windsor and Maidenhead	0·0095 (0·00602 to 0·0143)	0·00911 (0·00546 to 0·0141)
Wokingham	0·0116 (0·00753 to 0·0175)	0·0109 (0·00684 to 0·0166)
South West England	0·387 (0·276 to 0·547)	0·358 (0·251 to 0·495)
Bath and North East Somerset	0·0129 (0·00926 to 0·0179)	0·0119 (0·00817 to 0·0167)
Bournemouth	0·0117 (0·0075 to 0·018)	0·011 (0·00719 to 0·0163)
Bristol, City of	0·0402 (0·028 to 0·0574)	0·0371 (0·0253 to 0·0521)
Cornwall	0·042 (0·0317 to 0·0549)	0·0389 (0·0282 to 0·0529)
Devon	0·0446 (0·0311 to 0·0638)	0·0406 (0·0277 to 0·0572)
Dorset	0·0211 (0·0143 to 0·0307)	0·0196 (0·0127 to 0·0289)
Gloucestershire	0·0481 (0·0323 to 0·0707)	0·045 (0·0291 to 0·0663)
North Somerset	0·0119 (0·00828 to 0·017)	0·0109 (0·00741 to 0·0153)
Plymouth	0·0207 (0·0139 to 0·03)	0·0194 (0·0127 to 0·0281)
Poole	0·012 (0·00848 to 0·0167)	0·0112 (0·00769 to 0·0159)
Somerset	0·0267 (0·0175 to 0·0402)	0·0245 (0·0175 to 0·0347)
South Gloucestershire	0·0222 (0·0155 to 0·0318)	0·021 (0·0141 to 0·0302)
Swindon	0·0183 (0·012 to 0·0274)	0·0164 (0·0112 to 0·0232)
Torbay	0·0145 (0·0098 to 0·0216)	0·0136 (0·00876 to 0·0203)
Wiltshire	0·0397 (0·0275 to 0·0574)	0·037 (0·025 to 0·053)
West Midlands	0·636 (0·454 to 0·901)	0·584 (0·41 to 0·808)
Birmingham	0·14 (0·0967 to 0·207)	0·131 (0·0884 to 0·187)
Coventry	0·048 (0·034 to 0·0681)	0·0439 (0·0319 to 0·059)
Dudley	0·0372 (0·0234 to 0·0565)	0·0341 (0·0202 to 0·0531)
Herefordshire, County of	0·0111 (0·00781 to 0·0158)	0·00983 (0·00717 to 0·0134)
Sandwell	0·0485 (0·0343 to 0·0687)	0·0445 (0·0324 to 0·0605)
Shropshire	0·027 (0·0187 to 0·0396)	0·025 (0·0171 to 0·0354)
Solihull	0·021 (0·0148 to 0·0298)	0·0187 (0·0134 to 0·0256)
Staffordshire	0·0844 (0·0561 to 0·127)	0·0768 (0·048 to 0·116)
Stoke-on-Trent	0·0286 (0·0199 to 0·0418)	0·0258 (0·0181 to 0·0363)
Telford and Wrekin	0·0275 (0·0177 to 0·0419)	0·0254 (0·0159 to 0·0391)
Walsall	0·031 (0·02 to 0·0471)	0·0286 (0·018 to 0·0437)
Warwickshire	0·0411 (0·029 to 0·0583)	0·0372 (0·0266 to 0·0505)
Wolverhampton	0·0429 (0·0276 to 0·0651)	0·0395 (0·0247 to 0·0608)
Worcestershire	0·0476 (0·0337 to 0·0696)	0·0441 (0·031 to 0·062)
Yorkshire and the Humber	0·477 (0·34 to 0·675)	0·461 (0·323 to 0·638)
Barnsley	0·0329 (0·0227 to 0·0476)	0·032 (0·0209 to 0·0469)
Bradford	0·0589 (0·0397 to 0·0852)	0·0566 (0·0397 to 0·0795)
Calderdale	0·0224 (0·0148 to 0·0335)	0·0215 (0·0147 to 0·0306)
Doncaster	0·0229 (0·0153 to 0·0334)	0·0224 (0·0157 to 0·0312)
East Riding of Yorkshire	0·0205 (0·014 to 0·0294)	0·0198 (0·013 to 0·0288)
Kingston upon Hull, City of	0·0394 (0·0261 to 0·0581)	0·0378 (0·0239 to 0·0559)
Kirklees	0·037 (0·0256 to 0·0533)	0·0355 (0·0238 to 0·0509)
Leeds	0·0587 (0·0407 to 0·0845)	0·0565 (0·0378 to 0·0809)
North East Lincolnshire	0·00953 (0·00643 to 0·0138)	0·00898 (0·00631 to 0·0126)
North Lincolnshire	0·0165 (0·0115 to 0·023)	0·0159 (0·0106 to 0·0228)
North Yorkshire	0·0328 (0·0228 to 0·0472)	0·0313 (0·021 to 0·0448)
Rotherham	0·0322 (0·0216 to 0·0471)	0·0312 (0·0224 to 0·044)
Sheffield	0·0534 (0·0358 to 0·0784)	0·0535 (0·0339 to 0·0792)
Wakefield	0·0292 (0·0202 to 0·0421)	0·0281 (0·0188 to 0·0402)
York	0·0106 (0·00707 to 0·0155)	0·0102 (0·00644 to 0·0151)
Northern Ireland	0·201 (0·182 to 0·221)	0·195 (0·178 to 0·213)
Scotland	0·539 (0·5 to 0·579)	0·511 (0·476 to 0·545)
Wales	0·257 (0·184 to 0·364)	0·242 (0·17 to 0·335)
Latin America and Caribbean	291 (237 to 357)	283 (236 to 340)
Andean Latin America	23·1 (20·8 to 26·1)	22·8 (20·8 to 25·1)
Bolivia	6·58 (5·55 to 7·77)	6·46 (5·64 to 7·38)
Ecuador	3·92 (2·77 to 5·62)	3·77 (2·7 to 5·26)
Peru	12·6 (11·4 to 14)	12·6 (11·5 to 13·8)
Caribbean	36·5 (28·6 to 46)	35·5 (27·9 to 44·4)
Antigua and Barbuda	0·0225 (0·0157 to 0·0311)	0·023 (0·016 to 0·0316)
Barbados	0·0591 (0·0435 to 0·0787)	0·0574 (0·0423 to 0·0789)
Belize	0·191 (0·139 to 0·256)	0·189 (0·14 to 0·246)
Bermuda	0·0143 (0·0104 to 0·0195)	0·0136 (0·00979 to 0·0187)
Cuba	2·82 (2·66 to 2·99)	2·73 (2·59 to 2·88)
Dominica	0·0368 (0·0258 to 0·0512)	0·0374 (0·026 to 0·0513)
Dominican Republic	10 (7·03 to 14·3)	10·1 (7·06 to 14·5)
Grenada	0·069 (0·0503 to 0·0912)	0·0655 (0·0486 to 0·0853)
Guyana	1·09 (0·761 to 1·53)	1·06 (0·751 to 1·49)
Haiti	16·7 (11·5 to 23·6)	16 (11 to 22·2)
Jamaica	1·13 (0·846 to 1·48)	1·16 (0·893 to 1·48)
Puerto Rico	1·6 (1·11 to 2·26)	1·44 (1·01 to 2·02)
Saint Kitts and Nevis	0·0301 (0·0209 to 0·0417)	0·0282 (0·0196 to 0·039)
Saint Lucia	0·0847 (0·0726 to 0·0979)	0·0783 (0·0678 to 0·0901)
Saint Vincent and the Grenadines	0·039 (0·0276 to 0·0541)	0·0395 (0·0279 to 0·0529)
Suriname	0·533 (0·372 to 0·734)	0·515 (0·364 to 0·714)
The Bahamas	0·0798 (0·0593 to 0·105)	0·0811 (0·0622 to 0·103)
Trinidad and Tobago	0·699 (0·637 to 0·773)	0·672 (0·611 to 0·738)
Virgin Islands	0·066 (0·0458 to 0·0908)	0·0628 (0·0438 to 0·0887)
Central Latin America	138 (115 to 166)	136 (116 to 160)
Colombia	30·4 (21·3 to 42·7)	31 (21·8 to 45)
Costa Rica	0·713 (0·66 to 0·772)	0·681 (0·629 to 0·737)
El Salvador	3·25 (2·39 to 4·34)	3·25 (2·45 to 4·18)
Guatemala	41·3 (29·4 to 55·8)	39·6 (29·6 to 52·8)
Honduras	5·9 (4·36 to 7·95)	5·98 (4·45 to 7·99)
Mexico	43·7 (41·7 to 45·8)	43·5 (41·8 to 45·4)
Aguascalientes	0·441 (0·407 to 0·478)	0·423 (0·391 to 0·458)
Baja California	0·701 (0·649 to 0·763)	0·668 (0·616 to 0·723)
Baja California Sur	0·112 (0·101 to 0·123)	0·113 (0·102 to 0·125)
Campeche	0·327 (0·302 to 0·352)	0·325 (0·3 to 0·352)
Chiapas	2·1 (1·92 to 2·29)	2·12 (1·95 to 2·3)
Chihuahua	1·83 (1·7 to 1·97)	1·66 (1·55 to 1·79)
Coahuila	1·21 (1·13 to 1·29)	1·17 (1·09 to 1·25)
Colima	0·201 (0·187 to 0·215)	0·194 (0·181 to 0·209)
Durango	0·947 (0·824 to 1·07)	1·18 (1·06 to 1·32)
Guanajuato	2·41 (2·23 to 2·61)	2·43 (2·25 to 2·62)
Guerrero	1·83 (1·37 to 2·41)	1·86 (1·44 to 2·41)
Hidalgo	0·911 (0·847 to 0·975)	0·953 (0·883 to 1·03)
Jalisco	2·35 (2·16 to 2·55)	2·41 (2·2 to 2·62)
México	4·47 (4·03 to 4·95)	4·26 (3·88 to 4·65)
Mexico City	3·83 (2·68 to 5·32)	3·63 (2·53 to 4·98)
Michoacán de Ocampo	2·28 (2·12 to 2·46)	2·41 (2·24 to 2·6)
Morelos	0·574 (0·529 to 0·622)	0·646 (0·595 to 0·7)
Nayarit	0·292 (0·264 to 0·322)	0·322 (0·292 to 0·355)
Nuevo León	1·21 (1·1 to 1·32)	1·24 (1·14 to 1·35)
Oaxaca	1·3 (1·19 to 1·4)	1·33 (1·24 to 1·43)
Puebla	2·55 (2·37 to 2·74)	2·56 (2·39 to 2·74)
Querétaro	0·613 (0·57 to 0·656)	0·593 (0·55 to 0·64)
Quintana Roo	0·249 (0·232 to 0·266)	0·278 (0·26 to 0·298)
San Luis Potosí	1·6 (1·48 to 1·73)	1·5 (1·38 to 1·62)
Sinaloa	0·611 (0·579 to 0·647)	0·613 (0·584 to 0·645)
Sonora	0·604 (0·564 to 0·644)	0·64 (0·595 to 0·688)
Tabasco	0·964 (0·886 to 1·05)	0·965 (0·892 to 1·05)
Tamaulipas	1·18 (1·09 to 1·27)	1·09 (1·01 to 1·18)
Tlaxcala	0·34 (0·311 to 0·372)	0·343 (0·314 to 0·374)
Veracruz de Ignacio de la Llave	4·21 (3·93 to 4·5)	4·05 (3·78 to 4·33)
Yucatán	0·78 (0·709 to 0·856)	0·774 (0·706 to 0·851)
Zacatecas	0·688 (0·636 to 0·744)	0·737 (0·68 to 0·797)
Nicaragua	2·76 (2 to 3·75)	2·73 (1·96 to 3·65)
Panama	0·561 (0·404 to 0·768)	0·565 (0·403 to 0·762)
Venezuela	9·57 (7·42 to 12)	8·17 (6·6 to 10)
Tropical Latin America	92·8 (67·9 to 125)	88·8 (66·7 to 117)
Brazil	88 (63·4 to 121)	84·3 (62·1 to 113)
Acre	0·297 (0·209 to 0·432)	0·297 (0·208 to 0·422)
Alagoas	3·6 (2·51 to 5·28)	3·42 (2·38 to 4·9)
Amapá	0·0874 (0·0621 to 0·121)	0·0989 (0·0696 to 0·134)
Amazonas	1·06 (0·742 to 1·48)	1·09 (0·768 to 1·49)
Bahia	5·73 (4 to 7·94)	5·66 (3·98 to 7·7)
Ceará	4·25 (2·98 to 6·18)	4·25 (2·99 to 6·05)
Distrito Federal	0·624 (0·436 to 0·864)	0·617 (0·434 to 0·839)
Espírito Santo	1·17 (0·819 to 1·69)	1·11 (0·78 to 1·57)
Goiás	1·69 (1·18 to 2·34)	1·65 (1·16 to 2·25)
Maranhão	13·4 (9·24 to 20)	12·2 (8·39 to 17·7)
Mato Grosso	1·27 (0·883 to 1·77)	1·27 (0·89 to 1·74)
Mato Grosso do Sul	0·654 (0·46 to 0·948)	0·648 (0·456 to 0·918)
Minas Gerais	7·27 (5·02 to 10·3)	7·04 (4·91 to 9·83)
Pará	3·33 (2·36 to 4·61)	3·31 (2·34 to 4·51)

Paraíba	2·03 (1·42 to 2·96)	1·99 (1·4 to 2·83)	1·97 (1·39 to 2·81)	1·92 (1·33 to 2·72)	1·85 (1·31 to 2·58)	1·83 (1·31 to 2·48)	1·81 (1·33 to 2·42)	1·8 (1·34 to 2·33)	1·79 (1·45 to 2·2)	1·69 (1·5 to 1·88)	1·71 (1·6 to 1·84)	1·81 (1·69 to 1·94)	1·89 (1·76 to 2·03)	2·01 (1·87 to 2·15)	1·87 (1·75 to 2)	1·65 (1·55 to 1·76)	1·58 (1·48 to 1·68)	1·54 (1·45 to 1·64)	1·57 (1·48 to 1·67)	1·56 (1·47 to 1·66)	1·52 (1·44 to 1·63)	1·54 (1·45 to 1·64)	1·45 (1·37 to 1·54)	1·3 (1·22 to 1·38)	1·18 (1·11 to 1·26)	1·21 (1·14 to 1·28)	1·17 (1·1 to 1·25)	1·13 (1·06 to 1·21)	1·09 (1·03 to 1·16)	0·986 (0·927 to 1·05)	0·939 (0·848 to 1·04)	0·84 (0·692 to 1·03)
Paraná	6·61 (4·59 to 9·22)	6·33 (4·42 to 8·66)	5·78 (4·16 to 7·78)	5·71 (4·14 to 7·8)	5·82 (4·44 to 7·59)	5·91 (4·9 to 7·11)	5·03 (4·21 to 5·99)	3·64 (2·97 to 4·42)	3·14 (2·61 to 3·75)	2·62 (2·36 to 2·9)	2·4 (2·25 to 2·56)	2·4 (2·26 to 2·56)	2·37 (2·22 to 2·52)	2·34 (2·2 to 2·48)	2·07 (1·95 to 2·2)	1·81 (1·71 to 1·92)	1·79 (1·69 to 1·9)	1·88 (1·77 to 1·99)	1·88 (1·78 to 1·98)	1·87 (1·77 to 1·99)	1·89 (1·78 to 2)	1·82 (1·71 to 1·93)	1·75 (1·65 to 1·85)	1·49 (1·4 to 1·58)	1·45 (1·37 to 1·54)	1·59 (1·5 to 1·68)	1·42 (1·34 to 1·51)	1·26 (1·19 to 1·33)	1·16 (1·1 to 1·23)	1·08 (1·03 to 1·15)	0·996 (0·892 to 1·1)	0·954 (0·784 to 1·17)
Pernambuco	3·61 (2·52 to 5)	3·6 (2·53 to 4·9)	3·51 (2·54 to 4·71)	3·44 (2·51 to 4·68)	3·34 (2·56 to 4·34)	3·15 (2·61 to 3·79)	3·33 (2·8 to 3·95)	3·8 (3·09 to 4·63)	4·07 (3·32 to 4·88)	4·18 (3·67 to 4·71)	4·4 (4·05 to 4·77)	4·72 (4·34 to 5·13)	4·86 (4·48 to 5·29)	4·89 (4·52 to 5·29)	4·26 (3·94 to 4·57)	3·68 (3·42 to 3·94)	3·41 (3·19 to 3·66)	3·3 (3·07 to 3·52)	3·32 (3·1 to 3·56)	3·24 (3·03 to 3·49)	3·04 (2·83 to 3·26)	3·09 (2·88 to 3·31)	3·28 (3·06 to 3·52)	3·17 (2·97 to 3·4)	3·03 (2·83 to 3·23)	2·79 (2·6 to 2·99)	2·4 (2·24 to 2·57)	2·11 (1·96 to 2·26)	2·07 (1·93 to 2·23)	2 (1·86 to 2·13)	1·89 (1·67 to 2·11)	1·73 (1·42 to 2·11)
Piauí	1·66 (1·16 to 2·31)	1·65 (1·16 to 2·25)	1·63 (1·17 to 2·18)	1·6 (1·17 to 2·19)	1·53 (1·17 to 1·99)	1·45 (1·2 to 1·75)	1·57 (1·31 to 1·86)	1·87 (1·51 to 2·28)	2·11 (1·71 to 2·54)	2·4 (2·08 to 2·73)	2·52 (2·3 to 2·74)	2·53 (2·31 to 2·77)	2·26 (2·06 to 2·49)	2·04 (1·87 to 2·23)	1·94 (1·77 to 2·12)	1·98 (1·8 to 2·17)	2·16 (1·96 to 2·36)	2·02 (1·84 to 2·22)	1·8 (1·64 to 1·97)	1·74 (1·6 to 1·91)	1·72 (1·58 to 1·88)	1·71 (1·56 to 1·87)	1·74 (1·58 to 1·91)	1·63 (1·49 to 1·78)	1·02 (0·944 to 1·11)	0·675 (0·623 to 0·733)	0·694 (0·634 to 0·76)	0·698 (0·646 to 0·763)	0·751 (0·698 to 0·809)	0·658 (0·61 to 0·709)	0·578 (0·517 to 0·649)	0·498 (0·406 to 0·608)
Rio de Janeiro	7·22 (5·02 to 10)	6·82 (4·78 to 9·32)	6·94 (5 to 9·34)	7·11 (5·17 to 9·72)	7·47 (5·7 to 9·74)	7·26 (6 to 8·76)	6·86 (5·75 to 8·14)	5·77 (4·69 to 7·03)	4·84 (3·95 to 5·78)	4·23 (3·74 to 4·73)	3·91 (3·63 to 4·21)	3·54 (3·28 to 3·82)	3·37 (3·11 to 3·63)	3·37 (3·13 to 3·63)	2·77 (2·58 to 2·98)	2·21 (2·08 to 2·36)	2·23 (2·09 to 2·37)	2·26 (2·12 to 2·41)	2·07 (1·94 to 2·2)	1·95 (1·83 to 2·09)	2·02 (1·88 to 2·15)	2·04 (1·92 to 2·18)	1·99 (1·86 to 2·12)	1·77 (1·66 to 1·89)	1·78 (1·67 to 1·89)	2·22 (2·09 to 2·38)	2·58 (2·42 to 2·76)	2·53 (2·37 to 2·7)	2·48 (2·32 to 2·65)	2·43 (2·27 to 2·59)	2·38 (2·12 to 2·65)	2·25 (1·85 to 2·74)
Rio Grande do Norte	2·78 (1·94 to 4·07)	2·56 (1·79 to 3·67)	2·38 (1·67 to 3·42)	2·18 (1·53 to 3·12)	2·06 (1·46 to 2·87)	2·02 (1·44 to 2·75)	1·97 (1·41 to 2·68)	1·92 (1·41 to 2·52)	1·84 (1·44 to 2·31)	1·75 (1·48 to 2·03)	1·68 (1·5 to 1·88)	1·76 (1·57 to 1·95)	1·78 (1·61 to 1·97)	1·77 (1·6 to 1·97)	1·44 (1·31 to 1·58)	1·36 (1·24 to 1·48)	1·32 (1·21 to 1·46)	0·984 (0·905 to 1·07)	0·906 (0·829 to 0·991)	0·826 (0·759 to 0·899)	0·766 (0·701 to 0·835)	0·724 (0·664 to 0·788)	0·699 (0·641 to 0·769)	0·711 (0·651 to 0·776)	0·782 (0·715 to 0·855)	0·846 (0·775 to 0·915)	0·771 (0·707 to 0·843)	0·732 (0·666 to 0·796)	0·706 (0·648 to 0·768)	0·647 (0·592 to 0·706)	0·592 (0·517 to 0·67)	0·523 (0·421 to 0·651)
Rio Grande do Sul	2·86 (2 to 3·96)	3 (2·11 to 4·08)	2·72 (1·96 to 3·64)	2·66 (1·94 to 3·62)	2·67 (2·05 to 3·47)	2·8 (2·33 to 3·36)	2·59 (2·17 to 3·08)	2·14 (1·75 to 2·6)	2·05 (1·7 to 2·45)	1·76 (1·58 to 1·96)	1·76 (1·64 to 1·88)	1·65 (1·55 to 1·77)	1·54 (1·44 to 1·65)	1·53 (1·43 to 1·64)	1·38 (1·29 to 1·47)	1·18 (1·11 to 1·26)	1·16 (1·09 to 1·24)	1·11 (1·04 to 1·18)	1·05 (0·989 to 1·12)	0·99 (0·933 to 1·05)	0·995 (0·938 to 1·06)	0·997 (0·935 to 1·06)	0·98 (0·922 to 1·04)	1·01 (0·954 to 1·08)	1·01 (0·946 to 1·07)	0·996 (0·939 to 1·06)	0·986 (0·929 to 1·05)	0·965 (0·908 to 1·03)	0·921 (0·869 to 0·98)	0·901 (0·846 to 0·959)	0·842 (0·751 to 0·935)	0·848 (0·695 to 1·04)
Rondônia	1·02 (0·705 to 1·42)	0·99 (0·692 to 1·35)	0·954 (0·686 to 1·29)	0·929 (0·676 to 1·27)	0·935 (0·715 to 1·22)	1·02 (0·845 to 1·23)	0·995 (0·835 to 1·17)	0·865 (0·684 to 1·07)	0·768 (0·607 to 0·958)	0·679 (0·561 to 0·827)	0·623 (0·563 to 0·687)	0·549 (0·516 to 0·584)	0·49 (0·461 to 0·521)	0·469 (0·44 to 0·499)	0·409 (0·385 to 0·435)	0·372 (0·35 to 0·393)	0·379 (0·357 to 0·402)	0·375 (0·355 to 0·397)	0·356 (0·336 to 0·376)	0·348 (0·327 to 0·369)	0·342 (0·323 to 0·361)	0·31 (0·292 to 0·329)	0·305 (0·287 to 0·324)	0·324 (0·306 to 0·343)	0·33 (0·312 to 0·35)	0·336 (0·317 to 0·356)	0·333 (0·314 to 0·352)	0·333 (0·316 to 0·353)	0·344 (0·324 to 0·363)	0·312 (0·295 to 0·332)	0·284 (0·258 to 0·312)	0·198 (0·163 to 0·239)
Roraima	0·0966 (0·0679 to 0·14)	0·102 (0·0689 to 0·147)	0·106 (0·0748 to 0·151)	0·11 (0·0781 to 0·157)	0·115 (0·0835 to 0·161)	0·119 (0·0854 to 0·161)	0·119 (0·0852 to 0·161)	0·111 (0·0828 to 0·143)	0·109 (0·0883 to 0·134)	0·109 (0·0975 to 0·122)	0·109 (0·102 to 0·116)	0·103 (0·0966 to 0·11)	0·106 (0·0992 to 0·113)	0·134 (0·126 to 0·143)	0·11 (0·104 to 0·117)	0·0837 (0·0788 to 0·0888)	0·0844 (0·0795 to 0·0894)	0·08 (0·0756 to 0·0852)	0·085 (0·0802 to 0·0903)	0·0838 (0·079 to 0·089)	0·0921 (0·0867 to 0·0976)	0·105 (0·0987 to 0·112)	0·109 (0·103 to 0·116)	0·115 (0·108 to 0·122)	0·121 (0·109 to 0·133)	0·12 (0·104 to 0·138)	0·119 (0·108 to 0·131)	0·12 (0·113 to 0·128)	0·126 (0·114 to 0·138)	0·124 (0·113 to 0·136)	0·12 (0·107 to 0·133)	0·0986 (0·0803 to 0·121)
Santa Catarina	1·02 (0·716 to 1·47)	1·05 (0·741 to 1·49)	1·05 (0·742 to 1·49)	1·04 (0·726 to 1·46)	1·03 (0·729 to 1·43)	1·03 (0·737 to 1·39)	1·03 (0·76 to 1·37)	1·04 (0·778 to 1·34)	1·04 (0·844 to 1·27)	1·03 (0·923 to 1·14)	0·991 (0·928 to 1·06)	0·949 (0·888 to 1·02)	0·953 (0·894 to 1·02)	0·93 (0·87 to 0·993)	0·815 (0·767 to 0·866)	0·744 (0·7 to 0·789)	0·828 (0·78 to 0·882)	0·871 (0·817 to 0·929)	0·868 (0·812 to 0·925)	0·88 (0·824 to 0·939)	0·855 (0·798 to 0·913)	0·858 (0·801 to 0·915)	0·924 (0·865 to 0·984)	0·977 (0·914 to 1·04)	0·827 (0·778 to 0·877)	0·744 (0·701 to 0·79)	0·708 (0·667 to 0·752)	0·767 (0·725 to 0·818)	0·76 (0·716 to 0·805)	0·618 (0·583 to 0·656)	0·618 (0·56 to 0·684)	0·619 (0·511 to 0·756)
São Paulo	13 (9·15 to 18·9)	11·8 (8·33 to 16·8)	11·1 (7·88 to 15·9)	10·9 (7·64 to 15·5)	10·8 (7·64 to 15)	11 (7·84 to 14·8)	10·7 (7·93 to 14·3)	10·6 (7·87 to 13·6)	9·9 (8·03 to 12·2)	9·35 (8·32 to 10·4)	9·07 (8·46 to 9·69)	8·86 (8·24 to 9·53)	8·56 (7·95 to 9·18)	8·32 (7·76 to 8·92)	7·28 (6·81 to 7·79)	6·41 (6·02 to 6·83)	6·21 (5·83 to 6·61)	6·43 (6·04 to 6·84)	6·56 (6·17 to 6·97)	6·22 (5·83 to 6·63)	5·99 (5·65 to 6·41)	6·42 (6·03 to 6·83)	6·52 (6·12 to 6·92)	5·55 (5·23 to 5·88)	5·23 (4·92 to 5·56)	5·33 (5·02 to 5·66)	5·09 (4·77 to 5·42)	4·74 (4·44 to 5·05)	4·34 (4·09 to 4·62)	4·06 (3·81 to 4·33)	4·02 (3·63 to 4·46)	4·05 (3·33 to 4·95)
Sergipe	0·999 (0·7 to 1·45)	1·02 (0·685 to 1·46)	1·04 (0·736 to 1·49)	1·07 (0·74 to 1·51)	1·04 (0·729 to 1·45)	1·06 (0·752 to 1·44)	1·06 (0·757 to 1·43)	1·07 (0·784 to 1·39)	1·06 (0·832 to 1·32)	1·04 (0·902 to 1·2)	1·04 (0·946 to 1·16)	1·1 (0·995 to 1·21)	1·06 (0·954 to 1·17)	0·976 (0·886 to 1·07)	0·977 (0·887 to 1·07)	0·961 (0·866 to 1·06)	0·912 (0·822 to 1·01)	0·903 (0·817 to 0·993)	0·775 (0·709 to 0·849)	0·78 (0·714 to 0·848)	0·866 (0·784 to 0·953)	0·839 (0·763 to 0·927)	0·688 (0·632 to 0·755)	0·534 (0·495 to 0·582)	0·513 (0·472 to 0·558)	0·451 (0·416 to 0·494)	0·425 (0·38 to 0·476)	0·448 (0·401 to 0·495)	0·445 (0·409 to 0·48)	0·436 (0·403 to 0·473)	0·443 (0·39 to 0·5)	0·392 (0·316 to 0·486)
Tocantins	0·603 (0·423 to 0·877)	0·606 (0·426 to 0·862)	0·616 (0·435 to 0·88)	0·611 (0·425 to 0·865)	0·605 (0·428 to 0·842)	0·617 (0·44 to 0·836)	0·641 (0·472 to 0·854)	0·657 (0·486 to 0·855)	0·665 (0·527 to 0·822)	0·667 (0·584 to 0·762)	0·677 (0·62 to 0·741)	0·645 (0·591 to 0·699)	0·642 (0·586 to 0·701)	0·692 (0·632 to 0·757)	0·674 (0·616 to 0·736)	0·691 (0·631 to 0·753)	0·58 (0·536 to 0·628)	0·537 (0·496 to 0·582)	0·536 (0·494 to 0·579)	0·425 (0·396 to 0·456)	0·43 (0·4 to 0·463)	0·421 (0·392 to 0·452)	0·409 (0·382 to 0·441)	0·417 (0·389 to 0·45)	0·377 (0·349 to 0·406)	0·377 (0·351 to 0·405)	0·367 (0·342 to 0·396)	0·344 (0·318 to 0·37)	0·341 (0·316 to 0·367)	0·314 (0·292 to 0·336)	0·295 (0·261 to 0·329)	0·238 (0·193 to 0·294)
Paraguay	4·86 (4·11 to 5·69)	4·53 (3·76 to 5·39)	4·43 (3·62 to 5·38)	4·33 (3·46 to 5·42)	4·09 (3·22 to 5·16)	4·03 (3·14 to 5·05)	3·89 (3·06 to 4·9)	3·75 (2·93 to 4·78)	3·62 (2·85 to 4·55)	3·55 (2·82 to 4·42)	3·66 (2·95 to 4·47)	3·57 (2·95 to 4·24)	3·43 (2·91 to 4)	3·43 (3·02 to 3·86)	3·38 (3·05 to 3·71)	2·86 (2·62 to 3·12)	2·35 (2·16 to 2·56)	2·08 (1·89 to 2·29)	1·97 (1·74 to 2·22)	1·92 (1·64 to 2·23)	1·88 (1·55 to 2·27)	1·89 (1·51 to 2·34)	1·89 (1·47 to 2·4)	1·9 (1·45 to 2·42)	1·89 (1·43 to 2·45)	1·71 (1·27 to 2·25)	1·69 (1·23 to 2·23)	1·76 (1·29 to 2·34)	1·78 (1·32 to 2·42)	1·73 (1·28 to 2·37)	1·65 (1·21 to 2·27)	1·56 (1·12 to 2·12)
North Africa and Middle East	517 (422 to 633)	505 (420 to 613)	495 (412 to 595)	492 (412 to 589)	499 (422 to 598)	501 (425 to 596)	498 (427 to 584)	500 (431 to 587)	505 (439 to 584)	507 (446 to 582)	496 (437 to 570)	483 (425 to 554)	475 (421 to 543)	472 (420 to 532)	467 (417 to 524)	452 (404 to 508)	434 (387 to 491)	418 (371 to 475)	410 (364 to 468)	404 (355 to 460)	394 (345 to 451)	380 (332 to 438)	361 (313 to 418)	341 (297 to 396)	320 (277 to 372)	303 (261 to 354)	284 (244 to 332)	269 (231 to 316)	258 (220 to 305)	253 (215 to 300)	234 (197 to 281)	220 (184 to 266)
Afghanistan	36·3 (24·9 to 52)	36·3 (24·7 to 51·4)	37·1 (25·7 to 52·9)	40·8 (28·6 to 57·6)	46·4 (32·5 to 65·6)	50·5 (35·4 to 71·1)	52·3 (36·5 to 72·4)	52·9 (36·6 to 73·6)	53·2 (37·1 to 73·3)	53·4 (37·2 to 73·5)	53·1 (37 to 73·3)	52·1 (36·4 to 70·5)	50·6 (35·8 to 67·5)	50·5 (36·2 to 67·4)	51·6 (37·3 to 69·3)	51·7 (38·2 to 69·2)	51·8 (38·2 to 69·2)	52·1 (38·3 to 69·7)	51·7 (38 to 68·8)	50·8 (37 to 68·6)	50·6 (36·6 to 67·1)	50·9 (37·1 to 68·6)	51·6 (37·1 to 71·6)	52·8 (37·5 to 73·1)	53·8 (38·2 to 74·1)	53·7 (38·5 to 74·1)	52·4 (37·3 to 72·6)	51·3 (36·8 to 71·7)	50·4 (36·5 to 70·5)	49·5 (35·5 to 69·5)	48·3 (34·2 to 68·8)	47·7 (33·7 to 68·9)
Algeria	29 (26·2 to 32·3)	29·6 (23·6 to 36·1)	29·9 (22·7 to 39)	30 (21·9 to 39·5)	29·8 (21·8 to 40)	29·3 (21·6 to 38·7)	28·5 (21·9 to 36·6)	27·5 (22·3 to 33·8)	26·3 (23·7 to 29)	25·9 (24·5 to 27·4)	26 (24·5 to 27·7)	27·1 (25·5 to 28·7)	27·6 (26·1 to 29·2)	27 (25·4 to 28·6)	26·8 (25·3 to 28·4)	25·6 (24·1 to 27·1)	25·4 (24 to 26·9)	25·5 (24·1 to 27·1)	24·6 (23·3 to 26·1)	24·5 (23 to 26)	24·2 (22·8 to 25·7)	23·3 (22 to 24·7)	22·9 (21·6 to 24·3)	22·4 (21·1 to 23·8)	21·7 (20·6 to 23)	21 (19·8 to 22·3)	20·3 (19·1 to 21·5)	19·3 (18·2 to 20·5)	18·7 (17·7 to 19·7)	17·7 (15·9 to 19·6)	15·9 (12·8 to 19·4)	14·6 (11·3 to 18·8)
Bahrain	0·63 (0·443 to 0·88)	0·629 (0·44 to 0·862)	0·604 (0·425 to 0·837)	0·571 (0·408 to 0·775)	0·526 (0·374 to 0·703)	0·455 (0·326 to 0·607)	0·371 (0·272 to 0·491)	0·316 (0·235 to 0·417)	0·282 (0·212 to 0·368)	0·255 (0·193 to 0·33)	0·231 (0·176 to 0·294)	0·213 (0·164 to 0·266)	0·194 (0·153 to 0·241)	0·187 (0·148 to 0·236)	0·192 (0·153 to 0·237)	0·192 (0·151 to 0·236)	0·195 (0·155 to 0·242)	0·202 (0·16 to 0·251)	0·206 (0·163 to 0·261)	0·214 (0·17 to 0·27)	0·224 (0·178 to 0·283)	0·225 (0·18 to 0·281)	0·227 (0·18 to 0·285)	0·233 (0·184 to 0·292)	0·234 (0·184 to 0·297)	0·227 (0·177 to 0·286)	0·219 (0·166 to 0·283)	0·211 (0·157 to 0·278)	0·198 (0·146 to 0·262)	0·182 (0·134 to 0·244)	0·15 (0·111 to 0·206)	0·138 (0·0992 to 0·195)
Egypt	78·3 (57·1 to 107)	73·1 (53·4 to 99·4)	69·8 (50·7 to 93·2)	66·1 (48·6 to 87·2)	62 (45·6 to 81·9)	59·6 (44·2 to 79·1)	56·1 (41·9 to 75)	54·9 (40·9 to 73·2)	54·9 (40·7 to 72·4)	56·3 (42·4 to 73·1)	56·2 (42·1 to 73·1)	54·2 (40·9 to 70·3)	51·5 (39·1 to 67·3)	50·2 (37·8 to 64·9)	48·6 (36 to 62·9)	47·6 (35·1 to 61·4)	47·6 (35·2 to 61·7)	47·7 (35·7 to 62·4)	48·3 (36·2 to 63·1)	51·6 (38·2 to 67·3)	54·1 (39·7 to 71)	55·4 (40·5 to 73·6)	51·1 (37·6 to 67·7)	45·5 (33·7 to 60·9)	38·7 (28·6 to 52·7)	35·6 (25·8 to 48·5)	32·3 (23·2 to 44·4)	30·9 (22·1 to 43·5)	29·4 (21 to 40·9)	28·9 (20·4 to 39·6)	24·2 (17·1 to 33·9)	22·8 (16·2 to 32·3)
Iran	38·6 (26·9 to 52·4)	35·6 (24·8 to 49·3)	33·5 (23·5 to 46·5)	31·6 (22·1 to 44·1)	29·4 (20·4 to 40·7)	27·2 (19 to 38·7)	25·6 (17·6 to 36·6)	24·5 (17·2 to 35)	23·5 (16·5 to 33·5)	22·6 (15·9 to 32·2)	21·8 (15·2 to 31·1)	21·3 (14·9 to 30·8)	21 (14·8 to 30·2)	20·3 (14·4 to 28·8)	19·4 (13·8 to 27·1)	18·6 (13·3 to 25·6)	18·5 (13·1 to 25·3)	19 (13·4 to 26)	19·8 (13·9 to 27·1)	20·2 (14·2 to 27·7)	20·2 (14·2 to 27·6)	19·9 (14·1 to 26·8)	19·8 (14·2 to 26·5)	19·5 (13·9 to 26·5)	18·8 (13·5 to 25·3)	17 (12·3 to 23)	13·5 (9·83 to 18·4)	11·4 (8·1 to 15·3)	9·78 (6·96 to 13·3)	8·52 (6·09 to 11·7)	5·85 (4·22 to 8·07)	3·92 (2·83 to 5·35)
Alborz	0·502 (0·349 to 0·683)	0·48 (0·333 to 0·666)	0·466 (0·325 to 0·647)	0·45 (0·314 to 0·63)	0·428 (0·298 to 0·594)	0·404 (0·28 to 0·576)	0·388 (0·266 to 0·555)	0·378 (0·266 to 0·539)	0·369 (0·258 to 0·525)	0·362 (0·255 to 0·516)	0·357 (0·249 to 0·509)	0·357 (0·248 to 0·516)	0·36 (0·254 to 0·517)	0·356 (0·253 to 0·504)	0·347 (0·248 to 0·484)	0·339 (0·243 to 0·466)	0·34 (0·241 to 0·467)	0·35 (0·245 to 0·479)	0·365 (0·256 to 0·502)	0·372 (0·26 to 0·512)	0·373 (0·261 to 0·511)	0·369 (0·26 to 0·499)	0·367 (0·262 to 0·495)	0·363 (0·257 to 0·493)	0·348 (0·249 to 0·476)	0·313 (0·224 to 0·425)	0·25 (0·18 to 0·341)	0·213 (0·15 to 0·288)	0·185 (0·133 to 0·252)	0·163 (0·116 to 0·223)	0·0987 (0·0715 to 0·136)	0·0667 (0·048 to 0·0914)
Ardebil	1·17 (0·804 to 1·6)	[illegible]	[illegible]	[illegible]	[illegible]	[illegible]	[illegible]	[illegible]	[illegible]	[illegible]	[illegible]	[illegible]	[illegible]	[illegible]	[illegible]	[illegible]	[illegible]	[illegible]	[illegible]	[illegible]	[illegible]	[illegible]	[illegible]	[illegible]	[illegible]	[illegible]	[illegible]	[illegible]	[illegible]	[illegible]	[illegible]	[illegible]
Bushehr	0·527 (0·364 to 0·722)	[illegible]	[illegible]	[illegible]	[illegible]	[illegible]	[illegible]	[illegible]	[illegible]	[illegible]	[illegible]	[illegible]	[illegible]	[illegible]	[illegible]	[illegible]	[illegible]	[illegible]	[illegible]	[illegible]	[illegible]	[illegible]	[illegible]	[illegible]	[illegible]	[illegible]	[illegible]	[illegible]	[illegible]	[illegible]	[illegible]	[illegible]
Chahar Mahaal and Bakhtiari	0·591 (0·409 to 0·81)	[illegible]	[illegible]	[illegible]	[illegible]	[illegible]	[illegible]	[illegible]	[illegible]	[illegible]	[illegible]	[illegible]	[illegible]	[illegible]	[illegible]	[illegible]	[illegible]	[illegible]	[illegible]	[illegible]	[illegible]	[illegible]	[illegible]	[illegible]	[illegible]	[illegible]	[illegible]	[illegible]	[illegible]	[illegible]	[illegible]	[illegible]
East Azarbayejan	2·83 (1·96 to 3·89)	[illegible]	[illegible]	[illegible]	[illegible]	[illegible]	[illegible]	[illegible]	[illegible]	[illegible]	[illegible]	[illegible]	[illegible]	[illegible]	[illegible]	[illegible]	[illegible]	[illegible]	[illegible]	[illegible]	[illegible]	[illegible]	[illegible]	[illegible]	[illegible]	[illegible]	[illegible]	[illegible]	[illegible]	[illegible]	[illegible]	[illegible]
Fars	2·21 (1·53 to 3·02)	[illegible]	[illegible]	[illegible]	[illegible]	[illegible]	[illegible]	[illegible]	[illegible]	[illegible]	[illegible]	[illegible]	[illegible]	[illegible]	[illegible]	[illegible]	[illegible]	[illegible]	[illegible]	[illegible]	[illegible]	[illegible]	[illegible]	[illegible]	[illegible]	[illegible]	[illegible]	[illegible]	[illegible]	[illegible]	[illegible]	[illegible]
Gilan	1·16 (0·806 to 1·59)	[illegible]	[illegible]	[illegible]	[illegible]	[illegible]	[illegible]	[illegible]	[illegible]	[illegible]	[illegible]	[illegible]	[illegible]	[illegible]	[illegible]	[illegible]	[illegible]	[illegible]	[illegible]	[illegible]	[illegible]	[illegible]	[illegible]	[illegible]	[illegible]	[illegible]	[illegible]	[illegible]	[illegible]	[illegible]	[illegible]	[illegible]
Golestan	1·11 (0·765 to 1·51)	[illegible]	[illegible]	[illegible]	[illegible]	[illegible]	[illegible]	[illegible]	[illegible]	[illegible]	[illegible]	[illegible]	[illegible]	[illegible]	[illegible]	[illegible]	[illegible]	[illegible]	[illegible]	[illegible]	[illegible]	[illegible]	[illegible]	[illegible]	[illegible]	[illegible]	[illegible]	[illegible]	[illegible]	[illegible]	[illegible]	[illegible]
Hamadan	1·36 (0·942 to 1·87)	[illegible]	[illegible]	[illegible]	[illegible]	[illegible]	[illegible]	[illegible]	[illegible]	[illegible]	[illegible]	[illegible]	[illegible]	[illegible]	[illegible]	[illegible]	[illegible]	[illegible]	[illegible]	[illegible]	[illegible]	[illegible]	[illegible]	[illegible]	[illegible]	[illegible]	[illegible]	[illegible]	[illegible]	[illegible]	[illegible]	[illegible]
Hormozgan	0·762 (0·527 to 1·04)	[illegible]	[illegible]	[illegible]	[illegible]	[illegible]	[illegible]	[illegible]	[illegible]	[illegible]	[illegible]	[illegible]	[illegible]	[illegible]	[illegible]	[illegible]	[illegible]	[illegible]	[illegible]	[illegible]	[illegible]	[illegible]	[illegible]	[illegible]	[illegible]	[illegible]	[illegible]	[illegible]	[illegible]	[illegible]	[illegible]	[illegible]
Ilam	0·363 (0·251 to 0·498)	[illegible]	[illegible]	[illegible]	[illegible]	[illegible]	[illegible]	[illegible]	[illegible]	[illegible]	[illegible]	[illegible]	[illegible]	[illegible]	[illegible]	[illegible]	[illegible]	[illegible]	[illegible]	[illegible]	[illegible]	[illegible]	[illegible]	[illegible]	[illegible]	[illegible]	[illegible]	[illegible]	[illegible]	[illegible]	[illegible]	[illegible]
Isfahan	2·1 (1·45 to 2·87)	[illegible]	[illegible]	[illegible]	[illegible]	[illegible]	[illegible]	[illegible]	[illegible]	[illegible]	[illegible]	[illegible]	[illegible]	[illegible]	[illegible]	[illegible]	[illegible]	[illegible]	[illegible]	[illegible]	[illegible]	[illegible]	[illegible]	[illegible]	[illegible]	[illegible]	[illegible]	[illegible]	[illegible]	[illegible]	[illegible]	[illegible]
Kerman	0·806 (0·582 to 1·08)	[illegible]	[illegible]	[illegible]	[illegible]	[illegible]	[illegible]	[illegible]	[illegible]	[illegible]	[illegible]	[illegible]	[illegible]	[illegible]	[illegible]	[illegible]	[illegible]	[illegible]	[illegible]	[illegible]	[illegible]	[illegible]	[illegible]	[illegible]	[illegible]	[illegible]	[illegible]	[illegible]	[illegible]	[illegible]	[illegible]	[illegible]
Kermanshah	1·5 (1·03 to 2·06)	[illegible]	[illegible]	[illegible]	[illegible]	[illegible]	[illegible]	[illegible]	[illegible]	[illegible]	[illegible]	[illegible]	[illegible]	[illegible]	[illegible]	[illegible]	[illegible]	[illegible]	[illegible]	[illegible]	[illegible]	[illegible]	[illegible]	[illegible]	[illegible]	[illegible]	[illegible]	[illegible]	[illegible]	[illegible]	[illegible]	[illegible]
Khorasan-e-Razavi	4·33 (2·99 to 5·94)	[illegible]	[illegible]	[illegible]	[illegible]	[illegible]	[illegible]	[illegible]	[illegible]	[illegible]	[illegible]	[illegible]	[illegible]	[illegible]	[illegible]	[illegible]	[illegible]	[illegible]	[illegible]	[illegible]	[illegible]	[illegible]	[illegible]	[illegible]	[illegible]	[illegible]	[illegible]	[illegible]	[illegible]	[illegible]	[illegible]	[illegible]
Khuzestan	2 (1·38 to 2·73)	[illegible]	[illegible]	[illegible]	[illegible]	[illegible]	[illegible]	[illegible]	[illegible]	[illegible]	[illegible]	[illegible]	[illegible]	[illegible]	[illegible]	[illegible]	[illegible]	[illegible]	[illegible]	[illegible]	[illegible]	[illegible]	[illegible]	[illegible]	[illegible]	[illegible]	[illegible]	[illegible]	[illegible]	[illegible]	[illegible]	[illegible]
Kohgiluyeh and Boyer-Ahmad	0·431 (0·298 to 0·591)	[illegible]	[illegible]	[illegible]	[illegible]	[illegible]	[illegible]	[illegible]	[illegible]	[illegible]	[illegible]	[illegible]	[illegible]	[illegible]	[illegible]	[illegible]	[illegible]	[illegible]	[illegible]	[illegible]	[illegible]	[illegible]	[illegible]	[illegible]	[illegible]	[illegible]	[illegible]	[illegible]	[illegible]	[illegible]	[illegible]	[illegible]
Kurdistan	1·35 (0·927 to 1·86)	[illegible]	[illegible]	[illegible]	[illegible]	[illegible]	[illegible]	[illegible]	[illegible]	[illegible]	[illegible]	[illegible]	[illegible]	[illegible]	[illegible]	[illegible]	[illegible]	[illegible]	[illegible]	[illegible]	[illegible]	[illegible]	[illegible]	[illegible]	[illegible]	[illegible]	[illegible]	[illegible]	[illegible]	[illegible]	[illegible]	[illegible]
Lorestan	1·27 (0·878 to 1·74)	[illegible]	[illegible]	[illegible]	[illegible]	[illegible]	[illegible]	[illegible]	[illegible]	[illegible]	[illegible]	[illegible]	[illegible]	[illegible]	[illegible]	[illegible]	[illegible]	[illegible]	[illegible]	[illegible]	[illegible]	[illegible]	[illegible]	[illegible]	[illegible]	[illegible]	[illegible]	[illegible]	[illegible]	[illegible]	[illegible]	[illegible]
Markazi	0·765 (0·529 to 1·05)	[illegible]	[illegible]	[illegible]	[illegible]	[illegible]	[illegible]	[illegible]	[illegible]	[illegible]	[illegible]	[illegible]	[illegible]	[illegible]	[illegible]	[illegible]	[illegible]	[illegible]	[illegible]	[illegible]	[illegible]	[illegible]	[illegible]	[illegible]	[illegible]	[illegible]	[illegible]	[illegible]	[illegible]	[illegible]	[illegible]	[illegible]
Mazandaran	0·577 (0·46 to 0·715)	[illegible]	[illegible]	[illegible]	[illegible]	[illegible]	[illegible]	[illegible]	[illegible]	[illegible]	[illegible]	[illegible]	[illegible]	[illegible]	[illegible]	[illegible]	[illegible]	[illegible]	[illegible]	[illegible]	[illegible]	[illegible]	[illegible]	[illegible]	[illegible]	[illegible]	[illegible]	[illegible]	[illegible]	[illegible]	[illegible]	[illegible]
North Khorasan	0·653 (0·451 to 0·895)	[illegible]	[illegible]	[illegible]	[illegible]	[illegible]	[illegible]	[illegible]	[illegible]	[illegible]	[illegible]	[illegible]	[illegible]	[illegible]	[illegible]	[illegible]	[illegible]	[illegible]	[illegible]	[illegible]	[illegible]	[illegible]	[illegible]	[illegible]	[illegible]	[illegible]	[illegible]	[illegible]	[illegible]	[illegible]	[illegible]	[illegible]
Qazvin	0·611 (0·423 to 0·836)	[illegible]	[illegible]	[illegible]	[illegible]	[illegible]	[illegible]	[illegible]	[illegible]	[illegible]	[illegible]	[illegible]	[illegible]	[illegible]	[illegible]	[illegible]	[illegible]	[illegible]	[illegible]	[illegible]	[illegible]	[illegible]	[illegible]	[illegible]	[illegible]	[illegible]	[illegible]	[illegible]	[illegible]	[illegible]	[illegible]	[illegible]
Qom	0·397 (0·275 to 0·542)	[illegible]	[illegible]	[illegible]	[illegible]	[illegible]	[illegible]	[illegible]	[illegible]	[illegible]	[illegible]	[illegible]	[illegible]	[illegible]	[illegible]	[illegible]	[illegible]	[illegible]	[illegible]	[illegible]	[illegible]	[illegible]	[illegible]	[illegible]	[illegible]	[illegible]	[illegible]	[illegible]	[illegible]	[illegible]	[illegible]	[illegible]
Semnan	0·245 (0·17 to 0·336)	[illegible]	[illegible]	[illegible]	[illegible]	[illegible]	[illegible]	[illegible]	[illegible]	[illegible]	[illegible]	[illegible]	[illegible]	[illegible]	[illegible]	[illegible]	[illegible]	[illegible]	[illegible]	[illegible]	[illegible]	[illegible]	[illegible]	[illegible]	[illegible]	[illegible]	[illegible]	[illegible]	[illegible]	[illegible]	[illegible]	[illegible]
Sistan and Baluchistan	1·51 (1·05 to 2·08)	[illegible]	[illegible]	[illegible]	[illegible]	[illegible]	[illegible]	[illegible]	[illegible]	[illegible]	[illegible]	[illegible]	[illegible]	[illegible]	[illegible]	[illegible]	[illegible]	[illegible]	[illegible]	[illegible]	[illegible]	[illegible]	[illegible]	[illegible]	[illegible]	[illegible]	[illegible]	[illegible]	[illegible]	[illegible]	[illegible]	[illegible]
South Khorasan	0·668 (0·461 to 0·917)	[illegible]	[illegible]	[illegible]	[illegible]	[illegible]	[illegible]	[illegible]	[illegible]	[illegible]	[illegible]	[illegible]	[illegible]	[illegible]	[illegible]	[illegible]	[illegible]	[illegible]	[illegible]	[illegible]	[illegible]	[illegible]	[illegible]	[illegible]	[illegible]	[illegible]	[illegible]	[illegible]	[illegible]	[illegible]	[illegible]	[illegible]
Tehran	3·55 (2·47 to 4·85)	[illegible]	[illegible]	[illegible]	[illegible]	[illegible]	[illegible]	[illegible]	[illegible]	[illegible]	[illegible]	[illegible]	[illegible]	[illegible]	[illegible]	[illegible]	[illegible]	[illegible]	[illegible]	[illegible]	[illegible]	[illegible]	[illegible]	[illegible]	[illegible]	[illegible]	[illegible]	[illegible]	[illegible]	[illegible]	[illegible]	[illegible]
West Azarbayejan	2·04 (1·41 to 2·8)	[illegible]	[illegible]	[illegible]	[illegible]	[illegible]	[illegible]	[illegible]	[illegible]	[illegible]	[illegible]	[illegible]	[illegible]	[illegible]	[illegible]	[illegible]	[illegible]	[illegible]	[illegible]	[illegible]	[illegible]	[illegible]	[illegible]	[illegible]	[illegible]	[illegible]	[illegible]	[illegible]	[illegible]	[illegible]	[illegible]	[illegible]
Yazd	0·386 (0·268 to 0·528)	[illegible]	[illegible]	[illegible]	[illegible]	[illegible]	[illegible]	[illegible]	[illegible]	[illegible]	[illegible]	[illegible]	[illegible]	[illegible]	[illegible]	[illegible]	[illegible]	[illegible]	[illegible]	[illegible]	[illegible]	[illegible]	[illegible]	[illegible]	[illegible]	[illegible]	[illegible]	[illegible]	[illegible]	[illegible]	[illegible]	[illegible]
Zanjan	0·782 (0·539 to 1·07)	[illegible]	[illegible]	[illegible]	[illegible]	[illegible]	[illegible]	[illegible]	[illegible]	[illegible]	[illegible]	[illegible]	[illegible]	[illegible]	[illegible]	[illegible]	[illegible]	[illegible]	[illegible]	[illegible]	[illegible]	[illegible]	[illegible]	[illegible]	[illegible]	[illegible]	[illegible]	[illegible]	[illegible]	[illegible]	[illegible]	[illegible]
Iraq	22·1 (15·8 to 29·9)	[illegible]	[illegible]	[illegible]	[illegible]	[illegible]	[illegible]	[illegible]	[illegible]	[illegible]	[illegible]	[illegible]	[illegible]	[illegible]	[illegible]	[illegible]	[illegible]	[illegible]	[illegible]	[illegible]	[illegible]	[illegible]	[illegible]	[illegible]	[illegible]	[illegible]	[illegible]	[illegible]	[illegible]	[illegible]	[illegible]	[illegible]
Jordan	4·38 (3·17 to 5·91)	[illegible]	[illegible]	[illegible]	[illegible]	[illegible]	[illegible]	[illegible]	[illegible]	[illegible]	[illegible]	[illegible]	[illegible]	[illegible]	[illegible]	[illegible]	[illegible]	[illegible]	[illegible]	[illegible]	[illegible]	[illegible]	[illegible]	[illegible]	[illegible]	[illegible]	[illegible]	[illegible]	[illegible]	[illegible]	[illegible]	[illegible]
Kuwait	0·81 (0·594 to 1·13)	[illegible]	[illegible]	[illegible]	[illegible]	[illegible]	[illegible]	[illegible]	[illegible]	[illegible]	[illegible]	[illegible]	[illegible]	[illegible]	[illegible]	[illegible]	[illegible]	[illegible]	[illegible]	[illegible]	[illegible]	[illegible]	[illegible]	[illegible]	[illegible]	[illegible]	[illegible]	[illegible]	[illegible]	[illegible]	[illegible]	[illegible]
Lebanon	0·965 (0·677 to 1·33)	[illegible]	[illegible]	[illegible]	[illegible]	[illegible]	[illegible]	[illegible]	[illegible]	[illegible]	[illegible]	[illegible]	[illegible]	[illegible]	[illegible]	[illegible]	[illegible]	[illegible]	[illegible]	[illegible]	[illegible]	[illegible]	[illegible]	[illegible]	[illegible]	[illegible]	[illegible]	[illegible]	[illegible]	[illegible]	[illegible]	[illegible]
Libya	4·53 (3·15 to 6·32)	[illegible]	[illegible]	[illegible]	[illegible]	[illegible]	[illegible]	[illegible]	[illegible]	[illegible]	[illegible]	[illegible]	[illegible]	[illegible]	[illegible]	[illegible]	[illegible]	[illegible]	[illegible]	[illegible]	[illegible]	[illegible]	[illegible]	[illegible]	[illegible]	[illegible]	[illegible]	[illegible]	[illegible]	[illegible]	[illegible]	[illegible]
Morocco	97·6 (70·6 to 133)	[illegible]	[illegible]	[illegible]	[illegible]	[illegible]	[illegible]	[illegible]	[illegible]	[illegible]	[illegible]	[illegible]	[illegible]	[illegible]	[illegible]	[illegible]	[illegible]	[illegible]	[illegible]	[illegible]	[illegible]	[illegible]	[illegible]	[illegible]	[illegible]	[illegible]	[illegible]	[illegible]	[illegible]	[illegible]	[illegible]	[illegible]
Oman	1·13 (1·06 to 1·2)	[illegible]	[illegible]	[illegible]	[illegible]	[illegible]	[illegible]	[illegible]	[illegible]	[illegible]	[illegible]	[illegible]	[illegible]	[illegible]	[illegible]	[illegible]	[illegible]	[illegible]	[illegible]	[illegible]	[illegible]	[illegible]	[illegible]	[illegible]	[illegible]	[illegible]	[illegible]	[illegible]	[illegible]	[illegible]	[illegible]	[illegible]
Palestine	2·11 (1·5 to 2·89)	[illegible]	[illegible]	[illegible]	[illegible]	[illegible]	[illegible]	[illegible]	[illegible]	[illegible]	[illegible]	[illegible]	[illegible]	[illegible]	[illegible]	[illegible]	[illegible]	[illegible]	[illegible]	[illegible]	[illegible]	[illegible]	[illegible]	[illegible]	[illegible]	[illegible]	[illegible]	[illegible]	[illegible]	[illegible]	[illegible]	[illegible]
Qatar	0·286 (0·219 to 0·365)	[illegible]	[illegible]	[illegible]	[illegible]	[illegible]	[illegible]	[illegible]	[illegible]	[illegible]	[illegible]	[illegible]	[illegible]	[illegible]	[illegible]	[illegible]	[illegible]	[illegible]	[illegible]	[illegible]	[illegible]	[illegible]	[illegible]	[illegible]	[illegible]	[illegible]	[illegible]	[illegible]	[illegible]	[illegible]	[illegible]	[illegible]
Saudi Arabia	10·4 (9·83 to 11·1)	[illegible]	[illegible]	[illegible]	[illegible]	[illegible]	[illegible]	[illegible]	[illegible]	[illegible]	[illegible]	[illegible]	[illegible]	[illegible]	[illegible]	[illegible]	[illegible]	[illegible]	[illegible]	[illegible]	[illegible]	[illegible]	[illegible]	[illegible]	[illegible]	[illegible]	[illegible]	[illegible]	[illegible]	[illegible]	[illegible]	[illegible]
Sudan	47·3 (33·5 to 65·8)	[illegible]	[illegible]	[illegible]	[illegible]	[illegible]	[illegible]	[illegible]	[illegible]	[illegible]	[illegible]	[illegible]	[illegible]	[illegible]	[illegible]	[illegible]	[illegible]	[illegible]	[illegible]	[illegible]	[illegible]	[illegible]	[illegible]	[illegible]	[illegible]	[illegible]	[illegible]	[illegible]	[illegible]	[illegible]	[illegible]	[illegible]
Syria	14·6 (10·2 to 20·3)	[illegible]	[illegible]	[illegible]	[illegible]	[illegible]	[illegible]	[illegible]	[illegible]	[illegible]	[illegible]	[illegible]	[illegible]	[illegible]	[illegible]	[illegible]	[illegible]	[illegible]	[illegible]	[illegible]	[illegible]	[illegible]	[illegible]	[illegible]	[illegible]	[illegible]	[illegible]	[illegible]	[illegible]	[illegible]	[illegible]	[illegible]
Tunisia	5·93 (5·49 to 6·39)	[illegible]	[illegible]	[illegible]	[illegible]	[illegible]	[illegible]	[illegible]	[illegible]	[illegible]	[illegible]	[illegible]	[illegible]	[illegible]	[illegible]	[illegible]	[illegible]	[illegible]	[illegible]	[illegible]	[illegible]	[illegible]	[illegible]	[illegible]	[illegible]	[illegible]	[illegible]	[illegible]	[illegible]	[illegible]	[illegible]	[illegible]
Türkiye	73·4 (52·3 to 104)	[illegible]	[illegible]	[illegible]	[illegible]	[illegible]	[illegible]	[illegible]	[illegible]	[illegible]	[illegible]	[illegible]	[illegible]	[illegible]	[illegible]	[illegible]	[illegible]	[illegible]	[illegible]	[illegible]	[illegible]	[illegible]	[illegible]	[illegible]	[illegible]	[illegible]	[illegible]	[illegible]	[illegible]	[illegible]	[illegible]	[illegible]
United Arab Emirates	0·854 (0·604 to 1·22)	[illegible]	[illegible]	[illegible]	[illegible]	[illegible]	[illegible]	[illegible]	[illegible]	[illegible]	[illegible]	[illegible]	[illegible]	[illegible]	[illegible]	[illegible]	[illegible]	[illegible]	[illegible]	[illegible]	[illegible]	[illegible]	[illegible]	[illegible]	[illegible]	[illegible]	[illegible]	[illegible]	[illegible]	[illegible]	[illegible]	[illegible]
Yemen	47·4 (39 to 57·4)	[illegible]	[illegible]	[illegible]	[illegible]	[illegible]	[illegible]	[illegible]	[illegible]	[illegible]	[illegible]	[illegible]	[illegible]	[illegible]	[illegible]	[illegible]	[illegible]	[illegible]	[illegible]	[illegible]	[illegible]	[illegible]	[illegible]	[illegible]	[illegible]	[illegible]	[illegible]	[illegible]	[illegible]	[illegible]	[illegible]	[illegible]
South Asia	1710 (1340 to 2170)	[illegible]	[illegible]	[illegible]	[illegible]	[illegible]	[illegible]	[illegible]	[illegible]	[illegible]	[illegible]	[illegible]	[illegible]	[illegible]	[illegible]	[illegible]	[illegible]	[illegible]	[illegible]	[illegible]	[illegible]	[illegible]	[illegible]	[illegible]	[illegible]	[illegible]	[illegible]	[illegible]	[illegible]	[illegible]	[illegible]	[illegible]
Bangladesh	300 (216 to 402)	[illegible]	[illegible]	[illegible]	[illegible]	[illegible]	[illegible]	[illegible]	[illegible]	[illegible]	[illegible]	[illegible]	[illegible]	[illegible]	[illegible]	[illegible]	[illegible]	[illegible]	[illegible]	[illegible]	[illegible]	[illegible]	[illegible]	[illegible]	[illegible]	[illegible]	[illegible]	[illegible]	[illegible]	[illegible]	[illegible]	[illegible]
Bhutan	2·16 (1·47 to 3·09)	[illegible]	[illegible]	[illegible]	[illegible]	[illegible]	[illegible]	[illegible]	[illegible]	[illegible]	[illegible]	[illegible]	[illegible]	[illegible]	[illegible]	[illegible]	[illegible]	[illegible]	[illegible]	[illegible]	[illegible]	[illegible]	[illegible]	[illegible]	[illegible]	[illegible]	[illegible]	[illegible]	[illegible]	[illegible]	[illegible]	[illegible]
India	895 (677 to 1140)	[illegible]	[illegible]	[illegible]	[illegible]	[illegible]	[illegible]	[illegible]	[illegible]	[illegible]	[illegible]	[illegible]	[illegible]	[illegible]	[illegible]	[illegible]	[illegible]	[illegible]	[illegible]	[illegible]	[illegible]	[illegible]	[illegible]	[illegible]	[illegible]	[illegible]	[illegible]	[illegible]	[illegible]	[illegible]	[illegible]	[illegible]
Nepal	74·6 (53·8 to 105)	[illegible]	[illegible]	[illegible]	[illegible]	[illegible]	[illegible]	[illegible]	[illegible]	[illegible]	[illegible]	[illegible]	[illegible]	[illegible]	[illegible]	[illegible]	[illegible]	[illegible]	[illegible]	[illegible]	[illegible]	[illegible]	[illegible]	[illegible]	[illegible]	[illegible]	[illegible]	[illegible]	[illegible]	[illegible]	[illegible]	[illegible]
Pakistan	433 (298 to 617)	[illegible]	[illegible]	[illegible]	[illegible]	[illegible]	[illegible]	[illegible]	[illegible]	[illegible]	[illegible]	[illegible]	[illegible]	[illegible]	[illegible]	[illegible]	[illegible]	[illegible]	[illegible]	[illegible]	[illegible]	[illegible]	[illegible]	[illegible]	[illegible]	[illegible]	[illegible]	[illegible]	[illegible]	[illegible]	[illegible]	[illegible]
Azad Jammu & Kashmir	8·87 (5·56 to 13·5)	[illegible]	[illegible]	[illegible]	[illegible]	[illegible]	[illegible]	[illegible]	[illegible]	[illegible]	[illegible]	[illegible]	[illegible]	[illegible]	[illegible]	[illegible]	[illegible]	[illegible]	[illegible]	[illegible]	[illegible]	[illegible]	[illegible]	[illegible]	[illegible]	[illegible]	[illegible]	[illegible]	[illegible]	[illegible]	[illegible]	[illegible]
Balochistan	26·2 (14·3 to 44·8)	[illegible]	[illegible]	[illegible]	[illegible]	[illegible]	[illegible]	[illegible]	[illegible]	[illegible]	[illegible]	[illegible]	[illegible]	[illegible]	[illegible]	[illegible]	[illegible]	[illegible]	[illegible]	[illegible]	[illegible]	[illegible]	[illegible]	[illegible]	[illegible]	[illegible]	[illegible]	[illegible]	[illegible]	[illegible]	[illegible]	[illegible]
Gilgit-Baltistan	3·01 (1·48 to 5·58)	[illegible]	[illegible]	[illegible]	[illegible]	[illegible]	[illegible]	[illegible]	[illegible]	[illegible]	[illegible]	[illegible]	[illegible]	[illegible]	[illegible]	[illegible]	[illegible]	[illegible]	[illegible]	[illegible]	[illegible]	[illegible]	[illegible]	[illegible]	[illegible]	[illegible]	[illegible]	[illegible]	[illegible]	[illegible]	[illegible]	[illegible]
Islamabad Capital Territory	2·69 (1·29 to 5·06)	[illegible]	[illegible]	[illegible]	[illegible]	[illegible]	[illegible]	[illegible]	[illegible]	[illegible]	[illegible]	[illegible]	[illegible]	[illegible]	[illegible]	[illegible]	[illegible]	[illegible]	[illegible]	[illegible]	[illegible]	[illegible]	[illegible]	[illegible]	[illegible]	[illegible]	[illegible]	[illegible]	[illegible]	[illegible]	[illegible]	[illegible]
Khyber Pakhtunkhwa	40·9 (28·1 to 57·8)	[illegible]	[illegible]	[illegible]	[illegible]	[illegible]	[illegible]	[illegible]	[illegible]	[illegible]	[illegible]	[illegible]	[illegible]	[illegible]	[illegible]	[illegible]	[illegible]	[illegible]	[illegible]	[illegible]	[illegible]	[illegible]	[illegible]	[illegible]	[illegible]	[illegible]	[illegible]	[illegible]	[illegible]	[illegible]	[illegible]	[illegible]
Punjab	201 (138 to 284)	[illegible]	[illegible]	[illegible]	[illegible]	[illegible]	[illegible]	[illegible]	[illegible]	[illegible]	[illegible]	[illegible]	[illegible]	[illegible]	[illegible]	[illegible]	[illegible]	[illegible]	[illegible]	[illegible]	[illegible]	[illegible]	[illegible]	[illegible]	[illegible]	[illegible]	[illegible]	[illegible]	[illegible]	[illegible]	[illegible]	[illegible]
Sindh	151 (104 to 214)	[illegible]	[illegible]	[illegible]	[illegible]	[illegible]	[illegible]	[illegible]	[illegible]	[illegible]	[illegible]	[illegible]	[illegible]	[illegible]	[illegible]	[illegible]	[illegible]	[illegible]	[illegible]	[illegible]	[illegible]	[illegible]	[illegible]	[illegible]	[illegible]	[illegible]	[illegible]	[illegible]	[illegible]	[illegible]	[illegible]	[illegible]
Southeast Asia, east Asia, and Oceania	927 (681 to 1240)	[illegible]	[illegible]	[illegible]	[illegible]	[illegible]	[illegible]	[illegible]	[illegible]	[illegible]	[illegible]	[illegible]	[illegible]	[illegible]	[illegible]	[illegible]	[illegible]	[illegible]	[illegible]	[illegible]	[illegible]	[illegible]	[illegible]	[illegible]	[illegible]	[illegible]	[illegible]	[illegible]	[illegible]	[illegible]	[illegible]	[illegible]
East Asia	604 (422 to 840)	[illegible]	[illegible]	[illegible]	[illegible]	[illegible]	[illegible]	[illegible]	[illegible]	[illegible]	[illegible]	[illegible]	[illegible]	[illegible]	[illegible]	[illegible]	[illegible]	[illegible]	[illegible]	[illegible]	[illegible]	[illegible]	[illegible]	[illegible]	[illegible]	[illegible]	[illegible]	[illegible]	[illegible]	[illegible]	[illegible]	[illegible]
China	591 (413 to 823)	[illegible]	[illegible]	[illegible]	[illegible]	[illegible]	[illegible]	[illegible]	[illegible]	[illegible]	[illegible]	[illegible]	[illegible]	[illegible]	[illegible]	[illegible]	[illegible]	[illegible]	[illegible]	[illegible]	[illegible]	[illegible]	[illegible]	[illegible]	[illegible]	[illegible]	[illegible]	[illegible]	[illegible]	[illegible]	[illegible]	[illegible]
North Korea	10·2 (7·14 to 14·2)	[illegible]	[illegible]	[illegible]	[illegible]	[illegible]	[illegible]	[illegible]	[illegible]	[illegible]	[illegible]	[illegible]	[illegible]	[illegible]	[illegible]	[illegible]	[illegible]	[illegible]	[illegible]	[illegible]	[illegible]	[illegible]	[illegible]	[illegible]	[illegible]	[illegible]	[illegible]	[illegible]	[illegible]	[illegible]	[illegible]	[illegible]
Taiwan (province of China)	1·79 (1·25 to 2·47)	[illegible]	[illegible]	[illegible]	[illegible]	[illegible]	[illegible]	[illegible]	[illegible]	[illegible]	[illegible]	[illegible]	[illegible]	[illegible]	[illegible]	[illegible]	[illegible]	[illegible]	[illegible]	[illegible]	[illegible]	[illegible]	[illegible]	[illegible]	[illegible]	[illegible]	[illegible]	[illegible]	[illegible]	[illegible]	[illegible]	[illegible]
Oceania	8·78 (6·18 to 12·1)	[illegible]	[illegible]	[illegible]	[illegible]	[illegible]	[illegible]	[illegible]	[illegible]	[illegible]	[illegible]	[illegible]	[illegible]	[illegible]	[illegible]	[illegible]	[illegible]	[illegible]	[illegible]	[illegible]	[illegible]	[illegible]	[illegible]	[illegible]	[illegible]	[illegible]	[illegible]	[illegible]	[illegible]	[illegible]	[illegible]	[illegible]
American Samoa	0·0329 (0·0286 to 0·0378)	[illegible]	[illegible]	[illegible]	[illegible]	[illegible]	[illegible]	[illegible]	[illegible]	[illegible]	[illegible]	[illegible]	[illegible]	[illegible]	[illegible]	[illegible]	[illegible]	[illegible]	[illegible]	[illegible]	[illegible]	[illegible]	[illegible]	[illegible]	[illegible]	[illegible]	[illegible]	[illegible]	[illegible]	[illegible]	[illegible]	[illegible]
Cook Islands	0·00579 (0·00458 to 0·00725)	[illegible]	[illegible]	[illegible]	[illegible]	[illegible]	[illegible]	[illegible]	[illegible]	[illegible]	[illegible]	[illegible]	[illegible]	[illegible]	[illegible]	[illegible]	[illegible]	[illegible]	[illegible]	[illegible]	[illegible]	[illegible]	[illegible]	[illegible]	[illegible]	[illegible]	[illegible]	[illegible]	[illegible]	[illegible]	[illegible]	[illegible]
Federated States of Micronesia	0·0778 (0·0544 to 0·108)	[illegible]	[illegible]	[illegible]	[illegible]	[illegible]	[illegible]	[illegible]	[illegible]	[illegible]	[illegible]	[illegible]	[illegible]	[illegible]	[illegible]	[illegible]	[illegible]	[illegible]	[illegible]	[illegible]	[illegible]	[illegible]	[illegible]	[illegible]	[illegible]	[illegible]	[illegible]	[illegible]	[illegible]	[illegible]	[illegible]	[illegible]
Fiji	0·185 (0·131 to 0·252)	[illegible]	[illegible]	[illegible]	[illegible]	[illegible]	[illegible]	[illegible]	[illegible]	[illegible]	[illegible]	[illegible]	[illegible]	[illegible]	[illegible]	[illegible]	[illegible]	[illegible]	[illegible]	[illegible]	[illegible]	[illegible]	[illegible]	[illegible]	[illegible]	[illegible]	[illegible]	[illegible]	[illegible]	[illegible]	[illegible]	[illegible]
Guam	0·0852 (0·0591 to 0·119)	[illegible]	[illegible]	[illegible]	[illegible]	[illegible]	[illegible]	[illegible]	[illegible]	[illegible]	[illegible]	[illegible]	[illegible]	[illegible]	[illegible]	[illegible]	[illegible]	[illegible]	[illegible]	[illegible]	[illegible]	[illegible]	[illegible]	[illegible]	[illegible]	[illegible]	[illegible]	[illegible]	[illegible]	[illegible]	[illegible]	[illegible]
Kiribati	0·0822 (0·0574 to 0·115)	[illegible]	[illegible]	[illegible]	[illegible]	[illegible]	[illegible]	[illegible]	[illegible]	[illegible]	[illegible]	[illegible]	[illegible]	[illegible]	[illegible]	[illegible]	[illegible]	[illegible]	[illegible]	[illegible]	[illegible]	[illegible]	[illegible]	[illegible]	[illegible]	[illegible]	[illegible]	[illegible]	[illegible]	[illegible]	[illegible]	[illegible]
Marshall Islands	0·0291 (0·0207 to 0·0408)	[illegible]	[illegible]	[illegible]	[illegible]	[illegible]	[illegible]	[illegible]	[illegible]	[illegible]	[illegible]	[illegible]	[illegible]	[illegible]	[illegible]	[illegible]	[illegible]	[illegible]	[illegible]	[illegible]	[illegible]	[illegible]	[illegible]	[illegible]	[illegible]	[illegible]	[illegible]	[illegible]	[illegible]	[illegible]	[illegible]	[illegible]
Nauru	0·00739 (0·00517 to 0·0103)	[illegible]	[illegible]	[illegible]	[illegible]	[illegible]	[illegible]	[illegible]	[illegible]	[illegible]	[illegible]	[illegible]	[illegible]	[illegible]	[illegible]	[illegible]	[illegible]	[illegible]	[illegible]	[illegible]	[illegible]	[illegible]	[illegible]	[illegible]	[illegible]	[illegible]	[illegible]	[illegible]	[illegible]	[illegible]	[illegible]	[illegible]
Niue	0·00103 (0·00072 to 0·00143)	[illegible]	[illegible]	[illegible]	[illegible]	[illegible]	[illegible]	[illegible]	[illegible]	[illegible]	[illegible]	[illegible]	[illegible]	[illegible]	[illegible]	[illegible]	[illegible]	[illegible]	[illegible]	[illegible]	[illegible]	[illegible]	[illegible]	[illegible]	[illegible]	[illegible]	[illegible]	[illegible]	[illegible]	[illegible]	[illegible]	[illegible]
Northern Mariana Islands	0·0444 (0·0313 to 0·0638)	[illegible]	[illegible]	[illegible]	[illegible]	[illegible]	[illegible]	[illegible]	[illegible]	[illegible]	[illegible]	[illegible]	[illegible]	[illegible]	[illegible]	[illegible]	[illegible]	[illegible]	[illegible]	[illegible]	[illegible]	[illegible]	[illegible]	[illegible]	[illegible]	[illegible]	[illegible]	[illegible]	[illegible]	[illegible]	[illegible]	[illegible]
Palau	0·00511 (0·00366 to 0·00716)	[illegible]	[illegible]	[illegible]	[illegible]	[illegible]	[illegible]	[illegible]	[illegible]	[illegible]	[illegible]	[illegible]	[illegible]	[illegible]	[illegible]	[illegible]	[illegible]	[illegible]	[illegible]	[illegible]	[illegible]	[illegible]	[illegible]	[illegible]	[illegible]	[illegible]	[illegible]	[illegible]	[illegible]	[illegible]	[illegible]	[illegible]
Papua New Guinea	6·96 (4·82 to 9·76)	[illegible]	[illegible]	[illegible]	[illegible]	[illegible]	[illegible]	[illegible]	[illegible]	[illegible]	[illegible]	[illegible]	[illegible]	[illegible]	[illegible]	[illegible]	[illegible]	[illegible]	[illegible]	[illegible]	[illegible]	[illegible]	[illegible]	[illegible]	[illegible]	[illegible]	[illegible]	[illegible]	[illegible]	[illegible]	[illegible]	[illegible]
Samoa	0·0601 (0·0428 to 0·0839)	[illegible]	[illegible]	[illegible]	[illegible]	[illegible]	[illegible]	[illegible]	[illegible]	[illegible]	[illegible]	[illegible]	[illegible]	[illegible]	[illegible]	[illegible]	[illegible]	[illegible]	[illegible]	[illegible]	[illegible]	[illegible]	[illegible]	[illegible]	[illegible]	[illegible]	[illegible]	[illegible]	[illegible]	[illegible]	[illegible]	[illegible]
Solomon Islands	0·421 (0·294 to 0·587)	[illegible]	[illegible]	[illegible]	[illegible]	[illegible]	[illegible]	[illegible]	[illegible]	[illegible]	[illegible]	[illegible]	[illegible]	[illegible]	[illegible]	[illegible]	[illegible]	[illegible]	[illegible]	[illegible]	[illegible]	[illegible]	[illegible]	[illegible]	[illegible]	[illegible]	[illegible]	[illegible]	[illegible]	[illegible]	[illegible]	[illegible]
Tokelau	0·000665 (0·000465 to 0·000924)	[illegible]	[illegible]	[illegible]	[illegible]	[illegible]	[illegible]	[illegible]	[illegible]	[illegible]	[illegible]	[illegible]	[illegible]	[illegible]	[illegible]	[illegible]	[illegible]	[illegible]	[illegible]	[illegible]	[illegible]	[illegible]	[illegible]	[illegible]	[illegible]	[illegible]	[illegible]	[illegible]	[illegible]	[illegible]	[illegible]	[illegible]
Tonga	0·0683 (0·0472 to 0·0942)	[illegible]	[illegible]	[illegible]	[illegible]	[illegible]	[illegible]	[illegible]	[illegible]	[illegible]	[illegible]	[illegible]	[illegible]	[illegible]	[illegible]	[illegible]	[illegible]	[illegible]	[illegible]	[illegible]	[illegible]	[illegible]	[illegible]	[illegible]	[illegible]	[illegible]	[illegible]	[illegible]	[illegible]	[illegible]	[illegible]	[illegible]
Tuvalu	0·0132 (0·0092 to 0·0185)	[illegible]	[illegible]	[illegible]	[illegible]	[illegible]	[illegible]	[illegible]	[illegible]	[illegible]	[illegible]	[illegible]	[illegible]	[illegible]	[illegible]	[illegible]	[illegible]	[illegible]	[illegible]	[illegible]	[illegible]	[illegible]	[illegible]	[illegible]	[illegible]	[illegible]	[illegible]	[illegible]	[illegible]	[illegible]	[illegible]	[illegible]
Vanuatu	0·141 (0·0988 to 0·197)	[illegible]	[illegible]	[illegible]	[illegible]	[illegible]	[illegible]	[illegible]	[illegible]	[illegible]	[illegible]	[illegible]	[illegible]	[illegible]	[illegible]	[illegible]	[illegible]	[illegible]	[illegible]	[illegible]	[illegible]	[illegible]	[illegible]	[illegible]	[illegible]	[illegible]	[illegible]	[illegible]	[illegible]	[illegible]	[illegible]	[illegible]
Southeast Asia	314 (251 to 389)	[illegible]	[illegible]	[illegible]	[illegible]	[illegible]	[illegible]	[illegible]	[illegible]	[illegible]	[illegible]	[illegible]	[illegible]	[illegible]	[illegible]	[illegible]	[illegible]	[illegible]	[illegible]	[illegible]	[illegible]	[illegible]	[illegible]	[illegible]	[illegible]	[illegible]	[illegible]	[illegible]	[illegible]	[illegible]	[illegible]	[illegible]
Cambodia	18·3 (12·5 to 25·3)	[illegible]	[illegible]	[illegible]	[illegible]	[illegible]	[illegible]	[illegible]	[illegible]	[illegible]	[illegible]	[illegible]	[illegible]	[illegible]	[illegible]	[illegible]	[illegible]	[illegible]	[illegible]	[illegible]	[illegible]	[illegible]	[illegible]	[illegible]	[illegible]	[illegible]	[illegible]	[illegible]	[illegible]	[illegible]	[illegible]	[illegible]
Indonesia	134 (106 to 167)	[illegible]	[illegible]	[illegible]	[illegible]	[illegible]	[illegible]	[illegible]	[illegible]	[illegible]	[illegible]	[illegible]	[illegible]	[illegible]	[illegible]	[illegible]	[illegible]	[illegible]	[illegible]	[illegible]	[illegible]	[illegible]	[illegible]	[illegible]	[illegible]	[illegible]	[illegible]	[illegible]	[illegible]	[illegible]	[illegible]	[illegible]
Aceh	2·37 (1·68 to 3·26)	[illegible]	[illegible]	[illegible]	[illegible]	[illegible]	[illegible]	[illegible]	[illegible]	[illegible]	[illegible]	[illegible]	[illegible]	[illegible]	[illegible]	[illegible]	[illegible]	[illegible]	[illegible]	[illegible]	[illegible]	[illegible]	[illegible]	[illegible]	[illegible]	[illegible]	[illegible]	[illegible]	[illegible]	[illegible]	[illegible]	[illegible]
Bali	1·09 (0·786 to 1·51)	[illegible]	[illegible]	[illegible]	[illegible]	[illegible]	[illegible]	[illegible]	[illegible]	[illegible]	[illegible]	[illegible]	[illegible]	[illegible]	[illegible]	[illegible]	[illegible]	[illegible]	[illegible]	[illegible]	[illegible]	[illegible]	[illegible]	[illegible]	[illegible]	[illegible]	[illegible]	[illegible]	[illegible]	[illegible]	[illegible]	[illegible]
Bangka-Belitung Islands	1·16 (0·809 to 1·6)	[illegible]	[illegible]	[illegible]	[illegible]	[illegible]	[illegible]	[illegible]	[illegible]	[illegible]	[illegible]	[illegible]	[illegible]	[illegible]	[illegible]	[illegible]	[illegible]	[illegible]	[illegible]	[illegible]	[illegible]	[illegible]	[illegible]	[illegible]	[illegible]	[illegible]	[illegible]	[illegible]	[illegible]	[illegible]	[illegible]	[illegible]
Banten	8·06 (5·72 to 11·4)	[illegible]	[illegible]	[illegible]	[illegible]	[illegible]	[illegible]	[illegible]	[illegible]	[illegible]	[illegible]	[illegible]	[illegible]	[illegible]	[illegible]	[illegible]	[illegible]	[illegible]	[illegible]	[illegible]	[illegible]	[illegible]	[illegible]	[illegible]	[illegible]	[illegible]	[illegible]	[illegible]	[illegible]	[illegible]	[illegible]	[illegible]
Bengkulu	1·08 (0·762 to 1·49)	[illegible]	[illegible]	[illegible]	[illegible]	[illegible]	[illegible]	[illegible]	[illegible]	[illegible]	[illegible]	[illegible]	[illegible]	[illegible]	[illegible]	[illegible]	[illegible]	[illegible]	[illegible]	[illegible]	[illegible]	[illegible]	[illegible]	[illegible]	[illegible]	[illegible]	[illegible]	[illegible]	[illegible]	[illegible]	[illegible]	[illegible]
Gorontalo	0·693 (0·48 to 0·975)	[illegible]	[illegible]	[illegible]	[illegible]	[illegible]	[illegible]	[illegible]	[illegible]	[illegible]	[illegible]	[illegible]	[illegible]	[illegible]	[illegible]	[illegible]	[illegible]	[illegible]	[illegible]	[illegible]	[illegible]	[illegible]	[illegible]	[illegible]	[illegible]	[illegible]	[illegible]	[illegible]	[illegible]	[illegible]	[illegible]	[illegible]
Jakarta	5·29 (3·7 to 7·3)	[illegible]	[illegible]	[illegible]	[illegible]	[illegible]	[illegible]	[illegible]	[illegible]	[illegible]	[illegible]	[illegible]	[illegible]	[illegible]	[illegible]	[illegible]	[illegible]	[illegible]	[illegible]	[illegible]	[illegible]	[illegible]	[illegible]	[illegible]	[illegible]	[illegible]	[illegible]	[illegible]	[illegible]	[illegible]	[illegible]	[illegible]
Jambi	2·71 (1·98 to 3·64)	[illegible]	[illegible]	[illegible]	[illegible]	[illegible]	[illegible]	[illegible]	[illegible]	[illegible]	[illegible]	[illegible]	[illegible]	[illegible]	[illegible]	[illegible]	[illegible]	[illegible]	[illegible]	[illegible]	[illegible]	[illegible]	[illegible]	[illegible]	[illegible]	[illegible]	[illegible]	[illegible]	[illegible]	[illegible]	[illegible]	[illegible]
West Java	18·3 (13 to 25)	[illegible]	[illegible]	[illegible]	[illegible]	[illegible]	[illegible]	[illegible]	[illegible]	[illegible]	[illegible]	[illegible]	[illegible]	[illegible]	[illegible]	[illegible]	[illegible]	[illegible]	[illegible]	[illegible]	[illegible]	[illegible]	[illegible]	[illegible]	[illegible]	[illegible]	[illegible]	[illegible]	[illegible]	[illegible]	[illegible]	[illegible]
Central Java	12·8 (9·5 to 17·2)	[illegible]	[illegible]	[illegible]	[illegible]	[illegible]	[illegible]	[illegible]	[illegible]	[illegible]	[illegible]	[illegible]	[illegible]	[illegible]	[illegible]	[illegible]	[illegible]	[illegible]	[illegible]	[illegible]	[illegible]	[illegible]	[illegible]	[illegible]	[illegible]	[illegible]	[illegible]	[illegible]	[illegible]	[illegible]	[illegible]	[illegible]
East Java	26·5 (18·8 to 35·9)	[illegible]	[illegible]	[illegible]	[illegible]	[illegible]	[illegible]	[illegible]	[illegible]	[illegible]	[illegible]	[illegible]	[illegible]	[illegible]	[illegible]	[illegible]	[illegible]	[illegible]	[illegible]	[illegible]	[illegible]	[illegible]	[illegible]	[illegible]	[illegible]	[illegible]	[illegible]	[illegible]	[illegible]	[illegible]	[illegible]	[illegible]
West Kalimantan	4·44 (3·29 to 5·85)	[illegible]	[illegible]	[illegible]	[illegible]	[illegible]	[illegible]	[illegible]	[illegible]	[illegible]	[illegible]	[illegible]	[illegible]	[illegible]	[illegible]	[illegible]	[illegible]	[illegible]	[illegible]	[illegible]	[illegible]	[illegible]	[illegible]	[illegible]	[illegible]	[illegible]	[illegible]	[illegible]	[illegible]	[illegible]	[illegible]	[illegible]
South Kalimantan	2·9 (2·02 to 4·07)	[illegible]	[illegible]	[illegible]	[illegible]	[illegible]	[illegible]	[illegible]	[illegible]	[illegible]	[illegible]	[illegible]	[illegible]	[illegible]	[illegible]	[illegible]	[illegible]	[illegible]	[illegible]	[illegible]	[illegible]	[illegible]	[illegible]	[illegible]	[illegible]	[illegible]	[illegible]	[illegible]	[illegible]	[illegible]	[illegible]	[illegible]
Central Kalimantan	0·822 (0·582 to 1·13)	[illegible]	[illegible]	[illegible]	[illegible]	[illegible]	[illegible]	[illegible]	[illegible]	[illegible]	[illegible]	[illegible]	[illegible]	[illegible]	[illegible]	[illegible]	[illegible]	[illegible]	[illegible]	[illegible]	[illegible]	[illegible]	[illegible]	[illegible]	[illegible]	[illegible]	[illegible]	[illegible]	[illegible]	[illegible]	[illegible]	[illegible]
East Kalimantan	1·78 (1·31 to 2·4)	[illegible]	[illegible]	[illegible]	[illegible]	[illegible]	[illegible]	[illegible]	[illegible]	[illegible]	[illegible]	[illegible]	[illegible]	[illegible]	[illegible]	[illegible]	[illegible]	[illegible]	[illegible]	[illegible]	[illegible]	[illegible]	[illegible]	[illegible]	[illegible]	[illegible]	[illegible]	[illegible]	[illegible]	[illegible]	[illegible]	[illegible]
North Kalimantan	0·148 (0·101 to 0·206)	[illegible]	[illegible]	[illegible]	[illegible]	[illegible]	[illegible]	[illegible]	[illegible]	[illegible]	[illegible]	[illegible]	[illegible]	[illegible]	[illegible]	[illegible]	[illegible]	[illegible]	[illegible]	[illegible]	[illegible]	[illegible]	[illegible]	[illegible]	[illegible]	[illegible]	[illegible]	[illegible]	[illegible]	[illegible]	[illegible]	[illegible]
Riau Islands	0·62 (0·463 to 0·821)	[illegible]	[illegible]	[illegible]	[illegible]	[illegible]	[illegible]	[illegible]	[illegible]	[illegible]	[illegible]	[illegible]	[illegible]	[illegible]	[illegible]	[illegible]	[illegible]	[illegible]	[illegible]	[illegible]	[illegible]	[illegible]	[illegible]	[illegible]	[illegible]	[illegible]	[illegible]	[illegible]	[illegible]	[illegible]	[illegible]	[illegible]
Lampung	5·66 (3·91 to 7·92)	[illegible]	[illegible]	[illegible]	[illegible]	[illegible]	[illegible]	[illegible]	[illegible]	[illegible]	[illegible]	[illegible]	[illegible]	[illegible]	[illegible]	[illegible]	[illegible]	[illegible]	[illegible]	[illegible]	[illegible]	[illegible]	[illegible]	[illegible]	[illegible]	[illegible]	[illegible]	[illegible]	[illegible]	[illegible]	[illegible]	[illegible]
Maluku	0·966 (0·714 to 1·28)	[illegible]	[illegible]	[illegible]	[illegible]	[illegible]	[illegible]	[illegible]	[illegible]	[illegible]	[illegible]	[illegible]	[illegible]	[illegible]	[illegible]	[illegible]	[illegible]	[illegible]	[illegible]	[illegible]	[illegible]	[illegible]	[illegible]	[illegible]	[illegible]	[illegible]	[illegible]	[illegible]	[illegible]	[illegible]	[illegible]	[illegible]
North Maluku	0·575 (0·409 to 0·806)	[illegible]	[illegible]	[illegible]	[illegible]	[illegible]	[illegible]	[illegible]	[illegible]	[illegible]	[illegible]	[illegible]	[illegible]	[illegible]	[illegible]	[illegible]	[illegible]	[illegible]	[illegible]	[illegible]	[illegible]	[illegible]	[illegible]	[illegible]	[illegible]	[illegible]	[illegible]	[illegible]	[illegible]	[illegible]	[illegible]	[illegible]
West Nusa Tenggara	3·74 (2·65 to 5·15)	[illegible]	[illegible]	[illegible]	[illegible]	[illegible]	[illegible]	[illegible]	[illegible]	[illegible]	[illegible]	[illegible]	[illegible]	[illegible]	[illegible]	[illegible]	[illegible]	[illegible]	[illegible]	[illegible]	[illegible]	[illegible]	[illegible]	[illegible]	[illegible]	[illegible]	[illegible]	[illegible]	[illegible]	[illegible]	[illegible]	[illegible]
East Nusa Tenggara	1·97 (1·41 to 2·7)	[illegible]	[illegible]	[illegible]	[illegible]	[illegible]	[illegible]	[illegible]	[illegible]	[illegible]	[illegible]	[illegible]	[illegible]	[illegible]	[illegible]	[illegible]	[illegible]	[illegible]	[illegible]	[illegible]	[illegible]	[illegible]	[illegible]	[illegible]	[illegible]	[illegible]	[illegible]	[illegible]	[illegible]	[illegible]	[illegible]	[illegible]
Papua	0·69 (0·509 to 0·921)	[illegible]	[illegible]	[illegible]	[illegible]	[illegible]	[illegible]	[illegible]	[illegible]	[illegible]	[illegible]	[illegible]	[illegible]	[illegible]	[illegible]	[illegible]	[illegible]	[illegible]	[illegible]	[illegible]	[illegible]	[illegible]	[illegible]	[illegible]	[illegible]	[illegible]	[illegible]	[illegible]	[illegible]	[illegible]	[illegible]	[illegible]
West Papua	0·478 (0·34 to 0·652)	[illegible]	[illegible]	[illegible]	[illegible]	[illegible]	[illegible]	[illegible]	[illegible]	[illegible]	[illegible]	[illegible]	[illegible]	[illegible]	[illegible]	[illegible]	[illegible]	[illegible]	[illegible]	[illegible]	[illegible]	[illegible]	[illegible]	[illegible]	[illegible]	[illegible]	[illegible]	[illegible]	[illegible]	[illegible]	[illegible]	[illegible]
Riau	3·23 (2·35 to 4·28)	[illegible]	[illegible]	[illegible]	[illegible]	[illegible]	[illegible]	[illegible]	[illegible]	[illegible]	[illegible]	[illegible]	[illegible]	[illegible]	[illegible]	[illegible]	[illegible]	[illegible]	[illegible]	[illegible]	[illegible]	[illegible]	[illegible]	[illegible]	[illegible]	[illegible]	[illegible]	[illegible]	[illegible]	[illegible]	[illegible]	[illegible]
West Sulawesi	0·847 (0·599 to 1·15)	[illegible]	[illegible]	[illegible]	[illegible]	[illegible]	[illegible]	[illegible]	[illegible]	[illegible]	[illegible]	[illegible]	[illegible]	[illegible]	[illegible]	[illegible]	[illegible]	[illegible]	[illegible]	[illegible]	[illegible]	[illegible]	[illegible]	[illegible]	[illegible]	[illegible]	[illegible]	[illegible]	[illegible]	[illegible]	[illegible]	[illegible]
South Sulawesi	5·12 (3·77 to 6·83)	[illegible]	[illegible]	[illegible]	[illegible]	[illegible]	[illegible]	[illegible]	[illegible]	[illegible]	[illegible]	[illegible]	[illegible]	[illegible]	[illegible]	[illegible]	[illegible]	[illegible]	[illegible]	[illegible]	[illegible]	[illegible]	[illegible]	[illegible]	[illegible]	[illegible]	[illegible]	[illegible]	[illegible]	[illegible]	[illegible]	[illegible]
Central Sulawesi	3·2 (2·33 to 4·37)	[illegible]	[illegible]	[illegible]	[illegible]	[illegible]	[illegible]	[illegible]	[illegible]	[illegible]	[illegible]	[illegible]	[illegible]	[illegible]	[illegible]	[illegible]	[illegible]	[illegible]	[illegible]	[illegible]	[illegible]	[illegible]	[illegible]	[illegible]	[illegible]	[illegible]	[illegible]	[illegible]	[illegible]	[illegible]	[illegible]	[illegible]
Southeast Sulawesi	1·28 (0·913 to 1·76)	[illegible]	[illegible]	[illegible]	[illegible]	[illegible]	[illegible]	[illegible]	[illegible]	[illegible]	[illegible]	[illegible]	[illegible]	[illegible]	[illegible]	[illegible]	[illegible]	[illegible]	[illegible]	[illegible]	[illegible]	[illegible]	[illegible]	[illegible]	[illegible]	[illegible]	[illegible]	[illegible]	[illegible]	[illegible]	[illegible]	[illegible]
North Sulawesi	0·726 (0·544 to 0·944)	[illegible]	[illegible]	[illegible]	[illegible]	[illegible]	[illegible]	[illegible]	[illegible]	[illegible]	[illegible]	[illegible]	[illegible]	[illegible]	[illegible]	[illegible]	[illegible]	[illegible]	[illegible]	[illegible]	[illegible]	[illegible]	[illegible]	[illegible]	[illegible]	[illegible]	[illegible]	[illegible]	[illegible]	[illegible]	[illegible]	[illegible]
West Sumatra	3·1 (2·3 to 4·18)	[illegible]	[illegible]	[illegible]	[illegible]	[illegible]	[illegible]	[illegible]	[illegible]	[illegible]	[illegible]	[illegible]	[illegible]	[illegible]	[illegible]	[illegible]	[illegible]	[illegible]	[illegible]	[illegible]	[illegible]	[illegible]	[illegible]	[illegible]	[illegible]	[illegible]	[illegible]	[illegible]	[illegible]	[illegible]	[illegible]	[illegible]
South Sumatra	4·68 (3·3 to 6·49)	[illegible]	[illegible]	[illegible]	[illegible]	[illegible]	[illegible]	[illegible]	[illegible]	[illegible]	[illegible]	[illegible]	[illegible]	[illegible]	[illegible]	[illegible]	[illegible]	[illegible]	[illegible]	[illegible]	[illegible]	[illegible]	[illegible]	[illegible]	[illegible]	[illegible]	[illegible]	[illegible]	[illegible]	[illegible]	[illegible]	[illegible]
North Sumatra	6·23 (4·56 to 8·31)	[illegible]	[illegible]	[illegible]	[illegible]	[illegible]	[illegible]	[illegible]	[illegible]	[illegible]	[illegible]	[illegible]	[illegible]	[illegible]	[illegible]	[illegible]	[illegible]	[illegible]	[illegible]	[illegible]	[illegible]	[illegible]	[illegible]	[illegible]	[illegible]	[illegible]	[illegible]	[illegible]	[illegible]	[illegible]	[illegible]	[illegible]
Yogyakarta	0·908 (0·666 to 1·19)	[illegible]	[illegible]	[illegible]	[illegible]	[illegible]	[illegible]	[illegible]	[illegible]	[illegible]	[illegible]	[illegible]	[illegible]	[illegible]	[illegible]	[illegible]	[illegible]	[illegible]	[illegible]	[illegible]	[illegible]	[illegible]	[illegible]	[illegible]	[illegible]	[illegible]	[illegible]	[illegible]	[illegible]	[illegible]	[illegible]	[illegible]
Laos	11 (7·58 to 15·5)	[illegible]	[illegible]	[illegible]	[illegible]	[illegible]	[illegible]	[illegible]	[illegible]	[illegible]	[illegible]	[illegible]	[illegible]	[illegible]	[illegible]	[illegible]	[illegible]	[illegible]	[illegible]	[illegible]	[illegible]	[illegible]	[illegible]	[illegible]	[illegible]	[illegible]	[illegible]	[illegible]	[illegible]	[illegible]	[illegible]	[illegible]
Malaysia	4·98 (4·45 to 5·55)	[illegible]	[illegible]	[illegible]	[illegible]	[illegible]	[illegible]	[illegible]	[illegible]	[illegible]	[illegible]	[illegible]	[illegible]	[illegible]	[illegible]	[illegible]	[illegible]	[illegible]	[illegible]	[illegible]	[illegible]	[illegible]	[illegible]	[illegible]	[illegible]	[illegible]	[illegible]	[illegible]	[illegible]	[illegible]	[illegible]	[illegible]
Maldives	0·5 (0·364 to 0·694)	[illegible]	[illegible]	[illegible]	[illegible]	[illegible]	[illegible]	[illegible]	[illegible]	[illegible]	[illegible]	[illegible]	[illegible]	[illegible]	[illegible]	[illegible]	[illegible]	[illegible]	[illegible]	[illegible]	[illegible]	[illegible]	[illegible]	[illegible]	[illegible]	[illegible]	[illegible]	[illegible]	[illegible]	[illegible]	[illegible]	[illegible]
Mauritius	0·585 (0·542 to 0·631)	[illegible]	[illegible]	[illegible]	[illegible]	[illegible]	[illegible]	[illegible]	[illegible]	[illegible]	[illegible]	[illegible]	[illegible]	[illegible]	[illegible]	[illegible]	[illegible]	[illegible]	[illegible]	[illegible]	[illegible]	[illegible]	[illegible]	[illegible]	[illegible]	[illegible]	[illegible]	[illegible]	[illegible]	[illegible]	[illegible]	[illegible]

Myanmar	43·4 (30·9 to 59·9)	42 (30 to 57·8)	40·6 (28·8 to 56·4)	39·3 (27·8 to 54·1)	38·1 (26·6 to 53)	37 (25·8 to 51·3)	36 (25·1 to 50·3)	35·3 (24·4 to 50·1)	34·8 (24·6 to 49·9)	34·2 (24 to 49·3)	33·6 (23·9 to 48·3)	33 (23·4 to 47·7)	32·3 (22·8 to 46·2)	31·6 (22·5 to 44·7)	30·8 (21·9 to 43·3)	30·1 (21·5 to 41·4)	29·3 (20·9 to 39·8)	28·5 (20·4 to 38·6)	27·8 (19·9 to 37·6)	27 (19·6 to 35·8)	26 (19·1 to 34·4)	25·3 (18·6 to 33·2)	24·6 (18·5 to 31·9)	23·9 (18·3 to 31·2)	23·1 (17·3 to 30·7)	22·6 (16·7 to 29·6)	22·2 (16·1 to 29)	21·7 (15·8 to 28·6)	21·2 (15·3 to 28)	20·7 (14·9 to 27·7)	20·1 (14·4 to 27·1)	19·7 (14·3 to 26·6)
Philippines	31·6 (24·8 to 40·2)	30·5 (24·6 to 37·7)	30 (24·8 to 35·5)	29·1 (25 to 33·8)	26·5 (23·6 to 29·7)	25·1 (23·3 to 27·1)	24·6 (23·3 to 26)	25·9 (24·4 to 27·4)	26·5 (25 to 28·1)	25·9 (24·6 to 27·3)	26·3 (25 to 27·7)	26·6 (25·4 to 28)	27·1 (25·8 to 28·4)	27·6 (26·2 to 28·9)	28·2 (26·8 to 29·5)	28·1 (26·7 to 29·4)	26·7 (25·4 to 28·1)	25·8 (24·6 to 27·1)	25·3 (24·2 to 26·6)	25·6 (24·4 to 26·9)	25·5 (24·2 to 26·6)	25 (23·9 to 26·2)	24·1 (22·9 to 25·3)	23·4 (22·3 to 24·5)	23·3 (22·2 to 24·6)	23·3 (22·2 to 24·6)	22·9 (21·8 to 24·1)	22·8 (21·2 to 24·7)	22·8 (20·4 to 25·2)	23·8 (20·4 to 27·5)	23·3 (19·1 to 27·4)	25·4 (20·6 to 30·6)
Seychelles	0·0324 (0·0238 to 0·0441)	0·031 (0·0232 to 0·041)	0·0298 (0·0228 to 0·038)	0·0285 (0·0221 to 0·0356)	0·0269 (0·0218 to 0·0333)	0·0265 (0·0219 to 0·0325)	0·0243 (0·02 to 0·0298)	0·0206 (0·0165 to 0·0255)	0·0184 (0·0144 to 0·0232)	0·017 (0·0133 to 0·0215)	0·0156 (0·0122 to 0·0196)	0·0147 (0·0116 to 0·0181)	0·014 (0·0112 to 0·0171)	0·0131 (0·0108 to 0·0156)	0·0141 (0·0118 to 0·0165)	0·0146 (0·0122 to 0·0172)	0·014 (0·0116 to 0·0168)	0·0147 (0·0116 to 0·018)	0·015 (0·0117 to 0·0188)	0·0151 (0·0117 to 0·0192)	0·0152 (0·0118 to 0·0195)	0·0154 (0·0119 to 0·0197)	0·0155 (0·0122 to 0·0195)	0·0156 (0·0125 to 0·0194)	0·0156 (0·0129 to 0·0186)	0·0159 (0·0135 to 0·0186)	0·0163 (0·014 to 0·0191)	0·0158 (0·0135 to 0·0185)	0·0153 (0·0127 to 0·0182)	0·015 (0·0123 to 0·018)	0·014 (0·0115 to 0·0168)	0·0137 (0·0111 to 0·0166)
Sri Lanka	3·36 (2·6 to 4·29)	3·45 (2·59 to 4·49)	3·76 (2·76 to 5·09)	4·47 (3·27 to 6)	5·05 (3·69 to 6·76)	5·33 (3·9 to 7·11)	5·75 (4·19 to 7·67)	5·48 (4 to 7·27)	4·75 (3·54 to 6·26)	4·71 (3·61 to 6·08)	4·9 (3·8 to 6·3)	4·7 (3·66 to 6·01)	4·36 (3·31 to 5·63)	4·25 (3·25 to 5·46)	3·92 (3·07 to 4·98)	3·85 (3·05 to 4·78)	3·61 (2·93 to 4·39)	2·94 (2·47 to 3·47)	2·83 (2·61 to 3·07)	3·1 (2·95 to 3·27)	3 (2·74 to 3·28)	2·77 (2·33 to 3·29)	2·45 (1·99 to 2·94)	2·27 (1·86 to 2·73)	1·85 (1·62 to 2·12)	1·61 (1·43 to 1·82)	1·6 (1·39 to 1·84)	1·52 (1·35 to 1·72)	1·57 (1·37 to 1·81)	1·59 (1·28 to 1·96)	1·47 (1·12 to 1·94)	1·34 (0·99 to 1·83)
Thailand	17·9 (12·6 to 24·9)	16·8 (11·7 to 23)	15·8 (11 to 21·6)	14·9 (10·1 to 20·4)	14 (9·45 to 19·4)	13·2 (9 to 18·3)	12·2 (8·29 to 17·2)	11·2 (7·7 to 15·5)	10·4 (7·19 to 14·4)	9·59 (6·65 to 13·3)	8·83 (6·23 to 12·3)	8·17 (5·8 to 11·4)	7·57 (5·42 to 10·5)	7·03 (4·98 to 9·82)	6·56 (4·64 to 8·99)	6·15 (4·33 to 8·52)	5·7 (4·02 to 7·92)	5·23 (3·65 to 7·4)	4·82 (3·4 to 6·73)	4·54 (3·2 to 6·33)	4·22 (2·99 to 5·88)	3·89 (2·75 to 5·37)	3·7 (2·62 to 5·04)	3·44 (2·44 to 4·71)	3·39 (2·37 to 4·65)	3·34 (2·31 to 4·67)	3·37 (2·37 to 4·62)	3·12 (2·19 to 4·29)	3·08 (2·15 to 4·25)	2·85 (1·99 to 3·98)	2·52 (1·78 to 3·54)	2·43 (1·74 to 3·45)
Timor-Leste	1·92 (1·33 to 2·69)	1·83 (1·26 to 2·54)	1·75 (1·21 to 2·42)	1·67 (1·12 to 2·32)	1·59 (1·06 to 2·22)	1·5 (1·02 to 2·1)	1·43 (0·962 to 2·03)	1·37 (0·929 to 1·9)	1·3 (0·891 to 1·81)	1·26 (0·864 to 1·75)	1·18 (0·828 to 1·66)	1·12 (0·788 to 1·57)	1·05 (0·747 to 1·47)	0·986 (0·694 to 1·39)	0·926 (0·65 to 1·28)	0·872 (0·611 to 1·22)	0·824 (0·578 to 1·15)	0·785 (0·544 to 1·12)	0·752 (0·529 to 1·06)	0·722 (0·507 to 1·01)	0·694 (0·489 to 0·97)	0·674 (0·475 to 0·934)	0·654 (0·46 to 0·895)	0·632 (0·446 to 0·868)	0·619 (0·431 to 0·852)	0·622 (0·429 to 0·873)	0·621 (0·435 to 0·855)	0·615 (0·43 to 0·848)	0·617 (0·43 to 0·854)	0·621 (0·433 to 0·873)	0·629 (0·441 to 0·885)	0·635 (0·453 to 0·904)
Viet Nam	46·2 (32·4 to 63·6)	44·1 (31·5 to 60·6)	41·7 (30·2 to 56·6)	39·3 (28·9 to 52·6)	36·8 (27·2 to 49·1)	34·5 (26·1 to 45·6)	32·3 (24·7 to 41·8)	30·4 (23·3 to 38·9)	29 (22·5 to 36·8)	27·7 (22 to 34·6)	26·6 (21·2 to 32·8)	25·4 (21 to 30·7)	24·3 (20·4 to 28·6)	23·2 (20·1 to 26·7)	22·3 (19·9 to 25)	22·1 (20·2 to 24·2)	22·5 (20·7 to 24·4)	23·1 (21·1 to 25·2)	24·3 (21·6 to 27·1)	25·2 (21·7 to 29)	25·2 (20·9 to 29·8)	25·3 (20·4 to 30·9)	24·9 (19·5 to 31·3)	24·6 (18·8 to 31·2)	24·1 (18·2 to 31·3)	23·7 (17·6 to 31)	23·2 (17 to 31·4)	22·6 (16·3 to 30·7)	21·9 (16 to 30·2)	21 (15·1 to 28·7)	20·1 (14·4 to 27·6)	18·8 (13·6 to 26·2)
Sub-Saharan Africa	1260 (979 to 1630)	1270 (996 to 1640)	1280 (1010 to 1640)	1290 (1020 to 1620)	1290 (1030 to 1620)	1300 (1050 to 1620)	1310 (1070 to 1630)	1320 (1090 to 1630)	1330 (1110 to 1630)	1340 (1130 to 1620)	1350 (1140 to 1610)	1360 (1170 to 1600)	1370 (1190 to 1580)	1370 (1200 to 1560)	1380 (1220 to 1570)	1400 (1250 to 1570)	1410 (1270 to 1570)	1400 (1270 to 1550)	1400 (1280 to 1540)	1410 (1300 to 1550)	1430 (1310 to 1560)	1430 (1320 to 1560)	1430 (1320 to 1560)	1440 (1330 to 1560)	1450 (1350 to 1570)	1460 (1360 to 1580)	1470 (1370 to 1600)	1460 (1350 to 1600)	1450 (1320 to 1610)	1430 (1280 to 1620)	1400 (1230 to 1610)	1380 (1200 to 1600)
Central sub-Saharan Africa	121 (90·6 to 162)	122 (91·4 to 161)	123 (92·9 to 163)	125 (94·3 to 161)	126 (96·8 to 161)	127 (97·5 to 164)	128 (98·7 to 165)	129 (99·7 to 165)	131 (101 to 165)	132 (102 to 165)	133 (106 to 164)	134 (109 to 162)	136 (115 to 162)	139 (121 to 161)	142 (127 to 159)	146 (135 to 158)	151 (142 to 162)	158 (149 to 169)	161 (150 to 174)	161 (144 to 180)	159 (138 to 183)	155 (132 to 183)	151 (125 to 182)	149 (122 to 183)	147 (120 to 182)	147 (117 to 183)	149 (117 to 190)	150 (116 to 195)	150 (114 to 201)	147 (112 to 200)	141 (106 to 193)	137 (104 to 187)
Angola	23·6 (16·8 to 32·7)	23·9 (16·9 to 33·1)	24·2 (16·7 to 33·4)	24·5 (16·8 to 34·3)	24·8 (17·1 to 34·9)	25 (17·5 to 35·9)	25·1 (17·8 to 35·7)	25·3 (17·7 to 36·3)	25·4 (17·8 to 36·5)	25·2 (17·7 to 35·8)	24·8 (17·6 to 34·9)	24·5 (17·5 to 34·4)	24·2 (17·4 to 33·1)	23·8 (17·4 to 32·3)	23·5 (17·2 to 31·6)	23·2 (17·1 to 30·6)	23 (17·1 to 30·1)	22·9 (17·1 to 29·7)	22·6 (17·5 to 28·9)	22·3 (17·6 to 28)	22·1 (17·9 to 26·9)	21·8 (18·2 to 25·9)	21·4 (18·5 to 24·6)	20·7 (18·8 to 22·6)	20·1 (19·3 to 20·9)	19·8 (19·1 to 20·7)	19·7 (17·9 to 21·8)	19·7 (17·1 to 22·6)	19·6 (16·4 to 23·4)	19·6 (15·8 to 24)	19·3 (15·2 to 24·2)	19·4 (14·9 to 25)
Central African Republic	11·7 (7·98 to 16·6)	11·7 (7·96 to 16·4)	11·8 (8 to 16·5)	11·9 (7·89 to 16·8)	12 (7·89 to 17)	12·1 (8·08 to 17·2)	12·2 (8·06 to 17·6)	12·2 (8·15 to 17·2)	12·2 (8·23 to 17·2)	12·2 (8·3 to 17·4)	12·3 (8·49 to 17·5)	12·4 (8·6 to 17·7)	12·4 (8·69 to 17·6)	12·4 (8·61 to 17·7)	12·4 (8·61 to 17·4)	12·5 (8·64 to 17·8)	12·6 (8·69 to 17·9)	12·7 (8·65 to 18·4)	12·5 (8·69 to 17·9)	12·1 (8·36 to 17·2)	11·1 (7·69 to 15·7)	9·13 (6·38 to 12·8)	7·24 (5·06 to 9·97)	6·54 (4·6 to 9·03)	6·47 (4·48 to 8·96)	6·62 (4·54 to 9·35)	7·02 (4·88 to 9·73)	7·46 (5·18 to 10·4)	7·81 (5·4 to 10·9)	7·83 (5·41 to 11·1)	7·61 (5·3 to 10·8)	7·46 (5·27 to 10·7)
Congo (Brazzaville)	3·74 (2·6 to 5·24)	3·81 (2·64 to 5·27)	3·89 (2·68 to 5·35)	3·96 (2·67 to 5·48)	4·03 (2·69 to 5·62)	4·11 (2·79 to 5·77)	4·22 (2·84 to 5·99)	4·32 (2·94 to 6·02)	4·41 (3·01 to 6·14)	4·52 (3·11 to 6·33)	4·63 (3·23 to 6·51)	4·71 (3·31 to 6·65)	4·78 (3·39 to 6·7)	4·86 (3·41 to 6·86)	4·93 (3·45 to 6·82)	4·96 (3·46 to 6·95)	4·94 (3·45 to 6·93)	4·96 (3·43 to 7·1)	4·91 (3·44 to 6·93)	4·76 (3·33 to 6·71)	4·4 (3·09 to 6·18)	3·69 (2·59 to 5·12)	2·94 (2·07 to 4·02)	2·62 (1·85 to 3·59)	2·58 (1·79 to 3·55)	2·66 (1·83 to 3·73)	2·83 (1·98 to 3·9)	2·97 (2·07 to 4·1)	3·08 (2·14 to 4·27)	3·05 (2·12 to 4·3)	2·91 (2·04 to 4·11)	2·84 (2·02 to 4·05)
DR Congo	79·3 (56 to 110)	80·3 (56 to 111)	80·8 (56·4 to 113)	81·5 (58·2 to 113)	82·3 (59·2 to 110)	83·2 (59·4 to 112)	84 (60·5 to 113)	84·8 (62·4 to 111)	85·7 (64·4 to 111)	86·7 (66·9 to 110)	87·9 (69·7 to 109)	89·5 (72·6 to 108)	92 (78·2 to 109)	95 (83·2 to 108)	98·1 (89·2 to 108)	102 (98·1 to 106)	107 (104 to 111)	114 (111 to 118)	118 (114 to 123)	119 (108 to 132)	119 (102 to 139)	118 (97·6 to 143)	117 (93·6 to 145)	117 (91·4 to 148)	116 (89·7 to 148)	116 (87·8 to 151)	118 (86·8 to 154)	118 (85·9 to 159)	117 (84·1 to 163)	114 (81·8 to 162)	108 (76·5 to 155)	105 (75·1 to 149)
Equatorial Guinea	1·06 (0·731 to 1·48)	1·08 (0·746 to 1·5)	1·11 (0·76 to 1·53)	1·13 (0·758 to 1·57)	1·16 (0·769 to 1·62)	1·19 (0·805 to 1·68)	1·24 (0·83 to 1·76)	1·23 (0·835 to 1·72)	1·24 (0·848 to 1·74)	1·26 (0·864 to 1·77)	1·28 (0·894 to 1·81)	1·27 (0·89 to 1·79)	1·27 (0·901 to 1·78)	1·29 (0·904 to 1·82)	1·3 (0·912 to 1·81)	1·34 (0·937 to 1·89)	1·36 (0·947 to 1·91)	1·39 (0·957 to 1·99)	1·41 (0·986 to 2)	1·38 (0·962 to 1·94)	1·28 (0·899 to 1·8)	1·09 (0·763 to 1·51)	0·878 (0·617 to 1·2)	0·785 (0·554 to 1·08)	0·786 (0·547 to 1·08)	0·834 (0·574 to 1·17)	0·916 (0·64 to 1·26)	1·01 (0·701 to 1·39)	1·08 (0·75 to 1·5)	1·12 (0·776 to 1·58)	1·1 (0·766 to 1·55)	1·08 (0·77 to 1·55)
Gabon	1·54 (1·05 to 2·12)	1·54 (1·07 to 2·14)	1·54 (1·06 to 2·16)	1·55 (1·06 to 2·2)	1·56 (1·09 to 2·23)	1·57 (1·1 to 2·21)	1·58 (1·1 to 2·23)	1·61 (1·13 to 2·32)	1·63 (1·15 to 2·36)	1·67 (1·17 to 2·41)	1·7 (1·2 to 2·43)	1·7 (1·19 to 2·45)	1·71 (1·2 to 2·4)	1·72 (1·2 to 2·41)	1·73 (1·2 to 2·41)	1·74 (1·23 to 2·41)	1·77 (1·27 to 2·44)	1·79 (1·29 to 2·47)	1·81 (1·3 to 2·49)	1·82 (1·34 to 2·48)	1·8 (1·34 to 2·41)	1·71 (1·31 to 2·27)	1·58 (1·21 to 2·04)	1·5 (1·18 to 1·9)	1·46 (1·19 to 1·81)	1·42 (1·18 to 1·7)	1·37 (1·19 to 1·58)	1·34 (1·21 to 1·47)	1·31 (1·25 to 1·36)	1·26 (1·21 to 1·31)	1·18 (1·07 to 1·3)	1·12 (0·975 to 1·29)
Eastern sub-Saharan Africa	550 (421 to 704)	555 (431 to 710)	558 (439 to 710)	560 (447 to 704)	559 (451 to 694)	559 (455 to 688)	555 (459 to 674)	548 (458 to 658)	543 (464 to 645)	539 (467 to 630)	537 (472 to 618)	535 (471 to 610)	534 (475 to 603)	537 (480 to 610)	541 (485 to 611)	545 (489 to 614)	545 (490 to 612)	522 (473 to 586)	503 (460 to 554)	499 (465 to 544)	496 (464 to 534)	483 (451 to 523)	473 (441 to 511)	472 (440 to 511)	473 (441 to 512)	471 (436 to 513)	473 (431 to 522)	467 (413 to 528)	460 (399 to 532)	455 (390 to 539)	448 (378 to 542)	443 (369 to 547)
Burundi	18·9 (13·1 to 26·4)	19 (13·2 to 26·5)	19·1 (13·1 to 27·2)	19·1 (13·2 to 27·8)	18·5 (12·8 to 26·7)	18·2 (12·8 to 26·2)	18·4 (13 to 26·6)	18 (12·8 to 25·6)	17·5 (12·7 to 24·3)	17·3 (12·5 to 23·6)	17·3 (12·7 to 23·1)	17·4 (12·7 to 22·7)	17·4 (13 to 22·6)	17·5 (13·4 to 22·6)	17·7 (13·7 to 22·4)	17·9 (14·3 to 22·3)	18·1 (14·8 to 21·9)	18·1 (15·3 to 21·3)	18·1 (16 to 20·4)	18·4 (17·1 to 19·9)	19·3 (17·9 to 20·6)	20·4 (18·5 to 22·4)	21·4 (19 to 23·9)	22·3 (19·9 to 25)	23·3 (21 to 25·8)	24·1 (22·4 to 26)	24·1 (22·3 to 26)	23·4 (20·7 to 26·3)	22·8 (19·4 to 26·6)	22·4 (18·5 to 26·9)	21·9 (17·5 to 27)	21·5 (16·7 to 27·2)
Comoros	1·77 (1·21 to 2·51)	1·76 (1·2 to 2·47)	1·74 (1·18 to 2·43)	1·72 (1·14 to 2·41)	1·69 (1·11 to 2·39)	1·66 (1·11 to 2·36)	1·62 (1·07 to 2·33)	1·57 (1·05 to 2·21)	1·53 (1·03 to 2·15)	1·49 (1·01 to 2·11)	1·47 (1·01 to 2·09)	1·43 (0·997 to 2·05)	1·39 (0·979 to 1·97)	1·36 (0·944 to 1·94)	1·33 (0·921 to 1·86)	1·3 (0·899 to 1·84)	1·26 (0·872 to 1·79)	1·21 (0·829 to 1·75)	1·16 (0·804 to 1·65)	1·11 (0·77 to 1·58)	1·06 (0·738 to 1·5)	1·02 (0·708 to 1·42)	0·976 (0·679 to 1·35)	0·936 (0·654 to 1·3)	0·903 (0·622 to 1·26)	0·87 (0·594 to 1·23)	0·839 (0·582 to 1·17)	0·811 (0·562 to 1·13)	0·786 (0·543 to 1·1)	0·765 (0·528 to 1·09)	0·746 (0·519 to 1·06)	0·73 (0·516 to 1·05)
Djibouti	0·826 (0·571 to 1·16)	0·911 (0·626 to 1·27)	0·988 (0·677 to 1·37)	0·977 (0·653 to 1·36)	0·935 (0·619 to 1·31)	0·913 (0·616 to 1·29)	0·907 (0·607 to 1·3)	0·903 (0·61 to 1·26)	0·912 (0·62 to 1·28)	0·933 (0·638 to 1·31)	0·959 (0·667 to 1·35)	0·983 (0·689 to 1·39)	1 (0·708 to 1·41)	1·02 (0·712 to 1·44)	1·04 (0·722 to 1·44)	1·05 (0·73 to 1·48)	1·05 (0·729 to 1·47)	1·04 (0·714 to 1·49)	1·02 (0·713 to 1·45)	1·01 (0·702 to 1·42)	0·991 (0·694 to 1·4)	0·981 (0·687 to 1·37)	0·969 (0·678 to 1·34)	0·957 (0·672 to 1·32)	0·942 (0·652 to 1·31)	0·923 (0·633 to 1·3)	0·895 (0·623 to 1·24)	0·864 (0·601 to 1·2)	0·832 (0·576 to 1·16)	0·797 (0·553 to 1·13)	0·758 (0·53 to 1·07)	0·727 (0·516 to 1·04)
Eritrea	8·31 (5·75 to 11·7)	8·09 (5·56 to 11·2)	7·51 (5·15 to 10·4)	7 (4·69 to 9·74)	6·78 (4·51 to 9·5)	6·78 (4·59 to 9·54)	6·75 (4·52 to 9·6)	6·64 (4·5 to 9·27)	6·52 (4·45 to 9·09)	6·39 (4·38 to 8·95)	6·3 (4·4 to 8·86)	6·23 (4·38 to 8·81)	6·16 (4·37 to 8·64)	6·16 (4·32 to 8·71)	6·22 (4·35 to 8·61)	6·27 (4·37 to 8·79)	6·23 (4·35 to 8·75)	6·09 (4·21 to 8·72)	5·99 (4·19 to 8·46)	5·91 (4·13 to 8·32)	5·83 (4·09 to 8·19)	5·73 (4·02 to 7·97)	5·62 (3·94 to 7·73)	5·6 (3·94 to 7·73)	5·51 (3·82 to 7·62)	5·44 (3·74 to 7·66)	5·34 (3·72 to 7·37)	5·27 (3·67 to 7·29)	5·19 (3·6 to 7·21)	5·12 (3·55 to 7·21)	5·04 (3·52 to 7·12)	4·99 (3·54 to 7·13)
Ethiopia	170 (118 to 238)	172 (120 to 241)	175 (120 to 248)	176 (122 to 256)	176 (122 to 253)	176 (124 to 253)	175 (124 to 253)	172 (123 to 243)	170 (124 to 237)	168 (122 to 230)	166 (122 to 222)	163 (120 to 213)	160 (119 to 207)	157 (120 to 201)	153 (119 to 194)	150 (120 to 186)	145 (119 to 175)	139 (118 to 163)	132 (117 to 148)	125 (116 to 135)	117 (109 to 126)	109 (99·2 to 120)	103 (91·9 to 115)	97·5 (87 to 109)	92·5 (83·7 to 102)	88·4 (82·5 to 95·1)	86·3 (80 to 92·7)	85·5 (76 to 95·6)	85 (72·6 to 98·8)	84·8 (70·2 to 101)	84·6 (67·7 to 104)	84·9 (66·5 to 107)
Addis Ababa	4·1 (2·58 to 5·95)	4·04 (2·63 to 5·75)	3·97 (2·79 to 5·49)	3·89 (2·82 to 5·16)	3·81 (2·79 to 5·02)	3·77 (2·76 to 5·11)	3·75 (2·66 to 5·24)	3·67 (2·55 to 5·17)	3·64 (2·45 to 5·25)	3·64 (2·39 to 5·24)	3·55 (2·38 to 5·09)	3·52 (2·43 to 5)	3·46 (2·52 to 4·69)	3·33 (2·6 to 4·26)	3·19 (2·63 to 3·89)	2·91 (2·44 to 3·45)	2·42 (2·03 to 2·88)	1·98 (1·68 to 2·3)	1·56 (1·37 to 1·78)	1·2 (1·11 to 1·3)	1·03 (0·934 to 1·12)	0·965 (0·842 to 1·12)	0·953 (0·8 to 1·12)	0·943 (0·776 to 1·12)	0·909 (0·744 to 1·09)	0·857 (0·699 to 1·04)	0·819 (0·659 to 1)	0·789 (0·627 to 0·987)	0·757 (0·587 to 0·955)	0·726 (0·558 to 0·908)	0·695 (0·55 to 0·872)	0·666 (0·541 to 0·827)
Afar	2·38 (1·35 to 3·8)	2·73 (1·6 to 4·24)	3·08 (1·89 to 4·69)	3·42 (2·17 to 5·03)	3·76 (2·54 to 5·4)	4·08 (2·85 to 5·71)	4·34 (3·05 to 6·06)	4·52 (3·14 to 6·38)	4·66 (3·21 to 6·65)	4·72 (3·18 to 6·85)	4·74 (3·11 to 6·9)	4·71 (3·06 to 6·93)	4·62 (3·04 to 6·8)	4·54 (2·99 to 6·73)	4·45 (2·86 to 6·71)	4·3 (2·71 to 6·57)	4·1 (2·56 to 6·36)	3·85 (2·43 to 6·08)	3·54 (2·31 to 5·26)	3·16 (2·17 to 4·54)	2·88 (1·99 to 4·08)	2·83 (1·96 to 3·91)	2·79 (1·97 to 3·82)	2·78 (1·99 to 3·82)	2·63 (1·87 to 3·61)	2·56 (1·88 to 3·47)	2·49 (1·79 to 3·39)	2·44 (1·69 to 3·45)	2·38 (1·63 to 3·34)	2·32 (1·63 to 3·17)	2·26 (1·67 to 3)	2·2 (1·71 to 2·85)
Amhara	58·4 (37·6 to 87·4)	58·3 (36·8 to 89·2)	58·6 (36·4 to 92·7)	58·5 (36·4 to 92·7)	58·3 (36·5 to 91)	58·1 (37·1 to 88·2)	57·5 (38·2 to 87·6)	56·5 (38·2 to 83·4)	55·6 (39·1 to 79·4)	54·4 (39·2 to 74·4)	53 (39·2 to 70·2)	51·5 (38·7 to 66·9)	49·9 (38·8 to 63·8)	48·2 (38·6 to 59·6)	46·7 (38·8 to 56·5)	45·1 (38 to 53·8)	43·3 (36·9 to 50·7)	40·9 (35·2 to 47·1)	38·2 (33·4 to 43·3)	34 (31·2 to 37·1)	28·5 (26·1 to 30·8)	23·8 (21·2 to 26·5)	20·5 (18·1 to 23·1)	17·9 (15·7 to 20·2)	16·1 (14·1 to 18·1)	15 (13·1 to 17·1)	14·4 (12·3 to 16·7)	14·2 (11·6 to 16·9)	14·1 (10·8 to 17·5)	14·1 (10·2 to 18·4)	14·1 (9·79 to 19·5)	14·2 (9·3 to 20·3)
Benishangul-Gumuz	2·37 (1·48 to 3·45)	2·28 (1·48 to 3·25)	2·21 (1·55 to 3·08)	2·14 (1·54 to 2·86)	2·07 (1·51 to 2·74)	2·03 (1·48 to 2·76)	1·99 (1·41 to 2·78)	1·92 (1·33 to 2·71)	1·87 (1·26 to 2·7)	1·84 (1·21 to 2·65)	1·8 (1·21 to 2·59)	1·75 (1·21 to 2·49)	1·7 (1·24 to 2·31)	1·66 (1·3 to 2·12)	1·61 (1·33 to 1·97)	1·61 (1·35 to 1·91)	1·63 (1·37 to 1·94)	1·66 (1·41 to 1·94)	1·7 (1·5 to 1·93)	1·63 (1·51 to 1·77)	1·54 (1·4 to 1·69)	1·52 (1·32 to 1·76)	1·53 (1·27 to 1·8)	1·53 (1·26 to 1·83)	1·5 (1·22 to 1·82)	1·45 (1·17 to 1·76)	1·41 (1·13 to 1·73)	1·38 (1·09 to 1·75)	1·36 (1·05 to 1·73)	1·34 (1·03 to 1·69)	1·33 (1·04 to 1·67)	1·31 (1·06 to 1·64)
Dire Dawa	0·626 (0·373 to 0·947)	0·589 (0·367 to 0·864)	0·558 (0·366 to 0·792)	0·531 (0·366 to 0·729)	0·504 (0·364 to 0·686)	0·486 (0·357 to 0·66)	0·474 (0·338 to 0·663)	0·459 (0·312 to 0·667)	0·45 (0·303 to 0·672)	0·44 (0·283 to 0·664)	0·429 (0·269 to 0·646)	0·417 (0·258 to 0·621)	0·401 (0·254 to 0·59)	0·388 (0·257 to 0·555)	0·378 (0·256 to 0·53)	0·361 (0·265 to 0·483)	0·345 (0·267 to 0·441)	0·325 (0·265 to 0·398)	0·301 (0·258 to 0·35)	0·269 (0·246 to 0·295)	0·247 (0·222 to 0·272)	0·246 (0·204 to 0·289)	0·242 (0·194 to 0·294)	0·24 (0·191 to 0·294)	0·242 (0·198 to 0·291)	0·229 (0·194 to 0·268)	0·221 (0·187 to 0·258)	0·216 (0·182 to 0·255)	0·211 (0·176 to 0·252)	0·207 (0·169 to 0·252)	0·201 (0·163 to 0·248)	0·197 (0·159 to 0·242)
Gambella	0·546 (0·324 to 0·831)	0·533 (0·331 to 0·786)	0·525 (0·343 to 0·747)	0·519 (0·356 to 0·714)	0·51 (0·367 to 0·696)	0·504 (0·368 to 0·686)	0·501 (0·356 to 0·702)	0·498 (0·338 to 0·726)	0·496 (0·333 to 0·743)	0·492 (0·316 to 0·746)	0·488 (0·305 to 0·734)	0·479 (0·296 to 0·714)	0·467 (0·295 to 0·689)	0·456 (0·301 to 0·653)	0·446 (0·302 to 0·627)	0·434 (0·317 to 0·581)	0·414 (0·32 to 0·529)	0·386 (0·314 to 0·472)	0·356 (0·305 to 0·415)	0·318 (0·291 to 0·35)	0·291 (0·261 to 0·32)	0·28 (0·232 to 0·33)	0·283 (0·227 to 0·345)	0·282 (0·224 to 0·347)	0·273 (0·223 to 0·329)	0·259 (0·219 to 0·303)	0·252 (0·212 to 0·293)	0·248 (0·209 to 0·292)	0·245 (0·204 to 0·292)	0·243 (0·198 to 0·297)	0·241 (0·195 to 0·297)	0·24 (0·194 to 0·295)
Harari	0·429 (0·242 to 0·687)	0·421 (0·246 to 0·656)	0·415 (0·254 to 0·634)	0·411 (0·26 to 0·604)	0·4 (0·27 to 0·574)	0·393 (0·275 to 0·55)	0·389 (0·274 to 0·541)	0·383 (0·267 to 0·54)	0·382 (0·264 to 0·544)	0·378 (0·254 to 0·546)	0·372 (0·245 to 0·543)	0·365 (0·238 to 0·535)	0·358 (0·236 to 0·524)	0·354 (0·234 to 0·524)	0·35 (0·226 to 0·526)	0·348 (0·22 to 0·529)	0·338 (0·212 to 0·522)	0·317 (0·201 to 0·5)	0·297 (0·194 to 0·439)	0·27 (0·186 to 0·387)	0·247 (0·171 to 0·348)	0·238 (0·165 to 0·327)	0·239 (0·169 to 0·326)	0·24 (0·172 to 0·329)	0·237 (0·168 to 0·324)	0·227 (0·166 to 0·306)	0·219 (0·157 to 0·297)	0·212 (0·147 to 0·3)	0·205 (0·14 to 0·287)	0·197 (0·138 to 0·269)	0·192 (0·142 to 0·254)	0·187 (0·145 to 0·242)
Oromia	56 (37·1 to 83·4)	57·6 (36·8 to 86·7)	59·1 (36·4 to 91·6)	60 (36·6 to 95·2)	60 (36·4 to 96·6)	60 (36·5 to 96·8)	59·5 (36·6 to 96·4)	58·2 (36·5 to 91·8)	57 (36·8 to 86·8)	55·9 (37·5 to 82·9)	54·6 (37·7 to 78·7)	53·1 (37 to 73·6)	51·4 (37·1 to 71·1)	50 (36·9 to 67)	48·5 (36·3 to 64·8)	46·8 (34·9 to 61·8)	45·2 (34·8 to 58·1)	43·5 (35·2 to 53·5)	42·1 (36·5 to 48·1)	43·9 (40·2 to 48·2)	45·3 (41·6 to 48·9)	42·6 (38 to 47·7)	39·6 (34·5 to 44·6)	36·8 (32·1 to 41·5)	34·7 (30·2 to 39·4)	33 (28·4 to 37·1)	32·3 (27·7 to 37·2)	32·2 (26·4 to 38·2)	32·3 (24·7 to 40·2)	32·5 (23·5 to 42·5)	32·8 (22·6 to 44·2)	33·3 (21·7 to 47·2)
Somali	10·3 (6·08 to 15·7)	10·8 (6·72 to 16·1)	11·5 (7·47 to 16·4)	12 (8·23 to 16·6)	12·7 (9·09 to 17·4)	13·3 (9·66 to 18·2)	13·9 (9·82 to 19·6)	14·3 (9·66 to 21)	14·8 (9·84 to 22·2)	15·2 (9·7 to 23·1)	15·5 (9·61 to 23·4)	15·6 (9·61 to 23·4)	15·7 (9·83 to 23·2)	15·7 (10·3 to 22·5)	15·5 (10·5 to 22)	15·3 (11·1 to 20·6)	15 (11·6 to 19·3)	14·6 (11·9 to 17·9)	14·1 (12 to 16·4)	13·2 (12 to 14·5)	12·5 (11·2 to 13·7)	12·5 (10·3 to 14·8)	12·7 (10·1 to 15·5)	12·9 (10·2 to 15·9)	12·8 (10·4 to 15·5)	12·4 (10·4 to 14·6)	12·2 (10·2 to 14·3)	12 (10·1 to 14·3)	11·9 (9·8 to 14·2)	11·8 (9·51 to 14·4)	11·5 (9·26 to 14·3)	11·4 (9·16 to 14·1)
Southern Nations, Nationalities, and Peoples	21·7 (15·1 to 29·5)	21·4 (15·4 to 28)	21 (16·2 to 26·6)	20·6 (16·3 to 25·7)	20 (16·2 to 24·5)	19·6 (15·9 to 24·4)	19·3 (15·4 to 24·4)	18·8 (14·8 to 24·2)	18·7 (14·4 to 24·2)	18·9 (14 to 25·3)	19·3 (13·5 to 26·8)	19·8 (13·8 to 28·2)	20·3 (14·2 to 28·9)	21·1 (14·6 to 29·6)	21·8 (15·6 to 29·9)	22·3 (16·7 to 29·6)	22·6 (17·7 to 28·6)	22·5 (18·5 to 27·4)	21·8 (18·8 to 25·1)	20·1 (18·4 to 22)	18·5 (16·9 to 20·3)	18·2 (15·9 to 20·9)	18·1 (15·4 to 20·9)	18·1 (15·2 to 21·3)	17·5 (14·5 to 20·9)	17·1 (13·9 to 20·6)	16·9 (13·5 to 20·7)	16·8 (13·2 to 21·1)	16·8 (12·9 to 21·3)	16·7 (12·8 to 20·9)	16·6 (13 to 20·8)	16·6 (13·4 to 20·8)
Tigray	13·6 (7·97 to 20·9)	13·6 (8·38 to 20·3)	13·6 (8·83 to 19·6)	13·6 (9·27 to 18·8)	13·5 (9·68 to 18·6)	13·5 (9·75 to 18·5)	13·4 (9·4 to 18·8)	13·1 (8·81 to 19·2)	12·9 (8·56 to 19·3)	12·5 (8 to 19·1)	12·2 (7·54 to 18·4)	11·7 (7·21 to 17·5)	11·3 (7·09 to 16·7)	10·9 (7·14 to 15·7)	10·5 (7·05 to 14·8)	9·97 (7·26 to 13·4)	9·38 (7·23 to 12)	8·76 (7·12 to 10·7)	8·03 (6·87 to 9·36)	7·11 (6·5 to 7·81)	6·49 (5·82 to 7·15)	6·27 (5·19 to 7·39)	6·05 (4·84 to 7·38)	5·88 (4·66 to 7·26)	5·57 (4·54 to 6·73)	5·28 (4·45 to 6·2)	5·07 (4·27 to 5·94)	4·93 (4·15 to 5·83)	4·8 (3·97 to 5·73)	4·68 (3·8 to 5·72)	4·58 (3·7 to 5·66)	4·5 (3·63 to 5·54)
Kenya	44·5 (32·6 to 59·1)	45·1 (33·3 to 59·9)	46 (34·6 to 59·9)	46·8 (35·9 to 59·9)	47·4 (37·5 to 59·5)	48 (38·9 to 59)	48·5 (40·3 to 58·7)	49·2 (41·7 to 58·1)	50 (43·2 to 57·6)	51 (44·6 to 58·2)	52 (46·5 to 58·6)	52·9 (48·2 to 58·2)	53·7 (49·8 to 57·9)	53·9 (50·4 to 57·6)	53·4 (50·1 to 57·2)	52·9 (49·3 to 56·9)	52·4 (48·8 to 56·3)	51·9 (48·2 to 56·1)	51 (46·9 to 55·6)	49·6 (45·2 to 54·8)	47·9 (42·9 to 53·8)	46·4 (40·8 to 53)	45 (39 to 52·2)	43·6 (37·5 to 51·4)	42·6 (36·1 to 50·8)	41·4 (34·5 to 49·7)	40·2 (33 to 49·4)	39 (31·6 to 48·4)	37·7 (30·2 to 47·4)	36·4 (28·6 to 46·9)	34·8 (26·8 to 45·5)	33·7 (25·9 to 44·6)
Baringo	1·08 (0·75 to 1·53)	[illegible]	[illegible]	[illegible]	[illegible]	[illegible]	[illegible]	[illegible]	[illegible]	[illegible]	[illegible]	[illegible]	[illegible]	[illegible]	[illegible]	[illegible]	[illegible]	[illegible]	[illegible]	[illegible]	[illegible]	[illegible]	[illegible]	[illegible]	[illegible]	[illegible]	[illegible]	[illegible]	[illegible]	[illegible]	[illegible]	[illegible]
Bomet	0·675 (0·469 to 0·941)	[illegible]	[illegible]	[illegible]	[illegible]	[illegible]	[illegible]	[illegible]	[illegible]	[illegible]	[illegible]	[illegible]	[illegible]	[illegible]	[illegible]	[illegible]	[illegible]	[illegible]	[illegible]	[illegible]	[illegible]	[illegible]	[illegible]	[illegible]	[illegible]	[illegible]	[illegible]	[illegible]	[illegible]	[illegible]	[illegible]	[illegible]
Bungoma	1·51 (1·13 to 1·96)	[illegible]	[illegible]	[illegible]	[illegible]	[illegible]	[illegible]	[illegible]	[illegible]	[illegible]	[illegible]	[illegible]	[illegible]	[illegible]	[illegible]	[illegible]	[illegible]	[illegible]	[illegible]	[illegible]	[illegible]	[illegible]	[illegible]	[illegible]	[illegible]	[illegible]	[illegible]	[illegible]	[illegible]	[illegible]	[illegible]	[illegible]
Busia	1·18 (0·864 to 1·54)	[illegible]	[illegible]	[illegible]	[illegible]	[illegible]	[illegible]	[illegible]	[illegible]	[illegible]	[illegible]	[illegible]	[illegible]	[illegible]	[illegible]	[illegible]	[illegible]	[illegible]	[illegible]	[illegible]	[illegible]	[illegible]	[illegible]	[illegible]	[illegible]	[illegible]	[illegible]	[illegible]	[illegible]	[illegible]	[illegible]	[illegible]
Elgeyo Marakwet	0·522 (0·364 to 0·725)	[illegible]	[illegible]	[illegible]	[illegible]	[illegible]	[illegible]	[illegible]	[illegible]	[illegible]	[illegible]	[illegible]	[illegible]	[illegible]	[illegible]	[illegible]	[illegible]	[illegible]	[illegible]	[illegible]	[illegible]	[illegible]	[illegible]	[illegible]	[illegible]	[illegible]	[illegible]	[illegible]	[illegible]	[illegible]	[illegible]	[illegible]
Embu	0·593 (0·417 to 0·821)	[illegible]	[illegible]	[illegible]	[illegible]	[illegible]	[illegible]	[illegible]	[illegible]	[illegible]	[illegible]	[illegible]	[illegible]	[illegible]	[illegible]	[illegible]	[illegible]	[illegible]	[illegible]	[illegible]	[illegible]	[illegible]	[illegible]	[illegible]	[illegible]	[illegible]	[illegible]	[illegible]	[illegible]	[illegible]	[illegible]	[illegible]
Garissa	0·249 (0·173 to 0·346)	[illegible]	[illegible]	[illegible]	[illegible]	[illegible]	[illegible]	[illegible]	[illegible]	[illegible]	[illegible]	[illegible]	[illegible]	[illegible]	[illegible]	[illegible]	[illegible]	[illegible]	[illegible]	[illegible]	[illegible]	[illegible]	[illegible]	[illegible]	[illegible]	[illegible]	[illegible]	[illegible]	[illegible]	[illegible]	[illegible]	[illegible]
Homa Bay	1·7 (1·18 to 2·38)	[illegible]	[illegible]	[illegible]	[illegible]	[illegible]	[illegible]	[illegible]	[illegible]	[illegible]	[illegible]	[illegible]	[illegible]	[illegible]	[illegible]	[illegible]	[illegible]	[illegible]	[illegible]	[illegible]	[illegible]	[illegible]	[illegible]	[illegible]	[illegible]	[illegible]	[illegible]	[illegible]	[illegible]	[illegible]	[illegible]	[illegible]
Isiolo	0·178 (0·123 to 0·248)	[illegible]	[illegible]	[illegible]	[illegible]	[illegible]	[illegible]	[illegible]	[illegible]	[illegible]	[illegible]	[illegible]	[illegible]	[illegible]	[illegible]	[illegible]	[illegible]	[illegible]	[illegible]	[illegible]	[illegible]	[illegible]	[illegible]	[illegible]	[illegible]	[illegible]	[illegible]	[illegible]	[illegible]	[illegible]	[illegible]	[illegible]
Kajiado	0·507 (0·357 to 0·701)	[illegible]	[illegible]	[illegible]	[illegible]	[illegible]	[illegible]	[illegible]	[illegible]	[illegible]	[illegible]	[illegible]	[illegible]	[illegible]	[illegible]	[illegible]	[illegible]	[illegible]	[illegible]	[illegible]	[illegible]	[illegible]	[illegible]	[illegible]	[illegible]	[illegible]	[illegible]	[illegible]	[illegible]	[illegible]	[illegible]	[illegible]
Kakamega	2·49 (1·75 to 3·46)	[illegible]	[illegible]	[illegible]	[illegible]	[illegible]	[illegible]	[illegible]	[illegible]	[illegible]	[illegible]	[illegible]	[illegible]	[illegible]	[illegible]	[illegible]	[illegible]	[illegible]	[illegible]	[illegible]	[illegible]	[illegible]	[illegible]	[illegible]	[illegible]	[illegible]	[illegible]	[illegible]	[illegible]	[illegible]	[illegible]	[illegible]
Kericho	1·24 (0·865 to 1·72)	[illegible]	[illegible]	[illegible]	[illegible]	[illegible]	[illegible]	[illegible]	[illegible]	[illegible]	[illegible]	[illegible]	[illegible]	[illegible]	[illegible]	[illegible]	[illegible]	[illegible]	[illegible]	[illegible]	[illegible]	[illegible]	[illegible]	[illegible]	[illegible]	[illegible]	[illegible]	[illegible]	[illegible]	[illegible]	[illegible]	[illegible]
Kiambu	0·578 (0·408 to 0·801)	[illegible]	[illegible]	[illegible]	[illegible]	[illegible]	[illegible]	[illegible]	[illegible]	[illegible]	[illegible]	[illegible]	[illegible]	[illegible]	[illegible]	[illegible]	[illegible]	[illegible]	[illegible]	[illegible]	[illegible]	[illegible]	[illegible]	[illegible]	[illegible]	[illegible]	[illegible]	[illegible]	[illegible]	[illegible]	[illegible]	[illegible]
Kilifi	1·42 (1·05 to 1·86)	[illegible]	[illegible]	[illegible]	[illegible]	[illegible]	[illegible]	[illegible]	[illegible]	[illegible]	[illegible]	[illegible]	[illegible]	[illegible]	[illegible]	[illegible]	[illegible]	[illegible]	[illegible]	[illegible]	[illegible]	[illegible]	[illegible]	[illegible]	[illegible]	[illegible]	[illegible]	[illegible]	[illegible]	[illegible]	[illegible]	[illegible]
Kirinyaga	0·347 (0·242 to 0·484)	[illegible]	[illegible]	[illegible]	[illegible]	[illegible]	[illegible]	[illegible]	[illegible]	[illegible]	[illegible]	[illegible]	[illegible]	[illegible]	[illegible]	[illegible]	[illegible]	[illegible]	[illegible]	[illegible]	[illegible]	[illegible]	[illegible]	[illegible]	[illegible]	[illegible]	[illegible]	[illegible]	[illegible]	[illegible]	[illegible]	[illegible]
Kisii	0·973 (0·726 to 1·28)	[illegible]	[illegible]	[illegible]	[illegible]	[illegible]	[illegible]	[illegible]	[illegible]	[illegible]	[illegible]	[illegible]	[illegible]	[illegible]	[illegible]	[illegible]	[illegible]	[illegible]	[illegible]	[illegible]	[illegible]	[illegible]	[illegible]	[illegible]	[illegible]	[illegible]	[illegible]	[illegible]	[illegible]	[illegible]	[illegible]	[illegible]
Kisumu	3·2 (2·2 to 4·55)	[illegible]	[illegible]	[illegible]	[illegible]	[illegible]	[illegible]	[illegible]	[illegible]	[illegible]	[illegible]	[illegible]	[illegible]	[illegible]	[illegible]	[illegible]	[illegible]	[illegible]	[illegible]	[illegible]	[illegible]	[illegible]	[illegible]	[illegible]	[illegible]	[illegible]	[illegible]	[illegible]	[illegible]	[illegible]	[illegible]	[illegible]
Kitui	0·856 (0·594 to 1·18)	[illegible]	[illegible]	[illegible]	[illegible]	[illegible]	[illegible]	[illegible]	[illegible]	[illegible]	[illegible]	[illegible]	[illegible]	[illegible]	[illegible]	[illegible]	[illegible]	[illegible]	[illegible]	[illegible]	[illegible]	[illegible]	[illegible]	[illegible]	[illegible]	[illegible]	[illegible]	[illegible]	[illegible]	[illegible]	[illegible]	[illegible]
Kwale	0·94 (0·698 to 1·22)	[illegible]	[illegible]	[illegible]	[illegible]	[illegible]	[illegible]	[illegible]	[illegible]	[illegible]	[illegible]	[illegible]	[illegible]	[illegible]	[illegible]	[illegible]	[illegible]	[illegible]	[illegible]	[illegible]	[illegible]	[illegible]	[illegible]	[illegible]	[illegible]	[illegible]	[illegible]	[illegible]	[illegible]	[illegible]	[illegible]	[illegible]
Laikipia	0·176 (0·123 to 0·243)	[illegible]	[illegible]	[illegible]	[illegible]	[illegible]	[illegible]	[illegible]	[illegible]	[illegible]	[illegible]	[illegible]	[illegible]	[illegible]	[illegible]	[illegible]	[illegible]	[illegible]	[illegible]	[illegible]	[illegible]	[illegible]	[illegible]	[illegible]	[illegible]	[illegible]	[illegible]	[illegible]	[illegible]	[illegible]	[illegible]	[illegible]
Lamu	0·195 (0·134 to 0·276)	[illegible]	[illegible]	[illegible]	[illegible]	[illegible]	[illegible]	[illegible]	[illegible]	[illegible]	[illegible]	[illegible]	[illegible]	[illegible]	[illegible]	[illegible]	[illegible]	[illegible]	[illegible]	[illegible]	[illegible]	[illegible]	[illegible]	[illegible]	[illegible]	[illegible]	[illegible]	[illegible]	[illegible]	[illegible]	[illegible]	[illegible]
Machakos	1·37 (0·964 to 1·89)	[illegible]	[illegible]	[illegible]	[illegible]	[illegible]	[illegible]	[illegible]	[illegible]	[illegible]	[illegible]	[illegible]	[illegible]	[illegible]	[illegible]	[illegible]	[illegible]	[illegible]	[illegible]	[illegible]	[illegible]	[illegible]	[illegible]	[illegible]	[illegible]	[illegible]	[illegible]	[illegible]	[illegible]	[illegible]	[illegible]	[illegible]
Makueni	1·15 (0·802 to 1·61)	[illegible]	[illegible]	[illegible]	[illegible]	[illegible]	[illegible]	[illegible]	[illegible]	[illegible]	[illegible]	[illegible]	[illegible]	[illegible]	[illegible]	[illegible]	[illegible]	[illegible]	[illegible]	[illegible]	[illegible]	[illegible]	[illegible]	[illegible]	[illegible]	[illegible]	[illegible]	[illegible]	[illegible]	[illegible]	[illegible]	[illegible]
Mandera	0·601 (0·416 to 0·846)	[illegible]	[illegible]	[illegible]	[illegible]	[illegible]	[illegible]	[illegible]	[illegible]	[illegible]	[illegible]	[illegible]	[illegible]	[illegible]	[illegible]	[illegible]	[illegible]	[illegible]	[illegible]	[illegible]	[illegible]	[illegible]	[illegible]	[illegible]	[illegible]	[illegible]	[illegible]	[illegible]	[illegible]	[illegible]	[illegible]	[illegible]
Marsabit	0·372 (0·258 to 0·521)	[illegible]	[illegible]	[illegible]	[illegible]	[illegible]	[illegible]	[illegible]	[illegible]	[illegible]	[illegible]	[illegible]	[illegible]	[illegible]	[illegible]	[illegible]	[illegible]	[illegible]	[illegible]	[illegible]	[illegible]	[illegible]	[illegible]	[illegible]	[illegible]	[illegible]	[illegible]	[illegible]	[illegible]	[illegible]	[illegible]	[illegible]
Meru	1·86 (1·3 to 2·6)	[illegible]	[illegible]	[illegible]	[illegible]	[illegible]	[illegible]	[illegible]	[illegible]	[illegible]	[illegible]	[illegible]	[illegible]	[illegible]	[illegible]	[illegible]	[illegible]	[illegible]	[illegible]	[illegible]	[illegible]	[illegible]	[illegible]	[illegible]	[illegible]	[illegible]	[illegible]	[illegible]	[illegible]	[illegible]	[illegible]	[illegible]
Migori	1·93 (1·34 to 2·7)	[illegible]	[illegible]	[illegible]	[illegible]	[illegible]	[illegible]	[illegible]	[illegible]	[illegible]	[illegible]	[illegible]	[illegible]	[illegible]	[illegible]	[illegible]	[illegible]	[illegible]	[illegible]	[illegible]	[illegible]	[illegible]	[illegible]	[illegible]	[illegible]	[illegible]	[illegible]	[illegible]	[illegible]	[illegible]	[illegible]	[illegible]
Mombasa	0·827 (0·579 to 1·15)	[illegible]	[illegible]	[illegible]	[illegible]	[illegible]	[illegible]	[illegible]	[illegible]	[illegible]	[illegible]	[illegible]	[illegible]	[illegible]	[illegible]	[illegible]	[illegible]	[illegible]	[illegible]	[illegible]	[illegible]	[illegible]	[illegible]	[illegible]	[illegible]	[illegible]	[illegible]	[illegible]	[illegible]	[illegible]	[illegible]	[illegible]
Murang'a	0·788 (0·551 to 1·09)	[illegible]	[illegible]	[illegible]	[illegible]	[illegible]	[illegible]	[illegible]	[illegible]	[illegible]	[illegible]	[illegible]	[illegible]	[illegible]	[illegible]	[illegible]	[illegible]	[illegible]	[illegible]	[illegible]	[illegible]	[illegible]	[illegible]	[illegible]	[illegible]	[illegible]	[illegible]	[illegible]	[illegible]	[illegible]	[illegible]	[illegible]
Nairobi	1·1 (0·809 to 1·44)	[illegible]	[illegible]	[illegible]	[illegible]	[illegible]	[illegible]	[illegible]	[illegible]	[illegible]	[illegible]	[illegible]	[illegible]	[illegible]	[illegible]	[illegible]	[illegible]	[illegible]	[illegible]	[illegible]	[illegible]	[illegible]	[illegible]	[illegible]	[illegible]	[illegible]	[illegible]	[illegible]	[illegible]	[illegible]	[illegible]	[illegible]
Nakuru	1·57 (1·09 to 2·19)	[illegible]	[illegible]	[illegible]	[illegible]	[illegible]	[illegible]	[illegible]	[illegible]	[illegible]	[illegible]	[illegible]	[illegible]	[illegible]	[illegible]	[illegible]	[illegible]	[illegible]	[illegible]	[illegible]	[illegible]	[illegible]	[illegible]	[illegible]	[illegible]	[illegible]	[illegible]	[illegible]	[illegible]	[illegible]	[illegible]	[illegible]
[truncated: 516,941 more chars]
